# Cross-species genetic screens identify transglutaminase 5 as a regulator of polyglutamine-expanded ataxin-1

**DOI:** 10.1172/JCI156616

**Published:** 2022-05-02

**Authors:** Won-Seok Lee, Ismael Al-Ramahi, Hyun-Hwan Jeong, Youjin Jang, Tao Lin, Carolyn J. Adamski, Laura A. Lavery, Smruti Rath, Ronald Richman, Vitaliy V. Bondar, Elizabeth Alcala, Jean-Pierre Revelli, Harry T. Orr, Zhandong Liu, Juan Botas, Huda Y. Zoghbi

**Affiliations:** 1 Integrative Molecular and Biomedical Science Program, and; 2 Department of Molecular and Human Genetics, Baylor College of Medicine, Houston, Texas, USA.; 3 Jan and Dan Duncan Neurological Research Institute, Houston, Texas, USA.; 4 Department of Pediatrics-Neurology, and; 5 Department of Pathology and Immunology, Baylor College of Medicine, Houston, Texas, USA.; 6 Howard Hughes Medical Institute, Houston, Texas, USA.; 7 Exceptional Research Opportunities Program, Howard Hughes Medical Institute, Houston, Texas, USA.; 8 Institute for Translational Neuroscience, University of Minnesota, Minneapolis, Minnesota, USA.

**Keywords:** Genetics, Neuroscience, Genetic diseases, Molecular pathology, Neurodegeneration

## Abstract

Many neurodegenerative disorders are caused by abnormal accumulation of misfolded proteins. In spinocerebellar ataxia type 1 (SCA1), accumulation of polyglutamine-expanded (polyQ-expanded) ataxin-1 (ATXN1) causes neuronal toxicity. Lowering total ATXN1, especially the polyQ-expanded form, alleviates disease phenotypes in mice, but the molecular mechanism by which the mutant ATXN1 is specifically modulated is not understood. Here, we identified 22 mutant ATXN1 regulators by performing a cross-species screen of 7787 and 2144 genes in human cells and *Drosophila* eyes, respectively. Among them, transglutaminase 5 (TG5) preferentially regulated mutant ATXN1 over the WT protein. TG enzymes catalyzed cross-linking of ATXN1 in a polyQ-length–dependent manner, thereby preferentially modulating mutant ATXN1 stability and oligomerization. Perturbing Tg in *Drosophila* SCA1 models modulated mutant ATXN1 toxicity. Moreover, TG5 was enriched in the nuclei of SCA1-affected neurons and colocalized with nuclear ATXN1 inclusions in brain tissue from patients with SCA1. Our work provides a molecular insight into SCA1 pathogenesis and an opportunity for allele-specific targeting for neurodegenerative disorders.

## Graphical abstract

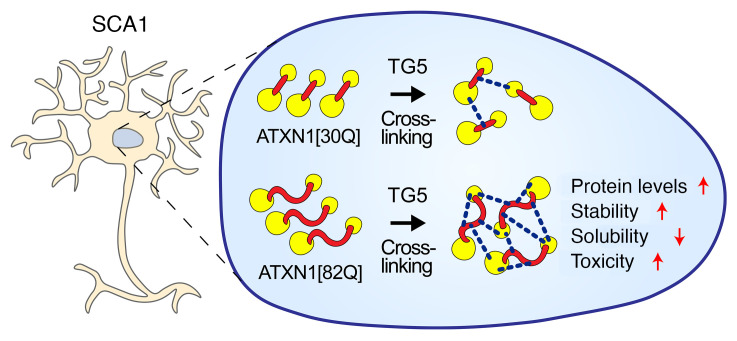

## Introduction

Neurodegenerative disorders are a common cause of morbidity and mortality in elderly people due to progressive loss of brain functions, including cognition, motor control, and bulbar function (1, 2). Though each neurodegenerative disorder is triggered by a specific disease-causing protein, a common pathological hallmark is the accumulation of specific misfolded proteins in both familial and sporadic cases (3, 4). For example, accumulation of β-amyloid (Aβ), α-synuclein (α-syn), and huntingtin (HTT) are found in Alzheimer’s disease (AD), Parkinson’s disease (PD), and Huntington’s disease (HD), respectively. Lowering levels of these proteins is beneficial and often rescues various disease phenotypes (5–7). Among the neurodegenerative proteinopathies, polyglutamine (polyQ) expansion disorders, such as HD and many spinocerebellar ataxias (SCAs), are caused by an accumulation of polyQ-expanded proteins encoded by dominantly inherited mutant alleles. Specific targeting of the mutant proteins is critical because some WT proteins have essential biological functions, raising concerns that targeting both WT and mutant proteins can induce deleterious side effects. For example, reducing HTT in HD and TATA-binding protein (TBP) in SCA17 can disturb neurogenesis and global transcription, respectively (8, 9).

SCA type 1 (SCA1) is a fatal neurodegenerative proteinopathy caused by an expansion of polyQ-encoding CAG repeats in the ataxin-1 (*ATXN1*) allele (10). The polyQ expansion stabilizes mutant ATXN1, resulting in its toxic accumulation in the nuclei of cerebellar and brainstem neurons (11, 12). Patients with SCA1 experience motor deficits and bulbar dysfunction, leading to difficulties in breathing and swallowing. Although previous work has shown that reducing both WT and mutant ATXN1 protein levels can rescue disease phenotypes in an SCA1 mouse model (13–15), it is critical to reduce mutant ATXN1 specifically for the following reasons: (1) loss of WT ATXN1 can potentiate AD by increasing Aβ deposition (16); (2) WT ATXN1 is protective for SCA1 (17); (3) selective reduction of mutant ATXN1 is more effective in rescuing disease phenotype than targeting both mutant and WT protein (18). Therefore, identifying mutant ATXN1-specific regulators is critical to understanding the molecular pathogenesis of SCA1 and to finding more effective therapeutic targets for the disease. However, to date, no such regulators have been found.

To identify regulators of mutant ATXN1, we performed a cross-species genetic screen in a human cell line and in *Drosophila*. We identified 22 posttranscriptional regulators of ATXN1 and focused our mechanistic studies on transglutaminase 5 (TG5) because of its preferential effect on mutant ATXN1 levels over the WT protein. TGs (gene name *TGM*) are calcium-dependent enzymes that can mediate intramolecular and intermolecular covalent cross-linking of proteins by forming an isopeptide bond between the side chains of glutamine and lysine residues (19). There are 8 genes that encode catalytically active TGs in the human genome. TG2 is ubiquitously expressed and has been implicated in many neurodegenerative disorders, including AD and PD (19, 20), as TG2 can cross-link Aβ and α-syn in vitro (21, 22). TG2 is also known to cross-link proteins with polyQ, such as HTT, androgen receptor, and ATXN1 in vitro (23–25). The contribution of other TGs to neurodegeneration is largely unknown, although some are considerably expressed in the brain. Moreover, it is largely unknown how TG-mediated cross-linking regulates the levels or stability of these neurodegeneration-driving proteins, even though their levels are critical to drive pathology (7, 26–28).

In this study, we found that TG5 can preferentially modulate mutant ATXN1 levels, stability, solubility, and oligomerization by catalyzing cross-links of ATXN1 in a polyQ-length–dependent manner. Tg regulates mutant ATXN1-mediated toxicity in *Drosophila* SCA1 models. Moreover, TG5 but not TG2 resides in the nuclei and colocalizes with ATXN1 nuclear inclusions (NIs) in the neurons of brain tissue from patients with SCA1. Our data suggest a potentially new therapeutic target for SCA1 and may provide insights into the selective lowering of mutant allele products in other neurodegenerative proteinopathies.

## Results

### Cross-species screen reveals regulators of mutant ATXN1 levels.

**Figure 1:**
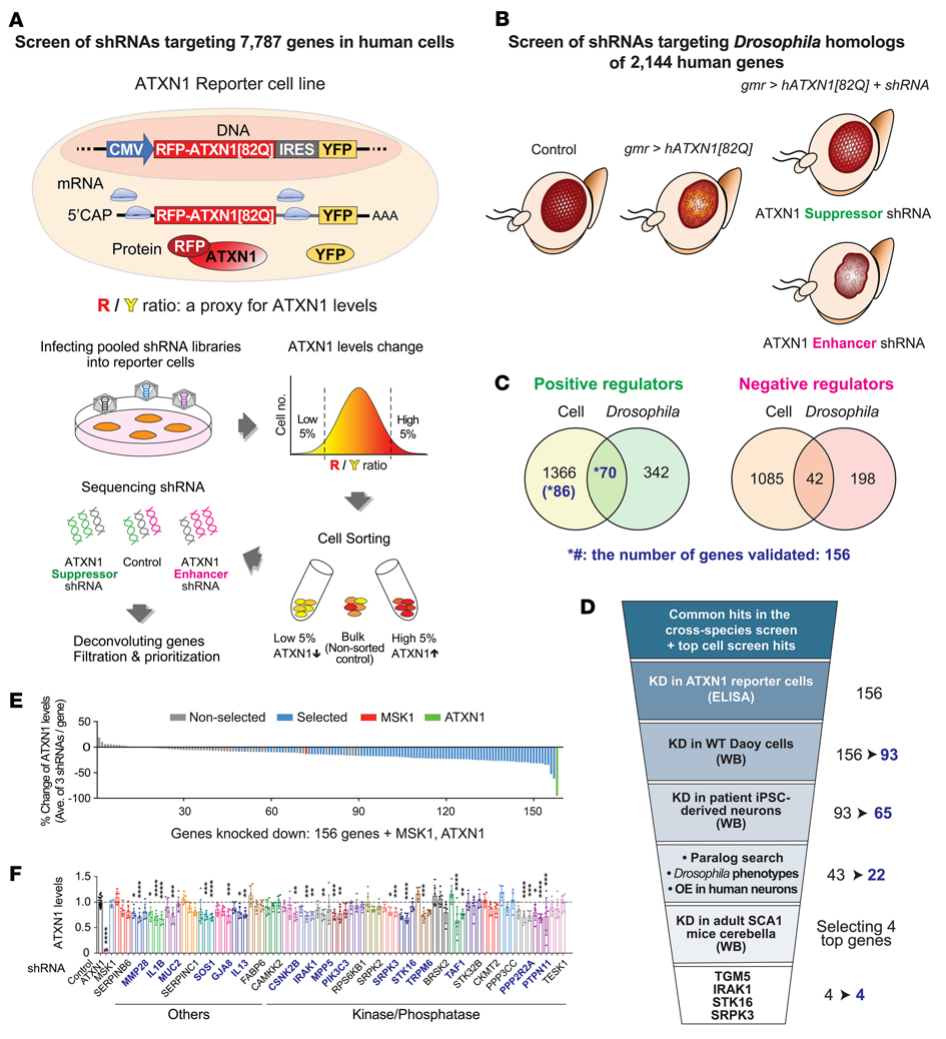
Cross-species screen of druggable genes reveals potential regulators of ATXN1. (**A**) Schematic representation of the cell-based screen. ATXN1 reporter cells produce RFP-ATXN1[82Q] and YFP from the same transcript through separate translation processes. ATXN1[82Q] levels are monitored by RFP/YFP ratio whereby YFP normalizes ATXN1 levels. After retroviral transduction of the reporter cells with pooled shRNA libraries targeting 7787 genes, cells were subjected to FACS to collect the cells with the lowest 5% and highest 5% RFP/YFP ratio. Genomic DNAs of these cells were extracted, and Illumina sequencing revealed relative enrichment of each shRNA in the sorted cells compared with the non-sorted bulk population. Identifying the genes targeted by the enriched or depleted shRNAs in each group revealed regulators of ATXN1 protein levels, which were filtered and prioritized (see Methods). (**B**) A diagram for modifier screen in *Drosophila*. Ectopic expression of human mutant ATXN1[82Q] in *Drosophila* eyes induces retinal degeneration. This fly was crossed with shRNA fly lines that target 1102 *Drosophila* genes corresponding to 2144 human homologs for identifying genes that suppress or exacerbate external eye phenotype. (**C**) The number of genes that overlapped between cell-based screen and *Drosophila*-based screen. The 156 positive regulators, including 70 genes that overlapped in the 2 screens and 86 top cell screen hits were selected for validation (See Methods for criteria applied). (**D**) A diagram for tiered validation of potential ATXN1 regulators. The number of genes before and after each validation step is displayed. (**E**) Summary of ATXN1[82Q] ELISA result presented as averaged percentage changes of ATXN1[82Q] levels after knockdown of 156 genes by 3 shRNAs in the ATXN1 reporter cells. MSK1 and ATXN1 were included as positive controls. (**F**) A representative ATXN1[82Q] ELISA result after knockdown of the genes that belong to the “others” and ”kinase/phosphatase” libraries. Individual bar displays ATXN1 levels of each shRNA. Blue-colored genes are selected for next validations. Data shown as mean ± SD, *P < 0.05, **P < 0.01, ***P < 0.001, ****P < 0.0001, 1-way ANOVA, post hoc Dunnett’s test.

To identify potential drug targets that posttranslationally regulate ATXN1 protein levels, we performed a cross-species screen of 7787 genes that encode potentially druggable targets in human cells and 1102 *Drosophila* genes corresponding to 2144 human homologs in *Drosophila* (Figure 1, A and B). In the cell-based screen, we used an ATXN1 reporter Daoy (human cerebellar medulloblastoma) cell line that expressed a bicistronic transgene (mRFP-ATXN1[82Q]-IRES-YFP) to monitor human mutant ATXN1[82Q] levels by measuring the red to yellow fluorescent protein (RFP/YFP) ratio (Figure 1A) (13). These reporter cells were transduced with a pooled library of shRNAs (~10 shRNAs/gene), and cells displaying a decrease (5% lowest) or increase (5% highest) in the RFP/YFP ratio were collected by FACS. After extracting genomic DNA and amplifying hairpin sequences, the relative abundance of individual shRNA in the sorted cells compared with the non-sorted bulk control was identified by next-generation sequencing. We classified the shRNAs into suppressors and enhancers — suppressors were the shRNAs enriched in the cells with the 5% lowest or depleted in the cells with the 5% highest RFP/YFP ratio, whereas enhancers were the shRNAs enriched in the cells with the 5% highest or depleted in the cells with the 5% lowest RFP/YFP ratio. For the screen in *Drosophila* eyes, we used a fly model that expressed human mutant ATXN1[82Q] in their eyes via the UAS/GAL4 system. These flies display degeneration of external eye structure in proportion to the expression levels of ATXN1[82Q] (29) (Figure 1B). We crossed these flies with *Drosophila* RNAi lines targeting 2144 human homologs. The shRNAs that ameliorated or exacerbated the eye degeneration were defined as suppressors and enhancers, respectively. Finally, deconvoluting the genes targeted by the suppressors and enhancers from the 2 independent screens revealed the positive and negative ATXN1 regulators, respectively. The regulators identified from the cell-based screen were filtered and ranked (see Methods, Supplemental Table 1; supplemental material available online with this article; https://doi.org/10.1172/JCI156616DS1), followed by overlap with the regulators identified from the *Drosophila* screen (Figure 1C and Supplemental Table 2). Among the 2 groups of ATXN1 regulators, we focused on positive regulators because their inhibition may provide a therapeutic opportunity for SCA1 by decreasing mutant ATXN1. We selected 156 genes for validation, including 70 genes that were shared in both screens and 86 genes that were the top scored hits in the cell-based screen.

**Figure 2:**
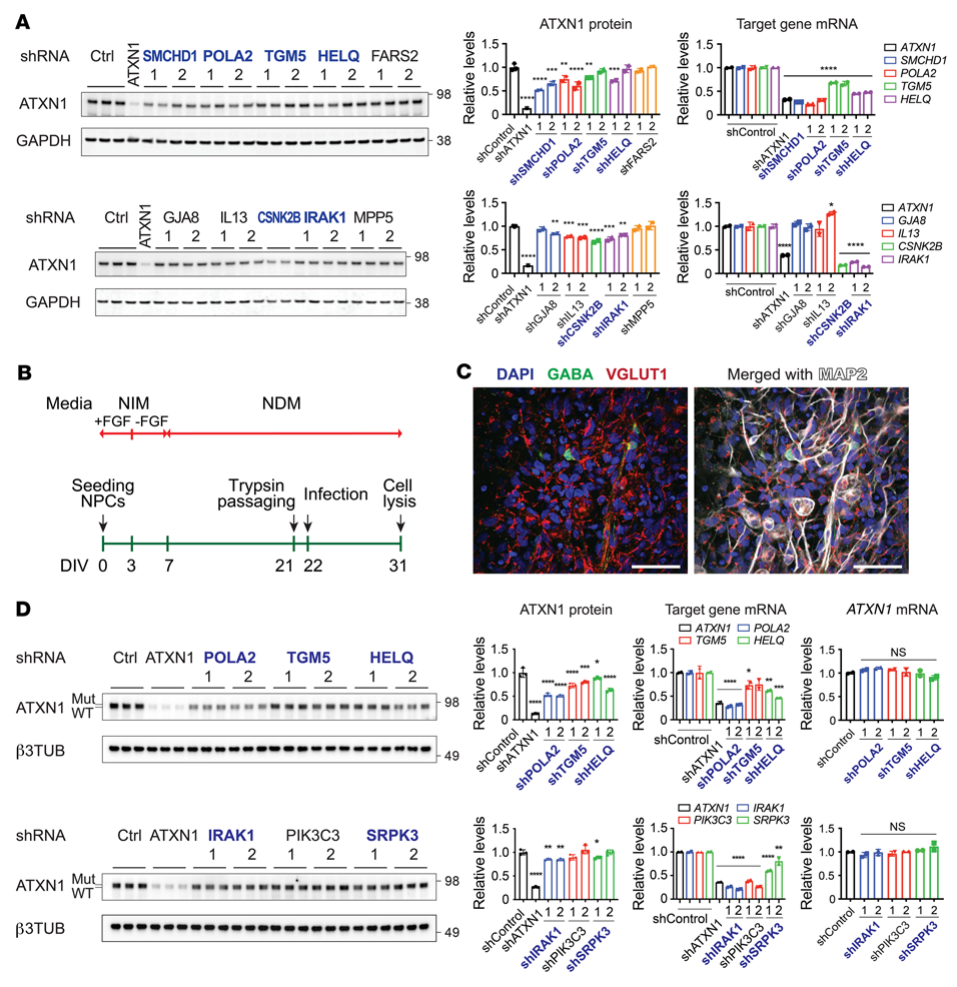
Validation of ATXN1 regulators in Daoy cells and iPSC-derived neurons from patients with SCA1. (**A**) Representative Western blot analysis of endogenous ATXN1 and qRT-PCR results of target genes after knockdown of 93 genes for 9 days in WT Daoy cells. ATXN1 shRNA was used as positive control. Data shown as mean ± SD, *P < 0.05, **P < 0.01, ***P < 0.001, ****P < 0.0001, 1-way ANOVA, post hoc Dunnett’s test for left graphs; Tukey’s test for right graphs. Blue-colored genes selected for further validation. (**B**) Culturing scheme of patient iPSC-derived neurons. iPSCs were first differentiated into NPCs, and then differentiated into neurons by incubating NPCs in NIM and NDM. (**C**) Immunofluorescent (IF) image of MAP2, VGLUT1, and GABA in the iPSC-derived neurons from patients with SCA1 after 3 weeks of differentiation. Scale bar: 50 μm. (**D**) Representative Western blot analysis of mutant and WT ATXN1, and qRT-PCR results of target genes and *ATXN1* after knockdown of ATXN1 regulators for 9 days in iPSC-derived neurons from patients with SCA1. Blue-colored genes are validated ATXN1 regulators. Data shown as mean ± SD, *P < 0.05, **P < 0.01, ***P < 0.001, ****P < 0.0001, 1-way ANOVA, post hoc Dunnett’s test for the left and right graphs; Tukey’s test for the middle graphs.

These 156 genes were validated in a stepwise manner (Figure 1D and Supplemental Table 3). First, we knocked down the 156 regulators individually in the ATXN1 reporter cells by transducing with 3 new shRNAs per gene (Supplemental Table 4) and measured the ATXN1[82Q] protein levels by ELISA (Figure 1E). As expected, most of the genes (143/156) behaved as positive ATXN1 regulators, i.e., their knockdown decreased ATXN1[82Q] levels. Encouragingly, knockdown of over half of them (85/156) decreased ATXN1[82Q] to a greater extent than knockdown of MSK1, a verified ATXN1 regulator that was used as a positive control (Figure 1E), revealing the soundness of our screen. We monitored the effect of individual shRNAs on ATXN1[82Q] levels and selected 93 genes that had at least one shRNA that significantly decreased ATXN1[82Q] and the other shRNA that showed a downward trend of ATXN1[82Q] levels (Figure 1F and Supplemental Figure 1). Next, these 93 genes were individually knocked down in WT Daoy cells via lentiviral transduction of shRNAs (2 shRNAs/gene, Supplemental Table 5). We measured endogenous ATXN1 levels by Western blot analysis and monitored target gene knockdown using quantitative real-time PCR (qRT-PCR) (Figure 2A and Supplemental Figure 2; see complete unedited blots in the supplemental material). Sixty-five genes were selected based on the following criteria: upon significant knockdown of target genes, (1) at least one shRNA significantly reduced ATXN1 or (2) two shRNAs showed a downward trend of ATXN1, with one of them decreasing ATXN1 by over 15%.

### TGM5, IRAK1, SRPK3, and STK16 regulate ATXN1 in iPSC-derived neurons from patients with SCA1 and SCA1 animal models.

To narrow down our candidate regulators, we investigated 43 out of the 65 genes based on absence of toxicity upon lentiviral transduction of shRNAs in iPSC-derived neurons from patients with SCA1, which expressed WT ATXN1[33Q] and mutant ATXN1[46Q]. The iPSCs were first differentiated into neural progenitor cells (NPCs), and then differentiated into neurons by incubating them serially in neural induction medium (NIM) and neural differentiation medium (NDM) (Figure 2B). Immunofluorescent (IF) staining revealed that most of the differentiated neurons were VGLUT1^+^ excitatory neurons (Figure 2C). After inhibiting target genes for 9 days by lentiviral transduction of shRNA (2 shRNA/gene, Supplemental Table 5), ATXN1 protein, mRNA, and target gene mRNA levels were measured. Given that the cell-based screen was designed to identify posttranslational regulators of ATXN1, we selected 22 genes that when inhibited, reduced total ATXN1 protein levels without changing *ATXN1* mRNA, but for 3 of these genes, the knockdown was not confirmed (Supplemental Table 6, Figure 2D, and Supplemental Figure 3; see complete unedited blots in the supplemental material).

**Figure 3:**
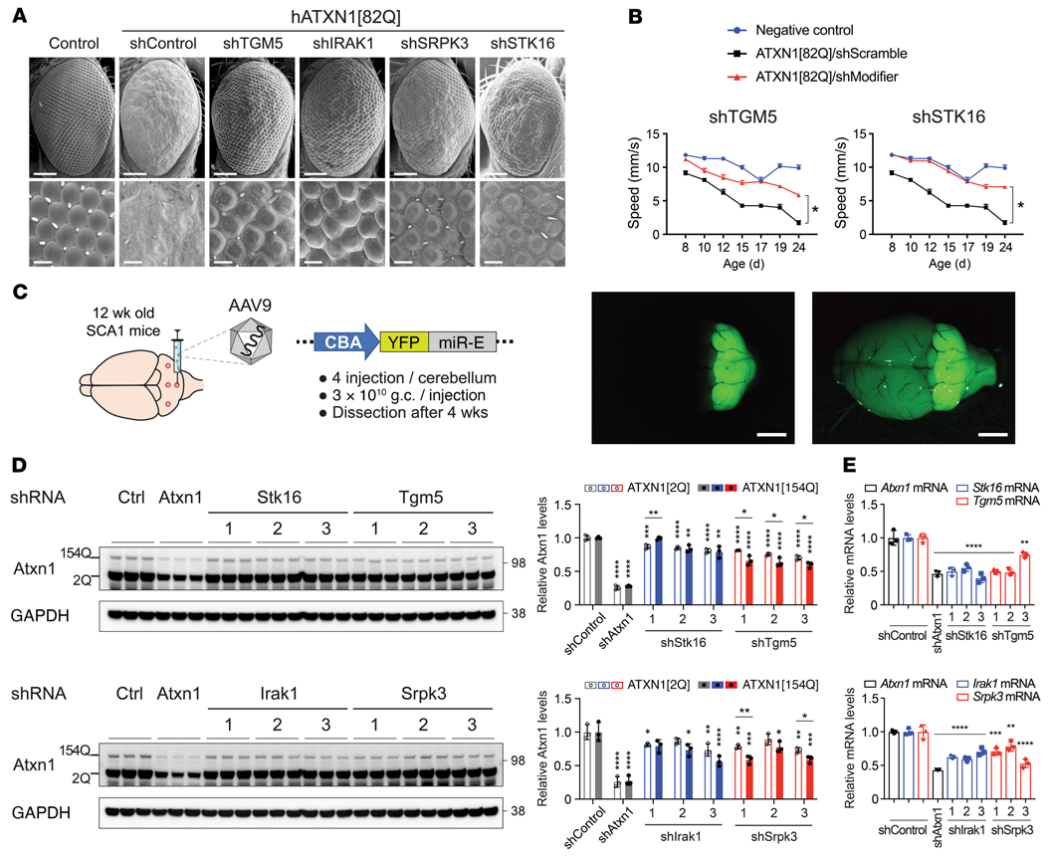
Validation of TGM5, IRAK1, SRPK3, and STK16 in SCA1 animal models. (**A**) Scanning electron microscopy images of *Drosophila* eyes expressing human ATXN1[82Q] with knockdown of *Drosophila* homologs of TGM5, IRAK1, SRPK3, or STK16. Scale bar: 100 μm in the top images; 10 μm in the bottom images. (**B**) Effect of TGM5 or STK16 knockdown on the motor performance of *Drosophila* SCA1 model expressing ATXN1[82Q] in the CNS. Data shown as mean ± SEM, *P < 0.05, linear mixed-effect model ANOVA. (**C**) Schematic representation for stereotaxic injection of adeno-associated virus serotype 9 (AAV9) carrying shRNAs into the cerebella of adult SCA1 mice (left), and representative fluorescence brain images with or without bright field taken after 4 weeks of the injection (right). Scale bar: 2 mm. (**D**) Western blot analysis of WT and mutant ATXN1 in the cerebella of SCA1 mice after the knockdown of *Stk16*, *Tgm5*, *Irak1*, or *Srpk3* using 3 different shRNAs. Data shown as mean ± SD, *P < 0.05, **P < 0.01, ***P < 0.001, ****P < 0.0001; 1-way ANOVA was performed in ATXN1[2Q] and ATXN1[154Q] separately. Post hoc Dunnett’s test; 2-tailed *t* test was used for comparing ATXN1[2Q] and ATXN1[154Q]. (**E**) qRT-PCR data of the mRNA levels of the 4 genes knocked down in **D**. Data shown as mean ± SD, **P < 0.01, ***P < 0.001, ****P < 0.0001, 1-way ANOVA, post hoc Tukey’s test.

To prioritize the 22 genes, we revisited and interrogated the original cell-based screen data to identify paralogs that are positive ATXN1 regulators, checked for phenotypic rescue in 2 different *Drosophila* SCA1 models, and measured increases of ATXN1 levels after overexpressing them in iPSC-derived neurons from patients with SCA1 (Supplemental Table 6, Figure 3, A and B, Supplemental Figure 4, A–C, and Supplemental Figure 5). Based on these approaches, we selected *TGM5* and 3 kinases (*IRAK1*, *SRPK3*, and *STK16*) given the consistency of their effects on ATXN1 levels in multiple assays.

We then investigated whether our prioritized targets regulate ATXN1 protein levels in the brain in an SCA1 mouse model. This mouse model has a polyQ-expanded *Atxn1* allele knocked into the endogenous locus (*Atxn1^154Q/2Q^*) and faithfully recapitulates features of SCA1, including motor deficits, breathing difficulties, and short lifespan (30). We performed stereotaxic injections of adeno-associated virus serotype 9 (AAV9) that carried shRNAs targeting each gene into the cerebella of the adult SCA1 mice. The virus was injected into 4 sites in the cerebellum to transduce the whole cerebellum, as visualized by the entire cerebellar YFP expression (Figure 3C). Individual knockdown of all 4 genes significantly decreased both mutant and WT ATXN1 protein levels (Figure 3, D and E). Notably, knockdown of *Tgm5* decreased mutant ATXN1[154Q] to a greater extent than WT ATXN1[2Q], suggesting that TG5 preferentially regulated the polyQ-expanded ATXN1 over the WT protein.

We selected TG5 for further mechanistic studies for the following reasons: (1) most of the TG5 paralogs were predicted to be potential positive regulators of ATXN1 (Supplemental Table 6); (2) knockdown of TG5 remarkably suppressed the ATXN1[82Q]-induced fly eye degeneration (Figure 3A) and significantly improved motor deficits in fruit flies that express human ATXN1[82Q] in the CNS (31), as evident by the increase in their climbing speed (Figure 3B); and (3) TG5 showed preferential regulation of mutant ATXN1 in the SCA1 mouse brain (Figure 3D; see complete unedited blots in the supplemental material). In addition, overexpression studies in iPSC-derived neurons from patients with SCA1 revealed that though *TGM5* was not successfully expressed, the overexpression of its paralogs *TGM2* and *TGM6* whose neuronal function and/or levels are well-known to contribute to polyQ-expansion disorders, such as Huntington’s disease or SCA35 (32–35), significantly increased total ATXN1 protein levels without changing mRNA levels, suggesting that *TGMs* regulated ATXN1 posttranslationally (Supplemental Figure 5; see complete unedited blots in the supplemental material).

### Catalytically active TGs directly regulate mutant ATXN1 levels and stability.

**Figure 4:**
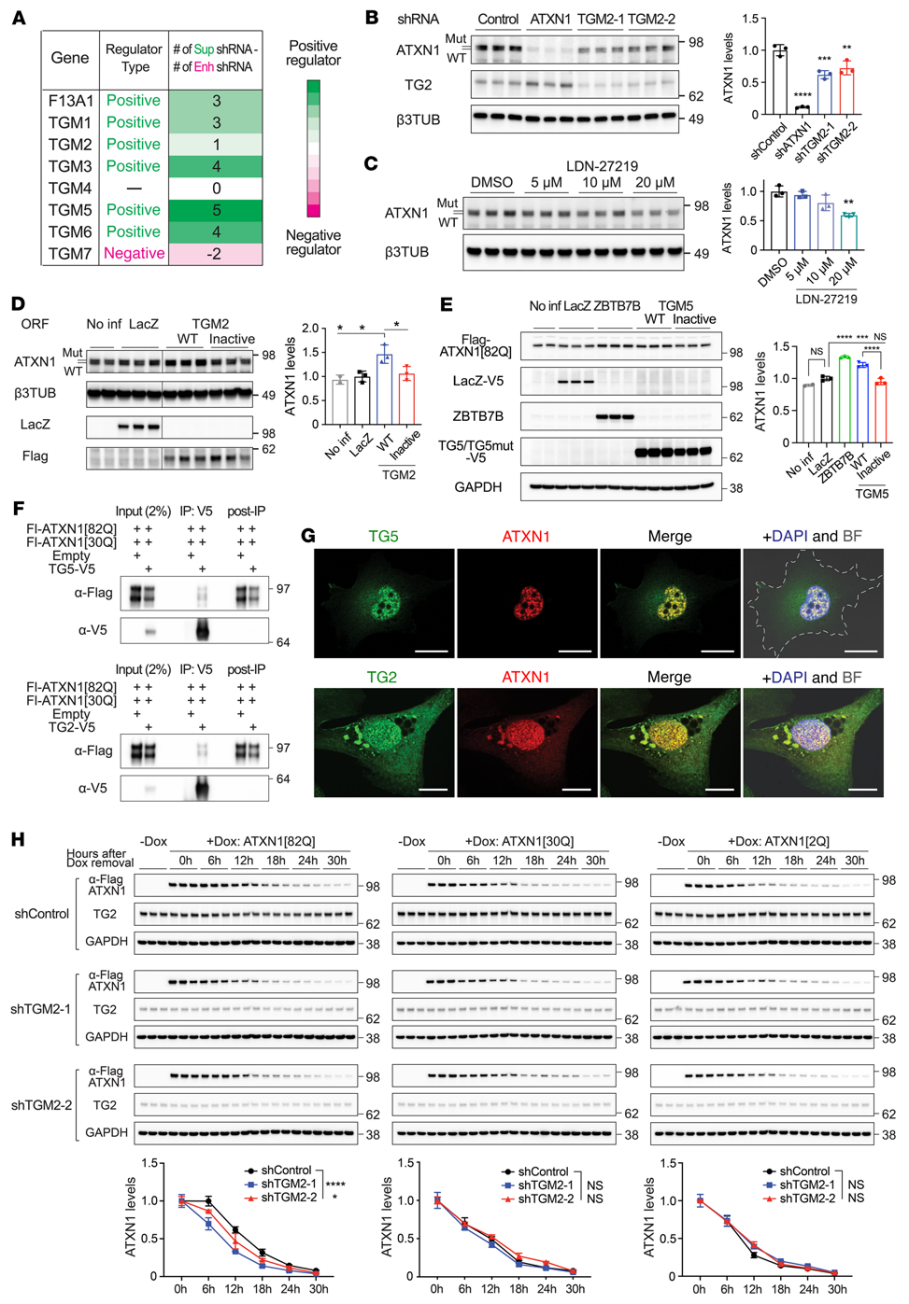
TGs regulate mutant ATXN1 levels and stability via their catalytic activity. (**A**) Cell screen data of the genes that encode catalytically active TG. Positive and negative regulators are colored with green and pink, respectively. Color scale is based on the net number of suppressor shRNAs and represented next to the table. (**B**) Western blot analysis of ATXN1 levels after knockdown of TG2 for 9 days in iPSC-derived neurons from patients with SCA1. Data shown as mean ±SD, **P < 0.01, ***P < 0.001, ****P < 0.0001, 1-way ANOVA, post hoc Dunnett’s test. (**C**) Western blot analysis of ATXN1 levels after a treatment with TG2 inhibitor (LDN-27219) for 3 days on iPSC-derived neurons from patients with SCA1. Data shown as mean ± SD, **P < 0.01, 1-way ANOVA, post hoc Dunnett’s test. (**D**) Western blot analysis of ATXN1 levels after overexpression of either WT or catalytically inactive mutant TG2 in patient iPSC-derived neurons for 9 days. Data shown as mean ± SD, *P < 0.05, 1-way ANOVA, post hoc Tukey’s test. (**E**) Western blot analysis of ATXN1 levels after overexpression of either WT or catalytically inactive mutant TG5 in ATXN1 reporter cells for 3 days. Data shown as mean ± SD, ***P < 0.001, ****P < 0.0001, 1-way ANOVA, post hoc Tukey’s test. (**F**) Co-IP results of ATXN1[82Q] and ATXN1[30Q] with TG5 (top) or TG2 (bottom) in the HEK293T cells co-overexpressing ATXN1 and TG. V5-tagged TG5 or TG2 were pulled down and flag-tagged ATXN1 was immunoblotted. (**G**) Representative IF images of ATXN1[82Q] and TG5 (top) or TG2 (bottom) in Daoy cells overexpressing ATXN1[82Q] and TG5/2. Note that the 2 proteins colocalize. Scale bar: 20 μm. (**H**) Stability assay of inducibly expressed ATXN1 with different polyQ-length in Daoy cells expressing shTGM2 or shControl. After a 48-hour doxycycline treatment for inducing ATXN1 expression, media was exchanged into growth media without doxycycline, and cells were collected every 6 hours until 30 hours after the media change. Data shown as mean ± SD, *P < 0.05, ****P < 0.0001, 2-way ANOVA, post hoc Dunnett’s test.

The human genome has 8 *TGMs* that encode catalytically active TGs. Given that 6 catalytically active TGs are predicted to be positive regulators of ATXN1 in our cell-based screen (Figure 4A), we hypothesized that the enzymatic activities of TGs regulate mutant ATXN1 levels. Among the 6 *TGM*s, *TGM5* had the highest net number of the suppressor shRNAs that reduced ATXN1 levels. We also studied *TGM2,* even though it had the lowest net number of the suppressor shRNA, because it is one of the most well-characterized TGs (20) and could cross-link ATXN1 (25). Notably, knockdown of TG2 or applying TG2 inhibitor LDN-27219 that blocks cross-linking activity (36) decreased ATXN1 levels in the patient iPSC-derived neurons (Figure 4, B and C; see complete unedited blots in the supplemental material). In addition, overexpression of WT TG2 and TG5, but not their catalytically inactive mutant C277S (37), increased ATXN1 in the patient iPSC-derived neurons and ATXN1 reporter cells, respectively (Figure 4, D and E; see complete unedited blots in the supplemental material). Altogether, these data suggest that not only TG5 but TG2 can regulate ATXN1 levels, and that this regulation is dependent on cross-linking activity of the enzymes. We next tested whether TG5 and TG2 could directly interact with ATXN1 using co-IP and IF. Both ATXN1[30Q] and ATXN1[82Q] were coimmunoprecipitated with TG5 or TG2 to a similar degree when they were coexpressed in HEK293T cells (Figure 4F; see complete unedited blots in the supplemental material). Moreover, overexpressed TG5 or TG2 colocalized with ATXN1[82Q] in the nuclei of Daoy cells expressing ATXN1[82Q], suggesting that the 2 TGs can physically interact with nuclear ATXN1 (Figure 4G).

We next asked whether TGs can regulate ATXN1 stability using Daoy cell lines that stably express shTGM2 or shTGM5 and inducibly express ATXN1[82Q], ATXN1[30Q], or ATXN1[2Q]. After inducing ATXN1 expression for 48 hours, we stopped the induction and measured ATXN1 protein levels every 6 hours. Interestingly, while mutant ATXN1[82Q] levels decreased faster in the cells expressing shTGM2 or shTGM5 than the cells expressing shControl, neither the WT ATXN1[30Q] nor the shorter polyQ ATXN1[2Q] did, suggesting that both TG2 and TG5 specifically regulated the polyQ-expanded ATXN1 stability (Figure 4H and Supplemental Figure 6A; see complete unedited blots in the supplemental material).

### TGs preferentially cross-link mutant ATXN1 and regulate its solubility and oligomerization.

**Figure 5:**
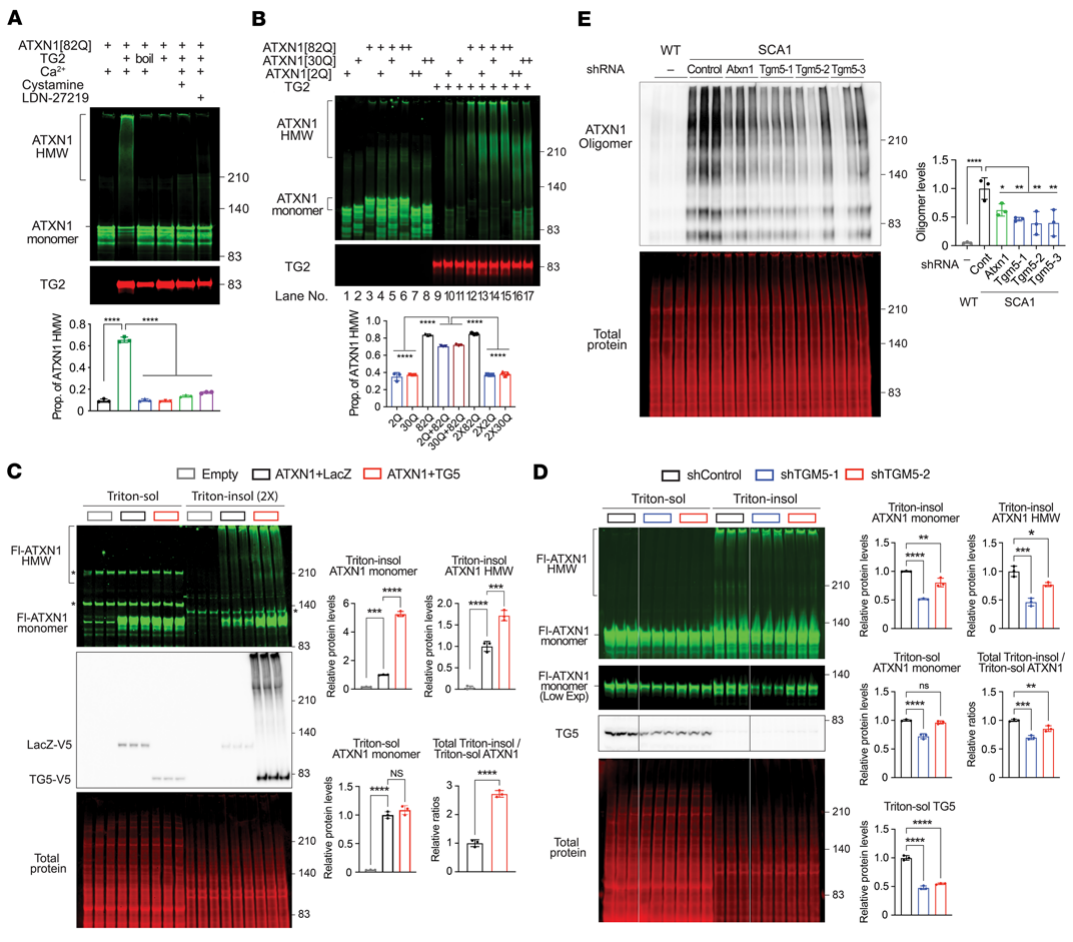
TGs preferentially cross-link mutant ATXN1 and regulate its solubility and oligomerization. (**A**) In vitro TG assay of ATXN1[82Q] with recombinant human TG2 under various conditions: with normal or heat-inactivated enzyme; with or without Ca^2+^ ion, pan-TG inhibitor cystamine (40 mM), or TG2-specific inhibitor LDN-27219 (2 mM). The proportion of ATXN1 high MW (HMW) species in each lane represented in bottom graph. Data shown as mean ± SD, ****P < 0.0001, 1-way ANOVA, post hoc Dunnett’s test. (**B**) In vitro TG assay of ATXN1[82Q], ATXN1[30Q], and ATXN1[2Q] with recombinant human TG2. Either 1× (+) dose or 2× (++) doses of ATXN1 was used for the reaction. The proportion of ATXN1 HMW species in lane 10–17 represented in bottom graph. Data shown as mean ± SD, ****P < 0.0001, 1-way ANOVA, post hoc Tukey’s test. (**C**) Western blot analysis of Triton X-100–soluble or –insoluble ATXN1 HMW species and monomer extracted from HEK293T cells overexpressing ATXN1[82Q] and TG5 or LacZ. Empty, empty vector; LacZ, overexpression control. Triton X-100–insoluble ATXN1 (HMW species + monomer) to Triton X-100–soluble ATXN1 (monomer) ratio in each overexpression group represented in bottom right graph. Asterisk indicates nonspecific bands. Data shown as mean ± SD, ***P < 0.001, ****P < 0.0001, 2-tailed *t* test for bottom right graph; 1-way ANOVA for other graphs with post hoc Dunnett’s test. (**D**) Western blot analysis of Triton X-100–soluble or –insoluble ATXN1 HMW species and monomer extracted from HEK293T cells overexpressing ATXN1[82Q] and shRNA against TG5. Triton X-100–insoluble ATXN1 (HMW species + monomer) to Triton X-100–soluble ATXN1 (monomer) ratio in each experimental group represented in middle right graph. Data shown as mean ± SD, *P < 0.05, **P < 0.01, ***P < 0.001, ****P < 0.0001, 1-way ANOVA, post hoc Dunnett’s test. (**E**) Western blot analysis of soluble ATXN1 oligomers in cerebellar lysate of WT mice and SCA1 mice expressing shTgm5 (same lysate in Figure 3D) using oligomer-specific F11G3 antibody. Data shown as mean ± SD, *P < 0.05, **P < 0.01, ****P < 0.0001, 1-way ANOVA, post hoc Dunnett’s test.

To investigate a mechanism by which the catalytic activity of TG regulates ATXN1, we tested whether TG could cross-link ATXN1 into high MW (HMW) species by performing an in vitro TG assay between the mutant ATXN1[82Q] purified from *E*. *coli* and recombinant human TG2 (Figure 5A and Supplemental Figure 6B; see complete unedited blots in the supplemental material). We found that ATXN1[82Q] was cross-linked into the HMW species when it was incubated with TG2 but not with the boiled inactive enzyme. In addition, depleting calcium that is required for cross-linking activity of TG or applying TG inhibitors, such as cystamine (pan-TG inhibitor) or LDN-27219 (TG2 inhibitor) (38), clearly reduced the ATXN1 HMW species, indicating that TG catalyzes ATXN1 cross-linking via its enzymatic activity. We next investigated whether this cross-linking is dependent on the polyQ length. In vitro TG assay of ATXN1 with different polyQ length clearly showed that ATXN1[82Q] produced a much larger proportion of HMW species (84%) than ATXN1[30Q] (37%) and ATXN1[2Q] (35%) (Figure 5B, lane 10–12 and lane 15–17; see complete unedited blots in the supplemental material), suggesting that the polyQ-expanded ATXN1[82Q] was more preferentially cross-linked by TG than the unexpanded ATXN1 proteins. To determine whether WT ATXN1 can suppress the cross-linking activity of TG on the polyQ-expanded ATXN1[82Q], we added the same amount of ATXN1[2Q] or ATXN1[30Q] onto ATXN1[82Q] (lane 13–14). Notably, the addition of WT ATXN1 significantly decreased the proportion of HMW species compared with the ATXN1[82Q] alone (lane 12 and 15), suggesting that in the context of the mutant ATXN1, WT ATXN1 can be protective against generation of the HMW species by TG.

Next, we tested whether TG-mediated cross-linking of ATXN1 can change its solubility within cells by performing a Western blot analysis of ATXN1 in Triton X-100–soluble and –insoluble protein fractions from HEK293T cells that overexpressed either TG5 or TG2 with ATXN1[82Q] (Figure 5C and Supplemental Figure 6C; see complete unedited blots in the supplemental material). Compared with the LacZ overexpression control, the overexpression of TG5 or TG2 significantly increased the Triton X-100–insoluble ATXN1 monomers and HMW species but not the Triton X-100–soluble ATXN1 monomers, thereby increasing the Triton X-100–insoluble to soluble ATXN1 ratio. Consistent with this, knockdown of TG5 in HEK293T cells overexpressing ATXN1[82Q] significantly decreased the Triton X-100–insoluble ATXN1 monomers and HMW species, with little or no changes in the levels of Triton X-100–soluble ATXN1 monomers (Figure 5D; see complete unedited blots in the supplemental material). Concomitantly, the ratio of total Triton X-100–insoluble to soluble ATXN1 decreased, suggesting that TG can transform the soluble ATXN1 monomers into the insoluble monomers and HMW species.

Mutant ATXN1 forms soluble oligomers that mediate neuronal toxicity (39, 40). These oligomers can be detected by the conformational anti-oligomer antibody F11G3 in human cells and mouse brain tissue. To investigate whether TG5 regulates mutant ATXN1[154Q]-driven oligomer levels in the SCA1 mouse cerebellum, we performed a Western blot of the oligomers in nonreducing condition using F11G3 antibody from the same cerebellar lysates in Figure 3D where the TG5 was knocked down. The levels of the ATXN1 oligomers were dramatically reduced upon knockdown of TG5, even to a greater extent than the knockdown of ATXN1, suggesting that TG5 is critical to mediate mutant ATXN1-driven protein oligomerization in the adult SCA1 mouse cerebellum (Figure 5E; see complete unedited blots in the supplemental material). Taken together, our data suggest that TG preferentially cross-linked mutant ATXN1 and regulated its solubility and oligomerization in vivo.

### Tg regulates mutant ATXN1 levels and toxicity in Drosophila.

**Figure 6:**
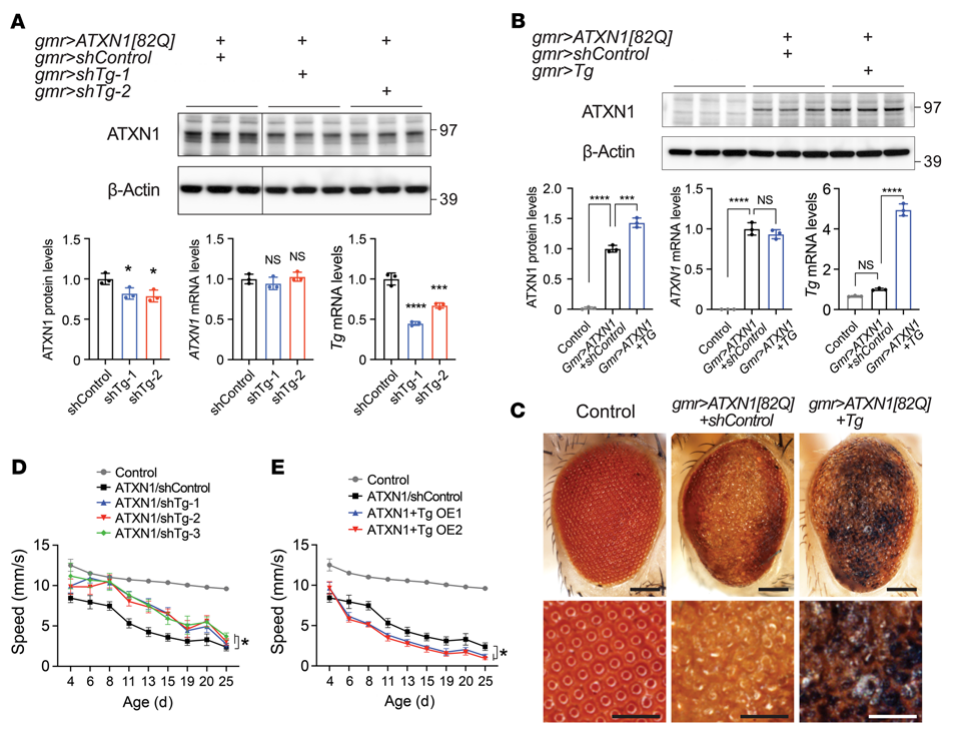
Tg modulates mutant ATXN1 and its toxicity in *Drosophila* SCA1 models. (**A**) Western blot and qRT-PCR analyses of ATXN1[82Q] protein and mRNA levels after the knockdown of Tg with 2 different shRNAs in *Drosophila* eyes expressing ATXN1[82Q]. Knockdown of Tg was confirmed by qRT-PCR (bottom right). Protein lysates were extracted from the pooled 16 fly heads per genotype. Data shown as mean ± SD, *P < 0.05, ***P < 0.001, ****P < 0.0001, 1-way ANOVA, post hoc Dunnett’s test. (**B**) Western blot and qRT-PCR analyses of ATXN1[82Q] protein and mRNA levels after overexpression of Tg in *Drosophila* eyes expressing ATXN1[82Q]. Overexpression of Tg was confirmed by qRT-PCR (bottom right). Protein lysates were extracted from the pooled 8 fly heads per genotype. Data shown as mean ± SD, ***P < 0.001, ****P < 0.0001, 1-way ANOVA, post hoc Dunnett’s test. (**C**) Representative images of *Drosophila* eyes showing external organization of the ommatidia from negative control and flies expressing ATXN1[82Q] together with Tg or control shRNA. Note the severely degenerated eye with black necrotic patches upon the coexpression of Tg and ATXN1[82Q]. Scale bar: 100 μm in the top images; 50 μm in the bottom images. (**D**) Effect of Tg knockdown and (**E**) effect of Tg overexpression on the motor performance of *Drosophila* SCA1 model expressing ATXN1[82Q] in the CNS. Data shown as mean ± SEM, *P < 0.05, linear mixed-effect model ANOVA.

In contrast to humans with 8 catalytically active *TGM* genes, *Drosophila* has only 1 *Tg* gene, making it a useful model organism to study genetic interaction between *Tg* and *ATXN1* by manipulating a single *Tg* without inducing any potential compensatory effect from other paralogs. Therefore, we investigated whether Tg could modulate mutant ATXN1 levels and its toxicity in *Drosophila* SCA1 models. Knockdown of Tg by 2 different shRNAs decreased ATXN1[82Q] protein levels in the *Drosophila* eyes that expressed ATXN1[82Q], whereas overexpression of the Tg increased the ATXN1[82Q] protein, without changing *ATXN1* mRNA levels in both cases (Figure 6, A and B; see complete unedited blots in the supplemental material). Consistent with the ATXN1 protein level changes, knockdown of Tg ameliorated ommatidial degeneration, whereas its overexpression exacerbated the degeneration by forming black necrotic patches extensively on the eye surface (Figure 3A and Figure 6C). In addition, knockdown of Tg rescued motor deficits by increasing climbing speed, whereas its overexpression reduced the climbing speed in *Drosophila* that expressed ATXN1[82Q] in the CNS (Figure 6, D and E). These results suggest that Tg regulated mutant ATXN1 levels and its toxicity in vivo, raising its potential as a therapeutic target for SCA1.

### TG5 resides in the nuclei of neurons and colocalizes with NIs of mutant ATXN1 in brain tissue from patients with SCA1.

**Figure 7:**
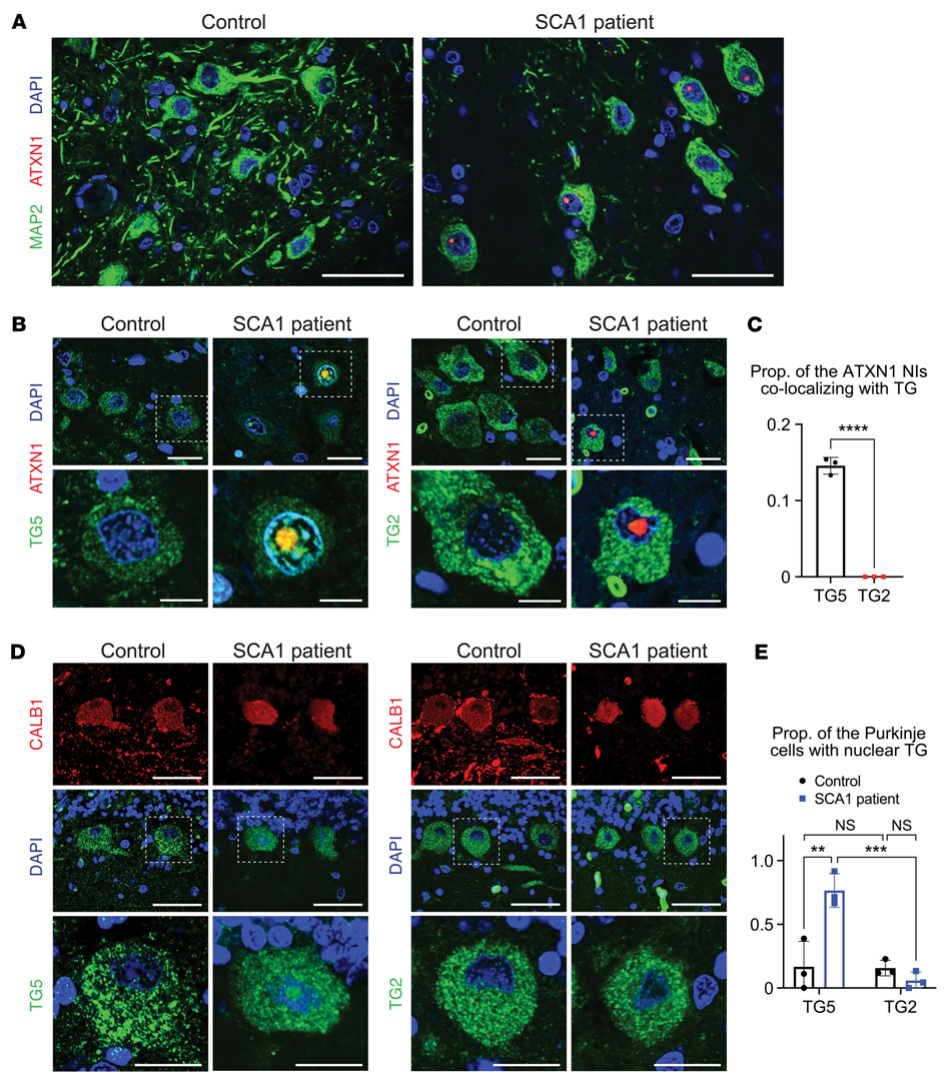
TG5 but not TG2 colocalizes with nuclear inclusions of ATXN1 in the pons of patients with SCA1. (**A**) Representative IF images of ATXN1 nuclear inclusions (NIs) in the MAP2^+^ neurons that have large nuclei in the control and SCA1 patient pons. Scale bar: 50 μm. (**B**) Representative IF images of TG5 (left) and TG2 (right) in the neurons that display ATXN1 NIs in the pons of patients with SCA1. Control pons were also stained for comparison. White dashed-line boxes in the top images are enlarged in the bottom. Scale bar: 25 μm in the top images; 10 μm in the bottom images. (**C**) Quantification of the proportion of ATXN1 NIs that colocalize with TG. Images were obtained from 3 controls and 3 patients with SCA1, and the proportion was calculated from 20–30 NIs in each individual. Data shown as mean ± SD, ****P < 0.0001, 2-tailed *t* test. (**D**) Representative IF images of TG5 (left) and TG2 (right) in the CALB1^+^ Purkinje cells in control and SCA1 patient cerebellum. White dashed-line boxes in the middle images are enlarged in the bottom. Scale bar: 50 μm in the top and middle images; 20 μm in the bottom images. (**E**) Quantification of the proportion of Purkinje cells with nuclear TG. Images were obtained from 3 controls and 3 patients with SCA1, and the proportion was calculated from 18–24 Purkinje cells in each individual. Data shown as mean ± SD, **P < 0.01, ***P < 0.001, 2-way ANOVA, post hoc Tukey’s test.

We investigated whether TG5 or TG2 could colocalize with NIs of ATXN1 in the SCA1 patient pons and cerebellum, the affected brain regions in SCA1. ATXN1 NIs were clearly stained by IF using ATXN1 antibody (11NQ) or polyglutamine antibody (1C2) in the specific population of MAP2^+^ neurons with large nuclei in the pons of patients with SCA1, but not in the control (Figure 7A). Both TG5 and TG2 were highly expressed in the soma of the pons neurons that showed ATXN1 NIs (Figure 7B). However, we observed colocalizations of the NIs only with TG5 (in 14.6% of NIs), but not with TG2, suggesting that TG5 may directly regulate ATXN1 NIs, whereas TG2 may not (Figure 7, B and C). In contrast to the pons, we could not find neurons that had ATXN1 NIs in the cerebellum of patients with SCA1 (data are not shown), which is consistent with a previous report (41). Instead, we investigated expression of the 2 TGs in the Purkinje cells that are one of the most vulnerable neuronal populations in SCA1. In the control cerebellum, both TG5 and TG2 were highly expressed in the calbindin 1^+^ Purkinje cells, where they mostly resided outside of the nuclei (Figure 7, D and E). However, in the cerebellum of patients with SCA1, TG5 and TG2 displayed different localization in that TG5 resided both inside and outside of the nuclei in most (76.6%) of the Purkinje cells, whereas TG2 remained outside of the nuclei. Considering that ATXN1 is a nuclear protein whose functions in the nucleus drive neuronal toxicity (42), these data collectively suggest that TG5 was more directly involved in modulating nuclear ATXN1 and SCA1 pathogenesis compared with TG2.

## Discussion

Reducing toxic protein levels is one of the promising therapeutic strategies for neurodegenerative disorders, with proven benefits across various neurodegenerative disorders. For example, decrease of amyloid precursor protein (APP), α-syn, and HTT rescues phenotypes of AD, PD, and HD in mice, respectively (5–7). This is also true for SCA1, as decreasing ATXN1 rescues disease phenotypes in mice (13–15), but specifically reducing mutant ATXN1 is more effective than targeting both WT and mutant ATXN1 (18). Because ATXN1 does not have enzymatic activity, it is not a feasible target for small molecule inhibition. Degradation of ATXN1 could be induced by using a bifunctional small molecule that recruits target proteins to endogenous proteolytic machinery, such as proteolysis-targeting chimera (PROTAC), but identifying the small molecule that specifically binds mutant ATXN1 can be challenging as its polyQ presumably has disordered structures (43, 44). Antisense oligonucleotides are able to decrease ATXN1, but act indiscriminately by decreasing both WT and mutant ATXN1 (14), unless the mutant allele has distinguished SNPs that can be exploited to RNAi strategy (45). Targeting mutant ATXN1-specific regulators is an alternative approach, but no such regulators have hitherto been found, limiting development of therapeutics for SCA1.

Our cross-species druggable genome screen revealed 22 regulators, including TG5 that preferentially regulates mutant ATXN1 over the WT protein in the cerebellum of the SCA1 mouse model. The cell screen data suggest that other catalytically active TGs are also potential ATXN1 regulators. Although ATXN1 is known to be cross-linked by TG2 (25), neither the degree of ATXN1 cross-linking with different polyQ-lengths nor the biological consequences of the cross-linking such as changing ATXN1 levels or toxicity are known. Our mechanistic studies showed that TG can directly cross-link ATXN1 in a polyQ-length–dependent manner, thereby preferentially regulating mutant ATXN1 protein levels, stability, solubility, and oligomerization. In addition, we showed the therapeutic potential of Tg inhibition using *Drosophila* SCA1 models and confirmed that TG5 colocalized with neuronal ATXN1 NIs in the pons of patients with SCA1, demonstrating that TG5 contributed to SCA1 pathogenesis by preferential regulation of mutant ATXN1.

Though our work identified TG5 as a therapeutic target for SCA1, the potential side effect of TG5 inhibition needs to be carefully evaluated given that missense mutations in one allele of *TGM6* (one of the transglutaminase paralogs) have been seen in families with SCA35 (35). In addition, knockout of another paralog, *TGM2*, can impair autophagy and the clearance of ubiquitinated oligomers (46). Given, however, that our in vivo data from adult SCA1 mice consistently showed decrease of ATXN1 monomers and oligomers, autophagy impairment, if any, may not be critical to regulate ATXN1 once SCA1 sets in. Autophagy dysfunction is also unlikely to hinder the clearance of ATXN1 in premanifest SCA1 for the following reasons: 1) ATXN1 levels consistently decreased upon TG5 inhibition across different cell lines and patient iPSC-derived neurons; 2) mutant ATXN1 is degraded by proteasome (11); and 3) autophagy does not regulate nuclear ATXN1, though it can regulate cytoplasmic ATXN1 (47). There is still the possibility, however, that TG5 inhibition could have negative effects on neurodegeneration by misregulating other autophagy targets and/or mitophagy (48).

Selective targeting of mutant alleles has been studied in other autosomal-dominant polyQ-expansion disorders, such as HD and SCA3 (45, 49–51). Mutant allele–specific inhibition is critical for preventing possible side effects arising from inhibiting essential biological functions of some WT alleles (50). Moreover, in SCA1, the presence of WT allele is protective for the disease in the context of the mutant allele (17), making allele-specific targeting more desirable to benefit patients with SCA1. Of note, however, in the absence of the mutant allele, increase of WT ATXN1 causes SCA1-like phenotypes (28, 29). Our in vitro TG assay data can give a possible explanation about this seemingly contradictory phenomenon. WT ATXN1 alone can generate HMW species by TG5, so its increase could lead to toxicity. However, because it allowed a relatively lower degree of cross-linking than the mutant ATXN1, WT ATXN1 reduced total intermolecular cross-linking in the context of the mutant protein, thereby decreasing overall ATXN1 stability. This was shown by the decreases of ATXN1 HMW species in Figure 5B. Therefore, our work not only provides a way to preferentially modulate mutant ATXN1, but also enhances our understanding of the context-dependent protective role of the WT ATXN1 in SCA1 pathogenesis.

TG regulated both insoluble ATXN1 HMW species and monomers (Figure 5, C and D). As the in vitro TG assay data showed, ATXN1 HMW species can be directly produced by TG-mediated cross-linking. In contrast, the size of insoluble ATXN1 monomers indicates that they are not cross-linked by TG, and hence some indirect mechanisms are involved. It remains to be investigated whether insoluble ATXN1 HMW species produced by TG sequester molecular chaperones that are required to maintain the solubility of mutant ATXN1 monomers.

TG can increase protein stability via either direct de novo polymerization of proteins into oligomers or “spotwelding” preformed oligomers that are reversibly assembled via noncovalent bond (19, 20). In our study, there were 2 different types of mutant ATXN1 product formed by TG — insoluble HMW species and soluble oligomers. Insoluble HMW species are likely to be produced by de novo polymerization of ATXN1, as they were directly generated from either in vitro TG assay of ATXN1 monomers or cells that overexpressed ATXN1 monomers by a transient transfection (Figure 5, A–D). This HMW species was not detected by the β-sheet conformationaloligomer antibody F11G3 (data not shown). In contrast, soluble ATXN1 oligomers that were regulated by TG5 in Figure 5E are possibly generated by the spotwelding mechanism because they maintain the F11G3-recognizable β-sheet structure formed by noncovalent bonds (52). Of the 2 types of TG-modified ATXN1 products, soluble oligomers are highly noteworthy as they are regarded to cause neuronal toxicity in SCA1 and many other neurodegenerative disorders (40, 53). Given that TG5 colocalizes with ATXN1 NIs in the postmortem pons of patients with SCA1, it is possible that TG5 initially produces and stabilizes ATXN1 oligomers that ultimately develop into NIs, which remains to be studied further.

Except for TG2, the function and expression pattern of other TGs in human brain are not well known. We confirmed that TG5 is highly expressed in the neurons that are pathologically important in SCA1 — the neurons in the pons that have ATXN1 NIs and the cerebellar Purkinje cells that are one of the most vulnerable neurons in SCA1 (41). This expression pattern and the colocalization with the NIs suggest that TG5 contributes to SCA1 pathogenesis by regulating nuclear ATXN1 aggregation. Although highly expressed in the soma of the aforementioned neurons, TG2 did not colocalize with ATXN1 NIs, possibly because TG2 is less localized in the nucleus compared with TG5 (Figure 7, D and E), suggesting a limited role of TG2 in modulating nuclear ATXN1.

In conclusion, our work reports 22 regulators of mutant ATXN1, including TG5, that preferentially modulate mutant ATXN1 by catalyzing its cross-linking. These regulators enhance our understanding of SCA1 pathogenesis and demonstrate that mutant proteins can be preferentially regulated over WT proteins via targeting a specific regulator, giving an insight into the development of an effective therapeutic strategy for SCA1 and other neurodegenerative proteinopathies.

## Methods

### Pooled shRNA screen in ATXN1 reporter cells.

Based on the functional categorization of target genes, the shRNAs targeting 7787 genes consist of 8 libraries referred to as chromatin, enzyme, GPCR, ion channel, transporter, ubiquitin, others, and kinase/phosphatase. shRNA screens of these libraries (except for the ubiquitin library) were performed in this study. The ubiquitin screen results were reanalyzed from Lee et al. (15) using a new bioinformatic pipeline together with the other 7 libraries as described below and compared with *Drosophila* screen hits. ATXN1 reporter cells that expressed a bicistronic transgene (mRFP-ATXN1[82Q]-IRES-YFP) were transduced in 150 mm dishes with retrovirus of each shRNA library at an MOI of 0.3 to give 1000× representation of the individual library of shRNA in the pool. After 24 hours, the transduced cells were selected by puromycin (1 μg/mL) for 6 days and further cultured for 3 days. The cells were trypsinized and sorted on a Sony SH800 sorter. The cells with the 5% highest or 5% lowest RFP/YFP ratio were collected into 15 mL conical tubes to over 100× representation and frozen. In parallel, non-sorted bulk cell populations were also collected by trypsinization and frozen. The above procedures were performed in quadruplicate and 12 samples (low 1–4, bulk 1–4, and high 1–4) in each library were prepared for Illumina sequencing. Genomic DNAs from these samples were extracted using the DNeasy blood and tissue kit (QIAGEN, 69506). The shRNA sequences in each sample were amplified with the ForAmp1 and RevAmp1 primers (See the Supplemental Table 7 for the sequences) and cleaned up using AMPure XP beads (Beckman Coulter, A63881) following the manufacturer’s protocol. Another round of PCR was performed to add indices to the amplified shRNA sequences of the 12 samples in each library using Nextera Index kit (Illumina, FC-121-1011), and then cleaned up. This amplicon (339 bp) was purified by Pippin prep (Saga Science). Illumina sequencing libraries were created using the Nextera XT DNA Library Prep kit and sequenced on a HiSeq 2500 System (Illumina). The sequencing data of the 7 shRNA libraries together with the data from ubiquitin library in Lee et al. (15) were analyzed through the standard-alone pipeline of CRISPRcloud (https://github.com/LiuzLab/CRISPRcloud-standalone) (54, 55). First, the index barcode sequences were quantified by using the parameter values: params.adapt = ′TAGTGAAGCCACAGATGTAT′, params.err_trim = 0.1, and params.mismatch = 2. Each sequence read was trimmed and aligned to the corresponding index barcode via cutadapt (doi:10.14806/ej.17.1.200) and bitap algorithm (doi:10.1145/135239.135244). This generated shRNA read count matrices (Supplemental Data 1) that were used for statistical analysis. The enrichment of every shRNA in “high” or “low” compared with the bulk control was tested by inverted beta-binomial test (56). Taking all shRNAs targeting each gene into account, we scored each gene’s potential to regulate ATXN1 by calculating hit ratio (the proportion of shRNAs that call their target gene a positive or negative regulator of ATXN1), directional score (the inter-shRNA consistency in enrichment vs. depletion), and conflict (the presence of enrichment or depletion in both the low and high populations) (54). The genes with hit ratio 0.2 or higher, directional score 0.7 or higher, and conflict 0.2 or less were filtered-in and prioritized based on the number of the enriched shRNAs in the low group. The top ranked genes in each library were selected for validation.

### Drosophila methods.

For the druggable genome screen in *Drosophila* eyes, we identified the *Drosophila* homologs of human genes using the Blast algorithm applied to protein sequences, applying a cutoff e-value of E-10 (1102 *Drosophila* genes corresponding to 2144 human homologs). We obtained all the available fly lines expressing inducible shRNA for each gene in the Vienna *Drosophila* Resource Center (VDRC) repository (http://stockcenter.vdrc.at/control/main). Animals were crossed and raised at 28°C. The fly line that expresses ATXN1[82Q] in the eyes [*w, UAS-ATXN1[82Q](F7); GMR-GAL4; +*] was used as previously described (13, 29). The shRNA screen of the 8 libraries except for the kinase/phosphatase library was performed in this study, and the screen hits of kinase/phosphatase in Park et al. (13) together with the hits from the 7 other libraries were compared with our cell-based screen hits. For *Tg* shRNA strains, we used fly lines 85403, 65086, and 15787 from the Bloomington *Drosophila* Stock Center (https://bdsc.indiana.edu/) in addition to the VDRC shRNA fly lines. The eyes were imaged either using a scanning electron microscope or a Leica MZ16 imaging system for fresh eyes as previously described (15). For the *Drosophila* motor performance assay, we used an automated data acquisition system and the fly line that expresses ATXN1[82Q] in the CNS [*w, UAS-ATXN1[82Q](F7); Nrv2-GAL4; +*] as previously described (15). Briefly, animals were tapped on a vial at 7-second intervals and videos were recorded while the animals were climbing. The videos were processed using a custom software program to calculate the average speed of the animals in each vial. This process was done 5 times per replicate in each day. Ten females were used in each replicate and 4 replicates were tested per genotype. For statistical analysis, linear mixed-effect model ANOVA was used across the indicated genotypes.

### Production, titration and infection of retrovirus, lentivirus, and AAV9.

The retrovirus used in the shRNA screen was produced in HEK293T cells by transfecting pMSCV-library, Gag/Pol, and VSV-G plasmids using *Trans*IT-293 transfection reagent (Mirus Bio LLC). Supernatants containing the virus were collected at 72 hours after the transfection and filtered by 0.45 μm polyethersulfone (PES) filter. For transducing the virus, polybrene (8 μg/mL) was added to the supernatants and the mixture was added on cell culture media dropwise. For titering the retrovirus, Daoy cells cultured in a 24-well plate were transduced with serially diluted virus. On the next day, the cells were selected by puromycin (1 μg/mL) for 3 days and the number of surviving cell colonies was counted.

Lentivirus was produced in HEK293T cells by transfecting lentiviral plasmids: pGIPZ for shRNA (Supplemental Table 4) and W118-1 (Addgene plasmid 17452) for overexpression and helper plasmids (pMD2.G and psPAX2) using *Trans*IT-293 reagent. Supernatants containing lentivirus were collected at 72 hours after transfection. After filtrating the supernatant with 0.45 μm PES filter, the virus was concentrated using Lenti-X concentrator (Takara Bio Inc.) following the manufacturer’s protocol. For transduction, lentivirus was added into media with an MOI of 5–10 and 20 for overexpression and knockdown, respectively. For titering the lentivirus, HEK293T cells were cultured in a 24-well plate and transduced with serially diluted virus. Three days later, the number of fluorescent colonies were counted.

AAV9 was produced as described (15). HEK293T cells were transfected with pAAV-CBA-YFP-miR-E, AAV9 packaging, and AAV helper plasmids using *Trans*IT-293 reagent and incubated for 72 hours. After scraping, the transfected cells and media were transferred together into a new tube and then separated by centrifugation. The virus in the media was pelleted by adding polyethylene glycol (final concentration 8%) and centrifugation and resuspended in PBS-MK buffer (1× PBS, 1 mM MgCl_2_, 2.5 mM KCl). The virus in the cell pellet was extracted by 2 cycles of freezing and thawing in freezing buffer (0.15 M NaCl; 50 mM Tris, pH 8.0). After removing cell debris by centrifugation, the virus extracted from the cells and media were merged and loaded onto iodixanol gradient (15%, 25%, 40%, and 60%) in an OptiSeal polypropylene tube (Beckman Coulter, 361625). The tube was ultracentrifuged and the 40% iodixanol layer was collected. The iodixanol was removed by adding PBS-MK buffer and centrifugal filtration using MWCO 100 kDa filters (MilliporeSigma, UFC910024). Titer of the virus was measured by qRT-PCR in which serially diluted pAAV-CBA-YFP-miR-E plasmids were used for standards.

### Protein extraction and Western blot.

A half of mouse cerebellum was immersed in 1 mL of cold cell lysis buffer (CLB; 0.5% Triton X-100, 50 mM Tris-HCl [pH 7.4], 150 mM NaCl) supplemented with protease inhibitor and phosphatase inhibitor cocktail (GenDEPOT, P3100-020 and P3200-020, respectively) and homogenized with 1 mL syringe fitted with a 22G needle and then with a 27G needle. After centrifugation at 16,000*g* at 4°C for 15 minutes, the supernatant was transferred into a new tube. For extracting proteins from fly heads, 50 μL of CLB was added to 8–16 fly heads from each genotype. The heads were ground by pellet pestle, spun down, and the supernatant was transferred into new tubes. Proteins from cultured cells were extracted by aspirating culture media and adding CLB onto cells (100, 200, 400 μL per well in a 24-, 12-, and 6-well plates, respectively). After scraping the cells, the lysate was transferred into new tubes and spun down. The supernatant was transferred into new test tubes. Protein lysates extracted from the 3 different sources were subjected to BCA assay using Pierce BCA Protein Assay kit (Thermo Fisher Scientific, 23225), and loading samples were made from the equal amount of protein from each lysate and NuPAGE LDS sample buffer supplemented with 5% 2-ME. The loading samples were then boiled at 95°C for 10 minutes and cooled down at room temperature. The protein samples were run in NuPAGE 4%–12% Bis-Tris protein gel with MES or MOPS running buffer or in 3%–8% Tris-acetate gels (Invitrogen) with tris-acetate running buffer, followed by wet transfer to a nitrocellulose membrane (0.2 μm) within Tris-glycine transfer buffer. If required, total protein was stained using the Revert 700 total protein stain kits (LI-COR, P/N 926), following the manufacturer’s protocol. After blocking the membrane in 5% skim milk in TBST, primary antibodies were applied as follows: ATXN1 (in-house, 11750 and 534), pS776 ATXN1 (in-house, PN1248), Flag (Sigma-Aldrich, F1804 and F7425), V5 (Invitrogen, R960-25), LacZ (DSHB, 40-1a), GAPDH (Advanced ImmunoChemical Inc., MAb 6C5), β3-TUB (Sigma-Aldrich, T8578), fly Actin (Thermo Fisher Scientific, ICN691001), ZBTB7B (Cell Signaling Technology, 13205S), TG2 (R&D Systems, AF4376), TG5 (Novus Biologicals, NBP2-94524), and oligomers (in-house, F11G3). After washing, HRP-conjugated secondary antibodies were applied in 5% skim milk in TBST and fluorophore-conjugated antibodies were applied in 3% BSA in TBST. Following wash, chemiluminescence was induced by ECL (GE Healthcare, RPN2236) and imaged by Image-Quant LAS 4000 imager (GE Healthcare), and fluorescence was scanned by Odyssey CLx imager (LI-COR).

### Custom ELISA.

Six biological replicates were performed for all procedures. Transgenic Daoy cells (2000 cells/well) were seeded in a 96-well plate in growth media (DMEM, 10% FBS, 1% antibiotic-antimycotic) and incubated for 20 hours until cells reached 30% confluency. The cells were transduced with the lentivirus listed in Supplemental Table 4 at the MOI of 20 and incubated for 24 hours, followed by puromycin selection (1 μg/mL) for 2 days. One-third of the cells were subcultured into a new 96-well plate in the growth media with puromycin (1 μg/mL) and incubated for 3 days, followed by another subculture into a new 96-well plate in media without puromycin and incubation for 3 days. CLB (50 μL/well) was added and the plate was orbitally shaken for 15 minutes on ice. Ten microliters of lysate from each well were used for BCA assay as described above, and the concentration of each lysate was adjusted to 5–10 ng/μL range by diluting 2^0^ to 2^5^-fold in a new round bottom 96-well plate. The ultra-sensitive ABC peroxidase rabbit IgG staining kit (Thermo Fisher Scientific, 32504) was used for ELISA. The diluted lysate (7 μL/well) and serially diluted ATXN1 standards (purified from *E*. *coli* as described below) were transferred into a flat-bottom 96-well plate containing antigen-coating buffer (50 mM NaHCO_3_, pH 9.6, 93 μL/well) and incubated at 4°C with gentle orbital shaking for overnight. The plate was emptied and washed 3 times with wash buffer (0.05% Tween 20 and 0.1% BSA in PBS, 200 μL/well). Wells were blocked for 1 hour with blocking buffer (1% normal goat serum in 1× PBS, 200 μL/well), and ATXN1 antibody was applied (affinity purified 11750 diluted into 1:6k in the blocking buffer, 200 μL/well) at room temperature for 1 hour. The plate was washed 3 times, followed by application of a biotinylated rabbit secondary antibody (45 μL in 10 mL of blocking buffer, 100 μL/well) incubated at room temperature for 45 minutes. Wells were washed 3 times and ABC reagent (prepared 30 min earlier) was added (100 μL/well) and incubated at room temperature for 30 minutes. Wells were washed 3 times and TMB ELISA substrate (Thermo Fisher Scientific, 34022, 100 μL/well) was added and incubated in the dark for 25 minutes to develop colors. Stop solution (1.5 M H_2_SO_4_, 100 μL/well) was then added and absorbance was measured using a microplate reader at 450 nm.

### Culturing iPSC-derived neurons from patients with SCA1.

iPSCs derived from patients with SCA1 were first induced into NPCs and differentiated into neurons (15). Briefly, NPCs were seeded in a Cultrex-coated T75 flask within NIM (1:1 mixture of DMEM/F12 and neurobasal with B27, N2, and GlutaMAX [2 mM]) supplemented with FGF2 (20 ng/mL) and incubated for 3 days. The cells were further incubated for 4 days within NIM without FGF2, and differentiated within NDM (Neurobasal with B27; GlutaMAX, 2 mM; BDNF, 20 ng/mL; GDNF, 10 ng/mL; NT-3, 10 ng/mL; db-cAMP, 100 μM; and ascorbic acid, 200 μM) for 14 days with exchange of media every 48 hours. The differentiated neurons were then passaged with trypsin into 24-well plates. To transduce neurons, virus was added into the media on the next day of passaging, and neurons were incubated for 9 days with full exchange of media in every 3 days. For inhibitor treatment, neurons were treated with LDN-27219 (Tocris, 4602) at 72 hours after passaging and incubated for 3 days.

### Immunostaining of patient iPSC-derived neurons and Daoy cells.

The patient iPSC-derived neurons were prepared by seeding NPCs in Cultrex-coated Millicell EZ Slide 8-well glass (EMD Millipore, PEZGS0816; 2,000 cells/well) and differentiating them for 3 weeks as described above. Daoy cells expressing mRFP-ATXN1 were plated in Millicell EZ Slide 8-well glass and transduced with lentivirus carrying TGM5-V5 or TGM2-V5 ORF for 72 hours. After fixing the cells with 4% PFA for 10 minutes at room temperature, the cells were washed, permeabilized with 0.3% PBST for 15 minutes, and blocked with 2% normal goat serum (NGS) or normal donkey serum (NDS) in 0.3% PBST for 1 hour. Primary antibodies for MAP2 (Abcam, ab5392, 1:5000), VGLUT1 (Sigma-Aldrich, AMAB91041, 1:200), GABA (Sigma-Aldrich, A2052, 1:200), V5 (Invitrogen, R960-25, 1:100), and/or TG2 (R&D Systems, AF4376, 1:500) were applied in blocking solution for 2 hours at room temperature and washed with 0.3% PBST. After incubating with secondary antibodies conjugated with fluorophores (Invitrogen, A-21449, A-21424, A-11008, A-11029, and/or A-11015 1:1000) for 1 hour at room temperature, the cells were washed and counterstained with DAPI (1 g/mL) for 5 minutes and mounted on a slide glass with Vectashield mounting medium (Vector laboratories, H-1400). Fluorescent images were taken with Nikon Eclipse Ti2-E confocal microscopy.

### Animal husbandry.

Mice were housed at room temperature with 12-hour light/12-hour dark cycle and were given water and standard rodent diet ad libitum. *Atxn1^154Q/2Q^* mice were previously described (30) and maintained on a C57BL/6 background.

### Stereotaxic injection of AAV9 into deep cerebellar nuclei.

The heads of 12-week-old SCA1 mice (mixed sex) anesthetized with isoflurane were fixed in stereotaxic frames. The skin on the head was disinfected and incised to expose the skull. Four spots above the cerebellum were marked and drilled to expose the cerebellum. AAV was loaded into 10 μL Hamilton syringe fitted with 32G needle and 1.5 μL (30 billion genome copies) of the virus was injected at a 0.15 μL/min flow rate into each of the 4 coordinates (x, y, z) derived from bregma: left/right (–/+1.75, –6.2, –2.1), middle anterior/posterior (0, –6.2/–6.8, –1.4). After the injection, the needle was held for 5 minutes before it was pulled out of the cerebellum. The incision was stitched, and the operated mouse was daily monitored. The mice were dissected 4 weeks after the injection, and fluorescence from the transduced cerebellum was imaged by Leica M80HD stereo microscope.

### ATXN1 stability assay.

Inducible transgenic Daoy cell lines expressing Fl-ATXN1[82Q], Fl-ATXN1[30Q], or Fl-ATXN1[2Q] were previously established (57). These cells were plated in a 6-well plate and transduced with lentivirus carrying shTGM2, shTGM5, or shControl, followed by puromycin selection (1 μg/mL) for 3 days. The selected cells were seeded in 24-well plates (20,000 cells/well) within growth media. The next day, ATXN1 expression was induced by applying doxycycline (300 ng/mL, Sigma-Aldrich, D9891) for 48 hours. The induction was terminated by exchanging the media into growth media without doxycycline, and cells were collected every 6 hours until 30 hours after the media change. Protein was extracted from the cells and ATXN1 levels were measured by Western blot with anti-flag antibody (Sigma-Aldrich, F1804).

### In vitro TG assay.

For the TG assay of ATXN1[82Q], 1 μg of purified human ATXN1[82Q] was mixed with 250 ng of normal or boiled (95°C for 3 minutes) recombinant human TG2 (R&D Systems, 4376) with or without cystamine (Tocris, 4981, final concentration: 40 mM) or LDN-27219 (Tocris, 4602, final concentration: 2 mM) to inhibit Tg activity in TG assay buffer (25 mM Tris-HCl, 5 mM CaCl_2_, pH 8) supplemented with protease inhibitor cocktail. For the TG assay of ATXN1 with different polyQ-length, 1 μg of purified human ATXN1[2Q], ATXN1[30Q], or ATXN1[82Q] was mixed with 250 ng of recombinant human TG2 in the TG assay buffer supplemented with protease inhibitor cocktail. Total volume of the reaction mixture was 50 μL, and the final concentration of urea was 300 mM. The reaction mixture was incubated at 30°C for an hour with gentle shaking. For terminating TG reaction, NuPAGE LDS sample buffer (4×) was added into reaction mixture and boiled at 95°C for 15 minutes. For Western blot, samples were cooled at room temperature, loaded onto 3%–8% Tris-acetate gels and steps were performed as described above.

### Western blot of ATXN1 HMW species from Triton X-100–soluble and –insoluble fraction after knockdown or overexpression of TG.

For overexpression of TG, WT HEK293T cells were plated in a 6-well plate (800,000 cells/well). The next day, 200 ng of pcDNA1.1 ATXN1[82Q] and W118-1 lacZ, TGM5, or TGM2 vectors were cotransfected via *Trans*IT-293 transfection reagent. For knockdown of TG, HEK293T cells were transduced with lentivirus generated from pGIPZ shTGM5 or shControl for 3 days and underwent puromycin selection (1 μg/mL) for 3 days. These cells were seeded in a 6-well plate (800,000 cells/well) and transfected with 500 ng of pcDNA1.1 ATXN1[82Q] vector using *Trans*IT-293 transfection reagent. Transfected cells were allowed to grow for 48 hours and lysed by applying 300 μL of CLB. The cells were then scraped, and the lysates were transferred into a Beckman Coulter VWR tube (BK357448), followed by ultracentrifugation at 110,000*g* with TLA-110 rotor for 30 minutes at 4°C. Triton X-100–soluble supernatant was carefully transferred and quantified by BCA assay as described above. Samples were used at equal concentration in NuPAGE LDS sample buffer with 2-ME and boiled at 95°C for 10 minutes. Triton X-100–insoluble pellets were washed with 1 mL of CLB and ultracentrifuged down as previously. After removing all the supernatant, 1× NuPAGE LDS sample buffer with 2-ME that was diluted with CLB from the original 4× concentration was added onto each pellet with the volume in proportion to the protein concentration of each corresponding Triton X-100–soluble supernatant (minimum volume was 400 μL). The pellets were dispersed by pipetting, boiled for 15 minutes, and probe-sonicated (40 pulses, output 2.5, duty cycle 30%) using a sonicator (Branson Sonifier 450). Both Triton X-100–soluble and –insoluble samples were loaded onto 3%–8% Tris-acetate gels into the 1:1 or 1:2 volume ratio, and Western blot was performed as described above.

### IF staining of human brain tissues.

The slides from paraffin-embedded postmortem human cerebellum and pons were deparaffinized and rehydrated by serially immersing them in xylene and 100%, 90%, and 80% ethanol. The slides were washed with PBS and antigens were retrieved in 10 mM sodium citrate buffer (pH 6.0) at 95°C in a steamer. The slides were washed again with PBS and blocked with a blocking solution (0.2% Triton X-100, 10% normal horse serum [NHS] in PBS) for 1 hour at room temperature. Primary antibodies were applied in 10% NGS in PBS (150 μL/slide) overnight at 4°C. The list of the primary antibodies and dilution ratios are as follows: anti-ATXN1 11NQ (in-house, 1:500), anti-polyglutamine 1C2 (EMD Millipore, MAB1574, 1:200), anti-TG2 (R&D Systems, AF4376, 1:500), anti-TG5 (Novus Biologicals, NBP2-94524, 1:50) anti-MAP2 (Abcam, ab5392, 1:5000), anti-calbindin D-28K (Swant, 300, 1:1000). After wash with PBS, fluorophore-conjugated secondary antibodies in PBS supplemented with 1× TrueBlack Plus Lipofuscin Autofluorescence Quencher (Biotium, 23014) were applied for 2 hours at room temperature. After wash with PBS, the slides were mounted with Vectashield mounting medium with DAPI (Vector laboratories, H-1800) and imaged with Nikon Eclipse Ti2-E confocal microscopy.

### Statistics.

Statistical tests for simple comparisons (2-tailed Student’s *t* test) and multiple comparisons (1-way or 2-way ANOVA) were performed using GraphPad Prism 9 software according to experimental design. For the post hoc analysis, either Dunnett’s test (comparing every mean to a control mean) or Tukey’s test (comparing every mean with every other mean) was used unless otherwise specified. *P* values of less than 0.05 were considered significant.

### Study approval.

All experiments performed with mice and postmortem human tissues were approved by the IACUC (AN-1013) and IRB (H-1020) at Baylor College of Medicine, respectively. The slides of paraffin-embedded cerebellum and pons derived from 3 patients with SCA1 and 3 individuals as the control group were obtained from the University of Florida and University of Michigan Brain Bank, respectively, following the protocols approved by the respective research ethics boards. Postmortem brain tissues were deposited in each brain bank with written, informed consent from individual donors or their legal representatives. Anonymity of donors was assured by the IRBs at the University of Florida (UF) and University of Michigan.

## Author contributions

WSL executed the project, performed the majority of experiments, analyzed data, and wrote the draft of manuscript. IAR performed the *Drosophila* screen and generated the majority of *Drosophila* data. HHJ analyzed the cell-based screen data and contributed to writing experimental methods. TL contributed to human tissue staining. CJA and RR contributed to ATXN1 purification and the in vitro transglutaminase assay. LAL, VVB, and JPR contributed to the cell-based screen. YJ, SR, and EA contributed to biochemical experiments. HTO, JB, and ZL provided resources, expertise, and scientific input for mouse, fly, and computational studies, respectively. HYZ, HTO, ZL, and JB edited the paper. HYZ conceived the project, interpreted the data, revised the manuscript, and secured funding.

## Supplementary Material

Supplemental data

Supplemental data set 1

Supplemental data set 2

Supplemental data set 3

Supplemental data set 4

Supplemental data set 5

Supplemental data set 6

Supplemental data set 7

Supplemental data set 8

Supplemental table 1

Supplemental table 2

Supplemental table 3

Supplemental table 4

Supplemental table 5

Supplemental table 7

## Version 1 05/02/2022

Electronic publication

## References

[1] Skovronsky DM (2006). Neurodegenerative diseases: new concepts of pathogenesis and their therapeutic implications. Annu Rev Pathol.

[2] Gan L (2018). Converging pathways in neurodegeneration, from genetics to mechanisms. Nat Neurosci.

[3] Soto C (2003). Unfolding the role of protein misfolding in neurodegenerative diseases. Nat Rev Neurosci.

[4] Ross CA, Poirier MA (2004). Protein aggregation and neurodegenerative disease. Nat Med.

[5] Farr SA (2014). Central and peripheral administration of antisense oligonucleotide targeting amyloid-β protein precursor improves learning and memory and reduces neuroinflammatory cytokines in Tg2576 (AβPPswe) mice. J Alzheimers Dis.

[6] Cole TA (2021). α-Synuclein antisense oligonucleotides as a disease-modifying therapy for Parkinson’s disease. JCI Insight.

[7] Kordasiewicz HB (2012). Sustained therapeutic reversal of Huntington’s disease by transient repression of huntingtin synthesis. Neuron.

[8] White JK (1997). Huntingtin is required for neurogenesis and is not impaired by the Huntington’s disease CAG expansion. Nat Genet.

[9] Martianov I (2002). RNA polymerase II transcription in murine cells lacking the TATA binding protein. Science.

[10] Orr HT (1993). Expansion of an unstable trinucleotide CAG repeat in spinocerebellar ataxia type 1. Nat Genet.

[11] Cummings CJ (1999). Mutation of the E6-AP ubiquitin ligase reduces nuclear inclusion frequency while accelerating polyglutamine-induced pathology in SCA1 mice. Neuron.

[12] Zoghbi HY, Orr HT (2009). Pathogenic mechanisms of a polyglutamine-mediated neurodegenerative disease, spinocerebellar ataxia type 1. J Biol Chem.

[13] Park J (2013). RAS-MAPK-MSK1 pathway modulates ataxin 1 protein levels and toxicity in SCA1. Nature.

[14] Friedrich J (2018). Antisense oligonucleotide-mediated ataxin-1 reduction prolongs survival in SCA1 mice and reveals disease-associated transcriptome profiles. JCI Insight.

[15] Lee WS (2021). Dual targeting of brain region-specific kinases potentiates neurological rescue in Spinocerebellar ataxia type 1. EMBO J.

[16] Suh J (2019). Loss of Ataxin-1 potentiates Alzheimer’s pathogenesis by elevating cerebral BACE1 transcription. Cell.

[17] Lim J (2008). Opposing effects of polyglutamine expansion on native protein complexes contribute to SCA1. Nature.

[18] Nitschke L (2021). Modulation of ATXN1 S776 phosphorylation reveals the importance of allele-specific targeting in SCA1. JCI Insight.

[19] Lorand L, Graham RM (2003). Transglutaminases: crosslinking enzymes with pleiotropic functions. Nat Rev Mol Cell Biol.

[20] Muma NA (2007). Transglutaminase is linked to neurodegenerative diseases. J Neuropathol Exp Neurol.

[21] Hartley DM (2008). Transglutaminase induces protofibril-like amyloid beta-protein assemblies that are protease-resistant and inhibit long-term potentiation. J Biol Chem.

[22] Junn E (2003). Tissue transglutaminase-induced aggregation of alpha-synuclein: Implications for Lewy body formation in Parkinson’s disease and dementia with Lewy bodies. Proc Natl Acad Sci U S A.

[23] Kahlem P (1998). Transglutaminase action imitates Huntington’s disease: selective polymerization of Huntingtin containing expanded polyglutamine. Mol Cell.

[24] Mandrusiak LM (2003). Transglutaminase potentiates ligand-dependent proteasome dysfunction induced by polyglutamine-expanded androgen receptor. Hum Mol Genet.

[25] D’Souza DR (2006). Tissue transglutaminase crosslinks ataxin-1: possible role in SCA1 pathogenesis. Neurosci Lett.

[26] Rovelet-Lecrux A (2006). APP locus duplication causes autosomal dominant early-onset Alzheimer disease with cerebral amyloid angiopathy. Nat Genet.

[27] Singleton AB (2003). alpha-Synuclein locus triplication causes Parkinson’s disease. Science.

[28] Gennarino VA (2015). Pumilio1 haploinsufficiency leads to SCA1-like neurodegeneration by increasing wild-type Ataxin1 levels. Cell.

[29] Fernandez-Funez P (2000). Identification of genes that modify ataxin-1-induced neurodegeneration. Nature.

[30] Watase K (2002). A long CAG repeat in the mouse Sca1 locus replicates SCA1 features and reveals the impact of protein solubility on selective neurodegeneration. Neuron.

[31] Al-Ramahi I (2007). dAtaxin-2 mediates expanded Ataxin-1-induced neurodegeneration in a Drosophila model of SCA1. PLoS Genet.

[32] Karpuj MV (1999). Transglutaminase aggregates huntingtin into nonamyloidogenic polymers, and its enzymatic activity increases in Huntington’s disease brain nuclei. Proc Natl Acad Sci U S A.

[33] Lesort M (1999). Tissue transglutaminase is increased in Huntington’s disease brain. J Neurochem.

[34] Guan WJ (2013). Transglutaminase 6 interacts with polyQ proteins and promotes the formation of polyQ aggregates. Biochem Biophys Res Commun.

[35] Tripathy D (2017). Mutations in TGM6 induce the unfolded protein response in SCA35. Hum Mol Genet.

[36] Case A, Stein RL (2007). Kinetic analysis of the interaction of tissue transglutaminase with a nonpeptidic slow-binding inhibitor. Biochemistry.

[37] Akimov SS (2000). Tissue transglutaminase is an integrin-binding adhesion coreceptor for fibronectin. J Cell Biol.

[38] Siegel M, Khosla C (2007). Transglutaminase 2 inhibitors and their therapeutic role in disease states. Pharmacol Ther.

[39] Lasagna-Reeves CA (2015). A native interactor scaffolds and stabilizes toxic ATAXIN-1 oligomers in SCA1. Elife.

[40] Lasagna-Reeves CA (2015). Ataxin-1 oligomers induce local spread of pathology and decreasing them by passive immunization slows Spinocerebellar ataxia type 1 phenotypes. Elife.

[41] Duyckaerts C (1999). Nuclear inclusions in spinocerebellar ataxia type 1. Acta Neuropathol.

[42] Klement IA (1998). Ataxin-1 nuclear localization and aggregation: role in polyglutamine-induced disease in SCA1 transgenic mice. Cell.

[43] https://citeseerx.ist.psu.edu/viewdoc/download?doi=10.1.1.1048.6341&rep=rep1&type=pdf.

[44] Adegbuyiro A (2017). Proteins containing expanded polyglutamine tracts and neurodegenerative disease. Biochemistry.

[45] Ostergaard ME (2013). Rational design of antisense oligonucleotides targeting single nucleotide polymorphisms for potent and allele selective suppression of mutant Huntingtin in the CNS. Nucleic Acids Res.

[46] D’Eletto M (2012). Type 2 transglutaminase is involved in the autophagy-dependent clearance of ubiquitinated proteins. Cell Death Differ.

[47] Iwata A (2005). Increased susceptibility of cytoplasmic over nuclear polyglutamine aggregates to autophagic degradation. Proc Natl Acad Sci U S A.

[48] Rossin F (2015). Transglutaminase 2 ablation leads to mitophagy impairment associated with a metabolic shift towards aerobic glycolysis. Cell Death Differ.

[49] Li Z (2019). Allele-selective lowering of mutant HTT protein by HTT-LC3 linker compounds. Nature.

[50] Miller VM (2003). Allele-specific silencing of dominant disease genes. Proc Natl Acad Sci U S A.

[51] Prudencio M (2020). Toward allele-specific targeting therapy and pharmacodynamic marker for spinocerebellar ataxia type 3. Sci Transl Med.

[52] Guerrero-Munoz MJ (2014). Amyloid-β oligomers as a template for secondary amyloidosis in Alzheimer’s disease. Neurobiol Dis.

[53] Kayed R (2003). Common structure of soluble amyloid oligomers implies common mechanism of pathogenesis. Science.

[54] Jeong HH (2017). CRISPRcloud: a secure cloud-based pipeline for CRISPR pooled screen deconvolution. Bioinformatics.

[55] Rousseaux MWC (2018). A druggable genome screen identifies modifiers of α-synuclein levels via a tiered cross-species validation approach. J Neurosci.

[56] Pham TV, Jimenez CR (2012). An accurate paired sample test for count data. Bioinformatics.

[57] Nitschke L (2020). miR760 regulates ATXN1 levels via interaction with its 5’ untranslated region. Genes Dev.

