# Supplementary material for: Cross-species genetic screens identify transglutaminase 5 as a regulator of polyglutamine-expanded ataxin-1
Source: J Clin Invest. 2022 May 2;132(9):e156616. doi: 10.1172/JCI156616 (PMC9057624; doi:10.1172/JCI156616)

Supplemental Table 6. Interrogation of the 22 genes whose knockdown reduced ATXN1 in SCA1 patient iPSC-derived neurons

Gene name	Library	Selected	Paralogs: positive ATXN1 regulators	*Drosophila* phenotype resue		Validation in the patient-derived neurons by overexpression
				Eye	Motor	
HDGFRP3	CH					
JADE2	CH					X
RAD54B	CH				X	
POLA2	EZ				X	
TGM5	EZ	X	F13A1, TGM1, TGM2, TGM3, TGM6	X	X	X (TGM2/6)
HELQ	EZ					X
QRFPR	GP			X	X	
MRGPRE	GP					
GABRA1	IC			X	X	
CATSPER2	IC				X	
SLC39A11	TP			X	X	
SLCO1B3	TP					
DNAJB2	UB				X	X
LBP	OT					
PLP2	OT					X
SEC62	OT					
TEKT4	OT					
IL1B	OT					X
MUC2	OT					
IRAK1	KP	X	IRAK2, IRAK3, IRAK4	X		X
SRPK3	KP	X	SRPK1, SRPK2	X		X
STK16	KP	X		X	X	X

CH: chromatin; EZ: enzyme; GP: GPCR; IC: ion channel; TP: transporter; UB: ubiquitin; OT: others; KP: kinase/phosphatase; Selected: genes selected for *in vivo* knockdown in the SCA1 mouse brain. The knockdown of targets except for LBP, TEKT4 and MUC2 were confirmed.

Supplemental Figure 1

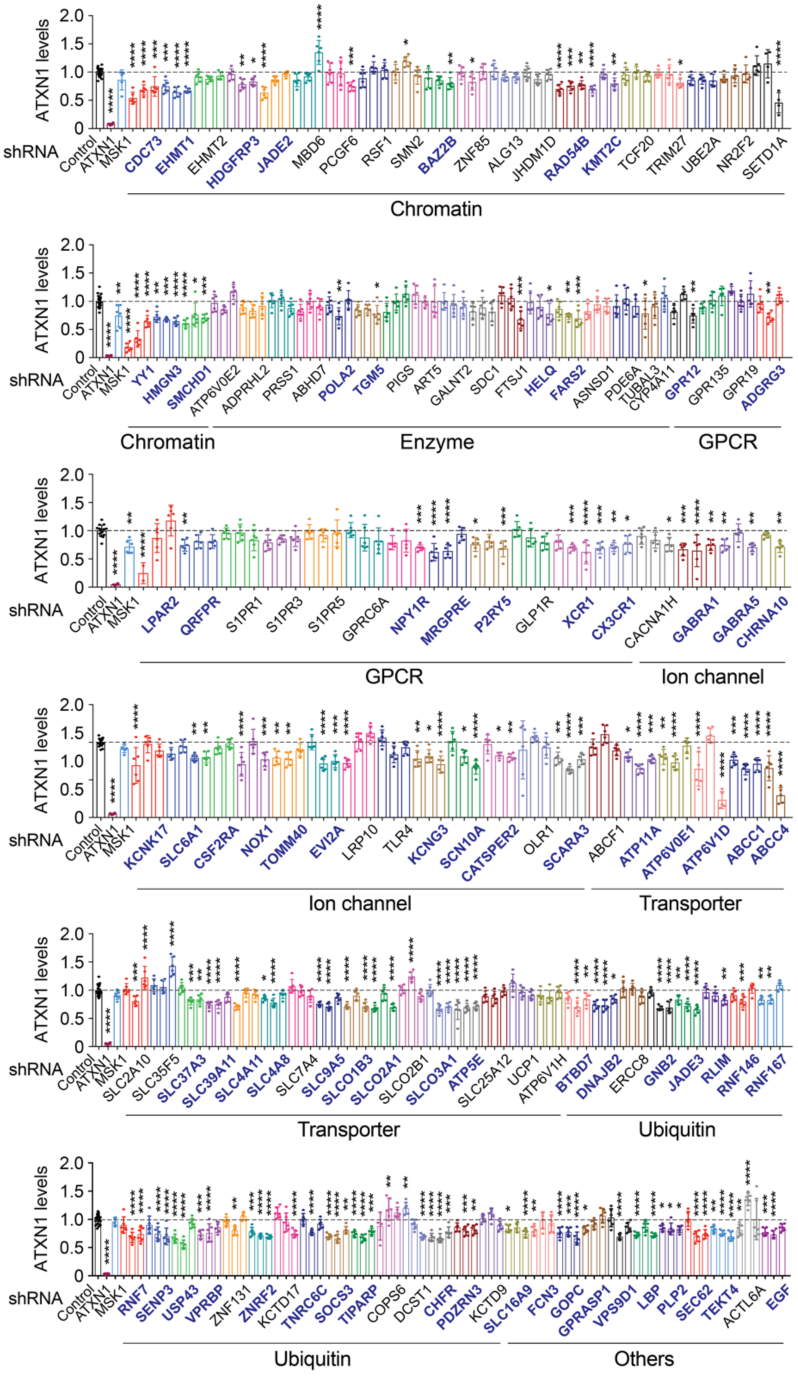

Supplemental Figure 1. ATXN1[82Q] ELISA results after knockdown of 156 genes in ATXN1 reporter cells. Individual bars display ATXN1 levels of each sample treated with different shRNAs. Genes whose names are colored in blue were selected for the next validations. The library in which individual gene belongs to is represented under each graph. Error bar: ±SD, *p<0.05, **p<0.01, ***p<0.001, ****p<0.0001, One-way ANOVA, post-hoc: Dunnett's test.

Supplemental Figure 2

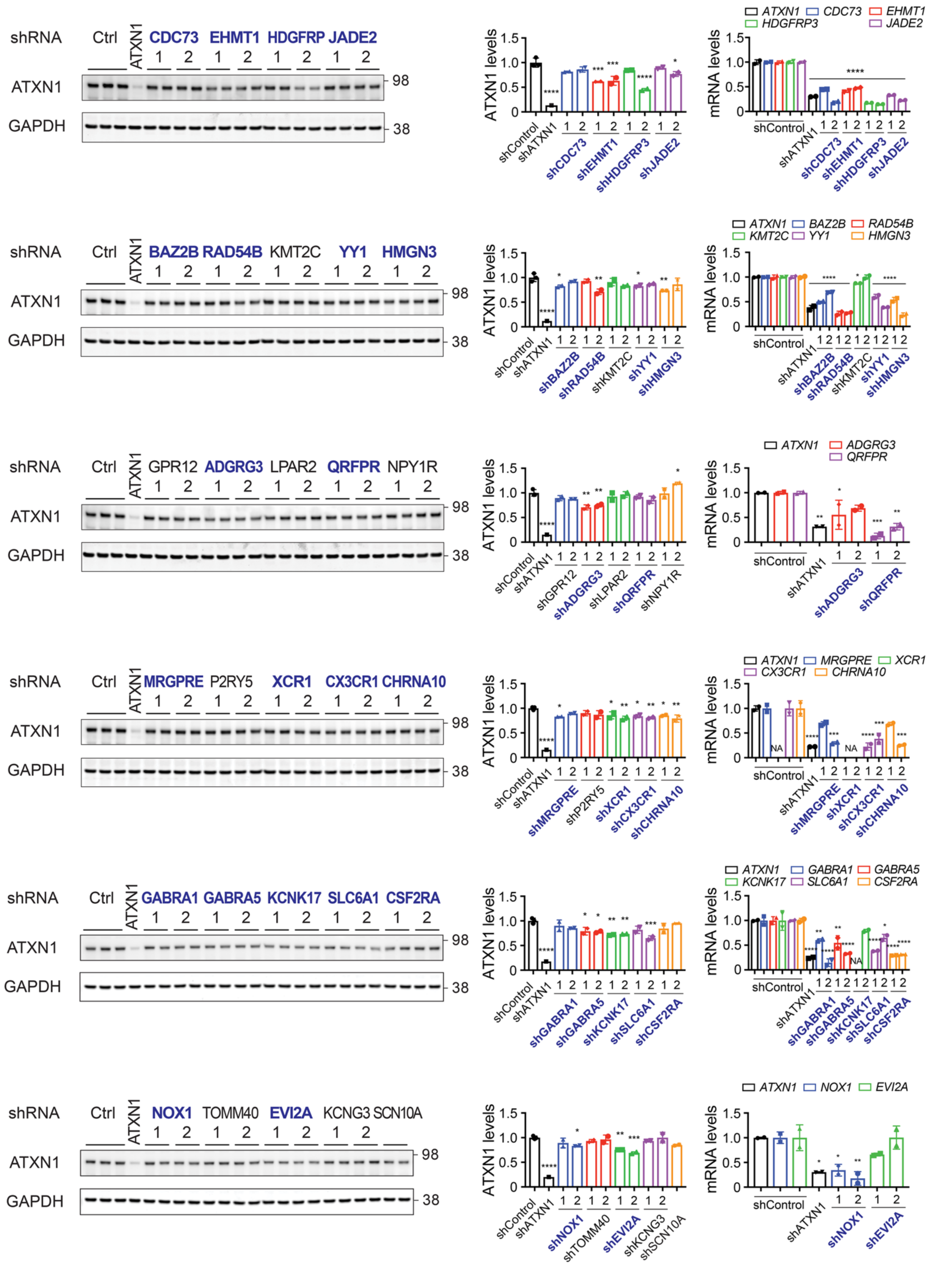

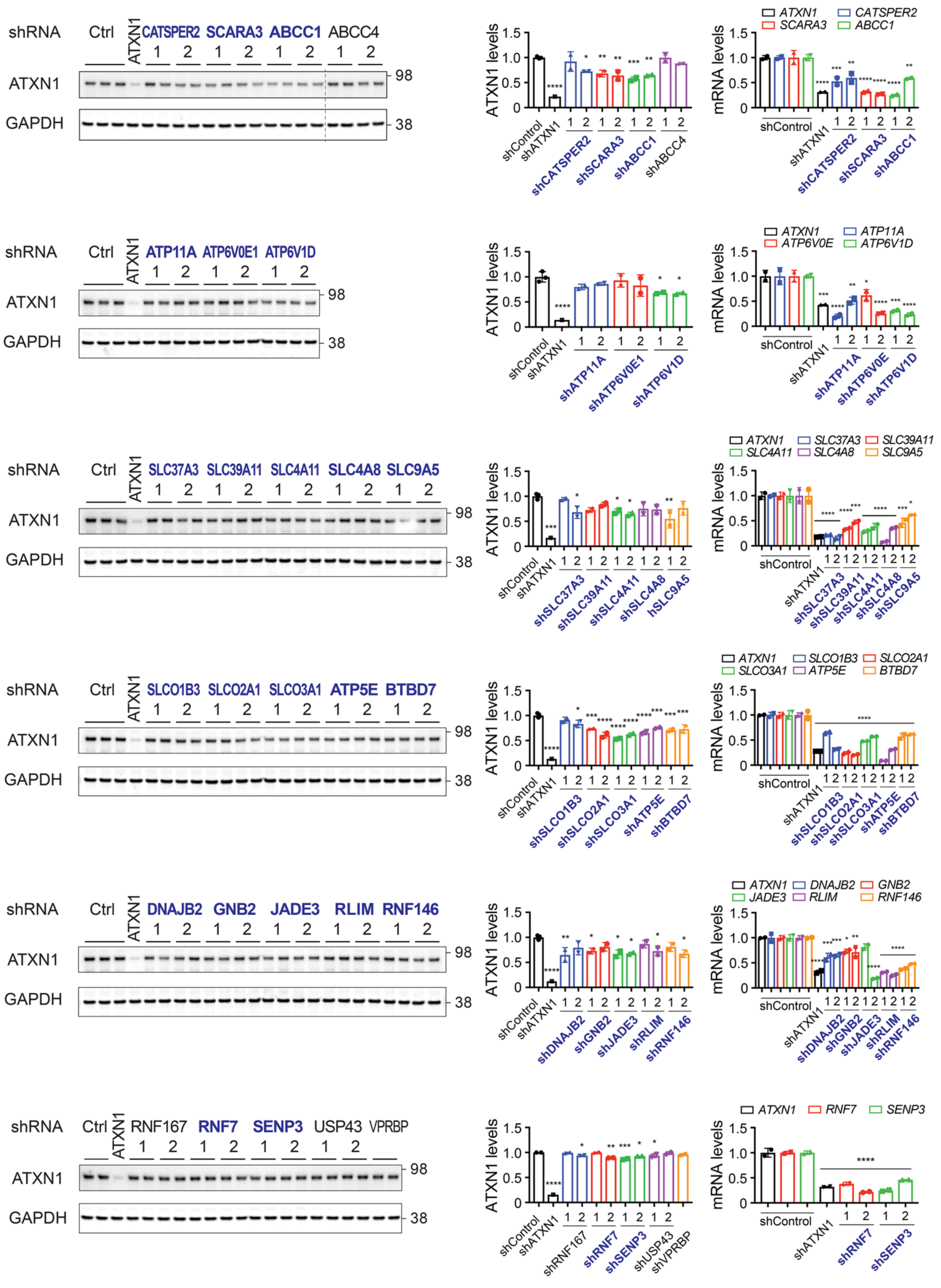
shRNA
Ctrl
ATXN1
CATSPER2 SCARA3 ABCC1 ABCC4
ATXN1
GAPDH
98
38
ATXN1 levels
shControl
shATXN1
shCATSPER2
shSCARA3
shABCC1
shABCC4
mRNA levels
ATXN1 CATSPER2 SCARA3 ABCC1
ATP11A ATP6V0E1 ATP6V1D
shATP11A
shATP6V0E1
shATP6V1D
ATXN1 ATP11A ATP6V0E ATP6V1D
SLC37A3 SLC39A11 SLC4A11 SLC4A8 SLC9A5
shSLC37A3
shSLC39A11
shSLC4A11
shSLC4A8
hSLC9A5
shSLC9A5
ATXN1 SLC37A3 SLC39A11 SLC4A11 SLC4A8 SLC9A5
SLCO1B3 SLCO2A1 SLCO3A1 ATP5E BTBD7
shSLCO1B3
shSLCO2A1
shSLCO3A1
shATP5E
shBTBD7
ATXN1 SLCO1B3 SLCO2A1 SLCO3A1 ATP5E BTBD7
DNAJB2 GNB2 JADE3 RLIM RNF146
shDNAJB2
shGNB2
shJADE3
shRLIM
shRNF146
ATXN1 DNAJB2 GNB2 JADE3 RLIM RNF146
RNF167 RNF7 SENP3 USP43 VPRBP
shRNF167
shRNF7
shSENP3
shUSP43
shVPRBP
ATXN1 RNF7 SENP3

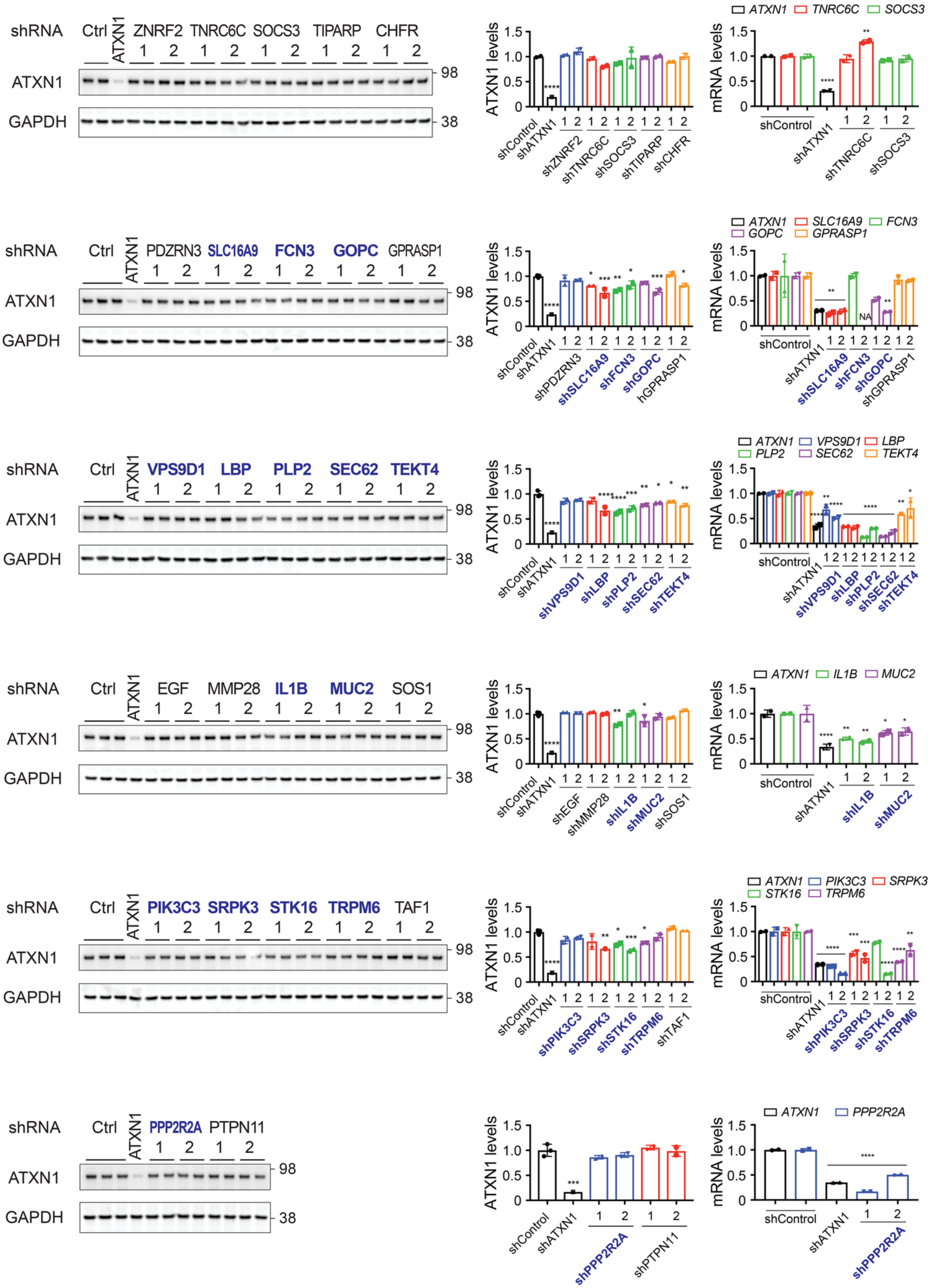
shRNA
Ctrl
ATXN1
ZNRF2
TNRC6C
SOCS3
TIPARP
CHFR
PDZRN3
SLC16A9
FCN3
GOPC
GPRASP1
VPS9D1
LBP
PLP2
SEC62
TEKT4
EGF
MMP28
IL1B
MUC2
SOS1
PIK3C3
SRPK3
STK16
TRPM6
TAF1
PPP2R2A
PTPN11
GAPDH
98
38
ATXN1 levels
mRNA levels
shControl
shATXN1
NA

Supplemental Figure 2. Validation of candidate ATXN1 regulators in wild-type Daoy cells. Western blot analysis of endogenous ATXN1 and qRT-PCR results of target genes after knockdown of 94 genes. Left graphs: densitometry of ATXN1 levels, right graphs: mRNA levels of genes targeted by each shRNA. Genes whose names are colored in blue were selected for the next validations. qPCR was performed for only the genes that show decrease of ATXN1 protein levels. Error bar: ±SD, *$p<0.05$, **$p<0.01$, ***$p<0.001$, ****$p<0.0001$, One-way ANOVA, post-hoc: Dunnett's test for the left graphs; Tukey's test for the right graphs.

Supplemental Figure 3

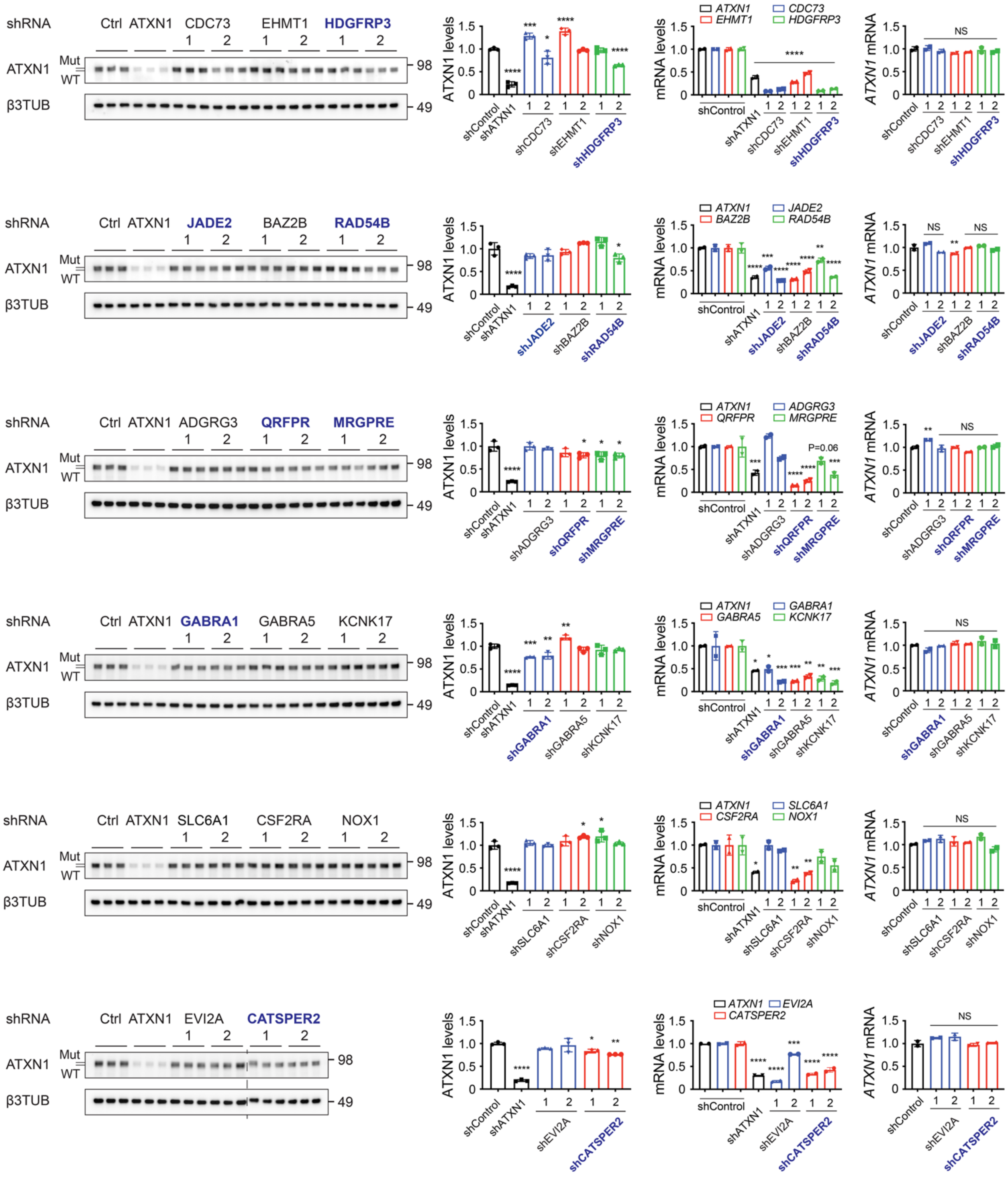

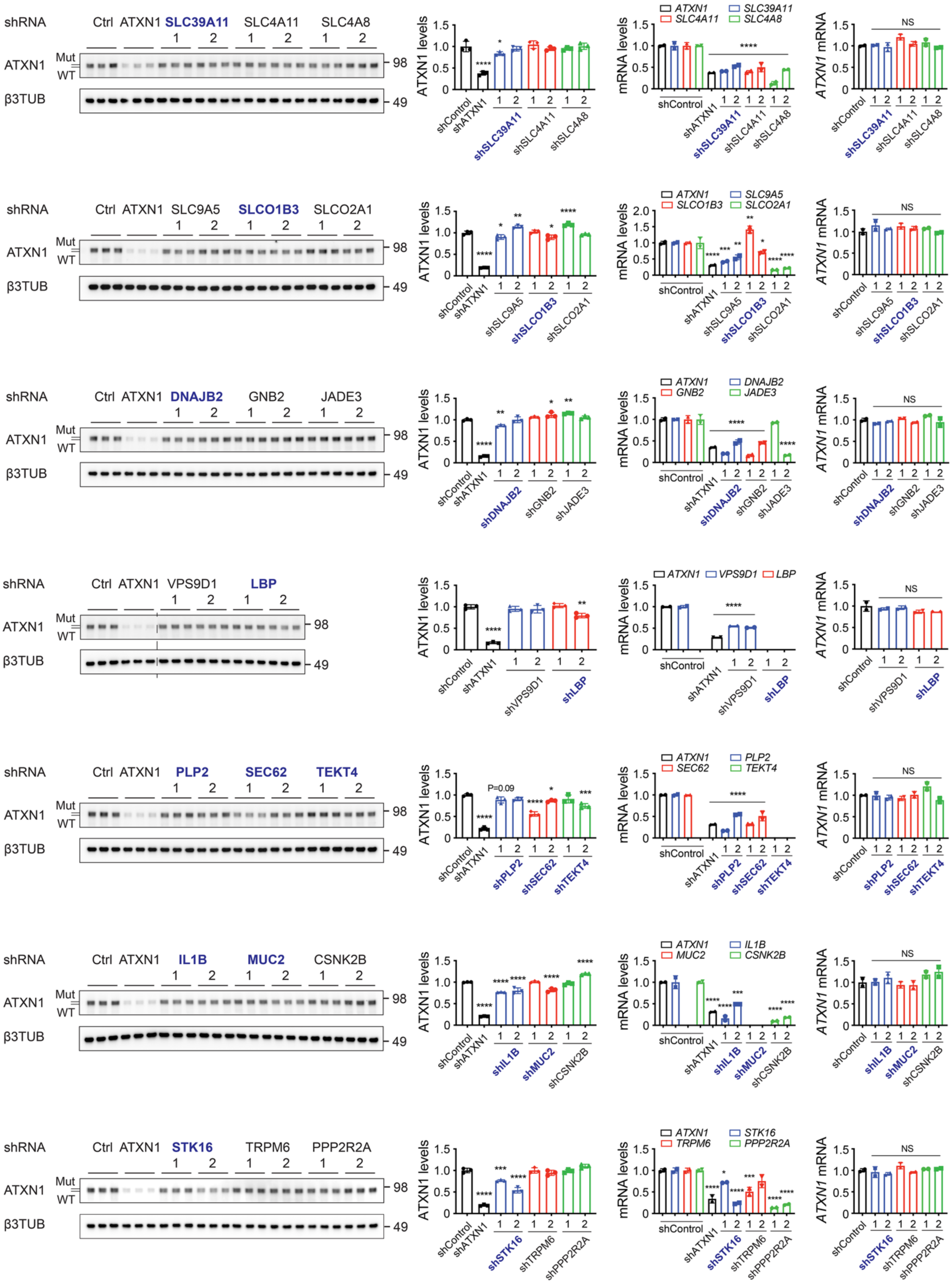
shRNA
Ctrl ATXN1 SLC39A11 SLC4A11 SLC4A8
ATXN1
Mut
WT
β3TUB
98
49
ATXN1 levels
mRNA levels
ATXN1 mRNA
shControl
shATXN1
shSLC39A11
shSLC4A11
shSLC4A8
Ctrl ATXN1 SLC9A5 SLCO1B3 SLCO2A1
shSLC9A5
shSLCO1B3
shSLCO2A1
Ctrl ATXN1 DNAJB2 GNB2 JADE3
shDNAJB2
shGNB2
shJADE3
Ctrl ATXN1 VPS9D1 LBP
shVPS9D1
shLBP
Ctrl ATXN1 PLP2 SEC62 TEKT4
P=0.09
shPLP2
shSEC62
shTEKT4
Ctrl ATXN1 IL1B MUC2 CSNK2B
shIL1B
shMUC2
shCSNK2B
Ctrl ATXN1 STK16 TRPM6 PPP2R2A
shSTK16
shTRPM6
shPPP2R2A
NS

Supplemental Figure 3. Validation of ATXN1 regulators by knocking them down in SCA1 patient iPSC-derived neurons. Western blot analysis of mutant and wild-type ATXN1 and qRT-PCR results of target genes and *ATXN1* mRNA levels after knockdown of candidate genes in SCA1 patient iPSC-derived neurons. Blue colored genes are validated ATXN1 regulators. Left graphs: densitometry of total ATXN1 levels; Middle graphs: mRNA levels of genes targeted by each shRNA; Right graphs: *ATXN1* mRNA levels. Error bar: ±SD, *$p<0.05$, **$p<0.01$, ***$p<0.001$, ****$p<0.0001$, One-way ANOVA, post-hoc: Dunnett's test for the left and right graphs; Tukey's test for the middle graphs.

Supplemental Figure 4

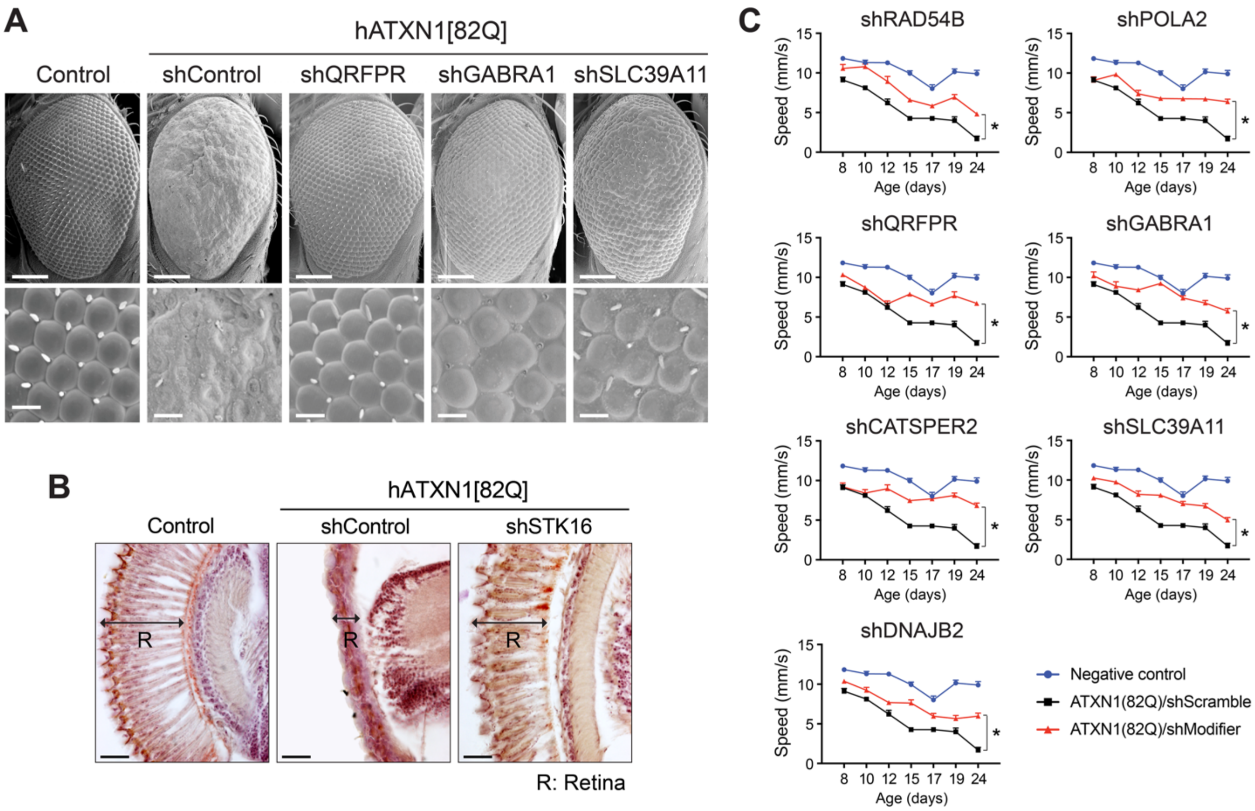

Supplemental Figure 4. Validation of ATXN1 regulators in *Drosophila* eyes and central nervous system that express ATXN1[82Q]. (A) Scanning electron microscopy images of *Drosophila* eyes expressing human ATXN1[82Q] with knockdown of *Drosophila* homologues of the indicated genes. The same control eye image in Figure 3A was used for comparison. Scale bar = 100 µm in the top images; 10 µm in the bottom images. **(B)** Histological evaluation of retinal thickness of *Drosophila* expressing human ATXN1[82Q] with knockdown of fly STK16 homologue. These data support the rescue of eye degeneration by shSTK16 in **Figure 3A**. Scale bar = 20 µm. **(C)** Effect of the knockdown of the indicated genes in the motor performance of *Drosophila* SCA1 model expressing ATXN1[82Q] in the central nervous system. Error bar: ±SEM, *$p < 0.05$, Linear mixed-effect model ANOVA.

Supplemental Figure 5

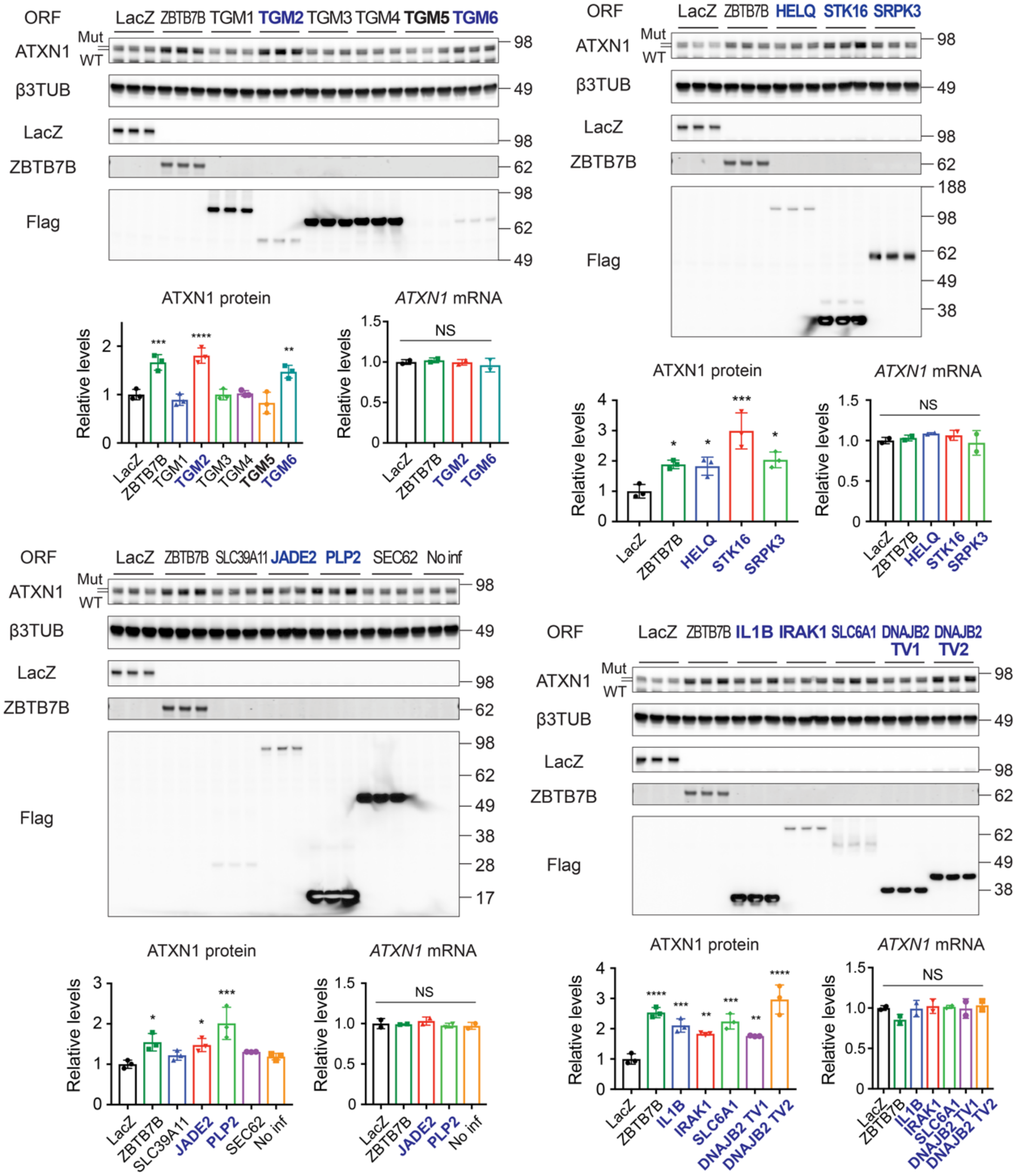

Supplemental Figure 5. Validation of ATXN1 regulators by overexpressing them in SCA1 patient iPSC-derived neurons. Western blot analysis of mutant and wild-type ATXN1 and qRT-PCR results of *ATXN1* mRNA levels after the overexpression of indicated genes for 9 days in the patient iPSC-derived neurons. ZBTB7B was used as a positive control. Genes whose overexpression increased ATXN1 protein levels are colored in blue. qPCR was performed for only the genes that show increase of ATXN1 protein levels. Overexpression of each gene was confirmed by flag antibody. Note that TGM5 was not expressed. Error bar: ±SD, *p<0.05, **p<0.01, ***p<0.001, ****p<0.0001, One-way ANOVA, post-hoc: Dunnett's test.

Supplemental Figure 6

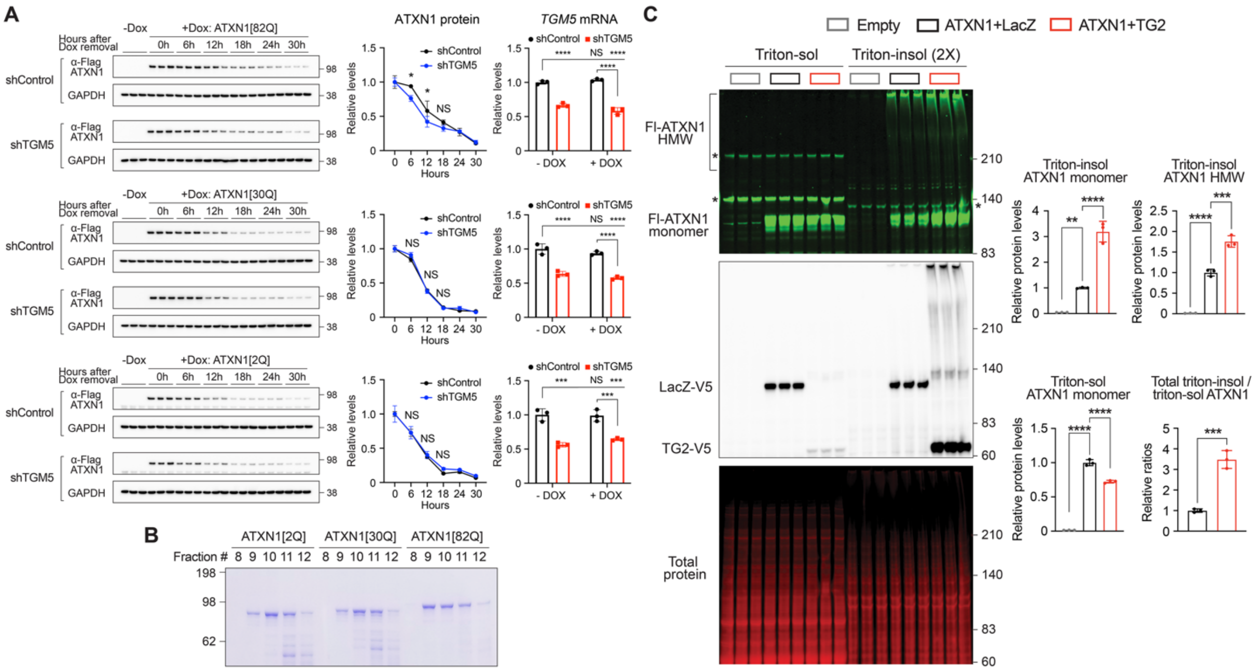

Supplemental Figure 6. TGs regulate mutant ATXN1 stability and insoluble HMW levels.
(A) Stability assay of inducibly expressed ATXN1 with different polyQ-length in Daoy cells expressing shTGM5 or shControl. After a 48-hour doxycycline treatment for inducing ATXN1 expression, media was exchanged into growth media without doxycycline, and cells were collected every 6 hours until 30 hours after the media change. Knockdown of *TGM5* by shTGM5 was confirmed by qRT-PCR. Error bar: ±SD, *p<0.05, **p<0.01, ***p<0.001, ****p<0.0001. Two-way ANOVA, post-hoc: Sidak's test for ATXN1 protein levels; Tukey's test for *TGM5* mRNA. **(B)** Coomassie staining of ATXN1[2Q], ATXN1[30Q], and ATXN1[82Q] that were purified from *E.coli*. Affinity purified proteins were subject to a size-exclusion chromatography (See Methods) and the fraction 8-12 were loaded on a gel. The fraction 9 or 10 of each protein was used for *in vitro* TG assay. **(C)** Western blot analysis of triton-soluble or -insoluble ATXN1 HMW and monomer that were extracted from the HEK293T cells overexpressing ATXN1[82Q] and TG2 or LacZ. Empty: empty vector; LacZ: overexpression control. Total triton-insoluble ATXN1 (HMW+monomer) to triton-soluble ATXN1 (monomer) ratio was calculated in each overexpression group and represented in the bottom right graph. Asterisks indicate non-specific bands. Error bar: ±SD, **p<0.01, ***p<0.001, ****p<0.0001, two-tailed t-test for the bottom right graph; one-way ANOVA for the other graphs with post-hoc Dunnett's test.

Supplemental Methods

Knockdown of candidate ATXN1 regulators in wild-type Daoy cells

The cell culture and lentiviral transduction of wild-type Daoy cells are the same with those of transgenic Daoy cells as described above. Daoy cells transduced with the lentivirus listed in **Supplemental Table 5** were selected by puromycin (1 μg/ml) for two days, and subcultured into 24-well plates in growth media supplemented with puromycin (1 μg/ml), followed by incubation for 3 days. Half of the cells were subcultured into new 24-well plates in media without puromycin and cultured for 3 more days. Protein was then extracted for western blot as described above.

Reverse transcription and qRT-PCR

TRIzol® reagent (Invitrogen) and RNeasy Kit (Qiagen) were used for extracting RNA from tissue and cells, respectively. SuperScript® III First-strand Synthesis kit (Invitrogen) was used to reverse-transcribe 150-2,000 ng of total RNA. Primers designed to span exons, cDNA (10-20 ng), PowerUp SYBR Green Master Mix (Fisher Scientific, A25778), and water were mixed in a total volume of 20 μl, and then run in C1000™ Thermal Cycler (BioRad). Relative gene expression was calculated by regular ΔΔCt method, where ACTB or GAPDH was used as a reference gene. The sequences of all primers are listed in **Supplemental Table 7**.

Producing ATXN1[2Q]/[30Q]/[82Q] in *E.coli*

Human ATXN1[2Q] and ATXN1[30Q] were cloned into pET-28a(+)-TEV vector, and N-terminal SUMO-conjugated ATXN1[82Q] was cloned into MCS1 region of pCDFDuet-1 vector (Novagen, 71340-3). These vectors were transformed into BL21(DE3) Competent E. coli (New England Biolabs, C2527H) and grown overnight in 10 ml of LB medium supplemented with kanamycin (50 μg/ml) and spectinomycin (50 μg/ml) for the pET-28a(+)-TEV and pCDFDuet-1 vector, respectively. The

cells were inoculated into 500 ml of LB media supplemented with the same antibiotics. When the cells reach 0.5 of OD_{600} value, they were induced to express proteins with 0.2% L-arabinose and 0.5 mM IPTG for 4 hours at 30°C for SUMO-ATXN1[82Q] and for 3 hours at 37°C for all the other proteins and then spun down at 6,000 rpm for 30 min at 4°C. The cells were resuspended in 10 ml of lysis buffer (6M urea, 20 mM 2-mercaptoethanol, 0.5 M NaCl, 30 mM Imidazole and 50 mM NaPO4, [pH 7.4]) and probe sonicated (total 100 pulses, output 3, duty cycle 40%). Samples were passed through a 10 ml syringe fitted with a 22G needle 3 times with and spun down at 10,000 rpm for 1 hour at 4 °C. The supernatant loaded onto Ni Sepharose High Performance beads (GE Healthcare, 17-5268-02) and passed through the column fitted with a 22G needle by gravity flow. The beads that bind SUMO-ATXN1[82Q] were washed four times serially with 6M, 2M, 1M, and 0.5M urea lysis buffer diluted in PBS, follow by elution with 300 mM imidazole in 0.4M Urea lysis buffer. The beads that bind ATXN1[2Q] or ATXN1[30Q] were washed four times with the 6M urea lysis buffer and eluted with 300 mM imidazole in the 6M lysis buffer. The SUMO-ATXN1[82Q] fraction with the highest protein concentration was incubated with SUMO hydrolase (50 μg) for 3 hours at RT. The fractions of ATXN1[30Q] and ATXN1[2Q] with the highest protein concentration were diluted into 4-fold with PBS (1.5 M urea). These deSUMOylated or diluted fractions were further subjected to size-exclusion chromatography. Briefly, 700 μl of the eluted protein was run on a Superdex S200 (Cytiva, 28-9909-44) in 1M Urea in PBS on an AKTA UPC 10 FPLC system (Cytiva). Fractions are eluted directly into a 100X protease inhibitor cocktail. Three MW markers [thyroglobulin (667 kDa), BSA (64 kDa), and cytochrome C (12 kDa)] were used to standardize the column. The size and purity of the purified proteins were confirmed by coomassie staining and the fraction 9 or 10 of each protein was used for *in vitro* TG assay.

Co-immunoprecipitation (Co-IP)

HEK293T cells were plated in a 6-well plate (800,000 cells/well). Next day, the cells were transfected with 500 ng of pcDNA1.1 ATXN1[82Q] and ATXN1[30Q] vector with 1 μg of W118-1 TG5-

flag or TG2-flag vector using *Trans*IT®-293 reagent and then incubated for 48 hours. For cell lysis, cold CLB (1 ml/well) were added onto the cells and the cell lysate was collected in a new tube. After centrifugation at 13,000 rpm for 15 min at 4°C, the supernatant was transferred into a new tube and 20 µl of the supernatant was saved for Input (pre-IP). Dynabeads™ Protein G (Invitrogen, 10004D) was transferred to a test tube (25 µl/IP sample), washed with PBS, and incubated with anti-V5 antibody (3 µg/IP sample; Invitrogen, R960-25) in PBS with 0.1% Tween-20 for an hour on a nutator. The dynabeads were then washed with CLB and added into each cell lysate, followed by overnight incubation with rotation at 4°C. After saving 20 µl of the lysates for post-IP, the rest of the lysates were discarded. The dynabeads were washed 4 times with CLB and boiled in 27 ul of 1X NuPAGE™ LDS Sample Buffer, together with the pre- and post-IP samples mixed with 7 ul of 4X sample buffer. The samples were run in 3-8% Tris-acetate gel, and the rest of western blot was performed.

***Drosophila* histology**

For histological staining of Drosophila paraffin sections, flies were fixed with 4% formaldehyde in 1X PBS and their heads were dehydrated in increasing concentrations of ethanol. After clearing in xylenes and embedding in paraffin overnight, the heads were sectioned at 10 µm thickness and mounted onto slides. The sections were rehydrated and stained with hematoxylin solution for 1 min. Histology images were captured using a Nikon Microphot-FXA Microscope equipped with Zeiss AxioCam MRc camera.

Uncropped western blot images

Figure 2A

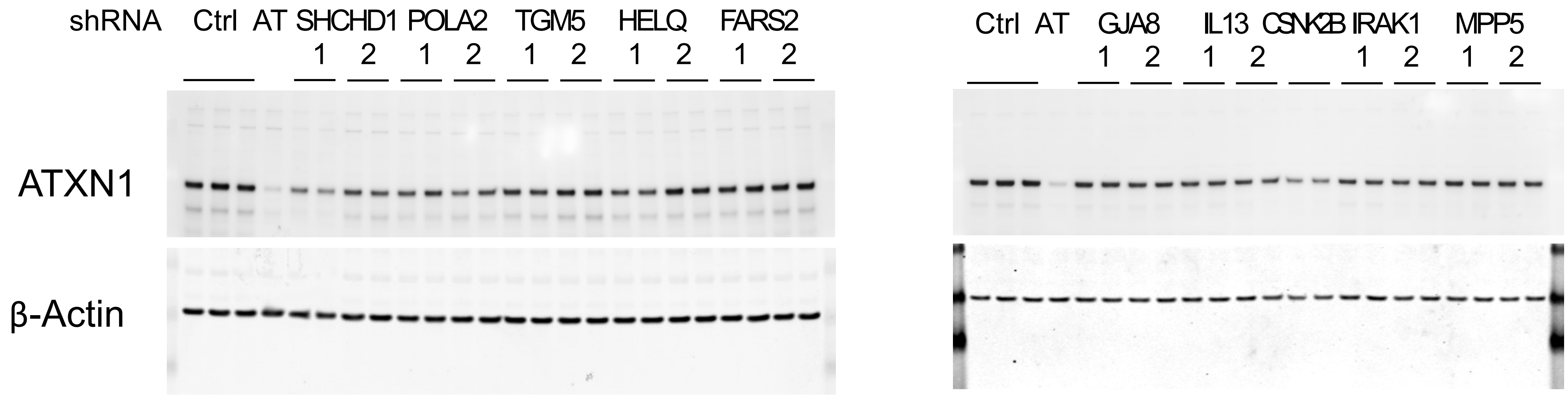

Figure 2D

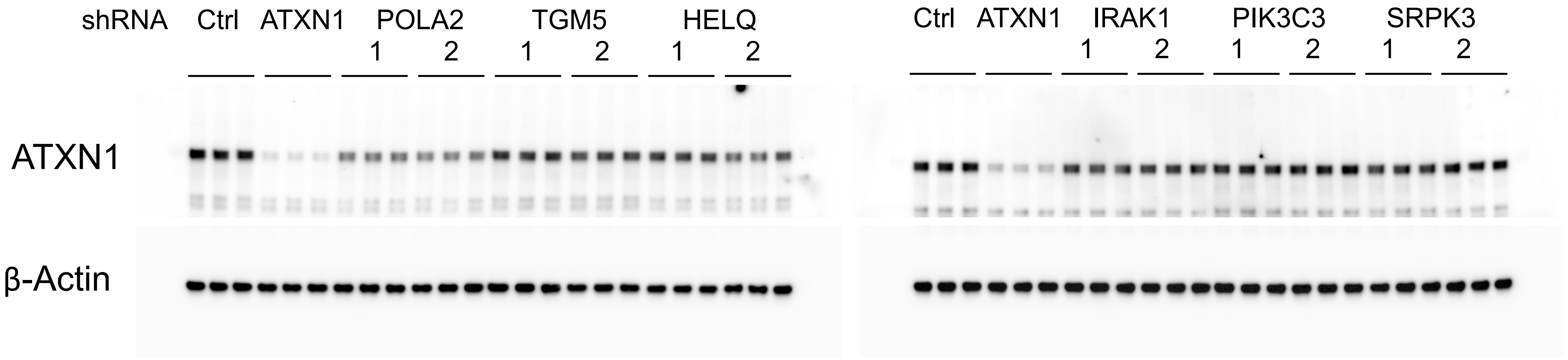

Figure 3D

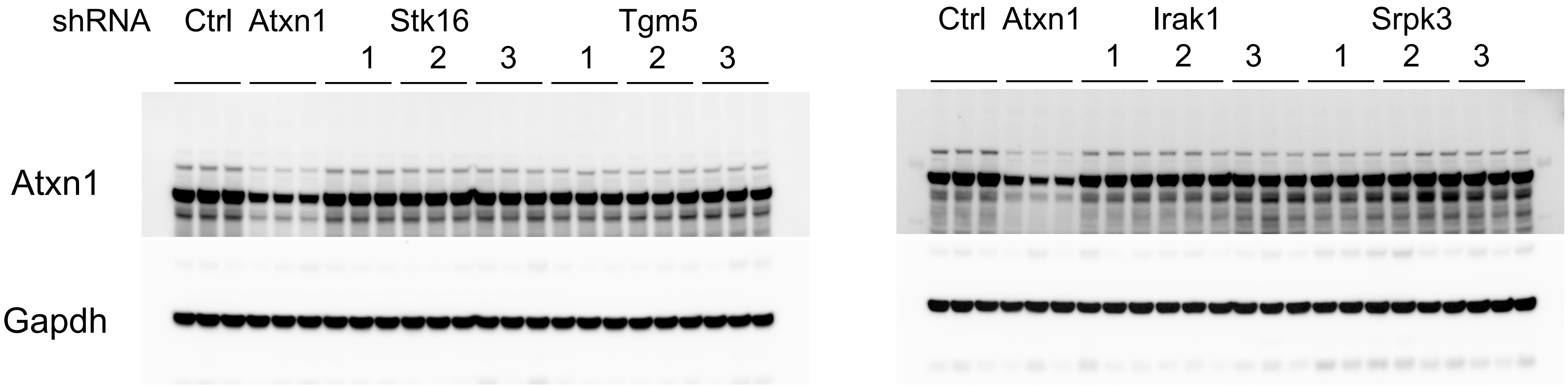
shRNA
Ctrl
Atxn1
Stk16
Tgm5
1
2
3
1
2
3
Ctrl
Atxn1
Irak1
Srpk3
1
2
3
1
2
3
Atxn1
Gapdh

Figure 4B

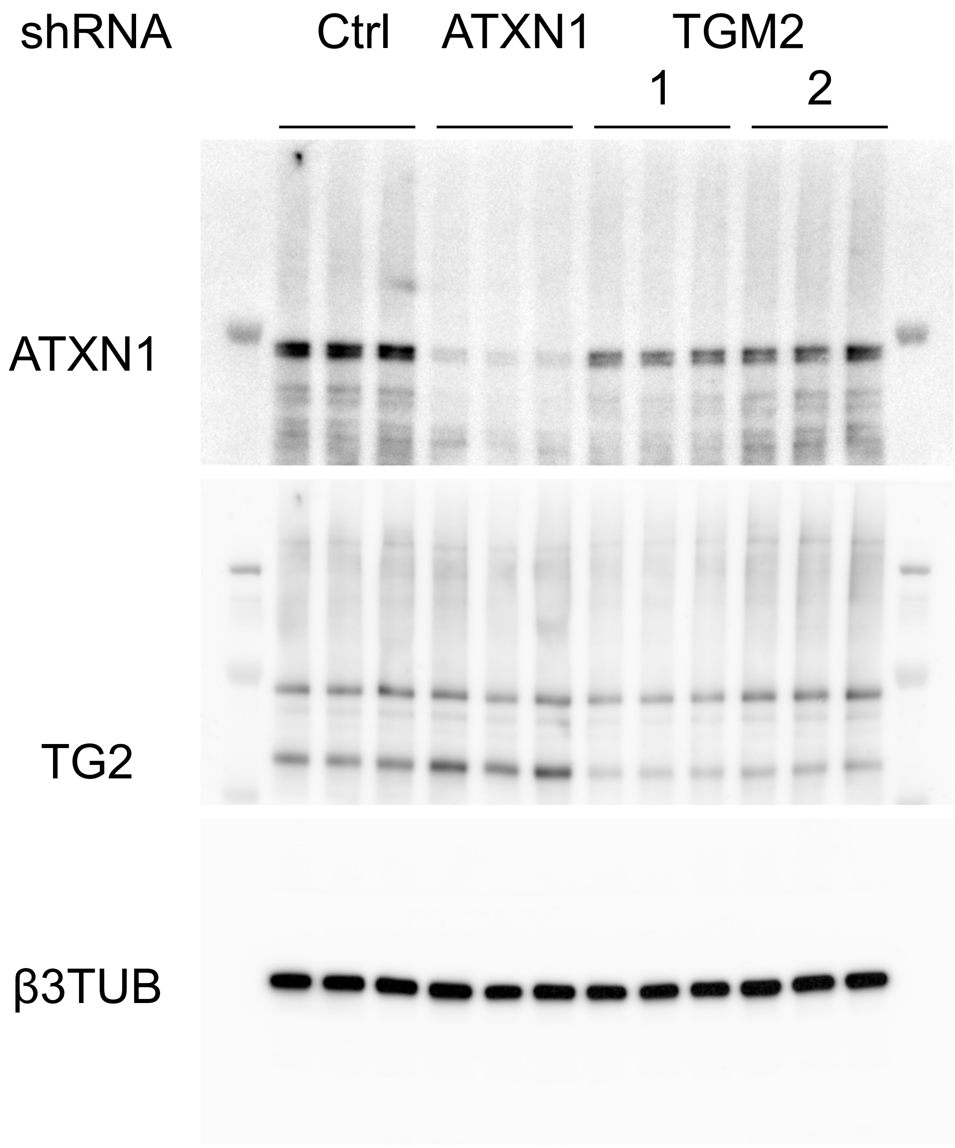

Figure 4C

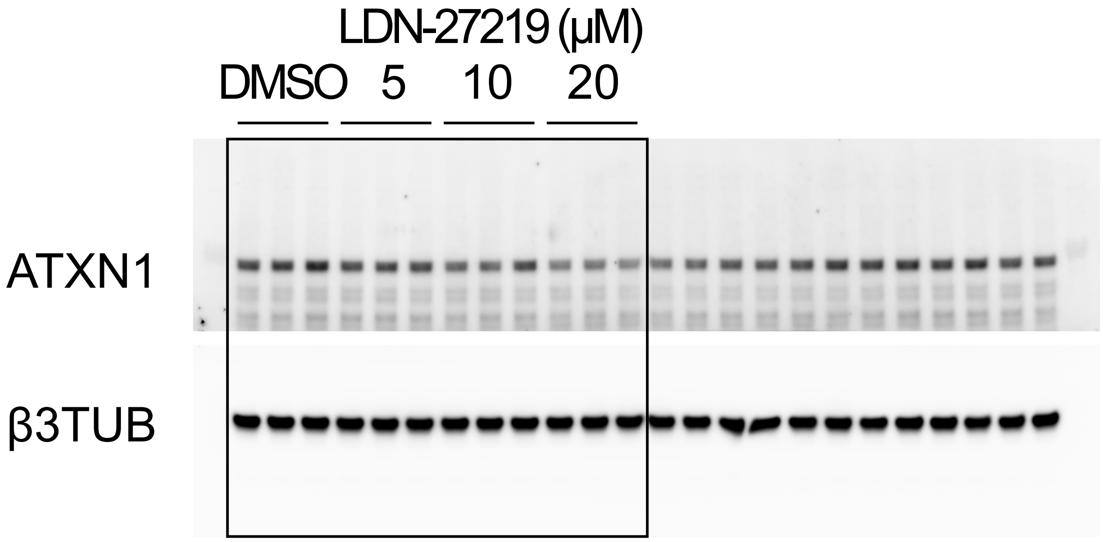

Figure 4D

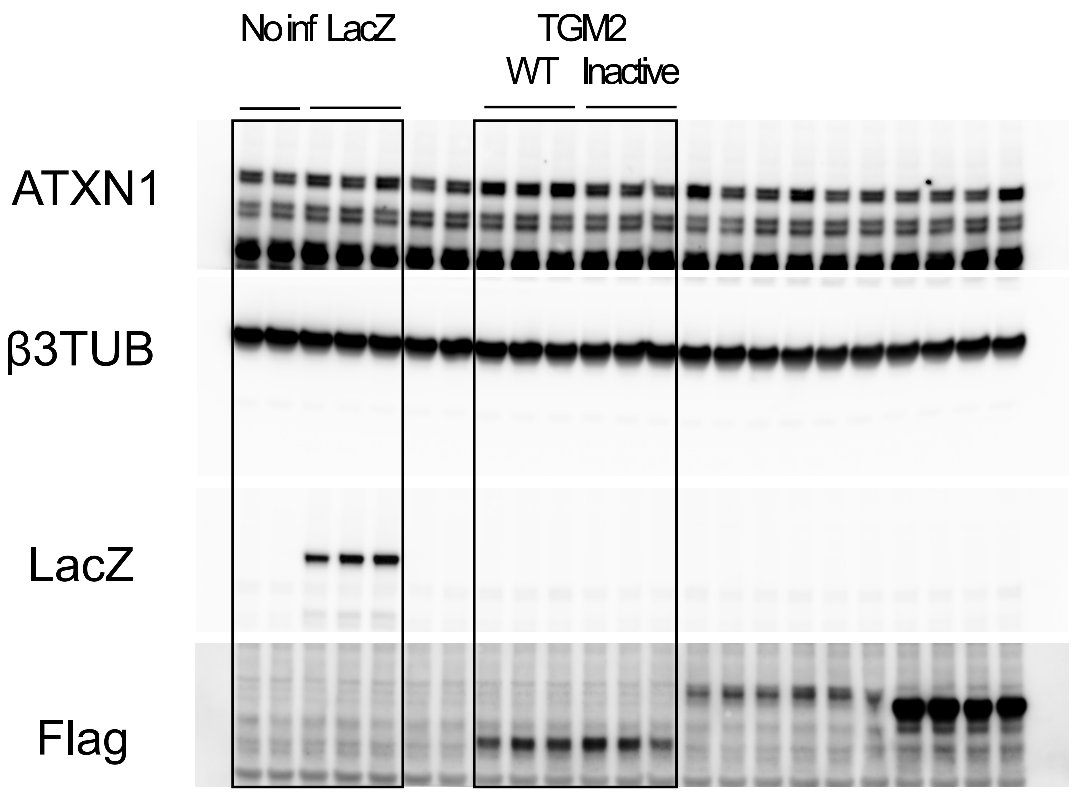

Figure 4E

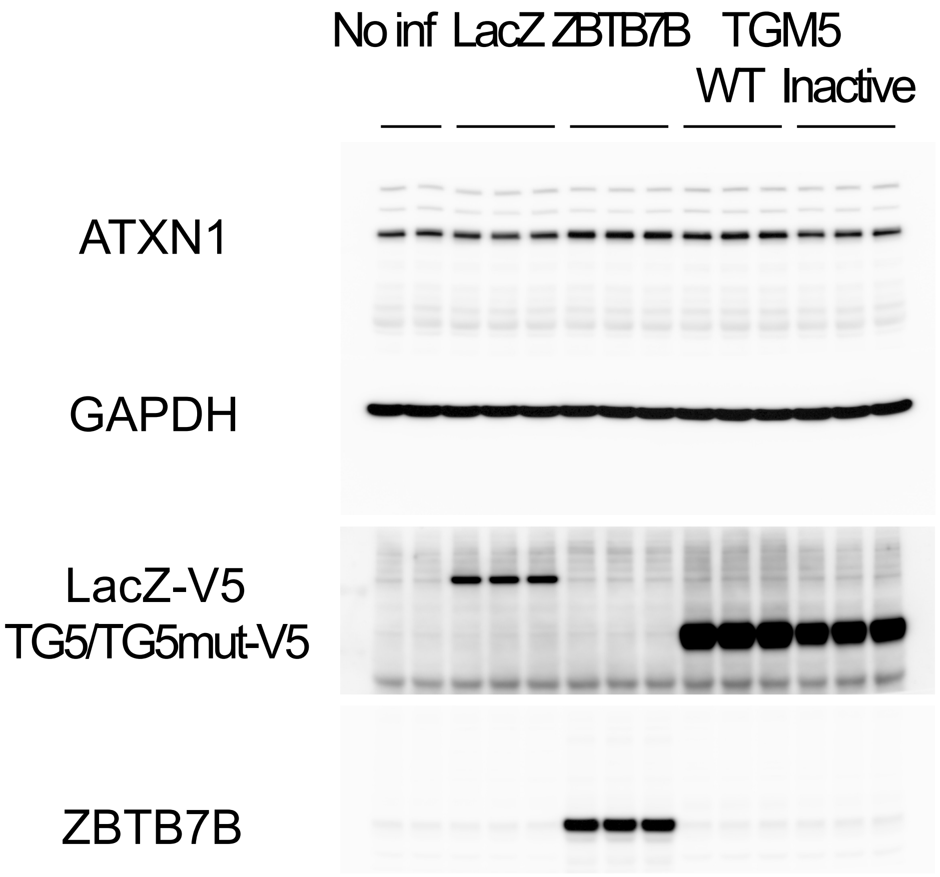

Figure 4F

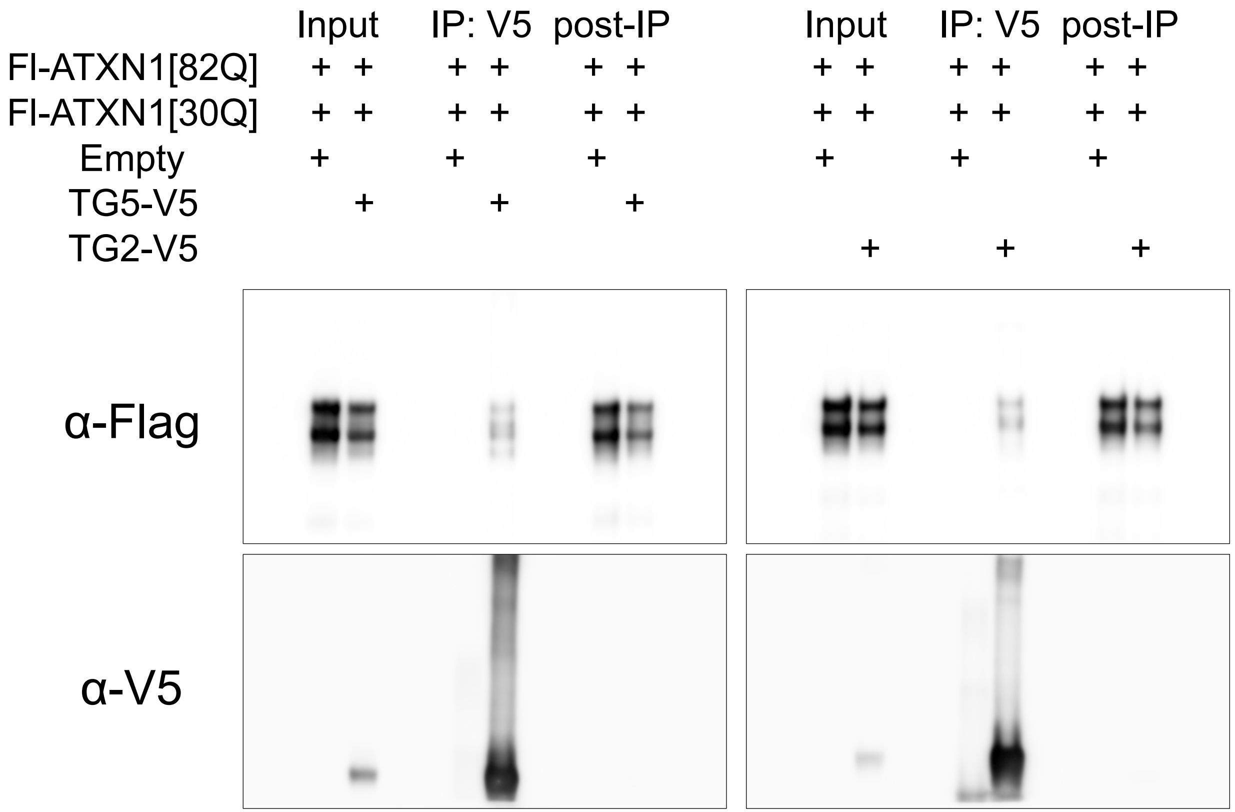

Input
IP: V5
post-IP
Fl-ATXN1[82Q]
Fl-ATXN1[30Q]
Empty
TG5-V5
TG2-V5
α-Flag
α-V5

Figure 4H

ATXN1[82Q]

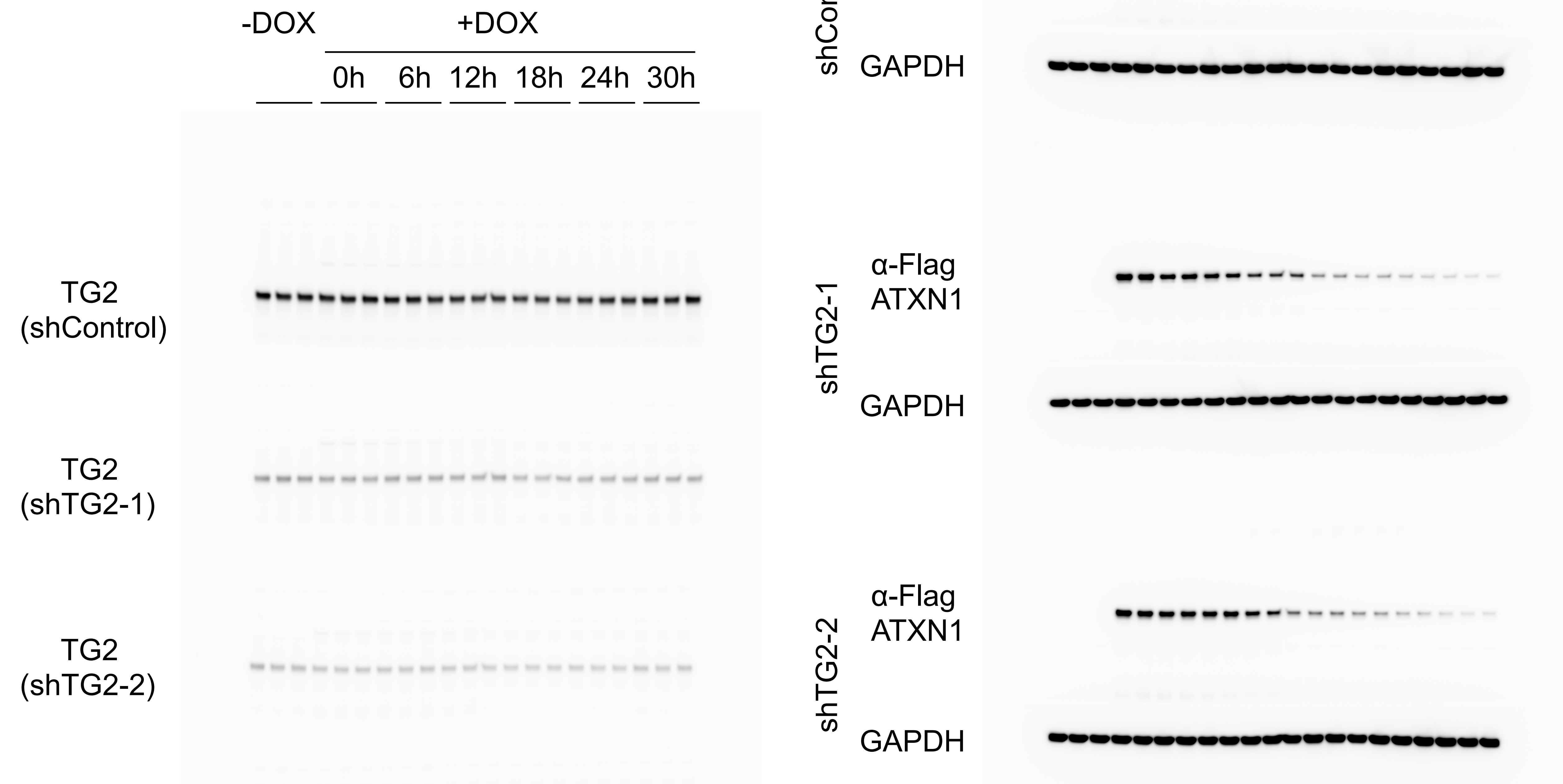

Figure 4H

ATXN1[30Q]

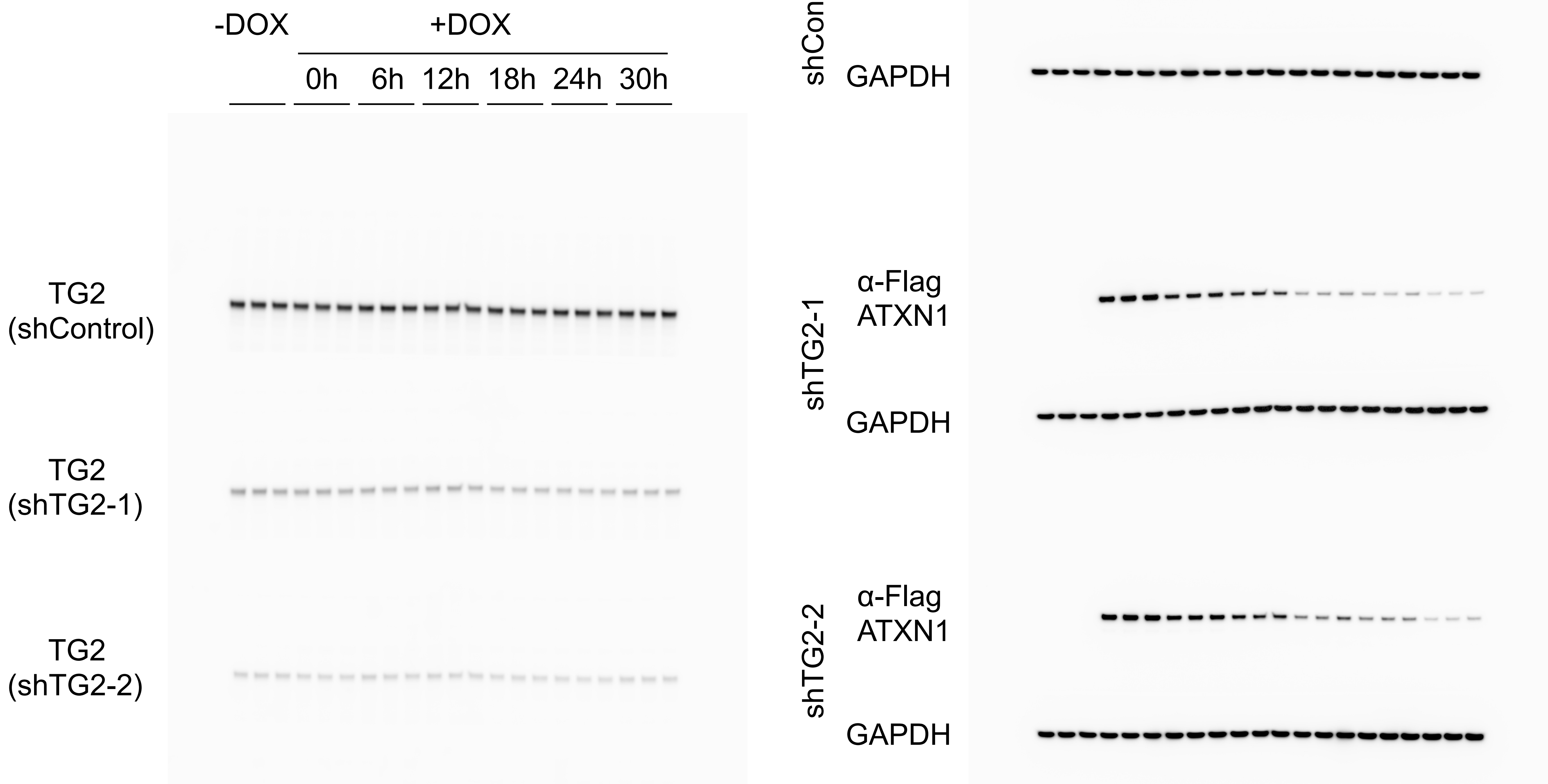

Figure 4H

ATXN1[2Q]

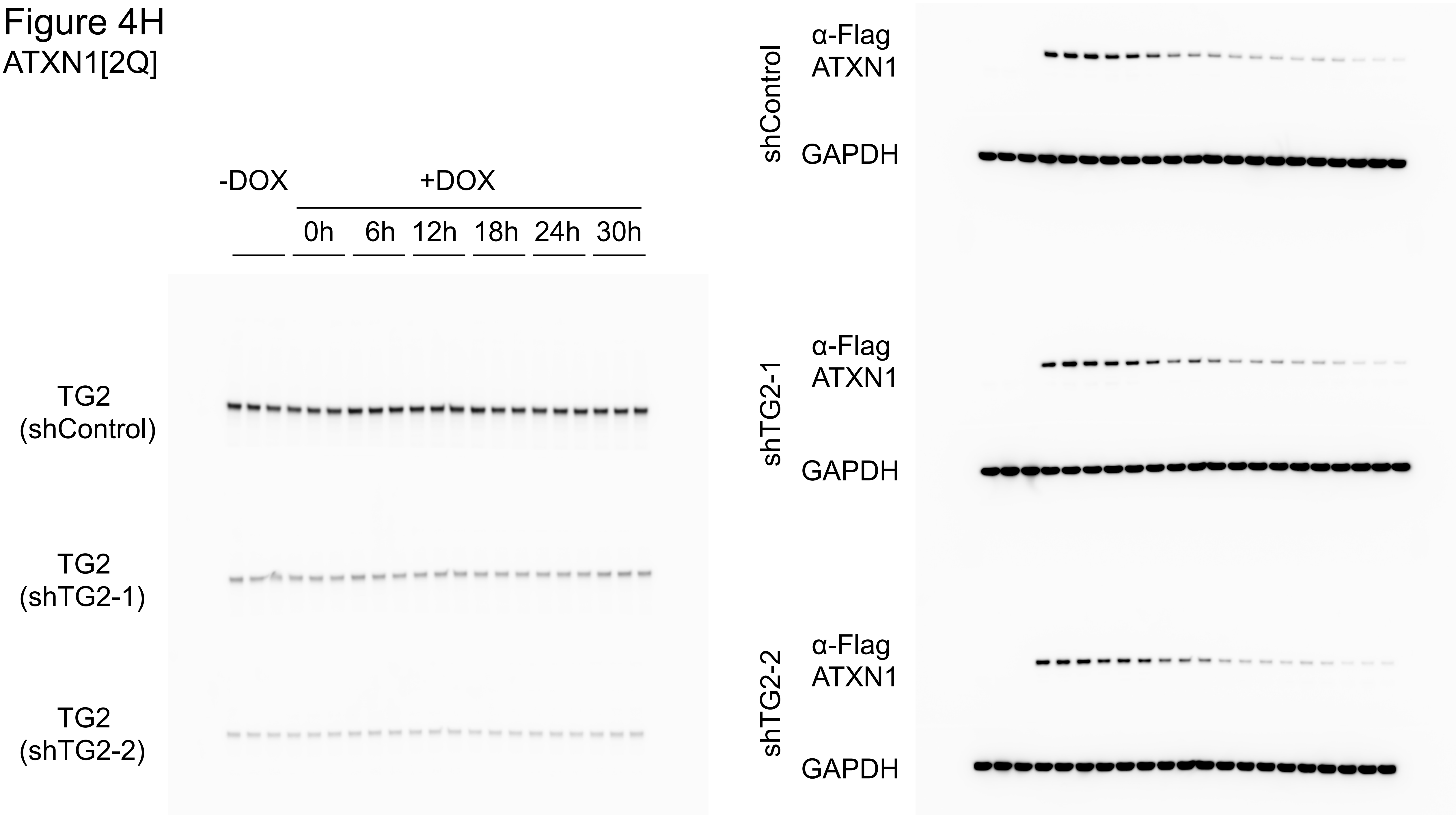

Figure 5A

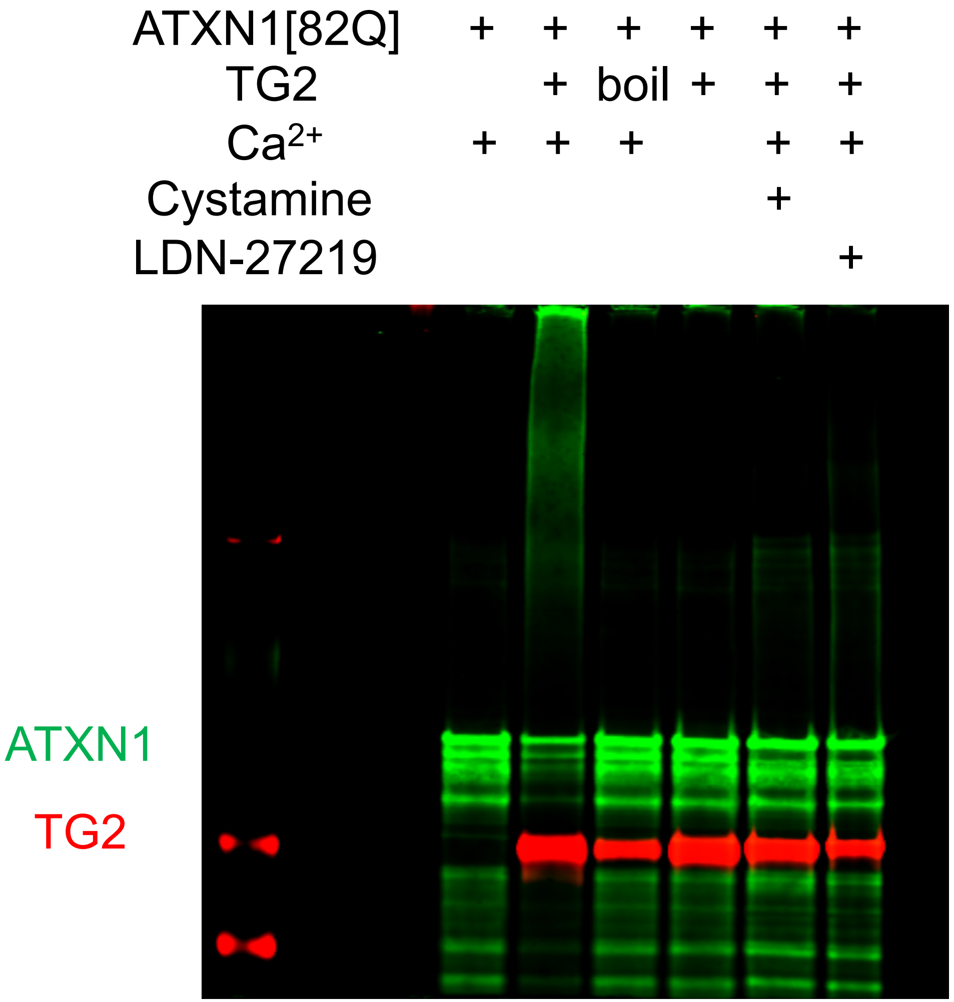

Figure 5B

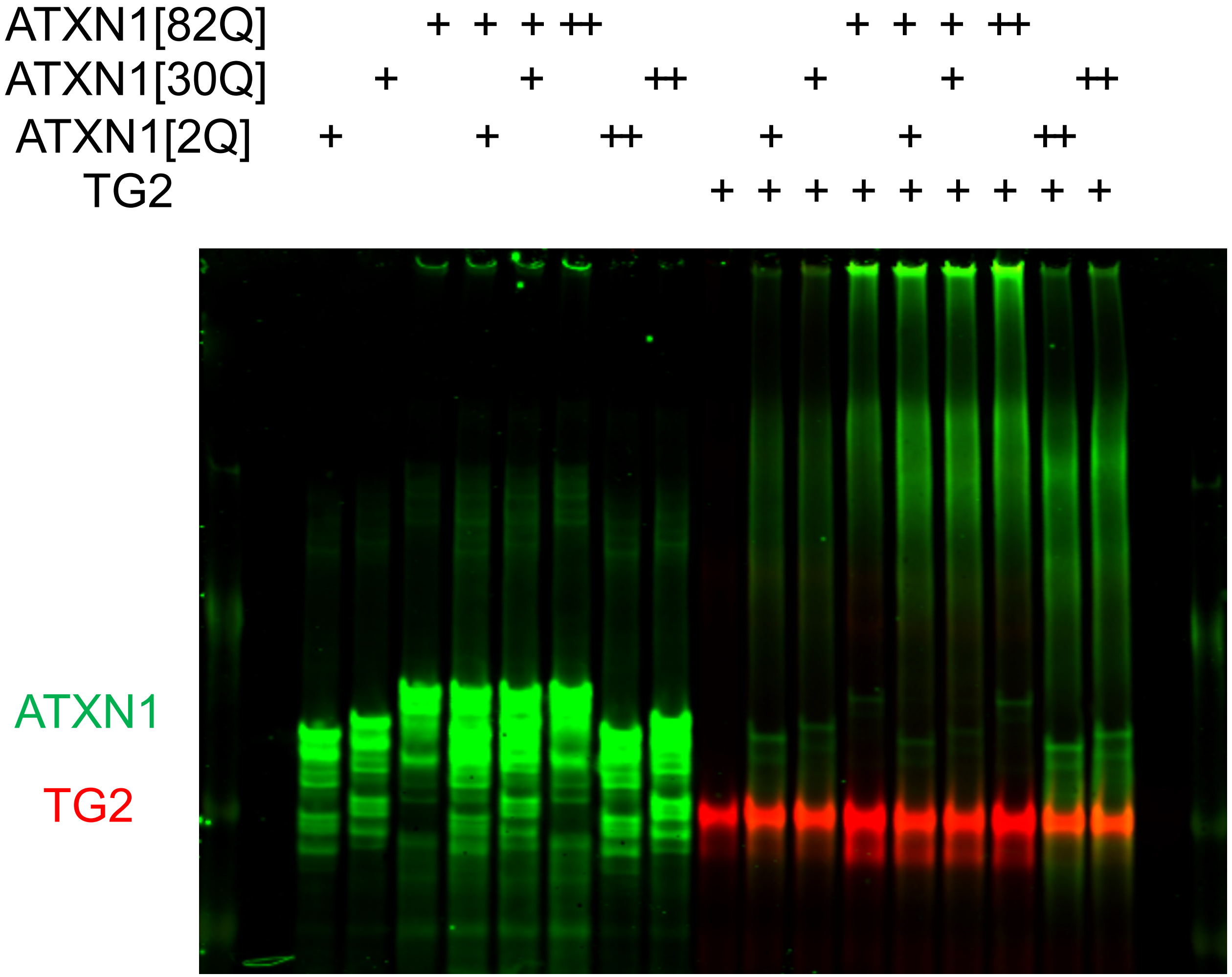

Figure 5C

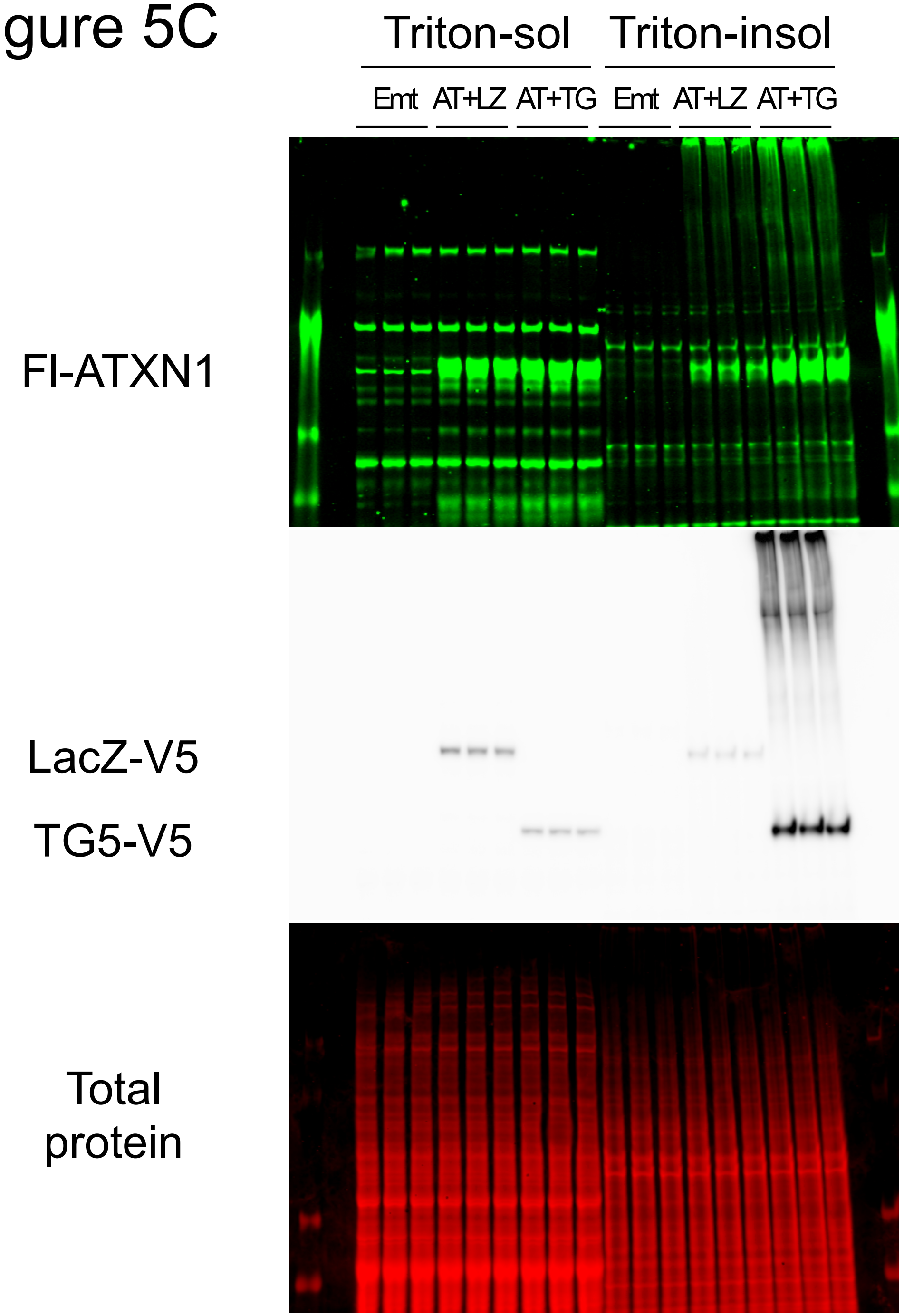

Figure 5D

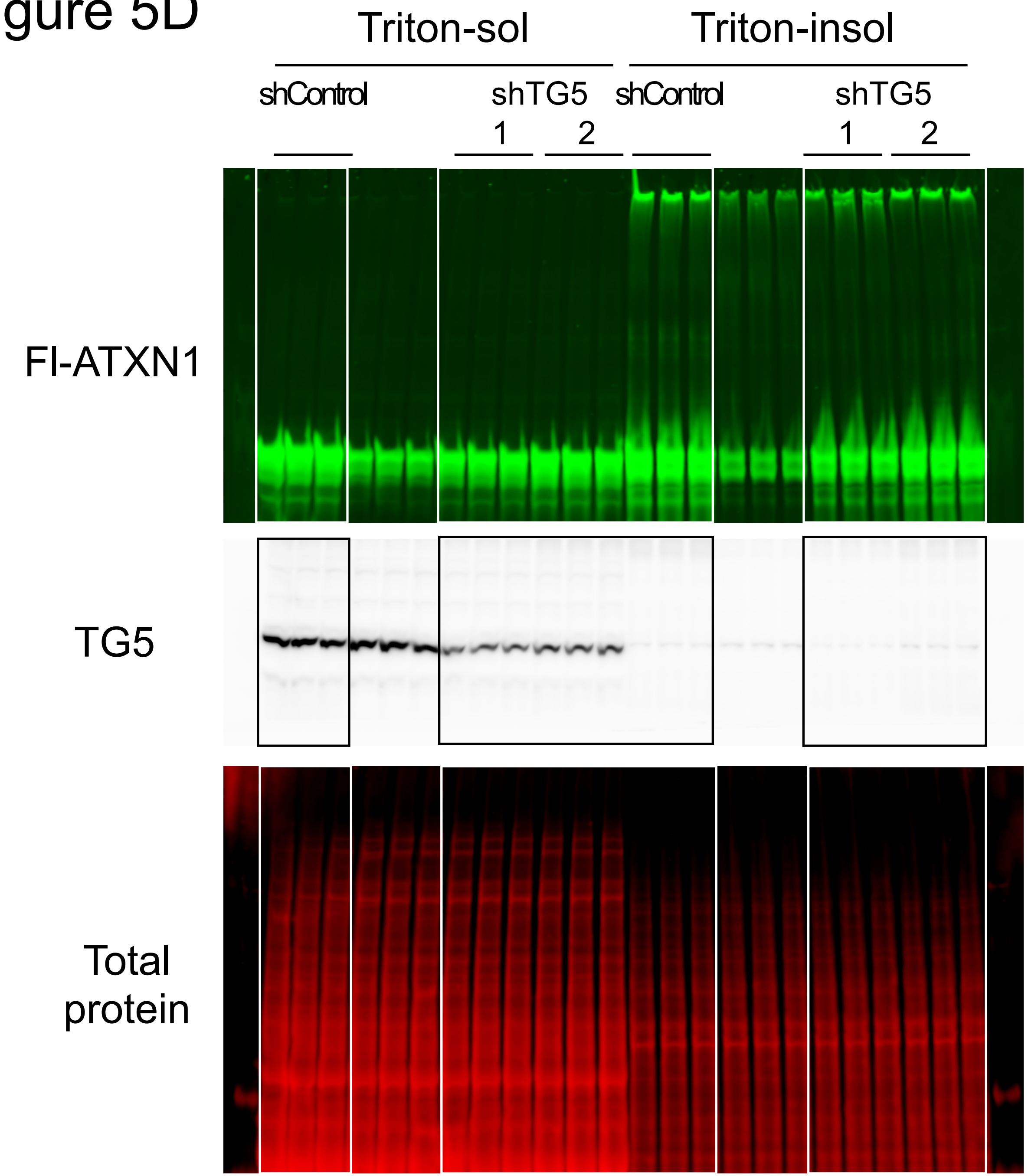

Figure 5E

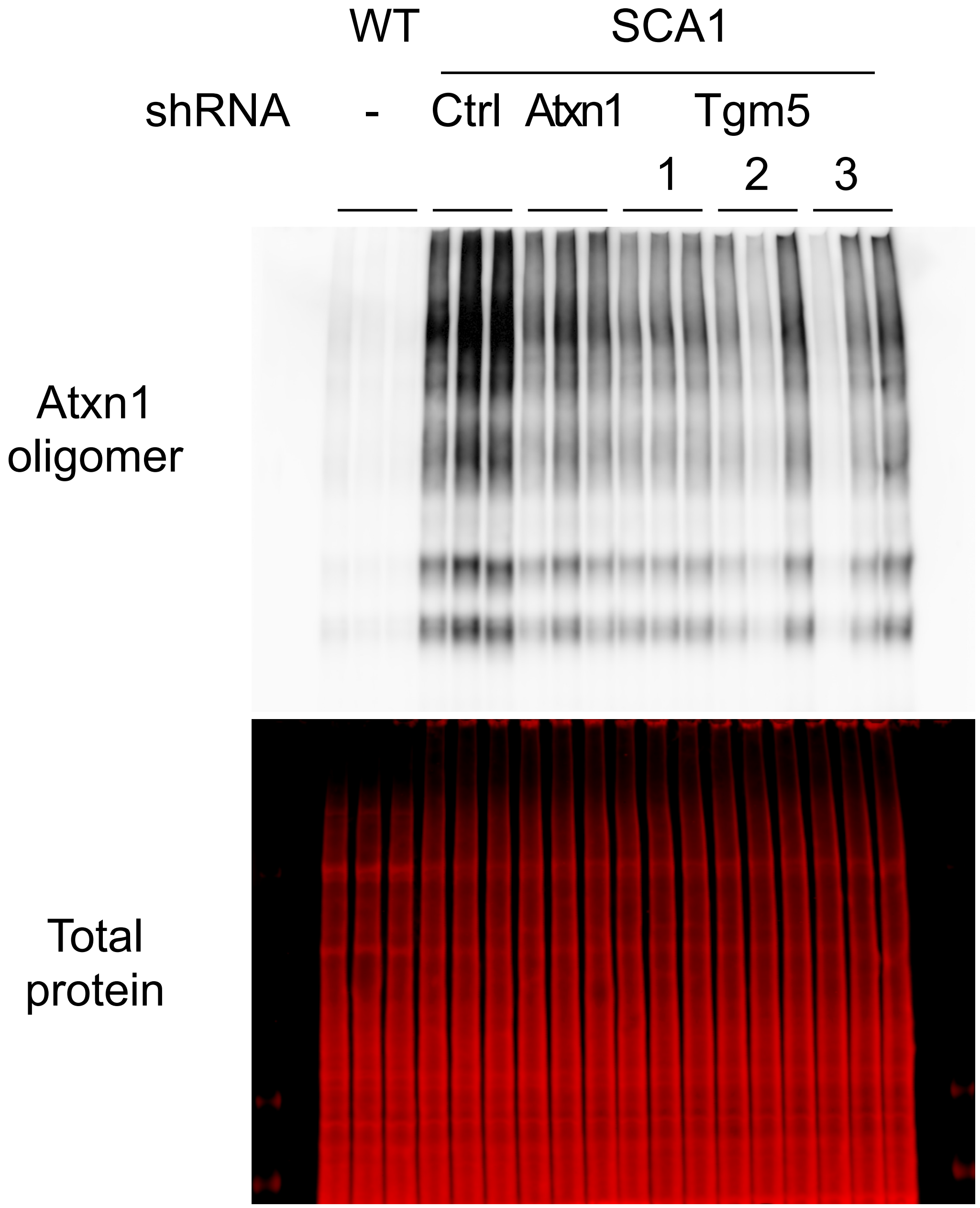
WT
SCA1
shRNA
-
Ctrl
Atxn1
Tgm5
1
2
3
Atxn1 oligomer
Total protein

Figure 6A

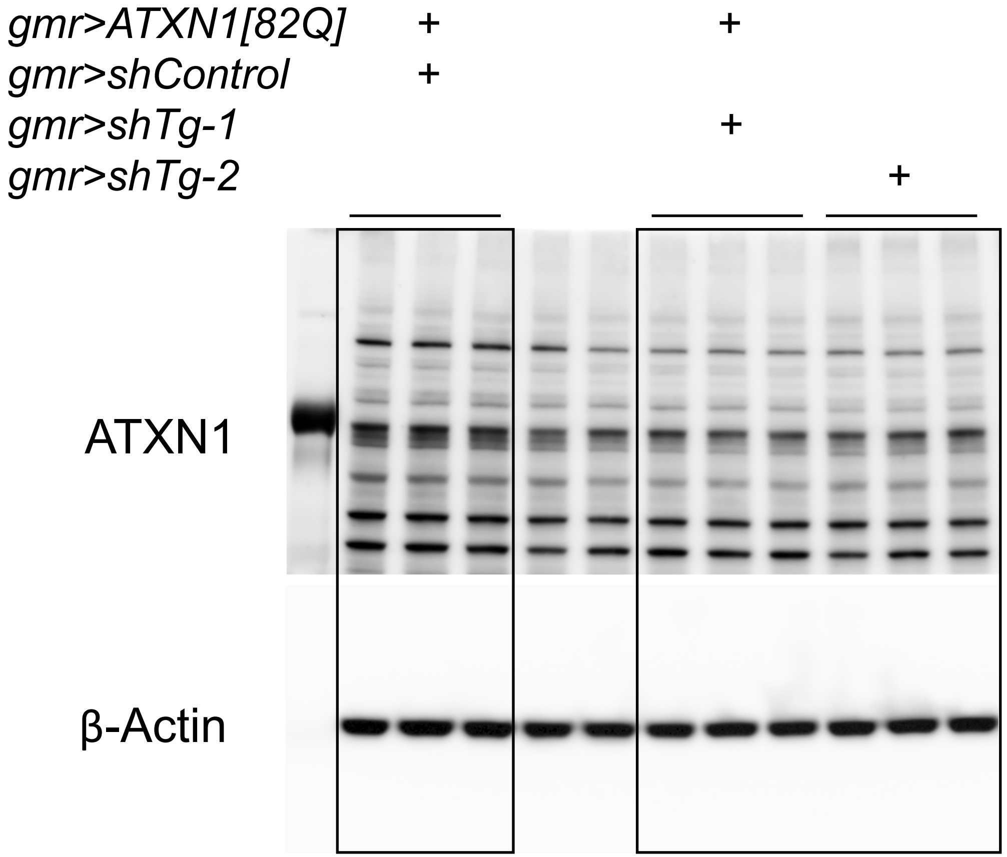

Figure 6B

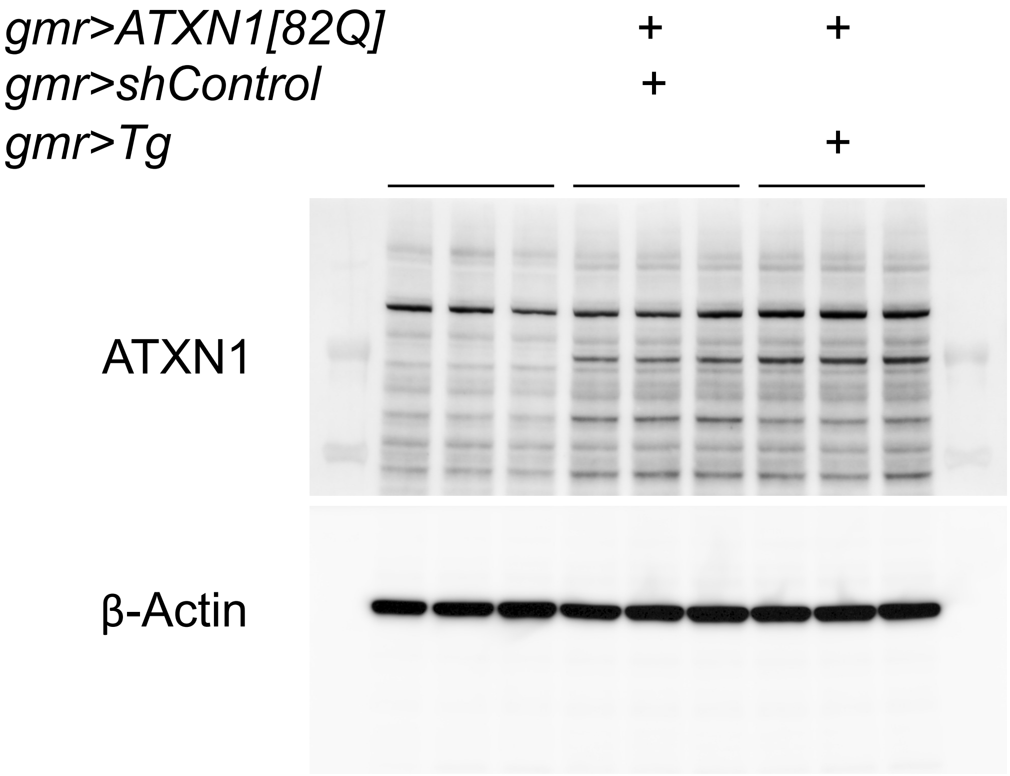

Supplemental Figure 2

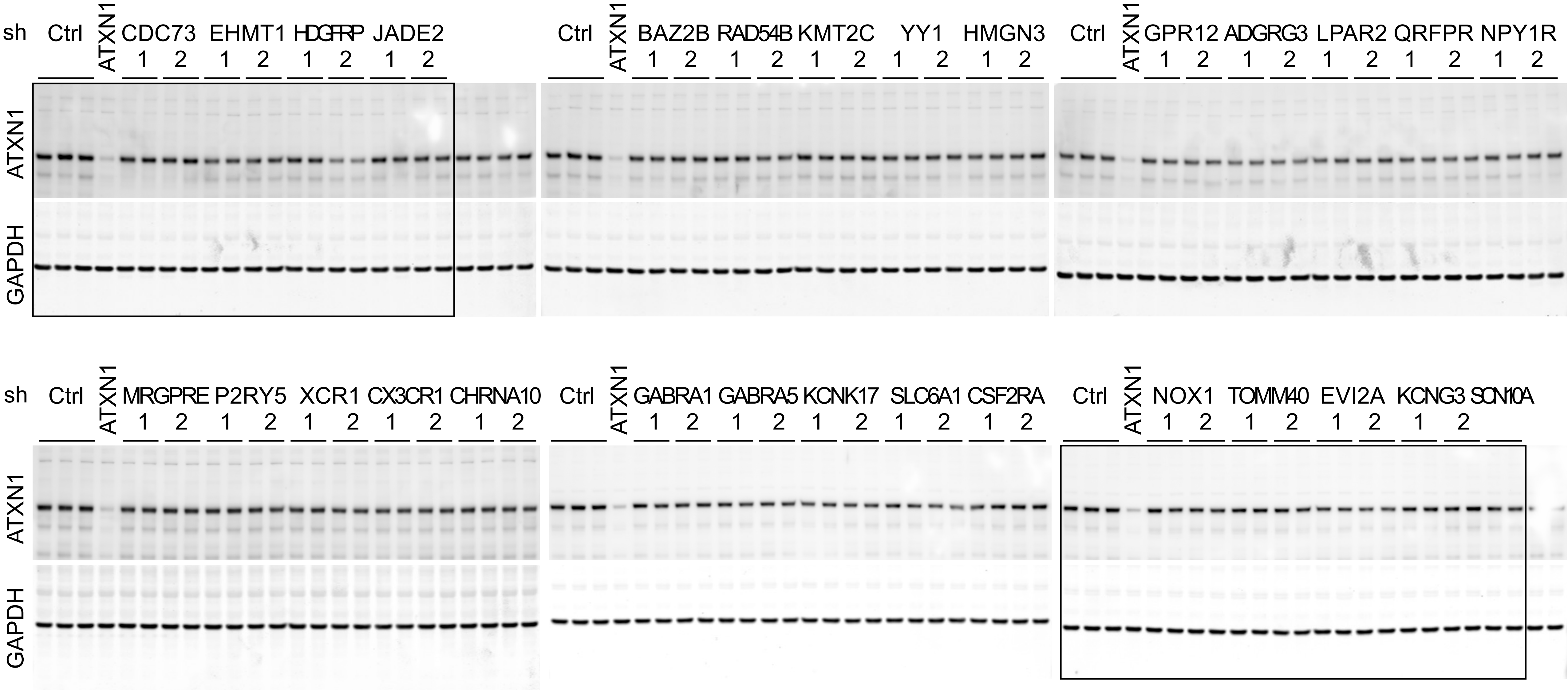

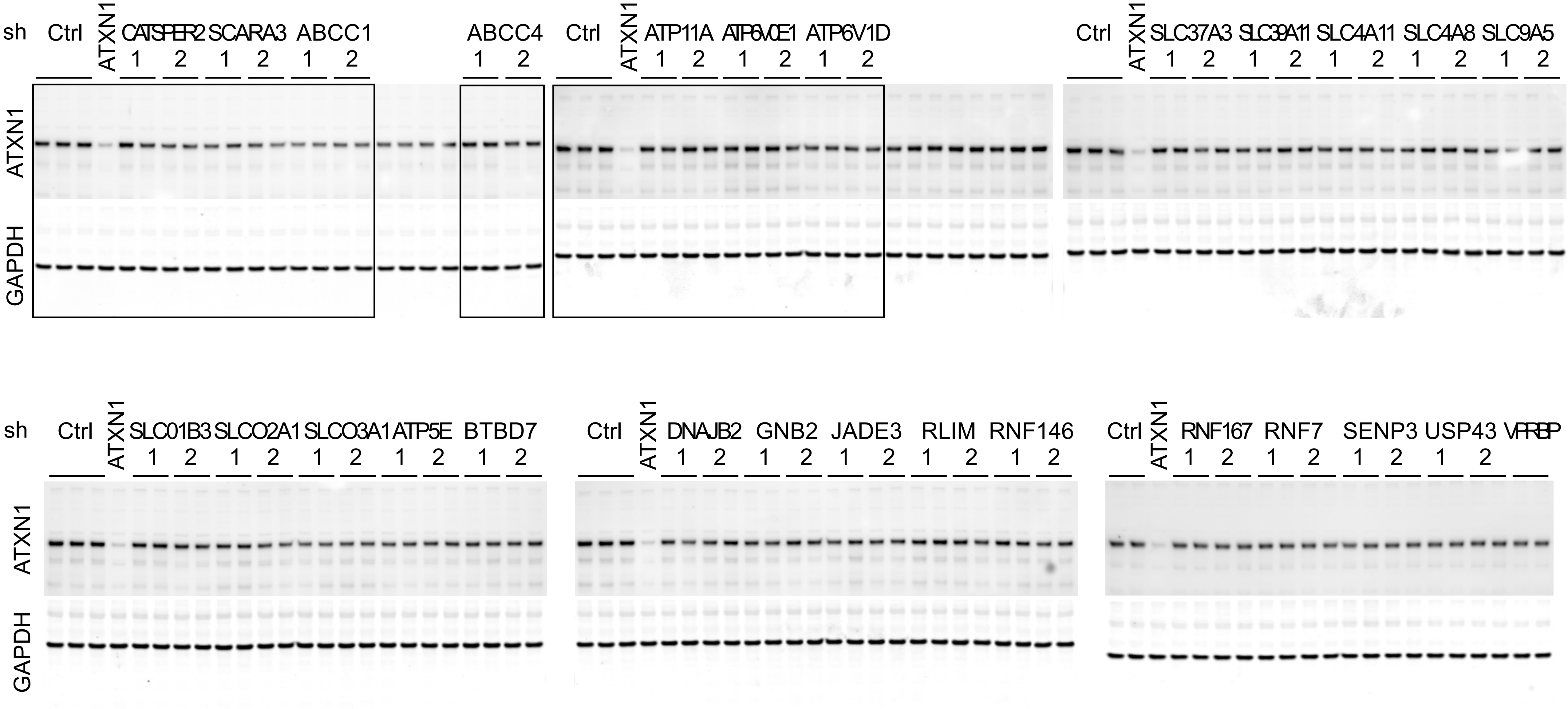
sh
Ctrl
ATXN1
CATSPER2
SCARA3
ABCC1
ABCC4
ATP11A
ATP6V0E1
ATP6V1D
SLC37A3
SLC39A11
SLC4A11
SLC4A8
SLC9A5
SLCO1B3
SLCO2A1
SLCO3A1
ATP5E
BTBD7
DNAJB2
GNB2
JADE3
RLIM
RNF146
RNF167
RNF7
SENP3
USP43
VPREB
ATXN1
GAPDH

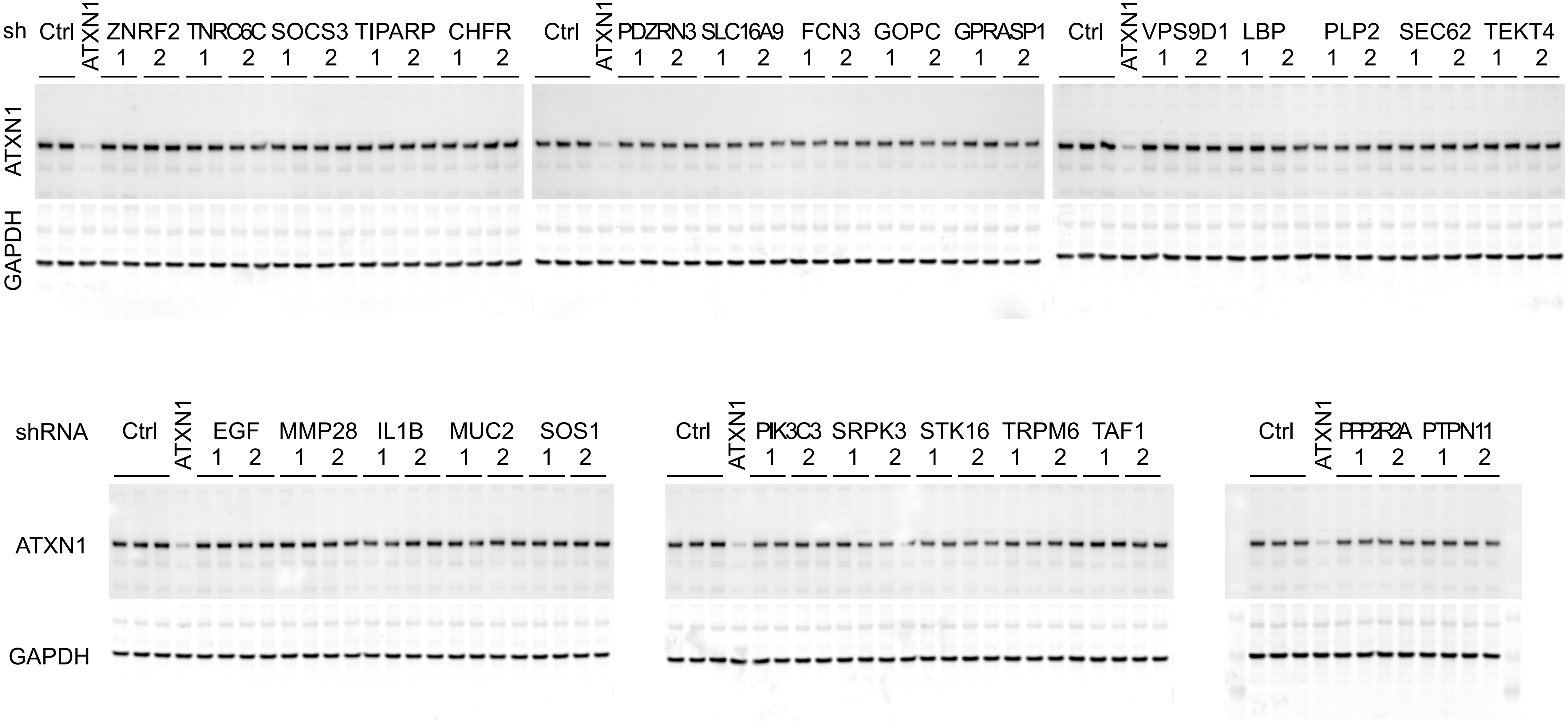
sh
Ctrl
ATXN1
ZNRF2
TNRC6C
SOCS3
TIPARP
CHFR
PDZRN3
SLC16A9
FCN3
GOPC
GPRASP1
VPS9D1
LBP
PLP2
SEC62
TEKT4
ATXN1
GAPDH
shRNA
EGF
MMP28
IL1B
MUC2
SOS1
PIK3C3
SRPK3
STK16
TRPM6
TAF1
PPP2R2A
PTPN11

Supplemental Figure 3

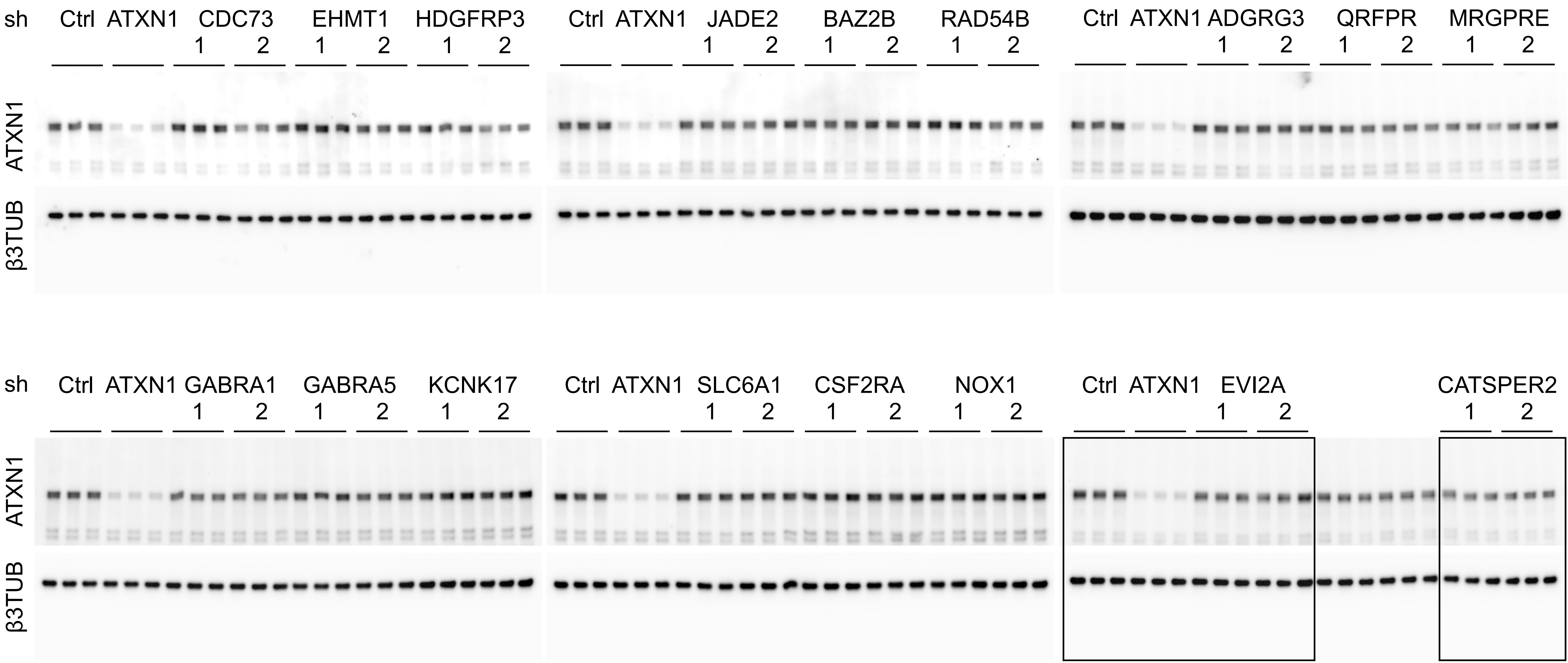

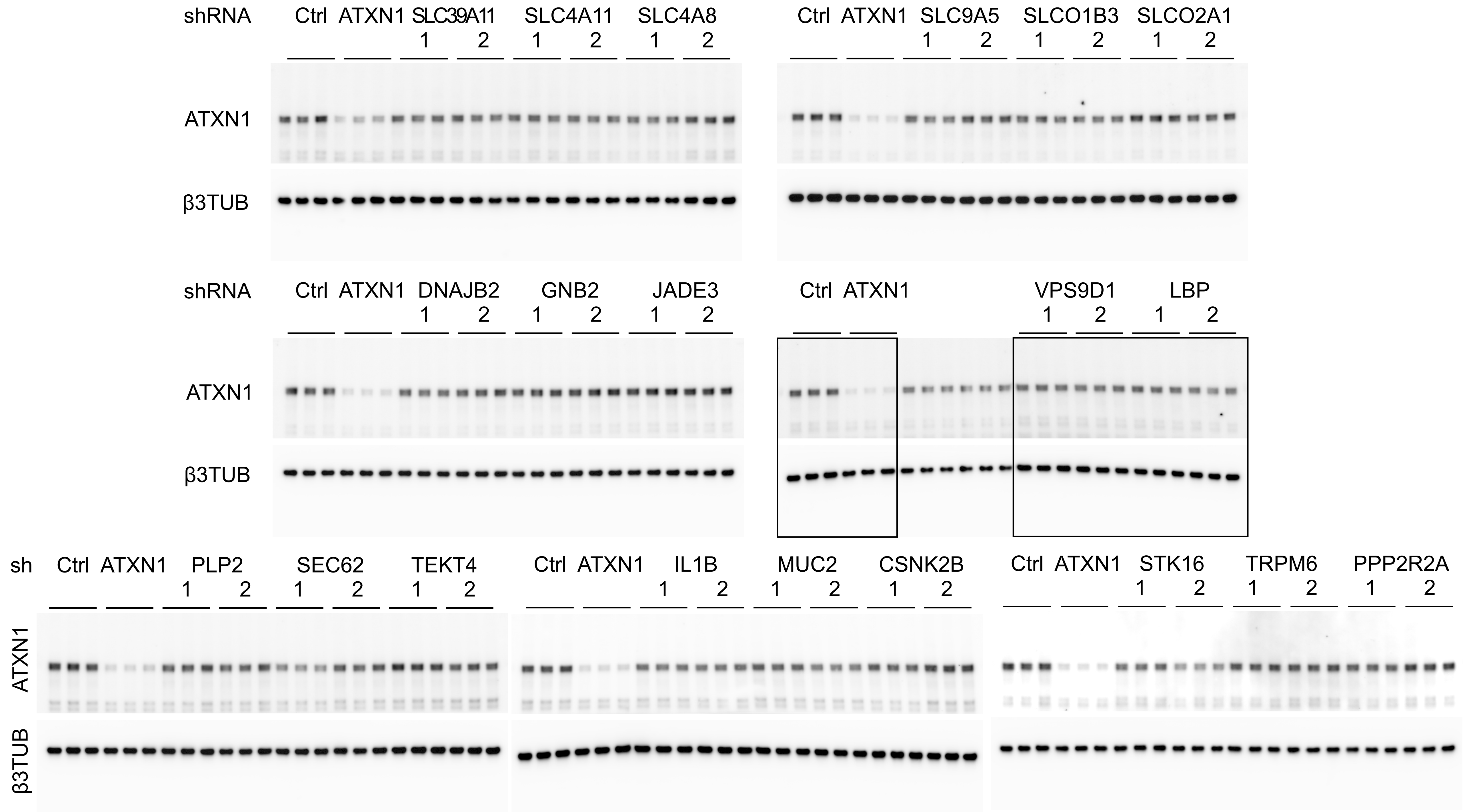
shRNA
Ctrl ATXN1 SLC39A11 SLC4A11 SLC4A8
1 2 1 2 1 2
ATXN1
β3TUB
Ctrl ATXN1 SLC9A5 SLCO1B3 SLCO2A1
1 2 1 2 1 2
shRNA
Ctrl ATXN1 DNAJB2 GNB2 JADE3
1 2 1 2 1 2
Ctrl ATXN1 VPS9D1 LBP
1 2 1 2
sh
Ctrl ATXN1 PLP2 SEC62 TEKT4
1 2 1 2 1 2
Ctrl ATXN1 IL1B MUC2 CSNK2B
1 2 1 2 1 2
Ctrl ATXN1 STK16 TRPM6 PPP2R2A
1 2 1 2 1 2

Supplemental Figure 5

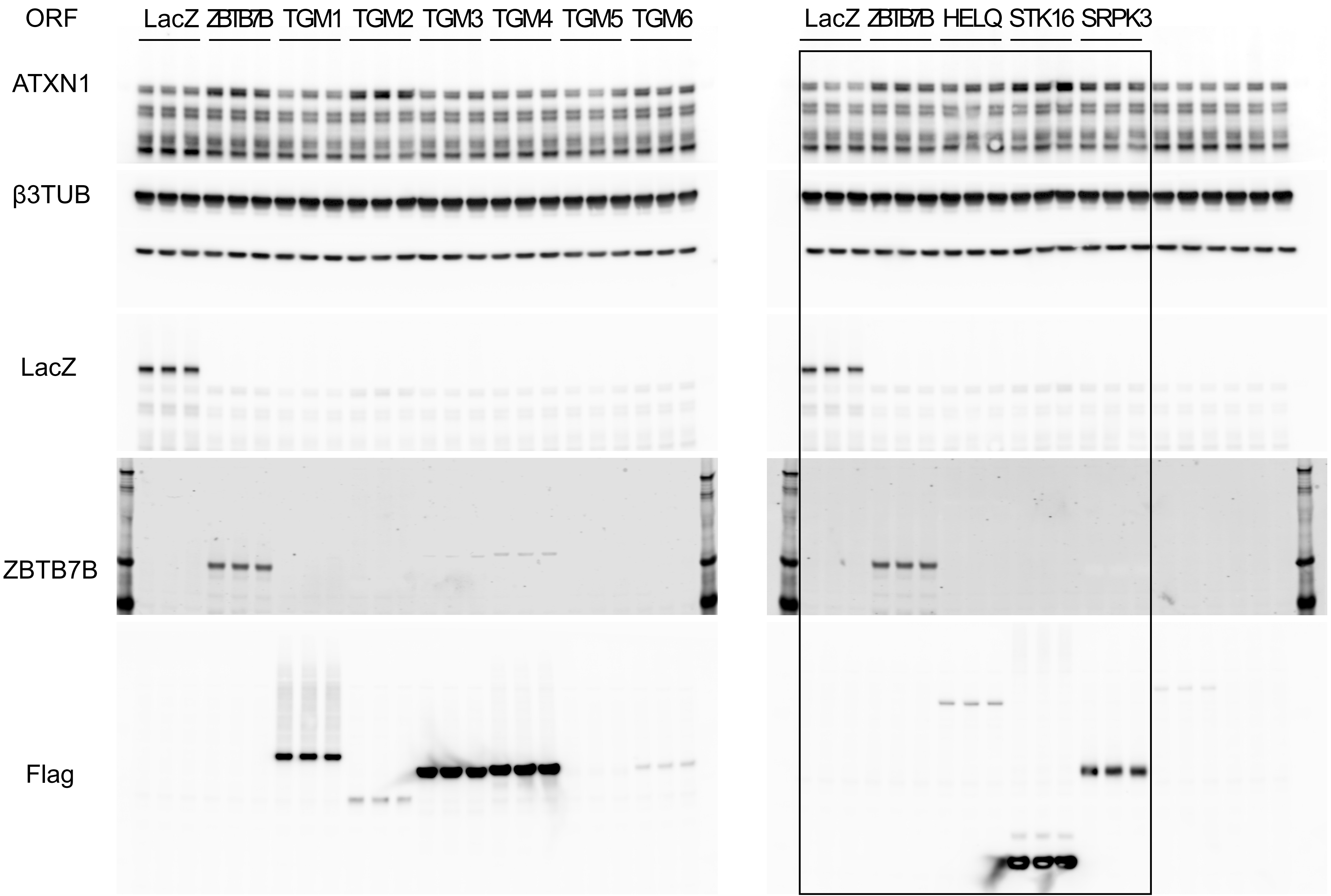

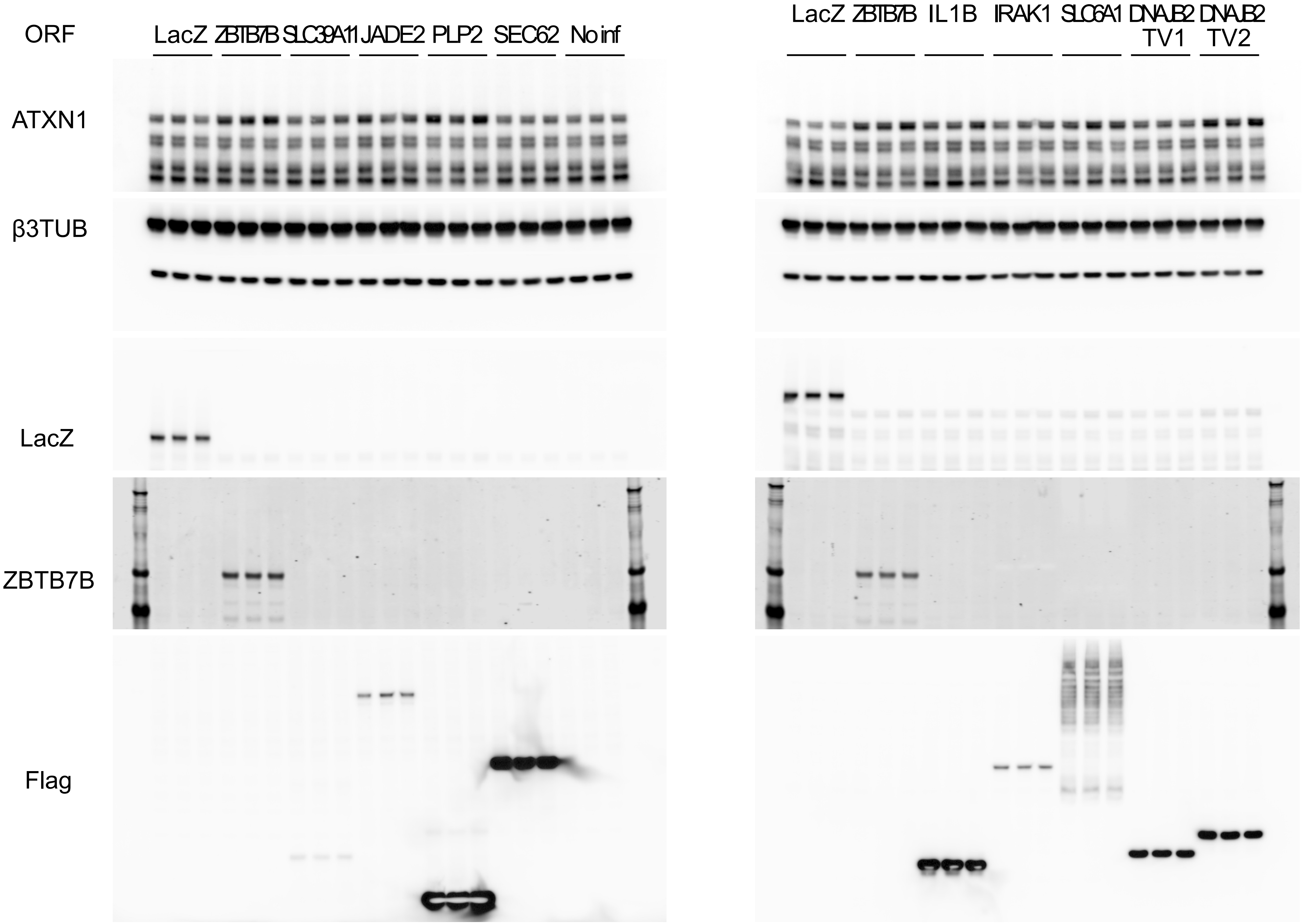

ORF
LacZ
ZBTB7B
SLC39A11
JADE2
PLP2
SEC62
No inf
LacZ
ZBTB7B
IL1B
IRAK1
SLC6A1
DNAJB2 TV1
DNAJB2 TV2
ATXN1
β3TUB
LacZ
ZBTB7B
Flag

Supplemental Figure 6A

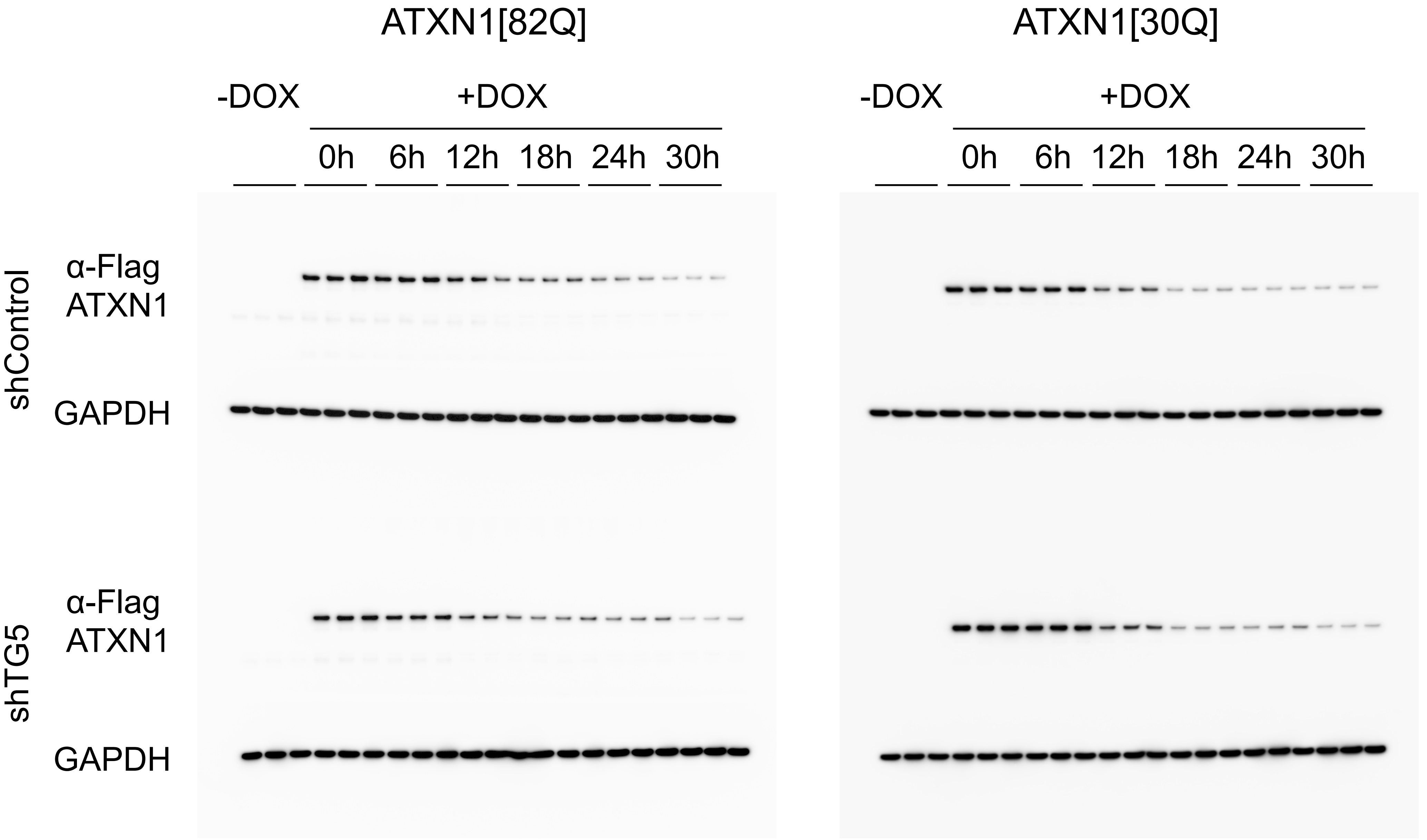

Supplemental Figure 6C

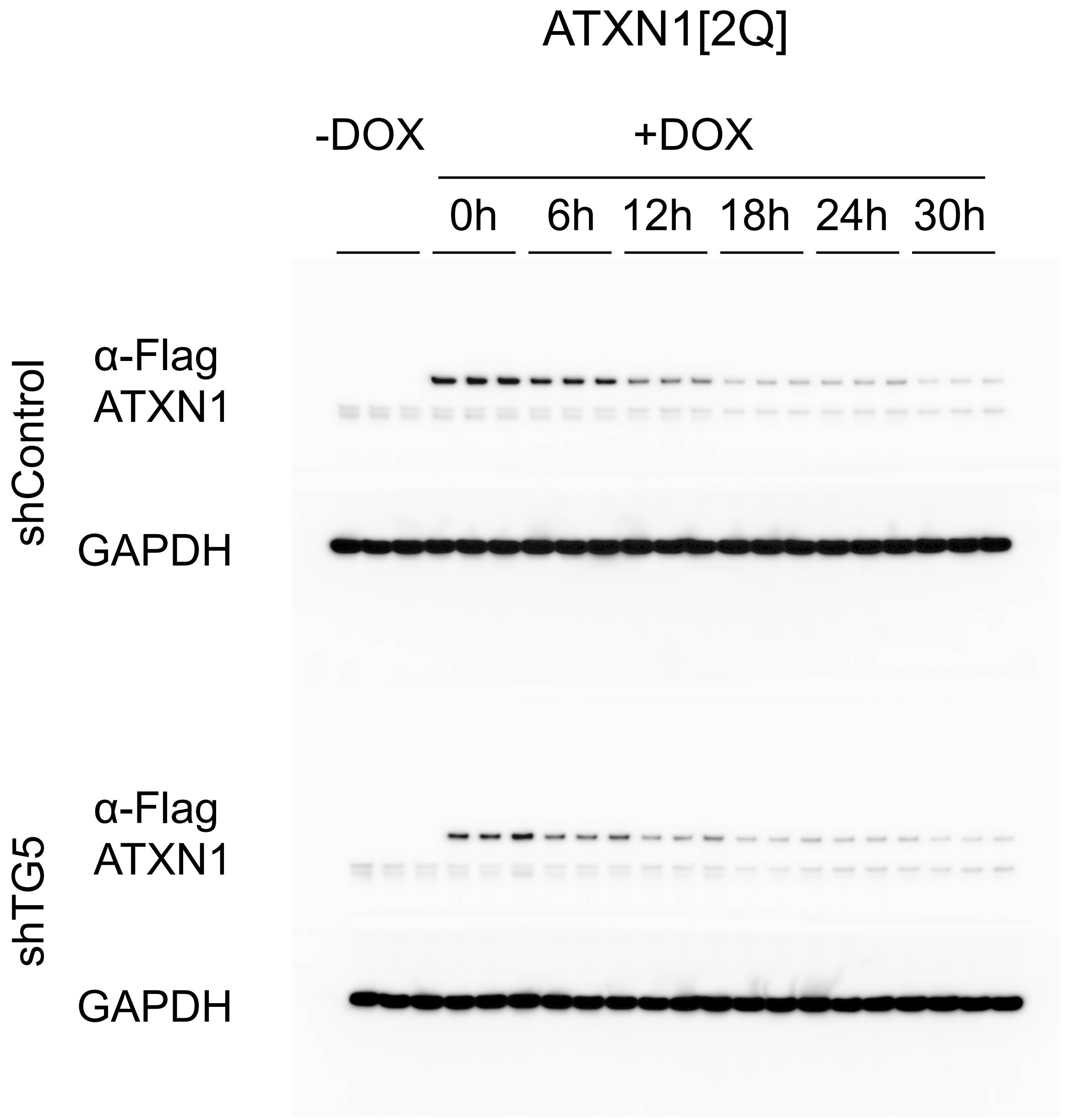

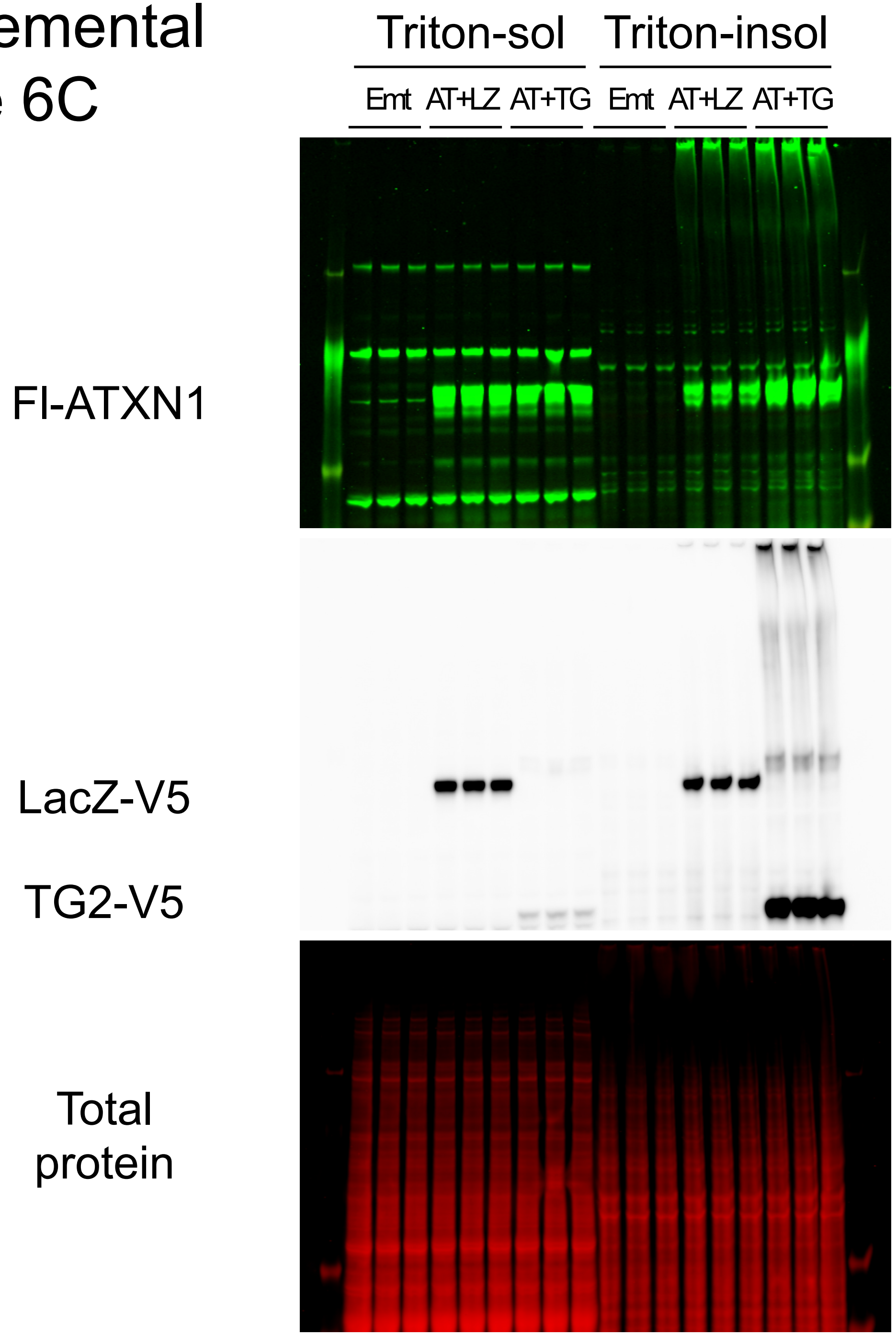

Supplement: Supplemental data [file jci-132-156616-s045.pdf]
