# Supplementary material for: Cross-species genetic screens identify transglutaminase 5 as a regulator of polyglutamine-expanded ataxin-1
Source: J Clin Invest. 2022 May 2;132(9):e156616. doi: 10.1172/JCI156616 (PMC9057624; doi:10.1172/JCI156616)
Supplement: Supplemental data set 1 [file jci-132-156616-s046.pdf]

sRNA,B1,B2,B3,B4,H1,H2,H3,H4,L1,L2,L3,L4
AHR_1_1,1470,1712,1115,1071,1296,1162,2729,710,670,1462,916,472
ALKBH1_1_2,622,1172,1262,1071,393,1944,1377,513,3877,689,2481,894
ALKBH3_1_3,896,985,1075,1495,782,898,2040,176,1578,680,1528,64
ARID2_1_4,1105,1250,1242,1089,1529,1292,959,1127,1131,1458,828,1133
ASF1A_1_5,7191,6777,6282,7457,8582,6385,9402,5381,6246,6018,8653,5756
ASF1B_1_6,1543,1549,1137,795,252,308,798,1314,2175,451,3520,277
ASH1L_1_7,990,622,1749,1008,776,1003,843,835,154,836,2059,791
ASXL2_1_8,2369,2696,2971,2049,3001,3578,2466,3312,5356,2472,1593,1329
ASXL3_1_9,1153,1517,1063,1135,536,1579,3053,537,2256,1105,1175,499
ASZ1_1_10,1580,1554,975,2218,3,1565,2158,431,432,931,634,296
ATAD2_1_11,496,434,571,618,886,417,0,603,1413,1016,0,80
ATF7IP_1_12,1467,2121,1705,1935,597,3363,2999,586,1071,1698,257,4965
AURKB_1_13,619,331,647,368,664,320,1125,313,400,163,614,1211
BAHCC1_1_14,325,235,644,210,705,392,229,43,205,193,466,232
BAHD1_1_15,298,399,450,357,687,521,50,0,235,109,18,367
BARD1_1_16,0,0,0,0,0,0,0,0,0,0,0,0
BAZ1B_1_17,8389,6879,10622,9072,9065,9911,8133,10680,6756,7318,7760,11
256
BAZ2A_1_18,609,386,765,1205,316,1320,398,899,194,363,1027,947
BAZ2B_1_19,3000,1480,2897,3652,2325,2888,3827,2792,3038,1680,2312,2755
BLM_1_20,13008,9754,14493,12419,17846,13286,12012,14279,14594,12247,10
178,14620
BMI1_1_21,1065,909,1573,1197,1100,2391,1903,1058,786,1498,475,1251
BRD1_1_22,914,937,1542,789,833,892,3240,1078,1475,1140,961,1653
BRD3_1_23,3310,2800,3160,2593,2362,1967,2426,2244,2069,3188,1918,2074
BRPF3_1_24,202,184,553,543,36,382,117,25,240,273,95,405
BRWD3_1_25,1143,1193,2166,2437,2409,2579,1171,1075,2,1183,2608,1684
C14orf169_1_26,652,992,908,943,689,1242,848,37,851,1363,760,1229
C20orf20_1_27,3054,2794,3396,2993,2489,4302,6405,3268,2070,3455,2403,2
264
CALR_1_28,3470,3184,3889,3586,2534,3009,6743,3055,4770,3142,1574,6219
CARM1_1_29,749,728,859,1080,1565,2158,1300,1519,1719,552,228,977
CBL_1_30,5501,5030,5808,5724,4756,7185,9281,5825,6991,4877,5532,6444
CBX4_1_31,646,428,633,807,346,1310,548,2141,1246,234,980,705
CBX6_1_32,689,557,649,290,2191,1209,1440,480,1339,535,1246,425
CBX7_1_33,721,592,741,756,758,579,541,273,38,469,613,395
CBX8_1_34,511,777,700,779,202,110,393,31,1349,309,838,1351
CCDC101_1_35,134,134,86,49,336,842,226,300,31,585,668,73
CCNE1_1_36,1423,1071,1731,1677,2894,2334,610,330,2739,1102,1414,1352
CCNT1_1_37,3057,3268,4007,4040,4431,3512,3644,3159,2908,4731,3622,2588
CDC73_1_38,4906,4385,4758,4864,5384,4582,5624,3960,5478,5000,7478,7809
CDK9_1_39,1219,1181,1214,1516,526,558,737,1128,995,1175,622,1110
CDY2A_1_40,8811,7896,10077,7638,10716,7445,9487,9773,7143,9054,6648,79
36
CDY2B_1_41,8811,7896,10077,7638,10716,7445,9487,9773,7143,9054,6648,79
36
CDYL2_1_42,1067,852,729,884,523,1671,1388,695,1479,805,1424,903
CECR2_1_43,5210,5856,6082,6164,9105,7705,11558,6108,6236,4975,5125,545
0

CHAF1A_1_44,547,501,349,538,609,319,932,257,1077,276,215,378
CHAF1B_1_45,133,157,203,82,329,41,183,0,250,7,228,35
CHD1_1_46,0,151,107,1,0,89,0,1,0,496,0,0
CHD1L_1_47,1601,1511,1197,2194,1678,1333,1289,679,2643,640,1648,5081
CHD4_1_48,895,982,1113,1125,321,1249,3706,1081,2612,265,85,878
CHD6_1_49,1771,2047,2353,1572,1646,2245,1513,2119,1706,1555,1735,3492
CHD7_1_50,3714,4535,4522,4312,5431,5334,4277,4738,4335,2355,3675,3245
CHD9_1_51,8612,9052,12107,9274,10327,10368,7772,7759,7676,10384,8161,7
719
CHMP1B_1_52,696,713,848,511,1524,945,2307,698,1537,668,895,312
CHMP4B_1_53,1559,1113,1439,1609,2670,1745,1536,953,576,1627,1232,2745
CHMP4C_1_54,12176,11556,14135,12788,12291,17624,15364,12017,7299,12078
,16459,17666
CHRAC1_1_55,1732,1422,2070,1958,712,1825,858,1471,874,1517,2465,2430
CLOCK_1_56,5005,4845,5842,6009,5881,5729,5928,4794,5140,3497,5836,4960
COPS5_1_57,2184,2469,1244,1877,2544,2014,4840,2288,2660,1633,1286,829
CRAMP1L_1_58,1362,1955,2328,825,1686,1762,1593,1934,1604,1083,3659,385
CTCFL_1_59,1166,1201,1529,1067,1451,1444,1157,1623,586,1336,2264,2751
DEAF1_1_60,1542,1401,1183,2445,909,2138,1203,454,2108,1391,740,3883
DNAJC1_1_61,899,586,859,578,170,869,733,367,229,525,212,660
DOT1L_1_62,854,837,753,1407,703,420,2475,2228,1307,608,145,1310
DPF2_1_63,3954,3365,3202,3448,7116,5005,3741,2086,4338,4067,2385,5459
DPF3_1_64,441,361,508,455,988,698,1070,517,439,311,2206,897
EIF4B_1_65,5035,5303,4553,4603,5339,4969,3856,4628,4689,3513,4611,5884
ELP3_1_66,5718,4987,6045,5768,4533,6888,4003,5738,3829,5224,4108,4397
EP300_1_67,4224,3454,4072,2803,2695,4639,5050,2425,2839,2762,7107,2316
EP400_1_68,4226,3985,3861,5219,5658,4664,7089,4818,5137,5930,5370,5695
EPC1_1_69,1198,729,1239,1397,1146,1845,1726,1983,2752,1587,1308,3254
EPC2_1_70,422,265,420,141,709,117,705,337,1,509,1524,0
ERCC6_1_71,947,1223,522,761,807,1121,598,702,803,1185,606,894
ESRRA_1_72,146,145,80,371,179,140,6,157,642,95,35,0
ESRRB_1_73,484,269,543,349,486,265,243,9,501,541,177,4
FANCM_1_74,4529,4997,4654,4731,3350,5209,5810,3771,5281,3355,6248,2967
FBXL19_1_75,115,6,18,51,17,0,17,154,0,1,162,448
FOS_1_76,4483,5640,6120,5297,8064,5335,6131,3399,10094,3667,9785,3664
FTO_1_77,3138,2805,3568,2614,4422,3545,3184,3311,2345,1463,3794,5127
FXR2_1_78,728,698,1040,447,506,1255,1275,843,9,457,944,86
G2E3_1_79,6598,5588,7206,4714,5577,7879,6406,4792,9209,4913,4535,9186
GATAD2A_1_80,2540,1667,2046,2448,1664,2424,2163,1761,1666,1695,3158,26
43
GLYR1_1_81,2111,1695,1800,2174,2180,1567,1924,1348,2406,2462,933,991
GMEB2_1_82,482,319,527,266,625,325,1495,1752,685,313,1,238
GSG2_1_83,1548,1140,1738,2380,1243,1120,1973,2185,1899,990,2484,531
GTF3C4_1_84,1571,1028,1049,933,674,2086,1706,909,730,1378,2162,522
HAT1_1_85,803,950,1066,750,10,871,1014,543,360,1112,351,880
HDAC1_1_86,1661,1131,1023,1658,1750,1384,1467,691,2319,1427,841,1813
HDAC2_1_87,1763,2097,2862,2936,2953,2527,2986,1945,6864,2144,1430,3113
HDAC3_1_88,3974,3110,3602,5252,2685,4805,3603,4625,5699,3500,3288,2432
HDAC4_1_89,428,315,779,187,141,1123,347,612,1339,609,951,746
HDAC6_1_90,4599,3413,3006,4089,5818,4678,6638,1961,2906,4535,3962,8535

HDGFL1_1_91,127,156,57,567,0,416,178,0,0,65,1859,0
HDGFRP3_1_92,471,191,377,168,45,461,56,604,300,331,16,1252
HELLS_1_93,3608,3533,2930,3958,4598,2592,2962,1280,6044,3077,2412,3364
HEMK1_1_94,1231,927,1239,1044,339,1904,3840,699,665,1456,2741,1165
HIF1AN_1_95,2063,2446,3283,3773,2184,3782,2151,3113,1237,2896,3556,299
1
HIRA_1_96,1548,1289,1980,980,2516,2545,2043,1296,2180,883,118,1242
HMG20B_1_97,247,508,408,425,101,144,363,460,746,221,500,1913
HMGN5_1_98,1256,986,1355,1510,2906,2562,1877,228,776,1081,1272,598
HNF4G_1_99,1860,1360,1827,2077,1933,2300,2858,1946,2594,1512,1854,958
HSPBAP1_1_100,467,542,385,1259,655,213,648,1352,648,389,1184,325
HUWE1_1_101,1719,1489,2083,784,1606,1205,54,985,2498,741,1005,73
ING5_1_102,886,564,1017,928,1004,1643,318,515,545,1262,738,1585
INO80_1_103,322,562,202,301,1389,557,650,464,75,237,1775,127
JARID2_1_104,374,226,345,356,286,109,419,507,363,430,969,53
JHDM1D_1_105,1385,1659,1271,2082,709,1472,159,2534,719,746,1203,4295
JMJD7_1_106,1691,2202,1592,1823,3116,2176,2142,1553,4829,805,748,5074
JMJD8_1_107,845,1092,1278,948,1740,1458,1064,146,787,1508,904,308
JUN_1_108,547,611,576,391,228,534,140,659,452,640,936,899
KAT2A_1_109,972,950,677,1318,738,550,685,924,2120,1113,1479,101
KAT2B_1_110,3629,2130,4978,5295,2272,3486,1863,4012,3221,3358,3003,387
4
KAT6B_1_111,2020,2524,2506,2217,3241,2685,2289,1714,1903,2008,838,724
KDM1B_1_112,2207,2736,3285,3512,2074,3139,5282,3299,2357,2051,6274,325
5
KDM2A_1_113,331,332,361,176,312,30,586,812,22,79,19,216
KDM3B_1_114,3046,3468,3905,3634,3881,3290,2468,1724,1552,2992,1678,342
9
KDM4A_1_115,348,902,839,970,528,877,714,464,118,430,1451,293
KDM4B_1_116,321,325,327,256,317,21,134,499,0,598,1126,206
KDM4D_1_117,3333,3384,4801,4071,2876,5722,4338,3108,3260,4115,2799,388
5
KDM5A_1_118,1547,1176,1131,1009,1591,554,1044,747,245,547,1017,1789
KDM5B_1_119,4199,2474,3960,3499,3786,4385,4805,4900,5817,2748,5778,520
2
KDM6A_1_120,1520,1385,1513,1936,1889,1523,3207,3620,3030,1659,1673,269
3
KDM6B_1_121,658,550,1143,1558,1530,848,296,483,764,917,476,3651
KIAA2026_1_122,5692,5937,8193,7768,7625,6361,8583,4903,3672,5917,4860,
6485
L3MBTL2_1_123,482,240,160,226,54,234,716,303,2,179,56,20
L3MBTL4_1_124,935,683,378,1068,1042,338,764,594,989,1301,1,2
MAEL_1_125,2026,2151,2390,1869,3445,1840,1509,5143,3273,1964,1445,2319
MBD3_1_126,1994,2378,2341,2819,1153,2616,2083,1934,3753,2872,1008,1554
MBD4_1_127,6881,6520,6525,7058,11027,9470,5244,4784,7926,4819,4076,673
7
MBD5_1_128,2781,2731,3640,2174,3510,3514,1912,2192,1828,1806,6787,2873
MBD6_1_129,170,48,100,63,0,0,61,38,185,17,0,0
MBTD1_1_130,3165,3668,4602,3686,2311,4628,4166,3592,4454,3247,4491,238
1

MDM2_1_131,4132,3969,4109,3909,4558,3123,5200,3780,5537,3850,1907,2752
MIER2_1_132,506,735,890,1021,204,1170,604,1372,372,350,266,530
MIER3_1_133,3236,2808,3651,4266,3712,4056,1850,3591,3258,3338,673,4572
MIS18BP1_1_134,5447,4966,7001,5669,3711,5499,8847,3887,4607,4657,3275,
5577
MKL1_1_135,1491,1599,1713,1560,1049,1766,845,995,106,1051,1606,2675
MLL2_1_136,2095,1537,1880,1565,961,1753,2802,720,982,1703,2958,2588
MLL3_1_137,8631,7490,9472,7757,9926,10244,13403,7392,6967,7180,11614,9
835
MLLT1_1_138,375,456,513,780,88,1516,188,369,184,418,42,894
MLLT3_1_139,2147,2486,2615,3001,2295,2791,4921,1901,1557,2622,1267,375
5
MLLT6_1_140,860,1499,1380,1953,2803,1875,3424,602,1576,692,1382,460
MPHOSPH8_1_141,1388,1167,1259,1563,2439,2051,870,373,1212,1027,486,129
MSH6_1_142,1605,1869,2369,2619,1513,1601,2873,2824,3067,1735,3311,1458
MSRB2_1_143,1066,883,1437,1264,376,966,112,1109,812,769,1187,1826
MTA2_1_144,1134,946,1682,1651,1007,1278,679,530,929,1352,3202,1519
MTA3_1_145,4510,3649,4107,4480,9000,6226,6059,5911,5414,3205,3693,5346
MYSM1_1_146,980,1587,1397,2503,3964,925,1465,981,1471,1590,584,921
NAP1L2_1_147,1496,1020,598,1316,2182,1365,629,1685,2762,625,738,370
NAP1L3_1_148,1105,1819,1957,1986,1722,1578,1470,2448,3393,1236,1027,22
73
NAP1L4_1_149,771,1038,693,892,870,543,408,919,2003,762,495,5021
NAP1L5_1_150,408,622,338,406,361,69,198,339,533,1305,0,929
NAT14_1_151,4,85,485,594,20,0,13,0,89,1,4,0
NAT8B_1_152,1147,1012,1052,975,1936,2095,655,666,1377,1025,1190,739
NAT8_1_153,2206,2227,1910,2255,1007,3730,2840,1408,2223,1126,995,1415
NAT8L_1_154,4036,3944,4038,3465,5829,5118,4669,4820,3286,5157,3482,398
6
NAT9_1_155,1718,1419,1831,1200,2946,2120,2174,4060,2799,1452,3154,989
NCOA2_1_156,617,696,584,182,1223,698,46,3,211,510,81,23
NPTXR_1_157,2321,2368,2979,2939,2021,2157,1954,1923,1126,2763,3093,101
3
NR0B1_1_158,2200,2276,2143,3047,2299,1885,1571,1892,3535,3395,2224,309
7
NR0B2_1_159,262,215,204,152,380,567,83,5,6,87,34,356
NR1D1_1_160,231,425,177,484,655,360,233,957,269,339,307,853
NR1H2_1_161,869,1033,1812,1398,722,871,3026,1247,1803,874,1768,792
NR2C2_1_162,1253,1673,1923,2186,2812,1160,1593,1299,2618,717,4275,682
NR2E1_1_163,2533,2625,2519,2828,4184,2416,3430,3769,6476,2240,2996,455
2
NR2F1_1_164,1009,1705,1464,1231,1127,2329,1039,2660,533,1322,25,1078
NR2F6_1_165,2210,1470,2130,2276,4465,3116,2938,1245,1512,2530,1246,139
6
NR4A2_1_166,1430,988,1237,1759,541,1883,1006,2038,1025,1892,872,2052
NR5A1_1_167,569,247,311,409,15,332,709,272,0,486,29,0
NRIP1_1_168,3347,3803,3648,4267,3549,5626,3508,3530,3708,4896,2131,655
5
PADI4_1_169,3040,3485,3739,3140,3792,5737,2005,2240,4813,2734,861,2543
PAWR_1_170,1901,1552,2011,1359,769,1843,1895,1402,1181,2260,1523,1951

PAX5_1_171,418,212,184,191,105,222,1597,833,190,224,5,26
PAXIP1_1_172,4613,5496,4964,4511,5550,6338,3377,2717,3582,4448,8135,39
85
PGRMC2_1_173,10213,9175,13763,11893,8141,9344,13874,8012,8922,10344,89
48,11317
PHB_1_174,1488,1449,2706,1516,118,2323,865,1917,2624,694,2986,4315
PHC3_1_175,1193,1481,1076,1760,2464,1447,4788,1195,1866,2157,3609,825
PHF13_1_176,2317,1750,2336,2023,3341,2917,3798,1777,733,1315,3784,1701
PHF14_1_177,2611,1882,3866,3072,4909,2295,6808,3970,770,3087,1255,2252
PHF15_1_178,1721,1996,3172,2562,2023,2936,3948,1335,2066,2877,2770,255
7
PHF20_1_179,1921,1493,1936,1299,2705,1630,824,2155,3023,1425,1338,3273
PHF23_1_180,621,755,678,725,407,842,2090,183,195,384,986,538
PHF2_1_181,584,742,716,838,1111,887,853,48,0,1335,336,2830
PHF3_1_182,3094,2102,2915,1810,2684,2213,2838,2695,1793,2354,4140,708
PHF5A_1_183,670,657,846,1069,1044,753,464,1227,2068,1570,498,669
PHIP_1_184,5449,6098,7256,6470,7518,5145,4992,6937,3450,5248,3014,3017
PHRF1_1_185,1461,899,1102,1481,1616,1230,833,1266,1923,2190,2198,2525
PIAS1_1_186,462,409,110,570,795,1028,1055,34,1342,642,190,294
PIWIL4_1_187,2119,2001,2439,1908,1979,2803,885,1438,1031,2255,2287,120
2
PPARGC1A_1_188,2993,2782,4034,3887,5491,5027,4248,3803,3406,1951,2915,
1952
PRDM11_1_189,3139,3905,2119,2249,2311,3831,3339,2034,145,1784,4447,613
PRDM12_1_190,42,104,47,21,31,70,28,7,0,105,0,318
PRDM13_1_191,532,520,759,652,325,422,1415,720,821,1028,37,962
PRDM14_1_192,4492,3185,4691,5013,4978,3032,4192,3755,2210,4476,6164,42
51
PRDM4_1_193,2159,2034,2120,2882,1980,1515,1149,1690,1696,2907,5271,282
5
PRDM5_1_194,2171,1076,1327,1909,4257,2093,2004,946,2046,1825,1122,1845
PRDM6_1_195,1682,3043,2010,2203,2706,2338,2761,1371,1260,1664,408,2081
PRDM9_1_196,811,980,1297,1134,922,832,1337,157,510,1163,810,1768
PRMT6_1_197,174,250,582,617,1355,229,902,627,1803,180,302,64
PRMT8_1_198,4512,5338,4933,5132,10812,6577,5238,4615,4770,4334,3062,41
71
PRPF6_1_199,3262,3004,2801,4885,3899,5728,3238,2185,1822,3568,3343,313
1
PYGO1_1_200,384,180,166,319,780,181,266,456,17,42,333,64
PYGO2_1_201,819,490,541,890,1540,951,1168,423,1162,803,779,407
RAG2_1_202,387,303,215,351,796,256,515,173,797,616,225,461
RAI1_1_203,623,771,853,741,1252,322,1636,6,626,882,134,34
RB1_1_204,9589,9170,9050,10796,12911,10957,14984,8309,9242,11856,8766,
12846
RCOR1_1_205,6000,5712,7011,7669,6768,6589,8273,6692,8834,6684,5081,836
3
RCOR2_1_206,418,501,299,451,271,373,336,562,559,931,494,1080
RECQL4_1_207,1615,1106,1761,1474,2404,1672,1237,1069,1670,1558,324,199
7
RELB_1_208,431,568,699,585,603,414,251,782,979,633,1047,3

REL_1_209,2450,2565,3692,3217,2041,3069,4065,2506,1689,1933,3218,1399
RING1_1_210,859,468,710,594,319,718,524,696,186,549,880,95
RNF20_1_211,1618,1479,1725,1774,3694,1779,1698,3133,2767,3012,2147,166
6
RNF25_1_212,1273,934,1448,664,1457,1422,2881,1967,3863,1178,478,1699
RNF2_1_213,2935,2758,3546,2694,4359,2454,1810,2206,2925,3722,5505,2842
RORB_1_214,601,324,493,381,1974,293,296,576,546,514,6,268
RSF1_1_215,4562,4876,5849,5146,5054,3555,6663,5897,5307,4824,5456,4300
RUVBL1_1_216,2028,1422,1388,1171,3864,3799,3055,1961,3783,2288,847,265
1
RUVBL2_1_217,692,312,687,584,188,776,327,293,880,276,713,1016
RXRA_1_218,180,439,159,908,550,1434,635,5,116,93,86,3
RXRB_1_219,1098,994,1182,874,731,650,1920,979,833,1974,1124,1580
RXRG_1_220,372,258,1212,893,110,398,893,945,39,511,17,211
SAP18_1_221,4110,4571,5379,4658,5677,3822,3167,4888,4234,4522,3034,273
9
SCML2_1_222,2827,2035,3923,2435,3078,1888,7311,4277,4074,4354,2842,246
8
SENP3_1_223,374,282,307,285,778,190,1943,12,1,224,350,883
SETD1A_1_224,2383,2475,2541,2055,1277,2695,1956,2952,1903,1871,4615,18
60
SETD1B_1_225,2014,1853,2689,2184,2387,2915,3336,837,2144,1300,1439,186
7
SETD2_1_226,4807,4481,4906,5249,3212,6465,9624,6600,4177,5044,4035,624
8
SETD5_1_227,3724,4947,4126,5616,3262,4734,3037,2247,4578,4102,5177,602
5
SETD7_1_228,2144,2455,2588,2841,3830,3600,4750,2590,6943,2032,4521,228
5
SETD8_1_229,2861,3543,2773,3750,4254,4460,3453,2815,2762,3814,4197,312
3
SETMAR_1_230,575,429,375,397,1196,605,799,734,72,630,306,708
SF3B3_1_231,926,874,1443,1415,880,973,1673,1190,1462,1041,720,419
SIAH2_1_232,4585,4613,5412,5154,7505,4008,3733,5689,4886,5599,4867,658
8
SIN3B_1_233,401,297,753,99,18,371,0,4,9,1,469,0
SIRT4_1_234,971,1061,1515,1489,592,792,1511,1359,1586,2193,388,2285
SIRT7_1_235,2649,1879,1908,1910,849,1959,1939,403,618,2402,1319,1316
SLC2A4RG_1_236,657,399,531,311,568,651,508,14,1689,694,131,1036
SMARCA5_1_237,6188,6398,6255,7048,7576,9567,13056,5537,10140,6385,5188
,9785
SMARCC1_1_238,2833,2628,2753,2757,3082,3449,2274,2134,3886,1454,1155,3
260
SMARCD2_1_239,7495,8436,8372,10044,10594,12076,7593,11672,5094,8629,57
12,10551
SMARCE1_1_240,2336,3075,4289,3661,2805,5834,914,2709,817,3653,2465,228
8
SMC1A_1_241,3038,2324,2158,3214,1092,2311,3295,3230,389,2827,2481,3206
SMC1B_1_242,5460,5876,5320,5294,3437,4549,7309,6204,10265,6221,4524,37
90

SMC3_1_243,1428,1361,1857,1600,1957,1491,550,2353,1346,2489,3415,1966
SMCHD1_1_244,5564,4842,6699,7636,6994,5059,7450,9929,5964,6713,4406,11
560
SMNDC1_1_245,2721,2853,2184,2581,5389,3136,2980,1881,1826,2755,988,220
8
SMYD1_1_246,1031,779,1442,902,770,1055,1171,837,3083,434,317,1049
SMYD2_1_247,118,309,579,604,677,135,123,177,1336,1487,455,1355
SMYD4_1_248,65,133,86,40,249,712,17,0,0,14,0,0
SMYD5_1_249,596,272,466,773,394,193,440,384,1297,364,706,270
SND1_1_250,1145,510,1850,1262,1777,2330,565,357,2050,2982,758,339
SP140L_1_251,3599,3953,4852,2785,5110,4495,5314,2080,6252,3934,432,374
9
SRCAP_1_252,362,117,232,118,15,268,590,166,0,275,1,128
STAT5B_1_253,295,678,337,23,32,39,235,1,279,346,350,850
SUDS3_1_254,3826,4172,4900,5191,4392,3477,5381,6378,2636,4012,4246,690
6
SUPT16H_1_255,2818,2924,2810,2926,3153,2082,4327,2143,2454,3015,783,40
05
SUPT4H1_1_256,2650,1853,1965,1738,1649,1436,2951,633,3545,2465,2557,11
81
SUPT6H_1_257,5519,4460,6651,4818,3964,3277,7010,2595,4545,5932,4604,92
19
SUPT7L_1_258,2806,2129,2770,2837,2875,2669,606,3577,853,1492,2053,2473
SUV39H1_1_259,496,511,950,766,595,928,651,429,343,509,500,1814
SUV420H2_1_260,629,618,362,902,568,280,1760,992,470,456,724,520
SUZ12_1_261,1774,1312,1453,1000,1255,1194,641,1779,1402,1318,125,351
TADA1_1_262,3910,4236,5947,5715,3869,4378,5734,9412,6365,3944,6020,724
9
TADA2B_1_263,1182,571,685,409,1368,474,356,380,14,1250,20,2247
TAF10_1_264,6010,4813,8386,6857,9127,6038,6716,6064,6133,4473,6888,883
4
TAF1L_1_265,2072,2081,1570,3073,4078,2294,3398,997,250,2779,46,2181
TAF3_1_266,298,501,498,378,107,290,328,64,50,156,83,328
TAF5_1_267,935,1316,703,901,421,271,3027,110,855,1143,700,1
TAF6L_1_268,1301,2049,1180,843,1421,2647,1877,691,2950,783,317,729
TAF8_1_269,973,959,1549,1174,2401,1591,2842,313,2293,664,1010,677
TCF7L1_1_270,1100,1751,1268,1220,1043,1418,927,608,1161,667,43,701
TDRD12_1_271,7833,6115,7492,5466,10123,6963,11155,4839,5516,6720,5537,
6121
TDRD1_1_272,712,767,858,952,1072,727,1224,646,717,773,707,1053
TDRD7_1_273,3804,4329,5073,3230,4471,3822,3504,4100,2566,3206,7323,454
0
TDRD9_1_274,743,978,965,1478,1316,1232,914,578,1320,1068,434,277
TERF2_1_275,2108,2494,2398,2840,1074,3303,1872,792,2275,4232,1541,1997
TET1_1_276,1087,1624,1648,1406,1939,2333,2045,1864,3412,2764,1491,2592
TET3_1_277,292,200,386,748,1530,1097,863,509,175,156,136,1
TLX2_1_278,273,13,246,76,25,807,312,34,1,6,0,103
TNRC18_1_279,3022,3361,3999,3935,4349,3871,7596,2697,5990,4672,1680,39
21
TRAF7_1_280,1041,493,913,445,1483,335,871,1612,957,650,278,889

TRDMT1_1_281,2657,1810,2800,2664,4265,2103,2605,739,1938,1512,5340,283
9
TRERF1_1_282,1820,1779,1840,1902,1629,785,1611,1234,2072,1926,455,633
TRIM25_1_283,21,226,51,196,3,0,323,2,128,1,6,722
TRIM27_1_284,153,149,152,97,437,109,214,0,320,370,794,157
TRIM28_1_285,1022,820,1598,1621,60,1888,957,1124,587,1219,531,4176
TRIM66_1_286,1871,1641,1295,2375,561,966,725,1028,753,1422,69,3099
TRRAP_1_287,1248,1493,1818,1081,227,2240,411,94,2803,740,990,67
TSG101_1_288,2663,3263,2754,2964,3819,3629,2812,3044,4172,2688,3416,15
42
TYW5_1_289,1919,2242,2516,1308,1925,2276,1586,2628,92,2471,514,1789
UBE2B_1_290,2469,1993,2709,2032,2394,1113,3819,704,1586,2189,2998,875
UBE2N_1_291,552,203,221,299,79,726,0,247,18,93,885,0
UBR7_1_292,4309,4839,4078,6003,8657,5823,3639,4847,2620,5388,3314,8500
UHRF2_1_293,3967,3250,6599,3554,3440,4721,4840,4676,4343,4463,3787,328
7
USP22_1_294,4668,4855,6395,6683,7597,5951,5811,3718,3862,4774,4887,506
2
YEATS2_1_295,754,520,276,447,98,694,2,220,179,930,0,409
YEATS4_1_296,8851,8725,12041,9136,11442,10480,10902,7433,9220,9604,137
58,13273
YY1_1_297,7286,5360,7932,6979,7393,12261,10820,5370,5947,8243,4664,115
03
ZAR1_1_298,1448,1154,1308,1338,1477,416,1949,2731,1011,1237,1714,1769
ZCWPW1_1_299,2795,2188,2426,2668,1634,2253,2883,777,2006,2262,757,1794
ZCWPW2_1_300,1493,1078,1994,1614,1373,2370,2985,1909,1438,1103,3136,93
6
ZFP57_1_301,2521,2490,3346,2048,1341,2311,1630,4182,1022,1765,6187,306
5
ZNF541_1_302,154,27,2,205,289,39,1,760,0,100,3,3
ZNF85_1_303,1501,1105,1674,1914,1709,1135,960,404,1078,1285,797,928
AANAT_1_304,177,477,483,45,10,397,16,509,177,232,22,0
AES_1_305,623,997,1141,678,1266,1195,298,1148,96,834,15,47
AIRE_1_306,150,117,195,352,396,125,208,14,35,79,554,326
AKAP1_1_307,1796,2089,3349,2290,1342,1207,2769,2079,2463,2169,1375,272
6
ALKBH2_1_308,122,361,193,37,60,309,83,151,894,475,35,118
ANKHD1_1_309,1712,1045,1186,1426,2965,1194,2408,1735,76,1810,607,822
ARID1A_1_310,977,835,900,1756,1184,552,4771,790,1247,1228,1433,940
ARID1B_1_311,805,776,1238,937,1386,1266,1273,583,1089,924,1609,128
ARID4A_1_312,5182,5682,7116,5448,4642,8216,10519,7199,6887,7498,7826,8
860
ARID4B_1_313,8807,8683,8122,9095,13754,11133,8921,6858,9542,10544,5121
,8487
ARRB1_1_314,926,974,1092,1564,1264,1512,924,1353,1825,612,1178,1726
ASH2L_1_315,625,430,698,736,765,1174,736,892,1099,391,242,731
ATAD2B_1_316,4615,5086,6323,5966,8546,4160,8324,5194,4225,7306,9540,54
08
ATAT1_1_317,3019,1687,3176,3064,2747,2050,1382,1997,5814,3217,2059,207
9

ATRX_1_318,3320,3118,3298,4477,5431,3967,4024,4892,1513,2570,4118,4988
AURKA_1_319,1106,1624,1616,1216,2429,1091,1279,1453,2403,2012,2457,101
6
AURKC_1_320,3527,3974,3240,3970,3006,2999,6916,3091,3845,4198,2293,485
0
BAZ1A_1_321,5925,7646,7941,6283,9282,5765,5991,7926,2976,6589,4437,637
9
BCOR_1_322,1632,1279,956,1309,624,1497,658,1427,532,1143,1523,283
BPTF_1_323,3627,3301,3322,3288,3285,1959,3460,2340,5290,5154,1700,6185
BRCA1_1_324,890,1024,1257,914,394,1409,1646,277,151,1005,1995,774
BRD2_1_325,646,595,698,395,381,353,205,91,96,224,246,632
BRD4_1_326,3219,2820,3120,2696,2345,2071,2436,2629,2083,3181,1927,2079
BRD7_1_327,42,153,288,164,58,284,281,565,21,25,5,1062
BRD8_1_328,1171,660,1427,942,1116,570,1208,1216,1068,1642,1019,774
BRD9_1_329,3452,5343,4358,3832,4975,3441,5790,4651,4011,3434,3056,4533
BRDT_1_330,2165,1453,2269,1661,4000,2928,3246,2277,3621,1567,881,1358
BRPF1_1_331,6257,4419,6702,5927,5468,6086,6946,7001,6834,7781,6225,673
5
BRWD1_1_332,2938,2418,2368,3028,3858,3596,6435,2829,1080,3517,2993,387
C14orf43_1_333,5470,4657,7003,5167,3948,6224,8256,8415,9783,7611,3776,
8723
CBX1_1_334,2104,2419,2399,3611,1540,1940,4135,1297,2558,1724,2570,1772
CBX3_1_335,3732,2940,3936,3784,3492,4062,4203,4577,2361,2448,3277,3461
CBX5_1_336,6421,6487,6562,5582,7754,7119,7551,5454,4703,5776,3540,4609
CCNT2_1_337,1197,666,932,1412,760,707,965,1144,148,785,280,827
CDY1B_1_338,8811,7896,10077,7638,10716,7445,9487,9773,7143,9054,6648,7
936
CDY1_1_339,8811,7896,10077,7638,10716,7445,9487,9773,7143,9054,6648,79
36
CDYL_1_340,1385,1251,623,1753,997,1604,932,751,272,738,881,881
CHD2_1_341,4884,4392,4738,3177,7540,4211,8062,3046,5467,4443,3865,3500
CHD3_1_342,3027,1482,1276,2646,2736,1819,1726,2830,4421,1177,1674,3315
CHD8_1_343,1739,1382,1382,1965,1217,3292,4095,1754,6077,1876,1151,837
CHMP2A_1_344,1449,1271,1406,1894,1547,883,1014,574,308,1188,1237,1251
CHMP5_1_345,1153,1420,2037,1599,783,2356,1056,1002,2771,2259,29,1286
COPS2_1_346,16244,15481,17005,18899,20333,22330,18840,14123,14041,2102
5,19478,16972
CPA4_1_347,1166,730,1099,588,1626,1837,2135,895,99,1006,871,695
CREB1_1_348,526,564,328,459,420,762,1191,252,91,670,119,34
CREBBP_1_349,0,0,0,0,0,0,0,0,0,0,0,0
CTCF_1_350,599,554,762,469,1719,504,49,8,2083,251,496,113
CTNNB1_1_351,1951,1506,1884,2433,1807,2059,694,1684,1042,1724,2544,279
8
CXXC1_1_352,395,639,310,396,21,295,218,133,712,545,78,1331
CYLD_1_353,3655,3923,3714,4789,3304,6398,3063,4767,3352,4599,3922,3446
DICER1_1_354,1489,1894,3061,2060,3665,3418,2127,1334,1486,2710,3370,44
37
DIDO1_1_355,143,673,868,397,398,118,256,3,6,168,88,1514
DMAP1_1_356,1576,1292,2045,1908,2288,1969,1225,1431,4518,959,2095,1703
DNAJC2_1_357,2436,1983,2282,1783,668,1231,4356,1250,1449,1792,2094,250

9
DNMT1_1_358,1267,1850,2033,1552,1061,2006,3646,2659,612,2709,2504,1751
DNMT3A_1_359,678,613,485,620,152,966,630,838,467,403,442,554
DNMT3B_1_360,256,346,846,378,269,503,868,0,546,113,1756,518
DNMT3L_1_361,3411,3547,3118,3208,3403,3835,1754,2009,6729,4596,4396,30
02
DPF1_1_362,1957,1314,1347,1226,1363,938,683,1377,669,1686,1057,1672
EED_1_363,391,244,435,324,226,129,41,263,387,38,21,196
EGR2_1_364,854,1113,547,1353,1293,1118,820,858,2084,461,243,1832
EHMT1_1_365,0,0,0,0,0,0,0,0,0,0,0,0
EHMT2_1_366,2792,3343,3245,2555,3839,3249,3566,1564,2981,3213,1370,311
7
ENO1_1_367,798,320,433,526,410,433,714,584,1530,252,219,250
ESR1_1_368,474,355,154,373,297,215,151,450,2828,580,461,793
ESR2_1_369,224,137,459,400,508,263,270,2,146,24,0,586
ESRRG_1_370,431,173,937,533,324,550,25,2,9,185,47,1029
EZH2_1_371,452,419,753,138,409,279,491,290,119,965,1043,2142
FBXO11_1_372,13837,12710,14580,15887,14835,13861,14668,14628,13294,162
48,15014,17347
FMR1_1_373,4354,3409,4037,3123,3746,4778,2082,1736,4928,3109,4257,3802
FXR1_1_374,3324,3366,2787,2813,2909,2742,3199,1645,2896,1688,4025,3666
GFI1B_1_375,618,324,410,332,485,869,20,444,0,620,0,25
GMEB1_1_376,2986,3331,4200,3769,4525,4655,4082,3278,1992,2227,1417,240
5
HDAC10_1_377,1865,1710,1731,2226,4277,2458,1880,1558,973,1076,457,1173
HDAC11_1_378,1166,660,516,664,993,545,217,865,137,569,354,464
HDAC5_1_379,639,498,465,575,1112,1187,1226,353,1438,339,1704,633
HDAC7_1_380,916,644,652,797,709,1385,254,152,864,1760,1757,978
HDAC9_1_381,4088,3711,4479,4202,5292,3806,3440,4298,4484,3757,3135,462
3
HDGF_1_382,422,850,547,1366,470,524,917,565,1286,152,0,374
HDGFRP2_1_383,460,204,395,356,824,798,470,610,0,405,1357,933
HLTF_1_384,6178,6245,7198,7495,8302,7819,8135,5827,9549,6398,8716,5258
HMGA1_1_385,544,405,436,1553,53,444,1940,131,811,769,240,733
HMGN3_1_386,2153,2445,2606,2055,5247,3119,1688,1370,382,1975,1970,1341
HNF4A_1_387,837,922,956,680,2863,1361,608,785,154,1107,69,1415
HPSE2_1_388,21,68,148,35,83,461,371,0,0,54,0,1
HR_1_389,6,59,152,186,0,0,0,490,0,2,0,1
ING1_1_390,2053,1093,1540,1509,1636,2538,815,273,2888,1258,312,518
ING4_1_391,1084,516,1203,1074,137,195,1284,473,2323,228,99,1390
INTS12_1_392,553,265,774,212,21,235,815,11,884,310,172,398
IRF4_1_393,1135,734,1217,1317,1270,914,1608,769,517,1064,1415,1730
JMJD1C_1_394,934,994,927,1462,479,1402,1507,1689,1227,1096,2001,867
JMJD4_1_395,1510,1842,2363,2642,1770,2500,3186,1719,1677,2377,1871,125
9
JMJD6_1_396,893,956,1011,787,752,851,549,249,894,519,46,231
KAT5_1_397,1443,1770,2373,1524,1528,1607,1405,1526,1597,1203,3821,3430
KAT6A_1_398,2071,1681,2806,1622,1077,1692,1392,1832,749,3799,2368,2781
KAT7_1_399,5810,5869,5514,6582,5683,6425,5796,6224,6748,5998,4149,6646
KAT8_1_400,543,826,697,566,762,679,718,476,577,806,481,273

KCTD1_1_401,15168,15015,18536,18734,13079,14485,23870,19103,17390,1452
4,13559,15026
KDM1A_1_402,5797,5505,5562,6581,8209,8217,7785,8013,6210,6579,7081,472
2
KDM2B_1_403,2228,1690,2201,2138,5219,3714,1573,2622,1712,2521,1723,203
2
KDM3A_1_404,1393,1366,1445,2339,2140,853,4087,2074,225,2308,625,1554
KDM4C_1_405,589,506,585,293,1191,110,90,1141,647,360,528,135
KDM5C_1_406,18149,16757,20311,17827,18478,22391,20388,11912,15748,1643
5,19420,21280
KDM5D_1_407,18318,16949,19622,18110,17646,23648,20378,11529,14801,1613
0,18986,19303
L3MBTL1_1_408,766,924,924,757,680,442,341,625,1010,297,559,490
L3MBTL3_1_409,1475,1048,1477,1494,1347,2235,1357,815,891,2362,597,1296
LBR_1_410,3188,3415,4169,2748,2778,2315,2846,3385,2200,2750,2862,3561
MAP3K12_1_411,324,138,404,491,816,81,940,0,0,75,140,0
MBD1_1_412,1660,1407,1378,1301,1694,1245,829,1297,805,1287,3867,6
MBD2_1_413,444,104,146,448,1,49,495,124,92,300,115,101
MECOM_1_414,3214,3314,3703,5106,2533,4297,3838,3743,6569,2992,4402,293
5
MECP2_1_415,773,537,523,801,2897,1415,935,714,1642,891,109,1812
MEN1_1_416,5233,4152,3999,4087,3224,5799,7467,5250,3838,4398,4976,4015
MGEA5_1_417,3969,3806,5601,4094,3645,4064,5160,4227,5014,3157,7272,646
5
MIB2_1_418,553,419,47,22,399,134,0,614,1,288,0,825
MIER1_1_419,2587,2690,3632,3276,6311,3201,4747,1738,5900,4738,4749,516
4
MINA_1_420,1718,1538,2361,1301,4275,1430,1079,1564,3407,1026,605,2039
MLL5_1_421,2789,2232,2619,4222,2057,3375,2492,3354,2105,3690,2315,6926
MLL_1_422,4053,4747,6773,4816,6463,7846,2970,5489,3066,4277,3697,6926
MORF4L1_1_423,6950,7183,7083,7603,6455,9510,10406,8412,8623,8124,6580,
7043
MSL3_1_424,1910,1385,2341,2063,2524,2079,4407,1072,769,2016,635,1256
MTA1_1_425,5098,4111,4918,4155,4099,4525,5011,5192,4593,3544,4954,6781
MTF2_1_426,4611,4106,2950,4381,5653,5957,3240,5052,6506,3496,4928,6067
NAA60_1_427,667,766,600,1251,1037,3153,678,748,941,933,1751,651
NAP1L1_1_428,4991,3815,4492,4527,6015,3946,6777,4839,4799,3866,5344,59
61
NAT10_1_429,1541,1284,1981,1533,3935,1875,1403,3090,1042,629,3086,2430
NCOA1_1_430,826,652,595,907,1222,2052,529,559,397,988,1973,275
NCOA3_1_431,8444,7458,7404,6641,10041,10132,8756,8463,11635,8646,9719,
9006
NCOA4_1_432,2880,1762,2195,2026,2382,3525,2607,1809,1462,1862,2023,115
2
NCOR1_1_433,762,834,1088,335,953,1065,154,666,233,110,1,296
NCOR2_1_434,1216,433,590,471,10,313,1422,696,1480,675,463,1
NFAT5_1_435,935,777,1339,1014,1341,1380,1895,1438,3687,1512,798,181
NFATC1_1_436,895,1274,1043,931,1286,1524,160,941,98,666,1213,419
NFATC3_1_437,3792,3367,4050,4347,3446,3976,2593,6681,2161,4019,3460,46
43

NFATC4_1_438,386,241,400,262,4,404,1057,11,135,540,0,101
NFKB1_1_439,6803,7934,8822,7719,8967,8328,9659,8056,6745,8509,6607,956
7
NFKB2_1_440,1305,1512,1281,1808,2423,1804,1178,751,1190,788,1441,2760
NPM1_1_441,25159,25434,28059,28650,32393,32381,31111,23123,25346,29234
,23093,29452
NR1D2_1_442,1473,1546,2770,1607,4019,1844,2205,834,945,2677,2337,2098
NR1H3_1_443,1473,1003,2049,2067,3189,831,2498,2370,1861,1430,667,3092
NR1H4_1_444,2616,2226,1911,2218,2733,4062,2651,889,2097,3636,4102,3385
NR1I2_1_445,512,712,400,725,639,763,524,1124,638,525,1446,269
NR1I3_1_446,922,681,867,1199,1696,543,1636,1094,1447,374,42,994
NR2C1_1_447,31144,27610,30704,32289,41294,40095,36228,27351,27090,3557
8,38568,29006
NR2E3_1_448,4722,5151,6099,5989,6190,4463,7440,4655,2808,6202,4753,590
9
NR2F2_1_449,1603,583,1129,1627,4988,2453,2007,2747,2847,1904,1418,1978
NR3C1_1_450,213,213,416,489,44,748,421,176,329,83,294,1284
NR3C2_1_451,1313,1448,1746,1984,1687,947,1207,2225,1130,2383,1019,1701
NR4A1_1_452,1378,2659,2456,2066,1520,3601,1890,2462,2687,2030,2131,243
5
NR4A3_1_453,473,455,1376,1350,614,761,700,322,510,581,589,185
NR5A2_1_454,2652,2043,1740,2150,3140,2141,4818,1940,3404,2068,1324,129
0
NR6A1_1_455,237,195,255,433,169,149,1205,516,16,122,346,88
NSD1_1_456,527,512,847,927,1052,559,613,579,46,784,888,764
PBRM1_1_457,3123,2561,3240,3609,4115,3362,4761,2288,5335,2625,5095,253
8
PCGF6_1_458,1197,996,667,975,2051,1795,2266,464,1087,952,2275,1097
PGR_1_459,1769,1499,1198,1638,1167,3590,1417,2660,1251,1093,2677,621
PHC2_1_460,44,124,147,114,30,104,1,15,11,0,12,19
PHF10_1_461,1989,3031,2753,2273,2409,4004,3640,3693,7257,1622,3667,333
8
PHF11_1_462,1926,1079,2259,1589,1833,1166,2369,1004,2474,848,648,699
PHF12_1_463,1876,890,1947,2300,2604,1254,2085,1299,1938,2647,931,1992
PHF16_1_464,1957,1920,2230,2758,1313,3665,2020,1792,639,1303,457,2171
PHF17_1_465,1363,1299,1679,1047,2209,2003,1130,398,288,802,259,320
PHF1_1_466,5015,5412,4446,5973,4836,6298,5411,3630,5954,4865,4809,1002
9
PHF20L1_1_467,6240,4822,7918,5799,8335,9962,7484,2809,7794,6665,4652,4
885
PHF21A_1_468,530,299,405,599,252,12,217,100,140,856,38,2292
PHF21B_1_469,1019,774,1544,1099,690,1516,3013,2185,186,1615,774,87
PHF6_1_470,1995,1522,2761,2252,2560,1962,2004,1690,1662,2252,1513,893
PHF7_1_471,1846,2050,1973,1379,2752,1776,2138,724,1693,779,1388,933
PHF8_1_472,1238,1140,1178,679,2045,994,1062,557,1062,1384,1637,275
PIAS2_1_473,2112,2413,2541,2944,3053,2739,4499,2606,2729,1996,1109,234
5
PICK1_1_474,481,722,537,334,543,289,325,494,827,392,1140,1294
PIWIL2_1_475,7644,5368,8880,7450,9164,9907,9982,5576,8574,5711,8087,72
95

PML_1_476,71,32,20,79,52,48,3,100,0,5,0,1
POLR1B_1_477,135,464,623,56,4,343,192,600,74,92,2097,66
PPARA_1_478,2971,2465,2103,2561,2630,2157,2757,3304,1954,2363,2679,240
8
PPARD_1_479,2634,2503,2980,2011,2635,2542,3387,2116,3731,1768,1051,341
PPARG_1_480,1464,1555,1764,1115,387,1280,1039,1235,2526,1409,872,2139
PRDM10_1_481,2871,2194,1726,1588,2096,2461,2558,868,2655,1059,1030,177
0
PRDM15_1_482,172,131,235,302,516,112,152,21,1,78,800,3
PRDM16_1_483,1956,1206,1930,2168,1828,1625,3414,1407,3431,1802,1750,23
95
PRDM1_1_484,435,311,591,109,210,64,232,144,1059,232,798,637
PRDM2_1_485,25927,25315,26892,28985,33395,28854,33605,24784,24264,2930
4,23923,28767
PRDM7_1_486,4088,4158,4527,4508,5336,6344,5800,4431,5006,4551,4682,431
6
PRDM8_1_487,1386,1497,1797,1290,1013,2248,1331,1789,854,1111,1630,2824
PRMT1_1_488,1025,610,455,463,1061,457,1599,899,1372,689,526,1208
PRMT2_1_489,2517,2285,2937,2896,3631,2747,3427,1901,1265,3040,2326,272
2
PRMT3_1_490,2749,3250,3694,3369,2942,5329,4389,3902,3177,1675,3697,223
0
PRMT5_1_491,1019,1407,1097,1149,2988,733,902,345,1058,507,1602,226
PRMT7_1_492,213,89,89,89,1,323,25,288,452,23,0,86
PSIP1_1_493,10097,9449,11253,11134,11287,11858,10485,10603,11753,9824,
11009,13118
PSMC5_1_494,272,134,533,179,53,228,29,4,18,228,226,318
PWWP2B_1_495,708,1011,824,399,614,612,491,265,81,450,466,597
RAD54B_1_496,7664,6579,8504,8226,11641,7576,6947,7338,8215,8100,7877,1
0429
RAD54L_1_497,365,99,207,417,190,33,352,123,0,331,0,13
RARA_1_498,3936,3377,3803,4343,3981,3816,3643,3138,4945,5999,3513,4042
RARB_1_499,2297,2346,2578,2361,4037,1435,624,3131,2644,2528,2293,2732
RARG_1_500,1571,1227,899,1874,1765,1103,3346,2282,1935,2203,441,1020
RBBP5_1_501,4028,4221,5234,4394,5639,3984,2167,3499,6171,3300,1968,275
7
RBCK1_1_502,179,321,259,217,300,214,186,221,214,443,53,1073
RBFOX2_1_503,244,412,395,434,360,379,209,60,255,100,8,10
RCC1_1_504,286,265,532,290,397,721,312,194,832,288,99,148
RCOR3_1_505,720,293,898,672,58,464,960,368,91,242,2366,899
RECQL5_1_506,6723,5548,5164,6083,4582,6373,3971,5478,2919,4563,5792,41
46
RECQL_1_507,6877,6364,7711,8589,6727,11562,7508,5395,7861,5689,5771,12
637
RELA_1_508,593,661,741,685,559,701,681,585,915,1535,737,117
RERE_1_509,19,6,11,0,0,53,0,0,0,210,0,0
RFC1_1_510,3804,3605,4548,4659,4872,6004,4357,3588,9310,2878,4486,2918
RNF14_1_511,904,1087,1290,1354,2270,2033,1244,1163,1188,986,2031,2218
RNF17_1_512,3151,3106,3187,2680,2121,5322,1930,3084,1154,3435,3378,204
6

RNF40_1_513,945,829,842,1193,1023,907,41,862,62,1448,1263,999
RNF8_1_514,1111,565,1204,1334,3187,807,1803,1519,1813,1179,2006,752
RORA_1_515,4092,3868,5297,4382,3168,5657,7776,3964,5865,3460,5886,5923
RORC_1_516,1200,2095,1764,1117,629,952,1596,1702,2255,2796,605,623
RPH3A_1_517,2143,920,2640,1816,1284,1265,3494,702,1002,1278,1036,907
RPS6KA5_1_518,3656,3639,4147,3192,5700,4825,5601,2733,2641,4334,6254,5
033
SATB1_1_519,2028,1955,2233,2215,2941,2499,2950,3139,1341,2637,1573,224
1
SATB2_1_520,10858,9516,9209,9993,13565,10225,12016,6919,8050,7126,5625
,8309
SCMH1_1_521,549,545,517,370,741,740,491,1565,856,905,351,74
SET_1_522,4665,5107,7359,5653,8490,3441,7195,7399,4566,7072,4804,7573
SETD3_1_523,1334,1218,1473,1703,841,1928,1462,1414,1175,806,3181,227
SETD4_1_524,1927,1632,1767,1345,1590,2288,2523,1217,736,2103,1081,2996
SETD6_1_525,549,447,1167,910,3135,700,667,2070,236,507,1571,318
SETDB1_1_526,4233,3363,4727,3176,6026,2984,1772,4788,6169,4720,3844,36
34
SETDB2_1_527,1046,668,885,745,350,1587,534,990,496,1020,1195,103
SFMBT1_1_528,1221,474,1451,1344,731,888,2607,486,1396,789,2083,921
SFMBT2_1_529,605,695,1066,659,314,790,1592,872,1331,785,56,1028
SHPRH_1_530,4902,4165,5788,5742,3543,3932,5396,4757,7917,6238,3694,370
6
SIN3A_1_531,6031,5637,5779,6062,6844,5861,5662,4780,4557,6675,3873,530
5
SIRT1_1_532,2995,3578,4007,3436,4329,3561,3024,4070,2184,3654,5074,572
4
SIRT2_1_533,307,313,283,457,560,180,126,577,5,1008,23,54
SIRT3_1_534,1462,976,1089,922,2739,1336,2302,983,652,841,1015,802
SIRT5_1_535,3263,3085,3853,3790,3193,2236,2600,3826,3282,3047,3949,114
7
SIRT6_1_536,209,198,645,296,431,395,9,14,37,1195,0,0
SLC38A1_1_537,4971,4650,5819,5280,6104,6848,7329,2928,6222,4109,3797,3
626
SMARCA1_1_538,3423,3104,3993,3869,3789,3760,3352,3451,2766,4012,4433,2
517
SMARCA2_1_539,5805,4969,4418,4511,6172,6561,5258,5252,5487,6413,4586,4
041
SMARCA4_1_540,1923,1978,2343,1765,3480,2191,2056,879,2824,2786,1974,38
7
SMARCAD1_1_541,1691,2181,1953,1909,2288,2193,471,1314,363,1583,1140,16
82
SMARCAL1_1_542,2872,2579,2974,2546,4816,3156,4945,1383,2450,3827,3855,
889
SMARCB1_1_543,910,852,1188,1437,1208,1077,616,273,1717,857,668,181
SMARCC2_1_544,4060,3137,4320,4309,7042,4891,3545,2560,5555,4058,4710,4
787
SMARCD1_1_545,4832,3800,4659,5070,4217,4619,4040,3049,5986,3341,3077,5
951
SMARCD3_1_546,245,143,47,12,0,1,6,4,0,13,3,18

SMC2_1_547,6356,4668,5936,6195,7660,6247,4812,6334,2201,6449,5361,9563
SMC4_1_548,1261,704,858,1083,1556,843,1267,524,627,581,1666,30
SMN1_1_549,2479,3119,3050,3563,4646,2341,3279,3537,224,2613,2227,2652
SMN2_1_550,2479,3119,3050,3563,4646,2341,3279,3537,224,2613,2227,2652
SMYD3_1_551,1202,1020,1053,1084,1394,1919,831,2923,869,1623,530,2629
SP100_1_552,6971,6094,6284,6037,7092,6840,9564,4139,8609,6716,7421,958
6
SP110_1_553,1182,1422,1280,1047,1459,1629,2300,1493,1648,1503,779,334
STK31_1_554,1547,834,668,1213,1941,1484,1286,661,925,640,2032,1375
SUPT3H_1_555,1316,845,1004,1439,786,1702,2030,725,1907,1533,1397,824
SUPT5H_1_556,1624,1581,1577,1646,731,1755,2841,1710,1592,880,3243,2665
SUV39H2_1_557,1289,651,611,894,1602,1106,1492,219,721,365,915,3
SUV420H1_1_558,714,734,1258,454,1122,559,446,306,336,889,604,137
TADA2A_1_559,3112,2739,2742,2318,2393,1693,5727,1584,2221,1447,2032,76
22
TADA3_1_560,393,211,264,329,867,1085,4,60,562,43,484,387
TAF12_1_561,2385,2020,1944,2306,3505,2630,2684,2696,1997,2739,2178,742
TAF15_1_562,7213,6212,8030,6967,7536,6511,13306,6839,3860,5466,4168,51
34
TAF1_1_563,2072,2081,1570,3073,4078,2294,3398,997,250,2779,46,2181
TAF5L_1_564,526,353,211,445,257,1006,69,781,1152,61,571,242
TCF19_1_565,231,199,119,668,279,108,481,97,969,631,327,145
TCF20_1_566,2059,1614,2636,1683,1245,3439,959,1904,1986,3291,3514,3962
TDRD10_1_567,722,722,594,825,2208,874,141,303,933,161,822,459
TDRD3_1_568,308,701,300,442,116,126,546,143,41,267,7,510
TDRD5_1_569,4118,3948,3844,4440,3896,7287,5497,2618,2247,4365,4554,376
9
TDRD6_1_570,3646,4719,4336,4476,5211,7193,5098,4269,6527,4844,3460,307
2
TDRKH_1_571,943,860,1536,1171,386,319,3860,518,2130,1277,1633,1018
TERF1_1_572,862,1234,1346,612,1335,1290,1730,1729,568,777,466,176
TET2_1_573,5848,5055,6883,5188,6127,5800,7366,5696,9640,7823,6766,8488
THRA_1_574,1486,1282,828,1586,2001,693,1654,1193,3647,1071,850,1530
THRB_1_575,256,196,128,64,55,440,243,177,0,442,13,10
TP53BP1_1_576,2720,3787,3373,4551,3978,2429,4008,2577,1402,3665,3505,4
345
TP53_1_577,2411,3200,3008,3233,3411,5149,4069,1954,3618,4382,4176,2728
TP73_1_578,606,1034,766,1248,906,211,657,1192,918,616,122,1266
TRIM24_1_579,2997,3143,2966,2577,4154,1228,5849,3016,3554,3134,2151,51
08
TRIM32_1_580,2527,2339,2337,2227,2934,1792,1125,1289,3365,1812,1345,13
90
TRIM33_1_581,1901,1150,2299,1632,3614,1696,2296,1785,3053,2291,2953,12
58
UBE2A_1_582,1783,1539,1908,1596,3767,2562,1521,1305,1857,1599,3158,165
4
UBE2E1_1_583,308,695,288,456,442,141,251,86,0,245,122,13
UBE2I_1_584,533,548,527,648,55,593,175,245,20,317,1266,177
UBE2K_1_585,1838,1465,2242,2599,3057,3275,1089,2693,4610,2406,2460,216
3

UBE2V1_1_586,3811,4918,4587,4807,4782,6096,6141,6316,5039,4120,4604,65
25
UHRF1_1_587,439,523,671,247,2122,536,841,1008,19,1057,63,47
USF2_1_588,358,841,337,464,720,108,294,1183,579,254,1137,181
UTY_1_589,1555,1282,1410,1590,2102,1494,3843,3645,3240,1693,517,2324
VDR_1_590,177,318,243,224,465,1273,544,19,93,847,1846,80
WDR5_1_591,748,347,353,184,12,617,67,63,480,31,170,1888
WHSC1_1_592,1480,1129,1318,1337,3271,919,1171,1327,563,1317,1440,1842
WHSC1L1_1_593,1037,1390,656,675,714,1205,217,332,546,810,310,154
WRB_1_594,240,261,174,293,10,379,8,528,514,43,1036,236
ZGPAT_1_595,1343,1303,2189,1469,761,1450,1702,2164,2771,782,570,1099
ZMYND11_1_596,1879,2052,1385,1555,1247,447,384,2769,610,2055,1831,1973
ZMYND8_1_597,1065,515,580,1127,1027,986,1062,617,202,503,66,496
ZNF451_1_598,1113,1249,856,1577,713,1841,827,433,1845,547,1273,513
ALG13_1_599,1365,1438,1071,1617,1239,1176,1605,1449,2086,1971,2175,253
6
ASXL1_1_600,1871,1453,2007,1070,2105,897,1030,1503,2949,1179,3453,430
CBX2_1_601,123,225,89,1,1,142,2,0,0,44,0,858
HDAC8_1_602,14654,12255,13635,16714,15293,18164,17919,13667,20003,1267
9,12656,15334
ING3_1_603,1954,1527,2789,2120,1633,2375,2477,2070,3447,1719,2403,7046
MLLT10_1_604,1993,2546,2129,2209,1440,2674,2541,1014,2872,1814,4184,47
21
PHF19_1_605,2290,1959,2257,2319,2973,1284,2662,3468,2344,2469,1131,311
3
RBM14_1_606,799,752,982,662,1294,1044,1413,628,1683,196,1147,282
SP140_1_607,3221,2301,3227,1993,3251,3069,2153,3332,2480,2383,3291,125
2
TAF9_1_608,15402,16319,18999,19270,16391,20676,19469,17086,15102,17955
,15469,19398
TAF9_1_609,6260,4475,5369,5346,5544,8045,5379,3866,7358,7195,4770,4379
AHR_1_610,1261,1314,1602,1441,1076,447,49,1157,1063,1454,1806,308
ALKBH1_1_611,601,1312,1521,579,2081,379,1212,74,2253,599,394,496
ALKBH3_1_612,673,726,667,759,665,1661,2384,843,200,324,138,412
ARID2_1_613,1410,1349,1149,1459,1326,1316,801,1062,1847,1510,730,1326
ASF1A_1_614,830,788,553,1021,467,2117,1398,611,716,806,25,630
ASF1B_1_615,451,398,744,499,830,369,284,62,364,502,32,904
ASH1L_1_616,1132,1187,1517,2048,1216,946,1593,1469,597,924,1696,1111
ASXL2_1_617,1442,1838,2126,1804,3013,2588,854,1089,1829,2268,2527,1192
ASXL3_1_618,2576,1203,1222,1812,2396,3486,827,931,1503,1432,1820,375
ASZ1_1_619,1381,882,1019,1471,896,2052,1080,686,2268,1665,1428,883
ATAD2_1_620,4265,4928,6598,4855,5548,6399,6870,2061,3298,5225,4592,201
6
ATF7IP_1_621,2615,1357,2156,1923,3514,1276,2957,3067,1577,1555,932,339
8
AURKB_1_622,584,715,1063,573,1106,660,1554,145,1829,734,1697,229
BAHCC1_1_623,295,377,917,231,25,179,1484,12,360,6,85,4
BAHD1_1_624,1015,963,1413,1561,2103,882,442,694,1053,1963,962,697
BARD1_1_625,1747,1897,2397,2171,2266,3631,1147,1326,2856,2418,1448,365
6

BAZ1B_1_626,2342,2072,1395,2505,2387,1354,2447,1695,913,787,1528,390
BAZ2A_1_627,1176,1561,1345,1337,1139,1419,592,944,232,1389,5408,1768
BAZ2B_1_628,2131,2451,2441,1854,3366,2359,2371,1626,3403,3189,2816,224
7
BLM_1_629,2400,1827,2846,1818,1440,1957,1026,2425,1314,1445,3195,1461
BMI1_1_630,2134,1771,3127,1441,1439,2079,4813,1759,2771,1563,3227,2140
BRD1_1_631,465,999,947,663,524,1088,342,316,0,676,0,1674
BRD3_1_632,682,1089,1059,1505,590,1247,1262,1301,661,742,346,239
BRPF3_1_633,589,594,988,580,1257,844,1032,255,887,602,1640,868
BRWD3_1_634,1744,1393,2555,2235,1113,916,4191,616,4079,2194,633,1877
C14orf169_1_635,883,1142,1141,1202,963,412,501,1752,1056,956,537,423
C20orf20_1_636,4810,3131,4421,4302,4850,5673,5085,3270,3326,4861,5233,
1727
CALR_1_637,117,249,182,242,47,12,36,260,0,157,12,0
CARM1_1_638,3418,3107,2762,3911,2639,4742,2178,1903,1246,2240,2359,450
0
CBL_1_639,559,733,408,1106,127,1581,333,1472,460,603,45,969
CBX4_1_640,998,1037,1121,893,1065,820,241,1331,1558,966,719,1744
CBX6_1_641,248,280,321,304,310,243,168,121,1,210,265,106
CBX7_1_642,500,549,624,1158,8,1236,1139,557,27,666,230,148
CBX8_1_643,591,655,954,430,1506,313,289,1136,2597,977,477,403
CCDC101_1_644,600,355,905,453,428,571,257,134,465,1419,100,359
CCNE1_1_645,1166,745,913,967,2843,947,19,514,1174,1300,632,374
CCNT1_1_646,801,553,413,442,1555,187,1440,248,271,552,55,1907
CDC73_1_647,4464,3756,3969,4764,7156,2779,3500,2894,2165,2826,4527,281
6
CDK9_1_648,2625,2695,3497,2015,4838,3603,2805,3308,2989,2203,2708,2887
CDY2A_1_649,1057,1061,1471,1564,372,601,966,3114,162,1364,1801,37
CDY2B_1_650,1057,1061,1471,1564,372,601,966,3114,162,1364,1801,37
CDYL2_1_651,1906,2003,2928,1939,2002,2428,2881,1598,1197,2027,681,1733
CECR2_1_652,3006,3568,3565,2509,5061,3358,4149,2752,1343,3056,2281,276
7
CHAF1A_1_653,334,102,351,264,78,119,281,89,98,283,10,4
CHAF1B_1_654,232,157,41,362,5,2,21,1,21,5,0,0
CHD1_1_655,4549,3898,5530,3692,5389,3941,7832,4157,8028,4813,5576,4458
CHD1L_1_656,900,1446,916,934,897,715,1801,486,1139,962,818,7
CHD4_1_657,1012,375,588,164,306,211,456,110,29,77,1,781
CHD6_1_658,0,0,0,0,0,0,0,0,0,0,0,0
CHD7_1_659,551,890,755,462,99,1164,2220,212,535,1495,491,50
CHD9_1_660,1739,1923,910,838,588,1074,2543,1295,160,1580,2584,1057
CHMP1B_1_661,1201,835,1132,1310,1778,1234,900,410,1311,1081,1715,858
CHMP4B_1_662,1566,1946,1651,1990,1421,1766,993,1844,3145,2099,606,280
CHMP4C_1_663,1488,3237,3155,2824,6082,2185,3981,2459,3519,1347,2522,14
53
CHRAC1_1_664,235,131,153,58,162,25,172,501,256,96,206,234
CLOCK_1_665,635,527,606,634,492,840,1923,947,977,689,656,437
COPS5_1_666,433,579,660,683,47,1004,2222,1006,439,1214,367,1151
CRAMP1L_1_667,357,256,435,259,171,56,712,625,423,320,92,220
CTCFL_1_668,3571,4073,3939,4500,4218,3421,3473,5448,4439,4787,3280,215
5

DEAF1_1_669,1013,657,569,1373,678,1553,798,345,509,298,951,1370
DNAJC1_1_670,8,26,19,15,0,13,0,21,0,5,209,34
DOT1L_1_671,2581,2627,3377,2297,1797,3399,5033,1481,3061,3712,3087,108
7
DPF2_1_672,952,822,1000,1196,1128,1125,633,286,779,670,875,992
DPF3_1_673,952,714,582,1046,389,629,1579,26,15,536,257,425
EIF4B_1_674,140,216,521,545,62,67,65,42,443,386,65,109
ELP3_1_675,5830,6376,5230,4369,10691,3655,4669,4837,5519,5615,3860,658
6
EP300_1_676,3101,2182,3560,2971,3980,2452,3808,3281,5563,4220,4434,256
3
EP400_1_677,311,429,198,439,239,129,32,240,83,645,473,615
EPC1_1_678,3713,3649,4037,2641,5684,4028,5573,1013,1283,3001,3229,2809
EPC2_1_679,3048,3347,4019,3025,5047,4507,2141,2297,1429,3051,881,3752
ERCC6_1_680,1554,1447,3189,1456,1311,1517,762,2029,644,1296,4908,1701
ESRRA_1_681,399,339,356,222,76,258,1379,16,444,362,16,148
ESRRB_1_682,115,256,162,349,1329,2,7,0,51,185,61,0
FANCM_1_683,296,25,93,262,459,111,28,18,3,2,8,459
FBXL19_1_684,686,801,611,447,62,421,1539,1075,190,321,138,211
FOS_1_685,4337,2991,3943,3552,4671,4121,3444,3906,3285,2779,4511,3625
FTO_1_686,1006,1054,2010,726,618,1200,585,757,1228,973,1840,774
FXR2_1_687,963,1036,907,613,209,1843,435,900,1206,1295,2583,844
G2E3_1_688,1952,1020,2294,2488,3735,2408,2957,2494,1050,3179,2602,3293
GATAD2A_1_689,866,509,832,536,109,837,263,325,187,465,98,437
GLYR1_1_690,4845,5424,6800,6722,7566,7759,5710,3736,5163,4724,2471,542
9
GMEB2_1_691,3778,4830,4867,6680,5290,5746,7498,3244,5213,3914,4785,429
4
GSG2_1_692,3622,3515,3701,3899,5046,2860,1904,2723,2808,4085,1483,1606
GTF3C4_1_693,1402,1081,1886,1726,958,1017,1451,2805,1769,803,2450,612
HAT1_1_694,1394,678,1528,946,1027,738,663,1640,501,805,1437,713
HDAC1_1_695,1236,803,1564,1249,2181,1596,1799,1552,2022,1248,545,912
HDAC2_1_696,694,613,1007,964,1492,678,1289,686,664,1739,683,498
HDAC3_1_697,1436,1794,1591,1249,1642,1773,2162,1849,1099,2113,701,1736
HDAC4_1_698,131,138,300,392,199,223,44,45,0,3,0,133
HDAC6_1_699,403,820,1139,592,2248,276,136,69,0,218,127,326
HDGFL1_1_700,448,831,1397,419,306,1489,887,82,46,114,344,626
HDGFRP3_1_701,1076,2084,2242,1142,1011,1279,1345,1016,1646,1610,2959,2
493
HELLS_1_702,2900,2706,2841,2247,3571,2998,765,2884,4299,1767,1505,1369
HEMK1_1_703,1527,1194,846,928,2222,1111,1013,814,1786,1522,1583,755
HIF1AN_1_704,3421,3277,3952,3889,5433,3016,9946,2934,2713,2651,4402,36
45
HIRA_1_705,640,1261,737,1196,85,1298,659,10,1180,1312,106,28
HMG20B_1_706,3737,3069,5453,3720,4460,3728,4660,3376,3629,1257,4028,48
23
HMGN5_1_707,736,861,1086,1210,796,907,454,998,1088,86,554,1016
HNF4G_1_708,1359,1434,1998,1527,1739,1178,1725,1010,1009,1763,2095,171
5
HSPBAP1_1_709,863,998,1491,906,1275,1599,1352,471,1828,1050,1204,936

HUWE1_1_710,3347,3764,5007,2954,4062,2198,2301,2641,4480,3709,1950,242
2
ING5_1_711,322,144,953,493,869,74,52,474,1263,716,2718,394
INO80_1_712,1171,2065,1800,1490,1775,875,1724,930,2,1694,123,578
JARID2_1_713,384,526,241,198,553,792,623,1870,0,21,143,2
JHDM1D_1_714,2089,1277,1181,1600,961,530,3913,1371,923,1866,732,2425
JMJD7_1_715,613,627,981,1158,888,544,1339,946,35,599,958,2308
JMJD8_1_716,1131,1094,1146,1073,416,451,1764,765,2711,1806,1497,28
JUN_1_717,753,501,599,533,116,291,518,138,472,386,731,94
KAT2A_1_718,860,684,933,1074,1316,352,1062,1256,529,396,2714,1216
KAT2B_1_719,2071,2479,2833,2206,2992,3480,2734,3153,3604,2669,4556,346
0
KAT6B_1_720,1837,1460,1190,2107,1971,2687,1539,615,1875,1913,4198,2037
KDM1B_1_721,1561,1110,2040,1804,575,1578,1602,998,1077,1928,255,449
KDM2A_1_722,42,7,69,172,683,172,0,1,14,0,255,7
KDM3B_1_723,627,317,462,537,210,793,6,168,2,544,15,254
KDM4A_1_724,3317,3846,3602,3361,5033,3655,5192,2297,6775,3335,5010,183
9
KDM4B_1_725,45,32,13,65,2,57,2,7,0,13,2,2
KDM4D_1_726,742,326,1146,425,68,180,180,833,707,419,738,333
KDM5A_1_727,1912,2451,2674,812,2344,2168,3222,1394,1569,2436,1474,1248
KDM5B_1_728,1153,1312,1108,1381,1246,1035,2055,641,2828,1384,1167,5113
KDM6A_1_729,6377,6459,8271,5872,6926,8924,8698,6562,5119,5867,9950,792
9
KDM6B_1_730,988,1174,696,1274,1570,2147,907,1983,753,1469,1309,559
KIAA2026_1_731,651,797,1065,787,1394,856,138,279,347,443,531,65
L3MBTL2_1_732,1675,1413,1499,1450,1235,1600,2459,2289,2707,986,2017,71
4
L3MBTL4_1_733,1250,1028,1325,1340,1283,3101,1801,1778,3538,2761,2416,1
081
MAEL_1_734,348,166,335,416,855,454,142,274,287,95,350,8
MBD3_1_735,570,690,756,978,1286,109,168,1844,1,386,366,104
MBD4_1_736,4550,4407,3924,4213,3420,5084,3592,1590,4331,2198,6599,4056
MBD5_1_737,1327,833,742,808,1216,1025,368,539,1610,801,1082,1818
MBD6_1_738,296,485,636,332,739,310,963,1441,60,283,229,3184
MBTD1_1_739,5578,4928,6736,5591,5141,5785,11928,6044,10008,5446,2399,1
1373
MDM2_1_740,1150,1408,1669,1174,1793,1400,2698,1662,2366,794,1441,1650
MIER2_1_741,332,312,210,271,127,545,505,473,1,129,10,580
MIER3_1_742,610,662,585,783,580,171,343,161,2,895,236,1178
MIS18BP1_1_743,10704,11310,9830,10287,12040,11727,9115,8036,8597,9896,
8530,5267
MKL1_1_744,438,803,688,516,223,533,1353,75,145,1002,743,1408
MLL2_1_745,335,235,275,312,62,211,896,205,9,389,1,780
MLL3_1_746,3523,1695,2237,2806,2766,2025,5153,2555,1201,3633,1089,1863
MLLT1_1_747,546,342,540,527,1951,753,529,582,380,1839,977,787
MLLT3_1_748,650,614,577,367,52,151,546,231,38,275,378,69
MLLT6_1_749,740,419,727,431,1741,816,712,912,943,632,1601,1125
MPHOSPH8_1_750,1109,1071,1270,1467,730,679,1088,248,1542,584,2445,551
MSH6_1_751,1994,1520,1943,1714,1333,2432,1145,806,245,983,1351,1473

MSRB2_1_752,2302,2082,1165,1791,2051,2128,1914,1299,1287,2854,3210,530
MTA2_1_753,1655,2522,3685,3158,2767,2187,2085,1763,4296,3755,1329,2554
MTA3_1_754,3404,4205,5124,4483,5128,5268,5961,6564,6064,2832,1649,3061
MYSM1_1_755,4835,4374,4627,5276,7810,4116,6003,4230,4864,3234,3954,765
1
NAP1L2_1_756,1126,890,1029,1375,330,776,2030,651,762,1738,1103,1512
NAP1L3_1_757,2952,3004,3138,3336,3822,3372,4013,4018,3653,2294,4162,33
98
NAP1L4_1_758,1564,1237,1868,2290,1667,2158,2008,1770,973,1702,1933,141
9
NAP1L5_1_759,117,319,206,335,188,112,583,62,22,689,620,0
NAT14_1_760,38,241,239,654,79,174,0,179,15,20,0,1952
NAT8B_1_761,1322,1260,1594,2097,1100,2385,1149,472,2054,1635,2712,1396
NAT8_1_762,1297,874,1514,1081,1904,1424,2380,843,1697,1362,1378,417
NAT8L_1_763,1128,430,1714,1450,315,343,2666,639,384,250,15,1119
NAT9_1_764,338,779,767,309,1071,879,1973,613,1984,1769,3,79
NCOA2_1_765,6556,6315,7184,6118,6206,5228,6521,4125,6825,5554,9609,907
9
NPTXR_1_766,455,373,239,637,886,140,1025,519,25,67,814,0
NR0B1_1_767,254,295,597,516,188,673,618,285,145,310,168,259
NR0B2_1_768,6253,6801,6798,7725,4857,6187,7726,8118,1829,6140,4857,570
6
NR1D1_1_769,1624,922,1088,1105,3838,487,2931,1862,1418,804,2650,807
NR1H2_1_770,1859,2774,3747,1987,2001,2070,3971,1301,2327,4009,1534,158
4
NR2C2_1_771,924,946,1147,849,1209,1079,256,1267,1028,1084,325,1550
NR2E1_1_772,1237,1033,1160,1514,1660,688,1886,1440,1304,754,1315,1459
NR2F1_1_773,1273,900,1209,952,1433,2526,1179,1105,1401,1422,271,547
NR2F6_1_774,1843,2282,1641,2105,977,1537,1825,693,2552,1473,1866,1036
NR4A2_1_775,398,450,643,375,1246,852,0,158,818,562,650,8
NR5A1_1_776,396,375,305,365,111,94,942,117,88,284,424,674
NRIP1_1_777,3759,2810,3423,4348,3735,5090,3051,3839,3601,4477,4948,482
8
PADI4_1_778,1896,1386,1386,2028,1309,1711,2737,1834,698,1059,1506,1757
PAWR_1_779,1149,1397,1279,1075,1998,1150,485,1146,1781,1685,617,642
PAX5_1_780,403,550,151,73,0,363,1,0,7,0,0,407
PAXIP1_1_781,1286,1013,1038,793,1149,1141,590,1248,1305,1245,1305,1402
PGRMC2_1_782,11994,11654,12858,13544,13501,12942,16356,11023,10768,106
73,12375,10193
PHB_1_783,1320,468,1341,522,1130,1042,116,1529,41,943,3148,1270
PHC3_1_784,1681,1085,1080,1369,1109,1338,1793,695,326,1701,1317,1737
PHF13_1_785,441,353,115,109,243,530,130,1,533,157,0,15
PHF14_1_786,1443,1184,1385,2340,978,423,1934,1093,859,249,979,4883
PHF15_1_787,167,318,292,533,153,170,219,79,701,180,16,399
PHF20_1_788,593,282,331,619,502,871,190,488,1431,545,41,718
PHF23_1_789,602,714,1062,586,510,2018,1013,270,1123,406,741,230
PHF2_1_790,92,168,22,62,1,61,0,0,0,0,0,0
PHF3_1_791,1011,859,1165,861,774,1758,488,76,2780,402,320,1232
PHF5A_1_792,5910,8026,9109,9644,6346,10774,5690,5934,7585,8061,6254,57
47

PHIP_1_793,1695,1074,686,678,556,1191,718,1797,14,280,403,320
PHRF1_1_794,835,894,997,1265,400,868,1599,413,125,885,416,2690
PIAS1_1_795,966,1207,2029,2123,2965,1816,1693,1852,1536,1225,1897,700
PIWIL4_1_796,5156,5578,6614,6795,8467,8320,7905,5469,2132,4070,5853,78
83
PPARGC1A_1_797,2278,2417,2453,4023,3794,1892,2240,2213,258,1906,2107,7
951
PRDM11_1_798,358,351,467,173,751,326,122,407,656,807,1,12
PRDM12_1_799,3479,2862,2938,2919,4355,3439,2429,3337,2632,3075,2496,24
85
PRDM13_1_800,706,377,590,1546,523,1400,1258,261,496,1100,134,870
PRDM14_1_801,337,417,719,32,1,674,283,38,426,86,2722,490
PRDM4_1_802,0,0,0,0,0,0,0,0,0,0,0,0
PRDM5_1_803,1584,1513,2095,2456,2183,2000,3689,1439,1175,1276,1044,186
5
PRDM6_1_804,2520,2213,2568,1482,1808,2588,1243,1995,1432,2352,2165,139
3
PRDM9_1_805,967,1231,1466,1995,1813,940,2233,464,2396,1091,613,753
PRMT6_1_806,856,802,928,847,850,1262,1404,1399,1814,298,2513,745
PRMT8_1_807,1837,1397,1713,1129,1744,2161,2134,2208,412,1047,2594,402
PRPF6_1_808,573,880,979,963,435,413,1099,72,497,673,720,600
PYGO1_1_809,758,1046,1222,828,1247,1003,736,1236,572,249,630,899
PYGO2_1_810,2501,1810,3589,2588,1695,2150,3092,732,3905,1637,2173,2400
RAG2_1_811,2221,2572,2120,2688,6376,3661,1174,2610,3068,2838,648,3329
RAI1_1_812,4710,4074,4068,4140,4289,2674,6729,4127,4014,3786,7633,1952
RB1_1_813,3541,2693,3385,2307,3447,3507,3826,3008,2372,2509,5336,1538
RCOR1_1_814,3953,2782,2962,2517,2495,3594,4197,2392,3077,1766,1816,431
2
RCOR2_1_815,2370,1513,2112,1486,3590,1541,1953,1237,1699,2976,1143,130
8
RECQL4_1_816,1314,2021,2502,1615,2646,1914,1469,1884,3351,1940,668,258
5
RELB_1_817,1092,982,2099,732,3273,653,953,1355,306,2769,1423,467
REL_1_818,1910,1582,1787,1360,3189,1144,2178,971,1420,1223,654,357
RING1_1_819,1248,1405,1500,1531,704,1967,3030,1172,1355,2049,1048,2348
RNF20_1_820,976,642,1734,493,67,421,1224,484,200,1212,500,269
RNF25_1_821,1813,1975,2348,1969,3903,2982,4893,581,3856,2257,497,1812
RNF2_1_822,1067,1765,1208,1047,624,2154,2669,2515,940,1366,904,889
RORB_1_823,2030,2123,3569,2663,2681,2611,5434,1708,2502,2815,891,4146
RSF1_1_824,1588,1357,1609,1180,1477,1336,514,1837,1257,1629,1064,676
RUVBL1_1_825,409,340,987,244,282,159,864,37,703,15,280,38
RUVBL2_1_826,321,586,152,302,1985,341,544,757,12,1362,22,76
RXRA_1_827,689,617,462,416,1496,244,187,595,330,522,315,609
RXRB_1_828,6295,4947,6619,5903,12066,6166,4681,5205,8480,7481,4231,759
1
RXRG_1_829,1021,812,1215,925,775,1501,312,1028,1284,538,2809,1782
SAP18_1_830,613,764,1708,1022,944,397,1005,155,335,378,242,519
SCML2_1_831,3308,1541,2649,2667,3052,2271,2503,1724,2276,2917,6102,599
SENP3_1_832,757,693,1258,1460,627,1173,2336,1594,1535,1027,6,1274
SETD1A_1_833,1400,930,1432,1848,1621,823,2599,2716,1769,1885,2146,3101

SETD1B_1_834,741,923,441,555,782,538,779,525,645,694,390,1
SETD2_1_835,619,547,1068,392,1171,636,709,1084,834,1019,466,233
SETD5_1_836,0,3,1,0,0,0,1,0,0,0,0,2
SETD7_1_837,1305,1339,1200,1735,1205,1705,2325,1379,985,910,1169,2582
SETD8_1_838,1255,1504,2464,1575,1933,1304,1307,241,583,1404,1401,414
SETMAR_1_839,18,66,24,31,0,298,9,1170,498,158,0,0
SF3B3_1_840,2210,1566,1618,2127,2834,2210,2739,1853,653,1725,1396,1156
SIAH2_1_841,142,569,636,429,17,638,659,55,87,36,13,134
SIN3B_1_842,431,737,974,759,1427,1425,613,779,648,830,1895,537
SIRT4_1_843,143,140,156,122,0,400,0,0,0,1,0,0
SIRT7_1_844,897,445,361,596,663,1049,11,871,704,330,349,0
SLC2A4RG_1_845,413,397,879,584,240,515,707,443,15,186,313,319
SMARCA5_1_846,2848,2713,3156,3227,4089,3209,5859,870,1442,3596,5332,15
04
SMARCC1_1_847,1395,1307,1789,1502,1204,1213,1038,2662,1479,1706,2187,1
502
SMARCD2_1_848,1500,706,867,655,4388,973,1475,1804,746,939,1559,757
SMARCE1_1_849,77,767,628,775,954,237,1362,119,240,23,54,1
SMC1A_1_850,1299,1379,1692,1666,3719,990,3150,2567,1721,920,116,972
SMC1B_1_851,5484,5731,6287,8106,10086,5826,10200,5281,7594,5623,7979,3
027
SMC3_1_852,1382,831,2608,891,909,767,3353,465,1348,687,929,1039
SMCHD1_1_853,2011,1791,2538,2250,4601,2912,1507,3269,3482,1536,2414,46
79
SMNDC1_1_854,877,549,1136,1400,550,1058,2949,260,604,912,1621,1353
SMYD1_1_855,1452,991,1227,1209,1377,1044,312,555,175,1240,1104,1306
SMYD2_1_856,781,487,1557,586,1074,641,2499,338,637,663,470,972
SMYD4_1_857,2653,1862,1930,2812,2989,1940,2416,1421,3976,2536,1421,450
0
SMYD5_1_858,2623,2600,2323,2409,5198,3986,1926,1504,1008,2661,3755,249
9
SND1_1_859,260,220,1044,340,115,1481,1011,615,169,804,1961,111
SP140L_1_860,7689,6664,6864,6434,7103,8076,14880,6329,4187,5437,6949,5
644
SRCAP_1_861,1767,1580,1619,1686,1543,785,3186,1989,1470,1020,874,2284
STAT5B_1_862,697,1209,562,547,700,1301,301,206,292,837,1925,799
SUDS3_1_863,942,1114,1161,1387,1322,2531,1799,1179,1593,704,817,1918
SUPT16H_1_864,460,507,60,675,52,176,1685,158,1291,54,0,90
SUPT4H1_1_865,326,176,319,242,993,139,30,76,481,427,1192,1354
SUPT6H_1_866,660,908,333,294,615,287,1528,953,720,924,1290,472
SUPT7L_1_867,1857,2382,3245,2693,3173,2841,4161,2316,1985,2006,2983,37
50
SUV39H1_1_868,370,256,416,155,747,373,169,149,71,683,103,86
SUV420H2_1_869,1334,1967,1615,2450,1316,1458,2496,1776,3161,1536,1776,
970
SUZ12_1_870,666,466,302,405,553,416,873,445,749,723,616,109
TADA1_1_871,1194,1201,1109,1933,826,1450,1334,826,1092,1066,201,1080
TADA2B_1_872,2908,2547,2630,2772,1674,2701,1722,2342,3684,2192,3079,34
64
TAF10_1_873,407,408,539,268,787,789,31,137,206,414,570,895

TAF1L_1_874,6491,4787,6638,6814,7233,8679,9746,8440,9242,6095,4001,773
3
TAF3_1_875,7317,7251,7266,7677,7674,7690,6859,8488,5089,6869,7295,1029
7
TAF5_1_876,8146,8855,9411,7578,13989,10872,11006,7713,7653,7790,7369,8
654
TAF6L_1_877,1106,1067,1319,1715,755,1318,921,2062,225,829,5381,1197
TAF8_1_878,533,1519,1063,1362,2581,1932,1619,457,1758,1137,992,168
TCF7L1_1_879,76,31,68,32,33,21,83,0,104,0,1064,0
TDRD12_1_880,1277,1275,1084,1425,1346,1443,2512,1858,2527,1878,870,102
2
TDRD1_1_881,3121,2298,3157,2622,3232,3610,3519,4101,2907,3462,3073,267
9
TDRD7_1_882,3837,3877,4734,4992,3647,5864,4649,3294,4538,4930,3359,379
7
TDRD9_1_883,1354,1404,918,1575,2157,899,935,1813,1805,593,1681,1662
TERF2_1_884,336,372,349,479,15,417,741,368,342,353,369,1
TET1_1_885,2997,2351,4008,3594,4260,4756,6864,4926,4035,3027,3510,3788
TET3_1_886,471,358,745,511,337,588,993,400,18,1360,203,219
TLX2_1_887,355,313,351,224,230,60,1568,60,390,199,9,0
TNRC18_1_888,422,908,470,595,292,545,714,1147,498,200,471,1406
TRAF7_1_889,198,120,113,159,358,428,403,19,71,92,0,64
TRDMT1_1_890,3248,3165,4408,2668,5587,3418,5184,1622,1787,1315,4209,21
42
TRERF1_1_891,465,798,639,557,1002,356,1577,258,484,787,1117,580
TRIM25_1_892,2253,1518,1937,1987,3504,1011,3393,938,1556,1532,3109,255
2
TRIM27_1_893,2290,2823,3101,3036,4178,2313,4463,3017,670,1994,3312,533
1
TRIM28_1_894,1021,1122,1374,688,1370,942,1010,242,1071,989,164,615
TRIM66_1_895,272,354,822,177,562,0,511,39,80,293,1,169
TRRAP_1_896,1701,1503,1410,1138,809,1183,1047,903,3186,512,14,998
TSG101_1_897,2068,1253,1394,1750,2106,1476,863,256,3343,1550,1925,392
TYW5_1_898,276,415,501,424,377,395,342,171,1890,431,24,626
UBE2B_1_899,716,1001,883,662,244,1545,146,2243,351,1080,557,96
UBE2N_1_900,5484,4689,5857,5449,10038,6243,6483,4340,5925,3187,8000,35
35
UBR7_1_901,489,486,926,661,463,550,471,499,525,586,3,686
UHRF2_1_902,2379,1791,2067,2600,586,497,2187,2119,1712,575,2371,1534
USP22_1_903,4535,5164,5809,5089,6658,4855,4518,4101,6374,3685,5891,602
8
YEATS2_1_904,2109,2019,2064,1809,4575,3123,1725,2336,2077,1435,2940,35
68
YEATS4_1_905,1049,1230,1396,1364,1012,753,5324,1520,2615,2053,2239,640
YY1_1_906,1104,1402,1306,2194,997,1419,2875,909,783,2165,272,2150
ZAR1_1_907,3076,4398,4808,4898,5281,5233,1812,2538,2803,3884,1335,5402
ZCWPW1_1_908,3392,3326,4964,4861,3831,8089,4100,2066,2369,3680,6486,42
55
ZCWPW2_1_909,7133,5831,8062,7192,8376,9212,4942,6881,6922,5214,7879,60
18

ZFP57_1_910,961,791,1054,910,637,1405,792,631,585,1783,2079,538
ZNF541_1_911,4529,3574,5127,4072,3879,5023,5397,3865,6765,5446,3032,36
83
ZNF85_1_912,2021,1838,1976,1962,3829,2318,1002,1237,968,2070,3920,1866
AANAT_1_913,619,846,439,760,1991,443,652,42,87,1568,420,923
AES_1_914,803,724,889,609,976,1028,996,564,165,674,7,344
AIRE_1_915,172,162,252,8,306,170,1325,0,1,10,18,19
AKAP1_1_916,1822,688,1027,1438,694,1478,2194,1855,836,2541,387,519
ALKBH2_1_917,1592,1620,1611,1632,528,1712,1265,1951,1264,2308,2113,154
6
ANKHD1_1_918,964,456,937,798,156,417,824,191,667,1271,1210,976
ARID1A_1_919,69,158,284,347,193,79,516,47,9,209,21,137
ARID1B_1_920,1431,884,1090,994,1887,1851,475,1834,119,1083,1302,592
ARID4A_1_921,257,292,244,301,575,627,94,333,16,97,429,1281
ARID4B_1_922,917,603,742,1148,263,911,362,1159,2375,2431,764,3058
ARRB1_1_923,87,211,266,139,23,17,3461,2,0,373,0,0
ASH2L_1_924,459,592,324,645,1442,1237,289,1077,38,1556,244,131
ATAD2B_1_925,3832,2911,4387,3074,5034,5168,3805,2401,3712,3288,2255,27
25
ATAT1_1_926,811,1242,710,749,734,1807,690,788,1344,646,174,896
ATRX_1_927,1590,1176,1316,1826,2173,784,889,1296,2259,827,956,1327
AURKA_1_928,2068,2000,2961,2894,2641,2563,3930,1900,1435,1607,3330,168
0
AURKC_1_929,466,710,1012,766,662,1135,151,205,88,236,939,219
BAZ1A_1_930,4252,3968,4258,3015,4783,5141,3867,3254,5796,3075,3539,386
9
BCOR_1_931,787,500,997,839,938,423,1110,1249,2357,643,805,1259
BPTF_1_932,664,716,496,971,1652,798,254,225,164,520,356,1326
BRCA1_1_933,697,707,1022,1253,520,1286,1505,909,912,1002,665,627
BRD2_1_934,1481,757,1654,1249,1630,1527,1051,1310,3172,1337,601,1528
BRD4_1_935,2918,3405,3895,3584,2281,4262,2984,3488,2662,3543,2440,3876
BRD7_1_936,236,247,565,111,10,359,95,403,720,984,552,0
BRD8_1_937,913,285,723,502,430,878,915,380,246,354,480,1273
BRD9_1_938,113,239,290,73,190,22,22,280,62,72,214,32
BRDT_1_939,1684,1417,2397,804,1472,1125,709,2291,1438,2155,2108,2266
BRPF1_1_940,3599,4293,2529,3194,3334,3346,902,1317,3304,2474,722,6369
BRWD1_1_941,775,679,531,810,169,458,1434,522,104,695,65,789
C14orf43_1_942,1768,1199,1542,1021,957,1174,1138,1197,401,971,1904,228
9
CBX1_1_943,991,611,801,1572,2063,972,1387,399,532,344,250,1189
CBX3_1_944,595,407,1043,758,1617,1238,1101,1247,949,524,690,468
CBX5_1_945,1646,1290,1468,2119,1105,1257,1419,1431,2072,759,311,357
CCNT2_1_946,968,967,1219,963,1347,1140,1978,2160,660,977,1458,1235
CDY1B_1_947,68988,63636,73874,67580,85858,84090,88424,58494,72450,6203
4,60779,85591
CDY1_1_948,68988,63636,73874,67580,85858,84090,88424,58494,72450,62034
,60779,85591
CDYL_1_949,386,266,649,843,611,206,371,230,595,946,457,2052
CHD2_1_950,3333,3443,5262,3808,5578,4907,2549,3050,2916,3242,3769,5105
CHD3_1_951,1806,2147,2350,1935,2990,2365,1765,1250,3173,1535,3167,3815

CHD8_1_952,2215,2075,1870,3181,1974,3026,3992,1979,1983,3009,828,3941
CHMP2A_1_953,1004,1384,2074,1393,1155,1367,3968,1451,496,1030,1013,513
CHMP5_1_954,758,746,1129,1733,726,1049,828,297,651,1506,69,2776
COPS2_1_955,322,582,320,478,895,676,489,318,989,632,486,651
CPA4_1_956,1266,1657,1133,730,1160,1074,1309,519,100,988,675,688
CREB1_1_957,680,672,573,1365,324,815,890,994,1980,877,1184,276
CREBBP_1_958,772,239,455,105,164,1155,185,25,7,304,1583,1180
CTCF_1_959,1273,1176,2130,1289,2171,2107,682,517,1562,1330,1395,994
CTNNB1_1_960,1747,1764,1777,2453,3103,2248,1738,765,1685,2239,3707,753
5
CXXC1_1_961,183,159,69,145,390,9,5,85,1114,23,76,54
CYLD_1_962,603,633,627,382,550,580,239,1434,442,381,1081,283
DICER1_1_963,3595,3586,4131,3776,8076,3202,3738,2838,2376,2405,3375,34
80
DIDO1_1_964,2263,2409,3037,3143,3587,3762,2356,1009,3302,1802,3900,194
8
DMAP1_1_965,2347,1817,1937,2197,2399,1318,1250,1803,1149,1872,1192,131
4
DNAJC2_1_966,2275,2104,2938,2384,2027,3181,1707,1080,3103,1977,1269,17
06
DNMT1_1_967,922,922,2228,1481,1805,717,1929,41,4068,1019,1575,2254
DNMT3A_1_968,216,124,990,251,239,1178,418,287,151,226,123,1359
DNMT3B_1_969,1238,823,1629,1230,2105,1419,914,574,1689,1513,24,2364
DNMT3L_1_970,777,773,1624,1804,519,1175,1062,770,579,738,1112,138
DPF1_1_971,56,103,233,123,15,177,30,202,13,26,29,16
EED_1_972,691,433,454,632,304,277,308,1071,1417,450,71,738
EGR2_1_973,998,655,785,510,2223,1227,1531,143,3589,443,302,924
EHMT1_1_974,4487,4444,4182,3671,5381,5547,5545,2988,4094,6028,6243,504
2
EHMT2_1_975,4399,5061,4364,4776,5321,5370,6000,2181,6661,3012,4345,569
3
ENO1_1_976,1100,668,745,889,806,641,165,536,1529,627,149,564
ESR1_1_977,2061,1629,1152,1760,742,1949,3336,1417,1908,1148,4216,3631
ESR2_1_978,2023,2331,3236,2722,5607,3492,3765,2067,3203,2333,3106,835
ESRRG_1_979,409,580,833,564,525,1466,2188,550,1370,613,1,643
EZH2_1_980,682,801,1647,902,795,1158,1221,475,528,761,525,2779
FBXO11_1_981,2131,2326,2157,2167,3038,3378,1216,1778,1423,1586,2127,18
02
FMR1_1_982,3265,3009,3734,3030,5463,2539,2386,1687,3493,4580,2885,5604
FXR1_1_983,3157,2452,2710,2275,2897,3345,1984,1240,2280,2519,3410,2062
GFI1B_1_984,1210,987,1771,1593,1140,1394,2466,231,419,833,1597,892
GMEB1_1_985,609,630,477,633,1308,380,582,398,798,686,1020,407
HDAC10_1_986,374,196,280,224,1032,468,221,0,3,205,15,248
HDAC11_1_987,5454,3827,4462,3597,7355,6098,2405,7361,3562,6617,3332,55
35
HDAC5_1_988,1623,1098,1140,1461,822,1159,1043,996,1517,746,1603,884
HDAC7_1_989,230,352,299,486,424,205,814,136,386,315,2591,48
HDAC9_1_990,1194,788,1255,1151,201,550,1161,119,562,238,102,828
HDGF_1_991,6783,5958,7551,7613,7795,11904,12395,6815,7611,7513,8500,79
93

HDGFRP2_1_992,358,842,1141,1266,1071,1822,1501,2038,1617,1435,225,85
HLTF_1_993,2525,3399,3619,4158,3335,4450,4147,3043,4918,3995,2129,3265
HMGA1_1_994,773,824,782,496,1707,1415,74,323,1037,925,532,620
HMGN3_1_995,850,589,1003,380,1146,459,1413,2364,1427,704,622,2161
HNF4A_1_996,748,623,424,513,663,363,1524,200,2357,255,319,73
HPSE2_1_997,1664,1583,2040,2919,2124,1785,1626,1478,2733,1428,1325,169
9
HR_1_998,414,202,412,337,191,279,45,313,327,212,810,64
ING1_1_999,155,247,232,108,255,5,52,235,420,103,110,205
ING4_1_1000,2724,2473,2891,3463,3485,5350,4035,1649,2749,3146,3647,338
3
INTS12_1_1001,1859,1702,1751,1934,2197,1957,1349,1049,1628,724,832,124
5
IRF4_1_1002,1478,2000,1769,1320,1903,1481,2034,840,2574,2240,1222,1532
JMJD1C_1_1003,1339,615,1253,570,573,308,674,1049,1000,484,1580,1314
JMJD4_1_1004,438,553,616,459,776,963,346,725,17,1157,774,325
JMJD6_1_1005,5574,5903,7419,6190,6682,6834,9073,5712,3315,5946,5345,67
77
KAT5_1_1006,5627,4411,5016,5247,6697,6631,4455,3692,6299,4041,5359,468
5
KAT6A_1_1007,556,981,429,1005,467,1679,453,714,109,442,17,1849
KAT7_1_1008,5985,6231,5578,7046,6002,6592,5821,6474,6749,5998,3388,667
6
KAT8_1_1009,871,815,1799,1003,381,1180,1572,1179,3192,1434,330,1131
KCTD1_1_1010,3527,3261,4772,3969,5267,3305,5748,3337,4242,3137,1849,25
60
KDM1A_1_1011,980,1090,1651,1440,1364,1522,2117,1098,2507,1363,254,1816
KDM2B_1_1012,1365,1360,1918,2128,1676,1203,2759,1171,950,951,1408,4031
KDM3A_1_1013,1594,1561,1668,1326,4162,1506,4079,390,1774,1114,766,2294
KDM4C_1_1014,6476,6518,9033,6833,9246,7417,9225,8203,7861,5240,6390,68
69
KDM5C_1_1015,366,35,653,110,794,63,612,477,481,29,384,68
KDM5D_1_1016,2272,2076,2356,2776,2820,1610,3062,2301,1103,946,1290,263
9
L3MBTL1_1_1017,378,483,1022,466,1088,1127,386,259,563,67,9,618
L3MBTL3_1_1018,1165,744,1022,602,671,853,466,558,1879,1475,540,253
LBR_1_1019,6040,5886,7808,7210,8590,7495,6886,6976,4748,7463,5489,9712
MAP3K12_1_1020,1748,1615,1516,2025,1959,2212,3438,844,1664,875,1434,10
93
MBD1_1_1021,567,1037,1348,1606,1396,927,1201,1162,2142,1058,3016,904
MBD2_1_1022,2183,1311,1493,2479,2613,1626,502,773,329,856,842,3508
MECOM_1_1023,274,222,287,223,793,197,213,110,1517,522,1063,1049
MECP2_1_1024,558,466,713,321,1294,280,387,258,1138,564,159,419
MEN1_1_1025,5227,4275,4076,4103,3943,5887,7978,5254,4707,4409,4466,399
0
MGEA5_1_1026,3355,3198,2957,3753,3094,5457,6082,1076,2287,3211,1310,29
69
MIB2_1_1027,625,388,326,426,667,328,898,125,1019,726,390,1114
MIER1_1_1028,743,787,1344,1306,2741,936,682,1336,252,2074,578,468
MINA_1_1029,995,1084,1155,1410,1654,2826,2326,2127,690,831,282,1942

MLL5_1_1030,824,1406,776,1882,778,1006,480,1151,216,1248,344,934
MLL_1_1031,6050,5099,6652,7473,5758,7906,7429,6147,13405,5802,2281,718
3
MORF4L1_1_1032,3501,2896,3863,3410,4260,2977,1294,2614,2847,3844,3786,
5199
MSL3_1_1033,1340,1445,2424,1688,1598,2927,5080,1192,1569,1126,738,3329
MTA1_1_1034,604,664,661,530,479,280,673,946,13,776,0,525
MTF2_1_1035,5067,4548,3450,4179,3961,6526,2161,5368,4073,2610,4557,480
9
NAA60_1_1036,460,723,455,507,258,798,1596,286,118,278,485,17
NAP1L1_1_1037,8433,8653,7693,8021,12127,7578,10203,10327,9754,9734,723
9,7848
NAT10_1_1038,1867,1230,1419,1402,2480,724,1805,1556,500,1781,1117,991
NCOA1_1_1039,608,651,232,863,584,1448,1341,715,28,143,151,295
NCOA3_1_1040,497,456,283,713,770,459,371,106,200,369,1158,26
NCOA4_1_1041,492,278,1140,281,1160,652,1290,872,2,926,0,1226
NCOR1_1_1042,3594,3421,3696,2616,3579,5151,3189,3714,8750,4019,2604,13
04
NCOR2_1_1043,186,80,85,405,75,42,2,88,214,417,4,7
NFAT5_1_1044,2487,2710,2210,3438,3050,1871,3107,598,2460,1600,869,2104
NFATC1_1_1045,737,421,984,677,56,314,166,1072,476,327,6,33
NFATC3_1_1046,1339,788,742,1277,2193,675,1827,355,1038,1129,162,1181
NFATC4_1_1047,360,511,722,426,242,586,513,385,263,362,354,364
NFKB1_1_1048,474,513,476,455,5,467,256,98,1,26,1233,0
NFKB2_1_1049,6036,6435,7535,5816,11537,7516,8993,3456,6198,6459,5792,5
733
NPM1_1_1050,3824,3531,3733,2251,5934,3643,1609,2197,4496,2484,2718,215
5
NR1D2_1_1051,1018,740,1678,1325,918,1535,616,1338,2521,1702,2555,1156
NR1H3_1_1052,497,468,542,371,654,1241,685,242,1422,368,1053,661
NR1H4_1_1053,6041,7168,7236,9094,7072,6037,5561,5713,4511,8637,8184,52
35
NR1I2_1_1054,670,305,860,214,131,357,571,1141,1225,360,19,72
NR1I3_1_1055,303,450,365,197,783,938,561,16,8,2,37,5
NR2C1_1_1056,2003,2205,2832,2813,1022,2723,1763,1763,1548,1594,1715,34
26
NR2E3_1_1057,1464,1454,2213,1595,2442,1459,1101,898,4561,1123,2036,721
NR2F2_1_1058,8576,7459,6656,6514,7804,8500,7459,7297,11418,8361,6859,6
569
NR3C1_1_1059,1576,1248,876,1365,590,1361,1221,2472,1414,1175,2349,1350
NR3C2_1_1060,1170,1580,1364,945,1942,1412,1730,1076,383,954,568,1272
NR4A1_1_1061,640,727,544,863,1174,164,659,348,1096,283,7,3782
NR4A3_1_1062,3639,2792,3826,4316,2500,2755,3563,4698,5830,4832,1757,23
58
NR5A2_1_1063,2305,2274,2336,1826,2029,1402,2042,269,2232,2426,1883,779
NR6A1_1_1064,6107,4762,6753,5333,6363,4995,6555,4757,6202,4989,6866,77
55
NSD1_1_1065,563,247,432,638,1244,172,89,474,587,611,21,750
PBRM1_1_1066,1921,1786,818,904,1392,646,1921,1769,1231,370,111,1822
PCGF6_1_1067,1156,1173,1139,1542,774,1301,1733,2914,1527,1144,1108,220

1
PGR_1_1068,5957,4844,6445,7086,5877,6628,5673,7554,1774,5207,4580,7912
PHC2_1_1069,406,432,242,217,530,158,169,121,193,13,9,5
PHF10_1_1070,1210,1795,1385,1657,5637,1714,1942,1220,153,701,1415,1790
PHF11_1_1071,1893,1847,2546,2336,2441,3393,2460,2274,695,1754,1929,651
2
PHF12_1_1072,849,1015,1014,1880,772,499,681,157,1106,1185,307,1098
PHF16_1_1073,1143,836,914,437,1465,564,1858,1925,1335,2209,357,1476
PHF17_1_1074,1452,2326,2490,2573,4537,2471,3426,2837,1895,2058,1652,11
49
PHF1_1_1075,234,477,646,399,415,260,22,10,108,250,1153,1319
PHF20L1_1_1076,1332,1989,1651,1438,479,749,2269,1144,1606,1915,850,162
9
PHF21A_1_1077,6799,5209,5026,7045,6603,7398,5929,4156,6553,4504,5477,3
074
PHF21B_1_1078,442,452,500,220,67,589,154,530,107,500,26,346
PHF6_1_1079,1558,2038,2661,2224,1360,2775,1177,2197,1606,1633,2784,207
5
PHF7_1_1080,2451,1561,2588,2898,1757,2552,1772,3795,4384,1734,2504,223
3
PHF8_1_1081,974,914,1259,471,123,1009,1700,2012,767,897,1778,1946
PIAS2_1_1082,1101,516,690,540,593,328,2148,280,245,501,668,39
PICK1_1_1083,132,179,82,190,51,172,54,62,570,79,15,16
PIWIL2_1_1084,1300,885,1782,1258,2402,1348,2543,1312,2309,831,1031,231
2
PML_1_1085,82,185,43,71,72,90,234,33,1,15,357,33
POLR1B_1_1086,2650,3295,3810,3485,2856,3178,4391,4180,1455,3180,868,66
40
PPARA_1_1087,1345,1169,1544,925,1188,1554,1320,2135,1711,1530,1439,557
PPARD_1_1088,263,163,214,372,134,22,316,377,0,51,530,1070
PPARG_1_1089,197,170,158,173,10,120,0,103,287,295,1,2
PRDM10_1_1090,1820,2130,2284,1976,2598,1853,2982,3524,1182,1804,564,13
64
PRDM15_1_1091,822,849,1043,515,1389,910,1133,266,84,601,332,371
PRDM16_1_1092,1013,1100,1586,1451,2976,700,648,1580,1014,2298,1311,531
PRDM1_1_1093,53,265,178,164,412,48,0,556,0,268,724,0
PRDM2_1_1094,688,706,742,1013,567,600,941,141,1343,914,956,400
PRDM7_1_1095,694,211,219,51,978,324,1660,0,138,119,0,0
PRDM8_1_1096,815,1182,982,1826,1449,1010,1158,916,1536,988,1430,551
PRMT1_1_1097,420,952,363,400,102,961,780,3,14,549,214,5
PRMT2_1_1098,1101,915,399,495,1532,173,364,146,516,387,116,2270
PRMT3_1_1099,338,281,416,427,32,219,1214,979,150,219,272,188
PRMT5_1_1100,695,655,669,583,623,978,1133,874,838,106,580,512
PRMT7_1_1101,6964,6330,8533,9432,8517,5752,10163,6277,10347,7626,10073
,7218
PSIP1_1_1102,1467,1275,1443,1081,524,2134,261,1033,176,345,271,500
PSMC5_1_1103,3779,3883,4005,5235,3762,6101,4719,3388,3154,3884,2884,48
62
PWWP2B_1_1104,92,151,177,395,484,346,92,537,126,526,2683,320
RAD54B_1_1105,1485,1612,796,647,292,961,2084,319,1280,2422,914,378

RAD54L_1_1106,2841,1664,1857,1755,1417,1999,1281,1803,1910,1847,1633,1
860
RARA_1_1107,1031,697,792,1102,2368,704,673,431,1881,596,1276,678
RARB_1_1108,784,842,795,692,1129,944,204,590,785,945,157,509
RARG_1_1109,659,543,940,507,761,826,1580,522,293,366,19,3167
RBBP5_1_1110,1159,762,805,551,823,651,519,941,599,420,236,792
RBCK1_1_1111,589,48,626,548,1041,2,1034,7,11,1038,484,1036
RBFOX2_1_1112,1971,2872,2439,2029,2075,3121,2374,1127,3502,2836,1870,2
934
RCC1_1_1113,246,182,353,51,358,297,387,768,0,35,773,242
RCOR3_1_1114,1001,671,757,856,204,804,406,139,193,734,118,192
RECQL5_1_1115,1991,3247,2532,3600,3763,2685,4111,2526,4661,2269,806,33
15
RECQL_1_1116,3084,2924,2667,3822,4009,4315,629,2323,4718,1850,1982,273
8
RELA_1_1117,1099,686,863,650,1728,768,2050,2176,165,958,279,424
RERE_1_1118,1123,753,1796,1448,1146,971,2118,733,757,534,146,1655
RFC1_1_1119,2704,2062,2289,2052,2848,2788,3362,2311,6145,2675,1508,228
8
RNF14_1_1120,255,493,168,88,33,261,138,0,0,567,0,0
RNF17_1_1121,1793,1988,1362,787,2376,2865,1000,3293,2372,2165,56,874
RNF40_1_1122,1031,580,1073,1085,1968,788,1755,305,761,987,248,1689
RNF8_1_1123,1431,1609,1934,1503,1131,2313,1702,2315,1514,743,1522,2758
RORA_1_1124,656,713,986,969,1322,594,422,632,84,560,420,396
RORC_1_1125,1056,504,1109,849,1125,613,566,303,468,500,935,349
RPH3A_1_1126,439,705,318,995,1,584,1206,0,306,445,92,54
RPS6KA5_1_1127,215,277,339,301,267,136,424,18,71,591,307,14
SATB1_1_1128,650,844,913,656,482,713,837,1365,564,659,1977,1199
SATB2_1_1129,1149,644,1293,1602,1070,1090,537,836,1506,1645,431,607
SCMH1_1_1130,909,842,430,517,732,1184,648,984,67,440,179,1863
SET_1_1131,1721,1040,1570,1432,1404,3334,2635,1156,2809,1595,1496,1097
SETD3_1_1132,1550,1696,2320,2097,2365,1207,2445,1543,3959,1099,4580,12
24
SETD4_1_1133,395,54,190,57,476,235,1,7,0,205,1610,398
SETD6_1_1134,268,81,253,247,83,0,22,208,723,11,44,12
SETDB1_1_1135,1730,1546,1339,1589,1711,1571,1165,1231,1774,1457,1476,9
51
SETDB2_1_1136,1970,2011,1684,1575,1728,1425,1826,1914,1933,1675,1069,3
428
SFMBT1_1_1137,566,625,673,331,76,630,1162,545,117,155,162,364
SFMBT2_1_1138,5161,4591,5887,5915,6000,4530,4715,2890,3429,5070,3800,6
780
SHPRH_1_1139,1911,1865,2343,2885,2027,2998,4019,2687,2291,2049,1589,91
8
SIN3A_1_1140,1021,677,1630,1260,636,607,408,730,1167,1139,1371,1582
SIRT1_1_1141,3999,3799,4696,4472,2798,3903,3533,4725,3764,3079,5640,26
20
SIRT2_1_1142,614,622,653,500,862,1009,98,265,698,532,506,977
SIRT3_1_1143,671,646,565,1000,823,751,1221,1048,553,612,9,27
SIRT5_1_1144,458,462,520,284,1591,88,625,597,905,727,1053,1066

SIRT6_1_1145,300,653,487,315,1845,155,263,115,141,277,1283,319
SLC38A1_1_1146,1617,1382,2202,1723,1762,1709,206,1641,2694,905,2097,12
22
SMARCA1_1_1147,1538,1281,2117,3236,3833,487,2924,821,2824,1840,1724,14
15
SMARCA2_1_1148,1637,1159,2016,1234,975,2486,2303,2388,274,1640,888,244
3
SMARCA4_1_1149,7634,6860,7989,7283,11272,7407,10387,3471,10014,8084,84
75,6138
SMARCAD1_1_1150,5406,5395,6383,4691,5293,5816,6870,3821,6659,3691,5651
,4011
SMARCAL1_1_1151,758,687,941,895,664,1218,1009,436,2809,323,220,1249
SMARCB1_1_1152,268,109,253,177,36,10,544,40,72,124,577,186
SMARCC2_1_1153,412,880,311,892,792,529,655,424,41,148,352,1
SMARCD1_1_1154,949,947,1736,1907,1386,839,1832,2492,1483,471,134,589
SMARCD3_1_1155,750,1023,1136,703,1340,612,602,696,88,298,652,1079
SMC2_1_1156,2465,1838,2258,2991,2151,1679,2148,3173,2640,2911,836,2368
SMC4_1_1157,1486,2110,1850,2114,3112,2778,1600,1511,743,1784,1536,2511
SMN1_1_1158,16077,15523,19679,18336,17056,18603,18696,13905,15260,1660
3,15708,23607
SMN2_1_1159,16077,15523,19679,18336,17056,18603,18696,13905,15260,1660
3,15708,23607
SMYD3_1_1160,1048,1240,1110,933,662,1012,387,965,807,1427,96,1546
SP100_1_1161,10782,11301,10244,9906,7427,10593,15835,9467,13240,9144,9
357,7297
SP110_1_1162,1738,1436,2291,1310,2514,1764,2280,482,3099,1744,406,3181
STK31_1_1163,1313,1200,1628,1592,1295,2741,2427,2014,903,1358,1034,185
0
SUPT3H_1_1164,1928,2496,1986,3289,3338,3595,3258,3794,4693,1883,2991,1
728
SUPT5H_1_1165,40,24,152,27,60,23,665,83,34,97,0,211
SUV39H2_1_1166,4510,5819,4078,5000,5331,4144,5581,1781,3169,4813,3087,
3975
SUV420H1_1_1167,3899,4853,4676,4752,7513,5359,4202,3913,6896,4083,2791
,9757
TADA2A_1_1168,360,669,695,480,849,1258,732,304,203,583,1824,932
TADA3_1_1169,387,698,738,1508,1067,641,1399,679,321,390,541,1827
TAF12_1_1170,1608,795,1524,1267,1690,1280,2798,297,2166,1066,679,1470
TAF15_1_1171,799,604,649,745,1591,1831,1260,1630,441,949,305,1734
TAF1_1_1172,1146,788,1472,1977,1110,1276,1063,719,942,1020,1510,1940
TAF5L_1_1173,497,969,785,946,834,432,4,67,999,937,6,476
TCF19_1_1174,439,408,477,797,434,719,410,182,653,411,19,1958
TCF20_1_1175,2069,1534,1829,2403,862,1371,1253,1038,259,2514,345,266
TDRD10_1_1176,2063,2275,2437,2282,1955,2671,2747,419,2703,1866,2438,21
84
TDRD3_1_1177,1095,937,1249,1045,829,2123,1977,455,493,316,724,335
TDRD5_1_1178,613,201,382,270,200,215,85,35,24,433,197,128
TDRD6_1_1179,2668,3069,2772,2568,3417,4273,4586,1584,1493,1811,3152,95
9
TDRKH_1_1180,1439,1244,1824,1950,2317,2028,2759,1168,30,1023,1607,1277

TERF1_1_1181,8970,8818,10336,10703,13459,12914,14522,11313,12197,10779
,8625,12200
TET2_1_1182,214,404,489,215,40,332,90,33,587,739,1253,405
THRA_1_1183,1203,1198,933,817,129,276,1424,359,426,645,556,768
THRB_1_1184,529,760,952,476,693,729,229,1611,893,846,615,833
TP53BP1_1_1185,1355,1269,1488,1496,2884,1592,2659,1739,425,1245,58,638
TP53_1_1186,361,439,573,650,294,709,1618,598,12,549,73,906
TP73_1_1187,440,421,403,333,130,1131,1135,1487,0,448,1,733
TRIM24_1_1188,0,54,207,0,0,0,0,0,0,0,0,0
TRIM32_1_1189,545,299,301,558,415,274,143,961,797,184,336,1
TRIM33_1_1190,659,719,1121,1445,1322,2505,822,870,149,545,937,377
UBE2A_1_1191,784,326,511,819,842,1037,78,816,499,452,917,506
UBE2E1_1_1192,13449,15263,16353,15517,22593,16710,14139,15812,14378,12
313,8808,15806
UBE2I_1_1193,5307,5786,8394,6774,5489,7872,6588,5810,7402,4003,3795,63
87
UBE2K_1_1194,7988,6480,8441,7371,11731,8822,12250,11009,8398,4607,8957
,7520
UBE2V1_1_1195,778,1198,743,719,399,419,841,1095,219,1567,435,1128
UHRF1_1_1196,239,107,187,445,207,493,693,26,3,238,81,38
USF2_1_1197,2275,2003,2308,1753,1783,2401,2836,2316,2047,1834,588,1510
UTY_1_1198,1781,1500,1223,1088,1483,716,2554,442,1425,1741,2484,744
VDR_1_1199,487,982,480,449,346,386,566,42,167,352,377,656
WDR5_1_1200,1025,1061,1289,885,1581,1429,1827,2085,60,1826,1273,22
WHSC1_1_1201,2253,2060,2269,1585,2241,3004,2094,1274,453,2689,2639,104
7
WHSC1L1_1_1202,1312,1169,874,1977,170,780,188,1431,744,887,1331,210
WRB_1_1203,1061,1812,1266,1587,297,857,3994,2741,748,1794,1024,1780
ZGPAT_1_1204,300,346,686,181,786,869,7,967,59,293,465,1
ZMYND11_1_1205,744,802,1391,953,279,1782,2559,242,3506,799,986,992
ZMYND8_1_1206,1941,1039,1261,1191,2351,1762,1641,662,30,1890,1478,1121
ZNF451_1_1207,4392,2409,3305,3238,3817,2814,3050,2318,5279,4927,2545,4
202
ALG13_1_1208,814,1542,1372,1403,823,797,247,1600,795,2332,0,630
ASXL1_1_1209,927,1440,1265,846,1727,968,421,1022,3886,1418,1284,344
CBX2_1_1210,346,131,151,276,8,343,84,219,71,590,13,10
HDAC8_1_1211,13760,12027,13731,15995,15239,18942,18425,13358,18996,123
11,13601,13874
ING3_1_1212,2279,2663,3472,2575,2589,2574,4871,3253,4018,2744,3793,565
2
MLLT10_1_1213,3768,4186,4186,4720,3616,3578,1629,2912,5567,2758,1069,1
459
PHF19_1_1214,4642,4769,5517,5561,7515,5371,6270,4492,4168,3351,5503,21
93
RBM14_1_1215,706,503,580,370,1546,550,642,833,811,843,710,2027
SP140_1_1216,2707,2311,2649,2221,2792,2417,2830,1550,2445,1047,2406,31
9
TAF9_1_1217,709,677,811,410,953,662,483,1915,775,338,93,8
TAF9_1_1218,234,562,502,706,413,146,1909,740,0,266,303,531
AHR_1_1219,6582,5167,5500,6545,8098,8867,4817,4550,5453,7162,5570,1003

5
ALKBH1_1_1220,117,157,142,367,164,166,745,316,1051,276,340,36
ALKBH3_1_1221,2085,1635,1522,1664,2143,1447,955,1738,2468,1979,1624,11
18
ARID2_1_1222,2029,1772,2860,1896,1482,2909,2349,1486,2524,1305,1752,10
95
ASF1A_1_1223,327,651,382,267,35,463,355,766,143,844,0,2
ASF1B_1_1224,616,927,1219,504,1130,711,414,536,835,767,848,774
ASH1L_1_1225,1342,1063,1062,1025,767,1413,912,870,762,648,630,1225
ASXL2_1_1226,142,141,17,29,130,93,77,39,6,245,0,1
ASXL3_1_1227,6352,4133,6118,6539,7326,7252,4267,4259,3246,4654,3973,73
77
ASZ1_1_1228,3993,4416,5234,4089,7738,5955,4992,4153,8256,4651,4734,298
2
ATAD2_1_1229,0,0,0,0,0,0,14,0,0,0,0,0
ATF7IP_1_1230,4095,4378,3621,3316,3517,2823,3171,4883,3429,4041,4610,1
955
AURKB_1_1231,855,832,920,759,25,1758,1279,582,196,385,579,914
BAHCC1_1_1232,610,97,112,72,0,192,0,0,1,2,20,0
BAHD1_1_1233,427,176,226,300,178,1069,133,551,562,333,190,1
BARD1_1_1234,4623,4656,3490,4553,2270,6386,4119,4924,6669,3991,3462,43
84
BAZ1B_1_1235,4013,3686,5329,7446,6413,3940,4832,3521,5528,2774,1971,63
75
BAZ2A_1_1236,2481,2620,2997,3883,4320,1498,2071,943,1779,3655,977,3428
BAZ2B_1_1237,5713,4328,6716,7691,4696,5363,6544,4809,7929,4599,9759,99
34
BLM_1_1238,1918,2705,2501,2456,993,4015,1883,2126,1364,2530,2339,2162
BMI1_1_1239,2325,1707,2070,1993,3158,1758,1035,344,1179,1656,3692,866
BRD1_1_1240,1657,1259,1905,749,2424,1897,2257,1821,714,1049,1376,512
BRD3_1_1241,799,770,1326,1000,2245,282,1302,380,2,401,212,1012
BRPF3_1_1242,1730,1480,1532,992,2902,2170,2218,1679,2001,1568,724,1111
BRWD3_1_1243,1315,396,917,748,2344,1297,2752,791,369,1042,91,41
C14orf169_1_1244,407,281,445,413,181,180,1263,70,154,428,1000,0
C20orf20_1_1245,152,156,194,179,1251,379,5,20,364,345,146,25
CALR_1_1246,376,114,309,309,307,416,47,400,533,617,2063,2
CARM1_1_1247,1729,1536,1689,2109,1783,1415,2902,2926,1729,3088,1929,14
99
CBL_1_1248,2674,3559,3701,3934,2854,4055,2845,2747,2818,4057,6127,3985
CBX4_1_1249,877,908,471,735,996,245,1034,1361,80,1773,113,341
CBX6_1_1250,605,480,1230,443,116,210,1353,992,287,463,0,333
CBX7_1_1251,37,194,748,313,1,185,0,599,59,244,54,0
CBX8_1_1252,19,48,84,274,67,556,113,55,13,16,4,9
CCDC101_1_1253,1728,1710,1061,1858,904,3004,3415,376,360,1982,1885,118
9
CCNE1_1_1254,2539,1709,2244,2662,2345,1960,3165,1677,3875,1634,3197,25
88
CCNT1_1_1255,872,796,1303,1082,1689,1370,1321,764,712,1879,1445,540
CDC73_1_1256,426,269,245,257,684,144,169,230,428,58,434,566
CDK9_1_1257,1226,1142,2630,1434,641,1759,437,1233,1936,1902,1847,416

CDY2A_1_1258,5462,4840,5495,7281,7773,4370,4555,5410,10074,5286,6446,4
489
CDY2B_1_1259,5462,4840,5495,7281,7773,4370,4555,5410,10074,5286,6446,4
489
CDYL2_1_1260,567,360,1103,458,124,546,833,291,9,474,10,104
CECR2_1_1261,617,776,675,563,1132,634,1033,407,299,1143,295,472
CHAF1A_1_1262,799,479,878,967,520,1359,708,1607,1823,166,205,423
CHAF1B_1_1263,213,142,154,188,671,366,983,920,123,147,27,213
CHD1_1_1264,3208,2148,3922,1765,2824,2318,3073,3039,1342,2720,1802,114
3
CHD1L_1_1265,651,425,245,135,382,858,93,661,14,471,0,57
CHD4_1_1266,916,625,1453,645,785,1088,1832,1427,504,1238,543,1403
CHD6_1_1267,1576,1570,1678,970,2053,2004,2720,2717,115,727,1589,1252
CHD7_1_1268,796,560,1486,821,505,780,1054,2770,331,607,219,1569
CHD9_1_1269,3096,2064,2478,3087,5380,1982,3509,3348,1186,2808,2270,205
9
CHMP1B_1_1270,2175,2221,1991,2153,1122,2937,2117,2371,1952,1464,3516,1
293
CHMP4B_1_1271,1899,2347,1819,2495,1436,2056,999,2228,3205,3043,3056,28
9
CHMP4C_1_1272,329,333,361,357,208,162,2222,934,217,201,485,1095
CHRAC1_1_1273,968,830,2024,1122,2217,551,913,746,2318,1233,1217,1062
CLOCK_1_1274,2424,2348,2102,1978,2414,2633,3184,3946,2845,2315,6502,42
78
COPS5_1_1275,1381,1117,1474,1075,1203,1353,944,1162,2016,1187,1625,728
CRAMP1L_1_1276,465,540,194,161,722,174,324,68,0,37,44,505
CTCFL_1_1277,3288,3213,3172,3099,1796,2261,1272,4537,4549,2249,2685,18
39
DEAF1_1_1278,2482,2224,1715,2026,1164,1066,1604,468,1152,1182,633,3288
DNAJC1_1_1279,517,368,775,557,401,707,1227,168,1600,276,554,56
DOT1L_1_1280,3599,5120,4737,5114,4097,5813,6930,2732,3806,4216,7122,53
17
DPF2_1_1281,315,710,386,498,828,525,180,1034,1957,341,165,62
DPF3_1_1282,1027,585,1048,869,1875,1125,1067,44,21,131,376,11
EIF4B_1_1283,258,197,639,215,671,593,829,57,110,262,559,724
ELP3_1_1284,3058,2749,3722,3449,3119,3703,6306,3730,2564,3603,2360,431
1
EP300_1_1285,4371,3997,5078,5148,3085,3908,4360,5334,5939,4157,5680,80
56
EP400_1_1286,3442,3910,3597,3101,2371,2659,4968,2467,5656,3163,6220,79
41
EPC1_1_1287,848,674,863,414,210,501,408,702,1684,224,1053,1062
EPC2_1_1288,3000,3758,3097,2884,5375,3333,3035,2780,2388,1832,2521,163
3
ERCC6_1_1289,508,86,353,221,893,0,0,0,0,0,0,0
ESRRA_1_1290,1909,1538,2078,2124,2500,2205,1398,1872,2183,1630,1619,16
61
ESRRB_1_1291,404,697,746,541,1347,268,92,27,91,97,1080,14
FANCM_1_1292,3174,2471,2556,3199,3413,3444,2324,2481,5170,2593,2452,20
41

FBXL19_1_1293,488,348,256,152,628,229,0,8,0,580,198,85
FOS_1_1294,1216,750,1299,1775,1026,1785,750,620,311,2472,1828,1047
FTO_1_1295,985,849,825,802,1145,1321,1130,37,599,860,252,353
FXR2_1_1296,722,777,816,1134,825,659,814,1047,377,1031,425,806
G2E3_1_1297,1746,1913,2056,2095,4020,3228,1153,1749,2855,1629,580,2768
GATAD2A_1_1298,34,45,127,175,487,14,0,141,126,0,1118,11
GLYR1_1_1299,305,411,229,734,1536,353,328,874,1145,332,55,311
GMEB2_1_1300,296,238,180,165,407,496,435,282,519,11,483,640
GSG2_1_1301,1279,760,1180,1225,847,1635,1409,2006,333,932,1740,2310
GTF3C4_1_1302,2265,1232,2655,2422,4042,2841,4438,730,634,537,2657,1214
HAT1_1_1303,1610,1619,1940,1447,1664,1317,384,1168,1075,1483,3035,1322
HDAC1_1_1304,1765,1316,2284,1877,3092,2575,1775,3346,2150,2608,3162,33
45
HDAC2_1_1305,806,967,1038,1565,700,688,986,1058,1999,1725,851,2295
HDAC3_1_1306,1482,880,1315,1997,1569,1903,2542,1028,211,2221,1355,4574
HDAC4_1_1307,1099,926,1290,1372,614,863,538,1919,124,1465,1606,325
HDAC6_1_1308,4875,3467,2970,4349,5250,4783,5906,2827,2895,4860,3955,78
34
HDGFL1_1_1309,641,876,1688,552,1074,1449,899,36,61,214,344,628
HDGFRP3_1_1310,118,213,496,461,0,798,89,255,166,241,146,162
HELLS_1_1311,2439,2201,1777,2156,2607,1564,663,1469,1440,3135,1915,904
HEMK1_1_1312,190,229,155,300,129,55,45,0,69,261,587,242
HIF1AN_1_1313,1605,1396,1179,986,1191,1551,1704,1458,1495,756,722,1982
HIRA_1_1314,233,755,769,583,361,81,1830,389,276,402,1095,889
HMG20B_1_1315,3217,2282,3018,3567,3325,5096,4958,4836,4307,2336,5102,4
313
HMGN5_1_1316,436,393,671,802,411,713,626,124,619,987,385,218
HNF4G_1_1317,173,350,145,315,14,30,28,531,4,92,362,412
HSPBAP1_1_1318,487,294,361,245,20,50,741,131,2,117,118,267
HUWE1_1_1319,934,680,1104,762,452,1151,772,1445,1109,1164,1117,766
ING5_1_1320,320,250,546,443,510,118,1682,1,0,38,2481,42
INO80_1_1321,902,632,531,1262,1596,555,238,774,373,408,346,208
JARID2_1_1322,419,393,404,525,615,456,969,296,333,671,1467,1013
JHDM1D_1_1323,3022,2715,3565,3012,3289,3530,4548,3934,1967,2949,370,64
26
JMJD7_1_1324,489,535,558,589,402,337,481,428,26,116,277,15
JMJD8_1_1325,3241,2804,3085,2971,4127,2614,2712,3041,5228,3872,1882,48
84
JUN_1_1326,699,1741,1301,1175,815,1540,1683,800,3195,1054,2220,105
KAT2A_1_1327,924,409,786,611,3699,1055,111,578,21,938,2,1178
KAT2B_1_1328,5986,5177,8345,5414,4604,5500,5609,4587,2824,5980,4999,76
14
KAT6B_1_1329,2726,2715,2511,2904,3597,3210,3086,3918,6740,1911,3425,19
08
KDM1B_1_1330,877,993,1429,1051,1580,1460,3115,192,1101,1459,1427,842
KDM2A_1_1331,184,112,38,220,7,96,0,90,141,1,0,126
KDM3B_1_1332,746,880,869,752,738,56,581,1079,179,1088,759,681
KDM4A_1_1333,83,423,288,241,791,1025,329,253,320,99,59,0
KDM4B_1_1334,3469,2840,5094,3057,4947,4561,1731,4552,2492,3177,3034,24
83

KDM4D_1_1335,448,677,1133,306,428,153,2037,326,310,16,396,365
KDM5A_1_1336,763,619,827,533,1225,1313,835,720,764,173,78,1188
KDM5B_1_1337,1517,1842,2110,2523,2228,2886,3182,2041,2012,1914,594,146
3
KDM6A_1_1338,21486,21820,27399,26168,28188,28442,31123,20404,18656,219
34,23023,29070
KDM6B_1_1339,1427,1491,1219,1528,484,573,1570,1003,1736,830,296,1007
KIAA2026_1_1340,1557,1651,1916,1443,1490,5639,1461,1369,1319,2090,1228
,711
L3MBTL2_1_1341,3137,2591,3606,2802,1913,2621,3490,2855,4357,1881,541,1
876
L3MBTL4_1_1342,3310,3644,3539,3568,3790,3527,4796,2761,3686,1909,6634,
3319
MAEL_1_1343,1844,1883,2394,1754,3148,1715,1501,4380,2843,1938,828,2315
MBD3_1_1344,24,30,15,164,56,167,300,146,0,6,2,1
MBD4_1_1345,2273,3571,2375,3692,3296,2792,4246,1981,3819,3610,1461,310
5
MBD5_1_1346,2178,1397,1473,2220,2538,1652,2238,1297,1344,2287,733,463
MBD6_1_1347,151,114,68,62,160,287,0,13,12,6,946,90
MBTD1_1_1348,469,361,1400,943,1123,829,105,469,478,658,316,81
MDM2_1_1349,1467,2037,2199,1302,2890,646,1830,1499,1160,1166,2047,594
MIER2_1_1350,259,311,188,355,556,420,732,177,181,193,777,1260
MIER3_1_1351,892,864,785,659,1214,545,262,375,285,424,45,561
MIS18BP1_1_1352,5406,5196,7088,6129,4134,5824,8950,4394,4634,5058,3293
,5614
MKL1_1_1353,105,134,24,148,787,406,6,55,0,94,0,102
MLL2_1_1354,712,751,383,730,1055,307,291,618,28,198,114,38
MLL3_1_1355,369,569,719,835,1,557,454,799,1,1040,4361,800
MLLT1_1_1356,550,254,599,283,206,397,160,146,535,262,41,55
MLLT3_1_1357,4243,3267,4580,3483,4604,3948,3300,3015,5034,2805,2500,53
18
MLLT6_1_1358,653,377,406,601,548,1124,1679,285,808,898,811,579
MPHOSPH8_1_1359,3445,2901,3510,3764,4829,3987,4193,2164,1776,2960,935,
3432
MSH6_1_1360,999,2092,1679,1937,1946,634,2126,1123,536,621,335,7
MSRB2_1_1361,750,876,849,418,1745,623,501,998,99,1300,133,637
MTA2_1_1362,857,694,370,383,441,514,533,527,99,954,1223,46
MTA3_1_1363,1566,1219,2453,1693,378,2006,5587,748,413,1493,1020,2066
MYSM1_1_1364,5052,4504,5428,4255,7372,5194,2401,3085,4446,4535,6462,45
36
NAP1L2_1_1365,852,747,283,548,1701,920,211,330,1483,824,275,198
NAP1L3_1_1366,1405,1851,1957,1920,1309,1594,1477,1897,2734,1241,1042,2
237
NAP1L4_1_1367,1640,1663,3884,2409,2869,2286,2826,2390,791,1826,3357,29
35
NAP1L5_1_1368,2325,1337,1822,1854,1878,2261,1769,2558,1451,840,1357,21
45
NAT14_1_1369,322,474,599,543,201,221,167,781,1247,511,40,344
NAT8B_1_1370,823,560,659,766,347,351,253,2170,246,604,1458,397
NAT8_1_1371,7556,7805,7697,9605,11386,8673,9425,6615,6256,6620,5367,12

228
NAT8L_1_1372,266,197,213,386,364,117,643,3,19,277,126,65
NAT9_1_1373,2133,1538,1943,1218,3320,2354,3166,4093,4336,1624,3151,978
NCOA2_1_1374,708,591,548,436,579,39,813,146,91,575,28,1417
NPTXR_1_1375,582,921,587,393,1109,1237,112,422,1104,392,5,990
NR0B1_1_1376,1430,1337,1891,1715,2579,1465,1592,969,2256,1206,834,2587
NR0B2_1_1377,4976,5572,6071,6322,4516,5429,5988,5719,1304,4813,3917,35
09
NR1D1_1_1378,746,749,827,644,643,1132,227,58,941,729,748,1276
NR1H2_1_1379,599,428,545,268,305,1091,1266,251,1248,22,86,808
NR2C2_1_1380,395,533,143,426,316,253,28,85,0,132,19,242
NR2E1_1_1381,2089,2520,2087,2011,1888,3224,1405,1113,2270,2302,1331,16
85
NR2F1_1_1382,1143,1800,1605,1176,1326,1994,1023,2463,513,1270,438,1661
NR2F6_1_1383,414,556,562,492,779,316,461,1029,0,622,839,291
NR4A2_1_1384,2974,1765,2418,1709,1118,2143,1945,2024,1670,3498,942,888
NR5A1_1_1385,444,121,121,358,743,234,6,651,783,676,350,266
NRIP1_1_1386,9384,7581,9981,9430,7298,9009,10558,7233,6546,7400,5093,1
1338
PADI4_1_1387,6082,6158,6103,6091,11026,7725,5989,4842,7894,5907,2565,6
592
PAWR_1_1388,1207,866,1157,975,487,350,377,1044,964,1570,827,884
PAX5_1_1389,1773,1436,1086,1673,603,1177,4775,655,381,1130,124,2459
PAXIP1_1_1390,1348,1149,1095,796,2261,721,985,840,1347,1638,228,532
PGRMC2_1_1391,2693,2698,3667,2018,881,3015,3048,1147,5408,2612,2004,48
39
PHB_1_1392,2145,1644,1668,1427,3537,958,2542,602,1484,1999,4998,932
PHC3_1_1393,3293,2549,3251,3230,1286,2198,4081,3228,3020,2856,1540,342
7
PHF13_1_1394,2368,1513,1560,2560,3322,1977,4317,1772,746,1137,3016,169
2
PHF14_1_1395,2208,1774,2182,2347,2605,2458,3971,1057,1318,2818,1988,22
51
PHF15_1_1396,983,1612,944,1633,226,1229,467,413,1217,693,528,2746
PHF20_1_1397,1476,1762,2289,697,1728,1467,494,614,2143,2174,1610,843
PHF23_1_1398,1473,788,1150,931,2745,1018,3348,856,1043,880,4022,2796
PHF2_1_1399,589,278,349,343,542,538,661,266,61,406,785,864
PHF3_1_1400,2148,1269,2161,3339,2093,2638,3117,2750,3479,1696,1276,110
3
PHF5A_1_1401,1524,1445,1524,1773,1717,1308,2235,3079,2241,1468,1507,28
26
PHIP_1_1402,4456,4830,3653,4479,6029,6332,3846,3581,3373,4872,8502,532
4
PHRF1_1_1403,1910,1276,1770,979,2334,1440,2264,862,67,1123,1649,166
PIAS1_1_1404,707,490,413,309,427,897,173,204,702,307,968,13
PIWIL4_1_1405,1938,1874,2615,2652,2404,2782,2720,1275,2471,1710,848,29
16
PPARGC1A_1_1406,1114,945,1781,1265,621,1151,2286,711,734,2260,324,2534
PRDM11_1_1407,512,1022,1029,1568,856,1319,65,348,2889,1381,22,3633
PRDM12_1_1408,1036,1250,1263,1444,1099,866,2063,1945,1291,1177,1269,95

9
PRDM13_1_1409,241,210,457,403,6,1576,351,168,30,147,1577,57
PRDM14_1_1410,705,877,919,670,401,1168,90,173,154,806,0,2384
PRDM4_1_1411,366,425,471,419,549,165,280,141,535,481,7,19
PRDM5_1_1412,198,231,201,71,3,182,62,381,546,345,75,572
PRDM6_1_1413,948,698,1129,1138,294,1833,2101,1135,104,500,38,1084
PRDM9_1_1414,579,511,406,478,1042,457,798,507,46,641,71,538
PRMT6_1_1415,362,323,545,564,294,239,1046,573,0,126,251,9
PRMT8_1_1416,1231,977,1538,1280,3016,251,1071,1463,3672,951,739,1048
PRPF6_1_1417,1856,1951,2358,1614,1189,2809,1360,1257,1861,2478,894,138
1
PYGO1_1_1418,251,101,250,144,1,146,638,164,5,156,460,0
PYGO2_1_1419,2805,3046,2965,2413,4156,2622,4555,3323,3287,1793,2149,31
24
RAG2_1_1420,6833,6352,7668,8159,7558,7630,9283,9311,7437,7480,3543,572
5
RAI1_1_1421,1669,2325,1875,2360,2669,1666,2162,3838,2432,1154,2221,270
5
RB1_1_1422,1385,1283,1623,1396,1008,1121,1337,1370,1630,1177,57,1546
RCOR1_1_1423,1005,1390,1438,1331,2307,462,911,1340,792,1512,787,573
RCOR2_1_1424,886,572,746,752,389,962,2973,813,406,1012,248,2114
RECQL4_1_1425,1175,988,517,1881,316,955,1145,873,1643,1174,1782,1310
RELB_1_1426,10,329,88,128,0,31,14,6,17,9,0,8
REL_1_1427,2764,2952,2620,2098,2699,3004,4197,1783,2229,3738,4522,2474
RING1_1_1428,599,607,864,316,802,964,2207,166,464,712,1016,370
RNF20_1_1429,2054,2091,1513,1858,3149,1240,2511,1942,483,2062,1493,155
0
RNF25_1_1430,1900,884,1173,1507,1459,1161,578,1901,1858,657,909,1845
RNF2_1_1431,853,1800,989,1182,1071,2320,2073,1141,349,1247,1158,1756
RORB_1_1432,2364,3878,2928,4155,2804,3038,372,1799,1305,2884,949,6182
RSF1_1_1433,5803,6455,5741,5595,9902,9182,7135,4289,7841,6823,9021,583
2
RUVBL1_1_1434,2205,2457,1689,2895,2157,2461,2439,2368,3608,988,2819,18
83
RUVBL2_1_1435,1155,884,861,1202,587,502,705,1528,736,813,712,1932
RXRA_1_1436,1500,1036,1423,1717,2239,1024,1911,1840,691,1158,3777,894
RXRB_1_1437,2026,1145,1865,1638,451,1431,4289,1727,3223,2124,635,2105
RXRG_1_1438,1375,1757,1731,2635,1916,2485,2187,2967,2705,1120,3119,149
9
SAP18_1_1439,157,28,177,75,49,30,37,278,0,295,11,10
SCML2_1_1440,1310,1587,2410,1852,2342,1508,873,1730,1065,1833,1089,349
SENP3_1_1441,797,552,1289,924,2070,1820,2110,518,176,1025,733,2097
SETD1A_1_1442,1156,652,730,928,1824,604,363,291,550,952,2227,1339
SETD1B_1_1443,568,578,460,889,395,456,730,72,43,413,100,80
SETD2_1_1444,8237,8723,8609,8416,13194,9856,8127,5923,11729,5858,10513
,6517
SETD5_1_1445,4113,3795,3947,4523,2747,4510,4477,5177,2967,4220,6564,61
23
SETD7_1_1446,353,220,210,417,114,117,5,18,27,10,11,9
SETD8_1_1447,1299,1078,939,1414,114,1182,624,1122,1687,1334,1310,447

SETMAR_1_1448,1154,1139,1233,1423,1146,3001,3137,663,1793,274,809,1008
SF3B3_1_1449,119,108,310,271,316,206,137,321,678,655,1,517
SIAH2_1_1450,4154,4025,4549,5276,7447,4356,3643,6036,4746,6422,3144,71
99
SIN3B_1_1451,1810,1568,1741,2134,1685,532,3055,1939,1939,1108,2834,495
SIRT4_1_1452,610,408,346,400,1719,599,20,537,53,684,398,1403
SIRT7_1_1453,2664,1873,2883,3407,4948,1814,3304,3401,3778,3136,1826,20
30
SLC2A4RG_1_1454,477,205,806,638,269,569,979,829,293,71,1114,47
SMARCA5_1_1455,3395,3072,2353,4370,3810,4173,3970,2919,7884,3604,3959,
6400
SMARCC1_1_1456,1504,1171,1070,1106,1707,1894,2858,1360,1535,2201,348,1
163
SMARCD2_1_1457,504,724,701,466,937,27,1171,465,818,719,265,14
SMARCE1_1_1458,651,478,346,572,10,719,1118,209,808,42,193,631
SMC1A_1_1459,1434,1687,1069,1275,1755,907,2125,394,1828,975,413,164
SMC1B_1_1460,751,564,704,459,250,429,725,1136,614,630,10,2637
SMC3_1_1461,1721,1256,2115,2688,2585,1365,3575,1619,1727,1508,3734,496
5
SMCHD1_1_1462,2414,1616,2963,2646,1054,3454,1778,3528,3114,1487,1767,3
107
SMNDC1_1_1463,14529,9986,17621,15810,15607,14706,19499,15581,14379,109
64,14792,13498
SMYD1_1_1464,180,101,333,632,449,161,308,54,1,294,3,49
SMYD2_1_1465,785,789,1267,1054,1115,613,782,546,54,650,1060,2242
SMYD4_1_1466,158,232,41,461,141,140,117,126,173,230,241,0
SMYD5_1_1467,935,1021,566,468,2632,967,462,1014,1,517,1221,0
SND1_1_1468,2085,1670,1689,1031,1091,1399,1958,3386,1337,1538,1485,672
SP140L_1_1469,681,627,1159,561,1175,1449,1120,508,31,1297,502,607
SRCAP_1_1470,349,468,290,588,1061,324,694,478,366,665,375,266
STAT5B_1_1471,245,39,233,157,257,56,18,1,628,291,195,18
SUDS3_1_1472,1157,823,2082,1139,1899,2402,945,219,2485,1010,2081,772
SUPT16H_1_1473,4203,3730,4654,5802,4831,7744,4473,5152,3440,4416,3880,
5395
SUPT4H1_1_1474,1844,2153,2254,2257,1299,1591,2603,4898,2034,1219,986,3
586
SUPT6H_1_1475,5991,4673,6036,6009,4530,5936,6614,5063,7807,7175,6310,6
097
SUPT7L_1_1476,1385,1316,1666,2311,3493,1138,600,1437,671,875,2147,472
SUV39H1_1_1477,1151,483,931,554,1040,886,741,1111,7,856,177,1348
SUV420H2_1_1478,155,253,426,491,170,502,714,476,301,112,983,248
SUZ12_1_1479,914,674,903,705,334,767,961,636,852,869,908,669
TADA1_1_1480,433,566,1033,180,45,601,400,60,20,1061,1,235
TADA2B_1_1481,160,54,71,27,9,219,14,78,283,99,107,3
TAF10_1_1482,2275,1465,2356,1845,2394,2280,1509,2261,1032,1814,837,154
3
TAF1L_1_1483,1365,2033,1725,1603,1446,1060,2242,884,1848,1779,2083,294
8
TAF3_1_1484,1417,1235,2054,1083,1343,288,566,769,575,2166,1412,1797
TAF5_1_1485,1477,1807,2140,1644,2241,1297,2379,2690,2843,2300,1548,135

7
TAF6L_1_1486,1662,1013,1258,2605,1204,1846,1747,386,317,1391,1327,1233
TAF8_1_1487,787,317,546,506,1165,910,672,933,937,418,1260,216
TCF7L1_1_1488,884,310,1181,911,188,1003,65,272,408,1008,2142,51
TDRD12_1_1489,6716,5653,8598,7373,7369,6147,8737,5861,7885,5282,4502,1
0257
TDRD1_1_1490,941,1168,981,865,2195,892,1913,752,1130,1511,837,427
TDRD7_1_1491,3615,3717,3755,3185,2715,6603,3945,3114,1713,4657,3772,39
82
TDRD9_1_1492,2101,2305,2895,2360,3454,2447,3006,1560,5386,3497,2279,28
30
TERF2_1_1493,810,268,720,681,434,676,343,156,9,1099,964,1414
TET1_1_1494,14943,14245,15213,18255,20960,17905,14817,14848,22829,1523
9,19146,22854
TET3_1_1495,2777,2337,2618,2471,2318,2308,5152,2337,1559,2161,2772,336
4
TLX2_1_1496,731,479,244,334,2090,427,17,761,388,54,13,157
TNRC18_1_1497,184,306,410,341,279,255,10,206,202,719,136,238
TRAF7_1_1498,1202,1406,873,1639,910,1272,407,261,993,1031,3592,2316
TRDMT1_1_1499,9840,8268,8012,11108,9047,6745,12233,9232,11109,10986,53
74,7507
TRERF1_1_1500,770,580,332,831,836,500,959,583,1057,979,114,420
TRIM25_1_1501,145,27,224,313,338,81,122,702,2,174,0,0
TRIM27_1_1502,1274,1446,2523,1628,986,2485,2466,841,199,1201,1421,1117
TRIM28_1_1503,661,312,478,430,1167,96,723,483,201,756,855,132
TRIM66_1_1504,595,496,418,583,90,205,1217,155,296,1138,26,192
TRRAP_1_1505,1669,1301,2251,1631,1541,1446,2374,2249,1562,1672,899,573
TSG101_1_1506,1375,852,1271,1663,2529,1728,778,2085,1258,1165,246,2411
TYW5_1_1507,4020,3825,4365,4415,4107,5474,4167,2853,1525,5404,2701,302
8
UBE2B_1_1508,5523,5061,6022,5906,6888,4922,7508,7044,6674,4072,5314,70
34
UBE2N_1_1509,403,667,714,676,208,1276,168,874,1164,876,705,136
UBR7_1_1510,3406,3753,4274,4021,7060,3384,5324,3745,4276,3089,5139,413
4
UHRF2_1_1511,1383,1798,2362,1913,2349,3123,999,753,2219,2329,2776,190
USP22_1_1512,1753,1649,1320,1952,1387,711,535,1427,861,1073,1286,1246
YEATS2_1_1513,556,365,609,1126,173,253,472,964,1843,521,1,0
YEATS4_1_1514,9336,8708,9871,9130,10397,7943,10555,5448,6911,7821,7025
,8431
YY1_1_1515,2741,2328,3040,2342,2888,2638,4540,3744,4173,5072,5533,2889
ZAR1_1_1516,4987,4830,4916,5502,4301,5806,4637,4272,5940,4970,8326,472
9
ZCWPW1_1_1517,1439,764,1026,988,936,957,1727,905,1610,1399,501,1114
ZCWPW2_1_1518,2252,1472,2244,1371,2304,2127,2171,1081,1817,1258,2888,1
616
ZFP57_1_1519,211,346,194,163,397,460,298,584,255,403,84,171
ZNF541_1_1520,315,419,278,201,144,1199,680,518,112,227,94,431
ZNF85_1_1521,1684,1274,1782,769,1941,1089,2025,1142,1921,857,1697,1763
AANAT_1_1522,356,225,82,260,437,67,396,132,0,401,291,0

AES_1_1523,545,125,385,156,14,79,336,322,78,166,272,175
AIRE_1_1524,517,548,603,800,217,253,852,8,75,14,5,1076
AKAP1_1_1525,4834,4295,4887,5089,6405,3468,4769,2399,1997,4324,3192,65
38
ALKBH2_1_1526,448,341,811,789,1081,222,437,871,1133,486,1330,309
ANKHD1_1_1527,2517,2711,3454,3406,923,4560,2438,2333,3181,1940,1926,21
39
ARID1A_1_1528,470,57,388,594,476,314,384,282,4,355,1178,50
ARID1B_1_1529,276,437,243,405,349,126,216,109,294,674,3028,18
ARID4A_1_1530,1,15,93,17,53,543,105,0,0,296,0,0
ARID4B_1_1531,4845,4114,4793,5047,3369,6631,5696,2593,3017,4453,4977,3
118
ARRB1_1_1532,1341,1287,713,1385,2657,997,858,391,579,1318,314,574
ASH2L_1_1533,2427,1748,2089,2079,2555,3260,3709,1660,3733,1889,987,118
8
ATAD2B_1_1534,2085,2260,1520,1266,1630,2182,919,2593,2144,868,803,3155
ATAT1_1_1535,4242,3378,4354,5803,5994,3674,5109,3746,5917,4044,4319,49
46
ATRX_1_1536,5639,6974,8001,7116,10472,8658,9230,5050,6152,7057,4543,38
03
AURKA_1_1537,8999,8126,8654,9194,9772,9440,9046,9603,7708,9513,8409,97
78
AURKC_1_1538,3666,3548,4431,2521,3263,4029,4948,989,2463,3620,2385,180
7
BAZ1A_1_1539,3584,3514,3081,3430,3427,3716,2194,4379,3441,2642,2321,31
85
BCOR_1_1540,942,337,361,473,477,672,637,504,38,475,467,336
BPTF_1_1541,1695,2124,2166,1453,2520,2438,2242,2958,747,1951,4530,2390
BRCA1_1_1542,1698,1030,1140,1951,3044,1487,846,1355,3179,1218,5584,282
0
BRD2_1_1543,1545,1585,2098,2088,1316,1589,1398,466,2528,1178,2599,423
BRD4_1_1544,1033,1023,1546,1444,3898,1869,2891,1290,3788,362,497,1420
BRD7_1_1545,1057,578,1121,1303,1626,887,597,683,151,1204,17,421
BRD8_1_1546,1851,1250,1753,1524,1434,2096,2550,2174,2038,881,143,2014
BRD9_1_1547,3880,4520,4202,3907,4533,4837,6155,3008,4671,2806,2416,451
5
BRDT_1_1548,2062,1918,1839,2138,1602,4034,1965,1959,2905,958,3049,2618
BRPF1_1_1549,414,103,124,49,217,76,0,458,297,66,646,5
BRWD1_1_1550,1427,784,1153,1043,871,320,1428,1333,909,1945,1800,242
C14orf43_1_1551,2712,3004,4190,3907,3070,4081,3685,5321,5336,4217,4114
,5215
CBX1_1_1552,1871,2356,2112,2215,2080,2855,2159,2741,2373,2007,1663,307
0
CBX3_1_1553,5294,4782,5647,6648,11457,5785,3818,3319,4255,5734,11076,7
384
CBX5_1_1554,2336,1893,2384,2496,1522,3954,1575,986,2103,3779,3574,3152
CCNT2_1_1555,1464,1518,2344,1584,1230,2119,2498,2606,2722,1751,2020,39
5
CDY1B_1_1556,5462,4840,5495,7281,7773,4370,4555,5410,10074,5286,6446,4
489

CDY1_1_1557,5462,4840,5495,7281,7773,4370,4555,5410,10074,5286,6446,44
89
CDYL_1_1558,1525,1960,1841,1634,2681,1091,2706,2266,4779,432,2575,211
CHD2_1_1559,2861,1999,2304,3503,1896,2065,3419,3280,5048,2399,3218,420
5
CHD3_1_1560,1935,983,2021,1451,2914,2317,1710,1662,776,1034,1781,1303
CHD8_1_1561,1395,1431,1591,1191,1825,1077,348,1506,2628,952,933,716
CHMP2A_1_1562,1277,915,1535,1347,1311,1704,2574,218,463,791,635,830
CHMP5_1_1563,2937,3028,3344,3190,4239,4222,4107,2335,3213,3879,3902,36
34
COPS2_1_1564,2874,2759,3033,2894,4451,3209,3247,2684,5309,4036,2250,12
21
CPA4_1_1565,1310,1004,1358,1291,3038,2157,1051,1847,843,1944,1591,2417
CREB1_1_1566,1075,788,1592,1974,2583,2959,2686,2272,1580,779,300,562
CREBBP_1_1567,1237,915,1811,1548,676,1323,973,1050,2781,1018,183,1823
CTCF_1_1568,3675,4469,4402,4360,5813,6023,4496,1198,4440,5155,5080,414
3
CTNNB1_1_1569,620,988,783,856,487,1256,1028,2582,1332,669,680,555
CXXC1_1_1570,308,301,436,676,414,511,139,788,0,719,87,886
CYLD_1_1571,1326,1380,954,1553,187,983,430,1323,1490,1310,1429,106
DICER1_1_1572,4503,4994,5121,4858,4990,6396,3453,7162,8505,2682,4002,4
041
DIDO1_1_1573,1621,1163,1431,1768,1136,403,2141,721,2090,927,583,2593
DMAP1_1_1574,2685,2052,2092,2381,2715,1797,1512,2280,2715,1992,1411,15
32
DNAJC2_1_1575,811,667,1200,1260,515,868,1026,462,1563,999,1332,782
DNMT1_1_1576,1866,1921,1830,1621,1029,1525,3600,2432,978,1543,2999,187
9
DNMT3A_1_1577,512,544,528,841,1125,405,458,483,146,388,2882,3096
DNMT3B_1_1578,77,118,115,313,20,15,34,1145,276,82,0,137
DNMT3L_1_1579,341,284,236,576,5,319,624,106,629,359,5,178
DPF1_1_1580,3347,3930,3518,2325,1672,3933,4787,1775,624,2753,2557,2682
EED_1_1581,1157,615,1176,230,3281,1908,1354,144,1030,819,324,287
EGR2_1_1582,1722,1456,1391,1628,761,1677,2594,3227,2228,902,409,1560
EHMT1_1_1583,6371,6464,6816,9324,11999,8081,7010,6359,5990,8688,8608,1
2027
EHMT2_1_1584,954,1261,1637,2079,1047,1175,2873,389,2088,581,198,1305
ENO1_1_1585,1565,1553,1451,1607,1164,2310,148,1470,1402,978,786,406
ESR1_1_1586,2684,2351,2620,2462,4306,1408,1546,3010,1693,3319,3478,795
ESR2_1_1587,465,167,540,468,622,1125,530,464,1074,624,45,38
ESRRG_1_1588,402,498,368,170,0,13,411,30,355,1,1436,0
EZH2_1_1589,1216,1061,1237,1900,661,1912,378,1133,754,939,720,1622
FBXO11_1_1590,5001,5886,6006,5912,8068,7183,4434,1082,2273,4041,4568,4
510
FMR1_1_1591,3838,3738,4302,3787,4352,5969,7499,6148,2498,4353,4939,441
5
FXR1_1_1592,13485,11556,14002,13014,20775,15226,19652,12314,16752,1381
4,10168,21066
GFI1B_1_1593,334,289,298,340,1076,184,79,92,13,115,43,97
GMEB1_1_1594,1808,1272,1640,985,1133,651,2444,2490,982,2096,3159,1342

HDAC10_1_1595,244,57,127,120,726,156,627,14,33,6,0,19
HDAC11_1_1596,485,730,879,571,905,767,193,395,303,215,353,762
HDAC5_1_1597,911,890,1722,1500,1924,1382,1023,2099,550,1430,1759,309
HDAC7_1_1598,521,272,987,1000,1040,313,1639,693,509,115,1637,713
HDAC9_1_1599,1311,1030,1199,1666,1421,1133,2154,296,1604,968,1370,826
HDGF_1_1600,918,1052,706,1224,350,636,807,1946,84,1088,3535,88
HDGFRP2_1_1601,567,895,899,530,515,684,1060,762,24,1419,93,1222
HLTF_1_1602,480,712,765,572,1414,421,354,870,0,757,146,781
HMGA1_1_1603,598,290,578,533,819,36,93,270,327,214,16,12
HMGN3_1_1604,1097,1567,2065,1074,1182,1867,2558,866,2936,1393,3248,307
4
HNF4A_1_1605,3406,2635,4144,3059,2558,4131,4018,1573,4053,3131,2780,21
89
HPSE2_1_1606,783,1159,530,889,70,545,1891,420,95,1852,1175,657
HR_1_1607,918,1267,1447,1235,2167,729,460,1196,1764,2043,697,414
ING1_1_1608,966,590,580,970,1597,1342,1253,499,648,1052,420,1547
ING4_1_1609,172,283,368,364,17,36,195,491,8,856,22,28
INTS12_1_1610,40,51,17,20,18,28,638,0,0,0,0,4
IRF4_1_1611,815,1045,1171,1021,2201,1688,195,93,2871,1298,1952,1038
JMJD1C_1_1612,2020,994,1305,3139,1189,1326,644,1792,520,1125,2762,422
JMJD4_1_1613,985,714,1422,934,1870,1486,1082,152,2555,1013,117,136
JMJD6_1_1614,353,356,241,229,327,52,58,330,319,279,33,423
KAT5_1_1615,826,799,694,1131,2631,531,292,1860,1434,1627,1365,173
KAT6A_1_1616,3896,3159,4123,3595,4216,3918,3793,4223,5985,3928,2142,55
46
KAT7_1_1617,2945,2640,3464,2749,3832,3215,1163,2030,4096,3054,1526,363
9
KAT8_1_1618,4874,4791,3392,5053,9173,5468,5855,4266,3634,3759,2714,648
5
KCTD1_1_1619,6503,6761,6578,6761,7586,7050,7994,6728,8059,5047,8377,10
974
KDM1A_1_1620,9128,7284,8839,9647,8210,7963,6893,6458,10353,7354,9144,7
502
KDM2B_1_1621,1844,2482,1684,2150,3112,2403,1750,1526,5523,1224,2151,17
59
KDM3A_1_1622,7871,8569,8862,8484,9304,11509,5901,8008,9325,6521,10404,
8680
KDM4C_1_1623,3986,4055,2778,4436,4561,4677,3681,4182,3230,4099,4398,18
75
KDM5C_1_1624,2240,1930,3152,3368,1378,3940,2969,997,2424,2009,1602,186
0
KDM5D_1_1625,5071,4028,5923,4308,5495,6571,5792,3332,6250,4053,4990,88
24
L3MBTL1_1_1626,1613,1392,1736,1376,3091,2108,1207,2899,535,2022,241,16
72
L3MBTL3_1_1627,3847,3846,4143,5566,8406,5631,1932,2559,4915,3619,3355,
4879
LBR_1_1628,2007,1072,1212,1308,1118,1305,1431,2081,514,1095,1834,1759
MAP3K12_1_1629,597,978,1092,466,1899,1071,1301,1302,3574,822,347,1046
MBD1_1_1630,1128,875,913,588,2272,870,1162,156,58,837,679,432

MBD2_1_1631,781,532,1068,265,750,901,454,417,3,1404,855,283
MECOM_1_1632,1687,1796,2111,1842,1967,2003,4193,2691,71,1461,3155,2637
MECP2_1_1633,1239,1002,798,1541,459,2488,1060,512,754,962,239,44
MEN1_1_1634,226,177,196,210,265,464,39,661,11,168,763,1093
MGEA5_1_1635,6835,7262,7498,6108,4759,8018,10255,5414,4143,5482,5452,7
334
MIB2_1_1636,476,123,317,249,217,488,785,1001,757,60,71,7
MIER1_1_1637,4718,4712,3455,3939,6424,4336,9635,4687,2686,5212,2950,38
94
MINA_1_1638,1711,1246,1316,1326,910,1359,3061,783,2719,1760,2078,1935
MLL5_1_1639,738,313,410,337,375,500,282,1273,1336,379,684,93
MLL_1_1640,1825,3011,2468,2320,2186,2539,3539,2373,2008,1973,1968,1690
MORF4L1_1_1641,1613,1219,870,1365,2225,1423,869,659,2201,1092,618,1022
MSL3_1_1642,6502,6586,5912,6109,8277,7365,6524,3531,4571,6059,11267,59
68
MTA1_1_1643,739,764,907,511,341,607,480,1057,333,340,213,2043
MTF2_1_1644,9037,6593,8668,7742,12398,8986,12603,7003,7736,7410,5693,6
707
NAA60_1_1645,498,840,418,386,812,629,164,4,118,611,89,316
NAP1L1_1_1646,2418,3567,3011,3638,2832,5326,2734,2591,4246,1938,2796,2
146
NAT10_1_1647,1120,1318,532,632,1611,1297,1019,1719,1181,865,49,237
NCOA1_1_1648,5699,5846,7028,4520,4786,7490,12200,3293,7923,6756,4014,6
962
NCOA3_1_1649,6161,5897,5930,4646,4221,6772,4083,5809,5484,5971,6617,71
93
NCOA4_1_1650,3031,2973,1995,3341,2844,3662,2510,1471,4015,3381,2248,15
99
NCOR1_1_1651,2846,2621,2089,2547,3128,4218,3790,2123,2022,2228,2401,39
58
NCOR2_1_1652,595,696,838,485,564,1309,6,541,1555,218,962,70
NFAT5_1_1653,3181,3538,3987,3103,5708,4065,2601,2491,4130,2636,5432,16
67
NFATC1_1_1654,469,169,286,290,109,695,758,178,38,87,1099,27
NFATC3_1_1655,1444,728,1241,1249,1938,1279,1939,295,989,1065,641,1155
NFATC4_1_1656,2430,3444,4219,4312,5220,2021,3534,4714,2111,3033,4700,2
477
NFKB1_1_1657,419,26,166,499,61,368,74,62,9,10,91,161
NFKB2_1_1658,5299,4244,4836,4567,3475,6200,7196,7708,3861,4651,3645,11
97
NPM1_1_1659,3996,4547,5747,4391,5334,6651,5729,4964,1552,3323,8166,408
6
NR1D2_1_1660,494,376,287,283,207,434,488,402,271,606,68,508
NR1H3_1_1661,165,90,80,50,137,628,272,113,0,107,0,25
NR1H4_1_1662,559,614,418,479,614,606,839,990,565,203,469,114
NR1I2_1_1663,1779,1681,1472,896,1281,1292,2463,1989,671,945,386,1207
NR1I3_1_1664,629,706,559,1087,383,1233,1172,1015,183,643,301,157
NR2C1_1_1665,1023,1031,731,695,1085,1025,1094,1460,1016,850,1125,651
NR2E3_1_1666,2481,1681,1763,1646,2969,2333,3104,620,1479,1199,2043,190
6

NR2F2_1_1667,1439,1664,1966,1102,2470,913,737,1116,1486,854,1110,4953
NR3C1_1_1668,16216,13755,21279,16091,21074,17752,14820,13981,10370,177
07,12502,19294
NR3C2_1_1669,2173,1930,1752,2094,2931,3368,1349,1740,2043,3269,980,186
9
NR4A1_1_1670,224,325,326,192,611,154,221,800,775,74,26,1
NR4A3_1_1671,584,69,651,59,0,36,15,1,45,186,1,1235
NR5A2_1_1672,1677,1392,1780,1730,2426,1264,1129,1587,88,1562,2484,2142
NR6A1_1_1673,2893,2592,3277,2777,1687,2995,4018,4324,2647,3234,2913,15
10
NSD1_1_1674,8797,7577,9064,8257,11994,13040,9171,7326,9667,7062,7277,7
200
PBRM1_1_1675,846,708,882,661,1104,1052,1036,1059,642,792,888,168
PCGF6_1_1676,2998,3031,3801,2784,2381,1674,1715,1514,2652,2460,3442,31
17
PGR_1_1677,1317,1018,1226,1037,1586,1320,477,634,322,1793,968,1759
PHC2_1_1678,992,673,797,684,372,307,1053,376,1421,513,775,717
PHF10_1_1679,1846,1814,1994,1934,3585,2505,1868,1822,1828,2248,2035,11
27
PHF11_1_1680,2283,1629,2046,2028,896,1340,1800,1157,1171,1098,3960,342
6
PHF12_1_1681,440,183,42,56,26,57,5,19,16,0,0,324
PHF16_1_1682,7250,5686,7184,7250,7112,7952,8743,5569,9677,6926,3476,66
02
PHF17_1_1683,4369,3566,3707,5118,3873,5498,4600,4135,5527,4388,4933,26
95
PHF1_1_1684,2043,1005,1593,860,1603,1032,370,584,1483,1022,791,1516
PHF20L1_1_1685,779,710,1023,1102,263,482,439,528,291,139,3260,547
PHF21A_1_1686,1467,682,1881,1401,1567,1019,3052,3180,387,1031,1882,132
5
PHF21B_1_1687,249,482,345,1119,0,175,680,619,621,1034,407,3659
PHF6_1_1688,7137,4758,7115,6501,6742,7248,7017,9888,8013,5531,5729,101
42
PHF7_1_1689,881,908,1425,1295,989,1076,1145,704,721,659,3484,718
PHF8_1_1690,1984,2498,3971,3603,3004,1591,2648,2530,2837,4148,2350,424
1
PIAS2_1_1691,252,213,145,443,2,119,42,2,1388,247,510,272
PICK1_1_1692,1369,1004,1668,1657,1804,1884,1728,874,2630,1929,1015,318
9
PIWIL2_1_1693,1355,1241,1162,750,183,1311,1402,1586,2263,974,625,1132
PML_1_1694,390,200,88,49,5,18,636,210,85,118,4,0
POLR1B_1_1695,2033,1612,1481,1332,1682,1551,1103,1870,2234,1049,1083,1
142
PPARA_1_1696,1876,2209,2423,2079,2185,2052,2969,2433,3816,1915,1458,70
3
PPARD_1_1697,587,435,697,594,1,1360,673,511,49,157,117,27
PPARG_1_1698,377,652,1718,847,837,6,2139,612,22,444,620,262
PRDM10_1_1699,562,79,194,321,768,529,146,706,291,193,137,3
PRDM15_1_1700,1230,877,977,1414,735,857,528,55,1381,1390,732,1820
PRDM16_1_1701,6323,7067,6990,5956,8019,5371,7335,7750,7236,2987,2577,3

301
PRDM1_1_1702,2102,1544,1383,2103,2276,2832,1321,1679,668,940,2590,1509
PRDM2_1_1703,4410,4320,3468,4506,4978,6373,4773,3095,7336,2554,3792,26
48
PRDM7_1_1704,1946,1026,3562,1655,3065,3097,2293,429,1836,1528,1927,927
PRDM8_1_1705,1360,1303,1754,860,2701,1151,2725,446,1042,284,1943,1070
PRMT1_1_1706,5305,4287,4875,5151,5200,4407,9044,2878,3169,4484,5191,50
12
PRMT2_1_1707,862,544,425,571,363,390,206,214,356,346,636,618
PRMT3_1_1708,2364,2503,3523,3017,3132,4421,6205,1453,2449,4078,4884,46
78
PRMT5_1_1709,1173,1196,999,734,1921,737,1676,1192,590,1190,700,866
PRMT7_1_1710,510,117,456,173,569,28,13,127,32,527,297,8
PSIP1_1_1711,1510,1701,2589,2085,1495,1371,2207,1084,584,744,936,366
PSMC5_1_1712,3171,2646,2576,3225,2737,4027,1621,2789,2943,1385,6384,62
56
PWWP2B_1_1713,673,698,964,715,473,377,24,292,7,982,156,1014
RAD54B_1_1714,8541,8381,9903,7476,8482,11099,10453,8802,15727,5864,290
2,8515
RAD54L_1_1715,717,496,434,613,1164,1118,829,1652,1377,818,871,57
RARA_1_1716,418,609,515,903,1193,1588,419,341,277,655,556,159
RARB_1_1717,162,96,110,117,464,488,76,1269,3,80,480,42
RARG_1_1718,3936,3377,3803,4343,3981,3816,3643,3138,4945,5999,3513,404
2
RBBP5_1_1719,829,909,1416,959,624,1443,746,985,1831,794,205,998
RBCK1_1_1720,62,147,75,108,142,60,2,36,206,119,24,252
RBFOX2_1_1721,963,639,977,815,665,1324,3086,165,590,1231,1456,854
RCC1_1_1722,2258,2693,3993,1923,2011,2963,3626,1146,4290,1563,2513,623
4
RCOR3_1_1723,3311,3071,4312,3683,2554,4037,3107,2486,2270,2211,2054,60
64
RECQL5_1_1724,1820,1677,2340,1589,1800,1948,2086,2470,1806,1457,2199,2
307
RECQL_1_1725,561,462,892,515,1371,976,526,162,2331,1000,400,24
RELA_1_1726,787,584,758,645,1581,645,23,218,512,568,535,1
RERE_1_1727,4136,4242,3499,4144,5193,3803,3549,3513,5430,3844,6233,499
6
RFC1_1_1728,2698,3402,2601,4061,3308,3344,4123,4169,1182,4206,2822,458
0
RNF14_1_1729,772,799,717,841,1323,816,867,1072,2478,672,1355,2290
RNF17_1_1730,2123,3182,2137,2400,2033,2889,4534,3165,2367,2327,2850,39
72
RNF40_1_1731,2079,2095,3156,2352,2921,3607,2622,1483,2877,4167,541,118
3
RNF8_1_1732,2588,1455,2589,2828,4379,2849,1476,3320,3093,2822,2661,314
4
RORA_1_1733,2340,1954,2496,2639,1687,1209,1736,2125,2048,3223,1458,133
6
RORC_1_1734,982,1279,1642,1466,1672,911,1245,865,1209,1879,1295,3343
RPH3A_1_1735,280,99,296,232,486,91,1301,190,210,257,279,88

RPS6KA5_1_1736,2221,1833,2465,2877,3340,2647,1365,3142,2289,2039,1938,
4435
SATB1_1_1737,1093,818,1399,1313,749,588,147,609,4926,588,1379,987
SATB2_1_1738,799,879,647,1114,1810,571,235,85,569,1081,24,509
SCMH1_1_1739,1360,1248,1363,856,874,2037,1225,909,543,523,1435,1367
SET_1_1740,15405,15732,15841,18640,22863,16884,20851,18103,13670,18859
,14070,16871
SETD3_1_1741,854,886,1066,1063,443,1946,1282,441,170,1639,3238,2798
SETD4_1_1742,42,157,290,325,997,197,38,208,74,122,374,497
SETD6_1_1743,666,627,823,1106,857,1251,327,1379,1,1419,1348,593
SETDB1_1_1744,2474,2860,3345,2505,3367,3341,4041,1422,2535,1386,3319,3
118
SETDB2_1_1745,770,439,890,403,352,869,3061,138,1164,653,17,168
SFMBT1_1_1746,1330,929,1073,665,918,489,790,102,1017,1118,494,782
SFMBT2_1_1747,1808,2299,1596,2624,1978,2946,2027,1558,719,2129,719,192
8
SHPRH_1_1748,4147,3749,4008,3835,3966,2631,3417,3235,2887,2621,2179,41
90
SIN3A_1_1749,3573,2665,2460,4244,4919,3151,2767,3427,1887,2727,789,269
4
SIRT1_1_1750,4756,5947,6649,6219,7317,6719,10259,7981,4852,5421,6446,8
612
SIRT2_1_1751,288,421,485,488,376,691,525,583,1578,1,765,173
SIRT3_1_1752,328,296,392,610,449,180,1833,385,374,143,35,300
SIRT5_1_1753,3289,2036,2380,2907,1283,2480,1108,2385,3610,3400,2326,13
66
SIRT6_1_1754,1401,1029,1159,2142,1594,1598,2490,1757,1694,1316,2183,36
0
SLC38A1_1_1755,11013,11573,14084,14100,18120,12800,15024,13689,9356,88
23,11659,11911
SMARCA1_1_1756,2091,1736,2119,1881,3409,1429,3952,1351,1179,1741,2757,
2405
SMARCA2_1_1757,3968,3686,4683,4206,3586,3607,3031,1276,1319,3740,2991,
4546
SMARCA4_1_1758,227,280,231,48,42,2,264,0,314,2,5,35
SMARCAD1_1_1759,3150,2065,2623,2996,2848,2302,3856,4034,4314,3317,3492
,635
SMARCAL1_1_1760,2381,2543,2925,2617,2824,3294,2362,1885,1916,2061,2513
,3433
SMARCB1_1_1761,1368,1077,1944,1023,1366,2080,354,2276,140,1419,1783,59
9
SMARCC2_1_1762,1414,1063,1237,1197,657,992,507,953,2142,744,1300,1562
SMARCD1_1_1763,1090,972,1115,1830,1235,1255,1505,909,2077,408,923,456
SMARCD3_1_1764,55,32,230,36,38,132,85,396,301,10,26,0
SMC2_1_1765,519,211,217,400,533,935,743,443,61,21,16,0
SMC4_1_1766,443,617,1081,622,801,784,441,232,912,630,400,1820
SMN1_1_1767,1247,1638,1355,1582,3683,670,1426,535,817,1719,1233,2055
SMN2_1_1768,1247,1638,1355,1582,3683,670,1426,535,817,1719,1233,2055
SMYD3_1_1769,1663,1421,1816,1807,831,2747,1906,837,788,1364,962,738
SP100_1_1770,1177,1743,2161,1693,1658,1445,1473,1498,5298,1419,2990,29

85
SP110_1_1771,850,533,659,975,346,384,1407,1355,1161,804,1541,1175
STK31_1_1772,952,453,358,679,349,389,349,781,53,337,12,870
SUPT3H_1_1773,4614,3896,5081,5633,5241,4241,3192,5233,4498,2598,3917,4
310
SUPT5H_1_1774,947,726,629,695,1047,903,185,1187,66,386,204,624
SUV39H2_1_1775,1720,1702,3040,2615,3161,2228,3301,2900,181,1526,2421,1
686
SUV420H1_1_1776,857,240,501,708,42,75,1906,151,32,531,11,771
TADA2A_1_1777,2728,1726,2670,2792,3686,4048,1957,1906,1490,1811,2615,2
901
TADA3_1_1778,290,217,320,452,140,114,386,295,307,29,291,214
TAF12_1_1779,3076,2761,3718,3610,2722,4532,4743,2213,4013,2648,4261,30
58
TAF15_1_1780,3444,3067,3199,2981,3692,2661,2802,3239,2465,2915,3116,62
89
TAF1_1_1781,7114,8642,11227,10572,9578,9448,9840,9768,11345,9736,8715,
7010
TAF5L_1_1782,1023,832,1551,1984,1460,1217,1557,743,710,917,2044,728
TCF19_1_1783,186,116,71,369,177,0,0,0,1,102,655,1186
TCF20_1_1784,5121,5150,6332,6296,9178,5414,4524,5544,7512,7953,6598,96
84
TDRD10_1_1785,147,165,315,314,22,5,1025,124,98,29,1,0
TDRD3_1_1786,3500,3140,3336,3513,3409,1801,4886,1126,2787,1214,1480,56
59
TDRD5_1_1787,366,456,342,261,181,317,116,77,896,563,10,4
TDRD6_1_1788,1478,1249,923,1454,689,1353,1555,1856,2006,1482,376,699
TDRKH_1_1789,776,555,463,516,603,335,639,170,1249,287,1,0
TERF1_1_1790,911,1346,845,549,887,2131,1754,734,309,1087,412,1444
TET2_1_1791,2015,1554,1379,2005,2386,1442,2183,1738,3927,1511,348,1281
THRA_1_1792,631,425,679,651,93,538,916,477,359,844,1051,327
THRB_1_1793,1113,1001,1261,1013,1389,587,713,494,1185,1252,2174,429
TP53BP1_1_1794,4333,4141,5083,5052,5255,5982,7350,3870,4423,3844,3548,
4829
TP53_1_1795,1605,867,948,784,3700,1112,845,1787,806,943,1335,356
TP73_1_1796,4554,3731,5295,3937,4956,4145,3653,2165,2700,5357,1592,267
9
TRIM24_1_1797,1021,1086,838,1277,828,412,373,419,1528,1723,996,1225
TRIM32_1_1798,924,907,983,1115,531,801,419,785,978,171,2078,93
TRIM33_1_1799,580,777,1109,1201,1547,1538,1379,308,672,940,937,91
UBE2A_1_1800,1989,2441,2575,2237,2009,2620,1961,997,1225,2479,2681,544
5
UBE2E1_1_1801,1242,1871,1633,1562,2096,2214,1375,1122,2153,432,2160,14
59
UBE2I_1_1802,1649,1894,2144,1329,2639,2959,2387,2808,2056,914,922,1436
UBE2K_1_1803,6733,5329,7594,6609,6425,6787,4255,6432,6059,4345,4263,11
791
UBE2V1_1_1804,2434,2172,2407,1648,3206,3081,4886,1858,1261,1995,2690,1
354
UHRF1_1_1805,367,632,259,130,1813,186,259,587,38,471,485,4

USF2_1_1806,277,414,66,374,68,1263,128,613,1,21,0,5
UTY_1_1807,21486,21820,27399,26168,28188,28442,31123,20404,18656,21934,23023,29070
VDR_1_1808,1001,726,1226,1292,720,597,888,826,1250,643,1451,808
WDR5_1_1809,2524,2080,2713,3893,2873,2536,3094,2978,3166,2384,2511,1981
WHSC1_1_1810,438,852,509,449,1097,1037,1880,714,1147,393,717,919
WHSC1L1_1_1811,472,1074,732,1335,2130,209,925,1288,438,994,384,78
WRB_1_1812,1514,1223,932,1169,2692,1328,2671,1110,1135,801,1486,1529
ZGPAT_1_1813,703,621,519,645,200,672,145,327,351,717,363,589
ZMYND11_1_1814,1014,1148,1142,718,460,981,1243,283,366,916,527,1108
ZMYND8_1_1815,2105,2258,1599,1361,2909,1576,702,740,2646,1237,2847,641
ZNF451_1_1816,1268,1570,2031,1214,1422,2295,274,1336,400,603,1758,554
ALG13_1_1817,1008,908,1888,1833,665,705,1279,382,1,154,2908,529
ASXL1_1_1818,1230,1122,864,644,417,1537,793,1988,590,353,111,2716
CBX2_1_1819,1284,1053,1932,1302,1582,477,1471,583,883,1129,1154,2616
HDAC8_1_1820,911,857,813,294,1894,963,845,221,1678,461,190,0
ING3_1_1821,1180,1103,1337,626,2120,508,1501,740,1027,1539,2020,935
MLLT10_1_1822,739,563,1015,1206,228,596,685,1136,2511,511,750,1555
PHF19_1_1823,3965,3026,4499,4359,6003,6836,2240,2350,3893,3546,2044,3657
RBM14_1_1824,931,1537,1793,1152,1329,922,823,1042,2249,793,666,880
SP140_1_1825,891,649,929,524,456,664,2085,364,274,821,459,510
TAF9_1_1826,4167,3833,4804,3413,3881,4411,5107,7241,6493,4309,5907,6492
TAF9_1_1827,814,1045,1288,1072,885,888,1356,1207,302,1284,345,437
AHR_1_1828,838,499,861,612,218,241,901,986,435,598,2072,684
ALKBH1_1_1829,659,536,513,565,872,782,1550,467,486,273,1368,2273
ALKBH3_1_1830,3853,3458,3462,4050,2742,4151,1766,2711,2740,4403,3323,2941
ARID2_1_1831,2867,2447,3158,2852,3860,3924,3255,1994,2328,1737,2671,1724
ASF1A_1_1832,2613,2765,3304,3913,2417,2406,1171,3785,4362,3981,2110,4796
ASF1B_1_1833,562,217,511,679,558,763,2143,391,0,446,211,2036
ASH1L_1_1834,581,492,1494,873,450,1015,2006,969,1792,643,1195,524
ASXL2_1_1835,2862,3335,3036,3375,4465,4234,3739,5560,866,2361,5934,6137
ASXL3_1_1836,2315,2045,2547,3744,2043,1835,1457,3251,2190,1111,6693,1939
ASZ1_1_1837,7695,6020,8419,7894,7128,9935,12884,5318,3200,4238,4765,9690
ATAD2_1_1838,18703,18786,20932,21774,29807,23303,17612,17632,15817,20857,19437,20299
ATF7IP_1_1839,1538,1699,2554,2050,2765,1990,1573,2135,1480,1958,1690,1744
AURKB_1_1840,3666,3548,4431,2521,3263,4029,4948,989,2463,3620,2385,1807
BAHCC1_1_1841,192,178,609,46,35,895,200,9,1747,11,0,8
BAHD1_1_1842,476,344,531,819,867,405,250,553,1003,682,154,549

BARD1_1_1843,2541,1431,2489,1851,2200,1351,1879,1315,2274,2667,5,626
BAZ1B_1_1844,8122,5788,8113,6935,9359,7200,9369,8058,5678,4665,5488,13
343
BAZ2A_1_1845,5328,5345,5158,5401,7572,5194,5846,3226,4328,5121,5802,37
51
BAZ2B_1_1846,25927,25315,26892,28985,33395,28854,33605,24784,24264,293
04,23923,28767
BLM_1_1847,2450,1608,2649,2148,1787,1169,1301,1079,2602,2160,1610,1978
BMI1_1_1848,4758,4479,4502,4532,4592,5212,2274,4067,3826,4737,4577,516
9
BRD1_1_1849,324,564,417,349,659,255,636,413,1015,363,673,343
BRD3_1_1850,1318,1636,1485,1298,4056,2005,1228,799,1355,878,439,1596
BRPF3_1_1851,480,729,227,469,333,597,805,161,21,437,1611,1009
BRWD3_1_1852,3280,4414,3338,4935,2295,3598,5853,4176,3739,2572,2487,35
93
C14orf169_1_1853,795,850,1018,819,1077,556,1901,2849,354,708,1478,192
C20orf20_1_1854,2276,2695,2291,1508,2694,2828,923,1664,621,2103,874,25
34
CALR_1_1855,337,574,423,336,13,338,495,1248,227,425,0,782
CARM1_1_1856,869,431,522,469,1310,1492,654,674,2217,449,693,56
CBL_1_1857,2355,2823,2771,3654,3428,1951,3460,2498,1978,1867,4091,1750
CBX4_1_1858,105,107,552,243,41,200,757,16,1,174,274,0
CBX6_1_1859,1390,981,535,894,868,1176,949,541,2548,746,1520,349
CBX7_1_1860,2788,2720,3097,3965,3702,2420,3731,2126,5920,3274,1991,340
5
CBX8_1_1861,896,646,916,397,1824,1288,1317,277,1642,523,1070,684
CCDC101_1_1862,1095,1026,1423,1609,1645,1139,570,1867,49,1093,2150,296
1
CCNE1_1_1863,1494,1119,1733,1519,2916,2435,616,416,2737,1111,1429,1359
CCNT1_1_1864,4007,4247,4087,4420,4115,4659,6044,5417,4080,3094,897,354
5
CDC73_1_1865,5063,4523,4881,4893,5419,4843,5629,3967,7233,5780,7431,77
55
CDK9_1_1866,1206,839,1009,1170,428,170,1230,695,615,778,257,667
CDY2A_1_1867,13302,14076,16755,15253,14336,13903,15304,14346,12698,137
97,14724,12498
CDY2B_1_1868,13302,14076,16755,15253,14336,13903,15304,14346,12698,137
97,14724,12498
CDYL2_1_1869,501,435,442,559,924,583,101,190,2190,305,6,68
CECR2_1_1870,794,1038,809,603,1368,727,620,850,586,619,1889,889
CHAF1A_1_1871,140,464,177,474,355,194,388,206,100,132,62,162
CHAF1B_1_1872,1759,889,2010,950,2937,2743,436,851,6500,747,2287,362
CHD1_1_1873,2928,2561,2797,2559,2449,3769,5492,2604,1983,2439,1173,211
4
CHD1L_1_1874,637,140,518,277,347,205,589,67,460,206,52,587
CHD4_1_1875,1703,2086,2248,2433,2286,1979,2464,2167,1003,1804,1521,170
5
CHD6_1_1876,434,605,812,548,414,365,2421,681,1321,1511,1089,1075
CHD7_1_1877,1308,900,821,1254,849,1528,841,1427,1288,1389,754,2900
CHD9_1_1878,1149,1006,1039,1156,588,770,3136,98,1161,733,1419,820

CHMP1B_1_1879,1241,243,471,507,164,817,988,432,401,1161,177,161
CHMP4B_1_1880,7201,6917,9792,8425,10279,6355,7979,10039,8708,6962,7128
,4568
CHMP4C_1_1881,3162,3511,3967,3085,2461,2836,3844,2622,2435,2347,2384,1
585
CHRAC1_1_1882,5652,5258,6842,5514,6989,6154,5796,3965,3594,6139,6658,7
552
CLOCK_1_1883,4108,4124,5271,3818,6460,3051,10124,2688,7216,5061,8091,2
843
COPS5_1_1884,1474,1045,2241,2024,2631,1673,3405,1538,668,1521,2516,629
CRAMP1L_1_1885,1341,1251,1140,1080,1558,1709,1022,1052,1024,803,1232,4
42
CTCFL_1_1886,1157,802,1077,857,1502,927,3238,1359,2049,573,1123,572
DEAF1_1_1887,355,443,586,582,280,468,4,5,129,346,687,520
DNAJC1_1_1888,5679,6168,6567,8106,7524,6072,5357,6349,5207,8018,3861,8
604
DOT1L_1_1889,1541,1024,904,1065,1009,1419,1755,1594,2004,857,1161,87
DPF2_1_1890,2339,1645,1985,2685,3101,3316,2791,2383,6019,1218,1824,178
6
DPF3_1_1891,37,0,6,59,0,0,1,1,0,6,0,0
EIF4B_1_1892,1178,2231,1308,1526,1575,959,3085,940,775,974,734,859
ELP3_1_1893,3436,2806,3106,2927,4117,3852,3304,1233,6333,3416,4468,291
6
EP300_1_1894,786,563,990,599,1082,703,452,50,860,121,4,1575
EP400_1_1895,142,699,260,421,396,38,200,159,19,230,716,1083
EPC1_1_1896,2596,1760,1989,3208,3116,1645,1127,1056,2105,2072,2209,326
4
EPC2_1_1897,1510,1374,1630,1523,1017,2063,1885,891,708,1685,3751,396
ERCC6_1_1898,199,138,425,1026,3,485,145,314,1259,301,587,211
ESRRA_1_1899,1053,1177,1445,1193,1717,1527,470,1536,1942,1211,1845,141
6
ESRRB_1_1900,778,614,897,422,1537,228,957,223,1239,612,281,1937
FANCM_1_1901,648,768,636,846,79,1074,3462,774,543,800,276,518
FBXL19_1_1902,933,642,892,600,1802,291,1397,233,659,771,1145,1289
FOS_1_1903,322,190,394,375,171,56,1021,100,1239,110,101,141
FTO_1_1904,3700,2814,2872,3657,4076,3784,3620,2497,2686,3777,2168,3798
FXR2_1_1905,1288,780,628,1364,1904,1199,622,757,310,1557,1027,1259
G2E3_1_1906,3180,4408,5835,4622,5911,6059,5591,3202,2636,4441,5355,440
4
GATAD2A_1_1907,604,187,246,343,20,368,77,295,229,70,86,167
GLYR1_1_1908,3638,3126,3220,3111,4520,3746,3948,2679,2316,3367,4867,52
83
GMEB2_1_1909,8,17,24,346,0,1,75,6,84,6,0,0
GSG2_1_1910,558,693,898,1027,818,154,1991,7,66,104,21,438
GTF3C4_1_1911,541,978,771,1010,1049,956,1032,1433,513,440,661,151
HAT1_1_1912,2024,1688,3246,1547,2287,2460,2881,3440,1060,2566,2910,150
7
HDAC1_1_1913,337,772,756,495,1795,548,915,309,353,1047,908,197
HDAC2_1_1914,2141,2036,2176,1507,412,1528,2116,724,614,1834,2420,3374
HDAC3_1_1915,38,106,45,119,158,27,20,33,3,137,81,146

HDAC4_1_1916,1837,1888,2495,1336,2909,2025,1383,2602,550,1456,3322,232
7
HDAC6_1_1917,0,0,0,304,0,2,0,324,0,1,0,0
HDGFL1_1_1918,443,1060,590,692,391,11,50,113,1643,306,0,74
HDGFRP3_1_1919,2134,1501,1830,1380,1836,1585,3499,2089,1386,918,347,21
06
HELLS_1_1920,4236,4559,4872,4063,5414,6204,4033,4871,6771,4527,5760,44
50
HEMK1_1_1921,1274,931,658,1235,512,1491,3088,580,560,671,760,1051
HIF1AN_1_1922,527,635,963,1631,10,294,3114,156,8,960,999,2668
HIRA_1_1923,4030,2968,4590,3936,4992,5110,4834,4274,3167,4378,2389,460
8
HMG20B_1_1924,648,350,367,487,1658,791,181,89,147,374,1,52
HMGN5_1_1925,939,679,720,561,2157,639,402,566,104,1027,2442,1920
HNF4G_1_1926,943,796,533,810,432,378,404,196,250,488,347,81
HSPBAP1_1_1927,2801,2218,1996,2789,2996,1799,3109,756,2989,1842,2178,3
454
HUWE1_1_1928,1259,1790,1023,2170,3448,2069,151,1387,651,2142,2109,2277
ING5_1_1929,2249,1318,2327,1890,3775,1389,696,1496,1437,1835,351,1933
INO80_1_1930,1885,1058,1785,2692,1605,1254,990,2300,1405,1535,1592,160
2
JARID2_1_1931,164,265,125,307,19,140,586,723,633,261,5,45
JHDM1D_1_1932,2261,1292,1930,2114,1972,2158,2411,707,3561,1072,581,625
JMJD7_1_1933,612,766,744,1150,1390,396,856,1403,75,610,880,929
JMJD8_1_1934,3551,3064,2909,3272,4749,2591,3089,3866,4402,3832,2084,45
18
JUN_1_1935,2165,2996,3142,3305,1594,3067,2132,1455,3620,3422,4208,6047
KAT2A_1_1936,735,265,467,449,734,401,738,132,999,531,410,1
KAT2B_1_1937,1951,2057,3081,1992,3034,4048,3090,1078,970,2329,72,1576
KAT6B_1_1938,1687,1759,2599,1687,1057,1426,2093,1978,964,3827,2372,277
0
KDM1B_1_1939,3300,3161,3822,2820,3207,5609,5009,2724,1708,2823,4214,30
32
KDM2A_1_1940,1345,1472,1342,934,2298,1499,1447,718,690,801,853,2292
KDM3B_1_1941,930,474,867,1206,1018,242,797,304,1005,415,2044,702
KDM4A_1_1942,545,726,492,948,662,951,411,711,322,775,807,365
KDM4B_1_1943,101,33,158,0,0,0,2,0,0,0,0,874
KDM4D_1_1944,999,566,639,602,604,591,955,1214,740,528,1277,793
KDM5A_1_1945,3398,3073,4650,3233,2940,3581,2187,2304,3544,2219,3526,18
49
KDM5B_1_1946,1199,589,759,686,376,844,939,1989,706,784,34,140
KDM6A_1_1947,2938,2964,3795,3068,6241,4162,5272,3846,4421,3029,5927,40
22
KDM6B_1_1948,1060,494,1477,1078,1007,64,793,364,433,374,86,57
KIAA2026_1_1949,739,773,630,689,644,1023,946,370,342,1068,757,1468
L3MBTL2_1_1950,755,292,140,362,1860,235,40,0,0,216,31,591
L3MBTL4_1_1951,2562,2283,1611,1350,2061,3333,1675,1573,390,1573,176,74
MAEL_1_1952,2676,1891,1921,2293,1050,2223,2303,1674,440,1644,1326,4224
MBD3_1_1953,473,695,607,467,623,646,530,1323,1123,352,3370,1
MBD4_1_1954,3941,2985,2622,3278,1495,1411,3070,1824,2762,1754,2769,427

9
MBD5_1_1955,8778,8602,8355,10720,9165,11540,12107,7457,11156,6273,6968
,10493
MBD6_1_1956,1780,913,2074,741,2290,1689,810,1724,2993,454,418,1024
MBTD1_1_1957,1371,1372,1324,1557,690,2221,1391,689,474,426,3180,256
MDM2_1_1958,4893,3438,4285,4055,4008,5848,5013,5317,3955,3742,4964,367
6
MIER2_1_1959,351,374,367,418,742,704,669,84,2014,94,615,24
MIER3_1_1960,2913,3719,4567,4813,2840,3497,2563,2488,3854,2728,4533,58
80
MIS18BP1_1_1961,1133,944,1474,1212,2089,961,651,1577,1061,890,936,1136
MKL1_1_1962,1069,576,1168,1016,1039,943,748,424,1070,1150,33,325
MLL2_1_1963,428,376,281,152,12,1706,943,163,1267,84,212,3
MLL3_1_1964,3251,2332,3900,3589,3011,2116,3891,2214,5239,3607,4094,371
6
MLLT1_1_1965,176,340,91,405,626,224,169,245,3,179,44,8
MLLT3_1_1966,2253,2473,2027,2771,3811,3915,2088,1601,2233,1650,643,117
2
MLLT6_1_1967,560,1020,917,1383,114,1223,1086,2431,759,834,777,1540
MPHOSPH8_1_1968,1833,1319,2568,1233,1459,2475,1134,1720,2041,818,1536,
141
MSH6_1_1969,1587,1553,1302,1966,715,2052,1158,2718,238,2008,1034,3374
MSRB2_1_1970,378,567,100,234,109,172,272,84,27,590,8,589
MTA2_1_1971,1223,934,1147,1431,2346,1230,1188,1662,610,893,1440,2644
MTA3_1_1972,2372,2505,2730,2245,2066,3378,2022,1153,1444,1347,2208,106
6
MYSM1_1_1973,4381,3503,4405,5607,1373,5605,6339,4948,3345,4817,3963,61
20
NAP1L2_1_1974,1728,1271,1311,1692,1049,957,2160,518,1492,2689,1218,261
3
NAP1L3_1_1975,2951,3452,3703,3546,5193,3606,2136,3149,3225,3261,4669,4
947
NAP1L4_1_1976,1168,1118,1356,1288,598,1354,1547,1709,1595,1046,1404,10
56
NAP1L5_1_1977,245,92,120,104,46,113,165,41,0,0,71,117
NAT14_1_1978,15,0,29,2,0,0,0,0,0,0,0,182
NAT8B_1_1979,5166,5078,6282,5131,3895,5229,7978,3095,6982,5853,8134,57
63
NAT8_1_1980,195,297,150,173,22,528,327,19,10,32,13,0
NAT8L_1_1981,18,95,398,13,0,1,16,387,110,87,0,0
NAT9_1_1982,442,337,281,221,127,389,194,462,286,66,86,279
NCOA2_1_1983,2121,1356,2898,3082,1221,2225,1959,2367,1733,2063,2142,45
20
NPTXR_1_1984,1031,1342,1388,696,1581,2332,1240,728,3,1206,1862,1260
NR0B1_1_1985,241,368,414,450,562,314,196,9,7,173,20,51
NR0B2_1_1986,5441,5579,5855,7057,4460,6530,7642,7658,1825,5704,4356,51
83
NR1D1_1_1987,250,445,1074,491,243,110,500,273,28,262,195,370
NR1H2_1_1988,1603,2031,2011,2462,1993,2834,1524,1903,1692,1293,1712,25
32

NR2C2_1_1989,759,754,1239,422,203,249,967,550,856,90,448,617
NR2E1_1_1990,635,1159,1230,1101,1407,374,1224,1680,1491,1359,2110,1519
NR2F1_1_1991,378,405,293,150,507,798,148,110,25,421,361,581
NR2F6_1_1992,1176,928,911,1516,457,1162,399,2289,373,739,1272,578
NR4A2_1_1993,30,237,411,139,1,19,6,0,21,40,171,0
NR5A1_1_1994,1417,1723,1217,1458,2809,571,1182,2113,2483,953,3976,2168
NRIP1_1_1995,804,657,182,570,810,951,713,403,662,709,1382,767
PADI4_1_1996,1462,1287,1368,1024,1320,1388,2839,722,1397,1089,979,433
PAWR_1_1997,453,428,350,99,58,126,102,162,437,563,947,1
PAX5_1_1998,2185,1392,2092,2014,1532,1314,2731,1702,2473,2069,1805,145
9
PAXIP1_1_1999,655,474,496,524,388,462,444,293,195,67,126,2427
PGRMC2_1_2000,1544,1521,1622,1671,1095,533,353,3474,539,2507,1949,265
PHB_1_2001,889,651,1163,632,247,200,712,176,2219,326,1987,30
PHC3_1_2002,1127,1351,1543,1551,637,1976,1338,2678,1398,1401,2782,3119
PHF13_1_2003,90,110,296,63,0,772,2,0,2156,354,11,6
PHF14_1_2004,1585,1460,2550,1623,971,1832,244,3224,3643,1934,1950,984
PHF15_1_2005,811,886,1360,1339,1132,639,1726,281,2362,294,950,991
PHF20_1_2006,3248,3057,3444,3413,4711,1635,3164,3204,2692,3592,1776,56
31
PHF23_1_2007,3935,4369,4619,5626,5301,4793,6178,4655,6027,5580,4276,56
14
PHF2_1_2008,1763,1385,1340,1862,736,2894,3218,641,2969,2992,3086,831
PHF3_1_2009,5715,4626,6313,4502,6647,6298,6999,5772,9636,3566,2747,631
6
PHF5A_1_2010,1224,1030,344,910,228,1490,140,427,778,249,1783,177
PHIP_1_2011,2702,3040,2630,3358,2662,2986,2437,2767,1827,3351,3131,555
4
PHRF1_1_2012,735,511,844,1163,1191,716,1596,1908,74,539,1355,101
PIAS1_1_2013,4403,4323,5187,5216,3651,2919,6329,6528,3739,2276,3935,86
04
PIWIL4_1_2014,746,933,573,832,1598,572,497,1016,474,765,408,930
PPARGC1A_1_2015,984,1245,1074,1030,3020,1448,2164,1055,789,574,36,1471
PRDM11_1_2016,924,769,628,595,1008,663,454,861,420,856,145,759
PRDM12_1_2017,155,149,486,144,26,29,4,3,149,191,13,11
PRDM13_1_2018,2032,1167,1098,1118,890,564,1865,711,3343,748,745,509
PRDM14_1_2019,1367,913,1408,1259,922,1394,1448,681,793,734,1710,754
PRDM4_1_2020,365,297,731,599,660,266,177,276,173,424,212,419
PRDM5_1_2021,2934,2267,2882,3418,3825,3013,2993,3552,7148,3996,3439,45
01
PRDM6_1_2022,499,429,496,756,312,1811,227,1924,384,391,1656,813
PRDM9_1_2023,1251,1140,1492,1517,616,1575,887,228,704,2004,2006,2432
PRMT6_1_2024,442,180,329,478,259,63,102,992,163,300,29,5
PRMT8_1_2025,1555,835,1348,1596,1400,1343,2586,1121,247,1547,2057,1093
PRPF6_1_2026,911,873,879,850,964,685,957,464,790,894,1020,2454
PYGO1_1_2027,1801,1091,1488,1563,1879,2883,1257,1721,664,1634,1210,242
8
PYGO2_1_2028,1543,1737,2281,1586,2195,3130,1367,1711,2033,1593,743,106
9
RAG2_1_2029,1878,1709,1952,1988,2985,3448,1662,596,3547,1188,718,2340

RAI1_1_2030,1741,1158,1818,2144,1837,1689,1306,2322,2413,1398,3484,186
5
RB1_1_2031,4213,4348,3477,4108,6308,6700,4785,3502,4852,2858,5690,9905
RCOR1_1_2032,2540,3041,2464,3076,1673,2084,1870,2989,3627,2491,3144,74
0
RCOR2_1_2033,132,74,126,122,935,0,63,88,347,175,0,0
RECQL4_1_2034,674,356,475,365,512,1121,1303,308,51,404,706,186
RELB_1_2035,493,735,668,827,753,367,235,286,287,437,266,246
REL_1_2036,909,682,936,985,618,663,418,227,1627,270,982,2448
RING1_1_2037,432,300,500,527,555,943,383,383,1177,783,2008,444
RNF20_1_2038,978,1203,1338,1145,473,1343,520,279,605,983,580,1577
RNF25_1_2039,222,52,10,14,2,77,31,0,0,35,0,22
RNF2_1_2040,530,603,994,1124,983,671,444,700,519,1459,681,430
RORB_1_2041,637,518,341,386,417,120,173,30,1493,1600,816,255
RSF1_1_2042,666,662,568,415,564,906,745,352,723,1870,1436,244
RUVBL1_1_2043,1789,1761,1483,2553,3118,923,1883,1868,842,1146,1140,270
5
RUVBL2_1_2044,814,836,674,1284,396,1448,597,941,1506,1251,1306,19
RXRA_1_2045,1022,883,1172,1525,2049,610,1412,1851,546,948,2960,1075
RXRB_1_2046,590,231,979,922,85,89,612,1270,53,390,34,1192
RXRG_1_2047,645,383,447,210,852,153,540,176,0,569,25,793
SAP18_1_2048,976,488,485,375,80,820,707,886,10,373,770,428
SCML2_1_2049,6635,4806,6710,5449,7017,5396,10493,6225,5069,5050,9820,5
438
SENP3_1_2050,1305,1018,776,750,1004,2191,778,1027,45,730,645,1734
SETD1A_1_2051,204,177,394,352,144,49,242,535,0,969,31,0
SETD1B_1_2052,3053,1823,1986,1946,3451,2919,2307,2233,1161,1654,2271,3
413
SETD2_1_2053,5658,6004,7521,7911,5637,6896,9002,7497,5764,5680,7982,12
499
SETD5_1_2054,678,418,428,713,997,501,817,232,234,615,802,53
SETD7_1_2055,644,429,461,685,1362,314,658,204,596,794,335,212
SETD8_1_2056,1732,1165,1455,1499,914,1042,1771,1086,1237,1054,859,2694
SETMAR_1_2057,2934,1973,2964,2499,4464,3017,2457,1151,3835,1611,1421,3
896
SF3B3_1_2058,218,541,1115,625,223,326,219,101,38,817,88,11
SIAH2_1_2059,298,323,364,59,147,457,442,0,363,258,0,60
SIN3B_1_2060,595,121,177,227,131,36,302,15,91,97,0,20
SIRT4_1_2061,929,1253,1707,1470,841,1110,1237,650,2499,1503,544,1616
SIRT7_1_2062,657,528,372,687,968,454,1969,725,246,1007,421,1433
SLC2A4RG_1_2063,995,901,578,771,134,1288,1127,38,612,494,725,1510
SMARCA5_1_2064,1227,888,1434,1131,2432,1595,750,670,334,1584,250,145
SMARCC1_1_2065,722,888,873,862,888,358,1322,663,3025,788,373,1857
SMARCD2_1_2066,1410,1051,1888,2056,2492,1666,3429,1687,619,2290,1108,1
252
SMARCE1_1_2067,389,50,145,144,16,668,0,14,35,106,0,122
SMC1A_1_2068,145,236,502,147,156,208,0,112,440,239,13,44
SMC1B_1_2069,1729,1279,1586,1590,2821,1768,440,1670,1291,3044,920,3865
SMC3_1_2070,331,146,417,390,86,126,124,520,59,55,67,379
SMCHD1_1_2071,6065,5307,6731,6482,8162,7518,4177,4840,2812,4255,6535,3

638
SMNDC1_1_2072,1946,1029,1227,1813,2325,2024,1494,894,1242,1231,1961,13
10
SMYD1_1_2073,2217,1984,1775,1624,3766,2866,1726,1994,5214,2536,1539,21
70
SMYD2_1_2074,1664,550,897,747,1127,2122,1710,1983,2654,1126,460,43
SMYD4_1_2075,1494,1105,1817,1435,1507,1765,1260,737,1018,2075,75,1028
SMYD5_1_2076,443,0,0,0,7,0,0,1,0,0,0,0
SND1_1_2077,1108,970,1265,1190,1801,1087,1324,758,894,1279,1503,609
SP140L_1_2078,2268,2116,3516,3026,3126,2268,1072,3712,1493,4004,1314,6
046
SRCAP_1_2079,955,823,926,973,1354,945,424,1659,1901,424,296,835
STAT5B_1_2080,2447,2203,2755,2846,2590,735,2588,1817,2874,1715,1829,11
41
SUDS3_1_2081,14479,16411,19800,16527,20152,16009,18475,16226,13709,163
61,16458,19636
SUPT16H_1_2082,5823,4253,6491,7450,4379,9728,4948,7888,4784,5252,3364,
7051
SUPT4H1_1_2083,1355,1181,605,1519,495,709,1766,2045,3088,710,851,1712
SUPT6H_1_2084,4346,4084,3552,3405,6730,3018,3347,5357,5399,2897,4423,6
864
SUPT7L_1_2085,869,1010,1195,903,639,1102,1210,575,1671,895,1006,747
SUV39H1_1_2086,2174,2282,1366,1857,2050,2690,1786,1304,1943,2034,2288,
1522
SUV420H2_1_2087,148,237,204,27,42,486,37,22,142,84,120,19
SUZ12_1_2088,1628,1138,2012,1883,4674,1927,1383,1781,3264,1511,2650,68
6
TADA1_1_2089,431,470,699,383,1076,563,811,6,723,306,0,1086
TADA2B_1_2090,5820,4175,6888,5246,9923,3931,7680,7339,5058,5210,3609,4
562
TAF10_1_2091,1910,1290,1609,855,3240,2028,1713,1337,2552,1490,311,1134
TAF1L_1_2092,2846,1986,2050,2195,3675,1885,3711,2521,4082,2084,2505,34
5
TAF3_1_2093,832,578,682,919,1383,695,368,292,225,2036,477,1802
TAF5_1_2094,0,0,0,0,0,0,0,0,0,0,0,0
TAF6L_1_2095,1746,612,1133,1469,2349,1577,2501,1022,465,661,482,1888
TAF8_1_2096,642,581,967,1070,1582,939,1239,1235,1527,1219,607,842
TCF7L1_1_2097,134,109,321,267,11,103,1214,1,43,175,0,2
TDRD12_1_2098,2343,2076,1892,1892,3107,2066,1880,1427,1446,1506,780,14
99
TDRD1_1_2099,161,70,175,582,846,692,206,17,0,336,39,471
TDRD7_1_2100,798,772,1214,1054,1294,1814,2074,1709,705,277,271,1135
TDRD9_1_2101,1881,2092,2205,2688,817,3135,1090,894,3209,2114,4481,1203
TERF2_1_2102,849,304,620,524,1082,443,340,154,865,153,79,1086
TET1_1_2103,937,484,1208,1028,510,524,415,1268,1099,322,1535,2799
TET3_1_2104,4683,4382,3952,7175,3089,5280,3314,5414,2152,6072,5256,305
3
TLX2_1_2105,1027,305,409,250,610,138,423,898,1,191,237,131
TNRC18_1_2106,3530,3209,4511,5078,3848,6085,3089,3998,5407,4409,4629,3
389

TRAF7_1_2107,3125,2625,3572,2343,3925,3049,4221,1289,2114,2545,2379,11
42
TRDMT1_1_2108,2292,2308,2654,2170,740,3082,1391,2141,2198,1427,864,301
0
TRERF1_1_2109,7914,8314,10536,9124,7991,8042,7742,10980,5043,7241,5919
,8258
TRIM25_1_2110,4388,4910,7030,6421,7391,8697,8819,4856,5358,5965,5796,6
050
TRIM27_1_2111,513,756,672,528,925,468,356,80,772,357,1137,229
TRIM28_1_2112,231,267,747,158,324,39,100,498,75,369,1361,335
TRIM66_1_2113,423,164,213,130,463,354,388,198,148,791,53,0
TRRAP_1_2114,2357,1729,2552,3216,5047,2132,4427,1159,2684,2374,3600,31
22
TSG101_1_2115,843,609,642,468,1047,576,1992,800,453,534,691,376
TYW5_1_2116,3179,2225,3305,2547,3022,2829,3254,1833,825,1326,4933,2922
UBE2B_1_2117,4984,4627,5307,5321,6514,3656,6682,6573,6655,3109,5500,70
15
UBE2N_1_2118,237,24,13,23,0,1,87,0,2,2,26,9
UBR7_1_2119,551,710,473,741,659,597,563,234,1640,880,385,284
UHRF2_1_2120,4738,4234,5456,5936,4861,3955,2867,3902,2541,4530,4393,38
50
USP22_1_2121,5264,5282,5691,5870,8016,5637,5291,3602,3878,4209,5562,50
76
YEATS2_1_2122,2669,3000,2915,3849,4224,3235,2129,3466,2419,2579,3166,5
187
YEATS4_1_2123,1461,1395,1343,1176,1077,1040,350,2555,893,1101,2891,208
0
YY1_1_2124,2742,1922,2762,2680,4037,3237,1913,1205,2273,1494,149,3470
ZAR1_1_2125,2638,1954,3681,2028,3054,4959,3149,3695,3416,1926,1144,229
8
ZCWPW1_1_2126,2925,2604,3851,3439,2965,4146,3792,3373,2739,3940,4212,2
634
ZCWPW2_1_2127,464,484,829,478,571,421,256,902,336,760,73,104
ZFP57_1_2128,1731,2553,2563,2415,2246,2826,2497,2419,2526,1108,2911,14
72
ZNF541_1_2129,295,506,522,395,304,457,901,475,124,105,439,181
ZNF85_1_2130,14131,13702,12250,14378,13340,11665,9042,9449,13292,9582,
11696,11966
AANAT_1_2131,62,182,84,129,31,33,1769,9,1,302,63,1
AES_1_2132,1638,1040,1943,992,1452,1074,2409,1911,1574,1110,466,983
AIRE_1_2133,464,39,34,326,5,186,15,5,1183,2,0,0
AKAP1_1_2134,2142,1426,1578,1836,1547,1516,1371,1347,3141,1639,3278,39
38
ALKBH2_1_2135,1489,1540,1360,1448,531,1608,1025,1664,859,2303,2067,155
0
ANKHD1_1_2136,621,743,864,873,282,786,589,906,977,1119,1567,1658
ARID1A_1_2137,1094,770,1457,554,1271,1438,1642,527,368,1054,341,1463
ARID1B_1_2138,685,443,709,496,531,823,1234,355,1487,490,134,920
ARID4A_1_2139,1386,1281,981,914,792,739,1094,488,742,1174,1708,510
ARID4B_1_2140,1455,958,807,1110,1810,449,1452,1234,1767,2232,2269,4245

ARRB1_1_2141,891,730,507,1432,728,480,1449,3101,2870,892,3,226
ASH2L_1_2142,11265,10215,13447,15556,19222,14308,7529,10818,10861,1102
5,8661,13571
ATAD2B_1_2143,1495,1827,2132,2325,1263,1439,2826,2793,3800,1905,3295,1
655
ATAT1_1_2144,635,778,876,665,932,459,724,1699,1595,475,1303,631
ATRX_1_2145,1073,936,911,529,713,2002,505,230,846,501,810,1002
AURKA_1_2146,309,286,419,436,6,199,1,46,322,482,302,2
AURKC_1_2147,3007,1931,3941,3345,1096,3265,1248,3266,2686,2885,497,355
0
BAZ1A_1_2148,11145,10810,12935,13377,17837,13137,15032,11255,15469,926
2,15121,12587
BCOR_1_2149,658,692,616,1152,1070,983,2272,322,878,1118,1187,462
BPTF_1_2150,3505,3781,3308,2489,3127,2805,3713,2845,2409,3325,2215,193
2
BRCA1_1_2151,2569,2178,2618,1591,1845,1308,1328,1362,755,2544,2949,329
0
BRD2_1_2152,1279,1382,2299,1697,2062,2692,1251,1327,3650,1393,722,4460
BRD4_1_2153,665,716,809,1105,705,1142,592,549,132,756,204,131
BRD7_1_2154,6373,6879,6335,6975,7007,5884,5223,4484,6749,4492,6530,538
9
BRD8_1_2155,1461,1366,2417,1162,2599,1970,1101,1131,993,1381,1568,2030
BRD9_1_2156,637,304,624,497,243,1114,1896,577,113,166,823,77
BRDT_1_2157,232,1160,946,751,220,871,837,381,327,691,268,571
BRPF1_1_2158,1086,788,836,502,1183,1377,2179,985,601,1162,167,204
BRWD1_1_2159,3637,3145,3194,3946,4019,4516,6800,3385,5237,4749,5548,30
81
C14orf43_1_2160,466,194,273,606,12,1404,952,220,392,242,296,253
CBX1_1_2161,1317,699,744,1029,701,607,1688,394,313,582,740,229
CBX3_1_2162,7551,8929,10102,8915,8579,7506,8073,9135,7176,10915,10926,
6898
CBX5_1_2163,4964,3586,4628,4121,1201,6993,4734,3936,5476,4126,2240,440
4
CCNT2_1_2164,1707,1943,1586,1922,2248,1896,2116,2152,1215,2488,494,203
7
CDY1B_1_2165,13302,14076,16755,15253,14336,13903,15304,14346,12698,137
97,14724,12498
CDY1_1_2166,13302,14076,16755,15253,14336,13903,15304,14346,12698,1379
7,14724,12498
CDYL_1_2167,3985,4337,4629,5469,6272,6019,4480,3165,3492,2901,2978,430
3
CHD2_1_2168,719,1149,1579,1049,865,434,461,714,100,1100,38,1859
CHD3_1_2169,2479,3113,3394,2860,2783,3550,4429,3403,5923,628,1790,4768
CHD8_1_2170,3803,3052,3841,4602,5938,3634,5071,2701,5155,2160,1759,501
1
CHMP2A_1_2171,6896,6391,8088,6588,7968,5862,5961,4694,5443,6312,7490,4
169
CHMP5_1_2172,1827,2223,2300,2818,3856,1494,1620,2835,1448,1371,340,255
1
COPS2_1_2173,2764,2955,1861,2789,1583,2988,3010,2349,5561,2413,1527,32

22
CPA4_1_2174,559,1151,1846,732,1361,669,861,40,1998,5,1537,6
CREB1_1_2175,1723,1317,1335,1027,684,1251,806,1328,1175,1444,469,1048
CREBBP_1_2176,3555,3221,3244,3506,4505,4892,3527,4256,2378,3039,3242,4
418
CTCF_1_2177,1610,2006,1839,1591,2430,1835,636,406,1046,1326,3324,1328
CTNNB1_1_2178,2972,2784,3315,3194,2946,2698,5048,2558,1789,4526,2405,2
315
CXXC1_1_2179,1289,1869,1155,1335,3330,3701,2928,277,754,3206,2269,1757
CYLD_1_2180,0,0,0,0,0,0,0,0,0,0,0,0
DICER1_1_2181,1639,846,1060,2502,729,1107,573,1755,176,2143,420,1131
DIDO1_1_2182,663,361,779,591,1238,323,208,278,40,886,775,261
DMAP1_1_2183,718,761,1077,306,731,933,1498,929,1190,288,4,64
DNAJC2_1_2184,2421,2830,3759,2876,2375,3552,2904,3269,4223,3068,2739,2
555
DNMT1_1_2185,1146,1214,1929,1453,2649,724,2009,1927,182,723,2186,803
DNMT3A_1_2186,1283,1548,1845,1985,2325,1287,2242,1878,352,1936,23,2709
DNMT3B_1_2187,1685,1673,2218,2361,4724,4004,1335,2683,1194,997,330,783
DNMT3L_1_2188,3187,3555,3220,3305,3419,3827,1988,2002,6736,4625,4413,2
970
DPF1_1_2189,3714,3468,2808,3032,5122,3753,4124,4392,1326,5180,2348,251
4
EED_1_2190,1842,2100,3090,2713,3083,4461,2701,3030,3152,1641,1447,933
EGR2_1_2191,1283,1321,2239,1479,2247,2474,1758,879,3405,1988,1778,836
EHMT1_1_2192,847,628,609,435,306,1235,617,407,1186,225,245,864
EHMT2_1_2193,542,477,210,435,614,72,17,234,36,310,80,4
ENO1_1_2194,350,339,476,109,353,1043,700,0,120,239,688,4
ESR1_1_2195,2172,1887,2368,2654,2520,2051,5181,1687,3039,1891,2153,323
3
ESR2_1_2196,1048,1637,1430,971,1393,794,1249,769,1206,1115,52,142
ESRRG_1_2197,241,21,118,285,858,110,53,270,0,37,806,150
EZH2_1_2198,3204,4053,4060,5396,4364,2942,2565,3906,3502,3390,4158,438
4
FBXO11_1_2199,7215,6686,8175,7947,5529,8545,10050,10310,7872,9482,4999
,5783
FMR1_1_2200,1237,1956,1981,1137,1543,2382,3242,536,2829,2845,2589,540
FXR1_1_2201,926,384,742,535,481,930,2242,1028,2250,1338,413,848
GFI1B_1_2202,655,350,1373,129,286,152,1071,328,1129,403,1594,501
GMEB1_1_2203,5837,5244,6405,5825,10361,7085,7855,6708,5781,5808,4998,7
685
HDAC10_1_2204,1488,1634,1559,1627,1112,2415,920,2163,1620,1543,2586,29
37
HDAC11_1_2205,628,591,332,686,1221,657,136,751,111,1195,918,214
HDAC5_1_2206,325,31,389,546,59,1,763,477,282,71,2585,0
HDAC7_1_2207,3631,3636,2904,4062,4657,2612,3126,3347,3411,4724,3653,47
85
HDAC9_1_2208,565,696,771,705,843,672,1119,702,899,314,79,875
HDGF_1_2209,620,599,720,602,255,1751,677,873,130,292,909,445
HDGFRP2_1_2210,221,322,17,294,0,35,188,2,19,464,0,994
HLTF_1_2211,2594,1992,2344,2776,2524,2789,5706,2415,2343,2531,1915,172

9
HMGA1_1_2212,726,793,640,332,124,1021,528,185,440,639,746,976
HMGN3_1_2213,173,464,936,86,102,457,204,243,246,13,178,228
HNF4A_1_2214,1581,1736,1241,1722,2647,1699,2653,1797,2311,1577,2222,11
82
HPSE2_1_2215,881,722,529,288,171,874,2618,961,1382,247,572,941
HR_1_2216,483,747,717,669,380,1375,1676,467,34,804,1229,497
ING1_1_2217,392,284,308,304,854,1,60,36,141,360,0,0
ING4_1_2218,2549,1707,3483,2988,3074,1876,4254,3310,5788,2268,4549,136
8
INTS12_1_2219,1953,2634,2333,2027,3272,1571,1877,3156,1848,2800,1487,1
880
IRF4_1_2220,1384,1426,1448,1625,2469,2486,3533,1627,875,980,936,1083
JMJD1C_1_2221,4143,4001,3676,4577,6582,6891,3878,2553,8804,2401,3680,5
006
JMJD4_1_2222,221,134,131,203,204,53,480,10,0,66,8,0
JMJD6_1_2223,571,360,377,712,454,497,66,949,178,700,741,1232
KAT5_1_2224,708,1321,985,1144,449,1240,394,1188,2204,787,2313,1060
KAT6A_1_2225,1876,1973,2579,2306,3916,1275,2995,1926,1234,3315,4524,18
09
KAT7_1_2226,1433,1282,609,1687,366,308,339,1212,316,937,130,1535
KAT8_1_2227,0,0,0,0,0,0,0,0,0,0,0,0
KCTD1_1_2228,1392,1258,1910,982,1789,1663,1079,839,1834,675,1932,1564
KDM1A_1_2229,14659,14480,15489,16183,16767,20213,23744,15548,23179,116
51,15045,12946
KDM2B_1_2230,815,168,462,131,738,173,533,545,0,292,383,1000
KDM3A_1_2231,1303,1379,1228,1454,2677,1761,993,2265,4,797,46,33
KDM4C_1_2232,2458,2096,1110,1737,2219,2506,1137,1143,3662,1972,2452,40
81
KDM5C_1_2233,2176,2303,3347,1836,2173,2123,3611,2569,2744,2349,2789,26
80
KDM5D_1_2234,121,37,3,25,6,12,16,60,203,38,152,27
L3MBTL1_1_2235,830,586,387,657,121,261,624,510,113,959,657,262
L3MBTL3_1_2236,699,515,897,612,1235,753,955,1014,520,440,240,2693
LBR_1_2237,1665,2086,1884,2197,1777,2407,485,3419,1634,1207,0,765
MAP3K12_1_2238,658,769,954,1203,1664,762,661,373,2110,664,943,252
MBD1_1_2239,883,693,276,854,1342,821,3005,494,637,1104,380,171
MBD2_1_2240,2585,2624,2771,3366,4118,3342,3833,1378,3407,3137,1624,114
7
MECOM_1_2241,3982,3873,4860,5418,5422,6125,3101,3366,8337,4683,2440,61
01
MECP2_1_2242,1428,2133,1996,2476,3720,976,1780,1159,1139,1462,2236,102
2
MEN1_1_2243,2055,1793,2168,2527,3255,1718,1954,1828,3239,2594,2726,100
8
MGEA5_1_2244,3073,2324,3720,2684,2946,2236,4187,3069,4255,1102,2900,13
23
MIB2_1_2245,68,200,270,408,1775,213,516,154,0,27,84,12
MIER1_1_2246,5450,6582,6702,7191,6940,8077,4799,6258,4985,6493,4165,63
14

MINA_1_2247,3535,4699,3420,4596,3163,2830,6739,3275,4232,3767,4179,510
3
MLL5_1_2248,1783,1933,2203,3079,3130,2910,3304,2255,2375,1594,1325,537
6
MLL_1_2249,1397,1645,1772,1235,1525,1571,2895,2708,1689,2050,943,1555
MORF4L1_1_2250,0,1,0,0,0,0,0,0,0,0,0,0
MSL3_1_2251,598,605,715,1042,1617,1762,384,487,1078,212,773,986
MTA1_1_2252,478,323,118,286,573,1036,56,8,1,384,270,41
MTF2_1_2253,3955,3278,3062,1685,2937,4525,4472,2011,1734,2326,2835,967
NAA60_1_2254,1165,954,1039,1074,1485,1109,2286,1110,639,1253,1849,1142
NAP1L1_1_2255,1507,821,1170,1663,2537,1596,2199,1713,3062,981,1788,301
NAT10_1_2256,1186,883,472,845,1322,791,551,1227,404,511,261,112
NCOA1_1_2257,2684,2329,3086,2063,2370,3262,1356,2427,611,2560,4625,228
1
NCOA3_1_2258,1516,2052,1628,1754,1581,1631,1914,972,1301,1859,2515,937
NCOA4_1_2259,2062,1334,1653,1971,2728,1734,946,985,1214,1894,447,5171
NCOR1_1_2260,974,1431,811,1270,1023,2606,2518,1354,2531,810,1281,444
NCOR2_1_2261,731,806,1136,766,915,1197,1417,888,221,197,131,316
NFAT5_1_2262,496,607,357,1078,186,1218,830,443,169,359,12,346
NFATC1_1_2263,634,303,528,465,225,263,1107,627,121,286,5,279
NFATC3_1_2264,4392,4743,5458,5388,6830,4906,4133,3143,4210,5830,4824,7
444
NFATC4_1_2265,155,59,208,117,0,264,82,535,59,126,3,162
NFKB1_1_2266,3931,3047,4681,3484,3718,4317,4393,2362,2187,2918,4501,32
74
NFKB2_1_2267,312,235,102,353,37,48,32,260,44,986,302,465
NPM1_1_2268,12078,14174,14630,16029,14438,16784,15835,13548,13749,1363
6,14607,14392
NR1D2_1_2269,2434,1595,1586,1914,3161,2578,2344,2303,2799,2412,880,129
3
NR1H3_1_2270,9278,10237,9963,12540,9672,11340,11506,9488,10004,11000,6
519,8531
NR1H4_1_2271,2377,1961,3094,2534,2347,3485,2374,1548,2406,2459,2407,23
28
NR1I2_1_2272,1153,538,1056,1989,1164,920,1309,599,807,156,1521,2338
NR1I3_1_2273,681,1689,1294,1433,408,718,2480,1233,1536,689,290,1089
NR2C1_1_2274,1069,1410,1517,1239,1340,914,1799,522,1741,1090,2008,66
NR2E3_1_2275,587,174,770,430,1313,657,594,1,1,355,344,754
NR2F2_1_2276,1009,1705,1464,1231,1127,2329,1039,2660,533,1322,25,1078
NR3C1_1_2277,2081,2066,2752,3074,2605,1137,2626,1620,1156,1326,2406,28
36
NR3C2_1_2278,1974,1864,1813,2302,2425,3256,2143,2815,1220,2877,1351,91
9
NR4A1_1_2279,546,615,248,468,551,838,525,2151,19,1411,86,637
NR4A3_1_2280,704,780,1149,346,727,469,837,273,498,479,824,1208
NR5A2_1_2281,6660,5655,6431,5086,6558,9792,7304,3727,6208,5126,4531,38
00
NR6A1_1_2282,761,1264,1249,1289,809,698,2224,1379,1505,1637,107,528
NSD1_1_2283,626,412,449,1076,94,300,26,206,1530,176,2074,640
PBRM1_1_2284,188,410,384,726,1274,625,258,766,1036,299,247,391

PCGF6_1_2285,687,539,601,238,1339,318,11,556,2090,290,213,487
PGR_1_2286,1235,761,1056,1010,1992,1999,1623,790,2410,1009,210,20
PHC2_1_2287,494,714,679,520,697,529,843,108,2384,1002,136,958
PHF10_1_2288,479,465,882,331,1176,333,504,209,904,350,2769,66
PHF11_1_2289,1381,1234,1138,1100,413,1206,700,1033,2655,1330,2658,756
PHF12_1_2290,893,900,1107,1272,547,1203,857,595,682,857,1332,688
PHF16_1_2291,1963,2236,1937,1483,2254,2486,2751,398,555,1019,593,2932
PHF17_1_2292,645,1076,1122,991,325,1964,301,624,27,1108,1091,848
PHF1_1_2293,232,232,31,227,49,85,782,90,0,578,0,332
PHF20L1_1_2294,2731,1771,2803,2621,2561,3329,4060,3275,3991,1606,2800,
3206
PHF21A_1_2295,1055,857,1275,960,2078,462,896,524,186,854,1294,461
PHF21B_1_2296,948,825,1120,1118,1528,1064,595,463,887,328,1335,229
PHF6_1_2297,2322,2819,1736,2213,2700,2845,3494,2299,878,2049,690,399
PHF7_1_2298,1623,855,1301,1151,1433,1767,1061,1094,253,1442,1317,249
PHF8_1_2299,460,202,324,470,832,246,514,5,192,230,669,3780
PIAS2_1_2300,1310,859,1553,1553,440,1693,1311,1151,261,1228,241,556
PICK1_1_2301,528,238,469,282,308,194,385,781,1586,447,434,648
PIWIL2_1_2302,1648,824,1255,1216,3381,2177,2924,2790,1290,1468,850,140
8
PML_1_2303,1279,1251,679,988,1281,1657,216,531,30,250,279,208
POLR1B_1_2304,1744,1190,1492,1837,2596,1649,2275,3019,302,1273,3461,29
99
PPARA_1_2305,908,421,658,679,702,610,189,59,154,482,1252,46
PPARD_1_2306,3767,3476,2689,3462,4729,4584,3683,4607,2967,2630,4403,44
02
PPARG_1_2307,3762,3309,2653,3615,4695,4404,3583,5313,2979,2706,4376,43
78
PRDM10_1_2308,3701,4551,4589,3714,4867,4672,3205,5050,956,4450,4048,34
69
PRDM15_1_2309,2833,2810,2891,2407,3363,4455,4198,1303,4685,2457,3082,3
209
PRDM16_1_2310,812,567,1531,767,841,909,1819,1181,643,461,652,2363
PRDM1_1_2311,4682,3857,3539,3576,3758,2574,4244,3793,3535,2307,3282,34
97
PRDM2_1_2312,2837,3014,3667,3477,3584,3314,3213,2980,2179,4056,2978,23
12
PRDM7_1_2313,1407,1485,2355,1410,1656,1833,490,2722,1061,793,1548,1053
PRDM8_1_2314,1972,2834,2856,2083,2459,2572,2095,2292,921,2740,2924,214
0
PRMT1_1_2315,1392,1181,2018,1356,4258,2235,2804,1126,952,1048,1339,126
4
PRMT2_1_2316,1175,647,1561,535,1140,803,1517,657,1968,1323,1332,2396
PRMT3_1_2317,3285,3265,4890,4758,2949,3159,5285,2850,3452,2330,6719,28
46
PRMT5_1_2318,4323,3884,4413,6113,5859,4503,4506,5239,5011,6466,7001,37
89
PRMT7_1_2319,655,800,828,812,954,1148,1463,439,280,496,2137,112
PSIP1_1_2320,2259,1639,2287,2304,2210,3244,1455,2987,822,2180,778,995
PSMC5_1_2321,1633,1515,1670,1125,2297,1302,1625,992,1333,1460,2157,254

7
PWWP2B_1_2322,213,208,210,311,829,562,16,65,1339,42,154,726
RAD54B_1_2323,2481,2312,3294,4240,1730,3735,787,3477,549,3332,800,3901
RAD54L_1_2324,1162,2212,1658,1962,1210,1847,1049,763,2296,1944,127,145
3
RARA_1_2325,2517,2215,1897,2557,2904,1986,2206,2799,3236,1255,1421,221
0
RARB_1_2326,2064,1325,1867,1470,1053,2604,2811,785,224,1585,1844,351
RARG_1_2327,758,943,783,727,917,1350,657,154,1122,189,1404,1251
RBBP5_1_2328,538,606,542,534,509,1004,828,43,326,613,506,113
RBCK1_1_2329,820,1696,964,1559,536,422,362,309,1655,681,2168,742
RBFOX2_1_2330,2079,2401,2070,2893,1999,2198,2455,2159,3363,1949,2581,3
480
RCC1_1_2331,471,706,666,530,1345,521,226,842,854,262,1,564
RCOR3_1_2332,630,514,851,796,1505,526,676,562,188,769,61,377
RECQL5_1_2333,1131,729,1674,849,1072,1040,655,1042,1005,883,150,1251
RECQL_1_2334,8162,9369,10180,10203,10319,8483,7987,10392,6823,11384,69
72,10931
RELA_1_2335,2040,1256,1775,1806,767,3094,947,2196,1243,1641,1855,1667
RERE_1_2336,478,1504,1288,933,2147,1103,1428,618,1203,1304,851,220
RFC1_1_2337,951,830,756,911,929,1430,889,688,2714,1109,462,307
RNF14_1_2338,610,981,1370,1078,155,1872,759,606,592,615,608,575
RNF17_1_2339,563,483,784,345,1381,864,1035,578,1931,21,902,1
RNF40_1_2340,990,819,875,1145,1858,1416,1627,1885,3529,380,88,2162
RNF8_1_2341,3477,2606,2561,2741,1206,2071,2155,2223,4270,2788,2866,195
9
RORA_1_2342,1707,1599,1242,1225,1347,2962,3523,916,2577,1872,1571,601
RORC_1_2343,1677,1513,1562,1292,665,2607,937,1186,1308,1297,1741,6356
RPH3A_1_2344,1563,1928,2384,1460,1923,2507,4091,1745,2457,1484,2334,15
57
RPS6KA5_1_2345,1351,2184,2217,2108,1366,1096,997,1985,1373,1608,2929,3
067
SATB1_1_2346,3697,5184,4898,4771,6309,5607,3628,2299,6649,4324,7126,61
59
SATB2_1_2347,1219,669,485,578,976,1210,2440,308,10,754,1366,454
SCMH1_1_2348,1045,570,1239,676,942,949,2496,137,826,1530,699,1861
SET_1_2349,4744,4502,5445,5784,8521,7484,6710,3968,4740,4712,4822,2886
SETD3_1_2350,65,98,240,279,0,36,47,1,2,265,0,19
SETD4_1_2351,2676,2203,4358,2465,2331,3213,3770,2516,3736,2888,1891,25
79
SETD6_1_2352,1237,1132,573,1234,3389,1184,416,67,3219,847,1297,1394
SETDB1_1_2353,982,262,557,815,76,903,308,456,1062,730,316,163
SETDB2_1_2354,2312,1570,2461,2461,2323,3350,1985,1288,2437,2539,1672,1
385
SFMBT1_1_2355,2131,2034,2596,2002,1697,3420,1733,1367,3898,2831,1651,1
990
SFMBT2_1_2356,57,22,28,28,28,1,20,0,48,68,47,0
SHPRH_1_2357,532,514,996,1386,754,1481,1458,342,456,1779,1673,1031
SIN3A_1_2358,2333,3146,2834,1922,3947,1791,4366,651,1547,1485,679,5009
SIRT1_1_2359,925,984,784,1051,2685,1790,373,777,1169,810,222,302

SIRT2_1_2360,2737,2406,2927,2868,3357,2093,2384,3563,2334,3433,1135,31
64
SIRT3_1_2361,1308,1898,1648,1884,1698,2132,2539,3158,2444,2629,409,235
SIRT5_1_2362,511,578,602,375,596,66,609,13,528,258,275,740
SIRT6_1_2363,255,63,250,334,504,275,14,50,11,14,0,1319
SLC38A1_1_2364,1120,1459,1503,1644,3201,1288,429,2091,944,838,127,547
SMARCA1_1_2365,0,0,0,0,0,0,1,0,0,0,0,0
SMARCA2_1_2366,2194,2751,2834,3114,4930,5111,4045,3433,1775,2449,2205,
4857
SMARCA4_1_2367,1163,1159,1473,822,635,363,1401,1278,551,1715,795,2003
SMARCAD1_1_2368,7577,6564,7245,8575,10581,7070,4391,3703,7375,5742,690
1,8413
SMARCAL1_1_2369,716,895,873,676,955,1244,816,354,4838,643,810,593
SMARCB1_1_2370,1067,652,688,1031,883,801,1076,338,11,578,1693,69
SMARCC2_1_2371,4794,4327,5885,5053,5355,5755,4435,3629,5442,6958,4232,
2409
SMARCD1_1_2372,636,153,143,424,238,33,575,1017,25,182,215,2
SMARCD3_1_2373,559,548,468,317,524,499,869,369,360,861,693,780
SMC2_1_2374,1243,1808,1762,1583,2012,771,3032,558,1222,1510,1240,2660
SMC4_1_2375,1013,1041,1537,1537,2371,561,1844,311,2623,738,2301,1012
SMN1_1_2376,4347,2342,4058,2296,2687,5201,2316,1452,1874,4394,1090,363
6
SMN2_1_2377,4347,2342,4058,2296,2687,5201,2316,1452,1874,4394,1090,363
6
SMYD3_1_2378,812,713,1141,979,240,1473,272,567,190,944,1758,504
SP100_1_2379,8905,7307,8342,8344,8140,8605,8283,8600,11677,7780,8052,8
194
SP110_1_2380,769,163,262,1078,93,299,475,3186,643,695,36,1509
STK31_1_2381,5917,4750,4637,4965,9361,6534,4855,4848,10417,4305,5749,5
212
SUPT3H_1_2382,1408,1091,765,1239,388,1187,483,673,1033,557,657,682
SUPT5H_1_2383,473,347,153,399,628,261,462,73,39,406,0,289
SUV39H2_1_2384,1035,801,1107,2070,402,568,795,478,638,485,890,732
SUV420H1_1_2385,2504,3255,4192,3703,3443,2848,5711,1575,3770,4170,2255
,2530
TADA2A_1_2386,2764,3053,3870,2963,1854,1942,1690,3574,677,2517,808,385
4
TADA3_1_2387,461,370,824,788,472,518,370,205,13,700,353,50
TAF12_1_2388,841,735,476,503,824,1011,453,1171,36,691,153,1405
TAF15_1_2389,2523,2444,2732,2437,2673,1877,2605,2499,2467,2189,2410,47
99
TAF1_1_2390,2846,1986,2050,2195,3675,1885,3711,2521,4082,2084,2505,345
TAF5L_1_2391,1140,1365,1198,950,1343,2077,2600,790,488,1183,8,2902
TCF19_1_2392,2164,1972,2620,2500,1654,1653,907,1440,3707,2168,510,3380
TCF20_1_2393,416,192,1241,780,215,214,956,323,584,629,223,518
TDRD10_1_2394,869,967,1581,1277,1957,1851,3072,1504,601,1173,1990,918
TDRD3_1_2395,289,560,267,175,0,988,0,0,0,606,491,0
TDRD5_1_2396,6518,5349,6658,6237,6852,6952,7046,3308,7085,6410,6277,43
79
TDRD6_1_2397,5987,5837,8434,8460,8636,3828,5730,8836,9296,6872,5862,71

51
TDRKH_1_2398,454,766,795,169,36,10,241,536,19,242,0,1101
TERF1_1_2399,5771,5848,7344,6440,6492,6583,8613,6777,6821,5589,7378,55
05
TET2_1_2400,2127,1197,1947,1769,1581,3970,1325,2231,3274,1089,2926,363
7
THRA_1_2401,178,88,78,201,290,444,2,12,10,14,62,369
THRB_1_2402,359,840,1046,693,460,244,847,1519,328,655,820,1749
TP53BP1_1_2403,699,651,1033,473,478,1351,318,347,201,437,136,422
TP53_1_2404,2816,3287,4182,3695,2323,2407,4343,3321,4697,2896,4332,341
0
TP73_1_2405,213,261,459,252,56,244,336,10,0,241,163,256
TRIM24_1_2406,4378,4377,4809,4724,4664,6491,4973,3955,4699,3262,3687,2
382
TRIM32_1_2407,904,2510,2064,3239,1540,1709,3500,2351,2016,2494,3323,34
86
TRIM33_1_2408,2279,1871,3695,1503,1260,1033,4196,1702,5023,2096,1035,2
607
UBE2A_1_2409,7535,6658,9647,7870,11674,7354,6949,5646,8583,7721,9320,7
863
UBE2E1_1_2410,675,808,1464,1177,1725,1536,844,678,398,178,426,4654
UBE2I_1_2411,3058,2787,3195,3144,9102,2301,2904,2568,3353,1785,2077,40
48
UBE2K_1_2412,3459,2946,2240,3019,3053,3980,1120,3578,1002,2784,1419,27
52
UBE2V1_1_2413,3435,2930,4379,3408,4477,2360,4258,3460,4695,3868,524,35
39
UHRF1_1_2414,1082,744,1509,1628,452,1684,499,291,739,1946,2601,864
USF2_1_2415,3257,2779,4221,3390,3121,2915,4032,3576,2217,3528,2203,131
2
UTY_1_2416,2316,1938,2206,1822,751,2490,2219,1749,344,1934,2544,876
VDR_1_2417,456,637,706,1325,383,1063,99,43,254,553,1461,1534
WDR5_1_2418,1567,1547,1408,1827,1167,1654,2702,2425,2487,1501,2004,413
WHSC1_1_2419,1226,1751,2229,1164,1416,1457,1413,1295,2218,1369,829,846
WHSC1L1_1_2420,949,851,816,789,437,742,3091,576,286,467,259,285
WRB_1_2421,4773,3988,4375,4195,6776,5299,3191,2952,10412,3523,3510,384
5
ZGPAT_1_2422,1093,1006,1489,887,1706,1650,1701,764,59,255,344,215
ZMYND11_1_2423,1701,894,1563,1879,876,1763,1461,1565,2593,1338,1026,47
01
ZMYND8_1_2424,1383,1742,1333,1416,2087,1327,792,540,889,2182,2050,352
ZNF451_1_2425,1781,1273,1550,1424,1346,367,5027,1321,1962,2481,1445,13
52
ALG13_1_2426,4701,4265,4413,5078,3017,6455,2105,4826,2140,4737,3141,36
71
ASXL1_1_2427,814,1279,783,1115,1272,1069,2405,657,1454,851,1035,1015
CBX2_1_2428,132,143,9,75,0,79,54,344,8,67,0,193
HDAC8_1_2429,8,76,37,0,0,42,260,15,0,68,93,1
ING3_1_2430,579,650,1093,773,906,286,440,745,5,409,2270,1
MLLT10_1_2431,97,157,180,208,274,191,208,410,1198,4,72,229

PHF19_1_2432,55,80,80,53,0,83,0,0,0,201,0,425
RBM14_1_2433,636,894,961,510,1066,1011,370,573,536,1225,596,196
SP140_1_2434,7862,6138,8323,6875,9763,8525,7609,7632,6288,4780,9324,52
87
TAF9_1_2435,3462,3326,3489,3640,5069,2230,3268,3900,3780,3171,1082,157
5
TAF9_1_2436,0,0,0,0,0,0,0,0,0,0,0,0
AHR_1_2437,365,500,319,357,378,337,1525,171,2,323,0,762
ALKBH1_1_2438,2423,1733,2674,3911,4494,3341,2386,1734,933,2714,3624,58
01
ALKBH3_1_2439,4157,3498,3141,3555,3153,4061,1777,2687,4337,1410,2371,4
054
ARID2_1_2440,390,301,432,441,18,847,236,1075,603,1094,401,842
ASF1A_1_2441,1235,1328,1004,1718,2090,4032,857,1374,888,2419,347,2086
ASF1B_1_2442,1886,1220,1555,2184,2906,3392,2267,1267,3535,2397,2456,26
51
ASH1L_1_2443,412,297,307,450,521,845,1054,597,1105,301,747,166
ASXL2_1_2444,1364,1042,1112,612,2042,666,264,1713,1,352,1078,679
ASXL3_1_2445,1048,874,1129,1445,986,1745,1583,420,1315,1147,969,2029
ASZ1_1_2446,5200,2797,4725,4483,3302,4120,5611,3274,2090,4551,6549,385
5
ATAD2_1_2447,2944,2641,3660,2835,2456,3502,2920,1043,2858,4084,2107,32
16
ATF7IP_1_2448,2218,1858,1471,2031,3665,2921,2899,2725,521,1482,2547,21
79
AURKB_1_2449,3096,2520,3088,3109,3492,3974,4845,4490,3767,3333,3639,93
57
BAHCC1_1_2450,521,891,1384,323,1547,333,493,540,740,319,978,31
BAHD1_1_2451,542,265,459,348,393,202,432,334,164,436,459,335
BARD1_1_2452,687,599,842,852,457,1210,69,268,142,1473,1050,1580
BAZ1B_1_2453,2676,2421,2339,2268,4856,4625,3407,3305,6370,3166,1189,15
06
BAZ2A_1_2454,7072,5570,7445,5844,5285,4480,5920,4823,5758,5128,3566,31
78
BAZ2B_1_2455,2072,2217,3219,2679,1384,1685,2429,1231,945,2587,944,1251
BLM_1_2456,2764,2276,2798,3309,3438,3324,3133,1326,2670,3052,2742,2040
BMI1_1_2457,1615,1489,1223,1554,1873,873,1062,1783,988,863,2137,1337
BRD1_1_2458,4386,3403,3739,4189,7176,5817,3346,3622,5612,4401,6882,389
3
BRD3_1_2459,4239,3398,3682,5253,3953,3903,3318,3236,3503,3704,3206,461
0
BRPF3_1_2460,473,463,211,460,567,256,1641,601,51,1486,214,27
BRWD3_1_2461,10358,8293,9960,9357,9688,8282,13553,9330,10752,8984,1132
9,14233
C14orf169_1_2462,378,227,581,620,1080,657,89,1,0,494,98,601
C20orf20_1_2463,830,1153,1052,1198,2362,1284,3984,658,915,286,1032,168
9
CALR_1_2464,164,318,566,80,14,895,379,22,69,850,30,642
CARM1_1_2465,489,133,273,118,7,343,2305,69,16,19,50,65
CBL_1_2466,566,473,352,324,499,47,759,567,0,886,554,10

CBX4_1_2467,292,531,311,375,746,360,365,537,398,201,444,75
CBX6_1_2468,548,695,1857,987,504,724,627,2523,17,679,1124,917
CBX7_1_2469,1959,1895,2055,2314,2461,2647,3788,1952,2503,1605,1880,131
5
CBX8_1_2470,1335,1386,1347,1147,2320,1585,1936,1114,363,1303,1396,5117
CCDC101_1_2471,1265,2692,1638,1234,2816,1292,1457,165,411,1383,1519,23
98
CCNE1_1_2472,1048,1305,1105,717,659,2916,838,445,1600,1693,2199,542
CCNT1_1_2473,1559,1208,1742,2724,4255,1098,1019,795,892,1446,737,1726
CDC73_1_2474,2237,1627,3230,2698,3669,2614,4630,1183,1544,2207,700,278
9
CDK9_1_2475,191,245,74,234,299,477,170,162,271,182,356,371
CDY2A_1_2476,2613,1976,3669,3335,2145,3960,3130,2542,1658,1976,2514,16
46
CDY2B_1_2477,2613,1976,3669,3335,2145,3960,3130,2542,1658,1976,2514,16
46
CDYL2_1_2478,2792,3663,3100,3804,5186,5599,3499,2409,3522,3137,2985,17
85
CECR2_1_2479,1585,548,414,669,32,181,860,1550,1574,380,24,722
CHAF1A_1_2480,449,298,154,214,599,309,575,261,931,262,207,367
CHAF1B_1_2481,2527,1791,2603,3059,3075,2625,496,3040,1421,3990,2225,36
08
CHD1_1_2482,4115,2628,4466,3625,2902,4776,2720,3178,6365,1983,8155,410
9
CHD1L_1_2483,798,1382,1475,1414,1574,1137,404,1410,799,858,5,2186
CHD4_1_2484,1006,927,492,759,810,1622,189,534,1132,605,267,269
CHD6_1_2485,882,927,927,1747,1291,780,1050,223,4323,687,213,1527
CHD7_1_2486,7298,6491,8862,8400,6629,5955,8578,6300,7488,8661,6822,753
3
CHD9_1_2487,3386,4029,3275,3539,3629,3747,3625,3070,4583,2490,2293,451
9
CHMP1B_1_2488,2395,1150,2791,1619,1886,1904,1611,3661,2942,1211,1157,2
189
CHMP4B_1_2489,844,532,1092,781,715,1110,727,408,1042,855,851,546
CHMP4C_1_2490,2079,1579,1670,1020,647,731,2060,1005,1059,1524,2046,663
CHRAC1_1_2491,1449,1028,2212,1207,3632,923,1301,769,2444,1149,1167,106
7
CLOCK_1_2492,4752,3531,5187,4114,6754,2215,3442,5386,5588,4930,5322,30
38
COPS5_1_2493,1521,1780,2245,2023,1840,2382,2233,3685,2257,1274,269,865
CRAMP1L_1_2494,165,170,214,262,295,33,116,39,0,271,0,5
CTCFL_1_2495,1398,1189,1404,1156,1015,1113,732,1486,743,493,1673,710
DEAF1_1_2496,3654,3720,4745,4554,4442,6997,7406,3771,6269,3540,3837,32
99
DNAJC1_1_2497,1936,1064,1823,1987,1720,1838,1829,1754,4550,1323,1802,1
530
DOT1L_1_2498,586,587,1336,1112,266,1590,1386,1515,1153,559,1373,1755
DPF2_1_2499,801,1199,1223,547,1801,1238,412,1768,1907,651,907,1580
DPF3_1_2500,2758,3047,2769,2535,3265,3051,4313,3553,3126,2796,5381,235
3

EIF4B_1_2501,2214,1503,2389,1944,2664,546,2068,547,1207,1777,1723,2609
ELP3_1_2502,2044,1794,2380,1259,1125,1971,2968,2181,2760,1651,3058,152
8
EP300_1_2503,373,252,339,364,192,64,449,89,9,60,1013,790
EP400_1_2504,431,498,382,429,14,546,106,49,16,340,289,732
EPC1_1_2505,1204,285,983,967,1034,1155,1398,79,698,1273,764,1645
EPC2_1_2506,2079,1290,1203,1631,2031,2661,2568,1406,973,1337,3122,2624
ERCC6_1_2507,903,480,1259,958,1758,766,820,546,1026,257,903,523
ESRRA_1_2508,464,327,71,562,215,140,406,1084,1305,613,222,458
ESRRB_1_2509,1274,1444,1108,1490,2220,718,849,524,768,2929,1387,2070
FANCM_1_2510,1395,1367,1270,1192,1377,1086,567,768,618,1888,316,1163
FBXL19_1_2511,250,371,123,376,259,325,530,0,0,1062,0,22
FOS_1_2512,1410,1025,1165,2003,1084,1175,2066,248,2298,1389,548,2172
FTO_1_2513,2554,2648,2447,2414,2698,2274,3078,1558,864,3082,4328,1724
FXR2_1_2514,7340,6842,8612,7345,12909,8598,6542,6301,6916,6309,6649,10
460
G2E3_1_2515,978,877,1400,1361,892,744,2909,670,474,583,1094,861
GATAD2A_1_2516,3759,4571,6201,5993,7193,5996,5480,4074,4040,4287,4966,
6038
GLYR1_1_2517,1598,569,1453,1190,1712,1303,1186,605,1938,867,470,1532
GMEB2_1_2518,585,289,178,190,788,331,1,676,1188,129,1,0
GSG2_1_2519,1598,1241,1475,1166,513,2130,2161,1801,1590,1150,291,1919
GTF3C4_1_2520,1171,341,895,664,218,686,3459,226,91,398,413,1538
HAT1_1_2521,2985,1777,2817,1623,2361,2076,1193,2407,695,1268,1365,1115
HDAC1_1_2522,3057,2077,2720,3038,1872,2183,3546,4128,2796,2427,3642,41
46
HDAC2_1_2523,8281,10049,12111,11521,11569,12817,8878,11619,13233,10822
,10322,7381
HDAC3_1_2524,97,257,83,139,370,0,42,109,0,605,0,134
HDAC4_1_2525,323,459,411,574,102,387,1124,419,81,167,201,409
HDAC6_1_2526,576,614,608,914,1616,1015,920,370,912,1066,298,436
HDGFL1_1_2527,594,964,1419,419,312,1256,883,36,62,172,342,667
HDGFRP3_1_2528,223,104,104,69,6,49,3,310,240,206,8,0
HELLS_1_2529,121,140,146,153,201,87,24,59,0,195,0,71
HEMK1_1_2530,2686,2215,3046,2376,3514,1758,3111,2810,2675,2329,4754,51
15
HIF1AN_1_2531,7541,6774,8003,7108,10096,6521,6629,6206,10338,8121,6043
,7706
HIRA_1_2532,3376,3172,3654,2589,2325,4097,3634,2245,2777,2866,6012,200
7
HMG20B_1_2533,678,742,439,621,908,1496,174,818,727,620,1754,153
HMGN5_1_2534,9662,8764,10639,9642,13987,9854,11558,5956,19734,10563,12
055,9995
HNF4G_1_2535,513,211,420,417,986,330,260,955,631,169,27,95
HSPBAP1_1_2536,2420,2138,2151,2248,2363,1800,804,1455,3111,2351,4742,2
267
HUWE1_1_2537,1430,404,641,1161,456,390,23,1154,351,1506,75,132
ING5_1_2538,734,512,608,804,848,395,1034,72,1155,751,277,276
INO80_1_2539,383,321,221,179,15,745,4,26,504,0,432,5
JARID2_1_2540,6766,7966,7245,7870,8771,10317,9917,6419,9678,5110,11404

,5883
JHDM1D_1_2541,724,897,450,415,953,796,2105,210,318,940,145,46
JMJD7_1_2542,3030,2560,2461,3391,452,2389,3338,2144,2812,2420,2055,135
6
JMJD8_1_2543,1369,569,1611,1004,908,1363,2638,702,1,1107,1072,5
JUN_1_2544,10,1,272,101,0,0,0,475,0,0,0,25
KAT2A_1_2545,1451,2021,1213,1976,1308,458,392,2447,1600,1732,2452,1606
KAT2B_1_2546,1230,647,1406,1646,1542,2416,2153,2353,1454,853,3292,309
KAT6B_1_2547,561,901,1172,583,943,388,578,601,847,645,22,336
KDM1B_1_2548,1145,1092,1050,697,1301,844,638,2505,977,1105,1098,1042
KDM2A_1_2549,1239,801,1358,1409,2481,1432,1015,1271,1414,1342,820,1725
KDM3B_1_2550,1023,471,789,652,1276,1925,565,250,957,252,266,1349
KDM4A_1_2551,608,490,1003,935,2714,690,615,451,785,269,2229,365
KDM4B_1_2552,1647,1117,2102,2277,2779,2863,1875,1414,2996,2012,1804,20
63
KDM4D_1_2553,3629,3283,5312,3192,5110,4694,1852,5029,2494,3149,3054,24
93
KDM5A_1_2554,0,0,0,0,0,219,0,0,0,0,0,0
KDM5B_1_2555,5236,4421,5039,4644,4941,5349,1939,3282,4339,4214,6866,25
65
KDM6A_1_2556,1338,1750,1464,2535,2227,2374,2952,855,21,767,969,3620
KDM6B_1_2557,149,568,430,286,675,165,70,0,8,11,656,0
KIAA2026_1_2558,147,304,442,312,444,41,213,4,487,178,2155,152
L3MBTL2_1_2559,2273,1747,3134,1971,2146,3201,3555,2606,1269,1659,3041,
3315
L3MBTL4_1_2560,3761,2570,2502,3101,3916,5315,5791,3195,3197,3059,4535,
2953
MAEL_1_2561,1182,692,1100,939,941,712,1310,301,461,894,289,13
MBD3_1_2562,399,345,239,182,594,848,347,251,29,263,0,113
MBD4_1_2563,2243,1515,1734,2238,2026,2060,3152,747,2384,2490,2859,1113
MBD5_1_2564,2479,2360,3347,2928,5270,1902,794,3282,1160,2024,1625,7320
MBD6_1_2565,1202,847,1115,1541,489,2106,916,1252,315,1485,279,457
MBTD1_1_2566,2059,1889,2420,2904,3942,2409,1959,1025,482,1396,1969,119
1
MDM2_1_2567,3318,3640,3678,4260,2238,4202,3015,4129,4074,3237,3717,696
4
MIER2_1_2568,829,1533,1476,1202,1360,1338,560,1178,720,780,122,2526
MIER3_1_2569,726,955,1201,1484,1639,1727,911,1022,2045,1106,511,154
MIS18BP1_1_2570,855,1873,1285,1158,1160,1397,1786,775,1508,1144,1638,3
264
MKL1_1_2571,462,862,386,1173,1557,404,172,121,1544,978,459,783
MLL2_1_2572,930,475,812,403,772,479,87,616,519,229,1392,157
MLL3_1_2573,2663,2390,3824,3034,2369,2437,3625,1371,3686,3260,2468,179
1
MLLT1_1_2574,1125,622,1366,675,557,185,591,1373,4,248,47,386
MLLT3_1_2575,833,716,1087,794,985,1470,1001,1366,1218,607,158,2493
MLLT6_1_2576,340,351,313,175,190,667,464,190,180,161,148,11
MPHOSPH8_1_2577,2640,3346,4560,2793,2355,3199,6379,2174,4297,3050,704,
2730
MSH6_1_2578,945,337,600,1451,768,376,1495,1178,4,453,600,1375

MSRB2_1_2579,291,269,78,322,295,201,0,134,25,117,132,0
MTA2_1_2580,514,346,694,374,644,284,404,324,1171,923,96,328
MTA3_1_2581,1267,1166,1240,836,1009,922,2467,309,937,270,456,1905
MYSM1_1_2582,1270,919,1590,691,1495,393,1406,185,3195,479,1709,511
NAP1L2_1_2583,2783,2623,2972,4402,3350,2099,4758,4878,681,1710,2567,13
01
NAP1L3_1_2584,5459,5039,5320,4258,6168,6044,5322,2849,4527,3977,3483,3
527
NAP1L4_1_2585,316,106,247,56,43,72,562,426,152,76,81,152
NAP1L5_1_2586,454,282,296,221,152,708,513,152,27,743,991,1346
NAT14_1_2587,116,43,37,41,0,187,0,20,1,92,0,0
NAT8B_1_2588,5959,5937,6565,8180,8659,6406,6694,5260,5864,5236,4688,10
450
NAT8_1_2589,1353,1336,1816,2191,1112,2792,1156,477,1888,1657,3038,1650
NAT8L_1_2590,141,110,202,121,45,299,96,335,0,583,17,80
NAT9_1_2591,1086,805,545,507,599,568,273,540,858,776,483,962
NCOA2_1_2592,1997,1261,1313,2063,1522,1190,1889,750,1313,454,976,4307
NPTXR_1_2593,1034,1342,2021,1216,1170,1653,4186,1451,1672,1442,5132,17
79
NR0B1_1_2594,1507,1831,1742,1893,1265,2481,909,205,1161,3345,2944,2466
NR0B2_1_2595,58,62,27,9,314,34,0,72,39,0,0,0
NR1D1_1_2596,707,416,1270,924,541,781,808,1113,616,1089,149,1187
NR1H2_1_2597,471,487,1197,528,611,1148,796,247,19,359,586,23
NR2C2_1_2598,374,106,249,340,95,143,59,478,468,152,11,38
NR2E1_1_2599,250,378,587,312,547,404,1813,60,324,333,9,1204
NR2F1_1_2600,649,251,535,818,1096,532,3,1,0,445,1,2605
NR2F6_1_2601,260,255,194,143,604,0,192,74,6,644,239,0
NR4A2_1_2602,170,89,148,190,50,31,346,47,13,2,2,6
NR5A1_1_2603,1616,1720,1455,1361,2593,577,1182,2138,2496,998,3998,2168
NRIP1_1_2604,2843,4282,3171,4194,4924,3471,5774,3493,3297,3930,1855,26
19
PADI4_1_2605,1268,482,1639,744,1614,866,865,669,1343,916,1238,458
PAWR_1_2606,480,735,221,314,1981,241,667,167,297,487,6,10
PAX5_1_2607,984,678,531,467,639,167,164,18,82,822,1808,987
PAXIP1_1_2608,4418,3130,3905,3654,3897,4856,3419,3371,5302,2718,3384,2
670
PGRMC2_1_2609,3660,4362,3945,4762,4683,4070,4959,3246,4222,5233,4784,6
059
PHB_1_2610,537,1092,750,645,1041,340,359,102,295,1640,278,187
PHC3_1_2611,264,129,265,118,614,90,377,1,84,140,64,722
PHF13_1_2612,1906,1101,1175,1396,304,3217,783,1292,1331,1760,1201,943
PHF14_1_2613,2056,2188,2073,2056,2639,3153,3900,1749,760,645,834,1865
PHF15_1_2614,936,877,1227,1019,837,1831,518,951,227,334,55,1496
PHF20_1_2615,714,431,1136,688,390,547,1187,556,633,273,1207,1221
PHF23_1_2616,2362,2486,2557,3664,3108,2695,2385,2559,4405,2840,934,405
5
PHF2_1_2617,299,200,435,680,346,270,587,1,21,108,14,0
PHF3_1_2618,1036,647,845,601,1076,976,405,99,2102,1078,310,205
PHF5A_1_2619,1513,1442,1569,1790,1961,1587,2410,3109,2244,1877,1517,28
45

PHIP_1_2620,528,767,184,511,1095,281,721,665,1045,226,733,2476
PHRF1_1_2621,181,601,205,162,619,274,1655,2,15,124,79,96
PIAS1_1_2622,2528,2054,1998,2280,1337,1992,2600,2491,1664,1157,1408,15
20
PIWIL4_1_2623,714,1097,1082,593,973,729,437,282,392,330,611,1173
PPARGC1A_1_2624,3882,3051,4431,3977,3673,4960,5367,2554,2329,3222,4450
,6140
PRDM11_1_2625,1169,1427,1229,1128,2801,1099,763,2058,40,447,1259,197
PRDM12_1_2626,835,830,594,732,582,818,1155,222,696,698,179,1
PRDM13_1_2627,689,418,382,52,11,117,1,86,286,340,42,28
PRDM14_1_2628,261,298,304,138,517,841,90,281,1231,282,4,775
PRDM4_1_2629,5,127,206,24,0,0,772,0,485,172,0,0
PRDM5_1_2630,1420,1990,1670,1033,2131,2060,1672,1255,1604,2193,1927,31
5
PRDM6_1_2631,3877,3719,4545,4445,7019,3682,2129,2953,5002,3769,4434,77
27
PRDM9_1_2632,3082,3328,2721,3709,3345,3581,2116,1852,2209,1658,1956,61
44
PRMT6_1_2633,583,868,789,596,969,820,998,678,1177,291,516,5
PRMT8_1_2634,0,0,0,0,0,0,0,0,0,0,0,0
PRPF6_1_2635,2434,1885,2169,2143,1416,1772,885,2051,4967,2199,1426,679
PYGO1_1_2636,1403,1318,1631,1158,2607,1505,1267,768,1902,1592,2436,211
PYGO2_1_2637,2858,1814,3747,2706,2960,2255,3985,1088,3921,2265,2945,32
96
RAG2_1_2638,2333,2902,4179,4178,2778,3227,2576,1293,4038,2499,1367,235
7
RAI1_1_2639,4289,3693,4505,5874,4063,6132,5902,4637,3295,3956,3354,322
4
RB1_1_2640,1771,1675,2126,1648,2469,387,4164,1835,804,1219,1217,2174
RCOR1_1_2641,6837,6193,7637,8154,7746,7072,9203,7110,9987,7004,5168,10
190
RCOR2_1_2642,378,443,499,566,843,381,868,260,26,507,556,196
RECQL4_1_2643,1565,795,917,1232,296,681,1226,1318,843,1337,1350,1506
RELB_1_2644,479,123,453,434,972,213,195,590,957,233,35,1905
REL_1_2645,1398,1639,1466,1491,2181,1942,1180,1923,2238,1378,1275,2703
RING1_1_2646,1973,1195,1821,1963,3825,953,1107,1518,758,497,2931,2372
RNF20_1_2647,211,271,135,506,288,493,433,61,0,182,68,103
RNF25_1_2648,631,781,1536,765,611,755,5815,917,1049,410,37,1170
RNF2_1_2649,3514,4592,5055,5226,5516,3835,6822,3585,3898,5256,5257,329
7
RORB_1_2650,756,946,818,1075,1652,1227,950,439,2722,516,35,681
RSF1_1_2651,3651,3561,4346,5726,3430,2556,3156,3260,2911,5108,4344,945
3
RUVBL1_1_2652,1280,1770,1213,1082,1021,944,660,471,1266,396,972,100
RUVBL2_1_2653,462,767,534,730,1009,504,517,560,227,243,2826,1333
RXRA_1_2654,562,315,669,26,235,139,451,397,944,258,327,23
RXRB_1_2655,2835,3164,3585,3746,4214,4847,4313,3223,3233,3228,3756,366
4
RXRG_1_2656,774,471,336,343,228,1060,85,165,150,581,499,98
SAP18_1_2657,2745,1684,3297,3192,3032,1774,3619,1751,3259,1870,1856,33

68
SCML2_1_2658,1585,1484,2436,1500,3013,1934,3648,939,1404,1325,1316,154
0
SENP3_1_2659,1166,750,735,639,852,823,1246,485,619,663,1533,264
SETD1A_1_2660,487,381,213,321,737,251,64,352,440,370,286,226
SETD1B_1_2661,1496,1610,1964,1331,1997,2697,654,453,2162,2494,2365,261
4
SETD2_1_2662,2651,3413,4172,3627,4051,4002,2222,2593,2941,4201,2796,40
79
SETD5_1_2663,965,632,701,1178,1688,866,670,19,207,902,148,980
SETD7_1_2664,3918,3836,4070,3452,4082,3895,3732,2589,4875,1641,3457,19
92
SETD8_1_2665,1919,1733,1959,2410,2863,2003,3439,1250,1502,2336,2679,24
73
SETMAR_1_2666,1805,1548,1357,2143,2641,904,2256,1397,1965,2147,2047,14
22
SF3B3_1_2667,440,658,653,495,85,1089,459,30,767,261,39,0
SIAH2_1_2668,1048,976,1121,1502,831,707,853,952,258,982,1284,1277
SIN3B_1_2669,831,227,397,690,418,1554,827,604,144,726,314,3
SIRT4_1_2670,2345,2162,1707,3003,2418,2736,1812,2455,2058,2345,2472,36
02
SIRT7_1_2671,2350,1885,1883,1913,846,1972,1944,401,620,2409,1329,1314
SLC2A4RG_1_2672,715,867,880,951,174,435,364,1998,982,322,10,366
SMARCA5_1_2673,4073,3741,4709,3714,6885,2764,5034,4616,7588,2473,2542,
4227
SMARCC1_1_2674,1141,572,1046,1127,1075,948,422,1509,908,1019,1926,2226
SMARCD2_1_2675,1951,1931,2123,2037,1834,2527,4788,2707,2221,1967,2321,
2480
SMARCE1_1_2676,116,235,385,97,576,270,279,275,0,532,564,40
SMC1A_1_2677,485,263,638,422,258,308,449,235,393,275,1318,21
SMC1B_1_2678,265,325,249,215,105,418,694,180,171,465,303,137
SMC3_1_2679,317,357,426,737,620,433,857,178,427,358,95,210
SMCHD1_1_2680,1916,2572,2865,1789,405,2471,3569,1822,572,1749,3912,100
3
SMNDC1_1_2681,4046,5111,4669,4914,5166,4524,6069,3667,9790,4612,6653,6
193
SMYD1_1_2682,576,735,471,491,149,348,184,704,1,378,147,876
SMYD2_1_2683,3074,2957,3276,1569,2438,2026,2565,2368,3106,1681,1118,28
79
SMYD4_1_2684,1163,557,1688,1068,650,1428,1723,266,1644,941,1350,1018
SMYD5_1_2685,3696,3030,5518,4421,6087,4956,4218,2808,591,2437,4687,306
8
SND1_1_2686,2013,1876,2203,1917,885,1454,6097,552,1629,3414,850,1502
SP140L_1_2687,6971,6094,6284,6037,7092,6840,9564,4139,8609,6716,7421,9
586
SRCAP_1_2688,1086,801,1313,963,2063,902,426,1541,1251,637,492,725
STAT5B_1_2689,1103,1697,1649,1445,564,1113,2880,1254,342,1365,1441,621
SUDS3_1_2690,990,492,1028,453,888,478,86,1491,43,254,237,125
SUPT16H_1_2691,2178,2656,2927,2230,4354,2584,2860,1705,3039,3680,2986,
1930

SUPT4H1_1_2692,530,881,477,410,659,478,2641,609,537,381,275,1065
SUPT6H_1_2693,1694,922,690,584,752,621,579,1117,1861,834,2227,766
SUPT7L_1_2694,785,622,1430,633,1747,929,1812,169,449,403,1125,1175
SUV39H1_1_2695,460,80,230,181,19,18,194,118,1724,28,2,75
SUV420H2_1_2696,904,975,1523,984,1563,725,1358,65,412,1434,1371,1333
SUZ12_1_2697,810,650,282,223,458,1554,2191,249,205,773,17,1051
TADA1_1_2698,1564,1763,1615,1377,979,1833,914,1598,2852,1417,239,1700
TADA2B_1_2699,1027,938,1908,979,793,613,1778,889,661,636,2384,1492
TAF10_1_2700,416,320,351,443,523,303,478,268,886,167,96,638
TAF1L_1_2701,683,453,772,1026,1231,239,363,145,1500,438,850,115
TAF3_1_2702,10431,9663,13133,11166,10325,12895,8502,11538,8241,9873,86
02,11288
TAF5_1_2703,2933,3010,4028,3107,4590,3541,4534,5607,1708,2534,3269,220
1
TAF6L_1_2704,1492,1655,1674,1396,872,1866,2025,739,433,4149,667,1306
TAF8_1_2705,166,496,733,545,492,524,1006,208,244,436,500,229
TCF7L1_1_2706,290,381,361,290,1107,136,17,933,0,19,36,0
TDRD12_1_2707,3837,3214,4333,5033,1922,2782,4642,3364,2171,3584,2735,5
759
TDRD1_1_2708,1813,1486,1418,2180,1141,2781,3989,2039,1842,1994,818,120
0
TDRD7_1_2709,780,445,312,497,1360,1522,2062,519,1015,445,223,14
TDRD9_1_2710,327,278,386,246,104,1045,626,5,73,440,54,761
TERF2_1_2711,1071,1960,2223,2200,1745,2826,5776,2222,649,933,333,2571
TET1_1_2712,1263,836,1213,1197,2125,1540,847,395,3823,719,2288,945
TET3_1_2713,1050,751,913,468,778,844,743,1747,1137,1053,939,92
TLX2_1_2714,631,499,603,631,456,398,652,83,285,1025,241,255
TNRC18_1_2715,1299,879,1232,1708,816,2245,3110,1662,427,1009,684,746
TRAF7_1_2716,548,413,506,330,581,23,84,2020,51,945,507,2
TRDMT1_1_2717,9880,8435,10534,9476,11338,11636,8712,7543,10069,9487,12
337,14483
TRERF1_1_2718,306,318,505,706,290,438,236,343,49,544,1112,1951
TRIM25_1_2719,178,379,452,189,0,1552,7,286,382,233,361,1106
TRIM27_1_2720,288,574,332,45,650,269,460,4,0,1223,0,0
TRIM28_1_2721,498,278,462,537,163,668,371,823,387,549,798,663
TRIM66_1_2722,2135,1709,2845,2470,3719,3702,1647,1920,3711,1533,869,27
92
TRRAP_1_2723,1353,1303,1995,1450,973,1299,644,1442,899,1543,2316,456
TSG101_1_2724,1523,1062,1541,2000,1786,1071,637,778,3359,650,1107,674
TYW5_1_2725,8162,8781,10054,8962,10292,7802,12097,7897,16748,8167,6222
,12248
UBE2B_1_2726,2793,2315,2534,3181,3964,3947,2298,1994,4906,2873,1399,58
41
UBE2N_1_2727,1770,1121,1878,1145,155,1362,1087,1338,1726,1549,582,1377
UBR7_1_2728,1440,1459,1043,1817,1599,535,2434,2649,1148,897,156,2636
UHRF2_1_2729,7854,7765,9465,8257,9379,6005,6670,7382,5846,5354,10036,1
3638
USP22_1_2730,1393,1446,1475,1696,1025,1795,712,1258,1609,1234,1601,197
2
YEATS2_1_2731,4074,3717,5066,3337,4770,3037,6907,2767,5281,4865,7395,6

116
YEATS4_1_2732,1374,1281,1274,1600,2639,2210,2687,1044,805,656,1447,216
7
YY1_1_2733,448,286,61,614,1588,85,185,900,65,473,1,537
ZAR1_1_2734,3602,3254,3076,3068,4992,5625,3711,4292,6832,4371,2720,736
1
ZCWPW1_1_2735,308,166,316,70,329,171,367,185,19,11,571,97
ZCWPW2_1_2736,8705,7321,6893,7727,4672,7844,15929,7794,9171,7449,10803
,11360
ZFP57_1_2737,1105,1088,1497,919,1131,1039,87,1108,1078,1222,1242,958
ZNF541_1_2738,355,228,407,160,273,199,4,389,188,225,1020,330
ZNF85_1_2739,3781,3249,4092,3830,3402,6061,2610,3961,3015,4235,1926,34
19
AANAT_1_2740,318,54,745,128,44,209,0,0,0,57,517,0
AES_1_2741,367,642,809,122,554,1114,213,231,337,240,16,0
AIRE_1_2742,5431,4663,6283,5842,5586,7914,3175,4636,9680,5397,8391,663
4
AKAP1_1_2743,780,719,1266,858,1257,1428,2635,507,670,979,764,203
ALKBH2_1_2744,1109,846,1684,1097,1387,626,2699,1687,986,1073,887,822
ANKHD1_1_2745,1248,1042,1554,2342,2623,916,1428,2026,2070,1565,4519,15
88
ARID1A_1_2746,1066,842,1044,848,936,1071,1410,595,860,906,888,809
ARID1B_1_2747,511,145,400,298,265,116,1525,119,292,88,217,39
ARID4A_1_2748,1752,1936,2528,2201,3698,2437,2196,994,2306,2396,3853,16
18
ARID4B_1_2749,2072,2124,2353,2872,2870,1290,1611,2862,1979,1365,2430,1
843
ARRB1_1_2750,356,416,597,233,235,79,180,21,851,424,111,126
ASH2L_1_2751,1052,884,1035,488,1429,1549,421,345,466,724,590,432
ATAD2B_1_2752,1064,586,1145,736,1512,790,324,102,785,1206,1944,24
ATAT1_1_2753,802,1193,1224,1174,740,1910,1227,771,1548,423,162,896
ATRX_1_2754,2020,1273,1200,1811,1623,1771,1131,2495,1732,1467,3860,390
AURKA_1_2755,827,832,1432,533,671,734,1024,584,914,676,1347,998
AURKC_1_2756,441,911,1254,891,885,1094,1041,806,4,845,899,620
BAZ1A_1_2757,3562,3075,3117,2980,4888,3532,3522,1300,4668,3131,1136,25
43
BCOR_1_2758,411,1078,987,480,905,772,675,2412,2832,968,276,681
BPTF_1_2759,3792,4356,3838,4931,5489,5884,8236,5682,4413,2820,4777,712
3
BRCA1_1_2760,248,100,204,241,688,110,340,67,4,80,1,641
BRD2_1_2761,673,805,701,551,749,400,1870,679,347,956,2878,168
BRD4_1_2762,1382,1654,1582,1247,3489,1677,787,998,1359,807,250,1547
BRD7_1_2763,9140,8734,9647,10019,8527,8004,13914,7470,5272,9859,12439,
8925
BRD8_1_2764,821,854,356,911,771,1247,497,576,217,998,22,219
BRD9_1_2765,54,486,374,130,687,778,180,0,8,4,4,1
BRDT_1_2766,2341,892,1792,1621,3047,738,1923,525,3414,901,1314,1602
BRPF1_1_2767,917,953,1379,1302,1555,2633,1362,358,460,985,725,2010
BRWD1_1_2768,3423,3885,3463,4279,2204,5086,1704,2343,849,3482,3257,462
7

C14orf43_1_2769,152,173,254,136,41,638,877,195,285,406,38,582
CBX1_1_2770,2463,1830,2207,1741,1704,3355,4076,1701,2237,1228,2159,153
3
CBX3_1_2771,4592,4225,3197,4202,6978,4755,3208,1927,2421,2621,3019,647
0
CBX5_1_2772,1686,1820,1170,1488,915,2313,1039,985,639,2721,1775,1216
CCNT2_1_2773,164,473,405,269,678,98,527,55,3,224,67,439
CDY1B_1_2774,2317,2725,2496,3608,2224,3280,3253,1648,1172,2507,3361,28
36
CDY1_1_2775,2317,2725,2496,3608,2224,3280,3253,1648,1172,2507,3361,283
6
CDYL_1_2776,4358,2987,3832,4680,7581,6902,6293,2642,5002,3365,3245,564
7
CHD2_1_2777,191,660,131,420,27,365,549,289,3,931,0,34
CHD3_1_2778,1188,914,1319,899,2125,1275,476,1463,803,1239,2424,2458
CHD8_1_2779,1447,787,1416,800,1773,1268,1848,223,198,864,706,2320
CHMP2A_1_2780,2392,1885,2738,2274,1876,1511,3082,2207,1566,2608,2439,2
368
CHMP5_1_2781,419,148,873,295,530,411,175,1084,216,644,1127,29
COPS2_1_2782,3130,2595,2616,3415,4325,2546,4615,3067,2487,2670,1552,51
37
CPA4_1_2783,473,357,250,504,351,1256,16,260,70,964,462,0
CREB1_1_2784,881,708,1092,1068,324,538,1751,1911,2983,1066,1627,251
CREBBP_1_2785,1182,1151,1208,946,946,1755,715,198,126,357,1640,1572
CTCF_1_2786,804,597,1369,1077,29,346,622,1506,69,710,136,1373
CTNNB1_1_2787,142,307,315,256,169,179,4,105,237,98,0,269
CXXC1_1_2788,2449,2061,1996,2458,2484,2762,2768,2451,2492,1023,2166,16
50
CYLD_1_2789,624,403,619,224,82,559,2777,306,1976,261,49,1435
DICER1_1_2790,10479,7383,9499,9874,13011,11408,10916,6472,14247,9189,1
1121,16260
DIDO1_1_2791,4885,4724,5482,5098,7904,6355,4506,3052,6811,3854,7013,33
78
DMAP1_1_2792,619,663,393,563,351,846,867,668,618,789,423,1694
DNAJC2_1_2793,3773,3426,5138,3969,5721,4251,5027,4492,2219,3942,2413,5
946
DNMT1_1_2794,1899,1656,2391,2491,3357,1534,1460,2628,1819,2780,1958,50
8
DNMT3A_1_2795,700,532,289,500,794,402,264,14,71,169,59,1333
DNMT3B_1_2796,472,496,353,560,691,99,186,614,1514,408,867,448
DNMT3L_1_2797,233,207,334,109,85,105,37,20,13,92,1413,8
DPF1_1_2798,667,1334,1361,682,589,581,736,79,1769,2568,268,1940
EED_1_2799,1547,1058,1750,1796,1626,1435,1305,2374,653,106,2127,826
EGR2_1_2800,2605,2029,3339,2439,830,797,424,1144,2331,1300,2694,3257
EHMT1_1_2801,0,0,0,0,0,0,0,0,0,0,0,0
EHMT2_1_2802,455,590,622,1333,254,401,1040,685,513,215,98,1190
ENO1_1_2803,725,1017,737,665,4120,869,1890,310,1105,826,61,766
ESR1_1_2804,1578,1407,2686,1938,3299,1151,1664,839,1655,2093,2237,788
ESR2_1_2805,125,91,337,148,807,144,67,250,314,216,0,0
ESRRG_1_2806,923,510,994,1016,703,304,292,768,925,1195,3594,862

EZH2_1_2807,5076,3833,4462,4511,5082,3658,2068,3890,6269,3989,3370,399
3
FBXO11_1_2808,590,774,650,1141,1867,1434,545,683,1068,226,1009,306
FMR1_1_2809,2134,1660,1653,2050,1670,2122,2229,2951,1423,1499,1744,159
9
FXR1_1_2810,1670,2131,2082,3684,2072,3462,3480,2305,2486,2322,2033,268
GFI1B_1_2811,741,656,426,674,1498,1401,890,1008,270,875,1083,630
GMEB1_1_2812,1835,1551,2523,2027,2784,3484,2764,3011,2213,1190,1577,23
86
HDAC10_1_2813,923,902,1400,843,931,1541,428,907,789,1447,1951,650
HDAC11_1_2814,1275,622,1120,636,2737,882,96,1629,283,461,484,1214
HDAC5_1_2815,93,73,74,117,41,125,29,120,0,32,6,52
HDAC7_1_2816,984,844,1407,1262,1871,1282,1027,1626,862,1338,21,228
HDAC9_1_2817,776,926,763,784,2052,762,851,283,1117,778,127,446
HDGF_1_2818,657,297,318,214,735,222,560,86,383,555,36,76
HDGFRP2_1_2819,1311,1532,1296,476,1187,1587,773,396,2918,1357,384,315
HLTF_1_2820,3098,2886,2612,3110,3218,1999,2116,2636,2486,4050,1435,512
3
HMGA1_1_2821,169,2,152,87,0,52,32,0,598,16,0,0
HMGN3_1_2822,1433,1756,2757,2364,3525,2439,3390,1977,1030,1340,3443,16
38
HNF4A_1_2823,262,435,323,360,329,1059,1024,57,74,201,64,1354
HPSE2_1_2824,48,230,289,223,322,103,190,65,539,246,184,3
HR_1_2825,1090,421,325,1110,549,1740,270,51,442,214,313,2793
ING1_1_2826,765,597,640,356,496,1224,487,1153,490,322,2568,897
ING4_1_2827,2399,2910,2372,2615,3820,3274,2007,3571,1367,2273,2599,270
5
INTS12_1_2828,1675,902,1867,2409,2890,1412,4558,1310,1859,3110,1042,15
43
IRF4_1_2829,397,685,217,768,575,360,31,452,106,926,1242,223
JMJD1C_1_2830,1493,1628,1475,1995,1610,1484,622,877,1026,1161,1124,230
2
JMJD4_1_2831,383,24,110,5,203,400,1279,0,5,19,487,35
JMJD6_1_2832,1614,1049,2384,1957,2003,2658,4277,1843,1342,2152,3277,30
33
KAT5_1_2833,1657,1270,2526,2387,3241,2058,3365,1167,2160,1769,3635,322
0
KAT6A_1_2834,1239,718,1952,1643,2520,654,315,1704,1065,1372,1390,1724
KAT7_1_2835,2031,2272,3751,3573,2914,2485,1544,2076,830,2162,2267,487
KAT8_1_2836,346,308,249,340,294,1010,943,196,424,710,94,32
KCTD1_1_2837,1347,632,1152,1602,1668,3307,857,1014,3216,823,322,2697
KDM1A_1_2838,3297,2721,3545,3626,3164,3613,4671,4581,2735,3277,2536,47
62
KDM2B_1_2839,593,134,248,671,1463,633,1163,557,95,314,156,13
KDM3A_1_2840,2053,721,1368,1603,1679,1535,1601,1138,444,1094,1314,838
KDM4C_1_2841,91,302,374,95,105,78,1257,17,1331,355,133,0
KDM5C_1_2842,1684,898,1811,1603,1557,1441,1387,1566,1151,1084,577,1392
KDM5D_1_2843,2525,2463,2746,1876,3131,3089,2679,1586,3866,2343,2935,31
12
L3MBTL1_1_2844,5709,3774,4019,4795,2690,5968,4730,4210,5479,4160,1807,

4365
L3MBTL3_1_2845,1490,1045,1543,1503,1366,2265,1363,827,897,2370,600,130
3
LBR_1_2846,2371,2394,2312,3208,2211,2507,2589,1021,1475,2741,2183,1480
MAP3K12_1_2847,545,907,1415,2244,201,462,2025,1442,1323,468,634,2566
MBD1_1_2848,1114,644,753,563,1155,1156,902,588,513,554,337,638
MBD2_1_2849,3467,1936,3584,2278,2781,2266,2918,2296,2909,2380,3116,282
1
MECOM_1_2850,1009,795,1526,1335,1963,1436,1981,3726,529,900,394,3011
MECP2_1_2851,607,494,1218,660,326,321,157,81,223,223,810,386
MEN1_1_2852,4677,4600,5218,3529,5745,4819,4482,6126,6455,7091,2805,205
5
MGEA5_1_2853,2571,1428,2956,2489,2568,2046,2467,1513,3442,2294,1329,12
89
MIB2_1_2854,176,214,427,211,240,398,181,180,18,433,692,13
MIER1_1_2855,1416,1427,1796,1952,1980,1198,1811,2168,2287,1149,1562,10
77
MINA_1_2856,661,645,141,756,559,1085,266,1192,56,414,7,9
MLL5_1_2857,2362,3077,2800,3860,4306,4052,3700,3737,3877,3577,1359,307
7
MLL_1_2858,326,247,233,160,165,563,121,176,15,223,0,23
MORF4L1_1_2859,766,580,785,815,1151,896,131,1531,250,1037,749,984
MSL3_1_2860,1506,1532,1541,1614,2409,1754,1724,785,1527,1100,1196,1513
MTA1_1_2861,205,457,428,276,89,388,29,22,0,130,51,317
MTF2_1_2862,1073,996,1570,1076,733,1631,2516,1228,5356,1324,793,552
NAA60_1_2863,1266,1069,1114,1112,730,1313,1179,656,618,1119,1878,365
NAP1L1_1_2864,623,325,697,626,788,161,1419,218,393,697,1476,941
NAT10_1_2865,1345,1639,1747,1014,1537,2243,2056,905,1732,1392,72,1079
NCOA1_1_2866,3889,4038,5007,4555,6744,5340,4860,3714,3815,3611,3693,57
32
NCOA3_1_2867,106,83,19,55,0,0,1,171,0,8,1,1
NCOA4_1_2868,564,827,833,1600,945,2352,907,1000,326,540,239,451
NCOR1_1_2869,1083,456,505,717,1041,117,1424,771,579,610,496,1026
NCOR2_1_2870,216,173,311,60,643,346,237,74,1131,62,143,24
NFAT5_1_2871,6745,6951,9562,9584,9901,8278,11011,4166,7695,6280,12767,
9140
NFATC1_1_2872,344,262,151,314,261,68,599,88,48,222,360,10
NFATC3_1_2873,1166,1148,1199,1300,1873,1580,945,236,2888,1319,821,1803
NFATC4_1_2874,3874,3047,4065,3475,4847,4270,4320,3191,4240,2167,4813,4
398
NFKB1_1_2875,756,908,847,436,1202,595,428,545,1358,494,1115,80
NFKB2_1_2876,1480,1135,1160,1044,2479,1719,983,1882,1729,601,2697,143
NPM1_1_2877,1746,1632,1697,977,2377,1215,1227,1565,610,1136,1612,2541
NR1D2_1_2878,1368,1030,1627,2865,3378,1279,2904,1736,2040,1277,1753,28
38
NR1H3_1_2879,43,67,11,23,0,175,0,9,3,108,45,525
NR1H4_1_2880,3314,2485,2969,3658,1935,6748,3927,4615,3159,3153,5126,13
89
NR1I2_1_2881,1044,1140,1116,1317,1022,1588,1236,363,2873,1823,1518,662
NR1I3_1_2882,1240,1291,1359,1122,1093,891,1754,1226,416,841,7,3286

NR2C1_1_2883,1142,1043,1564,1638,843,2679,3311,542,566,294,1704,3283
NR2E3_1_2884,1703,1279,2091,2164,1471,1835,3503,2082,1940,1536,2889,26
97
NR2F2_1_2885,841,816,731,744,373,618,910,22,257,2187,1045,510
NR3C1_1_2886,406,486,200,696,764,652,379,39,44,195,1234,418
NR3C2_1_2887,3237,3461,3050,4272,5204,3183,2834,3199,4891,2513,2763,33
72
NR4A1_1_2888,1425,1986,1821,2050,2159,2344,2252,1647,5149,1305,990,257
4
NR4A3_1_2889,117,292,359,171,73,89,324,0,52,212,194,258
NR5A2_1_2890,2150,1686,2638,2543,1845,668,2443,2895,324,2271,1761,2948
NR6A1_1_2891,2696,2216,3447,1799,1968,1892,2017,1182,4090,3184,2652,39
20
NSD1_1_2892,751,734,713,414,451,727,1974,992,1573,168,1800,353
PBRM1_1_2893,2464,3054,3785,2056,2866,3244,2339,2096,1077,2331,905,208
7
PCGF6_1_2894,790,755,326,598,2137,222,121,328,2112,1040,268,35
PGR_1_2895,1512,1683,1657,1796,782,3126,4282,2215,2041,1407,3352,2823
PHC2_1_2896,1037,1578,1508,1028,1363,1505,1040,1246,475,540,721,1565
PHF10_1_2897,2572,2483,2444,2753,2651,2117,3507,4132,4773,2039,4956,59
0
PHF11_1_2898,6452,5347,5943,7605,8718,5121,7753,6274,5582,5833,4295,66
99
PHF12_1_2899,792,838,652,670,822,499,1710,250,1331,486,749,1205
PHF16_1_2900,821,1150,1061,1327,1294,728,642,213,13,2397,523,477
PHF17_1_2901,936,877,1227,1019,837,1831,518,951,227,334,55,1496
PHF1_1_2902,547,257,347,120,1023,155,1021,567,271,356,0,136
PHF20L1_1_2903,2643,1661,3005,2238,3278,2157,4413,2236,4243,2678,2147,
4418
PHF21A_1_2904,163,115,345,493,706,45,775,172,705,2,97,1230
PHF21B_1_2905,316,399,738,24,8,1,661,38,2540,243,5,25
PHF6_1_2906,8031,5516,7213,6531,5800,6905,6176,10079,7878,4971,7153,12
192
PHF7_1_2907,2380,1843,1226,2237,1180,1226,1052,1071,2325,2818,1977,900
PHF8_1_2908,1027,1080,1229,978,1159,815,1659,549,688,1411,2679,3067
PIAS2_1_2909,2946,2834,4124,5652,5422,6702,3050,5037,2655,5757,2830,48
47
PICK1_1_2910,1418,515,1674,1507,1617,1356,741,1205,2610,1503,893,3337
PIWIL2_1_2911,1085,1089,1227,1051,1386,117,748,1572,80,434,811,40
PML_1_2912,1046,1005,787,876,1558,1408,2033,957,703,866,2903,1516
POLR1B_1_2913,921,1026,2086,1591,587,1601,2403,1981,3869,1158,387,887
PPARA_1_2914,751,473,421,429,85,1150,1523,118,1237,486,131,93
PPARD_1_2915,105,184,72,170,4,268,0,518,0,33,0,6
PPARG_1_2916,2716,2517,3543,2787,2257,2797,2391,2184,2696,1909,1474,38
11
PRDM10_1_2917,2337,2506,2807,2167,3347,2036,3018,2430,449,2759,1635,30
44
PRDM15_1_2918,1247,1183,610,1610,516,1015,1681,1548,747,1645,1522,927
PRDM16_1_2919,619,296,487,544,1389,789,2537,18,277,598,426,88
PRDM1_1_2920,1819,1127,1698,1743,1640,1485,496,583,852,2075,431,303

PRDM2_1_2921,1356,1092,1575,580,278,726,3174,876,1191,1039,1746,1026
PRDM7_1_2922,1033,1285,1024,610,899,945,510,110,1490,1087,271,682
PRDM8_1_2923,109,259,254,329,19,1651,0,345,0,368,140,814
PRMT1_1_2924,5227,3664,4088,4517,2814,6299,2488,1938,5201,3207,3863,44
43
PRMT2_1_2925,737,822,406,484,429,538,140,136,785,145,0,756
PRMT3_1_2926,3000,2860,2163,4241,3991,3924,5099,3752,4318,2251,5291,14
88
PRMT5_1_2927,5534,4902,4492,5969,5776,6168,6272,4756,7446,4505,5038,79
36
PRMT7_1_2928,428,419,131,152,3,0,150,411,9,550,0,873
PSIP1_1_2929,3024,3189,3138,2778,4215,2785,1680,2675,2432,4510,4819,24
64
PSMC5_1_2930,3126,2804,3898,2617,2400,5165,1924,3761,497,2226,3043,454
1
PWWP2B_1_2931,748,321,644,654,1047,1149,151,968,179,968,27,322
RAD54B_1_2932,1151,682,827,1005,1184,993,626,526,1256,755,1750,667
RAD54L_1_2933,99,253,195,266,128,269,383,383,291,52,86,18
RARA_1_2934,1403,1720,1405,1329,2676,881,420,1975,4296,1085,2053,591
RARB_1_2935,468,251,260,302,634,42,1090,332,1121,31,67,1436
RARG_1_2936,1727,1587,1540,1951,3583,1343,906,1521,3789,1365,4036,2049
RBBP5_1_2937,539,775,712,664,159,149,519,362,74,1076,0,0
RBCK1_1_2938,297,244,382,287,717,171,167,414,776,562,243,479
RBFOX2_1_2939,127,93,484,151,6,805,20,990,0,155,286,4
RCC1_1_2940,326,517,415,229,437,493,1713,126,933,147,143,626
RCOR3_1_2941,133,180,436,76,56,1051,416,42,27,157,7,1000
RECQL5_1_2942,484,505,1358,1014,1103,633,1871,1168,882,315,1949,1164
RECQL_1_2943,1134,1414,1482,1070,901,1679,1702,1079,1023,1185,352,2215
RELA_1_2944,835,782,1649,529,467,1529,712,565,132,979,49,1593
RERE_1_2945,415,147,430,542,124,773,568,714,39,133,61,465
RFC1_1_2946,4098,2801,3941,2805,2322,3619,4127,4533,4429,2846,5373,306
8
RNF14_1_2947,147,249,154,443,20,92,413,1008,207,466,163,49
RNF17_1_2948,1067,801,938,1285,1685,3180,876,1280,2400,758,2340,1134
RNF40_1_2949,38,344,41,517,0,535,7,0,0,0,0,0
RNF8_1_2950,776,814,665,432,1339,837,753,737,1524,344,526,2281
RORA_1_2951,4841,4394,4848,3835,4934,4022,5458,4989,4525,4246,5795,567
1
RORC_1_2952,2596,2178,2266,3147,2981,2909,2602,1522,3512,1619,2335,853
RPH3A_1_2953,539,905,1330,830,337,1227,290,725,195,725,306,342
RPS6KA5_1_2954,3857,4842,4459,4253,6386,4287,4859,2038,6865,4476,3059,
3564
SATB1_1_2955,4060,2521,3039,3459,4334,4152,4874,3549,2839,2790,4864,10
34
SATB2_1_2956,3549,5098,4628,4730,5848,5656,4281,2345,6979,4192,5808,62
42
SCMH1_1_2957,1298,781,1320,1185,2172,1889,1869,969,3381,813,1706,1379
SET_1_2958,2042,1489,1887,1804,3402,4037,4507,2249,1507,1500,1779,3679
SETD3_1_2959,1945,1289,1823,2334,2658,1904,1295,2644,2690,1807,1132,17
95

SETD4_1_2960,4349,4505,5983,6378,4584,5042,9733,3610,3663,3645,3215,11
95
SETD6_1_2961,582,457,563,936,597,379,869,396,747,68,332,2833
SETDB1_1_2962,2368,1521,1570,1918,3635,1266,2301,1374,1228,1393,1737,2
23
SETDB2_1_2963,1037,1358,1816,1479,620,1629,784,819,156,838,3524,905
SFMBT1_1_2964,799,595,302,134,712,124,167,1862,910,284,2399,711
SFMBT2_1_2965,4452,3702,3935,4148,5488,2753,3185,3067,3789,5177,3074,5
394
SHPRH_1_2966,3057,3218,3970,5270,6773,5757,4127,2184,3171,3792,1523,39
91
SIN3A_1_2967,1079,1171,2080,1518,1675,1260,2039,2095,985,407,434,229
SIRT1_1_2968,475,268,363,371,182,540,114,1749,1274,466,46,135
SIRT2_1_2969,1951,2340,2102,2067,2036,3793,2331,4025,1465,1126,3940,27
58
SIRT3_1_2970,1286,812,609,628,1312,526,404,259,1560,988,1521,469
SIRT5_1_2971,4026,3139,3727,3868,1967,5370,2815,4834,2491,3849,4411,37
66
SIRT6_1_2972,598,183,336,258,218,583,105,89,1206,66,0,68
SLC38A1_1_2973,1095,1391,1393,2022,1272,2509,287,1132,684,967,1294,215
4
SMARCA1_1_2974,3010,1975,3255,3192,3691,2103,2646,1931,4085,3387,366,1
452
SMARCA2_1_2975,3896,4110,3688,2753,7681,5225,7946,2476,5467,3616,2870,
2166
SMARCA4_1_2976,907,1319,1642,1210,1153,1625,1321,108,1370,2163,1263,86
0
SMARCAD1_1_2977,966,829,1275,842,758,756,447,1140,1642,631,8,539
SMARCAL1_1_2978,3446,2419,2494,2085,1446,2103,2022,1653,1366,2242,3113
,742
SMARCB1_1_2979,1455,1485,1706,1422,1948,2791,514,2158,826,3049,453,799
SMARCC2_1_2980,990,809,815,701,628,1126,714,1357,915,249,1722,1552
SMARCD1_1_2981,1605,547,1227,843,660,890,810,928,2160,894,1548,1407
SMARCD3_1_2982,3233,2647,2374,1806,3239,2504,2474,692,2759,2471,2133,3
132
SMC2_1_2983,5042,3852,5437,3371,3290,4330,4233,4470,3584,3118,2417,383
5
SMC4_1_2984,4788,4357,4941,3882,4784,4861,3751,3774,6551,4790,3328,731
6
SMN1_1_2985,11577,10085,10496,12196,11158,11131,13806,10306,15003,9950
,15860,14813
SMN2_1_2986,11577,10085,10496,12196,11158,11131,13806,10306,15003,9950
,15860,14813
SMYD3_1_2987,844,651,695,1069,390,716,1958,758,5013,557,1299,594
SP100_1_2988,981,1042,1517,874,891,1652,1237,900,1235,745,5,1382
SP110_1_2989,486,220,382,216,64,886,676,550,37,623,55,750
STK31_1_2990,6245,6745,6739,5775,6418,6251,8888,5110,5166,5141,6166,80
94
SUPT3H_1_2991,1716,1812,2106,2514,1770,1793,1981,2170,248,2319,507,145
7

SUPT5H_1_2992,459,925,497,689,865,181,168,108,36,1074,1182,657
SUV39H2_1_2993,1108,513,1681,1169,2040,1315,429,702,2331,1084,83,662
SUV420H1_1_2994,1159,1308,1129,966,1142,2215,1360,807,525,949,771,798
TADA2A_1_2995,3607,2392,3518,4155,5565,2271,1859,2905,3792,1240,984,26
44
TADA3_1_2996,1479,1382,1102,2291,884,1251,934,1207,119,1047,792,1675
TAF12_1_2997,439,623,514,602,704,578,1243,1086,716,1133,1466,217
TAF15_1_2998,1759,1316,1266,1518,2514,1299,449,750,1912,723,1268,4788
TAF1_1_2999,9243,7105,8357,8062,9085,6568,7443,8251,8724,8968,13440,73
67
TAF5L_1_3000,2674,2135,3147,3203,2448,3178,4596,1405,2347,2670,4663,39
57
TCF19_1_3001,370,591,353,654,319,877,1744,172,276,366,421,1934
TCF20_1_3002,4087,4032,2905,3484,2758,2305,4193,3118,6178,4108,4063,26
61
TDRD10_1_3003,909,992,1251,1166,2411,890,1135,1021,546,638,2708,2147
TDRD3_1_3004,4462,3903,6964,4835,6250,4565,5729,4842,6701,5207,3714,58
43
TDRD5_1_3005,2731,2367,3133,3185,3815,2833,2744,1671,1007,891,4719,199
8
TDRD6_1_3006,1454,1330,1605,1858,1107,2697,1539,1627,2249,1748,3249,19
25
TDRKH_1_3007,1089,790,974,1412,880,1083,641,792,586,484,709,2202
TERF1_1_3008,1018,1673,707,1635,2151,2271,1124,1530,4,1148,1062,1153
TET2_1_3009,2628,1658,2270,1956,3478,2109,3751,1202,4264,1963,1177,250
8
THRA_1_3010,2667,2460,3093,2491,2556,3667,4999,2804,3849,2223,2071,884
THRB_1_3011,707,898,1725,1226,720,1348,579,1287,1543,1285,733,2760
TP53BP1_1_3012,1828,1949,1876,2804,709,1989,2243,1691,1302,1612,1213,1
779
TP53_1_3013,2290,1945,2736,1616,1425,2245,1396,1749,3317,1454,4382,374
7
TP73_1_3014,90,302,263,17,18,497,2,167,0,538,3,1
TRIM24_1_3015,3781,5245,5142,4629,6009,5343,4409,6156,7720,3757,6420,2
739
TRIM32_1_3016,2435,2990,3197,2405,3576,4290,4446,2274,6128,1825,5709,2
507
TRIM33_1_3017,2409,2574,3183,3705,1807,4271,957,3644,4343,2522,4390,52
10
UBE2A_1_3018,1154,1297,1279,1093,1791,1118,862,929,949,2048,1301,3411
UBE2E1_1_3019,97,309,186,676,34,429,276,147,2,402,658,140
UBE2I_1_3020,258,186,336,328,16,182,96,408,0,182,30,94
UBE2K_1_3021,2979,2958,3794,4105,3719,4158,2762,1687,3902,2599,2236,51
51
UBE2V1_1_3022,2574,2257,2406,1820,3214,3073,4167,1868,1283,1993,2692,1
365
UHRF1_1_3023,252,64,181,57,126,36,3,83,131,138,2,205
USF2_1_3024,186,186,159,102,92,10,456,117,71,197,0,25
UTY_1_3025,17482,13849,16771,17996,19153,20418,19749,17989,16967,15735
,17704,16287

VDR_1_3026,170,212,210,272,20,22,194,62,91,26,191,52
WDR5_1_3027,474,648,1239,393,1384,476,259,443,919,967,286,772
WHSC1_1_3028,729,218,296,538,877,318,1094,839,426,573,1986,313
WHSC1L1_1_3029,890,1220,1339,555,766,1858,1647,1696,223,321,357,626
WRB_1_3030,478,52,155,14,15,930,46,5,0,0,1390,0
ZGPAT_1_3031,500,620,414,615,549,260,243,792,161,750,5,441
ZMYND11_1_3032,4751,4652,5382,6103,3924,4986,4462,4616,5040,5672,7932,
5687
ZMYND8_1_3033,566,190,199,501,423,104,80,203,723,239,531,156
ZNF451_1_3034,6110,4058,5455,4783,5641,3628,4008,4093,3383,3468,5448,2
728
ALG13_1_3035,537,383,160,332,403,429,3,272,282,203,44,308
ASXL1_1_3036,877,1027,852,455,545,499,1871,301,1317,849,321,1022
CBX2_1_3037,1156,1093,575,1064,440,1192,669,1498,1928,1026,238,29
HDAC8_1_3038,2632,2607,2444,2798,4092,1544,3252,2420,1711,835,756,1560
ING3_1_3039,321,346,476,695,976,418,72,27,16,923,490,1167
MLLT10_1_3040,199,99,235,247,405,100,1,501,0,5,14,1
PHF19_1_3041,8,68,3,229,4,0,0,0,0,0,0,160
RBM14_1_3042,189,175,115,275,5,151,321,725,165,10,0,0
SP140_1_3043,1122,871,1754,1831,1042,1565,4099,2064,246,1734,1341,1133
TAF9_1_3044,3944,4416,6349,5106,7981,5042,8461,2307,3366,4676,9367,381
4
TAF9_1_3045,8594,7292,8832,9073,8917,5931,10813,6587,12079,8181,8071,1
0293
AHR_1_3046,14110,13378,13424,15673,13836,14698,17279,14224,15560,14209
,17834,15205
ALKBH1_1_3047,1754,1579,2776,1628,2524,1495,157,2226,1730,2579,1313,41
9
ALKBH3_1_3048,8909,10329,12332,11523,11638,11052,13917,9767,8468,12413
,6915,9759
ARID2_1_3049,2018,1740,2290,2464,3086,1650,2214,698,824,1477,1032,1314
ASF1A_1_3050,3455,2823,3825,2109,5144,6644,1527,2736,4277,3101,3807,50
75
ASF1B_1_3051,2264,1681,2175,2382,1580,4330,3214,1582,1614,1444,1586,33
0
ASH1L_1_3052,1004,1328,957,1049,1207,889,1437,2259,1378,706,10,455
ASXL2_1_3053,2279,2608,2350,2452,3655,3757,2931,2529,1005,2279,3735,64
28
ASXL3_1_3054,1574,1521,2702,2060,1360,1332,1394,1578,3181,2456,127,197
6
ASZ1_1_3055,3109,3821,4010,3968,5224,4839,3912,2296,2963,2514,4942,108
1
ATAD2_1_3056,1407,1374,2122,1788,2324,1527,1588,1795,2385,830,2164,555
ATF7IP_1_3057,3279,2179,3563,3673,3437,4502,3666,2414,4831,1813,2577,3
39
AURKB_1_3058,586,352,239,184,865,189,193,282,26,405,428,155
BAHCC1_1_3059,1767,946,1712,1477,1859,1154,799,154,2827,1279,738,1557
BAHD1_1_3060,687,671,1400,512,2145,232,365,628,314,913,1109,296
BARD1_1_3061,939,1203,1311,1402,3647,885,1204,255,1152,432,630,1373
BAZ1B_1_3062,3512,3682,3711,4602,5225,3702,5510,2740,3723,2117,3166,28

50
BAZ2A_1_3063,1049,1838,1185,1772,1727,598,2395,1811,2690,708,334,2203
BAZ2B_1_3064,15141,13051,15058,16095,19936,15293,17281,13394,15369,166
76,18290,20824
BLM_1_3065,5217,5187,7312,6240,4388,8518,8230,6520,5629,5410,7075,1020
0
BMI1_1_3066,282,362,281,381,627,102,264,149,463,665,1313,183
BRD1_1_3067,106,149,308,278,207,177,164,59,384,33,672,3
BRD3_1_3068,3400,3414,4150,4114,1773,5848,2339,3027,5568,2991,2514,419
4
BRPF3_1_3069,1193,609,799,183,29,135,977,492,418,188,1,174
BRWD3_1_3070,652,676,787,576,576,679,1120,93,424,274,1,85
C14orf169_1_3071,1221,1145,1219,1199,1888,1254,1638,1543,679,1972,1508
,792
C20orf20_1_3072,1191,1379,1158,1249,681,1686,1143,1210,3765,1365,351,1
317
CALR_1_3073,947,1595,1898,1763,1382,823,1094,2043,387,2473,2698,1229
CARM1_1_3074,824,860,1174,946,2086,1894,1916,1016,2191,970,82,1483
CBL_1_3075,1654,1110,1408,1611,2519,1824,1847,1822,2166,1845,1540,1104
CBX4_1_3076,596,216,342,177,197,513,693,262,1614,564,28,143
CBX6_1_3077,333,253,346,333,677,436,459,488,1112,444,0,115
CBX7_1_3078,303,239,455,159,37,317,9,399,29,537,116,9
CBX8_1_3079,389,286,728,353,0,245,0,436,0,238,4,906
CCDC101_1_3080,1828,2419,1979,2613,3627,2474,1122,1602,3581,1135,115,1
957
CCNE1_1_3081,1256,474,492,915,246,1813,674,1055,1748,432,789,29
CCNT1_1_3082,2506,2826,2730,2422,2824,2846,2565,3046,3329,2782,2066,26
92
CDC73_1_3083,2857,2137,2135,1862,1423,3728,2806,1410,1780,3062,2691,25
88
CDK9_1_3084,1945,2326,2728,1616,2640,1345,3921,1495,1054,619,2563,1401
CDY2A_1_3085,6763,5256,5710,6562,10275,7802,5316,3404,5955,7507,7716,6
239
CDY2B_1_3086,6763,5256,5710,6562,10275,7802,5316,3404,5955,7507,7716,6
239
CDYL2_1_3087,586,518,851,944,872,465,722,1295,444,564,1208,758
CECR2_1_3088,492,266,649,292,1122,2100,97,6,12,702,1030,16
CHAF1A_1_3089,546,159,320,79,849,406,1970,1477,535,236,27,210
CHAF1B_1_3090,3503,3034,3718,4255,5411,5210,2715,2739,825,3245,2796,48
05
CHD1_1_3091,4635,4835,4592,4232,5290,6685,6178,4569,4980,4334,4769,281
2
CHD1L_1_3092,950,708,1214,486,212,139,1320,414,12,196,1026,454
CHD4_1_3093,388,692,598,595,1018,1027,1797,96,1979,1330,1,90
CHD6_1_3094,3849,2567,2342,2811,3400,1769,4600,2761,4533,2790,3370,232
9
CHD7_1_3095,7882,7787,8392,7890,10607,10697,7917,7467,8714,8137,7152,9
397
CHD9_1_3096,917,1164,1061,1487,566,1284,3426,1738,1221,965,669,1030
CHMP1B_1_3097,554,490,248,237,1250,398,294,474,357,397,46,185

CHMP4B_1_3098,410,939,588,892,1373,375,13,394,1133,354,452,1335
CHMP4C_1_3099,1318,475,1233,720,290,806,504,198,1444,495,957,479
CHRAC1_1_3100,294,188,613,520,934,1075,815,135,349,996,98,87
CLOCK_1_3101,299,924,903,1123,662,462,166,1133,915,448,182,11
COPS5_1_3102,3001,4596,4367,4927,3023,4312,3901,3482,1413,2980,6150,60
59
CRAMP1L_1_3103,2938,2858,3260,3057,3560,3986,4051,4312,3113,2305,1791,
1748
CTCFL_1_3104,564,511,1239,156,837,143,482,534,2185,1079,629,53
DEAF1_1_3105,1575,1344,958,1619,1587,905,984,1644,904,1632,633,2171
DNAJC1_1_3106,2205,2711,2215,1917,2193,2381,2003,2271,1423,2430,1944,2
111
DOT1L_1_3107,5978,5429,7480,5302,4233,10475,8856,4643,7349,6566,6450,8
890
DPF2_1_3108,1424,1369,1307,1954,2225,1476,1474,924,391,1010,1420,1576
DPF3_1_3109,409,136,825,755,1055,475,1500,185,61,482,744,339
EIF4B_1_3110,1803,935,1250,1405,547,697,951,1156,747,1534,48,415
ELP3_1_3111,2539,1764,1536,2310,2458,4004,2188,1325,2102,1216,1960,158
3
EP300_1_3112,2687,2830,4495,2846,3758,4410,4919,1785,1512,2667,1977,24
97
EP400_1_3113,482,294,417,163,561,586,182,39,15,678,158,1167
EPC1_1_3114,4663,4374,4735,3741,6903,3935,9876,6851,3918,4076,5737,458
4
EPC2_1_3115,708,419,793,676,403,1330,941,508,420,1431,898,1132
ERCC6_1_3116,631,570,789,974,959,1377,1616,870,1445,214,63,979
ESRRA_1_3117,3443,3344,4291,4118,3013,2541,2539,5022,4697,2265,3061,70
73
ESRRB_1_3118,2068,1601,1952,2320,1974,1235,1500,3033,2229,1629,1527,15
29
FANCM_1_3119,1295,1211,1445,1812,1415,1259,637,1466,403,1401,822,1232
FBXL19_1_3120,1225,891,1093,1174,154,763,397,1631,891,1663,386,844
FOS_1_3121,569,392,659,731,1306,1158,471,799,1658,1154,514,728
FTO_1_3122,13272,13923,14588,13711,18489,16970,13777,12057,13209,13513
,11019,20615
FXR2_1_3123,425,389,940,1123,827,919,2335,892,387,527,158,390
G2E3_1_3124,1965,1996,2375,2594,4523,4849,1654,1749,2831,2208,1291,273
8
GATAD2A_1_3125,3343,2894,3413,2402,3229,2954,1187,2560,5289,2877,2655,
2928
GLYR1_1_3126,1527,1357,1510,1317,3492,2148,1034,1139,1548,1338,733,364
6
GMEB2_1_3127,125,171,389,334,276,1,539,266,0,60,1,290
GSG2_1_3128,915,799,916,975,291,1546,425,363,26,858,1768,1050
GTF3C4_1_3129,2186,2612,4097,2475,1791,3524,2048,2411,1585,1529,889,24
65
HAT1_1_3130,6273,6035,8105,8131,9204,7183,10209,8669,5602,7183,7034,10
014
HDAC1_1_3131,753,1506,1101,1545,557,857,1519,228,317,792,2271,554
HDAC2_1_3132,9507,7607,8101,7914,13047,9968,10081,3578,7835,6130,9099,

12135
HDAC3_1_3133,264,230,683,323,132,753,836,137,260,381,884,635
HDAC4_1_3134,2075,2099,2019,1914,2262,1815,3619,2724,1214,1017,2019,23
00
HDAC6_1_3135,363,57,369,57,487,288,542,387,335,168,902,161
HDGFL1_1_3136,1144,1260,1750,1596,776,1257,1110,1219,1682,930,3035,101
5
HDGFRP3_1_3137,1151,1278,981,1442,951,1039,1618,722,758,1231,1284,625
HELLS_1_3138,969,1186,992,859,576,1200,1575,1439,50,1348,549,288
HEMK1_1_3139,2781,2070,3274,2538,3489,1758,3119,3193,2682,2338,4704,51
17
HIF1AN_1_3140,3543,2136,3427,3079,3707,4007,3332,3804,2267,2442,165,13
50
HIRA_1_3141,267,422,614,545,137,1925,454,0,346,32,182,1162
HMG20B_1_3142,277,111,732,140,1143,107,942,585,0,245,179,1
HMGN5_1_3143,2822,1923,1839,2185,1549,1617,2053,830,184,2620,2742,2511
HNF4G_1_3144,2815,3436,2813,4118,2074,3063,3990,5559,3608,2338,4399,34
65
HSPBAP1_1_3145,7076,6307,5398,6351,7154,8309,5020,4774,5386,9090,5528,
6871
HUWE1_1_3146,8818,6647,7364,5933,4166,6615,12390,3913,5188,5846,4238,6
054
ING5_1_3147,126,137,208,89,829,194,1,261,0,194,99,136
INO80_1_3148,1636,1193,2079,1250,2570,1599,1150,1144,493,3624,1380,199
6
JARID2_1_3149,948,695,727,860,2204,1747,775,813,815,864,592,140
JHDM1D_1_3150,3678,2455,4206,2975,4365,4013,2495,3031,4450,2570,4103,3
837
JMJD7_1_3151,304,267,266,273,325,377,1,432,150,827,836,198
JMJD8_1_3152,488,87,60,240,0,19,0,596,0,61,55,0
JUN_1_3153,409,777,1057,2064,1358,1120,236,212,2748,829,60,514
KAT2A_1_3154,436,726,649,951,314,243,1170,579,760,666,177,497
KAT2B_1_3155,254,126,223,273,84,257,64,226,305,125,15,3
KAT6B_1_3156,300,544,265,366,731,760,602,17,452,222,343,724
KDM1B_1_3157,159,241,331,220,263,90,317,287,60,64,1256,0
KDM2A_1_3158,870,553,484,238,183,432,346,929,92,254,341,8
KDM3B_1_3159,572,711,499,905,220,2059,876,810,1059,778,629,34
KDM4A_1_3160,998,279,1290,653,775,1130,1673,901,83,597,706,449
KDM4B_1_3161,937,557,908,997,507,1072,1187,838,694,1188,1471,2446
KDM4D_1_3162,3213,3292,4177,4044,2888,5456,4362,3101,3261,4095,2809,38
50
KDM5A_1_3163,237,119,95,133,178,336,6,83,0,210,9,31
KDM5B_1_3164,1675,1922,2296,1610,2316,2613,2402,2936,2161,1349,1844,96
8
KDM6A_1_3165,867,1517,1842,1036,2067,1045,514,520,1676,1286,1310,1621
KDM6B_1_3166,522,380,127,207,193,354,139,132,407,121,116,1186
KIAA2026_1_3167,488,955,1085,1262,753,1299,1704,744,970,791,97,1603
L3MBTL2_1_3168,284,681,146,307,1400,112,96,543,0,80,0,41
L3MBTL4_1_3169,1487,779,946,894,1165,2521,681,499,579,735,935,474
MAEL_1_3170,3905,4450,5348,5401,3313,4617,4827,2923,5138,4599,3519,498

8
MBD3_1_3171,545,637,676,706,1192,50,172,1833,1,167,362,103
MBD4_1_3172,283,225,274,496,100,465,271,685,148,543,136,154
MBD5_1_3173,905,933,1303,1123,707,439,405,4,1140,426,81,194
MBD6_1_3174,417,302,227,344,199,816,436,433,332,526,1080,7
MBTD1_1_3175,13333,14729,15782,15714,15156,13664,16726,10598,17129,143
31,13401,18006
MDM2_1_3176,3596,3573,3700,3569,3020,5841,4188,3107,6103,2396,2602,620
1
MIER2_1_3177,508,668,263,232,447,597,2444,647,571,393,414,112
MIER3_1_3178,2911,2000,2923,3363,3586,2754,3396,3074,1541,1355,1266,37
34
MIS18BP1_1_3179,1389,1314,1279,1473,755,2513,963,2777,2372,1783,928,23
67
MKL1_1_3180,438,309,475,531,520,1425,217,598,632,389,210,350
MLL2_1_3181,456,447,300,256,1167,426,728,185,11,106,980,116
MLL3_1_3182,5089,5365,5512,4971,10152,4366,3755,4327,1841,4704,2937,50
42
MLLT1_1_3183,442,429,192,92,867,183,1417,281,512,609,80,1274
MLLT3_1_3184,1605,2678,1487,1637,2619,2587,1170,1712,704,1131,711,3518
MLLT6_1_3185,899,892,1509,1601,340,1362,1631,522,264,915,560,620
MPHOSPH8_1_3186,1310,994,1328,1048,2074,682,949,1189,757,1152,1131,128
3
MSH6_1_3187,1950,1664,2813,2806,2831,2626,3068,1691,3155,1005,48,2441
MSRB2_1_3188,2347,1089,2271,1679,1041,2539,1510,665,2541,2005,1153,109
5
MTA2_1_3189,91,162,693,133,1030,5,174,36,3,50,7,356
MTA3_1_3190,389,550,337,376,160,121,180,562,831,201,915,110
MYSM1_1_3191,2321,1811,2106,2237,4199,2364,2049,1457,710,1699,1857,103
2
NAP1L2_1_3192,441,580,347,791,691,507,119,2,20,429,640,3734
NAP1L3_1_3193,5292,5356,7162,6410,7069,4524,7748,5270,5963,5799,6664,6
499
NAP1L4_1_3194,1883,1545,2259,2667,2342,2470,2794,1971,1550,2003,991,32
40
NAP1L5_1_3195,923,853,496,478,280,1354,808,1034,1,75,51,516
NAT14_1_3196,1044,1159,992,741,863,1104,739,398,374,119,771,181
NAT8B_1_3197,553,538,279,265,342,454,157,185,43,688,0,600
NAT8_1_3198,7189,7668,7608,9569,10846,8509,8485,6111,6294,5897,4895,12
638
NAT8L_1_3199,4065,4017,4128,3122,5072,5550,5388,4723,5103,5034,4230,42
13
NAT9_1_3200,336,224,437,77,1294,286,107,55,1032,373,397,292
NCOA2_1_3201,874,461,605,620,536,311,39,652,278,120,153,803
NPTXR_1_3202,545,725,943,681,525,1690,987,1015,793,1316,1754,542
NR0B1_1_3203,526,674,259,1237,441,355,731,523,572,196,322,7
NR0B2_1_3204,398,313,1007,33,729,283,77,1,39,419,133,1011
NR1D1_1_3205,1546,1536,1792,2087,2222,3071,2729,2010,1413,2067,1869,71
0
NR1H2_1_3206,413,193,230,210,123,322,517,153,207,117,11,0

NR2C2_1_3207,1699,872,896,1098,1925,1089,775,841,781,731,538,487
NR2E1_1_3208,1740,2392,1640,1422,1402,2198,3382,1141,2032,1277,2669,21
95
NR2F1_1_3209,532,819,753,921,1089,743,431,296,331,139,920,1223
NR2F6_1_3210,764,935,1097,619,633,1440,386,121,2758,549,2185,716
NR4A2_1_3211,571,743,767,797,1324,1306,1714,399,266,409,1483,773
NR5A1_1_3212,331,236,171,451,158,700,69,2,62,276,148,375
NRIP1_1_3213,3944,4127,5879,4786,6035,5637,3662,4312,6546,4185,4443,43
24
PADI4_1_3214,523,500,677,533,298,217,726,150,53,245,1470,161
PAWR_1_3215,451,629,846,562,24,865,713,58,32,116,159,374
PAX5_1_3216,1641,2068,2541,1305,3583,3146,2324,819,2257,2209,809,2085
PAXIP1_1_3217,976,1051,1783,800,489,1620,1268,1318,1101,1449,2186,1064
PGRMC2_1_3218,4014,2944,4850,3951,5772,4251,3801,2747,1890,3633,4579,3
520
PHB_1_3219,598,814,597,393,1576,474,1277,112,1719,1043,1039,174
PHC3_1_3220,471,220,620,208,380,426,1536,470,327,340,1339,670
PHF13_1_3221,3993,4056,4202,5510,5184,3757,5669,5095,5212,5482,4774,49
70
PHF14_1_3222,736,743,562,657,1748,1189,992,419,351,1133,525,2701
PHF15_1_3223,3677,5260,3681,5614,7264,4904,3538,5275,3428,4185,4341,58
17
PHF20_1_3224,1423,1307,1699,1143,2029,1327,1574,1849,2761,2608,353,148
6
PHF23_1_3225,712,531,958,531,1116,442,128,291,1,114,464,1726
PHF2_1_3226,999,738,1703,1278,632,712,570,286,3776,1212,1665,1573
PHF3_1_3227,66,269,206,143,195,1203,48,13,32,517,16,606
PHF5A_1_3228,437,152,1052,401,883,673,469,0,534,637,7,580
PHIP_1_3229,2621,2723,3290,3059,3312,3029,1884,3156,5122,3536,2437,430
7
PHRF1_1_3230,0,0,0,0,0,0,0,0,0,0,0,0
PIAS1_1_3231,1567,1467,1326,1665,2530,2879,1114,1932,1112,1464,3273,62
1
PIWIL4_1_3232,349,384,476,215,647,251,223,122,47,240,58,719
PPARGC1A_1_3233,14379,9361,17203,17642,14928,17160,17528,17023,15629,1
1545,14025,13739
PRDM11_1_3234,234,385,469,309,742,287,86,111,571,171,561,33
PRDM12_1_3235,155,160,138,146,10,91,342,439,1,48,0,26
PRDM13_1_3236,63,280,246,157,1,171,0,0,1,119,0,0
PRDM14_1_3237,513,292,459,417,10,162,613,18,63,608,99,106
PRDM4_1_3238,3002,2913,3010,3473,3156,4321,1617,1824,5749,2700,4861,48
17
PRDM5_1_3239,18396,20618,21236,21094,28855,23276,15870,17293,14639,200
66,22495,15272
PRDM6_1_3240,441,214,182,519,537,450,645,129,17,561,167,256
PRDM9_1_3241,4088,4158,4527,4508,5336,6344,5800,4431,5006,4551,4682,43
16
PRMT6_1_3242,260,352,190,389,43,919,367,281,512,435,1,104
PRMT8_1_3243,1018,826,930,1521,491,1195,655,965,196,1184,3194,609
PRPF6_1_3244,608,675,373,290,914,66,588,365,379,1011,1,1233

PYGO1_1_3245,368,428,521,680,885,191,1356,210,205,1050,109,58
PYGO2_1_3246,204,76,131,51,21,181,117,0,1,8,0,320
RAG2_1_3247,319,370,626,452,842,512,823,1442,107,224,183,948
RAI1_1_3248,434,615,479,392,162,844,1799,237,1,313,240,651
RB1_1_3249,3613,2515,4821,4665,5210,3480,2313,5267,2134,3658,4447,3679
RCOR1_1_3250,4063,3660,3673,4264,3593,4526,3305,4931,3144,3409,4657,59
43
RCOR2_1_3251,692,588,554,779,532,743,131,1285,0,755,877,416
RECQL4_1_3252,965,756,258,371,1577,881,161,484,3,278,856,1143
RELB_1_3253,216,918,573,710,328,638,1036,171,876,1199,0,0
REL_1_3254,247,353,497,295,250,300,34,67,751,336,318,813
RING1_1_3255,218,442,410,396,362,772,190,1289,0,10,0,5
RNF20_1_3256,3722,3937,5268,4174,3757,4491,3066,3268,2504,2591,5270,55
63
RNF25_1_3257,1323,791,2316,1323,2825,1969,1402,2282,2172,1987,806,2598
RNF2_1_3258,1342,1305,1494,1855,1854,1066,1226,840,819,1825,1478,77
RORB_1_3259,1006,1777,1716,1016,268,708,765,2282,1536,2218,587,964
RSF1_1_3260,2763,2920,2618,2407,4583,3192,3294,3254,3150,1611,2285,692
RUVBL1_1_3261,1025,1471,2058,1230,1466,1927,4600,1883,2197,1321,1252,8
90
RUVBL2_1_3262,608,865,537,689,2,729,553,529,1638,636,268,979
RXRA_1_3263,210,203,126,329,503,60,380,1,240,258,177,34
RXRB_1_3264,1289,1585,2046,1823,669,2822,3372,3371,2065,2718,2195,100
RXRG_1_3265,3017,2233,3455,1719,6674,5466,3484,3278,1907,2515,3590,251
8
SAP18_1_3266,4952,7036,6628,5317,6319,5429,4633,6000,3952,5491,5521,43
55
SCML2_1_3267,6647,5904,7316,6231,8575,6371,10954,6997,5277,6070,3918,1
1069
SENP3_1_3268,134,689,291,93,163,87,1,129,864,197,4,1059
SETD1A_1_3269,556,710,551,1412,606,1661,235,671,1786,796,59,724
SETD1B_1_3270,1094,1430,1558,1366,605,1399,561,889,1676,1751,1046,3417
SETD2_1_3271,844,1016,801,1624,651,238,2034,1326,1938,849,701,150
SETD5_1_3272,4243,3617,3963,3880,3793,3303,1499,2773,3096,2209,2336,31
24
SETD7_1_3273,1770,1384,2068,1579,4588,1714,1683,1631,366,1526,565,748
SETD8_1_3274,6425,5981,6849,6821,8618,6745,6668,5391,9800,7805,8918,41
91
SETMAR_1_3275,9499,11717,12464,10252,11155,12514,13913,8353,8497,11121
,13929,9044
SF3B3_1_3276,616,599,1133,1716,2621,583,1695,920,0,6,56,1711
SIAH2_1_3277,224,183,204,279,21,204,272,612,519,293,846,0
SIN3B_1_3278,1167,956,916,848,615,540,810,1201,414,1352,35,1811
SIRT4_1_3279,1463,1555,1578,1559,889,1638,1997,525,2846,1355,1365,1487
SIRT7_1_3280,214,54,139,18,0,88,91,45,28,94,543,1
SLC2A4RG_1_3281,218,254,380,197,229,725,0,142,409,120,0,0
SMARCA5_1_3282,18759,15668,22217,18942,20277,25740,22155,15883,28791,2
2620,17372,18873
SMARCC1_1_3283,1497,2704,2920,3328,1544,4740,3379,2025,3208,2129,5317,
1816

SMARCD2_1_3284,768,610,220,525,396,841,750,449,39,786,1750,1090
SMARCE1_1_3285,1419,1250,1425,1319,971,1754,1319,835,2174,402,1265,173
6
SMC1A_1_3286,1862,2375,1834,1920,3607,2059,1296,1797,3054,3313,1800,15
34
SMC1B_1_3287,3507,3280,4013,4061,3199,5073,2099,2414,3352,4464,5125,57
62
SMC3_1_3288,3958,3764,4606,4364,4009,5661,5926,3119,5013,3986,6993,682
0
SMCHD1_1_3289,5940,6836,6633,7292,10254,9390,4968,6187,5361,7081,4065,
6308
SMNDC1_1_3290,1744,1102,1068,2267,1551,2996,1437,648,1858,1209,1907,94
0
SMYD1_1_3291,3542,3587,4248,2577,4631,3897,3120,2948,2632,3514,1127,13
95
SMYD2_1_3292,1540,1398,1448,1089,1765,1939,1269,303,3021,1576,1135,126
SMYD4_1_3293,1139,1006,480,509,642,233,1839,1003,25,603,1554,373
SMYD5_1_3294,1499,832,1063,1640,1114,448,1132,361,925,2330,1175,340
SND1_1_3295,559,317,480,334,1012,8,443,673,87,893,533,39
SP140L_1_3296,1514,961,1519,1946,695,2165,1416,2741,758,1647,787,1230
SRCAP_1_3297,3335,1988,2060,2094,2506,1699,1150,2163,2586,1633,2727,15
34
STAT5B_1_3298,514,1195,426,375,905,1107,1362,412,1106,912,424,618
SUDS3_1_3299,1095,1160,1244,919,1062,590,602,874,242,560,1066,263
SUPT16H_1_3300,743,1459,1336,1670,1138,1125,435,599,1545,1739,884,761
SUPT4H1_1_3301,911,542,1521,846,1039,638,1193,1119,558,1109,566,2053
SUPT6H_1_3302,594,760,984,1057,1532,537,1276,1152,44,1398,358,220
SUPT7L_1_3303,2620,2735,2848,3170,1494,2367,1317,3272,3591,1443,808,28
05
SUV39H1_1_3304,228,189,111,125,1209,410,132,95,724,111,241,547
SUV420H2_1_3305,897,942,1515,1025,1571,721,1352,66,404,1429,1356,1312
SUZ12_1_3306,1365,1006,698,1886,1199,1425,2702,924,468,1099,149,3966
TADA1_1_3307,13027,11624,15260,12938,14130,18055,18223,12359,10924,128
71,16837,11613
TADA2B_1_3308,1137,984,1198,1005,2137,2234,489,589,1269,761,1131,257
TAF10_1_3309,601,201,494,798,825,77,618,394,33,851,36,43
TAF1L_1_3310,9243,7105,8357,8062,9085,6568,7443,8251,8724,8968,13440,7
367
TAF3_1_3311,2362,1944,2624,2978,3380,2311,1458,2654,2467,2204,1008,312
4
TAF5_1_3312,1436,1669,2905,1997,2525,3603,624,1158,1698,1613,2158,1165
TAF6L_1_3313,981,694,839,477,434,323,709,214,661,484,439,160
TAF8_1_3314,671,116,311,221,43,188,849,535,77,22,14,590
TCF7L1_1_3315,566,275,145,313,11,249,756,794,0,93,1142,30
TDRD12_1_3316,1497,1938,1855,2835,2862,1746,1007,1339,1768,2643,1478,6
47
TDRD1_1_3317,836,707,941,1289,967,1354,664,277,436,1697,327,898
TDRD7_1_3318,852,1513,1788,1053,1070,503,1292,715,522,632,2932,742
TDRD9_1_3319,5740,4587,5884,6336,6463,5947,8965,5082,1638,5240,5239,10
168

TERF2_1_3320,586,930,1043,1092,2192,1128,472,654,1124,1677,286,1677
TET1_1_3321,796,1462,1464,923,1383,707,834,975,1340,737,13,5538
TET3_1_3322,994,556,1117,1253,1300,670,330,1670,298,1072,3994,1155
TLX2_1_3323,169,544,32,90,42,554,1279,1,1,186,65,0
TNRC18_1_3324,238,201,1016,429,569,300,824,1277,1071,143,0,75
TRAF7_1_3325,1627,1549,1396,1780,1607,976,1211,1587,1350,1389,1991,127
6
TRDMT1_1_3326,3499,2569,3843,2943,1885,2721,4828,2011,4124,2418,5535,2
376
TRERF1_1_3327,5407,5394,7540,6066,7137,8520,7329,3253,3701,5326,5109,4
806
TRIM25_1_3328,4101,3386,3788,3883,2911,5252,4687,3023,3113,5060,3627,5
143
TRIM27_1_3329,852,867,1076,915,395,1173,970,1961,2576,973,476,2637
TRIM28_1_3330,1444,978,779,1436,1168,362,495,1282,805,1326,2663,3560
TRIM66_1_3331,458,186,383,325,316,529,277,1000,448,321,181,201
TRRAP_1_3332,2718,3321,3050,3540,3620,4546,2828,2717,4989,2874,2984,55
22
TSG101_1_3333,2851,3247,2658,2944,3505,3843,2613,2984,4852,2658,3409,1
546
TYW5_1_3334,6795,7565,8312,7467,4015,7510,6985,6062,5848,4874,1919,526
7
UBE2B_1_3335,628,651,1033,945,564,334,1662,987,904,528,332,609
UBE2N_1_3336,78,174,205,263,401,109,147,219,178,292,227,136
UBR7_1_3337,1443,1479,1260,569,1120,740,1536,513,2,838,2127,1532
UHRF2_1_3338,3498,3848,2558,3343,3963,5587,2820,2608,1959,3391,6515,23
01
USP22_1_3339,252,203,654,153,37,229,892,335,167,496,3,341
YEATS2_1_3340,585,1134,1405,936,862,379,2907,591,273,1309,555,1759
YEATS4_1_3341,1927,1489,2613,2766,3665,3452,2930,1474,3048,2086,5502,3
314
YY1_1_3342,13131,12486,13048,11245,13451,14945,17792,11512,21372,16483
,17117,23601
ZAR1_1_3343,3,29,128,6,2,31,0,0,30,8,0,0
ZCWPW1_1_3344,1271,1236,1106,1216,1358,1340,2927,498,1264,1021,2801,78
4
ZCWPW2_1_3345,518,337,452,247,845,322,1130,5,1241,590,0,38
ZFP57_1_3346,2294,2174,2761,1631,2701,2349,1851,2708,1906,1812,2850,31
39
ZNF541_1_3347,1230,1171,1600,1303,1196,1263,641,1385,206,1359,2606,214
3
ZNF85_1_3348,13220,13426,14416,14756,16758,17293,16849,11237,16537,149
38,14393,16842
AANAT_1_3349,2023,2416,2188,2435,2256,2328,4057,4001,916,2243,1412,310
9
AES_1_3350,802,198,279,114,932,45,707,126,72,438,599,1
AIRE_1_3351,141,198,352,213,0,327,12,35,16,250,2,88
AKAP1_1_3352,1259,1257,2029,1586,2026,949,1850,928,944,1928,2797,3038
ALKBH2_1_3353,222,117,110,45,58,75,585,0,1,45,59,4
ANKHD1_1_3354,677,870,732,941,1488,589,513,900,46,964,1812,693

ARID1A_1_3355,1083,982,1065,1799,1240,703,4939,823,1164,1220,1467,956
ARID1B_1_3356,593,481,374,1236,372,297,230,1356,129,111,773,421
ARID4A_1_3357,3106,3556,4034,3231,4283,5116,3251,1934,2730,3644,4260,1
610
ARID4B_1_3358,9756,8821,9809,8612,8986,10462,21821,6532,13016,8118,984
0,8737
ARRB1_1_3359,824,414,264,936,267,191,230,669,585,62,387,678
ASH2L_1_3360,952,647,1024,386,2610,754,1766,95,683,370,31,621
ATAD2B_1_3361,2651,2161,2923,1806,1481,1838,4809,2386,2975,1477,1266,2
852
ATAT1_1_3362,36,10,6,4,0,29,0,6,4,0,1,0
ATRX_1_3363,924,874,1163,715,689,789,733,235,1311,958,2101,1102
AURKA_1_3364,853,677,787,1112,1187,252,831,332,246,849,488,906
AURKC_1_3365,3714,3472,4467,2555,3333,4025,5014,1000,2479,3637,2410,18
20
BAZ1A_1_3366,3491,2883,3602,2980,4064,4482,285,2685,3090,2935,1739,411
4
BCOR_1_3367,823,845,610,955,996,543,70,124,114,206,206,849
BPTF_1_3368,576,754,857,1067,578,1106,1306,134,1819,690,1694,670
BRCA1_1_3369,888,1034,1048,1224,1362,2441,2374,277,1822,1956,1514,1758
BRD2_1_3370,496,530,503,325,431,422,301,23,432,642,1,1230
BRD4_1_3371,481,255,1146,379,1018,201,621,181,178,312,348,1170
BRD7_1_3372,1331,1296,1708,1602,1763,65,474,1069,58,796,255,771
BRD8_1_3373,1494,1623,1653,1401,2189,3494,3448,1562,906,1109,795,1835
BRD9_1_3374,914,948,698,945,1513,1644,454,409,1058,954,380,1080
BRDT_1_3375,2872,2760,3146,3322,2492,1770,3947,1007,3008,1972,4646,291
0
BRPF1_1_3376,2043,1803,2286,2506,3090,1732,1598,2368,5268,1304,1558,26
68
BRWD1_1_3377,562,458,505,694,1157,922,846,783,575,755,3354,831
C14orf43_1_3378,1087,839,647,1016,2750,1743,2211,1633,1541,1578,712,65
6
CBX1_1_3379,995,1300,1055,1671,1407,1723,1207,1605,1566,1253,278,2994
CBX3_1_3380,3712,3231,3261,2499,3339,3764,2586,5209,1249,1832,2529,162
4
CBX5_1_3381,3000,4273,3450,4109,4053,3274,5054,3315,3806,2950,3511,281
4
CCNT2_1_3382,8128,6747,8520,7895,10741,6873,12023,8732,8269,6835,8103,
5625
CDY1B_1_3383,2613,1976,3669,3335,2145,3960,3130,2542,1658,1976,2514,16
46
CDY1_1_3384,2613,1976,3669,3335,2145,3960,3130,2542,1658,1976,2514,164
6
CDYL_1_3385,426,723,419,1138,573,388,976,608,232,442,9,160
CHD2_1_3386,45,17,140,300,0,273,136,12,0,0,41,0
CHD3_1_3387,1561,1874,1703,1712,1777,2477,2263,1987,1084,1369,3674,321
0
CHD8_1_3388,4197,6116,5657,4700,6499,4708,3611,1987,6878,4149,5212,768
1
CHMP2A_1_3389,2562,1836,1981,2351,2585,1341,1707,1340,2682,2604,1007,1

631
CHMP5_1_3390,1136,1102,2209,1080,1751,2558,1018,1627,2530,2168,2166,32
38
COPS2_1_3391,1852,2477,2883,2117,3333,1621,1900,1939,1178,2772,754,459
5
CPA4_1_3392,807,807,972,1198,974,892,408,724,3850,253,18,1524
CREB1_1_3393,498,775,518,596,244,1622,1418,652,59,583,206,551
CREBBP_1_3394,470,503,995,1478,544,952,397,441,904,1578,51,797
CTCF_1_3395,383,839,688,1288,878,818,122,95,290,1431,1788,899
CTNNB1_1_3396,5267,4910,6240,4774,6059,6264,4952,4180,6922,6354,7551,4
513
CXXC1_1_3397,570,940,465,205,641,174,1019,152,1239,681,44,74
CYLD_1_3398,732,1051,784,882,590,504,711,37,29,955,335,1584
DICER1_1_3399,13690,13913,15215,13159,18848,18876,16502,13942,12055,14
940,9701,11270
DIDO1_1_3400,3317,3788,3181,4282,8795,4065,4914,3065,1746,2577,1648,25
34
DMAP1_1_3401,261,317,419,518,34,476,1,22,0,647,435,1735
DNAJC2_1_3402,800,871,1423,681,2696,1800,2171,955,579,468,666,1232
DNMT1_1_3403,846,1135,334,924,1283,843,2369,539,1,926,609,728
DNMT3A_1_3404,243,271,345,83,9,84,29,67,33,371,912,304
DNMT3B_1_3405,725,847,487,515,964,251,693,586,230,353,3,97
DNMT3L_1_3406,759,891,1149,1415,854,1913,1019,636,1171,1258,863,1522
DPF1_1_3407,1929,1823,1516,1528,3914,870,2224,2979,1615,1206,2898,5394
EED_1_3408,396,295,649,121,597,875,861,297,163,788,658,149
EGR2_1_3409,433,349,570,219,148,542,51,75,411,746,812,104
EHMT1_1_3410,378,314,674,608,21,143,83,268,0,212,0,465
EHMT2_1_3411,1211,1042,1236,985,902,773,1110,208,1945,1086,183,427
ENO1_1_3412,756,201,289,293,208,396,885,225,177,282,145,1277
ESR1_1_3413,587,523,757,255,380,601,80,189,35,21,11,1441
ESR2_1_3414,452,914,700,1483,645,1812,448,305,795,587,72,1706
ESRRG_1_3415,1370,1408,2424,1135,1711,1892,992,39,1420,1126,611,1338
EZH2_1_3416,2886,2994,2253,2745,1506,2407,2790,479,17,2524,2721,2773
FBXO11_1_3417,4055,4186,3436,3292,3535,4255,3396,2247,3718,5630,3658,5
700
FMR1_1_3418,2961,3145,2811,2795,3128,4614,3550,2787,2657,3399,2097,378
7
FXR1_1_3419,2705,2101,2702,1801,2869,1451,5081,1435,2508,1930,2636,219
1
GFI1B_1_3420,596,475,823,460,148,719,1484,621,243,343,9,632
GMEB1_1_3421,465,431,108,454,715,626,95,174,2,651,332,1407
HDAC10_1_3422,870,726,812,859,818,72,1095,1546,59,1265,1248,277
HDAC11_1_3423,5587,4126,4714,4132,7889,6094,2757,8239,3575,6870,3324,6
348
HDAC5_1_3424,1427,998,1406,1792,1182,721,1827,690,715,1330,1021,233
HDAC7_1_3425,318,155,323,448,269,650,809,86,887,609,1263,15
HDAC9_1_3426,376,248,191,319,44,830,629,5,22,233,366,1263
HDGF_1_3427,920,928,897,1282,1987,1063,2467,147,2140,1434,2859,1493
HDGFRP2_1_3428,1327,644,2104,983,2322,782,1829,1255,1829,627,494,752
HLTF_1_3429,1250,802,894,346,424,623,124,373,1897,874,165,162

HMGA1_1_3430,40,367,273,187,623,250,0,154,0,430,866,0
HMGN3_1_3431,1303,1561,1364,1624,2224,1424,1482,519,1930,1761,2388,214
9
HNF4A_1_3432,112,18,149,67,137,753,0,32,7,0,0,271
HPSE2_1_3433,918,819,1398,848,632,1099,1532,1531,602,1587,1385,544
HR_1_3434,882,407,563,350,764,243,865,1054,599,238,2885,718
ING1_1_3435,165,172,85,499,23,549,0,0,19,9,0,0
ING4_1_3436,313,302,853,446,60,958,886,800,15,72,0,63
INTS12_1_3437,3814,3158,3986,3215,4268,4006,3148,2977,4291,2975,4600,1
686
IRF4_1_3438,186,171,237,245,248,352,47,122,218,334,189,531
JMJD1C_1_3439,6845,7059,7050,7954,5329,8094,8442,6657,5597,6345,7109,6
999
JMJD4_1_3440,1351,1359,2427,1918,869,2476,1067,645,2640,1013,968,1118
JMJD6_1_3441,314,575,497,828,879,404,416,1501,351,659,248,435
KAT5_1_3442,682,1083,1358,671,297,706,135,391,392,565,177,1972
KAT6A_1_3443,5642,4261,5370,5094,8174,5678,5001,5710,10489,4245,5730,4
628
KAT7_1_3444,1179,1387,1266,1782,2312,1470,1725,1064,225,611,1066,939
KAT8_1_3445,159,511,430,576,482,809,1209,199,1400,905,250,93
KCTD1_1_3446,802,805,949,819,1318,504,112,375,876,526,1344,632
KDM1A_1_3447,726,551,789,546,630,517,866,1255,415,1131,833,934
KDM2B_1_3448,806,1413,1369,1362,1280,1142,2393,535,236,1763,232,717
KDM3A_1_3449,269,483,128,422,432,344,199,441,238,666,159,349
KDM4C_1_3450,1719,1392,1502,1287,1252,1149,2285,134,499,1165,3504,682
KDM5C_1_3451,154,134,23,87,252,254,9,74,54,208,82,676
KDM5D_1_3452,1684,898,1811,1603,1557,1441,1387,1566,1151,1084,577,1392
L3MBTL1_1_3453,43,83,145,203,8,81,1,150,14,302,0,2
L3MBTL3_1_3454,873,1409,930,1164,2027,1378,1806,1784,1347,1486,2227,96
2
LBR_1_3455,5962,5823,7792,7328,8560,7349,6860,6944,3955,7442,5527,9685
MAP3K12_1_3456,1586,1045,2070,1469,3148,2020,1375,3162,928,1481,1014,1
642
MBD1_1_3457,9628,8980,11160,10554,8508,9988,11279,9952,10021,12226,139
14,10347
MBD2_1_3458,1170,863,1436,1927,1122,1634,307,908,1543,1433,2376,1821
MECOM_1_3459,1355,2327,2410,1672,1423,2769,857,3417,2280,2463,1245,553
MECP2_1_3460,1306,1446,1803,1116,3358,1015,1360,1796,2627,813,364,797
MEN1_1_3461,143,363,265,577,1176,409,402,223,160,85,1165,3
MGEA5_1_3462,1355,1215,1438,1138,2556,636,170,1087,197,1519,1623,510
MIB2_1_3463,179,187,481,120,367,700,597,404,5,919,100,28
MIER1_1_3464,1285,1412,1223,1803,2007,808,1651,2434,2294,953,1848,418
MINA_1_3465,407,166,187,300,217,214,25,12,8,326,44,141
MLL5_1_3466,2019,1487,1381,766,2072,1186,601,206,1266,762,463,2277
MLL_1_3467,1223,1104,911,438,1153,794,925,952,2699,1489,855,273
MORF4L1_1_3468,3645,3356,3480,2747,4731,2910,2015,2224,4604,3659,1786,
7145
MSL3_1_3469,5464,4604,3965,4498,4902,5026,4870,2626,4716,4545,6120,484
4
MTA1_1_3470,1226,771,1193,823,1149,872,901,273,1081,671,1264,126

MTF2_1_3471,2734,2584,4756,4506,2162,1998,3645,1297,3008,3886,3058,211
8
NAA60_1_3472,1677,2337,1992,1982,1390,1255,3708,337,1850,1387,458,1470
NAP1L1_1_3473,2203,1883,1887,2732,3541,2979,3932,2484,5087,2209,2365,1
898
NAT10_1_3474,395,605,1120,899,870,262,375,1279,78,331,952,45
NCOA1_1_3475,14973,16817,19922,19905,16623,25260,23404,19606,19198,160
82,19231,17755
NCOA3_1_3476,1077,720,1166,598,1623,1360,1993,1014,102,1207,1157,655
NCOA4_1_3477,1188,1136,1138,1387,3036,2548,2361,1648,1088,2015,1500,29
95
NCOR1_1_3478,293,451,548,1406,294,388,1,200,181,750,964,1056
NCOR2_1_3479,605,1019,803,753,531,507,574,359,92,1419,1334,146
NFAT5_1_3480,530,616,622,426,627,1297,508,209,916,102,566,466
NFATC1_1_3481,1383,1364,1562,1911,2425,684,5471,926,731,718,1845,1459
NFATC3_1_3482,146,275,561,182,367,458,181,309,29,750,580,117
NFATC4_1_3483,208,185,59,235,12,16,201,14,100,376,597,10
NFKB1_1_3484,2323,2671,2019,2319,3991,2572,3557,2535,2402,2422,1486,17
32
NFKB2_1_3485,737,576,278,272,826,771,132,510,2100,202,834,146
NPM1_1_3486,6011,5340,5425,5272,8832,6053,2614,4090,6101,4104,5224,341
3
NR1D2_1_3487,767,553,1380,599,607,2154,569,107,711,1575,696,934
NR1H3_1_3488,407,448,223,349,388,234,1016,154,95,170,426,1016
NR1H4_1_3489,1800,1722,1531,2632,971,1556,2135,2268,2375,1941,1307,125
8
NR1I2_1_3490,700,637,387,944,24,633,347,656,303,697,3,624
NR1I3_1_3491,3981,3651,4663,4387,5737,4630,6133,5561,7326,3325,5509,37
57
NR2C1_1_3492,1280,1200,1598,1648,1599,1155,2047,1516,1218,1460,3115,26
18
NR2E3_1_3493,1640,1609,2153,2668,3053,1449,1494,891,4336,1012,2033,119
5
NR2F2_1_3494,8006,7362,6671,6497,7800,8518,7431,7308,9595,8361,6872,65
80
NR3C1_1_3495,2199,1878,2830,2501,2967,2635,1394,1645,458,2947,2219,352
5
NR3C2_1_3496,134,118,77,120,0,14,0,0,418,316,0,0
NR4A1_1_3497,770,389,885,700,284,222,418,1767,180,389,811,43
NR4A3_1_3498,4989,3760,5855,4884,5712,6998,4809,2505,3659,5269,4806,42
88
NR5A2_1_3499,408,100,297,699,285,754,1027,1083,429,203,27,203
NR6A1_1_3500,694,476,709,875,371,1266,1502,421,1240,583,987,1136
NSD1_1_3501,4105,3836,4459,4633,5891,6841,3806,3362,3882,3024,2128,205
0
PBRM1_1_3502,10138,10424,10407,10347,10932,13740,8936,13179,10481,8791
,5486,10777
PCGF6_1_3503,1040,872,809,1360,502,1190,1485,1205,3567,1541,1144,1645
PGR_1_3504,2821,2770,3479,2395,2172,3621,4168,2051,2342,2485,682,1772
PHC2_1_3505,45,5,1,50,0,15,0,0,0,0,0,0

PHF10_1_3506,545,307,444,178,829,478,376,589,684,550,3,2846
PHF11_1_3507,753,592,1632,1051,670,601,1781,1078,114,512,1305,633
PHF12_1_3508,1145,692,891,733,560,597,12,608,2515,1279,182,2561
PHF16_1_3509,464,204,534,349,1050,645,875,304,567,453,1130,289
PHF17_1_3510,1105,1111,1206,722,1354,2169,3470,1227,1082,558,1655,170
PHF1_1_3511,855,960,815,879,406,313,482,1060,131,476,520,1
PHF20L1_1_3512,2717,3552,3916,2945,3979,2438,4429,2056,2775,2656,4985,
3624
PHF21A_1_3513,2825,2610,2778,2657,4609,4378,2013,2740,770,1367,468,282
3
PHF21B_1_3514,443,449,525,242,462,740,1254,45,460,322,498,254
PHF6_1_3515,4236,3358,4815,3599,3618,3052,2867,2559,6090,3025,3073,576
1
PHF7_1_3516,3392,2292,4291,2782,3262,4919,3211,1412,1936,3339,1536,217
7
PHF8_1_3517,816,1228,1152,1360,924,1647,265,1242,383,1199,907,967
PIAS2_1_3518,1196,879,1263,832,448,551,2521,172,1880,699,7,157
PICK1_1_3519,822,730,503,477,940,260,635,1034,99,218,828,1251
PIWIL2_1_3520,6275,5859,5663,5267,9282,5973,5118,6871,5548,5423,7160,7
969
PML_1_3521,98,159,132,70,646,17,0,3,0,0,0,3
POLR1B_1_3522,149,97,227,130,246,456,188,53,268,24,14,14
PPARA_1_3523,1644,1706,1768,2422,1663,2368,2314,2070,1750,3231,740,115
9
PPARD_1_3524,449,59,29,51,67,53,200,106,62,345,125,12
PPARG_1_3525,2169,1503,2757,2608,4195,2309,632,652,1185,3532,3221,1909
PRDM10_1_3526,668,471,816,678,780,206,911,150,177,280,934,116
PRDM15_1_3527,889,331,705,517,955,530,265,737,0,1232,1849,0
PRDM16_1_3528,459,577,415,369,108,961,838,112,329,385,47,439
PRDM1_1_3529,1386,1309,1253,1090,1165,692,943,1002,733,421,3032,1374
PRDM2_1_3530,1390,1115,1015,953,427,819,754,1173,989,1708,686,1533
PRDM7_1_3531,1115,942,1226,1243,858,1493,3145,92,1199,585,1305,637
PRDM8_1_3532,6760,6357,7630,6657,6107,4984,4152,7288,5943,5815,4448,83
47
PRMT1_1_3533,258,227,191,187,807,554,52,29,4,712,525,68
PRMT2_1_3534,539,204,691,422,182,92,1186,40,3436,842,811,0
PRMT3_1_3535,7125,6220,8170,7674,8467,7851,7203,8612,6218,6951,6095,61
85
PRMT5_1_3536,1251,1216,1418,1278,579,2511,1003,960,2859,2053,78,525
PRMT7_1_3537,1650,1213,1571,2278,1579,1486,1950,1032,868,1692,1021,159
6
PSIP1_1_3538,781,681,1276,1156,538,995,932,691,472,1773,1364,3018
PSMC5_1_3539,1597,1289,2022,2296,1359,3033,3113,876,1804,2792,1416,933
PWWP2B_1_3540,449,206,153,91,450,92,33,6,48,44,1612,31
RAD54B_1_3541,4418,5473,7292,5059,4738,6981,4360,4890,5743,6346,4629,6
526
RAD54L_1_3542,783,1027,1174,1273,567,1493,3141,1758,13,643,379,153
RARA_1_3543,1179,695,693,940,846,256,580,768,1762,819,591,385
RARB_1_3544,2276,3248,2976,2582,3290,1812,4541,2504,1156,2909,5520,660
4

RARG_1_3545,1454,953,1110,987,583,1543,1959,693,691,793,163,640
RBBP5_1_3546,535,827,574,698,400,198,109,139,1837,606,12,745
RBCK1_1_3547,936,1057,1081,745,849,563,1512,1295,277,1203,521,1186
RBFOX2_1_3548,860,574,501,342,259,505,1275,2,29,1033,1193,112
RCC1_1_3549,169,102,137,32,25,26,363,33,130,26,211,0
RCOR3_1_3550,673,672,947,673,635,1120,330,309,312,58,718,786
RECQL5_1_3551,2233,1284,1934,2755,2411,2400,200,668,2101,1858,2344,365
9
RECQL_1_3552,746,722,541,378,1,149,786,591,98,474,5,40
RELA_1_3553,527,257,170,255,17,809,432,249,49,51,170,111
RERE_1_3554,1686,1347,2080,2012,2499,3486,1209,1888,670,2285,840,184
RFC1_1_3555,7651,6820,10551,7437,6840,8162,7362,7071,9449,6094,6411,64
55
RNF14_1_3556,706,301,605,499,916,1827,414,1,688,499,39,755
RNF17_1_3557,1325,1184,1520,1121,2577,2197,1125,612,1596,704,1053,1474
RNF40_1_3558,479,720,1531,585,1410,1522,2572,129,776,313,693,1742
RNF8_1_3559,1468,1449,2730,1862,2646,1339,1876,1306,1763,1700,175,839
RORA_1_3560,1118,953,1332,1498,1578,2330,1024,407,1384,776,1158,3242
RORC_1_3561,2195,2446,1774,2497,1274,3315,2097,1610,552,3031,1326,2155
RPH3A_1_3562,2523,1566,1524,2791,4058,3303,2152,1369,4007,1271,3147,64
9
RPS6KA5_1_3563,786,1299,1756,1029,1766,1023,907,1178,4232,1135,1877,21
53
SATB1_1_3564,10444,9407,10091,10696,13483,11257,10598,8371,7733,7409,7
784,8861
SATB2_1_3565,4097,2627,3146,3515,2869,3677,4701,2546,4624,4796,4811,51
38
SCMH1_1_3566,5320,4970,5991,5345,5072,10393,7931,4813,3200,4644,8250,5
439
SET_1_3567,27858,26403,29205,31962,34891,32179,33031,24745,24222,33535
,27134,32472
SETD3_1_3568,631,285,446,298,482,309,242,609,665,198,31,234
SETD4_1_3569,1988,2555,2763,2598,1561,2396,2715,4312,2802,3273,4241,14
90
SETD6_1_3570,764,503,765,644,963,515,508,198,58,652,2467,554
SETDB1_1_3571,1058,1839,1638,1305,1618,2823,3114,292,352,1374,2547,136
3
SETDB2_1_3572,1204,1606,1478,1546,967,968,642,1022,452,1892,98,1056
SFMBT1_1_3573,517,382,600,447,952,754,328,704,405,392,1646,85
SFMBT2_1_3574,2118,2251,2609,1515,2021,3927,2695,1603,2428,1634,3523,2
130
SHPRH_1_3575,703,1427,1449,574,532,1239,1791,136,1676,803,739,1176
SIN3A_1_3576,399,454,508,1230,143,505,706,240,284,758,785,341
SIRT1_1_3577,3125,2320,3485,3557,1951,6013,7245,2145,4582,3437,3464,40
91
SIRT2_1_3578,3556,2988,2996,3240,3537,2421,6495,3605,3702,3991,2206,33
89
SIRT3_1_3579,782,635,775,1046,219,759,1066,1075,738,850,380,491
SIRT5_1_3580,562,400,182,646,68,212,34,344,193,104,43,180
SIRT6_1_3581,403,154,255,140,0,18,0,475,106,312,23,1

SLC38A1_1_3582,278,504,560,722,936,826,426,1569,37,1312,18,1047
SMARCA1_1_3583,1406,1085,1738,1544,2603,1845,2462,1455,2614,1587,1669,
817
SMARCA2_1_3584,3991,3871,4432,3523,4632,4768,5080,4879,2279,3918,2831,
3462
SMARCA4_1_3585,2961,3295,3240,3457,3217,3212,1863,1605,2128,2461,2726,
5528
SMARCAD1_1_3586,1982,2168,2372,1213,2270,2967,3170,1616,2022,1456,2609
,415
SMARCAL1_1_3587,421,677,494,134,636,28,4,217,712,58,1,21
SMARCB1_1_3588,145,358,210,171,267,357,33,352,218,80,2,6
SMARCC2_1_3589,1465,1195,1437,1431,1780,1229,1884,1167,5077,596,1089,2
230
SMARCD1_1_3590,1671,2453,1473,1166,2271,1732,684,1517,241,652,3707,196
4
SMARCD3_1_3591,1425,1522,1806,2264,2730,1214,2704,2273,2627,2596,2057,
2546
SMC2_1_3592,3287,2935,4419,2831,3780,4042,4431,1191,3513,2291,6709,507
3
SMC4_1_3593,1761,2061,1923,2544,2413,1742,587,2203,168,1060,1434,441
SMN1_1_3594,1126,1183,1612,1275,1348,294,1332,1139,305,1297,540,1350
SMN2_1_3595,1126,1183,1612,1275,1348,294,1332,1139,305,1297,540,1350
SMYD3_1_3596,221,159,177,461,855,927,822,277,913,371,28,0
SP100_1_3597,1500,961,1511,1939,700,2141,1408,2738,768,1644,791,1235
SP110_1_3598,399,263,437,332,905,479,1591,382,10,431,825,79
STK31_1_3599,944,1177,1098,956,1394,782,2101,569,746,767,1169,1695
SUPT3H_1_3600,3568,2045,3947,3303,2803,3267,1642,4210,3447,3150,3247,2
463
SUPT5H_1_3601,422,276,628,240,212,0,968,841,2,416,1216,0
SUV39H2_1_3602,994,640,1421,1749,542,411,2152,947,2628,254,714,873
SUV420H1_1_3603,925,640,1130,649,1425,1472,1576,504,434,683,13,381
TADA2A_1_3604,2584,2158,3700,2226,2120,1857,3058,1731,1893,3640,2776,1
570
TADA3_1_3605,1377,1165,830,1962,1893,1162,1179,1323,879,835,1143,1739
TAF12_1_3606,400,506,984,934,514,268,1916,374,142,403,1768,362
TAF15_1_3607,870,896,671,843,2317,2247,689,2115,1281,1025,441,1721
TAF1_1_3608,182,138,415,436,73,337,1,696,0,56,118,0
TAF5L_1_3609,612,582,666,1079,774,1274,465,831,206,1124,324,629
TCF19_1_3610,256,493,423,909,203,922,175,125,182,381,833,336
TCF20_1_3611,1789,1551,2737,1619,2316,1668,1936,959,882,1397,3343,2332
TDRD10_1_3612,708,536,623,1181,836,1101,782,781,623,1003,339,715
TDRD3_1_3613,8885,7316,9750,10811,6103,10118,10345,10388,11972,6862,10
174,12519
TDRD5_1_3614,4801,6242,5912,6802,6102,7175,7581,5151,3902,5883,5615,96
59
TDRD6_1_3615,2310,1813,2765,1836,3761,1061,4003,2226,3615,1704,228,358
1
TDRKH_1_3616,561,439,725,222,1055,1082,28,1693,189,251,344,3
TERF1_1_3617,3374,3267,4387,3945,4304,4686,4828,2914,3335,4379,2647,24
17

TET2_1_3618,1147,606,1396,1037,4139,1259,3491,2415,1564,2013,1254,1598
THRA_1_3619,551,198,318,287,438,415,221,232,20,310,411,85
THRB_1_3620,1542,1581,1819,1789,3167,1362,2409,801,2038,604,436,232
TP53BP1_1_3621,819,1205,929,1074,1908,577,446,1007,4070,735,2420,1577
TP53_1_3622,438,825,392,475,470,839,87,1263,1448,375,891,198
TP73_1_3623,463,592,1091,615,517,1048,247,120,1813,983,1135,315
TRIM24_1_3624,1997,1469,1760,1623,2503,3126,1436,2039,1191,1761,1110,1
778
TRIM32_1_3625,1016,876,1511,1205,985,1346,3143,285,1162,1407,1374,218
TRIM33_1_3626,3211,3159,3702,3036,6203,4751,4737,1142,4923,3790,4535,3
431
UBE2A_1_3627,906,969,803,984,489,1082,497,512,1116,1497,644,1098
UBE2E1_1_3628,1582,1195,1646,2332,3293,922,2548,2287,1929,1816,2532,10
78
UBE2I_1_3629,355,395,607,658,1057,657,1576,1569,534,1057,133,1689
UBE2K_1_3630,165,211,103,278,174,260,415,61,195,239,1014,8
UBE2V1_1_3631,1424,1189,1740,1393,933,934,2618,906,872,956,759,279
UHRF1_1_3632,574,790,1014,1158,64,1298,405,1018,1029,325,540,145
USF2_1_3633,433,607,427,336,564,355,415,95,1256,868,801,1088
UTY_1_3634,4019,2702,3489,3830,3859,2616,3963,5791,3502,4881,739,3075
VDR_1_3635,2429,2579,3512,3063,5798,3080,945,2441,4210,2957,2122,1963
WDR5_1_3636,200,937,1657,751,41,887,0,1429,0,297,1044,458
WHSC1_1_3637,942,742,146,722,2,423,1862,1859,0,1206,1,153
WHSC1L1_1_3638,9245,8220,8045,7447,12159,13353,12430,10854,12250,7870,
8120,14103
WRB_1_3639,1944,1547,2229,1707,2015,2235,3056,481,3094,1332,1702,484
ZGPAT_1_3640,459,897,1646,968,64,901,139,132,2068,1183,356,40
ZMYND11_1_3641,1096,1007,1068,1195,1080,1376,1575,1629,576,394,752,112
7
ZMYND8_1_3642,677,1197,787,1190,490,1547,1319,888,222,861,71,1036
ZNF451_1_3643,2048,2779,1881,2635,3506,2831,386,2967,2611,1785,1792,23
45
ALG13_1_3644,857,1323,1817,1047,1953,985,1276,344,1763,1887,2236,1039
ASXL1_1_3645,1274,1098,1225,2267,2074,1307,3235,1412,2212,442,561,1816
CBX2_1_3646,2779,1858,5895,2541,3874,3405,2254,3758,1283,2945,4192,197
8
HDAC8_1_3647,1821,2042,898,2541,2582,2005,381,1537,826,1339,36,4984
ING3_1_3648,522,441,496,559,887,454,600,708,57,720,351,194
MLLT10_1_3649,934,1037,1401,687,1460,1651,966,978,1220,700,2610,161
PHF19_1_3650,896,936,776,527,910,1617,925,600,589,511,828,471
RBM14_1_3651,625,873,785,1428,156,641,891,32,1812,1702,801,3112
SP140_1_3652,3599,3953,4852,2785,5110,4495,5314,2080,6252,3934,432,374
9
TAF9_1_3653,1992,1622,2390,2076,2709,2067,5139,908,381,2035,2621,1480
TAF9_1_3654,318,135,238,424,793,564,285,9,203,595,1,5
AHR_1_3655,3048,2856,2910,1708,2765,2502,3674,1801,2458,2279,1865,2418
ALKBH1_1_3656,1832,1330,2279,2178,1291,2988,2502,1255,2360,1183,1431,1
541
ALKBH3_1_3657,1235,528,851,295,499,773,693,762,1800,1674,530,212
ARID2_1_3658,1415,1739,1183,924,1312,1980,838,1038,1410,686,1387,929

ASF1A_1_3659,593,307,728,1088,307,1523,1613,680,217,686,1185,3
ASF1B_1_3660,4529,3489,5205,4350,5509,2895,5145,4391,2660,2545,1871,26
75
ASH1L_1_3661,2975,1895,2617,1794,1603,3429,2475,2615,1011,1941,1091,21
40
ASXL2_1_3662,119,690,958,577,2,542,870,1627,348,41,267,1197
ASXL3_1_3663,1522,1135,1831,1211,2364,1777,1518,1018,1614,1347,1798,59
6
ASZ1_1_3664,9143,8833,9623,9407,9618,10608,5597,9310,7474,11486,8594,1
2930
ATAD2_1_3665,4822,4613,4847,4628,7405,4939,4916,4999,6110,4948,3013,50
73
ATF7IP_1_3666,4694,5013,5846,4647,7859,4458,4839,5438,3496,5639,2457,8
799
AURKB_1_3667,3714,3472,4467,2555,3333,4025,5014,1000,2479,3637,2410,18
20
BAHCC1_1_3668,1800,2213,2108,1788,1696,2309,3794,2316,1693,1637,1314,3
216
BAHD1_1_3669,371,629,1071,384,1026,669,928,657,206,455,124,2
BARD1_1_3670,1539,1035,1620,2470,1321,1255,1278,1720,3156,1153,857,238
4
BAZ1B_1_3671,2239,2067,2008,1643,1371,4052,1744,609,1146,1107,2624,152
7
BAZ2A_1_3672,200,493,317,97,83,100,105,454,911,484,625,594
BAZ2B_1_3673,1054,186,1458,667,567,847,77,27,229,410,913,2411
BLM_1_3674,6083,4417,5209,5190,5398,8538,5198,3889,2987,5282,2851,3372
BMI1_1_3675,1958,2447,3493,2840,3435,2061,1923,949,5373,2774,3421,1350
BRD1_1_3676,174,362,375,282,1072,565,520,350,355,474,20,1189
BRD3_1_3677,198,130,105,48,16,142,230,934,12,30,0,2
BRPF3_1_3678,1876,1862,1793,1175,1501,1118,1306,2252,1109,1584,403,130
1
BRWD3_1_3679,446,458,768,529,86,1674,157,386,57,538,790,2304
C14orf169_1_3680,808,576,534,1282,633,97,120,79,1253,717,2060,1528
C20orf20_1_3681,2221,2408,1755,1820,2502,2124,1881,1165,2437,1427,911,
2351
CALR_1_3682,681,784,1118,962,573,557,385,1138,64,1103,165,1615
CARM1_1_3683,1664,2202,1970,2291,2818,2110,1711,2955,1336,2004,697,207
3
CBL_1_3684,787,742,570,768,1037,45,613,596,256,439,1538,174
CBX4_1_3685,747,885,773,305,447,342,124,447,481,855,114,514
CBX6_1_3686,265,637,578,496,149,407,1360,175,408,1013,833,1911
CBX7_1_3687,1720,1739,3090,1721,3166,1810,2097,429,3331,2092,1903,3419
CBX8_1_3688,1263,796,664,852,795,1896,959,1461,255,1793,1497,1735
CCDC101_1_3689,60,45,149,23,77,215,255,179,16,26,47,0
CCNE1_1_3690,618,357,87,375,1691,738,310,712,49,616,816,176
CCNT1_1_3691,0,0,0,0,0,0,0,0,0,0,0,0
CDC73_1_3692,2376,2339,1753,2022,1661,3717,1412,1793,482,1710,685,4791
CDK9_1_3693,491,384,898,1182,770,1302,1419,561,1566,953,1911,1215
CDY2A_1_3694,3367,4625,4145,4224,5140,3272,6643,3571,5180,4390,2602,68
12

CDY2B_1_3695,3367,4625,4145,4224,5140,3272,6643,3571,5180,4390,2602,68
12
CDYL2_1_3696,1047,926,311,929,1185,447,1452,914,419,1379,49,447
CECR2_1_3697,2981,3367,3588,2737,2907,2799,3175,1802,2121,2264,3032,52
1
CHAF1A_1_3698,259,102,464,448,398,438,396,592,352,265,0,4
CHAF1B_1_3699,529,444,479,396,541,792,3813,424,1206,470,199,578
CHD1_1_3700,4223,3883,5157,3853,5925,2619,3211,2945,6662,4453,7618,395
0
CHD1L_1_3701,1441,1198,1139,1418,832,1071,1078,1045,1336,651,743,703
CHD4_1_3702,658,875,1025,1270,2780,887,1109,815,1215,1747,4,494
CHD6_1_3703,902,790,945,896,213,1186,2688,577,579,857,82,1034
CHD7_1_3704,1016,1518,1611,1147,1077,1127,4520,1919,2184,1495,1067,240
6
CHD9_1_3705,2805,2358,2757,2344,1834,3437,2379,425,1610,1753,1703,1327
CHMP1B_1_3706,235,517,412,267,8,483,238,84,292,149,55,4
CHMP4B_1_3707,1799,2305,1842,2355,1354,1980,983,2196,3167,2840,3100,28
0
CHMP4C_1_3708,644,318,688,579,571,956,650,670,835,876,153,14
CHRAC1_1_3709,690,308,304,617,356,767,889,289,862,273,358,576
CLOCK_1_3710,3118,3195,3754,2936,3946,3912,3783,4695,2094,4945,2841,37
93
COPS5_1_3711,749,478,511,900,1191,1002,411,496,753,817,479,99
CRAMP1L_1_3712,931,736,997,767,1092,242,731,61,107,730,609,767
CTCFL_1_3713,425,307,513,269,241,186,111,1055,84,561,57,253
DEAF1_1_3714,523,415,515,715,1490,472,525,490,39,487,5,1300
DNAJC1_1_3715,1445,554,1077,840,945,826,803,1461,993,1067,315,881
DOT1L_1_3716,2304,1626,2245,1922,3176,2265,3647,3850,3131,3156,1417,28
05
DPF2_1_3717,154,317,260,989,57,365,4,543,0,194,216,181
DPF3_1_3718,316,197,188,246,268,138,76,175,954,157,161,681
EIF4B_1_3719,2281,1995,1592,2189,2237,2553,2082,1324,999,3137,4402,144
9
ELP3_1_3720,1133,1250,1924,1814,795,1102,1219,2149,74,2177,1413,1794
EP300_1_3721,2197,2025,2330,3418,2513,4397,2041,2717,3204,2778,1564,18
25
EP400_1_3722,482,591,442,239,212,491,436,124,40,1491,1749,72
EPC1_1_3723,1291,871,759,1171,1372,1218,998,452,1891,561,558,845
EPC2_1_3724,2118,2276,2040,1900,2251,2152,1797,1459,787,2232,2669,2278
ERCC6_1_3725,2679,3138,2409,1796,6025,2560,2477,694,1693,2222,1873,664
ESRRA_1_3726,834,379,773,888,124,770,54,611,816,481,930,1090
ESRRB_1_3727,872,763,755,441,175,661,814,1070,1839,768,56,1525
FANCM_1_3728,5224,4643,5264,5828,7954,5179,5853,4843,5163,4159,4928,34
49
FBXL19_1_3729,172,71,393,193,68,62,815,9,0,203,1,28
FOS_1_3730,435,390,776,282,648,664,771,453,607,410,127,26
FTO_1_3731,1146,1218,1374,1616,1106,1632,4031,81,295,782,2342,1271
FXR2_1_3732,531,403,502,207,744,899,116,113,169,427,266,1731
G2E3_1_3733,368,670,827,807,951,570,946,574,288,383,424,1048
GATAD2A_1_3734,573,289,643,783,678,275,1052,310,881,662,333,878

GLYR1_1_3735,630,788,440,785,199,1147,236,6,599,745,1029,271
GMEB2_1_3736,810,686,443,664,365,555,637,351,1114,273,208,769
GSG2_1_3737,2455,2284,2307,1463,1181,3039,1423,902,2452,2508,1715,1425
GTF3C4_1_3738,277,381,44,371,54,601,25,341,12,206,219,0
HAT1_1_3739,6254,7168,8454,5657,8946,7848,6168,5636,8137,7954,7333,588
9
HDAC1_1_3740,1739,924,1328,1286,803,1329,1061,1470,1340,1527,1505,3008
HDAC2_1_3741,2595,1889,1795,2036,1890,2417,2349,1401,1630,1509,2070,21
95
HDAC3_1_3742,1046,888,587,629,2026,2159,2173,340,944,813,682,1360
HDAC4_1_3743,43,4,5,134,14,2,503,0,0,0,158,0
HDAC6_1_3744,586,774,766,596,1220,1392,1923,972,1,471,1758,1037
HDGFL1_1_3745,630,714,792,961,626,521,10,611,1721,1134,1417,1817
HDGFRP3_1_3746,1169,1670,1047,1446,1051,1070,679,2962,1923,866,1897,33
90
HELLS_1_3747,1600,1439,1712,1789,2978,2039,1265,1725,2590,831,2128,133
8
HEMK1_1_3748,877,420,535,931,0,492,226,299,109,960,337,702
HIF1AN_1_3749,87,160,215,150,0,77,301,321,363,130,0,44
HIRA_1_3750,1912,1568,2169,1490,2856,2048,1347,2179,2702,1066,2569,175
1
HMG20B_1_3751,4977,4271,5174,4176,11149,3781,5010,5623,5997,4773,4307,
5687
HMGN5_1_3752,2821,3571,3458,3570,6378,2908,5818,2160,4513,3256,3895,51
50
HNF4G_1_3753,2013,1548,2138,3186,2319,1721,1652,725,310,2385,912,2324
HSPBAP1_1_3754,1101,730,1589,952,1123,844,794,889,70,1193,203,1253
HUWE1_1_3755,1862,1897,2623,3302,2096,2197,1868,1464,2779,2048,2784,30
00
ING5_1_3756,91,470,496,462,1118,63,94,421,241,30,0,284
INO80_1_3757,5251,4486,5065,5248,6937,5438,4598,3068,3550,4900,5576,52
49
JARID2_1_3758,567,486,1011,625,1128,957,1015,305,618,1205,1187,41
JHDM1D_1_3759,3346,2229,3049,1705,3511,1918,3484,1618,3054,2745,775,31
27
JMJD7_1_3760,126,212,266,31,106,0,485,3,268,23,591,25
JMJD8_1_3761,829,603,1424,1161,1837,1460,843,1833,2048,902,132,646
JUN_1_3762,487,696,281,261,332,38,3,455,220,1706,172,366
KAT2A_1_3763,2264,2033,1659,2028,2541,2544,1460,3319,590,1733,4772,832
KAT2B_1_3764,5667,5858,6828,6184,6730,6007,5566,5527,8284,7356,4124,66
86
KAT6B_1_3765,2195,3165,2943,2360,1191,3433,802,2366,3032,1987,1499,342
7
KDM1B_1_3766,1276,1220,962,1707,1271,700,193,494,756,1374,434,1761
KDM2A_1_3767,135,119,65,78,142,469,212,489,2,1,0,237
KDM3B_1_3768,1015,1488,828,784,1254,524,1143,954,22,1181,1397,284
KDM4A_1_3769,431,240,345,408,44,455,57,228,445,348,836,4
KDM4B_1_3770,276,177,107,219,928,341,46,5,2,34,393,302
KDM4D_1_3771,224,356,384,268,415,68,942,93,1226,324,85,22
KDM5A_1_3772,302,756,999,784,370,931,264,418,0,1,1185,0

KDM5B_1_3773,2134,2112,2509,2151,1688,3637,3444,2610,2372,2226,2401,17
09
KDM6A_1_3774,841,688,445,893,1774,1308,29,696,434,313,1231,2291
KDM6B_1_3775,3117,2772,3125,3552,3149,3839,2936,1994,4601,4183,1958,12
99
KIAA2026_1_3776,1815,1842,2560,2883,1713,3344,1545,2612,1399,1309,2149
,2553
L3MBTL2_1_3777,2378,2200,2079,2764,2561,2141,5505,3210,2751,1536,676,2
524
L3MBTL4_1_3778,1119,447,548,847,796,181,1077,52,1685,428,312,153
MAEL_1_3779,6156,5875,7510,6936,7461,4511,8900,5458,6790,6209,4301,631
7
MBD3_1_3780,735,1264,1214,578,319,590,1361,565,1880,1465,310,1165
MBD4_1_3781,4034,3055,4693,4656,4741,3732,5968,1977,5094,4712,4273,231
2
MBD5_1_3782,892,1463,971,882,998,2004,788,997,690,890,2083,1710
MBD6_1_3783,2284,2337,2871,3186,4267,2834,2092,2469,4178,3031,2421,270
2
MBTD1_1_3784,2841,2378,4026,2682,1436,3828,3943,3155,4695,2635,893,66
MDM2_1_3785,3660,3108,4479,3236,3361,3944,3900,2476,2630,4277,5616,368
4
MIER2_1_3786,509,540,156,771,12,119,167,119,254,288,26,1343
MIER3_1_3787,906,705,757,759,1979,853,502,675,145,230,754,83
MIS18BP1_1_3788,1597,1792,1680,810,2494,1078,1933,1574,1007,1619,2732,
646
MKL1_1_3789,663,456,973,464,404,656,1667,557,1756,283,12,198
MLL2_1_3790,2718,2739,2652,3282,2786,3800,3279,4486,3011,3478,3042,274
9
MLL3_1_3791,2149,1467,3111,2807,2422,2642,914,2650,3196,1423,1226,2507
MLLT1_1_3792,899,534,889,580,449,452,1233,444,1109,838,721,664
MLLT3_1_3793,2449,2212,2535,1786,3657,2727,1532,1586,2462,2207,4884,10
55
MLLT6_1_3794,333,558,621,467,210,69,407,576,459,527,1233,26
MPHOSPH8_1_3795,6375,4782,5973,5897,4899,7438,9483,8495,4822,4089,5034
,6077
MSH6_1_3796,449,183,247,198,326,399,255,29,0,418,316,214
MSRB2_1_3797,1257,869,777,1276,1396,2185,1627,476,1756,596,2905,1416
MTA2_1_3798,2024,2811,4287,3681,2719,2309,4751,2304,4395,4425,1001,411
5
MTA3_1_3799,1790,1417,1436,1322,1914,2729,1889,1063,1177,2177,1364,275
1
MYSM1_1_3800,2154,2082,1535,1745,1714,1438,1819,1908,1753,1621,2474,32
7
NAP1L2_1_3801,4909,4469,5242,3838,5483,6025,5797,5864,3303,2718,5208,5
401
NAP1L3_1_3802,3166,2856,3135,3153,2154,3131,2336,2871,2644,3674,3196,4
513
NAP1L4_1_3803,2328,1703,2607,1763,3104,2135,1409,2133,2248,1365,1809,3
586
NAP1L5_1_3804,842,927,494,397,278,1343,806,1034,0,74,50,519

NAT14_1_3805,12,328,108,106,12,447,0,0,0,1,0,187
NAT8B_1_3806,1021,962,762,437,699,1686,880,768,811,322,714,120
NAT8_1_3807,5223,4734,6321,5058,3905,4428,8037,3120,7059,5690,6751,517
7
NAT8L_1_3808,4315,4290,4745,3740,5609,5622,5631,5218,4857,6067,4843,42
66
NAT9_1_3809,3528,3612,4021,4375,6718,2362,4052,3982,2643,2181,2430,504
9
NCOA2_1_3810,732,225,82,217,4,449,180,1,0,109,77,1165
NPTXR_1_3811,1123,700,652,923,2153,734,799,989,1029,1411,233,688
NR0B1_1_3812,3517,2592,2608,2189,3685,3981,2577,4296,4279,3140,1129,35
44
NR0B2_1_3813,894,538,883,821,1269,719,943,518,549,644,692,1051
NR1D1_1_3814,272,205,384,61,7,361,1602,369,9,518,405,53
NR1H2_1_3815,3572,3544,4174,4165,3463,2314,3522,3514,4977,3857,4117,60
8
NR2C2_1_3816,586,257,936,755,8,351,362,473,1,37,2001,990
NR2E1_1_3817,2073,2115,2352,2396,3542,2931,1273,1473,1298,1464,2549,37
41
NR2F1_1_3818,848,1007,1061,1820,659,1210,267,274,8,881,208,243
NR2F6_1_3819,647,626,615,449,126,837,337,322,10,265,332,393
NR4A2_1_3820,1590,967,1578,1644,753,1952,1278,2032,1031,2456,868,3077
NR5A1_1_3821,39,106,39,11,2,91,47,130,280,0,0,407
NRIP1_1_3822,1672,1604,1237,1675,1499,1376,615,393,2242,1561,1259,458
PADI4_1_3823,738,777,412,240,165,738,3570,264,33,171,381,297
PAWR_1_3824,2205,2395,2023,2978,3790,1902,3312,3192,1163,2444,1572,333
0
PAX5_1_3825,360,463,405,264,314,938,381,587,1736,390,43,1848
PAXIP1_1_3826,1927,1757,1112,1478,2634,2645,1482,1727,890,1424,1344,32
95
PGRMC2_1_3827,1052,1326,1371,1121,297,966,3016,1131,1721,1056,1022,424
PHB_1_3828,253,339,247,170,139,679,93,271,36,130,28,7
PHC3_1_3829,1127,608,1649,803,2097,852,526,510,800,1328,976,1218
PHF13_1_3830,451,375,414,611,204,619,260,1328,188,599,371,291
PHF14_1_3831,233,322,441,260,436,365,247,168,476,161,45,61
PHF15_1_3832,358,102,332,416,113,406,400,487,971,206,548,274
PHF20_1_3833,1158,1808,2026,2007,780,2807,2474,1769,494,2064,222,1123
PHF23_1_3834,2457,2025,1948,1514,1114,2956,3301,1734,1553,1447,2068,12
05
PHF2_1_3835,904,284,780,398,770,106,554,450,533,504,12,1
PHF3_1_3836,1329,971,1518,1264,1711,1211,518,905,2921,882,1000,1432
PHF5A_1_3837,1471,768,1086,684,381,822,362,1596,1786,848,2365,407
PHIP_1_3838,666,1072,1079,1007,1239,553,16,1608,961,261,535,2891
PHRF1_1_3839,562,746,448,463,54,245,234,73,33,1558,99,484
PIAS1_1_3840,1066,731,1048,775,927,885,1586,997,206,432,264,188
PIWIL4_1_3841,1948,2055,1442,607,1568,1446,1951,1040,1888,1129,2031,42
4
PPARGC1A_1_3842,1513,1397,1714,1911,1512,1101,1546,351,2031,1520,1470,
974
PRDM11_1_3843,84,211,481,251,1079,451,351,16,67,1,0,643

PRDM12_1_3844,687,518,385,689,677,242,1330,302,224,948,557,15
PRDM13_1_3845,185,242,76,293,29,15,639,92,0,755,1289,140
PRDM14_1_3846,1014,901,1386,1535,1191,1769,1310,1825,2301,1021,1164,18
92
PRDM4_1_3847,3558,4827,5337,5728,6951,4501,4088,3041,5170,3274,3999,40
58
PRDM5_1_3848,1632,1436,1693,1983,2008,2037,2447,1030,1463,2012,1793,11
95
PRDM6_1_3849,34,39,125,161,56,19,29,1,1,198,4,54
PRDM9_1_3850,745,861,851,882,703,355,2398,397,1094,469,812,1976
PRMT6_1_3851,4693,3916,6451,5168,6796,5979,4338,2331,4180,2702,6595,49
60
PRMT8_1_3852,791,505,726,654,2103,522,240,279,150,341,376,334
PRPF6_1_3853,1782,1569,1499,2380,1273,1515,1503,784,3337,902,1046,1437
PYGO1_1_3854,3687,3314,4441,4533,5481,3571,4586,3000,3275,4160,4441,40
58
PYGO2_1_3855,848,705,870,1186,272,1019,353,1365,1021,525,214,720
RAG2_1_3856,1195,886,825,1359,551,1197,1020,1756,745,859,381,1069
RAI1_1_3857,1122,969,1688,734,866,135,950,389,1978,1152,216,129
RB1_1_3858,680,1713,1380,1427,837,1905,1745,1641,1902,1135,1868,1903
RCOR1_1_3859,2085,1219,2680,1457,4763,1810,3865,1345,2924,2072,1671,17
36
RCOR2_1_3860,715,651,598,289,2,707,83,2417,95,606,2,54
RECQL4_1_3861,2474,1968,2424,2076,2411,2846,2776,2224,1830,2508,4732,1
370
RELB_1_3862,630,726,1045,613,979,580,935,432,956,771,37,1776
REL_1_3863,2285,2589,2703,2707,1832,3682,5061,1435,3931,4941,1792,1883
RING1_1_3864,1856,2185,2172,1967,1462,2454,1968,1579,3123,2749,2044,22
49
RNF20_1_3865,875,803,488,371,623,221,1102,470,47,434,50,1106
RNF25_1_3866,203,101,214,134,16,12,56,579,421,321,28,11
RNF2_1_3867,8537,11077,14664,10274,13800,11484,12013,7147,8965,10998,8
709,10767
RORB_1_3868,2170,1975,1892,2187,3221,1421,1279,1578,3336,2263,2942,165
0
RSF1_1_3869,338,391,1009,593,417,784,54,927,1998,1795,774,20
RUVBL1_1_3870,547,311,239,461,59,1317,214,421,1,1052,79,156
RUVBL2_1_3871,479,423,506,918,49,523,0,0,363,221,6,0
RXRA_1_3872,631,684,1287,928,814,1000,2595,579,276,1422,923,2100
RXRB_1_3873,2363,3477,3084,3103,3672,2896,2611,1114,2795,2186,916,2759
RXRG_1_3874,1188,1178,1794,1886,2175,1575,2296,1174,451,1034,1089,2056
SAP18_1_3875,1183,575,1343,890,218,508,1186,1016,132,1046,372,1012
SCML2_1_3876,1627,2076,1511,1601,2749,3332,1887,1127,1096,1413,1298,13
77
SENP3_1_3877,170,353,18,124,479,20,23,359,5,6,1,0
SETD1A_1_3878,3129,2514,2076,2324,3357,2699,2529,2914,1836,2226,1921,3
081
SETD1B_1_3879,3203,3042,2828,2288,2340,1849,4067,1361,5077,1404,1586,4
874
SETD2_1_3880,430,277,197,156,2344,584,1310,896,1287,368,443,1872

SETD5_1_3881,86,148,104,61,73,4,5,4,12,271,3,0
SETD7_1_3882,2053,1444,2044,1885,5013,1533,1698,1725,355,1768,1165,116
8
SETD8_1_3883,4071,3869,3085,2685,7480,2755,5494,3145,304,3965,4592,421
5
SETMAR_1_3884,3368,3917,3427,3326,1792,3347,1552,2514,2916,4373,3349,3
126
SF3B3_1_3885,861,587,1038,890,814,942,557,57,1611,1861,448,189
SIAH2_1_3886,1083,941,695,933,681,807,439,461,1970,1428,2146,403
SIN3B_1_3887,246,470,385,115,230,582,82,48,1138,190,621,178
SIRT4_1_3888,403,257,351,277,94,476,61,34,1,4,1845,1436
SIRT7_1_3889,484,256,944,695,736,411,495,137,250,420,261,43
SLC2A4RG_1_3890,52,96,70,183,5,684,0,20,24,15,0,217
SMARCA5_1_3891,1950,1242,1100,927,1104,2984,887,935,2410,2123,550,1298
SMARCC1_1_3892,536,924,852,805,1329,570,30,94,940,667,44,632
SMARCD2_1_3893,723,984,1286,677,734,1500,551,593,835,937,997,1246
SMARCE1_1_3894,1382,884,1803,1158,930,1176,601,1501,4701,1582,2962,807
SMC1A_1_3895,5663,4581,6157,5342,9039,5118,9284,4720,4250,5807,6111,99
37
SMC1B_1_3896,11570,10552,13302,13302,13827,15393,19531,9294,14049,1206
4,11583,9044
SMC3_1_3897,8692,9464,9102,10689,9227,9576,7741,9011,5144,10469,11131,
8464
SMCHD1_1_3898,159,15,322,83,0,0,0,734,0,1,0,0
SMNDC1_1_3899,1372,1677,1596,1524,1629,827,2524,600,778,1212,2041,1461
SMYD1_1_3900,3610,3589,4505,2471,4600,3891,3100,3009,2641,3846,1115,13
94
SMYD2_1_3901,675,526,583,573,475,1417,196,745,575,1035,1775,149
SMYD4_1_3902,986,910,937,729,517,2583,1180,444,2155,1687,1393,808
SMYD5_1_3903,611,487,362,352,225,541,844,1144,1337,175,107,46
SND1_1_3904,1761,2296,2895,1924,1923,1960,3177,2925,1633,1425,1020,246
9
SP140L_1_3905,14180,15110,13680,15050,14212,15025,15607,15062,6894,123
42,13603,14882
SRCAP_1_3906,5742,5685,6461,6206,5737,7567,4465,4326,6234,6067,6049,66
52
STAT5B_1_3907,563,522,207,683,621,783,79,1134,773,248,959,1208
SUDS3_1_3908,1120,567,1016,987,241,904,1321,897,76,555,261,1075
SUPT16H_1_3909,282,272,546,544,1,231,2862,529,1082,291,874,943
SUPT4H1_1_3910,36,9,164,5,7,74,0,0,11,90,1,0
SUPT6H_1_3911,581,658,524,367,925,320,328,788,8,66,1636,1097
SUPT7L_1_3912,3765,3539,4112,3447,6026,4143,4816,3143,4322,4090,1531,2
980
SUV39H1_1_3913,514,629,608,876,4,161,558,508,1410,368,9,117
SUV420H2_1_3914,414,117,481,260,653,492,622,56,47,160,302,1220
SUZ12_1_3915,1762,1790,2110,922,3088,894,917,1875,2881,1854,643,3091
TADA1_1_3916,736,931,2085,1312,156,436,1038,621,620,1030,742,389
TADA2B_1_3917,186,16,6,19,145,13,62,2,0,14,1,8
TAF10_1_3918,844,677,1072,1268,941,567,488,712,278,1542,71,1083
TAF1L_1_3919,7906,7515,9907,10409,6546,8030,7547,9231,5301,7223,11344,

10095
TAF3_1_3920,586,866,1542,851,616,1090,968,1544,140,420,884,1528
TAF5_1_3921,1866,605,1385,1135,2016,1169,2422,118,1247,541,303,1407
TAF6L_1_3922,2775,2643,3873,3175,3082,2811,3296,2876,1741,1997,1453,21
80
TAF8_1_3923,1084,114,391,163,274,476,157,149,689,114,41,2472
TCF7L1_1_3924,15,23,231,10,9,9,0,200,0,810,71,33
TDRD12_1_3925,838,665,513,584,1111,747,608,774,247,729,163,283
TDRD1_1_3926,2920,2835,3323,3686,2960,4639,1932,2906,2790,2072,1730,42
05
TDRD7_1_3927,559,841,722,755,568,593,68,135,1300,1051,470,270
TDRD9_1_3928,1371,871,833,433,1105,728,1436,521,670,829,675,123
TERF2_1_3929,1087,1254,930,1308,698,1016,442,229,617,1701,2306,1515
TET1_1_3930,2609,1819,1695,2110,3322,3116,2064,2327,3010,1711,822,1132
TET3_1_3931,2847,2395,2627,2558,2321,2304,5138,2438,1567,2164,2784,385
1
TLX2_1_3932,309,496,299,149,308,735,347,1050,1978,56,2,53
TNRC18_1_3933,91,166,210,228,1,7,135,200,0,91,0,29
TRAF7_1_3934,720,919,1013,761,2062,1992,853,543,12,80,556,1023
TRDMT1_1_3935,4905,4234,3693,4733,5755,4041,5636,3308,4620,6961,3988,9
992
TRERF1_1_3936,328,556,457,464,267,825,259,193,13,461,314,958
TRIM25_1_3937,1956,2225,3231,2226,5097,2708,2047,2661,2379,1943,4159,1
009
TRIM27_1_3938,288,417,535,306,316,130,74,102,1137,278,28,1016
TRIM28_1_3939,487,208,360,504,626,576,1467,477,288,150,16,2223
TRIM66_1_3940,735,389,783,547,61,950,938,433,2873,496,972,1724
TRRAP_1_3941,1176,1452,1980,1535,708,1564,1080,652,1792,1223,1100,445
TSG101_1_3942,4826,3317,5430,5587,5448,3190,5615,3170,4328,2903,5334,5
171
TYW5_1_3943,1846,3050,2764,2435,1123,990,4292,551,4260,695,392,156
UBE2B_1_3944,3466,2230,3244,2834,2550,4450,2350,2698,2947,1808,173,337
3
UBE2N_1_3945,646,811,1502,1022,1187,690,1524,1555,985,986,1722,1409
UBR7_1_3946,642,733,1156,1227,924,2887,748,462,675,272,2069,925
UHRF2_1_3947,2098,3116,2045,3018,3401,1529,3446,1352,3275,1777,2545,11
58
USP22_1_3948,500,568,601,412,66,307,0,52,817,296,1167,522
YEATS2_1_3949,243,387,608,208,63,533,149,125,476,458,21,2222
YEATS4_1_3950,1949,2262,2205,1332,837,3000,2158,1161,1548,1266,1038,14
70
YY1_1_3951,289,92,129,135,202,73,0,314,113,79,146,79
ZAR1_1_3952,195,349,210,404,592,450,7,265,24,328,0,125
ZCWPW1_1_3953,2831,1767,3043,2807,3471,2932,2047,3676,1568,3888,1520,1
949
ZCWPW2_1_3954,4267,3654,3331,4990,4451,2770,2247,2967,9719,4880,412,27
40
ZFP57_1_3955,695,453,316,603,1594,679,325,418,98,600,700,0
ZNF541_1_3956,1475,964,1308,442,845,777,1533,1322,1347,959,810,966
ZNF85_1_3957,1916,2187,1940,2595,1426,2910,1727,3343,2114,1410,3297,18

79
AANAT_1_3958,622,411,871,658,576,504,246,677,229,591,1445,96
AES_1_3959,275,269,317,218,378,180,67,124,227,484,844,85
AIRE_1_3960,5517,4890,6470,6226,6122,8087,3309,4193,9639,5690,8387,673
4
AKAP1_1_3961,402,343,261,74,23,862,513,724,2196,39,1310,10
ALKBH2_1_3962,124,75,57,1,7,5,0,0,1,139,0,43
ANKHD1_1_3963,1874,2264,2578,2208,2272,1807,1928,2201,2088,2765,5187,2
031
ARID1A_1_3964,200,452,373,197,497,431,597,109,508,253,653,2
ARID1B_1_3965,3903,2761,3559,4159,2636,4857,2773,2910,5263,4114,5209,3
093
ARID4A_1_3966,2076,2104,1399,1349,2162,2576,957,746,2090,2493,2129,349
2
ARID4B_1_3967,13068,13545,12937,15433,17669,17222,17195,14517,13772,18
760,8722,15725
ARRB1_1_3968,531,476,284,6,175,286,1739,74,1074,146,4,0
ASH2L_1_3969,1736,1169,2009,1898,1906,2747,332,685,3610,2201,1505,665
ATAD2B_1_3970,1939,2312,2409,2310,3313,3263,3592,1195,2019,2015,684,30
23
ATAT1_1_3971,2913,2475,4199,3301,2603,3076,3717,2191,6019,2455,2319,19
04
ATRX_1_3972,3374,4803,5114,5615,4580,5632,4504,6759,1258,4320,7260,489
5
AURKA_1_3973,877,991,769,1155,1104,218,721,134,135,523,182,492
AURKC_1_3974,1077,858,1092,778,1211,1043,2815,834,1141,1785,211,1396
BAZ1A_1_3975,671,1149,1420,776,2664,2020,276,1223,546,1244,643,2077
BCOR_1_3976,571,726,625,1022,880,701,695,660,1140,659,1790,151
BPTF_1_3977,1212,918,1137,981,994,2229,144,1054,2682,1235,754,787
BRCA1_1_3978,1406,1462,1381,933,968,2005,252,1429,1849,598,1042,884
BRD2_1_3979,232,118,237,357,344,494,340,321,90,895,553,864
BRD4_1_3980,860,848,1126,425,569,1286,1344,108,73,537,1159,1043
BRD7_1_3981,863,875,600,931,301,674,578,366,1292,62,224,720
BRD8_1_3982,2015,1488,2322,1594,3670,3250,1324,1105,2656,1660,4465,278
0
BRD9_1_3983,1173,914,1214,1748,789,1619,1310,1508,209,1344,634,1772
BRDT_1_3984,2280,1888,2837,3186,2800,3376,4308,2565,1835,2538,2128,150
9
BRPF1_1_3985,6717,5898,6262,7288,6313,6967,6739,8764,7804,8389,8365,65
83
BRWD1_1_3986,1086,1211,773,1156,1269,1600,1615,949,64,1188,1559,1015
C14orf43_1_3987,566,793,592,604,289,178,83,22,967,857,788,692
CBX1_1_3988,210,196,834,373,48,224,254,43,842,60,880,94
CBX3_1_3989,1290,1203,2221,994,858,1378,817,280,2662,901,1877,74
CBX5_1_3990,700,309,493,348,881,372,46,88,94,216,390,3
CCNT2_1_3991,3708,3955,4636,4565,4378,4562,2588,4458,3983,3812,5117,92
38
CDY1B_1_3992,1962,925,1546,2001,5535,1711,2647,1202,1524,1822,2132,109
5
CDY1_1_3993,1962,925,1546,2001,5535,1711,2647,1202,1524,1822,2132,1095

CDYL_1_3994,2120,2271,2391,2622,2031,2575,2268,2434,6348,2775,3375,121
4
CHD2_1_3995,192,599,472,368,241,60,54,297,2715,544,75,0
CHD3_1_3996,6482,5578,6286,7730,5496,6879,8032,5258,6981,4835,6790,523
6
CHD8_1_3997,990,378,648,584,1437,955,754,1082,166,864,965,1136
CHMP2A_1_3998,92,633,816,415,208,260,767,111,729,472,42,222
CHMP5_1_3999,793,450,880,423,1279,142,1650,742,1349,684,372,391
COPS2_1_4000,1322,1955,2789,1871,1291,1368,2052,2142,1747,1058,2624,14
50
CPA4_1_4001,2694,3008,3325,2805,4118,2357,4357,1745,2641,2869,2835,458
1
CREB1_1_4002,1676,1852,1634,1806,838,1929,1855,1619,1673,1976,2025,232
0
CREBBP_1_4003,2432,1752,2182,2037,2475,1717,933,2357,2314,1820,2645,87
7
CTCF_1_4004,1619,1037,1015,985,1910,556,1619,1352,2353,1486,395,1318
CTNNB1_1_4005,294,602,707,462,476,223,1285,729,329,1044,696,1945
CXXC1_1_4006,264,341,187,280,330,399,839,5,65,42,0,611
CYLD_1_4007,11573,11160,11497,12314,16148,15496,13096,12795,9578,12050
,9906,8764
DICER1_1_4008,2049,1268,1970,1857,1092,1977,1100,441,2192,1975,1470,12
39
DIDO1_1_4009,2769,1787,3002,2759,3692,3478,683,2487,2686,2708,2555,138
0
DMAP1_1_4010,2241,2150,2983,1628,4622,3772,2188,542,3642,1605,1765,328
4
DNAJC2_1_4011,3296,3095,3953,3883,4267,3136,5142,3741,4008,3239,2361,4
665
DNMT1_1_4012,778,736,879,696,493,1402,529,1355,4538,956,235,1780
DNMT3A_1_4013,1340,1551,1769,1479,2538,2276,3915,2472,1975,1372,1766,1
035
DNMT3B_1_4014,384,673,1085,1198,779,192,629,258,1371,1088,377,535
DNMT3L_1_4015,1815,1842,2162,2193,2585,1976,2128,2482,1347,2198,851,24
44
DPF1_1_4016,305,398,585,613,368,149,364,653,15,272,214,392
EED_1_4017,1965,1204,2176,2401,2537,2347,2229,2414,686,3644,1802,2070
EGR2_1_4018,458,905,645,494,127,1331,600,332,476,603,1480,77
EHMT1_1_4019,4309,4114,4053,5254,3253,4327,4343,3828,7138,4275,8194,71
94
EHMT2_1_4020,1433,924,2290,1372,1558,916,2677,1493,1260,1379,1985,3263
ENO1_1_4021,1128,509,792,799,464,735,1214,665,1216,925,665,492
ESR1_1_4022,5158,5206,5106,5545,5656,5000,3028,3245,4970,4056,5202,693
1
ESR2_1_4023,0,0,0,0,0,0,0,0,0,0,0,0
ESRRG_1_4024,2538,2887,3276,2429,4083,1609,4307,1917,6965,2071,1671,22
95
EZH2_1_4025,2342,1310,2156,2362,3280,2690,438,2098,1037,2168,5096,4158
FBXO11_1_4026,6182,5901,9222,7564,9042,6033,6819,3796,6140,6956,8443,8
079

FMR1_1_4027,1858,1156,1672,1562,2560,1780,1456,2758,1320,1839,1847,114
5
FXR1_1_4028,1376,1647,1008,705,586,1619,1618,912,183,1046,1639,2823
GFI1B_1_4029,446,675,642,559,733,1257,2130,591,4,1025,486,72
GMEB1_1_4030,5738,4710,5846,5527,8839,7345,7229,5709,6310,5730,6492,73
33
HDAC10_1_4031,356,318,267,367,661,1210,20,719,512,130,166,594
HDAC11_1_4032,367,138,105,263,240,317,307,5,0,399,26,13
HDAC5_1_4033,1576,2160,2349,1923,604,3464,2446,1947,1150,2466,1279,134
5
HDAC7_1_4034,533,576,881,1044,369,238,986,287,2994,233,1028,159
HDAC9_1_4035,1737,2198,1881,2523,2264,3075,4171,1211,2973,2185,1496,82
7
HDGF_1_4036,839,1406,1095,648,1406,1401,691,364,1605,1249,714,846
HDGFRP2_1_4037,1051,528,987,844,290,977,1678,1336,261,279,34,121
HLTF_1_4038,5099,5348,5158,6704,4615,4615,5975,3595,3522,4218,7056,538
3
HMGA1_1_4039,1713,998,1907,1477,4081,1340,2307,476,1035,919,372,3564
HMGN3_1_4040,4667,3601,5289,4574,4942,4881,4974,5938,2383,3527,4539,45
35
HNF4A_1_4041,200,405,277,233,552,577,428,1325,108,1020,31,463
HPSE2_1_4042,313,242,307,188,46,100,20,538,9,429,286,92
HR_1_4043,288,170,457,128,535,212,655,22,20,206,875,155
ING1_1_4044,876,1174,260,306,1068,303,33,449,1152,268,603,139
ING4_1_4045,2179,2885,4053,2646,4013,2159,4960,1923,2704,2236,1872,493
INTS12_1_4046,1727,1803,1088,1995,2431,2372,1002,1687,501,1621,870,762
IRF4_1_4047,651,123,399,15,38,532,0,1521,1239,285,2164,173
JMJD1C_1_4048,603,769,534,428,424,1020,135,570,460,454,7,2208
JMJD4_1_4049,519,483,579,603,973,945,931,173,181,1370,58,444
JMJD6_1_4050,440,405,75,43,552,520,415,65,251,156,56,3
KAT5_1_4051,2002,1373,2700,2850,4128,1940,1049,3736,2268,2766,2294,245
6
KAT6A_1_4052,489,622,863,734,497,1065,1517,292,35,732,3882,812
KAT7_1_4053,276,318,89,581,295,1389,0,6,0,77,0,0
KAT8_1_4054,2764,2218,3667,2696,3864,2039,6053,1387,3059,1816,214,3696
KCTD1_1_4055,4655,4837,6350,4801,5749,3976,4305,3485,4738,2555,5566,66
30
KDM1A_1_4056,7871,8571,10928,7990,10588,7838,6668,4860,6918,6720,8263,
9222
KDM2B_1_4057,594,134,242,679,1480,633,1030,557,95,312,156,0
KDM3A_1_4058,2132,2075,1859,1812,1601,1645,1166,979,1379,2045,1837,191
0
KDM4C_1_4059,5672,4763,5667,5081,6905,8127,7046,7037,5287,3911,6227,70
82
KDM5C_1_4060,614,703,309,646,337,690,191,599,389,618,689,957
KDM5D_1_4061,1441,1193,959,1024,836,1679,223,1553,1252,1408,1501,604
L3MBTL1_1_4062,491,426,567,691,584,719,1767,451,217,324,305,14
L3MBTL3_1_4063,4068,4228,4124,5629,3908,4254,8101,2519,3620,3870,4373,
6790
LBR_1_4064,630,545,492,1046,1372,1659,1641,1054,114,616,319,77

MAP3K12_1_4065,2379,2243,3536,3655,3416,3480,4070,2542,1294,1586,2997,
1755
MBD1_1_4066,9,64,322,68,0,176,213,322,0,46,0,523
MBD2_1_4067,784,1255,2091,1825,3126,1909,617,2593,1018,773,2145,2266
MECOM_1_4068,3141,4235,3671,2580,3778,3872,4641,2815,3583,3470,2449,21
56
MECP2_1_4069,583,469,316,277,829,500,182,293,910,866,659,73
MEN1_1_4070,404,413,214,357,782,1005,55,866,257,644,0,712
MGEA5_1_4071,3328,2264,5992,3840,3455,3132,1598,3218,1152,2076,3050,16
80
MIB2_1_4072,641,507,527,224,1204,693,799,304,330,875,1492,167
MIER1_1_4073,1260,708,1316,847,2215,1109,602,963,406,1153,1955,1925
MINA_1_4074,2095,1262,1564,1947,4099,2117,3238,1991,2772,2052,3173,418
7
MLL5_1_4075,3981,2994,3449,3845,4883,3327,4294,2912,4552,4758,3920,332
5
MLL_1_4076,649,718,449,564,2127,1753,259,185,867,441,1504,322
MORF4L1_1_4077,3821,3718,4346,3442,6126,2927,4211,2469,6217,3025,6039,
4723
MSL3_1_4078,1305,1708,2126,2704,3565,1721,1734,1982,1897,664,3528,1819
MTA1_1_4079,472,714,565,624,382,1086,312,1251,1136,430,183,4
MTF2_1_4080,668,607,651,415,801,1374,472,1565,184,697,936,33
NAA60_1_4081,2274,2175,2196,2428,2311,997,4103,996,1438,2182,767,2329
NAP1L1_1_4082,12001,12206,14663,15816,12525,14483,14802,15331,14102,13
182,12345,16914
NAT10_1_4083,1435,1694,1758,705,4061,2801,2299,773,525,1992,1100,791
NCOA1_1_4084,1140,929,771,730,601,1494,1111,261,1082,474,1564,232
NCOA3_1_4085,4059,3291,4340,4572,3404,5833,4399,2399,3797,5557,5516,70
22
NCOA4_1_4086,4329,5045,5944,6925,6030,6049,6945,5060,6182,7773,5747,71
30
NCOR1_1_4087,1219,1351,2090,1632,2334,1161,2385,605,1209,1608,786,1257
NCOR2_1_4088,97,495,240,319,822,118,336,161,750,7,80,0
NFAT5_1_4089,419,619,969,228,94,1562,17,1022,170,323,1294,481
NFATC1_1_4090,1615,2422,2024,1713,3275,2673,1901,3409,1021,2127,1187,1
429
NFATC3_1_4091,305,203,364,524,1157,333,516,702,472,175,551,206
NFATC4_1_4092,882,449,481,250,2053,688,164,255,764,1282,123,1191
NFKB1_1_4093,4660,4538,4302,4760,4493,2569,5018,4747,5480,4292,2894,81
94
NFKB2_1_4094,2259,2555,3312,2163,4242,2324,1558,2615,3208,2636,267,102
3
NPM1_1_4095,1079,1351,1895,1853,1443,1958,1892,1350,963,1230,1408,2538
NR1D2_1_4096,4058,2620,4073,3506,6431,4643,4447,756,2128,3700,932,1450
NR1H3_1_4097,150,36,60,87,169,43,1126,19,0,196,87,0
NR1H4_1_4098,1148,686,1167,835,297,674,384,444,325,866,1265,1569
NR1I2_1_4099,2443,2426,2695,2287,1816,1279,3380,2386,2263,1727,3108,19
77
NR1I3_1_4100,1903,2123,2143,2785,2273,2332,4344,2660,1463,2240,3321,11
94

NR2C1_1_4101,2189,1158,1651,1802,2303,1575,723,1595,1069,1319,915,2053
NR2E3_1_4102,419,296,231,350,22,113,730,625,122,19,1778,400
NR2F2_1_4103,1629,587,1135,1614,5033,2482,2034,2772,2868,1911,1411,195
9
NR3C1_1_4104,2970,3690,4525,4179,4426,3245,4058,4233,5499,4575,1267,46
22
NR3C2_1_4105,1118,1293,1065,1284,728,1581,2425,264,2226,396,1000,2985
NR4A1_1_4106,413,449,746,499,878,239,365,161,458,206,1199,211
NR4A3_1_4107,1610,1725,1960,2358,1421,2219,1235,1092,2028,1816,2656,87
8
NR5A2_1_4108,2170,2094,2399,1855,1336,1649,2028,975,1175,1707,2538,741
NR6A1_1_4109,410,102,251,98,204,52,52,11,81,220,2,570
NSD1_1_4110,2,0,0,0,0,0,0,0,0,1,0,0
PBRM1_1_4111,715,992,307,619,323,1021,592,992,28,1802,323,83
PCGF6_1_4112,2509,1826,2569,2377,3069,1659,2664,943,963,905,2395,1875
PGR_1_4113,1028,555,686,1021,835,581,299,1052,4,1752,686,1195
PHC2_1_4114,634,808,918,545,704,975,594,969,1207,817,735,1402
PHF10_1_4115,1434,2183,2310,1338,3300,2554,1339,1115,1319,2160,1503,11
4
PHF11_1_4116,1074,869,2086,1208,1275,2088,2125,674,473,1355,1990,82
PHF12_1_4117,1391,1111,1062,1800,2166,1235,1271,728,2340,1499,1542,231
3
PHF16_1_4118,1889,1259,1730,1920,2677,1853,841,2785,1458,1151,722,1470
PHF17_1_4119,519,455,822,533,347,850,1591,951,129,1136,556,209
PHF1_1_4120,539,976,508,298,1187,837,1087,424,1569,1295,16,1170
PHF20L1_1_4121,1425,1106,1767,1886,1090,3133,2197,1996,2559,1858,350,6
28
PHF21A_1_4122,221,132,161,69,275,928,25,550,0,473,541,323
PHF21B_1_4123,219,234,104,21,0,2,1019,61,235,74,1273,4
PHF6_1_4124,5793,4184,5612,5216,3329,7071,6481,4666,4027,4434,5622,534
9
PHF7_1_4125,562,299,504,946,905,89,36,933,969,343,943,787
PHF8_1_4126,2763,1896,3377,2155,3725,2845,4821,2047,5336,826,266,3567
PIAS2_1_4127,325,375,426,215,98,492,640,0,1105,106,30,0
PICK1_1_4128,1262,384,1461,1418,1616,1353,739,1214,1897,1559,897,3369
PIWIL2_1_4129,1510,1143,1968,1015,1169,1272,1774,476,1153,1920,727,542
PML_1_4130,181,27,127,20,0,32,197,68,0,87,0,0
POLR1B_1_4131,1587,990,2087,1989,1524,2225,1181,739,549,1699,3248,289
PPARA_1_4132,3754,2926,3186,3522,2367,3335,3988,3605,3907,3046,3781,12
28
PPARD_1_4133,299,355,423,816,426,85,308,628,815,188,226,52
PPARG_1_4134,639,1065,1196,997,937,489,1869,1122,38,774,845,936
PRDM10_1_4135,1141,843,1471,841,835,1104,3607,288,1056,479,880,477
PRDM15_1_4136,754,824,1011,705,550,463,1101,842,1733,474,906,24
PRDM16_1_4137,2510,2203,2270,3569,4207,2247,2740,2073,1853,2759,2053,2
048
PRDM1_1_4138,185,193,249,444,641,28,301,744,0,565,333,0
PRDM2_1_4139,10479,11024,12590,13762,11790,14342,16067,12287,10265,108
96,16163,14147
PRDM7_1_4140,2675,2821,2480,3313,3933,2175,1534,1776,2094,1492,2969,51

40
PRDM8_1_4141,7018,6456,8062,6790,6059,4998,4857,7901,5928,5827,4424,10
785
PRMT1_1_4142,210,149,12,134,75,12,1,0,0,166,27,0
PRMT2_1_4143,440,562,772,454,811,678,467,371,1820,763,596,1539
PRMT3_1_4144,2162,1923,1708,2278,3676,1811,3119,1588,1232,1759,3206,13
73
PRMT5_1_4145,1061,1051,1050,861,2191,1645,1697,1284,1455,398,1754,2473
PRMT7_1_4146,1249,1201,1386,1093,1720,1956,1618,1450,425,1207,961,1379
PSIP1_1_4147,5205,3825,6302,6200,5399,6158,7873,2336,2686,4523,3661,60
80
PSMC5_1_4148,2448,3192,2382,2297,1327,1551,347,2597,2043,1971,613,882
PWWP2B_1_4149,426,277,508,335,212,670,33,400,995,78,295,1571
RAD54B_1_4150,504,387,127,194,225,1082,46,40,492,719,191,362
RAD54L_1_4151,2602,2216,2902,2665,4404,2561,1669,2678,2403,2553,1798,1
332
RARA_1_4152,764,912,1033,763,991,1493,1007,342,1029,415,1386,1043
RARB_1_4153,948,870,792,1811,2038,1455,2889,440,2364,905,632,693
RARG_1_4154,2888,2287,2115,2808,4915,1800,1469,2730,1900,1616,1413,321
9
RBBP5_1_4155,2525,2549,3020,3520,2719,4024,1218,2163,1594,2520,2074,26
52
RBCK1_1_4156,634,494,531,712,478,658,2177,512,439,840,185,252
RBFOX2_1_4157,2812,2270,2153,2631,1238,2643,2530,1923,1006,1333,1001,4
078
RCC1_1_4158,816,384,630,441,901,669,39,227,46,580,346,300
RCOR3_1_4159,6431,5382,7191,5009,7857,5433,7309,3539,7238,5386,4781,34
37
RECQL5_1_4160,1097,713,848,899,1345,1529,65,103,1696,1351,498,106
RECQL_1_4161,2922,3316,3944,3643,5135,5239,1405,3672,5234,3628,2115,41
97
RELA_1_4162,423,428,774,311,490,197,862,519,251,799,1462,1867
RERE_1_4163,620,58,135,219,635,437,1090,0,672,89,0,281
RFC1_1_4164,4028,4089,3992,3605,4687,4729,5590,2010,4257,4215,2297,371
9
RNF14_1_4165,2194,1302,3058,3259,4122,3462,2144,2538,1796,3218,2627,18
53
RNF17_1_4166,1026,1083,684,874,588,1196,2199,220,1672,957,2549,1955
RNF40_1_4167,1199,821,1445,1023,1483,1442,1374,1025,407,299,434,1472
RNF8_1_4168,284,189,425,360,305,1897,551,310,241,479,760,299
RORA_1_4169,3558,3766,5086,4357,2923,3378,2813,2219,2887,2543,3108,514
1
RORC_1_4170,1022,1160,868,1125,941,1103,677,621,1006,437,372,420
RPH3A_1_4171,819,436,1144,734,2601,1269,2251,472,63,1264,852,1210
RPS6KA5_1_4172,693,494,298,312,894,0,0,0,0,0,570,1
SATB1_1_4173,1423,1179,1995,1627,2311,916,1814,243,1040,663,3443,3245
SATB2_1_4174,1083,718,366,1039,1228,1311,879,984,554,602,1744,376
SCMH1_1_4175,5381,5155,6092,5573,5522,10585,7890,4791,4451,4604,8249,5
342
SET_1_4176,4888,5185,5072,3475,7374,6474,8001,2759,6907,8278,2416,3543

SETD3_1_4177,3056,2682,3401,3215,3330,4146,3664,2845,4353,3486,7980,32
40
SETD4_1_4178,1206,1343,1126,730,2014,462,1521,679,1135,722,268,372
SETD6_1_4179,1298,1019,1298,896,1791,958,3200,825,1244,1219,746,1004
SETDB1_1_4180,999,929,1587,1825,2493,1167,1394,1902,2758,1707,2232,137
6
SETDB2_1_4181,2932,2923,4625,3442,1580,2190,3867,3249,4254,2214,2284,4
770
SFMBT1_1_4182,719,401,741,343,1181,711,33,901,504,845,1870,91
SFMBT2_1_4183,356,207,267,233,304,661,79,1089,700,132,0,463
SHPRH_1_4184,11136,9577,11940,9432,6365,8278,13747,7495,12006,7571,104
34,6103
SIN3A_1_4185,2536,1829,2563,1903,2686,2161,3617,1460,3682,1822,2384,21
01
SIRT1_1_4186,834,629,1156,642,253,960,2068,347,2304,1116,1627,969
SIRT2_1_4187,2153,2014,1456,2111,1986,3465,2473,2055,1612,2237,554,117
1
SIRT3_1_4188,416,324,160,33,28,144,393,135,0,38,6,57
SIRT5_1_4189,1170,1352,1362,2304,1440,1997,1331,1192,1544,2159,376,231
SIRT6_1_4190,190,239,676,194,280,529,28,163,675,684,292,165
SLC38A1_1_4191,369,526,316,231,6,1408,143,851,28,455,332,339
SMARCA1_1_4192,4876,3866,5604,4696,7984,7292,9763,5069,8556,4495,4413,
2463
SMARCA2_1_4193,2268,2622,2901,3041,4941,5392,4046,3452,1790,2457,2212,
4809
SMARCA4_1_4194,821,601,634,607,1377,665,887,226,1103,354,1205,377
SMARCAD1_1_4195,5419,6330,6839,7521,6572,8831,3432,6561,8104,4695,5580
,6410
SMARCAL1_1_4196,2482,2715,2144,2375,2038,3571,3788,968,1020,880,2459,2
716
SMARCB1_1_4197,2840,3153,1903,2002,2633,2010,1241,2472,1411,1795,2096,
565
SMARCC2_1_4198,300,407,166,199,286,450,716,131,19,1020,580,535
SMARCD1_1_4199,2570,2284,2979,3006,1890,2535,3355,2952,3866,2329,2411,
3223
SMARCD3_1_4200,1486,1921,1680,2013,1599,1831,4854,2401,3661,2716,3671,
2411
SMC2_1_4201,1350,1232,2043,1038,1455,2955,1627,990,915,456,828,561
SMC4_1_4202,1238,929,1151,1391,2380,1197,1591,821,21,568,465,526
SMN1_1_4203,2539,2485,3525,3034,3366,2629,1925,2087,1952,3795,4040,161
3
SMN2_1_4204,2539,2485,3525,3034,3366,2629,1925,2087,1952,3795,4040,161
3
SMYD3_1_4205,954,687,845,466,704,901,3353,222,394,1457,54,498
SP100_1_4206,6491,5292,7629,5901,5563,7854,10015,4904,6315,7745,7489,8
651
SP110_1_4207,310,488,739,317,608,1771,507,276,767,198,1412,356
STK31_1_4208,2264,1601,3127,2223,1996,1990,3854,2359,2518,2247,2524,11
51
SUPT3H_1_4209,793,791,883,1053,1276,60,207,595,2398,619,541,17

SUPT5H_1_4210,1035,867,1027,1411,270,1110,847,844,526,482,1221,512
SUV39H2_1_4211,2913,2772,2865,3331,3325,2835,3770,3149,1549,1970,2750,
2553
SUV420H1_1_4212,396,544,524,264,67,686,774,606,36,328,18,1329
TADA2A_1_4213,3674,2887,2982,3617,4787,3015,2530,1446,3172,2765,1064,2
665
TADA3_1_4214,2593,1706,2348,1693,2856,3565,4889,3131,206,1612,919,3263
TAF12_1_4215,9468,10284,12802,11567,8166,8402,7524,10004,11416,8904,11
323,11765
TAF15_1_4216,914,858,628,947,1629,2298,1269,2123,1306,1025,447,1749
TAF1_1_4217,1352,1041,2168,1576,1985,1627,689,482,1267,1228,713,708
TAF5L_1_4218,428,469,907,516,1074,773,1251,1428,1733,308,239,1429
TCF19_1_4219,467,324,568,628,412,633,446,821,875,701,854,449
TCF20_1_4220,463,347,840,780,246,853,1508,586,29,418,182,1229
TDRD10_1_4221,654,1033,748,990,1459,1190,1243,654,845,736,1333,755
TDRD3_1_4222,2503,3846,2798,2800,3016,3071,3432,2881,4217,2255,1206,22
21
TDRD5_1_4223,3720,4713,5910,6020,5392,5195,7296,3902,3531,4677,4127,41
28
TDRD6_1_4224,3381,4575,3669,4214,6574,4292,2196,3897,4108,2980,2036,24
73
TDRKH_1_4225,2579,2704,3297,3220,4778,3313,3170,3215,822,2387,1273,276
2
TERF1_1_4226,156,331,308,725,390,41,1077,512,74,1044,244,100
TET2_1_4227,540,364,370,635,687,797,462,381,386,147,72,689
THRA_1_4228,552,883,989,1415,607,634,663,1746,1278,924,69,339
THRB_1_4229,1097,1427,1616,2745,2577,2687,754,1704,380,874,1915,1609
TP53BP1_1_4230,2697,2482,2938,2394,1918,1926,2856,2127,2069,2539,3726,
2357
TP53_1_4231,1366,1304,1136,1152,3155,2382,2645,2140,1633,1600,1070,831
TP73_1_4232,1873,1646,1809,1484,1417,1300,1399,1164,1976,2166,3243,461
TRIM24_1_4233,1089,1117,1390,1420,2707,1659,312,1535,876,1425,1620,356
TRIM32_1_4234,3419,3515,4962,3403,6342,4906,7170,4336,3017,3307,6314,2
479
TRIM33_1_4235,3553,2787,3675,2747,1822,1920,2856,3502,3081,3003,1355,1
097
UBE2A_1_4236,7922,6792,10099,7932,11640,7444,7472,5683,8560,7768,9317,
8741
UBE2E1_1_4237,2091,2736,2305,2759,4582,2645,2525,3021,807,1910,1946,21
55
UBE2I_1_4238,715,669,515,566,179,391,1449,575,260,70,92,1185
UBE2K_1_4239,5436,3586,6014,4314,6939,5019,3949,5686,5914,3846,3777,96
60
UBE2V1_1_4240,8588,7305,7426,8604,6536,7591,11228,9363,5648,9469,7211,
5869
UHRF1_1_4241,397,389,520,203,2113,605,372,697,9,1013,17,44
USF2_1_4242,3869,2830,4397,4491,3326,3392,4616,3662,2878,3001,3177,152
8
UTY_1_4243,5700,5684,5019,5041,6180,4693,5570,5476,8674,3647,4058,6664
VDR_1_4244,129,469,609,20,189,193,49,15,445,175,540,36

WDR5_1_4245,10257,11752,10834,9371,11197,13463,9255,7847,13444,11082,9
074,11931
WHSC1_1_4246,435,324,305,364,4,346,402,122,9,778,120,35
WHSC1L1_1_4247,2501,2445,2553,2430,2900,2300,2473,1996,1709,2020,2157,
2296
WRB_1_4248,192,241,518,385,110,121,184,35,19,59,38,7
ZGPAT_1_4249,797,559,643,670,587,269,507,113,2357,148,156,1861
ZMYND11_1_4250,885,1573,733,1514,1531,1377,3062,2442,443,553,990,1321
ZMYND8_1_4251,2709,2955,3117,2687,3003,1213,1593,940,4881,2530,2923,11
29
ZNF451_1_4252,4235,4054,4800,4270,3514,3959,4963,4727,4912,3114,4718,2
372
ALG13_1_4253,744,420,487,339,490,512,131,138,460,547,525,377
ASXL1_1_4254,455,481,215,724,470,1105,2357,117,196,123,2,620
CBX2_1_4255,183,464,580,547,315,857,1020,787,306,893,730,1016
HDAC8_1_4256,675,432,987,492,1009,642,81,476,3089,301,230,1013
ING3_1_4257,4027,3147,3277,2493,2399,3722,5337,5409,2707,2592,4975,518
4
MLLT10_1_4258,123,27,46,168,29,18,916,358,3,65,94,260
PHF19_1_4259,2108,2788,2826,2921,5613,3322,2269,2036,1916,1728,2058,18
67
RBM14_1_4260,1408,609,1411,1071,2227,1316,1362,600,1822,1161,1546,1587
SP140_1_4261,2268,2116,3516,3026,3126,2268,1072,3712,1493,4004,1314,60
46
TAF9_1_4262,1551,1755,2150,2177,1932,2187,4354,1335,2133,1502,4144,406
TAF9_1_4263,728,961,1132,1032,824,736,942,970,212,1069,346,427
AHR_1_4264,1691,1245,1503,1553,1517,1233,2719,733,47,1453,2035,1334
ALKBH1_1_4265,3502,3563,4328,3448,4755,3645,5603,4047,3469,2135,3498,3
101
ALKBH3_1_4266,3280,2302,2322,2022,1722,2461,1708,1481,5726,1950,2790,1
455
ARID2_1_4267,269,202,300,116,362,57,86,0,142,79,15,1220
ASF1A_1_4268,465,297,387,227,1110,953,182,144,582,907,927,23
ASF1B_1_4269,809,652,1025,816,1408,772,269,201,89,1050,1786,719
ASH1L_1_4270,4337,5046,4607,3865,5824,5526,8637,2888,6209,3582,4303,39
30
ASXL2_1_4271,420,431,447,536,1272,25,330,574,5,761,560,1359
ASXL3_1_4272,896,607,496,319,119,126,14,1337,988,656,29,287
ASZ1_1_4273,520,672,365,303,972,277,175,235,320,478,443,442
ATAD2_1_4274,3101,2655,2096,3380,4211,1661,2927,3989,1134,2740,848,361
3
ATF7IP_1_4275,2051,2615,2419,2136,3144,1869,1355,2230,2194,1624,1421,1
595
AURKB_1_4276,413,218,68,449,739,653,662,8,0,452,278,936
BAHCC1_1_4277,468,235,529,358,322,80,861,1831,49,146,27,282
BAHD1_1_4278,355,303,697,152,524,1698,72,410,1646,179,0,924
BARD1_1_4279,1382,1741,1459,1498,1403,1611,325,1085,2144,1245,1161,135
4
BAZ1B_1_4280,1687,2058,2969,2208,716,1740,984,2352,2698,2073,2809,1996
BAZ2A_1_4281,1211,904,1077,626,1600,925,699,621,2646,225,588,1571

BAZ2B_1_4282,2425,3013,2468,2573,2630,2357,2564,3470,3571,1626,3951,41
87
BLM_1_4283,9655,7569,11279,9454,14724,10342,9131,11101,10059,8765,7705
,9704
BMI1_1_4284,1421,1409,1726,1199,708,1771,1064,1220,546,1790,1660,1875
BRD1_1_4285,415,303,1069,634,1599,382,782,630,175,1457,70,13
BRD3_1_4286,526,189,350,383,758,190,589,857,253,556,553,816
BRPF3_1_4287,2651,3354,3064,4204,4742,2381,3853,2186,4941,3519,1268,22
71
BRWD3_1_4288,1131,1304,541,837,1156,294,506,236,1282,290,1246,1411
C14orf169_1_4289,713,926,994,472,1440,299,2180,266,271,608,650,1229
C20orf20_1_4290,1821,1547,1065,1211,857,636,43,943,945,921,229,1314
CALR_1_4291,2322,2596,3333,2581,1611,1751,3807,3045,2358,1895,4428,930
CARM1_1_4292,1371,1812,2508,2297,505,3861,2102,1091,1553,1516,1808,140
7
CBL_1_4293,930,893,525,600,1325,718,453,373,980,515,1193,2158
CBX4_1_4294,7204,6142,7252,6897,8800,7914,6818,6002,7758,4410,4703,532
5
CBX6_1_4295,937,530,369,818,610,983,416,371,1570,511,941,731
CBX7_1_4296,420,600,692,488,400,509,891,198,133,349,118,977
CBX8_1_4297,1563,1364,1796,1352,1795,1044,1543,1306,556,1468,1159,3477
CCDC101_1_4298,1270,1070,754,1172,1174,854,1152,1672,1188,903,225,1422
CCNE1_1_4299,1604,1119,1370,1255,1534,1232,1709,658,578,1122,1001,624
CCNT1_1_4300,147,116,94,215,424,216,219,522,12,271,0,0
CDC73_1_4301,2587,2023,1537,2066,3805,1689,3038,1941,3054,2111,3625,67
78
CDK9_1_4302,76,70,182,319,142,94,262,39,2,61,236,297
CDY2A_1_4303,734,887,1257,520,1714,1118,2139,328,222,881,212,24
CDY2B_1_4304,734,887,1257,520,1714,1118,2139,328,222,881,212,24
CDYL2_1_4305,940,980,1584,966,570,662,131,491,1686,411,53,428
CECR2_1_4306,1155,1804,1744,2026,1297,3284,2686,2352,3191,2767,2531,30
41
CHAF1A_1_4307,3183,2460,2770,2632,3457,1297,4096,4658,2022,1354,1318,6
016
CHAF1B_1_4308,245,456,234,270,511,62,118,33,23,1191,0,82
CHD1_1_4309,2971,2484,2952,2828,4134,1628,3321,1450,4122,2464,1603,190
2
CHD1L_1_4310,3607,2477,2672,3223,2297,2280,3539,2788,1922,2747,3105,49
12
CHD4_1_4311,1061,1507,1806,1433,1146,1990,1666,1464,1624,1677,2161,568
CHD6_1_4312,1944,2164,1765,2163,2265,3504,2324,1674,2141,1670,1730,350
4
CHD7_1_4313,112,212,199,111,161,265,376,0,87,132,1635,5
CHD9_1_4314,2283,2685,2864,3067,1369,3916,3570,3628,1301,1329,349,2785
CHMP1B_1_4315,272,417,642,598,104,348,139,861,0,161,142,333
CHMP4B_1_4316,2442,2176,1167,1743,1088,928,3232,1926,1466,1069,1920,11
35
CHMP4C_1_4317,745,466,648,1088,279,578,544,909,287,508,395,400
CHRAC1_1_4318,5614,5504,6934,5603,7683,5961,5808,3962,3614,5944,6618,7
529

CLOCK_1_4319,363,455,402,352,337,327,300,362,535,607,519,1539
COPS5_1_4320,595,288,759,286,154,455,449,200,538,157,810,0
CRAMP1L_1_4321,551,683,945,468,1026,2255,349,2132,30,578,946,510
CTCFL_1_4322,381,276,471,383,324,355,961,583,580,215,212,114
DEAF1_1_4323,3230,3432,3923,4287,3933,5074,4314,3744,3925,5440,3752,55
50
DNAJC1_1_4324,2455,2310,3541,3797,3712,3166,3001,2553,1785,1881,4277,2
887
DOT1L_1_4325,523,1351,1287,860,314,1804,1168,1008,3,493,781,1045
DPF2_1_4326,3353,4002,4943,3557,4310,5385,5632,621,2418,6643,5769,7425
DPF3_1_4327,768,571,1411,823,1727,276,669,814,1108,482,151,121
EIF4B_1_4328,857,1620,1046,1451,1280,863,1096,698,2140,1997,1622,1181
ELP3_1_4329,661,705,664,482,346,406,769,1,110,377,1399,400
EP300_1_4330,1541,1475,2141,2095,623,1916,502,1220,3501,2281,1019,1381
EP400_1_4331,3741,3836,4310,4659,7741,4142,1469,2803,3114,3709,2594,21
73
EPC1_1_4332,5419,3282,2602,3133,3417,3483,4501,5792,5995,3829,3615,390
2
EPC2_1_4333,7660,9172,9201,8349,7881,8742,9577,9579,12016,7929,13888,1
0814
ERCC6_1_4334,3692,2791,3480,4169,5258,5193,5145,2440,2221,2998,4594,28
83
ESRRA_1_4335,92,58,77,124,1,93,120,836,365,73,2,403
ESRRB_1_4336,2757,2777,3740,3924,2692,3629,3116,3179,3003,2199,1333,74
9
FANCM_1_4337,234,294,323,153,234,346,180,237,892,658,299,240
FBXL19_1_4338,478,295,291,199,40,463,1,568,171,350,980,303
FOS_1_4339,538,158,251,263,1065,358,351,399,99,196,95,256
FTO_1_4340,11473,12396,12806,11821,16484,15786,11386,10998,11293,11340
,11057,17971
FXR2_1_4341,254,98,309,92,1085,155,6,384,257,300,182,151
G2E3_1_4342,577,494,859,816,600,84,1984,1442,199,334,172,495
GATAD2A_1_4343,941,746,499,558,797,736,850,510,3,350,295,887
GLYR1_1_4344,4390,5242,5598,4210,3366,2345,5227,2567,3098,3223,6403,44
94
GMEB2_1_4345,134,15,177,64,1,299,322,0,7,45,0,146
GSG2_1_4346,184,254,324,525,77,186,213,406,1398,122,9,344
GTF3C4_1_4347,398,1269,399,747,404,1013,619,1153,590,1163,5,141
HAT1_1_4348,1176,1514,1902,1863,3578,1151,973,2209,2334,1895,1601,2339
HDAC1_1_4349,815,1682,1223,1095,574,844,1522,680,681,877,2285,854
HDAC2_1_4350,5766,5981,7318,8504,4875,7571,6963,7636,6938,7768,6901,56
64
HDAC3_1_4351,624,451,637,342,246,46,1629,367,726,729,438,550
HDAC4_1_4352,2208,2130,1657,1536,3158,3214,1747,1896,580,3412,1295,142
8
HDAC6_1_4353,1825,940,2286,869,1604,1343,3776,1108,270,1079,2387,761
HDGFL1_1_4354,96,833,202,26,10,2,69,124,1,271,41,187
HDGFRP3_1_4355,743,974,952,712,282,1507,406,300,1674,579,285,3795
HELLS_1_4356,7706,7369,8145,9000,8675,7856,11000,5486,8344,8320,4008,1
0273

HEMK1_1_4357,1124,1103,1604,1057,1431,1080,249,469,51,1770,876,3392
HIF1AN_1_4358,4645,4149,5428,4518,4182,4675,4882,4565,7064,5411,4869,5
025
HIRA_1_4359,3,54,42,13,99,0,1,12,0,36,0,0
HMG20B_1_4360,434,223,314,284,79,661,1018,79,28,128,7,70
HMGN5_1_4361,2652,2450,3043,2913,2367,3013,5561,2296,6060,2127,2428,44
00
HNF4G_1_4362,1492,1357,1375,1007,1323,987,1512,269,1021,921,1986,1283
HSPBAP1_1_4363,434,483,847,390,184,346,119,528,4,405,239,658
HUWE1_1_4364,755,865,1152,363,408,50,2,951,2627,75,1629,765
ING5_1_4365,319,249,547,441,512,117,1686,1,0,38,2491,42
INO80_1_4366,968,1207,729,1010,1490,670,631,1411,531,510,1724,2110
JARID2_1_4367,616,135,628,440,1009,853,160,293,910,442,858,845
JHDM1D_1_4368,1768,915,1890,1080,1029,692,2649,1347,703,2259,521,846
JMJD7_1_4369,2677,3364,2958,3484,4540,2973,3066,4177,5573,1675,1212,62
02
JMJD8_1_4370,463,640,337,323,36,223,254,205,110,830,190,1336
JUN_1_4371,394,305,371,385,350,272,226,241,1399,529,68,19
KAT2A_1_4372,720,1065,1055,2294,1345,1599,259,1335,2915,607,906,1098
KAT2B_1_4373,3529,3846,4248,3411,5403,5351,4855,3927,9346,3785,4231,26
56
KAT6B_1_4374,142,404,92,79,44,475,13,45,24,111,313,384
KDM1B_1_4375,2170,1787,2209,1898,1589,1273,2966,840,3953,2006,1322,503
KDM2A_1_4376,760,1336,701,1139,1967,1420,516,814,718,554,1598,816
KDM3B_1_4377,1004,956,1341,1312,612,1728,730,379,3457,2460,2556,1722
KDM4A_1_4378,3353,3021,5093,3053,4966,4553,1731,4549,2491,3052,3036,24
91
KDM4B_1_4379,231,100,260,100,124,184,3,24,294,511,276,490
KDM4D_1_4380,9479,7662,9987,8799,12698,11447,8249,5521,12794,11319,922
6,9144
KDM5A_1_4381,3550,3197,4491,3921,4817,3875,2791,2298,3459,4564,8045,50
26
KDM5B_1_4382,521,311,791,768,506,377,769,288,1825,219,288,1164
KDM6A_1_4383,2386,1569,3146,2172,2501,2969,2876,2000,1132,1448,1954,99
7
KDM6B_1_4384,810,730,1146,1102,495,1510,41,1240,1324,31,868,1332
KIAA2026_1_4385,6970,5357,5193,7514,8627,5399,5122,7164,5154,7848,7963
,5213
L3MBTL2_1_4386,1397,1209,1641,1727,1026,2343,1642,763,1115,1159,1229,1
633
L3MBTL4_1_4387,675,373,166,53,95,372,916,542,350,463,28,777
MAEL_1_4388,1180,1718,1734,2297,2223,1759,994,1342,1524,1373,576,855
MBD3_1_4389,310,289,224,402,384,704,132,414,1178,91,600,207
MBD4_1_4390,741,872,945,1200,701,678,2392,398,1092,476,1451,1964
MBD5_1_4391,1005,1388,1370,1315,633,1543,2182,1056,1030,894,366,2603
MBD6_1_4392,1671,759,1976,742,2305,1690,807,1733,2997,464,1734,988
MBTD1_1_4393,2225,1963,1785,1518,2863,2511,3517,2349,2421,2133,4073,82
2
MDM2_1_4394,2427,2508,3410,4273,3688,4134,3395,4497,1838,3091,392,2820
MIER2_1_4395,650,769,1158,1056,1544,1906,269,1045,671,1083,595,2418

MIER3_1_4396,3171,2032,2769,3038,3593,2215,1886,1396,1190,2737,2972,4398
MIS18BP1_1_4397,9735,11288,10998,10357,11159,12573,14688,10348,10282,7241,7352,5256
MKL1_1_4398,491,307,328,574,309,451,356,1040,83,357,1838,1326
MLL2_1_4399,343,242,8,101,101,567,180,13,769,539,2,0
MLL3_1_4400,1111,878,1966,954,770,1046,740,1521,84,1162,976,1112
MLLT1_1_4401,767,421,654,1262,1035,706,330,772,18,257,1513,240
MLLT3_1_4402,428,535,336,551,756,446,331,223,1892,298,7,2160
MLLT6_1_4403,1813,1435,2574,2368,1585,2772,1346,891,3351,2078,2051,2551
MPHOSPH8_1_4404,761,860,611,352,888,439,1364,213,1497,957,953,1120
MSH6_1_4405,1032,897,899,1246,333,762,942,313,345,2124,1030,987
MSRB2_1_4406,340,163,112,158,35,161,1,342,201,106,96,10
MTA2_1_4407,750,631,1413,1048,101,666,1511,689,1201,880,114,1320
MTA3_1_4408,4970,4124,5737,6646,4207,6402,4963,3671,4292,6581,4913,6971
MYSM1_1_4409,1557,1160,2299,1525,1738,1557,1124,1113,1397,1084,1411,1928
NAP1L2_1_4410,1170,1371,2005,1397,1406,1942,1850,670,1380,831,148,3707
NAP1L3_1_4411,1610,1862,1617,1621,2811,2389,2126,1236,4839,1428,3023,1519
NAP1L4_1_4412,3658,3459,3428,2924,5060,3594,3190,1692,1554,2500,4748,2900
NAP1L5_1_4413,660,106,622,425,548,489,0,1,957,1,1,98
NAT14_1_4414,613,337,364,676,33,452,705,1346,686,713,346,525
NAT8B_1_4415,199,706,306,270,540,381,37,27,451,170,468,62
NAT8_1_4416,553,538,279,265,342,454,157,185,43,688,0,600
NAT8L_1_4417,239,297,74,35,523,43,187,119,288,224,0,67
NAT9_1_4418,1678,1572,2129,2269,1637,1764,2287,756,670,3398,1579,950
NCOA2_1_4419,1301,697,922,606,679,547,2338,1374,275,122,227,301
NPTXR_1_4420,1340,1282,1576,1082,1310,1694,1091,247,1153,1071,2819,1743
NR0B1_1_4421,134,137,4,98,0,0,5,0,0,4,0,0
NR0B2_1_4422,736,556,466,726,732,602,873,1,1180,436,448,1024
NR1D1_1_4423,1022,474,857,844,446,769,12,36,1973,804,66,254
NR1H2_1_4424,803,1311,446,974,990,620,123,1857,43,526,575,1759
NR2C2_1_4425,2232,1799,1934,1757,742,2633,2042,2020,4023,1371,747,1987
NR2E1_1_4426,537,626,549,582,2075,865,1573,883,1739,724,307,181
NR2F1_1_4427,181,621,270,362,904,882,41,90,70,304,1252,224
NR2F6_1_4428,23,94,1,0,0,0,0,0,0,6,0,0
NR4A2_1_4429,862,370,312,816,400,688,361,755,99,277,359,4
NR5A1_1_4430,136,278,88,155,642,15,28,973,0,169,64,261
NRIP1_1_4431,765,1298,1069,1548,1009,1559,1020,1877,330,441,21,2107
PADI4_1_4432,3041,2139,2830,2642,2147,1464,6520,2106,1752,1816,2742,1246
PAWR_1_4433,1517,1942,1474,2080,3058,1555,578,1441,2636,2170,1917,1165
PAX5_1_4434,1015,1270,1247,1518,1647,383,189,1780,428,1352,952,747
PAXIP1_1_4435,493,593,493,727,2655,1551,294,192,814,566,911,655
PGRMC2_1_4436,1255,1585,1518,1480,2418,1594,1761,1990,503,767,917,1255

PHB_1_4437,2049,1141,1648,1514,3550,917,2691,599,1468,1867,2054,957
PHC3_1_4438,301,260,410,137,1268,242,33,617,3,157,83,27
PHF13_1_4439,2571,2139,2552,2622,1836,2856,4362,2681,2299,1532,1851,84
0
PHF14_1_4440,1608,1299,1527,1285,1738,1766,2489,730,4709,2092,801,1057
PHF15_1_4441,757,1020,636,680,2928,1231,875,466,1655,316,313,2711
PHF20_1_4442,1842,2257,1848,2163,2555,1374,829,1356,1843,2227,198,1754
PHF23_1_4443,2052,2048,2584,2326,2881,1805,2076,2637,3829,2210,1681,25
12
PHF2_1_4444,497,296,128,550,1109,102,615,93,2,0,1,61
PHF3_1_4445,4928,4495,3783,4775,6795,4033,5093,4795,5036,4963,3276,301
3
PHF5A_1_4446,2040,2537,2993,1496,3235,3337,4732,1161,5189,1716,1266,22
82
PHIP_1_4447,370,217,503,525,679,400,240,92,1664,192,549,967
PHRF1_1_4448,323,209,211,388,4,264,6,28,499,21,1,151
PIAS1_1_4449,1539,1671,2079,1956,1707,2022,1729,1304,3451,1930,1099,90
9
PIWIL4_1_4450,648,420,380,744,2067,143,1117,636,1228,192,0,450
PPARGC1A_1_4451,2838,1588,1327,1829,2603,1677,1841,1238,1789,1837,2589
,2915
PRDM11_1_4452,265,667,883,211,75,1441,278,690,167,342,314,12
PRDM12_1_4453,3225,3085,2874,2698,3900,3946,2004,3318,1474,2796,2450,2
530
PRDM13_1_4454,795,703,1394,1084,1502,123,1132,564,806,914,114,1051
PRDM14_1_4455,578,909,778,586,528,662,1070,410,573,920,38,66
PRDM4_1_4456,664,199,700,337,459,276,903,734,0,405,4,101
PRDM5_1_4457,2229,2853,2778,2193,3367,1823,396,1782,1669,2308,1443,138
8
PRDM6_1_4458,43,348,179,195,0,76,0,0,0,35,0,50
PRDM9_1_4459,638,968,1030,432,791,1141,1393,1037,456,317,487,713
PRMT6_1_4460,201,502,69,189,0,488,0,129,554,344,0,500
PRMT8_1_4461,2495,2306,3681,2906,2095,2994,2543,2566,3655,2497,3530,25
17
PRPF6_1_4462,1198,1391,1434,307,1924,550,583,373,603,910,2322,202
PYGO1_1_4463,5281,5365,6746,5790,8285,4005,8808,3688,4902,7251,4823,10
242
PYGO2_1_4464,1594,1590,1490,1711,708,833,1468,1121,1992,1493,3074,528
RAG2_1_4465,515,649,854,520,442,707,650,66,1875,453,612,335
RAI1_1_4466,680,172,466,59,800,500,529,267,1,417,165,49
RB1_1_4467,278,398,118,450,284,101,29,84,820,45,17,5
RCOR1_1_4468,823,766,1121,259,669,285,324,3,33,1335,582,68
RCOR2_1_4469,710,404,351,172,968,18,1484,305,906,604,1,155
RECQL4_1_4470,1114,1294,756,1231,2082,361,926,367,1896,834,1173,348
RELB_1_4471,829,581,586,875,609,799,328,763,77,236,929,525
REL_1_4472,2625,2916,2603,2870,2961,3412,4555,1331,3774,3229,6885,3010
RING1_1_4473,5388,3848,4215,4883,8822,4364,5621,5759,4480,5093,7093,73
11
RNF20_1_4474,2999,2681,3468,2830,2124,3127,2987,2387,3553,3067,3217,59
95

RNF25_1_4475,2159,2877,1919,2044,2096,2736,4089,2611,3073,2044,2679,44
38
RNF2_1_4476,827,631,543,624,507,682,983,120,291,636,302,1011
RORB_1_4477,347,130,135,91,126,2,398,102,264,198,198,423
RSF1_1_4478,4437,3316,5305,4744,5608,2744,7972,2485,5715,4123,4636,538
3
RUVBL1_1_4479,2459,1519,1620,1991,5773,3790,3037,2004,4883,2213,1222,2
485
RUVBL2_1_4480,510,777,1039,1036,545,1309,285,1364,1893,564,324,185
RXRA_1_4481,2493,2502,2833,2660,2211,2928,3890,2351,1962,1955,2808,268
9
RXRB_1_4482,1732,2282,1158,1583,2724,1693,3129,1241,858,2267,1462,1247
RXRG_1_4483,637,504,724,465,738,750,771,255,4,512,216,560
SAP18_1_4484,407,613,632,539,2310,536,18,216,277,1507,211,8
SCML2_1_4485,4456,4407,4093,5059,5009,5774,2100,2693,2204,3389,1805,81
54
SENP3_1_4486,573,295,156,336,607,1177,617,8,12,309,176,61
SETD1A_1_4487,404,142,330,63,270,125,65,0,657,172,2,563
SETD1B_1_4488,1090,1450,1620,1408,600,1480,561,872,1672,1870,1028,3359
SETD2_1_4489,4472,4512,5823,4910,4140,8887,5573,3754,3362,3931,7059,58
38
SETD5_1_4490,1214,1064,765,1204,1172,1071,551,853,724,623,202,1062
SETD7_1_4491,6695,6793,6236,7579,7484,7485,5438,4557,6460,6004,6528,71
66
SETD8_1_4492,378,252,61,350,440,933,597,118,236,383,1,1425
SETMAR_1_4493,697,856,274,744,1019,681,996,914,1480,1241,1114,1612
SF3B3_1_4494,338,361,126,313,157,451,67,112,542,643,135,367
SIAH2_1_4495,1159,984,490,993,441,600,330,282,621,882,1268,541
SIN3B_1_4496,1742,571,1214,1223,629,1084,2326,1319,1165,1348,1081,539
SIRT4_1_4497,1787,3364,3090,4029,1861,2965,4042,1240,304,2499,1493,292
2
SIRT7_1_4498,242,138,142,188,3,242,61,4,4,95,171,0
SLC2A4RG_1_4499,294,236,517,255,398,200,31,0,334,665,10,1
SMARCA5_1_4500,2323,1893,2278,2747,3703,2126,3794,1718,4581,3042,2773,
2075
SMARCC1_1_4501,1526,1674,1851,2406,3044,1028,2890,602,3332,2004,5458,1
946
SMARCD2_1_4502,393,578,549,234,453,277,327,186,676,259,535,13
SMARCE1_1_4503,387,238,337,349,1651,718,74,39,5,157,150,6
SMC1A_1_4504,4879,4732,4685,5011,6264,7400,4554,3606,7166,4774,3818,43
71
SMC1B_1_4505,2011,1003,1691,2265,1925,680,1198,1042,2207,1047,3473,775
SMC3_1_4506,1941,1614,1890,1802,1163,735,1695,629,571,2273,1178,300
SMCHD1_1_4507,2152,1513,2193,2548,2225,1279,2595,4160,3459,1975,4259,2
692
SMNDC1_1_4508,302,354,688,429,221,1127,177,791,1228,719,441,58
SMYD1_1_4509,396,426,459,560,388,470,837,100,18,758,259,581
SMYD2_1_4510,1918,1161,1887,1525,1365,1842,919,298,1673,1727,1222,433
SMYD4_1_4511,866,787,897,919,1970,1050,579,1163,554,1031,426,629
SMYD5_1_4512,1218,2033,1769,2130,2057,2077,3278,3181,314,1556,1722,285

3
SND1_1_4513,1100,709,510,995,388,612,521,902,282,379,180,311
SP140L_1_4514,2707,2311,2649,2221,2792,2417,2830,1550,2445,1047,2406,3
19
SRCAP_1_4515,519,341,555,154,339,402,1888,41,0,855,19,1
STAT5B_1_4516,1073,846,833,690,355,539,760,420,378,485,2885,2308
SUDS3_1_4517,338,482,158,230,401,266,4,126,934,441,392,210
SUPT16H_1_4518,198,220,275,520,178,335,97,433,659,108,203,2238
SUPT4H1_1_4519,1072,1192,2417,1322,1452,1374,633,624,796,541,126,713
SUPT6H_1_4520,2715,2645,2924,3556,3460,2767,3383,1238,2394,2749,3678,4
941
SUPT7L_1_4521,2510,2266,2234,3395,2298,1221,1438,4342,3041,3015,1119,4
314
SUV39H1_1_4522,1628,2285,1982,1309,2070,3283,1380,1118,1375,1314,1339,
1437
SUV420H2_1_4523,484,768,765,1005,252,911,2275,1004,1477,384,372,534
SUZ12_1_4524,1682,2084,2693,1731,2257,1879,605,966,1476,484,754,145
TADA1_1_4525,2160,1636,2103,1881,1436,2455,934,2296,1918,1966,1718,231
4
TADA2B_1_4526,596,287,651,647,537,781,260,404,1614,361,12,636
TAF10_1_4527,245,577,108,156,159,306,16,135,835,457,1335,203
TAF1L_1_4528,860,541,643,706,1053,167,305,705,797,477,64,666
TAF3_1_4529,3180,3686,4634,3727,3591,5002,2893,3367,4778,4258,2676,521
0
TAF5_1_4530,1051,1242,1384,1518,1645,1628,535,754,549,1228,1948,691
TAF6L_1_4531,889,814,1124,1016,1274,2227,2402,667,937,315,1690,755
TAF8_1_4532,510,521,438,254,239,28,1407,211,37,235,1039,65
TCF7L1_1_4533,462,231,232,746,484,638,775,68,1177,271,10,381
TDRD12_1_4534,11913,10598,13619,12058,13102,11238,12634,12483,10683,11
817,11265,7172
TDRD1_1_4535,6022,7254,8362,7786,7299,8821,3375,4168,4338,7592,4941,57
70
TDRD7_1_4536,701,867,860,1958,64,476,1041,699,1184,602,2781,832
TDRD9_1_4537,1123,703,931,495,377,462,499,928,761,1513,685,335
TERF2_1_4538,162,259,211,447,416,211,470,1578,0,219,25,107
TET1_1_4539,1050,1109,2041,1000,1710,593,899,1769,787,628,1059,1399
TET3_1_4540,53,27,520,323,91,168,0,6,6,19,2,1
TLX2_1_4541,182,174,39,61,342,55,112,251,0,41,269,38
TNRC18_1_4542,0,0,0,0,0,0,0,0,0,0,0,0
TRAF7_1_4543,523,419,524,443,248,566,389,718,916,465,364,3
TRDMT1_1_4544,964,547,805,639,589,424,737,485,397,703,244,234
TRERF1_1_4545,688,317,584,740,94,292,824,332,302,245,533,345
TRIM25_1_4546,3660,4542,6063,5236,6824,10140,7403,4840,3738,4770,3935,
6673
TRIM27_1_4547,3923,4176,4697,5900,3245,4611,2681,3272,5384,3512,4090,3
565
TRIM28_1_4548,272,59,162,140,704,217,492,51,159,137,29,182
TRIM66_1_4549,0,0,0,0,0,0,0,0,0,4,0,0
TRRAP_1_4550,1226,928,1098,1127,3140,1109,826,1093,943,790,419,638
TSG101_1_4551,636,653,1482,485,2032,1164,2391,484,799,711,1557,1409

TYW5_1_4552,2469,1728,2881,2684,1929,1825,2516,1501,1060,2211,2763,193
8
UBE2B_1_4553,1420,1214,1674,1518,3236,917,2148,1189,407,1484,2114,2437
UBE2N_1_4554,1714,1796,1815,1489,2894,2428,1326,917,3400,1538,3101,260
2
UBR7_1_4555,4751,4317,6058,5101,4952,6351,9132,3659,6060,5160,3646,316
4
UHRF2_1_4556,3205,2801,4111,2828,3362,2843,3666,3998,2240,3496,1057,90
4
USP22_1_4557,1313,1395,1199,1409,1382,678,529,1409,856,1067,1268,852
YEATS2_1_4558,1567,1655,1624,2031,1156,3791,231,803,3194,1561,1614,120
9
YEATS4_1_4559,2210,2408,1949,2368,2436,4223,2570,3917,1608,2161,1086,3
169
YY1_1_4560,2349,2213,2313,2186,3035,2034,812,2024,877,2748,2730,1598
ZAR1_1_4561,3620,3246,3170,3558,5333,4783,2837,3550,6263,3215,3371,382
4
ZCWPW1_1_4562,22174,22028,26154,25615,30738,32869,24877,23032,26609,24
194,13289,26957
ZCWPW2_1_4563,2490,1884,2661,2141,4743,1601,1106,1385,3421,2292,2795,8
94
ZFP57_1_4564,1839,2006,2201,2171,2838,1271,1978,1992,2340,2346,1354,14
00
ZNF541_1_4565,4722,3870,6106,4524,3869,5045,5435,3858,6801,5532,3982,3
820
ZNF85_1_4566,9330,8394,7346,10536,10966,10641,12428,4498,7503,7393,100
37,8502
AANAT_1_4567,116,196,120,163,132,81,26,40,121,254,902,9
AES_1_4568,446,425,433,397,1062,794,434,118,524,363,2,414
AIRE_1_4569,4043,3656,5039,4526,3264,6031,2194,2819,7555,3993,5682,550
8
AKAP1_1_4570,801,727,959,849,1931,939,152,1195,1752,493,405,342
ALKBH2_1_4571,384,340,554,697,742,1300,1017,592,348,553,429,119
ANKHD1_1_4572,1451,1764,1639,1673,1853,2350,3374,2731,749,1549,1084,23
02
ARID1A_1_4573,480,224,79,641,836,454,99,377,988,622,340,3
ARID1B_1_4574,303,379,483,491,796,76,446,252,682,348,41,188
ARID4A_1_4575,2228,1275,1874,1477,1446,1484,1375,2626,697,1334,881,162
8
ARID4B_1_4576,2404,1360,2084,2553,2278,1451,2420,1236,2295,1903,660,22
00
ARRB1_1_4577,291,273,529,362,418,323,317,484,509,333,254,607
ASH2L_1_4578,418,293,615,390,331,743,693,943,220,775,815,20
ATAD2B_1_4579,6550,5087,6975,8061,6813,8570,5003,6361,8576,5613,8341,5
216
ATAT1_1_4580,4267,4040,5557,3866,5301,5366,5904,5313,1269,2271,5780,37
19
ATRX_1_4581,4308,3244,4893,5700,3174,4018,6156,2732,4633,2888,4372,173
8
AURKA_1_4582,1505,903,1390,1083,936,2106,362,1427,734,1841,456,814

AURKC_1_4583,4152,3268,3539,3554,3938,3000,8244,2152,4881,3421,4483,25
73
BAZ1A_1_4584,9323,8799,9969,8441,11924,8987,11112,8163,7887,9988,5431,
9459
BCOR_1_4585,1129,761,1816,1063,326,650,529,220,3283,1554,876,5
BPTF_1_4586,2224,2503,1857,2553,2099,3026,2519,2908,2226,4495,1240,511
9
BRCA1_1_4587,1196,511,965,874,996,580,1412,1030,2570,335,779,1077
BRD2_1_4588,1146,1874,1824,1466,2831,2108,1357,1819,2226,909,1629,928
BRD4_1_4589,962,279,689,648,148,1316,442,113,491,119,79,257
BRD7_1_4590,1310,1599,1672,2021,2493,1455,2923,1943,2493,1901,4515,142
5
BRD8_1_4591,503,351,412,336,405,1246,927,151,81,400,686,188
BRD9_1_4592,2473,1713,2055,2358,1788,3120,3264,715,1318,2185,2814,715
BRDT_1_4593,152,323,339,453,463,1318,674,0,145,257,940,1200
BRPF1_1_4594,1163,1944,1331,2248,2808,1093,575,1283,1885,1277,570,341
BRWD1_1_4595,4153,3558,4137,3891,5729,6495,5146,4312,6468,3861,3996,25
86
C14orf43_1_4596,8550,7570,9423,6181,10413,6754,7013,6260,9439,8847,110
72,6248
CBX1_1_4597,2629,2938,3099,4078,1948,4568,3677,2169,2360,3503,3281,298
7
CBX3_1_4598,1465,1801,2137,2086,3099,2155,1218,3395,2176,552,679,4131
CBX5_1_4599,593,1476,777,621,452,2630,352,421,85,964,212,1138
CCNT2_1_4600,416,457,942,992,444,1131,1273,979,418,621,373,774
CDY1B_1_4601,4769,5089,7809,4118,4156,5586,6910,1820,7610,3690,8174,50
00
CDY1_1_4602,4769,5089,7809,4118,4156,5586,6910,1820,7610,3690,8174,500
0
CDYL_1_4603,1466,1299,1337,1611,1259,874,1516,1227,2029,2082,1321,1008
CHD2_1_4604,571,743,481,323,571,1155,856,456,2024,894,0,859
CHD3_1_4605,1323,815,683,851,1353,1099,923,807,480,463,841,904
CHD8_1_4606,1267,1513,1554,812,1761,1645,1088,999,1408,1662,801,3966
CHMP2A_1_4607,746,808,405,435,398,891,586,291,670,951,897,1711
CHMP5_1_4608,4104,3281,5067,2855,3013,6121,3990,4789,4518,4733,3200,37
36
COPS2_1_4609,1239,1361,1935,2754,3318,1587,3039,2369,199,2154,1866,210
1
CPA4_1_4610,1281,1088,1261,2364,1948,1304,1099,932,105,1092,3250,2720
CREB1_1_4611,546,302,824,665,378,261,467,947,176,617,872,4782
CREBBP_1_4612,331,80,245,172,487,739,3,18,6,809,968,129
CTCF_1_4613,4002,4570,4027,4675,5710,4040,5699,6236,2341,3810,5005,435
8
CTNNB1_1_4614,1112,656,1953,1736,209,1549,1157,2085,663,1148,578,328
CXXC1_1_4615,1305,983,1349,1278,2014,1927,178,1336,3771,1553,1195,812
CYLD_1_4616,17051,16275,15991,17334,18478,18364,19189,12716,17920,1220
0,17662,19049
DICER1_1_4617,2433,1498,2411,2472,5431,3368,3498,3927,1029,1063,3483,1
752
DIDO1_1_4618,565,128,47,468,21,368,56,344,157,4,0,100

DMAP1_1_4619,2555,2016,1946,2355,2550,1688,1471,2204,2731,1874,1391,15
20
DNAJC2_1_4620,437,138,200,858,194,382,371,173,90,514,625,1269
DNMT1_1_4621,1068,891,916,1281,66,2090,1925,723,41,344,320,830
DNMT3A_1_4622,738,982,734,1674,693,1468,250,1258,291,938,180,71
DNMT3B_1_4623,2257,1448,2374,1993,2740,2186,1199,2563,593,1752,1382,39
76
DNMT3L_1_4624,480,703,924,1117,123,1361,810,1545,156,1117,1256,1876
DPF1_1_4625,476,860,540,676,1124,297,469,832,536,1090,207,52
EED_1_4626,5654,4698,5528,6255,7611,10576,5928,6031,7541,5923,5298,942
1
EGR2_1_4627,645,418,214,679,1626,526,313,953,617,388,1113,1201
EHMT1_1_4628,778,822,900,932,38,1313,508,1050,1491,872,1052,540
EHMT2_1_4629,867,688,350,292,279,1057,1508,189,1698,287,625,696
ENO1_1_4630,1837,1164,1159,1484,892,1981,3303,1355,644,1380,1119,723
ESR1_1_4631,1638,1809,1615,1423,1935,3589,2743,652,1204,1310,2145,926
ESR2_1_4632,1736,1899,1309,3248,1995,1247,2625,348,923,814,3486,2495
ESRRG_1_4633,422,763,439,389,480,1426,457,491,1128,949,1617,202
EZH2_1_4634,2032,1215,1589,1273,1492,1448,454,297,989,977,278,2432
FBXO11_1_4635,0,0,0,0,0,0,0,0,0,0,0,0
FMR1_1_4636,7785,5974,6282,7825,11903,9008,4585,4292,5056,5816,10029,6
512
FXR1_1_4637,1284,979,2362,2107,3364,1244,632,3601,1351,1346,1717,5180
GFI1B_1_4638,512,241,335,202,601,1101,393,5,974,587,73,0
GMEB1_1_4639,818,601,837,631,585,1003,476,702,184,898,841,682
HDAC10_1_4640,826,354,635,609,48,55,112,160,249,236,388,124
HDAC11_1_4641,305,217,84,221,197,23,0,1,131,48,3,439
HDAC5_1_4642,1262,1330,1490,988,2357,2228,2791,1083,580,2384,80,1017
HDAC7_1_4643,415,680,598,527,942,826,737,1106,0,1409,661,83
HDAC9_1_4644,859,644,918,603,205,463,972,269,838,752,298,266
HDGF_1_4645,598,251,387,957,738,1319,873,824,945,32,700,221
HDGFRP2_1_4646,241,103,252,218,37,152,5,82,0,463,0,37
HLTF_1_4647,1199,984,639,614,564,1630,450,635,1319,576,200,573
HMGA1_1_4648,67,46,55,175,4,129,51,32,96,9,10,2
HMGN3_1_4649,504,764,584,650,593,232,261,119,2157,884,1148,263
HNF4A_1_4650,1308,936,1201,786,1913,1349,1069,696,563,1140,2,1389
HPSE2_1_4651,493,878,616,775,1887,796,190,571,83,392,6,1771
HR_1_4652,21,8,26,8,22,12,707,6,450,0,3,24
ING1_1_4653,145,175,82,306,549,58,5,78,312,80,0,737
ING4_1_4654,907,1168,1690,874,1810,1853,1515,519,1160,768,329,312
INTS12_1_4655,754,367,468,792,539,1291,24,344,258,653,1080,309
IRF4_1_4656,2539,2254,2157,2903,2993,2952,4130,2358,1079,1868,1685,234
5
JMJD1C_1_4657,3001,3851,3960,3269,5611,4341,3813,2378,1453,1648,5493,5
331
JMJD4_1_4658,81,59,488,237,299,532,0,837,43,535,37,0
JMJD6_1_4659,2113,1615,2130,3183,1088,2367,2231,1003,2742,2744,842,154
7
KAT5_1_4660,739,733,539,701,615,1756,1168,318,859,516,119,1131
KAT6A_1_4661,5674,6203,5857,6409,4381,5725,6329,5779,3666,5884,6134,10

KAT7_1_4662,1825,1012,1457,504,493,1349,710,294,1049,1519,1445,1429
KAT8_1_4663,748,409,496,663,724,1006,378,808,141,544,287,326
KCTD1_1_4664,863,505,383,872,435,284,555,779,67,666,859,1324
KDM1A_1_4665,3163,2275,2180,2325,3060,4687,3306,1993,1018,1523,4057,37
30
KDM2B_1_4666,7020,6521,5533,6442,6560,8070,4003,4812,8406,8509,3070,66
41
KDM3A_1_4667,2768,2827,2862,2022,1735,2114,3435,965,1842,2433,4020,488
8
KDM4C_1_4668,499,411,475,335,299,409,189,340,195,288,607,335
KDM5C_1_4669,5864,4093,6587,4739,5170,6627,4967,2831,6285,4479,3491,92
63
KDM5D_1_4670,6647,6194,7041,5222,5227,7906,6327,5321,6501,6537,3247,10
276
L3MBTL1_1_4671,1751,1573,1514,1694,711,1169,2990,1144,2269,2296,1135,5
06
L3MBTL3_1_4672,4891,3029,4410,3696,5497,3902,6152,3944,2800,3498,3460,
1537
LBR_1_4673,3352,2769,2856,2498,3867,3109,558,3934,2100,3315,2206,2060
MAP3K12_1_4674,1117,1360,1924,1404,616,1992,1019,607,1964,1194,889,474
8
MBD1_1_4675,2475,2457,2892,3170,2426,3621,3168,2053,1954,2489,2823,208
7
MBD2_1_4676,1397,1038,994,812,1378,1761,1222,632,654,1029,1776,3820
MECOM_1_4677,446,1048,686,1140,365,1040,413,498,211,663,1332,799
MECP2_1_4678,293,637,686,738,57,491,596,0,2579,513,190,2013
MEN1_1_4679,0,0,0,0,0,0,0,0,0,0,0,0
MGEA5_1_4680,3554,3275,4903,4424,5842,4066,3150,3144,6766,2998,4419,24
38
MIB2_1_4681,796,1235,986,1713,1314,1364,1075,375,544,929,1517,1461
MIER1_1_4682,1310,1369,1097,1020,1359,3684,1359,989,2964,726,1612,1076
MINA_1_4683,5618,4213,5820,5058,5050,2501,5256,4118,6190,2407,6005,504
2
MLL5_1_4684,2835,2360,2654,2056,4917,3452,1695,1469,2557,1883,2809,467
4
MLL_1_4685,1345,336,981,1230,1377,725,757,434,792,604,1780,841
MORF4L1_1_4686,1253,995,1109,578,1938,672,514,975,1848,552,165,990
MSL3_1_4687,333,257,488,224,114,278,0,277,0,723,889,526
MTA1_1_4688,886,776,940,502,678,1193,404,127,1062,1289,360,941
MTF2_1_4689,1264,1100,2071,1436,1106,1244,2683,580,561,959,228,2218
NAA60_1_4690,1284,1319,1711,2474,1397,1396,1992,1729,453,1523,920,289
NAP1L1_1_4691,2717,2236,2302,2387,2757,4005,1393,712,354,2828,1585,260
8
NAT10_1_4692,463,302,382,481,562,339,128,6,56,68,7,425
NCOA1_1_4693,830,503,594,646,800,1204,343,366,383,161,1198,760
NCOA3_1_4694,1526,933,1346,652,1821,714,895,296,581,1206,2545,1041
NCOA4_1_4695,1364,1964,2153,705,1089,2752,1405,1280,830,477,890,575
NCOR1_1_4696,737,462,561,736,1779,62,229,321,116,872,1246,2174
NCOR2_1_4697,998,948,689,821,19,799,1091,120,1262,513,916,1108

NFAT5_1_4698,1803,1952,1376,1329,918,1340,2465,1310,1345,1607,978,1426
NFATC1_1_4699,450,375,128,197,52,48,1241,950,632,253,1163,18
NFATC3_1_4700,436,979,1111,852,595,1974,649,1990,74,64,483,46
NFATC4_1_4701,212,287,162,262,248,132,469,840,703,334,14,154
NFKB1_1_4702,5832,6184,5545,5876,7345,8497,5126,5390,7791,3452,4961,86
08
NFKB2_1_4703,71,487,572,287,98,122,1563,384,77,1131,1381,115
NPM1_1_4704,1451,1624,2013,1700,2259,1932,2504,772,1189,1206,5784,523
NR1D2_1_4705,4315,2306,4437,3506,2714,4810,5248,4154,3283,2478,3938,28
39
NR1H3_1_4706,1318,1398,2392,1633,2781,2671,2330,1301,2617,1308,60,2138
NR1H4_1_4707,2756,2513,3450,2557,2439,3169,4629,2582,3355,1738,4208,25
20
NR1I2_1_4708,1535,1742,1559,1794,2638,1277,2531,2036,703,1525,2657,451
1
NR1I3_1_4709,3264,3041,3930,4261,5074,3262,6310,1640,3620,2117,5358,34
31
NR2C1_1_4710,1341,1466,1538,1671,1798,2395,935,2342,3693,2822,662,1631
NR2E3_1_4711,3718,3886,3844,3797,5361,2941,2358,6643,3943,1966,4415,28
39
NR2F2_1_4712,1143,1800,1605,1176,1326,1994,1023,2463,513,1270,438,1661
NR3C1_1_4713,1866,2081,1411,1592,2845,2709,3291,2654,2843,1754,1503,87
6
NR3C2_1_4714,1230,749,963,1045,781,852,2155,73,2034,559,716,689
NR4A1_1_4715,25,338,114,324,51,104,1,5,0,294,872,0
NR4A3_1_4716,5599,3991,6303,5108,5734,6684,4810,2805,3681,5394,5514,43
12
NR5A2_1_4717,836,692,976,1155,909,1374,329,974,1188,589,354,154
NR6A1_1_4718,3358,3452,5003,4950,4402,4788,4651,3256,6531,3275,5072,43
57
NSD1_1_4719,1460,1255,2293,868,2185,577,1952,1377,755,714,1685,615
PBRM1_1_4720,426,1535,530,1001,1660,802,874,1188,46,1301,1890,330
PCGF6_1_4721,78,80,617,353,176,3,381,1,2075,83,1042,20
PGR_1_4722,2210,2088,2533,1612,2307,2398,1704,798,1513,1974,3171,4114
PHC2_1_4723,557,175,200,266,69,473,390,727,445,427,1074,25
PHF10_1_4724,10060,7770,10240,9722,13726,10866,8764,5190,5780,7888,912
9,7430
PHF11_1_4725,336,240,200,99,61,133,943,59,215,35,0,756
PHF12_1_4726,446,343,173,336,24,111,506,0,952,178,2,273
PHF16_1_4727,802,507,992,701,643,2114,780,206,1223,858,1659,1248
PHF17_1_4728,851,810,1313,1064,1177,1474,1059,956,734,1465,49,2005
PHF1_1_4729,714,981,417,866,303,280,2464,752,1346,489,143,1044
PHF20L1_1_4730,420,542,536,492,375,466,780,436,939,1333,410,21
PHF21A_1_4731,1036,846,1121,906,1279,1801,1377,779,1255,1166,234,765
PHF21B_1_4732,283,401,203,121,28,833,401,109,36,1023,929,1550
PHF6_1_4733,2670,2222,3431,2898,3262,1293,2047,2663,3648,2998,2548,107
4
PHF7_1_4734,784,1046,743,1291,1734,910,499,332,1132,1957,1507,733
PHF8_1_4735,5933,4066,6971,3973,6727,5226,4927,5195,4514,3381,6493,307
9

PIAS2_1_4736,1755,3362,2862,2078,1783,2649,1309,1358,2227,2842,3169,44
43
PICK1_1_4737,601,617,743,1201,298,410,84,0,399,295,0,5
PIWIL2_1_4738,651,174,959,900,325,673,1515,826,823,485,69,1750
PML_1_4739,191,201,276,177,389,394,82,151,0,653,848,164
POLR1B_1_4740,969,845,1596,498,829,858,237,83,830,590,81,174
PPARA_1_4741,488,136,423,251,74,26,163,13,74,679,73,4
PPARD_1_4742,489,371,340,145,46,27,0,368,0,219,351,153
PPARG_1_4743,559,636,839,578,172,133,641,1093,439,360,1075,661
PRDM10_1_4744,256,388,560,445,451,595,350,145,2458,272,341,1736
PRDM15_1_4745,503,714,821,888,1386,621,166,962,1316,610,627,548
PRDM16_1_4746,849,588,640,937,568,1200,2424,324,1194,337,103,773
PRDM1_1_4747,1086,1079,1120,1779,2144,1239,1504,1019,254,1330,157,257
PRDM2_1_4748,768,705,607,443,554,250,1485,627,691,654,844,1748
PRDM7_1_4749,1123,1020,1615,937,2557,2366,1218,605,461,1135,236,274
PRDM8_1_4750,4377,4624,4686,4353,3502,5185,3881,2962,5498,2548,5352,36
16
PRMT1_1_4751,806,392,630,610,273,1112,298,873,627,819,135,1209
PRMT2_1_4752,3334,2349,2540,2402,2610,2484,2017,909,3091,2228,3725,316
7
PRMT3_1_4753,1026,1445,1048,1116,1069,988,2690,1362,877,1356,1703,1513
PRMT5_1_4754,495,437,256,185,394,794,789,55,106,621,176,1034
PRMT7_1_4755,1031,304,770,1134,2178,739,680,76,916,200,745,301
PSIP1_1_4756,2838,1628,2917,2315,3276,2262,3821,2182,1134,2276,2096,26
77
PSMC5_1_4757,776,990,798,922,192,899,19,1174,1828,932,263,334
PWWP2B_1_4758,256,767,1145,450,1267,456,996,1238,270,93,128,373
RAD54B_1_4759,1728,1184,790,1017,1899,2354,3003,1591,1841,753,1791,125
0
RAD54L_1_4760,1606,1988,2399,2036,2099,2259,2311,1548,1824,1543,2790,3
095
RARA_1_4761,1309,876,1056,573,1748,1528,553,293,1945,1687,102,143
RARB_1_4762,3495,2577,4596,3202,4858,3962,5617,3175,4441,1770,3628,375
7
RARG_1_4763,1183,1367,208,432,964,1107,690,2062,569,219,262,80
RBBP5_1_4764,1894,1025,1909,1777,4237,2198,1947,1439,1059,1015,2118,89
4
RBCK1_1_4765,1829,1308,1699,2064,1121,1354,1544,1572,3607,1922,2482,25
43
RBFOX2_1_4766,2171,2511,2017,2866,2043,2661,1875,3160,3382,2087,2569,3
540
RCC1_1_4767,599,226,893,625,871,183,2143,37,42,248,1287,40
RCOR3_1_4768,1176,1265,1330,1345,3186,1358,1478,690,1077,1964,1640,164
1
RECQL5_1_4769,1489,1179,1724,1619,3433,1867,2225,956,1623,1311,1003,15
75
RECQL_1_4770,8519,7997,7712,9210,9884,8686,13590,9090,9077,8083,7256,1
0300
RELA_1_4771,1534,2111,2289,1751,2705,1219,3259,2609,1829,1739,1397,278
8

RERE_1_4772,3240,3538,4756,4132,3866,5531,5695,3773,5737,3962,3653,313
8
RFC1_1_4773,2247,1637,1899,2156,1279,1727,1152,1761,987,2996,2024,2200
RNF14_1_4774,550,785,775,575,1395,575,738,237,1,577,710,39
RNF17_1_4775,5489,6415,7251,5827,6628,4144,8050,4209,7377,5822,3451,66
65
RNF40_1_4776,152,172,286,252,128,171,84,67,171,484,402,105
RNF8_1_4777,3565,2576,2637,2582,6016,2300,2623,1926,1471,2993,2325,184
3
RORA_1_4778,1393,1582,1810,1728,2118,1281,665,417,1972,1218,2058,1023
RORC_1_4779,1226,1135,866,1164,1743,453,562,897,1610,1031,2318,724
RPH3A_1_4780,1870,1270,1043,1455,1268,1770,2090,2763,1597,1336,11,476
RPS6KA5_1_4781,1557,1614,2162,2139,1843,1805,1594,950,135,1576,3476,52
3
SATB1_1_4782,3132,3637,4642,3585,3312,2920,4557,4108,4583,4154,2836,42
55
SATB2_1_4783,531,609,163,533,535,760,259,1117,1358,505,15,12
SCMH1_1_4784,731,422,559,806,368,824,193,1372,1413,554,0,679
SET_1_4785,680,526,416,857,988,396,47,366,228,1522,15,500
SETD3_1_4786,2353,2571,3417,3101,2572,2222,4622,3570,3611,2519,2953,81
6
SETD4_1_4787,1099,554,486,890,1218,877,666,302,724,1345,505,564
SETD6_1_4788,1561,1170,1285,734,1089,1432,1243,864,1648,1602,508,1111
SETDB1_1_4789,90,125,170,11,264,39,0,0,0,18,0,0
SETDB2_1_4790,4434,3941,4253,4476,4367,4053,5355,2443,2652,2579,3380,1
786
SFMBT1_1_4791,2454,2448,2306,3159,3535,6061,1913,1534,1620,2267,2246,7
592
SFMBT2_1_4792,2263,1926,2203,2468,1404,849,1805,4058,1013,1498,2831,22
94
SHPRH_1_4793,1595,858,1162,1194,1976,1525,1423,1598,2382,1257,987,2792
SIN3A_1_4794,2294,2571,2648,2910,2049,1929,1929,1828,1844,3631,841,425
9
SIRT1_1_4795,1403,1907,2393,1664,1105,2639,2528,1299,1850,1576,2269,88
2
SIRT2_1_4796,1824,1278,2029,1215,1253,1552,2314,606,2215,1967,1732,808
SIRT3_1_4797,278,248,333,175,197,222,184,94,420,106,102,44
SIRT5_1_4798,4134,3137,4094,4404,7264,5260,5781,2494,8469,2738,7625,45
88
SIRT6_1_4799,366,338,637,886,407,361,159,334,434,293,379,1998
SLC38A1_1_4800,406,639,850,659,992,630,1017,255,412,1037,360,2900
SMARCA1_1_4801,1298,1115,1176,1185,137,1734,1796,1048,169,813,217,1227
SMARCA2_1_4802,544,543,1050,746,433,787,914,545,929,253,849,26
SMARCA4_1_4803,1077,1102,1610,1334,1600,1275,132,1646,2477,1559,457,54
0
SMARCAD1_1_4804,4809,4427,4129,4643,5639,4434,8582,4372,4577,4175,3828
,2219
SMARCAL1_1_4805,947,585,696,1472,966,450,869,3080,1097,526,1515,611
SMARCB1_1_4806,1960,2608,2561,2944,1541,3268,1191,4680,1406,2216,1517,
1705

SMARCC2_1_4807,1022,1332,1024,1387,1769,1469,1319,459,2161,2090,129,57
1
SMARCD1_1_4808,2694,2382,2642,3168,2670,3108,2286,1675,3340,2456,1848,
4459
SMARCD3_1_4809,1388,1751,2040,1303,1380,1892,1983,382,100,1419,1503,18
90
SMC2_1_4810,2478,2144,2159,1934,1569,2790,1600,2036,1974,1176,964,1845
SMC4_1_4811,476,329,353,466,90,1207,456,152,465,412,184,169
SMN1_1_4812,4363,4281,4634,5540,4103,3182,4393,5287,5827,4049,3614,574
7
SMN2_1_4813,4363,4281,4634,5540,4103,3182,4393,5287,5827,4049,3614,574
7
SMYD3_1_4814,1907,1029,1257,1337,3619,2087,2891,868,1196,1540,842,1722
SP100_1_4815,2237,2258,3505,3831,2285,2722,2669,2904,3265,3507,1157,14
78
SP110_1_4816,2411,2242,2566,3374,3072,3463,3100,1170,1834,2358,1313,12
85
STK31_1_4817,1060,1105,1408,1522,2449,1353,1441,711,1354,1293,1321,624
SUPT3H_1_4818,883,1075,1207,948,1195,809,1081,879,2926,943,613,1906
SUPT5H_1_4819,626,345,678,133,312,584,633,7,1230,24,108,30
SUV39H2_1_4820,4461,4534,5224,4827,3824,5521,6292,2628,8035,4615,3412,
4045
SUV420H1_1_4821,815,101,450,158,1,5,0,1,0,5,792,10
TADA2A_1_4822,1530,1465,1690,1691,2419,1711,464,1305,1614,852,141,1455
TADA3_1_4823,445,659,365,802,441,144,257,933,813,220,72,2144
TAF12_1_4824,6626,7367,7613,7236,6574,5092,10345,8426,8147,5075,3060,4
742
TAF15_1_4825,2998,2803,2632,2776,3382,2486,2726,3133,2415,2810,3029,61
67
TAF1_1_4826,2036,1382,1586,926,692,1072,1540,1154,1254,1398,959,1022
TAF5L_1_4827,1556,1232,1965,1189,859,1081,1868,590,1554,1431,1989,714
TCF19_1_4828,8394,7976,7894,8143,9113,8607,9063,4093,7672,9031,8728,90
80
TCF20_1_4829,2612,2454,2503,2975,1834,2678,2271,3011,3848,2286,3420,37
79
TDRD10_1_4830,311,312,270,265,260,106,557,70,870,167,474,52
TDRD3_1_4831,889,667,638,630,1862,309,1260,268,956,973,980,127
TDRD5_1_4832,893,744,1223,904,1099,1485,459,809,463,876,158,329
TDRD6_1_4833,2319,2032,2907,1665,2860,2752,3638,2012,1788,1954,2812,45
86
TDRKH_1_4834,517,345,392,420,1758,412,945,290,471,873,873,402
TERF1_1_4835,1552,1367,1719,1359,2661,2585,1535,1591,1675,1211,2502,31
09
TET2_1_4836,1777,851,1323,1478,1453,599,3092,1170,1227,768,1738,939
THRA_1_4837,557,484,367,1178,806,239,642,499,29,229,2,421
THRB_1_4838,684,548,250,588,281,1103,335,63,962,237,875,217
TP53BP1_1_4839,2468,2791,1744,1821,3657,2813,3242,1322,1665,1963,2702,
2610
TP53_1_4840,1061,671,198,831,805,1118,239,590,394,220,354,728
TP73_1_4841,459,541,465,1105,574,202,55,87,163,387,106,1048

TRIM24_1_4842,4084,3162,3319,2902,2891,2658,2870,2064,2027,1326,1069,2
945
TRIM32_1_4843,4845,3031,3977,5845,3854,5138,4651,4616,4627,5997,4247,3
969
TRIM33_1_4844,2214,2343,2688,2748,2268,3176,3429,1259,897,2129,2653,13
96
UBE2A_1_4845,883,1273,1019,648,2587,775,1878,149,594,438,782,1089
UBE2E1_1_4846,1381,1077,1412,1288,524,1692,1259,668,721,1291,765,1845
UBE2I_1_4847,1728,1410,1389,1464,2261,2132,925,1496,2639,2212,1560,145
3
UBE2K_1_4848,3246,2078,2092,2657,1639,3552,1329,3233,1574,2397,1477,19
31
UBE2V1_1_4849,698,765,1281,461,513,1435,1161,1613,1243,1515,244,1852
UHRF1_1_4850,1338,1093,1168,1671,1977,856,469,2287,3645,1540,2276,2044
USF2_1_4851,2352,1882,2247,2147,2175,2288,3536,2400,1719,2029,1127,109
0
UTY_1_4852,4070,3731,3211,4118,4789,4481,4447,3305,3744,2909,2137,4614
VDR_1_4853,1299,782,821,1166,971,869,219,395,6,901,145,694
WDR5_1_4854,1647,1702,1474,1902,1172,2456,2699,2766,3525,1509,2290,418
WHSC1_1_4855,1847,1769,2241,1004,2087,1746,1264,1318,2060,2498,2558,28
65
WHSC1L1_1_4856,1701,1863,2427,1779,2030,1949,2726,1304,1817,1407,762,1
366
WRB_1_4857,1140,1735,1445,2233,1503,2051,1224,827,124,1988,1381,917
ZGPAT_1_4858,1288,1364,1159,1345,574,2114,411,1753,728,1450,2306,2439
ZMYND11_1_4859,935,825,1522,1021,1308,1087,329,1245,372,392,1557,917
ZMYND8_1_4860,258,164,206,190,1674,24,455,133,25,98,44,57
ZNF451_1_4861,1664,1394,1906,995,1321,2341,2246,182,2487,1095,328,3822
ALG13_1_4862,1814,2153,2920,2249,2471,2964,1997,1718,2952,3025,1390,37
91
ASXL1_1_4863,2725,2723,2357,1973,2277,3948,1723,1318,845,2638,1321,134
6
CBX2_1_4864,473,188,32,541,537,870,795,8,1,321,621,556
HDAC8_1_4865,859,1207,771,1044,2146,1085,279,476,3112,818,19,2261
ING3_1_4866,6578,6242,5914,6964,6445,4670,4031,4789,3441,7032,3877,384
0
MLLT10_1_4867,2419,2600,3468,2998,2355,3603,2518,1267,2968,2679,4310,1
357
PHF19_1_4868,995,1123,709,918,2991,910,2281,612,774,716,663,2798
RBM14_1_4869,781,378,236,459,79,432,1366,678,212,773,1629,29
SP140_1_4870,14180,15110,13680,15050,14212,15025,15607,15062,6894,1234
2,13603,14882
TAF9_1_4871,982,419,560,754,297,623,699,902,300,1193,802,1108
TAF9_1_4872,266,204,60,201,34,216,250,368,137,119,477,426
AHR_1_4873,3434,4244,3897,4355,2649,6127,6034,1150,3608,3698,5782,3381
ALKBH1_1_4874,1935,1459,2228,1447,2283,863,447,1138,617,934,1512,703
ALKBH3_1_4875,466,236,222,816,632,303,249,220,286,640,1459,300
ARID2_1_4876,820,1055,2089,1153,651,735,2124,2414,698,1305,337,29
ASF1A_1_4877,1986,2245,1772,1431,1409,2639,1203,1215,5224,829,866,707
ASF1B_1_4878,7663,7484,7024,7171,8472,8834,7647,6101,9614,6216,9201,54

16
ASH1L_1_4879,720,745,610,752,314,1019,325,1228,1172,593,224,72
ASXL2_1_4880,373,155,268,182,171,153,927,513,442,580,59,1067
ASXL3_1_4881,1554,1267,1626,1588,2635,871,1509,613,431,135,2791,802
ASZ1_1_4882,3571,2930,3323,2985,4259,4130,3717,3015,2387,2451,2703,470
7
ATAD2_1_4883,793,450,383,1054,128,947,732,256,993,659,148,442
ATF7IP_1_4884,1142,1071,766,707,983,2267,205,111,783,1149,1448,1400
AURKB_1_4885,694,376,417,423,1641,321,175,468,35,1264,1,1
BAHCC1_1_4886,362,522,468,380,777,340,2012,272,384,824,526,943
BAHD1_1_4887,1491,820,964,884,827,352,1386,1090,2248,790,1244,2258
BARD1_1_4888,5294,4999,5218,6586,6077,7025,3275,4792,5066,3318,5231,58
05
BAZ1B_1_4889,711,794,1178,873,497,1487,1164,147,1614,1085,1025,924
BAZ2A_1_4890,2377,1877,2572,1440,2404,2839,2508,2052,658,1276,1969,270
2
BAZ2B_1_4891,1219,891,1636,1517,1835,1869,2603,1298,543,1056,973,1124
BLM_1_4892,5610,5615,5962,6182,8883,3212,3154,5067,4646,5294,4534,2612
BMI1_1_4893,4290,4210,6509,5702,3029,7064,8220,3874,5432,6890,1221,570
9
BRD1_1_4894,142,149,161,263,17,559,13,86,2,266,28,134
BRD3_1_4895,2516,2522,3263,2220,3027,2139,2048,3722,1131,2407,2565,541
BRPF3_1_4896,957,1121,1072,1386,745,962,284,1231,338,295,1007,945
BRWD3_1_4897,1359,784,966,976,301,486,1454,1159,29,475,2370,1157
C14orf169_1_4898,4257,2250,3377,3811,4668,5027,6004,3497,3795,2150,296
8,3836
C20orf20_1_4899,2833,3003,3641,3639,2231,3255,3280,2895,3182,2789,3137
,3870
CALR_1_4900,510,319,399,764,535,599,45,196,778,601,45,715
CARM1_1_4901,642,332,682,243,678,504,134,292,713,966,1736,113
CBL_1_4902,108,61,129,541,416,12,170,87,113,7,4,66
CBX4_1_4903,1578,1793,2044,2514,2383,2399,784,2855,1501,1847,2502,1196
CBX6_1_4904,1853,1255,716,1197,1417,1452,955,1337,3192,763,1079,826
CBX7_1_4905,957,1008,2306,1066,2216,382,694,260,700,1678,1702,1209
CBX8_1_4906,2148,2869,1814,1293,2574,2204,2125,2066,1348,2594,1293,205
3
CCDC101_1_4907,34,532,243,251,210,0,60,37,356,328,11,0
CCNE1_1_4908,1348,407,920,966,825,971,2432,107,25,1060,58,336
CCNT1_1_4909,94,158,119,313,298,100,101,150,0,169,0,279
CDC73_1_4910,7704,7284,7138,8826,7400,8194,4882,5734,10428,7059,4308,8
582
CDK9_1_4911,1882,1885,1520,2097,2266,1065,2876,2219,2524,1432,1392,607
CDY2A_1_4912,68988,63636,73874,67580,85858,84090,88424,58494,72450,620
34,60779,85591
CDY2B_1_4913,68988,63636,73874,67580,85858,84090,88424,58494,72450,620
34,60779,85591
CDYL2_1_4914,1219,1078,1323,884,1174,1985,1359,2705,1366,1567,534,1336
CECR2_1_4915,314,244,265,384,367,252,1275,252,570,926,10,295
CHAF1A_1_4916,1044,1080,1075,1086,4276,892,3576,1257,2011,1107,175,927
CHAF1B_1_4917,858,1334,976,800,987,748,1098,644,1538,329,597,938

CHD1_1_4918,1533,1906,2173,2317,2377,2995,1356,1243,1008,1354,2743,401
7
CHD1L_1_4919,542,286,321,556,1048,63,45,299,67,686,206,290
CHD4_1_4920,564,394,558,521,708,273,1,657,7,271,0,70
CHD6_1_4921,1945,1979,1592,1952,1902,3605,2305,606,1552,2230,1170,4200
CHD7_1_4922,5669,5603,5945,7577,7164,10739,7884,7730,6454,3567,6889,52
90
CHD9_1_4923,580,968,1522,415,953,1508,927,536,14,562,2481,1412
CHMP1B_1_4924,1841,1307,1802,1424,2397,1713,902,428,1344,1797,1752,857
CHMP4B_1_4925,1568,808,1502,1020,858,1739,499,2170,2382,794,1769,1061
CHMP4C_1_4926,866,1044,791,556,1143,1074,1578,182,956,251,527,232
CHRAC1_1_4927,1167,1016,1301,1019,350,1271,3200,556,925,950,1326,158
CLOCK_1_4928,283,863,340,604,298,216,169,356,906,395,181,418
COPS5_1_4929,2596,2099,2505,2513,3406,3757,2784,3338,1889,4553,2439,34
08
CRAMP1L_1_4930,1220,1214,1545,1603,2177,2061,1837,600,909,1660,989,887
CTCFL_1_4931,540,932,373,427,1762,399,1676,1,475,545,120,385
DEAF1_1_4932,533,282,812,1273,735,484,186,156,599,528,574,8
DNAJC1_1_4933,1240,693,926,974,2144,1313,1775,1169,249,1493,2067,1421
DOT1L_1_4934,189,241,119,183,554,1,27,0,0,34,0,56
DPF2_1_4935,14,47,146,24,323,10,318,1,0,0,0,12
DPF3_1_4936,3407,2367,3243,3163,3952,3429,3514,2342,4978,2834,1284,224
9
EIF4B_1_4937,2561,1859,3268,2304,2793,2078,2227,3323,893,2062,1540,150
3
ELP3_1_4938,2981,3224,2109,1792,2447,3194,3135,1056,2421,2923,2179,261
EP300_1_4939,2304,2826,2012,2328,3202,2492,2674,1005,1509,2926,1917,38
64
EP400_1_4940,269,238,414,320,411,920,2091,1961,921,74,399,1
EPC1_1_4941,2881,2633,3069,3558,3693,3093,3168,3655,4433,2357,4093,172
9
EPC2_1_4942,3721,3886,3642,4821,2396,4285,1941,4344,6057,3561,2598,254
5
ERCC6_1_4943,11558,12694,12030,10654,13667,11913,13722,9172,8698,10912
,11474,9173
ESRRA_1_4944,0,0,0,0,0,0,0,0,0,0,0,0
ESRRB_1_4945,69,44,14,112,119,37,52,3,3,44,0,0
FANCM_1_4946,544,481,214,336,30,0,756,9,2,310,6,3
FBXL19_1_4947,1310,1142,961,3151,2922,2110,1680,2249,1372,867,936,1377
FOS_1_4948,1934,1455,1601,1951,2513,2314,2256,3286,790,1535,2856,806
FTO_1_4949,2729,3432,2674,2500,3017,2818,2260,1668,2303,3434,2200,3476
FXR2_1_4950,2038,2811,2645,2330,2968,2369,1981,3031,3247,2040,1208,337
7
G2E3_1_4951,2723,1720,2399,2141,3011,1979,2637,1171,4329,1514,1859,113
9
GATAD2A_1_4952,413,316,377,471,1722,413,1002,516,607,343,1,1021
GLYR1_1_4953,724,947,496,709,528,259,336,186,2157,784,272,668
GMEB2_1_4954,1041,914,1148,990,1502,1667,599,980,14,920,514,368
GSG2_1_4955,173,3,254,42,234,253,96,0,0,77,336,171
GTF3C4_1_4956,1076,1384,887,1919,3177,1249,1437,489,1251,1945,516,1729

HAT1_1_4957,1661,940,1511,1318,1104,1727,2635,1949,1646,1734,1649,3619
HDAC1_1_4958,107,1,3,35,0,133,0,0,0,0,166,13
HDAC2_1_4959,2819,3108,3142,3066,4246,5071,2428,2116,4943,3834,893,404
5
HDAC3_1_4960,327,706,698,382,1828,251,1901,147,412,346,88,1469
HDAC4_1_4961,187,314,312,300,314,101,311,32,159,497,1,291
HDAC6_1_4962,588,749,74,170,274,20,123,215,41,108,1141,581
HDGFL1_1_4963,302,184,201,195,139,188,1481,38,283,616,194,276
HDGFRP3_1_4964,757,273,1122,671,128,805,925,38,38,389,35,0
HELLS_1_4965,2213,2257,2419,2615,2809,1880,2444,2072,1079,1995,2148,99
4
HEMK1_1_4966,2012,1827,2601,1481,2458,1047,2782,1216,1467,1968,427,209
3
HIF1AN_1_4967,2397,1508,1936,2222,2613,2002,2980,1051,3573,1280,1962,2
875
HIRA_1_4968,2109,1487,1240,1809,1623,1554,3741,1530,3355,1475,1487,123
7
HMG20B_1_4969,1308,1097,1171,1017,910,1174,733,1088,806,2053,1649,400
HMGN5_1_4970,1982,1918,2342,2221,2981,3006,3328,1420,1560,2255,1928,33
33
HNF4G_1_4971,734,735,917,711,2216,346,1168,142,920,1346,1079,456
HSPBAP1_1_4972,402,651,976,531,452,695,504,935,136,753,289,561
HUWE1_1_4973,895,700,874,915,719,1243,1080,776,734,719,593,1375
ING5_1_4974,90,2,34,58,386,0,2,0,14,8,0,0
INO80_1_4975,870,568,508,827,866,484,528,846,307,942,1828,314
JARID2_1_4976,7064,8835,8215,8305,9207,8087,4993,6410,9728,5327,10578,
7629
JHDM1D_1_4977,3040,1922,3071,2812,3194,3455,1280,3074,3631,3849,3763,3
870
JMJD7_1_4978,480,432,376,457,273,73,1195,670,1252,349,244,178
JMJD8_1_4979,354,289,720,353,38,50,6,75,4,19,427,0
JUN_1_4980,330,266,411,347,348,241,430,263,254,431,15,281
KAT2A_1_4981,188,157,112,14,151,71,0,0,0,139,2,0
KAT2B_1_4982,522,589,361,436,1151,183,422,558,1502,393,465,747
KAT6B_1_4983,594,408,584,457,1760,572,1958,613,60,1117,1609,180
KDM1B_1_4984,1264,1193,1323,870,756,876,2578,735,989,1654,355,1074
KDM2A_1_4985,2802,3291,4060,2124,4372,3908,4070,3437,3543,2326,2259,49
10
KDM3B_1_4986,1656,1271,1560,2483,3888,2301,1674,3770,2062,2083,3502,19
49
KDM4A_1_4987,702,460,538,685,687,932,714,545,74,572,1377,252
KDM4B_1_4988,1514,1346,1397,1772,1686,1208,1779,1582,1460,1030,3283,48
4
KDM4D_1_4989,2574,2001,3085,2476,5637,2971,3848,2053,1414,1920,1256,37
44
KDM5A_1_4990,1553,1945,1775,2193,1848,974,2780,2413,1978,1868,1952,170
0
KDM5B_1_4991,957,848,1299,1532,1442,1665,1012,1342,1316,992,514,269
KDM6A_1_4992,18123,14446,17291,17620,18814,19216,17495,16778,16888,137
74,17154,13962

KDM6B_1_4993,1465,616,1430,917,775,599,2583,1014,1223,1011,1115,28
KIAA2026_1_4994,1960,1965,3315,1811,2600,1273,1271,915,3195,2101,2563,
4000
L3MBTL2_1_4995,511,380,327,818,216,279,2,799,0,61,2378,163
L3MBTL4_1_4996,3909,3912,4619,4939,4319,4637,4646,2396,7522,4347,5197,
4716
MAEL_1_4997,2183,1778,2152,2946,2138,1897,1804,3205,2325,1786,3085,154
3
MBD3_1_4998,747,733,954,475,390,828,288,919,265,139,651,482
MBD4_1_4999,8596,9425,9701,10562,11775,11589,12577,7757,12979,10325,79
17,9163
MBD5_1_5000,549,957,884,709,1597,806,128,1291,737,669,40,376
MBD6_1_5001,730,541,1054,568,1125,276,2155,141,67,428,2096,770
MBTD1_1_5002,3997,3040,3041,2553,3266,2902,4853,4046,4412,2646,5423,49
59
MDM2_1_5003,3248,2044,2956,2733,3382,3173,1253,3162,5036,3441,2120,292
8
MIER2_1_5004,1558,2393,1055,1138,1849,1770,1133,2662,639,2014,264,421
MIER3_1_5005,1777,1016,2069,1759,800,2059,1976,729,688,1200,1448,521
MIS18BP1_1_5006,5308,6159,8263,6468,5525,6311,11237,3850,7343,5685,667
4,5277
MKL1_1_5007,1397,1697,1320,1555,594,2587,2837,658,3169,1378,1219,2122
MLL2_1_5008,1922,1748,759,1430,1006,1101,3828,343,503,889,4282,589
MLL3_1_5009,1355,1381,1397,1623,884,1915,2032,1874,1604,2311,1769,2695
MLLT1_1_5010,114,380,323,188,315,440,515,101,2,855,1,432
MLLT3_1_5011,3144,2312,2640,3181,4442,2631,2869,2526,2704,2423,5309,44
06
MLLT6_1_5012,137,47,68,130,60,12,142,65,0,3,23,38
MPHOSPH8_1_5013,1238,1783,939,1109,2270,875,718,1107,1532,1508,392,178
5
MSH6_1_5014,461,576,532,1034,686,733,104,1776,79,460,1257,728
MSRB2_1_5015,1511,1968,1611,1939,1301,2173,1971,193,505,791,1411,3857
MTA2_1_5016,1602,1168,1946,1650,2425,1031,714,933,523,1653,1971,1656
MTA3_1_5017,4151,3754,5724,5874,3390,5399,3398,2917,6280,4220,3114,514
0
MYSM1_1_5018,1759,938,1013,1150,762,2849,3480,1539,4502,1849,3255,1474
NAP1L2_1_5019,2246,2667,3262,2725,3492,2391,2272,2554,1396,2868,2722,5
005
NAP1L3_1_5020,1087,1591,1379,1194,1599,1839,595,251,433,1426,783,351
NAP1L4_1_5021,1659,2088,1561,1974,2529,2022,3498,1588,1281,2301,2239,5
801
NAP1L5_1_5022,245,776,592,458,504,471,1038,778,14,321,685,14
NAT14_1_5023,0,0,0,0,0,0,0,0,0,0,0,0
NAT8B_1_5024,1985,1412,1767,1527,1382,1503,706,2373,849,1393,8,2158
NAT8_1_5025,2237,2103,3191,2216,2527,1462,1976,1400,2823,2788,931,2898
NAT8L_1_5026,1,176,48,76,366,8,41,66,235,309,0,0
NAT9_1_5027,1928,716,1563,1424,1558,1455,2012,384,1146,2008,542,834
NCOA2_1_5028,370,352,334,729,589,632,183,281,103,540,107,1862
NPTXR_1_5029,991,993,1296,1063,877,1482,3348,1251,656,1511,3295,1321
NR0B1_1_5030,323,583,617,354,287,579,256,92,123,629,749,229

NR0B2_1_5031,611,858,603,245,296,261,683,453,568,714,831,1580
NR1D1_1_5032,493,549,520,451,566,491,44,144,985,1221,1320,258
NR1H2_1_5033,414,276,98,221,88,60,302,158,424,190,0,193
NR2C2_1_5034,362,699,696,825,1750,464,481,117,63,239,492,730
NR2E1_1_5035,6200,5734,7193,7550,5762,6054,9488,5214,9032,5800,5681,54
95
NR2F1_1_5036,768,571,268,1057,147,639,305,427,194,547,1091,186
NR2F6_1_5037,269,349,415,518,512,195,254,127,84,107,2,8
NR4A2_1_5038,805,562,440,632,1048,130,0,998,636,622,0,430
NR5A1_1_5039,135,66,183,220,258,37,9,49,1450,467,4,0
NRIP1_1_5040,1042,1059,1772,1547,1540,1961,3013,924,1161,818,612,3373
PADI4_1_5041,252,260,249,633,114,434,255,1013,1493,1011,164,187
PAWR_1_5042,5091,4027,5271,5485,6407,5371,2938,4959,2828,2651,7160,461
3
PAX5_1_5043,99,45,50,49,21,270,0,75,156,220,1,0
PAXIP1_1_5044,223,133,76,282,494,114,52,13,80,65,151,68
PGRMC2_1_5045,255,23,74,33,1,0,1,87,0,12,18,8
PHB_1_5046,776,636,1139,733,1596,585,844,1609,201,682,938,206
PHC3_1_5047,1396,894,975,957,1198,1025,1733,819,80,1449,1823,2501
PHF13_1_5048,1335,1257,1032,1013,1211,969,1481,1758,777,1433,1955,1450
PHF14_1_5049,1700,1237,1684,2148,1536,1767,1060,2331,2841,1990,1509,17
13
PHF15_1_5050,824,896,434,482,280,733,1325,918,51,623,1733,327
PHF20_1_5051,4926,3912,4973,3522,5703,5759,3928,6581,6535,4645,2607,65
03
PHF23_1_5052,429,280,259,722,327,390,761,22,126,1500,212,269
PHF2_1_5053,190,294,296,482,1500,167,159,584,567,242,221,394
PHF3_1_5054,2531,2027,1746,1924,1496,2520,1078,2322,2881,2113,2341,142
5
PHF5A_1_5055,5930,7903,9053,9992,6378,10863,5698,6720,6807,7595,5770,6
224
PHIP_1_5056,2832,2266,2344,3150,1736,2686,2613,2647,1957,1788,3335,153
9
PHRF1_1_5057,1531,1793,1316,1151,2377,3458,2010,1530,2367,1067,2211,24
00
PIAS1_1_5058,661,305,303,339,30,210,112,4,245,294,50,74
PIWIL4_1_5059,1697,1524,1397,1225,1216,1444,2172,1700,796,1144,2423,12
91
PPARGC1A_1_5060,2372,1746,1845,1997,2670,3016,1447,1545,1351,2590,1081
,1348
PRDM11_1_5061,309,514,455,806,233,386,193,614,310,351,4,16
PRDM12_1_5062,1541,884,849,1162,1395,1506,1999,1139,1309,1225,1163,68
PRDM13_1_5063,52,6,0,17,43,385,0,1,0,324,0,0
PRDM14_1_5064,861,1305,1257,1123,1166,1699,1287,781,1652,1513,1041,288
4
PRDM4_1_5065,2543,2294,3305,2706,3419,3466,3315,2245,5071,2275,2581,21
30
PRDM5_1_5066,537,412,402,428,157,420,1285,124,28,187,893,71
PRDM6_1_5067,984,1068,1326,1186,818,731,1628,2380,2837,328,901,393
PRDM9_1_5068,1305,823,1085,689,2878,2623,1090,169,1142,948,2068,1176

PRMT6_1_5069,1427,1417,1321,1204,1875,1121,1514,653,2560,760,1940,1737
PRMT8_1_5070,443,586,964,880,335,354,2593,531,182,374,817,91
PRPF6_1_5071,1786,1774,1372,1223,1852,1009,1149,1443,4059,1965,3703,15
73
PYGO1_1_5072,2277,1816,2473,2144,3576,1680,1144,789,3064,1277,1490,235
1
PYGO2_1_5073,231,420,474,393,180,453,162,0,624,6,115,96
RAG2_1_5074,1286,1236,1561,1061,1175,1787,728,353,2014,2179,693,1996
RAI1_1_5075,615,524,865,952,389,1103,905,2392,275,1070,100,91
RB1_1_5076,1241,1064,1490,1220,1018,932,1336,1368,1621,996,57,1561
RCOR1_1_5077,4206,3721,3629,5244,3896,4324,4037,3065,6945,4182,1542,70
84
RCOR2_1_5078,520,456,759,202,634,24,176,815,1673,89,402,21
RECQL4_1_5079,840,578,968,644,740,555,210,307,1871,535,1481,936
RELB_1_5080,912,728,1308,440,289,358,879,365,1946,246,293,766
REL_1_5081,3866,5197,6079,3903,2739,4146,3704,3839,6368,3573,3679,3530
RING1_1_5082,732,707,842,836,1804,2239,3680,505,1165,1427,1194,497
RNF20_1_5083,1449,1701,2973,1112,1615,1419,391,1251,2680,1495,2651,646
RNF25_1_5084,245,230,515,75,26,644,138,28,1099,117,5,713
RNF2_1_5085,1769,1586,1643,1481,2944,917,1118,176,4106,782,1661,1607
RORB_1_5086,3430,3108,3314,2749,4152,2574,3003,2563,4364,2745,1916,219
6
RSF1_1_5087,605,511,538,851,711,844,2045,875,273,778,291,55
RUVBL1_1_5088,491,347,775,505,593,338,423,39,465,399,1032,841
RUVBL2_1_5089,1572,2757,2252,1417,2411,1728,1736,3315,1584,575,852,142
5
RXRA_1_5090,14,7,0,1,0,0,65,0,1,3,0,0
RXRB_1_5091,710,660,608,886,826,1127,727,1578,1071,325,1390,1133
RXRG_1_5092,1474,1127,1803,2559,1817,1578,2317,1631,451,1641,1447,2049
SAP18_1_5093,483,170,506,680,204,266,342,98,10,223,0,0
SCML2_1_5094,3250,2019,2814,2796,3189,3456,2280,3853,2232,3774,1838,13
08
SENP3_1_5095,1058,1270,1299,1447,1154,1623,733,2195,295,1724,668,328
SETD1A_1_5096,1448,1333,853,1328,2430,915,1558,804,973,1397,1363,678
SETD1B_1_5097,1763,1305,2105,1518,1576,3154,2609,1260,1218,2050,715,38
38
SETD2_1_5098,2648,2847,4264,3332,4061,3689,5303,2853,5461,4273,3426,21
69
SETD5_1_5099,4132,4574,5751,5317,3468,4845,7616,8275,4946,4077,6524,68
04
SETD7_1_5100,1456,1447,1963,2270,2266,1146,1983,2423,3340,946,1385,864
SETD8_1_5101,2160,3167,2570,2514,4332,4616,2912,5010,1966,3810,2957,44
60
SETMAR_1_5102,10581,14188,14441,11193,12333,12943,16378,8018,10442,119
45,17180,9539
SF3B3_1_5103,455,402,382,536,1115,320,306,436,223,970,397,277
SIAH2_1_5104,320,804,588,310,759,388,812,251,360,421,175,15
SIN3B_1_5105,2461,2400,3394,2983,2571,3910,4288,2160,1221,3388,1131,29
90
SIRT4_1_5106,1288,1180,1859,1451,1641,1933,2650,522,1530,1794,708,691

SIRT7_1_5107,2555,2176,2316,1474,3081,1450,1916,2368,2716,2320,2869,35
50
SLC2A4RG_1_5108,407,248,305,151,524,227,1007,230,0,350,119,1
SMARCA5_1_5109,1015,1204,824,601,493,1820,3593,226,4,1896,2552,697
SMARCC1_1_5110,868,584,1142,769,361,1999,1159,1247,739,1275,374,1236
SMARCD2_1_5111,1038,841,989,1587,1699,470,1104,649,998,570,1434,646
SMARCE1_1_5112,1701,1277,2298,1138,1512,1845,1582,1292,645,1266,1720,2
252
SMC1A_1_5113,171,369,203,54,216,34,13,630,25,0,34,391
SMC1B_1_5114,1671,1552,924,1575,1117,1304,725,1140,384,1575,853,2084
SMC3_1_5115,5045,4703,5722,6900,7470,4873,7912,4447,6435,4409,5385,374
4
SMCHD1_1_5116,3518,3879,4235,5429,3816,5492,6372,2837,4773,4857,5680,6
681
SMNDC1_1_5117,323,299,828,147,1098,170,104,21,727,606,1,518
SMYD1_1_5118,2252,3337,3972,2801,3996,5188,4910,2813,2277,1297,2581,27
50
SMYD2_1_5119,3215,4135,5232,4204,6604,4434,4592,1951,7152,4808,3316,36
87
SMYD4_1_5120,876,968,747,732,167,831,364,157,1049,724,1543,1271
SMYD5_1_5121,550,917,1067,493,1970,306,1265,221,1172,541,4412,748
SND1_1_5122,2523,1989,1790,3262,639,2010,2732,2729,797,2693,1626,2488
SP140L_1_5123,393,245,914,813,539,937,1606,19,197,194,44,315
SRCAP_1_5124,757,727,924,603,641,1762,708,372,1035,448,1458,292
STAT5B_1_5125,236,451,260,236,485,429,253,367,2,364,423,665
SUDS3_1_5126,1069,1219,980,2443,1075,2514,765,1388,1308,685,849,3176
SUPT16H_1_5127,1282,1758,778,900,1496,1406,1387,923,1618,647,1250,1409
SUPT4H1_1_5128,1303,1369,2563,2031,2234,2775,4320,518,1234,1310,2221,5
271
SUPT6H_1_5129,988,574,955,597,578,848,1853,772,965,304,1040,767
SUPT7L_1_5130,1988,1691,1095,1883,1439,1350,1169,1803,1166,1728,2217,1
874
SUV39H1_1_5131,1793,1709,1751,1499,1527,2825,2292,1960,5317,4394,346,1
319
SUV420H2_1_5132,1111,905,1809,1254,1143,1558,1879,2189,3294,1471,1761,
1719
SUZ12_1_5133,1781,1498,1656,2561,2468,2440,2770,763,3747,1893,747,1611
TADA1_1_5134,1832,1382,2046,1698,1782,2473,1896,905,1104,1435,164,2956
TADA2B_1_5135,53,8,112,163,222,12,53,1,32,166,0,255
TAF10_1_5136,338,640,775,386,193,846,281,139,860,463,1372,217
TAF1L_1_5137,1280,1032,2175,1577,2106,1633,679,489,1416,1233,708,716
TAF3_1_5138,2867,2925,4760,3489,3302,3943,4305,4241,3702,3216,1373,435
5
TAF5_1_5139,978,992,1150,888,1633,1284,643,803,949,732,1088,1782
TAF6L_1_5140,1929,1967,1674,1723,2028,2143,3117,3403,2704,1802,88,4359
TAF8_1_5141,1670,1745,3004,2135,1897,2248,6106,1534,3338,825,2507,3578
TCF7L1_1_5142,740,967,481,415,1091,1700,230,1525,148,893,341,963
TDRD12_1_5143,1263,819,1428,1393,2086,1462,1431,326,717,907,278,1170
TDRD1_1_5144,4608,4383,5449,5111,6467,5951,5269,4766,4858,5923,3609,41
06

TDRD7_1_5145,3857,3002,3752,4284,4701,3436,5542,4343,4529,2754,1456,30
19
TDRD9_1_5146,5230,4404,5390,5492,6737,6346,4202,5804,5046,6021,6599,61
41
TERF2_1_5147,1156,854,653,1047,2534,1293,1000,1725,302,911,1248,367
TET1_1_5148,1041,559,978,982,1053,1689,237,502,1442,640,432,415
TET3_1_5149,1862,1175,1968,1253,2793,1183,338,726,2137,1239,655,1311
TLX2_1_5150,1575,1316,1595,1350,1618,2870,2836,1699,487,2674,1447,3019
TNRC18_1_5151,1139,852,1154,1384,1572,1548,930,820,936,1837,2227,1635
TRAF7_1_5152,37,43,172,3,23,284,70,319,594,41,3,226
TRDMT1_1_5153,2211,3653,3045,3475,5313,3465,3376,1150,2495,1892,2042,3
881
TRERF1_1_5154,463,623,405,285,962,1390,362,264,200,538,883,52
TRIM25_1_5155,719,471,586,512,631,555,475,760,306,1344,1768,1699
TRIM27_1_5156,2544,1951,2064,1690,3047,2568,2166,1202,4935,2395,2155,3
640
TRIM28_1_5157,905,1366,1737,1525,1665,1442,1188,719,1074,1623,706,205
TRIM66_1_5158,463,409,1072,498,1428,730,895,136,445,572,338,471
TRRAP_1_5159,1049,977,821,1171,1560,1347,2675,1662,742,1424,604,1062
TSG101_1_5160,442,354,402,196,547,315,189,981,249,271,718,395
TYW5_1_5161,1360,1175,1388,1190,2022,2032,1053,625,2308,1795,1884,1848
UBE2B_1_5162,711,396,488,462,327,721,46,1245,973,651,22,68
UBE2N_1_5163,1204,1008,1382,1268,3451,1211,917,515,827,949,719,901
UBR7_1_5164,2412,2359,2396,2699,3032,3981,4924,2057,4250,2355,2723,227
6
UHRF2_1_5165,1471,1802,2433,1334,3169,1444,2440,1411,3864,1537,1704,40
4
USP22_1_5166,2412,1064,2027,1751,2723,2735,3403,2929,4302,2042,887,337
0
YEATS2_1_5167,4116,5033,5793,4444,3053,3722,7378,2462,1752,4241,4054,3
092
YEATS4_1_5168,3386,3088,3813,2958,4762,2852,3681,4491,8404,2348,1807,3
588
YY1_1_5169,1104,1185,1072,1895,1116,1319,2872,1229,1341,1626,273,2181
ZAR1_1_5170,2184,1615,1559,1821,1461,2126,2631,1864,4630,1422,1780,728
ZCWPW1_1_5171,3001,2581,3533,3269,1356,3387,3564,2290,974,3224,1548,12
95
ZCWPW2_1_5172,1137,820,1583,760,1177,882,901,423,310,217,2961,1362
ZFP57_1_5173,759,1048,312,893,620,801,283,790,482,1613,14,1037
ZNF541_1_5174,4569,3283,4012,3307,4672,2950,7388,1083,6371,2260,3951,3
968
ZNF85_1_5175,13194,13418,14437,14529,16700,17132,16971,11220,16447,148
08,14123,17161
AANAT_1_5176,68,268,135,95,158,381,66,411,0,155,95,6
AES_1_5177,933,544,701,835,1129,737,969,722,571,866,186,9
AIRE_1_5178,390,538,790,388,185,609,853,142,15,873,178,5
AKAP1_1_5179,5,3,4,22,0,0,0,0,0,6,0,9
ALKBH2_1_5180,1965,1751,2289,1977,1783,2196,1043,1402,1594,911,1453,98
2
ANKHD1_1_5181,3114,3587,3840,2178,3092,4595,5118,2365,1038,2843,1060,1

686
ARID1A_1_5182,109,1,8,1,373,72,317,0,1,5,0,5
ARID1B_1_5183,2269,1856,2867,2865,2424,2539,2239,2749,2412,1626,2190,2
924
ARID4A_1_5184,3470,2727,2863,2362,5245,4075,2637,4078,1472,2516,387,30
18
ARID4B_1_5185,928,717,1593,1541,514,1204,1832,764,1208,878,1184,1671
ARRB1_1_5186,470,365,161,344,307,383,168,93,6,290,78,305
ASH2L_1_5187,2272,2357,2828,3870,558,2647,4570,2886,3420,1774,3288,498
9
ATAD2B_1_5188,3720,4268,5879,4749,5547,7184,4139,4560,3817,5593,8424,6
426
ATAT1_1_5189,4696,3702,4952,5925,6014,3785,5751,3749,5917,4301,5854,51
12
ATRX_1_5190,6731,6778,9190,7477,6582,6833,7992,9270,8646,3961,5936,964
5
AURKA_1_5191,1581,1616,2130,1490,1251,1323,1594,1042,2047,960,1362,206
2
AURKC_1_5192,1002,729,999,1367,807,1804,1589,979,773,2272,2850,363
BAZ1A_1_5193,11358,10147,13789,12731,12967,16654,13521,11442,12192,107
12,13863,15017
BCOR_1_5194,184,372,976,355,147,682,626,559,0,760,597,13
BPTF_1_5195,828,578,775,1076,2662,2952,1020,599,791,1013,863,2787
BRCA1_1_5196,3926,4488,5030,4325,2874,7322,3424,6027,6736,5066,3583,72
60
BRD2_1_5197,4037,3817,4266,5486,5159,4551,7829,5136,3484,4458,4718,666
1
BRD4_1_5198,882,1742,1183,1295,584,2433,1197,667,696,416,1062,0
BRD7_1_5199,974,748,1758,1157,1817,648,1809,1710,374,899,1839,1715
BRD8_1_5200,1587,1531,1492,1080,1250,714,1008,715,455,1048,243,290
BRD9_1_5201,817,735,646,925,754,2040,659,809,2326,897,905,1744
BRDT_1_5202,150,639,174,246,85,202,368,163,582,800,211,55
BRPF1_1_5203,1997,1659,2531,1403,2789,2372,3337,2868,2590,1848,1139,34
2
BRWD1_1_5204,1005,418,1086,498,447,53,373,373,377,643,1194,238
C14orf43_1_5205,419,172,267,222,163,707,999,242,123,484,117,154
CBX1_1_5206,1324,887,1066,1414,2987,1251,2240,2242,4019,1858,885,1270
CBX3_1_5207,2202,2957,3567,4030,3176,3710,3824,2882,2123,3636,1155,207
0
CBX5_1_5208,2041,1193,2040,1696,573,1816,3554,1136,2239,1241,1430,748
CCNT2_1_5209,1172,1288,1509,2122,538,2392,798,1727,916,1147,632,3013
CDY1B_1_5210,6763,5256,5710,6562,10275,7802,5316,3404,5955,7507,7716,6
239
CDY1_1_5211,6763,5256,5710,6562,10275,7802,5316,3404,5955,7507,7716,62
39
CDYL_1_5212,466,246,881,1023,193,446,14,83,219,1570,1921,699
CHD2_1_5213,3040,3163,3130,3899,3754,3443,4993,4080,4251,4420,2742,268
5
CHD3_1_5214,746,287,665,669,1080,476,549,628,416,279,534,1340
CHD8_1_5215,18,7,63,54,0,6,30,54,3,38,4,25

CHMP2A_1_5216,1229,781,1303,1627,457,784,1183,1279,989,994,1583,3272
CHMP5_1_5217,2896,1944,3283,2252,2977,2846,1838,2430,2410,2932,3102,51
21
COPS2_1_5218,14569,12516,14357,11469,19458,19142,19223,9387,14254,1322
7,15553,16623
CPA4_1_5219,499,825,1032,735,2037,1307,507,1871,1065,1417,1311,90
CREB1_1_5220,1284,1349,960,2024,2132,1673,2426,1169,2603,719,873,1333
CREBBP_1_5221,1451,1894,1763,1862,4823,1794,1137,1913,38,1518,2021,353
2
CTCF_1_5222,1211,2144,2135,1796,2333,2065,336,1534,957,1354,4342,520
CTNNB1_1_5223,1102,719,978,858,1389,1662,1903,683,4929,1247,3804,445
CXXC1_1_5224,1401,1394,1502,2072,3233,2211,1969,712,2288,2324,3584,341
9
CYLD_1_5225,2403,1852,2303,2338,2384,1391,1675,613,3874,1871,1319,930
DICER1_1_5226,4064,3332,5016,5135,4334,4201,3317,3566,4690,2589,2900,3
032
DIDO1_1_5227,76,114,122,73,252,6,159,2,25,138,47,344
DMAP1_1_5228,1881,1234,1183,2532,1584,2818,3307,2025,2792,2520,3658,30
73
DNAJC2_1_5229,2193,1575,1424,1874,2943,2273,834,1803,1679,2468,771,221
8
DNMT1_1_5230,2737,2589,2940,4024,4151,5160,6107,1301,1369,2971,4690,16
68
DNMT3A_1_5231,1659,1155,1184,1094,1696,2029,3153,924,2303,1428,650,217
3
DNMT3B_1_5232,1451,2161,2094,1323,2783,2734,1688,1540,838,1014,1912,13
08
DNMT3L_1_5233,1525,1197,1154,967,1289,1903,3021,674,1160,1336,623,2240
DPF1_1_5234,3714,3969,4442,4509,6159,5278,4403,2395,2524,3983,2153,294
4
EED_1_5235,496,616,479,587,352,188,48,263,97,135,535,203
EGR2_1_5236,767,581,465,735,842,1575,1587,911,736,442,1237,56
EHMT1_1_5237,675,350,159,194,608,717,2,29,791,30,386,201
EHMT2_1_5238,1517,1515,2357,988,1518,1958,2887,2599,2948,1604,3603,417
5
ENO1_1_5239,152,118,240,272,48,51,49,89,195,667,0,818
ESR1_1_5240,1195,514,915,760,325,476,721,503,626,1435,1803,34
ESR2_1_5241,6212,5384,4688,6065,6810,3759,11225,3494,4131,4804,2724,44
70
ESRRG_1_5242,334,215,362,605,799,565,496,1002,145,815,292,3
EZH2_1_5243,993,1019,1426,1235,3359,1358,2920,453,512,839,1832,814
FBXO11_1_5244,3577,3642,4591,4562,7070,4747,5217,3952,3097,3742,7025,4
906
FMR1_1_5245,4600,3646,4531,3544,4483,4691,5747,4894,4351,4687,3647,311
5
FXR1_1_5246,3795,4600,5640,4917,7340,4939,5747,2468,2023,6015,3119,347
6
GFI1B_1_5247,1176,961,1990,1777,1313,1390,2466,908,1298,621,1742,894
GMEB1_1_5248,1065,1193,1174,553,585,618,424,871,568,987,434,322
HDAC10_1_5249,42,150,2,49,1,149,0,27,6,306,0,0

HDAC11_1_5250,1292,1055,1561,1159,3690,1524,1924,1503,1603,1467,2481,1
249
HDAC5_1_5251,1430,1295,1726,1143,2017,2007,2987,1832,318,1743,826,21
HDAC7_1_5252,503,463,683,523,146,559,1317,128,137,109,736,299
HDAC9_1_5253,1152,1036,1002,2074,1517,1724,2779,1962,1932,1300,346,292
HDGF_1_5254,426,429,610,388,768,402,940,682,328,724,401,1060
HDGFRP2_1_5255,373,829,545,262,486,305,819,476,721,207,5,420
HLTF_1_5256,4044,2735,4058,3486,5496,1859,2953,2147,6461,3267,4578,329
0
HMGA1_1_5257,3124,3049,2740,3209,2662,2348,1519,2450,5291,4004,3515,45
83
HMGN3_1_5258,2338,2540,2655,2011,2843,1821,2100,1727,1594,2388,822,279
0
HNF4A_1_5259,369,350,419,468,574,818,1167,83,21,314,1064,509
HPSE2_1_5260,486,202,626,697,562,1007,45,12,842,284,332,18
HR_1_5261,700,1101,574,789,1411,2658,213,244,268,611,123,650
ING1_1_5262,1136,719,637,1314,2384,1160,1277,511,1512,909,186,2152
ING4_1_5263,2330,1551,2933,2620,3377,1812,4320,3555,6514,1896,4215,207
6
INTS12_1_5264,968,1066,842,1178,1336,624,1489,429,955,1037,1177,54
IRF4_1_5265,2308,1889,1944,2586,3043,2973,4448,2218,886,1206,2376,1517
JMJD1C_1_5266,1058,1031,2219,1576,1726,1796,900,515,1963,1440,1103,295
2
JMJD4_1_5267,1274,1738,2199,2513,2222,2492,2548,1571,1631,2286,1964,14
38
JMJD6_1_5268,1183,772,1204,1676,1358,1270,624,1537,563,902,1053,1180
KAT5_1_5269,233,251,50,654,772,214,183,730,1447,365,6,1156
KAT6A_1_5270,2370,1793,2929,2877,3151,3088,1460,4569,1139,2218,681,118
3
KAT7_1_5271,9309,7709,8132,7976,9181,12037,6559,9730,5966,8370,8229,90
85
KAT8_1_5272,76,38,189,266,0,11,358,174,0,35,2,184
KCTD1_1_5273,12022,12387,14335,14704,14492,13316,10359,13810,10541,143
08,10382,13635
KDM1A_1_5274,2441,3094,4440,2888,3509,2640,4345,3457,3130,3275,4099,28
52
KDM2B_1_5275,83,118,151,91,36,281,0,0,0,0,0,7
KDM3A_1_5276,1128,1506,1714,1256,958,1415,2736,2085,1432,2064,511,463
KDM4C_1_5277,4662,3771,4534,3465,3143,3639,5410,2697,6387,4176,6940,46
45
KDM5C_1_5278,429,357,720,852,320,1336,81,762,13,844,1164,0
KDM5D_1_5279,871,1315,1506,788,681,659,1384,586,419,719,642,131
L3MBTL1_1_5280,328,203,358,409,1152,604,1308,224,21,403,2,372
L3MBTL3_1_5281,3857,4001,4387,3341,5698,5574,6365,3219,2892,3947,4494,
2975
LBR_1_5282,5863,6376,6738,6675,7180,6524,9617,5967,4520,5748,7238,8808
MAP3K12_1_5283,622,829,991,1295,673,1195,584,1315,3285,959,422,709
MBD1_1_5284,318,372,573,518,287,596,527,289,434,333,942,665
MBD2_1_5285,3232,1941,3063,2508,3035,2278,2949,2320,2924,2395,3134,283
7

MECOM_1_5286,351,504,968,419,61,71,391,10,766,376,165,584
MECP2_1_5287,324,157,464,200,486,446,600,53,10,164,404,136
MEN1_1_5288,346,455,207,657,833,253,7,909,541,105,251,1
MGEA5_1_5289,5304,4692,6526,6748,7833,5475,4812,5024,5568,9409,9347,51
90
MIB2_1_5290,444,574,228,546,212,577,214,178,8,367,45,121
MIER1_1_5291,4229,4942,6480,5339,1663,4349,6923,4311,3805,5545,7150,61
00
MINA_1_5292,7113,7043,7765,5671,8371,9164,7155,4737,6300,6152,4858,604
6
MLL5_1_5293,1070,728,332,807,1032,933,2445,1068,57,1310,463,528
MLL_1_5294,571,299,926,503,313,969,782,8,467,1334,975,973
MORF4L1_1_5295,838,645,795,951,937,902,162,1532,249,942,825,1168
MSL3_1_5296,2927,2090,2787,2305,2873,4439,3065,1235,2406,1301,3774,210
5
MTA1_1_5297,1423,2292,2193,1375,3396,3069,1145,825,1112,2072,3105,1961
MTF2_1_5298,649,569,525,783,1196,666,2492,596,1175,1136,6,0
NAA60_1_5299,983,434,587,844,1221,260,469,945,3103,401,745,428
NAP1L1_1_5300,181,241,201,34,45,480,954,859,95,140,92,452
NAT10_1_5301,1038,941,1172,1334,1351,659,1740,661,849,1630,2259,1086
NCOA1_1_5302,457,447,424,262,229,632,261,95,95,493,470,33
NCOA3_1_5303,632,553,581,361,518,322,61,434,13,779,92,441
NCOA4_1_5304,2981,3447,3322,3403,2511,2384,5205,2831,3873,5172,1526,38
44
NCOR1_1_5305,38,86,779,148,152,40,112,0,0,140,6,13
NCOR2_1_5306,827,444,550,1319,215,1084,630,2320,1650,1106,588,654
NFAT5_1_5307,3007,2409,3631,2760,3201,2881,2382,2393,2966,2749,4888,32
91
NFATC1_1_5308,644,358,483,418,128,286,1329,28,0,585,901,1072
NFATC3_1_5309,650,434,608,574,288,884,517,826,114,364,752,483
NFATC4_1_5310,678,1582,1500,534,250,844,1538,811,125,1161,3,302
NFKB1_1_5311,7521,6269,8886,8458,8140,10994,11480,7818,10393,7723,8978
,7657
NFKB2_1_5312,894,1136,753,945,1575,867,1220,727,28,1477,1414,444
NPM1_1_5313,1615,1650,1571,980,1960,921,1365,2387,596,1140,1141,2539
NR1D2_1_5314,5601,6925,8732,8436,7646,10276,6634,7209,5112,6077,5262,6
384
NR1H3_1_5315,1348,1014,1436,1027,961,1373,404,92,1420,734,1004,618
NR1H4_1_5316,394,394,564,771,823,274,866,1860,782,999,107,731
NR1I2_1_5317,2567,2772,4262,2503,2556,2843,2265,2133,1888,1338,1586,41
96
NR1I3_1_5318,1148,561,1132,1038,811,1703,433,586,491,414,287,602
NR2C1_1_5319,709,751,788,995,1125,910,465,1424,358,1352,645,546
NR2E3_1_5320,1149,651,1134,645,755,1143,335,510,3642,1105,720,820
NR2F2_1_5321,1183,1259,1318,875,1106,705,812,1031,860,1852,966,2111
NR3C1_1_5322,7671,9562,11850,10528,13699,8871,12703,10600,9008,7559,12
777,13419
NR3C2_1_5323,1191,1786,1300,1526,1347,1079,1156,351,1207,985,1673,628
NR4A1_1_5324,458,261,601,352,505,181,1113,213,1159,268,1048,508
NR4A3_1_5325,4361,3585,5056,4648,5530,2287,5835,2678,6806,5838,6455,24

10
NR5A2_1_5326,2430,1754,2619,1975,2813,1665,3250,1416,914,1224,1417,256
9
NR6A1_1_5327,816,843,786,814,989,1002,1137,570,2826,350,281,158
NSD1_1_5328,2417,1975,2480,2506,4415,2071,4566,4235,2446,1747,3057,653
PBRM1_1_5329,403,362,856,382,353,977,491,239,78,1303,1485,581
PCGF6_1_5330,1959,1846,2537,2002,826,1784,2120,989,3670,2561,1145,1220
PGR_1_5331,2403,2635,1715,2477,2305,2158,2074,1506,1234,2299,1138,4633
PHC2_1_5332,252,190,162,29,467,765,126,125,49,7,0,523
PHF10_1_5333,2277,3548,3701,1574,2777,3407,1714,1851,2213,1974,2220,47
25
PHF11_1_5334,1205,736,914,638,970,2103,629,152,383,1070,493,1061
PHF12_1_5335,501,713,478,550,588,568,341,67,338,333,671,754
PHF16_1_5336,699,729,480,815,805,550,59,86,259,1517,378,171
PHF17_1_5337,1543,1641,2191,1532,1068,2235,1652,994,97,1250,1100,420
PHF1_1_5338,818,1307,1270,834,431,563,1119,260,1291,470,1105,2673
PHF20L1_1_5339,5410,4468,4630,4288,4286,5615,3073,4732,3253,5654,4144,
5303
PHF21A_1_5340,1004,1484,1337,1886,1503,825,658,920,399,1615,619,612
PHF21B_1_5341,201,140,26,44,3,167,0,228,3,11,573,13
PHF6_1_5342,1955,1576,1669,2270,1599,2137,2459,276,2635,2755,1532,591
PHF7_1_5343,1458,1134,1476,1342,2386,1626,2612,1330,2019,771,878,791
PHF8_1_5344,3397,4187,3939,3717,5447,2676,3640,4927,2955,2606,2374,230
3
PIAS2_1_5345,1875,1658,2124,2028,1546,1451,1509,1243,1188,3082,1636,23
58
PICK1_1_5346,773,1172,1382,1298,501,659,140,210,0,928,806,3495
PIWIL2_1_5347,3126,3170,2868,2497,4628,2991,2676,2728,4098,2204,5118,2
472
PML_1_5348,546,385,809,818,358,324,1814,575,674,695,313,816
POLR1B_1_5349,1195,1566,958,1506,1952,2525,899,678,1404,350,701,968
PPARA_1_5350,2601,3580,3477,3004,2621,3616,1882,2535,6174,1609,2220,19
37
PPARD_1_5351,158,446,458,428,217,485,205,430,10,330,502,77
PPARG_1_5352,1467,1226,1481,755,1569,981,2073,932,1218,658,1650,796
PRDM10_1_5353,594,454,526,761,1306,971,232,100,26,650,115,447
PRDM15_1_5354,0,2,0,0,0,0,147,0,0,0,0,0
PRDM16_1_5355,782,715,620,915,197,623,1232,338,1238,315,421,122
PRDM1_1_5356,4278,2714,4065,3578,4370,5232,3514,3852,2576,4136,2788,29
86
PRDM2_1_5357,27858,26403,29205,31962,34891,32179,33031,24745,24222,335
35,27134,32472
PRDM7_1_5358,2684,3375,3585,4375,3350,3373,4231,3864,2019,1400,977,294
7
PRDM8_1_5359,1670,1585,2311,1794,2327,1156,2687,715,1134,2120,1992,199
7
PRMT1_1_5360,1712,1321,1785,1423,2089,2414,3816,292,4879,1349,3029,327
4
PRMT2_1_5361,913,1012,679,1229,940,1369,338,945,332,265,1390,1340
PRMT3_1_5362,1849,1977,3101,1521,1428,2070,3599,2390,4206,2043,891,231

2
PRMT5_1_5363,302,726,1892,643,944,1008,505,9,1332,447,2534,1100
PRMT7_1_5364,595,585,448,362,1055,388,371,205,31,508,794,422
PSIP1_1_5365,341,232,397,1104,736,268,558,229,2,300,1379,1146
PSMC5_1_5366,2344,2803,1970,2120,1319,1021,872,2043,1895,1726,611,877
PWWP2B_1_5367,706,412,472,556,63,1613,873,633,939,388,496,256
RAD54B_1_5368,7757,6306,8299,8676,10467,7394,5580,6854,8175,7526,8817,
10438
RAD54L_1_5369,1740,2202,1606,880,2638,1779,1421,902,686,952,408,1778
RARA_1_5370,1196,1453,1393,2302,1723,1219,1159,1497,804,1834,1298,2391
RARB_1_5371,3307,1694,2487,2431,1973,2010,941,1119,1425,2360,2340,757
RARG_1_5372,391,185,574,374,495,185,148,116,44,434,231,701
RBBP5_1_5373,3947,3291,5725,4779,5354,5107,5688,4546,4071,4430,4307,23
27
RBCK1_1_5374,1703,1748,1333,1900,723,2473,2029,1723,1105,1719,3836,276
RBFOX2_1_5375,422,286,508,519,479,440,246,381,1312,241,155,29
RCC1_1_5376,511,710,828,934,665,496,42,47,117,942,359,758
RCOR3_1_5377,2858,4318,3572,4003,5831,4441,3231,2622,3763,3644,5335,47
60
RECQL5_1_5378,943,883,1432,1292,1901,1707,1915,1425,1994,262,1890,258
RECQL_1_5379,2592,2910,1866,3425,3333,3013,2494,1382,2171,2493,2520,24
28
RELA_1_5380,642,977,885,603,2403,613,470,348,1286,799,21,1028
RERE_1_5381,172,283,97,128,552,197,91,0,1,220,0,0
RFC1_1_5382,588,476,440,535,487,947,91,812,82,1023,204,1171
RNF14_1_5383,2738,3101,2273,3234,1893,2647,2412,4068,3387,3728,2065,21
63
RNF17_1_5384,2064,1912,1834,1855,1360,2442,1601,1303,3883,2136,2423,18
36
RNF40_1_5385,426,965,785,600,731,711,70,426,149,550,701,1773
RNF8_1_5386,989,1055,607,589,1140,2311,326,184,1756,1666,517,157
RORA_1_5387,1094,1488,1522,1183,2179,778,1120,836,3137,920,2375,1138
RORC_1_5388,3069,2479,2592,2517,2709,1508,2934,1949,1389,2633,1169,689
RPH3A_1_5389,375,533,483,482,1455,637,574,443,128,43,278,1
RPS6KA5_1_5390,6312,6189,7925,8062,7406,8407,6656,5804,5599,5023,6665,
5359
SATB1_1_5391,8670,9096,10211,8745,11719,11415,11878,7508,6163,8348,560
8,7616
SATB2_1_5392,95,34,67,4,206,174,11,0,0,261,6,0
SCMH1_1_5393,1599,1823,1655,1452,1586,1177,594,1287,540,1310,281,2932
SET_1_5394,1601,1326,1405,2165,3510,1945,2086,2489,1237,3082,657,3717
SETD3_1_5395,2410,2146,2889,3084,1288,2156,2131,3402,2360,3518,3955,20
59
SETD4_1_5396,1270,978,1006,725,2766,681,438,255,1141,513,44,1868
SETD6_1_5397,443,648,272,290,149,459,170,584,130,503,67,1152
SETDB1_1_5398,707,564,1015,361,1171,1366,731,832,433,755,164,408
SETDB2_1_5399,889,850,691,815,847,60,1312,550,26,1361,1571,1441
SFMBT1_1_5400,1210,1397,633,684,544,1711,3188,37,2130,546,1298,1733
SFMBT2_1_5401,1214,1397,1611,1466,1387,1251,287,818,2133,882,1938,2430
SHPRH_1_5402,2989,3591,4569,2818,4911,4450,2710,1722,2386,3902,3823,69

47
SIN3A_1_5403,6113,5799,7218,6216,8625,3935,6162,4439,9210,6995,9701,64
87
SIRT1_1_5404,751,1090,1383,635,1285,2408,1447,1005,1370,1312,408,220
SIRT2_1_5405,367,116,374,150,34,88,0,0,0,2,2,18
SIRT3_1_5406,842,865,1100,1048,2228,401,1350,1666,1403,747,1629,399
SIRT5_1_5407,789,1029,650,915,1548,549,729,1687,94,744,1,643
SIRT6_1_5408,2724,2043,2970,1747,1765,2257,563,703,2617,1239,2457,1193
SLC38A1_1_5409,2917,3495,3477,2717,3910,4770,4578,4303,4332,2108,2004,
3295
SMARCA1_1_5410,246,167,312,490,893,101,29,127,74,117,1094,12
SMARCA2_1_5411,294,264,523,268,233,233,222,250,45,258,37,126
SMARCA4_1_5412,85,45,263,320,207,143,637,88,42,19,12,84
SMARCAD1_1_5413,3352,3034,3650,3991,5515,3138,2547,2410,3523,2427,4934
,3754
SMARCAL1_1_5414,1358,1339,1429,1353,1254,2023,1201,928,2001,1155,3287,
3142
SMARCB1_1_5415,480,299,997,746,924,1758,954,2309,208,766,951,261
SMARCC2_1_5416,313,247,616,835,473,608,746,618,2760,559,497,10
SMARCD1_1_5417,1163,1677,1376,1373,1719,1787,1728,507,458,1645,754,375
SMARCD3_1_5418,372,485,651,708,105,900,455,933,117,390,1187,508
SMC2_1_5419,994,1925,967,1740,1011,1080,2186,744,1412,1271,40,4192
SMC4_1_5420,3735,3700,4994,3696,3684,4380,4183,3742,2802,2880,3290,424
1
SMN1_1_5421,2815,2300,3097,2864,3872,3218,2893,3415,1495,2267,1552,410
6
SMN2_1_5422,2815,2300,3097,2864,3872,3218,2893,3415,1495,2267,1552,410
6
SMYD3_1_5423,283,405,103,252,1875,280,270,313,44,20,1,5
SP100_1_5424,2399,1704,1756,1835,1095,3458,2676,901,2002,2602,2888,271
8
SP110_1_5425,1987,2595,1727,1864,2133,2091,538,826,2024,2385,1502,1981
STK31_1_5426,337,394,324,252,110,351,596,283,0,404,0,0
SUPT3H_1_5427,1596,2180,2357,1271,2762,2186,2028,416,1895,2062,750,276
1
SUPT5H_1_5428,222,582,248,271,543,487,338,234,87,51,113,56
SUV39H2_1_5429,3775,3758,4393,4022,3163,2961,3548,3713,5161,2801,2368,
6038
SUV420H1_1_5430,1194,1108,1779,1456,767,831,424,2730,554,458,1072,759
TADA2A_1_5431,952,1253,1437,1191,2342,1731,1003,1251,317,1217,30,986
TADA3_1_5432,173,255,65,149,359,264,7,0,10,168,1346,969
TAF12_1_5433,745,790,1119,594,704,1253,1587,586,193,694,96,1934
TAF15_1_5434,1096,1347,977,1588,518,1267,460,651,628,637,1805,1106
TAF1_1_5435,6414,4640,6645,6786,6628,8405,9163,7864,9201,5712,3957,767
9
TAF5L_1_5436,956,1301,686,1479,1719,1234,2083,1114,1722,768,1461,336
TCF19_1_5437,1668,1025,1613,2619,1627,1403,1857,934,3353,2195,2235,175
4
TCF20_1_5438,1324,1022,1304,1240,1808,980,1666,889,492,1415,2030,264
TDRD10_1_5439,132,118,364,97,15,522,880,52,342,40,229,7

TDRD3_1_5440,716,758,950,957,1263,789,2536,255,588,680,3516,730
TDRD5_1_5441,566,646,598,590,1900,1076,788,89,913,246,145,1160
TDRD6_1_5442,3419,3507,4504,2909,3365,5031,2050,2855,3663,2825,5789,53
21
TDRKH_1_5443,2561,2078,1728,2923,2600,3030,1251,1854,3267,2096,3499,16
14
TERF1_1_5444,268,1075,619,901,290,774,487,616,195,297,109,1209
TET2_1_5445,3250,3615,4186,3798,4534,4865,3375,3368,3635,2539,4193,426
3
THRA_1_5446,762,521,869,1381,1067,553,12,401,416,496,20,1369
THRB_1_5447,511,465,1453,1827,1169,860,951,118,371,802,217,2018
TP53BP1_1_5448,2185,1687,2930,2114,1773,1817,811,1857,1032,1262,453,14
42
TP53_1_5449,1608,1974,2037,2756,3291,1427,2313,1997,1978,1711,3410,232
2
TP73_1_5450,52,505,105,26,6,290,423,4,21,0,15,0
TRIM24_1_5451,1174,1325,1088,1023,647,1030,302,186,1700,674,935,864
TRIM32_1_5452,4460,3292,3869,5682,4229,5260,4668,3728,4579,5965,4264,4
715
TRIM33_1_5453,3244,4491,3882,3149,2909,4331,4378,4248,5494,2391,1612,2
510
UBE2A_1_5454,887,1810,1580,855,3174,1371,535,893,808,1088,476,1840
UBE2E1_1_5455,648,408,537,197,785,825,257,701,10,1059,157,847
UBE2I_1_5456,2361,2097,1763,2477,1054,2483,3224,1547,3505,1543,967,296
6
UBE2K_1_5457,4247,3566,4744,4390,6400,6650,6239,5768,4677,4897,1043,36
70
UBE2V1_1_5458,1324,1402,1492,2107,1493,1545,1730,972,2230,943,2090,186
7
UHRF1_1_5459,2120,2297,4185,2614,841,3109,3397,3839,3068,2323,2771,339
5
USF2_1_5460,157,118,196,24,68,184,254,36,106,112,95,16
UTY_1_5461,4223,4442,3898,5231,5357,4923,2199,2649,3602,2938,6037,4354
VDR_1_5462,772,266,582,628,766,538,171,117,972,200,798,899
WDR5_1_5463,869,1213,823,693,700,1826,2024,287,345,351,3027,1
WHSC1_1_5464,2078,830,1438,1737,2633,2200,1724,2013,1509,1853,1206,347
WHSC1L1_1_5465,217,442,668,233,48,54,38,52,1367,292,10,257
WRB_1_5466,2104,1998,2373,2733,4689,2719,2633,1357,4230,1587,693,1746
ZGPAT_1_5467,1759,2259,2502,1862,2307,2639,2265,1689,3314,2356,1690,21
17
ZMYND11_1_5468,7463,6200,9365,8986,9668,11221,10909,7322,9387,7842,691
7,8133
ZMYND8_1_5469,2695,1887,3378,2061,3247,2098,1071,4204,2879,2027,4259,4
368
ZNF451_1_5470,1992,2543,2580,2949,1964,2284,3797,4782,6382,2045,3995,4
221
ALG13_1_5471,3854,3329,3582,3526,2821,2519,2967,3074,4988,5062,5637,39
02
ASXL1_1_5472,125,543,137,223,421,83,902,17,137,575,444,19
CBX2_1_5473,516,202,296,361,539,600,34,1101,232,786,1053,304

HDAC8_1_5474,4212,4569,5194,4606,8016,4239,4149,4690,6348,3679,5723,31
85
ING3_1_5475,2640,2894,3756,3724,1483,1730,5825,4313,1308,3312,1269,185
4
MLLT10_1_5476,2616,2528,2889,1465,2760,3486,2498,2666,2496,2555,1919,2
500
PHF19_1_5477,965,827,1233,1800,1328,1676,2109,551,2349,1113,1093,1102
RBM14_1_5478,235,437,105,120,1266,55,78,10,0,23,47,322
SP140_1_5479,545,308,476,1445,2300,780,174,936,38,430,1874,229
TAF9_1_5480,1241,1669,2647,1891,1073,2084,2061,1992,3244,1587,689,3093
TAF9_1_5481,597,1144,703,388,1597,1148,566,231,1234,362,1266,182
AHR_1_5482,1593,1020,1869,2271,944,2031,1001,444,2025,2256,501,3111
ALKBH1_1_5483,693,1853,1331,2011,3142,1512,596,1330,2156,1566,1306,232
3
ALKBH3_1_5484,566,581,786,395,42,329,279,747,746,457,243,207
ARID2_1_5485,3180,2965,2438,3008,6657,2520,3111,1999,2101,2934,2551,24
10
ASF1A_1_5486,196,216,722,428,364,467,2052,90,310,455,90,149
ASF1B_1_5487,250,321,386,804,395,1101,101,43,42,674,3,588
ASH1L_1_5488,3094,2388,2722,2066,3046,2275,1976,1899,3341,3534,2625,51
22
ASXL2_1_5489,863,621,622,567,793,304,1134,258,694,621,1691,5
ASXL3_1_5490,2793,2342,2519,2816,4166,4696,3051,2323,2887,2498,2593,13
74
ASZ1_1_5491,138,1,98,266,0,5,1,0,0,234,0,44
ATAD2_1_5492,3682,3266,4203,4079,5523,4769,7119,4235,921,3472,2910,470
3
ATF7IP_1_5493,3695,3861,4755,4449,3841,4799,6252,3343,3627,2797,3492,5
140
AURKB_1_5494,468,1493,1247,902,2247,919,513,943,1832,486,480,1179
BAHCC1_1_5495,266,272,435,114,285,399,134,177,221,99,423,13
BAHD1_1_5496,622,377,815,792,548,314,1333,112,1438,386,4,664
BARD1_1_5497,1001,699,861,790,624,564,807,528,40,585,268,1566
BAZ1B_1_5498,2774,2433,3775,3800,1900,3050,6751,2324,5181,2637,2332,36
24
BAZ2A_1_5499,5841,5278,5915,5456,5222,6761,1675,3124,6693,4292,4074,59
00
BAZ2B_1_5500,1671,1363,2162,2527,1290,1536,1610,1519,1585,2472,982,621
BLM_1_5501,2584,2890,4201,3172,4072,4322,6001,2478,4987,4301,1471,3273
BMI1_1_5502,1787,2153,1746,1936,827,1326,3054,1608,842,1333,475,1073
BRD1_1_5503,1033,746,1157,752,199,997,826,530,909,1850,1471,830
BRD3_1_5504,3611,4195,4919,4211,2372,6756,1950,2944,4070,3481,3218,376
3
BRPF3_1_5505,288,608,587,536,69,364,106,108,284,93,79,233
BRWD3_1_5506,2031,1121,1580,1874,1547,1609,1410,936,3041,1735,3622,119
7
C14orf169_1_5507,364,359,1109,372,157,654,401,1086,388,216,138,0
C20orf20_1_5508,2137,2052,2989,2281,3703,1867,861,2077,1581,1889,1412,
3517
CALR_1_5509,622,872,644,644,1151,600,615,1607,885,885,1,627

CARM1_1_5510,1288,1957,2309,2497,1962,3014,1856,1606,2739,2547,823,152
7
CBL_1_5511,1487,1001,1077,1329,804,1079,2816,1371,480,687,1480,867
CBX4_1_5512,209,75,213,163,23,17,0,48,192,57,0,16
CBX6_1_5513,1407,1162,2224,934,1351,820,645,160,18,609,4945,286
CBX7_1_5514,3289,3118,3254,3783,2874,2822,4092,1610,5337,4064,2796,464
3
CBX8_1_5515,511,269,295,474,231,96,19,233,296,347,218,0
CCDC101_1_5516,2166,2278,2488,3134,2166,1943,1560,4520,2299,2094,3220,
3572
CCNE1_1_5517,1433,2103,2931,1711,2094,1834,1444,989,216,2017,1993,1130
CCNT1_1_5518,4558,5031,6147,5606,3245,4600,5055,3261,4124,4474,6797,39
14
CDC73_1_5519,3342,4092,3982,5949,2734,3853,3740,5439,5408,2592,4266,51
97
CDK9_1_5520,700,510,329,560,359,274,541,980,1423,670,1067,398
CDY2A_1_5521,3564,3310,3934,4331,3342,5219,5499,2437,6998,2280,3018,59
25
CDY2B_1_5522,3564,3310,3934,4331,3342,5219,5499,2437,6998,2280,3018,59
25
CDYL2_1_5523,964,612,2004,1562,562,1460,2990,1281,141,636,320,487
CECR2_1_5524,779,749,651,544,362,474,250,434,724,252,146,106
CHAF1A_1_5525,380,247,334,363,41,723,166,30,48,247,34,1033
CHAF1B_1_5526,2752,1180,1142,1067,544,854,821,3488,422,1915,2049,4874
CHD1_1_5527,959,911,1204,1337,1896,601,1601,245,1,750,2220,421
CHD1L_1_5528,642,545,919,1329,532,1168,781,512,1,424,242,39
CHD4_1_5529,1980,2255,2305,2041,2780,2273,850,4178,4049,1866,2131,1682
CHD6_1_5530,4866,4876,5878,5845,7232,4742,9090,7593,8318,4281,5122,744
1
CHD7_1_5531,3234,5399,4551,4641,5475,4896,3412,3157,4934,4768,4690,596
4
CHD9_1_5532,468,346,216,443,310,801,716,365,1795,423,23,4
CHMP1B_1_5533,760,754,946,587,553,1296,555,356,482,1324,460,761
CHMP4B_1_5534,857,1040,2014,1390,3087,1272,2263,1257,645,1748,3537,335
9
CHMP4C_1_5535,3308,2504,2816,2998,4529,1496,4284,4398,3372,2545,2749,3
091
CHRAC1_1_5536,4226,3549,3234,3304,6802,3236,6437,3121,4022,4732,3404,1
025
CLOCK_1_5537,40,49,282,129,0,119,0,0,0,1,0,0
COPS5_1_5538,1707,951,1530,2465,2539,2022,2069,2397,1880,1065,919,2132
CRAMP1L_1_5539,246,301,13,159,67,118,60,29,1649,251,0,3
CTCFL_1_5540,352,519,708,637,1097,1206,342,0,345,644,417,1889
DEAF1_1_5541,791,745,963,858,1559,1163,858,134,839,710,22,313
DNAJC1_1_5542,3912,3040,4378,4110,4240,6104,8249,2162,3244,3148,4783,3
102
DOT1L_1_5543,3113,2942,3130,4415,2711,2465,3764,3393,4538,3819,1205,30
81
DPF2_1_5544,1432,785,1006,627,1020,1481,492,441,2576,353,18,264
DPF3_1_5545,218,154,290,260,341,200,79,47,417,397,376,326

EIF4B_1_5546,1209,1153,548,1020,744,1063,1362,202,1417,1030,460,2291
ELP3_1_5547,7558,5502,7604,6342,6387,9371,9400,4802,7588,6683,5053,689
2
EP300_1_5548,1230,2117,1732,1916,1822,2694,1086,2143,1565,1824,1219,32
96
EP400_1_5549,483,277,680,176,559,284,1244,47,39,41,161,123
EPC1_1_5550,955,940,1393,1039,2928,2211,1628,1200,1903,714,238,1015
EPC2_1_5551,2328,2392,1922,3142,1779,3552,2251,2656,3020,1524,2537,200
0
ERCC6_1_5552,1359,1288,1269,1433,1558,1301,1565,370,3317,1013,377,1778
ESRRA_1_5553,419,488,252,176,1670,238,410,192,1,341,471,0
ESRRB_1_5554,349,178,371,236,26,283,0,85,319,261,0,0
FANCM_1_5555,9041,9380,9250,8011,6866,14166,11608,9179,9946,7608,11864
,10552
FBXL19_1_5556,930,475,1283,1122,1177,228,918,1343,147,1148,153,85
FOS_1_5557,850,994,969,1419,617,441,578,1348,1323,287,845,1452
FTO_1_5558,592,485,617,492,413,892,179,278,465,368,1041,576
FXR2_1_5559,1243,945,1256,864,935,1410,707,456,528,1465,686,548
G2E3_1_5560,3748,3971,4147,3235,2443,5187,3526,4338,2303,3356,1481,265
3
GATAD2A_1_5561,1184,1194,1099,1921,732,2286,1526,3543,6,2299,1469,89
GLYR1_1_5562,644,364,518,831,198,418,235,819,518,92,597,376
GMEB2_1_5563,527,543,811,654,533,513,783,548,1183,662,102,36
GSG2_1_5564,2613,3328,3859,2453,3145,4183,2529,2494,1809,3314,2772,239
0
GTF3C4_1_5565,2646,2778,2546,2290,802,2472,3755,1559,2696,1806,2194,10
49
HAT1_1_5566,486,606,1347,632,1460,1531,1362,1487,418,650,38,257
HDAC1_1_5567,2024,1497,2302,2102,801,1770,3299,853,1736,2821,1904,3176
HDAC2_1_5568,2875,4768,4251,4815,4104,6760,5033,2624,4685,2888,2597,55
64
HDAC3_1_5569,1061,883,889,1064,2552,1558,2531,202,72,1002,510,828
HDAC4_1_5570,145,302,212,259,159,74,521,72,110,4,1035,16
HDAC6_1_5571,1737,1181,1615,988,2161,1879,401,1594,1912,1557,1358,826
HDGFL1_1_5572,445,1085,594,702,391,121,51,114,1639,318,0,75
HDGFRP3_1_5573,3752,3558,4327,3755,7733,3968,5127,2422,4801,4470,2427,
3201
HELLS_1_5574,2002,1796,1985,1832,1458,2212,5119,1339,3251,941,1396,212
3
HEMK1_1_5575,2534,2661,3969,3042,5601,4761,6523,5972,2312,2969,1879,60
22
HIF1AN_1_5576,7157,5961,7585,6757,6834,8149,5167,5363,7538,7968,7267,6
570
HIRA_1_5577,2627,2564,2526,1758,3699,4127,1597,3478,2300,2702,991,3459
HMG20B_1_5578,698,963,1535,603,1395,1068,1556,76,7,1561,15,141
HMGN5_1_5579,2901,2032,2586,2577,1988,2552,2546,1618,2229,3016,3122,27
50
HNF4G_1_5580,1832,1766,2402,1963,692,1679,2347,1739,833,1526,423,1825
HSPBAP1_1_5581,5893,5782,6006,6518,7415,7581,4971,4375,4467,8555,6279,
6370

HUWE1_1_5582,1088,1124,1722,1069,1013,554,4516,2317,860,1133,79,681
ING5_1_5583,974,889,1319,1478,790,1357,329,618,22,1961,1069,633
INO80_1_5584,3701,3659,4158,5059,3379,6387,6464,2439,2147,3413,5034,51
24
JARID2_1_5585,4956,6531,6268,4948,5439,5133,6960,6015,5839,6659,3636,3
210
JHDM1D_1_5586,3150,1989,2207,2335,1602,2638,2122,1574,4151,2003,2393,2
652
JMJD7_1_5587,292,273,250,136,0,243,150,11,0,15,979,0
JMJD8_1_5588,874,1185,953,1207,694,1046,561,567,746,1051,2722,180
JUN_1_5589,1470,1558,1630,987,3213,2114,1905,1790,1290,1553,902,293
KAT2A_1_5590,7860,7447,9752,8262,10002,9470,11607,7050,6323,6404,9869,
11490
KAT2B_1_5591,6392,4267,5676,6178,6251,6807,6404,3388,2979,4888,6024,92
13
KAT6B_1_5592,601,648,1156,244,2301,568,942,605,6,593,107,60
KDM1B_1_5593,184,119,181,172,188,309,149,50,1,31,7,4
KDM2A_1_5594,1026,852,828,870,893,875,739,898,1182,708,893,798
KDM3B_1_5595,1348,2100,1316,1383,1796,1097,1787,1614,1194,1428,2767,24
8
KDM4A_1_5596,1076,1472,1700,937,1704,957,1577,2425,1806,1530,1370,967
KDM4B_1_5597,535,400,774,334,1130,630,750,157,126,902,26,2255
KDM4D_1_5598,746,400,968,456,1292,572,571,441,555,301,423,947
KDM5A_1_5599,7144,7031,8113,8264,11316,9965,7103,6157,8129,7010,12266,
6043
KDM5B_1_5600,395,163,600,930,981,246,193,284,14,624,1035,355
KDM6A_1_5601,2083,1556,1812,1774,1570,1006,1503,870,1733,1022,2105,289
0
KDM6B_1_5602,323,759,595,315,72,256,103,247,165,488,259,265
KIAA2026_1_5603,962,836,623,832,97,806,474,231,1094,607,473,920
L3MBTL2_1_5604,1051,1554,1262,1002,1284,396,462,532,1289,1470,1266,141
4
L3MBTL4_1_5605,3353,3753,4087,4094,3990,4354,5377,3793,4623,3189,6600,
3324
MAEL_1_5606,1525,1075,1293,1828,1553,244,1754,2636,2057,1712,1000,686
MBD3_1_5607,375,250,97,282,1471,415,118,227,535,487,21,0
MBD4_1_5608,5696,5389,6374,5968,7580,4836,9157,5800,4003,3985,7910,373
0
MBD5_1_5609,1951,2016,1422,957,1979,2269,1126,2237,1273,1614,890,2030
MBD6_1_5610,869,759,488,583,516,973,198,275,88,1486,1380,3198
MBTD1_1_5611,6845,7713,7565,7780,8632,5815,6436,7995,6898,3763,3395,60
44
MDM2_1_5612,3234,2031,2945,2394,3358,3161,1241,3152,5012,3200,1707,330
1
MIER2_1_5613,341,53,158,249,78,587,406,582,0,37,1555,1063
MIER3_1_5614,3994,4119,3488,4295,5404,5350,9942,1934,6272,3877,3910,17
80
MIS18BP1_1_5615,1404,1249,1346,557,2080,942,1474,1293,263,1475,52,916
MKL1_1_5616,936,731,1258,944,2297,458,1882,1792,775,701,339,1915
MLL2_1_5617,283,96,147,23,144,431,109,0,907,21,1,463

MLL3_1_5618,1537,1736,1520,2359,4333,1864,1320,2030,1305,2660,1989,154
1
MLLT1_1_5619,2258,1788,1668,2058,1267,2608,3132,1542,662,1662,1236,973
MLLT3_1_5620,3089,3536,4462,4038,2865,4158,3076,1180,3622,3700,5360,64
36
MLLT6_1_5621,503,388,822,738,704,540,214,581,1018,1772,47,48
MPHOSPH8_1_5622,379,236,133,201,947,200,201,221,1163,47,3,77
MSH6_1_5623,1155,1314,1810,1282,1167,1433,1601,1734,704,1441,1705,592
MSRB2_1_5624,1407,1960,1372,1460,3107,1125,2608,1390,3821,2374,2613,13
35
MTA2_1_5625,1569,920,1547,1272,1664,848,2696,811,301,1353,1830,378
MTA3_1_5626,1202,917,1153,760,1728,2575,1449,507,34,1403,2243,409
MYSM1_1_5627,802,1878,859,992,1033,659,1317,561,691,1030,195,2399
NAP1L2_1_5628,708,1027,998,647,624,353,629,928,1203,796,591,823
NAP1L3_1_5629,1364,1346,1203,1308,1086,1663,997,2561,2615,1787,541,876
NAP1L4_1_5630,2425,2096,2889,2475,3731,3335,2867,2753,2444,4105,1206,4
796
NAP1L5_1_5631,727,245,1122,772,530,734,790,561,2245,612,1896,244
NAT14_1_5632,1044,1241,995,745,866,1124,744,714,375,119,775,181
NAT8B_1_5633,3171,3289,4013,5120,5449,3531,4269,3728,4300,4579,7053,20
90
NAT8_1_5634,1094,368,814,1213,1365,1268,1039,1231,926,864,438,153
NAT8L_1_5635,420,66,48,566,117,66,1640,3,36,29,1564,0
NAT9_1_5636,1694,2265,1818,2137,2597,3132,2784,3883,1490,2090,2338,286
9
NCOA2_1_5637,3562,3188,4905,4105,3515,4549,4573,2879,4027,3196,6156,58
22
NPTXR_1_5638,574,793,767,456,481,1086,574,525,108,460,41,337
NR0B1_1_5639,3980,3092,4489,4189,3012,4064,2866,3944,4380,3840,4171,33
25
NR0B2_1_5640,290,165,98,200,106,386,14,246,2,32,158,45
NR1D1_1_5641,556,1270,515,677,570,489,45,148,989,1503,1324,259
NR1H2_1_5642,1008,952,1373,1102,201,1546,671,825,1018,967,1955,1496
NR2C2_1_5643,584,1076,605,744,303,773,558,790,885,375,225,36
NR2E1_1_5644,4772,4295,4950,5472,6056,3726,7741,5212,4478,3624,4819,60
97
NR2F1_1_5645,764,748,1406,893,893,1683,1320,1233,1262,1462,0,996
NR2F6_1_5646,64,209,389,1061,265,272,132,580,116,128,223,223
NR4A2_1_5647,3249,3630,3585,3678,3424,5550,4814,1931,2824,2915,3777,15
81
NR5A1_1_5648,111,208,67,53,9,818,52,29,233,140,18,552
NRIP1_1_5649,2018,1132,1621,1690,2174,1976,1343,1224,1033,1172,1620,14
43
PADI4_1_5650,5978,6121,6037,6074,10456,7464,6004,5163,7938,5470,2559,6
622
PAWR_1_5651,2415,2394,2416,2343,2500,3711,2249,3451,1777,1617,2170,167
9
PAX5_1_5652,1412,1123,1262,1549,2155,1496,698,616,3363,732,1322,914
PAXIP1_1_5653,3958,3184,4487,5020,3043,3646,4903,4321,1872,4194,6282,3
028

PGRMC2_1_5654,1248,1365,1285,1278,2391,1597,1165,1995,506,764,918,1262
PHB_1_5655,299,245,467,436,461,128,1230,152,189,152,325,16
PHC3_1_5656,456,734,577,702,1320,573,699,0,134,412,894,8
PHF13_1_5657,132,261,208,207,236,25,138,504,5,225,4,40
PHF14_1_5658,3056,2721,3692,2891,3767,1478,1216,2582,4060,2270,2010,80
71
PHF15_1_5659,732,625,891,416,80,922,535,17,1679,557,281,90
PHF20_1_5660,4472,3824,5511,3528,3221,6313,5557,5991,8687,2838,2155,18
19
PHF23_1_5661,1064,791,636,846,990,429,1133,592,989,130,889,0
PHF2_1_5662,1412,2059,2034,1924,1224,1089,2489,1847,1538,3399,1647,204
8
PHF3_1_5663,2033,1441,1779,3132,2507,3021,2077,2408,1378,1244,2573,186
6
PHF5A_1_5664,924,610,629,775,297,575,287,855,1326,540,874,319
PHIP_1_5665,2266,2206,1709,3252,2071,3005,2081,3591,4716,1701,2255,333
7
PHRF1_1_5666,1627,1191,902,1105,1520,833,1930,50,1114,617,1202,48
PIAS1_1_5667,588,462,470,229,2588,512,830,1621,611,507,465,110
PIWIL4_1_5668,13864,12242,15383,12528,16642,14766,18395,12525,9019,116
72,12655,12331
PPARGC1A_1_5669,1120,495,715,278,1199,1300,103,1075,48,551,1286,5
PRDM11_1_5670,2106,2068,2009,3138,2617,2826,1291,1648,2441,1812,1786,2
644
PRDM12_1_5671,851,659,366,257,756,416,121,839,31,513,362,706
PRDM13_1_5672,133,118,63,47,98,1,12,2,0,44,15,20
PRDM14_1_5673,1357,1056,1366,529,279,728,3138,870,1193,1025,1750,1030
PRDM4_1_5674,460,361,356,675,553,484,430,506,299,578,417,98
PRDM5_1_5675,2369,2690,1779,3401,2833,2787,2376,2551,3169,2715,1814,27
17
PRDM6_1_5676,1313,1160,1500,913,291,1700,1117,1355,1406,1130,1402,251
PRDM9_1_5677,400,727,638,730,799,1133,638,1008,140,663,478,291
PRMT6_1_5678,160,131,643,132,143,270,4,6,311,139,2,0
PRMT8_1_5679,673,910,444,779,1375,611,34,1099,6,624,462,58
PRPF6_1_5680,2385,1617,2030,2112,2376,3168,770,2118,3540,1807,3480,221
0
PYGO1_1_5681,8168,9353,11283,11343,9952,9009,10949,5488,10070,5472,106
27,12215
PYGO2_1_5682,1424,1653,2228,1601,1421,3145,1370,1716,2039,1599,745,107
1
RAG2_1_5683,493,149,696,432,371,598,56,572,96,342,471,47
RAI1_1_5684,1586,1636,2023,2937,2582,3604,2502,1864,3,1834,1476,1133
RB1_1_5685,1991,1658,2214,1433,2263,2736,1278,748,3601,1479,6195,3723
RCOR1_1_5686,660,982,973,823,1022,1525,455,304,350,1626,23,1318
RCOR2_1_5687,1436,1409,1082,652,3265,2298,726,994,2150,1661,420,1172
RECQL4_1_5688,144,129,110,83,164,545,47,97,1,0,70,344
RELB_1_5689,1394,1466,1400,1412,2405,1943,1371,1457,465,1213,1307,492
REL_1_5690,3534,3832,5493,5052,6372,4337,3866,5556,4124,4085,7210,2271
RING1_1_5691,2007,2207,2183,1973,1463,2402,1982,1579,3138,2760,2045,29
58

RNF20_1_5692,1074,832,1041,508,1660,924,427,107,947,330,1,562
RNF25_1_5693,24,143,190,29,41,6,560,6,13,389,3,24
RNF2_1_5694,3584,3581,3602,4139,4046,4618,2877,2491,2508,2326,5557,407
0
RORB_1_5695,4797,5969,6764,7101,6228,7913,5332,7329,6361,6192,6725,624
1
RSF1_1_5696,2292,2208,2419,1972,1774,1944,3284,1237,1562,1009,1823,260
5
RUVBL1_1_5697,2097,2741,2887,1828,3615,2283,2164,1764,1223,1194,1665,4
31
RUVBL2_1_5698,1528,2091,2234,1368,1633,1724,1722,3317,1572,572,841,142
9
RXRA_1_5699,3046,2917,3471,2966,1772,3822,1853,2729,879,3212,4391,3888
RXRB_1_5700,618,416,729,260,243,120,526,1038,73,159,71,65
RXRG_1_5701,389,373,557,398,7,57,810,363,89,88,5,76
SAP18_1_5702,492,242,489,266,860,266,207,218,247,365,814,722
SCML2_1_5703,2704,2721,2507,2672,2378,1947,3407,1352,2908,1936,1900,26
50
SENP3_1_5704,1532,1017,1206,1054,572,487,2837,3151,727,1269,1189,1375
SETD1A_1_5705,944,1295,1307,832,1304,1412,2311,671,1010,2088,1605,1408
SETD1B_1_5706,200,333,167,122,109,89,57,0,138,103,4,239
SETD2_1_5707,10251,11488,14279,13008,12169,12097,10334,10157,11695,123
05,11036,11417
SETD5_1_5708,811,801,1842,1345,1302,1254,490,42,1635,886,1073,156
SETD7_1_5709,2414,1621,2047,1885,1237,1128,2980,2228,3368,1368,4491,15
77
SETD8_1_5710,2168,2093,2088,2059,1386,1814,2765,1863,1600,1864,2379,16
42
SETMAR_1_5711,103,36,115,137,409,141,0,1,21,167,97,102
SF3B3_1_5712,1226,1008,1814,1049,941,1890,498,2127,2138,1178,1147,1518
SIAH2_1_5713,502,406,754,837,367,1462,456,561,32,538,105,2375
SIN3B_1_5714,162,4,116,269,0,90,2,540,111,156,13,0
SIRT4_1_5715,1095,1023,1495,743,765,1555,1951,234,614,828,1162,689
SIRT7_1_5716,814,626,527,662,550,598,686,151,1312,700,52,134
SLC2A4RG_1_5717,111,10,20,29,17,142,5,0,59,133,3,0
SMARCA5_1_5718,3092,2729,3972,3738,5507,4714,6469,5248,1742,3410,3240,
3433
SMARCC1_1_5719,708,267,640,860,614,74,955,1041,1016,140,6,102
SMARCD2_1_5720,1694,1955,2039,2199,2514,1804,1076,950,5486,1608,4508,3
216
SMARCE1_1_5721,1279,594,1057,1385,1067,1288,1619,928,1141,646,752,1234
SMC1A_1_5722,1388,1418,1600,993,1522,1215,1941,1693,1052,2100,567,3130
SMC1B_1_5723,6733,6880,6848,7375,10537,9221,10776,8407,5767,7416,8474,
5459
SMC3_1_5724,8968,9725,9229,10810,7835,9981,9469,9334,5128,11541,11124,
8453
SMCHD1_1_5725,1808,1244,2056,1448,3193,2268,2824,1034,1296,2131,359,59
0
SMNDC1_1_5726,7394,7182,10168,9087,9622,8461,8820,6366,14789,7126,6346
,9339

SMYD1_1_5727,2561,2689,2825,2259,1575,3912,2928,2738,1721,1349,900,103
9
SMYD2_1_5728,1982,2129,2123,1668,1815,1302,2932,2870,1590,2796,3148,29
09
SMYD4_1_5729,2388,1879,3481,3145,2463,3840,5029,1627,1158,3004,1296,32
51
SMYD5_1_5730,242,167,441,300,384,591,550,207,618,478,1693,368
SND1_1_5731,2191,1107,2596,1336,1327,1458,2545,2921,1420,1710,714,2965
SP140L_1_5732,2946,3730,4362,3959,3990,3461,4139,3018,3571,3100,4147,1
224
SRCAP_1_5733,855,676,1411,1564,1006,676,541,1044,1425,1547,283,2
STAT5B_1_5734,1822,2667,2359,2654,925,2530,3875,1045,2041,3048,1989,31
61
SUDS3_1_5735,14502,16377,19588,16831,20104,15982,18453,16779,13704,162
17,16501,19702
SUPT16H_1_5736,2078,804,1921,1231,2421,1904,2004,185,1459,1683,997,30
SUPT4H1_1_5737,1138,1287,1797,1717,1423,2238,3624,446,999,915,2183,438
6
SUPT6H_1_5738,2857,2562,2748,3599,3105,2776,3377,1224,2364,2591,2773,4
938
SUPT7L_1_5739,1786,749,1493,1321,2239,398,337,1323,1875,344,1239,535
SUV39H1_1_5740,1701,1860,2188,1658,3299,1154,1407,2714,4659,2758,1683,
5077
SUV420H2_1_5741,2015,1409,1720,1633,1855,2663,2108,1096,1434,2848,4402
,1829
SUZ12_1_5742,8519,8852,11867,10334,10469,10332,15098,8939,5556,7571,56
73,18287
TADA1_1_5743,1398,869,1219,1358,1053,545,625,707,1594,1198,421,793
TADA2B_1_5744,790,866,1178,1806,1130,1543,1397,1782,943,1319,2527,700
TAF10_1_5745,576,346,115,195,547,574,686,296,316,409,3,114
TAF1L_1_5746,1079,1769,1868,1345,1100,1894,1166,1108,477,1150,3497,229
6
TAF3_1_5747,1156,1819,2245,741,1338,1423,1979,2246,231,1845,826,703
TAF5_1_5748,1903,2850,2413,2535,5036,2883,3306,1352,2835,1856,1073,142
3
TAF6L_1_5749,1666,1701,1286,1291,1065,235,1083,2017,2489,2144,521,2842
TAF8_1_5750,2463,1575,2630,2349,1923,2870,4754,1480,3649,1172,2512,339
7
TCF7L1_1_5751,1888,1664,1541,1740,3475,1109,3611,1837,663,1523,656,352
8
TDRD12_1_5752,2158,3387,3739,3244,2767,2826,2412,2527,5511,1772,2482,5
140
TDRD1_1_5753,135,64,30,278,280,258,241,142,34,140,630,243
TDRD7_1_5754,849,572,791,1204,973,747,256,1019,762,365,296,1934
TDRD9_1_5755,2920,2230,3037,2493,2212,2450,2914,769,1098,3100,1780,198
2
TERF2_1_5756,2515,2299,3178,2725,3420,2142,5013,1040,2802,3658,3757,19
16
TET1_1_5757,685,655,533,562,2376,1153,724,1201,213,1887,2478,180
TET3_1_5758,103,61,323,79,183,65,0,86,0,78,17,153

TLX2_1_5759,131,196,125,14,0,21,101,42,340,25,151,28
TNRC18_1_5760,2443,2510,2054,2010,3018,4091,3462,2391,1549,2176,1955,1
477
TRAF7_1_5761,1635,1249,1841,1050,532,1000,498,799,715,687,659,1151
TRDMT1_1_5762,1149,901,1801,1430,1518,770,744,1017,1818,1708,512,919
TRERF1_1_5763,2359,3000,2483,1955,4842,2456,4100,3749,2508,1525,3566,1
220
TRIM25_1_5764,356,635,294,886,1335,413,390,53,5,683,1459,14
TRIM27_1_5765,2366,1460,2549,1962,2925,2471,2058,1100,2767,1468,2262,1
863
TRIM28_1_5766,122,548,306,535,310,64,36,474,9,147,0,63
TRIM66_1_5767,541,610,414,370,1909,458,33,94,281,397,16,67
TRRAP_1_5768,1296,661,1400,1321,1431,842,784,1219,2476,2116,703,2050
TSG101_1_5769,199,426,428,253,258,296,1836,1033,309,68,725,156
TYW5_1_5770,6978,6308,7446,5822,5724,6189,7804,6656,6279,6695,6744,402
4
UBE2B_1_5771,5029,4838,4278,5215,6330,5719,5625,6679,2471,4243,6112,63
74
UBE2N_1_5772,1260,1140,841,1022,1989,1255,1070,1043,1723,1230,529,750
UBR7_1_5773,2317,1484,3437,2851,2523,1881,3046,2129,2271,2343,1337,540
UHRF2_1_5774,1361,1807,1977,2030,1223,1222,606,1403,1787,1278,869,1726
USP22_1_5775,984,812,856,623,1252,1632,560,990,696,787,795,319
YEATS2_1_5776,233,196,83,519,458,173,192,101,98,991,202,64
YEATS4_1_5777,1048,1055,1005,854,671,780,714,805,942,1129,1726,917
YY1_1_5778,2371,1482,1997,1553,2358,1149,3067,1141,2983,1705,397,793
ZAR1_1_5779,4084,3667,2956,4030,4957,4784,3658,3699,7101,3743,4017,275
7
ZCWPW1_1_5780,1744,1625,1929,2363,786,2072,1986,2042,938,2532,1696,145
1
ZCWPW2_1_5781,602,557,724,811,166,710,490,267,620,405,641,2565
ZFP57_1_5782,1042,900,1540,1197,1005,1516,1247,1781,1267,594,1045,442
ZNF541_1_5783,862,309,870,884,267,557,1123,1700,634,437,943,1209
ZNF85_1_5784,2226,1762,3097,2185,2222,3206,3904,2826,2286,3284,6399,44
05
AANAT_1_5785,1249,1240,1667,1953,3022,1990,70,2150,308,913,1227,387
AES_1_5786,618,687,791,416,213,643,1297,156,225,773,810,44
AIRE_1_5787,345,634,631,589,585,868,442,146,922,546,1690,1106
AKAP1_1_5788,618,331,528,149,170,679,148,6,732,134,11,202
ALKBH2_1_5789,274,248,286,236,87,702,0,8,66,381,210,472
ANKHD1_1_5790,413,485,365,225,31,504,146,145,0,447,176,7
ARID1A_1_5791,382,497,720,656,572,759,692,345,351,719,347,120
ARID1B_1_5792,1411,1951,1462,1752,1116,2537,2513,1563,1587,1392,335,28
14
ARID4A_1_5793,2931,3693,3537,4094,1492,3597,3306,1586,2000,2836,3210,1
692
ARID4B_1_5794,6610,5452,6590,8713,7655,7896,7447,9762,7828,6345,5490,1
2737
ARRB1_1_5795,11056,8837,10759,10909,11330,11948,11620,11748,8878,9960,
11004,11147
ASH2L_1_5796,432,497,527,979,839,1023,1043,1885,111,429,202,960

ATAD2B_1_5797,636,404,612,704,486,716,1348,446,351,396,820,66
ATAT1_1_5798,108,326,149,229,1968,362,150,56,142,121,29,367
ATRX_1_5799,782,858,836,1111,1316,393,1493,993,2000,648,213,484
AURKA_1_5800,2279,967,1278,1521,1623,1993,1430,571,839,1778,1584,1646
AURKC_1_5801,722,717,379,797,346,639,212,119,99,631,329,680
BAZ1A_1_5802,440,339,116,273,1008,473,12,499,688,102,257,34
BCOR_1_5803,1115,1107,1363,574,1606,445,369,25,1104,1406,2063,343
BPTF_1_5804,579,494,634,354,663,509,207,275,1173,377,811,1466
BRCA1_1_5805,2017,1529,2406,2693,2468,1522,3647,1574,1488,1709,1118,11
78
BRD2_1_5806,1585,1317,2414,2403,1592,1043,1923,584,3571,2249,1002,1510
BRD4_1_5807,63,110,188,215,26,80,85,24,71,108,2,1109
BRD7_1_5808,2466,1872,2578,2876,2271,3094,4981,2026,2889,2957,2342,160
9
BRD8_1_5809,2165,1691,2462,2776,3085,2650,2291,1694,1841,3620,2056,264
2
BRD9_1_5810,802,1552,1273,1151,666,2058,969,285,513,1733,908,1517
BRDT_1_5811,3634,3958,5430,5265,6625,4638,3249,4296,4948,4588,1513,693
5
BRPF1_1_5812,790,239,494,873,464,1053,835,81,168,966,521,1551
BRWD1_1_5813,665,1077,1172,590,904,989,816,0,0,442,963,2
C14orf43_1_5814,5504,4853,7863,6039,4396,6955,8820,9252,11027,8411,431
9,8479
CBX1_1_5815,2589,2139,2619,2356,1661,3275,2710,1875,2573,2101,2699,342
7
CBX3_1_5816,3600,2794,3931,3707,3496,4055,4225,3896,2368,2661,3279,346
8
CBX5_1_5817,960,1376,931,1246,1010,1176,436,247,735,1697,1663,516
CCNT2_1_5818,3188,2636,3474,3309,3708,2600,1631,3348,2409,2869,1459,51
26
CDY1B_1_5819,734,887,1257,520,1714,1118,2139,328,222,881,212,24
CDY1_1_5820,734,887,1257,520,1714,1118,2139,328,222,881,212,24
CDYL_1_5821,1139,647,1351,1663,719,972,176,2849,1360,381,1923,63
CHD2_1_5822,771,1418,615,864,409,47,1146,913,385,919,176,1933
CHD3_1_5823,2309,2197,2474,2677,4072,2084,1416,1369,2040,2401,5476,333
0
CHD8_1_5824,928,1085,921,1639,993,682,541,221,3542,684,210,1533
CHMP2A_1_5825,5829,5737,6946,5829,8697,6523,7333,4661,7313,5602,6445,7
243
CHMP5_1_5826,265,273,397,98,409,226,342,213,9,63,0,0
COPS2_1_5827,1800,1585,1455,1570,2216,2057,1587,429,381,2362,2967,1708
CPA4_1_5828,1273,981,1291,1376,3027,2104,1050,1560,847,1933,1300,2417
CREB1_1_5829,4911,4147,3948,5445,7180,4155,3623,8164,1816,7235,5155,23
14
CREBBP_1_5830,3301,3548,4134,4161,4063,3697,4012,2951,3587,2974,3294,3
459
CTCF_1_5831,653,535,580,946,1495,576,1046,351,742,938,1286,671
CTNNB1_1_5832,302,342,383,669,484,456,568,661,181,225,701,540
CXXC1_1_5833,1502,1925,1762,2111,1212,1973,1596,2521,2006,1450,3403,18
90

CYLD_1_5834,499,373,889,771,392,233,945,767,1,343,13,1362
DICER1_1_5835,120,122,41,318,31,98,54,573,52,14,13,0
DIDO1_1_5836,301,304,595,256,429,74,204,9,805,285,2,5
DMAP1_1_5837,1185,631,374,390,64,678,45,138,175,359,233,0
DNAJC2_1_5838,2106,1754,2216,2117,3176,1875,1840,599,3034,1836,758,139
9
DNMT1_1_5839,1863,2049,1294,2256,3082,1429,1480,2430,506,2258,1213,210
7
DNMT3A_1_5840,917,547,399,1395,374,545,995,661,296,122,770,384
DNMT3B_1_5841,398,696,545,215,134,1100,37,772,2,436,84,961
DNMT3L_1_5842,396,621,452,733,1506,1386,119,284,703,683,1607,324
DPF1_1_5843,473,948,467,531,245,890,410,210,520,202,312,10
EED_1_5844,1239,1432,1290,1135,428,1682,571,669,385,584,175,1120
EGR2_1_5845,289,161,183,218,143,361,468,401,202,89,182,14
EHMT1_1_5846,826,531,766,1086,291,701,1349,508,971,1475,1370,1707
EHMT2_1_5847,1069,1193,2298,1795,928,739,2924,540,375,1882,1005,1096
ENO1_1_5848,451,618,429,308,121,389,134,795,274,426,568,1654
ESR1_1_5849,1428,926,2864,1382,2336,1716,3405,1454,1114,2162,1602,926
ESR2_1_5850,796,1206,1484,883,1062,1354,853,1141,1310,402,2017,2538
ESRRG_1_5851,3681,4350,3629,4812,2319,2252,4419,4317,1695,3189,3584,23
82
EZH2_1_5852,775,515,968,972,972,1248,1130,786,1192,407,2265,883
FBXO11_1_5853,409,304,502,351,1460,733,5,18,30,292,1126,2
FMR1_1_5854,0,0,0,86,0,0,0,0,0,0,0,0
FXR1_1_5855,870,1530,1319,1416,256,1440,244,2456,572,953,1432,3472
GFI1B_1_5856,583,652,964,867,324,410,1561,818,620,1079,889,128
GMEB1_1_5857,1619,1859,1632,2244,793,1370,2817,1460,1027,2232,651,577
HDAC10_1_5858,974,846,1160,1173,957,1417,195,989,279,1552,606,1370
HDAC11_1_5859,1412,2256,1497,2062,2529,1664,3384,1167,2674,2057,815,30
1
HDAC5_1_5860,269,541,457,409,1256,1496,1057,275,0,560,4,613
HDAC7_1_5861,1615,2163,2465,1962,607,3363,2443,1930,1140,2458,1266,134
5
HDAC9_1_5862,2000,1611,1772,1758,1970,2684,1808,1297,1666,1180,3048,11
65
HDGF_1_5863,1234,1293,1114,1250,768,394,1697,634,247,711,491,1008
HDGFRP2_1_5864,328,42,568,249,493,376,290,444,39,30,363,34
HLTF_1_5865,3933,3096,4607,4313,5539,1793,2814,1678,3128,3272,1828,287
7
HMGA1_1_5866,1397,765,784,473,1719,1926,1199,2432,1375,769,1246,1394
HMGN3_1_5867,332,376,627,230,1106,288,373,372,0,225,1396,9
HNF4A_1_5868,239,145,866,191,646,1207,845,504,105,515,402,60
HPSE2_1_5869,2051,2151,2730,2293,2093,2386,1813,3170,4154,2577,3813,34
91
HR_1_5870,187,239,149,552,13,342,0,22,55,0,0,4
ING1_1_5871,374,422,418,313,35,505,375,95,931,1109,45,8
ING4_1_5872,2007,2381,2925,2064,1554,3482,1319,1747,3204,1861,1944,362
4
INTS12_1_5873,974,841,822,932,1255,1061,667,2027,520,1159,672,987
IRF4_1_5874,822,906,900,1324,426,850,395,321,823,1573,976,914

JMJD1C_1_5875,2570,2204,2737,1793,3620,2864,4096,3113,3391,2608,2825,8
54
JMJD4_1_5876,4125,4718,4966,4056,5935,4910,4263,4303,2240,4529,3530,55
30
JMJD6_1_5877,2445,1071,1434,1424,1366,1046,2151,475,3026,695,1089,2962
KAT5_1_5878,31,644,366,373,355,343,225,91,1,1000,460,167
KAT6A_1_5879,500,299,457,524,357,234,444,4,889,165,56,387
KAT7_1_5880,1663,1854,2589,1911,1742,1164,2164,2042,1466,2165,1599,102
1
KAT8_1_5881,1646,2896,2528,3014,2304,3199,5574,3099,2254,3210,950,2232
KCTD1_1_5882,250,438,118,399,120,276,100,42,0,192,32,12
KDM1A_1_5883,1157,1017,1169,1018,684,1176,3298,740,1676,1543,992,3161
KDM2B_1_5884,1017,877,834,837,1576,333,1468,576,500,477,618,1182
KDM3A_1_5885,3998,4451,3840,3930,3844,4075,5103,3737,6610,3428,1913,23
03
KDM4C_1_5886,313,198,343,572,603,115,1758,205,246,412,829,802
KDM5C_1_5887,863,556,691,471,384,380,388,623,156,189,1515,1487
KDM5D_1_5888,550,1555,1258,1455,1797,1654,538,535,107,1604,1004,1026
L3MBTL1_1_5889,1726,1396,2146,1867,2351,1560,735,2057,2222,1472,1077,4
017
L3MBTL3_1_5890,2146,1754,2694,2074,2624,2646,2131,1535,650,2053,2056,4
312
LBR_1_5891,3431,2972,3122,3122,4674,3415,2867,2562,4612,3337,2939,2035
MAP3K12_1_5892,523,571,548,694,205,681,146,1173,235,602,448,282
MBD1_1_5893,1458,1095,1589,2013,1439,984,569,485,162,1398,230,1039
MBD2_1_5894,470,248,714,101,96,135,10,204,55,285,216,471
MECOM_1_5895,2092,1549,2777,2134,1411,1828,2343,1415,1017,913,3040,149
7
MECP2_1_5896,234,211,171,105,22,246,1469,0,8,21,4,0
MEN1_1_5897,487,778,545,793,1119,262,2867,460,682,573,213,1493
MGEA5_1_5898,3642,2235,2958,3893,4122,2248,3838,2554,3929,3060,2266,27
38
MIB2_1_5899,184,405,393,266,359,833,1274,3,0,369,1,1083
MIER1_1_5900,864,790,1237,597,952,1887,709,691,1624,557,110,323
MINA_1_5901,3004,2602,4042,2873,2777,3277,3530,2507,3771,1558,3160,284
1
MLL5_1_5902,2256,1714,2372,2353,1548,1304,863,1395,1385,1712,1555,1408
MLL_1_5903,813,1054,1101,1181,340,1567,1460,868,1147,915,709,963
MORF4L1_1_5904,187,79,359,122,2,790,0,326,2,563,1,1195
MSL3_1_5905,2795,1602,2039,2003,960,1397,4009,1869,2204,1381,1618,1099
MTA1_1_5906,251,35,146,62,70,45,301,998,82,1,0,137
MTF2_1_5907,411,128,760,242,485,443,638,531,6,0,1,166
NAA60_1_5908,4992,4035,5168,4345,6338,3296,5023,6066,3968,4615,4863,25
08
NAP1L1_1_5909,2784,1500,3250,2339,2766,3808,2988,2770,1737,2834,4571,2
811
NAT10_1_5910,2549,2188,1868,2299,2265,1784,5883,1603,4329,1343,724,320
8
NCOA1_1_5911,714,839,598,373,1140,1289,492,357,561,370,1378,669
NCOA3_1_5912,674,421,544,444,1557,1226,279,803,1210,64,0,0

NCOA4_1_5913,3803,2948,4405,4123,3893,3543,3352,4117,3270,3956,5018,33
17
NCOR1_1_5914,2360,2290,1755,1620,1521,2100,3188,1220,1420,1826,2217,17
91
NCOR2_1_5915,2762,915,1575,1206,2580,1566,2645,574,344,1164,2939,3738
NFAT5_1_5916,741,568,802,782,628,579,927,323,524,643,2220,312
NFATC1_1_5917,353,419,631,148,241,304,61,547,169,32,0,280
NFATC3_1_5918,551,759,500,572,481,150,536,242,205,770,4,245
NFATC4_1_5919,323,436,582,271,267,163,1082,43,29,411,0,2120
NFKB1_1_5920,3065,2778,4198,4036,3394,3716,2902,4544,6516,3216,1421,38
75
NFKB2_1_5921,2912,2088,2217,2562,3592,2133,3270,2160,1171,2380,4855,29
31
NPM1_1_5922,2002,1862,3281,2350,1960,2098,4798,956,972,2712,3562,1025
NR1D2_1_5923,1003,1099,940,1070,1326,452,1032,1570,2147,576,302,641
NR1H3_1_5924,341,165,224,240,11,402,506,779,152,42,112,124
NR1H4_1_5925,744,290,538,377,1143,904,766,75,3,54,315,663
NR1I2_1_5926,1806,1368,1261,1436,1272,1680,1662,741,2497,691,1383,1085
NR1I3_1_5927,569,728,517,1460,410,1278,336,1486,1011,1515,1568,783
NR2C1_1_5928,1442,1882,2454,2273,3190,2386,1639,1682,1494,2591,1030,25
14
NR2E3_1_5929,470,631,118,269,249,422,587,54,994,12,239,1020
NR2F2_1_5930,284,455,458,446,146,181,548,0,141,312,976,400
NR3C1_1_5931,2037,1904,2818,2341,1965,1607,2851,2028,1456,2424,2052,32
66
NR3C2_1_5932,1444,2254,1977,1310,1759,1571,3901,1251,3644,2220,2672,14
63
NR4A1_1_5933,859,661,618,715,613,1034,1511,415,294,254,1344,378
NR4A3_1_5934,746,301,560,782,287,128,0,600,50,162,1130,680
NR5A2_1_5935,3100,2208,2487,2263,3138,2804,5091,1197,2920,2308,2270,29
30
NR6A1_1_5936,3943,2677,3648,2978,3485,4875,5506,1920,3778,2207,1296,40
70
NSD1_1_5937,476,215,327,140,264,170,207,949,57,73,23,85
PBRM1_1_5938,3840,3824,6313,3820,4473,3323,8177,3535,4352,2717,4140,43
96
PCGF6_1_5939,2810,2181,3025,2875,4066,1383,3398,1968,1404,1655,2387,19
11
PGR_1_5940,74,381,320,331,237,2,3,685,151,4,287,921
PHC2_1_5941,596,785,514,865,665,714,198,488,187,957,756,65
PHF10_1_5942,4815,3949,5037,4236,3245,5657,6077,3292,3964,4240,6008,45
64
PHF11_1_5943,394,269,194,343,196,37,536,720,1092,455,1579,63
PHF12_1_5944,2092,2416,2805,2317,3864,1461,1822,3078,2064,1765,3902,24
86
PHF16_1_5945,433,468,432,262,941,170,243,176,204,31,0,32
PHF17_1_5946,1987,2038,2977,2168,2359,2743,2296,4916,2219,3369,1023,11
94
PHF1_1_5947,580,1209,272,204,1140,641,868,656,40,90,925,1705
PHF20L1_1_5948,902,1296,904,1153,2228,1176,2397,895,735,1100,2932,2221

PHF21A_1_5949,2966,2857,3379,3075,6295,4155,1579,3137,1320,2058,1137,4
017
PHF21B_1_5950,1160,670,1101,818,2936,656,1333,328,2157,666,1721,947
PHF6_1_5951,1969,1669,1888,2919,808,2081,3235,1567,2080,1155,3517,3118
PHF7_1_5952,2541,2188,2526,1606,3545,2249,2213,3487,2547,2302,1397,261
2
PHF8_1_5953,4969,5361,5014,6340,5062,5739,3933,3923,8319,6053,5053,169
1
PIAS2_1_5954,3666,4139,5954,4875,3185,3305,5475,4167,7245,4300,4074,38
60
PICK1_1_5955,83,228,272,115,704,282,1642,67,1,172,35,655
PIWIL2_1_5956,1200,1834,1327,1586,1321,2013,942,1353,757,1085,220,949
PML_1_5957,527,592,922,784,202,323,1817,576,675,686,318,818
POLR1B_1_5958,792,1283,2075,1459,439,2313,501,1013,441,1708,1415,1330
PPARA_1_5959,2365,2013,3018,2625,2105,3429,1578,3534,4498,2291,2761,47
34
PPARD_1_5960,139,47,253,192,0,88,561,52,255,43,40,9
PPARG_1_5961,2701,2428,2597,2311,3737,2800,2700,1868,4836,2915,1861,39
89
PRDM10_1_5962,852,574,566,748,649,621,184,6,9,784,77,300
PRDM15_1_5963,802,1457,2088,1509,948,2838,587,778,767,687,444,1491
PRDM16_1_5964,1583,938,1984,2078,1155,1338,2355,649,1893,1342,790,3590
PRDM1_1_5965,493,754,479,575,603,266,322,301,1203,1306,1149,595
PRDM2_1_5966,589,736,773,763,1270,1464,1611,342,1165,1051,553,479
PRDM7_1_5967,5132,5798,5274,4191,7564,4890,5872,2965,2875,5295,5851,37
43
PRDM8_1_5968,223,152,266,190,142,210,139,0,96,223,36,1325
PRMT1_1_5969,136,272,55,179,96,220,37,1,773,214,5,4
PRMT2_1_5970,27,49,123,93,5,6,1,0,439,0,0,0
PRMT3_1_5971,5832,6363,7918,6742,5612,8593,9096,6421,6247,5921,6159,10
901
PRMT5_1_5972,2313,1706,1522,1837,2840,1402,952,2097,2638,2259,1107,219
2
PRMT7_1_5973,1002,492,1214,1304,1028,802,744,1323,147,1081,898,1111
PSIP1_1_5974,1680,1635,2757,2963,2954,3540,2879,1662,3378,2040,3504,80
9
PSMC5_1_5975,2728,2502,2832,2939,1080,2883,2540,713,4912,2223,2907,442
1
PWWP2B_1_5976,514,90,494,605,684,635,0,1027,1,467,12,1
RAD54B_1_5977,3108,3203,4242,2616,3359,4284,4752,1631,1988,2496,2309,2
527
RAD54L_1_5978,2039,2174,2527,3011,2822,2571,1300,1821,2780,1038,4965,4
772
RARA_1_5979,1000,542,665,501,889,1137,363,1483,61,903,521,1311
RARB_1_5980,1482,1479,1147,663,508,1296,3165,997,798,1303,1566,1977
RARG_1_5981,1244,800,1687,890,1437,1207,2955,430,1334,852,1734,735
RBBP5_1_5982,519,595,270,744,76,801,703,819,1078,636,1182,474
RBCK1_1_5983,541,370,318,193,665,136,806,174,194,162,259,10
RBFOX2_1_5984,1768,1795,1268,1323,1664,1950,3629,2230,3813,1533,1777,1
126

RCC1_1_5985,894,741,520,357,21,514,181,306,169,596,132,18
RCOR3_1_5986,2073,1608,2232,2618,1038,2895,947,1640,802,2523,1266,2517
RECQL5_1_5987,432,445,820,591,1391,519,661,469,50,219,603,770
RECQL_1_5988,265,177,291,209,0,155,1,265,308,487,750,0
RELA_1_5989,2987,1514,2612,1498,2597,3135,2119,2307,740,297,1066,1573
RERE_1_5990,1052,1151,1160,1220,710,532,1004,2266,551,421,6,1102
RFC1_1_5991,1409,1073,1452,1416,2610,1477,1020,616,2238,1238,2117,1399
RNF14_1_5992,2566,2902,2811,2620,3375,1810,3365,2426,1445,1806,2659,84
5
RNF17_1_5993,890,621,848,845,1759,422,178,41,1221,567,1271,328
RNF40_1_5994,1342,1426,2195,1942,829,1433,3834,4819,198,960,1176,1974
RNF8_1_5995,719,437,823,896,729,1282,2630,942,390,363,452,1110
RORA_1_5996,1099,1015,967,1167,1293,439,760,1106,740,479,1239,702
RORC_1_5997,360,161,240,55,70,35,2,214,19,131,0,0
RPH3A_1_5998,474,251,553,666,268,1677,22,247,87,780,1482,126
RPS6KA5_1_5999,311,868,640,613,520,2387,1858,1316,180,1343,1369,39
SATB1_1_6000,1004,783,300,685,154,608,926,195,272,1203,315,1031
SATB2_1_6001,301,230,396,152,142,637,293,133,144,287,401,38
SCMH1_1_6002,1466,1856,1532,1915,1137,1005,2270,1703,1432,1298,786,150
7
SET_1_6003,1839,1649,1782,1914,2807,3152,3423,2477,1557,1829,1865,3707
SETD3_1_6004,385,172,354,357,260,319,62,229,170,243,1941,9
SETD4_1_6005,4774,5303,3881,3498,3497,6693,4887,2831,1907,4702,4424,71
33
SETD6_1_6006,333,511,526,440,198,287,140,301,1771,624,0,547
SETDB1_1_6007,936,1717,1773,1627,2357,2822,658,1921,648,1688,419,2180
SETDB2_1_6008,296,374,374,515,387,1327,265,108,419,324,1906,627
SFMBT1_1_6009,3716,3147,3664,3845,6378,3223,4552,3989,6387,2439,3678,4
806
SFMBT2_1_6010,2983,2098,2270,2805,2449,3037,2846,2580,2544,2574,3057,2
088
SHPRH_1_6011,285,268,593,243,478,279,421,246,1,668,690,259
SIN3A_1_6012,101,144,53,80,0,59,244,504,485,80,384,0
SIRT1_1_6013,429,395,223,217,56,10,550,0,32,4,144,606
SIRT2_1_6014,1502,894,1146,1440,1846,1235,1301,1665,461,1940,288,791
SIRT3_1_6015,0,55,23,15,0,0,0,0,0,367,0,0
SIRT5_1_6016,410,215,193,139,99,150,228,349,554,385,317,2
SIRT6_1_6017,3889,3450,5090,6292,4832,5802,5748,3472,4409,3374,6991,51
75
SLC38A1_1_6018,3066,3016,3462,3199,3072,2628,3925,1470,4457,2287,1009,
6350
SMARCA1_1_6019,3187,3084,2866,2520,3493,1964,3053,5011,4683,2291,1119,
1618
SMARCA2_1_6020,294,414,576,531,624,1162,1214,232,669,260,585,354
SMARCA4_1_6021,598,350,391,546,1068,554,329,92,1086,260,549,244
SMARCAD1_1_6022,1149,1652,709,1335,822,2005,2031,1350,333,894,858,25
SMARCAL1_1_6023,218,293,558,233,668,192,552,135,0,200,109,78
SMARCB1_1_6024,2081,2523,2156,2660,1725,3659,1222,1380,3783,1226,1106,
2772
SMARCC2_1_6025,462,813,1511,864,1039,337,1361,681,470,875,1085,2347

SMARCD1_1_6026,0,0,0,0,0,0,0,0,0,0,0,0
SMARCD3_1_6027,316,387,588,537,281,376,897,435,1170,269,43,72
SMC2_1_6028,1359,874,1250,1533,1536,1447,1273,564,1013,1424,1978,1745
SMC4_1_6029,3092,2498,2887,3271,1352,3197,2925,2701,1937,2771,2425,238
1
SMN1_1_6030,2944,2231,2509,2385,1266,2366,1624,1583,4908,3554,1593,265
7
SMN2_1_6031,2944,2231,2509,2385,1266,2366,1624,1583,4908,3554,1593,265
7
SMYD3_1_6032,3162,2120,3303,2774,2586,2017,3470,4023,3144,3130,2314,34
91
SP100_1_6033,8546,7223,8970,10185,7899,5843,8978,8894,10574,7999,5970,
7053
SP110_1_6034,1572,974,646,1270,1401,805,953,2070,1601,862,1145,538
STK31_1_6035,4231,4560,5195,3532,3318,6036,5701,4345,2864,4218,2297,32
00
SUPT3H_1_6036,9883,9991,11460,11521,12489,17108,13499,10061,7646,10636
,9277,20309
SUPT5H_1_6037,713,1505,1483,952,2167,2183,645,636,583,959,1250,1141
SUV39H2_1_6038,1509,803,1500,1719,598,506,2187,1002,2722,544,774,903
SUV420H1_1_6039,1302,1638,1353,1762,554,1978,293,1479,1592,2060,1191,1
979
TADA2A_1_6040,1669,857,1687,835,692,567,2486,452,1562,1037,551,2285
TADA3_1_6041,472,297,617,916,578,808,262,186,531,607,41,322
TAF12_1_6042,2729,2619,2797,2819,5362,2324,4376,2644,3634,1871,4757,27
28
TAF15_1_6043,1287,954,847,978,1274,2361,2511,774,2254,1879,219,126
TAF1_1_6044,1455,909,1560,1311,990,1386,3192,1168,2000,1059,307,814
TAF5L_1_6045,150,227,106,33,497,401,571,67,0,118,108,330
TCF19_1_6046,417,331,322,369,206,426,68,212,593,393,0,1878
TCF20_1_6047,414,191,1243,761,213,216,881,325,584,626,225,510
TDRD10_1_6048,1694,1257,2521,1841,4443,2683,374,2353,2082,1956,1845,26
53
TDRD3_1_6049,7352,5719,9039,6797,6133,7713,6176,5881,1463,5127,4561,85
35
TDRD5_1_6050,1423,788,1108,751,1206,1183,870,786,1667,720,994,916
TDRD6_1_6051,6453,7549,6617,6734,7609,8576,6410,7009,6750,5804,6307,57
67
TDRKH_1_6052,1309,1143,1183,1384,2575,1261,3273,478,1461,504,1183,542
TERF1_1_6053,1020,1691,714,1649,2141,2270,1126,1531,2,1153,1083,1159
TET2_1_6054,1495,745,2009,927,654,784,317,1213,554,1013,83,1137
THRA_1_6055,911,532,934,528,1107,1280,1131,1189,133,585,1513,224
THRB_1_6056,1093,842,1016,1083,1445,916,1515,1099,1684,414,1362,3068
TP53BP1_1_6057,2205,1829,1664,1915,1677,3161,4417,1603,1722,1381,2470,
2983
TP53_1_6058,1455,1836,1895,2309,3698,1317,1625,1757,2401,1498,2680,223
9
TP73_1_6059,611,652,1161,626,445,1046,246,121,1825,1124,1620,312
TRIM24_1_6060,852,724,662,594,1697,695,1458,149,188,484,282,794
TRIM32_1_6061,337,590,494,983,386,163,1240,783,160,880,589,11

TRIM33_1_6062,465,542,562,705,677,261,314,920,70,441,929,21
UBE2A_1_6063,2967,3033,2790,3008,3423,2625,3099,2738,1094,2095,2142,16
67
UBE2E1_1_6064,1800,1204,1614,2338,3269,924,3063,2276,1938,1830,2531,10
80
UBE2I_1_6065,467,668,817,768,1249,534,120,623,732,146,640,192
UBE2K_1_6066,2838,3042,3643,3925,2574,2484,5052,1821,4937,2567,5502,27
97
UBE2V1_1_6067,3052,2488,3722,2796,3487,2328,2870,4087,3700,2285,2939,1
343
UHRF1_1_6068,3128,1751,1997,2444,1309,2129,3602,548,1105,1927,2384,205
2
USF2_1_6069,816,1070,1401,790,708,1181,1509,2054,673,881,2042,1335
UTY_1_6070,1539,2683,2293,1719,2267,1919,1694,370,399,1803,540,166
VDR_1_6071,506,446,284,336,554,537,163,249,112,207,36,10
WDR5_1_6072,1663,1600,1613,1760,1779,1448,2073,767,3924,1306,2417,443
WHSC1_1_6073,2921,3172,4655,4464,4987,4881,3791,5173,5953,4431,1894,89
01
WHSC1L1_1_6074,6297,5013,5682,5786,5231,4403,4997,7414,5615,4559,5535,
6326
WRB_1_6075,398,271,614,356,1,370,912,107,602,71,0,0
ZGPAT_1_6076,99,213,147,110,59,99,343,351,18,105,2,18
ZMYND11_1_6077,226,354,197,252,260,424,695,1208,13,452,58,864
ZMYND8_1_6078,89,280,414,33,101,179,147,248,77,6,180,0
ZNF451_1_6079,3187,2629,3785,3269,2476,2712,2390,3261,1018,4603,7011,1
268
ALG13_1_6080,1816,2132,2644,1805,2788,2097,3256,1575,2489,3137,1108,38
78
ASXL1_1_6081,406,182,438,153,110,160,175,1160,150,0,149,1
CBX2_1_6082,580,686,1061,1331,164,879,1206,847,671,1130,350,3122
HDAC8_1_6083,430,1202,742,617,1223,2161,784,394,765,364,85,576
ING3_1_6084,455,963,903,428,558,616,342,73,1220,699,988,149
MLLT10_1_6085,1719,2508,2324,2141,2599,3585,4567,845,2171,1247,3339,41
16
PHF19_1_6086,4697,5248,6246,5599,8031,5001,6372,4548,3591,3434,5884,40
33
RBM14_1_6087,1348,1480,2186,2466,4361,1919,1557,1582,316,954,274,980
SP140_1_6088,3815,3050,3615,3664,4304,2023,5223,1193,4445,2254,3375,30
09
TAF9_1_6089,1489,1515,1383,1640,1618,1076,1647,1048,1171,2152,1708,200
0
TAF9_1_6090,734,909,1253,1111,513,785,864,1116,260,966,338,431
ARID4A_1_6091,949,637,665,704,233,466,254,1284,688,460,430,226
ARID4A_1_6092,55,68,338,65,3,5,606,4,13,37,0,0
ARID4A_1_6093,573,526,418,459,1428,368,168,587,1023,598,4,1044
ARID4A_1_6094,586,1115,761,950,372,841,635,1397,1348,1454,1686,757
ARID4A_1_6095,353,253,456,774,2572,314,232,914,33,344,249,132
ARID4A_1_6096,1753,1332,1764,1166,2216,2043,2615,1613,945,2039,516,154
8
ARID4A_1_6097,155,245,377,71,184,400,991,1,1,59,492,88

ARID4A_1_6098,393,42,335,128,1,86,74,91,123,395,411,0
ARID4A_1_6099,941,874,1440,1871,1339,1391,2094,894,777,1524,1322,921
ARID4A_1_6100,447,426,677,924,1220,503,913,895,571,377,148,919
BRIP1_1_6101,1113,1157,1361,1916,2667,1480,1555,738,477,1876,762,1193
BRIP1_1_6102,11617,12068,12278,12059,11742,11979,12804,9775,8420,11601
,10573,17464
BRIP1_1_6103,514,488,633,785,434,315,518,309,1028,500,374,510
BRIP1_1_6104,2004,1466,1063,813,1962,1373,965,1309,1079,392,1067,350
BRIP1_1_6105,2684,2786,3016,2882,2951,1472,3734,2984,2496,1499,1707,43
24
BRIP1_1_6106,2196,2134,2186,2351,4275,3012,8464,2557,1240,1555,2480,33
51
BRIP1_1_6107,2757,2696,2572,3220,3635,4413,2479,2380,3697,2137,2746,29
19
BRIP1_1_6108,904,739,724,719,364,390,1798,1843,0,268,168,12
BRIP1_1_6109,2317,2010,3242,2104,2803,3861,5795,2590,2593,2651,5366,21
46
BRIP1_1_6110,4213,3656,4743,4920,6204,3710,2261,3493,4566,4073,4223,31
86
CHAF1B_1_6111,913,613,1702,1804,482,961,2051,514,1621,2289,1765,930
CHAF1B_1_6112,963,1524,2280,1653,2667,2445,613,1872,1662,1646,1729,162
9
CHAF1B_1_6113,4044,4194,5964,5771,5241,6085,5500,4259,5034,4220,3470,3
816
CHAF1B_1_6114,1828,1822,2870,2228,2895,3008,3516,2471,2191,866,2351,16
19
CHAF1B_1_6115,258,517,368,493,1163,155,804,1102,2305,545,43,1
CHAF1B_1_6116,1507,915,2960,1433,2737,1002,1965,2129,1507,1781,2012,15
51
CHAF1B_1_6117,2826,2008,3087,2533,3912,1575,1744,3446,1557,2548,1332,3
086
CHAF1B_1_6118,1627,2261,1937,2115,3548,2164,1980,1045,2917,1688,2568,1
118
CHAF1B_1_6119,4008,3983,4749,5134,4644,5597,3884,3396,4615,4000,3476,3
232
CHAF1B_1_6120,9852,10068,11227,11015,13111,13948,14166,10676,9394,9644
,10802,9721
WRB_1_6121,1213,1073,1377,1532,1129,1620,2581,1513,1326,999,2613,2682
WRB_1_6122,4183,4480,5223,4956,4582,5052,3079,7419,5614,4915,3549,2346
WRB_1_6123,2967,2552,2540,3153,2675,1904,3717,960,2359,2384,5268,6213
WRB_1_6124,585,767,531,703,567,245,1398,536,1171,348,318,30
WRB_1_6125,2644,2915,3138,2095,2204,5607,1680,1940,2942,2842,4549,6603
WRB_1_6126,2046,1418,2257,2607,1123,2827,1516,2922,1477,2186,1348,1034
WRB_1_6127,1122,724,944,891,1084,2211,382,668,2973,711,84,1906
WRB_1_6128,402,578,532,801,1047,192,1273,783,531,895,10,104
WRB_1_6129,1187,1342,1579,1127,951,638,221,687,153,413,1837,77
WRB_1_6130,2039,1991,2077,1363,1765,1746,2059,1781,2327,885,2461,350
CHD6_1_6131,1213,1073,1377,1532,1129,1620,2581,1513,1326,999,2613,2682
CHD6_1_6132,4183,4480,5223,4956,4582,5052,3079,7419,5614,4915,3549,234
6

CHD6_1_6133,2967,2552,2540,3153,2675,1904,3717,960,2359,2384,5268,6213
CHD6_1_6134,585,767,531,703,567,245,1398,536,1171,348,318,30
CHD6_1_6135,2644,2915,3138,2095,2204,5607,1680,1940,2942,2842,4549,660
3
CHD6_1_6136,2046,1418,2257,2607,1123,2827,1516,2922,1477,2186,1348,103
4
CHD6_1_6137,1122,724,944,891,1084,2211,382,668,2973,711,84,1906
CHD6_1_6138,402,578,532,801,1047,192,1273,783,531,895,10,104
CHD6_1_6139,1187,1342,1579,1127,951,638,221,687,153,413,1837,77
CHD6_1_6140,2039,1991,2077,1363,1765,1746,2059,1781,2327,885,2461,350
COPS2_1_6141,1852,1556,2026,1261,4446,996,2439,1828,1068,1786,1287,149
1
COPS2_1_6142,58,142,85,335,355,59,60,66,164,1168,325,119
COPS2_1_6143,1062,641,1826,1060,780,1445,2974,556,68,1634,754,1525
COPS2_1_6144,2148,2644,2557,2153,2123,1421,5574,1790,2145,1857,4552,24
64
COPS2_1_6145,520,95,501,416,31,549,90,1455,1945,790,64,2032
COPS2_1_6146,2081,1319,2540,2342,3947,1556,3514,1412,2444,1976,2921,30
59
COPS2_1_6147,1824,1962,1583,1873,1079,531,3122,1495,1455,1439,2479,943
COPS2_1_6148,323,350,595,632,28,1079,1144,512,257,272,174,2
COPS2_1_6149,6297,5601,7710,6057,7134,4583,4867,2993,7313,4222,3713,48
94
COPS2_1_6150,838,411,499,801,694,1063,1515,1463,360,864,32,62
CPA4_1_6151,7146,4895,6539,5226,8175,6386,7868,5462,8628,5559,3889,530
6
CPA4_1_6152,4636,3135,3665,2599,5396,4458,5669,1636,3656,5233,4594,489
9
CPA4_1_6153,204,219,218,315,0,554,408,115,124,0,2,332
CPA4_1_6154,2183,2259,1470,2150,2473,436,1681,733,3467,1958,1790,3031
CPA4_1_6155,2004,1735,2568,1427,896,2088,5891,2154,1528,1939,4565,5867
CPA4_1_6156,8,12,168,542,1,109,14,0,0,12,0,0
CPA4_1_6157,135,553,950,376,546,752,7,472,1,50,262,948
CPA4_1_6158,6372,6960,6918,5807,5726,7462,5024,5452,4880,4113,8706,716
7
CPA4_1_6159,295,150,633,66,6,67,0,368,252,2,1944,4
CPA4_1_6160,6729,4639,6363,5465,8018,6657,6941,5455,8592,5472,3872,527
3
CXXC1_1_6161,91,27,195,328,458,131,3,0,0,204,0,384
CXXC1_1_6162,2402,2240,2676,1368,3309,2765,2667,2715,3174,2277,2592,38
83
CXXC1_1_6163,3803,3203,3775,4436,4961,4387,3634,3141,2875,3040,1715,40
55
CXXC1_1_6164,816,1016,1531,411,293,939,450,930,2023,205,90,659
CXXC1_1_6165,3353,2810,3258,4374,4178,3819,3746,3351,3322,2232,1512,34
45
CXXC1_1_6166,2523,2115,1791,1314,821,2529,1813,2307,1951,2220,1140,345
2
CXXC1_1_6167,1270,858,1208,426,1273,1147,941,828,995,598,977,158
CXXC1_1_6168,1504,907,2598,1482,926,1886,924,1147,5250,2475,1522,1734

CXXC1_1_6169,2013,1523,1318,1401,1961,630,1667,684,1270,1481,589,835
CXXC1_1_6170,1045,740,598,914,1643,265,671,2392,48,286,1208,397
DEAF1_1_6171,1029,581,1006,201,1874,1601,133,218,129,309,0,6
DEAF1_1_6172,818,920,1106,775,996,896,214,927,871,1277,28,547
DEAF1_1_6173,843,493,931,894,1300,279,448,804,865,140,939,1115
DEAF1_1_6174,270,122,106,369,279,716,424,13,188,441,308,112
DEAF1_1_6175,917,1126,995,877,992,990,213,931,877,1520,28,1150
DEAF1_1_6176,441,669,309,574,720,413,257,575,29,264,392,6
DEAF1_1_6177,2786,3362,3347,3206,3073,4145,6953,2041,2148,3418,2001,17
15
DEAF1_1_6178,384,787,788,579,532,493,1775,1151,147,1092,1835,387
DEAF1_1_6179,446,261,869,407,1028,535,335,656,835,108,1376,133
DEAF1_1_6180,717,837,537,742,237,353,155,1246,180,358,35,1702
DIDO1_1_6181,1761,1522,1675,2651,2256,2841,1066,1830,2929,2327,1821,18
78
DIDO1_1_6182,1559,2240,1623,1750,1466,2332,2151,2063,1574,1640,2036,12
84
DIDO1_1_6183,3482,4076,3288,4108,5821,4743,3306,3134,8815,3516,8684,49
33
DIDO1_1_6184,4763,5030,7180,5743,9196,5429,4840,2719,4031,4547,6898,44
35
DIDO1_1_6185,292,313,295,358,6,405,168,806,389,124,480,1305
DIDO1_1_6186,4696,4738,7016,6037,8675,5661,4817,2725,4032,4553,6917,44
89
DIDO1_1_6187,647,364,319,429,1001,524,182,211,464,352,232,2172
DIDO1_1_6188,2102,2258,2492,2321,1783,4803,1217,1664,3267,1940,2432,25
2
DIDO1_1_6189,1683,2311,3026,2724,1699,2366,2070,2775,2297,1900,2312,14
91
DIDO1_1_6190,2610,3123,3492,3743,2262,3626,2798,3355,4397,2506,2481,20
48
DMAP1_1_6191,3720,3542,4007,4367,6614,2255,4101,3486,2864,3581,2101,17
23
DMAP1_1_6192,1943,2049,2248,2383,1120,2077,857,1876,2106,1260,2713,129
3
DMAP1_1_6193,0,0,0,0,0,0,0,0,0,0,0,0
DMAP1_1_6194,805,1209,1693,1684,1637,1565,1719,1259,706,1000,2541,1970
DMAP1_1_6195,6626,6806,6282,7467,9900,6379,7155,7223,4086,7516,11889,4
379
DMAP1_1_6196,2213,1570,2372,1992,1648,2503,1435,2338,1053,1926,1045,99
6
DMAP1_1_6197,5548,4138,5968,4853,4435,6347,5827,5140,5981,3912,5068,95
14
DMAP1_1_6198,3485,2313,2772,2128,3565,2382,3939,2796,3835,2167,3776,22
38
DMAP1_1_6199,1767,1840,1933,2092,2957,1534,4030,2081,2819,1665,1671,16
52
DMAP1_1_6200,3372,3027,3772,3216,4334,2299,2770,2394,1821,4946,2331,45
65
EZH2_1_6201,1283,1040,2185,1229,1132,849,824,1005,12,527,327,438

EZH2_1_6202,408,406,329,43,421,49,294,10,0,442,70,51
EZH2_1_6203,1878,2104,1517,1663,1555,1065,856,2120,2730,1422,3764,1348
EZH2_1_6204,873,671,731,705,849,932,786,595,584,1418,2577,1031
EZH2_1_6205,2677,2698,4131,2553,4476,4300,5226,3317,5461,1785,2971,344
1
EZH2_1_6206,681,1335,639,1398,1011,601,986,446,260,638,768,2448
EZH2_1_6207,3474,4116,2448,2906,5394,4511,2321,2660,3235,2397,2211,383
0
EZH2_1_6208,2646,1828,2927,2609,2940,3647,5416,2056,4449,2618,844,2528
EZH2_1_6209,2223,1702,1613,1610,1307,1430,2374,2147,3812,1279,2891,210
3
EZH2_1_6210,3438,3268,4525,3479,2510,3212,2569,1506,3143,3751,3569,451
7
INGX_1_6211,413,322,1262,585,6,724,366,850,0,526,44,0
INGX_1_6212,641,506,789,484,115,428,774,162,485,548,179,43
INGX_1_6213,3899,3160,4209,3885,4300,5589,5743,2037,3706,3092,2931,441
9
INGX_1_6214,5431,5890,6078,6603,7828,9921,7771,6547,4106,6964,5010,762
7
INGX_1_6215,1596,2324,2160,2263,1335,2711,2387,2521,1300,1425,2033,320
0
INGX_1_6216,1135,2086,2262,1447,1714,1249,1242,957,1472,2115,2041,2879
INGX_1_6217,3291,2341,2774,2673,3995,2823,3554,1706,4378,2724,4275,849
INGX_1_6218,4718,2914,3956,4253,4716,2965,3168,4255,4189,3982,4586,508
4
INGX_1_6219,1519,1458,1360,1592,1191,2156,1769,2515,549,1317,1965,1805
INGX_1_6220,1928,1737,2141,2134,2548,1962,3719,923,3580,1726,1673,2327
IRF4_1_6221,481,444,625,785,75,307,148,267,45,663,862,1142
IRF4_1_6222,1466,2010,1667,2383,2694,1290,2519,1412,1356,2167,1286,157
4
IRF4_1_6223,332,53,120,20,31,23,0,0,0,0,225,2
IRF4_1_6224,2028,1746,1535,1837,1074,2156,2287,987,946,2058,1862,2086
IRF4_1_6225,223,211,239,323,77,344,1364,83,867,134,24,481
IRF4_1_6226,1025,1628,1149,1159,1587,1993,1298,1607,1,1158,2155,1648
IRF4_1_6227,1062,1056,1451,1373,1383,973,525,1361,1044,1432,579,1401
IRF4_1_6228,1750,2146,3174,2201,2303,2267,6192,3642,946,1213,917,1818
IRF4_1_6229,203,72,209,349,146,359,5,29,315,181,39,71
IRF4_1_6230,3989,3831,3675,4597,5415,4014,3205,3736,3176,3572,4849,242
6
KDM4E_1_6231,9479,7662,9987,8799,12698,11447,8249,5521,12794,11319,922
6,9144
KDM4E_1_6232,780,476,945,829,748,2118,371,650,257,684,1965,219
KDM4E_1_6233,3629,3283,5312,3192,5110,4694,1852,5029,2494,3149,3054,24
93
KDM4E_1_6234,444,675,1130,302,547,153,2032,325,310,17,398,367
KDM4E_1_6235,31,202,238,148,77,17,0,0,2,76,16,68
KDM4E_1_6236,182,503,192,115,229,106,7,0,455,63,18,0
KDM4E_1_6237,636,537,530,475,1438,82,603,600,1247,453,29,835
KDM4E_1_6238,321,321,432,304,162,359,57,49,211,438,229,14
KDM4E_1_6239,620,451,1124,1828,720,350,2685,636,2781,613,766,671

KDM4E_1_6240,3742,3860,4091,3035,1278,2651,3424,3643,5900,3758,4584,47
15
KDM8_1_6241,179,189,260,382,718,150,31,131,13,351,568,111
KDM8_1_6242,859,887,863,1332,1030,1089,214,134,2486,786,305,31
KDM8_1_6243,139,231,512,380,214,332,809,181,873,69,311,385
KDM8_1_6244,1104,471,742,530,2140,534,1004,536,990,1115,548,1308
KDM8_1_6245,403,451,242,565,253,591,1066,315,80,865,6,103
KDM8_1_6246,491,256,382,87,232,544,720,64,1105,56,252,180
KDM8_1_6247,579,849,1393,1101,1197,1578,712,2487,1519,1493,1303,1570
KDM8_1_6248,137,108,93,199,126,8,86,76,59,368,364,96
KDM8_1_6249,875,523,278,518,240,534,447,286,973,261,788,239
KDM8_1_6250,102,215,218,548,208,190,135,431,15,456,52,26
NCOA2_1_6251,413,309,241,331,404,454,1666,708,1649,825,705,96
NCOA2_1_6252,1547,1185,1488,1103,428,1468,811,1038,1436,952,1661,384
NCOA2_1_6253,2736,1891,4066,3391,2359,3268,1923,2584,1823,2882,2312,21
48
NCOA2_1_6254,1335,878,675,1527,1220,1505,1536,295,852,983,2629,359
NCOA2_1_6255,1662,1371,1363,1465,1995,1551,1300,1571,1546,1016,1442,10
89
NCOA2_1_6256,297,193,176,215,337,818,288,29,592,248,300,103
NCOA2_1_6257,5836,4393,5358,7596,4569,5942,10217,4099,5362,3457,3435,6
908
NCOA2_1_6258,2343,3396,3038,4971,2442,2979,4506,2293,3724,2717,2152,47
92
NCOA2_1_6259,1163,1045,980,1422,653,1063,1500,644,466,924,670,427
NCOA2_1_6260,1571,1661,1284,1583,1903,2694,1672,2613,4118,1451,2405,27
17
RNF2_1_6261,453,624,921,849,662,939,316,1005,1,1171,624,833
RNF2_1_6262,105,409,140,75,0,105,27,481,3,2,133,108
RNF2_1_6263,3738,3254,4069,4299,4075,4198,1788,3070,2860,2020,2990,391
0
RNF2_1_6264,860,627,1466,1117,597,475,1583,589,1046,1054,1179,1048
RNF2_1_6265,1137,1166,757,818,603,1956,346,666,322,791,1090,842
RNF2_1_6266,2204,2220,2679,2676,3999,2624,3167,4340,3401,3325,3769,314
3
RNF2_1_6267,3616,3125,4526,2336,4063,2294,1899,2268,2394,2945,3315,275
8
RNF2_1_6268,936,899,1367,663,1477,491,1476,463,771,1081,177,1698
RNF2_1_6269,974,288,511,486,382,1211,58,1410,401,555,55,203
RNF2_1_6270,516,526,357,281,105,428,477,346,1554,275,1948,1
MBD1_1_6271,5521,7411,7798,6609,7187,6626,7988,6818,2917,7955,7457,107
32
MBD1_1_6272,6289,6114,7369,8416,7716,6199,9162,8302,5996,6105,6897,700
3
MBD1_1_6273,1028,726,738,1213,1488,1477,1610,453,2591,1233,977,2899
MBD1_1_6274,7661,7186,9072,7254,8752,9174,6214,9120,6532,6748,4959,756
2
MBD1_1_6275,721,963,2080,907,537,582,1078,493,779,940,3158,275
MBD1_1_6276,2086,1329,2117,3407,809,4245,2391,5313,1595,2160,1078,1993
MBD1_1_6277,1097,515,976,970,2077,854,664,1101,870,1088,2232,529

MBD1_1_6278,1213,2038,1806,1339,980,1515,1603,446,1155,2017,1739,542
MBD1_1_6279,1658,1809,1357,1889,2163,1389,1641,1320,390,2133,1337,584
MBD1_1_6280,1120,1091,1207,887,4475,926,1362,867,1318,636,233,506
MBD4_1_6281,981,967,1601,688,919,1333,1349,562,2127,1635,2528,39
MBD4_1_6282,2375,1148,1582,1024,730,1596,1840,1068,1316,1837,1155,1052
MBD4_1_6283,3237,4275,4402,3249,6048,4038,4250,1476,3862,4400,3484,465
5
MBD4_1_6284,898,411,838,628,996,442,802,798,2690,678,216,748
MBD4_1_6285,740,655,537,772,747,1213,615,1608,1331,391,1055,1368
MBD4_1_6286,545,1014,616,549,1141,955,1441,579,57,692,1272,1263
MBD4_1_6287,4553,5283,4927,5177,6452,4415,3481,6293,6158,5759,2984,749
6
MBD4_1_6288,3901,4046,4792,4272,4294,5553,3848,4254,4664,4769,8498,518
1
MBD4_1_6289,1532,1409,1839,1046,1118,1207,1787,573,1688,1604,2117,2149
MBD4_1_6290,1040,1614,831,1442,985,1265,1256,1188,2327,695,279,1257
MKL1_1_6291,3010,2324,2301,2622,3373,2979,4864,3176,2164,2241,1987,166
7
MKL1_1_6292,820,1341,1232,1701,1253,978,728,98,1526,559,62,381
MKL1_1_6293,476,618,276,304,763,457,280,178,2,540,7,30
MKL1_1_6294,346,243,353,311,5,136,743,128,1,82,38,63
MKL1_1_6295,419,390,589,542,655,395,49,23,400,585,1231,206
MKL1_1_6296,904,752,668,469,560,515,552,632,1960,1045,1460,1692
MKL1_1_6297,273,139,109,319,353,11,141,134,19,710,1,30
MKL1_1_6298,1399,858,1039,1418,570,1149,870,1417,1976,721,28,364
MKL1_1_6299,3510,3125,4853,3636,4286,4498,2668,6804,4856,3913,3212,449
2
MKL1_1_6300,2651,2253,2142,2500,2722,2840,4823,3003,2160,2564,1988,166
2
MLL2_1_6301,1575,887,822,369,784,910,387,585,1558,863,147,182
MLL2_1_6302,587,154,247,255,50,183,1,19,33,406,516,66
MLL2_1_6303,1860,2547,2315,2138,1863,2881,2073,2360,1340,1317,1989,143
0
MLL2_1_6304,551,348,994,642,501,189,68,518,31,255,142,1037
MLL2_1_6305,502,371,1055,203,68,780,1268,227,703,951,287,1150
MLL2_1_6306,2236,2393,1717,2288,2617,2556,1474,1630,4867,1003,1951,147
8
MLL2_1_6307,217,103,68,50,28,327,58,142,2747,720,38,111
MLL2_1_6308,526,711,519,463,919,1261,130,1571,30,534,1266,1163
MLL2_1_6309,477,519,699,453,603,1429,225,211,574,566,2031,341
MLL2_1_6310,2229,1817,1892,2746,1980,2530,2747,2338,1742,1126,1832,268
4
NAT8_1_6311,14590,13862,14311,13824,20093,14673,14351,11742,10588,1137
4,13502,12860
NAT8_1_6312,4654,5567,4774,7119,5654,6243,5608,5522,4753,5021,3956,658
7
NAT8_1_6313,766,1000,489,1038,1202,1084,1361,442,1143,667,256,916
NAT8_1_6314,553,1080,1105,889,377,599,789,412,483,548,2791,902
NAT8_1_6315,3214,4380,4506,3409,5191,3655,5058,2686,3161,3955,4482,309
5

NAT8_1_6316,837,671,863,941,2049,810,261,727,1207,541,566,1785
NAT8_1_6317,942,1682,1540,1437,972,2513,1362,1222,1070,1374,145,922
NAT8_1_6318,1007,1879,590,1117,1337,1033,447,642,86,1731,2015,364
NAT8_1_6319,1578,1393,1575,1966,3023,2224,1550,1692,869,1365,2170,1770
NAT8_1_6320,670,658,979,677,316,1072,149,458,784,1042,36,655
NCOA3_1_6321,4448,4077,5912,4052,5514,4889,6144,3538,5293,3755,3371,28
81
NCOA3_1_6322,259,45,121,93,49,30,0,18,278,75,257,0
NCOA3_1_6323,1178,912,1813,1067,1382,827,1217,1819,2643,169,621,2663
NCOA3_1_6324,431,561,564,342,105,663,482,679,109,871,186,1270
NCOA3_1_6325,162,31,23,89,0,13,32,3,0,357,211,0
NCOA3_1_6326,1338,889,1746,847,1157,234,588,1593,2784,633,516,3145
NCOA3_1_6327,1764,1557,2005,2473,1977,1526,3297,1165,2848,2107,1020,62
2
NCOA3_1_6328,1378,947,1740,960,1389,454,1059,1613,2793,636,594,3231
NCOA3_1_6329,879,1352,251,675,1717,341,1776,1143,133,585,11,1035
NCOA3_1_6330,273,141,377,217,2,233,12,53,0,56,2,501
PHIP_1_6331,12175,10531,14523,13313,15192,12019,11210,11014,9007,8978,
8657,16822
PHIP_1_6332,1547,1269,1875,1355,4059,2729,1020,1641,2432,1573,1102,139
3
PHIP_1_6333,842,1172,1479,1237,753,543,559,1513,893,478,2673,1023
PHIP_1_6334,544,457,1609,513,659,847,1715,259,1468,1030,1694,167
PHIP_1_6335,12116,10553,14473,13353,14963,12036,11250,11029,9050,8886,
8662,16834
PHIP_1_6336,1236,1613,1132,1529,1232,2400,1250,1214,1133,945,2187,1709
PHIP_1_6337,649,719,752,1007,933,1172,1542,1903,244,1027,777,1186
PHIP_1_6338,639,1002,514,417,698,452,689,504,427,736,2218,1735
PHIP_1_6339,2097,2278,2946,2526,3592,2160,3135,2193,2811,2600,3158,376
9
PHIP_1_6340,9968,6560,9320,8319,9649,9622,13428,6831,13633,6878,9977,7
641
POLR1B_1_6341,957,816,1137,565,553,975,810,805,734,822,701,184
POLR1B_1_6342,469,277,721,591,157,622,482,8,35,279,1117,1912
POLR1B_1_6343,521,624,416,718,1122,350,670,355,443,122,1290,318
POLR1B_1_6344,2378,1896,4445,3119,2489,2059,2182,2909,2369,2514,2422,2
276
POLR1B_1_6345,3740,3028,2693,3634,3029,4820,5821,4175,5837,2979,2920,4
571
POLR1B_1_6346,1223,1565,2617,1954,834,1421,1860,553,1020,1571,3162,856
POLR1B_1_6347,160,223,268,154,0,0,0,457,0,0,0,0
POLR1B_1_6348,104,130,290,185,693,120,304,141,45,643,4,915
POLR1B_1_6349,1729,1351,2462,1752,1888,1631,1368,2221,349,1681,774,212
6
POLR1B_1_6350,114,271,199,591,551,552,172,106,0,190,341,621
RAI1_1_6351,2943,2276,2323,2713,1443,2452,1750,2279,1210,2539,2912,193
7
RAI1_1_6352,1051,743,963,1251,1142,981,1999,2222,2454,1327,1503,872
RAI1_1_6353,5164,5137,5123,7009,5938,9301,8202,4787,6713,3909,5609,415
8

RAI1_1_6354,1442,694,882,743,573,1202,358,565,298,1253,776,1171
RAI1_1_6355,1206,991,869,896,1553,1392,652,1161,2199,842,23,365
RAI1_1_6356,9191,7941,7634,9035,12723,9475,9920,10896,6101,10106,8716,
11158
RAI1_1_6357,3817,2122,4388,4512,1975,3129,3594,4562,4690,2611,2610,222
3
RAI1_1_6358,1106,1329,902,1404,1474,1836,1795,2949,624,1261,1384,856
RAI1_1_6359,4201,2806,3642,4075,7069,2977,5220,2849,1680,2609,3305,280
2
RAI1_1_6360,3973,3258,4463,4821,3362,3023,3266,4098,8369,5926,3155,550
2
RORA_1_6361,2237,2149,2162,618,2756,1017,785,1287,1656,442,1778,2208
RORA_1_6362,920,901,598,501,386,235,628,208,664,1032,80,1113
RORA_1_6363,8369,8171,8921,9795,9756,6698,10678,7826,11298,8134,8726,9
409
RORA_1_6364,626,422,430,311,96,691,861,616,795,668,1744,217
RORA_1_6365,8793,8519,9151,9566,10689,7463,11337,7856,11391,8354,8156,
9391
RORA_1_6366,1808,1021,918,1164,1419,1039,629,2148,1974,1808,1043,1448
RORA_1_6367,1282,2389,1845,2112,837,1545,1941,996,2794,1369,1015,1618
RORA_1_6368,2358,1633,2473,1660,5162,1653,912,1017,936,3280,1284,436
RORA_1_6369,3754,4192,4265,4398,4991,2065,8539,3786,6378,4052,4813,358
1
RORA_1_6370,6696,4988,5921,6995,6266,6018,7376,6077,4752,7037,5581,745
4
RORA_1_6371,333,222,477,423,139,330,2,440,1,540,6,222
RORA_1_6372,702,328,571,179,387,783,145,8,948,309,55,247
RORA_1_6373,624,858,916,635,1767,881,789,433,728,878,1995,263
RORA_1_6374,1586,978,1423,1333,2872,1423,807,323,1375,1115,2413,427
RORA_1_6375,311,445,436,584,3162,377,739,557,616,479,149,477
RORA_1_6376,1055,822,851,1038,389,693,2180,223,848,821,229,2488
RORA_1_6377,1106,1213,1219,1223,1276,1045,1672,275,1762,1365,978,1667
RORA_1_6378,6857,4369,6855,6449,9778,8966,7745,2875,7080,5461,7653,259
2
RORA_1_6379,450,666,565,1132,1219,246,716,766,330,508,1076,205
RORA_1_6380,499,706,1219,976,463,661,1291,553,968,1109,846,2086
SF3B3_1_6381,1510,1325,1140,1487,1172,2421,1195,1568,1020,1490,2174,15
34
SF3B3_1_6382,247,510,227,272,253,147,270,114,378,194,2,319
SF3B3_1_6383,441,128,242,392,597,549,10,487,922,299,430,347
SF3B3_1_6384,810,390,660,295,199,198,277,1332,146,1667,1450,149
SF3B3_1_6385,391,334,641,513,113,15,210,537,273,97,63,17
SF3B3_1_6386,2100,1732,1821,1824,1601,2461,2221,1049,2219,2528,1744,11
60
SF3B3_1_6387,570,153,361,402,893,521,50,93,2,324,1,926
SF3B3_1_6388,575,699,632,828,1091,584,434,1179,675,265,122,816
SF3B3_1_6389,662,1147,590,967,1185,613,341,557,132,1107,353,0
SF3B3_1_6390,306,287,257,133,23,876,1011,8,2089,530,0,1345
SLC38A1_1_6391,11011,9314,10903,10565,13307,12432,11892,7842,16302,898
1,6795,13291

SLC38A1_1_6392,828,670,1260,607,477,1296,1018,348,368,653,747,1384
SLC38A1_1_6393,2269,2114,2988,3089,2936,1946,3758,1621,1543,2164,1369,
1787
SLC38A1_1_6394,2800,2067,2336,3533,2513,2308,2069,3447,2257,3017,1721,
1818
SLC38A1_1_6395,1278,1182,2312,1360,1045,714,3342,576,1209,1532,1168,12
81
SLC38A1_1_6396,217,145,103,139,136,111,86,450,114,101,0,149
SLC38A1_1_6397,1185,1020,1518,625,1665,584,2813,445,1238,1676,2267,408
SLC38A1_1_6398,1731,2009,1539,1209,1614,1535,787,1419,2551,1084,2882,1
065
SLC38A1_1_6399,335,334,670,843,179,305,925,3,0,91,0,25
SLC38A1_1_6400,1863,1522,2510,2283,1804,821,2280,1892,4383,1424,3083,2
530
SLC38A1_1_6401,3730,3466,3710,5180,2079,5937,6024,2364,4601,4306,1798,
7697
SLC38A1_1_6402,2755,2382,2524,3656,2244,5711,673,866,2436,3364,3360,28
78
SLC38A1_1_6403,517,452,399,1223,1716,711,252,3,516,929,327,4
SLC38A1_1_6404,1649,1449,2282,2093,3911,1879,2829,2132,3482,1511,845,1
326
SLC38A1_1_6405,8881,7375,7688,9206,11108,13326,12087,8218,9042,7556,10
058,10882
SLC38A1_1_6406,426,453,555,825,261,412,661,152,655,75,357,22
SLC38A1_1_6407,1306,1050,1490,1823,2064,2332,1357,1269,824,1311,514,16
37
SLC38A1_1_6408,1188,882,742,1012,854,916,523,7,1692,1110,750,1213
SLC38A1_1_6409,1497,990,1292,1229,1695,2082,1755,1593,1617,1602,2312,6
4
SLC38A1_1_6410,1777,1285,863,1383,1761,1176,2426,495,1764,984,2006,412
SRCAP_1_6411,5432,4879,5831,5171,5060,6524,7192,1988,5295,5944,7676,21
97
SRCAP_1_6412,2804,3282,3373,1878,3002,1288,3072,2067,464,2841,1841,437
8
SRCAP_1_6413,2753,3310,3498,2210,3036,1974,3080,2079,458,2638,1837,438
5
SRCAP_1_6414,4625,3454,3388,3980,2849,4451,4481,1767,5224,2232,3630,29
50
SRCAP_1_6415,444,530,479,378,1752,196,844,1526,489,154,312,926
SRCAP_1_6416,366,641,255,547,115,114,94,109,0,435,220,14
SRCAP_1_6417,4389,2978,4700,5099,5183,4789,4860,3234,9377,5513,6638,33
60
SRCAP_1_6418,224,61,151,472,188,172,1352,4,64,114,55,198
SRCAP_1_6419,390,411,913,258,634,253,140,816,324,131,457,531
SRCAP_1_6420,322,151,730,489,459,309,2231,4,294,488,114,107
TCF7L1_1_6421,376,225,354,329,626,226,193,135,1778,188,1,226
TCF7L1_1_6422,935,245,410,592,210,309,366,382,909,301,73,2
TCF7L1_1_6423,201,362,865,323,568,336,405,159,1752,52,443,248
TCF7L1_1_6424,1936,2646,3378,2512,3613,2917,1885,483,2155,2443,4531,18
37

TCF7L1_1_6425,321,212,292,564,135,629,1521,832,1182,261,535,666
TCF7L1_1_6426,470,239,433,479,323,1384,488,119,1146,1121,121,505
TCF7L1_1_6427,1763,1662,1387,1941,976,1162,435,1694,2923,1378,2115,734
TCF7L1_1_6428,212,405,755,468,778,317,459,247,1064,54,400,258
TCF7L1_1_6429,3676,2847,4317,2425,3796,4570,4920,3332,754,2740,2295,16
03
TCF7L1_1_6430,373,281,448,221,301,244,194,95,346,592,30,876
