# Supplementary material for: Cross-species genetic screens identify transglutaminase 5 as a regulator of polyglutamine-expanded ataxin-1
Source: J Clin Invest. 2022 May 2;132(9):e156616. doi: 10.1172/JCI156616 (PMC9057624; doi:10.1172/JCI156616)
Supplement: Supplemental data set 2 [file jci-132-156616-s047.pdf]

```
sRNA,B1,B2,B3,B4,H1,H2,H3,H4,L1,L2,L3,L4
A4GALT_2_1,299,270,625,453,395,628,466,432,339,243,259,808
A4GNT_2_2,290,233,211,190,82,41,526,17,145,695,253,231
AACS_2_3,249,276,318,263,88,307,86,132,183,276,171,78
AADAC_2_4,501,439,630,471,1839,1551,539,980,321,465,1217,348
AARS2_2_5,503,398,370,540,57,780,795,268,173,0,449,444
AARS_2_6,685,677,579,472,831,362,530,1265,324,1567,562,282
AASDH_2_7,290,222,128,391,242,600,503,203,58,149,113,447
AASDHPPT_2_8,599,582,786,532,714,358,280,715,102,846,1605,611
AASS_2_9,359,308,351,319,198,313,757,483,133,460,113,456
ABHD14A_2_10,116,18,87,89,74,0,39,6,2,0,0,218
ABHD1_2_11,639,844,749,879,615,645,748,1503,343,361,792,236
ABHD3_2_12,296,308,211,228,479,244,17,101,450,532,235,468
ABHD5_2_13,417,231,500,381,2004,432,213,450,395,227,528,216
ABHD6_2_14,209,178,266,319,58,330,290,121,674,56,22,191
ABHD8_2_15,16,5,52,43,70,24,0,0,17,133,0,10
ABO_2_16,1284,1150,918,1078,1030,718,2233,287,1039,1060,930,1351
ABP1_2_17,1566,1058,1265,1961,1036,1637,1371,482,2220,1481,1521,1315
ACAA2_2_18,409,507,344,549,368,401,489,120,361,2,472,760
ACACB_2_19,171,398,277,306,1,262,26,127,476,27,321,173
ACAD11_2_20,263,218,249,185,258,118,97,431,316,271,315,417
ACAD8_2_21,649,692,653,817,425,613,161,597,660,822,841,1057
ACAD9_2_22,922,605,701,631,646,631,381,1061,805,257,930,433
ACADL_2_23,382,305,305,371,688,192,173,371,441,363,949,911
ACADSB_2_24,199,239,280,326,255,206,639,596,157,313,272,133
ACADS_2_25,420,335,252,301,264,114,902,188,519,459,423,69
ACAT1_2_26,1937,1404,1600,1843,2023,1995,1896,1302,1556,2023,1720,1876
ACER1_2_27,475,408,417,587,189,575,254,326,420,349,498,450
ACER2_2_28,522,299,239,345,1,569,376,150,37,955,0,108
ACER3_2_29,89,56,74,119,70,89,51,23,100,111,93,15
ACMSD_2_30,279,216,491,236,198,78,482,138,88,151,47,63
ACO1_2_31,3098,2189,3113,3134,2531,2751,4039,2564,1780,3301,3059,2011
ACO2_2_32,150,67,47,62,18,0,169,14,212,7,2,28
ACOT12_2_33,624,371,493,509,570,65,140,636,509,412,459,326
ACOT1_2_34,322,336,388,277,253,1052,715,165,73,408,31,376
ACOT2_2_35,322,336,388,277,253,1052,715,165,73,408,31,376
ACOT4_2_36,453,613,363,504,265,411,558,172,243,918,767,429
ACOT6_2_37,547,549,512,617,679,288,850,479,501,1414,1335,941
ACOT8_2_38,78,83,85,134,8,48,2,0,0,26,67,100
ACOX2_2_39,873,654,600,696,158,215,348,85,589,1076,490,761
ACOXL_2_40,659,554,597,623,542,642,1072,396,819,306,502,977
ACR_2_41,313,349,408,425,588,326,88,5,29,30,894,915
ACSBG2_2_42,175,89,236,191,139,233,499,625,54,174,100,256
ACSF2_2_43,619,623,474,475,50,428,229,357,1069,203,220,391
ACSL1_2_44,242,203,284,344,305,169,898,1,112,2,0,101
ACSM1_2_45,333,180,368,272,252,507,313,208,204,0,79,0
ACSM2A_2_46,69,101,59,64,3,86,0,109,5,83,0,32
ACSM4_2_47,183,167,132,155,6,518,389,0,33,64,67,179
ACSM5_2_48,1015,1065,1148,1241,1502,1241,343,1639,944,965,2375,1761
ACSS1_2_49,326,371,229,415,633,487,396,2,308,740,242,395
```

ACSS3_2_50,455,403,223,551,825,278,577,1576,1,1,663,340
ACTC1_2_51,241,280,276,371,179,141,459,233,69,182,624,406
ACY3_2_52,41,22,12,4,0,20,19,0,1,0,0,42
ACYP2_2_53,281,138,227,392,215,106,17,12,166,181,107,127
ADA_2_54,336,286,183,201,137,180,135,484,145,184,0,211
ADAM10_2_55,1235,1206,1474,1494,1425,1229,1641,887,779,2224,2216,1428
ADAM17_2_56,600,520,554,414,154,582,522,417,655,1100,134,529
ADAMTS4_2_57,73,54,102,88,142,20,33,512,218,40,3,0
ADARB2_2_58,485,448,437,405,89,578,1042,458,643,630,634,198
ADAT1_2_59,581,524,606,767,1394,339,759,644,606,737,265,892
ADAT2_2_60,104,63,92,139,101,223,1,26,52,20,9,0
ADC_2_61,43,96,50,160,0,25,2,13,27,135,28,163
ADCY1_2_62,88,46,74,108,0,30,23,0,28,51,83,3
ADCY2_2_63,215,225,257,170,0,84,344,396,468,174,326,359
ADCY7_2_64,356,246,293,416,185,335,421,1516,310,104,416,436
ADCY8_2_65,760,595,728,813,1161,391,488,686,927,9,677,716
ADCY9_2_66,553,533,465,435,1066,173,0,120,725,273,469,412
ADH1A_2_67,327,212,194,178,459,193,189,0,20,2,206,364
ADH1B_2_68,335,278,321,287,211,57,398,416,294,237,103,316
ADH1C_2_69,1341,1200,1131,1290,635,1493,1146,1159,847,2182,924,861
ADH4_2_70,54,7,21,37,3,96,14,3,0,0,67,40
ADH5_2_71,748,503,579,762,407,1056,258,356,814,334,191,809
ADHFE1_2_72,1026,602,611,969,716,1008,331,71,278,1193,594,445
ADI1_2_73,340,268,287,387,595,351,41,471,163,453,731,328
ADO_2_74,78,91,67,79,78,0,0,99,47,101,0,0
ADPRH_2_75,238,320,231,233,901,50,2,168,53,89,203,679
ADPRHL2_2_76,1432,904,1474,1180,1231,1398,1249,743,1371,1277,817,1455
ADSS_2_77,244,351,262,280,367,177,246,217,291,160,199,292
AEN_2_78,63,26,30,26,0,5,1,0,91,68,2,68
AGBL2_2_79,1312,787,1013,1027,678,1330,1507,420,730,2064,1072,921
AGMAT_2_80,79,78,98,68,370,5,8,151,70,0,28,75
AGMO_2_81,3895,2982,3293,3748,2224,3137,4515,2117,2363,2428,1978,5455
AGPAT4_2_82,163,169,238,217,60,64,427,44,368,385,10,180
AGPAT5_2_83,725,835,733,1074,179,695,1913,603,459,1481,408,1090
AGPAT6_2_84,185,184,95,206,165,82,15,435,189,425,29,318
AGPAT9_2_85,187,111,117,113,133,94,509,113,130,59,56,100
AGPS_2_86,239,250,420,272,155,167,321,16,38,27,474,496
AGXT2_2_87,2887,2661,2031,2791,1763,2530,2158,751,2486,4615,2497,1924
AGXT2L2_2_88,408,351,425,581,193,266,507,548,0,859,354,147
AGXT_2_89,132,119,148,113,45,0,146,684,26,197,13,273
AICDA_2_90,59,60,86,90,173,2,0,16,0,38,0,1
AKR1B10_2_91,132,143,92,117,167,4,383,253,109,44,2,38
AKR1B15_2_92,132,143,92,117,167,4,383,253,109,44,2,38
AKR1B1_2_93,71,47,47,69,171,41,19,74,31,0,0,4
AKR1C1_2_94,328,245,281,324,322,89,73,94,224,377,398,138
AKR1C3_2_95,638,506,405,466,39,602,699,25,515,157,499,769
AKR1C4_2_96,927,747,824,801,295,719,892,345,412,227,2012,1044
AKR1E2_2_97,229,218,164,264,359,96,40,324,277,237,32,273
AKR7A2_2_98,77,175,241,311,266,13,38,116,66,242,193,425
AKR7A3_2_99,734,549,656,773,612,539,658,60,491,1937,599,506

ALAD_2_100,146,94,114,130,68,168,211,28,158,33,87,103
ALDH1A1_2_101,640,885,861,799,192,321,814,760,762,358,1039,1393
ALDH1A3_2_102,589,318,265,360,844,518,488,414,890,96,178,405
ALDH1B1_2_103,149,84,86,138,125,45,287,98,61,76,8,116
ALDH1L1_2_104,738,498,594,613,252,385,268,909,534,852,1197,733
ALDH1L2_2_105,54,79,188,118,0,489,972,0,2,139,0,303
ALDH6A1_2_106,264,267,255,374,392,1506,91,196,376,579,180,341
ALDH9A1_2_107,1361,962,1114,1536,1144,720,849,1046,855,1145,664,1078
ALDOB_2_108,136,70,150,64,19,37,471,31,0,28,794,199
ALDOC_2_109,1571,1436,1542,1487,2521,1529,3225,1888,1466,1661,608,1895
ALG10_2_110,414,556,621,457,474,328,234,1003,281,668,360,719
ALG11_2_111,1167,1148,1184,1094,563,1927,378,604,757,817,1517,1228
ALG12_2_112,59,139,109,243,227,0,886,0,185,388,1194,232
ALG14_2_113,1023,656,672,955,607,1107,198,1043,1079,1613,620,489
ALG1_2_114,150,57,82,39,26,0,2,8,45,79,0,21
ALG2_2_115,183,298,436,382,58,606,229,353,772,401,0,605
ALG6_2_116,1315,792,1026,1540,1705,297,1929,1246,441,1609,1005,1064
ALKBH8_2_117,550,321,337,280,237,313,1040,203,203,464,511,459
ALLC_2_118,394,334,341,374,294,427,777,497,1050,204,338,438
ALOX12B_2_119,228,165,128,193,406,49,1,0,398,146,14,49
ALOX12_2_120,1384,968,977,930,363,398,548,1900,1060,856,1215,982
ALOX15_2_121,639,457,494,447,517,511,730,1346,449,129,227,968
ALOX5_2_122,48,21,6,60,149,0,2,0,0,1,8,15
AMDHD1_2_123,498,382,480,536,629,6,416,656,942,319,425,439
AMY1B_2_124,17017,14576,13584,15429,13055,10746,12810,17567,13688,1776
1,15061,20077
AMY1C_2_125,17017,14576,13584,15429,13055,10746,12810,17567,13688,1776
1,15061,20077
AMY2A_2_126,1043,1172,1054,1173,1697,706,1438,718,459,881,587,1551
AMY2B_2_127,17017,14576,13584,15429,13055,10746,12810,17567,13688,1776
1,15061,20077
ANPEP_2_128,572,626,451,558,934,32,477,648,848,147,538,568
AOC3_2_129,185,152,202,226,60,0,129,60,222,124,216,296
AOX1_2_130,404,501,580,634,857,343,700,856,475,424,1049,453
APEH_2_131,123,173,100,220,73,17,35,398,237,27,14,208
APEX2_2_132,234,156,129,247,125,203,0,242,47,364,408,513
APIP_2_133,92,87,82,58,0,1,409,4,116,452,65,0
APLF_2_134,595,486,590,506,601,1027,1514,263,49,487,528,61
APOBEC1_2_135,1220,1185,886,1189,551,1254,801,1490,618,1721,1736,1175
APOBEC2_2_136,362,217,108,330,633,20,339,337,122,200,458,83
APOBEC3B_2_137,342,473,449,341,87,647,516,461,182,366,720,700
APOBEC3C_2_138,741,670,688,665,1352,216,876,1044,478,999,683,337
APOBEC3G_2_139,481,421,305,455,313,45,180,91,451,245,473,428
ARF3_2_140,225,256,162,169,138,9,8,158,310,110,322,32
ARF4_2_141,448,194,259,292,110,280,392,185,395,3,67,412
ARG2_2_142,104,53,41,107,17,9,0,75,115,53,24,7
ARHGEF10_2_143,279,294,306,258,88,434,496,193,663,320,586,149
ARL3_2_144,415,174,225,340,18,42,60,18,486,219,80,379
ARL4C_2_145,275,105,279,297,132,443,243,231,223,105,48,310
ARL4D_2_146,176,86,107,125,139,1,20,545,16,201,33,6

ARL5B_2_147,1094,1306,1029,1116,1536,499,2383,1651,614,1188,976,944
ARL8A_2_148,1232,893,780,657,1183,273,373,1190,793,364,97,239
ARL8B_2_149,392,248,469,469,207,5,662,121,301,784,1250,123
ARSD_2_150,99,36,171,94,0,0,28,0,665,0,646,0
ARSE_2_151,294,221,302,372,261,239,312,355,688,116,299,122
ARSG_2_152,60,36,57,41,3,126,56,1,84,0,0,9
ARSH_2_153,313,238,207,280,269,420,388,0,541,48,1,206
ARSI_2_154,333,185,160,194,422,318,304,67,174,237,257,67
ARSJ_2_155,364,208,191,214,176,29,318,739,338,440,339,332
ARSK_2_156,597,658,435,698,536,891,526,486,1089,320,501,304
ART1_2_157,289,260,212,388,652,394,51,12,201,334,1011,205
ART4_2_158,182,92,418,142,44,85,250,29,276,329,611,0
AS3MT_2_159,389,440,348,360,20,53,360,260,541,302,184,638
ASNA1_2_160,299,346,377,436,465,132,93,338,234,445,176,453
ASNSD1_2_161,0,0,0,0,0,0,0,108,0,0,0,0
ASPG_2_162,150,156,150,285,0,0,697,258,0,11,0,180
ASPHD2_2_163,207,554,126,265,646,153,695,16,282,274,325,58
ATAD1_2_164,201,166,279,233,156,113,151,215,57,608,43,194
ATIC_2_165,746,656,549,547,1540,358,1505,776,691,182,1061,484
ATP2C2_2_166,207,199,209,224,27,101,306,33,65,172,52,213
AUH_2_167,759,956,930,1108,1094,870,1466,114,702,710,1867,1186
AWAT1_2_168,92,126,146,194,22,22,0,10,144,2,163,77
AWAT2_2_169,810,559,603,778,1326,128,886,1065,835,339,711,371
B3GALNT2_2_170,172,89,121,65,196,6,458,528,425,0,15,180
B3GALT1_2_171,1093,824,626,886,1077,1169,1190,514,1148,549,900,942
B3GALT2_2_172,1112,997,1141,1679,1513,1134,697,1119,1357,1115,1777,869
B3GALT4_2_173,268,255,308,337,381,209,688,589,472,637,254,709
B3GALT6_2_174,7,1,36,35,0,119,0,0,0,4,0,0
B3GAT2_2_175,373,263,483,434,361,18,699,97,223,346,923,908
B3GAT3_2_176,2416,2079,1911,2135,1580,702,2037,2185,1705,2985,1230,167
5
B3GNT2_2_177,128,191,212,134,119,335,200,164,88,5,51,25
B3GNT3_2_178,403,263,254,277,178,624,71,61,211,433,529,275
B3GNT4_2_179,88,168,135,126,140,2,19,46,263,3,5,216
B3GNT5_2_180,5959,4236,4121,5317,4329,2796,5387,6922,6346,5852,4215,54
32
B3GNT7_2_181,88,75,154,78,50,6,362,1,68,35,213,38
B3GNT9_2_182,446,376,408,590,877,345,556,491,570,1000,342,204
B3GNTL1_2_183,739,756,726,928,217,643,1351,217,946,219,927,609
B4GALNT1_2_184,98,171,144,199,486,570,97,111,80,76,4,256
B4GALNT3_2_185,186,143,187,194,171,782,87,160,131,87,69,51
B4GALNT4_2_186,53,19,0,9,0,0,0,2,0,0,1,30
B4GALT1_2_187,870,929,761,822,425,739,1393,224,844,586,382,647
B4GALT5_2_188,314,257,339,312,430,527,160,1,1,582,591,220
B4GALT6_2_189,533,529,327,442,281,504,784,397,308,663,363,537
B4GALT7_2_190,161,153,163,171,9,0,264,156,39,110,21,285
BBOX1_2_191,325,214,205,294,893,133,529,571,284,212,30,535
BCHE_2_192,74,117,103,22,53,2,11,62,166,0,0,0
BCMO1_2_193,254,79,215,86,229,9,50,345,32,0,2,75
BDH2_2_194,232,121,109,182,43,40,29,36,82,15,51,55

BHMT_2_195,89,54,105,88,36,0,7,427,190,3,4,35
BLM_2_196,2470,2298,2451,2551,2042,3423,2247,2941,2416,2272,1891,2609
BLMH_2_197,83,49,60,168,23,144,10,79,4,31,141,40
BLVRA_2_198,375,187,218,280,931,713,2,112,393,402,333,947
BLVRB_2_199,15,13,65,14,25,0,0,0,0,79,121,0
BPHL_2_200,1515,1603,1095,1358,943,1808,1308,1057,464,1160,903,926
BST1_2_201,292,319,180,505,499,269,1019,661,127,236,4,178
BTD_2_202,78,177,58,115,0,36,17,52,297,259,3,0
C12orf5_2_203,387,286,338,316,116,287,396,147,110,264,131,255
C16orf79_2_204,230,235,232,224,26,3,267,338,2,715,1727,801
C18orf56_2_205,46,107,63,65,5,27,116,36,12,9,79,7
C1GALT1_2_206,567,486,520,384,307,131,897,47,143,380,490,418
C1R_2_207,109,128,188,150,120,63,293,92,105,20,31,262
C22orf28_2_208,176,202,70,135,6,31,151,61,105,186,67,513
C2orf43_2_209,5,58,15,3,0,0,0,8,0,300,0,0
C5orf4_2_210,22,58,73,82,0,5,94,602,7,24,222,231
C6orf130_2_211,469,349,299,240,349,83,247,12,328,377,119,396
CA13_2_212,63,33,20,39,430,0,61,0,37,91,0,65
CA14_2_213,107,203,188,70,219,167,127,983,375,85,420,10
CA2_2_214,82,65,73,96,105,35,193,79,135,74,5,9
CA3_2_215,248,288,354,273,127,420,468,61,16,142,243,284
CA4_2_216,109,21,58,155,322,9,53,99,36,26,0,4
CA5A_2_217,0,0,0,0,0,0,0,0,0,0,0,0
CA5B_2_218,714,637,664,686,772,575,1247,972,1039,395,731,849
CA6_2_219,88,99,45,118,2,58,0,109,2,205,158,52
CA8_2_220,191,201,238,194,615,213,139,0,65,220,109,202
CA9_2_221,571,181,411,451,561,380,363,1359,50,396,243,428
CARS2_2_222,91,87,79,213,307,224,46,120,284,155,0,178
CASD1_2_223,967,979,791,1151,399,460,1596,616,1233,1480,1855,1725
CAT_2_224,1694,2015,1791,1702,2300,1638,687,841,2382,2594,1729,1781
CBR1_2_225,1098,1549,1162,1331,1189,923,1277,1679,1075,1287,819,760
CBR3_2_226,152,144,199,232,346,1,176,40,90,58,466,3
CBR4_2_227,742,616,544,873,982,178,569,344,773,821,890,877
CCDC88B_2_228,68,26,41,44,0,0,132,0,37,0,0,10
CCDC92_2_229,14,49,46,180,0,17,0,53,22,8,0,434
CCNO_2_230,47,15,27,36,0,0,0,0,0,0,0,0
CCS_2_231,82,11,95,136,3,1,0,0,180,137,2,1
CCT8_2_232,210,307,244,435,326,348,196,402,133,244,18,581
CD274_2_233,1182,1026,943,1167,1015,838,959,526,619,357,1729,891
CD38_2_234,95,106,181,172,24,4,73,129,22,6,840,9
CDA_2_235,1208,748,801,1217,1142,171,1918,1073,1686,512,886,601
CDIPT_2_236,829,866,870,994,830,1382,904,1768,1108,1287,1001,784
CDO1_2_237,228,216,303,279,152,220,587,160,124,311,830,831
CDS2_2_238,502,381,439,568,493,513,557,1066,269,502,530,474
CELA1_2_239,133,134,101,102,37,96,100,361,226,50,122,140
CELA2A_2_240,166,202,172,191,150,13,76,75,17,16,6,32
CELA2B_2_241,166,202,172,191,150,13,76,75,17,16,6,32
CELA3A_2_242,477,441,626,658,216,1513,108,1411,219,604,1150,445
CELA3B_2_243,477,441,626,658,216,1513,108,1411,219,604,1150,445
CEL_2_244,90,195,262,164,35,2,763,1,49,271,312,290

CETN1_2_245,12,3,21,7,11,30,10,0,38,25,527,1
CETN2_2_246,2855,2167,2294,2251,2103,1813,1994,2972,2662,2619,2193,196
0
CETP_2_247,520,501,315,409,350,245,545,415,362,816,592,652
CFB_2_248,123,76,132,131,0,209,102,357,63,171,37,247
CFD_2_249,29,36,10,30,0,1,306,1,8,48,0,53
CFI_2_250,49,26,26,53,43,0,1,0,76,50,0,118
CH25H_2_251,246,346,214,390,1022,101,126,299,234,16,464,524
CHDH_2_252,111,104,52,50,27,80,51,2,20,29,7,203
CHI3L1_2_253,160,98,116,113,238,255,57,235,51,64,145,227
CHIT1_2_254,23,16,68,13,2,4,0,2,3,298,0,0
CHML_2_255,2180,1814,1952,2311,3001,4564,1124,3088,2073,2057,2814,2481
CHPF2_2_256,205,273,230,240,203,353,109,9,298,288,1,3
CHPT1_2_257,1106,833,863,915,215,573,684,375,1221,1555,780,703
CHST10_2_258,75,13,60,31,46,39,10,0,34,150,2,5
CHST12_2_259,378,383,308,406,59,594,248,716,187,157,8,627
CHST13_2_260,623,443,452,596,653,167,937,708,480,667,912,822
CHST14_2_261,4,10,3,23,24,2,52,7,39,120,0,15
CHST1_2_262,120,124,159,151,517,130,217,30,423,32,459,202
CHST2_2_263,727,692,803,652,1399,218,780,1351,881,802,993,422
CHST3_2_264,398,278,177,291,331,52,980,83,781,726,8,381
CHST5_2_265,0,0,0,0,0,0,0,0,0,0,0,0
CHST6_2_266,664,780,512,636,530,265,145,42,429,1178,398,39
CHST7_2_267,19,61,25,32,0,21,0,3,26,25,55,0
CHST9_2_268,542,591,588,706,574,1115,337,541,386,1175,245,650
CHSY1_2_269,559,534,543,549,304,393,1096,41,94,259,383,534
CHSY3_2_270,1353,1576,1214,1544,1335,2241,2836,1381,1338,718,1915,607
CLPP_2_271,734,542,492,558,56,147,929,1217,529,472,256,516
CLPX_2_272,419,312,350,624,200,399,871,464,547,372,504,451
CLYBL_2_273,465,284,289,273,660,334,151,93,224,814,267,202
CMA1_2_274,429,509,285,340,598,294,323,172,288,405,228,278
CMAS_2_275,436,437,267,373,498,548,296,112,594,616,358,473
CMBL_2_276,499,372,263,377,855,333,57,140,253,164,266,369
CNDP1_2_277,129,144,88,108,182,43,39,60,26,78,11,46
CNTN6_2_278,45,145,50,76,3,106,233,0,238,1,60,88
COIL_2_279,336,321,251,388,236,330,728,185,296,149,127,390
COMTD1_2_280,120,94,26,42,16,0,2,0,2,0,0,0
COQ2_2_281,395,385,354,547,468,474,51,694,409,288,212,204
COQ3_2_282,280,223,203,393,306,32,302,668,321,17,84,1021
COQ5_2_283,343,339,295,258,251,3,29,58,370,739,289,65
COX10_2_284,103,88,91,122,66,1,357,95,138,36,44,49
COX17_2_285,1289,1525,1541,1612,672,1404,1564,1160,869,2783,1928,1555
COX4I1_2_286,112,75,123,157,298,57,0,32,585,75,10,75
COX4I2_2_287,3420,3663,3513,3640,2861,3060,4533,2298,2637,2852,4755,40
13
COX5A_2_288,268,225,158,215,265,58,124,517,90,333,9,198
COX5B_2_289,122,80,30,18,51,111,73,0,198,10,38,10
COX6A1_2_290,28,27,24,48,0,6,905,19,43,41,1,0
COX6A2_2_291,56,183,199,220,231,0,938,49,13,1021,687,14
COX6B1_2_292,210,83,131,179,266,30,9,180,1,234,1,5

COX6B2_2_293,73,60,59,43,111,33,0,185,0,41,7,25
COX6C_2_294,1767,1440,1808,1739,1464,613,1738,1244,1543,1704,3074,2193
COX7A1_2_295,105,239,242,285,89,0,491,4,6,34,423,23
COX7A2_2_296,558,651,602,568,201,405,655,376,656,478,522,1040
COX7A2L_2_297,474,387,566,633,146,1125,794,756,817,322,1063,188
COX7B2_2_298,6415,5257,5216,6374,6160,4468,6792,7038,4395,5970,5066,65
36
COX7B_2_299,212,128,120,121,151,41,238,30,283,245,116,330
COX7C_2_300,1643,1538,1407,1532,1696,1217,1185,1677,1526,1508,1974,119
8
COX8A_2_301,32,41,54,9,38,4,362,0,0,2,13,3
COX8C_2_302,649,709,425,346,58,208,172,215,197,121,62,137
CPA1_2_303,120,133,86,159,64,367,138,77,78,382,98,114
CPA2_2_304,142,175,136,74,9,660,112,0,315,91,84,7
CPB1_2_305,20,49,51,13,88,2,347,44,3,0,3,96
CP_2_306,1767,1883,1731,1930,921,1119,1704,2065,2282,1491,990,2427
CPE_2_307,626,457,640,650,355,695,430,880,994,520,418,453
CPOX_2_308,105,126,138,141,180,79,209,0,298,66,42,288
CPSF3_2_309,609,474,570,449,63,282,366,290,426,237,378,306
CPT2_2_310,209,249,304,312,97,2,595,672,517,138,473,598
CRAT_2_311,222,277,225,362,614,189,172,18,515,37,703,63
CREG2_2_312,709,498,635,534,627,579,954,361,935,723,1147,419
CRY1_2_313,135,117,123,115,43,0,295,10,264,88,70,42
CRYL1_2_314,15,15,33,50,37,140,327,0,0,0,16,5
CRYZL1_2_315,501,278,358,406,787,531,282,414,463,663,230,669
CSAD_2_316,389,107,245,347,766,0,0,389,147,379,136,61
CS_2_317,230,269,170,219,253,512,201,189,284,210,24,39
CSGALNACT2_2_318,515,546,514,610,61,842,278,281,398,549,780,453
CSRP2BP_2_319,187,244,126,224,124,92,14,716,299,186,66,477
CTBS_2_320,392,243,222,310,1003,9,362,511,567,80,3,156
CTRB1_2_321,366,289,284,357,8,145,843,313,226,89,181,166
CTRB2_2_322,366,289,284,357,8,145,843,313,226,89,181,166
CTRC_2_323,783,552,784,1178,442,552,936,1082,882,27,554,1256
CTSD_2_324,67,27,58,12,1,91,475,0,0,7,199,34
CTSF_2_325,94,58,249,143,379,40,351,2,69,46,0,33
CTSG_2_326,290,180,336,194,327,545,360,134,353,43,524,106
CTSH_2_327,1291,1202,1370,1783,815,1270,1449,1440,1200,1372,1193,2372
CTSK_2_328,814,588,564,501,419,586,720,401,893,199,331,561
CTSZ_2_329,485,540,654,687,1194,451,99,866,653,291,34,739
CWC27_2_330,1730,1213,1431,1998,1368,1193,2036,1787,1660,2326,711,2512
CXorf21_2_331,450,505,359,466,42,200,299,366,144,354,264,176
CYB561D2_2_332,75,30,15,76,90,1,0,40,9,1,0,126
CYB5B_2_333,977,509,917,887,1442,766,1181,1119,1335,1587,732,1128
CYB5R1_2_334,141,57,194,187,332,230,437,26,587,499,160,152
CYB5R2_2_335,339,156,130,189,8,0,118,4,485,12,191,158
CYB5R4_2_336,1196,1599,1269,1295,1614,1510,2976,1159,1066,782,772,1170
CYBA_2_337,205,191,139,152,12,49,186,279,285,367,64,174
CYBB_2_338,115,164,95,202,3,88,161,3,68,44,38,156
CYC1_2_339,59,61,44,61,3,24,104,92,101,40,274,1
CYCS_2_340,118,135,103,188,360,33,36,72,405,116,0,104

CYP11B2_2_341,425,376,443,270,0,0,1817,275,0,438,467,398
CYP17A1_2_342,82,174,153,192,14,242,15,300,11,120,0,120
CYP1A1_2_343,158,131,199,93,1,23,17,110,15,114,24,51
CYP1A2_2_344,117,42,54,99,181,13,147,0,68,52,6,133
CYP1B1_2_345,638,228,202,327,19,519,169,226,349,146,1040,205
CYP20A1_2_346,533,261,445,368,245,612,336,595,193,308,278,396
CYP26B1_2_347,0,0,7,8,0,0,0,0,38,0,0,0
CYP26C1_2_348,385,169,200,385,408,219,445,673,620,565,612,678
CYP27A1_2_349,19,20,23,42,0,57,0,1,143,25,0,5
CYP27B1_2_350,98,42,92,126,5,622,79,125,22,2,1,3
CYP2A13_2_351,343,466,288,439,309,230,17,359,71,632,447,89
CYP2A6_2_352,343,466,288,439,309,230,17,359,71,632,447,89
CYP2B6_2_353,95,133,143,164,36,3,106,34,58,121,1,206
CYP2C19_2_354,1063,1023,1152,1653,1389,1098,639,1001,433,1928,3948,125
3
CYP2C9_2_355,1063,1023,1152,1653,1389,1098,639,1001,433,1928,3948,1253
CYP2E1_2_356,245,217,249,362,0,194,250,300,752,219,636,208
CYP2F1_2_357,481,187,324,252,13,0,392,482,112,570,277,294
CYP2J2_2_358,708,638,829,883,272,357,670,1098,1398,473,65,382
CYP2R1_2_359,3086,2908,2533,3391,4366,3597,5469,2295,3207,2964,3610,18
92
CYP2S1_2_360,69,127,117,199,0,0,6,26,30,516,0,265
CYP2U1_2_361,276,179,330,352,144,19,416,29,169,199,1524,169
CYP2W1_2_362,91,264,347,287,272,316,785,0,152,387,97,166
CYP39A1_2_363,488,262,337,317,1023,0,61,78,448,518,217,230
CYP3A7_2_364,766,882,779,693,745,676,908,741,696,793,380,1317
CYP46A1_2_365,34,138,110,58,11,88,0,22,27,51,251,6
CYP4A11_2_366,2,0,0,0,0,0,0,0,0,77,0,0
CYP4A22_2_367,824,1082,1157,1309,826,788,1932,2032,601,754,1441,1267
CYP4F12_2_368,1125,1156,1479,1184,1338,835,1156,1435,1121,1538,2335,11
04
CYP4F22_2_369,119,108,101,98,21,4,447,45,115,11,182,37
CYP4F2_2_370,570,757,483,540,202,561,843,168,264,145,347,537
CYP4F8_2_371,28,28,9,68,9,0,295,88,314,267,0,110
CYP4V2_2_372,1144,1407,1118,1213,1203,623,1692,910,1071,1145,2219,1034
CYP4X1_2_373,1503,1435,1324,1812,706,1447,3620,976,1129,1233,2275,377
CYP4Z1_2_374,648,819,773,868,1280,536,941,1050,1129,329,607,979
CYP7A1_2_375,473,391,466,433,602,373,821,122,617,329,194,79
CYP7B1_2_376,546,786,399,641,632,1002,670,936,398,515,579,779
CYP8B1_2_377,1081,1040,920,1109,628,457,1146,579,212,866,1605,632
DAD1_2_378,79,51,30,93,340,68,0,92,112,21,34,24
DAGLA_2_379,129,104,105,141,26,547,4,293,223,7,2,23
DAK_2_380,101,87,116,240,115,226,10,727,145,167,6,75
DARS2_2_381,745,499,511,680,288,671,970,529,958,386,573,757
DARS_2_382,676,821,624,744,387,925,1025,812,566,399,1341,1070
DBH_2_383,511,544,479,647,206,375,1058,294,730,268,953,465
DBR1_2_384,90,87,230,257,484,456,469,390,24,29,1266,225
DBT_2_385,250,123,191,276,293,61,60,17,416,313,233,313
DCLRE1B_2_386,97,155,144,218,443,129,265,53,125,68,205,112
DCPS_2_387,141,167,78,67,88,0,17,0,0,220,21,1

DCTN6_2_388,894,961,751,1087,447,37,1674,709,630,1013,976,1023
DCTPP1_2_389,5,1,10,10,7,29,1,380,0,69,230,10
DDAH2_2_390,2,20,48,0,0,0,0,0,2,1,1,0
DDOST_2_391,36,28,11,15,26,24,1,209,98,4,12,262
DDX10_2_392,469,367,249,362,626,924,442,1243,234,64,597,546
DDX18_2_393,2098,2174,2335,2801,3871,2596,2140,3544,1701,1642,3483,207
9
DDX19A_2_394,328,107,257,153,65,2,11,38,43,196,20,123
DDX1_2_395,675,620,754,579,529,503,1037,102,1431,776,690,513
DDX20_2_396,853,847,661,1002,970,16,2362,563,1156,590,1324,813
DDX21_2_397,357,301,499,475,23,372,316,504,260,549,855,366
DDX23_2_398,217,285,295,364,418,101,57,328,140,247,59,111
DDX24_2_399,915,854,835,923,768,240,1612,1833,840,1016,1668,1384
DDX25_2_400,261,228,347,396,89,513,345,386,137,253,357,512
DDX27_2_401,561,608,482,632,844,882,1460,916,363,1019,399,561
DDX28_2_402,4,25,56,70,0,133,0,31,151,158,0,15
DDX39A_2_403,255,185,223,307,595,102,384,3,291,244,93,113
DDX41_2_404,157,150,112,103,86,122,116,277,185,76,187,73
DDX43_2_405,655,494,482,744,154,356,919,623,476,311,88,817
DDX46_2_406,446,539,431,571,305,294,506,506,926,123,555,423
DDX49_2_407,342,438,370,348,538,78,1020,1,73,102,0,224
DDX50_2_408,813,336,480,605,1859,152,152,1044,381,960,1353,1368
DDX51_2_409,146,125,118,260,10,1,67,409,7,26,0,734
DDX52_2_410,2734,2094,2019,2180,1748,1122,1884,1301,2615,2012,2936,188
7
DDX53_2_411,9,6,42,73,0,0,339,0,5,95,0,0
DDX55_2_412,1577,1460,1358,1371,652,1489,1369,1939,1985,1540,1312,1888
DDX56_2_413,57,67,23,101,105,158,774,14,6,115,0,43
DDX58_2_414,158,275,266,353,93,17,565,80,380,340,194,305
DDX59_2_415,1313,1501,1068,1215,2043,998,875,1219,1146,2213,2186,1569
DDX5_2_416,343,749,438,718,291,357,998,378,375,1348,295,187
DDX60_2_417,1528,1523,1521,1771,629,658,1667,2095,1101,1752,3593,1747
DDX6_2_418,133,111,143,100,156,98,126,68,82,157,10,79
DECR1_2_419,73,84,135,66,0,0,57,0,55,103,11,113
DECR2_2_420,237,194,93,145,42,0,36,20,55,43,4,204
DEGS1_2_421,245,433,277,347,795,245,649,413,370,857,727,482
DEGS2_2_422,26,69,33,93,18,96,40,9,0,6,1,101
DERA_2_423,1024,953,1005,992,763,1408,2214,1149,845,1066,1495,1387
DFFB_2_424,304,184,236,312,129,1,948,125,56,613,588,153
DGAT1_2_425,42,5,31,13,27,0,0,0,1,0,68,0
DGAT2_2_426,1475,1256,1150,1864,1072,1034,1276,1331,705,1077,963,906
DHCR24_2_427,1084,1362,954,1376,814,2416,511,1433,751,1139,599,1449
DHDH_2_428,7,5,23,37,1,0,0,0,78,20,0,0
DHFR_2_429,692,617,521,727,210,253,823,675,919,798,328,615
DHODH_2_430,269,350,358,380,476,567,160,239,219,156,450,539
DHRS13_2_431,7,50,20,29,50,20,4,4,1,306,9,0
DHRS3_2_432,126,172,107,145,213,102,280,427,442,76,74,314
DHRS4_2_433,1473,1411,1502,1536,1113,1886,1548,1269,2021,866,1606,1956
DHRS7_2_434,451,440,526,799,662,302,255,715,189,367,1008,823
DHRSX_2_435,211,84,95,163,25,85,51,2,13,25,9,184

DHTKD1_2_436,149,90,116,238,30,21,393,12,50,307,68,0
DHX15_2_437,300,203,202,222,473,337,1617,784,145,15,28,464
DHX29_2_438,106,171,31,119,8,2,592,0,0,130,2,249
DHX32_2_439,151,185,167,121,0,351,146,3,128,549,0,1
DHX34_2_440,260,308,177,219,0,546,49,26,168,248,63,166
DHX37_2_441,175,136,284,110,187,1,136,82,60,31,642,309
DHX38_2_442,362,280,193,298,125,65,154,3,606,65,100,74
DHX57_2_443,1186,800,983,1154,1380,1726,1683,983,1002,842,1284,1376
DHX58_2_444,7,8,16,9,0,35,4,0,0,0,9,9
DHX8_2_445,412,247,358,334,459,696,244,261,359,384,146,138
DHX9_2_446,295,308,319,449,216,356,348,134,397,890,1,481
DIO3_2_447,71,93,85,86,82,9,10,55,15,187,62,37
DIRAS1_2_448,95,60,50,50,0,0,237,42,0,0,0,0
DIRAS2_2_449,1132,793,925,964,573,1927,751,765,761,486,647,782
DIRAS3_2_450,565,555,763,637,503,698,685,1190,806,861,540,785
DIS3L2_2_451,102,123,104,81,129,146,45,85,50,852,6,123
DLAT_2_452,2187,1799,1655,2615,1538,1259,2234,1879,1630,2018,2958,2499
DLD_2_453,13,45,41,32,0,0,40,1,103,0,211,1
DLL1_2_454,58,127,18,99,138,0,85,173,6,56,9,178
DLST_2_455,1087,845,824,1193,564,338,609,343,782,1657,617,1179
DMGDH_2_456,470,609,353,515,18,947,447,33,81,731,424,26
DNA2_2_457,624,527,464,700,641,881,824,29,620,443,525,822
DNAH11_2_458,669,575,424,308,1008,265,648,455,739,677,365,584
DNAH3_2_459,168,137,111,111,0,420,384,0,287,244,162,0
DNAH5_2_460,437,322,353,471,931,84,183,547,639,145,523,827
DNAH8_2_461,887,715,610,735,575,288,798,15,1085,670,499,1163
DNAJA2_2_462,2063,1761,1674,2007,1840,1688,1895,1124,1611,1279,1523,28
38
DNAJB14_2_463,61,251,187,207,28,21,492,107,75,292,27,20
DNAJC10_2_464,1142,1026,831,979,744,1732,1778,1188,1328,1238,851,1128
DNAJC18_2_465,453,298,460,340,172,163,839,468,356,24,461,440
DNAL4_2_466,30,115,111,167,30,1,12,398,3,444,305,278
DNASE1_2_467,17,23,57,83,0,0,395,13,1,0,2,0
DNASE1L2_2_468,96,88,99,112,31,3,414,37,66,0,1,135
DNASE1L3_2_469,334,210,296,359,222,103,661,463,963,49,173,612
DNASE2_2_470,57,65,72,100,27,76,0,29,7,12,0,447
DNPEP_2_471,120,217,242,162,119,300,959,375,329,227,274,529
DPAGT1_2_472,1115,1370,1089,1507,1496,1344,685,1592,1236,1072,945,1244
DPEP2_2_473,194,263,160,228,242,137,140,129,167,70,101,329
DPM1_2_474,976,773,1085,1337,1053,891,1351,1379,1712,1250,1084,1132
DPM2_2_475,61,153,74,66,120,448,116,75,0,284,1,80
DPP4_2_476,920,1053,879,938,184,1385,1861,915,1062,960,1274,446
DPP7_2_477,61,116,94,233,0,3,23,424,188,137,35,11
DPP9_2_478,501,393,610,688,245,235,787,646,480,1156,331,1006
DPYS_2_479,262,270,256,219,300,16,566,634,608,365,112,242
DPYSL4_2_480,126,43,89,107,135,84,159,365,104,13,95,42
DPYSL5_2_481,657,836,770,886,1074,1435,1358,994,382,1060,1566,414
DQX1_2_482,274,168,307,272,521,203,144,707,182,2,233,97
DSEL_2_483,361,392,507,435,267,39,639,708,268,686,1126,603
DTD1_2_484,306,188,206,294,316,111,61,20,371,556,280,132

DUOX2_2_485,3063,2735,2873,3202,3068,3658,3193,2699,3922,4752,1822,334
9
DUPD1_2_486,185,110,121,206,480,0,239,330,191,1,98,241
DUSP28_2_487,43,94,47,34,0,0,0,0,93,0,0,251
EARS2_2_488,231,338,301,393,349,33,462,253,32,25,984,449
EBPL_2_489,141,200,235,252,415,140,428,216,25,90,303,389
ECH1_2_490,237,481,257,325,46,55,1154,16,24,223,3,289
ECHDC3_2_491,251,160,151,528,664,205,264,797,309,421,64,209
ECHS1_2_492,565,404,400,508,237,3,213,12,650,448,368,7
EDEM1_2_493,732,897,775,877,864,870,648,942,947,1293,954,687
EDEM3_2_494,1217,1061,1428,1505,1681,1611,2353,1703,941,1367,799,2169
EEF1A1_2_495,231,200,517,298,279,78,192,78,204,83,180,426
EEF1A2_2_496,89,69,105,89,101,22,6,14,19,153,0,93
EEF2_2_497,119,48,34,109,3,146,0,76,420,4,1,2
EGLN1_2_498,1525,1152,1253,1414,1724,1470,1646,1157,2178,1776,715,1804
EGLN3_2_499,103,90,61,121,236,0,2,6,88,328,3,77
EHD4_2_500,1041,657,962,858,726,963,1348,655,653,383,1309,735
EIF4A3_2_501,243,143,143,210,287,60,65,820,174,111,454,274
ELAC1_2_502,56,69,84,49,0,37,146,45,71,20,0,2
ELANE_2_503,72,65,79,75,130,14,13,137,1,10,49,12
ELOVL1_2_504,98,122,128,172,234,32,344,111,11,2,265,10
ELOVL2_2_505,2221,1728,1880,2172,1485,1040,2521,2080,1790,2023,1533,25
83
ELOVL3_2_506,59,72,40,138,17,1,36,2,0,19,199,36
ELOVL4_2_507,151,225,165,201,362,668,3,5,332,81,0,1
ENDOD1_2_508,216,104,202,314,267,199,59,2,68,72,140,201
ENDOG_2_509,101,188,168,116,137,377,265,234,22,118,21,251
ENGASE_2_510,391,245,130,177,84,0,0,611,0,137,6,108
ENO2_2_511,172,137,102,191,185,0,26,1,7,55,352,1
ENO4_2_512,94,91,204,222,0,0,287,0,167,0,40,0
ENOPH1_2_513,160,105,84,135,272,50,42,130,241,241,0,298
ENPEP_2_514,599,505,427,603,422,1011,207,568,307,837,774,694
ENPP1_2_515,528,384,470,542,415,112,144,99,423,1022,581,683
ENPP3_2_516,1014,877,958,1069,1637,386,1026,1442,725,1338,995,785
ENPP4_2_517,1491,1711,1262,1524,2399,2096,2340,1208,1467,1431,1677,214
6
ENPP5_2_518,443,380,343,490,727,253,103,2,536,806,520,395
ENPP6_2_519,297,166,139,147,287,14,572,190,227,30,2,145
ENPP7_2_520,460,308,374,447,817,28,808,271,395,799,595,346
ENTPD3_2_521,2725,1931,2167,2287,2229,2593,2184,1479,3138,2692,2828,35
34
ENTPD5_2_522,156,226,76,192,15,27,2,0,94,507,245,75
ENTPD7_2_523,112,114,136,426,402,50,1,203,0,0,431,462
EPHX2_2_524,776,547,592,569,471,262,633,211,387,507,1306,663
EPHX4_2_525,432,409,236,332,338,369,35,1107,405,1028,539,492
EPRS_2_526,947,786,809,965,580,431,526,1590,781,1499,876,789
EPT1_2_527,679,692,788,754,1575,89,594,226,659,688,356,774
EPX_2_528,307,135,226,234,704,170,279,3,46,310,94,66
ERAS_2_529,3,22,19,31,0,0,0,24,9,4,0,46
ERCC3_2_530,212,238,306,366,421,132,325,186,301,87,251,626

ERCC4_2_531,824,643,810,628,1447,593,545,769,486,932,625,1778
ERCC5_2_532,745,678,556,535,717,981,518,425,434,651,279,628
ERI1_2_533,390,329,237,157,189,649,115,339,286,496,366,407
ERO1L_2_534,1340,1402,1434,1467,1514,514,2754,2500,956,1257,1268,1610
ERP44_2_535,351,557,413,505,887,565,808,396,284,209,1211,488
ESD_2_536,46,31,49,127,15,3,3,0,118,13,1,357
ESPL1_2_537,46,38,21,160,0,0,0,460,0,0,50,19
ETFDH_2_538,583,705,627,813,1158,463,337,670,491,610,923,708
EXOSC1_2_539,50,3,15,127,0,0,0,0,0,0,0,0
EXOSC2_2_540,1352,1268,788,949,1064,1175,1398,1385,1356,1144,681,1338
EXOSC4_2_541,522,515,380,498,436,327,895,403,873,978,150,413
EXOSC5_2_542,21,50,97,81,33,0,54,141,133,188,1,38
EXOSC7_2_543,34,124,134,64,68,74,3,148,1,64,0,135
EXOSC8_2_544,424,425,409,466,472,586,1216,898,612,621,143,819
EXT1_2_545,417,246,424,473,210,215,179,716,593,250,128,486
EXTL1_2_546,427,363,291,312,107,66,393,419,180,3,266,541
EXTL3_2_547,230,187,284,366,215,198,151,10,43,253,376,454
F10_2_548,86,70,85,41,48,39,344,1,108,173,0,116
F12_2_549,128,37,34,33,3,135,0,9,308,3,46,18
F13A1_2_550,279,229,213,180,314,592,1192,360,592,720,109,241
F13B_2_551,305,308,212,377,416,206,466,101,155,123,54,108
F2_2_552,613,340,336,636,208,198,1174,446,494,755,195,629
F5_2_553,606,518,353,419,845,654,346,340,449,642,211,351
F9_2_554,205,435,342,281,69,853,278,300,298,387,282,428
FA2H_2_555,395,553,462,610,350,235,717,904,425,1005,1881,573
FAAH2_2_556,416,376,454,427,412,839,214,764,351,389,594,503
FADS1_2_557,5017,5130,4950,5510,4022,3924,4506,6141,3877,6128,4924,525
6
FADS2_2_558,863,658,521,789,974,898,1501,753,263,311,721,851
FADS3_2_559,207,120,258,169,25,505,580,614,0,1,355,581
FAHD2A_2_560,463,621,651,534,434,596,1730,1644,444,1327,406,1411
FAM108C1_2_561,236,234,239,250,234,281,356,283,344,38,13,250
FAM135B_2_562,727,497,424,730,876,75,849,988,717,642,888,594
FAM20B_2_563,517,571,606,679,1259,563,69,589,237,21,406,1154
FANCM_2_564,317,441,173,372,172,564,346,171,204,646,266,864
FAR1_2_565,581,313,395,528,1624,250,367,152,727,261,401,405
FAR2_2_566,790,530,588,635,968,482,398,1358,291,611,750,936
FARS2_2_567,45,125,107,51,7,10,72,3,148,19,45,156
FARSA_2_568,336,250,178,96,0,67,460,2,102,1,12,0
FARSB_2_569,766,920,831,1101,1051,603,919,1825,528,1230,586,1154
FASN_2_570,87,124,92,115,127,32,71,109,133,274,201,330
FDFT1_2_571,1188,1050,1331,1491,1318,345,1298,1532,2273,1242,839,1723
FEN1_2_572,417,250,253,180,683,0,511,6,298,147,5,74
FH_2_573,821,511,723,520,591,144,460,858,826,154,359,389
FIG4_2_574,230,217,143,152,0,227,190,55,9,0,492,83
FKBP10_2_575,580,208,365,402,5,262,605,198,241,754,131,356
FKBP14_2_576,91,183,179,213,178,8,5,1,11,0,206,23
FKBP15_2_577,189,58,121,107,492,24,290,31,86,433,0,228
FKBP3_2_578,41,23,137,55,72,3,135,0,175,0,196,13
FKBP4_2_579,167,298,176,347,1083,375,7,211,271,324,151,204

FKBP8_2_580,74,65,91,82,21,11,300,13,54,38,75,361
FMO1_2_581,259,230,238,229,256,225,438,119,177,638,127,444
FMO4_2_582,215,336,420,327,85,307,551,195,55,301,60,346
FNBP1_2_583,902,699,764,876,923,415,1100,1976,543,402,825,742
FNTA_2_584,640,824,413,519,545,937,354,574,85,1053,1766,581
FNTB_2_585,98,49,63,51,3,10,633,0,0,7,5,416
FTH1_2_586,47,31,47,53,59,53,4,0,0,67,56,3
FTMT_2_587,349,444,401,394,296,402,336,2067,658,585,1047,1085
FTSJ2_2_588,0,0,0,0,0,0,0,0,0,0,0,0
FTSJ3_2_589,379,268,286,408,240,108,422,938,557,362,221,864
FTSJD2_2_590,93,123,96,77,1,57,861,60,72,133,46,363
FUCA1_2_591,395,339,341,595,166,147,230,448,289,254,795,153
FUCA2_2_592,300,277,397,335,439,172,315,434,367,312,2278,1032
FURIN_2_593,146,212,272,205,0,162,0,0,6,0,0,1
FUT10_2_594,62,87,86,55,9,221,0,14,24,4,0,2
FUT11_2_595,1416,1620,1391,1738,1687,824,1504,1708,1028,1536,1658,1406
FUT1_2_596,321,178,158,216,35,223,386,1,36,103,197,398
FUT4_2_597,221,137,208,107,227,635,146,145,146,345,9,349
FUT5_2_598,481,282,349,440,398,487,805,276,332,80,36,472
FUT7_2_599,174,161,169,149,14,297,0,34,206,5,234,435
FUT9_2_600,377,487,277,506,556,617,323,84,426,182,497,284
GADL1_2_601,952,1062,972,1007,797,589,1594,183,763,781,483,1198
GAL3ST1_2_602,44,96,41,43,0,115,176,1,54,201,296,177
GAL3ST3_2_603,342,287,213,343,213,150,350,409,298,116,25,102
GAL3ST4_2_604,177,181,178,306,158,9,237,2,153,210,30,108
GALM_2_605,314,187,243,386,158,103,105,29,55,89,23,105
GALNS_2_606,180,139,115,169,190,0,282,0,153,168,552,143
GALNT10_2_607,138,135,58,62,602,0,80,15,53,0,537,38
GALNT11_2_608,101,92,55,51,89,31,138,85,386,63,5,0
GALNT12_2_609,886,585,656,854,463,558,1238,1108,570,497,703,998
GALNT13_2_610,2245,2283,1806,1976,3331,609,2240,2085,1514,2302,1691,26
87
GALNT14_2_611,312,472,253,324,43,39,854,91,242,118,27,262
GALNT1_2_612,1436,1428,1427,1352,699,1132,2216,1433,949,1823,902,1582
GALNT2_2_613,460,449,522,465,1210,405,995,35,632,253,587,907
GALNT3_2_614,584,517,380,512,505,363,773,254,520,1266,309,636
GALNT4_2_615,2217,1911,1896,2040,2133,2621,1846,2181,1706,1733,1655,26
43
GALNT5_2_616,2107,1605,1410,1913,410,2253,2092,1984,1060,967,1605,1738
GALNT6_2_617,212,249,283,317,303,233,558,134,0,0,1250,338
GALNT8_2_618,937,1014,689,1111,707,788,1202,877,78,933,1717,974
GALNTL2_2_619,1943,1900,1814,1785,3554,2023,1673,740,2472,1353,1051,13
83
GALNTL4_2_620,305,258,299,238,229,158,499,278,69,484,90,110
GALNTL5_2_621,288,185,263,280,647,44,581,48,344,100,7,83
GALNTL6_2_622,96,99,224,171,123,149,107,215,30,24,690,92
GALT_2_623,128,127,238,122,0,234,294,14,1,161,180,147
GANC_2_624,981,873,918,1105,1357,811,1806,499,732,1042,303,689
GAPDH_2_625,128,149,138,151,74,298,571,0,97,184,135,138
GAPDHS_2_626,1128,996,884,1167,732,981,1085,1177,573,241,1193,1632

GARS_2_627,256,404,282,443,144,194,282,257,396,416,76,391
GATC_2_628,271,346,281,302,165,21,269,143,218,424,674,322
GATM_2_629,112,139,68,87,134,44,756,7,102,4,247,133
GBA2_2_630,18,53,15,25,0,0,0,0,0,1,0,0
GBE1_2_631,359,324,344,519,845,3,190,1275,279,71,308,542
GBGT1_2_632,253,158,203,303,121,315,48,444,20,208,246,95
GBP1_2_633,583,439,383,515,333,348,516,585,256,800,109,687
GBP2_2_634,583,439,383,515,333,348,516,585,256,800,109,687
GBP3_2_635,583,439,383,515,333,348,516,585,256,800,109,687
GBP4_2_636,667,654,681,970,1057,421,1096,108,434,1108,631,268
GBP6_2_637,235,187,182,324,320,99,171,560,85,302,111,90
GBP7_2_638,134,114,131,291,59,109,176,58,50,106,116,109
GCLM_2_639,533,709,558,844,149,499,254,1092,518,516,142,958
GCNT3_2_640,1057,982,867,880,1144,1648,1591,547,901,461,845,702
GCNT4_2_641,591,764,618,760,16,483,94,370,475,1072,830,1084
GCSH_2_642,226,394,190,172,379,292,716,110,411,124,890,307
GDE1_2_643,1030,998,1014,1114,958,599,416,241,1089,1158,401,590
GDPD3_2_644,98,23,30,29,2,5,14,9,5,69,9,5
GFM1_2_645,1290,880,787,1028,1295,589,575,1262,1133,1191,928,591
GFOD2_2_646,532,244,235,441,228,403,158,413,500,243,18,519
GFPT1_2_647,78,59,61,61,0,2,719,216,390,237,0,501
GFPT2_2_648,340,246,274,216,49,192,492,207,585,68,249,542
GGH_2_649,728,456,529,829,375,162,858,490,202,618,235,392
GGPS1_2_650,67,124,50,84,59,87,108,123,154,57,140,317
GGT7_2_651,14,14,9,17,0,0,237,9,0,0,1,1
GGTLC2_2_652,161,267,142,219,519,44,23,738,148,138,8,881
GLCE_2_653,121,213,152,131,476,128,10,8,125,91,17,85
GLDC_2_654,60,39,135,88,0,44,1,35,26,0,396,2
GLO1_2_655,391,471,278,496,650,18,5,282,91,620,266,1027
GLOD4_2_656,201,275,157,257,10,216,64,33,392,20,56,6
GLS2_2_657,63,62,45,58,14,19,85,35,0,132,0,253
GLT1D1_2_658,202,117,234,264,0,2,1,238,387,83,223,279
GLT25D1_2_659,463,264,392,553,694,540,529,251,471,316,125,97
GLT25D2_2_660,464,244,275,522,752,557,587,7,323,40,219,109
GLT8D2_2_661,1112,906,778,1163,1664,419,1066,1599,815,1107,855,987
GLUD1_2_662,47,19,77,101,262,10,514,740,47,381,390,239
GLUD2_2_663,380,324,365,575,204,128,826,130,188,401,675,405
GMDS_2_664,310,347,289,339,311,64,352,194,834,45,28,321
GMIP_2_665,27,45,20,20,3,0,0,0,0,0,18,92
GMPR_2_666,1072,728,1179,1025,1220,327,884,2323,1162,740,1219,1151
GMPS_2_667,468,333,250,513,80,226,56,202,454,676,1191,212
GNA11_2_668,213,219,228,175,17,415,350,26,346,312,49,435
GNA12_2_669,238,201,367,272,225,312,200,258,198,657,20,283
GNA13_2_670,692,580,505,679,359,406,527,466,349,960,50,1075
GNA14_2_671,746,543,493,802,371,846,435,1043,540,588,446,507
GNA15_2_672,559,339,357,323,413,806,205,102,260,482,1045,715
GNAI1_2_673,1118,1057,1080,1142,773,646,1398,687,624,386,1858,1664
GNAI3_2_674,294,483,371,565,765,82,558,188,432,160,949,1044
GNAQ_2_675,2363,2086,2218,2726,1510,1285,3662,2387,2269,2138,2461,3233
GNAT2_2_676,1192,974,1079,983,1214,1129,818,148,741,1102,1924,688

GNAT3_2_677,346,264,333,314,201,398,482,383,492,91,320,56
GNAZ_2_678,1208,1155,876,866,2013,1027,1257,1300,1702,777,582,769
GNB1_2_679,274,400,160,259,562,585,479,669,74,250,9,492
GNB2L1_2_680,27,29,36,52,0,0,2,0,0,284,0,2
GNB3_2_681,539,544,609,835,563,1062,676,1391,395,312,1214,638
GNB4_2_682,356,540,418,644,575,175,301,29,78,9,994,292
GNG11_2_683,35,111,86,146,8,22,41,337,14,532,26,58
GNG12_2_684,17,27,15,68,19,0,0,0,3,267,0,70
GNG13_2_685,44,12,63,80,471,14,404,5,0,65,0,0
GNG2_2_686,336,250,183,282,481,5,68,37,326,299,326,70
GNG3_2_687,431,191,281,287,1025,63,96,250,154,540,25,238
GNG7_2_688,2480,2155,1899,2311,2345,2456,1449,1421,1937,2971,1406,2073
GNG8_2_689,35,41,51,2,2,0,0,1,13,12,0,130
GNGT1_2_690,670,839,714,871,699,940,701,783,600,590,190,386
GNL2_2_691,449,400,392,404,46,18,74,44,41,265,174,464
GNMT_2_692,175,265,98,195,19,0,46,4,150,97,21,23
GNPAT_2_693,154,156,231,170,72,273,336,277,61,224,218,171
GNPDA1_2_694,146,125,130,112,57,82,252,73,63,245,191,246
GNPDA2_2_695,210,149,224,334,42,25,368,113,149,340,0,92
GNPNAT1_2_696,1018,909,996,1115,1380,734,1114,1303,530,1258,1179,1147
GNPTAB_2_697,802,580,662,725,564,538,669,1060,390,282,1134,544
GNPTG_2_698,244,175,220,272,54,1,363,18,12,263,1,396
GNS_2_699,109,205,123,175,33,60,80,478,50,229,7,98
GOT2_2_700,618,384,439,714,252,0,159,782,486,527,610,550
GPAA1_2_701,59,60,12,52,110,0,14,211,6,18,292,93
GPAM_2_702,128,108,93,97,174,14,149,514,67,70,80,347
GPAT2_2_703,0,0,0,0,0,0,0,0,0,0,0,0
GPD1_2_704,928,853,796,813,53,979,1796,339,539,727,890,1196
GPD1L_2_705,347,295,312,261,483,160,560,85,192,96,82,322
GPX2_2_706,158,171,178,190,1,5,0,42,12,88,0,245
GPX3_2_707,580,472,401,463,509,390,358,419,644,768,698,594
GPX7_2_708,21,9,66,57,0,0,0,0,0,0,0,195
GPX8_2_709,717,793,742,946,486,453,963,929,356,257,349,501
GRHPR_2_710,79,115,67,100,127,1,673,149,20,8,92,7
GSTA1_2_711,850,703,577,653,1052,414,507,719,630,685,916,1047
GSTA2_2_712,179,105,193,150,0,236,519,11,23,0,3,477
GSTA3_2_713,985,983,728,1173,1620,881,472,961,1275,718,646,1458
GSTA4_2_714,985,901,888,973,805,986,381,688,640,324,672,1061
GSTA5_2_715,985,983,728,1173,1620,881,472,961,1275,718,646,1458
GSTM3_2_716,121,83,156,89,16,10,10,299,1,55,1,275
GSTM5_2_717,610,545,673,608,605,181,27,880,248,677,1171,532
GSTP1_2_718,141,151,213,96,117,38,578,97,178,184,648,37
GSTT1_2_719,44,15,9,22,86,0,0,0,32,0,2,0
GSTT2B_2_720,69,48,42,57,36,1,84,27,7,68,442,131
GSTT2_2_721,69,48,42,57,36,1,84,27,7,68,442,131
GTF2F2_2_722,2552,2166,2364,2488,1932,1589,2912,3298,2095,2768,4683,18
33
GTPBP1_2_723,31,90,77,64,55,63,172,11,24,25,26,4
GTPBP2_2_724,66,57,53,95,0,3,8,210,151,13,0,357
GTPBP4_2_725,1633,1385,1453,1572,2423,1512,2458,782,1407,1344,261,1301

GUCY1A2_2_726,0,0,0,0,0,0,0,0,0,0,0,0
GUCY1B3_2_727,142,189,162,340,482,165,497,116,67,205,80,167
GUSB_2_728,35,53,57,75,24,42,0,156,27,52,27,201
GYLTL1B_2_729,258,382,359,411,8,3,336,11,338,24,709,323
GYS2_2_730,597,619,677,672,864,1205,809,704,592,325,547,732
GZMA_2_731,508,424,384,476,347,197,187,161,468,585,1047,231
GZMB_2_732,114,420,201,209,77,40,13,74,149,126,86,483
H6PD_2_733,791,669,763,820,810,908,661,771,859,628,404,941
HAAO_2_734,137,104,109,175,0,19,292,0,87,113,35,615
HACL1_2_735,162,254,314,271,126,78,754,283,76,258,354,232
HADHA_2_736,408,343,473,540,204,609,496,702,328,294,420,284
HADHB_2_737,138,62,189,126,30,218,88,220,13,191,0,297
HAGHL_2_738,33,31,97,33,4,6,3,0,29,120,0,49
HAL_2_739,2202,2416,2467,2663,2644,879,4738,3172,1399,2616,1872,1855
HAO1_2_740,288,153,337,363,1540,68,0,526,74,626,301,328
HARS_2_741,467,337,317,322,490,461,399,601,651,485,504,430
HAS1_2_742,1356,1189,1179,1341,795,1358,1162,991,429,1558,1142,2459
HAS2_2_743,836,856,637,747,877,294,448,681,467,716,405,268
HDHD3_2_744,819,758,675,808,374,992,163,1477,676,145,308,345
HELB_2_745,17,9,5,17,0,41,0,39,6,1,37,0
HELQ_2_746,790,565,910,745,398,610,1393,899,1191,806,1298,924
HELZ_2_747,1641,1660,1325,1681,197,1394,1868,2188,1086,1133,1019,869
HEXA_2_748,180,205,218,131,188,236,1120,431,52,69,143,160
HEXB_2_749,392,281,311,497,743,238,660,92,193,446,439,263
HEXDC_2_750,291,389,211,169,320,178,563,1138,330,210,1,816
HGD_2_751,449,370,447,516,578,283,638,408,315,412,815,702
HGSNAT_2_752,422,305,319,333,301,327,169,211,178,483,492,508
HHATL_2_753,275,125,199,115,279,0,47,139,9,82,20,519
HIBADH_2_754,142,109,63,33,520,98,0,33,45,151,0,7
HINT1_2_755,1170,1615,1153,1340,1977,1720,1841,1501,1095,1632,1460,856
HMOX1_2_756,102,118,121,114,132,77,805,116,17,51,224,3
HPGDS_2_757,826,609,524,734,596,189,297,879,415,691,528,203
HPRT1_2_758,894,939,881,982,1614,1097,1505,432,882,2178,756,895
HS3ST1_2_759,28,37,53,44,265,69,7,1,60,13,12,81
HS3ST2_2_760,347,234,253,302,199,151,588,345,194,406,348,144
HS3ST3A1_2_761,169,53,54,95,236,4,83,68,125,73,3,33
HS3ST3B1_2_762,169,53,54,95,236,4,83,68,125,73,3,33
HS3ST4_2_763,631,543,652,489,994,692,138,222,577,600,628,827
HS3ST6_2_764,50,37,46,45,58,110,298,1,259,1,1,12
HS6ST1_2_765,1658,1397,1190,1745,1576,1331,1162,943,854,484,2348,2318
HS6ST3_2_766,264,138,357,458,84,298,13,58,45,167,178,584
HSD11B2_2_767,77,97,106,231,284,25,169,76,110,660,511,5
HSD17B11_2_768,1644,1334,1587,2124,2849,1296,1966,2769,835,1537,1215,2
102
HSD17B12_2_769,64,51,82,138,0,196,53,24,43,34,570,118
HSD17B14_2_770,940,858,691,930,1528,154,2148,511,1370,1259,1232,911
HSD17B1_2_771,1899,1615,1739,1696,2370,1491,2288,1967,2059,2207,1877,1
422
HSD17B2_2_772,70,63,49,43,0,21,91,249,80,67,2,99
HSD17B3_2_773,855,702,519,829,1117,1207,1671,682,694,587,1193,472

HSD17B6_2_774,125,38,129,126,72,5,83,121,31,1,246,305
HSD17B8_2_775,81,21,37,103,17,10,0,0,171,0,50,3
HSD3B1_2_776,1211,641,787,1029,1225,1099,755,365,1334,815,429,896
HSP90AB1_2_777,0,0,1,0,66,1,0,0,0,0,2,0
HSPA5_2_778,1047,742,658,726,317,380,1423,560,550,791,255,1083
HSPE1_2_779,551,506,524,520,14,1057,76,225,898,63,569,446
HSPG2_2_780,275,191,175,155,469,144,218,122,163,43,119,253
HYAL4_2_781,720,842,684,978,906,620,1806,688,347,461,115,1144
IARS2_2_782,506,512,380,668,164,802,617,345,288,789,97,341
ICMT_2_783,210,318,213,246,457,50,77,1,151,145,738,272
ICT1_2_784,147,180,176,146,538,34,520,841,111,191,180,303
IDH1_2_785,58,121,80,91,79,0,34,125,323,17,261,211
IDH2_2_786,316,462,478,462,295,210,571,254,666,349,353,301
IDH3A_2_787,811,497,297,589,325,376,633,1114,379,800,440,659
IDI1_2_788,1118,763,876,1114,668,886,435,970,915,1298,1233,924
IDI2_2_789,377,292,236,364,361,818,233,29,667,52,412,165
IDO1_2_790,2596,2639,2195,2200,1728,2428,1530,1716,1168,2782,1910,1660
IDO2_2_791,310,301,465,604,232,546,273,14,244,271,92,476
IDUA_2_792,137,41,32,121,6,129,6,361,55,2,21,5
IFI30_2_793,735,906,774,962,53,539,270,1264,785,1145,2518,1089
IFIH1_2_794,313,318,480,335,334,1019,236,58,40,539,645,215
IGHMBP2_2_795,120,204,183,224,149,163,51,79,20,41,118,110
IHH_2_796,451,249,224,352,623,385,435,203,215,388,194,399
ILVBL_2_797,533,311,384,417,377,341,0,176,274,158,437,221
IMPAD1_2_798,70,52,62,63,100,83,116,20,230,0,0,97
IMPDH2_2_799,144,225,250,122,174,638,5,20,126,1194,175,81
IRS1_2_800,86,101,86,82,174,2,234,18,162,114,1,112
IRS2_2_801,246,117,190,132,367,437,261,54,200,99,1,146
ISG20_2_802,10,66,43,164,124,0,22,1,7,18,196,0
ISG20L2_2_803,22,29,30,43,0,1,0,54,4,39,111,0
ISOC1_2_804,353,305,299,261,351,125,164,103,281,592,157,453
IWS1_2_805,16,68,31,37,6,61,114,22,4,285,23,2
KDSR_2_806,1738,1260,1375,1247,1252,1482,1705,782,1968,1598,954,1116
KHSRP_2_807,110,32,41,63,8,68,398,48,0,33,255,0
KIAA0317_2_808,672,715,911,1069,496,498,1369,888,638,1113,577,482
KIAA1279_2_809,805,1078,827,913,902,317,383,344,1156,749,1124,1101
KIAA2022_2_810,1089,1038,1082,1074,1136,1144,666,1307,815,665,1161,108
8
KIF18A_2_811,606,683,549,839,766,929,623,778,700,659,739,1963
KIF20B_2_812,2813,2056,1680,2485,2024,810,2851,1739,3607,1633,3174,328
6
KIF3A_2_813,268,428,314,286,305,284,241,265,183,191,1228,670
KLB_2_814,376,279,227,407,18,91,318,506,324,166,13,642
KLC3_2_815,192,228,129,109,9,1,93,125,170,6,0,0
KL_2_816,1280,1226,1246,1269,1027,587,1156,2107,1101,863,1272,859
KLK1_2_817,359,279,333,340,894,33,11,70,502,208,71,320
KLKB1_2_818,295,270,341,526,396,46,30,552,480,6,18,350
KMO_2_819,960,931,826,1100,1292,726,1069,405,2088,1090,520,778
KRTCAP2_2_820,241,314,226,380,252,450,80,323,319,308,97,342
L2HGDH_2_821,1695,1417,1428,1846,998,1845,548,2867,801,1293,1409,2144

LALBA_2_822,387,308,278,381,647,212,301,352,195,561,29,218
LAP3_2_823,316,286,408,340,306,414,1756,159,139,1012,19,569
LARS2_2_824,166,93,114,178,469,6,303,106,86,115,188,232
LARS_2_825,638,593,515,583,971,621,716,216,426,677,736,877
LCAT_2_826,306,183,115,168,552,374,0,156,154,316,118,259
LCMT2_2_827,511,437,448,419,561,625,838,422,56,529,616,341
LCT_2_828,292,165,205,209,319,391,1,337,279,427,276,734
LCTL_2_829,346,609,564,611,536,100,413,724,435,480,608,329
LDHAL6B_2_830,1875,2044,1743,1693,1529,1526,1624,4491,2607,1102,2267,1
968
LEPREL2_2_831,584,413,480,707,623,52,77,379,142,507,1053,3
LGALS13_2_832,354,149,394,306,304,79,140,493,376,378,3,78
LHFPL2_2_833,22,12,21,35,160,0,15,0,60,0,106,365
LIPC_2_834,1730,1535,1247,1143,1232,1425,1237,1316,1369,1299,891,725
LIPE_2_835,61,80,131,114,34,0,258,0,1,415,77,130
LIPG_2_836,50,51,102,136,15,0,0,0,21,138,0,0
LIPH_2_837,377,236,392,410,0,184,176,895,391,76,438,856
LIPI_2_838,720,624,635,830,467,0,0,1219,1107,2022,538,338
LIPM_2_839,428,308,298,286,21,716,241,421,1,235,705,252
LIPN_2_840,1061,1041,901,929,631,1029,675,1085,1074,874,1051,1548
LIPT2_2_841,241,407,404,320,133,340,739,263,114,424,485,258
LONP2_2_842,536,447,587,610,991,1089,857,546,717,686,565,327
LOXL1_2_843,101,110,116,175,362,374,582,214,191,76,558,417
LOXL2_2_844,52,34,31,22,0,1,18,0,19,31,0,12
LOXL3_2_845,142,66,55,129,44,421,28,16,31,0,155,84
LOXL4_2_846,147,92,127,179,124,100,131,36,185,32,0,51
LPCAT1_2_847,45,74,49,50,153,12,151,1,11,53,28,6
LPCAT2_2_848,2343,1632,1784,2119,3471,1347,1906,3112,2165,2224,858,130
7
LPCAT3_2_849,126,141,90,215,68,9,459,244,223,229,2,28
LPCAT4_2_850,78,118,113,58,11,2,1,19,113,69,264,191
LPIN1_2_851,310,331,431,351,67,150,0,695,254,632,709,350
LPIN2_2_852,76,37,23,43,4,6,0,23,18,107,13,115
LPIN3_2_853,85,113,71,110,201,51,22,23,183,79,7,18
LPL_2_854,557,323,382,472,854,328,5,0,791,208,340,262
LRAT_2_855,52,97,69,84,0,0,0,1,7,1,0,26
LTA4H_2_856,1293,1239,976,1064,805,448,1428,454,1412,1178,1293,474
LTC4S_2_857,10,215,70,94,273,52,7,54,268,244,4,4
LYG2_2_858,691,489,501,649,818,63,309,618,587,668,973,757
LYPLA1_2_859,200,246,256,181,438,14,491,248,23,232,91,127
LYPLA2_2_860,48,65,73,52,48,267,0,0,0,376,207,219
LYPLAL1_2_861,1055,943,988,973,724,909,562,624,832,292,876,1711
LYZ_2_862,443,361,439,698,370,354,176,60,185,175,591,114
LYZL1_2_863,877,491,533,515,1072,539,100,281,843,461,1446,613
LYZL2_2_864,374,314,432,445,423,221,182,528,488,393,1039,468
LYZL4_2_865,388,231,383,468,196,22,435,598,479,146,1334,342
MACROD1_2_866,48,12,37,72,0,0,1,0,31,8,8,6
MAGT1_2_867,3172,2679,2597,3440,2948,2305,3229,1937,3393,2984,3246,311
8
MAN1A1_2_868,147,383,179,170,16,374,138,314,915,835,7,75

MAN1A2_2_869,1004,1070,1262,1348,723,535,511,1338,1064,813,549,1208
MAN1B1_2_870,653,921,713,667,748,916,516,497,1364,302,570,131
MAN1C1_2_871,135,176,210,199,251,183,354,775,181,2,276,202
MAN2A1_2_872,245,89,130,157,258,52,299,281,79,291,57,79
MAN2A2_2_873,650,489,498,437,614,237,573,245,767,694,1308,406
MAN2B2_2_874,280,345,286,331,568,331,265,512,433,161,401,823
MAN2C1_2_875,472,506,458,645,131,1190,264,67,279,665,1067,76
MANBA_2_876,257,75,169,161,300,682,1,0,72,24,52,255
MANEA_2_877,657,582,534,843,1681,691,685,403,492,825,1398,1652
MAOA_2_878,143,88,126,164,22,21,262,414,136,445,106,127
MAOB_2_879,298,353,302,419,560,284,245,0,599,0,63,401
MAP1S_2_880,27,112,45,48,0,0,0,0,4,0,0,16
MAPRE3_2_881,548,302,301,408,392,260,109,114,434,492,656,578
MARS2_2_882,149,75,79,116,352,7,289,96,201,14,96,129
MARS_2_883,150,79,123,128,746,147,526,233,295,84,54,167
MAT1A_2_884,52,104,136,268,150,166,798,36,179,27,41,337
MAT2A_2_885,113,128,194,182,153,96,437,491,120,193,498,318
MBOAT1_2_886,1751,1584,1287,1790,1066,880,526,1316,1649,1549,1163,1274
MBOAT2_2_887,2695,2603,2294,2716,3148,1373,2642,2441,2196,1791,1786,18
03
MBOAT4_2_888,306,455,188,278,75,133,395,1,658,37,970,39
MBTPS1_2_889,1569,1112,1165,1278,759,2183,1196,1783,970,769,961,1244
MBTPS2_2_890,41,4,19,2,12,0,8,3,2,2,0,0
MCCC1_2_891,554,472,432,470,492,656,258,291,295,193,679,255
MCCC2_2_892,358,306,368,381,431,67,287,92,924,155,541,182
MCEE_2_893,2571,2037,1680,2628,2456,1681,2168,928,1802,1908,1312,1787
MCM3_2_894,707,614,476,704,337,628,1210,506,1064,1179,405,789
MCM5_2_895,95,85,65,107,19,27,41,518,74,54,25,92
MCM6_2_896,391,311,132,296,472,344,112,80,395,219,200,219
MDH1B_2_897,2393,2084,2227,2724,2131,1322,2745,1918,1709,1332,3228,178
8
MDH2_2_898,1212,1062,1059,1030,224,752,2593,1781,1723,461,555,1682
MEP1A_2_899,1299,1265,950,1463,558,842,760,1059,1327,899,901,1294
MEP1B_2_900,2140,1848,1651,2037,2010,1554,2423,1752,3051,917,2289,1470
METAP1_2_901,701,777,694,806,647,1108,1030,426,1010,1451,2819,670
METAP1D_2_902,489,318,330,383,302,366,396,745,315,456,85,440
METAP2_2_903,430,540,722,517,501,629,1317,545,474,636,318,213
METTL14_2_904,1117,755,919,1233,1271,1075,570,744,939,1133,1079,278
METTL22_2_905,418,390,375,562,539,380,160,1008,416,146,319,144
METTL2B_2_906,1512,1309,1485,1682,1451,1514,1745,1120,787,1284,1411,28
01
METTL3_2_907,609,584,505,576,135,449,417,847,815,726,567,454
METTL5_2_908,841,440,511,455,172,345,584,503,162,402,253,407
METTL6_2_909,1033,899,870,949,646,481,1167,1088,1136,994,1494,701
METTL7B_2_910,84,82,64,57,0,61,32,21,210,0,0,337
METTL8_2_911,333,506,521,340,188,100,461,676,233,501,233,72
MFN1_2_912,345,367,305,337,381,209,615,359,404,335,82,184
MGAM_2_913,770,800,716,830,719,281,656,1050,386,937,821,577
MGAT4C_2_914,591,547,478,804,1233,169,754,416,822,361,83,134
MGAT5_2_915,820,418,460,760,18,409,776,1184,691,361,386,737

MGMT_2_916,72,186,113,149,1,204,334,149,8,62,331,826
MGST3_2_917,886,933,985,819,1510,295,1545,505,800,791,360,430
MIOX_2_918,23,34,24,37,183,426,0,0,0,244,32,86
MIPEP_2_919,887,542,561,698,911,610,1870,468,819,1419,656,516
MLYCD_2_920,145,124,149,213,492,66,4,78,283,297,107,373
MMAB_2_921,14,42,24,36,0,0,0,0,13,13,0,6
MMEL1_2_922,299,362,243,313,175,572,23,358,775,453,27,376
MMP10_2_923,634,371,595,812,591,144,494,1174,649,659,704,609
MMP12_2_924,2416,2489,1994,2802,3127,1668,2948,2741,1489,1884,3950,215
2
MMP14_2_925,420,133,326,330,506,22,5,716,599,0,1136,212
MMP3_2_926,57,120,135,172,1,38,946,4,19,129,168,496
MMP7_2_927,138,147,69,200,19,91,9,360,1,43,0,0
MMP8_2_928,220,257,191,244,172,151,237,285,26,274,47,224
MMP9_2_929,181,107,109,86,2,409,153,206,66,108,109,30
MOCOS_2_930,479,619,778,555,516,826,2164,681,1167,734,822,606
MOGAT1_2_931,95,67,19,66,5,2,0,0,1,0,80,90
MOGAT2_2_932,163,199,170,183,416,608,120,259,224,92,13,132
MOGAT3_2_933,226,183,124,287,254,231,77,3,164,126,11,864
MOXD1_2_934,127,90,97,66,0,0,0,108,31,812,341,48
MPI_2_935,715,758,642,859,413,995,713,212,411,1776,1134,1114
MPO_2_936,188,218,168,276,601,0,0,429,0,163,243,11
MRPL37_2_937,1496,1430,1368,1694,369,2445,2030,1666,2582,1757,1693,202
4
MRPL44_2_938,931,614,809,627,684,1047,1445,480,774,366,960,286
MRPS30_2_939,69,180,81,72,162,31,33,11,183,88,469,4
MSH2_2_940,679,393,637,446,523,549,877,382,131,574,838,459
MSH3_2_941,661,734,825,772,468,490,962,295,644,313,614,704
MSH4_2_942,840,942,875,965,393,835,1846,621,551,584,421,1053
MTAP_2_943,649,575,597,523,1000,1033,0,728,1034,1253,523,777
MTFMT_2_944,282,351,443,480,84,113,992,564,86,327,256,275
MTHFD1_2_945,152,101,68,79,282,20,0,57,5,3,2,217
MTHFD2_2_946,290,340,339,359,370,6,4,39,182,190,264,81
MTHFD2L_2_947,2004,1676,1391,2229,2255,1093,1606,525,1885,2515,1049,16
15
MTHFR_2_948,241,83,122,114,72,19,207,233,182,143,9,317
MTPAP_2_949,3731,3111,3056,3346,4536,3384,4223,3549,3814,3297,2153,429
8
MTR_2_950,234,225,218,196,164,104,112,27,352,113,176,406
MUS81_2_951,481,280,258,315,460,146,229,134,1091,279,1,151
MUT_2_952,369,216,506,495,488,23,1511,326,1298,486,283,280
MVD_2_953,35,122,63,65,0,131,80,0,138,92,198,223
MX2_2_954,1887,1849,1814,2125,844,2420,3620,1675,1442,3595,3134,1234
MYH1_2_955,1006,635,632,751,866,863,1016,956,449,266,497,731
MYH3_2_956,259,442,228,256,217,4,70,99,86,267,42,171
MYH6_2_957,796,722,762,545,225,443,326,575,680,339,820,500
MYH7_2_958,644,493,600,440,217,451,320,498,579,305,818,370
MYH9_2_959,27,30,35,39,66,0,0,0,47,20,382,0
MYL7_2_960,74,68,34,71,1,76,642,0,64,14,72,18
MYO1E_2_961,635,398,687,691,482,715,723,1443,890,197,2169,631

MYO5B_2_962,68,34,63,154,0,0,39,1,0,3,0,100
MYO9A_2_963,556,643,390,620,285,654,1423,790,352,376,228,625
N6AMT2_2_964,2079,1561,1511,2084,2006,1589,1935,1382,1658,1484,1237,18
17
NAA10_2_965,925,690,762,786,523,652,563,701,439,677,458,1110
NAA11_2_966,811,786,715,734,203,628,826,218,382,518,479,556
NAA15_2_967,146,144,179,359,8,17,7,216,88,145,302,64
NAA30_2_968,111,131,91,57,2,32,3,0,130,11,92,26
NAA50_2_969,934,996,937,1511,1537,999,1970,1314,1307,775,977,1407
NAALAD2_2_970,2188,1461,1843,2135,3469,1680,2743,1230,1685,1830,1905,1
509
NAALADL1_2_971,240,261,137,324,205,14,29,160,17,167,0,321
NADSYN1_2_972,369,309,276,410,406,388,114,299,72,455,316,64
NAGA_2_973,28,23,47,88,110,1,64,28,64,83,0,52
NAGLU_2_974,11,183,119,220,0,1,2,0,280,1,0,181
NAGPA_2_975,397,344,284,501,152,199,252,279,300,289,347,256
NAMPT_2_976,45,29,20,31,13,242,0,1,2,3,0,0
NANP_2_977,939,731,802,720,452,527,385,874,387,708,783,1612
NANS_2_978,31,1,5,15,220,24,0,0,4,0,8,0
NAPRT1_2_979,150,138,116,100,0,5,26,88,21,17,222,1
NARS_2_980,135,197,100,148,2,520,2,153,184,12,200,823
NCF1_2_981,12,70,104,137,16,3,6,10,16,144,0,1
NDST1_2_982,101,102,86,119,11,0,1,491,169,3,186,207
NDST2_2_983,599,442,350,687,1241,768,12,723,98,100,469,761
NDST3_2_984,522,549,353,617,412,186,916,139,366,504,33,264
NDST4_2_985,1028,736,571,725,385,648,1551,976,605,393,949,914
NDUFA10_2_986,104,145,30,119,35,3,1,24,40,223,3,523
NDUFA12_2_987,2408,2149,2097,2519,1956,1707,2265,2495,1354,2164,2323,2
696
NDUFA13_2_988,154,209,175,110,17,169,187,456,40,6,2,303
NDUFA1_2_989,566,445,517,560,297,80,824,605,398,382,79,298
NDUFA3_2_990,156,211,235,332,74,0,13,7,0,60,602,127
NDUFA4_2_991,2728,2709,2223,2987,3526,1762,2514,2605,2715,2409,2595,29
80
NDUFA4L2_2_992,215,307,241,169,97,315,191,57,275,142,5,350
NDUFA5_2_993,310,387,574,482,826,319,531,184,236,486,296,489
NDUFA6_2_994,381,508,478,533,235,757,394,536,476,199,168,629
NDUFA7_2_995,403,217,284,163,100,1,963,9,376,7,416,104
NDUFA8_2_996,1917,1795,1532,2032,1912,1491,953,2036,1057,1644,1093,968
NDUFA9_2_997,649,360,375,483,305,633,13,189,345,717,390,605
NDUFAB1_2_998,4182,3917,3380,4005,4761,4160,6062,5364,3707,3933,2216,5
596
NDUFB10_2_999,585,340,446,404,346,346,1266,1175,229,441,580,838
NDUFB1_2_1000,63,59,39,57,43,2,2,0,32,36,0,3
NDUFB2_2_1001,298,253,333,287,151,286,44,241,102,398,390,311
NDUFB3_2_1002,578,552,594,558,457,494,1790,327,352,236,866,750
NDUFB7_2_1003,12,29,10,19,0,1,20,0,8,46,305,0
NDUFB8_2_1004,260,187,269,312,172,2,94,326,199,0,370,128
NDUFB9_2_1005,29,38,18,22,16,80,38,0,51,315,0,59
NDUFS3_2_1006,122,114,121,131,228,103,10,38,69,132,1,1

NDUFS4_2_1007,259,89,206,120,185,375,217,34,271,547,44,2
NDUFS6_2_1008,713,493,597,599,793,697,370,547,903,755,833,1586
NDUFS7_2_1009,238,314,398,362,662,347,161,660,308,0,420,63
NDUFS8_2_1010,517,458,513,398,90,914,0,132,677,82,527,482
NDUFV2_2_1011,184,194,159,122,133,85,40,362,259,254,83,596
NEDD8_2_1012,779,652,703,633,308,1099,1763,202,929,883,328,1217
NEIL1_2_1013,55,28,31,50,87,12,47,17,87,2,0,39
NEIL3_2_1014,81,63,45,64,34,12,536,20,36,180,4,13
NEU1_2_1015,225,217,101,158,221,299,68,143,246,232,290,144
NEU2_2_1016,742,575,521,678,1082,745,792,558,574,1482,221,858
NEU3_2_1017,215,329,240,313,598,678,56,361,224,288,736,1345
NHLRC2_2_1018,1263,1041,1140,1227,947,785,1360,1425,821,1717,1114,980
NIT2_2_1019,404,551,416,421,101,283,1133,361,632,807,236,567
NKIRAS1_2_1020,298,173,258,287,260,262,452,98,440,103,1,305
NLGN1_2_1021,158,152,97,233,408,149,49,199,322,415,172,282
NLGN2_2_1022,838,800,790,925,670,803,837,320,1066,631,837,735
NLN_2_1023,2032,1627,1492,1863,2291,1810,2196,1985,1726,1346,1750,2609
NME1-NME2_2_1024,17,22,51,51,27,51,109,0,8,0,0,185
NMNAT1_2_1025,186,298,264,154,34,2,43,41,58,104,6,350
NMT1_2_1026,433,933,580,746,1045,34,808,865,463,593,361,945
NMT2_2_1027,313,158,229,158,24,58,256,127,522,777,807,237
NNMT_2_1028,463,533,307,438,32,715,846,668,550,182,89,890
NOP58_2_1029,626,418,570,720,468,582,1352,942,589,831,394,486
NOX3_2_1030,549,351,667,493,506,301,839,12,196,946,440,295
NQO2_2_1031,125,71,105,119,663,0,299,29,23,84,0,126
NRAS_2_1032,548,556,554,724,1339,407,13,1324,268,958,979,1221
NSF_2_1033,271,157,174,292,228,82,148,108,485,204,136,58
NSUN6_2_1034,112,47,46,122,53,22,213,23,10,0,33,36
NTAN1_2_1035,766,812,868,741,1080,842,767,931,175,339,124,988
NTHL1_2_1036,164,146,166,247,112,22,347,371,11,196,9,294
NTPCR_2_1037,1421,933,809,881,1277,862,2268,556,367,1041,478,2088
NUDT10_2_1038,2826,2358,2271,2985,3028,1929,2628,3571,3073,1714,1524,2
462
NUDT11_2_1039,2826,2358,2271,2985,3028,1929,2628,3571,3073,1714,1524,2
462
NUDT12_2_1040,151,108,114,79,93,49,109,0,69,636,12,38
NUDT14_2_1041,910,732,761,719,467,521,583,419,1870,521,1129,939
NUDT3_2_1042,41,50,39,35,1,3,45,46,45,5,122,5
NUDT5_2_1043,317,204,285,244,124,63,109,156,101,395,165,208
NUDT7_2_1044,207,226,125,243,191,361,165,259,35,313,1,338
NXNL1_2_1045,814,607,651,673,703,37,576,361,599,892,853,813
OAS3_2_1046,128,126,150,138,423,252,130,101,65,285,93,241
OAZ1_2_1047,1960,1861,1849,2159,1533,2140,3522,2470,2472,2835,1768,185
7
OC90_2_1048,770,441,552,620,934,174,366,875,332,503,330,282
ODF3B_2_1049,63,91,44,25,42,22,50,84,25,140,5,37
OPLAH_2_1050,366,515,350,256,270,52,17,207,245,885,425,905
OSGEP_2_1051,207,285,391,349,415,197,249,790,654,270,2089,295
OSGEPL1_2_1052,146,158,82,146,94,91,261,197,244,128,2,153
OSTC_2_1053,174,178,155,173,127,193,656,8,147,0,146,323

OTC_2_1054,448,463,383,569,183,516,1012,381,303,816,712,518
OVGP1_2_1055,218,193,121,174,184,57,66,85,286,315,105,191
OXA1L_2_1056,438,502,576,484,961,316,341,45,622,564,810,379
OXCT1_2_1057,543,382,229,296,282,57,1200,8,424,207,2,639
OXCT2_2_1058,1623,1557,1374,1731,1241,747,1880,1631,1408,2446,1047,937
P4HA3_2_1059,453,418,371,383,751,380,236,384,343,646,92,595
P4HB_2_1060,134,73,61,97,331,39,0,0,133,370,22,416
PADI1_2_1061,43,44,49,21,1,23,1,0,0,0,47,136
PADI2_2_1062,569,392,580,707,124,523,614,566,121,492,279,600
PADI3_2_1063,34,24,30,32,12,0,6,0,33,10,0,4
PADI6_2_1064,392,363,281,371,254,702,1261,52,203,197,899,514
PAFAH2_2_1065,429,391,516,554,750,250,870,304,304,416,616,552
PAH_2_1066,217,282,394,423,327,297,489,501,117,453,327,140
PAPOLA_2_1067,975,1223,1339,1806,1223,1094,673,904,1550,723,1744,1706
PAPOLB_2_1068,1149,819,668,869,1692,418,268,722,1022,958,822,1195
PAPOLG_2_1069,1369,910,948,1245,2985,505,571,1021,1487,1322,477,1648
PAPPA_2_1070,109,77,167,241,42,1,171,8,98,61,24,83
PAPSS1_2_1071,593,909,438,786,158,369,108,850,1462,305,949,966
PARG_2_1072,1518,1472,1262,1770,1719,2094,2743,1189,1545,1762,1581,161
5
PARP1_2_1073,557,449,441,573,283,32,484,74,96,771,622,350
PARP4_2_1074,524,432,525,519,451,430,144,477,335,730,53,629
PARP6_2_1075,489,606,480,730,429,230,793,907,495,258,1318,204
PARS2_2_1076,933,582,703,838,906,1335,542,472,1179,1054,781,1398
PCBD1_2_1077,363,589,416,431,487,638,875,98,555,120,177,373
PCBD2_2_1078,774,503,431,565,697,1075,150,76,27,1065,1128,569
PCMT1_2_1079,609,668,607,491,499,343,131,412,176,719,281,797
PCMTD1_2_1080,282,305,300,240,161,304,459,373,577,283,116,375
PCYOX1_2_1081,180,240,202,209,31,34,130,0,65,197,660,59
PCYT1A_2_1082,1229,1215,974,1380,919,276,977,1534,1475,1717,1521,1613
PDCD1LG2_2_1083,62,125,107,160,355,220,1609,149,70,559,283,26
PDCL_2_1084,113,226,177,215,149,0,10,175,60,263,27,256
PDE3A_2_1085,208,94,130,152,253,80,42,3,103,636,23,83
PDE3B_2_1086,263,187,330,329,70,39,846,14,411,213,372,111
PDE6A_2_1087,87,48,61,114,0,24,0,34,10,87,124,49
PDE6C_2_1088,91,154,121,75,9,59,73,407,38,27,0,162
PDE6D_2_1089,840,854,677,783,1033,66,479,1304,626,713,1527,1387
PDE6G_2_1090,685,459,585,717,591,206,298,375,712,467,117,640
PDE6H_2_1091,271,246,196,195,0,199,536,604,131,215,271,323
PDE7B_2_1092,97,132,99,102,111,15,402,69,130,219,305,172
PDHA2_2_1093,1414,1317,1299,1905,1510,1669,1517,1480,1462,1330,1630,78
6
PDIA2_2_1094,224,318,154,198,85,280,150,136,163,65,418,187
PDIA3_2_1095,126,87,91,129,0,218,123,0,19,346,0,82
PDIA4_2_1096,183,114,88,134,154,24,294,187,75,176,345,68
PDIA5_2_1097,542,398,427,567,282,335,137,14,727,742,287,261
PDIA6_2_1098,352,204,368,429,33,93,33,426,590,379,287,381
PDPR_2_1099,109,40,80,53,0,88,36,59,13,124,1,81
PDSS1_2_1100,340,235,366,254,326,313,180,49,0,579,193,378
PDSS2_2_1101,190,113,182,156,0,0,47,6,550,281,381,26

PDXP_2_1102,72,85,56,95,26,47,0,1,11,24,0,71
PECR_2_1103,576,511,446,423,16,3,342,229,418,18,799,624
PELI1_2_1104,610,639,647,679,1263,751,675,1036,474,867,324,1228
PET117_2_1105,1397,1340,1205,1226,1427,800,748,900,906,967,664,2903
PEX1_2_1106,2181,2341,2248,2521,1889,1516,2026,3267,2108,2947,2143,224
8
PEX6_2_1107,216,140,160,182,240,294,333,114,439,145,536,63
PFAS_2_1108,169,319,288,387,111,8,1131,468,2,33,562,176
PGA3_2_1109,1121,925,976,984,1339,710,302,953,928,817,2759,447
PGA4_2_1110,1121,925,976,984,1339,710,302,953,928,817,2759,447
PGA5_2_1111,1121,925,976,984,1339,710,302,953,928,817,2759,447
PGAM4_2_1112,399,472,264,496,217,806,628,760,659,640,291,419
PGAP1_2_1113,447,351,434,515,354,792,633,601,366,401,364,694
PGAP3_2_1114,728,687,702,863,587,278,527,415,738,595,690,893
PGD_2_1115,325,411,358,460,17,270,357,212,90,503,562,504
PGGT1B_2_1116,671,422,572,725,487,342,647,1627,1341,902,1356,624
PGLS_2_1117,1088,815,620,1012,546,870,1888,1240,874,1703,1847,507
PGM2_2_1118,271,281,138,205,110,349,193,116,99,138,5,252
PGM2L1_2_1119,1037,759,911,902,610,1512,978,376,344,2073,274,768
PGM5_2_1120,258,418,353,318,527,345,96,495,157,63,41,226
PGPEP1_2_1121,711,439,638,834,515,347,601,2482,736,378,1093,874
PGS1_2_1122,116,91,115,160,1,0,232,0,13,57,83,146
PHGDH_2_1123,56,21,19,43,144,458,0,0,134,13,0,26
PHLPP1_2_1124,718,339,407,380,498,508,59,0,353,62,543,299
PHLPP2_2_1125,227,212,212,188,26,97,25,449,135,108,74,252
PIF1_2_1126,34,123,61,72,10,0,28,41,28,13,36,190
PIGB_2_1127,672,727,746,754,303,265,745,1375,454,2148,1070,813
PIGH_2_1128,118,150,134,123,18,8,0,0,46,4,49,215
PIGL_2_1129,256,185,179,201,642,387,123,13,42,240,9,370
PIGM_2_1130,1122,1046,1253,1046,1382,522,872,1144,297,996,623,1544
PIGS_2_1131,1002,638,506,544,88,430,882,953,465,609,612,683
PIGU_2_1132,583,563,668,607,642,321,445,1632,380,1143,1144,427
PIGW_2_1133,377,356,302,312,249,304,386,451,411,549,44,187
PIGZ_2_1134,81,57,194,225,45,127,606,32,3,1,10,5
PIN1_2_1135,336,536,598,579,1150,25,2,5,344,304,66,624
PIPOX_2_1136,85,112,108,125,12,0,150,1,134,0,0,482
PITPNM2_2_1137,190,188,212,307,7,50,170,146,256,115,50,285
PLA2G10_2_1138,161,330,312,277,101,343,461,274,161,280,71,34
PLA2G12A_2_1139,424,267,247,221,261,761,1,208,308,850,330,56
PLA2G12B_2_1140,303,334,364,405,653,2,0,1296,92,1,245,181
PLA2G15_2_1141,94,110,116,160,69,110,85,0,17,3,557,281
PLA2G1B_2_1142,44,73,45,85,2,12,183,0,0,0,0,245
PLA2G2C_2_1143,535,389,395,620,373,546,697,312,165,455,164,538
PLA2G2D_2_1144,2251,2490,1998,2523,3923,2512,2463,2379,1685,2285,1985,
1291
PLA2G2E_2_1145,232,180,166,216,415,273,119,440,41,108,185,80
PLA2G2F_2_1146,119,101,73,119,29,199,12,8,22,34,4,1
PLA2G3_2_1147,47,67,121,137,190,206,0,41,235,37,0,1
PLA2G4A_2_1148,596,380,385,351,57,818,742,822,158,83,1434,626
PLA2G4B_2_1149,1347,1510,1712,1568,1123,1232,2517,368,1163,1533,1736,2

PLA2G4D_2_1150,128,189,176,198,893,31,90,41,0,38,19,121
PLA2G4E_2_1151,484,311,337,362,450,337,288,52,154,505,457,203
PLA2G4F_2_1152,126,113,139,177,206,19,0,0,285,2,67,0
PLA2G5_2_1153,70,37,33,72,0,18,149,3,6,21,1,56
PLCB2_2_1154,424,373,532,582,1172,644,484,672,1109,662,214,760
PLCD3_2_1155,122,101,125,72,48,0,0,94,48,66,0,69
PLCD4_2_1156,748,800,800,799,1153,716,1035,231,911,1118,382,1254
PLCG2_2_1157,620,592,567,602,674,420,1006,543,561,1176,985,754
PLCH2_2_1158,95,57,133,235,26,17,0,18,8,124,163,287
PLCL1_2_1159,971,958,806,979,686,388,957,1783,1162,1764,798,1140
PLCXD1_2_1160,154,226,270,411,1,109,343,417,37,311,0,363
PLCZ1_2_1161,854,683,619,918,1523,656,521,403,492,866,1137,471
PLD4_2_1162,267,269,265,324,0,89,1040,55,146,586,345,497
PLOD1_2_1163,202,149,98,236,415,0,296,528,156,462,309,1
PLOD3_2_1164,103,126,77,85,66,125,331,47,123,64,202,0
PLSCR1_2_1165,995,761,953,1044,970,259,1102,465,1074,572,781,1077
PMM1_2_1166,3,4,4,3,28,110,0,0,2,2,0,0
PMM2_2_1167,599,412,511,358,674,50,1035,187,120,684,116,463
PMPCA_2_1168,16,8,22,23,0,0,21,99,0,0,1,48
PMPCB_2_1169,416,393,541,299,337,180,672,260,524,665,415,370
PNLIP_2_1170,95,97,73,135,49,20,170,37,73,241,26,20
PNLIPRP1_2_1171,516,566,511,561,1081,284,27,347,504,524,1016,322
PNLIPRP2_2_1172,1243,664,1053,986,1235,675,471,589,473,511,908,1075
PNLIPRP3_2_1173,751,646,542,730,904,270,654,1188,282,1132,999,667
PNMT_2_1174,80,39,47,113,30,150,352,99,1,0,2,38
PNPLA2_2_1175,34,67,22,38,22,0,2,1,25,68,0,12
PNPLA3_2_1176,333,119,229,78,0,1,2,346,168,276,0,613
PNPLA8_2_1177,548,506,457,529,335,184,718,393,611,796,278,482
PNPO_2_1178,17,72,49,76,7,3,349,0,25,21,1,107
PNPT1_2_1179,808,554,665,761,661,381,1404,702,564,456,1703,873
POLA1_2_1180,1685,1554,1357,1685,1779,2643,1683,1528,1971,2221,1292,14
51
POLA2_2_1181,181,160,178,199,211,73,240,131,205,195,293,366
POLB_2_1182,416,521,452,596,178,310,177,767,555,547,442,483
POLD4_2_1183,350,330,302,368,164,343,442,557,191,249,62,295
POLE3_2_1184,654,559,549,555,765,413,621,207,409,305,1153,785
POLE4_2_1185,360,775,549,539,64,587,1635,600,268,1702,158,123
POLE_2_1186,1559,1452,1471,1644,977,791,1747,2762,1729,755,2595,2878
POLG2_2_1187,40,80,67,114,1,26,171,0,2,0,103,228
POLI_2_1188,702,788,532,529,488,1196,408,816,631,629,815,1068
POLM_2_1189,314,179,141,281,64,0,130,87,140,465,310,266
POLN_2_1190,595,294,273,366,208,191,667,421,480,354,122,227
POLR1A_2_1191,1219,1774,1169,1278,751,1028,394,2704,1038,1841,1379,143
6
POLR1C_2_1192,450,301,298,338,202,367,497,1,646,143,266,241
POLR1E_2_1193,320,315,363,307,177,176,606,294,257,84,137,326
POLR2A_2_1194,180,140,150,156,130,255,4,312,243,43,50,308
POLR2B_2_1195,488,410,298,552,915,432,770,343,318,784,407,541
POLR2C_2_1196,242,196,206,222,78,33,1638,291,135,254,196,180

POLR2D_2_1197,1337,1246,1079,1230,960,2142,545,712,1184,1705,820,1180
POLR2E_2_1198,18,23,20,5,0,0,0,0,0,1,0,0
POLR2F_2_1199,176,175,170,154,52,12,350,0,28,11,19,326
POLR2G_2_1200,221,291,294,367,174,165,831,548,476,206,971,305
POLR2H_2_1201,1202,789,1176,1345,1353,1545,1191,600,1474,691,1833,943
POLR2J_2_1202,55,102,56,38,120,0,179,1,240,563,250,27
POLR2K_2_1203,138,180,176,306,622,153,1,2,90,134,432,423
POLR2L_2_1204,114,58,59,62,32,0,170,259,85,219,0,13
POLR3A_2_1205,96,41,105,121,257,223,0,1,196,237,0,100
POLR3C_2_1206,172,107,102,128,134,0,244,13,128,101,2,105
POLR3F_2_1207,403,172,148,261,890,15,57,4,76,482,83,391
POLR3G_2_1208,596,486,509,516,757,371,595,1240,901,651,692,449
POLRMT_2_1209,267,388,319,464,494,389,1,327,66,53,181,214
POMGNT1_2_1210,91,115,139,111,102,12,45,119,150,182,292,205
POMT2_2_1211,352,388,391,313,589,178,882,80,406,550,698,781
PON3_2_1212,682,330,501,585,422,12,660,64,273,287,583,583
POP4_2_1213,789,502,527,523,422,161,242,836,135,277,629,364
POP7_2_1214,860,937,737,900,883,420,372,47,832,835,252,473
POR_2_1215,511,414,415,504,992,48,163,417,288,56,23,846
PPA1_2_1216,254,151,214,163,33,524,379,369,200,11,214,67
PPCDC_2_1217,253,306,161,132,253,77,13,153,201,345,250,658
PPIA_2_1218,1179,1033,934,1186,974,694,323,585,979,1368,1356,1133
PPIAL4A_2_1219,1184,868,1220,1326,1086,593,920,3262,1085,1613,1374,985
PPIAL4B_2_1220,1184,868,1220,1326,1086,593,920,3262,1085,1613,1374,985
PPIAL4C_2_1221,1184,868,1220,1326,1086,593,920,3262,1085,1613,1374,985
PPIAL4E_2_1222,1184,868,1220,1326,1086,593,920,3262,1085,1613,1374,985
PPIAL4G_2_1223,1547,1568,1159,1453,1406,549,1758,1036,913,2199,982,173
8
PPIB_2_1224,286,153,116,222,446,236,0,130,126,2,3,140
PPIC_2_1225,533,558,852,730,1171,429,133,1450,302,1240,395,227
PPID_2_1226,988,957,1105,1179,876,484,2629,1524,619,421,370,659
PPIF_2_1227,1286,1152,1074,1096,1643,1399,1829,1289,1296,1120,1150,373
PPIG_2_1228,580,696,524,695,1825,71,619,761,435,593,287,642
PPIH_2_1229,230,162,206,210,500,4,0,0,169,202,276,48
PPIL1_2_1230,59,37,113,86,11,107,105,0,34,17,71,6
PPIL4_2_1231,623,859,773,827,607,215,489,1383,518,876,659,610
PPIP5K2_2_1232,944,804,798,598,421,553,746,557,368,557,432,639
PPM1H_2_1233,410,433,274,410,432,304,131,157,210,1032,361,589
PPM1J_2_1234,528,297,360,291,226,134,891,796,318,334,379,536
PPM1N_2_1235,467,416,443,480,247,166,773,255,411,736,907,278
PPME1_2_1236,1557,1262,1367,1458,1589,1211,1960,346,1054,1900,2904,219
0
PPWD1_2_1237,1629,1708,1392,1446,2879,366,2691,1160,1593,1716,2878,114
4
PRDX4_2_1238,884,794,588,741,904,134,2168,177,508,927,560,327
PREP_2_1239,36,10,22,103,1,23,0,0,1,166,8,0
PRHOXNB_2_1240,206,283,384,241,322,304,62,258,444,567,12,190
PRIM1_2_1241,803,568,601,763,913,738,1622,527,633,427,695,817
PRIM2_2_1242,1848,1639,1432,1454,1913,1468,1517,1876,2568,933,2425,167
6

PROC_2_1243,591,300,322,604,224,216,176,0,174,1133,432,727
PROSC_2_1244,475,462,523,517,167,320,267,414,277,284,330,143
PRR14L_2_1245,1027,771,903,935,771,450,1318,610,490,369,882,1276
PRSS1_2_1246,120,119,143,146,207,46,191,628,406,213,367,148
PRSS2_2_1247,113,314,221,235,238,105,13,19,405,167,15,249
PRTFDC1_2_1248,21,28,23,51,86,2,1,1,13,16,8,1
PRTN3_2_1249,245,71,122,177,117,222,3,45,19,85,20,196
PRUNE_2_1250,381,275,288,349,294,416,272,607,739,697,468,185
PSMA2_2_1251,1215,1686,1299,1549,971,1949,1239,1030,359,1355,1718,1034
PSMA6_2_1252,514,615,532,573,651,318,2082,560,551,1152,746,314
PSMA7_2_1253,147,208,168,239,132,767,223,40,110,261,306,126
PSMB10_2_1254,1069,929,965,886,1296,748,727,683,2088,1359,865,493
PSMB11_2_1255,220,34,78,38,591,84,0,0,194,1,210,6
PSMB1_2_1256,522,620,516,711,562,212,333,142,328,866,755,59
PSMB3_2_1257,141,171,57,188,165,196,97,14,80,82,0,496
PSMB4_2_1258,387,419,301,303,192,354,377,124,645,579,447,182
PSMB6_2_1259,337,281,301,255,8,6,205,11,135,2,237,328
PSMB7_2_1260,136,216,206,268,112,257,185,326,51,275,110,183
PSMB9_2_1261,27,9,7,24,52,0,0,0,0,0,0,37
PSMD6_2_1262,66,82,56,52,226,0,18,0,152,113,151,35
PTDSS1_2_1263,288,183,250,313,337,289,258,88,171,11,87,36
PTDSS2_2_1264,411,267,272,385,377,283,197,256,447,85,746,353
PTGDS_2_1265,147,181,172,238,15,3,215,51,70,42,370,27
PTGES2_2_1266,128,208,135,143,0,1,89,2,0,290,405,654
PTGES3_2_1267,876,929,1016,992,893,636,1580,1456,1304,637,160,1011
PTGES_2_1268,189,149,96,95,182,122,90,373,178,0,3,170
PTGIS_2_1269,277,246,244,265,20,652,3,1317,20,629,121,24
PTGS2_2_1270,523,436,686,683,506,347,697,41,401,875,1232,284
PTRH1_2_1271,27,36,78,26,0,41,3,87,5,27,0,11
PTRH2_2_1272,80,86,102,65,65,176,155,100,41,1,12,135
PTS_2_1273,238,181,175,320,185,417,562,382,91,168,60,83
PUS3_2_1274,171,133,180,219,529,231,432,134,203,445,496,43
PUSL1_2_1275,190,122,84,123,558,3,75,1,7,298,0,244
PXDN_2_1276,21,14,46,12,0,3,0,0,0,17,0,0
PXDNL_2_1277,354,407,413,563,7,391,441,1195,222,157,211,1388
PYCR2_2_1278,154,111,107,63,24,0,7,2,46,1,2,128
PYCRL_2_1279,792,649,688,889,434,851,1623,478,368,275,718,618
PYGB_2_1280,473,609,529,588,617,679,189,659,768,205,461,795
QDPR_2_1281,510,333,363,363,417,380,444,419,737,296,248,378
QPCT_2_1282,1080,696,1058,1268,1169,732,711,1892,1119,935,613,1202
QPRT_2_1283,105,67,102,114,7,82,168,111,104,31,63,459
QRSL1_2_1284,39,45,71,107,3,8,32,14,41,28,23,7
QSOX2_2_1285,213,168,239,232,151,35,203,124,33,313,641,142
QTRT1_2_1286,10,26,63,71,121,8,7,11,9,17,0,65
QTRTD1_2_1287,269,339,502,482,560,386,27,373,134,510,560,558
RAB10_2_1288,1719,1043,1170,1447,1197,1707,937,1602,991,1743,1147,1241
RAB12_2_1289,174,125,123,114,184,0,0,221,34,0,0,9
RAB13_2_1290,335,114,209,365,101,4,1052,36,305,1,480,514
RAB14_2_1291,2189,1783,1823,1829,2377,1314,2347,1148,856,1675,1669,192
8

RAB15_2_1292,287,251,159,263,45,180,433,116,233,816,104,224
RAB17_2_1293,59,128,90,44,16,112,158,0,0,402,29,214
RAB18_2_1294,143,92,85,91,1,6,4,265,25,30,5,8
RAB19_2_1295,184,63,102,174,184,48,313,199,65,17,24,46
RAB1B_2_1296,110,79,98,156,153,115,94,184,505,374,531,517
RAB20_2_1297,237,203,187,193,210,87,138,0,284,57,164,336
RAB21_2_1298,471,397,540,566,176,829,1835,300,529,522,265,519
RAB22A_2_1299,1115,897,876,1116,1494,968,2707,406,1316,490,1120,1563
RAB25_2_1300,900,769,700,954,734,330,1084,294,680,940,653,676
RAB26_2_1301,15,59,174,36,2,0,0,130,0,1,2,92
RAB27B_2_1302,438,101,192,199,286,39,48,0,242,52,223,271
RAB30_2_1303,317,349,355,371,746,469,459,86,263,606,292,95
RAB31_2_1304,621,741,471,738,732,702,1048,968,466,1581,220,211
RAB33A_2_1305,0,3,80,0,81,232,0,0,0,0,0,0
RAB33B_2_1306,1000,387,818,899,1032,540,1981,11,649,571,906,863
RAB36_2_1307,260,199,315,423,172,104,436,315,28,0,22,268
RAB38_2_1308,205,122,146,147,150,172,305,79,332,410,73,94
RAB39B_2_1309,257,319,183,272,179,462,136,354,153,355,157,374
RAB3A_2_1310,497,620,271,376,101,330,1135,52,213,673,1023,84
RAB3B_2_1311,240,195,176,183,535,27,26,433,88,205,40,247
RAB3C_2_1312,339,194,269,204,110,263,0,254,112,7,5,401
RAB3D_2_1313,1227,1127,975,984,808,1357,1170,716,1281,587,1016,891
RAB3GAP2_2_1314,425,510,398,424,302,149,1007,615,105,469,777,965
RAB4A_2_1315,571,531,398,507,1441,127,819,1102,575,897,280,140
RAB4B_2_1316,22,30,77,77,27,0,5,36,2,4,0,31
RAB5A_2_1317,994,762,960,893,1304,561,920,415,1764,393,989,787
RAB5B_2_1318,64,87,94,71,0,10,10,147,31,64,131,118
RAB6B_2_1319,207,145,137,153,172,52,20,80,2,27,84,159
RAB6C_2_1320,566,542,693,645,1056,400,1762,445,724,1001,611,294
RAB7A_2_1321,170,184,239,224,303,165,447,57,167,251,188,196
RAB8B_2_1322,238,194,269,200,284,270,78,0,267,302,264,304
RAB9B_2_1323,252,43,188,274,104,30,1,30,351,106,442,246
RABGGTB_2_1324,165,429,109,135,434,80,968,402,158,569,356,59
RABL3_2_1325,96,120,58,70,36,103,41,0,7,45,9,109
RAC2_2_1326,195,45,129,199,6,62,0,26,226,17,1,292
RAD50_2_1327,1391,1451,1180,1507,1307,1073,1388,981,1625,1284,1239,154
2
RAD54L2_2_1328,623,526,445,588,1053,139,434,290,312,430,442,349
RALA_2_1329,97,156,325,174,81,1256,114,24,27,47,92,1
RALB_2_1330,369,246,230,332,535,329,113,165,271,24,1114,411
RALBP1_2_1331,1317,1126,1140,1002,1927,867,528,704,811,1157,1450,1138
RANBP2_2_1332,463,283,335,410,135,317,256,427,457,392,690,347
RAN_2_1333,264,247,234,224,161,328,226,192,686,0,33,163
RAP2A_2_1334,9,25,29,24,1,0,0,1,76,2,1,17
RAP2B_2_1335,301,276,388,341,124,138,1061,570,67,365,494,352
RAP2C_2_1336,1003,996,774,976,667,1055,582,1035,1515,374,93,436
RARS2_2_1337,614,543,418,790,791,367,105,796,679,490,1446,513
RARS_2_1338,1214,1104,1075,1329,1087,1375,1817,1462,930,1586,747,774
RASD2_2_1339,149,127,140,186,0,175,330,133,494,51,376,556
RASL10B_2_1340,67,112,113,162,56,62,124,143,3,270,0,101

RASL11B_2_1341,371,348,312,358,474,712,127,308,384,146,343,410
RASL12_2_1342,48,35,162,153,35,0,141,1,3,642,2,468
RC3H1_2_1343,872,1379,1129,1450,1822,788,1102,1244,1429,481,1124,1482
RCL1_2_1344,77,92,189,164,35,0,35,39,200,178,483,48
RDH10_2_1345,920,885,803,903,759,598,890,467,340,1090,534,813
RDH11_2_1346,297,222,219,302,483,128,112,189,357,124,380,72
RDH12_2_1347,366,437,303,464,388,906,720,268,305,262,305,484
RDH14_2_1348,520,419,361,609,565,336,1343,337,227,261,581,337
RDH16_2_1349,139,67,141,65,330,0,435,65,135,0,101,149
RDH8_2_1350,15,19,32,14,2,0,0,1,5,224,382,0
RECQL4_2_1351,384,393,373,454,628,1017,61,1174,336,379,580,366
REM1_2_1352,103,106,62,114,447,37,187,42,127,117,3,157
REM2_2_1353,227,268,283,206,284,113,599,565,239,818,16,388
RENBP_2_1354,41,187,109,102,0,835,10,320,155,217,0,173
RETSAT_2_1355,763,904,666,694,974,49,713,1082,886,905,525,552
REV3L_2_1356,540,435,412,472,510,113,862,33,609,486,780,409
REXO1L1_2_1357,27,84,15,22,6,5,125,1,10,16,9,20
REXO2_2_1358,439,444,313,384,439,317,295,301,440,741,592,534
RFNG_2_1359,637,366,459,393,840,593,211,720,193,285,224,395
RFX6_2_1360,75,117,131,133,28,35,29,245,174,27,28,114
RGS7_2_1361,1002,1292,1203,1074,1280,842,1866,706,779,1245,819,1724
RHBDL1_2_1362,215,191,240,238,91,119,95,448,297,515,177,371
RHBDL2_2_1363,131,218,263,270,47,47,363,384,129,96,494,44
RHBDL3_2_1364,166,74,111,91,93,182,32,85,122,1,88,115
RHEBL1_2_1365,154,80,37,170,77,27,23,4,82,107,247,3
RHOA_2_1366,361,380,322,471,182,845,185,887,258,435,534,233
RHOB_2_1367,739,729,615,1058,909,637,428,303,452,236,1099,648
RHOD_2_1368,188,163,198,216,234,14,48,12,194,302,251,398
RHOF_2_1369,71,8,30,39,15,0,91,0,36,0,0,0
RHOG_2_1370,42,26,35,63,16,4,8,346,0,2,223,58
RHOH_2_1371,444,459,501,431,31,493,675,28,500,354,331,799
RHOJ_2_1372,721,588,705,669,24,26,1327,571,445,681,112,542
RHOQ_2_1373,943,696,728,830,305,244,1750,1133,490,1120,442,413
RHOT2_2_1374,915,1113,923,1410,697,395,2389,1436,1493,390,287,1232
RHOU_2_1375,430,397,248,409,227,187,242,478,117,549,413,67
RHOV_2_1376,840,590,624,826,518,984,1158,1457,1400,887,672,1399
RIT1_2_1377,747,557,506,499,587,90,1367,446,493,607,986,485
RIT2_2_1378,28,175,31,144,0,0,0,0,0,0,548,220
RNASE2_2_1379,17,34,3,74,0,14,1,10,18,0,0,263
RNASE3_2_1380,34,39,31,97,1,102,8,208,23,0,0,186
RNASE6_2_1381,1016,830,540,914,630,685,1466,815,637,1082,782,779
RNASE7_2_1382,2374,1705,1807,2265,2275,777,3134,2216,1221,2866,1464,15
94
RNASE8_2_1383,1048,851,867,781,655,380,1487,723,561,158,542,1410
RNASEH2A_2_1384,297,324,262,174,63,43,29,455,23,97,663,257
RNASET2_2_1385,781,793,509,839,528,462,469,679,271,780,904,284
RND1_2_1386,327,286,204,246,318,491,177,191,351,130,269,362
RND2_2_1387,1032,1004,895,1006,1012,1148,633,2205,626,1375,2267,1000
RND3_2_1388,314,211,214,187,446,88,129,97,240,106,1575,166
RNF148_2_1389,607,565,357,453,562,888,775,74,312,883,315,496

RNMT_2_1390,488,521,554,821,558,6,296,932,192,320,709,643
RNMTL1_2_1391,817,667,648,814,420,442,722,1077,811,669,575,1050
RNPEP_2_1392,18,85,59,23,291,1,0,0,2,0,2,12
RPAP2_2_1393,213,147,106,186,416,82,167,136,29,237,29,98
RPE65_2_1394,205,212,194,414,69,150,53,1,564,76,8,74
RPIA_2_1395,686,680,712,1126,987,247,829,249,546,1009,1859,1665
RPL4_2_1396,72,33,99,83,6,45,65,59,0,34,17,124
RPN1_2_1397,510,537,364,490,427,398,315,630,215,510,66,215
RPP25_2_1398,209,185,179,302,221,247,45,628,119,69,38,37
RPP40_2_1399,5013,4804,4166,5088,3099,3133,5032,4635,6016,6132,5554,83
88
RPS3_2_1400,9,14,39,25,0,0,229,3,1,0,0,54
RPUSD1_2_1401,58,127,77,59,0,16,6,0,0,168,355,33
RPUSD2_2_1402,257,191,111,286,99,2,48,82,215,130,136,110
RRAGA_2_1403,1593,1451,1316,1568,1333,900,1069,1193,1325,868,1382,1419
RRAGC_2_1404,63,44,39,58,130,109,2,25,11,63,1,110
RRAGD_2_1405,692,734,733,774,193,856,1232,459,701,1035,30,469
RRAS_2_1406,190,211,151,279,137,20,265,436,99,493,139,325
RRM1_2_1407,269,138,159,244,53,44,68,93,103,464,249,217
RRP8_2_1408,487,493,433,550,549,212,679,496,219,409,1037,119
RSAD2_2_1409,243,298,171,254,184,186,10,12,275,331,211,40
RTN4IP1_2_1410,338,402,345,486,336,202,219,0,653,559,29,864
SAMHD1_2_1411,182,145,229,130,5,2,69,26,26,44,768,179
SDF2_2_1412,135,145,145,135,0,579,0,171,66,413,198,52
SDHA_2_1413,192,161,186,185,297,330,80,386,168,25,32,286
SDHB_2_1414,1257,944,844,1090,2825,507,411,935,1377,447,2353,506
SDHD_2_1415,195,203,228,203,45,322,71,101,360,220,161,19
SDR16C5_2_1416,1816,1475,1481,1751,993,2034,1591,1023,1578,1508,2242,2
297
SDR42E1_2_1417,1255,917,876,1057,876,531,2152,391,816,2106,519,862
SDR9C7_2_1418,1017,1182,1138,1376,962,550,558,1844,533,1968,1827,1677
SDSL_2_1419,56,11,30,83,99,38,7,1,1,3,0,360
SEPSECS_2_1420,638,798,656,638,803,421,1179,611,210,1087,410,603
1-Sep_2_1421,790,680,583,669,1556,248,165,737,643,67,469,347
SEPW1_2_1422,899,722,578,517,1141,574,279,556,383,971,964,99
SERHL2_2_1423,179,175,244,192,31,318,168,13,46,356,623,51
SETX_2_1424,215,205,211,163,583,28,259,439,264,113,179,72
SGMS1_2_1425,1217,966,1068,1457,907,1111,1578,2590,1313,1736,1441,1522
SGPL1_2_1426,175,254,217,228,332,121,175,180,47,0,1152,317
SGSH_2_1427,213,119,217,190,332,193,175,136,173,0,86,221
SH3GL2_2_1428,303,230,134,165,16,7,245,79,758,121,433,95
SKIV2L2_2_1429,224,193,268,414,725,89,155,6,8,6,452,484
SLFN12_2_1430,1112,1311,1547,1500,2023,545,1060,909,641,399,1335,1592
SLFN12L_2_1431,468,525,535,679,851,183,756,1773,1235,995,272,764
SLFN13_2_1432,404,332,290,427,648,353,215,75,294,511,645,285
SLFN5_2_1433,614,605,612,682,710,181,978,1248,712,947,409,501
SLU7_2_1434,629,557,634,617,608,481,451,568,258,278,614,441
SMG8_2_1435,1204,1058,900,1473,767,1233,2470,399,544,998,1327,633
SMPD2_2_1436,84,14,42,22,0,1,0,0,8,7,53,0
SMPD3_2_1437,534,478,682,852,968,70,17,1217,1158,478,1023,864

SMPDL3A_2_1438,392,365,253,539,93,307,28,1,290,231,326,184
SMUG1_2_1439,178,89,138,180,51,162,130,220,348,16,445,9
SNF8_2_1440,188,219,167,251,408,648,109,26,699,97,8,173
SNRNP200_2_1441,315,297,280,297,61,0,0,7,166,316,82,502
SOAT1_2_1442,556,603,664,621,469,416,464,1185,391,551,963,1107
SOAT2_2_1443,274,364,327,378,16,152,417,202,282,476,1775,204
SOD1_2_1444,39,62,103,76,97,11,32,2,17,234,301,224
SOD3_2_1445,588,359,533,381,382,74,30,897,612,862,49,74
SORD_2_1446,519,615,411,638,603,250,84,798,1011,855,177,888
SPACA3_2_1447,23,28,66,74,79,212,219,123,106,2,0,316
SPACA5B_2_1448,240,284,325,438,473,20,76,573,504,60,1378,211
SPACA5_2_1449,240,284,325,438,473,20,76,573,504,60,1378,211
SPEM1_2_1450,513,258,247,367,490,390,296,295,426,295,157,297
SPTLC2_2_1451,529,410,322,509,735,127,452,67,624,546,602,327
SPTLC3_2_1452,677,553,642,833,204,131,700,440,673,404,407,645
SPTSSA_2_1453,152,242,164,117,174,94,768,126,179,352,543,301
SQLE_2_1454,312,385,292,520,430,308,379,16,79,271,470,80
SQRDL_2_1455,4872,5306,4885,5826,3055,2376,5640,5142,4151,5305,6508,42
67
SRD5A1_2_1456,216,97,115,116,586,115,483,0,37,0,4,42
SRD5A2_2_1457,2362,2378,1961,2798,2734,2026,3264,3553,2046,1493,1955,2
479
SRD5A3_2_1458,12,4,2,34,0,2,0,1,0,367,0,214
SRR_2_1459,536,430,531,499,310,95,745,182,536,360,1270,141
SRSF9_2_1460,488,412,606,595,757,537,957,697,567,1379,484,809
SRXN1_2_1461,119,75,67,148,6,26,0,36,60,23,0,120
SSB_2_1462,355,354,221,305,116,349,113,413,510,355,144,561
SSU72_2_1463,2034,1572,2020,2048,913,1137,4238,1490,1943,2516,1911,197
8
ST14_2_1464,132,97,72,83,23,11,12,7,399,106,82,34
ST3GAL2_2_1465,41,92,68,155,197,259,0,1,224,12,0,285
ST3GAL4_2_1466,1,0,16,6,2,0,0,0,0,0,0,0
ST3GAL6_2_1467,674,407,601,637,170,932,157,485,752,519,560,437
ST6GALNAC1_2_1468,175,76,144,99,2,99,0,0,212,0,87,130
ST6GALNAC2_2_1469,11,17,13,16,20,0,0,0,82,11,0,77
ST6GALNAC5_2_1470,1605,1405,1069,1216,1982,460,1599,1162,1378,1236,120
3,2051
ST6GALNAC6_2_1471,128,169,126,149,426,118,190,510,38,50,12,199
ST8SIA1_2_1472,184,186,150,166,186,31,183,360,317,238,32,239
ST8SIA2_2_1473,45,47,82,129,0,120,36,31,31,260,51,119
ST8SIA3_2_1474,1590,1777,1540,1825,773,699,2461,2137,1558,2703,2325,17
72
ST8SIA5_2_1475,401,256,241,395,827,404,367,915,351,586,294,423
ST8SIA6_2_1476,650,462,531,592,715,670,542,177,468,724,361,1019
STS_2_1477,354,333,492,295,306,350,238,22,65,10,83,68
STT3A_2_1478,386,430,389,355,331,94,109,1,231,552,105,700
STT3B_2_1479,1838,1241,1192,1249,1165,860,2483,1011,653,1257,1777,1061
SUCLA2_2_1480,722,863,771,718,683,436,413,1217,203,387,793,1670
SUCLG1_2_1481,59,93,57,178,92,5,4,287,17,48,0,105
SULT1A3_2_1482,438,399,229,363,131,107,72,90,273,363,132,633

SULT1A4_2_1483,438,399,229,363,131,107,72,90,273,363,132,633
SULT1B1_2_1484,437,240,139,214,160,184,471,640,172,293,532,136
SULT1C3_2_1485,322,260,335,211,73,757,196,5,217,113,282,376
SULT1C4_2_1486,419,418,612,622,4,1165,353,805,661,66,562,691
SULT1E1_2_1487,2336,2406,2102,2507,1990,1445,1646,2990,2163,1026,2832,
2241
SULT2A1_2_1488,252,219,352,252,404,488,786,1297,132,84,517,460
SULT4A1_2_1489,219,103,83,208,0,35,215,203,0,19,271,106
SUPV3L1_2_1490,336,246,204,317,256,520,769,182,154,424,73,157
SURF1_2_1491,29,70,55,99,24,16,0,10,1,4,341,13
TALDO1_2_1492,68,65,59,129,25,62,33,27,30,57,20,341
TARS2_2_1493,22,19,46,89,0,0,16,590,41,0,2,5
TARS_2_1494,642,592,478,750,535,465,1442,875,713,708,1712,539
TARSL2_2_1495,181,26,101,133,1,35,0,166,105,65,13,0
TAT_2_1496,534,719,489,696,526,990,1017,689,66,688,614,445
TBC1D10B_2_1497,465,740,650,797,1418,791,233,563,467,918,675,657
TBCC_2_1498,31,45,57,61,25,57,18,334,88,2,0,35
TDG_2_1499,427,323,275,330,292,1066,51,320,77,499,94,268
TDO2_2_1500,224,181,67,263,230,0,1,402,552,3,57,240
TECR_2_1501,480,532,329,451,221,329,174,510,410,211,822,489
TECTA_2_1502,407,252,372,597,324,79,230,455,446,802,229,332
TFB2M_2_1503,2362,1775,1735,1961,1934,1686,1994,2569,1472,1264,2268,24
47
TGDS_2_1504,1293,1222,1312,1225,1316,1206,2497,781,1596,1547,1312,988
TGM1_2_1505,173,115,134,158,96,7,3,585,15,18,12,225
TGM3_2_1506,56,45,257,298,0,2,0,291,12,80,195,97
TGM4_2_1507,442,340,331,484,623,42,535,596,427,407,4,289
TGM6_2_1508,4476,4245,4320,4258,3755,5065,3704,2602,4392,5087,3768,356
2
TGM7_2_1509,50,83,87,89,213,225,0,84,0,123,176,0
TGS1_2_1510,2401,2373,2243,2836,2892,3318,3598,1833,1894,3237,2794,256
3
THG1L_2_1511,623,477,526,438,306,168,617,1034,505,1683,86,437
THOP1_2_1512,137,383,246,338,82,544,0,36,104,477,233,400
THUMPD2_2_1513,932,1041,900,1089,1414,1305,510,467,1046,972,1045,1423
TKTL2_2_1514,296,299,353,222,117,210,415,75,229,636,700,172
TM7SF2_2_1515,568,472,506,486,474,145,331,274,725,205,1981,231
TMEM55A_2_1516,786,472,666,961,1217,1522,225,1126,437,270,1225,481
TMEM62_2_1517,421,449,372,492,1,725,293,991,1092,203,1279,34
TMEM86B_2_1518,93,160,141,226,18,284,0,7,0,0,2,18
TMPRSS15_2_1519,1433,1314,1038,1204,1174,1383,1704,1437,1121,531,1197,
1084
TMX1_2_1520,93,183,145,187,30,260,298,239,5,162,84,266
TMX3_2_1521,361,358,440,279,416,240,145,552,130,237,383,356
TMX4_2_1522,408,383,346,369,560,297,167,137,421,360,361,598
TNKS2_2_1523,2172,2148,1705,2140,2410,1647,2506,3169,3562,1856,1435,23
36
TNKS_2_1524,559,316,407,548,178,290,109,727,642,458,731,378
TOP1_2_1525,646,462,572,726,1467,136,347,613,677,847,879,308
TOP1MT_2_1526,318,382,419,504,76,432,1114,703,532,123,224,203

TOP2A_2_1527,181,130,116,221,304,4,0,378,274,33,62,105
TOP2B_2_1528,196,231,281,221,428,253,1117,41,460,382,512,208
TOP3A_2_1529,189,203,149,296,4,54,379,128,172,105,609,111
TOP3B_2_1530,113,37,42,51,1,0,0,13,0,86,32,239
TOR3A_2_1531,745,909,1109,1121,635,520,2000,1058,653,1601,552,775
TPH1_2_1532,253,259,324,377,92,289,1203,977,361,13,815,303
TPMT_2_1533,528,645,658,643,842,916,1121,1286,394,480,355,905
TPP1_2_1534,197,189,189,235,195,88,192,162,124,412,342,685
TPP2_2_1535,123,83,161,141,1,78,187,183,45,56,0,31
TPSAB1_2_1536,1093,1219,1040,1123,796,635,426,770,1445,1505,440,840
TPSB2_2_1537,444,491,482,591,688,246,782,321,234,447,429,225
TPSD1_2_1538,1093,1219,1040,1123,796,635,426,770,1445,1505,440,840
TPST1_2_1539,276,266,290,212,200,184,131,109,469,23,753,182
TREH_2_1540,596,520,492,612,191,883,551,761,1177,453,127,742
TREX2_2_1541,128,141,164,129,304,0,44,334,181,253,13,88
TRHDE_2_1542,1392,1181,981,1200,1087,590,1861,899,1198,1534,842,1243
TRIM21_2_1543,464,524,348,426,420,685,0,1529,52,501,39,606
TRIT1_2_1544,420,544,305,567,392,973,476,686,830,151,80,458
TRMT112_2_1545,282,248,215,251,112,229,624,22,184,268,282,254
TRMT61A_2_1546,37,7,15,27,0,0,0,0,18,27,0,0
TRMU_2_1547,325,290,290,324,326,156,48,539,424,139,153,155
TRNT1_2_1548,1600,1171,1084,1410,1382,543,1848,525,2470,1079,741,978
TRUB1_2_1549,222,180,244,325,0,108,15,61,48,314,98,224
TRUB2_2_1550,290,86,180,206,5,32,0,0,0,0,333,260
TSTA3_2_1551,27,40,60,71,7,25,340,40,63,16,17,236
TST_2_1552,576,848,511,670,125,382,261,1333,1333,526,279,590
TTLL13_2_1553,51,43,50,34,179,9,0,14,28,2,68,146
TTLL1_2_1554,6,9,0,3,1,0,8,0,5,1,0,0
TTLL3_2_1555,243,408,348,322,601,552,516,457,226,37,455,125
TTLL4_2_1556,245,183,168,124,315,1,76,214,30,570,148,123
TUFM_2_1557,1007,848,1105,958,1074,1373,1115,1228,824,1055,497,1495
TULP2_2_1558,858,1287,1028,1030,1429,633,1497,557,1108,434,1046,1233
TUT1_2_1559,1346,1362,1495,1581,1909,1200,2150,1217,2232,1872,1256,184
3
TXN2_2_1560,642,768,552,537,1212,355,117,763,522,547,1318,219
TXN_2_1561,806,480,746,864,1078,424,1110,894,255,276,725,603
TXNDC11_2_1562,622,419,388,411,275,548,586,171,513,291,630,148
TXNDC12_2_1563,2897,2914,2313,2625,3080,3346,4028,2423,3426,2541,3176,
2587
TXNDC15_2_1564,383,438,322,477,418,44,1157,202,142,103,618,221
TXNDC17_2_1565,437,423,543,525,622,588,392,346,78,544,204,514
TXNL1_2_1566,1366,1509,1293,1440,1197,991,1529,1310,1696,2382,1638,149
9
TXNL4A_2_1567,462,523,559,692,803,390,472,723,311,877,997,128
TXNRD2_2_1568,1128,1165,1051,1229,950,1242,1574,990,942,1134,719,1538
TYMS_2_1569,99,86,76,109,46,4,206,69,108,43,0,376
TYR_2_1570,706,361,480,498,253,158,268,678,435,699,72,536
TYRP1_2_1571,783,479,480,806,490,91,51,770,705,644,442,661
UAP1_2_1572,769,894,759,743,999,901,278,1760,1250,600,829,639
UAP1L1_2_1573,102,96,52,103,0,40,15,259,67,34,3,209

UBB_2_1574,174,48,156,184,154,304,107,91,181,11,136,13
UBIAD1_2_1575,194,271,158,162,0,39,0,2,159,880,282,3
UBL4A_2_1576,139,428,474,630,481,304,484,425,464,255,1160,1339
UFSP1_2_1577,2,109,53,51,89,0,240,0,0,0,245,116
UFSP2_2_1578,987,732,778,834,945,1199,1220,633,813,377,951,1233
UGCG_2_1579,1176,1228,1034,1052,811,287,911,457,1073,1075,910,1579
UGGT1_2_1580,41,47,15,30,80,0,0,0,43,88,1,0
UGGT2_2_1581,303,197,64,168,526,5,0,20,297,70,306,57
UGT1A10_2_1582,2679,2233,2503,2686,3382,2192,3271,4050,2534,3234,3602,
2561
UGT1A1_2_1583,2679,2233,2503,2686,3382,2192,3271,4050,2534,3234,3602,2
561
UGT1A3_2_1584,2679,2233,2503,2686,3382,2192,3271,4050,2534,3234,3602,2
561
UGT1A4_2_1585,2679,2233,2503,2686,3382,2192,3271,4050,2534,3234,3602,2
561
UGT1A5_2_1586,2679,2233,2503,2686,3382,2192,3271,4050,2534,3234,3602,2
561
UGT1A7_2_1587,2679,2233,2503,2686,3382,2192,3271,4050,2534,3234,3602,2
561
UGT1A8_2_1588,243,252,156,217,416,298,71,262,36,295,151,336
UGT1A9_2_1589,2679,2233,2503,2686,3382,2192,3271,4050,2534,3234,3602,2
561
UGT2A1_2_1590,705,654,702,1061,528,566,885,10,42,615,1282,459
UGT2A2_2_1591,1251,1109,1256,1359,2855,809,1161,2917,2340,1541,2049,14
30
UGT2A3_2_1592,79,50,156,153,155,0,47,227,218,248,5,288
UGT2B15_2_1593,1647,1238,1166,1314,344,920,1990,1033,869,884,987,1745
UGT2B17_2_1594,1647,1238,1166,1314,344,920,1990,1033,869,884,987,1745
UGT2B4_2_1595,601,461,488,536,819,91,556,568,924,544,713,509
UGT2B7_2_1596,601,461,488,536,819,91,556,568,924,544,713,509
UMPS_2_1597,337,398,276,350,188,767,249,32,107,881,661,371
UPB1_2_1598,416,202,175,391,29,239,45,86,43,638,1,482
UPF1_2_1599,15,0,9,7,9,0,0,0,0,0,0,1
UPRT_2_1600,443,583,477,445,333,66,755,1096,258,397,272,215
UQCR11_2_1601,59,117,87,95,88,217,1,116,94,17,80,155
UQCRC1_2_1602,1078,1064,895,978,1017,800,1662,860,528,818,145,1193
UQCRC2_2_1603,785,758,819,1055,531,988,211,851,1131,598,498,563
UQCRFS1_2_1604,82,89,156,134,31,394,673,16,353,63,394,35
UQCRH_2_1605,1115,748,767,946,835,438,2194,20,1102,1572,543,2305
UQCRQ_2_1606,1178,711,868,1081,763,1594,206,1784,929,860,1138,645
UROD_2_1607,242,103,252,169,142,295,438,143,0,426,202,576
UROS_2_1608,599,519,348,609,481,291,423,478,533,767,100,421
UST_2_1609,1052,1039,893,1071,774,931,1233,1220,1086,561,382,1216
UXS1_2_1610,1523,1635,1359,1601,1682,1767,1191,458,1133,1379,1595,1002
VARS_2_1611,174,132,141,187,5,267,424,69,179,272,114,111
VAT1L_2_1612,438,559,384,447,571,422,412,230,527,666,6,648
VCP_2_1613,356,190,169,262,354,551,1194,0,43,0,246,303
VNN1_2_1614,659,604,388,499,1611,442,1340,601,758,1476,1248,1021
WBSCR17_2_1615,82,50,23,48,19,31,9,26,60,45,0,39

WBSCR27_2_1616,151,82,86,69,14,174,161,30,45,25,389,85
WDFY3_2_1617,629,712,682,596,944,174,2353,780,533,519,522,385
WRN_2_1618,562,544,548,716,711,480,608,422,292,694,1351,776
XDH_2_1619,1265,707,643,996,955,379,949,404,853,687,340,670
XPNPEP2_2_1620,626,597,543,666,847,993,1272,799,185,1115,494,320
XRCC2_2_1621,288,319,277,172,0,184,106,0,271,920,129,854
XRCC5_2_1622,290,374,351,439,280,1248,865,792,172,498,1126,260
XRCC6_2_1623,1836,1437,989,1668,877,1064,1228,1775,690,2522,642,1443
XRN2_2_1624,118,178,41,56,379,194,1,0,57,65,0,12
XYLT1_2_1625,505,327,285,402,43,1105,1850,0,35,232,236,286
XYLT2_2_1626,46,59,80,110,3,9,0,21,63,13,99,42
YARS2_2_1627,387,198,290,206,287,217,121,170,87,236,32,229
YARS_2_1628,172,123,55,116,44,55,79,23,326,43,17,109
YKT6_2_1629,53,14,18,18,0,191,41,1,0,8,0,145
YPEL1_2_1630,40,64,55,63,0,9,1,288,157,33,11,8
ZADH2_2_1631,857,674,677,707,1008,350,209,1087,597,408,1481,879
ZCCHC4_2_1632,1991,1361,1370,1655,1252,1519,585,1129,1364,1498,1399,92
8
ZDHHC17_2_1633,1264,1177,1212,1162,1175,1166,1000,1727,1600,1388,769,5
54
ZDHHC18_2_1634,174,118,142,222,166,448,144,386,253,19,271,195
ZDHHC1_2_1635,181,202,252,281,132,282,86,93,397,113,255,242
ZDHHC21_2_1636,1511,1110,1291,1397,895,1232,633,1166,1586,686,1936,199
8
ZDHHC2_2_1637,877,745,536,908,242,648,1199,322,335,992,973,603
ZER1_2_1638,553,345,286,324,91,348,685,440,272,724,541,212
ZMPSTE24_2_1639,1746,1343,1177,1390,2093,1444,1410,327,1577,1045,1554,
1282
ZRANB3_2_1640,478,377,469,494,1078,146,915,39,719,246,725,739
A1CF_2_1641,306,341,394,501,205,968,691,314,372,160,692,244
A2LD1_2_1642,79,160,173,86,471,392,15,40,176,354,49,0
AADAT_2_1643,894,595,624,959,856,488,501,813,770,775,378,1218
AARSD1_2_1644,152,121,123,92,58,35,480,51,302,27,40,1
ABAT_2_1645,387,449,379,426,502,293,586,20,404,33,0,511
ABHD11_2_1646,271,203,259,315,333,88,299,394,211,99,232,425
ABHD12_2_1647,112,86,32,50,30,0,11,0,9,0,68,4
ABHD14B_2_1648,64,194,168,190,0,7,0,254,553,0,1026,18
ABHD2_2_1649,648,348,375,505,1391,373,376,181,283,283,1064,357
ACAA1_2_1650,38,20,37,31,0,0,377,0,0,0,132,4
ACACA_2_1651,2561,2333,2699,2441,1359,3122,3388,2890,1908,2598,3110,25
36
ACAD10_2_1652,105,192,54,80,123,4,0,0,77,426,67,384
ACADM_2_1653,77,142,283,178,13,169,71,91,9,51,530,118
ACADVL_2_1654,186,261,266,385,1068,271,482,604,9,130,0,134
ACCS_2_1655,377,441,338,395,401,741,27,132,561,18,162,221
ACE_2_1656,213,263,172,271,311,274,94,207,102,368,210,532
ACHE_2_1657,1187,1264,1147,1301,1842,1402,543,2185,1188,594,1252,1576
ACIN1_2_1658,288,186,272,354,155,8,1356,352,340,45,293,372
ACLY_2_1659,0,0,0,0,0,0,0,0,0,0,0,0
ACOT11_2_1660,3,13,6,13,1,0,40,6,1,18,1,2

ACOT13_2_1661,990,941,902,811,361,881,1092,1958,540,1052,244,1434
ACOT7_2_1662,205,163,206,139,235,33,72,261,0,57,164,246
ACOT9_2_1663,373,311,372,466,324,406,1361,540,492,90,160,134
ACOX1_2_1664,826,1076,813,918,1009,774,636,662,1031,736,870,735
ACOX3_2_1665,701,330,510,586,49,284,572,112,264,267,146,909
ACSBG1_2_1666,123,166,70,101,51,6,55,266,199,148,451,80
ACSF3_2_1667,61,84,69,122,85,15,3,165,116,42,38,37
ACSL3_2_1668,3740,3287,2834,3132,2988,2856,4735,2877,2919,2688,3497,36
99
ACSL4_2_1669,1121,894,1140,1182,981,1727,1689,1173,1388,843,1627,1928
ACSL5_2_1670,435,712,517,516,450,8,628,516,930,852,580,673
ACSL6_2_1671,1074,1357,818,1084,1512,1409,1100,661,447,1455,856,808
ACSM2B_2_1672,69,101,59,64,3,86,0,109,5,83,0,32
ACSM3_2_1673,208,139,280,298,493,33,420,161,159,0,1092,339
ACSS2_2_1674,210,230,255,227,27,108,395,1,214,228,40,265
ACY1_2_1675,116,20,12,27,0,67,12,0,19,5,318,15
ADAMTS2_2_1676,126,199,133,143,137,275,83,431,39,257,20,310
ADARB1_2_1677,653,523,456,675,653,367,474,913,304,303,82,598
ADAR_2_1678,398,309,251,388,434,0,413,907,120,254,600,162
ADCY10_2_1679,101,142,77,118,2,56,113,744,112,135,24,73
ADCY4_2_1680,850,739,756,761,1018,597,339,247,1647,278,1586,1574
ADCY5_2_1681,851,764,766,778,1009,600,379,246,1648,277,1364,1573
ADCY6_2_1682,243,64,139,100,169,7,1347,15,145,136,1,23
ADH6_2_1683,69,59,20,84,2,14,0,0,106,148,1,0
ADH7_2_1684,630,344,427,493,645,340,323,633,206,303,726,500
ADPRHL1_2_1685,10,0,1,2,23,1,0,0,0,11,0,0
ADSL_2_1686,108,118,91,84,5,98,183,0,3,0,17,68
ADSSL1_2_1687,155,86,116,64,1,3,0,358,145,1,122,2
AFMID_2_1688,172,161,220,201,41,51,230,236,145,28,1209,376
AGA_2_1689,750,590,745,877,531,498,376,237,585,836,1352,429
AGAP1_2_1690,112,18,26,48,6,5,0,24,255,0,1,101
AGAP2_2_1691,220,291,133,134,1,0,26,0,0,12,26,9
AGL_2_1692,1460,1207,1182,1516,1045,866,542,1648,1182,888,1944,1592
AGPAT1_2_1693,629,337,343,451,75,325,117,663,550,395,7,6
AGPAT2_2_1694,135,95,122,143,66,0,0,250,30,85,0,117
AGPAT3_2_1695,198,285,201,264,120,0,277,259,257,24,0,923
AGXT2L1_2_1696,2213,1869,1778,2482,2095,2609,1926,2034,2146,2409,2827,
1709
AHCY_2_1697,100,33,113,64,23,7,40,0,0,0,3,0
AHCYL1_2_1698,128,67,184,53,39,0,253,11,228,37,1,2
AHCYL2_2_1699,801,700,661,505,1301,736,811,890,948,767,766,787
AIFM1_2_1700,476,236,269,343,1122,462,108,616,637,289,485,796
AIFM2_2_1701,169,72,91,146,302,297,34,130,131,91,90,234
AIFM3_2_1702,96,114,148,96,40,0,173,5,91,106,329,127
AKIRIN1_2_1703,4934,5067,4608,4676,4861,5098,3854,3374,4226,5904,4062,
4832
AKR1A1_2_1704,472,405,176,435,597,269,768,8,393,434,30,36
AKR1C2_2_1705,330,248,280,321,226,90,73,95,227,380,401,134
AKR1D1_2_1706,1121,1199,1220,1465,1213,768,489,947,464,1409,1059,1895
ALAS1_2_1707,227,290,298,282,206,154,396,445,212,495,8,439

ALAS2_2_1708,138,104,93,142,98,61,427,99,106,47,203,210
ALDH16A1_2_1709,139,55,153,107,62,144,32,35,62,356,270,222
ALDH1A2_2_1710,729,688,617,604,796,875,1101,735,602,581,187,695
ALDH2_2_1711,93,94,57,91,39,4,378,0,7,292,13,1
ALDH3A1_2_1712,541,684,701,679,1040,466,636,865,479,619,195,511
ALDH3A2_2_1713,978,665,749,945,1260,543,1406,890,1592,566,864,844
ALDH3B1_2_1714,263,288,365,293,23,134,51,325,294,163,260,298
ALDH3B2_2_1715,403,351,307,462,164,11,119,304,151,300,337,177
ALDH4A1_2_1716,18,54,5,29,0,121,0,9,197,0,0,88
ALDH5A1_2_1717,609,327,332,403,688,471,959,89,350,83,574,488
ALDH7A1_2_1718,1287,858,973,1073,991,738,837,599,1028,1267,710,792
ALDH8A1_2_1719,232,171,332,288,83,401,1846,603,61,241,263,125
ALDOA_2_1720,2,19,19,32,0,0,0,0,7,10,0,91
ALG3_2_1721,471,304,320,266,218,101,904,162,260,205,81,111
ALG5_2_1722,1859,1777,1328,1923,2671,852,2196,2231,1532,2721,1044,1601
ALG8_2_1723,1640,1717,1767,1731,1269,2614,1753,1523,1226,1376,2192,148
3
ALG9_2_1724,474,286,367,499,98,812,273,690,126,444,248,371
ALOX15B_2_1725,62,45,128,168,0,4,5,2,78,4,778,288
ALOXE3_2_1726,169,458,315,284,73,313,310,745,132,317,2,469
AMACR_2_1727,94,133,99,190,2,193,221,551,88,352,15,87
AMD1_2_1728,746,549,696,821,1099,648,939,797,1153,786,429,191
AMDHD2_2_1729,119,58,74,146,105,88,0,416,117,42,0,111
AMPD1_2_1730,296,566,299,593,295,553,407,193,264,362,661,417
AMPD2_2_1731,24,149,72,130,0,1,1,30,88,222,304,0
AMPD3_2_1732,222,190,307,341,418,353,1487,554,162,282,940,54
AMT_2_1733,50,123,77,160,136,1,7,5,11,42,0,20
AMY1A_2_1734,17017,14576,13584,15429,13055,10746,12810,17567,13688,177
61,15061,20077
ANG_2_1735,194,188,148,165,244,228,45,231,474,332,216,623
AOAH_2_1736,649,692,600,883,652,475,494,865,494,375,251,943
AOC2_2_1737,15,20,29,54,1,0,274,40,3,5,69,0
APC_2_1738,2696,2359,2157,2265,2290,1297,1157,2360,2835,1566,1760,2428
APEX1_2_1739,166,157,190,268,34,387,118,28,108,120,271,99
APOBEC3A_2_1740,1572,1277,1463,1811,1376,574,1089,2654,1054,646,780,16
78
APOBEC3H_2_1741,535,868,801,833,395,1278,1297,341,317,343,2017,512
APRT_2_1742,138,279,264,136,46,127,78,152,169,294,20,58
ARF1_2_1743,157,115,76,148,65,233,191,13,73,526,631,1
ARFRP1_2_1744,38,72,112,128,28,139,5,5,67,15,20,32
ARHGAP5_2_1745,371,535,314,339,61,66,116,232,186,688,691,468
ARHGEF10L_2_1746,202,210,193,206,219,422,229,240,6,556,91,174
ARL2_2_1747,302,279,315,216,49,46,11,151,314,342,195,509
ARL4A_2_1748,843,888,895,863,288,1136,806,720,604,398,1695,1382
ARL5A_2_1749,159,212,206,190,8,184,370,16,47,60,2,149
ARSA_2_1750,8,15,3,41,22,17,1,144,3,0,0,26
ARSB_2_1751,259,240,262,421,40,0,13,329,691,254,9,536
ARSF_2_1752,3294,3321,3138,3808,4141,4213,2707,3482,2731,4129,3338,343
8
ART3_2_1753,1176,1034,992,1309,2790,933,1249,1042,927,768,1615,1070

ART5_2_1754,1161,1352,1547,1427,1604,822,3267,1459,1561,821,1002,549
ASAH1_2_1755,413,408,294,542,841,581,64,12,879,221,346,423
ASAH2_2_1756,1275,1667,1531,1907,1836,2238,1251,2273,2551,1899,2326,13
34
ASL_2_1757,1483,1287,1624,1739,1171,1254,877,2002,1054,854,3602,2222
ASMT_2_1758,385,432,300,532,450,318,601,86,278,885,124,376
ASMTL_2_1759,40,60,41,97,62,431,0,35,2,71,1,40
ASNS_2_1760,279,203,217,418,131,8,675,580,366,63,876,561
ASPA_2_1761,942,941,723,907,336,954,1132,1046,598,856,1321,585
ASRGL1_2_1762,366,353,285,331,581,83,219,499,243,1,832,246
ASS1_2_1763,391,598,437,514,340,169,0,429,67,324,1524,161
ATE1_2_1764,1982,2433,2436,2522,1586,2476,2861,2460,2686,2258,1247,234
2
ATL1_2_1765,532,414,401,536,269,263,212,504,350,465,1334,761
AURKAIP1_2_1766,283,122,235,134,582,0,1,362,227,0,6,20
AZIN1_2_1767,362,269,331,430,340,3,359,124,40,354,195,553
B3GALNT1_2_1768,305,100,342,261,352,9,173,151,403,148,394,234
B3GALT5_2_1769,340,171,201,239,168,335,255,167,154,234,118,285
B3GAT1_2_1770,103,38,64,134,0,31,141,0,329,8,8,45
B4GALNT2_2_1771,258,107,89,187,0,0,0,80,194,120,135,207
B4GALT2_2_1772,284,299,250,327,187,27,4,35,153,45,9,89
B4GALT3_2_1773,54,65,60,75,193,58,245,86,0,223,65,100
B4GALT4_2_1774,6151,5828,5545,6489,4119,4902,5473,4579,4224,5861,4648,
6243
BAAT_2_1775,53,86,89,83,316,0,0,71,34,352,240,93
BACE1_2_1776,323,339,303,315,5,311,330,371,84,219,431,126
BACE2_2_1777,553,181,198,328,441,164,80,254,528,201,0,345
BCAT1_2_1778,3560,3169,2888,3750,4041,3106,4633,6041,3201,3575,3487,30
91
BCAT2_2_1779,1010,890,1135,1125,1116,298,610,238,404,887,1205,590
BCKDHA_2_1780,86,92,89,192,200,278,139,0,55,4,47,194
BCKDHB_2_1781,307,391,348,327,336,25,142,443,396,210,73,360
BCO2_2_1782,545,372,426,490,1691,599,1460,1069,525,925,1054,550
BDH1_2_1783,632,572,286,688,392,593,399,769,229,596,233,302
BFSP1_2_1784,244,190,199,196,493,387,215,240,31,235,93,213
BHMT2_2_1785,268,112,276,251,476,287,446,77,120,78,290,227
BMP1_2_1786,655,638,424,590,833,1116,1212,1085,553,1356,603,183
C17orf101_2_1787,52,35,30,41,44,0,0,42,29,0,0,2
C1GALT1C1_2_1788,444,368,288,441,451,718,552,29,1122,350,262,1297
C1S_2_1789,124,122,88,188,0,45,35,25,238,54,0,110
C2_2_1790,123,160,143,200,24,20,0,62,94,0,3,498
CA10_2_1791,65,245,138,207,417,174,94,107,79,253,583,97
CA12_2_1792,308,326,266,300,253,0,682,102,390,375,701,195
CA1_2_1793,0,0,0,0,0,0,0,0,0,0,0,0
CA7_2_1794,773,741,577,654,363,1701,1824,457,115,293,651,319
CAB39_2_1795,1996,1517,1663,2012,948,1125,3570,1812,1303,2862,878,1917
CANT1_2_1796,52,21,21,9,25,0,0,0,6,0,34,0
CAPN1_2_1797,71,112,76,97,0,183,0,0,195,128,199,124
CAPN2_2_1798,394,283,292,450,214,334,162,536,192,26,33,505
CARNS1_2_1799,20,131,58,30,1,0,0,0,2,0,0,402

CARS_2_1800,1311,1064,968,1283,741,962,810,1137,978,881,947,747
CASP10_2_1801,413,511,368,537,660,340,238,431,484,237,153,437
CASP1_2_1802,855,578,457,590,1053,710,188,966,467,341,56,612
CASP2_2_1803,318,381,330,325,84,362,150,234,147,695,39,187
CASP3_2_1804,727,763,675,1136,1025,771,1852,1357,808,1065,448,791
CASP4_2_1805,3288,3068,3190,3488,2263,1407,3380,2883,3478,4056,1637,34
42
CASP5_2_1806,3263,3042,3187,3422,2248,1402,3366,2871,3458,4035,1624,34
32
CASP6_2_1807,694,487,466,496,561,585,790,619,384,732,908,608
CASP7_2_1808,557,648,428,543,775,70,1033,292,702,895,333,216
CASP8_2_1809,145,179,180,269,413,229,28,128,138,4,173,175
CASP9_2_1810,1143,642,704,644,988,327,1445,2300,1011,87,99,957
CASZ1_2_1811,91,37,69,72,82,12,263,35,275,129,91,36
CAV3_2_1812,66,75,104,94,306,182,0,24,47,1,11,111
CBS_2_1813,940,367,500,351,26,60,699,27,562,216,1132,337
CCBL1_2_1814,123,217,302,176,161,797,0,995,292,271,919,250
CCBL2_2_1815,1069,946,852,1207,1069,650,1845,1227,1312,572,700,1134
CDC42_2_1816,390,399,304,272,397,263,630,365,508,285,397,747
CDH16_2_1817,48,58,64,96,190,82,0,0,63,78,3,39
CECR1_2_1818,396,311,364,359,407,36,1,21,248,29,476,328
CEPT1_2_1819,907,490,679,772,2017,439,1992,111,1600,1185,539,864
CERS1_2_1820,112,59,110,79,90,286,5,7,10,577,0,42
CES1_2_1821,678,420,492,623,444,206,436,247,232,470,1102,510
CES3_2_1822,23,15,31,19,0,0,0,0,80,0,92,0
CES4A_2_1823,244,198,219,176,64,636,76,804,78,143,161,251
CES5A_2_1824,3424,2724,2613,2945,3838,2222,2748,3004,3264,3473,3157,36
82
CHAT_2_1825,194,85,104,106,34,137,434,39,76,207,8,64
CHI3L2_2_1826,95,25,62,72,240,30,115,206,287,38,2,2
CHIA_2_1827,149,277,160,242,0,312,133,647,71,81,20,15
CHPF_2_1828,164,141,167,100,450,0,0,5,46,231,6,0
CHST11_2_1829,887,1094,879,1010,439,908,1013,1120,997,954,1375,788
CHST15_2_1830,699,558,637,763,764,120,551,1110,774,440,586,1210
CHST4_2_1831,120,133,176,160,23,37,4,214,165,131,96,222
CHST8_2_1832,87,9,105,96,0,0,0,297,0,60,177,173
CLP1_2_1833,875,795,912,668,384,247,494,640,965,10,829,384
CNDP2_2_1834,82,55,77,82,1,0,8,0,262,5,6,25
CNTN1_2_1835,510,423,559,745,1428,549,1598,1053,975,538,510,523
CNTN4_2_1836,893,534,645,818,628,417,545,1255,981,858,784,347
COMT_2_1837,549,747,561,769,725,497,439,637,430,312,702,713
COQ6_2_1838,352,561,661,662,449,932,741,701,494,426,34,1160
COX11_2_1839,303,196,214,259,472,9,143,438,550,2,37,73
COX15_2_1840,224,245,203,200,85,531,853,171,392,336,305,48
CPA5_2_1841,820,864,1017,954,1522,919,2351,1097,768,553,1625,745
CPB2_2_1842,970,1130,934,1121,1136,674,1802,1446,851,1057,730,815
CPM_2_1843,3032,2219,2931,2743,2749,1912,2985,2208,3061,3368,3258,3478
CPPED1_2_1844,502,512,467,657,594,601,723,360,875,836,889,827
CPT1A_2_1845,123,150,179,141,247,32,91,0,152,62,20,15
CPT1B_2_1846,66,49,76,93,2,8,54,7,159,0,1112,31

CPT1C_2_1847,762,825,771,839,1675,1605,295,556,1011,521,1342,768
CRLS1_2_1848,363,302,309,439,97,174,482,370,413,360,411,316
CRMP1_2_1849,888,1003,1207,1462,1343,1036,1170,556,1636,1831,1623,1753
CROT_2_1850,329,191,298,250,492,759,0,530,520,554,386,245
CRY2_2_1851,86,84,89,165,71,25,0,0,225,199,27,158
CRYM_2_1852,59,182,127,96,318,41,176,115,155,66,305,215
CRYZ_2_1853,991,757,868,1168,791,970,1482,190,1229,364,900,936
CSDE1_2_1854,619,385,578,667,437,502,310,135,155,815,622,607
CSGALNACT1_2_1855,877,419,678,698,905,1333,409,403,1050,494,1710,651
CSMD3_2_1856,1891,1836,1407,1723,1506,2381,2333,1858,1580,2650,1309,16
91
CTAGE5_2_1857,853,340,687,658,1072,594,808,410,937,630,1025,691
CTBP1_2_1858,27,21,42,46,98,1,0,22,117,6,10,54
CTPS2_2_1859,372,351,368,516,33,319,277,992,1004,308,924,507
CTSA_2_1860,13,10,75,41,41,0,156,124,2,1,0,23
CTSB_2_1861,249,383,438,462,240,169,148,688,514,756,351,1012
CTSE_2_1862,276,290,186,292,408,166,64,210,191,521,197,70
CTSL1_2_1863,303,408,245,608,942,830,575,153,259,355,639,639
CTSL2_2_1864,235,201,194,338,8,705,14,425,38,50,351,457
CTSS_2_1865,258,360,351,345,4,2,443,1265,96,5,391,378
CYB561_2_1866,224,349,113,343,215,4,0,447,97,19,252,359
CYB5A_2_1867,370,324,295,264,399,89,52,275,164,546,187,263
CYB5R3_2_1868,193,282,367,308,226,660,766,47,187,514,142,353
CYP11A1_2_1869,1016,887,998,911,973,771,352,314,599,989,864,667
CYP11B1_2_1870,163,244,69,65,0,464,485,0,229,307,259,6
CYP19A1_2_1871,3485,3136,3072,3062,2894,2190,2786,3852,1785,3619,5054,
2751
CYP21A2_2_1872,117,159,183,131,311,10,119,46,194,171,575,293
CYP24A1_2_1873,145,101,88,197,91,311,279,83,9,36,0,36
CYP26A1_2_1874,169,45,119,122,351,0,12,87,87,284,2,75
CYP2A7_2_1875,276,146,108,194,72,128,486,14,290,149,40,93
CYP2C18_2_1876,469,799,597,747,551,1044,487,1070,1179,952,859,394
CYP2C8_2_1877,1063,1023,1152,1653,1389,1098,639,1001,433,1928,3948,125
3
CYP2D6_2_1878,74,59,123,95,0,39,309,1,253,4,0,3
CYP3A43_2_1879,49,267,148,199,30,55,160,0,3,345,372,213
CYP3A4_2_1880,461,340,327,350,235,476,1097,546,888,217,376,653
CYP4B1_2_1881,0,23,6,17,0,62,0,0,0,0,0,0
CYP4F11_2_1882,31,18,41,10,0,0,92,0,0,10,0,322
CYP4F3_2_1883,570,757,483,540,202,561,843,168,264,145,347,537
CYP51A1_2_1884,747,739,571,670,1007,353,857,79,485,595,618,1822
DAGLB_2_1885,441,260,340,427,673,28,39,257,158,181,1369,1031
DCLRE1C_2_1886,783,695,816,942,1705,772,1286,1405,424,1101,830,742
DCP2_2_1887,139,284,230,223,571,90,22,373,124,13,5,421
DCT_2_1888,276,245,202,173,74,255,228,292,222,180,270,290
DCTD_2_1889,237,261,153,203,356,188,51,532,668,304,48,730
DCXR_2_1890,167,195,130,138,174,4,109,157,66,198,609,107
DDAH1_2_1891,117,117,205,119,310,40,47,319,16,230,397,393
DDC_2_1892,182,110,224,182,16,109,114,35,600,436,221,102
DDHD1_2_1893,1953,1453,1685,1621,1808,1305,1038,1265,1833,1719,940,175

4
DDO_2_1894,277,231,209,240,94,194,596,207,371,265,798,295
DDT_2_1895,134,64,137,186,31,8,14,33,22,0,666,207
DDX11_2_1896,320,292,143,205,448,1,1,334,0,484,629,26
DDX17_2_1897,456,833,463,748,435,403,918,709,332,1345,328,502
DDX19B_2_1898,328,107,257,153,65,2,11,38,43,196,20,123
DDX31_2_1899,176,104,161,125,357,164,220,27,20,206,94,160
DDX39B_2_1900,0,0,0,0,0,0,0,0,0,0,0,0
DDX3X_2_1901,205,166,183,226,109,393,182,268,88,197,283,409
DDX3Y_2_1902,417,298,317,307,16,16,432,514,208,517,198,24
DDX42_2_1903,227,269,265,327,229,7,13,53,58,34,500,75
DDX47_2_1904,290,310,340,263,119,61,376,850,78,348,67,122
DDX4_2_1905,747,1011,827,757,81,483,1428,1093,386,426,516,1272
DDX54_2_1906,1143,670,1134,995,806,592,1553,940,1161,1112,943,564
DFFA_2_1907,480,554,475,733,932,289,322,446,756,722,479,285
DGCR8_2_1908,567,469,715,729,569,227,278,782,551,947,320,437
DHCR7_2_1909,109,71,128,171,88,0,829,395,287,127,176,20
DHDDS_2_1910,405,383,376,450,169,408,1180,236,91,193,882,626
DHFRL1_2_1911,316,256,216,294,153,113,852,197,189,458,301,76
DHPS_2_1912,209,77,106,107,39,79,40,7,1,165,0,98
DHRS1_2_1913,367,329,491,299,18,375,18,328,850,146,232,8
DHRS2_2_1914,137,167,197,174,3,52,289,57,8,229,29,194
DHRS9_2_1915,162,196,98,169,135,120,41,74,46,263,246,195
DHX16_2_1916,72,58,122,84,145,20,5,36,225,54,860,70
DHX30_2_1917,100,108,100,69,59,3,136,73,43,441,65,4
DHX33_2_1918,42,19,35,29,0,1,43,0,174,65,0,2
DHX35_2_1919,411,566,435,695,1933,67,750,89,1124,317,134,367
DHX36_2_1920,419,381,264,446,395,306,140,130,105,401,965,33
DHX40_2_1921,170,93,104,135,354,41,341,14,119,66,102,24
DIAPH3_2_1922,132,84,63,103,156,0,3,183,11,1,548,18
DIS3_2_1923,1502,1133,1253,1260,1527,1389,2851,941,1045,1580,940,970
DIS3L_2_1924,88,63,112,187,1,3,516,192,91,99,4,96
DKC1_2_1925,504,290,344,541,644,112,313,629,518,263,295,293
DNAJC27_2_1926,20,44,9,18,4,2,2,0,7,0,0,14
DNASE1L1_2_1927,123,118,53,183,318,147,59,172,38,124,16,201
DNM1_2_1928,240,134,102,240,131,169,0,52,13,108,31,152
DNM1L_2_1929,658,477,764,633,99,115,940,705,368,851,588,1526
DNM2_2_1930,1609,1669,1506,1617,1618,2531,1377,2199,1737,1397,2145,107
7
DNM3_2_1931,1351,947,995,1068,534,1354,1517,1567,699,1108,521,738
DNTT_2_1932,327,454,346,518,265,715,777,388,296,844,167,973
DOHH_2_1933,107,160,237,123,0,211,165,117,30,590,188,114
DOLPP1_2_1934,290,178,219,250,196,105,117,376,240,495,287,298
DPEP1_2_1935,8,24,18,11,1,2,2,0,28,4,48,2
DPEP3_2_1936,826,802,840,1227,770,374,2028,453,480,1907,813,1161
DPH5_2_1937,95,83,84,91,9,1,2,322,32,124,0,377
DPM3_2_1938,553,375,584,635,1012,914,615,977,646,303,223,785
DPP3_2_1939,812,755,701,538,118,212,117,605,406,843,1591,639
DPP8_2_1940,1108,1110,1478,1310,622,241,2096,1119,597,1047,1774,1012
DPYSL2_2_1941,1362,1094,1524,1489,1300,815,1745,1594,2091,870,641,2449

DPYSL3_2_1942,63,116,82,92,42,10,57,133,127,118,156,53
DROSHA_2_1943,301,310,304,215,311,534,182,380,280,479,0,108
DSE_2_1944,220,113,245,246,23,448,304,218,48,172,707,371
DUOX1_2_1945,3457,3227,3116,3640,3561,3776,3536,2749,4521,4928,1484,38
72
DUT_2_1946,1665,1734,2082,2376,2070,815,2226,1805,2359,807,2152,1664
ECE1_2_1947,107,122,117,193,8,17,2,633,10,36,516,268
ECE2_2_1948,219,104,174,242,495,269,447,255,194,461,41,374
ECI1_2_1949,98,94,159,122,72,151,10,0,186,4,199,70
ECI2_2_1950,460,344,407,472,496,651,785,361,559,368,500,253
EDEM2_2_1951,376,199,252,329,882,294,131,535,369,434,263,138
EFEMP1_2_1952,100,122,113,136,17,7,23,318,265,18,18,1
EFTUD2_2_1953,662,514,592,646,646,340,1210,513,753,726,828,1236
EGLN2_2_1954,238,236,199,244,677,578,191,145,103,448,69,330
EHHADH_2_1955,186,169,280,294,261,139,34,508,420,571,65,311
ELAC2_2_1956,103,130,88,110,468,9,561,160,67,2,1,545
ELOVL5_2_1957,682,537,584,597,18,205,879,613,771,235,592,517
ELOVL6_2_1958,194,204,172,250,112,185,31,89,157,441,118,194
ELOVL7_2_1959,2208,1832,2029,1725,923,2123,1683,1436,1107,1342,2181,24
38
ENDOV_2_1960,0,0,0,0,0,0,0,0,0,0,0,0
ENO3_2_1961,427,373,277,346,261,7,16,7,486,383,872,281
ENOX2_2_1962,137,224,120,171,24,731,752,502,239,98,50,279
ENPP2_2_1963,175,225,176,197,546,76,621,0,150,473,146,453
ENTPD1_2_1964,1447,1717,1531,2213,1781,1965,2488,1296,642,1770,1082,20
25
ENTPD2_2_1965,265,266,103,193,122,305,1,16,50,117,337,213
ENTPD4_2_1966,263,487,302,379,679,287,290,501,221,697,495,157
ENTPD6_2_1967,162,169,183,264,23,53,50,17,255,100,3,82
ENTPD8_2_1968,6,60,88,98,0,0,0,318,42,0,2,859
EPHX1_2_1969,85,182,125,149,7,368,0,33,232,84,36,243
EPHX3_2_1970,391,356,366,472,222,533,1505,908,689,155,60,244
ERCC1_2_1971,294,296,303,326,93,169,90,39,370,221,89,55
ERCC2_2_1972,448,269,295,482,483,115,574,211,328,348,708,64
EXO1_2_1973,209,89,136,133,203,43,355,139,363,422,632,7
EXOG_2_1974,1653,1671,1749,1867,1598,1403,1217,1520,1207,1367,3364,206
4
EXOSC3_2_1975,4775,3340,3744,4922,3024,2841,4739,3872,3176,5666,3971,4
275
EXOSC9_2_1976,289,344,240,385,258,235,449,528,289,321,336,107
EXTL2_2_1977,124,89,111,127,356,102,243,55,24,307,206,208
F7_2_1978,27,27,74,11,0,0,0,0,0,23,0,443
FAHD1_2_1979,365,460,561,369,418,571,26,35,80,658,370,506
FAM108A1_2_1980,159,71,173,153,57,99,99,5,9,0,96,36
FAM135A_2_1981,421,212,238,378,162,144,12,692,467,1024,319,247
FAN1_2_1982,1717,1516,1687,1712,1267,988,1565,2803,1475,2486,1803,1273
FDPS_2_1983,331,170,248,249,196,3,1081,262,191,220,23,410
FDXR_2_1984,557,487,421,632,535,514,1158,49,573,597,271,548
FECH_2_1985,142,144,198,200,40,67,243,286,149,225,251,159
FERMT3_2_1986,197,120,230,225,25,0,0,0,24,363,0,380

FHIT_2_1987,70,36,82,58,73,0,200,5,49,0,0,72
FIGNL1_2_1988,312,420,340,385,145,110,374,940,475,881,144,229
FKBP11_2_1989,143,174,126,120,333,280,201,52,168,114,57,68
FKBP1A_2_1990,509,724,595,670,131,137,582,707,536,640,604,458
FKBP1B_2_1991,495,476,518,607,593,577,882,601,598,677,459,476
FKBP2_2_1992,134,60,97,83,16,93,0,23,23,439,211,109
FKBP5_2_1993,110,251,155,176,371,325,105,140,68,5,410,185
FKBP7_2_1994,144,81,92,127,2,0,0,1,39,91,171,163
FLAD1_2_1995,265,169,202,300,61,11,499,429,33,492,79,317
FMO3_2_1996,128,88,206,151,200,2,640,23,438,25,6,267
FMO5_2_1997,496,366,338,486,824,84,191,366,447,369,1213,289
FN1_2_1998,951,889,999,722,630,1284,1233,774,630,535,445,1266
FOLH1_2_1999,275,260,113,226,50,45,90,21,996,202,104,371
FPGS_2_2000,67,25,39,36,134,1,23,157,0,74,113,0
FPGT_2_2001,1668,1566,1345,1872,809,941,1878,984,968,1368,1843,2106
FTCD_2_2002,77,292,117,162,156,63,40,19,175,60,55,264
FTSJ1_2_2003,346,332,275,317,200,401,103,456,367,187,311,704
FUT2_2_2004,92,133,218,239,0,265,468,95,0,0,227,138
FUT3_2_2005,475,210,261,281,353,478,795,275,282,67,36,501
FUT6_2_2006,234,148,120,125,132,59,194,340,170,3,118,370
FUT8_2_2007,3515,3522,3352,3664,3953,4373,2057,3479,2333,3358,4976,377
5
G3BP1_2_2008,135,126,134,112,180,57,2,247,17,96,51,211
G3BP2_2_2009,257,233,258,269,190,425,93,577,154,5,164,482
G6PD_2_2010,247,330,276,281,651,124,22,14,423,437,66,9
GAA_2_2011,21,10,7,32,0,226,0,21,48,0,0,0
GAD1_2_2012,626,643,627,871,194,282,1786,910,1523,907,1691,767
GAD2_2_2013,251,301,145,239,772,668,615,3,308,548,629,636
GALC_2_2014,262,149,218,235,143,2,51,22,2,608,517,50
GALE_2_2015,408,368,244,465,254,28,318,75,411,337,86,115
GALNT9_2_2016,1087,629,879,1048,772,801,1255,1256,485,479,416,654
GALNTL1_2_2017,353,276,272,443,156,112,103,24,117,288,0,621
GAMT_2_2018,355,492,431,366,378,436,1,812,441,38,766,417
GANAB_2_2019,38,67,59,76,92,57,6,11,47,0,70,88
GART_2_2020,110,27,140,112,55,2,21,1,138,76,17,65
GBA_2_2021,539,333,522,550,524,0,444,974,103,304,854,757
GBP5_2_2022,260,237,203,221,539,544,569,170,44,377,23,493
GCAT_2_2023,492,519,691,550,358,1009,833,891,1543,583,410,181
GCDH_2_2024,455,366,429,422,1,433,0,137,34,190,1011,307
GCH1_2_2025,261,234,152,89,341,597,24,78,8,126,0,196
GCLC_2_2026,435,275,261,154,596,289,346,416,268,201,195,98
GCNT1_2_2027,1200,801,746,1016,407,586,591,1284,802,1370,115,981
GDPD1_2_2028,927,864,763,995,899,916,2099,445,921,895,1253,1176
GDPD2_2_2029,461,411,429,466,330,899,356,198,281,545,27,943
GEM_2_2030,193,143,99,120,109,189,74,3,118,194,7,340
GEN1_2_2031,769,474,605,445,1205,532,1316,1213,335,928,252,1290
GFM2_2_2032,551,584,748,767,195,255,1346,488,306,885,452,842
GFOD1_2_2033,231,144,175,242,600,36,503,67,175,5,99,216
GGCT_2_2034,1319,937,954,1258,1181,444,1848,1281,967,1721,472,779
GGCX_2_2035,1056,943,964,801,2009,926,607,568,989,1488,935,1214

GGT1_2_2036,161,267,142,219,519,44,23,738,148,138,8,881
GGT5_2_2037,1352,1392,1236,1277,518,791,1261,1582,820,1072,1254,913
GGT6_2_2038,21,39,23,16,222,12,0,127,23,0,157,24
GGTLC1_2_2039,6116,5372,5081,5398,6463,5108,6733,7224,6524,4752,6647,6
122
GLB1_2_2040,550,467,440,543,461,172,278,302,630,50,514,639
GLRX2_2_2041,101,158,149,172,116,80,94,198,226,170,96,96
GLRX3_2_2042,582,346,375,493,279,353,334,680,218,384,867,527
GLRX_2_2043,1306,1136,985,1307,1199,732,951,1107,637,1119,1373,1201
GLT8D1_2_2044,161,112,98,96,54,66,325,89,81,40,58,27
GLUL_2_2045,498,522,509,416,562,451,802,1087,477,143,101,260
GM2A_2_2046,419,237,318,399,65,254,339,296,68,337,923,304
GMPPA_2_2047,91,9,83,28,66,0,12,183,0,43,0,4
GMPPB_2_2048,402,386,425,479,36,485,240,372,399,183,61,358
GMPR2_2_2049,1715,1920,1545,2124,1840,3442,2117,1900,2016,2375,2167,23
02
GNAI2_2_2050,329,506,388,564,762,156,455,191,497,151,818,1022
GNAL_2_2051,633,745,775,743,86,295,332,443,857,515,90,512
GNAO1_2_2052,174,164,159,107,56,147,90,397,171,72,39,101
GNAT1_2_2053,235,208,165,171,86,5,103,211,98,72,44,302
GNB5_2_2054,203,234,239,332,742,458,144,507,192,194,6,318
GNG10_2_2055,282,323,260,401,533,612,31,865,74,89,539,38
GNG4_2_2056,458,459,376,339,511,464,452,319,162,421,303,137
GNGT2_2_2057,704,738,794,848,145,932,1442,472,324,360,294,375
GPD2_2_2058,119,205,210,114,158,314,491,689,247,115,159,11
GPHN_2_2059,287,211,276,228,294,292,221,83,371,226,102,352
GPNMB_2_2060,169,90,58,114,171,291,53,109,66,6,368,0
GPT2_2_2061,296,303,204,226,207,146,225,166,119,6,13,210
GPX4_2_2062,63,106,74,80,1,18,0,28,49,9,0,199
GSR_2_2063,14,2,37,18,10,0,4,0,6,0,0,0
GSTCD_2_2064,200,391,325,392,39,581,1081,14,176,473,525,0
GSTK1_2_2065,0,9,65,92,0,136,0,0,0,0,151,186
GSTM1_2_2066,610,545,673,608,605,181,27,880,248,677,1171,532
GSTM2_2_2067,583,805,867,581,942,337,440,821,938,441,759,810
GSTM4_2_2068,1366,1368,1256,1578,1307,1363,1571,2444,1474,1334,742,168
7
GSTO1_2_2069,34,120,101,85,0,9,72,6,0,572,0,189
GSTO2_2_2070,712,773,826,832,284,1746,909,388,331,849,1461,614
GSTZ1_2_2071,252,112,272,213,17,23,38,0,103,83,388,2
GTPBP3_2_2072,338,434,291,376,379,1620,556,336,204,278,265,1046
GUCY1A3_2_2073,562,360,480,639,763,831,518,319,1458,783,651,448
GYG1_2_2074,350,247,338,392,310,161,792,37,24,49,946,510
GYG2_2_2075,213,255,110,440,159,32,1,566,155,321,474,86
GYS1_2_2076,101,56,64,72,12,128,11,0,32,19,0,58
HADH_2_2077,460,640,473,542,1194,1103,482,372,648,385,213,517
HAGH_2_2078,324,232,199,205,17,155,51,328,3,247,0,270
HAO2_2_2079,125,185,80,85,175,64,0,251,0,248,9,357
HAS3_2_2080,625,491,589,649,552,565,1319,831,724,551,253,1049
HCCS_2_2081,1390,1473,1358,1438,1550,1450,1064,193,1105,1544,1621,1430
HDHD1_2_2082,397,348,396,453,62,9,381,338,166,333,145,200

HENMT1_2_2083,1233,1063,848,1209,1107,1007,473,1265,1192,431,287,622
HHAT_2_2084,56,107,44,100,7,19,173,5,10,64,97,10
HIBCH_2_2085,548,502,468,556,751,790,304,115,385,279,465,569
HLCS_2_2086,109,30,116,123,12,12,867,0,3,0,299,11
HMBS_2_2087,282,428,248,170,250,96,415,179,142,496,265,330
HMGA2_2_2088,1870,1870,1406,1920,1889,1272,2106,2258,1942,1108,2235,20
67
HMGCL_2_2089,3609,3063,3306,3409,3679,4865,1992,2113,3785,3285,2516,41
34
HMGCLL1_2_2090,133,248,214,234,384,467,93,191,495,492,182,205
HMGCR_2_2091,927,962,796,1172,1038,691,1320,1414,391,484,1137,224
HMGCS1_2_2092,1342,1121,1173,1420,1340,1450,1768,698,1586,1392,1265,14
37
HMGCS2_2_2093,807,845,822,910,1413,987,1676,896,692,1114,879,646
HMOX2_2_2094,185,282,358,265,626,274,1,358,894,137,1058,674
HNRNPAB_2_2095,474,242,283,379,397,216,351,261,272,313,460,438
HPD_2_2096,30,30,82,41,0,0,0,2,218,180,0,3
HPGD_2_2097,345,565,455,413,900,475,21,312,537,232,414,364
HPSE2_2_2098,92,47,36,59,0,62,0,126,0,0,0,8
HPSE_2_2099,274,205,542,368,681,101,240,955,559,91,810,823
HRAS_2_2100,247,177,131,188,207,114,543,119,194,255,199,29
HS2ST1_2_2101,194,290,203,255,320,9,191,197,387,49,489,122
HS6ST2_2_2102,70,31,16,39,185,56,110,14,2,8,9,4
HSD11B1_2_2103,110,142,78,159,187,32,113,12,153,54,171,70
HSD17B10_2_2104,276,304,315,173,299,40,75,334,71,525,65,308
HSD17B13_2_2105,139,101,83,149,0,2,54,51,121,21,0,37
HSD17B4_2_2106,521,685,519,729,830,96,76,139,362,435,350,555
HSD3B2_2_2107,809,726,668,805,1070,33,927,264,173,861,862,898
HSD3B7_2_2108,217,96,144,219,0,517,0,703,187,516,557,202
HSDL1_2_2109,391,354,362,400,320,455,629,795,502,180,119,520
HSP90AA1_2_2110,324,680,427,580,1066,67,651,802,1455,465,237,716
HSPA8_2_2111,46,127,61,28,0,138,76,1,40,40,0,61
HSPD1_2_2112,807,526,724,589,436,739,399,794,583,960,419,542
HTRA2_2_2113,142,105,62,125,0,55,19,55,377,190,0,5
HYAL1_2_2114,37,222,291,313,402,280,1099,0,2,282,422,0
HYAL2_2_2115,612,184,388,290,330,64,119,1199,485,137,699,120
HYI_2_2116,759,782,488,744,692,268,69,201,662,536,1151,799
IARS_2_2117,600,517,605,450,537,756,319,286,249,382,126,1498
IDE_2_2118,1109,668,841,914,891,1974,883,1136,1497,637,875,1276
IDH3B_2_2119,14,60,65,50,0,268,0,0,203,6,0,4
IDH3G_2_2120,86,141,73,144,74,111,339,226,155,360,0,1
IDS_2_2121,539,480,565,498,753,473,1038,397,288,794,303,805
IFT27_2_2122,78,163,129,127,68,5,12,57,98,29,51,129
IL4I1_2_2123,98,99,46,61,204,30,69,0,40,38,99,175
IMPDH1_2_2124,169,246,176,296,455,223,747,32,338,98,361,232
INMT_2_2125,152,129,122,162,3,193,169,116,46,47,641,105
INPP5K_2_2126,15,6,5,37,2,0,2,141,2,0,0,79
INTS6_2_2127,1685,1135,1366,1962,1454,1702,1233,1047,1277,1775,1750,13
02
IPCEF1_2_2128,410,468,529,524,341,100,555,537,215,362,837,687

ISOC2_2_2129,694,553,516,785,145,423,1196,1741,112,671,225,1080
ISPD_2_2130,1057,966,1088,1143,2410,1755,1727,2421,693,1402,771,1129
ISYNA1_2_2131,82,84,136,95,46,21,375,59,103,40,49,9
ITPA_2_2132,499,393,384,384,381,5,611,301,627,249,348,514
IVD_2_2133,177,205,129,245,17,139,44,0,262,6,129,384
IYD_2_2134,1003,804,788,993,1396,499,1184,720,537,380,275,1322
JMJD7-
PLA2G4B_2_2135,1347,1510,1712,1568,1123,1232,2517,368,1163,1533,1736,2
462
KARS_2_2136,1123,1062,1357,1199,1571,955,1315,863,639,2134,2652,1591
KATNA1_2_2137,216,82,244,111,0,1,48,0,100,176,3,204
KATNAL1_2_2138,1608,1431,1104,1363,1352,834,1081,1547,1601,1211,1089,1
440
KIF16B_2_2139,276,147,183,304,57,66,431,382,96,289,438,226
KIF9_2_2140,308,245,312,446,719,535,539,536,292,290,300,1144
KIFC3_2_2141,28,71,91,62,0,58,437,169,0,42,423,0
KLK2_2_2142,16,25,27,45,0,261,13,0,118,99,0,33
KLK7_2_2143,19,16,23,4,0,0,7,0,99,0,0,50
KRAS_2_2144,545,524,493,601,193,513,319,385,343,398,303,361
KYNU_2_2145,545,542,487,631,551,260,1652,1103,534,973,569,436
LAMP2_2_2146,1135,934,1026,1000,1459,206,2056,657,1656,1685,330,1097
LARGE_2_2147,28,32,32,24,10,0,37,5,90,17,0,117
LCLAT1_2_2148,2041,1859,2065,2111,1811,2274,1896,2056,917,1230,3907,25
60
LCMT1_2_2149,28,8,48,69,0,0,2,4,0,391,296,0
LDHA_2_2150,5399,4978,5359,5907,7894,4059,8836,4130,5439,5052,5448,623
6
LDHAL6A_2_2151,913,612,625,913,580,620,146,401,1546,485,950,508
LDHB_2_2152,42,52,17,33,941,288,1,167,111,65,0,169
LDHC_2_2153,97,53,116,122,13,328,784,1,42,503,0,23
LDHD_2_2154,37,8,10,25,0,0,38,172,0,57,327,0
LEPRE1_2_2155,436,390,266,630,378,40,794,770,372,419,413,234
LEPREL1_2_2156,59,95,36,33,0,259,0,0,0,0,0,0
LFNG_2_2157,607,517,584,811,1113,437,332,880,1002,1029,1543,551
LGMN_2_2158,201,171,239,407,545,0,1246,249,0,259,951,237
LGSN_2_2159,909,814,776,1005,671,260,854,1128,442,859,159,1887
LIAS_2_2160,112,112,163,141,65,192,554,695,86,53,201,171
LIG3_2_2161,251,417,395,329,134,274,348,530,780,291,540,150
LIG4_2_2162,188,133,227,176,19,37,5,147,318,35,21,592
LIPF_2_2163,332,483,543,509,24,404,255,825,869,692,467,624
LIPT1_2_2164,448,417,665,557,559,144,597,231,250,1281,157,426
LMO7_2_2165,797,858,714,792,607,504,1389,546,1126,441,730,948
LNPEP_2_2166,1324,1277,1173,1218,1855,409,1900,986,2038,1058,415,1179
LOX_2_2167,296,369,265,340,2,162,385,76,179,194,264,253
LPO_2_2168,134,166,121,193,186,296,93,29,128,152,114,166
LRRC16A_2_2169,651,808,604,852,739,667,355,1048,489,646,714,554
LSS_2_2170,78,61,125,46,105,33,176,88,280,194,0,3
LYZL6_2_2171,1552,1142,1363,1093,619,933,832,708,1349,2459,1350,1499
MACF1_2_2172,442,369,259,294,996,67,1080,134,667,402,31,399
MACROD2_2_2173,248,231,272,337,227,311,254,313,232,72,0,126

MAD2L2_2_2174,223,472,268,131,476,285,0,31,1,270,44,366
MAN2B1_2_2175,18,49,56,115,1,4,0,2,43,0,49,59
MASP2_2_2176,77,10,38,22,5,0,55,11,0,2,0,0
MAT2B_2_2177,568,301,521,457,201,251,126,573,472,975,9,477
MCAT_2_2178,455,371,258,487,290,1,244,76,337,1242,689,431
MCM4_2_2179,288,171,294,339,270,120,58,55,291,3,306,737
MCM7_2_2180,132,37,71,161,60,240,183,122,17,30,29,12
MCM8_2_2181,0,0,0,0,0,0,0,0,0,0,0,0
MCM9_2_2182,102,68,83,111,18,24,74,211,4,114,9,4
MDH1_2_2183,362,340,229,397,328,345,312,47,260,417,82,612
MECR_2_2184,242,130,116,191,385,93,276,17,3,250,3,299
MEPCE_2_2185,621,524,435,561,556,628,611,84,788,420,731,507
METTL13_2_2186,428,256,315,302,90,127,1099,552,295,895,52,556
MFN2_2_2187,400,441,484,279,500,0,952,224,774,124,344,112
MFNG_2_2188,198,94,161,171,18,5,42,309,16,30,1436,77
MGAT3_2_2189,731,507,585,577,840,448,888,379,614,525,899,425
MGAT4A_2_2190,2192,1886,1597,2164,1608,863,2837,2637,2264,2766,1083,26
01
MGAT4B_2_2191,118,57,134,225,189,0,33,137,21,340,38,0
MGLL_2_2192,686,772,461,692,413,407,265,1470,753,265,1289,870
MGST1_2_2193,718,590,638,902,729,287,198,585,492,496,868,1265
MGST2_2_2194,424,360,390,451,78,537,32,156,503,363,797,566
MICAL1_2_2195,346,265,322,194,0,44,0,668,40,247,0,350
MLH1_2_2196,650,734,487,608,483,688,1292,1462,242,663,527,534
MME_2_2197,251,302,225,218,2,101,0,0,0,179,214,89
MMP1_2_2198,927,857,879,1011,580,1613,2553,480,1122,1029,1363,1118
MMP2_2_2199,189,70,92,155,138,119,145,9,94,195,192,149
MOCS1_2_2200,435,296,335,462,365,314,1185,602,739,310,709,765
MOGS_2_2201,530,244,354,506,861,502,1163,152,669,268,629,121
MOV10_2_2202,126,59,148,189,135,0,397,174,15,7,32,320
MOV10L1_2_2203,984,731,638,775,1082,758,389,84,949,286,1036,268
MPG_2_2204,378,395,346,406,6,318,369,77,388,465,567,944
MPPE1_2_2205,1009,1027,730,854,1333,341,704,1068,445,1167,989,953
MPST_2_2206,40,4,1,12,0,0,2,4,34,3,0,194
MRAS_2_2207,0,0,0,0,0,0,0,0,0,2,0,1
MRE11A_2_2208,576,504,479,415,535,541,539,845,828,1310,652,378
MRI1_2_2209,186,177,190,360,113,461,1028,92,74,308,721,309
MSH5_2_2210,690,492,624,537,266,396,514,267,496,866,453,977
MSRA_2_2211,358,302,208,203,32,23,683,31,288,418,34,415
MSRB3_2_2212,578,475,448,555,606,566,1372,42,309,1163,1021,289
MTHFD1L_2_2213,640,362,410,418,1011,1318,471,122,840,369,134,296
MTHFS_2_2214,1184,1260,1093,1218,725,236,1752,291,1880,1318,960,860
MTO1_2_2215,1234,1204,1091,1355,1389,1404,1380,1061,972,654,1550,760
MTRR_2_2216,913,582,827,1249,921,665,2302,2315,413,984,1132,1154
MUTYH_2_2217,1702,1471,1293,1744,1137,993,1350,2225,934,1234,1326,2120
MX1_2_2218,128,108,185,111,11,17,2,20,0,255,56,106
MYBBP1A_2_2219,195,183,90,109,126,3,417,86,69,4,165,60
MYH2_2_2220,746,726,666,513,223,442,322,478,612,379,821,354
MYO5A_2_2221,1376,1113,1470,1394,1282,1752,1212,1347,1439,1117,1371,16
15

MYO7A_2_2222,48,57,22,63,19,109,0,174,79,3,1,0
MYO9B_2_2223,662,780,718,810,523,940,619,218,216,1293,600,195
N6AMT1_2_2224,199,31,82,168,0,0,0,260,0,0,0,198
NAA16_2_2225,310,179,189,233,509,102,362,286,165,68,384,474
NAA20_2_2226,1342,948,830,1477,1973,523,659,1891,804,845,600,1804
NAAA_2_2227,304,159,194,290,43,637,16,384,521,729,680,0
NAPEPLD_2_2228,257,387,293,439,589,346,24,92,267,95,315,581
NARF_2_2229,319,401,525,514,442,410,718,339,207,537,391,1231
NARS2_2_2230,190,296,183,244,443,329,141,84,103,14,0,297
NAV1_2_2231,57,152,79,92,6,138,145,45,87,0,27,166
NCEH1_2_2232,2342,1976,1761,2042,2172,1600,1983,2302,2893,1462,1716,16
79
NCF2_2_2233,386,117,311,264,504,82,0,43,215,0,0,0
NCF4_2_2234,286,274,333,411,576,8,375,473,272,96,224,128
NDOR1_2_2235,656,845,561,894,485,403,688,1481,718,430,641,609
NDUFA2_2_2236,86,7,18,38,199,21,317,3,7,241,312,2
NDUFB11_2_2237,99,168,185,268,64,38,506,226,41,65,20,56
NDUFB4_2_2238,19,5,10,18,1,0,0,0,15,0,0,33
NDUFB5_2_2239,1667,1312,1143,1409,1297,1132,1135,673,1642,788,813,1319
NDUFB6_2_2240,574,466,515,749,643,654,816,889,39,501,830,765
NDUFC1_2_2241,873,670,852,970,638,750,1021,917,706,143,1102,1339
NDUFS1_2_2242,596,627,567,1034,1083,1026,206,887,582,427,566,2455
NDUFS2_2_2243,324,262,286,664,240,271,407,23,29,40,521,499
NDUFS5_2_2244,84,157,162,151,139,201,488,27,18,108,246,42
NDUFV1_2_2245,50,11,58,105,101,59,904,5,8,34,0,7
NEIL2_2_2246,625,654,782,576,635,103,391,48,266,1532,454,1233
NEU4_2_2247,1779,1400,1278,1583,3388,1462,1940,880,1459,1724,2124,2118
NFS1_2_2248,116,107,157,118,263,375,2,125,284,268,178,226
NGLY1_2_2249,110,55,50,174,0,222,95,16,56,355,24,328
NIPSNAP1_2_2250,430,287,314,289,284,544,103,1,360,50,15,647
NIT1_2_2251,173,293,240,238,3,181,24,49,561,159,69,370
NKIRAS2_2_2252,635,275,465,580,66,163,293,233,468,312,484,287
NLGN3_2_2253,114,147,62,63,2,0,3,82,349,126,82,87
NLGN4X_2_2254,1049,1066,943,943,804,1000,1348,1658,1195,792,518,1110
NLGN4Y_2_2255,1049,1066,943,943,804,1000,1348,1658,1195,792,518,1110
NMNAT2_2_2256,335,275,276,291,159,75,282,313,199,290,431,483
NNT_2_2257,1108,1237,1279,1190,714,1315,966,2079,1085,2327,632,801
NOX4_2_2258,737,594,624,844,358,228,367,574,1419,525,399,263
NPL_2_2259,386,169,157,368,30,181,629,372,29,158,123,499
NQO1_2_2260,128,160,132,109,68,171,84,385,104,293,51,35
NRD1_2_2261,1128,763,1101,1132,436,455,1137,806,802,510,576,897
NSDHL_2_2262,664,681,503,673,511,289,657,33,208,22,174,749
NSUN2_2_2263,409,411,331,336,615,326,153,231,355,547,196,700
NUDT2_2_2264,422,679,371,501,532,641,1452,450,784,404,536,166
NXN_2_2265,152,112,120,138,62,194,63,384,224,29,570,37
OAS1_2_2266,303,188,379,351,192,154,153,515,28,285,192,206
OAS2_2_2267,235,277,205,248,229,155,112,200,130,111,55,26
OASL_2_2268,40,23,7,22,0,10,3,0,35,82,0,0
OAT_2_2269,141,148,162,95,18,762,0,0,7,0,83,0
OGDH_2_2270,146,235,251,153,55,170,7,0,82,336,326,0

OGDHL_2_2271,433,357,361,521,257,538,267,390,1,536,368,579
OGG1_2_2272,274,405,381,439,209,308,592,128,63,139,541,324
OGT_2_2273,323,192,233,413,503,219,620,1002,227,10,89,129
OLAH_2_2274,582,518,498,629,189,18,410,662,669,532,761,291
P4HA1_2_2275,415,752,476,714,589,325,1269,456,774,867,203,770
P4HA2_2_2276,354,273,285,402,343,698,146,208,230,615,208,32
P4HTM_2_2277,123,75,121,119,173,303,1,115,1,155,34,295
PAFAH1B2_2_2278,2040,1784,1664,2093,2280,1137,1129,2084,1208,1193,1754
,1397
PAFAH1B3_2_2279,95,67,51,56,0,34,12,0,7,0,17,61
PAICS_2_2280,122,137,69,90,257,139,34,326,113,1,89,57
PAOX_2_2281,45,107,85,166,74,18,136,68,18,182,0,110
PAPD4_2_2282,2105,2045,1983,2282,1859,1644,3365,2425,1541,1461,2493,26
15
PAPD5_2_2283,69,108,98,176,6,79,119,24,5,49,526,282
PAPD7_2_2284,85,78,252,103,45,48,0,9,285,199,24,96
PAPSS2_2_2285,1758,1547,1360,1765,464,772,860,2090,924,1537,1814,1646
PARK7_2_2286,1164,960,901,1053,1100,1101,1558,662,817,1017,1377,905
PARL_2_2287,2496,1996,2506,2246,2482,1703,1485,2179,2128,2563,1419,148
4
PARN_2_2288,649,859,786,773,800,177,739,768,478,869,1678,653
PARP2_2_2289,676,408,791,889,788,282,1102,607,826,767,977,626
PARP3_2_2290,198,157,117,150,259,78,104,63,193,259,0,9
PARP8_2_2291,1006,788,840,727,534,1010,1015,446,706,965,928,1392
PBLD_2_2292,611,509,637,621,601,1006,417,502,418,639,350,919
PCCA_2_2293,302,114,278,236,746,77,548,299,179,213,77,99
PCCB_2_2294,1324,1395,1061,1478,2183,228,1203,1113,533,1350,1502,919
PCMTD2_2_2295,1130,814,824,1092,703,913,2023,314,1016,1255,768,1375
PCNA_2_2296,356,249,329,447,171,0,214,435,483,623,101,98
PCSK1_2_2297,227,123,139,405,164,0,184,12,18,24,16,125
PCSK2_2_2298,37,74,94,86,12,60,37,13,18,19,59,300
PCYT1B_2_2299,309,321,594,573,847,2,414,58,246,329,702,455
PCYT2_2_2300,798,627,907,891,1257,430,1799,837,692,642,449,441
PDE10A_2_2301,467,372,498,482,22,167,2,162,746,129,816,98
PDE11A_2_2302,712,1129,723,815,279,708,296,477,830,592,684,568
PDE1A_2_2303,944,1034,840,837,1377,711,854,478,869,1055,873,1399
PDE1B_2_2304,192,268,222,163,4,1,0,38,402,462,369,303
PDE1C_2_2305,64,40,34,25,6,0,49,1,3,1,8,129
PDE2A_2_2306,294,171,311,261,544,151,95,11,154,357,3,44
PDE4A_2_2307,1000,673,876,1109,846,607,219,483,1118,1007,1165,1565
PDE4B_2_2308,386,56,230,192,54,265,62,109,160,38,315,215
PDE4C_2_2309,128,77,166,104,24,104,44,68,135,167,10,111
PDE4D_2_2310,54,104,41,70,5,1,41,59,5,102,63,24
PDE5A_2_2311,116,75,130,113,192,10,409,161,6,161,0,22
PDE6B_2_2312,326,97,191,115,122,19,20,156,400,229,88,26
PDE7A_2_2313,548,352,355,602,576,326,550,609,662,908,170,172
PDE8A_2_2314,2,65,31,46,1,0,0,24,6,106,0,7
PDE8B_2_2315,277,260,239,307,703,486,86,394,421,302,173,294
PDE9A_2_2316,97,24,64,71,2,0,3,2,40,0,20,34
PDHA1_2_2317,142,85,45,98,0,1,0,258,0,360,141,0

PDHB_2_2318,218,222,228,348,45,3,3,94,121,151,575,347
PDHX_2_2319,1515,1438,1376,1714,1781,761,2161,1410,2025,1662,1696,1688
PEMT_2_2320,345,385,434,401,122,437,259,353,116,28,483,190
PEPD_2_2321,244,188,120,100,9,426,1,0,0,5,430,171
PGAM5_2_2322,400,179,208,349,4,5,339,78,185,307,11,277
PGBD1_2_2323,326,237,340,254,665,230,188,11,336,409,759,18
PGC_2_2324,5,8,22,21,0,0,275,39,40,93,0,0
PGM1_2_2325,112,50,69,150,11,254,17,12,107,0,0,0
PGM3_2_2326,648,659,557,637,87,589,1525,1175,292,902,629,589
PHOSPHO1_2_2327,2,6,17,58,0,0,0,0,0,0,0,9
PHOSPHO2_2_2328,3878,4288,3782,4720,3772,2707,2185,3053,3979,4833,4982
,3843
PHYH_2_2329,178,83,167,158,157,489,139,0,32,226,11,141
PIGA_2_2330,405,695,338,508,121,1142,187,109,219,358,483,687
PIGC_2_2331,112,112,54,81,0,8,0,263,54,1,0,35
PIGF_2_2332,922,681,722,776,294,754,536,286,642,198,1130,709
PIGG_2_2333,474,389,263,470,296,448,1311,415,9,168,266,301
PIGN_2_2334,406,210,270,186,351,334,243,164,562,49,641,187
PIGO_2_2335,328,417,314,410,18,40,28,27,18,1438,842,79
PIGP_2_2336,1316,1014,972,1193,1824,956,984,796,1915,716,849,1420
PIGQ_2_2337,125,105,173,168,583,21,185,66,336,320,131,35
PIGT_2_2338,406,528,360,463,251,246,478,361,197,485,493,267
PIGV_2_2339,72,51,135,198,74,15,566,0,53,5,402,12
PIN4_2_2340,544,359,600,826,637,816,582,771,330,1175,1084,538
PLA1A_2_2341,660,731,316,579,134,755,497,480,1306,409,839,487
PLA2G2A_2_2342,855,952,851,824,793,1169,780,786,471,1440,527,567
PLA2G4C_2_2343,440,233,169,366,110,197,0,153,307,4,93,229
PLA2G6_2_2344,388,415,559,628,513,67,994,463,76,48,252,328
PLA2G7_2_2345,1169,1307,1162,1396,1787,697,1037,1302,2028,1628,617,141
6
PLAT_2_2346,443,702,203,491,348,356,180,228,509,88,58,691
PLAU_2_2347,442,361,379,636,511,18,1487,811,297,476,287,771
PLB1_2_2348,635,342,460,580,271,355,655,433,854,1204,794,475
PLCB1_2_2349,240,239,269,215,0,4,609,113,42,800,474,171
PLCB3_2_2350,125,90,73,73,59,44,82,134,9,103,0,83
PLCB4_2_2351,321,219,159,269,346,1,1042,334,337,142,38,305
PLCD1_2_2352,467,387,546,402,1,162,1170,1,77,831,713,296
PLCE1_2_2353,250,213,210,277,533,108,257,350,232,143,507,359
PLCG1_2_2354,185,131,139,90,7,192,159,158,308,122,4,47
PLCH1_2_2355,715,561,490,760,1342,455,869,102,194,1576,1795,2032
PLCL2_2_2356,484,548,361,397,432,801,835,42,122,663,78,655
PLCXD2_2_2357,494,601,474,436,580,310,164,90,785,312,782,1161
PLD2_2_2358,389,319,553,477,58,449,790,289,110,187,296,430
PLD3_2_2359,271,144,148,191,298,489,295,70,225,110,482,269
PLOD2_2_2360,412,344,355,387,259,293,738,447,495,157,178,462
PLSCR3_2_2361,27,135,24,48,0,1,3,20,56,0,0,0
PLSCR4_2_2362,153,119,142,99,5,0,133,109,51,66,229,54
PMEL_2_2363,46,40,30,77,10,0,120,1,8,32,14,62
PMS1_2_2364,2294,1706,1758,1862,2125,1499,2509,1725,1958,1381,1604,801
PNPLA1_2_2365,1029,687,814,1072,1513,435,1205,1599,379,1547,627,931

PNPLA4_2_2366,99,60,70,120,263,21,2,261,200,59,351,0
PNPLA6_2_2367,973,638,852,911,657,382,1457,642,335,1757,413,925
PNPLA7_2_2368,363,478,540,551,334,667,947,349,1310,109,215,623
POFUT1_2_2369,57,31,64,80,121,0,57,1,113,0,8,0
POFUT2_2_2370,224,240,390,436,174,0,369,176,34,688,285,679
POGZ_2_2371,299,223,194,241,592,354,388,8,463,457,381,250
POLD2_2_2372,179,212,175,293,54,90,82,138,224,252,362,207
POLE2_2_2373,268,238,181,231,230,0,452,333,116,154,244,159
POLG_2_2374,0,0,0,0,0,0,0,0,0,0,0,0
POLL_2_2375,173,164,196,160,66,605,112,771,193,23,48,256
POLR1D_2_2376,357,456,267,263,427,32,198,594,214,115,216,305
POLR3B_2_2377,541,351,440,515,681,4,238,1370,779,505,401,507
POLR3H_2_2378,79,106,99,54,188,11,0,8,84,0,203,15
POMT1_2_2379,123,81,161,46,2,76,624,0,565,75,2,394
PON2_2_2380,2597,1637,2065,2468,2550,1242,989,417,892,2067,649,2451
POP5_2_2381,142,110,47,85,0,0,5,0,12,274,0,131
PPA2_2_2382,826,959,954,919,1926,651,1742,829,960,1557,1246,905
PPIE_2_2383,379,416,405,453,652,462,289,869,295,1339,478,118
PPIL2_2_2384,703,490,536,644,908,528,41,354,1087,481,354,1451
PPIL3_2_2385,489,383,305,416,271,903,476,76,288,281,412,138
PPIL6_2_2386,84,113,128,174,87,9,461,178,83,237,0,96
PPIP5K1_2_2387,198,96,101,111,133,0,623,68,7,296,887,110
PPT1_2_2388,198,109,155,279,448,25,359,224,124,154,39,1
PPT2_2_2389,223,82,92,150,15,7,0,19,28,11,0,311
PRCP_2_2390,738,723,577,664,408,880,479,864,1539,127,327,1280
PRDX1_2_2391,72,114,104,84,25,1,14,19,31,45,0,164
PRDX3_2_2392,115,133,126,224,40,245,167,22,114,280,182,19
PRDX5_2_2393,3267,3542,3315,3589,2206,2839,3809,3331,3342,3721,3457,33
02
PRKCSH_2_2394,1340,1384,1247,1592,1678,847,1078,746,1862,1336,2795,186
9
PRODH_2_2395,345,340,190,527,43,637,447,339,269,488,63,410
PRSS3_2_2396,237,235,217,198,361,5,147,527,562,253,29,495
PSAT1_2_2397,183,136,321,212,95,31,159,8,167,72,59,219
PSMA1_2_2398,387,364,428,482,186,197,226,1003,503,862,234,448
PSMA3_2_2399,55,6,2,46,0,0,0,0,0,0,0,0
PSMA4_2_2400,201,196,233,325,604,390,36,436,86,317,485,308
PSMA5_2_2401,852,937,976,1040,992,520,1431,350,970,817,848,465
PSMA8_2_2402,377,276,251,260,268,149,73,73,82,348,64,246
PSMB2_2_2403,202,66,111,114,169,138,5,6,223,32,0,306
PSMB5_2_2404,826,871,673,771,1028,238,182,1381,891,1093,419,703
PSMB8_2_2405,409,549,438,577,410,839,571,627,543,957,378,229
PTBP1_2_2406,538,372,230,311,87,7,0,40,21,395,4,251
PTER_2_2407,305,278,289,366,347,179,225,53,0,190,1142,223
PTGR1_2_2408,460,313,478,434,115,219,97,367,270,141,457,582
PTGR2_2_2409,274,168,96,107,0,16,0,5,0,12,0,84
PTGS1_2_2410,223,185,255,128,569,280,1,0,80,23,19,0
PUS1_2_2411,2949,2454,2067,2606,2485,1953,3346,2154,2115,2513,1032,191
5
PYCR1_2_2412,100,117,42,75,0,0,1,0,198,255,0,0

PYGL_2_2413,371,412,347,428,335,560,571,100,700,346,496,493
PYGM_2_2414,1342,1021,1138,1279,474,903,528,1874,1133,1036,1114,1129
QPCTL_2_2415,409,335,444,413,143,399,580,257,35,617,775,620
QSOX1_2_2416,76,22,33,85,17,77,1,0,80,0,0,0
RAB11A_2_2417,193,174,96,134,132,2,817,116,268,8,418,104
RAB1A_2_2418,990,845,772,1014,2026,959,1726,1130,1411,869,883,1104
RAB23_2_2419,556,649,628,628,359,865,1388,458,733,311,680,372
RAB27A_2_2420,13,23,8,47,0,9,69,0,2,164,0,1
RAB28_2_2421,97,47,84,100,0,29,8,90,1,92,7,181
RAB2A_2_2422,409,418,367,534,233,362,980,159,358,597,13,543
RAB2B_2_2423,211,98,48,56,0,5,0,1,216,273,0,57
RAB34_2_2424,517,388,340,368,536,642,217,578,397,499,72,315
RAB35_2_2425,141,67,18,76,0,37,0,11,28,30,182,229
RAB37_2_2426,131,391,162,312,7,664,8,916,375,432,81,271
RAB40C_2_2427,426,240,401,355,845,229,951,44,285,501,672,118
RAB5C_2_2428,85,107,72,68,0,13,196,0,188,288,0,0
RAB6A_2_2429,1146,1605,1658,1782,1954,828,1302,1614,926,2206,2285,2051
RAB7L1_2_2430,94,149,147,214,161,259,14,340,484,41,418,129
RAB9A_2_2431,126,125,124,277,50,123,196,49,312,14,463,78
RABGGTA_2_2432,234,300,212,385,660,353,271,364,230,549,317,247
RABL2A_2_2433,806,769,668,994,647,708,388,633,778,1528,535,1256
RABL2B_2_2434,806,769,668,994,647,708,388,633,778,1528,535,1256
RAC1_2_2435,793,659,856,806,817,329,245,585,1281,669,1961,1164
RAD51B_2_2436,89,124,86,84,41,145,7,36,250,72,13,130
RAD51_2_2437,181,84,95,181,243,0,150,281,92,239,11,186
RAD51D_2_2438,864,687,652,707,858,638,1572,814,1078,836,614,861
RAD54B_2_2439,1491,1560,1258,1660,1601,859,648,2759,1870,1360,788,2022
RAD54L_2_2440,22,15,48,27,0,73,90,0,56,0,3,0
RAD9A_2_2441,506,352,374,480,160,136,875,225,379,341,114,473
RAP1A_2_2442,257,126,151,143,54,90,176,258,15,227,11,90
RAP1B_2_2443,1622,832,1036,1505,1068,929,2691,1357,1125,964,1584,1787
RBBP8_2_2444,287,155,529,390,947,374,844,423,164,638,404,295
RDH13_2_2445,27,8,32,6,0,0,0,0,2,1,0,0
RDH5_2_2446,91,38,147,143,122,1,479,169,15,28,700,49
RECQL5_2_2447,1576,1746,1529,1886,1859,1612,2526,1511,1187,1158,1052,1
989
RECQL_2_2448,942,747,1089,1054,501,287,1409,221,2240,1571,1577,644
RERG_2_2449,185,67,92,83,169,0,0,175,828,1,0,225
REV1_2_2450,1082,1196,811,1198,877,1304,1379,1792,937,1642,769,1107
RFC3_2_2451,363,245,294,492,376,115,49,762,399,765,228,7
RFC5_2_2452,103,88,57,53,170,0,0,107,31,85,5,53
RGN_2_2453,374,324,245,290,326,248,486,568,180,108,524,164
RGS11_2_2454,357,269,288,243,397,294,365,55,960,584,498,192
RGS6_2_2455,72,103,116,122,164,378,217,10,64,245,44,98
RHOC_2_2456,212,283,261,272,757,131,68,39,345,301,341,601
RHOT1_2_2457,726,505,450,670,561,343,1222,746,439,290,151,478
RNASE1_2_2458,349,401,369,379,297,77,523,271,366,25,401,534
RNASE4_2_2459,260,243,300,302,283,374,571,239,68,156,440,481
RPAP3_2_2460,1999,1659,1828,2208,2434,929,2820,2559,1658,2495,1841,186
8

RPE_2_2461,1014,930,1088,1064,982,944,667,415,918,979,556,1040
RPN2_2_2462,96,446,265,278,259,1,0,14,347,267,368,13
RPP14_2_2463,183,118,54,92,65,18,4,10,23,64,3,75
RPP21_2_2464,396,294,360,392,475,300,329,625,447,1,196,180
RPP30_2_2465,1386,1380,1030,1718,1252,615,1448,1417,1492,898,1746,1365
RPP38_2_2466,553,458,336,471,251,283,2078,367,892,530,188,570
RPS27A_2_2467,213,169,131,125,20,126,234,121,81,207,328,94
RRAD_2_2468,197,84,196,102,47,5,9,37,252,0,0,215
RRAGB_2_2469,465,305,351,297,219,290,196,409,224,759,481,563
RRAS2_2_2470,411,607,475,342,395,519,58,336,339,47,1028,49
RRM2B_2_2471,389,351,530,683,941,942,481,324,555,414,512,324
RRM2_2_2472,133,124,125,113,4,108,539,1,63,38,20,38
RTEL1_2_2473,80,107,46,145,0,0,82,0,16,342,437,10
SAR1A_2_2474,1050,1040,984,1213,759,557,1810,556,223,2718,781,460
SAR1B_2_2475,1684,1992,1874,2041,2330,1819,2032,2180,1307,1927,2934,27
18
SARDH_2_2476,227,232,267,295,563,190,8,358,253,234,82,213
SARS2_2_2477,144,69,117,107,0,3,1,92,257,73,273,431
SBNO1_2_2478,733,539,694,896,1217,527,620,331,361,701,1087,491
SC5DL_2_2479,306,275,303,277,188,290,92,89,21,72,387,331
SCD5_2_2480,394,330,207,388,234,310,168,93,522,507,636,427
SDC1_2_2481,505,399,480,491,341,407,343,323,724,635,564,537
SDCBP_2_2482,677,806,821,951,320,1380,910,396,739,642,2579,758
SDHC_2_2483,622,267,382,504,153,761,196,357,19,58,1514,436
SEPHS1_2_2484,147,97,53,101,325,0,118,0,81,1,0,14
3-Sep_2_2485,94,73,90,76,150,74,12,246,93,207,47,60
4-Sep_2_2486,362,239,253,212,177,462,81,20,41,185,120,376
5-Sep_2_2487,80,82,102,112,44,118,112,18,23,20,88,12
9-Sep_2_2488,196,121,121,232,27,16,7,208,182,13,2,371
SGMS2_2_2489,263,276,200,327,684,709,248,603,125,389,151,403
SH3GLB1_2_2490,37,68,50,29,0,103,0,99,109,1,75,1
SHMT1_2_2491,100,82,123,276,276,234,435,9,6,260,250,334
SHMT2_2_2492,68,109,86,83,76,119,75,164,16,166,112,9
SIAE_2_2493,41,81,93,188,27,284,175,0,66,0,146,8
SLFN11_2_2494,1343,737,997,1349,589,768,1009,1576,978,558,345,1287
SMG6_2_2495,118,134,206,139,10,187,207,61,97,115,75,120
SMOX_2_2496,233,180,204,197,14,91,0,56,173,361,694,372
SMPD1_2_2497,50,127,87,137,55,242,28,12,66,98,2,207
SMPD4_2_2498,862,449,462,671,735,1032,571,117,387,358,107,235
SMPDL3B_2_2499,421,419,250,429,8,441,226,130,441,751,1835,417
SOD2_2_2500,138,131,136,108,104,57,391,113,121,77,176,25
SPAM1_2_2501,407,232,219,338,255,252,164,318,147,50,223,886
SPAST_2_2502,0,0,0,0,0,0,0,0,0,0,0,0
SPG21_2_2503,372,383,411,387,396,85,1340,191,703,893,214,611
SPO11_2_2504,191,418,197,367,121,89,1382,5,55,317,0,899
ST3GAL1_2_2505,84,77,37,71,9,37,0,0,0,0,13,4
ST3GAL5_2_2506,95,155,90,293,399,5,32,83,256,2,85,0
ST5_2_2507,38,89,26,0,0,0,0,0,0,0,0,0
ST6GAL1_2_2508,509,412,526,474,250,364,750,337,308,712,38,1097
ST6GAL2_2_2509,325,430,475,487,521,170,765,897,829,1221,375,259

ST6GALNAC3_2_2510,929,808,755,881,613,763,815,671,949,1091,589,1001
ST6GALNAC4_2_2511,229,136,149,259,49,237,189,373,534,275,300,192
ST8SIA4_2_2512,214,203,181,153,131,372,379,10,124,636,347,87
SUCLG2_2_2513,369,336,344,286,176,154,603,692,323,478,38,778
SULF1_2_2514,388,447,394,594,781,672,797,327,76,508,225,330
SULF2_2_2515,239,261,266,406,276,175,236,405,508,70,418,363
SULT1A1_2_2516,438,399,229,363,131,107,72,90,273,363,132,633
SULT1A2_2_2517,266,130,192,193,389,14,604,310,163,235,1036,420
SULT2B1_2_2518,705,695,591,690,289,421,845,646,616,378,166,518
SUOX_2_2519,101,81,116,86,6,0,39,345,0,2,3,301
SYTL1_2_2520,355,165,263,277,333,302,487,436,518,314,213,293
TAB1_2_2521,20,70,8,143,10,52,268,0,4,0,0,0
TBXAS1_2_2522,92,146,294,266,5,188,0,0,0,0,726,16
TCIRG1_2_2523,349,377,454,513,299,388,368,340,467,458,167,196
TDP1_2_2524,426,182,245,222,493,920,20,368,234,1,836,615
TERT_2_2525,203,259,253,393,494,276,169,361,242,33,473,227
TGM2_2_2526,197,217,149,177,11,657,706,523,182,301,323,425
TGM5_2_2527,679,489,412,585,187,1008,442,260,663,594,895,789
TH_2_2528,28,0,9,9,0,0,3,98,0,8,327,0
TIAM2_2_2529,759,542,657,759,1444,220,1710,589,548,279,368,730
TKTL1_2_2530,41,45,59,32,183,42,138,51,17,60,0,11
TMEM55B_2_2531,986,1026,1103,1056,1109,1528,2499,1276,1327,1713,676,94
3
TMLHE_2_2532,1022,1007,824,1064,659,1075,958,1491,595,939,131,723
TMOD1_2_2533,2073,2226,2146,2426,1900,1653,2867,1660,2322,2342,1914,30
67
TMX2_2_2534,569,356,421,430,66,268,693,212,199,325,75,428
TNNI2_2_2535,153,184,174,119,0,116,56,0,2,572,208,297
TP53I3_2_2536,258,82,314,262,2,234,192,7,23,104,636,48
TPI1_2_2537,124,133,144,158,109,69,0,106,43,175,121,239
TPST2_2_2538,735,695,671,879,867,995,1604,1394,650,697,1178,797
TREX1_2_2539,154,70,212,274,84,0,0,743,250,253,0,210
TRMT1_2_2540,146,183,167,283,208,1,86,0,53,228,42,240
TRMT1L_2_2541,352,257,266,385,1211,46,19,356,891,518,46,66
TRMT2B_2_2542,437,221,281,311,1090,48,223,306,548,246,196,484
TRPT1_2_2543,92,84,41,47,632,168,55,39,15,109,80,22
TSEN2_2_2544,40,47,60,28,15,9,0,74,34,5,37,17
TSEN34_2_2545,182,167,116,73,22,0,85,11,81,217,72,1
TTLL6_2_2546,386,206,226,303,113,299,895,439,352,147,112,68
TUBAL3_2_2547,936,598,753,867,230,1502,859,710,720,454,134,894
TUSC3_2_2548,588,544,448,505,348,260,527,583,421,811,123,300
TXNDC16_2_2549,563,886,688,690,516,564,31,699,966,1280,735,1186
TXNDC2_2_2550,113,65,94,92,38,36,0,54,76,15,6,36
TXNDC5_2_2551,399,411,265,292,76,225,243,519,293,639,119,1084
TXNL4B_2_2552,327,168,184,316,261,247,250,310,144,282,311,521
TXNRD1_2_2553,1538,2186,1447,1948,1483,2488,1696,1092,1278,2036,2471,1
807
TXNRD3_2_2554,621,484,583,796,646,409,568,506,501,567,398,870
TYMP_2_2555,132,225,189,238,343,328,305,127,253,636,96,139
UBA52_2_2556,302,284,294,295,18,204,707,121,249,211,800,278

UEVLD_2_2557,293,278,268,316,60,212,1166,412,524,481,184,191
UGDH_2_2558,267,324,317,455,144,810,618,689,250,301,461,106
UGP2_2_2559,486,466,411,540,140,298,692,305,852,186,293,421
UGT1A6_2_2560,2679,2233,2503,2686,3382,2192,3271,4050,2534,3234,3602,2
561
UGT2B10_2_2561,601,461,488,536,819,91,556,568,924,544,713,509
UGT2B28_2_2562,601,461,488,536,819,91,556,568,924,544,713,509
UGT3A1_2_2563,1048,506,546,569,430,853,524,1395,477,150,706,588
UGT8_2_2564,307,166,100,267,343,210,956,66,709,74,0,159
UNG_2_2565,267,353,295,214,310,46,0,1331,21,24,608,595
UPP1_2_2566,478,415,430,493,2,429,96,934,34,310,1054,12
UPP2_2_2567,1205,835,1189,1121,299,759,890,182,1361,326,341,1157
UQCR10_2_2568,498,465,406,518,587,142,707,448,779,403,175,494
UQCRB_2_2569,453,479,356,491,551,276,778,26,264,896,517,1383
UROC1_2_2570,137,168,99,127,151,20,15,238,62,57,20,29
VCL_2_2571,200,81,104,135,721,321,33,17,19,146,59,29
VNN2_2_2572,734,778,498,676,872,1186,1925,952,845,894,1056,949
VPS29_2_2573,302,165,209,323,82,24,351,314,47,101,75,227
WARS2_2_2574,741,563,341,664,1375,911,457,165,871,203,37,344
WARS_2_2575,1099,1123,977,1120,1483,964,1681,524,1387,1665,994,656
WBSCR22_2_2576,860,1007,911,855,806,671,1050,873,556,580,523,1319
WDR46_2_2577,52,291,153,249,121,36,125,305,25,234,758,224
WFS1_2_2578,139,158,130,231,129,0,44,72,181,203,236,232
WRNIP1_2_2579,880,805,975,1074,950,1006,1206,248,744,601,1024,854
WWOX_2_2580,225,193,202,153,237,420,494,49,34,30,14,208
XPNPEP1_2_2581,260,167,266,213,260,202,198,260,153,357,10,282
XRCC3_2_2582,437,301,308,761,543,227,254,348,638,459,0,507
XRN1_2_2583,99,67,102,84,22,4,183,15,83,22,12,58
YWHAZ_2_2584,287,270,201,474,377,405,224,234,830,473,391,77
ZBED1_2_2585,68,46,71,50,117,43,0,0,130,59,441,83
ZCCHC11_2_2586,99,208,84,155,167,75,290,93,68,60,15,141
ZCCHC6_2_2587,611,487,752,622,716,1122,663,1263,500,861,353,1158
ZDHHC15_2_2588,933,777,933,955,724,450,2388,1425,1060,1534,1048,634
ZDHHC7_2_2589,298,298,241,192,223,173,1244,65,180,94,77,168
ACYP1_2_2590,489,445,419,360,618,147,1435,513,513,670,441,441
APOBEC3F_2_2591,1954,1857,1856,2153,1812,2248,841,2009,630,2487,1861,1
671
ASCC3_2_2592,166,60,153,84,0,0,0,0,760,0,247,98
ASPH_2_2593,858,983,920,1156,1230,128,1182,999,1805,1084,989,1239
ATP6V0E2_2_2594,300,171,156,244,69,18,188,209,251,79,66,253
C10orf2_2_2595,91,94,123,229,36,162,0,0,0,16,255,343
CAPN3_2_2596,179,200,210,195,23,600,204,341,122,313,40,106
CHM_2_2597,2180,1814,1952,2311,3001,4564,1124,3088,2073,2057,2814,2481
CTSC_2_2598,1669,1585,1542,1879,1597,1544,2481,1048,1144,1935,1169,130
8
CYBRD1_2_2599,249,208,189,185,406,215,119,83,120,489,106,644
CYP3A5_2_2600,74,35,97,98,0,0,724,2,0,0,0,127
DIO2_2_2601,142,189,211,188,193,711,673,212,234,44,168,182
DNASE2B_2_2602,425,475,324,519,418,1223,316,535,366,279,362,519
DPYD_2_2603,286,364,435,431,163,45,1254,399,349,113,432,217

GBA3_2_2604,1349,902,1018,1247,1167,363,1063,1304,843,1094,646,504
GCNT2_2_2605,373,422,486,426,198,25,312,60,676,60,799,340
GLYAT_2_2606,1117,695,1059,1318,394,477,2356,510,953,973,1211,2051
GPLD1_2_2607,345,300,218,374,200,522,203,468,42,197,79,855
GPX1_2_2608,35,128,122,64,36,6,12,237,12,19,51,103
GPX5_2_2609,883,888,913,1071,461,649,676,431,1200,1611,1572,546
HNMT_2_2610,848,777,805,750,1109,1304,928,924,474,1019,824,912
HOGA1_2_2611,49,34,111,154,0,64,0,393,3,234,0,64
HYAL3_2_2612,182,154,86,213,118,501,1059,0,153,6,217,153
JPH2_2_2613,124,195,145,253,20,29,508,0,6,190,208,0
KLK8_2_2614,80,107,127,116,43,185,444,483,98,118,24,464
LAMA4_2_2615,619,697,562,635,265,345,641,1487,889,914,367,373
LRR1_2_2616,527,505,372,608,596,61,588,509,165,597,152,337
LRTOMT_2_2617,684,696,487,638,833,410,749,240,516,714,618,890
METTL1_2_2618,207,118,82,100,41,38,378,39,3,485,699,300
MOCS2_2_2619,356,538,591,412,105,630,168,203,305,117,1004,362
NDUFA11_2_2620,944,1031,828,1512,721,1723,1556,578,849,1591,1097,614
NDUFV3_2_2621,169,140,174,240,23,22,418,587,1,90,0,113
NMNAT3_2_2622,21,14,42,44,0,18,0,0,123,9,0,94
PDE4DIP_2_2623,571,492,431,616,1081,35,784,810,319,720,464,1100
PLG_2_2624,1507,1692,1385,1803,1687,353,562,844,1764,3234,930,1842
PPCS_2_2625,368,741,635,662,227,899,332,4,4,0,4,414
PRDX2_2_2626,2385,1492,1622,2211,2298,1801,2407,2476,1905,952,1057,332
6
RAD51C_2_2627,941,801,833,1159,1476,1038,407,460,620,917,1185,1189
RASD1_2_2628,394,460,315,557,114,567,0,1,257,0,0,1413
RASL10A_2_2629,84,9,42,64,1,101,13,2,447,0,9,0
SLX1A_2_2630,469,313,346,304,73,19,240,123,423,473,700,759
SLX1B_2_2631,469,313,346,304,73,19,240,123,423,473,700,759
SPTLC1_2_2632,74,101,61,78,0,2,55,12,23,162,45,84
ST3GAL3_2_2633,205,280,192,109,28,41,844,102,146,0,31,63
VKORC1_2_2634,41,50,57,135,20,54,70,6,21,186,377,133
XPNPEP3_2_2635,265,179,258,258,339,226,0,139,314,467,253,34
GNAS_2_2636,542,432,519,661,81,217,650,570,344,700,1377,748
GNAS_2_2637,110,12,140,99,0,35,0,937,238,71,0,127
GNAS_2_2638,54,0,15,20,0,0,0,0,10,0,0,0
A4GALT_2_2639,134,237,111,178,0,0,0,0,7,234,0,0
A4GNT_2_2640,379,260,287,364,833,253,807,236,14,81,553,874
AACS_2_2641,312,448,255,367,281,546,559,9,3,570,584,63
AADAC_2_2642,190,123,135,142,162,0,42,160,123,7,63,208
AARS2_2_2643,321,243,132,243,251,356,214,502,365,102,0,21
AARS_2_2644,5,27,89,49,13,78,45,46,0,4,315,1
AASDH_2_2645,352,327,194,187,322,296,26,225,20,34,147,147
AASDHPPT_2_2646,676,378,394,649,147,499,715,944,312,414,313,447
AASS_2_2647,739,582,572,709,715,343,1211,310,369,281,627,903
ABHD14A_2_2648,17,4,0,39,0,0,33,4,1,0,0,6
ABHD1_2_2649,265,250,159,182,69,295,62,211,176,74,16,169
ABHD3_2_2650,362,160,169,311,562,124,39,81,203,457,29,544
ABHD5_2_2651,309,339,423,349,289,381,197,780,86,437,272,227
ABHD6_2_2652,1048,887,855,1108,324,757,801,411,955,851,1643,645

ABHD8_2_2653,184,126,66,65,225,20,160,73,4,11,1,126
ABO_2_2654,432,287,460,370,224,11,604,51,327,308,100,435
ABP1_2_2655,156,19,217,168,183,0,0,131,167,24,532,67
ACAA2_2_2656,480,407,435,695,278,717,160,558,652,647,1374,1320
ACACB_2_2657,224,195,207,436,490,407,1573,1283,382,207,481,19
ACAD11_2_2658,253,119,303,153,1218,9,0,24,0,0,147,321
ACAD8_2_2659,641,502,637,841,337,367,643,182,727,263,405,855
ACAD9_2_2660,146,228,333,239,152,124,212,388,205,0,202,16
ACADL_2_2661,130,138,187,117,224,349,636,24,76,6,489,100
ACADSB_2_2662,568,483,493,462,599,713,1638,757,675,455,441,189
ACADS_2_2663,325,433,291,581,126,486,653,620,300,1430,459,897
ACAT1_2_2664,305,174,255,314,80,13,443,109,102,239,76,472
ACER1_2_2665,50,101,52,245,5,1,132,334,10,0,0,445
ACER2_2_2666,1712,1581,1734,1854,2001,1696,2154,1749,535,2222,1218,138
5
ACER3_2_2667,744,479,613,716,779,434,378,1192,689,106,1048,440
ACMSD_2_2668,537,389,245,507,614,42,28,7,1028,754,88,259
ACO1_2_2669,195,140,196,222,41,57,65,145,571,17,154,201
ACO2_2_2670,796,875,687,841,1140,789,920,372,432,1111,625,932
ACOT12_2_2671,299,233,215,295,934,107,420,621,535,166,283,653
ACOT1_2_2672,266,290,238,283,181,307,1005,594,79,135,464,6
ACOT2_2_2673,164,284,147,269,98,93,518,112,16,274,207,234
ACOT4_2_2674,86,106,46,36,77,0,2,104,250,19,70,14
ACOT6_2_2675,488,283,460,475,463,4,304,75,671,544,193,675
ACOT8_2_2676,67,14,62,74,22,397,5,23,0,3,0,9
ACOX2_2_2677,34,98,301,244,27,0,62,308,0,413,40,66
ACOXL_2_2678,0,0,4,1,0,0,0,0,0,0,0,0
ACR_2_2679,24,3,1,23,2,1,0,0,55,0,0,0
ACSBG2_2_2680,1075,1203,1218,1316,1799,263,1429,1083,605,777,2450,1313
ACSF2_2_2681,318,223,221,281,98,183,67,211,243,198,5,383
ACSL1_2_2682,64,91,48,123,124,44,10,56,99,115,3,220
ACSM1_2_2683,728,418,477,721,245,569,689,128,402,845,187,787
ACSM2A_2_2684,560,552,591,521,635,760,1107,352,742,1009,211,554
ACSM4_2_2685,746,548,609,866,509,524,757,205,733,1233,319,436
ACSM5_2_2686,557,467,677,527,481,843,479,589,639,823,310,737
ACSS1_2_2687,257,250,246,263,158,90,293,908,54,322,208,178
ACSS3_2_2688,151,164,155,244,10,43,63,10,326,180,1,23
ACTC1_2_2689,310,232,243,198,41,164,948,12,120,160,221,665
ACY3_2_2690,144,204,125,161,150,206,142,60,288,97,9,83
ACYP2_2_2691,361,247,184,270,259,15,27,30,142,137,212,77
ADA_2_2692,25,21,32,41,0,0,0,0,0,0,0,17
ADAM10_2_2693,818,457,325,757,985,686,385,933,1139,723,313,376
ADAM17_2_2694,558,701,519,627,75,528,1728,640,100,306,629,243
ADAMTS4_2_2695,188,158,114,153,3,83,156,3,153,197,22,21
ADARB2_2_2696,23,29,20,28,3,7,1,12,23,22,9,13
ADAT1_2_2697,10,5,14,9,1,0,2,0,14,11,0,3
ADAT2_2_2698,444,172,233,216,19,48,112,297,924,332,47,142
ADC_2_2699,273,164,255,256,343,46,97,1,66,50,47,489
ADCY1_2_2700,2142,2030,1988,2403,1862,1809,726,1951,2505,1821,2441,190
9

ADCY2_2_2701,572,765,451,926,673,393,105,415,266,620,927,547
ADCY7_2_2702,19,23,29,43,0,5,3,9,149,0,0,49
ADCY8_2_2703,484,401,616,478,475,754,866,1292,316,493,1018,435
ADCY9_2_2704,82,65,35,62,19,8,464,42,27,145,0,4
ADH1A_2_2705,388,383,343,478,623,392,561,737,251,529,108,386
ADH1B_2_2706,16,2,29,35,2,0,3,1,171,0,3,9
ADH1C_2_2707,943,1038,823,862,1345,1170,1162,975,1067,389,814,767
ADH4_2_2708,284,323,343,402,176,0,504,32,138,581,131,163
ADH5_2_2709,343,332,189,276,0,143,270,0,143,141,0,349
ADHFE1_2_2710,943,1015,847,962,926,229,1271,1270,888,2180,630,1084
ADI1_2_2711,598,431,419,651,728,440,795,614,315,816,792,460
ADO_2_2712,3,31,26,37,0,0,0,1,70,0,0,197
ADPRH_2_2713,141,45,61,106,86,61,8,20,0,49,14,31
ADPRHL2_2_2714,13,13,38,27,0,179,0,0,232,111,0,0
ADSS_2_2715,216,78,137,171,282,222,568,177,224,22,216,323
AEN_2_2716,196,137,30,92,2,0,313,0,301,1,0,0
AGBL2_2_2717,376,181,255,387,628,0,321,115,206,352,236,214
AGMAT_2_2718,309,189,293,268,545,45,387,330,294,194,116,215
AGMO_2_2719,301,631,337,574,101,527,5,14,472,645,558,50
AGPAT4_2_2720,600,499,440,476,801,346,418,759,353,178,244,889
AGPAT5_2_2721,712,575,562,687,834,206,898,931,599,323,1242,505
AGPAT6_2_2722,250,263,261,277,168,64,376,91,552,89,221,228
AGPAT9_2_2723,715,474,313,434,93,1282,305,786,483,705,593,59
AGPS_2_2724,75,86,96,235,5,75,239,139,144,263,13,140
AGXT2_2_2725,151,231,266,196,124,140,492,128,101,548,132,174
AGXT2L2_2_2726,796,611,473,642,628,332,496,167,599,770,131,699
AGXT_2_2727,431,199,224,308,22,116,15,261,95,376,742,1
AICDA_2_2728,1187,955,920,1191,2105,1343,1280,1149,1309,893,860,983
AKR1B10_2_2729,190,195,195,135,381,0,0,305,146,0,11,0
AKR1B15_2_2730,905,773,763,820,1492,415,571,1068,700,1582,2522,1152
AKR1B1_2_2731,227,175,238,219,210,170,229,127,36,56,0,297
AKR1C1_2_2732,693,641,758,837,1693,711,794,167,1175,554,1646,954
AKR1C3_2_2733,4545,3958,3762,4863,4236,2694,5834,5003,2837,4737,3560,4
471
AKR1C4_2_2734,357,282,365,539,322,446,368,393,873,1131,121,802
AKR1E2_2_2735,184,211,167,297,625,252,470,182,147,95,40,394
AKR7A2_2_2736,462,452,314,433,270,569,802,598,173,696,910,172
AKR7A3_2_2737,753,673,807,801,294,573,845,596,591,663,888,1332
ALAD_2_2738,471,405,365,320,135,212,761,180,583,938,250,814
ALDH1A1_2_2739,1254,1049,883,1268,1446,699,1101,503,565,402,1101,633
ALDH1A3_2_2740,548,781,741,932,1160,637,963,423,346,1162,2042,580
ALDH1B1_2_2741,257,203,187,340,672,87,250,154,143,286,85,407
ALDH1L1_2_2742,0,9,7,14,0,0,0,0,0,0,0,0
ALDH1L2_2_2743,105,106,25,168,0,0,0,29,38,55,25,246
ALDH6A1_2_2744,202,182,135,165,452,471,63,51,625,45,11,122
ALDH9A1_2_2745,116,180,195,147,102,0,0,474,20,121,1,70
ALDOB_2_2746,231,130,116,200,72,73,474,5,204,115,36,43
ALDOC_2_2747,380,348,342,211,377,2,13,467,290,368,142,12
ALG10_2_2748,131,190,152,245,49,978,168,24,19,448,761,58
ALG11_2_2749,1394,1657,1451,1499,1861,1402,1421,955,1610,1122,708,1387

ALG12_2_2750,484,245,353,385,327,163,284,85,327,629,391,132
ALG14_2_2751,526,484,530,700,1252,102,663,201,134,853,396,502
ALG1_2_2752,251,344,342,280,55,11,902,443,501,73,726,95
ALG2_2_2753,628,761,844,948,624,626,692,1390,1033,1340,848,1283
ALG6_2_2754,112,148,131,218,453,15,15,144,1029,166,184,316
ALKBH8_2_2755,425,285,409,338,470,274,328,315,487,773,13,704
ALLC_2_2756,474,391,356,384,494,396,1058,561,170,483,416,389
ALOX12B_2_2757,198,177,211,240,392,592,369,104,364,185,65,280
ALOX12_2_2758,313,316,243,387,365,92,596,275,65,482,35,242
ALOX15_2_2759,49,42,65,60,234,0,158,14,44,0,0,78
ALOX5_2_2760,81,77,91,144,214,0,3,32,183,19,0,175
AMDHD1_2_2761,1357,1507,1168,1645,581,1455,1299,1060,1492,898,1741,221
1
AMY1B_2_2762,12885,11074,11511,13873,9341,7552,13601,11922,10004,9866,
8929,12719
AMY1C_2_2763,12885,11074,11511,13873,9341,7552,13601,11922,10004,9866,
8929,12719
AMY2A_2_2764,207,290,313,219,100,60,703,292,458,204,202,408
AMY2B_2_2765,12885,11074,11511,13873,9341,7552,13601,11922,10004,9866,
8929,12719
ANPEP_2_2766,837,905,838,880,1314,324,1146,522,201,1016,362,823
AOC3_2_2767,471,488,317,471,358,517,24,279,835,443,709,384
AOX1_2_2768,586,416,562,790,503,145,641,52,366,439,1003,736
APEH_2_2769,134,218,202,221,107,387,469,360,134,64,4,99
APEX2_2_2770,1282,1252,1270,1821,1885,544,1138,958,1591,898,1291,1515
APIP_2_2771,684,522,553,549,444,317,571,87,555,562,129,860
APLF_2_2772,204,271,334,298,110,229,170,669,301,74,90,423
APOBEC1_2_2773,385,286,304,554,663,476,373,699,347,377,170,310
APOBEC2_2_2774,335,398,275,266,19,429,555,77,427,536,633,924
APOBEC3B_2_2775,876,921,811,935,822,579,1300,530,735,1669,414,584
APOBEC3C_2_2776,455,284,466,410,346,354,473,514,494,98,4,583
APOBEC3G_2_2777,446,639,380,758,20,152,629,131,248,463,329,479
ARF3_2_2778,1206,1192,1151,1409,1912,1493,1477,1779,1156,571,1066,1291
ARF4_2_2779,1977,1713,1918,1513,1533,1640,532,812,1373,1115,2505,1312
ARG2_2_2780,223,275,169,324,593,130,187,660,25,619,37,117
ARHGEF10_2_2781,418,301,416,311,190,52,470,265,139,314,319,183
ARL3_2_2782,31,36,42,21,4,116,45,0,62,17,13,44
ARL4C_2_2783,217,264,171,159,8,53,58,425,34,581,409,543
ARL4D_2_2784,395,447,399,358,276,158,346,464,672,469,166,231
ARL5B_2_2785,277,440,133,412,99,311,1,326,475,322,1,274
ARL8A_2_2786,362,293,340,264,213,364,372,477,209,915,358,571
ARL8B_2_2787,37,12,25,23,11,12,8,4,5,47,14,36
ARSD_2_2788,152,62,29,66,67,7,60,634,16,24,552,7
ARSE_2_2789,337,308,299,326,290,288,728,709,19,306,262,428
ARSG_2_2790,2,2,1,6,41,0,0,3,12,2,0,0
ARSH_2_2791,642,433,564,606,548,584,369,858,321,471,1593,804
ARSI_2_2792,21,26,45,56,9,124,174,92,54,136,1,67
ARSJ_2_2793,1386,1260,997,1366,966,715,1227,2433,1260,745,1085,1310
ARSK_2_2794,1055,797,690,859,1252,482,875,1215,861,803,1695,995
ART1_2_2795,648,424,449,446,991,558,1280,104,665,594,1223,825

ART4_2_2796,272,151,234,229,433,84,71,480,220,214,354,317
AS3MT_2_2797,164,124,292,254,369,262,233,512,378,10,660,106
ASNA1_2_2798,400,470,376,361,498,437,151,590,554,610,109,1017
ASNSD1_2_2799,647,697,748,796,830,720,436,447,399,117,1711,329
ASPG_2_2800,74,31,28,74,180,0,1,120,184,3,2,176
ASPHD2_2_2801,446,282,313,467,188,273,984,540,345,381,165,903
ATAD1_2_2802,253,203,263,332,483,219,281,419,491,158,382,546
ATIC_2_2803,545,494,444,587,535,368,1270,506,674,338,407,661
ATP2C2_2_2804,1055,670,717,1191,1029,36,1045,1373,761,1238,1366,1027
AUH_2_2805,368,350,141,468,384,235,177,88,0,277,50,52
AWAT1_2_2806,48,59,52,79,86,0,5,14,60,37,4,55
AWAT2_2_2807,233,320,283,325,322,264,322,0,81,348,398,567
B3GALNT2_2_2808,268,375,160,192,265,337,99,146,150,318,281,328
B3GALT1_2_2809,587,358,364,693,713,254,785,274,738,264,948,584
B3GALT2_2_2810,477,298,250,335,754,174,231,408,738,544,56,386
B3GALT4_2_2811,109,251,133,151,0,28,962,61,98,164,560,241
B3GALT6_2_2812,9,45,51,95,76,28,0,181,6,57,0,213
B3GAT2_2_2813,200,140,139,319,186,311,167,299,283,133,246,405
B3GAT3_2_2814,67,91,68,75,130,7,402,15,1,153,84,8
B3GNT2_2_2815,155,175,196,194,0,0,7,0,118,271,818,161
B3GNT3_2_2816,523,380,476,503,483,968,401,417,893,595,671,522
B3GNT4_2_2817,181,121,70,139,371,63,0,23,1,42,65,416
B3GNT5_2_2818,195,357,276,302,310,450,536,204,128,295,374,326
B3GNT7_2_2819,159,128,217,130,31,41,388,342,270,204,605,114
B3GNT9_2_2820,46,21,3,26,0,2,0,0,437,0,0,0
B3GNTL1_2_2821,92,59,69,42,0,56,0,0,0,56,75,39
B4GALNT1_2_2822,127,53,132,98,0,0,1,7,6,9,104,296
B4GALNT3_2_2823,160,154,118,122,260,142,60,31,13,71,15,278
B4GALNT4_2_2824,54,37,33,12,5,0,49,2,127,22,5,51
B4GALT1_2_2825,980,758,781,854,634,1285,295,904,436,852,359,1074
B4GALT5_2_2826,1306,1001,639,869,712,320,978,222,309,842,367,938
B4GALT6_2_2827,735,1043,1010,1035,558,172,1642,307,821,873,502,1335
B4GALT7_2_2828,64,157,178,138,229,596,0,283,227,1,0,1
BBOX1_2_2829,318,270,219,293,559,115,35,29,118,201,910,376
BCHE_2_2830,588,645,560,718,509,161,276,298,452,677,585,703
BCMO1_2_2831,136,121,278,170,2,1,3,2,308,118,0,229
BDH2_2_2832,304,439,442,367,393,267,790,66,668,310,851,243
BHMT_2_2833,263,220,234,275,183,235,51,71,264,481,163,265
BLM_2_2834,158,162,114,86,21,214,18,35,2,102,38,228
BLMH_2_2835,1238,1021,909,1125,647,777,409,870,1188,882,0,707
BLVRA_2_2836,385,199,248,311,280,300,397,396,435,377,369,597
BLVRB_2_2837,448,346,567,594,115,124,599,74,209,191,46,505
BPHL_2_2838,410,755,459,568,298,995,682,642,202,518,156,475
BST1_2_2839,435,464,377,422,72,380,771,44,579,654,222,234
BTD_2_2840,286,268,175,353,273,289,268,31,193,430,207,129
C12orf5_2_2841,623,302,266,391,11,42,591,17,1003,15,166,166
C16orf79_2_2842,132,198,254,370,188,1,569,153,44,314,194,158
C18orf56_2_2843,30,28,30,19,1,0,16,20,17,7,2,29
C1GALT1_2_2844,379,299,150,360,71,144,804,372,335,253,55,478
C1R_2_2845,581,336,510,485,244,981,10,708,559,685,63,372

C22orf28_2_2846,116,104,139,92,61,46,16,155,135,137,292,166
C2orf43_2_2847,968,1110,927,1099,1000,532,428,633,946,935,658,515
C5orf4_2_2848,76,77,90,120,92,147,1,1,0,79,1,35
C6orf130_2_2849,248,238,191,179,284,440,518,404,273,73,13,554
CA13_2_2850,420,466,255,407,79,216,507,128,7,205,891,558
CA14_2_2851,746,628,467,910,1399,844,831,148,683,1011,193,952
CA2_2_2852,294,318,376,442,406,102,295,565,282,398,88,650
CA3_2_2853,202,512,165,198,7,629,304,85,204,173,273,305
CA4_2_2854,373,243,349,316,55,216,294,102,191,230,341,451
CA5A_2_2855,198,124,84,94,56,251,104,49,218,233,226,194
CA5B_2_2856,306,446,369,564,1140,43,813,1009,392,645,1380,642
CA6_2_2857,0,0,0,0,0,0,0,0,0,0,0,0
CA8_2_2858,602,332,627,661,232,345,460,366,325,289,544,861
CA9_2_2859,205,226,323,271,279,337,248,734,519,118,130,183
CARS2_2_2860,50,82,19,47,21,93,0,65,161,8,0,4
CASD1_2_2861,292,155,191,124,801,9,5,66,192,20,527,18
CAT_2_2862,421,193,284,313,518,247,702,144,480,18,553,77
CBR1_2_2863,210,243,202,198,159,103,250,700,128,42,48,264
CBR3_2_2864,6,5,29,20,0,0,1,0,66,20,0,0
CBR4_2_2865,67,55,68,131,6,135,55,32,68,56,65,103
CCDC88B_2_2866,45,64,51,108,14,0,0,38,294,41,0,0
CCDC92_2_2867,565,592,426,582,218,682,992,395,139,334,175,834
CCNO_2_2868,389,327,245,386,155,176,19,24,462,187,531,661
CCS_2_2869,0,0,9,0,0,0,0,0,0,0,136,0
CCT8_2_2870,583,523,336,509,844,634,72,47,198,943,30,609
CD274_2_2871,944,709,935,1013,669,493,402,1892,733,328,1371,948
CD38_2_2872,864,653,708,753,753,679,776,1162,838,912,295,912
CDA_2_2873,728,708,569,666,728,757,768,904,572,795,904,361
CDIPT_2_2874,623,515,354,614,1084,383,170,228,751,808,103,491
CDO1_2_2875,1050,1116,1146,1164,441,1287,3708,2019,892,2917,2190,1123
CDS2_2_2876,1581,1712,1679,1462,817,1575,910,858,574,1031,2476,998
CELA1_2_2877,100,233,92,183,62,29,14,652,207,173,439,363
CELA2A_2_2878,322,149,544,377,51,126,454,89,218,238,511,104
CELA2B_2_2879,577,317,229,336,92,279,74,53,458,168,19,64
CELA3A_2_2880,76,81,75,58,33,13,152,24,215,3,8,38
CELA3B_2_2881,29,21,36,25,0,0,0,0,0,4,6,88
CEL_2_2882,81,156,137,240,0,0,0,0,200,0,0,2
CETN1_2_2883,208,250,102,71,32,20,6,362,163,23,89,83
CETN2_2_2884,208,198,310,291,4,33,1,306,201,2,42,91
CETP_2_2885,274,244,194,329,26,850,142,328,233,167,65,119
CFB_2_2886,685,488,440,509,440,625,179,1092,699,839,379,612
CFD_2_2887,100,7,12,8,51,0,0,49,0,42,0,0
CFI_2_2888,626,558,507,811,1298,55,463,1451,688,793,283,1037
CH25H_2_2889,176,168,73,220,208,51,1,0,1,160,0,174
CHDH_2_2890,311,341,266,434,138,1,41,354,396,202,2,123
CHI3L1_2_2891,161,222,262,179,611,271,8,0,348,0,82,1
CHIT1_2_2892,134,117,69,216,106,0,8,274,256,785,45,141
CHML_2_2893,500,642,523,691,345,351,883,1451,466,810,278,1261
CHPF2_2_2894,36,31,44,73,44,17,392,15,2,0,0,146
CHPT1_2_2895,776,688,675,620,411,538,529,354,705,521,249,503

CHST10_2_2896,323,311,257,327,132,243,161,534,220,519,2,57
CHST12_2_2897,2,3,25,24,0,0,0,0,2,139,0,0
CHST13_2_2898,27,17,37,8,1,48,0,0,10,0,10,71
CHST14_2_2899,31,54,19,13,309,6,9,0,63,0,0,244
CHST1_2_2900,24,10,57,60,0,0,85,0,0,22,3,80
CHST2_2_2901,205,129,104,114,10,52,100,63,699,60,218,43
CHST3_2_2902,1370,1113,1111,1358,1113,905,524,506,2215,2410,752,694
CHST5_2_2903,50,55,123,87,71,0,345,213,60,199,48,9
CHST6_2_2904,165,118,123,164,134,138,138,173,15,369,72,288
CHST7_2_2905,26,2,33,31,0,0,1,0,0,0,482,8
CHST9_2_2906,2332,2269,2455,2435,1673,1294,3434,1734,2873,2068,2209,27
26
CHSY1_2_2907,713,490,465,515,454,497,938,848,516,117,472,345
CHSY3_2_2908,278,381,413,392,383,283,745,723,338,545,15,273
CLPP_2_2909,42,20,42,107,39,33,2,0,205,23,0,16
CLPX_2_2910,281,282,248,284,213,217,60,174,85,140,250,642
CLYBL_2_2911,71,47,9,90,41,0,0,58,5,518,0,9
CMA1_2_2912,345,240,201,269,473,210,279,674,53,649,364,179
CMAS_2_2913,778,716,698,925,1367,943,436,322,499,921,1495,287
CMBL_2_2914,326,245,237,210,401,302,27,154,316,484,113,55
CNDP1_2_2915,872,660,768,857,153,1063,2151,817,369,163,948,595
CNTN6_2_2916,161,209,229,363,559,445,294,114,73,18,305,202
COIL_2_2917,278,236,161,463,0,543,320,276,1,185,0,192
COMTD1_2_2918,94,44,88,101,468,3,19,149,164,10,21,53
COQ2_2_2919,592,584,476,546,162,15,719,128,557,571,1410,570
COQ3_2_2920,81,23,21,157,245,2,110,3,7,0,89,33
COQ5_2_2921,1335,1018,1081,1118,1118,501,1269,1459,1031,1118,619,1207
COX10_2_2922,1772,1842,1381,1621,973,1205,2178,1176,1542,2577,172,1803
COX17_2_2923,509,278,365,491,267,366,489,150,687,393,833,289
COX4I1_2_2924,295,254,249,252,200,138,184,107,413,68,397,79
COX4I2_2_2925,78,33,54,42,0,0,44,26,15,3,0,0
COX5A_2_2926,496,567,594,447,711,421,802,1,172,382,656,323
COX5B_2_2927,73,11,47,32,76,1,28,0,0,4,0,7
COX6A1_2_2928,93,102,120,156,207,9,514,1,332,75,225,229
COX6A2_2_2929,111,120,54,47,0,1,169,0,0,0,237,0
COX6B1_2_2930,533,339,310,593,269,574,390,140,583,147,59,329
COX6B2_2_2931,140,84,142,187,316,37,28,2,9,4,0,208
COX6C_2_2932,305,196,106,294,33,452,625,208,238,109,373,166
COX7A1_2_2933,2,36,15,46,36,184,0,0,19,34,0,1
COX7A2_2_2934,736,773,744,733,204,565,1427,490,911,586,521,1343
COX7A2L_2_2935,533,398,351,461,60,330,192,6,238,343,18,172
COX7B2_2_2936,48,75,52,33,0,1,0,6,8,91,143,0
COX7B_2_2937,182,247,154,131,27,56,419,27,281,352,253,317
COX7C_2_2938,578,777,535,841,616,624,84,699,459,1259,67,1633
COX8A_2_2939,205,64,50,25,68,241,113,47,83,11,105,178
COX8C_2_2940,536,531,449,633,883,38,363,354,468,9,631,273
CPA1_2_2941,92,158,97,159,88,76,7,2,342,0,0,92
CPA2_2_2942,197,425,190,394,36,76,111,263,362,45,154,87
CPB1_2_2943,1236,1185,1331,1429,1625,1149,1830,2047,1617,264,913,1251
CP_2_2944,305,322,380,309,106,348,585,2,296,306,177,512

CPE_2_2945,89,114,124,49,312,39,66,60,75,23,2,87
CPOX_2_2946,129,112,158,194,36,393,31,96,74,119,47,34
CPSF3_2_2947,443,400,319,600,6,191,61,454,216,261,569,461
CPT2_2_2948,1073,991,1236,1491,589,1810,1777,1653,1208,774,347,1552
CRAT_2_2949,59,42,54,88,0,43,0,82,0,0,40,209
CREG2_2_2950,154,168,140,131,195,604,198,72,60,28,208,329
CRY1_2_2951,314,274,291,296,175,1,700,354,661,0,667,386
CRYL1_2_2952,387,324,318,407,42,3,132,464,176,233,279,293
CRYZL1_2_2953,209,154,218,198,297,16,858,303,73,174,128,79
CSAD_2_2954,152,149,171,155,331,3,880,0,251,123,1429,39
CS_2_2955,143,186,183,175,211,38,125,29,168,375,981,10
CSGALNACT2_2_2956,277,187,106,223,273,0,328,166,12,15,2,41
CSRP2BP_2_2957,209,198,207,261,277,317,70,471,127,513,512,172
CTBS_2_2958,29,179,100,127,23,0,439,3,0,0,870,1
CTRB1_2_2959,372,306,301,430,410,133,489,378,512,781,447,89
CTRB2_2_2960,692,545,975,1201,948,429,342,1148,60,650,805,1198
CTRC_2_2961,438,358,439,754,305,508,245,752,207,10,424,969
CTSD_2_2962,80,209,484,339,79,1348,105,59,56,169,5,75
CTSF_2_2963,1085,912,738,1132,661,519,763,1010,139,1025,628,628
CTSG_2_2964,134,191,96,198,6,32,0,67,15,100,18,105
CTSH_2_2965,155,181,109,162,21,117,555,90,313,236,527,97
CTSK_2_2966,101,38,34,56,12,0,0,0,0,62,0,11
CTSZ_2_2967,13,6,13,27,4,0,0,0,3,3,0,5
CWC27_2_2968,1668,1163,1483,1988,1371,1198,1903,1799,1676,2342,811,252
2
CXorf21_2_2969,443,427,271,593,218,282,393,758,113,99,674,605
CYB561D2_2_2970,730,704,787,669,575,320,1565,538,676,264,767,968
CYB5B_2_2971,1215,1160,866,1022,1147,481,349,809,270,1691,53,971
CYB5R1_2_2972,201,182,169,179,327,45,552,87,37,30,612,54
CYB5R2_2_2973,127,97,92,148,230,339,73,70,231,90,8,17
CYB5R4_2_2974,701,633,647,886,405,1432,286,437,526,657,758,1152
CYBA_2_2975,52,93,15,20,0,0,1,0,75,0,0,3
CYBB_2_2976,219,378,289,522,26,361,18,7,166,777,2,164
CYC1_2_2977,148,88,134,131,17,146,94,270,169,15,46,98
CYCS_2_2978,378,384,385,441,307,379,60,172,83,699,459,435
CYP11B2_2_2979,377,470,358,624,310,305,45,485,332,503,450,344
CYP17A1_2_2980,177,88,133,100,1,120,324,6,69,53,54,170
CYP1A1_2_2981,425,353,482,564,136,421,590,200,524,208,645,673
CYP1A2_2_2982,476,425,416,415,271,267,881,13,967,714,211,941
CYP1B1_2_2983,95,146,38,122,0,3,0,0,0,226,0,0
CYP20A1_2_2984,541,242,529,478,0,1,101,90,192,464,1636,397
CYP26B1_2_2985,43,31,62,29,42,63,16,25,1,130,312,154
CYP26C1_2_2986,109,99,79,148,13,223,10,172,4,17,902,196
CYP27A1_2_2987,22,88,63,90,207,0,41,294,129,54,157,0
CYP27B1_2_2988,601,462,494,588,899,638,614,893,146,1428,314,648
CYP2A13_2_2989,301,201,232,243,92,257,65,138,72,656,318,126
CYP2A6_2_2990,47,72,76,140,12,171,83,381,148,219,59,187
CYP2B6_2_2991,811,582,643,699,806,338,833,523,825,1016,823,1203
CYP2C19_2_2992,27,4,104,27,201,0,0,0,3,59,0,5
CYP2C9_2_2993,340,217,222,311,50,79,97,21,132,219,965,204

CYP2E1_2_2994,164,235,95,243,403,178,0,515,322,256,145,486
CYP2F1_2_2995,242,341,212,273,231,368,164,6,64,234,167,374
CYP2J2_2_2996,62,90,42,103,459,22,78,39,20,3,3,176
CYP2R1_2_2997,10,26,7,10,1,0,3,0,2,0,0,50
CYP2S1_2_2998,247,456,463,725,4,496,88,326,41,694,1731,1243
CYP2U1_2_2999,960,782,858,750,1134,470,571,304,1039,1012,863,460
CYP2W1_2_3000,37,79,7,58,4,0,4,152,7,279,0,107
CYP39A1_2_3001,211,314,411,334,70,86,494,253,580,442,494,168
CYP3A7_2_3002,1095,1311,1192,1290,392,1149,1182,1502,1005,1753,1688,18
74
CYP46A1_2_3003,172,105,105,119,230,223,28,50,461,125,16,195
CYP4A11_2_3004,201,432,296,278,10,216,89,36,27,77,574,272
CYP4A22_2_3005,489,506,489,534,558,950,558,311,75,683,106,537
CYP4F12_2_3006,499,627,628,564,1,351,1019,447,332,308,732,356
CYP4F22_2_3007,8,0,42,46,0,71,60,3,0,22,0,0
CYP4F2_2_3008,8,14,14,15,0,0,1,1,28,0,0,4
CYP4F8_2_3009,382,430,634,649,55,303,365,630,451,427,1631,818
CYP4V2_2_3010,163,124,129,152,472,1,34,239,126,79,162,159
CYP4X1_2_3011,43,54,53,76,167,0,0,0,37,0,0,13
CYP4Z1_2_3012,425,358,348,421,849,194,726,191,183,491,407,328
CYP7A1_2_3013,448,272,330,337,583,81,1345,1122,105,186,348,319
CYP7B1_2_3014,200,242,175,157,36,1,174,20,429,108,140,560
CYP8B1_2_3015,486,360,563,765,345,544,115,548,436,302,249,387
DAD1_2_3016,714,497,633,722,744,839,433,36,337,951,964,229
DAGLA_2_3017,96,39,75,74,118,0,37,8,243,69,0,217
DAK_2_3018,56,64,22,39,19,0,29,59,52,2,7,135
DARS2_2_3019,240,166,202,262,128,3,486,111,72,455,560,108
DARS_2_3020,1826,1069,1472,1777,460,370,270,2703,1496,1133,2582,1710
DBH_2_3021,180,144,221,185,203,178,8,11,0,269,613,174
DBR1_2_3022,476,295,348,371,634,355,0,115,145,330,103,501
DBT_2_3023,38,46,65,20,5,59,38,0,8,146,215,30
DCLRE1B_2_3024,474,334,262,321,276,536,20,151,263,78,3,49
DCPS_2_3025,587,406,664,535,199,50,96,43,357,250,674,741
DCTN6_2_3026,362,334,365,422,259,135,317,135,100,463,898,1072
DCTPP1_2_3027,1297,1209,1110,1371,2039,795,1510,1360,801,2522,703,618
DDAH2_2_3028,725,572,559,637,467,520,1928,420,728,814,750,756
DDOST_2_3029,328,65,246,256,1311,0,20,570,22,13,696,21
DDX10_2_3030,536,700,569,788,826,559,1000,931,456,942,404,1505
DDX18_2_3031,345,545,407,517,718,594,520,983,1057,454,271,901
DDX19A_2_3032,286,419,332,368,315,462,759,474,158,108,527,190
DDX1_2_3033,460,391,374,429,350,772,661,127,314,176,1168,482
DDX20_2_3034,493,368,414,477,235,312,131,381,255,470,535,547
DDX21_2_3035,1018,802,798,936,1043,303,1018,550,448,1603,1146,722
DDX23_2_3036,643,604,498,682,631,84,214,324,572,322,359,848
DDX24_2_3037,1009,1144,1183,1365,238,605,1904,1608,880,1735,517,1910
DDX25_2_3038,457,390,422,290,1226,381,69,173,580,0,0,657
DDX27_2_3039,900,835,710,824,489,814,756,919,803,383,653,896
DDX28_2_3040,136,248,260,283,94,104,449,357,168,153,385,360
DDX39A_2_3041,133,74,80,76,68,274,408,0,75,3,29,102
DDX41_2_3042,139,191,411,139,83,589,526,0,9,0,36,443

DDX43_2_3043,1038,872,966,1108,544,724,50,2556,322,2149,875,1368
DDX46_2_3044,225,183,162,146,227,30,331,126,372,143,434,480
DDX49_2_3045,267,447,414,427,912,142,693,377,184,137,426,159
DDX50_2_3046,64,84,43,11,34,122,0,0,9,0,23,0
DDX51_2_3047,19,7,35,20,132,166,0,0,203,0,165,0
DDX52_2_3048,132,180,178,181,241,91,13,274,91,0,1,460
DDX53_2_3049,1453,960,1009,1349,1396,1390,371,2215,1729,1023,854,1859
DDX55_2_3050,122,145,71,64,141,74,0,9,54,345,1,100
DDX56_2_3051,386,507,386,543,25,46,372,10,39,835,269,262
DDX58_2_3052,424,721,544,578,70,414,97,810,654,1864,906,572
DDX59_2_3053,1281,1608,1140,1355,1824,991,2199,1213,1267,1913,2302,156
4
DDX5_2_3054,227,362,294,268,395,75,128,176,144,313,714,38
DDX60_2_3055,722,511,611,735,226,307,628,171,307,664,367,108
DDX6_2_3056,566,618,283,566,281,584,351,1026,1040,686,229,783
DECR1_2_3057,203,304,181,326,210,201,240,309,135,169,129,151
DECR2_2_3058,1745,1574,1569,1596,1524,1530,1579,2121,1456,2144,1538,16
04
DEGS1_2_3059,71,66,47,43,358,3,0,25,66,105,54,178
DEGS2_2_3060,139,184,301,214,246,456,266,206,225,70,168,79
DERA_2_3061,257,398,208,304,103,54,49,5,398,91,198,114
DFFB_2_3062,95,40,41,79,22,19,11,76,57,43,266,122
DGAT1_2_3063,66,65,132,140,19,19,291,31,13,23,131,147
DGAT2_2_3064,148,123,95,133,212,29,144,208,111,149,239,68
DHCR24_2_3065,81,107,119,44,11,92,2,441,227,46,370,20
DHDH_2_3066,150,223,133,254,75,190,206,942,116,352,2,127
DHFR_2_3067,270,281,194,287,571,280,126,276,18,677,211,629
DHODH_2_3068,126,21,13,85,0,0,3,379,0,85,8,0
DHRS13_2_3069,369,325,333,394,205,177,174,825,123,289,47,126
DHRS3_2_3070,102,268,223,215,656,82,65,111,11,95,4,225
DHRS4_2_3071,175,81,118,97,37,49,37,52,62,184,1,3
DHRS7_2_3072,224,153,232,237,52,357,232,592,230,4,4,191
DHRSX_2_3073,211,171,148,241,80,0,167,90,148,228,403,162
DHTKD1_2_3074,112,18,15,49,0,106,0,0,31,21,0,0
DHX15_2_3075,60,114,98,34,103,72,0,162,41,5,85,105
DHX29_2_3076,340,328,259,281,243,266,277,677,612,343,80,219
DHX32_2_3077,287,374,306,241,16,126,94,275,331,162,576,10
DHX34_2_3078,23,48,6,10,0,0,0,20,0,7,0,19
DHX37_2_3079,69,77,48,89,133,0,122,272,202,131,2,28
DHX38_2_3080,369,395,404,655,175,10,1030,103,496,1291,150,436
DHX57_2_3081,1597,1509,1498,1900,2196,2672,3339,1307,1281,1871,2962,13
82
DHX58_2_3082,300,158,80,145,5,193,224,128,339,492,365,40
DHX8_2_3083,707,485,661,530,41,905,25,400,257,247,272,1043
DHX9_2_3084,200,168,116,219,3,2,632,97,324,5,431,218
DIO3_2_3085,52,45,393,166,5,1,292,4,0,85,793,146
DIRAS1_2_3086,259,168,216,305,45,52,449,404,213,0,138,4
DIRAS2_2_3087,146,93,122,131,36,149,0,34,240,78,309,69
DIRAS3_2_3088,58,166,53,250,0,0,0,11,211,0,0,3
DIS3L2_2_3089,23,90,64,96,245,0,23,2,0,30,0,259

DLAT_2_3090,484,348,444,595,583,429,478,1543,610,1144,391,944
DLD_2_3091,219,161,149,170,676,19,93,52,198,12,413,57
DLL1_2_3092,218,131,169,142,222,30,165,330,267,408,241,58
DLST_2_3093,265,501,143,321,60,439,0,262,3,289,615,292
DMGDH_2_3094,560,307,310,349,322,288,7,302,261,820,224,513
DNA2_2_3095,380,252,425,456,12,22,533,32,231,287,131,578
DNAH11_2_3096,221,295,294,175,48,275,0,418,165,147,619,268
DNAH3_2_3097,1502,1242,1294,1522,1082,1249,835,885,1300,1134,673,1509
DNAH5_2_3098,587,641,962,1125,1121,1596,1285,1268,947,891,759,1241
DNAH8_2_3099,175,207,238,443,382,609,329,783,252,125,408,674
DNAJA2_2_3100,417,353,383,521,303,439,450,411,455,353,45,731
DNAJB14_2_3101,20,5,31,26,104,8,0,0,0,0,0,7
DNAJC10_2_3102,658,635,565,634,302,490,585,158,937,913,332,1065
DNAJC18_2_3103,88,105,67,212,0,0,495,0,1,0,595,774
DNAL4_2_3104,233,111,66,152,0,148,238,153,68,14,0,344
DNASE1_2_3105,27,11,20,32,301,0,0,32,7,0,0,0
DNASE1L2_2_3106,58,41,77,63,27,27,149,10,85,40,178,32
DNASE1L3_2_3107,16,15,34,5,12,0,55,0,21,38,0,0
DNASE2_2_3108,167,33,42,95,1,283,46,2,6,7,0,21
DNPEP_2_3109,30,15,74,18,0,1,0,13,8,0,0,112
DPAGT1_2_3110,291,158,143,248,80,113,160,188,136,71,288,89
DPEP2_2_3111,2322,2110,2104,2318,2256,1282,2618,1716,1848,2954,2873,21
57
DPM1_2_3112,347,130,259,225,172,0,0,230,229,113,822,251
DPM2_2_3113,114,95,50,26,1,29,0,9,34,0,0,1
DPP4_2_3114,321,378,378,349,339,58,98,16,184,377,218,571
DPP7_2_3115,43,119,39,101,0,0,52,92,112,1,0,50
DPP9_2_3116,84,117,139,125,5,913,684,83,24,1,525,33
DPYS_2_3117,315,365,332,279,296,284,396,224,541,371,294,1010
DPYSL4_2_3118,1314,1035,872,985,1679,530,1190,1438,724,1309,447,977
DPYSL5_2_3119,156,144,113,145,0,0,30,71,339,460,0,83
DQX1_2_3120,553,474,390,494,112,400,707,218,310,97,480,541
DSEL_2_3121,387,518,332,408,244,108,68,918,383,310,1245,382
DTD1_2_3122,464,368,440,463,1674,682,631,464,54,126,710,24
DUOX2_2_3123,252,206,141,121,198,214,677,63,465,0,336,32
DUPD1_2_3124,33,222,86,91,182,57,49,0,0,2,404,249
DUSP28_2_3125,295,158,92,186,280,0,0,0,0,113,0,626
EARS2_2_3126,314,294,170,355,0,60,318,252,131,13,55,17
EBPL_2_3127,253,314,289,323,319,191,745,71,218,480,12,126
ECH1_2_3128,63,74,71,62,201,202,77,92,201,13,13,79
ECHDC3_2_3129,222,140,174,164,0,277,23,10,57,237,345,0
ECHS1_2_3130,438,335,332,432,296,360,568,898,221,352,76,490
EDEM1_2_3131,206,90,175,438,22,54,117,392,303,8,37,662
EDEM3_2_3132,88,110,96,82,103,11,51,100,42,32,14,353
EEF1A1_2_3133,224,190,228,159,104,325,17,289,53,211,57,155
EEF1A2_2_3134,49,101,44,124,32,0,0,0,0,7,186,0
EEF2_2_3135,1011,826,638,822,629,986,1483,782,736,889,1047,1596
EGLN1_2_3136,649,644,596,759,527,888,1080,502,136,216,576,607
EGLN3_2_3137,55,20,17,32,34,8,13,18,221,1,82,51
EHD4_2_3138,132,86,177,109,0,28,12,160,0,97,191,144

EIF4A3_2_3139,102,170,216,136,667,4,475,80,1,272,1,673
ELAC1_2_3140,88,67,61,59,48,15,57,0,10,294,31,112
ELANE_2_3141,331,359,434,554,1019,808,113,926,149,688,256,584
ELOVL1_2_3142,958,903,1109,1041,975,946,1639,894,980,405,240,1448
ELOVL2_2_3143,707,811,885,1162,228,805,82,1329,700,593,1817,974
ELOVL3_2_3144,52,113,124,78,165,31,29,25,109,0,0,465
ELOVL4_2_3145,533,361,380,472,240,44,15,51,564,452,564,585
ENDOD1_2_3146,62,71,54,26,2,1,415,20,0,4,16,81
ENDOG_2_3147,190,83,306,294,82,50,103,115,1,67,59,445
ENGASE_2_3148,343,241,230,194,164,293,265,0,11,886,577,99
ENO2_2_3149,469,364,315,490,209,210,199,503,543,576,704,420
ENO4_2_3150,1160,931,1012,1132,1579,457,1870,1120,1280,1614,1645,1405
ENOPH1_2_3151,565,599,526,635,1001,53,419,515,461,401,521,484
ENPEP_2_3152,94,55,32,56,64,22,15,54,137,121,280,50
ENPP1_2_3153,74,91,199,135,19,40,30,8,0,40,4,0
ENPP3_2_3154,109,228,132,119,76,54,194,9,60,4,79,275
ENPP4_2_3155,571,818,617,440,296,520,613,539,627,863,761,512
ENPP5_2_3156,915,1033,964,1022,1862,1238,671,1055,996,674,27,536
ENPP6_2_3157,493,290,265,391,240,475,75,241,640,182,806,560
ENPP7_2_3158,293,335,239,507,36,1430,74,501,522,441,226,418
ENTPD3_2_3159,562,280,367,444,426,459,381,878,55,354,384,216
ENTPD5_2_3160,311,270,210,303,21,187,454,225,137,651,297,166
ENTPD7_2_3161,649,507,328,400,1032,279,84,469,1,476,692,591
EPHX2_2_3162,311,328,323,360,932,566,268,657,487,10,29,364
EPHX4_2_3163,110,165,25,102,423,0,0,0,238,29,0,21
EPRS_2_3164,461,413,397,429,829,241,1198,743,981,271,277,1083
EPT1_2_3165,1584,1313,859,1414,468,839,528,205,1498,1315,1470,1369
EPX_2_3166,47,94,19,48,1,0,0,239,8,138,0,9
ERAS_2_3167,132,101,141,166,80,145,74,44,237,19,131,23
ERCC3_2_3168,352,341,327,210,11,10,348,6,242,136,72,303
ERCC4_2_3169,64,149,50,139,18,26,53,103,131,69,188,218
ERCC5_2_3170,56,145,77,158,2,258,0,0,6,1,12,26
ERI1_2_3171,321,421,383,415,552,246,411,684,326,377,350,348
ERO1L_2_3172,625,310,335,369,117,252,1148,518,636,460,247,663
ERP44_2_3173,1219,962,1435,1030,929,1447,1378,909,1657,1022,1239,1451
ESD_2_3174,2785,3241,2799,3538,2850,2947,1907,4305,1978,3636,3270,3562
ESPL1_2_3175,278,205,275,235,154,253,22,217,124,471,1106,77
ETFDH_2_3176,494,666,601,689,151,206,102,1049,425,901,195,410
EXOSC1_2_3177,200,158,225,160,61,111,388,229,92,112,381,95
EXOSC2_2_3178,827,802,1049,1139,842,1097,174,1119,668,1243,638,1100
EXOSC4_2_3179,6,0,7,32,0,0,1,0,0,2,0,0
EXOSC5_2_3180,397,184,217,279,274,383,77,162,220,639,4,109
EXOSC7_2_3181,19,33,4,33,0,0,0,0,28,15,31,0
EXOSC8_2_3182,256,135,222,215,142,420,194,58,274,275,159,238
EXT1_2_3183,133,97,64,76,187,35,58,154,43,51,42,43
EXTL1_2_3184,192,173,150,190,6,51,301,275,68,9,167,372
EXTL3_2_3185,63,19,13,56,1,144,66,10,0,46,1,62
F10_2_3186,284,165,134,183,19,619,191,30,144,291,47,241
F12_2_3187,327,331,262,297,57,293,464,491,105,272,125,446
F13A1_2_3188,104,81,30,92,18,38,0,257,17,50,241,17

F13B_2_3189,670,399,379,615,821,587,557,25,1037,362,547,527
F2_2_3190,645,533,373,765,857,692,891,483,366,851,261,371
F5_2_3191,256,284,361,371,153,567,289,783,189,109,398,321
F9_2_3192,665,385,428,516,399,264,488,344,398,109,230,315
FA2H_2_3193,61,242,157,153,120,0,236,28,153,524,423,122
FAAH2_2_3194,82,47,96,40,4,78,213,1,122,0,22,4
FADS1_2_3195,254,268,304,308,316,17,305,182,55,55,41,298
FADS2_2_3196,410,497,334,528,328,465,194,431,547,267,7,490
FADS3_2_3197,113,85,146,176,230,32,353,47,83,14,595,307
FAHD2A_2_3198,40,51,47,55,0,1,30,90,1,4,0,26
FAM108C1_2_3199,1053,1122,746,1135,1551,2107,1537,670,682,1781,1900,16
85
FAM135B_2_3200,476,456,430,587,163,516,1319,1106,823,278,18,615
FAM20B_2_3201,256,357,279,258,312,121,47,701,395,208,706,701
FANCM_2_3202,57,59,52,64,0,0,61,14,67,40,2,10
FAR1_2_3203,7000,5995,5825,6797,6529,5761,6589,5200,5652,7036,4390,459
5
FAR2_2_3204,434,239,356,344,310,344,1081,947,477,274,169,422
FARS2_2_3205,189,118,194,118,380,366,10,6,262,166,274,141
FARSA_2_3206,140,224,184,89,47,30,310,176,140,240,90,249
FARSB_2_3207,291,227,424,414,969,0,258,971,88,24,106,236
FASN_2_3208,84,117,133,187,10,292,184,91,7,32,541,174
FDFT1_2_3209,335,383,308,547,386,218,46,312,269,260,297,880
FEN1_2_3210,437,376,326,450,49,334,67,241,746,552,23,104
FH_2_3211,259,200,278,325,132,0,51,679,170,0,314,102
FIG4_2_3212,1846,1926,1946,1782,2157,1559,1507,1629,1764,1821,1529,223
9
FKBP10_2_3213,69,93,80,162,14,42,51,326,0,219,620,9
FKBP14_2_3214,307,198,189,362,367,48,219,118,390,430,209,325
FKBP15_2_3215,365,212,288,252,27,447,21,356,332,592,500,161
FKBP3_2_3216,24,17,3,56,0,31,127,0,44,0,5,0
FKBP4_2_3217,258,90,141,150,27,2,25,62,138,36,75,825
FKBP8_2_3218,186,140,191,117,17,293,5,124,474,15,473,126
FMO1_2_3219,1508,1267,1094,1454,1917,487,1076,2268,538,1404,1749,1267
FMO4_2_3220,586,488,542,872,199,614,2063,361,321,738,156,1159
FNBP1_2_3221,236,422,115,321,325,292,0,3,0,288,73,12
FNTA_2_3222,123,209,105,75,243,9,50,1,6,52,550,55
FNTB_2_3223,1080,800,685,832,1237,737,897,1227,1034,1087,334,108
FTH1_2_3224,25,7,17,0,0,14,0,0,66,26,1,3
FTMT_2_3225,213,268,248,236,173,23,38,54,607,169,131,125
FTSJ2_2_3226,0,0,0,0,0,0,0,0,0,0,0,0
FTSJ3_2_3227,388,253,323,482,420,333,137,451,445,341,209,1045
FTSJD2_2_3228,0,64,4,8,9,28,0,0,0,0,0,0
FUCA1_2_3229,129,147,88,144,259,6,83,43,295,121,212,337
FUCA2_2_3230,440,463,542,490,416,87,268,1227,246,426,558,825
FURIN_2_3231,236,225,268,266,521,366,12,0,336,174,171,311
FUT10_2_3232,1296,1379,1177,1508,1524,1290,343,590,1217,860,1745,1147
FUT11_2_3233,20,141,127,75,6,0,175,81,0,3,8,48
FUT1_2_3234,39,85,128,61,0,220,216,0,0,25,2453,0
FUT4_2_3235,9,23,93,33,3,0,0,0,37,14,263,1

FUT5_2_3236,37,23,68,102,17,23,0,340,1,1,53,96
FUT7_2_3237,135,120,46,60,69,261,11,137,118,83,0,295
FUT9_2_3238,4697,4728,4138,4676,2747,4888,5263,3663,3870,3557,5863,358
7
GADL1_2_3239,249,270,353,429,519,124,623,160,385,778,695,279
GAL3ST1_2_3240,121,48,103,158,3,0,0,326,1,0,75,0
GAL3ST3_2_3241,54,93,70,67,0,33,105,37,4,110,463,26
GAL3ST4_2_3242,15,17,45,101,0,32,9,80,0,0,5,71
GALM_2_3243,168,275,217,239,61,394,864,37,417,498,239,230
GALNS_2_3244,197,29,46,105,0,0,119,0,306,75,2,58
GALNT10_2_3245,782,843,715,1207,1069,884,371,688,934,838,1450,584
GALNT11_2_3246,457,426,437,559,210,419,1062,427,845,495,113,75
GALNT12_2_3247,126,17,101,110,0,0,0,229,0,0,0,75
GALNT13_2_3248,323,536,603,617,748,710,979,72,476,616,1446,696
GALNT14_2_3249,1040,851,819,801,1201,763,836,1121,1184,832,581,1062
GALNT1_2_3250,80,108,118,174,89,207,517,56,397,219,363,269
GALNT2_2_3251,457,274,447,417,380,473,750,577,543,788,1227,737
GALNT3_2_3252,633,378,373,650,1051,100,304,559,674,585,274,211
GALNT4_2_3253,105,199,134,228,19,1,101,0,79,0,7,65
GALNT5_2_3254,32,20,22,10,0,63,22,1,15,40,0,10
GALNT6_2_3255,61,81,78,91,1,33,109,70,130,46,342,312
GALNT8_2_3256,17,24,35,44,123,63,4,0,0,21,182,14
GALNTL2_2_3257,197,221,113,158,578,20,136,0,65,24,13,34
GALNTL4_2_3258,35,4,17,41,68,204,15,3,70,1,17,20
GALNTL5_2_3259,273,239,405,252,702,430,827,240,267,2,166,345
GALNTL6_2_3260,180,247,213,291,269,77,435,76,425,44,61,506
GALT_2_3261,352,201,250,343,146,296,528,242,511,353,819,601
GANC_2_3262,240,280,199,222,8,178,188,6,61,120,512,278
GAPDH_2_3263,85,57,151,124,121,169,0,3,130,3,348,36
GAPDHS_2_3264,128,295,206,298,99,220,842,55,407,298,470,13
GARS_2_3265,999,1180,1011,1333,1471,1165,700,356,1001,1378,1413,1174
GATC_2_3266,223,187,202,355,131,515,1044,563,243,141,10,108
GATM_2_3267,39,40,20,55,71,0,0,10,1,0,0,0
GBA2_2_3268,17,6,17,44,0,18,0,0,1,8,0,0
GBE1_2_3269,1547,1633,1262,1485,1946,2139,1116,1749,1150,746,1285,1783
GBGT1_2_3270,532,333,424,422,993,158,466,633,384,424,66,429
GBP1_2_3271,390,269,291,446,300,153,32,896,305,279,823,681
GBP2_2_3272,435,480,238,480,320,635,146,548,302,469,504,355
GBP3_2_3273,46,17,65,62,290,0,93,74,13,0,0,216
GBP4_2_3274,192,203,121,224,120,99,783,49,638,11,213,481
GBP6_2_3275,261,281,344,313,226,417,383,273,254,518,22,464
GBP7_2_3276,700,695,659,799,921,824,1872,805,383,442,237,945
GCLM_2_3277,105,94,155,211,357,267,131,125,67,258,0,126
GCNT3_2_3278,310,449,307,214,403,194,564,408,343,240,7,156
GCNT4_2_3279,77,33,42,86,0,0,68,0,12,0,0,62
GCSH_2_3280,6,123,9,3,0,17,0,0,0,1,0,168
GDE1_2_3281,542,477,354,464,930,275,172,486,979,381,201,353
GDPD3_2_3282,52,3,40,116,45,0,0,3,0,6,0,2
GFM1_2_3283,1019,1363,1083,1064,859,976,583,472,1011,1894,1855,863
GFOD2_2_3284,200,63,102,130,101,275,203,3,231,35,97,249

GFPT1_2_3285,204,49,104,131,0,0,1,37,68,0,0,133
GFPT2_2_3286,218,149,219,221,161,199,277,167,541,65,526,93
GGH_2_3287,507,665,370,554,765,547,145,1259,255,114,21,136
GGPS1_2_3288,222,169,98,166,72,159,167,18,88,124,165,280
GGT7_2_3289,85,30,129,74,26,0,0,151,6,9,45,5
GGTLC2_2_3290,31,46,20,12,0,4,232,37,78,18,0,21
GLCE_2_3291,190,171,140,183,45,302,52,5,159,39,32,209
GLDC_2_3292,22,20,18,13,21,0,10,0,0,0,0,8
GLO1_2_3293,296,242,234,302,104,240,218,516,199,265,0,142
GLOD4_2_3294,276,357,371,447,69,8,1064,1,243,533,201,224
GLS2_2_3295,485,416,403,532,156,425,173,639,342,170,246,1268
GLT1D1_2_3296,490,384,638,620,129,1,490,31,71,930,974,441
GLT25D1_2_3297,262,147,213,373,15,45,406,261,137,178,124,738
GLT25D2_2_3298,319,241,263,305,216,218,618,508,80,278,10,146
GLT8D2_2_3299,156,345,116,231,0,62,15,323,4,5,69,14
GLUD1_2_3300,1022,771,888,1017,2073,1829,1288,1481,799,1150,1174,1714
GLUD2_2_3301,1482,1519,1386,1550,1571,2193,1266,1259,1489,1221,974,284
1
GMDS_2_3302,653,337,305,412,695,751,301,700,296,416,318,361
GMIP_2_3303,125,142,205,120,182,455,0,135,735,840,308,182
GMPR_2_3304,332,274,535,347,350,318,847,375,497,378,327,569
GMPS_2_3305,67,28,47,62,0,0,6,1,26,70,0,0
GNA11_2_3306,195,530,188,377,3,330,0,306,649,785,30,0
GNA12_2_3307,210,464,416,329,326,1,780,25,35,238,765,92
GNA13_2_3308,1110,831,951,1519,1271,519,1160,1788,1097,1901,1399,392
GNA14_2_3309,408,278,357,353,16,15,142,60,34,342,825,230
GNA15_2_3310,9,15,25,18,1,0,0,0,1,60,0,61
GNAI1_2_3311,114,84,140,102,12,0,423,65,0,530,1322,2
GNAI3_2_3312,1638,1422,1600,1499,2225,1304,1672,2024,1132,1376,1749,16
98
GNAQ_2_3313,1104,678,704,949,399,341,792,734,922,760,983,1258
GNAT2_2_3314,158,337,239,279,57,390,122,145,156,126,308,519
GNAT3_2_3315,1631,1247,1361,1286,2307,444,883,506,1521,821,927,1567
GNAZ_2_3316,105,183,174,209,618,40,104,22,1,0,29,157
GNB1_2_3317,367,351,294,310,0,223,939,0,248,338,821,189
GNB2L1_2_3318,210,153,210,225,5,151,156,10,476,240,164,150
GNB3_2_3319,10,9,17,8,0,0,0,0,0,0,0,197
GNB4_2_3320,1432,712,674,904,1404,1282,66,848,1025,445,1266,517
GNG11_2_3321,403,332,285,415,288,524,126,5,486,359,95,508
GNG12_2_3322,281,225,141,307,399,41,53,521,325,143,620,237
GNG13_2_3323,242,322,290,276,194,184,61,793,338,174,513,179
GNG2_2_3324,188,94,30,168,213,26,62,601,68,337,0,314
GNG3_2_3325,335,373,224,435,258,0,0,293,396,280,392,617
GNG7_2_3326,1773,1734,1219,1993,1769,1270,1419,791,1280,2234,1146,1477
GNG8_2_3327,50,181,71,186,13,0,0,352,105,941,0,18
GNGT1_2_3328,699,455,536,607,599,253,509,259,423,264,976,394
GNL2_2_3329,705,567,544,529,543,907,1200,1029,840,349,134,302
GNMT_2_3330,56,127,24,37,9,404,470,0,5,0,0,76
GNPAT_2_3331,2422,2147,2287,2040,1389,1349,4290,1918,2369,1117,3140,25
62

GNPDA1_2_3332,300,138,161,203,188,301,706,66,138,25,311,119
GNPDA2_2_3333,887,746,552,768,1233,976,221,809,314,1158,1652,500
GNPNAT1_2_3334,173,262,284,169,164,66,202,0,114,153,131,259
GNPTAB_2_3335,511,477,732,494,861,699,1227,328,1067,978,620,388
GNPTG_2_3336,175,113,27,116,39,0,0,0,0,6,0,65
GNS_2_3337,209,306,257,365,317,678,550,138,117,225,11,53
GOT2_2_3338,199,116,167,163,236,121,80,126,79,20,14,383
GPAA1_2_3339,251,162,182,243,271,226,652,3,80,315,255,323
GPAM_2_3340,127,119,156,144,50,373,429,65,293,181,20,18
GPAT2_2_3341,25,30,23,1,44,0,0,0,0,25,0,1
GPD1_2_3342,1372,1289,1604,1433,348,2019,704,1801,897,1144,1077,1115
GPD1L_2_3343,61,49,75,69,80,1,58,0,291,116,55,97
GPX2_2_3344,436,226,191,273,553,484,21,250,257,257,180,193
GPX3_2_3345,55,22,126,82,42,5,93,3,20,0,0,30
GPX7_2_3346,52,67,68,10,24,0,18,0,151,736,160,0
GPX8_2_3347,779,763,799,921,590,1690,1017,1311,1102,952,1166,800
GRHPR_2_3348,19,56,19,34,3,4,115,131,132,175,0,47
GSTA1_2_3349,169,151,131,183,9,109,308,135,143,126,38,438
GSTA2_2_3350,2681,2360,2041,2636,2773,569,2434,1730,1899,2730,3556,190
1
GSTA3_2_3351,1403,1566,1603,1799,911,1269,2443,850,777,1408,1280,1756
GSTA4_2_3352,3318,2624,2776,2861,4438,2182,5298,2412,2494,3541,3260,35
61
GSTA5_2_3353,1095,717,701,913,1906,944,891,15,970,773,2053,457
GSTM3_2_3354,52,119,59,100,115,139,13,93,0,189,0,13
GSTM5_2_3355,522,534,411,532,204,976,699,462,684,147,339,422
GSTP1_2_3356,62,49,107,36,16,149,273,4,1,140,0,89
GSTT1_2_3357,30,0,29,16,1,7,39,168,0,13,42,179
GSTT2B_2_3358,1221,701,913,877,3007,288,814,538,637,1006,1103,1046
GSTT2_2_3359,1221,701,913,877,3007,288,814,538,637,1006,1103,1046
GTF2F2_2_3360,64,63,44,18,63,101,0,0,54,8,341,58
GTPBP1_2_3361,264,233,274,317,26,764,10,374,766,258,51,141
GTPBP2_2_3362,81,171,168,131,115,19,329,34,43,474,53,58
GTPBP4_2_3363,207,251,163,282,30,136,21,0,4,68,74,300
GUCY1A2_2_3364,849,1019,733,1102,851,829,294,176,1136,1049,2039,882
GUCY1B3_2_3365,279,351,250,242,200,12,21,0,259,13,245,0
GUSB_2_3366,124,108,73,108,309,280,50,232,206,187,48,176
GYLTL1B_2_3367,0,0,0,0,0,0,0,0,0,0,0,0
GYS2_2_3368,175,69,103,136,1310,25,112,0,13,67,2,1
GZMA_2_3369,88,21,59,61,2,13,0,68,9,144,3,132
GZMB_2_3370,360,504,321,515,525,288,85,646,208,435,8,583
H6PD_2_3371,75,60,195,70,118,286,0,0,139,2,90,168
HAAO_2_3372,506,326,343,392,737,727,229,1171,138,60,680,614
HACL1_2_3373,163,157,181,192,345,202,237,60,250,277,41,305
HADHA_2_3374,1287,1502,1030,1536,968,909,212,825,836,1396,806,1858
HADHB_2_3375,214,207,136,215,545,235,398,249,284,23,0,131
HAGHL_2_3376,135,129,188,165,97,198,200,185,200,2,155,368
HAL_2_3377,301,197,138,203,243,65,181,98,547,796,27,86
HAO1_2_3378,214,283,189,308,592,221,473,1040,58,812,582,317
HARS_2_3379,208,157,74,148,120,143,157,269,184,8,18,584

HAS1_2_3380,186,113,84,111,69,223,1089,1,0,195,0,156
HAS2_2_3381,0,0,0,0,0,0,0,0,0,0,0,0
HDHD3_2_3382,433,287,367,460,978,0,547,14,58,0,119,11
HELB_2_3383,470,598,452,489,742,622,432,267,169,21,112,733
HELQ_2_3384,0,0,0,0,0,0,0,0,0,0,0,0
HELZ_2_3385,876,942,711,1031,240,981,1322,2129,914,1679,1389,1754
HEXA_2_3386,210,262,294,437,381,47,32,363,133,258,0,552
HEXB_2_3387,1011,822,852,1094,898,942,1556,1177,1163,799,675,1284
HEXDC_2_3388,96,121,73,166,0,124,0,0,460,225,52,196
HGD_2_3389,1145,1148,744,1265,783,589,272,93,502,250,2682,1116
HGSNAT_2_3390,19,21,32,45,4,118,59,1,151,10,16,49
HHATL_2_3391,1065,1424,1123,1181,268,1026,451,689,1214,1894,778,1360
HIBADH_2_3392,349,513,770,417,1002,579,615,362,511,18,423,366
HINT1_2_3393,278,195,167,515,536,174,7,40,272,1,289,316
HMOX1_2_3394,650,413,389,460,464,754,841,196,911,792,747,264
HPGDS_2_3395,310,167,280,415,385,1,78,418,420,71,15,315
HPRT1_2_3396,1008,922,837,1306,549,2489,704,2649,1816,905,1719,1108
HS3ST1_2_3397,134,182,89,83,276,167,277,166,1,67,63,137
HS3ST2_2_3398,558,466,451,529,371,374,47,262,237,399,477,698
HS3ST3A1_2_3399,845,602,446,366,624,901,637,1156,179,826,124,536
HS3ST3B1_2_3400,144,99,81,153,387,9,4,273,4,234,353,68
HS3ST4_2_3401,515,484,458,535,168,420,298,444,439,633,361,697
HS3ST6_2_3402,27,26,18,63,0,7,2,2,4,63,7,7
HS6ST1_2_3403,94,21,99,49,9,37,0,120,4,90,5,162
HS6ST3_2_3404,122,55,140,114,98,50,31,0,170,9,0,273
HSD11B2_2_3405,92,48,63,131,46,0,191,20,56,2,0,316
HSD17B11_2_3406,1404,1294,1414,1514,610,577,2084,1278,1310,1600,1185,9
81
HSD17B12_2_3407,152,126,136,161,167,6,0,30,147,63,483,193
HSD17B14_2_3408,279,389,275,331,109,0,334,6,177,163,455,374
HSD17B1_2_3409,86,44,57,47,147,43,0,9,247,136,167,40
HSD17B2_2_3410,561,309,391,425,976,361,416,915,188,300,409,239
HSD17B3_2_3411,326,171,258,231,90,5,611,7,361,84,9,194
HSD17B6_2_3412,159,146,84,111,124,220,148,79,80,116,181,162
HSD17B8_2_3413,1180,664,923,1190,1076,338,886,650,953,521,2082,1110
HSD3B1_2_3414,530,371,380,489,433,816,391,194,653,72,802,724
HSP90AB1_2_3415,559,658,440,665,631,70,764,336,875,389,186,79
HSPA5_2_3416,603,355,450,447,732,53,403,25,358,312,137,44
HSPE1_2_3417,190,66,46,45,77,0,5,56,75,146,0,0
HSPG2_2_3418,375,292,285,445,82,301,270,207,1350,286,155,263
HYAL4_2_3419,214,208,231,159,169,357,0,29,166,143,362,122
IARS2_2_3420,421,552,314,597,483,243,1212,352,80,706,2026,279
ICMT_2_3421,406,293,297,363,414,29,517,330,472,279,556,102
ICT1_2_3422,1146,849,938,1206,1454,1378,1163,1255,309,757,1236,1046
IDH1_2_3423,1813,2037,1658,1977,1783,1317,979,1170,1509,2212,4274,753
IDH2_2_3424,87,58,63,69,325,342,63,0,37,3,12,226
IDH3A_2_3425,1108,1285,1053,1472,1354,1097,1080,912,1798,1241,1348,125
0
IDI1_2_3426,741,744,852,940,901,277,300,911,490,1066,829,295
IDI2_2_3427,887,378,431,785,534,338,1,360,591,676,816,386

IDO1_2_3428,43,62,19,40,39,7,105,4,13,74,0,9
IDO2_2_3429,57,37,17,27,3,0,0,8,0,6,310,19
IDUA_2_3430,465,331,566,547,890,810,1997,378,289,480,694,376
IFI30_2_3431,104,57,17,51,1,0,0,1,137,6,46,258
IFIH1_2_3432,1029,1056,1002,1087,1296,762,357,1251,149,1387,2660,1394
IGHMBP2_2_3433,74,25,42,41,210,5,219,54,1,0,421,348
IHH_2_3434,27,22,54,38,0,0,0,0,7,0,0,140
ILVBL_2_3435,401,352,303,316,402,94,171,19,302,307,392,334
IMPAD1_2_3436,474,231,263,311,931,283,225,297,587,91,3,46
IMPDH2_2_3437,18,168,66,60,70,312,258,54,61,81,299,18
IRS1_2_3438,497,574,517,516,680,211,817,942,849,214,533,669
IRS2_2_3439,380,368,430,666,591,352,529,199,427,451,316,846
ISG20_2_3440,54,67,115,88,111,271,0,27,15,701,22,0
ISG20L2_2_3441,91,102,43,49,0,0,64,3,32,0,0,0
ISOC1_2_3442,72,37,54,76,78,7,72,55,143,108,12,71
IWS1_2_3443,237,95,209,389,829,77,1012,686,910,1,85,540
KDSR_2_3444,880,459,682,848,700,310,1270,658,210,963,897,293
KHSRP_2_3445,185,89,129,308,6,9,3,282,6,1,3,5
KIAA0317_2_3446,1490,1303,1814,1877,1388,915,1585,1021,1395,1286,1669,
1455
KIAA1279_2_3447,185,85,107,187,2,6,51,52,205,55,1,299
KIAA2022_2_3448,1,9,10,23,4,0,1,0,87,461,0,0
KIF18A_2_3449,627,381,493,464,39,282,392,410,335,472,586,187
KIF20B_2_3450,10,9,24,16,2,0,43,18,0,0,0,0
KIF3A_2_3451,1846,1291,1082,1179,1029,1059,2596,1496,1233,1311,1087,10
83
KLB_2_3452,314,383,350,478,251,87,98,694,533,114,798,658
KLC3_2_3453,19,19,19,67,0,0,7,121,31,20,258,0
KL_2_3454,910,459,598,791,595,213,1136,264,399,238,567,537
KLK1_2_3455,43,40,44,75,0,50,96,8,66,54,208,204
KLKB1_2_3456,315,323,515,319,381,5,720,507,405,265,0,486
KMO_2_3457,336,113,194,205,6,93,107,11,112,106,0,423
KRTCAP2_2_3458,161,190,137,67,0,1,495,16,6,3,491,7
L2HGDH_2_3459,258,287,318,236,110,417,388,973,393,74,51,249
LALBA_2_3460,82,101,41,43,132,26,1,295,41,66,21,0
LAP3_2_3461,63,90,49,71,196,51,35,145,393,30,99,29
LARS2_2_3462,515,423,466,546,493,538,702,60,515,363,261,425
LARS_2_3463,361,429,382,387,53,289,23,138,580,219,557,357
LCAT_2_3464,10,63,0,9,0,0,0,0,0,0,0,0
LCMT2_2_3465,71,40,23,46,225,215,58,11,62,146,0,0
LCT_2_3466,126,188,97,106,218,59,159,2,192,257,100,620
LCTL_2_3467,1772,1632,1640,1589,1982,990,2089,1125,2571,1763,1520,1265
LDHAL6B_2_3468,4575,4208,3887,4271,4008,2835,5078,3223,4330,3632,3881,
4118
LEPREL2_2_3469,37,142,79,141,0,0,9,2,0,104,292,0
LGALS13_2_3470,179,185,102,170,1,2,37,30,256,37,9,326
LHFPL2_2_3471,809,687,530,691,502,919,1094,443,778,1115,1024,303
LIPC_2_3472,38,70,66,27,34,77,113,0,159,78,0,23
LIPE_2_3473,301,142,97,106,101,223,142,63,10,529,28,28
LIPG_2_3474,908,855,910,1125,252,628,1010,1750,856,1105,602,236

LIPH_2_3475,1449,1263,1535,1896,1978,792,566,1906,1715,1339,1090,2337
LIPI_2_3476,723,577,734,723,621,1254,1695,328,389,546,300,767
LIPM_2_3477,47,12,20,43,0,0,0,0,0,1,0,22
LIPN_2_3478,436,343,307,400,576,244,105,308,287,511,201,34
LIPT2_2_3479,275,161,281,385,7,422,132,0,73,434,1303,444
LONP2_2_3480,320,305,361,321,542,373,765,206,396,317,111,302
LOXL1_2_3481,154,81,139,118,64,52,484,206,27,112,0,50
LOXL2_2_3482,587,696,531,579,791,553,555,77,1184,437,114,226
LOXL3_2_3483,47,89,42,150,0,378,8,42,1,42,10,12
LOXL4_2_3484,67,63,103,91,52,17,12,52,2,1,325,66
LPCAT1_2_3485,408,245,307,375,9,217,317,334,294,4,227,222
LPCAT2_2_3486,158,170,76,191,10,344,103,5,205,61,19,99
LPCAT3_2_3487,642,373,575,533,909,323,734,171,692,565,95,237
LPCAT4_2_3488,25,82,57,76,0,21,37,64,25,244,137,207
LPIN1_2_3489,879,647,654,637,508,301,1074,1377,523,374,840,1689
LPIN2_2_3490,639,774,607,907,202,825,566,735,574,332,1279,1029
LPIN3_2_3491,39,68,28,60,291,16,96,84,129,34,85,89
LPL_2_3492,1036,931,917,860,642,934,326,1250,883,294,1415,1220
LRAT_2_3493,93,165,137,189,183,103,64,67,105,195,0,31
LTA4H_2_3494,124,59,117,116,28,19,53,148,11,139,0,37
LTC4S_2_3495,232,110,277,203,0,2,15,0,134,49,739,47
LYG2_2_3496,64,118,88,172,77,56,9,58,67,58,514,136
LYPLA1_2_3497,4,40,140,43,0,68,0,12,30,0,54,194
LYPLA2_2_3498,192,73,79,83,48,247,22,14,189,84,8,212
LYPLAL1_2_3499,192,387,399,308,177,371,226,2,36,373,504,868
LYZ_2_3500,510,510,459,565,1057,173,120,164,1004,212,74,389
LYZL1_2_3501,1046,1196,1157,1371,1133,1888,1976,1283,1238,632,1912,210
6
LYZL2_2_3502,661,470,595,628,122,816,520,673,41,225,575,559
LYZL4_2_3503,390,261,397,529,194,22,120,65,248,286,624,544
MACROD1_2_3504,39,145,190,181,3,0,511,10,531,412,240,208
MAGT1_2_3505,586,590,536,511,377,306,948,182,318,972,1048,459
MAN1A1_2_3506,1071,1057,991,1031,1813,946,483,1045,397,743,1523,1302
MAN1A2_2_3507,35,36,44,17,21,0,19,2,1,19,8,60
MAN1B1_2_3508,86,106,104,151,0,0,61,224,158,113,198,128
MAN1C1_2_3509,425,439,397,431,262,438,24,311,261,79,301,372
MAN2A1_2_3510,809,673,515,609,214,1234,931,271,1086,656,1236,1437
MAN2A2_2_3511,592,1025,648,943,143,414,400,1104,620,1758,1155,932
MAN2B2_2_3512,404,470,421,403,210,352,841,93,219,562,512,825
MAN2C1_2_3513,123,91,99,114,13,2,1461,0,142,79,53,1
MANBA_2_3514,257,112,192,212,349,245,2,393,207,0,250,371
MANEA_2_3515,1263,923,779,1243,1233,2489,1609,567,1133,1763,1347,907
MAOA_2_3516,1337,1391,1354,1871,1329,2607,638,203,1455,1054,1649,2663
MAOB_2_3517,1398,1100,982,1372,841,969,1121,1500,1179,1584,961,1028
MAP1S_2_3518,0,1,27,1,0,52,0,0,0,0,0,0
MAPRE3_2_3519,901,676,680,842,555,718,460,341,471,650,1518,820
MARS2_2_3520,187,83,136,112,94,124,0,0,0,0,71,174
MARS_2_3521,135,212,119,155,445,219,63,420,89,147,23,155
MAT1A_2_3522,338,159,215,337,271,60,206,25,534,446,214,134
MAT2A_2_3523,22,36,140,67,2,136,124,776,23,12,0,0

MBOAT1_2_3524,660,699,504,722,1573,595,847,1060,678,371,903,257
MBOAT2_2_3525,1492,971,1188,1357,1256,1418,1297,1279,1181,1518,1397,10
74
MBOAT4_2_3526,162,46,75,87,1,0,0,0,26,0,48,37
MBTPS1_2_3527,201,353,265,573,0,420,82,1102,340,76,707,326
MBTPS2_2_3528,1888,1600,2009,2183,1443,1346,1239,2156,1482,1843,1160,2
633
MCCC1_2_3529,1440,1151,1494,1455,708,206,723,691,1471,1669,1143,1321
MCCC2_2_3530,8,24,12,49,11,18,39,1,6,0,571,0
MCEE_2_3531,1239,1334,896,1186,1906,1274,827,1022,1386,735,1119,1263
MCM3_2_3532,216,81,140,182,813,2,98,0,288,281,19,75
MCM5_2_3533,128,97,161,121,0,0,369,0,6,3,106,586
MCM6_2_3534,451,329,369,376,692,55,50,673,326,249,229,370
MDH1B_2_3535,31,54,47,30,56,288,2,16,132,2,1,108
MDH2_2_3536,190,201,158,233,2,330,154,254,65,20,76,6
MEP1A_2_3537,358,339,306,237,361,38,721,222,274,34,366,355
MEP1B_2_3538,125,164,67,62,11,121,166,43,124,214,657,44
METAP1_2_3539,0,47,17,11,0,0,0,99,0,0,0,0
METAP1D_2_3540,226,137,196,302,708,568,446,54,136,271,216,315
METAP2_2_3541,107,134,297,108,33,0,501,1091,0,840,589,6
METTL14_2_3542,556,416,320,430,101,354,32,834,106,1104,192,633
METTL22_2_3543,212,177,172,268,168,1,55,182,316,259,14,154
METTL2B_2_3544,1804,1807,1569,2239,1608,985,3205,1463,1732,2489,1927,2
224
METTL3_2_3545,129,81,102,82,168,0,421,80,23,33,2,34
METTL5_2_3546,740,681,556,806,325,332,44,743,678,2366,41,449
METTL6_2_3547,865,738,710,659,808,403,463,410,1037,634,1107,616
METTL7B_2_3548,267,141,170,197,167,75,183,562,345,51,587,333
METTL8_2_3549,1236,992,785,1153,680,854,955,2281,1194,908,877,973
MFN1_2_3550,454,229,334,337,450,10,465,14,304,223,804,287
MGAM_2_3551,98,182,74,56,340,33,46,10,32,138,0,0
MGAT4C_2_3552,376,218,353,407,532,439,477,1514,448,830,76,565
MGAT5_2_3553,94,161,92,66,80,131,171,1,322,558,4,309
MGMT_2_3554,1564,1350,1278,1777,1844,1952,3072,1125,1790,1594,3161,182
5
MGST3_2_3555,1433,1565,1684,1790,809,742,1233,1544,1901,1480,1896,1108
MIOX_2_3556,89,54,2,81,764,0,0,0,192,495,2,0
MIPEP_2_3557,539,335,402,528,485,326,539,238,329,317,49,270
MLYCD_2_3558,185,182,97,123,549,375,268,681,85,46,44,6
MMAB_2_3559,178,219,232,399,93,201,149,68,147,625,158,149
MMEL1_2_3560,306,180,272,422,89,692,15,35,400,43,331,495
MMP10_2_3561,529,597,540,593,439,454,153,892,162,526,129,686
MMP12_2_3562,162,94,103,144,124,83,10,11,138,234,1,62
MMP14_2_3563,244,109,173,224,55,365,43,122,15,52,14,126
MMP3_2_3564,57,79,43,123,150,168,83,0,57,29,289,75
MMP7_2_3565,190,57,126,176,189,359,117,65,42,0,10,8
MMP8_2_3566,980,1030,887,1082,573,1309,840,1304,433,289,1127,689
MMP9_2_3567,160,74,65,114,16,195,101,15,10,0,5,297
MOCOS_2_3568,847,747,749,896,1553,635,1237,1479,525,1330,1270,1194
MOGAT1_2_3569,1929,2183,1494,2296,3661,1703,2133,2739,1466,2283,915,19

MOGAT2_2_3570,289,333,139,308,1,109,554,349,94,6,1,0
MOGAT3_2_3571,1219,1250,1193,1530,2093,1533,1723,1517,1863,1281,1683,1
720
MOXD1_2_3572,568,335,444,318,394,189,192,745,516,222,113,924
MPI_2_3573,360,241,281,285,106,267,162,641,30,564,28,635
MPO_2_3574,716,756,671,820,449,965,65,1848,622,745,297,700
MRPL37_2_3575,2120,2022,2181,2231,1141,2110,2172,2096,2263,2109,2021,2
177
MRPL44_2_3576,678,661,657,796,1197,545,396,1246,225,68,1722,854
MRPS30_2_3577,326,329,328,265,271,306,751,50,315,971,689,144
MSH2_2_3578,1302,891,1068,1045,1104,590,1876,716,897,1016,1965,688
MSH3_2_3579,203,53,152,170,524,414,10,2,1,171,61,421
MSH4_2_3580,237,166,202,244,45,24,101,301,262,25,172,89
MTAP_2_3581,273,388,418,342,468,363,155,0,584,289,860,695
MTFMT_2_3582,1512,1296,1314,1752,1401,1693,2315,758,714,2296,2583,1190
MTHFD1_2_3583,263,518,341,366,84,165,272,745,261,304,367,545
MTHFD2_2_3584,876,1170,847,1013,1136,218,329,688,907,711,1537,1456
MTHFD2L_2_3585,278,162,203,239,384,8,52,293,170,490,511,308
MTHFR_2_3586,45,9,99,19,203,86,100,0,1,0,556,1
MTPAP_2_3587,308,184,224,193,203,246,339,85,85,326,1124,29
MTR_2_3588,36,41,31,84,33,0,51,18,1,0,0,25
MUS81_2_3589,6,18,8,37,7,0,2,0,26,0,0,8
MUT_2_3590,346,241,277,359,325,18,615,354,322,591,70,372
MVD_2_3591,248,266,186,360,730,190,0,321,562,10,0,178
MX2_2_3592,780,673,579,556,485,291,313,238,916,599,393,514
MYH1_2_3593,70,47,128,98,1,34,110,369,83,826,6,29
MYH3_2_3594,96,72,15,94,58,0,14,774,17,1,0,15
MYH6_2_3595,165,187,248,318,276,23,24,0,127,0,162,123
MYH7_2_3596,15,2,16,12,0,0,0,193,9,0,0,27
MYH9_2_3597,102,86,67,121,65,30,259,0,121,71,14,59
MYL7_2_3598,111,62,90,169,13,420,1,52,85,15,9,17
MYO1E_2_3599,414,183,252,401,487,21,305,517,514,63,354,379
MYO5B_2_3600,1757,1237,1379,1438,974,1725,1201,1205,1497,1139,1674,106
1
MYO9A_2_3601,99,69,92,151,34,192,18,6,84,63,1,241
N6AMT2_2_3602,642,994,838,1190,952,1230,617,303,199,436,540,1229
NAA10_2_3603,180,26,66,155,11,258,15,9,6,6,1,391
NAA11_2_3604,115,124,41,31,31,135,34,14,119,118,83,165
NAA15_2_3605,1268,980,1000,1355,1170,863,850,1750,1111,397,1358,1519
NAA30_2_3606,275,174,220,228,748,45,251,5,293,50,306,379
NAA50_2_3607,1328,1131,1201,1294,1023,687,1122,1504,547,494,909,701
NAALAD2_2_3608,101,80,58,114,245,28,12,169,86,181,0,70
NAALADL1_2_3609,77,163,125,82,90,15,46,32,133,275,1,127
NADSYN1_2_3610,1149,933,938,1111,638,760,926,1030,496,1033,363,931
NAGA_2_3611,528,353,391,517,454,87,875,291,837,144,148,400
NAGLU_2_3612,859,374,387,831,468,1256,1643,75,408,172,788,320
NAGPA_2_3613,347,412,272,187,41,595,40,271,885,306,515,14
NAMPT_2_3614,1116,905,900,943,1419,1162,1793,1348,977,1623,763,1225
NANP_2_3615,100,112,89,55,1,24,38,123,192,144,335,119

NANS_2_3616,240,156,214,293,124,28,563,293,229,270,152,419
NAPRT1_2_3617,38,123,48,113,1,0,9,203,1,3,0,24
NARS_2_3618,103,147,110,71,49,1,8,0,38,16,26,0
NCF1_2_3619,94,17,17,24,0,23,134,0,47,201,0,0
NDST1_2_3620,496,621,539,581,671,854,711,1096,310,805,223,615
NDST2_2_3621,1004,1039,850,942,609,476,1132,346,885,1038,1162,1414
NDST3_2_3622,287,309,354,359,111,207,81,228,323,779,38,192
NDST4_2_3623,791,674,690,970,1351,333,360,348,1026,954,762,882
NDUFA10_2_3624,486,265,467,381,90,606,334,796,97,5,1934,41
NDUFA12_2_3625,181,196,184,207,188,114,121,391,284,7,229,129
NDUFA13_2_3626,48,268,207,252,0,1,572,0,150,0,1579,407
NDUFA1_2_3627,83,50,49,68,0,0,27,11,35,27,3,15
NDUFA3_2_3628,2,43,14,26,2,0,0,0,0,2,0,1
NDUFA4_2_3629,149,137,82,100,74,28,472,285,136,189,200,54
NDUFA4L2_2_3630,801,842,847,904,1218,756,1350,682,632,605,645,951
NDUFA5_2_3631,782,891,765,1254,1484,1047,738,1047,523,1096,501,568
NDUFA6_2_3632,504,450,323,524,195,194,375,99,474,495,308,520
NDUFA7_2_3633,114,214,146,37,477,176,0,47,5,0,7,22
NDUFA8_2_3634,160,124,220,199,16,4,372,49,37,107,202,18
NDUFA9_2_3635,61,55,54,61,226,82,399,142,5,193,0,49
NDUFAB1_2_3636,171,251,163,243,133,66,20,34,112,4,2,587
NDUFB10_2_3637,65,32,39,63,90,110,30,0,7,48,238,13
NDUFB1_2_3638,456,437,485,518,723,201,1537,1178,351,470,327,434
NDUFB2_2_3639,293,357,415,631,305,431,884,458,902,102,73,492
NDUFB3_2_3640,184,162,138,187,102,94,6,19,359,180,58,111
NDUFB7_2_3641,0,0,5,0,19,0,0,0,0,43,0,0
NDUFB8_2_3642,155,42,109,45,42,5,209,13,340,32,3,172
NDUFB9_2_3643,582,447,472,591,576,542,608,947,732,795,873,286
NDUFS3_2_3644,423,527,297,439,1081,470,668,709,235,205,36,152
NDUFS4_2_3645,306,318,318,365,389,378,171,609,475,18,91,349
NDUFS6_2_3646,379,275,274,267,558,615,187,139,21,901,0,90
NDUFS7_2_3647,145,41,131,143,2,399,329,11,180,4,349,130
NDUFS8_2_3648,366,306,347,356,229,57,454,1,517,341,6,656
NDUFV2_2_3649,197,255,296,350,458,435,507,191,122,114,293,235
NEDD8_2_3650,104,93,54,100,292,167,76,75,160,17,0,388
NEIL1_2_3651,222,102,83,145,70,2,37,164,12,209,105,16
NEIL3_2_3652,473,237,249,494,243,40,1071,23,571,186,305,501
NEU1_2_3653,16,16,8,40,294,9,0,16,0,47,0,2
NEU2_2_3654,29,63,75,65,431,46,32,8,32,19,239,87
NEU3_2_3655,149,334,161,299,139,477,65,307,333,395,780,52
NHLRC2_2_3656,345,473,464,499,0,87,115,2,431,256,374,263
NIT2_2_3657,113,112,83,170,25,10,84,5,189,152,39,217
NKIRAS1_2_3658,151,183,161,203,8,44,7,212,121,208,298,277
NLGN1_2_3659,2272,2412,1660,2347,2866,2468,2121,1901,2547,1967,2214,22
37
NLGN2_2_3660,254,220,269,540,108,50,214,67,114,29,188,143
NLN_2_3661,448,316,465,582,293,668,381,107,173,650,15,490
NME1-NME2_2_3662,284,390,307,449,1224,370,410,554,678,203,118,328
NMNAT1_2_3663,384,414,489,552,227,124,865,273,105,279,261,580
NMT1_2_3664,22,28,83,130,7,15,356,112,75,0,97,384

NMT2_2_3665,661,846,834,835,608,880,1268,139,656,1139,613,519
NNMT_2_3666,539,554,372,455,655,1013,108,636,395,11,970,363
NOP58_2_3667,6,15,16,53,0,2,81,327,0,8,0,0
NOX3_2_3668,723,804,517,859,971,933,823,1633,439,783,628,1056
NQO2_2_3669,686,612,428,685,533,487,485,140,786,227,828,511
NRAS_2_3670,18,28,20,45,0,39,36,0,0,0,0,22
NSF_2_3671,305,411,232,309,455,754,787,243,148,274,105,417
NSUN6_2_3672,767,940,471,795,378,254,261,546,532,422,697,412
NTAN1_2_3673,414,497,403,656,1032,811,1086,431,573,3,741,665
NTHL1_2_3674,181,222,71,191,11,478,408,11,37,52,0,606
NTPCR_2_3675,640,577,537,459,624,67,597,248,743,457,445,438
NUDT10_2_3676,96,57,153,165,224,27,11,0,40,39,98,4
NUDT11_2_3677,15,85,38,67,4,332,80,18,185,0,0,148
NUDT12_2_3678,137,87,117,297,381,34,52,51,6,44,793,176
NUDT14_2_3679,976,636,785,770,297,519,707,422,2185,512,867,829
NUDT3_2_3680,860,578,921,922,725,599,983,520,744,1212,505,910
NUDT5_2_3681,199,169,195,174,696,409,446,0,97,509,315,17
NUDT7_2_3682,520,444,328,331,454,1864,124,327,553,882,145,357
NXNL1_2_3683,136,110,83,111,21,32,49,76,197,550,86,135
OAS3_2_3684,209,224,99,188,248,538,1,1,185,46,207,44
OAZ1_2_3685,764,937,714,605,319,853,1639,1831,421,1345,1117,583
OC90_2_3686,633,600,506,540,1384,412,918,227,658,303,210,1261
ODF3B_2_3687,0,3,1,24,0,0,0,0,1,0,0,123
OPLAH_2_3688,127,132,139,192,104,35,816,8,66,292,6,104
OSGEP_2_3689,303,267,314,245,605,464,35,98,131,339,87,239
OSGEPL1_2_3690,752,630,532,609,837,370,382,588,597,304,374,351
OSTC_2_3691,116,71,113,151,4,394,35,17,71,162,24,222
OTC_2_3692,1148,1205,1081,1255,1798,845,4116,967,1085,1339,540,1019
OVGP1_2_3693,1489,1110,1071,1229,588,322,450,2008,1907,1250,471,1753
OXA1L_2_3694,760,815,952,1052,1931,273,719,615,1304,1130,506,1050
OXCT1_2_3695,496,286,181,239,246,56,1042,8,401,201,2,339
OXCT2_2_3696,9,17,34,62,0,19,0,0,43,39,0,1
P4HA3_2_3697,324,302,295,226,263,152,422,466,283,353,17,649
P4HB_2_3698,22,92,122,35,70,323,255,11,66,21,0,1
PADI1_2_3699,429,406,577,648,249,588,288,159,331,671,192,440
PADI2_2_3700,63,36,46,33,0,2,38,3,0,0,3,36
PADI3_2_3701,265,93,263,291,626,5,23,35,747,26,140,336
PADI6_2_3702,425,516,464,732,1370,644,518,541,105,552,369,395
PAFAH2_2_3703,527,484,449,527,369,182,746,319,710,980,57,308
PAH_2_3704,240,260,212,301,5,331,0,3,420,198,19,256
PAPOLA_2_3705,387,251,273,530,595,44,13,345,582,152,460,88
PAPOLB_2_3706,511,276,295,472,429,250,550,1704,12,344,53,248
PAPOLG_2_3707,286,193,232,247,151,100,166,52,573,270,201,407
PAPPA_2_3708,703,642,574,700,930,1364,905,940,874,310,199,808
PAPSS1_2_3709,574,564,281,274,690,744,375,615,357,1560,222,352
PARG_2_3710,113,41,81,59,67,301,0,1,35,15,71,66
PARP1_2_3711,832,923,848,1032,1024,966,280,1093,427,564,746,745
PARP4_2_3712,174,172,144,346,120,460,1401,758,134,341,0,110
PARP6_2_3713,629,742,707,895,342,304,1093,312,918,1260,774,792
PARS2_2_3714,325,341,258,487,136,290,255,306,514,435,248,446

PCBD1_2_3715,970,812,794,1303,1015,368,1278,700,925,302,2049,622
PCBD2_2_3716,95,28,87,114,79,0,0,0,79,0,32,78
PCMT1_2_3717,301,291,443,386,252,18,425,428,403,339,226,332
PCMTD1_2_3718,1187,668,988,1166,1114,889,733,699,1310,1472,639,1244
PCYOX1_2_3719,979,1112,1071,1265,582,651,138,1070,370,714,1403,1236
PCYT1A_2_3720,108,138,227,423,203,0,562,0,559,475,0,5
PDCD1LG2_2_3721,119,126,91,124,33,59,74,127,49,12,1,16
PDCL_2_3722,254,185,366,359,329,30,4,676,369,135,464,198
PDE3A_2_3723,55,22,43,48,24,15,0,20,141,25,3,1
PDE3B_2_3724,0,0,0,0,0,0,0,0,0,0,0,0
PDE6A_2_3725,330,406,378,521,1544,157,641,631,530,849,826,457
PDE6C_2_3726,83,166,130,86,267,197,896,0,12,2,244,22
PDE6D_2_3727,515,298,385,550,954,430,1071,878,290,384,212,813
PDE6G_2_3728,253,213,292,332,335,249,7,7,212,545,64,384
PDE6H_2_3729,233,286,260,396,619,476,227,229,213,335,284,221
PDE7B_2_3730,392,241,247,362,204,235,184,261,231,16,29,128
PDHA2_2_3731,62,15,16,30,2,34,104,64,29,45,0,327
PDIA2_2_3732,1266,1190,1010,1171,1694,787,2129,1187,470,1053,830,1595
PDIA3_2_3733,346,354,345,441,52,433,301,578,203,266,17,38
PDIA4_2_3734,209,291,369,424,671,195,237,4,104,278,306,403
PDIA5_2_3735,281,301,318,218,341,38,209,179,558,362,349,151
PDIA6_2_3736,675,604,519,661,1156,308,87,810,1028,376,674,525
PDPR_2_3737,835,673,767,830,643,807,1249,316,674,418,1893,538
PDSS1_2_3738,309,164,342,340,214,75,1503,128,638,133,19,513
PDSS2_2_3739,858,738,812,738,1151,580,658,942,452,322,791,592
PDXP_2_3740,377,248,219,220,489,47,643,150,423,179,384,580
PECR_2_3741,355,223,295,420,146,94,11,219,0,630,200,561
PELI1_2_3742,149,102,168,78,69,15,111,81,65,40,194,0
PET117_2_3743,485,514,414,467,586,498,133,817,611,448,0,330
PEX1_2_3744,269,323,388,375,306,368,298,50,189,54,594,275
PEX6_2_3745,144,81,71,132,137,25,43,24,86,0,0,325
PFAS_2_3746,427,401,306,295,602,0,0,320,722,29,624,406
PGA3_2_3747,390,214,375,320,259,91,235,79,338,131,386,329
PGA4_2_3748,390,214,375,320,259,91,235,79,338,131,386,329
PGA5_2_3749,390,214,375,320,259,91,235,79,338,131,386,329
PGAM4_2_3750,38,29,11,41,0,0,1,0,0,126,0,8
PGAP1_2_3751,599,554,681,784,275,243,758,854,720,751,643,536
PGAP3_2_3752,37,36,42,72,3,0,0,6,28,10,28,216
PGD_2_3753,130,275,168,371,6,38,150,644,28,188,961,47
PGGT1B_2_3754,431,325,237,502,452,422,63,569,411,384,355,399
PGLS_2_3755,365,298,578,568,0,40,734,29,220,450,710,965
PGM2_2_3756,214,86,141,344,107,58,744,30,53,50,290,332
PGM2L1_2_3757,1380,1160,1285,1384,2078,1194,2070,1809,809,1865,1370,11
60
PGM5_2_3758,1514,1004,1258,1529,1224,1377,2551,453,945,911,643,2010
PGPEP1_2_3759,80,67,87,151,107,3,16,29,71,36,17,85
PGS1_2_3760,400,332,307,370,384,333,309,177,118,205,553,750
PHGDH_2_3761,145,157,83,160,125,0,72,555,225,600,429,279
PHLPP1_2_3762,204,77,137,140,29,192,149,61,70,31,154,121
PHLPP2_2_3763,412,406,317,467,94,0,187,324,357,0,715,275

PIF1_2_3764,98,61,128,165,125,0,67,0,77,114,201,57
PIGB_2_3765,293,161,379,343,306,43,14,14,377,24,21,538
PIGH_2_3766,727,600,844,577,95,228,406,1282,755,1104,364,275
PIGL_2_3767,724,622,825,551,719,657,353,583,315,416,186,1207
PIGM_2_3768,1299,1279,1112,1315,830,833,2222,1219,1491,1539,1378,1807
PIGS_2_3769,116,176,197,162,205,16,20,301,170,273,278,184
PIGU_2_3770,497,491,387,547,12,475,281,581,187,748,439,420
PIGW_2_3771,809,943,761,1006,979,837,1247,411,1058,1368,730,1110
PIGZ_2_3772,39,9,37,39,1,0,251,0,1,0,0,70
PIN1_2_3773,266,387,539,479,780,3,2,4,201,317,102,224
PIPOX_2_3774,182,171,79,111,142,329,0,25,4,74,49,314
PITPNM2_2_3775,50,42,57,65,0,29,34,0,123,18,22,6
PLA2G10_2_3776,36,74,3,41,138,126,0,171,1,0,0,11
PLA2G12A_2_3777,541,627,439,525,1173,324,610,825,548,579,1,248
PLA2G12B_2_3778,493,294,323,424,977,420,680,237,616,106,906,223
PLA2G15_2_3779,117,41,86,75,0,397,14,403,289,1,186,8
PLA2G1B_2_3780,260,372,254,310,606,131,460,90,344,657,246,352
PLA2G2C_2_3781,575,544,594,434,1077,550,599,630,781,702,1395,783
PLA2G2D_2_3782,20,74,52,115,36,4,1,5,74,31,0,14
PLA2G2E_2_3783,122,63,89,35,57,0,31,29,43,40,0,2
PLA2G2F_2_3784,912,1204,627,1074,316,337,1772,1073,327,343,897,370
PLA2G3_2_3785,266,226,210,147,125,475,110,27,198,144,0,164
PLA2G4A_2_3786,486,458,308,355,48,461,20,385,320,129,175,340
PLA2G4B_2_3787,87,88,91,74,27,68,40,174,14,126,586,168
PLA2G4D_2_3788,381,95,106,208,113,17,78,216,71,29,34,4
PLA2G4E_2_3789,305,263,245,266,606,47,365,232,352,69,328,455
PLA2G4F_2_3790,319,148,197,284,262,1814,375,117,8,0,593,87
PLA2G5_2_3791,312,418,412,463,787,244,192,1001,239,423,302,316
PLCB2_2_3792,17,112,34,139,2,0,289,0,1,118,0,115
PLCD3_2_3793,125,250,82,141,71,332,186,32,140,24,1,184
PLCD4_2_3794,486,561,320,420,0,32,70,31,416,184,299,573
PLCG2_2_3795,388,425,239,465,877,933,440,93,98,78,10,661
PLCH2_2_3796,59,96,45,150,55,382,0,0,65,27,194,53
PLCL1_2_3797,456,585,296,340,662,258,1427,667,493,501,270,746
PLCXD1_2_3798,73,26,42,68,77,0,1,36,62,100,100,157
PLCZ1_2_3799,135,218,101,155,41,268,448,23,66,15,190,61
PLD4_2_3800,107,75,65,46,128,13,64,3,98,11,516,0
PLOD1_2_3801,200,178,254,206,48,390,235,311,234,141,408,251
PLOD3_2_3802,38,9,18,38,0,1,223,0,0,10,0,0
PLSCR1_2_3803,1535,1263,1202,1663,717,534,1638,741,1333,851,2835,1546
PMM1_2_3804,42,60,34,163,182,263,179,3,148,4,0,127
PMM2_2_3805,226,360,363,320,60,99,2,270,512,188,593,232
PMPCA_2_3806,203,225,135,278,122,76,490,134,628,95,91,304
PMPCB_2_3807,218,178,163,190,395,120,152,472,463,241,102,154
PNLIP_2_3808,1602,1370,1455,1796,1269,1837,1793,1860,1218,1166,2174,87
2
PNLIPRP1_2_3809,495,438,498,595,800,399,247,139,290,326,69,755
PNLIPRP2_2_3810,1014,969,636,730,1247,424,661,1484,1330,763,726,324
PNLIPRP3_2_3811,891,718,883,1060,505,546,587,46,1236,1002,2621,315
PNMT_2_3812,0,30,2,29,57,22,0,366,0,1,405,4

PNPLA2_2_3813,19,41,24,40,2,0,0,51,249,0,0,75
PNPLA3_2_3814,44,28,76,53,63,96,161,35,30,1,375,18
PNPLA8_2_3815,1261,679,852,832,462,1070,1292,291,195,774,740,558
PNPO_2_3816,0,0,40,20,0,0,0,0,0,0,0,0
PNPT1_2_3817,173,93,92,141,286,143,4,5,13,287,14,32
POLA1_2_3818,488,444,276,591,85,258,0,1,240,556,369,242
POLA2_2_3819,201,278,198,302,147,74,273,188,274,403,289,872
POLB_2_3820,145,213,155,120,101,357,364,20,378,94,28,138
POLD4_2_3821,15,18,17,0,0,0,39,0,20,45,0,0
POLE3_2_3822,3,15,4,40,3,1,0,0,24,0,0,0
POLE4_2_3823,329,529,208,195,1120,0,188,258,135,158,299,353
POLE_2_3824,227,203,225,172,349,404,3,125,154,124,221,318
POLG2_2_3825,2792,2065,2243,2419,1707,910,3013,2255,2084,2177,1807,258
5
POLI_2_3826,438,436,321,656,596,350,182,189,268,655,560,565
POLM_2_3827,42,23,49,100,0,0,0,3,5,149,27,1
POLN_2_3828,257,566,407,302,960,151,73,223,194,88,565,105
POLR1A_2_3829,751,736,646,880,680,677,1873,527,1124,585,400,975
POLR1C_2_3830,77,15,51,75,0,0,0,0,0,226,0,0
POLR1E_2_3831,316,124,339,324,212,379,106,100,128,29,323,224
POLR2A_2_3832,478,893,753,964,190,5,2349,729,316,344,790,1483
POLR2B_2_3833,182,195,176,258,331,42,358,785,131,327,173,434
POLR2C_2_3834,61,23,80,60,0,0,29,209,1,630,2,0
POLR2D_2_3835,207,111,100,105,197,343,94,61,339,35,24,88
POLR2E_2_3836,219,179,179,213,239,60,201,411,232,202,337,520
POLR2F_2_3837,225,226,283,261,317,189,193,245,317,353,102,215
POLR2G_2_3838,1084,956,876,922,462,1976,501,421,733,1222,449,518
POLR2H_2_3839,283,312,198,250,0,110,1071,1,4,1234,438,300
POLR2J_2_3840,407,576,339,329,560,409,131,450,30,19,826,461
POLR2K_2_3841,362,268,295,325,444,77,646,53,326,181,104,637
POLR2L_2_3842,216,98,205,217,157,233,549,104,667,208,11,163
POLR3A_2_3843,205,202,121,179,793,2,560,11,250,5,1,129
POLR3C_2_3844,239,153,157,279,314,23,1,677,46,99,68,378
POLR3F_2_3845,253,122,165,200,76,191,84,375,59,161,75,192
POLR3G_2_3846,74,60,69,112,109,4,144,118,43,49,392,68
POLRMT_2_3847,108,151,75,137,120,176,111,209,7,119,0,160
POMGNT1_2_3848,151,103,228,188,117,88,356,33,91,15,290,292
POMT2_2_3849,35,50,33,15,154,82,4,0,61,0,104,83
PON3_2_3850,1206,1015,996,976,811,414,998,709,2037,517,1186,573
POP4_2_3851,46,168,167,129,244,2,80,510,1,552,121,361
POP7_2_3852,534,468,541,500,216,807,782,359,615,661,341,374
POR_2_3853,21,56,20,4,0,0,0,27,0,3,0,0
PPA1_2_3854,242,167,145,128,172,144,271,169,182,9,154,6
PPCDC_2_3855,56,16,75,129,2,63,0,8,26,43,406,403
PPIA_2_3856,178,275,215,206,211,141,211,31,91,254,496,73
PPIAL4A_2_3857,705,601,537,604,269,313,497,459,719,494,258,661
PPIAL4B_2_3858,705,601,537,604,269,313,497,459,719,494,258,661
PPIAL4C_2_3859,705,601,537,604,269,313,497,459,719,494,258,661
PPIAL4E_2_3860,705,601,537,604,269,313,497,459,719,494,258,661
PPIAL4G_2_3861,7093,6616,6817,7219,4672,6436,8386,7382,8209,6322,8501,

5857
PPIB_2_3862,826,1036,712,913,986,866,1045,951,625,1305,464,606
PPIC_2_3863,137,308,124,234,0,395,25,43,215,322,0,649
PPID_2_3864,109,194,103,162,296,372,3,19,257,332,520,133
PPIF_2_3865,532,508,335,489,536,53,93,407,789,166,13,784
PPIG_2_3866,2308,1655,2418,2041,2450,1700,2289,1828,3073,1966,2057,208
3
PPIH_2_3867,214,213,220,223,268,183,218,338,179,268,278,57
PPIL1_2_3868,1143,993,1001,1037,872,199,2417,1343,123,589,730,1087
PPIL4_2_3869,516,413,453,762,1352,726,206,1072,202,180,837,1423
PPIP5K2_2_3870,138,210,139,98,0,476,0,117,28,254,8,0
PPM1H_2_3871,1210,1201,1428,1464,1344,1982,2751,2200,1601,943,1016,172
0
PPM1J_2_3872,335,215,281,217,18,35,26,84,239,340,412,78
PPM1N_2_3873,144,26,106,171,7,221,235,1,209,0,0,20
PPME1_2_3874,121,152,178,123,242,176,172,212,85,3,709,52
PPWD1_2_3875,434,347,389,579,512,243,371,67,158,383,368,557
PRDX4_2_3876,34,44,36,39,0,0,0,0,245,0,96,0
PREP_2_3877,4,18,32,21,0,116,0,6,0,0,26,222
PRHOXNB_2_3878,1755,1524,1484,1925,2262,615,2297,775,2037,1341,1837,19
09
PRIM1_2_3879,131,112,120,178,196,524,226,312,44,83,0,192
PRIM2_2_3880,982,1093,1063,1255,1260,864,831,659,649,636,1503,1634
PROC_2_3881,300,295,207,252,623,152,606,238,280,97,270,514
PROSC_2_3882,33,16,48,44,0,56,0,55,13,0,0,5
PRR14L_2_3883,292,422,331,476,395,610,847,205,369,244,589,424
PRSS1_2_3884,101,146,164,230,4,316,145,190,61,175,79,7
PRSS2_2_3885,0,0,0,0,0,0,0,0,0,0,0,0
PRTFDC1_2_3886,223,437,242,204,78,202,99,375,147,613,253,615
PRTN3_2_3887,192,97,163,124,2,0,5,351,3,2,505,0
PRUNE_2_3888,97,119,156,149,19,53,24,35,19,124,0,38
PSMA2_2_3889,135,232,166,221,58,4,65,6,1,201,27,129
PSMA6_2_3890,894,577,841,799,489,694,1266,683,1078,787,357,822
PSMA7_2_3891,849,1017,831,953,1153,480,550,2030,663,916,709,568
PSMB10_2_3892,123,35,199,131,580,0,273,448,1,649,545,7
PSMB11_2_3893,20,50,14,32,12,50,0,0,0,2,525,3
PSMB1_2_3894,2457,2063,2251,2755,2463,679,1952,1570,1778,2087,5071,291
6
PSMB3_2_3895,1,19,41,80,4,0,0,6,9,2,273,0
PSMB4_2_3896,111,121,197,241,80,189,905,326,107,47,638,165
PSMB6_2_3897,71,63,109,114,154,483,402,4,11,97,50,5
PSMB7_2_3898,250,191,166,283,362,250,464,219,247,269,1002,221
PSMB9_2_3899,48,14,11,12,136,0,0,88,0,39,0,15
PSMD6_2_3900,388,284,347,492,170,56,402,562,154,136,616,528
PTDSS1_2_3901,277,238,397,359,723,437,21,294,487,78,466,246
PTDSS2_2_3902,48,33,55,44,5,45,152,99,2,2,3,119
PTGDS_2_3903,17,11,23,33,2,48,7,0,100,5,0,0
PTGES2_2_3904,128,124,117,108,6,66,84,234,19,32,0,2
PTGES3_2_3905,1212,1109,1025,1342,1118,1182,1248,1000,1264,1522,1393,1
779

PTGES_2_3906,654,642,884,951,250,892,2413,462,914,803,1280,961
PTGIS_2_3907,0,0,0,0,0,0,0,0,0,0,0,0
PTGS2_2_3908,271,172,259,323,0,531,242,43,282,126,840,3
PTRH1_2_3909,69,48,170,149,0,576,473,0,0,0,157,239
PTRH2_2_3910,119,60,26,41,356,52,0,6,45,0,29,14
PTS_2_3911,114,100,57,73,7,271,0,728,1,175,286,2
PUS3_2_3912,1246,678,648,797,1452,60,1075,234,707,1082,1040,1073
PUSL1_2_3913,48,155,85,60,0,3,410,6,0,16,14,505
PXDN_2_3914,280,377,464,553,322,1401,550,1257,378,0,435,344
PXDNL_2_3915,143,105,82,104,224,63,0,14,1,24,0,34
PYCR2_2_3916,344,167,176,234,305,363,191,893,174,401,129,160
PYCRL_2_3917,112,93,60,117,222,52,12,34,274,151,172,188
PYGB_2_3918,275,199,190,508,53,0,378,638,413,99,3,418
QDPR_2_3919,1047,894,595,1245,1850,886,1447,367,2665,1516,273,636
QPCT_2_3920,246,120,115,197,131,287,19,103,157,5,192,151
QPRT_2_3921,56,107,51,118,13,5,0,1,134,0,0,45
QRSL1_2_3922,218,321,251,430,1065,35,96,272,53,15,248,113
QSOX2_2_3923,1618,1842,1621,1955,1035,728,2513,3197,1821,2117,1315,151
9
QTRT1_2_3924,0,0,0,0,0,0,0,0,0,0,0,0
QTRTD1_2_3925,179,147,206,177,314,46,55,281,182,188,9,108
RAB10_2_3926,49,73,68,60,26,44,605,3,107,55,0,1
RAB12_2_3927,229,308,156,289,203,224,1342,57,452,448,753,105
RAB13_2_3928,154,78,143,191,247,128,184,49,77,515,118,70
RAB14_2_3929,753,812,633,806,650,517,1173,1489,1242,538,988,773
RAB15_2_3930,285,175,301,378,135,38,376,6,77,0,98,6
RAB17_2_3931,827,683,625,778,553,401,756,787,547,939,273,802
RAB18_2_3932,745,708,572,683,1263,100,591,937,511,410,678,788
RAB19_2_3933,483,247,299,409,636,190,729,693,24,261,26,322
RAB1B_2_3934,131,120,132,116,233,184,75,119,224,207,152,95
RAB20_2_3935,98,56,79,106,57,16,10,0,112,26,0,135
RAB21_2_3936,112,136,124,200,8,522,28,49,42,21,43,182
RAB22A_2_3937,212,294,296,328,221,133,150,8,281,48,369,142
RAB25_2_3938,872,991,728,918,184,134,1107,526,1636,444,1357,625
RAB26_2_3939,165,111,138,173,380,6,242,85,136,0,16,2
RAB27B_2_3940,116,54,54,56,374,69,83,76,150,12,122,20
RAB30_2_3941,528,458,514,724,245,1033,796,852,684,250,643,508
RAB31_2_3942,328,357,208,321,217,342,162,356,99,804,148,551
RAB33A_2_3943,169,189,173,285,163,128,0,338,513,7,456,0
RAB33B_2_3944,442,401,340,462,915,25,116,1278,325,108,234,661
RAB36_2_3945,649,563,342,577,1285,38,231,209,485,383,263,71
RAB38_2_3946,267,52,193,164,21,280,126,27,190,305,18,817
RAB39B_2_3947,1,41,44,35,5,2,0,114,2,43,170,10
RAB3A_2_3948,171,227,259,149,299,0,523,0,326,0,0,412
RAB3B_2_3949,210,124,151,248,411,10,205,83,1,382,360,491
RAB3C_2_3950,143,214,53,269,59,0,25,15,1,4,0,205
RAB3D_2_3951,69,51,38,15,90,2,0,144,13,108,404,4
RAB3GAP2_2_3952,207,240,164,201,210,275,20,611,835,63,302,123
RAB4A_2_3953,9,23,12,4,95,0,0,0,0,1,10,0
RAB4B_2_3954,61,9,24,31,2,64,7,11,302,29,22,3

RAB5A_2_3955,117,160,135,160,164,7,145,23,30,48,325,224
RAB5B_2_3956,1693,1552,1310,1680,1201,1687,2496,647,1737,1355,2938,137
5
RAB6B_2_3957,415,315,227,331,364,44,26,311,142,454,649,812
RAB6C_2_3958,480,714,409,560,275,949,781,1174,260,756,466,689
RAB7A_2_3959,27,39,52,46,18,1,0,0,0,20,194,0
RAB8B_2_3960,57,128,111,114,488,55,0,24,59,1,24,17
RAB9B_2_3961,106,57,26,30,1,0,226,0,72,77,0,12
RABGGTB_2_3962,652,421,479,548,293,285,215,317,785,456,408,329
RABL3_2_3963,551,405,403,441,729,180,350,180,155,974,39,154
RAC2_2_3964,2,0,18,8,0,0,0,39,0,0,0,0
RAD50_2_3965,105,175,146,83,34,133,44,91,65,242,34,95
RAD54L2_2_3966,121,153,123,209,86,187,81,128,152,222,59,294
RALA_2_3967,370,338,365,458,991,354,952,250,562,62,795,230
RALB_2_3968,1107,766,871,1169,1620,650,2111,1215,1270,1509,996,1198
RALBP1_2_3969,183,201,164,165,35,0,3,14,69,12,35,56
RANBP2_2_3970,1741,1095,1155,1339,1760,1237,1252,1635,1049,1214,1203,1
216
RAN_2_3971,238,302,162,148,4,487,0,28,74,496,572,339
RAP2A_2_3972,342,157,403,478,428,632,273,873,432,444,208,288
RAP2B_2_3973,3,0,39,68,377,0,4,0,1,0,0,3
RAP2C_2_3974,1482,1292,875,1134,1927,1251,1377,997,1492,1118,1474,1133
RARS2_2_3975,654,655,776,694,2672,755,661,416,445,1107,834,648
RARS_2_3976,4794,4060,3913,4354,3850,3451,4190,3509,4912,4883,4178,634
1
RASD2_2_3977,132,155,129,70,337,0,896,0,255,125,2,0
RASL10B_2_3978,48,58,33,64,0,43,0,154,0,0,46,162
RASL11B_2_3979,101,90,110,76,321,18,330,180,54,58,64,194
RASL12_2_3980,126,25,114,111,272,0,0,350,0,33,0,0
RC3H1_2_3981,88,283,75,112,0,276,0,0,25,249,0,0
RCL1_2_3982,283,252,236,424,389,230,52,363,603,88,33,114
RDH10_2_3983,104,124,159,199,82,520,0,434,120,219,412,226
RDH11_2_3984,442,628,562,608,538,446,1066,1067,456,180,544,920
RDH12_2_3985,42,68,112,92,42,0,205,0,15,1,0,10
RDH14_2_3986,743,824,546,704,516,816,662,612,509,625,501,900
RDH16_2_3987,36,22,36,83,2,57,0,80,0,85,8,32
RDH8_2_3988,55,71,132,60,5,63,12,7,41,155,0,34
RECQL4_2_3989,308,493,392,456,279,302,1213,315,398,1262,287,593
REM1_2_3990,23,56,34,51,4,46,0,0,4,0,91,1
REM2_2_3991,217,163,113,119,51,464,44,33,56,114,3,82
RENBP_2_3992,26,32,18,27,312,13,0,21,0,2,0,3
RETSAT_2_3993,244,187,305,247,709,336,135,136,148,103,200,434
REV3L_2_3994,53,26,61,48,1,4,235,0,138,1,95,5
REXO1L1_2_3995,8,34,8,9,12,0,2,225,0,226,0,3
REXO2_2_3996,211,116,108,222,0,127,342,18,683,38,661,180
RFNG_2_3997,50,6,53,0,3,0,0,0,19,3,12,17
RFX6_2_3998,496,559,578,775,930,264,362,892,459,919,885,182
RGS7_2_3999,32,53,65,52,8,80,50,0,0,6,0,15
RHBDL1_2_4000,234,213,247,276,11,174,222,446,305,528,177,375
RHBDL2_2_4001,525,594,625,933,316,686,433,1491,742,385,861,1138

RHBDL3_2_4002,329,262,336,458,0,314,21,378,92,423,1462,127
RHEBL1_2_4003,707,426,467,535,506,19,1059,208,437,306,1268,652
RHOA_2_4004,264,231,368,280,236,18,263,417,187,56,465,177
RHOB_2_4005,139,64,155,65,2,26,313,592,4,153,307,41
RHOD_2_4006,6,8,8,107,0,0,0,0,74,0,0,0
RHOF_2_4007,101,94,76,107,5,18,86,103,133,341,74,53
RHOG_2_4008,776,562,434,614,971,585,839,19,137,395,540,585
RHOH_2_4009,937,1123,709,958,625,1313,1044,1283,897,1709,946,892
RHOJ_2_4010,106,204,99,116,184,70,122,349,37,86,63,35
RHOQ_2_4011,230,291,106,379,335,190,159,565,33,279,211,560
RHOT2_2_4012,582,617,602,560,433,1172,155,185,873,1246,561,943
RHOU_2_4013,181,136,115,128,0,210,3,149,7,533,4,2
RHOV_2_4014,734,506,728,815,944,192,763,887,144,499,709,798
RIT1_2_4015,368,290,314,453,261,713,347,296,314,298,194,496
RIT2_2_4016,304,401,283,418,647,192,90,784,116,252,632,304
RNASE2_2_4017,20,71,46,6,102,16,0,0,22,50,0,0
RNASE3_2_4018,902,771,663,853,1213,507,2275,913,442,85,730,755
RNASE6_2_4019,233,189,175,311,179,342,853,325,528,361,27,191
RNASE7_2_4020,273,203,209,224,118,98,37,11,252,19,374,369
RNASE8_2_4021,19,49,20,25,38,7,22,0,0,0,130,142
RNASEH2A_2_4022,39,75,40,70,0,145,9,17,211,53,2,337
RNASET2_2_4023,225,220,321,202,19,86,414,726,101,25,758,257
RND1_2_4024,95,15,81,87,20,2,445,306,473,1,0,45
RND2_2_4025,281,216,364,383,1116,434,11,396,515,207,66,652
RND3_2_4026,242,233,279,321,271,18,351,282,81,260,416,294
RNF148_2_4027,735,678,752,794,592,1180,1265,920,927,688,495,930
RNMT_2_4028,4,35,7,10,0,101,0,0,0,113,0,0
RNMTL1_2_4029,1022,1099,766,897,1107,600,834,1,425,321,1645,1402
RNPEP_2_4030,211,348,254,190,333,19,73,30,367,286,12,193
RPAP2_2_4031,1862,1619,1639,2027,2023,1018,1138,1826,1227,2222,2424,21
87
RPE65_2_4032,698,524,451,440,813,730,475,334,734,624,164,658
RPIA_2_4033,181,204,126,413,185,219,509,287,57,476,510,415
RPL4_2_4034,1455,1766,1270,1568,1452,1470,1698,697,570,1002,2084,2393
RPN1_2_4035,0,0,0,0,0,0,0,0,0,0,0,0
RPP25_2_4036,305,467,428,466,16,662,688,484,466,6,682,533
RPP40_2_4037,890,705,617,884,1662,757,827,491,690,1215,1312,832
RPS3_2_4038,429,434,427,433,125,632,817,820,202,338,778,572
RPUSD1_2_4039,47,84,31,29,11,653,27,6,136,0,0,4
RPUSD2_2_4040,186,245,199,361,554,116,372,8,256,378,158,460
RRAGA_2_4041,16,118,50,182,0,0,0,5,113,14,370,70
RRAGC_2_4042,757,623,575,687,1830,351,876,269,768,975,735,500
RRAGD_2_4043,160,225,186,212,83,91,327,166,265,83,267,364
RRAS_2_4044,639,565,686,596,759,562,554,236,463,536,406,963
RRM1_2_4045,111,72,118,148,0,2,2,77,66,4,0,4
RRP8_2_4046,1272,1256,1029,1399,1174,1664,1443,1143,1480,1092,2123,100
6
RSAD2_2_4047,487,578,439,439,18,27,3,0,153,179,746,23
RTN4IP1_2_4048,103,89,145,133,0,3,50,83,248,71,30,18
SAMHD1_2_4049,1027,1038,1095,1198,268,783,198,439,1248,1036,1500,1275

SDF2_2_4050,196,159,229,164,394,248,216,30,218,149,24,100
SDHA_2_4051,266,132,160,279,256,132,866,1172,407,9,404,152
SDHB_2_4052,290,384,210,271,436,88,279,217,114,477,9,178
SDHD_2_4053,356,134,212,315,63,614,200,103,16,96,598,459
SDR16C5_2_4054,182,242,224,251,8,126,637,143,154,89,743,140
SDR42E1_2_4055,112,92,78,140,89,56,0,493,638,470,28,13
SDR9C7_2_4056,697,538,589,592,486,328,514,216,475,448,161,454
SDSL_2_4057,76,55,19,21,203,1,0,98,0,11,15,20
SEPSECS_2_4058,207,119,181,239,5,100,762,233,183,164,70,28
1-Sep_2_4059,52,47,27,23,0,0,71,0,107,0,1,3
SEPW1_2_4060,330,272,204,291,208,208,210,261,305,222,188,310
SERHL2_2_4061,565,488,394,363,594,854,29,385,792,234,228,294
SETX_2_4062,93,63,97,43,0,13,120,149,66,0,354,120
SGMS1_2_4063,121,159,93,76,359,11,12,0,15,5,0,474
SGPL1_2_4064,227,173,131,189,285,20,5,185,82,277,0,283
SGSH_2_4065,34,86,107,29,259,5,0,0,298,367,2,214
SH3GL2_2_4066,135,51,96,107,297,0,1,175,188,142,3,19
SKIV2L2_2_4067,45,76,76,63,167,79,59,27,33,111,12,95
SLFN12_2_4068,152,152,112,108,5,89,4,30,407,261,34,0
SLFN12L_2_4069,801,674,646,704,1198,589,530,259,225,282,601,1408
SLFN13_2_4070,1458,1469,1066,1728,1791,1050,806,966,1064,979,1383,1338
SLFN5_2_4071,793,865,521,984,75,546,1506,1255,451,992,289,411
SLU7_2_4072,1102,921,977,997,852,319,1076,471,756,1899,776,685
SMG8_2_4073,1663,1198,1226,1447,1650,1965,1656,977,2332,1222,1317,2769
SMPD2_2_4074,189,361,394,306,15,225,1037,220,156,97,1383,201
SMPD3_2_4075,24,16,30,54,18,22,0,0,0,0,0,7
SMPDL3A_2_4076,1083,1065,905,985,1273,707,1290,561,1307,2464,547,357
SMUG1_2_4077,16,6,7,15,0,0,1,47,59,0,0,0
SNF8_2_4078,25,11,1,18,0,5,0,0,0,0,0,0
SNRNP200_2_4079,405,345,252,404,256,246,367,16,150,0,213,353
SOAT1_2_4080,2521,2517,2128,3092,1047,1649,3815,2605,1655,2950,2166,41
99
SOAT2_2_4081,184,311,209,287,316,234,32,237,139,421,164,517
SOD1_2_4082,164,143,172,188,87,43,246,48,34,111,79,421
SOD3_2_4083,150,145,121,96,4,0,252,2,26,231,6,11
SORD_2_4084,97,76,77,136,33,57,61,149,175,28,6,460
SPACA3_2_4085,0,0,4,12,0,0,0,0,0,0,0,0
SPACA5B_2_4086,455,478,542,770,67,18,732,342,550,335,755,1152
SPACA5_2_4087,455,478,542,770,67,18,732,342,550,335,755,1152
SPEM1_2_4088,35,11,37,35,2,0,48,2,0,38,0,14
SPTLC2_2_4089,1235,897,1008,868,930,259,1492,1122,576,1243,682,495
SPTLC3_2_4090,124,172,159,201,152,196,189,15,371,72,314,60
SPTSSA_2_4091,110,130,140,89,582,176,1,6,23,10,538,131
SQLE_2_4092,409,526,350,478,401,14,1036,606,274,622,31,446
SQRDL_2_4093,391,364,182,503,422,164,340,663,224,302,714,298
SRD5A1_2_4094,108,119,190,184,18,116,144,184,65,0,3,427
SRD5A2_2_4095,429,336,401,356,241,482,277,758,2,161,486,457
SRD5A3_2_4096,387,587,286,293,400,19,187,199,442,399,651,573
SRR_2_4097,1734,1384,1359,1524,1902,816,1012,788,1437,1347,2373,1767
SRSF9_2_4098,188,206,112,185,0,207,0,193,8,61,245,295

SRXN1_2_4099,424,258,330,304,107,167,480,227,160,225,208,991
SSB_2_4100,601,691,683,582,443,560,751,756,440,565,608,490
SSU72_2_4101,538,435,649,705,662,301,209,315,1060,10,318,1076
ST14_2_4102,293,180,250,315,286,1,281,175,166,317,0,10
ST3GAL2_2_4103,131,70,112,162,0,0,0,310,335,560,635,258
ST3GAL4_2_4104,208,230,248,278,80,11,0,0,153,273,331,547
ST3GAL6_2_4105,47,12,38,68,7,0,116,256,27,6,0,121
ST6GALNAC1_2_4106,142,76,212,102,31,110,227,2,107,17,0,12
ST6GALNAC2_2_4107,723,560,510,689,622,584,1008,0,901,65,1862,1157
ST6GALNAC5_2_4108,238,196,128,253,229,859,678,217,472,450,67,356
ST6GALNAC6_2_4109,85,36,47,73,1,26,25,37,19,5,149,16
ST8SIA1_2_4110,451,536,486,598,893,433,446,18,488,48,1167,282
ST8SIA2_2_4111,972,1250,802,835,1112,809,657,905,952,1965,1426,756
ST8SIA3_2_4112,1805,2203,2105,1767,2493,1089,3862,1358,1646,2115,1366,
1367
ST8SIA5_2_4113,469,558,497,666,614,737,1016,0,577,700,909,1603
ST8SIA6_2_4114,226,233,209,136,33,220,183,869,257,252,355,233
STS_2_4115,1332,1376,1267,1471,1560,1959,711,92,1533,1117,1378,1565
STT3A_2_4116,37,22,47,54,57,6,0,33,0,23,10,452
STT3B_2_4117,167,83,92,173,17,123,3,0,142,208,6,374
SUCLA2_2_4118,1285,1418,1141,1296,1771,1943,853,1214,848,884,894,1271
SUCLG1_2_4119,534,754,596,647,451,420,108,292,215,899,1076,885
SULT1A3_2_4120,609,446,409,615,655,503,565,609,184,728,215,251
SULT1A4_2_4121,609,446,409,615,655,503,565,609,184,728,215,251
SULT1B1_2_4122,739,659,771,839,353,1193,1056,663,1187,1017,697,995
SULT1C3_2_4123,583,779,421,665,911,371,713,584,175,1000,455,351
SULT1C4_2_4124,1188,1254,1261,1460,928,647,2530,1184,1262,1396,2287,13
67
SULT1E1_2_4125,305,295,268,459,36,409,474,347,177,43,332,49
SULT2A1_2_4126,74,61,73,79,80,0,125,17,230,0,0,211
SULT4A1_2_4127,107,161,99,115,12,195,10,0,0,0,64,177
SUPV3L1_2_4128,232,381,239,273,306,275,354,270,135,179,124,273
SURF1_2_4129,142,117,162,221,12,21,10,19,27,68,519,31
TALDO1_2_4130,101,71,123,110,611,76,29,53,100,122,20,191
TARS2_2_4131,22,66,36,22,0,58,67,58,1,149,61,7
TARS_2_4132,256,159,164,283,393,211,496,350,2,41,242,514
TARSL2_2_4133,493,770,516,716,243,945,1809,829,691,936,501,153
TAT_2_4134,420,418,286,441,688,535,92,485,330,211,437,371
TBC1D10B_2_4135,567,415,559,682,417,716,1178,338,936,116,1033,426
TBCC_2_4136,48,70,56,50,41,80,248,72,92,48,7,20
TDG_2_4137,640,408,631,548,221,587,898,1138,789,500,549,616
TDO2_2_4138,845,805,900,938,2174,1098,655,1167,1195,375,1836,645
TECR_2_4139,342,239,201,264,303,123,4,199,13,238,322,145
TECTA_2_4140,346,426,265,328,105,317,206,3,261,771,130,320
TFB2M_2_4141,580,503,526,858,673,753,837,293,293,1109,648,940
TGDS_2_4142,191,206,220,275,425,115,61,1,195,33,145,50
TGM1_2_4143,138,72,98,119,264,12,394,121,84,165,0,335
TGM3_2_4144,216,63,144,157,158,2,87,12,388,63,56,751
TGM4_2_4145,180,145,149,281,179,166,423,52,325,53,105,143
TGM6_2_4146,582,552,449,687,304,32,175,582,442,346,1093,413

TGM7_2_4147,1204,913,854,1204,982,487,1452,432,678,813,982,1935
TGS1_2_4148,815,919,781,817,1529,807,566,417,685,1337,975,854
THG1L_2_4149,399,424,633,631,725,481,494,669,395,715,536,512
THOP1_2_4150,19,41,28,55,175,0,0,4,1,45,0,212
THUMPD2_2_4151,416,179,213,431,295,345,219,547,348,309,883,522
TKTL2_2_4152,103,111,129,140,38,110,0,155,302,223,823,57
TM7SF2_2_4153,369,408,503,624,62,101,413,381,228,125,310,173
TMEM55A_2_4154,870,671,822,859,1107,812,614,829,640,898,319,618
TMEM62_2_4155,101,136,119,189,110,3,91,191,0,106,507,137
TMEM86B_2_4156,218,97,127,195,0,2,0,8,0,474,949,40
TMPRSS15_2_4157,253,347,199,331,15,48,427,83,364,143,492,34
TMX1_2_4158,507,274,305,340,321,118,1591,368,282,477,320,629
TMX3_2_4159,772,539,616,649,1121,199,1329,564,629,647,312,579
TMX4_2_4160,255,162,152,135,16,149,262,86,197,308,0,90
TNKS2_2_4161,435,418,366,385,550,285,4,25,678,432,1063,117
TNKS_2_4162,439,535,478,452,354,129,762,758,104,933,99,650
TOP1_2_4163,406,374,419,481,337,483,543,1028,388,6,260,357
TOP1MT_2_4164,282,227,333,359,150,2,327,270,101,247,443,146
TOP2A_2_4165,162,63,120,84,387,33,432,147,139,0,11,0
TOP2B_2_4166,725,899,689,873,1699,869,1332,292,1191,1474,1211,634
TOP3A_2_4167,1095,1075,1015,1102,699,894,2151,1591,834,1907,577,653
TOP3B_2_4168,348,229,364,333,97,93,154,127,549,756,846,79
TOR3A_2_4169,334,225,185,222,178,44,391,127,473,619,31,398
TPH1_2_4170,1404,1098,1039,997,1076,585,2117,1263,1715,1484,2061,1338
TPMT_2_4171,725,513,527,714,1137,303,332,1069,1234,792,486,686
TPP1_2_4172,95,131,92,88,1,3,0,57,65,147,25,282
TPP2_2_4173,976,717,406,752,1291,760,2200,1220,623,875,567,762
TPSAB1_2_4174,455,291,316,383,0,0,521,0,0,105,775,66
TPSB2_2_4175,455,291,316,383,0,0,521,0,0,105,775,66
TPSD1_2_4176,418,322,235,501,0,516,28,3,0,28,551,124
TPST1_2_4177,79,54,52,68,155,1,163,178,22,184,4,34
TREH_2_4178,280,141,171,264,112,360,49,16,420,8,400,126
TREX2_2_4179,102,105,79,179,8,193,173,0,202,0,21,187
TRHDE_2_4180,196,204,224,259,694,105,0,11,514,1,347,650
TRIM21_2_4181,132,94,36,82,184,18,212,4,7,22,24,0
TRIT1_2_4182,375,352,334,500,172,233,690,236,208,385,580,636
TRMT112_2_4183,1131,1124,905,978,1325,778,966,559,827,832,276,553
TRMT61A_2_4184,3,82,11,20,0,6,96,9,0,0,0,0
TRMU_2_4185,66,33,54,50,37,106,96,722,91,56,1,59
TRNT1_2_4186,419,682,643,591,1060,735,1194,339,448,376,229,459
TRUB1_2_4187,267,413,263,369,679,281,1058,410,269,230,589,547
TRUB2_2_4188,279,232,224,159,155,0,903,211,517,81,0,176
TSTA3_2_4189,425,378,456,284,839,620,303,37,721,226,21,90
TST_2_4190,108,38,60,118,0,1,0,49,77,152,0,103
TTLL13_2_4191,27,43,16,56,88,164,0,9,184,72,376,123
TTLL1_2_4192,1029,602,751,941,933,839,535,124,638,321,1977,987
TTLL3_2_4193,82,7,87,108,19,4,0,5,318,0,0,34
TTLL4_2_4194,202,128,142,230,31,90,501,485,49,77,2,7
TUFM_2_4195,3,8,4,52,162,0,0,1,1,261,0,1
TULP2_2_4196,335,190,226,291,170,306,151,17,206,231,250,72

TUT1_2_4197,27,16,47,34,0,10,135,3,0,52,142,0
TXN2_2_4198,314,304,288,340,476,185,401,352,189,360,1541,386
TXN_2_4199,798,605,523,738,737,736,106,591,337,800,623,973
TXNDC11_2_4200,575,416,372,433,391,246,137,618,287,1066,621,163
TXNDC12_2_4201,231,250,236,305,410,312,211,196,267,368,55,275
TXNDC15_2_4202,212,252,229,373,417,44,900,201,47,103,529,39
TXNDC17_2_4203,74,56,40,109,0,75,73,427,4,1,0,5
TXNL1_2_4204,2053,1209,1406,1496,1065,810,2369,1498,1776,1053,1083,264
5
TXNL4A_2_4205,716,813,762,884,1414,1313,1203,531,735,956,1376,1030
TXNRD2_2_4206,299,284,318,182,58,6,14,144,102,34,1408,345
TYMS_2_4207,372,281,182,342,210,54,533,38,589,438,0,492
TYR_2_4208,43,19,30,15,16,37,100,2,276,0,14,18
TYRP1_2_4209,947,701,902,944,624,212,1960,110,564,522,252,1698
UAP1_2_4210,170,58,72,105,1,0,187,8,11,34,7,32
UAP1L1_2_4211,85,92,147,171,312,9,60,85,144,57,1,18
UBB_2_4212,114,105,128,136,0,4,325,5,11,39,27,147
UBIAD1_2_4213,133,74,99,163,58,1,333,5,59,265,0,24
UBL4A_2_4214,77,21,51,43,389,11,1,0,1,244,50,0
UFSP1_2_4215,962,823,665,930,1049,166,1368,705,721,761,418,799
UFSP2_2_4216,915,831,689,1194,726,487,794,505,1138,637,457,878
UGCG_2_4217,27,27,38,17,0,197,0,37,0,0,0,0
UGGT1_2_4218,597,947,751,779,1024,536,1098,1013,655,683,1535,311
UGGT2_2_4219,699,610,522,511,733,975,298,259,531,1024,17,735
UGT1A10_2_4220,225,286,395,363,307,38,486,36,133,332,458,85
UGT1A1_2_4221,60,34,49,28,0,0,2,34,0,14,10,69
UGT1A3_2_4222,2594,2226,2599,2694,3338,1917,3275,4051,2537,3244,4252,2
741
UGT1A4_2_4223,88,178,115,132,56,122,187,59,263,120,65,527
UGT1A5_2_4224,2997,3149,2788,3124,3241,2141,3704,2931,2641,2558,2055,2
881
UGT1A7_2_4225,2594,2226,2599,2694,3338,1917,3275,4051,2537,3244,4252,2
741
UGT1A8_2_4226,93,66,45,92,206,50,4,57,4,1,130,16
UGT1A9_2_4227,897,529,687,753,574,1016,1439,179,956,539,703,1057
UGT2A1_2_4228,830,583,693,664,648,341,1943,851,869,729,315,1125
UGT2A2_2_4229,372,561,298,414,144,380,1528,674,454,370,637,588
UGT2A3_2_4230,125,61,36,222,471,92,0,54,0,485,1618,0
UGT2B15_2_4231,499,282,330,465,108,219,547,10,128,308,713,985
UGT2B17_2_4232,499,282,330,465,108,219,547,10,128,308,713,985
UGT2B4_2_4233,472,351,478,421,320,540,763,284,382,593,338,238
UGT2B7_2_4234,824,848,803,1045,2038,266,605,2066,1194,517,717,672
UMPS_2_4235,201,57,76,75,182,0,45,213,282,49,0,188
UPB1_2_4236,149,64,63,89,5,108,24,15,3,97,10,220
UPF1_2_4237,334,266,362,377,245,529,312,51,260,241,242,113
UPRT_2_4238,31,82,114,220,41,3,64,159,201,31,63,0
UQCR11_2_4239,81,24,43,70,0,0,81,8,4,0,0,5
UQCRC1_2_4240,473,410,397,456,493,284,255,600,503,478,768,528
UQCRC2_2_4241,60,85,55,73,45,72,153,219,97,8,5,259
UQCRFS1_2_4242,951,743,717,824,431,950,2025,861,1296,1697,558,880

UQCRH_2_4243,1400,1051,839,1143,916,459,2378,1079,685,1730,2881,827
UQCRQ_2_4244,365,356,343,174,3,250,382,31,637,66,157,404
UROD_2_4245,90,177,37,52,129,4,40,12,40,22,29,97
UROS_2_4246,265,342,184,179,275,430,339,264,825,783,191,626
UST_2_4247,53,50,15,35,0,341,256,6,4,16,0,0
UXS1_2_4248,1153,637,672,682,2036,1147,460,1277,1075,953,826,412
VARS_2_4249,112,101,157,98,1,46,383,404,116,49,21,292
VAT1L_2_4250,185,155,108,159,265,1,113,371,74,72,0,179
VCP_2_4251,108,58,134,84,70,129,0,2,0,11,0,252
VNN1_2_4252,418,438,364,474,45,1092,1133,262,509,990,528,278
WBSCR17_2_4253,53,25,6,20,8,0,0,0,12,13,0,1
WBSCR27_2_4254,105,61,58,23,198,0,68,68,250,243,0,91
WDFY3_2_4255,184,129,124,143,50,86,12,240,279,395,18,298
WRN_2_4256,355,303,291,397,777,240,475,270,70,490,0,192
XDH_2_4257,139,69,123,111,117,0,254,131,233,61,0,0
XPNPEP2_2_4258,4,50,94,85,218,83,3,0,1,8,2,0
XRCC2_2_4259,185,181,294,220,160,322,727,426,131,127,473,580
XRCC5_2_4260,431,423,400,302,287,236,784,384,220,907,86,244
XRCC6_2_4261,520,450,540,443,464,686,521,617,608,60,544,623
XRN2_2_4262,428,250,280,357,453,130,195,608,187,112,407,384
XYLT1_2_4263,23,19,26,30,0,0,0,0,112,0,0,0
XYLT2_2_4264,17,26,43,37,2,2,47,23,0,0,0,0
YARS2_2_4265,2263,1672,1896,2167,2097,1830,3086,1431,2363,2083,1396,16
64
YARS_2_4266,955,919,796,960,582,152,634,1047,638,1039,547,881
YKT6_2_4267,473,578,411,510,97,294,1039,46,314,325,219,803
YPEL1_2_4268,226,345,292,353,442,425,685,270,94,833,306,525
ZADH2_2_4269,19,54,35,67,149,3,24,0,0,0,0,0
ZCCHC4_2_4270,372,362,419,307,129,148,242,433,168,358,236,376
ZDHHC17_2_4271,192,194,187,126,0,131,1,273,176,19,5,47
ZDHHC18_2_4272,268,204,300,256,372,202,454,389,115,102,132,414
ZDHHC1_2_4273,160,67,95,126,309,116,143,41,107,0,10,239
ZDHHC21_2_4274,452,436,341,485,432,819,441,568,386,351,506,596
ZDHHC2_2_4275,889,883,896,953,1832,678,1663,643,747,1615,531,1093
ZER1_2_4276,99,96,113,64,16,8,322,391,124,54,440,8
ZMPSTE24_2_4277,2020,1952,1629,2015,1459,2269,994,2936,1098,1974,1662,
2094
ZRANB3_2_4278,353,575,324,578,633,1403,408,62,369,250,501,202
A1CF_2_4279,2164,1676,1555,1940,2305,1734,2035,1140,2416,2643,2247,189
1
A2LD1_2_4280,144,279,185,148,42,43,138,397,84,250,11,136
AADAT_2_4281,486,439,427,456,449,755,36,44,459,688,642,198
AARSD1_2_4282,116,107,128,144,112,67,183,9,72,155,0,164
ABAT_2_4283,331,386,271,286,248,556,337,332,271,3,47,223
ABHD11_2_4284,204,426,210,493,54,283,356,459,269,135,161,91
ABHD12_2_4285,57,33,48,85,51,10,361,5,40,22,0,85
ABHD14B_2_4286,599,392,322,510,141,343,190,465,358,136,439,305
ABHD2_2_4287,586,491,526,466,553,112,205,176,592,324,147,595
ACAA1_2_4288,82,137,203,260,3,430,1,359,44,60,24,316
ACACA_2_4289,325,297,314,333,553,134,560,817,307,256,128,300

ACAD10_2_4290,256,191,191,251,304,558,269,313,460,284,74,99
ACADM_2_4291,38,157,57,48,342,0,96,153,40,128,354,149
ACADVL_2_4292,593,498,305,671,505,107,277,701,473,295,1082,929
ACCS_2_4293,208,51,134,64,129,38,117,71,278,315,0,12
ACE_2_4294,67,34,37,134,0,0,1179,528,0,251,6,26
ACHE_2_4295,22,34,45,30,2,0,0,170,0,0,0,6
ACIN1_2_4296,431,824,473,540,683,615,835,837,73,272,970,502
ACLY_2_4297,193,83,86,115,0,159,3,3,0,19,506,135
ACOT11_2_4298,63,62,139,128,78,139,0,102,3,24,1,55
ACOT13_2_4299,175,403,368,216,280,41,773,189,496,242,306,289
ACOT7_2_4300,239,445,330,556,298,63,378,328,164,1,573,272
ACOT9_2_4301,215,107,165,103,179,1,754,0,77,385,34,0
ACOX1_2_4302,92,87,57,154,376,65,259,50,25,3,272,124
ACOX3_2_4303,89,57,36,48,7,14,382,220,2,8,362,6
ACSBG1_2_4304,485,547,345,293,180,155,1142,882,232,596,149,215
ACSF3_2_4305,23,103,65,67,5,2,1,6,25,17,98,20
ACSL3_2_4306,289,303,207,235,103,43,406,225,352,375,533,101
ACSL4_2_4307,646,800,858,673,475,1073,517,501,510,1502,763,1140
ACSL5_2_4308,422,434,500,363,645,465,811,215,521,291,292,808
ACSL6_2_4309,705,583,453,863,390,786,924,352,1124,337,1007,647
ACSM2B_2_4310,1961,1391,1776,2007,1302,972,1060,2639,2701,2161,886,276
8
ACSM3_2_4311,206,188,98,127,0,0,0,181,136,56,0,18
ACSS2_2_4312,59,69,49,72,52,0,999,0,8,436,0,0
ACY1_2_4313,107,105,55,74,297,0,616,35,24,157,164,481
ADAMTS2_2_4314,56,54,43,51,48,6,33,134,7,0,12,116
ADARB1_2_4315,162,281,206,263,28,277,1112,343,678,266,0,373
ADAR_2_4316,285,155,163,284,269,51,319,219,269,311,72,198
ADCY10_2_4317,793,569,659,558,263,790,562,345,394,1253,1259,409
ADCY4_2_4318,73,21,30,57,0,0,16,0,0,0,0,37
ADCY5_2_4319,297,245,279,207,470,181,375,370,304,810,68,449
ADCY6_2_4320,168,200,143,251,587,203,594,549,31,308,616,152
ADH6_2_4321,673,823,741,1283,627,764,1492,511,1022,1195,625,957
ADH7_2_4322,161,147,197,252,0,270,133,440,202,33,360,296
ADPRHL1_2_4323,52,38,42,83,40,0,154,8,48,69,0,96
ADSL_2_4324,147,89,75,107,77,3,532,168,65,10,419,313
ADSSL1_2_4325,243,177,140,97,193,9,505,0,4,68,6,289
AFMID_2_4326,264,270,145,282,141,405,193,464,26,35,185,12
AGA_2_4327,76,147,79,95,111,0,4,228,42,2,209,193
AGAP1_2_4328,145,80,67,95,117,157,52,59,108,141,115,174
AGAP2_2_4329,4,13,21,34,0,0,0,27,0,0,0,0
AGL_2_4330,282,241,239,243,139,1,137,311,371,110,137,295
AGPAT1_2_4331,67,142,210,123,3,10,4,181,121,176,0,255
AGPAT2_2_4332,101,83,85,156,107,4,103,368,48,138,3,248
AGPAT3_2_4333,260,145,208,282,712,35,587,1790,144,530,663,152
AGXT2L1_2_4334,2169,1810,1762,2468,2093,2603,1925,1687,2142,2110,2816,
1704
AHCY_2_4335,25,120,163,75,31,524,546,0,2,9,360,33
AHCYL1_2_4336,1129,846,1121,1168,1155,848,921,540,1192,1513,1184,811
AHCYL2_2_4337,412,475,311,290,391,72,532,116,308,70,166,811

AIFM1_2_4338,359,387,281,279,296,289,159,171,180,357,419,311
AIFM2_2_4339,258,105,91,206,232,0,227,0,78,0,1,1115
AIFM3_2_4340,654,745,482,726,747,1326,450,1326,653,576,821,259
AKIRIN1_2_4341,145,62,46,58,157,13,201,13,141,18,2,7
AKR1A1_2_4342,414,399,388,482,209,208,498,798,842,520,424,143
AKR1C2_2_4343,395,321,174,366,726,341,133,248,365,293,2,359
AKR1D1_2_4344,119,81,64,76,35,7,6,0,235,121,0,68
ALAS1_2_4345,922,773,662,668,259,603,1129,679,738,840,1333,176
ALAS2_2_4346,1540,1417,1345,1612,2737,1700,2599,1693,1856,1846,1990,93
3
ALDH16A1_2_4347,340,410,352,330,169,597,402,205,632,120,30,383
ALDH1A2_2_4348,54,28,62,60,2,0,70,22,118,70,22,1
ALDH2_2_4349,784,581,769,636,370,86,1784,1175,1058,872,570,1051
ALDH3A1_2_4350,1107,1195,1096,1073,1236,749,725,1664,1808,1075,1243,12
75
ALDH3A2_2_4351,149,156,255,175,33,51,1044,27,292,176,286,189
ALDH3B1_2_4352,101,41,24,30,1,1,2,7,130,0,0,32
ALDH3B2_2_4353,59,7,44,27,40,0,26,1,51,41,315,0
ALDH4A1_2_4354,276,119,123,145,35,100,0,3,132,93,496,37
ALDH5A1_2_4355,350,347,276,403,43,264,410,126,296,49,161,360
ALDH7A1_2_4356,3654,2724,2480,3483,4391,2250,2113,2460,3832,3095,2946,
3672
ALDH8A1_2_4357,1097,886,773,975,518,1681,667,1440,906,724,392,1042
ALDOA_2_4358,210,331,172,162,0,4,198,48,148,49,441,181
ALG3_2_4359,571,403,563,622,1112,193,622,587,805,787,340,137
ALG5_2_4360,805,394,620,657,414,365,230,360,668,296,137,786
ALG8_2_4361,79,23,28,66,0,590,5,0,0,77,11,167
ALG9_2_4362,105,189,167,203,339,181,851,190,111,175,312,6
ALOX15B_2_4363,43,167,111,222,19,111,166,0,6,238,0,381
ALOXE3_2_4364,37,10,7,29,8,221,89,1,53,6,0,9
AMACR_2_4365,1579,1195,1522,1702,1229,1183,1551,1057,1244,652,1253,111
5
AMD1_2_4366,1353,1086,910,992,1483,904,1302,1275,1384,1331,769,1627
AMDHD2_2_4367,237,88,95,113,10,20,0,0,5,176,0,276
AMPD1_2_4368,484,511,276,592,1073,1181,1255,261,745,422,65,704
AMPD2_2_4369,351,65,127,269,573,256,89,11,273,0,224,231
AMPD3_2_4370,268,187,181,249,594,277,123,204,429,189,362,465
AMT_2_4371,196,188,142,180,168,42,364,731,37,75,30,302
AMY1A_2_4372,12885,11074,11511,13873,9341,7552,13601,11922,10004,9866,
8929,12719
ANG_2_4373,1472,1363,1270,1502,792,517,2309,1112,1460,2858,643,1777
AOAH_2_4374,0,18,54,3,9,0,0,103,0,0,0,3
AOC2_2_4375,201,94,164,175,74,21,34,267,19,72,711,33
APC_2_4376,1116,738,907,842,1729,659,868,485,863,357,1115,516
APEX1_2_4377,941,911,757,899,741,886,816,345,576,2171,424,806
APOBEC3A_2_4378,69,75,154,187,10,269,0,0,706,1,97,0
APOBEC3H_2_4379,519,849,792,832,396,1268,1300,341,95,344,2005,513
APRT_2_4380,13,8,29,65,74,22,3,66,55,7,173,25
ARF1_2_4381,61,121,107,64,226,308,3,43,113,177,8,292
ARFRP1_2_4382,37,10,8,32,7,0,27,0,1,47,0,0

ARHGAP5_2_4383,541,577,490,314,93,325,400,1287,16,535,608,481
ARHGEF10L_2_4384,118,18,45,26,83,0,0,121,0,0,0,0
ARL2_2_4385,0,1,10,8,0,0,0,0,0,3,0,0
ARL4A_2_4386,218,155,96,200,196,213,48,184,140,497,127,97
ARL5A_2_4387,2928,2573,2588,2798,2502,1475,2655,2353,3010,2261,2646,29
02
ARSA_2_4388,25,24,38,2,0,0,11,0,27,13,7,0
ARSB_2_4389,161,201,174,166,129,116,33,60,105,445,72,189
ARSF_2_4390,85,153,233,115,44,43,0,966,248,0,324,395
ART3_2_4391,495,428,433,602,577,462,276,581,457,304,715,783
ART5_2_4392,242,247,332,301,279,136,393,485,281,354,392,415
ASAH1_2_4393,355,445,351,497,13,645,0,1,469,124,510,727
ASAH2_2_4394,258,234,264,217,676,133,309,15,238,183,126,346
ASL_2_4395,47,43,60,25,0,175,6,164,25,7,15,59
ASMT_2_4396,547,382,471,535,229,459,668,72,328,740,1106,359
ASMTL_2_4397,20,2,35,10,14,0,0,153,0,0,473,1
ASNS_2_4398,1260,809,704,969,227,772,250,817,773,1198,916,754
ASPA_2_4399,331,327,244,230,268,217,231,187,569,165,664,28
ASRGL1_2_4400,919,810,818,709,703,1696,1531,904,212,645,1066,1372
ASS1_2_4401,145,120,123,229,34,0,31,0,107,440,74,149
ATE1_2_4402,2131,1606,1628,1817,2066,1292,1111,1082,1439,1718,2980,142
5
ATL1_2_4403,204,108,90,135,17,287,269,52,129,69,492,161
AURKAIP1_2_4404,135,277,268,233,213,14,231,167,528,323,102,292
AZIN1_2_4405,94,76,90,89,4,307,16,211,37,138,19,23
B3GALNT1_2_4406,815,909,764,1113,450,574,1119,682,966,998,942,332
B3GALT5_2_4407,1074,1411,1192,1176,1345,82,2146,476,777,822,2716,1691
B3GAT1_2_4408,0,0,77,6,0,0,0,0,0,0,0,0
B4GALNT2_2_4409,88,62,91,102,79,4,28,213,46,18,0,11
B4GALT2_2_4410,99,63,137,119,91,144,2,133,186,0,542,7
B4GALT3_2_4411,81,74,73,60,0,246,61,5,26,77,0,58
B4GALT4_2_4412,607,606,374,446,447,356,518,723,222,783,43,286
BAAT_2_4413,12,63,55,70,84,3,0,328,4,17,0,135
BACE1_2_4414,319,222,225,351,442,146,396,248,394,645,299,285
BACE2_2_4415,143,124,134,124,4,101,783,293,98,93,47,32
BCAT1_2_4416,397,443,410,293,757,488,311,426,108,82,1305,235
BCAT2_2_4417,0,35,51,0,0,0,0,0,0,108,0,442
BCKDHA_2_4418,802,593,618,930,690,530,782,1034,922,1018,694,1178
BCKDHB_2_4419,1011,950,672,875,386,652,1890,604,428,771,423,1696
BCO2_2_4420,961,793,928,839,1520,596,1678,1015,241,1048,989,1101
BDH1_2_4421,329,412,320,361,96,211,587,487,310,693,151,223
BFSP1_2_4422,304,289,275,263,422,49,13,344,107,30,296,54
BHMT2_2_4423,121,134,164,123,157,4,0,0,33,1,31,5
BMP1_2_4424,227,123,143,74,7,0,0,1,160,192,0,194
C17orf101_2_4425,22,66,47,26,268,6,0,31,8,129,333,0
C1GALT1C1_2_4426,504,466,427,584,215,1290,98,852,295,0,183,517
C1S_2_4427,1763,1126,1385,1720,2215,1452,1347,2105,1540,2458,1525,1259
C2_2_4428,223,248,335,307,334,89,17,164,257,569,36,488
CA10_2_4429,113,189,186,126,237,0,0,69,48,0,402,336
CA12_2_4430,82,84,203,107,0,0,172,0,18,130,5,15

CA1_2_4431,108,188,129,260,0,96,0,0,135,1,462,168
CA7_2_4432,69,11,118,50,88,53,266,154,13,0,0,150
CAB39_2_4433,620,568,346,611,309,225,211,307,620,1506,1294,300
CANT1_2_4434,328,346,238,312,281,462,307,324,60,245,267,548
CAPN1_2_4435,463,298,407,426,376,28,630,374,448,353,149,616
CAPN2_2_4436,148,98,183,74,402,8,723,0,228,120,416,10
CARNS1_2_4437,1181,982,1004,1129,1031,1785,1051,640,825,1211,1256,458
CARS_2_4438,432,449,409,364,283,169,118,229,269,238,118,291
CASP10_2_4439,1239,1032,1089,1551,236,760,1229,646,1527,804,503,970
CASP1_2_4440,213,291,377,292,348,47,0,1221,551,493,234,464
CASP2_2_4441,47,69,53,43,77,0,7,82,10,103,8,1
CASP3_2_4442,308,322,270,409,377,271,511,363,211,137,24,328
CASP4_2_4443,568,425,518,492,79,155,924,1217,347,517,44,702
CASP5_2_4444,615,557,608,728,1222,248,967,1296,567,841,719,814
CASP6_2_4445,663,694,594,702,806,146,596,929,1234,746,268,510
CASP7_2_4446,937,875,727,801,1255,437,980,721,717,764,655,734
CASP8_2_4447,613,379,643,676,522,560,614,1085,197,1211,217,725
CASP9_2_4448,26,78,83,82,2,68,6,23,163,144,0,161
CASZ1_2_4449,95,92,71,154,2,0,57,0,162,0,0,48
CAV3_2_4450,274,430,211,285,85,864,2091,866,42,29,1054,798
CBS_2_4451,158,232,104,189,43,53,762,218,67,66,50,241
CCBL1_2_4452,110,51,132,82,63,0,0,4,230,242,0,0
CCBL2_2_4453,696,490,661,833,918,512,819,821,663,484,323,446
CDC42_2_4454,768,864,1074,665,1162,1108,796,237,858,692,671,1415
CDH16_2_4455,68,126,67,108,109,0,4,0,32,193,4,163
CECR1_2_4456,879,747,601,646,1128,447,1107,938,592,1368,825,774
CEPT1_2_4457,173,14,109,139,174,0,306,115,198,156,18,647
CERS1_2_4458,236,373,175,330,176,338,597,30,243,318,27,390
CES1_2_4459,232,182,257,312,901,69,287,66,482,166,190,4
CES3_2_4460,257,256,188,285,8,640,372,856,164,173,172,171
CES4A_2_4461,47,51,48,23,5,11,8,0,21,69,48,159
CES5A_2_4462,106,218,118,64,19,67,0,364,172,73,24,81
CHAT_2_4463,327,147,228,216,31,363,132,148,393,595,26,42
CHI3L2_2_4464,72,229,148,169,60,177,0,24,100,267,68,223
CHIA_2_4465,517,389,195,357,193,0,292,0,151,316,0,223
CHPF_2_4466,417,278,335,428,113,119,242,495,58,391,54,525
CHST11_2_4467,0,0,0,0,0,0,0,0,0,0,0,0
CHST15_2_4468,1660,1731,1227,1557,2216,73,1576,1747,531,2192,1943,1482
CHST4_2_4469,337,143,138,199,378,69,272,44,158,654,297,79
CHST8_2_4470,574,548,620,597,1017,724,178,853,654,417,1328,372
CLP1_2_4471,70,74,58,33,276,0,4,35,57,86,161,1
CNDP2_2_4472,29,14,145,66,0,5,0,23,131,212,4,88
CNTN1_2_4473,864,685,654,939,968,759,533,391,127,556,62,949
CNTN4_2_4474,167,106,88,212,29,2,61,315,61,92,207,526
COMT_2_4475,126,234,183,166,245,436,1011,207,163,174,32,153
COQ6_2_4476,80,94,201,189,0,0,44,22,17,459,150,82
COX11_2_4477,3312,2658,2508,2685,2330,2809,2512,2893,2274,2896,2265,29
30
COX15_2_4478,529,609,469,710,122,507,546,128,184,326,592,363
CPA5_2_4479,240,86,55,111,51,8,54,3,40,121,3,0

CPB2_2_4480,49,117,203,156,41,0,360,107,0,86,676,0
CPM_2_4481,770,687,717,884,970,1085,828,477,845,613,886,933
CPPED1_2_4482,2048,2014,2112,1942,3168,1687,1078,1293,1472,3481,1708,2
815
CPT1A_2_4483,557,326,327,562,206,473,104,433,1245,183,199,563
CPT1B_2_4484,122,114,177,163,3,169,492,45,26,13,0,41
CPT1C_2_4485,765,840,802,983,815,1359,703,860,629,579,593,687
CRLS1_2_4486,32,33,26,39,187,6,144,0,75,33,1,0
CRMP1_2_4487,1012,887,737,1072,228,1052,814,802,687,577,1028,1244
CROT_2_4488,2096,1800,1330,2184,1781,1407,2397,1581,1573,1402,1482,223
5
CRY2_2_4489,122,61,50,89,268,0,34,25,12,78,16,82
CRYM_2_4490,358,179,258,342,293,261,195,0,921,87,24,432
CRYZ_2_4491,260,500,438,619,490,840,456,283,808,787,494,476
CSDE1_2_4492,204,246,227,228,218,171,121,414,221,357,472,86
CSGALNACT1_2_4493,660,555,485,689,365,15,468,971,90,0,1283,910
CSMD3_2_4494,1040,1031,766,1080,472,1858,1969,692,444,1894,1459,603
CTAGE5_2_4495,583,678,567,519,654,403,63,230,163,563,591,345
CTBP1_2_4496,105,102,110,150,53,0,201,47,59,5,93,33
CTPS2_2_4497,274,312,320,297,464,536,490,684,505,66,202,225
CTSA_2_4498,35,91,81,107,1,251,275,4,4,183,0,244
CTSB_2_4499,326,309,399,320,466,764,371,596,102,144,763,520
CTSE_2_4500,53,57,99,83,25,201,85,52,1,41,45,13
CTSL1_2_4501,37,27,25,30,22,69,65,4,4,25,128,57
CTSL2_2_4502,294,184,229,322,79,264,1335,81,508,0,310,376
CTSS_2_4503,962,828,831,1338,898,814,1113,431,1744,979,1453,620
CYB561_2_4504,151,97,162,177,175,89,117,75,101,67,147,490
CYB5A_2_4505,149,112,67,156,340,0,177,0,470,336,0,296
CYB5R3_2_4506,72,162,58,84,0,4,0,0,11,115,0,84
CYP11A1_2_4507,672,751,537,580,790,710,973,1129,317,235,194,633
CYP11B1_2_4508,377,470,358,624,310,305,45,485,332,503,450,344
CYP19A1_2_4509,367,319,392,430,632,84,106,28,415,370,977,355
CYP21A2_2_4510,171,103,149,193,96,0,357,0,379,311,0,0
CYP24A1_2_4511,21,6,15,15,0,0,0,0,23,0,0,0
CYP26A1_2_4512,212,71,60,202,5,21,36,315,125,13,161,93
CYP2A7_2_4513,251,206,126,179,157,42,31,35,36,358,142,190
CYP2C18_2_4514,0,0,0,0,0,0,0,0,0,0,0,0
CYP2C8_2_4515,1132,771,816,1244,447,512,671,2830,1432,481,635,641
CYP2D6_2_4516,238,328,392,448,472,755,96,251,29,457,622,175
CYP3A43_2_4517,192,187,181,226,177,0,154,0,19,22,109,332
CYP3A4_2_4518,105,91,63,98,2,90,77,279,61,227,155,317
CYP4B1_2_4519,122,49,76,103,468,74,33,73,71,29,19,229
CYP4F11_2_4520,91,16,50,213,4,0,0,359,0,24,0,11
CYP4F3_2_4521,206,392,274,335,26,513,29,258,127,785,30,420
CYP51A1_2_4522,459,354,342,243,317,519,1033,383,315,641,411,316
DAGLB_2_4523,368,448,373,420,444,456,701,169,401,798,63,262
DCLRE1C_2_4524,995,857,808,997,1464,573,1097,328,426,761,1230,1332
DCP2_2_4525,212,176,115,177,319,437,166,165,144,406,13,67
DCT_2_4526,462,292,384,417,928,137,612,165,317,441,197,261
DCTD_2_4527,349,72,264,240,56,1,404,3,16,0,50,306

DCXR_2_4528,175,603,349,392,521,83,98,737,116,9,79,421
DDAH1_2_4529,824,899,908,768,641,463,1201,189,285,1431,855,1847
DDC_2_4530,614,703,792,961,1484,142,559,1272,233,1426,725,325
DDHD1_2_4531,363,263,371,395,425,108,181,148,250,401,877,1028
DDO_2_4532,211,418,301,364,163,574,0,1,88,51,81,256
DDT_2_4533,217,144,239,135,51,3,483,649,182,100,105,322
DDX11_2_4534,753,451,655,562,793,329,351,1812,629,692,467,714
DDX17_2_4535,931,605,855,1031,1557,1156,344,1003,609,736,2163,733
DDX19B_2_4536,217,204,193,234,18,257,774,41,8,16,1,417
DDX31_2_4537,1014,923,811,1048,506,1390,826,728,1758,1103,477,404
DDX39B_2_4538,115,135,81,229,631,28,0,0,0,8,8,265
DDX3X_2_4539,334,230,143,308,139,9,0,332,160,0,135,0
DDX3Y_2_4540,369,331,413,356,360,139,725,364,570,383,303,725
DDX42_2_4541,72,119,107,109,0,2,2,173,215,154,0,0
DDX47_2_4542,69,76,82,124,226,23,452,30,99,43,21,2
DDX4_2_4543,1233,988,1184,866,739,917,1253,509,1099,902,620,604
DDX54_2_4544,166,206,91,126,98,5,583,991,286,147,193,61
DFFA_2_4545,307,213,238,287,548,107,231,285,276,123,91,103
DGCR8_2_4546,665,572,536,773,514,522,822,517,343,1019,949,1374
DHCR7_2_4547,44,12,49,26,41,75,16,0,0,7,0,0
DHDDS_2_4548,5,13,23,48,0,0,0,0,0,0,0,0
DHFRL1_2_4549,317,174,184,268,159,254,491,11,55,292,106,54
DHPS_2_4550,20,1,16,22,0,0,59,9,176,0,0,13
DHRS1_2_4551,71,116,69,149,163,204,0,583,17,205,285,3
DHRS2_2_4552,322,548,458,635,420,303,358,499,1086,940,0,309
DHRS9_2_4553,35,38,43,46,0,22,146,26,0,7,0,12
DHX16_2_4554,1536,1355,1096,1313,1033,1647,1429,1085,1294,1569,218,133
3
DHX30_2_4555,52,60,113,129,69,48,96,135,121,28,550,46
DHX33_2_4556,34,73,107,141,22,0,0,169,7,3,1,248
DHX35_2_4557,70,110,83,24,19,44,0,164,289,93,83,18
DHX36_2_4558,1392,1028,1111,1430,1987,1527,222,1756,1115,1036,1230,109
4
DHX40_2_4559,975,606,437,570,5,801,7,149,692,674,1672,277
DIAPH3_2_4560,1077,658,825,844,1185,170,271,547,821,410,435,322
DIS3_2_4561,143,276,198,252,298,165,501,31,483,283,269,105
DIS3L_2_4562,414,209,216,252,42,368,50,337,55,5,1,34
DKC1_2_4563,1094,1019,1294,1049,831,1082,2245,1000,1140,889,1056,1962
DNAJC27_2_4564,838,636,738,655,483,736,1606,1033,419,386,71,922
DNASE1L1_2_4565,348,267,250,356,188,0,403,623,458,92,667,511
DNM1_2_4566,1290,865,864,1175,362,2095,1403,1725,1275,1442,647,1344
DNM1L_2_4567,1015,936,1016,999,1232,401,730,570,544,1746,573,858
DNM2_2_4568,181,280,244,391,33,504,31,483,234,212,334,50
DNM3_2_4569,624,460,495,631,424,673,218,1157,401,667,224,191
DNTT_2_4570,292,359,233,274,352,38,172,674,562,14,89,85
DOHH_2_4571,0,4,59,5,0,0,0,0,0,32,0,0
DOLPP1_2_4572,497,435,386,351,114,53,525,66,148,755,98,106
DPEP1_2_4573,2340,1920,2051,2463,2246,1894,2331,2866,1666,2804,4142,18
34
DPEP3_2_4574,815,527,444,848,663,70,433,1548,795,391,388,852

DPH5_2_4575,180,151,108,103,224,42,13,109,149,458,591,48
DPM3_2_4576,299,304,388,299,990,97,192,850,534,42,80,683
DPP3_2_4577,257,181,222,136,135,60,319,314,197,643,398,171
DPP8_2_4578,854,1102,982,955,1395,1854,824,1594,1157,1230,942,1648
DPYSL2_2_4579,23,4,14,37,2,0,0,0,0,0,0,6
DPYSL3_2_4580,6,26,26,59,0,0,31,3,0,53,11,0
DROSHA_2_4581,1472,1164,1035,1089,1305,555,1143,480,1509,1286,1004,653
DSE_2_4582,19,63,114,87,0,5,34,9,0,19,0,4
DUOX1_2_4583,336,328,300,481,397,406,507,93,271,470,614,55
DUT_2_4584,158,293,215,207,205,5,0,200,167,255,333,4
ECE1_2_4585,253,342,347,427,472,484,410,499,118,539,102,365
ECE2_2_4586,147,124,82,39,1,0,18,409,1,263,0,0
ECI1_2_4587,49,58,26,53,0,0,0,1,117,0,0,64
ECI2_2_4588,221,219,276,348,75,272,164,158,248,80,110,561
EDEM2_2_4589,255,197,138,141,532,0,411,144,270,7,185,323
EFEMP1_2_4590,4057,3776,3585,4122,2791,3585,3363,2775,3133,4399,3388,3
336
EFTUD2_2_4591,82,97,76,67,82,21,4,93,80,103,0,43
EGLN2_2_4592,1609,1144,1465,1574,896,784,1310,2195,1234,1230,2347,935
EHHADH_2_4593,789,739,756,755,519,207,854,299,661,819,976,769
ELAC2_2_4594,85,51,58,127,0,68,13,18,59,3,24,151
ELOVL5_2_4595,365,197,200,292,118,391,81,86,374,432,76,209
ELOVL6_2_4596,802,556,847,837,731,617,870,252,862,1244,540,807
ELOVL7_2_4597,665,1005,819,950,758,1099,926,518,1071,333,3404,1219
ENDOV_2_4598,106,82,81,115,54,102,73,0,49,149,28,198
ENO3_2_4599,29,43,19,34,22,4,1,131,502,1,0,0
ENOX2_2_4600,754,651,562,577,1101,617,710,1290,914,885,471,1726
ENPP2_2_4601,839,875,871,934,972,1154,949,582,480,356,317,581
ENTPD1_2_4602,561,593,404,684,769,584,234,1016,1059,626,773,685
ENTPD2_2_4603,171,178,257,196,77,277,305,280,433,226,95,241
ENTPD4_2_4604,266,266,199,319,145,425,51,408,565,83,154,189
ENTPD6_2_4605,582,507,543,341,64,556,570,401,90,109,390,824
ENTPD8_2_4606,240,314,300,281,293,96,353,244,686,13,185,281
EPHX1_2_4607,117,44,70,83,8,91,178,170,113,307,49,20
EPHX3_2_4608,949,791,618,726,855,445,839,1591,428,488,748,771
ERCC1_2_4609,259,135,234,132,192,198,0,0,1,454,79,24
ERCC2_2_4610,81,180,45,76,163,7,0,13,85,285,12,2
EXO1_2_4611,173,165,134,264,631,85,226,556,27,4,0,0
EXOG_2_4612,550,517,456,429,965,1198,207,454,187,319,723,645
EXOSC3_2_4613,579,652,557,601,1345,418,128,820,690,572,214,1449
EXOSC9_2_4614,241,267,311,511,412,88,507,154,163,277,233,0
EXTL2_2_4615,269,39,139,238,0,56,943,737,308,322,139,127
F7_2_4616,360,382,351,314,251,1070,664,29,210,200,164,361
FAHD1_2_4617,1164,677,884,895,529,603,1,731,435,679,1702,1215
FAM108A1_2_4618,7,6,0,12,0,0,0,5,26,0,0,2
FAM135A_2_4619,217,217,144,420,108,519,100,326,281,168,433,429
FAN1_2_4620,878,798,995,940,552,338,566,598,696,639,1389,819
FDPS_2_4621,490,420,474,402,273,113,726,110,298,534,267,848
FDXR_2_4622,35,59,87,74,13,13,4,6,2,0,0,35
FECH_2_4623,29,25,43,18,0,0,1,7,113,132,0,118

FERMT3_2_4624,59,38,21,43,34,0,0,87,10,94,43,125
FHIT_2_4625,38,12,4,59,5,0,0,0,27,0,0,40
FIGNL1_2_4626,48,18,41,45,162,14,0,1,156,59,0,50
FKBP11_2_4627,201,50,132,193,192,0,67,0,23,5,91,52
FKBP1A_2_4628,4833,4648,4297,4910,4932,2965,4460,3451,3953,5778,3933,4
371
FKBP1B_2_4629,103,73,103,135,182,340,47,1,113,0,85,218
FKBP2_2_4630,210,280,294,407,164,622,133,209,101,223,0,251
FKBP5_2_4631,254,237,311,299,17,557,250,86,115,331,223,283
FKBP7_2_4632,117,111,103,130,656,0,0,0,363,0,0,229
FLAD1_2_4633,522,417,438,473,282,479,32,865,254,76,343,712
FMO3_2_4634,69,196,123,157,2,374,0,0,9,155,282,1
FMO5_2_4635,237,421,168,250,198,149,218,344,86,60,113,468
FN1_2_4636,893,947,961,912,1422,862,1291,364,841,1120,488,1012
FOLH1_2_4637,186,228,107,281,263,988,12,73,19,124,378,147
FPGS_2_4638,145,71,169,95,15,643,15,290,63,39,40,16
FPGT_2_4639,417,386,269,304,148,54,451,51,785,786,277,182
FTCD_2_4640,77,294,118,162,156,63,40,19,175,60,55,260
FTSJ1_2_4641,91,146,102,148,5,452,371,6,211,250,446,212
FUT2_2_4642,47,46,40,54,39,16,0,0,3,44,1,0
FUT3_2_4643,428,295,309,294,418,63,105,59,446,286,287,266
FUT6_2_4644,428,295,309,294,418,63,105,59,446,286,287,266
FUT8_2_4645,452,117,269,270,455,4,8,64,323,1,186,171
G3BP1_2_4646,3,32,34,28,42,0,13,0,32,241,96,3
G3BP2_2_4647,1050,1057,932,850,303,927,820,470,619,978,888,1345
G6PD_2_4648,69,83,83,36,88,19,14,262,286,68,0,136
GAA_2_4649,16,32,16,14,83,0,13,41,0,12,23,98
GAD1_2_4650,717,575,711,894,225,17,1297,1067,1276,732,1702,881
GAD2_2_4651,337,361,218,253,1031,460,268,215,648,363,203,762
GALC_2_4652,157,110,96,151,88,164,7,17,175,85,176,12
GALE_2_4653,1514,1623,1579,1551,1471,1427,532,2717,1084,2714,1279,1844
GALNT9_2_4654,1132,1281,1125,1322,2404,1280,1516,1089,989,1195,2748,20
73
GALNTL1_2_4655,20,61,113,102,13,0,0,0,0,0,0,2
GAMT_2_4656,518,442,453,436,584,587,341,555,833,804,297,82
GANAB_2_4657,106,101,60,102,168,147,89,0,20,34,1,159
GART_2_4658,649,789,503,576,1013,730,574,407,655,278,303,1116
GBA_2_4659,309,324,318,426,203,238,286,281,322,505,204,434
GBP5_2_4660,1176,1022,1236,1118,273,453,2065,1362,927,1712,1476,1081
GCAT_2_4661,175,157,107,115,205,118,338,61,143,8,554,51
GCDH_2_4662,58,20,5,42,2,0,5,0,139,0,17,38
GCH1_2_4663,0,5,0,10,1,0,0,0,0,222,11,0
GCLC_2_4664,780,791,767,875,483,1285,1016,1412,761,554,1188,1115
GCNT1_2_4665,445,69,259,340,315,84,608,140,180,641,196,547
GDPD1_2_4666,1161,1022,1026,1077,1629,1218,1029,615,1795,1176,1122,158
8
GDPD2_2_4667,60,26,119,62,74,0,2,3,4,1,473,7
GEM_2_4668,31,67,23,21,13,5,7,0,14,2,0,0
GEN1_2_4669,1211,810,877,1027,818,797,632,1134,1690,1623,563,899
GFM2_2_4670,100,93,139,166,224,36,300,91,137,118,6,45

GFOD1_2_4671,30,61,75,48,0,9,0,0,35,0,0,1
GGCT_2_4672,353,181,157,204,142,333,234,23,167,164,66,339
GGCX_2_4673,90,61,28,27,1,4,936,15,40,0,145,5
GGT1_2_4674,257,122,248,318,1,201,924,0,0,41,184,212
GGT5_2_4675,138,58,109,67,337,0,132,19,30,118,82,256
GGT6_2_4676,128,116,71,118,94,0,6,1,1,35,491,21
GGTLC1_2_4677,168,276,148,202,762,45,18,726,149,136,11,878
GLB1_2_4678,103,15,80,115,0,337,395,0,1,0,433,3
GLRX2_2_4679,967,807,592,723,354,540,845,463,1332,803,123,621
GLRX3_2_4680,573,762,270,587,234,399,699,700,667,543,1170,177
GLRX_2_4681,1329,1127,951,1320,1004,731,1519,928,533,1109,1385,1372
GLT8D1_2_4682,29,47,40,76,0,0,0,6,0,48,0,5
GLUL_2_4683,470,368,562,578,293,429,192,720,309,587,16,702
GM2A_2_4684,539,695,617,590,571,904,695,367,1155,282,796,851
GMPPA_2_4685,198,285,188,238,373,274,107,336,252,255,311,31
GMPPB_2_4686,16,18,8,16,8,7,0,0,0,3,0,0
GMPR2_2_4687,147,88,95,83,39,213,42,123,0,4,500,148
GNAI2_2_4688,25,79,108,87,1,1,0,63,3,21,254,105
GNAL_2_4689,213,381,334,335,133,644,807,412,31,278,785,450
GNAO1_2_4690,1045,918,877,868,1304,382,714,1297,671,868,514,998
GNAT1_2_4691,281,234,118,255,347,80,101,170,283,44,18,472
GNB5_2_4692,29,25,21,135,22,58,0,0,1,0,3,283
GNG10_2_4693,875,1075,950,1188,1653,1110,758,2224,672,850,670,846
GNG4_2_4694,157,117,181,219,504,0,971,250,408,4,1,353
GNGT2_2_4695,1332,1097,1598,1604,1556,880,964,1807,1310,726,1581,889
GPD2_2_4696,1299,930,1447,1526,657,1063,504,641,1741,475,1529,2732
GPHN_2_4697,591,289,394,497,426,670,854,454,675,503,429,617
GPNMB_2_4698,216,206,376,235,146,134,517,0,111,37,272,325
GPT2_2_4699,60,42,27,13,0,0,0,2,23,2,198,0
GPX4_2_4700,91,97,97,135,8,7,103,153,14,0,0,43
GSR_2_4701,22,1,1,12,23,18,0,0,0,0,0,0
GSTCD_2_4702,583,383,439,568,287,29,315,741,584,125,602,628
GSTK1_2_4703,864,955,1080,902,655,793,778,698,1241,697,1761,703
GSTM1_2_4704,445,448,449,506,812,544,321,371,408,323,713,681
GSTM2_2_4705,554,499,442,513,229,547,319,1038,648,379,896,353
GSTM4_2_4706,178,210,284,356,140,36,131,119,216,139,812,105
GSTO1_2_4707,333,222,260,179,133,92,243,362,191,23,117,190
GSTO2_2_4708,393,389,600,353,426,545,104,344,50,8,1008,279
GSTZ1_2_4709,67,11,47,89,110,3,35,19,106,2,0,7
GTPBP3_2_4710,188,106,55,104,11,55,0,298,119,400,43,285
GUCY1A3_2_4711,176,189,253,159,58,102,65,118,12,105,289,298
GYG1_2_4712,214,128,128,230,599,22,0,314,195,371,3,39
GYG2_2_4713,405,303,231,382,586,202,169,882,326,706,118,294
GYS1_2_4714,576,527,604,404,420,122,643,474,450,433,690,277
HADH_2_4715,548,467,549,543,62,429,371,232,87,595,926,614
HAGH_2_4716,633,545,382,554,778,406,336,501,458,735,1364,624
HAO2_2_4717,520,316,539,397,117,226,30,331,410,385,334,73
HAS3_2_4718,413,346,315,340,101,81,1041,406,9,165,7,170
HCCS_2_4719,6,16,24,39,0,35,0,1,0,0,0,0
HDHD1_2_4720,328,131,88,158,470,180,4,702,178,17,20,84

HENMT1_2_4721,80,42,75,80,56,20,30,168,0,66,0,70
HHAT_2_4722,35,16,46,17,0,65,3,0,0,2,0,3
HIBCH_2_4723,33,69,29,147,78,0,29,13,200,255,95,86
HLCS_2_4724,725,588,671,645,796,575,457,419,351,550,782,691
HMBS_2_4725,36,41,24,56,218,187,0,2,44,0,8,130
HMGA2_2_4726,125,155,143,204,98,27,899,40,0,47,5,184
HMGCL_2_4727,87,84,128,89,19,0,2,94,109,6,68,100
HMGCLL1_2_4728,730,344,588,687,316,8,194,404,397,843,397,673
HMGCR_2_4729,68,8,14,55,0,0,149,10,32,63,0,17
HMGCS1_2_4730,1907,1655,1517,2097,1822,837,866,1488,1321,2550,2836,183
7
HMGCS2_2_4731,105,112,30,57,3,162,4,1,97,233,473,2
HMOX2_2_4732,86,59,67,29,26,77,4,43,23,114,0,60
HNRNPAB_2_4733,137,112,75,72,24,41,102,10,8,121,126,21
HPD_2_4734,332,437,345,516,48,423,562,861,8,30,269,549
HPGD_2_4735,1533,1042,996,1088,1703,435,1032,1101,887,749,1223,1510
HPSE2_2_4736,205,213,223,193,533,471,299,216,312,336,102,194
HPSE_2_4737,404,107,182,316,503,949,134,229,3,289,30,185
HRAS_2_4738,38,82,65,182,99,261,352,31,25,211,3,223
HS2ST1_2_4739,2,45,16,10,4,0,179,4,0,0,18,40
HS6ST2_2_4740,24,8,15,61,3,8,0,10,49,240,0,4
HSD11B1_2_4741,0,0,0,0,0,0,0,0,0,0,0,0
HSD17B10_2_4742,12,78,36,47,3,1,105,254,2,7,0,1
HSD17B13_2_4743,979,1081,965,1206,869,1490,1440,989,1301,1085,1086,946
HSD17B4_2_4744,181,49,81,47,465,0,467,175,64,0,0,158
HSD3B2_2_4745,474,509,441,538,835,321,473,604,68,1056,343,1111
HSD3B7_2_4746,105,109,169,225,72,36,0,0,91,193,1,7
HSDL1_2_4747,263,311,315,402,104,158,344,242,163,315,235,239
HSP90AA1_2_4748,913,1295,968,1048,1876,967,654,1262,529,1025,1654,1035
HSPA8_2_4749,59,35,93,68,20,0,42,0,0,310,164,168
HSPD1_2_4750,216,153,136,326,61,715,431,10,165,250,698,489
HTRA2_2_4751,1437,1263,1177,1277,1363,1535,1497,467,1238,1640,638,1512
HYAL1_2_4752,188,109,234,154,460,28,0,57,300,4,504,8
HYAL2_2_4753,318,114,154,266,493,325,212,1,195,186,2,234
HYI_2_4754,32,7,20,10,0,0,1,164,0,0,1,10
IARS_2_4755,821,607,494,527,509,877,993,447,802,849,915,292
IDE_2_4756,203,99,255,200,62,0,403,9,82,0,5,415
IDH3B_2_4757,254,217,143,142,203,234,250,607,274,105,59,68
IDH3G_2_4758,0,0,0,0,0,0,0,0,0,0,0,0
IDS_2_4759,192,197,178,211,400,458,10,137,135,20,22,355
IFT27_2_4760,632,800,905,755,137,732,2008,951,740,1042,1813,1175
IL4I1_2_4761,166,302,94,97,478,683,57,317,223,278,133,10
IMPDH1_2_4762,9,29,8,62,17,7,0,0,3,17,0,3
INMT_2_4763,284,133,160,196,320,116,563,315,84,261,93,143
INPP5K_2_4764,675,505,493,593,863,859,313,819,913,97,312,631
INTS6_2_4765,666,555,636,803,838,203,1214,495,424,335,662,208
IPCEF1_2_4766,127,36,100,114,134,0,789,10,156,286,12,25
ISOC2_2_4767,18,73,101,8,0,265,76,10,0,0,0,0
ISPD_2_4768,179,206,246,281,145,374,127,606,171,25,6,56
ISYNA1_2_4769,32,56,66,100,0,0,121,345,10,8,1,289

ITPA_2_4770,42,69,113,80,608,276,0,23,0,0,428,0
IVD_2_4771,85,97,92,139,25,9,0,0,0,190,0,373
IYD_2_4772,974,795,814,734,140,416,1754,791,690,453,471,479
JMJD7-
PLA2G4B_2_4773,1352,1481,1666,1558,1145,1342,2539,378,1157,1524,1727,2
464
KARS_2_4774,289,374,267,318,122,950,847,228,33,926,656,257
KATNA1_2_4775,522,526,574,721,846,1434,362,406,221,487,291,361
KATNAL1_2_4776,992,1107,899,1133,633,738,1195,1137,1057,1265,1184,887
KIF16B_2_4777,282,434,276,506,0,475,370,578,292,2,321,0
KIF9_2_4778,146,112,124,159,129,205,42,149,27,9,54,116
KIFC3_2_4779,11,3,46,65,2,59,6,0,0,0,18,0
KLK2_2_4780,202,121,82,161,243,167,17,18,13,159,0,29
KLK7_2_4781,786,661,504,728,757,834,1067,93,975,1053,767,728
KRAS_2_4782,101,44,163,167,236,0,193,105,127,125,241,144
KYNU_2_4783,933,929,718,898,685,1376,902,669,694,822,840,667
LAMP2_2_4784,840,971,946,1127,1758,982,531,1097,1258,1163,225,836
LARGE_2_4785,27,32,28,33,0,1,0,35,23,0,0,67
LCLAT1_2_4786,189,239,145,194,1,0,540,346,20,184,2,257
LCMT1_2_4787,919,781,529,801,481,219,45,821,318,57,768,666
LDHA_2_4788,2107,1710,1546,2300,1771,871,1913,3239,1795,1388,2781,982
LDHAL6A_2_4789,180,74,55,177,19,284,0,292,0,14,0,113
LDHB_2_4790,1053,813,879,701,1388,2061,365,1069,816,884,814,1493
LDHC_2_4791,382,181,111,208,34,67,35,238,60,16,27,282
LDHD_2_4792,11,15,23,43,0,0,0,0,13,147,0,1
LEPRE1_2_4793,398,315,303,380,152,32,58,68,339,254,231,166
LEPREL1_2_4794,21,138,12,85,2,1,0,930,93,0,0,13
LFNG_2_4795,79,55,196,188,20,32,581,0,182,353,565,244
LGMN_2_4796,213,235,175,254,64,472,226,214,8,247,10,207
LGSN_2_4797,425,637,448,612,1198,802,745,858,575,302,430,816
LIAS_2_4798,2162,1751,1793,1966,2349,1961,2129,1550,2258,642,1045,2191
LIG3_2_4799,169,96,107,139,22,88,335,9,121,157,1090,107
LIG4_2_4800,399,318,388,475,826,567,591,237,590,240,917,395
LIPF_2_4801,49,112,73,131,100,3,42,16,54,125,43,197
LIPT1_2_4802,66,98,69,133,187,3,30,6,171,22,2,16
LMO7_2_4803,87,12,85,46,1,134,2,10,54,8,700,53
LNPEP_2_4804,125,79,78,102,149,13,174,10,15,70,633,149
LOX_2_4805,752,755,595,798,1342,738,708,493,516,301,130,570
LPO_2_4806,379,494,401,526,615,394,676,43,189,1176,1663,293
LRRC16A_2_4807,163,110,90,136,84,15,0,10,245,0,13,35
LSS_2_4808,239,264,155,196,377,213,451,367,91,539,407,198
LYZL6_2_4809,214,75,143,194,59,157,59,67,246,136,0,169
MACF1_2_4810,490,533,448,569,235,186,584,393,379,617,209,410
MACROD2_2_4811,333,380,256,356,158,59,394,688,273,722,101,423
MAD2L2_2_4812,644,354,428,702,573,452,537,338,1063,440,169,509
MAN2B1_2_4813,113,158,114,168,89,22,19,8,137,24,101,232
MASP2_2_4814,602,490,625,455,318,354,80,458,437,401,589,338
MAT2B_2_4815,429,240,188,303,293,61,721,146,329,225,370,321
MCAT_2_4816,450,285,397,393,327,9,20,395,231,317,237,727
MCM4_2_4817,66,103,77,87,166,1,40,156,121,24,16,224

MCM7_2_4818,79,174,133,212,96,0,76,243,52,19,125,263
MCM8_2_4819,30,65,21,46,42,0,0,207,2,56,36,14
MCM9_2_4820,116,144,101,186,143,522,12,139,80,543,9,118
MDH1_2_4821,214,69,115,124,100,0,0,0,73,565,114,132
MECR_2_4822,173,498,303,295,141,736,402,74,531,8,1104,438
MEPCE_2_4823,29,6,9,46,1,0,937,4,4,1,587,52
METTL13_2_4824,280,293,384,468,622,721,264,236,517,485,669,18
MFN2_2_4825,166,228,229,245,248,0,269,7,397,766,381,14
MFNG_2_4826,338,321,361,507,511,378,304,160,537,583,137,399
MGAT3_2_4827,391,292,427,369,688,386,152,373,462,430,670,203
MGAT4A_2_4828,131,116,193,114,640,309,10,0,10,0,1,129
MGAT4B_2_4829,142,213,246,318,351,456,372,814,383,494,196,370
MGLL_2_4830,529,564,365,547,391,358,248,1436,303,263,1262,761
MGST1_2_4831,577,379,476,259,231,291,468,155,476,681,230,550
MGST2_2_4832,852,838,757,814,1236,69,1211,1559,1329,953,943,1064
MICAL1_2_4833,32,50,45,22,0,1,4,156,101,25,0,88
MLH1_2_4834,499,268,469,639,321,847,124,300,402,578,1344,480
MME_2_4835,156,162,143,145,44,149,110,26,280,40,172,305
MMP1_2_4836,156,211,386,292,392,49,98,586,137,0,78,123
MMP2_2_4837,20,63,82,110,73,396,4,0,52,16,169,0
MOCS1_2_4838,106,19,23,41,35,0,0,104,165,38,82,4
MOGS_2_4839,50,14,89,66,49,0,84,15,233,0,1,5
MOV10_2_4840,293,158,136,141,142,58,395,129,66,92,121,302
MOV10L1_2_4841,475,482,412,493,489,125,471,498,87,1286,367,654
MPG_2_4842,176,172,229,283,68,3,760,253,14,0,230,144
MPPE1_2_4843,207,269,167,213,125,267,94,485,214,431,130,63
MPST_2_4844,196,130,170,150,19,96,231,239,331,17,5,22
MRAS_2_4845,110,84,133,301,160,0,252,73,42,0,259,799
MRE11A_2_4846,230,187,153,159,240,206,43,241,199,302,153,91
MRI1_2_4847,33,11,8,68,191,0,77,0,6,5,14,4
MSH5_2_4848,199,73,64,95,246,74,0,0,0,0,0,57
MSRA_2_4849,288,342,372,369,280,830,2,967,61,528,766,557
MSRB3_2_4850,51,85,27,41,58,12,0,1,58,483,0,22
MTHFD1L_2_4851,402,377,418,480,355,156,638,811,357,629,297,478
MTHFS_2_4852,346,365,427,233,936,7,338,7,250,577,4,84
MTO1_2_4853,644,744,776,812,770,637,550,740,301,376,1370,904
MTRR_2_4854,360,314,287,478,279,126,971,1,428,74,605,821
MUTYH_2_4855,73,31,48,42,25,14,364,35,14,8,9,4
MX1_2_4856,287,190,242,280,606,118,62,43,166,29,19,323
MYBBP1A_2_4857,15,39,25,69,0,0,0,17,0,4,0,2
MYH2_2_4858,145,189,170,281,74,12,763,18,80,16,131,221
MYO5A_2_4859,101,75,90,91,180,0,39,0,100,119,0,179
MYO7A_2_4860,39,53,126,38,14,21,7,10,42,19,397,29
MYO9B_2_4861,65,75,48,75,5,170,41,31,0,32,0,0
N6AMT1_2_4862,634,351,252,427,152,206,218,389,3,257,182,252
NAA16_2_4863,498,365,393,489,569,115,758,332,282,273,202,526
NAA20_2_4864,1036,1146,999,1171,1500,1321,1067,567,1550,1061,774,1155
NAAA_2_4865,128,104,306,125,336,195,1107,278,195,73,393,89
NAPEPLD_2_4866,1439,1382,1386,1172,1488,1799,831,1920,1827,645,592,919
NARF_2_4867,85,96,140,110,13,241,18,17,124,10,12,45

NARS2_2_4868,766,669,847,872,291,1150,2031,1054,1201,474,585,233
NAV1_2_4869,585,669,501,993,627,763,281,818,909,522,115,451
NCEH1_2_4870,614,578,500,438,289,357,346,849,472,328,239,305
NCF2_2_4871,574,401,577,475,497,181,464,237,396,211,812,999
NCF4_2_4872,78,128,106,120,189,192,78,373,70,69,1,125
NDOR1_2_4873,1012,996,805,942,1146,903,496,350,429,247,556,897
NDUFA2_2_4874,333,254,330,445,894,129,92,624,51,190,1667,155
NDUFB11_2_4875,88,57,91,106,0,217,217,29,15,52,464,17
NDUFB4_2_4876,7,0,18,34,0,0,0,0,0,0,0,0
NDUFB5_2_4877,379,248,331,311,161,237,401,303,220,285,236,170
NDUFB6_2_4878,703,990,643,1064,472,1251,615,1512,339,928,485,1269
NDUFC1_2_4879,442,396,346,325,58,37,68,8,401,156,267,438
NDUFS1_2_4880,593,620,565,1029,1068,1020,205,883,576,429,559,2447
NDUFS2_2_4881,187,140,136,129,79,12,669,99,132,113,434,98
NDUFS5_2_4882,141,39,56,149,11,12,67,93,93,614,51,67
NDUFV1_2_4883,96,64,83,145,164,285,80,132,51,14,118,214
NEIL2_2_4884,644,653,804,851,1101,681,869,478,339,820,1190,312
NEU4_2_4885,20,82,15,17,0,19,0,0,0,45,0,0
NFS1_2_4886,81,200,130,95,189,216,0,51,376,2,72,231
NGLY1_2_4887,22,26,68,140,107,3,207,26,22,1,0,0
NIPSNAP1_2_4888,78,34,55,69,200,1,0,0,3,1,20,18
NIT1_2_4889,92,18,4,50,0,0,0,13,9,0,0,0
NKIRAS2_2_4890,1205,946,963,1125,1217,1454,555,702,987,744,582,717
NLGN3_2_4891,306,205,254,395,161,411,111,801,104,120,370,567
NLGN4X_2_4892,367,500,398,379,639,269,283,445,173,804,97,739
NLGN4Y_2_4893,1,74,147,5,0,0,0,0,0,3,286,2
NMNAT2_2_4894,302,186,157,246,491,72,152,204,73,331,208,211
NNT_2_4895,646,555,565,564,34,495,1746,448,127,376,1415,881
NOX4_2_4896,121,238,244,282,252,260,232,71,257,189,348,907
NPL_2_4897,132,168,228,185,318,371,34,133,63,167,429,49
NQO1_2_4898,590,289,161,331,908,1,327,637,412,131,847,0
NRD1_2_4899,290,352,172,447,108,220,103,1196,335,328,124,756
NSDHL_2_4900,79,75,60,165,12,69,0,281,48,93,150,467
NSUN2_2_4901,233,31,95,70,46,7,300,52,105,207,14,3
NUDT2_2_4902,18,49,45,49,22,188,809,25,0,0,39,57
NXN_2_4903,32,10,93,33,2,0,15,2,6,85,0,2
OAS1_2_4904,170,355,212,304,284,274,440,422,399,538,234,77
OAS2_2_4905,168,111,91,117,0,34,4,87,2,0,0,181
OASL_2_4906,1608,1325,1102,1478,2627,308,1788,791,1174,1721,814,1112
OAT_2_4907,82,101,65,37,107,72,100,0,135,4,100,171
OGDH_2_4908,59,130,81,103,1,244,0,317,7,371,861,366
OGDHL_2_4909,129,114,137,109,164,19,260,201,234,98,18,51
OGG1_2_4910,79,73,48,50,0,0,438,47,0,3,312,321
OGT_2_4911,118,132,95,101,76,29,74,305,143,121,18,67
OLAH_2_4912,1026,786,890,797,922,567,539,1853,669,1024,733,1309
P4HA1_2_4913,141,55,90,231,357,0,156,8,45,4,26,210
P4HA2_2_4914,306,363,447,340,1111,206,448,52,166,291,368,23
P4HTM_2_4915,26,11,48,9,0,1,3,0,17,8,0,0
PAFAH1B2_2_4916,260,104,200,193,523,62,49,298,116,171,677,240
PAFAH1B3_2_4917,11,3,33,50,0,0,1,0,1,0,168,23

PAICS_2_4918,1357,1459,854,1665,1431,827,1099,1791,1986,2116,874,1459
PAOX_2_4919,294,346,373,307,612,150,427,283,628,440,305,297
PAPD4_2_4920,1749,1381,1419,1559,1648,1519,3093,1741,1660,1162,1038,16
25
PAPD5_2_4921,448,407,325,539,1048,328,590,278,809,398,483,303
PAPD7_2_4922,1281,1305,955,1461,2034,898,783,1154,1297,1772,326,1078
PAPSS2_2_4923,330,589,481,622,477,253,703,3,161,840,513,229
PARK7_2_4924,183,151,82,79,79,169,0,72,257,73,114,125
PARL_2_4925,173,160,225,271,6,0,95,0,14,431,0,567
PARN_2_4926,337,321,268,316,309,88,442,90,500,172,228,233
PARP2_2_4927,1016,586,586,916,800,253,903,1053,725,508,72,1956
PARP3_2_4928,207,184,190,125,169,7,129,12,208,279,0,137
PARP8_2_4929,309,256,202,385,189,0,438,474,160,439,480,393
PBLD_2_4930,344,244,303,318,193,99,670,181,708,157,576,290
PCCA_2_4931,71,111,83,209,59,231,59,51,0,0,146,26
PCCB_2_4932,251,256,217,287,333,342,404,202,321,240,96,91
PCMTD2_2_4933,324,194,195,397,48,11,27,504,134,400,0,82
PCNA_2_4934,687,658,634,923,936,1216,450,640,760,1662,1324,274
PCSK1_2_4935,74,101,97,134,71,94,539,31,3,169,277,2
PCSK2_2_4936,13,41,28,9,0,0,32,0,319,0,0,0
PCYT1B_2_4937,66,20,79,44,109,0,249,43,13,66,24,4
PCYT2_2_4938,332,346,351,309,20,176,54,80,200,323,596,243
PDE10A_2_4939,25,25,8,4,131,14,0,0,25,34,0,17
PDE11A_2_4940,425,282,215,222,323,268,17,493,131,255,269,135
PDE1A_2_4941,273,311,240,319,396,430,180,427,52,299,140,642
PDE1B_2_4942,204,125,187,275,440,190,107,398,315,168,16,369
PDE1C_2_4943,137,86,41,148,160,24,0,410,128,66,5,21
PDE2A_2_4944,7,2,32,22,27,3,0,44,0,12,0,0
PDE4A_2_4945,116,220,113,187,437,197,395,0,215,230,171,146
PDE4B_2_4946,245,244,313,246,722,239,374,125,449,388,0,369
PDE4C_2_4947,4,5,4,0,21,0,1,0,10,0,0,1
PDE4D_2_4948,1872,1524,1333,1291,2211,855,2173,2512,1823,2569,1464,160
8
PDE5A_2_4949,41,23,54,67,23,57,0,210,20,20,17,212
PDE6B_2_4950,361,410,251,283,473,205,1139,328,0,595,0,213
PDE7A_2_4951,170,148,214,268,20,841,0,778,141,281,1,698
PDE8A_2_4952,111,65,78,82,143,0,109,44,151,20,42,168
PDE8B_2_4953,401,434,434,449,224,179,458,54,158,18,257,747
PDE9A_2_4954,902,1054,830,1185,920,1895,936,772,668,1094,841,1670
PDHA1_2_4955,272,222,173,297,527,3,250,110,479,414,202,301
PDHB_2_4956,68,52,149,138,63,13,4,4,18,208,560,696
PDHX_2_4957,1499,1067,1020,1246,1757,1105,797,2929,1003,2052,1042,1042
PEMT_2_4958,14,33,92,65,0,0,4,1,80,0,0,18
PEPD_2_4959,104,114,117,109,34,102,6,200,20,119,3,221
PGAM5_2_4960,30,43,19,7,0,0,0,0,0,0,0,0
PGBD1_2_4961,0,51,120,81,0,0,0,0,0,12,0,2
PGC_2_4962,658,386,383,490,1573,705,142,105,903,449,466,169
PGM1_2_4963,336,464,363,444,86,205,179,36,852,409,93,722
PGM3_2_4964,295,174,112,370,36,764,37,404,537,221,233,116
PHOSPHO1_2_4965,73,40,51,57,0,86,44,5,119,68,0,287

PHOSPHO2_2_4966,3218,2696,2393,2675,2921,2675,2444,2875,2556,2971,2521
,3126
PHYH_2_4967,270,193,254,463,0,68,192,116,764,368,44,372
PIGA_2_4968,2404,2325,1791,1968,1566,1784,3125,2037,860,2002,3026,1822
PIGC_2_4969,1051,1152,908,1208,870,907,894,1012,354,392,667,371
PIGF_2_4970,1640,1135,1179,1330,2556,529,1796,1053,1337,948,4803,1076
PIGG_2_4971,12,29,36,23,0,0,87,0,1,0,0,0
PIGN_2_4972,275,228,271,261,393,306,19,1426,292,292,232,100
PIGO_2_4973,91,192,68,147,0,0,1,0,232,553,177,385
PIGP_2_4974,892,932,757,747,525,1746,1435,1725,450,960,490,455
PIGQ_2_4975,112,85,108,165,37,33,0,6,11,42,0,47
PIGT_2_4976,26,60,11,62,264,5,328,13,0,510,336,3
PIGV_2_4977,87,66,64,136,121,82,204,202,2,0,330,137
PIN4_2_4978,874,1097,956,884,819,704,2233,1128,732,839,2246,809
PLA1A_2_4979,166,221,177,233,79,300,427,47,106,341,506,155
PLA2G2A_2_4980,419,238,313,453,394,173,472,70,314,282,2,334
PLA2G4C_2_4981,753,875,924,1115,693,944,1044,620,828,866,751,1381
PLA2G6_2_4982,44,17,46,11,1,59,3,0,144,16,267,5
PLA2G7_2_4983,1034,691,792,985,578,1347,700,234,1335,780,291,1558
PLAT_2_4984,85,252,171,116,0,11,5,198,2,85,66,45
PLAU_2_4985,11,9,6,5,16,0,0,0,0,0,0,84
PLB1_2_4986,559,402,446,411,680,200,341,49,589,368,608,339
PLCB1_2_4987,401,193,264,245,428,459,249,47,104,165,203,282
PLCB3_2_4988,354,221,337,448,249,319,53,974,328,59,849,643
PLCB4_2_4989,1118,972,822,724,1235,428,2029,245,1013,1686,421,1223
PLCD1_2_4990,461,445,670,522,1215,472,597,364,227,888,709,239
PLCE1_2_4991,150,115,92,110,160,0,59,44,344,37,58,209
PLCG1_2_4992,210,191,143,187,6,0,25,572,177,125,319,0
PLCH1_2_4993,1008,941,897,1142,1369,486,1332,1137,1021,325,225,1206
PLCL2_2_4994,2426,2515,2403,3369,2029,1738,934,1834,1829,4601,3040,335
9
PLCXD2_2_4995,501,461,231,373,1226,48,61,142,444,611,537,375
PLD2_2_4996,84,24,42,76,469,119,67,239,200,39,5,57
PLD3_2_4997,219,19,89,88,10,202,72,457,34,104,44,25
PLOD2_2_4998,1709,1918,1238,1764,930,1447,2059,1209,1183,2965,723,499
PLSCR3_2_4999,126,117,56,96,0,1,0,0,42,7,36,71
PLSCR4_2_5000,28,138,127,196,0,4,29,1,49,248,79,35
PMEL_2_5001,97,100,48,110,258,224,0,0,31,9,35,237
PMS1_2_5002,200,242,254,186,243,76,298,292,259,99,42,339
PNPLA1_2_5003,580,498,313,598,424,317,361,242,426,590,168,575
PNPLA4_2_5004,381,483,486,482,265,624,142,144,97,560,880,334
PNPLA6_2_5005,5,24,25,23,0,0,173,0,0,0,102,0
PNPLA7_2_5006,324,412,304,383,449,475,250,723,553,52,29,171
POFUT1_2_5007,554,448,466,562,454,106,438,532,375,581,1111,384
POFUT2_2_5008,166,146,331,178,0,43,0,297,0,230,374,183
POGZ_2_5009,50,81,43,51,0,127,6,0,72,53,18,90
POLD2_2_5010,160,158,200,203,229,102,197,113,34,165,339,248
POLE2_2_5011,2055,1735,1548,1792,1667,882,424,2600,1872,2887,2194,1521
POLG_2_5012,168,182,130,138,69,0,514,0,162,268,21,276
POLL_2_5013,89,93,57,44,12,26,5,9,140,31,245,113

POLR1D_2_5014,131,171,237,222,315,166,230,409,297,96,55,386
POLR3B_2_5015,272,229,182,345,174,411,529,174,344,114,776,269
POLR3H_2_5016,1535,1545,1272,1548,1177,1760,2588,1319,2178,1724,1706,1
539
POMT1_2_5017,49,32,28,51,2,49,39,293,3,0,0,63
PON2_2_5018,2243,2187,2164,2538,2581,1704,3086,2816,2048,1519,2804,265
3
POP5_2_5019,2276,1967,2055,2236,2787,1321,2350,3300,1742,3552,2932,243
8
PPA2_2_5020,572,431,520,598,198,496,58,827,78,50,838,384
PPIE_2_5021,237,199,231,174,177,60,881,453,0,1,20,97
PPIL2_2_5022,30,61,69,106,27,0,28,33,37,111,65,0
PPIL3_2_5023,2366,1846,1603,1920,1156,1351,1262,1540,1114,1874,2601,22
83
PPIL6_2_5024,1327,1411,1198,1530,1167,2336,947,181,950,1604,1526,1123
PPIP5K1_2_5025,705,728,490,542,1156,301,896,233,123,965,539,791
PPT1_2_5026,423,286,192,208,76,40,77,629,159,351,95,51
PPT2_2_5027,252,79,185,149,220,112,186,26,223,43,101,262
PRCP_2_5028,185,140,73,262,7,32,550,116,22,244,3,290
PRDX1_2_5029,177,254,98,205,167,272,165,273,196,69,1424,159
PRDX3_2_5030,159,164,159,249,206,49,164,55,392,123,12,43
PRDX5_2_5031,280,144,373,322,46,870,156,734,350,635,1134,149
PRKCSH_2_5032,90,16,185,31,0,86,92,1,2,20,0,87
PRODH_2_5033,111,66,193,148,14,79,2,5,6,208,249,278
PRSS3_2_5034,1014,945,887,875,1192,510,937,926,922,959,805,799
PSAT1_2_5035,467,520,422,396,993,17,207,476,804,328,613,283
PSMA1_2_5036,24,11,5,74,0,4,0,0,44,36,0,46
PSMA3_2_5037,246,90,151,167,105,311,160,7,9,153,234,321
PSMA4_2_5038,1176,861,757,876,446,1024,2783,1444,597,625,413,735
PSMA5_2_5039,659,290,244,673,144,905,381,169,578,103,283,372
PSMA8_2_5040,293,569,490,485,316,763,448,39,358,648,0,789
PSMB2_2_5041,930,796,657,880,761,358,576,219,335,793,1024,893
PSMB5_2_5042,89,95,131,149,92,15,473,115,439,295,309,249
PSMB8_2_5043,4,25,11,11,2,243,0,0,0,14,0,2
PTBP1_2_5044,20,21,23,27,0,0,0,0,0,0,0,644
PTER_2_5045,279,180,217,166,54,95,68,148,55,392,17,383
PTGR1_2_5046,1678,1277,1505,1790,1823,815,1819,3984,1979,1074,1309,173
4
PTGR2_2_5047,613,726,497,546,576,338,114,933,803,287,1165,815
PTGS1_2_5048,93,66,242,178,61,13,13,339,104,244,100,132
PUS1_2_5049,2748,2369,1969,2479,2261,1936,3329,1498,1900,2346,1433,192
3
PYCR1_2_5050,41,127,35,42,14,71,545,77,191,561,38,34
PYGL_2_5051,443,233,325,304,118,193,364,19,434,56,158,540
PYGM_2_5052,82,50,82,75,8,0,0,314,98,2,2,97
QPCTL_2_5053,523,447,307,527,1110,191,247,393,202,556,429,748
QSOX1_2_5054,99,92,58,43,259,2,163,56,66,6,10,191
RAB11A_2_5055,862,907,1041,968,348,359,1536,155,1299,1579,1071,683
RAB1A_2_5056,1287,1362,944,1081,942,1640,834,1597,1939,1100,150,812
RAB23_2_5057,1200,1200,1208,1486,1119,878,1771,1408,810,1803,1767,792

RAB27A_2_5058,534,332,542,485,569,422,805,1025,175,429,476,742
RAB28_2_5059,180,176,270,321,228,63,579,335,668,311,361,55
RAB2A_2_5060,91,92,38,111,211,42,109,2,0,94,127,34
RAB2B_2_5061,165,113,158,148,136,187,1251,145,246,135,354,26
RAB34_2_5062,363,336,154,301,1,403,4,336,565,247,749,174
RAB35_2_5063,440,303,400,368,395,116,840,468,273,386,721,386
RAB37_2_5064,118,61,27,30,7,23,3,41,26,4,6,3
RAB40C_2_5065,80,152,122,86,22,3,363,97,239,15,0,1
RAB5C_2_5066,240,443,220,463,5,4,366,0,308,523,199,468
RAB6A_2_5067,410,185,196,293,392,71,1037,535,293,130,461,274
RAB7L1_2_5068,278,172,100,138,85,268,0,198,519,7,190,141
RAB9A_2_5069,1353,1182,1491,1359,626,736,1207,1063,1086,742,848,1589
RABGGTA_2_5070,236,183,110,319,158,3,136,146,0,323,1026,230
RABL2A_2_5071,1822,1819,1941,1879,1671,1380,1586,1394,2206,1560,1990,2
507
RABL2B_2_5072,22,50,33,70,3,0,0,0,6,4,9,90
RAC1_2_5073,115,55,37,96,0,145,0,246,120,43,0,28
RAD51B_2_5074,40,158,70,78,279,19,61,0,95,121,0,180
RAD51_2_5075,371,380,568,674,83,504,125,474,404,203,298,1038
RAD51D_2_5076,162,150,158,420,8,1,379,81,208,282,357,261
RAD54B_2_5077,97,283,187,148,133,225,24,310,87,243,271,19
RAD54L_2_5078,342,405,269,436,579,146,899,45,158,946,63,731
RAD9A_2_5079,156,138,121,225,44,23,30,292,0,267,0,232
RAP1A_2_5080,102,99,77,162,199,618,29,118,17,606,250,57
RAP1B_2_5081,369,421,367,618,284,448,361,353,577,552,635,439
RBBP8_2_5082,799,692,744,948,1321,827,985,738,899,776,1061,1058
RDH13_2_5083,357,378,365,339,38,96,1261,307,528,109,361,66
RDH5_2_5084,6,28,36,41,0,0,0,0,7,6,0,129
RECQL5_2_5085,452,295,484,372,640,329,458,761,237,564,92,606
RECQL_2_5086,376,468,448,526,143,725,511,887,266,305,649,602
RERG_2_5087,1276,1175,981,1361,962,795,751,1614,1301,1223,2137,1560
REV1_2_5088,416,581,373,486,641,537,444,616,720,1461,878,323
RFC3_2_5089,128,38,184,142,366,209,92,269,194,7,92,210
RFC5_2_5090,103,131,80,118,125,63,109,189,185,105,44,58
RGN_2_5091,551,323,292,510,567,373,125,617,126,292,0,119
RGS11_2_5092,1043,761,884,964,350,1161,1268,931,598,1268,278,548
RGS6_2_5093,139,280,251,412,31,0,13,105,6,61,166,595
RHOC_2_5094,542,432,475,596,23,106,669,97,453,420,1468,672
RHOT1_2_5095,11,20,15,22,19,9,3,0,0,18,535,50
RNASE1_2_5096,90,165,124,154,132,75,67,320,63,129,513,93
RNASE4_2_5097,121,51,55,69,19,35,37,132,1,229,0,128
RPAP3_2_5098,291,132,152,174,263,9,95,33,230,258,10,48
RPE_2_5099,185,96,169,148,173,297,87,49,306,213,11,378
RPN2_2_5100,179,177,211,214,220,52,6,71,232,204,153,458
RPP14_2_5101,106,118,77,136,0,0,224,0,55,0,0,0
RPP21_2_5102,973,837,822,853,505,881,1434,381,999,702,536,759
RPP30_2_5103,5433,4673,4806,5460,5458,6334,6104,3869,5982,7469,3968,54
43
RPP38_2_5104,589,449,659,596,1115,641,601,596,543,77,719,791
RPS27A_2_5105,371,313,442,375,1426,307,465,157,403,500,854,7

RRAD_2_5106,24,26,106,63,0,0,218,0,241,0,377,12
RRAGB_2_5107,197,176,178,138,140,189,697,19,167,169,250,86
RRAS2_2_5108,62,77,29,125,39,0,0,0,42,1,131,1
RRM2B_2_5109,729,916,800,867,663,335,1071,395,878,704,1225,417
RRM2_2_5110,0,0,0,0,0,0,0,0,0,0,0,0
RTEL1_2_5111,743,554,557,668,812,325,417,406,359,500,182,801
SAR1A_2_5112,356,301,262,337,736,204,422,347,281,435,540,560
SAR1B_2_5113,862,1014,1005,1135,285,1004,456,1517,715,555,1573,1004
SARDH_2_5114,150,83,71,128,48,0,0,436,70,46,52,47
SARS2_2_5115,42,19,67,66,0,2,86,151,27,20,0,119
SBNO1_2_5116,273,327,182,240,110,82,311,274,181,122,169,200
SC5DL_2_5117,2210,1564,1709,2331,1440,2073,2684,3365,2442,1731,1435,32
64
SCD5_2_5118,129,156,88,160,100,111,408,182,541,234,10,459
SDC1_2_5119,137,104,133,85,46,68,471,48,72,158,180,51
SDCBP_2_5120,576,859,751,745,106,1453,720,613,55,725,1085,286
SDHC_2_5121,468,229,303,366,489,13,323,188,406,321,25,1041
SEPHS1_2_5122,57,82,58,94,433,364,0,0,4,36,0,68
3-Sep_2_5123,135,89,154,147,92,38,32,173,94,56,10,330
4-Sep_2_5124,450,271,438,397,646,45,434,464,701,754,110,486
5-Sep_2_5125,135,103,134,94,151,22,307,219,271,264,41,142
9-Sep_2_5126,326,179,237,313,357,63,269,589,449,739,16,447
SGMS2_2_5127,543,322,352,520,584,32,579,136,348,181,1085,353
SH3GLB1_2_5128,403,321,400,450,384,63,357,90,890,325,22,440
SHMT1_2_5129,1127,901,842,1425,550,842,258,1517,634,127,690,1187
SHMT2_2_5130,29,35,22,21,0,105,14,2,2,2,82,440
SIAE_2_5131,147,264,126,222,777,362,656,0,38,32,46,407
SLFN11_2_5132,82,107,115,121,103,14,37,109,76,0,20,174
SMG6_2_5133,217,229,109,28,821,391,10,31,126,234,6,9
SMOX_2_5134,218,206,277,225,295,323,22,220,0,44,391,60
SMPD1_2_5135,221,352,333,296,495,274,0,0,0,1040,1111,340
SMPD4_2_5136,841,796,867,802,1448,869,703,1402,533,841,572,2025
SMPDL3B_2_5137,86,90,69,59,52,10,0,110,24,101,80,14
SOD2_2_5138,105,62,109,125,59,19,0,447,49,113,123,56
SPAM1_2_5139,258,336,313,658,1183,784,203,182,65,447,23,437
SPAST_2_5140,557,633,428,537,675,194,211,986,695,182,580,212
SPG21_2_5141,441,513,607,577,478,185,701,512,366,249,629,667
SPO11_2_5142,259,338,243,282,468,959,127,150,125,368,22,253
ST3GAL1_2_5143,64,29,9,54,8,15,7,0,232,29,2,13
ST3GAL5_2_5144,454,344,498,704,347,326,241,514,824,319,1891,502
ST5_2_5145,53,74,66,46,9,10,50,19,47,271,0,35
ST6GAL1_2_5146,438,342,370,579,359,251,202,120,821,196,545,337
ST6GAL2_2_5147,401,366,404,430,290,158,136,305,561,108,0,193
ST6GALNAC3_2_5148,3400,2744,2625,3415,2790,2045,3930,5233,2713,2773,19
66,3800
ST6GALNAC4_2_5149,123,105,77,95,61,13,51,159,152,95,362,26
ST8SIA4_2_5150,1165,852,903,831,2124,429,1214,777,1359,850,891,439
SUCLG2_2_5151,1186,1251,1208,1229,734,322,344,2174,733,427,1021,1696
SULF1_2_5152,2480,2576,1989,3008,2231,1772,1669,1359,1491,2927,2171,25
50

SULF2_2_5153,0,1,0,20,0,4,7,0,1,231,0,0
SULT1A1_2_5154,307,314,460,357,701,506,542,669,272,460,392,418
SULT1A2_2_5155,2469,2163,1980,2272,2716,2656,2377,2251,1185,2696,3467,
2529
SULT2B1_2_5156,92,59,76,70,0,53,0,41,390,19,171,0
SUOX_2_5157,236,385,172,370,490,290,0,50,498,367,0,310
SYTL1_2_5158,25,21,66,10,0,11,15,1,0,0,47,38
TAB1_2_5159,381,497,505,336,113,111,404,19,505,911,670,835
TBXAS1_2_5160,623,686,799,966,771,517,377,535,703,653,1088,1186
TCIRG1_2_5161,227,265,181,343,215,1,94,104,131,368,30,416
TDP1_2_5162,279,328,246,262,1090,9,484,83,307,385,628,28
TERT_2_5163,100,93,84,154,151,261,224,475,2,32,0,21
TGM2_2_5164,348,184,221,348,130,163,443,55,151,722,507,330
TGM5_2_5165,332,431,281,374,416,269,488,344,512,197,170,88
TH_2_5166,419,431,612,550,606,399,204,134,456,141,432,965
TIAM2_2_5167,162,150,188,292,20,104,27,12,770,308,14,190
TKTL1_2_5168,180,333,348,397,25,152,196,37,284,10,3,338
TMEM55B_2_5169,114,144,39,93,0,0,0,0,0,125,115,181
TMLHE_2_5170,976,698,1020,908,516,796,862,1412,646,760,1765,1544
TMOD1_2_5171,882,765,726,882,570,1298,1030,1076,946,675,154,1840
TMX2_2_5172,92,17,61,125,5,0,0,25,3,4,98,26
TNNI2_2_5173,202,100,183,102,359,23,601,326,50,82,0,615
TP53I3_2_5174,1025,787,814,827,1337,619,383,1011,1021,891,1311,986
TPI1_2_5175,19,23,50,49,3,114,17,17,34,219,12,2
TPST2_2_5176,70,85,70,76,1,23,2,0,5,4,0,5
TREX1_2_5177,3,11,6,8,0,0,45,0,0,0,0,10
TRMT1_2_5178,295,217,153,375,343,5,320,231,96,301,1,8
TRMT1L_2_5179,142,40,189,141,476,6,207,46,6,88,25,195
TRMT2B_2_5180,147,216,232,117,0,346,666,145,176,32,7,41
TRPT1_2_5181,15,24,35,45,0,7,50,66,0,0,0,66
TSEN2_2_5182,67,99,112,104,0,61,77,19,58,188,71,17
TSEN34_2_5183,213,618,179,253,54,282,138,377,158,482,0,431
TTLL6_2_5184,13,93,21,55,60,331,3,32,1,1,0,42
TUBAL3_2_5185,231,105,173,123,43,19,287,217,80,144,65,333
TUSC3_2_5186,369,269,245,395,258,169,61,119,374,25,462,736
TXNDC16_2_5187,449,306,521,468,360,489,39,1019,625,105,432,520
TXNDC2_2_5188,92,104,125,90,4,7,31,0,45,2,95,92
TXNDC5_2_5189,129,106,46,35,7,97,449,0,49,23,1,181
TXNL4B_2_5190,390,228,269,159,0,0,0,601,731,344,427,449
TXNRD1_2_5191,834,479,548,689,473,568,1226,620,791,1185,88,322
TXNRD3_2_5192,98,88,73,156,2,24,87,23,4,154,0,419
TYMP_2_5193,11,45,10,48,3,63,105,120,0,25,0,0
UBA52_2_5194,514,277,272,513,734,148,1010,346,457,486,544,381
UEVLD_2_5195,550,518,401,633,332,652,289,796,37,562,369,607
UGDH_2_5196,271,121,392,280,453,643,153,271,188,114,142,91
UGP2_2_5197,363,330,240,330,162,191,353,152,419,387,216,423
UGT1A6_2_5198,227,216,179,152,433,209,276,120,360,293,1,299
UGT2B10_2_5199,479,328,417,491,459,3,414,207,284,203,102,575
UGT2B28_2_5200,1107,1104,984,1175,191,250,824,346,1435,1378,962,1491
UGT3A1_2_5201,434,418,310,334,351,693,235,259,505,1057,36,295

UGT8_2_5202,0,0,0,0,0,0,0,0,0,0,0,0
UNG_2_5203,1370,1208,1056,1293,952,469,1302,1259,896,2154,511,1724
UPP1_2_5204,536,419,493,485,502,377,1181,1437,449,120,830,739
UPP2_2_5205,312,290,235,337,663,23,208,383,277,223,324,167
UQCR10_2_5206,174,130,110,110,125,138,2,4,34,145,1,249
UQCRB_2_5207,261,227,254,387,34,679,272,131,526,265,256,1074
UROC1_2_5208,136,200,102,90,11,125,86,0,123,372,189,270
VCL_2_5209,325,261,305,377,505,39,271,404,121,63,579,273
VNN2_2_5210,1360,848,1071,1167,892,686,808,1469,1173,786,611,1129
VPS29_2_5211,364,370,353,364,69,463,654,55,507,526,412,506
WARS2_2_5212,610,473,419,653,82,166,463,917,106,365,624,256
WARS_2_5213,833,871,865,930,579,348,742,650,599,215,819,1194
WBSCR22_2_5214,10,69,43,80,0,23,0,173,27,72,5,5
WDR46_2_5215,749,593,680,740,894,1048,472,462,785,1116,637,565
WFS1_2_5216,219,260,126,269,174,204,286,84,39,217,238,185
WRNIP1_2_5217,64,8,1,10,28,0,0,0,9,0,0,0
WWOX_2_5218,237,194,148,202,638,72,143,121,148,110,75,359
XPNPEP1_2_5219,917,592,999,862,561,269,1728,417,170,240,565,444
XRCC3_2_5220,81,101,34,93,56,8,0,0,7,1,10,237
XRN1_2_5221,67,151,82,89,377,5,0,67,56,44,1,191
YWHAZ_2_5222,430,729,624,355,841,364,973,249,886,1229,336,335
ZBED1_2_5223,38,10,15,37,0,0,0,0,21,148,0,6
ZCCHC11_2_5224,720,855,685,921,759,1701,31,429,411,439,639,1033
ZCCHC6_2_5225,754,543,428,473,514,610,135,552,596,955,1112,440
ZDHHC15_2_5226,188,68,67,89,265,11,51,121,6,109,392,154
ZDHHC7_2_5227,625,589,674,703,653,59,569,219,362,898,577,632
ACYP1_2_5228,953,805,832,845,454,1380,506,1164,592,1274,1055,925
APOBEC3F_2_5229,873,931,764,921,698,561,1105,260,736,1630,510,585
ASCC3_2_5230,322,278,275,334,167,182,229,529,346,128,241,433
ASPH_2_5231,596,359,400,398,377,97,1198,524,766,186,175,538
ATP6V0E2_2_5232,98,143,118,235,0,337,4,0,535,0,308,59
C10orf2_2_5233,151,79,95,150,25,100,471,393,150,9,73,235
CAPN3_2_5234,1611,897,1079,1384,811,297,705,1111,906,1378,246,1813
CHM_2_5235,51,142,59,57,373,657,3,2,251,149,346,124
CTSC_2_5236,691,1029,746,834,381,2020,1161,1222,564,927,1163,428
CYBRD1_2_5237,2080,1868,2107,2598,1802,1916,1799,1267,2121,1833,1702,2
877
CYP3A5_2_5238,521,469,366,499,8,302,221,683,406,208,143,444
DIO2_2_5239,365,310,284,296,13,314,780,884,133,1038,409,368
DNASE2B_2_5240,301,291,361,307,902,69,239,160,242,464,279,95
DPYD_2_5241,451,478,372,446,183,384,104,164,106,234,753,861
GBA3_2_5242,115,83,106,164,20,41,57,195,28,302,153,0
GCNT2_2_5243,420,433,432,580,1085,290,368,941,694,731,266,376
GLYAT_2_5244,281,426,307,293,95,449,338,531,103,373,76,356
GPLD1_2_5245,136,40,20,34,50,0,0,118,0,0,0,194
GPX1_2_5246,0,0,0,0,0,0,0,0,0,0,0,0
GPX5_2_5247,204,156,275,383,37,168,902,103,201,517,146,347
HNMT_2_5248,347,250,145,223,172,244,415,381,57,7,0,423
HOGA1_2_5249,341,309,501,550,398,473,201,96,424,545,352,714
HYAL3_2_5250,43,68,0,8,0,0,2,0,254,0,0,0

JPH2_2_5251,169,176,86,207,6,210,192,167,224,202,153,142
KLK8_2_5252,32,30,17,42,1,28,0,83,29,0,0,86
LAMA4_2_5253,350,345,168,205,769,83,151,886,259,313,0,823
LRR1_2_5254,8,23,4,61,1,17,0,0,98,9,0,4
LRTOMT_2_5255,277,324,251,353,163,157,294,615,89,549,153,150
METTL1_2_5256,29,111,85,80,55,46,5,24,111,234,0,243
MOCS2_2_5257,510,496,555,415,424,269,165,202,493,369,996,346
NDUFA11_2_5258,103,89,59,79,133,38,53,0,43,79,1,363
NDUFV3_2_5259,1018,1050,1101,1013,1198,702,846,239,1321,1446,279,1131
NMNAT3_2_5260,371,389,662,672,556,243,129,323,782,656,1260,247
PDE4DIP_2_5261,325,292,378,530,863,118,352,661,109,123,835,138
PLG_2_5262,237,124,178,93,263,348,324,92,130,129,377,308
PPCS_2_5263,221,177,383,362,346,74,347,231,26,231,679,449
PRDX2_2_5264,2123,1400,1354,2080,1857,1337,3162,2588,1918,1184,1034,30
97
RAD51C_2_5265,1372,1488,1192,1550,964,994,1959,478,1446,1141,1916,1665
RASD1_2_5266,9,41,2,12,0,7,0,0,0,0,0,2
RASL10A_2_5267,146,200,139,170,125,70,115,234,105,133,432,576
SLX1A_2_5268,428,334,348,325,73,19,240,0,425,471,697,758
SLX1B_2_5269,428,334,348,325,73,19,240,0,425,471,697,758
SPTLC1_2_5270,2654,2356,2403,2339,3039,2121,1315,1886,1316,3218,1540,1
633
ST3GAL3_2_5271,269,182,287,316,325,384,0,71,67,6,869,231
VKORC1_2_5272,30,71,67,127,144,4,0,28,415,16,121,252
XPNPEP3_2_5273,1143,1096,911,1089,1520,887,374,57,991,519,476,487
GNAS_2_5274,107,106,122,156,74,362,0,72,160,0,9,325
GNAS_2_5275,434,435,479,505,885,577,596,521,476,302,512,558
GNAS_2_5276,32,47,38,79,1,233,0,161,0,19,565,168
A4GALT_2_5277,679,739,680,804,746,1035,1435,384,605,403,2039,201
A4GNT_2_5278,10,77,8,13,3,0,28,39,0,301,0,2
AACS_2_5279,15,10,12,19,5,31,36,1,4,0,372,59
AADAC_2_5280,200,89,161,207,0,0,0,0,19,0,0,406
AARS2_2_5281,36,41,75,101,50,346,53,4,30,24,56,385
AARS_2_5282,585,610,767,809,652,961,788,375,714,544,855,834
AASDH_2_5283,2006,1443,1601,1862,986,2068,2631,2883,1452,2245,1537,212
1
AASDHPPT_2_5284,2256,2048,2020,2128,1753,1498,1835,3925,2736,2267,2029
,2030
AASS_2_5285,732,719,776,702,1000,307,196,444,1270,822,156,738
ABHD14A_2_5286,403,228,198,427,163,408,13,201,154,377,677,255
ABHD1_2_5287,448,250,230,357,62,267,62,212,229,80,335,105
ABHD3_2_5288,851,741,747,831,1398,386,446,970,1630,1045,2473,789
ABHD5_2_5289,163,103,144,108,0,0,6,0,0,1,3,282
ABHD6_2_5290,190,119,185,259,93,8,549,544,475,262,265,13
ABHD8_2_5291,117,47,17,62,14,0,11,0,106,82,27,80
ABO_2_5292,46,5,10,97,0,70,0,2,0,1,0,0
ABP1_2_5293,823,727,690,871,1045,986,1143,945,1420,842,683,1118
ACAA2_2_5294,372,294,338,238,415,504,355,0,427,230,254,437
ACACB_2_5295,144,115,119,108,249,530,301,59,25,17,2,25
ACAD11_2_5296,1029,964,904,1262,843,671,466,792,1065,1356,1336,497

ACAD8_2_5297,811,448,671,752,562,285,610,61,987,525,470,1102
ACAD9_2_5298,15,21,13,49,0,1,31,205,0,0,554,4
ACADL_2_5299,276,403,276,410,430,47,51,34,88,801,299,153
ACADSB_2_5300,1290,1071,1095,1612,1558,1594,1388,2137,1518,1334,2130,1
206
ACADS_2_5301,382,519,448,521,96,316,117,797,953,803,387,422
ACAT1_2_5302,1015,732,560,798,1233,458,299,730,309,847,623,1241
ACER1_2_5303,1227,998,989,1226,845,1106,829,1049,777,915,345,1203
ACER2_2_5304,217,173,152,133,140,145,434,446,230,158,147,57
ACER3_2_5305,2144,2022,2276,2494,1712,2927,1712,1636,2390,1575,2408,17
84
ACMSD_2_5306,209,192,171,145,291,22,185,141,178,563,47,210
ACO1_2_5307,0,0,0,0,0,0,0,0,0,0,0,0
ACO2_2_5308,17,24,30,10,2,0,0,341,21,1,0,104
ACOT12_2_5309,332,242,236,281,298,87,452,611,159,24,8,336
ACOT1_2_5310,186,83,170,215,66,0,347,0,26,4,693,33
ACOT2_2_5311,186,83,170,215,66,0,347,0,26,4,693,33
ACOT4_2_5312,171,45,56,106,4,1,411,3,116,17,14,84
ACOT6_2_5313,729,625,531,874,538,296,232,946,930,852,603,1035
ACOT8_2_5314,204,310,159,303,483,0,347,610,309,261,66,0
ACOX2_2_5315,170,72,73,214,246,0,268,199,262,174,1,173
ACOXL_2_5316,111,121,42,101,20,0,0,3,16,116,0,293
ACR_2_5317,348,382,412,461,121,56,291,377,515,513,474,897
ACSBG2_2_5318,580,540,584,659,414,756,781,28,495,356,743,688
ACSF2_2_5319,78,60,31,27,105,89,3,51,16,8,350,23
ACSL1_2_5320,62,148,97,43,135,1,130,95,14,0,0,46
ACSM1_2_5321,1784,1267,1707,1902,1304,965,960,3127,2295,2164,722,2609
ACSM2A_2_5322,4770,4232,3866,4464,6505,4304,3413,5871,4423,3156,2245,5
235
ACSM4_2_5323,275,387,290,447,112,3,430,303,273,146,712,151
ACSM5_2_5324,0,108,114,71,0,0,6,0,6,30,0,75
ACSS1_2_5325,1478,1635,1512,1680,1668,1421,2231,1607,1158,1310,1149,12
93
ACSS3_2_5326,461,544,239,412,262,3,16,238,560,557,23,166
ACTC1_2_5327,709,791,500,721,324,1012,378,69,530,687,838,1175
ACY3_2_5328,421,487,328,373,289,416,234,56,439,486,428,714
ACYP2_2_5329,891,772,781,647,999,761,1055,820,786,757,803,792
ADA_2_5330,163,141,161,145,137,214,140,16,69,367,0,245
ADAM10_2_5331,65,34,54,80,0,2,144,0,87,0,15,169
ADAM17_2_5332,107,80,120,76,61,102,2,134,63,3,0,20
ADAMTS4_2_5333,808,1128,850,1068,1067,362,1536,486,217,215,2271,490
ADARB2_2_5334,78,147,134,48,0,0,40,9,256,540,402,218
ADAT1_2_5335,287,263,379,328,420,540,127,195,126,311,304,73
ADAT2_2_5336,862,717,985,857,861,183,168,352,936,485,378,1394
ADC_2_5337,60,101,62,182,2,29,2,22,27,137,28,164
ADCY1_2_5338,23,48,36,63,0,128,438,0,44,0,137,1
ADCY2_2_5339,472,608,532,395,1305,547,67,481,313,758,1527,446
ADCY7_2_5340,44,55,16,70,22,1,11,0,17,244,4,21
ADCY8_2_5341,448,368,511,459,476,754,875,1296,317,287,907,438
ADCY9_2_5342,358,514,347,375,83,508,1293,262,271,341,263,157

ADH1A_2_5343,334,284,322,288,210,55,400,417,294,237,105,317
ADH1B_2_5344,1882,1883,1931,1911,1777,2012,3314,1318,1904,1805,1663,21
20
ADH1C_2_5345,335,278,321,287,211,57,398,416,294,237,103,316
ADH4_2_5346,320,410,402,396,274,135,26,116,259,169,834,1297
ADH5_2_5347,401,217,186,297,246,69,896,442,499,1213,74,970
ADHFE1_2_5348,141,126,141,195,15,23,400,503,200,73,4,162
ADI1_2_5349,45,26,30,47,54,7,115,45,0,54,11,4
ADO_2_5350,868,702,822,753,1079,1137,1224,1307,1372,657,1114,1062
ADPRH_2_5351,78,113,55,75,5,57,20,128,96,68,103,143
ADPRHL2_2_5352,100,67,85,169,272,93,98,6,22,415,22,357
ADSS_2_5353,325,212,355,261,237,207,183,51,754,30,0,381
AEN_2_5354,49,66,130,38,0,0,560,86,1,421,0,4
AGBL2_2_5355,635,711,566,856,1223,647,97,626,802,642,871,1104
AGMAT_2_5356,329,121,141,149,457,125,80,305,331,127,14,297
AGMO_2_5357,2251,1940,1647,2141,3842,916,3854,1940,1709,1795,1235,1499
AGPAT4_2_5358,863,768,557,878,1155,362,590,829,546,429,828,584
AGPAT5_2_5359,300,308,361,309,555,526,543,337,209,204,875,497
AGPAT6_2_5360,528,352,417,493,752,60,414,644,215,430,384,181
AGPAT9_2_5361,575,352,581,591,608,861,1375,500,490,1242,207,446
AGPS_2_5362,563,519,433,620,377,816,591,299,733,296,207,663
AGXT2_2_5363,933,800,807,875,875,312,1492,1212,1211,769,1795,889
AGXT2L2_2_5364,83,194,92,71,8,1,369,180,180,2,187,213
AGXT_2_5365,721,847,678,816,574,826,916,399,731,926,935,293
AICDA_2_5366,171,359,192,280,362,310,0,0,73,183,180,302
AKR1B10_2_5367,656,582,512,674,711,235,335,107,647,740,581,504
AKR1B15_2_5368,885,842,701,832,792,700,532,278,817,1051,730,854
AKR1B1_2_5369,164,145,138,235,612,30,349,177,94,95,37,388
AKR1C1_2_5370,126,279,284,259,32,601,300,1,86,310,980,127
AKR1C3_2_5371,126,279,284,259,32,601,300,1,86,310,980,127
AKR1C4_2_5372,126,279,284,259,32,601,300,1,86,310,980,127
AKR1E2_2_5373,458,384,229,341,575,504,89,402,305,351,238,285
AKR7A2_2_5374,859,983,1141,1085,714,466,2511,1017,765,1349,521,666
AKR7A3_2_5375,235,423,348,368,620,410,291,33,293,452,21,306
ALAD_2_5376,46,43,69,105,6,0,7,111,11,30,0,198
ALDH1A1_2_5377,104,159,91,98,0,70,282,277,266,10,1,13
ALDH1A3_2_5378,613,603,416,892,703,500,760,1029,891,688,309,1110
ALDH1B1_2_5379,378,192,278,235,277,4,127,283,251,620,72,230
ALDH1L1_2_5380,22,26,16,125,0,178,385,0,114,0,19,94
ALDH1L2_2_5381,549,523,566,712,209,651,931,504,271,178,453,59
ALDH6A1_2_5382,380,439,365,410,396,571,451,143,492,104,363,350
ALDH9A1_2_5383,721,708,632,571,859,566,245,496,934,1014,142,950
ALDOB_2_5384,132,112,166,93,461,44,4,313,414,0,26,150
ALDOC_2_5385,205,156,133,97,189,29,18,21,104,49,10,119
ALG10_2_5386,222,217,196,215,0,305,252,0,0,0,105,1
ALG11_2_5387,641,662,604,680,1084,22,329,938,888,703,1373,377
ALG12_2_5388,163,92,105,151,72,215,38,375,95,28,0,156
ALG14_2_5389,119,53,50,79,176,1,176,110,232,1,0,335
ALG1_2_5390,0,0,0,0,0,0,0,0,0,0,0,0
ALG2_2_5391,501,429,252,317,170,872,554,18,992,502,126,459

ALG6_2_5392,2234,1963,1954,2186,2355,2085,1128,682,1914,1543,2213,1618
ALKBH8_2_5393,681,622,508,596,330,437,1513,1599,573,842,658,322
ALLC_2_5394,1488,1269,1059,1471,2137,1499,1358,974,1757,1413,1197,1634
ALOX12B_2_5395,263,377,270,303,363,117,1000,361,41,367,474,318
ALOX12_2_5396,203,143,269,248,401,271,30,36,476,149,154,129
ALOX15_2_5397,1,23,9,13,1,2,0,419,3,33,0,0
ALOX5_2_5398,273,162,123,210,168,369,478,330,15,2,546,44
AMDHD1_2_5399,631,588,605,496,586,745,657,540,1121,973,328,673
AMY1B_2_5400,8233,7337,7006,8193,10507,6219,9545,7924,7194,9149,5567,1
0898
AMY1C_2_5401,8233,7337,7006,8193,10507,6219,9545,7924,7194,9149,5567,1
0898
AMY2A_2_5402,16827,14399,13317,15147,12788,10710,13041,16530,13634,167
41,14899,19942
AMY2B_2_5403,1043,1172,1054,1173,1697,706,1438,718,459,881,587,1551
ANPEP_2_5404,8,15,15,9,3,32,314,0,1,0,0,0
AOC3_2_5405,1151,626,1024,1056,1674,1325,716,804,650,1935,2045,907
AOX1_2_5406,586,294,447,429,134,416,447,491,443,398,762,227
APEH_2_5407,73,93,74,88,203,31,412,81,67,159,16,66
APEX2_2_5408,232,152,128,243,17,44,1,72,78,205,649,586
APIP_2_5409,454,463,406,563,571,671,376,884,718,707,566,708
APLF_2_5410,190,237,245,420,439,14,728,30,83,6,24,606
APOBEC1_2_5411,2458,2415,1915,2227,2822,1529,1821,1786,1401,1508,3404,
1983
APOBEC2_2_5412,60,101,36,73,173,158,24,9,15,120,8,39
APOBEC3B_2_5413,863,966,736,801,903,646,605,1545,1609,610,1140,658
APOBEC3C_2_5414,1570,1169,1319,1615,1326,950,594,987,618,2010,834,1429
APOBEC3G_2_5415,1220,784,841,1360,1803,794,770,1290,1243,567,1638,1142
ARF3_2_5416,807,660,592,628,563,617,801,707,445,788,1296,508
ARF4_2_5417,451,232,367,343,139,280,391,500,871,134,194,297
ARG2_2_5418,264,194,204,312,84,60,246,31,317,56,106,127
ARHGEF10_2_5419,103,120,123,167,216,12,13,29,129,542,443,24
ARL3_2_5420,76,31,18,53,16,6,0,16,213,38,9,18
ARL4C_2_5421,37,62,39,74,23,19,402,333,13,51,0,133
ARL4D_2_5422,250,262,187,266,163,500,207,142,375,582,161,324
ARL5B_2_5423,269,292,343,311,155,161,1579,10,582,330,620,152
ARL8A_2_5424,136,147,138,101,231,59,50,2,6,294,293,238
ARL8B_2_5425,539,743,891,935,1398,560,900,533,351,352,2148,265
ARSD_2_5426,437,745,394,568,276,1507,411,1351,717,1596,359,765
ARSE_2_5427,186,244,236,262,365,287,204,322,297,266,101,225
ARSG_2_5428,1719,1673,1498,1729,1891,1259,2424,1539,2269,2192,2447,205
7
ARSH_2_5429,481,374,405,436,345,41,567,608,510,959,34,483
ARSI_2_5430,78,72,129,49,1,3,72,0,8,45,407,39
ARSJ_2_5431,137,113,93,112,172,0,16,7,133,255,38,100
ARSK_2_5432,780,467,506,652,1116,417,465,255,332,315,573,725
ART1_2_5433,177,199,154,288,214,302,64,548,231,223,414,314
ART4_2_5434,261,211,253,295,165,194,454,60,62,117,268,123
AS3MT_2_5435,887,772,611,777,1807,881,560,212,1304,1685,384,658
ASNA1_2_5436,89,170,141,183,74,39,17,0,0,5,205,391

ASNSD1_2_5437,34,31,16,33,36,119,127,61,7,7,0,3
ASPG_2_5438,0,1,25,26,0,0,0,0,0,0,0,0
ASPHD2_2_5439,99,90,69,121,7,14,353,0,5,110,362,1
ATAD1_2_5440,345,429,253,428,308,11,206,442,327,673,1006,485
ATIC_2_5441,167,212,100,102,159,205,284,1,162,258,27,22
ATP2C2_2_5442,33,24,61,21,0,6,38,0,30,0,0,63
AUH_2_5443,155,271,201,255,100,278,26,352,366,62,93,496
AWAT1_2_5444,853,822,599,871,660,683,1118,635,980,289,780,748
AWAT2_2_5445,368,219,332,240,454,163,454,496,357,327,20,216
B3GALNT2_2_5446,564,340,505,555,474,404,32,217,75,238,165,644
B3GALT1_2_5447,533,541,481,518,672,390,572,242,308,410,590,359
B3GALT2_2_5448,2092,2300,2097,2255,980,974,1356,2471,1730,2390,2564,31
15
B3GALT4_2_5449,854,660,716,744,524,531,1495,125,720,843,195,138
B3GALT6_2_5450,27,26,20,17,0,0,3,36,1,3,1,10
B3GAT2_2_5451,1289,1257,1333,1339,1659,829,925,542,1202,1683,1205,984
B3GAT3_2_5452,355,102,169,279,156,0,0,288,162,66,0,1
B3GNT2_2_5453,1896,1479,1610,1702,703,1406,1806,2000,799,1856,843,1610
B3GNT3_2_5454,66,63,83,108,205,0,64,10,134,40,237,0
B3GNT4_2_5455,124,172,55,219,19,11,67,67,127,16,1,100
B3GNT5_2_5456,700,496,602,764,1068,729,887,491,586,632,433,1020
B3GNT7_2_5457,37,27,45,49,1,0,69,0,203,1,0,92
B3GNT9_2_5458,434,423,294,410,660,371,220,86,449,129,118,436
B3GNTL1_2_5459,60,38,36,127,128,14,572,70,27,74,25,28
B4GALNT1_2_5460,62,50,66,185,58,11,1,19,18,10,470,134
B4GALNT3_2_5461,286,713,375,376,738,50,65,177,181,404,266,890
B4GALNT4_2_5462,343,334,262,323,521,168,475,118,322,608,277,192
B4GALT1_2_5463,702,700,851,784,1162,1286,777,1254,458,1235,541,941
B4GALT5_2_5464,212,106,287,139,75,340,0,194,43,47,558,165
B4GALT6_2_5465,269,275,391,403,90,107,585,1,114,74,406,234
B4GALT7_2_5466,250,235,209,213,565,37,154,367,169,439,29,71
BBOX1_2_5467,187,113,126,124,14,229,19,35,80,55,0,125
BCHE_2_5468,433,278,403,470,869,72,187,139,312,114,364,552
BCMO1_2_5469,1659,1264,1386,1611,2006,2514,1421,1157,1411,1096,1245,21
32
BDH2_2_5470,696,733,647,532,847,182,605,821,762,568,1222,640
BHMT_2_5471,71,102,91,173,111,4,338,191,243,16,30,55
BLM_2_5472,830,469,646,846,343,836,606,873,781,678,676,620
BLMH_2_5473,827,534,590,853,723,648,181,538,756,733,587,995
BLVRA_2_5474,471,363,371,429,245,197,48,480,668,711,173,282
BLVRB_2_5475,40,1,62,113,155,17,50,32,0,0,122,0
BPHL_2_5476,132,61,85,140,397,1,0,124,172,109,1,30
BST1_2_5477,53,136,73,187,0,0,104,39,46,348,0,34
BTD_2_5478,67,108,97,86,1,6,907,28,56,5,0,264
C12orf5_2_5479,379,279,335,314,116,286,399,149,110,118,132,259
C16orf79_2_5480,205,105,183,179,130,31,250,74,363,0,48,17
C18orf56_2_5481,108,103,100,78,0,0,0,103,49,99,6,119
C1GALT1_2_5482,972,1067,905,1068,312,1930,190,1393,1242,2069,818,1472
C1R_2_5483,80,40,94,108,3,17,332,347,267,0,0,10
C22orf28_2_5484,945,701,737,870,609,1347,881,976,871,600,852,787

C2orf43_2_5485,398,355,355,383,120,772,273,476,659,481,906,137
C5orf4_2_5486,65,27,112,40,83,0,0,186,0,0,0,127
C6orf130_2_5487,338,321,305,536,57,528,144,483,149,248,61,660
CA13_2_5488,277,287,230,348,387,88,181,471,493,778,825,188
CA14_2_5489,406,215,353,476,10,0,28,1092,1072,679,369,696
CA2_2_5490,65,179,208,121,369,17,138,107,49,0,797,391
CA3_2_5491,170,326,193,364,5,284,14,195,65,10,596,1191
CA4_2_5492,1383,1440,1477,1806,1710,2193,1529,914,816,2488,2006,2150
CA5A_2_5493,279,280,242,148,212,68,596,587,191,198,33,134
CA5B_2_5494,82,161,63,93,63,95,87,10,161,330,12,545
CA6_2_5495,2448,1948,1806,1950,1201,916,2115,2122,1609,1807,1759,933
CA8_2_5496,835,779,659,733,831,63,678,517,387,811,840,459
CA9_2_5497,124,187,205,165,132,59,334,137,288,145,221,3
CARS2_2_5498,883,1057,688,628,391,368,1236,257,888,1127,228,872
CASD1_2_5499,18,15,2,22,27,14,1,0,2,7,0,2
CAT_2_5500,1046,921,1055,979,722,1068,1219,458,573,792,2183,993
CBR1_2_5501,65,72,89,132,57,59,470,421,184,61,0,192
CBR3_2_5502,417,345,308,466,205,431,373,655,824,150,398,432
CBR4_2_5503,1027,664,723,772,661,716,1094,932,611,913,1355,272
CCDC88B_2_5504,124,121,80,144,390,14,29,296,28,291,0,138
CCDC92_2_5505,47,19,41,35,3,0,250,11,43,0,0,4
CCNO_2_5506,89,71,60,82,228,63,80,35,36,0,5,54
CCS_2_5507,62,48,50,34,32,0,70,0,37,2,24,172
CCT8_2_5508,1950,2013,2100,2299,1034,2169,2880,1354,1939,2159,2282,370
7
CD274_2_5509,453,493,489,502,230,243,791,613,347,354,407,808
CD38_2_5510,645,412,452,468,892,140,919,251,525,465,585,414
CDA_2_5511,373,296,469,354,740,439,1414,336,274,182,378,344
CDIPT_2_5512,292,219,208,305,164,219,54,154,244,172,34,219
CDO1_2_5513,822,525,578,707,288,939,303,2015,511,618,204,643
CDS2_2_5514,73,42,66,58,17,0,6,69,103,2,6,2
CELA1_2_5515,422,276,307,430,510,591,662,398,412,144,499,485
CELA2A_2_5516,132,127,112,88,49,243,267,20,113,570,60,183
CELA2B_2_5517,762,441,828,720,356,741,402,516,665,854,857,411
CELA3A_2_5518,78,86,30,119,24,5,103,56,67,284,61,57
CELA3B_2_5519,77,22,27,25,0,0,1,0,3,15,38,0
CEL_2_5520,614,386,469,591,624,536,638,364,604,821,267,566
CETN1_2_5521,414,381,388,460,317,36,375,9,184,506,98,258
CETN2_2_5522,472,453,306,522,288,881,469,430,247,458,351,320
CETP_2_5523,163,95,94,96,390,69,40,121,15,114,151,359
CFB_2_5524,56,61,21,60,33,0,12,61,0,0,44,0
CFD_2_5525,251,251,248,200,248,9,411,22,0,0,168,1
CFI_2_5526,116,130,122,103,32,41,1,2,204,1,87,176
CH25H_2_5527,85,117,120,163,0,49,0,26,254,307,1,82
CHDH_2_5528,697,508,378,535,743,212,392,1128,703,771,270,832
CHI3L1_2_5529,120,78,147,51,62,34,0,36,0,0,271,299
CHIT1_2_5530,18,15,22,12,2,0,246,3,39,0,2,2
CHML_2_5531,421,363,348,362,249,22,30,623,189,584,419,736
CHPF2_2_5532,273,167,193,320,250,73,147,331,373,205,695,175
CHPT1_2_5533,547,376,550,720,217,107,190,1,362,456,599,259

CHST10_2_5534,195,84,136,194,543,93,28,26,44,51,1,357
CHST12_2_5535,48,9,22,1,0,3,0,0,237,8,0,0
CHST13_2_5536,371,316,330,294,719,281,99,376,393,166,293,167
CHST14_2_5537,470,241,457,472,859,168,381,640,343,337,341,164
CHST1_2_5538,236,70,73,134,0,3,190,0,1,0,33,135
CHST2_2_5539,265,149,230,191,97,279,588,215,482,24,215,250
CHST3_2_5540,235,328,252,235,128,34,0,134,144,0,300,109
CHST5_2_5541,12,36,16,19,12,6,3,0,9,7,0,0
CHST6_2_5542,103,74,50,56,228,26,11,319,235,158,194,16
CHST7_2_5543,869,1306,763,1151,622,480,1218,387,620,946,1014,632
CHST9_2_5544,932,896,586,879,1124,430,412,299,490,1332,1404,58
CHSY1_2_5545,275,401,357,705,17,128,352,308,267,598,615,889
CHSY3_2_5546,197,299,163,459,741,119,4,224,43,272,47,841
CLPP_2_5547,226,191,134,246,284,7,2,3,8,39,284,589
CLPX_2_5548,790,689,717,1004,417,859,1150,754,662,750,355,683
CLYBL_2_5549,1048,801,844,739,458,875,1163,380,2133,694,786,629
CMA1_2_5550,165,78,31,160,37,2,31,20,399,45,25,1
CMAS_2_5551,703,592,349,657,121,384,1011,248,1780,450,1072,688
CMBL_2_5552,160,148,86,131,430,149,42,166,8,222,65,166
CNDP1_2_5553,1316,925,872,1101,356,913,557,1203,719,1010,381,818
CNTN6_2_5554,1278,815,880,1147,417,1699,1000,2472,1496,763,764,1393
COIL_2_5555,227,157,360,232,500,72,225,45,215,617,521,208
COMTD1_2_5556,132,152,178,160,134,21,4,178,157,55,81,11
COQ2_2_5557,469,388,348,541,381,334,1005,598,339,449,649,823
COQ3_2_5558,574,464,424,545,287,236,617,53,186,701,757,122
COQ5_2_5559,55,18,17,56,26,0,6,16,0,0,47,36
COX10_2_5560,60,186,67,158,177,11,56,153,63,103,130,296
COX17_2_5561,701,403,389,504,450,168,413,933,521,457,295,525
COX4I1_2_5562,96,120,126,133,81,148,35,24,0,9,391,3
COX4I2_2_5563,2318,2402,2242,2074,1088,1693,2146,1559,1910,1721,1685,2
212
COX5A_2_5564,220,189,244,289,294,288,219,41,476,471,6,473
COX5B_2_5565,117,169,179,178,7,22,9,411,11,140,646,542
COX6A1_2_5566,820,577,628,734,780,408,1342,963,685,414,695,1180
COX6A2_2_5567,141,56,91,199,24,147,55,731,164,6,0,122
COX6B1_2_5568,574,483,462,485,99,81,448,297,34,648,380,377
COX6B2_2_5569,158,138,183,129,30,215,378,31,175,46,372,384
COX6C_2_5570,817,700,563,761,1452,318,809,437,805,493,401,248
COX7A1_2_5571,33,11,21,59,1,511,0,25,2,4,0,7
COX7A2_2_5572,405,258,230,288,471,260,260,106,700,331,13,253
COX7A2L_2_5573,512,370,336,437,513,269,359,58,794,49,664,39
COX7B2_2_5574,962,714,816,804,823,646,1238,303,255,890,1427,1016
COX7B_2_5575,345,350,293,328,86,123,345,352,615,685,288,555
COX7C_2_5576,49,54,17,37,0,45,0,11,1,38,0,2
COX8A_2_5577,86,32,20,63,32,34,0,63,0,0,109,0
COX8C_2_5578,100,117,130,204,938,89,54,1092,25,255,115,72
CPA1_2_5579,61,65,94,72,68,61,38,101,95,73,10,89
CPA2_2_5580,301,230,159,212,231,104,278,128,20,77,25,71
CPB1_2_5581,730,317,420,436,1,245,1141,600,312,214,798,121
CP_2_5582,82,119,58,75,111,41,66,29,140,96,155,45

CPE_2_5583,53,52,93,97,32,658,72,84,49,1,48,179
CPOX_2_5584,236,214,173,157,498,583,23,395,477,693,92,346
CPSF3_2_5585,703,627,567,928,139,203,653,319,537,631,848,891
CPT2_2_5586,602,698,464,508,155,379,239,764,175,1338,642,661
CRAT_2_5587,136,134,66,65,129,57,49,1,2,359,10,97
CREG2_2_5588,935,1369,862,1167,1569,398,578,1221,1344,1011,1476,973
CRY1_2_5589,461,489,364,587,246,268,311,209,334,171,339,664
CRYL1_2_5590,196,327,206,243,368,304,508,0,229,588,329,218
CRYZL1_2_5591,479,605,738,873,258,471,1477,1421,481,808,805,1409
CSAD_2_5592,221,214,105,157,10,35,724,882,7,133,11,38
CS_2_5593,57,60,33,31,30,29,39,2,59,0,97,71
CSGALNACT2_2_5594,1906,1755,1754,2277,1574,1899,1399,1383,2835,860,158
7,1995
CSRP2BP_2_5595,142,122,99,219,265,2,125,122,8,10,45,271
CTBS_2_5596,457,254,322,313,560,280,297,248,671,200,137,190
CTRB1_2_5597,256,322,176,350,126,1794,537,0,9,220,382,13
CTRB2_2_5598,539,399,338,368,91,218,408,313,509,152,540,358
CTRC_2_5599,296,366,275,237,436,30,0,676,381,12,447,246
CTSD_2_5600,577,601,631,659,1433,368,1048,370,550,770,221,1026
CTSF_2_5601,73,148,26,43,142,1,14,45,8,144,77,197
CTSG_2_5602,1117,959,999,1143,1410,509,2321,1294,955,1099,1007,1109
CTSH_2_5603,1018,1020,864,978,643,1169,626,1509,706,1675,882,623
CTSK_2_5604,2353,1701,1979,2204,2020,1773,2046,1759,1896,1855,1378,242
6
CTSZ_2_5605,121,91,47,145,225,0,0,164,56,3,15,67
CWC27_2_5606,1080,976,1088,1363,1604,782,457,752,1457,1918,1791,283
CXorf21_2_5607,238,274,165,233,430,77,444,20,162,12,27,85
CYB561D2_2_5608,59,35,56,40,4,369,33,78,146,9,0,74
CYB5B_2_5609,591,662,707,885,432,1006,945,1164,1016,665,479,456
CYB5R1_2_5610,497,414,378,385,16,259,116,160,169,545,274,159
CYB5R2_2_5611,34,9,39,25,0,0,1,0,0,6,0,3
CYB5R4_2_5612,709,860,736,688,201,371,367,280,564,1199,453,996
CYBA_2_5613,101,109,131,197,51,45,106,2,16,89,128,216
CYBB_2_5614,854,740,766,765,281,553,883,232,881,296,1471,465
CYC1_2_5615,304,258,316,288,729,773,91,212,226,280,628,99
CYCS_2_5616,16,96,88,44,18,4,0,0,0,0,0,3
CYP11B2_2_5617,163,244,69,65,0,464,485,0,229,307,259,6
CYP17A1_2_5618,185,124,221,177,157,155,91,362,90,30,20,442
CYP1A1_2_5619,496,416,561,584,237,231,767,49,1055,308,33,611
CYP1A2_2_5620,50,156,113,112,220,90,15,1,54,1,50,1
CYP1B1_2_5621,43,19,16,108,43,0,0,13,15,8,346,5
CYP20A1_2_5622,477,405,438,498,276,25,465,796,106,152,673,933
CYP26B1_2_5623,221,177,141,128,174,1,430,0,389,274,781,324
CYP26C1_2_5624,82,88,86,121,323,0,407,0,690,282,0,59
CYP27A1_2_5625,97,127,63,129,0,0,0,1,21,34,1,1
CYP27B1_2_5626,146,169,261,121,0,69,732,10,12,1,4,47
CYP2A13_2_5627,46,44,80,94,74,8,56,147,52,0,624,49
CYP2A6_2_5628,46,44,80,94,74,8,56,147,52,0,624,49
CYP2B6_2_5629,384,527,307,276,366,272,56,609,272,386,0,274
CYP2C19_2_5630,265,450,251,289,13,507,19,709,294,141,45,61

CYP2C9_2_5631,1419,1092,1446,1520,848,1003,1194,1056,707,672,1273,942
CYP2E1_2_5632,1463,1077,1348,1353,1198,1208,2117,1030,1296,1498,1898,1
040
CYP2F1_2_5633,603,617,451,570,853,280,1252,1166,340,285,587,1076
CYP2J2_2_5634,829,863,817,1037,984,618,1282,1227,736,1103,1283,600
CYP2R1_2_5635,817,667,760,796,480,377,607,1271,449,1135,528,660
CYP2S1_2_5636,238,281,162,299,251,585,247,253,340,271,676,148
CYP2U1_2_5637,4,1,3,0,1,0,0,0,0,0,0,1
CYP2W1_2_5638,185,70,132,189,44,0,0,18,1,11,0,110
CYP39A1_2_5639,1050,877,1083,1257,1673,1089,2308,767,1336,1431,816,750
CYP3A7_2_5640,461,340,327,350,235,476,1097,546,888,217,376,653
CYP46A1_2_5641,133,280,263,327,55,1,311,671,14,85,40,229
CYP4A11_2_5642,839,1034,1134,1261,761,646,1931,2029,600,750,1413,1234
CYP4A22_2_5643,2,0,0,0,0,0,0,0,0,77,0,0
CYP4F12_2_5644,372,327,176,315,321,344,269,214,377,298,540,619
CYP4F22_2_5645,38,167,34,38,12,0,0,4,21,0,97,367
CYP4F2_2_5646,558,378,244,487,471,123,156,272,595,996,250,143
CYP4F8_2_5647,388,410,429,418,64,173,27,0,337,641,137,1026
CYP4V2_2_5648,776,921,704,912,653,170,223,19,930,818,872,664
CYP4X1_2_5649,577,1101,710,996,810,1158,1871,1682,718,1116,790,528
CYP4Z1_2_5650,918,1088,814,1118,1614,863,436,532,1320,744,276,744
CYP7A1_2_5651,104,232,222,130,121,102,72,81,32,223,107,275
CYP7B1_2_5652,1502,1511,1006,1622,731,1682,862,688,1094,1563,1038,1728
CYP8B1_2_5653,457,411,287,432,700,85,199,25,752,434,383,321
DAD1_2_5654,181,143,204,86,350,3,5,431,43,12,463,489
DAGLA_2_5655,163,319,190,235,177,471,510,361,470,94,2,260
DAK_2_5656,45,26,80,81,234,67,43,31,98,17,0,166
DARS2_2_5657,287,188,259,261,374,93,272,0,18,283,284,239
DARS_2_5658,205,123,166,95,235,43,156,179,168,34,11,77
DBH_2_5659,388,221,248,300,263,959,333,270,413,133,14,81
DBR1_2_5660,376,617,334,521,317,414,648,209,442,1621,205,279
DBT_2_5661,377,388,432,554,684,443,1654,44,493,951,340,208
DCLRE1B_2_5662,128,191,169,200,223,571,211,719,222,43,7,135
DCPS_2_5663,256,244,244,248,361,132,154,86,280,141,222,110
DCTN6_2_5664,445,399,320,502,510,665,1099,43,63,33,1713,782
DCTPP1_2_5665,99,206,163,155,0,174,320,7,4,57,141,117
DDAH2_2_5666,381,416,404,374,726,283,671,119,589,753,433,649
DDOST_2_5667,253,291,316,241,13,10,460,5,173,150,516,98
DDX10_2_5668,379,360,225,310,526,457,333,2,640,3,146,124
DDX18_2_5669,507,350,414,451,599,106,195,274,278,118,114,58
DDX19A_2_5670,1031,1218,1031,1281,1460,896,1546,222,82,1681,1478,604
DDX1_2_5671,516,375,382,356,400,789,597,125,147,178,1277,632
DDX20_2_5672,436,658,461,560,348,683,125,186,506,361,374,324
DDX21_2_5673,333,275,320,228,73,150,245,90,563,584,39,266
DDX23_2_5674,637,442,496,603,1199,56,581,337,763,1450,321,483
DDX24_2_5675,156,131,124,204,571,351,603,6,492,15,20,56
DDX25_2_5676,501,411,292,519,1042,408,113,1268,692,44,415,1043
DDX27_2_5677,278,316,180,333,75,255,0,119,362,269,310,295
DDX28_2_5678,560,441,526,549,538,602,116,732,339,1017,757,775
DDX39A_2_5679,287,213,237,262,190,7,792,48,180,467,231,215

DDX41_2_5680,77,77,129,125,32,374,14,101,138,81,580,35
DDX43_2_5681,1024,1238,804,1020,456,900,787,627,994,1413,722,1328
DDX46_2_5682,330,441,435,455,249,178,0,1865,146,822,643,318
DDX49_2_5683,51,34,61,59,217,50,361,10,98,117,0,0
DDX50_2_5684,10,29,13,4,2,87,1,2,10,200,0,9
DDX51_2_5685,2,0,44,0,1,183,0,0,2,0,0,0
DDX52_2_5686,171,324,320,306,463,638,405,373,697,466,518,303
DDX53_2_5687,786,924,999,955,386,1078,318,1228,1522,439,515,601
DDX55_2_5688,669,353,427,540,642,394,186,697,401,317,921,683
DDX56_2_5689,79,40,18,76,133,348,41,0,155,0,34,63
DDX58_2_5690,1052,532,657,858,675,207,409,129,861,101,332,1539
DDX59_2_5691,267,263,204,345,53,131,6,30,390,301,57,176
DDX5_2_5692,138,147,142,181,192,43,0,50,134,76,100,325
DDX60_2_5693,1414,1509,1216,1913,1650,519,1385,637,1145,2040,1216,2442
DDX6_2_5694,444,438,276,441,1014,388,88,661,764,201,569,1380
DECR1_2_5695,468,110,276,351,61,415,438,116,261,166,108,457
DECR2_2_5696,26,93,66,44,5,61,0,0,6,186,13,6
DEGS1_2_5697,1186,919,900,1090,1784,900,986,690,750,413,743,966
DEGS2_2_5698,47,44,19,48,174,0,17,92,5,8,0,0
DERA_2_5699,249,228,165,130,16,10,225,11,21,203,511,293
DFFB_2_5700,222,259,308,325,1,48,520,4,123,17,565,327
DGAT1_2_5701,87,91,93,73,3,3,0,43,72,64,4,234
DGAT2_2_5702,700,435,649,570,737,22,1083,26,825,1419,658,602
DHCR24_2_5703,64,86,69,91,64,1,366,312,80,46,0,198
DHDH_2_5704,4,4,1,13,0,0,0,0,0,0,0,35
DHFR_2_5705,584,409,596,633,137,342,449,848,173,454,507,905
DHODH_2_5706,145,162,225,210,38,261,0,70,27,187,3,211
DHRS13_2_5707,40,63,62,18,0,0,308,0,0,0,30,224
DHRS3_2_5708,130,192,116,151,216,103,284,437,449,76,76,322
DHRS4_2_5709,63,47,80,168,38,13,3,376,189,0,141,0
DHRS7_2_5710,1991,1838,1792,1896,2162,1612,3269,1358,3784,1638,2559,27
04
DHRSX_2_5711,1277,1073,1097,1178,1370,1412,2336,597,1058,1530,882,605
DHTKD1_2_5712,106,104,88,156,31,103,471,183,20,342,49,20
DHX15_2_5713,1293,1361,1512,1169,1394,1106,596,303,1459,1561,2554,1216
DHX29_2_5714,339,220,292,469,263,432,658,52,322,145,183,410
DHX32_2_5715,521,425,430,436,420,129,328,260,450,105,1714,330
DHX34_2_5716,51,93,36,38,24,22,42,31,6,35,0,0
DHX37_2_5717,33,29,37,32,39,40,2,48,127,0,0,66
DHX38_2_5718,527,452,322,337,940,605,752,160,77,694,47,412
DHX57_2_5719,1004,721,881,1018,1385,1493,1689,984,760,683,1235,1375
DHX58_2_5720,500,407,395,495,329,453,90,270,798,413,399,840
DHX8_2_5721,121,77,65,111,90,159,0,65,5,27,412,19
DHX9_2_5722,294,272,169,260,395,103,659,224,356,970,0,478
DIO3_2_5723,0,0,0,0,0,0,0,0,0,0,0,0
DIRAS1_2_5724,105,48,110,104,79,93,175,1,145,56,536,0
DIRAS2_2_5725,106,51,113,108,80,94,174,1,147,56,537,0
DIRAS3_2_5726,269,381,289,325,298,15,503,414,284,200,320,475
DIS3L2_2_5727,120,53,115,52,227,94,154,12,68,86,118,47
DLAT_2_5728,0,0,3,0,0,0,554,1,0,0,0,0

DLD_2_5729,165,123,162,135,357,13,39,268,87,670,87,712
DLL1_2_5730,0,0,0,0,0,0,0,0,0,0,0,0
DLST_2_5731,344,358,290,356,195,196,389,177,175,167,658,386
DMGDH_2_5732,3255,2707,2635,2712,2688,1430,5524,1586,2776,2781,2610,21
43
DNA2_2_5733,185,173,154,95,230,103,147,52,150,197,2,345
DNAH11_2_5734,837,1014,853,913,1197,1428,1061,1906,256,1534,1111,686
DNAH3_2_5735,324,140,264,197,582,72,185,468,72,206,276,518
DNAH5_2_5736,156,230,195,206,247,46,516,111,196,290,31,195
DNAH8_2_5737,2128,1747,2148,2184,1701,920,1946,833,1608,2469,1674,2122
DNAJA2_2_5738,3755,3832,3726,4062,3619,3534,5820,2985,3360,4450,5364,3
103
DNAJB14_2_5739,867,825,777,988,943,1258,949,362,1112,877,920,696
DNAJC10_2_5740,1609,1519,1525,1707,972,666,1751,1125,1677,684,2468,131
1
DNAJC18_2_5741,878,834,793,1124,924,841,2553,1613,363,633,273,1162
DNAL4_2_5742,507,501,348,483,560,447,1021,15,231,172,615,147
DNASE1_2_5743,45,13,38,7,0,0,449,0,33,1,61,1
DNASE1L2_2_5744,430,350,355,334,568,275,328,217,261,199,871,234
DNASE1L3_2_5745,145,74,119,209,312,78,10,232,151,46,174,168
DNASE2_2_5746,82,73,58,41,0,0,55,30,2,161,14,204
DNPEP_2_5747,0,4,34,19,0,0,0,0,0,0,3,0
DPAGT1_2_5748,306,278,319,348,1094,453,0,359,135,833,0,11
DPEP2_2_5749,686,553,590,601,501,21,718,395,2,235,503,397
DPM1_2_5750,230,288,195,178,105,145,233,84,117,293,407,7
DPM2_2_5751,91,208,126,166,181,0,89,326,95,377,127,226
DPP4_2_5752,636,422,441,470,980,287,80,825,780,291,113,582
DPP7_2_5753,115,114,84,67,0,11,115,13,4,43,0,163
DPP9_2_5754,23,88,40,95,17,218,286,0,26,20,16,451
DPYS_2_5755,75,86,64,145,0,196,1,32,58,59,185,41
DPYSL4_2_5756,115,60,143,123,186,82,8,56,116,43,398,145
DPYSL5_2_5757,519,526,374,356,203,727,381,26,778,556,9,243
DQX1_2_5758,149,89,106,123,27,30,446,39,57,186,11,23
DSEL_2_5759,505,414,318,544,616,358,1313,380,694,77,745,820
DTD1_2_5760,439,393,487,409,162,152,11,773,737,1203,430,353
DUOX2_2_5761,356,212,160,341,55,24,347,411,60,153,69,261
DUPD1_2_5762,162,211,234,171,297,78,14,283,233,94,277,20
DUSP28_2_5763,37,40,72,31,1,0,2,165,0,144,0,0
EARS2_2_5764,230,213,207,261,465,61,2,305,246,21,524,218
EBPL_2_5765,170,168,126,115,2,25,391,162,0,4,0,236
ECH1_2_5766,87,52,86,71,197,28,8,57,34,7,2,12
ECHDC3_2_5767,252,161,152,534,667,210,264,806,310,423,64,211
ECHS1_2_5768,475,621,607,704,263,420,667,989,573,752,377,770
EDEM1_2_5769,150,125,132,77,0,0,1,0,114,284,0,140
EDEM3_2_5770,1848,1375,1537,1884,695,1317,3076,1021,940,1082,2678,2013
EEF1A1_2_5771,331,148,243,275,164,11,598,468,90,731,167,29
EEF1A2_2_5772,434,80,161,296,352,0,4,1,0,545,493,470
EEF2_2_5773,60,65,127,112,0,156,28,2,17,146,3,38
EGLN1_2_5774,144,102,202,206,0,0,87,0,0,668,600,149
EGLN3_2_5775,138,172,142,175,208,309,266,158,84,211,46,502

EHD4_2_5776,96,67,165,242,418,1,369,0,4,0,0,2
EIF4A3_2_5777,545,555,525,662,1033,40,1361,420,263,664,153,1025
ELAC1_2_5778,151,57,74,147,269,0,19,0,3,1,1,264
ELANE_2_5779,107,136,152,191,205,76,381,219,72,231,67,133
ELOVL1_2_5780,377,265,237,199,249,118,378,0,549,326,16,186
ELOVL2_2_5781,260,186,236,241,248,277,0,67,138,32,358,128
ELOVL3_2_5782,148,137,84,246,966,0,462,600,1,240,54,2
ELOVL4_2_5783,881,777,656,679,752,274,946,679,918,282,2407,678
ENDOD1_2_5784,52,81,92,66,7,0,0,169,156,150,0,0
ENDOG_2_5785,98,204,134,148,182,182,270,543,119,247,93,106
ENGASE_2_5786,166,57,120,218,96,444,5,2,266,250,0,49
ENO2_2_5787,179,203,394,284,307,85,0,654,146,30,493,200
ENO4_2_5788,751,882,868,981,1045,729,205,891,1014,431,436,691
ENOPH1_2_5789,804,444,744,796,55,297,933,1465,241,270,1100,1087
ENPEP_2_5790,3813,2857,2630,3769,3597,1792,2576,2008,2930,3190,2270,17
39
ENPP1_2_5791,404,266,418,486,11,146,688,583,780,525,152,196
ENPP3_2_5792,1239,1167,1023,1331,1623,1062,399,1922,1306,1116,1275,113
0
ENPP4_2_5793,291,326,270,387,220,476,405,506,38,399,140,293
ENPP5_2_5794,670,713,543,575,482,303,1048,537,650,413,415,676
ENPP6_2_5795,1395,1249,996,1515,2176,1164,1650,1753,2019,1747,1319,142
7
ENPP7_2_5796,44,80,111,132,0,11,312,101,17,3,0,8
ENTPD3_2_5797,29,90,63,38,0,0,0,0,0,0,0,0
ENTPD5_2_5798,965,856,932,1157,706,1677,536,815,528,1412,519,853
ENTPD7_2_5799,317,140,204,246,200,4,1172,0,301,67,199,29
EPHX2_2_5800,415,520,390,537,233,192,337,568,661,339,806,724
EPHX4_2_5801,883,820,706,955,539,757,907,1840,831,1132,245,839
EPRS_2_5802,430,323,481,488,164,860,187,494,553,392,224,504
EPT1_2_5803,224,194,192,107,588,0,10,99,383,151,23,563
EPX_2_5804,7,3,9,4,0,7,0,0,14,0,0,0
ERAS_2_5805,57,55,85,126,0,9,74,1,42,51,1,4
ERCC3_2_5806,99,78,52,143,126,3,34,99,13,9,109,0
ERCC4_2_5807,209,212,228,295,47,222,173,292,472,0,393,262
ERCC5_2_5808,328,257,331,266,317,210,748,390,852,441,261,155
ERI1_2_5809,1602,1529,1611,1531,633,1522,763,609,1752,1868,2290,1513
ERO1L_2_5810,711,698,689,546,5,175,687,1193,150,238,415,549
ERP44_2_5811,264,504,471,508,504,46,211,2,36,207,457,676
ESD_2_5812,600,514,528,624,823,474,452,1128,280,1577,724,949
ESPL1_2_5813,406,308,236,243,504,367,8,270,253,539,164,123
ETFDH_2_5814,604,769,761,1041,723,330,177,481,757,908,276,1214
EXOSC1_2_5815,241,261,86,168,316,136,50,302,7,263,17,108
EXOSC2_2_5816,59,76,92,125,64,107,422,49,8,172,81,90
EXOSC4_2_5817,523,512,383,498,439,326,888,406,776,969,149,412
EXOSC5_2_5818,51,90,95,146,245,31,278,148,77,27,0,75
EXOSC7_2_5819,116,174,138,164,49,596,79,195,90,171,22,60
EXOSC8_2_5820,176,177,115,127,727,42,168,28,300,66,189,365
EXT1_2_5821,188,234,271,255,152,271,398,263,354,447,70,299
EXTL1_2_5822,208,134,346,344,156,253,672,424,66,110,1261,410

EXTL3_2_5823,303,174,132,220,98,221,779,45,130,6,40,156
F10_2_5824,75,103,118,204,108,25,272,0,3,222,287,248
F12_2_5825,308,280,207,254,404,253,689,99,241,395,359,223
F13A1_2_5826,300,255,364,399,174,218,175,265,318,132,122,367
F13B_2_5827,388,355,285,488,199,306,323,199,638,366,225,314
F2_2_5828,51,51,37,61,1,18,1,25,5,2,9,65
F5_2_5829,323,406,338,224,318,565,639,147,491,833,83,333
F9_2_5830,791,613,536,722,1064,728,446,250,818,752,1360,451
FA2H_2_5831,79,155,186,227,113,101,29,964,121,4,155,246
FAAH2_2_5832,370,356,509,436,830,317,499,1309,251,358,168,199
FADS1_2_5833,477,575,519,452,609,36,284,401,133,549,297,706
FADS2_2_5834,659,585,662,740,590,379,419,1589,1132,421,556,969
FADS3_2_5835,16,36,12,23,0,7,2,0,0,72,0,2
FAHD2A_2_5836,48,48,69,51,30,215,7,452,30,3,93,491
FAM108C1_2_5837,329,249,252,292,3,365,660,267,163,213,4,108
FAM135B_2_5838,105,65,109,44,0,67,247,599,32,0,52,57
FAM20B_2_5839,759,1076,838,898,397,1285,1334,1344,945,805,1541,1176
FANCM_2_5840,347,423,471,438,149,240,134,9,290,1234,361,977
FAR1_2_5841,479,544,343,473,1514,571,1354,508,320,501,945,46
FAR2_2_5842,1190,1095,861,1258,802,342,1334,1126,1560,2446,1351,1390
FARS2_2_5843,8,0,0,1,0,0,0,0,0,4,0,0
FARSA_2_5844,574,553,599,732,282,162,734,929,380,258,360,361
FARSB_2_5845,480,528,425,608,685,389,688,457,237,275,127,418
FASN_2_5846,317,226,246,416,211,1,1049,718,655,517,2,591
FDFT1_2_5847,360,201,176,292,367,62,723,402,202,409,267,295
FEN1_2_5848,11,12,72,6,0,0,18,0,10,0,0,120
FH_2_5849,99,75,171,165,202,13,15,532,10,90,185,358
FIG4_2_5850,1322,1137,1234,1377,513,1461,1737,1294,1096,1398,1638,766
FKBP10_2_5851,0,0,0,0,0,0,0,0,66,0,0,0
FKBP14_2_5852,204,105,157,159,66,16,165,40,215,219,223,122
FKBP15_2_5853,1426,1294,1370,1828,1473,618,1385,556,2000,462,3353,1721
FKBP3_2_5854,154,182,230,161,79,20,636,534,9,346,392,37
FKBP4_2_5855,225,164,199,89,250,152,177,66,235,53,1087,129
FKBP8_2_5856,432,506,549,558,356,549,625,673,544,390,398,331
FMO1_2_5857,572,476,675,900,1027,475,926,2270,391,667,758,933
FMO4_2_5858,406,227,133,164,231,1039,0,124,483,0,0,7
FNBP1_2_5859,114,94,127,88,0,168,40,104,279,152,0,3
FNTA_2_5860,1077,799,865,996,996,784,299,276,751,790,1110,548
FNTB_2_5861,1210,821,642,882,2130,815,900,784,720,829,1173,850
FTH1_2_5862,1077,1120,1298,1491,1436,1957,975,994,387,1033,2310,1448
FTMT_2_5863,80,136,103,74,192,191,106,221,65,351,0,5
FTSJ2_2_5864,254,124,195,123,260,110,230,4,277,63,349,170
FTSJ3_2_5865,634,474,586,689,169,106,1505,992,838,401,122,134
FTSJD2_2_5866,249,195,359,275,189,303,198,253,160,419,18,217
FUCA1_2_5867,569,453,294,266,214,578,157,242,296,506,459,646
FUCA2_2_5868,113,291,234,359,159,200,26,247,404,90,0,481
FURIN_2_5869,114,110,124,226,233,1,369,113,61,437,6,104
FUT10_2_5870,316,176,159,128,50,264,39,148,115,145,121,21
FUT11_2_5871,297,203,182,167,831,304,378,90,35,169,20,237
FUT1_2_5872,36,47,63,44,0,41,58,104,48,127,159,43

FUT4_2_5873,95,79,73,61,195,0,0,10,70,233,21,5
FUT5_2_5874,199,356,282,501,236,1109,292,634,248,39,1127,332
FUT7_2_5875,251,158,117,206,69,47,44,81,123,134,40,282
FUT9_2_5876,163,164,147,146,74,144,380,306,81,0,42,63
GADL1_2_5877,170,175,136,152,25,102,227,14,3,211,220,53
GAL3ST1_2_5878,406,411,238,506,483,504,33,12,93,118,255,68
GAL3ST3_2_5879,26,10,53,62,2,1,2,2,63,213,0,113
GAL3ST4_2_5880,481,648,427,580,308,219,396,328,515,10,1160,441
GALM_2_5881,502,367,718,649,709,588,128,2047,517,1455,654,869
GALNS_2_5882,550,310,453,432,730,1007,1664,833,384,419,474,523
GALNT10_2_5883,589,560,405,520,544,252,781,1173,393,550,706,354
GALNT11_2_5884,130,52,123,61,22,1,258,0,56,256,149,285
GALNT12_2_5885,278,380,347,397,702,386,888,1188,535,933,374,535
GALNT13_2_5886,3069,2710,3086,3196,4028,5227,4011,2760,3345,2849,2610,
3738
GALNT14_2_5887,10,43,6,17,0,175,0,0,0,0,0,54
GALNT1_2_5888,931,575,622,890,487,458,230,325,618,1211,1426,486
GALNT2_2_5889,250,610,432,595,292,534,791,560,243,804,747,811
GALNT3_2_5890,206,89,94,181,315,66,0,161,528,266,10,117
GALNT4_2_5891,82,36,36,31,99,1,77,1,182,27,9,0
GALNT5_2_5892,357,376,269,244,720,80,232,27,517,54,200,166
GALNT6_2_5893,111,144,197,179,115,0,479,56,12,311,0,201
GALNT8_2_5894,91,33,131,101,4,0,4,160,82,17,3,220
GALNTL2_2_5895,262,208,170,208,66,20,0,402,138,70,102,104
GALNTL4_2_5896,283,301,155,225,182,34,74,161,292,282,4,121
GALNTL5_2_5897,1606,1486,1679,1710,2097,555,1012,1297,1930,2459,2272,1
567
GALNTL6_2_5898,718,430,442,568,666,249,459,364,417,280,747,287
GALT_2_5899,563,520,366,494,229,125,17,593,703,1196,323,1056
GANC_2_5900,329,278,325,316,677,246,334,422,449,263,415,505
GAPDH_2_5901,35,68,69,58,8,0,20,6,22,20,0,2
GAPDHS_2_5902,142,184,165,305,45,28,537,0,0,80,68,579
GARS_2_5903,0,0,0,0,0,0,0,0,0,0,0,0
GATC_2_5904,102,171,180,127,185,30,140,1,352,162,161,72
GATM_2_5905,76,92,167,450,107,604,1,563,44,239,108,358
GBA2_2_5906,48,50,60,16,394,19,0,4,1,0,0,10
GBE1_2_5907,759,874,895,921,512,843,354,1210,873,1602,2237,899
GBGT1_2_5908,237,175,112,206,114,82,326,409,91,9,0,62
GBP1_2_5909,453,227,517,401,150,561,71,26,334,79,673,449
GBP2_2_5910,1103,797,841,887,477,679,366,453,1633,292,857,873
GBP3_2_5911,102,19,83,91,62,0,227,91,1,392,102,69
GBP4_2_5912,1348,904,918,1468,2094,1533,581,2025,1059,1093,2223,1028
GBP6_2_5913,966,1127,1226,1504,789,2426,1736,443,1085,988,818,991
GBP7_2_5914,308,235,205,424,266,46,31,538,303,128,384,663
GCLM_2_5915,176,399,349,508,683,181,764,422,124,271,1001,63
GCNT3_2_5916,119,105,88,77,361,14,438,157,84,1,0,290
GCNT4_2_5917,243,217,229,258,266,321,126,35,244,2,439,22
GCSH_2_5918,1471,1231,1156,1311,1299,664,656,1263,1285,1300,565,1652
GDE1_2_5919,287,250,198,246,126,101,392,145,412,113,199,320
GDPD3_2_5920,928,822,748,620,289,1142,1005,646,498,568,752,489

GFM1_2_5921,1122,786,1031,1107,401,693,1626,685,862,894,1724,1295
GFOD2_2_5922,24,0,1,3,46,0,0,1,0,0,250,0
GFPT1_2_5923,130,32,42,31,166,0,0,0,100,135,0,23
GFPT2_2_5924,414,216,271,437,802,163,255,333,115,81,312,439
GGH_2_5925,1337,1311,1197,1441,1576,1453,1882,643,1044,800,398,2053
GGPS1_2_5926,307,219,391,443,248,472,770,890,63,709,423,370
GGT7_2_5927,123,168,94,193,0,604,11,0,110,171,357,132
GGTLC2_2_5928,1054,1174,1121,1341,834,1340,2069,819,1380,1337,362,1613
GLCE_2_5929,1144,754,910,882,1231,716,858,948,1149,895,896,476
GLDC_2_5930,13,15,12,15,0,0,49,0,30,6,2,0
GLO1_2_5931,120,182,111,254,0,0,0,17,137,290,0,0
GLOD4_2_5932,150,157,209,158,215,74,167,99,372,638,304,72
GLS2_2_5933,239,163,179,233,223,93,104,121,213,233,106,204
GLT1D1_2_5934,661,620,488,840,565,1111,417,395,309,548,820,260
GLT25D1_2_5935,168,77,81,122,2,0,0,1,171,350,644,0
GLT25D2_2_5936,202,154,201,223,70,255,499,646,69,176,105,182
GLT8D2_2_5937,81,157,311,214,98,2,279,0,257,441,114,394
GLUD1_2_5938,271,329,339,585,165,559,3,4,318,312,1348,1143
GLUD2_2_5939,47,19,77,101,262,10,514,740,47,381,390,239
GMDS_2_5940,141,113,166,91,2,18,29,1,142,17,236,218
GMIP_2_5941,160,120,148,135,150,182,20,244,154,28,90,291
GMPR_2_5942,116,60,86,144,91,39,70,10,98,40,1,151
GMPS_2_5943,309,296,286,413,304,633,185,314,263,298,131,297
GNA11_2_5944,287,282,300,350,375,84,1050,581,15,447,3,259
GNA12_2_5945,265,247,255,265,147,710,260,154,106,501,99,174
GNA13_2_5946,2022,2043,1649,2208,1694,1764,231,1982,1165,2861,3351,123
8
GNA14_2_5947,267,176,378,327,355,293,8,216,220,352,390,205
GNA15_2_5948,415,270,339,286,353,483,26,353,411,469,1096,0
GNAI1_2_5949,79,59,50,59,2,9,162,1,58,71,2,59
GNAI3_2_5950,33,6,8,8,0,4,0,0,14,51,0,0
GNAQ_2_5951,837,803,794,871,948,1462,858,1222,532,893,588,1167
GNAT2_2_5952,2354,1662,2111,2273,2373,1800,2673,1572,2076,2608,1320,31
96
GNAT3_2_5953,0,0,0,0,0,0,0,0,0,0,0,0
GNAZ_2_5954,230,328,172,319,52,881,26,261,222,253,374,794
GNB1_2_5955,533,538,605,822,557,1051,676,1386,391,312,1212,634
GNB2L1_2_5956,152,149,152,244,897,106,35,257,35,7,45,145
GNB3_2_5957,568,425,562,564,176,774,818,191,820,607,371,625
GNB4_2_5958,274,289,220,283,359,199,503,53,210,259,41,248
GNG11_2_5959,1442,1482,1032,1662,1348,1219,1353,2816,1170,1075,2949,20
87
GNG12_2_5960,236,394,290,244,112,458,262,71,389,527,65,373
GNG13_2_5961,344,362,390,347,247,183,61,960,337,333,513,188
GNG2_2_5962,276,333,234,298,505,311,59,289,484,3,362,549
GNG3_2_5963,94,75,33,74,1,38,156,0,69,108,22,146
GNG7_2_5964,2520,2175,1797,2163,1801,2483,920,1134,1690,3448,1766,1945
GNG8_2_5965,56,184,73,192,24,3,13,346,115,958,0,17
GNGT1_2_5966,835,931,879,952,774,924,781,781,577,688,247,646
GNL2_2_5967,389,350,330,301,322,116,262,27,242,249,241,456

GNMT_2_5968,200,178,76,126,73,234,508,317,163,587,22,301
GNPAT_2_5969,204,244,260,261,0,152,1288,528,130,300,243,487
GNPDA1_2_5970,316,530,361,466,863,472,148,43,545,900,1,383
GNPDA2_2_5971,954,887,717,965,1115,308,1207,834,548,837,1002,668
GNPNAT1_2_5972,530,653,647,625,186,635,673,1087,444,677,347,757
GNPTAB_2_5973,1919,1759,1337,1918,2332,1009,3941,1268,1789,1594,1044,1
759
GNPTG_2_5974,619,777,613,903,547,998,706,518,315,194,642,880
GNS_2_5975,561,583,563,688,862,477,629,724,146,949,543,365
GOT2_2_5976,2849,2625,2821,3241,2773,2078,2922,4164,2833,2030,1787,324
3
GPAA1_2_5977,13,161,25,84,57,450,5,83,18,62,117,3
GPAM_2_5978,1080,984,1001,1214,972,472,1512,1207,827,562,1222,893
GPAT2_2_5979,483,205,204,417,130,93,99,153,165,355,501,238
GPD1_2_5980,986,875,832,868,55,1079,1804,301,621,708,884,1224
GPD1L_2_5981,0,25,0,32,0,0,0,0,0,0,43,0
GPX2_2_5982,236,193,112,235,370,254,0,246,276,503,159,157
GPX3_2_5983,484,754,641,825,220,364,590,1595,970,685,1286,384
GPX7_2_5984,246,232,275,293,34,248,364,100,341,172,704,150
GPX8_2_5985,262,234,381,368,73,772,1662,0,540,176,933,1140
GRHPR_2_5986,232,271,241,242,199,53,95,426,226,99,129,309
GSTA1_2_5987,1231,774,671,983,1256,1179,813,548,1447,627,572,542
GSTA2_2_5988,1234,773,672,1040,1264,1180,817,545,1436,631,564,542
GSTA3_2_5989,1773,1423,1558,1727,1351,2206,875,2175,1258,1482,1077,770
GSTA4_2_5990,251,375,225,302,466,336,687,3,300,411,115,447
GSTA5_2_5991,179,105,193,150,0,236,519,11,23,0,3,477
GSTM3_2_5992,511,511,722,591,783,1011,574,1386,792,490,157,627
GSTM5_2_5993,1613,1134,1173,1508,1526,1505,2034,2004,2053,889,1217,162
2
GSTP1_2_5994,19,42,22,82,1,0,88,0,14,1,0,23
GSTT1_2_5995,290,202,284,304,0,27,444,1252,10,109,486,201
GSTT2B_2_5996,1673,1523,1580,2012,1536,1580,2520,1970,1917,1922,877,20
48
GSTT2_2_5997,1673,1523,1580,2012,1536,1580,2520,1970,1917,1922,877,204
8
GTF2F2_2_5998,200,195,172,211,183,0,108,141,15,25,223,117
GTPBP1_2_5999,67,43,76,74,187,13,8,131,0,239,3,103
GTPBP2_2_6000,3,76,36,49,166,64,394,0,22,0,29,0
GTPBP4_2_6001,2191,1612,1841,1957,2706,865,1337,1358,1474,1111,2934,17
44
GUCY1A2_2_6002,980,784,670,1024,499,892,796,1187,1086,1018,1167,1148
GUCY1B3_2_6003,429,413,230,243,320,23,9,0,81,439,335,305
GUSB_2_6004,810,551,461,410,246,799,715,140,524,334,139,703
GYLTL1B_2_6005,0,0,7,0,304,0,0,77,0,0,0,0
GYS2_2_6006,100,114,120,112,177,41,312,134,55,76,2,69
GZMA_2_6007,128,175,114,121,30,24,521,416,30,248,6,104
GZMB_2_6008,1754,2124,1664,2042,2619,1964,1464,1574,1858,1602,666,1205
H6PD_2_6009,1075,936,883,893,537,208,368,475,371,822,1280,754
HAAO_2_6010,235,203,184,160,3,0,6,563,30,512,41,35
HACL1_2_6011,226,200,149,250,19,156,5,191,1029,386,180,154

HADHA_2_6012,203,149,218,283,127,76,122,97,295,200,247,280
HADHB_2_6013,365,220,194,305,505,95,1806,376,337,261,21,217
HAGHL_2_6014,0,0,0,0,0,0,0,0,0,0,0,0
HAL_2_6015,411,484,325,348,404,157,157,296,174,47,632,714
HAO1_2_6016,1108,988,1011,1386,1865,1536,2625,1334,1226,1726,1066,1586
HARS_2_6017,295,279,328,327,291,341,249,594,409,283,120,196
HAS1_2_6018,265,267,335,232,38,28,1004,50,123,340,9,393
HAS2_2_6019,342,328,570,525,116,323,612,359,839,242,0,372
HDHD3_2_6020,227,168,227,175,213,109,171,61,80,238,41,210
HELB_2_6021,1326,922,992,1127,938,876,1195,905,984,809,1389,1345
HELQ_2_6022,886,675,568,957,775,146,1192,266,1043,872,750,526
HELZ_2_6023,805,427,456,653,97,29,584,161,545,311,335,625
HEXA_2_6024,1096,876,853,873,1048,547,1340,413,910,807,882,720
HEXB_2_6025,1194,1090,959,1046,176,596,675,1490,889,1014,698,1064
HEXDC_2_6026,46,77,41,64,1,0,0,362,0,4,0,0
HGD_2_6027,452,373,450,516,583,284,641,409,316,413,817,705
HGSNAT_2_6028,446,418,443,653,654,483,1124,989,154,119,672,615
HHATL_2_6029,796,730,755,986,984,1306,555,518,317,206,141,1378
HIBADH_2_6030,29,64,50,27,0,8,64,48,8,34,3,7
HINT1_2_6031,1497,1596,1276,1561,1736,1846,2298,1452,1403,1638,1033,14
90
HMOX1_2_6032,88,185,100,204,65,5,0,5,19,117,0,243
HPGDS_2_6033,1513,1239,1394,1547,939,2273,1623,1946,2102,1757,3395,124
8
HPRT1_2_6034,672,609,614,889,238,895,938,340,719,92,1040,426
HS3ST1_2_6035,234,281,205,302,198,270,639,71,78,185,234,284
HS3ST2_2_6036,1238,1224,758,1054,829,651,1503,832,724,1710,614,1208
HS3ST3A1_2_6037,4781,3877,3602,4646,2504,4018,3474,3299,3479,3205,3434
,5220
HS3ST3B1_2_6038,111,110,95,113,342,692,38,9,113,333,91,325
HS3ST4_2_6039,51,109,71,115,169,281,148,24,34,17,303,66
HS3ST6_2_6040,276,180,166,299,277,82,344,1457,48,27,52,6
HS6ST1_2_6041,65,15,18,29,13,29,19,95,51,0,4,11
HS6ST3_2_6042,315,276,425,321,230,906,536,101,352,652,809,444
HSD11B2_2_6043,432,398,760,670,76,331,414,8,241,142,634,335
HSD17B11_2_6044,76,77,124,176,517,152,2,125,498,83,0,6
HSD17B12_2_6045,935,1233,757,1322,868,964,982,790,1624,1426,515,211
HSD17B14_2_6046,255,452,362,558,326,346,451,691,190,533,1,174
HSD17B1_2_6047,41,82,34,61,8,23,204,19,382,21,0,16
HSD17B2_2_6048,238,206,89,249,84,2,31,2,285,165,189,158
HSD17B3_2_6049,359,448,502,544,29,638,374,374,663,670,535,828
HSD17B6_2_6050,816,728,906,873,581,1449,669,664,723,511,1280,837
HSD17B8_2_6051,252,135,139,182,220,105,48,30,65,260,25,244
HSD3B1_2_6052,1136,1057,1262,1252,1210,230,900,299,531,1054,1344,1037
HSP90AB1_2_6053,620,407,506,415,370,498,21,524,186,813,400,686
HSPA5_2_6054,110,91,165,103,153,4,328,0,4,4,228,0
HSPE1_2_6055,1155,913,987,1018,735,1405,300,477,703,287,1227,620
HSPG2_2_6056,122,63,33,96,100,137,17,33,233,14,0,232
HYAL4_2_6057,1735,1850,1254,1877,1378,779,2172,1319,2034,2153,821,1106
IARS2_2_6058,1096,935,823,1152,816,413,557,495,1214,1329,750,1282

ICMT_2_6059,248,198,260,279,219,173,74,97,805,489,8,227
ICT1_2_6060,98,127,96,113,0,0,1,317,1,237,0,646
IDH1_2_6061,812,608,650,728,135,591,765,230,276,635,651,828
IDH2_2_6062,98,25,125,94,397,4,1,85,27,19,1,33
IDH3A_2_6063,445,598,419,447,801,836,445,362,599,456,470,413
IDI1_2_6064,2274,2315,1770,2435,1750,2930,1106,1621,2229,2663,1908,199
2
IDI2_2_6065,1837,1988,1550,2066,1723,2537,651,1578,2158,2431,1282,1745
IDO1_2_6066,411,220,224,159,464,135,104,328,247,668,46,205
IDO2_2_6067,145,127,70,176,194,0,325,39,132,226,47,157
IDUA_2_6068,177,356,350,216,470,1018,569,170,53,291,706,234
IFI30_2_6069,43,108,83,86,5,11,3,292,4,83,12,51
IFIH1_2_6070,21,38,42,56,0,2,24,194,2,91,0,133
IGHMBP2_2_6071,156,154,312,330,116,1033,337,341,244,337,497,234
IHH_2_6072,8,9,28,74,0,91,22,0,0,73,0,1
ILVBL_2_6073,535,312,395,421,377,342,0,178,274,158,442,222
IMPAD1_2_6074,560,511,438,454,507,559,1295,530,940,460,5,153
IMPDH2_2_6075,43,22,33,34,0,0,4,6,13,6,0,24
IRS1_2_6076,256,182,98,180,243,404,205,1009,147,99,303,157
IRS2_2_6077,403,307,375,287,203,41,760,299,1007,780,675,59
ISG20_2_6078,75,116,46,46,64,24,0,47,246,41,0,0
ISG20L2_2_6079,157,135,132,156,177,199,64,24,1,89,0,503
ISOC1_2_6080,225,363,219,252,204,263,187,23,302,355,146,0
IWS1_2_6081,311,311,149,266,336,260,99,364,233,104,261,211
KDSR_2_6082,60,73,76,108,0,11,0,0,14,117,1,470
KHSRP_2_6083,1375,934,1346,1400,1045,1138,1164,795,1560,1114,1769,949
KIAA0317_2_6084,527,406,543,525,625,890,459,194,667,130,659,504
KIAA1279_2_6085,1501,1379,1462,1673,1378,1389,2289,1328,1643,1623,1401
,1461
KIAA2022_2_6086,161,155,111,218,344,105,902,46,18,347,197,486
KIF18A_2_6087,124,58,87,65,589,116,35,0,90,130,225,101
KIF20B_2_6088,102,135,137,231,16,26,677,0,321,1,212,207
KIF3A_2_6089,158,205,219,256,57,158,3,217,53,168,305,201
KLB_2_6090,344,284,320,163,190,14,13,34,9,45,405,157
KLC3_2_6091,159,98,169,159,483,2,163,0,90,340,15,316
KL_2_6092,3138,2404,2201,2871,2972,1714,2065,2022,3473,2886,2191,2528
KLK1_2_6093,347,200,281,405,0,16,49,392,470,0,916,7
KLKB1_2_6094,647,584,762,576,740,1140,1033,1396,635,545,153,793
KMO_2_6095,62,13,28,54,14,4,52,0,373,100,0,6
KRTCAP2_2_6096,418,253,421,367,226,294,1,0,83,128,103,121
L2HGDH_2_6097,122,118,121,38,233,22,72,0,35,48,0,8
LALBA_2_6098,441,306,389,430,162,733,131,230,906,312,532,477
LAP3_2_6099,102,102,63,130,0,91,94,128,187,200,31,398
LARS2_2_6100,321,428,191,168,21,117,192,268,46,398,96,329
LARS_2_6101,699,608,386,912,1238,398,595,959,454,359,1021,451
LCAT_2_6102,103,146,156,94,342,128,87,79,202,35,38,40
LCMT2_2_6103,756,790,797,893,745,964,580,580,694,1224,1368,1078
LCT_2_6104,112,44,83,76,69,0,82,1,108,71,523,45
LCTL_2_6105,371,625,574,618,558,149,410,721,401,483,646,329
LDHAL6B_2_6106,888,769,709,972,907,533,270,666,1315,603,977,749

LEPREL2_2_6107,274,128,202,212,237,184,104,74,246,83,0,135
LGALS13_2_6108,456,187,151,146,562,349,39,10,0,186,22,293
LHFPL2_2_6109,236,107,226,189,237,124,175,220,430,125,8,789
LIPC_2_6110,257,210,311,219,93,267,12,466,224,173,164,200
LIPE_2_6111,2272,2307,2034,2219,2588,2456,2020,3353,3226,2626,1779,183
4
LIPG_2_6112,353,226,166,279,379,144,0,715,390,97,14,401
LIPH_2_6113,480,648,526,891,1311,712,196,1221,971,479,504,656
LIPI_2_6114,0,0,0,0,0,0,0,0,0,0,0,0
LIPM_2_6115,206,182,216,236,789,229,0,86,608,205,81,547
LIPN_2_6116,732,762,738,1063,1054,752,141,250,489,1072,306,510
LIPT2_2_6117,45,26,59,16,26,0,0,127,27,27,0,89
LONP2_2_6118,2638,2308,2445,2507,2050,1493,2298,2236,2673,2472,3156,22
90
LOXL1_2_6119,285,138,228,249,481,25,645,139,47,519,246,441
LOXL2_2_6120,153,249,152,274,0,834,407,330,305,315,0,2
LOXL3_2_6121,45,29,48,28,34,0,1,0,0,18,0,0
LOXL4_2_6122,225,179,175,244,163,270,581,51,392,180,306,183
LPCAT1_2_6123,186,86,92,275,3,74,0,0,323,226,0,216
LPCAT2_2_6124,747,485,580,501,723,327,384,823,875,173,1697,741
LPCAT3_2_6125,347,156,168,319,240,78,219,80,234,300,290,449
LPCAT4_2_6126,141,202,105,66,200,0,9,91,115,15,505,9
LPIN1_2_6127,126,101,115,99,390,39,3,91,2,137,102,15
LPIN2_2_6128,1861,1476,1268,1545,1425,2159,1001,1603,1024,1467,1836,22
37
LPIN3_2_6129,57,101,6,52,0,0,0,0,11,0,527,0
LPL_2_6130,241,155,218,282,250,43,125,437,210,308,454,172
LRAT_2_6131,164,150,150,180,393,305,187,99,386,42,192,210
LTA4H_2_6132,257,238,139,240,150,229,192,94,125,311,159,249
LTC4S_2_6133,51,124,31,224,0,263,44,185,0,5,853,19
LYG2_2_6134,341,338,386,538,409,242,134,914,711,243,546,140
LYPLA1_2_6135,486,938,578,637,707,1177,863,536,113,88,886,441
LYPLA2_2_6136,247,334,193,472,446,3,69,648,163,443,0,528
LYPLAL1_2_6137,1209,1261,893,1192,1126,1559,2496,456,830,1693,1563,984
LYZ_2_6138,264,162,195,342,262,91,18,1,163,376,240,149
LYZL1_2_6139,360,475,540,528,152,399,21,743,105,119,858,1000
LYZL2_2_6140,360,475,540,528,152,399,21,743,105,119,858,1000
LYZL4_2_6141,29,43,101,104,193,0,22,24,74,1,25,0
MACROD1_2_6142,745,601,849,802,394,559,1050,791,557,310,658,1238
MAGT1_2_6143,200,96,160,90,140,244,131,209,24,248,22,364
MAN1A1_2_6144,111,109,197,189,5,299,326,50,16,2,396,477
MAN1A2_2_6145,518,655,355,630,468,825,764,318,550,883,148,763
MAN1B1_2_6146,10,14,20,82,100,61,0,39,57,0,0,171
MAN1C1_2_6147,61,71,161,275,1,52,14,10,167,101,875,903
MAN2A1_2_6148,1605,1540,1411,1748,1472,461,1103,1976,1382,1330,1093,15
24
MAN2A2_2_6149,1151,840,683,844,456,271,236,765,379,536,2443,842
MAN2B2_2_6150,223,163,147,228,230,72,38,43,58,16,0,0
MAN2C1_2_6151,80,57,102,101,12,65,3,0,247,129,3,37
MANBA_2_6152,456,427,343,481,345,418,322,934,1537,705,219,578

MANEA_2_6153,1107,955,856,1119,1025,519,185,601,802,865,1282,560
MAOA_2_6154,51,43,49,31,0,432,6,0,1,536,0,0
MAOB_2_6155,257,146,362,238,23,246,387,92,147,1,1826,73
MAP1S_2_6156,56,16,16,56,16,33,3,0,229,0,7,59
MAPRE3_2_6157,244,192,175,282,66,133,143,306,258,69,312,236
MARS2_2_6158,279,186,155,175,637,61,216,323,352,13,63,83
MARS_2_6159,120,203,166,262,33,232,94,113,84,194,385,22
MAT1A_2_6160,107,267,139,174,658,21,356,52,135,161,329,30
MAT2A_2_6161,602,504,497,697,59,787,1167,132,1315,213,314,607
MBOAT1_2_6162,863,653,572,763,776,727,1099,195,1023,483,314,499
MBOAT2_2_6163,1029,761,798,728,549,553,272,2949,516,1207,1005,982
MBOAT4_2_6164,421,464,429,366,429,8,111,982,571,353,1715,933
MBTPS1_2_6165,470,628,457,673,750,315,1055,201,634,741,175,880
MBTPS2_2_6166,892,930,946,1010,492,799,962,1655,655,975,838,168
MCCC1_2_6167,93,108,91,91,1,120,226,419,88,20,1,334
MCCC2_2_6168,705,231,449,314,509,299,307,319,502,401,132,747
MCEE_2_6169,169,42,162,82,60,0,6,199,118,61,59,81
MCM3_2_6170,194,179,114,183,606,185,195,75,125,95,3,446
MCM5_2_6171,313,289,211,245,369,391,360,28,150,30,25,232
MCM6_2_6172,596,670,591,725,35,829,1506,32,911,593,590,952
MDH1B_2_6173,75,158,106,124,34,345,50,26,17,162,16,286
MDH2_2_6174,130,119,132,153,162,144,0,73,22,162,20,121
MEP1A_2_6175,729,667,396,643,907,210,752,143,673,144,692,409
MEP1B_2_6176,1337,897,1052,1138,817,693,1270,2468,807,787,1033,1447
METAP1_2_6177,668,541,554,409,1139,322,472,1476,586,113,330,495
METAP1D_2_6178,1,0,67,62,1,2,165,55,0,5,1,4
METAP2_2_6179,1001,1011,946,827,531,446,2012,778,306,753,534,397
METTL14_2_6180,826,516,771,868,1159,794,583,561,754,607,1308,961
METTL22_2_6181,327,287,306,329,230,368,238,712,115,647,333,566
METTL2B_2_6182,231,559,363,393,195,521,11,307,287,190,992,315
METTL3_2_6183,181,213,130,208,0,339,85,0,326,47,152,24
METTL5_2_6184,211,350,553,566,308,504,1,303,110,619,1633,680
METTL6_2_6185,359,399,496,499,891,435,44,425,757,321,592,131
METTL7B_2_6186,44,27,26,38,5,1,6,119,119,222,0,103
METTL8_2_6187,971,917,1017,1038,856,404,1148,576,856,368,1831,668
MFN1_2_6188,412,355,314,453,702,110,788,165,77,877,425,346
MGAM_2_6189,208,142,173,151,0,1,1221,84,138,8,5,33
MGAT4C_2_6190,1162,1111,1145,1365,2294,270,838,1091,1384,1681,3575,157
8
MGAT5_2_6191,383,183,228,345,153,13,21,295,48,134,35,305
MGMT_2_6192,210,466,288,315,112,65,166,392,406,432,331,155
MGST3_2_6193,66,67,63,53,0,0,233,154,96,116,513,28
MIOX_2_6194,127,101,233,123,393,0,227,1,0,0,1369,32
MIPEP_2_6195,190,341,200,380,85,117,218,30,63,154,0,341
MLYCD_2_6196,838,729,956,1088,547,1155,1993,430,1149,693,494,514
MMAB_2_6197,52,52,50,48,2,2,122,84,4,86,4,118
MMEL1_2_6198,316,358,321,540,53,353,242,403,79,177,515,175
MMP10_2_6199,325,255,301,398,373,195,508,113,315,136,207,59
MMP12_2_6200,788,606,681,849,749,246,437,399,331,652,1041,976
MMP14_2_6201,233,524,334,470,117,62,814,426,135,715,741,337

MMP3_2_6202,1122,1337,1157,1376,1853,1498,189,1142,715,1764,1336,1929
MMP7_2_6203,79,106,81,83,34,217,9,46,0,0,0,51
MMP8_2_6204,3096,2811,2480,3395,4023,3065,4001,3459,4523,3725,4781,400
2
MMP9_2_6205,31,39,35,59,88,42,17,0,8,177,0,25
MOCOS_2_6206,904,791,715,1033,1773,421,257,836,365,326,320,1175
MOGAT1_2_6207,926,731,800,984,1383,473,1283,106,492,623,846,1798
MOGAT2_2_6208,1053,713,788,821,1129,474,1122,854,883,280,224,1238
MOGAT3_2_6209,1190,1216,1040,920,800,518,377,632,1010,1601,759,1445
MOXD1_2_6210,6,10,20,17,16,0,48,0,11,73,0,2
MPI_2_6211,692,606,625,744,528,847,1058,73,923,581,806,860
MPO_2_6212,153,93,186,82,52,3,26,594,348,190,247,1
MRPL37_2_6213,52,79,62,113,6,44,71,2,0,72,0,0
MRPL44_2_6214,1472,1830,1694,1611,2428,1049,1042,1409,1749,2108,1761,1
457
MRPS30_2_6215,268,276,157,229,251,305,59,256,370,75,0,65
MSH2_2_6216,865,874,855,856,1075,1452,772,434,748,288,1180,485
MSH3_2_6217,1153,1081,801,1330,1331,1120,1234,644,1864,805,621,975
MSH4_2_6218,132,128,90,112,0,321,0,0,136,29,1,231
MTAP_2_6219,99,39,48,55,9,0,53,23,74,25,178,7
MTFMT_2_6220,262,266,209,300,681,331,1131,650,155,521,82,146
MTHFD1_2_6221,151,224,279,487,2,220,45,351,77,298,326,144
MTHFD2_2_6222,308,261,517,322,46,721,272,486,77,452,567,129
MTHFD2L_2_6223,721,589,656,533,36,115,1072,434,853,723,2414,518
MTHFR_2_6224,202,437,392,454,0,322,521,223,82,77,609,461
MTPAP_2_6225,286,318,85,133,648,4,1,360,440,61,680,58
MTR_2_6226,74,202,143,190,0,0,789,1,0,0,0,0
MUS81_2_6227,504,270,593,853,1268,39,454,0,620,339,1161,919
MUT_2_6228,230,375,128,337,14,0,437,0,0,14,1253,1
MVD_2_6229,255,362,308,377,207,29,531,98,219,177,0,633
MX2_2_6230,596,442,609,588,523,156,345,345,743,171,1200,612
MYH1_2_6231,655,498,581,459,217,444,322,477,609,308,847,446
MYH3_2_6232,398,459,363,314,492,73,1184,73,694,540,169,856
MYH6_2_6233,115,82,137,124,102,51,312,47,169,211,18,20
MYH7_2_6234,145,74,223,164,469,5,0,101,192,55,197,70
MYH9_2_6235,69,86,126,79,1,0,2,0,67,5,322,153
MYL7_2_6236,257,202,290,320,26,155,3,701,228,60,127,115
MYO1E_2_6237,245,227,218,262,405,20,2,52,22,234,424,269
MYO5B_2_6238,19,37,4,0,0,1,0,164,0,0,0,0
MYO9A_2_6239,848,814,732,1112,610,1019,973,1030,1328,761,705,938
N6AMT2_2_6240,1008,648,947,1182,1282,729,343,845,816,1173,357,1618
NAA10_2_6241,595,574,586,604,359,437,388,435,177,309,681,734
NAA11_2_6242,1299,1026,1208,1329,892,1481,1236,1078,1039,821,1052,1539
NAA15_2_6243,244,371,253,184,0,19,523,7,251,57,294,100
NAA30_2_6244,468,297,256,170,279,27,77,12,22,338,1649,113
NAA50_2_6245,111,54,85,149,10,90,40,61,70,15,15,25
NAALAD2_2_6246,776,826,837,725,330,944,502,347,1170,1507,1150,856
NAALADL1_2_6247,0,0,0,0,0,0,0,0,0,0,0,0
NADSYN1_2_6248,14,0,55,28,3,5,48,37,29,0,395,63
NAGA_2_6249,1356,1247,1405,1673,701,1262,1222,739,1627,2189,3370,2207

NAGLU_2_6250,93,124,73,85,101,2,180,0,57,0,5,26
NAGPA_2_6251,260,281,213,329,106,424,51,656,77,195,755,86
NAMPT_2_6252,943,946,755,849,1064,623,861,1117,751,1535,742,741
NANP_2_6253,694,502,613,787,1098,206,1365,1134,883,390,469,326
NANS_2_6254,519,446,563,614,365,640,1382,642,1014,335,838,1136
NAPRT1_2_6255,41,152,122,124,304,4,2,0,0,368,559,35
NARS_2_6256,80,16,40,91,329,0,0,0,27,65,43,62
NCF1_2_6257,46,102,65,92,37,60,182,582,47,193,0,101
NDST1_2_6258,212,366,227,255,10,39,513,28,63,497,419,418
NDST2_2_6259,521,736,604,799,558,744,1680,194,727,887,550,1013
NDST3_2_6260,4294,3526,3268,3636,4455,1614,4136,3598,4092,4719,3733,37
50
NDST4_2_6261,96,58,142,161,115,20,23,11,131,27,0,0
NDUFA10_2_6262,426,316,310,336,202,1,453,71,69,414,495,129
NDUFA12_2_6263,839,987,572,881,651,210,201,339,894,1789,626,556
NDUFA13_2_6264,203,201,174,200,280,94,200,2,143,160,1,99
NDUFA1_2_6265,3,51,7,13,12,0,0,2,0,165,0,28
NDUFA3_2_6266,89,131,112,130,34,124,597,5,46,28,154,131
NDUFA4_2_6267,184,102,169,84,47,231,253,55,107,9,4,81
NDUFA4L2_2_6268,1586,1466,1124,1407,1607,1752,1252,683,1289,1701,1248,
1779
NDUFA5_2_6269,127,26,68,111,0,46,1,296,735,180,43,0
NDUFA6_2_6270,2098,2103,2064,2674,2356,1953,2286,2637,1499,2213,1519,3
732
NDUFA7_2_6271,79,299,183,254,207,0,146,9,143,239,717,305
NDUFA8_2_6272,69,95,63,79,12,21,159,47,164,3,251,18
NDUFA9_2_6273,1172,911,934,1000,1105,657,562,983,815,1409,675,1144
NDUFAB1_2_6274,245,121,459,175,380,131,24,52,4,410,52,0
NDUFB10_2_6275,386,412,365,420,89,167,32,254,219,757,644,335
NDUFB1_2_6276,233,214,184,236,207,552,0,8,133,320,328,269
NDUFB2_2_6277,226,76,115,100,334,1,172,0,229,7,513,26
NDUFB3_2_6278,244,280,206,315,139,349,342,765,381,447,343,158
NDUFB7_2_6279,38,79,103,100,89,20,0,0,0,0,0,0
NDUFB8_2_6280,36,187,141,99,221,0,13,0,8,8,108,9
NDUFB9_2_6281,319,337,334,345,208,989,493,654,610,0,143,322
NDUFS3_2_6282,183,147,170,133,14,16,84,334,58,123,97,244
NDUFS4_2_6283,733,637,687,719,406,574,641,846,589,465,238,830
NDUFS6_2_6284,0,0,0,0,0,0,0,0,0,0,0,0
NDUFS7_2_6285,102,43,67,25,0,0,2,0,0,0,460,0
NDUFS8_2_6286,938,791,637,826,563,787,423,728,839,1498,783,426
NDUFV2_2_6287,2489,2296,2080,2468,1438,2803,619,2341,1527,1589,2983,27
07
NEDD8_2_6288,1872,2322,1883,2108,977,1544,2301,386,1897,2382,2007,2130
NEIL1_2_6289,54,92,43,57,1,1,57,180,0,0,4,319
NEIL3_2_6290,73,73,48,88,28,0,186,238,54,97,293,10
NEU1_2_6291,17,11,38,10,0,0,6,7,41,0,0,33
NEU2_2_6292,458,454,360,402,970,203,137,438,380,487,157,552
NEU3_2_6293,219,126,184,157,43,30,0,174,187,70,0,400
NHLRC2_2_6294,1235,891,734,995,825,769,728,1084,1244,1806,945,617
NIT2_2_6295,89,101,111,142,212,14,93,246,172,111,14,20

NKIRAS1_2_6296,369,211,313,295,263,261,408,314,440,290,73,500
NLGN1_2_6297,1078,751,916,974,1060,667,701,559,1088,895,571,754
NLGN2_2_6298,134,154,206,154,536,52,31,72,34,202,247,301
NLN_2_6299,1926,1698,1489,2124,1813,1598,973,3002,1910,2042,1329,2524
NME1-NME2_2_6300,1197,728,934,1223,1317,98,913,475,509,1485,41,1430
NMNAT1_2_6301,80,57,25,33,0,173,0,187,0,0,0,33
NMT1_2_6302,452,416,598,413,437,601,475,54,487,133,105,374
NMT2_2_6303,262,202,172,134,182,341,157,67,114,191,631,172
NNMT_2_6304,322,288,196,331,569,54,984,171,288,56,776,381
NOP58_2_6305,1084,880,977,1108,988,919,365,278,719,738,1040,1055
NOX3_2_6306,0,0,0,0,0,0,0,0,0,0,0,0
NQO2_2_6307,154,170,227,88,257,23,29,9,12,0,338,0
NRAS_2_6308,376,383,370,424,335,482,41,411,281,457,63,480
NSF_2_6309,265,287,355,335,244,104,565,736,114,265,833,5
NSUN6_2_6310,778,477,530,739,578,864,584,345,1229,361,240,672
NTAN1_2_6311,34,102,55,32,11,1,288,0,3,2,40,27
NTHL1_2_6312,276,391,328,323,577,453,224,157,518,316,759,226
NTPCR_2_6313,105,118,103,43,0,423,12,55,295,15,404,36
NUDT10_2_6314,35,80,88,82,107,34,9,50,377,19,46,56
NUDT11_2_6315,2407,2165,1899,2255,1771,1965,1547,3078,2223,924,1944,22
70
NUDT12_2_6316,135,87,160,360,249,35,50,109,6,291,429,232
NUDT14_2_6317,78,44,45,67,104,0,125,66,143,2,73,30
NUDT3_2_6318,247,397,154,194,552,0,6,410,223,383,58,289
NUDT5_2_6319,169,183,312,224,1,606,1066,4,0,19,321,138
NUDT7_2_6320,496,413,393,675,471,338,1210,436,493,861,623,365
NXNL1_2_6321,0,3,0,34,0,0,0,0,0,7,0,4
OAS3_2_6322,504,465,331,311,740,154,91,343,294,209,646,210
OAZ1_2_6323,201,199,315,250,72,196,593,32,30,149,431,171
OC90_2_6324,278,131,243,272,116,2,7,420,538,291,317,361
ODF3B_2_6325,36,77,39,106,53,0,234,0,52,84,19,26
OPLAH_2_6326,80,38,53,60,112,0,0,0,26,80,29,21
OSGEP_2_6327,197,107,156,130,83,40,1,19,403,68,5,166
OSGEPL1_2_6328,136,113,136,182,13,0,984,104,12,371,760,515
OSTC_2_6329,60,23,42,70,76,242,0,99,0,375,26,34
OTC_2_6330,764,669,690,753,371,221,715,324,465,559,1074,1464
OVGP1_2_6331,196,74,168,175,353,3,11,347,42,5,151,0
OXA1L_2_6332,404,222,192,212,136,433,648,485,87,257,54,459
OXCT1_2_6333,77,90,38,107,237,0,0,133,13,37,8,38
OXCT2_2_6334,1444,1180,1405,1187,2460,973,2313,2037,1099,1511,3056,112
3
P4HA3_2_6335,124,91,85,199,99,24,92,61,76,20,0,0
P4HB_2_6336,242,145,223,265,45,32,178,303,43,50,82,176
PADI1_2_6337,147,203,200,257,113,57,570,269,86,270,82,190
PADI2_2_6338,324,397,317,443,497,278,348,117,175,773,329,197
PADI3_2_6339,821,835,655,778,828,868,780,1595,956,602,815,1463
PADI6_2_6340,349,493,562,529,313,508,831,388,280,231,1733,395
PAFAH2_2_6341,922,845,673,992,1012,286,229,888,539,898,727,598
PAH_2_6342,260,190,203,330,331,52,16,205,128,513,405,143
PAPOLA_2_6343,250,209,330,313,233,391,1042,495,64,549,846,766

PAPOLB_2_6344,167,121,177,290,338,473,1221,111,190,194,361,409
PAPOLG_2_6345,386,281,396,416,371,223,148,646,222,456,862,255
PAPPA_2_6346,1174,1241,1080,1573,1792,1742,1219,746,1651,1060,1217,122
7
PAPSS1_2_6347,285,372,276,400,135,145,169,143,241,78,66,441
PARG_2_6348,314,349,319,342,235,623,222,220,190,319,442,292
PARP1_2_6349,43,8,10,58,92,28,5,3,1,1,1,0
PARP4_2_6350,3650,3226,3202,4163,3680,3264,3004,3231,2177,3821,2299,41
09
PARP6_2_6351,131,133,187,142,28,630,380,104,68,18,681,93
PARS2_2_6352,86,31,68,83,162,25,0,0,0,1,0,0
PCBD1_2_6353,19,19,73,49,1,55,216,136,2,0,88,581
PCBD2_2_6354,292,334,109,170,326,390,382,96,138,444,13,290
PCMT1_2_6355,228,235,174,199,99,246,461,286,184,28,0,139
PCMTD1_2_6356,435,274,161,379,239,407,179,136,289,222,254,187
PCYOX1_2_6357,2052,1702,1477,1556,2490,939,1326,1461,2361,2211,1769,13
54
PCYT1A_2_6358,125,110,176,111,113,85,15,88,158,86,15,202
PDCD1LG2_2_6359,107,151,217,313,117,0,2,307,55,122,266,160
PDCL_2_6360,44,89,71,119,156,14,104,0,138,107,1,149
PDE3A_2_6361,814,735,897,898,627,932,673,1361,838,1674,243,904
PDE3B_2_6362,405,337,340,245,679,123,482,32,251,118,502,271
PDE6A_2_6363,149,120,130,166,95,171,47,66,167,135,47,125
PDE6C_2_6364,273,301,286,310,298,182,54,82,228,380,363,232
PDE6D_2_6365,0,0,0,0,0,0,0,0,0,0,0,0
PDE6G_2_6366,413,227,434,469,37,88,752,553,238,11,283,37
PDE6H_2_6367,1145,876,991,950,636,927,206,394,1132,233,829,1069
PDE7B_2_6368,255,329,348,333,298,10,284,162,478,0,456,17
PDHA2_2_6369,88,20,65,37,650,30,16,15,0,0,5,0
PDIA2_2_6370,51,56,60,62,61,9,1,0,0,30,2,0
PDIA3_2_6371,711,483,365,442,719,131,271,350,351,748,282,128
PDIA4_2_6372,190,273,151,208,313,0,3,13,154,254,16,310
PDIA5_2_6373,50,105,39,67,95,0,0,786,82,0,2,49
PDIA6_2_6374,532,596,421,610,483,406,600,661,322,232,817,617
PDPR_2_6375,274,266,353,324,220,237,194,102,626,62,32,430
PDSS1_2_6376,742,665,510,658,1299,508,920,222,634,103,1012,967
PDSS2_2_6377,222,142,134,148,442,144,261,461,101,124,191,130
PDXP_2_6378,44,33,98,29,0,0,0,0,0,0,180,7
PECR_2_6379,176,106,53,80,8,11,60,164,77,97,204,0
PELI1_2_6380,731,551,748,788,344,590,689,490,685,767,1230,363
PET117_2_6381,595,610,576,744,671,149,1074,184,633,583,1217,930
PEX1_2_6382,2078,1838,1270,1846,1529,2130,2636,2884,1421,1215,1654,220
7
PEX6_2_6383,556,512,584,591,322,630,713,829,397,569,249,490
PFAS_2_6384,230,64,124,89,92,117,157,6,87,1,441,60
PGA3_2_6385,1996,1819,1327,1891,1125,925,2027,1412,2092,2687,1824,1451
PGA4_2_6386,1996,1819,1327,1891,1125,925,2027,1412,2092,2687,1824,1451
PGA5_2_6387,1996,1819,1327,1891,1125,925,2027,1412,2092,2687,1824,1451
PGAM4_2_6388,100,107,67,89,102,110,31,2,50,169,16,88
PGAP1_2_6389,280,343,245,343,124,266,25,140,219,601,5,288

PGAP3_2_6390,617,594,535,689,456,704,437,443,441,1281,671,723
PGD_2_6391,735,710,603,728,340,754,320,81,339,374,978,555
PGGT1B_2_6392,974,625,547,720,120,589,646,164,691,245,302,468
PGLS_2_6393,265,362,377,294,132,388,178,370,310,463,227,338
PGM2_2_6394,1434,1144,956,1085,1536,978,471,799,383,1278,2070,1460
PGM2L1_2_6395,397,413,400,336,312,339,48,328,154,472,242,198
PGM5_2_6396,100,76,64,26,315,130,138,161,108,330,112,16
PGPEP1_2_6397,62,42,75,55,362,12,195,200,2,57,234,82
PGS1_2_6398,339,223,214,183,223,65,149,110,193,350,26,432
PHGDH_2_6399,337,217,173,115,53,132,14,0,43,3,585,339
PHLPP1_2_6400,180,161,302,358,82,358,39,251,241,409,356,786
PHLPP2_2_6401,369,230,337,275,727,170,396,340,384,387,52,16
PIF1_2_6402,458,235,346,373,96,128,392,64,533,373,1000,177
PIGB_2_6403,393,351,273,332,104,0,216,669,409,1362,1945,207
PIGH_2_6404,1062,867,768,817,1797,1046,1049,720,1095,839,525,1494
PIGL_2_6405,292,186,221,432,1,209,483,792,225,240,516,36
PIGM_2_6406,2052,1409,1191,1326,1995,1388,1506,2493,1507,1660,1363,118
3
PIGS_2_6407,194,328,376,182,0,0,0,0,155,0,0,39
PIGU_2_6408,362,262,333,278,269,670,141,768,404,569,138,218
PIGW_2_6409,829,1131,602,973,1681,1191,538,944,504,756,524,1237
PIGZ_2_6410,259,257,155,242,272,66,360,157,318,214,171,219
PIN1_2_6411,15,7,39,36,9,0,0,0,49,26,4,6
PIPOX_2_6412,276,206,421,354,386,25,208,582,183,174,380,194
PITPNM2_2_6413,896,963,865,1130,559,535,1648,955,874,615,2665,944
PLA2G10_2_6414,40,32,3,21,1,0,0,0,4,0,3,0
PLA2G12A_2_6415,47,11,13,52,0,0,0,0,0,120,0,0
PLA2G12B_2_6416,227,161,204,228,2,72,0,281,352,832,135,74
PLA2G15_2_6417,129,108,62,184,117,23,236,5,317,94,148,142
PLA2G1B_2_6418,54,87,20,36,0,0,0,0,1,151,0,0
PLA2G2C_2_6419,293,79,103,239,117,390,105,143,224,262,21,96
PLA2G2D_2_6420,2969,3550,2634,2900,5154,3773,3201,2580,2339,2989,2833,
1680
PLA2G2E_2_6421,451,434,366,626,296,663,26,621,430,223,356,47
PLA2G2F_2_6422,39,31,61,19,0,0,1,0,26,0,0,0
PLA2G3_2_6423,394,356,177,432,637,533,604,158,560,514,965,336
PLA2G4A_2_6424,697,946,850,753,1042,419,410,389,552,606,94,665
PLA2G4B_2_6425,168,283,170,205,179,224,7,112,6,52,1,239
PLA2G4D_2_6426,234,210,146,245,246,445,104,12,401,297,93,168
PLA2G4E_2_6427,37,21,16,15,32,0,0,0,3,2,0,0
PLA2G4F_2_6428,89,127,125,68,0,23,38,180,252,0,23,8
PLA2G5_2_6429,149,50,97,148,148,8,549,22,9,404,10,0
PLCB2_2_6430,62,21,23,33,25,0,0,2,93,74,10,11
PLCD3_2_6431,8,2,16,12,0,0,1,0,0,0,0,0
PLCD4_2_6432,114,39,167,145,0,0,840,0,133,3,803,348
PLCG2_2_6433,517,596,591,493,487,129,616,1793,441,857,82,1216
PLCH2_2_6434,249,146,200,160,2,625,326,0,74,0,534,564
PLCL1_2_6435,262,615,330,352,274,305,1105,551,466,60,375,296
PLCXD1_2_6436,529,500,445,665,226,93,1149,196,871,207,348,741
PLCZ1_2_6437,299,130,208,306,187,60,503,292,169,22,4,162

PLD4_2_6438,152,240,185,185,310,163,439,204,30,16,14,232
PLOD1_2_6439,278,156,171,217,193,165,296,315,501,55,50,86
PLOD3_2_6440,295,302,192,244,200,139,132,604,533,67,106,143
PLSCR1_2_6441,139,193,82,164,436,339,116,28,79,13,31,242
PMM1_2_6442,68,75,113,122,131,18,28,236,83,21,0,35
PMM2_2_6443,370,601,413,726,317,186,292,510,166,152,322,487
PMPCA_2_6444,4,86,29,31,0,4,0,0,0,0,275,0
PMPCB_2_6445,98,47,89,132,19,6,196,205,66,20,64,230
PNLIP_2_6446,493,256,352,399,272,213,450,111,418,257,851,375
PNLIPRP1_2_6447,974,695,742,723,770,24,844,23,911,1281,1423,349
PNLIPRP2_2_6448,803,764,479,606,42,176,475,502,651,669,1522,382
PNLIPRP3_2_6449,1199,996,751,1026,1268,1845,632,632,1510,1250,1465,112
7
PNMT_2_6450,183,334,326,288,79,632,1568,15,436,564,105,252
PNPLA2_2_6451,23,66,47,60,0,30,304,135,8,153,248,0
PNPLA3_2_6452,278,224,120,188,436,2,154,1,85,297,38,112
PNPLA8_2_6453,2971,2581,2110,2557,2729,1563,2005,1957,3431,2976,1973,2
115
PNPO_2_6454,883,547,550,739,1325,970,1289,457,676,833,736,259
PNPT1_2_6455,186,380,335,218,0,195,0,447,0,383,762,99
POLA1_2_6456,628,406,412,326,607,213,232,166,284,653,979,500
POLA2_2_6457,258,481,271,261,573,40,138,0,408,372,338,246
POLB_2_6458,21,6,11,21,0,112,0,0,0,0,1,20
POLD4_2_6459,132,118,87,107,233,131,158,0,150,54,49,194
POLE3_2_6460,140,205,193,155,0,92,263,207,250,186,337,144
POLE4_2_6461,6,6,16,70,0,0,0,13,1,15,2,0
POLE_2_6462,77,66,86,100,500,1,0,521,193,0,0,0
POLG2_2_6463,39,27,57,25,0,98,52,20,1,206,1,60
POLI_2_6464,389,364,409,604,259,421,350,676,68,402,513,356
POLM_2_6465,1,29,8,24,0,0,0,14,56,269,0,0
POLN_2_6466,253,76,109,143,0,409,237,330,0,239,0,26
POLR1A_2_6467,99,78,64,79,140,64,580,324,66,73,276,257
POLR1C_2_6468,483,223,232,301,195,24,149,247,509,262,219,323
POLR1E_2_6469,314,356,422,376,322,881,409,7,3,310,264,35
POLR2A_2_6470,131,184,97,161,63,106,0,51,204,44,248,43
POLR2B_2_6471,400,654,308,397,229,570,9,545,94,541,65,394
POLR2C_2_6472,223,116,256,354,426,301,360,329,278,29,317,277
POLR2D_2_6473,140,104,135,80,77,1,5,7,104,280,11,11
POLR2E_2_6474,161,217,215,178,476,0,0,303,0,856,0,149
POLR2F_2_6475,66,57,55,28,170,612,8,0,53,0,28,3
POLR2G_2_6476,792,425,730,659,705,247,382,1095,635,110,283,182
POLR2H_2_6477,134,186,250,397,727,54,7,0,206,237,726,112
POLR2J_2_6478,991,918,870,1199,1014,965,1646,976,1233,786,940,1758
POLR2K_2_6479,584,294,242,248,354,240,339,303,555,360,490,409
POLR2L_2_6480,663,771,506,628,341,644,1033,191,367,1106,397,1175
POLR3A_2_6481,413,455,514,515,587,158,392,342,445,122,75,408
POLR3C_2_6482,74,18,31,83,11,61,0,8,55,78,2,15
POLR3F_2_6483,489,332,433,450,148,15,552,180,395,404,514,770
POLR3G_2_6484,571,566,535,492,79,224,440,222,595,373,72,410
POLRMT_2_6485,24,24,23,43,52,7,0,3,0,3,0,1

POMGNT1_2_6486,14,23,41,38,19,0,0,0,0,7,0,9
POMT2_2_6487,360,249,268,381,103,593,77,166,336,233,102,103
PON3_2_6488,135,144,32,238,258,0,477,360,218,249,0,592
POP4_2_6489,333,292,374,397,56,346,480,114,596,193,380,325
POP7_2_6490,315,348,383,391,571,29,837,662,545,353,440,171
POR_2_6491,43,14,15,7,0,0,74,0,0,19,0,33
PPA1_2_6492,286,326,241,570,135,157,187,0,200,558,634,100
PPCDC_2_6493,282,129,182,145,598,13,9,230,173,371,4,522
PPIA_2_6494,117,141,93,181,49,96,128,288,50,99,30,54
PPIAL4A_2_6495,1547,1573,1160,1458,1367,554,1761,1036,921,2043,985,174
7
PPIAL4B_2_6496,1547,1573,1160,1458,1367,554,1761,1036,921,2043,985,174
7
PPIAL4C_2_6497,1547,1573,1160,1458,1367,554,1761,1036,921,2043,985,174
7
PPIAL4E_2_6498,1547,1573,1160,1458,1367,554,1761,1036,921,2043,985,174
7
PPIAL4G_2_6499,1184,868,1220,1326,1086,593,920,3262,1085,1613,1374,985
PPIB_2_6500,882,758,743,1038,973,584,1644,503,568,291,933,786
PPIC_2_6501,176,379,611,375,918,498,44,35,27,18,1576,84
PPID_2_6502,244,206,156,295,208,255,306,3,91,889,581,677
PPIF_2_6503,20,23,67,17,0,2,73,0,16,0,0,8
PPIG_2_6504,209,116,92,151,233,107,98,116,233,189,567,89
PPIH_2_6505,0,0,0,0,0,0,0,0,0,0,0,0
PPIL1_2_6506,1042,537,698,1129,1230,435,346,1259,378,696,1636,398
PPIL4_2_6507,4247,3024,2488,3719,4390,3246,3438,3568,3473,3266,1148,34
18
PPIP5K2_2_6508,1998,2019,1962,1903,1943,1658,2666,829,2113,2393,2911,1
490
PPM1H_2_6509,78,148,147,101,0,753,0,214,0,73,0,259
PPM1J_2_6510,60,45,0,10,0,0,0,0,196,0,32,0
PPM1N_2_6511,493,454,503,499,246,366,771,258,410,732,528,278
PPME1_2_6512,112,120,106,103,83,55,220,6,183,14,84,59
PPWD1_2_6513,1863,1812,1690,2166,2372,1244,1537,1833,2017,1283,2613,17
84
PRDX4_2_6514,188,165,186,317,291,174,641,125,372,449,19,190
PREP_2_6515,574,640,662,721,372,980,1014,489,738,650,1075,1198
PRHOXNB_2_6516,126,25,96,66,12,0,70,0,4,66,0,41
PRIM1_2_6517,264,358,205,249,503,121,336,34,326,37,20,351
PRIM2_2_6518,472,453,539,408,351,686,239,62,304,1039,26,552
PROC_2_6519,57,92,100,168,5,366,362,0,35,504,35,6
PROSC_2_6520,93,106,86,111,3,8,205,214,79,302,6,128
PRR14L_2_6521,511,506,474,391,144,475,587,427,458,172,911,320
PRSS1_2_6522,121,118,143,148,208,46,192,627,408,213,368,149
PRSS2_2_6523,187,76,183,122,77,267,567,364,206,0,1048,16
PRTFDC1_2_6524,371,359,378,352,282,213,748,740,139,263,356,336
PRTN3_2_6525,283,271,169,245,129,337,40,393,395,439,26,152
PRUNE_2_6526,10,105,57,27,0,0,0,1,3,0,2,3
PSMA2_2_6527,205,138,312,226,14,0,257,98,47,114,21,212
PSMA6_2_6528,358,631,326,397,312,178,31,477,397,31,761,390

PSMA7_2_6529,249,265,208,281,269,315,981,159,115,221,1,213
PSMB10_2_6530,97,128,117,54,191,847,400,2,235,0,0,40
PSMB11_2_6531,990,714,665,1109,438,279,512,181,310,648,637,1113
PSMB1_2_6532,308,281,254,460,263,5,327,186,349,260,0,130
PSMB3_2_6533,42,28,50,55,20,7,4,49,115,66,75,1
PSMB4_2_6534,329,270,264,392,206,336,0,1087,394,419,896,498
PSMB6_2_6535,158,178,190,447,322,173,1,318,297,147,173,604
PSMB7_2_6536,438,380,321,256,268,254,423,9,759,25,39,141
PSMB9_2_6537,223,215,206,343,79,720,477,149,165,314,10,232
PSMD6_2_6538,402,615,392,406,964,678,1044,520,128,642,1449,569
PTDSS1_2_6539,409,387,410,456,169,503,722,475,296,136,191,345
PTDSS2_2_6540,865,512,544,870,942,278,28,474,188,461,197,349
PTGDS_2_6541,160,138,100,126,340,45,9,146,48,151,144,10
PTGES2_2_6542,125,198,237,312,501,31,35,368,307,77,326,2
PTGES3_2_6543,977,1136,807,942,1033,322,386,2008,837,889,1150,846
PTGES_2_6544,188,84,115,79,100,58,36,320,200,42,105,57
PTGIS_2_6545,53,65,72,116,37,2,206,559,80,170,48,43
PTGS2_2_6546,782,744,530,707,728,388,1284,760,597,302,1337,1626
PTRH1_2_6547,275,335,322,258,67,394,142,112,614,33,390,279
PTRH2_2_6548,76,34,93,129,29,13,119,7,79,20,0,41
PTS_2_6549,1116,1040,1129,1421,1073,685,1898,616,1091,2780,3550,1130
PUS3_2_6550,1718,1592,1383,2399,1389,2472,2686,1115,1258,2939,2064,165
6
PUSL1_2_6551,73,146,215,269,485,0,1,497,76,321,166,272
PXDN_2_6552,129,98,169,181,0,0,229,175,262,7,17,23
PXDNL_2_6553,766,789,650,656,652,230,327,1031,547,1288,917,506
PYCR2_2_6554,120,97,134,93,130,20,122,136,121,97,237,152
PYCRL_2_6555,40,187,142,97,205,381,210,0,89,1,240,5
PYGB_2_6556,298,277,320,464,347,332,449,58,227,328,673,214
QDPR_2_6557,87,43,206,115,63,0,2,164,53,4,509,243
QPCT_2_6558,514,221,352,397,470,284,561,668,310,240,133,402
QPRT_2_6559,294,150,112,205,6,80,0,71,61,468,87,250
QRSL1_2_6560,2615,2108,1848,1946,3595,1894,4185,2064,2963,2733,814,181
2
QSOX2_2_6561,304,290,272,459,319,11,357,370,351,113,41,539
QTRT1_2_6562,79,80,53,67,44,18,0,81,42,42,0,120
QTRTD1_2_6563,133,157,167,152,246,60,34,1,16,68,76,143
RAB10_2_6564,1210,977,925,1434,2040,1542,1630,1565,1139,865,1227,1371
RAB12_2_6565,200,144,153,277,405,101,477,0,289,30,11,420
RAB13_2_6566,744,678,683,907,752,815,782,794,858,759,191,877
RAB14_2_6567,179,216,471,366,4,57,92,443,259,251,701,425
RAB15_2_6568,30,42,19,13,1,12,916,0,0,0,1,0
RAB17_2_6569,346,270,346,431,415,18,8,299,18,383,4,782
RAB18_2_6570,193,195,157,156,84,72,449,65,37,455,33,145
RAB19_2_6571,1752,1736,1620,1904,1442,1637,3424,1066,1691,744,1080,275
9
RAB1B_2_6572,182,174,156,190,361,547,62,323,863,203,42,198
RAB20_2_6573,50,15,37,28,0,0,99,0,0,0,84,5
RAB21_2_6574,190,279,204,297,627,207,214,11,128,531,156,438
RAB22A_2_6575,614,366,395,673,286,431,602,185,494,803,366,184

RAB25_2_6576,73,35,51,73,59,40,0,83,40,199,0,29
RAB26_2_6577,113,43,72,54,56,37,156,10,61,50,1,23
RAB27B_2_6578,1033,651,670,1119,1026,690,377,983,824,672,1577,1535
RAB30_2_6579,829,579,547,624,371,358,1277,692,894,685,324,1094
RAB31_2_6580,939,1165,876,1157,1039,551,1605,800,287,808,795,726
RAB33A_2_6581,358,632,390,489,800,60,1853,426,496,194,180,585
RAB33B_2_6582,309,243,317,403,0,200,94,830,414,251,736,320
RAB36_2_6583,60,238,74,54,71,0,0,202,3,333,0,2
RAB38_2_6584,585,390,362,398,647,158,267,393,466,1071,318,533
RAB39B_2_6585,718,597,577,739,729,773,1047,837,212,650,950,1044
RAB3A_2_6586,489,610,564,650,1073,637,360,1001,516,660,511,1178
RAB3B_2_6587,203,248,143,181,34,180,73,3,116,22,379,73
RAB3C_2_6588,323,187,266,435,186,255,505,59,56,204,552,352
RAB3D_2_6589,105,104,51,145,16,17,0,5,5,2,0,23
RAB3GAP2_2_6590,103,123,89,179,34,230,0,113,25,0,93,32
RAB4A_2_6591,3811,3713,3360,3924,5222,2776,4153,1808,3189,3144,4105,47
45
RAB4B_2_6592,73,30,76,132,11,13,503,2,59,577,21,267
RAB5A_2_6593,135,122,122,147,86,211,298,123,83,98,170,141
RAB5B_2_6594,1461,1370,1089,1536,587,1839,1326,1438,1067,984,410,1523
RAB6B_2_6595,149,253,120,196,53,237,21,47,0,472,102,589
RAB6C_2_6596,1146,1605,1658,1782,1954,828,1302,1614,926,2206,2285,2051
RAB7A_2_6597,217,257,280,218,472,156,51,5,167,237,348,411
RAB8B_2_6598,453,277,376,348,502,474,669,574,186,242,157,1580
RAB9B_2_6599,293,291,260,217,334,170,109,198,547,520,83,14
RABGGTB_2_6600,564,437,603,427,74,507,597,996,450,1077,403,898
RABL3_2_6601,167,111,126,84,154,38,42,304,17,204,111,86
RAC2_2_6602,386,294,260,288,262,344,268,376,484,73,199,366
RAD50_2_6603,177,229,156,182,128,167,921,80,34,123,274,141
RAD54L2_2_6604,181,295,234,256,32,60,86,101,60,416,315,153
RALA_2_6605,2,0,1,0,0,0,0,1,0,0,0,0
RALB_2_6606,197,149,180,194,322,1,38,608,70,62,23,291
RALBP1_2_6607,107,83,83,54,242,24,53,1,5,51,2,38
RANBP2_2_6608,582,517,428,456,656,752,253,623,62,378,304,649
RAN_2_6609,0,0,0,0,0,0,0,0,0,0,0,0
RAP2A_2_6610,207,322,314,352,299,43,1007,51,388,160,498,364
RAP2B_2_6611,565,611,491,631,552,265,1698,727,191,275,374,553
RAP2C_2_6612,70,80,47,69,4,0,52,55,0,89,14,1
RARS2_2_6613,62,35,77,117,5,22,208,61,113,41,6,130
RARS_2_6614,430,301,343,311,286,291,828,59,379,177,152,341
RASD2_2_6615,201,244,157,324,47,178,194,30,118,59,135,237
RASL10B_2_6616,43,105,156,152,248,84,39,527,72,427,40,700
RASL11B_2_6617,514,500,551,467,149,276,4,82,368,293,926,671
RASL12_2_6618,278,283,179,304,510,347,0,834,0,496,439,309
RC3H1_2_6619,151,122,126,167,218,172,1120,68,252,210,10,59
RCL1_2_6620,153,180,140,141,270,41,151,50,235,3,209,123
RDH10_2_6621,379,278,260,333,145,323,637,282,420,290,165,407
RDH11_2_6622,4,74,99,5,39,0,364,0,0,85,0,6
RDH12_2_6623,1570,1498,1639,1485,1066,2333,1566,1084,2123,2170,2113,17
98

RDH14_2_6624,1256,899,952,1119,1985,688,842,1014,983,487,1004,736
RDH16_2_6625,33,8,32,34,27,0,79,0,126,0,26,37
RDH8_2_6626,188,122,156,124,364,763,22,0,32,126,0,804
RECQL4_2_6627,315,291,231,208,819,1,0,222,422,464,449,9
REM1_2_6628,15,64,25,66,1,1,0,0,0,12,0,0
REM2_2_6629,52,11,64,43,81,0,256,0,0,1,77,2
RENBP_2_6630,1721,1432,1306,1445,1087,1698,1446,1280,892,1099,2193,972
RETSAT_2_6631,219,186,191,328,141,233,679,162,257,71,0,99
REV3L_2_6632,781,430,631,554,60,703,1526,822,294,602,405,513
REXO1L1_2_6633,1,4,3,6,0,0,0,0,11,0,0,0
REXO2_2_6634,1030,974,1108,1086,848,1787,2707,408,602,1439,1246,755
RFNG_2_6635,34,92,81,117,30,50,5,73,17,91,51,25
RFX6_2_6636,211,195,182,157,105,212,554,54,45,290,302,29
RGS7_2_6637,927,952,858,893,497,1550,1297,1018,1435,684,1111,588
RHBDL1_2_6638,270,290,287,307,96,5,420,121,101,141,548,529
RHBDL2_2_6639,114,158,147,144,3,505,0,123,132,91,25,186
RHBDL3_2_6640,41,48,56,79,0,56,49,0,3,119,0,0
RHEBL1_2_6641,287,213,240,216,7,17,283,271,233,122,805,320
RHOA_2_6642,628,554,428,471,468,550,670,92,438,863,173,749
RHOB_2_6643,820,811,899,1056,977,636,430,340,623,237,1117,938
RHOD_2_6644,101,32,41,75,1,5,0,25,17,152,2,13
RHOF_2_6645,502,620,511,548,370,527,138,22,321,9,0,509
RHOG_2_6646,5,9,8,9,79,23,0,0,15,5,0,29
RHOH_2_6647,110,179,67,108,202,80,89,176,79,111,30,235
RHOJ_2_6648,482,310,305,622,738,277,760,587,726,540,53,667
RHOQ_2_6649,283,212,234,252,633,0,0,390,373,1,91,11
RHOT2_2_6650,170,109,125,163,65,38,140,53,38,193,27,379
RHOU_2_6651,118,12,73,47,129,13,196,19,25,70,238,1
RHOV_2_6652,101,19,93,169,497,173,223,24,2,57,0,67
RIT1_2_6653,76,0,3,15,0,0,0,0,17,0,0,0
RIT2_2_6654,190,192,251,335,209,74,163,169,107,239,53,500
RNASE2_2_6655,1634,1805,1446,1739,2524,1851,1186,674,1734,1660,1359,11
89
RNASE3_2_6656,1133,818,1044,1060,2063,1004,1157,726,619,670,1248,1100
RNASE6_2_6657,700,716,651,847,477,388,733,245,385,236,802,809
RNASE7_2_6658,233,45,40,67,271,633,103,3,2,41,2,1
RNASE8_2_6659,1750,1767,1441,1728,2062,1533,3880,2368,969,2303,1398,21
40
RNASEH2A_2_6660,141,106,91,224,265,339,0,305,25,192,3,155
RNASET2_2_6661,751,555,616,668,609,150,1001,526,668,1007,211,867
RND1_2_6662,43,41,112,104,176,0,13,102,1,45,0,32
RND2_2_6663,184,304,228,328,338,81,370,26,111,646,337,390
RND3_2_6664,188,108,229,261,758,82,294,69,217,137,158,142
RNF148_2_6665,381,137,516,541,262,339,0,37,188,323,814,398
RNMT_2_6666,177,262,148,222,649,119,0,0,551,682,131,100
RNMTL1_2_6667,138,183,133,152,0,8,449,337,15,65,0,142
RNPEP_2_6668,138,92,95,148,51,342,587,36,374,6,0,237
RPAP2_2_6669,484,673,422,632,699,337,205,746,475,849,73,449
RPE65_2_6670,356,203,215,257,170,294,424,1,211,394,318,218
RPIA_2_6671,173,227,187,162,15,482,17,1,268,85,13,205

RPL4_2_6672,188,88,133,185,6,235,0,100,0,0,0,0
RPN1_2_6673,148,147,160,287,332,528,385,82,126,335,95,354
RPP25_2_6674,7,50,49,68,0,271,0,101,408,10,0,20
RPP40_2_6675,1296,1123,1066,1054,479,684,1596,643,997,1423,1621,1413
RPS3_2_6676,140,139,121,147,162,287,11,122,27,36,408,108
RPUSD1_2_6677,49,23,35,48,0,36,0,67,22,26,0,80
RPUSD2_2_6678,372,411,407,341,875,486,769,0,948,274,9,325
RRAGA_2_6679,170,241,252,288,224,46,909,22,191,280,190,178
RRAGC_2_6680,62,77,47,98,332,0,0,707,7,3,0,90
RRAGD_2_6681,196,221,193,258,101,277,512,19,3,245,331,184
RRAS_2_6682,263,152,182,290,29,18,517,611,460,0,676,336
RRM1_2_6683,754,902,636,900,1045,198,1174,409,1063,2115,994,666
RRP8_2_6684,961,628,658,815,1315,656,1251,541,1171,441,1218,842
RSAD2_2_6685,19,18,7,13,0,0,0,0,1,0,0,0
RTN4IP1_2_6686,238,246,254,335,417,27,421,518,332,38,296,318
SAMHD1_2_6687,540,553,516,750,1164,356,424,346,809,320,72,743
SDF2_2_6688,499,711,561,597,39,267,696,59,856,940,615,218
SDHA_2_6689,174,171,204,84,235,307,481,236,153,52,92,162
SDHB_2_6690,1071,932,965,988,775,1042,1276,468,656,657,1892,414
SDHD_2_6691,434,298,335,306,505,529,199,208,43,376,311,85
SDR16C5_2_6692,345,186,266,266,389,71,475,148,460,337,230,508
SDR42E1_2_6693,189,104,187,263,2,523,2,355,320,21,78,231
SDR9C7_2_6694,703,546,594,594,489,328,514,215,476,449,163,457
SDSL_2_6695,147,162,45,92,2,53,0,88,261,4,6,44
SEPSECS_2_6696,669,470,551,644,591,128,1270,3,1110,467,1375,712
1-Sep_2_6697,912,816,674,870,1761,318,118,989,701,392,634,543
SEPW1_2_6698,320,303,246,294,201,134,259,260,313,350,988,355
SERHL2_2_6699,347,353,279,480,101,137,1294,564,151,76,279,506
SETX_2_6700,2532,2036,2063,2362,2761,2073,1829,2351,2183,1642,2158,195
8
SGMS1_2_6701,1283,1221,1144,1369,802,857,382,1441,1197,709,1409,1010
SGPL1_2_6702,403,437,232,471,399,620,286,105,299,379,330,423
SGSH_2_6703,19,60,27,34,0,0,36,57,38,118,16,65
SH3GL2_2_6704,101,133,128,152,307,414,0,39,26,78,99,521
SKIV2L2_2_6705,436,194,284,325,716,757,899,612,405,40,50,451
SLFN12_2_6706,504,449,614,586,415,678,448,253,465,325,569,540
SLFN12L_2_6707,1112,1311,1547,1500,2023,545,1060,909,641,399,1335,1592
SLFN13_2_6708,282,459,384,350,1,405,2,241,448,625,850,618
SLFN5_2_6709,1237,1502,1082,1500,884,1661,1094,554,1091,498,178,1756
SLU7_2_6710,487,522,392,573,485,324,1636,1228,224,1053,414,380
SMG8_2_6711,751,603,648,665,578,589,409,753,747,146,822,909
SMPD2_2_6712,39,14,39,25,0,67,5,3,16,23,0,147
SMPD3_2_6713,59,61,33,152,102,0,35,0,16,191,49,20
SMPDL3A_2_6714,449,508,407,476,388,69,383,65,416,271,999,248
SMUG1_2_6715,227,156,186,215,5,217,0,98,277,43,193,29
SNF8_2_6716,121,354,158,270,708,146,19,113,178,232,126,183
SNRNP200_2_6717,411,530,589,716,527,804,357,318,91,745,220,391
SOAT1_2_6718,222,205,183,260,0,85,39,25,53,153,144,252
SOAT2_2_6719,115,112,108,166,70,0,4,104,237,43,0,2
SOD1_2_6720,69,204,98,174,194,12,9,313,24,56,1,56

SOD3_2_6721,93,70,104,103,14,308,0,0,73,6,21,302
SORD_2_6722,14,25,4,24,0,16,1,0,13,12,0,14
SPACA3_2_6723,168,205,155,213,163,179,272,161,76,4,11,634
SPACA5B_2_6724,1063,872,917,872,1015,562,2083,1503,633,469,979,609
SPACA5_2_6725,1063,872,917,872,1015,562,2083,1503,633,469,979,609
SPEM1_2_6726,247,254,224,249,489,14,136,0,282,225,242,538
SPTLC2_2_6727,813,781,666,771,619,222,1467,995,486,907,1853,520
SPTLC3_2_6728,254,327,122,328,315,443,2072,445,252,103,8,289
SPTSSA_2_6729,348,341,335,389,398,266,316,289,382,625,522,277
SQLE_2_6730,1415,1233,1573,1402,1373,734,2114,1426,1410,872,426,1823
SQRDL_2_6731,61,66,52,76,325,141,2,6,56,33,10,17
SRD5A1_2_6732,2633,2271,2518,3021,3727,1915,1786,3954,1979,2004,3088,2
456
SRD5A2_2_6733,604,716,394,694,1310,1871,1002,368,639,826,183,126
SRD5A3_2_6734,510,558,395,437,596,216,173,761,1023,489,452,181
SRR_2_6735,395,365,502,394,873,883,661,1048,183,181,507,180
SRSF9_2_6736,187,171,113,181,33,15,109,147,165,507,140,226
SRXN1_2_6737,217,232,179,410,11,54,0,303,696,503,103,405
SSB_2_6738,282,271,272,274,191,157,995,459,289,797,545,339
SSU72_2_6739,553,591,427,515,437,808,1176,976,105,319,741,412
ST14_2_6740,96,10,51,107,0,0,0,7,29,0,68,222
ST3GAL2_2_6741,181,199,243,298,300,345,47,58,29,135,551,552
ST3GAL4_2_6742,231,206,198,170,170,328,81,0,19,294,162,96
ST3GAL6_2_6743,988,768,815,916,742,625,2100,1305,720,2160,2033,963
ST6GALNAC1_2_6744,220,72,84,123,28,28,547,179,88,60,3,179
ST6GALNAC2_2_6745,497,477,569,310,588,76,1123,178,763,441,372,196
ST6GALNAC5_2_6746,679,655,710,559,996,100,1364,583,962,1595,283,546
ST6GALNAC6_2_6747,89,57,88,53,110,0,70,0,0,3,1,29
ST8SIA1_2_6748,201,144,149,254,173,144,196,97,127,39,140,315
ST8SIA2_2_6749,752,711,758,799,297,1303,883,605,694,1219,332,254
ST8SIA3_2_6750,962,790,884,1127,805,293,546,1251,872,1258,677,1570
ST8SIA5_2_6751,219,158,201,269,123,219,296,198,155,0,0,289
ST8SIA6_2_6752,276,399,428,432,353,473,586,0,9,256,72,674
STS_2_6753,503,339,412,548,7,723,511,185,46,844,922,276
STT3A_2_6754,517,523,523,619,339,449,612,1059,309,706,142,235
STT3B_2_6755,829,711,617,695,760,853,728,397,441,673,144,561
SUCLA2_2_6756,663,989,770,1097,869,469,919,396,622,1004,1403,1387
SUCLG1_2_6757,162,155,93,196,5,18,21,313,174,57,69,64
SULT1A3_2_6758,130,113,106,155,338,96,13,374,102,262,35,106
SULT1A4_2_6759,130,113,106,155,338,96,13,374,102,262,35,106
SULT1B1_2_6760,517,395,521,396,92,774,998,205,493,348,946,416
SULT1C3_2_6761,52,75,86,56,95,117,13,87,15,115,446,129
SULT1C4_2_6762,2528,2357,2215,2705,1612,1970,2852,2735,3036,2774,3646,
3550
SULT1E1_2_6763,1,14,53,50,0,0,213,14,42,89,79,0
SULT2A1_2_6764,123,140,58,55,545,65,14,48,50,135,0,4
SULT4A1_2_6765,292,86,167,127,8,129,179,8,224,0,64,139
SUPV3L1_2_6766,84,96,145,133,55,3,218,1,36,183,8,501
SURF1_2_6767,149,199,170,231,133,150,6,477,183,59,7,243
TALDO1_2_6768,209,112,146,144,26,4,2,2,146,0,0,28

TARS2_2_6769,291,154,183,212,55,400,264,78,155,198,9,300
TARS_2_6770,304,278,221,255,310,0,95,215,728,252,196,316
TARSL2_2_6771,345,312,290,380,491,0,269,148,70,140,246,68
TAT_2_6772,145,126,155,178,249,108,129,198,166,243,0,77
TBC1D10B_2_6773,108,83,102,105,70,14,0,57,225,91,98,27
TBCC_2_6774,5,29,73,35,12,507,8,0,0,236,10,126
TDG_2_6775,794,693,813,1086,468,951,123,601,724,663,713,814
TDO2_2_6776,220,248,168,260,262,353,319,386,67,504,200,218
TECR_2_6777,241,149,159,157,323,140,201,112,250,183,9,380
TECTA_2_6778,158,32,162,196,0,410,27,129,5,315,4,423
TFB2M_2_6779,1785,2042,1490,1962,1437,1064,1587,1832,552,1756,1105,157
3
TGDS_2_6780,308,322,353,309,436,172,342,34,66,196,305,212
TGM1_2_6781,52,26,110,66,106,0,0,23,149,1,0,0
TGM3_2_6782,264,203,166,212,415,65,183,165,275,86,0,260
TGM4_2_6783,0,0,0,0,0,0,0,0,0,0,0,0
TGM6_2_6784,232,267,235,362,87,165,232,81,123,1245,27,504
TGM7_2_6785,409,251,166,414,1060,486,422,27,563,1087,12,627
TGS1_2_6786,786,659,805,645,796,189,1278,429,474,679,332,751
THG1L_2_6787,30,64,28,30,0,98,0,0,6,9,3,8
THOP1_2_6788,73,116,58,144,5,54,2,20,124,21,14,82
THUMPD2_2_6789,3076,2228,2109,2344,1576,2116,1981,2499,2979,2449,2406,
3045
TKTL2_2_6790,109,43,106,211,13,213,0,329,46,305,0,169
TM7SF2_2_6791,188,274,230,146,81,377,351,278,381,329,213,15
TMEM55A_2_6792,15,8,5,35,10,0,0,0,2,64,201,0
TMEM62_2_6793,739,831,495,760,883,535,782,523,1546,1294,409,1120
TMEM86B_2_6794,75,62,91,35,85,1,26,24,3,74,36,129
TMPRSS15_2_6795,146,24,125,140,21,128,300,25,52,94,66,191
TMX1_2_6796,170,265,155,292,344,73,1,140,319,171,184,610
TMX3_2_6797,455,416,426,426,776,195,1033,137,293,1175,806,210
TMX4_2_6798,277,195,296,222,32,2,0,283,167,107,505,501
TNKS2_2_6799,2867,2239,2755,3042,2358,2435,2957,2407,3235,2460,3203,26
64
TNKS_2_6800,45,371,284,391,945,310,440,1,93,0,360,160
TOP1_2_6801,963,798,502,578,766,634,926,276,1141,559,982,1015
TOP1MT_2_6802,59,25,42,59,26,19,26,100,30,65,162,9
TOP2A_2_6803,335,282,280,307,1152,378,586,61,506,357,380,195
TOP2B_2_6804,414,421,451,414,491,74,105,652,530,596,206,570
TOP3A_2_6805,164,111,102,202,259,29,26,31,142,100,94,10
TOP3B_2_6806,334,319,319,469,20,685,726,72,228,483,914,647
TOR3A_2_6807,187,163,137,191,1,7,103,392,7,370,232,79
TPH1_2_6808,93,83,30,81,62,147,41,34,174,6,6,10
TPMT_2_6809,674,949,650,1090,721,373,486,301,467,830,966,1309
TPP1_2_6810,182,190,189,234,196,88,193,162,123,412,344,687
TPP2_2_6811,254,277,206,393,546,204,402,231,215,238,0,232
TPSAB1_2_6812,444,491,482,591,688,246,782,321,234,447,429,225
TPSB2_2_6813,396,246,307,328,0,0,0,301,2,277,1563,106
TPSD1_2_6814,679,500,580,417,388,35,173,492,408,583,131,547
TPST1_2_6815,68,23,24,15,0,0,6,0,5,0,12,0

TREH_2_6816,49,163,94,82,20,91,134,1,12,0,48,3
TREX2_2_6817,77,1,13,77,313,0,0,0,123,0,355,0
TRHDE_2_6818,180,206,241,228,327,715,367,0,53,80,56,196
TRIM21_2_6819,42,2,51,13,11,0,34,0,106,0,21,59
TRIT1_2_6820,331,483,437,487,623,204,513,323,195,452,271,273
TRMT112_2_6821,253,229,198,251,140,94,112,65,217,334,1,182
TRMT61A_2_6822,238,189,173,178,597,278,1,304,97,6,279,38
TRMU_2_6823,63,66,42,60,217,159,18,4,0,53,145,47
TRNT1_2_6824,40,186,175,39,0,0,0,50,107,310,0,64
TRUB1_2_6825,252,404,228,307,394,102,758,84,267,230,541,273
TRUB2_2_6826,397,230,377,351,0,1,0,658,0,15,691,562
TSTA3_2_6827,83,35,19,68,41,0,34,3,56,178,71,0
TST_2_6828,97,173,181,106,571,0,19,0,0,2,80,9
TTLL13_2_6829,104,260,114,223,99,100,104,0,274,85,337,390
TTLL1_2_6830,506,427,452,611,406,639,842,97,646,756,735,255
TTLL3_2_6831,3310,3653,3754,4031,2703,4971,4351,3144,2465,4622,3961,33
11
TTLL4_2_6832,190,90,190,170,30,39,224,56,305,169,243,13
TUFM_2_6833,140,154,122,150,164,85,34,108,418,379,101,318
TULP2_2_6834,141,148,64,183,43,0,91,113,1,75,0,277
TUT1_2_6835,75,51,54,100,138,352,0,77,179,245,55,39
TXN2_2_6836,165,117,204,223,287,34,0,13,141,98,0,278
TXN_2_6837,811,770,556,675,854,637,70,869,762,790,877,916
TXNDC11_2_6838,2482,1950,1854,2308,2172,1750,1182,2678,1315,2418,2458,
2215
TXNDC12_2_6839,135,176,85,187,96,74,92,153,143,10,508,106
TXNDC15_2_6840,158,63,162,179,163,182,487,23,545,127,64,119
TXNDC17_2_6841,69,55,102,73,28,406,0,0,237,43,10,0
TXNL1_2_6842,1042,927,1038,911,434,724,432,372,1119,788,807,988
TXNL4A_2_6843,397,208,127,150,373,17,249,114,171,146,862,302
TXNRD2_2_6844,239,253,323,394,347,524,17,180,185,2,4,434
TYMS_2_6845,350,372,343,370,347,557,638,149,356,311,643,376
TYR_2_6846,653,458,392,363,47,117,1347,193,344,256,250,231
TYRP1_2_6847,362,401,388,355,562,107,206,96,278,65,280,167
UAP1_2_6848,1927,2174,1724,1756,2132,755,2056,1250,1629,1310,2865,1321
UAP1L1_2_6849,53,106,62,94,54,0,0,0,24,0,50,137
UBB_2_6850,295,292,152,149,1,97,71,270,11,43,9,138
UBIAD1_2_6851,1665,1314,1519,1735,2047,216,2245,2296,1606,1278,2733,11
94
UBL4A_2_6852,69,130,161,72,76,50,14,23,51,0,159,218
UFSP1_2_6853,35,137,120,90,0,67,0,0,163,508,384,542
UFSP2_2_6854,188,436,389,410,597,728,790,784,395,794,918,361
UGCG_2_6855,5,0,0,0,0,0,0,0,0,0,0,0
UGGT1_2_6856,1423,1040,1193,1572,1645,1384,651,1195,1722,1954,1188,195
4
UGGT2_2_6857,546,408,493,469,81,563,549,8,167,611,246,346
UGT1A10_2_6858,1365,951,858,1272,908,1492,1068,827,2205,1824,1305,1635
UGT1A1_2_6859,49,130,36,36,0,7,26,154,10,14,19,60
UGT1A3_2_6860,194,77,109,163,268,102,284,127,256,7,20,67
UGT1A4_2_6861,2431,1866,1948,2591,2024,1618,3042,1710,2924,1959,3480,2

208
UGT1A5_2_6862,167,105,113,179,66,95,63,258,171,36,988,37
UGT1A7_2_6863,794,514,447,717,997,164,524,1049,657,498,1155,696
UGT1A8_2_6864,2679,2233,2503,2686,3382,2192,3271,4050,2534,3234,3602,2
561
UGT1A9_2_6865,794,514,447,717,997,164,524,1049,657,498,1155,696
UGT2A1_2_6866,312,281,322,302,444,225,198,20,52,321,72,242
UGT2A2_2_6867,0,0,0,0,0,0,0,0,0,0,0,0
UGT2A3_2_6868,609,420,524,574,1156,581,488,99,162,88,221,589
UGT2B15_2_6869,53,42,70,99,0,327,160,42,3,3,1,0
UGT2B17_2_6870,53,42,70,99,0,327,160,42,3,3,1,0
UGT2B4_2_6871,2778,2889,2213,2821,1757,2889,2421,2227,1984,2846,1637,1
980
UGT2B7_2_6872,1647,1238,1166,1314,344,920,1990,1033,869,884,987,1745
UMPS_2_6873,1375,1060,1057,1533,735,1344,1134,1518,1981,845,1857,1714
UPB1_2_6874,289,333,138,307,274,634,90,384,134,135,792,507
UPF1_2_6875,48,46,40,133,106,74,0,10,0,530,0,129
UPRT_2_6876,97,135,135,146,9,188,38,2,1,39,4,109
UQCR11_2_6877,2695,2146,1849,2198,3540,2235,2284,2468,2441,2591,829,20
56
UQCRC1_2_6878,237,125,163,206,433,178,304,332,296,288,3,91
UQCRC2_2_6879,293,165,289,185,592,325,413,146,467,63,10,32
UQCRFS1_2_6880,353,489,332,439,351,179,640,864,142,375,535,498
UQCRH_2_6881,1132,934,795,895,1210,524,2213,1166,594,1414,2972,990
UQCRQ_2_6882,270,192,180,157,29,299,15,106,30,228,3,16
UROD_2_6883,220,113,190,206,69,35,202,55,269,56,1,12
UROS_2_6884,136,191,170,178,57,1,345,362,47,45,253,0
UST_2_6885,139,201,202,221,38,482,3,102,247,321,123,9
UXS1_2_6886,236,217,151,265,169,303,790,5,423,347,104,334
VARS_2_6887,410,211,438,460,780,551,497,5,327,53,6,576
VAT1L_2_6888,454,547,537,601,243,360,1094,215,364,660,251,546
VCP_2_6889,734,555,587,648,664,818,14,440,251,156,2837,232
VNN1_2_6890,151,132,94,198,236,20,279,51,106,110,125,8
WBSCR17_2_6891,166,64,79,48,0,0,138,383,21,1,330,9
WBSCR27_2_6892,28,7,38,7,10,0,0,2,0,6,0,38
WDFY3_2_6893,2643,2084,1911,2320,2783,1616,1639,2872,980,1841,3975,268
8
WRN_2_6894,1909,1612,1595,1715,665,2024,1716,944,1287,1667,1619,1645
XDH_2_6895,294,376,396,511,542,586,479,61,387,257,664,351
XPNPEP2_2_6896,590,860,700,863,980,219,663,1122,500,761,1185,1169
XRCC2_2_6897,1735,1503,1506,1356,1238,518,788,1316,1191,1363,2133,1173
XRCC5_2_6898,65,123,64,123,46,6,0,138,20,4,114,0
XRCC6_2_6899,137,57,39,29,115,48,181,271,193,115,0,5
XRN2_2_6900,2434,2014,2431,2341,1597,789,1610,1419,2018,3236,2715,1779
XYLT1_2_6901,94,174,82,53,56,1,23,91,69,85,29,143
XYLT2_2_6902,81,55,86,102,278,36,148,9,354,23,26,232
YARS2_2_6903,301,349,256,552,222,193,349,881,598,320,41,1239
YARS_2_6904,326,147,177,176,226,160,121,643,228,534,325,450
YKT6_2_6905,81,63,84,97,20,16,10,6,163,151,922,9
YPEL1_2_6906,349,222,271,334,98,34,388,23,217,54,298,125

ZADH2_2_6907,330,120,386,333,276,419,1089,401,196,523,522,198
ZCCHC4_2_6908,1036,974,1060,884,1632,755,800,1425,533,839,1516,987
ZDHHC17_2_6909,1908,1354,1728,1767,1380,1763,1176,1796,1798,994,1484,2
147
ZDHHC18_2_6910,136,91,192,234,477,783,242,278,210,161,43,418
ZDHHC1_2_6911,156,190,261,347,1,42,0,316,4,307,148,633
ZDHHC21_2_6912,241,455,354,221,2,277,221,768,30,701,0,14
ZDHHC2_2_6913,779,650,481,716,306,363,1209,317,172,875,849,473
ZER1_2_6914,43,227,126,276,9,2,2,0,42,161,0,45
ZMPSTE24_2_6915,1243,1408,1253,1525,1175,2417,1740,1377,1757,1528,1689
,1235
ZRANB3_2_6916,122,103,160,88,130,29,479,5,93,227,51,29
A1CF_2_6917,456,264,334,338,672,219,458,159,466,335,773,442
A2LD1_2_6918,120,34,69,102,222,1,146,54,279,35,50,60
AADAT_2_6919,347,415,525,534,271,7,169,527,952,399,55,819
AARSD1_2_6920,115,107,128,144,112,67,182,9,72,151,0,166
ABAT_2_6921,208,75,93,110,470,33,17,0,93,63,217,173
ABHD11_2_6922,101,292,184,284,0,1009,305,2,166,11,0,198
ABHD12_2_6923,124,66,73,80,372,55,643,4,0,25,12,219
ABHD14B_2_6924,67,35,83,116,0,0,0,0,32,404,0,50
ABHD2_2_6925,539,375,270,449,921,773,89,425,349,200,133,766
ACAA1_2_6926,183,240,184,206,26,0,68,2,71,539,394,130
ACACA_2_6927,112,108,127,188,123,100,35,115,97,90,53,80
ACAD10_2_6928,359,156,411,236,73,257,505,368,116,155,173,263
ACADM_2_6929,1101,1031,1158,1161,1158,1220,982,595,1176,1587,773,1873
ACADVL_2_6930,0,0,0,0,0,0,0,0,0,0,0,0
ACCS_2_6931,145,77,149,143,11,28,1245,306,473,106,118,166
ACE_2_6932,606,598,518,579,372,571,760,105,481,272,220,873
ACHE_2_6933,58,128,34,128,0,0,0,220,213,210,0,0
ACIN1_2_6934,6,61,11,48,0,0,1,0,0,0,0,0
ACLY_2_6935,223,501,462,627,232,305,737,251,8,163,1264,189
ACOT11_2_6936,1496,1131,1390,1370,1382,939,995,2338,1326,1118,704,1610
ACOT13_2_6937,516,492,567,509,355,289,435,599,0,0,1859,545
ACOT7_2_6938,227,343,119,356,447,561,57,822,503,530,0,41
ACOT9_2_6939,122,45,24,24,3,0,0,36,43,0,262,347
ACOX1_2_6940,819,783,697,992,827,383,1481,424,463,573,624,736
ACOX3_2_6941,131,312,182,193,265,71,633,221,210,133,9,172
ACSBG1_2_6942,65,12,10,8,0,0,3,0,11,50,195,41
ACSF3_2_6943,568,400,245,400,342,336,661,58,83,930,328,242
ACSL3_2_6944,2809,2907,2793,3297,3400,2214,4799,2307,2316,1975,2224,28
24
ACSL4_2_6945,164,180,163,132,132,145,251,15,404,118,1094,319
ACSL5_2_6946,1612,1903,1463,2036,661,1638,1447,1287,1870,1317,1848,265
1
ACSL6_2_6947,701,838,757,793,400,216,761,584,560,828,1764,595
ACSM2B_2_6948,345,258,371,370,402,368,1003,124,382,529,45,296
ACSM3_2_6949,314,221,405,201,339,10,153,157,449,253,861,562
ACSS2_2_6950,27,14,29,55,0,0,0,0,51,3,0,1
ACY1_2_6951,2277,2113,2036,2619,3014,1979,3041,2636,3122,3626,2980,315
7

ADAMTS2_2_6952,629,383,262,508,501,213,150,693,770,39,859,599
ADARB1_2_6953,1279,1315,1094,1660,1946,422,1292,1332,1789,961,908,802
ADAR_2_6954,0,0,0,0,0,0,0,0,0,0,0,0
ADCY10_2_6955,292,259,369,277,76,168,580,439,60,410,514,327
ADCY4_2_6956,36,64,111,118,107,92,4,0,43,76,96,292
ADCY5_2_6957,136,146,231,153,130,137,20,326,312,370,171,21
ADCY6_2_6958,127,48,136,163,447,30,189,2,51,60,37,118
ADH6_2_6959,632,679,508,662,727,755,879,474,1045,394,766,1165
ADH7_2_6960,685,510,365,541,749,606,494,524,443,232,577,709
ADPRHL1_2_6961,72,13,29,118,0,68,0,369,0,156,4,74
ADSL_2_6962,274,214,304,312,439,36,1042,376,259,191,428,326
ADSSL1_2_6963,256,342,279,444,335,32,1111,143,263,492,752,335
AFMID_2_6964,55,80,83,141,20,0,108,14,23,0,19,44
AGA_2_6965,665,595,411,791,638,372,1615,266,669,675,113,606
AGAP1_2_6966,0,0,1,12,0,0,0,0,0,0,0,5
AGAP2_2_6967,437,261,261,389,378,378,31,308,637,447,385,521
AGL_2_6968,1243,1035,1235,1002,536,931,680,499,1046,775,2045,387
AGPAT1_2_6969,877,537,740,784,320,850,623,482,486,708,958,718
AGPAT2_2_6970,242,230,304,291,496,297,77,534,269,270,618,213
AGPAT3_2_6971,109,174,206,315,96,355,491,661,137,142,258,21
AGXT2L1_2_6972,469,444,222,455,215,128,286,3,451,281,79,519
AHCY_2_6973,154,368,93,147,60,60,94,18,8,114,397,171
AHCYL1_2_6974,569,474,432,466,367,30,370,720,249,191,177,332
AHCYL2_2_6975,120,75,165,148,23,6,0,48,7,620,252,51
AIFM1_2_6976,183,128,148,321,70,163,424,113,495,67,5,171
AIFM2_2_6977,55,54,82,70,0,0,0,7,0,0,0,0
AIFM3_2_6978,346,205,366,386,354,42,2062,192,166,221,193,526
AKIRIN1_2_6979,558,232,188,205,99,56,576,0,429,1,239,159
AKR1A1_2_6980,29,75,16,12,5,0,0,0,0,0,0,248
AKR1C2_2_6981,126,279,284,259,32,601,300,1,86,310,980,127
AKR1D1_2_6982,201,405,274,251,89,711,297,618,87,461,371,264
ALAS1_2_6983,46,52,55,34,8,0,295,0,72,8,230,26
ALAS2_2_6984,52,163,73,99,5,758,207,360,88,0,269,1
ALDH16A1_2_6985,622,682,655,875,1160,839,559,593,397,226,700,422
ALDH1A2_2_6986,8,65,36,45,186,17,14,143,6,10,0,170
ALDH2_2_6987,569,506,462,431,712,38,438,516,601,706,540,464
ALDH3A1_2_6988,624,337,350,297,171,475,8,137,366,76,1156,32
ALDH3A2_2_6989,0,0,0,0,0,0,0,0,0,0,0,0
ALDH3B1_2_6990,149,223,175,260,30,11,118,304,2,159,68,62
ALDH3B2_2_6991,451,381,517,545,33,208,64,321,295,161,325,459
ALDH4A1_2_6992,149,149,83,130,86,114,1,290,0,5,55,164
ALDH5A1_2_6993,210,146,94,179,129,428,672,365,27,41,20,22
ALDH7A1_2_6994,235,293,213,221,1,78,707,53,105,293,65,289
ALDH8A1_2_6995,24,42,45,14,87,7,43,2,21,1,429,10
ALDOA_2_6996,292,174,163,220,198,74,480,9,186,155,68,43
ALG3_2_6997,394,341,287,349,88,325,514,219,262,66,479,182
ALG5_2_6998,1007,888,754,1051,527,897,545,1441,1103,628,288,775
ALG8_2_6999,754,551,626,729,404,42,1015,317,603,739,832,541
ALG9_2_7000,2632,2060,2443,2264,3118,1549,1449,770,1533,2418,2903,2394
ALOX15B_2_7001,294,170,132,175,126,76,232,121,32,680,73,252

ALOXE3_2_7002,18,12,31,8,13,0,2,29,459,11,0,91
AMACR_2_7003,288,215,257,540,718,284,463,28,247,24,20,296
AMD1_2_7004,495,670,423,469,307,34,369,475,434,836,833,771
AMDHD2_2_7005,626,252,405,419,694,457,179,100,124,73,1281,243
AMPD1_2_7006,1440,1434,1270,1467,784,1045,784,128,885,1412,1050,914
AMPD2_2_7007,66,46,60,70,45,0,18,13,307,49,223,13
AMPD3_2_7008,184,407,224,204,123,259,194,24,351,252,3,0
AMT_2_7009,441,389,451,380,394,452,446,417,410,574,216,439
AMY1A_2_7010,8233,7337,7006,8193,10507,6219,9545,7924,7194,9149,5567,1
0898
ANG_2_7011,225,313,302,291,89,351,549,132,55,78,158,402
AOAH_2_7012,218,160,143,134,21,206,201,28,9,29,48,80
AOC2_2_7013,147,93,110,208,0,0,111,0,99,0,47,257
APC_2_7014,182,129,138,188,158,369,313,241,154,33,27,52
APEX1_2_7015,193,343,226,292,429,276,44,0,220,11,331,477
APOBEC3A_2_7016,928,825,739,1075,318,27,674,898,557,216,1346,2074
APOBEC3H_2_7017,513,831,769,815,388,1266,1192,340,321,229,2009,503
APRT_2_7018,138,277,248,133,46,126,78,151,168,290,20,58
ARF1_2_7019,274,183,190,189,58,62,5,10,129,18,109,97
ARFRP1_2_7020,211,151,226,133,328,64,10,0,46,11,519,42
ARHGAP5_2_7021,334,395,450,380,670,450,327,310,277,824,122,357
ARHGEF10L_2_7022,32,12,5,56,5,0,0,2,1,0,10,4
ARL2_2_7023,3,11,40,28,0,0,0,1,0,0,264,1
ARL4A_2_7024,1613,1893,1253,1509,276,1415,1786,859,921,736,628,695
ARL5A_2_7025,1589,1070,1322,1229,930,1343,1036,1206,802,774,1509,1291
ARSA_2_7026,21,89,51,4,0,0,0,0,5,287,0,4
ARSB_2_7027,6188,5437,5425,5842,6812,5082,7087,4937,6966,6863,4123,636
3
ARSF_2_7028,1477,1453,1099,1226,1836,1183,1573,520,972,2380,2154,1342
ART3_2_7029,130,206,126,145,380,0,0,49,278,0,0,403
ART5_2_7030,950,559,853,829,677,1679,667,426,983,753,827,2322
ASAH1_2_7031,425,337,443,375,647,269,244,150,986,509,585,449
ASAH2_2_7032,1483,1483,1415,1946,1381,901,493,2769,1665,1914,1202,2443
ASL_2_7033,81,8,121,13,0,7,76,101,5,0,0,0
ASMT_2_7034,103,79,76,181,51,35,40,23,16,85,391,26
ASMTL_2_7035,212,284,330,353,196,144,742,216,229,508,499,436
ASNS_2_7036,579,285,388,384,120,110,55,490,444,570,169,260
ASPA_2_7037,662,390,520,402,223,344,307,488,684,664,1113,626
ASRGL1_2_7038,127,94,96,158,144,29,95,10,57,23,0,28
ASS1_2_7039,436,373,732,865,486,174,2020,585,542,548,1185,524
ATE1_2_7040,359,307,391,459,519,0,1167,1,176,240,614,187
ATL1_2_7041,621,467,491,463,296,257,248,840,195,573,347,399
AURKAIP1_2_7042,380,235,319,263,386,377,32,940,105,129,609,383
AZIN1_2_7043,204,227,217,383,455,39,42,219,198,118,385,292
B3GALNT1_2_7044,2975,2259,2318,2709,3289,2524,4331,1982,3002,2236,2589
,3182
B3GALT5_2_7045,895,648,580,595,1486,543,398,87,587,584,695,469
B3GAT1_2_7046,202,308,437,551,14,26,1105,182,80,490,771,546
B4GALNT2_2_7047,3,3,1,12,0,16,0,0,0,0,0,22
B4GALT2_2_7048,95,20,69,120,10,19,0,0,0,0,0,187

B4GALT3_2_7049,249,147,207,359,147,8,132,67,378,293,77,232
B4GALT4_2_7050,679,507,351,497,317,450,1692,777,397,610,674,396
BAAT_2_7051,775,667,549,724,93,904,1831,141,445,685,749,1165
BACE1_2_7052,259,199,173,243,440,98,380,248,367,644,296,286
BACE2_2_7053,0,0,0,0,0,0,0,0,0,0,0,0
BCAT1_2_7054,721,696,776,903,311,1087,708,734,419,1579,454,622
BCAT2_2_7055,145,116,131,153,99,781,74,243,3,8,0,88
BCKDHA_2_7056,345,447,366,198,500,66,0,177,365,668,0,589
BCKDHB_2_7057,105,106,12,223,0,0,359,823,0,12,30,16
BCO2_2_7058,961,1071,886,1034,1089,1410,1221,588,775,712,598,1158
BDH1_2_7059,5,4,26,35,0,73,96,17,116,0,0,43
BFSP1_2_7060,416,213,270,460,696,275,6,77,299,94,0,275
BHMT2_2_7061,25,63,68,165,12,1,27,77,387,213,3,331
BMP1_2_7062,256,162,195,196,9,0,41,633,0,58,24,43
C17orf101_2_7063,526,861,540,820,906,561,606,1570,686,1115,558,827
C1GALT1C1_2_7064,1510,1366,1157,1270,1057,1182,1750,1697,927,1133,1503
,2029
C1S_2_7065,49,26,15,59,0,0,33,2,8,5,1,21
C2_2_7066,26,106,40,21,0,0,1,56,10,10,105,10
CA10_2_7067,1411,1550,1335,1640,855,1219,1237,617,1672,974,1029,1613
CA12_2_7068,178,118,148,138,251,0,804,51,284,12,0,200
CA1_2_7069,201,341,205,222,2,100,51,376,112,88,657,315
CA7_2_7070,70,128,51,228,103,275,88,151,27,44,1485,6
CAB39_2_7071,82,21,78,62,12,0,0,45,360,56,1,24
CANT1_2_7072,78,101,81,85,1,123,4,0,166,0,0,42
CAPN1_2_7073,138,24,67,43,295,0,0,0,72,205,0,120
CAPN2_2_7074,0,0,0,0,0,0,0,0,0,0,0,0
CARNS1_2_7075,216,263,164,236,27,133,43,496,238,397,196,38
CARS_2_7076,1670,1586,1382,1924,1184,2218,965,2231,1716,2052,1824,2094
CASP10_2_7077,243,76,91,83,5,2,0,58,74,161,2,0
CASP1_2_7078,268,140,177,108,110,120,1060,250,119,133,127,36
CASP2_2_7079,58,60,81,48,33,1,58,0,28,578,15,2
CASP3_2_7080,773,459,369,547,314,790,687,536,223,463,395,515
CASP4_2_7081,748,573,438,666,739,129,1226,688,378,679,643,236
CASP5_2_7082,980,760,746,805,1180,1094,139,548,918,862,1400,1603
CASP6_2_7083,442,363,318,353,72,467,655,291,681,493,597,132
CASP7_2_7084,427,334,337,402,722,317,316,782,495,358,8,718
CASP8_2_7085,322,202,163,251,102,775,601,33,535,375,278,530
CASP9_2_7086,970,635,560,662,1206,626,688,953,686,108,1267,526
CASZ1_2_7087,78,21,53,83,93,0,20,0,52,46,1,15
CAV3_2_7088,71,150,43,66,0,0,0,0,0,0,2,23
CBS_2_7089,98,127,146,216,368,271,126,58,178,52,267,294
CCBL1_2_7090,58,6,0,9,0,0,36,6,47,0,0,2
CCBL2_2_7091,812,803,968,1069,917,728,1282,717,550,1033,818,533
CDC42_2_7092,203,278,141,316,311,0,4,392,10,57,72,91
CDH16_2_7093,176,40,86,97,0,10,195,0,616,0,365,3
CECR1_2_7094,854,589,662,562,898,997,267,474,313,392,501,735
CEPT1_2_7095,471,434,289,359,496,198,606,770,249,881,8,491
CERS1_2_7096,183,247,306,343,264,317,81,38,70,261,330,257
CES1_2_7097,168,337,317,334,105,3,91,106,202,207,705,114

CES3_2_7098,101,107,184,330,267,191,141,355,1,4,27,174
CES4A_2_7099,0,0,0,0,0,0,0,0,0,0,0,0
CES5A_2_7100,894,653,718,772,492,759,177,367,808,555,649,518
CHAT_2_7101,96,117,137,47,145,2,0,17,27,33,75,155
CHI3L2_2_7102,427,398,306,405,14,322,78,152,120,319,75,449
CHIA_2_7103,89,48,106,192,346,197,27,2,29,16,120,221
CHPF_2_7104,648,415,449,512,722,819,1790,129,747,365,261,313
CHST11_2_7105,419,426,441,546,1284,78,270,833,276,576,374,564
CHST15_2_7106,214,180,354,381,54,280,514,638,43,23,690,244
CHST4_2_7107,133,144,88,187,120,361,135,54,192,6,164,127
CHST8_2_7108,34,199,95,69,42,4,114,2,150,0,0,2
CLP1_2_7109,54,44,49,70,45,81,96,8,4,19,0,37
CNDP2_2_7110,45,56,33,83,3,291,204,14,210,284,55,27
CNTN1_2_7111,188,145,110,247,1,6,440,153,338,0,9,217
CNTN4_2_7112,511,354,401,461,624,245,518,144,707,406,888,1108
COMT_2_7113,148,68,107,217,0,6,0,1,57,259,454,0
COQ6_2_7114,143,49,162,237,462,79,1,224,112,30,91,401
COX11_2_7115,3249,3090,2568,3439,2982,2632,4026,4971,2863,4163,3077,26
50
COX15_2_7116,192,120,134,237,53,73,107,42,282,45,405,469
CPA5_2_7117,169,240,199,230,13,2,8,0,258,15,122,227
CPB2_2_7118,120,68,103,59,168,581,4,22,82,96,0,33
CPM_2_7119,6502,5005,5005,6774,5614,3342,6360,7253,3969,9165,6591,5914
CPPED1_2_7120,728,624,980,756,1720,316,1495,439,517,1213,1580,1006
CPT1A_2_7121,62,103,177,43,5,9,22,0,144,3,730,57
CPT1B_2_7122,128,110,110,97,1,5,0,97,12,95,175,179
CPT1C_2_7123,732,433,335,501,493,52,1253,313,664,27,512,77
CRLS1_2_7124,283,264,196,337,499,279,134,350,0,617,3,349
CRMP1_2_7125,47,78,61,39,0,249,11,48,0,3,0,39
CROT_2_7126,644,561,682,413,438,461,681,230,285,1065,1053,674
CRY2_2_7127,16,22,36,34,0,175,30,0,5,14,0,117
CRYM_2_7128,991,1064,905,978,823,1287,288,706,397,560,554,1228
CRYZ_2_7129,627,465,336,407,541,431,135,220,228,521,1561,269
CSDE1_2_7130,747,603,678,999,592,984,22,441,1260,1120,1559,1101
CSGALNACT1_2_7131,18,30,108,82,46,0,645,3,13,0,1385,58
CSMD3_2_7132,500,479,445,701,1348,661,80,335,922,665,344,313
CTAGE5_2_7133,79,77,38,165,0,13,191,0,471,267,5,0
CTBP1_2_7134,242,268,71,215,77,5,1,69,3,19,117,234
CTPS2_2_7135,192,169,120,146,752,58,2,146,220,341,0,286
CTSA_2_7136,191,277,226,463,7,0,315,622,149,391,219,596
CTSB_2_7137,157,193,146,264,59,9,100,9,144,3,592,294
CTSE_2_7138,435,357,411,362,22,281,34,0,481,31,366,163
CTSL1_2_7139,821,1024,703,938,1037,922,522,730,978,638,161,1500
CTSL2_2_7140,467,223,249,312,114,140,159,44,294,250,376,378
CTSS_2_7141,331,314,318,356,503,560,39,177,851,307,1125,264
CYB561_2_7142,119,119,33,84,13,98,465,2,11,365,10,297
CYB5A_2_7143,202,121,190,293,95,0,272,385,2,296,135,16
CYB5R3_2_7144,177,204,86,281,658,374,230,127,30,165,1,15
CYP11A1_2_7145,223,217,111,155,194,279,13,68,441,30,92,179
CYP11B1_2_7146,231,142,134,167,23,1,36,156,93,109,426,113

CYP19A1_2_7147,282,175,109,160,138,254,534,108,139,177,226,37
CYP21A2_2_7148,468,389,381,541,521,453,435,1235,604,211,402,726
CYP24A1_2_7149,447,484,609,490,1371,572,562,237,239,465,1152,296
CYP26A1_2_7150,99,142,40,36,0,7,6,139,250,0,1,40
CYP2A7_2_7151,705,569,658,581,630,879,220,224,843,319,116,1251
CYP2C18_2_7152,1063,1023,1152,1653,1389,1098,639,1001,433,1928,3948,12
53
CYP2C8_2_7153,513,524,468,499,508,624,972,789,474,586,81,857
CYP2D6_2_7154,75,72,39,106,0,69,5,329,177,78,3,268
CYP3A43_2_7155,635,682,688,724,1015,1145,2040,681,574,1687,686,218
CYP3A4_2_7156,7037,6543,6570,7472,7267,4369,8900,5315,5231,5741,7292,6
320
CYP4B1_2_7157,29,7,25,45,9,0,306,0,0,0,0,12
CYP4F11_2_7158,776,935,662,801,871,652,764,633,1795,1226,295,876
CYP4F3_2_7159,602,534,547,510,524,11,557,255,291,458,197,774
CYP51A1_2_7160,329,384,426,574,444,113,80,332,514,378,263,805
DAGLB_2_7161,155,173,86,233,253,0,8,186,289,0,28,12
DCLRE1C_2_7162,546,388,334,647,385,202,818,487,325,141,172,241
DCP2_2_7163,567,616,441,466,372,840,33,488,109,507,270,478
DCT_2_7164,316,378,255,248,501,170,177,330,175,660,881,806
DCTD_2_7165,58,6,28,17,0,220,6,0,0,6,0,0
DCXR_2_7166,148,144,156,191,119,46,160,590,319,79,306,80
DDAH1_2_7167,144,132,235,122,61,26,18,224,131,245,70,202
DDC_2_7168,814,633,798,1136,1453,450,962,766,594,166,1118,1045
DDHD1_2_7169,319,270,367,407,95,197,228,558,321,141,66,91
DDO_2_7170,681,541,531,552,296,725,261,868,893,604,98,372
DDT_2_7171,571,499,516,564,464,388,687,714,149,541,1201,801
DDX11_2_7172,70,87,102,62,109,0,542,66,25,19,12,86
DDX17_2_7173,908,970,798,897,554,235,482,800,785,612,1181,1049
DDX19B_2_7174,23,29,23,58,309,93,18,95,1,70,34,14
DDX31_2_7175,187,127,141,191,255,0,0,334,60,75,251,167
DDX39B_2_7176,139,138,111,210,191,178,67,0,9,401,3,391
DDX3X_2_7177,827,696,784,639,1070,609,1886,533,146,1160,431,407
DDX3Y_2_7178,110,101,172,194,143,33,0,170,138,79,2,142
DDX42_2_7179,405,377,353,419,626,387,266,736,334,1072,551,960
DDX47_2_7180,512,616,567,635,488,695,982,925,653,853,831,645
DDX4_2_7181,970,999,1013,1273,1132,1551,66,1630,382,1020,1033,622
DDX54_2_7182,40,25,72,8,161,0,34,14,162,2,23,56
DFFA_2_7183,344,294,331,498,417,472,505,406,600,646,779,679
DGCR8_2_7184,20,20,17,11,6,7,0,5,0,31,0,32
DHCR7_2_7185,987,842,656,983,181,459,45,1184,963,904,970,1360
DHDDS_2_7186,64,122,78,126,11,102,251,2,63,15,411,88
DHFRL1_2_7187,692,617,521,727,210,253,823,675,919,798,328,615
DHPS_2_7188,67,97,140,139,43,94,95,63,23,59,310,58
DHRS1_2_7189,526,457,458,536,238,591,717,490,323,635,196,390
DHRS2_2_7190,34,27,25,109,14,2,0,5,1,50,22,4
DHRS9_2_7191,238,314,315,254,708,60,251,297,468,448,154,348
DHX16_2_7192,56,75,82,140,67,357,158,23,78,48,68,373
DHX30_2_7193,169,388,170,314,283,265,25,0,27,135,181,5
DHX33_2_7194,82,165,91,153,196,49,96,0,44,35,0,6

DHX35_2_7195,213,157,175,141,60,465,0,496,203,304,137,258
DHX36_2_7196,256,248,139,167,54,409,328,429,133,411,468,4
DHX40_2_7197,204,175,181,148,369,256,164,337,21,294,37,384
DIAPH3_2_7198,584,347,448,353,351,190,285,630,333,5,130,239
DIS3_2_7199,173,183,280,153,176,60,565,383,17,124,78,101
DIS3L_2_7200,744,558,671,730,507,858,1370,1134,941,330,390,1275
DKC1_2_7201,248,289,272,406,532,757,936,104,102,382,66,100
DNAJC27_2_7202,175,190,281,241,377,175,93,142,189,261,70,173
DNASE1L1_2_7203,244,118,259,292,0,544,0,548,94,17,744,235
DNM1_2_7204,1076,1227,1104,1197,945,1236,949,328,401,1846,1914,2953
DNM1L_2_7205,347,405,369,450,0,29,394,552,593,282,14,317
DNM2_2_7206,224,344,277,471,33,497,10,638,63,218,330,52
DNM3_2_7207,2508,1812,1714,1978,1938,2821,2386,1700,1647,3392,1421,258
1
DNTT_2_7208,547,440,384,576,634,233,479,638,384,800,476,245
DOHH_2_7209,255,203,149,302,177,137,355,328,141,106,235,444
DOLPP1_2_7210,43,77,21,26,0,27,89,190,87,46,0,3
DPEP1_2_7211,237,206,287,265,344,215,55,618,246,87,237,231
DPEP3_2_7212,196,258,160,218,243,137,277,131,166,69,88,332
DPH5_2_7213,209,213,166,199,230,57,149,37,87,10,12,222
DPM3_2_7214,40,53,42,19,0,0,0,25,257,32,0,0
DPP3_2_7215,296,258,275,185,690,44,253,36,563,46,336,298
DPP8_2_7216,408,281,394,478,117,246,1038,209,410,369,108,978
DPYSL2_2_7217,95,127,137,256,0,1,3,451,358,274,3,2
DPYSL3_2_7218,496,558,362,370,426,29,1,583,161,144,0,340
DROSHA_2_7219,360,370,248,297,148,236,74,304,90,0,116,192
DSE_2_7220,368,191,373,327,24,633,398,353,281,240,959,613
DUOX1_2_7221,203,114,255,230,287,0,0,0,0,263,682,140
DUT_2_7222,294,264,181,189,1,0,329,102,0,260,186,489
ECE1_2_7223,519,343,473,588,366,33,294,208,960,226,1508,362
ECE2_2_7224,400,379,388,511,415,288,322,97,643,608,364,352
ECI1_2_7225,137,63,34,80,66,20,121,234,109,82,6,55
ECI2_2_7226,512,472,517,590,441,742,504,502,390,261,477,241
EDEM2_2_7227,832,722,641,914,854,425,297,405,878,739,1173,1071
EFEMP1_2_7228,272,192,320,169,350,78,343,183,283,252,87,416
EFTUD2_2_7229,312,351,332,501,412,10,434,425,294,461,390,85
EGLN2_2_7230,197,163,117,268,317,100,23,553,333,304,423,437
EHHADH_2_7231,383,257,310,701,271,228,770,213,687,496,942,567
ELAC2_2_7232,116,29,86,126,155,2,151,598,16,0,11,49
ELOVL5_2_7233,347,282,260,343,543,337,26,209,283,265,152,391
ELOVL6_2_7234,596,552,346,337,681,249,163,49,412,982,127,349
ELOVL7_2_7235,695,509,559,845,393,393,404,608,990,706,512,626
ENDOV_2_7236,16,72,66,34,0,1,0,17,123,581,2,257
ENO3_2_7237,590,656,534,736,721,680,724,879,692,1364,981,659
ENOX2_2_7238,292,303,241,259,268,170,467,567,390,292,656,664
ENPP2_2_7239,168,66,65,129,419,145,2,15,34,50,656,171
ENTPD1_2_7240,365,233,286,253,280,9,471,415,66,636,321,20
ENTPD2_2_7241,661,589,615,656,856,1246,218,1336,1166,467,733,1254
ENTPD4_2_7242,336,250,203,220,255,132,147,747,159,227,25,77
ENTPD6_2_7243,915,1067,1194,1543,513,1894,1460,1242,950,1374,559,2362

ENTPD8_2_7244,51,48,104,132,13,37,7,326,181,148,14,372
EPHX1_2_7245,196,164,194,239,28,170,11,11,209,184,2,57
EPHX3_2_7246,132,256,145,468,43,584,5,831,310,588,0,147
ERCC1_2_7247,129,218,185,120,127,0,202,44,52,51,38,251
ERCC2_2_7248,44,31,49,81,26,1,7,9,1,1,1,47
EXO1_2_7249,1256,1611,1287,1289,1216,1492,1748,1463,1076,2273,1451,738
EXOG_2_7250,276,144,219,237,160,293,53,536,204,216,155,71
EXOSC3_2_7251,762,1332,1137,1281,883,691,324,1066,545,844,1902,1059
EXOSC9_2_7252,117,122,67,182,0,171,12,14,0,38,214,47
EXTL2_2_7253,441,250,272,340,836,0,711,279,324,810,668,201
F7_2_7254,296,320,305,515,29,183,89,71,433,269,104,361
FAHD1_2_7255,85,67,147,94,19,51,358,8,152,5,1,118
FAM108A1_2_7256,12,95,7,0,0,0,0,0,0,0,0,15
FAM135A_2_7257,1523,1239,1456,1404,1001,761,1823,759,1742,1012,1210,19
01
FAN1_2_7258,1051,925,748,1033,486,317,517,549,906,875,782,764
FDPS_2_7259,0,0,0,0,0,0,0,0,0,0,0,0
FDXR_2_7260,53,40,141,56,19,1,4,11,44,304,341,91
FECH_2_7261,698,716,500,909,442,78,399,328,488,1781,733,603
FERMT3_2_7262,1023,946,601,964,1433,418,499,921,700,1140,201,697
FHIT_2_7263,613,408,461,551,403,794,898,364,1724,35,253,305
FIGNL1_2_7264,1026,890,787,1038,1288,465,994,1089,408,1240,755,839
FKBP11_2_7265,654,901,584,821,584,672,538,1265,823,421,188,1053
FKBP1A_2_7266,262,217,203,258,367,113,419,235,434,490,10,28
FKBP1B_2_7267,911,757,801,1187,1095,1193,902,1635,1161,1683,1352,1180
FKBP2_2_7268,167,155,221,337,407,96,771,624,375,7,548,85
FKBP5_2_7269,273,325,212,358,272,188,14,376,122,332,670,467
FKBP7_2_7270,1078,1339,873,1003,388,2185,809,1997,761,727,2319,744
FLAD1_2_7271,1068,833,975,961,1208,645,501,868,1293,243,508,375
FMO3_2_7272,215,171,236,183,146,408,274,175,342,640,118,198
FMO5_2_7273,765,650,750,731,246,1443,119,371,477,921,671,802
FN1_2_7274,102,201,318,172,141,35,675,0,299,7,644,50
FOLH1_2_7275,68,84,188,162,305,2,0,78,57,0,1072,645
FPGS_2_7276,237,206,230,261,500,298,21,187,62,363,0,615
FPGT_2_7277,423,525,396,432,1092,977,172,777,93,108,722,473
FTCD_2_7278,9,3,14,27,16,265,1,0,197,2,0,0
FTSJ1_2_7279,0,0,0,0,0,0,0,0,0,0,0,0
FUT2_2_7280,464,327,217,330,42,365,462,60,78,418,259,408
FUT3_2_7281,199,356,282,501,236,1109,292,634,248,39,1127,332
FUT6_2_7282,199,356,282,501,236,1109,292,634,248,39,1127,332
FUT8_2_7283,792,534,558,686,472,350,773,1006,97,680,167,1203
G3BP1_2_7284,295,137,229,293,345,125,445,3,254,42,604,121
G3BP2_2_7285,154,159,195,182,57,15,260,80,36,445,18,4
G6PD_2_7286,448,481,483,509,735,309,155,815,677,951,783,458
GAA_2_7287,1362,1553,1008,1246,958,508,500,2433,786,978,1808,1700
GAD1_2_7288,394,359,262,254,176,196,36,270,184,263,91,262
GAD2_2_7289,498,530,465,659,227,44,274,866,349,365,613,333
GALC_2_7290,1430,1647,1133,1526,1837,1627,1302,1739,744,1477,1183,1529
GALE_2_7291,5058,3359,4585,4782,5527,4660,2615,2248,5407,3773,6519,524
8

GALNT9_2_7292,55,43,68,73,92,53,49,2,0,0,42,17
GALNTL1_2_7293,56,50,32,63,162,0,396,159,91,0,108,79
GAMT_2_7294,183,169,242,240,161,112,97,147,89,326,174,149
GANAB_2_7295,260,126,245,233,199,218,149,1,64,129,0,361
GART_2_7296,2791,2209,2438,2630,2179,2800,2155,2041,2525,3433,3486,292
9
GBA_2_7297,335,308,187,243,187,212,67,119,218,123,602,208
GBP5_2_7298,74,40,18,42,0,1,6,1,296,0,0,0
GCAT_2_7299,560,627,524,757,724,921,781,609,902,590,89,220
GCDH_2_7300,229,244,268,172,86,151,53,353,92,42,480,71
GCH1_2_7301,233,246,191,328,7,340,0,321,499,11,110,908
GCLC_2_7302,540,577,596,764,101,152,1352,205,470,977,219,805
GCNT1_2_7303,73,103,174,148,591,13,617,4,42,616,89,101
GDPD1_2_7304,102,225,131,149,40,12,24,145,132,56,249,144
GDPD2_2_7305,463,412,437,469,334,903,175,202,285,549,28,944
GEM_2_7306,648,401,395,390,49,96,2116,117,75,243,625,211
GEN1_2_7307,241,218,301,292,552,81,524,157,398,333,8,65
GFM2_2_7308,404,291,250,223,251,400,176,456,335,599,3,372
GFOD1_2_7309,80,89,127,129,101,329,5,285,188,263,148,82
GGCT_2_7310,865,660,708,866,1438,1027,1065,447,793,636,675,943
GGCX_2_7311,650,841,661,762,531,361,1119,682,667,691,1314,220
GGT1_2_7312,1054,1174,1121,1341,834,1340,2069,819,1380,1337,362,1613
GGT5_2_7313,90,88,64,75,61,36,1,83,28,21,1,20
GGT6_2_7314,313,276,199,239,349,277,89,526,677,70,51,159
GGTLC1_2_7315,172,274,170,225,755,45,24,747,149,137,11,902
GLB1_2_7316,371,140,124,234,126,152,96,265,177,1,604,164
GLRX2_2_7317,619,426,531,294,0,577,168,379,411,409,326,278
GLRX3_2_7318,860,494,570,821,450,632,275,1127,556,298,381,701
GLRX_2_7319,899,849,760,1124,693,1634,874,844,818,1151,873,1314
GLT8D1_2_7320,1943,1847,2021,2103,2715,2099,1767,2895,1798,2432,2577,1
987
GLUL_2_7321,254,194,254,324,128,0,73,0,593,215,49,514
GM2A_2_7322,361,590,540,419,293,727,651,640,489,578,530,1088
GMPPA_2_7323,481,378,466,471,799,429,63,84,450,82,790,344
GMPPB_2_7324,177,100,212,83,75,107,132,88,38,0,111,383
GMPR2_2_7325,116,266,149,249,59,82,228,139,71,86,235,304
GNAI2_2_7326,2328,1697,2132,2269,2236,1800,2704,1593,2141,2584,1725,32
91
GNAL_2_7327,180,152,112,244,1,1,43,55,88,115,150,293
GNAO1_2_7328,55,139,204,224,3,2,101,347,161,58,260,108
GNAT1_2_7329,69,82,99,61,44,0,16,5,75,33,896,259
GNB5_2_7330,109,206,102,240,7,545,290,213,116,78,0,81
GNG10_2_7331,94,108,214,419,293,0,0,400,420,2,362,626
GNG4_2_7332,90,69,26,113,26,1,0,81,10,267,0,271
GNGT2_2_7333,500,457,550,405,289,463,29,1033,681,584,354,784
GPD2_2_7334,610,477,369,383,617,17,1024,528,764,216,1607,423
GPHN_2_7335,1518,1309,1434,1633,1565,773,1754,2494,1720,2118,654,2290
GPNMB_2_7336,298,346,201,384,30,356,563,310,369,290,197,269
GPT2_2_7337,157,217,298,279,272,56,63,361,87,290,158,344
GPX4_2_7338,271,321,264,348,0,22,132,649,543,296,184,532

GSR_2_7339,142,76,165,171,246,120,54,94,265,441,492,556
GSTCD_2_7340,505,636,275,529,1459,102,382,680,181,576,1137,293
GSTK1_2_7341,863,936,1084,894,652,793,774,690,1233,693,1769,702
GSTM1_2_7342,522,420,467,439,37,381,303,134,1066,1113,211,1238
GSTM2_2_7343,1613,1134,1173,1508,1526,1505,2034,2004,2053,889,1217,162
2
GSTM4_2_7344,1638,1134,1174,1527,1537,1514,2021,2011,2054,896,1221,161
9
GSTO1_2_7345,128,109,119,102,568,404,0,0,9,80,335,73
GSTO2_2_7346,241,230,187,317,113,375,453,193,505,174,261,167
GSTZ1_2_7347,869,763,817,1102,864,1443,669,847,887,1417,433,1252
GTPBP3_2_7348,752,570,450,533,656,1625,699,559,639,487,383,1125
GUCY1A3_2_7349,849,569,777,918,931,639,724,1413,809,366,950,314
GYG1_2_7350,184,174,165,195,142,0,377,315,208,433,394,430
GYG2_2_7351,138,102,76,240,338,52,832,300,7,23,498,233
GYS1_2_7352,92,75,73,92,47,0,326,1,98,178,0,190
HADH_2_7353,321,140,257,198,267,24,623,409,169,13,1307,19
HAGH_2_7354,42,28,16,85,56,0,0,59,46,10,15,2
HAO2_2_7355,91,142,135,161,76,26,18,146,139,0,199,118
HAS3_2_7356,339,471,303,345,206,466,352,221,641,301,380,325
HCCS_2_7357,362,174,223,220,84,254,123,213,102,359,872,352
HDHD1_2_7358,2,67,11,7,0,58,0,0,13,29,64,0
HENMT1_2_7359,713,628,765,596,770,348,1479,86,490,529,926,468
HHAT_2_7360,493,376,440,307,1033,763,368,0,304,48,212,767
HIBCH_2_7361,1666,1713,1909,2057,1770,1509,1489,1849,1218,1240,2081,20
61
HLCS_2_7362,329,294,137,222,6,119,481,193,231,496,137,220
HMBS_2_7363,58,112,156,134,45,532,102,64,77,12,27,156
HMGA2_2_7364,273,138,137,163,425,518,37,20,283,261,158,70
HMGCL_2_7365,832,590,461,662,878,691,679,46,650,788,421,623
HMGCLL1_2_7366,1171,1146,1013,908,720,668,1723,390,991,849,2181,946
HMGCR_2_7367,1698,1373,1251,1318,1734,898,1068,1866,1458,2013,1072,821
HMGCS1_2_7368,439,297,368,359,307,250,238,827,102,336,749,236
HMGCS2_2_7369,432,350,425,481,144,187,651,601,247,141,193,245
HMOX2_2_7370,155,124,115,178,127,10,607,5,47,384,0,374
HNRNPAB_2_7371,22,61,109,75,16,164,158,26,1,0,12,22
HPD_2_7372,210,302,221,294,95,262,154,483,116,444,211,363
HPGD_2_7373,1727,1714,1480,1507,754,1153,3154,2210,1274,780,1124,1316
HPSE2_2_7374,197,286,267,298,269,132,129,100,48,297,164,367
HPSE_2_7375,1736,1727,1263,2059,2387,1090,429,1636,703,2156,534,1951
HRAS_2_7376,221,461,303,348,65,701,999,0,13,83,458,242
HS2ST1_2_7377,490,436,418,604,575,513,461,897,210,3,1622,979
HS6ST2_2_7378,112,147,81,139,36,74,226,10,209,113,31,158
HSD11B1_2_7379,85,6,71,157,109,0,14,0,17,11,104,11
HSD17B10_2_7380,193,349,222,175,403,206,0,3,95,217,360,323
HSD17B13_2_7381,122,153,122,205,205,4,194,245,132,260,16,75
HSD17B4_2_7382,230,237,344,232,525,209,271,182,201,592,311,388
HSD3B2_2_7383,114,108,83,15,15,0,5,37,7,0,233,117
HSD3B7_2_7384,78,7,15,17,0,30,0,7,0,4,0,2
HSDL1_2_7385,13,5,7,40,36,0,31,12,15,0,0,17

HSP90AA1_2_7386,346,361,299,541,1318,558,465,44,61,99,985,616
HSPA8_2_7387,57,74,95,61,46,141,82,35,72,9,51,162
HSPD1_2_7388,460,435,502,570,254,20,145,302,227,299,847,569
HTRA2_2_7389,48,137,43,42,48,0,0,218,33,7,8,336
HYAL1_2_7390,364,116,316,497,148,34,175,443,328,346,344,325
HYAL2_2_7391,604,461,388,521,146,656,507,338,357,291,259,329
HYI_2_7392,21,9,34,62,2,28,0,0,0,490,0,70
IARS_2_7393,261,289,337,323,398,304,138,197,466,324,329,664
IDE_2_7394,947,742,622,1334,988,570,77,1091,561,638,671,1410
IDH3B_2_7395,164,124,203,191,253,116,668,39,101,279,322,159
IDH3G_2_7396,185,102,56,151,12,45,0,49,15,351,117,168
IDS_2_7397,455,492,508,485,174,340,423,574,532,528,1128,1192
IFT27_2_7398,66,51,94,96,32,0,0,1,448,0,0,128
IL4I1_2_7399,28,39,71,86,202,0,0,41,0,12,0,143
IMPDH1_2_7400,272,446,286,354,154,425,294,101,111,741,465,180
INMT_2_7401,151,70,102,122,1,101,0,0,36,5,5,0
INPP5K_2_7402,21,150,65,91,113,0,64,0,5,1,0,28
INTS6_2_7403,310,340,230,302,237,481,630,105,994,1,228,197
IPCEF1_2_7404,1415,1199,1432,1530,1119,1055,1151,2210,1347,1253,978,12
12
ISOC2_2_7405,57,71,84,75,7,0,529,7,12,33,171,8
ISPD_2_7406,760,728,607,735,1170,306,335,587,375,248,701,1284
ISYNA1_2_7407,22,28,59,89,128,35,23,75,20,24,1,5
ITPA_2_7408,431,404,435,447,1723,325,1754,232,204,434,125,647
IVD_2_7409,142,53,106,96,37,118,0,0,11,51,66,64
IYD_2_7410,659,718,525,527,81,198,299,563,567,1511,302,912
JMJD7-PLA2G4B_2_7411,168,283,170,205,179,224,7,112,6,52,1,239
KARS_2_7412,393,286,227,505,15,143,225,223,322,43,856,346
KATNA1_2_7413,254,269,175,220,227,11,531,574,68,221,825,197
KATNAL1_2_7414,1879,1579,1524,2081,2906,2009,3825,900,2099,2033,1306,3
570
KIF16B_2_7415,465,395,494,531,779,338,1385,50,931,727,786,311
KIF9_2_7416,214,248,168,180,116,871,725,103,62,16,565,17
KIFC3_2_7417,149,156,133,205,350,136,224,221,0,548,161,117
KLK2_2_7418,0,6,2,10,0,0,0,0,0,0,0,0
KLK7_2_7419,17,119,156,215,79,0,0,6,7,3,0,0
KRAS_2_7420,548,645,567,704,696,677,1587,941,549,653,933,716
KYNU_2_7421,1914,1907,1951,1914,1428,1662,2119,2057,976,2399,2553,1931
LAMP2_2_7422,1087,1152,926,1101,499,190,825,856,1196,1982,1572,1271
LARGE_2_7423,497,504,630,511,296,350,267,269,429,691,412,813
LCLAT1_2_7424,227,181,175,175,48,633,783,65,335,88,8,165
LCMT1_2_7425,53,42,102,108,194,111,370,0,181,0,296,4
LDHA_2_7426,908,454,620,735,290,469,521,1071,355,454,495,695
LDHAL6A_2_7427,284,111,110,192,291,157,28,7,240,364,253,248
LDHB_2_7428,165,160,81,127,202,115,122,18,645,160,40,297
LDHC_2_7429,443,273,271,369,261,148,105,52,264,5,254,532
LDHD_2_7430,31,0,21,9,0,0,0,0,0,0,0,0
LEPRE1_2_7431,229,470,209,329,0,11,237,268,343,8,460,343
LEPREL1_2_7432,44,110,37,105,39,126,135,127,208,156,22,88
LFNG_2_7433,430,323,373,490,990,293,234,763,534,657,1506,270

LGMN_2_7434,81,88,80,118,137,0,2,10,23,3,176,229
LGSN_2_7435,357,402,336,483,215,291,1491,401,910,3,1046,666
LIAS_2_7436,85,26,18,17,328,0,0,0,220,491,0,0
LIG3_2_7437,479,281,484,491,1183,188,319,307,253,477,1336,284
LIG4_2_7438,779,560,574,552,487,84,1575,426,831,1140,612,624
LIPF_2_7439,384,343,318,405,59,297,864,465,1038,361,231,769
LIPT1_2_7440,337,206,188,182,203,0,0,1,222,87,96,363
LMO7_2_7441,703,652,602,616,460,780,176,711,528,623,883,1796
LNPEP_2_7442,219,416,264,223,56,490,91,405,123,438,300,226
LOX_2_7443,801,810,648,664,451,369,1790,1415,931,882,527,261
LPO_2_7444,3,6,10,30,24,287,4,11,3,0,0,0
LRRC16A_2_7445,831,818,747,869,261,911,1180,963,629,596,1440,722
LSS_2_7446,61,83,30,82,99,0,53,366,43,123,0,268
LYZL6_2_7447,188,160,94,151,749,36,52,191,147,311,128,116
MACF1_2_7448,338,283,250,374,182,1118,85,452,177,654,813,132
MACROD2_2_7449,133,132,115,53,0,31,846,0,113,0,39,62
MAD2L2_2_7450,19,19,41,23,0,260,0,9,5,77,106,114
MAN2B1_2_7451,96,107,111,142,238,104,263,0,34,10,5,354
MASP2_2_7452,9,17,14,27,0,0,0,0,48,16,50,0
MAT2B_2_7453,187,170,195,189,365,139,816,163,6,349,106,337
MCAT_2_7454,39,22,116,19,0,16,103,2,0,82,13,0
MCM4_2_7455,166,150,115,148,275,19,58,145,153,116,12,115
MCM7_2_7456,144,168,143,215,26,35,380,182,271,275,16,147
MCM8_2_7457,327,283,186,261,307,17,510,146,187,229,326,745
MCM9_2_7458,160,70,60,119,356,1,1037,1032,139,30,11,30
MDH1_2_7459,62,44,60,70,18,11,20,147,12,75,180,29
MECR_2_7460,709,438,566,401,159,222,413,426,383,419,491,71
MEPCE_2_7461,901,935,970,758,471,1058,924,4,362,271,847,397
METTL13_2_7462,80,116,95,93,109,0,0,17,135,158,55,59
MFN2_2_7463,150,255,249,272,152,372,489,227,84,540,891,711
MFNG_2_7464,1172,1080,1138,1704,843,819,813,2263,1932,1038,1754,1105
MGAT3_2_7465,57,79,36,96,0,1,0,0,295,798,0,0
MGAT4A_2_7466,922,761,681,830,1265,46,967,551,1293,723,420,866
MGAT4B_2_7467,236,68,124,246,6,7,194,246,508,51,288,223
MGLL_2_7468,0,0,0,3,0,0,0,0,0,0,0,0
MGST1_2_7469,470,452,466,602,706,268,2234,321,250,299,598,437
MGST2_2_7470,61,67,78,45,44,43,0,50,8,70,0,4
MICAL1_2_7471,51,83,26,44,12,112,29,35,239,70,0,9
MLH1_2_7472,2141,1884,2129,1873,1855,762,1971,1101,1584,2531,1752,1589
MME_2_7473,1024,921,699,717,1104,1193,780,617,735,505,514,527
MMP1_2_7474,27,30,86,60,21,88,3,17,2,31,0,18
MMP2_2_7475,28,35,32,14,1,0,0,0,85,0,0,66
MOCS1_2_7476,98,100,96,123,181,39,8,82,182,129,1,76
MOGS_2_7477,511,325,260,539,609,1185,488,255,1085,533,601,203
MOV10_2_7478,437,342,384,515,754,117,451,514,1280,748,357,476
MOV10L1_2_7479,118,176,98,114,25,291,26,0,1,209,98,319
MPG_2_7480,309,400,190,436,603,219,223,221,529,552,66,331
MPPE1_2_7481,494,353,315,487,248,699,514,142,684,522,208,323
MPST_2_7482,30,58,16,10,0,0,0,0,0,214,0,0
MRAS_2_7483,123,70,293,124,102,176,138,33,4,116,1,258

MRE11A_2_7484,214,75,185,137,442,132,516,1180,78,128,116,17
MRI1_2_7485,24,67,10,46,14,0,0,0,0,6,0,198
MSH5_2_7486,107,154,315,324,194,647,653,96,321,10,132,672
MSRA_2_7487,603,262,548,249,186,1,694,510,178,384,648,310
MSRB3_2_7488,1088,848,787,921,654,781,247,289,1103,1598,1023,853
MTHFD1L_2_7489,157,181,167,193,136,90,59,103,122,94,148,210
MTHFS_2_7490,449,469,410,562,227,250,1808,451,458,581,476,33
MTO1_2_7491,11,28,61,24,1,102,7,9,1,20,0,0
MTRR_2_7492,580,533,514,628,84,401,750,508,857,919,1073,466
MUTYH_2_7493,51,42,34,40,129,107,84,22,7,124,42,137
MX1_2_7494,29,44,37,16,44,0,7,36,42,0,0,220
MYBBP1A_2_7495,49,98,29,56,254,27,309,47,5,1,0,20
MYH2_2_7496,430,518,437,577,1114,558,1000,664,269,469,357,1219
MYO5A_2_7497,222,233,288,320,391,810,43,198,564,174,270,144
MYO7A_2_7498,43,60,35,211,0,0,507,0,11,1,0,639
MYO9B_2_7499,346,162,258,341,284,97,405,18,269,354,206,395
N6AMT1_2_7500,7268,6599,5743,7022,5772,6871,7672,4363,6648,6828,6113,4
409
NAA16_2_7501,243,268,233,184,252,0,130,258,143,0,3,147
NAA20_2_7502,1408,1321,1110,1203,1832,1143,506,689,1153,1421,203,1197
NAAA_2_7503,537,400,389,438,606,111,379,228,305,436,490,555
NAPEPLD_2_7504,1145,1087,1215,1321,3016,434,2661,730,903,1532,1017,137
9
NARF_2_7505,193,68,81,97,44,0,599,5,65,75,3,156
NARS2_2_7506,168,386,219,210,492,136,248,1,375,47,945,176
NAV1_2_7507,480,398,333,256,104,42,590,161,558,126,619,66
NCEH1_2_7508,287,281,253,232,533,24,28,380,19,119,297,557
NCF2_2_7509,109,7,13,39,18,1,0,0,2,0,0,0
NCF4_2_7510,89,148,71,234,224,1,11,131,211,97,76,134
NDOR1_2_7511,172,238,161,173,0,243,0,0,143,15,21,75
NDUFA2_2_7512,121,154,164,279,1,147,997,367,63,9,68,103
NDUFB11_2_7513,580,638,478,1023,523,261,1825,391,13,667,1007,424
NDUFB4_2_7514,142,262,332,167,472,33,717,345,518,287,120,205
NDUFB5_2_7515,224,259,296,278,5,154,10,193,262,66,52,192
NDUFB6_2_7516,176,241,180,173,330,140,102,51,118,153,6,226
NDUFC1_2_7517,259,369,101,389,55,131,36,1,397,1180,591,77
NDUFS1_2_7518,358,399,211,386,506,35,420,160,185,587,978,624
NDUFS2_2_7519,588,523,656,598,92,664,115,567,776,482,1679,881
NDUFS5_2_7520,22,70,73,88,114,87,0,20,195,24,6,160
NDUFV1_2_7521,614,200,301,318,457,196,270,84,128,283,108,210
NEIL2_2_7522,35,5,43,36,0,60,154,0,42,17,6,0
NEU4_2_7523,179,136,134,211,731,18,1,69,35,68,457,226
NFS1_2_7524,672,539,486,1037,892,71,1542,1075,524,821,1193,288
NGLY1_2_7525,443,341,457,356,961,199,179,107,194,438,315,341
NIPSNAP1_2_7526,181,223,107,140,76,433,125,150,89,80,0,475
NIT1_2_7527,41,30,62,41,64,34,3,8,63,86,0,1
NKIRAS2_2_7528,106,116,66,93,294,85,61,28,91,53,422,107
NLGN3_2_7529,37,30,12,42,35,1,0,0,20,0,5,2
NLGN4X_2_7530,959,971,874,1024,631,804,1556,603,1011,1144,834,761
NLGN4Y_2_7531,959,971,874,1024,631,804,1556,603,1011,1144,834,761

NMNAT2_2_7532,245,360,280,305,520,424,52,316,339,565,531,312
NNT_2_7533,829,519,535,634,967,527,1083,736,567,758,344,345
NOX4_2_7534,175,167,203,238,133,218,198,99,77,427,0,78
NPL_2_7535,102,73,110,83,318,0,1,235,8,299,0,10
NQO1_2_7536,922,751,550,895,1080,1152,918,322,398,261,472,634
NRD1_2_7537,218,185,148,167,188,287,46,108,302,727,612,441
NSDHL_2_7538,57,78,112,73,140,3,59,227,57,116,2,257
NSUN2_2_7539,204,226,187,214,479,140,589,220,341,97,376,130
NUDT2_2_7540,183,77,174,180,52,12,229,145,173,25,241,154
NXN_2_7541,54,43,51,120,594,141,0,178,95,49,1,0
OAS1_2_7542,98,100,199,168,183,208,10,144,96,144,3,49
OAS2_2_7543,40,41,65,71,61,156,84,89,3,37,15,78
OASL_2_7544,18,10,20,17,22,1,0,0,25,0,3,176
OAT_2_7545,741,653,532,732,1335,369,922,0,332,243,1018,542
OGDH_2_7546,225,655,350,261,264,1683,582,356,297,1368,0,376
OGDHL_2_7547,53,36,45,29,0,3,0,0,3,2,0,60
OGG1_2_7548,220,215,241,249,240,27,423,277,205,300,554,72
OGT_2_7549,370,460,468,350,46,1309,347,942,629,388,282,821
OLAH_2_7550,547,644,797,917,676,291,1325,199,409,1105,842,959
P4HA1_2_7551,29,10,2,38,0,0,1,1,1,45,0,54
P4HA2_2_7552,269,362,300,331,86,184,12,4,213,449,1,450
P4HTM_2_7553,337,400,210,293,295,54,574,318,83,327,397,161
PAFAH1B2_2_7554,1001,590,631,513,856,323,730,8,501,578,22,406
PAFAH1B3_2_7555,100,47,44,65,99,0,0,458,30,0,64,282
PAICS_2_7556,640,427,366,625,585,318,569,318,606,462,329,149
PAOX_2_7557,545,611,587,905,124,363,464,231,737,914,247,904
PAPD4_2_7558,489,375,371,534,648,34,121,135,485,170,506,1189
PAPD5_2_7559,340,204,225,234,90,81,137,290,515,144,108,425
PAPD7_2_7560,526,430,578,535,247,186,594,1329,123,240,698,356
PAPSS2_2_7561,1759,1629,1685,1973,2089,1889,2244,1621,1303,2241,973,14
23
PARK7_2_7562,270,214,265,378,5,434,5,263,340,202,688,443
PARL_2_7563,570,803,614,786,1039,583,145,296,611,470,771,574
PARN_2_7564,118,80,152,135,152,301,0,691,166,0,63,26
PARP2_2_7565,824,528,634,846,1131,476,757,948,793,464,1087,870
PARP3_2_7566,331,421,339,572,653,355,286,275,638,438,149,277
PARP8_2_7567,199,323,300,443,149,9,119,23,128,219,2,10
PBLD_2_7568,305,308,346,407,127,314,122,394,533,321,3,57
PCCA_2_7569,1635,1343,1533,1869,827,739,1423,2607,1919,1936,1329,1395
PCCB_2_7570,15,16,18,67,4,1,2,6,0,7,0,5
PCMTD2_2_7571,240,114,93,116,198,101,95,63,114,48,8,253
PCNA_2_7572,294,613,370,611,38,84,3,381,11,349,585,760
PCSK1_2_7573,127,15,35,110,310,0,2,7,2,68,296,4
PCSK2_2_7574,228,344,233,440,737,119,167,557,246,119,534,473
PCYT1B_2_7575,217,141,139,128,22,35,178,201,199,113,42,256
PCYT2_2_7576,512,413,520,686,500,1191,893,1162,431,121,765,634
PDE10A_2_7577,678,833,776,896,1059,1437,1090,1099,410,1046,1567,437
PDE11A_2_7578,537,381,552,892,291,427,1038,879,353,482,825,504
PDE1A_2_7579,4691,3976,3869,4726,6374,2393,5656,4983,4184,3617,4086,37
85

PDE1B_2_7580,145,218,243,357,124,220,158,118,489,117,15,384
PDE1C_2_7581,231,180,219,194,290,311,50,566,23,180,13,276
PDE2A_2_7582,23,37,37,35,2,25,13,1,0,0,0,6
PDE4A_2_7583,439,484,372,517,344,218,28,274,823,295,593,388
PDE4B_2_7584,453,484,373,514,345,217,28,270,826,295,603,385
PDE4C_2_7585,102,23,28,60,233,51,0,0,0,56,0,118
PDE4D_2_7586,1881,1392,1341,1380,2194,855,1892,2512,1981,2053,1462,188
4
PDE5A_2_7587,153,66,247,148,76,7,1,331,146,253,271,47
PDE6B_2_7588,224,227,246,247,193,39,213,33,202,430,425,401
PDE7A_2_7589,254,305,221,223,270,324,1108,149,373,637,490,32
PDE8A_2_7590,65,154,38,121,75,15,314,12,24,194,14,36
PDE8B_2_7591,474,550,475,426,344,478,273,531,509,413,65,401
PDE9A_2_7592,94,82,126,117,74,3,549,141,85,155,47,279
PDHA1_2_7593,490,484,321,616,265,483,245,1129,96,90,26,266
PDHB_2_7594,41,29,57,46,186,0,16,39,44,147,0,31
PDHX_2_7595,2390,1538,1712,2172,1546,1885,2306,1895,1870,1511,2623,228
1
PEMT_2_7596,196,389,211,514,63,272,19,415,50,561,241,244
PEPD_2_7597,253,270,305,273,445,247,816,93,143,346,82,211
PGAM5_2_7598,20,73,51,63,1,308,63,3,12,0,0,0
PGBD1_2_7599,421,486,324,611,632,805,1469,486,370,859,1073,146
PGC_2_7600,88,73,31,93,0,23,0,0,17,144,1,85
PGM1_2_7601,369,568,231,473,209,590,325,219,149,347,159,369
PGM3_2_7602,2846,2348,2449,2341,2576,2296,1767,1326,2804,3024,3231,135
6
PHOSPHO1_2_7603,151,258,221,189,358,171,87,47,66,258,19,9
PHOSPHO2_2_7604,594,523,467,560,221,446,377,980,871,466,172,435
PHYH_2_7605,111,113,122,149,181,27,472,196,240,23,3,203
PIGA_2_7606,108,53,57,63,54,35,1,10,90,18,249,248
PIGC_2_7607,265,89,175,338,523,438,206,60,126,67,121,15
PIGF_2_7608,1336,971,1151,1343,1878,1109,2049,1647,1865,1275,1441,2132
PIGG_2_7609,1018,721,687,799,1298,593,1280,1413,881,1198,442,716
PIGN_2_7610,283,157,171,203,286,21,73,136,359,112,310,704
PIGO_2_7611,43,17,57,51,14,40,6,14,64,8,112,25
PIGP_2_7612,433,245,332,350,587,359,492,75,78,243,424,622
PIGQ_2_7613,112,85,108,168,37,33,1,7,98,42,0,174
PIGT_2_7614,37,18,40,45,57,8,24,60,166,0,0,6
PIGV_2_7615,98,90,84,138,416,1,5,71,52,218,69,31
PIN4_2_7616,1534,1440,1264,1354,1906,597,2653,1006,2255,1391,1332,1818
PLA1A_2_7617,739,812,350,617,191,799,504,480,1310,406,833,317
PLA2G2A_2_7618,969,445,606,616,676,964,242,237,1534,411,955,471
PLA2G4C_2_7619,474,484,368,342,677,361,491,743,747,402,566,111
PLA2G6_2_7620,51,124,29,145,0,89,27,289,3,26,18,401
PLA2G7_2_7621,760,905,550,1102,656,167,1046,373,521,1707,619,553
PLAT_2_7622,208,239,204,191,34,29,92,102,181,1,200,203
PLAU_2_7623,2107,1847,1982,2343,3372,1217,1822,2945,1746,2648,1073,220
4
PLB1_2_7624,229,237,284,340,143,465,3,630,1216,541,764,100
PLCB1_2_7625,255,139,102,172,204,188,497,256,235,354,146,195

PLCB3_2_7626,144,90,137,148,59,0,29,37,250,375,28,104
PLCB4_2_7627,265,159,153,179,141,151,369,91,245,295,1,322
PLCD1_2_7628,9,6,13,18,2,5,176,212,42,2,8,10
PLCE1_2_7629,858,750,673,824,636,906,771,1416,648,634,663,1048
PLCG1_2_7630,96,91,177,110,0,32,0,0,344,195,190,2
PLCH1_2_7631,1037,1253,1209,1452,1173,497,1491,886,591,1145,150,1325
PLCL2_2_7632,736,732,451,545,698,290,714,482,268,394,1948,434
PLCXD2_2_7633,689,710,639,773,422,634,627,1050,751,864,256,1211
PLD2_2_7634,387,306,259,380,68,153,350,177,69,945,11,635
PLD3_2_7635,179,206,178,218,826,171,25,237,39,25,0,96
PLOD2_2_7636,998,1270,975,1452,1054,987,652,921,199,1750,1434,1080
PLSCR3_2_7637,153,72,91,122,184,520,0,83,93,107,0,0
PLSCR4_2_7638,246,128,158,209,88,35,5,71,387,0,16,31
PMEL_2_7639,53,133,87,87,26,7,0,21,107,2,1,167
PMS1_2_7640,528,259,514,515,88,124,1070,0,165,184,134,349
PNPLA1_2_7641,280,178,167,293,112,108,0,13,225,427,382,69
PNPLA4_2_7642,277,157,175,218,283,462,12,166,214,0,881,158
PNPLA6_2_7643,131,12,25,106,2,4,84,22,0,4,2,10
PNPLA7_2_7644,60,53,46,51,0,0,0,0,0,0,13,0
POFUT1_2_7645,138,111,128,222,214,0,956,22,2,270,88,1
POFUT2_2_7646,102,125,179,385,177,1,12,333,179,21,12,1
POGZ_2_7647,1363,1686,1511,1727,1807,1316,2690,1461,1468,1834,1789,195
4
POLD2_2_7648,542,558,677,875,378,606,772,809,351,586,610,800
POLE2_2_7649,650,395,428,556,522,572,484,83,295,780,640,837
POLG_2_7650,2817,2401,1856,2573,2703,801,3245,2130,2364,2355,2184,1455
POLL_2_7651,220,256,206,165,136,275,344,216,171,52,9,158
POLR1D_2_7652,158,174,98,183,85,24,292,612,107,132,172,102
POLR3B_2_7653,1589,1376,1322,1479,1354,1409,1603,1564,1940,1671,469,17
78
POLR3H_2_7654,1628,1511,1483,1726,2030,334,2520,812,1393,1076,1417,955
POMT1_2_7655,148,177,224,143,278,303,145,6,126,39,96,47
PON2_2_7656,405,261,261,417,142,54,38,100,487,444,364,374
POP5_2_7657,79,43,76,138,11,160,47,3,38,1,2,30
PPA2_2_7658,971,888,893,1047,430,647,154,868,788,871,1217,896
PPIE_2_7659,509,266,172,216,743,335,555,291,213,97,274,150
PPIL2_2_7660,383,391,291,497,0,792,11,0,104,188,511,0
PPIL3_2_7661,2376,1909,1606,1945,1165,1358,1263,1551,1120,1868,2620,23
87
PPIL6_2_7662,394,163,306,295,530,185,733,371,119,330,96,261
PPIP5K1_2_7663,113,122,125,95,383,2,179,15,180,0,61,232
PPT1_2_7664,84,114,79,186,26,278,159,375,49,0,5,179
PPT2_2_7665,227,302,353,304,177,125,183,340,130,715,0,149
PRCP_2_7666,112,60,38,67,80,1,2,152,46,7,77,79
PRDX1_2_7667,1342,1111,976,1218,915,1913,451,1917,1662,1097,564,2240
PRDX3_2_7668,106,40,82,155,68,28,91,34,208,42,0,85
PRDX5_2_7669,596,646,616,493,294,945,471,464,69,579,582,285
PRKCSH_2_7670,123,150,113,265,12,200,309,337,164,208,1,365
PRODH_2_7671,416,526,199,483,704,594,95,318,176,271,726,456
PRSS3_2_7672,702,779,742,885,517,1142,1317,256,246,927,728,998

PSAT1_2_7673,1,9,6,43,0,0,0,0,0,0,0,0
PSMA1_2_7674,271,328,482,507,169,98,0,229,112,288,445,440
PSMA3_2_7675,357,152,190,326,51,525,342,0,336,36,160,171
PSMA4_2_7676,338,137,204,238,45,65,1244,815,40,69,67,273
PSMA5_2_7677,551,581,612,640,385,590,1411,396,288,1116,866,648
PSMA8_2_7678,578,390,283,521,672,201,163,55,357,1089,737,263
PSMB2_2_7679,2249,3112,2486,3352,2758,3408,4246,2021,2839,2152,3691,34
03
PSMB5_2_7680,358,236,145,221,40,0,88,347,430,7,683,20
PSMB8_2_7681,1147,562,607,842,837,600,416,1364,754,302,1137,683
PTBP1_2_7682,122,175,93,235,101,64,96,11,86,5,0,123
PTER_2_7683,958,727,797,1224,786,654,1186,1285,864,388,355,839
PTGR1_2_7684,422,285,499,437,515,345,62,314,269,102,450,564
PTGR2_2_7685,306,298,164,338,25,73,289,365,845,1296,432,116
PTGS1_2_7686,405,476,254,489,57,425,2,479,922,211,260,175
PUS1_2_7687,92,105,149,184,84,100,32,71,55,80,0,579
PYCR1_2_7688,346,146,164,226,269,357,190,762,142,386,16,96
PYGL_2_7689,333,182,230,274,768,258,748,165,184,66,94,815
PYGM_2_7690,226,72,84,222,528,0,199,500,12,76,0,522
QPCTL_2_7691,1352,850,897,1005,1410,255,457,372,944,743,1348,1117
QSOX1_2_7692,209,282,339,457,332,35,1,88,77,574,1036,15
RAB11A_2_7693,277,309,216,230,76,146,21,348,73,622,55,177
RAB1A_2_7694,1936,1874,1774,1899,1265,2126,2567,3255,1494,1889,995,287
4
RAB23_2_7695,421,450,334,413,249,108,143,816,245,584,689,590
RAB27A_2_7696,762,744,896,989,969,1560,441,730,947,751,1346,1327
RAB28_2_7697,664,366,623,583,549,112,623,415,603,589,1872,233
RAB2A_2_7698,403,400,370,530,233,355,749,160,361,606,13,538
RAB2B_2_7699,458,362,394,398,530,262,714,361,239,307,580,836
RAB34_2_7700,240,94,172,146,52,155,168,24,509,184,78,327
RAB35_2_7701,441,241,527,635,700,663,360,620,351,981,3,887
RAB37_2_7702,101,97,99,74,121,0,49,334,7,31,47,453
RAB40C_2_7703,76,88,67,111,124,15,70,0,0,21,12,0
RAB5C_2_7704,73,128,152,185,77,63,28,610,108,70,85,71
RAB6A_2_7705,744,933,558,857,698,700,846,694,480,1323,484,616
RAB7L1_2_7706,2224,1610,1632,2550,1027,1484,1843,1910,2332,1761,1674,2
955
RAB9A_2_7707,3733,3113,2972,3705,3643,3525,3518,4169,4696,3233,3594,29
82
RABGGTA_2_7708,202,41,85,91,2,163,2,0,21,420,102,120
RABL2A_2_7709,0,0,0,0,0,0,0,0,0,0,0,0
RABL2B_2_7710,0,0,0,0,0,0,0,0,0,0,0,0
RAC1_2_7711,686,600,665,551,1010,849,152,751,650,730,430,948
RAD51B_2_7712,133,110,98,214,170,442,57,0,89,8,16,188
RAD51_2_7713,260,182,227,267,386,275,1,431,257,292,319,398
RAD51D_2_7714,88,47,171,218,387,133,146,198,26,220,15,161
RAD54B_2_7715,3303,3149,3016,3422,2738,2328,3753,4650,2416,3009,1587,2
688
RAD54L_2_7716,207,147,166,181,152,330,128,14,80,184,92,71
RAD9A_2_7717,166,145,117,111,259,60,6,20,63,440,166,175

RAP1A_2_7718,941,566,655,903,715,700,1599,584,452,1372,770,595
RAP1B_2_7719,619,798,556,814,421,704,85,195,472,538,1593,398
RBBP8_2_7720,395,235,184,162,185,339,62,719,233,154,100,277
RDH13_2_7721,103,32,33,64,166,12,2,80,216,162,0,44
RDH5_2_7722,43,111,57,100,2,255,433,0,92,110,1,1
RECQL5_2_7723,406,248,176,229,136,470,316,138,682,220,71,319
RECQL_2_7724,73,50,24,46,170,6,55,43,30,119,0,149
RERG_2_7725,85,83,80,51,204,105,21,25,82,24,1,63
REV1_2_7726,2839,2766,2728,2627,1679,1420,2716,2760,2740,4449,1797,342
1
RFC3_2_7727,147,138,170,172,197,15,583,85,289,557,322,128
RFC5_2_7728,359,232,244,242,702,243,394,466,318,341,53,233
RGN_2_7729,350,116,188,276,309,3,0,356,136,15,312,291
RGS11_2_7730,728,615,720,760,393,1068,1030,584,231,942,258,401
RGS6_2_7731,763,772,606,801,407,992,639,425,657,832,580,765
RHOC_2_7732,99,48,121,82,1,22,0,0,0,0,0,282
RHOT1_2_7733,253,279,249,265,278,210,158,379,597,414,371,486
RNASE1_2_7734,276,139,145,216,193,85,0,562,182,239,0,164
RNASE4_2_7735,799,494,538,666,504,309,360,172,471,534,693,591
RPAP3_2_7736,699,381,668,518,888,48,243,416,233,189,435,252
RPE_2_7737,640,407,455,531,442,250,573,533,658,586,1018,272
RPN2_2_7738,309,227,233,291,535,1,368,97,70,359,875,238
RPP14_2_7739,759,663,563,455,587,590,435,270,546,290,340,737
RPP21_2_7740,54,165,56,135,0,306,1,270,28,274,51,40
RPP30_2_7741,983,767,697,1051,1035,602,841,1135,856,760,964,1324
RPP38_2_7742,483,419,356,352,1054,508,1602,499,328,28,268,259
RPS27A_2_7743,195,48,163,184,154,303,106,91,182,63,137,13
RRAD_2_7744,102,68,50,108,20,1,438,19,59,84,19,90
RRAGB_2_7745,713,573,723,763,894,112,1310,492,427,672,828,560
RRAS2_2_7746,123,54,61,192,0,42,1,0,15,0,4,36
RRM2B_2_7747,387,515,392,417,897,231,851,27,1384,498,70,284
RRM2_2_7748,976,882,920,861,1045,817,1047,1953,846,1227,981,1168
RTEL1_2_7749,142,297,276,236,17,143,4,148,141,181,646,738
SAR1A_2_7750,541,706,425,639,466,231,796,165,636,163,808,931
SAR1B_2_7751,1959,1700,1721,2009,1854,798,964,520,2389,620,1418,1504
SARDH_2_7752,216,166,116,148,194,30,331,47,121,94,2,462
SARS2_2_7753,324,215,231,258,155,118,143,36,240,254,412,521
SBNO1_2_7754,2899,2536,2694,3042,2950,2472,2293,3120,1810,1544,2412,31
75
SC5DL_2_7755,1020,1014,978,1157,1530,1012,788,2026,593,1324,560,1642
SCD5_2_7756,469,296,419,437,165,142,583,450,354,270,717,325
SDC1_2_7757,0,0,0,0,0,0,0,0,0,0,0,0
SDCBP_2_7758,577,330,500,632,1183,266,1504,558,677,114,1592,453
SDHC_2_7759,118,96,266,176,76,4,325,399,56,123,429,259
SEPHS1_2_7760,326,354,355,308,106,271,96,13,318,618,393,590
3-Sep_2_7761,92,144,124,105,197,10,49,333,13,707,165,148
4-Sep_2_7762,433,635,534,810,926,337,1013,1189,372,518,1126,318
5-Sep_2_7763,393,308,529,571,1160,0,923,925,499,0,366,400
9-Sep_2_7764,238,152,249,334,321,205,225,568,369,19,123,170
SGMS2_2_7765,394,486,283,297,292,49,1219,230,252,502,223,280

SH3GLB1_2_7766,793,732,651,807,1325,754,367,906,679,892,692,485
SHMT1_2_7767,64,34,93,34,8,49,112,49,21,316,37,0
SHMT2_2_7768,172,225,173,243,214,73,381,215,154,53,78,59
SIAE_2_7769,190,84,70,74,160,0,140,286,215,43,19,12
SLFN11_2_7770,282,459,384,350,1,405,2,241,448,625,850,618
SMG6_2_7771,46,84,22,59,136,249,0,0,84,81,1,44
SMOX_2_7772,118,180,47,102,9,0,260,218,151,108,34,86
SMPD1_2_7773,67,121,98,131,301,92,207,109,45,216,1,9
SMPD4_2_7774,52,54,80,61,32,0,176,341,7,79,0,60
SMPDL3B_2_7775,72,66,77,55,6,11,63,6,6,304,0,103
SOD2_2_7776,101,89,7,40,0,12,0,0,17,96,5,1
SPAM1_2_7777,901,681,882,1018,862,662,1265,955,480,1348,1183,628
SPAST_2_7778,691,546,390,627,564,358,606,924,325,679,602,379
SPG21_2_7779,137,157,126,112,3,57,41,64,75,66,0,293
SPO11_2_7780,1896,1932,1939,1904,1755,1583,2031,3492,2251,2560,2477,18
23
ST3GAL1_2_7781,160,188,211,258,179,211,598,309,98,209,211,224
ST3GAL5_2_7782,193,283,177,278,51,544,19,0,186,231,287,1498
ST5_2_7783,82,125,95,213,1,240,537,7,0,81,902,0
ST6GAL1_2_7784,398,515,286,357,95,411,383,126,293,409,724,541
ST6GAL2_2_7785,244,162,190,244,25,133,191,260,425,259,285,15
ST6GALNAC3_2_7786,109,61,71,75,139,70,78,0,87,10,28,45
ST6GALNAC4_2_7787,107,72,61,88,1,13,50,112,139,95,364,26
ST8SIA4_2_7788,181,141,137,191,40,174,521,369,9,87,0,445
SUCLG2_2_7789,172,156,171,215,27,164,24,28,79,475,6,14
SULF1_2_7790,296,283,259,230,42,6,680,262,119,344,387,136
SULF2_2_7791,62,145,36,22,0,0,0,70,51,418,0,0
SULT1A1_2_7792,2024,2023,2138,2284,2010,3328,3651,2470,2413,1412,2936,
2700
SULT1A2_2_7793,2024,2023,2138,2284,2010,3328,3651,2470,2413,1412,2936,
2700
SULT2B1_2_7794,400,337,221,381,24,240,57,719,30,210,177,114
SUOX_2_7795,102,66,28,48,7,16,0,0,114,54,0,191
SYTL1_2_7796,77,148,43,166,0,0,0,343,139,1,0,166
TAB1_2_7797,337,289,501,498,891,125,75,355,184,472,293,256
TBXAS1_2_7798,95,65,87,34,274,1,0,35,36,0,147,39
TCIRG1_2_7799,93,92,71,90,76,202,126,124,70,142,30,342
TDP1_2_7800,916,754,817,664,705,459,128,440,641,1023,190,1128
TERT_2_7801,24,58,41,49,0,59,20,23,117,181,5,11
TGM2_2_7802,343,286,281,318,454,0,1350,211,140,257,177,469
TGM5_2_7803,166,178,146,123,35,73,417,35,195,244,205,205
TH_2_7804,57,39,70,47,0,1,25,0,36,0,0,429
TIAM2_2_7805,386,321,285,382,227,54,168,0,205,66,346,414
TKTL1_2_7806,622,317,399,401,189,124,260,602,55,770,228,468
TMEM55B_2_7807,54,137,198,84,214,0,267,434,2,3,350,207
TMLHE_2_7808,2318,1981,1871,2383,1510,722,2267,1406,2303,1576,2523,128
2
TMOD1_2_7809,1695,1855,1799,2003,1094,1193,1391,1369,1855,2252,1909,21
25
TMX2_2_7810,166,126,116,109,168,148,409,277,120,41,810,76

TNNI2_2_7811,281,259,279,258,530,105,159,74,550,425,535,259
TP53I3_2_7812,463,205,339,410,19,254,58,58,873,555,912,0
TPI1_2_7813,257,144,313,185,210,601,546,523,605,301,231,676
TPST2_2_7814,96,152,101,157,292,91,424,440,146,505,1,339
TREX1_2_7815,100,110,134,165,18,81,0,0,111,24,381,1
TRMT1_2_7816,229,254,251,286,113,251,125,294,202,41,354,132
TRMT1L_2_7817,107,61,89,66,0,0,257,31,40,132,4,79
TRMT2B_2_7818,42,17,68,46,9,378,164,40,73,0,3,7
TRPT1_2_7819,137,84,70,103,1,0,52,0,0,0,1,64
TSEN2_2_7820,588,980,699,811,1327,1056,464,1811,614,522,856,712
TSEN34_2_7821,284,285,108,214,26,16,0,819,90,509,138,0
TTLL6_2_7822,100,101,131,111,490,14,70,92,124,357,351,93
TUBAL3_2_7823,217,184,90,178,2,392,1,16,99,76,354,494
TUSC3_2_7824,1195,1306,1040,1253,1117,672,639,1600,1580,1818,1033,1768
TXNDC16_2_7825,3655,3449,2914,3499,3728,2904,3958,2676,2307,3442,3641,
2884
TXNDC2_2_7826,46,82,23,53,203,2,3,0,32,0,4,61
TXNDC5_2_7827,113,40,108,88,90,42,17,0,13,98,44,6
TXNL4B_2_7828,790,777,740,926,891,649,888,790,1018,1208,599,900
TXNRD1_2_7829,226,170,119,188,217,71,136,226,21,219,188,96
TXNRD3_2_7830,557,771,695,758,2,25,1598,543,755,1180,946,1148
TYMP_2_7831,306,329,638,294,110,302,163,43,18,283,305,161
UBA52_2_7832,209,147,117,252,414,266,68,78,170,289,0,139
UEVLD_2_7833,526,239,274,429,45,214,167,655,85,426,1645,499
UGDH_2_7834,904,716,744,893,969,428,109,583,918,1079,1092,882
UGP2_2_7835,414,204,405,465,315,912,259,408,306,187,482,287
UGT1A6_2_7836,2431,1866,1948,2591,2024,1618,3042,1710,2924,1959,3480,2
208
UGT2B10_2_7837,2356,1596,1799,2372,2362,2863,2566,1585,1198,1815,2095,
1815
UGT2B28_2_7838,1092,1438,957,1294,1946,1237,632,1113,1599,1172,1423,14
48
UGT3A1_2_7839,713,650,663,733,947,440,655,898,425,139,372,652
UGT8_2_7840,670,669,486,744,266,421,930,830,556,867,164,332
UNG_2_7841,3,15,0,8,0,0,2,0,0,0,0,2
UPP1_2_7842,98,54,143,113,288,15,1,269,112,0,23,95
UPP2_2_7843,813,775,774,591,363,694,1217,467,366,375,418,929
UQCR10_2_7844,158,263,158,274,101,271,503,0,2,2,1,163
UQCRB_2_7845,210,165,123,192,237,74,459,220,26,47,230,293
UROC1_2_7846,334,546,320,577,287,191,652,835,277,360,594,677
VCL_2_7847,422,394,276,420,478,271,206,505,682,47,592,989
VNN2_2_7848,854,734,923,1002,412,222,301,451,646,590,400,345
VPS29_2_7849,310,343,332,375,192,200,775,992,13,156,476,132
WARS2_2_7850,1130,969,673,1122,421,701,1147,997,1465,986,1285,553
WARS_2_7851,97,46,56,69,181,7,32,0,62,37,364,65
WBSCR22_2_7852,1469,1358,1469,1794,748,1173,1448,665,1034,1189,1760,13
25
WDR46_2_7853,129,86,189,172,28,214,7,295,234,66,664,47
WFS1_2_7854,939,825,895,876,1267,1089,1206,312,600,1463,448,826
WRNIP1_2_7855,197,84,105,169,16,13,26,0,309,0,125,6

WWOX_2_7856,62,24,37,13,0,19,2,0,32,1,0,102
XPNPEP1_2_7857,190,106,76,137,113,19,276,35,197,47,19,235
XRCC3_2_7858,144,240,202,157,0,42,402,329,61,2,726,29
XRN1_2_7859,639,552,575,613,861,236,442,317,658,569,131,966
YWHAZ_2_7860,393,321,536,447,853,408,154,57,353,75,670,357
ZBED1_2_7861,655,672,588,478,69,231,41,293,596,698,552,464
ZCCHC11_2_7862,1256,1183,1021,1286,1147,610,1161,741,585,759,1040,1807
ZCCHC6_2_7863,378,498,411,445,325,255,5,311,9,170,589,201
ZDHHC15_2_7864,169,308,205,212,643,1,238,349,4,513,283,422
ZDHHC7_2_7865,552,735,587,791,595,666,22,263,674,989,142,570
ACYP1_2_7866,96,146,103,190,194,268,617,2,95,2,9,15
APOBEC3F_2_7867,2082,1644,1680,1818,1125,1535,987,1960,2188,2856,2019,
2135
ASCC3_2_7868,499,465,451,529,1,463,154,658,831,825,101,897
ASPH_2_7869,209,201,204,169,900,0,459,206,435,8,0,86
ATP6V0E2_2_7870,195,129,131,211,641,277,1,56,33,459,4,433
C10orf2_2_7871,107,121,120,189,284,182,23,58,179,8,119,43
CAPN3_2_7872,5,1,58,53,129,0,0,57,0,0,0,104
CHM_2_7873,350,300,205,330,628,380,634,80,242,436,314,644
CTSC_2_7874,186,259,149,214,7,506,139,156,205,100,222,135
CYBRD1_2_7875,196,271,233,174,233,1,108,51,313,149,88,282
CYP3A5_2_7876,9634,9127,8412,9754,9960,5425,10406,7419,7255,8133,9427,
9433
DIO2_2_7877,85,203,96,202,3,129,454,3,25,0,0,84
DNASE2B_2_7878,238,94,158,156,0,8,0,37,2,150,31,117
DPYD_2_7879,34,7,23,7,20,0,0,0,4,27,0,13
GBA3_2_7880,865,461,415,723,193,179,877,779,535,354,642,793
GCNT2_2_7881,1180,1090,1064,1550,1043,645,952,1824,1089,972,1034,1126
GLYAT_2_7882,492,333,401,508,104,222,477,192,378,438,469,446
GPLD1_2_7883,14,4,12,24,0,13,0,0,11,0,0,28
GPX1_2_7884,580,472,401,463,509,390,358,419,644,768,698,594
GPX5_2_7885,754,556,778,955,842,151,79,910,913,444,513,1315
HNMT_2_7886,280,203,304,228,92,387,160,214,147,222,151,417
HOGA1_2_7887,102,27,80,71,5,2,139,13,144,33,0,92
HYAL3_2_7888,228,232,254,292,8,9,198,319,246,232,177,573
JPH2_2_7889,115,104,85,170,9,21,0,211,92,100,659,122
KLK8_2_7890,676,392,564,1055,521,7,677,280,745,256,568,1043
LAMA4_2_7891,1604,1466,1198,1627,1779,190,1684,1534,1144,2100,995,729
LRR1_2_7892,704,254,364,417,532,667,205,0,348,1124,864,245
LRTOMT_2_7893,974,646,745,746,966,288,226,256,850,493,1867,936
METTL1_2_7894,167,133,173,177,109,0,143,357,95,113,0,20
MOCS2_2_7895,485,312,312,339,176,464,23,341,201,632,798,196
NDUFA11_2_7896,87,59,57,92,2,55,32,22,97,4,126,72
NDUFV3_2_7897,727,408,466,545,457,79,531,6,268,171,378,500
NMNAT3_2_7898,722,603,639,840,305,469,337,729,852,462,364,744
PDE4DIP_2_7899,149,220,64,157,72,12,474,13,272,392,145,93
PLG_2_7900,149,90,56,83,49,156,10,24,69,69,10,147
PPCS_2_7901,268,176,191,295,71,89,365,141,400,220,13,388
PRDX2_2_7902,26,52,27,59,41,1,312,0,1,11,36,5
RAD51C_2_7903,2283,1691,1886,1619,1585,2052,1892,2526,1820,2453,1325,1

644
RASD1_2_7904,80,49,150,119,0,14,42,1,1,101,8,209
RASL10A_2_7905,36,31,64,16,36,8,324,121,1,9,79,2
SLX1A_2_7906,598,649,389,589,538,654,1340,270,564,836,300,1780
SLX1B_2_7907,598,649,389,589,538,654,1340,270,564,836,300,1780
SPTLC1_2_7908,3147,3565,3197,3649,4433,3055,4719,3372,3258,4603,4213,3
807
ST3GAL3_2_7909,344,330,399,298,207,812,290,78,759,370,126,496
VKORC1_2_7910,1619,1439,1378,1627,1048,1371,1394,1200,1595,3189,670,12
52
XPNPEP3_2_7911,132,182,99,165,339,2,287,22,176,310,39,226
GNAS_2_7912,542,430,519,658,81,219,647,571,341,700,1374,745
GNAS_2_7913,548,477,512,528,918,631,598,520,624,300,630,661
GNAS_2_7914,79,42,26,100,3,0,0,37,0,0,0,1
A4GALT_2_7915,265,134,235,191,301,244,195,261,100,0,1639,217
A4GNT_2_7916,9,54,143,35,0,0,0,0,0,1,66,0
AACS_2_7917,251,279,276,312,489,116,41,158,347,205,678,238
AADAC_2_7918,502,404,668,545,101,470,1626,186,153,9,1364,200
AARS2_2_7919,346,320,265,310,126,455,404,631,687,212,43,256
AARS_2_7920,729,755,609,851,651,826,1557,1184,953,1767,1571,939
AASDH_2_7921,417,721,579,834,484,814,470,844,410,1096,88,494
AASDHPPT_2_7922,187,218,140,118,197,256,25,224,75,202,306,253
AASS_2_7923,152,136,161,200,282,68,83,190,148,12,7,279
ABHD14A_2_7924,37,43,19,54,2,95,0,47,14,58,0,38
ABHD1_2_7925,173,84,55,104,194,27,61,2,1,45,0,128
ABHD3_2_7926,88,120,115,124,22,3,177,57,275,2,33,403
ABHD5_2_7927,20,100,56,107,341,1,1050,14,98,138,0,493
ABHD6_2_7928,490,497,463,624,271,465,363,1127,393,392,221,396
ABHD8_2_7929,380,497,284,421,504,330,69,1041,260,583,772,293
ABO_2_7930,445,559,425,684,697,54,273,896,1597,337,374,916
ABP1_2_7931,1664,1163,1452,2219,1150,1635,1734,744,2557,1512,1598,1634
ACAA2_2_7932,287,317,343,307,180,216,274,730,520,330,0,79
ACACB_2_7933,1128,930,682,1138,1265,527,562,973,1292,1078,1136,1003
ACAD11_2_7934,278,281,227,220,433,640,0,385,460,651,104,100
ACAD8_2_7935,184,149,80,197,36,165,353,124,73,129,273,114
ACAD9_2_7936,417,593,380,558,462,487,26,218,377,118,299,417
ACADL_2_7937,140,102,62,82,263,100,0,734,26,80,10,16
ACADSB_2_7938,106,288,156,130,277,436,17,336,26,70,422,112
ACADS_2_7939,161,231,194,271,241,319,435,1053,359,179,47,179
ACAT1_2_7940,269,291,297,263,266,269,712,190,230,155,193,545
ACER1_2_7941,1132,1007,1016,1201,839,932,833,854,778,916,341,1202
ACER2_2_7942,228,268,161,116,291,204,0,1343,275,0,136,90
ACER3_2_7943,1264,1087,969,1241,1320,1038,253,645,1029,1050,1046,1417
ACMSD_2_7944,32,36,54,68,9,1,133,0,17,2,0,306
ACO1_2_7945,1401,1464,1246,1623,1540,2319,2627,800,1282,1807,920,1307
ACO2_2_7946,378,194,203,369,302,26,0,0,74,64,24,590
ACOT12_2_7947,388,242,147,191,26,145,285,565,19,0,875,1350
ACOT1_2_7948,1022,882,808,978,415,1632,1090,945,1069,1205,1696,1915
ACOT2_2_7949,169,105,96,216,220,1,0,991,148,0,61,14
ACOT4_2_7950,451,285,305,374,725,330,687,789,314,537,701,265

ACOT6_2_7951,191,221,184,235,81,30,39,27,274,29,12,245
ACOT8_2_7952,145,155,84,213,307,562,295,58,101,339,86,140
ACOX2_2_7953,476,550,462,359,1678,1118,675,495,265,562,575,455
ACOXL_2_7954,219,171,199,128,41,76,104,247,219,233,0,315
ACR_2_7955,871,859,657,875,1105,895,1277,197,1133,983,530,1349
ACSBG2_2_7956,152,69,84,197,98,66,268,3,5,55,11,225
ACSF2_2_7957,188,134,88,76,120,9,38,31,36,40,0,12
ACSL1_2_7958,191,176,86,139,105,451,30,6,353,0,0,112
ACSM1_2_7959,117,31,116,93,208,16,16,438,48,51,266,64
ACSM2A_2_7960,3279,2940,2935,3407,2637,2654,4813,2799,2694,3063,2479,3
699
ACSM4_2_7961,1807,1484,1518,1617,1787,1435,2054,1836,1283,1927,2029,25
85
ACSM5_2_7962,119,131,76,122,0,0,257,197,238,24,135,9
ACSS1_2_7963,113,41,78,97,36,40,231,482,35,1,0,17
ACSS3_2_7964,197,217,191,310,909,78,16,300,211,545,102,259
ACTC1_2_7965,317,483,327,587,510,103,278,392,415,278,415,661
ACY3_2_7966,307,296,424,462,417,74,176,0,412,764,389,615
ACYP2_2_7967,71,56,79,43,0,0,1,14,139,4,0,39
ADA_2_7968,49,80,27,53,140,77,81,263,129,207,0,4
ADAM10_2_7969,954,727,729,989,1187,449,1573,556,480,1313,1218,892
ADAM17_2_7970,260,176,264,279,337,361,283,163,626,340,66,655
ADAMTS4_2_7971,88,35,48,19,4,14,8,57,141,349,0,0
ADARB2_2_7972,292,486,592,189,746,0,8,0,0,2,736,617
ADAT1_2_7973,155,194,167,109,103,122,55,51,626,10,0,67
ADAT2_2_7974,88,275,101,105,608,22,6,23,55,89,513,23
ADC_2_7975,576,479,351,567,315,558,138,641,427,354,486,673
ADCY1_2_7976,141,78,138,225,506,10,4,4,73,16,1,39
ADCY2_2_7977,61,67,153,69,93,27,32,2,88,302,9,79
ADCY7_2_7978,363,247,334,422,186,337,671,1776,397,104,415,436
ADCY8_2_7979,200,89,140,241,18,158,30,421,37,0,1127,7
ADCY9_2_7980,107,19,24,52,15,45,832,12,172,5,21,44
ADH1A_2_7981,416,380,298,311,830,19,154,776,470,757,35,122
ADH1B_2_7982,1910,1875,2133,2175,2134,2256,3452,1368,1887,2065,2278,21
95
ADH1C_2_7983,59,51,81,127,1,175,216,162,1,0,0,0
ADH4_2_7984,481,300,348,504,752,75,342,263,129,268,392,141
ADH5_2_7985,138,193,118,127,0,0,0,3,541,131,0,238
ADHFE1_2_7986,982,874,780,835,298,638,1108,1326,455,1126,2618,1108
ADI1_2_7987,369,241,319,466,309,201,432,194,417,248,555,399
ADO_2_7988,34,64,125,153,92,0,184,10,160,134,7,0
ADPRH_2_7989,458,606,785,648,1010,587,119,419,204,906,417,311
ADPRHL2_2_7990,65,24,11,32,46,1,39,37,233,56,61,80
ADSS_2_7991,1038,1245,1045,1205,194,1496,675,880,517,569,1764,1024
AEN_2_7992,359,239,381,291,223,328,603,77,387,768,537,347
AGBL2_2_7993,219,232,212,244,6,349,139,655,209,265,815,4
AGMAT_2_7994,486,452,425,546,362,207,676,388,410,549,832,293
AGMO_2_7995,96,165,142,137,175,431,94,15,368,166,144,260
AGPAT4_2_7996,2036,1806,1834,1912,1420,1329,1545,1577,647,1740,2277,32
48

AGPAT5_2_7997,2102,1958,1743,1897,1154,655,3568,2001,2043,1506,1843,19
51
AGPAT6_2_7998,666,461,475,549,213,491,699,218,544,596,348,832
AGPAT9_2_7999,428,302,466,695,20,282,807,145,35,947,129,237
AGPS_2_8000,140,159,128,182,13,166,142,120,73,301,279,293
AGXT2_2_8001,0,0,1,0,0,0,0,0,0,0,0,0
AGXT2L2_2_8002,434,803,420,496,759,273,599,196,1098,696,258,61
AGXT_2_8003,105,83,105,125,5,1,98,43,118,277,49,217
AICDA_2_8004,160,73,163,146,55,81,38,119,178,81,15,228
AKR1B10_2_8005,1565,1492,1364,1804,1065,1048,1841,498,1159,1981,1639,9
87
AKR1B15_2_8006,1565,1492,1364,1804,1065,1048,1841,498,1159,1981,1639,9
87
AKR1B1_2_8007,155,180,146,123,147,40,119,14,358,79,9,189
AKR1C1_2_8008,717,576,463,559,69,611,955,25,619,223,497,782
AKR1C3_2_8009,200,107,89,178,71,210,299,175,148,138,0,194
AKR1C4_2_8010,444,515,352,470,332,638,890,142,53,371,280,142
AKR1E2_2_8011,303,215,285,453,177,340,248,684,297,424,473,823
AKR7A2_2_8012,278,261,312,609,7,403,0,343,506,226,0,625
AKR7A3_2_8013,907,1049,1169,1173,897,470,2514,1023,766,1349,1234,668
ALAD_2_8014,7,53,120,35,0,0,0,0,0,0,0,0
ALDH1A1_2_8015,139,102,147,310,34,375,556,383,53,78,585,689
ALDH1A3_2_8016,378,187,257,329,732,190,106,109,1091,340,890,401
ALDH1B1_2_8017,148,62,147,88,179,0,516,247,58,104,153,76
ALDH1L1_2_8018,31,2,47,51,0,36,3,0,0,2,0,3
ALDH1L2_2_8019,465,573,431,490,416,667,932,842,513,1324,251,237
ALDH6A1_2_8020,885,509,626,830,565,87,891,692,509,655,1114,501
ALDH9A1_2_8021,492,443,418,572,711,856,387,562,778,593,277,468
ALDOB_2_8022,337,253,248,349,306,14,87,362,424,536,365,287
ALDOC_2_8023,320,343,380,369,842,638,595,104,74,502,67,393
ALG10_2_8024,929,930,780,933,1210,892,642,1375,1356,1210,606,1383
ALG11_2_8025,401,556,589,529,257,386,1355,243,775,678,392,771
ALG12_2_8026,1375,1160,1297,1223,1038,1777,934,504,477,955,1697,1261
ALG14_2_8027,193,126,111,110,367,22,78,701,21,0,73,37
ALG1_2_8028,1175,1332,1554,1366,1209,2078,1559,1470,1492,1196,1614,781
ALG2_2_8029,721,499,662,627,1009,772,477,472,654,641,726,1103
ALG6_2_8030,1222,1129,855,1229,1383,112,1569,1323,1826,606,459,1662
ALKBH8_2_8031,280,333,209,261,26,132,470,0,182,467,34,321
ALLC_2_8032,178,137,174,175,19,90,58,31,132,69,159,242
ALOX12B_2_8033,135,134,170,83,319,64,176,67,143,72,16,64
ALOX12_2_8034,153,346,226,249,95,26,0,456,67,111,120,201
ALOX15_2_8035,405,365,350,462,406,188,604,155,926,303,216,1131
ALOX5_2_8036,45,108,88,75,198,22,48,19,3,23,18,38
AMDHD1_2_8037,595,481,396,586,223,399,792,874,494,602,500,698
AMY1B_2_8038,1300,1329,1106,1228,949,1335,2142,980,866,1074,2386,1604
AMY1C_2_8039,1300,1329,1106,1228,949,1335,2142,980,866,1074,2386,1604
AMY2A_2_8040,1300,1329,1106,1228,949,1335,2142,980,866,1074,2386,1604
AMY2B_2_8041,1310,1326,1089,1227,948,1338,2144,982,870,1075,2385,1600
ANPEP_2_8042,137,138,205,147,115,78,319,29,12,111,30,77
AOC3_2_8043,338,275,233,298,83,427,7,495,275,593,468,354

AOX1_2_8044,161,152,116,170,200,0,118,64,32,183,76,36
APEH_2_8045,141,118,64,82,58,79,126,319,47,47,0,175
APEX2_2_8046,817,612,697,881,457,159,319,164,767,526,1053,587
APIP_2_8047,185,276,149,258,356,229,1083,15,117,450,550,86
APLF_2_8048,1295,1351,1619,1348,1227,857,1516,1173,1224,1261,843,2303
APOBEC1_2_8049,1057,991,1035,1211,1538,1377,1623,928,593,968,1399,1663
APOBEC2_2_8050,202,510,359,374,19,478,756,416,155,1088,184,169
APOBEC3B_2_8051,2076,1642,1668,1818,1123,1540,989,1964,2187,2856,2024,
2139
APOBEC3C_2_8052,1954,1857,1856,2153,1812,2248,841,2009,630,2487,1861,1
671
APOBEC3G_2_8053,404,287,253,460,255,206,47,368,54,366,693,1113
ARF3_2_8054,58,32,28,48,10,310,0,0,19,0,120,0
ARF4_2_8055,781,539,618,815,972,654,486,314,360,473,903,950
ARG2_2_8056,525,469,301,341,63,163,34,118,558,321,45,617
ARHGEF10_2_8057,133,220,199,165,245,209,10,2,146,221,24,156
ARL3_2_8058,112,123,106,171,90,133,0,19,210,546,0,156
ARL4C_2_8059,166,92,91,116,141,2,21,456,13,204,4,2
ARL4D_2_8060,374,416,372,336,276,154,343,461,670,465,166,229
ARL5B_2_8061,536,300,386,509,328,376,465,139,838,517,485,541
ARL8A_2_8062,223,156,209,242,18,0,4,0,36,535,0,704
ARL8B_2_8063,100,48,54,57,3,0,0,4,284,53,4,30
ARSD_2_8064,335,458,302,563,3,737,133,360,164,449,331,354
ARSE_2_8065,467,290,298,453,720,252,171,155,223,403,892,222
ARSG_2_8066,226,91,171,148,0,13,45,550,354,104,14,167
ARSH_2_8067,117,123,173,236,150,314,407,23,255,173,125,63
ARSI_2_8068,1040,1003,907,949,1232,687,489,396,1283,810,1233,945
ARSJ_2_8069,401,317,283,403,393,145,455,365,262,769,77,756
ARSK_2_8070,397,573,333,618,137,977,187,482,270,643,88,370
ART1_2_8071,96,86,55,119,74,4,140,35,102,94,4,51
ART4_2_8072,645,472,587,522,698,853,1315,480,225,286,988,354
AS3MT_2_8073,35,41,50,90,143,0,215,0,1,403,278,33
ASNA1_2_8074,335,152,225,230,411,1,0,1025,187,307,379,50
ASNSD1_2_8075,818,807,694,778,891,444,1017,318,820,728,73,594
ASPG_2_8076,509,580,635,573,1618,184,252,581,316,1039,624,929
ASPHD2_2_8077,120,158,64,94,89,64,0,13,164,75,122,9
ATAD1_2_8078,361,413,459,433,12,1011,453,318,289,0,884,531
ATIC_2_8079,511,407,370,425,350,736,289,166,158,323,187,517
ATP2C2_2_8080,178,146,181,248,106,7,192,277,344,191,6,362
AUH_2_8081,296,336,312,212,386,30,411,279,95,136,280,353
AWAT1_2_8082,142,138,163,214,98,103,328,38,118,153,418,238
AWAT2_2_8083,89,77,123,167,1,225,4,492,150,23,1,126
B3GALNT2_2_8084,436,320,373,517,404,369,495,73,666,601,178,455
B3GALT1_2_8085,57,207,68,194,0,1,381,335,136,325,15,1
B3GALT2_2_8086,32,111,86,150,91,25,166,107,97,121,46,90
B3GALT4_2_8087,66,85,105,78,37,5,1,0,52,22,461,160
B3GALT6_2_8088,448,209,154,247,451,18,9,697,365,26,33,93
B3GAT2_2_8089,262,270,290,322,558,26,582,78,225,713,150,432
B3GAT3_2_8090,304,249,230,430,72,134,41,33,41,311,614,152
B3GNT2_2_8091,698,648,683,697,134,365,476,186,180,554,311,477

B3GNT3_2_8092,56,76,99,75,6,0,272,14,17,95,55,56
B3GNT4_2_8093,176,108,103,106,28,48,81,251,221,283,29,248
B3GNT5_2_8094,2469,1821,1670,2180,1292,2522,2864,1858,1802,1497,1232,3
275
B3GNT7_2_8095,126,66,143,108,55,139,93,71,127,15,707,135
B3GNT9_2_8096,286,224,209,290,983,281,8,43,3,99,76,24
B3GNTL1_2_8097,298,126,251,173,18,68,3,25,373,90,772,95
B4GALNT1_2_8098,274,163,143,203,0,954,1,541,5,44,139,13
B4GALNT3_2_8099,159,287,130,138,89,11,122,87,221,149,257,170
B4GALNT4_2_8100,229,132,173,231,0,17,1,66,51,73,0,9
B4GALT1_2_8101,166,246,240,190,342,0,0,108,228,253,361,402
B4GALT5_2_8102,821,813,683,605,1460,990,1157,682,540,620,366,903
B4GALT6_2_8103,162,84,103,231,159,25,135,201,457,747,0,250
B4GALT7_2_8104,105,109,100,98,377,0,39,6,234,0,0,1
BBOX1_2_8105,805,551,908,568,517,374,959,83,874,167,1841,543
BCHE_2_8106,465,504,592,847,750,820,590,906,656,734,573,226
BCMO1_2_8107,199,496,321,402,63,693,450,41,234,1382,420,87
BDH2_2_8108,28,54,55,58,4,0,1160,15,70,0,27,90
BHMT_2_8109,488,451,412,398,595,110,53,430,397,89,613,465
BLM_2_8110,322,323,232,219,659,156,281,17,255,558,26,213
BLMH_2_8111,725,586,565,734,806,867,1800,93,501,385,439,1133
BLVRA_2_8112,285,165,192,313,249,505,52,55,271,27,356,311
BLVRB_2_8113,78,85,24,48,6,4,357,1,25,2,1,38
BPHL_2_8114,1271,930,819,921,1925,1489,762,1341,781,877,1656,1523
BST1_2_8115,173,198,230,306,116,54,22,25,1,3,331,476
BTD_2_8116,602,437,515,517,300,78,1273,170,581,1049,619,610
C12orf5_2_8117,360,178,147,206,296,148,297,27,24,276,293,242
C16orf79_2_8118,252,133,295,333,237,86,26,231,351,249,251,404
C18orf56_2_8119,294,279,274,371,1,286,0,282,360,0,553,0
C1GALT1_2_8120,581,530,544,560,768,510,340,330,628,484,239,973
C1R_2_8121,396,323,251,352,0,455,835,104,487,785,336,284
C22orf28_2_8122,924,958,841,921,705,1082,1025,292,1053,1111,1606,706
C2orf43_2_8123,978,618,694,859,1327,827,945,1407,572,741,288,868
C5orf4_2_8124,308,319,354,303,36,168,326,485,354,362,218,193
C6orf130_2_8125,1108,886,825,1076,1134,794,898,1197,1277,831,629,1448
CA13_2_8126,80,62,199,228,254,26,577,0,431,246,17,412
CA14_2_8127,392,192,265,229,282,475,585,638,213,244,209,215
CA2_2_8128,330,140,233,327,164,130,15,167,107,474,80,167
CA3_2_8129,183,211,429,379,89,40,89,388,438,386,339,537
CA4_2_8130,45,54,70,88,178,10,1,118,149,249,8,82
CA5A_2_8131,117,129,99,194,10,18,149,178,130,1066,0,311
CA5B_2_8132,185,187,270,430,323,2,433,731,437,202,284,338
CA6_2_8133,1329,894,1002,1073,2315,699,851,1115,1388,1576,1477,1052
CA8_2_8134,611,473,508,640,111,393,568,418,526,236,1381,215
CA9_2_8135,452,274,405,290,211,34,90,28,202,730,3,54
CARS2_2_8136,33,64,79,71,3,460,40,82,206,30,45,81
CASD1_2_8137,383,339,528,546,34,77,922,153,149,704,922,1031
CAT_2_8138,1133,766,1359,1278,1406,909,124,927,882,1101,864,1143
CBR1_2_8139,212,167,133,133,191,188,139,0,82,32,2,392
CBR3_2_8140,1144,1166,954,1617,1026,1343,934,2092,1017,1467,1476,3002

CBR4_2_8141,1267,1009,1060,970,836,330,1257,111,1291,1066,374,821
CCDC88B_2_8142,378,576,619,678,44,941,20,1,618,15,1369,733
CCDC92_2_8143,42,60,126,151,7,42,183,74,58,59,157,219
CCNO_2_8144,84,75,38,36,2,0,0,412,2,870,658,0
CCS_2_8145,331,313,339,321,364,137,107,68,498,287,297,438
CCT8_2_8146,329,521,333,438,305,442,547,451,646,500,164,102
CD274_2_8147,744,679,541,580,1076,1021,962,1013,617,1050,194,947
CD38_2_8148,233,185,193,124,103,352,211,170,199,424,396,147
CDA_2_8149,1230,749,800,1198,1142,172,1913,1074,1695,512,890,603
CDIPT_2_8150,132,301,216,331,89,24,42,636,51,38,177,835
CDO1_2_8151,222,248,235,247,326,58,41,704,283,541,11,142
CDS2_2_8152,974,889,808,978,901,449,499,776,700,1444,694,994
CELA1_2_8153,71,48,27,72,12,0,0,0,4,63,0,0
CELA2A_2_8154,402,441,401,389,396,830,803,57,505,91,322,30
CELA2B_2_8155,132,127,112,88,49,243,267,20,113,570,60,183
CELA3A_2_8156,587,445,475,529,218,498,486,858,1024,0,423,585
CELA3B_2_8157,85,29,33,64,12,6,0,62,112,14,1,86
CEL_2_8158,27,62,18,18,0,3,3,0,1,0,0,1
CETN1_2_8159,37,76,108,40,530,14,26,0,265,267,102,88
CETN2_2_8160,7,12,10,7,0,0,0,0,43,25,0,0
CETP_2_8161,131,163,88,128,86,57,133,5,32,178,9,277
CFB_2_8162,206,215,143,360,346,499,1490,3,83,222,270,386
CFD_2_8163,73,5,3,8,0,0,0,6,53,0,0,4
CFI_2_8164,766,721,382,736,666,51,179,218,161,959,188,387
CH25H_2_8165,46,55,60,38,0,56,33,65,0,1,49,91
CHDH_2_8166,76,42,347,65,79,17,696,3,0,162,6,20
CHI3L1_2_8167,408,431,391,541,314,1,51,58,655,833,675,1343
CHIT1_2_8168,823,402,516,757,326,623,26,228,938,16,662,783
CHML_2_8169,416,95,261,312,304,249,26,36,591,92,110,139
CHPF2_2_8170,326,566,283,385,106,928,808,227,808,1512,697,354
CHPT1_2_8171,489,492,447,517,508,193,36,559,582,351,1485,520
CHST10_2_8172,155,129,67,116,160,130,0,1,4,19,334,142
CHST12_2_8173,283,231,302,431,379,51,637,0,20,181,809,445
CHST13_2_8174,875,780,654,819,768,375,949,1018,482,1531,1364,830
CHST14_2_8175,6,16,8,4,0,7,0,132,0,110,0,0
CHST1_2_8176,359,356,402,473,498,450,210,456,270,413,26,1411
CHST2_2_8177,768,634,538,497,676,456,302,696,407,698,650,501
CHST3_2_8178,94,81,173,146,173,102,105,108,447,227,3,218
CHST5_2_8179,661,775,506,626,528,246,144,42,428,1173,398,38
CHST6_2_8180,212,224,207,243,353,502,430,456,347,11,57,13
CHST7_2_8181,128,175,201,290,413,0,8,527,19,339,61,36
CHST9_2_8182,183,273,155,181,303,26,55,25,164,620,14,147
CHSY1_2_8183,110,89,46,179,175,7,0,1,0,75,12,1
CHSY3_2_8184,72,100,77,82,0,247,0,50,32,64,0,11
CLPP_2_8185,27,23,18,7,0,1,188,0,14,8,0,0
CLPX_2_8186,724,819,1170,921,568,633,417,731,383,978,1446,1284
CLYBL_2_8187,1009,760,983,856,1866,828,1495,1087,830,826,1283,1448
CMA1_2_8188,678,879,936,701,889,1008,1555,455,660,2088,1277,592
CMAS_2_8189,84,29,110,177,24,12,78,0,0,8,0,66
CMBL_2_8190,236,284,151,456,301,409,489,1310,295,377,472,364

CNDP1_2_8191,596,570,635,672,484,818,1819,1799,687,883,657,653
CNTN6_2_8192,405,272,332,369,467,609,378,107,154,13,304,60
COIL_2_8193,997,1099,1063,1047,846,1104,1525,1694,757,537,828,1473
COMTD1_2_8194,28,99,67,99,33,17,0,385,0,35,1,108
COQ2_2_8195,687,576,426,474,536,474,723,308,412,491,358,620
COQ3_2_8196,56,39,19,38,3,65,0,35,90,4,0,140
COQ5_2_8197,866,466,556,511,841,663,746,660,927,1062,802,785
COX10_2_8198,975,883,892,1102,563,1214,457,442,896,791,881,771
COX17_2_8199,1486,1157,1100,1379,864,749,516,1563,1444,1161,1425,1388
COX4I1_2_8200,77,10,31,34,13,0,0,21,0,0,0,42
COX4I2_2_8201,68,41,55,144,38,140,14,126,108,583,72,387
COX5A_2_8202,2,16,7,58,32,41,3,3,0,38,0,0
COX5B_2_8203,837,466,632,793,826,1874,1430,650,955,356,820,520
COX6A1_2_8204,519,558,369,793,313,8,1402,723,384,157,2,720
COX6A2_2_8205,310,317,235,256,320,183,36,79,639,233,73,243
COX6B1_2_8206,597,402,340,363,642,238,9,361,22,0,389,770
COX6B2_2_8207,429,298,285,370,36,268,698,137,353,113,437,563
COX6C_2_8208,417,509,755,746,585,139,1308,331,687,742,540,751
COX7A1_2_8209,97,55,111,77,4,75,16,54,57,32,109,128
COX7A2_2_8210,40,63,28,57,127,667,16,93,3,39,0,21
COX7A2L_2_8211,598,677,341,657,521,222,208,545,449,368,543,1258
COX7B2_2_8212,358,305,195,235,402,40,370,262,92,1048,173,480
COX7B_2_8213,164,198,232,234,271,23,68,44,48,68,453,222
COX7C_2_8214,137,104,80,252,598,50,23,108,5,136,32,32
COX8A_2_8215,113,3,35,46,0,7,85,36,6,1,0,6
COX8C_2_8216,570,614,512,687,750,431,794,848,655,880,880,567
CPA1_2_8217,152,47,35,36,2,0,0,72,137,0,1,64
CPA2_2_8218,494,391,402,535,889,592,42,648,257,177,328,1196
CPB1_2_8219,116,71,100,81,47,1,84,5,33,207,0,24
CP_2_8220,62,41,74,76,0,2,28,7,26,5,197,133
CPE_2_8221,220,211,301,630,112,362,333,589,650,343,350,647
CPOX_2_8222,184,84,159,311,1,101,0,46,43,15,0,123
CPSF3_2_8223,444,475,404,483,745,897,233,1140,828,319,152,167
CPT2_2_8224,29,28,48,45,0,3,51,0,52,0,0,0
CRAT_2_8225,270,282,244,248,70,763,38,33,0,267,37,149
CREG2_2_8226,511,346,236,443,4,321,5,723,309,453,97,418
CRY1_2_8227,204,145,206,166,144,33,513,217,59,197,2,24
CRYL1_2_8228,1179,970,784,1412,1094,1575,837,308,1670,1843,861,1317
CRYZL1_2_8229,858,687,731,947,839,1259,1733,814,737,1411,1697,1465
CSAD_2_8230,327,290,205,192,294,548,179,256,212,203,91,150
CS_2_8231,169,98,104,220,360,211,163,55,131,46,3,84
CSGALNACT2_2_8232,728,918,741,1054,807,1530,450,932,464,266,1294,1191
CSRP2BP_2_8233,365,477,460,488,209,373,840,32,239,243,533,59
CTBS_2_8234,272,179,200,197,30,2,10,94,119,382,83,111
CTRB1_2_8235,539,399,338,368,91,218,408,313,509,152,540,358
CTRB2_2_8236,441,281,503,283,597,343,351,176,180,239,832,500
CTRC_2_8237,120,115,120,94,1,18,2,3,226,2,70,88
CTSD_2_8238,616,355,388,321,548,349,520,532,218,426,146,62
CTSF_2_8239,253,278,258,355,262,255,511,663,47,319,876,73
CTSG_2_8240,46,67,31,79,3,2,2,0,2,17,0,15

CTSH_2_8241,824,506,732,624,477,399,710,185,784,229,411,584
CTSK_2_8242,190,125,228,122,285,36,411,338,624,212,5,205
CTSZ_2_8243,262,222,259,180,350,597,18,453,824,117,1124,240
CWC27_2_8244,584,636,515,525,923,242,508,720,541,187,387,990
CXorf21_2_8245,593,410,656,625,922,279,1204,614,281,218,330,315
CYB561D2_2_8246,259,209,148,141,117,64,135,749,231,351,0,144
CYB5B_2_8247,439,515,453,778,552,318,735,228,1127,468,717,1075
CYB5R1_2_8248,863,623,577,581,582,658,419,241,437,943,660,779
CYB5R2_2_8249,298,294,255,274,114,26,205,29,33,264,434,80
CYB5R4_2_8250,1017,743,859,949,528,957,1781,782,773,490,746,670
CYBA_2_8251,143,84,194,106,393,66,0,198,130,143,0,167
CYBB_2_8252,64,77,6,51,0,0,0,5,0,4,24,10
CYC1_2_8253,208,241,204,313,259,216,0,856,0,1,481,687
CYCS_2_8254,267,179,128,212,260,127,6,232,250,482,1,1158
CYP11B2_2_8255,679,534,613,679,745,488,1539,551,698,746,391,979
CYP17A1_2_8256,88,68,64,183,21,303,39,15,66,27,0,42
CYP1A1_2_8257,496,523,500,738,104,768,401,213,407,895,621,336
CYP1A2_2_8258,281,270,195,231,21,577,24,101,194,772,929,71
CYP1B1_2_8259,427,164,272,458,312,556,6,155,478,272,427,113
CYP20A1_2_8260,1319,1008,1348,1382,1012,419,1866,1037,753,1700,1351,13
54
CYP26B1_2_8261,60,196,150,146,8,8,85,1,15,186,8,9
CYP26C1_2_8262,46,21,72,64,1,1,1,0,170,0,0,0
CYP27A1_2_8263,7,17,39,15,0,0,0,8,79,0,0,6
CYP27B1_2_8264,39,17,45,25,71,35,7,41,133,12,15,1
CYP2A13_2_8265,654,376,488,703,343,319,1903,627,741,663,238,992
CYP2A6_2_8266,592,490,612,488,532,964,264,211,767,74,283,1104
CYP2B6_2_8267,145,76,209,116,60,0,85,1,400,151,1,293
CYP2C19_2_8268,461,346,309,391,190,480,5,214,771,278,405,216
CYP2C9_2_8269,265,450,251,289,13,507,19,709,294,141,45,61
CYP2E1_2_8270,254,114,208,222,159,176,691,108,301,270,722,0
CYP2F1_2_8271,340,201,323,380,51,26,133,26,277,63,100,215
CYP2J2_2_8272,1347,1272,946,1019,762,862,747,1209,1666,1174,899,1014
CYP2R1_2_8273,386,426,353,405,598,349,17,167,376,226,32,326
CYP2S1_2_8274,1,15,64,75,7,0,14,0,1,0,0,0
CYP2U1_2_8275,399,252,416,370,244,84,484,879,140,228,167,216
CYP2W1_2_8276,16,62,20,68,4,0,0,0,5,0,0,0
CYP39A1_2_8277,299,500,463,403,629,158,5,2,673,190,657,1294
CYP3A7_2_8278,7037,6543,6570,7472,7267,4369,8900,5315,5231,5741,7292,6
320
CYP46A1_2_8279,249,224,310,266,259,1,490,428,93,138,702,309
CYP4A11_2_8280,353,336,314,366,0,14,512,556,7,182,192,55
CYP4A22_2_8281,353,336,314,366,0,14,512,556,7,182,192,55
CYP4F12_2_8282,216,159,238,122,147,89,17,21,161,235,1,97
CYP4F22_2_8283,941,976,760,845,1355,1718,475,436,1302,1135,1272,1055
CYP4F2_2_8284,738,740,673,686,678,368,1135,480,355,543,271,883
CYP4F8_2_8285,1125,1156,1479,1184,1338,835,1156,1435,1121,1538,2335,11
04
CYP4V2_2_8286,707,734,610,610,392,456,673,265,230,568,1913,1440
CYP4X1_2_8287,108,74,221,148,157,36,1188,1,119,26,1709,10

CYP4Z1_2_8288,127,170,125,145,46,71,256,353,109,660,88,161
CYP7A1_2_8289,20,43,119,85,381,94,313,7,6,55,0,202
CYP7B1_2_8290,1217,1212,1145,1467,1228,1301,938,745,1089,1833,991,976
CYP8B1_2_8291,713,532,662,492,499,251,575,460,392,356,329,852
DAD1_2_8292,151,159,109,227,17,116,0,17,155,50,0,1
DAGLA_2_8293,431,342,434,411,72,237,239,106,35,216,328,392
DAK_2_8294,1581,997,872,1100,2373,1502,1004,1808,954,1428,1375,958
DARS2_2_8295,1,18,22,0,0,0,0,0,0,0,0,0
DARS_2_8296,349,443,338,414,15,86,17,555,43,502,211,612
DBH_2_8297,276,106,69,196,9,0,0,161,330,469,0,18
DBR1_2_8298,292,354,500,369,365,184,498,0,610,413,50,1057
DBT_2_8299,1628,1008,1081,1061,1735,724,483,2476,938,1573,1087,1728
DCLRE1B_2_8300,118,56,52,82,263,1,19,78,0,114,393,0
DCPS_2_8301,81,40,39,46,27,90,0,1,115,26,116,17
DCTN6_2_8302,681,840,569,807,1016,699,515,924,750,672,606,854
DCTPP1_2_8303,374,219,325,325,90,6,841,290,63,401,354,547
DDAH2_2_8304,76,53,48,137,0,5,32,6,168,274,17,1
DDOST_2_8305,911,796,735,951,741,1771,766,450,1475,581,1541,502
DDX10_2_8306,101,112,111,121,291,21,0,303,10,536,0,56
DDX18_2_8307,464,379,342,428,1344,238,454,510,531,155,5,581
DDX19A_2_8308,309,101,269,161,54,12,36,1,32,238,20,197
DDX1_2_8309,2290,1925,1613,1783,2216,1576,3643,820,1796,2001,2833,1759
DDX20_2_8310,836,754,644,823,391,120,206,45,876,161,444,897
DDX21_2_8311,245,259,275,408,608,359,261,0,230,476,273,578
DDX23_2_8312,365,203,296,216,362,89,221,89,295,421,285,294
DDX24_2_8313,1101,821,817,884,1345,481,639,42,970,1245,653,782
DDX25_2_8314,320,169,260,204,2,104,673,23,273,360,0,75
DDX27_2_8315,90,121,50,51,10,105,13,108,28,0,61,13
DDX28_2_8316,26,17,19,11,0,0,422,0,0,4,0,3
DDX39A_2_8317,974,1100,632,873,917,108,408,435,1523,175,2007,180
DDX41_2_8318,20,29,46,58,1,4,575,0,0,0,2,84
DDX43_2_8319,502,403,305,514,1044,406,113,1275,699,45,415,1047
DDX46_2_8320,352,260,215,292,209,206,23,1,10,264,98,629
DDX49_2_8321,381,331,344,380,239,169,334,576,205,312,375,404
DDX50_2_8322,1392,1366,1615,1322,383,858,1891,588,1186,529,832,1270
DDX51_2_8323,179,291,193,401,68,338,222,516,270,3,860,327
DDX52_2_8324,204,240,153,209,284,125,142,243,144,363,56,306
DDX53_2_8325,892,554,541,921,333,890,985,616,288,772,963,1501
DDX55_2_8326,1969,1651,1785,1774,1383,553,2412,1081,1835,1997,2290,203
2
DDX56_2_8327,142,82,83,98,88,35,4,52,49,27,95,294
DDX58_2_8328,215,154,110,93,6,14,828,440,246,148,7,125
DDX59_2_8329,203,161,158,210,18,17,229,46,26,126,696,400
DDX5_2_8330,80,115,131,160,17,99,458,72,51,199,0,36
DDX60_2_8331,1104,1066,1137,1356,315,1087,1547,1747,556,1464,927,1290
DDX6_2_8332,266,374,291,388,284,552,125,344,233,47,389,594
DECR1_2_8333,685,697,331,623,244,1205,390,720,1221,545,388,913
DECR2_2_8334,1754,1583,1575,1576,1531,1533,1586,2131,1467,2160,1546,16
16
DEGS1_2_8335,1253,1185,855,1129,1035,528,455,856,591,1124,1000,995

DEGS2_2_8336,101,67,38,130,623,0,209,185,12,0,0,189
DERA_2_8337,1030,901,1026,985,765,1408,2228,1008,843,907,1510,1121
DFFB_2_8338,22,16,22,39,24,21,0,110,20,87,0,0
DGAT1_2_8339,49,20,23,60,0,282,0,300,58,36,0,65
DGAT2_2_8340,261,343,65,188,2,0,0,0,0,134,169,183
DHCR24_2_8341,142,147,152,322,256,162,319,15,237,65,43,104
DHDH_2_8342,184,149,150,107,21,2,134,902,92,92,1,174
DHFR_2_8343,138,110,74,208,298,37,401,0,25,36,382,306
DHODH_2_8344,488,203,311,336,327,220,396,417,95,645,851,177
DHRS13_2_8345,180,168,196,152,6,3,158,338,116,90,1482,22
DHRS3_2_8346,238,186,220,289,43,278,357,454,117,159,310,303
DHRS4_2_8347,68,20,69,83,6,0,6,0,4,329,8,0
DHRS7_2_8348,306,441,337,508,224,142,485,261,368,282,239,11
DHRSX_2_8349,55,58,19,46,0,0,10,0,3,28,2,0
DHTKD1_2_8350,153,137,72,176,318,35,6,123,131,64,5,247
DHX15_2_8351,25,57,61,124,37,0,155,685,69,0,141,84
DHX29_2_8352,305,232,262,321,219,217,905,224,419,166,73,330
DHX32_2_8353,43,29,33,48,0,67,52,4,3,9,115,30
DHX34_2_8354,440,359,307,275,584,26,237,393,269,242,49,314
DHX37_2_8355,280,326,384,263,53,507,41,386,296,201,3,64
DHX38_2_8356,294,320,159,234,862,299,0,46,453,17,251,595
DHX57_2_8357,1079,928,920,823,1129,1240,844,357,846,1079,888,538
DHX58_2_8358,431,248,248,283,126,111,636,423,545,309,395,417
DHX8_2_8359,57,69,58,62,0,0,312,0,1,0,0,0
DHX9_2_8360,1509,1406,1543,1499,1099,650,1291,1872,1630,2021,2331,2248
DIO3_2_8361,99,158,74,206,109,93,8,0,130,225,0,144
DIRAS1_2_8362,221,102,240,269,45,51,740,391,212,1,135,5
DIRAS2_2_8363,260,161,244,368,340,3,388,170,119,293,479,18
DIRAS3_2_8364,288,362,336,319,192,180,640,583,202,309,502,240
DIS3L2_2_8365,241,397,391,400,628,138,1040,871,410,105,608,341
DLAT_2_8366,898,585,670,794,407,481,977,947,773,307,131,511
DLD_2_8367,226,329,293,310,457,168,166,161,172,132,690,292
DLL1_2_8368,119,129,225,153,61,231,123,1,123,23,138,318
DLST_2_8369,435,505,518,692,300,378,178,1026,317,719,775,490
DMGDH_2_8370,517,344,322,445,115,28,81,72,300,145,733,529
DNA2_2_8371,989,685,686,866,572,2277,582,593,704,921,941,351
DNAH11_2_8372,509,461,309,326,140,3,34,609,708,384,1112,592
DNAH3_2_8373,327,426,364,477,45,115,501,6,197,141,164,280
DNAH5_2_8374,1374,821,958,1346,655,577,552,1585,799,1417,1131,1069
DNAH8_2_8375,746,420,353,387,402,485,534,1052,340,389,1270,600
DNAJA2_2_8376,109,60,70,82,66,92,140,0,0,0,185,231
DNAJB14_2_8377,445,647,428,656,74,152,1076,136,510,408,147,1068
DNAJC10_2_8378,283,236,199,217,549,15,1,0,6,92,118,193
DNAJC18_2_8379,202,214,232,262,133,152,384,428,326,203,10,441
DNAL4_2_8380,421,421,496,350,145,217,452,778,178,964,893,720
DNASE1_2_8381,190,296,192,407,197,362,114,246,19,2,138,1013
DNASE1L2_2_8382,160,93,152,219,32,3,115,36,235,0,2,139
DNASE1L3_2_8383,188,263,239,220,188,730,136,294,561,193,0,195
DNASE2_2_8384,72,2,120,134,0,0,0,0,300,0,217,41
DNPEP_2_8385,10,21,1,21,11,3,0,0,4,1,0,145

DPAGT1_2_8386,31,21,52,18,6,11,2,7,128,46,0,19
DPEP2_2_8387,611,494,376,295,1147,207,2147,560,90,678,1083,852
DPM1_2_8388,60,93,55,79,105,120,74,97,28,0,70,21
DPM2_2_8389,251,298,305,329,215,101,694,66,32,13,275,833
DPP4_2_8390,137,153,127,121,122,506,18,63,10,40,22,250
DPP7_2_8391,14,29,15,63,1,6,0,510,10,44,144,75
DPP9_2_8392,177,281,331,401,216,23,1,776,178,203,1083,499
DPYS_2_8393,34,90,96,185,81,28,0,34,97,23,63,608
DPYSL4_2_8394,14,24,33,59,64,0,21,3,23,1,26,121
DPYSL5_2_8395,190,375,198,165,541,39,907,460,75,178,237,81
DQX1_2_8396,139,193,142,248,200,196,176,57,42,14,29,176
DSEL_2_8397,88,58,114,209,144,188,0,7,317,143,0,379
DTD1_2_8398,190,97,187,196,297,78,244,13,149,28,629,245
DUOX2_2_8399,234,186,228,453,499,410,1658,1309,389,211,484,1
DUPD1_2_8400,0,0,4,7,0,0,3,0,0,0,0,0
DUSP28_2_8401,139,118,273,298,45,479,679,16,123,151,0,402
EARS2_2_8402,82,128,118,147,0,136,54,0,21,58,0,106
EBPL_2_8403,387,511,435,503,332,88,954,236,628,707,485,496
ECH1_2_8404,51,203,67,145,18,478,34,122,6,0,43,30
ECHDC3_2_8405,224,423,332,315,199,218,521,322,205,255,446,42
ECHS1_2_8406,173,157,283,274,46,84,299,439,300,10,146,170
EDEM1_2_8407,51,97,53,74,3,116,0,80,8,3,0,24
EDEM3_2_8408,224,192,190,252,249,681,219,393,137,5,213,48
EEF1A1_2_8409,459,280,149,235,102,0,22,182,97,127,436,151
EEF1A2_2_8410,417,319,297,289,333,81,362,536,466,43,166,66
EEF2_2_8411,394,498,478,625,306,269,619,895,328,80,244,350
EGLN1_2_8412,359,411,431,409,73,414,1601,128,372,411,1069,97
EGLN3_2_8413,8,1,2,3,13,0,2,6,0,0,59,1
EHD4_2_8414,36,44,47,19,198,0,5,226,77,68,0,7
EIF4A3_2_8415,23,40,13,113,0,513,7,33,66,106,0,17
ELAC1_2_8416,372,558,353,400,317,682,5,291,243,463,216,348
ELANE_2_8417,26,35,142,53,80,0,98,46,36,0,0,83
ELOVL1_2_8418,101,138,61,66,42,168,0,0,182,250,0,70
ELOVL2_2_8419,784,981,849,926,953,576,758,613,500,822,667,438
ELOVL3_2_8420,673,669,576,797,542,301,389,374,727,434,168,1166
ELOVL4_2_8421,659,515,439,390,335,305,542,0,159,16,624,165
ENDOD1_2_8422,594,626,517,711,161,524,550,219,839,485,1052,281
ENDOG_2_8423,250,257,227,209,29,148,371,92,286,62,155,46
ENGASE_2_8424,220,138,188,224,577,240,316,98,222,0,173,440
ENO2_2_8425,94,73,48,115,355,0,18,2,113,47,14,25
ENO4_2_8426,52,105,124,177,106,49,1039,158,36,16,2,9
ENOPH1_2_8427,997,572,460,629,419,353,541,642,554,577,753,841
ENPEP_2_8428,901,379,483,687,658,1702,940,267,656,532,634,325
ENPP1_2_8429,1138,1253,930,1177,1807,1154,1834,643,1636,1208,690,1453
ENPP3_2_8430,3012,2454,2114,2540,2688,785,2984,4425,2248,3147,3359,216
8
ENPP4_2_8431,235,174,151,210,128,277,454,257,146,193,514,127
ENPP5_2_8432,0,0,0,0,0,0,0,0,0,0,0,0
ENPP6_2_8433,168,201,226,104,50,0,227,207,134,700,70,129
ENPP7_2_8434,67,153,96,84,242,47,5,121,23,308,32,199

ENTPD3_2_8435,171,200,229,417,0,14,432,82,117,170,31,343
ENTPD5_2_8436,42,36,27,26,6,33,42,1,21,11,0,17
ENTPD7_2_8437,778,772,871,752,1468,347,1868,1175,366,958,1158,1020
EPHX2_2_8438,325,89,118,157,439,3,58,9,8,4,225,6
EPHX4_2_8439,508,349,376,536,150,377,148,1,103,1117,412,601
EPRS_2_8440,1008,898,1077,1095,852,338,1129,913,1049,1539,1001,1017
EPT1_2_8441,1280,1311,1067,1554,808,1736,1838,943,1235,1917,1096,1408
EPX_2_8442,138,76,81,125,35,0,249,0,154,16,0,389
ERAS_2_8443,308,73,157,200,337,12,805,6,406,338,45,100
ERCC3_2_8444,139,75,65,81,17,0,2,47,0,8,57,99
ERCC4_2_8445,524,394,548,405,619,221,501,527,300,491,101,486
ERCC5_2_8446,280,195,260,276,68,429,148,17,172,378,35,324
ERI1_2_8447,552,573,579,834,187,272,979,591,854,25,847,311
ERO1L_2_8448,777,512,542,627,368,1247,1222,429,651,1086,713,245
ERP44_2_8449,123,285,205,100,0,24,493,392,3,49,202,188
ESD_2_8450,800,764,1076,1396,1202,665,723,1678,376,683,2290,699
ESPL1_2_8451,522,474,317,500,93,240,0,733,202,695,283,401
ETFDH_2_8452,575,444,434,555,1162,1009,315,466,529,173,439,241
EXOSC1_2_8453,133,123,47,155,225,119,146,66,79,207,0,346
EXOSC2_2_8454,95,116,50,33,10,97,5,2,45,106,251,62
EXOSC4_2_8455,168,109,225,189,14,98,9,17,436,79,2,25
EXOSC5_2_8456,53,13,51,11,0,0,0,8,0,11,0,0
EXOSC7_2_8457,450,222,519,406,925,82,37,117,317,366,735,2
EXOSC8_2_8458,293,472,230,186,204,217,658,495,331,137,489,163
EXT1_2_8459,52,44,10,30,0,24,5,36,304,0,0,0
EXTL1_2_8460,248,142,93,94,101,10,20,266,61,4,0,7
EXTL3_2_8461,110,104,86,145,500,272,13,1,79,12,17,279
F10_2_8462,489,483,601,746,1109,275,0,905,4,897,707,732
F12_2_8463,12,29,4,4,0,2,0,0,13,0,0,0
F13A1_2_8464,154,135,96,78,4,261,8,0,63,24,100,207
F13B_2_8465,848,829,676,688,337,572,716,539,382,334,1452,1317
F2_2_8466,176,231,224,181,191,200,177,52,162,99,0,171
F5_2_8467,0,0,0,0,0,0,0,0,0,0,0,0
F9_2_8468,246,244,229,274,289,461,766,0,331,273,0,77
FA2H_2_8469,392,443,339,384,655,355,1887,1397,165,275,427,1068
FAAH2_2_8470,362,424,285,221,599,202,439,42,11,578,262,414
FADS1_2_8471,655,581,670,728,583,377,414,1572,1122,418,555,998
FADS2_2_8472,634,422,493,581,539,557,131,317,672,320,1010,322
FADS3_2_8473,76,123,143,111,0,25,47,86,2,131,418,16
FAHD2A_2_8474,114,101,112,45,591,32,225,2,24,4,77,25
FAM108C1_2_8475,842,695,746,842,869,1782,921,794,935,972,1007,357
FAM135B_2_8476,465,360,610,409,349,147,934,47,151,300,419,90
FAM20B_2_8477,1034,769,837,764,827,300,634,234,1346,202,904,582
FANCM_2_8478,268,234,129,260,252,476,9,245,425,310,254,474
FAR1_2_8479,7711,6750,6162,7488,6444,6926,6603,5491,6196,7785,4692,558
5
FAR2_2_8480,826,946,524,793,300,148,1076,314,821,814,840,542
FARS2_2_8481,1407,1238,1427,1618,1352,2051,1417,1590,851,850,1079,2012
FARSA_2_8482,44,41,11,31,6,105,3,0,102,78,0,0
FARSB_2_8483,65,50,48,37,24,47,0,23,38,0,0,17

FASN_2_8484,224,161,106,143,94,0,0,174,435,175,5,148
FDFT1_2_8485,877,927,1010,924,633,691,616,976,772,2306,1223,1301
FEN1_2_8486,691,527,388,613,872,438,787,602,679,468,499,309
FH_2_8487,700,588,621,675,586,62,145,637,306,336,705,368
FIG4_2_8488,276,285,115,240,21,446,45,181,442,178,52,98
FKBP10_2_8489,5,16,2,32,0,0,0,0,9,136,0,0
FKBP14_2_8490,3793,2977,3392,3563,2356,3354,3121,3056,2282,2696,1849,4
645
FKBP15_2_8491,225,249,158,196,407,26,3,19,263,3,40,133
FKBP3_2_8492,1050,921,912,1037,1431,793,581,1049,877,1397,1317,820
FKBP4_2_8493,37,12,42,31,38,0,33,0,8,30,0,27
FKBP8_2_8494,434,513,546,560,326,544,629,679,554,389,398,325
FMO1_2_8495,325,362,315,267,395,307,587,65,282,146,302,193
FMO4_2_8496,1020,693,569,908,511,939,342,955,528,767,892,673
FNBP1_2_8497,6,64,62,22,0,1,0,2,0,0,143,44
FNTA_2_8498,1841,1480,1513,1967,1202,1015,1267,1208,953,1215,1833,1961
FNTB_2_8499,203,321,135,266,101,107,309,57,762,560,77,300
FTH1_2_8500,19,120,49,88,0,35,17,126,40,40,6,44
FTMT_2_8501,588,1098,684,1062,227,692,1492,322,381,1529,1543,452
FTSJ2_2_8502,172,127,184,219,96,16,68,156,341,24,79,19
FTSJ3_2_8503,863,831,697,883,423,818,1198,1461,1111,535,604,960
FTSJD2_2_8504,210,259,136,379,202,289,243,451,214,22,752,395
FUCA1_2_8505,509,442,303,509,219,670,3,1312,340,191,549,756
FUCA2_2_8506,152,133,190,397,494,260,1026,59,358,11,402,194
FURIN_2_8507,131,133,81,67,129,21,0,0,404,60,74,65
FUT10_2_8508,653,353,454,474,227,1038,1165,820,292,670,130,333
FUT11_2_8509,180,270,256,406,230,2,58,0,1,341,4,366
FUT1_2_8510,38,41,21,26,4,143,0,20,20,1,5,4
FUT4_2_8511,100,79,141,87,0,288,77,4,0,1,76,7
FUT5_2_8512,591,456,625,583,402,423,223,1590,252,321,427,584
FUT7_2_8513,506,469,467,481,600,593,630,921,672,508,86,772
FUT9_2_8514,749,434,419,783,275,320,171,217,751,1254,986,420
GADL1_2_8515,212,224,409,172,242,292,97,143,174,92,0,331
GAL3ST1_2_8516,170,172,160,132,388,0,0,1251,0,1359,293,39
GAL3ST3_2_8517,11,15,55,52,60,17,0,38,4,117,0,14
GAL3ST4_2_8518,19,37,67,42,39,0,0,0,7,0,0,1
GALM_2_8519,258,336,393,679,416,129,23,823,403,1043,373,308
GALNS_2_8520,139,114,216,99,16,3,281,33,199,109,684,259
GALNT10_2_8521,889,1044,1182,1339,894,1139,1144,1291,1653,957,1903,189
5
GALNT11_2_8522,15,12,30,36,0,14,0,0,10,8,0,30
GALNT12_2_8523,674,623,432,626,264,81,257,409,813,404,109,22
GALNT13_2_8524,486,669,448,469,11,106,540,523,353,414,676,674
GALNT14_2_8525,228,185,171,232,211,105,73,280,49,100,160,247
GALNT1_2_8526,352,236,450,322,152,391,366,233,147,613,29,328
GALNT2_2_8527,507,521,603,596,114,255,853,434,208,890,930,967
GALNT3_2_8528,177,182,167,250,141,1,16,0,137,103,0,82
GALNT4_2_8529,554,730,536,635,331,266,330,1007,1485,150,756,612
GALNT5_2_8530,182,202,254,272,54,212,185,3,1,254,8,434
GALNT6_2_8531,11,49,40,14,2,0,34,78,1,2,0,19

GALNT8_2_8532,130,81,208,215,9,74,14,326,498,1,14,100
GALNTL2_2_8533,30,300,103,124,0,500,62,0,0,281,36,23
GALNTL4_2_8534,0,0,0,0,0,0,0,0,0,0,0,0
GALNTL5_2_8535,1433,1450,1089,1209,1021,1097,379,1955,1285,1512,422,66
8
GALNTL6_2_8536,2289,2801,2246,2218,2648,1954,2041,715,2579,3291,1323,2
622
GALT_2_8537,137,275,185,224,144,25,166,269,90,160,461,223
GANC_2_8538,723,435,431,551,889,963,726,0,704,219,1276,567
GAPDH_2_8539,718,457,469,829,335,435,558,126,340,590,656,233
GAPDHS_2_8540,58,151,111,290,6,4,2,1108,0,0,0,35
GARS_2_8541,749,904,961,973,445,1526,899,1305,728,891,2694,1012
GATC_2_8542,70,75,87,94,73,0,44,8,136,67,244,9
GATM_2_8543,195,157,166,158,53,37,301,259,164,460,356,72
GBA2_2_8544,275,251,291,236,492,301,7,0,71,196,0,334
GBE1_2_8545,686,670,479,815,327,566,552,415,914,568,1267,1005
GBGT1_2_8546,404,244,209,314,114,41,0,355,725,70,722,349
GBP1_2_8547,155,94,84,161,352,13,31,54,65,176,110,229
GBP2_2_8548,622,524,536,692,528,461,256,567,355,1028,246,955
GBP3_2_8549,1103,797,841,887,477,679,366,453,1633,292,857,873
GBP4_2_8550,140,40,85,95,22,90,245,382,44,30,101,24
GBP6_2_8551,387,192,319,326,39,344,352,668,257,396,303,124
GBP7_2_8552,119,60,52,117,0,37,693,49,126,34,1,116
GCLM_2_8553,701,923,550,726,475,1667,1,1400,575,833,1697,619
GCNT3_2_8554,338,236,235,282,34,312,53,292,181,550,420,575
GCNT4_2_8555,818,604,417,493,382,635,599,311,412,653,465,257
GCSH_2_8556,404,526,441,375,396,158,1116,309,799,383,170,835
GDE1_2_8557,1073,1084,1161,1184,959,591,418,241,1073,1154,590,587
GDPD3_2_8558,163,214,122,52,386,0,27,256,32,23,151,212
GFM1_2_8559,42,41,64,40,1,0,116,42,42,74,40,11
GFOD2_2_8560,1282,1359,1156,1385,1333,1232,413,602,817,1866,1746,1565
GFPT1_2_8561,126,153,146,80,128,14,0,441,192,131,90,204
GFPT2_2_8562,72,67,38,95,36,112,152,0,1,20,6,1
GGH_2_8563,285,225,215,188,58,10,63,96,202,312,346,130
GGPS1_2_8564,487,487,558,542,145,282,929,664,697,553,216,696
GGT7_2_8565,469,383,294,438,833,325,446,222,685,132,252,335
GGTLC2_2_8566,472,335,398,664,795,114,750,509,282,12,814,660
GLCE_2_8567,524,853,919,753,729,434,977,742,1486,1069,326,765
GLDC_2_8568,512,586,475,540,140,141,654,413,627,1318,475,985
GLO1_2_8569,784,670,605,661,435,422,743,383,128,929,909,740
GLOD4_2_8570,93,50,113,158,75,557,322,26,2,175,353,99
GLS2_2_8571,186,131,97,106,29,4,4,6,37,236,0,1
GLT1D1_2_8572,153,281,306,458,545,268,556,16,128,82,100,705
GLT25D1_2_8573,558,693,522,646,17,354,738,347,108,323,894,314
GLT25D2_2_8574,337,353,371,311,58,134,1184,375,473,121,76,308
GLT8D2_2_8575,194,204,237,169,197,95,0,0,433,144,567,101
GLUD1_2_8576,221,170,193,286,0,5,125,126,466,223,226,138
GLUD2_2_8577,271,329,339,585,165,559,3,4,318,312,1348,1143
GMDS_2_8578,477,659,577,623,180,28,9,633,242,530,0,366
GMIP_2_8579,276,362,298,296,317,192,829,297,260,235,223,227

GMPR_2_8580,1246,1050,1193,1453,1500,1231,1157,644,947,1201,858,576
GMPS_2_8581,115,120,70,74,263,0,149,149,16,28,4,4
GNA11_2_8582,883,835,893,901,520,18,1172,632,585,901,1078,829
GNA12_2_8583,509,342,493,459,27,214,1187,22,756,648,256,606
GNA13_2_8584,60,7,53,21,211,0,0,3,21,1,0,1
GNA14_2_8585,660,456,517,429,1283,47,537,70,133,399,265,22
GNA15_2_8586,103,45,137,92,0,102,27,54,10,152,48,61
GNAI1_2_8587,146,182,173,246,12,14,625,104,40,57,28,242
GNAI3_2_8588,453,244,367,341,191,34,287,85,330,27,298,98
GNAQ_2_8589,686,671,545,711,1019,571,152,863,733,494,1045,940
GNAT2_2_8590,1715,1419,1294,1573,987,1423,1785,1045,1571,1328,2253,302
5
GNAT3_2_8591,1235,1172,1156,1152,1217,1139,820,418,758,1106,1949,747
GNAZ_2_8592,2354,1662,2111,2273,2373,1800,2673,1572,2076,2608,1320,319
6
GNB1_2_8593,113,88,97,194,228,169,82,353,165,15,93,10
GNB2L1_2_8594,294,208,177,208,209,33,249,247,75,273,186,196
GNB3_2_8595,433,223,324,346,73,390,437,275,72,320,604,118
GNB4_2_8596,461,413,203,263,177,255,223,280,410,348,130,99
GNG11_2_8597,989,1264,946,1191,1266,240,817,701,503,1936,267,434
GNG12_2_8598,507,736,554,618,790,1245,825,917,447,310,687,473
GNG13_2_8599,152,258,121,198,89,194,364,88,64,127,14,546
GNG2_2_8600,93,185,85,79,4,291,65,185,56,207,11,50
GNG3_2_8601,461,444,473,504,859,319,1333,125,910,358,373,106
GNG7_2_8602,163,132,129,124,48,68,430,125,86,147,81,388
GNG8_2_8603,496,467,521,406,995,675,687,433,1111,489,644,828
GNGT1_2_8604,36,160,88,140,0,14,68,130,52,246,483,11
GNL2_2_8605,88,77,98,72,165,193,146,10,170,119,66,100
GNMT_2_8606,279,348,234,224,223,313,86,32,40,512,507,0
GNPAT_2_8607,537,359,260,407,551,312,260,634,528,488,396,234
GNPDA1_2_8608,138,81,134,90,0,190,0,399,19,1,11,18
GNPDA2_2_8609,847,847,822,849,666,722,124,211,924,749,1783,205
GNPNAT1_2_8610,423,184,161,232,185,353,158,130,330,450,924,387
GNPTAB_2_8611,147,112,157,178,37,284,204,568,290,132,336,0
GNPTG_2_8612,96,60,46,138,0,24,2,224,4,441,332,448
GNS_2_8613,261,184,257,183,0,3,358,389,264,0,48,128
GOT2_2_8614,103,27,33,35,59,0,54,14,238,2,0,95
GPAA1_2_8615,546,515,537,441,779,469,596,41,355,497,287,749
GPAM_2_8616,1015,869,912,1027,970,466,1357,1189,815,326,1225,884
GPAT2_2_8617,335,143,346,288,122,100,843,22,242,123,583,462
GPD1_2_8618,280,266,176,462,213,258,0,492,362,58,395,459
GPD1L_2_8619,524,342,615,496,553,429,716,799,701,632,742,613
GPX2_2_8620,84,31,52,126,290,60,0,174,85,0,0,311
GPX3_2_8621,405,549,380,414,55,548,971,31,9,372,399,243
GPX7_2_8622,450,427,533,521,604,194,245,608,236,503,451,574
GPX8_2_8623,690,661,703,667,430,371,628,65,859,485,1120,223
GRHPR_2_8624,24,17,11,27,0,0,0,1,0,0,0,0
GSTA1_2_8625,1318,1563,1360,1401,1870,1369,927,982,1072,1234,891,1363
GSTA2_2_8626,645,575,627,668,1108,538,355,617,192,217,497,1495
GSTA3_2_8627,1155,1252,890,1260,1129,1177,1513,1303,1710,2118,1339,117

6
GSTA4_2_8628,591,582,640,823,849,346,623,1433,370,490,334,683
GSTA5_2_8629,850,703,577,653,1052,414,507,719,630,685,916,1047
GSTM3_2_8630,2498,2581,2076,2381,1676,2927,2627,1929,2418,2293,2453,19
39
GSTM5_2_8631,1181,1011,979,1074,1524,1857,726,592,804,1402,813,996
GSTP1_2_8632,9,55,31,77,11,0,21,10,0,20,4,0
GSTT1_2_8633,219,173,144,221,0,120,38,1352,299,147,558,556
GSTT2B_2_8634,361,223,328,585,87,1,2,4,408,217,26,715
GSTT2_2_8635,361,223,328,585,87,1,2,4,408,217,26,715
GTF2F2_2_8636,1690,1709,1558,1561,1159,977,2360,1167,1443,1648,1768,11
24
GTPBP1_2_8637,128,202,184,382,21,353,2,394,3,130,375,1
GTPBP2_2_8638,142,22,86,112,58,12,9,123,315,12,5,148
GTPBP4_2_8639,304,386,224,178,77,67,97,363,402,390,93,121
GUCY1A2_2_8640,307,108,248,161,325,157,204,29,142,396,174,302
GUCY1B3_2_8641,154,165,291,265,752,690,3,63,333,193,93,535
GUSB_2_8642,294,214,197,204,0,169,80,462,285,242,4,60
GYLTL1B_2_8643,257,382,359,411,8,3,336,11,340,24,709,323
GYS2_2_8644,342,402,336,347,310,321,1000,284,196,167,76,274
GZMA_2_8645,260,351,155,289,66,92,81,187,185,580,582,121
GZMB_2_8646,308,394,361,327,188,368,210,790,271,470,539,928
H6PD_2_8647,79,119,114,103,77,7,32,96,423,39,7,70
HAAO_2_8648,504,273,390,515,17,407,173,695,691,59,858,541
HACL1_2_8649,836,595,859,701,935,454,547,431,410,1199,638,936
HADHA_2_8650,1040,1100,858,1115,553,906,209,533,832,811,309,1495
HADHB_2_8651,122,74,146,88,125,0,48,374,46,116,0,161
HAGHL_2_8652,268,47,90,181,164,0,164,344,120,6,24,78
HAL_2_8653,707,741,539,847,534,428,969,98,970,781,179,625
HAO1_2_8654,291,203,397,389,341,379,51,251,237,584,355,162
HARS_2_8655,101,142,178,173,821,0,814,0,78,16,89,237
HAS1_2_8656,96,308,158,226,4,46,545,0,141,141,3,701
HAS2_2_8657,4017,3484,3412,3767,5016,2488,5185,3603,4700,3478,4136,495
5
HDHD3_2_8658,392,243,161,246,41,131,166,206,91,180,611,354
HELB_2_8659,770,544,508,744,622,834,1192,1013,563,1243,306,906
HELQ_2_8660,28,41,49,29,32,0,3,9,42,119,0,21
HELZ_2_8661,61,70,31,88,18,9,0,2,103,577,6,53
HEXA_2_8662,149,277,158,155,51,471,6,0,29,1,34,288
HEXB_2_8663,2053,2064,1804,1959,2319,2182,1802,2771,2211,3182,2191,172
9
HEXDC_2_8664,881,695,719,682,918,1173,2751,455,617,773,1223,871
HGD_2_8665,393,627,472,396,185,237,15,430,428,209,684,982
HGSNAT_2_8666,129,91,154,120,30,19,245,74,3,12,240,120
HHATL_2_8667,132,232,84,178,302,143,0,2,14,496,324,67
HIBADH_2_8668,746,499,350,565,292,338,194,525,343,1348,43,650
HINT1_2_8669,150,231,129,144,318,145,2,203,44,110,651,195
HMOX1_2_8670,2,9,15,0,0,12,14,0,0,2,0,0
HPGDS_2_8671,863,604,534,545,576,215,798,822,426,248,564,962
HPRT1_2_8672,483,509,613,548,361,369,439,221,83,752,487,1016

HS3ST1_2_8673,168,224,291,261,106,84,430,121,217,299,88,188
HS3ST2_2_8674,1333,1588,969,1218,1441,653,1721,833,728,1922,791,1506
HS3ST3A1_2_8675,68,107,123,109,22,160,7,54,17,184,137,92
HS3ST3B1_2_8676,4781,3877,3602,4646,2504,4018,3474,3299,3479,3205,3434
,5220
HS3ST4_2_8677,681,639,415,560,959,466,585,462,683,76,487,768
HS3ST6_2_8678,1917,1233,1279,1266,622,1205,1743,1667,1452,931,950,1661
HS6ST1_2_8679,244,187,175,267,164,503,382,148,257,20,388,418
HS6ST3_2_8680,79,111,65,146,31,21,0,22,29,425,612,32
HSD11B2_2_8681,150,155,185,307,91,5,114,55,193,97,175,51
HSD17B11_2_8682,1378,1327,1421,1533,614,573,2097,1280,1312,1613,1185,9
88
HSD17B12_2_8683,569,833,498,826,798,977,972,409,454,508,489,586
HSD17B14_2_8684,367,358,274,426,437,395,451,13,117,0,451,404
HSD17B1_2_8685,114,93,64,56,27,31,1,6,70,20,49,62
HSD17B2_2_8686,80,26,75,105,2,234,52,0,25,17,0,25
HSD17B3_2_8687,146,56,112,264,494,9,49,315,147,0,253,0
HSD17B6_2_8688,698,699,815,810,579,1454,669,436,718,512,1287,836
HSD17B8_2_8689,154,34,55,101,0,3,0,1,54,12,2,46
HSD3B1_2_8690,299,398,387,375,349,167,392,140,47,223,913,462
HSP90AB1_2_8691,210,198,180,246,430,121,98,358,114,37,352,232
HSPA5_2_8692,71,76,17,107,125,74,1,1,27,349,401,193
HSPE1_2_8693,361,242,280,265,895,216,430,93,724,286,5,757
HSPG2_2_8694,146,128,135,202,132,46,15,619,13,13,45,167
HYAL4_2_8695,1089,1144,837,1077,925,470,1241,1213,1143,1701,827,1123
IARS2_2_8696,415,318,388,459,188,56,244,85,41,397,5,250
ICMT_2_8697,280,199,258,245,290,134,221,216,179,193,0,346
ICT1_2_8698,169,112,140,199,9,0,639,2,201,338,1,83
IDH1_2_8699,327,260,356,569,226,197,1770,921,392,303,181,263
IDH2_2_8700,141,148,94,104,37,0,105,54,47,40,114,18
IDH3A_2_8701,1774,1515,1390,1611,855,1103,2563,1671,1339,1526,1561,284
0
IDI1_2_8702,1123,1031,1066,1247,537,827,2110,521,772,1391,1569,1490
IDI2_2_8703,344,241,299,466,562,572,243,938,589,375,32,199
IDO1_2_8704,236,243,332,303,250,536,31,23,8,55,871,8
IDO2_2_8705,162,344,197,150,78,1070,247,473,115,444,10,135
IDUA_2_8706,111,49,122,88,14,5,36,65,71,72,357,18
IFI30_2_8707,255,387,273,354,319,83,100,182,574,617,395,423
IFIH1_2_8708,32,48,59,96,9,0,660,1,0,0,0,0
IGHMBP2_2_8709,849,644,673,627,871,317,305,1020,994,797,684,677
IHH_2_8710,185,132,167,220,185,196,398,130,36,265,789,90
ILVBL_2_8711,330,331,333,479,29,2,909,142,214,606,809,452
IMPAD1_2_8712,609,658,637,814,264,1393,913,736,695,259,1685,1207
IMPDH2_2_8713,108,120,138,203,0,117,136,0,181,4,158,310
IRS1_2_8714,586,394,407,627,799,60,1014,215,649,335,748,101
IRS2_2_8715,110,78,186,121,52,59,513,121,57,68,34,209
ISG20_2_8716,34,26,33,50,12,0,47,0,29,0,1,0
ISG20L2_2_8717,794,577,665,657,700,408,1116,483,492,759,53,926
ISOC1_2_8718,4,78,82,54,0,0,16,0,0,40,0,1
IWS1_2_8719,163,161,133,243,311,174,333,4,57,374,338,175

KDSR_2_8720,346,171,195,259,42,1,830,83,37,71,28,70
KHSRP_2_8721,595,498,457,540,1041,1271,1636,621,229,931,499,433
KIAA0317_2_8722,55,100,41,81,99,13,8,10,466,3,38,27
KIAA1279_2_8723,210,123,120,117,6,29,152,52,231,2,83,238
KIAA2022_2_8724,775,936,787,857,877,1243,1508,695,1202,1795,906,925
KIF18A_2_8725,867,589,930,754,392,206,1038,1319,1015,312,3043,974
KIF20B_2_8726,724,707,576,774,1016,1339,1087,666,417,566,860,1270
KIF3A_2_8727,914,772,829,866,548,1194,279,762,922,355,1008,639
KLB_2_8728,192,153,121,150,367,62,223,4,246,362,106,188
KLC3_2_8729,387,441,346,402,595,218,509,802,464,514,292,388
KL_2_8730,350,298,293,335,645,117,294,1,750,82,134,233
KLK1_2_8731,343,226,423,392,609,706,272,27,412,84,11,768
KLKB1_2_8732,662,861,670,941,449,701,732,1117,222,1027,250,1790
KMO_2_8733,843,627,606,1031,634,222,891,649,130,76,1792,851
KRTCAP2_2_8734,354,487,602,776,185,677,116,123,180,446,1409,730
L2HGDH_2_8735,193,162,123,285,388,264,130,36,275,207,97,27
LALBA_2_8736,389,331,298,390,590,212,305,223,198,753,93,292
LAP3_2_8737,378,307,535,611,643,283,588,168,253,249,248,1453
LARS2_2_8738,69,23,61,127,1,0,135,200,2,77,39,27
LARS_2_8739,885,1109,1233,1609,664,524,1142,2196,1180,1154,1140,1716
LCAT_2_8740,131,66,115,86,159,16,285,120,111,19,56,51
LCMT2_2_8741,214,208,178,170,35,85,310,857,194,38,27,146
LCT_2_8742,114,91,138,105,40,73,63,179,0,0,414,2
LCTL_2_8743,245,226,212,151,472,141,28,332,111,46,3,285
LDHAL6B_2_8744,1120,1230,723,1122,1036,590,1243,1187,666,932,826,1435
LEPREL2_2_8745,100,7,27,42,11,0,37,1,252,1,0,28
LGALS13_2_8746,522,364,527,407,843,108,123,367,297,463,723,423
LHFPL2_2_8747,121,242,309,404,13,157,496,0,252,256,0,1188
LIPC_2_8748,107,89,168,124,101,0,1,0,155,376,215,8
LIPE_2_8749,2279,2295,2064,2235,2582,2450,2282,3351,3216,2626,1778,165
7
LIPG_2_8750,68,103,186,47,193,706,0,42,38,200,0,0
LIPH_2_8751,438,599,410,421,400,440,147,498,549,98,926,332
LIPI_2_8752,253,269,222,258,0,617,0,688,500,617,289,427
LIPM_2_8753,86,30,12,28,56,10,0,27,7,43,15,8
LIPN_2_8754,0,0,0,0,0,0,0,0,0,0,0,0
LIPT2_2_8755,4,18,0,45,0,0,0,1,0,0,0,125
LONP2_2_8756,427,330,385,376,505,273,1520,522,237,1204,602,676
LOXL1_2_8757,267,138,226,235,485,26,636,139,46,513,241,441
LOXL2_2_8758,307,268,308,320,80,158,446,49,58,47,615,180
LOXL3_2_8759,312,178,205,126,70,491,10,284,460,17,62,727
LOXL4_2_8760,227,174,179,260,44,375,588,58,256,232,312,209
LPCAT1_2_8761,25,21,52,38,9,3,4,86,53,45,81,2
LPCAT2_2_8762,105,10,44,67,0,99,5,9,0,29,0,33
LPCAT3_2_8763,536,531,435,554,32,600,752,144,285,174,180,87
LPCAT4_2_8764,312,238,189,445,65,250,30,182,285,251,282,287
LPIN1_2_8765,1212,802,710,945,816,1819,941,911,536,486,1104,1544
LPIN2_2_8766,253,217,222,312,430,189,470,96,469,309,275,284
LPIN3_2_8767,422,192,311,285,6,2,38,32,433,273,0,209
LPL_2_8768,155,131,93,172,26,176,64,68,236,0,52,194

LRAT_2_8769,447,472,474,442,653,282,213,1354,131,531,375,411
LTA4H_2_8770,581,538,682,828,1785,131,1466,1170,925,855,658,1203
LTC4S_2_8771,183,194,213,242,861,11,40,183,105,224,6,145
LYG2_2_8772,231,205,329,384,504,96,19,907,605,126,351,123
LYPLA1_2_8773,639,651,533,908,603,222,1055,922,1027,931,523,171
LYPLA2_2_8774,506,394,384,559,394,14,487,1,791,591,442,403
LYPLAL1_2_8775,480,609,434,457,744,1029,688,265,579,600,937,740
LYZ_2_8776,76,7,42,44,5,13,56,0,5,0,4,12
LYZL1_2_8777,203,180,104,242,234,422,2,514,178,338,137,527
LYZL2_2_8778,203,180,104,242,234,422,2,514,178,338,137,527
LYZL4_2_8779,140,139,125,30,452,84,53,1,140,76,0,17
MACROD1_2_8780,15,3,0,15,0,269,0,0,0,0,0,0
MAGT1_2_8781,241,254,391,345,308,883,460,737,196,258,98,499
MAN1A1_2_8782,87,78,81,60,25,437,5,119,60,18,368,249
MAN1A2_2_8783,147,202,156,270,127,219,277,83,129,182,53,278
MAN1B1_2_8784,310,211,160,425,18,10,17,87,894,335,484,98
MAN1C1_2_8785,259,527,294,305,208,423,666,340,353,318,75,314
MAN2A1_2_8786,1151,840,683,844,456,271,236,765,379,536,2443,842
MAN2A2_2_8787,148,66,62,80,816,19,459,71,362,2,21,19
MAN2B2_2_8788,151,89,145,110,266,15,372,82,420,253,502,21
MAN2C1_2_8789,61,34,81,146,128,2,1,19,145,9,0,187
MANBA_2_8790,436,468,264,452,568,293,307,856,370,531,570,367
MANEA_2_8791,696,398,524,572,301,288,890,93,259,699,422,201
MAOA_2_8792,134,82,110,77,441,58,195,45,151,63,731,63
MAOB_2_8793,0,0,9,1,0,0,0,0,0,0,0,0
MAP1S_2_8794,258,174,155,476,535,189,13,306,143,3,341,14
MAPRE3_2_8795,129,229,177,128,57,59,292,595,187,64,238,235
MARS2_2_8796,155,67,73,183,55,95,0,100,60,12,262,63
MARS_2_8797,109,125,136,161,213,26,1,120,76,238,0,140
MAT1A_2_8798,49,63,47,57,89,0,72,6,32,2,48,52
MAT2A_2_8799,149,150,139,148,274,259,320,9,16,412,65,7
MBOAT1_2_8800,388,479,368,433,707,278,273,284,355,725,217,524
MBOAT2_2_8801,54,11,23,16,5,0,41,0,1,0,14,1
MBOAT4_2_8802,106,130,140,106,205,76,75,108,65,138,20,28
MBTPS1_2_8803,660,767,442,609,340,131,991,457,283,543,320,244
MBTPS2_2_8804,1182,1013,854,1086,1566,530,656,979,509,1613,436,1346
MCCC1_2_8805,192,441,171,194,924,84,181,408,70,207,0,193
MCCC2_2_8806,166,27,39,58,29,0,21,109,691,48,0,17
MCEE_2_8807,632,664,618,609,322,55,997,92,361,421,127,105
MCM3_2_8808,213,208,226,208,378,120,216,247,16,120,7,15
MCM5_2_8809,48,88,19,32,2,0,6,197,4,9,1,13
MCM6_2_8810,2298,2387,2432,2580,1374,948,2573,1011,3164,1582,2447,2371
MDH1B_2_8811,873,885,537,849,700,423,1196,462,1675,1267,1276,949
MDH2_2_8812,325,219,319,398,15,54,100,540,130,467,37,294
MEP1A_2_8813,309,203,283,533,403,313,109,627,129,451,85,690
MEP1B_2_8814,720,610,498,534,579,752,157,726,703,982,419,888
METAP1_2_8815,341,289,217,308,35,638,120,101,335,169,533,103
METAP1D_2_8816,397,303,325,234,16,2,260,154,449,418,36,343
METAP2_2_8817,480,300,350,425,318,335,45,126,298,335,611,362
METTL14_2_8818,92,137,169,111,190,0,487,0,138,163,105,107

METTL22_2_8819,186,197,137,133,548,1,485,527,21,456,5,0
METTL2B_2_8820,31,42,67,59,37,160,26,16,28,120,0,35
METTL3_2_8821,345,169,266,328,160,86,210,646,667,520,698,669
METTL5_2_8822,460,320,127,438,109,61,17,5,145,291,86,497
METTL6_2_8823,57,185,161,194,158,259,0,197,281,175,46,32
METTL7B_2_8824,91,47,137,111,217,0,0,89,1,0,412,238
METTL8_2_8825,23,7,23,4,0,136,0,0,4,3,0,12
MFN1_2_8826,427,420,390,415,226,743,661,410,243,202,144,215
MGAM_2_8827,391,339,434,508,138,318,171,301,343,339,368,865
MGAT4C_2_8828,0,0,0,0,0,0,0,0,0,0,0,0
MGAT5_2_8829,1311,1428,1333,1289,1107,1441,968,1445,1852,1198,1515,140
4
MGMT_2_8830,109,312,256,254,121,405,297,349,542,435,336,454
MGST3_2_8831,915,886,812,711,556,96,1051,549,789,951,638,157
MIOX_2_8832,62,207,152,83,0,0,0,222,140,10,0,0
MIPEP_2_8833,354,208,315,300,42,127,380,208,292,843,495,176
MLYCD_2_8834,422,499,418,577,107,564,675,1161,328,960,686,634
MMAB_2_8835,335,370,337,496,162,267,239,578,199,292,815,264
MMEL1_2_8836,91,97,110,44,400,37,27,4,18,59,160,7
MMP10_2_8837,76,41,53,65,39,48,595,50,31,53,77,59
MMP12_2_8838,395,243,306,494,911,750,648,942,602,155,51,424
MMP14_2_8839,101,280,190,145,577,3,331,39,14,0,59,216
MMP3_2_8840,909,982,954,1205,902,969,1748,795,1273,962,671,505
MMP7_2_8841,283,156,325,323,717,161,0,530,311,406,59,466
MMP8_2_8842,655,493,465,501,129,147,850,107,710,191,275,983
MMP9_2_8843,281,460,285,299,521,102,687,121,544,418,148,574
MOCOS_2_8844,128,160,126,226,128,39,134,4,87,216,21,287
MOGAT1_2_8845,361,452,253,413,391,228,480,405,261,161,735,325
MOGAT2_2_8846,92,168,97,191,102,90,262,39,63,360,2,157
MOGAT3_2_8847,155,202,154,138,33,18,249,34,123,169,86,153
MOXD1_2_8848,1506,1336,1586,1312,1050,1493,2720,994,1882,2210,971,1229
MPI_2_8849,2,24,15,72,0,468,0,0,0,0,0,636
MPO_2_8850,134,84,147,57,0,269,0,0,0,37,68,17
MRPL37_2_8851,12,7,14,11,0,3,0,0,0,229,0,10
MRPL44_2_8852,969,780,808,1041,1263,581,790,624,736,907,706,912
MRPS30_2_8853,252,267,247,238,237,16,440,3,293,948,401,141
MSH2_2_8854,402,297,310,424,194,42,20,313,779,374,133,1216
MSH3_2_8855,260,180,235,184,133,43,22,0,10,93,425,184
MSH4_2_8856,319,317,268,258,197,249,1199,97,331,104,86,155
MTAP_2_8857,450,215,291,362,332,510,495,269,456,185,29,182
MTFMT_2_8858,215,322,143,211,723,0,367,312,149,1269,531,686
MTHFD1_2_8859,444,404,267,476,711,93,144,706,266,289,105,359
MTHFD2_2_8860,225,358,129,212,298,135,660,174,89,143,414,70
MTHFD2L_2_8861,775,844,690,959,662,541,1033,457,701,670,720,706
MTHFR_2_8862,74,46,102,108,102,138,582,21,128,99,484,74
MTPAP_2_8863,334,274,327,394,176,208,39,153,446,805,340,66
MTR_2_8864,172,206,224,219,241,51,135,93,15,162,78,417
MUS81_2_8865,349,346,279,335,82,14,520,491,305,545,930,472
MUT_2_8866,258,182,93,169,73,105,14,58,92,55,19,140
MVD_2_8867,109,298,240,306,374,126,378,264,38,589,78,221

MX2_2_8868,592,669,775,740,779,1018,604,948,478,1265,173,538
MYH1_2_8869,497,324,410,588,88,162,413,382,576,287,681,284
MYH3_2_8870,135,103,92,118,72,596,464,74,123,54,0,243
MYH6_2_8871,375,289,279,197,604,317,531,183,210,204,482,76
MYH7_2_8872,329,268,203,145,281,309,185,189,213,202,0,75
MYH9_2_8873,219,216,233,198,168,227,1115,291,45,576,156,184
MYL7_2_8874,201,277,217,187,292,255,0,310,6,0,446,133
MYO1E_2_8875,180,222,135,166,71,5,338,43,66,405,179,97
MYO5B_2_8876,568,366,289,461,369,271,565,1147,367,201,20,551
MYO9A_2_8877,1238,1216,1053,1427,1498,794,1745,1400,1610,1391,2123,151
4
N6AMT2_2_8878,2057,1616,1465,2057,2135,1577,1283,1372,1546,1692,1223,1
884
NAA10_2_8879,355,339,338,374,122,661,2,33,574,430,141,523
NAA11_2_8880,466,602,403,447,391,208,472,923,734,755,1411,854
NAA15_2_8881,907,807,759,1245,1290,1077,784,880,1549,801,237,814
NAA30_2_8882,268,254,211,230,492,199,626,301,324,130,224,325
NAA50_2_8883,263,226,174,224,315,711,0,1,240,242,137,397
NAALAD2_2_8884,655,508,428,570,279,1403,293,392,114,940,463,261
NAALADL1_2_8885,223,116,154,173,114,22,436,760,56,107,223,69
NADSYN1_2_8886,59,13,54,32,21,0,41,66,111,3,0,248
NAGA_2_8887,226,150,104,304,741,137,397,125,459,104,181,354
NAGLU_2_8888,132,49,66,111,21,193,46,140,71,61,145,132
NAGPA_2_8889,137,97,61,158,0,0,0,457,0,0,0,2
NAMPT_2_8890,475,306,274,536,503,424,165,40,389,97,55,463
NANP_2_8891,116,82,150,83,295,13,114,0,20,0,0,164
NANS_2_8892,190,299,264,484,279,12,0,29,0,431,362,367
NAPRT1_2_8893,160,320,78,144,530,129,73,569,107,283,316,12
NARS_2_8894,2822,1867,2303,2344,3555,1657,3276,2143,2768,1867,2628,281
5
NCF1_2_8895,78,15,40,44,0,0,0,0,4,0,0,1
NDST1_2_8896,138,185,195,213,31,213,170,0,129,39,436,305
NDST2_2_8897,271,284,249,413,339,103,351,399,195,239,298,251
NDST3_2_8898,62,31,135,112,292,44,59,0,0,27,0,51
NDST4_2_8899,925,739,681,796,399,628,1023,1281,1095,395,1007,1004
NDUFA10_2_8900,132,136,242,190,3,0,0,0,1,70,588,100
NDUFA12_2_8901,463,406,255,412,473,264,508,229,1236,1560,880,377
NDUFA13_2_8902,144,125,66,98,32,15,9,115,56,56,1,9
NDUFA1_2_8903,661,566,513,509,1315,191,624,438,503,339,81,1115
NDUFA3_2_8904,40,44,47,38,455,0,150,75,0,45,311,0
NDUFA4_2_8905,31,50,36,47,17,5,3,0,1,191,0,73
NDUFA4L2_2_8906,805,845,847,921,1224,764,1350,691,638,607,647,955
NDUFA5_2_8907,1848,1336,1451,1596,1328,1895,1095,708,1451,2253,1607,12
65
NDUFA6_2_8908,42,11,58,75,1,8,388,2,0,22,640,0
NDUFA7_2_8909,151,71,130,114,87,9,297,51,29,32,8,134
NDUFA8_2_8910,110,119,44,54,10,111,57,0,229,133,0,251
NDUFA9_2_8911,6,3,2,0,37,0,0,0,0,76,0,5
NDUFAB1_2_8912,47,113,77,95,7,8,605,104,134,168,274,28
NDUFB10_2_8913,262,206,108,194,796,139,43,48,426,85,9,144

NDUFB1_2_8914,523,336,373,583,453,12,14,506,504,796,266,832
NDUFB2_2_8915,277,127,195,216,539,210,257,31,172,4,929,219
NDUFB3_2_8916,606,472,503,616,680,1279,983,1122,655,502,711,1158
NDUFB7_2_8917,28,0,20,1,77,69,1,143,0,0,0,0
NDUFB8_2_8918,280,277,423,380,214,9,843,192,103,539,664,104
NDUFB9_2_8919,680,496,595,696,918,975,329,813,604,553,1056,947
NDUFS3_2_8920,334,424,315,406,547,476,519,167,285,616,143,218
NDUFS4_2_8921,295,285,182,245,60,268,943,17,77,21,664,83
NDUFS6_2_8922,0,0,0,15,0,0,0,0,0,0,0,0
NDUFS7_2_8923,59,96,90,124,203,108,68,112,75,24,556,150
NDUFS8_2_8924,785,817,626,749,884,232,30,805,1419,1020,317,1162
NDUFV2_2_8925,114,68,39,63,0,0,244,0,120,0,0,123
NEDD8_2_8926,213,301,299,489,492,346,435,15,372,10,508,614
NEIL1_2_8927,125,130,192,164,93,104,4,15,258,134,9,245
NEIL3_2_8928,516,428,568,718,342,156,1881,1104,239,782,503,772
NEU1_2_8929,551,510,295,441,455,846,117,547,1383,680,305,459
NEU2_2_8930,76,130,91,142,122,186,243,428,75,1,48,54
NEU3_2_8931,59,121,138,88,58,20,76,14,4,61,0,155
NHLRC2_2_8932,929,825,882,975,1306,1276,1777,881,758,787,902,921
NIT2_2_8933,954,983,937,1057,1042,245,1297,696,1154,939,1396,986
NKIRAS1_2_8934,1694,1788,1465,1873,1254,2578,1283,951,1295,1656,1110,1
659
NLGN1_2_8935,852,541,805,784,197,611,1381,514,386,751,993,839
NLGN2_2_8936,23,111,46,24,0,13,25,3,0,21,0,152
NLN_2_8937,499,484,613,535,21,504,101,99,320,826,469,860
NME1-NME2_2_8938,1085,696,908,1173,1314,98,915,473,512,1480,41,1190
NMNAT1_2_8939,0,0,0,0,0,0,0,0,0,0,0,0
NMT1_2_8940,69,28,62,54,65,0,64,79,118,26,154,104
NMT2_2_8941,30,138,24,99,8,0,0,0,10,323,0,184
NNMT_2_8942,89,73,114,24,55,5,0,1,155,0,0,79
NOP58_2_8943,839,594,590,717,601,590,672,347,170,966,48,774
NOX3_2_8944,1262,873,847,830,1476,608,1193,627,1021,1363,673,895
NQO2_2_8945,1480,1563,1534,1672,1944,1027,2234,926,1009,825,2114,925
NRAS_2_8946,503,712,890,713,459,85,683,474,127,91,2217,1142
NSF_2_8947,243,199,199,297,408,17,644,457,432,565,499,381
NSUN6_2_8948,539,358,509,342,324,215,464,180,616,334,1520,960
NTAN1_2_8949,152,164,105,114,0,516,262,34,16,14,280,59
NTHL1_2_8950,332,331,238,530,360,191,489,317,141,328,301,274
NTPCR_2_8951,1988,1377,1351,2039,1100,1189,1733,1841,1251,1168,952,127
4
NUDT10_2_8952,2407,2165,1899,2255,1771,1965,1547,3078,2223,924,1944,22
70
NUDT11_2_8953,69,66,88,68,19,33,834,43,28,265,273,202
NUDT12_2_8954,2839,2524,2214,2789,2308,1521,2849,2147,1447,2195,2134,2
409
NUDT14_2_8955,1148,1003,936,900,925,1270,798,424,2404,524,1757,1100
NUDT3_2_8956,115,53,96,92,0,4,134,2,70,37,186,37
NUDT5_2_8957,628,448,741,540,296,544,92,336,544,373,1688,459
NUDT7_2_8958,944,817,956,895,318,640,868,294,1831,1319,817,706
NXNL1_2_8959,838,1162,613,1183,878,766,521,633,498,1073,396,731

OAS3_2_8960,336,100,209,201,164,252,276,412,285,28,424,626
OAZ1_2_8961,111,178,192,144,49,161,36,868,3,399,247,197
OC90_2_8962,0,0,0,0,0,0,0,0,0,0,0,0
ODF3B_2_8963,15,43,93,4,0,0,0,0,0,0,362,0
OPLAH_2_8964,53,17,35,42,14,146,1,0,0,246,186,5
OSGEP_2_8965,377,321,341,341,652,390,519,576,72,396,503,407
OSGEPL1_2_8966,229,252,138,308,190,3,573,566,10,311,298,2
OSTC_2_8967,264,173,256,229,93,242,347,25,763,235,163,11
OTC_2_8968,256,361,332,416,295,58,37,126,66,746,872,434
OVGP1_2_8969,86,73,97,156,399,0,6,414,24,6,79,74
OXA1L_2_8970,92,198,171,207,23,27,0,346,202,305,72,199
OXCT1_2_8971,167,121,179,215,255,189,415,200,50,83,52,201
OXCT2_2_8972,249,362,304,374,7,231,31,665,672,140,528,522
P4HA3_2_8973,41,36,70,36,0,23,10,55,3,110,0,2
P4HB_2_8974,274,241,129,298,361,606,205,371,140,410,5,463
PADI1_2_8975,72,35,78,30,166,12,146,43,8,4,0,7
PADI2_2_8976,392,336,369,279,504,404,0,553,70,107,261,506
PADI3_2_8977,698,738,601,912,804,532,8,1201,198,557,742,1191
PADI6_2_8978,315,406,303,309,1144,499,110,263,678,294,80,442
PAFAH2_2_8979,1417,1330,1238,1619,913,1581,2223,1608,885,1102,1077,151
3
PAH_2_8980,304,233,322,280,398,355,359,188,391,343,51,15
PAPOLA_2_8981,30,108,90,114,1,0,0,350,80,78,387,2
PAPOLB_2_8982,358,318,374,343,526,422,181,425,193,64,240,529
PAPOLG_2_8983,652,728,481,796,1260,437,1023,153,683,1054,1481,971
PAPPA_2_8984,280,378,112,388,250,267,97,2,266,508,460,387
PAPSS1_2_8985,65,85,308,116,249,0,0,0,0,264,302,207
PARG_2_8986,630,810,862,843,612,301,1504,65,557,992,1554,1105
PARP1_2_8987,135,77,136,125,25,64,429,18,118,45,0,63
PARP4_2_8988,612,690,687,695,904,146,945,802,612,516,538,790
PARP6_2_8989,253,224,133,80,401,39,2,7,145,1028,123,182
PARS2_2_8990,169,275,124,136,106,58,18,28,46,84,231,10
PCBD1_2_8991,112,299,275,281,161,159,15,628,170,293,650,268
PCBD2_2_8992,0,0,0,0,0,0,0,0,0,0,0,0
PCMT1_2_8993,286,199,251,211,194,0,152,535,167,189,97,150
PCMTD1_2_8994,454,215,298,258,116,111,258,283,205,115,622,306
PCYOX1_2_8995,29,43,18,22,1,0,116,0,28,15,0,25
PCYT1A_2_8996,486,294,373,495,634,548,745,305,428,498,5,921
PDCD1LG2_2_8997,430,325,358,411,159,195,377,143,334,142,79,1037
PDCL_2_8998,104,134,134,99,171,113,482,191,149,50,55,55
PDE3A_2_8999,178,192,203,195,138,481,191,260,169,456,0,179
PDE3B_2_9000,695,691,768,697,1006,537,923,117,338,518,759,1319
PDE6A_2_9001,169,232,156,360,340,0,81,28,272,3,904,203
PDE6C_2_9002,475,332,372,417,720,425,629,463,92,150,31,814
PDE6D_2_9003,0,0,0,6,0,0,0,0,0,0,0,0
PDE6G_2_9004,98,118,81,152,133,779,418,5,1,0,455,0
PDE6H_2_9005,484,267,240,407,52,216,293,1415,405,917,0,215
PDE7B_2_9006,479,334,307,483,798,569,320,537,368,112,9,669
PDHA2_2_9007,1039,940,813,952,283,1206,1006,613,817,1172,1076,571
PDIA2_2_9008,759,744,663,1049,583,622,859,512,851,1116,1233,856

PDIA3_2_9009,979,978,1197,1228,371,1337,1202,1860,553,375,1331,1350
PDIA4_2_9010,898,794,618,831,320,530,260,914,847,533,612,598
PDIA5_2_9011,459,272,321,370,497,845,592,598,188,226,101,590
PDIA6_2_9012,53,137,87,97,0,0,2,18,136,222,200,331
PDPR_2_9013,180,157,161,194,598,31,166,139,162,249,82,92
PDSS1_2_9014,293,228,223,297,300,17,0,308,287,610,176,256
PDSS2_2_9015,107,100,81,157,7,82,207,71,29,2,0,8
PDXP_2_9016,92,44,105,100,25,93,223,298,97,5,58,7
PECR_2_9017,160,157,251,140,263,64,206,54,161,0,426,35
PELI1_2_9018,612,501,541,649,436,85,754,741,679,845,223,847
PET117_2_9019,1737,1604,1639,1762,1141,2752,1168,1204,2055,1987,1629,1
495
PEX1_2_9020,2198,1561,1696,2158,1468,1279,2807,1883,2037,2889,685,2302
PEX6_2_9021,183,119,138,97,600,58,334,127,538,0,7,33
PFAS_2_9022,510,418,429,533,372,630,105,655,625,320,2654,961
PGA3_2_9023,2544,2200,1862,2436,3045,2098,1597,1385,1411,2793,3455,243
5
PGA4_2_9024,2544,2200,1862,2436,3045,2098,1597,1385,1411,2793,3455,243
5
PGA5_2_9025,2544,2200,1862,2436,3045,2098,1597,1385,1411,2793,3455,243
5
PGAM4_2_9026,196,314,152,239,195,197,73,9,172,213,283,128
PGAP1_2_9027,610,798,511,636,735,836,474,376,495,500,400,653
PGAP3_2_9028,111,134,200,139,73,242,21,0,193,190,620,42
PGD_2_9029,272,101,195,234,419,232,556,266,267,16,263,108
PGGT1B_2_9030,47,66,7,39,55,205,82,0,0,9,15,9
PGLS_2_9031,87,121,154,165,460,148,1,127,13,456,0,84
PGM2_2_9032,154,246,163,214,315,61,462,101,283,663,0,112
PGM2L1_2_9033,848,689,697,846,1389,298,681,413,334,458,480,894
PGM5_2_9034,327,415,291,368,220,349,0,698,402,28,295,78
PGPEP1_2_9035,110,55,35,60,36,28,350,13,0,1,19,2
PGS1_2_9036,293,338,249,438,175,637,201,131,157,187,58,633
PHGDH_2_9037,245,270,216,233,73,263,1195,310,314,274,242,139
PHLPP1_2_9038,347,393,534,588,205,264,929,410,181,133,375,70
PHLPP2_2_9039,606,431,312,434,411,198,734,460,491,349,130,483
PIF1_2_9040,165,122,104,160,208,170,361,32,23,84,1,78
PIGB_2_9041,329,146,91,331,495,1007,0,418,0,355,0,11
PIGH_2_9042,342,280,176,338,18,5,37,348,59,460,441,454
PIGL_2_9043,120,91,52,95,215,0,6,67,20,113,0,273
PIGM_2_9044,181,167,104,186,539,47,0,130,575,131,463,121
PIGS_2_9045,439,453,539,574,669,382,845,466,785,86,99,600
PIGU_2_9046,1012,763,885,881,788,616,840,1097,795,831,160,623
PIGW_2_9047,232,142,167,283,438,297,205,94,103,658,280,81
PIGZ_2_9048,1072,1091,914,1301,800,447,423,1146,821,1375,1236,1498
PIN1_2_9049,64,42,30,103,96,236,0,0,70,277,393,38
PIPOX_2_9050,70,81,71,109,18,385,31,0,18,81,1,398
PITPNM2_2_9051,18,2,0,21,0,50,0,0,0,0,174,27
PLA2G10_2_9052,425,441,604,662,13,4,260,278,538,599,1124,971
PLA2G12A_2_9053,291,224,407,326,128,198,108,294,196,1337,412,85
PLA2G12B_2_9054,477,255,311,264,372,199,422,132,382,484,55,164

PLA2G15_2_9055,417,500,265,648,1147,688,173,128,168,281,153,682
PLA2G1B_2_9056,51,69,21,13,0,0,0,52,8,0,0,3
PLA2G2C_2_9057,191,128,138,115,170,21,21,157,117,303,146,329
PLA2G2D_2_9058,78,66,54,114,1,92,37,0,18,57,14,19
PLA2G2E_2_9059,342,370,293,354,504,315,613,312,479,592,470,346
PLA2G2F_2_9060,229,212,179,409,112,139,232,310,162,371,126,284
PLA2G3_2_9061,35,40,29,69,99,49,0,0,157,0,0,0
PLA2G4A_2_9062,849,1222,805,1078,821,1086,484,237,1015,1139,722,1378
PLA2G4B_2_9063,686,557,413,598,664,44,473,648,261,499,351,772
PLA2G4D_2_9064,236,191,106,146,254,85,108,26,145,40,26,188
PLA2G4E_2_9065,1268,1075,885,1198,999,324,1194,1357,1019,822,1225,868
PLA2G4F_2_9066,66,28,34,64,1,0,1,109,0,4,1,202
PLA2G5_2_9067,152,256,175,377,570,39,0,0,39,0,7,53
PLCB2_2_9068,1045,1182,1122,1261,1217,925,1628,724,1356,1825,1270,879
PLCD3_2_9069,190,159,246,193,144,137,204,598,449,38,15,186
PLCD4_2_9070,158,90,120,217,490,0,545,889,0,52,0,209
PLCG2_2_9071,95,161,123,189,406,37,0,49,271,278,508,134
PLCH2_2_9072,46,24,30,34,175,0,24,4,0,22,0,143
PLCL1_2_9073,1608,1291,1288,1788,2955,1146,1207,840,1411,1214,1109,274
0
PLCXD1_2_9074,85,92,35,97,1,0,0,284,134,0,0,0
PLCZ1_2_9075,957,905,659,1003,757,656,716,1875,848,721,671,956
PLD4_2_9076,132,62,164,138,49,45,199,12,116,152,12,64
PLOD1_2_9077,49,32,9,28,58,6,37,6,34,0,0,10
PLOD3_2_9078,145,107,15,45,2,0,0,87,233,91,0,0
PLSCR1_2_9079,1084,948,1149,1325,1361,366,1312,669,966,688,1267,1271
PMM1_2_9080,649,469,492,527,1178,548,1448,222,574,407,566,691
PMM2_2_9081,291,383,212,218,718,247,570,274,285,127,224,209
PMPCA_2_9082,56,33,35,26,0,29,1,0,15,1,0,41
PMPCB_2_9083,159,106,106,83,2,74,3,144,0,116,0,30
PNLIP_2_9084,209,212,142,296,108,218,303,75,430,29,434,60
PNLIPRP1_2_9085,1678,1242,1001,1204,1684,1418,2330,734,2251,1840,1052,
1018
PNLIPRP2_2_9086,119,97,49,75,149,20,1,77,156,93,38,107
PNLIPRP3_2_9087,372,123,144,284,298,225,1189,1059,30,416,133,269
PNMT_2_9088,102,135,52,61,40,145,0,62,72,60,22,41
PNPLA2_2_9089,199,447,141,309,69,148,398,227,66,217,81,165
PNPLA3_2_9090,581,354,321,388,1383,175,310,514,736,307,402,151
PNPLA8_2_9091,1874,2034,1478,2038,1174,1782,1093,2446,843,1366,1418,19
02
PNPO_2_9092,294,264,207,257,253,111,19,267,823,3,541,262
PNPT1_2_9093,857,829,756,865,552,606,440,931,459,1244,1209,1603
POLA1_2_9094,686,521,419,651,507,547,256,802,799,171,625,408
POLA2_2_9095,178,118,101,156,120,169,13,40,144,240,6,154
POLB_2_9096,822,730,861,1039,615,329,595,1588,1684,651,2274,252
POLD4_2_9097,370,333,275,368,161,210,350,555,119,440,62,293
POLE3_2_9098,4,15,15,53,0,0,0,105,0,0,0,8
POLE4_2_9099,73,48,87,118,44,26,168,12,289,158,387,135
POLE_2_9100,479,526,440,615,187,260,831,4,751,665,303,634
POLG2_2_9101,124,106,55,143,41,50,0,101,344,414,140,307

POLI_2_9102,29,7,37,68,1,1,0,2,0,0,0,0
POLM_2_9103,255,233,170,172,12,222,47,469,288,567,7,124
POLN_2_9104,1329,1347,1068,1398,1131,452,1224,829,1565,820,1377,996
POLR1A_2_9105,423,617,452,682,404,4,94,64,46,549,948,751
POLR1C_2_9106,241,347,298,352,144,8,6,681,39,368,458,392
POLR1E_2_9107,115,241,233,180,0,44,0,6,138,73,157,423
POLR2A_2_9108,268,225,348,199,47,0,1002,70,94,547,621,199
POLR2B_2_9109,173,117,123,132,135,282,477,699,5,55,292,85
POLR2C_2_9110,282,191,208,229,80,34,1651,214,133,260,198,241
POLR2D_2_9111,109,123,83,224,8,4,19,361,36,1,45,323
POLR2E_2_9112,121,151,142,135,259,260,181,28,63,0,75,30
POLR2F_2_9113,194,94,106,195,26,237,315,135,166,10,286,183
POLR2G_2_9114,419,373,473,639,197,91,312,598,591,103,1123,346
POLR2H_2_9115,525,516,547,435,285,583,368,203,980,762,309,216
POLR2J_2_9116,704,304,362,397,322,220,371,440,723,93,410,1485
POLR2K_2_9117,487,538,573,683,392,319,925,651,542,370,808,500
POLR2L_2_9118,609,557,448,588,306,594,968,190,199,573,389,1176
POLR3A_2_9119,105,108,67,106,20,43,16,202,245,20,0,29
POLR3C_2_9120,288,390,332,262,170,669,23,236,176,631,392,521
POLR3F_2_9121,797,723,619,780,957,986,1020,291,846,332,346,651
POLR3G_2_9122,452,333,403,399,174,218,494,103,265,122,264,195
POLRMT_2_9123,4,88,5,28,43,2,1,0,8,2,0,0
POMGNT1_2_9124,615,424,324,582,565,753,518,212,392,590,84,574
POMT2_2_9125,713,810,1005,958,496,640,830,282,593,1054,695,598
PON3_2_9126,423,519,508,650,1296,818,581,1226,1034,224,284,565
POP4_2_9127,451,267,300,514,503,40,83,219,453,103,182,543
POP7_2_9128,193,69,64,121,93,161,0,16,47,11,278,140
POR_2_9129,27,84,33,49,13,453,0,7,33,162,0,137
PPA1_2_9130,128,65,84,61,70,17,16,264,73,100,7,93
PPCDC_2_9131,638,700,741,628,1290,1058,738,1659,462,298,329,822
PPIA_2_9132,983,783,836,902,469,172,1900,718,1692,388,1947,803
PPIAL4A_2_9133,1184,1052,943,1189,978,695,320,586,977,1367,1385,1137
PPIAL4B_2_9134,1184,1052,943,1189,978,695,320,586,977,1367,1385,1137
PPIAL4C_2_9135,1184,1052,943,1189,978,695,320,586,977,1367,1385,1137
PPIAL4E_2_9136,1184,1052,943,1189,978,695,320,586,977,1367,1385,1137
PPIAL4G_2_9137,1184,1052,943,1189,978,695,320,586,977,1367,1385,1137
PPIB_2_9138,456,337,372,373,492,25,202,671,429,360,656,60
PPIC_2_9139,788,831,881,815,864,755,566,1649,380,764,972,1413
PPID_2_9140,300,147,214,142,0,327,369,0,19,514,316,441
PPIF_2_9141,0,0,0,0,0,0,0,0,0,0,0,0
PPIG_2_9142,980,971,707,1167,711,1005,1000,1229,502,812,705,637
PPIH_2_9143,3403,2549,2971,3244,3228,3745,2798,4393,3049,4265,2916,414
6
PPIL1_2_9144,659,660,566,705,70,83,1467,1221,474,336,528,505
PPIL4_2_9145,5732,4545,4898,5618,5158,3146,5766,4807,6592,4829,2565,64
32
PPIP5K2_2_9146,825,846,748,1092,517,561,45,795,753,535,1523,1385
PPM1H_2_9147,1459,1461,1736,1764,1579,2673,2961,2475,1833,1030,1653,23
58
PPM1J_2_9148,768,805,786,721,665,308,126,412,839,984,1298,721

PPM1N_2_9149,461,683,309,564,206,344,1259,149,276,1400,262,598
PPME1_2_9150,108,176,127,183,38,159,50,529,176,609,335,78
PPWD1_2_9151,257,540,276,463,317,1142,730,356,122,967,683,286
PRDX4_2_9152,1670,1527,1439,1752,1325,884,1250,2000,1259,1998,1045,167
7
PREP_2_9153,10,6,25,9,0,0,18,0,50,0,0,18
PRHOXNB_2_9154,0,0,0,0,0,0,0,0,0,0,0,0
PRIM1_2_9155,604,547,752,724,119,1893,660,469,1074,410,659,839
PRIM2_2_9156,88,172,304,305,160,48,440,667,194,365,490,232
PROC_2_9157,29,27,8,15,14,0,144,11,126,40,0,109
PROSC_2_9158,375,351,246,267,660,238,0,567,350,702,432,244
PRR14L_2_9159,392,397,325,313,237,63,338,133,120,827,393,345
PRSS1_2_9160,1130,1003,1009,755,1350,977,472,1191,1624,1556,813,847
PRSS2_2_9161,691,828,767,1049,602,1254,1434,419,509,1148,621,1369
PRTFDC1_2_9162,490,539,319,355,483,286,932,47,93,494,158,553
PRTN3_2_9163,63,42,70,98,9,0,146,221,40,91,0,272
PRUNE_2_9164,320,462,227,418,276,12,270,402,369,371,468,400
PSMA2_2_9165,88,252,285,390,427,12,119,22,134,37,17,3
PSMA6_2_9166,82,101,115,112,40,1,0,0,218,64,52,25
PSMA7_2_9167,259,128,131,184,237,0,312,235,138,0,144,2
PSMB10_2_9168,11,3,37,49,76,1,0,7,2,2,1,182
PSMB11_2_9169,54,145,34,66,0,72,0,182,11,0,107,42
PSMB1_2_9170,153,137,61,80,94,40,119,8,237,0,475,232
PSMB3_2_9171,197,208,225,187,347,156,125,149,94,17,632,436
PSMB4_2_9172,389,358,332,197,177,640,143,46,143,406,7,68
PSMB6_2_9173,1245,1272,1283,1356,850,1637,2456,1436,1276,932,2183,1081
PSMB7_2_9174,388,451,491,481,450,202,1019,653,355,582,470,427
PSMB9_2_9175,339,396,310,422,231,363,114,619,66,240,38,656
PSMD6_2_9176,207,440,324,446,307,314,542,640,288,226,366,637
PTDSS1_2_9177,183,64,156,124,5,176,51,0,293,74,0,122
PTDSS2_2_9178,350,514,468,574,350,121,11,161,482,436,694,802
PTGDS_2_9179,23,141,25,70,23,66,0,38,4,44,0,0
PTGES2_2_9180,47,115,52,47,8,134,67,101,68,62,0,5
PTGES3_2_9181,102,145,119,172,93,161,14,185,115,1,180,127
PTGES_2_9182,70,33,15,30,57,2,42,61,11,33,0,23
PTGIS_2_9183,228,117,229,289,157,269,448,322,583,35,176,518
PTGS2_2_9184,173,213,198,244,1,193,114,181,55,211,12,86
PTRH1_2_9185,142,30,37,68,0,11,28,178,6,1,4,98
PTRH2_2_9186,635,692,733,620,642,451,1294,959,430,453,2445,1198
PTS_2_9187,1247,1159,1270,1698,755,1758,1694,683,807,1353,2523,1603
PUS3_2_9188,352,205,154,305,278,109,208,32,304,307,589,546
PUSL1_2_9189,36,14,26,31,58,0,13,24,0,55,204,15
PXDN_2_9190,278,214,322,226,67,0,786,2,2,246,285,112
PXDNL_2_9191,157,178,139,248,124,79,200,100,39,54,420,163
PYCR2_2_9192,408,356,280,335,292,429,197,262,550,275,289,527
PYCRL_2_9193,22,15,21,20,0,0,36,13,3,0,27,1
PYGB_2_9194,0,0,0,0,0,0,0,0,0,0,0,0
QDPR_2_9195,0,0,0,0,0,0,0,0,0,0,0,0
QPCT_2_9196,82,109,62,149,427,296,15,30,3,53,14,338
QPRT_2_9197,168,114,152,158,48,82,0,394,183,211,8,3

QRSL1_2_9198,182,211,192,116,49,279,410,4,98,4,138,289
QSOX2_2_9199,529,501,677,450,488,284,314,956,345,201,684,686
QTRT1_2_9200,31,8,36,108,72,0,173,259,41,10,0,71
QTRTD1_2_9201,52,77,83,121,364,12,50,4,0,26,7,89
RAB10_2_9202,263,152,167,154,161,0,3,137,210,28,24,200
RAB12_2_9203,620,451,260,375,367,103,296,452,719,1002,229,494
RAB13_2_9204,279,133,107,147,218,0,356,203,73,172,5,123
RAB14_2_9205,2003,1476,1644,2174,2165,718,1864,555,1838,2331,1706,2314
RAB15_2_9206,1061,1027,1123,1205,456,1140,1173,929,716,648,1523,647
RAB17_2_9207,283,268,366,304,393,65,96,404,90,215,717,65
RAB18_2_9208,709,455,445,557,177,665,389,1032,431,763,240,762
RAB19_2_9209,1134,717,995,919,1656,690,521,469,1036,951,886,1050
RAB1B_2_9210,1297,959,961,1115,440,856,1083,670,1792,795,1461,965
RAB20_2_9211,75,67,45,150,1,0,318,0,1,0,23,0
RAB21_2_9212,43,66,79,60,10,71,200,105,66,502,175,151
RAB22A_2_9213,118,202,118,128,242,12,709,64,7,310,479,303
RAB25_2_9214,798,922,585,828,598,149,1104,861,1649,787,911,315
RAB26_2_9215,105,99,102,79,68,46,90,9,156,46,11,90
RAB27B_2_9216,2325,2148,2024,2508,4435,1428,2687,3652,2205,2207,1742,2
462
RAB30_2_9217,1003,969,826,1064,1101,793,313,240,1282,607,257,1307
RAB31_2_9218,1732,1855,1493,2011,1514,842,1423,1013,1453,2177,950,2156
RAB33A_2_9219,32,84,133,89,134,82,185,133,8,235,50,84
RAB33B_2_9220,103,178,38,131,0,1,22,203,0,241,1,2
RAB36_2_9221,516,440,370,450,693,574,70,85,222,818,622,475
RAB38_2_9222,253,374,352,476,48,350,399,172,295,384,83,676
RAB39B_2_9223,1274,1298,1054,1638,813,677,1048,2975,1041,1524,1033,142
0
RAB3A_2_9224,228,209,185,128,310,493,16,225,279,64,24,213
RAB3B_2_9225,4,14,24,35,9,0,377,49,0,0,0,10
RAB3C_2_9226,1600,1528,1679,1707,2722,1649,2171,1094,1969,1434,1197,12
82
RAB3D_2_9227,1102,820,757,1086,690,853,1137,438,923,1012,1257,1038
RAB3GAP2_2_9228,528,751,622,604,886,619,293,380,563,685,83,773
RAB4A_2_9229,453,161,213,316,7,0,0,3,264,0,222,433
RAB4B_2_9230,115,113,120,99,56,54,440,105,258,0,105,72
RAB5A_2_9231,1359,1186,1019,1064,2075,1364,1329,1264,1809,2045,1012,21
27
RAB5B_2_9232,512,369,314,312,215,320,100,524,1050,248,102,707
RAB6B_2_9233,254,258,191,289,272,125,110,218,23,391,142,391
RAB6C_2_9234,62,101,123,82,2,0,152,21,0,14,8,0
RAB7A_2_9235,109,112,93,203,0,6,499,3,186,62,266,567
RAB8B_2_9236,702,630,565,604,793,211,585,1087,557,269,454,775
RAB9B_2_9237,178,98,87,113,38,22,0,1,1,12,168,53
RABGGTB_2_9238,114,99,77,169,444,8,5,107,308,159,93,172
RABL3_2_9239,255,468,297,429,446,343,190,645,148,189,917,253
RAC2_2_9240,292,286,377,203,36,278,187,29,62,251,1299,295
RAD50_2_9241,575,647,565,729,369,1,3,0,1082,76,912,698
RAD54L2_2_9242,457,312,272,322,170,369,477,476,729,457,107,429
RALA_2_9243,103,118,159,130,87,8,147,184,56,129,276,252

RALB_2_9244,2018,2213,1736,2173,2083,1279,1548,1816,2371,2196,2184,309
8
RALBP1_2_9245,63,53,28,59,71,189,44,24,80,1,0,1
RANBP2_2_9246,1238,1461,1089,1546,1152,1636,786,755,999,944,877,2392
RAN_2_9247,13,72,68,62,54,12,0,86,37,0,0,14
RAP2A_2_9248,666,436,638,621,1396,319,1230,935,224,489,362,606
RAP2B_2_9249,218,382,330,334,9,40,408,42,52,332,322,502
RAP2C_2_9250,811,715,898,773,945,1643,576,405,515,464,532,842
RARS2_2_9251,639,507,512,498,1415,178,457,554,155,811,498,720
RARS_2_9252,551,511,453,938,942,576,1883,457,601,1075,718,213
RASD2_2_9253,49,142,20,28,0,246,180,0,1,0,3,28
RASL10B_2_9254,173,108,117,209,407,45,174,81,215,193,0,621
RASL11B_2_9255,426,585,266,492,255,103,1458,9,566,261,990,463
RASL12_2_9256,43,40,29,65,0,224,0,31,2,130,0,7
RC3H1_2_9257,369,268,156,259,5,151,41,299,52,466,164,275
RCL1_2_9258,278,258,87,370,102,92,2,66,155,50,66,488
RDH10_2_9259,263,380,430,418,152,530,1126,139,655,1319,139,590
RDH11_2_9260,359,302,220,308,552,117,63,261,71,103,250,323
RDH12_2_9261,59,52,42,105,0,62,53,117,113,0,0,4
RDH14_2_9262,358,373,493,605,285,296,17,764,243,187,1416,1340
RDH16_2_9263,202,172,104,150,477,34,185,0,39,86,283,206
RDH8_2_9264,335,106,184,304,316,102,159,354,131,62,55,46
RECQL4_2_9265,75,51,24,39,1,43,0,45,105,12,0,6
REM1_2_9266,197,223,164,332,453,123,387,281,246,374,95,598
REM2_2_9267,217,162,113,122,51,464,44,33,56,113,3,82
RENBP_2_9268,106,19,51,15,92,5,4,0,0,14,0,1
RETSAT_2_9269,1122,1154,1105,1091,596,1620,811,639,1015,945,1122,1959
REV3L_2_9270,731,531,593,697,1336,614,451,1112,1185,1030,185,448
REXO1L1_2_9271,504,480,397,542,348,239,54,12,264,329,358,328
REXO2_2_9272,444,329,425,582,343,352,448,491,438,285,101,1523
RFNG_2_9273,245,100,228,96,331,1,1,62,241,3,2,311
RFX6_2_9274,288,324,306,408,236,460,2,42,160,552,722,227
RGS7_2_9275,546,711,528,622,767,600,585,1275,535,974,370,894
RHBDL1_2_9276,87,74,62,101,103,20,111,37,6,366,5,54
RHBDL2_2_9277,329,358,180,277,69,417,264,107,682,117,31,263
RHBDL3_2_9278,197,144,119,150,152,130,2,272,125,58,98,616
RHEBL1_2_9279,556,370,276,369,329,62,115,553,296,286,4,171
RHOA_2_9280,190,85,129,186,120,317,394,67,113,260,276,223
RHOB_2_9281,0,0,0,5,0,0,0,0,0,0,0,0
RHOD_2_9282,258,234,227,273,352,162,625,483,50,222,750,6
RHOF_2_9283,12,10,12,53,4,1,0,9,2,0,6,10
RHOG_2_9284,378,303,426,282,36,401,923,99,62,132,1363,287
RHOH_2_9285,3,2,25,45,1,10,274,70,0,0,0,13
RHOJ_2_9286,389,167,157,335,134,117,406,265,266,185,49,309
RHOQ_2_9287,1499,1432,1338,1791,1906,1010,2388,2170,1343,867,828,2143
RHOT2_2_9288,201,193,265,177,275,30,43,131,80,27,1146,240
RHOU_2_9289,1143,924,895,1194,819,459,552,1134,1160,1268,831,1213
RHOV_2_9290,593,493,543,765,394,909,700,1261,1090,1033,449,1368
RIT1_2_9291,342,443,311,380,732,730,576,559,152,719,20,364
RIT2_2_9292,355,294,278,387,143,0,20,7,544,589,439,634

RNASE2_2_9293,362,523,345,662,732,135,468,1094,364,387,28,416
RNASE3_2_9294,94,111,139,217,177,122,4,0,0,325,301,13
RNASE6_2_9295,503,408,492,492,38,891,636,538,248,332,1065,622
RNASE7_2_9296,430,364,377,464,0,274,1524,9,38,659,185,546
RNASE8_2_9297,1065,869,874,796,663,380,1490,724,578,159,550,1471
RNASEH2A_2_9298,397,271,333,347,380,111,649,700,132,607,230,182
RNASET2_2_9299,564,660,470,746,420,738,549,474,265,374,260,556
RND1_2_9300,2195,2511,1992,2151,2026,2156,2825,2631,1281,1420,1122,998
RND2_2_9301,199,134,320,340,284,539,7,430,218,76,536,412
RND3_2_9302,181,163,156,99,130,0,412,53,174,279,47,304
RNF148_2_9303,327,346,402,589,314,178,872,776,8,157,13,733
RNMT_2_9304,1118,846,808,884,2020,979,520,1290,1009,804,632,2190
RNMTL1_2_9305,0,18,20,28,0,0,2,4,164,1,0,15
RNPEP_2_9306,133,80,96,58,74,245,2,39,681,223,1,46
RPAP2_2_9307,1284,849,1086,1366,1217,842,865,769,1348,1380,1803,1456
RPE65_2_9308,364,226,575,417,359,157,594,1274,551,254,763,493
RPIA_2_9309,14,17,0,3,0,2,0,0,0,0,0,3
RPL4_2_9310,366,212,203,265,983,176,868,4,341,228,257,491
RPN1_2_9311,387,306,308,267,502,90,3,112,314,112,76,1084
RPP25_2_9312,278,328,132,124,0,269,90,5,4,264,0,221
RPP40_2_9313,2626,2404,2435,2734,1453,2083,3365,2719,2905,3523,2240,42
24
RPS3_2_9314,868,756,647,986,1272,1270,922,565,727,626,244,621
RPUSD1_2_9315,67,54,23,22,21,1,5,0,10,6,0,1
RPUSD2_2_9316,592,392,630,601,1062,401,719,294,197,268,742,130
RRAGA_2_9317,31,32,29,45,42,48,61,53,273,0,0,129
RRAGC_2_9318,702,778,703,586,463,1309,1585,347,316,342,964,462
RRAGD_2_9319,786,640,577,707,1832,354,878,509,773,975,740,400
RRAS_2_9320,228,178,180,209,168,113,537,116,196,250,197,14
RRM1_2_9321,0,0,0,0,0,0,0,0,0,0,0,0
RRP8_2_9322,625,838,728,615,1081,1146,161,426,528,684,56,640
RSAD2_2_9323,77,56,57,137,94,15,152,272,99,88,2,114
RTN4IP1_2_9324,526,412,445,392,537,287,424,24,284,1348,44,352
SAMHD1_2_9325,242,356,182,275,21,6,32,70,96,421,796,446
SDF2_2_9326,187,157,216,164,395,246,222,30,217,147,24,28
SDHA_2_9327,1276,643,769,886,587,131,412,1689,1119,230,907,1251
SDHB_2_9328,973,1120,987,1236,530,522,678,2,1050,609,1510,956
SDHD_2_9329,122,171,150,257,29,222,970,9,26,28,399,110
SDR16C5_2_9330,499,811,686,529,446,245,703,608,901,705,187,458
SDR42E1_2_9331,215,77,226,164,79,0,0,307,211,281,13,285
SDR9C7_2_9332,231,119,89,142,103,80,138,9,0,165,13,118
SDSL_2_9333,206,160,166,96,65,175,18,258,19,117,473,6
SEPSECS_2_9334,607,687,518,577,490,347,285,368,805,787,256,874
1-Sep_2_9335,170,129,221,106,0,1,0,4,102,285,0,22
SEPW1_2_9336,202,36,86,11,118,28,44,278,92,47,111,4
SERHL2_2_9337,569,462,471,451,514,499,1920,936,350,272,500,199
SETX_2_9338,1445,1240,871,1448,335,793,2035,336,1417,1396,1946,894
SGMS1_2_9339,3331,3201,2900,3808,1978,1799,4738,3092,2830,3167,4026,23
17
SGPL1_2_9340,503,267,271,366,164,122,154,19,196,65,326,391

SGSH_2_9341,2,0,4,4,1,44,0,0,0,141,0,369
SH3GL2_2_9342,482,373,357,407,149,376,467,358,420,632,231,188
SKIV2L2_2_9343,714,519,713,584,1052,258,542,233,709,671,309,850
SLFN12_2_9344,2584,2162,2390,2492,1278,1453,3857,2185,2363,2935,2863,2
389
SLFN12L_2_9345,504,449,614,586,415,678,448,253,465,325,569,540
SLFN13_2_9346,1076,1074,884,927,951,987,2171,2625,885,786,1038,1278
SLFN5_2_9347,866,916,812,938,585,800,541,846,1085,301,633,1591
SLU7_2_9348,25,84,145,90,0,0,0,0,0,0,0,0
SMG8_2_9349,249,127,224,252,78,0,2,0,38,321,0,213
SMPD2_2_9350,142,82,63,101,33,15,10,24,98,35,0,58
SMPD3_2_9351,216,453,416,485,245,238,900,311,666,0,2590,392
SMPDL3A_2_9352,216,204,258,344,93,154,296,110,340,43,375,664
SMUG1_2_9353,234,213,175,251,138,397,202,263,532,0,140,303
SNF8_2_9354,2,5,0,45,0,0,5,0,0,0,0,0
SNRNP200_2_9355,24,42,19,43,16,0,31,0,73,0,0,35
SOAT1_2_9356,1880,1614,1490,1829,888,876,1217,1588,916,2013,1332,1410
SOAT2_2_9357,34,30,16,45,0,8,0,0,119,0,224,38
SOD1_2_9358,100,169,392,356,47,6,534,271,264,0,0,1
SOD3_2_9359,8,32,51,3,0,0,1,0,0,0,173,106
SORD_2_9360,54,95,29,53,105,0,21,0,0,78,0,45
SPACA3_2_9361,539,414,316,577,423,156,306,368,421,165,420,521
SPACA5B_2_9362,2031,1570,1659,1842,1782,982,2272,874,1462,1384,671,179
6
SPACA5_2_9363,2031,1570,1659,1842,1782,982,2272,874,1462,1384,671,1796
SPEM1_2_9364,556,286,280,380,630,420,471,285,418,297,300,299
SPTLC2_2_9365,78,292,199,200,63,169,63,169,224,499,185,181
SPTLC3_2_9366,279,241,428,260,70,274,279,61,2,247,176,297
SPTSSA_2_9367,157,180,172,129,622,185,92,11,32,21,552,146
SQLE_2_9368,0,0,0,0,0,0,0,0,0,0,0,0
SQRDL_2_9369,127,68,118,55,249,8,1,149,142,85,0,548
SRD5A1_2_9370,303,299,310,296,225,39,9,485,70,578,38,332
SRD5A2_2_9371,202,97,142,233,369,198,128,290,579,320,135,22
SRD5A3_2_9372,1012,1065,867,1273,944,183,752,1412,923,782,1732,636
SRR_2_9373,353,309,263,531,123,107,658,0,858,427,141,84
SRSF9_2_9374,469,413,351,449,752,543,513,246,828,640,718,355
SRXN1_2_9375,1065,1274,909,1079,2601,645,1125,87,931,1506,956,1173
SSB_2_9376,1754,1455,1640,1809,1406,1544,1736,730,1314,2933,1778,2459
SSU72_2_9377,160,226,295,538,298,455,259,517,149,4,287,312
ST14_2_9378,105,43,111,104,80,76,256,130,67,1,7,0
ST3GAL2_2_9379,178,279,156,172,55,12,233,211,354,308,8,285
ST3GAL4_2_9380,46,14,68,26,60,0,361,124,12,4,0,11
ST3GAL6_2_9381,32,68,107,25,0,0,1,25,185,85,0,70
ST6GALNAC1_2_9382,273,209,162,325,343,32,661,11,116,217,428,91
ST6GALNAC2_2_9383,544,296,288,433,305,448,649,61,570,151,174,537
ST6GALNAC5_2_9384,262,313,239,231,422,406,244,364,369,453,72,470
ST6GALNAC6_2_9385,90,91,77,280,13,9,730,0,1,0,0,15
ST8SIA1_2_9386,0,42,18,81,0,42,0,318,1,0,0,5
ST8SIA2_2_9387,840,624,579,974,1050,771,259,1326,100,543,1337,1926
ST8SIA3_2_9388,137,175,141,200,368,104,859,90,142,446,70,129

ST8SIA5_2_9389,404,306,556,418,607,352,568,193,749,267,387,919
ST8SIA6_2_9390,505,316,321,357,454,5,904,218,29,41,314,862
STS_2_9391,2786,2641,2380,3087,3233,2395,3553,3797,1373,3165,2329,2137
STT3A_2_9392,315,184,233,263,230,1072,406,291,105,558,235,113
STT3B_2_9393,605,547,565,749,198,142,1216,14,407,434,813,359
SUCLA2_2_9394,301,179,189,208,130,79,110,897,279,210,77,233
SUCLG1_2_9395,183,274,171,165,219,886,250,43,305,68,273,1
SULT1A3_2_9396,2024,2023,2138,2284,2010,3328,3651,2470,2413,1412,2936,
2700
SULT1A4_2_9397,2024,2023,2138,2284,2010,3328,3651,2470,2413,1412,2936,
2700
SULT1B1_2_9398,1193,1281,1010,1570,1899,1479,259,1614,891,1684,1967,17
03
SULT1C3_2_9399,473,259,276,372,28,88,243,404,321,456,116,468
SULT1C4_2_9400,1236,1201,1198,1313,1002,323,1065,1586,1100,2146,866,10
38
SULT1E1_2_9401,645,698,715,780,416,324,314,831,1028,228,683,576
SULT2A1_2_9402,146,80,109,171,21,124,512,3,35,396,19,211
SULT4A1_2_9403,569,505,472,403,239,816,64,52,337,320,418,241
SUPV3L1_2_9404,1107,933,723,873,1606,1239,1793,880,946,1520,1290,573
SURF1_2_9405,581,411,409,710,349,340,413,773,83,120,802,523
TALDO1_2_9406,68,68,60,131,25,61,33,27,30,57,20,343
TARS2_2_9407,41,23,109,75,27,11,284,0,64,0,54,23
TARS_2_9408,268,158,184,280,397,214,506,344,2,43,240,513
TARSL2_2_9409,342,296,482,407,279,512,304,1447,256,417,788,640
TAT_2_9410,214,95,161,115,160,442,1098,0,140,1,200,278
TBC1D10B_2_9411,0,0,2,0,0,0,0,0,0,0,0,0
TBCC_2_9412,163,67,102,212,204,0,149,199,373,40,41,5
TDG_2_9413,200,279,305,347,663,226,430,144,222,148,573,194
TDO2_2_9414,655,584,647,670,762,578,1153,748,850,563,1337,1763
TECR_2_9415,343,349,339,404,565,512,901,858,244,406,424,830
TECTA_2_9416,856,547,597,812,428,367,8,764,487,171,1444,752
TFB2M_2_9417,330,361,250,293,434,478,175,604,810,357,2,129
TGDS_2_9418,281,526,483,342,408,436,321,744,979,616,129,961
TGM1_2_9419,87,103,47,125,289,146,7,0,26,156,76,6
TGM3_2_9420,216,124,49,167,272,100,127,43,544,14,45,127
TGM4_2_9421,110,22,17,7,8,0,0,0,7,9,0,18
TGM6_2_9422,433,407,328,288,868,44,750,242,634,647,40,151
TGM7_2_9423,127,210,182,287,176,428,413,29,274,534,300,2
TGS1_2_9424,204,246,138,183,88,2,239,114,73,70,21,90
THG1L_2_9425,791,495,513,498,608,113,511,762,495,1490,152,95
THOP1_2_9426,205,277,180,197,162,1214,665,54,316,87,271,243
THUMPD2_2_9427,1539,1347,1307,1827,1917,772,2407,1449,625,697,1332,168
4
TKTL2_2_9428,518,416,506,675,436,100,780,1657,862,1191,1,111
TM7SF2_2_9429,10,15,27,25,0,0,0,39,42,0,0,29
TMEM55A_2_9430,2,14,19,21,3,6,0,1,11,7,0,0
TMEM62_2_9431,187,125,216,203,36,1,97,89,164,347,372,129
TMEM86B_2_9432,0,0,0,0,0,0,0,0,0,0,0,0
TMPRSS15_2_9433,60,16,126,42,1,16,1,0,0,112,2,1

TMX1_2_9434,481,496,403,616,232,280,360,730,652,404,881,151
TMX3_2_9435,1543,1683,1391,1468,2220,1273,1716,1948,1393,1100,1649,208
4
TMX4_2_9436,182,153,230,222,350,507,647,199,173,13,58,262
TNKS2_2_9437,347,329,443,531,109,11,1146,1157,512,180,29,532
TNKS_2_9438,646,683,456,733,632,540,460,1407,501,774,138,711
TOP1_2_9439,372,443,340,453,475,13,458,920,153,476,361,352
TOP1MT_2_9440,2,0,65,24,0,2,0,0,21,0,0,0
TOP2A_2_9441,441,399,301,415,214,274,224,323,278,458,52,292
TOP2B_2_9442,517,531,687,700,972,644,684,1409,1439,371,160,809
TOP3A_2_9443,13,50,38,63,126,1,22,0,27,515,0,63
TOP3B_2_9444,409,401,417,473,512,184,322,694,440,404,233,943
TOR3A_2_9445,87,66,64,127,0,526,136,41,76,21,68,37
TPH1_2_9446,253,300,214,213,134,181,302,709,274,113,70,116
TPMT_2_9447,396,360,416,421,76,384,838,585,571,223,724,609
TPP1_2_9448,353,234,316,237,2,1,0,652,20,1,621,4
TPP2_2_9449,964,429,515,746,393,204,458,334,445,799,338,935
TPSAB1_2_9450,396,246,307,328,0,0,0,301,2,277,1563,106
TPSB2_2_9451,445,494,485,583,691,249,1197,324,237,451,430,225
TPSD1_2_9452,396,246,307,328,0,0,0,301,2,277,1563,106
TPST1_2_9453,215,194,180,279,270,78,274,195,320,283,10,113
TREH_2_9454,114,90,76,102,27,38,581,14,123,79,65,243
TREX2_2_9455,88,110,98,170,1,4,207,71,18,7,70,14
TRHDE_2_9456,70,18,73,44,14,0,55,83,8,1,68,194
TRIM21_2_9457,321,232,330,437,216,161,186,294,421,191,190,386
TRIT1_2_9458,1758,1646,1375,2083,1543,1180,1520,1468,937,2121,2584,168
0
TRMT112_2_9459,255,235,204,251,269,99,113,66,219,334,255,182
TRMT61A_2_9460,17,37,9,44,0,0,122,51,0,112,69,0
TRMU_2_9461,420,601,425,440,179,1131,955,452,720,1158,221,254
TRNT1_2_9462,564,509,447,433,394,455,371,723,678,442,389,833
TRUB1_2_9463,746,768,868,760,531,764,1757,883,383,582,830,856
TRUB2_2_9464,288,250,337,341,168,335,691,223,158,308,143,145
TSTA3_2_9465,107,49,130,80,89,0,55,197,5,2,7,2
TST_2_9466,561,775,478,657,126,381,260,1335,1227,525,278,589
TTLL13_2_9467,111,109,134,148,19,18,572,82,161,153,967,72
TTLL1_2_9468,182,154,161,184,33,349,189,437,74,134,363,123
TTLL3_2_9469,1383,1319,1312,1684,1307,1539,875,2294,1248,1412,1839,997
TTLL4_2_9470,25,55,49,21,41,2,0,0,15,15,274,2
TUFM_2_9471,1744,967,1295,1656,1078,1768,2763,1542,1616,1455,1493,1945
TULP2_2_9472,519,401,405,761,756,491,593,14,735,475,150,361
TUT1_2_9473,30,46,61,52,12,0,0,87,24,74,0,56
TXN2_2_9474,192,105,106,147,5,161,24,901,77,4,279,347
TXN_2_9475,242,263,215,249,494,259,172,470,579,235,82,147
TXNDC11_2_9476,55,40,62,65,318,0,152,0,8,214,2,4
TXNDC12_2_9477,1255,991,900,1061,1619,655,581,2185,1111,638,703,529
TXNDC15_2_9478,124,113,159,128,95,94,0,204,49,0,990,102
TXNDC17_2_9479,2,67,55,41,4,40,48,0,5,13,23,0
TXNL1_2_9480,169,72,124,213,99,115,78,32,154,45,49,23
TXNL4A_2_9481,432,268,474,452,299,523,369,1116,92,554,436,483

TXNRD2_2_9482,1078,899,856,1232,1650,1084,1588,574,795,1248,1167,1306
TYMS_2_9483,86,166,108,145,70,108,206,17,33,210,56,5
TYR_2_9484,2283,1500,1463,2135,1604,2041,2078,1386,1440,2393,2456,2789
TYRP1_2_9485,1318,1098,1006,1200,555,966,551,1169,1108,1031,1215,1377
UAP1_2_9486,157,223,189,184,20,146,369,339,108,729,3,119
UAP1L1_2_9487,237,178,167,194,65,107,100,473,168,10,558,5
UBIAD1_2_9488,373,414,457,603,772,595,119,266,600,164,306,458
UBL4A_2_9489,45,34,36,5,0,0,11,0,56,4,221,89
UFSP1_2_9490,7,106,28,48,0,0,0,0,0,0,0,3
UFSP2_2_9491,2170,2148,2109,2112,2007,2370,714,2208,2054,1749,3950,142
6
UGCG_2_9492,3310,2593,2356,2546,2819,926,1522,1931,1525,2688,2499,2100
UGGT1_2_9493,525,408,325,397,251,552,28,720,79,1142,1435,287
UGGT2_2_9494,2504,2062,1680,2371,3930,1465,1880,1943,1359,1380,1179,29
32
UGT1A10_2_9495,782,503,456,716,998,165,522,1039,645,498,1136,691
UGT1A1_2_9496,2431,1866,1948,2591,2024,1618,3042,1710,2924,1959,3480,2
208
UGT1A3_2_9497,167,105,113,179,66,95,63,258,171,36,988,37
UGT1A4_2_9498,2878,2307,2748,3568,3157,1537,2130,2034,2707,3236,4247,3
192
UGT1A5_2_9499,2431,1866,1948,2591,2024,1618,3042,1710,2924,1959,3480,2
208
UGT1A7_2_9500,2431,1866,1948,2591,2024,1618,3042,1710,2924,1959,3480,2
208
UGT1A8_2_9501,794,514,447,717,997,164,524,1049,657,498,1155,696
UGT1A9_2_9502,2431,1866,1948,2591,2024,1618,3042,1710,2924,1959,3480,2
208
UGT2A1_2_9503,1097,677,545,507,579,441,337,583,636,927,1485,539
UGT2A2_2_9504,1001,1063,720,779,601,327,880,858,929,321,186,1675
UGT2A3_2_9505,1364,1406,1399,1501,2245,1031,1247,1919,1332,1237,1826,9
08
UGT2B15_2_9506,3031,2257,2159,2822,1818,2425,2243,2247,2532,2205,2133,
3075
UGT2B17_2_9507,3031,2257,2159,2822,1818,2425,2243,2247,2532,2205,2133,
3075
UGT2B4_2_9508,1647,1238,1166,1314,344,920,1990,1033,869,884,987,1745
UGT2B7_2_9509,1534,1015,1096,1506,2253,694,1463,1643,2028,942,2146,102
8
UMPS_2_9510,279,368,270,267,117,33,12,18,69,34,20,33
UPB1_2_9511,2,11,76,4,25,2,461,8,3,29,295,0
UPF1_2_9512,707,631,471,462,329,4,889,646,1048,536,211,555
UPRT_2_9513,196,241,211,235,571,268,44,407,108,128,190,308
UQCR11_2_9514,2935,2429,2000,2601,3565,2381,1676,2457,3171,2930,1208,2
136
UQCRC1_2_9515,349,247,189,231,129,11,157,120,334,351,553,178
UQCRC2_2_9516,1018,755,686,816,1253,576,348,1189,425,946,394,1032
UQCRFS1_2_9517,1640,1502,1324,1586,1918,1775,2637,543,1846,1551,1551,1
629
UQCRH_2_9518,427,473,537,654,265,320,330,362,438,141,203,895

UQCRQ_2_9519,943,686,600,804,717,1192,374,165,123,1031,942,601
UROD_2_9520,35,91,38,68,27,375,1,174,46,29,448,223
UROS_2_9521,585,647,473,728,102,635,86,0,592,221,231,887
UST_2_9522,77,19,28,9,0,0,0,10,45,16,0,30
UXS1_2_9523,214,179,224,330,74,106,737,93,20,107,494,106
VARS_2_9524,19,14,15,34,19,2,4,10,4,2,0,48
VAT1L_2_9525,67,56,33,34,3,312,0,48,9,396,96,9
VCP_2_9526,74,184,114,176,3,92,143,0,50,544,247,212
VNN1_2_9527,401,539,449,325,521,594,628,390,186,30,651,232
WBSCR17_2_9528,540,591,559,638,1465,714,888,573,375,847,1453,1618
WBSCR27_2_9529,177,103,153,287,70,288,804,395,58,475,13,164
WDFY3_2_9530,678,500,461,516,354,798,182,195,737,878,413,444
WRN_2_9531,531,745,700,700,264,1158,773,233,618,630,537,1087
XDH_2_9532,272,150,241,118,4,10,455,32,587,467,13,38
XPNPEP2_2_9533,236,130,175,185,122,358,2,2,245,53,61,0
XRCC2_2_9534,294,133,191,225,228,53,689,163,94,177,318,155
XRCC5_2_9535,67,53,118,37,37,2,0,112,1,97,404,120
XRCC6_2_9536,379,364,404,162,318,353,422,780,694,448,682,374
XRN2_2_9537,339,150,229,276,210,302,379,803,470,17,45,187
XYLT1_2_9538,72,135,118,231,61,0,1,193,264,65,0,335
XYLT2_2_9539,203,113,130,105,22,223,1,6,181,175,158,215
YARS2_2_9540,949,658,882,897,1012,1565,1533,382,1391,977,389,1677
YARS_2_9541,173,325,307,384,21,724,349,1638,177,318,922,463
YKT6_2_9542,781,540,480,634,844,43,431,395,229,437,1272,1315
YPEL1_2_9543,1291,1333,1431,1466,1223,1197,1839,2263,991,2194,308,1093
ZADH2_2_9544,965,721,656,610,489,499,530,525,713,826,649,376
ZCCHC4_2_9545,348,270,350,279,140,1010,329,145,329,447,168,180
ZDHHC17_2_9546,966,976,1048,997,276,1191,1699,471,650,755,1637,1057
ZDHHC18_2_9547,246,58,37,42,122,0,183,286,53,0,0,100
ZDHHC1_2_9548,55,99,65,133,183,4,9,0,142,7,64,105
ZDHHC21_2_9549,1239,1339,985,1337,1620,996,1676,2074,1901,1463,1200,16
65
ZDHHC2_2_9550,373,262,333,302,1315,367,427,137,246,288,2,132
ZER1_2_9551,1270,1102,1037,1091,998,640,1056,625,781,2291,1963,2204
ZMPSTE24_2_9552,565,413,482,464,1191,123,489,401,132,201,901,782
ZRANB3_2_9553,202,108,100,116,511,2,102,19,0,21,0,10
A1CF_2_9554,503,260,335,376,806,256,463,161,461,338,356,442
A2LD1_2_9555,9,4,37,42,0,6,0,51,31,0,0,0
AADAT_2_9556,375,359,435,597,606,362,454,268,98,722,504,627
AARSD1_2_9557,181,121,52,102,121,49,17,457,104,20,1,178
ABAT_2_9558,0,12,14,22,299,0,0,0,158,41,0,0
ABHD11_2_9559,370,408,291,413,983,237,356,1115,246,473,1049,316
ABHD12_2_9560,4372,3544,2875,3658,3767,1690,3280,3033,2759,4307,3538,3
844
ABHD14B_2_9561,62,187,68,90,67,126,574,492,138,229,0,30
ABHD2_2_9562,795,673,635,948,750,193,548,336,1733,1374,538,1112
ACAA1_2_9563,832,596,973,857,266,823,1171,2063,1092,747,547,707
ACACA_2_9564,523,352,356,406,570,111,894,222,466,860,470,230
ACAD10_2_9565,166,158,170,226,270,37,28,299,242,30,363,327
ACADM_2_9566,803,666,664,829,735,235,701,221,413,1035,294,549

ACADVL_2_9567,248,216,308,184,9,402,351,7,6,154,124,120
ACCS_2_9568,25,33,66,21,31,11,44,0,9,2,0,68
ACE_2_9569,171,238,236,236,280,214,515,487,139,305,780,280
ACHE_2_9570,794,547,753,682,295,470,1006,1531,498,481,500,1092
ACIN1_2_9571,260,147,267,189,230,52,1025,8,13,73,358,299
ACLY_2_9572,128,224,303,275,0,0,0,21,246,333,535,213
ACOT11_2_9573,90,126,249,220,304,531,31,0,322,0,333,359
ACOT13_2_9574,61,154,150,130,539,92,34,64,36,267,0,404
ACOT7_2_9575,58,5,16,29,0,0,0,0,0,74,0,0
ACOT9_2_9576,559,378,323,368,67,338,505,88,713,192,18,488
ACOX1_2_9577,500,442,397,411,527,1273,706,88,246,1102,437,388
ACOX3_2_9578,278,203,167,181,96,0,316,377,9,72,573,184
ACSBG1_2_9579,309,263,204,326,0,0,0,670,528,334,607,212
ACSF3_2_9580,186,301,252,167,441,564,525,21,174,656,71,181
ACSL3_2_9581,218,276,307,234,206,203,76,266,144,109,73,74
ACSL4_2_9582,753,746,595,536,1790,816,485,455,463,664,420,214
ACSL5_2_9583,126,144,195,119,27,106,18,74,125,85,129,104
ACSL6_2_9584,399,307,539,402,1101,214,44,78,315,445,70,1082
ACSM2B_2_9585,4770,4232,3866,4464,6505,4304,3413,5871,4423,3156,2245,5
235
ACSM3_2_9586,814,966,686,701,1274,227,544,913,495,592,782,902
ACSS2_2_9587,409,451,469,472,174,786,313,189,335,569,252,467
ACY1_2_9588,616,364,433,494,619,487,584,298,579,487,746,545
ADAMTS2_2_9589,777,582,341,658,498,202,649,694,772,40,860,868
ADARB1_2_9590,98,110,123,148,398,73,81,28,291,58,114,220
ADAR_2_9591,347,199,158,254,858,356,81,439,352,323,2,407
ADCY10_2_9592,1425,863,1016,1209,2030,860,1680,904,1427,1266,1147,814
ADCY4_2_9593,557,601,715,640,450,44,712,373,437,516,1360,1526
ADCY5_2_9594,427,317,268,475,952,90,8,651,397,722,225,581
ADCY6_2_9595,298,134,152,153,347,211,307,1,219,116,36,474
ADH6_2_9596,326,256,383,339,517,802,206,142,292,22,564,257
ADH7_2_9597,92,78,130,64,64,14,12,16,4,558,3,4
ADPRHL1_2_9598,202,115,322,281,0,0,590,0,278,3,401,362
ADSL_2_9599,301,271,220,270,298,237,51,52,180,196,85,444
ADSSL1_2_9600,112,376,258,276,2,402,327,132,46,19,1050,714
AFMID_2_9601,266,209,162,149,291,27,116,25,10,189,205,56
AGA_2_9602,1222,1652,1365,1718,1030,1794,980,884,1328,966,952,3224
AGAP1_2_9603,85,27,67,111,1,5,0,0,37,0,6,28
AGAP2_2_9604,187,440,362,529,252,394,0,352,16,631,546,444
AGL_2_9605,619,743,474,386,104,348,1025,206,873,598,634,264
AGPAT1_2_9606,985,615,952,918,323,1079,737,533,733,768,962,763
AGPAT2_2_9607,171,180,177,249,497,47,77,535,123,270,569,182
AGPAT3_2_9608,136,70,83,144,116,95,87,0,30,3,167,201
AGXT2L1_2_9609,880,1023,877,1018,807,837,899,513,1100,1057,946,1025
AHCY_2_9610,696,894,961,1062,177,620,1442,504,1039,90,479,701
AHCYL1_2_9611,284,210,222,206,156,98,628,492,138,151,152,242
AHCYL2_2_9612,448,374,356,321,304,479,200,300,1042,765,208,384
AIFM1_2_9613,1001,816,951,788,712,1063,1736,1513,1371,751,672,1056
AIFM2_2_9614,248,331,280,329,185,149,183,9,160,252,4,233
AIFM3_2_9615,60,85,113,80,292,24,137,114,23,82,0,0

AKIRIN1_2_9616,2508,2126,1795,2480,2363,1383,1932,1324,1135,2900,1890,
3033
AKR1A1_2_9617,198,134,258,131,61,105,0,49,0,43,32,52
AKR1C2_2_9618,717,576,463,559,69,611,955,25,619,223,497,782
AKR1D1_2_9619,1816,1647,1938,2059,531,822,1327,2508,2207,1590,1454,146
7
ALAS1_2_9620,185,128,228,177,41,21,79,615,248,1330,414,272
ALAS2_2_9621,90,130,86,90,1,90,0,311,85,19,122,87
ALDH16A1_2_9622,79,111,121,112,29,103,24,140,252,181,20,4
ALDH1A2_2_9623,121,90,96,112,8,245,182,0,98,59,2,13
ALDH2_2_9624,109,74,124,65,1,0,8,0,35,0,168,30
ALDH3A1_2_9625,144,212,167,272,69,0,5,208,1,102,161,34
ALDH3A2_2_9626,138,147,130,173,130,6,186,202,32,261,2,115
ALDH3B1_2_9627,34,26,32,25,0,50,0,7,0,0,66,0
ALDH3B2_2_9628,133,100,112,156,130,26,22,10,148,54,37,207
ALDH4A1_2_9629,148,73,71,71,4,0,0,108,72,10,19,87
ALDH5A1_2_9630,497,329,417,473,715,114,790,325,263,153,1022,222
ALDH7A1_2_9631,292,257,259,383,659,1,788,413,204,59,113,482
ALDH8A1_2_9632,213,307,364,297,122,432,504,175,534,0,987,391
ALDOA_2_9633,291,213,216,232,9,462,331,414,53,263,435,223
ALG3_2_9634,152,166,278,263,244,600,403,289,171,230,874,122
ALG5_2_9635,85,75,75,101,239,84,313,15,177,6,61,112
ALG8_2_9636,732,544,619,710,402,40,1013,317,600,728,826,537
ALG9_2_9637,974,722,705,1019,1687,220,813,539,526,542,788,535
ALOX15B_2_9638,871,1245,871,1000,471,1240,969,507,941,523,1262,1369
ALOXE3_2_9639,556,314,326,282,170,173,292,25,426,156,119,258
AMACR_2_9640,199,150,190,223,2,65,150,307,465,425,177,39
AMD1_2_9641,986,606,929,881,929,461,1736,461,1163,520,851,824
AMDHD2_2_9642,75,33,7,53,2,0,7,350,0,0,0,0
AMPD1_2_9643,817,969,1011,1205,929,751,1846,1217,1024,500,805,1365
AMPD2_2_9644,90,99,158,141,58,314,8,55,31,235,245,79
AMPD3_2_9645,66,46,60,70,45,0,18,13,307,49,223,13
AMT_2_9646,241,130,133,213,156,381,21,544,128,386,149,158
AMY1A_2_9647,1300,1329,1106,1228,949,1335,2142,980,866,1074,2386,1604
ANG_2_9648,628,496,512,584,655,416,527,549,286,798,282,635
AOAH_2_9649,108,101,193,111,314,187,27,53,28,393,380,141
AOC2_2_9650,94,172,72,90,131,40,25,14,382,188,412,50
APC_2_9651,495,581,401,787,396,10,790,592,595,222,458,314
APEX1_2_9652,329,464,203,474,211,3,498,66,2,470,220,635
APOBEC3A_2_9653,1953,1645,1758,2091,2025,781,1246,2848,1797,1032,665,2
378
APOBEC3H_2_9654,286,410,171,342,653,895,206,388,85,196,2,104
APRT_2_9655,133,118,130,188,0,0,0,147,44,260,451,1
ARF1_2_9656,326,287,252,237,462,222,322,148,249,438,247,154
ARFRP1_2_9657,1020,1035,790,985,834,852,650,1007,1445,923,1058,1192
ARHGAP5_2_9658,630,804,899,575,1232,518,942,1065,628,603,848,491
ARHGEF10L_2_9659,53,63,104,66,0,1,8,3,49,35,386,5
ARL2_2_9660,275,451,95,292,202,13,36,125,203,165,436,182
ARL4A_2_9661,206,187,138,134,0,42,141,218,0,96,73,2
ARL5A_2_9662,852,668,743,610,2445,72,1234,766,625,575,235,680

ARSA_2_9663,160,157,118,285,3,0,0,370,158,2,458,2
ARSB_2_9664,483,637,562,523,832,1012,310,497,255,401,349,363
ARSF_2_9665,3199,3217,3043,3638,4135,4221,2703,3493,2739,4130,3332,344
5
ART3_2_9666,179,360,360,232,91,92,86,425,338,233,312,195
ART5_2_9667,77,82,97,126,0,2,96,26,70,16,0,11
ASAH1_2_9668,197,114,209,198,154,233,34,50,120,62,258,212
ASAH2_2_9669,2185,1719,1541,1884,1693,1678,1573,1453,2212,1896,337,187
3
ASL_2_9670,360,545,403,526,369,897,1027,451,253,1576,94,440
ASMT_2_9671,56,39,47,27,0,1,82,7,50,2,0,16
ASMTL_2_9672,278,234,203,289,506,932,76,1071,219,278,75,609
ASNS_2_9673,76,128,92,98,70,176,217,10,44,5,122,173
ASPA_2_9674,837,751,755,1032,1410,893,1558,955,1115,1272,999,296
ASRGL1_2_9675,464,539,553,736,21,47,235,164,522,1058,1240,632
ASS1_2_9676,22,0,8,20,0,0,3,1,0,0,0,0
ATE1_2_9677,827,750,784,876,766,264,1194,503,658,780,1103,701
ATL1_2_9678,160,77,94,107,11,15,39,1,45,16,12,140
AURKAIP1_2_9679,38,16,26,6,116,0,0,0,0,5,1,48
AZIN1_2_9680,199,199,207,176,480,165,375,197,174,106,567,235
B3GALNT1_2_9681,2057,2024,1847,2247,1783,1808,1554,2543,1284,2735,3520
,2633
B3GALT5_2_9682,340,328,267,297,1084,319,662,64,116,178,502,222
B3GAT1_2_9683,69,70,26,43,23,2,2,35,23,167,22,131
B4GALNT2_2_9684,107,184,130,155,203,0,34,372,0,191,0,179
B4GALT2_2_9685,169,185,111,78,288,0,24,6,135,224,164,76
B4GALT3_2_9686,812,778,729,893,1039,722,1573,454,1204,831,489,1181
B4GALT4_2_9687,632,672,545,658,958,446,295,1752,91,638,714,500
BAAT_2_9688,295,271,186,236,362,29,353,0,111,959,136,420
BACE1_2_9689,29,8,34,37,75,66,38,105,97,6,0,80
BACE2_2_9690,55,119,69,86,48,18,0,30,288,189,56,169
BCAT1_2_9691,296,250,247,306,157,45,60,430,343,387,135,244
BCAT2_2_9692,840,978,627,871,637,944,284,584,282,772,742,396
BCKDHA_2_9693,101,167,80,199,132,133,46,95,218,66,124,10
BCKDHB_2_9694,1417,1513,986,1643,1198,1509,2586,1701,999,1366,867,2001
BCO2_2_9695,633,482,443,653,609,361,1753,1092,582,100,1388,198
BDH1_2_9696,13,67,20,36,2,0,0,103,0,13,0,0
BFSP1_2_9697,12,5,4,54,6,0,1,0,0,0,0,0
BHMT2_2_9698,408,339,494,436,191,50,388,503,185,310,334,378
BMP1_2_9699,717,646,537,722,1215,1130,1077,1051,497,1632,414,535
C17orf101_2_9700,67,72,59,97,251,0,124,54,0,1,0,51
C1GALT1C1_2_9701,793,528,569,719,1536,1387,230,2064,281,1052,642,865
C1S_2_9702,303,176,209,285,286,0,323,83,666,84,0,0
C2_2_9703,883,614,750,904,559,742,1106,490,1125,1234,1235,1182
CA10_2_9704,410,385,451,456,387,525,139,157,32,440,682,235
CA12_2_9705,279,229,86,254,477,267,559,1129,74,275,14,296
CA1_2_9706,297,128,147,278,48,5,598,71,493,220,33,66
CA7_2_9707,0,0,0,0,0,0,0,0,0,0,0,0
CAB39_2_9708,1377,1015,1260,1454,934,805,2550,1475,1192,1883,685,1132
CANT1_2_9709,1095,667,938,1119,524,78,894,193,898,491,215,1715

CAPN1_2_9710,55,27,173,134,41,0,148,0,7,13,388,238
CAPN2_2_9711,517,370,289,472,560,179,843,289,250,8,94,376
CARNS1_2_9712,67,177,268,410,7,285,0,4,0,58,716,234
CARS_2_9713,934,585,688,702,1983,398,148,1441,705,253,812,1025
CASP10_2_9714,400,285,329,417,134,40,411,73,777,164,34,635
CASP1_2_9715,914,796,776,1082,710,28,475,854,107,877,269,766
CASP2_2_9716,79,87,48,199,2,97,0,313,10,0,10,93
CASP3_2_9717,3583,3594,3068,3520,3744,1628,4966,3644,3369,4862,2784,65
47
CASP4_2_9718,1043,832,917,949,1361,1002,148,1190,1126,738,1480,1404
CASP5_2_9719,1125,939,969,957,1563,1368,150,937,1159,987,1518,1524
CASP6_2_9720,623,491,442,508,755,615,684,841,301,293,923,674
CASP7_2_9721,24,19,0,0,0,0,0,0,19,0,1,0
CASP8_2_9722,276,103,153,179,184,219,5,207,60,97,4,407
CASP9_2_9723,320,379,295,151,166,739,207,47,43,460,0,375
CASZ1_2_9724,7,79,41,95,1,12,23,0,222,60,0,1
CAV3_2_9725,524,332,383,230,200,93,409,165,221,139,1131,73
CBS_2_9726,34,49,7,30,0,3,0,0,2,2,0,0
CCBL1_2_9727,349,179,300,250,99,17,519,664,57,141,419,60
CCBL2_2_9728,452,529,394,333,1004,148,50,352,275,198,550,1042
CDC42_2_9729,1247,1315,1312,1400,1437,2133,4012,1114,1508,958,2607,120
8
CDH16_2_9730,64,115,129,45,32,401,160,57,0,0,15,190
CECR1_2_9731,181,139,162,206,591,296,0,107,290,461,281,96
CEPT1_2_9732,730,753,783,1068,734,926,1600,1045,970,612,508,952
CERS1_2_9733,50,28,58,58,1,98,93,21,83,3,1,4
CES1_2_9734,137,89,96,55,227,0,108,1,0,213,412,49
CES3_2_9735,67,51,61,41,16,316,7,31,110,4,3,99
CES4A_2_9736,306,243,465,317,137,512,137,775,79,46,546,405
CES5A_2_9737,374,464,514,473,879,483,1359,374,192,731,367,702
CHAT_2_9738,125,207,73,181,15,18,0,580,204,324,443,44
CHI3L2_2_9739,539,665,449,440,37,1444,610,846,224,1019,893,788
CHIA_2_9740,172,97,126,147,173,225,10,203,118,12,41,284
CHPF_2_9741,19,109,25,39,1,20,578,1,2,0,0,23
CHST11_2_9742,387,401,394,418,311,827,214,479,419,535,4,242
CHST15_2_9743,147,284,413,304,36,124,377,337,575,60,574,981
CHST4_2_9744,125,114,181,153,78,14,483,2,201,139,482,192
CHST8_2_9745,26,75,56,86,16,18,9,75,160,0,17,13
CLP1_2_9746,133,48,60,80,15,106,209,7,63,150,1,63
CNDP2_2_9747,258,367,310,324,422,232,251,281,88,1153,208,385
CNTN1_2_9748,712,633,465,498,739,500,1009,18,375,992,376,787
CNTN4_2_9749,661,757,713,836,184,335,590,1098,643,430,1338,1307
COMT_2_9750,407,332,266,298,274,71,357,109,112,72,179,268
COQ6_2_9751,393,685,650,702,875,722,473,540,368,769,1545,460
COX11_2_9752,120,14,65,185,97,0,0,1,263,4,121,161
COX15_2_9753,177,90,271,176,75,331,329,26,44,71,423,491
CPA5_2_9754,103,108,43,29,16,298,0,15,0,239,0,77
CPB2_2_9755,201,270,159,205,49,178,10,193,210,364,0,714
CPM_2_9756,378,363,363,467,495,178,212,294,369,339,46,371
CPPED1_2_9757,190,232,241,187,17,0,214,387,413,45,423,457

CPT1A_2_9758,1130,883,760,1161,667,347,958,915,1364,1202,1594,1243
CPT1B_2_9759,207,272,265,345,205,388,403,593,244,841,147,112
CPT1C_2_9760,28,59,30,20,0,50,0,5,119,71,0,50
CRLS1_2_9761,73,121,153,95,231,0,30,241,265,212,436,0
CRMP1_2_9762,210,205,99,170,336,47,27,299,155,0,117,40
CROT_2_9763,516,555,531,927,681,271,667,161,478,484,444,612
CRY2_2_9764,5,67,13,14,3,0,0,0,6,5,0,3
CRYM_2_9765,366,357,289,344,627,262,987,438,454,130,868,278
CRYZ_2_9766,130,158,180,85,25,42,5,34,2,9,1,48
CSDE1_2_9767,415,296,135,243,129,35,133,225,316,229,9,509
CSGALNACT1_2_9768,1097,1139,1098,1104,624,918,1462,996,964,552,1103,39
0
CSMD3_2_9769,302,296,300,255,116,441,315,499,80,197,48,123
CTAGE5_2_9770,1500,1287,1351,1109,1426,1389,2293,434,1386,1482,920,191
2
CTBP1_2_9771,115,147,108,171,86,66,0,20,26,338,617,1
CTPS2_2_9772,222,177,227,243,862,362,236,662,253,342,183,237
CTSA_2_9773,88,227,148,161,126,519,635,291,47,188,557,165
CTSB_2_9774,94,104,137,239,342,150,243,246,218,6,0,113
CTSE_2_9775,457,537,500,574,168,759,1117,681,575,247,150,246
CTSL1_2_9776,235,201,194,338,8,705,14,425,38,50,351,457
CTSL2_2_9777,0,0,0,0,0,0,0,0,0,0,0,0
CTSS_2_9778,610,720,524,511,726,1008,1140,1446,1067,558,1533,914
CYB561_2_9779,512,430,286,336,422,272,227,248,565,302,9,102
CYB5A_2_9780,202,252,392,426,2,357,20,20,596,284,756,624
CYB5R3_2_9781,40,42,133,115,23,0,1,0,3,0,22,15
CYP11A1_2_9782,100,230,218,295,0,40,222,259,145,80,107,144
CYP11B1_2_9783,458,501,400,465,5,371,746,538,522,279,659,660
CYP19A1_2_9784,141,266,263,252,229,686,32,1,244,396,473,65
CYP21A2_2_9785,86,228,142,211,173,0,116,128,32,86,185,52
CYP24A1_2_9786,14,117,54,219,0,29,31,0,1,25,0,0
CYP26A1_2_9787,135,257,151,164,97,490,114,32,41,488,6,103
CYP2A7_2_9788,1277,1072,1068,1436,985,925,1661,646,983,591,476,1675
CYP2C18_2_9789,263,135,271,264,151,499,91,100,472,210,171,324
CYP2C8_2_9790,679,743,482,682,423,329,438,950,728,1093,1615,1106
CYP2D6_2_9791,224,117,378,182,466,187,42,0,246,130,40,92
CYP3A43_2_9792,154,255,319,327,460,87,87,362,268,228,578,350
CYP3A4_2_9793,317,130,253,219,110,114,123,73,194,64,124,80
CYP4B1_2_9794,130,93,120,158,326,139,80,389,200,343,374,347
CYP4F11_2_9795,137,205,212,402,92,502,49,538,55,11,24,123
CYP4F3_2_9796,1189,1056,1241,1164,1765,783,1103,1255,750,1360,358,1658
CYP51A1_2_9797,396,403,452,581,288,274,490,1100,125,157,287,872
DAGLB_2_9798,1116,981,694,818,819,842,630,1489,635,1171,580,762
DCLRE1C_2_9799,2517,2822,2626,2923,3062,2014,3539,1957,2384,3135,4725,
2802
DCP2_2_9800,912,764,591,664,566,875,1411,281,951,1217,953,1023
DCT_2_9801,315,290,316,290,354,208,290,523,322,41,158,214
DCTD_2_9802,292,181,89,229,464,389,259,0,264,145,0,1
DCXR_2_9803,96,53,46,51,0,0,11,128,217,64,139,56
DDAH1_2_9804,885,813,939,996,854,1315,1157,455,722,984,406,699

DDC_2_9805,426,344,424,445,421,591,355,524,448,730,916,839
DDHD1_2_9806,30,37,55,58,292,0,0,0,41,7,0,0
DDO_2_9807,176,125,237,363,662,243,641,378,136,26,210,215
DDT_2_9808,960,744,775,753,1194,578,760,835,728,1160,569,751
DDX11_2_9809,616,432,719,720,800,555,372,244,151,195,955,648
DDX17_2_9810,1195,973,971,1240,517,659,1443,495,498,398,543,1054
DDX19B_2_9811,1031,1218,1031,1281,1460,896,1546,222,82,1681,1478,604
DDX31_2_9812,47,46,42,74,3,21,3,16,112,327,31,136
DDX39B_2_9813,1134,1178,1241,1236,1310,653,1996,986,975,352,648,800
DDX3X_2_9814,191,95,98,117,341,19,46,107,0,386,451,31
DDX3Y_2_9815,62,42,21,58,43,24,17,3,2,52,24,2
DDX42_2_9816,88,123,61,63,222,3,14,36,0,0,0,17
DDX47_2_9817,520,461,462,565,153,278,1826,367,685,708,260,634
DDX4_2_9818,93,111,110,203,7,6,384,0,3,201,3,50
DDX54_2_9819,123,20,32,26,8,0,3,0,332,250,70,0
DFFA_2_9820,89,112,93,150,58,0,0,704,127,106,335,29
DGCR8_2_9821,49,69,85,99,1,35,2,5,4,0,2,117
DHCR7_2_9822,254,151,216,140,171,197,5,322,27,73,174,340
DHDDS_2_9823,117,73,55,107,18,179,418,1,155,5,9,6
DHFRL1_2_9824,300,192,304,263,419,61,238,7,127,158,350,339
DHPS_2_9825,269,170,253,317,311,147,552,121,173,235,322,227
DHRS1_2_9826,90,59,50,101,35,125,254,320,92,0,0,98
DHRS2_2_9827,111,127,135,190,270,112,143,19,79,527,0,447
DHRS9_2_9828,166,84,127,109,60,74,538,110,25,170,0,61
DHX16_2_9829,7,13,40,7,0,0,213,1,2,2,243,0
DHX30_2_9830,576,485,402,398,517,286,635,423,295,398,218,358
DHX33_2_9831,68,65,32,46,7,30,3,23,50,2,0,312
DHX35_2_9832,2389,1981,1794,2057,1865,1928,2827,2502,1899,3407,1191,24
90
DHX36_2_9833,928,862,653,804,1060,554,1217,801,1062,567,1940,406
DHX40_2_9834,482,590,462,592,895,190,201,486,782,399,136,495
DIAPH3_2_9835,1414,1308,1218,1455,1949,714,942,1338,883,2879,1689,1073
DIS3_2_9836,17,25,34,4,4,35,6,0,25,0,0,0
DIS3L_2_9837,746,567,672,728,511,861,1386,1124,952,323,391,1288
DKC1_2_9838,216,493,248,383,0,348,0,10,372,202,223,489
DNAJC27_2_9839,636,713,753,686,458,226,907,142,425,436,618,956
DNASE1L1_2_9840,445,418,422,545,1037,143,828,865,417,682,556,1153
DNM1_2_9841,372,185,243,411,641,198,12,3,354,225,1,234
DNM1L_2_9842,82,45,38,67,1,163,53,14,150,169,2,0
DNM2_2_9843,88,31,123,84,2,0,88,173,11,118,283,23
DNM3_2_9844,719,724,664,703,324,699,145,563,1296,536,677,928
DNTT_2_9845,183,325,245,367,347,56,366,540,410,423,431,49
DOHH_2_9846,52,51,23,46,31,53,32,0,6,0,0,0
DOLPP1_2_9847,528,511,589,446,332,1103,57,898,594,324,340,189
DPEP1_2_9848,428,454,521,370,113,749,1076,234,513,188,1178,220
DPEP3_2_9849,495,354,305,355,547,675,97,227,389,441,617,353
DPH5_2_9850,522,424,379,354,330,18,161,403,169,422,143,442
DPM3_2_9851,96,88,107,135,6,0,0,617,23,28,0,0
DPP3_2_9852,117,28,104,58,86,0,2,0,0,14,2,0
DPP8_2_9853,61,47,50,58,69,197,13,68,114,63,2,87

DPYSL2_2_9854,467,183,346,447,273,0,538,1494,313,162,0,491
DPYSL3_2_9855,175,194,212,353,23,1,9,442,356,281,4,56
DROSHA_2_9856,180,262,107,223,10,1,121,5,101,45,0,59
DSE_2_9857,336,508,197,433,182,627,16,256,6,265,474,99
DUOX1_2_9858,114,166,118,89,50,107,179,31,11,209,102,275
DUT_2_9859,1997,2125,1974,1741,1989,1165,1950,769,1384,2606,2534,2155
ECE1_2_9860,203,185,91,122,337,534,55,0,570,254,727,0
ECE2_2_9861,36,40,26,42,37,18,179,41,1,61,1,8
ECI1_2_9862,1238,899,1202,1120,539,1219,561,1759,1682,905,973,1325
ECI2_2_9863,458,422,413,539,78,434,16,288,145,1003,466,1081
EDEM2_2_9864,1010,993,939,1243,1355,1114,478,649,1090,904,2415,1316
EFEMP1_2_9865,36,29,47,32,97,84,17,126,2,37,7,3
EFTUD2_2_9866,22,29,23,18,7,217,22,1,38,7,0,2
EGLN2_2_9867,35,30,35,79,3,155,5,0,59,56,2,0
EHHADH_2_9868,289,359,415,351,167,826,782,260,804,258,451,1004
ELAC2_2_9869,114,70,82,105,36,168,42,0,249,60,0,263
ELOVL5_2_9870,1168,722,716,863,973,1068,1296,968,385,908,1677,1188
ELOVL6_2_9871,484,385,360,507,187,175,265,145,930,499,434,408
ELOVL7_2_9872,177,318,272,308,121,112,1027,626,371,354,630,607
ENDOV_2_9873,235,138,165,161,60,23,25,81,59,88,129,217
ENO3_2_9874,82,155,87,68,18,2,295,157,86,77,3,36
ENOX2_2_9875,5,10,38,72,0,6,10,1,0,9,203,0
ENPP2_2_9876,297,279,294,314,272,366,280,94,308,481,217,275
ENTPD1_2_9877,371,441,390,406,103,275,1004,243,643,485,435,574
ENTPD2_2_9878,38,82,66,48,44,2,763,25,3,23,1,8
ENTPD4_2_9879,1087,1159,1030,1043,138,1330,1013,627,667,499,658,1017
ENTPD6_2_9880,273,391,272,314,413,290,885,295,160,172,99,232
ENTPD8_2_9881,411,278,335,561,103,138,70,265,270,432,402,929
EPHX1_2_9882,99,127,94,81,34,66,516,39,182,337,107,186
EPHX3_2_9883,953,823,622,730,852,445,835,1601,426,489,753,771
ERCC1_2_9884,24,72,69,84,143,107,87,6,28,2,285,0
ERCC2_2_9885,74,70,82,65,65,48,9,6,27,3,0,69
EXO1_2_9886,522,603,400,563,102,135,248,201,165,176,249,923
EXOG_2_9887,167,81,133,127,5,197,44,4,84,0,517,203
EXOSC3_2_9888,431,364,295,470,1528,60,185,542,368,708,159,475
EXOSC9_2_9889,219,181,156,232,102,25,358,256,236,282,319,260
EXTL2_2_9890,869,581,663,682,704,1862,1248,422,2186,1313,219,635
F7_2_9891,848,721,929,950,441,1059,1621,1184,715,997,544,2001
FAHD1_2_9892,76,22,158,84,86,340,278,7,120,33,106,39
FAM108A1_2_9893,64,25,28,39,5,505,75,144,0,104,38,65
FAM135A_2_9894,79,118,178,119,5,184,0,212,0,3,217,272
FAN1_2_9895,411,643,502,548,452,861,187,179,546,242,275,301
FDPS_2_9896,539,720,417,402,385,511,379,796,385,240,621,474
FDXR_2_9897,174,87,58,222,281,281,89,410,233,200,0,32
FECH_2_9898,165,190,169,213,28,149,90,184,132,42,35,293
FERMT3_2_9899,54,28,31,75,255,14,0,83,32,7,0,215
FHIT_2_9900,446,391,366,399,0,278,2,160,640,594,0,0
FIGNL1_2_9901,808,655,627,712,802,174,1329,686,1489,446,1090,821
FKBP11_2_9902,174,54,74,186,291,9,4,11,278,11,1,1
FKBP1A_2_9903,16,16,4,5,0,2,0,0,0,108,0,0

FKBP1B_2_9904,936,637,551,849,803,599,314,812,615,1080,139,1514
FKBP2_2_9905,89,37,1,20,0,23,159,4,228,11,5,2
FKBP5_2_9906,1264,976,1053,984,849,846,1194,1679,612,957,1209,1525
FKBP7_2_9907,780,651,735,767,1022,362,823,860,634,662,1306,1386
FLAD1_2_9908,497,276,283,365,845,434,196,189,111,206,107,468
FMO3_2_9909,64,120,56,108,14,225,464,127,212,122,105,219
FMO5_2_9910,634,606,575,578,198,786,369,1150,598,443,668,612
FN1_2_9911,112,68,79,84,111,58,96,1,30,146,51,24
FOLH1_2_9912,671,888,586,1073,597,152,344,53,406,1365,1385,995
FPGS_2_9913,108,110,195,62,152,119,11,112,43,55,332,54
FPGT_2_9914,464,403,327,424,277,41,13,39,31,454,302,590
FTCD_2_9915,37,2,6,8,0,36,0,7,0,4,0,0
FTSJ1_2_9916,522,481,614,572,671,0,2509,128,736,231,40,1087
FUT2_2_9917,216,157,108,82,10,0,159,0,21,145,51,53
FUT3_2_9918,3135,3111,3400,3509,2105,1838,4740,1512,3087,4375,4567,379
9
FUT6_2_9919,591,456,625,583,402,423,223,1590,252,321,427,584
FUT8_2_9920,955,730,764,651,217,962,1179,447,1018,525,1303,818
G3BP1_2_9921,88,78,132,119,146,313,26,40,48,203,29,469
G3BP2_2_9922,51,44,37,56,0,0,0,106,4,0,0,2
G6PD_2_9923,118,62,34,148,285,0,38,0,0,1,38,126
GAA_2_9924,79,121,198,79,8,56,388,2,56,18,186,112
GAD1_2_9925,85,100,49,83,6,393,17,18,62,51,1,63
GAD2_2_9926,104,57,122,182,52,25,194,12,8,258,62,23
GALC_2_9927,1630,1302,1035,1552,1931,1370,1282,1575,1245,879,722,800
GALE_2_9928,312,280,216,221,211,528,67,91,598,116,237,438
GALNT9_2_9929,16,70,44,44,23,0,212,2,74,55,2,26
GALNTL1_2_9930,474,385,243,470,320,345,152,730,1073,475,59,526
GAMT_2_9931,168,85,231,106,61,9,3,102,81,138,2,172
GANAB_2_9932,117,104,83,42,6,1,24,119,10,13,5,132
GART_2_9933,172,214,221,165,83,170,90,4,153,362,0,56
GBA_2_9934,109,78,142,81,18,5,203,778,157,11,61,26
GBP5_2_9935,21,106,187,167,0,5,0,0,20,133,0,18
GCAT_2_9936,16,22,38,42,6,2,133,70,0,62,2,24
GCDH_2_9937,7,46,6,18,0,0,27,117,0,0,7,63
GCH1_2_9938,34,130,69,94,16,236,133,37,225,276,50,53
GCLC_2_9939,1046,931,884,909,474,1229,991,348,1065,1553,353,1330
GCNT1_2_9940,198,298,269,294,582,433,185,39,249,188,223,86
GDPD1_2_9941,325,286,301,252,572,389,589,597,611,45,723,722
GDPD2_2_9942,176,253,275,186,154,103,159,38,110,313,722,181
GEM_2_9943,564,769,584,682,970,164,487,402,1089,697,49,659
GEN1_2_9944,36,47,84,54,31,3,134,138,10,193,107,26
GFM2_2_9945,172,91,109,251,15,35,290,107,244,37,0,134
GFOD1_2_9946,187,282,137,153,194,199,144,227,10,50,7,366
GGCT_2_9947,663,569,617,612,1173,1091,768,641,638,519,360,714
GGCX_2_9948,70,83,89,94,316,7,21,2,542,167,312,114
GGT1_2_9949,472,335,398,664,795,114,750,509,282,12,814,660
GGT5_2_9950,174,95,174,92,371,248,461,256,135,521,303,264
GGT6_2_9951,47,52,27,31,10,36,53,17,0,28,85,4
GGTLC1_2_9952,1054,1174,1121,1341,834,1340,2069,819,1380,1337,362,1613

GLB1_2_9953,208,38,43,82,0,16,70,130,213,10,9,368
GLRX2_2_9954,284,310,280,381,667,496,202,9,254,220,67,294
GLRX3_2_9955,185,198,250,296,33,103,0,197,497,183,651,626
GLRX_2_9956,500,327,476,367,359,82,196,40,412,1003,62,368
GLT8D1_2_9957,243,289,346,320,162,443,166,21,148,215,959,416
GLUL_2_9958,157,74,154,231,0,28,24,15,199,3,505,162
GM2A_2_9959,496,405,428,519,226,617,340,785,458,736,278,591
GMPPA_2_9960,270,113,78,287,419,187,343,355,0,39,71,267
GMPPB_2_9961,118,130,145,148,203,66,84,66,208,300,1,412
GMPR2_2_9962,188,206,216,224,12,129,291,41,268,0,378,295
GNAI2_2_9963,64,80,77,108,29,22,0,351,37,357,9,66
GNAL_2_9964,260,172,163,138,457,23,549,77,177,781,273,88
GNAO1_2_9965,99,136,131,134,133,3,3,18,37,231,156,92
GNAT1_2_9966,1444,925,1356,1484,870,680,1826,895,1286,1323,616,2394
GNB5_2_9967,123,26,119,217,28,44,68,7,259,25,606,206
GNG10_2_9968,89,55,43,150,0,43,71,395,10,43,33,149
GNG4_2_9969,1134,1025,1193,1219,1039,703,2468,752,774,1697,398,682
GNGT2_2_9970,363,272,312,391,117,370,231,183,34,417,705,334
GPD2_2_9971,1201,1020,1046,1026,704,364,425,897,1270,453,1528,692
GPHN_2_9972,385,161,264,192,156,107,51,970,364,202,100,377
GPNMB_2_9973,144,152,175,63,22,2,26,0,151,373,517,221
GPT2_2_9974,28,30,49,10,72,10,4,0,4,0,0,38
GPX4_2_9975,860,537,657,1036,537,674,972,541,670,656,1402,622
GSR_2_9976,148,62,84,124,11,22,90,217,40,71,63,80
GSTCD_2_9977,810,704,575,841,1379,423,677,773,379,643,1344,733
GSTK1_2_9978,1399,1531,1212,1494,180,623,1571,2422,553,1073,513,1468
GSTM1_2_9979,445,474,450,506,809,543,320,381,407,325,710,684
GSTM2_2_9980,711,490,653,689,204,675,616,401,205,225,661,192
GSTM4_2_9981,168,174,114,141,93,1,658,949,106,14,278,215
GSTO1_2_9982,136,168,137,152,11,334,61,524,105,531,479,298
GSTO2_2_9983,248,170,145,150,822,18,109,0,82,27,156,204
GSTZ1_2_9984,358,304,341,449,347,5,278,332,346,481,451,259
GTPBP3_2_9985,91,45,95,28,48,0,4,211,0,42,396,387
GUCY1A3_2_9986,207,419,284,208,268,2,419,455,214,52,438,19
GYG1_2_9987,225,139,213,320,259,214,540,83,45,237,985,484
GYG2_2_9988,1117,877,1041,950,2119,679,972,1081,971,963,1970,1518
GYS1_2_9989,241,298,274,272,108,316,255,174,68,231,265,340
HADH_2_9990,55,33,67,74,19,30,28,287,1,0,261,1
HAGH_2_9991,182,89,105,91,33,101,196,430,13,1,83,67
HAO2_2_9992,664,624,626,599,652,244,921,1043,739,540,1073,676
HAS3_2_9993,110,276,240,403,47,50,280,49,145,657,111,153
HCCS_2_9994,460,313,351,467,323,538,221,701,260,985,289,258
HDHD1_2_9995,1039,893,750,1010,1212,455,139,603,1112,1036,2533,466
HENMT1_2_9996,549,334,366,413,358,1049,895,18,379,291,344,351
HHAT_2_9997,233,114,140,172,441,3,301,402,18,111,5,146
HIBCH_2_9998,656,624,439,520,848,1182,397,790,277,385,230,925
HLCS_2_9999,233,203,111,171,42,280,184,17,9,358,0,555
HMBS_2_10000,160,185,197,200,113,219,123,54,184,206,436,67
HMGA2_2_10001,259,279,231,230,99,27,897,40,0,48,6,183
HMGCL_2_10002,156,87,65,116,0,24,166,1,9,11,0,142

HMGCLL1_2_10003,972,680,847,1090,727,1195,1471,528,578,1642,1276,1544
HMGCR_2_10004,403,409,281,444,296,84,110,305,976,136,235,76
HMGCS1_2_10005,366,370,321,178,147,373,30,384,372,31,123,211
HMGCS2_2_10006,1043,937,795,903,1388,173,1037,997,331,1510,635,1664
HMOX2_2_10007,1068,877,1012,1052,879,1154,954,526,1409,467,825,1129
HNRNPAB_2_10008,44,96,51,96,58,7,237,456,208,270,7,208
HPD_2_10009,289,274,277,259,518,267,109,0,179,158,805,100
HPGD_2_10010,69,11,11,45,13,4,2,86,0,4,0,1
HPSE2_2_10011,45,65,66,37,23,166,13,7,42,16,0,49
HPSE_2_10012,667,792,875,733,344,376,306,557,177,1291,974,1286
HRAS_2_10013,14,22,57,17,92,12,200,86,34,3,5,7
HS2ST1_2_10014,161,126,115,254,15,268,109,44,31,119,40,329
HS6ST2_2_10015,831,636,894,850,65,509,658,221,627,551,183,1058
HSD11B1_2_10016,826,864,571,686,1018,452,1176,668,789,554,572,657
HSD17B10_2_10017,47,95,107,50,52,0,0,107,92,3,2,6
HSD17B13_2_10018,763,866,634,777,1009,376,594,798,681,366,1177,184
HSD17B4_2_10019,554,587,579,559,977,259,640,536,1022,620,897,705
HSD3B2_2_10020,266,447,315,332,475,518,1205,69,712,1065,53,215
HSD3B7_2_10021,207,199,203,225,342,6,725,0,0,228,659,550
HSDL1_2_10022,321,108,194,242,26,0,0,272,2,0,392,201
HSP90AA1_2_10023,954,891,910,1302,708,648,974,580,1273,237,1039,1274
HSPA8_2_10024,455,525,451,392,462,299,661,583,292,331,376,960
HSPD1_2_10025,206,241,241,257,187,72,765,451,440,404,41,124
HTRA2_2_10026,280,203,251,213,24,50,305,1,12,276,2,301
HYAL1_2_10027,321,293,176,322,353,126,2092,1050,355,661,332,215
HYAL2_2_10028,373,322,261,314,458,118,254,310,247,333,207,567
HYI_2_10029,189,86,187,242,519,0,530,953,603,305,216,768
IARS_2_10030,741,805,814,967,782,737,542,377,861,744,695,600
IDE_2_10031,491,528,481,438,1103,114,300,99,670,601,340,763
IDH3B_2_10032,150,153,135,151,0,114,51,28,255,40,85,121
IDH3G_2_10033,0,4,13,8,0,0,4,0,0,5,0,4
IDS_2_10034,305,199,190,301,439,176,68,337,61,246,5,489
IFT27_2_10035,402,389,350,431,238,304,208,241,165,471,533,289
IL4I1_2_10036,163,9,53,190,0,2,9,90,0,1,0,296
IMPDH1_2_10037,43,122,40,118,0,101,32,490,1,127,406,309
INMT_2_10038,127,122,150,126,393,0,2,15,0,533,580,34
INPP5K_2_10039,1417,1367,1226,1292,1283,541,783,618,1309,857,1578,1577
INTS6_2_10040,523,412,336,396,365,341,513,414,171,301,992,46
IPCEF1_2_10041,214,214,310,235,91,311,774,9,307,224,131,170
ISOC2_2_10042,419,684,397,566,316,1140,284,762,215,13,911,683
ISPD_2_10043,775,692,649,722,1579,316,1013,451,477,314,1030,1463
ISYNA1_2_10044,57,62,27,79,2,0,0,0,97,0,0,21
ITPA_2_10045,144,312,387,355,342,106,839,44,229,412,236,917
IVD_2_10046,1621,1366,1157,1414,402,865,504,1851,880,2187,495,1613
IYD_2_10047,211,218,212,304,196,175,145,165,163,14,102,451
JMJD7-PLA2G4B_2_10048,686,557,413,598,664,44,473,648,261,499,351,772
KARS_2_10049,328,415,327,288,75,738,31,742,534,95,287,442
KATNA1_2_10050,24,92,66,94,9,0,10,0,11,9,1,11
KATNAL1_2_10051,511,465,427,553,198,605,338,317,857,437,279,279
KIF16B_2_10052,872,655,673,716,244,134,808,293,320,711,1061,372

KIF9_2_10053,336,371,195,238,177,67,132,747,95,216,635,249
KIFC3_2_10054,35,40,11,21,0,6,247,12,0,3,10,32
KLK2_2_10055,200,384,253,463,165,295,181,353,9,30,92,630
KLK7_2_10056,603,440,417,558,885,100,389,139,817,866,657,233
KRAS_2_10057,187,140,101,173,6,32,173,15,274,40,187,113
KYNU_2_10058,101,203,138,117,88,10,65,74,218,157,50,146
LAMP2_2_10059,271,141,206,191,381,223,146,468,107,183,1,404
LARGE_2_10060,23,4,8,29,0,0,0,57,3,0,8,0
LCLAT1_2_10061,54,139,63,54,0,0,3,95,29,7,32,9
LCMT1_2_10062,283,391,261,372,828,866,679,273,115,382,334,520
LDHA_2_10063,446,341,319,499,209,672,40,254,687,182,146,479
LDHAL6A_2_10064,1365,1492,1262,1435,409,996,1968,410,1308,1469,2324,14
60
LDHB_2_10065,799,526,521,719,649,725,532,293,435,33,1859,683
LDHC_2_10066,1501,1603,1831,1589,1769,1370,2648,1276,1037,1218,2221,54
8
LDHD_2_10067,397,326,370,402,163,89,307,438,507,821,213,293
LEPRE1_2_10068,280,206,202,176,187,53,317,145,8,33,0,291
LEPREL1_2_10069,467,218,274,556,330,394,415,837,301,538,250,487
LFNG_2_10070,793,665,617,727,727,666,250,795,562,981,881,1285
LGMN_2_10071,89,148,81,203,0,152,10,183,97,236,67,4
LGSN_2_10072,1434,1410,1182,1401,1380,620,361,961,1435,1882,1304,1115
LIAS_2_10073,2092,1610,1752,1880,2348,1585,2124,1551,2269,642,1045,203
9
LIG3_2_10074,199,140,125,90,0,75,17,18,0,3,18,138
LIG4_2_10075,167,278,179,326,145,529,39,306,4,66,657,232
LIPF_2_10076,1554,1149,1099,1599,1431,1608,1121,998,1528,1745,584,1318
LIPT1_2_10077,572,554,487,602,1248,70,1477,1215,295,248,1075,239
LMO7_2_10078,705,678,618,621,461,783,179,529,528,631,884,1805
LNPEP_2_10079,536,615,449,748,966,591,1029,328,649,1153,851,591
LOX_2_10080,82,133,50,177,33,382,80,144,54,193,10,90
LPO_2_10081,103,86,82,118,1,0,1,38,633,4,644,3
LRRC16A_2_10082,999,846,747,871,591,1031,212,204,1251,588,94,723
LSS_2_10083,67,35,67,40,28,21,0,20,83,6,2,2
LYZL6_2_10084,1462,1077,1232,1398,1362,1002,2112,649,1338,891,1256,250
4
MACF1_2_10085,161,273,166,143,0,101,681,134,52,5,23,217
MACROD2_2_10086,1414,1000,870,1107,1662,904,1395,873,1286,785,945,1467
MAD2L2_2_10087,90,45,50,93,46,2,0,390,170,1,0,0
MAN2B1_2_10088,75,115,73,112,1,146,362,21,146,189,176,107
MASP2_2_10089,0,29,6,3,0,0,0,0,0,6,0,0
MAT2B_2_10090,78,58,53,80,328,0,0,13,2,592,266,0
MCAT_2_10091,563,341,485,659,936,809,796,811,361,1079,1183,242
MCM4_2_10092,72,65,47,77,73,195,66,25,85,1,0,1
MCM7_2_10093,122,131,215,187,144,242,243,163,69,175,428,392
MCM8_2_10094,703,509,524,460,644,89,728,314,556,597,395,881
MCM9_2_10095,632,630,532,763,1044,230,710,132,39,745,636,509
MDH1_2_10096,522,502,471,549,551,277,582,858,572,555,590,869
MECR_2_10097,79,60,88,131,522,43,131,335,98,87,40,780
MEPCE_2_10098,25,19,19,24,11,256,27,29,3,0,83,156

METTL13_2_10099,116,118,110,60,130,70,181,73,42,49,440,247
MFN2_2_10100,83,58,28,96,35,0,0,0,2,165,11,0
MFNG_2_10101,367,413,495,410,38,207,0,966,269,249,17,267
MGAT3_2_10102,158,68,149,190,39,46,15,28,98,440,18,49
MGAT4A_2_10103,2973,2317,2278,2988,3209,2010,2565,3082,2158,2715,2264,
3255
MGAT4B_2_10104,44,38,46,40,18,101,27,127,6,16,30,76
MGLL_2_10105,38,69,91,12,3,0,3,1,1,0,0,393
MGST1_2_10106,463,438,469,605,701,130,2217,317,249,297,594,436
MGST2_2_10107,203,366,279,381,1,0,95,553,498,434,197,170
MICAL1_2_10108,109,90,47,120,2,12,0,638,68,52,107,38
MLH1_2_10109,574,489,554,569,483,1002,972,1157,21,369,854,509
MME_2_10110,92,142,88,117,309,93,242,197,57,272,432,246
MMP1_2_10111,202,170,187,207,729,235,63,24,487,173,106,102
MMP2_2_10112,952,903,700,1118,268,478,1812,2074,786,1941,1500,941
MOCS1_2_10113,363,410,490,569,489,271,447,401,303,177,825,177
MOGS_2_10114,317,268,290,451,236,5,67,633,500,347,328,680
MOV10_2_10115,725,597,559,657,753,486,348,896,113,852,649,981
MOV10L1_2_10116,550,643,503,502,53,574,394,602,304,1058,607,258
MPG_2_10117,394,466,334,575,437,201,377,445,574,1116,206,427
MPPE1_2_10118,131,137,92,229,170,23,15,337,51,129,85,172
MPST_2_10119,111,107,127,69,415,78,163,11,163,147,6,136
MRAS_2_10120,121,324,199,437,115,266,26,382,117,102,356,5
MRE11A_2_10121,303,211,211,314,50,251,214,59,490,961,350,202
MRI1_2_10122,27,40,54,83,0,304,0,311,0,0,4,0
MSH5_2_10123,289,230,202,256,380,62,151,306,95,273,84,172
MSRA_2_10124,208,217,204,259,274,28,0,15,1,692,113,316
MSRB3_2_10125,758,609,640,808,903,848,830,411,885,597,1145,584
MTHFD1L_2_10126,405,422,296,444,294,370,1171,473,582,138,755,262
MTHFS_2_10127,613,569,367,535,164,249,441,9,571,1013,138,887
MTO1_2_10128,127,186,204,197,374,262,105,204,133,29,375,140
MTRR_2_10129,3947,3697,3138,3876,3814,4189,4095,4124,2842,3726,1748,44
01
MUTYH_2_10130,1878,1547,1413,2021,1368,1406,1448,2153,982,1604,1375,26
65
MX1_2_10131,124,209,158,222,5,8,2,200,304,142,575,14
MYBBP1A_2_10132,201,87,172,157,106,15,0,25,100,69,520,84
MYH2_2_10133,317,398,264,365,429,381,908,345,280,656,367,707
MYO5A_2_10134,984,814,1046,952,738,1972,554,1639,1639,1089,1162,1412
MYO7A_2_10135,102,231,173,304,58,219,0,90,281,68,351,91
MYO9B_2_10136,143,217,120,248,0,0,0,0,299,251,0,98
N6AMT1_2_10137,367,462,328,289,205,1669,756,570,409,99,602,318
NAA16_2_10138,2043,1404,1654,1428,1723,1151,723,1464,2393,792,2066,195
8
NAA20_2_10139,381,387,362,466,347,102,332,382,424,206,217,270
NAAA_2_10140,101,105,94,180,0,27,748,3,12,144,0,674
NAPEPLD_2_10141,464,368,482,566,229,480,292,590,336,202,523,1055
NARF_2_10142,54,91,135,168,0,179,68,62,34,253,115,89
NARS2_2_10143,241,257,168,184,322,0,427,558,0,6,260,0
NAV1_2_10144,87,157,152,192,95,16,129,79,69,341,0,503

NCEH1_2_10145,2847,2471,2006,2671,1118,2405,2237,2387,2251,1960,1455,3
023
NCF2_2_10146,100,66,65,51,108,36,18,1,96,66,1,43
NCF4_2_10147,388,209,119,201,854,133,879,0,143,196,0,701
NDOR1_2_10148,7,0,17,0,0,0,0,0,0,0,0,0
NDUFA2_2_10149,589,776,646,654,1093,479,1690,381,36,766,1012,258
NDUFB11_2_10150,434,477,299,434,165,567,21,647,608,470,140,278
NDUFB4_2_10151,915,717,744,971,512,534,430,320,1456,791,484,1385
NDUFB5_2_10152,452,497,406,411,597,776,483,530,531,525,100,194
NDUFB6_2_10153,1589,1291,1087,1256,359,877,1829,667,824,1325,2209,984
NDUFC1_2_10154,434,543,476,478,233,743,428,416,200,435,265,509
NDUFS1_2_10155,168,166,101,123,0,10,13,296,40,3,0,1
NDUFS2_2_10156,740,602,619,630,48,279,71,1171,547,1785,552,801
NDUFS5_2_10157,1047,1016,1018,1133,1562,1266,1720,718,699,551,145,582
NDUFV1_2_10158,17,116,69,128,24,0,33,0,15,1,0,97
NEIL2_2_10159,53,77,30,63,0,0,543,26,57,0,0,65
NEU4_2_10160,132,198,173,82,52,98,146,110,14,444,171,152
NFS1_2_10161,367,475,484,406,244,1117,1470,486,193,469,643,513
NGLY1_2_10162,899,869,906,717,904,1102,1102,987,817,1399,1156,1227
NIPSNAP1_2_10163,503,400,346,491,48,648,1090,334,349,36,701,815
NIT1_2_10164,165,115,163,142,35,272,264,46,39,96,405,261
NKIRAS2_2_10165,226,124,131,156,2,148,129,140,28,39,141,90
NLGN3_2_10166,12,94,3,10,191,9,0,26,6,1,0,93
NLGN4X_2_10167,247,350,363,312,107,700,471,320,519,601,167,426
NLGN4Y_2_10168,685,646,538,730,110,928,486,541,434,968,629,445
NMNAT2_2_10169,202,267,224,405,355,169,306,185,104,437,417,323
NNT_2_10170,257,118,247,232,325,326,54,183,55,6,88,172
NOX4_2_10171,600,802,768,677,186,1653,454,327,1018,398,0,588
NPL_2_10172,54,41,104,16,7,1,0,2,223,7,1286,208
NQO1_2_10173,218,431,294,491,547,801,24,582,261,122,16,633
NRD1_2_10174,121,64,187,144,32,76,47,36,188,43,8,362
NSDHL_2_10175,564,657,486,655,755,350,464,32,252,149,176,729
NSUN2_2_10176,1771,1916,1507,1835,1837,1309,1610,1323,1065,1578,1505,2
533
NUDT2_2_10177,185,157,120,208,65,326,750,60,288,160,9,8
NXN_2_10178,332,334,377,346,547,174,212,557,211,483,116,152
OAS1_2_10179,105,203,174,133,240,54,290,424,210,201,100,147
OAS2_2_10180,387,276,269,347,269,67,166,435,283,292,478,189
OASL_2_10181,369,173,299,289,221,92,318,402,223,4,720,261
OAT_2_10182,116,201,132,152,508,39,342,16,232,52,173,68
OGDH_2_10183,139,110,109,73,139,12,14,76,87,75,236,122
OGDHL_2_10184,49,58,47,36,0,0,91,0,2,129,0,128
OGG1_2_10185,171,261,83,189,131,25,149,273,56,13,0,181
OGT_2_10186,41,57,35,51,0,133,2,6,113,144,63,107
OLAH_2_10187,285,169,275,261,217,103,43,382,291,599,24,567
P4HA1_2_10188,962,996,747,1178,964,468,1008,1226,477,968,2171,953
P4HA2_2_10189,114,99,80,114,75,156,49,22,122,255,74,26
P4HTM_2_10190,387,419,332,313,591,26,305,172,95,615,408,235
PAFAH1B2_2_10191,245,119,241,225,103,112,149,140,284,199,558,107
PAFAH1B3_2_10192,0,47,0,29,0,2,0,0,2,0,0,293

PAICS_2_10193,1183,1087,1098,1004,1214,1637,697,1012,996,1034,755,937
PAOX_2_10194,665,391,511,646,477,629,402,483,1506,333,592,653
PAPD4_2_10195,1992,1644,1931,2429,2896,1316,1224,1557,1511,1704,705,22
48
PAPD5_2_10196,817,938,845,916,461,721,505,962,1351,1268,593,1279
PAPD7_2_10197,715,663,706,851,450,256,861,697,850,1855,675,115
PAPSS2_2_10198,170,283,172,144,216,5,0,0,35,210,0,284
PARK7_2_10199,172,99,144,151,15,712,1,137,108,273,119,280
PARL_2_10200,256,199,215,303,288,419,116,138,255,81,39,387
PARN_2_10201,720,679,884,960,1140,798,457,852,690,202,647,812
PARP2_2_10202,34,27,41,41,3,23,18,0,18,0,0,0
PARP3_2_10203,299,169,305,196,486,17,566,0,0,192,227,60
PARP8_2_10204,324,150,105,216,98,363,36,107,145,228,316,239
PBLD_2_10205,223,226,194,219,624,16,98,102,193,119,251,611
PCCA_2_10206,441,535,491,504,495,343,153,939,686,642,273,412
PCCB_2_10207,172,310,205,334,216,1059,927,435,209,202,9,604
PCMTD2_2_10208,376,366,434,400,5,326,694,342,694,409,10,1059
PCNA_2_10209,84,31,44,24,299,58,0,332,11,3,0,0
PCSK1_2_10210,565,353,585,552,475,56,546,712,1182,86,124,569
PCSK2_2_10211,313,181,163,149,245,193,30,5,45,635,3,319
PCYT1B_2_10212,99,149,164,172,0,0,693,0,0,72,57,356
PCYT2_2_10213,1695,1187,1268,1390,1702,855,1288,1077,750,1677,1272,132
6
PDE10A_2_10214,206,189,113,125,132,69,340,12,59,239,94,23
PDE11A_2_10215,0,0,0,0,0,0,0,0,0,0,0,0
PDE1A_2_10216,641,507,537,515,361,575,363,218,775,729,26,441
PDE1B_2_10217,79,211,127,141,220,1,17,418,0,0,0,76
PDE1C_2_10218,8,16,37,54,29,0,21,300,6,4,0,0
PDE2A_2_10219,18,6,72,21,0,0,0,0,2,51,0,21
PDE4A_2_10220,379,142,156,203,246,571,667,487,46,51,145,63
PDE4B_2_10221,109,126,131,91,32,15,367,8,124,0,64,102
PDE4C_2_10222,185,142,161,120,151,93,228,234,73,46,1,102
PDE4D_2_10223,800,603,488,593,966,690,883,647,889,562,700,428
PDE5A_2_10224,507,468,499,478,886,651,937,94,477,423,1266,1178
PDE6B_2_10225,33,67,82,38,23,0,2,0,1,16,0,73
PDE7A_2_10226,139,106,144,146,2,0,396,428,26,0,0,138
PDE8A_2_10227,123,51,85,84,207,93,108,206,127,35,159,244
PDE8B_2_10228,832,522,669,799,119,502,296,489,1028,781,155,705
PDE9A_2_10229,294,263,170,186,170,32,499,277,402,81,551,52
PDHA1_2_10230,90,108,48,50,0,0,410,0,1,201,0,11
PDHB_2_10231,128,124,59,78,102,0,0,154,130,76,64,84
PDHX_2_10232,362,364,314,310,21,68,116,430,284,581,771,59
PEMT_2_10233,197,391,208,517,142,271,19,416,50,562,241,244
PEPD_2_10234,1374,1204,1227,1179,818,1264,1376,1791,1360,884,854,1883
PGAM5_2_10235,235,209,117,251,174,18,1,39,120,73,1,127
PGBD1_2_10236,219,248,185,331,167,325,128,0,30,66,61,152
PGC_2_10237,28,39,53,90,4,0,249,0,8,12,5,146
PGM1_2_10238,347,451,322,328,281,62,1133,1070,270,224,295,412
PGM3_2_10239,286,164,206,234,0,0,21,8,74,237,21,83
PHOSPHO1_2_10240,169,272,222,169,351,332,165,60,68,262,33,33

PHOSPHO2_2_10241,238,321,323,345,374,163,114,224,378,508,166,257
PHYH_2_10242,261,393,436,395,272,109,749,45,272,456,96,226
PIGA_2_10243,1212,814,881,1033,528,422,821,1650,1718,881,869,814
PIGC_2_10244,402,284,318,342,271,132,279,31,114,530,202,129
PIGF_2_10245,1365,1220,1021,1674,3203,1093,1632,2024,638,899,1450,1077
PIGG_2_10246,113,97,83,106,62,32,8,13,135,59,19,235
PIGN_2_10247,290,308,350,400,649,347,221,450,206,135,224,527
PIGO_2_10248,192,301,289,618,452,133,312,300,497,895,450,117
PIGP_2_10249,391,777,677,654,84,748,1525,397,523,1254,389,323
PIGQ_2_10250,83,222,82,223,74,203,3,554,602,273,481,483
PIGT_2_10251,167,112,197,259,661,770,425,317,156,28,583,0
PIGV_2_10252,40,74,38,42,0,0,0,0,2,185,1,1
PIN4_2_10253,1122,1316,973,1225,1090,790,1447,2315,1177,875,1681,473
PLA1A_2_10254,697,822,354,617,192,830,506,484,1316,415,841,501
PLA2G2A_2_10255,144,162,73,240,306,271,83,93,129,105,0,94
PLA2G4C_2_10256,876,1142,882,1047,825,449,651,44,1599,751,662,624
PLA2G6_2_10257,158,284,197,280,8,40,373,113,45,56,150,553
PLA2G7_2_10258,1033,677,781,984,577,1330,700,232,1328,777,283,1555
PLAT_2_10259,1384,941,839,1324,1243,459,639,301,781,476,2102,879
PLAU_2_10260,169,290,141,183,36,120,12,0,213,490,20,58
PLB1_2_10261,426,304,306,363,385,79,186,358,515,312,520,275
PLCB1_2_10262,191,93,98,126,271,104,57,104,327,236,251,140
PLCB3_2_10263,62,81,126,90,17,0,74,65,78,34,42,69
PLCB4_2_10264,491,633,537,610,321,820,904,913,491,1525,86,793
PLCD1_2_10265,123,112,138,51,135,0,308,296,257,235,303,1
PLCE1_2_10266,833,1108,931,1301,1044,412,1232,1203,734,1305,806,380
PLCG1_2_10267,479,479,370,602,442,220,611,421,104,126,630,773
PLCH1_2_10268,1303,1013,1237,1423,892,684,1693,930,404,1040,1241,1037
PLCL2_2_10269,899,783,867,778,872,1083,1227,268,835,1098,1184,706
PLCXD2_2_10270,184,213,245,235,0,250,97,139,190,288,87,190
PLD2_2_10271,60,142,237,124,1,0,6,304,0,92,210,480
PLD3_2_10272,249,271,454,358,769,53,2,358,94,90,7,1345
PLOD2_2_10273,350,393,352,327,223,61,66,215,291,1609,133,461
PLSCR3_2_10274,1349,1450,1645,1589,1298,2169,1961,1197,1595,1255,2166,
1860
PLSCR4_2_10275,380,326,352,496,154,271,169,817,260,561,72,222
PMEL_2_10276,738,507,660,688,987,210,1007,701,418,365,730,957
PMS1_2_10277,522,528,565,790,428,417,738,251,1409,726,104,636
PNPLA1_2_10278,46,63,158,41,42,3,3,1,115,4,133,111
PNPLA4_2_10279,67,136,208,135,159,385,313,208,11,118,13,166
PNPLA6_2_10280,564,442,416,480,638,376,262,229,263,311,488,636
PNPLA7_2_10281,199,359,141,259,364,322,366,71,64,0,0,1
POFUT1_2_10282,111,31,8,75,290,1,35,129,1,8,43,2
POFUT2_2_10283,286,224,109,240,12,184,278,458,61,254,581,392
POGZ_2_10284,238,236,256,482,0,654,594,35,127,52,213,156
POLD2_2_10285,265,201,89,300,0,9,113,61,472,92,614,550
POLE2_2_10286,242,165,204,375,260,223,37,366,156,326,314,141
POLG_2_10287,48,14,25,57,50,13,65,68,19,10,0,0
POLL_2_10288,366,454,235,328,1393,78,782,388,320,170,22,633
POLR1D_2_10289,121,169,163,243,44,26,136,254,150,366,61,72

POLR3B_2_10290,1206,860,1166,1176,1093,932,1898,1280,1285,516,362,919
POLR3H_2_10291,341,327,151,290,65,103,36,92,121,252,938,239
POMT1_2_10292,116,42,16,55,574,1,7,6,44,85,1,23
PON2_2_10293,293,228,241,160,183,816,237,95,276,129,598,60
POP5_2_10294,376,385,396,567,880,351,828,1186,459,267,1004,653
PPA2_2_10295,759,552,569,634,893,1432,795,656,1068,609,243,1011
PPIE_2_10296,230,175,206,130,635,0,51,0,175,542,217,499
PPIL2_2_10297,197,124,96,183,39,229,300,22,8,64,23,175
PPIL3_2_10298,1211,652,687,920,625,1114,643,503,746,590,56,1863
PPIL6_2_10299,460,715,653,817,274,372,324,700,216,922,536,1206
PPIP5K1_2_10300,28,40,49,69,0,0,0,3,4,6,232,20
PPT1_2_10301,475,405,297,445,175,299,466,264,715,850,135,235
PPT2_2_10302,211,126,237,216,189,285,4,88,57,342,76,130
PRCP_2_10303,560,446,351,729,229,199,339,40,481,941,417,498
PRDX1_2_10304,465,302,349,566,410,366,1665,679,391,412,435,913
PRDX3_2_10305,321,616,390,391,89,890,132,334,314,555,452,414
PRDX5_2_10306,31,48,39,198,357,0,9,261,165,22,155,2
PRKCSH_2_10307,264,133,272,209,458,72,226,26,630,26,0,232
PRODH_2_10308,131,202,309,254,40,3,0,378,586,678,0,109
PRSS3_2_10309,1070,1104,1035,1037,1381,720,711,628,908,1229,1405,1155
PSAT1_2_10310,313,243,442,281,26,82,236,195,709,133,4,392
PSMA1_2_10311,4,11,27,21,0,0,0,0,0,0,0,0
PSMA3_2_10312,1132,1185,1033,1225,706,591,1108,498,857,1422,1766,1533
PSMA4_2_10313,640,776,671,883,1089,284,405,979,142,506,1107,953
PSMA5_2_10314,499,420,413,494,641,579,267,926,1235,257,255,445
PSMA8_2_10315,333,344,306,452,211,27,797,168,164,314,1049,731
PSMB2_2_10316,145,175,226,267,101,952,1,47,274,134,147,67
PSMB5_2_10317,292,196,92,159,41,0,63,349,435,7,592,20
PSMB8_2_10318,265,222,90,123,567,229,115,6,78,27,33,9
PTBP1_2_10319,187,246,148,275,181,2,269,185,332,263,231,558
PTER_2_10320,660,1156,867,936,718,789,528,1495,911,1054,504,1216
PTGR1_2_10321,698,410,490,625,469,676,396,753,546,7,112,591
PTGR2_2_10322,471,249,311,252,8,276,0,33,270,275,457,638
PTGS1_2_10323,182,110,369,288,61,150,13,539,155,244,99,132
PUS1_2_10324,69,104,133,149,56,137,297,29,126,115,441,435
PYCR1_2_10325,126,126,39,127,0,2,0,380,164,479,101,6
PYGL_2_10326,778,995,695,826,313,1535,1852,468,809,731,1000,1490
PYGM_2_10327,594,507,488,384,1370,337,775,222,533,106,423,345
QPCTL_2_10328,300,379,186,205,100,26,600,281,53,360,448,216
QSOX1_2_10329,361,227,195,313,174,78,230,44,197,184,54,219
RAB11A_2_10330,170,137,181,167,146,2,9,404,191,309,53,85
RAB1A_2_10331,531,464,333,395,213,58,766,204,559,213,344,379
RAB23_2_10332,244,239,154,205,21,201,83,23,103,278,221,120
RAB27A_2_10333,1864,1736,1536,1601,2100,1301,1925,1965,1269,1291,3242,
1245
RAB28_2_10334,1061,1017,787,1117,1521,971,1747,685,318,469,1497,846
RAB2A_2_10335,805,971,876,1051,916,435,427,260,580,797,1742,1217
RAB2B_2_10336,56,90,88,117,114,161,211,93,77,39,0,17
RAB34_2_10337,0,0,0,0,0,0,0,0,0,0,0,0
RAB35_2_10338,54,178,176,90,2,0,49,173,1,23,1,0

RAB37_2_10339,157,160,126,195,3,35,295,150,114,50,117,353
RAB40C_2_10340,81,52,116,98,28,223,22,5,95,186,43,129
RAB5C_2_10341,1277,1165,926,1275,376,1632,986,1049,863,925,442,1309
RAB6A_2_10342,1094,755,836,939,1283,871,814,961,291,1524,907,1298
RAB7L1_2_10343,387,211,258,170,1078,695,69,34,462,329,472,247
RAB9A_2_10344,360,382,321,579,403,583,80,5,380,74,15,624
RABGGTA_2_10345,46,11,34,174,12,0,492,369,2,20,0,0
RABL2A_2_10346,43,96,83,112,53,0,128,89,69,235,0,99
RABL2B_2_10347,43,96,83,112,53,0,128,89,69,235,0,99
RAC1_2_10348,545,501,444,497,987,938,54,353,470,664,43,725
RAD51B_2_10349,113,83,129,144,5,140,148,78,242,108,9,190
RAD51_2_10350,239,229,263,335,101,394,314,74,267,327,151,158
RAD51D_2_10351,340,275,319,410,792,357,530,1,88,385,414,418
RAD54B_2_10352,380,430,295,358,360,433,481,3,140,671,509,585
RAD54L_2_10353,548,634,448,617,340,210,526,816,706,379,476,261
RAD9A_2_10354,736,725,816,798,1145,544,437,1225,118,101,433,383
RAP1A_2_10355,707,384,363,489,519,533,350,320,474,218,892,424
RAP1B_2_10356,623,797,742,773,24,793,1986,999,267,1132,720,1054
RBBP8_2_10357,412,351,462,431,618,182,422,272,357,456,94,488
RDH13_2_10358,16,37,9,28,0,1,12,0,5,285,0,26
RDH5_2_10359,93,139,44,42,0,0,35,278,70,106,34,18
RECQL5_2_10360,314,165,176,236,171,526,123,136,664,201,392,378
RECQL_2_10361,1044,812,811,1125,1094,1134,826,427,1010,1119,471,1119
RERG_2_10362,169,245,160,226,0,44,86,135,549,778,113,257
REV1_2_10363,127,178,240,227,104,27,347,103,20,241,525,228
RFC3_2_10364,191,157,158,168,23,436,93,42,129,280,184,86
RFC5_2_10365,1427,1070,1136,1291,1300,38,840,844,1176,1166,654,1151
RGN_2_10366,226,229,153,333,248,34,216,197,127,52,274,137
RGS11_2_10367,127,88,89,53,637,205,33,0,20,0,0,34
RGS6_2_10368,52,72,26,35,72,1,0,1,0,322,32,66
RHOC_2_10369,564,555,470,662,690,585,135,617,417,447,801,604
RHOT1_2_10370,298,150,229,259,461,32,221,65,311,381,19,11
RNASE1_2_10371,490,486,342,447,447,412,1355,31,566,1530,605,137
RNASE4_2_10372,66,132,73,81,13,4,18,0,17,18,48,15
RPAP3_2_10373,813,527,504,671,775,322,433,679,548,978,511,857
RPE_2_10374,772,468,744,915,536,667,330,201,627,262,912,820
RPN2_2_10375,124,37,22,118,80,0,1,18,0,61,0,7
RPP14_2_10376,663,709,592,431,589,590,443,496,548,289,342,533
RPP21_2_10377,94,256,164,220,203,111,1,521,648,136,127,69
RPP30_2_10378,465,125,278,287,274,157,67,128,38,14,44,122
RPP38_2_10379,168,132,83,171,310,0,334,13,409,20,404,62
RPS27A_2_10380,273,173,232,225,90,289,458,417,372,226,452,521
RRAD_2_10381,544,644,594,685,315,1001,700,819,583,291,614,1243
RRAGB_2_10382,0,0,9,26,0,1,0,0,6,1,0,232
RRAS2_2_10383,1038,858,819,1095,1133,948,1719,975,768,2029,2083,607
RRM2B_2_10384,1548,1168,1278,1293,382,2493,3277,594,1106,1133,1604,114
1
RRM2_2_10385,0,0,0,0,0,0,0,0,0,0,0,0
RTEL1_2_10386,579,612,454,629,447,1039,193,421,585,618,137,853
SAR1A_2_10387,1224,1539,1581,1508,1011,1619,1678,1887,1182,1416,1779,2

232
SAR1B_2_10388,618,393,477,552,354,79,440,678,529,336,490,636
SARDH_2_10389,126,156,114,131,459,824,107,0,289,165,71,343
SARS2_2_10390,17,27,13,31,0,23,15,59,3,0,0,0
SBNO1_2_10391,75,94,188,79,21,75,162,253,36,0,228,33
SC5DL_2_10392,327,412,425,557,469,37,889,295,245,208,0,944
SCD5_2_10393,302,284,200,453,174,45,151,826,758,824,361,551
SDC1_2_10394,262,240,294,173,202,146,421,256,543,220,28,198
SDCBP_2_10395,70,97,124,100,53,105,155,1137,129,6,1,205
SDHC_2_10396,91,94,59,93,24,43,270,69,12,57,21,3
SEPHS1_2_10397,859,717,702,788,645,619,1605,1407,1086,1472,1141,800
3-Sep_2_10398,117,166,137,227,57,370,5,766,168,316,122,104
4-Sep_2_10399,299,206,178,401,188,142,716,1,215,854,14,72
5-Sep_2_10400,88,163,105,152,94,146,156,250,119,9,78,70
9-Sep_2_10401,523,345,373,334,2,53,1450,2,146,0,175,385
SGMS2_2_10402,589,636,642,733,929,284,574,788,431,1107,288,341
SH3GLB1_2_10403,206,178,128,196,327,0,1,233,142,55,8,93
SHMT1_2_10404,154,181,90,57,399,44,774,372,133,83,276,199
SHMT2_2_10405,146,132,196,127,63,279,163,370,122,272,970,964
SIAE_2_10406,144,143,123,222,798,0,14,8,183,120,100,232
SLFN11_2_10407,128,159,94,153,7,8,410,23,162,394,0,51
SMG6_2_10408,335,268,256,287,153,497,531,0,132,7,220,301
SMOX_2_10409,203,117,94,113,470,11,19,419,447,33,59,39
SMPD1_2_10410,480,267,134,295,390,0,0,271,0,335,189,0
SMPD4_2_10411,40,85,133,188,238,16,0,0,75,4,0,251
SMPDL3B_2_10412,437,378,304,269,148,803,257,484,180,131,16,394
SOD2_2_10413,58,25,36,58,14,1,8,48,58,56,34,2
SPAM1_2_10414,1068,760,661,1020,430,956,935,825,1635,1004,1191,253
SPAST_2_10415,120,176,152,196,10,208,292,148,53,211,147,158
SPG21_2_10416,2492,2268,2126,2272,2863,1814,2685,3194,2080,3616,2347,1
985
SPO11_2_10417,1428,943,936,1069,2077,1403,503,918,1294,1389,1193,1017
ST3GAL1_2_10418,136,134,126,138,176,89,231,526,113,50,436,336
ST3GAL5_2_10419,933,529,725,779,882,972,915,461,1360,954,2146,1127
ST5_2_10420,376,300,197,295,724,113,5,119,89,76,126,57
ST6GAL1_2_10421,273,256,315,287,4,211,463,673,2,480,906,132
ST6GAL2_2_10422,488,586,486,423,455,829,1140,105,756,1058,435,476
ST6GALNAC3_2_10423,1237,975,1013,841,851,2031,1947,628,941,393,934,782
ST6GALNAC4_2_10424,428,427,367,478,1026,440,82,803,460,176,45,765
ST8SIA4_2_10425,1590,1526,1389,1616,1222,1525,2360,1149,2028,2786,966,
1790
SUCLG2_2_10426,185,100,152,137,296,167,541,405,70,15,290,57
SULF1_2_10427,6,13,22,26,0,0,2,0,0,14,0,2
SULF2_2_10428,568,329,427,557,611,776,549,181,1222,914,435,965
SULT1A1_2_10429,347,541,327,461,662,118,170,490,250,116,775,570
SULT1A2_2_10430,429,386,228,352,132,97,72,88,270,362,130,634
SULT2B1_2_10431,72,14,26,99,0,0,0,56,1,11,9,2
SUOX_2_10432,278,347,328,450,370,525,43,466,315,234,68,195
SYTL1_2_10433,167,248,184,185,2,0,0,0,6,0,440,129
TAB1_2_10434,47,27,50,43,144,0,2,24,6,8,276,4

TBXAS1_2_10435,548,452,608,461,1211,622,199,405,956,335,1070,392
TCIRG1_2_10436,730,537,654,942,314,375,2,542,1392,885,602,670
TDP1_2_10437,995,983,1132,987,2204,1547,1244,1098,1675,801,1656,1378
TERT_2_10438,1199,1033,995,1301,961,1214,1045,875,423,675,1663,808
TGM2_2_10439,301,145,198,254,1340,330,25,36,41,9,520,22
TGM5_2_10440,129,92,185,130,81,100,59,5,99,11,394,137
TH_2_10441,241,246,171,234,95,154,387,661,307,610,2,128
TIAM2_2_10442,124,119,49,139,11,246,0,0,53,16,0,1
TKTL1_2_10443,14,37,30,72,87,1,0,127,42,1,0,0
TMEM55B_2_10444,801,975,1040,917,258,870,722,736,1444,460,210,482
TMLHE_2_10445,127,74,62,100,70,6,0,233,58,47,773,446
TMOD1_2_10446,496,661,398,419,483,358,1236,501,805,336,610,400
TMX2_2_10447,48,16,32,37,59,0,11,225,112,200,18,0
TNNI2_2_10448,0,19,30,12,1,0,0,0,0,0,0,0
TP53I3_2_10449,424,192,425,381,52,233,404,17,34,106,656,536
TPI1_2_10450,111,34,63,80,101,0,150,29,36,0,0,40
TPST2_2_10451,10,11,28,52,0,190,0,10,20,15,273,1
TREX1_2_10452,24,66,35,32,3,155,144,0,17,2,0,206
TRMT1_2_10453,192,273,387,242,177,204,0,460,13,562,43,542
TRMT1L_2_10454,562,458,404,561,242,513,72,116,121,499,299,298
TRMT2B_2_10455,189,191,118,151,487,114,210,234,121,198,61,161
TRPT1_2_10456,124,99,95,237,55,509,0,9,38,14,0,84
TSEN2_2_10457,591,617,645,712,312,317,1971,912,378,811,335,720
TSEN34_2_10458,1,1,6,0,7,0,0,1,0,65,0,0
TTLL6_2_10459,1100,755,954,1244,795,585,966,1220,1474,1190,137,961
TUBAL3_2_10460,0,8,4,52,0,0,0,9,0,0,2,414
TUSC3_2_10461,278,151,124,168,171,236,23,1151,47,89,28,441
TXNDC16_2_10462,2919,2369,2475,2955,1733,905,2472,1840,2571,3689,3176,
2661
TXNDC5_2_10463,875,664,771,843,579,54,895,1242,693,386,51,1179
TXNL4B_2_10464,494,364,534,533,487,191,107,574,64,853,1566,553
TXNRD1_2_10465,900,535,746,666,292,741,1367,643,514,507,1122,496
TXNRD3_2_10466,267,313,431,544,425,281,107,638,193,271,1106,234
TYMP_2_10467,196,365,257,290,412,274,496,779,311,423,88,97
UBA52_2_10468,345,473,347,484,125,351,14,736,225,1078,172,688
UEVLD_2_10469,465,608,493,563,196,138,375,728,483,167,223,594
UGDH_2_10470,33,39,14,105,0,18,12,0,0,3,0,39
UGP2_2_10471,1302,1157,1155,1386,1248,221,2323,1011,1090,649,1154,1381
UGT1A6_2_10472,7380,7453,6654,7381,9006,6776,5932,5233,7222,7506,7058,
6773
UGT2B10_2_10473,1647,1238,1166,1314,344,920,1990,1033,869,884,987,1745
UGT2B28_2_10474,790,723,786,839,518,372,117,175,882,387,505,706
UGT3A1_2_10475,439,344,346,372,335,71,197,307,615,320,1529,495
UGT8_2_10476,776,549,653,545,813,1529,1254,1831,1060,1196,202,642
UNG_2_10477,334,169,474,317,291,222,359,625,216,632,528,229
UPP1_2_10478,545,622,638,745,264,1192,881,19,749,365,278,611
UPP2_2_10479,338,420,226,560,203,235,466,344,591,870,878,652
UQCR10_2_10480,52,26,13,44,13,9,43,1,5,5,87,0
UQCRB_2_10481,128,112,56,122,283,64,71,134,40,259,28,282
UROC1_2_10482,767,708,761,1152,581,549,1561,1248,656,947,1065,904

VCL_2_10483,1569,1151,1064,1237,1192,1322,1270,1281,1643,923,1608,1358
VNN2_2_10484,590,686,536,638,279,276,773,343,721,1027,211,708
VPS29_2_10485,314,278,339,235,828,198,18,55,285,331,536,360
WARS2_2_10486,65,15,42,32,5,0,1,0,0,0,0,38
WARS_2_10487,129,109,74,160,221,115,1,32,0,325,0,308
WBSCR22_2_10488,721,411,569,688,424,1247,512,376,370,264,398,531
WDR46_2_10489,118,94,55,162,439,7,138,95,1,259,1,183
WFS1_2_10490,18,14,42,101,6,73,167,86,211,0,4,0
WRNIP1_2_10491,282,273,282,322,86,1,174,242,154,306,826,116
WWOX_2_10492,12,16,49,12,95,3,0,23,0,0,0,0
XPNPEP1_2_10493,548,258,364,327,581,498,260,0,823,149,925,141
XRCC3_2_10494,58,65,45,59,25,255,561,1,104,18,0,132
XRN1_2_10495,1103,921,808,904,1090,723,841,732,1154,1053,632,428
YWHAZ_2_10496,190,128,141,173,267,67,59,228,195,114,65,42
ZBED1_2_10497,448,381,469,385,158,564,145,63,272,116,474,292
ZCCHC11_2_10498,254,217,229,344,430,163,183,95,106,293,0,95
ZCCHC6_2_10499,315,184,252,255,368,472,295,279,148,100,189,187
ZDHHC15_2_10500,2218,2092,1924,2340,2391,927,3148,1750,2654,2396,2509,
1632
ZDHHC7_2_10501,603,702,479,743,803,657,1145,883,485,265,862,735
ACYP1_2_10502,313,254,352,325,548,127,364,220,630,573,570,235
APOBEC3F_2_10503,1482,1127,1293,1578,1124,952,594,987,619,1972,456,143
0
ASCC3_2_10504,556,557,475,426,321,147,384,305,281,393,175,165
ASPH_2_10505,307,490,323,553,375,229,432,605,249,452,1887,226
ATP6V0E2_2_10506,21,37,61,27,21,0,208,609,1,28,0,216
C10orf2_2_10507,658,870,683,922,990,632,897,1,0,726,1602,114
CAPN3_2_10508,0,0,0,0,0,0,0,0,0,0,0,0
CHM_2_10509,332,336,266,426,331,344,251,325,339,53,353,188
CTSC_2_10510,238,69,221,189,76,6,46,91,354,147,35,90
CYBRD1_2_10511,1856,1846,1659,1863,810,2646,2107,2003,1118,2054,672,16
28
CYP3A5_2_10512,766,882,779,693,745,676,908,741,696,793,380,1317
DIO2_2_10513,2541,3027,2468,3032,2915,4659,1942,1803,2515,3516,2659,30
79
DNASE2B_2_10514,131,297,220,390,67,16,29,1189,73,263,180,192
DPYD_2_10515,450,208,394,409,877,141,383,226,44,439,95,312
GBA3_2_10516,567,386,308,369,407,435,189,267,233,346,476,27
GCNT2_2_10517,1206,1457,906,1377,1497,470,2712,948,1209,1848,198,1626
GLYAT_2_10518,390,412,264,432,663,298,256,161,244,923,483,939
GPLD1_2_10519,493,510,511,499,640,416,605,507,149,62,772,763
GPX1_2_10520,327,361,278,308,186,454,1082,440,330,119,366,155
GPX5_2_10521,495,517,435,585,234,546,328,187,692,753,1167,299
HNMT_2_10522,0,0,0,0,0,0,0,0,0,0,0,0
HOGA1_2_10523,72,66,30,152,318,0,6,3,1,15,0,128
HYAL3_2_10524,227,233,254,290,8,9,200,315,246,232,177,572
JPH2_2_10525,98,95,79,45,0,88,139,165,260,333,144,19
KLK8_2_10526,115,118,124,168,306,21,13,0,152,304,639,27
LAMA4_2_10527,108,178,215,185,1,200,452,0,0,1,538,73
LRR1_2_10528,37,42,18,63,0,2,0,1,4,0,0,0

LRTOMT_2_10529,272,303,257,320,163,140,289,455,62,468,153,149
METTL1_2_10530,49,36,38,124,151,0,31,14,25,15,17,160
MOCS2_2_10531,236,249,161,217,368,491,494,234,0,541,277,240
NDUFA11_2_10532,117,108,87,143,189,68,243,310,86,135,1,262
NDUFV3_2_10533,294,361,332,378,531,176,58,79,203,704,6,648
NMNAT3_2_10534,527,475,471,471,1066,212,89,551,200,812,154,691
PDE4DIP_2_10535,140,130,114,69,89,65,226,0,99,84,44,1
PLG_2_10536,91,103,101,146,4,112,134,537,69,63,9,7
PPCS_2_10537,958,717,995,980,922,698,825,559,1144,852,304,967
PRDX2_2_10538,72,114,55,72,92,1,884,5,1,289,0,0
RAD51C_2_10539,1139,909,832,933,951,671,1027,471,911,1006,1570,736
RASD1_2_10540,3,0,2,6,0,2,0,0,0,27,85,121
RASL10A_2_10541,58,32,49,65,18,126,0,63,4,8,0,92
SLX1A_2_10542,206,108,239,269,4,42,1,333,7,245,580,64
SLX1B_2_10543,206,108,239,269,4,42,1,333,7,245,580,64
SPTLC1_2_10544,381,323,166,410,0,599,140,0,587,128,0,33
ST3GAL3_2_10545,292,263,229,299,45,496,120,146,164,477,559,336
VKORC1_2_10546,386,371,412,542,8,281,1121,1063,292,5,1561,68
XPNPEP3_2_10547,79,64,54,61,144,259,101,304,29,0,0,89
GNAS_2_10548,227,239,277,226,1338,69,24,1,66,356,63,773
GNAS_2_10549,299,330,645,478,668,124,1076,639,473,40,176,299
GNAS_2_10550,556,283,346,256,62,9,766,242,493,537,28,314
A4GALT_2_10551,606,307,379,416,614,42,90,51,660,436,495,390
A4GNT_2_10552,273,320,153,283,240,482,164,42,302,612,93,88
AACS_2_10553,155,118,147,45,297,1,4,0,24,35,487,1
AADAC_2_10554,134,47,117,78,83,61,17,13,298,122,20,404
AARS2_2_10555,119,55,66,145,54,366,15,62,150,16,23,0
AARS_2_10556,99,75,39,151,4,1,0,258,83,9,0,67
AASDH_2_10557,2104,1428,1655,1952,1662,2050,2614,3072,1317,2237,456,21
03
AASDHPPT_2_10558,212,145,137,102,122,29,150,143,18,280,306,124
AASS_2_10559,331,141,169,214,251,358,96,58,122,457,673,240
ABHD14A_2_10560,185,140,107,160,268,75,165,106,193,141,123,134
ABHD1_2_10561,28,87,31,29,81,0,0,0,56,0,21,14
ABHD3_2_10562,194,123,168,246,91,169,70,68,446,257,18,109
ABHD5_2_10563,95,119,120,181,75,0,346,390,118,15,147,110
ABHD6_2_10564,178,100,172,230,394,4,89,221,73,93,45,62
ABHD8_2_10565,147,153,66,92,55,1,310,32,9,50,146,80
ABO_2_10566,192,148,171,132,248,137,144,503,356,429,175,107
ABP1_2_10567,980,987,902,1401,883,702,1898,538,1443,861,563,1165
ACAA2_2_10568,134,59,31,46,77,31,0,21,200,158,151,149
ACACB_2_10569,90,239,167,88,90,106,25,12,3,15,219,127
ACAD11_2_10570,788,897,678,687,437,168,747,633,1398,367,844,489
ACAD8_2_10571,580,319,373,401,696,375,441,666,182,802,613,587
ACAD9_2_10572,132,249,216,227,9,31,72,35,8,209,0,145
ACADL_2_10573,284,391,236,279,436,48,0,671,272,124,125,88
ACADSB_2_10574,1734,1211,1313,1399,1418,1151,2156,905,1288,1271,1133,1
394
ACADS_2_10575,402,352,312,388,159,437,103,472,192,336,136,318
ACAT1_2_10576,2127,2082,2198,2655,2107,2830,3154,1856,2273,1769,3446,1

695
ACER1_2_10577,243,161,228,214,100,625,112,1,4,29,393,224
ACER2_2_10578,604,353,649,1364,343,4,881,608,514,760,1924,834
ACER3_2_10579,198,263,141,191,34,29,62,285,140,86,490,347
ACMSD_2_10580,179,133,167,48,27,348,15,299,10,39,194,118
ACO1_2_10581,600,454,589,396,1063,221,517,379,221,726,958,497
ACO2_2_10582,587,301,479,369,354,465,582,2060,306,327,892,565
ACOT12_2_10583,39,33,54,40,109,52,19,12,1,12,26,28
ACOT1_2_10584,583,406,364,307,630,589,339,333,904,903,725,705
ACOT2_2_10585,633,782,783,780,316,403,610,371,1031,1286,968,591
ACOT4_2_10586,574,347,503,410,180,61,279,410,581,229,51,547
ACOT6_2_10587,362,277,327,261,325,51,439,24,316,289,750,571
ACOT8_2_10588,609,359,331,480,916,468,736,375,750,248,190,275
ACOX2_2_10589,1485,1004,998,1187,1117,1120,400,904,1950,1211,737,500
ACOXL_2_10590,396,417,297,193,35,281,266,50,181,130,348,131
ACR_2_10591,285,196,337,309,300,161,0,5,135,12,911,199
ACSBG2_2_10592,270,133,278,194,8,207,9,430,216,37,18,182
ACSF2_2_10593,109,80,103,173,209,70,509,19,56,241,142,89
ACSL1_2_10594,381,436,353,428,863,843,23,2,559,293,521,725
ACSM1_2_10595,351,332,355,335,373,254,230,85,325,135,284,427
ACSM2A_2_10596,253,245,165,236,14,18,403,316,151,184,0,611
ACSM4_2_10597,11,65,45,36,0,210,51,167,5,86,412,22
ACSM5_2_10598,143,16,89,49,0,252,5,30,192,0,116,0
ACSS1_2_10599,304,236,179,311,537,507,315,191,390,204,157,143
ACSS3_2_10600,296,239,325,428,30,394,261,228,312,645,268,744
ACTC1_2_10601,324,218,309,217,40,165,1062,12,167,163,18,726
ACY3_2_10602,293,413,327,587,798,987,81,472,477,562,554,402
ACYP2_2_10603,415,299,297,323,471,14,478,844,371,323,405,616
ADA_2_10604,49,72,74,36,15,2,1,2,11,19,527,11
ADAM10_2_10605,220,94,72,187,5,99,205,4,84,9,0,343
ADAM17_2_10606,252,397,234,288,4,810,723,86,217,317,276,536
ADAMTS4_2_10607,528,248,240,304,812,233,123,3,439,218,2,51
ADARB2_2_10608,22,26,34,12,0,489,378,7,247,0,2,14
ADAT1_2_10609,1138,1007,796,783,985,712,902,466,505,625,904,1001
ADAT2_2_10610,623,657,563,710,33,333,366,407,459,670,548,487
ADC_2_10611,60,42,41,189,1,0,0,0,0,273,0,152
ADCY1_2_10612,72,106,73,171,2,285,0,0,173,2,0,23
ADCY2_2_10613,287,293,196,286,543,98,751,760,335,239,45,232
ADCY7_2_10614,124,229,161,84,1,0,10,69,4,30,34,260
ADCY8_2_10615,43,99,48,131,109,87,170,157,0,188,70,2
ADCY9_2_10616,646,721,525,537,110,604,980,9,728,564,388,437
ADH1A_2_10617,102,78,68,81,214,105,36,173,282,214,5,10
ADH1B_2_10618,388,383,343,478,623,392,561,737,251,529,108,386
ADH1C_2_10619,1882,1883,1931,1911,1777,2012,3314,1318,1904,1805,1663,2
120
ADH4_2_10620,398,447,382,526,881,641,610,1012,251,1067,175,698
ADH5_2_10621,790,628,747,1055,1161,560,734,929,633,453,1113,332
ADHFE1_2_10622,339,135,157,250,6,0,132,8,158,61,417,132
ADI1_2_10623,120,49,42,90,22,0,331,9,31,57,0,51
ADO_2_10624,165,287,184,182,283,106,1,53,180,19,993,109

ADPRH_2_10625,174,92,100,70,434,3,77,180,16,377,354,36
ADPRHL2_2_10626,134,135,122,107,161,8,89,91,230,223,43,164
ADSS_2_10627,264,152,129,165,127,68,4,0,278,293,226,125
AEN_2_10628,51,58,25,72,0,3,12,41,0,14,0,115
AGBL2_2_10629,117,134,83,195,188,0,100,0,326,6,0,77
AGMAT_2_10630,870,667,688,638,693,419,458,737,1555,924,460,739
AGMO_2_10631,53,166,175,226,140,25,49,6,488,427,129,114
AGPAT4_2_10632,22,20,10,75,37,0,0,206,143,4,0,0
AGPAT5_2_10633,307,366,215,227,425,301,166,811,322,346,268,438
AGPAT6_2_10634,58,48,87,32,31,0,24,43,1,681,802,117
AGPAT9_2_10635,128,134,195,117,257,2,683,190,176,49,258,33
AGPS_2_10636,734,656,679,661,285,314,1132,1167,631,968,759,796
AGXT2_2_10637,298,280,187,217,245,546,425,196,522,8,268,238
AGXT2L2_2_10638,147,134,121,208,13,53,289,362,79,229,50,6
AGXT_2_10639,36,5,6,35,61,1,0,0,1,2,3,139
AICDA_2_10640,915,961,953,730,609,1009,1363,887,467,865,547,738
AKR1B10_2_10641,977,775,698,1016,602,218,1961,1117,403,817,2889,1360
AKR1B15_2_10642,1173,1605,1349,1549,496,1026,1132,146,532,857,1923,129
7
AKR1B1_2_10643,284,307,248,324,107,78,334,419,136,216,123,408
AKR1C1_2_10644,4597,3926,3781,4866,4258,2696,5859,5019,2843,4764,3593,
4495
AKR1C3_2_10645,977,1125,684,842,599,939,377,298,791,1645,512,594
AKR1C4_2_10646,371,410,359,400,263,433,479,322,539,301,574,247
AKR1E2_2_10647,241,310,332,445,175,17,158,241,273,739,464,388
AKR7A2_2_10648,165,329,272,313,401,321,207,63,8,305,0,330
AKR7A3_2_10649,13,13,29,20,56,0,0,0,10,25,0,78
ALAD_2_10650,293,143,148,236,279,641,305,93,626,118,415,215
ALDH1A1_2_10651,172,252,228,246,223,125,215,6,130,185,9,567
ALDH1A3_2_10652,83,124,158,59,13,248,38,356,0,64,84,136
ALDH1B1_2_10653,89,67,55,90,2,236,0,131,2,30,43,16
ALDH1L1_2_10654,876,773,624,723,997,299,1429,511,573,1349,1019,1134
ALDH1L2_2_10655,293,273,417,538,92,483,744,409,425,58,293,342
ALDH6A1_2_10656,277,326,285,312,69,159,304,10,785,90,47,377
ALDH9A1_2_10657,202,107,87,77,1,201,0,43,2,476,9,168
ALDOB_2_10658,288,253,256,314,180,272,757,610,195,167,174,180
ALDOC_2_10659,379,354,341,213,377,2,13,469,289,367,140,12
ALG10_2_10660,849,929,657,1012,786,1222,1299,1390,675,430,1760,760
ALG11_2_10661,1683,1925,1592,1567,772,954,2055,1189,1826,1012,669,1346
ALG12_2_10662,375,415,177,255,20,608,111,370,141,163,319,135
ALG14_2_10663,805,706,633,818,1097,1176,1846,1400,393,248,471,569
ALG1_2_10664,224,139,161,233,6,3,18,20,1,233,1533,1334
ALG2_2_10665,348,318,374,399,35,61,813,290,189,587,28,573
ALG6_2_10666,152,133,147,131,75,22,230,0,109,363,295,66
ALKBH8_2_10667,286,330,506,479,185,149,383,305,617,17,1011,111
ALLC_2_10668,7,2,7,12,0,0,1,0,15,0,0,0
ALOX12B_2_10669,87,59,98,55,31,14,17,77,9,127,0,118
ALOX12_2_10670,595,469,540,884,963,598,1025,1729,72,912,1062,1022
ALOX15_2_10671,219,333,361,367,227,51,206,293,41,65,809,420
ALOX5_2_10672,701,388,406,374,648,448,1281,96,355,143,374,247

AMDHD1_2_10673,239,104,88,213,0,93,676,150,7,48,226,24
AMY1B_2_10674,5393,4903,4355,5424,4695,5579,3973,5187,6084,3824,5183,5
693
AMY1C_2_10675,5393,4903,4355,5424,4695,5579,3973,5187,6084,3824,5183,5
693
AMY2A_2_10676,7924,6877,6344,7512,10237,4910,10171,7860,6175,8080,5898
,11180
AMY2B_2_10677,7924,6877,6344,7512,10237,4910,10171,7860,6175,8080,5898
,11180
ANPEP_2_10678,708,781,819,862,1033,306,975,446,210,839,358,832
AOC3_2_10679,204,169,139,246,192,48,126,333,210,49,2,443
AOX1_2_10680,305,341,269,359,456,332,310,618,412,280,128,471
APEH_2_10681,658,646,571,586,595,150,524,62,564,488,452,269
APEX2_2_10682,111,71,88,196,376,62,50,146,5,14,199,85
APIP_2_10683,250,370,304,316,790,506,79,263,172,995,491,141
APLF_2_10684,269,319,233,290,269,95,170,167,177,66,76,39
APOBEC1_2_10685,460,461,433,480,821,163,1642,433,177,419,399,792
APOBEC2_2_10686,250,430,275,326,277,24,5,434,66,17,464,621
APOBEC3B_2_10687,2640,2334,2582,3175,3987,1686,2273,4640,3135,2998,187
9,3399
APOBEC3C_2_10688,139,119,161,149,741,124,415,190,59,25,153,295
APOBEC3G_2_10689,1543,1362,1461,1877,3041,1237,1299,2988,2169,2627,130
9,2291
ARF3_2_10690,154,101,157,154,66,25,232,69,93,194,2,140
ARF4_2_10691,60,26,21,44,165,0,0,1,1,0,168,26
ARG2_2_10692,249,321,293,293,27,993,21,14,78,99,23,2
ARHGEF10_2_10693,338,328,215,547,223,97,384,491,452,128,382,434
ARL3_2_10694,89,83,116,103,787,179,367,5,0,203,0,282
ARL4C_2_10695,128,132,110,144,112,289,13,94,302,296,56,83
ARL4D_2_10696,17,46,20,3,5,36,149,46,4,6,0,0
ARL5B_2_10697,979,953,865,1159,416,396,2644,1651,1079,1882,1505,1431
ARL8A_2_10698,65,60,70,55,6,44,0,60,163,5,27,30
ARL8B_2_10699,519,408,423,472,525,603,618,224,952,812,35,677
ARSD_2_10700,144,542,235,220,120,70,373,0,113,82,0,146
ARSE_2_10701,23,15,9,35,0,242,10,44,9,8,0,1
ARSG_2_10702,172,146,159,139,710,328,45,21,151,217,207,93
ARSH_2_10703,262,418,259,489,296,1059,125,1103,464,968,88,530
ARSI_2_10704,603,604,630,593,906,139,43,662,417,381,1029,73
ARSJ_2_10705,1368,1413,1570,1605,1457,1490,1442,2203,1781,1262,2140,13
66
ARSK_2_10706,357,390,339,399,485,528,375,24,681,458,959,841
ART1_2_10707,138,126,170,179,4,1,54,289,46,71,1010,451
ART4_2_10708,695,587,884,765,1055,142,334,782,593,756,782,538
AS3MT_2_10709,306,318,382,630,912,1180,13,365,265,308,103,526
ASNA1_2_10710,232,254,141,180,25,86,310,16,17,49,779,504
ASNSD1_2_10711,291,207,283,210,0,1,0,142,54,0,355,166
ASPG_2_10712,50,58,19,136,0,0,0,360,42,126,173,109
ASPHD2_2_10713,439,280,302,340,891,496,495,370,287,231,142,272
ATAD1_2_10714,416,656,513,547,201,180,429,78,557,602,780,703
ATIC_2_10715,484,318,438,218,222,1175,298,334,147,253,1096,1177

ATP2C2_2_10716,79,127,70,114,184,90,179,105,115,18,21,22
AUH_2_10717,131,127,204,145,211,226,309,241,91,98,222,481
AWAT1_2_10718,165,269,276,281,117,100,1467,587,0,133,246,10
AWAT2_2_10719,271,312,296,265,292,283,0,0,42,324,298,367
B3GALNT2_2_10720,696,635,758,737,476,677,535,406,203,666,1708,816
B3GALT1_2_10721,225,191,194,178,267,24,134,0,55,236,657,0
B3GALT2_2_10722,628,358,462,592,344,473,256,26,315,411,85,578
B3GALT4_2_10723,44,115,114,54,13,111,85,57,172,51,16,74
B3GALT6_2_10724,24,106,37,30,0,0,803,315,38,0,0,0
B3GAT2_2_10725,141,105,41,90,10,5,117,193,92,1,2,22
B3GAT3_2_10726,122,179,293,358,55,0,538,108,47,214,710,381
B3GNT2_2_10727,988,697,520,778,355,1124,768,1462,539,1240,1531,1282
B3GNT3_2_10728,173,231,193,75,958,6,77,0,67,42,338,10
B3GNT4_2_10729,144,63,74,83,0,84,0,9,824,373,0,380
B3GNT5_2_10730,2008,1475,1896,2058,1197,1888,1648,2292,1958,2657,1708,
1467
B3GNT7_2_10731,89,80,126,152,280,25,298,8,78,86,2,103
B3GNT9_2_10732,371,221,382,570,414,191,361,489,448,505,718,229
B3GNTL1_2_10733,136,112,146,205,483,41,376,35,145,546,10,98
B4GALNT1_2_10734,86,231,178,232,53,54,0,0,199,1,440,511
B4GALNT3_2_10735,394,287,358,446,202,272,34,554,275,170,264,280
B4GALNT4_2_10736,83,51,50,89,0,74,18,15,113,0,0,33
B4GALT1_2_10737,1820,1835,1129,1667,705,1206,1341,2149,709,1493,2954,2
090
B4GALT5_2_10738,557,441,462,572,292,271,133,547,94,941,771,180
B4GALT6_2_10739,575,708,505,625,400,516,350,1017,416,398,452,669
B4GALT7_2_10740,342,278,284,344,139,345,11,344,6,262,1,231
BBOX1_2_10741,559,318,292,417,341,655,752,787,535,349,475,570
BCHE_2_10742,1363,1577,1410,1633,1395,1749,2170,1357,1815,1954,1538,14
86
BCMO1_2_10743,156,157,314,176,2,0,0,119,313,119,0,120
BDH2_2_10744,1801,1961,2073,2083,1708,2013,2805,1583,1577,2309,1771,22
21
BHMT_2_10745,200,278,291,383,68,713,745,0,144,893,696,430
BLM_2_10746,696,669,481,545,561,631,630,458,777,210,286,572
BLMH_2_10747,2,0,0,0,0,0,0,0,36,43,0,0
BLVRA_2_10748,123,88,79,52,58,0,85,23,355,17,90,486
BLVRB_2_10749,37,54,114,29,3,0,0,339,0,0,1,231
BPHL_2_10750,172,220,360,386,279,1,150,97,221,387,72,206
BST1_2_10751,459,264,288,401,678,333,177,468,256,141,279,245
BTD_2_10752,724,544,682,823,133,611,1662,1436,789,352,1220,403
C12orf5_2_10753,733,687,877,852,1002,869,133,1543,1167,1073,906,523
C16orf79_2_10754,111,159,78,200,8,138,12,683,9,4,463,26
C18orf56_2_10755,38,83,17,4,0,0,1,0,235,20,2,0
C1GALT1_2_10756,1300,1050,1049,1488,1072,577,1495,1170,849,1960,562,91
2
C1R_2_10757,505,561,522,477,1337,343,260,191,856,674,151,374
C22orf28_2_10758,193,276,199,245,4,30,193,58,632,441,218,670
C2orf43_2_10759,1390,1133,978,1346,802,787,486,861,784,806,851,1455
C5orf4_2_10760,425,330,428,434,327,171,209,29,475,177,529,605

C6orf130_2_10761,168,142,74,114,53,49,1356,0,91,10,612,246
CA13_2_10762,728,825,639,702,1584,336,2503,642,683,406,416,1203
CA14_2_10763,15,19,23,20,7,0,4,0,14,10,0,13
CA2_2_10764,747,513,677,758,404,1751,279,1029,1078,445,1150,1082
CA3_2_10765,136,87,102,156,21,0,20,53,68,0,243,21
CA4_2_10766,213,78,141,363,444,32,169,99,9,238,130,268
CA5A_2_10767,125,196,214,182,29,16,0,501,319,2,0,1
CA5B_2_10768,884,925,743,929,493,262,912,397,652,643,956,1217
CA6_2_10769,153,215,178,242,1,43,143,185,72,283,64,978
CA8_2_10770,782,882,960,971,1654,674,929,1532,348,743,1617,796
CA9_2_10771,92,16,77,48,28,0,3,18,0,0,0,0
CARS2_2_10772,216,92,72,168,0,0,0,561,154,0,1,1
CASD1_2_10773,383,202,273,376,69,131,49,90,30,89,534,138
CAT_2_10774,512,536,376,497,398,370,248,818,339,339,774,69
CBR1_2_10775,442,488,586,760,232,54,718,191,412,1089,890,627
CBR3_2_10776,145,128,246,154,326,23,1147,47,55,181,236,381
CBR4_2_10777,183,152,166,320,21,79,84,183,19,39,28,362
CCDC88B_2_10778,59,22,8,85,34,0,212,67,2,0,0,15
CCDC92_2_10779,241,212,144,199,195,246,172,481,391,306,249,11
CCNO_2_10780,309,126,160,149,39,533,0,108,61,128,71,178
CCS_2_10781,273,83,78,131,246,77,0,29,11,61,148,168
CCT8_2_10782,550,490,321,486,447,738,698,464,427,582,773,912
CD274_2_10783,108,108,95,218,186,27,65,9,199,2,33,364
CD38_2_10784,0,0,0,0,0,0,0,0,0,0,0,0
CDA_2_10785,1558,1176,1289,1422,1956,652,1598,1479,1413,1595,603,1862
CDIPT_2_10786,750,785,478,812,608,821,138,1542,1396,1595,851,635
CDO1_2_10787,183,168,104,165,6,465,613,120,13,166,464,83
CDS2_2_10788,672,367,419,388,953,777,1019,610,1008,809,281,597
CELA1_2_10789,507,409,513,638,211,0,75,539,325,540,576,700
CELA2A_2_10790,477,262,321,370,54,416,79,66,553,112,585,823
CELA2B_2_10791,402,441,401,389,396,830,803,57,505,91,322,30
CELA3A_2_10792,347,412,320,348,259,56,674,736,61,139,136,428
CELA3B_2_10793,430,291,230,256,630,255,451,31,84,332,506,300
CEL_2_10794,22,1,36,3,0,11,1,0,1,4,0,0
CETN1_2_10795,319,278,335,313,240,35,244,113,146,100,622,312
CETN2_2_10796,77,39,75,70,289,20,74,3,22,6,123,40
CETP_2_10797,48,174,50,119,0,0,77,41,163,338,166,11
CFB_2_10798,48,10,46,64,151,37,37,0,0,69,428,16
CFD_2_10799,141,86,97,69,81,125,0,30,401,3,51,6
CFI_2_10800,790,728,644,725,1058,544,569,846,870,332,472,965
CH25H_2_10801,8,50,82,98,0,0,0,133,0,102,0,0
CHDH_2_10802,648,444,561,379,557,0,0,57,265,145,1283,90
CHI3L1_2_10803,155,309,200,301,66,74,61,238,114,475,561,503
CHIT1_2_10804,68,56,66,56,0,273,0,0,0,0,0,0
CHML_2_10805,108,87,97,175,47,96,84,196,153,233,33,149
CHPF2_2_10806,239,140,115,278,240,182,13,34,145,30,503,65
CHPT1_2_10807,642,354,525,521,864,268,1152,574,609,764,186,288
CHST10_2_10808,119,119,169,167,282,482,84,150,85,168,0,0
CHST12_2_10809,87,135,92,95,7,145,1,66,364,85,39,83
CHST13_2_10810,592,696,484,625,493,738,409,744,161,1214,1233,537

CHST14_2_10811,103,114,79,48,0,0,96,31,0,54,101,19
CHST1_2_10812,64,53,55,71,156,0,0,158,147,0,0,502
CHST2_2_10813,252,713,245,477,444,635,735,486,456,734,321,663
CHST3_2_10814,1302,861,796,959,748,1532,566,531,713,702,309,856
CHST5_2_10815,372,163,199,302,430,216,953,665,658,168,123,284
CHST6_2_10816,64,66,58,88,3,0,12,4,10,132,20,184
CHST7_2_10817,32,56,36,17,29,0,3,2,0,49,0,70
CHST9_2_10818,1721,1778,1355,1707,1294,1781,1525,2206,1424,2345,1498,1
831
CHSY1_2_10819,1519,1197,1105,1336,684,1531,1501,558,2214,2099,1466,195
2
CHSY3_2_10820,359,308,366,349,299,181,37,341,129,450,7,350
CLPP_2_10821,222,180,131,221,284,7,1,126,8,39,286,503
CLPX_2_10822,331,231,226,314,638,138,970,165,31,211,200,467
CLYBL_2_10823,865,483,658,641,458,607,1161,296,1298,516,790,544
CMA1_2_10824,52,79,31,69,38,36,101,0,35,0,0,18
CMAS_2_10825,104,26,52,50,3,0,0,0,25,159,7,35
CMBL_2_10826,165,86,71,27,177,15,53,1,26,176,2,98
CNDP1_2_10827,177,145,187,193,218,322,91,26,10,36,2,195
CNTN6_2_10828,285,436,273,268,363,275,481,32,263,1102,718,998
COIL_2_10829,200,121,105,230,171,3,36,118,126,156,149,25
COMTD1_2_10830,32,10,11,25,3,343,0,0,0,84,0,4
COQ2_2_10831,357,401,462,475,513,493,143,971,384,180,1605,626
COQ3_2_10832,81,61,80,150,151,113,208,197,19,196,75,21
COQ5_2_10833,158,120,229,192,66,468,443,118,41,278,288,330
COX10_2_10834,992,1150,961,1303,717,515,610,912,429,594,1637,1052
COX17_2_10835,347,371,361,499,407,665,58,333,520,808,93,451
COX4I1_2_10836,177,92,142,298,12,166,79,146,120,136,157,79
COX4I2_2_10837,773,776,691,1118,384,695,1324,180,463,688,1160,343
COX5A_2_10838,2614,2236,1580,2007,2164,651,1106,2475,1910,3559,2157,24
30
COX5B_2_10839,233,217,262,227,100,220,17,27,154,227,2,347
COX6A1_2_10840,372,542,320,504,1199,890,0,671,0,535,754,391
COX6A2_2_10841,55,55,18,46,0,13,0,20,3,16,0,18
COX6B1_2_10842,58,21,108,80,0,59,5,64,220,168,50,26
COX6B2_2_10843,371,162,219,273,30,263,392,119,231,48,403,447
COX6C_2_10844,608,627,479,877,1812,506,385,349,326,1070,253,1044
COX7A1_2_10845,68,90,44,61,67,64,0,16,93,49,71,74
COX7A2_2_10846,204,170,135,205,159,172,915,146,54,198,95,279
COX7A2L_2_10847,404,338,191,470,7,501,340,64,332,385,24,194
COX7B2_2_10848,1063,742,875,921,949,537,1246,307,519,889,1497,1023
COX7B_2_10849,360,428,405,417,668,280,174,0,161,599,48,669
COX7C_2_10850,808,756,894,887,675,392,1109,513,688,596,2049,1283
COX8A_2_10851,18,6,14,13,38,40,1,0,3,370,0,1
COX8C_2_10852,652,694,432,350,58,208,171,213,182,120,287,137
CPA1_2_10853,87,141,67,114,228,264,109,327,332,321,0,0
CPA2_2_10854,41,12,2,32,198,0,0,0,0,0,0,0
CPB1_2_10855,413,407,387,454,494,594,671,155,278,652,211,234
CP_2_10856,468,274,413,527,1361,415,10,23,288,863,32,495
CPE_2_10857,55,75,73,89,1,1,1169,39,44,22,141,21

CPOX_2_10858,508,646,501,609,147,237,361,355,561,247,346,216
CPSF3_2_10859,743,875,604,961,419,643,1149,2133,1047,1149,672,1181
CPT2_2_10860,80,26,10,79,0,0,0,0,81,0,0,163
CRAT_2_10861,637,715,577,672,763,551,647,759,652,388,373,291
CREG2_2_10862,238,119,208,260,0,11,0,532,2,4,1,45
CRY1_2_10863,1348,1220,1341,1252,2193,1843,1716,1736,1650,922,2321,141
8
CRYL1_2_10864,131,184,92,147,58,269,3,4,311,161,71,18
CRYZL1_2_10865,154,20,18,34,18,0,131,71,100,62,0,0
CSAD_2_10866,137,116,107,148,157,153,55,48,4,73,0,131
CS_2_10867,257,327,233,408,260,775,429,221,203,216,5,261
CSGALNACT2_2_10868,735,583,823,886,499,1777,1025,513,387,378,1060,23
CSRP2BP_2_10869,112,111,123,215,28,17,347,0,154,0,8,378
CTBS_2_10870,1177,1614,1368,1032,543,924,896,2934,1132,1504,3719,539
CTRB1_2_10871,441,281,503,283,597,343,351,176,180,239,832,500
CTRB2_2_10872,369,446,566,400,141,573,638,626,610,728,4,474
CTRC_2_10873,274,152,206,387,163,719,73,9,57,336,87,131
CTSD_2_10874,310,124,277,311,628,80,162,3,245,268,351,222
CTSF_2_10875,60,149,148,131,41,46,327,88,18,416,39,111
CTSG_2_10876,16,19,18,25,9,58,0,1,0,106,52,62
CTSH_2_10877,354,451,399,364,344,1219,1462,359,275,253,733,366
CTSK_2_10878,264,254,247,379,836,289,403,630,374,411,191,432
CTSZ_2_10879,24,51,0,9,2,34,0,0,69,0,2,0
CWC27_2_10880,719,568,524,409,535,285,1040,360,141,156,371,381
CXorf21_2_10881,231,210,158,169,42,19,52,739,244,81,1305,113
CYB561D2_2_10882,214,156,192,311,61,639,420,140,167,70,486,134
CYB5B_2_10883,521,610,734,805,304,1057,951,1367,1038,673,496,465
CYB5R1_2_10884,220,183,275,208,402,10,128,443,24,3,872,166
CYB5R2_2_10885,237,126,135,250,83,144,202,438,128,253,118,378
CYB5R4_2_10886,62,88,99,43,0,37,5,131,85,552,99,12
CYBA_2_10887,0,12,1,11,0,0,0,0,0,0,0,0
CYBB_2_10888,200,144,98,185,350,166,173,177,212,34,103,132
CYC1_2_10889,0,0,0,0,0,0,0,0,0,0,0,0
CYCS_2_10890,214,81,91,164,666,688,4,146,4,204,57,59
CYP11B2_2_10891,72,191,142,213,19,4,310,179,0,43,64,206
CYP17A1_2_10892,857,806,638,662,766,316,1438,1118,937,1065,536,919
CYP1A1_2_10893,1167,1012,741,972,1179,802,1104,1343,473,826,286,1140
CYP1A2_2_10894,33,11,17,105,109,0,0,4,0,0,310,108
CYP1B1_2_10895,8,13,9,10,22,12,1,0,0,1,6,1
CYP20A1_2_10896,626,768,724,896,234,420,1521,807,1444,732,667,749
CYP26B1_2_10897,232,258,216,209,987,43,371,36,139,130,0,412
CYP26C1_2_10898,196,236,198,190,189,191,390,304,434,140,351,52
CYP27A1_2_10899,902,929,912,1063,757,487,702,1740,808,1809,1526,840
CYP27B1_2_10900,971,918,1082,1118,484,629,1206,522,710,1482,334,589
CYP2A13_2_10901,54,43,122,25,53,0,1,89,1,36,294,0
CYP2A6_2_10902,1277,1072,1068,1436,985,925,1661,646,983,591,476,1675
CYP2B6_2_10903,1392,1330,1089,1313,1990,2483,1904,2344,1162,871,967,10
40
CYP2C19_2_10904,116,58,113,99,9,2,17,47,47,29,7,6
CYP2C9_2_10905,1033,955,1070,1135,1179,1044,911,1487,531,618,915,1376

CYP2E1_2_10906,2228,2021,1851,1986,1584,1377,1572,2195,1810,3414,745,1
611
CYP2F1_2_10907,157,131,138,120,812,155,202,14,338,164,16,12
CYP2J2_2_10908,252,232,243,248,151,0,4,22,32,87,978,359
CYP2R1_2_10909,32,83,81,36,0,0,59,255,22,0,324,9
CYP2S1_2_10910,240,151,208,156,51,57,25,86,1,473,132,32
CYP2U1_2_10911,785,592,597,727,819,1133,538,1064,390,1022,241,881
CYP2W1_2_10912,83,72,67,178,0,209,0,0,56,206,1,620
CYP39A1_2_10913,54,49,11,17,60,0,222,0,0,0,0,6
CYP3A7_2_10914,317,130,253,219,110,114,123,73,194,64,124,80
CYP46A1_2_10915,600,251,251,380,485,7,151,447,177,970,577,385
CYP4A11_2_10916,122,237,168,109,358,262,234,9,120,376,184,279
CYP4A22_2_10917,122,237,168,109,358,262,234,9,120,376,184,279
CYP4F12_2_10918,210,110,47,139,119,177,148,222,387,91,0,152
CYP4F22_2_10919,27,39,82,66,10,22,0,0,13,7,0,330
CYP4F2_2_10920,1871,1200,1447,1724,854,1417,1835,981,1373,913,978,1680
CYP4F8_2_10921,329,220,203,202,0,284,0,0,29,9,1377,384
CYP4V2_2_10922,195,164,191,167,132,545,580,133,62,24,121,50
CYP4X1_2_10923,68,6,31,43,139,25,0,0,9,108,103,9
CYP4Z1_2_10924,320,239,254,370,10,16,544,207,268,1,1034,352
CYP7A1_2_10925,725,654,364,479,335,172,450,533,1008,542,261,989
CYP7B1_2_10926,349,435,377,532,1042,2002,734,485,268,91,695,604
CYP8B1_2_10927,769,586,574,469,446,269,554,432,854,556,662,774
DAD1_2_10928,441,660,467,628,559,303,266,120,108,1045,244,914
DAGLA_2_10929,287,225,379,446,344,338,1053,905,582,187,976,21
DAK_2_10930,1465,883,758,1018,1992,1307,1010,1294,801,1061,1299,947
DARS2_2_10931,1129,653,900,724,957,341,295,170,246,1405,634,607
DARS_2_10932,603,494,373,715,1006,664,596,477,404,323,115,533
DBH_2_10933,9,62,23,69,0,42,2,324,31,3,0,8
DBR1_2_10934,1073,1098,837,1095,1103,188,765,651,1037,1426,2343,860
DBT_2_10935,1263,1646,1399,2024,1691,1955,3444,1243,849,935,1369,1884
DCLRE1B_2_10936,227,261,202,220,183,429,313,0,550,0,36,254
DCPS_2_10937,137,26,128,103,0,2,95,2,63,0,0,20
DCTN6_2_10938,356,322,401,379,804,58,504,215,233,612,503,257
DCTPP1_2_10939,62,89,33,174,9,88,2,200,138,9,35,40
DDAH2_2_10940,1,1,55,116,0,0,0,330,17,0,0,0
DDOST_2_10941,102,144,137,81,135,20,14,37,178,39,54,162
DDX10_2_10942,1721,1392,1289,2102,1656,1938,1422,1782,1865,2375,2452,1
650
DDX18_2_10943,952,1053,870,1037,729,897,709,818,1264,666,370,771
DDX19A_2_10944,535,607,478,585,41,1058,683,550,772,546,698,1019
DDX1_2_10945,39,54,45,120,14,0,0,22,3,940,306,85
DDX20_2_10946,546,254,390,251,782,109,36,1166,479,132,151,903
DDX21_2_10947,380,411,324,596,225,307,131,363,900,357,141,501
DDX23_2_10948,77,80,62,107,13,34,375,16,301,23,0,96
DDX24_2_10949,265,128,166,226,238,35,176,161,439,158,0,21
DDX25_2_10950,1539,1603,1255,1451,716,1406,409,582,2282,1448,966,1672
DDX27_2_10951,440,493,545,565,610,281,423,1164,947,853,593,511
DDX28_2_10952,219,140,239,204,25,31,411,310,198,167,16,233
DDX39A_2_10953,1044,584,951,852,1046,1417,2013,1017,530,695,737,1163

DDX41_2_10954,148,144,168,184,312,108,110,484,300,281,171,178
DDX43_2_10955,72,25,117,108,10,2,43,279,46,6,20,143
DDX46_2_10956,2223,1826,1554,2003,1944,1627,789,1210,1760,2412,1728,21
42
DDX49_2_10957,87,42,83,108,0,11,190,3,87,0,0,385
DDX50_2_10958,196,229,215,201,255,107,515,119,355,238,530,231
DDX51_2_10959,190,153,169,204,97,352,192,140,105,78,343,106
DDX52_2_10960,487,649,682,772,508,765,656,446,831,171,382,597
DDX53_2_10961,152,159,273,116,498,0,146,0,0,0,0,190
DDX55_2_10962,64,39,60,30,3,0,0,32,20,1,110,0
DDX56_2_10963,65,65,81,107,32,133,15,21,186,300,21,92
DDX58_2_10964,277,96,139,196,679,142,154,709,147,131,88,71
DDX59_2_10965,170,140,214,195,232,231,325,50,7,308,0,173
DDX5_2_10966,131,58,107,168,95,0,1,10,1,221,2,170
DDX60_2_10967,1057,882,880,967,1107,665,1021,1574,1926,505,1084,1034
DDX6_2_10968,22,27,31,32,0,0,66,0,0,0,0,0
DECR1_2_10969,2212,2542,1768,2543,4759,2374,2736,1044,1573,1570,1781,2
240
DECR2_2_10970,367,228,287,581,13,784,476,288,12,37,1148,289
DEGS1_2_10971,224,211,313,211,603,60,181,224,238,219,559,474
DEGS2_2_10972,18,155,51,27,29,232,0,85,146,2,0,0
DERA_2_10973,14,84,46,20,0,0,0,0,23,14,19,0
DFFB_2_10974,63,182,49,125,0,154,107,386,4,6,322,59
DGAT1_2_10975,219,398,317,253,260,282,715,0,31,0,742,273
DGAT2_2_10976,164,255,186,166,245,124,678,231,83,318,98,228
DHCR24_2_10977,84,136,137,119,126,172,676,347,22,85,0,60
DHDH_2_10978,239,302,309,349,185,361,583,8,124,781,14,266
DHFR_2_10979,1133,1096,829,1276,1996,893,1502,1568,1959,1165,602,1107
DHODH_2_10980,208,96,141,175,324,16,320,1,203,64,287,174
DHRS13_2_10981,32,52,7,20,0,0,8,43,0,0,0,245
DHRS3_2_10982,337,291,199,382,34,480,240,365,294,133,279,337
DHRS4_2_10983,868,661,817,962,859,1228,1581,1727,697,1332,878,1344
DHRS7_2_10984,1422,1019,929,1007,548,1941,1055,652,1132,1265,517,1225
DHRSX_2_10985,223,169,120,160,217,13,16,42,321,724,322,125
DHTKD1_2_10986,195,231,212,110,260,307,864,146,52,409,184,161
DHX15_2_10987,373,550,434,646,764,345,676,188,642,471,177,492
DHX29_2_10988,127,151,151,216,1,59,1,100,4,211,352,35
DHX32_2_10989,515,584,471,505,417,693,449,429,417,1005,187,361
DHX34_2_10990,49,79,63,63,64,15,251,1,161,17,31,340
DHX37_2_10991,577,955,490,800,845,941,35,631,491,880,354,761
DHX38_2_10992,141,155,235,269,52,75,290,337,24,291,199,211
DHX57_2_10993,238,83,265,213,62,32,497,94,200,49,62,418
DHX58_2_10994,197,256,212,303,52,204,553,188,214,350,44,147
DHX8_2_10995,307,212,228,291,556,88,144,137,225,298,302,217
DHX9_2_10996,390,432,342,385,38,49,510,391,312,441,266,539
DIO3_2_10997,632,669,540,751,781,623,144,479,757,175,297,901
DIRAS1_2_10998,153,35,188,174,2,399,0,396,191,4,545,174
DIRAS2_2_10999,166,204,293,303,258,679,871,32,60,422,11,235
DIRAS3_2_11000,379,215,223,346,158,460,67,240,165,88,18,102
DIS3L2_2_11001,446,399,340,336,179,93,958,255,544,162,219,396

DLAT_2_11002,515,194,285,402,171,228,391,620,514,285,156,606
DLD_2_11003,0,0,0,0,0,0,0,0,0,0,0,0
DLL1_2_11004,385,398,313,298,275,871,238,4,465,918,1637,586
DLST_2_11005,163,157,171,256,178,1,149,239,138,651,374,126
DMGDH_2_11006,669,694,653,698,755,292,1192,526,750,374,1030,262
DNA2_2_11007,3403,3577,3448,3327,2802,2835,4620,3959,4735,4186,3480,28
52
DNAH11_2_11008,863,580,897,940,1004,283,1100,537,711,1468,1395,257
DNAH3_2_11009,407,291,471,512,431,651,990,486,540,80,59,337
DNAH5_2_11010,323,233,281,407,491,155,432,112,163,422,290,310
DNAH8_2_11011,756,883,802,891,799,776,1631,1071,693,2309,1353,1417
DNAJA2_2_11012,1358,1466,1264,1316,1134,1490,987,2210,711,879,1991,103
8
DNAJB14_2_11013,503,462,416,385,436,1151,543,10,777,367,606,548
DNAJC10_2_11014,109,192,170,166,352,151,114,3,219,253,47,49
DNAJC18_2_11015,117,152,202,103,27,42,153,0,219,8,81,106
DNAL4_2_11016,1,13,66,42,0,47,5,28,0,53,472,1
DNASE1_2_11017,16,40,53,16,0,70,293,0,131,0,0,45
DNASE1L2_2_11018,240,260,178,249,620,31,184,488,250,679,116,69
DNASE1L3_2_11019,285,150,237,255,45,250,431,494,127,0,209,92
DNASE2_2_11020,847,795,890,982,795,703,1098,1718,887,886,337,1377
DNPEP_2_11021,114,186,37,14,0,697,0,1,0,259,23,13
DPAGT1_2_11022,239,157,167,187,0,49,696,220,257,158,0,192
DPEP2_2_11023,329,340,262,367,247,288,703,174,306,186,1107,354
DPM1_2_11024,38,38,47,94,0,67,0,1,1,80,32,52
DPM2_2_11025,144,274,178,242,0,18,60,109,223,75,1,51
DPP4_2_11026,1480,1093,813,1116,1045,1158,1083,1849,1073,802,1185,2300
DPP7_2_11027,1417,846,741,898,603,327,1550,326,514,419,396,1242
DPP9_2_11028,672,758,537,883,576,209,1369,1298,410,182,217,1949
DPYS_2_11029,397,323,373,551,639,588,765,97,626,304,6,274
DPYSL4_2_11030,48,23,53,105,5,50,159,59,0,1,1,8
DPYSL5_2_11031,482,444,371,514,195,336,937,466,676,601,336,555
DQX1_2_11032,197,178,116,139,235,171,76,39,50,83,0,244
DSEL_2_11033,442,558,412,466,113,211,114,42,184,431,173,284
DTD1_2_11034,159,153,97,128,0,11,277,270,197,79,14,290
DUOX2_2_11035,41,35,55,104,15,113,184,144,65,0,0,191
DUPD1_2_11036,399,253,234,324,223,669,209,740,281,337,39,334
DUSP28_2_11037,139,119,271,298,43,478,680,16,122,151,0,402
EARS2_2_11038,240,390,230,436,248,65,0,346,84,535,637,361
EBPL_2_11039,676,601,504,742,177,833,527,659,225,721,1249,1246
ECH1_2_11040,1511,1265,1189,1505,1658,1559,2086,733,935,1569,670,1399
ECHDC3_2_11041,487,273,422,420,329,245,750,621,736,318,822,515
ECHS1_2_11042,322,236,298,321,517,377,1272,396,692,80,163,276
EDEM1_2_11043,425,591,727,494,1454,495,289,740,257,66,964,250
EDEM3_2_11044,878,693,693,875,472,702,498,1719,548,962,328,753
EEF1A1_2_11045,1020,1144,1039,998,1061,762,944,1474,1046,2040,2499,103
0
EEF1A2_2_11046,14,42,57,35,108,4,0,0,2,108,1,0
EEF2_2_11047,45,23,43,59,4,7,73,48,18,0,1,1
EGLN1_2_11048,171,162,166,147,212,259,113,148,13,7,415,143

EGLN3_2_11049,54,113,72,125,0,5,4,18,232,0,0,250
EHD4_2_11050,527,548,540,592,504,441,486,436,138,1287,1146,552
EIF4A3_2_11051,607,407,508,658,683,462,515,68,643,178,173,672
ELAC1_2_11052,34,34,48,73,0,11,12,0,71,33,697,37
ELANE_2_11053,104,130,150,192,204,31,359,219,72,228,67,134
ELOVL1_2_11054,793,685,671,684,541,817,568,282,2419,782,1552,862
ELOVL2_2_11055,651,570,665,656,526,1287,456,461,480,781,528,1036
ELOVL3_2_11056,1132,1138,867,1032,1354,634,758,573,831,1441,1062,808
ELOVL4_2_11057,118,116,161,97,220,0,67,11,87,23,1,158
ENDOD1_2_11058,2080,1850,1698,2193,1398,960,2394,1373,1726,1608,671,27
11
ENDOG_2_11059,9,0,2,16,0,0,0,0,0,0,0,0
ENGASE_2_11060,87,182,122,143,2,104,791,62,79,0,116,6
ENO2_2_11061,68,48,59,50,1,84,50,0,1,165,0,229
ENO4_2_11062,205,111,330,309,91,163,697,428,158,267,82,38
ENOPH1_2_11063,563,600,778,564,617,461,1127,952,502,279,530,906
ENPEP_2_11064,58,87,95,104,0,0,0,1,664,84,0,5
ENPP1_2_11065,11,44,31,54,3,368,4,2,0,18,0,78
ENPP3_2_11066,1462,1185,1294,1719,2163,950,2081,2805,1313,2712,1229,20
11
ENPP4_2_11067,725,474,408,451,542,275,80,62,722,409,1245,662
ENPP5_2_11068,65,62,41,137,249,6,489,43,57,36,0,50
ENPP6_2_11069,1536,1397,1078,1717,2474,1133,872,1776,1658,1055,753,104
6
ENPP7_2_11070,13,3,7,20,0,0,0,0,0,0,0,0
ENTPD3_2_11071,224,167,233,258,105,114,456,178,1,166,81,202
ENTPD5_2_11072,138,125,67,97,320,6,51,372,128,288,194,124
ENTPD7_2_11073,50,19,35,19,3,193,0,0,0,70,1,16
EPHX2_2_11074,366,156,230,241,251,373,27,515,230,1,70,229
EPHX4_2_11075,256,224,175,292,69,386,281,39,249,308,63,224
EPRS_2_11076,1529,1269,1205,1556,2122,1270,1271,1338,859,1918,630,1294
EPT1_2_11077,122,133,222,154,65,13,0,19,8,274,290,0
EPX_2_11078,199,405,249,318,2,82,723,8,52,0,484,229
ERAS_2_11079,31,55,108,38,2,0,0,27,0,4,0,23
ERCC3_2_11080,487,326,374,507,581,394,41,465,126,415,217,354
ERCC4_2_11081,593,556,601,728,422,460,1250,1186,1004,997,1533,571
ERCC5_2_11082,241,336,349,370,415,1267,2381,12,317,515,354,353
ERI1_2_11083,504,445,268,509,551,24,167,30,311,212,204,1272
ERO1L_2_11084,326,167,241,175,160,650,711,128,109,76,6,231
ERP44_2_11085,94,97,86,31,0,23,180,78,3,49,0,42
ESD_2_11086,2750,3125,2780,3506,2842,2418,1898,4282,1973,3619,3259,355
4
ESPL1_2_11087,95,175,69,411,153,34,495,282,279,0,766,185
ETFDH_2_11088,574,442,432,540,1167,1013,317,465,527,178,440,240
EXOSC1_2_11089,168,126,195,115,69,274,427,240,92,359,48,286
EXOSC2_2_11090,363,353,347,456,280,241,550,101,110,354,134,600
EXOSC4_2_11091,389,310,338,200,553,241,327,10,192,330,68,178
EXOSC5_2_11092,1738,1225,1234,1376,1390,1668,123,921,1197,1447,680,878
EXOSC7_2_11093,309,367,356,376,103,638,308,6,412,1,25,662
EXOSC8_2_11094,136,100,122,174,22,54,6,457,5,73,210,264

EXT1_2_11095,45,170,80,131,421,32,0,583,134,0,268,6
EXTL1_2_11096,79,162,105,196,72,87,180,190,200,13,28,20
EXTL3_2_11097,243,126,153,214,4,331,1,0,528,187,114,516
F10_2_11098,374,661,441,576,488,140,595,975,866,901,178,429
F12_2_11099,196,40,116,92,24,1,107,7,535,75,46,3
F13A1_2_11100,260,239,224,445,54,258,24,299,283,10,55,400
F13B_2_11101,598,314,355,394,438,435,467,493,102,200,363,242
F2_2_11102,201,238,247,254,203,418,264,304,76,244,51,290
F5_2_11103,0,0,0,0,0,0,0,0,0,0,0,0
F9_2_11104,116,86,56,86,33,83,0,341,0,28,11,358
FA2H_2_11105,18,0,0,3,0,0,10,0,0,0,0,1
FAAH2_2_11106,212,233,217,233,117,701,41,381,38,11,207,206
FADS1_2_11107,153,157,158,334,160,0,1,53,62,180,355,52
FADS2_2_11108,5134,5275,5104,6090,4570,4147,6279,5963,5471,7034,5284,5
209
FADS3_2_11109,493,418,536,614,62,18,894,1031,124,258,977,532
FAHD2A_2_11110,77,80,87,166,3,0,0,6,433,31,0,177
FAM108C1_2_11111,61,1,74,77,0,1,0,0,0,3,0,0
FAM135B_2_11112,152,130,204,122,339,175,112,188,196,379,429,91
FAM20B_2_11113,527,611,448,627,267,705,467,863,643,820,411,743
FANCM_2_11114,215,89,68,168,76,73,310,107,192,398,291,39
FAR1_2_11115,225,59,221,280,98,2,234,34,13,308,178,221
FAR2_2_11116,440,249,192,228,189,91,393,440,181,409,250,298
FARS2_2_11117,1112,859,1118,1167,1168,1111,1421,1267,845,532,1080,1531
FARSA_2_11118,139,173,265,116,35,0,1,111,4,20,508,47
FARSB_2_11119,663,459,537,602,1384,203,413,797,273,813,619,979
FASN_2_11120,35,55,58,47,19,136,0,175,34,0,0,30
FDFT1_2_11121,188,195,122,238,67,102,14,27,224,231,27,302
FEN1_2_11122,105,66,103,131,60,12,129,0,65,306,43,44
FH_2_11123,1458,1382,1304,1641,1513,2211,1466,1327,1122,1921,1462,1419
FIG4_2_11124,360,239,390,374,553,488,206,367,403,655,181,925
FKBP10_2_11125,356,277,249,340,563,455,304,1,557,68,19,216
FKBP14_2_11126,779,595,691,895,514,599,411,1305,235,460,655,1286
FKBP15_2_11127,279,229,289,263,85,21,392,137,169,171,219,363
FKBP3_2_11128,49,82,43,47,1,344,147,0,38,0,0,26
FKBP4_2_11129,18,77,11,59,2,0,0,0,37,0,0,40
FKBP8_2_11130,190,232,197,219,0,0,193,558,370,1,95,539
FMO1_2_11131,683,466,628,619,406,957,937,391,488,644,586,707
FMO4_2_11132,163,118,154,218,75,513,91,46,140,217,399,252
FNBP1_2_11133,763,512,709,951,483,103,919,302,950,325,1099,364
FNTA_2_11134,159,65,132,127,206,43,0,89,142,393,40,356
FNTB_2_11135,269,295,336,544,291,427,511,227,209,557,215,527
FTH1_2_11136,327,341,284,246,188,869,333,336,586,221,42,315
FTMT_2_11137,110,138,133,99,200,262,430,334,67,506,3,26
FTSJ2_2_11138,97,50,29,125,1,0,24,0,169,142,64,408
FTSJ3_2_11139,441,391,473,450,1044,763,35,278,1131,542,221,670
FTSJD2_2_11140,435,227,403,276,129,262,393,578,264,146,523,365
FUCA1_2_11141,430,223,503,440,426,456,175,1158,125,133,52,421
FUCA2_2_11142,487,380,292,353,737,120,243,170,435,326,42,788
FURIN_2_11143,207,291,273,248,657,113,837,227,34,335,255,520

FUT10_2_11144,707,724,529,724,648,1440,1433,309,807,973,932,197
FUT11_2_11145,154,150,145,179,70,216,80,361,48,378,509,407
FUT1_2_11146,223,195,137,215,378,73,227,186,106,441,9,233
FUT4_2_11147,637,562,510,600,265,398,476,1159,642,199,0,1410
FUT5_2_11148,3011,3040,3169,3449,2102,2165,4733,1749,2422,3913,4290,33
60
FUT7_2_11149,250,177,150,210,95,48,176,82,119,90,89,49
FUT9_2_11150,800,677,876,910,904,592,1026,362,1219,1777,1242,1171
GADL1_2_11151,98,145,82,103,784,39,1,201,100,15,308,52
GAL3ST1_2_11152,211,199,95,162,67,245,97,0,142,286,28,31
GAL3ST3_2_11153,172,168,143,225,93,231,622,127,256,27,394,305
GAL3ST4_2_11154,74,85,68,218,0,0,500,76,20,23,379,32
GALM_2_11155,145,207,107,92,79,20,4,159,100,246,9,127
GALNS_2_11156,149,88,98,144,52,4,242,0,38,98,90,25
GALNT10_2_11157,674,390,431,548,524,398,1264,243,583,791,0,709
GALNT11_2_11158,827,621,741,818,786,423,1073,84,632,336,259,465
GALNT12_2_11159,270,292,288,208,388,588,53,47,152,350,811,381
GALNT13_2_11160,252,254,186,220,23,39,377,5,148,684,216,56
GALNT14_2_11161,96,92,43,104,59,17,177,6,68,41,0,202
GALNT1_2_11162,324,286,195,144,386,279,170,421,422,75,135,155
GALNT2_2_11163,98,45,82,113,97,15,0,43,1,2,201,270
GALNT3_2_11164,332,254,429,294,151,299,641,781,219,654,862,339
GALNT4_2_11165,207,159,129,260,330,5,21,65,694,97,77,416
GALNT5_2_11166,760,766,667,857,604,399,248,546,507,1734,3564,1448
GALNT6_2_11167,277,318,289,291,395,16,812,207,181,226,94,340
GALNT8_2_11168,403,292,328,345,1054,47,635,2,331,78,477,155
GALNTL2_2_11169,17,40,17,3,0,0,9,0,0,0,0,0
GALNTL4_2_11170,407,379,346,320,125,372,30,28,314,104,488,737
GALNTL5_2_11171,360,212,216,274,338,168,137,370,454,596,429,197
GALNTL6_2_11172,490,448,418,579,767,194,1181,988,279,293,364,460
GALT_2_11173,28,49,0,7,0,0,0,0,0,76,0,0
GANC_2_11174,15,75,75,54,74,65,143,3,9,0,0,12
GAPDH_2_11175,106,52,22,10,18,9,0,347,15,0,0,24
GAPDHS_2_11176,76,107,43,116,8,10,309,36,7,10,0,69
GARS_2_11177,970,1104,1030,1344,1458,763,545,354,995,1095,1358,1079
GATC_2_11178,1169,1016,771,783,1014,405,1175,756,1566,568,1696,795
GATM_2_11179,87,44,38,34,26,74,0,0,15,0,5,19
GBA2_2_11180,123,47,35,30,0,0,0,0,0,0,0,0
GBE1_2_11181,1377,1431,1563,1485,1480,1154,1106,1313,1161,1391,2368,11
64
GBGT1_2_11182,528,668,577,805,790,68,198,819,749,1247,665,648
GBP1_2_11183,414,396,315,362,287,388,697,437,46,499,800,330
GBP2_2_11184,444,396,372,523,411,686,575,623,567,690,325,329
GBP3_2_11185,453,227,517,401,150,561,71,26,334,79,673,449
GBP4_2_11186,0,0,0,0,0,0,0,0,0,0,0,0
GBP6_2_11187,817,895,713,991,574,439,340,967,346,1229,920,468
GBP7_2_11188,362,569,404,343,714,24,1143,849,468,541,671,66
GCLM_2_11189,534,553,391,517,1035,477,481,998,98,208,319,563
GCNT3_2_11190,887,997,774,797,873,1630,1614,601,968,598,1990,836
GCNT4_2_11191,407,503,580,419,672,465,1091,616,686,372,159,251

GCSH_2_11192,1423,1373,1039,1468,808,620,1154,907,1052,1095,1297,990
GDE1_2_11193,132,134,165,286,3,23,257,382,0,233,364,28
GDPD3_2_11194,765,671,571,648,982,481,1583,227,693,892,437,376
GFM1_2_11195,775,644,543,751,248,9,229,258,1222,924,196,914
GFOD2_2_11196,289,360,625,687,228,229,912,308,639,960,253,476
GFPT1_2_11197,546,376,360,531,69,464,391,793,319,561,1911,428
GFPT2_2_11198,454,402,242,293,442,356,833,407,8,763,287,341
GGH_2_11199,645,534,504,516,651,577,410,542,1086,489,301,278
GGPS1_2_11200,222,100,67,144,0,1,340,0,0,37,0,172
GGT7_2_11201,98,125,87,137,53,119,70,104,74,93,0,177
GGTLC2_2_11202,947,770,674,676,827,346,1073,647,651,1202,903,332
GLCE_2_11203,1954,1814,1629,1911,2024,2814,2668,1317,1273,1895,2095,17
53
GLDC_2_11204,295,147,189,193,358,98,549,77,233,111,199,422
GLO1_2_11205,768,733,663,884,929,387,793,564,379,613,477,1358
GLOD4_2_11206,1610,1329,1254,1667,1260,1212,1913,1627,1120,2157,2773,1
443
GLS2_2_11207,510,361,331,405,88,194,564,475,605,164,725,861
GLT1D1_2_11208,131,88,44,108,45,0,24,17,38,39,62,16
GLT25D1_2_11209,1152,1229,1223,1734,1640,1602,989,1457,1482,1069,1196,
786
GLT25D2_2_11210,277,242,371,337,536,162,1382,1504,66,428,545,243
GLT8D2_2_11211,1121,1180,848,1065,579,544,1038,1105,2385,700,992,989
GLUD1_2_11212,882,765,838,774,301,1149,566,589,599,990,1075,1340
GLUD2_2_11213,221,170,193,286,0,5,125,126,466,223,226,138
GMDS_2_11214,586,279,260,350,575,69,179,695,236,412,234,320
GMIP_2_11215,345,212,235,365,14,171,76,150,289,743,226,628
GMPR_2_11216,395,580,578,542,403,355,71,2,67,720,1801,42
GMPS_2_11217,570,748,501,798,387,331,955,793,425,1072,471,858
GNA11_2_11218,476,579,443,432,616,147,419,1167,940,708,364,1024
GNA12_2_11219,60,61,120,103,264,85,378,0,137,47,97,327
GNA13_2_11220,795,838,666,859,260,197,701,693,2119,750,1023,782
GNA14_2_11221,255,322,306,217,761,285,45,180,319,740,124,250
GNA15_2_11222,59,81,24,99,159,46,0,1,12,15,52,47
GNAI1_2_11223,725,341,469,498,957,63,10,244,605,410,280,517
GNAI3_2_11224,545,366,397,430,773,674,90,643,291,456,316,317
GNAQ_2_11225,251,212,213,179,14,373,471,16,327,279,25,522
GNAT2_2_11226,385,422,457,369,247,49,494,603,382,410,595,279
GNAT3_2_11227,842,750,813,736,794,356,2249,728,318,1134,1033,238
GNAZ_2_11228,75,29,135,78,0,0,0,0,209,10,0,0
GNB1_2_11229,797,664,671,865,414,225,705,1261,597,667,74,677
GNB2L1_2_11230,1115,945,752,1048,1250,218,690,951,946,510,491,993
GNB3_2_11231,11,7,0,3,24,0,0,0,0,0,2,2
GNB4_2_11232,822,762,826,1095,1168,269,594,453,426,247,1377,607
GNG11_2_11233,69,96,121,115,142,0,11,8,91,215,0,235
GNG12_2_11234,50,48,118,114,53,351,91,28,173,3,302,39
GNG13_2_11235,12,8,28,32,0,0,0,0,0,3,0,0
GNG2_2_11236,281,259,316,385,154,107,210,134,292,41,0,268
GNG3_2_11237,437,226,255,356,454,129,118,276,401,46,33,593
GNG7_2_11238,904,969,729,971,666,447,1723,380,671,1270,1282,1919

GNG8_2_11239,110,52,78,85,36,9,1,202,85,1,304,14
GNGT1_2_11240,99,90,96,148,0,134,177,6,64,41,275,649
GNL2_2_11241,56,69,41,85,80,25,37,33,12,2,0,72
GNMT_2_11242,1062,687,550,667,392,505,1598,1224,1580,298,1093,577
GNPAT_2_11243,154,154,144,142,38,33,109,80,26,478,62,113
GNPDA1_2_11244,2,16,22,43,2,0,0,0,0,0,3,0
GNPDA2_2_11245,190,163,130,164,334,22,237,314,297,36,22,83
GNPNAT1_2_11246,561,605,568,571,472,490,253,141,779,97,457,961
GNPTAB_2_11247,110,57,96,86,80,61,23,79,26,11,118,228
GNPTG_2_11248,36,44,137,113,0,0,377,131,0,20,0,0
GNS_2_11249,715,784,786,976,1067,602,165,956,332,976,338,1062
GOT2_2_11250,452,357,621,375,820,180,508,288,565,590,459,573
GPAA1_2_11251,263,333,308,278,249,209,149,182,463,62,153,47
GPAM_2_11252,532,503,361,710,420,354,1305,293,606,1455,220,434
GPAT2_2_11253,273,381,224,425,149,261,183,226,208,276,684,551
GPD1_2_11254,173,159,202,185,16,45,251,569,110,197,89,241
GPD1L_2_11255,284,343,241,301,680,375,897,516,854,48,353,264
GPX2_2_11256,274,182,225,142,235,260,254,201,419,59,110,40
GPX3_2_11257,21,54,14,18,40,0,0,0,0,13,1,30
GPX7_2_11258,822,510,453,669,362,252,215,261,207,362,1040,307
GPX8_2_11259,428,350,642,489,162,187,158,192,433,154,11,385
GRHPR_2_11260,118,152,158,183,48,37,58,17,13,7,54,197
GSTA1_2_11261,1613,1628,1345,1600,1585,1108,1948,1856,2330,2524,1786,1
257
GSTA2_2_11262,406,361,402,425,539,159,319,343,269,232,536,496
GSTA3_2_11263,774,596,420,465,429,331,440,606,442,101,400,603
GSTA4_2_11264,2205,1819,1415,1851,1878,1472,2274,2321,1145,1046,949,21
37
GSTA5_2_11265,407,360,402,404,539,159,318,344,268,232,536,493
GSTM3_2_11266,148,171,213,154,289,136,367,700,32,45,27,54
GSTM5_2_11267,811,764,601,670,1294,1204,1509,617,986,498,337,436
GSTP1_2_11268,239,175,237,284,36,25,87,46,69,0,676,60
GSTT1_2_11269,83,97,79,106,9,0,561,664,16,130,0,15
GSTT2B_2_11270,297,388,425,423,396,241,236,502,49,256,895,920
GSTT2_2_11271,297,388,425,423,396,241,236,502,49,256,895,920
GTF2F2_2_11272,436,499,430,520,586,555,402,685,56,288,363,635
GTPBP1_2_11273,443,289,519,494,156,259,401,365,722,171,416,293
GTPBP2_2_11274,474,182,470,547,0,350,923,299,870,295,342,5
GTPBP4_2_11275,623,271,478,657,505,382,341,626,22,630,524,345
GUCY1A2_2_11276,850,443,781,907,687,1081,3,957,197,293,710,731
GUCY1B3_2_11277,103,206,106,207,38,65,14,104,100,114,52,292
GUSB_2_11278,287,266,250,223,186,249,43,190,312,140,88,482
GYLTL1B_2_11279,69,146,90,139,13,45,52,5,123,36,3,158
GYS2_2_11280,203,105,114,153,0,0,9,32,286,1,5,119
GZMA_2_11281,458,669,645,777,630,384,440,872,186,1437,955,321
GZMB_2_11282,1216,1633,1163,1456,956,1099,560,1709,1333,1438,890,1802
H6PD_2_11283,228,153,216,243,131,5,58,860,144,137,303,49
HAAO_2_11284,581,576,354,565,203,980,655,944,266,662,65,422
HACL1_2_11285,833,994,813,902,694,781,2507,784,543,460,662,882
HADHA_2_11286,403,339,383,383,648,250,826,745,181,494,217,580

HADHB_2_11287,386,279,231,191,332,381,131,263,364,315,181,360
HAGHL_2_11288,0,0,0,0,0,0,0,0,0,0,0,0
HAL_2_11289,1199,1066,909,1383,728,501,1100,1205,2001,515,1247,2003
HAO1_2_11290,881,529,745,505,823,490,110,329,548,666,1244,313
HARS_2_11291,51,54,28,40,20,0,0,0,0,3,0,203
HAS1_2_11292,921,894,886,940,627,1038,1162,680,317,1316,1137,1514
HAS2_2_11293,310,386,583,425,80,505,70,344,470,419,1246,668
HDHD3_2_11294,245,229,133,212,0,20,539,50,788,6,302,344
HELB_2_11295,179,70,122,267,259,155,54,3,43,52,0,1007
HELQ_2_11296,480,278,443,489,190,720,507,263,138,252,63,247
HELZ_2_11297,376,269,467,458,361,62,309,643,170,70,520,508
HEXA_2_11298,233,115,91,135,56,203,146,291,129,66,10,18
HEXB_2_11299,113,214,83,163,47,1,700,627,50,74,0,46
HEXDC_2_11300,60,86,143,51,0,25,1,74,153,55,6,18
HGD_2_11301,455,511,478,567,171,225,427,949,747,359,1,671
HGSNAT_2_11302,1068,930,998,1373,1625,875,1115,1621,745,738,520,805
HHATL_2_11303,298,94,150,94,222,214,25,246,342,136,23,47
HIBADH_2_11304,217,318,253,231,371,203,105,865,146,332,54,23
HINT1_2_11305,1276,1062,1048,1112,372,1191,1410,909,902,840,738,1152
HMOX1_2_11306,217,134,233,287,46,15,0,20,120,129,286,459
HPGDS_2_11307,360,204,228,337,112,529,203,93,153,477,422,221
HPRT1_2_11308,0,0,0,0,0,0,0,0,0,0,0,0
HS3ST1_2_11309,238,280,128,210,456,335,20,232,310,88,194,376
HS3ST2_2_11310,478,182,284,324,180,171,456,328,331,278,654,144
HS3ST3A1_2_11311,364,382,550,361,114,455,252,342,390,639,184,208
HS3ST3B1_2_11312,68,107,123,109,22,160,7,54,17,184,137,92
HS3ST4_2_11313,807,744,519,530,933,597,581,503,776,222,856,536
HS3ST6_2_11314,29,42,75,66,0,5,26,0,6,119,0,8
HS6ST1_2_11315,769,362,539,519,948,251,678,348,387,873,636,264
HS6ST3_2_11316,704,648,432,556,1451,266,1,1586,522,503,370,533
HSD11B2_2_11317,29,2,30,22,0,0,0,0,0,33,0,15
HSD17B11_2_11318,635,558,471,782,1212,599,965,1171,654,912,581,746
HSD17B12_2_11319,268,186,270,210,134,378,50,130,140,171,73,269
HSD17B14_2_11320,191,183,224,262,44,168,375,67,269,272,6,500
HSD17B1_2_11321,471,257,167,245,3,526,334,140,178,300,29,69
HSD17B2_2_11322,248,168,303,437,341,144,3,13,150,483,470,371
HSD17B3_2_11323,371,263,289,145,736,5,127,263,297,1,0,166
HSD17B6_2_11324,85,147,118,114,2,0,239,256,278,273,0,88
HSD17B8_2_11325,870,685,799,773,1376,720,310,930,907,578,965,1029
HSD3B1_2_11326,296,494,334,351,471,523,1208,69,955,1086,54,216
HSP90AB1_2_11327,722,652,568,859,326,645,246,1191,420,712,208,1357
HSPA5_2_11328,263,238,209,334,157,360,975,68,187,967,273,361
HSPE1_2_11329,444,492,337,476,470,170,506,169,258,1198,283,1102
HSPG2_2_11330,47,82,65,45,236,5,0,53,2,3,0,612
HYAL4_2_11331,665,510,329,689,417,656,282,179,1111,452,533,156
IARS2_2_11332,706,573,570,677,899,739,238,304,355,655,452,1012
ICMT_2_11333,1288,1347,1333,1304,1754,1236,1692,932,1126,1468,553,2421
ICT1_2_11334,0,0,0,0,0,0,0,0,0,0,0,0
IDH1_2_11335,1000,760,756,820,271,678,855,679,341,695,205,975
IDH2_2_11336,163,64,120,47,53,160,23,141,243,27,36,20

IDH3A_2_11337,591,596,621,851,1167,728,1400,1069,609,1159,466,504
IDI1_2_11338,1402,916,1098,1239,512,827,1292,696,697,2183,866,764
IDI2_2_11339,585,500,543,705,1029,588,412,1153,1161,380,33,338
IDO1_2_11340,255,100,233,197,205,75,9,581,0,283,316,349
IDO2_2_11341,54,82,119,88,0,18,165,62,89,244,43,108
IDUA_2_11342,19,20,14,33,37,5,0,101,0,114,0,0
IFI30_2_11343,73,10,86,56,0,6,48,0,254,27,41,22
IFIH1_2_11344,358,526,268,393,584,346,813,194,262,176,17,461
IGHMBP2_2_11345,529,165,149,269,98,0,435,28,232,233,37,118
IHH_2_11346,16,76,67,75,1,0,0,5,0,350,0,0
ILVBL_2_11347,238,89,175,88,310,80,302,58,178,22,165,108
IMPAD1_2_11348,424,454,397,470,314,8,959,2075,731,12,3,434
IMPDH2_2_11349,549,405,631,669,304,287,467,1256,425,548,1063,675
IRS1_2_11350,458,414,226,380,1085,277,99,336,423,534,177,360
IRS2_2_11351,97,71,144,179,134,88,17,144,103,51,421,95
ISG20_2_11352,258,112,204,245,195,165,11,34,223,292,6,254
ISG20L2_2_11353,127,117,114,357,5,2,50,124,58,26,0,288
ISOC1_2_11354,441,616,678,634,207,627,876,283,865,918,1376,655
IWS1_2_11355,111,138,102,52,120,36,0,259,154,397,218,2
KDSR_2_11356,427,392,422,453,71,54,253,755,534,53,643,1064
KHSRP_2_11357,104,90,73,79,69,45,28,204,108,66,76,10
KIAA0317_2_11358,500,563,402,526,664,195,336,309,22,395,8,312
KIAA1279_2_11359,300,261,256,182,104,159,392,210,276,348,947,202
KIAA2022_2_11360,1757,1212,1161,1378,1286,1348,1837,2114,1457,1136,934
,1910
KIF18A_2_11361,1255,980,840,988,344,141,1312,310,326,1083,1025,305
KIF20B_2_11362,260,351,225,318,239,291,31,50,481,674,0,348
KIF3A_2_11363,360,399,400,331,655,177,73,265,346,398,142,31
KLB_2_11364,208,156,263,192,77,30,300,374,505,7,402,58
KLC3_2_11365,1000,945,613,1069,577,472,693,943,496,1349,557,1453
KL_2_11366,93,85,214,168,473,0,141,1,9,524,896,424
KLK1_2_11367,356,418,467,351,148,1,555,245,610,1,1054,416
KLKB1_2_11368,612,679,514,750,982,693,503,355,1002,1028,1093,270
KMO_2_11369,186,137,129,247,27,233,262,393,177,132,99,98
KRTCAP2_2_11370,14,3,16,3,0,0,0,0,21,0,0,10
L2HGDH_2_11371,126,63,67,93,10,46,2,6,48,54,10,136
LALBA_2_11372,1812,1180,1009,1290,1415,1166,1824,1403,1558,1988,861,96
5
LAP3_2_11373,51,59,24,109,0,95,93,130,7,198,0,370
LARS2_2_11374,343,452,616,492,484,166,891,1183,507,1010,25,356
LARS_2_11375,338,369,283,289,217,69,242,448,216,402,78,194
LCAT_2_11376,56,131,108,86,10,2,0,34,1,0,1,208
LCMT2_2_11377,2379,1893,1305,2166,4143,1470,2254,1054,2498,1335,1802,1
420
LCT_2_11378,16,57,86,35,43,2,6,8,202,15,3,0
LCTL_2_11379,836,494,603,606,387,552,201,642,474,788,594,672
LDHAL6B_2_11380,148,203,80,204,253,29,74,57,98,39,138,218
LEPREL2_2_11381,19,1,128,40,1,0,33,338,1,33,0,0
LGALS13_2_11382,102,96,93,122,292,0,81,0,18,230,1,46
LHFPL2_2_11383,269,223,172,284,18,50,47,81,228,358,312,172

LIPC_2_11384,969,1122,863,1219,878,754,1192,197,1095,884,889,713
LIPE_2_11385,23,36,14,36,0,0,6,0,0,47,122,0
LIPG_2_11386,144,114,96,160,97,0,91,395,49,161,0,24
LIPH_2_11387,401,355,292,310,489,164,107,119,382,93,123,299
LIPI_2_11388,65,58,48,68,18,94,29,2,0,5,0,196
LIPM_2_11389,456,323,392,355,950,89,224,55,166,143,212,557
LIPN_2_11390,513,474,562,527,761,350,1138,50,587,788,812,473
LIPT2_2_11391,510,186,354,426,302,138,1596,654,103,233,723,920
LONP2_2_11392,523,552,486,538,695,504,1074,25,414,684,209,902
LOXL1_2_11393,152,83,129,135,65,23,484,207,26,114,0,49
LOXL2_2_11394,460,448,514,528,463,172,245,475,388,650,706,644
LOXL3_2_11395,0,0,0,0,0,0,0,0,0,0,0,0
LOXL4_2_11396,111,62,47,104,11,589,31,16,30,0,150,63
LPCAT1_2_11397,1015,1036,928,1152,610,1153,1002,2935,457,740,964,882
LPCAT2_2_11398,775,545,622,558,718,1188,625,829,876,327,1991,737
LPCAT3_2_11399,116,62,133,109,47,31,203,0,95,524,27,112
LPCAT4_2_11400,318,95,241,266,303,111,40,336,237,118,15,191
LPIN1_2_11401,453,365,368,566,747,959,396,523,1078,367,525,577
LPIN2_2_11402,19,14,131,22,0,3,6,0,0,10,0,0
LPIN3_2_11403,559,466,576,574,142,341,650,373,933,677,489,427
LPL_2_11404,495,564,540,422,693,569,266,117,242,341,283,734
LRAT_2_11405,463,214,212,288,711,343,52,289,228,76,354,470
LTA4H_2_11406,443,387,395,537,190,6,559,24,735,360,820,199
LTC4S_2_11407,498,287,420,409,1287,698,1378,792,342,302,753,239
LYG2_2_11408,456,278,430,339,515,212,309,380,229,316,65,133
LYPLA1_2_11409,320,271,324,313,20,0,599,696,478,206,597,434
LYPLA2_2_11410,38,117,38,79,218,68,0,188,6,135,0,40
LYPLAL1_2_11411,101,66,82,115,0,3,3,105,0,0,2,196
LYZ_2_11412,1215,927,911,1498,2073,947,598,618,1194,1475,1066,1124
LYZL1_2_11413,298,315,376,499,179,57,709,532,304,25,22,124
LYZL2_2_11414,122,0,30,38,6,3,15,5,0,31,0,2
LYZL4_2_11415,4110,3882,3394,3916,4580,4255,3094,2472,4394,2895,4904,2
745
MACROD1_2_11416,19,5,12,27,0,0,0,100,26,1,0,1
MAGT1_2_11417,98,148,58,110,120,17,2,21,8,281,272,509
MAN1A1_2_11418,178,157,121,218,26,136,23,74,6,253,763,52
MAN1A2_2_11419,991,1034,1247,1332,717,530,512,1338,1064,801,546,1205
MAN1B1_2_11420,47,16,14,18,222,1,8,43,0,0,300,11
MAN1C1_2_11421,170,219,65,242,179,377,69,220,48,403,134,957
MAN2A1_2_11422,141,232,191,184,2,234,237,6,8,140,1,202
MAN2A2_2_11423,11,0,6,47,0,0,0,0,5,5,0,0
MAN2B2_2_11424,226,313,232,370,585,324,216,106,755,37,718,161
MAN2C1_2_11425,26,8,27,29,0,27,2,0,120,0,1,78
MANBA_2_11426,426,478,349,419,219,222,179,475,265,339,474,751
MANEA_2_11427,669,686,766,900,510,1257,521,687,954,433,669,457
MAOA_2_11428,882,744,819,709,1252,417,549,350,696,971,499,292
MAOB_2_11429,999,1177,648,881,121,697,579,344,308,989,272,957
MAP1S_2_11430,40,24,282,76,0,13,182,1,2,0,0,20
MAPRE3_2_11431,1002,817,1064,1356,190,358,2110,148,1003,1486,1855,1272
MARS2_2_11432,120,128,82,72,277,21,83,106,147,562,160,450

MARS_2_11433,35,59,20,31,9,1,8,29,0,0,55,0
MAT1A_2_11434,145,338,262,302,123,1,0,335,149,206,420,200
MAT2A_2_11435,424,576,460,466,1498,24,116,232,150,90,196,65
MBOAT1_2_11436,128,101,79,157,276,374,3,51,173,150,16,265
MBOAT2_2_11437,299,287,389,317,373,574,889,395,89,313,322,92
MBOAT4_2_11438,517,442,394,422,478,275,428,578,854,255,802,685
MBTPS1_2_11439,100,185,108,82,97,143,28,50,27,129,2,36
MBTPS2_2_11440,386,344,305,361,190,401,298,438,382,415,876,57
MCCC1_2_11441,723,617,560,620,623,634,533,342,408,618,781,739
MCCC2_2_11442,113,87,102,151,181,325,136,100,199,36,10,242
MCEE_2_11443,407,594,256,757,515,369,161,1059,125,1651,879,740
MCM3_2_11444,8,6,12,12,0,0,0,0,2,0,29,0
MCM5_2_11445,255,353,296,434,311,245,0,302,278,0,351,394
MCM6_2_11446,316,336,177,550,143,212,302,318,837,304,275,379
MDH1B_2_11447,212,103,71,199,31,45,9,609,163,21,2,173
MDH2_2_11448,890,535,500,758,253,414,662,146,428,293,434,700
MEP1A_2_11449,625,979,410,669,1091,566,652,41,708,855,925,355
MEP1B_2_11450,358,279,308,458,329,107,185,250,93,45,358,349
METAP1_2_11451,888,853,764,865,793,847,1603,978,482,1013,1103,503
METAP1D_2_11452,113,153,108,170,222,260,215,1,202,0,98,6
METAP2_2_11453,1308,1237,1391,1188,2437,692,1893,935,667,1105,2484,630
METTL14_2_11454,342,213,228,165,111,143,15,361,166,1,44,268
METTL22_2_11455,1102,937,1024,1172,1354,776,1559,283,889,1233,1138,148
5
METTL2B_2_11456,357,126,234,163,79,272,93,108,613,91,0,270
METTL3_2_11457,501,320,338,493,1051,329,177,210,820,276,82,582
METTL5_2_11458,1479,1117,844,1422,1316,1191,935,1067,556,1056,306,888
METTL6_2_11459,701,422,303,495,146,372,457,643,294,26,454,494
METTL7B_2_11460,231,178,276,232,525,62,188,60,381,44,1198,99
METTL8_2_11461,66,78,82,62,136,29,312,44,129,5,61,235
MFN1_2_11462,264,143,250,220,187,264,295,4,59,238,41,177
MGAM_2_11463,72,86,54,102,16,48,192,310,23,79,210,98
MGAT4C_2_11464,101,203,75,90,5,469,98,64,44,273,0,245
MGAT5_2_11465,32,22,13,56,0,17,210,27,4,10,0,34
MGMT_2_11466,97,188,157,143,1,22,14,116,130,37,0,155
MGST3_2_11467,555,394,337,430,270,1413,110,475,650,1123,632,25
MIOX_2_11468,272,206,241,204,270,21,167,77,321,465,220,167
MIPEP_2_11469,196,34,111,100,188,0,0,0,0,34,0,19
MLYCD_2_11470,37,40,57,33,29,3,41,10,205,1,0,16
MMAB_2_11471,117,169,267,102,0,0,0,74,506,471,348,8
MMEL1_2_11472,23,14,70,48,67,0,87,15,1,347,267,73
MMP10_2_11473,28,1,51,23,10,26,45,0,39,46,0,2
MMP12_2_11474,26,97,58,59,4,45,368,178,295,49,183,132
MMP14_2_11475,504,536,505,557,715,377,179,125,609,611,399,351
MMP3_2_11476,315,477,323,349,568,619,384,501,131,711,113,335
MMP7_2_11477,180,187,128,142,165,32,508,1,69,111,166,62
MMP8_2_11478,576,565,546,633,252,1030,225,399,849,220,1252,56
MMP9_2_11479,80,213,88,109,31,98,3,0,31,232,40,112
MOCOS_2_11480,833,592,630,711,385,185,1228,1235,492,572,541,301
MOGAT1_2_11481,168,81,145,200,143,3,40,32,8,259,91,216

MOGAT2_2_11482,168,238,256,227,157,56,59,376,116,474,2,511
MOGAT3_2_11483,599,451,660,702,840,602,673,556,425,897,239,1847
MOXD1_2_11484,1170,936,703,1198,969,655,545,1039,1272,793,480,1610
MPI_2_11485,644,499,475,565,169,206,198,666,833,1214,501,217
MPO_2_11486,90,133,146,175,60,28,5,33,153,121,825,85
MRPL37_2_11487,204,288,238,229,274,241,12,159,124,298,415,79
MRPL44_2_11488,313,443,508,537,458,432,837,322,592,419,395,648
MRPS30_2_11489,381,391,342,327,1203,417,591,864,548,506,190,638
MSH2_2_11490,541,417,485,660,1000,408,796,280,247,1108,1077,443
MSH3_2_11491,1395,1078,1476,1208,1720,1100,1273,1617,819,1238,1594,108
7
MSH4_2_11492,418,456,483,592,502,113,693,601,277,713,955,928
MTAP_2_11493,180,182,145,120,15,58,155,774,12,53,1029,72
MTFMT_2_11494,283,425,189,270,404,126,199,191,452,269,534,370
MTHFD1_2_11495,140,116,112,190,93,98,7,86,90,503,49,78
MTHFD2_2_11496,465,236,199,282,2,273,355,109,225,759,192,357
MTHFD2L_2_11497,186,105,96,131,289,414,165,299,365,583,116,31
MTHFR_2_11498,243,246,163,354,196,892,501,692,267,200,59,357
MTPAP_2_11499,274,259,201,310,198,0,619,27,185,31,0,51
MTR_2_11500,264,302,203,268,5,904,5,12,66,194,342,305
MUS81_2_11501,85,46,68,84,16,26,0,47,2,6,15,1
MUT_2_11502,601,539,633,701,1749,887,639,917,355,489,1021,837
MVD_2_11503,220,240,119,248,81,220,410,1349,73,302,129,402
MX2_2_11504,30,49,30,82,33,336,0,1,2,0,0,0
MYH1_2_11505,430,518,437,577,1114,558,1000,664,269,469,357,1219
MYH3_2_11506,1031,1064,1167,925,797,1820,311,525,1348,852,976,1042
MYH6_2_11507,571,485,546,541,643,534,406,591,444,95,475,577
MYH7_2_11508,236,157,293,285,41,234,202,173,1,330,38,777
MYH9_2_11509,33,72,65,94,16,0,67,1,49,44,0,275
MYL7_2_11510,103,195,104,81,659,7,13,35,91,105,58,57
MYO1E_2_11511,184,157,140,147,38,22,364,192,131,120,108,448
MYO5B_2_11512,172,54,43,63,17,60,192,19,15,0,362,19
MYO9A_2_11513,133,100,112,180,24,255,182,156,807,32,267,137
N6AMT2_2_11514,70,40,52,106,32,185,116,5,0,0,51,65
NAA10_2_11515,379,331,421,464,116,303,132,466,95,276,382,303
NAA11_2_11516,11,11,3,21,0,0,0,0,0,2,0,0
NAA15_2_11517,2151,1628,1908,1792,1293,1436,891,1557,2777,1244,2125,19
84
NAA30_2_11518,33,59,10,4,0,6,30,0,49,17,0,22
NAA50_2_11519,1133,992,1008,954,528,591,1072,1418,1198,2081,1723,1312
NAALAD2_2_11520,793,838,596,661,425,766,656,1295,504,971,1602,599
NAALADL1_2_11521,233,193,263,230,187,265,553,82,62,156,272,171
NADSYN1_2_11522,55,41,57,97,39,6,1,3,93,48,0,111
NAGA_2_11523,618,467,555,763,286,991,185,882,1236,200,51,638
NAGLU_2_11524,369,390,301,456,336,161,789,141,239,460,41,168
NAGPA_2_11525,309,289,242,159,41,593,40,118,526,310,514,14
NAMPT_2_11526,79,143,60,198,44,80,0,1,100,25,0,100
NANP_2_11527,323,315,311,332,242,799,142,322,271,316,66,292
NANS_2_11528,1057,1116,855,863,944,617,736,1293,466,1138,416,2032
NAPRT1_2_11529,152,84,44,70,647,11,439,0,327,9,0,92

NARS_2_11530,480,315,309,492,502,13,1192,311,984,73,183,260
NCF1_2_11531,204,121,147,223,105,182,354,33,137,265,55,250
NDST1_2_11532,298,171,193,325,728,368,83,106,332,264,131,545
NDST2_2_11533,29,41,34,32,34,0,65,1,3,309,0,12
NDST3_2_11534,4140,3391,3200,3487,4431,1613,4115,3577,3998,4424,3466,3
355
NDST4_2_11535,723,319,343,424,838,450,742,96,1377,684,92,279
NDUFA10_2_11536,16,23,78,46,0,41,36,56,0,32,0,0
NDUFA12_2_11537,858,1076,940,1016,794,195,1280,1449,404,1611,1421,568
NDUFA13_2_11538,759,964,910,1096,1197,852,1593,655,587,1141,1466,1006
NDUFA1_2_11539,843,587,486,612,520,377,1162,551,802,916,293,686
NDUFA3_2_11540,305,327,310,375,86,72,615,77,273,638,58,96
NDUFA4_2_11541,176,213,258,361,320,263,221,219,383,359,449,617
NDUFA4L2_2_11542,1557,1296,1512,1448,2335,1663,705,2665,2460,1639,2030
,1382
NDUFA5_2_11543,700,730,491,636,281,509,226,306,610,899,581,619
NDUFA6_2_11544,1078,992,743,1030,1562,1154,1643,894,1113,1097,986,2307
NDUFA7_2_11545,130,114,83,124,129,170,172,1,168,115,36,238
NDUFA8_2_11546,605,625,462,866,155,242,202,532,252,444,249,25
NDUFA9_2_11547,747,668,595,776,903,382,939,752,561,617,493,582
NDUFAB1_2_11548,195,130,148,113,9,0,2,0,96,3,1,14
NDUFB10_2_11549,121,78,82,184,154,285,84,478,57,145,54,461
NDUFB1_2_11550,222,228,338,355,132,327,35,153,317,846,193,657
NDUFB2_2_11551,258,145,290,219,239,317,26,44,196,570,349,570
NDUFB3_2_11552,1034,811,1082,1158,2281,1224,1019,1909,913,1154,1454,90
0
NDUFB7_2_11553,7,1,4,29,14,1,0,0,1,0,0,28
NDUFB8_2_11554,244,247,319,284,3,140,272,532,23,221,37,418
NDUFB9_2_11555,202,40,103,81,461,16,204,228,233,281,12,171
NDUFS3_2_11556,42,17,41,45,101,0,230,0,108,42,1,27
NDUFS4_2_11557,127,62,67,90,71,31,139,91,146,168,0,35
NDUFS6_2_11558,730,787,535,548,809,939,987,881,1107,394,91,727
NDUFS7_2_11559,386,425,278,463,645,22,66,37,324,346,129,322
NDUFS8_2_11560,232,273,126,314,161,176,332,218,313,240,285,293
NDUFV2_2_11561,1005,1026,798,1152,384,949,1382,441,852,38,621,1364
NEDD8_2_11562,691,514,549,762,823,643,181,554,573,311,2689,732
NEIL1_2_11563,124,102,57,101,46,50,99,90,0,175,2,130
NEIL3_2_11564,852,875,758,962,1685,953,237,977,669,953,969,1446
NEU1_2_11565,107,112,113,150,168,19,0,58,146,101,0,1
NEU2_2_11566,149,116,68,139,0,175,0,340,0,271,0,114
NEU3_2_11567,473,437,568,592,212,247,980,435,283,257,456,749
NHLRC2_2_11568,217,281,331,269,88,94,642,513,294,413,49,271
NIT2_2_11569,170,239,84,73,9,11,35,75,76,221,2,19
NKIRAS1_2_11570,301,489,331,321,23,656,77,236,241,870,573,417
NLGN1_2_11571,238,209,182,278,360,907,148,560,170,103,632,66
NLGN2_2_11572,82,67,117,45,4,38,30,0,32,0,258,40
NLN_2_11573,618,514,527,639,718,769,168,598,348,255,251,254
NME1-NME2_2_11574,135,159,69,108,34,133,83,150,4,393,13,427
NMNAT1_2_11575,608,376,382,320,666,139,484,1506,524,436,752,405
NMT1_2_11576,818,630,529,640,1407,704,635,342,984,267,786,681

NMT2_2_11577,483,359,403,403,932,378,601,380,233,1024,301,402
NNMT_2_11578,145,257,231,360,363,44,313,7,47,80,385,349
NOP58_2_11579,106,94,90,191,12,0,378,57,237,257,9,135
NOX3_2_11580,261,239,301,239,373,222,550,439,252,158,277,365
NQO2_2_11581,155,90,133,150,0,354,151,0,0,61,327,32
NRAS_2_11582,124,89,116,103,189,47,14,144,127,22,68,276
NSF_2_11583,200,134,233,174,143,61,55,224,642,344,376,166
NSUN6_2_11584,414,304,238,240,300,572,50,406,299,589,352,157
NTAN1_2_11585,332,265,257,334,301,91,359,52,144,228,255,116
NTHL1_2_11586,528,509,329,383,569,150,100,336,673,300,692,420
NTPCR_2_11587,636,579,563,460,620,67,599,250,751,457,443,436
NUDT10_2_11588,225,275,199,197,154,807,120,23,32,321,123,401
NUDT11_2_11589,225,275,199,197,154,807,120,23,32,321,123,401
NUDT12_2_11590,987,1002,998,1000,683,422,1393,848,472,2203,1136,1112
NUDT14_2_11591,101,80,83,56,6,88,18,102,45,26,0,57
NUDT3_2_11592,1180,750,1092,1083,1481,637,1180,668,1071,1343,785,906
NUDT5_2_11593,263,517,449,563,78,200,91,114,252,319,22,241
NUDT7_2_11594,822,914,768,913,840,284,1131,910,680,1326,1735,771
NXNL1_2_11595,860,633,708,773,864,698,43,876,714,805,37,1025
OAS3_2_11596,137,124,130,82,597,0,4,39,10,55,0,189
OAZ1_2_11597,125,190,200,147,18,169,34,813,5,376,284,195
OC90_2_11598,482,478,398,424,1022,555,399,891,637,682,357,769
ODF3B_2_11599,394,312,86,236,655,290,318,657,426,176,175,358
OPLAH_2_11600,257,140,169,179,164,131,6,270,41,612,22,133
OSGEP_2_11601,35,31,27,47,13,36,31,10,11,28,0,0
OSGEPL1_2_11602,443,511,633,462,701,24,20,473,430,627,488,837
OSTC_2_11603,3185,2991,3316,3488,3997,1513,3905,3890,2502,2695,3209,38
43
OTC_2_11604,451,235,236,304,535,414,188,196,45,1035,435,445
OVGP1_2_11605,383,244,451,572,438,330,624,51,963,234,540,910
OXA1L_2_11606,93,82,95,60,58,112,12,95,423,168,38,191
OXCT1_2_11607,0,0,0,0,0,0,0,0,0,0,0,0
OXCT2_2_11608,140,131,77,92,3,322,1,327,59,541,8,14
P4HA3_2_11609,303,220,189,343,266,170,0,402,289,351,210,428
P4HB_2_11610,706,932,611,609,780,1018,1197,726,300,828,1592,1133
PADI1_2_11611,207,125,86,118,19,162,46,199,8,148,55,113
PADI2_2_11612,138,96,176,223,14,3,41,0,9,312,26,66
PADI3_2_11613,348,264,163,399,271,4,419,10,667,29,197,5
PADI6_2_11614,273,151,163,356,227,140,284,296,388,106,270,465
PAFAH2_2_11615,1,1,29,0,0,0,0,6,0,0,0,0
PAH_2_11616,442,298,338,327,515,8,144,149,343,413,742,367
PAPOLA_2_11617,790,444,492,620,514,122,696,95,507,146,1253,329
PAPOLB_2_11618,983,765,791,991,696,123,700,333,721,145,2107,418
PAPOLG_2_11619,207,147,98,118,210,13,0,26,0,75,14,153
PAPPA_2_11620,389,441,439,499,769,573,20,748,740,224,197,286
PAPSS1_2_11621,34,91,46,115,15,75,1,0,35,0,0,59
PARG_2_11622,79,89,57,143,27,30,0,8,52,6,6,65
PARP1_2_11623,161,312,186,130,679,277,67,297,112,299,13,279
PARP4_2_11624,960,921,763,1058,811,1690,1085,419,648,773,476,858
PARP6_2_11625,185,137,83,112,11,24,69,0,235,190,220,278

PARS2_2_11626,26,63,32,69,67,59,5,56,0,0,22,8
PCBD1_2_11627,139,119,166,257,174,27,0,285,224,3,387,223
PCBD2_2_11628,57,74,31,73,32,9,4,25,32,378,0,56
PCMT1_2_11629,166,134,153,60,16,172,373,2,13,10,0,258
PCMTD1_2_11630,160,101,164,142,277,81,556,14,480,102,22,219
PCYOX1_2_11631,781,802,568,714,233,441,823,353,743,262,312,886
PCYT1A_2_11632,93,135,73,165,0,140,0,650,6,330,5,51
PDCD1LG2_2_11633,849,648,321,555,597,1537,91,474,1048,289,679,53
PDCL_2_11634,372,327,273,438,436,384,330,543,605,397,115,419
PDE3A_2_11635,823,781,723,749,318,969,1485,554,915,491,336,569
PDE3B_2_11636,114,167,114,188,75,389,0,134,199,36,0,164
PDE6A_2_11637,339,408,381,521,1542,159,645,634,532,851,830,457
PDE6C_2_11638,151,377,164,231,195,380,172,73,19,159,119,352
PDE6D_2_11639,693,823,598,726,476,1150,1036,648,712,275,990,679
PDE6G_2_11640,121,132,94,140,137,47,0,78,327,65,303,122
PDE6H_2_11641,898,916,943,921,353,934,530,393,944,232,555,1069
PDE7B_2_11642,539,551,431,535,44,252,1362,0,1330,100,228,643
PDHA2_2_11643,16,183,68,33,6,158,89,26,1,89,0,422
PDIA2_2_11644,0,0,0,0,0,0,0,0,0,0,0,0
PDIA3_2_11645,1127,577,674,735,1467,645,146,1365,1185,1092,590,901
PDIA4_2_11646,91,68,31,47,10,48,188,50,27,15,0,1
PDIA5_2_11647,1922,1792,1769,1927,1547,1492,2043,947,1242,2089,916,195
4
PDIA6_2_11648,456,588,459,601,39,326,965,1256,475,521,439,746
PDPR_2_11649,19,0,12,8,0,0,1,1,0,18,0,0
PDSS1_2_11650,546,750,343,676,815,487,404,790,844,274,1050,906
PDSS2_2_11651,1023,709,738,1022,681,902,892,735,977,740,1332,799
PDXP_2_11652,135,31,98,71,0,0,50,13,232,43,0,68
PECR_2_11653,605,459,740,710,155,342,1555,595,410,305,204,861
PELI1_2_11654,139,32,117,95,29,3,304,0,37,306,695,109
PET117_2_11655,680,571,481,689,2090,272,26,529,499,393,285,571
PEX1_2_11656,1068,769,920,767,1122,654,696,232,1012,491,2421,1145
PEX6_2_11657,418,443,289,322,864,342,2,380,359,301,268,305
PFAS_2_11658,79,25,60,50,119,0,154,10,222,3,64,95
PGA3_2_11659,2782,2101,2333,2453,3156,1502,1620,3594,2462,1338,2628,28
90
PGA4_2_11660,2782,2101,2333,2453,3156,1502,1620,3594,2462,1338,2628,28
90
PGA5_2_11661,2782,2101,2333,2453,3156,1502,1620,3594,2462,1338,2628,28
90
PGAM4_2_11662,241,144,228,172,292,7,70,367,34,1,0,218
PGAP1_2_11663,365,315,429,349,130,135,1072,405,408,340,560,280
PGAP3_2_11664,127,123,114,197,104,0,22,251,55,42,0,128
PGD_2_11665,341,212,315,358,145,214,59,332,280,319,215,229
PGGT1B_2_11666,344,489,398,513,143,304,695,117,9,38,23,134
PGLS_2_11667,231,176,76,185,91,170,320,0,17,329,607,104
PGM2_2_11668,571,547,544,647,432,147,615,719,488,302,842,270
PGM2L1_2_11669,36,171,39,45,150,24,141,0,70,0,9,0
PGM5_2_11670,200,229,142,312,334,334,0,5,274,4,445,74
PGPEP1_2_11671,164,212,157,342,8,368,125,305,13,54,507,481

PGS1_2_11672,85,140,143,162,45,264,515,0,99,4,13,53
PHGDH_2_11673,552,405,435,483,370,41,328,210,260,249,878,414
PHLPP1_2_11674,46,171,194,229,6,312,0,12,0,566,0,2
PHLPP2_2_11675,438,378,417,350,268,238,196,551,581,305,546,382
PIF1_2_11676,7,6,0,35,3,0,4,0,8,57,0,3
PIGB_2_11677,2105,1840,1531,1637,1548,1955,2712,1114,1711,2179,1736,17
94
PIGH_2_11678,345,483,353,479,0,206,674,448,234,288,62,445
PIGL_2_11679,384,519,593,548,414,18,586,521,485,711,509,203
PIGM_2_11680,137,185,121,255,79,254,85,210,331,129,154,18
PIGS_2_11681,1,3,13,50,359,0,11,5,104,0,0,64
PIGU_2_11682,35,46,39,40,13,0,1,0,51,109,2,3
PIGW_2_11683,3244,2809,2900,3302,2273,1027,3618,3276,3261,3311,2478,26
76
PIGZ_2_11684,596,257,552,625,107,390,1199,981,380,439,1119,364
PIN1_2_11685,334,533,604,587,1158,25,2,4,342,346,102,619
PIPOX_2_11686,1655,1623,1593,1753,1552,887,2329,1496,1825,2301,1756,30
45
PITPNM2_2_11687,113,174,126,180,258,0,235,246,202,150,26,290
PLA2G10_2_11688,14,55,35,45,0,0,238,7,20,1,11,127
PLA2G12A_2_11689,660,470,415,702,364,187,995,277,1376,128,373,825
PLA2G12B_2_11690,151,80,192,149,184,110,48,0,537,10,15,34
PLA2G15_2_11691,20,5,5,45,0,0,0,0,2,0,0,0
PLA2G1B_2_11692,622,729,561,596,767,735,2182,177,847,118,747,506
PLA2G2C_2_11693,464,575,411,688,660,321,554,343,297,329,478,635
PLA2G2D_2_11694,229,410,522,342,11,102,213,728,18,137,1479,197
PLA2G2E_2_11695,374,346,289,371,482,304,461,348,436,598,449,394
PLA2G2F_2_11696,480,452,692,610,528,328,156,655,285,847,630,334
PLA2G3_2_11697,456,458,487,649,217,289,231,547,815,881,965,377
PLA2G4A_2_11698,145,112,129,105,20,1,479,44,12,30,14,13
PLA2G4B_2_11699,351,466,300,453,274,32,1503,986,367,507,1,636
PLA2G4D_2_11700,575,649,674,807,266,866,349,192,1340,480,1067,799
PLA2G4E_2_11701,242,151,134,191,210,165,64,135,1,74,190,118
PLA2G4F_2_11702,363,98,88,189,78,146,42,22,447,8,615,203
PLA2G5_2_11703,2566,2738,2603,2704,1388,2273,4764,2138,1483,2430,2232,
2921
PLCB2_2_11704,697,919,725,875,804,606,522,321,562,1062,666,425
PLCD3_2_11705,44,4,27,26,0,0,36,52,5,18,0,57
PLCD4_2_11706,90,204,142,199,237,335,23,52,119,40,6,176
PLCG2_2_11707,5,4,3,5,0,12,80,0,0,19,2,0
PLCH2_2_11708,134,74,222,312,26,17,0,18,9,186,163,473
PLCL1_2_11709,56,16,19,66,39,0,66,51,0,0,0,45
PLCXD1_2_11710,116,22,33,58,194,216,34,51,12,4,0,16
PLCZ1_2_11711,144,176,73,134,48,65,125,117,41,268,80,213
PLD4_2_11712,83,55,39,108,8,3,0,0,19,131,0,0
PLOD1_2_11713,841,686,784,829,172,1080,1038,343,1025,217,1724,782
PLOD3_2_11714,134,241,214,286,41,258,144,825,457,1421,0,36
PLSCR1_2_11715,259,209,270,291,249,399,313,284,167,125,8,670
PMM1_2_11716,190,121,154,169,50,65,0,1,114,292,0,13
PMM2_2_11717,1532,1202,1105,1593,770,1552,1341,481,961,1215,657,1405

PMPCA_2_11718,442,391,333,397,516,50,10,753,872,262,1258,96
PMPCB_2_11719,1149,893,1029,1070,758,185,573,557,325,1585,189,1227
PNLIP_2_11720,474,177,418,523,321,226,28,149,499,571,903,608
PNLIPRP1_2_11721,109,132,90,113,20,19,3,252,24,231,0,137
PNLIPRP2_2_11722,33,27,33,63,142,18,48,0,32,5,0,66
PNLIPRP3_2_11723,12,104,59,44,19,153,0,3,9,2,104,35
PNMT_2_11724,133,47,125,35,274,269,79,47,101,0,27,214
PNPLA2_2_11725,112,94,55,48,43,0,21,5,69,111,0,16
PNPLA3_2_11726,1117,663,823,1013,1057,852,1115,1044,1058,991,370,1074
PNPLA8_2_11727,636,474,491,572,1421,326,1039,756,655,1212,63,728
PNPO_2_11728,100,194,161,167,29,3,4,492,78,0,0,18
PNPT1_2_11729,23,54,23,43,9,1,2,14,43,60,0,0
POLA1_2_11730,40,41,39,64,21,0,19,0,43,1,12,0
POLA2_2_11731,274,299,161,217,210,214,8,82,583,560,449,230
POLB_2_11732,1543,1485,1437,1771,645,549,1296,1404,969,1166,1986,796
POLD4_2_11733,305,317,275,297,777,224,58,291,75,2,1014,243
POLE3_2_11734,912,1065,935,930,509,236,2203,386,1482,874,334,385
POLE4_2_11735,52,184,133,130,36,154,0,25,112,203,161,194
POLE_2_11736,39,29,52,69,2,0,0,26,15,76,0,58
POLG2_2_11737,309,241,299,257,362,219,0,452,182,342,93,106
POLI_2_11738,693,795,643,806,803,347,1603,396,977,1074,458,1083
POLM_2_11739,188,266,110,211,150,244,81,297,26,23,30,311
POLN_2_11740,282,250,262,315,159,570,41,15,51,244,249,121
POLR1A_2_11741,164,88,61,180,121,505,47,42,19,42,93,382
POLR1C_2_11742,204,225,209,224,468,241,81,122,68,266,283,98
POLR1E_2_11743,278,221,183,323,125,116,163,890,190,414,187,166
POLR2A_2_11744,517,379,588,558,1062,602,448,579,971,840,77,810
POLR2B_2_11745,228,289,173,414,249,209,435,264,302,8,126,160
POLR2C_2_11746,395,247,371,312,279,670,491,120,717,387,251,266
POLR2D_2_11747,1200,1222,994,1171,765,2122,541,713,889,1695,832,1094
POLR2E_2_11748,106,67,34,118,4,61,125,83,15,73,0,48
POLR2F_2_11749,191,141,122,156,209,74,32,305,127,34,860,51
POLR2G_2_11750,1034,484,741,1049,549,519,828,1018,954,309,213,1173
POLR2H_2_11751,1055,1017,1201,1094,950,1211,1367,933,384,706,611,1905
POLR2J_2_11752,385,219,321,446,34,23,1999,42,406,677,676,300
POLR2K_2_11753,646,588,586,665,636,250,1199,413,302,587,873,257
POLR2L_2_11754,619,505,497,600,542,366,972,505,3,520,1270,505
POLR3A_2_11755,347,472,395,262,50,982,428,168,451,340,939,530
POLR3C_2_11756,1261,884,1059,1346,1853,1944,912,1268,773,968,1011,1658
POLR3F_2_11757,913,834,1170,999,273,727,1078,1273,113,582,681,446
POLR3G_2_11758,405,335,701,393,947,107,1813,102,395,241,257,386
POLRMT_2_11759,79,57,39,32,0,0,138,160,89,12,0,7
POMGNT1_2_11760,42,40,52,109,22,39,557,1,21,450,364,34
POMT2_2_11761,101,126,122,59,4,79,115,118,160,130,5,59
PON3_2_11762,386,285,307,342,164,95,85,596,505,321,624,130
POP4_2_11763,312,222,212,266,321,9,280,0,203,447,0,202
POP7_2_11764,377,404,249,314,312,31,143,91,298,304,120,228
POR_2_11765,247,292,284,245,970,179,74,297,729,396,543,69
PPA1_2_11766,1734,1395,1612,1615,612,1016,1075,2109,707,2059,1978,2483
PPCDC_2_11767,549,647,554,505,1161,1056,630,1384,38,298,208,438

PPIA_2_11768,1047,1014,1047,1310,1204,678,1124,1164,1007,371,920,1825
PPIAL4A_2_11769,953,647,898,899,1616,514,1057,463,1236,605,224,520
PPIAL4B_2_11770,953,647,898,899,1616,514,1057,463,1236,605,224,520
PPIAL4C_2_11771,953,647,898,899,1616,514,1057,463,1236,605,224,520
PPIAL4E_2_11772,953,647,898,899,1616,514,1057,463,1236,605,224,520
PPIAL4G_2_11773,953,647,898,899,1616,514,1057,463,1236,605,224,520
PPIB_2_11774,179,87,139,150,0,264,27,0,370,6,335,163
PPIC_2_11775,520,487,555,433,697,164,250,1118,608,736,973,346
PPID_2_11776,347,324,246,638,480,140,334,12,259,577,94,492
PPIF_2_11777,1179,1063,1039,1037,1292,805,1398,1189,1150,1155,1124,420
PPIG_2_11778,48,130,84,101,128,19,100,31,27,44,0,24
PPIH_2_11779,1322,1180,1179,1326,1029,1522,807,1444,1330,1040,856,976
PPIL1_2_11780,775,640,453,720,225,378,693,48,740,1110,148,150
PPIL4_2_11781,25,33,69,82,102,1,0,585,287,3,0,18
PPIP5K2_2_11782,2986,2920,2649,3258,3061,3413,3642,3524,3000,4521,1811
,3127
PPM1H_2_11783,580,638,535,651,462,867,986,0,878,534,926,372
PPM1J_2_11784,194,102,76,165,25,155,135,75,89,258,52,203
PPM1N_2_11785,214,460,249,357,889,686,452,1168,210,33,496,291
PPME1_2_11786,368,259,215,280,284,142,1,79,253,662,407,378
PPWD1_2_11787,1561,1589,1342,1353,2353,368,2702,1154,1397,1709,2871,10
50
PRDX4_2_11788,0,0,0,0,0,0,0,0,0,0,0,0
PREP_2_11789,113,103,111,76,3,24,0,47,0,145,73,16
PRHOXNB_2_11790,431,534,318,498,209,125,524,429,527,503,430,584
PRIM1_2_11791,514,418,521,463,385,53,350,30,812,448,377,163
PRIM2_2_11792,197,145,156,157,267,1,77,276,22,258,362,110
PROC_2_11793,247,410,324,222,360,0,1152,33,226,683,706,520
PROSC_2_11794,237,102,93,167,189,100,194,1,113,27,0,218
PRR14L_2_11795,215,188,174,291,486,205,339,187,11,883,40,97
PRSS1_2_11796,246,152,176,185,55,159,22,118,294,180,23,199
PRSS2_2_11797,65,173,87,69,141,58,29,19,99,4,0,23
PRTFDC1_2_11798,1606,1602,1500,1677,2748,2177,1410,2107,2384,2893,1286
,2242
PRTN3_2_11799,542,540,680,477,98,325,175,1031,369,322,414,311
PRUNE_2_11800,618,471,387,489,576,247,1088,255,276,287,515,888
PSMA2_2_11801,175,255,167,294,351,410,171,504,141,193,108,181
PSMA6_2_11802,89,50,124,189,41,76,42,65,0,4,0,334
PSMA7_2_11803,113,259,62,267,18,281,46,131,617,259,21,171
PSMB10_2_11804,182,174,149,244,15,144,142,50,374,296,3,141
PSMB11_2_11805,28,22,39,46,0,0,0,12,0,16,0,47
PSMB1_2_11806,268,124,147,256,452,12,92,321,97,30,89,320
PSMB3_2_11807,18,0,0,2,0,0,0,0,0,0,0,0
PSMB4_2_11808,678,456,433,483,132,206,376,648,478,449,352,371
PSMB6_2_11809,721,541,372,321,126,498,109,760,294,79,961,291
PSMB7_2_11810,145,118,111,211,374,3,443,426,363,235,30,213
PSMB9_2_11811,30,48,42,34,31,8,190,4,196,70,12,4
PSMD6_2_11812,170,290,253,407,50,68,522,1,29,456,604,392
PTDSS1_2_11813,645,374,595,679,833,938,489,290,302,1052,879,690
PTDSS2_2_11814,310,621,342,505,707,520,959,510,234,869,315,1026

PTGDS_2_11815,527,384,430,525,245,884,354,590,365,710,392,373
PTGES2_2_11816,551,449,444,660,545,707,600,307,235,253,1284,1209
PTGES3_2_11817,466,352,340,363,448,749,84,0,430,562,1664,598
PTGES_2_11818,183,94,126,111,177,120,135,359,189,2,3,193
PTGIS_2_11819,707,439,519,702,253,256,345,277,617,155,564,853
PTGS2_2_11820,2710,2357,2597,2967,2562,2361,1645,705,2128,2629,2101,23
22
PTRH1_2_11821,160,61,167,165,71,1,163,166,145,0,84,43
PTRH2_2_11822,179,114,142,92,143,140,495,75,89,261,176,216
PTS_2_11823,1040,1130,1117,1358,1612,768,2467,620,1191,1721,2695,970
PUS3_2_11824,537,238,314,380,386,236,48,622,241,219,282,436
PUSL1_2_11825,92,322,180,156,380,19,49,556,64,200,0,140
PXDN_2_11826,50,24,47,52,64,45,6,314,0,0,1,349
PXDNL_2_11827,72,48,114,162,0,0,7,57,0,0,0,8
PYCR2_2_11828,380,226,289,330,868,235,655,0,104,347,371,348
PYCRL_2_11829,304,244,276,236,317,82,477,20,302,390,120,213
PYGB_2_11830,166,76,158,161,141,253,34,23,66,58,23,16
QDPR_2_11831,54,26,35,41,0,16,240,0,59,2,1,32
QPCT_2_11832,627,413,664,651,166,380,932,1489,583,409,991,885
QPRT_2_11833,311,205,194,316,374,41,500,123,166,285,860,370
QRSL1_2_11834,403,271,393,306,1038,708,39,52,653,363,261,699
QSOX2_2_11835,126,158,140,158,250,181,267,47,50,141,157,23
QTRT1_2_11836,118,47,108,131,88,46,432,38,143,15,2,29
QTRTD1_2_11837,195,186,53,113,412,45,76,156,4,0,388,6
RAB10_2_11838,75,193,46,146,0,1,66,389,0,0,363,0
RAB12_2_11839,1310,1192,1021,1199,1137,767,853,1584,283,1587,882,665
RAB13_2_11840,347,370,331,394,295,162,836,269,438,274,615,294
RAB14_2_11841,384,183,204,158,531,1,859,147,370,5,555,327
RAB15_2_11842,432,374,348,393,461,345,380,315,459,104,417,813
RAB17_2_11843,315,290,222,287,559,256,288,19,252,284,188,41
RAB18_2_11844,324,327,363,389,876,646,456,1063,168,746,428,205
RAB19_2_11845,2038,1705,1536,2031,2074,2782,3548,3371,1483,2830,1906,1
670
RAB1B_2_11846,75,64,26,42,10,0,172,261,0,310,0,480
RAB20_2_11847,297,277,423,299,372,269,1258,295,16,362,12,479
RAB21_2_11848,674,607,659,774,797,352,1122,942,537,541,900,493
RAB22A_2_11849,22,34,67,35,0,16,0,66,0,0,381,23
RAB25_2_11850,26,55,110,76,50,22,0,0,0,0,238,0
RAB26_2_11851,104,165,256,119,40,154,236,565,114,0,400,402
RAB27B_2_11852,351,171,233,235,368,30,47,423,476,127,32,254
RAB30_2_11853,136,20,133,171,121,8,147,17,2,409,20,35
RAB31_2_11854,604,515,580,664,146,1230,211,525,104,188,878,663
RAB33A_2_11855,127,109,86,262,150,110,172,101,97,26,8,299
RAB33B_2_11856,243,353,364,280,15,455,10,165,90,706,216,418
RAB36_2_11857,8,6,31,27,0,0,0,2,0,170,0,0
RAB38_2_11858,716,547,494,686,596,416,82,359,451,989,638,1427
RAB39B_2_11859,89,222,231,238,1,469,143,55,11,106,307,9
RAB3A_2_11860,145,163,79,115,0,23,803,118,176,162,1,72
RAB3B_2_11861,301,445,365,382,1113,583,297,288,436,45,12,567
RAB3C_2_11862,382,375,539,523,370,388,900,198,629,238,416,472

RAB3D_2_11863,88,167,128,195,325,124,119,53,162,45,349,893
RAB3GAP2_2_11864,536,621,458,599,242,635,246,486,750,841,897,465
RAB4A_2_11865,243,353,364,280,15,455,10,165,90,706,216,418
RAB4B_2_11866,678,625,502,653,353,599,452,138,1419,322,469,525
RAB5A_2_11867,8,53,1,9,0,0,0,0,1,73,0,0
RAB5B_2_11868,1727,1557,1313,1681,1603,1684,2495,644,1732,1352,2930,13
78
RAB6B_2_11869,579,433,535,616,522,207,2491,221,804,406,434,93
RAB6C_2_11870,1094,755,836,939,1283,871,814,961,291,1524,907,1298
RAB7A_2_11871,80,72,128,67,140,339,227,146,2,206,401,145
RAB8B_2_11872,114,258,134,180,0,0,360,129,214,268,253,17
RAB9B_2_11873,146,52,105,179,459,46,66,173,39,10,1,22
RABGGTB_2_11874,1225,800,1004,1084,1177,666,841,657,1160,724,1258,965
RABL3_2_11875,413,191,437,308,1019,250,411,662,446,719,221,801
RAC2_2_11876,53,51,162,154,0,0,0,4,85,358,89,18
RAD50_2_11877,272,274,196,208,177,193,397,1,661,290,0,640
RAD54L2_2_11878,45,53,136,72,103,120,42,23,406,30,45,107
RALA_2_11879,165,24,124,56,100,219,1,141,52,39,17,9
RALB_2_11880,462,491,428,519,725,880,963,435,99,645,69,726
RALBP1_2_11881,289,243,383,376,681,61,569,870,443,215,469,227
RANBP2_2_11882,384,369,377,363,222,49,78,446,29,710,664,210
RAN_2_11883,0,0,0,0,0,0,0,0,0,0,0,0
RAP2A_2_11884,449,305,296,392,423,223,334,307,496,856,595,209
RAP2B_2_11885,105,97,152,235,240,0,153,165,112,210,143,255
RAP2C_2_11886,340,247,257,339,462,411,177,400,340,47,475,230
RARS2_2_11887,1545,1027,1244,1362,2229,1133,1482,1649,771,475,1157,101
1
RARS_2_11888,126,164,126,132,224,62,629,44,311,93,135,508
RASD2_2_11889,12,84,40,60,0,158,0,45,0,211,12,205
RASL10B_2_11890,218,186,230,262,9,508,423,304,34,49,0,11
RASL11B_2_11891,177,101,236,143,159,40,165,83,200,99,489,365
RASL12_2_11892,152,60,61,69,346,8,95,0,0,4,7,51
RC3H1_2_11893,254,304,203,305,136,303,170,519,415,265,278,530
RCL1_2_11894,347,295,140,261,361,2,112,188,390,230,164,427
RDH10_2_11895,2287,1882,1901,2219,2098,1344,2778,1632,2284,2554,1211,1
414
RDH11_2_11896,385,358,279,445,452,112,33,692,197,9,317,1135
RDH12_2_11897,114,172,101,125,109,318,0,1,215,1,1,66
RDH14_2_11898,867,516,613,693,1165,488,802,879,834,61,351,523
RDH16_2_11899,123,54,47,56,13,51,3,4,49,10,0,55
RDH8_2_11900,227,121,309,126,72,454,204,6,140,69,661,375
RECQL4_2_11901,92,107,90,43,34,8,3,64,38,445,1,591
REM1_2_11902,180,29,53,162,99,128,170,4,63,12,5,375
REM2_2_11903,252,447,190,295,159,59,1,256,273,417,782,36
RENBP_2_11904,1,3,0,13,0,2,0,14,0,0,1,13
RETSAT_2_11905,41,28,54,39,17,10,33,32,19,5,0,6
REV3L_2_11906,346,191,249,336,315,246,43,121,286,13,314,351
REXO2_2_11907,393,283,97,231,220,254,160,666,172,325,588,120
RFNG_2_11908,3,40,13,35,1,16,26,0,41,0,4,0
RFX6_2_11909,127,122,146,163,140,52,145,44,456,30,28,75

RGS7_2_11910,550,558,374,453,353,430,862,377,223,385,616,617
RHBDL1_2_11911,190,240,162,164,53,421,462,215,93,307,352,223
RHBDL2_2_11912,140,206,113,271,187,76,206,220,142,327,206,397
RHBDL3_2_11913,1132,796,702,803,1034,290,173,457,790,1121,1467,800
RHEBL1_2_11914,215,179,144,141,146,7,686,3,153,33,104,125
RHOA_2_11915,338,460,407,432,878,517,43,274,345,211,138,511
RHOB_2_11916,280,132,454,218,374,104,450,640,366,481,367,331
RHOD_2_11917,38,53,23,31,0,0,0,16,0,127,46,127
RHOF_2_11918,390,282,311,304,201,235,414,473,169,604,397,20
RHOG_2_11919,251,331,343,241,300,51,295,45,554,93,280,225
RHOH_2_11920,525,459,439,578,44,0,42,168,151,524,220,199
RHOJ_2_11921,249,193,248,432,660,273,469,709,526,543,5,499
RHOQ_2_11922,421,396,335,599,543,343,1113,178,499,265,676,538
RHOT2_2_11923,419,342,277,269,322,170,157,497,169,509,142,183
RHOU_2_11924,139,89,131,121,29,63,758,10,19,18,26,304
RHOV_2_11925,259,412,191,549,5,2,7,30,1181,0,1464,523
RIT1_2_11926,348,294,243,374,110,0,91,7,537,569,426,625
RIT2_2_11927,188,71,191,218,279,0,0,16,0,384,0,7
RNASE2_2_11928,320,692,533,797,186,174,1601,389,257,1,807,385
RNASE3_2_11929,1516,1414,1149,1347,1654,1360,1857,2083,808,797,1152,12
81
RNASE6_2_11930,339,248,238,168,630,269,504,96,94,134,65,27
RNASE7_2_11931,75,84,87,91,76,26,1,171,75,677,7,59
RNASE8_2_11932,468,450,298,432,478,162,439,395,107,347,167,433
RNASEH2A_2_11933,334,272,250,142,73,0,0,24,207,150,82,161
RNASET2_2_11934,1216,856,794,888,1536,464,555,1626,605,697,690,2276
RND1_2_11935,184,193,277,241,120,328,205,286,114,305,599,103
RND2_2_11936,114,130,315,277,586,0,568,197,68,54,88,98
RND3_2_11937,408,328,214,392,703,193,14,303,290,266,479,145
RNF148_2_11938,738,571,444,743,1904,151,310,1290,455,410,738,1394
RNMT_2_11939,2113,1672,2012,2314,2511,1496,2804,1416,1466,1342,2669,27
24
RNMTL1_2_11940,870,654,523,703,376,248,207,290,1100,706,863,787
RNPEP_2_11941,287,260,281,440,186,343,1008,0,234,250,308,601
RPAP2_2_11942,547,843,450,473,772,0,0,624,795,467,134,302
RPE65_2_11943,426,338,424,600,420,211,474,87,257,298,80,801
RPIA_2_11944,409,435,302,400,371,266,273,362,156,230,1211,323
RPL4_2_11945,173,255,275,295,361,4,612,60,301,4,76,449
RPN1_2_11946,1254,1540,1344,1640,1020,917,1148,892,1091,959,3753,1429
RPP25_2_11947,37,46,26,115,3,0,0,0,0,34,0,23
RPP40_2_11948,377,326,270,310,614,550,375,17,390,561,295,206
RPS3_2_11949,802,463,649,659,1551,244,996,1624,777,487,1236,233
RPUSD1_2_11950,140,194,91,131,50,10,239,26,38,22,212,419
RPUSD2_2_11951,982,992,864,1019,1229,1061,451,837,841,2344,690,351
RRAGA_2_11952,43,134,166,128,44,0,0,0,66,244,1,285
RRAGC_2_11953,758,546,612,645,346,492,415,754,1368,510,498,80
RRAGD_2_11954,766,866,799,936,1425,609,2025,426,795,934,370,671
RRAS_2_11955,133,167,98,233,1,293,158,24,329,4,13,7
RRM1_2_11956,233,189,213,248,886,12,205,221,155,535,136,204
RRP8_2_11957,382,345,262,299,35,287,580,367,47,6,61,439

RSAD2_2_11958,84,127,205,145,28,0,12,0,78,146,0,84
RTN4IP1_2_11959,61,55,26,39,2,0,41,23,47,92,2,14
SAMHD1_2_11960,687,502,581,572,605,130,324,420,314,666,458,675
SDF2_2_11961,1018,663,835,899,1264,1075,863,202,1059,344,1090,958
SDHA_2_11962,307,411,485,554,41,153,169,645,552,467,164,621
SDHB_2_11963,844,592,473,818,159,444,388,696,649,754,984,528
SDHD_2_11964,46,26,15,54,0,69,0,215,0,0,62,2
SDR16C5_2_11965,281,193,196,441,658,88,216,86,72,84,183,44
SDR42E1_2_11966,211,331,252,536,20,311,44,347,241,921,206,503
SDR9C7_2_11967,28,38,35,72,15,157,60,108,47,58,19,92
SDSL_2_11968,1474,1180,1042,1249,170,701,1398,3091,1314,1040,1189,1273
SEPSECS_2_11969,1029,946,752,1143,937,1219,812,2150,1462,961,2204,759
1-Sep_2_11970,435,632,531,798,927,337,1010,1176,369,514,1121,316
SEPW1_2_11971,832,797,805,1187,35,967,1092,1760,836,368,770,2374
SERHL2_2_11972,27,105,94,54,0,16,32,349,145,16,0,81
SETX_2_11973,390,310,323,273,443,561,223,987,376,464,455,456
SGMS1_2_11974,401,485,437,455,505,577,813,3,452,223,320,59
SGPL1_2_11975,125,203,231,224,182,315,288,8,47,661,370,139
SGSH_2_11976,537,575,562,678,587,79,1189,642,281,82,238,983
SH3GL2_2_11977,1904,1399,1353,1522,1044,1538,1928,2190,1661,2344,1050,
1687
SKIV2L2_2_11978,219,210,218,283,159,117,429,106,810,427,325,169
SLFN12_2_11979,366,123,298,444,385,594,874,708,31,200,23,282
SLFN12L_2_11980,2584,2162,2390,2492,1278,1453,3857,2185,2363,2935,2863
,2389
SLFN13_2_11981,1103,1385,1065,1463,1304,821,986,1090,1229,977,1764,149
6
SLFN5_2_11982,511,264,318,343,528,555,0,540,328,634,73,300
SLU7_2_11983,2121,1848,1553,1831,2018,1666,3030,945,2321,1047,1847,192
6
SMG8_2_11984,206,556,240,269,379,334,409,255,1,133,208,371
SMPD2_2_11985,119,228,106,251,26,10,0,392,108,333,1,98
SMPD3_2_11986,909,683,544,845,368,933,1545,760,907,352,1297,490
SMPDL3A_2_11987,373,350,204,297,315,541,527,110,178,83,87,436
SMUG1_2_11988,568,376,362,539,258,109,590,278,137,197,1120,578
SNF8_2_11989,206,110,156,191,204,183,36,89,256,151,208,104
SNRNP200_2_11990,60,61,24,68,0,71,1,26,0,120,0,71
SOAT1_2_11991,0,0,0,3,0,173,0,0,0,0,0,0
SOAT2_2_11992,262,272,299,233,148,109,394,194,112,483,381,276
SOD1_2_11993,90,38,22,61,0,0,25,8,64,11,0,8
SOD3_2_11994,107,116,120,134,102,23,132,6,2,508,161,213
SORD_2_11995,302,184,208,163,181,230,384,0,0,130,1199,0
SPACA3_2_11996,345,147,173,279,942,82,133,335,81,106,70,79
SPACA5B_2_11997,466,392,458,532,562,548,405,269,119,181,1144,508
SPACA5_2_11998,466,392,458,532,562,548,405,269,119,181,1144,508
SPEM1_2_11999,409,448,419,494,1047,307,994,157,185,162,103,677
SPTLC2_2_12000,724,644,606,551,404,230,1596,786,922,474,641,826
SPTLC3_2_12001,303,187,243,429,680,102,1088,415,280,447,919,144
SPTSSA_2_12002,302,322,216,266,142,378,205,42,176,0,183,264
SQLE_2_12003,521,263,336,331,380,775,286,416,757,499,717,664

SQRDL_2_12004,447,265,335,304,53,206,89,101,207,407,339,241
SRD5A1_2_12005,32,117,76,33,24,0,132,112,0,0,0,4
SRD5A2_2_12006,690,675,863,915,585,1556,1037,706,624,493,1078,916
SRD5A3_2_12007,108,76,124,80,65,0,158,40,41,114,1,40
SRR_2_12008,53,23,51,29,51,0,10,36,63,13,0,1
SRSF9_2_12009,797,763,648,710,338,415,156,132,518,2009,857,826
SRXN1_2_12010,39,37,13,42,0,266,0,0,165,0,1,0
SSB_2_12011,221,237,192,148,37,7,3,39,460,168,439,653
SSU72_2_12012,369,204,179,209,16,264,1,0,347,491,227,876
ST14_2_12013,142,98,80,92,40,56,12,2,394,111,85,13
ST3GAL2_2_12014,46,38,40,34,13,0,17,24,0,19,23,1
ST3GAL4_2_12015,201,139,141,103,5,105,121,640,168,23,56,47
ST3GAL6_2_12016,265,439,296,516,417,320,208,364,181,349,2129,545
ST6GALNAC1_2_12017,58,91,39,63,24,154,12,162,115,34,15,62
ST6GALNAC2_2_12018,615,343,375,404,773,902,283,605,788,790,1017,323
ST6GALNAC5_2_12019,265,580,423,434,781,950,1635,56,1,282,514,136
ST6GALNAC6_2_12020,173,199,143,227,105,122,22,89,129,53,66,837
ST8SIA1_2_12021,396,511,486,601,898,431,447,19,488,48,1169,282
ST8SIA2_2_12022,74,49,31,48,144,7,263,2,2,0,0,7
ST8SIA3_2_12023,362,281,333,404,125,296,320,274,237,78,157,578
ST8SIA5_2_12024,248,223,171,257,236,336,180,353,113,186,263,416
ST8SIA6_2_12025,1045,744,955,1101,632,1514,693,1067,1323,988,1086,971
STS_2_12026,24,0,5,7,84,0,51,1,5,0,0,41
STT3A_2_12027,249,332,243,340,18,379,312,51,126,142,645,649
STT3B_2_12028,369,291,325,419,407,680,807,588,108,216,1408,352
SUCLA2_2_12029,447,424,517,666,227,145,776,778,259,216,1083,542
SUCLG1_2_12030,470,328,266,657,315,385,651,0,549,570,431,320
SULT1A3_2_12031,378,236,468,458,200,117,1235,18,689,436,146,382
SULT1A4_2_12032,378,236,468,458,200,117,1235,18,689,436,146,382
SULT1B1_2_12033,643,612,689,788,361,1175,1049,660,1179,1016,886,992
SULT1C3_2_12034,202,111,114,131,125,113,130,36,344,5,106,121
SULT1C4_2_12035,419,459,254,549,9,197,326,119,188,208,5,100
SULT1E1_2_12036,36,15,21,72,0,22,0,20,13,19,399,261
SULT2A1_2_12037,648,711,720,720,415,575,1532,1217,275,737,459,357
SULT4A1_2_12038,146,155,245,282,16,668,37,4,1,90,0,247
SUPV3L1_2_12039,230,174,101,117,142,259,109,391,72,338,63,146
SURF1_2_12040,281,184,229,150,81,299,627,2,146,60,695,189
TALDO1_2_12041,236,97,188,194,60,200,53,68,296,200,363,138
TARS2_2_12042,50,64,126,42,3,165,511,26,200,0,195,86
TARS_2_12043,610,620,683,777,533,583,1161,397,485,315,800,433
TARSL2_2_12044,449,165,244,239,28,433,383,177,244,197,144,323
TAT_2_12045,718,601,484,693,895,689,1279,610,644,652,1033,749
TBC1D10B_2_12046,18,105,49,51,9,490,218,15,165,296,572,91
TBCC_2_12047,7,10,17,29,0,0,12,0,1,0,0,0
TDG_2_12048,190,253,152,232,17,358,133,196,2,236,418,84
TDO2_2_12049,170,249,244,245,126,38,56,53,330,493,507,299
TECR_2_12050,57,100,100,125,0,0,5,4,286,0,440,234
TECTA_2_12051,347,223,257,331,16,595,212,303,195,333,59,200
TFB2M_2_12052,534,321,405,527,617,252,349,353,234,293,257,564
TGDS_2_12053,1614,1369,1447,1947,1079,610,1705,2336,1211,1358,1129,194

8
TGM1_2_12054,469,633,359,411,431,138,861,27,714,446,494,354
TGM3_2_12055,317,340,270,312,140,30,267,84,287,47,7,424
TGM4_2_12056,470,271,315,419,560,482,180,207,214,423,306,664
TGM6_2_12057,892,626,573,933,457,794,238,1086,828,1158,1596,520
TGM7_2_12058,737,1134,942,980,1506,720,1260,689,496,1838,527,580
TGS1_2_12059,1059,707,674,878,1013,619,55,565,1260,517,1051,558
THG1L_2_12060,145,251,300,212,137,191,138,149,45,135,279,280
THOP1_2_12061,93,40,54,65,200,176,155,118,107,341,61,1
THUMPD2_2_12062,416,542,237,395,376,226,689,232,250,225,1047,106
TKTL2_2_12063,650,488,491,641,393,161,1027,287,576,501,859,1206
TM7SF2_2_12064,199,255,185,160,49,857,15,0,147,526,1103,332
TMEM55A_2_12065,86,120,111,165,133,17,0,121,0,196,0,342
TMEM62_2_12066,904,1208,1113,1339,401,544,34,1355,927,1397,2439,1390
TMEM86B_2_12067,348,371,401,402,231,288,55,49,216,37,307,54
TMPRSS15_2_12068,855,764,739,800,615,850,406,415,724,526,2110,454
TMX1_2_12069,852,815,961,779,1554,594,421,794,93,717,458,316
TMX3_2_12070,905,880,660,1074,2367,1527,677,664,414,814,624,1637
TMX4_2_12071,230,131,92,91,154,1,24,14,151,16,0,15
TNKS2_2_12072,585,306,481,578,169,163,141,615,602,559,387,751
TNKS_2_12073,159,174,154,127,186,2,0,62,222,106,0,0
TOP1_2_12074,319,382,155,281,66,489,1064,202,3,140,1,353
TOP1MT_2_12075,235,363,327,313,140,81,464,75,300,209,581,612
TOP2A_2_12076,972,757,673,983,665,901,810,987,680,1389,693,1053
TOP2B_2_12077,469,648,619,424,727,1070,80,160,426,145,344,224
TOP3A_2_12078,199,148,185,188,533,1,6,10,388,3,0,636
TOP3B_2_12079,366,298,219,380,3,787,25,156,82,557,260,235
TOR3A_2_12080,74,4,33,70,12,2,1,63,96,0,0,74
TPH1_2_12081,420,465,384,599,168,30,391,117,614,673,479,491
TPMT_2_12082,1116,858,784,768,1040,938,734,737,1284,415,751,689
TPP1_2_12083,123,158,110,123,204,518,362,246,0,64,995,46
TPP2_2_12084,132,42,119,23,1,246,0,4,0,0,94,82
TPSAB1_2_12085,445,494,485,583,691,249,1197,324,237,451,430,225
TPSB2_2_12086,17,9,63,72,0,0,0,5,15,0,213,0
TPSD1_2_12087,151,68,62,175,6,13,96,911,317,245,136,134
TPST1_2_12088,1194,1283,1693,1530,1526,744,1124,1326,893,777,1812,1523
TREH_2_12089,169,27,41,90,357,45,5,149,0,1,149,146
TREX2_2_12090,174,140,184,231,12,7,10,47,119,293,30,16
TRHDE_2_12091,1026,1298,1128,1348,1526,65,1407,993,910,1435,739,1573
TRIM21_2_12092,179,160,242,193,164,0,5,0,354,228,19,77
TRIT1_2_12093,35,1,12,12,3,0,0,0,10,29,0,14
TRMT112_2_12094,0,0,0,0,0,0,0,0,0,0,0,0
TRMT61A_2_12095,309,279,285,360,157,104,522,71,222,171,151,311
TRMU_2_12096,1646,2006,1425,1763,1000,2170,1037,1825,1054,1644,2060,11
87
TRNT1_2_12097,693,526,662,999,364,637,26,145,383,552,2665,1054
TRUB1_2_12098,321,364,471,443,234,405,268,840,258,93,130,406
TRUB2_2_12099,166,154,177,148,131,25,357,43,126,94,370,261
TSTA3_2_12100,5,18,3,78,0,0,10,9,1,101,6,135
TST_2_12101,459,269,334,364,8,423,2,1234,130,84,396,694

TTLL13_2_12102,154,181,138,206,312,21,614,80,294,155,985,203
TTLL1_2_12103,252,213,243,186,541,229,216,266,72,42,197,127
TTLL3_2_12104,206,214,179,302,0,50,61,892,82,712,275,158
TTLL4_2_12105,285,282,162,353,556,20,13,19,581,114,13,839
TUFM_2_12106,161,77,174,179,653,10,322,75,265,19,301,160
TULP2_2_12107,477,416,440,719,758,423,595,129,731,482,149,361
TUT1_2_12108,2,20,23,25,11,88,27,0,0,12,0,0
TXN2_2_12109,278,191,263,288,108,13,494,410,217,489,948,85
TXN_2_12110,257,243,189,143,362,212,330,177,0,96,423,231
TXNDC11_2_12111,123,82,108,142,93,13,203,55,60,136,38,170
TXNDC12_2_12112,41,57,60,34,13,22,598,35,35,68,28,6
TXNDC15_2_12113,716,651,716,666,537,34,296,557,918,252,1886,512
TXNDC17_2_12114,119,70,148,190,207,0,0,174,0,0,107,62
TXNL1_2_12115,230,272,299,213,1,3,305,610,67,304,364,235
TXNL4A_2_12116,129,172,137,165,13,172,618,16,2,388,320,101
TXNRD2_2_12117,828,381,344,563,888,11,1189,160,57,38,416,555
TYMS_2_12118,46,43,13,50,0,0,59,22,82,11,0,29
TYR_2_12119,685,901,764,652,601,783,304,1010,848,319,1635,174
TYRP1_2_12120,545,504,375,531,439,776,162,340,474,736,369,555
UAP1_2_12121,526,466,398,546,56,82,394,958,1020,382,595,462
UAP1L1_2_12122,630,406,412,641,2397,570,50,305,644,748,158,154
UBIAD1_2_12123,29,6,46,58,22,0,0,14,0,1,165,0
UBL4A_2_12124,211,184,185,155,94,69,109,11,33,363,0,8
UFSP1_2_12125,237,159,150,65,427,2,0,0,322,240,195,132
UFSP2_2_12126,420,292,367,353,836,802,349,520,407,635,1351,273
UGCG_2_12127,453,364,429,636,232,229,434,360,99,228,417,490
UGGT1_2_12128,173,103,130,127,501,4,105,97,274,269,0,20
UGGT2_2_12129,595,752,359,522,191,490,511,918,841,206,493,705
UGT1A10_2_12130,2431,1866,1948,2591,2024,1618,3042,1710,2924,1959,3480
,2208
UGT1A1_2_12131,7380,7453,6654,7381,9006,6776,5932,5233,7222,7506,7058,
6773
UGT1A3_2_12132,2431,1866,1948,2591,2024,1618,3042,1710,2924,1959,3480,
2208
UGT1A4_2_12133,7380,7453,6654,7381,9006,6776,5932,5233,7222,7506,7058,
6773
UGT1A5_2_12134,2878,2307,2748,3568,3157,1537,2130,2034,2707,3236,4247,
3192
UGT1A7_2_12135,7380,7453,6654,7381,9006,6776,5932,5233,7222,7506,7058,
6773
UGT1A8_2_12136,1365,951,858,1272,908,1492,1068,827,2205,1824,1305,1635
UGT1A9_2_12137,7380,7453,6654,7381,9006,6776,5932,5233,7222,7506,7058,
6773
UGT2A1_2_12138,3031,2257,2159,2822,1818,2425,2243,2247,2532,2205,2133,
3075
UGT2A2_2_12139,3031,2257,2159,2822,1818,2425,2243,2247,2532,2205,2133,
3075
UGT2A3_2_12140,365,185,366,317,282,432,199,338,330,62,396,92
UGT2B15_2_12141,955,1148,1064,1384,1760,1415,2252,1702,1582,823,1802,2
654

UGT2B17_2_12142,955,1148,1064,1384,1760,1415,2252,1702,1582,823,1802,2
654
UGT2B4_2_12143,3031,2257,2159,2822,1818,2425,2243,2247,2532,2205,2133,
3075
UGT2B7_2_12144,41,68,118,91,199,1,51,69,20,34,648,284
UMPS_2_12145,197,125,183,113,3,0,0,0,207,0,0,10
UPB1_2_12146,517,351,285,549,181,0,234,295,328,381,263,963
UPF1_2_12147,268,190,222,224,164,567,137,375,169,430,910,428
UPRT_2_12148,1081,629,653,552,923,94,1290,238,149,243,227,282
UQCR11_2_12149,84,33,93,53,79,0,0,2,79,0,0,0
UQCRC1_2_12150,207,100,178,139,266,267,6,48,164,324,9,32
UQCRC2_2_12151,702,712,726,633,486,533,226,389,958,1321,889,1057
UQCRFS1_2_12152,1453,1088,916,905,922,569,956,251,1198,520,1293,1323
UQCRH_2_12153,897,534,590,854,1317,657,676,330,1064,593,250,508
UQCRQ_2_12154,371,389,454,544,376,187,667,1320,509,215,548,473
UROD_2_12155,282,228,216,280,308,102,714,220,656,277,239,95
UROS_2_12156,2490,2133,2128,2351,2676,1922,815,2383,1062,2638,3770,186
9
UST_2_12157,1563,1721,1827,1937,1392,579,2170,2569,816,2621,2587,1774
UXS1_2_12158,134,201,176,152,282,92,148,255,82,563,354,91
VARS_2_12159,80,66,66,92,122,9,166,96,46,309,0,10
VAT1L_2_12160,260,175,200,445,192,618,537,389,212,38,346,253
VCP_2_12161,108,146,74,72,163,233,62,444,25,27,10,115
VNN1_2_12162,208,266,245,194,48,560,529,377,420,124,206,345
WBSCR17_2_12163,456,322,287,336,264,884,203,15,297,511,264,131
WBSCR27_2_12164,287,308,367,346,6,293,616,361,467,349,204,288
WDFY3_2_12165,406,626,555,502,137,499,167,63,671,9,629,335
WRN_2_12166,393,308,349,326,728,148,306,1,398,321,383,127
XDH_2_12167,221,212,85,146,1,1,287,263,124,273,2,0
XPNPEP2_2_12168,41,11,20,35,0,4,0,75,16,46,42,0
XRCC2_2_12169,511,361,395,643,419,40,57,16,101,84,436,291
XRCC5_2_12170,751,486,653,750,775,1019,1014,408,11,607,233,1199
XRCC6_2_12171,429,392,330,561,650,781,494,124,748,21,38,1047
XRN2_2_12172,26,5,13,36,16,0,12,0,0,32,0,1
XYLT1_2_12173,650,622,500,690,521,1,180,508,839,542,735,236
XYLT2_2_12174,44,15,45,42,15,0,1,359,18,0,0,1
YARS2_2_12175,356,322,457,371,178,67,333,479,316,621,327,396
YARS_2_12176,794,800,709,734,780,576,418,892,1173,489,489,188
YKT6_2_12177,378,424,194,386,382,259,339,379,130,472,432,308
YPEL1_2_12178,71,28,55,41,6,0,0,3,82,5,54,48
ZADH2_2_12179,170,178,218,251,128,233,76,71,89,146,118,356
ZCCHC4_2_12180,642,541,504,744,63,288,263,612,773,1104,1132,527
ZDHHC17_2_12181,1806,1309,1178,1356,984,2027,1804,1773,1296,1964,895,9
83
ZDHHC18_2_12182,329,255,132,205,512,336,0,0,5,129,0,57
ZDHHC1_2_12183,0,0,0,0,0,0,0,0,0,0,0,0
ZDHHC21_2_12184,0,0,3,3,0,0,0,0,0,0,0,0
ZDHHC2_2_12185,386,341,307,516,729,98,759,208,317,254,1495,42
ZER1_2_12186,1274,1108,1009,1093,1001,642,1056,625,785,2297,1969,2213
ZMPSTE24_2_12187,1460,1444,1237,1523,1682,1895,1331,2043,673,923,1555,

985
ZRANB3_2_12188,108,329,353,276,573,1063,326,130,20,35,222,371
A1CF_2_12189,226,193,176,220,126,74,601,666,66,72,440,909
A2LD1_2_12190,132,70,68,146,274,33,251,191,181,0,0,24
AADAT_2_12191,653,418,463,546,606,274,516,370,592,288,1311,759
AARSD1_2_12192,1124,1130,1097,1295,1188,798,261,778,731,1195,927,1243
ABAT_2_12193,329,264,227,239,4,274,529,791,251,326,585,460
ABHD11_2_12194,146,198,196,220,214,96,38,0,68,164,151,6
ABHD12_2_12195,438,324,227,347,1,282,384,96,12,757,80,658
ABHD14B_2_12196,149,31,9,44,35,3,130,116,129,20,0,17
ABHD2_2_12197,687,519,348,668,281,94,355,165,616,58,566,206
ACAA1_2_12198,49,9,61,35,227,9,0,76,38,52,16,34
ACACA_2_12199,299,324,434,509,58,96,179,850,161,226,370,476
ACAD10_2_12200,360,310,424,323,159,266,474,367,50,267,5,696
ACADM_2_12201,322,113,274,332,212,240,148,356,342,144,948,18
ACADVL_2_12202,370,267,272,248,59,274,294,369,286,30,1,86
ACCS_2_12203,18,94,5,37,0,5,0,0,27,2,0,33
ACE_2_12204,1376,1198,1083,1685,1517,754,1282,1604,1301,886,464,843
ACHE_2_12205,456,308,434,427,888,164,809,411,348,770,39,93
ACIN1_2_12206,524,470,441,356,582,421,769,42,726,186,310,153
ACLY_2_12207,81,72,73,123,2,89,64,0,77,77,444,122
ACOT11_2_12208,425,367,367,613,359,643,584,534,322,631,215,237
ACOT13_2_12209,637,460,532,418,1801,47,618,1,327,298,178,143
ACOT7_2_12210,246,220,158,244,99,237,1,25,190,297,246,18
ACOT9_2_12211,979,969,850,1130,1014,652,572,994,588,965,333,604
ACOX1_2_12212,203,213,362,281,279,699,485,303,125,590,807,413
ACOX3_2_12213,985,618,862,917,1232,1029,917,420,247,1400,1541,713
ACSBG1_2_12214,101,184,111,157,179,440,44,222,174,200,118,122
ACSF3_2_12215,251,92,162,214,394,31,117,79,157,262,261,203
ACSL3_2_12216,130,188,130,154,84,328,246,128,65,388,474,187
ACSL4_2_12217,176,306,156,83,270,78,470,4,88,0,20,370
ACSL5_2_12218,1181,760,879,1195,1374,1002,1188,589,719,1514,764,941
ACSL6_2_12219,342,313,289,357,786,144,380,142,54,414,332,326
ACSM2B_2_12220,3279,2940,2935,3407,2637,2654,4813,2799,2694,3063,2479,
3699
ACSM3_2_12221,403,574,333,395,457,633,212,703,365,540,61,696
ACSS2_2_12222,67,88,170,118,56,90,32,315,464,10,0,74
ACY1_2_12223,407,507,312,372,415,705,270,706,270,1204,1796,1582
ADAMTS2_2_12224,303,235,205,260,395,5,0,1118,61,471,0,670
ADARB1_2_12225,76,98,172,175,7,114,445,8,13,88,0,273
ADAR_2_12226,50,13,33,108,1,5,0,0,38,0,0,0
ADCY10_2_12227,825,556,517,795,1082,660,603,357,402,389,555,615
ADCY4_2_12228,183,201,165,184,167,487,204,566,435,118,464,378
ADCY5_2_12229,115,48,136,163,453,30,189,0,51,36,37,61
ADCY6_2_12230,56,92,63,77,11,0,6,24,22,110,4,267
ADH6_2_12231,154,96,135,158,253,105,1,10,52,167,171,0
ADH7_2_12232,218,276,257,206,4,3,71,147,33,83,556,265
ADPRHL1_2_12233,269,382,332,361,369,754,420,389,417,454,964,529
ADSL_2_12234,400,285,338,407,460,541,343,849,566,362,224,712
ADSSL1_2_12235,789,648,486,600,344,929,90,0,197,802,379,428

AFMID_2_12236,115,192,98,154,151,103,70,262,223,228,119,73
AGA_2_12237,46,127,50,65,0,214,222,32,172,31,138,2
AGAP1_2_12238,300,189,248,192,335,277,266,51,355,248,650,68
AGAP2_2_12239,530,288,491,339,105,313,161,411,405,886,284,286
AGL_2_12240,414,506,481,603,876,297,857,134,376,290,481,263
AGPAT1_2_12241,140,151,126,163,1,281,22,26,133,99,120,486
AGPAT2_2_12242,75,258,146,187,0,78,0,2,19,443,484,28
AGPAT3_2_12243,346,290,281,301,335,232,45,17,51,555,696,339
AGXT2L1_2_12244,271,263,203,237,192,51,88,6,358,6,564,93
AHCY_2_12245,54,39,54,61,6,0,179,34,21,23,88,80
AHCYL1_2_12246,70,40,115,118,162,31,224,14,40,0,9,415
AHCYL2_2_12247,2,1,4,32,0,0,0,0,29,102,0,17
AIFM1_2_12248,206,139,84,178,382,120,169,157,246,156,215,22
AIFM2_2_12249,37,97,48,98,125,388,0,0,27,216,1,562
AIFM3_2_12250,47,98,33,107,163,45,155,121,47,47,0,168
AKIRIN1_2_12251,432,392,242,473,573,441,458,8,418,40,0,224
AKR1A1_2_12252,35,54,100,100,1,60,318,4,115,78,3,74
AKR1C2_2_12253,933,674,820,815,296,779,891,347,414,227,1857,1046
AKR1D1_2_12254,394,380,478,589,480,913,927,297,1173,695,254,752
ALAS1_2_12255,357,196,377,345,378,1319,62,1216,267,121,254,402
ALAS2_2_12256,218,121,101,105,484,0,0,283,486,248,83,87
ALDH16A1_2_12257,461,481,410,442,168,737,1042,205,909,349,264,385
ALDH1A2_2_12258,20,51,7,35,53,32,30,21,1,5,0,36
ALDH2_2_12259,386,307,225,424,227,84,0,0,301,616,381,579
ALDH3A1_2_12260,30,71,70,56,94,39,1,280,46,457,0,2
ALDH3A2_2_12261,269,260,205,361,2,263,396,95,206,318,626,939
ALDH3B1_2_12262,237,208,298,362,104,1,33,223,70,60,0,1648
ALDH3B2_2_12263,237,208,298,362,104,1,33,223,70,60,0,1648
ALDH4A1_2_12264,188,204,131,183,757,1079,1171,0,0,0,405,0
ALDH5A1_2_12265,280,285,243,436,25,52,1,13,31,277,74,742
ALDH7A1_2_12266,353,269,354,435,360,306,396,330,997,19,624,66
ALDH8A1_2_12267,400,192,254,191,170,42,18,156,483,1,126,576
ALDOA_2_12268,105,16,11,30,13,3,6,0,2,0,0,0
ALG3_2_12269,113,58,46,44,0,26,0,0,0,1,0,71
ALG5_2_12270,136,235,104,235,369,357,57,244,30,589,18,224
ALG8_2_12271,233,254,143,239,315,772,38,18,76,29,82,187
ALG9_2_12272,167,83,100,140,2,223,80,133,215,64,14,21
ALOX15B_2_12273,96,296,175,291,14,181,26,236,161,0,291,144
ALOXE3_2_12274,120,224,123,229,15,54,6,10,15,39,0,82
AMACR_2_12275,174,121,133,150,213,14,43,121,242,93,0,98
AMD1_2_12276,458,398,322,623,295,926,202,390,314,264,387,523
AMDHD2_2_12277,38,39,49,81,85,0,0,106,22,50,0,3
AMPD1_2_12278,546,439,480,457,833,213,380,709,507,368,363,1406
AMPD2_2_12279,24,52,58,67,2,17,0,4,53,3,49,141
AMPD3_2_12280,128,111,112,158,105,13,455,0,4,0,302,208
AMT_2_12281,371,439,449,444,409,547,34,0,497,293,1813,533
AMY1A_2_12282,5393,4903,4355,5424,4695,5579,3973,5187,6084,3824,5183,5
693
ANG_2_12283,316,263,122,385,330,55,54,256,198,341,5,360
AOAH_2_12284,43,32,24,8,95,10,59,18,23,187,13,54

AOC2_2_12285,262,174,193,153,142,156,83,0,516,273,63,246
APC_2_12286,529,603,366,611,369,18,237,62,717,658,223,374
APEX1_2_12287,49,107,52,74,0,0,4,0,54,4,3,10
APOBEC3A_2_12288,914,748,690,1036,317,26,555,848,555,197,1348,2071
APOBEC3H_2_12289,400,277,306,456,167,890,231,505,481,410,421,386
APRT_2_12290,277,225,163,272,38,57,428,581,7,195,437,439
ARF1_2_12291,18,3,20,7,49,0,6,3,0,0,0,22
ARFRP1_2_12292,52,3,103,74,37,0,24,0,510,0,17,0
ARHGAP5_2_12293,1558,1812,1598,1751,1191,1545,1721,1408,1324,2317,2294
,1853
ARHGEF10L_2_12294,117,73,178,84,0,0,0,0,100,0,1033,1
ARL2_2_12295,237,256,176,197,102,797,47,411,65,126,51,540
ARL4A_2_12296,2821,2378,2599,2652,1843,2502,1996,1833,2060,933,2422,20
96
ARL5A_2_12297,929,723,792,935,471,1115,447,583,1176,812,1277,463
ARSA_2_12298,341,235,255,343,150,420,93,88,197,287,7,254
ARSB_2_12299,328,229,172,216,177,7,239,22,145,273,11,334
ARSF_2_12300,269,135,147,157,31,206,84,126,316,206,653,19
ART3_2_12301,1280,864,821,909,774,803,354,445,1344,472,807,609
ART5_2_12302,213,297,266,309,365,217,300,523,1088,552,438,472
ASAH1_2_12303,1155,1138,961,1306,789,926,563,1373,1822,1755,1465,1664
ASAH2_2_12304,744,949,955,894,990,310,1149,1601,755,891,363,1853
ASL_2_12305,251,178,320,322,275,243,0,108,255,242,254,96
ASMT_2_12306,72,19,24,17,3,9,0,18,0,5,0,236
ASMTL_2_12307,171,190,140,222,237,152,27,509,242,79,97,290
ASNS_2_12308,359,401,306,326,55,1187,141,239,683,446,385,153
ASPA_2_12309,275,503,464,349,191,447,316,148,655,399,136,220
ASRGL1_2_12310,80,72,51,77,0,0,31,51,1,77,25,489
ASS1_2_12311,275,106,143,197,264,263,116,240,108,260,4,564
ATE1_2_12312,454,200,443,288,288,300,130,419,266,472,122,129
ATL1_2_12313,93,181,202,160,586,485,387,229,28,60,0,144
AURKAIP1_2_12314,381,233,319,261,389,377,32,939,105,129,612,385
AZIN1_2_12315,127,151,202,280,149,187,100,438,15,36,208,42
B3GALNT1_2_12316,522,370,416,366,620,621,135,35,320,414,1021,1265
B3GALT5_2_12317,234,221,170,268,469,109,220,297,174,58,41,271
B3GAT1_2_12318,15,24,14,5,0,36,0,8,26,0,0,7
B4GALNT2_2_12319,37,37,39,50,279,0,375,0,0,3,4,60
B4GALT2_2_12320,187,196,252,245,251,287,342,1,114,3,26,86
B4GALT3_2_12321,254,148,209,380,146,8,132,68,377,294,77,232
B4GALT4_2_12322,741,493,442,550,489,1001,528,769,482,498,425,287
BAAT_2_12323,542,549,571,612,457,210,720,1071,505,567,333,464
BACE1_2_12324,163,111,160,203,280,48,14,163,512,231,0,50
BACE2_2_12325,268,252,212,247,87,578,631,42,297,235,70,129
BCAT1_2_12326,31,49,16,22,0,232,1,446,3,0,7,15
BCAT2_2_12327,639,633,496,592,1290,802,164,535,696,208,574,838
BCKDHA_2_12328,951,492,499,858,602,642,2024,639,702,545,319,969
BCKDHB_2_12329,368,351,376,449,635,142,930,420,442,309,95,704
BCO2_2_12330,472,419,333,408,108,512,285,201,242,106,576,427
BDH1_2_12331,94,65,161,92,5,176,289,16,163,23,12,115
BFSP1_2_12332,515,597,540,605,170,203,444,729,405,1130,791,461

BHMT2_2_12333,340,144,226,274,263,138,256,11,281,596,331,124
BMP1_2_12334,26,131,93,95,6,3,21,0,9,332,0,62
C17orf101_2_12335,35,36,43,28,0,21,0,1,2,55,512,7
C1GALT1C1_2_12336,739,736,345,931,607,257,290,245,738,635,186,677
C1S_2_12337,737,568,537,831,931,1540,908,375,710,745,1503,644
C2_2_12338,871,615,753,908,565,745,1109,653,1138,1239,1249,1193
CA10_2_12339,15,56,9,4,0,10,0,0,3,0,6,0
CA12_2_12340,237,184,128,75,0,0,270,9,137,10,0,24
CA1_2_12341,742,584,496,589,459,882,1237,319,139,960,361,127
CA7_2_12342,461,319,415,368,79,333,14,411,474,51,148,240
CAB39_2_12343,645,801,550,608,970,186,657,595,547,951,240,1154
CANT1_2_12344,97,32,104,53,1,9,1,0,18,0,0,30
CAPN1_2_12345,698,613,471,710,772,512,756,741,1122,343,152,1101
CAPN2_2_12346,92,43,100,85,0,0,309,8,0,0,23,303
CARNS1_2_12347,1156,888,852,1099,909,1162,1052,90,813,795,961,455
CARS_2_12348,258,203,231,353,56,236,611,110,137,86,7,131
CASP10_2_12349,854,1101,905,1005,708,721,631,7,84,46,1410,966
CASP1_2_12350,121,39,140,113,244,8,907,106,80,88,15,37
CASP2_2_12351,1079,1492,1260,1706,642,1864,1056,2899,599,1593,1865,141
2
CASP3_2_12352,1449,1004,1041,963,1201,608,1761,191,1227,1031,1014,1007
CASP4_2_12353,162,88,126,173,144,43,832,104,0,123,76,32
CASP5_2_12354,531,472,316,591,324,1,1053,565,203,558,619,217
CASP6_2_12355,517,334,319,444,454,421,359,168,413,154,77,187
CASP7_2_12356,98,177,225,220,114,34,134,7,3,169,557,148
CASP8_2_12357,328,182,196,233,288,132,136,6,446,35,551,410
CASP9_2_12358,35,66,97,48,337,181,69,220,73,33,17,41
CASZ1_2_12359,27,66,143,52,0,0,0,3,0,4,0,1
CAV3_2_12360,168,112,155,172,20,10,1,256,5,85,343,205
CBS_2_12361,483,369,217,378,697,51,195,50,14,139,24,179
CCBL1_2_12362,159,132,68,210,414,0,15,337,6,1,2,645
CCBL2_2_12363,1000,1203,954,1387,1376,1135,881,1220,1014,1059,2404,429
CDC42_2_12364,328,367,364,537,632,272,439,188,175,85,298,261
CDH16_2_12365,64,109,76,93,88,65,144,15,134,193,13,155
CECR1_2_12366,1149,1211,960,1116,447,1636,1072,640,769,675,1617,877
CEPT1_2_12367,962,704,853,962,504,430,392,701,567,973,783,1196
CERS1_2_12368,361,262,274,364,265,985,140,689,295,101,697,480
CES1_2_12369,157,170,167,341,713,81,356,0,119,179,83,279
CES3_2_12370,80,130,81,152,5,0,25,0,3,0,213,22
CES4A_2_12371,42,97,59,51,93,129,31,100,139,0,12,54
CES5A_2_12372,164,78,133,211,0,392,30,0,0,11,368,135
CHAT_2_12373,134,144,179,100,40,103,391,210,130,1,7,73
CHI3L2_2_12374,1672,1257,1078,1387,1897,752,917,1540,1241,702,2077,616
CHIA_2_12375,54,100,157,83,245,64,31,357,144,11,2,119
CHPF_2_12376,143,263,134,219,0,369,0,0,32,14,3,65
CHST11_2_12377,360,188,232,303,188,109,381,81,152,519,298,178
CHST15_2_12378,638,560,535,606,614,122,497,1103,710,437,587,1200
CHST4_2_12379,99,84,102,63,158,0,0,29,5,0,364,252
CHST8_2_12380,262,231,512,385,97,76,135,418,604,367,307,425
CLP1_2_12381,61,111,61,107,378,208,0,43,18,5,0,141

CNDP2_2_12382,413,256,396,381,400,475,304,377,634,195,46,43
CNTN1_2_12383,258,347,227,325,251,50,62,20,196,722,117,106
CNTN4_2_12384,82,25,69,46,55,12,115,83,21,3,2,0
COMT_2_12385,11,24,16,77,0,29,412,0,0,519,203,4
COQ6_2_12386,360,567,364,497,215,773,450,425,236,230,970,765
COX11_2_12387,369,334,261,369,493,211,893,235,542,444,165,1055
COX15_2_12388,392,158,116,272,28,131,193,49,174,56,357,297
CPA5_2_12389,212,238,171,170,62,97,512,191,123,75,164,327
CPB2_2_12390,1427,1735,1551,1494,1336,1359,1754,1306,1266,2091,2082,18
79
CPM_2_12391,320,260,245,388,30,387,216,208,136,216,210,347
CPPED1_2_12392,609,570,557,661,783,728,795,358,1119,1017,658,491
CPT1A_2_12393,107,18,16,34,83,21,60,105,67,38,13,87
CPT1B_2_12394,182,226,255,442,88,523,433,19,91,254,215,107
CPT1C_2_12395,219,256,221,274,277,21,1,40,297,47,113,180
CRLS1_2_12396,658,732,689,977,431,1401,821,795,490,927,906,868
CRMP1_2_12397,31,34,150,155,39,103,124,0,28,25,0,799
CROT_2_12398,588,227,457,338,236,375,103,476,502,241,51,918
CRY2_2_12399,294,300,231,347,254,241,366,218,223,510,92,225
CRYM_2_12400,134,237,190,134,885,105,491,68,544,127,1,267
CRYZ_2_12401,250,261,273,290,377,85,344,824,266,461,61,524
CSDE1_2_12402,440,677,462,686,265,114,494,292,334,772,561,267
CSGALNACT1_2_12403,224,188,319,336,281,126,412,351,372,262,13,503
CSMD3_2_12404,92,54,55,80,63,299,137,310,67,382,360,99
CTAGE5_2_12405,527,492,570,464,733,88,1010,257,813,309,654,572
CTBP1_2_12406,131,122,75,52,3,239,13,471,3,472,3,63
CTPS2_2_12407,1103,1535,1201,1485,783,853,1976,431,780,1874,1393,1485
CTSA_2_12408,193,90,199,202,92,144,33,123,139,684,0,204
CTSB_2_12409,453,364,529,574,649,193,1182,577,470,302,308,720
CTSE_2_12410,423,389,391,735,324,359,1282,594,704,179,241,1602
CTSL1_2_12411,106,60,131,103,362,6,19,331,156,43,0,546
CTSL2_2_12412,221,494,345,403,625,180,420,260,226,1366,2140,228
CTSS_2_12413,114,134,114,167,0,12,1,349,7,7,0,78
CYB561_2_12414,56,19,12,178,0,9,0,243,443,0,0,9
CYB5A_2_12415,107,46,148,80,19,15,18,7,30,1,155,89
CYB5R3_2_12416,205,183,128,198,104,344,66,381,220,474,215,191
CYP11A1_2_12417,439,547,452,709,162,130,921,234,845,294,544,1316
CYP11B1_2_12418,679,534,613,679,745,488,1539,551,698,746,391,979
CYP19A1_2_12419,702,735,609,781,344,508,399,892,341,432,7,1401
CYP21A2_2_12420,214,186,251,205,116,209,73,334,241,832,423,233
CYP24A1_2_12421,535,568,518,508,888,569,568,237,38,503,1150,68
CYP26A1_2_12422,115,231,92,162,60,40,10,15,59,571,1,268
CYP2A7_2_12423,526,308,555,427,523,39,928,691,275,677,761,469
CYP2C18_2_12424,1269,962,1011,1020,1489,1229,1438,1177,230,2173,816,14
72
CYP2C8_2_12425,631,483,445,513,389,245,480,400,954,462,496,469
CYP2D6_2_12426,262,287,156,154,470,18,0,51,90,12,23,62
CYP3A43_2_12427,695,709,741,1091,640,215,1863,619,784,637,447,695
CYP3A4_2_12428,2697,2496,2450,3155,2198,2704,3004,4159,2754,1827,3962,
1795

CYP4B1_2_12429,14,37,38,57,24,0,9,1,1,73,1,64
CYP4F11_2_12430,610,183,306,395,527,4,636,260,529,90,376,554
CYP4F3_2_12431,1097,908,892,1022,928,994,1210,1112,1151,637,848,859
CYP51A1_2_12432,164,233,342,247,35,15,0,250,121,33,494,230
DAGLB_2_12433,212,246,291,326,588,388,539,151,550,1255,196,317
DCLRE1C_2_12434,212,164,112,373,43,1,73,0,136,164,192,58
DCP2_2_12435,122,58,121,123,52,143,512,179,132,172,20,57
DCT_2_12436,550,431,426,673,381,771,584,264,269,409,401,414
DCTD_2_12437,39,37,24,17,0,0,0,30,0,0,3,4
DCXR_2_12438,143,46,87,167,23,91,14,4,6,15,0,2
DDAH1_2_12439,446,319,335,300,377,121,102,11,251,188,698,333
DDC_2_12440,589,677,734,873,1155,149,341,1211,240,1370,731,177
DDHD1_2_12441,85,51,79,113,251,21,6,176,140,0,0,14
DDO_2_12442,222,50,101,32,3,110,74,8,602,187,0,134
DDT_2_12443,55,122,99,120,0,52,7,0,20,203,0,56
DDX11_2_12444,288,271,245,224,38,163,635,84,302,655,86,8
DDX17_2_12445,75,106,81,90,9,14,31,12,121,174,483,21
DDX19B_2_12446,309,101,269,161,54,12,36,1,32,238,20,197
DDX31_2_12447,68,50,108,64,4,0,3,36,3,0,0,45
DDX39B_2_12448,101,104,164,138,0,0,400,343,95,230,0,159
DDX3X_2_12449,727,646,653,896,919,445,186,329,371,564,722,289
DDX3Y_2_12450,430,224,288,217,357,19,482,487,330,405,343,312
DDX42_2_12451,145,99,121,182,15,301,0,383,0,266,293,198
DDX47_2_12452,209,175,239,171,527,21,318,84,502,9,80,171
DDX4_2_12453,57,71,94,64,48,64,114,118,61,74,1,294
DDX54_2_12454,17,80,34,52,0,166,22,152,0,121,13,0
DFFA_2_12455,211,165,155,231,67,577,444,280,78,156,376,734
DGCR8_2_12456,296,341,494,465,733,455,424,599,194,18,667,421
DHCR7_2_12457,448,397,425,314,202,72,1241,622,220,491,1401,417
DHDDS_2_12458,173,159,124,106,33,289,432,45,152,363,71,146
DHFRL1_2_12459,138,110,74,208,298,37,401,0,25,36,382,306
DHPS_2_12460,1107,545,651,873,304,472,185,1106,646,1180,737,713
DHRS1_2_12461,31,24,39,21,16,21,0,3,9,41,102,82
DHRS2_2_12462,194,87,124,143,364,3,467,123,92,231,4,1
DHRS9_2_12463,133,154,146,191,100,312,811,337,572,23,52,120
DHX16_2_12464,288,289,168,280,23,32,709,492,388,491,110,540
DHX30_2_12465,512,395,400,354,270,9,256,260,382,761,68,144
DHX33_2_12466,138,71,165,152,167,10,0,0,5,360,73,25
DHX35_2_12467,464,327,511,472,582,520,692,137,301,238,229,707
DHX36_2_12468,237,297,233,414,110,106,243,73,175,235,691,232
DHX40_2_12469,101,25,101,63,170,0,333,348,35,19,11,66
DIAPH3_2_12470,127,106,155,142,498,8,200,647,75,96,14,163
DIS3_2_12471,357,698,336,442,104,801,389,8,305,22,476,579
DIS3L_2_12472,2785,2035,2189,2478,1613,2688,3120,1088,3166,1763,1499,2
107
DKC1_2_12473,80,27,72,61,37,0,29,193,194,0,0,117
DNAJC27_2_12474,719,792,784,770,699,1049,572,970,1011,569,201,930
DNASE1L1_2_12475,36,32,44,23,21,0,190,0,14,1,230,6
DNM1_2_12476,90,44,102,112,95,0,5,0,101,0,271,64
DNM1L_2_12477,292,489,360,438,612,76,11,223,43,497,851,193

DNM2_2_12478,87,179,148,225,0,13,464,0,130,194,515,72
DNM3_2_12479,278,297,233,570,253,171,321,301,466,16,184,289
DNTT_2_12480,270,360,385,312,33,8,222,626,389,192,2,242
DOHH_2_12481,201,289,410,322,40,8,241,37,164,823,232,280
DOLPP1_2_12482,26,46,123,49,0,6,1,11,49,0,197,122
DPEP1_2_12483,77,38,82,66,273,0,50,227,0,103,381,142
DPEP3_2_12484,685,553,593,605,502,21,719,398,2,235,504,398
DPH5_2_12485,564,511,499,553,353,930,205,1094,798,345,394,660
DPM3_2_12486,169,59,59,55,3,0,7,333,227,0,0,14
DPP3_2_12487,408,502,444,483,174,689,1063,14,287,325,640,274
DPP8_2_12488,153,214,171,139,146,22,200,138,331,163,257,479
DPYSL2_2_12489,510,674,416,531,110,687,124,540,375,447,512,450
DPYSL3_2_12490,158,95,184,142,238,0,648,206,320,7,0,787
DROSHA_2_12491,194,123,92,232,36,551,79,191,341,237,414,34
DSE_2_12492,932,890,837,1256,874,357,376,1071,851,1697,829,1386
DUOX1_2_12493,897,694,1036,880,704,876,230,1014,749,875,1188,1188
DUT_2_12494,520,440,292,680,349,12,678,767,211,103,111,571
ECE1_2_12495,38,8,8,15,2,0,131,0,5,0,0,9
ECE2_2_12496,701,615,497,650,775,879,699,1045,616,766,268,1117
ECI1_2_12497,486,282,463,421,314,413,433,340,385,250,1735,470
ECI2_2_12498,318,383,470,465,211,183,861,151,134,600,7,473
EDEM2_2_12499,635,410,506,458,544,706,926,244,533,489,480,801
EFEMP1_2_12500,92,67,60,80,25,0,2,0,81,116,19,85
EFTUD2_2_12501,217,337,394,448,410,265,414,273,245,906,24,181
EGLN2_2_12502,194,50,83,119,1,53,421,304,189,77,822,89
EHHADH_2_12503,492,394,260,527,224,97,125,304,444,861,478,925
ELAC2_2_12504,122,128,141,160,2,34,457,3,10,486,419,3
ELOVL5_2_12505,390,426,368,495,297,148,245,719,432,1145,646,654
ELOVL6_2_12506,1784,1878,1303,1610,2609,1394,1390,1462,2436,1667,1550,
1469
ELOVL7_2_12507,429,368,561,282,31,4,162,186,708,495,472,274
ENDOV_2_12508,34,4,2,44,0,1,130,0,35,0,283,0
ENO3_2_12509,334,211,229,278,49,278,301,9,509,50,275,427
ENOX2_2_12510,744,587,838,775,712,282,460,282,565,508,1038,491
ENPP2_2_12511,842,886,834,934,340,1208,747,1735,958,1151,487,2045
ENTPD1_2_12512,1625,1616,1368,1598,1289,2044,1436,2230,1584,1459,1110,
1083
ENTPD2_2_12513,917,982,768,819,1301,1804,214,1046,1729,1139,972,1206
ENTPD4_2_12514,1332,1503,1258,1384,1060,1433,1144,731,1557,1759,2145,1
483
ENTPD6_2_12515,1130,972,983,1032,542,403,617,1871,746,581,535,1089
ENTPD8_2_12516,25,8,32,12,0,137,323,0,38,73,0,0
EPHX1_2_12517,276,260,214,307,169,284,632,1207,188,356,795,262
EPHX3_2_12518,0,0,7,2,0,0,0,0,0,0,0,1
ERCC1_2_12519,162,88,95,113,126,2,87,8,38,125,4,389
ERCC2_2_12520,1,127,42,1,0,0,4,0,0,0,2,34
EXO1_2_12521,2749,2340,2072,2656,1151,2737,1742,1481,2067,1843,2034,22
79
EXOG_2_12522,1237,1394,1087,1323,1333,1106,600,1042,1417,1400,797,1799
EXOSC3_2_12523,321,343,257,370,1024,342,707,8,230,448,589,469

EXOSC9_2_12524,149,145,156,130,9,120,77,187,162,1,689,187
EXTL2_2_12525,519,493,594,554,1154,1771,225,682,871,548,807,955
F7_2_12526,10,6,5,21,4,0,26,0,26,30,0,90
FAHD1_2_12527,1136,720,855,1030,1410,579,831,1297,993,636,331,1325
FAM108A1_2_12528,145,178,131,152,54,40,138,85,14,11,11,244
FAM135A_2_12529,543,799,471,847,590,448,684,813,410,858,81,716
FAN1_2_12530,87,34,110,189,37,278,906,29,71,92,147,88
FDPS_2_12531,822,724,653,947,372,751,443,662,607,595,232,1056
FDXR_2_12532,88,141,65,48,103,0,1,1,166,224,0,136
FECH_2_12533,1882,1398,1372,2162,1671,2059,1373,1480,2852,2354,1176,17
30
FERMT3_2_12534,959,674,534,820,1289,411,360,542,817,1099,493,559
FHIT_2_12535,806,705,642,786,2,256,976,762,997,566,872,1145
FIGNL1_2_12536,868,561,501,540,821,758,1070,518,247,840,257,494
FKBP11_2_12537,59,18,39,111,0,187,158,5,85,12,14,12
FKBP1A_2_12538,172,84,102,149,260,106,114,37,700,1,3,62
FKBP1B_2_12539,748,622,636,984,1089,1184,895,961,903,727,951,1173
FKBP2_2_12540,34,62,31,31,4,25,293,0,16,1,0,38
FKBP5_2_12541,594,399,393,411,541,522,1085,394,714,248,63,549
FKBP7_2_12542,384,326,498,517,253,548,416,207,300,281,319,691
FLAD1_2_12543,59,54,64,38,0,133,2,39,42,0,1,42
FMO3_2_12544,367,255,255,316,334,199,439,266,112,641,446,412
FMO5_2_12545,844,828,751,780,1809,589,1518,515,1644,986,1390,1071
FN1_2_12546,782,613,510,677,775,229,965,305,857,624,1112,961
FOLH1_2_12547,22,29,24,60,0,363,193,56,11,49,0,79
FPGS_2_12548,166,55,82,177,13,64,118,32,197,92,8,56
FPGT_2_12549,702,608,614,662,453,181,714,501,466,616,1101,831
FTCD_2_12550,32,26,15,88,1,0,0,0,0,13,0,15
FTSJ1_2_12551,520,479,614,570,671,0,2508,128,734,232,40,1086
FUT2_2_12552,247,125,205,198,1312,218,64,635,85,5,96,132
FUT3_2_12553,732,827,930,945,499,697,584,2227,579,335,410,1572
FUT6_2_12554,3011,3040,3169,3449,2102,2165,4733,1749,2422,3913,4290,33
60
FUT8_2_12555,507,256,477,478,376,98,457,917,342,20,397,721
G3BP1_2_12556,1246,1044,1052,1124,1060,515,165,832,1162,1262,1426,1160
G3BP2_2_12557,31,33,53,47,0,2,7,51,2,129,82,11
G6PD_2_12558,65,44,48,75,27,9,130,0,31,26,0,135
GAA_2_12559,262,312,189,251,91,22,1124,21,237,151,182,467
GAD1_2_12560,8,5,33,19,0,0,0,0,13,1,8,11
GAD2_2_12561,3,4,5,17,0,0,0,0,29,0,48,1
GALC_2_12562,40,128,59,130,116,4,0,4,1,241,5,286
GALE_2_12563,107,145,90,110,0,35,442,98,39,275,0,146
GALNT9_2_12564,74,95,81,91,278,371,193,65,4,7,480,16
GALNTL1_2_12565,898,908,716,814,921,749,863,1253,745,848,418,462
GAMT_2_12566,79,41,136,131,1,4,128,434,294,203,636,1
GANAB_2_12567,51,56,81,75,17,0,40,316,45,5,39,5
GART_2_12568,57,12,27,18,58,11,4,0,1,73,0,16
GBA_2_12569,24,59,136,134,18,73,211,15,91,30,0,0
GBP5_2_12570,435,358,439,514,82,745,513,652,461,737,1247,832
GCAT_2_12571,41,5,81,54,70,0,127,59,22,63,57,0

GCDH_2_12572,62,48,48,29,66,0,19,103,50,35,0,36
GCH1_2_12573,504,326,388,448,843,256,633,38,65,689,758,502
GCLC_2_12574,73,81,69,74,183,0,12,22,15,0,0,211
GCNT1_2_12575,158,192,258,259,84,84,13,7,10,757,133,125
GDPD1_2_12576,97,91,75,95,293,0,230,16,234,86,230,133
GDPD2_2_12577,101,353,366,376,250,164,72,0,1153,1,6,612
GEM_2_12578,177,306,161,205,44,0,231,228,491,270,442,18
GEN1_2_12579,46,36,99,66,19,0,517,46,36,11,141,194
GFM2_2_12580,818,962,625,692,96,207,1121,409,1037,796,941,1559
GFOD1_2_12581,48,27,37,72,1,117,1,109,21,0,0,0
GGCT_2_12582,17,70,18,44,17,0,16,107,19,5,129,11
GGCX_2_12583,308,191,221,215,349,472,421,67,180,83,136,414
GGT1_2_12584,94,82,92,101,0,138,488,44,64,19,0,118
GGT5_2_12585,101,89,58,86,30,70,7,352,82,35,21,131
GGT6_2_12586,52,7,106,59,1,41,0,0,106,629,0,168
GGTLC1_2_12587,481,327,399,671,795,115,747,507,281,12,814,663
GLB1_2_12588,175,185,133,96,20,332,304,121,37,16,89,509
GLRX2_2_12589,124,135,139,119,221,26,146,238,157,499,242,476
GLRX3_2_12590,64,130,113,64,178,30,334,1,11,0,179,78
GLRX_2_12591,977,926,822,1233,1193,825,869,876,712,1318,1201,1417
GLT8D1_2_12592,271,217,346,375,22,212,298,495,600,244,735,534
GLUL_2_12593,124,105,133,274,432,275,108,3,2,182,208,245
GM2A_2_12594,902,815,979,1002,696,209,656,802,1202,762,345,395
GMPPA_2_12595,252,303,199,280,827,199,672,177,196,456,145,498
GMPPB_2_12596,44,44,50,77,4,0,66,45,0,7,0,83
GMPR2_2_12597,236,160,278,239,24,184,24,99,168,106,553,122
GNAI2_2_12598,781,604,698,887,1114,621,1307,1049,959,439,555,1111
GNAL_2_12599,223,256,150,346,217,248,379,382,67,61,152,570
GNAO1_2_12600,791,638,584,620,671,350,463,1161,563,390,342,468
GNAT1_2_12601,1999,1602,1440,1827,1208,1726,2084,1430,1800,1601,2778,3
330
GNB5_2_12602,180,259,195,241,2,421,0,100,252,233,535,108
GNG10_2_12603,39,25,16,61,4,54,1,0,54,92,0,3
GNG4_2_12604,34,17,21,27,61,0,64,0,0,0,87,0
GNGT2_2_12605,0,0,0,0,0,0,0,0,0,0,0,0
GPD2_2_12606,535,613,556,671,160,1130,146,773,472,441,788,1163
GPHN_2_12607,412,300,265,426,502,43,283,284,201,290,694,902
GPNMB_2_12608,611,541,608,638,564,128,247,678,579,191,554,1034
GPT2_2_12609,173,79,168,102,111,63,100,246,75,236,345,113
GPX4_2_12610,1,89,14,66,167,16,0,2,22,0,0,0
GSR_2_12611,349,341,531,348,24,124,853,666,755,48,270,584
GSTCD_2_12612,159,281,341,358,673,54,46,160,25,135,136,216
GSTK1_2_12613,454,297,380,338,1183,348,340,110,155,501,10,271
GSTM1_2_12614,571,380,660,556,842,598,217,934,271,303,598,551
GSTM2_2_12615,570,380,644,555,841,604,217,935,273,300,600,550
GSTM4_2_12616,25,27,40,31,1,320,537,0,58,1,0,69
GSTO1_2_12617,419,299,500,308,406,90,155,414,234,15,603,352
GSTO2_2_12618,958,666,870,897,712,0,487,492,927,710,1157,1392
GSTZ1_2_12619,74,33,47,69,0,0,0,23,0,71,0,0
GTPBP3_2_12620,0,0,0,0,0,0,0,0,0,0,0,0

GUCY1A3_2_12621,73,11,90,38,2,0,35,0,5,0,39,9
GYG1_2_12622,281,176,262,370,280,403,512,95,105,246,1017,485
GYG2_2_12623,20,90,92,67,71,369,10,193,2,31,61,83
GYS1_2_12624,185,267,122,132,292,121,0,25,195,330,525,303
HADH_2_12625,1926,1570,1337,1412,1815,1297,1764,1191,1486,1900,2151,18
49
HAGH_2_12626,293,189,270,378,581,175,924,377,223,51,428,214
HAO2_2_12627,132,75,62,56,505,77,0,0,0,65,21,50
HAS3_2_12628,344,166,125,185,80,252,204,90,355,228,13,197
HCCS_2_12629,102,68,53,64,0,31,24,247,108,12,13,103
HDHD1_2_12630,489,352,504,437,229,294,446,194,344,685,296,244
HENMT1_2_12631,47,103,153,185,37,0,2,45,161,110,7,2
HHAT_2_12632,1198,1282,1075,1390,776,1008,613,1701,1127,814,1142,1401
HIBCH_2_12633,195,291,165,163,315,28,858,703,12,59,46,141
HLCS_2_12634,84,53,166,168,40,0,0,28,0,16,0,280
HMBS_2_12635,483,581,684,766,623,351,35,644,294,830,569,564
HMGA2_2_12636,22,85,74,64,84,36,316,6,4,1,9,83
HMGCL_2_12637,761,570,457,591,889,542,680,47,622,924,354,638
HMGCLL1_2_12638,249,189,118,256,172,686,0,264,248,755,140,146
HMGCR_2_12639,171,71,106,233,457,37,83,417,34,21,0,131
HMGCS1_2_12640,693,657,518,747,686,1449,1256,1580,481,832,751,1042
HMGCS2_2_12641,57,46,85,110,28,148,53,364,3,1,1,90
HMOX2_2_12642,126,150,72,22,9,13,246,11,81,67,0,113
HNRNPAB_2_12643,140,163,228,185,0,278,425,213,230,286,118,123
HPD_2_12644,2253,1795,1880,1962,1856,781,1863,1736,2102,2923,1695,1163
HPGD_2_12645,112,53,64,81,0,0,20,14,4,1,0,105
HPSE2_2_12646,52,24,60,55,120,14,0,0,4,24,0,22
HPSE_2_12647,1,60,6,14,5,2,0,0,40,1,0,0
HRAS_2_12648,8,6,18,23,0,0,0,2,0,0,21,0
HS2ST1_2_12649,994,873,768,1013,1315,2169,200,532,604,530,831,877
HS6ST2_2_12650,382,197,284,341,166,2,987,535,63,381,285,178
HSD11B1_2_12651,569,332,398,390,109,520,19,252,120,442,618,897
HSD17B10_2_12652,528,345,373,543,21,139,244,219,275,239,1134,799
HSD17B13_2_12653,761,919,632,990,1006,901,1020,793,680,367,1187,456
HSD17B4_2_12654,691,616,459,533,216,358,527,176,629,840,459,326
HSD3B2_2_12655,458,532,326,476,382,145,1022,370,919,303,94,409
HSD3B7_2_12656,82,50,43,66,0,3,7,10,31,2,0,494
HSDL1_2_12657,344,170,183,228,193,130,298,377,151,301,3,255
HSP90AA1_2_12658,356,273,337,366,834,385,244,142,368,540,669,616
HSPA8_2_12659,881,568,652,841,605,1411,166,944,1033,1498,1552,900
HSPD1_2_12660,186,275,305,304,188,231,1139,446,443,451,635,219
HTRA2_2_12661,485,572,646,563,418,240,531,177,563,1189,1556,354
HYAL1_2_12662,1220,1123,1218,1438,1634,641,2978,548,903,266,1427,2116
HYAL2_2_12663,643,202,405,335,513,64,119,1204,491,141,702,121
HYI_2_12664,290,123,132,115,598,240,20,299,168,429,98,353
IARS_2_12665,942,731,604,774,525,1951,691,677,792,789,287,520
IDE_2_12666,127,137,103,151,29,15,197,0,43,23,348,97
IDH3B_2_12667,345,253,298,391,240,268,20,90,423,250,24,155
IDH3G_2_12668,182,110,182,160,101,193,430,342,468,27,70,85
IDS_2_12669,232,164,240,163,8,754,3,0,193,5,23,0

IFT27_2_12670,303,409,306,259,438,287,460,53,88,232,434,421
IL4I1_2_12671,173,220,141,298,353,10,714,64,51,354,47,624
IMPDH1_2_12672,94,57,67,142,125,30,118,319,180,22,20,336
INMT_2_12673,266,185,222,244,351,637,230,246,149,95,146,419
INPP5K_2_12674,1403,1425,1011,1221,1247,729,548,1062,1350,2392,955,169
2
INTS6_2_12675,300,94,201,179,102,235,7,78,133,11,1,71
IPCEF1_2_12676,438,535,296,577,552,722,965,444,240,403,376,246
ISOC2_2_12677,73,78,44,34,8,0,11,0,2,1,18,51
ISPD_2_12678,1602,1013,1045,1100,2433,577,1774,1632,716,1074,799,2045
ISYNA1_2_12679,85,69,23,9,159,0,0,11,0,138,0,0
ITPA_2_12680,11,1,18,1,0,1,0,0,25,44,0,27
IVD_2_12681,659,759,533,805,700,635,544,1068,552,277,504,449
IYD_2_12682,456,579,287,590,547,610,1721,752,669,1234,244,748
JMJD7-PLA2G4B_2_12683,351,466,300,453,274,32,1503,986,367,507,1,636
KARS_2_12684,176,397,321,251,288,2,295,288,93,950,4,264
KATNA1_2_12685,1314,556,956,840,271,798,458,509,1257,1104,1056,468
KATNAL1_2_12686,1885,1363,1589,1966,1876,478,2553,2354,2164,1032,2384,
1834
KIF16B_2_12687,72,47,115,83,0,206,0,0,0,661,16,0
KIF9_2_12688,583,455,367,383,467,158,537,245,389,428,345,332
KIFC3_2_12689,146,151,120,189,9,605,1,378,165,34,199,296
KLK2_2_12690,53,30,51,42,6,0,68,33,0,3,0,26
KLK7_2_12691,2,2,38,11,0,0,16,1,0,69,0,17
KRAS_2_12692,110,391,280,197,292,954,71,52,354,69,37,384
KYNU_2_12693,820,858,717,896,381,811,817,924,726,345,1586,1043
LAMP2_2_12694,378,388,275,304,229,443,711,37,315,230,601,748
LARGE_2_12695,1019,756,1035,854,775,162,627,1191,1311,1387,2069,710
LCLAT1_2_12696,364,313,303,354,68,319,319,307,164,331,136,206
LCMT1_2_12697,77,49,100,187,25,23,30,112,54,7,0,79
LDHA_2_12698,155,179,279,278,268,540,454,276,58,158,473,215
LDHAL6A_2_12699,277,643,363,456,60,339,148,222,664,32,1100,1318
LDHB_2_12700,145,127,150,144,594,788,10,157,311,57,0,65
LDHC_2_12701,2043,1908,1848,2140,1435,2289,1827,1702,1459,1992,2909,18
08
LDHD_2_12702,4,22,85,7,0,0,0,0,0,171,0,0
LEPRE1_2_12703,9,4,8,32,38,157,0,0,30,2,0,82
LEPREL1_2_12704,165,156,140,228,85,37,40,491,147,315,297,324
LFNG_2_12705,186,172,213,277,123,317,292,802,10,37,0,112
LGMN_2_12706,156,53,89,66,0,3,11,27,42,136,7,24
LGSN_2_12707,124,177,97,120,7,96,47,54,227,267,5,17
LIAS_2_12708,35,9,5,23,0,1,0,0,10,2,0,32
LIG3_2_12709,241,247,415,495,168,212,545,312,33,411,106,140
LIG4_2_12710,112,135,129,116,10,109,450,464,103,3,18,83
LIPF_2_12711,285,398,314,365,282,15,639,9,65,78,323,166
LIPT1_2_12712,393,384,329,400,299,150,786,0,632,599,669,141
LMO7_2_12713,509,297,389,473,668,701,624,494,394,547,406,598
LNPEP_2_12714,125,118,70,72,228,44,391,271,3,250,21,38
LOX_2_12715,113,165,93,146,77,206,30,8,127,52,154,99
LPO_2_12716,1234,1031,891,1137,1279,1263,533,1028,1015,816,713,1154

LRRC16A_2_12717,471,390,459,461,29,202,490,248,714,386,103,448
LSS_2_12718,50,42,28,79,296,0,305,23,26,49,0,84
LYZL6_2_12719,290,204,317,360,481,356,825,416,246,624,779,435
MACF1_2_12720,62,72,118,73,0,0,10,300,115,0,309,0
MACROD2_2_12721,109,190,175,65,8,6,404,179,137,2,334,146
MAD2L2_2_12722,188,64,42,124,23,10,134,76,231,314,0,19
MAN2B1_2_12723,120,349,226,170,237,263,349,1,244,67,1,281
MASP2_2_12724,66,36,67,50,0,31,170,2,39,0,58,37
MAT2B_2_12725,498,465,459,620,389,160,783,391,248,225,1299,539
MCAT_2_12726,106,102,55,104,42,1,70,42,4,11,0,155
MCM4_2_12727,53,68,41,42,0,38,0,7,0,0,161,228
MCM7_2_12728,264,370,111,190,14,15,0,3,314,252,0,0
MCM8_2_12729,290,221,267,270,147,130,855,478,336,211,496,247
MCM9_2_12730,117,172,156,272,127,1,571,1,52,120,60,125
MDH1_2_12731,595,672,518,825,501,1424,987,466,299,712,218,422
MECR_2_12732,37,12,21,41,47,0,5,14,7,0,0,33
MEPCE_2_12733,597,538,419,541,545,629,608,84,611,671,730,454
METTL13_2_12734,120,81,102,107,43,64,130,198,60,24,11,7
MFN2_2_12735,78,117,86,53,8,144,180,0,15,58,0,10
MFNG_2_12736,92,37,61,99,0,36,0,68,71,6,1,0
MGAT3_2_12737,149,99,104,114,10,353,86,18,134,66,1082,80
MGAT4A_2_12738,3081,3513,2643,3104,2231,2200,2698,2690,2605,2745,3125,
3222
MGAT4B_2_12739,142,109,130,124,281,0,0,365,0,426,386,74
MGLL_2_12740,154,41,50,59,0,118,0,0,53,0,0,1
MGST1_2_12741,339,333,256,463,27,158,606,295,475,400,51,489
MGST2_2_12742,81,58,49,95,41,37,152,81,2,65,168,63
MICAL1_2_12743,709,295,359,452,408,496,376,285,1197,213,247,354
MLH1_2_12744,178,218,161,192,32,32,0,585,422,425,414,158
MME_2_12745,92,177,302,250,160,0,739,297,70,268,190,633
MMP1_2_12746,598,390,377,363,469,463,852,360,267,432,834,405
MMP2_2_12747,494,277,316,520,942,208,300,3,94,441,3,843
MOCS1_2_12748,1140,1201,1137,1383,799,1433,100,400,728,1285,4906,1172
MOGS_2_12749,66,94,36,90,4,76,131,39,29,8,2,383
MOV10_2_12750,45,75,110,62,0,0,4,324,105,0,858,209
MOV10L1_2_12751,3,1,13,3,71,0,19,0,0,0,0,0
MPG_2_12752,178,49,86,134,17,22,143,6,50,22,2,23
MPPE1_2_12753,16,46,47,61,0,60,42,0,27,3,12,2
MPST_2_12754,139,44,86,70,10,1,117,76,81,15,86,37
MRAS_2_12755,200,312,209,139,0,199,233,15,46,0,1386,72
MRE11A_2_12756,1198,867,993,1225,809,685,200,535,1663,826,640,1308
MRI1_2_12757,321,207,28,226,784,1,354,10,524,518,0,280
MSH5_2_12758,123,117,93,96,20,0,21,396,32,13,1,166
MSRA_2_12759,9,25,39,58,0,0,0,0,0,0,73,1
MSRB3_2_12760,881,730,587,999,430,837,1210,670,67,1449,732,748
MTHFD1L_2_12761,66,114,112,108,97,301,271,7,185,26,1,165
MTHFS_2_12762,1170,1258,1090,1218,724,235,1739,291,1872,1318,956,860
MTO1_2_12763,92,95,81,92,15,127,0,95,46,10,9,350
MTRR_2_12764,177,286,277,264,411,408,1015,430,51,30,2,92
MUTYH_2_12765,77,250,124,242,25,125,2,79,121,57,0,855

MX1_2_12766,438,139,180,174,777,128,53,446,608,6,325,93
MYBBP1A_2_12767,101,39,90,75,184,71,148,49,27,3,82,73
MYH2_2_12768,349,301,280,451,187,442,276,577,132,624,70,135
MYO5A_2_12769,639,569,591,1045,977,741,880,1393,455,1007,528,509
MYO7A_2_12770,53,48,35,34,158,27,51,3,69,103,0,31
MYO9B_2_12771,82,52,122,116,363,209,3,492,0,29,0,288
N6AMT1_2_12772,321,493,347,471,687,179,76,379,520,738,546,671
NAA16_2_12773,288,157,173,165,7,170,644,19,182,15,78,413
NAA20_2_12774,36,109,100,56,0,6,6,0,105,9,13,53
NAAA_2_12775,563,415,477,597,436,307,1706,656,834,247,781,385
NAPEPLD_2_12776,591,713,551,645,237,499,108,726,414,613,643,749
NARF_2_12777,88,110,82,123,4,290,11,4,249,138,3,43
NARS2_2_12778,59,76,49,94,3,0,0,0,0,6,0,167
NAV1_2_12779,15,70,55,16,0,7,42,0,5,0,13,59
NCEH1_2_12780,844,742,635,1060,1156,225,32,970,1263,813,79,1062
NCF2_2_12781,159,170,210,306,0,190,80,68,196,88,1566,737
NCF4_2_12782,227,306,147,132,316,18,4,76,49,30,111,32
NDOR1_2_12783,556,789,541,853,700,666,1024,1490,463,1073,1488,648
NDUFA2_2_12784,330,253,328,443,895,127,93,624,13,190,1662,156
NDUFB11_2_12785,366,292,308,278,855,434,639,140,266,166,12,276
NDUFB4_2_12786,388,327,352,739,0,478,552,649,90,3,3,586
NDUFB5_2_12787,972,675,780,938,773,910,917,968,1048,566,615,1013
NDUFB6_2_12788,33,83,58,55,2,0,127,0,31,202,0,136
NDUFC1_2_12789,605,538,428,676,150,690,114,127,298,99,13,173
NDUFS1_2_12790,405,328,416,477,304,8,696,94,257,725,995,752
NDUFS2_2_12791,455,210,254,315,1,231,686,0,495,558,617,742
NDUFS5_2_12792,483,383,436,402,522,149,568,144,661,681,344,919
NDUFV1_2_12793,11,40,10,55,1,5,3,1,4,143,0,216
NEIL2_2_12794,464,533,427,495,709,163,119,1079,620,39,381,412
NEU4_2_12795,2816,2713,2033,2491,1590,1691,2510,2198,2637,3335,3224,27
04
NFS1_2_12796,104,149,148,136,102,59,296,620,195,341,74,21
NGLY1_2_12797,124,94,156,175,69,12,246,21,78,259,74,20
NIPSNAP1_2_12798,968,718,413,847,443,71,831,1078,491,224,447,851
NIT1_2_12799,767,664,807,688,712,952,1622,896,802,663,333,1032
NKIRAS2_2_12800,894,1040,823,846,577,493,449,58,1100,931,886,921
NLGN3_2_12801,249,336,132,167,115,362,679,35,339,276,220,290
NLGN4X_2_12802,589,408,432,645,178,410,393,544,399,942,94,234
NLGN4Y_2_12803,589,408,432,645,178,410,393,544,399,942,94,234
NMNAT2_2_12804,116,54,41,67,119,0,74,11,137,84,0,1
NNT_2_12805,27,65,2,29,5,0,0,298,13,5,0,2
NOX4_2_12806,698,538,377,385,517,264,149,478,1192,409,304,532
NPL_2_12807,353,246,256,322,364,28,1127,62,16,426,156,200
NQO1_2_12808,43,43,144,94,13,32,416,96,1,112,59,24
NRD1_2_12809,201,212,155,229,310,299,113,182,383,408,102,130
NSDHL_2_12810,841,725,651,804,331,378,2506,73,564,1219,1053,399
NSUN2_2_12811,49,71,117,30,71,319,0,111,0,0,0,62
NUDT2_2_12812,391,640,356,474,493,640,1448,448,783,405,514,166
NXN_2_12813,216,361,297,261,154,446,1069,802,277,903,163,280
OAS1_2_12814,132,20,98,75,3,0,0,637,5,0,0,301

OAS2_2_12815,282,242,112,149,91,177,148,231,253,129,564,58
OASL_2_12816,1811,1545,1243,1682,2652,439,1784,788,1177,1891,833,1281
OAT_2_12817,255,208,164,192,14,199,451,209,73,111,114,28
OGDH_2_12818,296,242,213,281,672,524,811,214,321,202,188,497
OGDHL_2_12819,89,145,66,148,0,0,0,4,3,0,0,235
OGG1_2_12820,253,60,262,180,141,121,10,92,60,4,37,348
OGT_2_12821,1265,1202,888,1070,442,1363,1208,500,834,858,1514,1108
OLAH_2_12822,408,321,393,363,477,417,177,213,471,442,68,361
P4HA1_2_12823,176,260,246,287,12,227,10,23,358,35,12,281
P4HA2_2_12824,23,78,70,35,0,9,2,66,67,350,93,4
P4HTM_2_12825,176,102,49,116,0,1,0,264,361,123,489,236
PAFAH1B2_2_12826,743,1026,762,776,1093,876,682,763,1376,649,798,1052
PAFAH1B3_2_12827,114,103,75,116,0,28,139,15,19,59,328,32
PAICS_2_12828,363,352,301,339,45,197,495,189,384,266,658,384
PAOX_2_12829,553,441,513,642,647,455,634,838,275,1675,159,1128
PAPD4_2_12830,875,663,770,883,1197,1414,1973,685,325,1812,552,1425
PAPD5_2_12831,1481,1594,1189,1513,948,1875,3203,981,367,1167,795,1587
PAPD7_2_12832,78,40,99,40,52,22,950,54,81,30,0,111
PAPSS2_2_12833,462,510,576,789,569,464,319,861,945,292,689,448
PARK7_2_12834,225,317,400,214,313,299,453,212,343,99,15,205
PARL_2_12835,136,175,279,355,200,268,195,115,214,187,549,172
PARN_2_12836,37,25,61,34,0,1,311,62,32,23,0,29
PARP2_2_12837,853,631,744,973,1451,628,763,1492,917,467,1091,872
PARP3_2_12838,339,295,364,370,191,152,254,271,214,918,260,411
PARP8_2_12839,61,32,29,42,44,0,0,7,14,0,0,93
PBLD_2_12840,16,13,18,25,0,13,22,79,3,0,151,17
PCCA_2_12841,18,40,26,56,0,1,30,0,244,0,0,170
PCCB_2_12842,111,124,130,83,2,26,30,361,52,44,8,280
PCMTD2_2_12843,1102,1299,957,1178,1164,1460,816,1183,1153,1118,477,107
8
PCNA_2_12844,364,718,505,721,105,338,216,385,48,291,619,710
PCSK1_2_12845,115,49,29,101,320,27,57,40,70,229,18,283
PCSK2_2_12846,356,382,224,403,0,506,0,550,656,237,974,569
PCYT1B_2_12847,979,929,955,1033,728,958,1328,1029,1089,998,511,1073
PCYT2_2_12848,65,86,59,49,212,0,239,25,12,1,190,1
PDE10A_2_12849,29,23,26,109,261,0,4,328,0,34,0,2
PDE11A_2_12850,211,240,184,190,388,8,331,206,198,328,157,150
PDE1A_2_12851,494,437,468,516,1058,183,861,524,308,629,516,1107
PDE1B_2_12852,22,62,8,64,265,0,39,391,0,224,0,167
PDE1C_2_12853,876,1003,831,1075,1677,1091,875,609,1606,551,371,822
PDE2A_2_12854,394,466,514,668,610,1072,151,1,323,820,1624,1783
PDE4A_2_12855,84,40,37,73,0,0,0,0,7,0,131,3
PDE4B_2_12856,487,458,305,435,117,115,601,227,227,446,50,251
PDE4C_2_12857,1,0,7,7,0,1,0,0,0,0,0,6
PDE4D_2_12858,4521,4429,4149,5018,4247,3563,3047,6379,4077,5360,3213,5
917
PDE5A_2_12859,100,30,58,99,173,53,6,90,113,8,30,1
PDE6B_2_12860,65,5,63,115,3,0,0,0,1,23,0,56
PDE7A_2_12861,310,187,125,140,0,431,260,263,12,425,280,199
PDE8A_2_12862,521,440,493,504,173,243,337,260,620,443,124,550

PDE8B_2_12863,2247,1973,1649,1724,2069,2379,2026,1114,2491,1500,3073,1
614
PDE9A_2_12864,352,596,606,460,324,417,481,472,308,167,76,419
PDHA1_2_12865,1505,918,1173,1165,1497,1018,1075,1996,1268,953,1703,954
PDHB_2_12866,901,468,728,729,714,285,1928,379,941,164,346,1106
PDHX_2_12867,219,281,226,357,412,785,1444,40,211,536,2023,393
PEMT_2_12868,211,171,113,214,579,38,124,0,20,208,2,405
PEPD_2_12869,30,23,1,7,2,3,14,0,1,47,0,89
PGAM5_2_12870,635,768,855,705,470,797,466,366,1415,737,1505,1223
PGBD1_2_12871,682,701,450,755,406,1103,512,562,531,560,402,506
PGC_2_12872,438,407,449,446,0,6,6,30,440,298,339,482
PGM1_2_12873,119,125,114,166,2,44,327,761,213,274,71,57
PGM3_2_12874,641,505,525,588,2133,610,174,7,1233,1211,481,1075
PHOSPHO1_2_12875,21,60,57,63,0,2,1,1,0,32,0,253
PHOSPHO2_2_12876,408,221,224,388,264,51,541,256,585,311,35,734
PHYH_2_12877,375,310,251,285,211,193,33,109,574,394,199,405
PIGA_2_12878,1062,860,1019,1077,2481,1063,1327,815,752,574,956,1000
PIGC_2_12879,286,199,168,298,325,234,0,15,0,30,24,947
PIGF_2_12880,486,439,267,485,4,571,113,341,278,121,162,289
PIGG_2_12881,264,198,136,197,84,58,22,11,38,12,237,124
PIGN_2_12882,296,238,365,424,317,93,819,114,231,604,696,395
PIGO_2_12883,7,19,4,51,0,0,0,0,0,0,0,2
PIGP_2_12884,243,418,259,532,91,109,1012,379,183,448,260,328
PIGQ_2_12885,199,199,106,233,109,132,234,59,526,62,304,398
PIGT_2_12886,14,1,17,6,31,24,0,18,0,0,0,52
PIGV_2_12887,167,210,224,232,0,395,13,7,5,874,235,8
PIN4_2_12888,484,267,421,489,512,313,965,608,246,502,489,673
PLA1A_2_12889,524,428,391,466,345,183,152,202,589,199,606,687
PLA2G2A_2_12890,0,10,10,133,0,0,29,0,0,0,4,0
PLA2G4C_2_12891,216,81,183,297,5,278,1137,634,221,74,0,199
PLA2G6_2_12892,48,37,35,25,20,0,0,0,17,170,1,42
PLA2G7_2_12893,0,0,0,0,0,0,0,0,0,0,0,0
PLAT_2_12894,109,166,105,276,2,58,442,228,20,243,37,482
PLAU_2_12895,329,431,428,490,461,39,633,547,188,1014,867,327
PLB1_2_12896,469,408,419,486,639,17,1084,307,495,571,480,338
PLCB1_2_12897,240,146,179,158,595,81,61,148,202,72,126,263
PLCB3_2_12898,819,473,495,720,1199,396,867,252,1505,871,973,515
PLCB4_2_12899,274,236,319,398,20,1243,63,2034,213,13,39,632
PLCD1_2_12900,34,30,61,114,2,96,114,2,35,4,79,76
PLCE1_2_12901,659,462,467,682,843,364,63,141,304,511,458,636
PLCG1_2_12902,68,29,37,68,168,85,0,53,59,71,2,42
PLCH1_2_12903,467,479,254,512,291,568,785,1085,640,699,11,320
PLCL2_2_12904,210,241,122,162,572,376,48,33,149,198,364,334
PLCXD2_2_12905,413,366,369,355,336,62,616,315,104,224,644,303
PLD2_2_12906,373,267,249,424,262,141,75,615,403,94,382,117
PLD3_2_12907,40,6,22,21,7,0,0,8,123,0,1,45
PLOD2_2_12908,957,915,719,967,310,933,1534,934,274,1179,992,1131
PLSCR3_2_12909,733,680,612,913,747,548,475,775,849,973,405,997
PLSCR4_2_12910,309,505,247,483,803,881,550,454,265,1354,513,266
PMEL_2_12911,327,226,248,280,5,96,40,6,231,376,483,157

PMS1_2_12912,337,580,441,489,189,60,770,660,336,674,759,597
PNPLA1_2_12913,6,52,85,39,0,0,0,0,0,83,13,3
PNPLA4_2_12914,31,7,3,27,26,0,0,0,27,0,0,9
PNPLA6_2_12915,4,8,2,14,44,56,0,0,0,0,0,0
PNPLA7_2_12916,191,135,94,134,42,26,252,113,88,49,8,73
POFUT1_2_12917,228,223,193,341,134,305,435,118,128,418,338,414
POFUT2_2_12918,2,150,64,71,58,20,0,0,170,0,561,0
POGZ_2_12919,120,119,123,246,196,206,3,650,518,44,92,126
POLD2_2_12920,36,21,39,22,0,64,0,0,2,5,0,2
POLE2_2_12921,350,199,372,407,57,135,157,467,174,52,66,332
POLG_2_12922,0,0,0,0,0,0,0,0,0,1,0,0
POLL_2_12923,180,237,265,279,516,435,0,1,158,266,0,0
POLR1D_2_12924,541,505,484,647,338,680,1070,2,1057,88,249,1420
POLR3B_2_12925,320,259,245,350,409,52,875,252,288,342,878,940
POLR3H_2_12926,247,317,292,372,56,375,213,479,21,659,152,893
POMT1_2_12927,13,66,43,27,13,0,23,1,274,54,0,92
PON2_2_12928,141,91,153,133,535,214,14,173,169,398,538,9
POP5_2_12929,24,5,29,39,0,0,0,0,117,34,0,0
PPA2_2_12930,645,527,394,555,701,707,604,464,276,720,62,578
PPIE_2_12931,575,364,391,458,141,941,109,13,30,127,185,974
PPIL2_2_12932,123,167,76,206,47,117,179,31,47,22,0,111
PPIL3_2_12933,128,178,197,181,25,192,91,160,227,677,9,409
PPIL6_2_12934,110,89,62,149,0,34,6,0,1,92,16,330
PPIP5K1_2_12935,160,150,191,172,573,20,396,73,263,864,0,327
PPT1_2_12936,414,192,308,343,188,283,332,253,142,997,364,314
PPT2_2_12937,457,565,534,682,246,717,1054,287,806,138,937,393
PRCP_2_12938,97,70,55,85,5,30,106,16,35,0,3,14
PRDX1_2_12939,261,153,127,136,308,155,306,52,113,4,0,213
PRDX3_2_12940,623,593,676,862,832,360,594,1103,1094,559,275,523
PRDX5_2_12941,122,179,99,195,10,122,0,0,218,98,98,83
PRKCSH_2_12942,39,146,205,176,76,270,0,0,19,530,0,85
PRODH_2_12943,12,7,22,34,15,0,6,191,0,35,0,0
PRSS3_2_12944,7,7,7,0,15,0,2,0,8,0,2,0
PSAT1_2_12945,228,184,192,296,83,172,51,24,11,208,543,219
PSMA1_2_12946,268,203,154,248,20,185,224,410,395,178,175,479
PSMA3_2_12947,901,1008,878,1023,734,32,2367,783,791,1190,544,1132
PSMA4_2_12948,1694,1384,1853,2144,2409,832,1769,2201,1558,1158,1876,18
38
PSMA5_2_12949,455,280,233,406,314,1,108,122,602,401,89,581
PSMA8_2_12950,1304,914,1310,1359,1518,470,1338,1099,1037,1029,2023,100
0
PSMB2_2_12951,211,66,116,141,169,138,5,6,223,32,0,308
PSMB5_2_12952,43,86,99,175,3,227,0,8,18,43,0,379
PSMB8_2_12953,275,166,75,252,100,0,0,652,139,7,25,3
PTBP1_2_12954,194,77,163,112,141,70,71,6,27,9,0,149
PTER_2_12955,1539,1110,1330,1592,1398,564,2338,1246,1415,1592,1201,135
6
PTGR1_2_12956,143,60,76,87,260,42,161,197,42,25,0,111
PTGR2_2_12957,766,343,462,760,801,221,1005,704,469,359,452,869
PTGS1_2_12958,58,71,82,138,0,162,87,90,230,117,0,82

PUS1_2_12959,974,1042,946,1020,472,677,1778,786,1205,1148,732,1144
PYCR1_2_12960,272,191,160,183,179,306,0,186,0,434,461,188
PYGL_2_12961,64,85,52,44,0,34,0,16,4,148,308,86
PYGM_2_12962,200,251,111,286,453,23,121,0,30,308,15,433
QPCTL_2_12963,35,39,40,212,25,21,0,45,17,63,287,80
QSOX1_2_12964,185,317,119,104,11,303,18,6,103,457,526,12
RAB11A_2_12965,315,276,312,478,280,13,0,0,1003,2,49,513
RAB1A_2_12966,85,24,46,27,69,0,1,0,39,1,0,76
RAB23_2_12967,50,50,76,92,43,11,56,29,36,72,0,327
RAB27A_2_12968,736,723,821,946,958,1570,442,739,783,761,1113,1335
RAB28_2_12969,501,357,309,388,467,479,261,306,413,146,125,260
RAB2A_2_12970,727,624,757,665,435,444,734,565,710,690,226,586
RAB2B_2_12971,170,160,110,222,33,414,0,452,401,40,729,97
RAB34_2_12972,0,0,0,0,0,0,0,0,0,0,0,0
RAB35_2_12973,141,232,226,354,367,316,200,14,114,500,645,525
RAB37_2_12974,69,6,2,26,1,0,7,89,0,0,0,0
RAB40C_2_12975,161,147,155,132,32,66,87,263,119,136,350,300
RAB5C_2_12976,156,163,106,134,76,231,305,19,38,14,192,561
RAB6A_2_12977,1169,1300,782,1277,1524,1031,1371,904,646,948,1363,1229
RAB7L1_2_12978,54,102,34,58,10,6,297,37,82,159,46,32
RAB9A_2_12979,400,483,250,338,275,111,183,41,469,845,299,645
RABGGTA_2_12980,237,202,120,323,158,3,135,144,0,322,1032,232
RABL2A_2_12981,18,53,133,128,50,1,16,0,70,52,11,39
RABL2B_2_12982,18,53,133,128,50,1,16,0,70,52,11,39
RAC1_2_12983,385,294,261,290,265,339,270,375,483,72,199,366
RAD51B_2_12984,834,608,592,798,1236,395,1760,610,979,919,449,683
RAD51_2_12985,559,609,554,522,247,242,888,155,394,568,189,310
RAD51D_2_12986,46,24,67,43,76,0,51,9,19,0,0,62
RAD54B_2_12987,168,129,289,190,81,114,1,2,8,253,29,400
RAD54L_2_12988,41,17,17,26,0,0,0,6,104,37,12,2
RAD9A_2_12989,107,116,115,184,235,3,66,26,385,41,9,14
RAP1A_2_12990,651,835,814,886,24,825,1964,1042,414,1193,723,1211
RAP1B_2_12991,639,643,519,592,950,435,1137,844,616,916,413,579
RBBP8_2_12992,424,340,209,407,0,504,0,314,0,3,0,211
RDH13_2_12993,29,25,64,25,285,8,97,90,0,0,1,137
RDH5_2_12994,134,44,70,202,41,99,257,89,19,16,90,175
RECQL5_2_12995,280,272,453,440,514,525,971,1,130,390,51,406
RECQL_2_12996,512,459,613,742,332,587,218,86,681,524,370,566
RERG_2_12997,131,49,102,76,14,636,20,58,212,3,258,104
REV1_2_12998,332,343,341,393,130,452,520,949,399,825,440,222
RFC3_2_12999,202,159,244,245,622,275,300,1119,79,424,0,447
RFC5_2_13000,274,172,209,185,11,62,29,49,273,51,116,355
RGN_2_13001,584,469,630,713,902,1261,627,380,181,313,311,163
RGS11_2_13002,513,411,311,331,331,147,486,729,365,158,57,724
RGS6_2_13003,233,336,177,233,219,147,144,393,477,73,158,117
RHOC_2_13004,415,893,933,735,848,53,1595,754,777,603,395,354
RHOT1_2_13005,192,118,223,122,110,76,491,13,97,1,0,276
RNASE1_2_13006,69,188,153,107,111,14,2,116,0,556,0,19
RNASE4_2_13007,24,3,47,40,4,0,0,206,74,1,2,0
RPAP3_2_13008,415,314,263,419,142,30,408,26,200,88,515,298

RPE_2_13009,358,364,187,369,240,345,170,542,253,27,2,346
RPN2_2_13010,314,235,228,256,472,130,430,151,11,237,29,311
RPP14_2_13011,457,270,433,435,178,4,1431,336,286,60,95,527
RPP21_2_13012,55,64,90,82,0,0,18,5,2,17,0,303
RPP30_2_13013,470,245,317,563,4,241,711,609,130,0,495,298
RPP38_2_13014,241,307,251,249,181,336,483,1104,597,163,512,466
RPS27A_2_13015,392,298,451,402,1420,306,504,159,401,498,847,7
RRAD_2_13016,170,216,166,84,64,23,139,90,80,377,349,114
RRAGB_2_13017,872,584,837,775,688,998,721,573,1031,1495,432,1735
RRAS2_2_13018,525,467,405,372,192,126,701,28,198,479,0,291
RRM2B_2_13019,370,609,448,517,58,111,627,1022,463,12,633,296
RRM2_2_13020,48,24,16,37,113,0,0,0,0,424,0,8
RTEL1_2_13021,97,106,75,132,472,195,20,97,2,107,8,181
SAR1A_2_13022,75,47,40,59,7,0,134,382,63,0,35,42
SAR1B_2_13023,464,366,374,437,329,72,468,612,256,853,330,699
SARDH_2_13024,136,174,200,164,315,38,97,248,209,369,135,491
SARS2_2_13025,181,112,203,103,602,488,201,7,219,130,365,33
SBNO1_2_13026,26,71,37,59,2,0,0,12,3,0,0,203
SC5DL_2_13027,566,459,509,595,1228,538,705,535,445,254,829,396
SCD5_2_13028,801,577,622,719,753,495,347,452,881,568,526,785
SDC1_2_13029,151,173,45,153,1,3,1,68,0,2,0,143
SDCBP_2_13030,348,330,363,382,126,454,128,304,172,557,563,189
SDHC_2_13031,691,636,478,794,967,162,441,1534,594,479,588,1189
SEPHS1_2_13032,31,58,5,7,0,2,0,1,13,0,0,19
3-Sep_2_13033,47,75,68,168,33,11,20,163,101,33,3,29
4-Sep_2_13034,103,45,68,121,11,43,98,3,37,126,3,24
5-Sep_2_13035,106,86,101,124,45,12,40,538,57,180,188,163
9-Sep_2_13036,573,508,652,1031,451,946,420,636,45,8,920,1470
SGMS2_2_13037,241,157,125,141,102,69,27,124,88,42,5,175
SH3GLB1_2_13038,341,378,430,408,368,394,359,464,603,274,281,105
SHMT1_2_13039,267,256,193,331,284,640,540,503,305,292,288,285
SHMT2_2_13040,0,1,6,20,0,7,0,0,0,0,0,0
SIAE_2_13041,1111,1199,1363,1792,1194,751,1580,692,660,1388,1239,2397
SLFN11_2_13042,77,92,106,205,10,9,278,57,56,66,25,47
SMG6_2_13043,132,117,95,121,41,41,14,515,24,511,20,152
SMOX_2_13044,208,111,176,105,207,0,114,2,172,48,21,256
SMPD1_2_13045,325,223,321,251,66,9,522,60,189,245,70,442
SMPD4_2_13046,113,147,153,90,41,503,262,47,7,11,0,157
SMPDL3B_2_13047,333,187,230,457,307,172,619,69,577,54,205,8
SOD2_2_13048,227,218,169,182,345,28,124,335,209,80,340,159
SPAM1_2_13049,1903,1739,1750,1947,2070,996,2134,1419,1492,1574,2955,23
95
SPAST_2_13050,774,832,797,958,950,102,1648,1845,667,931,528,971
SPG21_2_13051,245,151,147,192,178,1,156,41,60,816,161,42
SPO11_2_13052,954,815,806,903,461,427,187,1044,726,1256,926,1072
ST3GAL1_2_13053,33,24,49,4,0,0,11,0,0,0,98,23
ST3GAL5_2_13054,393,637,654,841,626,296,773,1086,274,169,106,254
ST5_2_13055,234,150,156,269,119,75,510,209,459,254,146,152
ST6GAL1_2_13056,488,287,325,514,209,256,415,262,428,184,877,343
ST6GAL2_2_13057,613,491,609,485,1061,344,822,616,227,758,331,580

ST6GALNAC3_2_13058,2222,1791,2028,2243,2327,1684,2078,1851,3267,1289,2496,2695
ST6GALNAC4_2_13059,161,101,41,110,309,0,17,2,4,528,73,63
ST8SIA4_2_13060,371,416,271,417,244,2,868,123,444,706,544,264
SUCLG2_2_13061,366,114,120,363,192,69,264,1030,0,263,122,534
SULF1_2_13062,118,44,47,126,265,1,93,0,1,77,125,101
SULF2_2_13063,24,88,28,47,36,2,176,6,38,4,0,0
SULT1A1_2_13064,2419,2432,2415,2709,2539,5091,2834,2646,3121,2215,3178,3090
SULT1A2_2_13065,347,541,327,461,662,118,170,490,250,116,775,570
SULT2B1_2_13066,42,22,4,27,0,0,1,0,0,2,21,209
SUOX_2_13067,75,177,35,103,0,0,0,3,1,0,437,0
SYTL1_2_13068,25,77,66,47,0,0,8,12,98,30,3,47
TAB1_2_13069,417,356,212,363,96,65,87,42,763,897,10,196
TBXAS1_2_13070,822,647,759,934,523,591,470,488,644,358,1203,603
TCIRG1_2_13071,69,31,52,32,52,0,7,12,9,0,49,35
TDP1_2_13072,218,390,157,255,755,765,532,0,465,17,849,6
TERT_2_13073,14,17,30,35,12,0,70,9,21,95,14,24
TGM2_2_13074,112,110,164,205,291,75,754,250,326,0,22,15
TGM5_2_13075,574,622,564,609,902,831,11,363,719,1042,838,867
TH_2_13076,166,108,90,109,150,17,176,6,3,452,960,46
TIAM2_2_13077,173,154,244,252,87,173,119,160,334,39,523,252
TKTL1_2_13078,49,55,69,129,266,9,14,0,62,18,542,75
TMEM55B_2_13079,1539,1604,1647,1598,1818,2911,2666,1733,1653,2154,1202,939
TMLHE_2_13080,273,370,406,561,1231,0,0,299,141,0,979,218
TMOD1_2_13081,2103,1639,1815,1914,2272,2299,3577,1304,2092,965,952,3005
TMX2_2_13082,537,329,517,439,401,186,1298,343,647,702,400,714
TNNI2_2_13083,359,246,250,350,149,227,22,640,64,120,12,419
TP53I3_2_13084,245,155,200,290,92,135,260,31,22,449,326,3
TPI1_2_13085,332,237,343,522,251,18,494,473,392,339,163,207
TPST2_2_13086,105,177,104,117,332,230,0,330,16,10,367,66
TREX1_2_13087,51,17,40,64,0,1,10,38,28,22,0,64
TRMT1_2_13088,698,537,658,499,276,1466,969,365,626,425,3087,688
TRMT1L_2_13089,45,38,15,38,41,1,332,5,85,25,0,28
TRMT2B_2_13090,265,269,276,359,104,389,111,36,393,250,246,266
TRPT1_2_13091,516,645,431,747,3,520,118,604,539,1573,458,351
TSEN2_2_13092,704,897,688,698,426,1471,78,511,361,1192,565,951
TSEN34_2_13093,557,980,676,806,535,1129,671,744,313,953,1380,532
TTLL6_2_13094,156,176,147,167,160,1,132,836,95,162,22,468
TUBAL3_2_13095,154,368,316,303,271,186,315,225,223,235,914,997
TUSC3_2_13096,545,754,849,984,527,283,386,812,1028,710,1930,1038
TXNDC16_2_13097,706,1009,717,986,700,966,679,1381,545,1533,483,907
TXNDC5_2_13098,249,75,254,272,7,6,173,12,48,0,2,170
TXNL4B_2_13099,32,32,17,24,18,57,49,1,53,64,0,2
TXNRD1_2_13100,1005,924,807,929,772,1711,1799,1197,654,1449,408,874
TXNRD3_2_13101,1911,1723,1661,1480,1644,1775,1110,2229,2590,1238,1810,1242
TYMP_2_13102,606,524,459,499,358,280,206,387,499,493,200,420

UBA52_2_13103,301,285,294,296,18,206,703,121,156,211,798,277
UEVLD_2_13104,42,67,36,42,8,81,0,0,21,298,0,25
UGDH_2_13105,55,32,67,72,416,5,0,5,125,55,154,112
UGP2_2_13106,483,349,390,733,898,377,61,242,677,603,349,409
UGT1A6_2_13107,2262,2364,2645,2734,3398,1101,2548,2834,2048,3123,3115,
2144
UGT2B10_2_13108,1603,1101,1205,1548,2314,804,1494,1756,2009,1023,2223,
1150
UGT2B28_2_13109,658,526,671,485,758,560,801,719,833,602,152,591
UGT3A1_2_13110,209,150,327,238,471,20,620,306,271,312,184,31
UGT8_2_13111,115,128,79,146,80,19,31,0,306,101,404,87
UNG_2_13112,66,46,28,53,31,214,0,242,4,0,0,150
UPP1_2_13113,130,220,254,380,2,1,0,432,366,396,814,165
UPP2_2_13114,120,73,42,63,2,39,28,51,262,3,45,69
UQCR10_2_13115,226,85,88,88,26,12,12,0,114,12,124,1
UQCRB_2_13116,356,461,421,487,178,804,319,326,333,393,329,866
UROC1_2_13117,69,239,241,136,0,17,21,67,0,60,25,117
VCL_2_13118,29,37,2,21,0,0,22,0,0,9,0,2
VNN2_2_13119,1185,865,668,1202,681,941,850,1133,1476,597,1541,1320
VPS29_2_13120,1427,1278,1441,1949,1236,839,1249,797,1612,2092,1505,207
0
WARS2_2_13121,428,367,278,426,589,332,328,457,1342,194,396,296
WARS_2_13122,306,365,203,345,155,185,210,321,23,129,184,350
WBSCR22_2_13123,301,382,301,349,49,371,668,499,358,387,465,390
WDR46_2_13124,398,706,422,520,532,518,490,675,446,254,258,706
WFS1_2_13125,199,302,295,416,234,237,367,314,319,721,386,244
WRNIP1_2_13126,567,697,534,562,203,447,1423,270,130,258,377,122
WWOX_2_13127,102,59,75,150,419,0,158,9,3,44,212,43
XPNPEP1_2_13128,509,439,505,529,312,801,423,492,537,741,747,323
XRCC3_2_13129,94,29,84,66,36,60,5,127,83,132,7,18
XRN1_2_13130,202,300,194,291,255,561,66,214,104,701,509,537
YWHAZ_2_13131,228,163,213,203,244,45,700,435,509,86,5,226
ZBED1_2_13132,372,390,355,534,491,361,0,723,146,23,1927,546
ZCCHC11_2_13133,820,673,567,658,842,428,783,1223,1127,664,1375,1278
ZCCHC6_2_13134,512,524,466,502,203,346,602,917,918,1002,621,483
ZDHHC15_2_13135,315,157,203,236,213,9,104,173,641,7,14,243
ZDHHC7_2_13136,125,152,86,155,26,2,118,16,27,43,219,13
ACYP1_2_13137,596,419,382,285,547,138,1334,508,442,630,404,252
APOBEC3F_2_13138,1220,784,841,1360,1803,794,770,1290,1243,567,1638,114
2
ASCC3_2_13139,285,156,250,356,380,22,165,506,0,115,643,627
ASPH_2_13140,488,241,332,490,321,354,3,816,48,38,993,952
ATP6V0E2_2_13141,157,201,108,132,35,0,29,48,61,109,718,9
C10orf2_2_13142,135,129,87,98,1,1,100,492,88,31,543,60
CAPN3_2_13143,295,138,235,180,11,147,0,0,72,0,454,247
CHM_2_13144,158,267,209,202,14,203,379,167,303,40,0,0
CTSC_2_13145,338,543,573,501,516,668,676,445,54,761,529,686
CYBRD1_2_13146,878,870,891,949,780,1012,591,1164,908,1675,1940,575
CYP3A5_2_13147,1950,1735,1797,2647,2376,1362,1062,1708,2979,1332,1795,
1918

DIO2_2_13148,232,158,200,308,111,14,112,279,80,113,280,112
DNASE2B_2_13149,751,717,740,982,1638,583,1466,1418,116,1338,405,296
DPYD_2_13150,177,154,120,185,104,73,233,35,346,78,678,482
GBA3_2_13151,148,120,135,191,356,371,130,32,222,53,425,463
GCNT2_2_13152,188,86,62,107,60,71,93,59,200,21,0,83
GLYAT_2_13153,290,265,174,194,262,186,966,10,209,45,21,176
GPLD1_2_13154,180,197,132,189,131,87,33,0,209,3,0,26
GPX1_2_13155,0,0,0,0,0,0,0,0,0,0,0,0
GPX5_2_13156,2705,2137,2178,2876,2529,1144,1854,2470,3042,2192,2799,23
20
HNMT_2_13157,156,117,31,108,10,0,330,11,23,2,92,179
HOGA1_2_13158,234,308,271,150,564,12,64,29,0,136,523,10
HYAL3_2_13159,75,148,49,147,0,0,87,271,168,14,116,149
JPH2_2_13160,31,47,69,38,0,6,0,0,0,1,0,4
KLK8_2_13161,464,494,395,398,1566,336,475,205,271,250,775,726
LAMA4_2_13162,30,23,33,79,2,17,183,70,78,239,66,7
LRR1_2_13163,520,407,559,341,518,453,984,64,724,126,62,331
LRTOMT_2_13164,279,138,206,245,77,377,587,333,210,189,100,263
METTL1_2_13165,333,394,163,452,301,299,354,104,467,346,53,448
MOCS2_2_13166,753,839,647,1100,694,1312,490,591,608,349,1123,806
NDUFA11_2_13167,8,21,34,52,128,0,24,0,8,4,0,26
NDUFV3_2_13168,85,58,183,39,6,304,43,18,56,291,0,33
NMNAT3_2_13169,131,118,205,230,179,243,754,55,138,55,469,117
PDE4DIP_2_13170,68,48,132,77,23,5,49,1,20,0,0,32
PLG_2_13171,417,779,551,653,829,1061,4,469,420,504,155,1197
PPCS_2_13172,915,693,1001,834,928,697,829,558,1009,850,305,823
PRDX2_2_13173,73,48,67,55,350,29,247,4,15,44,17,189
RAD51C_2_13174,741,434,342,783,609,113,250,449,467,501,433,694
RASD1_2_13175,47,43,34,26,40,86,0,0,4,0,5,302
RASL10A_2_13176,6,88,50,66,586,1,0,350,156,6,0,2
SLX1A_2_13177,552,233,271,660,65,330,73,214,496,43,0,1022
SLX1B_2_13178,552,233,271,660,65,330,73,214,496,43,0,1022
SPTLC1_2_13179,978,978,901,885,816,854,2838,996,1231,2143,651,623
ST3GAL3_2_13180,108,119,64,66,59,23,21,14,12,176,171,5
VKORC1_2_13181,2340,2031,2089,2157,2387,2057,1716,1521,2625,1873,2176,
1651
XPNPEP3_2_13182,1547,1835,1616,1474,1410,913,1755,1220,910,1830,1691,1
314
GNAS_2_13183,108,106,122,156,74,362,0,72,159,0,9,326
GNAS_2_13184,643,394,413,528,278,432,823,471,539,491,244,335
GNAS_2_13185,12,35,17,87,16,0,0,56,0,36,21,12
A4GALT_2_13186,64,106,109,169,123,0,165,288,196,22,98,143
A4GNT_2_13187,478,342,498,429,374,487,543,313,471,738,126,525
AACS_2_13188,8,40,42,4,0,0,0,108,37,2,391,17
AADAC_2_13189,515,435,463,416,103,582,549,794,752,594,175,681
AARS2_2_13190,294,91,212,190,414,42,243,0,134,163,6,226
AARS_2_13191,704,651,662,792,1308,769,389,992,653,1766,447,491
AASDH_2_13192,165,217,157,174,28,106,694,163,114,305,2,7
AASDHPPT_2_13193,308,179,163,244,30,17,36,520,111,87,603,8
AASS_2_13194,444,372,435,335,113,581,199,705,372,317,229,279

ABHD14A_2_13195,44,209,186,289,0,298,0,179,132,0,817,294
ABHD1_2_13196,122,130,108,97,2,8,28,391,158,263,83,18
ABHD3_2_13197,331,550,589,502,607,35,630,785,742,18,846,441
ABHD5_2_13198,997,673,784,1015,844,548,431,1313,493,1423,345,726
ABHD6_2_13199,841,856,889,1080,994,289,658,862,808,1629,1218,1239
ABHD8_2_13200,77,11,107,29,3,318,48,3,0,137,0,90
ABO_2_13201,396,252,428,328,247,11,642,51,249,268,105,395
ABP1_2_13202,429,553,495,365,207,667,76,162,145,173,575,640
ACAA2_2_13203,188,385,230,253,293,47,0,567,276,280,196,772
ACACB_2_13204,159,238,171,228,263,284,0,274,23,156,2,128
ACAD11_2_13205,430,255,337,238,941,272,454,20,292,261,270,78
ACAD8_2_13206,26,55,52,14,7,0,14,0,74,0,0,0
ACAD9_2_13207,404,271,290,380,351,407,256,315,387,595,841,586
ACADL_2_13208,1474,1122,1576,1665,1292,952,3141,1455,1412,1348,2192,18
47
ACADSB_2_13209,2389,2556,1947,2157,2676,2162,2500,1657,2620,2563,1464,
1526
ACADS_2_13210,53,153,20,66,22,0,52,2,3,0,6,13
ACAT1_2_13211,399,234,203,270,221,266,96,261,332,476,32,579
ACER1_2_13212,255,97,63,275,1,0,2,24,10,316,0,257
ACER2_2_13213,543,419,292,473,565,257,641,3,659,75,729,407
ACER3_2_13214,742,621,586,677,816,975,356,851,714,1267,137,236
ACMSD_2_13215,912,731,953,915,554,896,371,372,813,1154,1953,1040
ACO1_2_13216,0,0,0,0,0,0,0,0,0,0,0,0
ACO2_2_13217,300,194,167,220,42,100,18,203,134,629,102,329
ACOT12_2_13218,87,88,98,77,163,3,96,49,46,93,477,162
ACOT1_2_13219,192,412,334,391,78,189,1126,382,15,512,418,203
ACOT2_2_13220,744,663,609,805,291,1300,1070,472,742,991,1074,1854
ACOT4_2_13221,774,824,823,716,554,1318,2288,635,917,957,536,644
ACOT6_2_13222,970,895,824,1080,559,832,519,1165,1117,2068,414,568
ACOT8_2_13223,64,33,22,83,142,8,630,235,46,54,5,191
ACOX2_2_13224,380,405,366,420,708,610,819,681,69,135,291,781
ACOXL_2_13225,1709,1428,1410,1353,1487,1368,2404,634,2077,1544,1840,14
20
ACR_2_13226,112,95,83,171,17,177,328,119,2,220,247,112
ACSBG2_2_13227,548,398,595,646,328,251,1052,666,549,237,1482,421
ACSF2_2_13228,1086,924,786,835,1017,681,628,854,1020,600,932,1007
ACSL1_2_13229,81,26,48,27,0,97,86,104,4,0,0,0
ACSM1_2_13230,85,110,95,49,61,62,103,30,26,279,14,315
ACSM2A_2_13231,1518,1588,1223,1471,1057,719,2379,906,1915,943,1649,225
3
ACSM4_2_13232,463,749,409,802,145,520,1249,115,763,69,969,325
ACSM5_2_13233,483,487,458,582,426,117,295,758,183,508,581,431
ACSS1_2_13234,25,17,34,41,1,46,0,0,67,19,2,179
ACSS3_2_13235,66,29,116,79,2,75,88,70,88,163,3,106
ACTC1_2_13236,129,112,214,164,252,130,342,27,343,92,141,179
ACY3_2_13237,90,241,149,254,5,198,286,0,108,289,0,106
ACYP2_2_13238,140,228,151,213,170,18,78,10,182,261,107,223
ADA_2_13239,5,0,3,0,0,0,0,0,0,0,0,0
ADAM10_2_13240,942,578,901,972,846,313,1532,263,467,818,1163,737

ADAM17_2_13241,0,0,0,0,0,0,0,0,0,0,0,0
ADAMTS4_2_13242,23,53,34,52,0,0,1,5,1,0,3,0
ADARB2_2_13243,96,363,132,207,79,484,127,215,44,594,5,186
ADAT1_2_13244,825,793,843,931,1397,340,759,640,707,1185,495,892
ADAT2_2_13245,1602,1376,1313,1642,1588,540,2349,842,1488,1880,706,2243
ADC_2_13246,157,66,151,226,78,204,372,384,2,137,50,293
ADCY1_2_13247,252,154,178,165,233,74,222,34,198,192,353,38
ADCY2_2_13248,1884,1491,1406,2016,2065,1585,2148,1766,1394,2145,2075,2
468
ADCY7_2_13249,451,276,349,413,94,519,1255,888,283,91,900,410
ADCY8_2_13250,1052,738,948,1033,1176,481,445,1078,772,665,851,837
ADCY9_2_13251,181,201,186,175,301,470,502,217,96,4,118,330
ADH1A_2_13252,1440,1329,1199,1480,641,1881,1141,1325,857,2274,922,870
ADH1B_2_13253,1430,980,1276,1215,423,1510,2067,718,1189,1203,891,1301
ADH1C_2_13254,1910,1875,2133,2175,2134,2256,3452,1368,1887,2065,2278,2
195
ADH4_2_13255,1337,1408,1300,1528,451,849,860,547,1438,2419,1618,1898
ADH5_2_13256,209,165,304,182,109,701,428,166,122,20,275,439
ADHFE1_2_13257,529,464,441,589,364,508,1508,1420,311,1428,428,970
ADI1_2_13258,365,223,295,424,315,201,433,82,414,236,557,388
ADO_2_13259,537,128,315,223,358,189,91,296,326,141,1082,939
ADPRH_2_13260,546,510,532,782,692,201,242,984,100,231,459,769
ADPRHL2_2_13261,224,371,391,342,16,773,488,19,423,716,608,411
ADSS_2_13262,395,367,387,407,164,93,346,370,334,415,23,576
AEN_2_13263,162,193,188,237,236,21,64,274,123,569,783,234
AGBL2_2_13264,345,351,173,275,97,208,551,258,229,66,227,495
AGMAT_2_13265,372,180,241,304,296,0,801,53,467,60,293,377
AGMO_2_13266,217,137,163,152,2,601,19,81,398,460,66,197
AGPAT4_2_13267,466,505,517,492,573,689,1176,686,486,848,959,174
AGPAT5_2_13268,765,998,783,1165,812,195,130,381,212,1146,2526,2443
AGPAT6_2_13269,69,80,72,50,133,51,402,1,8,2,119,45
AGPAT9_2_13270,547,679,620,490,639,998,1429,29,490,481,782,954
AGPS_2_13271,1902,1687,1585,1726,2073,1315,1377,2011,1505,2493,1477,18
42
AGXT2_2_13272,3014,2407,2651,2507,5042,2886,2636,2475,2283,3120,2304,2
694
AGXT2L2_2_13273,330,351,296,322,336,321,782,237,716,528,141,505
AGXT_2_13274,264,304,373,354,351,135,1042,342,340,137,213,134
AICDA_2_13275,50,48,136,212,11,172,0,441,49,23,70,36
AKR1B10_2_13276,84,98,70,119,0,65,356,44,190,154,199,93
AKR1B15_2_13277,525,612,496,488,654,610,420,319,791,535,226,628
AKR1B1_2_13278,30,7,11,23,0,0,0,6,39,0,0,1
AKR1C1_2_13279,933,674,820,815,296,779,891,347,414,227,1857,1046
AKR1C3_2_13280,1112,803,614,1102,451,830,992,494,1089,775,418,983
AKR1C4_2_13281,301,323,265,337,550,770,10,0,260,217,74,382
AKR1E2_2_13282,151,118,137,130,225,191,149,146,288,194,1,8
AKR7A2_2_13283,127,180,147,146,0,554,67,20,37,6,0,73
AKR7A3_2_13284,1129,888,745,1305,900,899,1017,1039,1208,1382,1083,1358
ALAD_2_13285,123,147,224,274,129,539,449,297,0,45,0,69
ALDH1A1_2_13286,173,57,15,92,4,0,81,7,0,5,0,3

ALDH1A3_2_13287,408,318,295,335,171,463,44,63,700,696,183,296
ALDH1B1_2_13288,470,362,447,526,21,298,874,381,129,331,432,1180
ALDH1L1_2_13289,162,323,110,212,749,287,79,44,4,408,141,174
ALDH1L2_2_13290,266,221,238,256,112,25,331,208,170,47,26,234
ALDH6A1_2_13291,40,37,26,16,58,194,4,20,14,17,393,8
ALDH9A1_2_13292,288,214,263,305,293,405,42,372,225,587,669,300
ALDOB_2_13293,1223,1450,1147,1405,1886,1394,1846,1587,1407,1405,2084,1
132
ALDOC_2_13294,352,345,633,537,196,200,286,68,271,1061,1882,362
ALG10_2_13295,975,809,981,867,1178,282,1086,16,641,317,722,1008
ALG11_2_13296,821,475,545,581,418,362,891,543,433,1038,397,538
ALG12_2_13297,93,83,56,72,10,269,53,71,245,0,519,0
ALG14_2_13298,1242,994,1146,1018,1792,1004,1815,817,541,1621,2062,1595
ALG1_2_13299,134,96,125,76,15,1,15,74,5,44,44,89
ALG2_2_13300,67,80,71,56,4,3,4,100,19,531,12,37
ALG6_2_13301,197,172,289,289,279,2,159,0,262,363,130,229
ALKBH8_2_13302,1377,1617,1555,1948,2368,596,2703,3534,1812,488,1455,16
51
ALLC_2_13303,313,302,338,405,228,408,347,200,239,96,145,775
ALOX12B_2_13304,1487,1028,988,1192,1222,1180,1582,1417,904,1495,1187,1
267
ALOX12_2_13305,43,68,39,84,0,0,0,14,0,22,0,65
ALOX15_2_13306,257,204,192,416,10,380,91,396,295,37,0,100
ALOX5_2_13307,610,704,708,746,1032,25,835,255,448,409,394,771
AMDHD1_2_13308,1105,1203,1107,1050,635,490,1908,2463,1126,343,1221,159
2
AMY1B_2_13309,732,610,604,644,949,588,1960,447,585,357,578,1321
AMY1C_2_13310,732,610,604,644,949,588,1960,447,585,357,578,1321
AMY2A_2_13311,1387,1484,1462,1771,1906,2356,1206,2071,1268,3235,2346,2
383
AMY2B_2_13312,1387,1484,1462,1771,1906,2356,1206,2071,1268,3235,2346,2
383
ANPEP_2_13313,0,96,47,88,0,4,254,18,43,0,0,3
AOC3_2_13314,2864,2459,2834,2898,1454,1341,4278,2317,2466,2567,3809,19
30
AOX1_2_13315,995,601,656,991,762,1203,866,904,706,349,1209,887
APEH_2_13316,111,27,50,48,113,3,2,91,347,288,101,69
APEX2_2_13317,56,12,45,67,0,1,0,51,21,21,89,25
APIP_2_13318,331,364,355,347,354,336,296,582,173,569,620,490
APLF_2_13319,383,290,328,302,481,158,46,214,556,216,16,914
APOBEC1_2_13320,247,334,212,246,122,4,73,0,41,424,571,142
APOBEC2_2_13321,25,3,14,29,94,84,74,34,38,204,17,5
APOBEC3B_2_13322,666,354,436,716,868,264,340,497,1009,315,403,643
APOBEC3C_2_13323,66,60,77,46,7,12,0,21,9,120,18,13
APOBEC3G_2_13324,1139,775,1020,1272,1835,984,1701,1286,1003,767,1419,8
44
ARF3_2_13325,11,12,77,22,0,132,73,1,24,4,0,69
ARF4_2_13326,1438,995,1238,1133,2017,660,1523,1051,1606,1491,468,642
ARG2_2_13327,454,381,469,654,327,230,148,474,718,522,304,105
ARHGEF10_2_13328,1463,1278,813,1171,1333,149,223,860,1044,476,1219,858

ARL3_2_13329,271,352,348,386,285,1102,156,87,773,825,116,445
ARL4C_2_13330,65,24,129,61,14,0,0,3,20,89,293,0
ARL4D_2_13331,14,17,15,26,146,14,0,3,94,14,0,61
ARL5B_2_13332,727,362,474,574,328,551,522,139,841,593,489,734
ARL8A_2_13333,23,3,2,105,1,0,0,7,0,0,175,1
ARL8B_2_13334,776,467,638,775,1013,310,881,765,607,460,2268,476
ARSD_2_13335,93,82,139,127,99,182,8,30,205,20,52,208
ARSE_2_13336,371,390,411,346,179,295,452,60,65,617,57,313
ARSG_2_13337,122,70,77,70,0,49,250,446,77,8,139,195
ARSH_2_13338,317,293,120,183,412,628,271,150,698,640,332,594
ARSI_2_13339,80,141,152,106,237,73,588,519,30,284,0,253
ARSJ_2_13340,1081,945,1009,1125,559,1655,370,1363,616,821,2011,1495
ARSK_2_13341,194,139,198,133,48,56,124,43,1,0,347,336
ART1_2_13342,493,423,406,441,584,394,143,288,423,649,243,509
ART4_2_13343,554,436,385,446,965,469,454,408,228,222,951,361
AS3MT_2_13344,579,567,629,753,525,189,1104,427,593,283,1242,643
ASNA1_2_13345,669,550,434,435,586,181,405,73,1050,1153,231,524
ASNSD1_2_13346,691,449,425,586,543,558,440,393,937,789,624,774
ASPG_2_13347,0,1,70,36,9,0,0,0,0,207,0,57
ASPHD2_2_13348,40,98,100,57,21,15,32,250,6,31,181,115
ATAD1_2_13349,75,38,40,44,0,3,0,2,128,60,0,29
ATIC_2_13350,158,137,75,94,72,35,25,11,6,90,456,0
ATP2C2_2_13351,219,103,143,179,11,65,99,248,26,212,286,224
AUH_2_13352,1041,885,1127,1155,1075,25,629,1302,841,573,1091,576
AWAT1_2_13353,448,605,796,514,160,218,898,50,601,659,1099,200
AWAT2_2_13354,309,175,233,268,368,131,12,153,16,195,884,408
B3GALNT2_2_13355,75,165,113,111,147,0,274,28,85,381,10,7
B3GALT1_2_13356,618,465,508,514,509,733,435,234,388,533,784,64
B3GALT2_2_13357,723,466,476,550,860,624,1276,494,446,698,585,785
B3GALT4_2_13358,673,509,433,586,693,668,404,707,1110,304,388,450
B3GALT6_2_13359,208,67,55,113,0,332,21,1,98,0,0,6
B3GAT2_2_13360,1504,1287,1428,1407,1662,829,925,539,1201,1681,1205,112
8
B3GAT3_2_13361,303,251,230,428,72,133,40,33,42,309,613,152
B3GNT2_2_13362,142,45,94,54,158,0,4,0,0,0,0,53
B3GNT3_2_13363,15,73,59,41,46,272,0,0,0,20,0,17
B3GNT4_2_13364,196,320,154,358,236,142,288,808,76,92,349,357
B3GNT5_2_13365,3085,2825,2772,2987,2850,1302,2311,4334,2144,2817,1760,
2187
B3GNT7_2_13366,28,60,17,22,1,0,6,6,86,7,0,20
B3GNT9_2_13367,978,701,885,1184,725,206,997,650,927,1313,303,381
B3GNTL1_2_13368,54,49,34,69,22,44,155,148,22,17,0,100
B4GALNT1_2_13369,133,115,108,99,0,217,237,117,11,0,126,227
B4GALNT3_2_13370,252,158,206,287,370,368,481,287,379,367,533,40
B4GALNT4_2_13371,155,244,256,407,0,24,298,413,48,265,622,1
B4GALT1_2_13372,358,214,481,328,64,544,364,795,738,52,693,708
B4GALT5_2_13373,698,552,502,669,628,68,830,97,854,327,562,780
B4GALT6_2_13374,2056,1231,1530,1622,1393,1423,2075,1405,826,1992,548,9
98
B4GALT7_2_13375,6,10,29,32,0,0,0,4,0,0,0,98

BBOX1_2_13376,165,255,172,255,405,454,152,111,83,741,110,343
BCHE_2_13377,261,248,178,214,56,154,835,436,504,147,301,223
BCMO1_2_13378,10,8,25,47,19,1,8,0,89,0,0,229
BDH2_2_13379,2374,2871,2407,2972,2016,1579,2322,3756,2395,3026,2240,20
04
BHMT_2_13380,224,43,144,121,250,17,2,48,759,138,22,210
BLM_2_13381,1417,918,1224,1648,2793,771,527,2456,932,1212,1417,1771
BLMH_2_13382,99,100,69,90,56,21,7,100,10,54,7,82
BLVRA_2_13383,114,100,77,61,507,0,0,17,111,37,312,180
BLVRB_2_13384,29,57,20,47,15,0,10,0,10,13,0,65
BPHL_2_13385,0,0,0,15,0,0,0,0,0,0,0,0
BST1_2_13386,556,382,520,444,812,227,668,349,404,12,288,692
BTD_2_13387,41,23,53,24,15,0,0,148,17,49,0,371
C12orf5_2_13388,330,150,208,216,234,43,925,234,191,82,647,197
C16orf79_2_13389,1158,1261,1035,1202,353,1628,1646,1152,959,1906,862,6
46
C18orf56_2_13390,159,231,66,185,193,107,326,160,40,356,208,206
C1GALT1_2_13391,548,381,378,568,745,279,128,96,547,164,1175,178
C1R_2_13392,9,33,36,22,0,0,0,18,0,0,0,31
C22orf28_2_13393,675,495,681,854,1097,438,533,655,546,1174,629,709
C2orf43_2_13394,233,332,293,256,843,101,498,0,331,618,152,81
C5orf4_2_13395,393,243,105,301,100,89,325,446,30,161,392,160
C6orf130_2_13396,186,230,203,413,294,71,0,389,285,527,65,389
CA13_2_13397,492,416,511,501,159,110,675,104,833,320,1518,681
CA14_2_13398,689,449,650,819,848,1459,728,700,605,535,310,875
CA2_2_13399,140,113,118,101,8,170,138,304,11,59,0,38
CA3_2_13400,212,101,194,106,5,4,0,237,1,137,25,524
CA4_2_13401,116,62,94,124,183,243,27,853,11,7,23,19
CA5A_2_13402,679,832,701,678,770,166,1140,845,297,793,1065,515
CA5B_2_13403,1183,904,939,1241,967,925,1513,1520,1772,1099,1443,1573
CA6_2_13404,6977,6053,6002,6261,5710,6472,5336,7488,5278,7214,6422,533
1
CA8_2_13405,195,173,172,148,341,98,561,102,338,292,34,114
CA9_2_13406,1,36,33,9,0,337,0,0,53,0,0,0
CARS2_2_13407,320,283,197,159,215,271,190,441,263,560,2,331
CASD1_2_13408,141,70,99,115,0,96,0,147,208,61,19,56
CAT_2_13409,203,187,198,159,84,29,43,210,44,48,64,582
CBR1_2_13410,1343,1641,1294,1496,1444,1132,1651,1988,1200,1341,1136,93
7
CBR3_2_13411,1512,880,949,1213,1599,787,1235,862,989,1246,674,876
CBR4_2_13412,240,171,231,331,380,515,467,257,248,67,0,358
CCDC88B_2_13413,30,86,53,84,50,237,29,0,1,67,2,209
CCDC92_2_13414,0,0,0,0,0,0,0,0,0,0,0,0
CCNO_2_13415,49,163,54,66,0,0,0,82,26,1,0,0
CCS_2_13416,11,67,11,45,166,0,46,31,3,67,0,0
CCT8_2_13417,895,633,823,899,471,223,142,1480,965,934,590,1010
CD274_2_13418,398,459,634,460,139,509,585,350,408,769,268,785
CD38_2_13419,213,402,274,239,22,1026,505,21,238,437,433,181
CDA_2_13420,318,283,519,348,766,390,1329,162,254,275,523,523
CDIPT_2_13421,255,294,185,224,24,29,621,341,36,710,149,549

CDO1_2_13422,29,2,13,66,0,0,0,16,49,0,0,214
CDS2_2_13423,300,346,455,367,19,10,225,367,126,361,285,585
CELA1_2_13424,175,96,67,113,49,284,129,156,377,27,0,152
CELA2A_2_13425,1,1,8,29,0,0,0,1,0,72,0,2
CELA2B_2_13426,104,112,105,79,124,31,427,0,7,250,0,98
CELA3A_2_13427,428,290,230,249,627,254,343,31,85,330,505,300
CELA3B_2_13428,347,412,320,348,259,56,674,736,61,139,136,428
CETN1_2_13429,680,430,318,518,271,436,476,790,144,33,186,105
CETN2_2_13430,278,160,238,165,67,418,176,266,463,275,220,146
CETP_2_13431,429,557,480,596,862,382,294,356,902,1183,462,983
CFB_2_13432,210,51,64,136,301,8,406,5,83,10,4,39
CFD_2_13433,140,81,43,63,3,46,183,32,118,0,77,100
CFI_2_13434,455,444,391,751,322,677,134,768,831,422,440,359
CH25H_2_13435,90,139,176,169,1,22,0,11,214,24,169,43
CHDH_2_13436,1,9,0,7,0,0,0,219,0,0,0,134
CHI3L1_2_13437,150,153,120,101,384,252,0,25,7,253,317,147
CHIT1_2_13438,150,156,169,217,121,245,12,11,339,65,94,392
CHML_2_13439,837,1222,840,764,874,632,789,1716,1041,842,944,488
CHPF2_2_13440,675,422,403,640,1222,310,643,262,206,112,856,26
CHPT1_2_13441,424,245,333,327,404,507,380,89,513,238,23,130
CHST10_2_13442,240,397,310,382,775,526,266,45,0,61,454,624
CHST12_2_13443,11,1,2,0,0,0,0,0,0,0,0,0
CHST13_2_13444,364,420,339,374,341,249,332,666,340,27,219,89
CHST14_2_13445,241,292,299,339,447,204,367,536,273,657,210,464
CHST1_2_13446,232,151,154,161,604,170,183,69,424,114,206,202
CHST2_2_13447,334,286,315,218,392,362,742,149,332,516,8,198
CHST3_2_13448,140,166,198,156,183,0,160,102,230,512,48,194
CHST5_2_13449,1441,1188,1075,1548,645,1549,1724,960,966,1163,1930,1251
CHST6_2_13450,206,106,148,129,2,2,504,343,76,15,425,154
CHST7_2_13451,3,0,10,24,0,0,0,0,0,0,0,0
CHST9_2_13452,2329,2266,2430,2441,1676,1284,3431,1741,2873,2072,2203,2
720
CHSY1_2_13453,586,377,251,347,198,1281,60,304,403,430,646,679
CHSY3_2_13454,275,279,268,217,267,150,1397,334,315,508,1034,377
CLPP_2_13455,42,20,42,107,39,33,2,0,205,23,0,16
CLPX_2_13456,749,681,676,625,315,1004,554,500,936,951,265,582
CLYBL_2_13457,73,54,72,163,46,15,0,118,36,31,0,332
CMA1_2_13458,23,38,58,80,6,25,21,34,158,60,0,21
CMAS_2_13459,462,383,483,511,844,63,555,930,494,561,951,582
CMBL_2_13460,66,63,32,90,124,0,37,8,16,4,0,106
CNDP1_2_13461,276,231,255,289,936,82,69,265,495,371,67,177
CNTN6_2_13462,822,741,595,665,191,190,281,73,702,678,296,852
COIL_2_13463,15,54,22,35,21,0,3,0,17,45,0,125
COMTD1_2_13464,161,287,210,267,239,462,97,326,48,127,150,511
COQ2_2_13465,2616,2186,1752,2811,3060,2821,3315,1833,1455,3574,2299,13
23
COQ3_2_13466,1139,772,766,972,801,971,707,176,719,461,1164,826
COQ5_2_13467,241,242,152,222,411,18,188,155,167,342,61,89
COX10_2_13468,16,17,40,54,49,2,0,11,281,5,1,2
COX17_2_13469,1117,1007,904,1182,714,737,568,784,1521,849,1084,1088

COX4I1_2_13470,423,408,378,424,306,236,333,300,408,247,407,342
COX4I2_2_13471,190,151,211,330,651,410,3,275,451,237,340,290
COX5A_2_13472,155,134,132,155,51,91,27,212,20,84,687,400
COX5B_2_13473,336,171,181,183,23,0,33,411,418,23,390,766
COX6A1_2_13474,36,37,74,48,10,69,120,13,38,24,0,44
COX6A2_2_13475,86,187,214,243,267,5,967,50,13,1038,702,131
COX6B1_2_13476,185,132,137,177,187,109,144,308,170,212,0,200
COX6B2_2_13477,95,78,49,55,0,126,2,1,14,77,2,170
COX6C_2_13478,320,501,368,473,368,878,325,650,406,609,386,483
COX7A1_2_13479,613,515,372,407,552,52,116,1473,373,505,196,571
COX7A2_2_13480,24,94,89,75,2,50,45,1,95,134,2,537
COX7A2L_2_13481,46,50,39,1,4,18,0,13,28,0,311,0
COX7B2_2_13482,648,570,561,728,1492,229,395,1421,275,664,705,426
COX7B_2_13483,836,852,708,731,193,767,565,436,1225,1541,814,390
COX7C_2_13484,1539,1486,1351,1478,1616,917,1183,1677,1530,1346,1983,11
04
COX8A_2_13485,16,5,17,13,0,4,0,1,3,23,8,1
COX8C_2_13486,549,639,474,662,755,429,783,848,654,875,874,563
CPA1_2_13487,121,135,85,135,63,369,138,75,79,380,97,115
CPA2_2_13488,58,50,72,90,7,446,49,508,174,466,113,192
CPB1_2_13489,620,863,657,678,967,300,999,1441,157,296,1249,1304
CP_2_13490,1641,1122,1662,1597,1554,1287,2025,1345,565,1307,2367,1770
CPE_2_13491,481,394,579,315,217,259,230,50,455,540,671,97
CPOX_2_13492,1454,1838,1693,1747,2221,769,2104,1057,578,545,2317,1586
CPSF3_2_13493,902,633,701,891,1068,194,169,44,470,637,1014,411
CPT2_2_13494,1130,1321,805,1009,968,737,368,2787,730,1318,887,1221
CRAT_2_13495,43,41,16,60,98,0,0,0,5,108,0,138
CREG2_2_13496,32,24,17,16,0,95,0,122,127,0,0,40
CRY1_2_13497,249,307,194,369,411,326,497,427,99,137,117,1
CRYL1_2_13498,47,67,54,51,3,106,3,261,0,34,366,161
CRYZL1_2_13499,29,31,46,18,2,150,0,16,1,0,21,0
CSAD_2_13500,178,160,126,232,13,0,87,407,350,530,242,104
CS_2_13501,527,235,234,227,26,105,198,81,470,385,0,258
CSGALNACT2_2_13502,864,631,759,746,999,453,690,362,1606,983,581,907
CSRP2BP_2_13503,160,190,119,195,87,14,1180,317,435,222,207,127
CTBS_2_13504,139,54,138,141,158,27,488,11,0,213,501,40
CTRB1_2_13505,369,446,566,400,141,573,638,626,610,728,4,474
CTRB2_2_13506,194,77,112,99,143,0,1,21,46,0,372,16
CTRC_2_13507,62,101,38,83,30,4,100,0,14,20,0,337
CTSD_2_13508,35,18,1,9,0,33,3,0,37,0,0,65
CTSF_2_13509,82,1,11,6,442,0,0,0,87,0,57,0
CTSG_2_13510,505,558,404,694,938,438,691,92,246,595,682,1194
CTSH_2_13511,605,476,236,293,18,621,268,128,188,309,581,397
CTSK_2_13512,188,133,163,133,284,59,75,287,68,166,23,108
CTSZ_2_13513,419,446,339,292,382,711,508,485,959,119,1478,578
CWC27_2_13514,1356,1311,1136,1295,896,1492,3639,1437,638,993,975,1661
CXorf21_2_13515,461,253,337,429,350,210,633,705,406,599,317,949
CYB561D2_2_13516,226,174,231,292,61,630,503,204,164,70,369,207
CYB5B_2_13517,191,245,215,191,490,59,381,411,93,264,82,209
CYB5R1_2_13518,250,184,137,208,87,385,169,6,137,183,287,244

CYB5R2_2_13519,52,76,73,12,0,0,2,511,195,266,256,38
CYB5R4_2_13520,618,499,394,481,476,391,268,621,841,345,687,354
CYBA_2_13521,67,57,114,128,0,1,422,52,13,0,305,60
CYBB_2_13522,54,192,47,242,99,1,0,0,45,0,378,330
CYC1_2_13523,93,212,167,258,4,685,522,44,3,169,192,45
CYCS_2_13524,64,77,39,41,339,53,404,0,107,0,6,12
CYP11B2_2_13525,203,206,130,270,151,9,81,7,56,21,687,197
CYP17A1_2_13526,82,64,79,69,0,0,0,74,30,31,198,294
CYP1A1_2_13527,281,270,195,231,21,577,24,101,194,772,929,71
CYP1A2_2_13528,448,382,317,483,54,385,663,555,445,584,1546,318
CYP1B1_2_13529,290,216,160,207,377,61,41,1,199,359,0,228
CYP20A1_2_13530,868,832,718,891,1584,1038,1184,787,830,401,211,771
CYP26B1_2_13531,192,108,121,154,74,107,90,125,59,152,242,132
CYP26C1_2_13532,463,285,557,443,270,294,201,415,546,716,201,400
CYP27A1_2_13533,266,217,249,225,629,47,261,321,36,246,56,437
CYP27B1_2_13534,605,467,487,540,261,638,614,889,143,1776,312,537
CYP2A13_2_13535,1277,1072,1068,1436,985,925,1661,646,983,591,476,1675
CYP2A6_2_13536,452,286,357,315,264,787,912,337,369,115,69,327
CYP2B6_2_13537,293,268,436,340,490,696,608,198,65,1053,0,642
CYP2C19_2_13538,415,316,325,419,290,172,585,406,111,691,13,508
CYP2C9_2_13539,265,133,199,219,159,27,975,2,46,285,246,440
CYP2E1_2_13540,947,705,566,919,937,601,531,868,531,1058,279,1321
CYP2F1_2_13541,259,180,254,225,280,438,228,215,15,778,644,701
CYP2J2_2_13542,774,647,607,580,163,619,507,487,817,506,362,501
CYP2R1_2_13543,329,433,384,330,579,77,425,1401,361,576,396,514
CYP2S1_2_13544,45,19,6,13,152,2,58,0,3,8,177,30
CYP2U1_2_13545,1028,1312,1032,1462,527,1078,1672,777,1112,877,2114,105
9
CYP2W1_2_13546,125,35,37,106,0,28,0,0,1,0,0,20
CYP39A1_2_13547,945,738,675,983,556,892,557,1622,836,845,63,635
CYP3A7_2_13548,2697,2496,2450,3155,2198,2704,3004,4159,2754,1827,3962,
1795
CYP46A1_2_13549,78,35,75,144,0,62,0,0,44,37,48,2
CYP4A11_2_13550,1006,673,638,1071,332,578,1087,719,1169,1016,1064,1197
CYP4A22_2_13551,1006,673,638,1071,332,578,1087,719,1169,1016,1064,1197
CYP4F12_2_13552,971,1004,845,935,1097,1311,1221,671,1450,1093,1393,951
CYP4F22_2_13553,157,156,192,343,311,100,930,203,314,358,54,79
CYP4F2_2_13554,1218,1078,1252,1167,1765,789,1108,1260,749,1366,358,166
3
CYP4F8_2_13555,205,394,274,334,26,515,29,258,127,787,32,419
CYP4V2_2_13556,423,131,350,286,288,324,807,35,71,200,589,128
CYP4X1_2_13557,260,238,327,241,126,30,490,143,225,333,154,703
CYP4Z1_2_13558,2406,1930,1621,2203,2322,1226,1094,1303,1824,2616,986,1
232
CYP7A1_2_13559,122,97,88,153,68,151,0,132,24,381,35,59
CYP7B1_2_13560,555,608,500,597,557,475,456,757,481,563,160,606
CYP8B1_2_13561,111,60,78,125,81,144,264,11,49,35,0,94
DAD1_2_13562,460,402,342,636,8,61,261,12,838,516,161,560
DAGLA_2_13563,176,134,329,197,186,29,637,226,6,1,0,204
DAK_2_13564,108,148,129,170,49,0,731,14,407,33,31,134

DARS2_2_13565,863,916,912,1014,967,598,858,503,1169,719,607,1367
DARS_2_13566,657,895,871,909,1029,1092,753,405,763,751,980,1789
DBH_2_13567,10,6,15,9,0,3,0,0,0,0,0,0
DBR1_2_13568,741,629,623,722,554,373,1283,423,179,537,606,872
DBT_2_13569,2,26,47,40,0,0,1,347,22,0,0,0
DCLRE1B_2_13570,1421,1707,1754,1514,1226,1379,1107,2013,1034,2509,1154
,2215
DCPS_2_13571,255,291,294,177,63,9,45,613,157,46,3,6
DCTN6_2_13572,332,226,203,266,206,248,260,39,182,274,481,320
DCTPP1_2_13573,184,205,355,163,114,196,0,41,1,317,52,5
DDAH2_2_13574,498,526,429,465,244,467,364,982,156,171,146,143
DDOST_2_13575,307,418,365,462,67,276,385,712,448,292,415,460
DDX10_2_13576,21,62,27,42,2,22,0,2,20,0,0,0
DDX18_2_13577,391,399,247,291,651,314,489,166,116,184,144,482
DDX19A_2_13578,219,139,239,384,78,383,4,359,350,159,435,216
DDX1_2_13579,575,499,632,461,609,117,447,230,677,553,556,504
DDX20_2_13580,3229,2874,2500,3371,3309,2047,1421,3640,4939,4270,2977,2
566
DDX21_2_13581,88,134,105,124,25,34,471,4,91,44,1,22
DDX23_2_13582,26,29,11,23,1,11,106,7,28,0,5,0
DDX24_2_13583,182,358,195,324,79,543,358,255,422,27,0,148
DDX25_2_13584,0,0,0,0,0,0,0,0,0,0,0,0
DDX27_2_13585,1216,1135,1089,1616,2010,2544,2185,1544,915,1489,1268,21
64
DDX28_2_13586,108,179,164,143,4,26,50,50,61,10,269,195
DDX39A_2_13587,1123,941,1097,1057,868,1594,2909,482,1048,1287,1271,176
4
DDX41_2_13588,33,18,82,63,87,227,339,306,74,0,0,94
DDX43_2_13589,562,330,402,310,587,365,730,563,1131,1080,4,338
DDX46_2_13590,1385,706,1069,1072,630,1475,1463,1075,1293,1436,758,618
DDX49_2_13591,195,284,171,211,328,76,372,1,73,102,0,58
DDX50_2_13592,1274,777,885,1092,457,219,770,1462,856,661,2752,1080
DDX51_2_13593,827,665,879,1045,473,733,289,843,326,317,242,354
DDX52_2_13594,109,116,130,230,7,340,174,304,488,23,50,572
DDX53_2_13595,287,326,315,325,244,127,267,793,217,533,320,145
DDX55_2_13596,35,31,42,90,30,47,150,66,128,0,0,65
DDX56_2_13597,686,395,495,772,743,377,580,667,888,331,312,755
DDX58_2_13598,666,764,761,641,1448,687,966,746,968,897,384,822
DDX59_2_13599,143,118,201,220,107,50,9,17,50,20,142,65
DDX5_2_13600,605,590,614,828,792,265,283,618,775,758,1390,919
DDX60_2_13601,273,300,262,346,72,274,198,24,55,6,131,246
DDX6_2_13602,994,894,720,1061,852,1096,628,867,750,1024,134,956
DECR1_2_13603,477,328,441,678,234,196,411,619,202,22,892,615
DECR2_2_13604,174,263,161,367,121,37,37,290,100,382,0,77
DEGS1_2_13605,445,385,322,410,430,212,156,329,551,664,104,128
DEGS2_2_13606,199,65,65,73,21,0,61,592,33,4,0,67
DERA_2_13607,141,179,148,116,5,1,80,76,308,48,1,170
DFFB_2_13608,1107,853,912,1086,586,1426,1114,313,960,1335,1177,1000
DGAT1_2_13609,213,231,235,292,291,9,300,485,127,205,0,274
DGAT2_2_13610,304,334,288,375,115,230,514,651,235,727,596,847

DHCR24_2_13611,864,595,686,707,256,453,91,192,376,464,2097,328
DHDH_2_13612,206,218,290,353,188,177,584,8,124,570,18,100
DHFR_2_13613,848,701,660,899,833,614,971,771,346,569,1002,782
DHODH_2_13614,55,24,27,65,1,48,15,0,8,0,351,3
DHRS13_2_13615,54,73,51,190,22,0,467,2,59,0,469,4
DHRS3_2_13616,113,92,17,51,16,0,0,0,11,46,0,40
DHRS4_2_13617,80,37,95,83,268,3,8,44,49,17,198,131
DHRS7_2_13618,204,293,176,104,324,354,747,29,366,743,288,360
DHRSX_2_13619,418,484,479,668,858,147,865,532,223,301,188,421
DHTKD1_2_13620,447,246,243,267,158,92,209,454,586,177,386,149
DHX15_2_13621,282,173,218,268,116,499,550,13,46,1,39,133
DHX29_2_13622,59,72,109,98,123,213,47,0,115,106,14,154
DHX32_2_13623,186,214,193,252,405,673,352,38,252,217,42,212
DHX34_2_13624,6,64,28,65,1,0,0,0,0,0,0,54
DHX37_2_13625,1278,834,861,1183,1161,237,516,1305,1711,648,531,1589
DHX38_2_13626,368,309,339,366,565,499,67,332,229,728,531,335
DHX57_2_13627,51,75,99,67,63,51,11,127,236,551,0,132
DHX58_2_13628,212,62,110,242,309,0,0,264,258,0,544,14
DHX8_2_13629,573,436,619,393,462,73,1259,465,474,229,267,208
DHX9_2_13630,855,936,714,840,708,1015,571,641,275,1476,1144,2051
DIO3_2_13631,4,7,14,10,3,0,0,24,52,0,0,0
DIRAS1_2_13632,122,66,85,83,239,12,268,0,36,29,0,0
DIRAS2_2_13633,153,81,109,141,0,114,9,0,2,240,324,607
DIRAS3_2_13634,1085,1021,808,970,274,315,336,2653,750,529,589,936
DIS3L2_2_13635,294,190,327,290,586,5,74,112,381,182,271,568
DLAT_2_13636,1235,1175,1054,1393,1155,1797,1554,1105,866,313,1365,1287
DLD_2_13637,73,107,68,109,18,210,162,26,64,138,57,40
DLL1_2_13638,32,18,49,31,32,27,6,6,0,58,457,2
DLST_2_13639,1,4,19,15,0,0,123,0,0,0,1,0
DMGDH_2_13640,157,241,289,233,34,172,613,216,429,599,484,453
DNA2_2_13641,1619,1305,1281,1463,1665,1180,1289,1512,1593,2659,887,696
DNAH11_2_13642,1161,1172,1258,1221,1425,1046,562,246,965,654,1555,341
DNAH3_2_13643,395,358,399,583,1025,26,1,318,6,5,1,38
DNAH5_2_13644,3567,3267,3721,3722,2938,2082,5244,4368,2859,3793,3773,4
144
DNAH8_2_13645,447,579,515,573,92,730,1062,898,506,504,781,385
DNAJA2_2_13646,78,63,62,80,29,0,15,22,212,7,110,23
DNAJB14_2_13647,1037,851,823,1029,861,665,796,961,646,692,947,1040
DNAJC10_2_13648,81,75,125,114,3,2,0,0,289,77,8,186
DNAJC18_2_13649,631,404,550,723,232,113,1970,596,356,793,227,605
DNAL4_2_13650,17,24,8,15,0,0,0,0,0,6,0,0
DNASE1_2_13651,153,101,167,71,26,242,349,8,25,3,16,132
DNASE1L2_2_13652,282,487,495,822,36,1,252,500,544,0,3,652
DNASE1L3_2_13653,934,809,870,897,1386,973,2003,657,852,1164,737,483
DNASE2_2_13654,158,209,118,196,262,4,62,0,224,0,58,118
DNPEP_2_13655,76,48,14,51,1,0,174,0,45,60,0,41
DPAGT1_2_13656,109,41,65,66,118,42,594,2,410,2,1,0
DPEP2_2_13657,102,31,65,64,1,4,0,268,0,3,0,21
DPM1_2_13658,0,0,0,0,1,0,0,0,0,0,0,0
DPM2_2_13659,66,79,53,106,9,252,236,0,0,161,204,11

DPP4_2_13660,191,295,202,169,104,384,11,306,431,4,3,85
DPP7_2_13661,150,227,106,125,183,246,364,236,256,132,87,74
DPP9_2_13662,0,32,30,29,0,0,0,30,0,0,0,0
DPYS_2_13663,211,195,222,165,312,5,1,47,321,540,61,53
DPYSL4_2_13664,53,49,40,97,0,0,23,686,118,5,89,94
DPYSL5_2_13665,49,72,29,4,72,114,44,0,8,58,10,179
DQX1_2_13666,2791,2598,2290,2982,2142,1079,3845,3556,2015,3259,2189,29
77
DSEL_2_13667,287,235,218,356,609,310,644,352,58,99,171,458
DTD1_2_13668,89,63,46,60,55,65,2,0,8,132,21,29
DUOX2_2_13669,342,491,373,508,164,1,1617,281,963,830,166,485
DUPD1_2_13670,161,125,227,209,132,13,107,244,138,34,327,26
DUSP28_2_13671,155,54,78,200,114,51,15,0,173,5,44,316
EARS2_2_13672,213,319,194,263,110,398,390,231,383,123,204,376
EBPL_2_13673,412,544,495,525,330,280,939,530,613,744,482,418
ECH1_2_13674,452,538,348,456,134,332,8,637,363,20,971,45
ECHDC3_2_13675,699,465,384,465,671,767,92,832,1114,1328,688,269
ECHS1_2_13676,59,185,187,191,219,0,598,227,69,2,0,157
EDEM1_2_13677,969,797,677,674,513,1354,1522,393,289,491,1623,600
EDEM3_2_13678,351,214,216,197,596,172,12,147,431,426,8,225
EEF1A1_2_13679,134,43,172,124,163,4,0,14,1,352,528,43
EEF1A2_2_13680,92,86,279,122,18,118,97,55,125,215,265,61
EEF2_2_13681,620,613,362,423,1099,865,953,0,344,539,172,380
EGLN1_2_13682,1096,882,965,954,1271,1117,1374,993,1312,1613,709,1445
EGLN3_2_13683,1141,1033,870,1006,922,573,849,599,498,823,1253,700
EHD4_2_13684,250,380,363,423,211,235,487,143,391,426,39,429
EIF4A3_2_13685,635,542,580,548,1278,602,594,520,345,305,797,332
ELAC1_2_13686,149,108,197,169,98,183,359,183,46,40,694,103
ELANE_2_13687,49,50,19,17,0,20,81,0,15,242,0,272
ELOVL1_2_13688,716,816,667,687,460,1393,482,1189,969,780,1046,861
ELOVL2_2_13689,317,132,381,317,201,95,263,131,132,165,876,202
ELOVL3_2_13690,58,33,97,80,31,129,11,95,41,56,0,89
ELOVL4_2_13691,1891,1679,1876,2641,2089,2264,932,1690,1630,2113,2157,2
129
ENDOD1_2_13692,1751,1364,1393,1746,1570,1956,2590,2165,1817,1857,1846,
1467
ENDOG_2_13693,59,152,117,119,99,182,270,501,71,185,93,77
ENGASE_2_13694,73,67,50,86,56,2,38,3,94,15,0,94
ENO2_2_13695,565,433,590,598,804,1246,681,602,454,475,191,567
ENO4_2_13696,729,654,716,864,1304,0,217,242,1222,637,443,613
ENOPH1_2_13697,364,312,239,420,101,0,918,386,457,501,116,591
ENPEP_2_13698,434,308,444,370,378,101,1384,120,190,424,462,559
ENPP1_2_13699,485,454,419,787,0,908,0,1214,459,540,646,366
ENPP3_2_13700,647,620,527,644,542,380,414,681,526,406,78,664
ENPP4_2_13701,137,237,203,221,294,0,58,409,403,142,95,52
ENPP5_2_13702,1224,809,1087,1025,1563,1547,1725,1177,1037,713,1398,906
ENPP6_2_13703,76,137,109,98,198,0,168,0,0,44,317,284
ENPP7_2_13704,167,154,156,159,119,91,197,279,479,17,88,379
ENTPD3_2_13705,450,346,310,404,282,809,892,285,322,694,0,333
ENTPD5_2_13706,25,249,160,111,353,37,34,0,12,248,0,197

ENTPD7_2_13707,255,294,282,302,24,625,318,1,96,304,76,436
EPHX2_2_13708,177,202,237,256,23,23,27,660,66,232,526,58
EPHX4_2_13709,486,193,386,245,197,41,191,191,81,372,41,179
EPRS_2_13710,501,421,427,413,27,531,302,1121,485,273,378,425
EPT1_2_13711,108,53,76,95,148,0,591,183,9,172,1,28
EPX_2_13712,143,167,82,106,145,59,120,196,377,2,46,202
ERAS_2_13713,10,17,56,74,0,0,479,0,37,1,0,2
ERCC3_2_13714,374,285,292,301,235,200,516,108,123,694,132,470
ERCC4_2_13715,205,127,243,208,723,244,112,97,22,34,14,111
ERCC5_2_13716,51,56,53,63,293,154,52,4,79,39,325,1
ERI1_2_13717,314,238,349,297,145,43,111,14,551,133,3,89
ERO1L_2_13718,168,179,191,252,737,85,17,61,181,236,203,190
ERP44_2_13719,374,234,182,202,20,286,484,773,200,309,77,138
ESD_2_13720,121,216,208,191,42,6,0,96,44,758,321,113
ESPL1_2_13721,993,1042,676,817,738,804,996,1474,550,1086,781,1133
ETFDH_2_13722,77,114,60,92,5,111,0,257,1,64,283,1
EXOSC1_2_13723,0,0,0,0,0,0,0,0,0,0,0,0
EXOSC2_2_13724,265,244,167,292,99,128,8,92,107,397,5,624
EXOSC4_2_13725,160,224,216,113,182,134,463,420,133,21,444,89
EXOSC5_2_13726,15,16,27,44,0,0,10,101,45,13,106,0
EXOSC7_2_13727,308,368,354,373,100,637,310,6,412,1,25,655
EXOSC8_2_13728,541,551,565,784,912,477,578,354,297,90,277,658
EXT1_2_13729,688,876,480,746,431,720,361,562,422,584,942,492
EXTL1_2_13730,157,198,158,151,555,714,35,68,12,189,49,44
EXTL3_2_13731,1036,814,795,917,964,478,1340,378,886,745,615,579
F10_2_13732,122,53,54,137,0,0,0,7,38,270,9,215
F12_2_13733,66,11,27,68,11,18,0,0,52,441,0,76
F13A1_2_13734,478,504,407,604,585,318,1594,377,244,409,272,631
F13B_2_13735,233,204,164,313,146,226,660,236,257,141,235,228
F2_2_13736,125,222,86,159,179,28,25,13,265,333,509,61
F5_2_13737,200,216,219,209,165,247,385,465,127,849,196,238
F9_2_13738,309,205,277,318,363,85,394,250,349,93,439,292
FA2H_2_13739,24,39,44,103,15,24,0,41,71,17,1,36
FAAH2_2_13740,604,310,439,700,461,432,561,928,417,529,202,258
FADS1_2_13741,192,98,97,112,290,356,0,0,374,52,37,56
FADS2_2_13742,21,31,47,60,23,3,2,22,6,17,105,140
FADS3_2_13743,154,161,232,217,56,150,36,38,127,13,226,465
FAHD2A_2_13744,1708,1625,1358,1853,1886,989,1427,1203,860,2451,2094,18
25
FAM108C1_2_13745,123,71,171,126,100,172,31,233,8,161,0,112
FAM135B_2_13746,257,145,246,272,5,28,207,4,477,723,104,123
FAM20B_2_13747,99,152,177,181,62,117,307,377,19,507,609,127
FANCM_2_13748,116,137,87,104,158,83,230,28,77,140,2,18
FAR1_2_13749,514,386,414,358,1135,283,445,522,626,355,626,722
FAR2_2_13750,1125,1206,1005,1388,1136,1495,596,1516,1285,559,1445,1495
FARS2_2_13751,1219,1162,1296,1159,589,709,1818,1842,1251,1311,766,913
FARSA_2_13752,63,22,61,72,59,11,82,26,6,8,945,8
FARSB_2_13753,380,551,574,647,846,20,932,378,280,202,773,579
FASN_2_13754,119,55,42,180,161,159,69,260,77,496,2,8
FDFT1_2_13755,99,61,70,67,47,95,171,13,51,12,397,4

FEN1_2_13756,250,44,136,187,394,1,73,1,173,30,479,19
FH_2_13757,1529,1382,1186,1686,1048,836,1770,1292,952,2256,884,1163
FIG4_2_13758,932,1000,1049,1052,865,941,1237,286,835,537,1196,1275
FKBP10_2_13759,103,29,105,100,152,6,406,41,37,32,2,242
FKBP14_2_13760,115,98,70,139,228,3,31,67,114,90,4,34
FKBP15_2_13761,383,391,286,314,179,355,185,159,395,246,199,594
FKBP3_2_13762,777,641,547,851,190,889,502,496,663,822,257,899
FKBP4_2_13763,106,57,111,130,14,8,43,42,267,332,149,466
FKBP8_2_13764,151,77,149,258,80,83,0,0,0,100,26,410
FMO1_2_13765,130,123,215,80,351,55,237,44,533,40,106,684
FMO4_2_13766,2151,2228,1932,2572,2903,1949,1860,1497,2449,3756,1651,16
46
FNBP1_2_13767,233,95,105,122,165,1,3,100,272,0,102,1
FNTA_2_13768,1854,1506,1551,1987,1207,1016,1273,1217,964,1223,1844,196
0
FNTB_2_13769,340,423,245,293,121,72,137,375,353,239,204,250
FTH1_2_13770,40,49,69,52,1,0,64,6,55,2,64,37
FTMT_2_13771,331,281,316,276,2,425,0,12,235,15,350,228
FTSJ2_2_13772,332,252,171,315,191,332,634,0,295,215,959,223
FTSJ3_2_13773,178,133,132,211,178,0,315,15,101,500,10,63
FTSJD2_2_13774,320,237,330,385,3,322,1153,47,214,389,1093,332
FUCA1_2_13775,111,54,74,126,62,0,40,45,132,20,60,75
FUCA2_2_13776,99,150,125,138,40,339,457,14,110,386,272,143
FURIN_2_13777,1410,1003,1312,1330,1509,347,2246,1040,564,1627,1225,169
2
FUT10_2_13778,797,467,569,638,1019,702,126,778,796,872,165,440
FUT11_2_13779,267,183,297,373,102,32,345,122,430,422,37,52
FUT1_2_13780,244,165,122,144,32,92,25,156,6,20,15,247
FUT4_2_13781,399,541,478,495,265,397,476,1000,446,50,0,1249
FUT5_2_13782,147,225,119,244,401,10,188,421,124,184,189,142
FUT7_2_13783,19,22,29,4,0,107,0,50,0,147,26,2
FUT9_2_13784,0,0,0,0,0,0,0,0,0,0,0,0
GADL1_2_13785,118,104,42,98,0,39,43,0,0,249,87,142
GAL3ST1_2_13786,517,288,305,359,243,9,486,737,219,175,1163,291
GAL3ST3_2_13787,147,88,132,223,155,39,199,154,219,16,497,140
GAL3ST4_2_13788,342,320,253,440,374,621,207,247,55,56,175,307
GALM_2_13789,231,152,157,90,152,0,94,13,190,444,174,216
GALNS_2_13790,0,7,7,0,0,0,0,0,0,0,0,0
GALNT10_2_13791,330,279,279,316,25,116,210,387,143,434,753,402
GALNT11_2_13792,204,150,93,147,161,59,40,301,312,401,3,68
GALNT12_2_13793,711,731,744,887,1877,595,1330,1408,824,583,1227,1081
GALNT13_2_13794,399,386,419,378,107,156,482,223,760,408,392,347
GALNT14_2_13795,27,248,127,83,103,977,9,104,31,458,0,75
GALNT1_2_13796,624,607,484,517,798,631,1082,190,234,975,937,674
GALNT2_2_13797,476,293,317,455,689,216,381,362,147,1013,1054,342
GALNT3_2_13798,3016,3060,2702,3005,3559,3033,3145,3815,2227,3691,1289,
3378
GALNT4_2_13799,700,580,587,691,306,192,462,848,625,376,656,432
GALNT5_2_13800,821,536,591,639,842,68,712,520,885,183,721,681
GALNT6_2_13801,63,44,126,80,221,0,225,64,179,7,0,68

GALNT8_2_13802,590,397,500,620,344,592,1222,793,517,898,497,438
GALNTL2_2_13803,26,42,25,73,43,2,0,87,35,30,7,99
GALNTL4_2_13804,97,48,25,87,0,1,0,309,0,235,0,0
GALNTL5_2_13805,218,113,178,112,14,0,0,101,68,243,0,206
GALNTL6_2_13806,1111,700,797,942,1491,707,201,1024,1018,654,357,1023
GALT_2_13807,302,386,338,398,18,272,1032,679,378,159,119,460
GANC_2_13808,490,745,672,983,632,450,1322,392,456,407,1167,384
GAPDH_2_13809,3,6,51,10,7,0,18,60,0,13,0,50
GAPDHS_2_13810,266,148,189,135,32,3,550,2,161,37,234,10
GARS_2_13811,1024,1188,1218,1410,1758,1118,2544,350,667,1530,1079,1727
GATC_2_13812,314,226,201,421,85,396,961,822,549,142,849,483
GATM_2_13813,1044,1215,852,1026,1995,1091,972,1338,1064,754,1936,1401
GBA2_2_13814,219,162,98,207,602,31,1,400,71,440,225,117
GBE1_2_13815,485,529,479,545,580,375,177,235,902,151,500,810
GBGT1_2_13816,334,244,226,244,249,171,395,275,168,552,2,622
GBP1_2_13817,107,54,109,71,265,0,25,0,86,78,1,6
GBP2_2_13818,390,269,291,446,300,153,32,896,305,279,823,681
GBP3_2_13819,636,609,540,533,377,412,247,587,609,444,507,224
GBP4_2_13820,394,189,319,321,39,356,354,670,258,398,310,126
GBP6_2_13821,328,280,279,283,20,240,862,314,493,16,376,29
GBP7_2_13822,58,12,30,30,0,0,0,35,249,1,9,18
GCLM_2_13823,266,146,197,166,54,120,180,353,104,159,18,250
GCNT3_2_13824,70,46,51,96,32,1,0,192,7,1,5,290
GCNT4_2_13825,152,87,204,251,1277,110,303,371,187,462,519,1
GCSH_2_13826,50,48,97,112,45,1,6,7,0,56,0,305
GDE1_2_13827,727,581,688,580,849,451,1744,915,830,839,783,769
GDPD3_2_13828,307,154,147,304,91,150,306,15,189,323,70,330
GFM1_2_13829,476,414,402,468,39,189,186,875,141,665,84,889
GFOD2_2_13830,45,85,49,35,1,669,0,0,0,0,287,0
GFPT1_2_13831,204,159,146,249,99,30,393,570,152,179,364,81
GFPT2_2_13832,10,42,15,3,0,17,0,0,0,9,0,0
GGH_2_13833,62,101,68,75,202,0,479,0,1,168,237,154
GGPS1_2_13834,552,632,295,323,106,78,249,17,304,473,24,431
GGT7_2_13835,27,112,127,99,0,0,816,0,1,0,396,2
GGTLC2_2_13836,845,613,630,582,1106,620,747,305,784,272,254,309
GLCE_2_13837,563,401,442,520,106,589,617,837,396,110,359,492
GLDC_2_13838,135,69,145,139,0,87,0,11,0,40,10,19
GLO1_2_13839,1888,1662,1591,2061,970,1410,1211,1604,1108,1794,1462,132
3
GLOD4_2_13840,1644,1402,1665,1520,2583,1645,645,1203,1224,1041,2586,17
91
GLS2_2_13841,473,465,396,334,385,318,102,1178,199,229,137,227
GLT1D1_2_13842,199,78,170,99,39,1,146,274,1,5,0,31
GLT25D1_2_13843,43,22,61,58,35,15,14,0,44,32,0,72
GLT25D2_2_13844,143,153,186,211,117,101,0,73,54,341,1,182
GLT8D2_2_13845,253,341,267,359,218,354,1052,575,245,490,553,813
GLUD1_2_13846,382,291,198,414,672,877,1331,682,460,163,16,808
GLUD2_2_13847,1951,2335,1703,2237,2911,2228,2277,1613,1431,2089,1383,2
503
GMDS_2_13848,382,560,323,455,266,1054,131,397,327,242,20,358

GMIP_2_13849,208,206,270,235,117,412,0,0,137,271,346,355
GMPR_2_13850,207,85,118,120,25,221,44,15,4,26,701,171
GMPS_2_13851,555,501,536,545,145,239,504,87,495,1574,786,520
GNA11_2_13852,91,43,119,132,43,14,192,667,556,0,248,53
GNA12_2_13853,90,29,224,104,8,345,699,6,4,1,399,155
GNA13_2_13854,636,728,797,818,189,376,841,494,1607,356,1830,485
GNA14_2_13855,571,587,433,455,867,524,181,3,538,576,636,455
GNA15_2_13856,108,165,147,186,139,115,0,15,63,324,164,179
GNAI1_2_13857,1244,1191,1102,1675,783,1743,786,504,491,1573,696,1169
GNAI3_2_13858,347,553,414,596,758,81,558,97,546,472,943,1103
GNAQ_2_13859,76,3,95,85,1,0,0,0,20,70,0,181
GNAT2_2_13860,1007,689,723,958,729,177,1049,999,1379,1056,2018,882
GNAT3_2_13861,461,471,429,587,391,100,663,482,317,349,429,229
GNAZ_2_13862,242,186,249,312,437,232,600,27,243,11,264,28
GNB1_2_13863,9,30,49,15,0,19,1,113,24,152,0,0
GNB2L1_2_13864,16,59,30,39,18,71,0,0,27,0,0,21
GNB3_2_13865,0,0,10,0,0,0,46,0,0,0,457,1
GNB4_2_13866,1530,1230,1463,1604,1610,859,1727,1727,898,1115,1361,1290
GNG11_2_13867,2250,2240,1765,2498,2698,1672,1594,3187,1639,1641,3802,3
119
GNG12_2_13868,681,502,463,563,87,578,85,530,485,425,245,490
GNG13_2_13869,25,37,21,51,2,56,0,0,0,12,0,141
GNG2_2_13870,4,2,28,40,2,1,382,3,0,5,0,3
GNG3_2_13871,214,281,268,276,12,191,709,270,129,303,9,605
GNG7_2_13872,120,112,135,84,391,4,259,173,0,12,0,8
GNG8_2_13873,481,381,359,404,721,335,1296,83,405,47,310,524
GNGT1_2_13874,294,198,316,264,452,39,183,170,216,492,190,280
GNL2_2_13875,134,143,103,122,1,0,74,68,29,36,38,72
GNMT_2_13876,184,96,86,99,133,145,582,182,119,2,3,183
GNPAT_2_13877,514,514,423,434,560,497,242,92,532,462,407,196
GNPDA1_2_13878,642,743,667,697,935,947,712,674,193,1215,1731,809
GNPDA2_2_13879,458,442,412,387,644,303,419,244,480,729,174,507
GNPNAT1_2_13880,253,354,312,281,93,5,1061,5,26,873,1081,417
GNPTAB_2_13881,710,830,758,1214,1022,953,805,378,336,1227,223,285
GNPTG_2_13882,1483,1239,1248,1263,441,1250,1364,1918,1679,2393,2286,17
15
GNS_2_13883,314,277,287,229,188,99,531,211,326,212,547,270
GOT2_2_13884,463,297,432,412,377,642,223,167,537,150,369,284
GPAA1_2_13885,13,58,41,32,1,219,1,0,35,9,12,0
GPAM_2_13886,337,282,266,418,186,365,870,981,128,1,921,1133
GPAT2_2_13887,109,79,118,149,18,112,5,155,44,28,11,182
GPD1_2_13888,138,90,89,133,34,53,12,23,4,186,88,37
GPD1L_2_13889,356,334,377,386,106,546,167,850,460,326,280,530
GPX2_2_13890,539,637,422,661,829,31,409,642,653,392,825,405
GPX3_2_13891,487,719,379,408,296,783,721,450,354,175,561,530
GPX7_2_13892,200,323,210,279,366,28,698,61,38,158,22,242
GPX8_2_13893,2688,2560,2209,2698,1314,1134,2862,1459,1826,1485,2076,28
19
GRHPR_2_13894,210,163,123,105,59,0,454,219,201,214,199,108
GSTA1_2_13895,577,695,511,807,721,1675,212,598,789,1656,239,560

GSTA2_2_13896,1233,1482,1271,1316,1816,1212,860,566,1040,893,893,1352
GSTA3_2_13897,2579,2373,2001,2562,2774,567,2170,1317,1686,2731,3511,18
32
GSTA4_2_13898,77,37,46,106,0,0,0,0,222,40,0,0
GSTA5_2_13899,1318,1563,1360,1401,1870,1369,927,982,1072,1234,891,1363
GSTM3_2_13900,827,716,804,645,2012,811,1274,1018,921,1283,912,1350
GSTM5_2_13901,5185,5006,4088,5100,5889,4918,4834,2892,3719,5583,5489,4
577
GSTP1_2_13902,116,95,91,65,0,22,0,96,1,0,0,64
GSTT1_2_13903,229,118,161,148,70,94,229,404,332,37,140,222
GSTT2B_2_13904,269,101,221,180,169,4,64,8,285,520,0,16
GSTT2_2_13905,269,101,221,180,169,4,64,8,285,520,0,16
GTF2F2_2_13906,928,875,728,1408,512,528,222,626,1033,1467,171,548
GTPBP1_2_13907,419,455,340,405,30,306,314,642,392,978,1279,313
GTPBP2_2_13908,56,104,56,90,210,135,283,2,0,519,66,4
GTPBP4_2_13909,201,185,253,277,289,0,0,46,2,632,629,158
GUCY1A2_2_13910,821,521,650,929,22,112,1108,120,520,862,62,1086
GUCY1B3_2_13911,612,728,330,680,348,578,113,0,487,770,1191,179
GUSB_2_13912,367,316,374,500,302,68,499,877,283,211,553,366
GYLTL1B_2_13913,102,101,204,166,0,0,25,397,0,120,241,36
GYS2_2_13914,286,189,161,175,28,241,185,131,494,80,476,27
GZMA_2_13915,409,232,397,357,164,159,938,357,358,231,196,395
GZMB_2_13916,293,236,232,265,117,12,21,108,128,383,135,272
H6PD_2_13917,41,44,20,15,62,16,0,52,9,13,28,50
HAAO_2_13918,867,490,511,526,364,268,1030,115,164,664,1162,510
HACL1_2_13919,233,254,466,442,3,314,74,486,586,87,777,630
HADHA_2_13920,450,145,210,277,12,592,70,36,35,214,125,10
HADHB_2_13921,64,117,116,188,34,30,67,323,2,115,98,130
HAGHL_2_13922,151,259,106,135,247,6,445,477,645,580,13,91
HAL_2_13923,586,615,878,636,725,876,669,25,641,1108,339,952
HAO1_2_13924,273,211,215,289,351,5,399,356,244,180,497,250
HARS_2_13925,372,263,276,333,227,541,209,506,431,230,186,118
HAS1_2_13926,126,67,46,99,12,40,2,11,2,75,532,232
HAS2_2_13927,70,115,101,76,61,227,472,29,4,7,228,2
HDHD3_2_13928,40,26,0,19,0,0,0,0,0,11,0,0
HELB_2_13929,322,226,273,273,380,27,7,347,147,259,191,92
HELQ_2_13930,496,516,575,702,387,19,938,0,524,905,1094,282
HELZ_2_13931,354,249,285,359,302,283,745,104,265,229,1438,483
HEXA_2_13932,260,269,196,221,136,227,0,433,199,456,12,223
HEXB_2_13933,637,736,557,684,3,190,314,1039,287,738,1100,440
HEXDC_2_13934,86,48,100,48,118,0,50,308,63,0,0,3
HGD_2_13935,215,332,391,391,171,464,486,312,224,1,424,467
HGSNAT_2_13936,454,288,311,424,10,56,761,473,567,446,624,571
HHATL_2_13937,109,175,110,147,87,183,415,282,199,88,215,153
HIBADH_2_13938,156,217,135,77,284,56,25,254,64,31,7,94
HINT1_2_13939,393,311,289,336,240,810,916,572,880,177,343,701
HMOX1_2_13940,1203,684,928,908,967,375,1102,1394,1240,795,677,1541
HPGDS_2_13941,498,415,366,566,205,152,857,442,568,603,955,577
HPRT1_2_13942,404,290,288,333,448,541,184,723,535,558,541,319
HS3ST1_2_13943,102,143,146,112,132,76,0,1,188,47,258,209

HS3ST2_2_13944,779,535,389,704,1150,327,587,558,1223,348,100,683
HS3ST3A1_2_13945,111,108,159,154,31,1,58,225,178,461,0,215
HS3ST3B1_2_13946,364,382,550,361,114,455,252,342,390,639,184,208
HS3ST4_2_13947,308,354,167,309,265,352,21,658,149,307,94,186
HS3ST6_2_13948,218,162,138,149,400,95,196,39,40,241,25,249
HS6ST1_2_13949,296,256,288,256,352,1,93,233,15,83,295,55
HS6ST3_2_13950,737,688,774,682,916,779,1320,1211,345,1417,791,720
HSD11B2_2_13951,199,428,293,291,0,167,0,163,454,441,41,545
HSD17B11_2_13952,163,371,326,431,506,70,853,105,637,32,1663,192
HSD17B12_2_13953,2233,2035,1550,2607,1744,2372,2966,3104,2381,4814,192
7,2887
HSD17B14_2_13954,111,149,79,117,3,0,331,11,20,275,0,337
HSD17B1_2_13955,189,89,258,187,209,155,28,233,188,406,5,238
HSD17B2_2_13956,141,337,135,123,120,64,57,185,332,75,5,565
HSD17B3_2_13957,392,519,400,706,478,605,378,53,700,78,1026,285
HSD17B6_2_13958,219,205,156,208,713,130,1052,0,283,61,0,477
HSD17B8_2_13959,190,193,173,288,36,361,11,292,459,287,169,105
HSD3B1_2_13960,643,519,446,495,561,574,1347,135,556,512,324,816
HSP90AB1_2_13961,612,400,344,322,262,235,442,684,305,494,3,105
HSPA5_2_13962,214,231,185,182,54,329,170,475,232,84,0,361
HSPE1_2_13963,88,195,114,224,0,204,0,63,0,0,9,121
HSPG2_2_13964,139,51,118,156,249,0,16,0,0,0,322,28
HYAL4_2_13965,773,676,803,875,1005,657,824,624,937,463,461,555
IARS2_2_13966,199,240,250,230,211,161,253,8,106,50,13,157
ICMT_2_13967,1328,1341,1352,1366,1750,1232,1686,922,1209,1458,549,2435
ICT1_2_13968,114,171,214,303,406,60,22,42,233,76,157,1133
IDH1_2_13969,580,363,477,593,445,426,198,1783,765,212,115,350
IDH2_2_13970,223,158,319,151,361,243,406,85,184,157,172,51
IDH3A_2_13971,3,52,11,35,0,0,4,4,17,38,0,99
IDI1_2_13972,0,0,0,0,0,0,0,0,0,0,0,0
IDI2_2_13973,2490,1898,2047,2509,2090,1303,1924,1439,1769,1583,2298,15
43
IDO1_2_13974,687,862,712,930,315,665,380,505,736,1831,376,1318
IDO2_2_13975,215,314,187,279,364,151,334,0,227,51,227,32
IDUA_2_13976,580,960,616,641,1751,393,769,298,829,677,214,1283
IFI30_2_13977,197,371,79,274,917,666,0,37,39,517,334,174
IFIH1_2_13978,279,214,129,250,312,0,2,87,520,46,59,164
IGHMBP2_2_13979,328,484,350,458,587,61,424,192,369,109,94,368
IHH_2_13980,58,100,48,60,519,368,19,105,100,119,6,0
ILVBL_2_13981,70,34,42,48,42,45,1,0,9,213,3,27
IMPAD1_2_13982,106,42,71,90,97,2,75,38,81,35,84,233
IMPDH2_2_13983,371,321,322,441,496,142,607,35,413,247,36,93
IRS1_2_13984,416,238,315,308,221,590,472,301,183,133,266,408
IRS2_2_13985,403,387,454,694,608,349,557,417,507,490,316,830
ISG20_2_13986,148,108,113,118,632,553,0,25,9,25,3,1
ISG20L2_2_13987,547,346,324,532,636,844,1193,658,480,408,128,276
ISOC1_2_13988,629,561,334,437,447,413,89,101,475,265,340,165
IWS1_2_13989,604,404,339,600,222,374,399,413,624,444,557,637
KDSR_2_13990,1443,1516,1044,1285,1524,2342,1497,404,790,2182,1279,757
KHSRP_2_13991,272,297,132,316,215,390,473,220,74,219,27,347

KIAA0317_2_13992,147,257,290,300,281,1011,376,53,2,145,30,351
KIAA1279_2_13993,100,77,102,94,5,364,1,62,37,3,83,65
KIAA2022_2_13994,522,303,497,554,519,284,554,245,1034,359,1,479
KIF18A_2_13995,1163,726,943,1092,735,381,738,602,924,1180,555,806
KIF20B_2_13996,397,306,365,411,840,490,168,200,91,442,181,294
KIF3A_2_13997,852,771,721,691,766,670,994,515,551,941,1364,874
KLB_2_13998,1751,1595,1403,1557,1598,1125,2330,1206,1217,2114,918,1477
KLC3_2_13999,211,295,225,225,211,0,100,171,401,166,1133,293
KL_2_14000,479,432,312,422,330,151,107,227,368,320,337,1005
KLK1_2_14001,100,20,56,87,1,122,25,6,155,168,118,32
KLKB1_2_14002,457,284,448,345,208,229,335,255,231,351,86,596
KMO_2_14003,224,224,155,121,129,274,78,142,118,626,1,154
KRTCAP2_2_14004,159,141,108,192,191,260,36,29,138,71,32,144
L2HGDH_2_14005,1075,1023,878,1202,1036,298,1602,390,1182,1608,809,874
LALBA_2_14006,228,213,261,275,314,85,173,1000,201,362,6,38
LAP3_2_14007,720,681,563,696,1097,1007,181,235,701,533,812,633
LARS2_2_14008,460,333,352,458,316,12,317,216,836,397,444,753
LARS_2_14009,378,430,607,675,180,398,1519,488,468,474,25,30
LCAT_2_14010,29,65,39,66,131,26,11,40,5,63,7,30
LCMT2_2_14011,194,112,125,144,20,1,134,4,32,38,1,141
LCT_2_14012,154,206,142,215,1,346,75,403,68,499,26,434
LCTL_2_14013,553,484,440,653,313,661,1034,536,1117,522,145,667
LDHAL6B_2_14014,484,303,462,423,432,156,1229,30,763,384,283,166
LEPREL2_2_14015,465,451,432,595,641,537,434,44,706,456,324,199
LGALS13_2_14016,229,120,201,174,89,12,315,82,305,28,373,28
LHFPL2_2_14017,20,73,18,60,12,0,0,0,0,21,0,102
LIPC_2_14018,125,83,146,200,304,66,305,332,52,389,1065,101
LIPE_2_14019,802,761,624,600,1351,598,352,46,200,1051,200,788
LIPG_2_14020,21,33,18,43,17,0,350,153,51,0,0,72
LIPH_2_14021,256,268,182,241,349,587,308,8,156,480,69,262
LIPI_2_14022,604,405,527,505,835,402,807,266,881,691,285,586
LIPM_2_14023,298,307,248,276,105,221,630,51,201,36,15,322
LIPN_2_14024,72,60,84,61,150,108,0,155,0,152,0,121
LIPT2_2_14025,97,53,76,63,4,23,81,23,111,127,224,160
LONP2_2_14026,793,524,582,668,64,423,407,696,430,427,693,166
LOXL1_2_14027,195,171,228,151,623,15,63,332,303,258,139,36
LOXL2_2_14028,112,73,59,42,139,77,27,237,111,51,177,17
LOXL3_2_14029,66,115,72,59,31,97,257,42,0,13,0,89
LOXL4_2_14030,74,139,219,95,3,0,227,2,92,8,49,642
LPCAT1_2_14031,32,29,16,29,5,4,0,18,0,27,0,13
LPCAT2_2_14032,187,239,237,431,207,42,55,245,212,156,370,137
LPCAT3_2_14033,15,74,35,91,42,5,131,0,104,141,0,27
LPCAT4_2_14034,10,18,61,44,0,5,17,0,3,13,0,95
LPIN1_2_14035,21,18,79,27,0,0,1,5,60,194,21,16
LPIN2_2_14036,822,863,766,878,347,945,1004,336,445,704,528,640
LPIN3_2_14037,240,356,378,417,1,27,137,7,42,40,397,100
LPL_2_14038,432,448,383,525,162,17,13,345,388,297,150,1236
LRAT_2_14039,621,585,325,649,264,143,1,96,545,323,747,268
LTA4H_2_14040,96,92,74,222,16,0,0,84,13,1,36,51
LTC4S_2_14041,158,166,196,225,765,11,89,74,103,237,6,149

LYG2_2_14042,188,240,198,131,74,101,24,0,206,229,526,64
LYPLA1_2_14043,162,94,131,179,43,20,395,153,339,40,14,120
LYPLA2_2_14044,37,32,4,13,0,32,4,54,0,17,0,0
LYPLAL1_2_14045,266,369,307,372,317,253,248,173,392,108,13,389
LYZ_2_14046,645,512,522,513,1208,176,116,486,1124,332,51,403
LYZL1_2_14047,195,334,170,354,330,315,131,89,542,157,46,545
LYZL2_2_14048,195,334,170,354,330,315,131,89,542,157,46,545
LYZL4_2_14049,275,268,195,355,468,212,22,530,991,270,251,285
MACROD1_2_14050,1369,915,1432,1528,460,635,1066,1063,777,522,1068,2498
MAGT1_2_14051,288,138,271,200,13,3,2,156,404,395,355,427
MAN1A1_2_14052,151,235,236,315,552,122,175,232,213,102,31,110
MAN1A2_2_14053,17,17,12,16,25,0,2,1,1,0,175,21
MAN1B1_2_14054,0,5,0,0,0,0,0,0,0,0,0,0
MAN1C1_2_14055,96,93,127,157,69,0,393,57,18,10,54,108
MAN2A1_2_14056,2327,1721,1884,2431,2139,1252,3576,1662,3432,4205,3097,
2972
MAN2A2_2_14057,204,208,213,257,476,279,4,180,358,8,220,7
MAN2B2_2_14058,161,49,46,103,295,1,9,238,43,231,1,120
MAN2C1_2_14059,70,20,102,101,4,0,1,53,38,1,23,45
MANBA_2_14060,286,214,137,339,2,30,233,167,171,342,180,70
MANEA_2_14061,105,138,122,167,250,156,213,44,73,1,57,8
MAOA_2_14062,884,847,764,877,1217,1257,456,495,1019,540,1467,590
MAOB_2_14063,357,239,386,329,1055,157,473,440,387,618,46,179
MAP1S_2_14064,0,4,8,39,1,0,0,34,12,24,0,0
MAPRE3_2_14065,194,276,244,194,331,120,42,157,467,512,111,449
MARS2_2_14066,291,353,367,339,30,39,7,333,183,822,466,771
MARS_2_14067,70,78,100,75,203,193,66,199,52,79,4,64
MAT1A_2_14068,12,62,92,41,0,364,97,1,152,280,0,0
MAT2A_2_14069,192,202,54,276,40,611,44,32,11,100,736,71
MBOAT1_2_14070,604,616,557,715,1118,310,273,689,281,656,559,945
MBOAT2_2_14071,699,877,766,734,1291,851,1536,836,1107,1399,324,208
MBOAT4_2_14072,46,59,83,49,171,0,39,705,0,9,108,95
MBTPS1_2_14073,258,148,138,206,2,84,60,4,108,232,29,429
MBTPS2_2_14074,129,130,165,150,3,126,330,177,27,26,560,62
MCCC1_2_14075,139,148,110,147,52,8,7,302,114,7,0,68
MCCC2_2_14076,92,95,133,215,28,140,48,33,75,63,0,285
MCEE_2_14077,94,45,82,59,36,18,0,78,77,106,298,198
MCM3_2_14078,15,8,8,58,4,153,0,18,0,0,4,4
MCM5_2_14079,248,264,155,242,78,242,181,611,117,152,37,492
MCM6_2_14080,422,667,624,916,1123,198,2193,409,1093,597,1185,1281
MDH1B_2_14081,10,0,9,37,0,0,0,0,0,57,27,13
MDH2_2_14082,117,59,80,183,303,253,856,2,0,1,7,280
MEP1A_2_14083,668,610,586,347,471,427,1464,479,613,514,871,487
MEP1B_2_14084,224,115,285,186,32,448,0,0,68,51,2,551
METAP1_2_14085,789,681,535,672,930,1360,1040,471,407,1197,369,1133
METAP1D_2_14086,200,160,174,271,581,276,244,138,76,124,161,205
METAP2_2_14087,471,437,475,604,716,434,638,47,238,363,175,397
METTL14_2_14088,518,598,490,726,170,704,573,1678,485,769,263,1584
METTL22_2_14089,549,536,633,694,397,652,530,317,579,911,580,492
METTL2B_2_14090,1396,1326,1253,1504,1227,2249,2157,1012,706,1716,1182,

2896
METTL3_2_14091,141,99,193,251,166,1,506,148,6,88,266,94
METTL5_2_14092,261,112,175,179,68,218,302,849,267,57,8,678
METTL6_2_14093,198,169,145,174,4,206,709,172,43,254,2,669
METTL7B_2_14094,251,146,190,218,42,17,139,336,59,137,89,74
METTL8_2_14095,779,572,653,790,648,379,339,1425,135,702,684,656
MFN1_2_14096,967,873,881,847,524,755,842,206,1440,380,1055,1149
MGAM_2_14097,72,87,64,121,16,48,197,308,23,79,209,98
MGAT4C_2_14098,733,617,681,663,449,541,608,452,852,1020,433,310
MGAT5_2_14099,333,268,278,334,366,591,1244,439,144,330,288,393
MGMT_2_14100,1091,495,789,642,227,441,1056,527,800,797,587,99
MGST3_2_14101,158,172,137,140,384,48,48,84,263,151,108,395
MIOX_2_14102,487,584,481,379,956,1110,269,576,233,403,901,876
MIPEP_2_14103,140,100,178,194,325,156,436,110,185,61,377,248
MLYCD_2_14104,42,30,50,17,0,24,6,0,0,1,0,0
MMAB_2_14105,35,91,71,84,1,0,0,0,0,0,100,335
MMEL1_2_14106,34,13,15,26,0,0,0,2,0,0,0,12
MMP10_2_14107,908,983,950,1203,903,964,1737,794,1264,960,673,508
MMP12_2_14108,166,49,107,177,0,146,0,21,5,20,116,10
MMP14_2_14109,138,86,138,173,24,86,481,64,16,15,293,18
MMP3_2_14110,10,23,41,34,0,0,85,0,0,0,384,17
MMP7_2_14111,1528,1574,1716,1781,1867,1087,1496,712,1101,937,2014,1734
MMP8_2_14112,958,1171,957,994,688,546,1572,757,540,1369,291,819
MMP9_2_14113,43,16,51,90,6,53,6,5,6,96,99,9
MOCOS_2_14114,53,50,21,18,4,6,0,3,93,0,2,5
MOGAT1_2_14115,200,255,228,273,88,114,52,67,653,10,33,216
MOGAT2_2_14116,411,510,497,644,133,580,401,624,400,102,670,134
MOGAT3_2_14117,577,603,499,721,1023,282,304,203,358,368,454,1215
MOXD1_2_14118,98,107,84,51,1,52,162,161,3,7,2,104
MPI_2_14119,299,122,324,233,289,188,49,6,243,40,119,278
MPO_2_14120,50,56,63,81,5,81,27,5,252,18,51,63
MRPL37_2_14121,505,458,528,509,593,561,5,773,47,1462,5,688
MRPL44_2_14122,1095,824,1239,847,2324,1195,1253,1030,1058,1997,388,591
MRPS30_2_14123,320,530,325,382,74,610,39,295,28,257,391,598
MSH2_2_14124,677,567,449,752,196,42,21,519,890,374,726,1197
MSH3_2_14125,512,569,394,355,634,484,1454,289,212,652,163,405
MSH4_2_14126,247,264,174,194,675,4,33,220,201,173,414,2
MTAP_2_14127,254,211,141,211,421,0,0,124,399,65,0,293
MTFMT_2_14128,325,387,594,586,427,375,585,104,98,743,408,1129
MTHFD1_2_14129,715,691,691,835,759,296,2465,487,485,611,234,156
MTHFD2_2_14130,202,310,167,344,190,423,312,0,465,40,48,396
MTHFD2L_2_14131,0,0,0,0,0,0,1,0,0,0,0,0
MTHFR_2_14132,57,16,31,40,5,0,22,0,32,18,0,6
MTPAP_2_14133,548,523,391,444,6,462,825,323,38,283,28,188
MTR_2_14134,98,40,28,40,167,2,156,30,2,67,0,4
MUS81_2_14135,56,98,60,74,0,0,0,36,260,179,146,35
MUT_2_14136,439,357,307,544,359,155,368,47,306,441,284,183
MVD_2_14137,120,306,266,313,382,120,375,239,80,601,78,228
MX2_2_14138,3505,3533,3351,3698,2927,2243,2681,2761,3056,3915,4755,283
6

MYH1_2_14139,101,78,130,192,68,188,609,13,36,617,118,140
MYH3_2_14140,361,456,652,794,18,125,447,1,755,616,571,244
MYH6_2_14141,145,74,223,164,469,5,0,101,192,55,197,70
MYH7_2_14142,500,651,607,559,828,499,414,691,357,1479,8,703
MYH9_2_14143,111,91,101,172,15,539,63,46,223,106,0,96
MYL7_2_14144,218,329,188,211,73,483,293,7,137,54,1,133
MYO1E_2_14145,545,454,360,436,349,352,807,746,92,221,174,406
MYO5B_2_14146,32,51,58,23,36,0,184,0,2,26,0,4
MYO9A_2_14147,564,581,397,391,165,209,732,701,596,230,263,334
N6AMT2_2_14148,26,120,118,61,166,4,200,2,36,223,0,74
NAA10_2_14149,551,649,463,581,907,301,707,1003,900,745,1545,882
NAA11_2_14150,148,195,176,104,578,336,417,679,57,45,307,152
NAA15_2_14151,152,221,184,200,132,5,4,185,426,381,0,244
NAA30_2_14152,627,527,502,773,922,374,51,395,96,547,917,536
NAA50_2_14153,3308,2577,2475,2970,4276,2731,2387,1612,2461,2818,3279,2
391
NAALAD2_2_14154,294,475,260,452,387,107,844,22,435,185,373,607
NAALADL1_2_14155,80,169,97,230,588,85,129,20,101,93,76,0
NADSYN1_2_14156,70,16,46,53,51,7,81,199,49,0,19,33
NAGA_2_14157,10,0,18,49,41,8,335,0,2,20,0,2
NAGLU_2_14158,85,230,230,208,272,409,37,256,123,329,210,56
NAGPA_2_14159,35,54,72,74,164,156,31,30,22,19,66,35
NAMPT_2_14160,1835,1423,1502,1399,1750,1327,1778,229,615,1011,2979,187
8
NANP_2_14161,1067,1025,1283,1312,648,810,937,1927,1214,896,1447,1263
NANS_2_14162,91,34,64,123,98,250,128,291,423,63,19,168
NAPRT1_2_14163,73,19,25,39,292,1,228,138,207,246,0,1
NARS_2_14164,495,326,205,384,25,115,367,708,43,188,0,307
NCF1_2_14165,294,400,225,146,44,157,10,2,222,16,32,394
NDST1_2_14166,287,319,193,438,299,3,0,228,286,204,195,155
NDST2_2_14167,564,413,343,504,246,513,828,533,275,243,10,674
NDST3_2_14168,925,739,681,796,399,628,1023,1281,1095,395,1007,1004
NDST4_2_14169,287,309,354,359,111,207,81,228,323,779,38,192
NDUFA10_2_14170,309,154,234,146,215,293,552,95,301,52,208,424
NDUFA12_2_14171,88,48,60,80,103,3,49,16,93,61,0,53
NDUFA13_2_14172,142,66,212,92,1,34,0,10,0,1,0,130
NDUFA1_2_14173,256,273,335,266,339,674,9,108,136,39,408,105
NDUFA3_2_14174,229,108,146,156,11,128,135,1,94,112,1,57
NDUFA4_2_14175,389,649,478,697,17,194,239,1840,557,773,565,814
NDUFA4L2_2_14176,191,188,155,160,182,170,179,68,39,240,27,213
NDUFA5_2_14177,304,421,386,386,296,344,702,464,410,153,341,522
NDUFA6_2_14178,695,524,499,559,662,415,795,510,472,1009,469,480
NDUFA7_2_14179,33,80,79,97,209,7,553,82,215,195,0,118
NDUFA8_2_14180,472,528,522,444,1530,225,406,821,626,535,211,244
NDUFA9_2_14181,92,55,60,97,77,65,145,14,332,80,69,3
NDUFAB1_2_14182,152,197,116,131,97,44,157,261,142,60,16,300
NDUFB10_2_14183,261,311,229,377,69,104,68,54,237,425,457,203
NDUFB1_2_14184,876,873,908,1343,507,730,1351,587,1039,311,790,824
NDUFB2_2_14185,184,165,148,209,454,313,195,503,638,339,21,205
NDUFB3_2_14186,615,571,584,669,541,193,760,581,618,620,846,653

NDUFB7_2_14187,100,49,44,63,0,0,2,0,1,2,0,16
NDUFB8_2_14188,27,29,83,59,0,196,0,0,87,1,0,5
NDUFB9_2_14189,65,34,78,63,41,6,147,1,5,62,93,27
NDUFS3_2_14190,299,284,288,306,33,243,1023,337,562,140,137,296
NDUFS4_2_14191,211,157,144,223,762,64,239,31,157,20,627,54
NDUFS6_2_14192,649,504,562,522,789,696,365,557,909,848,837,1273
NDUFS7_2_14193,34,142,112,101,179,335,0,0,0,2,4,38
NDUFS8_2_14194,420,392,276,506,556,230,30,621,731,550,31,396
NDUFV2_2_14195,185,238,291,265,358,96,575,517,353,130,136,212
NEDD8_2_14196,269,181,157,249,337,134,410,436,149,128,547,136
NEIL1_2_14197,50,69,46,92,45,0,57,2,30,136,311,305
NEIL3_2_14198,731,1158,926,1154,547,478,1330,1368,893,1612,1372,979
NEU1_2_14199,48,17,61,85,30,12,51,102,88,88,0,42
NEU2_2_14200,198,31,154,126,33,68,0,0,27,9,4,74
NEU3_2_14201,14,9,10,13,38,7,5,121,0,4,0,99
NHLRC2_2_14202,529,657,602,714,480,37,704,163,291,270,117,1063
NIT2_2_14203,191,130,195,240,190,203,363,120,221,151,2,340
NKIRAS1_2_14204,445,486,324,453,479,208,16,1094,851,921,227,935
NLGN1_2_14205,169,190,119,174,396,170,259,175,161,187,122,299
NLGN2_2_14206,8,10,16,24,0,2,4,2,19,0,0,0
NLN_2_14207,1056,819,898,1008,639,556,788,1692,1530,781,551,1019
NME1-NME2_2_14208,402,430,283,484,83,162,119,850,506,275,1119,232
NMNAT1_2_14209,429,112,131,209,316,448,125,1,536,140,26,63
NMT1_2_14210,70,107,79,185,7,67,48,354,85,322,13,36
NMT2_2_14211,71,119,88,108,0,2,0,37,4,0,755,1
NNMT_2_14212,113,137,105,107,72,20,61,86,37,39,7,172
NOP58_2_14213,441,394,395,493,539,494,978,729,448,833,333,245
NOX3_2_14214,181,247,99,176,0,401,0,321,179,218,1,105
NQO2_2_14215,257,338,109,249,56,60,167,252,235,288,23,35
NRAS_2_14216,363,241,270,399,41,255,2075,8,563,521,228,294
NSF_2_14217,669,558,467,776,854,681,473,1209,231,1055,370,696
NSUN6_2_14218,236,71,97,130,504,233,66,194,34,35,13,75
NTAN1_2_14219,193,26,132,77,97,129,0,211,91,85,215,86
NTHL1_2_14220,242,301,318,276,572,130,224,156,516,210,762,224
NTPCR_2_14221,537,585,453,674,288,487,179,3,752,6,109,65
NUDT10_2_14222,3073,2532,2288,3144,3164,2457,2451,3614,3034,1647,1712,
2423
NUDT11_2_14223,3073,2532,2288,3144,3164,2457,2451,3614,3034,1647,1712,
2423
NUDT12_2_14224,445,326,277,512,137,179,621,69,352,552,273,179
NUDT14_2_14225,49,39,23,52,7,0,21,84,115,1,0,0
NUDT3_2_14226,731,392,501,456,847,465,704,329,988,674,207,656
NUDT5_2_14227,411,294,355,510,862,316,197,108,821,0,50,322
NUDT7_2_14228,95,79,91,163,90,213,515,112,37,220,88,81
NXNL1_2_14229,50,119,49,62,8,20,278,82,63,1,0,91
OAS3_2_14230,503,498,545,370,188,177,379,364,608,237,834,348
OAZ1_2_14231,2007,1670,1692,2093,1645,1971,3672,2235,2714,2401,2334,19
10
OC90_2_14232,558,474,447,541,409,277,77,397,353,990,128,345
ODF3B_2_14233,42,42,54,102,0,50,5,5,29,1,0,106

OPLAH_2_14234,11,40,5,3,0,288,1,1,69,0,430,0
OSGEP_2_14235,46,19,64,33,86,0,70,32,0,83,18,32
OSGEPL1_2_14236,58,116,189,96,20,1,0,12,0,494,137,1
OSTC_2_14237,76,44,169,75,27,0,313,35,81,99,0,70
OTC_2_14238,425,314,292,426,420,11,0,141,63,594,96,342
OVGP1_2_14239,738,787,703,1060,164,552,938,718,345,177,969,770
OXA1L_2_14240,237,293,254,263,11,17,453,132,200,433,414,453
OXCT1_2_14241,252,166,153,262,98,115,498,48,153,262,302,167
OXCT2_2_14242,28,3,2,18,0,0,0,0,0,147,9,33
P4HA3_2_14243,376,380,416,396,91,496,975,211,252,182,317,763
P4HB_2_14244,140,160,159,219,124,542,11,346,299,92,143,201
PADI1_2_14245,235,653,368,476,186,84,194,636,270,366,1125,351
PADI2_2_14246,95,79,101,139,56,281,188,88,78,8,3,484
PADI3_2_14247,303,183,312,297,256,89,315,607,351,456,511,149
PADI6_2_14248,120,132,132,46,69,6,54,2,58,39,32,44
PAFAH2_2_14249,24,23,31,20,2,0,0,0,10,5,0,2
PAH_2_14250,353,433,399,528,455,176,543,718,574,465,432,525
PAPOLA_2_14251,656,522,442,514,217,124,477,326,249,793,239,462
PAPOLB_2_14252,2868,2294,2157,2905,3382,2782,2587,2374,1990,1549,2145,
2513
PAPOLG_2_14253,378,308,164,260,734,225,0,1,289,535,0,539
PAPPA_2_14254,266,267,414,445,36,138,454,1169,266,47,1447,828
PAPSS1_2_14255,474,399,298,400,346,409,483,1115,19,482,145,375
PARG_2_14256,1776,1869,1431,1568,1560,912,1074,1300,1710,1939,1352,206
8
PARP1_2_14257,400,402,329,486,451,379,465,409,328,404,93,387
PARP4_2_14258,84,69,66,126,22,3,236,0,21,7,42,33
PARP6_2_14259,1854,1535,1266,1808,1201,1066,1680,2072,1344,2407,1809,2
559
PARS2_2_14260,182,83,112,228,0,0,159,1,131,44,374,174
PCBD1_2_14261,217,166,230,228,179,240,2,0,240,102,603,435
PCBD2_2_14262,170,148,123,214,230,13,45,140,212,25,3,50
PCMT1_2_14263,86,32,23,85,0,14,74,89,16,97,0,2
PCMTD1_2_14264,231,146,175,211,66,15,41,43,96,272,285,25
PCYOX1_2_14265,52,7,1,42,8,2,37,0,0,4,19,13
PCYT1A_2_14266,610,1015,770,962,215,830,672,131,691,946,206,279
PDCD1LG2_2_14267,1730,1167,1431,1617,1336,679,1791,2335,1516,1305,1941
,1864
PDCL_2_14268,320,315,273,415,436,384,246,545,600,333,96,416
PDE3A_2_14269,31,5,1,45,104,1,0,0,0,17,361,223
PDE3B_2_14270,1514,867,1129,1287,1134,1084,960,902,1088,729,1379,717
PDE6A_2_14271,378,157,206,136,63,9,17,174,479,290,85,26
PDE6C_2_14272,2168,1957,1891,1881,2063,1577,2502,1778,3479,2515,2874,1
832
PDE6D_2_14273,193,345,262,288,424,2,96,489,438,531,500,21
PDE6G_2_14274,138,108,73,173,248,37,0,297,3,174,380,71
PDE6H_2_14275,1106,998,986,884,226,972,2299,1303,549,738,1157,654
PDE7B_2_14276,1271,1498,1270,1613,1648,2571,1885,1498,886,1266,1178,59
0
PDHA2_2_14277,1505,918,1173,1165,1497,1018,1075,1996,1268,953,1703,954

PDIA2_2_14278,1105,1069,882,1041,1393,787,2123,773,471,1054,833,1594
PDIA3_2_14279,417,268,251,428,477,375,725,208,272,828,203,335
PDIA4_2_14280,89,130,149,213,60,71,51,63,120,528,230,37
PDIA5_2_14281,396,417,510,501,1204,1524,573,574,142,898,423,259
PDIA6_2_14282,31,81,51,72,1,119,177,7,131,0,181,45
PDPR_2_14283,17,17,82,29,2,4,0,370,1,0,0,20
PDSS1_2_14284,308,185,173,195,853,358,79,106,44,65,16,210
PDSS2_2_14285,506,463,351,397,195,16,441,489,501,768,102,382
PDXP_2_14286,382,274,247,269,506,85,636,148,421,178,383,642
PECR_2_14287,527,456,855,730,156,342,1811,592,829,512,252,654
PELI1_2_14288,184,302,169,199,98,266,0,0,85,0,191,235
PET117_2_14289,655,573,482,687,2014,267,101,594,490,423,465,592
PEX1_2_14290,1428,1584,1557,1560,1766,1875,2373,1144,1292,1537,1328,14
96
PEX6_2_14291,486,631,405,466,987,130,1006,401,423,716,344,257
PFAS_2_14292,280,148,131,163,89,382,154,6,259,0,395,176
PGA3_2_14293,152,179,337,190,114,69,693,141,196,484,53,156
PGA4_2_14294,152,179,337,190,114,69,693,141,196,484,53,156
PGA5_2_14295,152,179,337,190,114,69,693,141,196,484,53,156
PGAM4_2_14296,152,250,176,159,169,432,136,180,145,47,72,161
PGAP1_2_14297,935,992,1051,1139,904,565,1080,846,723,1315,1395,1008
PGAP3_2_14298,161,147,227,335,0,1,712,0,238,510,102,10
PGD_2_14299,270,286,190,300,158,110,274,401,147,426,1,370
PGGT1B_2_14300,1512,1243,1279,1638,1157,1010,1233,824,1233,2063,1772,2
493
PGLS_2_14301,92,59,98,92,369,0,26,0,34,27,380,3
PGM2_2_14302,199,141,125,233,179,11,148,1,149,72,8,150
PGM2L1_2_14303,739,718,483,967,688,381,698,993,330,745,83,558
PGM5_2_14304,481,264,456,467,584,407,125,888,523,6,17,234
PGPEP1_2_14305,136,17,55,50,99,13,0,0,0,17,0,183
PGS1_2_14306,122,91,78,142,34,26,58,2,154,188,33,100
PHGDH_2_14307,211,291,197,312,679,237,0,661,407,1,300,170
PHLPP1_2_14308,76,28,21,51,37,0,0,26,24,35,103,101
PHLPP2_2_14309,154,318,395,240,345,1057,65,538,66,415,453,230
PIF1_2_14310,41,77,89,99,301,0,1,479,80,18,39,18
PIGB_2_14311,155,312,303,192,10,544,1315,23,122,220,462,234
PIGH_2_14312,114,142,140,151,158,0,141,4,484,0,20,371
PIGL_2_14313,282,250,164,274,416,211,1270,181,334,155,272,632
PIGM_2_14314,297,207,148,248,7,5,129,9,803,266,0,598
PIGS_2_14315,189,87,183,160,30,267,60,456,223,321,499,323
PIGU_2_14316,1028,775,816,803,1305,467,804,768,1126,865,932,763
PIGW_2_14317,409,320,313,323,441,745,427,1015,537,566,607,51
PIGZ_2_14318,121,27,55,33,6,0,0,0,164,3,0,0
PIN1_2_14319,155,211,70,187,145,1,105,206,0,518,0,416
PIPOX_2_14320,1175,725,896,1187,2801,463,951,592,1104,830,1091,1334
PITPNM2_2_14321,674,650,747,629,549,398,362,232,107,361,992,788
PLA2G10_2_14322,203,209,161,91,15,8,386,159,188,253,0,93
PLA2G12A_2_14323,712,468,520,762,300,144,48,110,387,608,159,664
PLA2G12B_2_14324,887,594,527,654,1282,645,826,1106,888,373,549,276
PLA2G15_2_14325,231,303,338,227,114,64,777,507,419,561,340,309

PLA2G1B_2_14326,363,170,233,305,337,42,892,0,145,0,1276,681
PLA2G2C_2_14327,596,555,622,467,1192,546,589,621,767,603,1389,811
PLA2G2D_2_14328,343,317,263,318,347,355,0,452,6,175,35,175
PLA2G2E_2_14329,118,287,87,74,0,19,2,253,132,217,630,189
PLA2G2F_2_14330,268,244,195,205,181,445,606,211,58,170,312,281
PLA2G3_2_14331,17,29,39,11,9,132,0,27,1,0,494,0
PLA2G4A_2_14332,216,262,289,393,602,2,846,24,514,351,314,269
PLA2G4B_2_14333,246,178,222,150,618,199,135,16,273,233,28,146
PLA2G4D_2_14334,166,50,91,71,0,1,383,0,13,198,0,1
PLA2G4E_2_14335,345,265,209,198,6,898,38,165,215,445,224,303
PLA2G4F_2_14336,847,662,584,440,689,820,1243,924,560,442,304,584
PLA2G5_2_14337,396,462,356,414,432,1303,449,439,10,380,121,313
PLCB2_2_14338,162,235,208,335,5,252,528,0,439,486,0,137
PLCD3_2_14339,805,527,569,800,1003,109,1614,167,917,962,128,912
PLCD4_2_14340,39,27,87,79,34,19,8,63,68,8,0,10
PLCG2_2_14341,103,26,21,13,0,3,3,123,47,116,91,167
PLCH2_2_14342,48,123,42,24,0,6,14,20,30,17,684,6
PLCL1_2_14343,510,500,440,533,416,129,678,845,702,456,1432,4
PLCXD1_2_14344,101,190,115,203,53,506,0,778,3,237,10,56
PLCZ1_2_14345,2331,2028,1627,2197,3401,1866,1478,1912,2129,1995,2554,2
746
PLD4_2_14346,69,119,141,118,162,6,0,291,99,0,691,101
PLOD1_2_14347,44,108,67,70,0,9,1,39,63,10,0,15
PLOD3_2_14348,13,12,22,12,17,18,21,0,73,0,4,14
PLSCR1_2_14349,225,250,194,253,574,175,93,72,682,511,320,48
PMM1_2_14350,1,14,6,1,0,0,0,0,3,0,1,101
PMM2_2_14351,132,107,143,98,92,225,0,28,83,195,190,399
PMPCA_2_14352,0,0,0,0,0,0,0,0,0,0,0,0
PMPCB_2_14353,68,43,19,12,11,29,72,0,6,127,11,221
PNLIP_2_14354,639,556,744,763,289,263,473,329,396,895,390,260
PNLIPRP1_2_14355,760,480,423,486,443,804,523,488,501,648,377,376
PNLIPRP2_2_14356,530,519,630,739,699,99,480,236,180,480,363,580
PNLIPRP3_2_14357,539,309,326,463,161,507,147,644,345,757,88,876
PNMT_2_14358,84,29,85,170,102,441,0,0,17,215,13,23
PNPLA2_2_14359,232,142,159,135,70,11,355,60,58,347,268,243
PNPLA3_2_14360,938,512,460,672,128,531,1851,266,706,651,182,568
PNPLA8_2_14361,187,196,276,209,508,0,422,193,432,300,26,22
PNPO_2_14362,35,9,43,42,0,112,3,0,46,37,121,2
PNPT1_2_14363,632,629,526,412,838,484,713,542,283,234,604,689
POLA1_2_14364,302,297,207,513,371,556,157,837,51,44,116,277
POLA2_2_14365,464,559,495,470,1061,322,777,481,387,391,1180,516
POLB_2_14366,483,431,432,392,393,118,929,681,450,687,491,736
POLD4_2_14367,201,109,144,408,299,276,171,355,42,5,460,367
POLE3_2_14368,204,112,171,104,108,18,515,29,228,52,552,16
POLE4_2_14369,291,138,125,166,23,143,0,221,170,208,210,186
POLE_2_14370,41,2,15,20,0,0,0,1,0,12,349,0
POLG2_2_14371,193,164,69,232,41,31,488,3,308,33,81,182
POLI_2_14372,393,334,371,343,14,402,54,481,133,195,983,383
POLM_2_14373,33,11,58,48,73,4,416,10,6,22,409,8
POLN_2_14374,1009,944,669,1029,387,129,2596,1013,877,1191,565,681

POLR1A_2_14375,468,306,403,384,610,77,24,140,508,352,307,563
POLR1C_2_14376,328,186,219,294,659,91,60,324,339,70,264,114
POLR1E_2_14377,104,405,210,214,135,70,137,190,284,492,36,133
POLR2A_2_14378,125,116,63,102,111,225,0,42,46,64,261,103
POLR2B_2_14379,909,1001,852,858,1676,1087,225,433,1155,1255,605,1652
POLR2C_2_14380,14,19,18,35,0,0,3,73,0,14,0,0
POLR2D_2_14381,15,34,21,48,0,0,0,12,30,0,60,159
POLR2E_2_14382,87,92,62,103,143,21,102,275,129,65,120,310
POLR2F_2_14383,202,122,168,145,275,250,54,53,216,245,209,72
POLR2G_2_14384,138,175,129,260,378,390,256,16,291,3,29,89
POLR2H_2_14385,467,147,225,198,0,1,294,852,130,175,0,523
POLR2J_2_14386,273,269,225,368,176,260,143,26,6,791,369,91
POLR2K_2_14387,85,115,75,231,1,5,0,21,1,166,154,36
POLR2L_2_14388,666,774,513,640,354,647,1044,191,365,1105,397,1179
POLR3A_2_14389,151,155,209,165,246,48,50,380,195,180,27,437
POLR3C_2_14390,177,158,110,239,49,31,68,49,540,55,0,57
POLR3F_2_14391,237,169,235,189,362,546,103,1,379,254,488,808
POLR3G_2_14392,1684,1405,1033,1315,1738,2407,1774,2204,1117,2913,2722,
1964
POLRMT_2_14393,56,132,164,219,21,214,0,93,5,225,0,176
POMGNT1_2_14394,130,173,199,159,143,274,351,46,15,16,184,19
POMT2_2_14395,214,208,159,222,62,183,301,119,255,180,403,571
PON3_2_14396,352,400,366,458,490,78,405,224,257,627,285,234
POP4_2_14397,137,252,262,222,0,560,32,408,37,4,178,227
POP7_2_14398,168,153,123,194,1,204,0,376,170,44,448,0
POR_2_14399,99,83,74,95,340,0,78,37,258,214,0,152
PPA1_2_14400,272,179,253,257,307,17,110,0,120,201,124,4
PPCDC_2_14401,183,95,125,75,67,10,186,15,380,162,41,186
PPIA_2_14402,6805,6136,6289,6978,6528,5500,6742,5869,6015,8164,5095,83
28
PPIAL4A_2_14403,6805,6136,6289,6978,6528,5500,6742,5869,6015,8164,5095
,8328
PPIAL4B_2_14404,6805,6136,6289,6978,6528,5500,6742,5869,6015,8164,5095
,8328
PPIAL4C_2_14405,6805,6136,6289,6978,6528,5500,6742,5869,6015,8164,5095
,8328
PPIAL4E_2_14406,6805,6136,6289,6978,6528,5500,6742,5869,6015,8164,5095
,8328
PPIAL4G_2_14407,6805,6136,6289,6978,6528,5500,6742,5869,6015,8164,5095
,8328
PPIB_2_14408,118,141,74,262,203,1,0,0,0,26,0,0
PPIC_2_14409,174,224,209,229,231,119,105,26,20,100,159,229
PPID_2_14410,141,138,234,294,17,158,52,208,109,98,53,208
PPIF_2_14411,338,237,283,175,95,466,386,317,270,506,206,441
PPIG_2_14412,382,337,225,267,381,247,201,381,216,639,129,618
PPIH_2_14413,3560,2867,3246,3591,3498,3554,2570,4390,3162,4797,3099,40
26
PPIL1_2_14414,630,572,495,600,573,380,19,643,816,449,14,316
PPIL4_2_14415,1427,1554,1358,1904,972,793,2063,469,2027,2497,3149,2170
PPIP5K2_2_14416,3142,2143,2169,2750,3412,1814,2019,1413,3305,2261,3494

,2165
PPM1H_2_14417,1362,1346,1511,1575,1393,1788,3801,1807,2076,1238,1279,1
565
PPM1J_2_14418,92,187,45,123,175,37,45,439,0,18,0,4
PPM1N_2_14419,1077,944,622,1019,1144,891,745,978,714,1403,1677,816
PPME1_2_14420,146,123,343,274,440,33,72,566,713,26,4,545
PPWD1_2_14421,716,820,630,714,1161,448,930,362,388,716,636,678
PRDX4_2_14422,702,821,745,785,558,343,591,560,350,624,1673,252
PREP_2_14423,1585,1180,1078,1616,1607,449,411,1485,1007,822,464,1804
PRHOXNB_2_14424,259,276,214,259,164,382,990,732,67,824,5,75
PRIM1_2_14425,273,311,224,422,178,329,611,177,106,374,425,715
PRIM2_2_14426,1083,724,720,922,284,776,660,922,306,971,209,887
PROC_2_14427,879,864,738,948,401,911,622,989,609,1256,1789,776
PROSC_2_14428,460,287,428,434,585,645,110,199,474,597,295,34
PRR14L_2_14429,251,266,332,354,13,1,182,185,88,464,339,52
PRSS1_2_14430,285,396,301,458,270,814,220,659,377,181,117,222
PRSS2_2_14431,422,506,399,366,118,323,771,16,653,1,727,162
PRTFDC1_2_14432,1638,1290,1103,1637,494,132,912,2364,577,1119,1728,854
PRTN3_2_14433,235,64,124,176,115,239,3,0,13,83,19,191
PRUNE_2_14434,559,433,358,467,459,252,1035,72,210,277,520,892
PSMA2_2_14435,2217,2177,2119,2372,3045,2220,2594,1291,1047,2995,2893,2
992
PSMA6_2_14436,48,104,95,65,84,104,354,0,49,120,23,36
PSMA7_2_14437,484,537,534,510,958,669,1297,669,332,741,649,917
PSMB10_2_14438,322,162,359,251,490,219,207,273,744,147,229,110
PSMB11_2_14439,30,55,85,132,7,16,46,71,113,11,0,135
PSMB1_2_14440,91,106,189,99,61,76,247,241,0,55,9,280
PSMB3_2_14441,910,694,782,943,526,939,75,397,1146,257,732,378
PSMB4_2_14442,54,50,64,78,0,4,47,122,0,126,659,2
PSMB6_2_14443,246,147,40,154,0,0,1,0,331,480,294,15
PSMB7_2_14444,147,46,44,69,23,0,37,210,178,39,144,64
PSMB9_2_14445,51,98,125,125,19,0,163,302,172,271,0,9
PSMD6_2_14446,539,614,334,504,45,83,0,961,589,764,303,538
PTDSS1_2_14447,108,156,313,225,18,84,343,47,47,8,1064,31
PTDSS2_2_14448,16,6,16,64,20,0,0,0,83,102,0,0
PTGDS_2_14449,54,44,163,115,1,122,6,19,145,23,22,119
PTGES2_2_14450,207,122,162,213,121,135,171,653,151,94,87,174
PTGES3_2_14451,1204,1525,1084,1355,1159,309,805,969,1323,1571,784,1659
PTGES_2_14452,648,382,547,678,632,296,375,276,652,508,940,688
PTGIS_2_14453,1724,1357,1338,1763,1133,796,3202,794,1686,2599,2523,114
5
PTGS2_2_14454,208,162,81,235,84,445,80,210,21,179,306,210
PTRH1_2_14455,18,62,81,65,96,14,0,14,7,74,0,2
PTRH2_2_14456,730,652,466,770,1026,926,1154,326,1062,1241,344,979
PTS_2_14457,62,90,72,81,1,10,284,113,242,43,103,89
PUS3_2_14458,421,466,333,553,354,940,319,27,391,525,553,279
PUSL1_2_14459,114,69,74,295,220,4,1,33,2,35,158,6
PXDN_2_14460,315,267,301,329,298,162,29,531,241,164,240,345
PXDNL_2_14461,125,208,172,236,31,26,53,465,166,10,411,163
PYCR2_2_14462,83,56,43,20,236,0,633,0,0,48,0,0

PYCRL_2_14463,412,259,311,336,266,94,634,23,297,165,114,228
PYGB_2_14464,181,151,247,251,152,312,349,105,231,130,70,301
QDPR_2_14465,0,0,0,0,0,0,0,0,0,0,0,0
QPCT_2_14466,38,32,69,74,235,0,4,0,34,0,0,3
QPRT_2_14467,235,177,254,230,210,562,11,54,378,273,337,548
QRSL1_2_14468,372,479,421,449,511,321,119,308,462,563,614,112
QSOX2_2_14469,197,136,160,129,8,93,12,375,2,68,7,278
QTRT1_2_14470,73,84,54,71,103,5,20,18,135,83,67,33
QTRTD1_2_14471,272,386,469,411,84,236,222,377,309,208,607,236
RAB10_2_14472,174,141,178,243,307,1,40,669,89,334,271,235
RAB12_2_14473,276,533,252,394,628,542,852,11,116,516,297,386
RAB13_2_14474,142,56,87,85,65,195,11,232,21,31,41,33
RAB14_2_14475,506,397,384,531,865,20,474,51,423,793,218,367
RAB15_2_14476,12,10,35,4,0,0,49,0,0,0,266,0
RAB17_2_14477,831,659,630,802,558,406,761,793,556,949,279,810
RAB18_2_14478,53,64,64,64,183,35,202,152,16,213,55,59
RAB19_2_14479,62,98,136,47,2,0,50,0,39,25,1,0
RAB1B_2_14480,486,313,347,304,185,228,231,334,7,251,333,630
RAB20_2_14481,222,246,208,346,462,253,178,18,192,303,896,203
RAB21_2_14482,501,539,403,532,557,209,0,572,818,1401,180,261
RAB22A_2_14483,946,487,678,597,1398,448,1202,1257,819,479,427,273
RAB25_2_14484,144,33,136,33,0,550,0,0,0,20,0,194
RAB26_2_14485,44,99,22,52,8,226,0,350,136,0,6,8
RAB27B_2_14486,1566,1410,1584,1801,660,2648,1774,2304,1651,1149,2545,1
200
RAB30_2_14487,240,167,107,199,105,1,14,0,318,34,6,147
RAB31_2_14488,8,14,3,5,0,0,0,0,62,0,0,11
RAB33A_2_14489,647,644,564,738,478,583,401,1537,774,822,870,882
RAB33B_2_14490,138,146,152,138,108,63,116,99,313,116,166,3
RAB36_2_14491,108,71,90,70,0,0,40,303,0,0,110,21
RAB38_2_14492,27,110,11,74,141,0,0,2,13,0,0,48
RAB39B_2_14493,7,2,18,2,0,0,335,0,0,0,0,6
RAB3A_2_14494,127,138,196,244,475,68,472,76,313,191,139,780
RAB3B_2_14495,142,154,334,221,486,220,1,4,268,8,393,704
RAB3C_2_14496,381,515,448,508,75,1221,6,247,272,205,1199,466
RAB3D_2_14497,385,215,250,313,537,0,205,448,1,1039,363,506
RAB3GAP2_2_14498,131,133,182,112,28,18,152,2,120,105,92,179
RAB4A_2_14499,673,546,481,733,53,259,373,1166,682,578,649,988
RAB4B_2_14500,193,286,200,200,287,68,219,136,709,89,320,396
RAB5A_2_14501,128,172,122,232,0,0,0,0,0,0,0,0
RAB5B_2_14502,96,154,109,232,20,10,13,37,126,494,0,263
RAB6B_2_14503,559,495,530,442,294,443,164,308,246,249,845,595
RAB6C_2_14504,262,246,214,329,327,415,177,137,179,175,1736,611
RAB7A_2_14505,214,212,126,261,60,16,8,144,300,325,3,104
RAB8B_2_14506,345,266,231,272,433,163,92,0,389,562,76,249
RAB9B_2_14507,88,91,96,84,4,332,497,103,239,11,55,67
RABGGTB_2_14508,1157,1004,1207,1377,1175,61,693,1524,1818,1519,802,211
3
RABL3_2_14509,271,203,240,386,833,19,109,277,606,57,32,278
RAC2_2_14510,129,159,97,190,240,361,289,193,172,186,0,310

RAD50_2_14511,388,168,243,335,100,134,261,179,477,572,78,623
RAD54L2_2_14512,298,414,497,381,887,97,25,27,76,117,31,303
RALA_2_14513,0,0,0,0,0,0,0,0,0,0,0,0
RALB_2_14514,1104,739,833,1111,1464,652,2122,1218,1132,1510,973,1203
RALBP1_2_14515,98,75,9,96,3,6,0,0,9,159,0,236
RANBP2_2_14516,1390,1130,1123,1253,545,1449,779,837,1486,1587,898,1522
RAN_2_14517,62,51,100,63,54,7,298,2,332,60,13,1
RAP2A_2_14518,168,130,139,122,112,43,15,178,223,257,50,90
RAP2B_2_14519,548,611,492,632,552,263,1690,725,194,274,368,550
RAP2C_2_14520,107,80,54,80,0,176,65,31,31,2,0,142
RARS2_2_14521,178,124,114,184,321,130,322,219,177,18,460,55
RARS_2_14522,1633,1421,1346,1735,1403,1602,1269,1452,2603,1720,1293,24
72
RASD2_2_14523,69,106,93,233,0,2,455,0,0,0,0,0
RASL10B_2_14524,0,2,2,12,3,0,0,0,0,0,1,0
RASL11B_2_14525,108,10,22,92,7,16,505,0,55,4,2,0
RASL12_2_14526,343,217,77,184,49,1,131,85,0,453,0,24
RC3H1_2_14527,184,167,145,244,47,335,295,258,397,85,35,345
RCL1_2_14528,140,140,144,141,152,205,111,380,135,56,156,362
RDH10_2_14529,200,252,184,171,420,376,419,494,0,0,0,94
RDH11_2_14530,466,468,377,439,846,326,345,1409,762,279,282,798
RDH12_2_14531,211,163,220,254,452,358,319,151,270,424,80,440
RDH14_2_14532,640,793,586,987,196,1054,659,666,270,1193,420,555
RDH16_2_14533,365,366,277,236,780,127,1243,137,327,910,233,294
RDH8_2_14534,625,489,528,720,267,376,182,259,498,376,600,508
RECQL4_2_14535,81,129,35,62,314,2,1,141,5,6,0,142
REM1_2_14536,134,123,117,89,250,3,46,0,7,34,46,14
REM2_2_14537,86,111,45,118,4,6,289,3,4,108,16,137
RENBP_2_14538,1807,1519,1806,1628,828,1711,1429,1229,713,1612,3651,128
0
RETSAT_2_14539,1216,1093,806,1214,1336,1341,1354,461,438,1528,877,414
REV3L_2_14540,292,262,287,314,35,167,8,629,534,276,386,67
REXO2_2_14541,1331,1332,1380,1383,1315,1503,2291,796,710,1984,2856,132
6
RFNG_2_14542,458,239,290,451,253,89,553,700,307,47,376,648
RFX6_2_14543,2003,1679,1850,2212,2307,1591,1363,1619,2234,731,2770,166
8
RGS7_2_14544,444,458,358,356,394,424,984,490,382,295,674,298
RHBDL1_2_14545,286,175,368,223,493,694,463,33,735,376,787,71
RHBDL2_2_14546,511,636,643,735,442,1080,791,1044,559,254,471,1455
RHBDL3_2_14547,706,414,403,552,218,4,122,257,379,521,678,477
RHEBL1_2_14548,38,33,52,74,0,0,1,0,2,15,8,2
RHOA_2_14549,239,413,364,210,94,705,20,0,615,610,470,200
RHOB_2_14550,487,511,442,420,1105,475,417,284,440,592,101,160
RHOD_2_14551,61,90,35,47,32,1,27,0,0,220,0,155
RHOF_2_14552,13,33,5,15,123,203,0,11,19,0,0,0
RHOG_2_14553,100,74,135,21,562,20,5,9,7,1,417,509
RHOH_2_14554,105,38,122,171,72,2,27,2,0,276,498,240
RHOJ_2_14555,44,32,14,59,0,2,0,125,9,11,20,3
RHOQ_2_14556,237,343,119,403,333,190,157,563,33,280,211,558

RHOT2_2_14557,308,249,142,368,66,0,215,349,687,546,77,259
RHOU_2_14558,1093,614,770,891,1209,624,1065,110,787,473,85,521
RHOV_2_14559,511,330,490,564,519,197,93,526,150,326,591,573
RIT1_2_14560,492,156,215,319,229,53,208,6,211,177,132,180
RIT2_2_14561,201,217,128,135,9,38,7,132,150,85,23,220
RNASE2_2_14562,80,100,191,154,0,0,5,16,0,474,0,131
RNASE3_2_14563,580,662,547,663,376,470,1264,224,579,39,675,659
RNASE6_2_14564,216,151,127,162,281,66,226,91,248,429,227,106
RNASE7_2_14565,88,26,77,108,18,1,2,119,262,7,197,0
RNASE8_2_14566,375,337,450,487,421,148,330,1872,377,489,343,576
RNASEH2A_2_14567,230,128,299,270,205,267,63,120,205,74,0,317
RNASET2_2_14568,947,932,975,752,665,858,2541,1316,1231,1237,621,747
RND1_2_14569,193,177,191,180,85,471,468,205,82,89,200,199
RND2_2_14570,163,146,167,136,64,9,40,52,81,72,147,283
RND3_2_14571,204,186,200,287,36,228,190,75,131,99,190,243
RNF148_2_14572,576,525,526,677,537,134,132,217,822,430,181,702
RNMT_2_14573,613,532,530,527,337,395,1182,406,496,1337,1008,558
RNMTL1_2_14574,919,991,1039,900,1214,1094,2374,661,939,2500,1052,451
RNPEP_2_14575,469,244,241,308,466,236,328,503,230,468,349,268
RPAP2_2_14576,317,145,273,363,538,374,426,328,525,165,93,465
RPE65_2_14577,339,112,185,303,170,381,54,507,617,141,127,124
RPIA_2_14578,264,210,160,326,1172,278,738,91,408,3,200,332
RPL4_2_14579,53,188,102,167,154,294,129,319,0,151,95,54
RPN1_2_14580,327,253,226,446,94,665,0,668,145,362,313,872
RPP25_2_14581,6,0,16,8,0,0,0,0,0,0,0,0
RPP40_2_14582,3827,4009,3045,3858,3450,1713,5658,3132,5221,3288,4737,5
349
RPS3_2_14583,1322,1497,1589,1659,718,1509,2081,380,1069,827,1569,632
RPUSD1_2_14584,79,129,200,212,1,219,0,19,14,156,226,41
RPUSD2_2_14585,150,270,184,152,529,119,29,203,138,55,5,513
RRAGA_2_14586,882,824,652,714,226,940,1736,260,1104,780,731,762
RRAGC_2_14587,418,340,487,275,238,236,147,640,151,687,346,391
RRAGD_2_14588,1142,1041,1053,787,1413,911,647,562,875,1367,410,1690
RRAS_2_14589,273,373,266,502,219,403,533,22,690,426,320,835
RRM1_2_14590,9,86,10,43,10,0,0,0,0,19,0,0
RRP8_2_14591,150,30,93,52,0,0,1,189,343,163,779,118
RSAD2_2_14592,336,275,214,179,8,145,149,56,427,352,303,209
RTN4IP1_2_14593,77,26,27,24,25,0,0,29,0,0,0,46
SAMHD1_2_14594,355,193,391,345,808,473,375,125,258,39,755,241
SDF2_2_14595,341,240,338,377,32,524,177,222,549,245,647,144
SDHA_2_14596,84,118,124,58,129,222,15,23,10,58,5,56
SDHB_2_14597,2299,2535,2150,2395,2754,3415,3048,3032,993,3533,2913,152
1
SDHD_2_14598,103,99,138,98,167,496,1,15,0,293,0,27
SDR16C5_2_14599,870,974,888,1179,1306,530,429,837,1322,751,160,886
SDR42E1_2_14600,1082,899,910,1081,1474,810,777,793,797,839,1400,1156
SDR9C7_2_14601,181,241,206,176,1366,262,1,9,322,18,677,0
SDSL_2_14602,236,143,219,205,3,101,1,5,3,138,149,154
SEPSECS_2_14603,938,810,1200,989,352,1111,963,882,1405,425,352,878
1-Sep_2_14604,13,32,33,5,0,51,45,1,19,4,1,0

SEPW1_2_14605,1426,1180,1256,1418,1884,1123,1261,486,1220,1698,1743,14
17
SERHL2_2_14606,634,927,557,603,896,55,874,744,316,1525,1121,646
SETX_2_14607,743,666,725,809,207,11,453,512,708,180,1081,774
SGMS1_2_14608,1847,1247,1531,1703,1705,2008,2151,1067,1187,881,2690,72
1
SGPL1_2_14609,393,565,500,600,433,125,686,172,452,41,164,712
SGSH_2_14610,106,167,151,140,19,9,0,359,30,183,5,189
SH3GL2_2_14611,1115,964,983,1264,790,658,1715,1134,753,969,611,929
SKIV2L2_2_14612,15,25,20,25,0,0,0,18,1,1,0,165
SLFN12_2_14613,1149,951,950,1643,202,941,426,2354,987,1213,1353,2010
SLFN12L_2_14614,1149,951,950,1643,202,941,426,2354,987,1213,1353,2010
SLFN13_2_14615,103,108,65,77,66,85,3,112,82,146,0,171
SLFN5_2_14616,379,454,398,415,392,273,809,379,551,113,275,449
SLU7_2_14617,323,295,275,267,675,299,9,151,489,479,956,260
SMG8_2_14618,965,844,701,1178,669,1357,2225,395,533,1039,1273,701
SMPD2_2_14619,97,82,55,83,126,53,2,49,73,22,0,13
SMPD3_2_14620,107,37,61,74,0,1,11,180,212,34,1,66
SMPDL3A_2_14621,196,93,151,143,93,0,11,4,22,118,407,56
SMUG1_2_14622,166,50,39,50,14,0,0,4,316,0,93,0
SNF8_2_14623,271,150,191,158,390,209,169,11,54,444,104,43
SNRNP200_2_14624,168,153,217,165,571,159,27,0,1,77,211,12
SOAT1_2_14625,46,87,87,90,2,12,134,70,32,56,15,48
SOAT2_2_14626,21,28,10,14,0,75,7,48,0,0,269,50
SOD1_2_14627,199,116,66,128,1,241,337,3,113,147,0,376
SOD3_2_14628,572,501,480,459,104,74,30,900,398,872,50,381
SORD_2_14629,56,144,119,112,0,126,3,8,4,479,121,243
SPACA3_2_14630,226,182,155,218,400,3,0,298,39,22,346,90
SPACA5B_2_14631,216,150,242,223,138,4,8,161,199,26,485,138
SPACA5_2_14632,216,150,242,223,138,4,8,161,199,26,485,138
SPEM1_2_14633,174,196,126,187,739,138,39,620,39,397,187,356
SPTLC2_2_14634,277,133,424,307,374,492,45,382,110,942,1166,43
SPTLC3_2_14635,389,335,233,294,860,265,513,365,150,254,1741,376
SPTSSA_2_14636,126,168,130,97,652,0,15,285,169,192,0,169
SQLE_2_14637,286,168,182,170,358,86,309,42,54,432,41,72
SQRDL_2_14638,91,83,79,107,170,124,63,1,32,11,164,76
SRD5A1_2_14639,261,390,372,342,4,32,1255,265,437,731,2379,236
SRD5A2_2_14640,406,372,273,512,297,839,273,502,311,622,17,56
SRD5A3_2_14641,1196,1279,1128,1198,852,613,2058,644,928,1109,938,990
SRR_2_14642,128,105,73,111,180,0,0,36,77,102,136,28
SRSF9_2_14643,233,259,164,280,84,191,15,328,58,75,357,83
SRXN1_2_14644,76,177,248,114,0,27,484,6,0,4,1033,46
SSB_2_14645,191,310,151,203,36,134,901,570,380,138,58,419
SSU72_2_14646,557,583,482,513,720,460,296,313,581,838,692,329
ST14_2_14647,629,484,366,403,11,103,648,173,906,315,358,50
ST3GAL2_2_14648,149,286,173,402,22,394,604,61,382,353,750,440
ST3GAL4_2_14649,262,304,372,300,168,0,300,788,6,323,1099,642
ST3GAL6_2_14650,336,316,345,459,298,250,55,796,238,844,192,532
ST6GALNAC1_2_14651,304,195,185,277,425,352,99,180,188,73,302,677
ST6GALNAC2_2_14652,618,354,444,465,1508,267,954,307,357,944,743,274

ST6GALNAC5_2_14653,364,123,196,195,95,155,599,0,84,141,256,254
ST6GALNAC6_2_14654,158,54,89,78,59,91,12,215,94,136,5,190
ST8SIA1_2_14655,788,492,356,523,815,580,1460,77,756,488,198,529
ST8SIA2_2_14656,693,892,673,871,1197,739,948,188,844,467,354,450
ST8SIA3_2_14657,188,264,239,389,155,67,0,275,163,954,102,600
ST8SIA5_2_14658,100,153,158,158,0,221,383,71,31,0,0,195
ST8SIA6_2_14659,210,196,101,260,262,3,44,150,85,208,0,18
STS_2_14660,251,91,179,191,45,21,284,128,218,74,48,120
STT3A_2_14661,414,416,377,397,551,40,1,167,112,472,448,417
STT3B_2_14662,0,0,0,0,0,0,0,0,0,0,0,0
SUCLA2_2_14663,961,619,681,736,296,888,1375,518,689,1617,1205,726
SUCLG1_2_14664,117,51,130,36,169,147,6,0,4,4,285,119
SULT1A3_2_14665,347,541,327,461,662,118,170,490,250,116,775,570
SULT1A4_2_14666,347,541,327,461,662,118,170,490,250,116,775,570
SULT1B1_2_14667,913,697,616,599,368,825,694,183,1169,150,392,1143
SULT1C3_2_14668,604,878,593,968,794,330,1267,515,934,737,497,818
SULT1C4_2_14669,556,432,485,568,960,323,924,132,811,489,205,627
SULT1E1_2_14670,1087,797,933,1144,1743,720,1149,1560,955,1346,851,1536
SULT2A1_2_14671,199,106,117,160,376,42,153,397,204,25,51,176
SULT4A1_2_14672,3939,3978,3425,3807,3573,3124,3871,3432,4121,4281,3655
,4698
SUPV3L1_2_14673,610,454,339,368,82,1223,791,0,427,63,592,233
SURF1_2_14674,504,431,417,575,452,79,1044,545,204,179,328,1228
TALDO1_2_14675,592,499,374,595,1115,514,1343,587,543,828,296,1197
TARS2_2_14676,79,71,83,81,2,2,38,57,31,32,407,119
TARS_2_14677,526,206,337,427,461,263,773,55,945,140,65,126
TARSL2_2_14678,3204,2910,2466,2891,3377,2041,3787,3592,2374,2422,908,2
396
TAT_2_14679,71,33,32,62,198,1,5,0,59,0,1,182
TBC1D10B_2_14680,17,22,15,138,87,1,84,1,5,0,0,0
TBCC_2_14681,65,53,6,125,30,0,0,46,0,16,0,1
TDG_2_14682,1090,867,701,1109,1899,721,1124,1326,404,1210,852,831
TDO2_2_14683,53,32,28,36,174,11,263,9,2,0,209,37
TECR_2_14684,228,232,261,309,563,820,498,115,23,161,93,350
TECTA_2_14685,186,92,109,54,3,74,0,13,167,61,0,11
TFB2M_2_14686,84,92,154,262,89,55,18,51,9,0,678,49
TGDS_2_14687,1187,1008,883,1074,1431,1191,971,1790,1137,1998,1070,1110
TGM1_2_14688,549,669,660,560,409,1039,483,769,327,645,487,1044
TGM3_2_14689,21,2,14,39,0,0,0,0,63,0,0,87
TGM4_2_14690,56,148,124,159,34,149,323,509,63,36,344,383
TGM6_2_14691,1379,1157,933,1411,605,1416,479,1474,1996,1326,2107,897
TGM7_2_14692,124,32,86,97,8,0,11,167,9,3,0,1
TGS1_2_14693,1388,1669,1252,1365,1309,872,1890,475,1398,1405,492,1210
THG1L_2_14694,383,145,312,426,294,140,564,396,794,403,1,312
THOP1_2_14695,55,16,27,33,72,172,0,2,0,15,75,0
THUMPD2_2_14696,1578,1261,1304,1810,1609,1005,3045,1324,628,560,1336,1
690
TKTL2_2_14697,588,417,424,620,303,278,1074,444,678,177,721,1119
TM7SF2_2_14698,517,644,597,836,1036,1054,1340,1125,351,1186,1397,519
TMEM55A_2_14699,977,1529,1147,1248,346,974,809,1376,1306,967,583,957

TMEM62_2_14700,734,548,497,564,344,415,509,872,526,648,457,448
TMEM86B_2_14701,32,26,26,12,7,0,3,0,0,0,0,0
TMPRSS15_2_14702,136,137,189,137,134,54,582,15,7,138,292,190
TMX1_2_14703,895,563,711,803,1621,1018,174,308,698,946,892,950
TMX3_2_14704,229,315,312,263,50,39,32,468,100,255,24,478
TMX4_2_14705,318,363,307,258,449,573,613,456,444,476,30,284
TNKS2_2_14706,694,350,538,499,1229,254,752,137,427,819,212,798
TNKS_2_14707,644,869,752,693,668,570,330,981,906,513,722,698
TOP1_2_14708,413,306,469,470,631,155,268,673,496,258,731,527
TOP1MT_2_14709,28,17,33,18,0,47,0,106,3,1,330,0
TOP2A_2_14710,169,205,238,238,256,253,1079,84,350,496,663,339
TOP2B_2_14711,1209,787,965,1168,1313,261,570,2290,797,870,1408,1070
TOP3A_2_14712,0,0,0,0,0,0,0,0,0,0,0,0
TOP3B_2_14713,286,337,230,411,213,73,7,377,424,690,217,61
TOR3A_2_14714,64,34,106,91,0,118,120,1,1,109,56,88
TPH1_2_14715,1204,916,1188,1032,2586,73,1115,784,2491,669,768,1654
TPMT_2_14716,239,295,211,448,342,920,321,284,66,581,323,473
TPP1_2_14717,637,484,518,407,321,248,409,511,410,747,2535,721
TPP2_2_14718,144,88,53,218,150,50,281,219,199,167,240,3
TPSAB1_2_14719,17,9,63,72,0,0,0,5,15,0,213,0
TPSB2_2_14720,207,92,264,181,23,555,347,782,169,346,588,485
TPSD1_2_14721,13,30,5,3,0,0,0,40,27,0,0,10
TPST1_2_14722,150,223,232,193,82,683,45,359,647,390,246,258
TREH_2_14723,60,36,49,37,492,0,26,13,52,0,0,0
TREX2_2_14724,123,147,149,142,305,25,25,339,183,248,16,35
TRHDE_2_14725,1581,1186,1322,1515,1538,1128,1970,937,1202,1466,1905,69
7
TRIM21_2_14726,115,125,93,119,775,0,17,115,559,0,630,42
TRIT1_2_14727,486,427,463,519,244,428,1279,252,984,707,2132,745
TRMT112_2_14728,952,851,909,934,1094,770,1293,555,822,574,926,683
TRMT61A_2_14729,3,1,103,28,0,25,0,0,22,0,49,0
TRMU_2_14730,61,215,163,139,12,0,10,611,2,618,4,226
TRNT1_2_14731,659,820,669,1042,1486,289,1388,1042,1138,705,336,1213
TRUB1_2_14732,217,167,105,160,278,121,651,192,23,91,401,272
TRUB2_2_14733,241,240,201,166,127,245,310,115,202,63,122,117
TSTA3_2_14734,27,60,23,41,22,9,5,0,0,46,0,0
TST_2_14735,128,110,109,47,695,73,0,52,94,450,0,37
TTLL13_2_14736,284,186,250,229,221,604,283,281,652,88,15,63
TTLL1_2_14737,208,171,110,181,171,62,121,145,94,418,52,336
TTLL3_2_14738,349,219,348,449,253,323,53,1013,330,60,698,647
TTLL4_2_14739,255,214,61,187,318,2,0,1,13,246,544,0
TUFM_2_14740,282,185,166,159,297,161,624,10,127,461,9,62
TULP2_2_14741,44,111,165,233,261,11,408,0,90,308,454,434
TUT1_2_14742,166,145,170,156,51,0,859,47,375,443,155,478
TXN2_2_14743,192,128,246,141,0,0,334,10,37,236,855,62
TXN_2_14744,758,485,750,822,1080,426,1107,893,256,282,734,607
TXNDC11_2_14745,167,172,140,104,255,8,90,556,10,93,156,73
TXNDC12_2_14746,430,350,315,544,127,343,215,860,177,169,216,1056
TXNDC15_2_14747,409,162,332,356,15,375,209,419,198,36,244,437
TXNDC17_2_14748,144,79,141,114,331,0,1,64,3,0,748,391

TXNL1_2_14749,531,613,633,643,689,355,653,512,249,785,620,433
TXNL4A_2_14750,257,155,211,224,50,234,462,621,536,305,1,12
TXNRD2_2_14751,202,119,134,200,73,0,664,321,414,460,886,355
TYMS_2_14752,666,585,529,766,695,582,783,657,383,897,619,1082
TYR_2_14753,1129,958,1333,1334,1377,970,1589,1828,1428,853,832,1613
TYRP1_2_14754,211,196,287,208,267,185,236,221,239,248,440,224
UAP1_2_14755,384,459,519,421,379,1056,742,57,689,1020,443,444
UAP1L1_2_14756,165,165,181,271,171,61,94,7,188,472,1,13
UBB_2_14757,159,344,223,245,53,122,182,230,224,286,1,665
UBIAD1_2_14758,543,543,323,416,233,422,641,131,835,212,410,690
UBL4A_2_14759,91,166,89,113,80,14,88,100,300,62,180,72
UFSP1_2_14760,962,822,664,936,1048,168,1370,702,733,826,418,808
UFSP2_2_14761,2121,1957,1875,1767,2176,1695,1995,1978,2530,1905,2095,1
552
UGCG_2_14762,378,337,259,406,381,324,108,266,153,509,4,523
UGGT1_2_14763,2013,1855,1724,1789,2321,1752,1856,1592,1743,657,1324,18
41
UGGT2_2_14764,64,116,54,60,27,0,335,28,147,0,0,48
UGT1A10_2_14765,7380,7453,6654,7381,9006,6776,5932,5233,7222,7506,7058
,6773
UGT1A1_2_14766,525,386,544,423,505,292,100,1391,176,281,388,660
UGT1A3_2_14767,7380,7453,6654,7381,9006,6776,5932,5233,7222,7506,7058,
6773
UGT1A4_2_14768,181,119,101,112,422,105,311,34,63,33,235,109
UGT1A5_2_14769,7380,7453,6654,7381,9006,6776,5932,5233,7222,7506,7058,
6773
UGT1A7_2_14770,1476,1527,1398,1725,1801,1199,2374,1593,1244,1299,1096,
1372
UGT1A8_2_14771,2431,1866,1948,2591,2024,1618,3042,1710,2924,1959,3480,
2208
UGT1A9_2_14772,0,0,0,0,0,0,0,0,0,0,0,0
UGT2A1_2_14773,1100,1170,1101,1264,1810,1106,1139,2053,1255,1241,1226,
760
UGT2A2_2_14774,1100,1170,1101,1264,1810,1106,1139,2053,1255,1241,1226,
760
UGT2A3_2_14775,531,363,459,439,347,771,115,35,474,144,241,417
UGT2B15_2_14776,1834,1348,1588,1964,1367,557,2167,1900,1841,1060,1151,
1539
UGT2B17_2_14777,1834,1348,1588,1964,1367,557,2167,1900,1841,1060,1151,
1539
UGT2B4_2_14778,91,126,83,114,97,20,75,56,255,232,0,64
UGT2B7_2_14779,3031,2257,2159,2822,1818,2425,2243,2247,2532,2205,2133,
3075
UMPS_2_14780,1079,1182,1020,1314,1636,616,2151,698,1408,966,676,2009
UPB1_2_14781,172,41,134,136,742,44,0,132,222,63,348,59
UPF1_2_14782,145,113,120,62,167,287,163,90,0,0,478,0
UPRT_2_14783,69,136,107,135,0,1,0,119,33,227,14,499
UQCR11_2_14784,3433,2819,2391,2858,4022,2538,2548,2641,3538,3336,1546,
2287
UQCRC1_2_14785,1084,1251,816,1071,1016,803,1672,860,530,816,134,1199

UQCRC2_2_14786,1887,1427,1358,1854,2105,2069,1235,1707,540,1588,2432,1
092
UQCRFS1_2_14787,65,34,98,187,5,230,570,380,42,183,0,62
UQCRH_2_14788,423,455,411,561,262,313,328,362,437,141,201,607
UQCRQ_2_14789,334,403,294,376,637,242,269,1217,240,915,664,97
UROD_2_14790,15,8,3,21,0,0,0,0,11,0,0,0
UROS_2_14791,1358,1782,1514,1955,581,2522,1814,1919,602,1000,1380,1990
UST_2_14792,536,593,795,620,998,74,805,466,542,465,54,320
UXS1_2_14793,394,278,291,356,34,139,34,274,211,276,92,373
VARS_2_14794,278,333,152,320,258,267,680,680,51,86,573,143
VAT1L_2_14795,89,230,180,231,235,1,0,7,315,3,0,12
VCP_2_14796,278,210,123,235,12,208,531,910,441,165,128,78
VNN1_2_14797,152,157,188,143,152,12,0,26,6,12,0,22
WBSCR17_2_14798,375,373,357,353,90,286,21,107,204,248,195,96
WBSCR27_2_14799,210,120,132,228,4,231,0,278,150,412,255,1
WDFY3_2_14800,94,77,87,101,37,226,84,219,13,18,1,157
WRN_2_14801,730,524,411,843,618,162,335,320,159,344,298,10
XDH_2_14802,153,241,106,180,347,312,0,279,355,104,19,91
XPNPEP2_2_14803,391,282,457,295,437,0,0,46,50,260,1053,179
XRCC2_2_14804,366,342,272,257,437,39,478,282,607,77,127,875
XRCC5_2_14805,338,374,164,353,627,49,762,98,344,397,582,668
XRCC6_2_14806,234,442,271,304,7,1722,1156,687,226,157,475,733
XRN2_2_14807,450,477,579,579,1201,413,209,2,986,469,870,350
XYLT1_2_14808,627,686,501,537,828,729,664,760,682,479,255,85
XYLT2_2_14809,7,3,45,35,0,0,0,2,0,6,0,1
YARS2_2_14810,55,177,22,60,0,0,0,0,25,0,6,289
YARS_2_14811,35,47,9,108,37,0,27,0,0,1,187,5
YKT6_2_14812,350,427,344,265,379,329,55,1,108,929,296,330
YPEL1_2_14813,667,466,369,415,1128,396,717,300,424,285,858,685
ZADH2_2_14814,217,502,272,360,140,203,83,366,158,101,467,426
ZCCHC4_2_14815,106,77,97,97,54,94,0,181,43,4,0,114
ZDHHC17_2_14816,170,206,253,343,12,495,174,148,71,344,225,136
ZDHHC18_2_14817,282,305,237,235,122,554,492,41,191,509,678,288
ZDHHC1_2_14818,477,320,501,359,331,351,185,302,798,292,654,755
ZDHHC21_2_14819,220,173,164,279,3,402,980,20,13,0,680,415
ZDHHC2_2_14820,0,0,0,0,0,0,0,0,0,0,0,0
ZER1_2_14821,1733,1208,1605,1559,1095,1068,3537,1546,1931,1270,1612,20
62
ZMPSTE24_2_14822,498,374,266,538,146,589,771,176,401,468,275,202
ZRANB3_2_14823,911,903,945,818,891,336,948,1180,1172,926,991,497
A1CF_2_14824,721,543,615,719,660,621,328,884,414,968,220,984
A2LD1_2_14825,154,39,93,129,223,0,145,54,287,35,49,64
AADAT_2_14826,238,365,335,558,227,248,873,515,405,131,616,766
AARSD1_2_14827,118,30,107,200,2,0,0,13,2,361,0,44
ABAT_2_14828,247,182,188,258,291,205,148,92,0,338,40,237
ABHD11_2_14829,50,42,12,50,4,0,0,0,0,0,87,13
ABHD12_2_14830,269,244,190,246,477,206,518,168,269,79,51,209
ABHD14B_2_14831,13,27,85,20,14,3,0,0,8,95,26,13
ABHD2_2_14832,143,150,46,88,0,104,7,0,0,244,106,17
ACAA1_2_14833,7,29,29,14,0,218,0,0,0,0,0,0

ACACA_2_14834,553,443,577,707,482,555,1319,486,445,1025,883,1554
ACAD10_2_14835,296,179,143,205,247,18,419,323,63,232,17,201
ACADM_2_14836,356,273,374,281,203,641,963,734,134,251,70,81
ACADVL_2_14837,431,308,223,513,196,255,368,910,418,357,460,806
ACCS_2_14838,53,84,266,169,6,12,26,9,208,252,0,1
ACE_2_14839,15,20,32,13,1,21,0,0,32,35,1,0
ACHE_2_14840,1131,1212,1102,1231,1748,1183,465,2017,1107,595,936,1544
ACIN1_2_14841,748,827,765,796,254,720,820,830,529,741,1307,439
ACLY_2_14842,95,37,161,132,10,368,568,10,1,1,509,1
ACOT11_2_14843,1529,1163,1460,1435,1373,932,1246,2317,1711,1113,728,16
15
ACOT13_2_14844,379,459,450,475,386,521,1514,991,286,501,1,636
ACOT7_2_14845,7,41,30,62,109,6,61,6,0,0,97,23
ACOT9_2_14846,977,1017,898,1237,937,1651,569,1725,1072,1207,1434,1881
ACOX1_2_14847,594,485,443,538,574,405,433,78,136,300,813,538
ACOX3_2_14848,976,988,997,1332,996,744,662,630,1511,1011,1198,1370
ACSBG1_2_14849,41,28,82,71,108,5,468,121,4,137,8,52
ACSF3_2_14850,197,163,241,179,77,48,184,84,60,147,220,3
ACSL3_2_14851,476,454,320,318,615,5,148,308,282,1153,452,521
ACSL4_2_14852,871,969,910,866,886,375,1614,858,952,372,1810,750
ACSL5_2_14853,1173,1586,1070,1297,2132,2803,1235,1528,791,1367,1200,10
05
ACSL6_2_14854,111,103,117,132,188,26,53,128,224,250,96,470
ACSM2B_2_14855,253,245,165,236,14,18,403,316,151,184,0,611
ACSM3_2_14856,414,167,291,325,445,241,564,277,393,0,304,669
ACSS2_2_14857,416,369,330,329,660,200,677,236,130,401,373,160
ACY1_2_14858,434,511,311,419,570,978,268,705,268,724,1787,1579
ADAMTS2_2_14859,527,458,306,309,624,94,654,442,144,136,877,52
ADARB1_2_14860,547,716,370,711,563,915,918,815,234,1331,920,1072
ADAR_2_14861,50,27,83,47,101,0,7,0,20,0,0,0
ADCY10_2_14862,850,971,749,823,332,503,38,785,1121,760,363,1159
ADCY4_2_14863,242,115,260,356,0,1,0,125,0,214,140,0
ADCY5_2_14864,286,520,485,295,263,222,1332,414,348,605,12,205
ADCY6_2_14865,286,243,267,202,469,183,361,372,306,812,68,452
ADH6_2_14866,84,73,71,107,207,35,271,3,131,26,26,327
ADH7_2_14867,131,30,81,187,81,134,258,11,127,0,121,34
ADPRHL1_2_14868,638,349,296,411,636,498,669,311,302,391,180,476
ADSL_2_14869,711,742,819,693,917,344,552,108,700,385,492,859
ADSSL1_2_14870,135,144,291,119,545,273,131,781,93,370,196,47
AFMID_2_14871,272,280,209,342,1,337,312,470,158,96,2,303
AGA_2_14872,688,769,617,733,738,758,240,414,210,858,1490,647
AGAP1_2_14873,108,68,101,132,53,20,339,356,1,6,140,14
AGAP2_2_14874,75,118,47,49,284,210,0,0,0,0,0,0
AGL_2_14875,822,605,687,836,418,327,1002,598,1040,1093,575,534
AGPAT1_2_14876,967,846,622,756,480,530,531,110,499,210,1167,728
AGPAT2_2_14877,207,150,288,243,34,285,309,179,106,382,361,186
AGPAT3_2_14878,264,145,210,283,711,35,585,1792,162,531,660,152
AGXT2L1_2_14879,1584,1320,1360,1445,2800,1233,2446,1032,1644,2000,3170
,1694
AHCY_2_14880,170,99,90,70,95,7,103,334,32,430,2,87

AHCYL1_2_14881,311,279,285,507,709,206,571,150,610,371,1149,642
AHCYL2_2_14882,0,0,0,0,0,0,0,0,0,0,0,0
AIFM1_2_14883,997,807,909,1016,506,222,703,889,1295,656,565,294
AIFM2_2_14884,22,75,32,11,0,0,1,0,137,20,0,0
AIFM3_2_14885,45,98,33,106,163,45,156,121,47,48,0,176
AKIRIN1_2_14886,410,398,447,386,152,426,828,288,201,62,255,609
AKR1A1_2_14887,117,135,147,158,36,3,30,684,75,114,77,138
AKR1C2_2_14888,330,412,258,499,434,595,297,9,344,636,471,546
AKR1D1_2_14889,561,435,484,680,485,917,930,297,1180,695,254,751
ALAS1_2_14890,1263,1370,1078,1099,769,1073,1102,1340,925,1208,1619,139
2
ALAS2_2_14891,1116,730,1122,964,1699,637,1371,955,800,1039,2279,985
ALDH16A1_2_14892,97,23,42,123,285,8,15,27,36,3,1,0
ALDH1A2_2_14893,22,165,42,141,20,0,34,0,110,1,191,7
ALDH2_2_14894,390,307,208,502,322,324,276,312,573,196,49,484
ALDH3A1_2_14895,527,461,539,397,80,661,363,796,104,28,427,400
ALDH3A2_2_14896,705,408,664,629,203,451,1193,436,674,562,1649,602
ALDH3B1_2_14897,99,101,124,109,99,85,250,379,194,129,58,188
ALDH3B2_2_14898,327,381,507,352,582,63,3,35,1286,69,574,323
ALDH4A1_2_14899,11,31,35,34,0,0,0,0,30,0,0,0
ALDH5A1_2_14900,1080,1226,1376,1287,1571,518,1227,1315,1183,814,1264,1
226
ALDH7A1_2_14901,186,153,125,161,83,42,77,27,83,363,2,6
ALDH8A1_2_14902,44,20,40,21,0,0,0,0,308,80,0,4
ALDOA_2_14903,29,115,74,48,33,0,15,50,2,0,8,0
ALG3_2_14904,228,182,117,72,269,2,0,0,0,66,0,169
ALG5_2_14905,808,984,715,1031,802,1143,289,1646,1485,656,98,872
ALG8_2_14906,178,89,129,136,605,29,765,107,103,54,0,130
ALG9_2_14907,232,139,325,367,395,50,126,102,279,306,1,122
ALOX15B_2_14908,147,61,159,74,30,218,63,342,178,215,62,8
ALOXE3_2_14909,159,319,145,266,0,282,202,58,156,182,285,447
AMACR_2_14910,98,229,220,290,230,142,670,31,285,456,1,107
AMD1_2_14911,421,166,410,564,361,1,607,217,642,498,158,292
AMDHD2_2_14912,347,344,319,325,53,542,615,88,456,120,119,90
AMPD1_2_14913,189,97,148,196,185,54,546,53,145,73,466,83
AMPD2_2_14914,520,388,347,358,581,215,29,457,122,99,71,320
AMPD3_2_14915,610,342,466,458,847,485,697,428,139,611,1456,421
AMT_2_14916,1290,1462,1492,1866,1166,1376,2188,3243,1323,987,1009,1754
AMY1A_2_14917,732,610,604,644,949,588,1960,447,585,357,578,1321
ANG_2_14918,306,415,404,407,344,357,122,84,120,1126,82,573
AOAH_2_14919,594,563,431,710,829,387,597,344,433,542,81,883
AOC2_2_14920,50,18,66,56,76,33,0,1,62,1,0,39
APC_2_14921,470,535,485,424,829,205,651,474,465,136,339,154
APEX1_2_14922,6,9,6,15,6,2,0,0,0,0,0,65
APOBEC3A_2_14923,243,442,187,295,695,22,167,1022,219,265,557,29
APOBEC3H_2_14924,562,761,635,629,405,1133,788,319,486,821,908,722
APRT_2_14925,125,91,155,147,129,36,309,0,13,14,360,31
ARF1_2_14926,305,397,429,642,324,514,638,930,569,435,270,337
ARFRP1_2_14927,150,250,141,112,193,6,284,3,92,260,9,69
ARHGAP5_2_14928,724,839,754,710,1459,1165,434,1411,1238,904,390,1168

ARHGEF10L_2_14929,280,311,167,172,448,340,883,506,207,33,85,125
ARL2_2_14930,280,348,298,400,253,455,126,79,106,54,571,557
ARL4A_2_14931,939,657,725,967,726,271,321,539,512,876,740,721
ARL5A_2_14932,90,169,164,92,151,21,890,83,206,97,761,321
ARSA_2_14933,36,103,104,251,123,6,38,0,182,163,7,4
ARSB_2_14934,528,391,506,545,310,173,329,938,381,318,460,339
ARSF_2_14935,671,427,326,579,667,157,248,211,398,617,494,353
ART3_2_14936,291,178,152,310,381,150,1280,455,56,120,290,436
ART5_2_14937,1108,1349,1508,1395,1602,818,4144,1453,1419,808,995,549
ASAH1_2_14938,111,211,96,155,19,83,103,181,0,1,12,28
ASAH2_2_14939,805,1076,823,1095,726,783,834,463,794,1088,422,1295
ASL_2_14940,24,33,28,100,35,0,0,0,9,0,0,45
ASMT_2_14941,312,246,227,269,797,73,30,210,509,50,119,500
ASMTL_2_14942,74,55,62,183,2,18,23,29,100,44,0,42
ASNS_2_14943,1092,1149,843,1136,1883,762,936,590,2059,768,553,989
ASPA_2_14944,70,220,112,98,196,55,3,871,2,5,78,124
ASRGL1_2_14945,858,688,480,596,730,222,697,204,745,574,1164,425
ASS1_2_14946,1104,1241,1163,1461,1004,1468,1125,1887,470,1297,2262,141
9
ATE1_2_14947,529,538,676,653,538,409,769,217,391,611,251,554
ATL1_2_14948,399,429,376,448,2,210,129,195,11,229,350,710
AURKAIP1_2_14949,174,167,154,161,605,9,0,3,298,482,190,25
AZIN1_2_14950,855,724,558,780,495,247,926,284,441,1179,389,817
B3GALNT1_2_14951,463,422,501,513,1065,688,328,76,1035,778,425,344
B3GALT5_2_14952,239,98,198,362,143,517,0,398,118,20,340,116
B3GAT1_2_14953,179,230,256,222,567,44,843,341,243,314,615,369
B4GALNT2_2_14954,861,766,758,621,1185,344,1506,481,560,560,373,423
B4GALT2_2_14955,323,369,305,430,541,311,81,367,706,236,35,288
B4GALT3_2_14956,64,93,81,163,32,3,145,245,215,3,29,167
B4GALT4_2_14957,385,243,333,344,127,176,72,9,221,146,392,197
BAAT_2_14958,492,263,392,425,31,614,349,220,388,136,572,142
BACE1_2_14959,188,64,103,167,0,35,30,0,5,6,0,340
BACE2_2_14960,227,180,109,129,122,253,118,154,140,179,22,142
BCAT1_2_14961,68,79,51,86,6,14,18,31,68,174,8,112
BCAT2_2_14962,234,91,184,146,128,552,527,373,285,107,134,78
BCKDHA_2_14963,152,9,124,123,939,0,3,56,1,366,0,0
BCKDHB_2_14964,707,349,431,550,727,322,54,1649,495,385,14,110
BCO2_2_14965,692,862,493,782,576,700,159,1166,529,297,1382,652
BDH1_2_14966,192,180,168,140,303,422,323,563,251,144,85,206
BFSP1_2_14967,747,960,1121,1040,143,130,1895,2106,521,763,3305,1411
BHMT2_2_14968,2085,2076,1670,1913,1243,2252,3345,1668,2160,2437,1810,1
773
BMP1_2_14969,67,58,42,19,16,18,207,1,18,100,17,90
C17orf101_2_14970,232,128,149,243,24,37,976,287,524,172,213,573
C1GALT1C1_2_14971,590,324,366,274,171,178,532,166,660,509,267,35
C1S_2_14972,329,347,253,301,135,285,119,302,465,307,623,234
C2_2_14973,813,513,614,1007,946,981,1513,729,584,1,958,454
CA10_2_14974,67,247,145,217,417,173,94,107,83,253,578,98
CA12_2_14975,224,81,126,215,144,39,238,295,648,22,173,253
CA1_2_14976,8,49,47,33,0,0,0,0,0,210,0,0

CA7_2_14977,417,423,387,483,618,419,3,210,1084,521,586,902
CAB39_2_14978,1153,632,717,1056,972,445,230,835,1071,754,51,1102
CANT1_2_14979,44,16,4,34,5,0,0,0,227,11,0,0
CAPN1_2_14980,155,171,276,262,108,83,158,3,64,144,502,313
CAPN2_2_14981,30,20,22,39,0,1,0,17,0,31,0,0
CARNS1_2_14982,10,35,59,30,17,4,207,11,84,0,0,22
CARS_2_14983,88,95,73,78,24,306,102,314,75,72,39,10
CASP10_2_14984,88,59,132,120,26,138,0,0,36,174,201,46
CASP1_2_14985,174,210,219,246,312,264,433,380,227,221,295,226
CASP2_2_14986,191,175,156,238,294,70,9,607,362,193,79,303
CASP3_2_14987,117,63,49,43,207,12,538,0,56,1,14,2
CASP4_2_14988,346,333,239,408,151,306,390,474,61,136,285,806
CASP5_2_14989,337,194,116,258,49,103,81,89,138,267,68,363
CASP6_2_14990,208,246,293,351,347,43,498,242,522,147,20,21
CASP7_2_14991,7,5,22,22,36,1,340,0,0,0,432,0
CASP8_2_14992,64,39,65,26,76,0,8,38,1,8,0,96
CASP9_2_14993,897,612,653,550,853,668,771,748,626,551,1091,1327
CASZ1_2_14994,18,71,25,49,1,394,7,1,24,302,5,59
CAV3_2_14995,391,123,291,256,231,82,1305,220,120,277,264,214
CBS_2_14996,360,300,177,196,127,72,267,732,681,121,226,349
CCBL1_2_14997,235,434,195,280,136,454,144,41,254,4,769,242
CCBL2_2_14998,182,127,116,145,118,73,365,21,13,413,304,114
CDC42_2_14999,464,735,506,775,824,729,826,320,663,1018,801,181
CDH16_2_15000,1309,822,1075,1140,863,988,870,279,417,460,1082,810
CECR1_2_15001,1146,1219,954,1112,435,1639,1072,639,757,631,1608,872
CEPT1_2_15002,1652,1207,1582,1500,1883,1234,983,1819,602,1211,1207,203
6
CERS1_2_15003,82,72,137,246,188,9,32,492,200,76,0,45
CES1_2_15004,232,179,116,185,102,4,392,382,201,116,3,232
CES3_2_15005,66,113,177,195,213,39,182,517,25,222,117,104
CES4A_2_15006,276,169,192,241,391,207,90,436,8,57,109,450
CES5A_2_15007,1,17,8,4,0,0,0,15,0,154,0,0
CHAT_2_15008,353,403,269,353,599,303,207,16,182,79,188,167
CHI3L2_2_15009,244,489,435,379,317,66,513,650,21,299,117,302
CHIA_2_15010,220,256,160,212,53,0,340,54,204,10,113,254
CHPF_2_15011,73,77,13,29,0,215,0,0,0,0,600,0
CHST11_2_15012,440,391,499,498,196,604,398,21,290,141,1733,240
CHST15_2_15013,54,49,30,49,208,181,223,36,50,2,41,19
CHST4_2_15014,290,142,378,221,564,297,1032,13,169,16,31,631
CHST8_2_15015,38,8,6,44,3,8,0,0,0,0,0,20
CLP1_2_15016,897,795,940,672,387,247,495,641,964,10,827,383
CNDP2_2_15017,266,585,257,358,569,0,583,417,170,1048,590,638
CNTN1_2_15018,170,388,61,194,138,127,43,48,102,10,0,401
CNTN4_2_15019,516,458,387,406,631,370,196,219,156,349,594,510
COMT_2_15020,63,146,28,86,56,150,349,54,27,15,6,28
COQ6_2_15021,31,4,4,25,0,251,0,33,0,0,0,0
COX11_2_15022,349,175,208,184,293,308,479,61,237,219,440,138
COX15_2_15023,129,208,81,100,17,0,573,221,93,147,260,237
CPA5_2_15024,311,401,262,365,52,745,1434,269,92,233,375,262
CPB2_2_15025,844,815,657,737,429,384,116,535,567,704,39,169

CPM_2_15026,249,282,388,361,521,619,16,497,260,66,396,254
CPPED1_2_15027,47,68,33,66,119,0,251,138,77,29,49,3
CPT1A_2_15028,73,104,125,137,88,31,2,148,117,126,5,6
CPT1B_2_15029,73,109,137,162,3,171,73,47,22,16,0,41
CPT1C_2_15030,683,538,547,504,509,102,617,712,1112,156,155,1000
CRLS1_2_15031,537,627,479,679,617,747,1255,721,737,494,1328,365
CRMP1_2_15032,37,60,31,40,0,68,97,0,32,122,9,147
CROT_2_15033,51,60,76,44,23,0,0,3,115,256,38,22
CRY2_2_15034,210,100,82,172,206,0,2,0,6,3,0,0
CRYM_2_15035,156,140,133,150,658,0,116,10,13,303,496,51
CRYZ_2_15036,228,249,375,213,258,126,717,0,290,3,132,549
CSDE1_2_15037,499,200,227,318,228,447,656,332,768,395,396,156
CSGALNACT1_2_15038,270,148,268,254,10,167,317,2,13,113,131,263
CSMD3_2_15039,459,521,295,491,542,124,11,1235,183,851,528,347
CTAGE5_2_15040,330,167,133,213,316,107,291,252,99,338,182,312
CTBP1_2_15041,403,460,322,414,552,182,284,397,180,537,2135,635
CTPS2_2_15042,1171,695,703,804,1415,524,1119,384,476,1184,233,882
CTSA_2_15043,277,208,237,189,344,363,83,148,224,250,36,249
CTSB_2_15044,156,76,54,60,72,5,158,400,565,108,0,43
CTSE_2_15045,34,35,26,66,4,31,0,90,60,0,138,0
CTSL1_2_15046,28,93,27,17,53,0,137,0,1,126,0,18
CTSL2_2_15047,95,63,80,149,0,3,77,34,65,32,32,442
CTSS_2_15048,178,107,154,145,36,0,288,9,0,49,216,1
CYB561_2_15049,202,133,181,229,644,146,314,26,118,223,126,54
CYB5A_2_15050,708,803,837,770,307,247,1809,919,484,877,1708,1115
CYB5R3_2_15051,236,277,138,257,108,21,171,152,192,132,189,574
CYP11A1_2_15052,354,452,458,644,206,129,929,7,848,300,560,1313
CYP11B1_2_15053,72,191,142,213,19,4,310,179,0,43,64,206
CYP19A1_2_15054,393,525,475,441,179,201,406,371,437,157,759,112
CYP21A2_2_15055,220,279,242,300,214,145,7,702,45,490,19,142
CYP24A1_2_15056,240,205,194,174,20,94,277,51,206,50,14,230
CYP26A1_2_15057,244,230,73,439,1,275,1,99,381,527,519,330
CYP2A7_2_15058,452,286,357,315,264,787,912,337,369,115,69,327
CYP2C18_2_15059,529,739,626,634,107,242,929,1253,434,861,457,703
CYP2C8_2_15060,529,739,626,634,107,242,929,1253,434,861,457,703
CYP2D6_2_15061,142,321,333,325,187,13,466,470,1,436,14,494
CYP3A43_2_15062,48,230,120,138,31,49,27,15,3,266,362,24
CYP3A4_2_15063,1097,724,856,794,1110,538,799,704,1037,493,748,1178
CYP4B1_2_15064,18,92,41,22,8,1007,0,0,4,3,149,65
CYP4F11_2_15065,182,165,216,262,392,192,9,0,191,109,210,44
CYP4F3_2_15066,140,87,138,148,57,101,0,186,176,23,614,272
CYP51A1_2_15067,902,337,379,734,608,987,65,760,643,257,111,469
DAGLB_2_15068,61,35,75,53,9,0,1,18,73,108,1,43
DCLRE1C_2_15069,813,786,637,1109,108,779,1593,1894,1321,811,2123,743
DCP2_2_15070,833,869,907,772,547,803,680,710,712,1309,1574,763
DCT_2_15071,226,151,237,180,100,7,327,214,503,3,173,90
DCTD_2_15072,95,149,50,150,0,1,9,110,193,164,12,108
DCXR_2_15073,41,59,53,108,123,0,6,18,133,32,0,8
DDAH1_2_15074,297,208,288,373,9,39,878,47,47,244,2,38
DDC_2_15075,523,384,314,412,600,3,1915,1447,168,66,1796,113

DDHD1_2_15076,220,310,176,261,160,217,801,334,212,364,196,408
DDO_2_15077,764,678,723,921,1302,953,1300,870,1041,1260,836,618
DDT_2_15078,711,582,568,604,463,389,684,717,148,543,1542,795
DDX11_2_15079,171,156,133,201,155,123,1,10,429,75,5,171
DDX17_2_15080,223,220,227,186,166,279,0,0,244,7,377,69
DDX19B_2_15081,535,607,478,585,41,1058,683,550,772,546,698,1019
DDX31_2_15082,242,359,209,233,88,222,270,127,296,241,11,55
DDX39B_2_15083,6,30,4,40,0,175,0,1,3,0,0,15
DDX3X_2_15084,112,142,124,125,187,31,55,30,190,293,259,396
DDX3Y_2_15085,811,754,577,748,529,1,738,19,518,972,1440,411
DDX42_2_15086,1049,779,871,1112,466,391,1177,630,822,922,643,707
DDX47_2_15087,888,787,733,780,619,502,1762,253,138,974,1320,862
DDX4_2_15088,1508,1745,1418,1793,1829,1547,3850,2510,878,1767,2843,342
DDX54_2_15089,342,320,309,447,340,29,437,337,386,581,332,710
DFFA_2_15090,80,129,36,116,192,172,42,463,7,86,0,22
DGCR8_2_15091,0,0,0,0,0,0,0,0,0,0,0,0
DHCR7_2_15092,79,42,52,58,0,28,2,224,41,0,80,50
DHDDS_2_15093,541,317,396,658,461,756,206,391,70,762,1191,856
DHFRL1_2_15094,1133,1096,829,1276,1996,893,1502,1568,1959,1165,602,110
7
DHPS_2_15095,142,116,81,81,0,28,146,73,84,392,2,27
DHRS1_2_15096,829,427,499,852,409,52,39,547,402,330,352,500
DHRS2_2_15097,299,305,301,365,388,187,66,787,174,404,961,41
DHRS9_2_15098,138,112,191,111,274,18,57,198,291,381,540,50
DHX16_2_15099,1299,1090,1181,1365,467,1403,881,664,1091,1501,1186,1398
DHX30_2_15100,287,257,87,258,363,389,247,243,205,496,55,41
DHX33_2_15101,57,47,23,44,20,0,0,38,0,3,11,1
DHX35_2_15102,438,285,300,310,358,313,106,0,45,422,377,743
DHX36_2_15103,1827,1581,1778,1873,1034,1603,1854,1293,2221,2273,1588,1
663
DHX40_2_15104,64,134,143,128,45,29,11,169,20,374,2,252
DIAPH3_2_15105,66,100,70,92,155,81,16,6,36,5,0,128
DIS3_2_15106,494,625,765,713,549,972,1029,537,305,733,596,606
DIS3L_2_15107,335,318,398,402,250,555,332,362,724,565,737,404
DKC1_2_15108,247,135,152,151,279,311,59,28,168,27,50,142
DNAJC27_2_15109,400,433,343,336,97,285,78,2,410,814,246,208
DNASE1L1_2_15110,208,240,176,260,327,304,19,160,1,311,250,124
DNM1_2_15111,682,691,463,563,836,231,635,884,389,890,312,780
DNM1L_2_15112,746,627,576,921,1070,655,1332,624,379,1391,989,1580
DNM2_2_15113,436,507,507,480,483,972,1797,250,1051,395,967,662
DNM3_2_15114,350,203,202,262,526,9,536,3,302,277,890,172
DNTT_2_15115,747,508,508,609,159,713,606,1136,429,816,381,826
DOHH_2_15116,128,199,122,164,109,0,175,13,5,165,2,143
DOLPP1_2_15117,1083,781,581,799,345,1230,315,546,846,502,25,512
DPEP1_2_15118,65,65,123,162,1,30,66,3,27,0,743,46
DPEP3_2_15119,643,273,442,400,305,317,9,267,329,309,515,311
DPH5_2_15120,100,75,144,157,89,5,96,250,95,20,0,46
DPM3_2_15121,136,114,135,178,230,1,4,384,140,249,0,323
DPP3_2_15122,840,557,783,572,30,1108,511,704,602,742,656,484
DPP8_2_15123,191,216,245,211,188,166,144,674,38,25,54,193

DPYSL2_2_15124,92,125,134,140,6,17,20,30,142,3,53,320
DPYSL3_2_15125,7,41,36,55,2,0,6,1,7,27,10,0
DROSHA_2_15126,102,99,92,77,0,94,414,222,106,66,16,13
DSE_2_15127,47,47,9,54,266,8,17,9,64,38,158,2
DUOX1_2_15128,8,73,34,40,1,0,0,0,30,0,22,70
DUT_2_15129,1079,804,895,901,1622,415,562,1641,1089,1151,620,1795
ECE1_2_15130,91,36,43,99,146,84,110,0,35,13,0,13
ECE2_2_15131,208,129,43,186,182,0,163,267,8,29,39,8
ECI1_2_15132,53,46,76,94,1,156,54,0,3,13,1,2
ECI2_2_15133,187,173,196,174,226,9,30,155,53,100,1046,9
EDEM2_2_15134,801,1005,668,754,328,465,128,472,569,215,535,767
EFEMP1_2_15135,171,190,160,267,111,237,25,44,154,57,770,2
EFTUD2_2_15136,410,652,396,320,699,54,798,732,461,1222,973,189
EGLN2_2_15137,135,245,141,237,59,53,2,6,323,265,14,241
EHHADH_2_15138,392,588,525,786,1105,388,426,799,284,208,249,152
ELAC2_2_15139,158,116,178,211,590,0,519,433,0,89,153,51
ELOVL5_2_15140,727,701,822,718,780,660,730,364,914,642,310,398
ELOVL6_2_15141,175,283,199,285,169,35,147,10,344,407,123,97
ELOVL7_2_15142,1370,1257,813,1343,1563,755,1710,953,840,1218,1031,1309
ENDOV_2_15143,255,412,137,301,71,22,52,1,244,358,459,86
ENO3_2_15144,14,104,55,58,41,4,7,363,106,12,64,41
ENOX2_2_15145,126,205,134,136,169,42,232,40,61,13,0,4
ENPP2_2_15146,74,41,66,34,32,4,0,16,118,74,83,1
ENTPD1_2_15147,193,127,147,351,159,103,172,23,96,2,111,233
ENTPD2_2_15148,54,23,34,32,207,11,94,59,67,71,41,193
ENTPD4_2_15149,979,911,762,1001,1264,768,1267,1144,505,891,1303,598
ENTPD6_2_15150,299,221,111,149,3,388,0,39,7,83,571,413
ENTPD8_2_15151,223,336,232,260,1,657,269,3,0,425,370,423
EPHX1_2_15152,492,681,448,671,391,419,331,509,175,672,133,509
EPHX3_2_15153,131,257,145,468,42,584,4,838,308,592,0,147
ERCC1_2_15154,164,90,95,112,127,2,87,9,38,125,4,392
ERCC2_2_15155,141,270,447,395,551,104,511,633,279,277,227,491
EXO1_2_15156,128,139,120,141,419,310,2,251,105,9,160,167
EXOG_2_15157,205,95,210,205,571,0,0,262,180,385,0,72
EXOSC3_2_15158,57,123,64,174,137,298,40,0,68,129,272,230
EXOSC9_2_15159,1277,1268,1117,1338,1955,948,3090,791,1114,1585,3394,18
67
EXTL2_2_15160,796,1056,868,807,954,1422,622,20,665,857,657,406
F7_2_15161,33,89,85,89,0,14,342,30,117,277,235,0
FAHD1_2_15162,1116,722,868,1064,1404,585,839,1298,998,754,333,1330
FAM108A1_2_15163,11,11,122,27,54,0,0,75,58,402,0,96
FAM135A_2_15164,175,180,196,250,177,340,399,93,128,40,415,411
FAN1_2_15165,107,158,396,233,90,579,484,388,104,249,664,298
FDPS_2_15166,79,91,85,107,126,24,27,94,47,36,2,259
FDXR_2_15167,263,379,264,364,240,57,325,425,164,60,47,334
FECH_2_15168,61,54,55,51,3,58,24,84,147,99,38,40
FERMT3_2_15169,9,28,24,21,0,0,0,1,34,0,1,0
FHIT_2_15170,87,32,76,64,240,0,0,0,45,84,2,23
FIGNL1_2_15171,633,1036,676,827,221,442,936,692,498,1455,430,767
FKBP11_2_15172,408,314,297,468,360,195,281,740,274,416,119,392

FKBP1A_2_15173,161,70,88,112,247,104,114,37,696,1,3,60
FKBP1B_2_15174,496,471,509,602,592,574,881,569,597,678,462,477
FKBP2_2_15175,48,33,47,88,24,0,15,1,142,0,1,169
FKBP5_2_15176,758,748,663,751,392,304,1290,413,430,2153,311,733
FKBP7_2_15177,665,667,730,728,1286,122,1395,772,368,650,922,1305
FLAD1_2_15178,65,43,79,37,6,0,14,62,14,6,27,234
FMO3_2_15179,1609,1363,1337,1788,2089,1306,955,1275,1342,1796,2271,234
1
FMO5_2_15180,850,671,821,1163,1000,976,632,1192,1041,1339,819,1061
FN1_2_15181,378,196,381,426,780,0,529,196,217,227,174,411
FOLH1_2_15182,662,837,638,712,490,630,1607,1197,731,1295,799,489
FPGS_2_15183,261,411,162,129,0,0,587,0,256,306,1426,205
FPGT_2_15184,454,233,305,217,310,55,241,268,489,537,101,503
FTCD_2_15185,62,156,45,46,0,14,8,22,0,8,0,27
FTSJ1_2_15186,308,514,379,533,162,213,479,918,349,136,310,599
FUT2_2_15187,42,1,18,28,3,10,0,4,17,3,43,2
FUT3_2_15188,116,167,112,133,81,46,8,35,116,106,727,208
FUT6_2_15189,147,225,119,244,401,10,188,421,124,184,189,142
FUT8_2_15190,551,766,589,784,605,759,1022,469,552,215,895,937
G3BP1_2_15191,349,165,253,266,783,576,540,22,55,485,899,537
G3BP2_2_15192,1472,977,1061,1498,922,764,1382,1082,2029,1090,217,569
G6PD_2_15193,198,273,177,179,169,285,489,93,91,582,231,97
GAA_2_15194,131,199,148,136,491,98,19,39,249,0,101,51
GAD1_2_15195,534,441,404,603,568,334,4,64,131,369,427,689
GAD2_2_15196,2496,2301,2030,2151,3150,2245,2642,1353,1972,2018,2539,20
65
GALC_2_15197,544,351,578,420,514,1046,1276,1038,355,188,291,72
GALE_2_15198,61,37,71,118,0,0,0,125,551,433,0,188
GALNT9_2_15199,25,101,9,52,21,42,0,343,17,0,0,8
GALNTL1_2_15200,693,467,722,561,150,1027,308,499,100,357,1018,184
GAMT_2_15201,5,18,8,4,0,0,0,38,0,0,0,41
GANAB_2_15202,445,289,305,328,42,32,735,30,503,441,54,184
GART_2_15203,49,62,89,102,1,10,120,140,10,24,1,66
GBA_2_15204,206,201,258,182,990,225,362,385,71,230,81,197
GBP5_2_15205,584,478,663,951,60,887,948,280,294,455,1203,677
GCAT_2_15206,41,18,16,29,0,0,0,0,0,0,0,0
GCDH_2_15207,406,375,360,354,295,713,879,150,469,1179,273,297
GCH1_2_15208,678,681,675,852,833,809,670,425,711,437,363,900
GCLC_2_15209,123,129,182,184,119,91,747,100,270,184,10,130
GCNT1_2_15210,588,361,427,359,211,252,421,293,121,660,306,137
GDPD1_2_15211,920,950,925,1288,1057,703,452,2629,1893,1145,971,726
GDPD2_2_15212,352,179,272,344,178,267,318,107,414,127,118,786
GEM_2_15213,986,814,876,1092,1008,888,838,814,915,1627,728,535
GEN1_2_15214,338,203,401,329,142,20,53,200,280,308,371,536
GFM2_2_15215,258,494,407,779,90,479,414,1742,1138,721,346,618
GFOD1_2_15216,213,315,165,334,383,8,1572,71,640,372,185,25
GGCT_2_15217,363,210,259,223,419,112,630,107,230,886,682,180
GGCX_2_15218,339,246,295,316,439,647,458,28,616,989,315,667
GGT1_2_15219,315,405,280,404,442,486,422,5,35,185,1098,582
GGT5_2_15220,79,67,69,92,29,5,210,60,97,79,65,50

GGT6_2_15221,312,108,210,222,118,40,26,254,225,120,482,701
GGTLC1_2_15222,947,770,674,676,827,346,1073,647,651,1202,903,332
GLB1_2_15223,258,298,172,130,18,46,175,425,249,7,448,413
GLRX2_2_15224,1298,1469,1420,1647,896,922,2818,1546,814,2794,815,1988
GLRX3_2_15225,381,313,254,284,536,639,145,151,564,362,287,622
GLRX_2_15226,358,394,389,486,347,0,542,592,632,207,735,815
GLT8D1_2_15227,1950,1882,2026,2111,2726,2117,1773,2903,1810,2669,2595,
1994
GLUL_2_15228,152,130,176,142,14,0,450,334,134,151,51,191
GM2A_2_15229,859,828,712,786,572,892,210,1323,325,516,3075,677
GMPPA_2_15230,158,171,131,88,322,585,115,22,483,121,7,16
GMPPB_2_15231,8,11,16,11,0,0,24,0,0,0,0,0
GMPR2_2_15232,37,40,40,44,0,11,19,0,2,1,70,95
GNAI2_2_15233,257,351,278,152,302,306,271,698,274,254,15,57
GNAL_2_15234,135,119,160,240,152,71,41,1,194,164,610,541
GNAO1_2_15235,896,816,759,782,1081,386,931,1292,603,616,348,553
GNAT1_2_15236,112,124,167,142,377,8,40,78,23,193,313,482
GNB5_2_15237,239,264,258,301,586,34,585,34,207,680,1159,545
GNG10_2_15238,223,191,149,229,376,99,87,103,176,300,309,8
GNG4_2_15239,100,70,92,102,6,134,225,74,27,60,121,252
GNGT2_2_15240,0,0,0,0,0,0,0,0,0,0,0,0
GPD2_2_15241,217,138,167,108,264,7,126,70,148,341,0,469
GPHN_2_15242,2214,1334,1561,1656,2322,666,1288,1496,3094,1054,2105,166
1
GPNMB_2_15243,904,656,783,888,847,315,1686,703,645,511,502,1112
GPT2_2_15244,195,124,206,133,7,128,7,291,18,92,37,130
GPX4_2_15245,464,333,331,255,331,441,744,68,15,15,263,216
GSR_2_15246,580,469,390,571,81,264,294,395,206,198,435,276
GSTCD_2_15247,91,31,65,68,112,10,330,72,5,6,0,13
GSTK1_2_15248,380,521,311,483,493,11,17,633,79,769,905,396
GSTM1_2_15249,5185,5006,4088,5100,5889,4918,4834,2892,3719,5583,5489,4
577
GSTM2_2_15250,5185,5006,4088,5100,5889,4918,4834,2892,3719,5583,5489,4
577
GSTM4_2_15251,610,545,673,608,605,181,27,880,248,677,1171,532
GSTO1_2_15252,876,597,633,632,577,500,261,789,914,748,796,1552
GSTO2_2_15253,102,54,105,153,13,93,7,0,34,8,59,142
GSTZ1_2_15254,83,74,73,73,112,81,114,1,47,36,0,143
GTPBP3_2_15255,982,749,864,706,282,967,560,640,405,1123,926,373
GUCY1A3_2_15256,343,280,337,403,208,484,479,252,541,598,235,397
GYG1_2_15257,346,218,336,496,625,264,993,197,261,466,1039,425
GYG2_2_15258,583,514,680,575,839,731,265,703,370,379,2307,546
GYS1_2_15259,479,439,432,463,698,506,866,396,440,334,105,425
HADH_2_15260,109,242,74,25,24,33,12,2,83,124,571,71
HAGH_2_15261,427,314,361,296,68,306,213,289,164,815,235,371
HAO2_2_15262,132,87,60,70,177,36,254,32,134,116,0,3
HAS3_2_15263,45,19,36,84,6,0,3,43,1,0,54,0
HCCS_2_15264,197,374,366,638,530,275,1,0,292,528,1306,335
HDHD1_2_15265,158,181,181,212,415,20,81,33,200,32,545,50
HENMT1_2_15266,77,33,15,72,314,2,79,140,236,0,0,351

HHAT_2_15267,203,156,190,148,120,186,16,85,237,20,566,5
HIBCH_2_15268,455,324,338,366,282,182,80,0,433,551,191,315
HLCS_2_15269,10,17,4,7,0,0,4,18,0,0,0,0
HMBS_2_15270,206,232,299,295,359,316,1,359,18,952,1042,159
HMGA2_2_15271,317,310,247,238,433,504,39,286,270,257,192,74
HMGCL_2_15272,21,124,68,71,126,197,0,72,93,122,1,17
HMGCLL1_2_15273,1333,1080,1197,1398,1320,788,1270,1806,1119,795,624,11
43
HMGCR_2_15274,1083,924,903,1231,525,472,1411,1113,108,1775,527,1152
HMGCS1_2_15275,30,16,1,7,0,0,0,0,0,0,0,0
HMGCS2_2_15276,603,543,587,466,888,340,508,143,508,96,211,1157
HMOX2_2_15277,1398,1102,876,1144,1472,1218,1217,1287,1082,1227,609,126
1
HNRNPAB_2_15278,233,239,197,350,787,160,96,516,72,1,573,83
HPD_2_15279,387,353,508,524,344,245,447,916,782,354,231,612
HPGD_2_15280,318,201,223,207,665,183,245,405,172,372,35,334
HPSE2_2_15281,210,115,159,99,67,82,83,200,312,338,26,294
HPSE_2_15282,131,157,200,96,374,188,474,2,492,263,526,48
HRAS_2_15283,105,255,176,242,263,276,304,87,127,202,238,339
HS2ST1_2_15284,894,863,483,802,899,959,658,118,1076,412,681,580
HS6ST2_2_15285,797,743,673,1111,729,557,1567,231,1028,623,520,220
HSD11B1_2_15286,645,875,589,711,889,592,619,837,433,654,1082,795
HSD17B10_2_15287,503,442,382,628,691,576,1860,486,151,675,656,412
HSD17B13_2_15288,28,17,21,24,4,0,0,384,4,1,7,5
HSD17B4_2_15289,2981,1863,1815,2211,1842,1910,2203,2802,3016,3426,1454
,2938
HSD3B2_2_15290,984,880,923,1226,2127,1705,1188,2317,1288,1101,895,1404
HSD3B7_2_15291,134,114,198,251,2,268,345,2,103,40,219,28
HSDL1_2_15292,172,236,194,151,286,406,75,688,99,49,72,20
HSP90AA1_2_15293,151,214,131,113,9,186,138,36,208,113,120,58
HSPA8_2_15294,197,128,180,171,168,132,917,218,137,2,427,125
HSPD1_2_15295,473,288,369,578,494,127,722,910,262,717,10,983
HTRA2_2_15296,1532,515,923,1038,388,130,2383,1079,937,311,2819,1722
HYAL1_2_15297,1382,1327,1353,1704,1813,808,3008,747,1499,176,1434,2361
HYAL2_2_15298,23,25,30,30,0,0,0,1,0,0,0,61
HYI_2_15299,177,97,128,139,2,167,0,412,4,26,218,272
IARS_2_15300,72,103,108,123,205,77,415,0,223,8,152,123
IDE_2_15301,346,304,295,261,97,134,1287,53,479,126,171,558
IDH3B_2_15302,44,72,91,64,1,1,0,326,6,65,611,62
IDH3G_2_15303,119,100,82,76,7,32,51,135,519,156,0,32
IDS_2_15304,66,22,29,104,0,13,3,0,4,282,697,276
IFT27_2_15305,172,176,184,216,161,149,110,492,172,311,559,203
IL4I1_2_15306,16,24,24,39,0,57,239,125,29,104,249,3
IMPDH1_2_15307,149,95,102,207,0,0,4,5,3,96,345,54
INMT_2_15308,558,404,300,467,1281,4,537,160,599,500,758,123
INPP5K_2_15309,213,258,289,193,83,505,89,421,76,211,498,519
INTS6_2_15310,672,428,484,529,1449,116,521,1108,708,407,760,357
IPCEF1_2_15311,220,143,165,182,107,204,20,323,163,292,2,454
ISOC2_2_15312,73,0,30,52,28,36,3,2,0,0,23,0
ISPD_2_15313,250,146,246,300,188,40,327,321,90,8,438,182

ISYNA1_2_15314,150,71,98,61,9,20,0,0,12,27,13,129
ITPA_2_15315,676,794,814,821,716,271,1524,375,720,541,771,1489
IVD_2_15316,277,270,236,334,189,472,1111,1076,309,235,336,514
IYD_2_15317,193,258,164,281,257,22,63,409,106,615,298,636
JMJD7-PLA2G4B_2_15318,246,178,222,150,618,199,135,16,273,233,28,146
KARS_2_15319,70,53,115,106,1,55,294,19,47,126,487,26
KATNA1_2_15320,163,151,146,99,71,4,154,4,59,161,61,216
KATNAL1_2_15321,146,229,143,215,48,12,837,100,117,764,0,492
KIF16B_2_15322,46,42,45,127,2,3,10,1,59,157,144,17
KIF9_2_15323,670,583,623,836,321,195,1114,282,403,385,812,347
KIFC3_2_15324,157,248,177,395,32,0,1421,60,485,289,986,622
KLK2_2_15325,247,226,125,168,51,234,1,54,255,1166,220,222
KLK7_2_15326,781,659,501,720,755,829,1045,92,970,1037,1133,727
KRAS_2_15327,165,106,105,191,68,46,70,159,341,181,113,78
KYNU_2_15328,1114,813,1044,1270,1315,435,736,1341,827,634,1141,667
LAMP2_2_15329,421,332,275,442,593,509,371,242,696,356,639,493
LARGE_2_15330,172,190,150,204,661,1,1,516,105,296,175,69
LCLAT1_2_15331,405,356,368,309,685,108,168,329,435,98,359,686
LCMT1_2_15332,232,257,151,307,212,90,4,19,228,310,43,247
LDHA_2_15333,215,113,112,186,91,14,259,0,28,185,142,0
LDHAL6A_2_15334,825,703,557,607,685,1092,859,575,1227,287,1065,724
LDHB_2_15335,349,323,267,365,2,481,527,525,391,555,100,49
LDHC_2_15336,585,576,517,475,626,584,278,278,472,38,718,554
LDHD_2_15337,142,143,271,245,100,181,7,296,57,51,213,85
LEPRE1_2_15338,614,375,466,562,351,198,1723,550,262,239,354,155
LEPREL1_2_15339,1599,1330,1218,2177,1509,638,2911,1270,1066,2181,1263,
2394
LFNG_2_15340,70,37,103,58,91,0,66,112,23,137,0,45
LGMN_2_15341,99,27,49,30,0,73,0,0,30,130,0,3
LGSN_2_15342,166,242,181,190,67,450,26,1,261,107,400,203
LIAS_2_15343,1302,1102,1033,1120,1415,376,900,1271,697,1306,1284,797
LIG3_2_15344,338,250,265,359,183,282,134,310,67,502,59,55
LIG4_2_15345,1332,1132,1086,1415,668,1120,771,915,1166,2223,1234,1279
LIPF_2_15346,1022,1188,1175,923,973,1145,599,586,575,1524,1058,1058
LIPT1_2_15347,182,161,138,242,23,321,198,52,88,0,8,210
LMO7_2_15348,343,303,266,181,206,282,278,53,185,254,401,202
LNPEP_2_15349,381,163,144,189,67,0,89,142,708,692,5,606
LOX_2_15350,189,174,145,248,186,71,425,18,204,33,10,279
LPO_2_15351,336,242,236,260,423,12,162,51,144,226,74,248
LRRC16A_2_15352,1859,1798,1225,1681,1533,2129,1646,1261,1476,1800,1361
,1486
LSS_2_15353,561,269,369,400,276,111,427,580,301,218,369,337
LYZL6_2_15354,292,202,315,363,263,356,823,419,246,622,780,434
MACF1_2_15355,336,285,252,358,187,1118,88,453,178,654,815,133
MACROD2_2_15356,757,526,822,612,292,399,2149,282,1094,941,510,371
MAD2L2_2_15357,394,248,485,522,515,266,28,1180,935,357,505,1178
MAN2B1_2_15358,16,17,23,11,0,0,216,21,0,0,1,24
MASP2_2_15359,152,128,191,263,449,105,392,0,227,73,348,57
MAT2B_2_15360,433,309,298,339,250,89,292,282,242,501,700,597
MCAT_2_15361,403,339,379,425,104,361,352,1521,172,483,568,571

MCM4_2_15362,291,89,148,95,0,277,450,24,120,2,0,617
MCM7_2_15363,135,157,240,178,117,209,194,159,54,340,446,287
MCM8_2_15364,620,309,416,564,313,66,783,628,344,896,800,455
MCM9_2_15365,770,1312,753,1356,641,774,1393,327,758,1512,876,1638
MDH1_2_15366,151,127,171,212,353,277,386,105,133,122,96,194
MECR_2_15367,422,335,174,168,278,231,511,191,135,327,312,252
MEPCE_2_15368,480,153,216,274,367,6,262,433,534,103,3,418
METTL13_2_15369,108,195,271,304,384,89,110,708,151,163,3,311
MFN2_2_15370,1099,1086,1049,1230,1127,879,1445,955,1125,1432,1060,900
MFNG_2_15371,365,410,370,438,608,65,43,16,97,26,538,848
MGAT3_2_15372,685,430,332,472,389,205,313,649,422,946,388,347
MGAT4A_2_15373,585,881,575,871,368,332,656,171,1657,256,676,549
MGAT4B_2_15374,125,118,96,150,119,62,2,10,161,73,59,518
MGLL_2_15375,55,73,119,40,102,10,0,0,14,83,88,36
MGST1_2_15376,342,147,265,167,9,229,229,276,105,133,8,227
MGST2_2_15377,98,117,85,53,17,20,438,7,10,1,0,50
MICAL1_2_15378,287,265,317,272,32,368,91,576,71,474,48,325
MLH1_2_15379,103,27,82,30,0,1,14,276,0,0,127,142
MME_2_15380,247,112,153,205,617,69,42,132,106,27,118,38
MMP1_2_15381,220,219,379,213,161,4,615,13,183,151,319,390
MMP2_2_15382,166,245,202,262,8,680,29,227,5,495,486,593
MOCS1_2_15383,308,146,265,182,394,108,188,204,104,654,98,253
MOGS_2_15384,178,111,263,357,18,126,614,273,5,42,30,308
MOV10_2_15385,374,120,245,221,93,17,11,318,273,265,173,291
MOV10L1_2_15386,11,26,33,31,0,0,43,8,43,0,6,3
MPG_2_15387,662,359,485,680,0,4,19,334,659,319,834,4
MPPE1_2_15388,261,267,391,289,210,18,301,0,145,73,474,411
MPST_2_15389,172,147,41,203,0,0,59,10,43,0,2,0
MRAS_2_15390,418,313,324,521,331,591,313,44,337,427,310,233
MRE11A_2_15391,566,352,465,488,237,880,779,504,249,429,1033,26
MRI1_2_15392,94,21,13,83,36,0,32,6,43,0,235,328
MSH5_2_15393,56,46,72,45,0,267,0,1,129,243,371,104
MSRA_2_15394,429,286,362,375,543,460,67,5,544,481,913,527
MSRB3_2_15395,248,278,220,212,592,270,144,199,111,261,160,79
MTHFD1L_2_15396,150,175,97,226,0,26,0,326,34,0,0,14
MTHFS_2_15397,364,281,313,383,456,583,790,22,246,306,338,469
MTO1_2_15398,1413,1065,1072,1523,1362,1768,1864,1286,1130,1473,841,909
MTRR_2_15399,104,153,225,176,15,46,29,84,7,292,21,451
MUTYH_2_15400,27,43,46,72,43,2,2,0,6,2,0,23
MX1_2_15401,134,128,184,81,244,38,16,87,93,345,0,472
MYBBP1A_2_15402,256,252,174,133,166,61,161,993,107,79,14,1016
MYH2_2_15403,178,223,145,146,42,313,338,10,243,3,0,190
MYO5A_2_15404,47,81,86,72,8,28,31,391,100,81,15,113
MYO7A_2_15405,156,79,74,106,34,21,227,1,65,325,57,449
MYO9B_2_15406,380,164,329,142,187,431,19,633,209,223,484,64
N6AMT1_2_15407,276,251,157,274,250,258,347,813,237,605,59,17
NAA16_2_15408,367,345,342,384,349,488,1296,53,429,142,1197,380
NAA20_2_15409,218,224,157,233,186,30,176,150,111,140,24,96
NAAA_2_15410,407,554,542,481,709,144,600,59,874,664,440,556
NAPEPLD_2_15411,69,83,49,66,0,0,163,0,4,39,0,0

NARF_2_15412,769,693,785,712,261,207,415,1102,290,1960,662,767
NARS2_2_15413,28,101,85,78,33,14,9,95,4,92,0,26
NAV1_2_15414,84,75,84,36,4,67,9,90,7,0,44,3
NCEH1_2_15415,119,31,84,133,82,19,2,48,109,0,12,8
NCF2_2_15416,389,405,737,777,218,274,1105,232,125,894,1675,548
NCF4_2_15417,167,96,104,164,33,18,59,35,104,23,5,72
NDOR1_2_15418,629,706,659,603,820,557,815,934,284,827,560,367
NDUFA2_2_15419,492,446,517,537,784,141,471,299,548,893,216,719
NDUFB11_2_15420,32,58,44,120,81,539,128,53,41,129,41,166
NDUFB4_2_15421,564,467,425,560,397,175,249,557,525,674,710,495
NDUFB5_2_15422,823,568,658,731,761,861,954,736,1452,432,545,1031
NDUFB6_2_15423,540,484,532,553,565,0,365,827,167,400,797,3
NDUFC1_2_15424,459,432,371,500,818,630,487,291,690,284,303,407
NDUFS1_2_15425,268,223,241,282,21,17,346,100,600,671,143,164
NDUFS2_2_15426,166,189,145,192,321,193,53,15,287,5,603,162
NDUFS5_2_15427,423,398,372,568,433,287,1536,10,375,472,535,457
NDUFV1_2_15428,129,19,64,56,11,0,106,29,23,50,66,53
NEIL2_2_15429,318,740,514,594,593,936,883,1015,518,311,1576,275
NEU4_2_15430,43,10,74,21,3,5,0,0,13,163,0,0
NFS1_2_15431,212,100,95,184,0,60,22,12,0,0,239,5
NGLY1_2_15432,136,145,77,135,36,24,56,2,4,187,135,170
NIPSNAP1_2_15433,64,41,124,170,2,0,446,16,11,0,160,34
NIT1_2_15434,721,658,509,533,202,527,1154,696,803,833,308,474
NKIRAS2_2_15435,383,162,145,303,84,162,414,239,473,104,613,335
NLGN3_2_15436,14,23,20,57,0,2,0,8,0,5,0,84
NLGN4X_2_15437,196,463,300,588,517,266,0,251,318,194,200,359
NLGN4Y_2_15438,631,601,492,788,731,534,994,550,871,847,1802,577
NMNAT2_2_15439,215,159,293,295,208,0,128,2,497,151,390,50
NNT_2_15440,900,563,601,932,468,904,227,1026,440,418,155,377
NOX4_2_15441,301,112,97,180,210,14,327,621,94,504,91,132
NPL_2_15442,1034,985,927,936,555,842,695,749,310,1187,207,667
NQO1_2_15443,40,98,81,54,0,16,95,6,51,165,231,99
NRD1_2_15444,2063,1567,1705,1849,1025,1192,1797,1139,1741,1410,1968,15
71
NSDHL_2_15445,908,1000,930,1136,873,1155,1446,491,360,1422,397,1422
NSUN2_2_15446,871,617,662,649,726,239,431,442,1246,988,1456,605
NUDT2_2_15447,158,169,147,225,57,142,265,206,78,117,321,403
NXN_2_15448,280,325,253,159,381,37,144,38,362,796,1257,352
OAS1_2_15449,1566,1338,1210,1471,2947,1245,1548,1650,1043,2094,1555,82
0
OAS2_2_15450,614,576,623,635,283,1138,1782,884,899,232,559,902
OASL_2_15451,94,51,74,106,74,166,5,232,36,0,364,92
OAT_2_15452,49,63,89,20,0,0,0,0,2,0,0,0
OGDH_2_15453,587,800,686,623,513,138,621,1129,447,456,684,541
OGDHL_2_15454,1046,1112,850,1625,2504,351,546,442,841,414,869,997
OGG1_2_15455,368,270,198,423,513,88,301,54,503,301,137,257
OGT_2_15456,884,859,854,813,681,207,1782,877,1216,510,618,1186
OLAH_2_15457,671,427,542,563,1374,623,901,1658,482,584,234,785
P4HA1_2_15458,346,309,193,301,35,453,184,43,213,265,153,277
P4HA2_2_15459,33,57,52,54,4,48,0,2,0,0,0,46

P4HTM_2_15460,100,123,70,46,316,327,58,239,324,1,1,0
PAFAH1B2_2_15461,159,102,195,136,126,199,159,11,74,113,35,258
PAFAH1B3_2_15462,76,61,70,90,0,365,61,0,0,0,98,14
PAICS_2_15463,876,853,593,845,979,1176,903,496,564,1888,651,1418
PAOX_2_15464,851,621,645,656,767,1572,380,928,1560,558,818,510
PAPD4_2_15465,126,108,156,115,83,97,296,118,53,129,70,148
PAPD5_2_15466,363,229,264,236,346,13,305,462,893,78,219,269
PAPD7_2_15467,140,108,139,120,0,0,18,138,189,208,173,128
PAPSS2_2_15468,2003,1629,1348,2281,2594,711,1384,2820,2116,2048,1563,1
180
PARK7_2_15469,151,133,54,244,154,22,0,51,360,67,1153,220
PARL_2_15470,1213,841,1072,1407,1139,903,450,1103,362,777,1211,1009
PARN_2_15471,152,231,233,107,277,1,0,12,0,279,396,18
PARP2_2_15472,1199,1075,1065,1289,1253,669,781,1344,979,1618,707,796
PARP3_2_15473,169,191,119,205,83,133,0,12,117,222,23,856
PARP8_2_15474,561,450,505,568,242,387,591,92,562,507,406,564
PBLD_2_15475,157,281,170,162,99,448,24,345,211,203,396,68
PCCA_2_15476,860,545,750,764,222,469,1096,123,337,482,1430,561
PCCB_2_15477,354,288,249,318,13,330,93,52,90,84,1124,182
PCMTD2_2_15478,225,122,106,118,225,116,345,182,160,257,266,95
PCNA_2_15479,135,127,123,141,328,47,96,48,0,3,37,371
PCSK1_2_15480,222,302,215,161,55,132,94,85,197,931,53,73
PCSK2_2_15481,16,40,15,32,0,5,0,88,1,1,162,1
PCYT1B_2_15482,353,280,187,378,337,70,29,102,80,82,1015,300
PCYT2_2_15483,797,619,852,888,1261,429,1792,835,686,642,310,441
PDE10A_2_15484,146,118,41,129,163,345,59,2,225,0,1,118
PDE11A_2_15485,130,191,129,243,61,93,6,340,27,56,563,24
PDE1A_2_15486,270,295,230,491,674,418,415,34,290,265,465,196
PDE1B_2_15487,69,123,112,25,0,3,0,1,3,0,210,203
PDE1C_2_15488,265,142,254,305,183,0,380,5,332,432,398,76
PDE2A_2_15489,335,197,262,490,318,676,178,454,141,491,264,594
PDE4A_2_15490,322,165,254,182,171,205,159,300,1207,141,508,102
PDE4B_2_15491,119,71,70,31,23,247,434,90,39,24,1,4
PDE4C_2_15492,123,172,145,77,13,198,2,449,193,563,532,91
PDE4D_2_15493,76,81,97,62,123,317,22,22,1,260,0,57
PDE5A_2_15494,2431,2183,1929,2543,1783,2262,3263,1366,1816,2016,1680,2
307
PDE6B_2_15495,1841,1604,1741,1733,2191,2057,1479,2207,1096,2472,1548,2
023
PDE7A_2_15496,77,191,88,156,89,71,25,36,13,83,37,375
PDE8A_2_15497,370,459,562,530,911,381,286,267,316,202,437,189
PDE8B_2_15498,95,53,99,120,3,26,83,19,92,264,54,46
PDE9A_2_15499,371,439,312,431,227,247,1370,123,765,895,996,1282
PDHA1_2_15500,1234,1009,1069,1385,638,1202,843,1860,719,1353,798,1083
PDHB_2_15501,966,881,1010,957,1102,1053,1042,727,439,1497,1069,842
PDHX_2_15502,175,247,361,391,273,341,419,81,80,5,287,1041
PEMT_2_15503,111,148,214,143,275,588,0,33,2,148,11,576
PEPD_2_15504,193,17,81,207,0,18,47,336,1,147,0,259
PGAM5_2_15505,140,219,229,182,35,116,759,105,76,415,141,266
PGBD1_2_15506,145,203,265,155,7,23,2,53,505,7,231,263

PGC_2_15507,438,408,481,449,0,6,6,30,442,300,340,484
PGM1_2_15508,149,369,262,234,267,25,519,19,1,765,118,662
PGM3_2_15509,1251,813,585,980,580,235,724,375,630,1444,969,1247
PHOSPHO1_2_15510,53,51,62,46,12,292,0,0,0,115,0,0
PHOSPHO2_2_15511,114,150,211,81,412,7,113,340,341,60,460,81
PHYH_2_15512,623,477,459,548,490,566,496,515,368,301,157,718
PIGA_2_15513,626,687,514,525,343,99,480,654,489,592,448,452
PIGC_2_15514,398,418,316,304,232,198,606,328,421,557,56,1021
PIGF_2_15515,1299,899,867,1305,1966,643,761,1692,1023,1062,686,1570
PIGG_2_15516,643,718,731,1010,281,1420,667,1786,575,849,294,285
PIGN_2_15517,1280,1189,980,1140,1654,848,1140,579,1268,1726,1908,1639
PIGO_2_15518,855,519,712,941,1775,55,2027,33,592,264,1076,1077
PIGP_2_15519,1374,1110,1118,1132,266,1870,1676,1858,819,878,1028,1371
PIGQ_2_15520,87,30,60,85,694,15,172,17,100,88,27,85
PIGT_2_15521,653,939,761,891,615,673,1121,491,988,683,825,1217
PIGV_2_15522,5876,5300,4621,6072,4500,3393,4160,5088,5844,6212,4721,48
11
PIN4_2_15523,285,282,191,282,185,284,0,225,242,51,288,200
PLA1A_2_15524,898,678,628,939,1095,1312,549,836,766,1078,464,1052
PLA2G2A_2_15525,283,347,263,221,184,376,0,1,139,286,493,156
PLA2G4C_2_15526,932,868,854,1013,304,866,1395,475,407,343,1708,1439
PLA2G6_2_15527,514,317,312,342,25,641,0,151,466,427,924,83
PLA2G7_2_15528,387,349,313,403,84,593,8,0,598,526,163,165
PLAT_2_15529,67,16,50,59,147,14,52,18,1,14,11,3
PLAU_2_15530,2100,1823,1948,2333,3214,1214,1829,2783,1754,2651,1047,22
05
PLB1_2_15531,277,283,265,288,258,977,330,328,266,159,808,153
PLCB1_2_15532,878,1222,985,827,477,1975,280,590,1542,1486,2087,975
PLCB3_2_15533,209,167,140,236,222,134,220,38,157,455,108,135
PLCB4_2_15534,194,122,182,135,263,167,584,152,159,37,14,123
PLCD1_2_15535,107,162,119,88,0,27,1,359,4,1,0,3
PLCE1_2_15536,212,179,167,222,90,0,227,48,307,162,11,194
PLCG1_2_15537,124,99,208,128,3,9,366,281,0,2,216,196
PLCH1_2_15538,259,314,367,385,249,359,294,744,621,668,34,329
PLCL2_2_15539,96,70,74,87,18,6,6,203,1,5,0,14
PLCXD2_2_15540,31,15,7,75,80,0,20,41,89,3,4,0
PLD2_2_15541,30,24,24,14,46,0,77,14,23,12,0,8
PLD3_2_15542,120,133,99,78,216,5,452,247,21,44,0,39
PLOD2_2_15543,99,62,64,111,117,5,239,183,18,151,456,78
PLSCR3_2_15544,71,55,43,26,0,14,95,185,7,25,0,18
PLSCR4_2_15545,227,182,334,405,503,193,1028,396,122,1,676,231
PMEL_2_15546,95,88,137,66,108,67,255,260,44,203,11,204
PMS1_2_15547,147,208,279,234,69,201,56,234,390,510,467,119
PNPLA1_2_15548,22,90,116,83,15,104,42,82,0,14,124,239
PNPLA4_2_15549,1036,710,699,934,560,461,980,573,641,711,635,745
PNPLA6_2_15550,321,235,234,250,89,23,58,0,104,18,315,12
PNPLA7_2_15551,0,0,0,0,0,0,0,0,0,0,0,0
POFUT1_2_15552,513,639,497,596,995,280,9,154,295,968,386,249
POFUT2_2_15553,187,125,176,288,0,231,348,58,132,36,400,133
POGZ_2_15554,812,790,710,796,741,544,545,395,654,305,342,1106

POLD2_2_15555,199,167,141,153,484,0,351,47,46,240,136,69
POLE2_2_15556,79,44,34,46,106,3,112,6,113,65,122,323
POLG_2_15557,13,17,69,25,2,57,0,317,0,0,1,4
POLL_2_15558,262,174,91,142,257,116,11,370,12,423,0,84
POLR1D_2_15559,95,147,124,105,192,54,156,180,160,126,3,21
POLR3B_2_15560,365,306,285,415,362,275,524,893,269,193,531,98
POLR3H_2_15561,349,358,313,286,214,387,233,318,241,412,385,575
POMT1_2_15562,215,406,288,364,209,647,312,96,290,366,225,355
PON2_2_15563,304,271,325,401,447,124,611,608,312,217,462,209
POP5_2_15564,533,609,489,437,538,191,216,503,622,214,593,646
PPA2_2_15565,459,488,429,549,1038,202,456,975,249,1327,1171,421
PPIE_2_15566,312,137,136,200,164,189,92,725,395,115,21,249
PPIL2_2_15567,200,86,123,143,175,1,49,61,351,24,15,247
PPIL3_2_15568,179,73,142,224,8,73,32,45,4,118,2,500
PPIL6_2_15569,727,417,537,761,812,469,683,286,655,294,265,861
PPIP5K1_2_15570,123,157,113,95,397,16,202,8,51,0,0,36
PPT1_2_15571,250,238,172,226,96,574,306,82,652,313,318,145
PPT2_2_15572,173,37,26,47,121,0,172,151,7,409,0,0
PRCP_2_15573,90,69,110,70,21,648,273,0,61,7,121,3
PRDX1_2_15574,327,179,265,260,222,157,292,71,122,20,505,494
PRDX3_2_15575,156,97,169,120,215,57,518,50,27,70,388,70
PRDX5_2_15576,155,187,42,143,16,1,409,12,0,0,867,0
PRKCSH_2_15577,91,93,113,119,0,156,174,0,368,117,58,184
PRODH_2_15578,78,198,23,245,1,190,185,92,108,27,276,0
PRSS3_2_15579,223,315,250,245,55,110,119,290,47,885,312,526
PSAT1_2_15580,60,35,55,122,108,22,668,0,5,43,462,177
PSMA1_2_15581,67,106,75,78,13,216,0,26,11,216,76,187
PSMA3_2_15582,1018,1012,1003,912,1175,1081,890,1333,617,1046,939,960
PSMA4_2_15583,654,692,694,899,631,1793,1474,441,669,480,957,1277
PSMA5_2_15584,488,336,512,475,169,210,292,368,201,199,124,1088
PSMA8_2_15585,104,129,131,156,98,13,2,133,219,429,0,32
PSMB2_2_15586,38,72,9,73,17,0,0,294,5,263,0,0
PSMB5_2_15587,51,12,13,12,0,0,0,81,10,0,0,61
PSMB8_2_15588,1496,1161,1195,1141,807,1383,1614,2536,1683,1478,896,756
PTBP1_2_15589,165,170,148,197,5,0,1,1,65,39,843,182
PTER_2_15590,478,561,474,527,712,422,475,655,213,67,659,328
PTGR1_2_15591,278,103,189,244,110,6,864,262,243,28,272,20
PTGR2_2_15592,421,316,574,512,359,304,397,825,495,212,1210,782
PTGS1_2_15593,703,709,707,740,321,894,145,1332,992,730,269,703
PUS1_2_15594,473,570,385,462,287,623,636,565,593,715,1233,392
PYCR1_2_15595,26,14,58,32,0,0,0,0,0,0,0,255
PYGL_2_15596,136,269,133,109,22,161,103,305,165,49,93,179
PYGM_2_15597,214,151,128,182,101,5,184,65,63,101,43,115
QPCTL_2_15598,130,150,114,118,35,146,536,232,195,27,0,119
QSOX1_2_15599,162,23,32,51,147,0,2,0,131,27,0,1
RAB11A_2_15600,758,637,643,675,1098,214,400,641,311,546,1058,636
RAB1A_2_15601,1309,1138,955,1197,581,648,1793,2063,1187,1110,1590,642
RAB23_2_15602,571,574,997,845,1458,681,598,1244,684,991,1224,1216
RAB27A_2_15603,643,539,479,648,268,96,169,86,863,905,111,844
RAB28_2_15604,150,134,269,312,329,23,120,48,299,114,514,140

RAB2A_2_15605,751,845,794,940,571,29,634,675,953,792,2038,622
RAB2B_2_15606,127,115,115,199,327,25,512,24,79,36,13,175
RAB34_2_15607,0,0,0,0,0,0,0,0,0,0,0,0
RAB35_2_15608,369,277,163,493,70,0,26,132,220,27,0,619
RAB37_2_15609,159,221,87,201,52,7,22,253,146,56,88,390
RAB40C_2_15610,93,115,82,45,434,154,2,57,97,3,202,251
RAB5C_2_15611,40,164,39,58,2,89,7,0,79,515,0,2
RAB6A_2_15612,293,303,421,356,1318,301,477,231,35,62,447,150
RAB7L1_2_15613,88,86,54,58,199,0,0,3,69,0,0,140
RAB9A_2_15614,86,174,132,168,119,150,16,341,120,2,13,261
RABGGTA_2_15615,74,7,137,93,0,0,0,0,159,0,420,0
RABL2A_2_15616,951,582,985,1256,773,522,1575,461,890,687,2051,1155
RABL2B_2_15617,951,582,985,1256,773,522,1575,461,890,687,2051,1155
RAC1_2_15618,249,69,131,196,25,134,193,249,487,131,54,167
RAD51B_2_15619,348,281,330,448,381,69,372,374,188,372,45,287
RAD51_2_15620,103,134,171,214,0,239,151,134,623,92,1,439
RAD51D_2_15621,443,396,205,351,1123,352,304,183,539,160,313,424
RAD54B_2_15622,661,569,706,644,112,466,484,379,1332,1294,723,1893
RAD54L_2_15623,249,190,227,162,209,237,98,630,95,343,94,129
RAD9A_2_15624,79,101,195,215,319,8,333,223,323,145,426,69
RAP1A_2_15625,275,318,228,349,272,76,66,354,237,197,216,402
RAP1B_2_15626,771,714,648,637,968,274,1096,724,583,1320,491,615
RBBP8_2_15627,141,12,178,65,0,0,0,0,2,10,60,0
RDH13_2_15628,96,36,106,101,578,0,433,0,10,0,0,2
RDH5_2_15629,554,716,491,624,783,352,539,83,392,1738,47,673
RECQL5_2_15630,642,491,564,628,811,709,497,478,508,480,509,404
RECQL_2_15631,321,382,222,159,620,159,0,33,173,39,214,84
RERG_2_15632,1510,1125,1576,1618,1748,1803,1197,2144,1443,700,2335,120
2
REV1_2_15633,831,737,508,846,660,129,341,372,1112,1136,271,737
RFC3_2_15634,941,704,832,730,989,935,1307,1740,534,1500,1008,2159
RFC5_2_15635,280,288,298,371,322,256,236,103,258,204,33,358
RGN_2_15636,730,1071,639,713,569,606,448,745,495,647,998,491
RGS11_2_15637,38,76,26,27,0,0,164,11,23,14,0,5
RGS6_2_15638,112,118,134,120,91,199,5,149,74,34,2,44
RHOC_2_15639,822,596,890,897,408,600,645,1112,641,516,1065,614
RHOT1_2_15640,331,506,358,478,12,589,456,725,463,537,656,554
RNASE1_2_15641,266,249,407,364,466,777,463,432,366,164,163,429
RNASE4_2_15642,76,137,78,67,12,5,18,0,17,18,49,44
RPAP3_2_15643,306,120,301,150,181,0,133,589,266,204,42,85
RPE_2_15644,134,111,126,171,132,135,196,92,468,92,11,97
RPN2_2_15645,1598,1340,1232,1250,704,685,2241,1627,1460,1277,1596,1276
RPP14_2_15646,264,211,250,255,254,200,488,2,91,216,297,345
RPP21_2_15647,74,30,28,48,0,0,0,111,0,238,8,355
RPP30_2_15648,323,262,164,270,54,242,1,352,106,276,51,412
RPP38_2_15649,473,744,751,752,1545,4,1,791,226,633,498,1149
RPS27A_2_15650,221,170,225,211,34,287,458,420,370,228,450,510
RRAD_2_15651,72,113,145,163,111,114,11,59,44,83,54,158
RRAGB_2_15652,386,311,392,581,115,242,613,268,501,272,1415,372
RRAS2_2_15653,117,62,160,161,135,0,354,697,136,10,9,43

RRM2B_2_15654,385,472,458,500,919,187,628,781,158,266,932,125
RRM2_2_15655,2074,1806,1945,2252,1596,1418,3935,2341,768,1366,1659,198
3
RTEL1_2_15656,104,121,124,145,46,279,0,338,3,39,0,7
SAR1A_2_15657,412,363,461,438,28,161,653,325,485,212,808,582
SAR1B_2_15658,1655,1951,1881,2003,2271,1820,2038,2182,1305,1924,2937,2
731
SARDH_2_15659,529,454,254,533,101,98,631,16,396,55,2,419
SARS2_2_15660,455,346,306,351,40,447,771,413,360,238,120,98
SBNO1_2_15661,742,798,634,552,454,193,257,485,305,359,552,653
SC5DL_2_15662,601,736,624,676,376,1169,282,1180,418,1387,1227,511
SCD5_2_15663,171,113,217,270,2,113,195,22,61,0,34,38
SDC1_2_15664,219,188,212,301,126,38,106,58,117,115,605,478
SDCBP_2_15665,636,495,603,545,440,481,194,275,217,82,69,423
SDHC_2_15666,265,136,208,164,362,243,41,0,65,397,314,266
SEPHS1_2_15667,267,107,232,103,46,69,466,360,15,15,1064,242
3-Sep_2_15668,14,21,29,7,146,0,0,25,14,0,0,85
4-Sep_2_15669,569,384,328,350,482,822,336,783,397,217,316,392
5-Sep_2_15670,524,377,429,565,276,996,691,718,1025,285,581,320
9-Sep_2_15671,416,379,392,316,452,160,308,362,837,50,662,304
SGMS2_2_15672,342,221,505,448,276,157,47,234,98,74,341,416
SH3GLB1_2_15673,682,509,466,614,709,640,637,851,146,392,461,494
SHMT1_2_15674,38,83,59,150,146,119,32,6,94,246,33,124
SHMT2_2_15675,51,42,29,66,9,1,23,46,1,125,1,42
SIAE_2_15676,198,297,235,343,664,345,657,199,234,407,521,226
SLFN11_2_15677,676,452,402,702,399,251,329,877,707,889,287,182
SMG6_2_15678,454,258,331,402,4,105,0,163,594,250,352,24
SMOX_2_15679,127,253,202,149,126,266,17,128,1,0,0,0
SMPD1_2_15680,19,11,7,11,0,2,0,0,17,22,0,1
SMPD4_2_15681,266,315,163,245,198,416,10,32,449,51,0,960
SMPDL3B_2_15682,1416,1159,1016,1195,632,1351,926,252,1055,1279,1691,11
52
SOD2_2_15683,443,443,361,414,534,439,519,777,463,875,396,226
SPAM1_2_15684,1489,878,922,1222,1426,1358,649,1192,1058,952,441,1513
SPAST_2_15685,148,178,134,200,37,58,52,32,36,599,407,404
SPG21_2_15686,701,569,685,855,243,609,610,459,633,911,250,285
SPO11_2_15687,134,147,292,140,5,0,812,796,52,26,102,158
ST3GAL1_2_15688,121,178,136,224,115,0,443,18,254,386,63,525
ST3GAL5_2_15689,293,248,176,354,25,309,275,10,349,99,10,301
ST5_2_15690,99,182,80,133,249,18,10,6,37,34,201,68
ST6GAL1_2_15691,626,463,502,575,710,435,227,7,2,82,429,573
ST6GAL2_2_15692,711,454,481,500,576,476,376,910,578,718,363,1247
ST6GALNAC3_2_15693,262,184,243,229,62,475,82,6,20,81,97,257
ST6GALNAC4_2_15694,86,78,167,141,212,83,17,254,16,46,774,13
ST8SIA4_2_15695,156,88,144,207,243,12,2,368,343,71,193,147
SUCLG2_2_15696,708,781,693,753,1101,440,241,420,545,988,830,904
SULF1_2_15697,18,67,123,100,19,270,473,2,15,20,14,302
SULF2_2_15698,0,0,0,0,0,0,0,0,0,0,0,0
SULT1A1_2_15699,609,446,409,615,655,503,565,609,184,728,215,251
SULT1A2_2_15700,291,137,266,259,16,649,0,147,1,29,1,139

SULT2B1_2_15701,46,7,21,6,86,0,1,0,2,0,0,0
SUOX_2_15702,58,262,47,69,61,3,410,56,26,578,114,207
SYTL1_2_15703,130,221,157,249,564,293,0,0,60,221,0,284
TAB1_2_15704,1838,1535,1890,2353,2835,644,2454,924,2045,1717,1094,2571
TBXAS1_2_15705,298,299,421,281,0,935,0,0,324,489,225,394
TCIRG1_2_15706,214,150,236,172,6,61,37,527,371,26,366,45
TDP1_2_15707,749,562,601,511,858,708,36,133,855,269,1235,951
TERT_2_15708,550,436,348,466,857,738,1086,238,338,724,321,627
TGM2_2_15709,280,168,309,213,42,1,813,35,212,233,654,160
TGM5_2_15710,23,91,123,80,192,188,26,18,69,93,19,73
TH_2_15711,297,125,265,245,12,0,319,585,167,350,77,572
TIAM2_2_15712,1054,1061,805,996,1814,832,984,1355,1247,506,740,604
TKTL1_2_15713,580,241,334,303,189,122,256,600,55,776,227,469
TMEM55B_2_15714,448,505,515,664,638,450,318,388,267,514,298,1229
TMLHE_2_15715,108,35,66,194,186,0,0,25,27,4,41,6
TMOD1_2_15716,215,150,42,181,157,2,4,97,105,0,0,355
TMX2_2_15717,542,330,514,442,400,186,1300,350,647,705,402,717
TNNI2_2_15718,182,61,62,96,87,182,77,48,156,214,7,279
TP53I3_2_15719,105,63,42,85,0,8,1257,124,104,52,2,22
TPI1_2_15720,225,216,207,361,389,277,14,0,74,32,361,7
TPST2_2_15721,611,593,427,546,589,1189,1162,418,359,389,799,763
TREX1_2_15722,122,178,121,152,0,290,0,1,0,24,425,0
TRMT1_2_15723,101,144,160,152,108,175,528,3,0,162,216,6
TRMT1L_2_15724,110,275,300,270,9,262,173,573,556,54,247,153
TRMT2B_2_15725,65,40,7,39,35,1057,0,0,203,152,0,0
TRPT1_2_15726,353,263,371,400,17,742,230,32,374,711,956,421
TSEN2_2_15727,440,509,583,756,742,401,956,341,209,288,456,1135
TSEN34_2_15728,104,100,47,141,116,3,56,53,23,309,30,1
TTLL6_2_15729,291,304,206,309,292,213,340,345,376,363,92,455
TUBAL3_2_15730,198,95,140,100,148,0,365,85,203,1,169,138
TUSC3_2_15731,701,534,515,647,354,538,295,329,590,918,456,586
TXNDC16_2_15732,176,101,224,158,76,6,2,462,58,54,0,62
TXNDC2_2_15733,477,184,342,463,231,108,312,96,211,533,356,476
TXNDC5_2_15734,1292,1310,1475,1234,1299,1709,475,2298,836,1663,1149,14
92
TXNL4B_2_15735,657,614,670,769,515,742,36,557,882,414,609,759
TXNRD1_2_15736,1188,1160,906,1112,1020,1856,1825,1216,962,1506,920,959
TXNRD3_2_15737,236,225,224,162,349,323,142,437,282,246,192,313
TYMP_2_15738,63,104,143,154,114,22,22,6,2,170,209,407
UBA52_2_15739,16,13,10,16,0,0,475,0,5,0,0,31
UEVLD_2_15740,1702,1738,1695,1641,1138,1855,1862,1827,2555,2958,2430,1
522
UGDH_2_15741,38,33,20,51,315,0,0,1,8,0,0,118
UGP2_2_15742,665,631,666,745,723,573,238,16,467,861,323,669
UGT1A6_2_15743,2594,2226,2599,2694,3338,1917,3275,4051,2537,3244,4252,
2741
UGT2B10_2_15744,3031,2257,2159,2822,1818,2425,2243,2247,2532,2205,2133
,3075
UGT2B28_2_15745,875,968,985,1126,1154,1863,1327,1647,600,1660,1477,479
UGT3A1_2_15746,701,982,827,949,1624,560,274,698,595,1157,466,426

UGT8_2_15747,106,192,249,210,508,774,9,61,164,5,413,97
UNG_2_15748,1165,1754,1195,1546,1976,1745,758,1016,2089,1292,2546,1861
UPP1_2_15749,129,69,67,86,6,92,34,244,56,135,235,5
UPP2_2_15750,297,266,326,275,662,70,392,665,300,164,405,95
UQCR10_2_15751,455,449,427,487,579,141,89,197,518,602,44,548
UQCRB_2_15752,366,443,434,502,176,795,308,321,453,386,330,901
UROC1_2_15753,151,89,59,114,26,96,89,79,120,38,1,177
VCL_2_15754,8,19,6,50,239,20,0,0,0,0,0,8
VNN2_2_15755,486,558,532,456,647,166,35,168,242,1496,1523,155
VPS29_2_15756,714,534,721,660,771,593,325,595,494,995,735,833
WARS2_2_15757,421,316,247,449,521,331,330,453,1056,191,397,421
WARS_2_15758,158,201,220,307,131,163,41,276,129,67,175,128
WBSCR22_2_15759,20,13,23,22,111,12,20,0,0,22,0,3
WDR46_2_15760,239,314,243,231,490,37,290,466,180,468,155,40
WFS1_2_15761,731,833,537,805,913,161,785,442,610,977,802,713
WRNIP1_2_15762,161,144,142,165,308,263,0,74,193,315,0,34
WWOX_2_15763,155,151,186,109,75,167,371,42,324,29,118,18
XPNPEP1_2_15764,73,142,199,112,10,1,5,23,171,201,8,128
XRCC3_2_15765,57,66,55,51,34,0,31,13,91,33,1,1
XRN1_2_15766,900,1155,783,1048,1205,507,1089,2039,951,2452,1371,534
YWHAZ_2_15767,1577,1038,1215,1344,1050,1076,1288,769,1842,2183,881,165
7
ZBED1_2_15768,19,17,73,58,0,3,393,28,2,5,0,10
ZCCHC11_2_15769,814,1102,788,1127,1291,807,380,1245,678,905,341,1369
ZCCHC6_2_15770,242,157,127,150,67,70,172,350,339,52,0,208
ZDHHC15_2_15771,143,185,355,183,201,15,857,233,217,394,541,123
ZDHHC7_2_15772,231,223,195,213,69,243,16,105,177,56,1,92
ACYP1_2_15773,1611,1240,1295,1277,1259,932,1155,2150,1306,1550,1096,20
91
APOBEC3F_2_15774,242,163,202,227,142,3,225,69,251,39,973,22
ASCC3_2_15775,2809,2388,1994,2985,4409,2380,4011,2326,3457,2655,2899,2
289
ASPH_2_15776,127,113,78,171,279,0,0,267,306,265,1,287
ATP6V0E2_2_15777,0,1,2,31,31,0,0,0,0,12,0,46
C10orf2_2_15778,387,524,423,462,328,389,542,1065,438,351,1126,476
CAPN3_2_15779,2019,1658,1496,2077,1551,746,488,1425,2499,2305,2379,155
6
CHM_2_15780,263,135,296,145,572,163,0,186,159,95,307,1
CTSC_2_15781,54,83,33,102,87,53,34,26,122,48,140,8
CYBRD1_2_15782,1765,1324,1478,1616,1006,1340,762,1284,1959,1360,2641,1
007
CYP3A5_2_15783,461,341,326,345,236,473,1092,551,885,215,377,653
DIO2_2_15784,1340,1024,842,1320,1459,687,595,1112,1153,1984,1221,1069
DNASE2B_2_15785,968,753,662,861,1760,442,208,1066,1276,788,890,1873
DPYD_2_15786,1236,951,840,990,1012,569,600,730,922,1557,651,811
GBA3_2_15787,27,36,82,67,20,1,11,181,95,15,183,44
GCNT2_2_15788,441,504,379,566,0,195,7,0,394,45,1511,869
GLYAT_2_15789,400,386,319,374,38,39,4,504,743,194,292,202
GPLD1_2_15790,787,777,841,1113,802,529,2604,810,1075,865,895,377
GPX1_2_15791,4,2,4,38,183,0,0,0,27,2,0,0

GPX5_2_15792,181,159,159,286,368,0,81,1180,429,26,323,4
HNMT_2_15793,243,647,387,469,74,194,146,1899,374,247,781,251
HOGA1_2_15794,22,54,49,58,12,108,0,0,108,0,96,0
HYAL3_2_15795,172,180,119,230,300,184,401,3,437,124,0,103
JPH2_2_15796,7,11,25,83,252,0,0,154,1,0,43,0
KLK8_2_15797,440,514,901,677,38,331,372,219,467,497,495,483
LAMA4_2_15798,314,243,343,354,1,0,173,137,56,95,1,445
LRR1_2_15799,520,406,421,541,281,537,588,245,347,814,45,304
LRTOMT_2_15800,35,41,31,16,21,0,42,1,41,3,21,5
METTL1_2_15801,55,23,72,108,1,31,0,1,0,163,0,42
MOCS2_2_15802,724,818,643,1080,690,1312,491,589,589,313,1122,804
NDUFA11_2_15803,153,382,167,180,0,155,5,137,68,121,328,184
NDUFV3_2_15804,1049,1196,861,959,304,595,537,504,443,864,585,1084
NMNAT3_2_15805,189,67,85,80,32,7,26,14,54,21,560,1
PDE4DIP_2_15806,54,61,133,62,2,63,580,0,11,7,8,175
PLG_2_15807,546,304,297,511,278,143,181,247,77,667,330,175
PPCS_2_15808,429,215,212,314,346,581,381,125,321,160,46,572
PRDX2_2_15809,261,523,440,356,326,59,815,200,18,1044,571,153
RAD51C_2_15810,1010,506,587,944,1121,325,175,582,729,160,1350,453
RASD1_2_15811,0,32,0,0,0,0,0,0,0,0,0,0
RASL10A_2_15812,324,275,238,370,216,741,303,411,391,194,133,574
SLX1A_2_15813,519,562,353,565,851,213,840,1319,988,294,131,421
SLX1B_2_15814,519,562,353,565,851,213,840,1319,988,294,131,421
SPTLC1_2_15815,1434,950,1087,1241,951,1286,2849,563,463,496,1173,1699
ST3GAL3_2_15816,176,303,325,435,3,289,469,700,82,110,642,444
VKORC1_2_15817,632,563,520,679,543,301,283,559,509,364,315,490
XPNPEP3_2_15818,463,447,561,484,238,275,257,68,557,966,658,484
GNAS_2_15819,62,54,101,294,11,95,0,0,32,18,508,25
GNAS_2_15820,119,64,125,141,63,159,98,157,71,159,0,194
GNAS_2_15821,144,146,96,99,83,0,0,0,0,141,114,804
A4GALT_2_15822,149,156,93,260,2,0,354,356,2,16,0,69
A4GNT_2_15823,22,3,11,28,25,76,0,0,0,0,0,15
AACS_2_15824,164,242,231,246,408,0,955,58,21,76,280,396
AADAC_2_15825,2800,2913,2556,3294,4363,2241,2439,3140,1793,2566,2354,3
270
AARS2_2_15826,354,286,162,255,71,552,139,268,325,209,205,127
AARS_2_15827,781,716,616,897,307,914,1660,1076,723,953,1272,993
AASDH_2_15828,257,289,268,371,238,368,0,413,251,918,19,126
AASDHPPT_2_15829,86,56,56,90,101,196,26,7,172,86,0,40
AASS_2_15830,1164,1159,1238,1234,781,893,1273,632,855,821,1169,2426
ABHD14A_2_15831,182,112,160,159,0,659,2,0,12,0,676,21
ABHD1_2_15832,481,328,366,457,191,2,524,2,512,712,739,389
ABHD3_2_15833,1179,1211,1181,1042,512,261,1043,734,865,1660,1342,1622
ABHD5_2_15834,416,423,555,453,601,753,2050,812,204,356,427,118
ABHD6_2_15835,77,76,57,101,2,1,190,8,36,53,21,0
ABHD8_2_15836,155,114,122,188,130,25,529,35,205,30,0,135
ABO_2_15837,489,381,401,496,639,356,260,591,300,106,618,230
ABP1_2_15838,225,247,233,198,76,85,171,265,210,73,44,2
ACAA2_2_15839,219,130,208,295,220,262,187,61,179,122,478,118
ACACB_2_15840,338,365,381,481,211,96,25,348,154,431,310,249

ACAD11_2_15841,531,251,335,484,78,1,18,511,278,60,78,121
ACAD8_2_15842,184,276,116,217,258,162,4,122,290,0,0,102
ACAD9_2_15843,541,586,787,657,698,84,216,918,133,782,565,449
ACADL_2_15844,2087,2802,1910,2563,1866,2907,3726,2125,2222,3285,1384,2
554
ACADSB_2_15845,665,541,453,619,505,430,1588,964,264,1240,647,397
ACADS_2_15846,119,96,74,88,220,12,243,19,21,24,0,16
ACAT1_2_15847,113,35,107,63,107,0,244,106,18,52,0,66
ACER1_2_15848,195,127,109,200,22,153,92,33,39,239,90,116
ACER2_2_15849,708,628,537,612,472,97,1371,454,1128,231,259,625
ACER3_2_15850,111,140,159,169,1,4,27,338,52,116,17,132
ACMSD_2_15851,124,78,128,127,305,111,0,67,14,1,119,44
ACO1_2_15852,3228,2387,3473,3476,3055,2745,4023,2765,2489,3425,3049,21
20
ACO2_2_15853,126,86,168,205,27,0,209,10,92,75,202,0
ACOT12_2_15854,269,189,181,165,140,45,286,116,425,64,312,259
ACOT1_2_15855,271,311,237,263,152,153,6,219,612,257,19,523
ACOT2_2_15856,583,406,364,307,630,589,339,333,904,903,725,705
ACOT4_2_15857,119,233,65,139,38,297,34,69,87,21,0,77
ACOT6_2_15858,414,152,116,244,28,69,300,14,26,30,0,332
ACOT8_2_15859,205,222,62,187,0,243,21,0,236,181,0,0
ACOX2_2_15860,1475,1006,996,1193,1118,1119,397,902,2003,1210,740,499
ACOXL_2_15861,743,409,598,542,627,112,681,607,518,455,236,248
ACR_2_15862,300,259,285,340,117,549,193,310,598,289,284,758
ACSBG2_2_15863,497,409,246,414,668,207,458,406,165,502,163,250
ACSF2_2_15864,438,579,453,692,484,180,354,332,359,300,1338,307
ACSL1_2_15865,460,509,332,417,132,293,483,98,821,472,598,526
ACSM1_2_15866,156,82,42,104,276,1,0,120,27,3,0,155
ACSM2A_2_15867,428,359,270,284,92,606,436,33,209,344,846,257
ACSM4_2_15868,1710,1430,1383,1469,1796,1435,1678,1834,1275,1923,2009,2
585
ACSM5_2_15869,411,429,289,269,482,307,957,565,454,679,156,468
ACSS1_2_15870,75,95,143,79,2,172,15,118,96,7,972,0
ACSS3_2_15871,78,19,19,89,8,0,0,217,39,0,0,12
ACTC1_2_15872,397,410,209,449,1239,447,28,449,366,284,760,293
ACY3_2_15873,483,295,437,310,494,173,650,53,170,213,1,208
ACYP2_2_15874,1383,1169,1004,1214,1851,1507,617,605,1320,1191,737,1323
ADA_2_15875,164,92,183,82,1,0,31,4,77,294,5,0
ADAM10_2_15876,1106,966,1009,1119,2182,797,645,1173,662,1383,1077,1970
ADAM17_2_15877,752,517,391,410,709,433,618,1383,432,986,239,611
ADAMTS4_2_15878,103,102,80,194,96,42,251,103,61,361,6,88
ADARB2_2_15879,58,75,14,88,106,0,0,0,0,3,0,7
ADAT1_2_15880,675,624,843,812,1427,371,431,931,1222,604,541,895
ADAT2_2_15881,281,286,165,148,345,0,249,0,278,297,0,40
ADC_2_15882,95,65,30,188,34,34,0,50,57,190,0,15
ADCY1_2_15883,244,262,274,184,382,101,22,141,173,219,5,413
ADCY2_2_15884,369,369,172,211,452,181,121,220,187,993,118,189
ADCY7_2_15885,240,262,281,249,310,0,0,513,195,353,236,221
ADCY8_2_15886,120,147,69,105,110,0,80,248,150,86,54,68
ADCY9_2_15887,98,71,152,110,245,8,173,2,411,231,16,87

ADH1A_2_15888,59,51,100,125,1,175,216,162,1,0,0,0
ADH1B_2_15889,610,457,478,743,584,255,577,812,358,535,586,523
ADH1C_2_15890,344,372,244,418,216,33,129,499,497,1634,40,277
ADH4_2_15891,179,140,288,264,0,67,159,608,553,156,269,691
ADH5_2_15892,490,498,304,590,339,97,613,35,818,589,1387,446
ADHFE1_2_15893,541,422,413,432,119,533,0,1316,66,578,702,29
ADI1_2_15894,239,82,91,69,15,105,4,10,268,102,31,355
ADO_2_15895,886,723,792,783,1127,1136,1288,1250,1179,657,1128,1197
ADPRH_2_15896,24,30,30,30,141,2,0,0,52,2,0,1
ADPRHL2_2_15897,1412,717,1107,1000,1343,822,995,810,1051,1061,333,1118
ADSS_2_15898,178,290,188,298,4,535,52,2,152,431,174,41
AEN_2_15899,116,15,105,70,477,3,154,0,39,80,160,94
AGBL2_2_15900,128,116,88,122,76,397,6,3,9,2,0,65
AGMAT_2_15901,70,64,96,104,17,0,65,0,298,258,0,43
AGMO_2_15902,488,372,480,402,597,160,355,366,265,231,704,503
AGPAT4_2_15903,228,161,136,219,7,291,9,98,444,43,271,199
AGPAT5_2_15904,44,54,46,40,0,0,0,0,103,66,5,67
AGPAT6_2_15905,114,120,94,122,3,0,5,113,119,0,2,70
AGPAT9_2_15906,344,398,305,339,227,447,107,84,91,513,241,443
AGPS_2_15907,1959,1117,1225,1585,1604,1005,847,996,1521,1579,1412,1847
AGXT2_2_15908,340,330,386,411,49,264,7,493,52,57,1195,690
AGXT2L2_2_15909,126,66,152,186,410,135,195,59,161,33,61,260
AGXT_2_15910,141,1,131,114,37,34,0,0,1,13,114,2
AICDA_2_15911,299,101,75,280,565,116,0,3,261,226,604,0
AKR1B10_2_15912,174,108,124,199,28,458,323,89,126,107,4,170
AKR1B15_2_15913,596,511,482,708,358,928,137,492,344,422,841,441
AKR1B1_2_15914,306,129,73,115,614,0,261,2,435,426,0,0
AKR1C1_2_15915,330,412,258,499,434,595,297,9,344,636,471,546
AKR1C3_2_15916,87,61,108,36,1,2,37,0,43,39,143,54
AKR1C4_2_15917,401,417,346,419,64,148,124,176,168,97,150,433
AKR1E2_2_15918,73,123,145,220,234,21,23,192,54,447,1,323
AKR7A2_2_15919,1146,903,747,1316,887,903,1021,1042,1236,1396,1089,1358
AKR7A3_2_15920,75,147,105,87,0,246,0,0,64,265,14,5
ALAD_2_15921,146,74,112,131,68,168,212,28,158,33,86,103
ALDH1A1_2_15922,6,14,28,70,0,0,0,26,0,0,0,0
ALDH1A3_2_15923,382,345,324,530,116,50,104,188,89,337,32,7
ALDH1B1_2_15924,0,0,0,0,0,0,0,0,0,0,0,0
ALDH1L1_2_15925,713,645,793,814,647,609,523,60,325,196,1944,1062
ALDH1L2_2_15926,1064,1268,1314,1562,489,2122,250,1322,1710,1370,1130,2
073
ALDH6A1_2_15927,157,285,138,207,25,6,19,1283,2,335,235,228
ALDH9A1_2_15928,315,229,345,575,233,63,210,266,312,78,217,201
ALDOB_2_15929,298,282,309,582,30,81,16,1271,109,278,3,147
ALDOC_2_15930,522,489,526,455,525,246,682,1080,59,641,1362,480
ALG10_2_15931,148,84,94,117,177,160,191,364,38,566,21,138
ALG11_2_15932,154,257,177,252,440,272,24,4,343,359,711,489
ALG12_2_15933,115,171,140,140,39,205,0,111,55,268,9,633
ALG14_2_15934,77,39,50,53,29,0,51,173,1,8,1,57
ALG1_2_15935,86,51,53,63,0,1,1,37,38,1,0,76
ALG2_2_15936,283,309,254,315,141,121,197,400,646,91,914,27

ALG6_2_15937,607,434,819,588,269,444,724,1074,211,576,889,727
ALKBH8_2_15938,570,970,758,965,647,987,766,710,834,850,1741,1497
ALLC_2_15939,63,4,36,55,13,79,6,236,2,0,0,4
ALOX12B_2_15940,1011,1049,1058,1306,1115,822,1137,1550,663,770,964,789
ALOX12_2_15941,206,235,315,236,136,94,366,59,720,478,150,163
ALOX15_2_15942,302,322,260,225,56,53,319,32,132,129,586,210
ALOX5_2_15943,142,16,52,56,15,15,12,57,6,1,0,27
AMDHD1_2_15944,80,137,160,150,76,48,305,73,33,125,90,310
AMY1B_2_15945,13219,11353,11716,14121,10096,7505,13600,11952,10104,988
3,10997,12637
AMY1C_2_15946,13219,11353,11716,14121,10096,7505,13600,11952,10104,988
3,10997,12637
AMY2A_2_15947,433,365,243,541,551,107,292,636,69,338,114,337
AMY2B_2_15948,149,202,190,198,187,179,170,476,257,478,60,33
ANPEP_2_15949,47,37,58,48,4,30,0,1,15,10,55,2
AOC3_2_15950,1214,1249,862,1536,747,1603,2173,1411,1087,1533,542,1799
AOX1_2_15951,704,593,654,864,225,375,304,946,429,1102,471,1954
APEH_2_15952,446,322,403,597,667,5,573,597,490,220,301,324
APEX2_2_15953,128,197,143,104,282,0,3,259,9,220,6,54
APIP_2_15954,2858,3473,3064,3380,1901,2345,3214,2726,3336,4078,2297,23
74
APLF_2_15955,781,490,461,603,571,624,668,764,156,285,196,439
APOBEC1_2_15956,32,49,80,63,3,4,0,5,102,110,1,1
APOBEC2_2_15957,305,449,289,345,313,24,5,494,66,17,469,632
APOBEC3B_2_15958,194,74,88,118,88,208,303,102,111,86,1,85
APOBEC3C_2_15959,242,163,202,227,142,3,225,69,251,39,973,22
APOBEC3G_2_15960,554,609,379,635,272,73,398,378,779,986,837,686
ARF3_2_15961,482,404,437,529,17,107,512,50,85,512,1010,376
ARF4_2_15962,123,115,236,262,124,141,854,1,315,6,62,483
ARG2_2_15963,1209,1393,1121,1121,1603,819,651,890,1002,1529,991,1170
ARHGEF10_2_15964,255,290,167,251,82,387,163,45,91,684,0,524
ARL3_2_15965,523,536,445,558,472,486,163,234,275,1107,360,1810
ARL4C_2_15966,58,109,96,111,0,0,52,195,46,177,0,244
ARL4D_2_15967,26,88,36,63,12,2,39,0,21,434,95,164
ARL5B_2_15968,355,323,419,395,819,2,508,415,90,15,604,932
ARL8A_2_15969,213,204,167,222,7,1,507,130,17,0,77,14
ARL8B_2_15970,594,599,333,558,193,863,126,532,292,57,893,734
ARSD_2_15971,100,91,28,75,0,0,0,256,0,210,14,57
ARSE_2_15972,307,233,191,325,416,278,3,9,717,337,29,324
ARSG_2_15973,469,240,279,375,1006,250,181,405,335,1145,110,451
ARSH_2_15974,49,276,123,122,28,2,0,5,109,306,388,275
ARSI_2_15975,410,345,318,185,150,344,254,254,100,184,466,367
ARSJ_2_15976,204,130,136,208,270,10,195,87,313,46,235,90
ARSK_2_15977,153,37,77,151,225,11,22,71,59,30,399,94
ART1_2_15978,436,434,354,511,602,1286,322,240,228,86,1775,231
ART4_2_15979,341,393,331,496,262,114,4,64,477,351,887,529
AS3MT_2_15980,656,955,523,617,730,851,1080,341,308,414,353,516
ASNA1_2_15981,432,517,351,523,307,254,292,972,521,710,693,580
ASNSD1_2_15982,1439,1319,1103,1333,786,963,1090,1036,1186,1802,1599,15
27

ASPG_2_15983,464,448,680,520,1641,188,12,636,381,1045,506,431
ASPHD2_2_15984,650,359,528,496,866,921,951,388,543,438,197,430
ATAD1_2_15985,216,251,322,253,440,455,70,305,229,280,34,386
ATIC_2_15986,703,391,665,799,128,295,646,786,278,249,976,511
ATP2C2_2_15987,291,308,211,297,420,35,369,222,79,88,86,628
AUH_2_15988,423,603,516,467,395,423,369,893,236,978,502,828
AWAT1_2_15989,89,74,134,136,5,2,179,6,50,161,469,260
AWAT2_2_15990,114,139,205,264,10,370,3,44,901,234,0,31
B3GALNT2_2_15991,465,324,352,392,229,263,340,586,72,385,402,894
B3GALT1_2_15992,154,170,170,246,136,16,146,40,340,1,70,60
B3GALT2_2_15993,1306,1222,1552,1536,1115,380,1391,1296,1174,1476,890,2
334
B3GALT4_2_15994,293,204,199,236,354,78,45,252,48,137,47,397
B3GALT6_2_15995,76,40,77,66,5,0,2,242,65,70,8,86
B3GAT2_2_15996,252,241,226,245,95,1,92,655,72,282,0,242
B3GAT3_2_15997,192,115,213,241,19,179,768,263,148,298,0,96
B3GNT2_2_15998,132,179,155,87,72,90,37,210,191,89,58,220
B3GNT3_2_15999,6,54,35,71,1,316,19,2,0,65,0,2
B3GNT4_2_16000,1,13,1,19,0,0,0,0,0,0,0,0
B3GNT5_2_16001,813,978,760,744,978,356,767,1787,579,224,2279,982
B3GNT7_2_16002,86,67,34,96,243,48,83,0,107,134,64,38
B3GNT9_2_16003,296,134,152,187,55,304,55,304,134,64,71,65
B3GNTL1_2_16004,1338,1150,1034,1172,1308,624,1532,1314,1643,1054,2322,
1516
B4GALNT1_2_16005,294,285,282,270,280,163,180,692,217,212,324,333
B4GALNT3_2_16006,26,50,9,39,17,1,0,5,158,3,0,43
B4GALNT4_2_16007,178,329,122,396,329,72,263,32,21,313,411,265
B4GALT1_2_16008,333,300,269,276,569,408,238,48,597,912,58,237
B4GALT5_2_16009,50,103,64,117,69,0,51,4,129,0,0,228
B4GALT6_2_16010,259,233,199,246,35,97,5,11,11,35,0,204
B4GALT7_2_16011,19,13,51,39,0,9,0,0,0,0,283,21
BBOX1_2_16012,43,5,12,53,94,0,10,21,51,21,0,54
BCHE_2_16013,540,428,448,475,441,707,373,153,363,916,336,246
BCMO1_2_16014,528,558,597,543,585,386,854,232,317,601,331,1103
BDH2_2_16015,2133,1701,1795,2683,1592,1553,2665,2032,2002,1715,1640,21
73
BHMT_2_16016,616,718,429,923,570,861,945,668,812,393,551,930
BLM_2_16017,2361,2025,1661,2401,2466,1207,2036,2869,1397,1758,1897,227
0
BLMH_2_16018,0,0,0,0,2,0,1,0,1,0,0,0
BLVRA_2_16019,405,161,279,420,31,4,516,252,334,723,100,4
BLVRB_2_16020,459,407,593,611,115,122,598,88,209,191,47,506
BPHL_2_16021,180,102,111,158,347,155,131,166,96,39,37,69
BST1_2_16022,111,109,131,105,89,2,1,80,289,52,5,172
BTD_2_16023,644,398,345,479,630,206,327,156,224,527,208,715
C12orf5_2_16024,17,19,16,14,10,59,303,298,43,0,0,640
C16orf79_2_16025,78,23,82,112,0,282,2,367,27,18,169,35
C18orf56_2_16026,56,21,12,38,230,0,90,0,5,7,7,54
C1GALT1_2_16027,821,509,504,801,1062,130,524,1585,814,109,926,664
C1R_2_16028,57,22,74,128,0,3,0,0,84,6,3,217

C22orf28_2_16029,422,722,498,576,472,426,282,200,261,653,546,964
C2orf43_2_16030,49,41,21,80,19,52,66,167,23,1,15,78
C5orf4_2_16031,622,879,552,784,278,331,419,775,870,1317,766,259
C6orf130_2_16032,306,205,274,242,28,39,8,307,109,565,11,104
CA13_2_16033,136,154,106,130,1,123,6,298,171,41,628,290
CA14_2_16034,35,21,47,32,13,12,124,28,112,17,15,0
CA2_2_16035,102,59,106,70,0,170,0,18,34,8,14,178
CA3_2_16036,499,340,542,397,380,402,414,522,423,572,228,456
CA4_2_16037,1450,1499,1563,1897,1720,2200,1527,853,813,2571,2053,2168
CA5A_2_16038,219,177,202,254,539,353,29,441,180,90,271,254
CA5B_2_16039,1140,841,874,1157,967,882,1501,1159,1682,1060,1069,1344
CA6_2_16040,7197,6192,6228,6633,5887,6478,5467,7958,5273,7352,6430,549
3
CA8_2_16041,810,959,897,1076,1647,672,804,1213,372,614,1501,919
CA9_2_16042,324,298,160,249,107,2,408,256,35,145,330,926
CARS2_2_16043,810,1042,839,1220,925,160,687,1094,747,1070,1457,1550
CASD1_2_16044,155,159,138,133,239,44,14,180,77,61,169,91
CAT_2_16045,480,650,545,667,339,419,419,311,377,676,1350,312
CBR1_2_16046,200,258,280,178,70,19,18,136,291,197,70,211
CBR3_2_16047,1636,1156,1302,1421,2082,1365,2008,1081,800,1507,1742,101
4
CBR4_2_16048,145,212,182,235,311,325,463,119,22,216,343,308
CCDC88B_2_16049,22,53,3,7,24,0,75,0,2,55,0,123
CCDC92_2_16050,609,596,608,677,1197,489,752,984,326,117,432,616
CCNO_2_16051,17,49,63,29,0,0,0,0,0,0,0,57
CCS_2_16052,345,106,194,170,588,54,130,40,316,20,0,135
CCT8_2_16053,136,127,128,215,257,225,435,248,12,232,127,210
CD274_2_16054,609,503,444,395,1,449,397,284,214,1234,123,754
CD38_2_16055,633,699,740,705,1488,386,401,805,143,309,643,1307
CDA_2_16056,266,170,325,291,585,409,393,462,30,513,101,121
CDIPT_2_16057,298,311,198,215,220,48,1,247,617,507,324,74
CDO1_2_16058,476,178,424,272,561,98,497,201,370,783,386,412
CDS2_2_16059,1518,1511,1466,1771,1677,1300,1698,1680,1165,1946,1620,14
13
CELA1_2_16060,216,290,273,246,390,0,686,0,19,267,326,1
CELA2A_2_16061,42,29,38,25,0,0,0,38,9,35,100,0
CELA2B_2_16062,477,262,321,370,54,416,79,66,553,112,585,823
CELA3A_2_16063,78,64,74,74,29,1,1,3,252,383,0,58
CELA3B_2_16064,78,64,74,74,29,1,1,3,252,383,0,58
CEL_2_16065,63,56,67,101,124,17,42,139,155,12,0,158
CETN1_2_16066,350,264,234,375,13,35,366,14,183,187,100,157
CETN2_2_16067,132,69,122,129,39,556,4,35,327,8,11,92
CETP_2_16068,218,244,114,158,62,143,102,7,195,150,51,73
CFB_2_16069,607,495,566,799,1125,154,636,1197,1639,669,684,233
CFD_2_16070,76,196,96,171,0,287,3,362,320,0,395,360
CFI_2_16071,462,404,468,561,812,82,108,347,572,206,333,676
CH25H_2_16072,305,371,300,473,23,0,8,962,470,275,27,163
CHDH_2_16073,336,149,146,227,331,33,297,0,180,528,501,362
CHI3L1_2_16074,70,83,18,97,0,250,68,10,22,263,1,116
CHIT1_2_16075,20,61,20,62,17,187,231,10,26,83,0,49

CHML_2_16076,1815,1359,1257,1739,1224,1202,1556,799,1017,1602,1139,102
2
CHPF2_2_16077,274,172,204,333,253,75,273,332,514,207,693,175
CHPT1_2_16078,330,281,351,380,461,584,560,646,476,1,289,512
CHST10_2_16079,34,22,34,45,1,59,0,0,124,0,814,5
CHST12_2_16080,53,108,104,130,102,32,0,288,47,115,299,6
CHST13_2_16081,0,4,1,22,0,0,0,0,0,0,0,0
CHST14_2_16082,33,43,32,33,74,1,0,41,9,61,91,0
CHST1_2_16083,108,363,246,231,0,753,609,296,219,7,263,261
CHST2_2_16084,99,72,92,110,12,160,37,260,11,100,18,171
CHST3_2_16085,0,5,8,23,0,3,7,0,0,0,0,129
CHST5_2_16086,53,130,23,80,17,2,120,11,3,478,85,6
CHST6_2_16087,287,297,207,294,97,424,252,430,21,701,161,134
CHST7_2_16088,151,64,21,153,0,3,0,318,250,3,6,32
CHST9_2_16089,211,139,162,164,135,61,601,53,126,185,73,209
CHSY1_2_16090,144,159,315,246,677,115,209,463,113,70,108,392
CHSY3_2_16091,415,471,429,358,111,502,702,14,494,444,1025,230
CLPP_2_16092,119,144,89,194,25,129,440,413,7,265,0,8
CLPX_2_16093,209,80,58,154,270,24,8,42,160,23,94,53
CLYBL_2_16094,145,102,153,206,147,43,1,371,15,124,105,218
CMA1_2_16095,317,201,339,376,58,36,432,248,197,402,243,313
CMAS_2_16096,1725,1634,1481,1692,2381,982,1865,1819,1947,1191,1799,242
0
CMBL_2_16097,430,394,411,430,151,515,563,252,290,479,160,778
CNDP1_2_16098,87,132,56,70,242,47,191,68,201,11,0,38
CNTN6_2_16099,566,245,358,472,714,2,563,363,319,1,718,131
COIL_2_16100,1065,960,878,1230,218,786,1140,811,803,825,956,1223
COMTD1_2_16101,146,114,159,183,162,11,6,406,75,214,0,161
COQ2_2_16102,679,532,502,536,933,417,1039,506,819,590,269,984
COQ3_2_16103,75,87,2,17,1,36,155,178,4,0,0,0
COQ5_2_16104,884,459,579,576,488,435,744,663,928,898,800,857
COX10_2_16105,141,49,103,136,591,56,666,46,257,13,0,20
COX17_2_16106,1312,1532,1537,1607,674,1406,1471,1172,890,2778,1943,156
9
COX4I1_2_16107,137,105,117,91,332,131,0,11,331,110,11,6
COX4I2_2_16108,96,113,92,100,93,0,1,41,3,121,1,1
COX5A_2_16109,611,474,460,698,686,470,908,1498,276,437,615,540
COX5B_2_16110,2095,1946,1999,1997,2019,2058,3322,844,2115,1898,2238,20
50
COX6A1_2_16111,78,150,118,97,5,340,59,24,122,303,4,33
COX6A2_2_16112,507,400,366,442,568,528,329,378,337,470,239,556
COX6B1_2_16113,58,146,162,196,0,2,0,0,0,0,540,279
COX6B2_2_16114,256,177,154,137,490,151,141,189,182,221,53,31
COX6C_2_16115,853,698,568,778,1452,321,811,438,815,496,401,427
COX7A1_2_16116,359,483,375,350,1054,472,435,465,432,399,803,544
COX7A2_2_16117,880,624,793,612,539,849,98,350,788,867,2230,759
COX7A2L_2_16118,193,106,200,153,258,115,488,217,83,103,126,20
COX7B2_2_16119,418,300,197,246,143,10,285,458,543,191,162,128
COX7B_2_16120,235,111,100,209,740,27,29,0,423,0,1,615
COX7C_2_16121,285,229,190,296,209,150,129,291,304,39,12,219

COX8A_2_16122,171,353,187,295,75,39,187,1,274,157,18,168
COX8C_2_16123,793,784,590,931,707,403,753,537,363,1097,726,1504
CPA1_2_16124,201,172,141,175,583,0,368,0,44,142,5,271
CPA2_2_16125,207,208,171,175,242,145,170,290,444,358,67,185
CPB1_2_16126,543,663,354,468,535,163,1261,343,218,321,90,388
CP_2_16127,1,17,42,17,0,49,7,0,3,1,51,2
CPE_2_16128,1090,751,868,935,846,944,337,1409,1644,1252,819,525
CPOX_2_16129,369,408,298,359,424,662,465,363,212,542,38,442
CPSF3_2_16130,515,216,423,479,344,94,13,0,541,212,358,60
CPT2_2_16131,693,513,624,764,684,420,2110,398,486,364,430,716
CRAT_2_16132,117,122,74,124,11,152,65,466,127,110,145,172
CREG2_2_16133,34,16,13,24,78,76,30,25,1,0,54,1
CRY1_2_16134,80,53,72,74,9,59,0,238,13,189,0,250
CRYL1_2_16135,205,160,196,309,309,254,84,74,174,135,132,136
CRYZL1_2_16136,149,117,192,151,124,41,7,282,217,37,1,147
CSAD_2_16137,252,298,228,341,993,234,572,328,317,76,397,518
CS_2_16138,663,825,574,643,734,1070,1311,384,1029,1148,732,320
CSGALNACT2_2_16139,904,702,561,642,146,677,59,343,703,531,955,587
CSRP2BP_2_16140,75,16,39,103,32,0,216,0,0,0,0,29
CTBS_2_16141,201,157,111,187,189,2,266,3,422,79,40,81
CTRB1_2_16142,194,77,112,99,143,0,1,21,46,0,372,16
CTRB2_2_16143,26,140,155,95,168,11,0,0,50,234,864,69
CTRC_2_16144,3,10,3,39,0,0,0,0,2,0,0,0
CTSD_2_16145,78,99,79,79,144,132,6,72,1,1,127,108
CTSF_2_16146,1087,905,740,1144,658,527,765,1015,139,1022,628,627
CTSG_2_16147,471,669,535,725,735,72,674,0,2,704,502,489
CTSH_2_16148,391,242,440,312,578,137,193,243,344,302,185,180
CTSK_2_16149,285,88,286,191,137,6,616,1,240,66,295,268
CTSZ_2_16150,224,350,278,204,382,329,492,357,271,91,1233,583
CWC27_2_16151,204,137,161,276,259,242,1505,149,262,194,25,289
CXorf21_2_16152,469,576,423,530,21,165,610,492,455,1281,825,527
CYB561D2_2_16153,9,8,14,14,218,0,0,0,0,0,0,28
CYB5B_2_16154,31,158,40,68,0,381,25,9,2,67,7,0
CYB5R1_2_16155,768,788,667,979,501,757,1435,942,1291,1396,169,1754
CYB5R2_2_16156,1012,767,781,1054,714,345,284,615,965,942,728,1225
CYB5R4_2_16157,85,63,156,178,209,2,0,379,82,13,340,5
CYBA_2_16158,71,47,77,98,49,1,69,2,15,80,0,217
CYBB_2_16159,663,463,498,663,225,796,939,1004,503,288,595,262
CYC1_2_16160,262,119,284,99,1,0,45,42,14,2,776,130
CYCS_2_16161,661,443,432,891,807,793,416,650,394,1042,236,984
CYP11B2_2_16162,775,534,430,434,675,754,94,578,689,183,1227,710
CYP17A1_2_16163,141,135,130,104,81,15,108,8,450,29,4,106
CYP1A1_2_16164,988,1161,1015,1237,1562,237,1034,1143,1451,1026,2410,84
2
CYP1A2_2_16165,156,109,138,170,147,456,407,3,14,356,54,138
CYP1B1_2_16166,217,73,139,121,217,22,65,130,116,76,250,259
CYP20A1_2_16167,496,644,415,683,1092,384,406,334,314,202,838,831
CYP26B1_2_16168,8,30,11,24,0,1,0,5,1,0,1,8
CYP26C1_2_16169,30,11,37,4,14,0,0,1,7,17,15,4
CYP27A1_2_16170,711,818,754,955,1389,863,312,1257,650,422,454,920

CYP27B1_2_16171,244,111,280,329,26,229,962,1033,1,6,167,402
CYP2A13_2_16172,782,623,718,678,744,966,266,231,840,315,287,1249
CYP2A6_2_16173,202,212,134,144,74,222,231,1,80,104,304,176
CYP2B6_2_16174,337,239,278,432,693,319,327,49,77,362,286,312
CYP2C19_2_16175,170,158,75,172,359,94,54,18,16,110,11,78
CYP2C9_2_16176,1126,771,643,783,1235,1098,1046,656,448,574,444,875
CYP2E1_2_16177,1335,1231,867,1474,978,667,862,1236,1214,1416,1350,1591
CYP2F1_2_16178,592,615,452,564,851,279,1254,1149,341,285,588,1065
CYP2J2_2_16179,57,24,51,37,319,12,0,0,2,143,6,198
CYP2R1_2_16180,425,210,212,358,150,6,438,0,479,392,1454,305
CYP2S1_2_16181,0,0,0,0,0,0,0,0,0,0,0,0
CYP2U1_2_16182,318,232,394,477,18,465,155,565,259,245,617,250
CYP2W1_2_16183,130,53,108,93,75,23,60,65,4,235,0,307
CYP39A1_2_16184,1191,807,776,1115,1226,370,831,2055,1024,281,977,847
CYP3A7_2_16185,1097,724,856,794,1110,538,799,704,1037,493,748,1178
CYP46A1_2_16186,86,61,64,59,28,0,0,0,242,132,11,46
CYP4A11_2_16187,459,386,381,409,397,318,807,408,469,466,468,383
CYP4A22_2_16188,28,57,31,86,0,81,1,0,0,0,0,3
CYP4F12_2_16189,9,38,25,31,36,8,0,382,27,22,0,1
CYP4F22_2_16190,112,119,124,172,400,33,15,92,240,49,0,547
CYP4F2_2_16191,1097,908,892,1022,928,994,1210,1112,1151,637,848,859
CYP4F8_2_16192,1097,908,892,1022,928,994,1210,1112,1151,637,848,859
CYP4V2_2_16193,1058,1314,1274,1161,1559,1812,1766,1492,1316,1156,1524,
1502
CYP4X1_2_16194,224,273,207,183,285,201,631,189,237,165,275,105
CYP4Z1_2_16195,149,49,48,59,20,13,161,8,5,16,150,36
CYP7A1_2_16196,147,179,168,257,186,40,311,294,106,109,4,342
CYP7B1_2_16197,785,611,659,783,759,1213,390,1459,1047,911,138,938
CYP8B1_2_16198,52,158,81,119,5,48,587,14,491,21,67,4
DAD1_2_16199,204,435,158,250,183,770,20,23,415,116,483,324
DAGLA_2_16200,154,177,159,154,195,19,412,173,79,200,32,38
DAK_2_16201,32,26,4,7,2,4,0,6,0,0,0,11
DARS2_2_16202,482,313,342,465,266,289,1056,1019,1016,511,358,1161
DARS_2_16203,1837,1115,1498,1798,651,799,438,2694,1750,1138,2511,1843
DBH_2_16204,301,198,117,272,518,2,0,31,25,22,370,119
DBR1_2_16205,802,670,767,1172,748,139,430,616,745,661,1094,1469
DBT_2_16206,365,221,386,365,202,45,231,290,725,525,290,149
DCLRE1B_2_16207,402,434,445,451,646,393,356,1128,309,352,397,554
DCPS_2_16208,458,700,415,746,260,662,433,1089,525,789,899,1035
DCTN6_2_16209,363,366,364,451,253,187,394,385,87,524,890,1252
DCTPP1_2_16210,137,179,189,386,141,561,15,670,103,0,756,323
DDAH2_2_16211,200,119,176,61,10,49,22,0,1,56,0,16
DDOST_2_16212,32,46,15,20,104,0,78,82,21,0,237,11
DDX10_2_16213,448,483,432,391,192,213,532,955,194,576,848,374
DDX18_2_16214,383,405,535,512,586,15,442,63,21,659,127,308
DDX19A_2_16215,763,778,713,868,624,590,1249,658,647,1153,1656,699
DDX1_2_16216,215,135,127,139,39,63,134,11,329,213,347,451
DDX20_2_16217,993,1095,1146,1503,1264,892,2377,1697,1239,1585,1354,142
6
DDX21_2_16218,60,62,64,70,130,11,73,105,3,141,104,207

DDX23_2_16219,407,489,386,336,360,363,122,1316,518,1058,666,762
DDX24_2_16220,443,289,287,356,446,57,197,274,116,670,182,641
DDX25_2_16221,929,1203,936,1207,1139,481,699,1431,788,1532,1092,1296
DDX27_2_16222,464,340,293,552,433,895,863,189,301,238,852,753
DDX28_2_16223,336,219,166,353,324,325,53,795,614,162,15,449
DDX39A_2_16224,318,380,128,344,16,115,289,261,182,611,187,715
DDX41_2_16225,127,219,72,127,237,329,19,8,79,382,0,98
DDX43_2_16226,359,311,369,390,491,85,459,436,570,232,124,224
DDX46_2_16227,202,112,264,277,239,0,27,579,149,606,148,27
DDX49_2_16228,173,223,210,320,173,45,860,434,260,9,179,356
DDX50_2_16229,596,685,463,513,975,382,561,141,618,438,1340,880
DDX51_2_16230,96,55,52,69,58,0,131,189,2,62,251,35
DDX52_2_16231,992,727,634,734,805,752,929,278,493,1054,654,423
DDX53_2_16232,560,404,435,482,621,64,529,810,898,223,148,456
DDX55_2_16233,292,326,280,258,162,0,142,90,455,799,331,282
DDX56_2_16234,36,76,119,121,138,17,310,5,51,0,116,276
DDX58_2_16235,908,527,710,811,1193,1086,952,2043,462,1027,913,596
DDX59_2_16236,354,425,357,424,496,565,370,63,196,162,186,499
DDX5_2_16237,624,430,456,688,366,296,75,787,636,268,967,1084
DDX60_2_16238,604,681,705,566,1789,928,438,1155,725,609,1671,636
DDX6_2_16239,0,0,0,0,1,0,0,0,0,0,0,1
DECR1_2_16240,487,419,356,354,155,399,421,548,497,1209,293,993
DECR2_2_16241,26,9,21,29,1,0,0,0,50,0,50,0
DEGS1_2_16242,391,323,344,476,173,407,594,619,99,418,442,305
DEGS2_2_16243,9,53,35,24,0,0,0,0,0,1,2,8
DERA_2_16244,276,245,318,254,474,235,427,198,487,104,11,29
DFFB_2_16245,67,41,105,98,19,94,6,152,64,14,244,44
DGAT1_2_16246,57,42,31,126,55,481,0,28,34,0,215,471
DGAT2_2_16247,71,38,72,93,177,23,1,0,95,1,34,65
DHCR24_2_16248,78,66,154,60,14,3,294,0,156,0,149,148
DHDH_2_16249,325,592,427,489,741,599,519,1040,460,782,1975,443
DHFR_2_16250,0,0,86,56,0,0,0,0,0,0,0,0
DHODH_2_16251,614,515,503,496,345,721,260,81,35,614,2294,891
DHRS13_2_16252,46,83,42,162,7,9,5,104,142,0,0,70
DHRS3_2_16253,330,435,361,359,784,554,746,17,49,770,102,294
DHRS4_2_16254,201,195,204,253,224,20,54,184,152,335,136,101
DHRS7_2_16255,1226,661,744,1148,1512,238,2935,596,1422,212,630,197
DHRSX_2_16256,631,709,789,1030,1631,655,933,950,351,866,942,965
DHTKD1_2_16257,298,404,340,468,154,210,174,149,567,212,782,590
DHX15_2_16258,263,266,239,313,560,729,837,1247,278,766,135,389
DHX29_2_16259,320,490,288,313,324,434,1111,160,171,498,237,218
DHX32_2_16260,539,440,397,515,448,2,31,853,619,660,234,4
DHX34_2_16261,228,124,198,272,193,188,687,302,102,115,101,131
DHX37_2_16262,3,10,0,4,10,40,0,0,0,27,0,0
DHX38_2_16263,270,210,227,212,216,156,180,323,338,389,1,352
DHX57_2_16264,76,27,73,93,0,48,253,40,210,13,0,309
DHX58_2_16265,22,13,25,9,0,15,0,0,2,58,15,0
DHX8_2_16266,415,387,416,412,262,534,249,345,375,774,36,412
DHX9_2_16267,5,39,5,19,7,6,0,10,0,23,0,0
DIO3_2_16268,10,28,23,36,5,0,17,0,132,15,0,0

DIRAS1_2_16269,363,135,387,116,0,262,0,0,424,232,587,0
DIRAS2_2_16270,280,307,356,384,330,273,808,508,505,141,58,310
DIRAS3_2_16271,200,336,231,274,321,42,417,19,27,232,245,326
DIS3L2_2_16272,252,342,132,294,274,18,133,807,210,126,34,143
DLAT_2_16273,596,610,506,522,293,46,437,619,339,1433,590,935
DLD_2_16274,727,576,834,632,764,635,1070,288,659,280,655,783
DLL1_2_16275,1147,1045,867,1107,1147,697,1296,933,939,997,925,881
DLST_2_16276,984,900,755,1122,761,745,1156,531,1096,897,808,539
DMGDH_2_16277,323,179,331,382,112,83,937,580,346,492,471,99
DNA2_2_16278,98,165,106,168,2,10,0,0,0,159,132,248
DNAH11_2_16279,947,974,621,780,1344,364,258,385,616,124,118,1108
DNAH3_2_16280,427,512,610,368,371,657,481,189,208,366,880,275
DNAH5_2_16281,521,618,771,993,252,72,1129,613,736,635,1902,269
DNAH8_2_16282,255,141,213,117,214,81,173,189,160,207,32,620
DNAJA2_2_16283,12,22,13,12,11,13,8,14,15,0,0,23
DNAJB14_2_16284,332,227,268,275,274,443,381,2,200,77,230,393
DNAJC10_2_16285,272,324,145,277,234,0,14,153,486,265,570,355
DNAJC18_2_16286,45,36,37,66,0,18,0,3,243,42,0,17
DNAL4_2_16287,158,159,157,157,465,9,254,8,33,115,2,340
DNASE1_2_16288,137,292,95,185,6,303,6,0,196,405,0,209
DNASE1L2_2_16289,99,82,77,60,237,155,1,1,0,164,23,51
DNASE1L3_2_16290,1127,1083,1170,1288,1563,1592,1971,1228,987,1348,925,
573
DNASE2_2_16291,74,60,61,114,9,0,44,0,37,23,5,2
DNPEP_2_16292,234,305,163,249,68,111,5,122,92,12,0,206
DPAGT1_2_16293,715,607,490,473,492,492,224,1215,599,707,2317,487
DPEP2_2_16294,755,921,746,711,723,674,816,461,660,981,1338,698
DPM1_2_16295,144,77,218,122,2,11,0,32,80,70,0,269
DPM2_2_16296,125,105,164,123,172,150,146,63,116,131,51,136
DPP4_2_16297,93,34,65,57,32,72,90,61,178,3,694,22
DPP7_2_16298,47,22,35,35,0,58,49,29,11,101,0,127
DPP9_2_16299,187,118,155,213,96,186,210,9,2,120,318,28
DPYS_2_16300,118,52,102,40,47,5,244,10,36,153,4,213
DPYSL4_2_16301,839,730,757,815,583,1370,54,51,366,265,372,0
DPYSL5_2_16302,409,388,317,580,311,83,307,752,96,81,89,1396
DQX1_2_16303,194,219,236,417,127,341,35,197,166,795,262,76
DSEL_2_16304,870,445,551,973,836,740,503,471,1569,257,194,371
DTD1_2_16305,41,10,17,8,0,2,0,210,2,0,55,1
DUOX2_2_16306,80,132,31,107,0,1,68,2,1,130,67,1
DUPD1_2_16307,606,403,574,444,1168,393,95,465,496,338,329,108
DUSP28_2_16308,1208,1163,915,788,713,1628,1589,408,520,354,2473,1539
EARS2_2_16309,134,47,215,100,202,39,129,28,314,41,249,104
EBPL_2_16310,283,115,237,341,466,12,668,4,343,327,476,667
ECH1_2_16311,167,75,109,91,175,74,296,912,205,140,140,149
ECHDC3_2_16312,62,42,84,46,30,38,2,178,5,22,49,1
ECHS1_2_16313,106,86,96,89,103,135,127,27,9,387,329,104
EDEM1_2_16314,383,511,304,640,290,871,1186,702,391,725,435,427
EDEM3_2_16315,540,802,640,631,929,539,564,550,822,356,958,716
EEF1A1_2_16316,281,241,238,317,150,122,0,702,457,460,291,281
EEF1A2_2_16317,1413,1036,1045,1091,1635,1270,1821,870,997,2029,543,114

1
EEF2_2_16318,2114,2566,2281,1939,2353,923,1763,2137,2340,2641,4021,141
8
EGLN1_2_16319,56,49,65,88,0,1,28,156,15,268,164,28
EGLN3_2_16320,113,89,151,277,23,348,166,438,8,560,402,202
EHD4_2_16321,186,195,173,204,163,183,99,204,87,138,104,210
EIF4A3_2_16322,245,104,112,149,350,0,0,0,43,7,1623,200
ELAC1_2_16323,143,86,83,95,134,66,80,19,565,36,18,13
ELANE_2_16324,169,87,104,72,123,375,0,654,110,63,0,82
ELOVL1_2_16325,88,22,49,135,0,0,13,0,0,3,565,334
ELOVL2_2_16326,1944,1729,1828,1563,976,2329,1224,1434,1101,1128,1111,1
853
ELOVL3_2_16327,514,571,532,712,397,251,220,375,632,416,156,996
ELOVL4_2_16328,1134,907,759,1103,898,575,1360,468,1684,1382,763,1092
ENDOD1_2_16329,343,404,243,445,269,239,484,437,156,343,1060,529
ENDOG_2_16330,376,218,186,490,218,481,0,279,237,286,713,609
ENGASE_2_16331,76,48,88,67,0,0,506,1,19,25,528,0
ENO2_2_16332,388,240,246,301,27,50,101,196,207,112,597,92
ENO4_2_16333,104,58,104,17,0,0,1,6,14,0,12,0
ENOPH1_2_16334,267,73,89,50,386,303,2,63,44,52,0,489
ENPEP_2_16335,425,249,267,360,164,111,616,251,324,141,763,96
ENPP1_2_16336,132,116,200,192,270,321,0,136,40,214,679,117
ENPP3_2_16337,126,144,107,173,282,192,215,646,110,349,74,217
ENPP4_2_16338,802,618,726,783,458,225,730,443,305,1986,404,657
ENPP5_2_16339,458,649,494,474,600,998,382,141,636,1440,934,382
ENPP6_2_16340,537,263,393,462,429,77,419,672,516,34,523,574
ENPP7_2_16341,560,661,519,598,870,127,213,360,927,774,854,717
ENTPD3_2_16342,1068,891,735,1018,1650,404,1645,1403,671,854,473,1026
ENTPD5_2_16343,38,76,11,57,0,0,0,3,179,6,76,0
ENTPD7_2_16344,534,703,657,590,960,356,1128,198,740,405,618,367
EPHX2_2_16345,1,54,21,4,0,0,0,0,3,0,0,0
EPHX4_2_16346,1587,1531,1538,1647,2646,972,1487,2275,2361,1549,2934,12
45
EPRS_2_16347,740,745,616,798,269,367,902,722,394,1169,1270,843
EPT1_2_16348,120,114,82,119,307,24,133,65,239,75,1,32
EPX_2_16349,80,39,76,104,0,74,130,43,22,36,2,14
ERAS_2_16350,83,166,66,149,7,287,21,0,254,0,0,384
ERCC3_2_16351,175,194,149,205,431,291,296,148,398,3,11,486
ERCC4_2_16352,178,145,133,224,76,347,1,3,85,13,325,61
ERCC5_2_16353,649,512,761,860,650,620,1712,1,968,792,828,571
ERI1_2_16354,950,602,647,957,693,965,425,640,513,479,367,602
ERO1L_2_16355,144,272,192,240,1,288,385,60,234,326,34,423
ERP44_2_16356,376,208,260,459,373,481,285,220,680,75,469,402
ESD_2_16357,371,239,190,221,70,84,75,51,127,159,205,235
ESPL1_2_16358,139,131,113,113,243,1,0,112,553,40,1,317
ETFDH_2_16359,319,270,266,227,366,468,356,188,588,400,1,333
EXOSC1_2_16360,24,36,26,50,0,2,180,0,1,0,0,0
EXOSC2_2_16361,577,451,437,599,147,294,326,61,1241,218,253,187
EXOSC4_2_16362,196,24,221,255,57,42,2,31,20,52,31,340
EXOSC5_2_16363,11,4,84,22,0,0,34,0,1,4,465,0

EXOSC7_2_16364,194,109,186,162,0,33,42,322,4,163,48,13
EXOSC8_2_16365,302,309,246,335,16,0,470,200,180,200,887,378
EXT1_2_16366,523,522,513,493,531,98,37,353,256,130,99,323
EXTL1_2_16367,199,90,145,114,27,209,12,24,153,26,138,0
EXTL3_2_16368,246,91,329,132,405,35,545,196,60,65,58,178
F10_2_16369,242,376,427,373,31,549,358,151,257,196,705,565
F12_2_16370,105,148,42,18,0,506,0,21,2,234,0,7
F13A1_2_16371,585,397,523,503,366,189,338,138,443,368,426,910
F13B_2_16372,769,533,278,504,377,283,20,165,205,375,13,681
F2_2_16373,889,712,902,1145,441,558,1627,1237,453,1186,1783,1102
F5_2_16374,136,164,129,328,102,268,182,273,3,1,118,34
F9_2_16375,170,324,285,217,61,193,115,78,159,284,408,115
FA2H_2_16376,5,11,10,25,15,57,0,271,86,0,0,99
FAAH2_2_16377,636,716,457,729,545,500,501,438,1084,883,836,539
FADS1_2_16378,12,80,54,122,198,0,45,195,0,33,67,221
FADS2_2_16379,40,61,1,12,0,0,8,1,0,211,0,2
FADS3_2_16380,69,14,39,18,37,0,74,85,9,7,450,14
FAHD2A_2_16381,73,101,73,90,228,1,30,90,23,4,0,126
FAM108C1_2_16382,4,5,47,15,10,0,0,1,0,0,0,0
FAM135B_2_16383,2136,2115,2095,2781,1154,2269,3316,3640,1445,3002,4114
,2261
FAM20B_2_16384,201,206,211,242,199,282,259,314,178,216,356,477
FANCM_2_16385,1294,1400,1050,1405,416,654,867,982,1911,1575,1238,1475
FAR1_2_16386,451,335,326,345,226,219,451,474,213,589,380,664
FAR2_2_16387,93,94,51,40,110,43,35,162,99,50,1,15
FARS2_2_16388,413,230,244,240,22,162,1174,89,206,309,287,170
FARSA_2_16389,159,129,109,83,10,1,549,98,76,190,5,182
FARSB_2_16390,57,105,155,143,1,209,10,272,258,207,2,185
FASN_2_16391,194,101,190,332,809,178,14,15,994,376,279,470
FDFT1_2_16392,962,971,1062,1043,786,555,1415,893,897,822,1354,1473
FEN1_2_16393,155,138,131,80,188,5,166,0,13,311,136,45
FH_2_16394,1322,1031,983,1166,942,679,450,892,729,685,1355,521
FIG4_2_16395,177,194,223,163,74,431,185,0,275,405,1011,174
FKBP10_2_16396,156,64,107,117,12,76,80,1,85,343,5,75
FKBP14_2_16397,17,51,32,35,0,0,181,28,6,256,0,99
FKBP15_2_16398,16,6,4,4,0,0,0,0,0,0,0,0
FKBP3_2_16399,708,471,472,433,1047,578,536,106,306,434,792,244
FKBP4_2_16400,132,40,161,132,1,514,444,0,281,94,0,922
FKBP8_2_16401,460,577,601,776,396,620,95,513,314,39,2000,571
FMO1_2_16402,701,488,535,576,355,770,1195,1095,265,17,673,211
FMO4_2_16403,1634,1286,1423,1609,764,1252,1408,544,2099,942,2011,1918
FNBP1_2_16404,77,79,61,32,164,88,12,265,0,33,1,25
FNTA_2_16405,504,352,407,336,1352,755,1038,24,674,819,65,371
FNTB_2_16406,277,365,332,327,49,178,3,13,665,74,239,139
FTH1_2_16407,679,561,639,849,227,539,435,872,264,344,604,754
FTMT_2_16408,346,355,266,448,862,333,121,666,482,394,385,362
FTSJ2_2_16409,2468,1738,1651,1921,1944,1342,2477,1171,2365,1855,1674,2
251
FTSJ3_2_16410,68,36,79,78,0,34,217,1,69,13,49,9
FTSJD2_2_16411,459,435,335,157,62,3,17,78,9,11,1,24

FUCA1_2_16412,1254,1323,1257,1656,1802,549,1619,1858,1409,1178,656,107
7
FUCA2_2_16413,114,76,107,84,139,4,18,14,15,18,0,86
FURIN_2_16414,45,28,21,13,14,0,27,103,57,23,0,0
FUT10_2_16415,151,179,46,154,196,11,163,12,455,23,20,7
FUT11_2_16416,147,248,140,190,310,21,307,202,3,221,186,374
FUT1_2_16417,248,180,185,163,32,92,25,157,6,19,14,246
FUT4_2_16418,93,14,98,216,2,126,0,0,0,0,18,653
FUT5_2_16419,393,372,390,290,348,235,579,413,296,217,489,615
FUT7_2_16420,8,37,1,34,0,0,0,0,0,0,0,1
FUT9_2_16421,467,441,447,605,88,179,536,621,892,250,553,537
GADL1_2_16422,776,849,991,813,579,100,376,88,530,481,828,1589
GAL3ST1_2_16423,69,30,85,37,0,195,3,75,0,288,0,109
GAL3ST3_2_16424,117,207,212,96,19,36,3,82,23,118,47,24
GAL3ST4_2_16425,152,135,148,159,14,115,248,97,400,698,171,186
GALM_2_16426,143,248,116,116,285,62,67,0,5,37,223,394
GALNS_2_16427,0,17,8,3,0,32,72,0,66,8,0,1
GALNT10_2_16428,840,923,841,1060,1236,782,1048,1391,1139,1143,671,1315
GALNT11_2_16429,518,737,533,637,315,1317,1243,212,416,738,400,426
GALNT12_2_16430,34,28,44,33,0,13,0,53,14,18,65,13
GALNT13_2_16431,589,810,754,738,218,602,1092,824,391,675,203,731
GALNT14_2_16432,31,56,59,49,22,221,41,3,221,71,270,39
GALNT1_2_16433,716,491,448,754,218,454,157,319,641,944,1438,349
GALNT2_2_16434,137,43,41,105,16,0,71,16,268,5,403,369
GALNT3_2_16435,1508,1234,1170,1281,2254,480,858,1142,1016,1622,410,133
2
GALNT4_2_16436,543,370,475,652,572,224,60,551,565,114,1604,737
GALNT5_2_16437,414,459,397,526,250,521,891,706,527,334,260,699
GALNT6_2_16438,222,115,102,111,13,50,521,20,74,355,14,94
GALNT8_2_16439,6516,5587,5437,5440,4721,5077,7039,5049,4965,6060,6446,
5885
GALNTL2_2_16440,704,677,597,861,1101,1400,652,431,428,869,264,1005
GALNTL4_2_16441,441,418,374,441,838,32,284,311,490,332,132,610
GALNTL5_2_16442,150,89,131,145,29,196,540,381,82,4,109,12
GALNTL6_2_16443,175,261,234,257,844,139,178,255,153,101,548,44
GALT_2_16444,189,144,154,233,58,64,0,84,141,1476,174,460
GANC_2_16445,1404,1281,1157,1414,819,72,847,1382,1577,1387,1806,1109
GAPDH_2_16446,329,202,89,328,275,112,13,443,9,455,460,219
GAPDHS_2_16447,34,11,9,43,23,0,0,20,96,0,0,0
GARS_2_16448,230,69,117,84,93,52,159,8,378,184,21,248
GATC_2_16449,1089,1002,908,1225,2199,676,728,985,508,1230,735,637
GATM_2_16450,0,0,0,0,0,0,0,0,0,0,0,0
GBA2_2_16451,431,389,355,603,451,107,100,363,707,360,810,310
GBE1_2_16452,67,152,103,105,2,167,21,0,1,3,304,8
GBGT1_2_16453,0,0,0,0,0,0,0,0,0,0,0,0
GBP1_2_16454,386,381,377,380,510,1233,731,395,390,335,228,535
GBP2_2_16455,620,345,567,309,389,109,192,1148,378,313,1161,394
GBP3_2_16456,625,522,484,670,528,465,257,571,358,1035,246,956
GBP4_2_16457,2205,2040,1885,1946,1602,1534,2388,2842,2146,3102,2045,23
59

GBP6_2_16458,178,231,307,317,639,854,504,282,291,296,196,73
GBP7_2_16459,817,895,713,991,574,439,340,967,346,1229,920,468
GCLM_2_16460,108,125,106,220,278,18,38,134,7,27,0,167
GCNT3_2_16461,1752,1843,1487,1765,1464,1708,1591,2929,1374,2140,1080,1
695
GCNT4_2_16462,24,47,32,11,8,0,107,0,0,19,0,39
GCSH_2_16463,755,657,630,910,833,91,662,674,839,587,867,418
GDE1_2_16464,729,507,749,989,759,373,755,1674,521,903,1042,1005
GDPD3_2_16465,172,62,70,171,1,106,48,547,24,41,22,9
GFM1_2_16466,109,91,155,224,260,154,23,43,200,130,2,264
GFOD2_2_16467,270,196,146,119,7,76,2,269,363,10,183,179
GFPT1_2_16468,92,55,68,123,480,202,7,0,71,122,1,588
GFPT2_2_16469,2178,1796,1532,1631,1382,911,2713,1403,1744,2062,1383,19
07
GGH_2_16470,1351,1149,790,900,397,743,1052,801,562,1378,1019,2225
GGPS1_2_16471,384,216,409,558,248,474,770,889,63,709,427,347
GGT7_2_16472,116,50,87,141,77,321,124,17,18,242,68,300
GGTLC2_2_16473,74,70,120,90,3,0,0,372,1,59,0,1
GLCE_2_16474,1030,997,862,987,530,621,1164,224,1233,1127,766,455
GLDC_2_16475,554,497,453,601,683,968,1062,128,307,363,423,572
GLO1_2_16476,1594,1511,1587,1313,2790,1822,2380,1546,1165,2464,829,163
7
GLOD4_2_16477,378,237,403,330,286,524,444,159,209,193,30,1056
GLS2_2_16478,201,60,185,240,1,83,48,313,161,17,401,300
GLT1D1_2_16479,711,1221,1009,1066,744,450,983,964,1110,320,713,1929
GLT25D1_2_16480,690,612,419,663,1,6,176,737,163,147,1111,178
GLT25D2_2_16481,838,772,1070,1105,498,432,1254,666,873,1248,2694,1468
GLT8D2_2_16482,366,359,309,357,111,761,3,137,3,559,255,109
GLUD1_2_16483,425,321,357,432,292,1050,766,1231,826,151,162,787
GLUD2_2_16484,882,765,838,774,301,1149,566,589,599,990,1075,1340
GMDS_2_16485,306,457,396,551,359,324,135,413,76,278,351,510
GMIP_2_16486,88,100,174,146,0,27,0,349,162,0,183,0
GMPR_2_16487,106,282,179,186,459,241,168,205,431,73,2,161
GMPS_2_16488,13,19,26,18,45,2,69,45,98,60,0,5
GNA11_2_16489,581,565,467,337,393,1195,280,522,186,184,901,154
GNA12_2_16490,26,167,155,87,9,28,1,255,220,0,0,0
GNA13_2_16491,434,188,217,223,267,163,898,0,156,121,1128,55
GNA14_2_16492,429,294,301,439,318,155,356,544,202,339,213,331
GNA15_2_16493,124,72,139,67,331,2,320,1124,253,29,0,2
GNAI1_2_16494,652,686,686,811,784,407,359,1754,106,1805,1381,403
GNAI3_2_16495,75,169,87,223,216,103,30,77,168,304,13,119
GNAQ_2_16496,498,380,303,318,898,115,451,400,200,314,740,369
GNAT2_2_16497,1338,948,1126,957,838,1579,1230,247,1034,1285,1157,1171
GNAT3_2_16498,716,562,598,656,806,908,645,526,1017,317,850,582
GNAZ_2_16499,1715,1419,1294,1573,987,1423,1785,1045,1571,1328,2253,302
5
GNB1_2_16500,321,294,269,351,102,222,460,264,23,194,121,573
GNB2L1_2_16501,85,50,70,72,62,2,50,0,8,29,5,74
GNB3_2_16502,2,20,19,53,0,0,0,35,274,0,0,0
GNB4_2_16503,577,630,739,637,1142,352,1289,366,428,994,1754,725

GNG11_2_16504,356,65,161,246,196,6,308,8,235,32,125,73
GNG12_2_16505,636,554,346,585,733,1294,355,728,187,877,947,660
GNG13_2_16506,6,26,17,12,77,0,4,12,0,24,16,5
GNG2_2_16507,12,3,48,42,0,3,0,0,137,0,4,1
GNG3_2_16508,1127,897,1056,975,1414,602,851,948,279,515,1695,441
GNG7_2_16509,352,256,207,209,83,425,579,332,187,272,151,436
GNG8_2_16510,469,394,397,419,714,335,1768,31,416,274,321,530
GNGT1_2_16511,926,553,432,602,529,620,844,863,1065,1209,211,698
GNL2_2_16512,348,264,198,296,795,100,171,389,455,109,355,935
GNMT_2_16513,507,526,504,639,448,124,388,23,436,514,495,801
GNPAT_2_16514,73,38,86,89,99,53,158,8,19,327,25,74
GNPDA1_2_16515,41,40,34,6,0,0,288,3,0,0,0,28
GNPDA2_2_16516,896,560,540,791,604,516,72,1571,778,627,148,1055
GNPNAT1_2_16517,1686,1300,1665,1580,828,1511,1291,2216,1092,1439,1127,
1091
GNPTAB_2_16518,25,67,117,106,8,11,12,228,33,29,0,58
GNPTG_2_16519,44,45,137,115,0,0,377,131,0,20,0,0
GNS_2_16520,348,465,256,400,805,607,656,139,487,138,749,1059
GOT2_2_16521,267,198,240,220,112,319,187,130,121,430,162,147
GPAA1_2_16522,149,135,79,148,0,0,1,18,0,3,0,317
GPAM_2_16523,521,349,384,456,619,270,7,147,170,481,383,861
GPAT2_2_16524,336,426,336,504,129,262,461,232,185,449,706,638
GPD1_2_16525,434,478,179,411,78,298,421,256,483,57,288,406
GPD1L_2_16526,523,482,384,454,447,203,229,254,517,818,454,81
GPX2_2_16527,83,113,59,47,312,0,0,0,15,9,8,49
GPX3_2_16528,270,212,257,291,270,178,628,292,362,262,526,335
GPX7_2_16529,171,122,79,59,21,480,0,0,1,0,0,33
GPX8_2_16530,1655,1576,1495,1540,1091,749,862,1347,1434,2064,1302,1393
GRHPR_2_16531,163,288,276,338,0,878,227,1,30,104,2422,495
GSTA1_2_16532,907,769,547,647,687,525,1042,654,1032,192,198,946
GSTA2_2_16533,1613,1628,1345,1600,1585,1108,1948,1856,2330,2524,1786,1
257
GSTA3_2_16534,1647,1625,1340,1608,1392,1211,1970,1847,2325,2529,1785,1
413
GSTA4_2_16535,3362,2621,2904,2932,4416,2186,5285,2412,2490,3583,3260,3
623
GSTA5_2_16536,981,780,645,1028,544,263,2021,1114,1280,370,1516,717
GSTM3_2_16537,487,510,718,590,778,1006,570,1373,576,491,156,624
GSTM5_2_16538,1497,1143,1065,1174,2145,997,1675,1989,1577,1088,864,761
GSTP1_2_16539,322,521,280,423,353,0,273,51,451,2,8,34
GSTT1_2_16540,750,818,603,773,668,991,505,726,171,581,771,541
GSTT2B_2_16541,65,128,109,99,2,0,10,0,38,1,0,84
GSTT2_2_16542,65,128,109,99,2,0,10,0,38,1,0,84
GTF2F2_2_16543,1373,1010,979,976,1361,584,1734,283,820,1333,1995,835
GTPBP1_2_16544,49,29,68,72,12,1,71,5,65,203,11,86
GTPBP2_2_16545,234,250,391,339,869,18,151,235,402,60,614,56
GTPBP4_2_16546,394,629,900,776,606,521,1075,511,1136,219,813,558
GUCY1A2_2_16547,381,320,399,455,934,304,239,742,608,423,111,277
GUCY1B3_2_16548,352,320,321,250,763,202,705,150,417,462,137,497
GUSB_2_16549,120,71,17,64,151,6,33,84,16,1,0,1

GYLTL1B_2_16550,432,453,514,449,482,816,3,512,394,926,478,168
GYS2_2_16551,111,175,84,238,602,8,66,88,196,712,12,192
GZMA_2_16552,416,647,594,557,265,429,460,815,146,1356,170,292
GZMB_2_16553,206,198,336,372,385,12,741,1,233,119,458,311
H6PD_2_16554,477,441,420,411,796,51,83,774,366,216,167,428
HAAO_2_16555,179,224,159,243,549,225,11,44,259,479,127,187
HACL1_2_16556,71,1,41,107,1,24,390,0,0,1,0,242
HADHA_2_16557,1535,1687,1065,2076,1996,1323,1638,1728,1426,1011,1130,1
699
HADHB_2_16558,463,309,308,286,85,615,381,510,268,172,159,678
HAGHL_2_16559,688,616,411,643,575,640,337,494,453,1218,1378,620
HAL_2_16560,1442,1393,1410,1526,424,642,1713,867,1322,1169,1662,2358
HAO1_2_16561,431,288,300,369,395,44,544,350,245,170,506,191
HARS_2_16562,344,404,455,522,841,1238,801,839,527,261,828,506
HAS1_2_16563,1389,1324,1314,1568,797,1497,1168,991,431,1577,1361,2481
HAS2_2_16564,4362,3740,3434,3778,4324,3181,4610,2940,2580,2806,4306,38
98
HDHD3_2_16565,84,57,101,99,6,71,2,2,8,230,162,129
HELB_2_16566,61,24,45,32,0,0,185,0,3,174,0,0
HELQ_2_16567,1639,1168,1308,1228,1461,1425,984,2020,1027,1142,705,1530
HELZ_2_16568,944,1053,971,1117,1232,326,877,1350,729,783,554,1756
HEXA_2_16569,0,5,26,30,0,2,0,116,122,4,0,76
HEXB_2_16570,1212,1257,1041,1332,1831,341,590,590,1181,1417,1596,1412
HEXDC_2_16571,215,261,204,154,329,113,137,30,59,148,93,517
HGD_2_16572,425,223,221,272,322,0,303,56,215,513,311,188
HGSNAT_2_16573,76,68,90,73,50,2,125,267,23,189,0,205
HHATL_2_16574,1053,1067,1026,1060,414,493,1587,1587,822,574,3437,1168
HIBADH_2_16575,1377,1179,1146,1218,740,935,1235,1599,1265,1093,1475,59
2
HINT1_2_16576,375,211,253,214,524,153,76,552,374,266,332,232
HMOX1_2_16577,246,289,279,222,1,120,679,23,17,65,343,22
HPGDS_2_16578,1565,1333,1375,1631,1047,2276,1619,1943,2050,868,2742,17
22
HPRT1_2_16579,304,156,368,450,47,0,310,692,107,59,52,222
HS3ST1_2_16580,1331,1269,1165,1242,1100,1480,922,1085,689,1975,1194,12
80
HS3ST2_2_16581,374,298,288,490,453,626,554,751,321,246,687,666
HS3ST3A1_2_16582,203,144,186,225,151,299,51,370,226,284,376,391
HS3ST3B1_2_16583,45,26,79,24,0,0,0,357,0,256,0,74
HS3ST4_2_16584,198,172,169,267,25,226,151,182,229,144,13,366
HS3ST6_2_16585,42,93,22,152,9,11,17,479,0,71,0,0
HS6ST1_2_16586,533,525,515,641,194,826,561,291,187,1057,442,602
HS6ST3_2_16587,946,708,971,780,858,1227,1419,1021,1176,1115,1368,1074
HSD11B2_2_16588,83,17,44,188,75,61,14,22,220,44,0,4
HSD17B11_2_16589,322,241,118,303,37,11,65,268,353,34,627,141
HSD17B12_2_16590,138,121,103,192,33,329,177,10,21,94,56,78
HSD17B14_2_16591,173,114,18,217,21,21,47,312,125,349,129,207
HSD17B1_2_16592,21,56,116,81,0,0,2,49,2,0,0,40
HSD17B2_2_16593,52,28,9,25,0,74,0,14,2,0,129,0
HSD17B3_2_16594,347,289,282,373,72,401,918,19,310,464,136,371

HSD17B6_2_16595,585,632,573,667,1440,336,611,640,537,702,315,333
HSD17B8_2_16596,50,20,9,58,88,106,32,79,20,8,0,257
HSD3B1_2_16597,1464,853,1005,1249,1575,1110,1246,395,1579,981,832,1741
HSP90AB1_2_16598,572,267,346,290,606,314,472,3,654,300,477,510
HSPA5_2_16599,97,63,104,39,96,6,4,47,49,22,0,107
HSPE1_2_16600,740,763,544,805,711,874,125,359,1065,518,575,754
HSPG2_2_16601,8,7,2,33,7,1,0,0,0,0,0,0
HYAL4_2_16602,323,474,322,492,269,580,877,40,211,315,71,293
IARS2_2_16603,33,10,71,16,15,0,0,5,89,28,27,2
ICMT_2_16604,151,86,157,134,167,60,214,111,10,87,101,230
ICT1_2_16605,906,915,655,1084,1977,502,1349,828,813,1097,1555,1656
IDH1_2_16606,759,918,788,846,326,1126,1182,989,382,502,898,418
IDH2_2_16607,45,18,73,65,97,0,501,2,152,3,0,117
IDH3A_2_16608,400,400,329,359,537,141,400,174,762,413,33,240
IDI1_2_16609,1589,1223,1319,1375,2295,873,1528,693,1307,1307,1295,1010
IDI2_2_16610,400,583,554,488,287,522,584,884,175,436,426,306
IDO1_2_16611,131,104,149,168,177,18,88,817,305,120,139,37
IDO2_2_16612,516,436,469,769,1338,276,0,21,181,735,469,525
IDUA_2_16613,51,17,85,120,23,0,0,57,38,352,0,392
IFI30_2_16614,175,206,272,428,15,199,195,1,957,341,714,294
IFIH1_2_16615,378,327,290,445,378,346,132,213,282,66,45,524
IGHMBP2_2_16616,236,120,181,170,283,167,411,55,122,53,158,237
IHH_2_16617,11,23,19,15,0,10,121,6,0,7,0,73
ILVBL_2_16618,233,195,195,210,14,64,249,332,1,311,1,813
IMPAD1_2_16619,10,6,14,92,1,0,0,21,10,2,0,0
IMPDH2_2_16620,928,994,811,954,1076,756,557,1387,1115,833,308,1802
IRS1_2_16621,348,280,399,350,480,202,530,574,177,378,287,123
IRS2_2_16622,64,91,67,73,0,349,109,140,12,54,63,0
ISG20_2_16623,98,59,67,78,53,151,318,61,25,196,10,63
ISG20L2_2_16624,100,82,71,197,52,89,0,81,210,28,11,504
ISOC1_2_16625,1093,964,776,890,181,917,633,849,734,1424,293,962
IWS1_2_16626,1731,1970,1610,2082,2004,2612,1586,2138,1071,1101,1299,17
96
KDSR_2_16627,167,273,171,255,19,186,83,86,108,120,160,114
KHSRP_2_16628,91,36,56,75,20,52,14,246,57,4,0,45
KIAA0317_2_16629,164,323,115,395,114,58,232,1103,7,233,285,0
KIAA1279_2_16630,1171,1064,860,920,678,779,752,182,666,991,1847,924
KIAA2022_2_16631,535,274,349,389,415,336,76,352,712,272,359,425
KIF18A_2_16632,1596,1414,1550,1713,1252,1741,2677,756,1252,2701,2445,2
073
KIF20B_2_16633,434,398,370,501,253,645,283,184,479,206,799,346
KIF3A_2_16634,250,302,301,348,52,691,333,57,2,227,219,321
KLB_2_16635,438,431,526,583,681,19,155,298,21,135,81,271
KLC3_2_16636,14,71,55,30,18,0,0,86,0,0,0,1
KL_2_16637,285,184,187,204,413,158,148,166,138,58,107,326
KLK1_2_16638,376,205,286,269,92,2,610,349,365,599,312,224
KLKB1_2_16639,77,125,38,83,150,161,56,91,238,239,11,158
KMO_2_16640,21,81,31,26,0,1,6,3,2,0,5,2
KRTCAP2_2_16641,390,339,284,346,157,320,555,612,194,319,483,102
L2HGDH_2_16642,326,365,237,339,174,67,67,47,129,466,1551,43

LALBA_2_16643,517,760,501,614,300,789,794,158,1517,701,691,625
LAP3_2_16644,611,613,634,571,241,547,528,2060,288,1547,782,695
LARS2_2_16645,1407,961,1019,1340,1498,1744,1365,641,1176,1856,1030,106
6
LARS_2_16646,271,294,247,173,221,32,162,121,985,437,83,230
LCAT_2_16647,212,163,188,245,49,34,348,95,132,134,23,201
LCMT2_2_16648,253,296,353,345,145,235,361,2,151,18,6,696
LCT_2_16649,376,273,243,231,294,40,610,815,229,76,223,395
LCTL_2_16650,555,348,341,298,375,193,644,391,706,118,422,298
LDHAL6B_2_16651,3051,2520,2420,2618,2348,3147,2806,2487,2179,4822,2206
,2415
LEPREL2_2_16652,389,458,234,401,614,1110,195,141,504,231,195,229
LGALS13_2_16653,998,787,663,853,1233,97,473,1095,1173,479,943,1094
LHFPL2_2_16654,204,133,153,95,126,230,62,0,475,84,0,117
LIPC_2_16655,112,53,100,110,198,62,11,2,3,71,551,105
LIPE_2_16656,43,14,7,12,0,0,7,16,21,0,0,19
LIPG_2_16657,45,5,15,135,1,0,0,0,0,49,258,111
LIPH_2_16658,1019,881,861,824,447,980,1454,1017,647,831,1883,921
LIPI_2_16659,2366,2423,1954,2448,3628,1308,3177,2110,2421,2260,2032,13
62
LIPM_2_16660,0,0,3,0,0,0,0,0,3,2,0,0
LIPN_2_16661,608,492,508,465,71,177,483,309,308,153,338,314
LIPT2_2_16662,29,49,19,47,1,0,74,16,0,0,0,19
LONP2_2_16663,633,379,388,439,1097,412,775,491,338,214,464,340
LOXL1_2_16664,153,33,90,308,10,42,47,152,23,10,34,199
LOXL2_2_16665,181,174,296,211,81,68,61,9,168,92,93,233
LOXL3_2_16666,83,93,79,142,0,0,0,49,416,240,0,73
LOXL4_2_16667,19,0,8,6,2,0,0,0,1,0,1,0
LPCAT1_2_16668,779,649,520,644,537,237,628,1999,467,625,527,625
LPCAT2_2_16669,821,455,455,666,657,207,201,751,244,742,666,577
LPCAT3_2_16670,93,81,103,45,0,0,101,0,118,7,199,42
LPCAT4_2_16671,382,458,316,484,279,753,0,330,341,63,49,415
LPIN1_2_16672,593,505,445,464,518,371,265,163,1167,1149,228,786
LPIN2_2_16673,263,148,242,272,276,59,488,123,12,11,1084,193
LPIN3_2_16674,66,23,111,107,70,24,75,1,137,294,14,52
LPL_2_16675,72,76,104,111,28,13,100,5,167,14,324,20
LRAT_2_16676,1884,1856,1977,2041,2567,1312,1645,1525,1925,1865,2043,17
44
LTA4H_2_16677,143,90,84,90,109,122,3,0,51,32,115,83
LTC4S_2_16678,5,14,50,23,0,0,0,0,0,1,0,0
LYG2_2_16679,83,57,35,94,27,4,18,263,107,0,0,66
LYPLA1_2_16680,1,10,8,5,33,0,0,0,0,2,0,0
LYPLA2_2_16681,10,39,10,30,13,9,0,15,56,5,0,29
LYPLAL1_2_16682,1296,1320,1150,1315,840,1228,2731,778,1268,1273,1964,1
938
LYZ_2_16683,405,263,267,339,125,135,875,228,366,33,287,264
LYZL1_2_16684,2,4,10,0,7,0,0,0,0,1,0,0
LYZL2_2_16685,361,231,205,295,389,26,687,373,297,678,434,480
LYZL4_2_16686,507,685,673,778,1229,589,260,115,387,630,1115,285
MACROD1_2_16687,37,40,46,57,92,41,206,197,91,246,19,60

MAGT1_2_16688,264,172,146,195,347,182,291,128,161,449,77,235
MAN1A1_2_16689,463,572,561,459,467,232,399,552,323,921,1179,703
MAN1A2_2_16690,1061,784,692,949,1053,488,987,822,816,880,267,961
MAN1B1_2_16691,142,144,105,131,99,23,167,197,128,162,2,199
MAN1C1_2_16692,194,138,180,178,135,188,179,124,542,26,118,209
MAN2A1_2_16693,108,160,170,189,98,123,93,548,145,160,323,301
MAN2A2_2_16694,489,388,331,472,601,6,998,4,570,312,80,760
MAN2B2_2_16695,58,106,157,210,7,88,120,0,0,59,294,158
MAN2C1_2_16696,70,51,18,35,0,1,0,8,1,6,0,79
MANBA_2_16697,1465,1278,1179,1405,2011,1450,1817,571,1033,1386,898,105
7
MANEA_2_16698,2246,1918,2121,2545,1847,1836,2357,2906,1978,2167,2247,2
423
MAOA_2_16699,300,379,296,268,101,16,382,82,372,53,28,47
MAOB_2_16700,392,398,230,276,887,761,275,897,220,499,318,190
MAP1S_2_16701,132,101,63,98,224,141,219,21,308,247,6,144
MAPRE3_2_16702,644,516,633,548,1004,198,28,97,546,461,1679,398
MARS2_2_16703,106,62,203,96,210,258,1,1,214,15,422,152
MARS_2_16704,349,254,159,327,203,200,163,605,282,21,1519,269
MAT1A_2_16705,476,702,510,501,1477,24,119,232,147,302,203,67
MAT2A_2_16706,380,938,640,711,445,134,403,552,622,667,281,901
MBOAT1_2_16707,0,0,0,0,0,0,0,0,0,0,0,0
MBOAT2_2_16708,973,1145,811,964,982,584,1769,1710,761,507,350,680
MBOAT4_2_16709,380,338,309,473,33,114,978,138,513,217,821,246
MBTPS1_2_16710,0,0,0,0,0,0,0,0,0,0,0,0
MBTPS2_2_16711,413,404,645,567,327,604,601,569,492,519,983,272
MCCC1_2_16712,673,545,635,765,1048,1100,701,134,611,848,723,732
MCCC2_2_16713,666,672,613,634,917,475,1082,1210,285,556,997,795
MCEE_2_16714,2632,2346,1807,2710,2374,1648,2347,1106,1341,1972,1535,15
65
MCM3_2_16715,245,164,346,390,728,11,819,305,3,46,343,276
MCM5_2_16716,14,105,61,34,3,3,6,0,1,2,0,175
MCM6_2_16717,367,298,327,306,311,55,466,66,312,685,436,189
MDH1B_2_16718,514,360,247,279,145,79,90,325,726,428,34,212
MDH2_2_16719,180,100,79,133,436,169,88,3,35,302,93,28
MEP1A_2_16720,1670,1156,915,1505,910,712,525,1504,1248,1136,1797,1327
MEP1B_2_16721,272,205,159,294,362,193,64,52,145,107,649,26
METAP1_2_16722,494,689,447,601,27,543,505,250,389,402,290,825
METAP1D_2_16723,486,319,329,387,301,362,387,739,314,454,84,438
METAP2_2_16724,355,478,206,395,1234,183,70,438,355,595,328,882
METTL14_2_16725,1542,1182,995,928,1531,1007,206,1033,1222,539,322,1399
METTL22_2_16726,448,302,340,457,436,395,389,352,467,34,162,208
METTL2B_2_16727,703,474,569,804,1533,1307,819,381,562,927,1192,628
METTL3_2_16728,359,247,366,481,346,590,15,24,119,578,366,420
METTL5_2_16729,469,443,560,696,554,48,679,449,206,669,390,872
METTL6_2_16730,4977,3342,3636,4767,2671,2803,6031,3432,3712,3500,3016,
3523
METTL7B_2_16731,192,40,96,174,110,447,639,0,103,283,0,0
METTL8_2_16732,571,269,294,415,389,328,336,400,643,432,741,658
MFN1_2_16733,274,179,198,329,532,324,181,6,135,48,244,426

MGAM_2_16734,322,388,463,372,510,337,248,220,393,518,114,406
MGAT4C_2_16735,361,448,269,246,445,186,361,56,324,517,351,378
MGAT5_2_16736,314,332,256,161,119,51,188,924,295,184,379,83
MGMT_2_16737,879,667,722,614,1631,778,456,401,993,1102,413,319
MGST3_2_16738,4134,4347,4143,4088,3732,2602,4257,2038,4566,5922,4604,3
985
MIOX_2_16739,883,511,697,782,556,123,1939,930,455,682,939,165
MIPEP_2_16740,212,103,66,148,51,13,49,309,23,4,192,44
MLYCD_2_16741,520,806,453,641,384,352,1599,717,345,2162,175,422
MMAB_2_16742,260,109,166,204,326,381,257,56,660,166,1,182
MMEL1_2_16743,91,30,44,30,21,3,0,0,5,2,2,161
MMP10_2_16744,515,400,461,431,413,144,548,1033,198,286,729,42
MMP12_2_16745,241,235,209,231,66,170,145,57,422,237,298,148
MMP14_2_16746,694,645,679,797,407,736,1172,1025,1321,188,604,984
MMP3_2_16747,1376,1024,1064,1244,905,923,314,1463,647,503,2066,922
MMP7_2_16748,1815,1482,1485,1549,1064,918,2231,638,1838,1438,3276,1771
MMP8_2_16749,545,454,566,641,672,227,178,781,436,341,350,590
MMP9_2_16750,26,73,97,74,336,10,12,41,8,0,451,3
MOCOS_2_16751,42,18,56,84,2,0,9,100,12,133,8,82
MOGAT1_2_16752,300,231,380,360,182,0,34,144,841,424,513,270
MOGAT2_2_16753,970,1241,1049,1398,922,947,1311,715,1907,1841,1325,1329
MOGAT3_2_16754,291,279,331,396,93,534,508,451,447,405,1430,619
MOXD1_2_16755,1021,1036,933,1027,1170,404,1063,685,898,1995,938,1881
MPI_2_16756,166,96,152,198,11,207,144,283,29,140,40,139
MPO_2_16757,47,82,37,113,10,65,0,407,205,0,54,77
MRPL37_2_16758,97,118,114,167,16,57,45,32,45,0,15,84
MRPL44_2_16759,341,256,243,222,46,142,127,27,220,354,197,133
MRPS30_2_16760,287,452,295,364,131,546,176,292,199,496,477,455
MSH2_2_16761,2377,2229,2006,2427,2371,1359,3191,2781,1569,1549,1531,18
26
MSH3_2_16762,969,787,687,1028,162,1019,224,593,362,442,1356,697
MSH4_2_16763,145,150,163,164,27,222,81,5,276,317,200,157
MTAP_2_16764,87,71,73,92,0,3,79,0,0,0,5,0
MTFMT_2_16765,96,43,67,53,456,0,9,41,205,0,0,21
MTHFD1_2_16766,27,51,66,22,56,1,5,226,0,17,0,5
MTHFD2_2_16767,1660,1292,1497,1846,1373,1830,582,1201,795,1446,1610,13
86
MTHFD2L_2_16768,178,217,184,179,140,14,15,8,363,34,138,172
MTHFR_2_16769,282,371,320,367,11,92,277,44,676,518,95,674
MTPAP_2_16770,1487,1138,1013,1475,1057,574,2653,1801,1351,1760,1324,18
15
MTR_2_16771,293,105,116,203,992,0,72,6,382,85,147,90
MUS81_2_16772,32,44,40,60,151,95,0,0,20,1,4,0
MUT_2_16773,289,189,324,396,147,389,280,9,119,736,217,195
MVD_2_16774,133,193,9,41,0,3,0,2,7,518,575,0
MX2_2_16775,385,382,326,354,336,161,1169,141,306,994,436,557
MYH1_2_16776,349,301,280,451,187,442,276,577,132,624,70,135
MYH3_2_16777,201,200,213,244,436,51,166,84,236,552,131,212
MYH6_2_16778,510,797,707,759,524,623,2004,222,258,832,542,716
MYH7_2_16779,54,37,16,41,21,3,17,60,30,69,21,24

MYH9_2_16780,169,44,137,142,45,94,643,57,115,226,267,18
MYL7_2_16781,113,85,119,125,205,29,328,120,25,113,5,2
MYO1E_2_16782,52,30,64,50,0,2,0,135,0,0,0,7
MYO5B_2_16783,42,114,29,79,0,113,0,0,0,1,0,22
MYO9A_2_16784,1505,1268,893,1230,886,987,770,1686,1264,1188,1777,1488
N6AMT2_2_16785,308,140,51,144,0,4,0,369,27,160,122,561
NAA10_2_16786,286,331,225,377,60,16,51,1014,144,252,100,182
NAA11_2_16787,630,583,811,701,188,352,980,807,1240,561,255,527
NAA15_2_16788,627,440,553,566,652,1019,845,567,672,609,224,273
NAA30_2_16789,108,74,99,80,82,7,0,30,6,3,166,242
NAA50_2_16790,2771,2634,2222,2627,2869,2327,2140,2386,2743,2479,2723,2
053
NAALAD2_2_16791,165,108,164,155,1,58,360,340,3,260,0,47
NAALADL1_2_16792,0,0,0,0,0,0,0,0,0,0,0,0
NADSYN1_2_16793,137,96,145,273,296,70,71,2,9,172,203,0
NAGA_2_16794,603,512,383,497,678,259,669,873,1078,344,41,363
NAGLU_2_16795,180,258,184,176,116,236,288,121,287,352,130,129
NAGPA_2_16796,190,212,340,275,153,219,149,27,79,261,415,39
NAMPT_2_16797,155,119,63,99,167,264,504,693,281,141,17,82
NANP_2_16798,95,138,198,189,281,88,264,74,2,426,0,31
NANS_2_16799,402,536,339,447,260,458,287,175,469,107,410,172
NAPRT1_2_16800,30,36,83,69,0,13,254,99,11,221,0,41
NARS_2_16801,100,117,105,197,404,34,7,0,373,203,19,207
NCF1_2_16802,263,249,203,224,653,135,235,133,206,451,421,223
NDST1_2_16803,672,823,901,806,788,946,2289,907,693,636,996,634
NDST2_2_16804,427,688,362,394,24,542,1008,15,84,715,0,772
NDST3_2_16805,197,222,162,156,423,450,61,190,32,60,546,383
NDST4_2_16806,45,45,59,49,0,1,568,0,26,80,18,116
NDUFA10_2_16807,130,293,237,341,133,114,469,0,8,35,544,267
NDUFA12_2_16808,43,46,26,7,4,0,0,0,34,42,0,8
NDUFA13_2_16809,951,1073,994,1183,1365,867,1457,698,1065,1200,1490,104
3
NDUFA1_2_16810,254,272,335,264,338,670,9,108,136,39,410,103
NDUFA3_2_16811,160,74,100,192,36,50,4,172,52,159,505,447
NDUFA4_2_16812,2197,1412,1532,1754,1447,1886,2832,1070,1137,1027,1666,
1782
NDUFA4L2_2_16813,586,646,605,847,916,681,599,858,522,412,1149,974
NDUFA5_2_16814,574,835,600,619,480,433,630,198,1122,213,642,918
NDUFA6_2_16815,232,129,309,242,724,93,874,9,441,15,836,53
NDUFA7_2_16816,47,73,27,74,3,159,0,0,201,2,0,26
NDUFA8_2_16817,273,243,174,183,58,3,21,186,64,340,64,57
NDUFA9_2_16818,157,93,86,50,9,55,3,48,59,59,11,263
NDUFAB1_2_16819,1939,1562,1815,1981,1919,2125,3192,934,2075,1723,2721,
2446
NDUFB10_2_16820,261,207,107,192,798,137,43,49,426,85,9,141
NDUFB1_2_16821,567,202,279,522,776,180,41,322,494,523,173,431
NDUFB2_2_16822,32,32,33,20,5,15,6,26,20,62,0,132
NDUFB3_2_16823,465,401,496,404,133,491,690,327,205,238,872,455
NDUFB7_2_16824,104,41,64,154,50,20,10,0,0,0,0,15
NDUFB8_2_16825,209,175,227,275,2,530,538,1287,271,1,1345,47

NDUFB9_2_16826,876,746,734,1030,1534,1267,690,750,721,880,1553,1156
NDUFS3_2_16827,44,48,74,63,0,10,0,61,0,88,12,1
NDUFS4_2_16828,841,616,518,661,1071,339,689,846,1059,364,1077,753
NDUFS6_2_16829,42,120,27,72,75,11,76,0,50,0,0,27
NDUFS7_2_16830,207,192,162,300,191,206,680,51,40,177,222,295
NDUFS8_2_16831,9,41,6,19,1,0,0,0,0,113,0,9
NDUFV2_2_16832,1777,2127,1631,2531,2514,2272,1388,2905,1994,2630,3253,
2462
NEDD8_2_16833,596,619,622,480,693,416,660,262,570,955,358,658
NEIL1_2_16834,0,0,5,18,12,0,1,0,1,0,0,0
NEIL3_2_16835,189,152,117,250,728,111,0,14,77,295,0,98
NEU1_2_16836,52,87,21,21,64,86,24,31,1,20,61,0
NEU2_2_16837,39,57,22,76,190,111,24,5,165,13,0,55
NEU3_2_16838,387,398,202,307,140,570,102,81,164,292,334,605
NHLRC2_2_16839,208,244,309,326,37,168,145,343,275,27,428,40
NIT2_2_16840,146,93,157,111,18,0,9,482,249,34,61,336
NKIRAS1_2_16841,328,197,171,182,157,310,0,0,82,97,15,118
NLGN1_2_16842,182,288,134,250,14,208,5,756,116,280,50,141
NLGN2_2_16843,530,361,312,524,4,106,5,1809,540,216,0,341
NLN_2_16844,126,111,172,138,191,0,0,0,19,37,156,339
NME1-NME2_2_16845,270,218,164,195,166,324,38,1,100,334,412,514
NMNAT1_2_16846,247,274,377,236,804,14,292,146,896,458,64,394
NMT1_2_16847,422,403,406,561,200,40,1091,444,924,129,47,446
NMT2_2_16848,325,400,253,337,546,118,537,702,190,840,31,194
NNMT_2_16849,195,77,108,142,0,62,59,0,116,164,0,275
NOP58_2_16850,794,529,591,814,847,609,869,701,778,971,573,924
NOX3_2_16851,343,211,412,370,27,122,220,52,468,70,505,277
NQO2_2_16852,269,425,421,542,699,233,591,452,619,416,2306,722
NRAS_2_16853,55,25,20,47,0,0,0,20,1,24,320,31
NSF_2_16854,354,373,283,362,357,423,1073,264,367,191,14,99
NSUN6_2_16855,0,1,0,0,0,0,0,0,0,0,0,0
NTAN1_2_16856,470,395,344,470,981,171,723,823,181,246,427,1126
NTHL1_2_16857,1128,826,1222,853,811,1199,942,519,741,935,1090,672
NTPCR_2_16858,94,59,33,90,106,32,124,225,88,0,5,53
NUDT10_2_16859,1840,1802,1602,1349,737,1959,1143,1323,1269,1179,1804,1
832
NUDT11_2_16860,1840,1802,1602,1349,737,1959,1143,1323,1269,1179,1804,1
832
NUDT12_2_16861,742,786,727,929,486,437,877,937,921,1411,1306,1417
NUDT14_2_16862,29,100,95,71,20,43,90,7,27,119,105,3
NUDT3_2_16863,118,53,96,92,0,4,135,2,70,37,186,37
NUDT5_2_16864,185,142,114,229,81,148,91,24,325,564,205,39
NUDT7_2_16865,386,322,207,261,454,1377,123,325,258,873,96,356
NXNL1_2_16866,68,108,51,97,124,39,0,165,7,433,7,2
OAS3_2_16867,235,43,41,89,137,11,33,104,39,382,2,2
OAZ1_2_16868,218,397,552,402,515,107,609,352,415,514,485,831
OC90_2_16869,244,159,157,325,536,5,124,1038,58,250,1,168
ODF3B_2_16870,425,653,315,638,341,801,756,1711,1112,138,1028,380
OPLAH_2_16871,14,48,12,19,120,0,3,0,0,14,0,118
OSGEP_2_16872,209,264,292,174,382,3,458,470,409,285,510,154

OSGEPL1_2_16873,270,152,104,190,173,0,450,30,5,44,7,9
OSTC_2_16874,3451,2633,2687,2963,2588,1602,4954,3516,4041,3214,2462,46
61
OTC_2_16875,1131,1222,1078,1251,1790,847,4092,968,1083,1330,536,1020
OVGP1_2_16876,845,589,701,974,295,38,1274,727,1338,398,550,824
OXA1L_2_16877,18,37,30,54,0,0,15,0,0,0,141,0
OXCT1_2_16878,540,581,659,692,20,621,438,82,261,1313,1239,783
OXCT2_2_16879,399,185,269,283,89,70,245,109,81,224,18,563
P4HA3_2_16880,182,109,250,163,91,392,3,448,36,218,392,0
P4HB_2_16881,223,320,333,362,0,306,682,148,5,197,1365,111
PADI1_2_16882,601,497,688,859,298,589,285,245,337,889,190,506
PADI2_2_16883,884,874,729,1096,325,234,478,1241,195,1479,1310,624
PADI3_2_16884,132,93,112,93,85,20,508,79,247,3,56,367
PADI6_2_16885,347,170,197,217,296,290,33,77,402,299,69,180
PAFAH2_2_16886,934,909,934,1142,479,696,168,1568,858,1317,1571,1407
PAH_2_16887,280,425,289,264,126,318,140,27,129,80,0,816
PAPOLA_2_16888,387,261,240,420,1,1,795,12,129,595,0,301
PAPOLB_2_16889,2149,2610,2042,2466,1768,2277,2549,1304,1671,2527,2711,
2444
PAPOLG_2_16890,102,49,56,122,62,81,141,0,60,188,7,111
PAPPA_2_16891,1178,1241,1081,1574,1788,1739,1220,746,1640,1057,1212,12
21
PAPSS1_2_16892,58,61,105,34,0,0,1,17,45,428,5,0
PARG_2_16893,421,308,322,358,1072,576,937,390,347,648,479,148
PARP1_2_16894,133,366,127,271,62,0,218,352,0,336,6,15
PARP4_2_16895,238,80,150,162,351,8,28,5,18,405,474,17
PARP6_2_16896,150,245,197,243,262,404,45,160,56,43,663,657
PARS2_2_16897,84,121,57,200,91,93,0,233,1,1,0,117
PCBD1_2_16898,224,202,131,247,204,97,6,142,244,113,42,315
PCBD2_2_16899,123,201,185,211,301,432,500,138,367,11,196,77
PCMT1_2_16900,28,4,21,31,17,0,21,527,0,0,1,162
PCMTD1_2_16901,144,150,81,154,139,145,180,329,310,149,196,98
PCYOX1_2_16902,1072,1188,1093,1350,582,863,139,1071,369,716,1517,1362
PCYT1A_2_16903,107,74,158,103,12,1,447,358,16,62,232,37
PDCD1LG2_2_16904,521,519,358,548,98,203,336,1474,364,173,524,545
PDCL_2_16905,501,295,366,671,393,353,548,881,370,931,394,579
PDE3A_2_16906,289,238,175,194,173,535,160,36,118,258,204,242
PDE3B_2_16907,259,246,199,276,584,15,327,100,342,127,263,330
PDE6A_2_16908,32,20,0,63,0,0,0,288,0,185,0,0
PDE6C_2_16909,1036,1284,1018,1319,421,1164,1422,55,981,1443,1400,904
PDE6D_2_16910,517,307,373,560,1023,430,290,876,290,382,212,834
PDE6G_2_16911,187,91,185,235,361,283,111,213,211,297,1,189
PDE6H_2_16912,244,203,228,222,47,628,14,0,57,133,17,65
PDE7B_2_16913,217,184,284,526,559,500,0,174,367,449,802,485
PDHA2_2_16914,1383,1101,1091,1496,642,1200,839,1992,721,1346,796,1084
PDIA2_2_16915,599,378,485,439,328,147,1308,352,134,106,403,371
PDIA3_2_16916,110,12,139,65,63,3,47,0,57,65,0,383
PDIA4_2_16917,167,115,155,180,0,293,67,673,154,19,226,23
PDIA5_2_16918,233,283,158,354,77,876,12,600,47,187,137,385
PDIA6_2_16919,1706,1460,1606,1556,1907,998,1251,1289,1302,1668,2999,20

01
PDPR_2_16920,802,831,962,1129,707,727,378,274,1045,891,986,227
PDSS1_2_16921,1021,989,911,968,535,1119,1522,205,881,337,1702,1639
PDSS2_2_16922,2478,2054,1915,2631,1554,2542,3279,991,2072,3262,2104,23
76
PDXP_2_16923,177,22,217,116,3,560,238,3,33,300,414,174
PECR_2_16924,159,69,103,152,664,19,558,77,63,355,30,1
PELI1_2_16925,218,177,260,250,578,86,294,703,175,25,172,128
PET117_2_16926,63,5,54,91,136,0,80,12,21,34,11,233
PEX1_2_16927,260,301,347,391,0,545,337,149,427,226,343,141
PEX6_2_16928,280,341,261,215,867,55,2,333,256,301,268,247
PFAS_2_16929,81,102,46,74,79,39,259,71,24,27,0,10
PGA3_2_16930,1242,1101,1265,1359,407,1655,1316,1260,525,58,1032,1660
PGA4_2_16931,1242,1101,1265,1359,407,1655,1316,1260,525,58,1032,1660
PGA5_2_16932,1242,1101,1265,1359,407,1655,1316,1260,525,58,1032,1660
PGAM4_2_16933,46,89,101,37,0,0,0,0,20,0,379,7
PGAP1_2_16934,2737,1658,1570,1901,1672,1701,2332,2813,2360,2934,1152,2
289
PGAP3_2_16935,126,109,231,98,20,167,69,254,18,264,0,24
PGD_2_16936,5,24,17,29,4,0,6,2,98,0,0,34
PGGT1B_2_16937,1439,1343,1175,1525,2089,671,445,2958,1231,1140,972,250
1
PGLS_2_16938,68,20,33,36,1,1,0,0,0,0,509,0
PGM2_2_16939,252,302,227,244,123,130,226,1,241,109,50,240
PGM2L1_2_16940,637,578,425,576,226,933,542,47,321,263,122,539
PGM5_2_16941,1022,554,530,710,620,285,1027,1250,771,505,369,294
PGPEP1_2_16942,809,535,671,889,640,504,921,2485,780,898,1275,876
PGS1_2_16943,99,142,135,250,3,220,115,5,24,1,28,521
PHGDH_2_16944,260,256,224,266,243,286,129,292,208,399,293,281
PHLPP1_2_16945,236,236,271,253,279,1,28,89,238,51,250,13
PHLPP2_2_16946,174,109,43,98,255,0,0,7,180,164,0,10
PIF1_2_16947,2,106,23,0,0,265,0,0,0,0,0,0
PIGB_2_16948,513,592,301,450,788,550,394,644,393,646,163,229
PIGH_2_16949,439,347,203,358,354,325,121,419,452,31,584,279
PIGL_2_16950,74,78,97,65,11,85,84,2,12,38,282,31
PIGM_2_16951,220,114,208,233,44,45,53,104,522,243,107,476
PIGS_2_16952,330,347,328,386,554,595,265,277,429,778,117,441
PIGU_2_16953,605,710,696,915,553,857,602,316,389,1363,316,818
PIGW_2_16954,1312,983,1234,1284,819,572,1524,1238,1510,802,1739,1611
PIGZ_2_16955,106,98,86,119,89,187,103,22,108,1,26,134
PIN1_2_16956,37,17,52,25,0,1,0,3,10,9,295,163
PIPOX_2_16957,782,535,518,596,297,720,902,595,392,141,148,824
PITPNM2_2_16958,188,264,241,162,19,4,1,387,42,0,0,3
PLA2G10_2_16959,165,109,145,212,53,28,125,6,105,34,465,94
PLA2G12A_2_16960,244,305,270,347,48,0,2,0,48,746,775,188
PLA2G12B_2_16961,4,2,1,1,0,0,0,9,0,0,0,0
PLA2G15_2_16962,282,205,177,141,32,173,499,140,246,29,514,5
PLA2G1B_2_16963,27,22,49,16,0,0,0,0,0,0,2,0
PLA2G2C_2_16964,94,163,51,131,175,33,0,73,17,0,2,120
PLA2G2D_2_16965,3424,2451,2715,3158,2881,2424,4808,3536,2243,2818,3584

,1735
PLA2G2E_2_16966,655,473,569,606,452,902,26,622,432,225,530,47
PLA2G2F_2_16967,178,121,166,71,60,37,53,231,323,70,251,35
PLA2G3_2_16968,90,58,131,129,1,224,169,225,333,14,0,23
PLA2G4A_2_16969,280,180,152,277,94,94,135,354,257,88,457,261
PLA2G4B_2_16970,295,266,740,646,32,553,592,503,892,415,465,818
PLA2G4D_2_16971,258,116,241,265,199,99,638,160,459,102,124,231
PLA2G4E_2_16972,124,116,103,231,18,81,40,323,85,106,34,19
PLA2G4F_2_16973,604,653,440,730,828,577,143,457,329,324,1175,851
PLA2G5_2_16974,70,42,54,76,41,0,401,51,0,33,44,56
PLCB2_2_16975,108,143,236,149,331,56,411,2,157,0,591,31
PLCD3_2_16976,275,250,339,358,278,241,0,574,459,395,179,707
PLCD4_2_16977,75,86,94,47,40,61,23,19,45,36,213,121
PLCG2_2_16978,126,177,100,105,179,126,354,36,91,96,113,204
PLCH2_2_16979,430,143,155,153,294,175,147,14,525,338,0,7
PLCL1_2_16980,268,204,265,271,138,595,428,552,383,205,872,426
PLCXD1_2_16981,281,352,320,478,85,429,332,364,36,316,2,363
PLCZ1_2_16982,0,84,4,0,21,0,0,0,8,0,0,0
PLD4_2_16983,181,152,155,118,26,122,92,104,65,458,40,103
PLOD1_2_16984,424,298,254,348,269,57,284,112,237,380,405,115
PLOD3_2_16985,89,43,46,86,30,23,73,18,78,17,84,229
PLSCR1_2_16986,1036,836,896,988,681,423,1658,824,1116,436,1608,1379
PMM1_2_16987,111,102,118,178,2,12,125,128,0,0,125,37
PMM2_2_16988,57,67,54,75,19,14,0,213,219,49,501,2
PMPCA_2_16989,0,0,0,0,0,0,0,0,0,0,0,0
PMPCB_2_16990,134,52,33,191,240,0,147,0,144,133,0,0
PNLIP_2_16991,157,136,114,221,108,331,11,57,88,633,239,14
PNLIPRP1_2_16992,275,147,167,295,359,93,304,37,125,159,171,834
PNLIPRP2_2_16993,41,70,64,65,17,0,14,114,34,1,226,2
PNLIPRP3_2_16994,1106,1096,1086,1306,1529,1068,901,924,843,731,1850,61
4
PNMT_2_16995,251,160,261,229,282,220,9,141,334,354,48,318
PNPLA2_2_16996,181,127,114,163,10,43,182,355,0,37,0,77
PNPLA3_2_16997,403,219,171,172,277,476,98,3,147,7,94,421
PNPLA8_2_16998,905,1034,1038,1036,844,895,963,870,1297,393,2604,973
PNPO_2_16999,9,6,45,25,15,7,34,40,0,251,358,39
PNPT1_2_17000,846,782,683,877,1403,321,486,1252,614,652,753,941
POLA1_2_17001,257,423,295,277,0,418,30,12,152,761,668,261
POLA2_2_17002,403,450,380,333,842,104,772,476,254,201,1128,362
POLB_2_17003,40,23,38,82,101,10,48,355,18,15,0,161
POLD4_2_17004,159,95,108,139,0,423,0,0,0,0,528,828
POLE3_2_17005,447,353,491,464,804,116,707,305,128,1,298,323
POLE4_2_17006,200,165,162,194,562,23,15,103,101,144,53,109
POLE_2_17007,1,140,138,100,3,0,869,0,2,250,391,157
POLG2_2_17008,2771,2055,2209,2414,1701,912,3015,2246,2075,2163,1805,26
84
POLI_2_17009,129,281,182,138,6,391,199,400,148,280,317,359
POLM_2_17010,65,48,18,125,66,2,0,0,138,1,0,7
POLN_2_17011,60,49,24,95,3,5,116,89,107,9,1,64
POLR1A_2_17012,156,157,102,137,3,75,735,0,21,49,25,121

POLR1C_2_17013,128,147,174,134,67,110,501,27,19,25,72,168
POLR1E_2_17014,279,210,233,268,304,0,17,47,465,366,46,78
POLR2A_2_17015,797,852,697,823,825,1,412,551,1199,332,224,665
POLR2B_2_17016,507,563,571,542,451,258,60,240,572,243,1574,368
POLR2C_2_17017,221,225,373,407,264,193,421,659,0,431,587,237
POLR2D_2_17018,140,106,135,80,79,1,5,7,106,281,11,11
POLR2E_2_17019,1031,643,802,970,1258,566,1344,1059,874,632,775,1079
POLR2F_2_17020,88,12,104,49,1,0,7,31,1,20,36,25
POLR2G_2_17021,796,1109,914,1271,665,726,913,1038,1300,849,1986,1368
POLR2H_2_17022,1482,1545,1366,1536,1227,1729,936,1580,971,1518,1830,18
18
POLR2J_2_17023,967,959,680,812,350,527,1452,485,854,914,451,769
POLR2K_2_17024,124,260,164,250,19,959,680,348,28,35,0,32
POLR2L_2_17025,95,105,106,101,19,3,74,106,126,53,56,151
POLR3A_2_17026,490,431,266,404,164,6,236,65,530,1018,222,243
POLR3C_2_17027,378,213,324,430,425,0,74,800,17,110,21,697
POLR3F_2_17028,28,7,29,37,0,0,2,375,0,0,0,3
POLR3G_2_17029,160,112,96,206,93,192,16,6,20,213,0,119
POLRMT_2_17030,29,0,20,15,5,0,1,0,0,0,0,11
POMGNT1_2_17031,34,29,126,96,26,10,227,149,0,4,0,10
POMT2_2_17032,145,240,209,293,56,25,118,25,35,163,126,521
PON3_2_17033,263,174,137,287,47,27,375,23,216,214,46,111
POP4_2_17034,629,446,527,634,1192,387,835,796,765,855,662,438
POP7_2_17035,395,488,355,583,460,502,479,149,282,209,496,658
POR_2_17036,469,311,576,394,390,186,671,407,614,426,767,288
PPA1_2_17037,122,17,32,31,188,0,0,0,3,77,0,143
PPCDC_2_17038,620,321,488,438,328,317,292,401,867,21,113,414
PPIA_2_17039,259,230,228,300,78,164,41,539,27,81,253,216
PPIAL4A_2_17040,880,622,432,656,209,636,854,702,628,500,787,764
PPIAL4B_2_17041,880,622,432,656,209,636,854,702,628,500,787,764
PPIAL4C_2_17042,880,622,432,656,209,636,854,702,628,500,787,764
PPIAL4E_2_17043,3983,3668,3981,4304,4530,3078,4423,3003,3434,4002,5928
,3906
PPIAL4G_2_17044,143,88,106,121,2,87,238,134,7,139,139,230
PPIB_2_17045,20,13,18,66,0,0,23,0,4,27,152,2
PPIC_2_17046,1043,963,972,1180,1131,697,988,1485,1153,687,1283,1186
PPID_2_17047,132,65,170,74,173,0,0,0,28,0,88,31
PPIF_2_17048,250,220,129,238,122,0,9,200,18,167,213,204
PPIG_2_17049,335,494,534,456,466,249,438,155,593,280,25,15
PPIH_2_17050,49,94,59,101,152,0,0,41,19,31,0,132
PPIL1_2_17051,492,364,321,415,926,106,709,873,734,576,608,491
PPIL4_2_17052,1041,1131,1100,1151,1182,1143,724,748,448,1125,1062,1339
PPIP5K2_2_17053,396,258,327,250,46,232,1015,175,157,625,10,324
PPM1H_2_17054,16,31,26,30,0,6,111,126,3,0,79,0
PPM1J_2_17055,40,14,30,81,0,9,0,2,2,0,17,194
PPM1N_2_17056,354,323,343,307,81,75,725,176,240,311,275,673
PPME1_2_17057,33,88,90,159,0,0,404,0,44,0,2,330
PPWD1_2_17058,395,180,300,319,796,947,226,73,567,55,34,149
PRDX4_2_17059,415,413,423,548,621,70,144,285,172,868,590,415
PREP_2_17060,938,708,604,920,976,57,893,536,1353,221,735,1104

PRHOXNB_2_17061,135,90,58,28,51,0,0,0,12,5,0,6
PRIM1_2_17062,276,278,360,379,426,542,545,1152,279,161,164,690
PRIM2_2_17063,903,706,667,887,285,776,354,1115,720,795,244,465
PROC_2_17064,20,290,93,129,0,5,357,0,231,92,264,35
PROSC_2_17065,102,103,82,43,0,3,90,77,45,0,0,80
PRR14L_2_17066,247,260,422,490,674,3,216,7,25,8,498,384
PRSS1_2_17067,789,952,830,1099,602,1255,1447,438,505,1149,790,1370
PRSS2_2_17068,0,0,0,1,0,0,0,0,1,0,0,0
PRTFDC1_2_17069,1283,1319,1146,1304,2586,1595,2004,684,2010,1809,897,1
650
PRTN3_2_17070,23,20,8,59,73,0,23,275,67,5,96,9
PRUNE_2_17071,899,774,630,743,2345,547,1023,661,991,307,595,720
PSMA2_2_17072,1787,1671,1878,1917,3037,1284,2341,1007,795,1982,2312,24
84
PSMA6_2_17073,2315,2060,1846,2331,1503,2019,1492,1023,1808,2563,2390,2
876
PSMA7_2_17074,757,728,967,1084,444,484,533,379,884,854,1639,1031
PSMB10_2_17075,725,682,718,881,501,608,1778,2413,596,934,400,1233
PSMB11_2_17076,162,97,76,165,7,144,30,149,218,534,4,191
PSMB1_2_17077,157,161,186,410,362,70,389,4,94,356,800,537
PSMB3_2_17078,56,39,77,75,57,55,0,0,242,8,522,195
PSMB4_2_17079,66,42,39,56,23,112,81,0,1,51,1,21
PSMB6_2_17080,201,248,288,177,217,0,637,150,24,574,84,31
PSMB7_2_17081,631,307,351,509,1582,0,112,0,393,631,965,45
PSMB9_2_17082,1550,1531,969,1330,963,1374,1675,1482,1902,2877,1079,131
7
PSMD6_2_17083,82,157,60,109,203,23,0,164,32,142,0,192
PTDSS1_2_17084,32,24,21,30,0,0,0,42,20,4,16,4
PTDSS2_2_17085,50,19,37,77,192,4,0,8,2,7,0,9
PTGDS_2_17086,99,78,51,91,104,41,136,0,371,94,164,279
PTGES2_2_17087,40,43,21,57,0,0,0,0,2,40,0,0
PTGES3_2_17088,1152,891,1031,1224,549,955,1496,892,1368,843,846,823
PTGES_2_17089,87,99,94,138,70,0,0,65,1,287,0,0
PTGIS_2_17090,629,376,345,389,515,226,869,564,715,564,28,789
PTGS2_2_17091,127,150,88,184,15,0,810,8,267,0,22,73
PTRH1_2_17092,129,285,180,202,16,361,102,105,602,4,377,139
PTRH2_2_17093,1019,1069,795,1141,1458,1241,2041,1047,748,497,4065,1158
PTS_2_17094,859,675,859,971,1412,561,1166,636,522,439,1467,994
PUS3_2_17095,1089,955,841,962,477,783,627,915,657,929,1207,597
PUSL1_2_17096,456,432,227,435,774,359,0,198,552,655,194,663
PXDN_2_17097,114,110,106,272,2,43,0,3,83,507,1,3
PXDNL_2_17098,130,77,35,69,9,0,2,46,69,1,1,0
PYCR2_2_17099,27,22,56,54,66,133,0,14,0,78,911,42
PYCRL_2_17100,502,310,352,490,333,161,25,25,399,274,142,265
PYGB_2_17101,58,31,50,46,0,0,0,79,0,132,5,7
QDPR_2_17102,140,110,133,123,51,55,6,271,62,40,118,9
QPCT_2_17103,253,426,311,441,202,524,469,148,454,338,568,64
QPRT_2_17104,117,110,200,114,34,226,96,469,42,260,28,230
QRSL1_2_17105,199,115,145,163,53,5,268,238,289,102,36,212
QSOX2_2_17106,67,109,31,54,3,2,0,4,8,2,30,4

QTRT1_2_17107,59,65,163,133,325,0,0,647,336,31,248,17
QTRTD1_2_17108,1405,968,1047,1262,2251,885,975,1109,845,1767,32,1221
RAB10_2_17109,1143,935,803,1126,1963,1473,1574,534,984,680,877,1306
RAB12_2_17110,511,364,494,633,433,6,78,441,238,142,646,428
RAB13_2_17111,358,159,188,359,36,50,642,31,345,277,26,279
RAB14_2_17112,613,292,516,383,71,347,615,436,252,553,308,545
RAB15_2_17113,53,63,90,47,321,12,145,28,100,66,0,32
RAB17_2_17114,78,61,76,103,2,0,25,114,4,1,0,122
RAB18_2_17115,544,349,393,662,358,598,994,1153,157,597,112,287
RAB19_2_17116,212,431,323,299,168,1115,187,108,21,520,26,270
RAB1B_2_17117,115,43,88,143,0,139,84,41,11,7,0,129
RAB20_2_17118,431,82,225,411,197,235,25,200,100,255,501,46
RAB21_2_17119,452,432,505,415,32,197,606,199,339,189,712,1129
RAB22A_2_17120,282,446,482,480,231,481,155,27,288,334,371,142
RAB25_2_17121,129,69,28,85,0,205,4,0,247,105,0,424
RAB26_2_17122,24,39,83,38,0,0,0,2,43,4,481,84
RAB27B_2_17123,348,150,218,421,285,82,1182,592,733,778,296,380
RAB30_2_17124,260,237,186,251,173,173,104,296,118,181,60,418
RAB31_2_17125,136,207,167,164,128,0,509,212,346,107,321,99
RAB33A_2_17126,98,48,79,72,112,186,0,48,72,12,12,50
RAB33B_2_17127,88,57,91,142,370,57,18,23,78,39,1,139
RAB36_2_17128,34,16,55,20,1,24,33,1,10,7,5,7
RAB38_2_17129,264,158,176,260,10,13,439,559,42,393,14,39
RAB39B_2_17130,1522,1620,1396,1169,596,1593,2874,870,1622,1647,2583,14
48
RAB3A_2_17131,266,355,377,287,835,204,227,866,389,580,159,604
RAB3B_2_17132,109,141,142,218,107,11,18,179,247,6,26,221
RAB3C_2_17133,295,193,240,295,23,535,490,6,291,167,49,355
RAB3D_2_17134,61,95,134,45,2,0,49,0,38,25,1,0
RAB3GAP2_2_17135,1271,1366,1214,1483,1297,946,1212,1256,1300,1629,1500
,840
RAB4A_2_17136,779,633,558,640,399,895,154,988,777,242,1529,816
RAB4B_2_17137,1000,920,1224,1001,1020,378,1793,880,292,474,1489,271
RAB5A_2_17138,455,514,557,510,291,851,272,760,444,406,1892,884
RAB5B_2_17139,950,768,842,1059,1399,504,978,714,1467,627,1007,931
RAB6B_2_17140,1855,1443,1600,2183,1394,2153,885,1564,1850,1831,1706,24
83
RAB6C_2_17141,1169,1300,782,1277,1524,1031,1371,904,646,948,1363,1229
RAB7A_2_17142,889,717,728,741,455,266,262,706,639,415,550,486
RAB8B_2_17143,911,544,660,831,1026,261,1052,845,687,448,907,665
RAB9B_2_17144,110,214,205,136,0,0,0,296,0,63,74,111
RABGGTB_2_17145,1462,1044,1327,1200,1296,861,697,1240,1372,1516,1513,1
183
RABL3_2_17146,102,193,144,141,133,36,50,30,68,103,54,3
RAC2_2_17147,24,50,5,48,2,7,79,0,1,4,145,1
RAD50_2_17148,668,439,745,942,1024,525,1033,286,923,1,1236,844
RAD54L2_2_17149,82,167,153,119,67,57,149,154,267,30,25,108
RALA_2_17150,1622,1367,1098,1363,657,1277,423,2047,945,2257,1169,970
RALB_2_17151,519,373,363,357,290,763,939,357,340,28,412,711
RALBP1_2_17152,546,514,534,461,595,299,595,641,98,215,152,127

RANBP2_2_17153,4,1,9,0,0,6,0,1,9,0,0,0
RAN_2_17154,61,16,11,56,9,0,0,3,46,38,0,9
RAP2A_2_17155,27,0,4,37,0,1,0,0,11,1,0,1
RAP2B_2_17156,135,187,184,192,117,38,99,36,121,139,506,148
RAP2C_2_17157,1191,873,965,1295,668,1300,434,2023,1098,235,1490,1278
RARS2_2_17158,2019,1792,1309,1789,1353,947,1719,1523,1529,526,382,1342
RARS_2_17159,432,432,626,506,686,697,468,1094,400,473,41,379
RASD2_2_17160,25,104,40,71,10,155,0,45,0,308,12,257
RASL10B_2_17161,49,126,143,124,6,247,343,262,28,915,29,163
RASL11B_2_17162,52,63,70,95,0,44,49,29,190,153,0,8
RASL12_2_17163,301,403,528,356,23,0,1443,808,85,586,139,369
RC3H1_2_17164,499,808,393,606,857,289,55,1235,623,540,523,237
RCL1_2_17165,679,741,732,848,276,645,70,495,440,512,658,942
RDH10_2_17166,76,90,41,91,122,11,0,25,144,0,0,58
RDH11_2_17167,214,171,170,179,24,115,160,80,467,295,598,294
RDH12_2_17168,454,323,300,317,658,260,753,873,92,327,67,932
RDH14_2_17169,1039,846,916,1139,418,748,784,470,364,1633,921,727
RDH16_2_17170,441,359,363,521,52,265,274,497,375,394,242,27
RDH8_2_17171,209,314,133,233,115,372,376,327,257,160,132,45
RECQL4_2_17172,614,655,470,861,159,113,667,1745,531,171,2146,806
REM1_2_17173,110,50,89,140,147,19,1,261,40,246,4,70
REM2_2_17174,403,395,322,453,713,351,584,68,253,340,117,1706
RENBP_2_17175,57,27,10,9,1,0,1,1,1,0,102,0
RETSAT_2_17176,604,551,656,576,363,491,1352,32,13,217,425,410
REV3L_2_17177,821,735,755,709,308,1074,70,485,549,439,153,985
REXO1L1_2_17178,294,309,205,286,66,262,357,395,462,282,174,237
REXO2_2_17179,164,199,142,201,294,21,249,44,5,19,35,159
RFNG_2_17180,1030,1135,1106,1494,697,499,979,1130,2448,1383,1549,814
RFX6_2_17181,20,27,9,30,0,63,134,82,46,0,19,9
RGS7_2_17182,73,207,133,108,59,373,518,4,3,120,32,19
RHBDL1_2_17183,334,504,252,313,54,925,567,273,305,331,374,351
RHBDL2_2_17184,861,880,824,1134,442,247,1488,950,240,798,2128,756
RHBDL3_2_17185,243,148,154,230,28,100,251,573,134,104,2,462
RHEBL1_2_17186,1375,1058,1404,1330,299,1388,1645,2036,620,1522,1666,85
1
RHOA_2_17187,73,4,7,32,13,0,18,0,2,0,0,8
RHOB_2_17188,159,196,192,95,36,718,2,59,101,271,27,78
RHOD_2_17189,371,263,434,523,336,301,24,1067,60,293,576,202
RHOF_2_17190,29,39,37,112,52,129,89,0,8,8,15,108
RHOG_2_17191,18,1,18,14,0,0,0,0,1,3,0,0
RHOH_2_17192,954,973,845,1065,310,976,1162,965,981,536,573,1296
RHOJ_2_17193,104,44,110,170,115,10,1,155,86,1,687,16
RHOQ_2_17194,350,279,306,356,199,753,379,788,641,18,285,1000
RHOT2_2_17195,0,24,23,4,29,66,0,0,96,70,0,5
RHOU_2_17196,287,382,296,368,679,117,105,42,136,284,9,147
RHOV_2_17197,67,116,135,97,174,86,47,2,94,122,50,11
RIT1_2_17198,518,276,339,417,708,1008,590,809,422,430,1011,663
RIT2_2_17199,934,665,783,1018,442,262,607,537,90,1401,1335,955
RNASE2_2_17200,601,447,582,638,740,1106,258,294,293,362,596,718
RNASE3_2_17201,586,447,573,638,739,1107,256,294,323,359,596,718

RNASE6_2_17202,1227,1177,921,1232,1094,908,1234,1173,862,2225,1032,224
7
RNASE7_2_17203,116,152,198,179,69,78,733,204,14,108,441,106
RNASE8_2_17204,565,438,378,421,387,51,344,375,447,176,593,262
RNASEH2A_2_17205,57,22,39,43,0,0,2,22,0,10,0,69
RNASET2_2_17206,233,167,174,244,157,114,296,135,271,358,96,301
RND1_2_17207,431,477,705,722,837,839,716,206,468,295,3082,449
RND2_2_17208,271,164,311,339,323,534,8,288,205,76,523,397
RND3_2_17209,1088,917,714,852,1357,833,1149,1591,1240,234,1049,577
RNF148_2_17210,1073,877,711,1092,1165,497,790,297,745,559,1364,638
RNMT_2_17211,1151,851,826,938,2031,981,519,1580,1015,799,1149,2189
RNMTL1_2_17212,181,218,185,260,85,448,16,118,176,91,164,622
RNPEP_2_17213,130,144,278,230,618,216,101,438,463,347,63,176
RPAP2_2_17214,263,261,346,273,632,256,450,13,331,337,17,143
RPE65_2_17215,193,209,373,220,50,342,1,166,0,365,171,351
RPIA_2_17216,152,168,237,173,166,163,271,56,197,28,58,43
RPL4_2_17217,885,958,900,902,1136,857,654,528,545,425,1655,1379
RPN1_2_17218,513,465,366,450,169,152,740,337,782,554,241,548
RPP25_2_17219,193,185,166,298,218,247,45,628,119,43,38,24
RPP40_2_17220,2070,1416,1478,1480,931,1048,1286,1550,1504,2908,1110,20
17
RPS3_2_17221,48,100,113,124,49,232,561,55,27,48,59,16
RPUSD1_2_17222,75,46,105,70,38,19,11,144,0,17,0,17
RPUSD2_2_17223,278,142,329,284,570,181,188,403,264,123,0,737
RRAGA_2_17224,355,172,125,309,109,821,20,402,15,52,243,183
RRAGC_2_17225,223,299,258,363,498,4,340,242,599,10,568,3
RRAGD_2_17226,707,287,332,582,240,27,823,123,234,19,300,543
RRAS_2_17227,278,231,209,262,165,194,387,106,96,415,108,335
RRM1_2_17228,2529,2595,2050,3427,1526,1551,4047,3208,2755,3614,2493,34
15
RRP8_2_17229,185,204,195,231,220,148,229,126,67,149,9,92
RSAD2_2_17230,46,87,92,93,133,391,107,128,20,68,0,213
RTN4IP1_2_17231,431,332,357,252,643,279,539,596,269,634,573,202
SAMHD1_2_17232,238,264,309,280,426,352,361,373,132,22,322,208
SDF2_2_17233,1361,1114,1008,1255,1272,1762,1618,203,1216,550,1456,1025
SDHA_2_17234,678,489,711,847,42,633,197,699,375,1849,1154,1010
SDHB_2_17235,1050,873,845,965,911,1159,1045,222,722,1264,1769,617
SDHD_2_17236,98,133,194,188,0,127,9,741,74,0,0,238
SDR16C5_2_17237,528,511,304,412,135,37,148,332,748,842,284,944
SDR42E1_2_17238,275,514,321,545,389,103,45,817,217,675,1251,265
SDR9C7_2_17239,6,55,8,8,0,3,15,0,0,0,0,0
SDSL_2_17240,25,31,34,20,20,0,71,98,114,8,12,14
SEPSECS_2_17241,917,862,744,849,851,1022,1687,1214,381,1582,685,397
1-Sep_2_17242,483,311,441,431,132,765,904,881,906,266,532,298
SEPW1_2_17243,227,136,146,193,161,0,331,515,176,305,9,0
SERHL2_2_17244,581,805,553,671,475,961,757,833,532,597,521,327
SETX_2_17245,1071,1047,1048,1344,917,300,976,867,363,1129,712,1359
SGMS1_2_17246,390,508,596,817,334,273,1021,42,170,484,183,865
SGPL1_2_17247,1076,858,799,1066,267,721,425,997,1068,726,712,834
SGSH_2_17248,278,317,218,280,247,2,0,77,3,1,531,715

SH3GL2_2_17249,352,227,264,411,264,305,268,130,285,3,301,364
SKIV2L2_2_17250,810,901,822,932,1154,879,813,464,1137,182,799,824
SLFN12_2_17251,573,816,388,651,683,600,1224,685,906,817,610,496
SLFN12L_2_17252,351,722,286,477,406,395,1213,19,706,366,500,443
SLFN13_2_17253,2746,2760,2064,2545,1655,2633,1760,2193,2311,1362,2332,
2199
SLFN5_2_17254,1741,1172,1563,1617,1328,1344,2576,888,745,1476,1778,112
6
SLU7_2_17255,308,212,298,277,159,186,272,145,333,553,400,446
SMG8_2_17256,330,204,238,300,1,789,483,168,222,379,1123,279
SMPD2_2_17257,211,273,290,421,60,450,0,775,232,281,285,694
SMPD3_2_17258,341,317,535,633,89,1,61,800,220,44,1333,180
SMPDL3A_2_17259,1138,1119,1144,1033,904,888,1791,831,1060,686,2440,136
8
SMUG1_2_17260,677,930,700,814,612,556,168,1363,814,490,1094,1295
SNF8_2_17261,128,124,104,78,113,1,241,68,36,230,9,189
SNRNP200_2_17262,131,101,64,102,193,11,0,292,0,0,40,142
SOAT1_2_17263,2308,1844,1599,2054,858,1325,2809,1372,1538,1558,3190,21
53
SOAT2_2_17264,272,136,363,450,190,117,65,425,16,160,1050,341
SOD1_2_17265,171,324,190,229,215,70,234,443,280,210,124,210
SOD3_2_17266,90,63,123,93,0,21,0,0,169,0,281,224
SORD_2_17267,54,46,11,108,6,0,0,19,208,20,0,29
SPACA3_2_17268,92,84,111,57,0,2,331,0,0,0,91,2
SPACA5B_2_17269,448,523,477,911,699,1176,634,116,256,778,687,822
SPACA5_2_17270,448,523,477,911,699,1176,634,116,256,778,687,822
SPEM1_2_17271,108,93,70,192,171,152,8,113,166,0,0,151
SPTLC2_2_17272,24,50,36,50,0,0,0,1,8,0,0,0
SPTLC3_2_17273,13,7,17,1,0,0,0,1,0,0,0,0
SPTSSA_2_17274,223,206,105,215,475,10,0,1,12,0,334,60
SQLE_2_17275,239,487,258,317,366,131,641,581,466,76,117,419
SQRDL_2_17276,497,572,491,645,334,1144,242,181,601,97,331,336
SRD5A1_2_17277,604,527,626,632,632,604,161,953,593,494,330,692
SRD5A2_2_17278,318,366,226,333,205,229,190,219,658,489,537,172
SRD5A3_2_17279,1195,1199,1176,1586,516,668,2349,1290,845,578,1536,940
SRR_2_17280,457,365,341,434,195,208,177,164,446,348,335,196
SRSF9_2_17281,125,23,70,52,92,1,65,29,65,20,609,0
SRXN1_2_17282,149,5,66,29,12,4,0,27,9,43,10,12
SSB_2_17283,163,282,254,306,468,0,30,482,510,264,0,693
SSU72_2_17284,210,364,269,402,319,227,806,93,594,61,0,477
ST14_2_17285,164,183,313,329,169,623,123,0,159,254,62,222
ST3GAL2_2_17286,309,183,140,239,71,96,76,91,644,48,3,266
ST3GAL4_2_17287,30,27,85,72,42,6,261,48,16,8,0,6
ST3GAL6_2_17288,382,347,252,456,261,583,63,408,302,272,50,552
ST6GALNAC1_2_17289,243,220,148,243,571,306,50,20,89,162,478,487
ST6GALNAC2_2_17290,22,41,21,18,0,7,72,0,160,0,0,2
ST6GALNAC5_2_17291,684,575,640,824,383,513,46,348,474,605,1429,774
ST6GALNAC6_2_17292,54,46,91,19,519,5,1,364,109,43,38,55
ST8SIA1_2_17293,513,318,396,564,672,319,307,326,594,460,138,358
ST8SIA2_2_17294,1275,907,1051,1053,544,1401,2874,322,1741,610,1131,135

0
ST8SIA3_2_17295,301,200,210,264,291,310,322,278,284,182,354,58
ST8SIA5_2_17296,541,338,396,477,1016,122,241,305,1345,1179,752,162
ST8SIA6_2_17297,364,409,346,411,687,598,1171,347,181,601,118,340
STS_2_17298,49,29,15,52,92,0,0,45,67,71,23,0
STT3A_2_17299,210,182,130,148,1,100,354,472,147,45,703,375
STT3B_2_17300,6686,6701,6034,6715,5264,5691,6547,3660,6727,7276,7816,5
675
SUCLA2_2_17301,202,171,246,283,273,18,1139,113,601,294,0,463
SUCLG1_2_17302,23,53,16,55,0,0,0,0,16,13,1,272
SULT1A3_2_17303,305,508,443,317,195,1,9,337,663,10,165,615
SULT1A4_2_17304,305,508,443,317,195,1,9,337,663,10,165,615
SULT1B1_2_17305,3395,2772,2677,3611,3244,1561,4224,2510,2601,1956,2698
,4384
SULT1C3_2_17306,2456,2181,1955,2369,1646,2356,1965,2360,3236,3000,1823
,2606
SULT1C4_2_17307,197,230,126,199,113,57,75,16,82,39,5,378
SULT1E1_2_17308,500,576,689,811,547,460,1963,383,857,74,257,364
SULT2A1_2_17309,1213,1010,724,1021,2913,377,1808,247,520,363,1753,888
SULT4A1_2_17310,3975,4057,3432,3871,3767,3113,3853,3425,4118,4683,3668
,4685
SUPV3L1_2_17311,302,208,139,181,71,372,7,132,236,258,34,113
SURF1_2_17312,160,79,45,116,74,21,89,13,55,0,239,0
TALDO1_2_17313,288,162,472,417,341,103,213,142,462,376,123,438
TARS2_2_17314,397,215,309,448,425,333,420,705,517,173,540,497
TARS_2_17315,503,257,291,347,253,457,397,483,418,201,60,1156
TARSL2_2_17316,223,155,140,145,601,1,0,0,0,326,361,479
TAT_2_17317,1762,1642,1626,1608,1353,1579,931,2292,1939,2655,2043,1051
TBC1D10B_2_17318,27,66,27,37,166,0,3,0,2,0,0,0
TBCC_2_17319,213,363,204,277,183,11,59,393,177,159,123,180
TDG_2_17320,377,284,289,335,65,420,132,156,429,131,334,376
TDO2_2_17321,1551,984,1046,1051,753,1230,1401,571,664,1307,814,1322
TECR_2_17322,83,51,99,120,396,0,115,0,40,66,1,32
TECTA_2_17323,200,169,121,351,19,224,8,366,88,29,0,120
TFB2M_2_17324,480,558,556,399,439,187,282,9,87,808,211,303
TGDS_2_17325,1473,1325,1283,1269,1483,1324,2649,665,1648,1602,1464,102
2
TGM1_2_17326,436,297,360,465,0,51,425,57,493,478,52,535
TGM3_2_17327,357,377,244,428,644,405,729,20,329,25,1133,727
TGM4_2_17328,494,160,173,264,48,1,4,32,90,5,3,2
TGM6_2_17329,182,116,109,166,2,161,61,59,44,227,27,170
TGM7_2_17330,368,395,369,580,710,577,644,886,173,272,137,527
TGS1_2_17331,188,237,206,280,104,92,1,54,35,115,0,28
THG1L_2_17332,252,344,294,342,255,168,136,544,283,76,1,364
THOP1_2_17333,6,13,19,20,1,25,44,0,1,11,7,40
THUMPD2_2_17334,406,358,284,367,112,592,316,56,465,434,303,230
TKTL2_2_17335,173,236,256,312,301,171,510,256,124,173,43,81
TM7SF2_2_17336,186,147,103,149,0,1,252,0,544,21,480,201
TMEM55A_2_17337,67,90,13,150,1,2,1,1,10,217,0,208
TMEM62_2_17338,416,374,267,425,396,1278,361,237,1128,302,31,363

TMEM86B_2_17339,96,72,111,128,0,38,57,8,146,162,14,249
TMPRSS15_2_17340,4211,3611,3931,4245,3007,4786,4026,4815,3817,2880,4896,4719
TMX1_2_17341,1227,1136,814,943,754,1301,1262,898,1529,1082,1312,1073
TMX3_2_17342,392,414,459,486,309,357,296,515,762,468,76,98
TMX4_2_17343,1326,1666,1629,1678,1627,1979,3099,853,1390,1343,1409,1702
TNKS2_2_17344,181,93,118,88,51,163,197,108,10,699,334,97
TNKS_2_17345,111,137,81,154,701,23,58,105,148,452,318,113
TOP1_2_17346,220,279,110,169,33,247,0,8,314,1292,3,0
TOP1MT_2_17347,2991,2856,2971,3629,2506,3682,2816,3010,3250,3493,3202,2907
TOP2A_2_17348,2253,2103,1850,2057,2757,1735,2280,2712,2287,3146,1582,3083
TOP2B_2_17349,2245,1877,1936,2257,2299,828,1917,2233,1471,4259,1428,3347
TOP3A_2_17350,90,18,35,37,0,91,8,16,150,6,301,23
TOP3B_2_17351,8,34,40,40,4,1,1,6,4,10,0,10
TOR3A_2_17352,453,592,481,298,353,1162,481,439,241,385,229,585
TPH1_2_17353,393,699,524,592,539,60,412,743,227,492,542,553
TPMT_2_17354,928,770,692,698,1164,179,1136,286,143,631,1059,816
TPP1_2_17355,264,131,145,262,5,207,21,78,236,604,353,191
TPP2_2_17356,1047,693,953,891,726,493,1344,1322,865,105,2190,558
TPSAB1_2_17357,207,92,264,181,23,555,347,782,169,346,588,485
TPSB2_2_17358,151,68,62,175,6,13,96,911,317,245,136,134
TPSD1_2_17359,13,16,11,9,13,8,0,14,19,0,416,0
TPST1_2_17360,1030,1076,1038,917,1326,632,610,365,916,745,377,1494
TREH_2_17361,273,257,342,446,101,420,7,795,347,46,110,318
TREX2_2_17362,93,70,72,78,78,107,48,301,29,56,0,103
TRHDE_2_17363,889,547,571,691,1231,491,2236,1355,892,632,1097,570
TRIM21_2_17364,1549,1401,1370,1425,1496,643,2092,1136,827,1590,787,1709
TRIT1_2_17365,1041,1049,971,974,1186,446,923,279,444,941,1044,1281
TRMT112_2_17366,732,522,651,822,322,1041,1744,974,677,535,489,623
TRMT61A_2_17367,31,22,51,12,349,0,2,172,21,19,2,0
TRMU_2_17368,1031,841,959,1034,539,412,1784,519,948,1154,1983,940
TRNT1_2_17369,319,264,358,391,80,762,552,100,196,150,225,343
TRUB1_2_17370,2135,1746,1501,2129,2040,1494,2611,1281,1570,1221,2877,2143
TRUB2_2_17371,79,64,116,137,0,54,0,31,31,130,48,91
TSTA3_2_17372,64,111,107,175,22,4,63,0,118,24,29,23
TST_2_17373,53,51,42,15,0,78,0,0,1,111,55,18
TTLL13_2_17374,249,557,396,499,108,59,220,68,7,374,193,291
TTLL1_2_17375,155,200,84,147,0,1,225,112,90,380,132,12
TTLL3_2_17376,97,99,55,63,96,408,0,8,38,20,678,81
TTLL4_2_17377,1273,1080,1195,1111,546,398,2110,1564,2044,1015,1163,1675
TUFM_2_17378,159,162,137,182,407,83,34,104,418,169,108,324
TULP2_2_17379,332,78,173,178,41,350,116,414,504,141,0,32
TUT1_2_17380,146,127,124,231,280,25,434,3,37,82,34,119

TXN2_2_17381,61,108,69,49,40,320,21,13,7,83,0,5
TXN_2_17382,82,98,178,125,95,42,113,273,159,56,385,120
TXNDC11_2_17383,204,217,289,108,27,122,12,277,308,99,120,97
TXNDC12_2_17384,844,747,960,810,635,1258,1944,438,1588,121,415,1219
TXNDC15_2_17385,1000,525,911,968,673,1591,2047,504,695,1050,1388,1054
TXNDC17_2_17386,171,167,78,159,165,81,18,364,71,254,3,49
TXNL1_2_17387,335,299,237,373,51,102,113,197,246,741,632,439
TXNL4A_2_17388,678,788,540,530,868,965,472,135,289,429,898,630
TXNRD2_2_17389,372,258,224,380,324,32,14,374,248,702,126,15
TYMS_2_17390,693,514,676,448,1463,296,216,556,508,722,573,183
TYR_2_17391,220,227,158,154,29,70,416,380,396,419,196,90
TYRP1_2_17392,123,154,96,154,52,68,2,98,257,463,385,209
UAP1_2_17393,557,686,443,677,1077,169,1247,207,386,276,731,499
UAP1L1_2_17394,115,81,89,221,196,185,63,73,165,510,266,247
UBIAD1_2_17395,233,364,192,329,15,436,151,50,254,13,178,203
UBL4A_2_17396,77,21,51,43,386,11,1,0,1,246,50,0
UFSP1_2_17397,342,333,397,284,170,70,659,379,534,408,405,178
UFSP2_2_17398,500,356,363,360,695,161,66,163,376,297,516,501
UGCG_2_17399,58,293,141,145,1,0,0,593,27,395,520,0
UGGT1_2_17400,419,191,365,304,124,87,761,149,93,370,339,278
UGGT2_2_17401,587,427,437,549,606,367,614,323,669,664,179,541
UGT1A10_2_17402,1476,1527,1398,1725,1801,1199,2374,1593,1244,1299,1096
,1372
UGT1A1_2_17403,2262,2364,2645,2734,3398,1101,2548,2834,2048,3123,3115,
2144
UGT1A3_2_17404,870,570,578,696,208,1039,27,603,691,512,386,648
UGT1A4_2_17405,837,785,848,893,598,850,1254,1250,273,507,451,534
UGT1A5_2_17406,837,785,848,893,598,850,1254,1250,273,507,451,534
UGT1A7_2_17407,775,743,758,885,559,252,490,787,644,1278,863,587
UGT1A8_2_17408,7380,7453,6654,7381,9006,6776,5932,5233,7222,7506,7058,
6773
UGT1A9_2_17409,775,743,758,885,559,252,490,787,644,1278,863,587
UGT2A1_2_17410,1446,1468,1439,1404,2388,1315,1236,1487,1680,1653,1888,
908
UGT2A2_2_17411,671,720,771,750,1331,1071,585,553,812,534,961,848
UGT2A3_2_17412,1164,1071,1092,1199,1567,533,888,1498,814,1204,280,1482
UGT2B15_2_17413,1889,1666,1554,1740,1397,985,1508,3094,1608,824,1979,2
242
UGT2B17_2_17414,1889,1666,1554,1740,1397,985,1508,3094,1608,824,1979,2
242
UGT2B4_2_17415,313,255,190,205,214,41,130,545,202,17,212,48
UGT2B7_2_17416,790,723,786,839,518,372,117,175,882,387,505,706
UMPS_2_17417,593,380,491,656,319,295,24,651,670,354,602,378
UPB1_2_17418,86,101,154,199,156,248,12,81,699,13,693,188
UPF1_2_17419,386,410,241,346,40,1108,1,64,72,234,254,765
UPRT_2_17420,195,457,83,264,3,452,159,2,18,46,119,0
UQCR11_2_17421,18,28,13,33,0,4,2,7,6,1,0,42
UQCRC1_2_17422,394,643,504,489,0,283,428,1194,406,182,768,221
UQCRC2_2_17423,204,101,161,98,131,23,230,38,87,133,70,402
UQCRFS1_2_17424,1046,772,630,1005,194,859,1019,686,1058,56,725,1637

UQCRH_2_17425,492,647,410,557,528,299,703,118,322,806,176,666
UQCRQ_2_17426,244,185,184,228,211,133,268,53,290,50,1,409
UROD_2_17427,7,3,5,16,20,19,126,71,0,25,0,10
UROS_2_17428,1540,1694,1577,2102,1651,868,2963,2406,2142,2075,1667,172
8
UST_2_17429,329,191,458,386,694,6,1109,603,268,43,1446,410
UXS1_2_17430,61,81,37,98,79,19,85,137,51,120,0,49
VARS_2_17431,151,213,149,271,2,2,421,89,160,40,7,66
VAT1L_2_17432,277,243,216,159,393,427,246,25,180,502,51,171
VCP_2_17433,291,293,331,373,1094,176,515,0,445,33,1031,541
VNN1_2_17434,1611,1060,1284,1360,1252,1071,1375,1852,1552,893,993,1322
WBSCR17_2_17435,143,120,132,171,0,10,216,223,30,379,31,317
WBSCR27_2_17436,99,114,95,97,109,81,36,12,46,160,294,30
WDFY3_2_17437,929,881,1368,1230,671,2066,2839,1068,1255,634,748,680
WRN_2_17438,1248,958,932,1329,1101,562,1133,680,628,1221,910,781
XDH_2_17439,245,314,212,487,1382,479,138,280,374,538,362,476
XPNPEP2_2_17440,255,451,254,565,482,36,66,988,81,449,459,209
XRCC2_2_17441,1023,741,517,744,670,453,861,269,506,554,416,1031
XRCC5_2_17442,477,472,457,661,1180,625,1412,1085,716,647,658,810
XRCC6_2_17443,928,668,725,1082,1207,1125,929,654,370,711,465,587
XRN2_2_17444,1397,1382,1372,1379,1656,1007,1211,1447,1322,1195,2033,12
88
XYLT1_2_17445,80,170,135,57,0,2,2,44,193,233,261,0
XYLT2_2_17446,424,214,128,339,367,297,170,346,264,18,316,672
YARS2_2_17447,164,177,140,82,327,151,1,34,127,3,24,135
YARS_2_17448,653,693,682,716,341,678,1006,1092,956,1219,334,530
YKT6_2_17449,460,577,432,523,458,295,1034,46,314,323,137,804
YPEL1_2_17450,180,196,42,176,2,8,0,39,463,1,95,236
ZADH2_2_17451,92,138,132,103,279,187,4,67,219,80,125,624
ZCCHC4_2_17452,70,255,73,141,0,0,682,41,1,1,0,14
ZDHHC17_2_17453,129,72,91,183,299,12,38,44,2,3,0,316
ZDHHC18_2_17454,247,358,211,310,379,369,494,638,270,332,33,419
ZDHHC1_2_17455,40,37,74,51,17,17,45,29,86,12,0,64
ZDHHC21_2_17456,936,821,932,916,1242,191,1875,713,1341,148,1924,914
ZDHHC2_2_17457,54,61,36,18,47,44,18,32,136,103,0,82
ZER1_2_17458,78,61,113,101,112,334,296,15,58,334,121,73
ZMPSTE24_2_17459,524,462,608,409,325,704,842,608,841,382,408,31
ZRANB3_2_17460,442,398,235,598,723,262,887,0,64,802,9,734
A1CF_2_17461,49,28,81,55,0,0,139,2,1,14,193,0
A2LD1_2_17462,46,2,1,33,0,0,0,0,0,265,0,0
AADAT_2_17463,156,120,114,97,221,90,49,22,93,76,42,10
AARSD1_2_17464,1,38,292,54,0,12,0,0,0,11,140,65
ABAT_2_17465,686,664,723,623,432,460,479,309,1093,1590,401,487
ABHD11_2_17466,259,564,224,353,421,734,39,346,169,237,25,543
ABHD12_2_17467,609,689,535,608,231,875,326,145,440,304,194,614
ABHD14B_2_17468,113,150,224,190,656,0,448,113,45,102,34,638
ABHD2_2_17469,86,178,78,134,174,189,60,0,258,0,2,341
ACAA1_2_17470,21,52,69,86,19,0,47,7,2,93,36,83
ACACA_2_17471,318,415,335,369,629,759,319,48,274,720,544,148
ACAD10_2_17472,235,190,280,360,1,531,345,30,172,49,10,50

ACADM_2_17473,90,51,88,59,94,228,103,65,67,189,98,44
ACADVL_2_17474,316,288,318,407,842,632,448,681,162,390,512,150
ACCS_2_17475,123,152,104,133,1,72,21,313,500,453,0,247
ACE_2_17476,658,543,633,765,624,590,445,598,433,657,480,549
ACHE_2_17477,235,276,179,409,549,500,131,419,478,107,368,586
ACIN1_2_17478,23,45,39,44,61,3,0,6,10,1,7,57
ACLY_2_17479,273,294,622,532,564,653,729,59,416,78,537,286
ACOT11_2_17480,908,984,782,1079,434,517,900,881,477,1446,827,824
ACOT13_2_17481,663,473,502,590,533,547,785,424,485,403,481,261
ACOT7_2_17482,111,131,139,171,127,26,544,186,154,96,506,191
ACOT9_2_17483,52,120,70,17,8,3,0,0,12,47,9,1
ACOX1_2_17484,56,119,68,80,343,39,1,242,29,0,13,6
ACOX3_2_17485,264,113,312,148,454,36,52,118,95,6,27,376
ACSBG1_2_17486,564,604,743,620,280,1078,625,290,956,294,1545,204
ACSF3_2_17487,17,74,54,72,0,1,0,21,0,105,0,0
ACSL3_2_17488,797,624,493,991,361,685,631,230,685,982,874,408
ACSL4_2_17489,281,375,252,246,174,217,236,306,318,190,30,175
ACSL5_2_17490,59,42,68,51,62,2,5,0,168,35,9,19
ACSL6_2_17491,602,462,549,574,695,749,446,879,206,853,510,544
ACSM2B_2_17492,1518,1588,1223,1471,1057,719,2379,906,1915,943,1649,225
3
ACSM3_2_17493,317,411,600,383,264,123,1433,475,274,232,615,63
ACSS2_2_17494,273,273,272,193,56,353,257,165,167,175,156,482
ACY1_2_17495,67,15,126,79,0,3,0,280,12,0,0,7
ADAMTS2_2_17496,1,52,6,43,0,0,0,8,0,88,0,0
ADARB1_2_17497,116,16,33,73,0,16,0,0,0,0,0,0
ADAR_2_17498,567,428,365,611,578,632,325,107,385,187,448,1018
ADCY10_2_17499,1276,1036,1019,1121,1809,1255,2262,613,649,747,529,1043
ADCY4_2_17500,804,558,612,732,841,1153,391,396,479,1009,750,1040
ADCY5_2_17501,403,228,138,433,27,369,452,186,223,47,265,375
ADCY6_2_17502,137,36,98,73,5,124,0,60,239,0,29,35
ADH6_2_17503,1044,813,852,686,784,614,1094,812,956,541,523,431
ADH7_2_17504,546,342,348,440,737,776,425,406,678,399,560,370
ADPRHL1_2_17505,37,6,82,17,74,39,0,48,2,60,0,9
ADSL_2_17506,873,414,505,432,285,202,1028,331,975,514,677,680
ADSSL1_2_17507,296,225,296,345,165,178,550,215,253,91,420,62
AFMID_2_17508,2434,1630,1724,1958,2326,1545,1773,2320,1705,2005,1627,1
649
AGA_2_17509,78,51,55,65,0,306,1,243,2,0,0,86
AGAP1_2_17510,10,5,3,22,0,0,9,7,46,17,94,59
AGAP2_2_17511,389,198,347,293,284,360,11,624,290,374,104,338
AGL_2_17512,1686,1105,1089,1077,2468,1145,1822,1121,1165,1411,1397,148
9
AGPAT1_2_17513,412,458,591,596,101,601,355,113,1096,779,481,1519
AGPAT2_2_17514,290,483,292,263,477,4,104,700,58,482,347,475
AGPAT3_2_17515,72,60,33,34,1,15,4,27,99,1,0,246
AGXT2L1_2_17516,1508,1667,1214,1170,1490,666,1332,1515,1431,927,1570,1
945
AHCY_2_17517,153,44,24,49,49,53,54,6,119,34,30,47
AHCYL1_2_17518,340,645,551,681,120,1319,409,182,366,260,1028,170

AHCYL2_2_17519,359,382,503,509,234,1151,501,228,364,139,529,255
AIFM1_2_17520,41,40,21,32,240,160,0,0,60,0,0,41
AIFM2_2_17521,39,29,45,29,0,0,0,0,0,0,0,4
AIFM3_2_17522,104,168,181,238,12,26,0,4,0,41,25,260
AKIRIN1_2_17523,5019,5138,4620,4730,5114,5114,3863,3389,4622,5913,4119,4843
AKR1A1_2_17524,817,909,898,1007,1254,824,1478,777,925,1597,750,658
AKR1C2_2_17525,1051,759,896,725,1366,542,1365,1666,369,676,595,1037
AKR1D1_2_17526,283,387,238,283,112,230,0,252,10,161,378,9
ALAS1_2_17527,94,42,82,37,1,0,9,9,0,0,0,12
ALAS2_2_17528,564,357,326,395,957,64,253,1369,508,129,268,427
ALDH16A1_2_17529,103,100,69,82,0,133,254,144,322,442,0,194
ALDH1A2_2_17530,470,324,497,554,546,402,858,15,694,248,932,672
ALDH2_2_17531,218,99,107,145,240,140,143,49,227,124,0,142
ALDH3A1_2_17532,170,172,93,200,20,24,22,398,270,223,0,164
ALDH3A2_2_17533,525,380,363,524,387,516,747,462,624,170,320,370
ALDH3B1_2_17534,28,21,37,39,18,0,30,2,13,28,0,26
ALDH3B2_2_17535,108,117,113,107,14,329,47,0,24,20,72,28
ALDH4A1_2_17536,36,27,6,77,0,61,0,0,52,20,6,2
ALDH5A1_2_17537,78,98,100,73,228,88,364,99,32,117,278,1
ALDH7A1_2_17538,269,74,107,150,122,11,233,85,499,384,31,19
ALDH8A1_2_17539,1491,1167,908,984,1398,583,786,856,744,351,545,2111
ALDOA_2_17540,1416,1337,1425,1350,2410,1294,2613,1830,1309,1555,550,1772
ALG3_2_17541,22,25,24,14,68,0,4,0,0,6,1,0
ALG5_2_17542,242,339,167,256,400,5,27,381,147,73,558,548
ALG8_2_17543,929,766,714,910,1248,562,1674,361,729,575,395,598
ALG9_2_17544,176,214,206,149,14,209,35,47,96,57,275,52
ALOX15B_2_17545,595,753,677,907,414,582,586,1365,770,1164,1275,1024
ALOXE3_2_17546,36,19,13,9,194,10,45,24,0,10,1,27
AMACR_2_17547,515,336,347,433,241,734,428,150,254,180,55,429
AMD1_2_17548,154,304,152,135,123,476,6,134,12,43,416,70
AMDHD2_2_17549,412,289,378,287,369,13,267,657,584,192,1,251
AMPD1_2_17550,794,886,765,924,1288,356,453,1422,1497,1267,1067,666
AMPD2_2_17551,124,70,81,100,0,82,29,357,44,31,79,33
AMPD3_2_17552,696,498,443,675,230,87,589,50,104,589,90,948
AMT_2_17553,112,131,146,148,282,140,193,5,90,129,140,146
AMY1A_2_17554,13219,11353,11716,14121,10096,7505,13600,11952,10104,9883,10997,12637
ANG_2_17555,312,415,408,443,351,337,180,14,193,970,69,605
AOAH_2_17556,655,694,603,887,655,476,494,865,497,374,253,949
AOC2_2_17557,162,87,91,180,198,43,488,465,234,122,1,539
APC_2_17558,1985,1990,1749,2057,1441,898,1993,860,1377,1743,1208,2281
APEX1_2_17559,233,86,54,74,345,1,10,2,112,23,0,289
APOBEC3A_2_17560,446,639,380,758,20,152,629,131,248,463,329,479
APOBEC3H_2_17561,1087,660,684,609,1100,500,1122,930,834,427,734,828
APRT_2_17562,5,48,2,46,0,0,0,0,0,0,0,0
ARF1_2_17563,59,99,102,103,332,9,31,23,231,188,4,58
ARFRP1_2_17564,40,101,79,168,110,14,47,371,61,29,96,264
ARHGAP5_2_17565,620,512,316,456,204,317,214,387,473,723,305,408

ARHGEF10L_2_17566,126,143,71,89,70,0,9,240,103,0,0,74
ARL2_2_17567,200,101,192,227,95,70,406,69,81,16,148,199
ARL4A_2_17568,154,364,223,223,172,131,253,678,283,353,82,877
ARL5A_2_17569,331,205,391,351,658,595,192,17,183,154,102,299
ARSA_2_17570,150,252,167,285,684,389,138,1007,0,490,296,361
ARSB_2_17571,147,36,96,175,346,173,30,8,30,349,3,105
ARSF_2_17572,151,143,169,170,45,0,40,392,80,437,13,300
ART3_2_17573,0,0,0,0,0,0,0,0,0,0,0,0
ART5_2_17574,86,141,57,93,76,64,10,6,4,70,423,497
ASAH1_2_17575,590,798,883,710,650,258,1455,1518,334,809,2603,453
ASAH2_2_17576,2278,1776,1659,1914,2309,1676,1557,1451,2211,1886,521,18
74
ASL_2_17577,39,106,100,168,266,131,0,0,0,0,173,3
ASMT_2_17578,778,550,664,782,347,752,596,388,981,578,316,712
ASMTL_2_17579,411,632,378,581,477,310,140,208,658,186,95,537
ASNS_2_17580,61,44,52,39,102,14,22,8,72,2,88,70
ASPA_2_17581,1888,1447,1343,1554,2167,830,275,1405,1372,1642,696,1574
ASRGL1_2_17582,340,197,152,198,26,44,250,336,1336,170,2,38
ASS1_2_17583,175,207,229,201,29,6,11,160,48,63,135,1
ATE1_2_17584,1023,658,1015,1111,1205,588,901,345,744,1412,217,856
ATL1_2_17585,110,53,44,68,77,1,140,91,18,5,0,4
AURKAIP1_2_17586,233,337,449,299,22,1068,628,341,162,4,518,12
AZIN1_2_17587,75,33,87,35,0,0,0,0,42,0,468,34
B3GALNT1_2_17588,238,205,176,202,100,163,89,609,52,115,4,489
B3GALT5_2_17589,540,479,547,313,427,760,360,448,723,511,851,347
B3GAT1_2_17590,91,30,18,62,157,1,77,51,0,3,51,1
B4GALNT2_2_17591,531,388,687,741,611,577,435,1001,682,931,766,456
B4GALT2_2_17592,194,345,217,131,219,175,168,0,845,543,1,434
B4GALT3_2_17593,665,574,611,682,708,509,1451,1269,942,867,372,1092
B4GALT4_2_17594,725,536,582,648,433,298,1790,195,192,794,20,827
BAAT_2_17595,1827,1681,1938,2042,1514,1995,2866,1712,2438,2401,1135,25
49
BACE1_2_17596,786,981,777,938,438,1452,893,623,632,1266,804,800
BACE2_2_17597,2067,2180,2052,2018,1172,1598,1013,1079,1504,3560,385,14
33
BCAT1_2_17598,696,903,724,903,226,1250,1367,1166,1129,331,528,427
BCAT2_2_17599,163,332,260,428,730,0,45,869,104,167,41,552
BCKDHA_2_17600,154,110,164,229,5,111,15,27,64,13,333,1
BCKDHB_2_17601,665,259,559,492,812,305,1118,619,145,144,764,624
BCO2_2_17602,237,181,276,128,195,192,294,131,248,24,266,186
BDH1_2_17603,546,323,626,570,637,13,93,572,484,587,579,1072
BFSP1_2_17604,480,557,449,499,229,276,1084,65,174,249,746,529
BHMT2_2_17605,260,86,225,255,468,223,130,74,119,79,22,229
BMP1_2_17606,87,37,40,68,78,1,17,0,16,1,6,97
C17orf101_2_17607,299,77,91,51,235,31,7,24,3,15,0,0
C1GALT1C1_2_17608,8,25,77,34,0,0,0,0,31,0,0,17
C1S_2_17609,461,279,322,377,161,463,448,6,230,956,471,241
C2_2_17610,35,110,26,96,169,68,851,86,57,9,27,66
CA10_2_17611,74,81,77,52,22,0,90,110,9,0,9,8
CA12_2_17612,128,89,126,53,94,110,59,275,266,198,308,0

CA1_2_17613,87,103,175,175,14,41,0,0,0,25,1,0
CA7_2_17614,242,107,152,209,188,57,478,161,13,296,329,162
CAB39_2_17615,591,525,592,701,420,597,709,600,671,102,613,746
CANT1_2_17616,203,106,149,184,108,28,63,329,169,16,89,42
CAPN1_2_17617,210,357,329,239,42,100,511,441,36,63,1540,65
CAPN2_2_17618,112,120,133,329,78,66,75,100,537,43,11,243
CARNS1_2_17619,129,104,117,99,286,176,5,20,468,42,18,120
CARS_2_17620,1588,1285,1235,1427,2203,748,1669,1228,864,834,2456,1295
CASP10_2_17621,3819,3189,3339,4005,5822,3285,2597,3747,2952,3341,3461,
2529
CASP1_2_17622,281,367,300,242,688,48,576,301,716,237,200,160
CASP2_2_17623,1061,1489,1277,1701,640,1869,1055,2889,600,1594,1861,141
1
CASP3_2_17624,172,237,137,207,50,311,430,268,207,32,5,257
CASP4_2_17625,9,1,3,0,1,0,0,0,0,0,0,0
CASP5_2_17626,475,450,576,612,166,1058,380,623,481,601,478,510
CASP6_2_17627,60,58,58,87,143,66,39,4,4,0,3,0
CASP7_2_17628,170,224,150,282,224,292,298,35,23,90,38,77
CASP8_2_17629,663,1165,852,1093,1055,679,1090,444,1253,1542,2464,238
CASP9_2_17630,780,586,731,737,668,749,815,657,780,1204,295,581
CASZ1_2_17631,18,7,94,50,0,29,114,27,3,2,611,1
CAV3_2_17632,25,36,45,26,7,65,1,66,4,0,1,273
CBS_2_17633,42,20,33,10,1,0,13,0,45,0,76,0
CCBL1_2_17634,28,27,10,16,0,1,371,0,0,19,251,9
CCBL2_2_17635,535,520,396,738,305,1510,2419,7,815,626,120,478
CDC42_2_17636,2670,2282,2271,3320,2297,2703,3850,3692,2235,1994,4321,2
373
CDH16_2_17637,283,187,273,267,347,12,481,908,135,116,713,372
CECR1_2_17638,129,55,204,180,35,3,49,33,63,141,48,45
CEPT1_2_17639,755,1109,894,1281,0,611,125,272,143,371,1104,567
CERS1_2_17640,243,309,161,299,281,848,162,55,47,30,522,349
CES1_2_17641,13,22,17,86,0,0,0,0,0,0,0,0
CES3_2_17642,93,133,193,347,248,227,142,357,1,14,27,173
CES4A_2_17643,324,553,452,561,376,1299,26,759,879,445,845,288
CES5A_2_17644,658,604,584,643,1124,399,533,1145,402,553,720,900
CHAT_2_17645,224,236,194,388,205,114,290,227,301,186,292,420
CHI3L2_2_17646,390,378,271,413,276,91,73,328,182,551,364,174
CHIA_2_17647,90,73,190,131,122,0,615,272,52,392,23,9
CHPF_2_17648,637,347,448,494,725,449,1345,129,752,304,263,316
CHST11_2_17649,847,633,715,875,1104,623,1170,1089,915,900,1411,1115
CHST15_2_17650,121,213,196,195,217,170,518,0,374,472,47,112
CHST4_2_17651,252,315,302,254,1027,296,54,310,125,325,36,80
CHST8_2_17652,492,485,585,676,183,485,639,422,615,825,704,789
CLP1_2_17653,20,17,17,54,157,1,0,40,2,104,0,3
CNDP2_2_17654,57,79,53,56,235,1,13,41,11,22,53,179
CNTN1_2_17655,801,485,564,580,535,202,898,457,903,350,259,390
CNTN4_2_17656,363,116,178,301,273,9,374,555,213,734,601,428
COMT_2_17657,308,252,217,200,278,567,507,164,0,96,290,165
COQ6_2_17658,143,50,168,238,463,79,1,224,113,30,91,400
COX11_2_17659,3236,3043,2552,3415,2980,2614,4012,4934,2783,4050,3053,2

638
COX15_2_17660,1432,938,949,1306,830,895,2045,1294,2693,1166,432,514
CPA5_2_17661,107,133,122,116,602,0,0,0,37,0,0,22
CPB2_2_17662,1262,1600,1503,1351,1353,1104,1635,1095,1370,1908,1511,17
01
CPM_2_17663,277,283,315,336,876,99,853,456,71,414,42,272
CPPED1_2_17664,888,707,968,854,1064,811,1538,132,1190,1333,389,708
CPT1A_2_17665,1717,1645,1284,1941,1060,1288,689,1264,1811,572,1365,210
5
CPT1B_2_17666,32,74,67,68,6,12,0,0,56,111,0,0
CPT1C_2_17667,46,62,47,77,21,233,8,0,23,100,16,42
CRLS1_2_17668,795,897,758,749,476,517,625,693,1436,450,1733,355
CRMP1_2_17669,313,199,325,294,87,650,240,21,295,366,57,121
CROT_2_17670,689,650,452,795,426,397,101,402,409,403,798,1034
CRY2_2_17671,917,750,789,1059,1179,839,1850,510,403,604,1400,806
CRYM_2_17672,142,182,116,104,16,79,388,557,0,198,40,171
CRYZ_2_17673,241,237,281,289,71,0,136,131,25,13,3,624
CSDE1_2_17674,2732,2005,1944,2485,2625,1013,1903,2710,2602,1763,2120,3
038
CSGALNACT1_2_17675,192,210,142,191,0,0,0,363,308,3,79,504
CSMD3_2_17676,1145,994,787,1195,917,1739,186,1552,833,1429,1905,1030
CTAGE5_2_17677,1710,1376,1610,1684,1012,1534,1544,1229,1538,2880,2520,
1777
CTBP1_2_17678,272,138,123,184,67,196,30,63,82,51,219,335
CTPS2_2_17679,241,312,227,139,734,64,2,311,219,13,69,206
CTSA_2_17680,1524,905,978,1109,852,1596,1872,739,1085,1443,476,1133
CTSB_2_17681,234,250,84,234,10,129,7,197,157,66,9,60
CTSE_2_17682,355,343,405,315,462,81,777,242,162,14,1743,241
CTSL1_2_17683,650,429,427,591,1373,1057,0,312,338,920,0,882
CTSL2_2_17684,242,184,224,138,5,6,51,6,173,321,491,73
CTSS_2_17685,125,143,70,90,147,51,136,82,92,292,7,45
CYB561_2_17686,335,362,249,441,963,122,322,52,466,816,216,420
CYB5A_2_17687,126,147,226,177,357,398,128,39,0,7,79,292
CYB5R3_2_17688,101,58,55,127,217,21,0,15,114,266,44,14
CYP11A1_2_17689,202,141,157,180,255,342,367,47,126,103,112,223
CYP11B1_2_17690,203,206,130,270,151,9,81,7,56,21,687,197
CYP19A1_2_17691,0,0,0,0,0,0,0,0,0,0,0,0
CYP21A2_2_17692,15,134,37,44,3,1,0,0,26,34,0,2
CYP24A1_2_17693,401,349,338,357,113,586,839,135,138,459,256,174
CYP26A1_2_17694,168,214,228,241,380,19,36,85,74,413,22,33
CYP2A7_2_17695,683,473,522,471,683,274,234,326,317,484,554,867
CYP2C18_2_17696,158,88,152,161,0,0,4,45,6,115,1,161
CYP2C8_2_17697,1070,1136,995,883,1117,671,560,521,752,1723,1276,1298
CYP2D6_2_17698,629,611,572,742,841,1542,964,675,504,787,595,1013
CYP3A43_2_17699,4939,4815,4237,4852,4867,3483,4104,3860,3057,4536,4628
,5285
CYP3A4_2_17700,1330,1063,1090,1273,257,1422,699,1560,1072,523,1185,999
CYP4B1_2_17701,1373,918,852,1176,883,947,518,269,603,1016,1110,658
CYP4F11_2_17702,1040,688,766,695,613,325,439,1087,462,1121,725,1055
CYP4F3_2_17703,4374,3939,3515,3885,3496,2628,4625,3352,2600,3183,4857,

3842
CYP51A1_2_17704,485,774,469,466,650,212,387,679,222,287,665,382
DAGLB_2_17705,269,382,263,462,263,212,1069,360,589,127,636,245
DCLRE1C_2_17706,775,1049,912,927,1429,1050,937,1064,588,560,1529,905
DCP2_2_17707,3,2,13,8,0,1,0,0,125,0,43,0
DCT_2_17708,719,504,463,553,282,361,447,419,361,92,315,771
DCTD_2_17709,951,844,1225,887,1675,627,4583,782,638,1031,993,751
DCXR_2_17710,31,36,25,24,1,0,0,12,0,24,53,41
DDAH1_2_17711,148,67,92,99,75,83,671,361,355,92,44,291
DDC_2_17712,728,627,651,763,545,1155,284,622,688,421,637,531
DDHD1_2_17713,1523,1729,1529,1978,1794,917,4968,1713,1309,2049,1549,21
10
DDO_2_17714,122,52,103,39,580,0,0,0,157,0,447,7
DDT_2_17715,120,3,0,37,822,0,0,0,0,0,0,1
DDX11_2_17716,147,71,173,170,20,29,224,380,239,283,59,388
DDX17_2_17717,61,57,67,25,233,0,0,62,35,4,2,25
DDX19B_2_17718,219,139,239,384,78,383,4,359,350,159,435,216
DDX31_2_17719,906,667,816,985,674,332,352,734,1320,491,256,420
DDX39B_2_17720,424,913,598,767,612,674,768,1772,278,506,1660,481
DDX3X_2_17721,877,689,555,842,569,192,799,322,603,503,272,396
DDX3Y_2_17722,220,373,273,302,361,99,137,0,515,199,63,64
DDX42_2_17723,1019,1315,1286,1318,1348,1483,1490,1997,940,1052,1770,14
48
DDX47_2_17724,310,262,239,285,171,563,235,433,130,111,261,107
DDX4_2_17725,862,703,556,842,567,193,798,323,617,503,271,398
DDX54_2_17726,1154,704,1217,1083,628,919,1170,1233,1193,1774,671,855
DFFA_2_17727,1324,1284,1517,1398,1941,1537,1428,1646,1034,1387,806,455
DGCR8_2_17728,79,97,85,133,53,272,27,92,70,25,148,11
DHCR7_2_17729,17,15,11,18,300,0,0,25,0,1,0,4
DHDDS_2_17730,1099,1128,1136,874,1244,880,1492,1214,1234,905,557,993
DHFRL1_2_17731,412,396,417,631,379,283,1013,398,245,370,414,253
DHPS_2_17732,300,255,476,374,166,214,357,176,33,287,628,14
DHRS1_2_17733,125,206,191,211,285,172,42,472,80,427,139,414
DHRS2_2_17734,230,62,103,131,3,4,1,236,283,156,390,401
DHRS9_2_17735,252,210,284,256,109,134,126,59,90,683,188,133
DHX16_2_17736,211,137,121,364,50,244,23,102,389,172,0,21
DHX30_2_17737,544,671,451,756,470,309,208,321,239,503,695,436
DHX33_2_17738,17,9,7,17,0,0,10,0,5,0,0,0
DHX35_2_17739,1044,900,803,1295,450,1072,312,1185,783,1455,680,1011
DHX36_2_17740,1026,841,736,952,699,1475,1354,1309,1080,158,1078,361
DHX40_2_17741,1999,2246,1827,1761,1460,1580,1861,1742,707,644,1585,160
0
DIAPH3_2_17742,757,723,590,871,1068,802,704,492,513,866,271,758
DIS3_2_17743,91,111,111,138,54,72,6,52,47,312,191,213
DIS3L_2_17744,253,240,256,198,363,248,965,238,136,275,1,364
DKC1_2_17745,385,635,619,594,774,201,185,521,97,481,462,416
DNAJC27_2_17746,177,199,286,244,374,177,93,141,186,261,70,173
DNASE1L1_2_17747,413,447,429,580,1032,141,832,859,417,678,548,1154
DNM1_2_17748,24,13,63,45,13,233,0,126,39,0,0,0
DNM1L_2_17749,76,46,92,73,17,28,233,60,198,197,1,13

DNM2_2_17750,117,187,225,237,376,793,0,208,146,74,655,81
DNM3_2_17751,110,108,80,112,156,6,20,18,58,239,8,282
DNTT_2_17752,15,0,5,3,276,0,3,80,0,92,0,0
DOHH_2_17753,121,116,80,62,311,27,313,0,159,104,1,35
DOLPP1_2_17754,26,18,37,30,2,1,8,181,23,41,0,110
DPEP1_2_17755,246,233,283,284,346,215,54,619,247,88,236,231
DPEP3_2_17756,329,340,262,367,247,288,703,174,306,186,1107,354
DPH5_2_17757,299,192,336,224,240,215,639,57,224,380,492,258
DPM3_2_17758,124,73,104,163,26,142,56,66,301,106,109,68
DPP3_2_17759,271,99,163,224,186,284,263,294,0,70,253,335
DPP8_2_17760,648,467,739,683,600,285,600,583,159,362,995,627
DPYSL2_2_17761,38,72,119,52,38,0,4,54,238,33,21,0
DPYSL3_2_17762,82,85,44,80,146,532,7,282,192,16,210,224
DROSHA_2_17763,137,225,146,191,547,515,0,168,285,343,0,44
DSE_2_17764,998,738,664,1144,576,665,829,799,1342,1181,737,1226
DUOX1_2_17765,197,162,223,207,58,355,423,104,103,326,229,255
DUT_2_17766,155,180,137,76,172,203,185,44,29,210,4,13
ECE1_2_17767,1472,1505,1356,1609,1925,1131,3380,1273,1962,1367,1170,10
72
ECE2_2_17768,306,85,66,189,91,44,572,41,132,0,396,316
ECI1_2_17769,299,271,142,385,35,397,3,588,2,10,50,285
ECI2_2_17770,293,422,186,388,6,239,1,607,250,0,411,218
EDEM2_2_17771,56,173,107,129,34,25,4,10,124,30,177,164
EFEMP1_2_17772,1088,817,990,1165,1192,665,2003,1018,659,432,716,1388
EFTUD2_2_17773,812,538,518,748,368,283,1075,626,478,1471,954,1325
EGLN2_2_17774,1087,870,784,1146,1678,768,655,514,890,799,147,1226
EHHADH_2_17775,9,5,7,4,0,0,154,16,0,0,0,0
ELAC2_2_17776,119,111,140,166,17,0,0,34,0,18,56,130
ELOVL5_2_17777,313,337,201,153,673,184,141,182,62,185,953,120
ELOVL6_2_17778,592,590,501,507,282,295,140,269,438,777,134,630
ELOVL7_2_17779,596,511,662,780,707,487,201,322,216,818,224,776
ENDOV_2_17780,113,148,168,194,221,254,0,0,0,17,577,19
ENO3_2_17781,570,679,673,786,182,474,574,378,289,891,582,1037
ENOX2_2_17782,403,349,283,311,701,48,558,464,640,1095,9,135
ENPP2_2_17783,463,248,457,694,730,479,623,992,939,129,911,313
ENTPD1_2_17784,227,183,155,217,287,51,0,78,201,329,0,228
ENTPD2_2_17785,219,292,200,263,259,17,35,302,138,133,572,591
ENTPD4_2_17786,843,847,990,1026,1256,1108,1909,389,542,545,1011,761
ENTPD6_2_17787,65,153,68,143,63,22,50,50,181,95,144,20
ENTPD8_2_17788,364,194,214,366,7,490,1042,65,131,642,66,496
EPHX1_2_17789,108,195,78,98,96,114,393,9,24,55,64,59
EPHX3_2_17790,7,15,16,8,0,0,398,0,0,0,0,1
ERCC1_2_17791,875,997,833,1008,484,258,1115,711,1074,1203,1047,847
ERCC2_2_17792,291,197,192,316,1,0,153,19,320,64,572,139
EXO1_2_17793,177,122,315,287,88,243,118,69,299,331,334,424
EXOG_2_17794,35,86,105,47,4,1,92,1,3,5,0,8
EXOSC3_2_17795,102,157,57,201,135,9,301,0,20,2,372,174
EXOSC9_2_17796,774,718,992,790,1224,980,534,644,968,246,891,635
EXTL2_2_17797,1383,1141,1036,1388,845,1012,982,463,1813,1283,1942,1735
F7_2_17798,571,413,325,404,451,236,764,470,383,456,355,244

FAHD1_2_17799,41,35,17,32,71,142,70,121,1,0,0,0
FAM108A1_2_17800,154,25,124,180,52,31,33,362,321,64,275,421
FAM135A_2_17801,347,201,163,201,191,370,116,236,36,264,51,166
FAN1_2_17802,1740,1524,1687,1723,1271,990,1789,2828,1484,2491,1821,128
5
FDPS_2_17803,891,877,671,1157,374,722,442,656,598,595,230,1287
FDXR_2_17804,98,47,15,24,9,43,12,52,1,38,0,70
FECH_2_17805,60,24,25,83,24,23,65,0,21,24,0,26
FERMT3_2_17806,52,31,57,38,31,75,239,238,71,107,159,8
FHIT_2_17807,376,429,188,354,641,151,746,285,150,513,80,361
FIGNL1_2_17808,1662,1782,1751,1902,2457,2844,1136,1214,2593,1739,1226,
2188
FKBP11_2_17809,51,66,54,92,309,30,414,1,70,184,0,77
FKBP1A_2_17810,38,41,39,57,24,2,7,19,5,69,0,21
FKBP1B_2_17811,707,589,433,696,543,81,382,44,504,148,239,1078
FKBP2_2_17812,312,327,343,613,193,614,106,207,34,211,0,671
FKBP5_2_17813,91,55,77,153,15,253,0,0,0,0,836,0
FKBP7_2_17814,332,665,542,888,316,959,197,2094,660,853,1279,686
FLAD1_2_17815,207,198,121,200,325,7,73,0,71,506,0,44
FMO3_2_17816,596,341,404,404,682,357,415,296,281,222,1002,638
FMO5_2_17817,631,499,568,633,681,276,342,443,437,898,698,704
FN1_2_17818,84,70,73,59,246,158,17,46,11,94,5,107
FOLH1_2_17819,98,105,107,76,240,329,96,126,166,70,0,180
FPGS_2_17820,81,14,16,56,71,53,0,22,6,0,4,5
FPGT_2_17821,163,124,115,188,113,33,464,2,37,161,37,310
FTCD_2_17822,271,440,453,480,89,206,0,146,309,212,3,287
FTSJ1_2_17823,243,279,272,382,584,166,769,21,496,655,556,400
FUT2_2_17824,7,3,7,13,0,65,441,0,1,1,0,137
FUT3_2_17825,146,178,119,229,401,10,188,422,123,184,189,140
FUT6_2_17826,0,38,6,28,0,0,0,0,0,0,0,8
FUT8_2_17827,3141,3254,2924,3374,3778,4141,2062,3213,1927,3123,4240,36
72
G3BP1_2_17828,114,14,69,147,300,81,66,9,23,32,210,4
G3BP2_2_17829,178,221,190,171,109,5,47,2,244,185,92,224
G6PD_2_17830,177,149,209,285,21,71,237,693,92,322,265,625
GAA_2_17831,76,96,68,115,57,31,42,537,79,189,364,229
GAD1_2_17832,472,328,394,384,160,182,0,27,322,676,193,315
GAD2_2_17833,1850,1565,1546,1816,2464,2530,1560,1716,1384,1359,1001,18
71
GALC_2_17834,747,503,364,599,280,287,164,347,274,522,237,749
GALE_2_17835,384,126,331,312,1587,231,59,695,12,1,221,429
GALNT9_2_17836,1033,633,845,1046,776,801,1256,1259,482,479,416,648
GALNTL1_2_17837,131,298,136,129,77,137,289,75,345,167,21,209
GAMT_2_17838,1,73,0,28,0,0,0,0,0,0,311,0
GANAB_2_17839,151,206,195,76,31,131,1026,0,3,297,230,0
GART_2_17840,78,157,238,155,258,297,272,20,50,29,227,109
GBA_2_17841,721,964,770,789,961,1256,101,646,727,738,2179,879
GBP5_2_17842,221,280,267,488,7,820,0,502,270,146,86,134
GCAT_2_17843,15,46,54,14,0,4,0,0,0,14,0,31
GCDH_2_17844,125,69,120,193,4,8,4,0,290,296,471,186

GCH1_2_17845,267,85,168,241,583,406,502,815,533,144,64,102
GCLC_2_17846,168,39,47,109,32,24,2,175,0,96,13,340
GCNT1_2_17847,64,29,26,25,2,10,310,17,0,36,3,66
GDPD1_2_17848,567,993,607,896,329,472,1090,328,360,453,271,793
GDPD2_2_17849,597,284,251,378,338,314,184,58,113,652,1,1227
GEM_2_17850,297,153,164,165,285,111,85,49,314,49,261,338
GEN1_2_17851,362,177,217,303,1023,59,558,314,546,248,174,362
GFM2_2_17852,117,63,64,55,7,1,0,15,46,57,26,136
GFOD1_2_17853,26,64,32,21,14,5,28,2,155,0,0,159
GGCT_2_17854,643,552,527,666,1089,577,1461,205,479,651,365,310
GGCX_2_17855,19,21,62,18,21,265,91,62,0,0,0,0
GGT1_2_17856,947,770,674,676,827,346,1073,647,651,1202,903,332
GGT5_2_17857,83,188,69,127,6,0,0,527,220,239,220,268
GGT6_2_17858,146,217,93,70,64,0,0,50,0,1,0,111
GGTLC1_2_17859,845,613,630,582,1106,620,747,305,784,272,254,309
GLB1_2_17860,200,303,240,215,253,315,146,3,244,65,171,441
GLRX2_2_17861,339,532,392,450,313,300,1365,1084,415,565,532,790
GLRX3_2_17862,294,251,230,241,420,688,127,389,408,256,221,717
GLRX_2_17863,524,392,322,471,158,759,369,1038,142,1002,160,309
GLT8D1_2_17864,126,209,135,275,129,34,233,180,141,55,26,370
GLUL_2_17865,240,310,182,489,888,49,177,65,340,543,8,102
GM2A_2_17866,598,460,543,402,0,215,772,208,0,489,850,360
GMPPA_2_17867,114,184,192,59,299,506,0,1,100,155,10,26
GMPPB_2_17868,423,230,291,315,721,13,663,0,134,258,1339,352
GMPR2_2_17869,211,224,248,162,143,502,365,66,100,804,17,101
GNAI2_2_17870,227,181,270,338,410,282,270,236,110,1,371,105
GNAL_2_17871,890,765,804,921,758,501,762,548,786,576,1567,1493
GNAO1_2_17872,768,668,737,843,841,1063,1483,772,571,893,1277,995
GNAT1_2_17873,0,0,0,0,0,0,0,0,0,0,0,0
GNB5_2_17874,243,113,93,167,132,21,176,135,49,346,401,99
GNG10_2_17875,180,72,134,158,290,0,259,291,217,444,511,77
GNG4_2_17876,790,792,661,810,438,655,1078,379,1539,759,1229,699
GNGT2_2_17877,475,380,449,505,517,397,272,171,233,659,864,516
GPD2_2_17878,53,220,183,132,0,0,86,0,69,381,26,235
GPHN_2_17879,1012,1318,1032,1256,604,1187,1016,1283,829,1631,521,1442
GPNMB_2_17880,178,106,167,227,170,287,261,20,329,62,190,27
GPT2_2_17881,300,206,265,418,449,218,171,557,288,822,713,108
GPX4_2_17882,176,349,223,272,138,327,152,4,175,426,1278,64
GSR_2_17883,102,110,197,165,70,386,521,84,106,11,69,14
GSTCD_2_17884,1347,919,986,1072,1872,377,664,989,1108,894,1090,961
GSTK1_2_17885,17,50,52,27,0,0,0,0,0,312,0,0
GSTM1_2_17886,97,133,158,138,135,560,120,25,139,667,281,151
GSTM2_2_17887,462,358,153,325,462,436,912,265,243,471,339,67
GSTM4_2_17888,149,126,242,107,0,0,789,0,1,0,90,153
GSTO1_2_17889,336,327,162,442,58,311,97,287,5,16,86,46
GSTO2_2_17890,1734,1533,1359,1424,1264,801,1406,934,1825,2218,1905,126
6
GSTZ1_2_17891,135,107,100,137,59,124,5,43,84,52,2,43
GTPBP3_2_17892,558,276,254,226,412,109,169,395,595,497,536,163
GUCY1A3_2_17893,451,751,499,565,1001,449,216,905,394,384,2320,1330

GYG1_2_17894,329,346,114,334,895,843,463,645,431,89,336,207
GYG2_2_17895,238,392,337,521,249,578,270,659,343,480,22,497
GYS1_2_17896,212,267,201,150,0,227,21,94,56,102,1,240
HADH_2_17897,134,138,84,34,3,154,399,381,310,14,6,232
HAGH_2_17898,35,49,39,61,36,0,0,1,35,245,5,10
HAO2_2_17899,743,566,670,591,408,602,602,709,424,310,259,851
HAS3_2_17900,272,178,264,273,393,194,13,163,570,52,141,496
HCCS_2_17901,1181,1144,877,1020,805,523,850,488,333,929,303,1921
HDHD1_2_17902,104,67,57,114,8,151,1,18,164,28,0,130
HENMT1_2_17903,120,135,225,179,409,0,350,1,242,8,27,271
HHAT_2_17904,436,341,283,351,263,380,575,57,125,459,364,201
HIBCH_2_17905,204,45,40,54,2,5,84,56,273,15,2,139
HLCS_2_17906,123,40,77,94,73,147,130,0,95,52,20,53
HMBS_2_17907,469,343,352,380,535,290,176,1052,194,182,336,255
HMGA2_2_17908,673,724,656,967,1028,402,924,388,762,253,641,301
HMGCL_2_17909,688,629,487,853,511,363,657,529,364,256,412,1313
HMGCLL1_2_17910,246,306,228,446,5,76,375,102,273,132,1,357
HMGCR_2_17911,124,87,42,96,33,0,70,204,232,186,0,112
HMGCS1_2_17912,93,143,102,148,0,1,11,287,14,241,401,125
HMGCS2_2_17913,1406,1231,1090,1294,1283,380,1763,1967,912,2474,707,185
0
HMOX2_2_17914,68,62,10,66,241,42,0,0,0,41,166,0
HNRNPAB_2_17915,150,119,122,164,90,175,58,53,110,307,367,85
HPD_2_17916,131,91,154,176,0,199,402,0,90,0,277,279
HPGD_2_17917,960,706,828,755,233,401,451,1541,1164,705,747,744
HPSE2_2_17918,154,88,90,168,169,131,263,134,189,78,150,44
HPSE_2_17919,252,145,258,191,366,92,445,37,224,143,574,250
HRAS_2_17920,459,357,328,303,792,107,541,342,201,447,107,57
HS2ST1_2_17921,9,11,17,81,5,0,45,0,28,0,0,0
HS6ST2_2_17922,101,52,59,79,0,27,489,11,351,6,3,48
HSD11B1_2_17923,1989,1722,1605,2012,2234,534,2980,547,1858,2832,1319,1
796
HSD17B10_2_17924,481,463,366,560,369,544,419,174,903,561,766,821
HSD17B13_2_17925,681,382,370,521,195,170,883,413,203,731,545,435
HSD17B4_2_17926,0,0,20,0,0,0,0,0,0,0,0,0
HSD3B2_2_17927,612,659,657,566,810,819,1212,708,563,557,1074,863
HSD3B7_2_17928,70,79,49,121,109,127,5,110,27,1,0,114
HSDL1_2_17929,136,184,88,142,19,98,1,256,59,425,142,190
HSP90AA1_2_17930,256,197,266,337,610,277,527,610,0,251,199,93
HSPA8_2_17931,227,162,116,101,398,14,26,376,30,0,15,357
HSPD1_2_17932,473,385,326,591,511,823,804,546,530,688,340,629
HTRA2_2_17933,148,47,97,86,669,51,22,69,60,32,26,104
HYAL1_2_17934,257,170,243,147,24,69,370,6,217,334,182,97
HYAL2_2_17935,339,375,596,661,5,69,80,145,182,35,610,1287
HYI_2_17936,177,97,128,140,2,167,0,411,4,26,218,272
IARS_2_17937,150,153,79,119,194,1,296,114,103,6,19,269
IDE_2_17938,593,527,626,615,385,121,812,190,1179,1224,452,1712
IDH3B_2_17939,294,226,128,152,310,254,1,641,249,34,62,119
IDH3G_2_17940,144,145,36,76,200,40,356,47,400,8,438,186
IDS_2_17941,159,64,116,111,60,13,326,36,106,0,93,238

IFT27_2_17942,143,208,217,136,22,88,30,283,68,14,475,95
IL4I1_2_17943,41,12,3,62,332,0,0,1,0,0,0,5
IMPDH1_2_17944,0,0,0,0,0,0,0,0,0,0,0,0
INMT_2_17945,41,30,34,21,2,0,0,67,18,2,0,56
INPP5K_2_17946,63,76,74,68,107,0,4,156,18,37,5,93
INTS6_2_17947,58,83,121,113,98,97,78,48,95,76,266,56
IPCEF1_2_17948,255,196,267,237,134,193,723,0,431,199,32,104
ISOC2_2_17949,312,267,127,346,6,152,31,143,116,14,0,625
ISPD_2_17950,568,511,655,661,855,580,620,919,163,567,1093,478
ISYNA1_2_17951,213,209,159,182,462,1,16,52,451,203,142,22
ITPA_2_17952,261,175,171,245,315,151,793,208,480,324,101,187
IVD_2_17953,61,67,113,111,209,45,317,94,143,62,0,110
IYD_2_17954,581,661,542,637,487,449,300,604,559,1243,564,839
JMJD7-PLA2G4B_2_17955,295,266,740,646,32,553,592,503,892,415,465,818
KARS_2_17956,74,38,20,25,5,0,0,0,0,15,0,0
KATNA1_2_17957,598,813,560,762,89,619,268,9,1100,379,162,1523
KATNAL1_2_17958,1926,2422,1959,2406,1830,1750,1168,3266,1004,3113,1402
,2234
KIF16B_2_17959,1923,1489,1723,2177,2628,2201,1598,1918,1740,1589,1419,
1597
KIF9_2_17960,183,159,46,103,189,140,20,311,7,57,14,121
KIFC3_2_17961,198,89,124,153,1,246,0,314,9,18,1,1
KLK2_2_17962,698,601,722,680,376,151,1225,552,528,800,1744,603
KLK7_2_17963,137,128,157,195,230,1,7,0,355,42,25,246
KRAS_2_17964,144,331,435,274,319,193,241,622,74,53,491,182
KYNU_2_17965,173,255,118,180,1,0,510,324,4,0,4,154
LAMP2_2_17966,224,88,202,174,381,37,1,145,307,152,0,234
LARGE_2_17967,593,719,700,1457,412,559,2486,253,877,1291,1410,165
LCLAT1_2_17968,321,264,301,280,73,319,320,305,175,334,134,221
LCMT1_2_17969,749,516,536,592,1345,693,220,233,765,632,1212,235
LDHA_2_17970,5405,4963,5345,5863,7861,4068,8815,4130,5179,5040,5658,59
16
LDHAL6A_2_17971,4575,4208,3887,4271,4008,2835,5078,3223,4330,3632,3881
,4118
LDHB_2_17972,1405,990,857,1118,932,611,538,1609,802,501,702,2156
LDHC_2_17973,70,32,50,33,121,135,28,123,8,91,83,23
LDHD_2_17974,67,49,27,54,111,185,71,104,0,7,0,49
LEPRE1_2_17975,29,83,48,84,2,0,159,0,8,32,0,19
LEPREL1_2_17976,400,463,464,387,102,513,202,54,464,796,1039,339
LFNG_2_17977,45,35,56,9,27,118,0,50,22,0,0,59
LGMN_2_17978,389,132,181,226,529,242,454,472,192,509,30,1
LGSN_2_17979,792,869,633,990,639,807,1275,1117,894,999,1029,885
LIAS_2_17980,161,315,387,110,1,465,246,1,10,2,513,986
LIG3_2_17981,84,98,117,142,179,332,757,2,3,74,50,96
LIG4_2_17982,530,549,570,486,1143,1268,436,609,360,1039,256,539
LIPF_2_17983,288,418,320,367,285,121,642,9,67,78,330,167
LIPT1_2_17984,117,88,93,111,344,84,0,183,325,35,4,128
LMO7_2_17985,123,93,124,167,418,15,0,1,9,0,86,3
LNPEP_2_17986,354,114,139,236,127,8,71,23,604,115,291,26
LOX_2_17987,307,201,340,350,465,41,541,106,105,495,4,109

LPO_2_17988,47,25,19,52,5,1,0,155,3,69,0,0
LRRC16A_2_17989,691,757,795,780,171,469,677,421,771,1047,528,1048
LSS_2_17990,337,351,413,428,913,98,359,714,72,371,359,50
LYZL6_2_17991,1572,1143,1363,1085,617,933,828,708,1335,2454,1354,1498
MACF1_2_17992,138,26,139,37,163,1,1,2,26,49,328,6
MACROD2_2_17993,731,622,775,565,1145,179,573,29,471,919,605,1057
MAD2L2_2_17994,200,146,88,103,642,0,278,1,5,46,58,148
MAN2B1_2_17995,187,349,333,250,3,74,35,322,121,599,18,440
MASP2_2_17996,40,61,38,95,127,1,0,0,70,22,318,633
MAT2B_2_17997,228,252,206,152,935,477,362,282,233,228,2,88
MCAT_2_17998,245,179,457,231,38,45,385,8,0,0,122,163
MCM4_2_17999,1324,1258,1270,1233,1330,789,907,947,1343,748,979,650
MCM7_2_18000,807,590,542,736,432,476,382,794,1104,844,245,1222
MCM8_2_18001,932,1062,778,1127,734,622,609,620,621,788,1275,881
MCM9_2_18002,603,433,581,723,1071,558,932,337,466,100,470,582
MDH1_2_18003,361,317,465,487,302,652,292,261,564,472,168,322
MECR_2_18004,102,93,189,135,355,1,560,3,11,9,344,11
MEPCE_2_18005,419,524,524,347,604,5,937,799,762,939,866,150
METTL13_2_18006,168,179,157,199,175,24,96,306,151,166,135,244
MFN2_2_18007,777,398,572,737,159,153,203,983,263,500,1115,683
MFNG_2_18008,185,154,132,177,290,136,2,163,434,38,272,253
MGAT3_2_18009,37,27,9,5,60,2,0,35,13,3,32,31
MGAT4A_2_18010,235,261,223,256,29,208,792,10,120,239,64,467
MGAT4B_2_18011,143,127,115,119,39,188,257,58,161,126,164,228
MGLL_2_18012,442,622,358,576,104,317,90,595,525,894,888,417
MGST1_2_18013,220,401,219,255,191,470,216,66,311,196,297,165
MGST2_2_18014,50,31,10,53,41,0,43,20,14,270,0,2
MICAL1_2_18015,111,36,87,147,85,0,0,82,74,7,1,37
MLH1_2_18016,694,561,757,686,1038,252,1196,635,1049,1188,1293,318
MME_2_18017,0,0,0,0,0,0,0,0,0,0,0,0
MMP1_2_18018,116,37,88,90,120,43,58,46,66,45,189,3
MMP2_2_18019,798,639,737,624,903,547,1006,707,434,229,366,187
MOCS1_2_18020,78,85,129,73,213,0,21,4,98,20,30,127
MOGS_2_18021,297,157,187,309,38,20,0,92,141,355,81,561
MOV10_2_18022,478,321,484,529,450,643,460,351,545,591,536,725
MOV10L1_2_18023,269,305,320,467,231,243,313,425,270,523,277,325
MPG_2_18024,287,187,239,422,36,18,199,248,101,738,661,236
MPPE1_2_18025,217,233,196,313,75,13,36,331,278,387,199,137
MPST_2_18026,72,51,48,77,2,15,1,260,33,75,49,5
MRAS_2_18027,177,79,118,160,70,50,443,30,269,2,42,107
MRE11A_2_18028,1362,1204,1165,1397,1268,886,1144,834,956,1437,548,757
MRI1_2_18029,124,117,100,57,0,219,0,0,297,102,296,218
MSH5_2_18030,406,412,394,638,575,406,2,832,102,278,992,89
MSRA_2_18031,654,618,421,541,79,656,275,133,532,325,178,775
MSRB3_2_18032,24,14,61,12,39,0,17,0,3,0,0,19
MTHFD1L_2_18033,269,255,254,415,347,85,147,218,61,225,9,156
MTHFS_2_18034,363,444,342,526,971,67,291,120,473,676,358,478
MTO1_2_18035,358,429,297,277,380,503,364,666,403,90,218,454
MTRR_2_18036,1400,1093,1039,1075,1286,1054,1120,1414,1136,1425,1144,10
22

MUTYH_2_18037,1174,946,1231,1274,1143,659,708,1348,1055,434,2426,544
MX1_2_18038,338,343,358,458,67,261,393,560,72,1349,1837,715
MYBBP1A_2_18039,20,15,17,14,0,0,0,0,0,23,0,1
MYH2_2_18040,426,512,353,369,151,152,618,342,423,430,697,533
MYO5A_2_18041,3,32,30,41,0,0,0,0,0,0,0,0
MYO7A_2_18042,58,47,53,97,14,3,180,37,32,138,11,43
MYO9B_2_18043,128,24,65,48,234,22,693,0,104,0,1115,167
N6AMT1_2_18044,634,333,270,426,155,207,224,381,3,257,182,251
NAA16_2_18045,132,74,85,64,146,145,120,123,17,60,2,99
NAA20_2_18046,160,118,65,51,69,2,0,1,107,8,13,124
NAAA_2_18047,0,0,13,0,0,0,0,0,0,2,0,28
NAPEPLD_2_18048,453,505,363,639,668,1022,122,396,475,396,295,208
NARF_2_18049,141,239,249,160,104,232,0,219,150,174,200,13
NARS2_2_18050,711,856,580,806,463,831,1177,776,300,1187,1184,802
NAV1_2_18051,31,12,33,8,3,1,0,103,8,0,1,34
NCEH1_2_18052,257,221,410,398,91,223,23,7,135,46,667,211
NCF2_2_18053,67,50,55,112,1,20,25,82,134,16,1,27
NCF4_2_18054,618,294,502,391,430,458,643,260,636,338,80,879
NDOR1_2_18055,627,652,642,582,832,464,816,932,284,824,555,367
NDUFA2_2_18056,194,264,177,215,1036,138,152,0,601,26,212,175
NDUFB11_2_18057,38,50,33,20,19,253,36,140,92,75,3,46
NDUFB4_2_18058,163,41,54,127,4,2,155,62,2,72,11,13
NDUFB5_2_18059,134,43,71,73,3,26,8,0,85,104,1,8
NDUFB6_2_18060,602,484,432,438,619,319,349,937,661,285,1021,422
NDUFC1_2_18061,137,106,239,247,12,37,42,347,365,37,693,693
NDUFS1_2_18062,235,188,179,266,3,21,52,7,82,504,133,444
NDUFS2_2_18063,262,329,360,328,1000,107,247,602,70,185,642,438
NDUFS5_2_18064,1137,992,1179,1056,1765,788,657,2737,1275,606,482,2078
NDUFV1_2_18065,27,27,32,25,120,0,30,154,31,2,1,53
NEIL2_2_18066,73,97,90,50,17,102,96,115,52,2,73,163
NEU4_2_18067,574,505,713,697,982,252,691,474,295,466,781,377
NFS1_2_18068,376,282,432,415,230,471,149,152,471,457,193,221
NGLY1_2_18069,72,24,27,40,311,54,0,15,302,0,0,38
NIPSNAP1_2_18070,472,705,555,519,410,173,268,923,251,949,197,706
NIT1_2_18071,873,803,863,766,939,956,1623,1061,1015,805,459,1028
NKIRAS2_2_18072,122,142,135,131,13,0,106,35,233,4,169,85
NLGN3_2_18073,250,338,229,274,503,293,528,226,314,67,458,260
NLGN4X_2_18074,631,601,492,788,731,534,994,550,871,847,1802,577
NLGN4Y_2_18075,355,169,287,323,710,40,466,427,48,323,246,397
NMNAT2_2_18076,0,0,0,0,0,0,0,0,0,0,0,0
NNT_2_18077,692,648,812,894,202,642,312,1611,217,404,135,972
NOX4_2_18078,142,135,115,94,764,90,156,24,345,4,126,520
NPL_2_18079,927,799,529,834,1215,461,914,812,1045,1168,1049,877
NQO1_2_18080,53,152,59,89,0,90,0,19,2,12,83,109
NRD1_2_18081,137,91,72,71,12,266,116,13,404,2,31,102
NSDHL_2_18082,323,327,367,376,478,1452,1067,458,392,755,566,1233
NSUN2_2_18083,210,308,260,232,434,572,17,8,221,328,6,233
NUDT2_2_18084,128,141,154,141,11,190,561,77,67,319,644,118
NXN_2_18085,224,155,143,188,594,0,7,290,199,24,21,151
OAS1_2_18086,425,349,445,321,188,179,377,366,614,239,243,351

OAS2_2_18087,626,619,578,591,278,1141,1192,879,897,234,345,897
OASL_2_18088,1718,1320,983,1445,2518,415,1187,515,881,1479,830,1014
OAT_2_18089,724,636,544,717,1334,371,922,0,334,242,1005,536
OGDH_2_18090,971,845,752,1172,1320,1102,1604,1286,1077,1054,851,1045
OGDHL_2_18091,2,0,9,18,0,0,0,0,0,0,1,0
OGG1_2_18092,1743,1814,1680,2361,1384,332,2181,1342,557,2630,1636,2542
OGT_2_18093,138,77,56,112,185,260,4,281,44,49,0,48
OLAH_2_18094,254,459,458,599,12,243,105,4,202,370,410,296
P4HA1_2_18095,714,710,481,882,607,203,1093,753,1085,1150,1241,659
P4HA2_2_18096,55,100,26,88,0,0,4,543,109,0,64,1
P4HTM_2_18097,135,184,172,191,66,456,163,48,56,19,54,274
PAFAH1B2_2_18098,266,114,168,85,194,29,20,218,138,208,19,395
PAFAH1B3_2_18099,183,195,103,127,82,98,202,138,138,632,3,99
PAICS_2_18100,5,2,12,19,0,0,0,0,25,60,1,0
PAOX_2_18101,182,195,91,297,262,142,70,146,121,657,265,280
PAPD4_2_18102,340,649,472,478,826,110,33,404,377,331,634,130
PAPD5_2_18103,1406,1531,1263,1637,2722,1159,1463,1405,1261,2406,1392,1
539
PAPD7_2_18104,1154,998,1127,1076,1518,469,366,1877,1441,1573,561,1009
PAPSS2_2_18105,824,828,776,1051,1251,595,826,2092,859,339,1470,2083
PARK7_2_18106,158,218,259,155,188,4,94,260,2,362,333,105
PARL_2_18107,1305,1287,1314,1474,454,1802,1298,1323,1740,1248,1329,121
8
PARN_2_18108,469,217,386,261,524,347,466,126,496,265,397,166
PARP2_2_18109,195,202,209,372,0,418,963,142,0,134,1,372
PARP3_2_18110,640,469,471,611,837,557,730,247,683,478,828,712
PARP8_2_18111,765,828,668,752,740,248,880,1408,535,477,286,1093
PBLD_2_18112,299,248,305,234,404,318,579,55,241,345,585,563
PCCA_2_18113,85,114,67,167,8,380,3,129,1,307,134,472
PCCB_2_18114,208,48,128,154,115,0,46,176,141,42,143,111
PCMTD2_2_18115,282,193,120,132,247,382,96,625,430,75,90,148
PCNA_2_18116,1277,1254,968,1247,1480,1026,907,1334,1421,1137,1356,1194
PCSK1_2_18117,20,73,41,75,0,0,33,66,0,5,85,3
PCSK2_2_18118,350,170,143,215,75,221,354,286,175,211,37,95
PCYT1B_2_18119,518,430,433,562,883,157,7,918,674,211,428,203
PCYT2_2_18120,610,399,320,342,32,352,119,191,95,454,621,225
PDE10A_2_18121,73,71,58,56,53,0,1,206,0,80,0,83
PDE11A_2_18122,131,171,85,53,305,3,654,1,206,51,98,0
PDE1A_2_18123,23,26,84,69,24,48,37,35,41,53,0,21
PDE1B_2_18124,59,60,58,14,94,1,118,38,6,0,4,33
PDE1C_2_18125,78,85,153,110,5,61,240,0,80,173,37,31
PDE2A_2_18126,94,142,60,184,66,218,37,4,78,5,242,285
PDE4A_2_18127,165,61,43,152,57,5,1,721,148,276,73,239
PDE4B_2_18128,352,408,204,242,269,367,35,246,327,358,0,201
PDE4C_2_18129,44,54,86,187,26,114,10,39,25,17,0,54
PDE4D_2_18130,136,76,78,160,108,4,199,254,46,316,120,26
PDE5A_2_18131,729,901,769,541,56,408,1008,396,767,1837,1048,1389
PDE6B_2_18132,240,155,122,215,66,8,7,144,346,17,187,170
PDE7A_2_18133,3276,3397,3261,3506,2389,3583,3697,2741,2784,4718,2884,3
753

PDE8A_2_18134,502,453,448,382,281,296,417,428,207,343,527,381
PDE8B_2_18135,443,427,524,528,976,553,1139,558,834,1230,148,289
PDE9A_2_18136,135,174,204,247,372,122,484,293,19,137,13,203
PDHA1_2_18137,241,124,118,253,67,247,33,239,199,87,134,334
PDHB_2_18138,603,523,399,646,460,277,535,236,385,643,110,1103
PDHX_2_18139,197,405,412,358,376,331,696,496,187,1,236,465
PEMT_2_18140,197,208,153,144,30,544,173,0,229,185,262,26
PEPD_2_18141,205,31,76,85,69,0,1,700,834,296,0,0
PGAM5_2_18142,82,116,109,80,60,49,1,23,41,9,510,102
PGBD1_2_18143,1110,801,593,733,1242,289,1650,502,526,585,868,1691
PGC_2_18144,172,37,44,88,40,0,2,44,5,245,11,127
PGM1_2_18145,435,387,452,637,501,673,292,277,830,688,580,910
PGM3_2_18146,0,0,0,0,0,0,0,0,0,0,0,0
PHOSPHO1_2_18147,22,11,20,19,87,1,1,0,30,225,4,0
PHOSPHO2_2_18148,193,147,127,200,3,28,6,199,79,213,118,188
PHYH_2_18149,928,746,537,689,561,315,80,2159,387,425,1122,529
PIGA_2_18150,1278,840,936,1052,773,415,818,1644,1710,880,866,961
PIGC_2_18151,644,442,283,461,563,589,494,188,591,553,156,248
PIGF_2_18152,72,37,43,77,3,2,18,20,1,15,0,155
PIGG_2_18153,136,64,96,71,144,0,20,0,31,18,502,0
PIGN_2_18154,1210,1189,926,1193,750,342,1591,172,1021,2320,470,822
PIGO_2_18155,114,110,78,93,21,88,162,151,194,32,24,9
PIGP_2_18156,453,224,288,299,143,116,86,662,133,142,112,114
PIGQ_2_18157,810,816,969,958,927,207,1011,1391,335,598,317,837
PIGT_2_18158,197,231,196,297,61,526,170,800,231,314,240,106
PIGV_2_18159,510,228,372,417,712,506,652,393,469,225,325,556
PIN4_2_18160,74,76,44,44,150,357,276,32,188,177,33,56
PLA1A_2_18161,112,87,99,62,0,284,252,57,92,190,0,14
PLA2G2A_2_18162,385,397,603,418,0,346,1,515,478,277,166,105
PLA2G4C_2_18163,217,182,293,360,178,128,512,119,417,84,143,364
PLA2G6_2_18164,289,271,228,158,269,0,17,227,22,72,609,130
PLA2G7_2_18165,985,981,809,1197,332,1515,539,608,1657,1580,665,611
PLAT_2_18166,81,46,66,28,3,0,351,10,0,1,11,11
PLAU_2_18167,396,643,356,462,512,662,534,128,603,153,1040,203
PLB1_2_18168,70,75,52,147,0,38,7,2,12,453,106,236
PLCB1_2_18169,38,24,57,15,105,0,0,0,11,108,1,80
PLCB3_2_18170,82,73,119,102,300,0,1,0,16,5,0,214
PLCB4_2_18171,2171,2091,1837,2357,1932,2699,1684,2802,1695,1884,1596,3
063
PLCD1_2_18172,466,385,549,405,0,162,1172,18,54,833,713,273
PLCE1_2_18173,538,630,496,744,1474,174,492,498,403,1160,343,535
PLCG1_2_18174,94,75,106,97,342,1,18,226,29,667,9,1
PLCH1_2_18175,210,366,250,147,431,410,139,478,222,49,730,259
PLCL2_2_18176,505,349,349,435,478,556,285,171,454,366,759,809
PLCXD2_2_18177,647,432,465,600,635,9,424,534,619,1382,95,213
PLD2_2_18178,455,503,548,696,351,392,753,596,611,613,285,655
PLD3_2_18179,142,125,79,105,546,38,1,119,63,21,0,403
PLOD2_2_18180,2557,2616,2054,2303,2670,1863,2422,1568,2473,3176,2389,1
408
PLSCR3_2_18181,380,316,442,363,165,16,869,205,537,359,425,995

PLSCR4_2_18182,292,159,231,181,180,0,170,638,375,245,100,70
PMEL_2_18183,1038,583,988,1061,1642,613,1874,366,1018,265,672,1422
PMS1_2_18184,617,637,497,771,174,167,642,518,602,675,249,779
PNPLA1_2_18185,687,544,504,587,491,1063,1026,668,727,447,824,600
PNPLA4_2_18186,797,658,577,725,429,196,265,1104,389,885,166,862
PNPLA6_2_18187,292,208,261,287,550,459,346,209,74,147,154,90
PNPLA7_2_18188,21,4,6,23,18,3,41,21,26,2,98,150
POFUT1_2_18189,150,145,80,77,182,2,13,60,42,94,270,8
POFUT2_2_18190,361,307,226,241,423,165,358,627,271,98,54,63
POGZ_2_18191,70,125,59,50,0,0,0,13,163,56,108,74
POLD2_2_18192,100,62,30,39,133,0,0,0,164,4,70,0
POLE2_2_18193,45,36,27,51,171,0,124,17,2,0,0,2
POLG_2_18194,121,73,68,143,247,40,14,101,27,226,12,452
POLL_2_18195,276,347,240,306,396,240,601,425,483,501,287,464
POLR1D_2_18196,425,544,516,689,266,468,36,1171,247,604,2076,356
POLR3B_2_18197,1210,851,901,892,468,406,888,1687,1091,1328,764,699
POLR3H_2_18198,27,20,19,43,2,1,4,0,20,0,0,6
POMT1_2_18199,897,1072,1288,1214,586,1207,168,265,664,1304,1182,1052
PON2_2_18200,1733,1631,1538,1866,2906,1755,1466,1056,1469,2368,1294,12
88
POP5_2_18201,145,123,58,142,152,2,93,67,42,23,0,65
PPA2_2_18202,68,72,103,105,2,78,6,264,156,175,25,122
PPIE_2_18203,81,12,45,36,66,0,24,63,160,146,1,46
PPIL2_2_18204,515,279,386,429,599,346,511,358,197,283,478,45
PPIL3_2_18205,1103,720,918,1184,538,589,774,1166,1348,1439,763,790
PPIL6_2_18206,1176,975,839,1132,566,1239,1280,703,1174,1308,892,2538
PPIP5K1_2_18207,122,95,145,233,173,25,67,808,6,235,20,504
PPT1_2_18208,632,486,482,710,644,383,577,394,940,806,570,686
PPT2_2_18209,191,186,146,136,205,515,336,89,272,357,93,45
PRCP_2_18210,986,1216,1015,1258,1666,1014,621,177,892,1922,554,1283
PRDX1_2_18211,13,3,39,71,0,16,0,4,0,0,87,406
PRDX3_2_18212,1134,942,1104,1333,1503,875,1143,1689,1058,713,1217,1306
PRDX5_2_18213,285,381,442,386,502,276,325,743,249,386,376,432
PRKCSH_2_18214,311,494,400,452,158,498,240,240,81,567,459,388
PRODH_2_18215,169,78,27,67,0,0,0,374,0,0,0,0
PRSS3_2_18216,88,31,67,88,24,232,0,13,258,146,0,27
PSAT1_2_18217,1277,862,1259,1491,643,448,1693,244,1307,1428,1880,1652
PSMA1_2_18218,161,143,118,173,22,33,201,356,103,349,1,84
PSMA3_2_18219,667,862,756,941,734,191,700,1143,744,1176,466,710
PSMA4_2_18220,245,265,246,297,280,243,117,12,279,165,17,273
PSMA5_2_18221,391,204,263,270,674,341,646,103,643,169,445,212
PSMA8_2_18222,489,532,701,904,151,882,442,8,729,1126,1356,738
PSMB2_2_18223,1154,1300,807,979,1440,494,729,113,861,1408,1407,976
PSMB5_2_18224,73,5,33,40,560,1,48,32,4,0,1,10
PSMB8_2_18225,642,681,822,833,382,286,936,658,822,306,809,952
PTBP1_2_18226,183,69,143,253,5,5,2,239,83,243,55,202
PTER_2_18227,187,148,187,189,0,0,7,328,242,227,0,38
PTGR1_2_18228,473,293,399,518,346,229,720,310,578,241,321,322
PTGR2_2_18229,92,64,165,136,39,70,3,5,1,23,0,20
PTGS1_2_18230,322,181,231,208,309,553,275,297,86,203,183,160

PUS1_2_18231,11,23,28,39,0,0,0,0,32,113,4,148
PYCR1_2_18232,9,45,19,14,45,0,0,0,20,9,0,6
PYGL_2_18233,482,269,277,393,349,496,186,608,311,62,191,987
PYGM_2_18234,111,108,89,86,126,124,22,16,127,8,16,53
QPCTL_2_18235,17,3,14,39,57,0,16,326,2,8,0,0
QSOX1_2_18236,638,528,530,583,365,528,205,1264,585,534,186,525
RAB11A_2_18237,355,368,368,387,437,179,352,501,144,422,640,663
RAB1A_2_18238,990,818,735,755,782,648,1046,35,1012,466,575,702
RAB23_2_18239,645,514,433,662,1050,600,209,152,472,532,550,727
RAB27A_2_18240,180,22,56,13,1,20,658,126,27,0,1,3
RAB28_2_18241,278,162,277,325,77,579,634,93,101,675,641,456
RAB2A_2_18242,121,371,183,276,392,6,109,152,63,124,0,59
RAB2B_2_18243,499,431,333,556,181,96,369,676,345,663,244,77
RAB34_2_18244,0,0,0,0,0,0,0,0,0,0,0,0
RAB35_2_18245,38,14,10,18,0,93,0,50,0,0,0,0
RAB37_2_18246,45,139,92,35,4,227,0,553,0,19,0,236
RAB40C_2_18247,112,174,192,196,149,149,455,209,67,119,4,54
RAB5C_2_18248,127,109,138,242,116,12,14,632,97,106,9,51
RAB6A_2_18249,448,410,311,372,309,118,460,225,185,337,237,703
RAB7L1_2_18250,664,350,491,807,999,52,722,670,487,590,106,737
RAB9A_2_18251,107,103,68,108,116,299,1,273,79,16,0,101
RABGGTA_2_18252,300,240,276,222,44,472,149,101,66,1,144,580
RABL2A_2_18253,734,617,769,1124,696,228,508,737,842,1708,393,930
RABL2B_2_18254,734,617,769,1124,696,228,508,737,842,1708,393,930
RAC1_2_18255,5563,5022,4715,6010,4414,3916,4377,6084,5088,7234,4284,68
67
RAD51B_2_18256,24,17,34,12,3,53,1,0,0,0,0,0
RAD51_2_18257,407,559,647,614,252,773,622,370,460,462,63,275
RAD51D_2_18258,94,20,1,22,5,161,0,49,0,0,0,0
RAD54B_2_18259,222,56,102,157,4,51,35,36,80,0,98,202
RAD54L_2_18260,724,505,599,757,375,949,448,911,1247,858,1200,303
RAD9A_2_18261,574,620,556,786,503,530,338,709,211,166,341,934
RAP1A_2_18262,709,535,519,715,741,497,431,246,690,108,451,684
RAP1B_2_18263,488,351,384,519,74,178,2214,190,264,769,961,373
RBBP8_2_18264,446,367,483,644,405,166,313,108,371,178,289,465
RDH13_2_18265,668,568,737,813,654,529,974,985,662,1270,549,629
RDH5_2_18266,125,51,113,73,288,93,17,352,295,189,166,139
RECQL5_2_18267,311,274,307,415,354,207,334,474,354,768,74,374
RECQL_2_18268,1145,928,997,938,1375,755,1546,209,2045,884,717,650
RERG_2_18269,246,308,263,268,165,471,418,284,548,750,140,200
REV1_2_18270,2849,2771,2737,2626,1690,1419,2718,2767,2748,4458,1803,34
23
RFC3_2_18271,0,0,0,0,0,0,0,0,0,0,0,0
RFC5_2_18272,213,383,479,363,648,229,672,175,555,5,1032,292
RGN_2_18273,4555,4064,3286,3605,3206,2125,5611,3705,3418,2014,4229,304
0
RGS11_2_18274,144,153,155,244,0,322,91,0,51,45,261,377
RGS6_2_18275,439,540,360,696,709,517,14,73,663,495,145,892
RHOC_2_18276,842,710,734,1045,1120,1207,760,1008,615,450,1535,697
RHOT1_2_18277,573,560,508,606,1308,559,358,394,688,682,126,527

RNASE1_2_18278,188,199,121,315,160,181,302,931,30,426,394,124
RNASE4_2_18279,43,57,76,66,108,53,2,56,258,156,0,10
RPAP3_2_18280,520,593,389,445,304,360,605,821,855,526,869,583
RPE_2_18281,415,491,387,331,322,557,261,34,376,352,983,388
RPN2_2_18282,9,12,81,28,0,0,56,0,146,6,0,25
RPP14_2_18283,358,239,274,317,178,4,1438,338,64,60,95,149
RPP21_2_18284,55,64,89,81,0,0,19,5,2,17,1,303
RPP30_2_18285,1318,1269,978,1579,1259,613,1458,1421,1244,908,1650,1266
RPP38_2_18286,90,270,127,349,117,84,0,1,0,60,329,53
RPS27A_2_18287,47,90,65,22,122,0,10,119,33,12,0,34
RRAD_2_18288,435,572,305,608,756,394,533,646,161,25,488,1039
RRAGB_2_18289,559,437,580,660,122,570,827,133,579,994,496,321
RRAS2_2_18290,905,719,665,698,1548,468,1779,514,683,932,649,702
RRM2B_2_18291,604,526,437,511,710,442,435,1057,551,1489,276,861
RRM2_2_18292,387,193,452,241,105,191,502,81,294,22,497,401
RTEL1_2_18293,400,335,400,482,773,0,413,0,867,1027,913,298
SAR1A_2_18294,1040,825,617,683,797,1169,963,545,868,659,790,766
SAR1B_2_18295,561,582,592,704,413,365,1407,317,850,528,871,164
SARDH_2_18296,325,370,291,417,207,1010,1,352,938,410,221,223
SARS2_2_18297,978,688,992,1251,1405,2055,927,1429,744,1919,461,1775
SBNO1_2_18298,739,624,648,676,801,824,728,556,618,1006,268,524
SC5DL_2_18299,322,177,197,481,260,368,305,60,360,55,320,244
SCD5_2_18300,472,308,448,435,161,142,573,448,367,269,715,323
SDC1_2_18301,0,0,0,0,0,0,0,0,0,0,0,0
SDCBP_2_18302,477,207,311,367,530,149,84,5,222,44,89,951
SDHC_2_18303,1124,713,853,903,1049,250,1177,812,538,745,2132,550
SEPHS1_2_18304,544,391,335,407,541,654,106,506,75,256,179,179
3-Sep_2_18305,277,232,257,285,479,400,85,74,342,77,75,159
4-Sep_2_18306,502,473,593,344,257,459,838,491,743,450,434,488
5-Sep_2_18307,88,150,88,152,90,144,155,249,117,9,77,70
9-Sep_2_18308,921,725,621,642,986,800,1290,936,376,1259,747,1129
SGMS2_2_18309,733,683,597,701,342,528,1275,437,284,679,586,407
SH3GLB1_2_18310,265,224,222,289,1051,0,909,0,7,351,0,579
SHMT1_2_18311,30,35,76,69,63,23,0,0,28,12,6,355
SHMT2_2_18312,50,42,50,53,0,8,198,4,320,176,62,95
SIAE_2_18313,272,248,150,273,128,216,320,311,590,46,88,269
SLFN11_2_18314,1076,1074,884,927,951,987,2171,2625,885,786,1038,1278
SMG6_2_18315,291,240,218,221,657,249,120,147,92,648,141,89
SMOX_2_18316,312,204,234,415,138,4,294,703,367,473,197,349
SMPD1_2_18317,166,72,78,91,34,142,294,332,24,2,0,272
SMPD4_2_18318,212,40,69,112,57,9,0,44,50,119,5,100
SMPDL3B_2_18319,999,871,1032,1132,1657,1788,2111,544,1052,1211,1038,41
9
SOD2_2_18320,322,467,533,411,873,708,823,304,20,905,1112,409
SPAM1_2_18321,1282,1433,1012,1342,1157,808,2539,944,1211,1059,1770,386
SPAST_2_18322,221,429,341,443,145,227,459,170,117,335,520,278
SPG21_2_18323,186,193,174,218,1,162,0,0,111,11,686,346
SPO11_2_18324,255,343,331,461,357,44,147,13,510,83,16,386
ST3GAL1_2_18325,63,82,81,68,3,0,377,0,1,2,368,102
ST3GAL5_2_18326,346,516,424,282,96,23,1,78,455,402,328,271

ST5_2_18327,77,52,30,98,50,0,39,206,36,11,0,0
ST6GAL1_2_18328,521,530,644,662,251,716,257,679,628,288,1351,622
ST6GAL2_2_18329,485,618,540,439,455,830,1132,104,750,1056,435,475
ST6GALNAC3_2_18330,213,129,241,475,297,48,74,272,20,12,471,471
ST6GALNAC4_2_18331,151,398,240,240,234,436,65,34,23,152,437,78
ST8SIA4_2_18332,273,275,185,278,1,297,574,5,335,271,505,48
SUCLG2_2_18333,0,0,0,0,0,0,0,0,0,0,0,0
SULF1_2_18334,20,7,32,79,1,8,68,10,85,1,0,0
SULF2_2_18335,485,421,447,600,532,190,68,65,783,600,310,811
SULT1A1_2_18336,1178,1270,864,1060,495,859,1543,218,1043,1171,1040,134
1
SULT1A2_2_18337,2419,2432,2415,2709,2539,5091,2834,2646,3121,2215,3178
,3090
SULT2B1_2_18338,665,678,610,713,293,424,850,647,566,380,70,520
SUOX_2_18339,503,517,623,305,422,934,300,747,374,478,459,141
SYTL1_2_18340,83,55,169,126,10,0,0,0,30,133,0,0
TAB1_2_18341,3,28,33,11,0,36,0,0,0,3,0,0
TBXAS1_2_18342,1134,695,884,1133,1523,965,602,930,1255,1012,1047,1156
TCIRG1_2_18343,98,154,93,146,65,270,0,344,94,218,9,103
TDP1_2_18344,883,966,649,953,954,293,774,217,1901,951,1655,1136
TERT_2_18345,146,200,232,281,25,3,36,770,18,3,346,467
TGM2_2_18346,131,138,104,100,372,30,301,1,325,174,7,228
TGM5_2_18347,1097,676,1013,1002,1536,897,1068,571,705,924,1649,1062
TH_2_18348,258,96,252,299,69,2,153,121,168,284,350,212
TIAM2_2_18349,317,284,306,401,326,348,0,15,857,137,245,43
TKTL1_2_18350,389,307,301,222,592,32,270,293,200,285,404,134
TMEM55B_2_18351,1240,1240,1259,1201,1592,2171,1359,1177,1140,1272,1353
,1493
TMLHE_2_18352,167,136,85,116,164,77,332,169,147,155,82,148
TMOD1_2_18353,135,154,153,187,183,0,264,0,124,3,14,317
TMX2_2_18354,1950,1752,1593,2282,944,1760,1263,1265,890,1817,2272,1399
TNNI2_2_18355,189,291,393,326,24,358,448,1,462,254,638,384
TP53I3_2_18356,109,181,127,156,18,0,0,0,328,382,413,32
TPI1_2_18357,121,49,69,70,118,10,6,0,216,301,0,74
TPST2_2_18358,72,32,39,54,1,32,161,24,26,48,0,20
TREX1_2_18359,15,0,6,10,0,0,9,0,100,0,3,0
TRMT1_2_18360,136,52,79,133,0,2,13,52,4,383,0,111
TRMT1L_2_18361,471,383,457,569,648,100,44,139,404,634,1103,847
TRMT2B_2_18362,16,35,39,83,199,0,1,0,0,74,423,1
TRPT1_2_18363,126,183,108,217,198,115,9,66,50,7,198,177
TSEN2_2_18364,361,253,338,354,284,58,62,686,280,106,658,551
TSEN34_2_18365,37,238,124,125,125,5,2,82,164,45,0,203
TTLL6_2_18366,24,6,21,20,0,0,137,57,10,5,1,1
TUBAL3_2_18367,253,298,196,201,688,0,581,303,347,284,118,552
TUSC3_2_18368,445,308,355,441,328,191,940,189,135,768,917,215
TXNDC16_2_18369,878,700,559,620,997,582,787,609,1028,650,72,253
TXNDC2_2_18370,196,181,174,203,189,397,276,588,215,154,52,180
TXNDC5_2_18371,1121,1020,979,1074,898,700,1419,538,1329,930,761,1167
TXNL4B_2_18372,838,799,872,949,1214,644,895,780,886,1055,599,904
TXNRD1_2_18373,253,179,178,210,128,158,24,55,40,381,9,42

TXNRD3_2_18374,327,209,317,409,257,653,178,533,22,0,0,310
TYMP_2_18375,324,236,315,298,505,1,419,22,149,303,0,700
UBA52_2_18376,296,248,277,249,63,350,75,122,5,250,263,737
UEVLD_2_18377,920,932,987,895,761,901,717,539,1269,1361,963,745
UGDH_2_18378,339,448,391,358,412,685,49,515,427,550,93,168
UGP2_2_18379,722,593,487,709,1037,56,207,89,337,898,818,1075
UGT1A6_2_18380,2360,1851,1903,2334,2071,1590,2963,958,2378,2240,2125,2
152
UGT2B10_2_18381,790,723,786,839,518,372,117,175,882,387,505,706
UGT2B28_2_18382,290,269,397,346,258,48,357,557,19,68,275,243
UGT3A1_2_18383,428,252,500,257,281,455,964,139,235,441,357,449
UGT8_2_18384,204,353,227,239,334,0,361,25,89,423,290,101
UNG_2_18385,643,594,580,594,703,319,394,869,1072,706,738,1259
UPP1_2_18386,89,132,137,218,123,319,47,18,15,18,0,33
UPP2_2_18387,133,108,73,127,21,5,636,34,95,66,16,77
UQCR10_2_18388,18,3,29,20,5,0,1,0,24,0,127,13
UQCRB_2_18389,496,618,578,413,423,105,100,13,582,285,828,795
UROC1_2_18390,537,610,696,617,557,285,559,443,389,708,70,571
VCL_2_18391,619,446,551,344,651,108,1455,447,206,319,556,449
VNN2_2_18392,233,211,274,315,185,99,193,146,706,106,158,99
VPS29_2_18393,451,352,368,385,411,76,529,40,266,139,310,392
WARS2_2_18394,252,279,169,185,3,117,8,249,14,145,351,10
WARS_2_18395,1225,1537,1274,1205,983,2414,1464,1165,1410,1472,1622,972
WBSCR22_2_18396,159,126,163,218,63,101,91,274,200,127,38,264
WDR46_2_18397,112,238,171,334,129,329,109,337,272,458,345,524
WFS1_2_18398,347,302,377,320,54,131,69,332,83,142,400,592
WRNIP1_2_18399,265,253,441,290,367,69,223,6,112,279,733,2
WWOX_2_18400,100,76,126,65,300,172,7,183,21,93,28,15
XPNPEP1_2_18401,92,71,94,125,116,150,8,105,312,96,11,171
XRCC3_2_18402,59,55,81,208,0,37,0,6,9,0,0,142
XRN1_2_18403,118,260,100,163,236,267,398,398,17,1,18,590
YWHAZ_2_18404,255,289,223,215,298,220,879,0,291,226,249,313
ZBED1_2_18405,97,228,75,156,2,12,462,29,14,43,25,77
ZCCHC11_2_18406,108,101,101,150,0,45,77,1,300,11,472,195
ZCCHC6_2_18407,192,154,168,171,443,14,42,99,110,84,188,144
ZDHHC15_2_18408,217,218,203,253,242,104,162,254,428,38,376,214
ZDHHC7_2_18409,467,236,358,484,278,516,1281,516,737,317,270,512
ACYP1_2_18410,280,354,269,377,78,26,13,422,244,329,233,212
APOBEC3F_2_18411,66,60,76,46,7,12,0,21,9,120,18,13
ASCC3_2_18412,338,391,346,329,633,141,96,176,305,260,265,321
ASPH_2_18413,184,148,206,179,37,427,53,49,53,20,75,17
ATP6V0E2_2_18414,94,106,119,133,137,750,1,0,186,82,260,132
C10orf2_2_18415,1044,1133,1034,1169,1078,862,1363,2081,1195,999,1402,1
880
CAPN3_2_18416,180,134,128,118,1,185,30,274,253,326,474,0
CHM_2_18417,340,378,486,520,509,157,132,1536,197,688,174,438
CTSC_2_18418,306,172,159,253,515,88,4,31,389,338,280,181
CYBRD1_2_18419,58,59,48,45,1,0,2,8,15,6,37,319
CYP3A5_2_18420,162,182,115,194,173,157,1,460,8,23,79,69
DIO2_2_18421,21,59,39,65,0,147,0,0,22,125,143,0

DNASE2B_2_18422,186,86,120,183,97,472,1,1,113,44,112,148
DPYD_2_18423,1750,2039,1426,1709,1353,836,1879,2234,1176,2259,473,1524
GBA3_2_18424,613,668,827,744,1218,257,477,309,726,425,1021,1038
GCNT2_2_18425,109,152,63,97,261,420,109,19,107,0,115,1
GLYAT_2_18426,226,160,230,261,326,337,144,282,138,201,150,325
GPLD1_2_18427,2645,2634,2152,2664,2106,1313,3478,4324,988,2925,2493,38
41
GPX1_2_18428,37,16,28,85,165,0,31,158,0,6,6,6
GPX5_2_18429,292,100,144,199,307,1,340,406,451,59,34,408
HNMT_2_18430,14,9,77,22,21,1,27,12,0,6,0,40
HOGA1_2_18431,53,29,86,98,0,317,0,29,0,0,145,2
HYAL3_2_18432,78,104,99,89,13,0,7,19,3,1,0,119
JPH2_2_18433,227,242,217,297,294,307,369,445,121,336,193,280
KLK8_2_18434,110,102,194,112,216,86,4,447,140,16,3,396
LAMA4_2_18435,248,311,380,315,167,739,83,28,139,250,178,548
LRR1_2_18436,3982,3434,3637,3556,2849,3224,3169,3129,3409,2663,3939,32
64
LRTOMT_2_18437,51,9,79,89,47,0,196,0,3,72,418,173
METTL1_2_18438,238,175,160,240,339,205,79,3,174,5,481,513
MOCS2_2_18439,669,542,487,516,447,792,300,372,583,921,1216,866
NDUFA11_2_18440,413,458,483,512,53,515,353,231,121,473,34,589
NDUFV3_2_18441,188,199,167,288,229,82,149,225,119,22,186,150
NMNAT3_2_18442,163,62,85,68,3,7,41,0,21,7,545,1
PDE4DIP_2_18443,907,1218,877,1339,862,1239,337,892,313,1185,1721,569
PLG_2_18444,922,611,690,636,349,334,373,961,864,474,357,751
PPCS_2_18445,161,258,187,145,150,373,40,86,178,29,229,313
PRDX2_2_18446,64,27,24,66,2,0,0,34,83,7,164,182
RAD51C_2_18447,260,86,203,142,188,145,366,59,52,89,90,64
RASD1_2_18448,0,0,0,0,0,0,0,0,0,0,0,0
RASL10A_2_18449,47,46,166,29,2,8,0,184,58,139,8,0
SLX1A_2_18450,201,165,492,390,39,378,967,139,330,439,602,310
SLX1B_2_18451,201,165,492,390,39,378,967,139,330,439,602,310
SPTLC1_2_18452,176,127,162,167,50,686,231,193,11,193,41,6
ST3GAL3_2_18453,12,77,66,47,0,0,50,0,2,531,0,0
VKORC1_2_18454,457,440,463,492,315,509,945,670,95,957,378,298
XPNPEP3_2_18455,482,293,248,279,122,149,382,108,323,262,175,294
GNAS_2_18456,206,55,80,101,14,1,27,7,0,51,93,0
GNAS_2_18457,198,102,186,182,79,210,156,26,352,96,1,647
GNAS_2_18458,15,27,61,16,171,11,0,80,3,0,0,0
A4GALT_2_18459,486,264,313,368,579,17,89,52,490,368,429,347
A4GNT_2_18460,281,292,338,334,113,224,92,60,664,980,704,365
AACS_2_18461,24,3,42,11,0,6,224,20,2,90,3,38
AADAC_2_18462,419,549,235,257,164,328,419,106,89,924,38,423
AARS2_2_18463,200,124,177,208,51,74,199,149,170,115,334,419
AARS_2_18464,1033,1033,1086,1156,881,919,196,611,966,875,760,1391
AASDH_2_18465,247,303,387,579,139,909,475,300,341,125,69,192
AASDHPPT_2_18466,2178,2146,1971,2085,1771,1490,1738,4327,2669,2070,214
0,2176
AASS_2_18467,98,26,53,76,132,4,150,60,186,1,284,285
ABHD14A_2_18468,611,636,351,402,707,163,524,0,479,549,1558,156

ABHD1_2_18469,248,345,242,267,99,304,466,437,86,595,121,218
ABHD3_2_18470,697,411,575,584,849,519,559,437,569,461,1215,454
ABHD5_2_18471,34,2,39,33,29,0,49,2,3,42,59,0
ABHD6_2_18472,663,482,854,653,422,401,1195,468,1479,357,1395,1116
ABHD8_2_18473,116,123,261,165,98,164,848,137,52,249,47,88
ABO_2_18474,145,79,112,37,597,0,0,37,176,22,315,35
ABP1_2_18475,240,294,468,313,556,297,120,267,291,546,131,681
ACAA2_2_18476,1362,976,1412,1286,1656,1156,1623,2306,1996,511,1148,107
8
ACACB_2_18477,108,37,109,93,60,86,23,311,23,29,0,22
ACAD11_2_18478,208,245,216,301,69,116,74,493,478,286,0,54
ACAD8_2_18479,699,744,563,693,640,880,922,654,793,574,613,992
ACAD9_2_18480,157,151,101,63,83,0,102,19,28,22,487,67
ACADL_2_18481,238,277,201,475,2,129,264,2,190,281,129,332
ACADSB_2_18482,514,616,680,682,679,522,1999,866,544,120,497,924
ACADS_2_18483,264,348,344,332,530,77,10,107,434,401,933,328
ACAT1_2_18484,597,541,756,766,422,676,590,458,215,789,1095,430
ACER1_2_18485,41,50,66,24,254,27,37,0,150,325,0,6
ACER2_2_18486,7,88,13,43,41,0,0,24,21,3,0,25
ACER3_2_18487,763,591,450,597,235,330,1221,459,1131,709,414,374
ACMSD_2_18488,536,389,246,389,0,109,537,0,1271,1268,0,166
ACO1_2_18489,691,372,422,658,282,72,1072,214,294,370,1388,617
ACO2_2_18490,76,81,119,87,222,114,55,57,4,342,9,56
ACOT12_2_18491,526,423,334,322,551,175,88,456,528,470,454,170
ACOT1_2_18492,833,625,674,959,475,540,560,140,1303,621,541,526
ACOT2_2_18493,192,412,334,391,78,189,1126,382,15,512,418,203
ACOT4_2_18494,148,76,103,64,21,0,32,46,131,27,43,14
ACOT6_2_18495,95,96,70,55,84,103,4,309,182,87,57,16
ACOT8_2_18496,243,155,177,236,71,379,150,484,162,1103,328,113
ACOX2_2_18497,1114,1064,911,1155,1018,748,560,702,918,114,627,1402
ACOXL_2_18498,659,548,598,620,540,639,1072,400,814,308,498,981
ACR_2_18499,576,276,484,462,148,371,107,695,782,676,494,495
ACSBG2_2_18500,349,469,381,435,661,502,0,233,287,694,346,335
ACSF2_2_18501,140,116,157,234,415,87,284,25,30,0,289,493
ACSL1_2_18502,467,671,452,654,82,348,874,960,166,759,215,404
ACSM1_2_18503,69,46,57,133,23,18,19,45,66,19,55,397
ACSM2A_2_18504,534,351,389,526,193,9,24,746,1106,492,325,143
ACSM4_2_18505,761,787,761,688,1014,1137,474,787,173,446,678,782
ACSM5_2_18506,115,154,175,154,117,118,85,132,271,205,60,76
ACSS1_2_18507,81,73,42,9,0,0,0,0,37,2,0,0
ACSS3_2_18508,650,544,583,846,611,377,198,745,1013,386,108,1222
ACTC1_2_18509,786,725,775,751,714,1016,515,569,613,728,1190,1477
ACY3_2_18510,290,204,282,307,302,102,810,115,44,810,62,152
ACYP2_2_18511,43,103,26,107,0,84,88,29,31,46,78,17
ADA_2_18512,28,1,29,16,0,1,2,15,0,2,0,43
ADAM10_2_18513,408,240,320,290,301,156,564,73,456,557,1042,294
ADAM17_2_18514,120,172,196,221,136,98,262,48,70,552,541,24
ADAMTS4_2_18515,348,248,124,235,4,551,375,14,139,422,282,5
ADARB2_2_18516,264,328,413,449,186,302,231,128,296,623,603,164
ADAT1_2_18517,338,223,95,249,56,12,0,10,147,66,289,72

ADAT2_2_18518,369,173,162,165,599,405,72,59,351,161,297,309
ADC_2_18519,109,55,93,90,456,0,5,0,126,0,395,203
ADCY1_2_18520,715,704,644,820,920,496,235,528,1093,937,426,1210
ADCY2_2_18521,285,234,286,299,262,9,471,518,275,572,261,110
ADCY7_2_18522,248,212,166,203,326,113,232,92,145,159,684,241
ADCY8_2_18523,1482,1143,1190,1609,1884,972,975,1565,1831,1021,353,2199
ADCY9_2_18524,0,0,0,14,0,0,0,0,0,0,0,65
ADH1A_2_18525,1882,1883,1931,1911,1777,2012,3314,1318,1904,1805,1663,2
120
ADH1B_2_18526,868,709,914,818,1089,566,1573,634,663,1006,710,1188
ADH1C_2_18527,1745,1161,1480,1654,424,2218,2222,711,1360,1687,1147,137
7
ADH4_2_18528,436,433,390,386,565,157,911,108,240,264,660,677
ADH5_2_18529,406,440,400,429,356,517,91,317,373,262,104,556
ADHFE1_2_18530,87,60,97,44,0,1,27,4,173,31,0,306
ADI1_2_18531,501,296,329,491,655,408,794,493,184,386,131,198
ADO_2_18532,965,790,859,846,1137,1154,1288,1313,1246,664,1316,1197
ADPRH_2_18533,131,105,120,148,286,194,0,3,138,26,31,134
ADPRHL2_2_18534,387,275,253,254,25,266,950,452,489,347,607,354
ADSS_2_18535,89,70,66,132,13,296,35,6,430,430,0,141
AEN_2_18536,44,63,67,64,96,50,0,1,5,115,0,207
AGBL2_2_18537,164,33,73,149,133,10,18,81,53,85,1,126
AGMAT_2_18538,1491,1588,1200,1520,1704,1082,1027,1819,2217,919,964,176
5
AGMO_2_18539,525,683,931,969,1533,456,1566,378,255,1493,1193,878
AGPAT4_2_18540,2521,1902,1830,1908,3217,3114,2713,3044,2028,4081,1914,
1921
AGPAT5_2_18541,216,280,166,228,82,45,0,216,561,111,65,326
AGPAT6_2_18542,459,627,418,491,1076,252,267,221,212,452,2052,1083
AGPAT9_2_18543,201,139,106,145,223,78,37,208,299,577,0,415
AGPS_2_18544,549,607,532,689,378,0,1465,198,165,767,1268,704
AGXT2_2_18545,2278,1856,1768,1981,3141,1886,1792,2180,1794,3334,713,23
33
AGXT2L2_2_18546,473,390,453,659,331,455,564,777,157,380,290,355
AGXT_2_18547,146,135,123,239,3,232,733,77,79,684,296,36
AICDA_2_18548,593,599,587,583,534,369,234,532,836,753,1161,1247
AKR1B10_2_18549,905,773,763,820,1492,415,571,1068,700,1582,2522,1152
AKR1B15_2_18550,92,76,85,141,37,0,187,220,54,33,5,57
AKR1B1_2_18551,163,110,120,195,28,460,239,89,95,107,5,125
AKR1C1_2_18552,1051,759,896,725,1366,542,1365,1666,369,676,595,1037
AKR1C3_2_18553,181,129,121,146,124,368,456,125,113,0,254,542
AKR1C4_2_18554,1051,759,896,725,1366,542,1365,1666,369,676,595,1037
AKR1E2_2_18555,319,291,172,232,313,777,686,57,31,96,736,197
AKR7A2_2_18556,0,20,25,10,0,0,0,0,0,0,0,0
AKR7A3_2_18557,214,166,312,224,4,0,117,92,294,0,361,475
ALAD_2_18558,171,170,145,151,235,463,0,25,28,664,0,1
ALDH1A1_2_18559,36,31,43,12,0,0,443,0,2,0,1,1
ALDH1A3_2_18560,352,425,187,393,109,2,370,20,68,160,25,425
ALDH1B1_2_18561,1194,936,1020,1095,397,1338,687,2581,444,1082,1737,177
8

ALDH1L1_2_18562,462,238,327,234,363,120,266,260,470,173,338,418
ALDH1L2_2_18563,4,0,2,2,0,0,0,0,0,0,0,0
ALDH6A1_2_18564,1492,1377,1400,1757,1228,1055,1390,1466,1833,2025,1625
,2394
ALDH9A1_2_18565,380,464,401,473,498,680,611,1021,284,813,515,410
ALDOB_2_18566,837,737,1078,803,862,26,982,559,227,994,1450,1193
ALDOC_2_18567,124,81,81,210,0,498,234,29,10,154,18,136
ALG10_2_18568,908,779,954,940,775,493,2083,384,1034,799,1313,994
ALG11_2_18569,1510,1305,953,1283,255,1673,691,1706,1758,3243,1085,1249
ALG12_2_18570,668,654,608,494,732,1050,804,1155,817,914,188,1977
ALG14_2_18571,339,497,558,614,922,210,254,464,662,1050,410,319
ALG1_2_18572,139,146,217,279,649,31,508,266,155,111,143,168
ALG2_2_18573,832,620,686,760,587,909,237,1930,715,652,1018,894
ALG6_2_18574,406,418,363,562,900,752,287,860,682,776,353,323
ALKBH8_2_18575,585,465,615,832,781,480,742,1501,616,857,606,911
ALLC_2_18576,632,667,574,590,997,346,581,752,798,715,111,791
ALOX12B_2_18577,945,891,550,977,1823,1915,1786,297,565,1579,670,1054
ALOX12_2_18578,74,68,97,79,93,20,120,0,32,151,1,92
ALOX15_2_18579,67,54,133,128,86,18,395,442,21,48,0,278
ALOX5_2_18580,89,95,113,178,243,128,1237,13,18,0,99,1
AMDHD1_2_18581,292,393,301,369,292,39,326,772,454,84,912,826
AMY1B_2_18582,3036,2508,2713,2976,3054,2180,3086,3192,2485,2429,1368,2
293
AMY1C_2_18583,3036,2508,2713,2976,3054,2180,3086,3192,2485,2429,1368,2
293
AMY2A_2_18584,13219,11353,11716,14121,10096,7505,13600,11952,10104,988
3,10997,12637
AMY2B_2_18585,433,365,243,541,551,107,292,636,69,338,114,337
ANPEP_2_18586,115,137,111,162,7,2,249,20,274,0,2,671
AOC3_2_18587,1160,629,1028,1076,1676,1346,718,813,656,1950,2044,911
AOX1_2_18588,217,281,286,426,240,272,480,273,228,442,147,877
APEH_2_18589,19,87,87,79,34,19,50,21,4,0,0,637
APEX2_2_18590,102,49,60,104,11,4,18,0,87,9,0,29
APIP_2_18591,276,198,135,118,297,93,15,333,166,3,18,251
APLF_2_18592,74,108,109,77,76,79,2,121,145,26,316,137
APOBEC1_2_18593,407,252,436,322,621,23,1708,926,371,10,74,932
APOBEC2_2_18594,223,300,197,360,529,120,250,36,138,308,277,475
APOBEC3B_2_18595,36,50,64,46,22,0,5,0,197,18,322,12
APOBEC3C_2_18596,143,161,100,145,186,302,41,303,227,236,89,167
APOBEC3G_2_18597,358,314,248,439,0,54,1420,719,238,968,263,303
ARF3_2_18598,298,240,206,210,146,208,305,14,198,101,0,144
ARF4_2_18599,191,273,53,40,1,0,0,299,0,1,0,219
ARG2_2_18600,8,84,83,5,0,0,1,30,16,0,453,5
ARHGEF10_2_18601,110,76,107,109,1,6,376,290,91,41,59,108
ARL3_2_18602,775,772,820,1060,1953,1122,1552,133,604,1056,659,716
ARL4C_2_18603,227,214,158,187,151,139,411,97,155,586,56,316
ARL4D_2_18604,16,38,29,62,156,94,0,36,0,35,0,107
ARL5B_2_18605,53,81,88,22,70,96,70,5,4,445,14,8
ARL8A_2_18606,197,153,264,188,357,7,0,0,330,0,253,630
ARL8B_2_18607,781,697,547,548,359,471,524,205,596,467,464,530

ARSD_2_18608,340,227,322,300,260,287,448,256,396,153,48,381
ARSE_2_18609,141,113,134,200,13,128,0,0,34,138,0,6
ARSG_2_18610,37,81,43,58,17,25,0,0,96,41,0,48
ARSH_2_18611,102,134,127,130,0,29,243,0,0,0,0,0
ARSI_2_18612,113,91,21,131,117,58,0,0,48,36,2,83
ARSJ_2_18613,63,73,53,68,0,0,0,17,0,28,0,124
ARSK_2_18614,832,506,966,832,992,1442,1499,258,964,814,991,1178
ART1_2_18615,66,113,81,104,330,13,843,126,18,401,292,207
ART4_2_18616,1040,762,897,820,476,456,440,333,1068,1011,744,1364
AS3MT_2_18617,941,678,627,785,1814,963,559,215,1069,1507,387,661
ASNA1_2_18618,19,6,1,30,0,0,0,0,98,0,0,0
ASNSD1_2_18619,268,357,316,710,541,161,360,949,322,431,204,369
ASPG_2_18620,348,100,214,166,99,227,267,15,0,112,117,52
ASPHD2_2_18621,586,325,475,421,817,754,926,442,429,414,420,311
ATAD1_2_18622,277,291,352,372,536,451,271,395,242,90,32,401
ATIC_2_18623,23,16,26,47,0,186,8,0,0,0,0,34
ATP2C2_2_18624,1097,1014,1021,814,410,69,1318,603,1100,716,1261,1138
AUH_2_18625,59,300,140,276,169,310,260,0,7,25,1174,860
AWAT1_2_18626,237,166,164,213,142,275,49,20,171,172,317,145
AWAT2_2_18627,28,13,36,51,12,19,1,114,44,20,121,354
B3GALNT2_2_18628,456,290,307,506,195,383,590,627,108,252,614,299
B3GALT1_2_18629,449,325,387,302,203,135,697,689,472,304,438,464
B3GALT2_2_18630,1418,1099,1281,2014,1791,1712,1155,1227,1908,1187,2860
,922
B3GALT4_2_18631,5,18,16,30,2,0,0,0,0,2,0,85
B3GALT6_2_18632,0,2,0,0,0,0,0,0,0,0,0,0
B3GAT2_2_18633,1356,894,981,1130,1154,620,1435,1080,1001,2009,1594,104
1
B3GAT3_2_18634,401,165,158,328,344,1,183,369,219,48,605,1015
B3GNT2_2_18635,744,807,558,879,719,724,1329,785,818,1486,874,1247
B3GNT3_2_18636,57,33,3,156,288,191,0,311,13,0,278,397
B3GNT4_2_18637,661,369,298,416,147,644,712,386,272,284,1508,321
B3GNT5_2_18638,1758,1265,1459,1672,1328,783,787,995,1663,2155,1250,253
8
B3GNT7_2_18639,190,228,244,296,244,68,9,11,54,966,72,244
B3GNT9_2_18640,230,198,268,308,53,92,8,133,83,267,401,193
B3GNTL1_2_18641,1027,808,890,975,289,913,1151,270,1377,218,1502,754
B4GALNT1_2_18642,61,101,109,98,98,139,10,12,39,111,4,183
B4GALNT3_2_18643,148,119,87,123,142,0,64,1,64,446,1,11
B4GALNT4_2_18644,57,50,45,60,0,0,1,1,47,367,0,6
B4GALT1_2_18645,146,238,243,299,152,148,577,210,518,733,1,378
B4GALT5_2_18646,170,23,154,245,50,6,22,94,346,46,4,337
B4GALT6_2_18647,144,187,83,126,5,63,100,49,751,142,0,323
B4GALT7_2_18648,96,51,106,220,232,0,128,25,3,153,23,477
BBOX1_2_18649,66,40,44,33,79,0,0,23,184,9,9,41
BCHE_2_18650,2561,1964,2505,2428,1575,1580,1982,1623,1549,2825,2442,20
18
BCMO1_2_18651,79,39,33,38,0,0,187,60,18,10,0,2
BDH2_2_18652,3633,3330,3206,3187,4263,3260,3193,3847,3898,3706,4192,38
18

BHMT_2_18653,650,620,549,693,820,1060,167,1104,90,484,2159,1064
BLM_2_18654,1555,1590,1762,1676,1231,2505,1031,1281,1988,1590,2245,164
4
BLMH_2_18655,96,67,98,38,222,0,5,152,262,22,309,2
BLVRA_2_18656,1104,1061,955,1372,778,1709,2183,1177,1543,863,999,783
BLVRB_2_18657,47,9,54,38,5,15,6,6,8,22,130,1
BPHL_2_18658,232,356,288,357,164,24,315,388,782,15,142,654
BST1_2_18659,132,355,161,208,0,1,0,264,74,211,0,434
BTD_2_18660,13,37,7,75,115,16,4,1,0,78,2,254
C12orf5_2_18661,1497,1300,1389,1320,1414,2070,1122,1205,1393,1918,1216
,1136
C16orf79_2_18662,36,5,51,46,31,0,2,0,0,2,0,0
C18orf56_2_18663,426,149,165,211,1,199,176,305,39,83,167,8
C1GALT1_2_18664,564,829,623,776,297,88,1056,825,1422,1491,660,1197
C1R_2_18665,57,264,113,202,149,327,175,49,134,0,303,214
C22orf28_2_18666,1482,1013,1043,1241,1022,1245,1848,463,1019,661,1505,
501
C2orf43_2_18667,923,715,591,499,1165,339,947,1156,325,807,921,389
C5orf4_2_18668,581,766,534,864,529,340,616,236,861,928,753,1478
C6orf130_2_18669,763,781,718,767,793,948,894,834,989,816,214,1241
CA13_2_18670,641,374,529,672,932,535,705,365,203,1342,250,861
CA14_2_18671,131,133,91,145,138,236,57,41,411,218,0,57
CA2_2_18672,32,48,7,98,9,0,12,265,41,46,85,5
CA3_2_18673,266,212,296,355,348,165,435,1,157,371,76,625
CA4_2_18674,141,198,94,218,1152,0,13,111,384,0,193,39
CA5A_2_18675,79,60,91,78,69,14,170,30,82,51,69,58
CA5B_2_18676,1700,2103,2112,2170,4325,893,2896,2248,1165,3678,2535,169
9
CA6_2_18677,0,0,0,0,0,0,0,0,0,0,0,0
CA8_2_18678,933,1095,732,1042,793,952,1242,267,976,1259,202,1496
CA9_2_18679,81,71,86,128,9,12,0,764,198,9,585,192
CARS2_2_18680,736,538,630,688,420,470,261,361,335,138,108,917
CASD1_2_18681,166,98,142,182,422,43,136,232,34,266,0,47
CAT_2_18682,1286,962,1279,1311,1194,959,1410,628,644,1132,1659,886
CBR1_2_18683,819,646,714,669,627,557,836,908,512,851,378,591
CBR3_2_18684,1472,992,1212,1316,1941,1280,1997,1010,766,1345,1580,984
CBR4_2_18685,1287,1300,1010,1372,2251,382,1537,550,1226,1326,787,884
CCDC88B_2_18686,42,33,47,83,54,5,1,2,3,105,275,87
CCDC92_2_18687,317,336,276,372,275,193,11,747,193,131,346,148
CCNO_2_18688,199,233,124,235,317,286,4,0,192,341,0,431
CCS_2_18689,32,16,29,15,1,25,2,6,50,0,4,72
CCT8_2_18690,200,166,155,90,387,113,280,229,7,81,161,132
CD274_2_18691,1414,1656,1462,1552,827,1194,2041,1955,857,1278,1651,133
1
CD38_2_18692,111,219,199,227,411,0,380,69,125,205,44,335
CDA_2_18693,56,72,48,52,1,0,0,0,0,129,192,17
CDIPT_2_18694,632,541,425,653,1107,379,170,229,751,809,235,504
CDO1_2_18695,2190,1899,1567,1746,1541,2553,1114,1477,1977,1552,1421,12
30
CDS2_2_18696,131,146,98,163,63,9,49,579,41,54,184,11

CELA1_2_18697,125,257,61,98,23,71,268,71,255,88,322,146
CELA2A_2_18698,70,62,75,43,33,30,24,0,7,47,12,94
CELA2B_2_18699,563,629,341,684,319,129,600,31,115,403,97,338
CELA3A_2_18700,23,50,25,22,25,33,20,3,25,2,0,0
CELA3B_2_18701,38,42,5,29,26,72,0,0,3,5,0,41
CEL_2_18702,34,37,42,52,0,1,87,215,7,120,4,33
CETN1_2_18703,61,55,58,74,84,9,2,51,7,39,13,38
CETN2_2_18704,36,18,44,25,304,1,0,2,40,17,38,3
CETP_2_18705,170,81,207,185,100,86,26,0,329,314,15,146
CFB_2_18706,240,164,177,271,447,28,79,608,72,218,707,92
CFD_2_18707,10,127,147,95,0,0,0,340,2,1,0,0
CFI_2_18708,2960,2884,2329,2681,4456,2344,1526,3410,2091,2924,2522,219
9
CH25H_2_18709,159,15,18,54,288,0,39,235,145,20,120,7
CHDH_2_18710,31,50,27,72,22,0,6,0,356,31,26,123
CHI3L1_2_18711,715,537,529,687,1172,399,1788,113,165,215,1163,1078
CHIT1_2_18712,111,100,124,49,5,27,136,83,187,313,42,143
CHML_2_18713,715,671,566,803,566,569,945,681,718,812,528,406
CHPF2_2_18714,158,205,157,208,154,0,5,18,4,654,0,262
CHPT1_2_18715,771,683,671,619,410,539,531,354,708,518,249,504
CHST10_2_18716,111,100,86,93,0,263,0,0,164,97,499,114
CHST12_2_18717,29,36,31,41,0,0,41,0,43,3,0,0
CHST13_2_18718,247,146,230,329,8,349,0,394,87,588,669,393
CHST14_2_18719,29,16,68,45,6,51,42,168,66,30,17,3
CHST1_2_18720,0,51,25,135,0,0,0,0,0,0,1004,31
CHST2_2_18721,292,279,206,241,390,0,9,368,176,351,3,548
CHST3_2_18722,78,70,85,56,3,0,40,0,11,120,89,102
CHST5_2_18723,133,111,152,189,321,257,55,126,152,38,560,42
CHST6_2_18724,36,76,62,91,464,0,0,11,310,3,186,13
CHST7_2_18725,149,168,102,253,5,11,257,141,322,57,489,197
CHST9_2_18726,21,25,40,23,0,0,0,324,14,0,42,29
CHSY1_2_18727,847,942,775,935,1580,932,1070,1507,544,251,1637,987
CHSY3_2_18728,101,128,172,148,382,118,1053,127,4,202,454,50
CLPP_2_18729,19,38,36,30,211,3,344,86,9,7,378,146
CLPX_2_18730,1459,1269,992,1464,1288,830,940,2216,988,494,875,2192
CLYBL_2_18731,635,698,571,842,932,216,1221,843,635,719,661,701
CMA1_2_18732,163,320,192,199,42,70,171,442,576,12,580,411
CMAS_2_18733,54,68,54,73,0,0,68,5,131,5,49,8
CMBL_2_18734,173,175,162,203,15,0,106,237,238,397,282,305
CNDP1_2_18735,1227,1162,1498,1255,947,1224,977,758,1515,728,686,1446
CNTN6_2_18736,111,67,52,76,54,196,51,5,198,26,72,44
COIL_2_18737,372,207,182,225,2,245,945,62,612,29,1,23
COMTD1_2_18738,33,9,10,22,3,346,0,0,0,83,0,136
COQ2_2_18739,854,763,557,911,651,640,497,1088,1021,901,393,1068
COQ3_2_18740,137,55,95,16,0,12,336,404,290,6,0,105
COQ5_2_18741,835,694,639,716,933,596,676,805,829,1485,865,412
COX10_2_18742,7,5,33,11,2,42,0,0,0,0,1,18
COX17_2_18743,521,460,551,557,18,314,525,38,418,1001,1170,841
COX4I1_2_18744,163,68,88,110,9,19,63,54,261,9,28,50
COX4I2_2_18745,252,75,190,256,110,70,61,26,591,35,685,87

COX5A_2_18746,526,431,385,507,975,958,360,797,184,648,713,574
COX5B_2_18747,2068,1972,1989,1991,2007,2043,3330,842,2281,2153,2238,16
68
COX6A1_2_18748,68,35,153,58,70,0,1,0,3,0,93,249
COX6A2_2_18749,649,605,507,592,829,546,341,453,784,544,288,651
COX6B1_2_18750,110,1,86,51,0,0,1,0,9,30,76,9
COX6B2_2_18751,528,331,471,452,467,143,128,444,135,439,148,840
COX6C_2_18752,2417,2314,2059,2596,1091,918,3029,2759,1894,1719,4097,36
26
COX7A1_2_18753,34,39,18,44,122,195,0,0,20,36,0,1
COX7A2_2_18754,118,97,32,174,101,0,0,6,1,4,0,0
COX7A2L_2_18755,35,48,39,75,36,5,17,78,32,6,0,15
COX7B2_2_18756,990,614,766,904,832,588,2095,912,692,2135,857,619
COX7B_2_18757,77,55,47,44,29,49,1,10,42,66,35,122
COX7C_2_18758,285,372,338,348,448,340,212,470,535,332,140,335
COX8A_2_18759,156,316,161,270,75,39,187,1,234,17,18,167
COX8C_2_18760,185,216,184,244,353,275,962,210,149,86,366,130
CPA1_2_18761,45,81,51,59,32,14,63,36,11,143,1,140
CPA2_2_18762,123,113,222,142,158,75,447,253,103,515,63,107
CPB1_2_18763,59,9,57,35,0,0,19,550,4,0,0,0
CP_2_18764,831,884,591,831,438,901,115,0,769,701,1925,1548
CPE_2_18765,446,699,441,495,439,63,905,334,615,109,133,806
CPOX_2_18766,77,122,54,107,176,0,129,0,19,66,26,109
CPSF3_2_18767,330,326,310,389,370,390,193,372,231,686,749,579
CPT2_2_18768,5679,4268,4554,5523,5186,4348,5577,4140,5223,5418,6242,52
09
CRAT_2_18769,6,20,3,14,0,0,0,0,0,0,0,0
CREG2_2_18770,451,347,268,275,720,95,52,290,173,101,208,404
CRY1_2_18771,761,1007,555,868,639,1191,203,904,826,1564,285,1439
CRYL1_2_18772,1293,1367,792,987,796,67,671,472,694,948,1742,918
CRYZL1_2_18773,1870,2176,1813,2051,1524,854,3112,2020,1676,1803,1424,1
307
CSAD_2_18774,115,136,135,92,250,77,53,0,263,75,160,65
CS_2_18775,40,76,102,43,29,128,153,166,37,3,76,53
CSGALNACT2_2_18776,1382,1330,1622,1518,2560,1334,1467,2903,336,1518,18
50,2117
CSRP2BP_2_18777,494,352,408,569,131,3,464,29,810,1202,565,318
CTBS_2_18778,456,180,226,237,11,5,1,6,227,105,266,305
CTRB1_2_18779,26,140,155,95,168,11,0,0,50,234,864,69
CTRB2_2_18780,387,336,337,461,419,136,638,377,632,1016,456,89
CTRC_2_18781,120,55,39,69,71,99,440,0,28,11,40,68
CTSD_2_18782,909,900,863,859,1726,400,1419,642,484,1672,362,1351
CTSF_2_18783,229,314,144,138,281,588,83,209,408,197,196,271
CTSG_2_18784,1124,964,998,1150,1407,508,2326,1308,956,1112,1009,1118
CTSH_2_18785,301,220,270,290,466,98,42,224,202,224,98,187
CTSK_2_18786,720,748,733,783,43,794,211,594,287,71,782,695
CTSZ_2_18787,234,175,185,183,49,34,384,332,56,361,4,535
CWC27_2_18788,1055,1243,942,1175,1935,902,2123,2428,1820,1352,987,1059
CXorf21_2_18789,440,291,255,458,691,839,177,423,367,317,383,381
CYB561D2_2_18790,66,76,47,92,33,46,190,7,46,105,280,56

CYB5B_2_18791,87,69,95,133,81,67,14,0,15,39,89,163
CYB5R1_2_18792,198,171,282,207,395,12,124,386,2,118,860,191
CYB5R2_2_18793,641,433,401,566,147,257,603,579,387,435,1564,630
CYB5R4_2_18794,1775,1673,1425,1818,1488,1188,1396,2204,1754,981,1785,1
517
CYBA_2_18795,90,122,169,258,224,31,397,0,0,0,351,156
CYBB_2_18796,68,110,82,218,1,62,160,3,63,16,0,155
CYC1_2_18797,88,127,113,175,276,212,130,147,37,76,12,370
CYCS_2_18798,55,22,54,137,3,14,142,21,0,0,0,20
CYP11B2_2_18799,74,153,164,114,18,114,283,522,120,110,9,378
CYP17A1_2_18800,328,297,586,472,0,44,882,31,465,789,1127,141
CYP1A1_2_18801,166,72,231,115,172,188,335,37,11,344,1111,26
CYP1A2_2_18802,643,370,391,536,424,211,68,300,590,786,544,425
CYP1B1_2_18803,492,485,400,403,317,346,464,19,297,186,452,328
CYP20A1_2_18804,178,149,218,157,32,2,138,355,471,15,262,358
CYP26B1_2_18805,82,131,151,126,150,134,131,170,214,54,90,110
CYP26C1_2_18806,47,19,47,18,12,234,80,114,0,2,6,8
CYP27A1_2_18807,474,342,260,303,276,546,220,925,426,444,676,272
CYP27B1_2_18808,202,67,177,201,142,14,153,177,92,4,108,60
CYP2A13_2_18809,452,286,357,315,264,787,912,337,369,115,69,327
CYP2A6_2_18810,43,61,54,69,302,0,502,56,129,26,0,95
CYP2B6_2_18811,1516,1130,1227,1311,1046,696,1698,2225,1326,813,1687,19
59
CYP2C19_2_18812,529,739,626,634,107,242,929,1253,434,861,457,703
CYP2C9_2_18813,170,158,75,172,359,94,54,18,16,110,11,78
CYP2E1_2_18814,296,248,305,345,56,342,0,1046,490,598,736,608
CYP2F1_2_18815,175,143,184,117,312,109,122,18,141,294,24,207
CYP2J2_2_18816,112,59,84,117,22,110,34,72,85,120,118,185
CYP2R1_2_18817,678,624,364,698,105,744,223,252,495,1068,449,360
CYP2S1_2_18818,154,258,180,316,434,74,100,101,134,163,70,154
CYP2U1_2_18819,242,323,268,275,626,940,170,127,73,100,269,295
CYP2W1_2_18820,193,181,286,216,772,240,105,348,166,39,136,36
CYP39A1_2_18821,1527,1030,1529,1075,1406,1458,1131,1784,1133,1296,1259
,1267
CYP3A7_2_18822,589,495,633,921,464,122,1531,625,703,306,275,683
CYP46A1_2_18823,452,202,293,211,761,506,1,375,121,253,64,193
CYP4A11_2_18824,0,0,0,0,0,0,0,0,0,0,0,0
CYP4A22_2_18825,459,386,381,409,397,318,807,408,469,466,468,383
CYP4F12_2_18826,902,640,480,630,1651,179,269,1012,641,1178,1674,511
CYP4F22_2_18827,1083,883,998,1158,237,391,1893,118,182,660,2881,1294
CYP4F2_2_18828,140,87,138,148,57,101,0,186,176,23,614,272
CYP4F8_2_18829,135,240,142,223,387,11,59,374,226,82,41,270
CYP4V2_2_18830,1010,1167,1475,1663,668,813,409,1160,1696,1563,1538,208
2
CYP4X1_2_18831,397,275,168,247,200,146,200,67,426,244,113,272
CYP4Z1_2_18832,812,588,678,639,580,304,268,366,178,887,900,386
CYP7A1_2_18833,1024,761,808,849,705,993,2024,385,234,470,413,859
CYP7B1_2_18834,245,163,166,364,60,262,90,495,186,283,43,221
CYP8B1_2_18835,138,115,124,227,537,286,1438,596,605,17,417,505
DAD1_2_18836,608,573,405,638,510,680,143,49,559,452,1441,339

DAGLA_2_18837,178,179,177,181,226,0,107,419,328,71,0,3
DAK_2_18838,67,167,112,82,251,10,155,4,64,2,3,88
DARS2_2_18839,2276,2430,1354,2472,1970,1620,2864,542,1646,2033,1823,22
96
DARS_2_18840,348,275,372,327,392,284,332,797,271,269,382,364
DBH_2_18841,414,141,97,132,7,41,13,561,205,21,12,608
DBR1_2_18842,476,503,603,410,463,489,247,164,11,248,175,690
DBT_2_18843,623,459,428,703,417,258,648,478,230,535,561,182
DCLRE1B_2_18844,197,174,293,485,166,140,247,417,283,357,950,254
DCPS_2_18845,83,96,193,105,414,70,26,0,145,30,58,130
DCTN6_2_18846,612,596,452,704,832,500,76,1133,614,1125,378,354
DCTPP1_2_18847,2,14,39,146,0,0,0,3,0,176,0,0
DDAH2_2_18848,499,412,260,488,360,258,92,762,137,167,157,261
DDOST_2_18849,178,210,188,199,177,223,645,0,0,216,518,190
DDX10_2_18850,56,39,18,83,0,0,0,0,1,0,0,170
DDX18_2_18851,1287,666,879,952,1351,995,1007,1314,1133,777,308,1070
DDX19A_2_18852,1029,913,823,1230,1479,402,806,1169,918,1513,1153,572
DDX1_2_18853,397,360,267,412,232,69,281,149,736,799,285,197
DDX20_2_18854,417,254,119,175,0,168,423,0,717,598,387,217
DDX21_2_18855,272,134,337,273,60,0,161,475,45,204,636,699
DDX23_2_18856,209,123,132,201,246,58,21,2,100,137,310,190
DDX24_2_18857,526,374,444,702,537,120,926,355,116,378,733,1053
DDX25_2_18858,180,83,97,138,8,13,21,113,161,6,8,100
DDX27_2_18859,114,88,92,67,83,7,21,16,31,10,355,90
DDX28_2_18860,350,77,190,299,54,296,146,101,108,41,705,517
DDX39A_2_18861,257,123,207,340,216,218,178,172,286,508,11,276
DDX41_2_18862,1131,1298,971,1344,269,1592,2424,441,281,1632,784,1023
DDX43_2_18863,110,50,85,78,176,7,15,21,68,29,13,44
DDX46_2_18864,0,0,0,0,0,0,0,0,0,0,0,0
DDX49_2_18865,923,725,663,838,418,214,1523,674,746,984,594,1245
DDX50_2_18866,184,161,297,218,67,252,256,414,349,486,24,236
DDX51_2_18867,47,41,53,76,1,4,601,0,0,0,2,87
DDX52_2_18868,433,454,425,562,847,186,425,1203,244,2,900,391
DDX53_2_18869,77,29,22,98,50,126,69,0,36,168,14,2
DDX55_2_18870,704,489,572,627,631,238,437,653,571,315,323,1057
DDX56_2_18871,103,77,250,110,137,31,115,26,331,57,21,342
DDX58_2_18872,604,643,513,584,455,266,172,539,349,524,516,618
DDX59_2_18873,24,88,53,95,2,5,1,107,0,0,0,4
DDX5_2_18874,119,355,195,279,24,683,81,188,351,27,93,93
DDX60_2_18875,621,427,529,559,451,326,139,314,1104,290,75,320
DDX6_2_18876,464,233,226,279,373,269,1019,299,230,221,111,194
DECR1_2_18877,193,127,160,205,18,78,65,112,142,146,3,290
DECR2_2_18878,107,92,114,104,316,61,132,3,340,22,18,183
DEGS1_2_18879,529,344,268,402,337,340,552,295,153,454,394,829
DEGS2_2_18880,232,76,195,285,99,416,600,117,206,110,551,1378
DERA_2_18881,381,363,511,490,324,289,508,636,609,317,143,628
DFFB_2_18882,367,429,317,375,529,99,641,602,715,443,457,164
DGAT1_2_18883,361,529,413,519,428,226,194,1279,435,81,158,289
DGAT2_2_18884,31,47,32,98,0,34,67,294,212,8,0,131
DHCR24_2_18885,153,147,109,210,199,1,0,2,36,13,0,203

DHDH_2_18886,186,157,160,211,274,295,2,45,225,227,33,292
DHFR_2_18887,0,0,1,1,0,0,0,0,0,0,0,0
DHODH_2_18888,109,96,59,35,320,320,40,60,34,0,32,57
DHRS13_2_18889,37,27,116,120,9,0,409,259,2,222,77,243
DHRS3_2_18890,1,10,10,32,7,0,0,10,0,240,0,67
DHRS4_2_18891,70,67,83,42,86,1,168,43,320,49,246,12
DHRS7_2_18892,166,226,121,238,65,386,129,102,416,91,19,64
DHRSX_2_18893,119,134,56,99,4,145,236,173,263,123,129,75
DHTKD1_2_18894,472,494,630,494,634,682,1041,97,675,819,109,867
DHX15_2_18895,261,492,373,386,123,670,66,66,88,58,448,145
DHX29_2_18896,411,442,359,405,241,93,132,540,124,742,69,870
DHX32_2_18897,199,188,401,345,256,588,415,144,494,328,3,163
DHX34_2_18898,259,212,126,212,262,15,13,97,40,162,174,138
DHX37_2_18899,114,27,59,94,249,8,165,119,11,69,1,38
DHX38_2_18900,292,317,202,411,19,70,369,435,422,273,181,303
DHX57_2_18901,516,1253,921,1329,529,551,544,625,7,262,377,1772
DHX58_2_18902,5,23,3,7,0,0,0,0,0,0,0,48
DHX8_2_18903,572,437,609,394,462,73,1261,469,474,227,269,208
DHX9_2_18904,1528,1612,1540,1889,1606,1674,2463,1321,1575,2174,1760,16
37
DIO3_2_18905,22,109,75,42,158,187,282,21,210,242,0,15
DIRAS1_2_18906,2,67,19,5,55,0,0,0,0,0,0,4
DIRAS2_2_18907,94,12,51,95,0,1,39,8,287,28,2,15
DIRAS3_2_18908,91,51,62,90,0,174,0,0,70,0,0,169
DIS3L2_2_18909,454,253,433,422,3,98,608,16,248,77,235,244
DLAT_2_18910,527,386,154,219,440,155,614,41,62,134,85,90
DLD_2_18911,100,83,249,131,41,0,0,1048,153,8,0,932
DLL1_2_18912,2384,2127,2182,2373,2433,2792,1200,1988,3303,1812,2221,19
10
DLST_2_18913,91,156,139,149,202,0,96,86,206,143,314,111
DMGDH_2_18914,111,44,51,71,26,0,0,0,53,135,0,7
DNA2_2_18915,605,510,523,737,670,208,670,235,299,442,386,1326
DNAH11_2_18916,1745,1383,1494,1527,1993,405,1748,651,1128,1065,1703,60
9
DNAH3_2_18917,107,93,106,153,8,1,128,175,102,117,2,444
DNAH5_2_18918,863,518,393,476,184,339,252,205,530,504,383,544
DNAH8_2_18919,187,164,119,146,5,222,142,60,112,143,30,297
DNAJA2_2_18920,69,29,95,131,86,204,0,499,34,285,53,34
DNAJB14_2_18921,82,30,47,15,19,0,0,4,10,14,0,26
DNAJC10_2_18922,423,340,309,453,1185,179,287,292,422,946,196,367
DNAJC18_2_18923,400,200,362,208,43,5,226,322,361,429,239,304
DNAL4_2_18924,610,597,611,706,696,954,930,1180,933,874,177,640
DNASE1_2_18925,180,177,169,340,98,0,0,243,5,162,114,709
DNASE1L2_2_18926,217,259,115,190,10,77,16,1081,559,357,237,451
DNASE1L3_2_18927,276,232,358,306,454,744,344,163,157,115,28,1073
DNASE2_2_18928,46,18,35,56,0,0,0,25,0,373,0,0
DNPEP_2_18929,448,433,639,598,465,323,215,418,220,1068,370,483
DPAGT1_2_18930,360,345,428,550,356,280,571,768,498,34,396,45
DPEP2_2_18931,6,2,94,16,1,163,0,0,0,14,0,0
DPM1_2_18932,145,51,96,135,82,19,276,372,46,136,165,178

DPM2_2_18933,177,169,130,247,205,767,221,336,28,182,19,117
DPP4_2_18934,204,180,203,166,116,73,30,61,454,529,641,230
DPP7_2_18935,148,203,173,242,260,74,0,3,2,527,975,496
DPP9_2_18936,94,119,55,145,78,128,0,87,441,1,0,147
DPYS_2_18937,206,166,206,251,424,446,136,588,327,406,445,112
DPYSL4_2_18938,319,195,270,326,393,280,143,276,237,557,19,462
DPYSL5_2_18939,32,4,4,0,0,0,0,0,0,0,0,0
DQX1_2_18940,152,182,161,141,298,265,9,102,108,82,441,166
DSEL_2_18941,295,234,326,603,151,183,550,61,691,3,1049,324
DTD1_2_18942,201,230,184,344,157,100,308,0,296,19,304,301
DUOX2_2_18943,897,694,1036,880,704,876,230,1014,749,875,1188,1188
DUPD1_2_18944,0,48,27,22,0,0,0,12,40,2,0,0
DUSP28_2_18945,206,201,248,264,209,23,0,1,434,0,906,496
EARS2_2_18946,0,0,0,0,0,0,0,0,0,0,0,0
EBPL_2_18947,140,269,235,230,464,333,125,362,25,30,67,16
ECH1_2_18948,112,99,168,128,26,156,119,11,603,46,442,263
ECHDC3_2_18949,261,214,138,254,406,1,397,0,0,0,161,186
ECHS1_2_18950,7,0,4,3,0,0,0,0,0,0,0,0
EDEM1_2_18951,729,767,600,608,396,508,195,624,430,1073,611,93
EDEM3_2_18952,39,60,65,74,48,1,50,24,0,22,32,16
EEF1A1_2_18953,185,107,166,132,923,392,431,4,131,71,89,259
EEF1A2_2_18954,75,5,33,20,460,17,0,6,68,4,9,5
EEF2_2_18955,289,235,373,350,24,1,33,324,143,222,424,127
EGLN1_2_18956,1772,1419,1438,1571,1796,1124,1700,1467,2763,1861,1188,1
857
EGLN3_2_18957,423,327,156,265,310,407,309,966,144,799,56,393
EHD4_2_18958,536,459,482,357,244,374,938,466,442,415,147,326
EIF4A3_2_18959,675,524,664,590,0,1008,686,0,501,404,610,221
ELAC1_2_18960,266,281,310,376,134,225,599,438,429,285,462,347
ELANE_2_18961,29,5,60,25,123,0,0,0,2,0,556,0
ELOVL1_2_18962,523,536,441,584,750,899,249,213,241,921,825,505
ELOVL2_2_18963,459,439,461,443,103,397,669,393,593,1209,172,624
ELOVL3_2_18964,51,64,169,127,48,53,81,0,5,261,51,223
ELOVL4_2_18965,169,132,245,199,481,35,237,196,0,180,43,335
ENDOD1_2_18966,828,951,665,1096,791,1161,887,728,302,1449,793,1338
ENDOG_2_18967,43,59,29,23,0,4,39,0,0,0,132,0
ENGASE_2_18968,89,32,19,17,0,0,0,9,0,0,0,0
ENO2_2_18969,67,75,153,79,456,81,156,649,13,77,201,2
ENO4_2_18970,104,122,82,162,0,0,114,71,174,36,2,60
ENOPH1_2_18971,301,241,364,320,160,126,320,320,256,120,351,203
ENPEP_2_18972,703,687,476,621,563,49,1224,1004,349,310,608,744
ENPP1_2_18973,91,88,52,69,0,152,31,0,4,1,0,0
ENPP3_2_18974,1211,879,780,947,966,598,1335,347,1052,892,147,1812
ENPP4_2_18975,415,279,236,340,95,768,1122,462,29,202,145,192
ENPP5_2_18976,0,0,0,0,0,0,0,0,0,0,0,0
ENPP6_2_18977,1399,1198,1008,1509,2179,1163,1651,1746,2012,1743,951,14
25
ENPP7_2_18978,22,7,0,1,0,0,0,0,0,0,0,0
ENTPD3_2_18979,450,348,312,405,282,808,890,285,327,696,1,334
ENTPD5_2_18980,0,0,0,0,0,0,0,0,0,0,0,0

ENTPD7_2_18981,27,27,32,22,0,1,0,0,3,85,18,9
EPHX2_2_18982,111,158,109,106,76,111,16,85,39,44,58,308
EPHX4_2_18983,633,516,467,721,456,337,370,386,766,855,512,917
EPRS_2_18984,9,70,45,129,66,0,2,0,0,40,82,22
EPT1_2_18985,368,280,293,378,149,568,47,863,635,964,110,165
EPX_2_18986,108,172,399,438,80,0,113,22,290,301,169,256
ERAS_2_18987,10,93,5,39,1,0,47,0,1,0,0,11
ERCC3_2_18988,287,329,285,296,735,339,323,1,194,177,36,281
ERCC4_2_18989,499,385,276,435,334,524,1449,431,141,287,328,451
ERCC5_2_18990,1112,1165,949,1131,1737,1833,1298,43,886,1811,561,1420
ERI1_2_18991,98,159,189,236,567,340,250,4,620,416,246,179
ERO1L_2_18992,334,614,673,662,1,451,1114,562,533,1638,58,310
ERP44_2_18993,0,0,0,0,0,0,0,0,0,0,0,0
ESD_2_18994,2553,2175,2074,2458,1571,1280,1695,2077,2307,2632,1484,218
2
ESPL1_2_18995,31,88,37,77,0,0,2,0,0,4,58,105
ETFDH_2_18996,319,261,225,154,255,162,273,570,382,310,278,268
EXOSC1_2_18997,543,446,389,450,469,291,853,42,662,777,515,175
EXOSC2_2_18998,486,511,640,504,1024,1185,1945,380,378,583,1244,320
EXOSC4_2_18999,879,664,890,953,1298,794,632,1523,933,914,987,1019
EXOSC5_2_19000,114,245,160,83,181,10,0,0,19,241,916,111
EXOSC7_2_19001,616,521,659,760,579,423,1138,374,274,399,150,832
EXOSC8_2_19002,437,448,499,561,386,610,1347,39,618,301,785,779
EXT1_2_19003,1335,1441,1176,1599,500,1401,2199,1682,1651,1345,1237,879
EXTL1_2_19004,271,370,331,308,819,69,430,412,313,44,272,356
EXTL3_2_19005,353,279,208,284,157,170,271,259,147,84,195,228
F10_2_19006,194,66,162,287,166,283,38,627,163,52,398,21
F12_2_19007,118,154,196,212,411,0,257,0,1,0,0,147
F13A1_2_19008,110,86,65,92,1,22,3,0,27,141,37,23
F13B_2_19009,604,513,616,757,376,181,557,64,360,722,264,448
F2_2_19010,22,19,32,34,1,0,59,18,3,0,0,0
F5_2_19011,319,324,397,349,798,392,45,436,387,235,396,377
F9_2_19012,1880,1621,1688,2015,1834,1913,2248,1572,1140,2097,1659,3229
FA2H_2_19013,65,16,25,109,79,211,20,86,27,2,627,81
FAAH2_2_19014,70,189,123,127,157,243,190,28,74,96,339,190
FADS1_2_19015,258,211,231,376,1133,1,95,518,585,425,2,178
FADS2_2_19016,74,44,49,28,144,0,0,0,5,0,5,11
FADS3_2_19017,208,94,133,123,72,66,96,16,48,305,359,276
FAHD2A_2_19018,28,2,3,3,0,0,0,0,7,0,0,0
FAM108C1_2_19019,137,179,129,144,54,40,138,85,14,12,11,249
FAM135B_2_19020,334,231,268,338,115,9,259,46,25,305,0,213
FAM20B_2_19021,341,187,284,336,101,140,247,386,44,10,840,134
FANCM_2_19022,1476,1378,1221,1276,765,1105,570,1853,1475,2279,629,1254
FAR1_2_19023,592,528,453,515,552,376,51,708,746,451,1014,32
FAR2_2_19024,337,459,443,372,877,240,1242,28,665,197,907,536
FARS2_2_19025,206,144,157,174,440,345,0,0,59,1,241,295
FARSA_2_19026,54,51,94,72,0,0,127,0,0,52,0,1
FARSB_2_19027,155,77,149,124,263,99,52,18,159,16,220,70
FASN_2_19028,169,205,135,201,49,226,131,65,36,300,22,4
FDFT1_2_19029,259,356,503,412,709,176,1155,295,36,71,154,537

FEN1_2_19030,73,21,82,109,103,3,68,373,36,189,2,83
FH_2_19031,866,656,689,859,1320,369,1714,198,789,190,569,887
FIG4_2_19032,210,314,231,156,30,64,78,284,121,107,11,119
FKBP10_2_19033,50,76,46,72,230,61,0,436,11,95,43,82
FKBP14_2_19034,3076,3084,3142,3153,3020,3015,3393,2311,1625,3161,2631,
3062
FKBP15_2_19035,85,113,149,104,151,66,7,0,323,22,3,44
FKBP3_2_19036,529,444,292,409,973,305,182,823,465,260,380,498
FKBP4_2_19037,233,173,359,113,29,401,10,0,116,247,359,436
FKBP8_2_19038,69,82,108,168,309,0,6,5,5,20,84,61
FMO1_2_19039,1178,1263,995,978,764,366,636,536,1182,796,1531,579
FMO4_2_19040,499,430,496,501,657,426,964,795,952,618,157,136
FNBP1_2_19041,428,477,523,600,319,259,472,182,458,485,293,861
FNTA_2_19042,77,72,68,51,132,44,158,24,70,167,6,108
FNTB_2_19043,171,114,99,269,216,119,298,194,109,108,31,238
FTH1_2_19044,76,11,6,41,84,12,0,0,119,7,0,259
FTMT_2_19045,286,306,270,296,276,66,924,70,456,0,370,146
FTSJ2_2_19046,106,142,46,115,113,111,3,222,147,2,34,8
FTSJ3_2_19047,459,441,337,398,705,505,41,595,198,17,188,491
FTSJD2_2_19048,77,106,53,234,39,148,1,1,0,71,48,204
FUCA1_2_19049,960,887,823,1031,781,561,983,578,1117,612,460,1310
FUCA2_2_19050,415,805,567,711,508,344,677,56,24,497,46,605
FURIN_2_19051,271,191,281,504,9,295,3,75,219,225,1684,245
FUT10_2_19052,469,502,447,410,516,908,679,1075,238,723,646,642
FUT11_2_19053,404,445,437,268,219,200,500,298,432,651,416,121
FUT1_2_19054,9,21,51,0,0,0,0,0,2,0,0,0
FUT4_2_19055,43,24,76,84,5,0,4,0,0,1,1023,0
FUT5_2_19056,409,271,327,391,400,479,663,273,324,80,36,331
FUT7_2_19057,186,146,72,113,23,2,579,338,2,12,6,206
FUT9_2_19058,2758,2748,2360,3072,2809,1220,3999,1634,3651,2657,1369,43
93
GADL1_2_19059,612,466,381,715,799,394,561,446,515,366,640,993
GAL3ST1_2_19060,324,231,128,189,67,465,161,0,145,286,538,266
GAL3ST3_2_19061,382,346,176,201,310,52,949,397,452,475,4,396
GAL3ST4_2_19062,190,90,150,176,246,0,245,84,57,21,56,526
GALM_2_19063,13,7,15,25,23,19,0,5,5,0,0,0
GALNS_2_19064,205,45,105,175,113,41,84,0,116,1,0,48
GALNT10_2_19065,72,21,63,115,105,43,245,29,55,0,0,51
GALNT11_2_19066,892,956,768,872,1475,566,491,470,639,591,1114,563
GALNT12_2_19067,344,539,463,490,598,539,919,218,272,251,246,385
GALNT13_2_19068,12,10,16,10,0,0,0,33,0,0,57,0
GALNT14_2_19069,366,318,635,526,3,659,556,84,738,573,1286,280
GALNT1_2_19070,429,612,412,306,263,516,741,21,605,803,314,466
GALNT2_2_19071,1022,592,779,837,397,440,556,320,998,333,423,710
GALNT3_2_19072,169,86,181,214,55,51,386,128,54,26,0,175
GALNT4_2_19073,407,277,391,332,256,275,105,114,557,405,206,796
GALNT5_2_19074,255,203,217,252,5,434,143,162,215,235,283,119
GALNT6_2_19075,663,414,370,622,1107,329,237,400,784,301,318,1287
GALNT8_2_19076,388,454,423,530,365,617,205,514,546,487,280,379
GALNTL2_2_19077,404,299,323,380,448,118,105,178,301,938,181,432

GALNTL4_2_19078,121,84,142,225,12,4,0,894,9,1,59,109
GALNTL5_2_19079,280,463,257,303,175,991,626,401,134,269,369,226
GALNTL6_2_19080,377,349,470,393,757,235,898,187,223,157,367,373
GALT_2_19081,12,7,27,34,15,0,0,82,24,11,0,26
GANC_2_19082,877,516,674,533,1102,1286,234,405,843,776,412,529
GAPDH_2_19083,39,16,22,3,96,12,235,0,59,0,0,0
GAPDHS_2_19084,226,206,188,285,70,1,15,35,19,352,59,352
GARS_2_19085,848,527,667,807,613,694,992,1844,824,564,1113,816
GATC_2_19086,105,1,11,81,0,0,0,0,4,0,0,19
GATM_2_19087,83,146,134,132,13,79,28,1,210,33,194,87
GBA2_2_19088,194,246,225,218,29,400,35,157,252,657,646,166
GBE1_2_19089,109,72,56,121,6,0,29,0,108,149,195,20
GBGT1_2_19090,104,78,111,148,531,0,154,135,19,76,91,89
GBP1_2_19091,146,212,140,182,293,41,209,55,316,0,98,234
GBP2_2_19092,242,200,344,177,101,68,263,2,436,392,99,298
GBP3_2_19093,430,402,332,374,292,505,699,452,75,505,770,330
GBP4_2_19094,817,895,713,991,574,439,340,967,346,1229,920,468
GBP6_2_19095,373,344,158,252,507,5,278,2,157,135,4,206
GBP7_2_19096,1207,1204,1341,1209,880,940,989,1478,1064,630,1219,1271
GCLM_2_19097,13,27,24,41,14,0,7,116,0,5,0,0
GCNT3_2_19098,115,131,161,195,18,154,37,202,186,236,393,308
GCNT4_2_19099,141,116,50,79,98,116,601,0,0,89,106,81
GCSH_2_19100,148,108,119,55,0,6,0,0,2,0,0,456
GDE1_2_19101,456,284,344,555,127,393,1,505,520,243,428,20
GDPD3_2_19102,74,82,101,123,18,3,80,774,196,121,11,217
GFM1_2_19103,899,396,393,611,207,1054,1331,323,388,133,494,284
GFOD2_2_19104,74,86,110,137,55,0,515,31,95,83,25,4
GFPT1_2_19105,152,173,120,69,395,0,105,182,414,142,351,193
GFPT2_2_19106,294,246,284,281,224,75,1330,94,167,64,116,156
GGH_2_19107,687,607,513,669,759,485,475,218,680,1435,247,463
GGPS1_2_19108,17,28,34,70,70,2,14,0,254,10,0,56
GGT7_2_19109,131,188,116,183,0,598,11,0,130,173,371,118
GGTLC2_2_19110,294,462,383,692,246,87,280,458,250,1027,221,1073
GLCE_2_19111,425,324,312,391,67,210,651,773,728,471,61,70
GLDC_2_19112,127,17,63,94,176,7,1,33,72,58,0,199
GLO1_2_19113,98,143,78,57,79,505,23,129,105,240,0,20
GLOD4_2_19114,305,302,253,214,970,60,219,264,349,38,1204,42
GLS2_2_19115,351,267,388,424,443,3,348,398,84,84,1,120
GLT1D1_2_19116,477,341,600,580,128,1,492,32,71,930,924,243
GLT25D1_2_19117,84,96,86,101,9,2,1,59,15,256,365,0
GLT25D2_2_19118,227,169,254,251,499,255,39,38,229,222,116,165
GLT8D2_2_19119,104,58,51,56,149,256,17,0,40,0,0,203
GLUD1_2_19120,1980,2330,1772,2236,2889,2230,2265,1623,1261,2091,1363,2
503
GLUD2_2_19121,382,291,198,414,672,877,1331,682,460,163,16,808
GMDS_2_19122,373,382,403,435,567,1097,14,47,541,919,403,315
GMIP_2_19123,36,21,36,7,26,0,63,1,0,2,0,342
GMPR_2_19124,132,57,50,155,27,0,0,20,157,295,0,202
GMPS_2_19125,38,9,16,39,0,0,0,1,0,0,0,0
GNA11_2_19126,87,47,77,76,283,106,10,10,3,9,1,0

GNA12_2_19127,235,151,114,94,196,389,5,335,195,118,8,42
GNA13_2_19128,617,282,325,396,1056,162,840,44,256,427,117,331
GNA14_2_19129,462,288,343,511,452,4,515,370,316,1,1339,5
GNA15_2_19130,1,0,0,0,0,0,0,0,0,0,0,0
GNAI1_2_19131,213,152,190,260,24,69,156,32,54,202,3,40
GNAI3_2_19132,95,162,163,149,415,390,272,136,86,28,23,55
GNAQ_2_19133,416,533,345,481,495,780,483,372,549,356,492,395
GNAT2_2_19134,1289,1284,1536,1669,250,866,1765,1303,552,2626,4015,604
GNAT3_2_19135,1480,1195,1392,1581,1077,1183,2719,1723,1066,1552,2393,9
92
GNAZ_2_19136,616,530,619,514,576,295,530,1064,521,716,417,743
GNB1_2_19137,736,632,727,789,947,678,837,488,627,218,790,681
GNB2L1_2_19138,262,188,145,171,197,508,743,12,100,300,57,174
GNB3_2_19139,146,91,90,81,304,201,52,263,156,10,0,36
GNB4_2_19140,68,147,60,81,66,307,327,12,107,13,0,65
GNG11_2_19141,381,230,584,621,1,453,527,690,219,623,229,292
GNG12_2_19142,735,709,853,1130,963,745,894,1259,310,195,596,1358
GNG13_2_19143,21,20,17,18,0,0,0,1,19,0,0,0
GNG2_2_19144,180,187,83,278,165,241,0,116,121,0,25,216
GNG3_2_19145,290,187,196,297,120,2,6,493,351,533,557,498
GNG7_2_19146,212,73,106,170,111,11,10,42,315,0,178,41
GNG8_2_19147,57,187,74,199,24,3,13,352,117,955,0,18
GNGT1_2_19148,149,72,150,86,497,84,93,2,11,43,192,27
GNL2_2_19149,129,143,131,157,175,44,103,109,77,64,7,47
GNMT_2_19150,211,179,182,186,308,417,3,350,99,74,88,124
GNPAT_2_19151,332,495,330,505,162,257,336,371,121,540,605,528
GNPDA1_2_19152,574,427,505,746,518,25,185,633,633,131,18,623
GNPDA2_2_19153,345,274,320,422,1075,7,143,229,476,396,1,982
GNPNAT1_2_19154,1268,1526,1210,1708,1413,1578,2124,966,1601,2940,894,1
548
GNPTAB_2_19155,324,322,369,478,1115,294,37,510,151,784,34,610
GNPTG_2_19156,126,58,120,155,280,275,3,974,78,711,12,1
GNS_2_19157,179,161,139,321,4,41,614,269,54,114,160,315
GOT2_2_19158,44,51,56,61,0,0,7,0,41,35,3,138
GPAA1_2_19159,390,384,362,498,11,244,3,861,15,56,153,745
GPAM_2_19160,279,398,227,382,275,206,352,666,414,283,265,346
GPAT2_2_19161,50,30,33,42,21,0,10,41,0,0,0,248
GPD1_2_19162,109,98,185,326,303,116,87,299,30,94,9,38
GPD1L_2_19163,164,186,222,180,153,11,170,313,672,82,71,432
GPX2_2_19164,487,223,286,324,121,218,37,117,543,850,233,554
GPX3_2_19165,77,16,26,172,55,54,19,67,182,36,0,284
GPX7_2_19166,36,47,84,123,23,72,0,27,49,118,5,72
GPX8_2_19167,329,311,276,406,10,171,185,460,399,607,111,163
GRHPR_2_19168,32,193,27,100,2,75,13,0,59,4,1,0
GSTA1_2_19169,997,1223,1150,1356,828,1677,863,1004,703,1472,1421,976
GSTA2_2_19170,132,87,106,173,6,137,61,4,0,82,44,0
GSTA3_2_19171,1125,1232,1041,1151,1278,957,927,981,693,789,867,1038
GSTA4_2_19172,564,570,555,654,1338,490,1090,938,472,918,339,1156
GSTA5_2_19173,110,9,90,86,156,198,0,0,554,15,1,32
GSTM3_2_19174,2425,2491,1921,2202,1674,2542,2652,1932,2321,2288,2232,1

696
GSTM5_2_19175,1324,1093,980,1181,1931,858,946,1277,1252,443,455,1669
GSTP1_2_19176,136,149,214,109,118,38,578,94,179,184,646,37
GSTT1_2_19177,45,48,22,40,2,3,0,11,13,18,0,3
GSTT2B_2_19178,177,228,196,321,8,2,157,323,10,3,1207,84
GSTT2_2_19179,177,228,196,321,8,2,157,323,10,3,1207,84
GTF2F2_2_19180,1793,1529,1414,1886,2166,1472,1604,2328,2372,1807,2673,
2112
GTPBP1_2_19181,186,211,148,53,201,37,0,74,13,2,178,117
GTPBP2_2_19182,208,147,281,243,244,1,272,274,416,1103,163,330
GTPBP4_2_19183,102,19,50,178,30,132,29,139,89,92,0,119
GUCY1A2_2_19184,205,155,162,202,162,242,164,328,272,81,24,380
GUCY1B3_2_19185,759,570,587,583,236,930,205,423,717,220,1369,625
GUSB_2_19186,18,22,20,48,124,0,93,0,15,9,197,0
GYLTL1B_2_19187,606,431,680,935,415,321,425,423,1624,707,712,702
GYS2_2_19188,1065,699,703,768,177,574,2704,405,577,765,227,754
GZMA_2_19189,31,79,57,66,1,0,5,31,32,1,0,118
GZMB_2_19190,230,104,131,165,112,2,1,0,32,291,129,129
H6PD_2_19191,41,27,47,115,132,2,264,0,0,1,0,0
HAAO_2_19192,605,289,413,600,61,501,357,723,536,158,784,703
HACL1_2_19193,112,74,119,124,125,36,117,152,5,0,8,4
HADHA_2_19194,648,543,904,923,646,728,1252,1157,707,312,745,972
HADHB_2_19195,487,330,349,431,393,168,853,460,228,293,306,306
HAGHL_2_19196,1,28,7,5,0,0,3,0,5,0,0,10
HAL_2_19197,244,226,348,288,226,144,94,198,85,46,475,220
HAO1_2_19198,79,51,84,115,14,50,88,598,24,67,535,842
HARS_2_19199,378,277,257,367,551,406,153,69,457,389,140,368
HAS1_2_19200,143,108,140,185,308,58,8,101,131,0,0,174
HAS2_2_19201,274,165,254,199,252,10,321,314,447,324,290,251
HDHD3_2_19202,557,275,209,499,404,66,146,339,303,20,159,484
HELB_2_19203,85,42,30,6,0,0,313,0,0,0,0,1
HELQ_2_19204,387,371,476,452,960,179,7,903,724,577,661,670
HELZ_2_19205,247,347,253,466,425,243,247,347,507,340,184,302
HEXA_2_19206,72,163,95,176,549,266,0,9,101,269,122,304
HEXB_2_19207,781,560,485,632,490,280,595,524,1061,702,419,319
HEXDC_2_19208,1549,1233,975,1433,617,561,1356,1303,1342,909,1306,765
HGD_2_19209,48,48,91,72,3,136,642,281,80,13,0,43
HGSNAT_2_19210,44,43,54,72,1,158,0,7,5,0,161,45
HHATL_2_19211,149,58,106,168,5,68,178,0,0,276,208,26
HIBADH_2_19212,606,583,601,658,312,1181,1134,584,755,478,232,1269
HINT1_2_19213,228,46,62,143,22,218,200,7,21,84,30,377
HMOX1_2_19214,150,128,120,166,122,75,1,106,387,68,74,66
HPGDS_2_19215,286,203,279,289,327,170,166,65,163,125,271,213
HPRT1_2_19216,208,238,127,222,70,495,111,36,440,8,256,22
HS3ST1_2_19217,94,82,133,132,255,387,0,229,41,35,6,162
HS3ST2_2_19218,4578,3621,3249,4282,2381,4021,3205,3079,3196,3032,3100,
4795
HS3ST3A1_2_19219,401,308,374,364,525,109,168,388,356,750,90,326
HS3ST3B1_2_19220,401,308,374,364,525,109,168,388,356,750,90,326
HS3ST4_2_19221,267,257,278,335,796,319,336,357,385,421,127,466

HS3ST6_2_19222,845,602,446,366,624,901,637,1156,179,826,124,536
HS6ST1_2_19223,147,142,125,94,0,1,243,56,344,158,207,22
HS6ST3_2_19224,106,144,75,42,167,0,0,77,158,0,37,74
HSD11B2_2_19225,161,239,284,170,165,608,346,59,223,193,115,286
HSD17B11_2_19226,383,471,359,617,220,83,388,202,793,395,102,377
HSD17B12_2_19227,1191,730,950,1085,573,471,1860,1031,1819,375,324,1264
HSD17B14_2_19228,380,415,244,461,159,328,0,1712,403,675,366,292
HSD17B1_2_19229,99,30,17,79,9,2,65,137,1,0,519,218
HSD17B2_2_19230,366,446,258,327,456,313,1111,117,331,433,73,294
HSD17B3_2_19231,48,151,109,296,20,3,0,0,1,90,287,400
HSD17B6_2_19232,829,684,783,823,667,338,706,463,764,980,1093,441
HSD17B8_2_19233,69,93,44,107,180,102,0,0,79,4,26,0
HSD3B1_2_19234,61,79,64,61,13,17,0,118,7,119,17,71
HSP90AB1_2_19235,702,674,694,684,790,1484,601,137,725,219,314,822
HSPA5_2_19236,365,278,354,294,286,369,641,733,521,651,693,174
HSPE1_2_19237,530,334,239,422,147,3,409,186,109,181,153,320
HSPG2_2_19238,10,12,19,66,0,0,0,4,52,1,0,206
HYAL4_2_19239,511,339,295,449,516,447,393,730,178,327,616,465
IARS2_2_19240,460,590,477,860,263,391,281,1417,285,436,579,654
ICMT_2_19241,1854,1530,1396,1547,1628,1186,1943,791,1446,1080,1040,126
2
ICT1_2_19242,0,0,0,0,0,0,0,0,0,0,0,0
IDH1_2_19243,109,78,145,122,191,29,20,5,103,158,0,191
IDH2_2_19244,801,911,878,854,1139,116,109,1745,544,592,2402,1351
IDH3A_2_19245,866,842,851,1145,1221,797,1440,1160,435,1041,668,1159
IDI1_2_19246,880,665,376,595,236,197,347,190,606,657,256,549
IDI2_2_19247,187,120,117,138,124,0,433,0,0,168,57,358
IDO1_2_19248,192,178,231,301,11,12,414,668,124,28,7,39
IDO2_2_19249,207,210,170,223,73,322,8,172,145,101,98,394
IDUA_2_19250,337,447,308,334,291,1037,172,109,246,336,235,149
IFI30_2_19251,85,94,85,140,0,42,10,11,133,32,2,76
IFIH1_2_19252,534,325,250,454,311,27,437,831,285,605,214,838
IGHMBP2_2_19253,0,0,0,0,0,0,0,0,0,0,0,0
IHH_2_19254,488,797,699,621,493,703,1184,225,51,454,343,403
ILVBL_2_19255,16,0,22,0,0,0,0,0,0,1,0,0
IMPAD1_2_19256,334,284,166,335,795,21,1,36,19,291,0,61
IMPDH2_2_19257,2,1,5,0,0,0,0,88,6,0,0,6
IRS1_2_19258,257,173,243,234,201,78,450,114,1022,395,672,49
IRS2_2_19259,54,129,188,75,1,0,1388,4,8,151,407,377
ISG20_2_19260,519,359,609,393,488,177,173,567,409,918,143,469
ISG20L2_2_19261,120,175,200,68,23,147,225,222,4,24,1,72
ISOC1_2_19262,231,154,251,274,122,98,466,509,345,221,181,364
IWS1_2_19263,391,385,378,349,669,203,551,215,673,589,140,477
KDSR_2_19264,2803,2749,2245,2963,2794,1653,3011,2265,2024,3089,1884,18
36
KHSRP_2_19265,219,111,136,151,13,140,631,499,117,79,310,541
KIAA0317_2_19266,227,175,138,217,76,175,311,80,179,99,472,209
KIAA1279_2_19267,246,258,233,209,19,25,11,388,527,542,190,341
KIAA2022_2_19268,190,248,313,207,135,62,4,2,170,321,107,386
KIF18A_2_19269,611,327,548,462,467,29,53,300,319,309,667,895

KIF20B_2_19270,829,667,764,879,1158,980,740,74,824,356,537,1128
KIF3A_2_19271,271,133,154,168,398,111,8,3,192,55,108,467
KLB_2_19272,271,363,162,640,4,138,38,68,64,467,164,70
KLC3_2_19273,0,64,25,29,0,0,0,331,0,0,0,0
KL_2_19274,475,453,451,712,644,100,1052,84,345,657,757,183
KLK1_2_19275,701,614,720,609,525,327,434,770,337,518,344,749
KLKB1_2_19276,620,508,465,614,894,301,1337,573,650,353,449,517
KMO_2_19277,3591,3340,2538,3219,3119,1773,2333,2451,2329,3552,3465,304
3
KRTCAP2_2_19278,85,102,200,147,113,12,23,4,96,31,309,278
L2HGDH_2_19279,91,103,93,172,504,264,350,219,51,61,112,340
LALBA_2_19280,217,109,261,198,192,395,43,422,125,115,395,36
LAP3_2_19281,469,260,226,415,230,20,468,643,162,342,0,561
LARS2_2_19282,962,839,825,806,1090,1167,1183,973,1053,551,846,822
LARS_2_19283,208,144,230,205,12,62,98,10,11,38,5,79
LCAT_2_19284,262,313,218,374,294,614,100,0,345,2,34,156
LCMT2_2_19285,472,379,529,595,449,188,358,190,887,978,1823,313
LCT_2_19286,622,510,519,427,460,643,1059,403,368,447,545,547
LCTL_2_19287,336,232,273,204,673,29,544,98,347,302,402,329
LDHAL6B_2_19288,565,485,292,518,399,936,590,354,219,99,336,452
LEPREL2_2_19289,37,43,40,70,4,24,227,18,86,16,0,46
LGALS13_2_19290,508,830,567,827,49,1156,419,644,507,250,684,685
LHFPL2_2_19291,857,948,851,1137,852,773,2172,469,506,840,214,709
LIPC_2_19292,815,1090,1171,1168,1259,372,300,374,997,640,1144,1221
LIPE_2_19293,807,765,626,604,1355,603,353,46,200,1054,200,790
LIPG_2_19294,368,252,259,443,279,577,66,0,632,461,460,556
LIPH_2_19295,417,472,391,441,561,540,478,349,1244,204,279,277
LIPI_2_19296,965,788,847,1020,284,481,721,711,882,256,1285,802
LIPM_2_19297,411,674,447,796,157,474,699,1,225,290,390,220
LIPN_2_19298,106,96,81,61,164,0,403,86,74,0,22,585
LIPT2_2_19299,484,251,330,482,626,13,56,735,441,138,968,524
LONP2_2_19300,177,79,25,12,0,130,37,84,19,1,0,497
LOXL1_2_19301,410,243,296,267,173,45,193,538,695,222,193,182
LOXL2_2_19302,188,346,370,311,282,2,0,3,470,1,745,47
LOXL3_2_19303,931,566,675,664,151,1246,1051,362,942,974,105,501
LOXL4_2_19304,147,170,99,205,0,9,733,97,330,1,0,342
LPCAT1_2_19305,14,2,35,25,3,1,0,0,95,7,191,340
LPCAT2_2_19306,572,228,477,239,221,132,988,389,151,354,40,352
LPCAT3_2_19307,507,805,646,761,535,885,979,870,600,422,321,1044
LPCAT4_2_19308,335,351,300,410,245,763,0,334,249,56,49,410
LPIN1_2_19309,180,191,114,200,404,358,464,73,380,363,99,173
LPIN2_2_19310,554,503,600,645,526,431,1340,1090,619,1110,2,741
LPIN3_2_19311,33,58,51,6,98,1,6,3,0,44,414,0
LPL_2_19312,185,107,208,144,79,67,126,283,140,19,92,165
LRAT_2_19313,100,124,127,97,0,141,326,50,47,119,89,81
LTA4H_2_19314,830,831,704,787,1704,42,1008,539,985,272,283,755
LTC4S_2_19315,196,278,186,310,204,257,235,188,61,621,567,266
LYG2_2_19316,38,9,35,13,120,3,19,11,2,0,0,16
LYPLA1_2_19317,107,45,71,119,156,35,180,106,81,94,16,72
LYPLA2_2_19318,875,966,858,963,541,318,467,375,1053,1731,442,1208

LYPLAL1_2_19319,135,144,99,178,2,628,75,0,69,14,59,154
LYZ_2_19320,326,281,242,344,103,573,65,86,302,205,63,558
LYZL1_2_19321,723,588,529,841,445,547,702,155,408,557,941,502
LYZL2_2_19322,1046,1196,1157,1371,1133,1888,1976,1283,1238,632,1912,21
06
LYZL4_2_19323,235,225,258,332,129,133,1034,211,58,180,9,327
MACROD1_2_19324,153,69,80,200,155,30,10,242,19,47,2,465
MAGT1_2_19325,198,197,85,85,410,153,1,1,92,21,19,92
MAN1A1_2_19326,280,142,182,208,142,78,224,427,103,526,0,56
MAN1A2_2_19327,779,576,719,511,481,772,611,821,638,750,589,921
MAN1B1_2_19328,58,50,29,52,25,826,21,119,338,70,4,217
MAN1C1_2_19329,31,23,112,77,47,218,0,19,21,17,15,54
MAN2A1_2_19330,1366,1595,1409,1466,1327,1041,1653,745,1237,1149,916,15
23
MAN2A2_2_19331,64,57,59,70,19,4,0,2,97,12,0,36
MAN2B2_2_19332,54,126,78,171,163,21,274,3,69,0,182,81
MAN2C1_2_19333,287,226,305,323,54,286,21,347,16,118,900,489
MANBA_2_19334,864,1242,821,1045,662,860,817,214,859,399,1998,764
MANEA_2_19335,280,383,158,236,8,444,0,407,48,10,30,792
MAOA_2_19336,153,141,98,103,32,265,293,0,75,118,34,269
MAOB_2_19337,88,49,53,64,0,0,749,10,21,20,0,296
MAP1S_2_19338,52,25,20,118,224,0,6,397,34,3,0,0
MAPRE3_2_19339,580,344,355,356,540,259,417,516,555,1066,166,488
MARS2_2_19340,36,88,85,63,86,0,0,23,0,0,281,0
MARS_2_19341,632,596,540,652,436,555,300,572,651,523,925,411
MAT1A_2_19342,41,53,92,62,32,165,350,399,0,7,39,13
MAT2A_2_19343,369,456,405,403,1316,0,1,222,146,74,0,2
MBOAT1_2_19344,219,103,255,250,260,260,70,189,422,162,80,79
MBOAT2_2_19345,964,980,822,925,738,897,1386,988,1208,419,528,1023
MBOAT4_2_19346,45,11,80,30,2,17,3,184,23,0,0,0
MBTPS1_2_19347,448,268,745,586,177,238,1407,610,1214,233,503,429
MBTPS2_2_19348,59,84,66,73,472,0,36,0,0,0,41,4
MCCC1_2_19349,166,80,157,117,2,17,264,275,16,544,1,270
MCCC2_2_19350,404,245,218,413,282,122,465,1032,266,158,30,644
MCEE_2_19351,659,547,383,481,494,476,586,194,636,228,713,266
MCM3_2_19352,45,68,57,67,0,0,3,0,0,10,301,3
MCM5_2_19353,30,8,23,13,0,0,0,4,0,0,0,1
MCM6_2_19354,44,25,49,58,0,0,0,20,117,0,0,0
MDH1B_2_19355,277,240,186,233,114,41,292,104,328,521,125,67
MDH2_2_19356,29,32,56,38,0,39,16,191,49,0,1,101
MEP1A_2_19357,49,30,30,19,0,0,0,34,0,28,0,386
MEP1B_2_19358,162,153,175,151,437,25,85,88,667,381,5,665
METAP1_2_19359,1337,1336,1170,1345,956,1475,1883,1286,1053,2211,1187,1
471
METAP1D_2_19360,687,620,549,687,205,699,894,666,876,228,453,310
METAP2_2_19361,26,40,53,33,0,103,76,0,5,37,4,1
METTL14_2_19362,1378,1207,1193,1568,679,1120,1398,1260,1738,1112,818,2
596
METTL22_2_19363,1046,717,1112,1008,1062,1332,2621,755,937,797,1834,404
METTL2B_2_19364,1557,1261,1056,1378,993,933,1679,1273,2096,1930,1523,2

677
METTL3_2_19365,479,281,326,411,72,207,722,344,257,402,1359,626
METTL5_2_19366,1059,1112,1201,1386,1490,1640,1689,1588,839,1585,2059,8
73
METTL6_2_19367,424,301,394,373,282,351,696,715,338,395,4,476
METTL7B_2_19368,384,229,145,247,174,75,182,471,680,368,408,272
METTL8_2_19369,388,359,459,490,763,611,96,279,538,1088,914,378
MFN1_2_19370,586,431,519,465,268,375,511,202,1299,419,473,410
MGAM_2_19371,62,38,57,38,54,0,0,175,21,31,0,38
MGAT4C_2_19372,435,515,413,622,476,577,698,117,573,131,266,591
MGAT5_2_19373,351,145,151,356,9,188,178,19,353,242,405,119
MGMT_2_19374,228,262,210,251,476,52,132,228,10,11,1,156
MGST3_2_19375,414,270,450,453,74,296,770,608,192,116,8,755
MIOX_2_19376,57,81,55,148,22,6,0,0,0,345,0,0
MIPEP_2_19377,660,415,480,481,909,602,1511,683,193,1412,656,515
MLYCD_2_19378,398,217,435,300,532,290,1046,605,280,211,125,200
MMAB_2_19379,73,68,123,92,4,128,0,0,204,517,171,103
MMEL1_2_19380,50,65,143,71,11,4,13,5,231,171,139,59
MMP10_2_19381,1053,725,916,1185,1220,743,2412,509,1076,531,977,1632
MMP12_2_19382,0,21,0,0,2,0,0,0,0,0,0,0
MMP14_2_19383,444,270,425,372,421,54,464,377,309,341,717,965
MMP3_2_19384,201,79,89,115,202,235,30,176,6,136,52,107
MMP7_2_19385,212,112,271,218,159,80,44,29,314,205,367,54
MMP8_2_19386,2174,2188,1786,2561,1697,1131,1788,2091,1436,2375,2233,21
51
MMP9_2_19387,16,5,42,45,0,0,430,1,1,4,69,0
MOCOS_2_19388,318,239,206,334,277,258,899,30,331,43,216,277
MOGAT1_2_19389,950,714,828,824,1321,249,660,532,771,745,1329,714
MOGAT2_2_19390,315,403,317,363,172,702,643,73,555,436,890,475
MOGAT3_2_19391,263,341,212,294,704,605,0,73,315,637,243,152
MOXD1_2_19392,196,31,88,39,9,0,250,18,65,0,22,9
MPI_2_19393,79,103,72,157,0,5,216,20,41,2,85,184
MPO_2_19394,259,184,272,196,518,0,483,189,109,1,164,171
MRPL37_2_19395,6,2,12,14,0,0,15,12,0,1,0,0
MRPL44_2_19396,90,133,119,181,32,104,92,564,128,25,39,394
MRPS30_2_19397,108,71,73,118,469,4,175,405,206,49,2,183
MSH2_2_19398,444,375,347,627,67,1104,120,0,114,381,768,573
MSH3_2_19399,49,26,8,35,0,0,0,0,29,9,1,0
MSH4_2_19400,2479,2187,1963,2269,2797,2562,1062,1982,2836,3384,1469,27
53
MTAP_2_19401,515,393,406,494,336,623,506,269,466,256,29,182
MTFMT_2_19402,638,436,602,613,673,439,164,346,461,1157,705,967
MTHFD1_2_19403,0,0,32,16,0,0,158,0,0,1,0,0
MTHFD2_2_19404,461,501,482,506,446,70,92,749,456,1269,1768,566
MTHFD2L_2_19405,778,852,696,967,670,547,1046,458,701,676,722,708
MTHFR_2_19406,120,157,80,140,31,191,158,406,139,223,290,38
MTPAP_2_19407,403,208,295,329,187,298,0,329,431,22,0,84
MTR_2_19408,1154,1069,1017,1219,2550,359,306,294,920,1258,1097,1499
MUS81_2_19409,79,24,50,77,0,36,1,0,135,2,93,1
MUT_2_19410,1983,2107,1935,2060,1618,1672,1685,611,2185,2817,2252,2086

MVD_2_19411,87,195,106,135,162,262,81,0,191,0,3,104
MX2_2_19412,196,181,170,168,235,0,198,27,141,209,1,315
MYH1_2_19413,112,166,145,270,74,13,759,19,90,13,131,222
MYH3_2_19414,1076,673,1057,972,1406,607,1363,394,940,1173,470,1168
MYH6_2_19415,945,644,685,1034,284,799,390,339,989,386,413,547
MYH7_2_19416,390,439,385,261,413,353,227,392,490,220,308,152
MYH9_2_19417,248,235,278,421,319,227,117,793,40,0,671,37
MYL7_2_19418,107,232,136,126,102,25,0,17,0,0,0,350
MYO1E_2_19419,47,45,94,144,79,36,15,26,58,4,24,379
MYO5B_2_19420,263,221,165,228,91,219,119,581,124,339,202,127
MYO9A_2_19421,220,81,178,125,400,102,265,142,328,342,56,57
N6AMT2_2_19422,650,394,582,672,447,30,510,1252,547,942,1222,762
NAA10_2_19423,34,32,9,4,64,0,0,0,22,28,98,0
NAA11_2_19424,145,137,112,166,174,18,35,54,13,19,7,309
NAA15_2_19425,895,772,1076,967,1298,594,505,554,228,1059,548,1450
NAA30_2_19426,105,29,85,127,85,47,110,90,468,20,232,141
NAA50_2_19427,584,674,551,772,627,432,332,1728,543,344,581,608
NAALAD2_2_19428,1097,1094,693,963,863,590,786,872,835,925,763,1213
NAALADL1_2_19429,313,150,181,257,84,733,1026,377,205,1,10,186
NADSYN1_2_19430,217,232,202,251,289,675,300,163,192,293,37,195
NAGA_2_19431,455,279,271,416,531,701,597,529,278,194,61,588
NAGLU_2_19432,139,144,96,212,1,51,540,36,1,225,498,259
NAGPA_2_19433,12,20,12,22,25,82,99,1,77,4,0,4
NAMPT_2_19434,1254,990,1091,1111,888,274,1140,1045,1019,827,817,1149
NANP_2_19435,1075,815,957,1099,590,587,871,1178,987,1402,1180,1047
NANS_2_19436,517,446,565,614,370,637,1385,645,1015,329,836,1135
NAPRT1_2_19437,189,117,131,154,294,140,122,655,194,243,4,104
NARS_2_19438,177,72,187,47,98,0,325,46,78,535,0,159
NCF1_2_19439,74,0,104,13,0,0,131,1,0,0,0,0
NDST1_2_19440,108,124,73,82,140,0,15,0,39,17,230,54
NDST2_2_19441,747,833,929,821,954,897,2461,671,585,650,272,1492
NDST3_2_19442,62,344,184,187,0,389,0,0,368,1044,183,182
NDST4_2_19443,596,918,702,675,527,1379,1296,925,491,597,379,1128
NDUFA10_2_19444,469,333,379,374,27,112,385,716,211,93,220,269
NDUFA12_2_19445,306,258,271,282,387,184,378,1031,122,430,103,291
NDUFA13_2_19446,52,105,78,268,104,4,44,174,52,469,900,326
NDUFA1_2_19447,53,30,37,83,2,0,439,0,223,0,0,43
NDUFA3_2_19448,8,5,20,12,0,29,0,0,0,1,1,0
NDUFA4_2_19449,108,143,216,260,8,708,4,32,442,36,0,17
NDUFA4L2_2_19450,443,452,329,502,277,589,246,11,1167,537,890,352
NDUFA5_2_19451,104,104,124,169,13,9,144,45,70,263,3,302
NDUFA6_2_19452,2328,1514,1728,1907,1150,1353,2310,1636,1699,2712,3179,
1338
NDUFA7_2_19453,32,78,79,97,208,7,557,82,215,193,0,117
NDUFA8_2_19454,99,61,281,274,1,125,152,325,38,3,0,95
NDUFA9_2_19455,141,96,166,176,424,58,106,634,258,218,179,199
NDUFAB1_2_19456,884,758,750,1008,592,164,1779,837,863,1054,1556,1126
NDUFB10_2_19457,273,179,202,282,491,129,169,491,203,691,26,525
NDUFB1_2_19458,844,807,901,1196,332,741,1347,486,803,310,566,824
NDUFB2_2_19459,58,54,78,64,17,49,3,69,26,144,3,2

NDUFB3_2_19460,102,89,146,118,210,210,0,7,0,1,0,88
NDUFB7_2_19461,314,340,307,308,12,13,696,466,303,57,171,261
NDUFB8_2_19462,303,369,754,329,41,376,265,1363,0,428,154,277
NDUFB9_2_19463,31,12,26,98,0,0,422,0,0,0,0,0
NDUFS3_2_19464,240,163,188,152,198,14,207,405,383,156,59,298
NDUFS4_2_19465,837,554,601,589,213,424,859,578,744,111,646,424
NDUFS6_2_19466,105,150,115,258,66,506,22,364,11,282,2,169
NDUFS7_2_19467,89,30,39,9,0,1,7,0,0,64,2,20
NDUFS8_2_19468,398,363,421,290,247,780,221,315,110,669,1008,174
NDUFV2_2_19469,971,972,871,954,1028,1735,1142,1181,1169,1177,525,1105
NEDD8_2_19470,735,606,507,595,62,520,646,870,1175,587,340,890
NEIL1_2_19471,316,159,235,152,284,22,4,0,243,33,392,385
NEIL3_2_19472,651,261,499,456,416,573,616,76,223,231,1229,179
NEU1_2_19473,11,31,48,47,2,36,17,0,19,16,14,25
NEU2_2_19474,554,751,553,731,556,830,509,914,372,648,290,323
NEU3_2_19475,1074,920,692,805,773,865,940,716,1293,1304,1096,754
NHLRC2_2_19476,1059,870,691,1075,1060,1015,1196,310,666,656,767,1375
NIT2_2_19477,411,415,433,356,274,346,1021,334,234,530,150,798
NKIRAS1_2_19478,867,514,487,507,360,939,283,751,140,361,585,344
NLGN1_2_19479,347,355,300,398,501,712,156,203,223,680,447,116
NLGN2_2_19480,165,380,235,312,43,72,165,16,292,431,594,875
NLN_2_19481,708,789,781,1083,2322,221,921,270,1860,784,1208,1176
NME1-NME2_2_19482,166,171,72,97,108,93,23,32,224,361,137,256
NMNAT1_2_19483,92,130,57,16,130,171,18,229,86,28,69,7
NMT1_2_19484,15,14,12,1,0,0,0,0,0,0,0,20
NMT2_2_19485,1027,948,929,1110,962,969,1061,504,1101,1358,686,1648
NNMT_2_19486,101,95,51,75,16,950,0,0,186,227,0,210
NOP58_2_19487,516,306,423,380,45,799,594,57,424,321,49,286
NOX3_2_19488,585,452,530,625,655,122,718,221,564,1152,1130,826
NQO2_2_19489,319,265,443,285,55,86,91,419,444,212,554,332
NRAS_2_19490,265,293,393,395,50,118,1452,158,386,64,792,408
NSF_2_19491,136,184,66,128,315,213,0,0,26,7,33,230
NSUN6_2_19492,63,32,22,57,7,47,78,2,18,0,50,127
NTAN1_2_19493,331,264,256,329,301,91,358,52,147,226,255,116
NTHL1_2_19494,307,286,212,481,358,181,454,315,132,305,303,236
NTPCR_2_19495,46,81,100,105,12,217,171,215,11,11,42,130
NUDT10_2_19496,245,117,177,285,149,395,193,152,59,19,72,214
NUDT11_2_19497,245,117,177,285,149,395,193,152,59,19,72,214
NUDT12_2_19498,202,274,95,193,448,134,431,253,250,29,104,241
NUDT14_2_19499,188,76,72,29,2,0,11,6,25,1,254,198
NUDT3_2_19500,663,378,362,623,70,563,815,520,473,220,55,1222
NUDT5_2_19501,621,672,710,912,841,1093,550,632,685,774,293,442
NUDT7_2_19502,568,449,377,360,860,353,1021,430,323,58,187,628
NXNL1_2_19503,82,69,82,48,426,0,0,5,1,228,170,0
OAS3_2_19504,82,67,44,87,236,24,0,4,33,4,136,237
OAZ1_2_19505,74,47,23,67,11,0,2,0,270,22,0,239
OC90_2_19506,88,66,133,172,2,0,29,0,203,275,823,580
ODF3B_2_19507,160,293,271,164,2,263,14,48,351,484,344,59
OPLAH_2_19508,126,254,150,205,47,373,101,262,0,0,265,31
OSGEP_2_19509,288,253,230,238,106,0,755,95,180,148,282,391

OSGEPL1_2_19510,1068,901,753,866,1753,1128,1903,834,650,1375,853,1207
OSTC_2_19511,2857,2634,2769,2983,3698,1506,3472,2650,2496,2692,2315,35
62
OTC_2_19512,384,411,679,610,57,743,489,147,152,444,1213,892
OVGP1_2_19513,150,209,229,176,56,83,17,26,245,94,12,219
OXA1L_2_19514,599,303,567,427,570,274,91,752,415,131,436,863
OXCT1_2_19515,1274,1126,1083,1174,807,456,1157,194,962,964,1871,1170
OXCT2_2_19516,17,19,8,40,88,25,0,0,22,0,0,2
P4HA3_2_19517,169,107,87,153,235,25,31,214,35,10,20,108
P4HB_2_19518,78,101,118,125,3,270,26,0,320,306,499,31
PADI1_2_19519,244,170,284,416,55,504,0,90,11,322,30,218
PADI2_2_19520,538,354,522,514,438,620,152,1537,148,390,534,789
PADI3_2_19521,168,85,146,210,101,5,0,304,444,168,15,146
PADI6_2_19522,74,18,61,63,220,128,260,39,131,5,2,28
PAFAH2_2_19523,175,96,85,114,266,133,271,1,80,59,110,439
PAH_2_19524,337,375,213,223,174,224,388,0,137,253,226,216
PAPOLA_2_19525,910,956,696,856,1758,1299,612,680,1139,955,451,950
PAPOLB_2_19526,154,106,192,181,360,278,54,71,60,45,421,323
PAPOLG_2_19527,266,375,192,270,633,279,359,418,289,149,228,69
PAPPA_2_19528,647,575,480,603,912,797,903,857,782,262,197,754
PAPSS1_2_19529,2243,1267,1596,1614,2141,2446,631,563,1493,2500,1657,13
77
PARG_2_19530,1527,1472,1276,1765,1747,2104,2729,1184,1538,1773,1589,16
03
PARP1_2_19531,33,90,77,63,40,17,85,120,41,17,0,0
PARP4_2_19532,581,529,600,712,463,96,570,128,403,686,473,956
PARP6_2_19533,196,285,109,220,193,251,459,64,2,730,217,362
PARS2_2_19534,64,2,35,43,241,1,0,0,131,16,0,134
PCBD1_2_19535,21,56,90,24,0,1,0,0,1,0,408,285
PCBD2_2_19536,382,380,397,447,489,218,295,281,334,121,648,750
PCMT1_2_19537,302,296,341,349,352,52,301,294,292,203,207,243
PCMTD1_2_19538,498,832,693,559,888,564,597,852,346,753,853,1181
PCYOX1_2_19539,188,232,133,297,5,103,250,193,28,586,134,44
PCYT1A_2_19540,181,423,237,326,2,731,379,21,558,234,1924,883
PDCD1LG2_2_19541,483,551,460,614,781,412,144,172,278,253,13,483
PDCL_2_19542,96,69,124,139,25,0,579,5,166,248,0,335
PDE3A_2_19543,923,991,959,972,779,886,2276,923,673,599,702,897
PDE3B_2_19544,705,574,662,685,578,469,466,491,587,221,537,771
PDE6A_2_19545,48,73,92,155,123,7,1057,37,4,70,6,2
PDE6C_2_19546,1534,1831,1494,1504,1577,556,698,822,2640,1742,2688,1945
PDE6D_2_19547,228,454,254,393,569,243,560,502,211,85,262,331
PDE6G_2_19548,118,55,77,76,62,18,155,51,2,2,307,85
PDE6H_2_19549,238,204,102,176,480,0,0,306,276,1,0,342
PDE7B_2_19550,189,141,225,227,283,321,356,463,265,168,33,187
PDHA2_2_19551,613,657,529,720,733,416,228,366,484,453,594,1399
PDIA2_2_19552,5,18,13,2,9,0,0,0,0,0,0,0
PDIA3_2_19553,400,252,468,554,326,313,863,342,458,443,1104,375
PDIA4_2_19554,228,225,418,297,381,652,658,1,233,1550,240,380
PDIA5_2_19555,0,0,1,0,0,0,0,16,0,0,0,0
PDIA6_2_19556,168,190,229,331,195,61,34,702,10,243,2,256

PDPR_2_19557,411,309,596,347,237,352,396,651,204,155,1052,458
PDSS1_2_19558,229,51,58,65,155,361,8,0,19,25,5,10
PDSS2_2_19559,679,818,810,911,710,703,495,1140,792,944,67,585
PDXP_2_19560,99,41,57,106,58,11,0,0,42,2,35,141
PECR_2_19561,80,44,54,71,143,0,198,0,0,11,9,36
PELI1_2_19562,82,129,48,140,26,568,145,1054,143,89,289,22
PET117_2_19563,1443,1435,1084,1479,857,372,1666,830,1045,1586,1565,223
0
PEX1_2_19564,683,724,709,637,383,458,1008,697,742,290,1167,436
PEX6_2_19565,101,162,71,88,0,0,0,0,0,212,768,129
PFAS_2_19566,68,57,62,177,190,1,37,0,27,73,12,160
PGA3_2_19567,2610,2424,2026,2260,3028,2297,2138,1088,1837,1056,4067,23
95
PGA4_2_19568,2610,2424,2026,2260,3028,2297,2138,1088,1837,1056,4067,23
95
PGA5_2_19569,2610,2424,2026,2260,3028,2297,2138,1088,1837,1056,4067,23
95
PGAM4_2_19570,55,110,66,41,45,13,78,141,0,258,0,186
PGAP1_2_19571,341,453,282,395,602,245,394,368,225,139,297,455
PGAP3_2_19572,89,72,93,70,0,232,95,22,152,95,2,48
PGD_2_19573,2394,2379,1864,2627,1372,1369,2446,1934,1770,1567,2799,166
5
PGGT1B_2_19574,140,121,112,104,66,180,2,0,32,183,2,0
PGLS_2_19575,328,228,392,528,390,42,113,169,377,228,322,525
PGM2_2_19576,315,399,574,406,429,397,907,490,389,912,368,618
PGM2L1_2_19577,74,74,49,83,48,82,0,0,25,302,0,14
PGM5_2_19578,113,90,174,76,135,1,181,8,46,65,35,207
PGPEP1_2_19579,65,7,35,106,5,0,0,0,6,15,1,118
PGS1_2_19580,1618,1687,1526,1794,535,2229,2910,2059,1122,1288,2576,181
9
PHGDH_2_19581,24,22,49,93,13,1,132,0,30,24,22,160
PHLPP1_2_19582,279,52,177,105,0,1,291,5,0,1,65,30
PHLPP2_2_19583,505,487,342,519,497,193,649,487,299,616,202,425
PIF1_2_19584,110,48,141,98,173,111,455,38,28,61,0,7
PIGB_2_19585,6031,4900,4801,6608,4007,5927,6762,5110,4426,5022,4039,59
85
PIGH_2_19586,319,214,152,351,94,366,37,112,30,184,460,553
PIGL_2_19587,18,48,8,11,1,0,0,0,3,122,0,7
PIGM_2_19588,1252,985,939,1353,758,869,1402,819,1063,1808,1046,1059
PIGS_2_19589,11,89,15,106,16,0,0,0,6,0,0,1
PIGU_2_19590,1947,1527,1800,2077,2102,1525,857,1713,2171,1998,2370,159
7
PIGW_2_19591,426,274,377,430,469,66,239,368,342,303,244,440
PIGZ_2_19592,184,202,254,177,279,205,315,219,246,121,681,240
PIN1_2_19593,159,9,64,102,846,0,1921,0,0,242,299,206
PIPOX_2_19594,38,98,138,46,0,91,211,52,7,64,1,362
PITPNM2_2_19595,88,81,94,117,370,243,0,40,94,137,2,65
PLA2G10_2_19596,163,271,162,109,66,358,0,119,480,388,413,48
PLA2G12A_2_19597,300,212,144,128,462,1,27,4,108,110,441,478
PLA2G12B_2_19598,309,537,398,459,270,1056,484,306,941,220,1315,806

PLA2G15_2_19599,369,250,343,538,233,385,156,150,34,373,520,955
PLA2G1B_2_19600,151,129,155,215,17,177,52,25,17,89,14,212
PLA2G2C_2_19601,716,656,626,568,51,568,1472,361,573,811,134,348
PLA2G2D_2_19602,3083,2393,2658,3264,2670,2234,4785,3526,2170,2748,3481
,1563
PLA2G2E_2_19603,177,177,220,266,294,95,13,324,18,49,0,248
PLA2G2F_2_19604,52,42,0,114,22,0,0,0,0,0,0,0
PLA2G3_2_19605,59,72,38,124,15,0,4,126,2,114,273,31
PLA2G4A_2_19606,559,625,430,526,485,389,737,170,285,805,733,230
PLA2G4B_2_19607,113,65,117,135,41,107,135,0,277,72,206,83
PLA2G4D_2_19608,59,77,40,72,162,0,0,0,0,0,0,107
PLA2G4E_2_19609,880,759,711,795,920,486,1512,696,833,745,782,1296
PLA2G4F_2_19610,53,32,103,73,89,1,44,12,8,85,459,10
PLA2G5_2_19611,2021,2165,2194,2162,1405,1994,4061,1736,1302,1216,2079,
2249
PLCB2_2_19612,1608,1409,1538,1798,1501,664,2188,904,1476,1531,1891,119
3
PLCD3_2_19613,217,128,199,266,320,310,380,172,279,55,67,366
PLCD4_2_19614,838,421,717,551,1151,487,626,431,494,341,709,446
PLCG2_2_19615,210,166,113,184,22,166,343,202,158,624,753,62
PLCH2_2_19616,59,36,111,84,31,0,0,333,101,372,2,62
PLCL1_2_19617,172,100,311,99,283,327,21,0,134,23,718,165
PLCXD1_2_19618,24,27,6,75,0,401,0,0,0,0,0,1
PLCZ1_2_19619,338,209,343,436,66,124,452,334,135,104,159,556
PLD4_2_19620,154,130,179,316,1026,51,92,35,330,297,4,615
PLOD1_2_19621,0,16,37,67,0,0,0,0,0,0,1,132
PLOD3_2_19622,169,181,110,159,193,20,344,300,267,0,772,15
PLSCR1_2_19623,1477,1430,1656,2009,1990,1528,2138,681,1791,987,2298,22
09
PMM1_2_19624,43,75,69,56,0,3,0,4,9,424,8,1
PMM2_2_19625,29,2,19,13,6,105,0,0,0,36,1,4
PMPCA_2_19626,355,191,190,379,188,232,63,831,277,270,167,293
PMPCB_2_19627,426,230,403,429,254,184,355,144,297,599,519,236
PNLIP_2_19628,842,586,686,626,451,631,1513,677,341,485,1302,370
PNLIPRP1_2_19629,626,326,431,590,581,145,776,1113,398,472,808,664
PNLIPRP2_2_19630,589,439,426,610,692,316,1522,1044,253,1140,508,1072
PNLIPRP3_2_19631,1045,937,1058,1275,633,1020,341,1157,1161,2141,505,72
7
PNMT_2_19632,311,314,353,344,600,254,17,265,284,0,212,108
PNPLA2_2_19633,97,131,73,70,40,11,27,0,237,0,0,7
PNPLA3_2_19634,108,74,137,105,66,0,29,0,0,202,168,104
PNPLA8_2_19635,222,250,182,295,501,307,579,76,271,175,203,218
PNPO_2_19636,36,67,42,39,3,10,3,1,25,137,0,4
PNPT1_2_19637,348,450,422,583,596,7,304,431,622,80,490,739
POLA1_2_19638,711,690,644,793,203,779,1151,682,557,186,1606,217
POLA2_2_19639,248,382,278,249,476,135,354,207,390,246,91,33
POLB_2_19640,930,517,573,659,1207,233,893,221,290,711,1844,499
POLD4_2_19641,200,177,145,233,617,0,82,588,48,132,0,142
POLE3_2_19642,59,92,91,211,0,0,3,214,0,0,721,870
POLE4_2_19643,21,40,22,71,0,0,0,1,0,1,0,0

POLE_2_19644,216,201,314,380,27,214,188,244,207,76,769,119
POLG2_2_19645,451,391,512,607,113,267,344,555,320,169,97,452
POLI_2_19646,141,180,179,144,15,1,758,61,74,3,267,177
POLM_2_19647,55,26,39,75,7,108,133,1,45,89,366,103
POLN_2_19648,1105,777,652,931,1132,476,909,918,466,1184,1318,1221
POLR1A_2_19649,103,135,159,137,187,76,252,87,58,207,211,358
POLR1C_2_19650,142,48,100,40,41,139,84,0,199,98,0,12
POLR1E_2_19651,157,83,182,103,9,236,107,40,257,109,99,46
POLR2A_2_19652,61,59,59,39,59,10,0,88,34,0,2,30
POLR2B_2_19653,735,680,565,562,154,629,2137,609,507,536,89,766
POLR2C_2_19654,399,396,368,475,656,1012,251,166,517,223,398,699
POLR2D_2_19655,744,559,630,597,866,126,66,1214,283,1047,48,273
POLR2E_2_19656,117,193,190,215,845,4,22,387,0,3,236,211
POLR2F_2_19657,2,0,2,0,0,0,0,0,0,0,0,0
POLR2G_2_19658,455,490,403,430,150,378,409,10,647,357,249,293
POLR2H_2_19659,1488,1542,1405,1536,1231,1708,934,1561,971,1520,1828,18
26
POLR2J_2_19660,452,289,461,503,0,22,1968,42,234,1176,1368,299
POLR2K_2_19661,858,767,883,654,1298,209,1192,1103,702,868,238,1013
POLR2L_2_19662,10,23,98,19,6,0,219,24,122,85,0,1
POLR3A_2_19663,58,85,43,61,50,15,132,240,3,99,209,88
POLR3C_2_19664,722,645,517,531,983,388,1170,316,543,539,132,856
POLR3F_2_19665,651,754,588,633,791,634,334,1388,928,1116,75,374
POLR3G_2_19666,2498,2429,1781,2303,2423,1870,2627,1007,2611,2376,1701,
2723
POLRMT_2_19667,11,38,17,7,5,0,0,16,46,33,2,27
POMGNT1_2_19668,1468,1106,1305,1684,1298,885,538,613,867,931,1968,979
POMT2_2_19669,85,31,80,60,14,144,82,241,8,39,20,125
PON3_2_19670,580,353,379,494,120,462,549,406,266,225,486,234
POP4_2_19671,162,128,62,66,3,38,185,561,0,75,18,116
POP7_2_19672,421,507,404,371,921,94,306,97,410,369,579,403
POR_2_19673,92,79,56,85,9,0,66,0,20,145,0,20
PPA1_2_19674,286,204,266,242,787,516,112,708,371,728,0,505
PPCDC_2_19675,113,153,144,153,275,66,109,546,160,77,83,226
PPIA_2_19676,335,244,364,423,966,208,1530,346,880,229,33,303
PPIAL4A_2_19677,6743,6195,6305,7258,5727,6259,7081,5978,7018,4999,7362
,6485
PPIAL4B_2_19678,6743,6195,6305,7258,5727,6259,7081,5978,7018,4999,7362
,6485
PPIAL4C_2_19679,6743,6195,6305,7258,5727,6259,7081,5978,7018,4999,7362
,6485
PPIAL4E_2_19680,370,244,321,434,965,207,1535,349,880,229,34,301
PPIAL4G_2_19681,3983,3668,3981,4304,4530,3078,4423,3003,3434,4002,5928
,3906
PPIB_2_19682,472,370,439,466,25,313,8,282,189,809,811,731
PPIC_2_19683,262,159,106,154,282,14,71,322,128,47,20,24
PPID_2_19684,5075,4687,4547,5138,4140,3581,4612,4340,4980,5974,4458,55
72
PPIF_2_19685,118,204,80,206,852,513,601,361,142,231,1242,4
PPIG_2_19686,691,1109,1081,1190,328,594,920,507,1398,1526,847,1112

PPIH_2_19687,334,237,397,221,269,196,882,0,60,722,1,512
PPIL1_2_19688,79,46,169,115,2,109,4,99,2,11,63,103
PPIL4_2_19689,983,858,819,845,422,1020,1600,1066,568,1898,2089,671
PPIP5K2_2_19690,519,514,391,470,1414,161,435,288,240,487,706,375
PPM1H_2_19691,181,127,48,105,206,4,136,176,1,7,106,305
PPM1J_2_19692,191,113,19,78,0,0,0,4,4,61,5,6
PPM1N_2_19693,841,841,914,1095,757,163,546,679,137,233,2314,643
PPME1_2_19694,456,517,479,455,1140,164,471,283,669,452,84,579
PPWD1_2_19695,754,674,547,565,772,639,841,1264,1466,284,1401,1157
PRDX4_2_19696,83,143,196,192,357,0,0,0,0,58,0,17
PREP_2_19697,400,467,528,565,401,107,553,784,275,274,1264,553
PRHOXNB_2_19698,246,260,216,257,164,379,987,734,15,821,4,75
PRIM1_2_19699,455,301,528,739,374,346,554,463,237,224,404,510
PRIM2_2_19700,377,258,380,368,79,334,346,11,331,112,483,488
PROC_2_19701,254,420,327,226,363,1,1165,53,229,671,709,524
PROSC_2_19702,848,927,681,1168,761,837,790,524,901,821,952,843
PRR14L_2_19703,391,405,292,469,196,211,174,14,345,186,415,1085
PRSS1_2_19704,477,275,416,462,224,40,359,393,413,603,1461,710
PRSS2_2_19705,98,19,75,38,514,0,1,121,36,16,0,82
PRTFDC1_2_19706,60,47,60,27,5,0,48,16,91,44,562,2
PRTN3_2_19707,47,49,13,77,0,0,0,0,0,0,0,41
PRUNE_2_19708,21,3,6,14,0,0,2,0,0,0,25,0
PSMA2_2_19709,1017,980,853,812,714,434,1637,1190,82,975,708,672
PSMA6_2_19710,885,575,840,801,488,691,1264,682,1076,788,357,830
PSMA7_2_19711,54,169,124,150,10,108,120,186,107,121,36,531
PSMB10_2_19712,675,498,706,544,1035,530,475,325,1026,845,509,313
PSMB11_2_19713,240,306,318,221,5,591,269,464,0,3,561,0
PSMB1_2_19714,518,532,403,459,493,555,809,946,828,287,562,373
PSMB3_2_19715,388,370,400,492,98,99,373,867,34,286,319,187
PSMB4_2_19716,957,838,947,1189,936,1011,760,498,776,589,179,769
PSMB6_2_19717,155,215,253,169,22,17,74,92,287,173,807,137
PSMB7_2_19718,1089,941,701,1089,1060,1205,1440,1247,1220,600,519,1587
PSMB9_2_19719,39,30,49,61,71,0,111,21,0,0,358,11
PSMD6_2_19720,562,549,479,881,1154,70,685,2003,404,284,1248,1633
PTDSS1_2_19721,357,284,361,345,135,76,764,397,184,1421,79,1088
PTDSS2_2_19722,119,114,138,190,103,42,180,57,75,233,19,361
PTGDS_2_19723,2,38,30,19,0,293,80,1,35,949,0,112
PTGES2_2_19724,61,142,44,80,69,22,138,11,1,0,0,1
PTGES3_2_19725,420,256,208,314,71,55,678,85,432,48,123,491
PTGES_2_19726,33,97,43,42,0,17,5,12,10,5,0,0
PTGIS_2_19727,4,2,30,19,0,4,0,10,69,2,0,150
PTGS2_2_19728,165,185,233,248,266,141,9,43,45,117,468,193
PTRH1_2_19729,278,104,179,207,0,0,2,0,552,12,67,205
PTRH2_2_19730,214,203,129,171,64,358,0,415,774,0,307,351
PTS_2_19731,1328,994,918,1179,815,1015,415,1005,1326,1272,1162,731
PUS3_2_19732,1496,1110,1010,1325,1302,1471,1835,1249,1511,669,1062,148
3
PUSL1_2_19733,369,219,195,299,467,32,365,111,316,406,26,148
PXDN_2_19734,154,155,105,109,0,605,0,0,29,16,4,2
PXDNL_2_19735,1288,1289,952,1174,1253,1507,1358,1591,958,926,1985,1083

PYCR2_2_19736,52,65,112,101,2,33,23,38,128,0,0,7
PYCRL_2_19737,298,76,331,194,5,386,209,366,74,131,255,305
PYGB_2_19738,817,749,740,792,1005,621,869,740,300,1340,775,451
QDPR_2_19739,53,88,109,137,0,13,113,12,105,73,30,238
QPCT_2_19740,693,903,742,894,746,385,2389,692,337,562,604,486
QPRT_2_19741,127,297,115,125,2,4,217,0,223,172,11,7
QRSL1_2_19742,425,615,646,437,797,1114,769,81,671,1301,1498,675
QSOX2_2_19743,733,463,674,816,124,16,826,808,779,1099,1079,1096
QTRT1_2_19744,24,85,67,82,0,2,112,0,75,8,3,5
QTRTD1_2_19745,319,235,180,274,76,12,20,15,103,30,37,179
RAB10_2_19746,578,654,518,667,503,1188,224,453,933,221,676,783
RAB12_2_19747,84,62,140,93,50,124,92,0,241,365,187,81
RAB13_2_19748,3547,2798,3074,3055,3531,1256,3896,2902,3423,4566,2962,5
414
RAB14_2_19749,2101,1536,1690,2217,2177,724,1868,556,2094,2782,1718,232
5
RAB15_2_19750,554,605,535,653,1148,439,878,897,706,622,806,613
RAB17_2_19751,21,45,32,38,186,135,10,0,0,0,380,0
RAB18_2_19752,241,148,210,234,658,282,145,418,82,256,325,199
RAB19_2_19753,215,106,52,125,54,13,441,382,3,15,0,6
RAB1B_2_19754,193,233,261,316,250,507,193,433,132,216,567,797
RAB20_2_19755,1345,1163,1470,1709,1450,868,245,3027,713,1087,607,2237
RAB21_2_19756,731,357,547,502,793,714,1169,2,532,717,1651,1127
RAB22A_2_19757,756,1092,650,662,728,1178,236,525,300,386,21,1026
RAB25_2_19758,66,7,21,77,9,4,0,31,0,0,1,19
RAB26_2_19759,24,65,37,174,109,0,4,661,20,34,651,11
RAB27B_2_19760,1043,631,672,1136,954,686,358,1043,813,670,1547,1520
RAB30_2_19761,642,538,684,731,972,654,248,1361,587,557,793,762
RAB31_2_19762,186,133,115,273,243,41,271,179,441,123,8,169
RAB33A_2_19763,364,250,251,409,185,189,752,1063,529,489,760,1133
RAB33B_2_19764,52,31,86,131,22,166,74,65,18,21,49,55
RAB36_2_19765,72,151,143,134,33,164,418,0,0,13,88,41
RAB38_2_19766,390,331,312,425,614,581,1061,157,468,690,437,395
RAB39B_2_19767,48,36,18,42,0,43,4,453,88,46,37,10
RAB3A_2_19768,36,21,38,68,173,0,8,0,0,0,0,10
RAB3B_2_19769,137,61,105,45,10,41,0,105,184,32,296,88
RAB3C_2_19770,265,304,140,319,328,374,76,869,239,817,186,278
RAB3D_2_19771,1128,930,1000,1164,692,1100,1110,369,1207,560,1662,1416
RAB3GAP2_2_19772,1559,1681,1636,2057,1500,2235,1218,1601,1335,1920,183
9,2551
RAB4A_2_19773,901,686,838,781,305,1084,1445,310,282,603,1743,557
RAB4B_2_19774,424,326,203,159,468,168,103,137,707,223,167,211
RAB5A_2_19775,484,469,511,596,369,1483,232,4,179,375,82,988
RAB5B_2_19776,610,572,563,668,110,677,695,172,789,376,595,1359
RAB6B_2_19777,1662,1292,1465,1908,1094,1752,581,1680,1788,1326,1652,22
60
RAB6C_2_19778,293,303,421,356,1318,301,477,231,35,62,447,150
RAB7A_2_19779,107,233,314,261,144,430,146,160,28,513,70,105
RAB8B_2_19780,593,271,231,397,289,93,2,325,191,134,439,378
RAB9B_2_19781,57,84,61,78,0,17,569,0,4,5,0,0

RABGGTB_2_19782,981,1048,861,974,692,567,856,767,1189,1351,861,890
RABL3_2_19783,268,224,298,279,476,98,353,284,153,593,304,43
RAC2_2_19784,617,261,294,281,114,12,458,210,731,0,0,377
RAD50_2_19785,1115,726,900,1060,669,269,407,796,834,1165,926,1346
RAD54L2_2_19786,792,531,748,955,605,1179,669,728,865,1037,818,1040
RALA_2_19787,108,137,140,164,289,255,2,5,11,115,31,56
RALB_2_19788,677,525,563,619,541,835,1213,573,503,111,672,707
RALBP1_2_19789,283,130,179,174,259,164,481,290,189,353,4,169
RANBP2_2_19790,821,1083,579,836,849,741,26,817,943,1261,1983,1215
RAN_2_19791,187,200,150,272,554,104,0,0,168,85,214,143
RAP2A_2_19792,465,313,312,394,418,223,333,309,515,865,650,207
RAP2B_2_19793,307,537,260,214,0,283,709,908,73,304,411,410
RAP2C_2_19794,208,333,215,334,301,107,1319,38,299,139,14,284
RARS2_2_19795,1446,1435,1433,1556,1660,1048,1325,1334,2094,2206,2859,1
407
RARS_2_19796,961,906,989,1032,1127,581,641,1370,995,1455,921,926
RASD2_2_19797,270,141,137,91,333,321,235,4,276,125,5,213
RASL10B_2_19798,3,102,179,195,287,215,0,18,0,0,497,638
RASL11B_2_19799,32,206,44,79,0,309,280,0,0,0,0,42
RASL12_2_19800,48,32,45,117,0,27,24,0,1,35,0,9
RC3H1_2_19801,2597,2069,1503,1888,2470,1339,576,2450,1880,3035,999,184
9
RCL1_2_19802,407,449,290,372,602,281,124,210,566,291,359,321
RDH10_2_19803,76,78,87,101,4,152,66,3,45,11,0,64
RDH11_2_19804,113,140,175,162,158,10,49,3,142,25,6,140
RDH12_2_19805,110,46,57,102,261,54,0,46,209,34,1,23
RDH14_2_19806,542,625,483,660,80,116,492,536,233,1066,130,824
RDH16_2_19807,142,212,116,139,0,15,80,0,286,459,0,150
RDH8_2_19808,187,86,101,132,48,10,57,59,290,48,169,1
RECQL4_2_19809,79,42,66,64,125,43,351,0,88,26,0,483
REM1_2_19810,44,30,67,23,0,0,0,43,0,38,0,0
REM2_2_19811,293,247,188,154,2,13,269,111,17,202,95,168
RENBP_2_19812,35,87,65,119,174,3,0,0,289,0,11,545
RETSAT_2_19813,1425,1442,1341,1203,599,1066,703,719,1234,1428,1693,159
7
REV3L_2_19814,581,701,889,986,775,472,813,671,712,611,1398,963
REXO1L1_2_19815,449,247,418,453,766,99,710,507,510,339,513,234
REXO2_2_19816,175,175,207,314,62,23,173,431,489,480,172,517
RFNG_2_19817,126,137,111,155,280,113,202,80,115,3,28,7
RFX6_2_19818,2697,2143,2296,2714,4079,2089,2107,3682,1415,3469,2952,22
56
RGS7_2_19819,99,98,69,139,16,8,175,100,51,79,9,368
RHBDL1_2_19820,108,70,127,179,133,98,14,326,128,70,0,15
RHBDL2_2_19821,369,176,267,301,683,201,519,210,541,633,831,566
RHBDL3_2_19822,114,58,57,66,63,257,13,97,1,2,84,180
RHEBL1_2_19823,294,132,359,459,567,66,266,539,523,190,75,350
RHOA_2_19824,162,68,154,80,79,124,36,0,187,90,33,246
RHOB_2_19825,69,38,81,57,38,17,7,16,23,4,8,69
RHOD_2_19826,326,407,231,300,109,687,1466,392,330,799,333,445
RHOF_2_19827,503,361,511,422,402,111,343,143,244,24,0,162

RHOG_2_19828,91,8,25,59,255,0,0,4,0,1,0,27
RHOH_2_19829,986,974,849,1071,312,975,1164,967,980,541,577,1303
RHOJ_2_19830,159,121,136,190,534,30,70,906,405,122,16,211
RHOQ_2_19831,260,291,292,277,0,246,769,0,523,229,289,658
RHOT2_2_19832,171,158,141,131,164,8,77,98,352,323,261,180
RHOU_2_19833,132,92,48,62,99,45,97,74,318,0,82,145
RHOV_2_19834,456,195,576,481,1020,370,188,384,179,745,1676,488
RIT1_2_19835,0,29,41,18,304,0,0,0,2,16,0,56
RIT2_2_19836,63,33,67,86,0,38,0,0,31,66,6,31
RNASE2_2_19837,274,240,217,231,154,19,499,106,156,146,527,57
RNASE3_2_19838,29,0,25,40,7,0,1,55,6,14,0,5
RNASE6_2_19839,1695,1593,1579,1523,732,1139,2506,2368,1540,2376,1367,1
636
RNASE7_2_19840,2439,1786,1872,2317,2288,772,3130,2220,1714,2863,1475,1
593
RNASE8_2_19841,847,682,897,820,1270,345,868,686,826,1097,21,570
RNASEH2A_2_19842,246,321,284,245,776,384,36,57,11,343,633,409
RNASET2_2_19843,1331,1084,1123,1265,1696,39,1812,1303,1264,2110,741,13
00
RND1_2_19844,961,809,670,737,980,529,1051,1029,1034,329,694,1093
RND2_2_19845,400,395,328,531,154,41,79,929,1182,599,227,245
RND3_2_19846,233,215,154,275,513,257,0,27,83,56,319,241
RNF148_2_19847,67,79,66,95,146,0,667,104,126,365,0,150
RNMT_2_19848,532,333,300,501,81,214,121,7,393,364,300,42
RNMTL1_2_19849,793,828,713,745,595,380,817,380,531,1730,1414,830
RNPEP_2_19850,147,123,134,257,274,257,21,330,215,9,10,0
RPAP2_2_19851,594,255,355,456,511,516,256,600,491,812,138,422
RPE65_2_19852,120,89,91,179,110,47,34,1,120,9,34,277
RPIA_2_19853,96,58,88,103,85,3,113,200,61,4,0,205
RPL4_2_19854,39,58,31,43,0,85,197,64,22,0,0,334
RPN1_2_19855,1214,826,989,1038,404,874,1621,1111,916,712,895,1070
RPP25_2_19856,435,784,363,644,474,1052,587,1113,337,444,629,955
RPP40_2_19857,293,156,188,152,87,254,207,451,11,334,0,165
RPS3_2_19858,143,84,216,246,421,337,639,30,634,0,96,26
RPUSD1_2_19859,36,83,50,61,10,2,14,41,55,36,128,15
RPUSD2_2_19860,0,76,15,12,131,43,0,0,2,197,0,6
RRAGA_2_19861,323,142,231,452,111,178,644,93,332,3,1156,336
RRAGC_2_19862,288,246,201,251,89,258,353,149,484,303,270,179
RRAGD_2_19863,289,404,245,387,346,200,163,385,490,85,962,4
RRAS_2_19864,101,31,102,74,2,0,0,0,0,285,0,0
RRM1_2_19865,349,168,229,230,179,356,455,820,268,205,782,328
RRP8_2_19866,511,273,316,361,190,255,29,445,484,261,120,399
RSAD2_2_19867,234,250,280,219,155,322,348,318,185,663,482,409
RTN4IP1_2_19868,763,663,788,649,216,316,827,742,825,785,712,940
SAMHD1_2_19869,1083,933,1117,1131,455,716,2984,862,1160,2094,1231,1594
SDF2_2_19870,373,343,217,401,273,178,770,312,238,455,162,455
SDHA_2_19871,38,46,19,39,4,18,6,0,13,22,1,112
SDHB_2_19872,283,203,255,392,88,23,425,223,290,700,194,408
SDHD_2_19873,10,8,8,10,2,0,1,73,25,1,0,0
SDR16C5_2_19874,532,513,310,412,137,37,148,336,745,847,283,949

SDR42E1_2_19875,104,188,135,165,410,302,6,523,447,0,845,435
SDR9C7_2_19876,1024,930,726,1117,1113,903,933,998,1303,1061,769,1215
SDSL_2_19877,34,131,21,114,360,337,0,0,33,0,0,46
SEPSECS_2_19878,785,581,783,917,1442,222,1026,418,872,347,776,565
1-Sep_2_19879,263,136,138,179,164,102,18,163,346,74,0,143
SEPW1_2_19880,147,91,120,258,223,272,6,26,2,154,15,30
SERHL2_2_19881,685,660,730,588,71,739,746,424,547,675,1213,523
SETX_2_19882,229,239,264,266,81,162,321,39,130,423,231,104
SGMS1_2_19883,497,425,378,409,570,21,406,5,497,271,190,22
SGPL1_2_19884,314,243,436,372,120,366,836,148,454,1323,393,170
SGSH_2_19885,14,42,29,33,6,4,0,348,11,0,425,55
SH3GL2_2_19886,213,196,101,154,138,1,426,19,251,126,4,208
SKIV2L2_2_19887,395,409,341,257,919,354,526,211,221,349,821,278
SLFN12_2_19888,1127,1379,1306,1168,1528,1467,1115,1189,1050,963,705,88
8
SLFN12L_2_19889,1117,1380,1301,1164,1522,1465,1111,1191,1053,966,708,8
84
SLFN13_2_19890,997,1217,1116,1222,938,835,798,591,692,1245,1920,2466
SLFN5_2_19891,123,76,39,44,1,7,0,129,96,177,0,13
SLU7_2_19892,2420,1913,2276,2223,1981,1546,3328,2154,2663,2620,3003,19
00
SMG8_2_19893,44,38,59,25,41,0,0,41,2,3,0,29
SMPD2_2_19894,269,249,159,241,316,296,245,80,124,94,176,6
SMPD3_2_19895,342,466,359,404,417,250,69,41,63,238,736,469
SMPDL3A_2_19896,305,253,168,264,261,160,717,637,334,573,27,427
SMUG1_2_19897,176,269,210,247,227,98,892,665,283,48,15,426
SNF8_2_19898,53,74,57,77,7,23,1,0,0,3,0,0
SNRNP200_2_19899,117,133,39,79,0,0,2,219,371,2,0,121
SOAT1_2_19900,1880,1456,1321,1680,2216,1573,1208,775,1363,2793,1874,18
20
SOAT2_2_19901,121,149,223,172,370,198,289,300,62,171,494,254
SOD1_2_19902,986,544,831,881,1038,267,1106,610,1503,331,1544,680
SOD3_2_19903,85,119,114,127,102,21,98,7,2,484,161,218
SORD_2_19904,351,384,415,514,956,413,472,364,550,556,747,126
SPACA3_2_19905,919,1285,1145,1372,454,1559,923,795,1218,708,1443,852
SPACA5B_2_19906,2034,1578,1661,1840,1778,988,2266,871,1461,1386,668,17
95
SPACA5_2_19907,2034,1578,1661,1840,1778,988,2266,871,1461,1386,668,179
5
SPEM1_2_19908,1487,1075,1172,1372,1076,422,856,863,1583,1184,2388,1144
SPTLC2_2_19909,57,120,121,104,366,62,54,8,36,0,4,0
SPTLC3_2_19910,229,226,177,223,233,163,241,35,155,291,637,97
SPTSSA_2_19911,51,87,59,137,34,0,1,139,106,41,41,4
SQLE_2_19912,734,867,800,611,223,343,869,1094,1072,843,536,522
SQRDL_2_19913,632,373,400,357,511,477,417,495,840,734,342,207
SRD5A1_2_19914,720,531,598,785,641,525,163,1269,602,652,470,700
SRD5A2_2_19915,746,606,765,732,450,377,188,1639,1166,1063,642,565
SRD5A3_2_19916,205,148,178,271,1,20,34,117,137,12,348,531
SRR_2_19917,732,625,675,701,1474,295,153,286,207,220,1058,469
SRSF9_2_19918,363,355,361,426,112,15,6,3,157,421,609,5

SRXN1_2_19919,452,278,313,300,108,232,500,210,166,45,209,962
SSB_2_19920,2162,2308,2499,2380,1147,1200,2866,3172,2150,2493,2512,300
6
SSU72_2_19921,633,588,473,693,857,96,234,270,1301,210,321,1359
ST14_2_19922,10,46,24,14,0,2,0,0,23,0,0,5
ST3GAL2_2_19923,303,203,380,295,334,415,360,8,52,176,1354,396
ST3GAL4_2_19924,188,203,220,230,275,118,379,363,163,120,238,348
ST3GAL6_2_19925,60,29,44,86,36,67,76,12,55,39,3,11
ST6GALNAC1_2_19926,393,385,139,399,259,233,82,468,6,161,540,491
ST6GALNAC2_2_19927,33,17,44,20,99,14,470,9,5,2,220,3
ST6GALNAC5_2_19928,220,280,219,211,421,404,241,226,355,451,66,470
ST6GALNAC6_2_19929,162,109,269,227,123,0,149,277,154,384,32,472
ST8SIA1_2_19930,537,433,566,473,738,560,764,765,377,565,190,552
ST8SIA2_2_19931,43,62,36,65,0,0,5,34,118,444,3,10
ST8SIA3_2_19932,823,499,622,749,485,211,1991,1060,391,741,142,753
ST8SIA5_2_19933,60,46,54,39,2,7,1,11,0,206,93,155
ST8SIA6_2_19934,774,861,865,1343,953,1299,799,1756,766,1015,535,1336
STS_2_19935,222,325,216,275,35,265,471,451,80,341,138,198
STT3A_2_19936,289,308,209,373,491,881,181,85,806,122,372,197
STT3B_2_19937,255,268,289,230,222,94,310,1,221,92,136,6
SUCLA2_2_19938,124,197,124,207,237,2,1,219,160,161,14,122
SUCLG1_2_19939,743,495,523,816,681,868,324,1483,210,192,303,624
SULT1A3_2_19940,336,362,326,376,476,397,419,364,194,837,277,310
SULT1A4_2_19941,336,362,326,376,476,397,419,364,194,837,277,310
SULT1B1_2_19942,900,632,776,709,966,631,817,4,321,970,718,1015
SULT1C3_2_19943,2578,2197,2066,2404,1640,2345,1968,2606,3358,2991,1971
,2330
SULT1C4_2_19944,578,473,500,763,999,561,488,1966,1300,380,92,360
SULT1E1_2_19945,258,261,234,303,361,267,924,47,97,379,798,464
SULT2A1_2_19946,1040,1005,880,1205,1478,529,428,1380,504,1175,2117,984
SULT4A1_2_19947,247,117,119,137,115,3,698,237,90,285,22,81
SUPV3L1_2_19948,1655,1238,1449,1949,1999,932,2020,992,1744,3477,1604,2
073
SURF1_2_19949,498,418,284,274,215,268,59,616,221,155,1131,173
TALDO1_2_19950,16,19,29,36,46,0,0,40,0,23,0,6
TARS2_2_19951,1351,1184,1250,1361,880,803,611,770,1258,1411,1704,1978
TARS_2_19952,2929,2843,2375,2666,1918,2408,2989,2173,1727,2332,2096,18
97
TARSL2_2_19953,441,315,306,411,128,46,87,233,330,531,1062,671
TAT_2_19954,96,94,142,155,1,120,101,70,290,17,332,83
TBC1D10B_2_19955,256,106,156,72,96,5,0,329,187,11,94,285
TBCC_2_19956,818,696,656,681,616,893,1225,16,1056,414,177,527
TDG_2_19957,2390,2015,1728,2142,2299,2528,2007,4104,1649,1850,1207,173
9
TDO2_2_19958,491,750,742,761,149,691,935,354,484,1025,566,325
TECR_2_19959,77,33,0,96,0,0,0,0,198,0,0,0
TECTA_2_19960,73,36,7,70,1,9,0,3,14,387,126,28
TFB2M_2_19961,426,306,220,365,17,20,510,350,128,29,386,285
TGDS_2_19962,605,568,803,747,872,456,1631,960,876,967,1204,504
TGM1_2_19963,101,63,73,74,0,0,35,5,0,239,0,3

TGM3_2_19964,200,349,235,338,577,1,0,336,2,559,190,364
TGM4_2_19965,1263,1053,1205,1453,1452,1150,670,1123,1607,1698,1422,452
TGM6_2_19966,198,291,245,185,489,33,571,0,565,80,119,196
TGM7_2_19967,533,318,303,422,431,299,520,398,206,383,819,471
TGS1_2_19968,268,131,124,247,129,0,1121,70,610,1157,348,442
THG1L_2_19969,1757,1381,1223,1383,1684,838,2039,2251,1450,2099,1193,14
73
THOP1_2_19970,135,89,218,218,135,0,110,24,50,62,397,83
THUMPD2_2_19971,251,198,297,270,78,485,451,64,203,260,581,276
TKTL2_2_19972,383,534,423,355,340,553,57,92,685,642,436,207
TM7SF2_2_19973,250,227,208,388,46,515,314,421,326,227,328,92
TMEM55A_2_19974,323,287,219,273,184,69,581,249,193,564,33,233
TMEM62_2_19975,166,202,276,233,49,60,568,93,265,154,0,150
TMEM86B_2_19976,73,37,24,76,0,0,25,0,0,0,0,7
TMPRSS15_2_19977,1128,970,904,890,1174,1414,1272,1244,738,1227,179,112
6
TMX1_2_19978,901,549,743,800,1017,745,644,921,971,727,116,1372
TMX3_2_19979,1098,917,1110,1305,760,921,1881,1121,1088,1389,1339,599
TMX4_2_19980,636,433,626,551,607,42,74,529,763,211,603,628
TNKS2_2_19981,0,0,0,0,0,0,0,0,0,0,0,0
TNKS_2_19982,1284,1528,904,1148,1054,510,1224,1549,1042,1360,2075,2439
TOP1_2_19983,507,482,427,481,526,390,328,78,239,561,300,81
TOP1MT_2_19984,75,160,91,122,1,4,6,310,58,82,45,387
TOP2A_2_19985,699,819,495,652,1005,250,594,570,502,592,717,404
TOP2B_2_19986,679,641,442,732,382,278,394,1209,1342,422,344,619
TOP3A_2_19987,98,31,92,116,48,0,364,26,161,27,0,8
TOP3B_2_19988,83,52,77,104,140,15,918,121,30,18,95,38
TOR3A_2_19989,208,249,231,213,146,323,294,258,284,48,150,490
TPH1_2_19990,1145,1421,1355,1738,909,660,657,2892,1236,787,1556,860
TPMT_2_19991,834,713,504,833,277,812,2216,1063,920,229,1153,459
TPP1_2_19992,492,371,279,306,130,607,244,86,381,496,98,294
TPP2_2_19993,19,8,6,0,0,0,0,0,0,135,0,0
TPSAB1_2_19994,151,68,62,175,6,13,96,911,317,245,136,134
TPSB2_2_19995,1083,1255,1039,1128,799,636,426,769,1442,1511,439,856
TPSD1_2_19996,494,414,507,375,388,35,174,494,412,759,128,455
TPST1_2_19997,564,342,434,386,405,710,423,645,314,21,158,567
TREH_2_19998,927,876,1094,1169,2143,483,680,3564,853,486,2115,1479
TREX2_2_19999,32,80,9,50,0,2,221,56,197,1,1,1
TRHDE_2_20000,676,406,739,744,498,840,1323,763,968,1031,834,942
TRIM21_2_20001,266,221,224,232,511,228,147,48,244,496,67,480
TRIT1_2_20002,767,500,478,516,572,408,1251,117,521,586,162,242
TRMT112_2_20003,342,341,258,341,351,70,640,229,289,173,378,263
TRMT61A_2_20004,39,9,6,21,0,0,0,0,4,0,0,0
TRMU_2_20005,354,437,364,567,998,76,434,290,95,656,282,476
TRNT1_2_20006,526,537,477,756,753,733,100,285,360,264,591,434
TRUB1_2_20007,958,1043,1038,1125,479,995,1924,859,641,1180,960,991
TRUB2_2_20008,31,21,53,63,16,0,9,0,4,2,0,199
TSTA3_2_20009,91,35,77,41,1,0,8,1,3,232,5,161
TST_2_20010,572,507,545,570,245,561,913,87,121,295,719,790
TTLL13_2_20011,182,85,90,58,95,1,22,156,161,5,17,37

TTLL1_2_20012,11,43,20,12,0,1,0,33,1,3,0,0
TTLL3_2_20013,317,261,461,333,399,245,630,326,71,496,204,741
TTLL4_2_20014,662,498,373,634,134,162,399,344,231,473,551,899
TUFM_2_20015,459,330,425,410,203,412,1295,1282,416,210,204,618
TULP2_2_20016,506,497,311,303,1261,381,68,580,88,1224,1273,605
TUT1_2_20017,452,305,456,442,211,509,1417,539,171,1052,261,718
TXN2_2_20018,328,324,318,379,795,185,401,356,316,360,1543,580
TXN_2_20019,274,147,147,109,288,102,384,412,8,301,386,135
TXNDC11_2_20020,529,473,420,337,452,339,15,254,54,652,274,305
TXNDC12_2_20021,104,283,137,221,50,527,274,141,22,186,265,67
TXNDC15_2_20022,1509,1039,1288,1084,847,919,1087,974,1507,1018,1518,19
91
TXNDC17_2_20023,436,430,539,530,625,605,388,347,77,556,209,528
TXNL1_2_20024,836,854,846,778,598,798,527,259,795,598,961,678
TXNL4A_2_20025,4280,4060,3461,3756,4075,1717,2602,3176,3690,5836,5526,
4395
TXNRD2_2_20026,273,157,209,337,246,109,194,381,75,185,204,84
TYMS_2_20027,755,486,742,573,489,281,737,844,871,122,474,717
TYR_2_20028,321,266,274,376,126,544,75,451,1166,96,111,620
TYRP1_2_20029,63,73,68,62,0,1,307,669,206,164,42,103
UAP1_2_20030,144,222,205,278,259,287,213,335,127,129,124,124
UAP1L1_2_20031,39,28,17,39,0,0,15,1,26,0,3,85
UBIAD1_2_20032,560,377,523,472,568,195,998,52,270,293,306,938
UBL4A_2_20033,28,31,29,19,0,6,0,57,73,3,0,81
UFSP1_2_20034,195,229,284,291,171,478,28,354,189,90,454,300
UFSP2_2_20035,547,361,364,466,245,390,405,485,717,635,129,285
UGCG_2_20036,1147,1084,1078,1205,1280,1400,134,882,1315,1314,293,684
UGGT1_2_20037,24,20,3,24,43,0,92,35,40,166,0,40
UGGT2_2_20038,305,238,221,341,148,92,818,284,113,70,94,123
UGT1A10_2_20039,861,818,847,1033,973,1185,624,300,710,1663,1021,818
UGT1A1_2_20040,2594,2226,2599,2694,3338,1917,3275,4051,2537,3244,4252,
2741
UGT1A3_2_20041,2262,2364,2645,2734,3398,1101,2548,2834,2048,3123,3115,
2144
UGT1A4_2_20042,480,478,442,557,792,448,1437,1104,752,1234,110,843
UGT1A5_2_20043,2262,2364,2645,2734,3398,1101,2548,2834,2048,3123,3115,
2144
UGT1A7_2_20044,900,794,863,1041,970,824,622,301,709,1667,1026,818
UGT1A8_2_20045,1476,1527,1398,1725,1801,1199,2374,1593,1244,1299,1096,
1372
UGT1A9_2_20046,900,794,863,1041,970,824,622,301,709,1667,1026,818
UGT2A1_2_20047,128,58,35,202,478,98,0,54,0,494,718,0
UGT2A2_2_20048,1446,1468,1439,1404,2388,1315,1236,1487,1680,1653,1888,
908
UGT2A3_2_20049,208,239,197,208,164,108,735,914,210,229,231,258
UGT2B15_2_20050,1364,1406,1399,1501,2245,1031,1247,1919,1332,1237,1826
,908
UGT2B17_2_20051,1364,1406,1399,1501,2245,1031,1247,1919,1332,1237,1826
,908
UGT2B4_2_20052,133,55,72,154,180,122,7,19,90,187,60,120

UGT2B7_2_20053,408,503,482,583,373,489,33,329,381,552,822,826
UMPS_2_20054,344,424,468,444,796,123,990,593,82,279,311,515
UPB1_2_20055,319,366,154,313,297,640,92,391,213,338,1123,516
UPF1_2_20056,119,186,95,135,73,139,14,13,157,239,50,163
UPRT_2_20057,414,298,304,341,240,246,41,22,406,188,1590,754
UQCR11_2_20058,682,643,640,925,421,45,217,1293,779,647,442,1560
UQCRC1_2_20059,199,308,361,475,32,131,631,731,421,420,439,48
UQCRC2_2_20060,526,316,229,601,80,6,90,24,187,18,601,147
UQCRFS1_2_20061,554,561,639,400,198,603,1276,347,424,792,850,562
UQCRH_2_20062,1536,1220,967,1303,1217,524,2654,1377,912,1816,3106,1032
UQCRQ_2_20063,374,295,222,424,486,470,836,509,35,418,101,225
UROD_2_20064,474,436,369,485,257,437,861,22,413,282,892,821
UROS_2_20065,307,428,305,208,105,309,233,43,186,385,203,183
UST_2_20066,256,132,121,113,182,152,118,119,160,25,13,114
UXS1_2_20067,374,287,183,291,620,356,320,233,103,272,23,118
VARS_2_20068,188,261,204,320,330,432,497,211,25,232,383,374
VAT1L_2_20069,794,934,964,1166,810,584,3590,274,706,1019,779,1166
VCP_2_20070,236,138,124,260,673,66,88,86,425,84,455,168
VNN1_2_20071,1404,1176,903,1379,457,1586,153,1557,1688,1741,969,1572
WBSCR17_2_20072,74,48,155,115,16,187,145,8,106,115,154,315
WBSCR27_2_20073,250,67,127,217,26,0,5,208,5,0,375,647
WDFY3_2_20074,314,312,252,316,321,174,626,1,312,218,42,403
WRN_2_20075,239,239,165,362,37,171,112,1515,118,382,177,318
XDH_2_20076,97,77,113,78,183,18,198,346,49,8,633,387
XPNPEP2_2_20077,394,384,366,514,185,229,126,53,317,1002,703,1003
XRCC2_2_20078,101,65,92,159,31,50,31,64,6,350,0,140
XRCC5_2_20079,292,465,250,450,403,370,274,659,422,390,1152,426
XRCC6_2_20080,470,505,491,523,503,200,924,1145,339,101,1155,411
XRN2_2_20081,569,460,422,580,1137,192,859,775,619,1271,352,646
XYLT1_2_20082,114,117,139,189,198,5,19,387,111,151,10,62
XYLT2_2_20083,224,368,307,289,163,763,77,669,57,17,3,406
YARS2_2_20084,656,832,808,927,1132,429,1239,827,1111,711,353,869
YARS_2_20085,74,54,48,39,0,107,363,1,137,8,58,102
YKT6_2_20086,410,293,328,354,68,356,68,147,346,323,210,236
YPEL1_2_20087,594,531,446,733,1237,1105,1526,724,787,1003,263,1033
ZADH2_2_20088,65,40,82,105,71,94,58,486,107,27,0,95
ZCCHC4_2_20089,141,120,70,69,0,0,1,0,1,207,2,1
ZDHHC17_2_20090,5899,5540,4414,5789,4282,3958,4559,6190,4926,4518,7282
,5096
ZDHHC18_2_20091,68,41,17,35,14,0,176,10,2,26,3,69
ZDHHC1_2_20092,329,472,327,655,502,467,814,121,474,159,383,325
ZDHHC21_2_20093,393,321,330,528,259,280,153,718,277,332,15,376
ZDHHC2_2_20094,11,68,16,31,27,39,0,0,0,19,0,3
ZER1_2_20095,76,27,35,62,0,143,0,0,0,0,0,51
ZMPSTE24_2_20096,480,484,626,685,546,35,1707,1372,907,192,1159,849
ZRANB3_2_20097,246,179,205,267,140,215,309,5,380,273,301,255
A1CF_2_20098,226,195,168,228,126,74,602,665,66,72,440,915
A2LD1_2_20099,6,2,39,30,0,6,0,52,0,0,0,0
AADAT_2_20100,997,824,614,700,587,906,708,161,401,798,728,393
AARSD1_2_20101,79,121,120,194,40,13,252,248,237,269,30,75

ABAT_2_20102,610,365,413,425,419,179,972,25,386,750,362,548
ABHD11_2_20103,376,435,253,389,385,70,906,410,335,139,617,625
ABHD12_2_20104,478,468,498,639,424,941,362,975,469,376,164,697
ABHD14B_2_20105,55,3,12,77,1,0,2,4,0,37,3,94
ABHD2_2_20106,239,95,304,263,537,37,71,134,89,78,384,102
ACAA1_2_20107,58,31,28,59,76,0,406,181,89,10,0,1
ACACA_2_20108,453,160,216,393,190,679,266,134,330,128,516,269
ACAD10_2_20109,322,189,300,325,566,518,127,247,664,298,196,707
ACADM_2_20110,50,32,50,38,102,117,8,12,89,10,2,62
ACADVL_2_20111,877,903,830,1083,418,736,523,346,309,693,1198,957
ACCS_2_20112,287,411,432,496,6,515,37,632,481,476,963,210
ACE_2_20113,419,365,248,350,614,888,6,875,184,549,1,570
ACHE_2_20114,80,162,36,128,500,21,101,184,5,204,0,135
ACIN1_2_20115,1410,1328,1244,1561,958,1178,1980,912,966,1444,1201,1615
ACLY_2_20116,83,43,86,112,293,126,238,58,96,192,141,30
ACOT11_2_20117,77,24,81,87,1,1,0,0,0,1,2,288
ACOT13_2_20118,304,180,120,186,486,51,683,259,203,392,24,645
ACOT7_2_20119,499,437,288,336,89,292,3,256,92,5,68,380
ACOT9_2_20120,17,6,18,15,1,0,0,0,4,0,0,0
ACOX1_2_20121,249,282,262,389,55,46,0,0,79,71,331,345
ACOX3_2_20122,158,64,150,108,4,44,227,478,170,31,517,51
ACSBG1_2_20123,541,452,236,405,8,83,408,449,230,908,223,514
ACSF3_2_20124,60,74,59,56,78,50,0,1,88,0,24,52
ACSL3_2_20125,4026,3932,3859,4186,4829,3101,3619,2272,4140,4403,3776,4
707
ACSL4_2_20126,509,508,443,331,495,1061,364,362,503,542,1003,794
ACSL5_2_20127,104,32,104,161,402,892,1061,378,0,0,324,210
ACSL6_2_20128,100,127,131,87,83,55,175,23,0,122,61,36
ACSM2B_2_20129,428,359,270,284,92,606,436,33,209,344,846,257
ACSM3_2_20130,503,514,717,581,437,122,2275,699,277,423,617,195
ACSS2_2_20131,89,60,37,52,52,144,155,37,2,22,11,12
ACY1_2_20132,156,67,58,127,2,92,1,55,239,8,2,133
ADAMTS2_2_20133,288,134,198,201,1149,2,0,0,74,567,0,3
ADARB1_2_20134,158,68,221,240,201,399,743,59,60,0,670,841
ADAR_2_20135,286,217,283,295,0,193,433,6,350,84,24,116
ADCY10_2_20136,2148,1954,1738,2079,2116,1291,3955,1782,1905,2074,2722,
3939
ADCY4_2_20137,48,122,21,11,0,0,0,55,0,82,279,3
ADCY5_2_20138,303,523,542,788,623,275,996,76,485,730,2226,714
ADCY6_2_20139,154,47,81,147,87,0,160,47,23,540,3,0
ADH6_2_20140,45,65,107,86,45,48,5,16,52,15,93,2
ADH7_2_20141,505,616,543,429,186,315,194,625,569,805,334,499
ADPRHL1_2_20142,273,268,429,396,23,277,685,580,579,480,912,211
ADSL_2_20143,1363,1119,1207,1249,1380,1794,2198,1632,1025,2251,2268,15
41
ADSSL1_2_20144,335,310,356,383,538,172,28,534,475,282,2,412
AFMID_2_20145,86,19,78,97,0,0,0,0,0,0,0,32
AGA_2_20146,108,70,69,93,55,45,2,154,31,61,82,86
AGAP1_2_20147,49,14,48,15,1,0,0,0,217,6,0,191
AGAP2_2_20148,312,246,257,464,703,583,113,292,333,539,343,270

AGL_2_20149,699,771,653,635,581,420,1043,374,261,1357,732,699
AGPAT1_2_20150,1012,624,953,956,324,1109,734,535,724,787,966,762
AGPAT2_2_20151,563,231,411,507,15,756,12,1105,82,675,574,625
AGPAT3_2_20152,212,284,103,211,65,0,132,95,159,149,255,33
AGXT2L1_2_20153,164,192,121,184,0,112,1352,0,189,260,54,192
AHCY_2_20154,272,319,229,458,511,257,445,769,160,447,136,422
AHCYL1_2_20155,288,168,165,362,361,189,119,427,250,216,38,414
AHCYL2_2_20156,409,473,311,288,425,72,525,115,303,70,166,811
AIFM1_2_20157,383,521,556,730,304,886,199,553,292,518,656,412
AIFM2_2_20158,246,243,100,184,108,34,100,44,9,12,0,82
AIFM3_2_20159,487,583,359,460,689,759,361,311,413,600,0,705
AKIRIN1_2_20160,355,492,339,404,461,195,550,544,326,400,1353,402
AKR1A1_2_20161,30,66,49,64,58,406,13,0,5,109,0,222
AKR1C2_2_20162,1845,2139,1489,1882,1896,1369,2398,1150,1277,2240,445,1
190
AKR1D1_2_20163,123,79,75,66,36,7,28,0,231,38,0,31
ALAS1_2_20164,112,287,194,205,58,55,210,109,142,307,98,149
ALAS2_2_20165,292,227,171,283,104,7,38,27,130,279,338,512
ALDH16A1_2_20166,253,257,170,362,79,50,286,689,351,16,1,73
ALDH1A2_2_20167,445,714,652,643,880,1154,445,377,559,589,586,409
ALDH2_2_20168,392,456,441,451,793,541,367,227,301,438,410,586
ALDH3A1_2_20169,86,70,48,61,66,50,60,4,125,1,14,92
ALDH3A2_2_20170,0,0,0,0,0,0,0,0,0,0,0,0
ALDH3B1_2_20171,25,15,20,39,0,50,1,20,0,0,230,17
ALDH3B2_2_20172,250,318,236,332,19,9,1337,411,74,291,0,194
ALDH4A1_2_20173,71,14,89,76,36,28,0,25,8,3,0,14
ALDH5A1_2_20174,116,113,154,197,11,27,579,0,0,13,0,15
ALDH7A1_2_20175,319,364,279,281,209,114,464,276,496,739,516,355
ALDH8A1_2_20176,614,939,723,659,207,638,1004,261,1058,161,821,703
ALDOA_2_20177,353,250,281,308,308,71,486,104,267,185,190,457
ALG3_2_20178,344,240,272,324,80,326,369,230,229,63,476,138
ALG5_2_20179,280,345,323,280,920,616,449,443,220,653,880,605
ALG8_2_20180,875,1160,1145,1171,557,747,882,1316,1416,795,1507,1354
ALG9_2_20181,321,245,300,262,121,0,620,28,144,76,673,173
ALOX15B_2_20182,175,235,178,196,441,108,153,3,62,169,84,25
ALOXE3_2_20183,208,496,242,302,71,280,400,594,241,329,20,438
AMACR_2_20184,237,176,255,366,7,394,126,0,0,4,0,259
AMD1_2_20185,479,333,226,349,75,422,45,768,723,46,97,252
AMDHD2_2_20186,346,310,390,487,239,22,288,61,705,212,408,284
AMPD1_2_20187,123,39,32,66,194,0,122,35,35,40,0,69
AMPD2_2_20188,64,58,31,72,0,226,15,79,4,97,2,3
AMPD3_2_20189,91,80,95,135,8,38,130,163,163,78,182,321
AMT_2_20190,450,307,427,441,530,0,692,510,527,627,255,442
AMY1A_2_20191,3036,2508,2713,2976,3054,2180,3086,3192,2485,2429,1368,2
293
ANG_2_20192,568,410,478,567,610,365,513,544,351,618,249,664
AOAH_2_20193,586,498,512,831,512,377,1681,809,1130,1280,994,1166
AOC2_2_20194,702,608,487,573,361,151,1030,830,261,894,887,628
APC_2_20195,846,745,676,762,417,1160,813,311,619,749,197,926
APEX1_2_20196,186,193,280,457,490,7,0,357,573,0,699,694

APOBEC3A_2_20197,528,582,434,502,974,238,771,36,364,492,263,492
APOBEC3H_2_20198,570,795,629,631,406,1129,790,325,486,880,399,722
APRT_2_20199,154,51,203,149,273,55,13,36,126,63,19,126
ARF1_2_20200,254,172,207,105,329,1,34,53,132,300,0,0
ARFRP1_2_20201,53,46,55,35,3,1,0,0,21,10,0,0
ARHGAP5_2_20202,2351,2256,2236,2531,3471,3234,4478,1962,2005,2205,2531,1570
ARHGEF10L_2_20203,26,142,131,57,7,0,0,0,224,253,0,494
ARL2_2_20204,90,113,148,242,9,88,11,145,14,4,1,3
ARL4A_2_20205,1669,1414,1437,1770,1850,1197,1463,1096,929,1876,1209,1223
ARL5A_2_20206,502,468,420,559,1145,538,288,282,456,559,405,729
ARSA_2_20207,569,664,709,839,541,256,109,997,159,651,870,566
ARSB_2_20208,93,198,159,209,19,67,0,368,52,59,2,447
ARSF_2_20209,474,307,280,372,436,482,352,1026,163,275,42,26
ART3_2_20210,1165,990,977,1241,2779,937,902,1042,922,770,1615,1070
ART5_2_20211,437,122,485,248,0,224,331,308,119,613,0,1176
ASAH1_2_20212,974,1008,982,1010,615,1507,1155,393,464,957,1534,1198
ASAH2_2_20213,1606,1311,1118,1429,2162,1419,1272,1285,1508,1940,1013,1339
ASL_2_20214,1313,1190,1485,1603,535,1187,759,1978,1035,662,3312,2000
ASMT_2_20215,41,79,68,78,43,254,0,11,45,10,144,117
ASMTL_2_20216,0,0,0,0,0,0,0,0,0,0,0,0
ASNS_2_20217,1933,1757,1286,1974,1971,2747,1662,1899,1043,3637,2112,1679
ASPA_2_20218,145,128,116,116,467,179,6,3,197,21,228,18
ASRGL1_2_20219,302,384,463,346,59,422,189,420,399,603,595,117
ASS1_2_20220,41,44,179,55,77,0,3,13,22,6,23,153
ATE1_2_20221,384,235,277,267,493,18,0,0,0,0,955,605
ATL1_2_20222,339,216,405,129,284,255,395,48,420,209,80,294
AURKAIP1_2_20223,245,164,152,212,144,35,715,486,81,635,11,248
AZIN1_2_20224,874,675,377,581,40,1236,823,122,870,229,300,285
B3GALNT1_2_20225,53,37,24,52,9,3,347,0,5,0,542,55
B3GALT5_2_20226,1636,1273,1153,1615,1491,1231,1826,2645,1202,1090,978,1529
B3GAT1_2_20227,151,148,249,295,377,20,769,76,48,229,572,346
B4GALNT2_2_20228,501,385,412,481,708,149,403,891,139,276,398,251
B4GALT2_2_20229,53,117,54,128,41,0,68,40,17,25,29,30
B4GALT3_2_20230,141,73,61,135,13,6,41,76,0,62,126,32
B4GALT4_2_20231,101,225,237,236,0,0,85,286,222,400,155,353
BAAT_2_20232,105,109,194,243,147,79,197,214,108,59,8,787
BACE1_2_20233,905,1359,1146,1194,664,2090,901,1744,676,1157,1403,1741
BACE2_2_20234,1326,999,859,1062,563,878,1968,465,1122,616,1268,766
BCAT1_2_20235,438,397,383,482,85,139,95,427,108,623,288,787
BCAT2_2_20236,236,372,212,298,200,711,1698,1311,35,217,229,197
BCKDHA_2_20237,479,156,134,214,304,400,0,425,86,9,768,113
BCKDHB_2_20238,52,138,96,112,0,205,15,4,80,130,3,6
BCO2_2_20239,321,224,188,199,277,2,140,0,16,139,29,370
BDH1_2_20240,219,140,165,128,203,166,257,171,201,202,21,132
BFSP1_2_20241,12,4,2,3,0,1,0,0,1,0,0,0

BHMT2_2_20242,57,49,52,58,178,5,0,25,51,21,11,120
BMP1_2_20243,338,177,185,219,189,134,391,227,206,54,68,126
C17orf101_2_20244,470,539,640,1055,1054,30,546,383,680,175,979,771
C1GALT1C1_2_20245,890,694,673,964,1006,398,922,246,311,128,2225,1078
C1S_2_20246,252,189,185,297,257,751,341,236,133,11,162,373
C2_2_20247,678,489,547,490,438,622,178,789,693,843,376,612
CA10_2_20248,34,21,32,55,0,0,0,1,48,1,0,1
CA12_2_20249,0,0,8,2,0,0,0,1,0,0,0,10
CA1_2_20250,85,93,57,107,13,148,1,0,129,49,141,130
CA7_2_20251,126,190,195,224,26,48,805,407,347,60,63,407
CAB39_2_20252,1571,1184,1275,1654,516,1235,2752,1439,927,2323,671,1479
CANT1_2_20253,336,329,416,456,40,52,304,433,154,273,462,166
CAPN1_2_20254,45,142,84,109,30,487,172,12,96,155,143,66
CAPN2_2_20255,353,556,328,579,2,236,50,294,814,313,6,257
CARNS1_2_20256,43,12,31,36,46,0,2,0,1,0,6,153
CARS_2_20257,652,489,384,507,2227,405,147,577,416,253,547,663
CASP10_2_20258,1050,892,798,854,810,878,467,294,1201,1639,598,1077
CASP1_2_20259,761,1028,961,1014,945,677,745,1141,978,1039,576,964
CASP2_2_20260,35,51,112,104,11,32,101,222,99,4,5,24
CASP3_2_20261,744,694,647,1128,1025,772,1854,1318,660,837,447,791
CASP4_2_20262,273,227,239,281,492,197,198,0,10,144,57,269
CASP5_2_20263,162,88,126,173,144,43,832,104,0,123,76,32
CASP6_2_20264,99,103,108,182,74,217,347,77,72,159,96,617
CASP7_2_20265,4,4,16,16,246,0,0,7,3,1,0,22
CASP8_2_20266,108,116,79,144,121,197,0,162,323,315,21,0
CASP9_2_20267,79,68,52,95,155,0,150,1,15,322,21,76
CASZ1_2_20268,3,13,15,0,0,0,0,0,4,0,0,223
CAV3_2_20269,312,175,288,225,327,234,252,64,391,19,299,472
CBS_2_20270,22,54,61,94,0,31,48,10,142,0,0,0
CCBL1_2_20271,10,18,15,35,0,0,146,0,10,0,163,4
CCBL2_2_20272,234,106,105,127,93,51,4,8,127,97,76,116
CDC42_2_20273,1862,1807,1609,1585,1048,967,2930,1252,2303,1505,1686,12
11
CDH16_2_20274,157,180,187,153,8,157,14,158,27,383,138,316
CECR1_2_20275,11,11,49,39,0,29,239,0,0,246,36,109
CEPT1_2_20276,1350,1250,865,987,907,634,812,2529,1874,804,921,453
CERS1_2_20277,186,45,118,60,733,17,423,34,0,8,493,49
CES1_2_20278,7,13,43,66,1,103,50,20,25,9,0,20
CES3_2_20279,109,69,98,59,191,2,261,0,64,31,419,313
CES4A_2_20280,164,76,231,169,458,170,124,1,163,13,587,143
CES5A_2_20281,196,97,53,143,7,53,213,11,52,403,43,63
CHAT_2_20282,23,24,24,23,1,0,122,9,11,491,0,17
CHI3L2_2_20283,263,235,211,360,465,305,21,385,222,82,1054,188
CHIA_2_20284,856,689,520,597,341,524,387,755,276,436,334,485
CHPF_2_20285,161,106,144,315,390,303,26,0,14,518,55,8
CHST11_2_20286,570,561,510,828,413,68,800,250,312,441,1141,555
CHST15_2_20287,322,264,304,277,290,107,1296,314,289,370,364,807
CHST4_2_20288,117,25,144,132,52,188,2,40,18,12,227,33
CHST8_2_20289,371,206,197,246,256,329,321,56,782,615,42,169
CLP1_2_20290,228,150,150,181,694,22,0,152,137,136,185,249

CNDP2_2_20291,55,97,75,34,297,128,7,11,26,564,206,93
CNTN1_2_20292,308,233,314,370,117,740,643,139,181,287,996,111
CNTN4_2_20293,142,41,54,179,46,211,0,1,234,60,9,0
COMT_2_20294,395,285,170,149,317,260,12,405,542,581,234,170
COQ6_2_20295,84,135,129,93,209,195,6,391,20,133,0,217
COX11_2_20296,1269,1901,1725,1682,752,1751,1905,917,2506,1580,2491,104
5
COX15_2_20297,366,362,399,253,228,113,338,562,148,71,111,63
CPA5_2_20298,543,556,453,700,798,745,471,1373,330,742,550,610
CPB2_2_20299,179,264,208,409,150,5,0,0,3,510,102,0
CPM_2_20300,890,724,666,912,685,648,303,5,523,937,971,427
CPPED1_2_20301,843,754,816,772,920,890,970,319,1243,919,816,633
CPT1A_2_20302,27,49,67,106,0,0,5,2,66,212,0,0
CPT1B_2_20303,756,829,815,1057,814,1357,707,871,636,577,595,691
CPT1C_2_20304,1270,1100,1063,1174,1142,940,710,840,1444,927,2615,1352
CRLS1_2_20305,245,136,170,236,90,6,97,265,142,120,650,236
CRMP1_2_20306,77,81,122,86,343,190,38,8,8,94,81,1
CROT_2_20307,139,196,190,221,278,108,446,50,207,80,462,113
CRY2_2_20308,551,548,297,684,535,596,0,0,225,17,541,446
CRYM_2_20309,653,518,307,467,738,498,222,504,482,320,439,193
CRYZ_2_20310,108,44,39,158,61,184,54,0,41,164,49,19
CSDE1_2_20311,403,150,144,266,169,286,529,441,484,201,339,242
CSGALNACT1_2_20312,286,292,342,279,343,144,130,193,143,365,100,196
CSMD3_2_20313,93,63,166,168,412,173,39,173,0,72,553,550
CTAGE5_2_20314,95,101,111,77,204,181,0,155,144,46,0,0
CTBP1_2_20315,91,88,113,85,140,28,47,110,105,202,1,62
CTPS2_2_20316,309,364,326,364,240,45,369,499,266,380,177,407
CTSA_2_20317,245,278,225,149,268,179,485,240,78,147,681,81
CTSB_2_20318,1,0,35,1,0,0,535,22,0,0,0,0
CTSE_2_20319,677,890,592,941,366,332,550,56,603,716,1449,1099
CTSL1_2_20320,554,434,487,540,272,169,359,665,716,413,512,615
CTSL2_2_20321,292,259,288,286,216,156,17,107,742,745,16,310
CTSS_2_20322,804,856,740,738,902,572,718,719,408,1133,711,744
CYB561_2_20323,207,184,48,185,131,117,4,102,190,0,0,268
CYB5A_2_20324,283,55,141,88,20,128,11,0,15,64,0,500
CYB5R3_2_20325,337,400,333,325,210,660,419,515,286,285,360,252
CYP11A1_2_20326,48,68,94,63,0,12,49,294,7,0,0,74
CYP11B1_2_20327,74,153,164,114,18,114,283,522,120,110,9,378
CYP19A1_2_20328,188,59,49,82,148,196,5,26,9,189,1,375
CYP21A2_2_20329,70,2,9,20,0,0,0,8,0,5,0,0
CYP24A1_2_20330,1060,660,766,637,956,572,635,261,978,408,281,662
CYP26A1_2_20331,183,267,187,160,272,506,328,111,273,468,71,108
CYP2A7_2_20332,126,146,93,72,16,97,0,373,75,28,116,249
CYP2C18_2_20333,143,158,197,105,455,253,385,314,18,320,48,90
CYP2C8_2_20334,252,154,70,99,109,1,7,101,317,20,0,78
CYP2D6_2_20335,143,146,92,106,67,1,1,3,93,91,4,37
CYP3A43_2_20336,75,84,116,77,268,0,7,75,146,0,8,292
CYP3A4_2_20337,548,514,478,484,355,469,730,538,748,707,409,389
CYP4B1_2_20338,295,232,309,319,255,374,153,144,440,36,386,335
CYP4F11_2_20339,144,95,38,42,240,9,23,28,22,29,29,26

CYP4F3_2_20340,21,72,62,88,0,0,1,696,0,350,0,40
CYP51A1_2_20341,545,400,474,470,194,403,504,163,330,630,117,473
DAGLB_2_20342,147,204,270,154,69,16,10,412,33,14,251,234
DCLRE1C_2_20343,273,536,311,505,111,307,101,174,344,326,328,227
DCP2_2_20344,428,289,363,354,215,24,355,491,372,445,516,240
DCT_2_20345,63,92,125,64,95,2,10,71,31,257,0,346
DCTD_2_20346,128,116,79,126,75,75,51,76,75,0,121,110
DCXR_2_20347,801,480,697,849,204,613,123,980,1288,384,253,702
DDAH1_2_20348,891,868,762,660,867,53,903,1209,1069,1748,640,1038
DDC_2_20349,424,186,159,340,420,355,52,262,368,47,563,116
DDHD1_2_20350,169,419,259,271,291,260,1696,18,192,200,265,480
DDO_2_20351,549,392,525,315,667,165,720,933,297,175,562,284
DDT_2_20352,1548,1741,1003,1894,1131,1201,2780,1913,1903,1859,1401,142
5
DDX11_2_20353,240,252,280,350,345,329,144,2,121,500,344,470
DDX17_2_20354,172,229,256,247,138,123,376,397,392,20,286,76
DDX19B_2_20355,763,778,713,868,624,590,1249,658,647,1153,1656,699
DDX31_2_20356,147,150,179,122,16,376,26,228,88,28,49,11
DDX39B_2_20357,489,215,289,454,546,319,378,216,163,295,240,515
DDX3X_2_20358,196,74,155,196,9,66,350,248,49,122,525,152
DDX3Y_2_20359,221,203,289,253,804,471,72,29,135,376,112,442
DDX42_2_20360,652,471,508,438,516,133,1283,136,614,1053,474,374
DDX47_2_20361,317,342,229,305,249,69,283,121,682,563,163,104
DDX4_2_20362,840,808,845,886,726,179,324,719,544,905,434,252
DDX54_2_20363,247,170,187,180,336,618,14,308,193,50,277,385
DFFA_2_20364,1122,839,1011,1060,1132,824,2122,659,772,673,1823,721
DGCR8_2_20365,36,61,72,71,397,0,1,0,0,0,94,0
DHCR7_2_20366,281,182,227,135,54,110,0,0,17,133,0,4
DHDDS_2_20367,1405,1328,975,1394,943,1395,1300,888,836,1835,301,1251
DHFRL1_2_20368,848,701,660,899,833,614,971,771,346,569,1002,782
DHPS_2_20369,470,283,465,507,208,698,129,1064,570,168,299,209
DHRS1_2_20370,386,251,286,395,477,436,545,806,179,284,234,522
DHRS2_2_20371,2001,1550,1280,1472,1646,1903,2559,941,900,2358,1256,144
3
DHRS9_2_20372,1198,1304,1267,1409,760,851,345,1076,1332,381,2331,1768
DHX16_2_20373,551,829,654,921,162,291,934,544,497,91,446,955
DHX30_2_20374,343,460,245,370,21,20,634,658,314,418,700,851
DHX33_2_20375,3,142,169,41,217,0,0,1,0,291,532,0
DHX35_2_20376,396,460,507,672,1,671,763,53,440,313,1612,316
DHX36_2_20377,271,219,390,273,632,0,587,413,66,331,531,269
DHX40_2_20378,1029,698,578,869,540,666,320,690,490,665,10,1096
DIAPH3_2_20379,242,303,369,319,538,235,46,524,168,88,426,826
DIS3_2_20380,845,945,1263,1506,1363,935,1786,672,899,641,379,983
DIS3L_2_20381,1001,938,940,1010,887,479,2949,575,987,237,2803,1175
DKC1_2_20382,562,787,642,671,222,481,1,320,70,329,2718,745
DNAJC27_2_20383,517,374,304,566,995,58,504,456,714,566,605,417
DNASE1L1_2_20384,316,298,226,276,220,272,335,453,632,344,80,633
DNM1_2_20385,0,0,0,0,0,0,0,0,0,0,0,0
DNM1L_2_20386,1574,1595,1276,1672,806,680,1584,920,1355,987,2092,1797
DNM2_2_20387,371,279,317,279,673,233,795,358,364,334,435,261

DNM3_2_20388,917,670,720,871,545,777,237,1412,497,956,224,458
DNTT_2_20389,868,695,799,701,905,534,887,1703,502,352,177,493
DOHH_2_20390,115,56,48,68,0,106,113,66,67,4,0,37
DOLPP1_2_20391,35,47,51,10,275,6,0,41,39,2,0,22
DPEP1_2_20392,132,91,99,264,337,92,13,170,87,294,255,126
DPEP3_2_20393,207,93,136,222,17,25,9,220,281,21,810,114
DPH5_2_20394,305,329,276,254,314,386,74,288,220,108,73,72
DPM3_2_20395,24,20,0,21,0,0,0,0,0,0,0,5
DPP3_2_20396,188,292,145,148,53,0,38,78,18,126,24,256
DPP8_2_20397,2024,1866,1699,1834,1030,1044,976,1660,1657,1890,2053,102
3
DPYSL2_2_20398,326,362,411,253,364,283,258,275,86,137,1031,122
DPYSL3_2_20399,245,149,261,258,16,111,2,125,20,262,178,172
DROSHA_2_20400,415,337,244,385,39,561,356,408,14,824,293,467
DSE_2_20401,7,1,3,8,18,12,0,1,1,98,0,12
DUOX1_2_20402,28,68,31,44,48,0,53,0,53,0,0,21
DUT_2_20403,1659,1604,1504,1433,2205,2269,1652,524,1519,1027,1231,1819
ECE1_2_20404,232,140,149,151,513,2,8,144,562,219,82,171
ECE2_2_20405,954,831,601,818,1643,1039,355,633,837,1130,699,786
ECI1_2_20406,46,43,12,29,0,1,0,1,0,0,334,11
ECI2_2_20407,1601,1206,1418,1381,582,757,1285,1436,1707,1897,1726,1942
EDEM2_2_20408,972,618,727,623,590,399,854,701,254,36,1754,261
EFEMP1_2_20409,418,233,242,330,16,21,575,148,60,323,104,224
EFTUD2_2_20410,514,374,415,577,206,628,1379,847,311,1462,42,577
EGLN2_2_20411,513,388,422,460,357,114,89,270,356,142,231,377
EHHADH_2_20412,33,15,22,37,1,2,1,327,117,5,0,212
ELAC2_2_20413,93,115,130,108,0,0,0,0,187,0,10,0
ELOVL5_2_20414,923,693,628,685,796,1103,1303,783,273,905,1136,1036
ELOVL6_2_20415,291,532,359,363,336,545,270,517,327,450,753,383
ELOVL7_2_20416,403,380,392,574,480,71,624,144,403,318,136,850
ENDOV_2_20417,398,76,73,141,14,0,2,0,0,1,8,176
ENO3_2_20418,74,55,53,88,0,31,70,71,84,7,9,81
ENOX2_2_20419,360,129,119,182,36,147,205,95,152,40,9,67
ENPP2_2_20420,141,49,59,91,38,108,21,236,19,22,7,20
ENTPD1_2_20421,1281,1100,1039,1103,1077,809,1900,851,715,877,1496,977
ENTPD2_2_20422,418,643,444,483,394,232,191,1417,612,5,17,591
ENTPD4_2_20423,2154,1183,1396,2147,2035,2076,2159,1230,2336,1602,1122,
1455
ENTPD6_2_20424,210,136,147,173,136,12,453,89,356,58,18,123
ENTPD8_2_20425,36,22,92,194,39,130,0,74,0,237,0,215
EPHX1_2_20426,320,275,386,374,626,99,810,85,127,361,314,420
EPHX3_2_20427,114,134,74,112,0,0,0,8,0,2,0,443
ERCC1_2_20428,196,126,246,177,54,17,401,132,338,371,10,69
ERCC2_2_20429,36,44,51,119,0,0,272,59,0,1,0,234
EXO1_2_20430,85,112,107,138,35,1,26,50,249,116,0,42
EXOG_2_20431,1770,1413,1542,1961,2271,1330,1850,1783,1700,2428,1546,21
98
EXOSC3_2_20432,912,901,847,833,1264,1432,711,1362,1212,464,894,709
EXOSC9_2_20433,730,611,330,459,741,432,109,565,581,105,699,933
EXTL2_2_20434,838,638,456,725,263,20,598,398,175,280,20,192

F7_2_20435,193,145,57,83,323,0,338,28,264,2,112,4
FAHD1_2_20436,320,313,267,271,356,96,271,89,368,476,359,507
FAM108A1_2_20437,184,88,194,195,88,97,100,6,125,5,292,52
FAM135A_2_20438,129,145,61,91,3,170,272,75,19,1,44,5
FAN1_2_20439,248,280,185,337,122,303,230,368,141,1296,1253,22
FDPS_2_20440,194,91,159,273,44,163,20,299,265,173,72,436
FDXR_2_20441,411,324,325,330,507,285,475,48,564,607,227,535
FECH_2_20442,1843,1432,1464,2210,1887,2071,1368,1478,2753,2355,1166,15
22
FERMT3_2_20443,88,0,131,50,12,0,0,0,0,0,0,0
FHIT_2_20444,130,142,124,240,61,477,243,13,58,121,19,478
FIGNL1_2_20445,862,636,606,755,784,852,472,584,380,316,764,661
FKBP11_2_20446,368,247,373,361,0,248,20,410,248,476,35,808
FKBP1A_2_20447,1,8,15,12,10,0,0,0,2,0,0,49
FKBP1B_2_20448,1055,741,734,825,526,900,899,609,350,471,1048,1188
FKBP2_2_20449,334,383,353,647,197,622,163,220,99,287,0,668
FKBP5_2_20450,976,656,741,725,1035,314,1612,942,629,1202,152,910
FKBP7_2_20451,2195,1508,1699,2056,1279,1208,2626,2262,1748,2467,1698,1
538
FLAD1_2_20452,64,60,99,77,18,0,6,301,139,1,0,148
FMO3_2_20453,956,949,890,1108,961,511,1458,1107,813,216,1593,1084
FMO5_2_20454,452,604,522,712,310,324,548,770,739,1091,790,540
FN1_2_20455,361,633,253,479,219,423,347,153,478,236,49,772
FOLH1_2_20456,534,490,535,426,739,271,1387,795,109,386,1358,607
FPGS_2_20457,54,123,76,136,294,245,13,3,94,244,47,117
FPGT_2_20458,1510,1220,1224,1809,1666,567,937,1023,1077,2829,582,1593
FTCD_2_20459,148,204,69,103,316,97,0,77,124,290,3,143
FTSJ1_2_20460,81,184,94,121,169,340,118,292,2,0,0,260
FUT2_2_20461,91,99,65,68,164,0,0,8,184,0,97,199
FUT3_2_20462,393,372,390,290,348,235,579,413,296,217,489,615
FUT6_2_20463,176,108,130,186,120,286,13,381,3,96,27,128
FUT8_2_20464,775,572,725,833,1417,476,814,447,1043,695,297,1452
G3BP1_2_20465,41,20,30,73,46,9,1,0,25,115,0,130
G3BP2_2_20466,540,750,583,1063,939,1398,508,404,443,959,981,1220
G6PD_2_20467,29,112,100,87,0,0,393,52,0,18,1,2
GAA_2_20468,319,369,231,350,508,33,30,11,435,78,526,238
GAD1_2_20469,201,251,219,231,76,125,1034,163,59,400,117,138
GAD2_2_20470,261,261,433,298,475,343,142,71,91,690,242,463
GALC_2_20471,107,91,146,233,72,39,657,41,298,47,30,3
GALE_2_20472,1099,984,1142,1182,1005,840,832,805,898,806,262,1172
GALNT9_2_20473,270,214,159,243,210,0,512,42,138,101,113,164
GALNTL1_2_20474,42,87,83,75,18,122,193,33,41,3,45,81
GAMT_2_20475,151,62,131,157,68,174,94,372,31,10,0,88
GANAB_2_20476,152,174,199,197,337,32,148,38,200,119,6,362
GART_2_20477,183,150,244,221,63,851,140,59,117,77,281,380
GBA_2_20478,185,250,329,264,63,265,480,144,170,479,303,330
GBP5_2_20479,340,236,274,413,301,145,32,851,291,149,833,681
GCAT_2_20480,202,191,255,252,360,5,274,0,41,215,89,597
GCDH_2_20481,31,36,46,39,0,5,0,0,41,0,597,0
GCH1_2_20482,176,66,62,94,0,1,89,3,18,28,1,53

GCLC_2_20483,374,300,277,414,354,66,343,152,192,644,260,497
GCNT1_2_20484,480,288,425,359,963,364,191,404,518,322,323,571
GDPD1_2_20485,1236,1467,1268,1536,1338,1809,1073,363,1340,1491,1038,75
3
GDPD2_2_20486,149,134,215,181,0,22,64,50,6,27,0,173
GEM_2_20487,2460,1891,1846,2650,1660,1509,1972,1555,2151,2263,2133,312
9
GEN1_2_20488,369,414,444,351,59,951,610,273,179,280,308,213
GFM2_2_20489,77,214,150,145,0,0,338,7,1,171,189,203
GFOD1_2_20490,83,106,6,97,356,1,30,52,171,42,1,2
GGCT_2_20491,183,121,53,78,184,79,8,6,273,37,2,88
GGCX_2_20492,429,369,458,400,981,1145,448,190,755,67,33,419
GGT1_2_20493,155,67,190,102,0,1,4,0,193,0,1073,0
GGT5_2_20494,53,2,42,14,0,0,17,28,0,98,11,11
GGT6_2_20495,313,276,198,240,348,277,89,526,677,70,51,159
GGTLC1_2_20496,74,70,120,90,3,0,0,372,1,59,0,1
GLB1_2_20497,140,133,96,103,0,124,405,0,70,2,35,204
GLRX2_2_20498,385,264,629,563,13,155,269,25,820,351,1864,803
GLRX3_2_20499,1386,1183,1208,1793,945,1360,2041,2073,1029,651,623,1363
GLRX_2_20500,71,98,62,88,4,14,18,435,1,7,26,254
GLT8D1_2_20501,326,265,200,223,61,76,82,0,143,1,263,462
GLUL_2_20502,385,429,375,494,407,408,668,21,669,454,159,232
GM2A_2_20503,289,335,212,308,359,245,509,159,845,128,684,263
GMPPA_2_20504,442,345,472,566,424,375,81,89,716,276,172,488
GMPPB_2_20505,110,191,136,194,210,1,139,2,4,123,604,289
GMPR2_2_20506,511,374,331,369,230,798,166,444,184,1020,14,595
GNAI2_2_20507,1999,1602,1440,1827,1208,1726,2084,1430,1800,1601,2778,3
330
GNAL_2_20508,227,271,226,348,218,249,379,381,67,61,489,573
GNAO1_2_20509,443,351,347,461,451,58,399,516,510,584,217,473
GNAT1_2_20510,220,175,57,200,306,3,102,73,199,44,17,474
GNB5_2_20511,324,226,289,335,760,54,596,112,137,829,668,342
GNG10_2_20512,206,108,161,199,648,27,9,327,1,0,0,374
GNG4_2_20513,66,76,53,241,236,0,169,475,1,377,107,28
GNGT2_2_20514,712,741,802,850,146,936,1455,474,327,366,300,378
GPD2_2_20515,874,773,617,972,1668,779,820,1175,1052,820,179,927
GPHN_2_20516,164,174,63,142,420,119,50,20,124,176,101,192
GPNMB_2_20517,716,533,451,424,1602,352,910,303,1184,616,450,587
GPT2_2_20518,61,70,84,24,15,4,7,15,323,17,0,1
GPX4_2_20519,864,521,661,1043,535,679,932,543,685,640,1414,631
GSR_2_20520,953,883,786,1078,1277,713,376,534,1116,1048,470,851
GSTCD_2_20521,362,405,350,545,176,383,843,1159,677,35,31,706
GSTK1_2_20522,22,35,28,52,0,55,0,2,0,2,1,0
GSTM1_2_20523,1497,1143,1065,1174,2145,997,1675,1989,1577,1088,864,761
GSTM2_2_20524,1497,1143,1065,1174,2145,997,1675,1989,1577,1088,864,761
GSTM4_2_20525,570,380,644,555,841,604,217,935,273,300,600,550
GSTO1_2_20526,1396,1273,1369,1331,2435,1639,1994,2287,561,1787,1144,16
18
GSTO2_2_20527,1262,1327,1499,1698,375,639,1391,2206,1646,1403,2335,191
3

GSTZ1_2_20528,482,397,352,495,302,832,493,166,731,425,479,140
GTPBP3_2_20529,90,143,53,79,0,148,376,0,37,156,91,254
GUCY1A3_2_20530,757,839,641,1003,358,638,293,807,674,785,313,766
GYG1_2_20531,530,604,466,532,393,430,615,141,147,1608,1173,1228
GYG2_2_20532,196,127,211,330,63,120,58,74,218,151,0,278
GYS1_2_20533,62,30,105,72,63,1,84,14,12,8,25,68
HADH_2_20534,266,143,259,434,467,850,23,764,434,357,341,642
HAGH_2_20535,315,151,239,213,386,82,388,161,148,256,311,347
HAO2_2_20536,71,86,106,135,9,232,253,1,84,191,674,14
HAS3_2_20537,251,430,221,275,367,2,161,387,241,444,564,350
HCCS_2_20538,289,131,254,395,784,0,4,43,392,112,38,4
HDHD1_2_20539,158,182,180,213,416,20,80,33,200,31,546,50
HENMT1_2_20540,317,131,272,320,576,416,813,13,232,60,165,296
HHAT_2_20541,0,11,11,59,330,19,260,14,0,59,0,1
HIBCH_2_20542,283,191,235,408,383,141,108,5,55,143,57,6
HLCS_2_20543,71,25,48,53,146,1,0,8,455,52,2,0
HMBS_2_20544,138,93,74,118,8,38,218,126,52,211,1,15
HMGA2_2_20545,774,606,775,1064,1193,830,362,793,1388,244,1559,402
HMGCL_2_20546,124,67,33,46,40,119,2,12,22,12,0,30
HMGCLL1_2_20547,952,558,730,722,1107,1187,962,895,605,792,268,353
HMGCR_2_20548,527,375,402,651,357,547,492,503,175,457,158,1046
HMGCS1_2_20549,399,291,296,359,131,1080,280,0,400,167,119,203
HMGCS2_2_20550,335,170,358,216,1,22,71,32,676,21,765,595
HMOX2_2_20551,813,602,642,813,231,356,350,618,658,462,89,766
HNRNPAB_2_20552,461,218,218,368,395,215,360,263,317,314,468,437
HPD_2_20553,643,932,850,858,295,578,1108,772,803,739,1023,594
HPGD_2_20554,51,159,115,110,16,115,12,0,550,0,0,1
HPSE2_2_20555,26,11,57,47,21,0,1,15,5,18,73,3
HPSE_2_20556,41,21,31,13,0,0,37,0,2,92,0,15
HRAS_2_20557,114,2,68,168,0,342,3,65,14,305,0,113
HS2ST1_2_20558,220,156,266,287,5,159,355,44,300,298,30,431
HS6ST2_2_20559,27,35,51,71,0,295,0,110,4,135,0,21
HSD11B1_2_20560,1832,1688,1571,1877,2219,535,2973,554,1854,2508,1316,1
786
HSD17B10_2_20561,631,657,603,708,697,458,339,395,647,427,119,667
HSD17B13_2_20562,618,725,395,469,380,262,500,901,134,770,288,1360
HSD17B4_2_20563,3086,2205,1973,2403,1842,1915,2212,3163,3028,3434,1459
,3059
HSD3B2_2_20564,178,410,333,262,543,27,779,42,160,457,216,399
HSD3B7_2_20565,13,57,29,28,0,0,176,0,8,0,0,37
HSDL1_2_20566,1098,870,764,1176,896,1043,2787,1128,1250,785,858,1195
HSP90AA1_2_20567,444,612,516,522,570,640,1188,208,1014,429,1055,539
HSPA8_2_20568,61,78,53,54,152,3,46,65,147,24,14,54
HSPD1_2_20569,154,127,129,163,68,30,0,342,8,288,16,81
HTRA2_2_20570,39,6,101,137,5,0,0,0,3,0,0,10
HYAL1_2_20571,344,455,199,401,108,87,949,391,1,102,405,243
HYAL2_2_20572,566,300,333,309,71,619,1019,517,55,369,368,440
HYI_2_20573,85,86,31,61,83,0,306,178,315,2,49,5
IARS_2_20574,604,600,709,657,641,90,10,246,283,690,566,480
IDE_2_20575,2,72,141,106,0,4,0,18,2,253,382,15

IDH3B_2_20576,261,128,68,57,0,195,279,45,34,206,0,0
IDH3G_2_20577,71,216,47,94,81,572,27,8,180,460,172,1
IDS_2_20578,416,342,350,315,628,194,611,239,654,367,386,629
IFT27_2_20579,93,91,196,243,15,5,0,4,82,9,0,111
IL4I1_2_20580,124,124,183,147,433,455,518,212,47,424,2,344
IMPDH1_2_20581,2,27,9,38,0,0,0,15,0,0,0,0
INMT_2_20582,20,22,2,99,38,0,236,0,0,2,0,505
INPP5K_2_20583,40,20,6,26,182,0,0,0,0,1,0,4
INTS6_2_20584,591,563,452,657,565,310,426,327,79,892,490,564
IPCEF1_2_20585,187,318,261,313,11,228,6,307,127,312,291,334
ISOC2_2_20586,760,407,282,474,919,6,4,392,175,161,379,112
ISPD_2_20587,475,367,333,324,888,338,139,364,203,130,288,71
ISYNA1_2_20588,27,122,12,11,0,218,16,0,245,0,0,0
ITPA_2_20589,1284,1511,1208,1711,998,1200,2581,1903,1217,1474,1308,589
IVD_2_20590,136,168,150,134,165,244,100,105,272,212,47,112
IYD_2_20591,197,153,173,153,40,52,206,240,107,269,3,331
JMJD7-PLA2G4B_2_20592,113,65,117,135,41,107,135,0,277,72,206,83
KARS_2_20593,1486,1647,1519,1925,1705,1586,1864,802,1993,1228,1584,181
1
KATNA1_2_20594,494,348,422,367,919,29,303,434,98,121,301,612
KATNAL1_2_20595,1272,783,715,823,249,515,902,561,1048,890,541,957
KIF16B_2_20596,102,178,62,129,160,61,1,0,100,157,128,100
KIF9_2_20597,753,635,585,793,579,546,407,844,956,738,125,780
KIFC3_2_20598,367,152,161,376,1,123,251,8,223,914,489,392
KLK2_2_20599,30,98,61,63,7,72,105,17,30,18,15,23
KLK7_2_20600,495,541,521,529,170,516,14,256,476,362,1655,434
KRAS_2_20601,459,469,358,448,1047,303,183,256,746,678,406,502
KYNU_2_20602,1078,937,958,977,1209,232,2328,649,1263,938,1042,1157
LAMP2_2_20603,1585,1195,1372,1553,1256,716,1403,1807,1163,2550,1623,14
26
LARGE_2_20604,168,234,305,254,355,0,49,22,256,74,898,27
LCLAT1_2_20605,274,203,126,80,81,87,419,257,109,79,477,378
LCMT1_2_20606,1088,1352,917,1880,600,1188,1541,2512,1638,1220,1093,853
LDHA_2_20607,294,277,333,228,581,405,599,16,182,371,303,546
LDHAL6A_2_20608,284,175,313,252,815,172,16,90,152,288,298,370
LDHB_2_20609,1417,1039,858,1127,931,612,538,1604,827,499,707,2162
LDHC_2_20610,437,399,422,458,563,677,1005,608,127,1035,73,223
LDHD_2_20611,13,7,80,38,344,0,0,134,24,0,5,0
LEPRE1_2_20612,74,87,67,78,62,6,38,18,3,65,36,68
LEPREL1_2_20613,532,347,589,431,99,290,801,116,196,1054,416,664
LFNG_2_20614,19,25,22,61,47,0,58,27,72,0,0,1
LGMN_2_20615,1237,574,707,760,640,408,417,979,812,723,809,1017
LGSN_2_20616,514,351,452,360,400,347,160,353,171,462,99,263
LIAS_2_20617,521,420,567,663,744,41,696,111,575,612,458,677
LIG3_2_20618,47,25,19,12,15,0,0,4,0,7,0,35
LIG4_2_20619,5894,4049,4033,4190,4257,3354,3406,2365,4009,4676,4167,37
69
LIPF_2_20620,240,115,180,146,22,0,270,595,171,178,0,88
LIPT1_2_20621,355,296,230,311,21,339,18,279,353,169,412,450
LMO7_2_20622,270,509,310,374,96,144,762,629,265,21,46,258

LNPEP_2_20623,1189,1036,881,1203,892,261,943,175,274,1412,1568,1294
LOX_2_20624,390,389,317,416,633,471,779,748,366,444,685,239
LPO_2_20625,281,310,272,346,33,290,6,89,107,169,2,252
LRRC16A_2_20626,693,641,489,759,380,247,751,37,369,558,430,569
LSS_2_20627,69,14,67,42,10,0,0,0,67,0,27,100
LYZL6_2_20628,186,457,443,664,115,756,1455,391,473,46,58,698
MACF1_2_20629,2124,1537,1586,1825,2064,512,1693,1051,1989,808,1827,213
4
MACROD2_2_20630,252,278,280,239,27,46,134,11,527,301,365,155
MAD2L2_2_20631,272,495,351,359,45,79,37,308,48,532,52,252
MAN2B1_2_20632,32,23,11,12,2,1,0,28,4,0,0,0
MASP2_2_20633,201,244,110,210,0,0,0,8,22,205,326,0
MAT2B_2_20634,687,437,604,590,862,133,115,1302,766,416,375,434
MCAT_2_20635,38,0,7,0,0,0,0,2,0,0,0,0
MCM4_2_20636,299,206,292,239,6,56,835,295,189,233,412,20
MCM7_2_20637,109,50,13,71,2,46,0,7,2,0,18,227
MCM8_2_20638,53,15,118,78,0,61,0,1,65,35,0,0
MCM9_2_20639,807,746,743,841,665,603,1087,561,847,1041,539,479
MDH1_2_20640,1066,1133,991,1193,651,786,1591,774,682,930,876,948
MECR_2_20641,1510,1175,1168,1291,1503,806,876,1300,1727,820,752,1110
MEPCE_2_20642,44,66,81,39,1,0,44,0,0,146,71,8
METTL13_2_20643,312,337,288,498,365,118,125,471,524,1079,1066,351
MFN2_2_20644,163,141,95,242,78,78,206,131,153,105,1,126
MFNG_2_20645,156,87,53,128,1,2,0,0,221,245,2,45
MGAT3_2_20646,350,159,156,230,259,335,37,28,71,450,232,178
MGAT4A_2_20647,1901,1223,1355,1471,1350,572,1918,3052,1741,986,1624,10
62
MGAT4B_2_20648,160,167,83,130,0,21,652,339,154,0,1,20
MGLL_2_20649,76,82,36,88,63,140,3,70,8,13,0,7
MGST1_2_20650,1467,1552,1289,1621,2100,737,1568,2654,1287,1530,1551,13
03
MGST2_2_20651,709,539,611,595,520,227,861,559,566,578,858,597
MICAL1_2_20652,749,624,553,681,368,634,250,603,833,747,1845,724
MLH1_2_20653,77,91,136,128,13,44,0,3,23,31,0,360
MME_2_20654,201,214,139,242,140,471,26,14,47,507,3,91
MMP1_2_20655,216,112,125,109,0,18,2,271,0,237,57,235
MMP2_2_20656,79,57,39,66,0,230,8,122,35,53,13,25
MOCS1_2_20657,158,239,89,255,302,83,248,529,35,65,1,185
MOGS_2_20658,515,542,544,659,887,240,91,350,291,1033,980,719
MOV10_2_20659,2,2,49,5,0,2,0,0,0,0,121,3
MOV10L1_2_20660,3061,2224,3009,3211,3077,836,2609,3915,1989,2340,4384,
3244
MPG_2_20661,271,299,243,203,265,181,338,204,246,297,7,183
MPPE1_2_20662,267,350,206,335,84,809,763,102,373,91,475,109
MPST_2_20663,64,245,137,175,175,1,58,76,18,47,371,15
MRAS_2_20664,1016,745,829,933,909,1078,1346,409,854,513,888,662
MRE11A_2_20665,11,65,6,118,0,57,44,1,671,1,285,14
MRI1_2_20666,45,12,3,22,0,0,0,0,0,1,0,1
MSH5_2_20667,593,462,553,513,845,302,221,296,53,607,221,352
MSRA_2_20668,619,610,411,535,79,653,275,135,529,323,178,762

MSRB3_2_20669,365,434,395,561,95,602,578,284,128,692,800,610
MTHFD1L_2_20670,136,141,157,169,227,19,604,378,23,51,46,304
MTHFS_2_20671,341,366,277,460,544,252,634,725,105,679,280,748
MTO1_2_20672,9,5,2,17,0,0,16,0,34,0,0,0
MTRR_2_20673,430,599,418,437,25,211,502,12,171,238,996,33
MUTYH_2_20674,233,188,269,240,0,6,424,149,19,81,370,0
MX1_2_20675,218,150,130,102,227,64,7,7,71,12,22,75
MYBBP1A_2_20676,160,46,111,113,27,91,616,502,470,183,587,80
MYH2_2_20677,86,149,171,183,145,586,186,372,275,178,1,2
MYO5A_2_20678,93,201,170,153,24,25,230,0,274,58,2,312
MYO7A_2_20679,145,105,119,208,677,10,226,374,198,108,576,154
MYO9B_2_20680,464,421,375,493,389,0,638,354,582,582,1234,260
N6AMT1_2_20681,448,550,528,627,468,928,848,703,862,290,347,369
NAA16_2_20682,2271,2192,1833,2336,2083,1457,2077,1415,1276,3128,2472,1
961
NAA20_2_20683,648,682,872,784,1324,740,2848,1083,909,694,1429,1017
NAAA_2_20684,637,384,395,623,559,446,911,261,659,461,715,270
NAPEPLD_2_20685,608,649,497,433,484,371,225,372,955,756,1099,544
NARF_2_20686,108,116,122,73,127,1,319,31,27,45,99,219
NARS2_2_20687,74,140,126,105,123,14,130,541,143,57,74,201
NAV1_2_20688,1619,1808,1524,1924,1792,1346,1324,1061,1525,2183,1201,12
42
NCEH1_2_20689,207,136,124,187,34,202,166,220,113,20,88,521
NCF2_2_20690,274,198,307,361,699,266,883,88,127,415,302,721
NCF4_2_20691,429,497,327,518,1020,533,255,475,230,424,337,753
NDOR1_2_20692,103,51,50,52,8,50,47,92,22,25,4,94
NDUFA2_2_20693,41,8,17,14,4,0,0,0,22,0,0,29
NDUFB11_2_20694,378,303,273,294,411,528,222,107,604,448,72,206
NDUFB4_2_20695,713,468,535,921,978,463,55,1389,226,435,1,803
NDUFB5_2_20696,185,497,177,360,120,107,249,530,9,1095,36,327
NDUFB6_2_20697,444,553,539,448,608,816,1096,122,343,1039,689,757
NDUFC1_2_20698,1419,902,1018,925,1249,1042,1889,1612,1316,532,563,906
NDUFS1_2_20699,381,423,323,480,167,356,1146,298,769,340,227,71
NDUFS2_2_20700,1732,1331,1220,1594,1326,1389,1628,507,1567,1824,1814,1
429
NDUFS5_2_20701,393,399,307,401,570,179,213,544,425,358,197,819
NDUFV1_2_20702,565,592,534,804,323,1173,1268,549,616,489,799,1074
NEIL2_2_20703,173,199,167,246,300,168,272,122,1,772,696,466
NEU4_2_20704,2010,1997,1864,2277,1958,1765,1734,1241,2638,3250,913,148
1
NFS1_2_20705,1575,1823,1422,1902,1249,864,2028,595,1427,1878,1416,2012
NGLY1_2_20706,38,123,33,34,51,9,38,23,7,21,0,64
NIPSNAP1_2_20707,207,132,156,161,165,383,58,190,659,193,0,213
NIT1_2_20708,1221,1098,985,1179,758,687,694,1615,1177,908,682,1120
NKIRAS2_2_20709,30,86,28,122,335,20,3,67,31,25,153,41
NLGN3_2_20710,47,74,78,98,508,116,209,9,128,12,33,231
NLGN4X_2_20711,355,169,287,323,710,40,466,427,48,323,246,397
NLGN4Y_2_20712,414,384,535,460,445,467,435,0,536,324,1062,258
NMNAT2_2_20713,181,145,190,224,2,86,147,0,155,140,73,220
NNT_2_20714,2402,2240,1791,2234,2147,849,1588,1450,1719,2829,1996,2428

NOX4_2_20715,37,228,72,186,0,41,265,210,178,428,76,32
NPL_2_20716,419,323,251,268,549,44,454,632,662,220,233,2
NQO1_2_20717,362,327,390,468,543,335,424,275,729,30,410,352
NRD1_2_20718,1640,1290,1220,1223,1681,766,1855,1539,1629,1858,1660,133
9
NSDHL_2_20719,924,991,865,1118,864,1112,1395,490,357,1424,397,1418
NSUN2_2_20720,711,656,395,546,481,1141,1218,0,315,995,253,308
NUDT2_2_20721,0,11,86,75,11,2,414,0,0,2,343,0
NXN_2_20722,134,112,242,187,8,318,833,23,45,147,295,115
OAS1_2_20723,1665,1578,1330,1604,3082,1426,2063,1630,1047,2181,2194,12
67
OAS2_2_20724,151,230,193,134,211,25,53,46,474,190,105,241
OASL_2_20725,151,90,123,168,24,8,3,385,1,224,253,44
OAT_2_20726,134,19,69,40,279,2,22,0,0,78,0,10
OGDH_2_20727,348,256,212,277,229,57,72,363,152,226,399,555
OGDHL_2_20728,26,26,9,91,0,0,0,0,7,0,16,12
OGG1_2_20729,492,458,379,584,18,747,13,373,252,13,278,1054
OGT_2_20730,196,249,146,217,72,24,31,0,324,315,112,105
OLAH_2_20731,1374,1219,1221,1086,1119,952,739,1280,1195,611,941,1538
P4HA1_2_20732,571,286,401,541,587,24,190,197,106,444,1759,404
P4HA2_2_20733,86,232,116,152,36,46,531,628,368,26,35,166
P4HTM_2_20734,1236,905,914,1029,666,1431,766,797,952,894,263,714
PAFAH1B2_2_20735,800,956,920,712,1218,119,2578,102,1052,1291,1626,981
PAFAH1B3_2_20736,97,83,61,62,178,20,2,0,4,6,87,8
PAICS_2_20737,231,135,129,158,190,307,9,98,256,103,147,148
PAOX_2_20738,257,223,237,232,558,139,444,377,145,141,137,500
PAPD4_2_20739,2451,2442,1953,2476,1116,1249,446,1168,1937,4043,4060,21
17
PAPD5_2_20740,106,70,98,129,31,2,16,10,145,77,42,34
PAPD7_2_20741,134,189,140,134,56,271,0,1,254,42,20,233
PAPSS2_2_20742,315,224,324,484,104,179,127,873,272,486,4,446
PARK7_2_20743,833,494,488,722,1295,889,692,911,774,465,443,418
PARL_2_20744,39,88,19,107,5,5,0,184,12,8,0,5
PARN_2_20745,182,184,158,195,218,459,148,97,60,2,36,182
PARP2_2_20746,1310,1170,1192,1750,1535,433,910,480,1454,1921,1564,1346
PARP3_2_20747,402,266,441,599,549,561,633,12,521,987,1,344
PARP8_2_20748,927,763,942,756,2151,784,1093,727,661,1173,983,248
PBLD_2_20749,90,59,112,225,98,5,358,1,6,185,389,385
PCCA_2_20750,708,686,449,570,676,15,216,459,886,470,33,1351
PCCB_2_20751,328,225,240,245,0,283,907,215,145,112,151,25
PCMTD2_2_20752,5,105,50,51,3,26,20,94,4,341,0,10
PCNA_2_20753,219,377,271,284,113,520,159,594,377,300,123,589
PCSK1_2_20754,756,624,686,828,454,488,986,730,590,931,546,387
PCSK2_2_20755,263,285,256,449,633,149,268,922,706,309,410,329
PCYT1B_2_20756,245,232,539,442,790,2,670,55,332,314,716,330
PCYT2_2_20757,141,98,158,215,36,17,12,63,106,2,0,124
PDE10A_2_20758,166,180,241,254,419,363,275,24,213,197,207,85
PDE11A_2_20759,198,108,92,148,0,382,264,229,28,29,493,92
PDE1A_2_20760,1236,888,942,1264,1037,621,1198,643,665,1459,1903,1217
PDE1B_2_20761,417,97,193,223,5,111,31,329,116,266,49,16

PDE1C_2_20762,561,503,550,762,924,652,546,1003,1565,479,147,1252
PDE2A_2_20763,0,0,0,0,0,0,0,0,0,0,0,0
PDE4A_2_20764,96,146,76,134,55,292,52,8,44,1,40,20
PDE4B_2_20765,119,70,63,32,23,248,433,90,39,24,1,3
PDE4C_2_20766,2180,1503,1657,2193,1456,1286,1852,1089,983,2197,1887,18
35
PDE4D_2_20767,310,343,355,611,106,428,882,41,352,385,1009,420
PDE5A_2_20768,195,173,250,388,94,535,115,23,205,54,70,135
PDE6B_2_20769,96,128,94,157,101,221,27,66,63,79,109,158
PDE7A_2_20770,353,187,271,206,347,234,368,21,148,190,140,349
PDE8A_2_20771,9,131,74,45,0,0,39,95,0,0,0,158
PDE8B_2_20772,428,380,364,584,623,185,350,322,354,420,360,149
PDE9A_2_20773,330,311,264,242,721,386,5,312,163,274,313,493
PDHA1_2_20774,46,26,11,39,0,0,0,21,2,0,0,125
PDHB_2_20775,489,491,669,635,578,305,617,400,597,532,651,869
PDHX_2_20776,588,332,437,487,696,341,629,441,777,682,601,672
PEMT_2_20777,291,233,224,471,84,86,597,8,128,599,21,277
PEPD_2_20778,1036,854,976,811,583,1237,1451,934,919,498,588,1519
PGAM5_2_20779,763,828,1021,769,471,794,538,366,1410,905,1502,1220
PGBD1_2_20780,115,143,45,48,177,0,0,41,165,121,0,1
PGC_2_20781,61,235,73,105,91,0,0,0,2,230,0,40
PGM1_2_20782,341,420,401,412,959,386,140,793,587,596,237,466
PGM3_2_20783,410,283,345,470,863,110,770,34,416,103,4,518
PHOSPHO1_2_20784,165,99,125,82,426,33,54,4,157,65,10,108
PHOSPHO2_2_20785,887,1239,829,1090,532,1141,1468,1109,676,985,645,946
PHYH_2_20786,732,501,751,702,1355,557,1271,400,491,385,853,686
PIGA_2_20787,1126,946,1289,1235,455,1084,2036,977,1086,1077,1263,728
PIGC_2_20788,47,31,26,29,34,10,186,20,3,18,2,3
PIGF_2_20789,792,922,802,769,782,1063,387,284,913,692,642,1036
PIGG_2_20790,765,809,516,788,948,1076,687,642,143,828,1173,672
PIGN_2_20791,684,595,495,694,589,211,72,250,607,437,355,685
PIGO_2_20792,319,253,399,353,126,439,490,361,411,377,197,995
PIGP_2_20793,241,210,197,267,80,67,233,4,28,704,84,181
PIGQ_2_20794,241,579,258,295,359,4,230,360,350,18,348,430
PIGT_2_20795,35,62,40,58,1,0,165,82,0,4,1,2
PIGV_2_20796,218,159,265,300,52,13,181,89,7,79,159,496
PIN4_2_20797,872,1091,938,858,819,701,2212,1125,725,838,2239,808
PLA1A_2_20798,166,205,309,313,350,511,675,6,65,423,762,525
PLA2G2A_2_20799,22,28,80,82,1,2,0,80,33,77,0,92
PLA2G4C_2_20800,423,433,419,551,417,540,132,195,835,993,676,651
PLA2G6_2_20801,119,60,102,83,55,27,308,44,54,38,1,105
PLA2G7_2_20802,449,278,246,294,21,250,36,296,348,493,313,139
PLAT_2_20803,203,223,197,187,35,7,91,101,183,1,199,209
PLAU_2_20804,66,54,72,57,26,35,0,32,8,0,664,81
PLB1_2_20805,277,254,376,323,627,101,128,572,65,342,447,203
PLCB1_2_20806,386,295,265,364,139,303,391,531,982,257,55,52
PLCB3_2_20807,349,274,315,468,522,1286,271,105,461,572,454,824
PLCB4_2_20808,246,233,314,210,196,3,65,117,149,409,153,185
PLCD1_2_20809,89,180,184,127,0,370,51,50,41,53,206,255
PLCE1_2_20810,153,448,317,416,45,729,391,124,299,397,472,537

PLCG1_2_20811,86,17,20,63,0,0,286,28,0,16,0,0
PLCH1_2_20812,148,170,122,196,171,0,60,206,83,259,741,78
PLCL2_2_20813,629,488,591,619,303,650,199,289,817,1038,1054,994
PLCXD2_2_20814,746,703,539,757,1060,507,1194,361,398,302,493,580
PLD2_2_20815,129,34,210,204,40,0,350,96,29,68,390,12
PLD3_2_20816,273,234,295,186,2,362,770,15,1,73,180,199
PLOD2_2_20817,1026,915,722,687,654,107,1832,872,403,629,1051,1520
PLSCR3_2_20818,86,133,26,25,1,250,4,19,0,0,2,115
PLSCR4_2_20819,326,184,224,287,225,97,349,302,418,206,246,224
PMEL_2_20820,102,49,81,18,26,15,436,308,317,22,14,149
PMS1_2_20821,894,865,739,867,1104,601,749,806,969,1091,365,671
PNPLA1_2_20822,0,0,0,0,0,0,0,0,0,0,0,0
PNPLA4_2_20823,642,577,574,637,400,123,248,802,365,844,165,808
PNPLA6_2_20824,139,59,53,61,0,0,0,0,5,46,0,92
PNPLA7_2_20825,267,389,244,440,533,209,812,936,334,915,91,772
POFUT1_2_20826,125,106,152,187,698,331,537,0,130,13,255,26
POFUT2_2_20827,46,31,49,31,0,0,0,0,25,0,7,14
POGZ_2_20828,515,476,460,675,473,619,432,119,372,631,435,656
POLD2_2_20829,125,206,145,212,245,414,499,13,255,1406,578,356
POLE2_2_20830,852,1044,960,1207,303,617,2135,1122,1078,825,719,913
POLG_2_20831,557,599,633,582,42,907,1395,339,548,250,1348,388
POLL_2_20832,736,616,701,773,404,319,1439,432,867,958,438,1603
POLR1D_2_20833,495,599,676,701,277,349,502,1507,565,535,2091,416
POLR3B_2_20834,1360,1332,1364,1462,905,707,3179,1883,1461,1577,2087,21
58
POLR3H_2_20835,453,295,300,375,137,35,41,205,119,337,1010,435
POMT1_2_20836,2872,2509,2621,2825,3224,2297,3456,1740,3620,2010,3422,2
936
PON2_2_20837,164,30,105,69,287,87,115,106,184,19,1,52
POP5_2_20838,224,314,147,202,53,160,156,227,307,160,13,1105
PPA2_2_20839,543,584,458,397,347,470,725,250,424,175,475,637
PPIE_2_20840,677,663,534,686,210,588,1038,1002,275,1026,1062,886
PPIL2_2_20841,522,416,452,543,664,75,41,354,1090,506,340,1260
PPIL3_2_20842,1212,652,681,920,621,1114,634,494,743,587,56,1867
PPIL6_2_20843,507,523,509,475,822,1162,642,597,302,444,656,789
PPIP5K1_2_20844,774,352,580,603,822,353,289,254,1089,140,1461,191
PPT1_2_20845,87,23,103,107,277,1,19,61,126,30,0,32
PPT2_2_20846,125,47,157,124,162,75,471,98,97,7,241,18
PRCP_2_20847,311,191,294,196,187,516,155,18,280,204,412,905
PRDX1_2_20848,172,196,180,162,37,62,844,60,158,161,237,258
PRDX3_2_20849,116,131,141,224,40,245,166,22,114,280,184,19
PRDX5_2_20850,3178,3269,3123,3327,1940,2834,3818,3085,3184,3479,3266,2
728
PRKCSH_2_20851,33,82,50,71,21,58,59,0,2,37,105,9
PRODH_2_20852,112,90,177,148,9,50,337,242,43,370,351,16
PRSS3_2_20853,476,272,445,508,352,472,553,390,442,548,1452,717
PSAT1_2_20854,1060,949,999,1117,938,1276,774,634,885,889,2041,672
PSMA1_2_20855,1634,2180,1472,1868,1544,742,2213,628,1469,2534,1884,164
1
PSMA3_2_20856,688,522,490,597,429,317,285,313,1121,708,253,688

PSMA4_2_20857,297,635,525,695,346,451,914,289,75,1307,623,842
PSMA5_2_20858,480,498,359,383,185,526,505,851,432,26,558,154
PSMA8_2_20859,87,32,97,100,42,13,2,93,218,17,0,29
PSMB2_2_20860,56,7,106,78,65,29,49,26,43,24,4,5
PSMB5_2_20861,215,92,221,217,141,9,829,159,197,57,63,482
PSMB8_2_20862,490,636,464,508,331,372,680,1061,875,875,141,498
PTBP1_2_20863,249,293,244,261,300,599,411,86,276,34,20,207
PTER_2_20864,547,449,410,447,514,376,533,615,287,395,231,469
PTGR1_2_20865,644,555,671,523,787,390,643,496,288,623,1149,343
PTGR2_2_20866,434,310,414,340,461,62,500,311,264,377,161,813
PTGS1_2_20867,107,122,70,166,328,0,652,2,7,0,604,356
PUS1_2_20868,523,413,456,539,904,172,387,20,230,1034,785,577
PYCR1_2_20869,136,81,105,97,2,225,179,0,144,30,66,4
PYGL_2_20870,121,52,85,85,235,55,0,33,78,3,4,98
PYGM_2_20871,69,53,68,28,160,46,99,0,6,3,1,1
QPCTL_2_20872,65,59,145,103,15,0,82,0,8,4,500,15
QSOX1_2_20873,164,208,266,199,427,27,753,47,3,282,114,216
RAB11A_2_20874,1369,1605,1402,1346,824,1759,1782,1643,1267,1276,1215,1
314
RAB1A_2_20875,289,120,219,208,577,121,54,80,100,185,120,103
RAB23_2_20876,427,537,324,514,649,511,520,250,807,572,201,284
RAB27A_2_20877,113,153,58,160,353,17,23,156,30,138,26,0
RAB28_2_20878,1211,1032,1203,1278,1018,521,2785,1868,1547,393,819,1217
RAB2A_2_20879,987,1232,1031,1257,867,523,614,1248,1067,901,1077,712
RAB2B_2_20880,269,62,78,191,0,31,8,611,0,1,0,0
RAB34_2_20881,520,510,644,773,1021,711,638,136,218,563,179,699
RAB35_2_20882,371,435,480,377,370,1553,29,100,43,111,209,58
RAB37_2_20883,349,387,326,299,351,362,1076,329,436,290,439,673
RAB40C_2_20884,301,123,180,351,456,5,27,345,0,272,149,2
RAB5C_2_20885,293,441,282,380,6,304,54,18,80,488,1919,118
RAB6A_2_20886,866,1056,978,1040,2152,1064,1046,1620,471,931,693,559
RAB7L1_2_20887,731,762,809,774,292,928,600,150,991,720,303,342
RAB9A_2_20888,1228,1057,951,1356,629,1594,385,1492,735,825,263,791
RABGGTA_2_20889,54,19,102,17,90,0,6,11,0,0,0,0
RABL2A_2_20890,1853,1581,1190,1376,375,3123,2368,1583,1106,2348,818,69
1
RABL2B_2_20891,621,479,285,374,52,773,21,14,345,786,742,794
RAC1_2_20892,77,104,42,116,22,0,0,1,51,74,8,6
RAD51B_2_20893,525,583,613,717,960,150,440,360,392,675,203,823
RAD51_2_20894,294,127,227,230,18,99,586,90,126,91,45,30
RAD51D_2_20895,218,190,242,265,268,1079,0,10,531,41,5,297
RAD54B_2_20896,270,238,255,299,377,378,434,267,437,54,274,400
RAD54L_2_20897,434,238,222,335,94,0,79,249,602,503,200,192
RAD9A_2_20898,237,77,169,68,30,102,659,49,116,4,214,19
RAP1A_2_20899,70,89,124,137,12,3,4,5,1,260,0,381
RAP1B_2_20900,364,385,490,483,158,994,997,521,440,201,599,855
RBBP8_2_20901,469,542,635,770,372,182,455,34,414,1034,656,582
RDH13_2_20902,694,517,695,750,676,560,1259,959,851,1111,553,379
RDH5_2_20903,200,324,378,380,30,3,0,308,716,54,421,492
RECQL5_2_20904,948,662,521,861,737,834,2086,1144,584,20,1386,1316

RECQL_2_20905,1832,1580,1444,1859,882,2250,769,1842,944,1140,1273,2257
RERG_2_20906,325,129,315,252,355,725,459,174,218,218,259,218
REV1_2_20907,191,410,291,286,358,263,408,558,359,492,213,186
RFC3_2_20908,25,52,51,31,8,27,28,34,0,27,2,9
RFC5_2_20909,752,444,472,657,220,253,249,706,1069,311,1125,807
RGN_2_20910,244,262,242,207,105,179,146,76,2,64,449,144
RGS11_2_20911,40,51,46,68,11,19,1,125,9,136,5,23
RGS6_2_20912,250,221,239,368,349,63,4,80,119,395,11,276
RHOC_2_20913,56,70,54,144,1,0,3,43,41,86,0,82
RHOT1_2_20914,143,67,75,98,91,5,64,23,40,25,110,323
RNASE1_2_20915,136,108,144,145,449,24,127,0,19,18,53,105
RNASE4_2_20916,17,39,50,46,12,0,0,0,0,0,0,18
RPAP3_2_20917,364,217,229,286,327,296,6,780,253,292,244,575
RPE_2_20918,146,220,126,274,190,35,2,21,240,720,216,346
RPN2_2_20919,349,376,186,257,105,58,586,40,146,59,105,181
RPP14_2_20920,9,2,5,12,4,0,49,0,1,8,28,6
RPP21_2_20921,163,164,79,257,65,137,29,106,53,130,309,414
RPP30_2_20922,308,246,421,373,361,204,481,598,646,405,189,623
RPP38_2_20923,867,424,654,718,882,516,606,399,538,503,198,1403
RPS27A_2_20924,493,347,333,572,136,0,563,492,497,210,1220,1146
RRAD_2_20925,452,356,204,382,368,500,402,3,506,0,4,61
RRAGB_2_20926,505,331,269,354,252,118,44,322,448,236,494,401
RRAS2_2_20927,93,67,50,23,0,0,375,128,41,40,0,1
RRM2B_2_20928,206,250,489,499,480,766,165,48,309,389,361,369
RRM2_2_20929,254,232,126,269,334,118,339,363,403,158,85,403
RTEL1_2_20930,99,42,83,104,5,41,2,52,4,5,6,43
SAR1A_2_20931,427,258,431,272,388,276,495,155,580,445,378,330
SAR1B_2_20932,1260,1053,959,1212,402,452,1035,731,1527,1023,1024,981
SARDH_2_20933,6,36,6,10,0,0,20,0,0,0,0,4
SARS2_2_20934,407,480,432,542,379,295,1146,307,819,305,375,479
SBNO1_2_20935,191,381,285,256,104,292,560,0,132,366,341,538
SC5DL_2_20936,510,323,536,670,418,141,530,684,219,511,207,993
SCD5_2_20937,543,428,531,564,543,450,218,838,398,637,39,848
SDC1_2_20938,735,826,700,948,339,664,778,421,767,937,844,560
SDCBP_2_20939,977,744,673,876,850,720,628,319,1235,1007,315,743
SDHC_2_20940,87,117,53,65,28,74,52,0,63,86,381,91
SEPHS1_2_20941,71,36,39,70,2,1,0,541,45,38,1,62
3-Sep_2_20942,1149,1334,1097,1489,809,899,289,1253,1196,500,1485,3153
4-Sep_2_20943,421,363,388,601,363,572,428,626,724,1062,200,582
5-Sep_2_20944,173,109,132,161,37,77,113,18,32,20,112,9
9-Sep_2_20945,41,39,39,46,0,4,0,87,18,6,58,35
SGMS2_2_20946,272,353,238,372,88,247,179,0,488,297,337,201
SH3GLB1_2_20947,2156,1726,1390,1708,2247,1527,2654,2882,1420,1326,2055
,2784
SHMT1_2_20948,62,50,37,28,5,3,4,124,54,108,0,53
SHMT2_2_20949,427,533,384,533,537,1191,1796,947,7,997,599,415
SIAE_2_20950,17,28,34,10,3,0,8,27,6,0,0,136
SLFN11_2_20951,1148,1389,1120,1549,1315,819,974,1090,1370,973,1755,149
4
SMG6_2_20952,489,468,365,369,943,255,84,245,319,988,388,429

SMOX_2_20953,5,5,3,6,0,0,0,1,46,0,8,1
SMPD1_2_20954,4,30,19,33,0,0,0,0,0,0,0,0
SMPD4_2_20955,359,499,506,395,311,43,330,313,577,295,107,818
SMPDL3B_2_20956,91,208,143,124,15,559,0,255,8,182,1,488
SOD2_2_20957,292,264,148,282,832,120,126,170,257,156,2,500
SPAM1_2_20958,134,135,146,94,276,73,0,94,18,71,0,233
SPAST_2_20959,357,443,276,204,281,21,779,344,0,127,946,338
SPG21_2_20960,81,80,22,92,3,129,36,352,1,47,305,0
SPO11_2_20961,369,221,243,337,721,95,447,89,73,313,79,228
ST3GAL1_2_20962,114,68,63,92,4,197,3,16,63,12,346,28
ST3GAL5_2_20963,188,191,266,381,3,168,2,680,236,130,160,142
ST5_2_20964,64,27,42,88,3,286,7,777,234,0,0,103
ST6GAL1_2_20965,353,430,405,533,248,341,573,256,317,556,428,135
ST6GAL2_2_20966,276,167,234,260,25,134,191,262,430,259,284,15
ST6GALNAC3_2_20967,479,354,501,530,779,261,541,619,3,557,761,334
ST6GALNAC4_2_20968,75,132,79,196,0,0,223,8,2,522,0,184
ST8SIA4_2_20969,1184,858,924,845,2130,432,1215,779,1189,858,895,440
SUCLG2_2_20970,949,912,736,1043,722,605,639,1515,156,1811,213,852
SULF1_2_20971,1326,1586,1238,1295,1098,606,755,1011,1018,1346,1718,115
5
SULF2_2_20972,292,208,251,212,449,62,248,55,154,835,22,859
SULT1A1_2_20973,232,159,53,106,10,861,19,38,97,258,397,526
SULT1A2_2_20974,609,446,409,615,655,503,565,609,184,728,215,251
SULT2B1_2_20975,64,48,74,16,154,0,183,0,4,1,2762,76
SUOX_2_20976,194,433,350,390,811,203,43,33,206,286,18,543
SYTL1_2_20977,59,71,98,110,77,2,2,7,152,55,520,55
TAB1_2_20978,46,21,26,23,0,1,0,23,14,0,0,0
TBXAS1_2_20979,312,147,330,394,396,523,242,257,157,135,0,386
TCIRG1_2_20980,10,34,5,47,1,1,0,0,0,11,0,15
TDP1_2_20981,305,499,290,418,210,36,299,293,591,503,137,214
TERT_2_20982,312,266,300,278,498,0,1151,194,0,177,393,1
TGM2_2_20983,397,473,422,385,2,19,1097,453,0,224,13,620
TGM5_2_20984,325,299,305,241,928,79,296,30,146,75,374,314
TH_2_20985,266,123,79,166,305,89,1,0,109,420,0,155
TIAM2_2_20986,938,1215,890,1049,1838,670,982,1357,1447,506,886,416
TKTL1_2_20987,44,85,75,93,152,0,329,30,10,263,80,2
TMEM55B_2_20988,339,361,257,335,219,201,426,753,278,6,7,297
TMLHE_2_20989,491,552,424,485,791,1,176,877,104,620,415,217
TMOD1_2_20990,1444,1696,1671,1833,1089,1172,1758,1057,1665,1646,1730,2
008
TMX2_2_20991,29,19,15,8,0,0,0,0,16,0,0,0
TNNI2_2_20992,237,114,179,403,39,307,987,320,111,769,241,531
TP53I3_2_20993,58,38,65,90,41,45,14,74,42,124,0,72
TPI1_2_20994,593,437,734,489,159,573,551,580,404,904,1087,752
TPST2_2_20995,386,268,184,417,614,348,161,124,321,523,104,315
TREX1_2_20996,252,208,245,405,20,46,267,328,257,394,567,2
TRMT1_2_20997,189,45,76,32,8,1,230,0,107,280,71,199
TRMT1L_2_20998,526,556,485,514,794,64,638,576,143,592,692,350
TRMT2B_2_20999,189,134,172,195,89,76,133,254,161,74,479,86
TRPT1_2_21000,911,646,844,926,648,280,807,683,778,1205,1385,414

TSEN2_2_21001,238,156,230,137,42,10,508,407,0,600,1445,12
TSEN34_2_21002,38,38,122,85,19,0,327,64,170,0,1,25
TTLL6_2_21003,407,428,377,488,592,507,1099,516,271,729,171,616
TUBAL3_2_21004,237,344,453,501,487,837,119,255,540,641,1617,717
TUSC3_2_21005,139,49,66,7,1,0,0,0,0,161,0,0
TXNDC16_2_21006,201,179,123,224,87,222,59,233,78,120,16,200
TXNDC2_2_21007,236,126,226,205,340,263,904,516,470,161,919,261
TXNDC5_2_21008,65,65,21,102,60,0,27,62,7,226,6,9
TXNL4B_2_21009,5137,5253,4983,5998,3478,3938,7201,4542,5003,5148,5331,
3637
TXNRD1_2_21010,207,147,186,196,297,168,563,168,30,13,157,478
TXNRD3_2_21011,461,407,438,614,363,651,833,639,294,12,421,631
TYMP_2_21012,511,556,333,597,39,43,5,7,58,962,69,539
UBA52_2_21013,148,133,64,64,0,0,231,0,15,258,0,24
UEVLD_2_21014,918,908,709,1108,922,847,1070,844,797,1147,493,858
UGDH_2_21015,1328,1377,1300,1586,2458,3137,1781,1741,1488,1605,1187,26
95
UGP2_2_21016,691,837,572,683,561,560,601,294,919,576,682,795
UGT1A6_2_21017,26,18,70,49,11,41,116,121,134,65,0,115
UGT2B10_2_21018,764,739,707,989,934,306,672,319,471,778,1655,549
UGT2B28_2_21019,231,397,272,429,342,507,253,696,538,1071,333,430
UGT3A1_2_21020,657,271,443,515,1089,415,1325,804,689,729,791,243
UGT8_2_21021,142,104,124,106,7,6,0,167,29,265,242,473
UNG_2_21022,186,104,129,189,99,244,67,346,248,539,105,223
UPP1_2_21023,89,82,49,174,88,28,24,38,131,1,22,18
UPP2_2_21024,100,70,49,148,136,0,53,105,77,187,1,132
UQCR10_2_21025,12,7,20,43,0,0,2,0,59,0,25,18
UQCRB_2_21026,178,107,131,127,324,36,6,60,143,115,50,18
UROC1_2_21027,62,74,107,92,25,1,205,493,59,0,0,1
VCL_2_21028,203,106,130,158,299,2,357,50,68,56,167,216
VNN2_2_21029,334,189,226,187,831,27,14,0,297,75,12,854
VPS29_2_21030,542,491,631,744,492,199,1198,1477,152,1383,840,1003
WARS2_2_21031,152,199,191,265,434,70,80,562,124,188,590,156
WARS_2_21032,422,348,470,424,1278,186,126,336,132,632,270,20
WBSCR22_2_21033,152,71,83,197,19,11,0,686,400,2,6,126
WDR46_2_21034,246,271,207,204,442,37,568,456,168,457,153,6
WFS1_2_21035,49,47,67,121,67,38,0,257,0,19,0,114
WRNIP1_2_21036,36,77,16,99,0,0,0,1,0,0,0,0
WWOX_2_21037,64,41,62,134,402,0,116,9,0,43,212,0
XPNPEP1_2_21038,581,609,676,683,246,1323,351,1265,464,335,1333,587
XRCC3_2_21039,171,82,116,256,265,290,5,227,187,92,363,141
XRN1_2_21040,1595,1541,1214,1785,1373,785,2418,1730,2043,1555,1458,134
2
YWHAZ_2_21041,292,346,294,408,415,537,854,205,566,325,90,388
ZBED1_2_21042,630,671,608,471,69,231,41,294,597,696,552,465
ZCCHC11_2_21043,340,221,251,236,264,73,315,123,434,113,20,218
ZCCHC6_2_21044,48,85,74,167,37,101,14,552,33,11,1,288
ZDHHC15_2_21045,213,207,208,243,237,105,96,233,424,38,379,194
ZDHHC7_2_21046,687,681,778,821,1358,395,1122,1192,463,286,696,1176
ACYP1_2_21047,374,188,287,204,173,47,148,154,98,162,520,212

APOBEC3F_2_21048,1139,775,1020,1272,1835,984,1701,1286,1003,767,1419,8
44
ASCC3_2_21049,917,672,978,987,742,1005,2402,1418,425,1215,1267,1190
ASPH_2_21050,717,500,444,450,411,575,634,629,507,995,751,1330
ATP6V0E2_2_21051,1112,1370,958,1161,1652,909,1490,1194,2281,1018,1134,
808
C10orf2_2_21052,372,457,444,373,486,378,67,261,343,300,50,896
CAPN3_2_21053,109,40,54,64,12,168,0,0,281,0,99,45
CHM_2_21054,210,260,109,235,22,37,743,85,264,161,3,403
CTSC_2_21055,335,548,584,503,514,702,674,448,54,766,530,684
CYBRD1_2_21056,606,694,636,653,1016,236,190,70,339,630,324,531
CYP3A5_2_21057,747,721,747,1113,641,217,1862,630,786,684,449,700
DIO2_2_21058,175,215,164,148,385,113,343,262,2,274,556,19
DNASE2B_2_21059,232,93,157,155,0,8,0,37,2,150,31,116
DPYD_2_21060,449,343,371,397,790,378,749,178,253,617,1483,331
GBA3_2_21061,487,299,326,318,214,220,47,404,239,404,402,200
GCNT2_2_21062,398,634,620,755,1,53,1399,34,389,277,972,650
GLYAT_2_21063,69,39,125,59,9,318,0,1,2,8,644,0
GPLD1_2_21064,1288,922,1134,1119,1048,616,1440,702,634,1472,440,1507
GPX1_2_21065,6,12,29,3,8,6,93,0,0,126,104,103
GPX5_2_21066,473,553,659,555,375,1121,1064,1052,703,1092,715,860
HNMT_2_21067,1163,870,776,1080,854,730,986,1056,483,866,879,738
HOGA1_2_21068,160,195,254,241,20,113,286,63,39,48,10,158
HYAL3_2_21069,386,332,271,504,384,0,489,62,4,12,433,553
JPH2_2_21070,130,114,159,151,9,249,0,0,329,423,66,305
KLK8_2_21071,54,84,124,119,80,234,0,63,7,0,0,83
LAMA4_2_21072,1614,1583,1480,1474,436,1402,1280,1918,924,3276,450,2128
LRR1_2_21073,741,475,394,659,599,313,575,55,715,353,671,372
LRTOMT_2_21074,161,309,135,295,3,0,803,164,0,538,769,15
METTL1_2_21075,366,472,492,688,462,19,655,358,862,515,455,884
MOCS2_2_21076,551,299,451,497,827,255,179,136,495,294,421,304
NDUFA11_2_21077,389,489,306,421,122,10,490,743,310,324,127,393
NDUFV3_2_21078,170,54,103,145,141,14,4,692,28,46,371,210
NMNAT3_2_21079,149,105,131,144,4,58,0,343,11,0,0,259
PDE4DIP_2_21080,340,340,247,377,133,344,430,243,172,198,489,247
PLG_2_21081,29,4,26,31,0,0,0,0,0,0,0,0
PPCS_2_21082,311,449,380,338,0,3,51,54,121,590,254,306
PRDX2_2_21083,293,147,224,231,3,163,263,33,193,493,879,0
RAD51C_2_21084,93,141,150,194,12,50,85,0,62,430,105,221
RASD1_2_21085,58,49,60,50,0,0,19,66,0,0,311,37
RASL10A_2_21086,302,197,191,197,245,64,709,138,570,285,146,692
SLX1A_2_21087,70,79,111,56,647,1,0,34,336,3,540,1
SLX1B_2_21088,70,79,111,56,647,1,0,34,336,3,540,1
SPTLC1_2_21089,397,625,494,583,205,106,581,187,119,532,599,416
ST3GAL3_2_21090,183,85,120,108,257,8,121,78,204,198,19,383
VKORC1_2_21091,1981,1583,1569,2090,1841,1388,1397,1212,1665,3349,1292,
1651
XPNPEP3_2_21092,262,97,147,271,125,45,428,2,221,39,70,44
GNAS_2_21093,18,48,41,30,101,3,2,13,0,2,1,88
GNAS_2_21094,81,24,17,42,12,0,0,1,28,1,0,5

GNAS_2_21095,89,239,320,283,87,97,302,223,283,74,322,254
A4GALT_2_21096,162,210,241,212,56,125,350,155,31,221,695,791
A4GNT_2_21097,288,109,54,184,17,548,20,0,0,0,87,13
AACS_2_21098,159,178,92,134,141,41,57,177,354,127,0,38
AADAC_2_21099,440,262,247,269,143,102,90,182,235,99,516,199
AARS2_2_21100,194,54,265,191,978,120,0,164,1210,315,1035,138
AARS_2_21101,322,363,290,663,189,12,1077,130,478,106,1223,319
AASDH_2_21102,4033,3266,2834,3887,2897,4847,2694,3519,3951,3916,3954,4
638
AASDHPPT_2_21103,69,80,52,22,47,0,1,0,19,85,70,157
AASS_2_21104,1446,1239,1248,1542,501,1059,702,3701,1321,2573,1540,2237
ABHD14A_2_21105,50,89,62,74,74,0,198,493,296,170,0,161
ABHD1_2_21106,240,188,127,157,379,412,34,76,236,130,7,242
ABHD3_2_21107,240,121,192,238,403,410,122,116,39,2,87,58
ABHD5_2_21108,210,173,493,610,120,1198,561,669,2,1,618,578
ABHD6_2_21109,305,227,134,215,206,282,249,146,0,0,215,123
ABHD8_2_21110,128,53,78,126,1,263,41,1,35,6,5,32
ABO_2_21111,31,28,27,22,52,20,6,0,33,0,0,6
ABP1_2_21112,269,326,277,259,605,510,473,6,111,1583,839,462
ACAA2_2_21113,98,142,106,150,93,43,446,196,102,257,5,79
ACACB_2_21114,973,795,703,988,1055,518,555,633,1210,947,1110,1023
ACAD11_2_21115,819,729,836,782,611,781,776,335,717,876,320,1381
ACAD8_2_21116,296,239,220,253,669,279,0,0,10,9,2,701
ACAD9_2_21117,344,281,167,222,119,232,388,262,0,447,0,279
ACADL_2_21118,400,373,254,389,175,87,18,118,104,122,9,990
ACADSB_2_21119,520,334,364,436,426,529,331,448,448,387,316,689
ACADS_2_21120,446,343,253,301,361,7,1363,257,440,459,467,61
ACAT1_2_21121,582,544,473,599,221,574,567,202,244,449,251,375
ACER1_2_21122,314,191,251,253,99,703,112,1,130,175,560,219
ACER2_2_21123,99,129,92,213,22,37,77,200,18,153,37,79
ACER3_2_21124,574,479,410,531,635,140,562,577,485,354,114,437
ACMSD_2_21125,945,744,721,780,706,361,1306,693,1146,814,964,509
ACO1_2_21126,299,517,266,223,469,16,225,197,624,367,5,340
ACO2_2_21127,497,808,400,574,1543,1009,85,380,16,1069,2279,644
ACOT12_2_21128,831,779,718,770,365,964,629,1054,404,457,484,960
ACOT1_2_21129,1351,1457,1237,1553,1108,1277,1316,478,1081,770,1700,165
8
ACOT2_2_21130,271,311,237,263,152,153,6,219,612,257,19,523
ACOT4_2_21131,660,297,359,513,85,379,595,494,728,219,261,216
ACOT6_2_21132,866,1077,665,834,886,475,576,41,741,1038,666,1115
ACOT8_2_21133,102,143,146,252,19,4,66,7,37,1,93,10
ACOX2_2_21134,50,139,65,113,8,118,1,273,0,55,2,5
ACOXL_2_21135,814,735,709,880,1356,847,813,854,786,706,55,1129
ACR_2_21136,122,62,125,167,284,44,44,60,31,110,444,14
ACSBG2_2_21137,232,123,130,124,155,63,293,62,273,129,432,5
ACSF2_2_21138,1156,1105,835,1044,2474,743,349,424,687,1832,1280,1001
ACSL1_2_21139,210,172,129,243,327,727,5,8,398,270,113,297
ACSM1_2_21140,1276,945,1214,1101,1914,549,1178,441,1161,508,1274,1601
ACSM2A_2_21141,1961,1391,1776,2007,1302,972,1060,2639,2701,2161,886,27
68

ACSM4_2_21142,428,359,270,284,92,606,436,33,209,344,846,257
ACSM5_2_21143,612,692,564,715,660,405,1017,605,143,1481,361,757
ACSS1_2_21144,1478,1632,1511,1683,1670,1417,2234,1599,1165,1308,1148,1
295
ACSS3_2_21145,287,145,82,226,1380,184,429,461,38,60,101,460
ACTC1_2_21146,65,66,45,43,0,2,0,215,0,1,0,85
ACY3_2_21147,6,41,60,54,6,0,0,3,9,183,454,3
ACYP2_2_21148,1337,1201,1156,1231,1627,1227,1630,1122,971,1686,880,118
6
ADA_2_21149,81,73,48,90,26,64,24,202,166,90,158,20
ADAM10_2_21150,1,27,14,30,1,0,0,34,27,0,0,6
ADAM17_2_21151,937,1061,761,1003,1320,704,660,870,438,1190,539,1198
ADAMTS4_2_21152,66,59,68,43,1,204,9,0,0,0,9,0
ADARB2_2_21153,465,148,264,210,415,297,343,53,186,210,274,53
ADAT1_2_21154,37,55,58,92,252,136,564,157,249,29,12,426
ADAT2_2_21155,1306,705,1087,921,1143,1010,1518,3133,820,1187,1408,642
ADC_2_21156,74,143,75,62,434,122,47,52,157,333,0,61
ADCY1_2_21157,102,47,165,91,70,280,7,1,35,40,0,20
ADCY2_2_21158,411,591,340,398,215,399,132,532,272,227,202,537
ADCY7_2_21159,849,758,751,824,1006,598,340,247,1645,277,1466,1568
ADCY8_2_21160,34,35,44,57,52,0,0,424,318,4,0,22
ADCY9_2_21161,52,57,157,84,295,16,0,130,222,323,2,5
ADH1A_2_21162,1910,1875,2133,2175,2134,2256,3452,1368,1887,2065,2278,2
195
ADH1B_2_21163,943,1038,823,862,1345,1170,1162,975,1067,389,814,767
ADH1C_2_21164,316,130,265,242,60,375,218,19,200,439,296,668
ADH4_2_21165,651,705,614,897,1001,786,499,824,1732,961,541,1021
ADH5_2_21166,290,238,226,243,450,75,329,9,650,297,295,155
ADHFE1_2_21167,128,88,76,66,13,372,274,48,45,49,137,1
ADI1_2_21168,64,43,18,157,1,166,23,42,6,86,0,311
ADO_2_21169,98,43,59,159,386,157,0,11,0,439,0,398
ADPRH_2_21170,17,13,29,36,13,0,0,0,2,0,0,224
ADPRHL2_2_21171,231,344,403,316,15,483,539,99,420,705,610,406
ADSS_2_21172,546,632,636,618,1337,574,900,667,713,442,150,337
AEN_2_21173,67,100,45,199,0,9,427,688,2,169,105,11
AGBL2_2_21174,496,925,602,710,555,524,353,2837,119,261,1656,1247
AGMAT_2_21175,100,202,131,169,46,220,24,15,78,190,12,122
AGMO_2_21176,539,407,283,456,195,547,192,251,161,33,178,453
AGPAT4_2_21177,282,139,298,378,160,3,216,365,228,302,4,68
AGPAT5_2_21178,699,569,525,678,835,209,889,924,600,324,1233,505
AGPAT6_2_21179,14,44,7,20,0,0,1,0,60,0,0,3
AGPAT9_2_21180,55,88,37,18,111,26,21,257,20,1,11,228
AGPS_2_21181,1069,669,769,780,896,46,1258,1332,1394,356,103,824
AGXT2_2_21182,199,263,189,285,199,24,1256,261,645,409,47,182
AGXT2L2_2_21183,60,48,46,55,0,0,7,0,352,50,0,0
AGXT_2_21184,237,182,255,318,18,7,1,0,0,403,36,265
AICDA_2_21185,506,577,549,720,404,335,1621,1647,1032,1004,603,579
AKR1B10_2_21186,365,466,355,319,285,635,17,307,355,399,374,428
AKR1B15_2_21187,493,389,449,646,398,574,139,378,384,200,66,601
AKR1B1_2_21188,348,533,402,352,453,494,96,341,474,423,560,434

AKR1C1_2_21189,743,677,606,956,908,68,194,744,633,878,548,1513
AKR1C3_2_21190,933,674,820,815,296,779,891,347,414,227,1857,1046
AKR1C4_2_21191,743,677,606,956,908,68,194,744,633,878,548,1513
AKR1E2_2_21192,182,159,160,194,313,190,149,150,287,229,32,68
AKR7A2_2_21193,564,272,469,460,154,139,533,60,467,670,845,232
AKR7A3_2_21194,83,38,39,31,0,22,0,0,0,51,18,0
ALAD_2_21195,52,26,45,61,0,80,107,137,11,0,22,124
ALDH1A1_2_21196,365,328,284,270,193,459,469,272,187,540,276,277
ALDH1A3_2_21197,140,59,107,229,212,0,151,6,1,138,173,5
ALDH1B1_2_21198,38,143,164,153,319,33,5,3,140,145,475,112
ALDH1L1_2_21199,265,241,244,283,63,197,98,274,119,167,35,555
ALDH1L2_2_21200,123,106,122,128,103,11,37,183,306,23,43,72
ALDH6A1_2_21201,1523,1467,1416,1768,1232,1062,1404,1697,1840,2044,1641
,2509
ALDH9A1_2_21202,1018,802,683,1011,513,415,1563,671,857,812,203,903
ALDOB_2_21203,85,160,83,95,14,110,129,5,305,6,55,0
ALDOC_2_21204,1559,1435,1460,1522,2169,1524,2653,2093,1214,1605,605,18
84
ALG10_2_21205,2083,1854,1589,2083,2025,1983,526,1639,1851,2300,1506,19
29
ALG11_2_21206,5,25,6,33,16,0,0,0,2,4,0,16
ALG12_2_21207,47,50,41,64,111,10,7,1,2,2,0,12
ALG14_2_21208,21,20,92,84,1,1,222,0,251,24,0,0
ALG1_2_21209,1316,1287,1754,1508,1203,1491,1543,1459,1378,1359,1780,11
45
ALG2_2_21210,1095,1072,1036,1365,980,1333,1224,1680,1334,1319,811,1616
ALG6_2_21211,1245,1329,1235,1377,963,1915,438,723,1146,1224,1014,1250
ALKBH8_2_21212,642,799,455,542,269,67,43,93,472,285,207,112
ALLC_2_21213,179,149,118,169,147,128,212,117,121,525,1,126
ALOX12B_2_21214,1294,1109,1038,969,762,665,2010,1387,753,833,675,755
ALOX12_2_21215,55,22,92,100,0,45,0,3,136,3,88,70
ALOX15_2_21216,79,121,92,81,139,340,163,0,48,60,23,288
ALOX5_2_21217,199,181,231,189,307,8,69,2,21,0,0,348
AMDHD1_2_21218,652,270,599,504,754,550,230,474,191,686,910,660
AMY1B_2_21219,2286,1999,2213,2459,2993,2483,2547,2260,3514,3495,1812,3
902
AMY1C_2_21220,2286,1999,2213,2459,2993,2483,2547,2260,3514,3495,1812,3
902
AMY2A_2_21221,3036,2508,2713,2976,3054,2180,3086,3192,2485,2429,1368,2
293
AMY2B_2_21222,13219,11353,11716,14121,10096,7505,13600,11952,10104,988
3,10997,12637
ANPEP_2_21223,108,54,43,98,29,0,106,9,0,133,0,132
AOC3_2_21224,165,133,178,160,56,129,212,17,120,7,2,143
AOX1_2_21225,34,13,55,83,0,0,0,4,56,0,0,115
APEH_2_21226,255,342,228,284,444,4,392,330,672,198,329,296
APEX2_2_21227,307,338,232,364,12,0,0,186,1,264,564,529
APIP_2_21228,310,365,417,516,655,243,217,596,162,66,460,646
APLF_2_21229,1764,1226,1404,1521,927,1451,1504,1101,1705,1497,2782,144
2

APOBEC1_2_21230,226,135,158,245,128,0,392,16,43,372,204,394
APOBEC2_2_21231,220,351,205,418,534,121,250,37,140,574,277,477
APOBEC3B_2_21232,831,442,434,629,1186,164,515,957,636,327,1227,1145
APOBEC3C_2_21233,652,1023,696,709,218,661,1188,251,303,877,587,1357
APOBEC3G_2_21234,44,73,76,60,52,40,153,10,8,205,54,13
ARF3_2_21235,38,39,68,66,1,0,0,39,186,18,0,0
ARF4_2_21236,302,191,153,247,52,95,519,491,368,257,39,289
ARG2_2_21237,170,120,199,192,21,333,172,0,498,1,31,190
ARHGEF10_2_21238,47,87,47,50,0,19,11,42,26,123,15,9
ARL3_2_21239,123,140,91,68,69,18,60,82,51,27,9,251
ARL4C_2_21240,388,427,441,419,341,751,399,428,194,887,583,321
ARL4D_2_21241,24,122,79,156,67,7,0,2,141,15,0,51
ARL5B_2_21242,1034,1029,1269,1103,1143,1408,542,1056,956,998,1130,1038
ARL8A_2_21243,58,72,40,91,27,1,78,41,1,0,2,7
ARL8B_2_21244,426,243,276,236,116,75,582,155,469,701,22,133
ARSD_2_21245,90,78,137,119,100,174,8,29,204,20,52,203
ARSE_2_21246,408,237,302,341,182,310,33,628,382,648,436,324
ARSG_2_21247,211,126,101,230,158,76,210,457,431,282,210,69
ARSH_2_21248,336,364,316,376,289,281,1014,707,19,307,259,428
ARSI_2_21249,29,63,55,46,0,0,124,0,52,46,13,38
ARSJ_2_21250,517,695,489,606,243,634,308,1051,543,584,668,262
ARSK_2_21251,796,638,447,514,511,350,344,678,621,835,331,788
ART1_2_21252,278,137,88,157,239,0,91,303,308,168,332,0
ART4_2_21253,33,86,154,116,598,39,0,258,262,94,370,50
AS3MT_2_21254,106,155,139,170,263,12,247,0,199,0,14,227
ASNA1_2_21255,290,256,177,292,252,58,347,528,330,53,188,387
ASNSD1_2_21256,870,999,855,851,765,767,1798,1019,453,494,847,1476
ASPG_2_21257,275,330,320,410,69,166,203,374,310,199,77,923
ASPHD2_2_21258,29,32,100,13,7,2,67,0,1,31,4,45
ATAD1_2_21259,160,228,161,161,124,79,757,74,195,452,200,295
ATIC_2_21260,58,52,6,16,7,0,97,0,0,24,0,0
ATP2C2_2_21261,359,334,209,252,109,210,87,236,589,492,540,135
AUH_2_21262,16,189,57,63,1,0,6,1,14,268,0,267
AWAT1_2_21263,356,469,474,570,13,279,820,737,0,419,905,435
AWAT2_2_21264,658,542,684,616,1709,92,25,401,427,1152,611,671
B3GALNT2_2_21265,265,239,134,152,367,103,22,452,555,216,276,384
B3GALT1_2_21266,865,1107,1008,903,776,1059,2192,1272,1102,1511,1424,10
45
B3GALT2_2_21267,221,196,168,168,78,662,215,216,301,85,123,175
B3GALT4_2_21268,6,51,19,53,33,171,3,74,0,0,0,64
B3GALT6_2_21269,10,26,16,2,0,0,0,11,0,0,0,168
B3GAT2_2_21270,691,591,596,881,414,499,1162,2124,467,558,223,549
B3GAT3_2_21271,7,18,24,32,0,0,0,7,0,79,0,19
B3GNT2_2_21272,345,180,162,276,76,167,23,1,10,634,92,399
B3GNT3_2_21273,98,110,101,182,234,83,370,58,82,8,3,271
B3GNT4_2_21274,413,290,412,544,419,473,249,224,646,884,580,620
B3GNT5_2_21275,577,575,553,628,565,384,340,339,447,1421,402,550
B3GNT7_2_21276,17,1,12,35,0,0,0,65,1,0,2,42
B3GNT9_2_21277,272,134,134,181,81,306,55,301,133,64,71,65
B3GNTL1_2_21278,629,701,456,609,1090,102,733,1117,298,932,826,802

B4GALNT1_2_21279,92,73,96,181,1,177,154,0,3,289,43,75
B4GALNT3_2_21280,328,244,302,274,0,166,1160,2,1,265,19,16
B4GALNT4_2_21281,175,150,122,138,140,61,755,213,335,0,7,75
B4GALT1_2_21282,91,145,232,165,0,12,137,49,44,167,167,313
B4GALT5_2_21283,201,244,212,254,33,214,55,446,54,1073,4,842
B4GALT6_2_21284,43,22,41,154,79,2,0,1,111,484,1,56
B4GALT7_2_21285,12,45,14,44,0,0,1,0,81,14,295,1
BBOX1_2_21286,325,272,266,273,321,325,378,239,222,16,824,290
BCHE_2_21287,105,91,131,145,29,254,4,123,123,94,125,103
BCMO1_2_21288,22,29,82,68,10,21,0,26,71,1,0,14
BDH2_2_21289,342,285,330,418,866,762,447,1,836,246,150,87
BHMT_2_21290,25,63,62,153,12,1,27,77,396,209,3,331
BLM_2_21291,931,1067,994,1302,88,318,2333,1077,1466,324,728,1637
BLMH_2_21292,237,114,101,134,180,351,54,43,181,168,165,351
BLVRA_2_21293,68,127,87,102,0,103,570,205,33,216,67,148
BLVRB_2_21294,224,191,107,162,53,0,504,26,192,46,5,296
BPHL_2_21295,321,230,441,450,172,15,565,80,249,198,252,423
BST1_2_21296,994,504,675,801,1175,254,1746,807,581,245,1198,1013
BTD_2_21297,260,128,216,221,277,258,224,67,277,90,397,610
C12orf5_2_21298,642,578,383,505,282,186,656,397,389,704,407,412
C16orf79_2_21299,85,213,166,108,120,9,173,67,441,22,6,371
C18orf56_2_21300,31,0,0,84,0,0,0,0,0,0,0,0
C1GALT1_2_21301,198,255,135,288,212,226,198,242,196,82,435,105
C1R_2_21302,85,46,66,61,5,53,0,4,100,0,1,110
C22orf28_2_21303,942,699,735,865,607,1254,877,969,871,599,758,783
C2orf43_2_21304,609,759,419,691,665,1272,695,576,719,1093,954,813
C5orf4_2_21305,526,774,649,481,905,699,742,242,545,541,280,431
C6orf130_2_21306,230,100,189,238,146,10,6,173,60,65,145,405
CA13_2_21307,531,524,599,706,1021,476,114,870,318,351,260,954
CA14_2_21308,808,416,429,567,1008,288,125,908,7,586,827,1050
CA2_2_21309,275,177,273,265,184,346,34,167,70,436,68,164
CA3_2_21310,182,281,195,494,190,651,44,61,350,2,379,279
CA4_2_21311,290,240,174,235,109,31,191,205,200,156,8,231
CA5A_2_21312,104,43,36,90,461,0,0,139,98,0,0,20
CA5B_2_21313,11,27,36,73,0,12,33,112,5,1,11,13
CA6_2_21314,47,3,58,43,0,13,0,6,0,2,156,71
CA8_2_21315,531,369,257,490,597,74,267,421,540,336,403,274
CA9_2_21316,142,169,276,278,124,286,394,38,5,206,342,625
CARS2_2_21317,455,256,452,459,54,223,605,503,202,533,178,732
CASD1_2_21318,1097,717,775,841,478,637,703,189,2029,956,774,1228
CAT_2_21319,236,164,173,224,197,44,113,88,448,355,159,24
CBR1_2_21320,302,388,315,237,70,272,18,169,291,270,70,196
CBR3_2_21321,247,272,202,271,195,104,825,622,9,122,760,45
CBR4_2_21322,937,748,747,752,1405,668,438,1178,713,598,541,766
CCDC88B_2_21323,27,26,16,45,49,45,408,2,3,129,2,135
CCDC92_2_21324,295,183,163,143,243,77,473,0,225,56,0,21
CCNO_2_21325,15,1,0,54,1,9,0,0,0,67,0,5
CCS_2_21326,60,47,107,25,0,0,0,0,0,0,0,4
CCT8_2_21327,80,88,89,142,98,16,24,44,94,103,9,92
CD274_2_21328,424,340,501,550,331,514,165,790,230,675,716,478

CD38_2_21329,182,145,100,126,49,38,210,329,104,109,573,41
CDA_2_21330,639,587,469,488,666,335,768,289,425,484,170,520
CDIPT_2_21331,293,232,275,380,541,136,47,116,460,566,223,317
CDO1_2_21332,165,239,84,208,0,0,2,1,29,1,0,0
CDS2_2_21333,150,56,72,141,19,11,77,3,116,47,0,31
CELA1_2_21334,99,68,59,89,0,36,0,0,174,300,61,63
CELA2A_2_21335,70,112,21,113,378,591,486,266,120,0,59,407
CELA2B_2_21336,576,531,608,643,673,831,438,555,687,505,360,337
CELA3A_2_21337,99,59,103,138,245,20,179,38,26,35,4,34
CELA3B_2_21338,23,49,25,15,25,33,19,3,25,2,0,0
CEL_2_21339,228,234,188,163,91,180,733,202,119,20,34,140
CETN1_2_21340,44,17,62,48,334,0,0,8,14,75,145,23
CETN2_2_21341,218,138,107,197,260,18,234,1,412,37,481,82
CETP_2_21342,292,448,526,690,1215,566,577,35,101,326,907,927
CFB_2_21343,584,627,512,529,300,795,520,98,302,122,56,1567
CFD_2_21344,3,6,18,1,2,0,46,0,7,0,1,0
CFI_2_21345,420,387,449,533,578,82,108,366,579,206,337,676
CH25H_2_21346,22,10,14,43,3,20,1,68,0,3,0,8
CHDH_2_21347,1761,1108,1380,1672,1079,627,2306,2337,681,874,737,1776
CHI3L1_2_21348,198,115,205,182,73,373,531,16,229,23,0,64
CHIT1_2_21349,616,421,471,533,457,298,103,407,473,127,393,117
CHML_2_21350,562,667,540,718,636,444,376,851,159,558,940,468
CHPF2_2_21351,528,760,463,497,697,494,40,1503,615,337,312,268
CHPT1_2_21352,1183,840,939,1014,405,576,683,379,1229,1570,990,705
CHST10_2_21353,405,241,295,504,476,89,519,457,174,1184,374,109
CHST12_2_21354,88,82,46,62,61,267,158,32,0,6,132,37
CHST13_2_21355,131,147,156,110,230,64,537,193,82,2,15,369
CHST14_2_21356,25,30,26,129,0,0,96,346,498,73,0,244
CHST1_2_21357,148,46,88,129,216,0,0,0,8,0,275,1
CHST2_2_21358,128,104,74,200,10,25,0,140,1,337,1,1
CHST3_2_21359,40,29,37,36,29,22,36,58,2,5,21,68
CHST5_2_21360,206,106,148,129,2,2,504,343,76,15,425,154
CHST6_2_21361,108,252,292,206,46,205,528,159,17,7,420,231
CHST7_2_21362,56,19,58,80,50,1,157,23,107,0,8,237
CHST9_2_21363,139,160,133,116,110,209,347,38,82,125,5,192
CHSY1_2_21364,219,92,113,188,73,160,297,0,94,259,3,52
CHSY3_2_21365,648,498,382,517,656,418,364,518,465,247,954,1226
CLPP_2_21366,67,36,34,83,2,21,0,132,78,0,21,172
CLPX_2_21367,431,180,282,340,15,297,634,372,305,647,698,249
CLYBL_2_21368,1343,1116,810,1063,1342,1032,1235,81,847,320,827,1127
CMA1_2_21369,535,746,627,797,1421,1258,325,796,302,795,365,546
CMAS_2_21370,869,517,547,688,444,308,926,6,735,587,268,631
CMBL_2_21371,513,696,429,395,903,375,1823,882,346,523,296,397
CNDP1_2_21372,118,36,93,85,1,169,89,174,0,125,180,173
CNTN6_2_21373,1023,992,995,995,678,871,775,775,695,1798,2080,926
COIL_2_21374,1950,1737,1638,1572,996,631,3814,424,2268,1801,3958,2354
COMTD1_2_21375,340,201,262,430,28,250,2,22,507,149,348,335
COQ2_2_21376,251,143,221,186,281,44,0,6,33,136,1,70
COQ3_2_21377,315,375,267,523,458,8,588,549,140,795,1267,382
COQ5_2_21378,894,732,767,941,360,908,919,1313,995,1066,772,1298

COX10_2_21379,80,129,125,57,92,8,246,19,115,152,14,47
COX17_2_21380,399,327,394,502,202,187,477,86,138,293,910,382
COX4I1_2_21381,396,231,388,354,234,202,306,438,357,320,162,472
COX4I2_2_21382,141,176,94,227,1,0,1097,4,30,7,2,332
COX5A_2_21383,86,38,57,24,3,13,0,0,103,0,118,37
COX5B_2_21384,219,300,258,226,0,56,169,541,46,228,36,442
COX6A1_2_21385,259,88,112,164,0,28,7,0,186,466,0,277
COX6A2_2_21386,310,230,309,348,316,549,1140,25,64,492,593,315
COX6B1_2_21387,70,94,43,50,19,41,1,11,8,44,338,38
COX6B2_2_21388,445,254,359,281,496,46,145,54,120,457,44,919
COX6C_2_21389,2821,2671,2393,2784,2056,1034,3165,3280,2314,2301,4522,3
677
COX7A1_2_21390,31,61,88,81,0,28,5,0,0,5,0,287
COX7A2_2_21391,14,2,0,0,0,0,0,0,0,0,0,2
COX7A2L_2_21392,466,430,551,607,145,1128,795,760,698,324,1055,187
COX7B2_2_21393,631,532,597,653,677,255,639,1144,875,518,723,952
COX7B_2_21394,235,112,100,209,742,27,29,0,425,0,1,618
COX7C_2_21395,135,104,79,252,601,50,23,106,5,136,32,32
COX8A_2_21396,58,48,98,143,184,144,32,14,20,213,167,1
COX8C_2_21397,286,328,304,358,151,36,358,44,229,74,566,39
CPA1_2_21398,218,249,192,285,376,135,7,19,0,20,318,340
CPA2_2_21399,322,405,338,295,91,98,2,163,418,52,37,451
CPB1_2_21400,77,75,28,96,6,77,6,40,3,286,0,1
CP_2_21401,288,326,253,368,251,0,2,350,365,125,54,458
CPE_2_21402,1097,731,799,948,839,783,435,1004,1646,925,722,626
CPOX_2_21403,263,250,224,355,127,112,44,236,355,176,759,187
CPSF3_2_21404,302,220,225,403,0,42,544,827,306,276,126,264
CPT2_2_21405,89,74,40,81,0,0,2,0,54,0,0,0
CRAT_2_21406,578,656,518,594,71,457,336,293,199,294,787,474
CREG2_2_21407,178,157,385,240,155,55,19,314,558,137,296,252
CRY1_2_21408,543,541,668,626,922,307,763,1630,362,322,304,540
CRYL1_2_21409,609,402,322,356,789,219,310,454,738,417,257,270
CRYZL1_2_21410,439,461,269,480,360,201,333,140,68,557,100,862
CSAD_2_21411,88,67,92,111,97,74,36,40,157,70,135,107
CS_2_21412,64,20,19,7,3,0,21,1,12,6,0,0
CSGALNACT2_2_21413,786,614,585,557,1080,122,1071,1043,371,394,198,987
CSRP2BP_2_21414,271,102,213,199,607,110,1,54,82,12,0,118
CTBS_2_21415,1568,1127,1043,1172,711,900,1861,1129,738,927,1713,1021
CTRB1_2_21416,387,336,337,461,419,136,638,377,632,1016,456,89
CTRB2_2_21417,411,445,444,349,596,502,1658,879,1532,1043,131,509
CTRC_2_21418,231,448,278,384,230,9,38,2,407,163,9,791
CTSD_2_21419,807,854,827,891,1580,371,1237,678,592,1325,339,1313
CTSF_2_21420,176,200,149,310,21,43,2,520,547,28,0,21
CTSG_2_21421,43,3,6,15,0,9,116,0,0,0,17,96
CTSH_2_21422,364,308,440,343,297,144,280,205,381,77,206,514
CTSK_2_21423,374,343,543,482,354,93,577,1237,349,463,110,269
CTSZ_2_21424,237,285,207,310,362,74,34,935,361,445,337,234
CWC27_2_21425,1332,1376,1307,1412,1069,939,413,2042,1046,1071,1964,118
2
CXorf21_2_21426,70,191,145,215,0,11,159,85,0,14,172,8

CYB561D2_2_21427,194,220,154,132,302,54,129,4,97,215,297,271
CYB5B_2_21428,220,273,210,346,96,144,383,150,220,105,228,208
CYB5R1_2_21429,62,5,60,15,11,17,2,0,0,5,17,34
CYB5R2_2_21430,245,220,257,406,27,299,176,65,447,63,529,68
CYB5R4_2_21431,1764,1707,1422,1864,1680,1189,1387,2216,1733,1237,1782,
1660
CYBA_2_21432,38,35,20,30,0,1,315,5,2,0,0,0
CYBB_2_21433,133,102,107,141,50,4,37,229,152,43,290,476
CYC1_2_21434,524,883,555,644,1170,356,506,231,170,940,1650,417
CYCS_2_21435,218,200,162,166,43,5,446,70,741,128,99,8
CYP11B2_2_21436,506,438,342,375,946,1,363,670,287,0,660,697
CYP17A1_2_21437,394,333,313,554,136,408,32,474,453,655,219,968
CYP1A1_2_21438,383,332,472,187,387,96,1721,187,230,312,649,214
CYP1A2_2_21439,26,12,38,32,10,24,1,54,22,0,0,20
CYP1B1_2_21440,168,138,278,219,0,196,198,184,60,12,34,223
CYP20A1_2_21441,144,17,40,78,1,1,10,8,197,13,1,17
CYP26B1_2_21442,34,34,54,143,36,0,0,0,0,0,370,0
CYP26C1_2_21443,269,357,291,421,325,7,65,795,943,299,135,314
CYP27A1_2_21444,344,290,183,214,44,288,396,551,255,197,23,222
CYP27B1_2_21445,976,1119,781,934,1452,1112,524,364,959,887,992,1387
CYP2A13_2_21446,683,473,522,471,683,274,234,326,317,484,554,867
CYP2A6_2_21447,1059,949,999,875,1630,1280,782,305,822,1544,874,675
CYP2B6_2_21448,247,320,251,367,444,181,451,1,324,404,64,868
CYP2C19_2_21449,498,758,429,606,400,242,340,803,656,561,1464,717
CYP2C9_2_21450,529,739,626,634,107,242,929,1253,434,861,457,703
CYP2E1_2_21451,1070,882,715,1216,612,1007,727,603,727,720,1075,1089
CYP2F1_2_21452,86,90,62,122,2,466,2,47,114,131,44,45
CYP2J2_2_21453,227,218,260,264,440,391,57,3,183,436,182,395
CYP2R1_2_21454,2019,2240,1918,2388,1804,555,2268,3221,1942,1950,2134,2
211
CYP2S1_2_21455,26,28,24,76,26,9,0,74,11,49,0,286
CYP2U1_2_21456,658,537,448,690,326,681,600,1030,352,371,844,648
CYP2W1_2_21457,24,135,71,26,20,623,0,68,50,4,0,13
CYP39A1_2_21458,99,113,85,45,92,518,1,70,7,42,0,145
CYP3A7_2_21459,549,493,478,484,357,466,731,537,746,710,408,378
CYP46A1_2_21460,371,392,200,329,177,1095,37,21,52,682,7,367
CYP4A11_2_21461,153,106,119,98,559,4,24,190,477,156,265,304
CYP4A22_2_21462,93,174,104,140,58,219,111,371,163,37,6,335
CYP4F12_2_21463,1097,908,892,1022,928,994,1210,1112,1151,637,848,859
CYP4F22_2_21464,14,12,28,1,3,37,4,0,14,1,0,28
CYP4F2_2_21465,4374,3939,3515,3885,3496,2628,4625,3352,2600,3183,4857,
3842
CYP4F8_2_21466,592,324,260,592,92,256,743,385,465,457,725,123
CYP4V2_2_21467,954,686,669,902,646,559,405,253,678,810,2296,1733
CYP4X1_2_21468,1421,1356,1329,1464,1958,609,1414,2261,1847,1393,1204,1
538
CYP4Z1_2_21469,37,36,9,24,30,107,0,9,7,1,1,20
CYP7A1_2_21470,57,111,74,66,43,46,4,53,77,280,0,105
CYP7B1_2_21471,432,497,497,517,411,337,86,818,668,301,579,285
CYP8B1_2_21472,189,153,57,94,5,56,39,79,329,31,269,118

DAD1_2_21473,214,233,206,165,639,429,205,325,239,109,153,144
DAGLA_2_21474,9,7,42,46,128,0,0,0,0,0,0,36
DAK_2_21475,392,371,372,336,675,170,131,378,427,303,65,472
DARS2_2_21476,529,356,509,551,267,678,757,279,276,878,1,217
DARS_2_21477,306,317,274,403,1002,24,82,189,380,114,234,256
DBH_2_21478,126,166,115,143,57,300,2,155,367,364,5,203
DBR1_2_21479,478,351,411,485,684,377,48,90,881,143,36,672
DBT_2_21480,794,769,815,895,704,997,784,1141,162,1409,1163,556
DCLRE1B_2_21481,8,31,28,19,3,0,0,0,1,45,0,65
DCPS_2_21482,362,318,392,528,351,223,244,447,216,143,16,160
DCTN6_2_21483,1600,1177,1314,1347,751,1140,1143,1262,1668,1701,1245,15
98
DCTPP1_2_21484,25,56,61,83,0,0,4,0,0,0,0,0
DDAH2_2_21485,211,261,311,420,171,626,303,13,478,655,94,607
DDOST_2_21486,630,478,618,694,494,83,852,350,326,398,536,563
DDX10_2_21487,212,178,114,125,118,557,85,778,68,588,1,22
DDX18_2_21488,1223,997,1041,1116,1034,1367,870,966,1309,487,1435,1726
DDX19A_2_21489,310,670,393,557,926,795,222,1000,296,546,895,201
DDX1_2_21490,64,129,81,82,67,27,145,114,118,15,895,1
DDX20_2_21491,911,922,958,1013,105,841,803,513,271,469,1345,406
DDX21_2_21492,297,349,356,332,120,214,749,416,91,726,53,94
DDX23_2_21493,127,144,109,162,138,74,283,64,83,253,8,150
DDX24_2_21494,1447,1066,1267,1529,1571,716,1459,2864,942,966,1439,1119
DDX25_2_21495,275,338,312,302,689,790,561,169,410,355,421,470
DDX27_2_21496,39,44,32,43,48,1,4,18,64,0,2,0
DDX28_2_21497,134,152,140,175,62,8,1440,17,82,130,46,445
DDX39A_2_21498,1503,1126,1402,1294,1474,1756,2735,1054,943,1078,1768,1
951
DDX41_2_21499,9,4,8,9,9,0,15,0,0,62,0,0
DDX43_2_21500,208,161,134,278,45,82,746,229,16,379,186,63
DDX46_2_21501,71,199,126,123,240,0,2,13,4,199,115,345
DDX49_2_21502,1739,1367,1487,1604,1817,1254,837,1358,1186,1429,1314,19
45
DDX50_2_21503,170,114,115,82,29,111,456,8,98,217,97,19
DDX51_2_21504,79,104,182,78,7,213,93,0,4,282,7,238
DDX52_2_21505,937,1031,740,994,1338,624,644,1354,1006,1701,2027,908
DDX53_2_21506,114,71,39,69,15,9,116,83,37,31,100,0
DDX55_2_21507,1985,1747,1848,1800,1384,553,2686,1085,1832,2001,2373,20
42
DDX56_2_21508,324,240,177,280,641,75,281,103,159,650,328,266
DDX58_2_21509,1765,1261,1357,1364,1274,796,842,1512,1421,1050,557,1955
DDX59_2_21510,1263,917,834,882,1634,687,624,349,937,584,795,965
DDX5_2_21511,228,175,189,186,14,105,128,400,231,0,205,158
DDX60_2_21512,463,422,296,438,540,54,436,70,97,794,1144,507
DDX6_2_21513,201,258,265,257,314,391,231,83,181,14,985,115
DECR1_2_21514,220,107,85,160,47,130,117,7,369,15,2,90
DECR2_2_21515,277,199,181,482,17,880,149,8,26,7,1142,243
DEGS1_2_21516,310,210,388,108,25,311,470,0,0,351,0,312
DEGS2_2_21517,413,480,403,368,294,458,453,635,524,361,157,649
DERA_2_21518,1032,859,856,741,264,841,201,1037,190,1045,1167,1123

DFFB_2_21519,135,157,178,149,305,57,65,181,104,164,401,314
DGAT1_2_21520,18,38,39,97,248,1,0,637,54,32,654,109
DGAT2_2_21521,152,203,131,314,62,917,21,353,208,290,593,54
DHCR24_2_21522,391,383,213,327,87,352,1626,143,296,54,360,329
DHDH_2_21523,2,6,23,19,0,2,19,0,0,4,0,0
DHFR_2_21524,455,488,321,552,209,313,246,609,626,634,279,503
DHODH_2_21525,206,121,100,150,28,0,81,270,58,0,323,59
DHRS13_2_21526,198,286,262,451,239,181,29,299,31,460,80,671
DHRS3_2_21527,189,159,332,220,1,0,23,331,138,1,445,35
DHRS4_2_21528,861,649,796,955,859,1220,1562,1719,692,1327,871,1327
DHRS7_2_21529,53,3,12,0,0,0,0,0,0,0,0,79
DHRSX_2_21530,98,97,88,132,343,27,37,1,27,0,0,166
DHTKD1_2_21531,1172,984,628,1312,2099,1211,1264,734,1221,1976,1328,104
2
DHX15_2_21532,2576,1947,2065,2211,2497,3039,1489,3277,1760,2345,3123,1
926
DHX29_2_21533,95,199,126,109,319,41,104,293,50,314,19,50
DHX32_2_21534,856,622,796,893,1015,596,203,1115,852,1167,706,1226
DHX34_2_21535,158,261,238,482,105,265,69,107,94,959,93,118
DHX37_2_21536,739,590,451,656,489,660,113,559,593,1121,578,497
DHX38_2_21537,59,148,77,89,301,52,1,67,243,197,14,103
DHX57_2_21538,253,139,302,426,282,0,31,972,392,149,570,386
DHX58_2_21539,12,4,19,15,0,0,0,0,163,151,0,31
DHX8_2_21540,164,211,99,141,139,23,258,315,230,0,640,54
DHX9_2_21541,2652,2958,2642,2657,4051,2824,4190,1752,3022,2479,2310,29
59
DIO3_2_21542,373,269,319,369,250,126,262,665,283,329,270,805
DIRAS1_2_21543,970,642,774,800,497,1346,680,526,889,561,637,634
DIRAS2_2_21544,53,95,92,133,4,25,291,104,45,315,29,361
DIRAS3_2_21545,110,84,203,105,251,0,53,73,0,947,260,1
DIS3L2_2_21546,317,268,393,293,436,696,12,0,0,0,482,354
DLAT_2_21547,95,19,142,76,6,132,0,31,0,0,0,11
DLD_2_21548,279,147,195,178,213,9,295,698,122,93,15,46
DLL1_2_21549,0,0,0,0,0,0,0,0,0,0,0,0
DLST_2_21550,37,118,6,129,0,1,18,0,12,0,0,0
DMGDH_2_21551,208,157,212,212,750,125,263,0,133,531,233,29
DNA2_2_21552,866,738,603,694,235,616,879,13,568,272,504,367
DNAH11_2_21553,722,736,743,698,581,347,328,889,237,1190,945,428
DNAH3_2_21554,45,18,11,62,0,143,0,0,188,77,0,0
DNAH5_2_21555,632,624,353,575,468,437,1729,812,337,1149,132,265
DNAH8_2_21556,494,405,342,401,1294,279,511,331,94,382,92,444
DNAJA2_2_21557,12,33,55,7,0,0,0,9,68,10,0,101
DNAJB14_2_21558,388,218,486,327,421,1,120,0,0,764,203,426
DNAJC10_2_21559,270,412,251,392,134,670,48,943,101,221,191,71
DNAJC18_2_21560,174,157,161,159,52,1,187,18,406,81,31,672
DNAL4_2_21561,389,468,610,355,450,218,434,780,205,919,909,893
DNASE1_2_21562,346,422,191,267,21,144,265,1519,518,54,216,433
DNASE1L2_2_21563,496,466,396,841,346,526,296,935,316,161,343,926
DNASE1L3_2_21564,336,200,248,202,2,0,67,710,185,166,302,33
DNASE2_2_21565,89,108,108,131,132,19,8,40,279,4,1,37

DNPEP_2_21566,225,304,165,245,69,111,5,121,92,12,0,207
DPAGT1_2_21567,313,387,248,337,68,1248,169,728,399,653,149,72
DPEP2_2_21568,111,84,85,147,468,26,272,0,80,0,92,42
DPM1_2_21569,346,462,213,331,211,0,189,44,273,145,302,382
DPM2_2_21570,865,803,693,1072,441,1009,1053,1448,721,1049,173,618
DPP4_2_21571,699,540,547,613,233,537,369,941,385,324,825,535
DPP7_2_21572,182,253,172,230,72,367,4,412,0,213,0,176
DPP9_2_21573,115,87,68,249,217,0,122,353,117,200,255,443
DPYS_2_21574,1154,991,937,1209,939,831,1518,668,836,1469,1402,1594
DPYSL4_2_21575,110,39,75,93,135,47,161,366,101,11,74,41
DPYSL5_2_21576,402,276,345,236,320,478,708,408,301,179,477,248
DQX1_2_21577,213,284,319,414,72,788,418,162,81,142,101,124
DSEL_2_21578,1980,1586,1761,1953,1328,2525,2115,2480,2195,1697,937,111
7
DTD1_2_21579,432,424,521,454,522,199,433,131,395,1039,319,319
DUOX2_2_21580,352,358,314,315,196,283,579,475,233,277,708,144
DUPD1_2_21581,464,351,253,447,718,497,104,700,191,532,56,251
DUSP28_2_21582,16,59,35,94,239,28,0,8,12,40,2,201
EARS2_2_21583,44,36,55,62,11,10,47,8,22,11,3,68
EBPL_2_21584,310,178,224,192,492,25,30,81,193,287,244,106
ECH1_2_21585,461,251,301,503,63,2,129,27,117,160,607,1134
ECHDC3_2_21586,130,202,134,183,147,495,0,2,98,0,13,25
ECHS1_2_21587,617,508,378,427,144,158,630,412,547,605,320,520
EDEM1_2_21588,813,658,486,847,253,323,877,370,241,354,410,745
EDEM3_2_21589,361,177,212,278,150,8,133,18,86,78,315,189
EEF1A1_2_21590,325,375,282,388,488,29,202,345,238,486,48,24
EEF1A2_2_21591,572,474,296,436,334,448,318,398,443,78,458,472
EEF2_2_21592,110,109,39,164,21,326,35,5,525,208,0,17
EGLN1_2_21593,70,17,25,39,3,0,0,16,208,3,0,16
EGLN3_2_21594,33,25,54,15,0,92,494,0,1,6,199,95
EHD4_2_21595,734,704,730,1002,919,1521,691,410,1023,774,276,686
EIF4A3_2_21596,110,145,132,206,311,29,91,96,72,195,0,372
ELAC1_2_21597,251,27,76,40,17,372,3,0,32,29,41,0
ELANE_2_21598,46,62,11,26,519,53,274,132,0,7,8,485
ELOVL1_2_21599,2343,2391,2405,2568,2789,2626,2694,1822,2254,1412,2487,
2206
ELOVL2_2_21600,329,196,259,271,449,1,693,3,470,0,39,636
ELOVL3_2_21601,84,61,84,50,142,3,1,23,74,31,13,99
ELOVL4_2_21602,699,629,630,910,279,374,829,591,593,280,1091,961
ENDOD1_2_21603,224,308,243,323,223,516,161,23,272,32,190,72
ENDOG_2_21604,135,30,65,102,26,2,319,70,74,34,117,57
ENGASE_2_21605,323,375,180,216,435,24,81,475,28,501,157,263
ENO2_2_21606,457,405,385,524,971,16,708,1139,419,224,130,258
ENO4_2_21607,458,170,340,229,213,180,42,95,63,477,0,11
ENOPH1_2_21608,243,215,319,336,337,248,324,70,436,353,367,8
ENPEP_2_21609,403,288,284,279,0,137,352,131,357,156,303,147
ENPP1_2_21610,702,516,514,785,617,264,298,337,361,265,506,203
ENPP3_2_21611,101,86,217,90,22,117,0,25,771,56,0,20
ENPP4_2_21612,828,932,723,967,489,1456,1839,342,877,597,897,706
ENPP5_2_21613,231,117,169,98,60,70,41,361,206,157,50,105

ENPP6_2_21614,630,427,457,531,251,722,819,861,270,673,241,443
ENPP7_2_21615,39,20,6,19,91,0,26,45,8,33,0,5
ENTPD3_2_21616,21,50,66,60,0,192,153,90,62,9,0,45
ENTPD5_2_21617,3927,3105,2560,2871,3003,1355,2541,2662,2863,3093,3012,
3678
ENTPD7_2_21618,512,525,416,602,276,215,277,901,193,402,329,748
EPHX2_2_21619,398,283,291,326,177,298,61,331,201,409,193,447
EPHX4_2_21620,458,404,625,666,342,447,975,387,268,342,229,651
EPRS_2_21621,1752,1100,1430,1694,2457,1372,500,1650,960,872,1185,1251
EPT1_2_21622,350,540,362,516,449,245,395,543,165,79,20,461
EPX_2_21623,88,43,105,239,251,0,0,23,50,299,6,57
ERAS_2_21624,208,207,376,640,0,596,242,265,433,315,475,589
ERCC3_2_21625,377,250,269,415,159,327,432,217,368,539,181,261
ERCC4_2_21626,121,184,204,171,123,176,269,320,202,312,117,490
ERCC5_2_21627,5,16,28,25,1,0,275,41,140,94,61,4
ERI1_2_21628,0,0,0,0,0,0,0,0,0,0,0,0
ERO1L_2_21629,887,694,697,717,1043,80,677,152,531,662,615,542
ERP44_2_21630,435,745,521,691,977,647,354,328,563,801,678,1120
ESD_2_21631,322,281,180,257,336,705,93,520,158,111,367,482
ESPL1_2_21632,84,46,69,45,0,126,331,279,34,27,10,4
ETFDH_2_21633,268,217,234,197,169,453,145,184,542,242,1,342
EXOSC1_2_21634,168,126,190,114,67,275,426,238,94,356,48,285
EXOSC2_2_21635,479,300,281,318,305,94,357,19,171,431,49,557
EXOSC4_2_21636,1458,1299,1253,1490,2355,707,694,1751,1721,1450,581,117
2
EXOSC5_2_21637,136,190,82,156,43,334,34,573,180,490,324,404
EXOSC7_2_21638,3,10,14,27,20,0,0,0,179,1,0,0
EXOSC8_2_21639,367,197,202,341,85,362,189,123,817,101,0,499
EXT1_2_21640,440,437,284,225,70,284,535,462,538,387,422,346
EXTL1_2_21641,776,965,617,911,155,587,405,992,887,117,2655,769
EXTL3_2_21642,798,393,401,459,534,483,581,363,664,164,690,411
F10_2_21643,0,29,12,14,0,0,0,0,0,43,0,234
F12_2_21644,506,257,199,531,880,0,505,429,74,272,250,672
F13A1_2_21645,83,41,79,105,233,275,57,99,93,290,4,33
F13B_2_21646,1166,909,903,977,609,730,953,2042,1698,970,1138,768
F2_2_21647,706,401,562,640,418,624,778,147,305,412,799,1091
F5_2_21648,143,210,152,269,40,329,600,709,171,463,4,38
F9_2_21649,270,383,397,451,398,457,891,556,93,402,652,375
FA2H_2_21650,202,51,110,136,216,0,39,186,31,392,258,134
FAAH2_2_21651,555,610,448,726,535,498,500,164,743,696,836,537
FADS1_2_21652,459,370,303,383,303,392,122,638,105,209,87,1196
FADS2_2_21653,206,163,262,280,71,210,235,13,250,612,283,194
FADS3_2_21654,41,40,38,74,4,0,0,398,58,7,0,0
FAHD2A_2_21655,356,548,353,551,335,489,117,179,134,439,677,360
FAM108C1_2_21656,252,70,203,176,100,167,744,218,8,245,0,43
FAM135B_2_21657,251,262,321,282,6,265,140,63,345,327,139,112
FAM20B_2_21658,326,458,384,464,663,238,605,117,433,458,193,417
FANCM_2_21659,18,0,10,29,0,0,0,13,17,69,6,0
FAR1_2_21660,1156,956,1090,1375,1270,1135,435,1203,900,1155,430,1339
FAR2_2_21661,76,186,102,119,5,13,254,3,0,50,6,23

FARS2_2_21662,87,58,39,61,22,263,74,6,24,41,14,136
FARSA_2_21663,26,105,118,129,243,0,268,252,10,33,361,72
FARSB_2_21664,474,359,438,587,406,484,366,440,195,702,293,443
FASN_2_21665,1137,974,771,1020,1099,1464,700,1577,667,1151,1139,1077
FDFT1_2_21666,793,630,705,870,470,591,957,398,828,596,1643,376
FEN1_2_21667,32,61,11,16,40,26,0,7,0,117,3,0
FH_2_21668,143,251,143,223,5,58,429,58,147,76,927,442
FIG4_2_21669,1834,1561,1712,1594,1606,1843,1663,1755,1651,1836,2394,29
07
FKBP10_2_21670,693,683,551,643,378,790,155,115,518,1018,306,468
FKBP14_2_21671,109,40,99,151,1,0,189,3,59,65,0,5
FKBP15_2_21672,132,97,94,209,4,178,362,79,36,324,54,82
FKBP3_2_21673,191,76,144,138,433,113,159,184,145,28,17,300
FKBP4_2_21674,147,108,356,392,290,10,3,638,4,2,262,0
FKBP8_2_21675,109,212,158,209,532,155,79,150,3,155,0,40
FMO1_2_21676,136,61,85,245,8,10,921,31,29,208,364,1
FMO4_2_21677,3177,2300,2634,2813,2416,2644,4126,2190,2788,2013,3286,22
30
FNBP1_2_21678,20,62,9,1,0,0,0,1,0,1,0,64
FNTA_2_21679,200,169,106,276,188,1,411,394,372,508,356,123
FNTB_2_21680,275,149,81,98,4,0,7,0,14,245,0,343
FTH1_2_21681,9,0,2,7,0,0,0,1,1,0,0,5
FTMT_2_21682,18,38,63,76,81,3,68,1,72,0,0,200
FTSJ2_2_21683,16,28,7,168,0,0,48,0,0,0,67,1
FTSJ3_2_21684,5,2,34,43,2,0,0,0,0,0,7,24
FTSJD2_2_21685,580,422,450,622,353,287,1739,445,440,439,214,340
FUCA1_2_21686,553,700,795,719,434,565,1971,247,605,1358,1008,1220
FUCA2_2_21687,97,85,98,106,88,12,80,678,363,164,135,211
FURIN_2_21688,149,167,143,341,2,313,195,33,396,171,6,311
FUT10_2_21689,192,213,246,233,32,405,45,795,370,565,41,87
FUT11_2_21690,3099,2498,2482,3028,3008,1971,2642,2032,4374,3499,2174,2
896
FUT1_2_21691,33,0,19,51,0,0,0,0,24,4,0,14
FUT4_2_21692,5,11,8,31,0,0,74,2,1,0,0,0
FUT5_2_21693,134,35,54,87,0,1,29,0,0,58,159,0
FUT7_2_21694,263,62,169,305,484,166,365,122,395,147,758,78
FUT9_2_21695,424,368,386,460,564,0,121,0,0,510,516,514
GADL1_2_21696,409,370,405,243,440,406,453,200,308,215,220,411
GAL3ST1_2_21697,25,63,55,24,11,50,88,28,69,38,0,8
GAL3ST3_2_21698,76,88,63,61,113,80,209,69,127,36,43,13
GAL3ST4_2_21699,843,797,550,823,440,639,606,972,543,860,590,1129
GALM_2_21700,45,23,44,81,77,33,0,0,1,6,0,52
GALNS_2_21701,2758,2387,2034,2529,3747,2238,2277,1168,2039,2266,2638,2
433
GALNT10_2_21702,54,166,266,251,24,276,481,0,26,30,686,326
GALNT11_2_21703,1163,1110,1100,1472,1465,791,1484,758,1095,1648,820,15
76
GALNT12_2_21704,886,583,656,849,462,559,1239,1114,569,499,707,1007
GALNT13_2_21705,533,328,461,397,784,854,19,546,832,273,254,384
GALNT14_2_21706,281,357,128,126,3,752,33,132,199,262,1622,181

GALNT1_2_21707,34,70,49,89,150,14,59,128,44,66,94,241
GALNT2_2_21708,501,444,541,589,943,706,84,144,5,459,520,964
GALNT3_2_21709,633,604,549,904,575,899,239,1087,272,616,1046,1500
GALNT4_2_21710,121,107,49,87,1,116,1,79,92,471,69,256
GALNT5_2_21711,218,167,160,168,696,77,185,107,544,271,235,110
GALNT6_2_21712,169,225,171,237,391,0,53,11,16,77,484,57
GALNT8_2_21713,218,389,72,220,416,313,316,0,1,0,0,13
GALNTL2_2_21714,156,251,176,198,9,296,283,671,382,243,153,436
GALNTL4_2_21715,435,420,361,423,849,32,285,310,489,334,133,617
GALNTL5_2_21716,23,5,10,29,0,90,0,0,0,0,0,0
GALNTL6_2_21717,540,306,270,377,241,32,4,337,671,561,332,128
GALT_2_21718,34,118,62,92,9,224,1,21,125,445,6,102
GANC_2_21719,9,76,51,35,0,0,15,2,0,0,0,9
GAPDH_2_21720,84,88,87,155,26,136,27,650,139,149,36,19
GAPDHS_2_21721,45,16,21,11,6,0,0,0,145,0,1,1
GARS_2_21722,128,122,158,144,4,61,0,5,101,3,1110,25
GATC_2_21723,133,219,136,135,5,118,1,34,43,7,561,172
GATM_2_21724,142,283,251,125,898,182,10,69,260,135,17,119
GBA2_2_21725,231,245,229,264,139,120,6,11,68,125,30,443
GBE1_2_21726,332,270,364,383,153,442,586,417,190,48,22,470
GBGT1_2_21727,43,85,65,80,646,23,470,0,21,0,261,20
GBP1_2_21728,386,559,458,578,415,251,54,106,220,324,2331,293
GBP2_2_21729,160,351,209,296,268,100,137,74,55,508,199,319
GBP3_2_21730,2658,2429,2282,2645,2928,2223,2481,2953,3385,3330,3258,25
93
GBP4_2_21731,606,764,794,733,517,742,319,547,629,626,540,1271
GBP6_2_21732,539,412,292,313,97,279,157,178,56,686,732,325
GBP7_2_21733,380,671,517,389,821,110,1412,890,504,602,678,230
GCLM_2_21734,262,213,274,174,21,117,487,265,361,147,44,189
GCNT3_2_21735,208,239,246,202,533,61,54,234,71,139,541,375
GCNT4_2_21736,93,78,82,131,94,1,33,148,0,15,54,92
GCSH_2_21737,317,198,268,210,123,181,150,32,195,156,251,436
GDE1_2_21738,607,528,592,739,1074,728,521,896,576,692,517,869
GDPD3_2_21739,64,23,51,46,50,0,0,0,90,0,20,0
GFM1_2_21740,64,156,196,96,47,316,0,33,0,396,194,60
GFOD2_2_21741,416,180,255,416,830,287,387,122,288,229,117,435
GFPT1_2_21742,757,543,602,764,717,501,1002,1414,674,901,442,599
GFPT2_2_21743,514,505,515,572,210,450,446,666,552,874,411,469
GGH_2_21744,930,766,692,778,600,252,407,625,584,546,1383,1042
GGPS1_2_21745,145,335,348,384,826,451,59,214,261,229,68,958
GGT7_2_21746,172,316,103,101,884,348,394,0,0,23,1,0
GGTLC2_2_21747,410,334,382,566,117,581,660,381,471,458,50,386
GLCE_2_21748,0,0,0,0,0,0,0,0,0,0,0,0
GLDC_2_21749,67,87,94,31,52,9,288,0,88,0,571,77
GLO1_2_21750,507,600,526,428,655,0,285,0,17,281,259,1055
GLOD4_2_21751,1131,1071,1047,1239,2269,1113,2541,1146,538,888,1211,138
5
GLS2_2_21752,2613,2327,1917,2796,2495,2157,2047,2170,2952,2254,3090,16
41
GLT1D1_2_21753,162,96,113,153,72,9,23,15,64,138,386,205

GLT25D1_2_21754,556,374,396,460,703,436,1016,208,850,435,69,320
GLT25D2_2_21755,202,211,323,249,903,116,366,652,362,48,131,336
GLT8D2_2_21756,113,152,138,127,23,80,67,13,5,6,1,81
GLUD1_2_21757,216,90,139,93,50,103,19,503,241,0,126,72
GLUD2_2_21758,425,321,357,432,292,1050,766,1231,826,151,162,787
GMDS_2_21759,1334,1090,1040,1070,2181,927,761,932,470,1083,1392,1090
GMIP_2_21760,20,28,12,13,0,22,2,9,1,0,0,16
GMPR_2_21761,52,34,28,76,66,55,51,12,0,56,196,9
GMPS_2_21762,579,431,446,369,552,873,223,156,299,202,1116,158
GNA11_2_21763,295,205,308,293,246,293,138,88,187,296,246,207
GNA12_2_21764,112,82,109,52,24,0,4,40,15,73,27,347
GNA13_2_21765,38,165,178,135,0,0,0,0,7,172,0,132
GNA14_2_21766,1299,919,841,996,426,588,264,257,1597,1164,836,721
GNA15_2_21767,19,7,52,67,23,10,0,0,1,0,5,0
GNAI1_2_21768,277,265,192,282,194,1,90,324,656,224,477,280
GNAI3_2_21769,1105,658,729,734,1094,605,409,1379,1167,887,795,738
GNAQ_2_21770,1225,1143,1429,1308,2205,800,1108,1516,914,1043,1097,1159
GNAT2_2_21771,22,19,58,44,0,0,36,0,27,129,91,296
GNAT3_2_21772,308,435,286,364,118,455,576,294,378,443,101,579
GNAZ_2_21773,402,414,366,337,173,604,186,127,315,411,209,723
GNB1_2_21774,1183,1203,1189,1078,2425,1670,1365,910,1550,950,746,1296
GNB2L1_2_21775,341,275,227,167,55,4,4,105,96,498,662,204
GNB3_2_21776,113,95,111,103,0,0,497,7,0,44,130,4
GNB4_2_21777,36,57,41,40,31,94,30,381,115,255,1,17
GNG11_2_21778,837,902,786,1086,769,232,1240,162,1374,754,1169,1879
GNG12_2_21779,64,76,57,37,1,6,0,0,0,1,0,6
GNG13_2_21780,108,37,66,143,72,18,7,25,178,1,11,0
GNG2_2_21781,507,423,486,516,766,1266,265,370,256,390,16,584
GNG3_2_21782,1234,1246,1177,1168,1436,643,983,1165,280,819,1725,614
GNG7_2_21783,211,309,357,519,590,563,0,43,0,215,135,152
GNG8_2_21784,309,249,243,317,206,0,385,74,371,135,32,309
GNGT1_2_21785,192,115,99,177,103,194,57,108,344,167,3,50
GNL2_2_21786,210,116,148,210,1,417,49,418,164,859,3,139
GNMT_2_21787,253,121,174,189,153,91,110,211,38,214,65,542
GNPAT_2_21788,397,312,339,363,910,93,431,726,221,104,1550,111
GNPDA1_2_21789,218,116,175,170,88,59,197,1,192,18,3,235
GNPDA2_2_21790,40,98,51,53,0,0,0,101,17,247,4,15
GNPNAT1_2_21791,434,490,468,500,101,623,654,237,244,1120,513,513
GNPTAB_2_21792,360,293,300,357,1,430,0,547,41,434,488,83
GNPTG_2_21793,38,31,23,57,0,0,64,210,3,129,75,12
GNS_2_21794,168,218,151,174,138,4,150,181,339,49,31,95
GOT2_2_21795,877,1179,864,1187,756,677,958,898,568,1571,847,661
GPAA1_2_21796,177,83,147,112,214,243,205,0,36,48,56,201
GPAM_2_21797,323,294,152,113,530,367,0,0,1,696,1,801
GPAT2_2_21798,143,130,110,140,2,14,25,18,84,3,8,73
GPD1_2_21799,171,282,245,294,455,8,1271,314,310,143,490,323
GPD1L_2_21800,1769,1202,995,1805,976,508,2429,1662,1302,1746,1931,1853
GPX2_2_21801,81,99,104,105,0,54,389,32,14,244,4,345
GPX3_2_21802,916,945,867,714,353,318,0,567,479,1091,1316,1048
GPX7_2_21803,641,465,430,668,1167,658,779,410,182,1218,243,27

GPX8_2_21804,790,763,797,926,588,1691,1016,1317,1101,955,1161,796
GRHPR_2_21805,269,293,248,374,246,183,284,287,83,1058,507,407
GSTA1_2_21806,519,701,434,432,384,297,564,553,643,623,910,512
GSTA2_2_21807,577,695,511,807,721,1675,212,598,789,1656,239,560
GSTA3_2_21808,577,695,511,807,721,1675,212,598,789,1656,239,560
GSTA4_2_21809,195,158,156,232,24,69,87,4,170,452,2,521
GSTA5_2_21810,2277,2052,1864,2390,1902,461,1671,1201,1672,2125,2934,15
02
GSTM3_2_21811,19,23,97,5,284,0,1,1,0,0,0,4
GSTM5_2_21812,186,204,164,284,181,2,235,308,437,613,247,286
GSTP1_2_21813,61,79,190,196,35,33,86,45,1,5,194,6
GSTT1_2_21814,212,101,251,198,646,147,95,577,0,0,502,470
GSTT2B_2_21815,176,241,97,281,303,16,461,79,112,45,352,521
GSTT2_2_21816,176,241,97,281,303,16,461,79,112,45,352,521
GTF2F2_2_21817,2787,2224,2536,2789,1807,1469,4195,3607,2156,3285,4293,
2597
GTPBP1_2_21818,123,123,122,143,9,59,200,50,104,305,0,141
GTPBP2_2_21819,231,254,214,362,121,321,807,622,35,87,75,191
GTPBP4_2_21820,163,213,332,313,4,162,172,1023,258,393,1120,20
GUCY1A2_2_21821,181,77,159,145,19,63,330,27,65,117,2,166
GUCY1B3_2_21822,1107,623,1189,1171,1502,884,1654,779,472,601,193,1305
GUSB_2_21823,300,265,248,245,125,219,43,176,291,295,86,486
GYLTL1B_2_21824,415,620,356,411,7,458,467,427,229,122,426,400
GYS2_2_21825,716,765,1035,955,341,540,552,1127,752,577,273,1593
GZMA_2_21826,243,274,134,175,362,285,137,59,109,75,15,287
GZMB_2_21827,241,171,248,287,177,21,196,144,275,326,219,215
H6PD_2_21828,193,112,141,169,213,127,3,20,106,373,0,40
HAAO_2_21829,876,485,523,525,364,267,1089,114,165,694,1157,520
HACL1_2_21830,0,0,0,0,0,0,0,0,0,0,0,0
HADHA_2_21831,262,460,364,243,263,204,514,0,483,373,336,213
HADHB_2_21832,614,573,781,963,0,1087,1113,706,775,1443,1127,1410
HAGHL_2_21833,105,108,176,155,96,198,198,185,198,2,154,366
HAL_2_21834,123,105,63,119,8,19,27,210,81,571,76,26
HAO1_2_21835,84,153,121,66,0,16,190,105,313,0,24,98
HARS_2_21836,15,35,67,21,27,0,0,18,79,56,169,53
HAS1_2_21837,347,283,414,504,1158,0,869,60,450,182,31,300
HAS2_2_21838,312,381,417,330,135,753,1,257,202,384,0,811
HDHD3_2_21839,61,17,46,25,0,0,0,0,3,36,0,0
HELB_2_21840,96,90,95,96,23,0,0,310,2,24,8,10
HELQ_2_21841,273,147,228,314,105,85,250,95,26,123,742,29
HELZ_2_21842,329,184,329,339,387,51,353,24,630,236,150,549
HEXA_2_21843,156,106,110,111,109,78,1,59,12,178,188,348
HEXB_2_21844,81,127,82,157,16,24,0,163,110,412,69,74
HEXDC_2_21845,947,532,607,863,560,800,543,1269,401,413,767,540
HGD_2_21846,92,94,101,71,124,5,67,10,7,13,3,74
HGSNAT_2_21847,849,392,482,543,1394,687,1961,521,784,500,1333,388
HHATL_2_21848,84,51,26,69,15,3,29,1,74,0,4,6
HIBADH_2_21849,103,112,207,169,234,50,62,238,153,267,37,156
HINT1_2_21850,446,398,373,481,302,121,556,129,196,415,525,517
HMOX1_2_21851,1170,689,840,884,631,374,1096,1450,1233,803,678,1470

HPGDS_2_21852,90,176,145,140,17,131,253,63,132,186,203,248
HPRT1_2_21853,1060,865,776,1223,642,2104,900,2067,1298,630,1716,1388
HS3ST1_2_21854,496,423,439,593,595,159,194,114,340,747,1129,478
HS3ST2_2_21855,83,51,95,155,5,176,61,40,100,124,0,0
HS3ST3A1_2_21856,792,712,798,783,892,826,297,456,1235,548,1328,866
HS3ST3B1_2_21857,47,58,130,35,69,0,0,0,0,0,0,0
HS3ST4_2_21858,379,299,220,261,12,761,0,0,648,504,0,8
HS3ST6_2_21859,152,108,145,169,177,53,267,176,338,114,72,214
HS6ST1_2_21860,42,70,34,19,0,5,74,0,12,270,12,2
HS6ST3_2_21861,49,49,29,73,3,0,0,0,43,31,0,0
HSD11B2_2_21862,0,0,0,0,0,0,0,0,0,0,0,0
HSD17B11_2_21863,469,372,202,310,892,890,159,427,423,1011,274,310
HSD17B12_2_21864,279,200,293,273,269,449,51,133,258,274,73,332
HSD17B14_2_21865,185,189,204,120,272,9,47,551,223,723,302,5
HSD17B1_2_21866,26,71,16,104,4,21,5,2,10,0,91,3
HSD17B2_2_21867,27,15,54,97,0,0,1,86,192,4,267,2
HSD17B3_2_21868,338,151,125,314,550,0,587,0,367,529,0,184
HSD17B6_2_21869,121,119,109,216,23,197,156,0,368,130,14,183
HSD17B8_2_21870,844,516,598,642,537,706,636,701,828,396,610,690
HSD3B1_2_21871,458,532,326,476,382,145,1022,370,919,303,94,409
HSP90AB1_2_21872,1391,1209,1174,1150,905,1849,2049,1817,1727,1884,1381
,1815
HSPA5_2_21873,566,641,618,916,526,360,501,78,342,1035,754,610
HSPE1_2_21874,2169,1933,1792,2181,1826,1756,4047,1595,2294,2844,2294,2
014
HSPG2_2_21875,36,0,24,23,0,0,0,2,165,0,0,0
HYAL4_2_21876,732,723,626,955,962,1083,378,1330,1381,1225,706,1270
IARS2_2_21877,132,139,203,155,28,443,147,0,41,244,126,1
ICMT_2_21878,239,265,581,360,365,302,168,284,238,1774,125,334
ICT1_2_21879,994,392,703,636,1304,598,360,1082,356,997,845,507
IDH1_2_21880,275,248,315,397,43,336,38,533,179,153,72,824
IDH2_2_21881,47,104,25,121,0,0,2,0,10,232,9,138
IDH3A_2_21882,219,45,78,160,180,10,40,14,296,67,393,0
IDI1_2_21883,98,182,150,161,0,295,144,17,10,27,0,5
IDI2_2_21884,2420,2082,2182,2604,2027,1828,2529,1343,1848,1952,2987,17
20
IDO1_2_21885,797,816,683,819,541,318,1336,411,1105,1031,1497,641
IDO2_2_21886,374,361,305,294,18,525,1699,692,493,403,754,430
IDUA_2_21887,367,314,228,211,407,321,275,88,324,269,549,106
IFI30_2_21888,332,199,207,237,10,131,466,45,70,53,421,239
IFIH1_2_21889,1090,886,696,1267,1479,1015,965,1365,1232,806,1967,760
IGHMBP2_2_21890,1303,1461,1111,1250,434,627,1224,1276,1160,1578,1810,1
048
IHH_2_21891,0,0,0,44,0,0,0,0,0,0,0,2
ILVBL_2_21892,258,114,145,167,579,10,162,187,256,196,77,304
IMPAD1_2_21893,643,562,458,499,1087,111,1495,1008,423,547,307,218
IMPDH2_2_21894,677,443,395,633,190,737,351,911,783,1104,180,564
IRS1_2_21895,82,86,106,118,152,6,139,22,210,23,86,66
IRS2_2_21896,324,345,245,289,82,37,57,0,93,61,98,252
ISG20_2_21897,486,289,554,468,402,263,177,354,444,484,215,464

ISG20L2_2_21898,83,60,53,48,33,9,151,383,75,355,29,284
ISOC1_2_21899,126,89,153,146,24,179,84,144,195,12,163,103
IWS1_2_21900,743,589,619,513,0,942,555,1018,1031,691,484,632
KDSR_2_21901,1352,1053,1195,1660,1612,493,978,483,1283,1257,1459,1264
KHSRP_2_21902,93,66,83,113,68,2,267,29,95,149,165,45
KIAA0317_2_21903,336,89,174,78,0,1,0,162,53,316,470,127
KIAA1279_2_21904,1276,1457,1387,1661,184,891,1564,711,925,2237,1681,11
87
KIAA2022_2_21905,151,220,256,386,511,623,1174,838,253,761,376,637
KIF18A_2_21906,625,679,480,482,652,391,165,394,202,1580,245,847
KIF20B_2_21907,624,400,374,375,532,84,470,421,438,303,330,248
KIF3A_2_21908,107,100,129,123,34,37,0,180,373,500,3,0
KLB_2_21909,838,501,508,533,907,266,1443,95,649,1479,29,823
KLC3_2_21910,259,236,205,202,204,0,22,171,323,1,1149,271
KL_2_21911,836,882,690,740,471,1215,498,1079,942,1003,347,1769
KLK1_2_21912,59,26,39,55,94,0,24,2,0,41,0,0
KLKB1_2_21913,135,199,186,120,68,159,593,12,69,243,1039,11
KMO_2_21914,1214,1069,837,1288,1774,734,497,1159,1178,674,747,1193
KRTCAP2_2_21915,20,26,37,19,21,145,13,0,5,10,0,170
L2HGDH_2_21916,886,912,784,998,1008,1155,251,697,331,1024,771,925
LALBA_2_21917,3,1,5,35,0,0,0,6,0,0,0,0
LAP3_2_21918,18,98,36,35,111,1,41,10,88,711,0,107
LARS2_2_21919,825,551,468,746,957,1706,1076,453,516,356,607,377
LARS_2_21920,861,1137,809,1112,879,1153,36,781,535,1207,1746,907
LCAT_2_21921,305,355,518,419,4,68,69,77,111,1019,507,57
LCMT2_2_21922,0,0,0,0,0,0,0,0,0,0,0,0
LCT_2_21923,409,296,260,262,452,448,305,382,516,315,140,320
LCTL_2_21924,325,208,280,271,176,278,87,56,502,401,302,346
LDHAL6B_2_21925,204,170,180,337,47,516,392,304,161,53,12,137
LEPREL2_2_21926,54,77,66,87,0,1260,0,0,75,0,1,0
LGALS13_2_21927,121,134,101,145,99,62,566,0,164,92,47,0
LHFPL2_2_21928,88,50,130,93,237,78,176,180,200,3,323,488
LIPC_2_21929,272,466,625,488,961,75,26,268,444,136,580,558
LIPE_2_21930,9,10,50,34,0,113,48,0,45,0,0,0
LIPG_2_21931,78,104,158,289,7,67,8,7,0,90,0,5
LIPH_2_21932,345,384,352,331,553,612,564,238,74,299,51,718
LIPI_2_21933,1715,1358,1333,1154,1917,240,2249,2082,1391,1475,1528,127
3
LIPM_2_21934,1736,1456,1789,2238,1414,1393,1441,2650,921,1361,2627,164
0
LIPN_2_21935,530,443,391,374,239,454,113,538,992,452,155,688
LIPT2_2_21936,171,116,81,79,22,503,0,122,110,289,166,16
LONP2_2_21937,3197,2878,2976,3165,2331,1757,3502,2745,3409,3047,3258,2
520
LOXL1_2_21938,151,86,58,83,213,11,0,5,22,59,7,11
LOXL2_2_21939,108,65,126,205,305,0,8,180,1,16,0,563
LOXL3_2_21940,159,204,113,199,72,8,526,0,273,0,158,34
LOXL4_2_21941,236,283,214,364,213,532,494,418,43,144,456,272
LPCAT1_2_21942,280,328,528,528,85,19,142,717,216,7,772,304
LPCAT2_2_21943,113,219,252,295,207,175,4,626,260,129,167,131

LPCAT3_2_21944,99,135,83,141,75,1,141,28,62,45,47,242
LPCAT4_2_21945,82,32,28,85,46,42,1,25,1,6,121,44
LPIN1_2_21946,172,235,178,264,153,7,441,68,52,27,51,574
LPIN2_2_21947,406,256,250,391,189,302,265,231,661,472,34,20
LPIN3_2_21948,276,281,445,507,311,396,582,437,635,640,1437,358
LPL_2_21949,407,360,445,443,185,380,1126,454,949,293,473,314
LRAT_2_21950,207,181,229,353,39,102,445,67,117,41,26,302
LTA4H_2_21951,485,371,413,588,1090,678,382,369,641,1024,393,328
LTC4S_2_21952,468,250,389,384,1284,691,1361,633,285,234,445,241
LYG2_2_21953,106,39,64,80,0,98,7,37,90,188,27,488
LYPLA1_2_21954,332,407,451,332,3,368,216,121,324,177,790,227
LYPLA2_2_21955,877,968,863,959,536,316,471,372,1053,1737,441,1202
LYPLAL1_2_21956,974,975,851,919,971,292,764,284,669,844,784,1124
LYZ_2_21957,86,95,91,112,37,27,12,155,168,260,7,102
LYZL1_2_21958,359,232,216,307,390,26,803,387,297,603,433,490
LYZL2_2_21959,877,494,536,514,1073,540,100,284,841,464,1452,615
LYZL4_2_21960,29,43,101,103,189,0,22,24,74,1,25,0
MACROD1_2_21961,45,1,69,39,11,19,0,0,86,0,473,0
MAGT1_2_21962,79,109,103,188,133,7,91,119,338,124,0,254
MAN1A1_2_21963,729,724,505,692,782,629,1498,135,360,1262,658,679
MAN1A2_2_21964,270,182,190,300,60,396,455,109,160,139,65,364
MAN1B1_2_21965,292,189,272,365,219,11,73,0,57,473,160,66
MAN1C1_2_21966,425,647,397,643,548,483,980,1626,535,381,217,857
MAN2A1_2_21967,1243,1204,965,921,490,1560,1928,362,564,774,113,1318
MAN2A2_2_21968,504,431,625,576,293,116,503,1020,317,283,454,557
MAN2B2_2_21969,105,54,72,85,3,377,5,2,0,0,308,165
MAN2C1_2_21970,109,61,67,30,337,0,3,0,0,1,0,0
MANBA_2_21971,129,134,225,241,117,0,343,134,379,909,110,175
MANEA_2_21972,707,419,545,478,252,992,542,588,318,219,811,480
MAOA_2_21973,700,712,645,776,61,691,1547,381,782,493,649,605
MAOB_2_21974,118,183,126,98,202,208,0,1,323,108,37,2
MAP1S_2_21975,295,100,163,191,0,0,0,0,147,22,370,18
MAPRE3_2_21976,295,128,172,199,155,266,1,74,253,95,59,7
MARS2_2_21977,822,701,592,729,1190,579,1080,369,779,1019,68,387
MARS_2_21978,508,572,598,656,838,303,610,539,966,1163,252,1184
MAT1A_2_21979,292,240,326,341,401,454,98,780,296,131,0,124
MAT2A_2_21980,597,539,499,767,672,323,521,1316,561,1181,298,863
MBOAT1_2_21981,33,87,45,65,4,0,36,33,96,75,0,36
MBOAT2_2_21982,517,449,391,614,1123,122,709,559,163,455,482,703
MBOAT4_2_21983,102,309,179,185,349,337,436,259,1,329,19,51
MBTPS1_2_21984,222,211,121,135,359,851,1,110,89,7,41,274
MBTPS2_2_21985,232,140,109,146,271,75,109,824,130,306,47,232
MCCC1_2_21986,1687,1684,1478,1711,1113,605,1714,2076,699,755,2493,1915
MCCC2_2_21987,398,369,404,265,48,149,47,6,173,4,713,316
MCEE_2_21988,1032,1063,1009,1450,2112,580,1768,811,1417,979,893,2353
MCM3_2_21989,327,386,220,350,89,554,340,23,181,435,768,493
MCM5_2_21990,144,95,155,171,413,196,211,12,95,234,56,249
MCM6_2_21991,783,720,948,1099,998,219,828,639,650,790,920,636
MDH1B_2_21992,245,188,176,262,194,141,412,614,112,271,138,327
MDH2_2_21993,9,46,22,43,11,0,0,20,18,0,179,105

MEP1A_2_21994,157,235,145,206,272,132,0,118,234,0,17,112
MEP1B_2_21995,77,102,151,141,5,14,449,379,106,81,195,11
METAP1_2_21996,666,604,671,543,543,1,342,1069,486,1178,512,294
METAP1D_2_21997,208,89,153,127,163,13,178,25,706,33,614,255
METAP2_2_21998,106,88,78,94,35,7,6,86,141,56,117,74
METTL14_2_21999,951,1032,805,917,1233,647,962,362,1016,1320,1220,804
METTL22_2_22000,321,224,378,282,253,117,547,73,224,340,521,455
METTL2B_2_22001,129,199,207,152,0,663,110,175,98,178,209,31
METTL3_2_22002,847,593,614,640,952,130,587,95,91,437,299,825
METTL5_2_22003,1206,882,810,1004,1761,587,553,1476,479,602,250,785
METTL6_2_22004,53,13,35,12,5,4,6,32,0,9,112,40
METTL7B_2_22005,120,36,15,37,5,6,1,0,40,93,380,126
METTL8_2_22006,357,296,406,355,593,370,360,1281,117,96,975,802
MFN1_2_22007,325,456,178,215,282,216,18,205,182,551,33,135
MGAM_2_22008,333,268,295,366,757,59,141,185,93,226,201,694
MGAT4C_2_22009,1604,1231,1380,1674,1175,993,1738,1866,1350,993,911,219
8
MGAT5_2_22010,524,511,379,587,182,556,249,231,152,290,424,637
MGMT_2_22011,74,170,161,291,48,4,7,334,209,122,467,259
MGST3_2_22012,2134,1968,1516,1929,2227,1639,1461,3072,1928,2104,1443,2
206
MIOX_2_22013,27,65,41,12,0,0,86,0,0,0,6,0
MIPEP_2_22014,654,503,386,651,1305,142,560,683,492,970,1097,129
MLYCD_2_22015,52,36,84,61,203,88,2,0,47,47,1,0
MMAB_2_22016,375,258,180,244,221,186,269,44,588,2,61,429
MMEL1_2_22017,155,39,43,31,98,0,4,7,1,171,0,4
MMP10_2_22018,1040,1065,943,1030,1818,1415,474,844,695,1419,1319,1725
MMP12_2_22019,2278,1781,1887,2285,2912,980,2635,1950,2467,943,4181,119
1
MMP14_2_22020,214,213,202,272,851,426,1085,207,478,541,264,179
MMP3_2_22021,70,20,26,40,132,18,30,0,85,28,294,81
MMP7_2_22022,1372,1223,1197,1513,1207,1149,1264,708,1114,1489,1281,168
9
MMP8_2_22023,1489,1337,1544,1616,1311,2030,1971,2007,1539,1026,691,128
6
MMP9_2_22024,87,61,47,109,247,292,18,17,22,67,0,279
MOCOS_2_22025,599,346,553,391,487,420,901,627,118,512,904,225
MOGAT1_2_22026,1116,664,1050,974,456,883,717,1655,926,1117,491,1028
MOGAT2_2_22027,19,47,57,54,84,22,32,37,4,22,22,53
MOGAT3_2_22028,362,243,222,303,0,0,819,8,228,0,841,609
MOXD1_2_22029,1353,993,866,1229,979,982,678,748,1542,1495,1145,891
MPI_2_22030,48,81,63,44,18,1,61,1,24,353,547,142
MPO_2_22031,250,158,104,387,30,41,25,71,231,51,781,965
MRPL37_2_22032,75,47,76,64,20,90,84,32,47,118,8,59
MRPL44_2_22033,187,197,199,299,226,145,198,299,396,151,143,578
MRPS30_2_22034,189,266,276,319,172,995,325,327,16,38,243,90
MSH2_2_22035,2406,2306,2207,2417,2197,1719,1376,1853,2181,1688,2646,10
69
MSH3_2_22036,524,387,433,517,605,52,65,754,257,679,70,590
MSH4_2_22037,522,494,504,596,200,670,790,144,613,528,222,541

MTAP_2_22038,38,139,154,85,3,216,16,0,1,338,0,182
MTFMT_2_22039,620,668,509,570,505,394,676,273,475,1111,133,391
MTHFD1_2_22040,225,276,231,340,417,142,822,358,125,297,180,313
MTHFD2_2_22041,210,228,114,272,24,16,349,76,291,455,31,373
MTHFD2L_2_22042,191,133,165,177,390,31,232,184,199,126,85,278
MTHFR_2_22043,87,45,47,97,12,0,0,0,55,8,0,0
MTPAP_2_22044,801,700,642,857,753,895,222,278,888,738,645,1175
MTR_2_22045,452,206,269,311,106,351,130,1018,112,507,149,776
MUS81_2_22046,354,243,217,264,60,695,506,207,76,430,237,152
MUT_2_22047,239,258,223,183,1122,14,705,141,33,167,233,270
MVD_2_22048,33,131,121,102,0,29,0,3,124,47,33,0
MX2_2_22049,339,371,312,419,644,529,376,327,7,345,1,356
MYH1_2_22050,335,590,338,453,314,201,43,116,409,1304,1681,569
MYH3_2_22051,321,401,344,241,414,354,226,288,491,154,309,149
MYH6_2_22052,666,614,752,1030,287,834,984,368,371,773,995,1028
MYH7_2_22053,70,94,100,68,34,230,42,1,214,12,316,26
MYH9_2_22054,336,163,202,239,39,112,336,249,8,65,224,364
MYL7_2_22055,103,169,79,56,90,7,11,136,69,356,13,97
MYO1E_2_22056,0,0,0,0,0,0,0,0,0,0,0,0
MYO5B_2_22057,66,39,72,68,17,3,0,329,24,32,19,5
MYO9A_2_22058,360,351,352,373,73,162,725,516,138,155,334,193
N6AMT2_2_22059,177,115,168,101,58,136,235,316,202,297,36,131
NAA10_2_22060,622,509,793,706,187,371,986,805,1111,561,255,524
NAA11_2_22061,373,262,320,316,675,54,542,3,679,117,372,159
NAA15_2_22062,357,204,343,310,513,191,583,645,444,240,274,597
NAA30_2_22063,347,231,251,282,84,168,403,445,463,191,210,746
NAA50_2_22064,584,674,544,770,627,432,332,1722,543,342,583,606
NAALAD2_2_22065,155,297,406,274,639,43,202,655,329,536,1437,993
NAALADL1_2_22066,25,93,51,11,128,0,10,0,23,0,337,1
NADSYN1_2_22067,630,525,460,762,391,644,322,410,449,420,271,203
NAGA_2_22068,164,133,143,140,155,128,50,581,42,261,91,211
NAGLU_2_22069,71,161,81,169,117,60,167,36,83,29,0,34
NAGPA_2_22070,126,126,106,105,58,2,147,432,453,84,0,186
NAMPT_2_22071,914,1174,830,1176,1176,972,1122,1242,1016,609,1015,2109
NANP_2_22072,149,206,121,252,19,111,76,5,69,235,40,86
NANS_2_22073,235,121,189,178,812,639,151,143,112,281,2,324
NAPRT1_2_22074,46,52,29,52,48,0,0,7,0,3,0,132
NARS_2_22075,52,38,56,75,594,77,130,17,14,47,32,81
NCF1_2_22076,46,46,29,23,0,0,0,4,35,292,2,2
NDST1_2_22077,205,337,299,348,176,539,470,91,5,4,3,281
NDST2_2_22078,430,517,494,358,801,586,3,313,183,235,198,36
NDST3_2_22079,1320,1088,1265,1302,2965,889,1600,1602,1398,977,966,1755
NDST4_2_22080,411,310,337,356,140,260,233,92,364,258,344,540
NDUFA10_2_22081,87,67,150,83,14,25,35,51,17,266,55,0
NDUFA12_2_22082,74,102,81,92,43,17,47,22,50,28,120,25
NDUFA13_2_22083,173,153,209,122,599,0,4,0,0,0,577,18
NDUFA1_2_22084,25,8,16,7,0,0,5,0,8,0,0,0
NDUFA3_2_22085,955,1091,1083,1323,931,1410,1471,353,966,593,786,1064
NDUFA4_2_22086,366,412,285,410,248,7,1120,38,222,737,1383,269
NDUFA4L2_2_22087,1424,1454,1112,1242,1464,1119,2467,745,1129,1591,588,

1583
NDUFA5_2_22088,213,95,155,194,206,123,3,99,23,122,5,114
NDUFA6_2_22089,146,137,199,211,49,330,1,16,205,0,0,4
NDUFA7_2_22090,144,125,99,106,14,19,1114,139,432,242,10,294
NDUFA8_2_22091,250,221,168,157,260,352,49,65,206,373,386,129
NDUFA9_2_22092,109,31,59,70,32,94,298,533,138,1,28,210
NDUFAB1_2_22093,295,116,92,215,43,10,0,228,310,454,103,109
NDUFB10_2_22094,134,78,68,79,131,3,29,144,31,379,554,31
NDUFB1_2_22095,135,79,82,173,10,104,94,0,26,25,161,108
NDUFB2_2_22096,299,127,243,247,540,210,257,32,172,4,933,333
NDUFB3_2_22097,626,605,590,673,543,390,770,582,820,622,859,657
NDUFB7_2_22098,24,27,3,3,0,6,0,25,0,0,0,52
NDUFB8_2_22099,384,365,330,487,224,301,670,557,296,65,658,324
NDUFB9_2_22100,95,133,233,73,676,6,0,99,284,60,880,19
NDUFS3_2_22101,449,483,351,346,1048,420,472,803,265,338,50,179
NDUFS4_2_22102,272,147,223,212,29,523,142,226,136,38,382,223
NDUFS6_2_22103,116,72,45,69,27,2,655,278,17,60,43,83
NDUFS7_2_22104,11,9,15,42,0,6,0,0,2,2,0,0
NDUFS8_2_22105,368,200,144,214,3,93,8,44,287,109,254,197
NDUFV2_2_22106,302,337,196,287,448,468,0,0,370,805,1283,465
NEDD8_2_22107,214,203,149,212,6,29,67,372,302,204,617,107
NEIL1_2_22108,95,190,239,186,23,783,0,27,1,70,123,181
NEIL3_2_22109,806,578,642,599,776,682,872,526,893,291,934,391
NEU1_2_22110,542,478,706,727,325,440,1681,670,734,980,79,615
NEU2_2_22111,43,153,54,122,6,17,12,348,3,0,0,0
NEU3_2_22112,307,261,279,288,391,12,377,525,548,45,0,675
NHLRC2_2_22113,139,41,64,142,9,8,161,191,92,0,22,146
NIT2_2_22114,402,397,392,402,559,543,64,615,297,448,364,524
NKIRAS1_2_22115,334,280,329,270,134,64,272,340,427,234,79,497
NLGN1_2_22116,670,614,586,840,250,250,204,992,663,455,840,588
NLGN2_2_22117,115,114,241,110,193,38,44,138,71,78,25,102
NLN_2_22118,1004,762,989,721,461,776,996,673,1360,1957,1048,904
NME1-NME2_2_22119,606,436,515,421,387,525,113,379,178,415,640,552
NMNAT1_2_22120,414,216,521,618,21,201,56,376,372,24,1043,949
NMT1_2_22121,300,139,317,264,209,65,23,1,150,464,253,92
NMT2_2_22122,834,700,666,812,1087,569,1342,147,736,599,613,787
NNMT_2_22123,30,4,11,3,0,7,0,0,1,6,13,0
NOP58_2_22124,1172,1095,1066,1164,864,843,1587,476,1245,1485,2104,1895
NOX3_2_22125,345,186,136,203,156,811,353,342,69,104,148,262
NQO2_2_22126,1739,1858,1663,1658,2339,1110,1871,1003,1294,1282,2125,11
29
NRAS_2_22127,730,619,531,593,1124,724,597,176,487,898,249,365
NSF_2_22128,405,564,388,522,745,525,457,252,225,995,100,575
NSUN6_2_22129,73,62,51,99,322,26,1,118,83,81,0,73
NTAN1_2_22130,193,221,147,218,372,373,470,180,168,261,50,238
NTHL1_2_22131,65,43,27,17,50,0,20,196,76,43,0,68
NTPCR_2_22132,106,157,74,78,423,98,617,82,63,81,96,137
NUDT10_2_22133,68,64,94,68,124,5,847,42,21,256,226,205
NUDT11_2_22134,1829,1290,1420,1257,1878,1507,1704,453,1858,1282,911,21
14

NUDT12_2_22135,1162,1175,1141,1210,1608,953,1835,1235,1204,1587,928,14
47
NUDT14_2_22136,154,143,148,131,17,90,21,269,71,37,168,63
NUDT3_2_22137,143,194,316,297,59,6,1557,47,304,369,246,2
NUDT5_2_22138,511,590,702,828,508,933,1374,648,683,812,876,366
NUDT7_2_22139,739,868,779,733,826,323,1422,1123,942,1688,220,423
NXNL1_2_22140,734,914,443,908,639,342,522,445,430,823,239,575
OAS3_2_22141,922,971,1253,867,786,165,1429,505,629,406,1170,553
OAZ1_2_22142,174,350,486,348,515,106,559,351,412,449,448,659
OC90_2_22143,666,441,303,564,71,426,52,280,193,345,730,221
ODF3B_2_22144,27,50,25,51,46,32,15,175,10,15,0,12
OPLAH_2_22145,83,102,67,83,0,3,26,0,0,5,0,0
OSGEP_2_22146,331,464,350,587,1055,494,303,215,205,398,0,480
OSGEPL1_2_22147,302,383,313,399,194,572,296,414,196,158,307,218
OSTC_2_22148,250,210,202,234,182,216,230,36,188,38,114,84
OTC_2_22149,3049,2449,2611,3352,4347,1978,2938,4434,3778,3586,2209,369
8
OVGP1_2_22150,151,61,160,106,129,169,162,0,417,270,6,298
OXA1L_2_22151,780,721,773,819,1029,471,885,374,1125,770,274,629
OXCT1_2_22152,1322,1408,899,1200,2358,1333,1592,1674,1404,413,1410,114
6
OXCT2_2_22153,173,136,132,160,53,28,319,121,250,83,97,201
P4HA3_2_22154,60,62,135,30,46,2,0,22,43,0,0,10
P4HB_2_22155,145,101,201,131,4,12,112,0,2,379,0,1
PADI1_2_22156,313,149,309,220,27,100,33,776,467,78,26,218
PADI2_2_22157,75,57,29,85,0,1,106,1,17,50,109,34
PADI3_2_22158,421,332,299,373,246,470,238,139,564,372,111,312
PADI6_2_22159,427,469,333,401,499,302,978,551,377,463,71,527
PAFAH2_2_22160,1410,1397,1238,1624,914,1595,2235,1601,1039,1493,1085,1
413
PAH_2_22161,87,57,66,171,195,78,221,197,265,110,19,59
PAPOLA_2_22162,603,587,546,705,239,561,229,991,506,606,158,953
PAPOLB_2_22163,1056,956,892,1227,769,569,1002,989,861,1670,795,923
PAPOLG_2_22164,289,257,233,302,511,6,207,647,296,73,19,165
PAPPA_2_22165,378,363,278,453,93,180,151,560,274,200,392,285
PAPSS1_2_22166,190,181,217,334,172,80,469,393,204,186,28,297
PARG_2_22167,283,296,203,312,337,62,9,11,52,485,131,41
PARP1_2_22168,167,171,102,166,118,0,251,395,204,104,21,471
PARP4_2_22169,290,282,263,393,581,531,304,371,176,419,9,633
PARP6_2_22170,124,121,127,94,31,96,353,1,63,75,79,296
PARS2_2_22171,397,286,279,368,96,61,1,92,260,213,708,128
PCBD1_2_22172,182,170,198,200,60,82,53,0,170,104,621,459
PCBD2_2_22173,406,591,377,636,474,182,187,750,252,329,969,271
PCMT1_2_22174,308,182,131,200,275,26,351,208,202,198,38,10
PCMTD1_2_22175,391,382,243,219,202,314,321,311,509,99,3,535
PCYOX1_2_22176,871,727,580,698,819,76,962,645,1043,770,629,521
PCYT1A_2_22177,1681,1510,1277,1932,1103,1186,987,2631,1972,2206,1928,2
637
PDCD1LG2_2_22178,348,395,238,381,269,138,191,585,396,120,166,267
PDCL_2_22179,881,639,736,954,405,764,308,2015,620,344,718,1161

PDE3A_2_22180,76,88,146,253,213,270,517,5,12,97,52,235
PDE3B_2_22181,1466,1446,1270,1627,1657,1349,1995,1316,740,977,686,1347
PDE6A_2_22182,381,248,268,309,452,196,416,190,262,452,97,381
PDE6C_2_22183,683,923,837,856,667,321,632,643,349,699,669,671
PDE6D_2_22184,248,117,165,143,188,122,96,158,15,96,90,72
PDE6G_2_22185,698,491,624,722,751,206,298,377,708,627,113,642
PDE6H_2_22186,437,385,595,519,4,362,122,1462,69,969,568,401
PDE7B_2_22187,293,412,266,408,853,95,164,33,255,1231,129,724
PDHA2_2_22188,339,269,267,228,166,170,265,504,307,130,108,772
PDIA2_2_22189,65,27,21,44,5,10,151,0,10,1,0,0
PDIA3_2_22190,494,407,281,377,463,1325,1687,274,40,138,26,211
PDIA4_2_22191,84,87,87,122,10,231,14,111,153,18,19,46
PDIA5_2_22192,0,0,0,0,0,0,0,0,0,0,0,0
PDIA6_2_22193,643,692,599,865,840,962,660,336,1211,516,962,2215
PDPR_2_22194,451,349,416,342,357,272,1425,5,188,431,140,510
PDSS1_2_22195,69,96,62,119,428,265,46,184,33,92,35,75
PDSS2_2_22196,350,413,265,346,259,610,140,1012,230,20,219,648
PDXP_2_22197,105,9,52,88,674,6,0,38,9,49,0,17
PECR_2_22198,526,447,327,450,839,323,86,505,19,364,152,276
PELI1_2_22199,496,468,407,387,907,394,330,300,240,37,497,377
PET117_2_22200,1810,1856,1609,1991,920,1932,1901,1708,2006,1887,1395,1
843
PEX1_2_22201,990,929,1010,732,1197,1163,2041,538,970,720,769,1229
PEX6_2_22202,329,389,362,551,175,110,1057,906,361,236,226,137
PFAS_2_22203,50,23,48,93,90,249,222,0,12,9,35,39
PGA3_2_22204,278,265,269,309,214,901,404,173,69,503,220,458
PGA4_2_22205,278,265,269,309,214,901,404,173,69,503,220,458
PGA5_2_22206,278,265,269,309,214,901,404,173,69,503,220,458
PGAM4_2_22207,3,9,67,28,100,408,63,0,18,0,662,0
PGAP1_2_22208,437,313,681,627,478,279,422,774,497,71,1190,706
PGAP3_2_22209,387,548,417,646,264,4,420,358,296,498,684,691
PGD_2_22210,504,250,313,302,387,254,0,71,268,450,44,374
PGGT1B_2_22211,57,140,62,65,79,133,325,0,0,87,218,0
PGLS_2_22212,182,155,150,217,106,26,502,749,216,45,268,138
PGM2_2_22213,1608,1247,1732,1499,1405,1125,1502,1811,1508,1377,441,164
4
PGM2L1_2_22214,567,681,425,435,558,175,35,227,1076,256,712,311
PGM5_2_22215,205,341,340,235,326,181,491,304,250,38,163,764
PGPEP1_2_22216,473,169,308,222,336,1,90,254,749,104,285,402
PGS1_2_22217,22,63,120,26,0,2,0,19,9,0,0,69
PHGDH_2_22218,693,931,860,1159,1049,319,621,1022,437,833,319,915
PHLPP1_2_22219,79,20,80,118,8,73,0,190,0,2,179,0
PHLPP2_2_22220,1018,961,1167,1004,185,341,1440,872,542,880,1346,176
PIF1_2_22221,234,126,123,164,79,0,0,124,195,66,64,80
PIGB_2_22222,830,800,894,957,718,173,878,723,743,147,894,580
PIGH_2_22223,1032,753,655,730,1359,1052,1108,516,1064,2338,593,1133
PIGL_2_22224,62,45,54,60,105,40,200,25,71,0,1,81
PIGM_2_22225,243,73,110,110,287,0,264,48,0,1,530,37
PIGS_2_22226,375,185,280,311,603,62,72,226,418,334,496,318
PIGU_2_22227,258,535,318,435,114,314,116,25,332,320,748,604

PIGW_2_22228,413,597,485,538,322,7,734,68,341,817,3,484
PIGZ_2_22229,37,38,13,40,89,0,0,6,16,15,0,23
PIN1_2_22230,318,187,219,356,86,20,63,227,95,34,348,302
PIPOX_2_22231,158,184,213,252,106,229,416,373,40,2,579,200
PITPNM2_2_22232,70,75,45,43,14,139,12,7,225,0,0,63
PLA2G10_2_22233,163,47,124,109,151,0,107,34,101,57,210,186
PLA2G12A_2_22234,631,597,418,573,1264,184,697,763,589,676,0,570
PLA2G12B_2_22235,4,12,29,6,6,212,109,0,411,39,0,0
PLA2G15_2_22236,212,111,133,189,103,3,0,171,22,44,89,461
PLA2G1B_2_22237,136,88,104,143,199,8,276,140,79,9,43,135
PLA2G2C_2_22238,754,654,569,908,585,776,489,728,589,1701,194,745
PLA2G2D_2_22239,136,193,113,213,13,340,159,76,486,516,285,251
PLA2G2E_2_22240,2,5,17,0,0,0,0,0,0,0,0,0
PLA2G2F_2_22241,76,30,31,36,232,6,47,0,59,0,148,7
PLA2G3_2_22242,224,237,78,324,55,88,243,414,72,335,1,15
PLA2G4A_2_22243,826,645,558,660,446,597,965,1517,186,501,532,344
PLA2G4B_2_22244,866,641,646,649,742,580,971,711,1430,811,225,730
PLA2G4D_2_22245,494,603,366,474,617,682,7,0,431,617,536,7
PLA2G4E_2_22246,211,154,106,282,267,362,468,50,458,701,4,134
PLA2G4F_2_22247,2,3,104,107,0,0,238,1,4,1,0,0
PLA2G5_2_22248,251,711,529,805,433,881,999,41,50,235,2839,557
PLCB2_2_22249,359,159,249,217,125,608,76,3,36,20,9,8
PLCD3_2_22250,157,56,32,81,31,86,0,20,17,204,2,4
PLCD4_2_22251,114,154,137,87,44,24,159,124,83,271,139,240
PLCG2_2_22252,14,8,16,17,0,0,160,0,1,65,92,0
PLCH2_2_22253,140,207,362,500,410,45,149,14,394,644,1368,1099
PLCL1_2_22254,84,109,92,105,12,60,151,51,115,472,693,7
PLCXD1_2_22255,526,480,424,663,226,93,1150,196,864,210,348,742
PLCZ1_2_22256,995,780,718,969,1361,1347,1410,884,1025,1825,975,1073
PLD4_2_22257,432,220,407,203,222,6,500,313,594,640,0,154
PLOD1_2_22258,0,0,0,0,0,0,0,0,0,0,0,0
PLOD3_2_22259,198,315,270,375,19,145,110,312,126,261,1,745
PLSCR1_2_22260,1296,1074,1105,1226,682,476,1136,1307,298,1158,1743,649
PMM1_2_22261,253,224,334,482,118,33,324,508,227,109,485,165
PMM2_2_22262,2719,2254,2596,2918,3045,1862,2533,4753,2508,1890,1790,15
99
PMPCA_2_22263,387,460,411,464,145,85,271,465,471,285,0,197
PMPCB_2_22264,67,180,204,240,133,143,366,200,423,673,0,300
PNLIP_2_22265,518,465,368,504,646,228,342,269,867,323,337,765
PNLIPRP1_2_22266,630,424,456,511,630,557,636,956,500,135,59,120
PNLIPRP2_2_22267,5,41,44,31,0,148,89,1,101,14,0,60
PNLIPRP3_2_22268,325,314,246,546,159,681,541,266,126,425,27,377
PNMT_2_22269,757,914,607,753,804,299,475,1249,194,1009,803,904
PNPLA2_2_22270,715,852,656,846,651,633,1052,281,1212,834,1344,829
PNPLA3_2_22271,76,51,46,58,50,0,238,419,76,2,2,11
PNPLA8_2_22272,1112,923,741,928,542,462,1250,602,565,911,406,1238
PNPO_2_22273,121,123,115,123,8,90,204,92,153,117,211,119
PNPT1_2_22274,1490,1488,1054,1255,902,849,717,1348,1067,1782,608,941
POLA1_2_22275,316,332,286,262,768,4,43,860,406,185,246,620
POLA2_2_22276,68,53,47,51,2,18,3,0,20,2,73,20

POLB_2_22277,345,189,216,282,240,118,3,128,59,284,140,210
POLD4_2_22278,74,117,163,303,20,53,743,0,110,18,22,24
POLE3_2_22279,83,89,112,105,35,3,69,36,140,56,73,78
POLE4_2_22280,22,23,8,13,8,118,112,145,12,25,101,7
POLE_2_22281,418,341,195,449,167,413,189,690,165,207,0,291
POLG2_2_22282,249,313,448,355,208,345,213,634,534,472,426,164
POLI_2_22283,737,752,727,1070,367,173,1885,823,534,729,81,937
POLM_2_22284,83,30,46,80,72,113,4,144,97,222,0,105
POLN_2_22285,0,4,7,9,0,1,0,0,11,73,2,0
POLR1A_2_22286,106,100,42,124,28,13,0,5,1,314,0,333
POLR1C_2_22287,680,694,569,515,530,454,275,646,456,331,515,105
POLR1E_2_22288,350,345,437,451,324,340,129,160,144,113,328,338
POLR2A_2_22289,460,387,396,429,832,725,730,1,20,134,1461,377
POLR2B_2_22290,463,329,356,385,211,243,63,1046,30,275,326,318
POLR2C_2_22291,195,285,273,309,298,720,249,113,550,336,7,340
POLR2D_2_22292,196,271,258,137,308,392,117,23,10,265,430,142
POLR2E_2_22293,85,50,52,108,1,289,0,6,207,74,322,12
POLR2F_2_22294,629,549,516,754,914,424,360,353,440,974,1687,520
POLR2G_2_22295,119,41,112,45,38,62,19,154,248,147,47,81
POLR2H_2_22296,1492,1059,1172,1503,1082,1739,1439,990,715,1103,1407,12
79
POLR2J_2_22297,932,894,803,1101,1011,813,1652,658,1233,788,942,1760
POLR2K_2_22298,918,954,1024,768,889,321,898,1213,411,1245,915,1022
POLR2L_2_22299,114,88,98,179,178,100,344,21,237,55,32,426
POLR3A_2_22300,241,244,149,114,81,3,337,24,101,175,5,146
POLR3C_2_22301,265,172,176,155,152,48,55,1,341,105,27,241
POLR3F_2_22302,396,572,535,453,528,245,507,1220,0,695,358,461
POLR3G_2_22303,240,240,368,228,582,102,331,23,485,178,252,173
POLRMT_2_22304,31,14,34,139,0,9,0,0,0,4,0,0
POMGNT1_2_22305,466,331,359,461,147,158,657,75,254,114,120,268
POMT2_2_22306,173,113,141,197,134,125,134,542,181,104,35,300
PON3_2_22307,1376,1264,1266,1503,794,1146,897,1227,641,823,2706,1170
POP4_2_22308,163,79,166,322,185,91,155,630,31,23,3,512
POP7_2_22309,164,141,169,211,10,21,0,184,43,1051,18,416
POR_2_22310,89,49,45,72,19,105,53,46,311,121,892,43
PPA1_2_22311,56,87,54,113,135,308,274,1,31,60,126,129
PPCDC_2_22312,7,80,123,31,0,2,0,1,271,301,0,0
PPIA_2_22313,60,16,79,36,0,120,0,13,169,35,9,6
PPIAL4A_2_22314,1577,1432,1254,1550,1580,478,1635,1865,1887,1210,1185,
1929
PPIAL4B_2_22315,1577,1432,1254,1550,1580,478,1635,1865,1887,1210,1185,
1929
PPIAL4C_2_22316,1577,1432,1254,1550,1580,478,1635,1865,1887,1210,1185,
1929
PPIAL4E_2_22317,7093,6616,6817,7219,4672,6436,8386,7382,8209,6322,8501
,5857
PPIAL4G_2_22318,370,244,321,434,965,207,1535,349,880,229,34,301
PPIB_2_22319,742,589,624,791,1160,1007,1307,737,532,219,705,768
PPIC_2_22320,1052,945,1010,1271,620,797,827,770,1546,638,588,245
PPID_2_22321,657,592,558,809,435,567,413,0,425,1020,355,582

PPIF_2_22322,309,213,237,286,12,73,198,53,101,92,23,388
PPIG_2_22323,291,344,242,225,255,561,466,142,578,484,289,188
PPIH_2_22324,277,221,268,243,162,95,14,464,423,406,52,456
PPIL1_2_22325,93,86,36,91,244,2,0,0,37,34,0,18
PPIL4_2_22326,636,604,746,821,710,67,547,241,1182,815,413,421
PPIP5K2_2_22327,1244,945,925,1214,1076,1846,1823,1082,715,2049,939,122
6
PPM1H_2_22328,579,567,502,593,1006,247,671,466,1343,1096,352,1258
PPM1J_2_22329,33,11,110,17,0,61,0,0,0,92,5,51
PPM1N_2_22330,1019,1052,1025,1268,756,1024,1596,136,1083,1195,1007,133
1
PPME1_2_22331,445,435,263,370,335,348,198,389,313,16,45,194
PPWD1_2_22332,15,22,17,11,39,3,2,0,5,0,0,4
PRDX4_2_22333,190,352,265,415,782,137,536,0,392,134,347,3
PREP_2_22334,1240,1165,1142,1118,1279,611,1799,1841,1096,758,1084,845
PRHOXNB_2_22335,590,441,658,575,326,97,827,378,266,1163,221,1276
PRIM1_2_22336,312,386,321,478,289,143,232,126,845,101,367,722
PRIM2_2_22337,935,880,412,871,514,414,1050,559,396,546,565,1607
PROC_2_22338,14,91,185,189,209,0,355,151,1,0,74,2
PROSC_2_22339,77,127,72,143,65,34,79,380,39,16,2,368
PRR14L_2_22340,719,347,386,754,595,190,114,635,494,258,1010,833
PRSS1_2_22341,2,34,2,11,0,0,0,0,0,0,901,0
PRSS2_2_22342,464,319,397,401,186,467,534,261,357,299,324,351
PRTFDC1_2_22343,517,670,489,441,737,42,381,363,704,128,723,594
PRTN3_2_22344,106,182,66,195,108,134,2,22,81,33,297,22
PRUNE_2_22345,387,267,516,397,355,418,139,7,49,64,266,328
PSMA2_2_22346,344,428,475,512,214,349,220,202,177,551,559,374
PSMA6_2_22347,172,260,167,202,112,234,116,225,139,61,31,435
PSMA7_2_22348,224,154,158,217,310,330,23,0,11,252,428,381
PSMB10_2_22349,1213,1052,1072,965,1359,843,941,678,2100,1383,1018,688
PSMB11_2_22350,0,8,0,19,0,0,34,0,0,0,0,0
PSMB1_2_22351,657,517,598,511,1442,377,284,82,378,798,99,744
PSMB3_2_22352,381,207,294,322,190,100,676,574,550,595,430,83
PSMB4_2_22353,758,654,713,915,301,525,751,1235,582,704,542,763
PSMB6_2_22354,939,756,740,794,846,791,1235,891,874,360,1748,689
PSMB7_2_22355,173,112,148,130,87,155,232,403,163,137,83,172
PSMB9_2_22356,289,298,272,267,20,56,0,196,252,4,81,392
PSMD6_2_22357,1307,1163,1201,1326,786,946,1717,690,1267,811,2335,1606
PTDSS1_2_22358,566,807,448,654,1076,588,739,607,202,764,930,527
PTDSS2_2_22359,56,159,38,55,44,41,51,133,94,45,5,70
PTGDS_2_22360,222,98,127,233,75,222,25,350,319,498,0,294
PTGES2_2_22361,40,29,59,151,1,0,60,22,7,22,6,10
PTGES3_2_22362,769,449,498,1047,747,172,755,820,296,792,234,722
PTGES_2_22363,33,5,19,19,2,2,1,11,15,28,0,256
PTGIS_2_22364,298,300,199,361,235,279,35,312,258,111,142,119
PTGS2_2_22365,1324,1071,915,1348,1049,1427,805,600,1016,743,2337,1476
PTRH1_2_22366,30,138,109,166,183,275,765,122,253,0,1,195
PTRH2_2_22367,459,279,213,200,514,236,107,12,257,45,76,670
PTS_2_22368,809,615,585,750,798,687,413,738,704,952,368,622
PUS3_2_22369,786,510,742,967,897,217,724,571,833,1159,948,583

PUSL1_2_22370,45,13,54,19,113,0,0,49,49,0,0,0
PXDN_2_22371,135,104,106,146,33,510,86,331,93,30,83,286
PXDNL_2_22372,304,216,230,196,209,30,131,693,216,120,388,484
PYCR2_2_22373,252,196,209,238,144,21,314,135,136,98,236,592
PYCRL_2_22374,255,230,371,243,94,29,155,278,180,407,28,274
PYGB_2_22375,84,157,359,347,1,302,1269,494,196,163,1866,346
QDPR_2_22376,875,614,821,927,517,576,572,1177,806,844,591,621
QPCT_2_22377,950,824,1037,1259,654,1529,1708,1556,721,1327,2103,1111
QPRT_2_22378,19,47,89,53,6,219,4,267,4,192,12,41
QRSL1_2_22379,2546,2513,1952,2520,2650,2208,4956,2201,2263,3888,2247,3
015
QSOX2_2_22380,838,835,631,974,845,914,277,448,955,649,749,846
QTRT1_2_22381,33,6,2,10,55,2,0,9,16,0,0,1
QTRTD1_2_22382,362,380,461,506,373,421,0,1755,414,495,1638,400
RAB10_2_22383,1798,1505,1482,1718,1894,1727,2103,1166,3344,1224,1430,2
326
RAB12_2_22384,305,332,370,437,433,7,78,443,256,140,188,278
RAB13_2_22385,55,58,40,90,60,1,1,352,1,62,0,14
RAB14_2_22386,267,366,366,290,33,245,274,18,133,303,644,155
RAB15_2_22387,235,239,292,310,126,254,0,145,252,326,289,258
RAB17_2_22388,205,250,191,233,110,140,115,67,10,4,226,344
RAB18_2_22389,109,208,86,93,333,62,4,139,358,3,4,244
RAB19_2_22390,186,111,141,206,194,268,223,202,44,194,117,254
RAB1B_2_22391,245,91,207,195,79,138,332,186,189,111,374,156
RAB20_2_22392,197,107,130,134,2,2,261,147,331,297,232,289
RAB21_2_22393,439,391,426,392,380,178,1042,923,560,457,682,644
RAB22A_2_22394,191,374,375,401,306,588,807,16,398,128,867,1332
RAB25_2_22395,157,200,187,232,236,290,292,520,90,266,324,184
RAB26_2_22396,44,44,36,45,13,30,75,0,18,15,72,13
RAB27B_2_22397,963,865,765,932,634,565,539,730,703,1137,1639,973
RAB30_2_22398,152,82,41,78,10,108,42,11,230,54,0,13
RAB31_2_22399,193,395,391,416,326,590,769,316,464,148,878,1343
RAB33A_2_22400,268,158,177,249,18,206,205,148,223,13,220,56
RAB33B_2_22401,1105,1144,817,1319,976,1043,986,945,1167,2140,676,1011
RAB36_2_22402,132,103,155,53,66,262,4,0,44,314,0,80
RAB38_2_22403,165,85,296,194,99,1,223,50,170,87,171,53
RAB39B_2_22404,176,356,288,305,5,193,801,9,318,10,509,37
RAB3A_2_22405,94,87,268,212,204,85,703,78,291,124,126,695
RAB3B_2_22406,525,518,365,361,170,361,989,53,547,511,590,376
RAB3C_2_22407,459,288,400,295,963,287,387,674,587,293,341,238
RAB3D_2_22408,623,588,489,693,473,854,1383,234,362,820,1229,822
RAB3GAP2_2_22409,227,258,211,235,266,352,816,80,152,261,52,412
RAB4A_2_22410,635,436,479,645,794,343,1314,265,774,18,2609,346
RAB4B_2_22411,24,13,46,30,124,48,114,194,9,9,0,0
RAB5A_2_22412,421,441,229,254,199,334,373,304,651,81,281,170
RAB5B_2_22413,571,314,266,329,79,14,601,839,106,69,747,90
RAB6B_2_22414,546,486,526,473,291,302,162,310,246,248,824,596
RAB6C_2_22415,5,1,23,18,85,0,7,12,0,0,0,0
RAB7A_2_22416,450,290,408,301,397,261,0,191,464,37,483,249
RAB8B_2_22417,779,925,746,757,321,593,1252,51,439,887,381,650

RAB9B_2_22418,175,90,100,105,138,0,650,23,160,122,16,5
RABGGTB_2_22419,4,6,69,50,2,27,0,0,14,0,0,6
RABL3_2_22420,1275,1354,1117,1527,557,861,448,1414,363,2064,2357,1796
RAC2_2_22421,73,56,54,103,0,226,0,3,196,3,0,3
RAD50_2_22422,1141,858,968,772,658,620,315,1386,1003,560,822,655
RAD54L2_2_22423,83,20,51,85,98,0,10,2,22,77,31,12
RALA_2_22424,259,152,214,307,886,60,337,94,1046,1,104,198
RALB_2_22425,796,691,441,844,429,633,1319,195,444,564,435,426
RALBP1_2_22426,117,125,151,238,41,510,326,223,90,349,279,486
RANBP2_2_22427,1416,1199,1096,1346,408,902,1415,1100,1570,1531,583,291
1
RAN_2_22428,367,234,351,275,450,19,51,18,115,649,98,46
RAP2A_2_22429,105,97,152,235,240,0,153,165,112,210,143,255
RAP2B_2_22430,286,242,351,350,72,156,1039,384,137,65,442,9
RAP2C_2_22431,258,171,218,284,195,72,77,127,670,98,408,168
RARS2_2_22432,721,465,561,585,263,264,550,503,366,715,409,700
RARS_2_22433,1382,1231,1188,1671,1639,1647,286,1682,165,1500,2864,1935
RASD2_2_22434,255,341,296,351,2,100,173,21,0,347,96,134
RASL10B_2_22435,49,125,140,125,6,243,342,262,28,911,29,163
RASL11B_2_22436,69,96,129,79,1,0,0,3,8,0,187,0
RASL12_2_22437,100,20,24,137,5,13,0,361,106,1,415,0
RC3H1_2_22438,197,44,119,150,46,78,0,803,80,65,73,299
RCL1_2_22439,273,262,175,257,308,19,788,263,70,66,426,206
RDH10_2_22440,954,733,653,637,878,417,1019,290,965,459,858,865
RDH11_2_22441,247,170,244,194,98,115,160,81,147,354,748,371
RDH12_2_22442,161,121,160,124,17,327,1,0,33,36,0,38
RDH14_2_22443,255,104,184,159,11,45,0,1138,88,505,63,325
RDH16_2_22444,403,328,361,301,458,115,80,335,373,143,76,535
RDH8_2_22445,66,87,136,61,5,63,12,8,41,232,0,34
RECQL4_2_22446,38,36,27,28,393,12,3,103,77,0,326,63
REM1_2_22447,537,465,408,474,213,345,1661,522,55,245,20,702
REM2_2_22448,216,265,303,350,11,395,478,698,359,0,203,296
RENBP_2_22449,67,128,150,127,4,146,798,172,4,0,0,113
RETSAT_2_22450,27,55,47,98,16,2,353,19,181,0,314,117
REV3L_2_22451,759,639,523,644,625,1101,632,795,806,296,509,196
REXO1L1_2_22452,183,106,85,37,7,0,758,3,224,1,2,0
REXO2_2_22453,50,161,103,150,265,84,38,69,10,127,35,145
RFNG_2_22454,643,332,428,433,308,269,435,536,787,467,578,290
RFX6_2_22455,426,467,254,354,731,571,142,118,0,686,184,215
RGS7_2_22456,609,782,594,605,818,357,212,970,666,787,390,386
RHBDL1_2_22457,29,127,34,180,0,375,0,298,0,6,0,194
RHBDL2_2_22458,340,524,462,613,189,506,634,1149,234,629,1497,452
RHBDL3_2_22459,156,118,185,132,188,4,106,19,196,60,475,339
RHEBL1_2_22460,368,292,207,264,231,597,176,181,245,224,104,347
RHOA_2_22461,1076,1033,889,888,1244,985,756,964,1893,553,731,390
RHOB_2_22462,43,49,15,83,0,51,154,39,357,64,0,339
RHOD_2_22463,190,131,153,220,375,28,293,30,139,5,77,365
RHOF_2_22464,152,94,131,229,80,141,0,349,147,315,122,81
RHOG_2_22465,193,289,341,309,571,11,14,175,394,5,547,290
RHOH_2_22466,324,84,139,165,192,2,159,17,197,52,8,73

RHOJ_2_22467,923,698,863,1013,1284,790,634,131,1873,1213,793,1660
RHOQ_2_22468,890,748,744,769,1197,843,530,817,832,807,122,1160
RHOT2_2_22469,390,351,492,380,117,215,483,1047,167,825,199,56
RHOU_2_22470,526,305,307,337,374,617,559,98,259,416,31,308
RHOV_2_22471,401,222,445,470,662,301,413,323,209,631,466,469
RIT1_2_22472,46,97,104,171,67,46,1,7,175,7,0,98
RIT2_2_22473,502,437,550,361,243,297,917,424,595,647,509,1132
RNASE2_2_22474,1736,1425,1175,1800,1625,1177,1167,2998,1436,1143,695,1
823
RNASE3_2_22475,1736,1425,1175,1800,1625,1177,1167,2998,1436,1143,695,1
823
RNASE6_2_22476,333,543,339,613,342,426,329,803,84,2,710,518
RNASE7_2_22477,27,9,28,12,0,14,0,1,0,45,204,0
RNASE8_2_22478,300,221,277,308,12,643,179,410,49,798,323,2
RNASEH2A_2_22479,535,512,619,542,409,553,255,423,346,775,896,639
RNASET2_2_22480,705,683,673,725,577,455,0,707,969,687,1126,798
RND1_2_22481,90,81,130,127,16,2,9,0,0,11,5,2
RND2_2_22482,185,141,111,160,281,64,394,358,404,439,390,134
RND3_2_22483,136,23,98,52,73,0,7,0,0,123,10,0
RNF148_2_22484,184,171,170,283,142,113,291,280,121,174,486,307
RNMT_2_22485,695,615,753,894,356,701,1190,760,985,324,458,854
RNMTL1_2_22486,246,136,111,237,321,121,252,40,283,262,76,332
RNPEP_2_22487,75,66,120,99,2,16,1,0,576,14,191,234
RPAP2_2_22488,166,97,153,277,319,123,93,534,228,0,229,713
RPE65_2_22489,344,259,286,249,462,226,0,8,357,39,390,99
RPIA_2_22490,87,62,136,74,0,0,37,443,71,0,0,13
RPL4_2_22491,33,39,64,95,0,65,78,91,1,76,0,188
RPN1_2_22492,831,838,665,896,635,590,1130,814,527,997,909,1192
RPP25_2_22493,98,40,67,87,0,50,169,142,584,154,0,46
RPP40_2_22494,284,236,155,240,133,71,344,1,316,197,239,179
RPS3_2_22495,45,39,2,31,0,0,0,0,0,7,0,0
RPUSD1_2_22496,59,13,53,54,0,0,1012,10,0,116,25,0
RPUSD2_2_22497,171,212,186,249,656,0,474,94,5,0,0,31
RRAGA_2_22498,1248,1010,1066,1273,1329,1303,1000,1031,788,911,928,689
RRAGC_2_22499,3187,3399,2945,2923,3333,2431,3902,2353,1639,2971,2967,4
391
RRAGD_2_22500,1856,1378,1398,1470,2004,1290,1193,1523,1360,783,1029,17
12
RRAS_2_22501,69,71,84,94,274,341,18,103,383,208,94,375
RRM1_2_22502,177,250,229,250,26,213,72,41,20,181,129,150
RRP8_2_22503,1194,1046,993,1193,1123,537,524,1227,1318,971,1751,1131
RSAD2_2_22504,780,611,375,784,204,2,331,1012,92,521,172,332
RTN4IP1_2_22505,412,172,219,260,11,48,47,26,177,672,107,561
SAMHD1_2_22506,347,157,267,321,50,410,124,239,353,241,476,487
SDF2_2_22507,199,321,157,222,671,279,929,0,203,1,2,45
SDHA_2_22508,580,638,389,669,383,184,41,578,644,376,939,511
SDHB_2_22509,1951,1457,1189,1252,1387,1554,1822,1051,1206,1308,1617,22
31
SDHD_2_22510,153,194,125,165,211,0,0,0,119,850,4,77
SDR16C5_2_22511,816,805,847,792,304,483,508,970,290,616,1184,727

SDR42E1_2_22512,414,321,210,338,103,54,12,29,29,1063,607,69
SDR9C7_2_22513,6,27,3,18,140,0,2,33,0,0,0,136
SDSL_2_22514,26,40,84,88,0,149,361,43,0,33,1,226
SEPSECS_2_22515,202,313,136,208,592,521,491,1,11,109,0,61
1-Sep_2_22516,194,312,101,144,18,122,0,238,122,425,57,130
SEPW1_2_22517,303,248,326,410,906,116,407,83,263,51,690,198
SERHL2_2_22518,219,182,246,400,4,552,320,41,201,392,818,535
SETX_2_22519,182,153,149,242,6,2,124,179,175,435,236,138
SGMS1_2_22520,626,439,540,912,739,358,759,1816,874,850,1642,715
SGPL1_2_22521,521,826,510,729,377,728,300,678,8,966,114,89
SGSH_2_22522,545,596,596,680,582,135,1236,721,282,64,135,1092
SH3GL2_2_22523,202,190,282,409,822,110,0,174,148,4,567,566
SKIV2L2_2_22524,334,375,482,439,382,152,403,285,318,302,204,457
SLFN12_2_22525,369,244,230,217,61,224,746,579,358,556,22,368
SLFN12L_2_22526,1241,859,1066,1212,672,912,1450,1293,1327,1019,1020,18
62
SLFN13_2_22527,1399,1029,1030,1324,1167,1097,776,2126,917,612,1575,817
SLFN5_2_22528,111,200,41,143,396,0,322,0,13,0,0,177
SLU7_2_22529,560,495,644,705,966,740,186,542,341,1050,679,544
SMG8_2_22530,87,69,91,61,236,64,115,359,10,160,0,353
SMPD2_2_22531,617,350,286,410,947,627,905,213,7,1184,1799,596
SMPD3_2_22532,263,393,236,345,466,419,0,389,454,251,14,30
SMPDL3A_2_22533,1327,1575,1281,1493,2369,1312,683,1404,873,1300,140,54
6
SMUG1_2_22534,1029,674,659,1022,520,579,928,10,415,948,1094,1336
SNF8_2_22535,129,131,182,252,441,769,254,258,32,118,60,221
SNRNP200_2_22536,95,75,138,131,0,0,67,42,0,28,0,144
SOAT1_2_22537,627,595,552,637,412,898,258,37,520,1205,264,551
SOAT2_2_22538,195,174,194,237,262,7,176,154,117,612,52,467
SOD1_2_22539,545,491,369,623,903,314,493,828,84,204,295,789
SOD3_2_22540,127,43,131,86,289,1,6,8,240,0,0,11
SORD_2_22541,195,179,210,210,388,1,291,67,51,514,142,65
SPACA3_2_22542,91,123,109,189,62,166,309,6,139,186,385,300
SPACA5B_2_22543,733,680,505,714,570,44,136,612,114,140,1182,291
SPACA5_2_22544,733,680,505,714,570,44,136,612,114,140,1182,291
SPEM1_2_22545,332,427,313,325,76,136,46,92,169,125,329,51
SPTLC2_2_22546,645,479,486,660,908,845,1132,1045,509,1025,523,1155
SPTLC3_2_22547,1209,1045,1108,1279,1156,542,1071,705,977,1025,1003,136
4
SPTSSA_2_22548,28,31,91,78,11,1,185,17,29,35,1,41
SQLE_2_22549,211,169,284,204,210,428,213,39,811,4,71,258
SQRDL_2_22550,1610,1298,1214,1883,2995,960,3205,716,1391,1255,1066,137
1
SRD5A1_2_22551,710,717,649,792,731,231,232,1437,259,91,1428,493
SRD5A2_2_22552,174,99,64,110,102,64,5,17,27,8,0,308
SRD5A3_2_22553,761,936,745,929,878,1275,979,1113,1002,1735,784,905
SRR_2_22554,41,32,29,46,7,62,122,777,16,39,615,16
SRSF9_2_22555,588,548,574,576,723,1358,1730,163,381,28,157,1107
SRXN1_2_22556,1839,1749,1962,1980,1698,1439,2273,1145,1552,2311,1387,1
466

SSB_2_22557,296,249,250,364,251,20,6,338,119,337,260,93
SSU72_2_22558,0,0,0,0,0,0,0,0,0,0,0,0
ST14_2_22559,180,136,132,204,255,551,0,0,32,4,0,171
ST3GAL2_2_22560,293,190,157,139,337,48,710,413,262,0,129,81
ST3GAL4_2_22561,161,109,162,196,478,128,698,771,4,298,127,194
ST3GAL6_2_22562,333,396,211,457,258,580,62,409,300,269,28,553
ST6GALNAC1_2_22563,78,117,94,157,5,193,10,9,235,0,33,81
ST6GALNAC2_2_22564,114,79,52,106,73,0,4,10,58,109,5,4
ST6GALNAC5_2_22565,259,186,183,313,162,871,1211,215,664,453,68,356
ST6GALNAC6_2_22566,92,152,56,105,139,14,1,127,159,0,259,10
ST8SIA1_2_22567,419,355,243,324,345,340,449,491,385,149,152,227
ST8SIA2_2_22568,751,702,767,867,298,1312,885,609,697,1226,335,255
ST8SIA3_2_22569,443,359,365,487,329,867,438,185,102,453,582,723
ST8SIA5_2_22570,544,339,407,478,1020,121,239,308,1337,1182,753,161
ST8SIA6_2_22571,666,787,569,624,926,230,1211,967,497,420,1000,1129
STS_2_22572,167,90,164,94,42,7,196,3,46,1,0,81
STT3A_2_22573,2639,2568,1988,2516,3253,3315,3304,3296,2193,3451,3324,3
994
STT3B_2_22574,57,88,34,60,319,1,19,3,50,103,276,30
SUCLA2_2_22575,622,410,442,462,496,33,229,332,469,1441,334,911
SUCLG1_2_22576,1034,750,882,984,815,682,1253,969,1374,851,1779,580
SULT1A3_2_22577,121,132,127,194,23,9,82,108,25,571,10,163
SULT1A4_2_22578,121,132,127,194,23,9,82,108,25,571,10,163
SULT1B1_2_22579,227,306,215,222,0,407,477,1,68,326,184,251
SULT1C3_2_22580,1247,1072,1051,1297,912,1677,662,1264,1257,959,1058,15
31
SULT1C4_2_22581,2300,2078,2039,2270,1458,1968,2858,2729,2768,2772,3007
,3189
SULT1E1_2_22582,374,279,490,572,141,465,11,49,691,407,676,298
SULT2A1_2_22583,1353,1120,932,1349,1133,1016,897,1313,788,1809,1045,11
87
SULT4A1_2_22584,150,92,116,63,10,79,136,9,49,107,96,10
SUPV3L1_2_22585,1068,1658,1338,1801,1467,1792,1837,624,1117,2385,2028,
624
SURF1_2_22586,494,374,321,454,609,65,963,185,171,168,335,896
TALDO1_2_22587,1027,893,880,913,500,685,1761,363,479,1346,537,1357
TARS2_2_22588,4,9,22,23,0,59,0,0,14,4,0,476
TARS_2_22589,2951,2585,2450,2661,1283,1177,2500,1804,2094,2584,2752,18
72
TARSL2_2_22590,381,477,499,613,639,559,491,603,776,301,314,386
TAT_2_22591,298,132,248,267,596,211,0,658,782,138,98,100
TBC1D10B_2_22592,72,105,98,95,0,139,1,0,249,1,1,4
TBCC_2_22593,575,457,401,475,553,808,1150,14,861,375,171,325
TDG_2_22594,239,184,241,295,377,120,521,148,316,129,527,194
TDO2_2_22595,123,137,154,168,41,90,205,8,86,292,103,146
TECR_2_22596,403,366,264,314,1,7,176,281,404,0,752,2
TECTA_2_22597,334,255,254,264,1004,206,41,70,654,7,308,345
TFB2M_2_22598,453,383,186,259,22,339,1143,424,361,380,6,48
TGDS_2_22599,1264,1109,1370,1458,1968,1060,1483,817,992,1592,1093,1535
TGM1_2_22600,37,101,24,45,32,0,0,0,24,0,4,0

TGM3_2_22601,163,165,150,200,41,62,269,17,203,96,10,151
TGM4_2_22602,885,672,740,914,1613,963,869,459,605,1786,329,1053
TGM6_2_22603,460,307,330,519,750,14,36,214,227,856,177,0
TGM7_2_22604,327,480,423,553,348,197,137,86,122,435,271,541
TGS1_2_22605,779,575,582,809,397,373,522,672,564,1029,320,1137
THG1L_2_22606,282,294,264,266,42,1192,485,339,37,384,722,126
THOP1_2_22607,84,175,95,200,0,0,0,0,161,125,302,236
THUMPD2_2_22608,524,598,547,414,596,1256,237,833,317,431,878,641
TKTL2_2_22609,501,309,415,255,586,76,507,295,300,290,736,146
TM7SF2_2_22610,164,236,160,160,49,822,5,0,143,444,1086,309
TMEM55A_2_22611,60,49,66,59,49,23,5,276,282,11,61,25
TMEM62_2_22612,1116,1372,1042,1191,500,1157,1999,1395,647,894,2498,130
3
TMEM86B_2_22613,340,408,347,470,462,175,273,298,162,375,1035,667
TMPRSS15_2_22614,845,843,757,935,892,124,252,759,1174,1479,549,1320
TMX1_2_22615,176,162,34,81,148,353,0,62,129,17,2,91
TMX3_2_22616,177,242,151,193,73,20,64,271,84,163,136,129
TMX4_2_22617,441,273,234,229,142,104,658,263,337,288,205,215
TNKS2_2_22618,339,302,404,268,350,50,396,389,312,824,1419,307
TNKS_2_22619,267,265,223,194,43,49,118,13,139,196,144,300
TOP1_2_22620,1096,1264,1114,1076,963,1310,1603,1479,659,1251,1934,1069
TOP1MT_2_22621,2975,2889,3032,3622,2524,3209,2849,2855,3273,3528,3229,
2923
TOP2A_2_22622,664,678,818,902,214,734,1623,1395,333,1095,1509,882
TOP2B_2_22623,106,116,122,171,204,13,353,112,238,45,137,155
TOP3A_2_22624,405,237,195,311,241,107,163,238,49,421,244,147
TOP3B_2_22625,57,0,14,71,0,0,0,0,0,0,0,0
TOR3A_2_22626,108,81,130,127,29,222,17,3,0,57,17,13
TPH1_2_22627,107,93,150,90,6,228,350,220,127,12,540,72
TPMT_2_22628,1108,1042,868,1126,927,2023,745,1685,1157,1120,1075,1198
TPP1_2_22629,1004,1228,1254,1420,527,310,1056,2159,1292,1225,2017,824
TPP2_2_22630,627,634,696,634,1406,137,507,829,1013,288,983,265
TPSAB1_2_22631,148,297,243,202,235,541,159,442,43,90,344,240
TPSB2_2_22632,148,297,243,202,235,541,159,442,43,90,344,240
TPSD1_2_22633,73,88,101,101,37,5,275,1,62,466,59,353
TPST1_2_22634,80,66,56,77,0,201,130,19,229,44,0,50
TREH_2_22635,2,28,21,33,8,0,0,0,366,1,236,190
TREX2_2_22636,1351,744,1327,1732,376,1099,791,586,1716,875,2741,1075
TRHDE_2_22637,527,466,367,619,1070,282,228,673,814,371,326,294
TRIM21_2_22638,1613,1236,1032,1230,934,861,1116,1998,710,967,1585,1522
TRIT1_2_22639,233,194,127,259,73,337,834,289,48,498,627,272
TRMT112_2_22640,3,41,7,10,0,0,1,0,4,0,0,73
TRMT61A_2_22641,394,420,465,495,1212,499,828,483,119,76,132,224
TRMU_2_22642,141,207,219,199,199,66,475,1321,379,269,267,94
TRNT1_2_22643,39,54,58,32,3,86,66,43,77,94,15,64
TRUB1_2_22644,465,267,369,377,205,120,301,589,147,697,434,1428
TRUB2_2_22645,32,80,57,168,25,0,245,55,4,61,190,6
TSTA3_2_22646,127,200,217,306,360,36,0,44,8,245,59,72
TST_2_22647,331,352,241,398,51,40,382,227,511,388,22,218
TTLL13_2_22648,181,245,143,141,240,234,269,100,267,108,22,150

TTLL1_2_22649,1779,1899,1830,2254,2138,1636,3264,1903,1173,1485,2623,1
418
TTLL3_2_22650,55,43,66,35,4,0,0,67,1,0,15,1
TTLL4_2_22651,178,146,299,196,668,3,233,0,16,0,45,443
TUFM_2_22652,121,142,331,274,51,347,658,18,15,0,493,170
TULP2_2_22653,684,1049,838,897,522,1429,935,837,571,1156,213,1058
TUT1_2_22654,300,245,242,304,145,211,353,298,177,512,280,558
TXN2_2_22655,124,70,140,140,0,11,1,0,14,0,797,3
TXN_2_22656,110,65,155,105,55,56,32,58,65,78,47,29
TXNDC11_2_22657,485,664,468,627,388,1098,1086,282,664,416,787,992
TXNDC12_2_22658,82,148,103,98,380,96,18,12,142,75,19,10
TXNDC15_2_22659,324,287,265,411,205,573,1242,271,168,144,363,45
TXNDC17_2_22660,86,75,42,102,1,0,18,41,0,253,3,28
TXNL1_2_22661,240,105,71,65,208,88,435,216,51,260,992,132
TXNL4A_2_22662,1082,931,693,942,337,830,440,952,882,233,2998,567
TXNRD2_2_22663,225,234,208,242,255,47,358,288,113,148,89,465
TYMS_2_22664,3220,3335,2810,3323,3352,2258,2976,4294,2688,4124,2721,31
96
TYR_2_22665,949,638,450,712,1029,87,276,155,870,532,209,817
TYRP1_2_22666,1157,1051,1353,1341,457,657,641,1298,510,1390,1854,1344
UAP1_2_22667,149,95,92,96,135,15,45,38,71,83,111,97
UAP1L1_2_22668,45,140,82,81,46,98,1,1,5,302,1,21
UBB_2_22669,111,99,126,129,0,4,322,5,11,29,27,145
UBIAD1_2_22670,495,661,330,423,233,609,642,131,831,519,409,690
UBL4A_2_22671,260,207,281,185,88,18,95,195,48,272,61,263
UFSP1_2_22672,112,170,70,147,238,39,239,42,26,17,0,7
UFSP2_2_22673,1331,1294,1483,1484,942,954,1811,935,1174,2484,2570,1266
UGCG_2_22674,910,1059,733,1114,612,297,220,949,757,917,224,812
UGGT1_2_22675,342,322,308,570,178,277,258,449,84,86,1312,626
UGGT2_2_22676,364,359,532,549,440,585,101,231,268,353,7,515
UGT1A10_2_22677,775,743,758,885,559,252,490,787,644,1278,863,587
UGT1A1_2_22678,2360,1851,1903,2334,2071,1590,2963,958,2378,2240,2125,2
152
UGT1A3_2_22679,2847,2262,2745,3473,3157,1535,2122,2017,2701,2789,4217,
3172
UGT1A4_2_22680,2262,2364,2645,2734,3398,1101,2548,2834,2048,3123,3115,
2144
UGT1A5_2_22681,45,67,17,87,27,74,6,49,11,0,216,5
UGT1A7_2_22682,1555,1253,1073,1365,1119,1146,1015,751,1083,534,994,133
3
UGT1A8_2_22683,0,0,0,0,0,0,0,0,0,0,0,0
UGT1A9_2_22684,221,287,399,365,309,38,485,36,132,331,457,85
UGT2A1_2_22685,93,237,157,154,0,26,105,9,8,465,108,248
UGT2A2_2_22686,970,1108,839,1246,1333,582,827,1769,795,763,469,2380
UGT2A3_2_22687,822,720,627,750,864,141,758,40,745,1431,1302,270
UGT2B15_2_22688,365,508,280,535,1405,259,330,423,302,748,279,50
UGT2B17_2_22689,365,508,280,535,1405,259,330,423,302,748,279,50
UGT2B4_2_22690,1616,1454,1383,1369,1000,805,1557,989,1335,2867,2320,18
92
UGT2B7_2_22691,528,345,475,508,159,413,387,299,463,466,539,696

UMPS_2_22692,270,400,329,365,44,416,169,1428,206,197,4,0
UPB1_2_22693,49,54,231,99,25,82,143,37,256,5,1,0
UPF1_2_22694,302,305,336,310,179,265,9,862,227,261,283,323
UPRT_2_22695,301,601,388,331,59,1,0,9,569,250,523,529
UQCR11_2_22696,0,45,0,9,3,0,0,0,0,0,195,0
UQCRC1_2_22697,107,89,122,100,331,11,30,10,42,58,97,0
UQCRC2_2_22698,255,445,253,287,303,0,598,485,393,492,366,90
UQCRFS1_2_22699,1937,1118,1632,1479,880,843,1067,843,2401,1341,2293,18
65
UQCRH_2_22700,606,588,679,667,562,589,168,336,507,642,1512,1084
UQCRQ_2_22701,114,64,50,96,203,2,0,0,195,3,2,2
UROD_2_22702,143,119,176,150,10,238,94,552,147,51,127,447
UROS_2_22703,460,461,518,587,625,34,1301,424,407,224,397,511
UST_2_22704,171,244,255,269,739,297,203,37,800,391,12,6
UXS1_2_22705,153,159,48,129,348,111,1,737,93,2,7,26
VARS_2_22706,8,1,14,2,1,1,161,0,5,1,0,3
VAT1L_2_22707,231,68,85,138,86,172,247,132,102,89,0,258
VCP_2_22708,285,394,278,519,279,256,533,558,580,801,830,109
VNN1_2_22709,599,443,372,502,1654,152,249,108,923,787,629,822
WBSCR17_2_22710,145,169,93,104,71,98,519,16,146,52,512,72
WBSCR27_2_22711,72,82,80,36,0,94,719,10,99,5,12,31
WDFY3_2_22712,482,508,489,587,419,594,448,473,187,1028,858,789
WRN_2_22713,273,215,361,412,230,385,229,354,78,456,976,368
XDH_2_22714,116,240,258,255,183,40,666,311,575,393,297,57
XPNPEP2_2_22715,197,180,156,246,61,449,40,40,410,12,494,27
XRCC2_2_22716,1268,1082,827,1014,1192,316,1195,2515,277,1653,1012,1192
XRCC5_2_22717,256,360,172,212,1210,215,52,273,91,486,579,424
XRCC6_2_22718,306,222,226,227,28,53,191,171,23,372,114,130
XRN2_2_22719,412,453,575,503,412,447,451,1067,250,283,1614,434
XYLT1_2_22720,225,204,222,367,108,137,5,850,290,210,79,379
XYLT2_2_22721,180,120,70,175,183,145,34,80,169,220,299,117
YARS2_2_22722,405,206,335,258,560,44,399,170,161,181,28,252
YARS_2_22723,722,761,575,626,704,768,308,533,420,566,1055,432
YKT6_2_22724,453,348,566,546,108,403,643,2,171,631,417,983
YPEL1_2_22725,22,3,38,0,0,0,0,31,0,5,0,0
ZADH2_2_22726,858,703,666,758,1009,558,211,1089,600,411,1484,880
ZCCHC4_2_22727,6,48,25,22,7,0,646,0,8,22,0,439
ZDHHC17_2_22728,19,74,49,80,0,150,23,0,0,95,0,48
ZDHHC18_2_22729,55,138,125,77,350,137,37,1,88,258,22,314
ZDHHC1_2_22730,1649,1140,1185,1252,1423,1663,1411,690,1172,1196,2062,1
783
ZDHHC21_2_22731,489,435,460,612,475,145,490,761,414,542,51,634
ZDHHC2_2_22732,215,148,126,130,426,95,1101,2,96,5,0,136
ZER1_2_22733,1053,644,682,906,325,562,1468,121,1148,812,1051,468
ZMPSTE24_2_22734,571,571,459,604,598,1628,801,274,954,783,34,603
ZRANB3_2_22735,208,82,256,231,45,26,62,37,340,570,447,54
A1CF_2_22736,56,26,28,57,14,22,47,117,8,2,0,3
A2LD1_2_22737,45,133,226,100,5,5,25,54,13,148,483,1
AADAT_2_22738,481,190,303,256,31,15,697,396,190,222,249,455
AARSD1_2_22739,757,756,482,690,525,1700,656,968,743,705,1598,342

ABAT_2_22740,331,385,275,288,251,553,337,335,272,3,46,223
ABHD11_2_22741,272,170,254,325,332,88,299,390,211,99,234,425
ABHD12_2_22742,36,67,27,69,115,23,24,10,26,98,0,53
ABHD14B_2_22743,342,153,142,90,384,33,0,0,412,56,1,19
ABHD2_2_22744,95,116,194,116,0,0,0,2,0,0,0,3
ACAA1_2_22745,64,47,30,50,55,0,0,343,0,1,0,12
ACACA_2_22746,1169,783,912,788,733,664,544,1016,1241,1594,1027,1224
ACAD10_2_22747,54,11,42,28,50,0,4,0,18,10,0,31
ACADM_2_22748,99,110,161,117,265,45,6,297,156,406,38,0
ACADVL_2_22749,554,404,320,423,155,353,858,280,222,712,108,161
ACCS_2_22750,169,177,294,195,149,4,120,86,140,239,3,336
ACE_2_22751,139,185,121,145,5,1,223,0,154,919,9,286
ACHE_2_22752,617,469,641,573,560,241,338,943,521,771,448,667
ACIN1_2_22753,877,1125,988,1161,657,246,1184,285,1332,1478,821,866
ACLY_2_22754,164,139,151,143,0,97,61,0,98,77,108,0
ACOT11_2_22755,162,270,317,250,16,290,681,236,234,441,160,0
ACOT13_2_22756,53,87,24,43,126,18,22,370,26,288,10,46
ACOT7_2_22757,70,150,58,141,44,1,0,0,121,343,101,0
ACOT9_2_22758,198,302,326,333,858,0,1211,650,251,336,484,728
ACOX1_2_22759,476,444,421,454,761,35,175,320,179,520,284,42
ACOX3_2_22760,210,125,172,252,502,791,488,79,194,228,750,110
ACSBG1_2_22761,131,192,133,218,23,66,27,375,342,570,0,339
ACSF3_2_22762,749,620,630,784,31,324,648,625,82,692,553,338
ACSL3_2_22763,164,177,162,98,103,0,0,273,98,214,74,211
ACSL4_2_22764,151,51,132,171,184,256,60,601,362,290,15,437
ACSL5_2_22765,760,505,460,682,164,93,679,129,443,453,350,733
ACSL6_2_22766,640,564,565,618,284,592,223,461,321,477,689,667
ACSM2B_2_22767,534,351,389,526,193,9,24,746,1106,492,325,143
ACSM3_2_22768,852,983,709,682,1280,228,541,916,496,625,987,909
ACSS2_2_22769,372,265,183,304,337,198,276,354,231,242,191,444
ACY1_2_22770,54,89,39,36,2,0,4,22,17,79,0,0
ADAMTS2_2_22771,12,15,4,26,31,0,35,32,3,1,0,146
ADARB1_2_22772,1488,997,1003,1086,1985,1347,183,1091,1698,582,458,529
ADAR_2_22773,15,75,45,106,49,12,11,15,19,19,0,367
ADCY10_2_22774,262,253,282,284,8,186,71,103,206,406,484,44
ADCY4_2_22775,178,241,272,228,178,123,238,656,39,270,80,76
ADCY5_2_22776,230,256,267,295,11,644,594,120,288,316,1199,230
ADCY6_2_22777,16,32,15,21,7,14,2,0,0,0,63,81
ADH6_2_22778,1035,824,954,1291,1688,740,732,1128,1131,994,270,1193
ADH7_2_22779,17,12,3,4,0,45,0,0,0,0,62,6
ADPRHL1_2_22780,9,50,16,28,17,0,0,1,1,17,35,52
ADSL_2_22781,222,212,184,152,160,104,169,52,325,120,481,246
ADSSL1_2_22782,170,191,139,211,10,220,757,6,28,8,274,236
AFMID_2_22783,16,11,20,13,2,0,0,9,235,53,0,11
AGA_2_22784,634,472,601,483,217,346,130,73,642,854,509,873
AGAP1_2_22785,18,10,37,16,0,0,0,5,0,0,57,2
AGAP2_2_22786,207,150,149,170,519,363,174,66,189,93,366,120
AGL_2_22787,1172,1403,1140,1343,1653,934,573,1166,560,1408,171,1239
AGPAT1_2_22788,257,258,141,345,14,252,243,1,321,81,512,372
AGPAT2_2_22789,428,475,217,349,297,41,1343,396,222,685,89,644

AGPAT3_2_22790,23,7,75,54,59,26,0,149,27,193,0,2
AGXT2L1_2_22791,697,610,672,660,517,458,1142,447,356,897,1070,1091
AHCY_2_22792,280,247,170,304,509,812,546,894,85,3,2,201
AHCYL1_2_22793,401,510,464,425,657,323,241,377,820,507,125,591
AHCYL2_2_22794,135,195,66,147,3,0,0,0,108,2,0,0
AIFM1_2_22795,104,48,32,76,0,0,360,35,96,1,0,1
AIFM2_2_22796,246,329,177,242,142,70,228,373,119,546,31,522
AIFM3_2_22797,106,131,129,137,0,0,0,10,15,1,152,105
AKIRIN1_2_22798,418,526,379,445,661,198,551,615,559,409,1479,441
AKR1A1_2_22799,396,418,452,456,43,380,42,0,6,773,72,727
AKR1C2_2_22800,693,641,758,837,1693,711,794,167,1175,554,1646,954
AKR1D1_2_22801,573,487,625,419,666,92,110,600,885,657,96,1160
ALAS1_2_22802,674,896,1071,901,572,672,518,1086,220,859,955,427
ALAS2_2_22803,180,127,111,69,3,18,63,171,276,63,55,64
ALDH16A1_2_22804,67,327,93,57,553,194,1160,0,508,2,0,288
ALDH1A2_2_22805,812,683,633,925,610,198,742,424,514,398,1037,504
ALDH2_2_22806,107,62,43,94,99,1,0,14,20,2,12,276
ALDH3A1_2_22807,678,594,521,698,332,465,1122,289,513,311,242,361
ALDH3A2_2_22808,345,368,470,417,985,16,30,239,334,331,56,300
ALDH3B1_2_22809,14,39,33,12,4,0,0,0,1,0,0,122
ALDH3B2_2_22810,169,133,90,165,26,169,1,0,120,209,0,49
ALDH4A1_2_22811,376,330,336,550,199,580,756,341,259,343,40,440
ALDH5A1_2_22812,86,70,137,82,94,0,25,1,21,38,303,142
ALDH7A1_2_22813,80,38,62,70,133,2,1,63,38,45,7,63
ALDH8A1_2_22814,240,124,212,365,372,75,877,1449,137,150,325,235
ALDOA_2_22815,217,196,360,229,234,36,775,548,1,43,654,591
ALG3_2_22816,906,989,1031,1124,1622,1067,938,1812,1261,867,673,860
ALG5_2_22817,108,64,108,41,134,41,114,0,7,22,0,13
ALG8_2_22818,658,395,418,487,354,511,998,214,382,529,919,248
ALG9_2_22819,142,305,209,332,6,464,112,3,429,49,488,277
ALOX15B_2_22820,191,195,236,284,421,447,393,440,9,36,52,168
ALOXE3_2_22821,281,297,335,372,0,502,537,310,157,1,1148,29
AMACR_2_22822,247,215,219,195,204,116,536,212,355,266,290,134
AMD1_2_22823,411,540,429,476,372,410,1706,230,438,514,362,253
AMDHD2_2_22824,91,43,60,19,117,60,0,8,40,31,2,178
AMPD1_2_22825,1603,1266,1371,1643,1723,1266,2147,1395,1484,1605,1949,1
452
AMPD2_2_22826,323,119,257,281,76,59,258,449,530,192,84,143
AMPD3_2_22827,520,388,347,358,581,215,29,457,122,99,71,320
AMT_2_22828,88,89,63,134,48,76,259,85,137,81,104,306
AMY1A_2_22829,2286,1999,2213,2459,2993,2483,2547,2260,3514,3495,1812,3
902
ANG_2_22830,594,365,435,571,352,220,130,744,367,253,366,1096
AOAH_2_22831,83,30,155,76,7,8,251,1,78,10,2,114
AOC2_2_22832,39,30,27,67,6,3,2,1,130,0,0,0
APC_2_22833,805,788,848,950,376,815,1381,850,589,882,1083,765
APEX1_2_22834,91,121,62,106,41,43,3,4,41,28,137,76
APOBEC3A_2_22835,254,306,411,364,126,392,45,43,273,54,1246,3
APOBEC3H_2_22836,33,104,91,63,58,0,215,2,1,7,0,367
APRT_2_22837,44,8,1,24,38,26,56,0,4,29,0,187

ARF1_2_22838,346,435,516,688,324,676,504,1359,568,434,394,393
ARFRP1_2_22839,35,11,59,56,0,0,0,0,1,0,0,1
ARHGAP5_2_22840,320,261,427,322,127,313,660,502,303,666,610,259
ARHGEF10L_2_22841,351,155,138,311,534,211,25,507,110,114,2,498
ARL2_2_22842,109,70,161,338,55,6,0,564,31,36,4,220
ARL4A_2_22843,1009,981,971,1052,1299,1472,1616,799,1192,1479,444,969
ARL5A_2_22844,323,354,223,535,103,57,138,46,188,373,64,418
ARSA_2_22845,12,34,40,84,0,1,89,256,6,0,31,9
ARSB_2_22846,258,188,197,311,93,0,222,0,37,379,240,36
ARSF_2_22847,756,437,518,691,867,533,140,947,329,1300,190,503
ART3_2_22848,111,163,105,273,533,210,10,26,203,370,553,300
ART5_2_22849,1081,923,895,1232,695,996,775,433,1056,1010,1449,1697
ASAH1_2_22850,974,906,1027,1247,938,1005,561,536,1098,473,213,1601
ASAH2_2_22851,96,178,67,226,0,369,0,0,60,0,0,189
ASL_2_22852,1350,1209,1606,1632,933,1074,878,1876,1055,817,3723,2061
ASMT_2_22853,107,110,149,301,0,14,56,115,8,504,8,45
ASMTL_2_22854,235,167,172,302,50,491,323,294,512,347,0,293
ASNS_2_22855,2085,2057,2015,2622,1014,2573,1957,1500,1877,1741,2596,16
27
ASPA_2_22856,161,123,86,94,309,8,395,41,144,23,0,56
ASRGL1_2_22857,15,16,12,83,0,0,97,0,4,57,2,6
ASS1_2_22858,219,206,171,276,294,68,83,49,287,147,540,768
ATE1_2_22859,82,121,60,156,138,99,15,292,21,93,38,42
ATL1_2_22860,238,112,174,217,496,891,109,144,0,233,193,355
AURKAIP1_2_22861,206,155,119,170,351,35,20,746,89,188,719,292
AZIN1_2_22862,75,68,125,90,233,17,73,106,74,20,63,27
B3GALNT1_2_22863,516,495,585,423,456,740,247,220,859,414,333,457
B3GALT5_2_22864,521,418,412,529,574,13,297,323,301,445,1009,258
B3GAT1_2_22865,24,163,229,121,111,1,0,0,452,13,0,9
B4GALNT2_2_22866,172,244,224,212,113,7,4,336,213,453,480,2
B4GALT2_2_22867,177,149,145,130,94,316,157,280,53,109,32,131
B4GALT3_2_22868,118,148,254,328,191,22,0,373,128,167,249,372
B4GALT4_2_22869,198,171,219,180,235,264,218,6,208,26,59,466
BAAT_2_22870,122,74,136,90,178,43,138,64,139,34,18,206
BACE1_2_22871,87,154,30,172,124,6,0,30,130,258,13,86
BACE2_2_22872,65,94,123,108,31,21,0,194,16,200,53,144
BCAT1_2_22873,327,453,520,431,1743,0,480,104,346,656,481,629
BCAT2_2_22874,216,120,135,203,0,0,534,327,204,117,0,0
BCKDHA_2_22875,1,2,3,8,0,77,0,0,1,1,0,155
BCKDHB_2_22876,93,45,71,106,165,0,0,275,232,0,2,3
BCO2_2_22877,1704,1484,1166,1608,1496,1867,1044,2077,1635,1531,818,876
BDH1_2_22878,239,223,164,254,266,479,90,385,177,282,202,370
BFSP1_2_22879,440,389,213,423,134,258,888,463,619,488,232,976
BHMT2_2_22880,59,59,68,60,80,0,0,0,0,0,0,89
BMP1_2_22881,166,170,188,272,35,0,487,39,70,8,143,42
C17orf101_2_22882,412,534,480,568,219,16,87,164,1,422,823,905
C1GALT1C1_2_22883,149,256,177,214,104,7,0,0,58,11,396,12
C1S_2_22884,121,116,77,123,114,81,218,76,405,24,38,253
C2_2_22885,382,215,438,374,423,1177,280,111,211,158,153,453
CA10_2_22886,194,136,146,222,145,37,529,125,250,22,324,99

CA12_2_22887,178,179,319,182,110,4,0,354,191,493,0,175
CA1_2_22888,305,419,277,352,207,150,550,200,168,321,236,108
CA7_2_22889,94,157,158,133,123,106,729,138,42,120,173,49
CAB39_2_22890,360,396,480,560,398,64,197,291,473,74,129,179
CANT1_2_22891,266,455,254,298,36,178,1428,298,604,163,755,211
CAPN1_2_22892,222,118,115,104,2,1,10,41,34,3,183,323
CAPN2_2_22893,146,167,238,196,19,9,0,33,163,26,195,245
CARNS1_2_22894,45,51,51,54,35,0,0,0,3,0,0,3
CARS_2_22895,269,263,224,270,108,24,199,72,366,165,427,234
CASP10_2_22896,114,237,262,201,372,305,0,1103,116,216,395,59
CASP1_2_22897,1718,1398,1182,1456,2658,74,715,600,1252,1108,1578,1048
CASP2_2_22898,382,353,293,493,677,381,397,170,326,423,125,220
CASP3_2_22899,2279,2117,2278,2201,926,1941,3141,1918,2296,4433,3480,30
35
CASP4_2_22900,271,337,305,305,239,1178,265,115,423,335,96,130
CASP5_2_22901,363,300,227,286,175,209,390,30,134,136,284,539
CASP6_2_22902,205,244,164,193,29,11,253,75,10,23,17,130
CASP7_2_22903,92,135,62,80,0,82,224,388,78,13,69,259
CASP8_2_22904,333,350,360,327,624,2,100,39,358,544,394,294
CASP9_2_22905,986,780,575,637,342,394,13,1498,642,613,393,381
CASZ1_2_22906,18,2,16,64,0,3,0,24,10,0,3,121
CAV3_2_22907,734,544,688,932,783,480,475,566,375,1191,264,527
CBS_2_22908,221,145,214,212,17,341,1,1,368,204,0,361
CCBL1_2_22909,237,39,231,314,129,421,29,296,32,2,0,267
CCBL2_2_22910,432,435,496,496,883,797,422,172,475,704,344,662
CDC42_2_22911,280,225,244,238,133,322,1040,248,422,111,360,312
CDH16_2_22912,68,28,75,73,6,0,0,2,43,85,1,1
CECR1_2_22913,161,234,250,217,546,0,750,2,450,0,228,394
CEPT1_2_22914,676,816,663,925,582,1121,860,1443,901,1935,177,616
CERS1_2_22915,82,72,137,247,188,9,32,495,202,77,0,46
CES1_2_22916,2579,1993,1748,2327,1689,1292,1993,465,1829,2687,3246,149
2
CES3_2_22917,194,259,159,265,230,4,92,36,8,206,232,321
CES4A_2_22918,420,582,497,601,276,162,529,348,351,244,368,319
CES5A_2_22919,250,259,253,422,203,68,59,287,262,413,81,244
CHAT_2_22920,111,102,152,161,135,45,23,32,152,118,1,406
CHI3L2_2_22921,91,99,62,68,13,39,45,0,157,104,381,13
CHIA_2_22922,102,9,17,69,18,0,0,4,0,0,0,144
CHPF_2_22923,466,354,272,326,347,139,7,0,622,343,248,65
CHST11_2_22924,99,117,202,152,6,163,163,14,230,196,23,339
CHST15_2_22925,174,293,301,219,75,32,0,175,282,596,20,237
CHST4_2_22926,279,299,497,690,247,293,78,362,675,456,510,657
CHST8_2_22927,166,247,114,150,118,1,0,0,75,86,11,397
CLP1_2_22928,254,218,82,124,61,6,2,2,81,92,1,226
CNDP2_2_22929,255,371,267,399,733,855,176,591,120,658,354,654
CNTN1_2_22930,841,1090,1132,835,732,453,1318,623,809,702,2007,654
CNTN4_2_22931,1636,1190,1166,1644,989,869,1353,816,2062,2378,1009,973
COMT_2_22932,34,49,86,111,20,2,0,1,1,5,307,109
COQ6_2_22933,321,351,471,369,230,921,699,368,236,230,286,807
COX11_2_22934,808,720,494,802,511,150,1289,411,375,205,665,351

COX15_2_22935,0,0,0,0,0,0,0,0,0,0,0,0
CPA5_2_22936,97,180,89,108,153,30,27,0,3,10,1,106
CPB2_2_22937,343,342,314,351,175,536,398,221,304,278,92,230
CPM_2_22938,390,285,336,390,289,1088,377,458,542,175,693,219
CPPED1_2_22939,606,686,664,738,566,218,372,405,302,1771,657,806
CPT1A_2_22940,41,62,58,85,5,39,0,4,157,61,112,60
CPT1B_2_22941,71,73,70,202,38,0,79,354,0,34,0,104
CPT1C_2_22942,410,466,434,504,238,932,631,962,757,582,439,285
CRLS1_2_22943,633,564,596,1031,734,660,501,569,623,445,756,367
CRMP1_2_22944,180,208,257,277,40,44,152,285,8,110,172,289
CROT_2_22945,205,369,292,309,16,238,16,937,319,372,48,402
CRY2_2_22946,48,3,18,58,16,0,0,3,143,0,0,116
CRYM_2_22947,156,83,144,170,83,27,88,18,213,53,215,89
CRYZ_2_22948,585,780,832,795,809,420,681,781,783,1384,445,1236
CSDE1_2_22949,238,66,134,68,343,8,710,0,22,250,5,16
CSGALNACT1_2_22950,465,419,266,506,218,158,252,541,626,355,275,465
CSMD3_2_22951,38,36,40,46,27,0,30,25,31,8,369,104
CTAGE5_2_22952,378,325,383,352,93,168,510,126,520,316,324,717
CTBP1_2_22953,194,178,143,151,98,68,174,2,2,158,0,227
CTPS2_2_22954,221,179,302,286,146,113,407,987,107,47,125,40
CTSA_2_22955,183,187,294,212,196,0,235,144,309,0,144,158
CTSB_2_22956,115,147,97,209,354,46,64,283,123,94,19,169
CTSE_2_22957,108,82,91,98,10,8,178,206,15,115,1,53
CTSL1_2_22958,354,271,219,321,206,105,214,366,792,273,656,397
CTSL2_2_22959,147,76,108,147,101,32,149,380,242,106,61,115
CTSS_2_22960,66,56,72,131,32,199,1058,0,98,13,13,194
CYB561_2_22961,24,74,53,40,0,1,0,0,8,298,3,14
CYB5A_2_22962,3,37,12,10,0,0,0,0,0,0,0,0
CYB5R3_2_22963,80,63,68,67,11,5,31,13,26,42,5,39
CYP11A1_2_22964,169,261,267,222,112,151,55,528,167,291,106,351
CYP11B1_2_22965,138,137,64,86,359,31,22,573,17,303,2,25
CYP19A1_2_22966,1833,1626,1220,1310,2615,2321,1201,808,656,3021,1580,1
818
CYP21A2_2_22967,850,635,655,736,1070,1935,368,839,389,469,148,1054
CYP24A1_2_22968,175,178,282,323,0,0,132,282,108,661,923,326
CYP26A1_2_22969,188,298,204,279,96,733,119,33,42,483,23,198
CYP2A7_2_22970,1059,949,999,875,1630,1280,782,305,822,1544,874,675
CYP2C18_2_22971,994,1040,1054,984,1351,813,1271,1637,658,589,1854,1054
CYP2C8_2_22972,994,1040,1054,984,1351,813,1271,1637,658,589,1854,1054
CYP2D6_2_22973,148,117,112,162,5,30,122,0,117,230,7,51
CYP3A43_2_22974,548,514,478,484,355,469,730,538,748,707,409,389
CYP3A4_2_22975,1095,1311,1192,1290,392,1149,1182,1502,1005,1753,1688,1
874
CYP4B1_2_22976,137,157,147,216,40,31,111,0,71,353,77,80
CYP4F11_2_22977,42,62,56,76,37,7,4,132,175,132,0,10
CYP4F3_2_22978,6,6,42,18,0,0,0,0,0,1,20,7
CYP51A1_2_22979,532,718,493,780,989,338,179,1171,578,1290,208,921
DAGLB_2_22980,17,22,12,38,3,4,0,0,0,13,0,7
DCLRE1C_2_22981,1802,1646,1655,1771,1114,1655,1114,867,859,1282,3099,1
059

DCP2_2_22982,424,310,416,383,439,424,508,1053,825,873,390,80
DCT_2_22983,451,485,390,414,479,241,128,406,194,12,610,636
DCTD_2_22984,149,114,103,111,111,80,379,114,26,26,9,10
DCXR_2_22985,130,53,56,61,166,116,0,4,156,81,58,121
DDAH1_2_22986,293,321,324,324,102,17,448,386,363,526,30,452
DDC_2_22987,274,548,372,380,494,352,850,1225,692,159,1087,195
DDHD1_2_22988,257,266,232,333,35,477,256,261,18,75,651,247
DDO_2_22989,369,197,249,333,327,652,387,616,207,42,116,232
DDT_2_22990,46,39,105,137,3,22,0,0,0,138,0,110
DDX11_2_22991,486,548,386,487,643,112,414,196,86,706,1203,765
DDX17_2_22992,222,395,205,307,326,35,228,51,137,302,26,464
DDX19B_2_22993,1486,1054,1031,1442,1271,1246,1882,356,1219,945,2043,20
90
DDX31_2_22994,923,996,954,873,920,1161,1097,814,1137,555,1603,1074
DDX39B_2_22995,186,48,163,71,149,9,20,40,26,48,54,47
DDX3X_2_22996,99,137,54,140,50,72,1,276,91,6,204,294
DDX3Y_2_22997,1161,1114,1039,954,1978,779,1395,440,1720,876,1420,1430
DDX42_2_22998,191,115,169,106,0,1,284,117,2,325,146,184
DDX47_2_22999,2033,1252,1310,2095,1619,1009,2180,1013,1826,708,870,198
8
DDX4_2_23000,96,119,107,153,196,40,91,289,7,49,134,38
DDX54_2_23001,192,181,235,173,227,189,331,28,330,267,235,493
DFFA_2_23002,7,38,44,14,68,2,0,151,72,6,16,38
DGCR8_2_23003,121,141,79,67,0,30,3,505,4,143,298,2
DHCR7_2_23004,832,969,902,1031,1421,1177,1875,904,1032,659,683,509
DHDDS_2_23005,466,462,457,646,790,184,13,343,227,537,158,602
DHFRL1_2_23006,653,383,553,667,436,218,242,545,587,362,925,247
DHPS_2_23007,61,41,14,16,13,3,0,2,30,0,0,38
DHRS1_2_23008,829,411,491,842,404,52,39,542,394,325,352,498
DHRS2_2_23009,226,209,169,197,30,0,0,0,0,5,186,283
DHRS9_2_23010,0,3,9,3,0,7,0,0,0,0,0,0
DHX16_2_23011,667,553,586,618,464,272,315,948,607,539,574,734
DHX30_2_23012,216,241,226,265,443,33,397,290,96,539,187,210
DHX33_2_23013,341,235,305,303,966,608,110,144,50,621,290,1106
DHX35_2_23014,187,120,204,152,203,76,0,8,177,63,2,250
DHX36_2_23015,423,713,516,680,702,423,1507,619,621,1124,624,597
DHX40_2_23016,961,984,695,650,508,2036,363,1857,1191,719,729,743
DIAPH3_2_23017,116,212,185,336,411,35,344,23,272,150,58,311
DIS3_2_23018,240,368,154,349,223,644,225,598,30,245,625,116
DIS3L_2_23019,808,955,710,713,331,668,431,250,638,1663,310,846
DKC1_2_23020,551,395,498,449,541,573,439,109,716,204,114,285
DNAJC27_2_23021,15,55,25,49,21,2,14,19,38,28,5,0
DNASE1L1_2_23022,240,342,238,509,319,492,280,292,421,650,72,572
DNM1_2_23023,216,155,295,227,59,189,661,360,625,22,474,342
DNM1L_2_23024,201,276,189,293,138,391,100,394,88,508,11,86
DNM2_2_23025,343,401,380,503,794,34,579,69,646,297,104,236
DNM3_2_23026,78,47,36,12,0,0,0,64,0,11,0,85
DNTT_2_23027,52,269,204,144,3,0,13,338,9,200,79,29
DOHH_2_23028,70,9,21,24,0,18,202,15,1,0,0,0
DOLPP1_2_23029,531,410,527,717,1005,1295,60,1006,166,263,11,383

DPEP1_2_23030,15,176,89,82,35,101,78,48,88,81,31,92
DPEP3_2_23031,62,55,105,78,76,38,19,39,29,60,128,11
DPH5_2_23032,1042,811,611,975,503,1041,939,1412,437,1255,1068,739
DPM3_2_23033,125,73,103,165,26,142,57,66,301,106,127,125
DPP3_2_23034,8,14,34,21,1,0,0,0,1,1,0,35
DPP8_2_23035,558,408,490,655,152,221,1272,372,264,646,83,289
DPYSL2_2_23036,457,295,180,354,150,493,297,491,40,355,215,714
DPYSL3_2_23037,897,683,596,853,915,307,963,1035,1087,872,272,1135
DROSHA_2_23038,335,254,290,557,356,285,1123,821,266,42,447,157
DSE_2_23039,361,227,303,205,560,977,764,76,354,191,218,31
DUOX1_2_23040,74,57,59,70,162,2,312,144,342,10,14,111
DUT_2_23041,47,32,36,47,8,62,198,6,6,46,543,107
ECE1_2_23042,1009,816,975,945,975,1269,1012,477,647,919,1389,901
ECE2_2_23043,454,439,338,507,225,507,423,384,958,104,235,222
ECI1_2_23044,913,746,951,855,309,999,817,1301,1139,762,372,885
ECI2_2_23045,801,508,779,648,920,794,739,850,718,1248,408,1035
EDEM2_2_23046,80,41,32,56,1,14,0,170,5,238,63,61
EFEMP1_2_23047,1160,917,936,1052,742,873,1628,798,903,510,726,1307
EFTUD2_2_23048,2,0,10,10,0,3,0,23,187,0,0,0
EGLN2_2_23049,331,218,218,243,342,10,2,2,441,197,0,79
EHHADH_2_23050,1654,1813,1441,1924,2504,933,2154,1769,919,1624,2302,25
25
ELAC2_2_23051,3090,3092,3000,3308,3683,2637,2686,734,2788,2011,4431,26
35
ELOVL5_2_23052,360,420,395,415,137,251,208,750,318,1307,539,482
ELOVL6_2_23053,86,41,42,100,68,118,205,161,29,0,57,136
ELOVL7_2_23054,1331,1173,804,1285,1558,762,1714,951,567,1212,1030,1066
ENDOV_2_23055,35,92,21,75,9,155,0,212,26,58,0,1
ENO3_2_23056,517,270,308,501,487,70,925,274,506,513,1913,417
ENOX2_2_23057,15,4,53,14,0,61,0,23,2,0,0,1
ENPP2_2_23058,211,271,257,161,1,22,283,0,501,175,9,55
ENTPD1_2_23059,187,123,177,219,209,52,509,178,282,33,80,18
ENTPD2_2_23060,141,51,97,113,648,1,888,41,8,16,9,149
ENTPD4_2_23061,338,150,176,216,68,123,251,1,147,530,1,178
ENTPD6_2_23062,629,480,574,356,64,556,570,398,91,109,507,827
ENTPD8_2_23063,18,69,16,15,2,0,0,0,0,0,7,5
EPHX1_2_23064,11,9,43,31,0,38,8,0,63,249,157,0
EPHX3_2_23065,34,79,25,79,155,185,108,63,141,62,45,0
ERCC1_2_23066,39,20,32,48,153,71,6,50,213,161,6,9
ERCC2_2_23067,275,239,320,295,94,290,403,104,165,403,122,277
EXO1_2_23068,224,196,193,247,35,268,811,75,142,147,37,100
EXOG_2_23069,361,396,411,433,525,783,72,146,607,145,173,805
EXOSC3_2_23070,807,890,862,834,1528,1436,569,1366,1169,464,899,710
EXOSC9_2_23071,52,46,42,50,16,67,43,52,25,0,27,37
EXTL2_2_23072,60,71,51,195,0,0,0,0,116,0,0,0
F7_2_23073,422,457,386,434,709,226,259,112,228,503,1107,1014
FAHD1_2_23074,34,87,51,48,0,0,0,0,0,0,0,0
FAM108A1_2_23075,171,168,179,287,427,13,214,1131,18,243,0,163
FAM135A_2_23076,418,234,265,352,212,845,317,7,422,293,144,597
FAN1_2_23077,327,143,185,193,209,682,958,529,535,241,62,74

FDPS_2_23078,126,104,78,134,58,2,7,23,35,263,35,31
FDXR_2_23079,201,97,78,234,352,282,87,411,231,200,0,31
FECH_2_23080,464,550,366,555,177,64,227,304,88,637,688,293
FERMT3_2_23081,68,64,54,112,80,37,398,82,91,48,173,424
FHIT_2_23082,309,206,285,289,253,141,47,142,341,329,303,152
FIGNL1_2_23083,1,16,1,6,372,0,1,151,4,1,0,0
FKBP11_2_23084,167,73,62,179,0,0,1,0,88,67,557,211
FKBP1A_2_23085,453,242,117,240,326,679,470,14,133,167,175,270
FKBP1B_2_23086,75,72,70,77,18,41,105,119,17,3,0,15
FKBP2_2_23087,66,26,21,120,118,422,62,147,4,32,0,31
FKBP5_2_23088,967,974,785,1057,878,99,1117,1938,735,1340,603,1147
FKBP7_2_23089,3,149,166,103,294,0,0,798,0,66,4,10
FLAD1_2_23090,119,87,95,77,92,190,18,0,1,74,0,8
FMO3_2_23091,549,486,336,541,249,444,320,244,312,360,0,431
FMO5_2_23092,137,64,170,164,6,0,0,86,168,2,156,101
FN1_2_23093,63,50,97,143,108,26,99,6,135,81,61,331
FOLH1_2_23094,342,223,176,221,173,393,429,134,158,163,336,160
FPGS_2_23095,17,10,39,34,11,162,0,0,0,3,0,132
FPGT_2_23096,596,692,495,703,990,290,2429,330,708,363,808,122
FTCD_2_23097,319,289,207,204,243,477,783,3,232,68,477,459
FTSJ1_2_23098,335,407,393,470,159,171,33,210,129,755,163,172
FUT2_2_23099,28,34,15,25,0,28,9,0,0,0,0,0
FUT3_2_23100,134,35,54,87,0,1,29,0,0,58,159,0
FUT6_2_23101,203,168,243,282,406,283,581,727,139,286,15,139
FUT8_2_23102,329,232,359,257,202,132,0,52,427,248,40,298
G3BP1_2_23103,174,239,196,207,353,286,39,0,143,695,74,143
G3BP2_2_23104,842,737,676,723,577,361,932,799,198,377,664,653
G6PD_2_23105,376,192,329,392,399,73,319,621,503,134,156,453
GAA_2_23106,454,349,323,477,340,443,563,665,136,334,321,685
GAD1_2_23107,303,367,260,321,334,334,922,74,253,387,485,246
GAD2_2_23108,1686,1500,1537,1643,2654,2619,1578,1491,1269,1120,872,202
5
GALC_2_23109,342,345,303,404,88,318,857,214,341,397,84,276
GALE_2_23110,42,15,36,100,17,44,14,3,115,0,0,4
GALNT9_2_23111,444,461,359,504,570,12,410,631,239,213,70,564
GALNTL1_2_23112,18,14,12,30,0,118,0,0,0,0,4,37
GAMT_2_23113,264,182,181,305,24,67,34,145,25,196,73,687
GANAB_2_23114,86,70,99,76,158,0,82,172,324,35,99,92
GART_2_23115,487,553,449,481,482,301,1108,237,777,532,153,368
GBA_2_23116,331,513,672,586,74,2,1011,167,105,643,332,405
GBP5_2_23117,65,31,53,68,94,130,44,108,59,217,110,15
GCAT_2_23118,987,827,688,929,442,669,561,662,971,1821,295,1486
GCDH_2_23119,330,207,112,356,0,10,0,258,261,814,843,381
GCH1_2_23120,69,55,69,117,16,6,213,59,12,26,15,7
GCLC_2_23121,1531,1231,885,1212,1515,1125,1360,1605,2802,2385,1216,730
GCNT1_2_23122,157,192,258,260,84,84,13,7,10,757,133,125
GDPD1_2_23123,36,48,31,132,0,0,0,130,559,0,0,493
GDPD2_2_23124,764,934,1149,898,319,832,1810,471,749,686,459,1002
GEM_2_23125,567,771,587,685,981,164,485,402,1090,697,49,659
GEN1_2_23126,759,965,733,903,1198,654,509,1964,608,1312,633,689

GFM2_2_23127,278,196,134,146,16,471,147,85,84,247,9,95
GFOD1_2_23128,216,117,155,242,599,25,404,45,173,5,87,110
GGCT_2_23129,143,80,86,92,132,46,7,1,0,17,489,217
GGCX_2_23130,79,138,145,120,99,3,236,0,3,0,5,0
GGT1_2_23131,13,41,61,92,0,0,0,0,2,25,0,0
GGT5_2_23132,12,35,54,26,4,2,0,13,14,12,0,6
GGT6_2_23133,14,39,12,12,1,7,0,0,25,4,0,0
GGTLC1_2_23134,341,250,285,368,117,297,659,382,470,458,50,386
GLB1_2_23135,216,244,440,218,465,59,330,297,274,3,427,870
GLRX2_2_23136,90,76,63,59,0,4,0,15,0,13,0,44
GLRX3_2_23137,812,507,541,602,581,550,336,932,252,602,202,529
GLRX_2_23138,19,10,15,15,0,0,0,22,89,1,0,2
GLT8D1_2_23139,425,440,391,537,62,285,194,491,223,1008,839,347
GLUL_2_23140,278,174,235,288,668,28,39,367,47,100,284,138
GM2A_2_23141,399,537,587,417,411,228,87,756,467,476,183,389
GMPPA_2_23142,300,134,242,241,60,27,193,477,326,73,1175,363
GMPPB_2_23143,257,74,173,123,43,8,91,8,319,0,225,91
GMPR2_2_23144,13,20,12,18,0,535,0,0,172,2,2,0
GNAI2_2_23145,500,434,427,508,257,743,1011,639,294,229,543,492
GNAL_2_23146,223,339,258,244,280,3,27,648,398,64,0,36
GNAO1_2_23147,10,37,26,39,22,0,0,0,52,46,0,270
GNAT1_2_23148,862,832,807,1117,408,587,821,387,478,638,1861,945
GNB5_2_23149,17,15,41,40,0,0,0,6,64,114,0,3
GNG10_2_23150,948,1008,1148,1358,1592,1506,1943,901,257,966,2564,1264
GNG4_2_23151,239,162,154,363,242,264,0,710,180,4,360,47
GNGT2_2_23152,91,281,195,210,2,151,90,127,30,109,348,128
GPD2_2_23153,1079,896,959,841,705,381,424,497,906,449,1274,704
GPHN_2_23154,3,55,12,48,0,47,0,344,34,0,0,81
GPNMB_2_23155,563,511,631,617,372,541,243,229,1019,444,421,556
GPT2_2_23156,52,48,25,120,0,31,60,43,0,80,1,71
GPX4_2_23157,1,11,9,36,0,0,0,9,0,0,0,235
GSR_2_23158,67,99,127,200,69,2,84,779,180,51,6,15
GSTCD_2_23159,884,824,779,671,400,993,920,243,881,1282,1376,689
GSTK1_2_23160,88,109,113,119,469,35,2,41,11,48,557,64
GSTM1_2_23161,84,86,170,129,144,24,78,69,58,173,1,225
GSTM2_2_23162,712,511,653,687,207,866,617,402,207,225,667,193
GSTM4_2_23163,5185,5006,4088,5100,5889,4918,4834,2892,3719,5583,5489,4
577
GSTO1_2_23164,1,29,43,95,161,64,61,2,59,3,7,19
GSTO2_2_23165,295,293,326,323,292,617,388,351,199,896,129,254
GSTZ1_2_23166,230,173,123,180,509,462,270,0,368,166,253,113
GTPBP3_2_23167,340,372,377,287,25,243,406,39,122,275,169,209
GUCY1A3_2_23168,1616,1002,1402,1314,728,1245,2590,686,397,714,2743,140
1
GYG1_2_23169,496,491,495,659,744,407,90,338,749,258,88,1450
GYG2_2_23170,1464,1586,1358,1608,2532,1265,1217,1636,1694,2032,2128,15
46
GYS1_2_23171,545,520,598,427,414,122,642,473,449,429,688,275
HADH_2_23172,713,510,717,744,725,1305,532,433,824,520,706,831
HAGH_2_23173,54,229,94,161,302,97,1,0,8,91,3,424

HAO2_2_23174,684,746,775,705,897,244,917,1032,738,790,1057,673
HAS3_2_23175,612,596,752,757,914,559,595,326,918,753,1051,1078
HCCS_2_23176,314,123,275,359,406,80,303,90,524,49,536,374
HDHD1_2_23177,132,75,140,213,10,57,668,251,190,18,148,143
HENMT1_2_23178,239,217,205,206,33,177,353,654,63,393,511,145
HHAT_2_23179,32,18,30,16,0,52,6,42,3,2,1,0
HIBCH_2_23180,249,222,168,315,480,221,131,303,309,298,188,488
HLCS_2_23181,425,663,329,360,435,1046,42,200,214,474,0,195
HMBS_2_23182,160,55,65,52,210,51,80,186,115,145,0,75
HMGA2_2_23183,375,433,325,535,122,332,942,77,252,162,450,402
HMGCL_2_23184,3677,3158,3337,3518,3685,4853,1981,2105,3950,3274,2519,4
290
HMGCLL1_2_23185,112,155,201,125,6,130,321,42,116,204,11,168
HMGCR_2_23186,142,74,132,97,34,147,0,470,349,359,290,87
HMGCS1_2_23187,366,199,496,474,108,322,127,289,320,513,294,499
HMGCS2_2_23188,1301,1256,988,1149,1026,400,982,1171,972,1497,756,1354
HMOX2_2_23189,211,261,124,267,349,197,55,106,37,50,2,273
HNRNPAB_2_23190,130,108,141,183,78,198,67,52,114,244,374,91
HPD_2_23191,136,184,164,173,35,483,978,304,200,249,84,225
HPGD_2_23192,305,174,253,286,30,203,699,172,52,231,1187,456
HPSE2_2_23193,118,98,73,104,242,4,1,9,0,5,0,0
HPSE_2_23194,72,136,45,89,375,130,7,0,88,57,0,73
HRAS_2_23195,301,234,239,210,30,418,73,509,260,143,214,177
HS2ST1_2_23196,1367,1374,1132,1532,1917,495,979,575,1418,1781,1199,148
5
HS6ST2_2_23197,167,103,62,177,3,149,6,53,560,63,331,206
HSD11B1_2_23198,956,616,765,690,773,2066,239,375,769,36,256,853
HSD17B10_2_23199,308,132,122,153,410,4,654,26,115,0,22,318
HSD17B13_2_23200,767,1002,847,1100,871,1493,1443,986,1145,1089,1091,77
5
HSD17B4_2_23201,215,56,123,106,551,38,175,23,16,0,0,131
HSD3B2_2_23202,1166,1002,989,1185,2216,1134,1009,1824,1425,575,975,196
6
HSD3B7_2_23203,77,48,33,61,0,46,4,6,0,2,0,486
HSDL1_2_23204,633,490,438,458,985,538,166,544,427,745,163,615
HSP90AA1_2_23205,372,379,378,415,387,104,313,307,123,108,620,207
HSPA8_2_23206,1432,1535,1205,1812,1226,853,1113,1295,1194,2022,1727,13
65
HSPD1_2_23207,323,392,334,426,252,201,463,827,509,1004,149,425
HTRA2_2_23208,152,187,180,145,221,10,112,353,157,641,208,800
HYAL1_2_23209,71,52,25,50,25,1,0,23,598,1,0,87
HYAL2_2_23210,299,154,178,438,186,155,116,127,202,307,43,982
HYI_2_23211,131,70,79,32,11,0,23,0,0,0,0,10
IARS_2_23212,1725,1553,1221,1449,1894,1266,2087,1734,1900,758,1193,200
6
IDE_2_23213,382,208,335,292,32,400,140,1125,580,280,801,127
IDH3B_2_23214,258,363,102,237,55,1398,169,380,249,847,114,92
IDH3G_2_23215,28,3,9,9,4,1,0,1,17,112,5,15
IDS_2_23216,102,183,87,127,18,54,247,40,359,47,52,32
IFT27_2_23217,29,18,28,35,2,0,5,223,108,168,5,54

IL4I1_2_23218,31,12,5,27,6,0,0,1,91,0,0,0
IMPDH1_2_23219,38,82,82,114,6,57,11,11,42,0,1,9
INMT_2_23220,19,8,52,85,209,0,2,0,26,0,276,79
INPP5K_2_23221,155,101,95,165,34,117,177,199,92,7,313,23
INTS6_2_23222,793,842,720,1193,1329,1193,1871,1148,849,636,520,630
IPCEF1_2_23223,620,457,420,707,95,333,648,1906,503,705,909,952
ISOC2_2_23224,26,65,75,41,234,8,590,118,23,9,60,327
ISPD_2_23225,1896,1559,1572,1410,1624,1100,1237,1096,1544,892,1436,211
9
ISYNA1_2_23226,30,24,4,30,0,0,73,0,0,62,0,13
ITPA_2_23227,201,96,177,193,371,85,675,0,348,46,124,292
IVD_2_23228,216,242,240,311,663,503,0,0,0,192,1043,154
IYD_2_23229,917,1093,770,1421,1277,1210,1797,1028,1094,2191,497,403
JMJD7-PLA2G4B_2_23230,866,641,646,649,742,580,971,711,1430,811,225,730
KARS_2_23231,185,132,188,226,28,612,107,111,7,117,8,86
KATNA1_2_23232,353,405,348,440,99,219,39,137,251,488,820,102
KATNAL1_2_23233,781,840,651,748,499,595,1342,636,906,383,1294,1152
KIF16B_2_23234,63,142,47,161,4,149,200,162,27,121,52,60
KIF9_2_23235,135,129,157,115,57,8,69,33,11,71,134,169
KIFC3_2_23236,103,2,43,5,0,1,510,0,0,510,0,0
KLK2_2_23237,235,262,123,182,241,759,11,92,317,217,154,321
KLK7_2_23238,155,146,154,209,228,20,9,25,150,46,33,243
KRAS_2_23239,571,475,521,315,163,807,235,623,70,344,322,163
KYNU_2_23240,1637,1098,1291,1533,2261,1082,1363,761,1026,1465,1054,134
3
LAMP2_2_23241,296,230,189,193,545,323,27,92,387,195,2,228
LARGE_2_23242,53,86,77,30,40,0,0,51,5,2,0,140
LCLAT1_2_23243,400,425,298,369,663,89,475,137,137,243,397,301
LCMT1_2_23244,357,149,165,269,201,3,199,453,205,92,101,154
LDHA_2_23245,474,319,237,459,296,709,206,215,817,135,289,459
LDHAL6A_2_23246,54,49,34,101,8,117,0,93,62,78,0,46
LDHB_2_23247,574,484,561,670,588,56,883,294,664,1266,1182,554
LDHC_2_23248,32,37,64,46,9,133,0,0,123,4,0,0
LDHD_2_23249,56,102,107,159,0,31,20,0,393,2,193,56
LEPRE1_2_23250,507,557,448,575,208,124,486,294,252,138,516,623
LEPREL1_2_23251,226,301,145,246,496,11,20,175,173,150,181,242
LFNG_2_23252,212,271,366,421,200,413,418,178,359,102,855,335
LGMN_2_23253,954,578,664,913,808,982,1167,281,1070,613,761,606
LGSN_2_23254,301,312,392,356,549,0,185,481,266,513,562,313
LIAS_2_23255,124,98,165,173,167,12,0,395,41,44,0,88
LIG3_2_23256,154,122,262,235,61,89,180,316,140,133,88,337
LIG4_2_23257,2739,2228,2061,2898,2477,1873,2904,2237,1167,2760,3326,27
23
LIPF_2_23258,40,102,70,126,100,3,42,16,39,84,43,197
LIPT1_2_23259,883,664,648,846,92,130,1306,1143,1305,972,792,889
LMO7_2_23260,56,13,67,85,3,0,0,0,15,170,0,260
LNPEP_2_23261,383,145,220,229,174,63,1390,705,55,474,787,116
LOX_2_23262,212,149,167,307,166,42,795,215,350,165,7,181
LPO_2_23263,430,523,298,345,249,835,496,269,234,1076,36,344
LRRC16A_2_23264,329,206,223,372,162,670,40,369,46,49,1227,507

LSS_2_23265,524,409,458,535,425,204,88,17,385,473,152,890
LYZL6_2_23266,456,660,635,575,881,104,1092,235,300,786,310,721
MACF1_2_23267,1786,2174,1643,1970,1133,1164,1853,3073,1940,2317,932,19
90
MACROD2_2_23268,697,801,729,806,405,556,1124,990,513,2045,1197,753
MAD2L2_2_23269,585,548,533,584,1321,628,135,621,423,806,599,740
MAN2B1_2_23270,136,160,103,65,123,80,85,161,64,20,6,116
MASP2_2_23271,606,492,631,457,316,355,199,457,436,403,597,337
MAT2B_2_23272,760,606,628,694,1022,723,968,744,776,731,486,1362
MCAT_2_23273,427,318,388,391,263,14,15,529,232,311,233,728
MCM4_2_23274,109,112,51,86,130,113,74,8,16,686,140,48
MCM7_2_23275,790,588,512,735,430,476,381,794,1101,851,245,1221
MCM8_2_23276,168,90,93,168,71,19,23,10,70,23,92,82
MCM9_2_23277,366,193,315,261,538,564,96,95,54,142,297,233
MDH1_2_23278,91,28,35,65,25,6,260,2,4,43,68,29
MECR_2_23279,25,58,12,3,0,0,0,0,8,0,0,1
MEPCE_2_23280,15,62,31,37,5,1,3,32,46,39,0,3
METTL13_2_23281,501,530,601,576,247,471,230,755,579,594,825,666
MFN2_2_23282,147,251,340,356,81,0,15,401,0,271,24,400
MFNG_2_23283,275,123,179,366,114,0,0,106,152,0,41,304
MGAT3_2_23284,21,145,26,31,158,2,185,29,107,204,0,81
MGAT4A_2_23285,16,8,25,37,0,0,0,44,0,38,0,0
MGAT4B_2_23286,214,190,154,171,52,217,43,73,141,182,358,183
MGLL_2_23287,77,33,95,75,0,12,1,54,61,396,130,73
MGST1_2_23288,191,191,164,142,172,88,54,142,152,129,253,430
MGST2_2_23289,565,343,349,448,409,125,421,642,384,81,750,389
MICAL1_2_23290,256,157,172,274,53,75,821,355,302,32,0,218
MLH1_2_23291,71,43,97,8,6,42,36,3,10,23,240,8
MME_2_23292,2602,2146,2102,2666,2540,2223,2147,2253,2017,1247,1629,291
6
MMP1_2_23293,198,248,235,260,105,181,38,30,173,14,462,124
MMP2_2_23294,1472,1016,1098,1214,717,391,732,1154,548,1173,1171,740
MOCS1_2_23295,76,85,129,72,211,0,21,4,98,20,30,126
MOGS_2_23296,0,0,0,0,0,5,0,0,15,0,0,0
MOV10_2_23297,50,64,25,30,7,20,1,63,216,81,0,18
MOV10L1_2_23298,1205,1085,839,1133,537,1228,428,1164,1364,673,1372,843
MPG_2_23299,320,439,357,388,775,150,677,259,268,33,65,267
MPPE1_2_23300,70,66,57,127,357,201,9,28,0,1,26,263
MPST_2_23301,14,8,17,42,47,0,83,5,18,168,0,39
MRAS_2_23302,1712,1436,1765,2002,799,848,1107,1265,1375,1534,885,1378
MRE11A_2_23303,323,151,167,177,899,1,6,22,300,474,130,321
MRI1_2_23304,0,14,21,22,0,0,62,0,0,0,0,39
MSH5_2_23305,903,540,869,790,348,463,2479,525,709,1043,865,1093
MSRA_2_23306,180,227,262,133,59,20,184,67,45,535,542,23
MSRB3_2_23307,212,122,256,153,87,286,190,0,188,14,0,392
MTHFD1L_2_23308,417,370,248,395,440,162,902,333,319,205,472,85
MTHFS_2_23309,1300,1022,1050,1448,1392,1348,1100,1128,1107,1497,1706,1
364
MTO1_2_23310,132,245,179,220,361,127,215,631,207,51,98,376
MTRR_2_23311,446,147,260,234,50,254,17,381,380,131,238,42

MUTYH_2_23312,110,110,211,138,59,18,201,0,36,1,76,35
MX1_2_23313,489,342,309,369,1153,2,416,102,662,625,706,564
MYBBP1A_2_23314,39,13,17,10,6,0,0,2,0,5,0,5
MYH2_2_23315,0,0,0,0,0,0,0,0,0,0,0,0
MYO5A_2_23316,25,44,17,113,27,309,11,0,146,2,0,24
MYO7A_2_23317,63,47,65,46,187,182,0,0,0,35,191,12
MYO9B_2_23318,54,7,26,10,65,3,3,0,37,3,225,42
N6AMT1_2_23319,299,228,282,290,241,394,7,154,739,69,125,159
NAA16_2_23320,164,76,140,89,323,0,168,0,15,49,2,331
NAA20_2_23321,195,226,141,232,204,24,204,219,345,136,24,121
NAAA_2_23322,0,9,21,46,0,0,25,0,0,0,0,0
NAPEPLD_2_23323,257,200,346,560,496,221,685,66,416,238,195,339
NARF_2_23324,106,203,39,122,34,1,1,57,5,6,0,0
NARS2_2_23325,331,202,218,195,626,97,206,1,444,147,101,169
NAV1_2_23326,303,132,137,229,132,382,22,421,123,411,396,84
NCEH1_2_23327,802,952,877,1152,872,484,522,316,1036,1595,1152,1270
NCF2_2_23328,251,368,243,240,225,250,68,399,270,234,136,187
NCF4_2_23329,613,684,545,698,298,493,2301,681,850,595,1163,751
NDOR1_2_23330,64,42,43,105,1,127,0,0,1,0,0,0
NDUFA2_2_23331,194,263,176,233,1035,137,154,0,601,27,212,175
NDUFB11_2_23332,486,526,358,446,165,801,21,977,608,461,365,275
NDUFB4_2_23333,100,53,105,107,50,3,218,9,4,6,0,66
NDUFB5_2_23334,174,225,58,137,13,177,59,22,20,330,72,82
NDUFB6_2_23335,1944,1848,1920,2011,1242,1002,623,1425,1649,2486,2385,1
217
NDUFC1_2_23336,612,473,577,639,157,82,763,812,466,517,573,597
NDUFS1_2_23337,1087,944,976,1146,1565,630,474,664,844,1165,97,1019
NDUFS2_2_23338,542,662,646,645,594,1553,1741,12,786,374,322,435
NDUFS5_2_23339,1241,1179,1420,1133,1463,1435,1613,1752,1742,986,725,12
26
NDUFV1_2_23340,169,101,246,125,8,268,693,391,39,150,617,255
NEIL2_2_23341,40,40,35,43,1,83,0,154,0,6,2,33
NEU4_2_23342,25,32,19,52,1,124,0,51,14,160,0,34
NFS1_2_23343,552,382,462,662,864,237,228,181,36,409,430,1309
NGLY1_2_23344,396,287,182,226,87,331,172,24,177,348,75,441
NIPSNAP1_2_23345,851,887,823,717,378,564,899,1251,515,873,2046,845
NIT1_2_23346,62,85,51,108,0,125,89,25,20,227,0,15
NKIRAS2_2_23347,268,220,214,281,21,244,193,297,12,4,38,205
NLGN3_2_23348,65,49,77,198,493,138,135,3,29,33,0,190
NLGN4X_2_23349,414,384,535,460,445,467,435,0,536,324,1062,258
NLGN4Y_2_23350,367,500,398,379,639,269,283,445,173,804,97,739
NMNAT2_2_23351,182,41,33,47,0,0,0,70,0,162,0,198
NNT_2_23352,868,437,606,819,307,65,860,464,344,425,194,828
NOX4_2_23353,1404,1188,921,1473,2675,1870,1830,2907,1237,1276,2060,124
7
NPL_2_23354,233,376,178,238,176,453,132,59,19,296,51,202
NQO1_2_23355,478,427,358,436,99,234,42,242,211,307,103,755
NRD1_2_23356,37,82,39,42,0,114,88,23,42,0,0,2
NSDHL_2_23357,17,77,83,129,0,0,333,4,10,92,6,0
NSUN2_2_23358,1118,1145,915,861,1685,891,805,818,572,1224,771,1161

NUDT2_2_23359,89,51,143,45,37,0,41,7,143,189,76,22
NXN_2_23360,70,15,35,16,12,0,35,0,0,21,0,10
OAS1_2_23361,249,108,113,204,85,0,829,118,188,149,107,13
OAS2_2_23362,350,809,586,820,694,864,625,392,688,179,380,477
OASL_2_23363,541,388,440,427,679,83,715,285,243,427,664,988
OAT_2_23364,1021,862,964,1384,347,1471,1363,1115,1595,1213,1172,509
OGDH_2_23365,133,147,40,59,55,21,18,0,111,115,179,69
OGDHL_2_23366,40,67,73,115,238,72,0,44,1,9,202,0
OGG1_2_23367,246,459,429,306,126,1,517,385,97,4,1800,287
OGT_2_23368,488,407,640,510,1220,213,120,536,307,762,733,1433
OLAH_2_23369,795,756,810,594,1201,539,210,90,1216,456,513,528
P4HA1_2_23370,131,247,233,278,76,412,307,205,314,490,715,411
P4HA2_2_23371,580,478,536,591,568,230,493,483,724,39,290,556
P4HTM_2_23372,280,295,303,307,85,455,265,44,295,20,191,263
PAFAH1B2_2_23373,436,280,390,354,479,361,740,359,410,564,133,204
PAFAH1B3_2_23374,501,547,398,489,252,477,237,454,609,493,407,746
PAICS_2_23375,505,414,665,542,802,838,375,639,308,936,251,559
PAOX_2_23376,32,22,105,58,202,2,159,25,87,4,22,21
PAPD4_2_23377,169,182,202,265,0,90,19,334,99,3,7,117
PAPD5_2_23378,301,282,318,516,603,260,289,414,322,480,49,284
PAPD7_2_23379,573,594,514,715,1247,806,360,440,296,772,1121,623
PAPSS2_2_23380,108,86,116,129,140,1,10,140,52,40,3,449
PARK7_2_23381,58,34,60,83,5,0,143,24,29,115,0,82
PARL_2_23382,36,76,96,266,642,1,1,364,61,67,1,101
PARN_2_23383,525,501,656,581,163,215,1016,1350,228,23,1343,694
PARP2_2_23384,0,0,0,0,0,0,0,0,0,0,0,0
PARP3_2_23385,42,23,18,26,1,36,0,105,84,17,0,35
PARP8_2_23386,455,462,327,379,208,17,1052,129,830,662,88,282
PBLD_2_23387,176,132,101,174,307,67,0,85,171,93,30,233
PCCA_2_23388,196,194,322,350,32,148,162,489,256,163,214,22
PCCB_2_23389,533,400,497,515,720,441,172,284,619,271,735,443
PCMTD2_2_23390,1662,1410,1325,2185,3258,1055,1695,2694,1854,2243,2215,
1063
PCNA_2_23391,65,29,34,131,10,4,0,72,6,42,0,46
PCSK1_2_23392,2054,1581,1596,1876,1021,2434,1845,3014,1882,2371,2085,2
493
PCSK2_2_23393,391,431,410,419,642,227,188,361,308,1017,1990,911
PCYT1B_2_23394,374,348,221,316,528,375,253,311,334,493,60,417
PCYT2_2_23395,1766,1565,1684,1622,2464,1080,1276,1167,761,1455,1917,14
26
PDE10A_2_23396,666,545,677,859,856,188,621,325,410,490,1649,388
PDE11A_2_23397,311,254,149,220,24,148,371,330,490,285,259,479
PDE1A_2_23398,204,368,310,302,419,236,346,10,186,273,466,232
PDE1B_2_23399,735,504,567,779,235,505,489,1521,1352,524,689,628
PDE1C_2_23400,411,432,265,371,26,66,176,315,269,289,488,398
PDE2A_2_23401,206,156,71,144,95,392,369,3,29,54,0,185
PDE4A_2_23402,210,252,215,318,8,68,460,285,300,346,607,287
PDE4B_2_23403,227,179,176,271,117,290,79,25,210,5,425,32
PDE4C_2_23404,140,163,65,156,537,282,14,5,146,173,645,108
PDE4D_2_23405,567,388,494,494,116,417,324,625,397,448,222,512

PDE5A_2_23406,233,300,184,219,72,63,9,847,168,54,440,414
PDE6B_2_23407,64,19,148,127,49,0,6,2,62,2,505,0
PDE7A_2_23408,459,423,586,575,544,32,436,205,122,961,36,519
PDE8A_2_23409,133,149,277,199,161,30,662,157,218,466,6,44
PDE8B_2_23410,410,376,351,420,527,527,14,157,573,801,256,322
PDE9A_2_23411,43,23,34,41,30,0,0,0,1,3,0,3
PDHA1_2_23412,71,48,77,80,99,191,6,13,14,154,0,14
PDHB_2_23413,753,510,604,845,1533,653,176,441,279,980,983,776
PDHX_2_23414,1504,1065,1025,1263,1762,1113,793,2930,1001,2060,1042,104
2
PEMT_2_23415,302,250,254,487,84,151,598,10,128,250,21,383
PEPD_2_23416,5,29,37,7,0,0,0,0,2,3,0,1
PGAM5_2_23417,47,68,80,97,1,68,20,160,84,101,0,8
PGBD1_2_23418,242,293,195,197,135,677,78,47,319,337,1122,131
PGC_2_23419,254,159,217,259,508,113,705,85,187,163,196,307
PGM1_2_23420,230,332,186,80,318,0,237,0,3,0,410,244
PGM3_2_23421,271,167,105,160,18,3,303,34,0,61,9,102
PHOSPHO1_2_23422,84,48,46,57,82,8,6,100,2,0,13,25
PHOSPHO2_2_23423,551,356,378,517,649,1031,1003,610,438,275,1075,658
PHYH_2_23424,1195,1289,965,1007,1434,563,1243,950,615,1641,981,589
PIGA_2_23425,492,614,337,638,49,618,961,139,162,297,288,484
PIGC_2_23426,161,257,155,160,194,89,154,322,133,201,336,179
PIGF_2_23427,1018,630,869,959,509,809,366,375,733,894,1092,929
PIGG_2_23428,357,249,246,258,314,1087,306,81,118,270,135,313
PIGN_2_23429,100,142,90,277,27,6,124,137,312,208,498,231
PIGO_2_23430,4,6,14,42,0,0,0,0,0,0,0,222
PIGP_2_23431,1414,800,901,1275,851,949,1546,479,939,1875,1956,2146
PIGQ_2_23432,7,14,12,45,0,2,18,19,0,1,14,1
PIGT_2_23433,212,139,161,254,110,189,300,3,25,6,501,466
PIGV_2_23434,174,206,250,190,533,125,4,381,344,104,0,246
PIN4_2_23435,319,271,188,312,431,282,0,229,224,2,238,200
PLA1A_2_23436,529,671,645,766,832,702,279,441,506,250,838,419
PLA2G2A_2_23437,29,39,51,37,3,0,18,21,40,181,0,507
PLA2G4C_2_23438,88,22,89,55,276,1,61,81,71,262,1,0
PLA2G6_2_23439,26,116,27,85,10,0,0,7,392,198,0,0
PLA2G7_2_23440,1336,1134,1090,1271,942,573,857,1115,995,1027,1329,1541
PLAT_2_23441,4,28,22,82,21,0,0,0,193,17,0,5
PLAU_2_23442,896,960,1019,932,1360,869,823,585,1020,1797,1639,624
PLB1_2_23443,74,202,78,105,130,52,46,152,0,0,9,187
PLCB1_2_23444,633,722,591,499,1567,540,623,628,686,1104,505,868
PLCB3_2_23445,61,60,28,28,0,0,0,12,27,0,0,56
PLCB4_2_23446,273,235,319,397,20,1242,63,2033,214,13,39,630
PLCD1_2_23447,58,57,16,9,0,0,8,0,0,262,0,0
PLCE1_2_23448,909,686,823,743,1569,996,81,25,640,1368,661,346
PLCG1_2_23449,65,50,105,128,17,1,0,0,38,4,2,5
PLCH1_2_23450,173,87,83,141,380,37,66,25,24,30,5,130
PLCL2_2_23451,1305,1488,1057,1463,746,350,1160,823,574,1764,3599,1487
PLCXD2_2_23452,1185,1180,1160,1304,706,441,992,1465,756,768,1285,739
PLD2_2_23453,24,14,13,11,18,269,127,0,1,2,0,74
PLD3_2_23454,470,250,274,530,532,0,549,291,246,694,126,434

PLOD2_2_23455,0,0,1,0,0,0,0,0,0,0,0,0
PLSCR3_2_23456,53,56,49,50,0,119,2,2,179,0,0,1
PLSCR4_2_23457,1260,1299,1010,1805,1929,939,1463,664,1600,1149,1657,13
49
PMEL_2_23458,224,328,221,385,280,4,293,38,151,37,0,485
PMS1_2_23459,979,1154,988,1057,595,433,941,1555,1796,877,904,1401
PNPLA1_2_23460,448,317,539,340,11,835,851,0,354,164,1204,658
PNPLA4_2_23461,96,93,153,127,29,78,90,86,17,23,41,29
PNPLA6_2_23462,65,39,44,46,0,0,0,0,0,3,94,3
PNPLA7_2_23463,32,20,52,62,0,4,8,3,3,22,15,5
POFUT1_2_23464,288,285,295,250,231,216,559,1116,4,241,842,22
POFUT2_2_23465,190,92,91,216,219,210,210,74,167,314,137,443
POGZ_2_23466,17,61,11,21,0,0,8,4,39,0,2,25
POLD2_2_23467,196,231,320,346,10,199,318,162,365,494,122,170
POLE2_2_23468,859,678,514,842,355,201,626,730,765,714,251,1533
POLG_2_23469,218,205,277,233,230,30,7,320,167,197,42,62
POLL_2_23470,235,182,185,179,579,304,88,149,90,172,116,56
POLR1D_2_23471,2454,2483,2270,2389,2896,2002,1909,3172,1754,3686,1886,
1486
POLR3B_2_23472,540,644,605,659,428,1104,194,95,572,935,911,587
POLR3H_2_23473,311,192,146,251,100,2,6,143,100,247,173,315
POMT1_2_23474,33,48,19,72,52,54,17,0,151,0,0,36
PON2_2_23475,490,397,537,499,703,593,279,781,591,435,364,405
POP5_2_23476,2132,1966,1951,2125,2560,1413,3359,2934,1638,3288,2474,25
57
PPA2_2_23477,1048,710,792,964,1629,736,635,918,371,1005,352,1457
PPIE_2_23478,1553,1359,1578,1394,1647,1588,466,1371,1824,2875,1354,185
4
PPIL2_2_23479,2660,2379,2389,3261,2084,1584,2568,2137,2042,2149,2078,2
087
PPIL3_2_23480,227,159,154,241,134,140,4,392,201,154,55,73
PPIL6_2_23481,151,395,238,370,233,119,506,600,116,333,509,25
PPIP5K1_2_23482,168,130,123,135,11,177,82,6,201,69,75,100
PPT1_2_23483,978,699,879,870,995,626,988,125,1233,1094,520,890
PPT2_2_23484,202,301,228,235,273,276,176,204,387,385,293,497
PRCP_2_23485,2666,2059,2391,2862,2448,2245,2572,1472,2932,1728,2560,21
73
PRDX1_2_23486,484,354,356,503,291,373,124,129,785,520,292,783
PRDX3_2_23487,404,383,314,295,20,956,834,262,452,106,54,619
PRDX5_2_23488,287,164,367,346,38,784,155,732,346,560,1137,272
PRKCSH_2_23489,290,283,237,321,33,611,784,14,138,24,684,229
PRODH_2_23490,135,139,102,113,182,0,0,5,158,0,102,271
PRSS3_2_23491,222,138,246,219,306,501,360,66,174,611,91,151
PSAT1_2_23492,399,381,295,378,156,473,741,154,190,92,32,241
PSMA1_2_23493,383,375,510,574,549,842,51,253,372,914,1296,616
PSMA3_2_23494,203,126,171,211,138,6,0,3,51,228,44,367
PSMA4_2_23495,28,23,35,118,0,238,0,0,3,0,0,2
PSMA5_2_23496,13,0,1,10,0,0,0,0,0,0,0,0
PSMA8_2_23497,318,191,214,385,130,397,464,8,582,717,574,621
PSMB2_2_23498,1796,1603,1565,1806,2204,1217,1137,1302,1203,1368,2239,1

272
PSMB5_2_23499,358,355,339,292,373,1069,56,12,246,274,80,585
PSMB8_2_23500,641,690,827,838,381,285,933,659,825,305,814,955
PTBP1_2_23501,243,108,232,325,5,5,330,236,83,437,54,850
PTER_2_23502,1054,862,1030,1064,891,633,989,1112,634,756,672,1097
PTGR1_2_23503,698,478,512,612,455,380,724,627,467,462,1117,910
PTGR2_2_23504,445,363,483,436,167,75,569,599,167,513,38,442
PTGS1_2_23505,73,30,82,95,74,213,84,0,1,95,0,158
PUS1_2_23506,340,584,430,572,287,484,89,332,408,465,320,723
PYCR1_2_23507,11,38,23,29,0,0,0,0,2,2,306,4
PYGL_2_23508,1490,1419,1172,1192,1334,2193,1422,1825,1445,1193,1869,11
84
PYGM_2_23509,101,208,120,277,81,391,657,86,97,113,0,231
QPCTL_2_23510,134,110,112,217,185,82,0,0,111,191,2,8
QSOX1_2_23511,165,223,186,293,283,169,206,296,263,22,18,117
RAB11A_2_23512,1112,770,896,1115,1727,868,404,847,731,1083,739,958
RAB1A_2_23513,289,204,151,185,1494,53,341,62,120,175,98,693
RAB23_2_23514,1466,1160,1245,1210,1615,616,1248,2089,1698,1013,907,131
7
RAB27A_2_23515,152,166,200,226,320,66,369,85,36,259,0,88
RAB28_2_23516,149,211,218,368,235,3,237,1,71,206,948,351
RAB2A_2_23517,350,292,393,495,586,474,28,308,669,504,567,301
RAB2B_2_23518,240,237,235,212,170,412,162,349,433,73,266,442
RAB34_2_23519,101,69,162,191,1,428,1087,205,44,1,1,134
RAB35_2_23520,190,203,101,87,392,0,104,21,304,0,475,4
RAB37_2_23521,22,3,1,14,0,31,0,0,16,0,3,0
RAB40C_2_23522,197,40,41,141,2,47,156,33,190,63,0,24
RAB5C_2_23523,1123,894,1034,1092,2237,928,1216,1416,1221,1441,756,1549
RAB6A_2_23524,435,224,313,300,213,67,486,205,239,963,315,584
RAB7L1_2_23525,126,236,241,321,918,0,568,95,169,2,42,96
RAB9A_2_23526,2933,2477,2502,2814,2364,3022,2363,2870,1836,2793,2100,2
403
RABGGTA_2_23527,46,45,76,57,0,166,0,1,1,0,3,0
RABL2A_2_23528,209,398,231,248,349,60,57,405,513,786,66,299
RABL2B_2_23529,1822,1819,1941,1879,1671,1380,1586,1394,2206,1560,1990,
2507
RAC1_2_23530,616,261,295,282,115,12,458,210,735,0,0,378
RAD51B_2_23531,305,360,439,378,244,318,503,218,385,178,98,283
RAD51_2_23532,466,283,247,409,811,35,239,359,86,133,531,299
RAD51D_2_23533,59,84,69,124,80,0,0,0,7,39,51,8
RAD54B_2_23534,1486,1575,1208,1688,1591,853,648,2758,1765,1343,779,175
0
RAD54L_2_23535,77,81,101,82,20,23,6,107,50,88,15,18
RAD9A_2_23536,79,74,61,47,41,16,6,169,162,17,14,182
RAP1A_2_23537,286,246,335,293,82,19,93,31,316,217,99,617
RAP1B_2_23538,178,198,174,234,156,840,29,118,15,506,661,277
RBBP8_2_23539,484,244,387,379,291,644,281,342,761,308,324,616
RDH13_2_23540,69,8,37,77,182,3,0,0,3,1,34,22
RDH5_2_23541,170,89,38,48,171,146,64,1,364,34,2,39
RECQL5_2_23542,529,703,768,778,391,212,794,952,556,446,262,622

RECQL_2_23543,910,724,720,963,823,1019,701,1514,490,244,917,501
RERG_2_23544,380,383,262,332,267,425,98,433,931,141,830,813
REV1_2_23545,156,174,207,275,40,193,249,308,103,54,14,215
RFC3_2_23546,212,221,266,245,458,29,180,444,337,314,37,134
RFC5_2_23547,225,110,169,58,225,30,126,61,89,119,408,207
RGN_2_23548,234,202,299,288,0,2,173,470,239,265,59,651
RGS11_2_23549,126,125,134,205,0,326,0,0,9,11,215,299
RGS6_2_23550,525,266,343,443,380,655,482,279,347,290,409,282
RHOC_2_23551,5277,4646,4381,5759,4739,3690,4181,6174,4726,6507,4464,69
78
RHOT1_2_23552,511,405,373,419,537,412,890,899,691,1018,608,301
RNASE1_2_23553,251,642,229,423,627,240,791,629,576,611,40,70
RNASE4_2_23554,1224,1250,1083,1314,1469,559,1846,611,575,1759,870,962
RPAP3_2_23555,428,477,273,577,549,8,547,556,440,447,505,525
RPE_2_23556,467,305,424,600,74,1,1050,368,509,423,1385,1558
RPN2_2_23557,27,81,169,118,42,14,0,316,6,25,97,1
RPP14_2_23558,240,226,178,245,0,0,836,0,257,0,8,897
RPP21_2_23559,231,359,90,207,11,1,9,277,2066,7,342,267
RPP30_2_23560,686,530,498,555,777,355,707,827,1056,250,842,180
RPP38_2_23561,778,469,435,845,1011,193,761,579,408,493,790,1079
RPS27A_2_23562,865,769,874,1135,1096,805,1914,813,417,1112,555,1279
RRAD_2_23563,245,331,158,381,281,71,231,108,137,45,500,287
RRAGB_2_23564,172,216,294,260,101,120,461,108,168,180,38,264
RRAS2_2_23565,186,192,157,159,130,20,194,358,516,115,115,244
RRM2B_2_23566,66,46,12,58,51,12,0,0,1,0,0,8
RRM2_2_23567,123,107,78,100,49,379,218,290,0,2,0,0
RTEL1_2_23568,957,710,455,507,421,747,154,921,405,288,918,208
SAR1A_2_23569,1603,1491,1383,1622,1497,757,693,424,2432,438,1299,1197
SAR1B_2_23570,152,174,182,174,227,0,293,760,150,21,0,68
SARDH_2_23571,45,2,5,16,0,0,232,0,0,23,198,0
SARS2_2_23572,43,45,47,60,23,159,0,0,3,218,76,190
SBNO1_2_23573,419,223,234,213,232,516,655,161,113,277,181,448
SC5DL_2_23574,1109,903,1027,1105,317,827,1191,260,1253,1022,880,1356
SCD5_2_23575,487,242,286,285,295,210,142,91,492,412,161,475
SDC1_2_23576,546,520,509,511,342,410,678,320,772,715,719,560
SDCBP_2_23577,749,608,682,700,64,1409,572,255,424,344,832,985
SDHC_2_23578,1105,708,785,853,1086,248,1251,567,426,608,2079,640
SEPHS1_2_23579,309,254,174,213,221,582,0,472,220,174,91,285
3-Sep_2_23580,46,32,20,28,2,24,16,29,9,24,0,132
4-Sep_2_23581,361,369,269,313,343,383,293,376,278,231,109,322
5-Sep_2_23582,94,66,122,85,63,0,0,192,71,63,188,5
9-Sep_2_23583,1149,1334,1097,1489,809,899,289,1253,1196,500,1485,3153
SGMS2_2_23584,134,142,164,117,129,281,444,20,87,75,29,184
SH3GLB1_2_23585,813,1068,652,810,823,460,1070,490,699,293,1108,1108
SHMT1_2_23586,282,349,150,359,84,6,156,87,228,551,411,156
SHMT2_2_23587,46,27,2,19,32,0,28,1,7,50,25,2
SIAE_2_23588,282,178,316,357,593,409,291,750,166,649,48,392
SLFN11_2_23589,2746,2760,2064,2545,1655,2633,1760,2193,2311,1362,2332,
2199
SMG6_2_23590,250,229,283,292,199,76,400,1004,314,222,107,206

SMOX_2_23591,49,7,23,26,0,0,14,43,149,4,0,0
SMPD1_2_23592,316,280,507,606,318,86,879,828,343,421,1,537
SMPD4_2_23593,263,251,151,286,143,31,270,155,10,75,154,1
SMPDL3B_2_23594,237,184,148,138,1,321,191,108,124,165,89,292
SOD2_2_23595,126,136,124,144,196,6,0,347,292,0,165,15
SPAM1_2_23596,115,148,246,168,550,196,137,163,65,651,360,35
SPAST_2_23597,682,625,575,534,641,306,1561,667,747,1060,404,635
SPG21_2_23598,1732,1795,1668,1897,1076,1876,1169,3032,2050,2386,920,18
84
SPO11_2_23599,211,191,184,192,58,135,43,70,217,371,411,201
ST3GAL1_2_23600,411,369,347,463,641,473,690,124,81,483,287,81
ST3GAL5_2_23601,233,289,118,131,166,384,306,41,72,40,50,55
ST5_2_23602,361,420,250,321,194,308,98,726,389,100,4,842
ST6GAL1_2_23603,402,279,259,352,194,12,629,661,52,382,40,61
ST6GAL2_2_23604,319,250,162,369,126,17,0,223,99,286,138,438
ST6GALNAC3_2_23605,1682,1388,1508,1482,612,1504,1779,1328,1510,1065,18
17,2529
ST6GALNAC4_2_23606,774,1116,1035,1058,604,522,1320,450,1108,892,670,91
7
ST8SIA4_2_23607,1318,1242,1446,1279,840,297,2645,1115,1172,3644,556,14
58
SUCLG2_2_23608,237,168,238,412,0,0,2,373,47,448,913,503
SULF1_2_23609,293,300,212,255,3,249,355,20,152,482,391,185
SULF2_2_23610,46,47,131,40,250,370,12,3,0,0,0,42
SULT1A1_2_23611,2469,2163,1980,2272,2716,2656,2377,2251,1185,2696,3467
,2529
SULT1A2_2_23612,57,30,81,150,78,0,1,10,6,0,102,1
SULT2B1_2_23613,78,53,83,85,0,107,184,158,20,96,0,54
SUOX_2_23614,235,168,169,150,216,9,69,201,202,38,128,297
SYTL1_2_23615,461,317,403,653,898,822,144,167,46,773,87,763
TAB1_2_23616,21,38,18,61,0,0,0,0,74,0,0,40
TBXAS1_2_23617,281,284,206,270,37,37,488,544,489,845,113,130
TCIRG1_2_23618,120,137,107,38,3,0,28,48,157,0,0,0
TDP1_2_23619,771,976,669,1055,218,1661,410,2419,1049,1142,1045,1069
TERT_2_23620,81,101,97,167,562,232,0,0,0,32,186,274
TGM2_2_23621,93,65,92,148,1,0,0,171,125,0,54,44
TGM5_2_23622,35,7,8,8,33,0,4,1,0,3,47,74
TH_2_23623,182,60,98,169,25,1,276,10,199,238,17,216
TIAM2_2_23624,79,137,113,175,136,2,0,41,68,37,23,0
TKTL1_2_23625,81,46,27,102,57,46,160,109,104,253,12,199
TMEM55B_2_23626,325,95,272,154,254,159,726,353,44,256,0,442
TMLHE_2_23627,697,875,540,812,739,203,373,545,620,909,897,1111
TMOD1_2_23628,1802,1236,1294,1441,1947,1585,3009,1264,1310,592,628,232
7
TMX2_2_23629,385,594,685,488,424,557,622,7,96,591,278,961
TNNI2_2_23630,446,240,378,380,411,295,986,61,417,449,111,300
TP53I3_2_23631,160,185,147,117,81,45,1,0,392,44,1,372
TPI1_2_23632,1034,1105,617,854,337,724,917,779,337,956,274,970
TPST2_2_23633,344,181,151,201,0,76,307,54,173,0,186,278
TREX1_2_23634,75,158,53,56,254,79,13,153,255,42,5,133

TRMT1_2_23635,107,24,84,46,1,0,2,405,0,289,1,0
TRMT1L_2_23636,49,110,264,120,1,0,24,0,1,2,0,393
TRMT2B_2_23637,234,294,209,256,138,422,328,10,137,64,44,95
TRPT1_2_23638,34,27,25,11,20,0,0,0,0,0,0,8
TSEN2_2_23639,123,55,91,213,83,0,539,15,143,5,284,163
TSEN34_2_23640,109,103,122,151,30,100,86,86,56,22,13,22
TTLL6_2_23641,527,373,360,352,316,52,410,60,329,459,547,1025
TUBAL3_2_23642,133,197,146,232,143,134,11,2,295,114,0,235
TUSC3_2_23643,254,259,255,244,203,70,184,42,116,107,664,249
TXNDC16_2_23644,661,402,320,567,391,74,308,94,996,660,109,932
TXNDC2_2_23645,57,27,41,83,121,1,347,0,6,11,336,23
TXNDC5_2_23646,200,156,143,242,286,66,57,354,459,0,192,186
TXNL4B_2_23647,280,322,210,114,235,42,597,17,552,141,9,74
TXNRD1_2_23648,683,586,760,697,665,371,1249,345,760,964,390,989
TXNRD3_2_23649,245,228,220,260,53,1,919,171,152,106,212,333
TYMP_2_23650,136,85,83,116,256,101,18,6,66,210,68,149
UBA52_2_23651,48,109,137,134,176,0,72,306,86,188,9,74
UEVLD_2_23652,208,487,364,419,379,387,850,579,173,203,472,306
UGDH_2_23653,782,766,554,732,1154,889,815,1208,834,887,314,890
UGP2_2_23654,806,843,752,1018,853,1305,593,369,415,1184,615,884
UGT1A6_2_23655,876,756,637,868,316,553,833,521,792,1179,652,1337
UGT2B10_2_23656,275,365,362,467,329,228,54,5,252,434,276,318
UGT2B28_2_23657,854,647,724,875,1384,134,800,467,451,1403,738,567
UGT3A1_2_23658,517,366,396,516,1042,464,1004,220,754,256,1054,339
UGT8_2_23659,741,363,441,526,1056,712,550,351,387,804,77,253
UNG_2_23660,108,78,86,102,44,210,71,12,26,427,51,45
UPP1_2_23661,401,270,299,316,37,626,337,1014,36,277,58,472
UPP2_2_23662,487,488,434,641,492,1046,506,647,494,1119,376,300
UQCR10_2_23663,0,0,0,32,0,0,0,0,0,0,0,0
UQCRB_2_23664,461,534,400,471,585,283,1054,62,314,883,520,1280
UROC1_2_23665,101,84,122,77,15,5,141,403,287,233,0,360
VCL_2_23666,201,154,139,212,51,389,59,201,319,231,181,24
VNN2_2_23667,2592,2031,1914,2402,2678,1584,2375,2243,2534,2094,2044,24
29
VPS29_2_23668,390,373,384,481,170,393,1555,387,421,213,573,387
WARS2_2_23669,931,1020,977,830,679,0,648,186,357,1813,652,328
WARS_2_23670,155,224,220,180,320,0,443,774,410,7,0,5
WBSCR22_2_23671,331,305,375,465,197,72,99,59,295,657,86,397
WDR46_2_23672,869,737,657,726,1104,549,749,186,675,581,294,454
WFS1_2_23673,21,32,93,9,0,10,82,0,0,38,208,57
WRNIP1_2_23674,149,243,218,180,290,402,426,349,311,64,220,248
WWOX_2_23675,284,112,201,169,412,214,110,199,1200,536,116,44
XPNPEP1_2_23676,448,256,278,318,85,72,1050,32,394,458,34,560
XRCC3_2_23677,59,70,41,62,78,137,0,1,0,12,0,1
XRN1_2_23678,1009,980,766,1038,261,795,1363,472,1447,72,772,751
YWHAZ_2_23679,442,483,470,525,407,83,864,235,172,1426,105,633
ZBED1_2_23680,43,12,61,45,2,135,339,13,1,2,202,2
ZCCHC11_2_23681,823,809,739,612,377,633,362,238,509,594,490,1090
ZCCHC6_2_23682,2243,1828,1365,1961,845,2845,1465,1210,1258,1124,1932,2
008

ZDHHC15_2_23683,350,350,374,420,694,366,5,521,760,604,366,960
ZDHHC7_2_23684,688,683,777,821,1360,387,1121,1187,462,283,697,1180
ACYP1_2_23685,173,121,187,115,85,1,136,128,256,120,121,261
APOBEC3F_2_23686,554,609,379,635,272,73,398,378,779,986,837,686
ASCC3_2_23687,243,169,253,172,349,526,401,54,188,683,47,253
ASPH_2_23688,824,797,1047,1113,712,1691,862,89,766,803,991,617
ATP6V0E2_2_23689,1102,1244,892,1141,1466,901,1519,1191,2274,1019,1047,
736
C10orf2_2_23690,36,39,38,19,0,1,17,49,0,314,0,158
CAPN3_2_23691,643,678,725,833,573,1752,1766,628,1435,598,731,966
CHM_2_23692,230,333,120,150,2,21,351,11,323,281,6,257
CTSC_2_23693,42,71,55,15,16,0,0,0,0,510,0,0
CYBRD1_2_23694,2087,2268,1900,2438,1353,1610,2680,1453,1532,3027,1954,
2677
CYP3A5_2_23695,93,79,17,123,109,5,0,56,105,0,0,0
DIO2_2_23696,269,312,369,446,517,86,219,304,274,644,229,165
DNASE2B_2_23697,315,136,143,169,0,398,14,22,166,288,599,104
DPYD_2_23698,110,217,193,367,50,353,0,360,205,179,186,491
GBA3_2_23699,1243,1076,1323,1369,1161,885,617,1939,2451,1785,987,1082
GCNT2_2_23700,102,49,101,77,2,6,41,6,217,56,8,12
GLYAT_2_23701,452,373,377,313,521,5,42,316,543,95,662,618
GPLD1_2_23702,431,264,371,535,316,116,331,290,129,420,557,139
GPX1_2_23703,372,393,288,332,360,682,1082,447,331,270,364,155
GPX5_2_23704,222,33,30,59,0,0,40,0,141,92,2,1
HNMT_2_23705,272,263,283,370,126,66,0,394,560,819,39,451
HOGA1_2_23706,104,127,234,187,120,66,193,367,89,197,16,327
HYAL3_2_23707,46,91,49,64,242,86,68,345,134,1,4,74
JPH2_2_23708,245,190,307,221,10,814,171,178,227,138,5,461
KLK8_2_23709,14,33,74,57,22,0,16,25,155,20,12,6
LAMA4_2_23710,461,538,578,640,406,499,10,431,1162,540,505,437
LRR1_2_23711,171,81,155,289,189,17,90,307,259,113,214,167
LRTOMT_2_23712,21,19,51,39,19,12,93,3,10,0,0,9
METTL1_2_23713,106,141,243,91,97,0,175,2,1,247,2781,0
MOCS2_2_23714,94,41,216,258,86,0,11,1,113,50,4,196
NDUFA11_2_23715,452,464,503,515,446,93,553,1059,182,520,910,445
NDUFV3_2_23716,178,259,186,269,261,272,510,0,58,604,451,309
NMNAT3_2_23717,99,276,156,175,33,246,7,698,74,214,0,385
PDE4DIP_2_23718,493,281,382,409,577,288,630,278,203,481,172,143
PLG_2_23719,177,161,227,191,92,38,0,320,49,137,51,26
PPCS_2_23720,171,234,118,182,0,8,578,27,90,8,0,239
PRDX2_2_23721,403,384,354,515,608,412,687,406,295,441,137,997
RAD51C_2_23722,13,6,7,6,0,0,0,0,2,0,0,0
RASD1_2_23723,40,35,46,8,7,1,0,2,0,0,0,16
RASL10A_2_23724,104,103,143,197,182,60,122,579,44,191,948,182
SLX1A_2_23725,79,27,52,126,83,0,260,6,103,62,18,103
SLX1B_2_23726,79,27,52,126,83,0,260,6,103,62,18,103
SPTLC1_2_23727,615,736,743,759,592,555,836,188,119,754,596,415
ST3GAL3_2_23728,250,299,344,300,610,77,361,158,225,104,277,453
VKORC1_2_23729,225,113,235,195,67,0,7,16,204,751,0,147
XPNPEP3_2_23730,536,582,475,512,483,223,466,397,232,1505,826,736

GNAS_2_23731,183,288,280,349,427,174,335,101,291,534,83,268
GNAS_2_23732,288,261,280,192,479,525,338,471,0,245,507,3
GNAS_2_23733,114,64,32,74,11,12,847,0,25,118,7,122
A4GALT_2_23734,170,142,92,93,38,239,155,33,281,491,25,108
A4GNT_2_23735,177,82,120,110,267,69,604,46,144,106,210,207
AACS_2_23736,245,399,275,300,294,269,620,482,330,664,420,463
AADAC_2_23737,831,772,852,859,895,50,721,468,67,914,945,671
AARS2_2_23738,525,514,292,490,189,636,312,111,182,696,35,688
AARS_2_23739,1124,479,529,792,852,586,14,583,648,902,595,942
AASDH_2_23740,526,450,479,587,789,593,710,491,607,243,411,229
AASDHPPT_2_23741,0,0,0,0,0,0,0,0,0,0,0,0
AASS_2_23742,454,365,269,273,137,310,146,275,160,159,46,105
ABHD14A_2_23743,3,4,39,12,0,11,0,28,0,20,2,60
ABHD1_2_23744,256,288,225,218,110,269,337,289,645,224,119,68
ABHD3_2_23745,820,771,712,854,166,1078,935,729,143,568,764,1221
ABHD5_2_23746,267,185,116,173,105,122,25,206,22,340,232,38
ABHD6_2_23747,884,906,973,1248,991,290,656,1303,808,1711,1212,1237
ABHD8_2_23748,2,6,10,39,0,6,0,189,4,6,0,119
ABO_2_23749,41,87,37,76,31,0,0,0,4,0,72,3
ABP1_2_23750,57,38,48,35,68,0,10,3,141,10,0,0
ACAA2_2_23751,177,217,182,193,7,2,12,150,43,324,17,79
ACACB_2_23752,44,35,67,106,0,286,0,8,0,26,4,91
ACAD11_2_23753,1805,1652,1380,2110,1585,755,603,1752,1389,1404,921,177
0
ACAD8_2_23754,140,160,85,132,3,3,7,0,30,126,0,24
ACAD9_2_23755,886,569,557,944,1201,90,120,1527,258,470,609,757
ACADL_2_23756,1163,1376,999,1063,1504,1501,807,1490,2208,1156,419,1570
ACADSB_2_23757,170,170,169,348,29,261,414,1194,148,49,692,506
ACADS_2_23758,1232,1195,1184,990,307,1227,1439,884,1492,990,1391,1302
ACAT1_2_23759,229,170,300,287,585,0,3,1,177,499,0,852
ACER1_2_23760,231,224,198,250,138,148,0,832,93,48,30,462
ACER2_2_23761,156,132,70,110,293,29,4,3,9,411,0,72
ACER3_2_23762,88,64,103,152,83,21,11,18,20,11,6,65
ACMSD_2_23763,185,134,143,158,308,83,94,0,177,293,62,168
ACO1_2_23764,0,0,0,0,0,0,0,0,0,0,0,0
ACO2_2_23765,367,471,275,441,327,569,688,149,173,39,36,446
ACOT12_2_23766,123,26,69,130,16,32,194,12,9,1,23,0
ACOT1_2_23767,164,284,147,269,98,93,518,112,16,274,207,234
ACOT2_2_23768,833,625,674,959,475,540,560,140,1303,621,541,526
ACOT4_2_23769,198,417,353,401,99,285,1136,392,15,518,422,204
ACOT6_2_23770,79,39,91,106,65,55,390,42,0,4,1,29
ACOT8_2_23771,78,181,86,141,124,243,204,563,78,45,54,107
ACOX2_2_23772,264,236,91,167,347,285,166,360,709,58,142,13
ACOXL_2_23773,291,252,276,346,66,136,520,456,81,314,21,466
ACR_2_23774,28,18,13,44,6,328,1,20,0,12,0,0
ACSBG2_2_23775,532,439,396,420,383,961,226,776,365,689,291,357
ACSF2_2_23776,54,30,53,21,99,18,213,18,3,68,46,62
ACSL1_2_23777,705,551,647,673,972,360,652,417,444,670,395,727
ACSM1_2_23778,579,784,416,847,211,716,1219,185,750,102,959,571
ACSM2A_2_23779,284,290,423,201,641,94,234,211,140,126,224,4

ACSM4_2_23780,1303,924,901,1180,667,876,1068,1125,1675,1185,962,1219
ACSM5_2_23781,109,103,71,40,261,0,0,0,2,0,0,96
ACSS1_2_23782,554,613,529,573,31,36,209,276,240,643,454,662
ACSS3_2_23783,375,334,339,368,429,44,210,400,266,25,178,220
ACTC1_2_23784,133,303,253,218,578,145,322,134,117,109,19,382
ACY3_2_23785,344,249,175,266,199,54,163,652,249,293,162,507
ACYP2_2_23786,5,20,54,107,0,0,0,0,0,23,0,0
ADA_2_23787,504,430,577,577,674,216,104,326,4,543,55,153
ADAM10_2_23788,586,505,397,513,1358,336,1415,745,858,482,420,913
ADAM17_2_23789,1111,1235,1038,1236,990,2774,1508,653,1571,1543,813,101
1
ADAMTS4_2_23790,278,335,173,266,387,488,629,0,546,450,73,122
ADARB2_2_23791,41,67,51,27,14,9,5,1,1,35,4,39
ADAT1_2_23792,162,120,155,116,0,483,472,0,111,615,209,77
ADAT2_2_23793,1577,1394,1318,1658,1574,535,2387,834,1586,1867,716,2238
ADC_2_23794,172,255,191,232,0,175,226,23,213,444,368,205
ADCY1_2_23795,26,31,71,37,20,9,4,16,67,12,135,6
ADCY2_2_23796,63,73,149,82,2,0,184,37,33,114,126,13
ADCY7_2_23797,160,198,185,198,38,42,114,2,118,184,374,124
ADCY8_2_23798,129,148,70,50,1,0,0,163,24,61,14,86
ADCY9_2_23799,588,670,763,676,669,703,1249,656,1065,805,590,441
ADH1A_2_23800,344,374,248,420,216,33,129,502,496,1637,40,278
ADH1B_2_23801,1397,1201,1063,1287,626,1479,1130,1155,997,1916,902,886
ADH1C_2_23802,868,709,914,818,1089,566,1573,634,663,1006,710,1188
ADH4_2_23803,575,402,616,563,912,730,40,881,716,683,15,22
ADH5_2_23804,165,224,93,60,4,0,154,296,229,118,199,343
ADHFE1_2_23805,1136,777,1039,1039,2048,520,1365,1085,651,1529,1599,704
ADI1_2_23806,668,645,671,754,929,566,1018,805,472,134,680,480
ADO_2_23807,0,78,3,1,0,0,1,0,0,12,0,0
ADPRH_2_23808,14,7,10,22,0,0,1,0,242,80,0,0
ADPRHL2_2_23809,5,4,70,9,0,0,0,2,41,0,352,2
ADSS_2_23810,948,574,837,948,521,383,807,130,111,500,2213,1814
AEN_2_23811,396,440,417,457,221,242,748,203,271,496,353,441
AGBL2_2_23812,983,1084,819,1121,494,762,815,1667,1042,719,743,1648
AGMAT_2_23813,133,88,82,87,156,0,29,79,32,0,146,127
AGMO_2_23814,727,591,560,660,209,517,930,46,880,535,1589,1019
AGPAT4_2_23815,27,72,118,187,107,0,52,6,300,156,168,128
AGPAT5_2_23816,558,732,585,838,305,193,129,385,209,698,1246,1391
AGPAT6_2_23817,817,731,928,1113,953,1297,888,1126,661,1310,697,1597
AGPAT9_2_23818,791,672,744,870,2034,986,972,694,647,697,549,943
AGPS_2_23819,103,38,7,25,30,0,5,371,4,3,0,62
AGXT2_2_23820,393,240,300,283,255,413,1,881,47,56,188,6
AGXT2L2_2_23821,514,440,477,722,327,568,415,722,165,343,289,318
AGXT_2_23822,436,442,764,856,589,191,715,261,170,243,463,627
AICDA_2_23823,1220,1003,976,1231,1671,1022,1277,1151,1608,899,1105,118
9
AKR1B10_2_23824,228,194,214,305,66,38,31,320,181,148,852,138
AKR1B15_2_23825,84,98,70,119,0,65,356,44,190,154,199,93
AKR1B1_2_23826,313,418,378,488,506,76,328,411,502,433,122,520
AKR1C1_2_23827,1845,2139,1489,1882,1896,1369,2398,1150,1277,2240,445,1

190
AKR1C3_2_23828,141,137,139,188,0,0,312,90,123,126,10,92
AKR1C4_2_23829,210,124,123,135,434,239,210,105,296,150,9,98
AKR1E2_2_23830,183,186,181,182,18,0,435,76,189,127,7,99
AKR7A2_2_23831,461,457,319,459,271,568,805,593,175,694,909,178
AKR7A3_2_23832,66,171,103,143,20,20,32,21,77,40,496,5
ALAD_2_23833,31,70,83,37,48,352,0,24,6,0,0,8
ALDH1A1_2_23834,128,62,100,87,47,67,362,0,112,58,181,2
ALDH1A3_2_23835,561,563,368,843,473,498,753,1019,665,682,305,1106
ALDH1B1_2_23836,475,345,451,529,21,298,875,385,131,332,432,1180
ALDH1L1_2_23837,307,252,398,374,745,357,2,343,331,274,335,259
ALDH1L2_2_23838,1519,1447,1388,1628,1331,2098,745,1290,1972,2319,2033,
1676
ALDH6A1_2_23839,36,12,26,55,0,0,2,0,0,0,0,23
ALDH9A1_2_23840,72,311,244,307,73,41,477,534,241,306,969,382
ALDOB_2_23841,510,434,426,516,290,125,77,394,1155,509,224,240
ALDOC_2_23842,353,345,639,536,198,200,285,68,276,1057,1890,359
ALG10_2_23843,529,401,432,413,227,191,343,133,224,553,306,731
ALG11_2_23844,360,321,208,300,193,366,776,306,303,333,145,45
ALG12_2_23845,1162,1044,1012,1317,389,654,3613,2497,987,1215,910,2221
ALG14_2_23846,3270,3593,2306,3064,4483,1947,2361,2528,2965,5599,2276,4
069
ALG1_2_23847,126,65,86,76,17,80,48,73,10,18,0,39
ALG2_2_23848,103,95,14,85,472,226,85,7,73,57,9,7
ALG6_2_23849,1069,944,920,829,599,851,0,640,435,3,425,868
ALKBH8_2_23850,1202,1479,1355,1249,779,684,1061,894,1412,1987,1046,105
1
ALLC_2_23851,300,219,269,344,298,136,256,81,331,371,264,200
ALOX12B_2_23852,215,108,291,185,148,57,874,26,244,21,222,299
ALOX12_2_23853,821,497,596,654,72,462,237,288,868,996,743,670
ALOX15_2_23854,371,371,458,392,666,299,227,9,154,106,247,309
ALOX5_2_23855,241,125,113,83,0,2,0,160,168,375,1,1
AMDHD1_2_23856,159,163,182,330,1,0,201,736,266,4,11,335
AMY1B_2_23857,5003,4822,4727,5303,5984,4027,5641,4409,4590,6551,3209,6
376
AMY1C_2_23858,5003,4822,4727,5303,5984,4027,5641,4409,4590,6551,3209,6
376
AMY2A_2_23859,12885,11074,11511,13873,9341,7552,13601,11922,10004,9866
,8929,12719
AMY2B_2_23860,3036,2508,2713,2976,3054,2180,3086,3192,2485,2429,1368,2
293
ANPEP_2_23861,370,465,234,271,2,74,299,441,323,431,681,460
AOC3_2_23862,267,170,235,192,302,7,1830,26,9,87,39,6
AOX1_2_23863,734,772,848,855,2397,190,965,1915,1108,1133,424,863
APEH_2_23864,182,160,131,247,80,223,98,63,58,79,65,177
APEX2_2_23865,470,353,383,307,549,71,233,461,145,620,789,241
APIP_2_23866,140,85,85,77,19,114,1,3,121,28,29,28
APLF_2_23867,415,129,139,161,100,53,13,470,160,66,238,258
APOBEC1_2_23868,742,755,796,846,693,627,333,1298,1118,1367,1979,1184
APOBEC2_2_23869,39,3,2,51,9,0,1,0,11,1,0,40

APOBEC3B_2_23870,110,123,111,54,19,158,182,111,320,189,131,190
APOBEC3C_2_23871,30,16,32,33,1,12,0,64,42,0,0,0
APOBEC3G_2_23872,1008,710,733,964,403,585,640,633,991,637,812,1208
ARF3_2_23873,87,110,48,92,0,455,25,0,349,0,3,0
ARF4_2_23874,1421,995,1047,1357,632,736,1397,829,1628,1329,1063,1036
ARG2_2_23875,158,173,163,124,400,304,355,19,196,452,185,126
ARHGEF10_2_23876,23,23,10,50,40,0,0,90,23,1,0,82
ARL3_2_23877,1198,1054,1237,1156,738,978,1223,1808,582,983,1313,1666
ARL4C_2_23878,37,61,39,73,23,18,402,332,13,50,0,133
ARL4D_2_23879,476,499,389,564,754,104,310,1007,227,582,1347,551
ARL5B_2_23880,387,495,332,394,361,500,418,716,203,630,91,528
ARL8A_2_23881,264,253,215,285,305,221,44,117,136,285,23,88
ARL8B_2_23882,47,32,35,29,125,107,6,711,0,3,13,22
ARSD_2_23883,67,32,47,49,161,0,0,0,88,6,12,0
ARSE_2_23884,288,163,201,225,95,48,383,203,55,291,102,218
ARSG_2_23885,471,241,270,375,1005,252,182,405,334,1145,110,454
ARSH_2_23886,124,174,64,124,2,115,213,379,15,107,104,405
ARSI_2_23887,192,195,82,84,18,118,331,88,348,379,222,107
ARSJ_2_23888,391,274,315,393,59,181,1169,361,202,231,40,343
ARSK_2_23889,1492,1235,1314,1517,2109,1292,992,870,1114,1623,1095,1902
ART1_2_23890,203,151,144,238,651,344,51,12,140,22,959,101
ART4_2_23891,619,755,747,654,745,202,1631,0,181,35,1802,554
AS3MT_2_23892,513,343,336,405,127,621,218,934,534,469,0,303
ASNA1_2_23893,307,318,342,328,492,444,303,387,565,608,109,847
ASNSD1_2_23894,906,519,846,1112,779,460,485,473,1048,1344,1091,1585
ASPG_2_23895,31,13,27,70,357,0,0,29,0,10,0,4
ASPHD2_2_23896,311,169,234,228,670,400,323,246,669,207,394,214
ATAD1_2_23897,1429,1031,1225,1363,1668,607,3664,385,699,782,724,1385
ATIC_2_23898,145,118,139,103,165,358,78,120,292,144,367,190
ATP2C2_2_23899,143,54,165,183,150,15,64,33,216,49,40,5
AUH_2_23900,353,286,219,324,11,416,1136,277,347,78,271,169
AWAT1_2_23901,433,522,434,615,252,71,1765,715,476,438,1317,434
AWAT2_2_23902,303,260,221,249,94,175,408,290,414,108,197,341
B3GALNT2_2_23903,535,580,245,490,774,534,259,6,491,146,93,262
B3GALT1_2_23904,1333,965,1184,1540,992,995,594,1356,660,1305,805,1539
B3GALT2_2_23905,979,736,818,1048,1005,1656,611,579,620,638,645,933
B3GALT4_2_23906,114,57,169,146,240,0,90,220,36,310,1,398
B3GALT6_2_23907,3,19,12,21,0,0,10,0,0,0,67,1
B3GAT2_2_23908,140,146,180,109,6,160,151,165,220,156,70,6
B3GAT3_2_23909,88,159,243,347,55,0,479,107,47,213,593,369
B3GNT2_2_23910,213,216,211,357,294,195,334,304,67,290,328,141
B3GNT3_2_23911,361,475,356,287,542,363,487,285,561,669,124,82
B3GNT4_2_23912,772,549,751,621,779,1426,430,2176,745,943,343,257
B3GNT5_2_23913,152,127,75,71,0,3,8,0,89,1,296,258
B3GNT7_2_23914,229,142,230,134,32,40,725,584,270,204,611,260
B3GNT9_2_23915,577,510,387,312,1000,663,667,329,228,313,353,535
B3GNTL1_2_23916,330,305,213,272,489,427,94,335,829,278,29,441
B4GALNT1_2_23917,39,108,67,65,5,44,81,0,327,239,77,6
B4GALNT3_2_23918,288,714,361,378,742,50,65,176,181,399,263,891
B4GALNT4_2_23919,21,20,15,22,0,155,0,0,26,3,0,327

B4GALT1_2_23920,37,78,89,71,2,34,10,5,83,6,432,36
B4GALT5_2_23921,599,455,441,554,294,273,133,372,94,941,772,182
B4GALT6_2_23922,2580,2559,2307,2399,2212,1755,1862,2675,2549,4669,3136
,1711
B4GALT7_2_23923,62,34,30,91,96,104,50,524,245,95,0,6
BBOX1_2_23924,299,131,245,241,102,32,381,15,316,150,125,37
BCHE_2_23925,450,296,298,384,161,79,31,1163,322,457,216,178
BCMO1_2_23926,126,144,128,141,240,0,0,211,171,87,49,109
BDH2_2_23927,284,255,165,246,160,315,460,63,188,727,132,87
BHMT_2_23928,0,0,42,15,12,0,3,2,2,0,0,0
BLM_2_23929,64,121,150,286,0,476,10,0,326,0,0,453
BLMH_2_23930,536,633,625,608,968,285,56,791,716,903,584,331
BLVRA_2_23931,161,41,107,173,30,40,358,275,30,60,11,33
BLVRB_2_23932,176,231,105,178,130,122,219,443,360,46,158,517
BPHL_2_23933,519,236,136,204,576,264,503,0,225,229,60,518
BST1_2_23934,665,358,487,435,856,636,605,1960,729,244,746,701
BTD_2_23935,222,193,459,318,184,489,2,95,189,453,297,439
C12orf5_2_23936,1404,1597,1671,1798,399,508,734,990,1348,1881,943,1140
C16orf79_2_23937,175,102,170,161,128,29,0,43,359,0,3,7
C18orf56_2_23938,25,82,3,17,0,0,0,0,0,18,0,0
C1GALT1_2_23939,689,381,463,742,718,370,284,1170,516,567,394,328
C1R_2_23940,181,43,58,68,6,0,381,8,196,2,7,464
C22orf28_2_23941,181,391,210,381,39,252,351,2,127,569,820,898
C2orf43_2_23942,973,567,715,853,1402,1157,939,1390,563,620,284,863
C5orf4_2_23943,479,681,341,636,903,436,236,448,368,1100,552,293
C6orf130_2_23944,39,63,80,121,57,126,11,63,305,29,1,46
CA13_2_23945,143,210,130,223,0,0,1,0,262,0,0,223
CA14_2_23946,74,45,109,119,105,50,167,99,102,342,251,170
CA2_2_23947,176,40,63,176,358,1,3,97,315,22,1,41
CA3_2_23948,65,107,48,32,38,0,0,0,29,68,0,25
CA4_2_23949,173,210,97,258,1136,0,13,2,380,0,0,0
CA5A_2_23950,139,76,150,255,38,2,0,45,6,0,0,294
CA5B_2_23951,276,126,293,256,111,280,536,1,214,5,147,318
CA6_2_23952,477,389,518,527,525,535,692,828,151,450,414,438
CA8_2_23953,167,209,80,124,8,2,41,0,49,355,0,2
CA9_2_23954,44,15,35,56,9,178,0,0,0,2,26,12
CARS2_2_23955,152,142,97,81,48,96,120,1,196,61,67,321
CASD1_2_23956,415,331,505,478,147,151,799,523,422,262,847,577
CAT_2_23957,851,607,617,850,1295,386,518,673,920,715,1195,745
CBR1_2_23958,846,736,684,806,783,556,699,804,527,918,377,588
CBR3_2_23959,1603,1037,1226,1299,2077,1361,1770,1074,799,1496,1738,119
8
CBR4_2_23960,856,913,601,1082,406,816,1488,381,907,474,290,799
CCDC88B_2_23961,132,112,95,120,138,67,783,517,0,0,183,110
CCDC92_2_23962,650,281,336,505,393,231,46,137,525,311,1231,504
CCNO_2_23963,86,236,106,133,71,138,220,8,51,2,97,108
CCS_2_23964,275,202,350,243,178,54,319,332,559,304,291,238
CCT8_2_23965,691,491,533,522,59,210,567,100,361,565,510,760
CD274_2_23966,270,268,211,198,28,8,153,97,233,365,49,80
CD38_2_23967,742,545,595,759,675,633,1160,253,1312,1135,377,1024

CDA_2_23968,1821,1535,1421,1582,2327,656,2065,1492,1489,1509,1251,1933
CDIPT_2_23969,294,296,186,225,26,29,624,341,40,709,150,551
CDO1_2_23970,816,526,572,705,288,928,299,2008,508,612,204,650
CDS2_2_23971,721,458,683,780,494,421,563,1383,169,557,670,796
CELA1_2_23972,111,93,131,129,7,0,99,123,158,59,166,67
CELA2A_2_23973,716,836,714,590,893,188,464,372,310,998,617,445
CELA2B_2_23974,543,363,571,486,356,486,2,429,661,225,497,291
CELA3A_2_23975,8,80,20,0,0,0,0,0,0,0,0,0
CELA3B_2_23976,38,11,32,71,16,0,0,12,15,5,21,4
CEL_2_23977,102,69,170,138,75,81,202,13,30,295,155,19
CETN1_2_23978,41,58,106,49,448,85,0,0,11,59,1,16
CETN2_2_23979,730,500,564,774,1069,936,1354,920,470,1395,327,832
CETP_2_23980,64,56,39,46,226,23,92,27,11,0,0,39
CFB_2_23981,413,366,268,396,415,42,205,100,44,88,115,248
CFD_2_23982,107,58,43,48,3,3,5,32,0,0,13,16
CFI_2_23983,842,699,762,819,531,565,68,469,429,796,1087,719
CH25H_2_23984,698,669,614,576,112,356,1157,346,712,728,716,748
CHDH_2_23985,243,290,368,322,115,635,170,609,52,349,2,91
CHI3L1_2_23986,280,266,124,214,43,64,66,415,252,56,11,329
CHIT1_2_23987,2475,2292,1910,2047,2138,2451,1393,1459,1912,2193,1130,1
424
CHML_2_23988,1780,1925,1671,2016,1590,2835,1386,1061,1531,1615,2726,13
47
CHPF2_2_23989,109,105,139,178,16,60,176,162,337,25,20,143
CHPT1_2_23990,965,649,858,1033,1122,897,1647,454,847,641,305,901
CHST10_2_23991,203,476,331,600,0,0,284,135,36,57,935,378
CHST12_2_23992,59,12,19,1,0,0,0,459,0,0,0,0
CHST13_2_23993,415,370,301,355,1088,421,268,557,497,609,295,199
CHST14_2_23994,14,10,3,57,0,0,265,0,0,134,0,4
CHST1_2_23995,237,70,73,136,0,3,190,0,1,0,35,135
CHST2_2_23996,32,47,29,17,28,0,16,15,1,4,0,478
CHST3_2_23997,1081,751,724,791,748,1484,562,530,710,691,281,841
CHST5_2_23998,164,233,139,169,129,1,108,724,142,497,128,583
CHST6_2_23999,4,21,27,12,0,0,0,0,0,0,0,15
CHST7_2_24000,9,22,25,31,0,0,0,44,2,0,0,52
CHST9_2_24001,525,375,441,559,298,667,298,935,203,422,391,721
CHSY1_2_24002,727,752,593,840,1428,483,270,598,837,374,528,1100
CHSY3_2_24003,475,626,546,829,470,94,1234,821,434,189,720,877
CLPP_2_24004,726,537,490,553,56,146,836,1212,527,469,256,513
CLPX_2_24005,1090,834,585,811,661,693,263,1897,692,377,384,1473
CLYBL_2_24006,243,468,223,366,194,0,1400,0,60,25,824,9
CMA1_2_24007,177,63,76,118,101,95,191,106,219,108,24,56
CMAS_2_24008,592,495,350,540,1130,387,748,716,691,558,210,712
CMBL_2_24009,46,69,114,171,1,0,159,13,9,99,0,746
CNDP1_2_24010,195,119,177,162,118,103,586,158,225,391,7,133
CNTN6_2_24011,1335,1021,998,1401,1086,676,590,2190,982,950,607,1602
COIL_2_24012,272,314,345,303,206,596,264,134,799,139,251,248
COMTD1_2_24013,34,30,16,0,4,0,1,0,0,5,0,157
COQ2_2_24014,352,294,382,343,210,487,855,880,222,410,107,159
COQ3_2_24015,212,188,249,306,293,78,9,506,238,7,651,461

COQ5_2_24016,195,71,244,240,99,27,162,338,11,19,607,130
COX10_2_24017,524,508,510,485,376,592,275,329,993,659,117,563
COX17_2_24018,1727,1438,1302,1480,790,1318,541,1885,1942,1159,1934,156
4
COX4I1_2_24019,253,184,220,231,253,0,0,887,222,175,183,126
COX4I2_2_24020,1697,2006,1892,2138,2322,1693,2955,1420,1751,1980,2862,
3278
COX5A_2_24021,161,190,239,255,602,24,74,49,85,481,16,438
COX5B_2_24022,98,67,148,152,166,152,0,204,138,917,208,225
COX6A1_2_24023,229,280,175,160,150,86,474,193,390,203,14,130
COX6A2_2_24024,103,100,80,167,108,12,87,1,61,33,15,27
COX6B1_2_24025,284,219,176,219,291,2,16,2,204,3,0,165
COX6B2_2_24026,54,32,99,92,2,0,10,0,53,0,401,9
COX6C_2_24027,36,21,89,117,41,19,0,14,9,6,810,126
COX7A1_2_24028,96,53,101,69,4,74,16,54,1,20,102,59
COX7A2_2_24029,376,209,354,416,45,232,428,462,241,477,412,287
COX7A2L_2_24030,164,219,117,168,145,2,11,119,15,144,0,158
COX7B2_2_24031,103,82,89,89,56,51,1,28,161,123,0,96
COX7B_2_24032,933,540,724,612,371,336,1354,1398,382,118,794,687
COX7C_2_24033,286,232,189,300,209,149,129,294,305,40,12,220
COX8A_2_24034,48,35,25,46,1,0,0,42,0,1,0,86
COX8C_2_24035,86,71,38,120,328,3,253,2,115,155,14,50
CPA1_2_24036,508,576,466,446,526,238,543,645,445,519,624,1366
CPA2_2_24037,1676,1344,1330,1625,922,1461,1661,916,2462,1515,575,1696
CPB1_2_24038,68,152,84,182,60,236,0,231,0,23,32,187
CP_2_24039,358,304,172,313,368,278,67,108,326,3,377,326
CPE_2_24040,126,71,212,131,51,115,24,210,113,46,333,153
CPOX_2_24041,343,543,533,692,1006,216,990,331,679,1221,446,454
CPSF3_2_24042,1358,1251,1082,1189,1048,666,1528,1206,1552,1135,939,158
6
CPT2_2_24043,245,195,257,318,83,60,144,423,122,197,627,148
CRAT_2_24044,168,70,119,145,77,2,1,327,69,3,3,317
CREG2_2_24045,282,390,243,243,362,513,823,140,334,218,68,222
CRY1_2_24046,348,359,370,438,160,237,54,181,354,279,280,994
CRYL1_2_24047,198,219,237,174,178,368,535,376,247,192,287,37
CRYZL1_2_24048,222,205,165,483,224,180,148,1013,117,129,7,424
CSAD_2_24049,1352,1401,1640,1481,1668,968,1666,1112,1358,2150,2308,183
5
CS_2_24050,295,263,108,240,86,33,58,21,73,311,206,204
CSGALNACT2_2_24051,720,763,803,723,1342,831,1544,160,1121,967,1009,919
CSRP2BP_2_24052,197,64,165,163,62,3,358,231,72,49,14,23
CTBS_2_24053,1246,994,1013,1029,706,1129,913,1322,858,1663,412,788
CTRB1_2_24054,411,445,444,349,596,502,1658,879,1532,1043,131,509
CTRB2_2_24055,372,306,301,430,410,133,489,378,512,781,447,89
CTRC_2_24056,191,61,110,174,13,0,0,354,32,2,0,0
CTSD_2_24057,85,69,130,157,662,206,20,70,33,78,33,101
CTSF_2_24058,47,64,26,58,390,22,4,51,54,2,0,118
CTSG_2_24059,120,155,202,147,207,59,17,275,69,274,0,56
CTSH_2_24060,265,284,194,250,1095,0,362,27,212,838,62,416
CTSK_2_24061,175,66,48,74,4,169,280,19,38,42,1,56

CTSZ_2_24062,310,248,148,405,949,218,444,10,358,764,29,485
CWC27_2_24063,757,975,819,1299,672,1377,1518,1333,973,917,1009,1787
CXorf21_2_24064,81,198,112,189,221,45,107,0,53,134,403,4
CYB561D2_2_24065,40,161,35,25,68,0,58,57,30,47,3,90
CYB5B_2_24066,462,386,423,518,160,57,461,338,655,1090,595,805
CYB5R1_2_24067,168,93,122,70,150,13,351,21,374,322,904,190
CYB5R2_2_24068,1166,897,926,1287,797,384,613,1048,1205,855,2182,1377
CYB5R4_2_24069,1106,829,1148,1234,988,1366,1802,1229,884,1391,891,483
CYBA_2_24070,797,605,436,708,663,309,660,1147,169,1101,497,269
CYBB_2_24071,739,825,553,819,858,548,617,243,478,861,259,936
CYC1_2_24072,412,368,331,264,202,48,514,50,31,173,554,500
CYCS_2_24073,1370,1306,1260,1613,1393,1482,1242,1828,1649,1284,1618,19
69
CYP11B2_2_24074,48,52,43,5,256,1,22,57,0,37,25,27
CYP17A1_2_24075,473,399,498,512,825,195,1815,343,573,626,1324,717
CYP1A1_2_24076,140,124,81,114,36,0,78,0,33,102,445,7
CYP1A2_2_24077,423,366,372,468,484,199,331,197,325,450,652,474
CYP1B1_2_24078,371,526,517,714,30,547,2,0,393,907,1333,186
CYP20A1_2_24079,243,218,222,215,151,61,1,297,199,292,207,629
CYP26B1_2_24080,24,9,9,32,0,0,0,0,21,4,0,201
CYP26C1_2_24081,263,359,291,406,371,7,65,794,945,299,135,315
CYP27A1_2_24082,380,381,594,671,284,651,249,127,80,404,308,20
CYP27B1_2_24083,536,421,529,685,507,195,1078,180,332,261,1265,295
CYP2A13_2_24084,1030,687,514,797,499,490,755,669,421,356,1512,886
CYP2A6_2_24085,246,51,140,247,53,139,2,489,9,266,95,199
CYP2B6_2_24086,334,459,269,348,123,16,80,98,148,25,442,155
CYP2C19_2_24087,544,431,618,478,115,579,791,367,782,634,711,847
CYP2C9_2_24088,544,431,618,478,115,579,791,367,782,634,711,847
CYP2E1_2_24089,1459,1071,1344,1351,1204,1202,2121,1029,1292,1489,1907,
1036
CYP2F1_2_24090,45,47,65,54,0,0,172,147,18,105,30,10
CYP2J2_2_24091,2754,2496,2307,2922,3535,3475,2782,3917,2447,3476,1144,
3235
CYP2R1_2_24092,1050,1185,1075,1339,1036,117,1956,1583,899,1441,3294,14
60
CYP2S1_2_24093,173,68,143,181,345,23,27,94,157,2,470,129
CYP2U1_2_24094,908,702,621,877,1254,514,1165,433,431,783,526,777
CYP2W1_2_24095,40,124,103,124,8,1,0,0,0,0,2,0
CYP39A1_2_24096,309,333,464,360,89,24,506,316,437,197,440,591
CYP3A7_2_24097,1330,1063,1090,1273,257,1422,699,1560,1072,523,1185,999
CYP46A1_2_24098,55,28,54,85,0,59,1,9,147,1,105,336
CYP4A11_2_24099,392,388,416,452,583,10,364,354,524,769,703,1115
CYP4A22_2_24100,0,0,0,0,0,0,0,0,0,0,0,0
CYP4F12_2_24101,39,19,1,3,6,37,5,0,0,0,0,126
CYP4F22_2_24102,449,642,521,600,568,880,357,427,535,502,565,933
CYP4F2_2_24103,276,156,234,172,271,92,150,30,99,123,121,158
CYP4F8_2_24104,633,693,582,857,555,1310,1303,490,1153,737,596,1211
CYP4V2_2_24105,1609,837,1213,1147,1095,1362,1656,1497,1493,1095,830,10
17
CYP4X1_2_24106,489,322,301,546,868,383,135,346,459,368,239,725

CYP4Z1_2_24107,219,196,209,197,343,74,73,400,306,262,0,195
CYP7A1_2_24108,246,167,213,272,101,590,746,364,1,130,236,168
CYP7B1_2_24109,1461,1486,962,1592,665,1668,730,688,1097,1563,1030,1727
CYP8B1_2_24110,620,501,347,722,311,237,781,1246,463,782,77,389
DAD1_2_24111,475,399,345,674,8,76,433,45,758,627,161,654
DAGLA_2_24112,28,33,17,53,117,0,0,0,10,44,193,2
DAK_2_24113,447,218,157,530,289,145,125,31,467,171,1171,197
DARS2_2_24114,0,0,0,0,0,0,0,0,0,0,0,1
DARS_2_24115,95,198,160,103,68,377,154,106,175,119,2,93
DBH_2_24116,123,66,71,121,0,18,40,115,273,135,453,1
DBR1_2_24117,3877,3217,3172,3515,2522,3288,2921,2742,3087,5127,5261,32
72
DBT_2_24118,637,625,713,1109,755,556,757,121,898,366,1460,1023
DCLRE1B_2_24119,972,684,656,902,943,444,346,1343,540,636,648,1083
DCPS_2_24120,571,383,391,491,834,848,992,54,243,590,404,415
DCTN6_2_24121,906,956,766,1127,296,196,2147,483,674,1223,976,1107
DCTPP1_2_24122,20,41,62,44,3,8,21,0,76,32,0,4
DDAH2_2_24123,13,35,69,10,11,0,0,39,0,5,322,62
DDOST_2_24124,21,152,115,103,90,19,0,0,191,0,234,16
DDX10_2_24125,468,431,355,358,384,492,165,423,629,553,451,397
DDX18_2_24126,531,726,431,710,486,700,311,1038,836,398,166,843
DDX19A_2_24127,350,282,276,251,336,75,79,192,243,470,3,526
DDX1_2_24128,275,345,204,314,279,172,42,435,614,55,273,135
DDX20_2_24129,351,201,265,311,190,532,117,719,100,214,85,396
DDX21_2_24130,247,154,166,185,288,56,48,212,268,339,0,55
DDX23_2_24131,756,538,521,764,635,716,535,210,464,286,1850,679
DDX24_2_24132,232,153,114,325,172,69,34,121,66,341,49,40
DDX25_2_24133,679,720,656,847,143,1338,389,0,304,776,1472,1001
DDX27_2_24134,46,110,99,200,34,296,172,517,38,107,1,67
DDX28_2_24135,1141,734,796,849,1049,595,395,564,531,790,1119,788
DDX39A_2_24136,223,177,131,302,96,32,183,180,309,450,5,221
DDX41_2_24137,25,1,13,56,38,0,5,124,21,15,0,0
DDX43_2_24138,800,646,667,572,872,279,688,336,775,199,810,561
DDX46_2_24139,1089,635,760,735,740,132,1240,1325,843,378,1324,1127
DDX49_2_24140,51,91,81,134,0,0,129,46,0,457,0,0
DDX50_2_24141,136,50,79,100,142,185,897,313,1,64,287,117
DDX51_2_24142,3,9,5,7,42,0,0,301,0,0,0,1
DDX52_2_24143,408,445,379,536,302,385,441,612,181,282,1676,609
DDX53_2_24144,127,159,252,177,333,230,111,711,115,168,36,225
DDX55_2_24145,1002,945,844,1173,1453,400,1180,383,1155,1175,985,746
DDX56_2_24146,378,369,465,520,379,292,888,721,657,246,363,350
DDX58_2_24147,155,200,187,179,20,298,532,241,156,1,446,23
DDX59_2_24148,232,165,200,194,14,0,163,87,449,74,423,116
DDX5_2_24149,555,589,597,673,480,739,545,414,435,379,461,1060
DDX60_2_24150,3015,3370,3000,3156,3502,2630,4450,4247,2084,5521,2767,4
188
DDX6_2_24151,21,9,67,30,14,11,138,0,74,0,1,1
DECR1_2_24152,448,424,348,361,304,361,320,708,76,483,517,203
DECR2_2_24153,17,23,17,50,0,0,1,142,52,0,0,12
DEGS1_2_24154,7966,6835,6301,7772,6446,4686,6091,8162,5526,7976,6256,8

376
DEGS2_2_24155,22,12,38,12,0,0,0,0,0,0,0,0
DERA_2_24156,406,678,605,972,548,62,345,110,136,287,22,693
DFFB_2_24157,15,20,23,50,0,0,0,50,0,0,0,32
DGAT1_2_24158,33,35,32,12,52,8,29,9,0,2,0,1
DGAT2_2_24159,839,626,758,940,774,159,385,951,1644,504,1610,803
DHCR24_2_24160,175,160,143,235,336,402,64,263,886,39,0,155
DHDH_2_24161,64,7,15,59,1,0,372,0,13,7,36,207
DHFR_2_24162,1134,1169,1138,1637,859,851,335,1415,1077,1238,1720,977
DHODH_2_24163,54,58,89,105,49,191,42,9,142,171,1,44
DHRS13_2_24164,84,67,174,74,161,34,112,18,235,277,0,255
DHRS3_2_24165,7,11,23,42,0,0,9,0,0,0,0,75
DHRS4_2_24166,110,54,123,75,391,16,319,3,26,0,252,112
DHRS7_2_24167,197,220,236,231,1,340,325,368,38,14,15,275
DHRSX_2_24168,99,185,96,162,508,149,344,336,116,222,66,278
DHTKD1_2_24169,511,387,259,361,486,149,878,83,1015,641,336,253
DHX15_2_24170,447,378,380,569,365,57,201,434,391,236,64,835
DHX29_2_24171,309,142,237,221,403,197,344,290,279,226,83,559
DHX32_2_24172,11,0,50,1,0,0,0,0,0,0,0,0
DHX34_2_24173,68,56,67,55,2,3,0,17,16,48,443,70
DHX37_2_24174,518,454,361,431,413,359,596,488,423,365,277,235
DHX38_2_24175,86,102,73,112,2,10,0,404,31,104,45,89
DHX57_2_24176,178,474,416,444,223,256,268,273,228,316,373,750
DHX58_2_24177,238,187,114,224,149,136,170,332,135,89,164,168
DHX8_2_24178,33,64,103,73,50,0,0,0,50,2,397,8
DHX9_2_24179,175,85,117,172,134,115,241,48,1,355,357,181
DIO3_2_24180,457,382,392,378,82,0,422,417,89,165,391,678
DIRAS1_2_24181,1,23,11,8,0,2,0,0,0,0,0,11
DIRAS2_2_24182,159,134,85,145,134,18,93,217,51,232,175,209
DIRAS3_2_24183,261,117,139,165,35,150,98,3,51,5,0,311
DIS3L2_2_24184,71,21,47,49,31,0,31,5,128,0,5,0
DLAT_2_24185,316,245,307,366,86,454,723,32,195,579,904,191
DLD_2_24186,289,198,243,253,56,162,159,518,197,329,157,199
DLL1_2_24187,136,105,153,123,79,42,456,56,85,29,64,221
DLST_2_24188,486,567,410,594,355,555,191,685,657,478,660,912
DMGDH_2_24189,17,21,53,15,0,0,10,5,75,0,86,0
DNA2_2_24190,181,134,179,184,67,162,296,284,128,385,11,43
DNAH11_2_24191,1881,2146,1600,2150,1143,1499,3266,1604,2843,2015,1602,
1777
DNAH3_2_24192,325,153,228,277,472,105,63,11,169,15,322,669
DNAH5_2_24193,366,218,330,526,143,27,220,725,253,816,48,29
DNAH8_2_24194,86,63,115,65,129,57,9,172,471,3,2,67
DNAJA2_2_24195,249,207,130,167,94,184,0,1,105,30,12,235
DNAJB14_2_24196,284,357,275,420,78,319,194,229,635,167,186,122
DNAJC10_2_24197,1381,1165,1119,1433,1031,927,2195,2179,1149,1222,1478,
1851
DNAJC18_2_24198,139,106,264,183,505,371,563,361,196,133,221,184
DNAL4_2_24199,149,280,93,198,0,3,308,285,396,18,114,2
DNASE1_2_24200,35,26,24,178,0,9,81,86,0,152,0,26
DNASE1L2_2_24201,218,256,108,185,10,76,16,872,560,356,237,451

DNASE1L3_2_24202,502,470,403,851,353,529,477,942,318,161,343,936
DNASE2_2_24203,241,242,172,180,295,92,17,16,23,79,138,154
DNPEP_2_24204,109,56,35,73,133,14,8,37,34,29,526,27
DPAGT1_2_24205,1214,1065,904,928,981,417,1312,1529,1341,1065,2750,1381
DPEP2_2_24206,337,463,374,507,669,561,272,114,292,391,456,622
DPM1_2_24207,376,258,393,344,286,595,338,246,617,606,913,219
DPM2_2_24208,212,287,458,408,688,2,832,270,184,712,1527,117
DPP4_2_24209,20,0,3,0,0,0,0,0,0,58,0,0
DPP7_2_24210,19,13,69,68,0,0,205,4,174,0,0,48
DPP9_2_24211,137,42,45,94,3,4,63,1,60,294,5,77
DPYS_2_24212,527,201,259,399,259,343,410,83,478,93,17,264
DPYSL4_2_24213,14,11,18,35,19,4,26,0,16,4,28,5
DPYSL5_2_24214,149,65,114,120,7,138,40,58,13,154,2,22
DQX1_2_24215,270,323,185,316,244,290,1124,613,358,904,119,620
DSEL_2_24216,502,355,465,517,27,576,394,467,579,221,271,351
DTD1_2_24217,119,104,46,53,72,104,15,43,202,10,4,48
DUOX2_2_24218,197,162,223,207,58,355,423,104,103,326,229,255
DUPD1_2_24219,394,254,169,252,428,177,26,284,207,194,0,233
DUSP28_2_24220,479,482,503,455,186,46,1609,728,464,325,148,703
EARS2_2_24221,132,256,204,207,230,3,25,2,0,227,516,49
EBPL_2_24222,84,190,306,428,3,7,277,2,153,502,401,616
ECH1_2_24223,44,13,30,44,71,22,0,59,88,10,5,208
ECHDC3_2_24224,870,523,411,515,812,1043,92,860,1206,1388,713,320
ECHS1_2_24225,65,21,46,54,24,22,33,0,31,162,0,0
EDEM1_2_24226,103,196,143,153,564,75,127,84,78,405,35,24
EDEM3_2_24227,605,269,421,402,131,169,536,10,40,308,81,351
EEF1A1_2_24228,778,351,728,811,191,207,553,1559,345,379,1025,612
EEF1A2_2_24229,204,118,299,334,173,0,173,599,153,562,203,128
EEF2_2_24230,2236,2714,2420,2112,2468,927,1776,2847,2519,2639,4375,187
8
EGLN1_2_24231,774,571,453,780,660,666,731,589,136,214,586,760
EGLN3_2_24232,687,587,707,632,905,724,661,487,292,422,184,593
EHD4_2_24233,732,849,780,571,248,540,1250,587,469,666,697,585
EIF4A3_2_24234,35,95,88,182,41,0,67,361,43,0,0,13
ELAC1_2_24235,104,131,185,157,65,120,132,225,49,24,680,41
ELANE_2_24236,51,178,51,90,0,0,347,0,82,297,121,0
ELOVL1_2_24237,506,460,556,652,395,1420,712,84,267,588,1153,775
ELOVL2_2_24238,1671,1267,1079,1437,1318,1756,1507,851,1817,1611,1335,7
88
ELOVL3_2_24239,354,258,175,223,163,80,165,285,690,341,102,343
ELOVL4_2_24240,227,132,107,113,675,22,149,39,45,122,7,191
ENDOD1_2_24241,634,382,399,495,1172,528,578,571,744,347,1062,134
ENDOG_2_24242,236,261,192,188,33,150,358,47,240,63,154,116
ENGASE_2_24243,147,178,56,152,321,47,83,0,42,1,73,109
ENO2_2_24244,28,45,55,120,10,1,0,28,36,64,0,20
ENO4_2_24245,320,650,462,448,582,504,283,277,505,1603,62,399
ENOPH1_2_24246,946,761,834,1036,538,1763,932,952,967,700,1970,1464
ENPEP_2_24247,145,161,99,197,146,10,44,228,62,624,332,1
ENPP1_2_24248,706,519,436,541,346,1034,73,277,684,65,283,65
ENPP3_2_24249,259,317,207,242,143,333,51,682,164,51,5,125

ENPP4_2_24250,432,579,244,442,198,3,91,1062,121,324,1070,349
ENPP5_2_24251,326,458,338,275,197,394,388,299,573,331,599,396
ENPP6_2_24252,346,537,299,388,666,389,1080,773,196,222,405,572
ENPP7_2_24253,273,77,275,439,0,22,256,16,71,15,1,628
ENTPD3_2_24254,535,633,537,594,344,1121,697,18,313,623,1383,760
ENTPD5_2_24255,11,117,27,56,0,0,0,10,100,278,0,0
ENTPD7_2_24256,32,14,39,39,0,2,0,113,2,26,0,0
EPHX2_2_24257,635,626,735,696,421,481,2282,269,346,1324,483,854
EPHX4_2_24258,461,404,502,452,379,30,3,566,703,648,707,884
EPRS_2_24259,545,409,557,459,563,520,633,218,602,816,135,489
EPT1_2_24260,53,35,19,45,193,50,2,0,18,1,0,35
EPX_2_24261,418,237,390,393,330,830,689,568,254,139,494,301
ERAS_2_24262,131,116,153,214,80,360,79,44,241,20,135,54
ERCC3_2_24263,525,503,367,539,472,1016,292,252,796,423,353,400
ERCC4_2_24264,314,304,272,198,137,213,261,302,309,262,666,378
ERCC5_2_24265,66,136,191,117,0,0,0,0,0,0,319,280
ERI1_2_24266,878,713,748,727,164,608,758,1042,946,900,701,678
ERO1L_2_24267,1124,1557,1307,1356,2158,1184,2000,1164,1055,610,1898,13
76
ERP44_2_24268,880,774,1093,1223,941,844,1058,577,682,731,1002,1277
ESD_2_24269,517,681,663,711,433,1387,725,972,463,1330,664,1208
ESPL1_2_24270,224,111,415,271,15,0,117,0,0,5,715,458
ETFDH_2_24271,227,156,204,131,114,182,517,162,201,206,92,21
EXOSC1_2_24272,246,275,185,246,146,177,719,191,107,622,411,527
EXOSC2_2_24273,579,453,437,603,144,292,325,59,1241,218,258,187
EXOSC4_2_24274,92,109,139,211,405,217,29,451,598,346,1,483
EXOSC5_2_24275,181,135,60,113,97,0,144,104,18,822,29,38
EXOSC7_2_24276,860,652,509,729,1167,198,548,50,1180,291,1440,191
EXOSC8_2_24277,455,266,183,383,248,34,1910,434,1004,516,64,357
EXT1_2_24278,67,51,32,85,1,0,105,0,28,158,0,16
EXTL1_2_24279,211,162,102,100,152,152,77,18,21,523,45,54
EXTL3_2_24280,178,102,199,128,31,186,380,24,76,203,709,386
F10_2_24281,184,75,122,201,321,248,235,56,123,512,128,118
F12_2_24282,507,318,345,430,487,879,498,255,239,287,54,795
F13A1_2_24283,1406,1348,1399,1382,579,1356,1342,629,1297,1465,2074,117
4
F13B_2_24284,42,264,45,150,230,13,3,479,207,3,0,220
F2_2_24285,319,148,373,366,265,249,842,547,212,29,492,650
F5_2_24286,102,56,35,80,336,42,103,49,168,2,74,199
F9_2_24287,263,259,385,386,391,218,463,234,12,384,449,290
FA2H_2_24288,477,298,257,290,149,170,133,403,328,45,72,1283
FAAH2_2_24289,1958,1919,1661,2247,1825,1454,1755,1333,1372,1347,1698,1
706
FADS1_2_24290,470,643,407,655,420,697,235,537,369,964,497,314
FADS2_2_24291,724,629,625,1056,1251,1276,182,738,250,617,1312,774
FADS3_2_24292,163,146,121,212,241,109,157,75,148,565,0,10
FAHD2A_2_24293,449,579,617,528,326,346,1730,1382,447,1288,255,1229
FAM108C1_2_24294,267,279,310,374,315,481,139,454,337,197,177,448
FAM135B_2_24295,337,430,323,495,170,966,114,649,174,199,51,944
FAM20B_2_24296,105,105,76,154,66,168,0,205,131,16,399,102

FANCM_2_24297,2257,1745,1599,2359,1812,1887,1385,825,985,1244,1423,272
1
FAR1_2_24298,119,86,157,155,302,3,298,4,39,0,204,634
FAR2_2_24299,1012,769,913,1000,784,236,677,703,612,449,933,543
FARS2_2_24300,449,319,264,284,318,61,253,248,803,512,306,750
FARSA_2_24301,189,316,283,297,219,287,265,20,63,6,544,258
FARSB_2_24302,1129,883,747,838,1155,549,861,262,726,636,513,1135
FASN_2_24303,226,146,160,188,647,609,571,186,381,141,279,131
FDFT1_2_24304,390,241,344,269,1052,86,659,193,162,404,31,601
FEN1_2_24305,385,216,236,401,205,58,1258,380,463,136,112,378
FH_2_24306,1077,889,929,1051,523,596,2163,709,670,1713,1595,1034
FIG4_2_24307,2045,2079,1882,1839,1874,1760,2228,1987,2056,2454,1153,13
84
FKBP10_2_24308,241,96,179,156,623,490,292,1027,398,175,379,104
FKBP14_2_24309,1207,982,1255,1276,501,1020,1105,1246,1186,371,2238,125
4
FKBP15_2_24310,117,206,162,127,20,307,133,142,36,24,572,210
FKBP3_2_24311,869,613,861,880,872,230,869,868,301,464,598,721
FKBP4_2_24312,125,87,109,83,416,161,139,0,92,18,172,0
FKBP8_2_24313,447,461,403,450,946,553,307,789,279,1186,125,746
FMO1_2_24314,115,131,258,227,0,7,574,173,298,63,207,167
FMO4_2_24315,697,1028,573,864,1441,291,273,55,770,293,393,666
FNBP1_2_24316,546,471,583,409,256,210,843,381,313,539,570,438
FNTA_2_24317,257,172,250,353,817,661,617,139,695,487,801,145
FNTB_2_24318,206,299,131,251,9,107,310,39,718,285,76,310
FTH1_2_24319,197,203,218,349,231,272,810,270,42,543,444,281
FTMT_2_24320,592,622,532,555,472,470,356,79,54,296,361,947
FTSJ2_2_24321,93,78,153,23,0,367,0,36,9,0,5,2
FTSJ3_2_24322,175,251,171,203,142,2,162,79,158,2,320,155
FTSJD2_2_24323,105,60,93,55,2,0,221,0,16,158,663,72
FUCA1_2_24324,432,224,503,438,427,457,174,1157,125,133,51,422
FUCA2_2_24325,1937,1635,1955,2154,953,782,2067,1341,2079,3456,2199,239
4
FURIN_2_24326,287,76,225,234,1174,0,0,0,46,231,495,246
FUT10_2_24327,1308,1374,1140,1476,1520,1290,341,590,1215,857,1735,1147
FUT11_2_24328,25,93,32,55,9,183,1,3,38,0,0,0
FUT1_2_24329,52,31,54,54,7,58,8,62,27,53,25,164
FUT4_2_24330,796,558,574,477,518,377,2008,666,472,293,1238,306
FUT5_2_24331,428,295,309,294,418,63,105,59,446,286,287,266
FUT7_2_24332,27,140,177,234,0,107,69,0,158,248,0,137
FUT9_2_24333,256,290,126,249,4,289,118,142,444,197,0,72
GADL1_2_24334,428,414,392,452,343,297,806,169,90,331,741,795
GAL3ST1_2_24335,191,251,152,268,108,150,155,7,66,85,120,256
GAL3ST3_2_24336,23,2,11,69,25,0,0,42,34,0,0,102
GAL3ST4_2_24337,41,26,38,64,0,0,0,0,26,12,0,595
GALM_2_24338,135,143,82,243,374,38,100,13,121,83,17,179
GALNS_2_24339,36,4,36,31,11,41,0,108,0,98,0,107
GALNT10_2_24340,150,123,136,146,4,12,253,265,15,50,271,85
GALNT11_2_24341,291,301,177,313,212,171,341,152,91,74,189,305
GALNT12_2_24342,24,116,41,37,0,67,3,92,1,0,7,51

GALNT13_2_24343,8,5,20,4,119,35,0,187,16,8,0,0
GALNT14_2_24344,21,8,10,27,0,142,7,0,0,0,0,7
GALNT1_2_24345,690,718,596,688,946,1044,539,1590,543,464,1355,447
GALNT2_2_24346,921,924,917,964,1156,1140,598,125,1451,1156,2962,1192
GALNT3_2_24347,1953,1851,1769,2288,2140,1469,2519,1491,2113,1835,1143,
2255
GALNT4_2_24348,541,470,445,488,181,384,659,150,329,972,415,530
GALNT5_2_24349,331,247,231,222,352,183,1070,580,394,304,455,621
GALNT6_2_24350,277,260,290,281,391,16,813,209,184,226,94,339
GALNT8_2_24351,15,5,6,11,0,0,0,17,0,0,0,0
GALNTL2_2_24352,184,123,56,206,41,201,163,1056,52,155,0,254
GALNTL4_2_24353,199,379,291,446,190,0,541,272,0,265,6,771
GALNTL5_2_24354,1060,624,757,995,808,222,220,808,1367,671,844,1111
GALNTL6_2_24355,200,288,129,234,47,86,21,29,50,24,0,40
GALT_2_24356,444,644,546,610,201,510,1625,805,616,290,1189,315
GANC_2_24357,238,264,248,356,657,87,169,590,822,3,574,1465
GAPDH_2_24358,231,236,301,468,420,394,159,543,739,317,37,832
GAPDHS_2_24359,334,310,480,455,11,415,53,58,359,35,1404,474
GARS_2_24360,335,266,252,235,111,359,272,338,176,41,520,469
GATC_2_24361,1058,876,795,915,938,556,306,1700,796,604,1099,866
GATM_2_24362,22,6,13,13,12,0,0,0,0,16,4,0
GBA2_2_24363,393,228,266,355,413,97,192,425,297,81,34,279
GBE1_2_24364,495,510,319,462,191,120,388,894,396,268,67,486
GBGT1_2_24365,126,37,61,126,10,71,533,38,63,93,2,8
GBP1_2_24366,69,215,78,178,0,445,30,217,7,267,40,247
GBP2_2_24367,94,82,79,55,72,98,193,13,191,139,336,52
GBP3_2_24368,386,381,377,380,510,1233,731,395,390,335,228,535
GBP4_2_24369,715,723,846,761,574,269,756,897,1154,901,690,940
GBP6_2_24370,809,664,1010,981,1030,396,741,1029,1087,394,1246,558
GBP7_2_24371,687,724,817,751,577,267,758,896,1160,907,692,940
GCLM_2_24372,351,268,183,432,364,23,303,66,127,397,119,172
GCNT3_2_24373,160,214,295,309,442,12,22,32,362,422,272,205
GCNT4_2_24374,859,668,686,833,542,385,638,1041,1039,409,709,1284
GCSH_2_24375,71,86,83,118,57,0,262,156,7,10,1,172
GDE1_2_24376,120,145,155,128,285,161,1,131,201,0,55,8
GDPD3_2_24377,117,98,90,81,165,667,235,106,7,139,225,48
GFM1_2_24378,1016,952,1023,1209,1090,99,2485,1738,1289,1328,1714,1113
GFOD2_2_24379,157,101,187,141,263,75,281,255,41,3,581,104
GFPT1_2_24380,79,125,56,85,37,148,105,53,232,180,0,0
GFPT2_2_24381,663,586,915,662,368,74,1890,454,752,859,667,1039
GGH_2_24382,109,119,118,118,2,422,2,359,5,28,311,139
GGPS1_2_24383,257,252,138,62,13,621,15,419,46,264,482,14
GGT7_2_24384,61,63,25,126,339,0,0,197,245,0,0,0
GGTLC2_2_24385,116,125,23,120,0,211,0,147,15,90,369,78
GLCE_2_24386,247,160,201,305,97,13,478,135,186,194,0,195
GLDC_2_24387,321,322,200,292,743,102,540,634,572,222,161,668
GLO1_2_24388,53,137,100,88,428,119,139,336,162,167,102,208
GLOD4_2_24389,736,612,701,839,1255,425,973,846,1288,770,476,555
GLS2_2_24390,406,418,439,487,135,481,872,1204,130,945,602,411
GLT1D1_2_24391,65,67,66,145,158,1,254,225,60,229,8,208

GLT25D1_2_24392,217,241,303,547,1027,124,22,6,0,304,148,5
GLT25D2_2_24393,387,231,408,362,428,0,313,23,550,270,27,621
GLT8D2_2_24394,270,390,402,344,1095,243,127,90,372,584,436,454
GLUD1_2_24395,1482,1519,1386,1550,1571,2193,1266,1259,1489,1221,974,28
41
GLUD2_2_24396,103,142,89,124,12,183,200,5,55,175,0,113
GMDS_2_24397,2442,1981,1980,2130,1935,1161,1521,1748,1893,2732,1439,31
76
GMIP_2_24398,106,86,118,236,0,53,117,0,82,0,1,25
GMPR_2_24399,1691,1399,1201,1361,829,1167,1840,1583,1211,1956,1369,114
5
GMPS_2_24400,128,267,198,286,391,6,99,101,61,91,1,502
GNA11_2_24401,113,94,74,93,4,104,2,31,52,49,0,90
GNA12_2_24402,19,9,20,20,2,0,0,0,0,2,0,178
GNA13_2_24403,87,45,102,53,96,0,396,195,8,147,0,15
GNA14_2_24404,91,69,83,182,0,28,3,15,419,100,0,423
GNA15_2_24405,414,275,345,290,355,488,26,352,411,471,1212,0
GNAI1_2_24406,1101,781,647,1246,1119,788,1627,1837,1203,1300,958,1451
GNAI3_2_24407,211,100,145,170,357,0,379,338,1,159,5,154
GNAQ_2_24408,2557,2170,2236,2903,2215,1241,3047,2820,2836,1590,2830,31
86
GNAT2_2_24409,511,288,659,642,1130,517,868,260,451,5,1119,383
GNAT3_2_24410,181,236,214,292,85,126,14,46,91,85,154,245
GNAZ_2_24411,78,66,72,83,18,203,48,113,50,102,0,111
GNB1_2_24412,1258,860,694,1191,652,1193,671,1741,699,694,849,524
GNB2L1_2_24413,18,66,33,26,131,11,0,0,18,34,0,31
GNB3_2_24414,63,63,82,107,55,11,597,99,11,61,84,27
GNB4_2_24415,124,31,114,124,203,83,98,16,65,20,0,252
GNG11_2_24416,82,100,194,125,54,60,52,0,34,146,37,146
GNG12_2_24417,384,270,239,351,317,56,92,382,240,263,290,236
GNG13_2_24418,159,74,93,155,0,16,249,51,79,18,943,676
GNG2_2_24419,41,28,29,81,19,1,16,263,150,304,0,6
GNG3_2_24420,108,74,56,70,2,245,0,342,167,8,1,34
GNG7_2_24421,211,348,363,327,47,288,24,126,89,489,2,186
GNG8_2_24422,573,466,551,471,998,848,703,432,1121,573,643,977
GNGT1_2_24423,710,513,622,687,604,255,517,260,432,266,1366,404
GNL2_2_24424,340,229,314,425,252,351,318,115,423,345,289,335
GNMT_2_24425,148,224,189,293,9,448,292,181,75,636,585,543
GNPAT_2_24426,282,280,160,236,513,489,11,3,513,746,689,156
GNPDA1_2_24427,483,498,444,615,497,325,96,38,121,970,212,513
GNPDA2_2_24428,441,213,380,471,206,105,407,0,408,29,21,1127
GNPNAT1_2_24429,1975,1352,1738,1755,1307,1139,1424,2300,1549,1866,1520
,1326
GNPTAB_2_24430,412,413,313,366,866,11,195,57,557,232,756,710
GNPTG_2_24431,40,16,8,13,0,37,1,1,198,0,0,60
GNS_2_24432,182,291,231,419,203,26,255,44,63,184,399,265
GOT2_2_24433,152,212,108,240,131,360,213,87,54,640,194,741
GPAA1_2_24434,204,223,117,131,13,35,4,320,236,21,27,401
GPAM_2_24435,243,513,246,384,422,177,451,758,6,448,172,268
GPAT2_2_24436,475,385,499,441,65,78,301,476,892,286,1502,710

GPD1_2_24437,472,319,368,381,492,357,478,303,299,635,272,79
GPD1L_2_24438,342,353,306,304,52,381,420,271,279,21,96,24
GPX2_2_24439,509,282,433,443,173,21,628,353,375,337,652,528
GPX3_2_24440,67,32,125,84,42,5,32,101,52,0,0,27
GPX7_2_24441,12,4,32,13,3,0,18,0,0,109,0,3
GPX8_2_24442,2880,2751,2325,3417,1649,2029,900,5168,2132,3908,1996,268
1
GRHPR_2_24443,59,42,29,74,100,0,0,0,7,2,0,216
GSTA1_2_24444,470,375,522,514,885,299,219,602,463,624,294,1104
GSTA2_2_24445,872,906,993,1065,680,1681,557,675,710,1273,1320,949
GSTA3_2_24446,330,321,211,178,227,10,227,2,66,262,204,125
GSTA4_2_24447,805,888,924,953,1486,666,1358,411,643,1071,615,1286
GSTA5_2_24448,577,695,511,807,721,1675,212,598,789,1656,239,560
GSTM3_2_24449,201,283,311,312,652,208,375,282,106,42,101,763
GSTM5_2_24450,535,668,559,414,28,485,543,67,1101,275,625,483
GSTP1_2_24451,237,314,176,178,109,2,10,0,56,21,385,32
GSTT1_2_24452,82,20,0,30,9,5,0,131,22,0,0,0
GSTT2B_2_24453,1502,1354,1430,1687,1538,1474,1759,1504,1427,1924,573,1
804
GSTT2_2_24454,1502,1354,1430,1687,1538,1474,1759,1504,1427,1924,573,18
04
GTF2F2_2_24455,237,213,117,240,280,195,363,695,78,76,172,35
GTPBP1_2_24456,443,290,520,495,156,179,404,362,723,170,415,293
GTPBP2_2_24457,132,152,154,211,0,1,2,399,12,0,1,0
GTPBP4_2_24458,59,34,113,95,2,0,119,0,5,111,54,0
GUCY1A2_2_24459,1419,1037,1003,1155,1326,742,1661,666,772,949,811,1502
GUCY1B3_2_24460,324,237,154,151,238,390,106,123,426,261,390,77
GUSB_2_24461,94,72,38,43,343,1,0,410,83,413,0,0
GYLTL1B_2_24462,98,70,153,93,97,262,3,0,353,0,324,243
GYS2_2_24463,27,46,23,54,0,0,164,1,1,32,0,60
GZMA_2_24464,436,327,327,446,171,501,1250,266,345,736,972,291
GZMB_2_24465,411,197,211,265,217,18,16,86,228,23,155,21
H6PD_2_24466,480,373,381,504,552,2,520,0,183,0,621,226
HAAO_2_24467,92,71,62,73,8,73,191,100,23,36,91,205
HACL1_2_24468,1287,1089,1435,1200,960,829,1821,373,356,1384,1749,1104
HADHA_2_24469,487,372,388,342,196,68,348,957,133,97,617,473
HADHB_2_24470,57,129,172,87,115,0,32,42,352,39,1,257
HAGHL_2_24471,2,5,3,0,65,0,489,0,0,0,0,0
HAL_2_24472,727,548,539,508,976,281,1308,993,587,691,337,952
HAO1_2_24473,49,23,48,108,28,19,235,8,23,138,22,0
HARS_2_24474,402,423,382,269,163,337,123,358,286,283,108,262
HAS1_2_24475,43,0,7,3,5,0,0,0,0,0,0,0
HAS2_2_24476,34,43,51,47,367,4,19,34,83,104,115,73
HDHD3_2_24477,177,135,198,161,313,10,0,203,918,24,351,231
HELB_2_24478,137,133,131,136,165,7,209,0,82,125,190,181
HELQ_2_24479,206,214,147,219,186,412,250,284,141,238,112,84
HELZ_2_24480,138,127,128,126,171,231,0,645,358,164,132,149
HEXA_2_24481,2492,1828,2114,2079,2677,1508,1170,920,1816,2883,2463,184
2
HEXB_2_24482,913,729,1045,1070,1640,490,1154,329,388,335,321,1492

HEXDC_2_24483,1144,975,929,1031,1287,443,247,815,404,598,450,548
HGD_2_24484,483,608,511,843,350,264,196,575,1052,319,350,280
HGSNAT_2_24485,270,195,300,380,146,206,696,391,180,360,152,243
HHATL_2_24486,75,56,88,43,2,36,0,0,107,3,25,116
HIBADH_2_24487,68,6,15,72,130,351,2,0,2,0,0,0
HINT1_2_24488,125,53,67,61,11,9,5,164,50,390,610,86
HMOX1_2_24489,211,409,595,378,60,230,293,297,1550,510,897,109
HPGDS_2_24490,726,656,688,883,356,894,1384,553,979,494,785,1365
HPRT1_2_24491,26,7,12,18,0,0,0,11,55,14,0,0
HS3ST1_2_24492,95,133,180,263,222,365,1,0,215,156,12,12
HS3ST2_2_24493,85,90,54,39,0,1,17,3,1,208,6,30
HS3ST3A1_2_24494,108,109,96,119,339,689,38,9,116,333,92,370
HS3ST3B1_2_24495,433,516,428,358,754,498,406,753,612,657,222,359
HS3ST4_2_24496,189,217,104,256,159,40,155,474,336,224,625,505
HS3ST6_2_24497,2,53,44,30,0,0,0,1,0,8,0,18
HS6ST1_2_24498,219,182,165,268,164,501,1,148,254,20,380,417
HS6ST3_2_24499,629,682,647,615,906,781,1322,1071,279,1408,782,715
HSD11B2_2_24500,385,228,302,339,291,67,283,232,22,105,0,237
HSD17B11_2_24501,919,599,788,909,879,345,592,654,872,1100,1189,821
HSD17B12_2_24502,1224,1065,1096,1315,645,1580,411,794,301,1365,1370,99
5
HSD17B14_2_24503,462,358,291,327,229,225,401,97,756,223,16,95
HSD17B1_2_24504,41,22,6,28,0,2,15,23,1,2,0,0
HSD17B2_2_24505,64,11,29,24,135,0,0,0,27,16,0,29
HSD17B3_2_24506,268,398,181,374,35,23,430,281,352,388,878,565
HSD17B6_2_24507,20,17,32,61,37,121,99,0,0,78,0,342
HSD17B8_2_24508,96,98,122,152,44,40,1,0,16,19,449,344
HSD3B1_2_24509,956,1000,967,1355,1314,1652,1555,2103,1426,1098,783,189
1
HSP90AB1_2_24510,560,811,520,734,634,859,204,1921,754,718,218,729
HSPA5_2_24511,1108,913,867,838,1002,620,699,1350,1271,444,1026,503
HSPE1_2_24512,27,33,43,8,40,13,0,45,86,1,18,27
HSPG2_2_24513,345,642,436,592,40,0,1041,209,455,285,996,350
HYAL4_2_24514,200,183,269,297,501,187,456,597,411,323,1471,147
IARS2_2_24515,245,199,136,325,44,185,523,1123,375,419,1118,294
ICMT_2_24516,1368,1189,1075,1685,1225,1915,637,2462,626,1261,2248,1415
ICT1_2_24517,95,125,94,112,0,0,1,307,1,234,0,639
IDH1_2_24518,1161,1467,1292,1757,1052,1647,1243,2512,1987,1594,993,159
2
IDH2_2_24519,500,231,326,380,414,228,307,111,244,558,116,1087
IDH3A_2_24520,206,236,254,293,438,18,136,408,515,47,2,375
IDI1_2_24521,1532,1409,1412,1546,821,897,1028,543,975,2025,1691,1829
IDI2_2_24522,317,170,228,226,307,620,118,205,359,253,126,215
IDO1_2_24523,23,46,23,10,0,0,0,3,0,0,2,21
IDO2_2_24524,283,241,423,360,154,252,296,734,136,213,224,530
IDUA_2_24525,98,112,137,134,5,0,0,225,17,228,312,27
IFI30_2_24526,166,97,200,106,3,44,0,306,228,347,1,580
IFIH1_2_24527,811,685,615,1030,1848,86,454,901,953,1252,891,35
IGHMBP2_2_24528,151,66,112,267,152,20,148,331,76,70,262,20
IHH_2_24529,4,9,29,78,0,89,0,0,0,74,0,1

ILVBL_2_24530,96,162,92,107,34,114,38,31,0,217,0,459
IMPAD1_2_24531,895,1194,1020,967,703,981,60,1678,348,1649,2167,863
IMPDH2_2_24532,464,275,324,386,224,64,25,146,78,44,51,493
IRS1_2_24533,90,135,255,300,18,4,498,3,135,259,2,119
ISG20_2_24534,9,3,82,88,156,167,0,0,0,0,431,1
ISG20L2_2_24535,542,396,308,325,188,243,1430,8,311,8,197,76
ISOC1_2_24536,135,192,285,333,242,257,602,1,285,569,527,694
IWS1_2_24537,104,205,161,114,159,0,7,40,14,0,286,6
KDSR_2_24538,32,85,81,49,7,43,166,751,279,0,371,414
KHSRP_2_24539,30,0,7,12,4,0,0,0,0,2,4,0
KIAA0317_2_24540,182,197,143,117,77,60,334,40,294,770,314,215
KIAA1279_2_24541,478,534,531,710,765,352,877,126,779,668,1989,112
KIAA2022_2_24542,40,24,25,66,176,57,2,0,1,88,0,263
KIF18A_2_24543,841,379,486,519,369,325,922,299,632,331,124,472
KIF20B_2_24544,971,885,503,931,169,467,708,467,1761,495,1436,596
KIF3A_2_24545,573,469,608,640,847,138,959,117,557,584,242,925
KLB_2_24546,739,523,524,528,890,1045,584,310,219,1317,315,459
KLC3_2_24547,158,160,98,185,0,292,358,2,45,113,0,70
KL_2_24548,634,334,397,623,800,424,947,569,555,364,551,506
KLK1_2_24549,223,315,222,433,272,87,191,520,418,56,392,88
KLKB1_2_24550,675,742,620,675,1482,359,1028,1027,869,423,959,678
KMO_2_24551,130,159,108,161,199,111,341,267,21,577,20,107
KRTCAP2_2_24552,1541,1090,1151,1285,815,1205,590,1571,1751,1123,1761,1
688
L2HGDH_2_24553,0,2,0,0,0,0,0,4,0,0,0,0
LALBA_2_24554,299,350,315,377,47,260,340,344,202,489,218,455
LAP3_2_24555,40,132,81,30,1,30,0,0,16,104,0,93
LARS2_2_24556,89,244,230,270,6,442,374,4,280,0,0,48
LARS_2_24557,1366,932,1224,1283,989,1038,1572,1686,1221,1299,506,522
LCAT_2_24558,282,277,225,350,261,36,348,94,92,137,3,402
LCMT2_2_24559,21,32,76,92,73,0,0,0,183,354,6,89
LCT_2_24560,132,70,161,452,26,18,283,345,12,9,2,138
LCTL_2_24561,103,53,2,52,0,0,0,16,1,0,2,0
LDHAL6B_2_24562,44,27,47,137,7,4,56,69,195,33,115,109
LEPREL2_2_24563,101,107,79,189,3,0,0,1,3,483,0,1
LGALS13_2_24564,433,388,363,435,197,104,272,614,389,621,146,196
LHFPL2_2_24565,151,191,147,297,95,133,343,169,11,23,269,459
LIPC_2_24566,86,65,18,56,11,129,55,55,284,1,16,242
LIPE_2_24567,932,912,758,997,584,1849,2170,1186,470,694,1525,325
LIPG_2_24568,61,82,86,100,107,101,0,122,32,146,98,237
LIPH_2_24569,491,452,437,502,166,167,563,290,355,804,87,278
LIPI_2_24570,891,849,966,1033,768,282,916,433,332,1311,1517,1033
LIPM_2_24571,1041,759,712,1009,1612,522,792,895,1479,210,997,1411
LIPN_2_24572,153,646,287,350,299,217,221,312,106,190,41,211
LIPT2_2_24573,207,149,113,60,67,237,76,157,110,216,543,46
LONP2_2_24574,396,370,357,519,642,326,552,411,685,231,40,314
LOXL1_2_24575,227,286,267,506,11,54,250,441,240,113,1844,499
LOXL2_2_24576,83,97,17,82,14,56,0,1,15,69,0,2
LOXL3_2_24577,2067,1940,1556,1952,1661,288,1588,1857,1986,2098,1730,27
68

LOXL4_2_24578,245,290,214,363,211,531,496,410,43,144,456,328
LPCAT1_2_24579,313,334,281,460,219,241,319,4,655,251,482,83
LPCAT2_2_24580,252,349,389,310,667,270,388,371,225,18,625,704
LPCAT3_2_24581,238,229,170,164,59,132,804,24,91,99,92,308
LPCAT4_2_24582,342,47,219,197,1055,0,31,123,174,9,1448,322
LPIN1_2_24583,250,385,288,252,902,792,364,508,110,349,0,621
LPIN2_2_24584,2807,2679,2770,3437,2458,1800,2607,1701,3203,2189,1443,3
179
LPIN3_2_24585,247,200,245,281,2,0,509,7,2,511,495,143
LPL_2_24586,263,175,217,224,629,769,47,39,301,6,117,6
LRAT_2_24587,6,12,20,20,5,0,0,0,88,0,0,99
LTA4H_2_24588,315,279,299,268,682,227,93,273,181,190,367,328
LTC4S_2_24589,4,0,18,4,0,0,0,0,0,0,0,0
LYG2_2_24590,0,0,0,0,0,0,0,0,0,0,0,0
LYPLA1_2_24591,32,88,63,86,131,27,18,78,79,109,97,152
LYPLA2_2_24592,141,216,112,307,10,233,171,605,83,29,149,107
LYPLAL1_2_24593,1392,1668,1163,1409,1189,1678,779,1167,1051,211,349,12
41
LYZ_2_24594,162,66,159,90,18,37,26,59,196,129,132,169
LYZL1_2_24595,136,240,352,321,299,69,186,170,451,312,677,84
LYZL2_2_24596,108,266,252,181,295,220,360,654,162,257,270,70
LYZL4_2_24597,419,470,302,433,215,48,90,231,357,596,497,666
MACROD1_2_24598,764,771,730,773,588,95,1335,208,325,561,1228,717
MAGT1_2_24599,0,0,0,0,0,0,0,0,0,0,0,0
MAN1A1_2_24600,188,427,261,495,104,50,223,155,76,616,56,331
MAN1A2_2_24601,1319,1578,1361,1712,1512,403,794,1200,1817,1833,1835,13
58
MAN1B1_2_24602,37,28,42,55,70,0,0,18,28,127,0,58
MAN1C1_2_24603,102,98,96,135,33,426,248,1574,191,32,7,284
MAN2A1_2_24604,1383,997,1191,1454,610,966,2951,812,1810,866,1557,861
MAN2A2_2_24605,759,1000,865,984,864,1060,447,292,438,1990,1417,1666
MAN2B2_2_24606,31,89,23,26,23,208,88,149,39,0,122,116
MAN2C1_2_24607,105,65,66,68,37,347,0,5,201,70,79,348
MANBA_2_24608,242,213,313,295,100,231,21,247,154,873,262,111
MANEA_2_24609,49,66,37,91,19,0,267,35,67,211,0,91
MAOA_2_24610,0,3,48,7,273,0,0,0,0,0,0,0
MAOB_2_24611,324,262,349,356,375,244,145,141,307,597,625,131
MAP1S_2_24612,182,126,170,138,1,195,0,0,49,133,202,143
MAPRE3_2_24613,602,645,572,783,277,826,319,340,504,660,352,597
MARS2_2_24614,79,38,52,84,47,4,1178,0,3,12,1,7
MARS_2_24615,240,180,280,238,248,511,220,791,95,768,308,1487
MAT1A_2_24616,237,99,184,209,263,56,57,235,359,126,20,83
MAT2A_2_24617,1408,1032,1125,1213,1689,505,1789,2761,1916,1164,1176,10
86
MBOAT1_2_24618,13,1,4,0,5,121,0,0,0,0,0,18
MBOAT2_2_24619,131,142,147,114,209,11,272,146,111,75,240,108
MBOAT4_2_24620,157,195,221,177,119,343,170,75,108,167,215,310
MBTPS1_2_24621,91,62,158,205,29,28,196,7,169,11,352,92
MBTPS2_2_24622,1079,1021,1027,1184,2340,1103,2130,1108,634,1752,549,14
92

MCCC1_2_24623,304,390,301,366,268,273,451,82,68,152,293,543
MCCC2_2_24624,141,83,21,104,2,0,0,0,0,14,1291,149
MCEE_2_24625,59,90,69,92,25,27,295,9,60,86,30,42
MCM3_2_24626,264,193,195,187,170,299,722,25,72,72,667,16
MCM5_2_24627,39,63,15,29,0,0,0,1,0,0,0,0
MCM6_2_24628,435,521,394,576,536,236,300,663,505,597,791,878
MDH1B_2_24629,68,151,149,95,65,300,35,309,40,361,61,39
MDH2_2_24630,191,138,199,310,405,125,54,66,62,361,299,12
MEP1A_2_24631,916,935,589,733,704,1837,1239,770,248,1222,1162,597
MEP1B_2_24632,74,75,103,91,102,139,3,1,4,58,0,33
METAP1_2_24633,461,161,228,215,260,0,304,97,143,302,322,366
METAP1D_2_24634,302,334,416,427,288,561,570,69,824,387,256,462
METAP2_2_24635,329,383,185,297,192,99,747,240,550,208,606,481
METTL14_2_24636,299,382,491,432,488,24,639,115,202,378,902,356
METTL22_2_24637,916,452,654,707,619,960,347,95,777,1316,728,119
METTL2B_2_24638,2036,1517,1801,2402,1641,2352,3111,1855,2075,2314,2390
,2137
METTL3_2_24639,166,135,86,141,301,88,378,207,133,0,336,221
METTL5_2_24640,159,205,195,114,162,293,868,712,10,0,533,413
METTL6_2_24641,277,245,215,329,124,341,193,116,303,275,350,213
METTL7B_2_24642,242,378,429,552,937,64,0,302,0,4,1157,203
METTL8_2_24643,354,333,407,356,591,372,366,1287,119,95,973,803
MFN1_2_24644,213,108,146,148,97,3,103,78,49,47,15,187
MGAM_2_24645,7,30,46,51,0,38,96,8,7,19,1,14
MGAT4C_2_24646,447,365,426,422,355,492,412,91,54,361,446,539
MGAT5_2_24647,409,780,607,440,376,1548,1427,614,869,497,441,13
MGMT_2_24648,56,31,34,30,0,0,0,26,14,138,0,0
MGST3_2_24649,145,142,249,356,0,0,556,106,35,34,3,220
MIOX_2_24650,1285,1099,942,1224,600,2128,622,465,830,95,1143,1475
MIPEP_2_24651,684,837,620,794,323,470,329,1515,312,662,1058,666
MLYCD_2_24652,121,163,184,188,210,1,87,187,266,6,541,13
MMAB_2_24653,447,298,275,399,160,549,207,611,55,708,2,408
MMEL1_2_24654,415,294,348,385,493,1462,233,207,257,322,313,630
MMP10_2_24655,51,71,37,108,95,0,19,48,164,26,13,292
MMP12_2_24656,2113,2510,1853,2605,3112,1665,3287,2726,1670,1613,3344,2
147
MMP14_2_24657,69,99,71,57,36,4,6,39,14,0,59,12
MMP3_2_24658,315,191,244,272,154,110,205,751,436,517,15,145
MMP7_2_24659,1691,1558,1551,1835,1139,1609,1590,1057,1167,1842,2261,20
16
MMP8_2_24660,52,92,37,23,277,161,0,1,48,168,59,175
MMP9_2_24661,256,143,132,222,13,35,594,22,25,28,530,103
MOCOS_2_24662,136,113,232,307,7,50,700,485,309,427,772,489
MOGAT1_2_24663,2097,1640,1473,1921,1300,1413,1050,1699,1473,3769,3013,
1727
MOGAT2_2_24664,183,363,126,306,264,663,58,60,65,303,0,97
MOGAT3_2_24665,171,142,290,197,284,22,383,116,19,153,0,18
MOXD1_2_24666,1906,2070,1853,2087,2133,3041,903,828,1959,2229,2589,294
8
MPI_2_24667,60,31,52,24,129,17,31,0,0,20,0,0

MPO_2_24668,166,152,171,219,780,41,334,77,22,0,152,271
MRPL37_2_24669,1559,1550,1508,1785,366,2451,2369,1777,2604,2269,2212,2
024
MRPL44_2_24670,805,562,705,557,678,367,1449,481,770,366,926,288
MRPS30_2_24671,69,182,84,67,161,31,33,11,185,88,470,4
MSH2_2_24672,312,373,292,335,68,134,0,0,59,251,0,275
MSH3_2_24673,1027,907,876,785,530,321,819,933,617,1170,1490,722
MSH4_2_24674,1600,1770,1391,1922,1070,1297,2893,416,1406,2005,3831,965
MTAP_2_24675,177,321,114,182,476,0,59,378,3,173,137,157
MTFMT_2_24676,869,839,709,910,813,1950,1162,493,164,1532,61,604
MTHFD1_2_24677,228,179,108,156,231,13,0,613,10,0,327,540
MTHFD2_2_24678,444,611,662,517,171,731,480,9,703,449,166,696
MTHFD2L_2_24679,476,347,345,392,641,184,131,308,660,792,611,712
MTHFR_2_24680,669,469,625,698,1085,74,998,1406,107,1271,432,568
MTPAP_2_24681,245,179,315,292,670,33,14,6,86,371,1726,135
MTR_2_24682,482,512,509,609,98,242,518,285,371,743,1062,1065
MUS81_2_24683,49,40,10,89,0,0,0,1,10,24,0,0
MUT_2_24684,646,668,656,819,585,154,1033,94,219,1102,658,746
MVD_2_24685,1,33,47,36,1,0,4,17,0,0,32,0
MX2_2_24686,196,190,257,154,67,266,540,241,5,246,25,236
MYH1_2_24687,7,3,30,50,7,0,0,0,0,4,0,131
MYH3_2_24688,500,651,607,559,828,499,414,691,357,1479,8,703
MYH6_2_24689,500,651,607,559,828,499,414,691,357,1479,8,703
MYH7_2_24690,43,60,45,70,220,3,72,166,63,0,172,116
MYH9_2_24691,322,328,281,493,449,56,347,285,122,601,755,798
MYL7_2_24692,175,169,181,322,0,124,1089,8,573,0,0,14
MYO1E_2_24693,185,283,265,384,77,32,45,201,125,579,633,365
MYO5B_2_24694,704,441,527,624,216,933,1006,641,773,283,250,846
MYO9A_2_24695,160,121,97,118,7,161,440,79,42,81,16,170
N6AMT2_2_24696,321,303,265,341,586,461,129,50,135,276,183,294
NAA10_2_24697,254,169,316,293,160,230,212,644,205,509,116,463
NAA11_2_24698,104,18,72,95,117,16,0,28,14,45,9,67
NAA15_2_24699,801,660,751,771,1805,890,1045,1057,1047,1077,722,1190
NAA30_2_24700,320,207,378,404,750,45,251,5,535,50,307,391
NAA50_2_24701,80,110,103,195,247,267,370,67,97,440,34,158
NAALAD2_2_24702,158,192,273,274,24,177,42,8,19,305,98,431
NAALADL1_2_24703,41,57,18,3,9,1,14,7,48,0,145,60
NADSYN1_2_24704,516,353,387,482,278,768,285,167,390,556,543,593
NAGA_2_24705,456,416,235,319,258,176,63,356,383,587,487,602
NAGLU_2_24706,357,353,299,529,611,0,805,239,1,190,424,354
NAGPA_2_24707,391,148,258,298,9,144,293,42,293,278,54,144
NAMPT_2_24708,434,476,436,572,131,872,841,18,262,190,559,408
NANP_2_24709,28,51,83,59,14,3,340,3,18,49,32,0
NANS_2_24710,242,371,219,255,252,291,234,70,259,264,70,220
NAPRT1_2_24711,152,82,44,71,648,10,444,0,324,9,0,91
NARS_2_24712,164,169,137,201,1,0,349,208,147,362,201,186
NCF1_2_24713,0,0,27,11,44,0,0,0,1,0,18,1
NDST1_2_24714,10,8,41,33,24,6,1,501,19,0,0,0
NDST2_2_24715,815,636,674,674,795,319,1127,250,704,1055,936,1440
NDST3_2_24716,352,621,427,453,758,177,268,618,301,23,605,200

NDST4_2_24717,655,389,481,379,167,122,145,729,698,1294,397,771
NDUFA10_2_24718,87,67,147,83,14,25,35,51,17,267,55,0
NDUFA12_2_24719,69,139,151,201,158,19,22,306,40,53,175,272
NDUFA13_2_24720,86,2,23,3,0,0,1,0,0,66,0,0
NDUFA1_2_24721,5,14,30,15,0,0,0,69,0,0,0,0
NDUFA3_2_24722,976,1127,1123,1302,921,1391,1459,346,954,589,779,1059
NDUFA4_2_24723,79,48,31,50,132,13,9,324,4,1,5,109
NDUFA4L2_2_24724,2043,1246,1448,1616,1083,1339,2807,687,1065,1023,1499
,1501
NDUFA5_2_24725,805,663,889,974,598,581,1134,129,724,766,980,563
NDUFA6_2_24726,109,72,77,132,0,13,1,15,57,7,202,425
NDUFA7_2_24727,84,108,63,139,6,0,36,454,117,306,318,55
NDUFA8_2_24728,1805,1709,1419,1883,1481,1420,890,1711,1030,1618,1088,9
19
NDUFA9_2_24729,60,29,70,49,369,0,84,0,2,93,0,44
NDUFAB1_2_24730,4155,3910,3381,3997,4756,4151,5338,5337,3685,3918,2207
,5578
NDUFB10_2_24731,94,105,139,192,169,62,4,50,31,21,17,175
NDUFB1_2_24732,562,230,292,548,507,178,41,318,488,522,173,430
NDUFB2_2_24733,107,68,59,147,34,437,327,90,98,23,380,29
NDUFB3_2_24734,597,431,373,522,351,205,546,578,46,632,430,416
NDUFB7_2_24735,53,79,106,54,0,36,20,6,0,3,2,0
NDUFB8_2_24736,252,256,328,304,3,140,275,527,23,221,37,412
NDUFB9_2_24737,97,73,214,183,88,473,1,281,0,0,78,0
NDUFS3_2_24738,50,162,102,227,27,8,83,274,39,291,122,366
NDUFS4_2_24739,210,93,148,153,165,200,432,146,109,23,11,461
NDUFS6_2_24740,4,11,63,13,0,0,0,0,2,0,0,0
NDUFS7_2_24741,828,1123,752,928,1741,633,391,88,621,900,1119,1330
NDUFS8_2_24742,15,11,14,8,1,0,0,0,11,0,389,0
NDUFV2_2_24743,567,509,590,746,3,1117,225,236,667,1403,742,1076
NEDD8_2_24744,1475,1326,1202,1418,1445,2371,1418,1727,1751,1175,2623,9
21
NEIL1_2_24745,177,128,74,114,125,3,364,23,26,169,0,36
NEIL3_2_24746,730,480,603,725,832,114,484,470,387,817,822,968
NEU1_2_24747,487,473,610,670,167,352,1558,671,17,440,21,347
NEU2_2_24748,588,476,374,551,679,248,87,81,693,1201,225,384
NEU3_2_24749,31,91,92,39,165,1,11,64,15,0,158,69
NHLRC2_2_24750,1479,1695,1574,1772,1055,1519,952,955,559,1521,2028,275
2
NIT2_2_24751,1143,972,869,1150,758,1889,419,761,624,618,708,1063
NKIRAS1_2_24752,2687,2223,2097,2454,2245,2235,1636,287,2284,2619,1841,
1858
NLGN1_2_24753,11,7,2,39,2,0,0,0,81,40,5,0
NLGN2_2_24754,3,22,21,29,41,1,0,0,5,55,44,13
NLN_2_24755,35,23,53,138,55,0,0,321,0,23,0,40
NME1-NME2_2_24756,364,440,350,337,524,812,367,142,589,44,141,338
NMNAT1_2_24757,366,268,195,370,341,415,273,590,337,185,472,133
NMT1_2_24758,192,192,183,81,78,35,235,177,39,260,325,375
NMT2_2_24759,580,348,327,413,331,420,569,134,472,463,360,738
NNMT_2_24760,47,107,47,64,4,0,211,12,251,217,56,43

NOP58_2_24761,52,85,77,34,247,5,2,38,9,106,34,43
NOX3_2_24762,193,255,213,209,189,368,49,517,257,502,0,95
NQO2_2_24763,32,9,159,60,0,607,281,2,3,37,0,275
NRAS_2_24764,133,286,407,259,323,10,236,634,76,51,500,186
NSF_2_24765,90,207,140,188,1681,21,5,242,256,8,300,149
NSUN6_2_24766,142,106,109,72,64,167,0,157,57,19,7,0
NTAN1_2_24767,420,384,524,688,484,423,401,1761,482,552,46,624
NTHL1_2_24768,2,0,12,0,0,0,0,0,0,0,0,0
NTPCR_2_24769,940,540,842,853,1476,696,429,1471,551,552,1740,803
NUDT10_2_24770,1829,1290,1420,1257,1878,1507,1704,453,1858,1282,911,21
14
NUDT11_2_24771,96,57,153,165,224,27,11,0,40,39,98,4
NUDT12_2_24772,124,215,160,167,3,95,0,153,68,465,29,552
NUDT14_2_24773,867,510,724,657,119,462,508,382,1982,440,859,810
NUDT3_2_24774,135,49,54,51,463,178,292,20,80,15,41,145
NUDT5_2_24775,513,579,668,574,590,379,1089,1,591,311,77,150
NUDT7_2_24776,337,338,336,436,421,65,15,508,273,362,78,200
NXNL1_2_24777,862,637,712,780,868,703,43,877,724,810,39,1026
OAS3_2_24778,36,13,36,31,1,0,0,0,114,0,0,111
OAZ1_2_24779,28,33,13,28,104,0,17,0,66,0,0,70
OC90_2_24780,245,142,132,255,317,218,13,286,88,245,258,382
ODF3B_2_24781,40,54,243,152,0,176,2,76,0,0,127,12
OPLAH_2_24782,460,434,434,344,693,928,310,113,247,259,855,340
OSGEP_2_24783,7,39,13,49,152,0,0,0,68,0,1,33
OSGEPL1_2_24784,48,116,209,343,114,296,9,50,287,144,0,0
OSTC_2_24785,3441,2606,2678,2922,2577,1608,4949,3515,4028,3223,2450,46
50
OTC_2_24786,523,280,386,486,639,302,387,1331,851,232,328,608
OVGP1_2_24787,1144,1175,1131,1272,913,1187,1157,531,917,1675,2085,1207
OXA1L_2_24788,27,13,0,2,0,0,0,0,1,0,0,0
OXCT1_2_24789,203,214,216,206,279,247,59,36,241,197,77,361
OXCT2_2_24790,48,78,26,189,0,1,131,1,15,233,48,86
P4HA3_2_24791,71,22,56,71,0,50,256,44,65,4,0,0
P4HB_2_24792,107,80,33,53,0,159,78,0,310,52,763,24
PADI1_2_24793,99,283,297,336,111,56,178,15,47,140,26,190
PADI2_2_24794,83,85,89,129,312,4,212,64,5,65,802,66
PADI3_2_24795,163,201,174,232,138,383,91,202,212,167,175,287
PADI6_2_24796,281,325,230,269,269,528,0,1,385,336,47,457
PAFAH2_2_24797,66,93,137,109,68,1,213,141,56,45,105,48
PAH_2_24798,325,222,262,417,242,39,687,236,833,9,219,119
PAPOLA_2_24799,295,160,268,307,212,23,174,530,156,207,131,393
PAPOLB_2_24800,2495,2511,2179,2918,3449,2750,1525,3996,2639,1642,2428,
2584
PAPOLG_2_24801,1171,1034,1123,1406,1327,99,1009,19,1149,1605,1323,1211
PAPPA_2_24802,47,85,66,182,323,0,132,4,0,240,0,191
PAPSS1_2_24803,290,210,154,305,403,0,488,213,203,182,66,216
PARG_2_24804,318,377,326,521,317,839,165,1178,418,451,48,543
PARP1_2_24805,492,264,388,383,432,621,1121,431,227,514,336,332
PARP4_2_24806,949,706,623,1026,1291,619,1317,799,609,921,273,981
PARP6_2_24807,1016,731,992,1095,857,817,1440,725,925,1281,2722,758

PARS2_2_24808,25,7,18,10,2,5,0,0,5,4,0,2
PCBD1_2_24809,206,132,177,167,93,25,27,165,70,199,62,111
PCBD2_2_24810,502,391,695,559,352,760,403,640,494,484,1136,463
PCMT1_2_24811,366,174,184,243,521,355,308,124,403,68,419,206
PCMTD1_2_24812,823,675,540,862,751,996,632,835,577,182,344,459
PCYOX1_2_24813,1088,1352,1352,1747,1438,1313,1308,1933,432,1494,1909,1
036
PCYT1A_2_24814,1054,844,875,1170,664,735,301,1062,566,754,551,817
PDCD1LG2_2_24815,506,408,402,432,491,85,291,682,644,278,249,502
PDCL_2_24816,289,307,265,254,219,186,154,271,131,731,133,53
PDE3A_2_24817,370,387,300,450,248,307,13,6,247,46,72,776
PDE3B_2_24818,1,3,4,1,3,6,0,0,0,0,0,0
PDE6A_2_24819,333,315,193,545,265,169,468,462,2,58,852,661
PDE6C_2_24820,798,488,638,622,334,704,315,993,841,346,418,410
PDE6D_2_24821,0,0,0,5,0,0,0,0,0,0,0,0
PDE6G_2_24822,47,117,143,132,12,0,227,0,85,295,0,32
PDE6H_2_24823,651,689,443,698,686,157,730,243,421,783,649,482
PDE7B_2_24824,1148,1099,893,1250,1000,634,1336,2086,763,1729,1963,788
PDHA2_2_24825,22,37,3,21,254,104,0,0,0,54,0,0
PDIA2_2_24826,21,28,24,29,0,0,21,54,40,0,1,0
PDIA3_2_24827,425,599,554,857,286,508,279,525,689,589,395,514
PDIA4_2_24828,9,114,94,45,0,4,0,0,0,0,0,1
PDIA5_2_24829,209,127,114,125,227,54,165,105,404,451,1,2
PDIA6_2_24830,10,13,18,17,0,0,0,0,0,0,124,0
PDPR_2_24831,99,189,100,119,196,361,50,16,0,760,0,60
PDSS1_2_24832,524,587,461,588,222,1420,645,23,55,164,451,303
PDSS2_2_24833,1007,1237,905,934,527,510,919,1927,235,1068,2108,1042
PDXP_2_24834,17,13,10,25,0,11,83,1,31,3,0,52
PECR_2_24835,1092,689,636,810,1352,154,431,355,382,842,1104,1057
PELI1_2_24836,403,493,379,365,205,93,224,904,169,158,185,89
PET117_2_24837,1309,1266,972,1299,854,371,1393,830,1023,1594,1581,1767
PEX1_2_24838,201,344,178,157,65,222,498,141,692,324,95,173
PEX6_2_24839,59,71,261,122,17,3,22,61,25,46,733,19
PFAS_2_24840,163,4,37,3,0,0,47,0,10,0,0,0
PGA3_2_24841,25,55,70,37,22,0,0,17,89,0,540,18
PGA4_2_24842,25,55,70,37,22,0,0,17,89,0,540,18
PGA5_2_24843,25,55,70,37,22,0,0,17,89,0,540,18
PGAM4_2_24844,577,558,602,812,189,37,519,275,365,616,1936,739
PGAP1_2_24845,943,996,1053,1146,913,568,1083,856,727,1328,1402,1012
PGAP3_2_24846,692,691,654,842,593,279,525,421,739,604,697,912
PGD_2_24847,778,783,696,706,615,389,1346,837,1044,428,608,448
PGGT1B_2_24848,1686,1348,1451,1435,1334,1190,1570,1482,1873,1329,623,1
525
PGLS_2_24849,227,189,211,328,783,146,68,356,81,241,9,279
PGM2_2_24850,34,23,25,48,92,0,0,0,0,2,0,78
PGM2L1_2_24851,272,114,229,237,444,36,532,384,130,410,411,285
PGM5_2_24852,1031,557,529,715,617,285,1025,1264,771,502,370,296
PGPEP1_2_24853,286,188,236,237,197,15,58,56,271,288,139,511
PGS1_2_24854,265,227,336,337,146,47,79,483,263,556,209,414
PHGDH_2_24855,232,251,280,267,267,190,178,239,367,70,182,149

PHLPP1_2_24856,217,299,334,427,175,75,358,415,107,219,283,614
PHLPP2_2_24857,197,41,67,212,0,1,0,428,363,0,362,0
PIF1_2_24858,61,39,37,72,335,10,7,170,40,44,2,93
PIGB_2_24859,1484,1064,1004,1168,1387,507,1434,1091,670,1212,1060,1222
PIGH_2_24860,160,257,281,259,354,612,895,37,315,151,581,255
PIGL_2_24861,65,39,15,23,18,0,0,0,60,0,0,0
PIGM_2_24862,68,99,74,68,64,33,205,0,186,215,1,0
PIGS_2_24863,583,428,370,586,653,214,37,405,80,943,321,660
PIGU_2_24864,505,396,438,365,141,225,149,565,796,87,470,165
PIGW_2_24865,1020,1166,834,1202,303,0,1322,346,1267,950,126,1119
PIGZ_2_24866,364,384,545,427,403,573,322,274,255,259,281,412
PIN1_2_24867,10,44,30,62,1,0,69,40,0,0,93,17
PIPOX_2_24868,116,135,195,122,151,315,18,183,33,15,80,38
PITPNM2_2_24869,66,7,8,52,0,1,192,1,1,0,0,81
PLA2G10_2_24870,598,516,504,518,516,882,1377,320,380,570,557,278
PLA2G12A_2_24871,168,147,348,267,94,17,49,337,368,303,158,186
PLA2G12B_2_24872,697,780,629,811,458,299,515,974,670,527,615,563
PLA2G15_2_24873,315,193,128,221,167,98,242,145,136,421,254,182
PLA2G1B_2_24874,353,307,396,418,44,198,521,461,492,576,167,312
PLA2G2C_2_24875,35,2,29,22,0,191,2,0,0,0,0,6
PLA2G2D_2_24876,83,33,33,59,0,19,26,162,0,0,1,6
PLA2G2E_2_24877,1216,1024,902,1018,1657,1654,2329,607,909,1471,698,886
PLA2G2F_2_24878,206,236,157,322,299,0,0,130,1,0,0,44
PLA2G3_2_24879,625,269,304,501,43,332,947,656,359,1015,1459,999
PLA2G4A_2_24880,1757,1586,1986,1971,2070,1744,2058,1124,2391,1614,1439
,2543
PLA2G4B_2_24881,753,648,844,706,1632,775,3,654,291,433,146,1008
PLA2G4D_2_24882,319,254,285,440,176,209,826,359,697,331,121,258
PLA2G4E_2_24883,77,61,99,180,44,145,298,62,273,95,21,50
PLA2G4F_2_24884,254,152,165,169,268,315,522,1,361,208,438,165
PLA2G5_2_24885,20,72,113,70,9,0,255,0,73,143,515,125
PLCB2_2_24886,120,177,146,146,1004,111,352,488,225,63,16,176
PLCD3_2_24887,88,15,59,100,138,11,61,0,13,19,0,139
PLCD4_2_24888,479,285,235,474,322,1047,25,277,186,200,197,240
PLCG2_2_24889,33,86,89,41,0,0,5,40,23,20,8,24
PLCH2_2_24890,17,6,37,20,0,0,11,0,60,165,0,37
PLCL1_2_24891,480,366,437,484,242,181,713,78,761,882,96,332
PLCXD1_2_24892,46,41,53,51,105,0,0,11,59,21,147,122
PLCZ1_2_24893,1143,999,676,962,648,789,694,1878,854,754,707,973
PLD4_2_24894,330,267,161,281,423,115,97,123,348,18,54,188
PLOD1_2_24895,334,349,256,339,310,0,166,0,179,459,1179,0
PLOD3_2_24896,44,48,40,82,0,99,0,76,2,76,0,207
PLSCR1_2_24897,1071,821,800,1017,1256,709,1491,1401,538,631,728,1326
PMM1_2_24898,1430,1115,1456,1296,875,1013,1387,2023,1540,911,2434,1680
PMM2_2_24899,60,46,83,42,0,115,4,439,13,2,0,26
PMPCA_2_24900,274,184,178,269,124,115,3,30,357,367,87,108
PMPCB_2_24901,1671,1080,1144,1354,1046,537,1151,1266,1137,2003,1499,22
15
PNLIP_2_24902,32,0,45,49,2,2,0,0,18,19,1,284
PNLIPRP1_2_24903,59,123,257,119,106,0,1,0,153,174,601,260

PNLIPRP2_2_24904,430,432,432,488,548,149,325,36,310,803,195,130
PNLIPRP3_2_24905,126,173,194,127,32,286,202,128,218,195,364,313
PNMT_2_24906,20,18,18,15,0,0,183,2,2,0,0,2
PNPLA2_2_24907,1092,1234,870,1224,621,1041,1363,676,1156,761,321,1159
PNPLA3_2_24908,75,21,70,61,36,13,0,0,176,0,0,1
PNPLA8_2_24909,211,222,331,305,456,6,250,1,363,588,543,382
PNPO_2_24910,29,7,29,11,2,1,0,1,0,0,0,94
PNPT1_2_24911,354,211,164,224,0,347,0,0,0,574,988,558
POLA1_2_24912,322,241,367,418,222,143,323,0,33,293,1217,156
POLA2_2_24913,500,376,434,421,131,265,1346,224,674,171,51,953
POLB_2_24914,70,53,49,100,259,5,58,26,254,136,1,76
POLD4_2_24915,209,99,111,414,363,294,84,362,40,0,443,364
POLE3_2_24916,10,14,18,50,0,0,0,426,10,0,95,0
POLE4_2_24917,69,21,31,121,27,51,1,160,2,442,321,65
POLE_2_24918,543,476,296,321,139,366,201,929,612,626,147,127
POLG2_2_24919,180,148,67,114,269,181,11,254,295,320,554,135
POLI_2_24920,628,457,376,652,794,627,647,370,1167,1073,139,913
POLM_2_24921,26,4,9,3,0,0,0,0,0,0,0,0
POLN_2_24922,1282,1254,1189,1414,1142,1203,3742,1000,1489,1526,1235,68
7
POLR1A_2_24923,40,18,82,51,9,12,1,140,22,0,9,55
POLR1C_2_24924,1467,1088,1171,1177,410,784,1933,742,967,2168,1537,726
POLR1E_2_24925,93,5,118,71,0,34,6,3,404,3,469,0
POLR2A_2_24926,484,930,758,970,191,5,2356,730,315,342,792,1522
POLR2B_2_24927,190,157,155,156,368,432,125,258,203,13,22,141
POLR2C_2_24928,175,150,129,143,261,63,2,218,76,24,32,36
POLR2D_2_24929,1060,1208,1023,1679,959,1855,2425,1067,1422,829,193,269
5
POLR2E_2_24930,285,241,426,491,287,1339,336,141,23,290,5,416
POLR2F_2_24931,155,76,124,149,135,1,588,2,0,198,0,55
POLR2G_2_24932,128,65,133,48,38,152,105,153,249,146,273,82
POLR2H_2_24933,7,12,27,44,2,210,0,24,4,0,10,15
POLR2J_2_24934,70,216,100,67,73,18,131,264,13,19,42,7
POLR2K_2_24935,271,237,331,231,286,78,324,373,360,233,0,380
POLR2L_2_24936,191,197,193,158,185,326,78,198,426,49,4,375
POLR3A_2_24937,422,404,323,370,7,96,162,381,366,98,1079,114
POLR3C_2_24938,166,142,137,193,5,77,256,120,147,126,315,323
POLR3F_2_24939,552,482,494,602,334,710,247,623,482,829,204,615
POLR3G_2_24940,104,86,122,78,429,57,24,89,86,126,252,205
POLRMT_2_24941,72,14,27,52,10,11,5,8,0,45,0,6
POMGNT1_2_24942,468,298,371,377,435,75,684,95,514,190,779,430
POMT2_2_24943,106,84,73,174,1,200,597,188,106,2,6,66
PON3_2_24944,542,502,371,482,397,296,477,1211,928,231,194,349
POP4_2_24945,84,62,126,71,84,257,0,105,34,30,3,7
POP7_2_24946,6,20,120,75,0,121,151,4,0,119,673,35
POR_2_24947,38,28,33,110,27,1,6,0,94,12,1,15
PPA1_2_24948,554,590,500,591,397,650,2,0,64,0,708,260
PPCDC_2_24949,56,16,75,130,2,63,0,8,26,43,403,402
PPIA_2_24950,212,182,186,180,135,139,159,0,21,145,163,235
PPIAL4A_2_24951,7882,7081,7705,8198,6145,6352,9907,8364,8248,7046,8701

,7496
PPIAL4B_2_24952,7882,7081,7705,8198,6145,6352,9907,8364,8248,7046,8701
,7496
PPIAL4C_2_24953,7882,7081,7705,8198,6145,6352,9907,8364,8248,7046,8701
,7496
PPIAL4E_2_24954,1577,1432,1254,1550,1580,478,1635,1865,1887,1210,1185,
1929
PPIAL4G_2_24955,880,622,432,656,209,636,854,702,628,500,787,764
PPIB_2_24956,179,175,306,371,4,0,230,0,305,136,51,91
PPIC_2_24957,85,127,56,108,119,128,77,3,29,144,3,94
PPID_2_24958,703,683,628,529,423,407,147,819,531,585,1182,779
PPIF_2_24959,1526,1407,1286,1308,1739,1494,2202,1309,1399,1202,1528,42
8
PPIG_2_24960,839,694,678,735,588,485,2136,244,1072,584,11,723
PPIH_2_24961,1168,1026,1265,947,1630,743,1145,721,833,746,1475,1052
PPIL1_2_24962,1168,1091,1052,967,1645,666,1773,1643,570,975,559,2229
PPIL4_2_24963,151,127,117,225,166,110,44,197,30,302,417,162
PPIP5K2_2_24964,216,297,232,305,1,0,50,645,168,72,91,389
PPM1H_2_24965,227,364,234,348,172,196,0,4,698,662,267,157
PPM1J_2_24966,228,294,348,218,1300,0,65,248,1,0,0,775
PPM1N_2_24967,146,100,145,102,80,131,160,196,261,16,79,42
PPME1_2_24968,1562,1265,1369,1478,1598,1217,1980,347,1057,1911,2917,22
05
PPWD1_2_24969,1710,1803,1371,1920,1256,2019,2743,2168,1948,2891,2448,1
714
PRDX4_2_24970,55,36,120,129,271,125,2,2,93,26,98,0
PREP_2_24971,91,96,273,229,368,368,2,42,74,232,16,262
PRHOXNB_2_24972,543,572,363,535,365,125,522,428,634,499,533,583
PRIM1_2_24973,829,482,524,690,459,1023,851,753,574,1022,867,407
PRIM2_2_24974,267,398,364,484,308,86,32,1593,17,52,171,317
PROC_2_24975,297,216,209,252,59,90,545,125,371,467,61,212
PROSC_2_24976,374,310,271,251,363,655,543,772,662,156,1073,117
PRR14L_2_24977,270,185,180,409,286,2,511,283,120,264,254,35
PRSS1_2_24978,0,0,0,1,0,0,0,0,1,0,0,0
PRSS2_2_24979,373,326,548,674,2,20,71,116,350,660,503,314
PRTFDC1_2_24980,52,72,68,44,16,49,233,73,98,278,395,97
PRTN3_2_24981,140,66,112,206,27,0,28,0,36,55,0,47
PRUNE_2_24982,3178,2281,2475,2838,2082,2327,1913,796,4094,2266,3058,45
11
PSMA2_2_24983,349,256,326,246,101,361,205,655,601,10,705,175
PSMA6_2_24984,192,116,90,128,1,12,0,341,162,250,6,281
PSMA7_2_24985,844,675,636,960,800,29,560,565,294,475,1906,520
PSMB10_2_24986,65,86,114,134,43,151,11,2,1,3,0,99
PSMB11_2_24987,12,5,30,43,0,180,3,77,32,4,0,0
PSMB1_2_24988,565,534,458,652,1212,1039,574,262,343,861,426,992
PSMB3_2_24989,303,311,269,209,208,109,163,1386,668,182,263,396
PSMB4_2_24990,105,106,124,89,21,18,70,108,13,98,14,54
PSMB6_2_24991,78,150,114,177,595,114,54,578,183,43,245,84
PSMB7_2_24992,99,161,195,186,36,191,369,438,438,39,1,104
PSMB9_2_24993,274,558,352,416,132,437,22,263,323,458,107,303

PSMD6_2_24994,497,598,531,869,388,848,1283,143,564,1128,221,840
PTDSS1_2_24995,3619,3176,2843,3383,2885,2411,4236,2591,3266,2734,2581,
3636
PTDSS2_2_24996,191,98,169,173,79,709,22,42,2,428,56,378
PTGDS_2_24997,156,190,159,229,35,39,97,18,41,71,31,288
PTGES2_2_24998,171,314,238,237,376,2,795,405,41,271,201,352
PTGES3_2_24999,74,104,82,26,5,189,67,188,1,427,14,47
PTGES_2_25000,709,383,555,680,636,299,379,274,652,511,953,688
PTGIS_2_25001,60,112,75,86,44,81,3,67,105,203,180,80
PTGS2_2_25002,285,229,221,245,327,115,503,579,31,327,418,306
PTRH1_2_25003,222,310,294,235,68,386,79,31,555,33,336,284
PTRH2_2_25004,155,116,146,123,91,98,4,145,126,301,72,156
PTS_2_25005,424,700,307,570,283,798,277,450,110,282,516,523
PUS3_2_25006,363,373,355,492,411,379,280,465,148,546,281,480
PUSL1_2_25007,160,147,107,182,163,24,7,214,403,4,0,176
PXDN_2_25008,207,201,188,178,185,266,1015,249,237,2,496,144
PXDNL_2_25009,83,93,67,31,0,72,102,167,6,16,266,168
PYCR2_2_25010,25,33,15,29,3,5,0,10,81,0,116,0
PYCRL_2_25011,1150,751,722,973,584,931,1653,487,420,182,802,816
PYGB_2_25012,267,126,101,135,176,6,313,207,82,160,188,394
QDPR_2_25013,0,0,0,0,0,0,0,0,0,0,0,0
QPCT_2_25014,636,899,701,684,1049,890,132,1095,659,1109,1293,313
QPRT_2_25015,122,79,185,100,31,222,152,571,43,69,16,231
QRSL1_2_25016,239,159,212,198,501,63,40,162,92,672,36,482
QSOX2_2_25017,100,25,16,86,6,6,4,38,6,6,7,7
QTRT1_2_25018,116,132,130,153,296,1,265,644,193,35,209,199
QTRTD1_2_25019,342,306,216,284,569,768,159,657,35,436,1,398
RAB10_2_25020,99,120,90,128,123,24,302,199,171,291,54,5
RAB12_2_25021,18,12,8,62,0,5,0,1,0,0,0,1
RAB13_2_25022,800,652,569,1063,570,1488,743,885,461,776,1211,744
RAB14_2_25023,509,381,401,406,1043,1020,137,582,340,681,968,839
RAB15_2_25024,217,158,135,98,133,71,23,218,204,5,4,177
RAB17_2_25025,31,108,53,67,100,6,278,34,108,10,8,12
RAB18_2_25026,189,136,255,133,18,135,390,435,146,105,12,10
RAB19_2_25027,252,240,167,376,537,187,627,953,79,114,2,42
RAB1B_2_25028,191,53,130,75,346,403,81,240,66,170,16,27
RAB20_2_25029,5,36,12,28,13,0,0,0,0,0,0,35
RAB21_2_25030,547,411,296,436,83,504,453,166,911,425,92,575
RAB22A_2_25031,147,129,117,137,51,39,3,61,32,194,64,86
RAB25_2_25032,101,51,54,53,0,47,72,101,125,300,75,161
RAB26_2_25033,337,341,253,395,250,125,344,345,576,681,92,515
RAB27B_2_25034,379,245,194,281,199,192,972,14,366,94,439,376
RAB30_2_25035,633,537,341,638,253,143,264,0,955,300,294,965
RAB31_2_25036,176,211,400,229,0,115,466,663,82,0,1,494
RAB33A_2_25037,231,247,265,238,569,226,1207,197,244,89,121,302
RAB33B_2_25038,1147,1505,1303,1416,1558,705,2112,1028,689,1315,1011,84
3
RAB36_2_25039,297,298,258,350,695,373,69,85,149,818,288,319
RAB38_2_25040,279,488,335,360,593,355,780,480,485,537,264,578
RAB39B_2_25041,289,380,359,422,475,321,175,771,348,571,484,820

RAB3A_2_25042,45,37,95,46,271,0,123,4,0,0,284,0
RAB3B_2_25043,45,41,152,43,4,134,0,54,122,191,4,0
RAB3C_2_25044,55,124,128,149,127,136,0,13,51,182,0,81
RAB3D_2_25045,441,509,182,296,106,442,959,126,148,437,523,110
RAB3GAP2_2_25046,1075,689,664,786,1046,567,240,1444,428,325,785,659
RAB4A_2_25047,221,332,289,233,15,498,5,29,102,667,171,244
RAB4B_2_25048,1082,890,1040,1256,1234,603,1887,1205,1027,851,457,727
RAB5A_2_25049,93,71,95,191,0,2,333,205,54,63,4,106
RAB5B_2_25050,1426,1339,1015,1494,575,1601,1164,1438,1064,972,409,1481
RAB6B_2_25051,14,12,12,14,42,0,16,1,30,1,0,12
RAB6C_2_25052,448,410,311,372,309,118,460,225,185,337,237,703
RAB7A_2_25053,317,288,331,422,586,289,528,288,502,328,171,505
RAB8B_2_25054,583,509,697,720,1123,831,654,412,451,700,500,732
RAB9B_2_25055,178,107,91,162,63,145,272,205,20,282,5,131
RABGGTB_2_25056,1706,1876,1642,1648,2874,2019,3374,1295,2210,1843,3463
,1834
RABL3_2_25057,1430,1235,1376,1748,817,767,1088,1151,1768,1364,2836,115
2
RAC2_2_25058,182,332,198,128,2,774,238,148,115,206,575,129
RAD50_2_25059,3213,2730,2570,3397,2093,4355,3662,3149,2332,3041,2040,3
975
RAD54L2_2_25060,1029,1188,986,1487,1792,1946,507,1032,1371,749,1455,15
11
RALA_2_25061,291,402,346,376,218,163,13,445,164,382,757,791
RALB_2_25062,179,125,92,116,170,0,137,87,171,40,99,324
RALBP1_2_25063,1974,2096,1655,1820,477,2486,2660,479,2408,2211,1410,25
92
RANBP2_2_25064,657,862,694,882,94,357,632,1329,543,178,1562,1202
RAN_2_25065,234,238,385,430,3,432,224,810,246,269,147,140
RAP2A_2_25066,79,196,143,230,198,38,174,528,0,2,0,323
RAP2B_2_25067,63,111,111,129,45,0,1056,52,123,128,0,7
RAP2C_2_25068,284,183,210,342,43,207,721,300,190,134,288,21
RARS2_2_25069,113,73,88,106,13,186,95,603,14,2,107,22
RARS_2_25070,0,0,0,0,0,0,0,0,0,0,0,0
RASD2_2_25071,414,294,251,344,526,4,533,355,574,413,540,376
RASL10B_2_25072,57,105,126,131,48,0,571,67,6,223,0,42
RASL11B_2_25073,0,8,0,4,0,9,33,2,0,0,0,0
RASL12_2_25074,84,94,123,141,21,0,22,0,63,3,257,20
RC3H1_2_25075,312,324,507,530,146,610,658,467,616,530,1115,461
RCL1_2_25076,644,231,213,371,623,94,690,68,0,278,68,451
RDH10_2_25077,288,301,230,275,945,11,11,77,363,64,424,96
RDH11_2_25078,312,241,143,165,41,417,44,377,133,33,284,352
RDH12_2_25079,594,472,657,871,324,758,1077,1283,121,939,1101,956
RDH14_2_25080,87,101,125,78,0,510,6,9,23,35,0,265
RDH16_2_25081,204,142,305,287,742,19,805,215,65,129,1864,687
RDH8_2_25082,369,344,351,289,326,184,700,426,534,250,143,147
RECQL4_2_25083,249,47,195,211,6,295,0,263,2,2,307,0
REM1_2_25084,7,0,3,4,0,0,0,0,0,65,0,0
REM2_2_25085,7,43,14,9,0,0,299,0,6,0,0,0
RENBP_2_25086,177,98,151,162,504,45,549,54,18,162,205,201

RETSAT_2_25087,1894,1173,1037,1932,1501,1000,2134,855,1792,510,781,161
7
REV3L_2_25088,1297,1145,1219,1248,709,761,1369,1103,1714,2157,987,1024
REXO1L1_2_25089,25,10,32,51,25,26,0,0,0,7,112,0
REXO2_2_25090,43,40,66,120,188,97,2,53,4,23,1,528
RFNG_2_25091,197,144,58,98,11,493,193,0,65,10,0,0
RFX6_2_25092,215,116,200,255,25,140,1005,318,28,16,78,47
RGS7_2_25093,123,93,80,124,6,295,76,7,118,320,93,298
RHBDL1_2_25094,225,274,132,205,53,626,122,225,401,52,30,134
RHBDL2_2_25095,1673,1685,1394,1926,1897,419,464,1347,1126,3557,1750,15
08
RHBDL3_2_25096,266,187,178,285,62,66,4,149,40,219,101,392
RHEBL1_2_25097,203,30,93,78,38,1,106,1,12,0,9,16
RHOA_2_25098,1057,1021,890,894,1237,986,750,688,1531,548,735,390
RHOB_2_25099,180,205,238,260,214,13,182,1228,200,414,248,245
RHOD_2_25100,63,104,130,47,114,135,288,229,145,28,17,19
RHOF_2_25101,36,8,11,32,42,1,0,64,0,0,0,0
RHOG_2_25102,21,18,21,37,8,0,25,0,0,116,0,4
RHOH_2_25103,447,486,507,469,171,479,684,28,450,353,329,799
RHOJ_2_25104,433,409,394,392,127,519,465,347,878,183,962,264
RHOQ_2_25105,1118,788,838,955,393,780,2436,813,595,1115,411,596
RHOT2_2_25106,32,46,30,126,125,7,0,25,65,4,230,194
RHOU_2_25107,371,306,326,329,6,262,142,301,504,362,295,312
RHOV_2_25108,161,197,167,113,102,7,13,106,70,317,239,570
RIT1_2_25109,54,5,11,35,0,10,0,9,0,8,1,0
RIT2_2_25110,982,949,768,1037,446,869,196,1295,792,1287,1183,876
RNASE2_2_25111,975,850,1000,1000,1082,936,514,938,447,690,487,1310
RNASE3_2_25112,791,470,472,607,408,293,576,42,412,41,916,912
RNASE6_2_25113,409,285,231,367,224,266,304,60,483,32,265,747
RNASE7_2_25114,53,19,8,55,70,1,0,80,136,5,0,0
RNASE8_2_25115,545,434,337,383,342,51,117,350,452,181,591,262
RNASEH2A_2_25116,1,0,7,0,50,0,0,0,0,0,0,2
RNASET2_2_25117,457,590,423,515,817,703,1049,266,579,660,390,393
RND1_2_25118,29,315,111,237,15,266,26,50,4,253,23,3
RND2_2_25119,186,493,242,381,111,125,1007,239,77,123,429,890
RND3_2_25120,13,82,12,24,65,22,0,41,20,31,374,72
RNF148_2_25121,303,263,260,360,522,56,223,208,377,151,351,268
RNMT_2_25122,867,1153,1226,1017,1522,1368,1553,563,1039,2293,344,1404
RNMTL1_2_25123,267,391,241,417,419,628,0,277,126,149,545,184
RNPEP_2_25124,969,698,959,1066,107,1253,621,661,1049,207,650,597
RPAP2_2_25125,963,974,1040,741,1687,897,1775,1031,1629,1302,573,865
RPE65_2_25126,1125,825,930,716,1340,408,2316,664,480,605,400,482
RPIA_2_25127,1213,886,944,1428,894,1988,3053,1224,1440,1474,1009,1076
RPL4_2_25128,165,188,458,264,93,74,395,120,146,126,727,653
RPN1_2_25129,1408,1424,1028,1595,2372,564,741,1879,1489,1627,1188,2193
RPP25_2_25130,10,2,22,3,0,0,0,0,0,0,0,0
RPP40_2_25131,1257,1080,1194,1328,2204,825,858,2417,1526,685,1395,1693
RPS3_2_25132,626,625,400,454,920,1569,729,361,825,591,521,533
RPUSD1_2_25133,88,17,12,66,0,0,0,13,14,0,4,74
RPUSD2_2_25134,350,114,252,122,1164,34,0,225,316,194,1115,37

RRAGA_2_25135,245,246,246,182,165,314,691,20,251,189,257,323
RRAGC_2_25136,26,30,141,143,639,6,9,5,20,17,2,51
RRAGD_2_25137,106,50,181,120,0,173,7,0,0,0,228,572
RRAS_2_25138,168,44,106,64,100,120,0,16,11,25,0,3
RRM1_2_25139,126,200,225,271,29,227,523,351,205,66,882,375
RRP8_2_25140,699,741,492,470,587,375,1334,422,323,632,187,484
RSAD2_2_25141,1518,1656,1309,1709,1930,1735,1894,461,524,1590,386,3229
RTN4IP1_2_25142,112,47,123,201,357,247,430,67,64,55,351,117
SAMHD1_2_25143,41,44,49,65,7,0,5,23,80,1,508,15
SDF2_2_25144,93,123,122,195,0,6,78,492,33,45,35,199
SDHA_2_25145,106,131,50,89,3,467,15,111,30,313,429,0
SDHB_2_25146,208,149,241,240,173,9,270,14,162,77,29,689
SDHD_2_25147,353,122,218,303,62,614,196,103,16,96,595,408
SDR16C5_2_25148,173,106,179,105,310,89,371,156,22,59,196,365
SDR42E1_2_25149,949,829,967,1608,702,721,874,1749,1205,1149,1677,1235
SDR9C7_2_25150,1128,852,877,1205,1544,649,935,591,1257,1063,481,1143
SDSL_2_25151,381,312,470,387,8,622,391,122,437,310,45,173
SEPSECS_2_25152,471,683,542,702,1262,390,1046,692,283,645,2054,541
1-Sep_2_25153,1266,1218,1085,855,810,1118,1757,1437,1089,1710,706,950
SEPW1_2_25154,971,585,709,813,828,681,740,1291,822,1238,627,236
SERHL2_2_25155,712,658,583,738,651,215,897,1072,298,583,662,712
SETX_2_25156,1964,1536,2208,2202,2209,1090,2528,2228,1084,2674,2201,22
23
SGMS1_2_25157,294,216,178,302,30,34,66,401,274,139,416,689
SGPL1_2_25158,817,613,645,650,769,37,538,646,368,623,320,1382
SGSH_2_25159,274,322,195,276,246,0,22,11,3,116,595,712
SH3GL2_2_25160,15,61,34,33,21,0,3,0,1,1,227,8
SKIV2L2_2_25161,431,397,550,486,619,523,398,496,483,759,201,455
SLFN12_2_25162,794,1000,707,874,498,73,260,549,834,1284,903,1389
SLFN12L_2_25163,460,239,278,231,134,157,125,135,199,139,151,362
SLFN13_2_25164,335,353,343,476,1203,175,426,133,92,329,531,198
SLFN5_2_25165,1050,1170,1256,1120,1327,1336,761,1746,1158,473,1100,167
4
SLU7_2_25166,3177,2843,2508,3205,2599,2296,2659,3121,3194,3366,3382,29
99
SMG8_2_25167,2596,2612,2683,3220,3211,1011,3588,3773,2422,2386,3066,31
01
SMPD2_2_25168,206,132,123,227,244,594,0,33,8,144,1,48
SMPD3_2_25169,262,316,364,392,416,856,243,197,185,1072,352,434
SMPDL3A_2_25170,175,254,134,256,209,830,511,644,129,188,53,212
SMUG1_2_25171,74,258,141,206,0,159,25,186,18,362,288,19
SNF8_2_25172,579,463,614,588,24,613,14,727,418,237,14,468
SNRNP200_2_25173,259,206,230,141,65,68,332,171,514,265,123,347
SOAT1_2_25174,188,195,209,279,160,53,200,32,72,61,96,53
SOAT2_2_25175,427,469,430,466,404,245,477,249,358,553,1636,503
SOD1_2_25176,730,700,451,623,614,512,567,420,576,679,614,19
SOD3_2_25177,8,3,14,8,20,0,0,0,0,0,0,0
SORD_2_25178,41,26,41,34,0,50,0,70,0,1,0,54
SPACA3_2_25179,554,372,333,571,419,180,305,368,641,166,418,461
SPACA5B_2_25180,283,264,248,226,178,1,932,233,368,319,9,203

SPACA5_2_25181,283,264,248,226,178,1,932,233,368,319,9,203
SPEM1_2_25182,125,148,126,145,0,0,0,0,164,0,493,1
SPTLC2_2_25183,819,814,670,777,631,218,1472,1002,485,921,1866,524
SPTLC3_2_25184,149,74,159,327,92,4,122,39,569,67,59,83
SPTSSA_2_25185,54,38,62,38,82,43,4,14,0,120,0,56
SQLE_2_25186,732,645,616,674,779,328,714,514,1268,616,1266,247
SQRDL_2_25187,11,60,10,48,0,0,0,0,71,14,0,0
SRD5A1_2_25188,369,337,370,446,118,564,185,264,429,499,462,570
SRD5A2_2_25189,608,716,395,699,1304,1864,1003,364,638,823,183,127
SRD5A3_2_25190,413,562,351,476,496,542,627,455,185,636,411,525
SRR_2_25191,1650,1830,1961,2429,2021,1860,2799,1909,2038,1848,781,2067
SRSF9_2_25192,729,567,498,512,727,1223,623,167,406,23,200,1207
SRXN1_2_25193,1712,1792,1706,1697,2887,1751,1168,573,1261,2273,1419,15
06
SSB_2_25194,1006,1023,827,1188,631,692,2157,574,1684,1205,1167,595
SSU72_2_25195,36,77,112,138,146,223,254,42,42,4,190,0
ST14_2_25196,632,484,367,406,11,104,650,172,903,315,359,50
ST3GAL2_2_25197,37,11,19,29,17,0,11,31,13,1,0,18
ST3GAL4_2_25198,106,97,214,248,2,1,1,292,41,77,40,136
ST3GAL6_2_25199,1056,1045,1048,1157,1006,627,2572,2000,946,3217,2272,1
185
ST6GALNAC1_2_25200,631,394,499,457,763,557,490,508,596,498,319,510
ST6GALNAC2_2_25201,118,132,139,92,263,0,357,69,140,205,41,169
ST6GALNAC5_2_25202,996,986,746,928,510,1056,448,1512,489,1110,1103,136
4
ST6GALNAC6_2_25203,123,219,268,252,136,455,162,132,290,192,25,104
ST8SIA1_2_25204,521,489,358,385,155,125,74,617,73,953,29,767
ST8SIA2_2_25205,186,209,349,346,82,188,78,447,248,362,1396,323
ST8SIA3_2_25206,633,735,583,682,531,888,1166,280,151,527,620,721
ST8SIA5_2_25207,709,651,395,636,760,406,539,680,471,877,1113,744
ST8SIA6_2_25208,46,71,72,42,6,15,672,141,42,9,0,150
STS_2_25209,99,26,120,66,20,0,34,18,97,16,439,69
STT3A_2_25210,329,313,220,240,258,289,136,61,210,182,451,333
STT3B_2_25211,1517,1684,1670,2177,1484,740,2791,1723,2533,1650,1561,21
82
SUCLA2_2_25212,1206,1308,1066,1457,977,352,253,636,859,1653,2233,1900
SUCLG1_2_25213,33,35,13,45,103,0,0,62,6,30,0,14
SULT1A3_2_25214,2419,2432,2415,2709,2539,5091,2834,2646,3121,2215,3178
,3090
SULT1A4_2_25215,2419,2432,2415,2709,2539,5091,2834,2646,3121,2215,3178
,3090
SULT1B1_2_25216,632,590,654,617,914,529,521,422,848,171,815,662
SULT1C3_2_25217,818,762,724,863,538,171,339,427,669,372,204,505
SULT1C4_2_25218,345,278,446,241,230,681,1460,9,390,37,149,98
SULT1E1_2_25219,297,219,285,245,130,83,0,343,120,235,11,84
SULT2A1_2_25220,505,414,413,426,536,114,206,801,125,711,786,30
SULT4A1_2_25221,383,184,202,243,43,93,19,69,40,287,304,111
SUPV3L1_2_25222,91,75,97,66,4,364,0,36,81,138,12,131
SURF1_2_25223,69,284,181,135,0,151,343,298,7,450,310,58
TALDO1_2_25224,408,295,315,328,516,0,382,0,429,271,505,40

TARS2_2_25225,354,341,293,557,128,881,216,458,442,232,364,404
TARS_2_25226,329,245,335,403,672,357,567,273,700,59,36,274
TARSL2_2_25227,65,75,113,52,25,42,0,0,50,16,12,238
TAT_2_25228,461,318,447,455,611,706,1542,359,438,468,350,968
TBC1D10B_2_25229,576,428,463,544,424,24,579,236,745,372,238,554
TBCC_2_25230,356,247,269,269,288,170,367,306,146,558,745,220
TDG_2_25231,1311,1562,1740,1926,1226,798,2300,1272,1419,2015,919,1494
TDO2_2_25232,717,761,625,751,940,13,774,656,542,517,788,1011
TECR_2_25233,123,130,35,100,470,136,0,24,1,7,6,97
TECTA_2_25234,195,141,163,185,557,238,50,30,332,285,189,21
TFB2M_2_25235,653,496,488,528,968,828,430,409,876,707,294,857
TGDS_2_25236,1460,1118,1155,1756,900,1114,1270,2315,1186,1157,798,1103
TGM1_2_25237,230,97,168,316,269,15,203,15,133,236,156,539
TGM3_2_25238,53,52,53,40,172,193,33,24,1,16,90,57
TGM4_2_25239,631,692,536,692,427,43,407,350,301,1024,708,110
TGM6_2_25240,16,46,60,45,28,275,239,0,59,23,2,224
TGM7_2_25241,96,63,116,53,71,0,905,310,15,325,0,90
TGS1_2_25242,252,176,194,231,279,82,42,3,60,56,13,302
THG1L_2_25243,490,488,475,466,1,487,139,449,65,141,669,323
THOP1_2_25244,357,325,414,425,467,264,453,8,117,215,379,361
THUMPD2_2_25245,53,57,75,45,5,7,180,6,0,16,19,5
TKTL2_2_25246,152,171,122,119,175,76,48,0,277,11,0,1
TM7SF2_2_25247,1037,1358,756,999,519,384,240,1257,1161,1157,1662,764
TMEM55A_2_25248,106,75,99,65,19,11,368,0,120,0,58,142
TMEM62_2_25249,820,590,568,669,657,555,885,354,744,147,404,993
TMEM86B_2_25250,494,513,381,571,463,229,68,460,357,244,1331,1243
TMPRSS15_2_25251,148,170,109,172,402,21,216,69,103,131,34,202
TMX1_2_25252,2265,1935,1562,2198,894,1546,1860,1191,2213,2923,2112,186
7
TMX3_2_25253,199,248,186,223,89,7,5,95,18,0,352,11
TMX4_2_25254,0,0,0,3,0,92,0,0,120,0,0,85
TNKS2_2_25255,344,89,237,101,144,494,331,110,243,97,57,52
TNKS_2_25256,984,753,688,881,1560,826,1146,1365,1036,433,636,1215
TOP1_2_25257,463,432,421,308,414,659,969,265,698,140,532,562
TOP1MT_2_25258,696,442,607,540,838,689,816,643,24,1392,2274,975
TOP2A_2_25259,413,360,457,325,472,426,341,135,295,817,421,249
TOP2B_2_25260,221,186,149,249,348,32,325,95,109,248,131,516
TOP3A_2_25261,1242,1033,974,1302,1256,693,1817,853,937,1115,1116,1307
TOP3B_2_25262,493,543,507,501,673,560,476,207,206,82,256,911
TOR3A_2_25263,27,26,72,55,0,1,0,29,8,0,588,37
TPH1_2_25264,1426,1223,1038,1071,1411,1155,2138,1205,2175,1554,2090,13
57
TPMT_2_25265,772,538,629,734,1566,302,452,1067,1315,792,484,688
TPP1_2_25266,886,435,563,748,817,2,1373,399,1355,465,507,521
TPP2_2_25267,382,499,459,892,185,1064,613,494,653,498,314,384
TPSAB1_2_25268,236,136,182,311,412,23,576,428,43,124,8,569
TPSB2_2_25269,236,136,182,311,412,23,576,428,43,124,8,569
TPSD1_2_25270,31,55,121,92,365,5,6,153,0,282,0,82
TPST1_2_25271,927,793,998,1010,516,274,571,1073,1136,581,1603,977
TREH_2_25272,379,645,350,600,1017,517,550,250,465,152,426,579

TREX2_2_25273,117,83,6,36,17,0,274,0,0,61,308,159
TRHDE_2_25274,160,113,236,374,365,139,113,284,87,126,22,482
TRIM21_2_25275,737,547,477,756,1363,794,1015,223,378,725,746,454
TRIT1_2_25276,347,354,371,387,262,142,658,82,369,422,105,1089
TRMT112_2_25277,364,203,196,157,335,278,77,5,301,300,15,155
TRMT61A_2_25278,178,218,128,229,83,72,105,97,260,255,463,21
TRMU_2_25279,135,171,220,148,174,21,20,0,0,187,0,97
TRNT1_2_25280,132,121,147,134,504,255,598,74,137,5,19,26
TRUB1_2_25281,31,109,19,55,62,0,56,0,23,82,0,19
TRUB2_2_25282,384,348,354,521,23,217,82,210,359,549,735,888
TSTA3_2_25283,321,258,142,421,4,2,2,3,396,364,0,236
TST_2_25284,29,71,54,28,320,0,0,0,2,278,0,5
TTLL13_2_25285,508,767,562,687,656,1188,243,569,858,369,1278,602
TTLL1_2_25286,28,17,40,17,9,38,20,1,5,572,0,1
TTLL3_2_25287,252,142,362,436,162,42,0,0,0,185,778,0
TTLL4_2_25288,129,75,180,93,7,24,3,417,163,278,157,7
TUFM_2_25289,188,253,197,295,130,160,7,0,211,711,368,93
TULP2_2_25290,107,141,128,81,68,108,18,13,154,139,290,22
TUT1_2_25291,118,158,148,181,109,93,329,5,249,145,6,341
TXN2_2_25292,104,123,144,72,7,49,158,58,202,41,7,19
TXN_2_25293,548,499,449,403,220,42,42,529,444,553,746,647
TXNDC11_2_25294,351,336,336,495,630,331,379,246,335,799,357,336
TXNDC12_2_25295,1368,981,920,1137,1687,656,657,2190,1149,701,702,617
TXNDC15_2_25296,90,142,65,119,208,145,175,216,122,72,75,53
TXNDC17_2_25297,19,47,91,72,15,9,46,90,1,44,627,20
TXNL1_2_25298,346,194,225,201,231,237,427,191,315,202,317,204
TXNL4A_2_25299,300,171,310,304,210,211,753,278,371,119,666,301
TXNRD2_2_25300,734,721,470,716,603,611,687,400,453,760,336,873
TYMS_2_25301,39,94,54,55,25,18,2,0,269,7,4,9
TYR_2_25302,467,527,431,798,747,214,1292,357,168,367,105,776
TYRP1_2_25303,919,575,761,761,469,510,936,338,315,1313,1156,1012
UAP1_2_25304,656,819,785,833,1115,345,30,734,553,700,489,857
UAP1L1_2_25305,7,1,62,0,0,1,0,0,8,0,0,0
UBIAD1_2_25306,91,131,23,45,5,0,4,333,453,17,297,0
UBL4A_2_25307,194,154,119,143,347,47,11,173,71,8,149,161
UFSP1_2_25308,84,41,66,98,5,8,0,467,64,101,51,60
UFSP2_2_25309,1544,1135,1045,1554,2126,877,1318,237,1345,1358,1659,257
4
UGCG_2_25310,1490,1083,898,1244,1226,464,1800,1400,759,872,1411,904
UGGT1_2_25311,15,64,95,75,7,0,227,1,155,483,219,159
UGGT2_2_25312,663,490,505,673,607,473,191,161,336,301,674,655
UGT1A10_2_25313,93,66,45,92,206,50,4,57,4,1,130,16
UGT1A1_2_25314,177,177,238,252,2,192,0,1,28,226,0,359
UGT1A3_2_25315,162,77,89,166,19,89,361,33,225,57,740,46
UGT1A4_2_25316,650,816,859,832,379,324,917,839,431,476,231,947
UGT1A5_2_25317,2594,2226,2599,2694,3338,1917,3275,4051,2537,3244,4252,
2741
UGT1A7_2_25318,2262,2364,2645,2734,3398,1101,2548,2834,2048,3123,3115,
2144
UGT1A8_2_25319,775,743,758,885,559,252,490,787,644,1278,863,587

UGT1A9_2_25320,2262,2364,2645,2734,3398,1101,2548,2834,2048,3123,3115,
2144
UGT2A1_2_25321,199,141,412,282,76,2,266,204,90,722,1,276
UGT2A2_2_25322,128,58,35,202,478,98,0,54,0,494,718,0
UGT2A3_2_25323,288,113,267,233,204,115,39,106,292,124,202,52
UGT2B15_2_25324,1848,1570,1676,2084,1421,1092,2353,561,1381,1762,1291,
1468
UGT2B17_2_25325,1848,1570,1676,2084,1421,1092,2353,561,1381,1762,1291,
1468
UGT2B4_2_25326,122,130,117,105,341,108,34,0,143,262,188,7
UGT2B7_2_25327,971,586,458,729,844,296,257,594,160,545,668,476
UMPS_2_25328,371,404,473,442,805,123,647,270,82,278,313,515
UPB1_2_25329,298,329,336,379,83,392,181,174,397,415,225,147
UPF1_2_25330,35,9,23,26,0,39,169,0,0,3,1,48
UPRT_2_25331,942,737,1059,994,1274,1187,2266,1673,1073,1424,463,697
UQCR11_2_25332,1984,1402,1348,1376,2659,1149,1898,1439,1912,1638,979,1
306
UQCRC1_2_25333,366,261,236,262,480,19,532,657,224,329,1705,508
UQCRC2_2_25334,1786,1275,1582,1466,1906,1301,1754,343,1288,1643,476,12
89
UQCRFS1_2_25335,314,184,129,250,509,281,228,243,53,94,5,76
UQCRH_2_25336,223,265,200,199,41,127,17,31,347,324,45,219
UQCRQ_2_25337,486,412,544,603,226,582,511,202,669,442,935,505
UROD_2_25338,369,227,240,231,380,155,997,140,164,1,456,7
UROS_2_25339,1499,1342,1479,1338,1647,2250,1030,667,1226,1168,1483,129
1
UST_2_25340,435,606,712,489,539,726,274,603,765,288,367,373
UXS1_2_25341,420,276,249,310,324,284,186,472,36,307,408,163
VARS_2_25342,36,69,85,40,17,14,1,0,1,18,98,18
VAT1L_2_25343,165,57,78,61,23,13,0,0,139,103,77,250
VCP_2_25344,819,657,753,445,107,451,162,643,714,279,819,425
VNN1_2_25345,1205,1549,1116,1373,987,730,1476,73,1212,890,1196,850
WBSCR17_2_25346,58,29,27,38,1,3,1,53,35,125,0,5
WBSCR27_2_25347,181,110,98,161,9,0,20,0,607,293,1,220
WDFY3_2_25348,307,401,345,330,780,39,1279,101,186,21,538,409
WRN_2_25349,605,494,348,542,629,323,7,91,59,314,619,356
XDH_2_25350,249,158,199,210,684,121,653,174,260,113,276,545
XPNPEP2_2_25351,403,292,219,354,15,93,235,36,179,1089,189,305
XRCC2_2_25352,342,319,283,303,226,47,858,327,423,346,121,167
XRCC5_2_25353,304,186,242,245,236,143,196,129,347,141,84,244
XRCC6_2_25354,98,73,80,107,90,89,39,226,31,20,6,37
XRN2_2_25355,415,415,268,286,111,485,202,256,327,636,230,118
XYLT1_2_25356,503,328,285,402,43,1105,1853,0,35,232,239,285
XYLT2_2_25357,449,354,382,500,138,1075,457,693,230,263,183,264
YARS2_2_25358,587,446,425,582,897,549,79,2,837,1255,1342,184
YARS_2_25359,2075,1211,1596,2074,1454,1273,434,2169,1612,751,1232,1888
YKT6_2_25360,49,135,94,58,305,14,1,0,1,673,16,117
YPEL1_2_25361,77,148,108,85,43,217,1,339,179,60,143,8
ZADH2_2_25362,604,489,380,529,196,377,139,251,954,23,384,503
ZCCHC4_2_25363,17,97,54,91,61,21,246,271,55,108,81,0

ZDHHC17_2_25364,316,252,364,335,224,4,510,225,430,108,563,635
ZDHHC18_2_25365,174,151,104,120,193,119,206,219,228,45,235,276
ZDHHC1_2_25366,1791,1315,1297,1415,1692,2292,1372,728,1313,1493,1986,2
101
ZDHHC21_2_25367,260,203,183,247,284,288,379,154,218,275,26,338
ZDHHC2_2_25368,33,54,52,16,41,8,0,45,1,1,51,125
ZER1_2_25369,37,22,50,36,190,0,0,254,6,13,0,39
ZMPSTE24_2_25370,1238,859,917,982,906,507,496,791,953,1108,757,1261
ZRANB3_2_25371,361,383,347,394,1274,533,572,367,361,229,46,589
A1CF_2_25372,33,105,34,47,0,0,0,0,0,0,0,0
A2LD1_2_25373,4,0,12,41,4,0,0,0,0,0,0,4
AADAT_2_25374,441,438,360,405,441,505,271,509,357,205,310,318
AARSD1_2_25375,534,370,569,500,379,587,185,331,411,591,578,277
ABAT_2_25376,98,121,131,145,463,292,238,253,551,162,2,382
ABHD11_2_25377,414,537,421,505,507,537,28,446,806,630,664,589
ABHD12_2_25378,220,277,178,378,5,0,0,2,219,3,357,162
ABHD14B_2_25379,41,16,23,15,0,0,0,0,78,0,123,0
ABHD2_2_25380,194,107,77,187,241,60,0,0,168,268,175,82
ACAA1_2_25381,99,236,93,205,0,24,20,31,303,46,338,384
ACACA_2_25382,363,325,258,252,1,283,10,363,246,328,125,254
ACAD10_2_25383,432,408,442,586,417,153,122,392,387,645,1196,498
ACADM_2_25384,553,503,504,528,188,313,1538,295,1096,372,1080,775
ACADVL_2_25385,232,94,88,274,0,0,8,335,264,0,311,131
ACCS_2_25386,217,143,167,146,135,351,404,269,115,202,290,140
ACE_2_25387,54,58,100,126,0,24,18,237,1,0,12,335
ACHE_2_25388,52,37,29,20,0,0,0,0,20,0,0,6
ACIN1_2_25389,433,345,355,478,479,120,506,189,301,462,1144,333
ACLY_2_25390,137,162,46,123,2,6,4,40,1,25,66,114
ACOT11_2_25391,31,46,33,59,0,0,354,9,0,8,0,11
ACOT13_2_25392,195,168,179,118,45,47,252,108,45,140,93,117
ACOT7_2_25393,335,195,175,253,57,64,0,85,76,538,10,25
ACOT9_2_25394,247,151,134,250,227,11,232,297,190,213,90,321
ACOX1_2_25395,1118,1073,973,1006,2361,1590,1184,880,993,736,631,1365
ACOX3_2_25396,339,164,262,259,117,96,41,246,395,378,425,404
ACSBG1_2_25397,661,666,619,881,340,337,974,475,184,422,276,109
ACSF3_2_25398,17,1,3,6,0,0,0,0,120,1,0,1
ACSL3_2_25399,4056,4038,3883,4309,4817,3275,3609,2270,4136,4595,3785,4
863
ACSL4_2_25400,52,89,124,31,325,43,10,219,58,43,26,3
ACSL5_2_25401,22,16,18,77,7,156,8,32,47,4,17,3
ACSL6_2_25402,300,129,205,177,657,49,212,153,388,162,14,80
ACSM2B_2_25403,278,230,226,301,475,474,793,96,170,271,113,196
ACSM3_2_25404,266,240,148,302,232,179,51,1343,28,89,0,162
ACSS2_2_25405,49,65,46,42,14,8,132,192,119,26,467,130
ACY1_2_25406,337,337,381,617,421,73,27,1122,402,647,306,92
ADAMTS2_2_25407,115,172,198,214,207,23,54,97,74,106,87,6
ADARB1_2_25408,1105,840,852,793,1521,801,1071,1501,372,484,510,878
ADAR_2_25409,804,715,469,685,540,612,789,1188,155,1218,305,545
ADCY10_2_25410,608,640,546,707,701,300,397,1072,661,152,103,181
ADCY4_2_25411,126,43,148,198,6,4,5,8,41,16,4,39

ADCY5_2_25412,337,288,338,350,287,798,39,56,232,523,26,759
ADCY6_2_25413,367,454,282,381,232,642,0,237,283,202,373,114
ADH6_2_25414,762,624,511,785,531,579,384,1285,1551,1246,1335,568
ADH7_2_25415,889,731,834,1025,2582,542,421,320,375,1055,606,667
ADPRHL1_2_25416,18,36,46,82,207,52,382,101,105,100,96,4
ADSL_2_25417,148,66,167,245,277,0,0,31,1,0,306,0
ADSSL1_2_25418,13,29,77,27,153,0,0,1,18,0,269,0
AFMID_2_25419,363,172,340,401,326,97,340,45,81,299,426,275
AGA_2_25420,334,143,276,292,593,236,273,93,379,413,199,226
AGAP1_2_25421,142,118,172,207,0,244,1,45,466,821,0,5
AGAP2_2_25422,257,62,167,287,466,473,368,52,276,27,6,235
AGL_2_25423,947,618,635,1039,1466,434,997,517,214,880,236,558
AGPAT1_2_25424,350,431,269,290,427,326,10,102,187,219,459,166
AGPAT2_2_25425,38,23,18,48,0,23,93,11,3,2,92,4
AGPAT3_2_25426,302,196,265,398,279,295,310,74,410,153,343,45
AGXT2L1_2_25427,1243,829,957,1090,922,226,474,811,862,488,1463,439
AHCY_2_25428,45,28,56,17,7,0,15,15,131,96,173,34
AHCYL1_2_25429,106,130,144,141,86,17,0,61,187,206,70,23
AHCYL2_2_25430,1034,758,963,1042,1157,527,349,488,1115,461,1192,1999
AIFM1_2_25431,47,39,22,61,2,1,158,53,1,23,0,14
AIFM2_2_25432,69,168,126,108,2,305,39,7,211,333,0,244
AIFM3_2_25433,68,79,44,40,4,29,109,3,213,3,60,7
AKIRIN1_2_25434,21,26,111,20,20,0,245,0,0,0,0,0
AKR1A1_2_25435,533,322,408,480,192,221,557,111,433,1103,0,69
AKR1C2_2_25436,35,20,68,25,0,0,0,0,3,0,513,418
AKR1D1_2_25437,391,358,427,463,1092,422,69,421,409,136,489,760
ALAS1_2_25438,111,136,42,62,16,32,18,106,62,54,1,8
ALAS2_2_25439,189,218,167,157,75,277,170,180,196,62,112,57
ALDH16A1_2_25440,257,257,257,315,305,347,0,268,370,379,264,177
ALDH1A2_2_25441,335,517,480,511,303,121,102,369,635,576,205,356
ALDH2_2_25442,199,103,181,210,214,91,204,195,264,280,35,113
ALDH3A1_2_25443,1090,1200,1038,1042,1235,750,723,1419,1794,856,1253,12
74
ALDH3A2_2_25444,887,831,754,929,573,499,567,1247,441,712,288,640
ALDH3B1_2_25445,324,374,540,352,259,63,3,36,1296,69,570,323
ALDH3B2_2_25446,329,534,354,430,98,848,784,21,741,144,317,135
ALDH4A1_2_25447,25,14,3,6,0,0,0,0,0,0,0,15
ALDH5A1_2_25448,179,236,179,167,132,333,51,107,242,20,100,358
ALDH7A1_2_25449,998,685,699,824,1000,970,258,201,709,1314,507,871
ALDH8A1_2_25450,41,96,47,61,6,552,71,14,8,0,2,0
ALDOA_2_25451,107,20,97,85,13,549,2,25,0,0,563,0
ALG3_2_25452,162,217,137,207,669,5,72,391,191,0,23,1
ALG5_2_25453,110,9,57,64,150,0,0,0,0,0,0,0
ALG8_2_25454,1780,1361,1563,1863,708,1477,1797,2557,1530,1574,1144,159
5
ALG9_2_25455,248,351,187,240,252,496,178,39,51,413,23,310
ALOX15B_2_25456,65,204,167,141,94,39,131,22,15,26,14,680
ALOXE3_2_25457,947,792,790,1175,1188,1365,1460,2462,710,1041,1442,913
AMACR_2_25458,290,283,258,273,222,133,26,0,177,422,617,108
AMD1_2_25459,190,156,150,138,352,329,85,279,112,49,21,167

AMDHD2_2_25460,33,59,48,76,68,9,0,0,6,0,26,14
AMPD1_2_25461,33,31,41,21,0,16,0,1,21,1,31,127
AMPD2_2_25462,490,360,412,620,87,109,122,357,484,498,916,419
AMPD3_2_25463,185,133,107,129,384,8,62,105,55,27,0,65
AMT_2_25464,42,15,42,47,0,0,0,2,0,0,76,18
AMY1A_2_25465,5003,4822,4727,5303,5984,4027,5641,4409,4590,6551,3209,6
376
ANG_2_25466,358,132,261,373,1019,1,21,418,249,28,950,641
AOAH_2_25467,559,487,506,889,509,370,1670,797,1126,1282,993,1163
AOC2_2_25468,148,97,55,87,1,135,24,33,194,114,7,201
APC_2_25469,513,524,530,424,651,553,41,368,240,550,544,353
APEX1_2_25470,66,45,83,41,170,15,1,284,70,2,88,0
APOBEC3A_2_25471,1158,646,903,847,2009,297,1706,515,1194,972,766,784
APOBEC3H_2_25472,230,175,311,297,144,274,760,10,471,270,73,119
APRT_2_25473,433,266,610,529,27,153,0,367,541,424,1545,25
ARF1_2_25474,66,32,40,132,5,160,74,2,93,26,182,104
ARFRP1_2_25475,61,65,67,67,0,268,0,0,4,2,0,0
ARHGAP5_2_25476,1202,1330,1064,1322,1806,1658,1131,907,728,1660,426,64
4
ARHGEF10L_2_25477,246,121,147,221,131,168,0,52,39,115,30,152
ARL2_2_25478,342,300,182,330,250,277,39,260,182,156,496,530
ARL4A_2_25479,334,234,337,398,783,220,842,104,905,360,553,231
ARL5A_2_25480,479,384,419,384,1011,337,581,391,464,158,642,423
ARSA_2_25481,341,242,158,286,373,353,18,1,286,343,0,209
ARSB_2_25482,78,92,61,90,0,0,0,65,11,31,0,436
ARSF_2_25483,162,168,89,109,1,151,311,297,247,58,283,336
ART3_2_25484,364,361,238,200,289,467,1541,25,223,732,37,172
ART5_2_25485,484,466,580,591,712,286,554,513,401,835,569,301
ASAH1_2_25486,1448,1567,1112,1838,1259,1842,1580,1754,1035,909,2888,85
5
ASAH2_2_25487,144,203,319,250,217,231,1353,542,744,232,487,571
ASL_2_25488,435,295,337,510,360,121,492,521,143,187,576,371
ASMT_2_25489,291,63,164,118,4,205,320,0,81,77,98,196
ASMTL_2_25490,56,48,53,70,3,0,0,0,31,19,81,30
ASNS_2_25491,1277,1076,980,1636,746,942,1270,701,1597,1487,780,1539
ASPA_2_25492,314,293,94,147,53,26,6,33,203,458,716,19
ASRGL1_2_25493,52,60,33,28,44,0,0,97,1,8,2,34
ASS1_2_25494,249,111,358,242,123,88,789,317,117,118,138,11
ATE1_2_25495,363,309,333,476,274,252,466,456,340,716,79,178
ATL1_2_25496,337,371,407,318,214,291,723,538,333,209,300,375
AURKAIP1_2_25497,135,282,269,247,213,14,232,167,533,324,102,296
AZIN1_2_25498,641,594,531,633,477,443,357,816,862,512,1119,96
B3GALNT1_2_25499,919,1085,1065,1122,2041,546,1170,793,1496,2000,519,91
4
B3GALT5_2_25500,119,95,47,211,164,68,0,0,1,0,38,57
B3GAT1_2_25501,354,143,118,358,0,0,0,25,83,0,423,313
B4GALNT2_2_25502,270,148,251,147,0,364,771,327,6,610,324,1
B4GALT2_2_25503,236,122,223,251,105,51,515,606,139,11,216,514
B4GALT3_2_25504,742,810,698,778,1665,647,259,814,699,248,980,1116
B4GALT4_2_25505,64,81,40,144,401,0,296,3,239,233,0,42

BAAT_2_25506,203,186,298,185,64,154,202,51,470,41,268,171
BACE1_2_25507,202,263,136,223,327,1,410,151,146,287,0,237
BACE2_2_25508,111,154,174,157,22,581,1,8,187,297,0,320
BCAT1_2_25509,423,509,465,636,322,301,843,798,224,1037,329,718
BCAT2_2_25510,273,216,245,274,110,46,409,267,758,155,62,141
BCKDHA_2_25511,1421,1193,1144,1241,1123,264,848,1173,1515,1843,797,115
6
BCKDHB_2_25512,426,350,520,516,50,820,1526,577,540,282,720,766
BCO2_2_25513,1181,881,1065,1132,874,297,625,128,828,515,742,1339
BDH1_2_25514,41,24,59,102,62,208,0,105,6,35,3,43
BFSP1_2_25515,1474,1545,1444,1522,1233,675,1394,1431,1437,1018,2136,16
77
BHMT2_2_25516,157,78,139,132,4,8,13,1,15,244,0,0
BMP1_2_25517,24,7,1,21,0,0,20,0,0,22,0,0
C17orf101_2_25518,110,75,96,108,73,43,11,3,31,27,202,20
C1GALT1C1_2_25519,246,183,170,127,134,93,481,99,56,327,3,249
C1S_2_25520,15,22,35,23,19,0,0,10,0,0,0,0
C2_2_25521,979,769,479,850,561,779,1071,1138,751,513,168,816
CA10_2_25522,503,570,402,393,437,176,515,346,605,708,329,547
CA12_2_25523,76,3,46,43,1,0,172,516,106,0,0,69
CA1_2_25524,302,179,213,204,204,228,515,35,222,95,335,697
CA7_2_25525,79,42,52,58,22,208,59,1,167,215,0,4
CAB39_2_25526,233,279,272,220,365,49,187,29,345,283,178,128
CANT1_2_25527,203,99,139,182,110,28,63,329,172,16,89,43
CAPN1_2_25528,237,175,170,263,415,125,266,87,360,40,216,471
CAPN2_2_25529,154,185,149,168,141,163,494,99,207,533,49,91
CARNS1_2_25530,1093,859,824,1020,826,1170,928,129,812,802,1253,449
CARS_2_25531,7,30,37,47,1,2,2,140,36,263,2,29
CASP10_2_25532,446,340,303,351,248,184,130,168,633,310,483,586
CASP1_2_25533,1032,890,893,1002,911,1049,1447,862,1044,1055,597,249
CASP2_2_25534,99,29,53,90,212,62,0,5,0,31,66,4
CASP3_2_25535,389,245,292,278,34,813,770,204,127,384,14,347
CASP4_2_25536,52,50,129,143,34,170,295,104,20,7,18,84
CASP5_2_25537,698,596,621,1039,575,72,102,562,148,342,342,803
CASP6_2_25538,346,269,255,260,232,182,683,303,477,226,138,318
CASP7_2_25539,401,378,426,520,605,377,506,775,335,390,426,699
CASP8_2_25540,360,406,320,346,732,320,608,143,575,367,84,326
CASP9_2_25541,151,293,200,186,33,220,215,357,163,96,457,39
CASZ1_2_25542,321,203,199,256,449,107,80,297,591,89,24,89
CAV3_2_25543,84,55,59,131,0,1,102,21,48,59,3,53
CBS_2_25544,60,105,48,81,11,187,271,0,1,331,440,436
CCBL1_2_25545,133,49,62,61,3,31,23,35,11,28,649,4
CCBL2_2_25546,259,261,179,167,1,327,13,189,1,75,27,1
CDC42_2_25547,279,223,243,238,133,322,1034,251,421,107,360,309
CDH16_2_25548,60,37,54,112,41,6,29,0,14,91,1,133
CECR1_2_25549,824,651,488,713,109,359,1259,827,410,536,685,834
CEPT1_2_25550,54,36,45,40,14,0,49,4,60,86,74,144
CERS1_2_25551,7,61,9,9,0,0,0,0,0,9,0,0
CES1_2_25552,25,19,16,72,42,1,0,0,0,10,0,0
CES3_2_25553,154,94,91,113,257,2,309,336,103,125,0,287

CES4A_2_25554,341,434,365,277,670,103,888,201,412,131,224,140
CES5A_2_25555,3958,2949,2686,3071,4937,1006,3469,3352,3092,3467,3321,3
506
CHAT_2_25556,11,36,30,30,0,4,0,47,31,0,0,0
CHI3L2_2_25557,1179,1164,935,1328,2165,553,383,1034,1476,808,1385,970
CHIA_2_25558,457,232,293,428,6,8,286,123,169,282,151,37
CHPF_2_25559,319,827,418,523,283,430,86,134,39,720,507,677
CHST11_2_25560,159,85,92,40,27,1,18,25,0,1,20,60
CHST15_2_25561,734,585,673,540,1318,525,899,65,1766,365,636,255
CHST4_2_25562,82,122,104,119,75,26,2,16,14,75,180,83
CHST8_2_25563,884,701,719,931,346,529,1199,584,1126,1439,895,983
CLP1_2_25564,512,296,478,388,444,337,264,514,476,490,151,596
CNDP2_2_25565,309,304,312,365,147,310,90,202,237,394,909,365
CNTN1_2_25566,765,654,647,731,495,333,129,488,325,677,287,1342
CNTN4_2_25567,85,55,104,121,21,89,53,201,272,18,54,9
COMT_2_25568,89,88,29,88,23,10,4,344,46,43,0,280
COQ6_2_25569,321,311,233,196,16,60,229,136,45,10,49,78
COX11_2_25570,120,93,100,91,164,5,26,31,143,6,26,55
COX15_2_25571,165,181,191,115,19,26,32,53,201,27,41,324
CPA5_2_25572,180,132,137,247,44,376,78,133,20,183,383,206
CPB2_2_25573,98,205,114,194,0,216,284,75,67,109,14,241
CPM_2_25574,1938,1299,1467,1423,1348,717,2577,1328,2231,827,739,1324
CPPED1_2_25575,52,53,56,60,39,16,243,0,3,117,0,133
CPT1A_2_25576,1205,817,997,1068,998,620,264,1194,1107,837,447,1140
CPT1B_2_25577,135,167,153,124,633,1008,105,52,10,130,11,36
CPT1C_2_25578,496,456,468,490,806,50,75,79,822,820,90,627
CRLS1_2_25579,2881,2583,2691,2593,1929,2505,2833,801,2275,3610,1466,30
78
CRMP1_2_25580,250,186,250,409,360,386,157,56,260,51,48,527
CROT_2_25581,431,194,54,233,40,293,1,0,287,1,0,32
CRY2_2_25582,168,180,165,184,38,25,48,98,102,17,269,304
CRYM_2_25583,19,20,21,24,87,20,0,12,136,9,33,0
CRYZ_2_25584,589,782,832,798,810,426,685,779,783,1418,446,1242
CSDE1_2_25585,457,239,316,311,156,165,51,597,507,359,991,491
CSGALNACT1_2_25586,180,126,124,158,72,137,2,1,123,244,24,185
CSMD3_2_25587,330,327,282,290,415,274,881,272,183,545,249,219
CTAGE5_2_25588,64,46,92,42,149,159,608,420,40,48,68,139
CTBP1_2_25589,79,190,54,81,0,0,0,1,0,193,0,0
CTPS2_2_25590,49,22,80,57,89,137,19,217,0,11,0,0
CTSA_2_25591,224,189,290,228,215,47,212,239,223,15,140,243
CTSB_2_25592,255,201,170,206,125,128,15,82,572,141,479,255
CTSE_2_25593,660,539,500,643,454,666,836,1014,377,909,733,895
CTSL1_2_25594,193,154,213,116,126,87,49,391,265,99,212,33
CTSL2_2_25595,203,79,95,123,731,119,1,3,7,119,385,178
CTSS_2_25596,176,138,65,104,0,10,303,1,0,424,0,65
CYB561_2_25597,23,0,34,82,0,0,0,0,0,0,476,0
CYB5A_2_25598,187,19,104,148,35,11,0,196,365,105,358,52
CYB5R3_2_25599,180,49,130,293,971,91,774,8,376,0,0,857
CYP11A1_2_25600,416,355,269,484,1340,588,113,1054,273,752,393,802
CYP11B1_2_25601,242,163,232,212,162,195,48,67,131,244,184,507

CYP19A1_2_25602,788,750,466,717,363,233,1292,764,255,788,972,1405
CYP21A2_2_25603,70,69,57,13,12,267,218,12,18,0,252,121
CYP24A1_2_25604,257,237,350,307,87,256,377,1077,103,302,228,144
CYP26A1_2_25605,443,305,313,261,131,235,300,684,91,259,0,882
CYP2A7_2_25606,246,51,140,247,53,139,2,489,9,266,95,199
CYP2C18_2_25607,269,255,166,279,56,172,290,54,580,207,492,649
CYP2C8_2_25608,121,186,134,191,142,106,116,149,37,70,43,331
CYP2D6_2_25609,67,50,92,125,5,0,1,28,0,56,118,165
CYP3A43_2_25610,517,473,523,745,516,351,444,436,614,613,129,779
CYP3A4_2_25611,1435,1387,1012,1370,1730,1328,1397,720,1177,1761,552,78
7
CYP4B1_2_25612,116,103,66,110,3,37,211,8,66,3,0,2
CYP4F11_2_25613,238,306,257,347,556,684,67,35,245,434,835,324
CYP4F3_2_25614,319,116,142,314,301,43,542,674,180,192,629,113
CYP51A1_2_25615,485,261,407,363,483,858,436,375,231,447,222,445
DAGLB_2_25616,376,246,296,304,45,5,337,381,356,226,288,719
DCLRE1C_2_25617,2306,2097,1764,2543,1760,1429,3795,2154,2084,1801,2390
,1384
DCP2_2_25618,1973,2192,1888,1829,2836,1818,2258,1425,2753,1139,1340,27
12
DCT_2_25619,72,323,93,97,78,0,4,55,1,48,493,126
DCTD_2_25620,1684,1520,1143,1492,1933,719,2537,776,1312,905,620,1518
DCXR_2_25621,62,95,34,59,2,0,264,18,0,3,9,63
DDAH1_2_25622,313,368,380,426,271,306,167,429,447,110,470,141
DDC_2_25623,428,344,423,446,421,590,342,521,448,674,914,834
DDHD1_2_25624,118,95,222,131,172,71,123,10,12,9,11,793
DDO_2_25625,239,146,179,204,107,1,97,397,204,263,133,168
DDT_2_25626,2,18,10,51,1,0,0,57,0,51,0,4
DDX11_2_25627,252,56,130,148,587,73,0,206,6,465,0,204
DDX17_2_25628,58,77,53,56,2,4,8,0,5,5,1,7
DDX19B_2_25629,310,670,393,557,926,795,222,1000,296,546,895,201
DDX31_2_25630,460,240,265,232,407,120,697,55,368,185,86,510
DDX39B_2_25631,914,829,1091,1023,1043,948,426,360,775,972,909,1286
DDX3X_2_25632,1034,933,723,1029,1071,826,417,542,677,1412,918,1792
DDX3Y_2_25633,429,325,302,294,347,5,553,488,312,657,237,303
DDX42_2_25634,228,332,273,140,49,0,153,24,774,128,101,128
DDX47_2_25635,271,322,329,469,76,878,184,180,783,573,9,377
DDX4_2_25636,240,255,285,289,457,59,225,56,112,140,94,288
DDX54_2_25637,44,6,2,16,0,0,18,0,3,0,2,5
DFFA_2_25638,1542,1338,1381,1845,3362,1135,1071,1386,615,1040,3100,993
DGCR8_2_25639,875,847,870,949,1064,417,1515,513,203,552,442,776
DHCR7_2_25640,263,170,130,248,0,211,318,306,21,22,0,46
DHDDS_2_25641,894,718,371,539,333,497,244,1407,764,347,1098,496
DHFRL1_2_25642,130,108,97,113,244,17,99,42,162,35,5,154
DHPS_2_25643,30,48,80,79,11,204,297,0,85,361,61,56
DHRS1_2_25644,842,746,890,1037,1672,707,2750,749,597,811,781,350
DHRS2_2_25645,54,25,73,106,0,0,8,176,1138,1,0,0
DHRS9_2_25646,46,102,48,88,235,3,1,292,12,168,1,149
DHX16_2_25647,555,551,377,450,263,83,903,750,569,639,1094,720
DHX30_2_25648,123,80,213,159,320,58,184,7,243,84,341,34

DHX33_2_25649,740,748,639,923,331,265,871,616,1237,1263,156,1594
DHX35_2_25650,280,236,250,255,562,125,99,2,334,223,1274,609
DHX36_2_25651,325,369,517,416,326,215,313,185,489,10,152,701
DHX40_2_25652,210,264,182,180,83,1,301,32,135,166,11,290
DIAPH3_2_25653,10,30,42,25,10,92,162,6,0,54,12,56
DIS3_2_25654,342,339,361,444,179,2,366,383,418,590,288,383
DIS3L_2_25655,0,2,0,0,0,0,0,0,0,0,0,0
DKC1_2_25656,561,472,451,433,575,860,1080,472,573,711,502,366
DNAJC27_2_25657,379,352,330,306,43,257,104,342,906,391,152,540
DNASE1L1_2_25658,610,708,603,644,1002,1251,1129,917,560,163,487,442
DNM1_2_25659,528,336,417,397,174,181,118,628,503,605,78,367
DNM1L_2_25660,355,315,208,279,370,300,349,304,217,437,30,233
DNM2_2_25661,386,354,152,216,308,162,239,406,472,292,0,254
DNM3_2_25662,129,103,167,234,14,339,78,1,292,0,33,139
DNTT_2_25663,1024,818,771,888,1188,932,1582,949,658,486,619,669
DOHH_2_25664,89,144,128,191,0,4,0,7,0,564,9,35
DOLPP1_2_25665,958,610,513,672,346,1221,311,543,842,301,25,508
DPEP1_2_25666,155,67,229,185,0,0,0,0,0,1,75,0
DPEP3_2_25667,160,117,144,106,163,99,478,129,0,259,251,1
DPH5_2_25668,386,181,220,489,502,33,32,348,359,0,1036,655
DPM3_2_25669,585,420,651,670,1292,915,615,1024,912,302,271,788
DPP3_2_25670,870,917,662,693,608,849,1083,679,1009,1249,1045,1006
DPP8_2_25671,229,146,112,154,18,139,139,690,203,177,14,385
DPYSL2_2_25672,105,173,170,122,1,0,564,144,106,1,108,326
DPYSL3_2_25673,614,843,709,912,1040,1057,2877,901,480,812,1413,1026
DROSHA_2_25674,1610,1859,1600,1627,747,840,1417,1521,2223,3988,638,107
4
DSE_2_25675,524,248,417,597,682,622,881,406,265,117,259,633
DUOX1_2_25676,135,395,276,329,14,66,361,53,152,3,762,160
DUT_2_25677,408,242,163,319,408,169,121,134,157,350,405,165
ECE1_2_25678,433,466,558,464,632,58,240,273,412,312,208,355
ECE2_2_25679,258,245,295,425,51,435,278,320,121,418,98,489
ECI1_2_25680,7,4,43,23,0,0,43,0,2,0,0,292
ECI2_2_25681,1003,648,862,871,1109,949,979,324,1449,1017,798,1013
EDEM2_2_25682,0,0,0,0,0,0,0,0,0,0,0,0
EFEMP1_2_25683,1214,1022,921,1213,1868,668,1614,1446,1539,1217,978,143
8
EFTUD2_2_25684,280,656,386,513,336,558,226,54,607,1182,22,228
EGLN2_2_25685,0,3,0,0,0,0,0,0,0,0,0,0
EHHADH_2_25686,394,593,528,833,1100,388,428,804,284,208,250,151
ELAC2_2_25687,8,29,17,16,0,0,2,1,23,0,0,0
ELOVL5_2_25688,939,1002,1108,1014,766,1275,418,1266,689,885,653,824
ELOVL6_2_25689,194,202,165,251,113,184,31,88,158,439,118,193
ELOVL7_2_25690,45,56,107,138,360,239,26,0,94,34,21,500
ENDOV_2_25691,20,24,46,34,8,0,2,14,4,14,13,27
ENO3_2_25692,805,313,402,515,377,134,79,280,612,47,737,227
ENOX2_2_25693,1162,1007,797,1070,689,546,1281,1513,825,851,2032,1317
ENPP2_2_25694,46,77,29,142,2,208,425,15,47,11,2,39
ENTPD1_2_25695,701,644,657,655,81,821,720,1357,712,448,130,323
ENTPD2_2_25696,138,284,262,447,380,218,1,30,5,146,20,375

ENTPD4_2_25697,459,249,280,515,556,354,27,439,392,347,484,283
ENTPD6_2_25698,126,147,214,165,1159,20,9,237,175,268,26,161
ENTPD8_2_25699,85,101,72,96,29,29,8,0,99,258,25,173
EPHX1_2_25700,110,69,72,61,44,47,1,181,299,61,7,148
EPHX3_2_25701,77,148,75,62,63,2,1,223,21,43,19,76
ERCC1_2_25702,242,156,116,100,17,0,651,1,932,112,0,441
ERCC2_2_25703,343,256,231,400,476,52,380,203,382,104,924,63
EXO1_2_25704,173,225,109,315,479,147,83,197,338,318,324,175
EXOG_2_25705,75,90,63,71,128,130,0,28,207,368,6,32
EXOSC3_2_25706,5184,3828,3940,5016,3699,4086,5007,3324,3291,5156,5521,
4393
EXOSC9_2_25707,209,337,225,297,246,231,241,136,268,218,372,195
EXTL2_2_25708,71,12,86,17,0,0,278,23,149,0,0,85
F7_2_25709,247,228,130,268,84,153,634,308,130,0,441,104
FAHD1_2_25710,39,178,65,108,174,3,6,267,42,183,77,1
FAM108A1_2_25711,149,173,156,191,49,11,40,108,123,25,217,182
FAM135A_2_25712,542,542,445,572,139,737,842,309,1351,391,731,664
FAN1_2_25713,931,940,780,960,935,697,280,1009,962,572,398,913
FDPS_2_25714,67,61,27,46,10,25,22,52,34,11,0,17
FDXR_2_25715,29,38,64,117,2,63,9,96,12,56,65,21
FECH_2_25716,35,69,25,20,3,48,42,313,29,9,1,76
FERMT3_2_25717,42,29,52,47,0,47,24,0,6,0,0,7
FHIT_2_25718,420,427,217,362,643,149,739,286,148,510,80,527
FIGNL1_2_25719,218,65,114,68,34,188,0,89,130,1,0,2
FKBP11_2_25720,285,293,182,273,370,354,244,152,478,462,249,156
FKBP1A_2_25721,3,17,39,75,340,1,0,1,1,146,0,1
FKBP1B_2_25722,68,37,57,52,19,0,3,117,22,0,0,12
FKBP2_2_25723,229,225,122,149,191,106,182,0,16,133,54,148
FKBP5_2_25724,74,65,66,92,103,298,147,36,23,54,0,60
FKBP7_2_25725,509,753,691,681,1032,1793,834,438,557,955,958,1022
FLAD1_2_25726,23,18,7,12,54,0,0,0,0,0,0,18
FMO3_2_25727,352,325,469,298,632,216,70,47,124,872,449,141
FMO5_2_25728,80,80,89,103,42,48,141,188,78,263,66,23
FN1_2_25729,93,56,69,50,4,12,0,94,66,178,175,64
FOLH1_2_25730,0,0,0,0,0,0,0,0,0,0,0,0
FPGS_2_25731,97,71,39,63,181,0,21,249,14,5,161,0
FPGT_2_25732,235,152,247,268,15,45,272,19,262,250,338,281
FTCD_2_25733,33,141,134,113,6,0,0,1,0,0,1,28
FTSJ1_2_25734,89,103,103,81,0,290,14,2,4,253,487,94
FUT2_2_25735,174,135,115,268,41,105,250,0,233,411,760,88
FUT3_2_25736,25,98,40,63,4,9,214,1,34,75,0,120
FUT6_2_25737,393,372,390,290,348,235,579,413,296,217,489,615
FUT8_2_25738,370,224,267,297,143,45,36,18,12,76,18,329
G3BP1_2_25739,167,339,291,262,278,314,81,397,15,609,955,389
G3BP2_2_25740,1354,1169,1254,1323,1475,219,299,831,841,931,2522,726
G6PD_2_25741,416,457,359,548,49,507,99,826,381,905,47,301
GAA_2_25742,71,15,88,29,31,0,92,0,24,2,0,0
GAD1_2_25743,282,304,233,226,75,120,1016,171,47,375,101,310
GAD2_2_25744,123,129,91,132,188,1,201,144,37,138,2,153
GALC_2_25745,148,122,173,77,568,574,616,215,220,36,388,237

GALE_2_25746,125,102,61,119,69,51,14,736,6,14,15,96
GALNT9_2_25747,297,284,177,307,57,174,181,68,236,385,60,264
GALNTL1_2_25748,684,866,737,801,1328,999,448,748,727,675,1223,486
GAMT_2_25749,334,348,323,417,88,37,398,488,196,277,40,268
GANAB_2_25750,70,69,83,138,43,43,0,90,1,250,259,252
GART_2_25751,1124,1006,873,1201,1036,809,1223,1921,529,1275,683,1819
GBA_2_25752,61,8,71,79,10,37,0,382,49,4,6,11
GBP5_2_25753,328,212,346,388,468,85,23,477,64,745,450,247
GCAT_2_25754,192,227,101,123,210,119,340,61,145,134,555,10
GCDH_2_25755,187,242,269,161,83,145,53,345,92,42,482,70
GCH1_2_25756,657,869,506,806,382,727,1011,747,592,789,1335,836
GCLC_2_25757,1258,892,956,1079,1455,611,1398,1037,1072,1129,1443,1407
GCNT1_2_25758,103,149,120,197,13,46,115,797,188,130,7,3
GDPD1_2_25759,137,112,77,62,130,17,0,0,87,192,0,74
GDPD2_2_25760,953,779,767,786,1143,445,754,407,796,232,897,846
GEM_2_25761,990,820,888,1117,1012,892,839,818,919,1619,744,536
GEN1_2_25762,698,942,895,1066,2196,935,819,204,1516,2112,513,933
GFM2_2_25763,355,310,285,327,405,248,16,205,418,271,865,232
GFOD1_2_25764,67,20,20,30,1,136,100,19,45,2,0,137
GGCT_2_25765,146,189,345,300,315,138,7,2,15,284,0,274
GGCX_2_25766,161,407,258,361,2,142,1177,258,43,1,91,263
GGT1_2_25767,135,170,167,261,257,121,227,70,91,152,287,568
GGT5_2_25768,121,509,411,273,128,839,371,109,0,178,1396,0
GGT6_2_25769,35,61,69,72,0,0,158,176,0,0,0,0
GGTLC1_2_25770,116,125,23,120,0,211,0,147,15,90,369,78
GLB1_2_25771,72,164,101,87,31,36,554,0,173,7,786,22
GLRX2_2_25772,1052,899,701,773,352,346,844,1163,1330,928,418,614
GLRX3_2_25773,1467,1597,1609,1845,1603,1159,1332,2954,1117,999,1804,19
06
GLRX_2_25774,278,274,308,319,118,26,369,373,619,26,716,527
GLT8D1_2_25775,110,102,97,56,0,1,0,209,158,41,500,33
GLUL_2_25776,89,54,32,57,28,0,0,66,129,276,4,1
GM2A_2_25777,180,85,57,133,47,4,36,227,19,41,57,59
GMPPA_2_25778,281,272,168,309,246,113,271,2,407,86,0,3
GMPPB_2_25779,159,228,211,337,6,55,113,841,107,237,470,390
GMPR2_2_25780,232,203,158,172,30,695,363,197,114,37,66,213
GNAI2_2_25781,763,587,624,848,1117,614,1305,1048,951,326,549,1107
GNAL_2_25782,633,856,455,907,115,339,357,406,948,48,1045,871
GNAO1_2_25783,140,94,43,63,141,17,0,0,127,82,0,671
GNAT1_2_25784,45,11,23,52,18,0,51,0,2,0,0,28
GNB5_2_25785,339,308,276,340,779,575,240,379,104,322,126,272
GNG10_2_25786,1199,1387,1214,1628,2340,1368,2060,2319,773,1187,2552,13
47
GNG4_2_25787,325,186,215,313,1,22,542,51,4,7,0,93
GNGT2_2_25788,397,321,208,437,382,707,1290,580,142,313,54,258
GPD2_2_25789,386,376,305,391,740,600,306,111,174,39,504,432
GPHN_2_25790,244,376,211,303,396,86,353,19,343,828,139,158
GPNMB_2_25791,84,131,45,143,82,130,12,29,110,6,67,33
GPT2_2_25792,262,187,167,306,7,90,176,49,58,62,559,317
GPX4_2_25793,63,81,86,61,9,97,86,13,25,41,1,53

GSR_2_25794,500,477,419,503,533,806,435,1369,436,758,529,1084
GSTCD_2_25795,82,63,75,41,0,22,1,38,5,19,0,459
GSTK1_2_25796,489,360,369,597,383,354,345,113,317,170,14,161
GSTM1_2_25797,172,82,189,172,141,0,359,251,57,167,21,62
GSTM2_2_25798,1375,1387,1262,1640,1306,1363,1566,2433,1467,1334,748,16
82
GSTM4_2_25799,1497,1143,1065,1174,2145,997,1675,1989,1577,1088,864,761
GSTO1_2_25800,279,321,206,381,50,33,2,368,523,160,113,214
GSTO2_2_25801,195,276,257,162,263,4,41,0,46,745,381,181
GSTZ1_2_25802,471,389,299,423,408,105,795,924,793,553,127,1095
GTPBP3_2_25803,961,711,852,727,282,971,559,912,404,841,720,547
GUCY1A3_2_25804,243,361,267,350,167,231,93,17,65,168,254,353
GYG1_2_25805,812,691,725,925,406,797,2012,1165,382,220,1929,262
GYG2_2_25806,63,68,33,42,375,0,60,7,37,37,26,7
GYS1_2_25807,45,27,25,27,3,36,261,6,57,34,58,177
HADH_2_25808,944,621,668,699,801,714,1085,696,733,676,264,869
HAGH_2_25809,9,3,26,14,11,235,13,0,0,0,0,0
HAO2_2_25810,520,301,349,374,438,227,611,123,554,277,489,518
HAS3_2_25811,282,321,306,363,233,417,637,681,540,102,165,737
HCCS_2_25812,569,492,384,397,62,0,253,1204,441,457,119,98
HDHD1_2_25813,190,170,103,216,157,556,81,485,117,6,138,43
HENMT1_2_25814,213,187,188,226,202,95,138,151,218,137,207,287
HHAT_2_25815,131,172,91,122,96,0,330,0,62,121,95,158
HIBCH_2_25816,1163,955,1060,1036,1084,1286,1061,600,1306,1143,515,1134
HLCS_2_25817,33,54,28,31,97,0,12,199,43,1,8,40
HMBS_2_25818,82,46,29,81,42,8,8,0,2,72,7,25
HMGA2_2_25819,1841,1855,1359,1826,1890,1280,1927,2270,1959,1034,2231,1
754
HMGCL_2_25820,1174,952,929,912,1743,674,442,900,739,384,892,792
HMGCLL1_2_25821,534,430,326,386,80,241,17,590,1087,138,1140,351
HMGCR_2_25822,436,459,370,570,328,45,330,170,215,261,137,390
HMGCS1_2_25823,2354,2185,1798,2477,1677,1624,3230,2384,2191,2813,2028,
1868
HMGCS2_2_25824,720,574,602,671,1103,448,31,175,599,1675,858,996
HMOX2_2_25825,1191,993,1192,1252,710,1350,1452,1927,1497,1501,885,1024
HNRNPAB_2_25826,505,384,414,452,261,328,626,201,295,406,162,480
HPD_2_25827,21,15,32,18,0,0,73,4,0,5,27,0
HPGD_2_25828,6,6,92,8,55,0,57,16,6,0,0,0
HPSE2_2_25829,965,471,482,710,426,716,402,465,750,943,517,999
HPSE_2_25830,421,250,371,410,428,325,1076,264,529,628,1196,200
HRAS_2_25831,447,205,329,170,185,0,29,313,21,0,1061,98
HS2ST1_2_25832,962,914,558,862,1478,1225,909,119,1075,412,1261,780
HS6ST2_2_25833,248,262,114,221,70,49,102,490,53,206,57,65
HSD11B1_2_25834,79,52,92,43,94,36,82,133,87,13,8,49
HSD17B10_2_25835,391,388,399,465,35,468,699,266,623,836,747,411
HSD17B13_2_25836,643,304,397,493,573,432,315,442,1302,312,50,891
HSD17B4_2_25837,705,636,535,512,388,562,31,746,482,441,834,224
HSD3B2_2_25838,358,200,258,329,470,26,177,317,128,213,615,484
HSD3B7_2_25839,66,52,121,48,92,87,40,29,4,0,42,33
HSDL1_2_25840,2298,2819,2446,2361,2441,2563,3548,1919,1940,2931,2433,1

941
HSP90AA1_2_25841,288,238,372,355,10,528,28,1334,129,167,258,287
HSPA8_2_25842,68,134,123,92,9,316,12,105,834,84,14,378
HSPD1_2_25843,77,151,144,121,208,10,401,133,107,35,19,35
HTRA2_2_25844,344,297,405,498,187,149,97,1207,622,652,1041,1012
HYAL1_2_25845,461,148,404,496,1026,18,46,701,328,293,172,464
HYAL2_2_25846,642,764,816,1049,1256,683,978,836,568,1188,1634,1101
HYI_2_25847,200,261,141,255,36,461,12,8,99,14,180,49
IARS_2_25848,86,75,62,94,0,32,10,53,32,10,14,18
IDE_2_25849,988,1245,981,990,699,780,1200,723,836,371,445,1235
IDH3B_2_25850,48,13,3,39,0,0,0,10,47,10,0,3
IDH3G_2_25851,18,14,45,51,18,0,0,0,161,0,0,30
IDS_2_25852,563,993,571,663,1008,1088,1039,880,324,978,480,554
IFT27_2_25853,19,13,22,75,0,0,10,198,31,10,0,0
IL4I1_2_25854,47,25,31,63,0,137,0,51,0,1,5,0
IMPDH1_2_25855,594,349,319,330,300,405,167,104,497,570,1126,160
INMT_2_25856,95,230,146,236,489,0,9,2,143,5,58,417
INPP5K_2_25857,43,86,103,103,0,22,2,58,14,0,360,30
INTS6_2_25858,1403,1292,1171,1391,1052,1311,1889,502,793,2166,1330,184
1
IPCEF1_2_25859,26,5,44,47,27,29,9,856,27,43,0,103
ISOC2_2_25860,789,623,595,915,409,352,549,1719,112,674,225,1382
ISPD_2_25861,427,337,496,323,209,223,922,0,849,492,844,192
ISYNA1_2_25862,4,51,40,10,75,0,0,36,0,0,282,0
ITPA_2_25863,396,326,346,338,186,3,512,300,422,241,301,506
IVD_2_25864,158,200,221,218,41,643,159,124,150,111,153,114
IYD_2_25865,651,566,432,591,314,380,498,1079,521,590,44,571
JMJD7-PLA2G4B_2_25866,87,88,91,74,27,68,40,174,14,126,586,168
KARS_2_25867,43,76,124,34,38,0,254,0,7,52,491,317
KATNA1_2_25868,1900,2304,1900,2362,1816,1729,1145,3015,988,3116,1405,2
258
KATNAL1_2_25869,1327,789,882,851,249,517,911,566,1025,892,533,955
KIF16B_2_25870,353,205,256,173,39,124,801,367,117,263,65,56
KIF9_2_25871,672,583,610,840,321,197,1124,283,409,388,813,351
KIFC3_2_25872,50,68,100,101,262,0,13,344,196,0,32,26
KLK2_2_25873,72,74,115,103,168,0,60,0,23,7,0,86
KLK7_2_25874,84,62,50,52,137,79,0,59,42,51,0,54
KRAS_2_25875,931,541,864,794,601,370,519,1086,713,805,1232,769
KYNU_2_25876,953,902,779,1066,965,1259,1001,1041,532,217,1746,620
LAMP2_2_25877,456,495,695,703,342,176,618,1253,463,795,425,598
LARGE_2_25878,648,671,768,1413,383,1073,2317,231,751,1385,1199,325
LCLAT1_2_25879,482,375,479,653,1466,212,1704,719,335,60,634,207
LCMT1_2_25880,182,96,122,159,90,21,171,35,429,304,333,153
LDHA_2_25881,153,183,199,201,296,915,77,0,45,2,10,81
LDHAL6A_2_25882,88,29,17,81,221,14,0,89,230,0,0,0
LDHB_2_25883,507,361,480,739,182,313,28,67,125,180,149,464
LDHC_2_25884,548,474,380,431,449,313,854,585,611,290,686,471
LDHD_2_25885,119,110,116,127,333,482,19,1005,257,111,18,206
LEPRE1_2_25886,332,180,169,307,7,1,118,320,437,141,1,60
LEPREL1_2_25887,1872,1647,1850,1766,2617,1608,850,1354,1381,2384,1867,

1378
LFNG_2_25888,770,663,591,725,722,662,248,790,563,984,881,1274
LGMN_2_25889,727,605,526,491,807,230,470,490,192,665,190,1367
LGSN_2_25890,334,356,245,330,61,440,509,515,217,0,261,348
LIAS_2_25891,237,125,148,79,136,308,0,0,253,132,288,23
LIG3_2_25892,453,307,266,330,575,257,126,717,336,256,647,325
LIG4_2_25893,500,353,255,363,502,31,345,420,843,333,8,533
LIPF_2_25894,908,990,684,1061,1167,1569,787,1001,787,1302,656,831
LIPT1_2_25895,241,364,295,206,236,248,399,399,198,686,201,367
LMO7_2_25896,966,897,1123,857,552,528,680,1630,1362,981,1430,875
LNPEP_2_25897,5,9,107,54,41,0,9,0,0,14,0,4
LOX_2_25898,1211,1458,1064,1586,1597,797,589,1492,2072,1409,1833,1816
LPO_2_25899,215,164,295,401,208,8,255,70,677,72,522,290
LRRC16A_2_25900,1341,1293,1380,1464,780,1079,2098,476,819,802,2048,159
9
LSS_2_25901,232,268,284,285,254,136,787,480,69,122,295,252
LYZL6_2_25902,169,136,157,194,39,176,418,68,178,87,34,340
MACF1_2_25903,36,59,20,72,2,23,1,0,18,141,29,127
MACROD2_2_25904,415,476,584,626,407,72,512,228,443,1055,394,661
MAD2L2_2_25905,44,45,83,50,0,0,0,127,89,20,34,0
MAN2B1_2_25906,149,233,106,166,222,99,104,531,90,36,0,206
MASP2_2_25907,26,35,1,19,0,0,0,1,0,0,0,0
MAT2B_2_25908,302,275,397,388,276,18,915,636,366,134,732,691
MCAT_2_25909,411,429,359,537,225,565,1000,1072,242,221,1049,613
MCM4_2_25910,1814,1587,1706,1940,1982,1119,2128,1727,1487,1230,2671,15
64
MCM7_2_25911,148,133,135,163,7,38,121,7,209,44,0,163
MCM8_2_25912,28,24,4,13,0,0,83,6,7,111,0,7
MCM9_2_25913,1734,1712,1736,1939,1130,1623,1909,684,2813,1533,803,2797
MDH1_2_25914,279,164,149,220,1,207,0,256,120,1110,93,546
MECR_2_25915,52,76,27,70,0,164,82,11,1,32,0,12
MEPCE_2_25916,28,46,79,63,90,0,94,0,29,1,7,23
METTL13_2_25917,315,465,357,464,190,458,594,279,26,430,869,267
MFN2_2_25918,48,27,34,52,0,83,2,4,97,1,0,1
MFNG_2_25919,294,252,246,543,734,587,360,91,288,676,20,197
MGAT3_2_25920,30,0,25,8,1,0,0,0,0,14,0,0
MGAT4A_2_25921,3935,4658,3929,4229,4062,2515,3858,2794,3296,4473,8158,
4014
MGAT4B_2_25922,814,1287,1180,1386,3645,267,2657,2,1157,969,1808,1326
MGLL_2_25923,361,278,243,257,266,311,845,1090,660,104,5,201
MGST1_2_25924,805,871,660,734,981,733,558,595,763,895,1262,624
MGST2_2_25925,199,113,105,290,0,554,0,0,127,217,476,281
MICAL1_2_25926,372,205,248,227,227,33,146,125,485,338,0,367
MLH1_2_25927,1288,1350,706,1176,758,1183,574,1845,927,1096,1210,851
MME_2_25928,1479,1223,1136,1779,2286,589,904,888,2129,1523,740,1688
MMP1_2_25929,863,948,708,1081,499,855,1061,1260,481,561,821,847
MMP2_2_25930,529,589,577,564,502,863,960,233,790,1360,205,644
MOCS1_2_25931,156,191,190,300,395,201,27,465,262,14,1224,53
MOGS_2_25932,33,62,81,81,39,2,0,324,66,89,58,23
MOV10_2_25933,310,280,187,319,353,141,335,534,223,285,833,308

MOV10L1_2_25934,823,819,728,751,804,86,789,1063,505,739,343,875
MPG_2_25935,67,59,74,125,0,0,127,298,0,0,275,22
MPPE1_2_25936,89,94,129,92,18,32,88,112,59,218,15,188
MPST_2_25937,71,51,48,77,2,15,1,259,33,73,48,5
MRAS_2_25938,1759,1438,1824,2061,1070,857,1560,1275,1299,1540,888,1433
MRE11A_2_25939,87,77,99,135,125,173,266,20,11,118,210,190
MRI1_2_25940,123,45,64,117,150,15,14,47,16,0,520,27
MSH5_2_25941,475,283,308,334,574,956,698,762,555,105,10,692
MSRA_2_25942,331,684,531,341,547,529,334,1202,0,272,317,217
MSRB3_2_25943,344,309,448,267,117,339,894,593,498,732,85,406
MTHFD1L_2_25944,422,410,287,222,122,27,607,228,226,702,347,305
MTHFS_2_25945,621,514,586,693,704,329,1253,755,398,901,862,287
MTO1_2_25946,681,388,561,710,217,658,148,302,97,982,1551,1054
MTRR_2_25947,752,413,510,484,261,274,657,160,400,406,528,727
MUTYH_2_25948,114,36,25,53,36,0,0,9,165,0,0,9
MX1_2_25949,398,381,280,351,373,424,62,82,398,515,447,442
MYBBP1A_2_25950,167,35,92,51,157,49,21,130,0,205,28,117
MYH2_2_25951,81,71,103,114,66,30,99,32,86,71,30,22
MYO5A_2_25952,40,44,78,132,1,140,85,137,22,48,275,186
MYO7A_2_25953,150,285,131,337,244,0,1,153,118,358,274,152
MYO9B_2_25954,30,15,18,19,0,0,13,0,0,0,0,146
N6AMT1_2_25955,367,465,330,287,206,1665,760,579,411,99,604,319
NAA16_2_25956,12,6,3,5,0,0,0,0,4,0,0,0
NAA20_2_25957,333,142,233,252,65,133,97,31,158,458,573,523
NAAA_2_25958,66,52,32,81,73,2,109,1,18,63,15,363
NAPEPLD_2_25959,636,456,392,743,238,415,726,851,441,257,1676,872
NARF_2_25960,72,65,103,132,387,9,252,410,62,66,0,141
NARS2_2_25961,836,663,730,670,686,653,1265,1291,869,718,394,659
NAV1_2_25962,444,307,544,287,195,83,806,353,540,215,518,434
NCEH1_2_25963,390,538,601,525,692,205,917,670,544,845,895,659
NCF2_2_25964,1296,1173,1393,1215,995,1491,1727,1691,782,987,863,1389
NCF4_2_25965,199,136,129,84,18,148,336,2,212,137,175,99
NDOR1_2_25966,1,17,2,38,0,0,0,98,1,29,1,0
NDUFA2_2_25967,447,466,562,545,785,143,465,299,453,895,215,721
NDUFB11_2_25968,20,43,22,28,0,302,1,367,0,157,3,10
NDUFB4_2_25969,380,501,397,382,232,1025,258,84,859,201,234,429
NDUFB5_2_25970,1837,1700,1339,1617,1924,1331,2151,1376,1895,1999,2292,
1452
NDUFB6_2_25971,125,138,173,139,5,430,71,70,174,65,207,162
NDUFC1_2_25972,532,525,425,641,154,688,115,127,298,101,13,172
NDUFS1_2_25973,658,376,582,488,325,742,69,348,560,476,1333,289
NDUFS2_2_25974,1341,1186,1383,1708,1773,868,1944,1373,1562,1595,1171,1
447
NDUFS5_2_25975,92,47,73,111,0,1,348,0,11,149,0,196
NDUFV1_2_25976,565,470,531,803,525,430,859,991,756,381,648,208
NEIL2_2_25977,84,91,72,109,47,96,3,200,138,285,0,1
NEU4_2_25978,198,180,179,262,1,0,449,0,5,0,11,174
NFS1_2_25979,431,683,361,571,303,279,508,437,929,270,421,231
NGLY1_2_25980,186,196,164,200,416,98,26,59,43,577,16,283
NIPSNAP1_2_25981,4,16,6,0,0,0,0,3,9,0,0,9

NIT1_2_25982,543,534,458,418,675,850,485,241,452,710,537,617
NKIRAS2_2_25983,168,187,198,233,38,10,62,1,78,214,9,96
NLGN3_2_25984,168,134,109,138,182,24,174,5,4,133,0,608
NLGN4X_2_25985,93,25,71,26,97,151,0,0,418,1,3,28
NLGN4Y_2_25986,600,560,607,638,423,627,91,447,387,991,1323,791
NMNAT2_2_25987,210,155,209,265,96,132,72,344,125,54,214,58
NNT_2_25988,306,276,266,378,132,9,1,11,68,546,82,285
NOX4_2_25989,1803,1193,1352,1494,1587,1048,2309,1423,1199,1596,1919,11
01
NPL_2_25990,191,95,280,257,103,0,273,8,2,1,65,131
NQO1_2_25991,242,154,265,404,138,305,257,299,148,170,28,220
NRD1_2_25992,716,593,589,643,513,624,1379,431,777,662,280,1150
NSDHL_2_25993,126,59,102,86,9,75,1,19,92,233,1,41
NSUN2_2_25994,36,27,15,33,0,1,23,20,9,1,2,12
NUDT2_2_25995,89,39,112,135,2,34,39,70,398,16,24,3
NXN_2_25996,122,33,1,14,0,51,0,7,3,0,1,9
OAS1_2_25997,441,389,536,315,121,694,615,422,76,1011,25,697
OAS2_2_25998,69,63,72,64,104,331,778,169,15,13,4,49
OASL_2_25999,198,98,116,86,0,20,35,0,0,3,247,61
OAT_2_26000,930,750,661,766,1195,665,1440,689,675,532,786,912
OGDH_2_26001,223,302,269,144,152,0,171,10,414,248,1,14
OGDHL_2_26002,172,82,101,156,79,22,402,399,597,30,5,215
OGG1_2_26003,325,77,321,239,521,69,5,210,44,179,37,424
OGT_2_26004,864,617,866,824,1245,987,148,1512,705,1077,994,555
OLAH_2_26005,1128,713,699,927,779,1132,758,565,591,569,352,885
P4HA1_2_26006,1168,741,1002,1126,595,1179,1368,788,921,1044,768,1438
P4HA2_2_26007,23,13,5,8,0,0,3,0,1,167,0,6
P4HTM_2_26008,85,56,38,29,24,193,0,9,24,12,0,187
PAFAH1B2_2_26009,297,339,213,259,0,105,164,215,241,91,84,230
PAFAH1B3_2_26010,120,210,64,99,524,20,2,125,4,288,1,238
PAICS_2_26011,925,911,607,938,992,1191,914,853,578,1945,660,1442
PAOX_2_26012,2,23,31,113,0,1,3,0,147,16,0,36
PAPD4_2_26013,500,396,563,463,417,721,396,1498,488,262,984,641
PAPD5_2_26014,1689,1437,1221,1750,2255,202,2170,706,1483,2336,976,1702
PAPD7_2_26015,99,185,135,133,142,49,112,4,62,85,2,65
PAPSS2_2_26016,753,697,705,820,981,310,656,1471,742,339,1471,2088
PARK7_2_26017,354,328,364,481,126,736,329,509,447,654,694,426
PARL_2_26018,298,566,400,536,617,467,152,178,429,105,582,135
PARN_2_26019,150,111,142,153,7,196,146,8,3,293,19,8
PARP2_2_26020,362,339,389,455,62,284,421,779,253,570,200,267
PARP3_2_26021,246,107,87,247,219,10,750,212,286,169,28,252
PARP8_2_26022,307,195,275,232,430,25,448,431,453,29,170,505
PBLD_2_26023,289,476,353,514,957,261,174,1222,408,421,6,766
PCCA_2_26024,2028,1562,1417,1488,2454,1485,1436,2506,1634,1604,605,993
PCCB_2_26025,1339,1776,1507,1725,1423,972,1864,1303,1164,1867,1239,156
8
PCMTD2_2_26026,257,633,466,335,438,756,641,254,800,10,260,248
PCNA_2_26027,216,270,281,414,13,908,18,652,144,525,371,504
PCSK1_2_26028,646,695,694,796,1250,690,622,953,562,802,358,507
PCSK2_2_26029,1911,2273,1516,1950,2124,825,2522,1779,1490,1755,2690,11

77
PCYT1B_2_26030,646,764,673,729,488,731,1779,694,444,510,13,1160
PCYT2_2_26031,11,15,7,56,0,0,0,0,0,12,0,1
PDE10A_2_26032,541,619,644,605,173,434,390,337,218,363,685,703
PDE11A_2_26033,2183,2007,2045,2550,2120,2540,2547,1955,1454,2905,2728,
2632
PDE1A_2_26034,1336,1285,1067,1286,1763,1719,971,809,1030,1374,1548,701
PDE1B_2_26035,149,172,132,235,0,134,0,26,208,657,2,220
PDE1C_2_26036,374,268,255,492,150,482,345,32,69,204,346,700
PDE2A_2_26037,438,407,424,486,705,702,546,1661,463,1166,100,637
PDE4A_2_26038,304,402,532,341,499,80,827,497,301,294,156,371
PDE4B_2_26039,1876,1625,1310,1530,1752,1500,3908,1701,1900,2264,2142,9
02
PDE4C_2_26040,60,196,100,157,129,570,6,0,69,44,0,310
PDE4D_2_26041,34,10,32,31,13,0,14,0,0,40,0,0
PDE5A_2_26042,558,816,624,753,543,111,71,882,525,656,396,648
PDE6B_2_26043,396,291,440,478,510,169,40,69,582,175,30,1123
PDE7A_2_26044,2233,2034,1949,2563,1337,1445,2403,1867,2322,2318,1414,2
733
PDE8A_2_26045,764,562,552,747,104,183,1556,915,560,181,18,530
PDE8B_2_26046,502,289,243,244,1222,360,319,589,768,876,800,364
PDE9A_2_26047,303,232,338,322,142,401,326,395,355,461,857,856
PDHA1_2_26048,539,490,358,746,321,590,139,1051,228,113,134,552
PDHB_2_26049,393,296,226,338,221,46,445,10,0,3,22,73
PDHX_2_26050,78,157,138,186,275,38,12,25,40,49,478,11
PEMT_2_26051,210,205,183,321,72,40,627,193,112,16,1128,102
PEPD_2_26052,514,395,483,354,645,414,435,150,290,565,1478,431
PGAM5_2_26053,160,269,112,258,604,248,236,625,250,355,83,223
PGBD1_2_26054,170,89,113,130,372,24,0,118,670,251,207,115
PGC_2_26055,2,20,18,28,0,0,1,0,0,0,3,0
PGM1_2_26056,535,597,661,804,472,404,680,208,586,659,885,604
PGM3_2_26057,88,156,100,254,90,98,257,464,276,37,378,2
PHOSPHO1_2_26058,91,97,128,153,78,0,309,0,221,736,0,183
PHOSPHO2_2_26059,297,493,331,276,458,120,29,316,139,338,71,359
PHYH_2_26060,669,466,573,647,559,218,716,223,731,792,161,525
PIGA_2_26061,74,122,80,76,20,0,0,2,37,90,106,0
PIGC_2_26062,697,585,511,606,872,679,67,537,521,657,662,307
PIGF_2_26063,114,111,101,100,25,254,213,475,0,1,226,252
PIGG_2_26064,10,64,109,21,0,111,0,0,1,125,1,0
PIGN_2_26065,1892,1736,1520,1611,2045,792,2284,1616,2387,1602,1904,219
4
PIGO_2_26066,654,629,504,788,414,707,521,360,308,524,509,250
PIGP_2_26067,789,730,613,844,1025,236,1405,867,973,966,784,1203
PIGQ_2_26068,168,45,91,91,219,208,0,14,5,5,31,173
PIGT_2_26069,364,409,271,288,232,98,658,420,51,29,623,213
PIGV_2_26070,277,76,141,203,249,120,5,212,789,1,585,299
PIN4_2_26071,255,204,413,427,350,351,122,692,194,423,474,885
PLA1A_2_26072,612,575,385,465,46,114,85,486,469,451,34,612
PLA2G2A_2_26073,173,333,253,314,460,157,128,0,168,487,466,125
PLA2G4C_2_26074,627,729,749,1037,433,947,1044,619,624,573,1054,1323

PLA2G6_2_26075,55,48,99,34,9,2,1,127,0,0,2,384
PLA2G7_2_26076,1007,1102,1062,1272,1276,1045,1564,820,1490,1458,713,12
20
PLAT_2_26077,263,152,307,264,246,186,281,271,40,271,858,167
PLAU_2_26078,274,132,74,197,175,101,11,1,302,274,30,56
PLB1_2_26079,132,96,82,139,39,69,138,45,117,0,146,181
PLCB1_2_26080,96,245,351,267,3,774,239,1,110,0,649,212
PLCB3_2_26081,99,174,66,151,0,17,3,269,31,122,92,2
PLCB4_2_26082,1665,1307,1544,1573,1741,967,695,607,1994,1520,2070,1107
PLCD1_2_26083,297,305,335,368,480,214,305,270,380,90,474,411
PLCE1_2_26084,137,260,176,228,332,236,129,133,511,125,9,122
PLCG1_2_26085,197,281,259,522,893,4,63,414,2,236,1020,345
PLCH1_2_26086,959,980,673,902,870,386,942,1425,1227,1198,270,824
PLCL2_2_26087,1033,1000,1205,1065,848,946,499,445,915,853,1328,1546
PLCXD2_2_26088,660,830,620,771,147,62,577,1798,908,283,531,941
PLD2_2_26089,303,263,226,333,68,13,261,177,69,948,11,636
PLD3_2_26090,183,247,257,307,52,116,391,66,84,200,336,125
PLOD2_2_26091,1138,1384,1031,1289,2364,536,1025,1145,876,873,904,987
PLSCR3_2_26092,15,25,21,45,82,0,0,13,0,54,6,96
PLSCR4_2_26093,289,459,223,284,63,146,0,531,414,150,3,190
PMEL_2_26094,211,333,265,320,152,221,696,540,169,265,464,246
PMS1_2_26095,539,855,515,630,280,653,80,248,260,856,1478,1072
PNPLA1_2_26096,150,173,160,85,6,583,38,35,38,262,446,253
PNPLA4_2_26097,120,110,91,130,62,112,863,0,116,118,320,115
PNPLA6_2_26098,59,39,41,17,0,0,0,51,13,29,22,0
PNPLA7_2_26099,38,0,1,0,0,0,0,0,3,0,0,0
POFUT1_2_26100,548,573,605,432,571,1051,578,553,428,372,618,735
POFUT2_2_26101,184,283,190,407,2,381,0,92,335,394,38,173
POGZ_2_26102,491,354,400,591,213,340,979,230,341,521,109,352
POLD2_2_26103,499,524,825,825,388,680,640,821,303,436,622,889
POLE2_2_26104,113,24,121,77,47,37,12,12,543,6,37,18
POLG_2_26105,589,569,815,747,370,131,1669,930,411,743,1198,1058
POLL_2_26106,156,118,215,220,125,46,513,0,36,156,7,161
POLR1D_2_26107,154,169,196,214,403,173,227,400,359,125,56,378
POLR3B_2_26108,626,482,415,399,530,156,1265,212,331,489,1519,440
POLR3H_2_26109,248,282,180,318,279,120,860,346,345,69,128,816
POMT1_2_26110,2759,2344,2518,2688,3233,2034,3451,1459,3410,1755,3445,2
925
PON2_2_26111,64,55,32,56,182,7,0,0,18,75,0,8
POP5_2_26112,447,389,434,421,1064,256,873,261,522,728,41,590
PPA2_2_26113,625,580,498,634,413,369,374,393,671,1398,891,388
PPIE_2_26114,1627,1358,1590,1397,1652,1590,694,1380,1823,2944,1355,202
3
PPIL2_2_26115,692,435,554,648,411,811,389,1118,379,349,727,549
PPIL3_2_26116,639,502,605,739,911,453,528,159,1172,717,145,391
PPIL6_2_26117,464,403,347,547,328,578,705,491,418,213,335,324
PPIP5K1_2_26118,171,39,57,29,40,0,60,61,2,0,22,334
PPT1_2_26119,343,348,229,347,347,215,194,99,75,87,6,184
PPT2_2_26120,75,90,76,130,143,9,117,243,125,13,76,194
PRCP_2_26121,744,728,577,668,405,895,484,869,1543,127,331,1290

PRDX1_2_26122,525,488,401,549,1178,1273,552,600,654,447,409,347
PRDX3_2_26123,1068,962,1159,1391,1239,965,2443,877,1504,531,1209,1127
PRDX5_2_26124,37,13,68,57,0,0,0,139,1,247,0,0
PRKCSH_2_26125,262,178,250,318,694,814,65,495,341,70,97,348
PRODH_2_26126,123,162,300,200,0,2,0,341,553,659,0,46
PRSS3_2_26127,254,217,353,315,81,7,667,254,115,108,458,301
PSAT1_2_26128,263,244,311,290,487,37,581,746,371,537,992,666
PSMA1_2_26129,1030,1032,1118,1099,349,939,959,1417,1770,1944,2212,1656
PSMA3_2_26130,406,388,506,481,162,345,82,226,711,875,974,334
PSMA4_2_26131,202,189,162,189,230,406,74,71,561,187,4,116
PSMA5_2_26132,556,671,452,791,273,1634,489,584,343,413,327,1055
PSMA8_2_26133,1175,1315,1120,1100,1481,1191,1040,2092,1071,1160,1113,1
448
PSMB2_2_26134,659,740,687,735,769,626,276,1004,587,1290,524,1438
PSMB5_2_26135,606,393,422,342,551,404,772,219,481,429,290,670
PSMB8_2_26136,448,285,405,549,756,202,188,567,235,439,233,542
PTBP1_2_26137,529,438,346,443,264,18,28,83,309,596,408,805
PTER_2_26138,1014,700,737,1077,1024,544,743,456,1150,1817,322,350
PTGR1_2_26139,418,335,402,376,141,953,1045,260,172,303,109,529
PTGR2_2_26140,1538,1175,1058,1609,1230,694,470,1064,1076,660,2013,709
PTGS1_2_26141,95,73,123,96,56,17,196,8,95,46,289,15
PUS1_2_26142,311,349,266,259,212,249,21,117,292,480,51,546
PYCR1_2_26143,7,18,65,70,1,1,2,3,3,37,5,166
PYGL_2_26144,300,220,400,490,301,293,1978,126,602,188,1311,211
PYGM_2_26145,231,155,192,268,58,292,74,1015,571,15,285,373
QPCTL_2_26146,403,649,419,316,174,475,212,373,121,573,1059,68
QSOX1_2_26147,454,396,595,463,185,145,320,538,688,355,915,44
RAB11A_2_26148,18,23,123,91,0,3,346,0,42,3,17,98
RAB1A_2_26149,267,85,164,200,348,198,292,30,184,37,34,41
RAB23_2_26150,545,741,706,811,716,341,1371,461,719,847,1182,677
RAB27A_2_26151,89,79,76,75,36,238,147,8,37,21,0,62
RAB28_2_26152,500,361,312,393,463,484,263,303,414,153,125,263
RAB2A_2_26153,532,676,475,671,198,842,802,238,649,926,286,544
RAB2B_2_26154,114,93,84,93,4,0,13,31,224,0,0,11
RAB34_2_26155,390,414,232,371,131,361,612,153,609,95,432,81
RAB35_2_26156,230,127,174,236,563,18,745,0,222,596,40,34
RAB37_2_26157,74,58,97,73,120,1,702,15,116,272,13,78
RAB40C_2_26158,310,353,291,386,737,234,407,286,746,67,33,133
RAB5C_2_26159,1272,966,1128,1323,2142,1534,2578,1478,2114,1811,1665,23
42
RAB6A_2_26160,475,373,335,466,426,783,47,189,294,244,1718,822
RAB7L1_2_26161,165,137,197,186,9,280,0,3,0,32,0,6
RAB9A_2_26162,891,717,783,855,863,280,1495,1796,128,860,874,743
RABGGTA_2_26163,7,41,26,19,0,0,19,0,0,176,0,48
RABL2A_2_26164,621,479,285,374,52,773,21,14,345,786,742,794
RABL2B_2_26165,33,55,38,3,11,0,0,0,0,0,0,33
RAC1_2_26166,599,386,402,484,823,241,274,646,426,247,352,698
RAD51B_2_26167,128,69,77,86,126,61,33,98,26,3,368,0
RAD51_2_26168,187,115,192,221,44,2,336,43,240,185,66,79
RAD51D_2_26169,125,196,163,114,96,0,288,205,18,42,0,161

RAD54B_2_26170,389,328,203,254,612,410,104,683,245,161,261,1
RAD54L_2_26171,314,271,287,335,455,281,615,594,254,303,195,112
RAD9A_2_26172,33,18,52,39,8,3,29,0,109,8,0,195
RAP1A_2_26173,1069,546,565,790,1555,378,278,597,716,331,851,765
RAP1B_2_26174,1722,1741,1715,2133,2419,1617,766,1705,1335,1876,822,207
3
RBBP8_2_26175,391,356,163,270,62,385,528,13,23,185,61,74
RDH13_2_26176,217,285,328,407,145,137,89,198,245,385,60,460
RDH5_2_26177,134,127,156,147,0,7,21,178,57,112,19,19
RECQL5_2_26178,230,236,210,275,90,2,1266,139,202,420,7,392
RECQL_2_26179,203,253,196,307,227,211,0,364,231,142,446,142
RERG_2_26180,140,131,149,118,306,76,85,64,587,96,0,95
REV1_2_26181,842,896,758,1051,1788,841,739,192,1022,500,1037,834
RFC3_2_26182,755,1000,715,896,319,1262,586,1013,451,827,935,837
RFC5_2_26183,568,397,425,607,601,260,181,149,507,833,264,671
RGN_2_26184,453,489,388,436,126,330,201,92,349,806,1386,116
RGS11_2_26185,3,2,14,22,0,22,0,0,2,0,0,0
RGS6_2_26186,288,418,342,429,72,37,1044,642,150,296,496,140
RHOC_2_26187,191,238,255,268,103,479,674,528,749,218,687,21
RHOT1_2_26188,50,101,43,87,208,202,145,33,113,0,0,45
RNASE1_2_26189,433,357,336,403,792,45,762,426,327,874,111,413
RNASE4_2_26190,144,271,138,195,125,282,347,65,418,177,19,169
RPAP3_2_26191,244,140,164,198,65,6,4,94,117,129,294,148
RPE_2_26192,638,413,475,556,442,249,574,529,659,581,1190,272
RPN2_2_26193,701,747,753,587,634,1007,1544,194,1176,711,660,606
RPP14_2_26194,2184,2217,1966,2362,3433,2389,2005,2480,3652,2513,2303,2
013
RPP21_2_26195,969,872,810,887,502,873,1582,369,938,772,569,648
RPP30_2_26196,331,272,430,435,303,220,400,69,483,377,1658,368
RPP38_2_26197,305,139,275,292,353,6,121,384,399,15,1266,501
RPS27A_2_26198,900,746,607,831,828,480,63,870,616,518,432,759
RRAD_2_26199,224,88,88,74,0,114,0,137,289,0,3,8
RRAGB_2_26200,201,196,100,148,69,55,418,156,431,237,197,315
RRAS2_2_26201,34,7,1,6,10,66,127,0,42,13,0,0
RRM2B_2_26202,1007,904,919,1004,987,941,1529,995,1026,999,1718,713
RRM2_2_26203,147,188,174,123,30,62,756,366,28,120,18,353
RTEL1_2_26204,118,29,58,71,0,188,236,99,208,31,35,13
SAR1A_2_26205,183,84,144,149,33,100,75,73,486,201,668,180
SAR1B_2_26206,821,773,569,632,1022,745,457,553,502,59,723,518
SARDH_2_26207,217,147,196,197,312,38,136,220,548,368,108,472
SARS2_2_26208,42,31,43,97,19,66,64,0,6,0,100,96
SBNO1_2_26209,96,144,93,127,1,98,327,7,291,14,4,113
SC5DL_2_26210,729,901,772,1369,898,60,1005,1321,478,979,419,1105
SCD5_2_26211,176,159,159,115,236,322,288,6,0,187,174,1
SDC1_2_26212,108,74,97,54,23,30,542,47,99,54,175,24
SDCBP_2_26213,111,58,60,23,10,580,0,0,3,120,2,31
SDHC_2_26214,349,208,236,279,142,41,194,355,30,57,497,447
SEPHS1_2_26215,140,160,283,241,75,70,297,329,141,758,531,127
3-Sep_2_26216,265,317,228,479,27,202,1019,426,106,45,985,285
4-Sep_2_26217,524,377,429,565,276,996,691,718,1025,285,581,320

5-Sep_2_26218,70,21,99,65,160,103,0,254,20,8,151,14
9-Sep_2_26219,809,545,668,718,999,700,876,910,511,781,381,472
SGMS2_2_26220,137,236,114,89,120,1,77,1,119,9,10,87
SH3GLB1_2_26221,1086,877,1015,955,472,935,1306,1112,864,1030,1575,812
SHMT1_2_26222,1930,1070,1381,1634,2600,2551,596,642,857,716,1432,1214
SHMT2_2_26223,824,644,680,786,244,760,860,785,606,429,404,1197
SIAE_2_26224,553,654,475,817,310,330,1545,1562,432,255,644,665
SLFN11_2_26225,693,847,724,761,508,1564,568,323,541,580,846,645
SMG6_2_26226,43,84,65,121,0,219,0,0,269,0,0,0
SMOX_2_26227,261,123,104,257,32,182,0,101,168,108,0,24
SMPD1_2_26228,107,87,109,158,89,208,628,31,155,192,22,34
SMPD4_2_26229,924,963,995,1111,508,766,3948,761,876,2089,604,920
SMPDL3B_2_26230,256,423,305,308,21,789,669,569,1196,471,678,284
SOD2_2_26231,69,47,111,117,0,36,0,160,66,0,61,195
SPAM1_2_26232,886,950,777,1008,950,890,517,772,440,811,1151,1785
SPAST_2_26233,1527,1338,992,1297,2465,1481,615,1311,1865,1462,781,1491
SPG21_2_26234,384,315,282,323,323,600,250,171,458,28,0,329
SPO11_2_26235,615,564,633,414,350,615,834,926,609,862,588,1472
ST3GAL1_2_26236,71,35,7,34,39,0,2,1,19,2,0,1
ST3GAL5_2_26237,151,257,312,267,147,0,211,386,84,236,0,396
ST5_2_26238,415,158,175,172,109,19,765,244,264,685,265,222
ST6GAL1_2_26239,353,432,406,530,248,341,579,256,316,556,428,135
ST6GAL2_2_26240,626,483,349,369,119,404,202,638,387,211,175,224
ST6GALNAC3_2_26241,804,557,728,733,683,857,631,1287,1296,589,322,654
ST6GALNAC4_2_26242,581,523,387,513,1015,440,143,731,688,147,21,821
ST8SIA4_2_26243,215,205,225,175,74,156,146,137,58,106,56,156
SUCLG2_2_26244,1130,1173,1051,1338,948,852,811,942,688,1573,1002,1361
SULF1_2_26245,46,10,17,84,10,3,24,0,226,0,69,5
SULF2_2_26246,46,49,138,45,250,371,12,3,0,0,2,44
SULT1A1_2_26247,153,184,178,184,125,102,70,106,20,25,153,205
SULT1A2_2_26248,1178,1270,864,1060,495,859,1543,218,1043,1171,1040,134
1
SULT2B1_2_26249,274,254,134,255,448,434,790,583,102,208,17,14
SUOX_2_26250,150,110,143,95,0,0,91,0,146,188,0,563
SYTL1_2_26251,132,130,127,136,486,9,5,0,117,91,0,118
TAB1_2_26252,12,11,30,62,0,0,0,0,0,0,0,153
TBXAS1_2_26253,237,330,344,304,196,282,331,113,409,379,626,464
TCIRG1_2_26254,209,163,154,152,24,0,424,1,269,469,29,44
TDP1_2_26255,94,125,89,223,51,3,66,98,8,170,2,47
TERT_2_26256,149,99,106,151,282,150,19,79,8,72,27,41
TGM2_2_26257,607,238,430,491,1362,422,1,312,239,455,1513,643
TGM5_2_26258,348,309,308,506,182,41,3,74,260,62,17,576
TH_2_26259,141,150,102,168,451,375,2,130,30,56,107,580
TIAM2_2_26260,207,208,200,221,369,815,892,666,203,215,94,146
TKTL1_2_26261,356,239,390,577,158,159,71,164,749,208,970,663
TMEM55B_2_26262,523,746,794,675,248,524,574,699,1031,378,208,476
TMLHE_2_26263,1954,1529,1264,1898,2416,856,1859,982,1582,1661,1580,622
TMOD1_2_26264,260,199,237,305,510,221,806,786,316,306,293,296
TMX2_2_26265,46,64,59,69,0,47,64,0,77,238,0,170
TNNI2_2_26266,336,203,171,209,175,9,234,406,42,521,72,291

TP53I3_2_26267,226,291,162,267,531,284,288,139,202,343,40,164
TPI1_2_26268,478,478,463,491,1021,362,266,641,326,1469,854,87
TPST2_2_26269,59,197,62,50,2,418,4,181,49,44,6,14
TREX1_2_26270,30,0,0,55,0,0,1,0,4,7,0,0
TRMT1_2_26271,29,38,38,142,152,4,158,0,0,5,906,0
TRMT1L_2_26272,327,449,310,513,0,868,767,537,134,244,252,372
TRMT2B_2_26273,119,91,67,79,198,43,51,157,163,74,202,6
TRPT1_2_26274,160,267,123,251,54,0,360,466,115,49,304,437
TSEN2_2_26275,389,308,400,301,372,371,65,386,270,187,425,305
TSEN34_2_26276,85,86,21,20,0,1,0,29,0,89,0,2
TTLL6_2_26277,91,69,134,100,25,32,81,277,34,218,288,137
TUBAL3_2_26278,306,470,570,721,1,168,897,345,369,592,725,754
TUSC3_2_26279,397,472,468,524,248,177,582,1047,462,771,923,183
TXNDC16_2_26280,302,292,317,397,166,627,787,554,727,328,271,337
TXNDC2_2_26281,319,148,253,296,231,105,312,96,211,280,356,217
TXNDC5_2_26282,198,130,62,257,74,294,5,0,114,106,484,272
TXNL4B_2_26283,491,365,527,454,155,759,180,414,666,715,944,696
TXNRD1_2_26284,358,408,401,396,0,359,21,258,10,1092,114,430
TXNRD3_2_26285,1289,1351,1102,997,768,736,198,561,989,2550,1457,1698
TYMP_2_26286,25,22,24,40,0,22,1,4,0,2,112,8
UBA52_2_26287,110,105,131,138,0,4,322,5,11,28,27,146
UEVLD_2_26288,405,156,327,270,0,4,0,5,20,826,113,125
UGDH_2_26289,265,239,266,219,658,125,13,574,507,153,28,324
UGP2_2_26290,573,596,459,500,756,454,754,471,70,684,400,588
UGT1A6_2_26291,822,675,597,821,315,550,553,522,797,1161,652,1339
UGT2B10_2_26292,1284,1040,1134,1188,1589,1075,1734,856,1051,614,1472,8
21
UGT2B28_2_26293,151,63,232,161,28,0,95,4,15,718,545,349
UGT3A1_2_26294,338,446,360,528,574,556,670,206,382,318,213,244
UGT8_2_26295,587,616,596,658,748,359,282,366,350,17,193,625
UNG_2_26296,50,55,173,253,201,18,318,45,0,19,327,246
UPP1_2_26297,226,220,161,261,254,214,387,70,248,365,16,308
UPP2_2_26298,558,707,456,550,430,1023,226,206,391,936,764,915
UQCR10_2_26299,3,3,3,1,1,3,0,1,2,0,0,0
UQCRB_2_26300,604,591,546,611,1042,859,858,225,437,1192,667,1001
UROC1_2_26301,106,133,107,150,0,0,0,323,122,0,72,238
VCL_2_26302,165,145,151,239,69,27,160,144,11,250,228,358
VNN2_2_26303,415,420,353,398,251,639,775,994,390,263,97,187
VPS29_2_26304,1701,1410,1657,1860,1805,821,1648,1726,1882,2125,1797,27
68
WARS2_2_26305,157,217,193,265,436,56,80,562,124,188,592,175
WARS_2_26306,60,246,221,327,450,90,6,204,10,831,68,229
WBSCR22_2_26307,6,3,14,12,2,36,6,0,1,119,0,0
WDR46_2_26308,184,253,171,310,164,39,246,615,214,251,135,181
WFS1_2_26309,969,894,1111,1016,1007,1021,181,1555,798,1338,422,1892
WRNIP1_2_26310,60,39,77,15,1,137,376,6,37,42,12,0
WWOX_2_26311,228,85,250,333,0,0,88,3,331,159,27,163
XPNPEP1_2_26312,132,92,151,144,46,26,404,7,29,154,236,286
XRCC3_2_26313,94,74,64,128,296,12,364,165,307,17,41,237
XRN1_2_26314,760,510,598,579,59,1041,695,270,72,1545,142,379

YWHAZ_2_26315,100,97,71,165,170,172,11,33,171,146,117,10
ZBED1_2_26316,144,189,116,148,446,123,234,0,400,199,3,6
ZCCHC11_2_26317,1687,1495,1296,1288,1320,2132,2169,744,1122,2515,850,1
361
ZCCHC6_2_26318,935,921,733,1239,942,1081,1031,708,897,634,785,140
ZDHHC15_2_26319,800,1170,739,1067,1580,732,681,351,617,1355,899,444
ZDHHC7_2_26320,102,64,101,144,14,29,0,6,17,10,281,24
ACYP1_2_26321,2188,1686,1372,1677,1864,1785,2734,649,2672,2547,701,102
1
APOBEC3F_2_26322,652,1023,696,709,218,661,1188,251,303,877,587,1357
ASCC3_2_26323,489,300,327,536,1273,1215,481,597,1015,822,1483,406
ASPH_2_26324,1308,902,1134,1152,793,1221,1599,479,619,998,800,827
ATP6V0E2_2_26325,100,65,65,53,8,26,42,2,2,1,16,139
C10orf2_2_26326,661,654,506,595,309,486,188,261,435,1242,374,357
CAPN3_2_26327,699,738,704,937,575,1764,1827,631,1860,837,1162,945
CHM_2_26328,623,375,435,578,241,266,47,38,723,437,1619,844
CTSC_2_26329,98,164,147,214,77,101,265,22,277,7,0,73
CYBRD1_2_26330,2135,2228,1873,2439,1354,1678,2555,1729,1368,3074,1972,
2764
CYP3A5_2_26331,549,493,478,484,357,466,731,537,746,710,408,378
DIO2_2_26332,2602,3071,2544,3081,2926,4660,1944,1808,2526,3524,2720,31
19
DNASE2B_2_26333,143,176,148,197,141,53,68,777,239,35,0,3
DPYD_2_26334,557,481,429,556,432,259,795,632,479,1009,682,1408
GBA3_2_26335,1852,2054,2195,2325,2121,1078,799,2411,1951,1943,1019,196
1
GCNT2_2_26336,137,157,102,107,357,68,421,1,134,67,49,427
GLYAT_2_26337,757,885,642,787,612,879,1012,731,1040,1237,567,950
GPLD1_2_26338,238,161,127,140,53,197,16,115,91,138,13,156
GPX1_2_26339,200,142,118,215,127,37,16,160,161,164,78,159
GPX5_2_26340,2037,1543,1673,2206,1888,1029,1083,1668,1911,1625,2236,21
64
HNMT_2_26341,747,621,744,834,612,893,1761,55,86,831,999,672
HOGA1_2_26342,11,2,13,14,7,0,7,1,76,3,0,32
HYAL3_2_26343,621,904,781,774,298,447,2079,777,436,163,436,793
JPH2_2_26344,115,51,49,75,0,681,0,345,37,0,28,262
KLK8_2_26345,56,22,38,109,0,0,0,0,89,5,0,2
LAMA4_2_26346,1053,970,866,969,1062,1035,536,813,537,655,496,955
LRR1_2_26347,1224,1016,889,906,985,663,1164,810,624,499,418,732
LRTOMT_2_26348,7,57,21,24,0,0,73,45,2,0,269,4
METTL1_2_26349,1131,1250,970,1138,1051,1123,827,571,1535,1570,399,1425
MOCS2_2_26350,1347,1113,1079,1248,779,746,520,1751,880,1095,906,1514
NDUFA11_2_26351,455,467,499,511,447,93,556,1062,182,524,914,441
NDUFV3_2_26352,1015,1039,1101,1003,1193,698,844,239,1320,1446,277,1132
NMNAT3_2_26353,436,254,420,483,106,661,735,566,329,722,2,337
PDE4DIP_2_26354,155,237,148,208,285,76,527,2,0,55,12,280
PLG_2_26355,253,107,309,323,124,442,652,315,332,281,10,142
PPCS_2_26356,182,238,175,145,149,100,40,83,177,29,225,314
PRDX2_2_26357,69,27,8,53,0,0,2,2,0,1,0,7
RAD51C_2_26358,792,729,683,758,981,910,584,759,568,544,310,848

RASD1_2_26359,33,16,116,137,3,0,1,0,8,135,0,178
RASL10A_2_26360,10,37,27,31,176,85,77,10,0,0,0,9
SLX1A_2_26361,155,131,102,181,1,1,408,6,26,38,102,414
SLX1B_2_26362,155,131,102,181,1,1,408,6,26,38,102,414
SPTLC1_2_26363,2149,1800,1298,1659,1399,1358,2574,1817,2834,1987,1793,
1525
ST3GAL3_2_26364,143,383,227,255,1,0,300,346,2,75,55,214
VKORC1_2_26365,742,761,622,1025,52,799,1561,1681,416,211,1539,463
XPNPEP3_2_26366,662,607,494,598,429,675,1325,819,953,577,165,601
GNAS_2_26367,78,13,27,9,47,17,0,0,0,0,326,0
GNAS_2_26368,65,44,45,63,4,44,0,14,55,6,0,35
GNAS_2_26369,289,248,303,428,688,256,55,1156,328,455,392,376
ABP1_2_26370,189,236,218,263,149,222,729,253,118,87,60,268
ABP1_2_26371,222,219,204,237,232,369,100,534,493,308,62,376
ABP1_2_26372,77,75,64,129,10,61,7,0,232,65,18,82
ABP1_2_26373,724,803,736,769,1412,1061,327,1131,589,751,557,904
ABP1_2_26374,8,21,53,18,14,0,0,0,0,0,78,12
ABP1_2_26375,101,78,25,38,367,1,24,204,73,39,143,96
ABP1_2_26376,129,175,120,126,86,17,0,58,35,92,3,24
ABP1_2_26377,76,166,65,108,1,229,0,345,0,0,214,43
ABP1_2_26378,318,359,344,342,605,626,177,102,140,5,610,747
ABP1_2_26379,220,358,231,357,63,106,395,113,171,258,214,275
ADCY8_2_26380,17,2,17,29,0,0,2,0,0,0,17,0
ADCY8_2_26381,21,6,21,57,7,0,3,0,1,45,0,3
ADCY8_2_26382,675,555,625,826,1464,657,593,415,206,264,1379,717
ADCY8_2_26383,230,294,250,298,259,146,902,365,538,105,444,95
ADCY8_2_26384,1004,888,689,1005,735,485,993,552,507,847,1031,775
ADCY8_2_26385,271,238,262,322,75,135,614,520,366,24,409,134
ADCY8_2_26386,234,96,185,337,790,55,88,488,16,132,3,404
ADCY8_2_26387,319,368,337,553,566,174,410,75,24,556,651,112
ADCY8_2_26388,221,274,266,369,673,32,107,251,258,605,1,423
ADCY8_2_26389,146,181,156,164,460,30,22,112,394,195,521,118
AGXT_2_26390,388,358,315,505,9,523,191,0,68,437,1108,241
AGXT_2_26391,155,110,185,184,309,13,41,0,122,0,396,246
AGXT_2_26392,714,659,714,613,1034,160,672,229,448,438,284,886
AGXT_2_26393,1006,1000,975,1193,1056,1865,262,288,958,773,1419,1157
AGXT_2_26394,545,581,375,645,1250,404,812,918,272,928,749,580
AGXT_2_26395,61,22,34,34,0,0,0,0,92,9,0,34
AGXT_2_26396,41,65,89,26,360,0,68,0,31,13,0,515
AGXT_2_26397,103,56,92,135,119,144,21,37,140,120,104,159
AGXT_2_26398,626,341,461,498,42,265,11,282,676,644,33,516
AGXT_2_26399,59,141,165,92,21,4,6,2,53,11,0,17
AIFM2_2_26400,136,152,212,212,747,199,145,118,58,53,206,139
AIFM2_2_26401,250,297,107,215,0,0,253,318,0,270,0,276
AIFM2_2_26402,130,42,25,57,3,6,13,10,0,342,9,0
AIFM2_2_26403,925,707,751,839,881,930,256,1229,535,575,888,828
AIFM2_2_26404,73,115,87,64,147,0,7,79,320,98,3,16
AIFM2_2_26405,48,108,39,88,20,165,0,4,1,78,0,0
AIFM2_2_26406,85,70,125,123,3,0,0,18,12,0,233,253
AIFM2_2_26407,271,277,328,405,264,207,341,119,374,367,108,125

AIFM2_2_26408,11,52,16,50,1,36,6,174,6,21,0,18
AIFM2_2_26409,137,118,134,133,3,1,17,177,23,494,0,604
AKR1B1_2_26410,221,152,225,230,20,14,20,225,171,509,217,981
AKR1B1_2_26411,43,12,17,11,0,0,0,0,1,558,28,3
AKR1B1_2_26412,5,20,6,55,421,25,16,11,0,0,248,4
AKR1B1_2_26413,154,179,151,157,71,105,4,4,95,34,522,314
AKR1B1_2_26414,235,152,178,206,396,382,284,247,362,123,163,166
AKR1B1_2_26415,57,52,26,55,1,154,2,14,13,0,0,25
AKR1B1_2_26416,17,32,10,69,0,0,0,31,0,0,0,2
AKR1B1_2_26417,92,86,77,34,273,49,159,0,127,0,124,117
AKR1B1_2_26418,468,416,257,419,528,514,1191,76,274,258,75,523
AKR1B1_2_26419,182,238,243,189,171,2,132,265,224,425,500,312
AKR1B10_2_26420,543,568,781,626,83,392,1,215,479,288,374,1058
AKR1B10_2_26421,397,663,406,658,16,221,136,565,430,849,26,819
AKR1B10_2_26422,229,179,173,219,732,129,17,393,45,580,194,371
AKR1B10_2_26423,53,7,33,16,0,17,20,9,0,0,105,4
AKR1B10_2_26424,302,267,207,280,117,124,366,109,208,121,298,65
AKR1B10_2_26425,1714,1074,875,1249,1030,425,1224,1082,1081,1082,1340,1
387
AKR1B10_2_26426,46,147,63,99,4,0,31,0,0,121,102,7
AKR1B10_2_26427,215,373,250,326,389,497,163,278,293,248,289,351
AKR1B10_2_26428,723,706,768,893,115,1223,421,695,428,574,120,364
AKR1B10_2_26429,219,144,87,104,34,10,95,14,4,0,181,15
APEX1_2_26430,2309,1832,1985,2163,1636,2446,2171,2200,990,2376,1383,11
62
APEX1_2_26431,226,158,193,208,675,52,157,433,51,458,160,274
APEX1_2_26432,551,659,656,740,292,107,178,719,1162,399,593,484
APEX1_2_26433,922,1107,1096,1165,540,512,1540,1098,907,1058,711,470
APEX1_2_26434,221,180,202,119,345,87,10,3,92,460,216,99
APEX1_2_26435,182,188,131,199,216,22,0,55,163,82,0,135
APEX1_2_26436,1127,1201,1215,1512,1141,299,1251,609,449,1096,1047,1190
APEX1_2_26437,104,59,197,160,80,17,41,0,1,36,0,136
APEX1_2_26438,2342,1827,2075,2164,1528,2581,2158,2028,991,2380,1279,14
46
APEX1_2_26439,60,49,117,70,109,13,220,384,0,0,98,28
ART4_2_26440,320,491,276,433,188,381,2,355,717,72,914,803
ART4_2_26441,323,509,296,445,201,388,49,348,670,97,900,819
ART4_2_26442,1116,1016,764,1070,662,713,1922,1365,888,1144,930,782
ART4_2_26443,327,241,293,380,1090,52,351,183,32,283,925,270
ART4_2_26444,87,110,61,22,76,40,0,0,18,54,0,16
ART4_2_26445,220,128,192,211,11,190,57,202,79,256,56,29
ART4_2_26446,1159,1169,723,1162,1041,903,2421,1426,962,1661,920,868
ART4_2_26447,139,234,177,206,54,1073,399,2,566,64,411,374
ART4_2_26448,116,116,102,129,21,591,112,0,15,319,2,43
ART4_2_26449,223,76,176,176,301,0,3,116,252,0,358,578
AURKAIP1_2_26450,57,124,76,76,0,3,50,36,126,231,0,23
AURKAIP1_2_26451,60,129,122,96,99,90,15,147,4,84,26,55
AURKAIP1_2_26452,29,39,7,65,3,8,1,355,0,251,0,153
AURKAIP1_2_26453,503,358,415,506,732,672,735,710,383,467,269,691
AURKAIP1_2_26454,45,17,53,33,39,0,8,0,0,65,5,0

AURKAIP1_2_26455,51,59,119,32,152,0,232,0,0,1,155,0
AURKAIP1_2_26456,68,51,60,49,46,0,0,25,2,1,0,121
AURKAIP1_2_26457,80,58,91,48,22,0,1321,79,81,177,2,15
AURKAIP1_2_26458,133,125,185,117,0,0,536,0,0,0,365,24
AURKAIP1_2_26459,36,96,144,106,0,116,488,3,60,35,0,28
B3GNT1_2_26460,74,46,46,83,5,17,359,159,206,0,0,34
B3GNT1_2_26461,190,33,97,103,0,0,0,1,146,0,53,316
B3GNT1_2_26462,31,20,27,46,3,2,1,0,7,56,0,115
B3GNT1_2_26463,0,0,0,129,0,0,0,392,0,0,0,0
B3GNT1_2_26464,13,114,91,120,0,6,49,19,1,14,3,93
B3GNT1_2_26465,184,15,27,129,0,0,0,0,0,0,0,0
B3GNT1_2_26466,7,1,27,0,0,0,0,0,0,0,0,0
B3GNT1_2_26467,66,51,34,23,0,0,8,195,262,0,44,63
B3GNT1_2_26468,16,4,6,8,0,0,111,0,1,34,0,2
B3GNT1_2_26469,32,32,104,134,0,1,258,0,0,2,0,24
B3GNT2_2_26470,250,314,459,445,1432,619,799,67,153,528,477,336
B3GNT2_2_26471,930,864,693,598,802,386,1263,1073,984,575,430,1189
B3GNT2_2_26472,936,1005,758,1026,676,729,1511,1972,1896,1395,770,897
B3GNT2_2_26473,16,104,95,75,57,0,19,0,84,0,0,166
B3GNT2_2_26474,259,111,183,114,182,123,313,46,102,117,52,10
B3GNT2_2_26475,203,282,213,203,695,0,11,14,309,276,153,96
B3GNT2_2_26476,1208,1167,1209,1356,287,319,842,1233,959,2042,3029,1747
B3GNT2_2_26477,781,949,963,1087,231,291,691,961,809,1688,3019,996
B3GNT2_2_26478,118,82,28,83,152,8,0,354,224,1,2,382
B3GNT2_2_26479,160,9,27,24,9,1,0,0,1,33,426,0
B3GNTL1_2_26480,0,31,35,13,0,0,0,0,0,0,0,0
B3GNTL1_2_26481,98,102,140,55,106,64,138,179,18,142,3,156
B3GNTL1_2_26482,19,31,115,26,310,9,318,7,24,0,31,3
B3GNTL1_2_26483,130,200,149,176,728,147,375,185,0,144,163,349
B3GNTL1_2_26484,596,581,552,860,269,792,1279,885,421,260,897,740
B3GNTL1_2_26485,212,153,163,214,468,0,33,24,158,108,589,7
B3GNTL1_2_26486,173,87,80,180,109,305,23,241,22,402,11,403
B3GNTL1_2_26487,101,89,60,75,2,366,0,1,4,204,122,91
B3GNTL1_2_26488,538,561,551,821,269,772,1277,885,421,260,1001,738
B3GNTL1_2_26489,107,184,166,263,32,7,0,2,68,565,353,2
BCAT2_2_26490,244,299,255,300,49,493,11,155,26,465,99,558
BCAT2_2_26491,106,5,41,18,0,0,15,0,319,0,0,1
BCAT2_2_26492,92,136,83,83,0,0,122,0,51,36,23,343
BCAT2_2_26493,238,117,116,157,59,6,495,18,53,148,6,156
BCAT2_2_26494,15,4,26,18,7,0,1,0,0,0,23,0
BCAT2_2_26495,105,145,127,99,1,0,136,0,54,39,23,345
BCAT2_2_26496,58,49,30,52,0,3,0,0,0,1,0,0
BCAT2_2_26497,29,38,107,57,50,12,0,0,0,0,0,0
BCAT2_2_26498,149,29,142,117,271,2,135,218,52,3,146,128
BCAT2_2_26499,915,753,1144,956,1418,457,1052,1426,830,675,1316,517
GNAI2_2_26500,482,646,423,703,640,694,390,311,316,1024,934,552
GNAI2_2_26501,278,281,208,338,496,53,126,29,307,291,12,484
GNAI2_2_26502,508,585,384,694,342,229,238,132,406,324,263,851
GNAI2_2_26503,309,293,171,230,142,86,55,374,102,785,0,254
GNAI2_2_26504,498,609,458,817,326,539,287,489,366,951,375,928

GNAI2_2_26505,391,320,262,338,615,68,137,284,120,392,93,303
GNAI2_2_26506,312,102,148,229,545,720,0,294,6,488,1,561
GNAI2_2_26507,494,609,454,809,326,538,284,488,366,947,369,926
GNAI2_2_26508,162,74,121,221,158,64,167,197,27,53,281,71
GNAI2_2_26509,479,293,277,294,909,322,826,32,596,229,21,49
CES1_2_26510,154,203,195,225,40,150,730,315,176,458,144,252
CES1_2_26511,525,522,696,620,1006,397,260,250,947,1422,115,491
CES1_2_26512,172,110,70,253,542,3,324,6,18,14,2,295
CES1_2_26513,11,11,51,27,0,102,89,0,40,48,0,0
CES1_2_26514,583,403,503,515,307,924,632,606,540,306,511,367
CES1_2_26515,116,11,101,45,58,280,0,0,56,95,172,26
CES1_2_26516,559,482,519,452,1035,699,279,819,859,283,453,602
CES1_2_26517,21,8,52,71,0,0,0,1,85,4,0,3
CES1_2_26518,429,445,253,193,649,3,13,243,218,34,578,101
CES1_2_26519,264,365,219,207,724,436,61,1,174,223,1032,104
CNTN1_2_26520,597,511,498,672,434,190,349,423,339,275,266,236
CNTN1_2_26521,290,493,178,418,48,519,382,167,137,581,357,388
CNTN1_2_26522,911,703,541,682,948,1059,282,440,913,1326,542,291
CNTN1_2_26523,476,419,464,410,488,502,411,190,233,93,90,217
CNTN1_2_26524,524,658,551,816,1239,1259,598,143,211,937,304,159
CNTN1_2_26525,991,744,609,809,959,1076,362,439,914,1344,551,305
CNTN1_2_26526,414,220,186,264,623,493,127,95,313,310,143,313
CNTN1_2_26527,1040,795,799,973,752,503,438,537,638,514,295,278
CNTN1_2_26528,433,304,334,289,468,506,233,207,112,44,87,209
CNTN1_2_26529,897,731,851,813,473,409,686,853,1138,902,419,1209
CYP11B1_2_26530,468,53,66,160,0,8,5,2,13,265,336,10
CYP11B1_2_26531,104,214,300,238,23,7,385,68,52,283,244,264
CYP11B1_2_26532,5,5,17,67,32,1,99,33,3,5,6,7
CYP11B1_2_26533,178,105,124,124,18,31,8,0,0,93,193,8
CYP11B1_2_26534,96,60,70,89,0,22,54,84,382,328,0,685
CYP11B1_2_26535,249,228,199,285,228,121,78,183,162,252,33,200
CYP11B1_2_26536,33,21,47,15,23,25,41,1,41,74,0,1
CYP11B1_2_26537,459,606,629,579,295,73,127,714,847,726,819,540
CYP11B1_2_26538,19,11,13,23,20,0,1,8,1,2,6,0
CYP11B1_2_26539,205,172,203,187,361,150,290,173,307,473,57,385
CYP11B2_2_26540,338,191,274,246,607,201,69,256,160,409,232,264
CYP11B2_2_26541,227,217,242,346,16,87,674,235,124,81,547,210
CYP11B2_2_26542,3,8,43,21,0,19,0,0,45,2,0,37
CYP11B2_2_26543,246,282,216,391,281,546,464,221,592,58,513,178
CYP11B2_2_26544,57,55,14,44,225,2,0,0,8,29,65,2
CYP11B2_2_26545,474,464,406,462,582,137,461,368,318,120,230,637
CYP11B2_2_26546,63,93,61,93,137,133,63,55,0,81,0,0
CYP11B2_2_26547,190,333,382,326,31,149,378,354,300,175,1276,91
CYP11B2_2_26548,73,122,55,133,127,0,23,16,153,196,671,0
CYP11B2_2_26549,100,63,56,98,223,2,434,29,8,43,0,3
CPOX_2_26550,662,528,612,832,544,358,306,924,749,1033,1093,970
CPOX_2_26551,481,484,326,373,174,489,481,385,24,340,99,430
CPOX_2_26552,191,163,250,305,184,8,167,0,182,179,297,233
CPOX_2_26553,357,204,427,444,281,241,935,62,646,81,36,260
CPOX_2_26554,109,59,73,35,0,0,30,0,30,292,155,146

CPOX_2_26555,122,304,212,233,10,452,72,354,601,222,0,0
CPOX_2_26556,1412,1212,1531,1439,837,1449,1219,1197,851,1076,881,1594
CPOX_2_26557,708,690,771,738,1056,130,954,450,835,814,658,1291
CPOX_2_26558,511,336,266,409,545,507,455,540,248,98,744,633
CPOX_2_26559,369,198,228,408,44,21,502,40,1093,349,134,146
CTSK_2_26560,262,117,182,226,162,55,16,8,124,233,56,269
CTSK_2_26561,1176,793,857,791,1174,473,971,872,1038,1308,701,762
CTSK_2_26562,93,10,57,36,69,7,269,56,152,6,0,0
CTSK_2_26563,563,343,392,387,744,502,604,586,265,173,77,316
CTSK_2_26564,637,646,638,614,718,663,203,1286,556,1363,724,693
CTSK_2_26565,336,199,125,273,592,2,126,583,193,112,0,134
CTSK_2_26566,451,458,484,511,598,362,620,786,573,461,234,460
CTSK_2_26567,657,730,471,570,1085,348,1046,176,418,1027,491,855
CTSK_2_26568,0,0,0,0,0,0,0,0,0,0,0,0
CTSK_2_26569,0,48,12,0,0,0,63,0,0,0,0,0
CYP2A6_2_26570,1547,1453,1420,1640,1013,1243,1831,2405,2596,1207,1840,
1865
CYP2A6_2_26571,441,486,422,510,542,418,296,651,247,998,1426,1183
CYP2A6_2_26572,351,199,324,357,242,291,670,403,628,238,168,417
CYP2A6_2_26573,59,28,77,56,2,34,44,11,39,7,130,8
CYP2A6_2_26574,833,486,570,668,714,90,810,293,699,331,267,1224
CYP2A6_2_26575,661,660,492,879,209,523,723,1081,658,1291,234,510
CYP2A6_2_26576,139,87,81,165,191,7,25,146,148,149,433,209
CYP2A6_2_26577,417,406,248,363,383,280,368,538,261,501,419,547
CYP2A6_2_26578,4036,3306,3193,4115,4518,2995,2047,3820,2993,3028,2278,
4078
CYP2A6_2_26579,440,252,370,397,492,286,171,128,530,681,97,396
CYP21A2_2_26580,123,57,110,186,109,91,463,33,1,323,0,8
CYP21A2_2_26581,195,103,141,144,481,572,0,269,192,445,1,139
CYP21A2_2_26582,223,331,215,232,4,153,75,476,250,450,263,563
CYP21A2_2_26583,687,832,491,603,689,167,356,1111,90,662,642,650
CYP21A2_2_26584,346,367,595,523,281,131,57,384,85,56,40,188
CYP21A2_2_26585,228,118,174,203,373,24,125,122,194,194,196,149
CYP21A2_2_26586,118,116,69,92,3,34,45,42,157,98,5,421
CYP21A2_2_26587,380,344,428,661,216,292,327,198,545,392,78,427
CYP21A2_2_26588,434,925,497,483,1186,1165,404,366,206,842,47,1332
CYP21A2_2_26589,556,713,438,722,173,145,106,515,231,357,388,528
DDOST_2_26590,26,7,7,24,162,12,89,85,1,0,0,0
DDOST_2_26591,107,16,125,40,0,90,499,48,25,40,0,67
DDOST_2_26592,380,432,477,478,370,199,124,587,802,288,372,191
DDOST_2_26593,58,128,78,258,0,54,0,0,0,1,231,283
DDOST_2_26594,198,42,22,55,3,33,5,0,11,22,0,3
DDOST_2_26595,111,44,94,81,72,0,2,98,254,13,3,64
DDOST_2_26596,17,0,0,8,0,0,0,0,0,0,0,109
DDOST_2_26597,168,273,149,134,212,515,16,15,7,0,347,12
DDOST_2_26598,474,357,311,169,796,78,424,383,527,122,1724,329
DDOST_2_26599,492,458,493,480,374,198,125,587,812,287,381,327
DDX11_2_26600,10,12,52,59,0,0,0,0,0,0,0,0
DDX11_2_26601,95,130,67,53,6,171,55,19,50,49,35,165
DDX11_2_26602,192,124,126,130,114,0,458,0,44,43,83,87

DDX11_2_26603,188,199,211,104,4,35,151,248,24,182,0,486
DDX11_2_26604,348,241,255,455,104,0,458,84,215,281,1,578
DDX11_2_26605,395,384,361,480,559,167,939,306,680,263,228,396
DDX11_2_26606,317,281,271,273,216,59,39,277,164,253,26,38
DDX11_2_26607,603,469,445,594,389,242,135,671,304,343,437,418
DDX11_2_26608,741,849,1005,841,472,1645,835,237,194,1349,1392,691
DDX11_2_26609,2837,2897,2471,2879,3255,2804,4107,1692,2136,2885,2086,3
013
DFFB_2_26610,105,43,56,61,3,24,40,23,24,27,248,391
DFFB_2_26611,444,352,304,304,91,228,712,501,719,203,244,249
DFFB_2_26612,1,11,4,0,0,0,0,2,17,0,0,37
DFFB_2_26613,168,96,39,167,325,0,246,212,17,10,0,64
DFFB_2_26614,460,280,259,341,230,197,444,101,293,488,68,525
DFFB_2_26615,152,206,180,162,27,2,1,922,8,2,100,6
DFFB_2_26616,3,0,42,43,6,0,0,10,0,0,0,0
DFFB_2_26617,97,156,114,160,1,68,45,27,27,5,536,257
DFFB_2_26618,564,550,452,669,354,567,393,339,544,832,293,620
DFFB_2_26619,170,111,110,89,23,353,46,41,538,9,19,40
DHX8_2_26620,53,24,15,25,0,0,0,207,20,0,0,111
DHX8_2_26621,125,203,61,62,259,3,17,73,166,62,0,71
DHX8_2_26622,216,251,166,234,584,63,3,415,130,214,130,31
DHX8_2_26623,677,655,666,891,1019,1065,59,1212,405,993,590,856
DHX8_2_26624,589,471,406,487,348,92,48,965,661,457,604,531
DHX8_2_26625,208,102,84,88,166,382,18,189,53,47,14,35
DHX8_2_26626,246,247,305,201,264,238,44,193,284,201,112,240
DHX8_2_26627,687,579,662,868,412,524,541,545,110,902,925,627
DHX8_2_26628,104,82,137,148,39,74,64,83,185,542,296,97
DHX8_2_26629,1515,1368,1010,1256,1058,1668,514,1241,1446,1854,545,1320
DHX15_2_26630,112,183,142,144,117,227,215,6,19,1,483,205
DHX15_2_26631,75,195,86,222,2,15,43,202,150,60,67,100
DHX15_2_26632,353,159,219,354,212,473,0,18,133,174,551,168
DHX15_2_26633,4,13,29,25,0,0,0,0,51,0,109,27
DHX15_2_26634,172,214,205,292,0,0,7,45,20,37,875,226
DHX15_2_26635,207,216,188,323,304,95,581,232,635,271,57,312
DHX15_2_26636,16,107,87,37,364,120,2,0,10,0,388,43
DHX15_2_26637,223,276,184,397,262,305,118,8,34,138,0,329
DHX15_2_26638,580,649,623,647,1227,795,1579,519,365,1076,323,279
DHX15_2_26639,431,544,535,543,728,304,428,130,515,291,10,120
DIMT1_2_26640,253,90,201,113,396,0,355,0,485,219,103,122
DIMT1_2_26641,2165,1875,1674,2197,1763,2195,1676,619,1493,3951,1870,67
1
DIMT1_2_26642,650,571,697,761,643,433,94,746,469,166,478,984
DIMT1_2_26643,517,442,304,478,87,129,169,43,67,384,32,412
DIMT1_2_26644,200,355,164,326,28,5,629,165,88,39,720,251
DIMT1_2_26645,135,158,160,203,98,141,96,131,36,37,789,9
DIMT1_2_26646,791,585,846,704,629,389,432,931,481,137,222,1130
DIMT1_2_26647,29,37,85,137,247,0,0,0,0,5,0,0
DIMT1_2_26648,218,133,154,256,313,60,0,53,423,95,62,133
DIMT1_2_26649,189,158,141,207,234,102,257,51,142,246,19,145
DNPEP_2_26650,128,79,106,99,33,143,7,0,116,77,3,79

DNPEP_2_26651,488,431,464,599,10,23,841,309,772,799,791,298
DNPEP_2_26652,676,803,627,665,1185,696,686,626,446,806,517,424
DNPEP_2_26653,147,35,49,70,202,0,9,120,53,11,31,95
DNPEP_2_26654,185,238,246,260,536,143,321,29,201,728,362,471
DNPEP_2_26655,359,397,235,336,131,531,485,338,242,653,75,345
DNPEP_2_26656,44,55,60,52,2,2,42,0,7,46,0,0
DNPEP_2_26657,106,83,116,114,206,3,282,1,0,0,119,196
DNPEP_2_26658,226,125,201,113,237,383,291,30,302,194,52,379
DNPEP_2_26659,173,209,185,145,382,83,4,423,221,17,10,78
DPAGT1_2_26660,115,299,124,125,0,181,157,0,64,109,0,170
DPAGT1_2_26661,98,97,62,94,17,229,0,0,1,1,269,390
DPAGT1_2_26662,0,0,0,0,0,0,0,0,0,0,0,0
DPAGT1_2_26663,0,0,0,0,0,0,0,0,0,0,0,0
DPAGT1_2_26664,59,38,125,57,30,0,2,121,9,79,0,10
DPAGT1_2_26665,54,60,67,72,262,20,0,506,82,1,122,153
DPAGT1_2_26666,85,37,91,92,79,1,77,0,147,208,72,22
DPAGT1_2_26667,307,189,57,211,69,143,178,0,290,167,468,148
DPAGT1_2_26668,255,287,193,238,103,171,346,42,43,133,403,91
DPAGT1_2_26669,185,107,83,119,0,171,555,349,172,85,7,81
DPYSL2_2_26670,708,724,389,632,373,386,58,18,538,194,295,297
DPYSL2_2_26671,135,370,159,281,100,263,1,156,110,267,353,848
DPYSL2_2_26672,404,286,161,278,589,89,6,0,348,108,1058,150
DPYSL2_2_26673,27,53,62,50,0,17,0,257,0,1,0,1
DPYSL2_2_26674,280,168,289,459,477,45,214,401,257,308,668,577
DPYSL2_2_26675,345,238,186,346,1157,474,356,167,160,333,146,23
DPYSL2_2_26676,338,108,220,238,687,0,0,492,219,3,121,11
DPYSL2_2_26677,110,77,41,115,6,3,32,37,151,125,0,4
DPYSL2_2_26678,159,166,95,140,218,123,218,194,106,189,79,288
DPYSL2_2_26679,173,151,150,187,0,0,21,25,3,11,8,329
DTD1_2_26680,228,247,118,183,94,29,503,202,139,95,117,96
DTD1_2_26681,386,479,427,420,230,463,453,546,191,485,99,117
DTD1_2_26682,70,63,52,93,105,30,3,1,35,55,0,89
DTD1_2_26683,354,293,313,326,265,237,450,725,286,421,121,507
DTD1_2_26684,596,469,526,503,481,461,451,893,711,446,1183,665
DTD1_2_26685,632,401,440,469,379,427,782,613,509,492,127,366
DTD1_2_26686,206,122,105,149,547,22,127,137,49,37,274,145
DTD1_2_26687,6,4,3,9,0,0,0,0,0,0,0,0
DTD1_2_26688,73,76,74,119,2,87,3,44,106,371,26,113
DTD1_2_26689,147,101,195,114,36,276,316,317,63,24,3,72
DUPD1_2_26690,1388,1234,1232,1134,1169,759,1513,899,1189,818,1178,931
DUPD1_2_26691,207,154,178,226,68,121,251,311,134,489,8,432
DUPD1_2_26692,274,160,203,250,9,403,351,629,60,300,295,303
DUPD1_2_26693,1327,1150,955,1254,998,1134,1135,598,399,857,875,888
DUPD1_2_26694,213,113,89,230,141,0,144,234,400,165,69,181
DUPD1_2_26695,176,117,137,172,10,83,27,4,22,205,196,172
DUPD1_2_26696,42,24,8,47,16,1,0,97,10,9,0,6
DUPD1_2_26697,316,372,326,349,140,376,164,239,285,330,286,281
DUPD1_2_26698,60,29,30,51,0,120,0,0,94,139,0,2
DUPD1_2_26699,889,698,742,1026,579,527,371,582,1147,654,298,760
DUSP28_2_26700,144,99,125,126,148,3,263,14,194,399,23,114

DUSP28_2_26701,340,392,304,524,39,62,697,1593,648,64,267,416
DUSP28_2_26702,12,0,5,54,0,0,0,0,1,0,0,0
DUSP28_2_26703,82,71,129,58,59,6,0,0,37,18,34,14
DUSP28_2_26704,833,840,819,1137,389,622,484,944,734,858,1147,721
DUSP28_2_26705,413,134,213,237,163,35,625,6,455,39,187,304
DUSP28_2_26706,210,588,461,460,254,1037,59,8,709,540,1305,376
DUSP28_2_26707,57,63,68,134,44,1,0,27,6,129,0,46
DUSP28_2_26708,96,69,100,60,43,13,49,79,33,4,26,29
DUSP28_2_26709,187,639,467,395,274,472,433,240,735,183,893,619
EPRS_2_26710,251,194,148,226,290,346,250,75,37,72,226,145
EPRS_2_26711,234,167,175,269,0,0,36,0,0,4,74,140
EPRS_2_26712,1158,692,651,933,937,784,913,1137,1169,719,849,626
EPRS_2_26713,879,798,741,919,590,1282,1194,1197,1183,1089,421,628
EPRS_2_26714,95,120,87,165,38,0,126,441,24,0,106,104
EPRS_2_26715,45,26,78,31,38,38,19,0,116,3,509,46
EPRS_2_26716,30,38,33,45,131,23,1,17,55,106,2,50
EPRS_2_26717,618,505,690,565,480,879,504,955,802,663,605,644
EPRS_2_26718,60,14,22,20,3,144,0,3,14,9,36,1
EPRS_2_26719,11,43,42,16,0,0,0,0,0,104,32,169
EPX_2_26720,295,435,300,213,356,1482,189,643,16,169,210,39
EPX_2_26721,226,121,262,317,210,160,833,229,79,389,6,199
EPX_2_26722,619,865,576,826,371,468,216,636,807,761,804,599
EPX_2_26723,46,8,26,44,19,0,40,41,39,1,5,0
EPX_2_26724,853,735,707,688,794,518,72,319,405,294,769,969
EPX_2_26725,60,44,166,146,2,54,281,539,432,189,0,365
EPX_2_26726,133,147,156,131,135,158,359,13,55,46,22,377
EPX_2_26727,166,124,210,123,159,216,393,117,522,5,184,129
EPX_2_26728,181,172,163,139,223,159,359,13,55,47,22,377
EPX_2_26729,1224,1333,933,1302,1617,984,1032,488,918,1061,878,455
ERCC2_2_26730,167,113,273,421,2,224,15,355,8,129,567,37
ERCC2_2_26731,163,258,183,274,200,237,177,7,41,289,250,45
ERCC2_2_26732,7,17,26,16,2,0,0,1,10,80,75,71
ERCC2_2_26733,1197,883,759,1000,930,634,450,750,1387,1343,1181,368
ERCC2_2_26734,11,9,0,21,90,0,0,102,0,0,0,0
ERCC2_2_26735,88,100,72,108,31,81,0,51,79,92,0,562
ERCC2_2_26736,129,164,166,151,428,489,134,82,251,0,0,28
ERCC2_2_26737,27,53,29,56,0,60,0,32,16,10,1,280
ERCC2_2_26738,209,248,168,110,417,5,1,4,348,38,587,162
ERCC2_2_26739,93,171,107,127,59,56,911,132,236,37,91,132
ERCC4_2_26740,340,176,153,265,200,22,326,16,714,44,651,9
ERCC4_2_26741,456,651,596,664,1686,283,231,94,451,380,417,609
ERCC4_2_26742,767,709,454,828,507,655,321,299,761,543,613,783
ERCC4_2_26743,329,153,243,286,60,0,250,933,194,2,4,160
ERCC4_2_26744,512,468,614,569,642,504,571,65,356,350,369,805
ERCC4_2_26745,468,493,467,560,559,0,962,501,472,300,255,202
ERCC4_2_26746,656,455,552,440,748,382,525,664,275,286,667,55
ERCC4_2_26747,331,227,167,375,327,82,298,267,230,727,575,302
ERCC4_2_26748,40,44,11,83,405,16,142,6,46,0,0,24
ERCC4_2_26749,298,381,343,268,488,249,965,107,80,314,453,352
FA2H_2_26750,84,81,39,37,8,77,7,16,16,15,0,1

FA2H_2_26751,606,389,354,396,525,617,137,30,852,865,205,940
FA2H_2_26752,160,235,182,257,406,4,681,89,66,540,95,203
FA2H_2_26753,99,49,82,122,37,0,50,7,17,51,0,78
FA2H_2_26754,17,7,21,47,18,7,99,185,4,158,0,22
FA2H_2_26755,7,3,75,16,0,0,16,126,19,0,0,7
FA2H_2_26756,53,59,16,58,0,377,133,203,0,123,33,122
FA2H_2_26757,950,877,817,826,1942,602,273,398,614,1019,1562,260
FA2H_2_26758,44,64,24,119,140,1,173,54,1,6,28,6
FA2H_2_26759,130,189,170,203,193,201,77,2,203,114,181,82
FADS1_2_26760,370,393,391,453,44,445,455,264,383,499,423,520
FADS1_2_26761,125,173,40,96,0,0,0,415,14,3,0,30
FADS1_2_26762,300,279,297,453,3,740,440,1,369,225,565,589
FADS1_2_26763,179,61,51,105,272,5,604,2,79,74,15,44
FADS1_2_26764,224,280,181,339,148,634,359,0,3,519,15,2
FADS1_2_26765,190,190,206,288,175,13,66,0,245,116,0,594
FADS1_2_26766,782,1070,998,1024,897,1367,1113,986,867,820,1759,1219
FADS1_2_26767,168,73,83,66,0,163,132,78,123,5,83,0
FADS1_2_26768,274,208,223,200,268,261,334,487,162,497,1509,599
FADS1_2_26769,229,186,167,125,0,0,144,0,130,10,390,206
FAM213B_2_26770,150,145,185,370,481,2,469,0,102,0,625,322
FAM213B_2_26771,13,34,14,38,0,0,0,27,298,0,0,0
FAM213B_2_26772,370,340,106,335,27,13,10,213,251,866,431,38
FAM213B_2_26773,60,15,77,59,139,5,0,16,17,147,54,0
FAM213B_2_26774,206,149,145,176,90,445,115,162,372,93,48,349
FAM213B_2_26775,528,438,559,800,444,199,353,634,203,0,1482,779
FAM213B_2_26776,496,185,285,305,78,6,0,87,6,0,862,400
FAM213B_2_26777,101,7,92,44,4,0,0,0,37,0,34,4
FAM213B_2_26778,1,0,0,5,0,0,0,0,0,0,0,36
FAM213B_2_26779,84,151,81,103,68,347,1,200,51,68,8,172
FASN_2_26780,1937,1817,1529,1923,1330,1095,2418,2142,1120,1395,1806,22
47
FASN_2_26781,404,442,434,605,355,235,1024,787,1255,182,339,892
FASN_2_26782,732,785,745,710,563,514,832,431,623,579,2012,402
FASN_2_26783,605,348,451,456,675,42,212,303,487,434,1061,576
FASN_2_26784,232,278,246,241,269,194,61,5,72,8,0,163
FASN_2_26785,146,172,155,278,419,75,137,24,20,174,45,350
FASN_2_26786,369,332,167,204,118,23,159,125,631,511,23,273
FASN_2_26787,35,67,73,141,1,0,79,909,146,87,0,18
FASN_2_26788,296,179,227,271,302,385,773,302,445,719,80,422
FASN_2_26789,254,134,235,250,10,66,282,101,125,198,461,382
FKBP1B_2_26790,189,40,106,68,2,53,255,56,101,20,124,185
FKBP1B_2_26791,420,495,319,377,1083,447,99,103,392,173,508,402
FKBP1B_2_26792,164,97,123,135,160,143,0,8,347,37,1,207
FKBP1B_2_26793,137,92,90,133,250,222,4,0,38,86,0,116
FKBP1B_2_26794,727,398,382,695,204,96,504,43,455,553,469,478
FKBP1B_2_26795,122,99,114,140,6,48,2,151,203,63,88,24
FKBP1B_2_26796,462,455,436,354,578,271,1423,303,29,6,535,271
FKBP1B_2_26797,214,92,149,124,331,91,11,41,294,42,11,91
FKBP1B_2_26798,779,560,721,739,1028,465,1451,720,266,1449,679,774
FKBP1B_2_26799,276,350,160,244,468,326,225,15,246,198,126,224

FKBP10_2_26800,129,213,163,246,207,0,50,379,63,256,0,354
FKBP10_2_26801,64,168,206,189,0,0,237,0,0,165,730,1
FKBP10_2_26802,59,48,110,145,81,0,410,202,112,115,1,13
FKBP10_2_26803,295,105,254,340,108,77,474,303,160,249,442,275
FKBP10_2_26804,244,200,166,187,8,448,479,11,121,14,93,12
FKBP10_2_26805,274,170,303,342,0,166,1,28,571,278,66,0
FKBP10_2_26806,403,347,439,450,322,310,370,335,170,380,117,164
FKBP10_2_26807,667,617,611,678,1035,466,2,444,753,465,2,743
FKBP10_2_26808,1575,1499,1411,1881,1771,865,1016,1379,1976,1724,1413,1
273
FKBP10_2_26809,548,558,551,565,680,321,33,146,309,45,543,219
FMO4_2_26810,199,80,195,173,0,558,2,1,1,0,572,269
FMO4_2_26811,779,783,741,828,1232,429,1364,1068,344,1766,795,984
FMO4_2_26812,374,461,516,445,378,305,395,428,323,259,134,361
FMO4_2_26813,258,227,236,230,255,224,435,119,175,635,128,437
FMO4_2_26814,732,707,831,1055,422,178,396,515,325,1562,1691,1026
FMO4_2_26815,70,108,87,144,470,2,57,29,96,1,0,177
FMO4_2_26816,92,115,89,130,14,1,59,6,164,23,342,29
FMO4_2_26817,415,347,247,521,89,79,117,167,358,224,26,135
FMO4_2_26818,299,125,267,321,577,324,150,307,246,502,113,226
FMO4_2_26819,1725,1487,1290,1596,1263,891,585,553,1488,1488,2055,1740
GAL3ST3_2_26820,371,472,459,492,408,715,217,167,623,448,84,439
GAL3ST3_2_26821,516,630,548,698,887,863,223,224,627,448,670,518
GAL3ST3_2_26822,573,676,562,728,1217,280,431,1131,504,353,793,269
GAL3ST3_2_26823,342,492,405,496,731,504,147,218,269,624,569,203
GAL3ST3_2_26824,123,165,124,264,59,43,0,2,5,41,42,130
GAL3ST3_2_26825,190,171,252,242,7,49,0,313,1013,40,3,230
GAL3ST3_2_26826,23,1,11,12,0,0,0,0,357,0,0,0
GAL3ST3_2_26827,311,170,299,397,149,474,59,968,263,73,13,453
GAL3ST3_2_26828,251,326,191,302,336,151,22,779,733,1,32,528
GAL3ST3_2_26829,118,59,100,58,238,335,0,154,190,22,37,95
GALNTL2_2_26830,638,469,618,525,826,158,449,331,858,531,739,997
GALNTL2_2_26831,189,223,114,212,394,195,19,9,190,11,395,227
GALNTL2_2_26832,460,488,305,510,284,162,38,271,0,334,853,7
GALNTL2_2_26833,1826,2500,1628,2065,790,1146,121,1384,1049,5124,2015,2
352
GALNTL2_2_26834,931,1000,967,1035,952,1012,1044,1272,837,1050,2135,791
GALNTL2_2_26835,503,664,431,671,827,741,234,914,5,795,160,590
GALNTL2_2_26836,254,229,329,252,27,183,101,412,19,377,428,130
GALNTL2_2_26837,110,68,83,120,4,54,381,5,53,183,521,32
GALNTL2_2_26838,2,2,28,37,0,41,364,0,228,14,0,7
GALNTL2_2_26839,380,402,439,360,194,21,506,28,178,234,261,239
GARS_2_26840,658,723,566,539,920,416,954,414,968,846,899,628
GARS_2_26841,235,410,283,317,373,1,2,120,55,538,35,617
GARS_2_26842,724,708,633,747,235,547,432,712,448,802,850,896
GARS_2_26843,168,75,88,149,16,52,156,2,432,0,295,1
GARS_2_26844,1798,1893,1626,1998,1125,1402,369,2002,2666,2714,732,1639
GARS_2_26845,376,214,242,489,79,275,360,186,421,33,460,525
GARS_2_26846,1236,1126,1303,1026,705,493,1599,803,296,1470,1078,1023
GARS_2_26847,386,294,279,365,724,698,143,38,659,448,643,854

GARS_2_26848,338,171,233,206,311,485,0,170,174,227,2,198
GARS_2_26849,1050,1063,1143,1067,1379,1073,1650,958,1814,610,2083,896
HSD17B4_2_26850,329,296,279,320,93,506,291,4,324,1175,1445,104
HSD17B4_2_26851,249,118,90,119,117,416,14,251,198,68,1,128
HSD17B4_2_26852,1476,1374,1518,1642,1493,958,2943,1883,2009,1268,1009,
2059
HSD17B4_2_26853,50,7,15,81,502,27,0,171,0,0,6,0
HSD17B4_2_26854,105,7,37,3,1,83,0,175,329,0,0,0
HSD17B4_2_26855,1637,1493,1377,1742,1955,1490,2482,2028,1810,1619,1002
,1904
HSD17B4_2_26856,121,51,44,25,356,237,43,0,223,11,1,103
HSD17B4_2_26857,29,0,7,55,0,0,0,0,0,0,0,0
HSD17B4_2_26858,328,277,270,313,93,508,288,4,324,1168,1444,55
HSD17B4_2_26859,1733,1628,1487,1889,1944,1484,2534,2485,1804,1753,1032
,1958
GLB1_2_26860,494,739,478,432,913,1428,201,146,247,591,678,264
GLB1_2_26861,76,47,173,115,357,196,731,0,0,232,514,11
GLB1_2_26862,290,145,137,123,12,61,6,54,137,1,133,17
GLB1_2_26863,194,60,96,176,73,64,167,296,121,0,293,67
GLB1_2_26864,130,44,83,130,42,1,6,95,98,0,0,2
GLB1_2_26865,18,42,53,35,6,0,0,0,0,2,1,1
GLB1_2_26866,78,127,69,125,37,4,110,22,83,61,291,38
GLB1_2_26867,84,42,149,75,135,106,9,0,134,115,39,307
GLB1_2_26868,48,27,51,32,20,62,24,0,24,2,1,9
GLB1_2_26869,595,449,357,447,94,249,738,611,303,704,727,321
GLS2_2_26870,3002,2134,2373,2602,4042,2828,2222,1619,3720,3346,2380,38
55
GLS2_2_26871,241,269,247,261,62,77,686,364,100,616,404,159
GLS2_2_26872,345,256,229,477,239,172,142,111,3,115,36,382
GLS2_2_26873,782,677,710,643,1325,953,784,429,651,340,656,735
GLS2_2_26874,387,429,274,302,1038,602,14,84,219,269,20,92
GLS2_2_26875,480,425,339,521,104,526,269,578,185,7,1319,531
GLS2_2_26876,631,390,451,406,552,762,1562,574,345,376,88,145
GLS2_2_26877,710,743,478,542,512,188,1002,971,464,1136,198,391
GLS2_2_26878,841,686,755,685,1074,817,780,439,696,401,734,770
GLS2_2_26879,206,90,144,167,249,102,240,562,239,16,119,156
GNPDA1_2_26880,187,106,178,111,89,187,130,95,20,39,4,256
GNPDA1_2_26881,198,454,302,271,64,1112,586,490,378,169,88,48
GNPDA1_2_26882,207,163,270,219,415,158,3,374,394,153,179,1047
GNPDA1_2_26883,42,19,38,87,11,6,30,0,13,241,0,37
GNPDA1_2_26884,243,238,279,270,185,546,570,269,74,80,325,227
GNPDA1_2_26885,485,563,411,666,994,641,579,263,256,575,161,423
GNPDA1_2_26886,356,286,252,294,202,301,444,676,14,369,445,125
GNPDA1_2_26887,197,290,320,435,276,263,0,705,63,71,162,397
GNPDA1_2_26888,83,50,52,125,20,13,156,43,36,81,0,169
GNPDA1_2_26889,41,55,41,112,20,0,302,12,1,0,188,0
GPI_2_26890,1394,1189,1481,1460,1664,414,1734,466,1176,1782,1101,1340
GPI_2_26891,163,89,135,72,0,0,179,0,157,0,16,2
GPI_2_26892,22,77,83,109,0,15,55,297,6,15,0,0
GPI_2_26893,1327,1245,929,1300,979,1374,785,1206,1217,1460,1258,709

GPI_2_26894,380,175,224,293,68,178,942,36,747,226,115,374
GPI_2_26895,1171,1281,1008,1296,1683,505,1226,964,1410,1295,2032,1285
GPI_2_26896,779,765,645,725,827,783,1078,326,662,586,952,1039
GPI_2_26897,1048,994,770,710,77,1273,1403,1776,1240,540,259,613
GPI_2_26898,150,196,221,144,46,1508,27,16,123,384,487,358
GPI_2_26899,36,83,21,74,0,0,81,0,1,2,0,0
GPNMB_2_26900,155,165,250,263,179,36,68,32,122,511,208,55
GPNMB_2_26901,339,279,312,347,600,153,172,385,470,111,335,162
GPNMB_2_26902,331,224,212,274,9,9,577,459,316,272,339,576
GPNMB_2_26903,233,118,159,130,638,44,481,153,65,1,85,38
GPNMB_2_26904,239,468,259,397,374,319,2,353,832,597,205,424
GPNMB_2_26905,367,317,347,388,601,155,175,406,492,116,506,163
GPNMB_2_26906,420,364,336,392,477,189,174,406,421,116,507,159
GPNMB_2_26907,492,390,382,468,774,235,374,185,653,578,4,447
GPNMB_2_26908,10,59,1,28,4,3,63,1,0,43,0,28
GPNMB_2_26909,31,48,20,2,0,0,0,0,0,0,0,0
GPX7_2_26910,361,443,452,576,583,82,873,267,596,884,411,755
GPX7_2_26911,605,581,502,635,579,711,439,374,162,288,138,334
GPX7_2_26912,346,127,270,327,91,196,695,419,716,36,147,1124
GPX7_2_26913,1608,1560,1468,1938,1040,1190,875,1560,1677,1017,2600,203
4
GPX7_2_26914,185,306,318,268,94,572,849,50,49,438,1579,60
GPX7_2_26915,431,538,437,409,292,110,213,155,198,511,154,276
GPX7_2_26916,139,120,111,245,104,60,0,323,8,87,37,5
GPX7_2_26917,713,645,547,526,699,36,663,304,298,899,340,912
GPX7_2_26918,483,562,392,710,880,241,622,252,723,207,1128,101
GPX7_2_26919,125,109,104,84,23,54,288,117,443,98,179,598
GTPBP4_2_26920,185,120,180,151,125,111,259,101,2,158,1,144
GTPBP4_2_26921,35,10,16,57,8,0,48,1,0,0,0,170
GTPBP4_2_26922,52,58,88,93,41,90,64,0,0,7,6,2
GTPBP4_2_26923,105,198,129,26,0,18,0,17,0,0,0,0
GTPBP4_2_26924,130,76,212,163,0,2,36,374,108,80,330,418
GTPBP4_2_26925,35,38,47,25,25,0,153,0,1,32,960,22
GTPBP4_2_26926,25,39,59,46,13,6,83,45,9,7,0,1
GTPBP4_2_26927,267,133,144,158,12,145,386,211,107,274,273,130
GTPBP4_2_26928,389,240,335,395,686,923,290,780,303,44,22,269
GTPBP4_2_26929,287,257,315,306,710,915,110,835,289,57,5,292
HS3ST6_2_26930,352,284,283,268,509,422,765,62,220,161,404,118
HS3ST6_2_26931,820,454,555,626,881,492,986,382,374,223,370,646
HS3ST6_2_26932,2764,2122,1937,2718,2567,1738,2875,2707,3139,2782,1399,
1945
HS3ST6_2_26933,249,147,209,210,7,10,3,67,244,0,49,115
HS3ST6_2_26934,560,1067,523,901,867,1554,539,258,651,1114,2368,1063
HS3ST6_2_26935,2101,1988,1845,2320,1316,2651,4143,3915,2331,2312,3693,
621
HS3ST6_2_26936,558,1015,524,890,870,1554,540,255,646,1112,2370,803
HS3ST6_2_26937,1773,1146,1242,1730,1008,962,1177,1608,1281,263,725,786
HS3ST6_2_26938,76,85,57,52,13,106,7,11,19,7,5,23
HS3ST6_2_26939,881,1019,832,873,815,1153,1256,1142,1426,687,212,1373
HYAL1_2_26940,78,64,71,103,0,181,108,100,290,13,26,28

HYAL1_2_26941,283,305,252,355,306,80,120,288,212,65,198,336
HYAL1_2_26942,48,0,62,57,246,0,76,0,0,0,505,228
HYAL1_2_26943,101,107,63,149,278,2,4,15,86,42,41,43
HYAL1_2_26944,78,84,11,96,22,0,91,15,5,21,330,81
HYAL1_2_26945,445,493,508,605,613,85,527,338,754,499,164,547
HYAL1_2_26946,40,66,86,22,191,7,231,42,0,111,630,12
HYAL1_2_26947,444,428,557,680,365,207,154,309,819,172,205,609
HYAL1_2_26948,55,67,43,108,14,0,8,5,54,7,156,211
HYAL1_2_26949,741,696,691,813,216,200,351,980,487,647,1556,358
KHSRP_2_26950,70,119,77,41,165,28,145,65,87,98,155,71
KHSRP_2_26951,29,44,23,52,0,0,4,3,189,98,5,4
KHSRP_2_26952,712,602,553,564,280,678,719,476,90,452,937,382
KHSRP_2_26953,3,13,22,2,75,0,0,13,0,0,34,0
KHSRP_2_26954,224,121,200,177,286,246,524,5,70,236,21,262
KHSRP_2_26955,54,124,43,73,13,10,712,20,318,2,0,219
KHSRP_2_26956,126,80,94,122,133,55,1,395,13,47,14,152
KHSRP_2_26957,995,695,534,641,387,429,655,1157,477,814,276,1368
KHSRP_2_26958,140,233,296,251,480,6,124,59,418,163,1784,569
KHSRP_2_26959,28,13,37,45,0,0,0,0,23,11,0,162
KIF20B_2_26960,382,465,347,483,412,534,632,948,266,1192,204,171
KIF20B_2_26961,153,82,303,232,24,21,390,4,52,276,908,189
KIF20B_2_26962,57,114,47,34,0,8,1,0,57,24,0,53
KIF20B_2_26963,18,78,76,35,217,19,57,41,3,132,0,81
KIF20B_2_26964,747,715,698,894,579,1228,813,659,707,363,540,1487
KIF20B_2_26965,302,222,113,120,612,255,342,20,465,241,324,178
KIF20B_2_26966,203,300,207,222,245,205,26,1,7,313,7,74
KIF20B_2_26967,92,115,135,64,217,18,7,188,85,32,213,98
KIF20B_2_26968,87,125,66,73,290,80,8,29,58,159,12,216
KIF20B_2_26969,394,225,140,322,104,4,101,179,17,287,0,277
KLKB1_2_26970,50,25,51,199,0,3,0,162,1,58,0,59
KLKB1_2_26971,145,127,116,142,150,232,572,179,96,348,46,81
KLKB1_2_26972,94,163,78,102,64,16,22,281,10,171,0,91
KLKB1_2_26973,84,44,55,83,206,0,187,18,292,189,0,0
KLKB1_2_26974,134,120,73,175,0,128,0,109,178,328,44,139
KLKB1_2_26975,752,799,727,872,189,548,1042,564,430,1376,537,1277
KLKB1_2_26976,72,74,115,103,168,0,60,0,23,7,0,86
KLKB1_2_26977,107,55,63,77,0,0,185,415,364,2,452,347
KLKB1_2_26978,57,3,28,34,2,0,25,13,96,52,0,8
KLKB1_2_26979,207,290,288,268,186,50,488,137,158,411,273,75
LAMA4_2_26980,145,209,150,311,0,122,714,295,29,0,1036,351
LAMA4_2_26981,105,73,19,193,69,8,20,57,68,0,181,2
LAMA4_2_26982,125,133,102,149,10,96,352,19,84,566,456,374
LAMA4_2_26983,612,405,333,360,287,209,444,991,502,312,94,194
LAMA4_2_26984,26,8,14,41,13,0,0,54,0,15,0,0
LAMA4_2_26985,439,343,354,376,112,121,490,201,425,839,28,516
LAMA4_2_26986,76,155,87,264,43,81,1,2,0,55,1012,14
LAMA4_2_26987,656,315,413,414,1312,577,39,73,501,635,247,359
LAMA4_2_26988,65,111,91,124,55,59,8,84,1,2,1,106
LAMA4_2_26989,87,96,61,122,18,0,17,258,16,18,17,56
LGALS13_2_26990,553,367,419,377,204,201,537,242,754,684,586,558

LGALS13_2_26991,487,512,515,474,1317,321,1072,382,15,294,578,253
LGALS13_2_26992,1361,1325,1226,1558,1409,1605,174,2248,1659,1873,1340,
1865
LGALS13_2_26993,144,46,38,49,83,111,36,129,104,40,0,29
LGALS13_2_26994,10,17,43,14,99,3,41,10,1,0,0,0
LGALS13_2_26995,36,20,78,188,4,99,435,301,0,0,11,132
LGALS13_2_26996,694,808,654,594,1283,558,1275,581,171,715,1836,288
LGALS13_2_26997,206,136,93,129,63,135,241,72,356,0,65,149
LGALS13_2_26998,902,798,928,1045,1068,911,170,1359,999,1165,1141,1455
LGALS13_2_26999,791,895,810,777,1888,564,1317,652,170,474,1956,322
MIPEP_2_27000,48,39,30,15,45,45,0,0,102,2,15,158
MIPEP_2_27001,828,573,645,791,1109,319,242,919,654,707,210,611
MIPEP_2_27002,107,13,52,24,14,8,1,0,0,0,230,36
MIPEP_2_27003,349,229,339,198,178,1,434,73,40,88,954,747
MIPEP_2_27004,336,224,296,392,206,78,637,382,88,390,81,251
MIPEP_2_27005,44,46,23,40,89,68,3,76,13,16,2,0
MIPEP_2_27006,258,297,222,211,199,179,63,251,51,0,126,72
MIPEP_2_27007,635,492,619,717,637,322,242,630,538,718,210,615
MIPEP_2_27008,391,226,297,393,628,79,898,383,89,388,81,253
MIPEP_2_27009,493,425,549,573,138,1031,247,292,828,593,179,543
1-Mar_2_27010,853,1043,846,1149,692,1164,289,1939,831,1151,825,844
1-Mar_2_27011,131,43,101,32,0,20,9,20,0,146,0,156
1-Mar_2_27012,103,85,89,87,130,127,326,298,153,269,7,46
1-Mar_2_27013,630,560,486,595,480,251,104,18,1402,276,26,1095
1-Mar_2_27014,287,135,56,283,3,265,0,130,250,51,2,3
1-Mar_2_27015,794,626,622,856,213,638,571,603,1004,325,505,807
1-Mar_2_27016,419,332,309,344,221,730,421,256,258,557,671,494
1-Mar_2_27017,1555,889,1036,1295,1083,1441,272,1819,1330,854,977,1912
1-Mar_2_27018,294,250,365,427,258,150,190,432,226,217,179,333
1-Mar_2_27019,358,234,305,292,671,58,365,84,300,321,242,374
2-Mar_2_27020,331,405,284,435,320,16,783,378,265,83,357,256
2-Mar_2_27021,45,48,77,131,398,34,7,53,50,0,14,417
2-Mar_2_27022,266,171,201,244,122,559,246,436,78,245,70,141
2-Mar_2_27023,92,13,16,37,0,0,0,157,24,0,0,0
2-Mar_2_27024,388,273,339,303,1,970,543,393,310,373,1152,218
2-
Mar_2_27025,1520,1434,1322,1305,1066,1664,1773,1052,1168,1017,1076,106
5
2-Mar_2_27026,46,26,152,41,124,0,33,58,23,31,11,168
2-Mar_2_27027,5,6,56,4,6,0,0,0,19,0,373,2
2-Mar_2_27028,156,163,216,212,14,64,323,346,197,73,498,153
2-Mar_2_27029,22,24,18,22,0,277,0,38,122,0,0,19
MCM2_2_27030,127,88,124,113,207,25,71,206,240,16,32,61
MCM2_2_27031,266,97,147,144,445,100,2,91,224,355,24,118
MCM2_2_27032,588,399,330,440,175,444,43,508,736,538,147,4
MCM2_2_27033,446,306,326,306,550,355,227,399,403,611,1319,322
MCM2_2_27034,617,950,607,802,525,483,385,158,833,341,115,1269
MCM2_2_27035,117,95,166,109,50,0,398,165,38,56,68,81
MCM2_2_27036,61,9,21,28,0,125,0,15,37,81,22,0
MCM2_2_27037,274,180,219,286,123,37,883,124,53,622,597,94

MCM2_2_27038,613,481,421,472,1130,309,367,0,170,1048,608,327
MCM2_2_27039,288,559,271,432,0,1,880,11,395,581,234,0
MCM7_2_27040,25,72,35,27,4,0,13,2,0,114,0,230
MCM7_2_27041,203,318,319,364,106,390,64,372,228,241,432,214
MCM7_2_27042,65,33,63,45,31,0,4,1,2,3,50,60
MCM7_2_27043,337,467,357,540,129,504,855,1580,94,828,26,687
MCM7_2_27044,5,8,47,22,0,0,0,0,368,0,0,0
MCM7_2_27045,75,61,130,181,0,1,153,649,54,120,45,27
MCM7_2_27046,152,227,202,271,309,48,347,468,319,263,24,484
MCM7_2_27047,111,41,126,245,0,0,449,1,280,0,0,0
MCM7_2_27048,119,165,104,159,391,265,365,52,28,10,73,26
MCM7_2_27049,63,55,65,206,55,160,277,32,78,18,0,3
METAP1_2_27050,96,217,72,165,457,726,27,0,1,0,0,0
METAP1_2_27051,47,36,43,31,39,0,2,0,6,6,0,6
METAP1_2_27052,3,5,2,22,0,23,0,0,0,6,0,3
METAP1_2_27053,187,123,106,176,11,187,61,362,57,821,159,6
METAP1_2_27054,70,86,77,74,2,6,35,51,53,34,7,11
METAP1_2_27055,145,316,137,250,8,65,0,384,78,812,30,181
METAP1_2_27056,164,102,47,120,35,13,30,0,111,114,604,171
METAP1_2_27057,246,300,306,264,460,222,660,507,230,87,132,388
METAP1_2_27058,302,213,171,244,228,176,241,1291,115,184,27,328
METAP1_2_27059,1394,1021,986,1283,943,982,718,593,1677,540,1031,923
METAP2_2_27060,136,174,170,57,532,40,52,308,44,531,0,3
METAP2_2_27061,26,25,42,20,0,13,0,0,12,0,550,27
METAP2_2_27062,709,706,487,475,388,556,880,913,1201,512,134,771
METAP2_2_27063,197,178,121,176,105,26,41,77,197,35,3,400
METAP2_2_27064,121,154,113,125,63,0,40,176,3,10,6,62
METAP2_2_27065,0,0,0,0,0,0,0,0,0,0,0,0
METAP2_2_27066,224,257,336,344,12,293,119,357,9,0,770,209
METAP2_2_27067,197,177,115,176,105,27,41,77,199,35,3,400
METAP2_2_27068,0,0,0,0,0,0,0,0,0,0,0,0
METAP2_2_27069,110,277,177,288,457,161,425,108,51,302,57,83
MGAM_2_27070,702,849,629,733,384,245,1382,1603,489,250,3481,1128
MGAM_2_27071,1639,1703,1519,1729,910,2231,2528,1828,1876,2782,897,1164
MGAM_2_27072,34,44,42,158,179,0,0,5,71,0,19,111
MGAM_2_27073,117,117,48,49,17,173,11,0,209,348,0,17
MGAM_2_27074,1572,1571,1175,1411,1516,1211,1119,1757,1541,2273,1255,19
98
MGAM_2_27075,726,707,580,725,630,681,746,1682,759,906,1891,848
MGAM_2_27076,2907,2522,2183,2741,2808,2518,2819,3215,2003,2233,1978,16
60
MGAM_2_27077,231,105,48,79,0,0,345,3,41,156,0,187
MGAM_2_27078,823,698,676,549,258,1052,697,1841,903,690,140,851
MGAM_2_27079,1527,1258,1224,1597,1444,943,1388,1144,626,2559,834,1665
MIF_2_27080,416,486,267,416,830,369,604,303,304,647,83,1058
MIF_2_27081,428,353,476,367,926,293,489,223,184,543,869,903
MIF_2_27082,1326,993,1029,1130,685,875,1402,671,819,1960,894,742
MIF_2_27083,121,37,18,62,1,0,1,112,445,0,8,4
MIF_2_27084,407,648,389,477,38,448,321,462,332,494,616,276
MIF_2_27085,167,82,52,78,84,52,237,0,25,25,3,205

MIF_2_27086,386,173,143,188,366,189,0,300,35,12,422,5
MIF_2_27087,640,406,336,718,576,236,153,770,127,884,745,1165
MIF_2_27088,15,106,51,55,73,92,3,29,7,0,0,53
MIF_2_27089,61,75,51,90,14,3,16,117,76,15,3,113
MOCOS_2_27090,50,76,37,42,15,1,0,14,5,34,86,29
MOCOS_2_27091,284,253,179,324,470,135,708,164,159,780,0,392
MOCOS_2_27092,441,546,454,457,1001,927,885,302,609,578,622,570
MOCOS_2_27093,381,327,586,422,785,439,1035,59,385,130,5,477
MOCOS_2_27094,437,568,490,390,55,434,63,561,371,351,282,706
MOCOS_2_27095,543,755,596,762,993,785,981,845,866,685,922,997
MOCOS_2_27096,21,0,0,0,0,0,0,0,0,0,0,0
MOCOS_2_27097,19,45,49,77,5,10,1,32,123,8,0,0
MOCOS_2_27098,101,65,65,117,34,60,0,96,4,19,9,145
MOCOS_2_27099,189,208,266,214,381,13,66,50,125,133,4,88
MOGAT1_2_27100,678,893,794,810,863,942,218,216,540,471,1017,216
MOGAT1_2_27101,107,108,109,184,2,147,70,889,28,21,385,20
MOGAT1_2_27102,1106,908,963,958,233,713,766,1894,1054,382,422,680
MOGAT1_2_27103,102,71,182,193,26,0,504,93,62,588,5,214
MOGAT1_2_27104,404,450,456,610,384,377,173,346,397,238,678,163
MOGAT1_2_27105,303,292,208,386,267,641,639,423,333,2,520,522
MOGAT1_2_27106,232,113,118,307,156,186,165,67,274,67,396,173
MOGAT1_2_27107,129,158,138,216,129,313,0,1,1,290,130,162
MOGAT1_2_27108,1104,909,965,954,234,712,768,1898,1056,385,425,683
MOGAT1_2_27109,230,113,118,298,156,187,164,67,278,67,397,176
MOGAT2_2_27110,463,285,226,279,211,177,624,317,725,158,55,336
MOGAT2_2_27111,753,825,687,915,1118,1468,452,469,892,477,344,635
MOGAT2_2_27112,1765,1138,1192,1599,2077,1279,1135,1665,2028,1725,1587,
304
MOGAT2_2_27113,618,402,510,511,950,266,556,0,164,849,258,460
MOGAT2_2_27114,542,345,490,501,414,498,509,385,589,636,1206,701
MOGAT2_2_27115,1661,1527,1703,1959,1395,2520,2551,2576,1628,1711,1813,
2187
MOGAT2_2_27116,644,818,558,833,646,584,741,83,567,687,43,1048
MOGAT2_2_27117,455,517,317,642,527,387,635,468,452,553,678,554
MOGAT2_2_27118,260,236,181,221,1029,439,6,77,115,270,458,180
MOGAT2_2_27119,2226,1873,1834,2213,881,1744,3114,2149,1901,3926,1537,1
225
MSMO1_2_27120,26,30,23,41,0,1,9,304,8,0,23,0
MSMO1_2_27121,845,428,660,729,387,297,903,1054,1128,1161,1866,659
MSMO1_2_27122,524,511,562,488,739,779,405,542,856,435,301,410
MSMO1_2_27123,224,120,132,111,137,60,233,101,285,72,663,232
MSMO1_2_27124,1506,917,1331,1178,1866,695,1174,1509,1449,1408,2233,134
7
MSMO1_2_27125,918,975,783,919,1346,609,691,472,879,765,1109,834
MSMO1_2_27126,926,543,466,729,847,459,403,390,986,579,491,658
MSMO1_2_27127,854,720,829,868,780,351,299,1337,374,551,533,117
MSMO1_2_27128,257,256,309,375,112,779,919,498,211,41,410,507
MSMO1_2_27129,161,194,86,167,63,0,111,29,74,513,7,208
MYO1E_2_27130,140,64,192,170,474,1,0,1,196,134,705,44
MYO1E_2_27131,23,40,13,28,13,0,1,7,14,2,65,11

MYO1E_2_27132,358,227,211,510,223,285,337,314,79,180,996,185
MYO1E_2_27133,26,22,29,35,4,20,13,1,144,8,2,10
MYO1E_2_27134,218,263,166,201,496,336,76,7,58,199,0,123
MYO1E_2_27135,175,215,135,222,342,485,17,29,95,249,37,75
MYO1E_2_27136,528,519,443,429,365,341,1289,48,592,779,431,203
MYO1E_2_27137,388,312,291,337,807,3,195,973,69,455,90,598
MYO1E_2_27138,167,216,221,263,805,341,374,587,443,9,1034,83
MYO1E_2_27139,235,220,185,404,130,0,55,83,212,0,39,213
RDH14_2_27140,632,514,568,666,303,401,825,458,375,722,324,713
RDH14_2_27141,640,648,618,748,875,740,742,514,885,965,578,645
RDH14_2_27142,500,563,528,706,712,438,124,196,349,455,99,955
RDH14_2_27143,414,366,430,489,1111,164,333,210,220,713,361,753
RDH14_2_27144,79,88,75,103,7,35,0,254,24,427,259,0
RDH14_2_27145,221,185,138,296,537,30,228,85,5,10,160,12
RDH14_2_27146,242,158,166,200,9,148,0,101,246,8,11,281
RDH14_2_27147,90,74,125,114,113,3,271,68,206,338,316,0
RDH14_2_27148,87,78,106,95,156,0,204,0,28,254,0,5
RDH14_2_27149,113,55,78,59,0,0,0,0,0,431,27,4
NME9_2_27150,83,131,87,104,14,14,296,0,78,124,52,70
NME9_2_27151,286,331,466,400,95,21,155,908,569,30,888,387
NME9_2_27152,415,511,425,421,117,129,363,20,276,453,855,207
NME9_2_27153,633,669,554,718,141,561,196,840,498,262,557,808
NME9_2_27154,723,616,644,807,550,569,549,538,715,798,535,519
NME9_2_27155,317,455,246,220,713,324,194,43,79,457,69,290
NME9_2_27156,192,119,276,153,80,20,32,382,7,190,1,106
NME9_2_27157,2857,2753,3437,3451,2755,3535,2933,1220,1591,2906,3691,34
46
NME9_2_27158,317,273,328,274,420,448,182,232,310,57,147,231
NME9_2_27159,838,730,679,656,554,1082,518,1062,649,1160,551,904
PAPD7_2_27160,0,0,0,7,0,0,0,0,0,0,0,0
PAPD7_2_27161,432,384,359,423,28,312,114,63,85,441,1570,635
PAPD7_2_27162,443,627,655,503,619,1062,506,1042,336,604,509,341
PAPD7_2_27163,677,631,565,655,637,412,37,1298,429,989,370,1190
PAPD7_2_27164,2936,2875,2560,2907,4135,2915,2005,1979,1442,4134,2919,3
160
PAPD7_2_27165,956,1086,1073,1126,1085,1712,1242,1603,676,933,1400,1339
PAPD7_2_27166,552,211,325,445,209,48,757,371,527,270,60,538
PAPD7_2_27167,4662,3975,3637,3847,4477,2852,2689,4228,3985,4683,2534,3
088
PAPD7_2_27168,573,716,696,677,360,504,385,925,234,636,949,168
PAPD7_2_27169,193,179,250,191,164,162,422,354,73,137,81,97
PDHX_2_27170,31,78,68,99,0,0,1,0,166,0,0,562
PDHX_2_27171,215,299,208,284,227,7,597,55,486,16,270,171
PDHX_2_27172,77,35,70,80,7,17,246,278,94,49,39,50
PDHX_2_27173,193,182,201,217,216,277,762,94,88,168,76,351
PDHX_2_27174,67,136,167,154,11,563,6,0,128,552,65,69
PDHX_2_27175,1,47,19,15,0,97,0,0,0,80,0,23
PDHX_2_27176,35,35,59,55,0,0,0,0,57,35,82,0
PDHX_2_27177,177,151,275,254,208,4,28,168,71,24,3,242
PDHX_2_27178,143,120,235,198,85,4,27,78,60,24,3,217

PDHX_2_27179,93,135,274,282,0,217,190,116,184,75,86,55
PGAM4_2_27180,196,313,152,239,214,196,73,9,203,213,283,122
PGAM4_2_27181,181,53,56,107,35,1,0,135,9,145,257,28
PGAP3_2_27182,149,43,62,100,227,0,17,0,85,135,438,1
PGAP3_2_27183,59,65,129,75,100,44,268,0,15,200,0,240
PGAP3_2_27184,335,230,248,330,197,95,241,575,78,360,156,140
PGAP3_2_27185,540,371,421,392,1127,275,316,259,356,524,132,467
PGAP3_2_27186,184,127,186,189,190,257,2,4,21,14,434,220
PGAP3_2_27187,21,11,13,28,0,0,5,0,0,2,1,9
PGAP3_2_27188,461,562,331,440,389,38,26,71,310,705,71,1019
PGAP3_2_27189,53,32,73,71,27,4,229,15,84,538,306,85
PGAP3_2_27190,97,78,42,67,1,143,103,0,9,6,8,37
PGAP3_2_27191,441,272,294,216,15,201,761,1022,188,0,541,286
PGPEP1_2_27192,262,271,359,347,637,450,350,391,71,228,439,388
PGPEP1_2_27193,39,97,37,28,201,119,17,47,31,105,12,15
PGPEP1_2_27194,13,58,42,78,0,0,48,0,51,0,3,0
PGPEP1_2_27195,231,270,325,297,636,310,349,389,71,228,436,331
PGPEP1_2_27196,278,391,225,349,323,328,162,168,112,491,186,477
PGPEP1_2_27197,49,60,54,78,3,98,1,4,2,4,64,92
PGPEP1_2_27198,0,146,12,30,0,1,0,0,0,6,0,0
PGPEP1_2_27199,69,84,121,66,0,74,51,270,93,25,11,41
PGPEP1_2_27200,65,36,35,69,7,0,38,0,0,25,0,113
PGPEP1_2_27201,482,363,422,365,529,336,307,423,374,301,336,209
PIGU_2_27202,878,771,656,909,544,385,744,849,568,854,712,541
PIGU_2_27203,169,211,194,214,242,75,60,450,150,93,71,67
PIGU_2_27204,193,218,183,334,792,329,8,493,385,40,0,70
PIGU_2_27205,2085,1815,1722,2023,1600,2369,4023,2422,2017,1260,1905,19
67
PIGU_2_27206,770,516,400,706,324,437,508,477,476,697,736,347
PIGU_2_27207,241,127,69,78,137,110,96,0,169,18,13,85
PIGU_2_27208,354,523,298,425,377,744,136,34,207,123,80,469
PIGU_2_27209,1304,966,928,1520,1005,1030,512,2266,1177,337,965,1765
PIGU_2_27210,2,23,0,15,0,0,0,0,0,0,0,0
PIGU_2_27211,702,612,569,565,785,1010,1050,308,405,470,1027,343
PMEL_2_27212,301,434,369,466,540,62,463,148,217,922,423,950
PMEL_2_27213,858,595,597,838,414,174,68,763,739,830,655,546
PMEL_2_27214,0,1,0,0,0,0,0,0,0,0,0,0
PMEL_2_27215,842,666,623,788,1077,918,371,1381,1247,1078,669,1032
PMEL_2_27216,133,183,69,244,0,120,140,50,109,677,136,184
PMEL_2_27217,272,194,156,187,65,11,6,65,434,793,82,957
PMEL_2_27218,322,205,222,203,194,256,347,343,333,141,213,205
PMEL_2_27219,227,223,235,276,514,133,105,6,180,511,103,208
PMEL_2_27220,56,162,163,194,15,2,5,5,2,110,6,76
PMEL_2_27221,258,245,239,296,388,250,607,96,406,11,216,464
PMPCA_2_27222,2505,2110,1617,2353,1502,1543,2456,2814,2924,1809,654,32
98
PMPCA_2_27223,221,147,109,204,27,296,82,154,187,950,230,194
PMPCA_2_27224,21,75,90,122,71,8,0,0,0,117,0,276
PMPCA_2_27225,10,11,24,16,0,0,0,0,0,0,0,0
PMPCA_2_27226,160,154,87,66,37,275,92,76,300,46,90,66

PMPCA_2_27227,616,619,410,438,252,224,223,1370,221,873,225,384
PMPCA_2_27228,1552,1312,1386,1372,966,789,939,1842,1958,2124,1519,1361
PMPCA_2_27229,145,152,75,98,76,54,161,85,5,18,128,143
PMPCA_2_27230,101,227,116,175,71,0,94,371,0,363,125,654
PMPCA_2_27231,4,12,32,64,240,0,534,0,0,386,176,161
POLK_2_27232,170,198,123,170,440,178,0,890,78,0,280,345
POLK_2_27233,1581,1303,1347,1211,2438,1800,4020,636,1042,919,860,802
POLK_2_27234,1759,1776,1488,1767,1068,1924,1568,831,1339,1916,1417,771
POLK_2_27235,73,28,70,148,0,5,65,21,10,0,6,62
POLK_2_27236,137,127,48,68,371,181,22,145,72,2,28,41
POLK_2_27237,293,270,313,483,1,526,170,161,244,206,295,231
POLK_2_27238,829,680,709,871,987,1011,854,759,589,878,144,1372
POLK_2_27239,1058,806,740,819,876,961,671,463,671,914,1073,1109
POLK_2_27240,246,50,185,97,382,0,0,75,44,335,152,54
POLK_2_27241,305,585,282,470,749,643,368,307,244,489,1234,297
POLQ_2_27242,196,284,157,307,438,80,1,363,209,1042,260,124
POLQ_2_27243,767,836,821,927,2213,1234,372,1170,504,1060,844,937
POLQ_2_27244,768,677,687,875,967,748,712,507,191,1140,730,1516
POLQ_2_27245,895,735,933,934,314,664,1152,639,1234,274,868,605
POLQ_2_27246,135,220,340,469,393,556,180,419,517,358,1188,184
POLQ_2_27247,196,237,59,169,126,415,57,262,46,402,230,72
POLQ_2_27248,398,143,166,189,341,150,115,27,516,33,97,90
POLQ_2_27249,248,247,187,215,114,6,243,168,170,135,225,51
POLQ_2_27250,32,61,71,50,5,4,97,189,68,6,1,38
POLQ_2_27251,428,341,329,628,151,241,542,58,19,421,73,444
POLR1A_2_27252,74,49,105,93,316,95,0,88,250,3,0,176
POLR1A_2_27253,158,237,243,361,262,856,237,513,180,351,12,91
POLR1A_2_27254,69,59,58,48,0,0,39,0,140,84,0,415
POLR1A_2_27255,386,268,320,346,123,43,809,313,339,96,60,316
POLR1A_2_27256,429,347,521,543,882,392,338,739,504,82,245,549
POLR1A_2_27257,480,262,273,410,441,242,1358,229,223,499,1091,259
POLR1A_2_27258,106,43,139,157,0,6,1,12,277,0,47,0
POLR1A_2_27259,867,1433,1014,1158,1485,566,910,741,836,1063,524,1114
POLR1A_2_27260,464,503,503,492,180,320,447,1096,592,1193,1554,629
POLR1A_2_27261,487,637,638,567,468,645,1201,565,453,776,783,883
POLR2D_2_27262,701,677,761,673,379,474,1575,828,645,805,1369,214
POLR2D_2_27263,761,787,888,870,759,316,1196,1142,490,714,974,940
POLR2D_2_27264,742,787,826,851,766,316,1198,1146,495,715,721,941
POLR2D_2_27265,92,70,34,65,23,16,9,0,30,1,84,35
POLR2D_2_27266,27,54,52,56,2,0,0,0,47,137,4,105
POLR2D_2_27267,513,802,633,625,478,732,658,773,643,929,713,459
POLR2D_2_27268,369,644,556,546,477,592,650,294,509,778,128,459
POLR2D_2_27269,102,144,49,74,0,29,210,71,48,121,0,60
POLR2D_2_27270,684,528,348,604,31,754,1267,714,423,93,346,423
POLR2D_2_27271,96,73,25,52,0,54,283,85,29,0,126,19
PRDX5_2_27272,64,103,124,155,103,5,0,206,107,193,42,41
PRDX5_2_27273,668,784,721,741,218,324,1601,205,1076,1881,601,755
PRDX5_2_27274,543,570,573,622,344,212,790,424,267,241,719,281
PRDX5_2_27275,1787,2205,1658,1970,1321,2095,1185,1965,2133,2156,2295,1
883

PRDX5_2_27276,121,51,133,206,141,99,102,187,32,7,33,10
PRDX5_2_27277,508,551,336,807,611,231,511,1508,789,524,1,207
PRDX5_2_27278,181,135,114,132,2,142,504,178,31,72,361,570
PRDX5_2_27279,1600,2089,1611,2043,1419,2960,1204,1943,1710,2325,2459,1
973
PRDX5_2_27280,226,337,212,293,43,71,543,755,147,254,16,316
PRDX5_2_27281,545,569,578,618,344,213,790,423,269,241,730,281
PRDX6_2_27282,75,35,85,72,61,23,0,102,25,0,11,26
PRDX6_2_27283,374,377,686,430,66,269,807,380,311,577,2757,18
PRDX6_2_27284,528,422,416,641,398,303,462,562,325,363,1303,103
PRDX6_2_27285,176,167,202,394,158,85,213,362,83,2,0,868
PRDX6_2_27286,498,564,459,633,340,401,546,645,862,354,766,471
PRDX6_2_27287,569,667,387,603,436,198,51,205,780,530,203,572
PRDX6_2_27288,554,238,336,444,220,165,246,241,714,1125,279,419
PRDX6_2_27289,733,918,1062,884,1113,1201,655,1823,501,473,1182,433
PRDX6_2_27290,44,92,70,69,0,161,5,6,6,151,0,122
PRDX6_2_27291,57,71,65,54,0,383,101,0,51,370,155,93
PRODH_2_27292,228,181,178,188,0,27,12,305,123,61,516,299
PRODH_2_27293,676,621,488,662,1308,69,151,546,625,656,1151,863
PRODH_2_27294,286,366,334,368,169,153,37,527,99,410,821,178
PRODH_2_27295,366,287,332,319,440,373,471,476,386,318,280,603
PRODH_2_27296,1056,581,1067,1174,484,1262,2193,741,900,630,1135,906
PRODH_2_27297,860,668,553,743,1598,70,115,651,1170,653,1143,756
PRODH_2_27298,309,260,121,150,0,0,0,3,0,177,65,0
PRODH_2_27299,80,12,19,46,290,0,0,10,0,2,0,68
PRODH_2_27300,54,96,74,131,0,212,0,0,6,3,4,89
PRODH_2_27301,95,48,75,84,476,49,0,0,40,62,228,432
PSMA7_2_27302,919,563,611,652,563,171,245,749,453,523,966,899
PSMA7_2_27303,465,404,328,457,20,252,284,423,530,147,778,309
PSMA7_2_27304,375,536,524,414,153,429,387,1145,967,281,56,542
PSMA7_2_27305,398,499,348,477,528,387,536,3,422,301,415,489
PSMA7_2_27306,732,608,670,710,481,223,120,374,407,582,107,130
PSMA7_2_27307,201,235,186,215,98,12,195,94,120,428,7,359
PSMA7_2_27308,360,446,269,316,255,331,603,358,278,497,628,343
PSMA7_2_27309,256,160,247,325,3,149,322,95,186,347,111,177
PSMA7_2_27310,748,647,637,688,454,771,14,75,182,1186,1190,979
PSMA7_2_27311,147,167,132,167,199,100,817,699,76,63,83,38
PTRH2_2_27312,11,45,66,55,8,185,648,267,0,42,3,42
PTRH2_2_27313,165,147,155,157,181,269,1,202,107,128,250,14
PTRH2_2_27314,43,74,30,54,0,0,169,0,0,0,100,819
PTRH2_2_27315,24,6,18,23,0,1,6,9,0,61,0,25
PTRH2_2_27316,119,136,18,61,0,8,82,0,376,30,0,0
PTRH2_2_27317,502,537,614,528,709,42,456,341,641,593,443,78
PTRH2_2_27318,2,0,7,34,0,0,0,0,134,0,0,0
PTRH2_2_27319,65,173,205,120,16,0,0,364,0,495,513,0
PTRH2_2_27320,506,520,615,517,713,42,456,341,506,597,428,79
PTRH2_2_27321,135,110,119,135,101,230,0,200,25,82,37,12
PXDN_2_27322,83,59,101,83,13,73,0,1,55,2,1,22
PXDN_2_27323,816,562,537,614,634,884,893,573,534,374,334,189
PXDN_2_27324,132,98,119,86,79,14,353,0,129,124,0,117

PXDN_2_27325,980,689,764,888,1359,586,1432,1501,1261,319,356,555
PXDN_2_27326,147,192,172,236,203,34,379,479,509,395,347,35
PXDN_2_27327,419,697,483,609,1847,906,273,315,246,281,691,660
PXDN_2_27328,316,273,460,441,167,322,335,356,99,73,562,137
PXDN_2_27329,550,397,452,408,841,16,559,932,434,959,1097,839
PXDN_2_27330,749,606,566,842,1279,494,751,995,860,799,1004,1185
PXDN_2_27331,256,271,181,185,157,59,337,391,202,391,39,147
PXDN_2_27332,104,104,209,225,339,234,296,53,173,734,248,268
PXDN_2_27333,246,248,152,85,364,237,288,182,292,153,65,173
PXDN_2_27334,78,119,177,118,178,59,39,443,52,1,0,434
PXDN_2_27335,102,169,120,132,42,203,2,6,36,114,15,364
PXDN_2_27336,417,301,374,234,303,315,617,1042,582,113,125,827
PXDN_2_27337,301,326,225,330,825,279,1,240,71,568,1347,337
PXDN_2_27338,137,191,196,249,68,15,114,414,197,88,622,262
PXDN_2_27339,114,73,107,145,239,135,21,165,181,227,12,240
PXDN_2_27340,17,21,20,46,0,0,1,0,0,0,2,8
PXDN_2_27341,489,404,434,381,182,46,420,486,793,213,229,267
RAB34_2_27342,52,51,22,80,45,75,84,0,44,15,0,16
RAB34_2_27343,2911,3204,2927,3141,1614,3104,2160,2641,1543,2828,2161,2
652
RAB34_2_27344,3411,3100,3171,3207,4365,2345,5466,3073,1770,3244,2986,3
104
RAB34_2_27345,784,499,459,595,700,864,0,176,1481,797,366,106
RAB34_2_27346,1861,1649,1412,2200,1795,809,2152,1013,1456,1075,1282,11
58
RAB34_2_27347,905,683,905,884,650,669,2539,765,820,1376,394,909
RAB34_2_27348,451,404,258,418,82,522,533,536,317,375,63,627
RAB34_2_27349,1790,1617,1634,1991,524,1661,2034,1707,1802,1592,2143,15
60
RAB34_2_27350,1555,1431,1248,1622,1802,1751,1062,1825,1100,1438,1433,1
562
RAB34_2_27351,1249,901,1047,1052,796,213,1533,1633,1149,1004,2346,1109
RAB39A_2_27352,52,51,22,80,45,75,84,0,44,15,0,16
RAB39A_2_27353,2911,3204,2927,3141,1614,3104,2160,2641,1543,2828,2161,
2652
RAB39A_2_27354,3411,3100,3171,3207,4365,2345,5466,3073,1770,3244,2986,
3104
RAB39A_2_27355,784,499,459,595,700,864,0,176,1481,797,366,106
RAB39A_2_27356,1861,1649,1412,2200,1795,809,2152,1013,1456,1075,1282,1
158
RAB39A_2_27357,905,683,905,884,650,669,2539,765,820,1376,394,909
RAB39A_2_27358,451,404,258,418,82,522,533,536,317,375,63,627
RAB39A_2_27359,1790,1617,1634,1991,524,1661,2034,1707,1802,1592,2143,1
560
RAB39A_2_27360,1555,1431,1248,1622,1802,1751,1062,1825,1100,1438,1433,
1562
RAB39A_2_27361,1249,901,1047,1052,796,213,1533,1633,1149,1004,2346,110
9
REXO2_2_27362,71,98,48,99,53,231,87,333,27,10,118,35
REXO2_2_27363,240,341,256,285,122,80,0,84,486,169,452,470

REXO2_2_27364,4,27,12,43,0,111,29,0,0,208,0,0
REXO2_2_27365,15,45,73,44,0,5,0,0,0,1,139,426
REXO2_2_27366,743,620,706,618,494,294,1766,1183,1342,1197,753,1488
REXO2_2_27367,11,39,72,40,0,0,0,0,0,0,140,419
REXO2_2_27368,331,327,411,278,265,761,421,253,165,359,121,136
REXO2_2_27369,420,329,302,228,598,576,13,460,424,621,939,312
REXO2_2_27370,424,369,303,270,599,664,13,461,423,626,946,312
REXO2_2_27371,162,99,53,155,1,2,363,0,52,22,0,0
SARS2_2_27372,63,182,224,228,21,3,4,15,7,1271,28,312
SARS2_2_27373,51,26,123,93,245,223,1,186,136,513,0,0
SARS2_2_27374,103,66,102,122,16,201,14,137,78,68,41,61
SARS2_2_27375,1271,786,780,1210,1546,611,761,1334,866,770,856,1379
SARS2_2_27376,270,165,130,146,101,359,600,0,106,148,171,102
SARS2_2_27377,330,442,304,448,395,44,301,241,251,92,171,676
SARS2_2_27378,45,31,79,83,21,89,34,8,28,89,154,2
SARS2_2_27379,480,651,588,681,490,417,112,197,362,281,1000,908
SARS2_2_27380,1113,871,754,998,923,946,268,273,178,1005,760,425
SARS2_2_27381,457,511,503,375,249,299,1536,545,90,300,323,908
SEPSECS_2_27382,184,207,198,256,534,466,337,180,128,28,0,270
SEPSECS_2_27383,796,827,857,743,346,893,1137,65,370,77,1754,816
SEPSECS_2_27384,463,382,379,526,103,363,526,113,634,981,53,229
SEPSECS_2_27385,1204,1239,1072,1201,1305,611,792,732,740,2123,1883,114
0
SEPSECS_2_27386,292,288,231,315,310,332,138,357,412,373,645,212
SEPSECS_2_27387,795,869,882,754,345,886,1135,65,367,119,1745,813
SEPSECS_2_27388,236,412,334,274,298,112,714,328,407,12,209,545
SEPSECS_2_27389,124,87,37,94,894,1,1,146,6,36,51,95
SEPSECS_2_27390,66,85,89,152,33,444,153,74,0,431,11,83
SEPSECS_2_27391,117,29,76,95,28,166,0,0,144,20,0,31
SMOX_2_27392,80,62,103,91,28,29,34,130,44,6,8,0
SMOX_2_27393,230,103,109,106,96,87,180,174,158,70,42,345
SMOX_2_27394,508,471,418,324,455,198,938,180,139,272,447,288
SMOX_2_27395,285,225,202,164,597,173,67,1,87,500,612,693
SMOX_2_27396,155,169,206,166,7,82,11,112,370,80,322,76
SMOX_2_27397,150,79,69,132,105,140,3,240,220,0,3,254
SMOX_2_27398,729,837,744,574,960,15,461,1094,513,514,637,385
SMOX_2_27399,606,807,504,717,67,1356,4,471,564,807,549,266
SMOX_2_27400,69,97,54,49,123,99,0,31,106,2,0,6
SMOX_2_27401,91,69,49,124,86,214,152,156,12,0,63,100
SOAT2_2_27402,199,322,279,265,310,95,536,22,352,39,0,107
SOAT2_2_27403,116,168,133,230,312,13,203,13,74,227,138,143
SOAT2_2_27404,18,1,5,1,0,0,15,0,0,15,0,0
SOAT2_2_27405,222,415,372,268,566,363,63,645,33,678,890,253
SOAT2_2_27406,327,342,382,442,302,555,813,164,305,189,64,439
SOAT2_2_27407,82,99,83,44,126,25,12,90,261,0,0,183
SOAT2_2_27408,51,18,73,107,0,0,0,10,84,1,283,341
SOAT2_2_27409,1240,1081,1228,1253,912,318,1865,1359,1100,992,1464,2128
SOAT2_2_27410,104,157,136,255,259,201,161,67,266,435,21,187
SOAT2_2_27411,87,79,127,90,171,202,0,295,90,0,61,13
SULT1C4_2_27412,600,334,325,539,877,650,321,63,504,806,718,474

SULT1C4_2_27413,1435,1242,1076,1383,1199,652,987,555,2029,1611,1275,65
4
SULT1C4_2_27414,326,428,322,366,121,74,192,398,132,350,304,184
SULT1C4_2_27415,17,13,48,44,0,0,0,9,20,9,10,6
SULT1C4_2_27416,377,381,338,358,491,569,3,0,272,949,951,314
SULT1C4_2_27417,751,404,516,585,592,470,1054,1323,829,637,726,248
SULT1C4_2_27418,173,141,169,163,95,72,91,97,91,3,41,271
SULT1C4_2_27419,131,82,128,117,99,209,6,213,43,64,36,358
SULT1C4_2_27420,108,70,64,97,49,57,27,0,113,36,2,62
SULT1C4_2_27421,92,152,198,173,85,0,2,42,168,76,488,170
TDO2_2_27422,202,425,266,281,177,214,100,227,162,272,48,33
TDO2_2_27423,346,424,547,830,269,70,888,819,12,820,746,289
TDO2_2_27424,349,450,299,306,631,754,63,431,402,134,591,481
TDO2_2_27425,216,107,120,246,286,43,8,162,21,215,164,319
TDO2_2_27426,305,347,143,342,358,284,247,619,960,288,525,670
TDO2_2_27427,285,363,282,216,691,63,696,34,66,33,720,26
TDO2_2_27428,102,84,140,114,36,22,120,58,53,105,20,67
TDO2_2_27429,1404,942,985,1334,1992,530,829,1061,996,1146,665,1582
TDO2_2_27430,254,292,216,474,523,371,38,26,65,267,754,367
TDO2_2_27431,49,7,25,54,25,0,0,0,7,86,3,1
UGT2B4_2_27432,373,478,251,482,467,51,65,267,581,513,372,337
UGT2B4_2_27433,790,723,786,839,518,372,117,175,882,387,505,706
UGT2B4_2_27434,1178,1494,1122,1386,1945,1315,829,1114,1598,1181,1569,1
449
UGT2B4_2_27435,635,528,664,474,758,559,802,720,719,298,153,590
UGT2B4_2_27436,1896,1286,1565,1919,1796,1825,2069,1267,1172,1497,907,1
253
UGT2B4_2_27437,128,58,35,202,478,98,0,54,0,494,718,0
UGT2B4_2_27438,1630,1148,1207,1626,2175,840,1552,1773,2058,1032,2267,1
170
VARS_2_27439,54,26,41,63,28,31,32,352,20,1,0,37
VARS_2_27440,260,307,235,373,173,887,998,503,137,440,414,206
VARS_2_27441,137,90,49,80,11,27,117,0,304,24,245,2
VARS_2_27442,30,7,15,29,217,0,5,0,2,0,0,5
VARS_2_27443,162,9,84,43,240,0,164,0,187,9,0,4
VARS_2_27444,133,149,133,207,1,0,24,25,104,144,0,502
VARS_2_27445,229,224,204,218,308,160,142,640,560,483,110,184
VARS_2_27446,166,34,85,36,129,46,319,110,569,21,212,82
VARS_2_27447,197,133,94,115,42,73,91,139,94,0,0,133
VARS_2_27448,141,302,282,367,0,233,721,529,169,0,1193,192
