# Supplementary material for: Cross-species genetic screens identify transglutaminase 5 as a regulator of polyglutamine-expanded ataxin-1
Source: J Clin Invest. 2022 May 2;132(9):e156616. doi: 10.1172/JCI156616 (PMC9057624; doi:10.1172/JCI156616)
Supplement: Supplemental data set 3 [file jci-132-156616-s048.pdf]

```
shRNA,B1,B2,B3,B4,H1,H2,H3,H4,L1,L2,L3,L4
ADORA2A_3_1,1015,1311,1458,1294,1613,1634,1852,1108,1048,683,150,110
ADORA2B_3_2,733,2314,1603,2309,2149,3061,269,1192,1736,960,1570,2867
ADRA1B_3_3,4394,3625,3356,4696,6686,1168,3852,2610,3035,4486,3401,4647
ADRA1D_3_4,353,707,419,246,0,2,207,519,19,15,1162,189
ADRA2A_3_5,2286,1992,2391,3706,4850,1739,2702,5173,3536,1313,1654,5648
ADRA2B_3_6,4476,6246,6098,7370,4675,6100,3715,7210,5183,6592,4902,6510
ADRA2C_3_7,1067,1179,1079,746,1302,438,274,1110,1052,181,220,397
ADRB1_3_8,1998,2758,1886,3160,3988,6929,4504,4500,2101,2880,2138,4300
ADRB2_3_9,944,472,926,901,617,888,603,206,522,1217,1451,1420
ADRB3_3_10,1389,3551,2947,3284,2950,5868,3641,2149,3975,1097,747,3675
AGTR2_3_11,1298,1588,1934,3099,1335,2048,1414,3874,228,2857,1622,2647
APLNR_3_12,56,578,656,282,605,68,410,15,505,1217,32,515
AVPR1A_3_13,1247,1196,912,1361,3600,702,3125,2088,887,1742,1693,1590
AVPR1B_3_14,737,1010,751,1677,752,224,774,2484,1128,1476,2432,859
BAI1_3_15,469,581,212,93,137,78,323,6,955,41,373,882
BAI2_3_16,686,1754,1547,3326,1500,2268,1166,2336,675,4470,747,6461
BAI3_3_17,11614,10653,11052,16052,6458,13787,12514,18461,12533,10068,11876,17259
BDKRB1_3_18,2972,2537,3342,4738,3162,6632,3134,2454,6520,3121,4962,7315
BDKRB2_3_19,1046,1861,2208,2045,1961,1061,1090,1340,1306,381,2528,678
BRS3_3_20,4986,4575,4314,4075,3700,7661,6021,2516,5260,5973,5158,2547
C3AR1_3_21,4291,4276,3613,4630,3509,5667,8039,1856,2818,3356,3498,6178
C5AR1_3_22,2126,2180,3174,2542,805,5601,4517,5665,1168,2180,1443,4163
CALCRL_3_23,1749,2289,2107,1854,223,1202,1823,3825,3180,3072,3122,3431
CCKAR_3_24,2593,2901,4035,4613,2496,1661,1704,1715,3943,1997,3998,5801
CCKBR_3_25,766,537,741,373,376,270,619,854,1457,13,34,704
CCR1_3_26,1850,2413,2943,3497,1610,6027,4157,4347,2142,6403,1459,5309
CCR7_3_27,672,639,570,1007,1428,1103,1217,128,642,1769,174,1838
CCR8_3_28,316,658,334,918,1216,686,131,572,521,56,1528,484
CELSR1_3_29,1752,1601,692,2801,794,4882,2753,3293,4028,2740,3688,3593
CELSR2_3_30,776,1105,1193,2743,347,1429,1374,2000,1119,3031,290,1892
CELSR3_3_31,1120,1453,1529,1367,970,1051,254,117,678,1138,870,704
CHRM1_3_32,184,605,451,319,12,333,380,121,301,1749,3,62
CHRM3_3_33,487,326,256,253,565,1322,1518,248,665,2002,80,14
CHRM4_3_34,1371,1404,750,1434,478,670,717,3338,826,824,1098,1980
CHRM5_3_35,5981,5714,5017,6549,5064,5260,6792,11021,10255,5459,4735,7916
CXCR1_3_36,639,649,528,290,431,1537,71,1928,169,1294,1004,273
CXCR6_3_37,1944,3511,2417,4948,4248,2455,2801,5168,1510,2508,4206,2097
CXCR7_3_38,1419,798,975,2645,1083,1027,2021,409,1358,2107,1990,1382
CYSLTR1_3_39,10076,10204,11496,14368,14477,14775,13585,12997,12359,9348,16875,11645
CYSLTR2_3_40,1271,1207,1729,2738,1503,681,711,564,1031,1383,736,1668
DRD1_3_41,2201,2631,2650,5446,2389,4818,4096,3512,2818,3088,3638,3055
DRD4_3_42,1475,1064,1289,3814,1013,2253,1484,1808,830,2656,1170,3749
DRD5_3_43,1171,722,1696,982,2561,2246,1479,3170,1495,1501,1213,1038
ELTD1_3_44,1736,1559,1473,3036,1700,1413,2674,2736,1206,3027,4895,2287
EMR1_3_45,871,1543,2186,1922,494,714,3270,440,2460,3073,1755,2553
```

EMR3_3_46,388,279,600,802,574,996,12,2197,527,1274,903,212
F2R_3_47,7791,8060,5874,5802,11359,8076,7419,8322,9351,6783,5465,6205
F2RL1_3_48,246,397,531,1234,969,332,209,133,1289,1087,47,296
F2RL2_3_49,1146,1844,2403,3242,3886,1054,990,2216,1139,2607,710,5747
F2RL3_3_50,473,140,450,403,135,140,1336,323,310,660,1022,115
FFAR1_3_51,599,298,609,580,353,842,397,869,90,161,882,1650
FFAR2_3_52,2660,3069,2918,2351,3333,1473,3177,1238,5351,4456,5067,2970
FFAR3_3_53,1180,989,1258,2136,565,2777,1986,4247,362,3069,1633,1835
FZD10_3_54,1879,2404,2013,3763,387,3149,1465,1684,2261,1809,1575,1695
FZD1_3_55,2349,2259,2722,3765,952,2832,1933,2174,2741,4405,2610,3778
FZD2_3_56,386,394,830,415,1014,22,921,111,256,96,665,626
FZD4_3_57,5076,7160,5589,5168,6055,8643,6456,6251,4541,2613,8000,4432
FZD5_3_58,1351,1335,1272,2627,589,2247,1645,1973,200,3105,2608,3222
FZD7_3_59,2349,2259,2722,3765,952,2832,1933,2174,2741,4405,2610,3778
FZD8_3_60,625,1850,1218,1781,2244,5146,3216,714,1729,1313,535,1931
FZD9_3_61,2075,2436,1940,2893,2781,3927,1482,2793,1134,3718,2732,940
GABBR2_3_62,2056,2614,2987,2698,972,2597,5358,2408,2089,3429,2604,3387
GALR1_3_63,5682,4936,5929,6293,4455,6299,7284,12990,4192,5143,8593,554
2
GALR2_3_64,720,675,2477,1424,696,689,2644,1388,121,190,2608,673
GALR3_3_65,900,280,379,433,545,68,768,33,33,1105,703,1089
GCGR_3_66,440,881,1003,1052,281,1047,523,1851,541,1957,554,1227
GHRHR_3_67,1734,2947,1368,1853,1989,1582,874,3021,1166,2493,2586,2354
GIPR_3_68,1210,1012,1437,1381,1093,188,1436,3094,1368,504,241,315
GLP1R_3_69,1046,901,1481,1373,2229,1053,984,2704,1710,3151,645,1873
GLP2R_3_70,1630,1398,1546,2080,824,1330,1465,1234,2114,805,3426,311
GPR101_3_71,147,136,230,633,669,4,214,131,0,1603,73,619
GPR108_3_72,3661,3307,3407,4687,946,2225,5918,2430,3223,2275,4509,2087
GPR111_3_73,795,1680,1348,1033,180,882,942,2250,697,527,917,1288
GPR112_3_74,2055,2747,3251,3079,2853,4648,2826,3538,4245,2554,1200,250
1
GPR114_3_75,2551,3879,3659,5531,5080,6130,4098,5445,1998,3680,5147,446
2
GPR115_3_76,4855,5432,8441,7300,6022,11821,6499,4527,5773,8330,5135,75
95
GPR119_3_77,3392,3991,4596,7052,4108,10490,3158,7712,6561,4956,7788,70
53
GPR123_3_78,6971,5580,6715,9756,6501,7013,6671,6457,12005,5919,14405,8
946
GPR124_3_79,818,215,130,381,1016,572,54,514,748,37,79,816
GPR125_3_80,11852,15731,15132,14398,12713,25141,19825,23141,12846,1017
4,14746,14358
GPR128_3_81,4007,3942,5208,6205,3091,5837,4067,5363,7061,5004,9596,482
3
GPR12_3_82,2763,3871,4849,4777,2485,3602,4267,4612,3014,7029,1649,5108
GPR132_3_83,768,383,419,354,169,913,1711,40,364,177,983,83
GPR133_3_84,2754,3718,4441,4155,4053,4889,6127,3687,4537,2952,5475,676
3
GPR135_3_85,648,1135,899,1678,370,794,711,2154,1028,664,2339,700
GPR139_3_86,964,1002,881,2066,751,556,2893,261,896,342,148,2150

GPR141_3_87,4853,4520,3940,2373,3182,9670,5157,2126,6196,5219,2967,3546
GPR142_3_88,1362,1570,2408,2670,2455,1581,4928,1798,1946,3202,306,3209
GPR143_3_89,1495,2666,1259,1589,2421,2071,2347,4621,998,7062,1175,1687
GPR144_3_90,719,1344,1197,1686,1630,1791,759,1151,386,1175,1112,1332
GPR146_3_91,2503,1959,2149,2611,2761,3754,1948,2274,2103,843,3158,1741
GPR148_3_92,1274,1669,1202,1029,750,870,1920,2111,1446,405,230,753
GPR149_3_93,426,564,3,152,120,1664,0,8,0,1776,46,14
GPR150_3_94,596,1496,129,449,59,1054,1836,233,232,9,124,119
GPR151_3_95,1439,2521,1497,3120,1550,1377,1827,2803,2217,1815,3287,2186
GPR152_3_96,345,239,1114,1021,253,2433,86,287,143,1109,99,600
GPR153_3_97,709,514,491,707,503,129,57,1969,464,452,863,839
GPR157_3_98,2328,2297,3538,5682,1063,892,3065,5266,1608,879,5279,3400
GPR158_3_99,11645,10876,10511,14390,8431,13469,10153,15874,9835,11063,11961,18749
GPR15_3_100,887,2206,1967,1813,4058,3071,1125,945,3121,1803,1317,1452
GPR160_3_101,3057,2979,4184,4512,1817,4515,3012,4951,1120,4813,3163,3933
GPR161_3_102,2041,2264,2183,3730,1410,2826,1650,4347,1262,2814,2530,1507
GPR171_3_103,1894,1142,740,1855,775,3065,1806,1612,5,665,186,332
GPR173_3_104,5394,4624,5713,5561,3954,2840,4767,5559,5388,8805,6124,5815
GPR174_3_105,3350,3568,2206,3570,4775,1784,2423,1190,4454,3248,2783,4470
GPR176_3_106,567,818,248,966,323,796,354,83,559,233,1314,568
GPR179_3_107,6775,6823,9138,10954,7553,13624,12032,9509,7010,10467,15168,12209
GPR182_3_108,424,1103,573,1479,567,1800,420,1111,408,543,1390,2959
GPR183_3_109,611,261,70,1144,514,1043,659,4141,28,145,303,811
GPR19_3_110,3963,4416,4645,4866,4742,9591,5652,6989,3687,9317,13925,3923
GPR20_3_111,1774,1983,3165,4204,2351,3658,2368,2250,2895,9257,3177,2701
GPR21_3_112,1400,1284,1708,2098,1609,3163,4260,708,3454,934,4098,3704
GPR22_3_113,1408,1916,2749,3236,2565,2208,3247,4734,1742,2218,5671,5482
GPR25_3_114,522,352,697,637,496,2944,1093,208,892,641,1476,862
GPR27_3_115,800,819,544,945,511,317,1931,94,0,15,429,937
GPR31_3_116,1305,1663,644,996,653,813,1955,948,579,242,3086,1958
GPR32_3_117,1275,2591,2896,2486,2031,2331,1835,2734,1174,2039,3404,3513
GPR37_3_118,2055,1243,3413,3213,557,3167,1919,9801,1070,4030,6572,967
GPR37L1_3_119,42,14,2,263,5,418,0,4,2,1,6,0
GPR39_3_120,0,0,0,0,0,0,0,0,0,0,0,0
GPR3_3_121,8246,10015,11072,15714,11683,12562,11171,15343,9564,8138,15549,13071
GPR45_3_122,1527,1896,983,1729,1190,1434,1296,806,1263,1886,450,2343
GPR4_3_123,124,558,1358,459,246,942,2638,873,36,1366,358,1670

GPR50_3_124,1217,1976,3109,4692,2216,4384,3678,550,1308,1674,3526,3109
GPR52_3_125,2891,2206,3778,3643,5286,3044,6752,2953,2033,1686,6869,244
3
GPR55_3_126,3542,4468,4888,3586,7574,6358,3754,4904,4867,9005,3914,408
4
GPR61_3_127,609,1432,2282,654,1858,3879,978,1255,642,2654,3223,751
GPR62_3_128,1188,1055,1241,1460,3743,235,1260,2301,2154,3461,3791,1198
GPR65_3_129,12182,13897,13635,20815,12445,22931,15600,20944,17864,2246
2,15470,19802
GPR6_3_130,8246,10015,11072,15714,11683,12562,11171,15343,9564,8138,15
549,13071
GPR75_3_131,19341,21359,27425,30873,32296,26342,33878,31389,23827,2190
9,29206,27953
GPR77_3_132,1847,2209,2373,4276,5750,5062,1731,6177,1894,1135,692,846
GPR78_3_133,799,1027,933,1233,1577,295,980,1934,1760,1278,411,1870
GPR82_3_134,10560,9379,6894,12869,10266,13358,5514,5494,10119,13004,17
051,11349
GPR83_3_135,3179,5396,5081,6835,3139,4973,4733,4635,5890,2513,10048,65
30
GPR84_3_136,2072,2134,1640,2220,1170,3100,2453,224,1729,1696,288,2107
GPR87_3_137,3284,3404,2648,5404,4433,4010,736,2308,2241,5000,5992,1969
GPR88_3_138,357,302,47,1040,222,18,94,715,68,680,428,183
GPR97_3_139,18540,21910,21392,26365,18511,17038,24917,26326,28267,2932
2,21641,29846
GPR98_3_140,6405,8883,10071,10902,9366,15999,9922,17852,7469,6240,7335
,10669
GPRC5A_3_141,1051,795,1217,1675,725,1019,1217,865,56,1980,4744,212
GPRC5B_3_142,3066,3702,4346,3245,3964,3650,5083,4732,2638,3974,2967,24
38
GPRC5D_3_143,1053,672,1230,1313,1363,1349,2317,4189,1814,257,161,73
GPRC6A_3_144,3135,2815,4023,3141,3270,2699,7325,1434,2901,2330,4413,10
02
GRM3_3_145,2276,2327,1781,2774,4461,3936,1735,6216,2163,2526,1648,1739
GRM4_3_146,0,0,0,0,0,0,0,0,0,0,0,0
GRM6_3_147,1053,1339,1485,1977,1044,891,625,177,102,3622,576,1031
GRPR_3_148,1822,2420,1619,2450,1064,5128,3097,2154,3743,1988,3232,2521
HCAR1_3_149,1203,1815,1900,2556,392,2200,1151,1068,1108,246,691,2035
HCAR2_3_150,6282,6160,8406,8571,7509,5824,9390,12005,6770,7224,13371,5
708
HCAR3_3_151,6282,6160,8406,8571,7509,5824,9390,12005,6770,7224,13371,5
708
HCRTR1_3_152,443,771,245,900,615,4657,1635,1668,1026,421,850,805
HRH3_3_153,1057,1705,2346,2914,1332,1317,2564,2337,1009,4306,1955,1462
HTR1A_3_154,1331,621,664,1429,568,984,887,2548,390,456,262,513
HTR1B_3_155,101,497,313,737,680,52,688,572,37,276,226,740
HTR1D_3_156,117,207,65,56,8,0,204,554,341,2357,21,229
HTR1E_3_157,16122,21289,21924,25380,18174,20471,23826,28912,24184,2962
8,26794,24503
HTR1F_3_158,493,725,237,536,1028,781,639,0,1665,1373,203,1
HTR2B_3_159,1576,2783,2217,3478,4151,3305,2538,4940,5535,6755,2776,185

9
HTR2C_3_160,2438,2936,5047,3288,2090,2644,5733,4252,4481,4779,2078,577
3
HTR5A_3_161,2826,1391,1971,3423,3149,103,1616,5736,3170,1308,2418,4186
HTR6_3_162,506,152,1032,316,864,66,116,220,258,25,172,198
KISS1R_3_163,212,924,913,1157,750,55,570,777,948,408,634,825
LGR4_3_164,1521,3939,2491,4511,2456,1136,3746,4340,3152,3163,4524,1342
LGR5_3_165,6068,5651,6441,10129,4346,9009,4898,7849,5942,10938,8291,90
42
LHCGR_3_166,2152,1665,1362,2636,2523,4065,1729,1686,1825,1278,3852,304
0
LPAR2_3_167,1880,2010,2786,1950,1866,2293,1588,3245,4330,2702,1028,548
7
LPAR3_3_168,1554,1730,2002,1881,2775,1091,2556,2716,791,1503,529,1055
LPAR4_3_169,876,1060,1154,1760,404,3238,470,1557,1929,1654,537,3728
LPHN2_3_170,1449,1755,1585,3226,2660,2218,1891,7413,956,3238,1045,3172
LPHN3_3_171,1438,1521,2933,1520,1034,1292,1061,867,2381,1402,1502,2065
MAS1_3_172,1379,2872,2174,2352,2698,3110,1644,4864,2464,1822,1631,2197
MAS1L_3_173,721,351,679,109,14,371,263,337,876,16,1402,32
MC1R_3_174,1126,1402,1824,1905,97,829,2066,3084,920,331,4039,2190
MC2R_3_175,5533,5685,6415,7540,6555,13456,8656,9780,6927,7529,6702,502
5
MC3R_3_176,562,896,864,622,1171,767,1171,2111,141,856,3758,708
MC4R_3_177,3863,6541,6800,4733,6690,7753,6099,5836,4771,3116,5612,5947
MC5R_3_178,3629,3978,2986,3971,5568,2338,3024,3563,4525,3661,3310,4870
MCHR1_3_179,642,880,264,1383,1425,2548,33,465,81,15,1065,766
MLNR_3_180,673,1031,982,402,1194,1802,456,51,742,52,805,894
MRGPRD_3_181,1137,1767,2128,2872,2424,3005,2906,1928,2078,1751,1730,25
35
MRGPRE_3_182,254,601,366,29,18,117,3106,110,7,806,337,887
MRGPRG_3_183,897,1583,1650,1521,561,2154,371,500,1548,2411,1366,1379
MRGPRX1_3_184,255,834,719,435,793,533,882,536,681,86,307,710
MRGPRX2_3_185,1179,879,650,1173,615,1490,956,703,1250,1304,286,2126
MRGPRX3_3_186,3806,5122,4316,4949,5855,5644,3278,4861,6267,4888,3312,4
083
MRGPRX4_3_187,3781,5242,3280,4573,5216,4493,3484,2590,5716,4445,3248,2
925
MTNR1A_3_188,2223,2249,1931,1825,1529,2883,2530,1673,1746,2002,1389,13
45
MTNR1B_3_189,641,2058,696,859,1537,519,1454,560,1209,2047,142,1478
NMBR_3_190,4632,3487,6448,5616,5358,3904,6846,8789,5169,3715,7701,8068
NMUR1_3_191,592,390,173,403,169,86,148,100,143,1115,539,0
NMUR2_3_192,2735,1944,2761,5691,2615,1525,6633,6375,8183,2041,4759,528
2
NPBWR1_3_193,2103,1402,2000,2123,2527,1190,2543,902,915,2858,900,1592
NPBWR2_3_194,628,536,809,614,517,1436,190,826,840,218,484,963
NPFFR1_3_195,315,945,574,886,891,794,552,452,1984,2117,536,110
NPY1R_3_196,1866,3121,1787,2696,2870,5007,1926,2034,2577,8162,3005,322
0
NPY2R_3_197,3584,2748,3576,3772,2809,2217,3001,3130,714,2107,4647,4951

NPY5R_3_198,4036,5851,5065,5298,2443,6601,10160,5587,3317,1751,8131,2965
NTSR1_3_199,541,1045,797,496,461,262,62,276,128,955,3229,1504
NTSR2_3_200,1508,856,2333,2472,749,2948,947,1791,2308,2655,2295,3981
OMG_3_201,4914,4771,4903,3906,7341,4948,5868,3566,6034,3439,2971,4504
OPN1LW_3_202,6837,7467,6556,8342,5184,13138,4060,7897,12848,10341,10284,9541
OPN1MW2_3_203,6837,7467,6556,8342,5184,13138,4060,7897,12848,10341,10284,9541
OPN1MW_3_204,6837,7467,6556,8342,5184,13138,4060,7897,12848,10341,10284,9541
OPN1SW_3_205,3071,3924,4719,6144,4597,3973,6103,7482,3865,2579,4510,6979
OPN3_3_206,1012,1290,1759,2149,1149,4403,894,1717,3397,5800,153,1974
OPN5_3_207,230,1122,561,908,365,331,1563,513,1415,1316,553,883
OPRD1_3_208,393,1365,780,1068,268,1742,2963,275,1167,58,213,2650
OPRK1_3_209,3625,3659,4246,4171,3070,3438,2889,4262,3910,1877,3541,3959
OXER1_3_210,852,908,351,1238,330,469,833,2603,492,1388,139,473
OXGR1_3_211,1002,3186,2822,3159,3667,4905,3109,888,2779,7052,4454,2749
OXTR_3_212,3061,3424,3137,4257,7805,3331,4547,5339,5213,6576,2311,3877
P2RY11_3_213,1206,1574,1435,989,601,1055,310,735,1732,186,734,879
P2RY13_3_214,1428,1340,1339,2832,914,866,1779,4152,959,1223,2032,1804
P2RY1_3_215,3040,4380,4100,6501,2870,5556,6610,5173,4386,6339,2176,7755
P2RY4_3_216,3392,3804,2677,6351,2652,6189,6594,4675,2952,4176,3239,7327
P2RY8_3_217,609,1302,1226,274,102,286,454,2978,505,55,272,173
PPYR1_3_218,269,863,762,1199,2,4,1436,636,96,10,146,426
PRLHR_3_219,203,689,926,34,940,109,1143,270,617,1058,3686,7
PROKR1_3_220,919,1110,1268,878,283,2127,1221,499,1477,908,810,878
PROKR2_3_221,9137,9117,10237,10571,7657,10165,10776,9136,7139,7696,16418,6450
PTGDR_3_222,270,1215,651,399,795,1263,267,2489,423,1159,135,36
PTGER1_3_223,322,906,178,1292,116,68,271,509,656,371,259,1186
PTGER2_3_224,2153,1616,2810,4180,3017,1840,3826,3491,1690,1216,4187,2467
PTGER4_3_225,1243,1594,1148,1806,3303,3064,678,1562,1861,1086,2212,852
PTGIR_3_226,284,268,378,693,1027,78,967,153,531,693,15,284
PTH2R_3_227,1159,581,539,985,3405,510,1433,1414,504,451,1017,766
QRFPR_3_228,8573,9464,8208,11962,10111,14605,15303,11781,9912,13573,6549,10798
RHO_3_229,1160,1310,858,1841,1486,1261,863,1665,540,1538,684,1873
RRH_3_230,4922,6763,6870,8473,4216,14582,15409,9585,3401,4960,4404,10508
RXFP1_3_231,7251,6986,7928,7376,7334,7892,8518,3741,4489,5511,8177,5817
RXFP3_3_232,3374,3590,4016,4284,3070,2206,5494,3661,4062,4323,3822,6887
RXFP4_3_233,641,659,173,812,696,995,1570,299,38,9,38,479

S1PR1_3_234,3031,3988,3991,4653,4772,8864,2285,9754,6116,2108,2264,661
7
S1PR2_3_235,377,23,164,6,5,1,52,0,388,3,236,165
S1PR3_3_236,2216,1329,1588,2770,412,710,1108,1923,2560,1983,4255,1569
S1PR4_3_237,2017,2969,2600,3663,3049,3422,3589,3425,1850,2019,4271,240
2
SCTR_3_238,203,20,133,235,1210,414,448,4,71,58,57,1640
SSTR1_3_239,2919,4298,4553,5354,632,4756,3732,3248,4472,9120,6318,4612
SSTR2_3_240,568,575,558,1013,1201,460,1219,2406,427,404,891,671
SSTR3_3_241,640,877,685,761,975,1559,3160,736,776,897,377,20
SSTR4_3_242,33,13,39,17,3,470,25,0,4,5,47,114
SUCNR1_3_243,2751,3910,2871,4948,1309,4391,9139,1710,4597,3979,8371,62
48
TAAR1_3_244,8202,5419,7537,9373,8589,12449,8188,10102,7973,8599,3169,7
133
TAAR5_3_245,518,1071,1385,1147,646,263,595,3030,1496,3171,420,1439
TAAR6_3_246,4107,5296,3894,5865,4322,7663,6016,4248,5795,3084,2137,731
9
TAAR8_3_247,4239,3837,6000,7879,9764,11503,3845,8129,8184,5624,15216,1
0113
TAAR9_3_248,2367,2418,2751,4083,3447,2190,2242,6320,2715,4587,899,4016
TACR2_3_249,1509,1382,1877,1374,195,2475,1296,1731,743,2206,3107,919
TACR3_3_250,1376,1786,1012,1980,2203,2885,2672,2625,952,2069,1152,3100
TAPT1_3_251,1242,1689,1326,2779,1724,1423,928,1806,1176,1602,2915,3562
TAS1R2_3_252,1580,1521,2078,3671,3193,1544,1944,6075,2347,4240,827,136
4
TAS1R3_3_253,21,966,321,487,1319,1559,763,890,2,1746,86,1591
TAS2R10_3_254,17391,20767,24936,27019,20517,30634,28526,26325,31377,19
877,15177,34146
TAS2R13_3_255,4644,6172,6767,8980,5515,11773,8234,9798,8038,6364,8138,
8118
TAS2R14_3_256,661,386,797,1183,1919,1324,697,1192,420,961,449,770
TAS2R16_3_257,1364,1479,1218,1744,606,949,2640,427,2188,203,4137,938
TAS2R1_3_258,17119,19198,21497,26537,25578,23646,23294,32450,31804,207
40,37735,31267
TAS2R20_3_259,21461,25496,24956,35243,23805,29966,38792,32346,27881,31
617,43913,31542
TAS2R31_3_260,31499,36923,36625,37146,33641,37214,40751,48929,28881,34
539,44421,28668
TAS2R38_3_261,3390,5316,7323,6265,7168,7321,10248,4330,9426,8231,5933,
5717
TAS2R3_3_262,2035,3564,2814,4852,3672,2603,810,4768,3336,2144,2772,480
1
TAS2R40_3_263,9689,8308,7522,6739,7473,8947,8043,8312,9924,12609,11060
,10388
TAS2R41_3_264,649,972,1140,717,1746,395,1718,714,524,1544,801,1805
TAS2R46_3_265,31499,36923,36625,37146,33641,37214,40751,48929,28881,34
539,44421,28668
TAS2R4_3_266,4002,3755,5182,3071,5274,4909,3342,6001,5059,1446,7370,52
18

TAS2R5_3_267,8434,11692,9844,14508,8695,6779,14833,7323,11313,3985,887
3,17649
TAS2R60_3_268,704,1270,719,530,1354,1233,1296,1300,1847,291,304,1278
TAS2R7_3_269,1369,1808,1290,2078,1341,2705,1619,2390,1398,1575,2611,13
86
TAS2R8_3_270,29486,32829,33197,39994,31872,50077,37236,41079,26524,420
61,56053,36434
TAS2R9_3_271,2229,1888,3179,2169,2958,6655,2858,2654,3436,899,2198,359
4
TM2D1_3_272,3145,2788,3100,3955,4468,7183,2187,4191,4505,11869,8263,67
68
TMEM11_3_273,1790,3091,2908,2532,1010,63,3702,2615,1081,1819,1750,1100
TRHR_3_274,1762,3345,2767,4929,5240,4051,2662,4300,1827,2225,1297,4637
UTS2R_3_275,509,59,350,912,183,0,1042,614,546,1044,2027,179
VIPR1_3_276,88,142,567,203,498,478,107,159,160,44,34,6
VIPR2_3_277,2702,3109,2489,4872,2478,2559,3507,1070,2852,5083,2684,393
0
VN1R1_3_278,10972,11989,12869,16228,13636,16606,16376,11581,17487,1289
5,11216,17484
VN1R2_3_279,4171,3644,4057,4848,4843,5207,4320,5714,4797,3390,13032,25
67
VN1R4_3_280,2559,2088,2957,4557,1577,2875,3748,9426,2986,5028,4123,333
8
ADCYAP1R1_3_281,2696,5778,4043,4551,5326,3738,4825,4581,5054,10641,343
3,3512
ADORA1_3_282,2175,1633,2337,2868,1416,931,1593,3966,1952,4184,2463,179
5
ADORA3_3_283,2218,4052,2441,2729,3187,3410,1521,4265,2746,5295,3757,35
92
AGTR1_3_284,12617,13562,13890,18815,12296,17388,15476,17336,10318,1458
8,12594,28019
AGTRAP_3_285,482,487,1799,822,196,259,1286,1065,91,27,697,483
AVPR2_3_286,443,772,230,1106,195,91,215,274,556,1237,447,485
CALCR_3_287,6041,7322,7010,6857,9751,9905,5347,5871,4673,6464,6113,452
2
CASR_3_288,4157,5049,6270,7978,3872,1589,8680,9658,6960,5777,3822,4657
CCR2_3_289,1246,1541,2110,1818,126,984,2686,2740,984,1626,6565,935
CCR3_3_290,10799,9815,11098,12097,15872,13077,7748,11951,9730,13032,17
798,13275
CCR5_3_291,2972,2224,2294,3566,2458,3373,1248,2942,1468,2866,3484,6657
CCR6_3_292,2574,1759,2321,2052,1733,854,4411,3044,1096,981,1481,3580
CCRL2_3_293,3046,4316,3730,6912,2988,4689,5115,7966,4701,3401,1765,600
5
CD97_3_294,507,391,463,488,508,645,431,517,410,388,251,669
CHRM2_3_295,1938,4079,2923,4426,1867,1957,3477,4286,3054,4512,4574,338
3
CMKLR1_3_296,5271,7474,7603,8708,3765,9149,8681,5957,6248,5049,6826,89
88
CRHR1_3_297,1017,527,915,799,305,935,673,491,391,1020,916,599
CRHR2_3_298,1537,1748,1434,2645,92,719,1453,949,2173,825,562,155

CX3CR1_3_299,2254,1865,2403,2853,2274,3658,2607,5869,3845,2773,3685,30
82
CXCR2_3_300,5703,6941,5330,9171,5255,9129,5798,8957,7195,7442,14006,74
18
CXCR3_3_301,1390,1120,2481,1616,1599,826,1793,886,1256,683,808,574
CXCR4_3_302,1481,1998,1604,1866,2121,1024,1766,233,2720,1784,1619,1168
CXCR5_3_303,2090,3139,2605,2710,1904,1696,4054,2647,3346,5626,3282,432
0
DARC_3_304,932,489,383,569,1016,36,161,380,552,652,110,321
DRD2_3_305,3438,5095,2748,3791,4995,6398,5033,4385,4351,4852,3318,2616
DRD3_3_306,1105,2246,1848,1377,1514,1078,3338,2058,1935,1336,913,1028
EDNRB_3_307,2215,1767,3130,2193,1538,993,5752,2841,1905,1107,2124,2892
EMR2_3_308,1932,2041,1541,1402,2640,2624,1007,809,776,2603,891,2380
FPR1_3_309,551,1181,530,1274,296,221,835,846,1388,1019,2461,1233
FSHR_3_310,220,557,945,670,104,0,855,744,1,22,1703,1067
FZD6_3_311,1183,819,1377,1491,2335,1552,1874,1973,1661,3082,582,3432
GABBR1_3_312,627,1583,882,1689,3607,661,2688,161,1315,1649,794,997
GHSR_3_313,3351,2071,2263,3251,1854,1909,2485,3320,2953,2300,5746,4535
GNRHR_3_314,5759,8644,7135,9612,8671,10844,9087,11775,8556,8002,11946,
9731
GPBAR1_3_315,2009,1850,1759,1984,1077,1771,2923,3019,1995,3257,866,142
1
GPER_3_316,3129,2901,2286,2901,2226,3442,4776,2186,3597,3004,3741,1591
GPR107_3_317,1917,4414,2568,2516,3585,3752,5675,6304,6791,3281,3816,63
88
GPR110_3_318,5193,6654,7297,10510,4927,8203,10333,5390,6673,4975,5572,
8567
GPR113_3_319,2690,2260,2500,6278,5669,3456,4081,2139,2082,3367,6029,40
42
GPR116_3_320,17205,22468,22089,31240,15526,25893,24895,24429,17233,258
04,31143,27741
GPR126_3_321,4872,4974,8159,7988,3856,8042,10910,7185,9343,5284,16624,
9173
GPR155_3_322,6364,6395,5590,7316,5396,8444,6241,11949,5212,7143,10957,
4417
GPR156_3_323,1177,1586,1716,2639,892,1122,3159,2928,428,3532,1780,1025
GPR162_3_324,1425,584,1306,915,1965,2981,2682,494,3009,945,1082,1115
GPR17_3_325,1176,1702,1698,1840,801,1486,1528,4491,469,1599,2097,1355
GPR18_3_326,142,89,1496,328,841,271,969,1,301,101,648,12
GPR1_3_327,5884,9659,10046,8342,8372,6615,12825,9270,5363,13778,11579,
5087
GPR34_3_328,5594,6674,7514,8479,6774,14620,6159,4763,7661,10867,12545,
9194
GPR35_3_329,162,301,573,616,587,99,913,623,546,450,307,373
GPR56_3_330,0,0,0,97,0,0,0,0,0,0,0,851
GPR63_3_331,2996,3570,3075,6274,2255,5616,2793,5264,4530,5498,6621,557
7
GPR64_3_332,8906,9826,10546,11710,7866,20783,11589,9780,6911,11163,160
49,15881
GPR68_3_333,8944,9548,9593,10057,6173,11959,10517,13839,11610,15088,93

05,11802
GPR85_3_334,947,1739,3325,2811,1833,4069,4085,2279,1735,499,2174,1846
GPRC5C_3_335,1437,2403,1338,1155,2307,3059,919,2983,2097,3127,466,1800
GRM1_3_336,2364,2133,1675,1430,3291,2385,1192,2528,2697,748,693,2621
GRM2_3_337,967,1672,1082,3206,389,1011,877,104,2240,888,265,1773
GRM5_3_338,5227,5805,4058,6782,6118,9824,5368,6924,5533,6126,4325,4810
GRM7_3_339,1030,1805,640,1125,198,138,2456,1362,934,1152,1814,775
GRM8_3_340,3674,4813,3442,5138,2926,4760,5847,4381,6936,7557,5337,4100
HRH4_3_341,4513,4320,6700,5770,4623,1895,4503,3868,4813,5526,6936,9360
HTR2A_3_342,285,574,542,1254,535,147,726,1398,320,1932,62,1382
HTR4_3_343,2623,2096,2821,2053,4945,2294,4386,1663,5629,1428,1294,1479
HTR7_3_344,640,293,1406,887,1201,468,2103,536,235,1143,2543,2007
LGR6_3_345,258,178,38,72,383,680,0,582,834,0,5,65
LPAR1_3_346,436,1116,590,1097,569,1718,325,1199,1009,1762,1024,2776
LPAR5_3_347,855,468,759,652,814,812,8,897,632,217,36,674
LPAR6_3_348,4394,4975,5808,5910,6129,7313,7541,6652,6902,9407,6770,499
7
LPHN1_3_349,2487,3703,2968,1254,3982,5098,4359,3532,3037,1946,2207,142
8
LTB4R2_3_350,540,1001,986,1445,222,842,1309,131,60,1182,115,1776
LTB4R_3_351,434,265,642,113,36,804,44,24,1599,17,134,119
LYPD1_3_352,536,486,111,558,304,428,210,556,952,243,679,2781
MCHR2_3_353,21614,20821,26179,26219,33482,30606,35099,34274,27529,2771
2,27042,21648
MRGPRF_3_354,702,1102,1350,1089,2262,1009,550,2591,415,2523,294,243
NPFFR2_3_355,312,1009,588,1054,905,797,332,546,2052,1307,217,144
NPSR1_3_356,4491,3886,5518,5913,3526,4379,6380,3515,4452,4907,5867,494
5
O3FAR1_3_357,1522,625,1253,1906,940,1562,2187,915,290,1245,1522,677
OPN4_3_358,1018,601,1006,1040,966,887,318,94,2247,1272,154,142
OPRL1_3_359,1080,444,1203,1229,1211,1870,2046,1109,948,70,582,909
OPRM1_3_360,2568,3678,3297,4138,1693,1524,4958,2526,454,1582,4102,1432
P2RY10_3_361,278,341,230,600,164,162,50,1243,138,560,53,545
P2RY12_3_362,2001,4232,4053,5034,2451,4453,6222,9302,2220,2810,6391,42
81
P2RY14_3_363,1018,1934,975,1535,2063,9,1066,953,1658,4176,2700,1167
P2RY2_3_364,1564,956,607,2450,958,2237,779,3542,316,814,1185,1476
P2RY6_3_365,249,219,1025,297,599,232,294,71,444,1730,384,364
PTAFR_3_366,445,630,589,138,2,319,338,43,36,7,19,86
PTGER3_3_367,283,365,799,790,652,1693,1145,143,184,758,1001,177
PTGFR_3_368,2950,3532,5383,5692,3864,6393,5267,9786,2921,2849,12037,19
66
PTH1R_3_369,618,802,1211,1813,923,141,448,884,123,1137,853,35
RGR_3_370,276,753,330,203,953,852,89,773,514,153,23,80
RXFP2_3_371,1043,1353,621,1550,974,3042,710,760,802,788,5323,410
S1PR5_3_372,558,662,905,1940,171,1319,2243,748,1214,1024,2432,3289
SIGMAR1_3_373,5721,7066,6266,8729,6811,5951,6240,9809,6904,7770,14400,
12555
SSTR5_3_374,381,1369,947,1672,618,525,501,1232,1227,1046,4142,861
TAAR2_3_375,5400,4877,4942,11588,5531,9554,11126,4544,3810,9192,5440,7

695
TACR1_3_376,639,279,690,1047,50,483,993,1206,755,346,74,3128
TAS1R1_3_377,1663,2221,2361,3412,1756,645,3584,4952,4646,1996,5806,384
3
TBXA2R_3_378,301,900,117,1170,116,68,265,508,486,367,253,1178
TPRA1_3_379,158,161,370,280,7,2415,0,544,8,9,5,0
TSHR_3_380,42139,49050,47245,57069,66001,67948,59434,70217,50447,55945
,57344,47179
XCR1_3_381,1576,1795,2694,1763,2504,2265,3624,3218,2509,5307,6412,1746
XPR1_3_382,2352,3199,2331,6659,6402,1548,2440,626,4387,3489,3387,4799
ADORA2A_3_383,426,758,231,838,766,294,96,59,432,1692,971,1099
ADORA2B_3_384,241,1107,514,558,77,57,518,1000,255,14,34,118
ADRA1B_3_385,2062,2636,2545,3597,3507,5920,5204,1380,1418,1929,4301,34
95
ADRA1D_3_386,763,415,611,885,173,448,265,426,1624,970,5379,411
ADRA2A_3_387,2632,2258,2631,3993,5201,2306,2843,3058,3816,1688,1878,47
65
ADRA2B_3_388,728,631,826,699,69,345,1250,768,884,15,712,433
ADRA2C_3_389,2693,1412,1612,4899,1345,2601,2938,2151,910,2424,828,3351
ADRB1_3_390,903,202,197,977,840,550,901,681,2,1200,22,1054
ADRB2_3_391,566,778,818,748,615,1965,1065,139,170,1828,836,321
ADRB3_3_392,1152,915,1944,963,4028,579,684,1055,1334,146,283,641
AGTR2_3_393,2365,3987,3123,3705,3107,5408,4479,6355,1585,4237,2400,163
2
APLNR_3_394,2103,2807,3220,3140,1229,5933,3328,4635,3536,2739,1504,246
3
AVPR1A_3_395,1136,1235,1984,2431,1423,1642,2093,2392,1447,2435,2908,12
37
AVPR1B_3_396,8316,8053,8695,12891,14397,7246,10209,11033,9653,11060,11
520,14744
BAI1_3_397,247,997,541,732,741,1267,1446,94,630,1018,160,660
BAI2_3_398,4017,4177,6527,7201,4221,5290,6452,7323,5157,4823,5553,6730
BAI3_3_399,1905,2494,2763,2561,1852,3068,2660,3992,1692,621,1866,4110
BDKRB1_3_400,1002,741,1235,516,1184,1127,411,263,895,756,2400,1288
BDKRB2_3_401,672,1311,1927,2111,1417,552,411,1692,2128,417,3051,1369
BRS3_3_402,2454,4375,3267,3106,2774,7137,7636,3439,2958,2336,3304,3289
C3AR1_3_403,1069,2032,1709,1808,398,1595,912,1603,1976,2076,2865,2651
C5AR1_3_404,802,746,1461,1586,419,69,419,771,1126,861,349,1844
CALCRL_3_405,864,833,1345,588,333,1304,1522,586,1046,399,3915,1214
CCKAR_3_406,259,344,283,130,17,386,188,258,1073,31,20,138
CCKBR_3_407,2671,1944,1470,1817,2629,952,942,2116,452,1636,1814,1521
CCR1_3_408,1954,844,1480,1025,960,212,1152,3233,2336,731,1994,1341
CCR7_3_409,4834,5017,5999,4583,4767,2203,5946,3890,5430,7635,7908,4693
CCR8_3_410,6239,5823,6374,8610,5645,6409,8018,7785,10914,3317,6047,988
0
CELSR1_3_411,1381,583,978,1985,760,813,975,516,815,485,535,1025
CELSR2_3_412,231,439,531,473,372,1392,18,888,76,967,228,54
CELSR3_3_413,44,527,188,204,75,2600,0,13,94,135,595,7
CHRM1_3_414,791,413,209,319,252,1001,202,259,204,673,1180,343
CHRM3_3_415,2106,1419,809,1823,696,2204,3406,1323,1359,1753,3680,907

CHRM4_3_416,1590,2062,936,2025,2745,473,492,2494,1382,2543,1204,2030
CHRM5_3_417,3440,4722,5749,6794,5563,12337,3819,3433,7512,5157,2893,46
01
CXCR1_3_418,776,2058,1628,3614,737,2301,2779,2133,3210,1446,3247,742
CXCR6_3_419,2038,1604,2705,3017,3356,7044,3023,4496,2591,2994,1870,649
5
CXCR7_3_420,2020,1935,1757,2213,819,2076,1502,1621,2289,2007,1170,1613
CYSLTR1_3_421,2059,2806,2927,4168,1001,4512,1786,4341,4152,1089,3502,1
370
CYSLTR2_3_422,1769,2418,2216,2834,1060,2247,3909,1228,4521,6053,2173,5
386
DRD1_3_423,427,486,232,1493,398,80,2,52,16,105,2260,0
DRD4_3_424,961,684,510,2836,1430,771,490,2172,886,661,1105,656
DRD5_3_425,1255,666,1234,1611,1095,969,1602,196,1661,2365,1082,1203
ELTD1_3_426,708,1136,486,1372,979,1866,603,369,136,1805,322,176
EMR1_3_427,894,938,518,2227,677,1257,828,1122,2594,2888,967,1290
EMR3_3_428,1339,2552,3587,2006,1576,1899,746,542,370,1854,4005,4421
F2R_3_429,1090,1183,1276,1523,481,1358,667,1073,2059,943,1067,3898
F2RL1_3_430,5209,6050,7283,7513,6382,10681,7780,5206,4267,5091,5951,79
92
F2RL2_3_431,2015,1574,2964,1946,780,2159,1894,1184,2644,2788,6344,4212
F2RL3_3_432,806,723,796,760,303,40,286,423,829,1963,176,2578
FFAR1_3_433,53,290,479,320,121,0,1195,1,255,23,24,9
FFAR2_3_434,326,731,638,1585,675,245,1110,818,925,4160,382,2323
FFAR3_3_435,933,924,582,1546,2083,859,123,44,1204,691,328,378
FZD10_3_436,113,881,276,647,68,0,1561,488,14,25,1445,136
FZD1_3_437,1576,2027,1659,2339,2438,2338,2327,1503,2548,1605,2050,1325
FZD2_3_438,1096,1401,1415,1218,548,2678,707,3935,879,314,3458,1778
FZD4_3_439,1229,432,1057,1677,1134,265,1229,1743,1247,1128,729,2206
FZD5_3_440,92,101,55,243,119,3,2,88,66,306,13,284
FZD7_3_441,584,204,540,755,229,582,189,463,77,153,234,450
FZD8_3_442,568,1017,1101,1358,306,1026,2272,1871,1138,990,3976,781
FZD9_3_443,655,1434,529,687,283,1835,240,637,54,800,471,750
GABBR2_3_444,330,232,428,438,1042,1986,1065,79,0,268,384,8
GALR1_3_445,1699,1779,1029,1929,992,2532,1566,2687,1780,1607,533,1788
GALR2_3_446,47,203,148,343,255,0,4,1,16,1435,105,0
GALR3_3_447,462,850,699,682,2535,171,1937,918,2066,314,174,839
GCGR_3_448,1031,1600,1403,2196,1675,1549,227,1378,3707,2630,2131,930
GHRHR_3_449,89,452,120,901,32,1946,781,1,272,392,1,830
GIPR_3_450,2756,5604,6566,7784,1734,4347,6553,5181,5062,6019,2503,6632
GLP1R_3_451,294,337,543,422,670,25,1020,879,44,23,187,371
GLP2R_3_452,3282,5488,3929,5074,3331,7231,2285,3901,3521,12178,2058,55
58
GPR101_3_453,1815,2582,3601,2827,3434,4351,2089,4623,2302,3003,5322,33
18
GPR108_3_454,1439,1474,1906,2262,824,2551,1216,1412,2356,1110,2352,225
9
GPR111_3_455,877,1415,773,1380,421,1881,1493,607,1192,933,545,1107
GPR112_3_456,215,0,52,0,0,0,0,0,0,0,13,0
GPR114_3_457,593,1045,469,578,1773,1097,2070,1178,169,3510,4777,715

GPR115_3_458,251,706,779,810,746,1310,980,5350,346,19,27,61
GPR119_3_459,721,1507,625,1902,1484,793,1010,2546,2774,1439,165,3313
GPR123_3_460,390,672,758,1036,1,1833,931,1014,393,2470,534,715
GPR124_3_461,223,1145,309,586,90,315,313,2,1876,3,95,729
GPR125_3_462,15027,18649,20437,21300,19486,34330,18205,20874,18148,182
60,20084,17571
GPR128_3_463,5359,7737,6820,9017,9080,12625,7643,13343,7443,9668,6519,
10282
GPR12_3_464,3838,4610,4707,5947,4348,2681,6481,7173,3778,4094,5815,106
42
GPR132_3_465,1327,3052,2125,2442,2072,1902,3599,1559,2378,3273,1302,27
69
GPR133_3_466,449,903,355,393,1453,180,165,730,64,1840,111,693
GPR135_3_467,2388,2713,2201,3506,1536,2494,2354,2077,3678,2967,3153,24
47
GPR139_3_468,651,177,1442,872,41,1407,1800,343,260,460,39,1735
GPR141_3_469,1748,1525,2583,2891,969,691,1253,3220,4313,3896,6205,5548
GPR142_3_470,3308,4577,3943,7888,2088,4477,5014,6187,3194,4906,5548,69
11
GPR143_3_471,1266,1849,1274,1340,1434,1982,1535,3308,599,1560,3448,505
GPR144_3_472,1561,1547,1124,1520,2477,1604,3696,1738,2128,647,1266,311
0
GPR146_3_473,5339,4944,3868,5891,4439,5170,6392,5088,7060,5166,11484,4
544
GPR148_3_474,1912,2182,2438,2797,3883,2343,1046,797,4294,3729,823,2876
GPR149_3_475,3719,3942,2876,4146,4484,4673,4048,3772,4070,5093,3280,55
76
GPR150_3_476,873,898,413,555,500,331,370,84,966,1555,353,1950
GPR151_3_477,1715,1300,3183,2328,1439,324,2089,2830,1597,2071,964,4891
GPR152_3_478,103,13,8,104,40,0,42,552,0,1,0,298
GPR153_3_479,1463,1940,1538,4108,1833,384,1470,2005,1861,3978,1437,116
1
GPR157_3_480,388,692,973,387,1220,1957,542,436,603,331,1843,596
GPR158_3_481,1308,1785,1042,2568,1363,1195,655,4339,888,1125,1506,3026
GPR15_3_482,1051,507,518,1472,1514,1222,611,645,1750,1018,176,73
GPR160_3_483,2743,3156,2101,2996,2205,3198,4025,1005,1862,1864,1348,28
65
GPR161_3_484,529,2063,655,865,1438,1066,544,130,2023,1378,256,1044
GPR171_3_485,2974,4017,3249,3635,5289,1179,3701,2053,2467,1422,5549,35
15
GPR173_3_486,963,612,1034,1173,295,339,815,1517,1763,1516,2219,1869
GPR174_3_487,1063,1108,1081,3086,2291,96,2750,2088,2069,4667,2611,5504
GPR176_3_488,441,414,359,807,708,212,337,91,358,23,316,889
GPR179_3_489,1000,2531,951,2163,981,4640,2550,1292,2392,1694,4972,1479
GPR182_3_490,5596,6854,7040,7148,7063,7996,4969,6699,6650,5688,12258,6
186
GPR183_3_491,3192,8049,6901,10420,7824,8150,9080,8828,7653,5635,8439,1
0051
GPR19_3_492,518,255,546,1654,256,2,1101,525,812,902,1705,578
GPR20_3_493,9,460,288,1529,869,1543,299,1064,1434,874,150,10

GPR21_3_494,2901,3081,2983,2055,1232,1925,1343,4216,2263,1928,3371,436
1
GPR22_3_495,3242,4141,2414,3612,2310,4101,946,9194,1334,3038,2473,4002
GPR25_3_496,0,0,0,0,0,0,0,0,0,0,0,0
GPR27_3_497,1,130,119,171,659,0,0,0,0,1,0,504
GPR31_3_498,742,785,1169,1835,1618,1696,1129,1519,1639,1123,297,1757
GPR32_3_499,878,769,839,1677,733,309,622,437,480,3170,5888,2490
GPR37_3_500,611,1006,1626,1387,179,1275,1892,1696,929,184,2988,2614
GPR37L1_3_501,653,695,1251,2778,2077,1236,2086,728,706,1928,2931,1135
GPR39_3_502,773,1998,2348,3375,1899,2336,1662,1303,838,3599,3633,3320
GPR3_3_503,1798,2051,1988,1897,467,4716,2278,2086,2931,3738,1332,2914
GPR45_3_504,108,725,580,1693,483,664,528,2053,4,159,130,505
GPR4_3_505,2641,3262,3630,8684,3699,3812,4393,4724,2662,2225,9332,6760
GPR50_3_506,5061,6154,5771,9579,5502,4679,7865,7177,5018,5489,11419,10
476
GPR52_3_507,788,1181,1389,1247,932,1687,1143,444,277,389,385,350
GPR55_3_508,1658,2805,3330,3090,2070,6614,3061,2617,1827,3756,1947,435
9
GPR61_3_509,3095,3399,3276,3257,4664,3227,2940,2136,4670,5205,4976,772
1
GPR62_3_510,181,138,76,147,359,64,383,389,76,1,568,14
GPR65_3_511,8550,8231,11921,12524,12678,16743,10115,9654,12372,13629,1
6358,13674
GPR6_3_512,75,564,449,1,1,0,922,548,647,155,9,399
GPR75_3_513,2959,4434,4042,4884,1287,6798,2827,4219,1407,2946,9174,852
7
GPR77_3_514,638,299,220,735,128,961,660,339,252,271,798,356
GPR78_3_515,707,147,254,328,0,549,23,1548,691,48,623,4
GPR82_3_516,1034,1055,589,584,680,823,901,516,692,140,407,1117
GPR83_3_517,3130,3710,4808,5652,3693,1659,7980,4465,3866,8579,5173,844
7
GPR84_3_518,185,333,144,677,143,34,743,299,430,30,40,0
GPR87_3_519,6431,9578,6997,10984,13731,14858,16294,8867,7604,12927,136
01,8365
GPR88_3_520,176,98,79,580,433,11,2,987,2,21,6,340
GPR97_3_521,3288,2767,3565,4749,2796,2182,4027,3339,3003,5244,3822,503
0
GPR98_3_522,246,327,63,322,732,0,448,371,2,712,17,432
GPRC5A_3_523,3554,5023,3840,5011,5975,3920,6345,6906,5804,3063,5967,71
14
GPRC5B_3_524,637,129,386,728,601,133,2603,853,503,236,1148,20
GPRC5D_3_525,653,589,782,1333,144,2398,151,2068,819,1363,15,1006
GPRC6A_3_526,4309,5945,5725,7844,8764,7475,6490,5208,7346,7688,13781,8
816
GRM3_3_527,512,284,583,543,107,2585,1809,194,9,664,89,1398
GRM4_3_528,1225,619,2294,2566,1687,4463,2487,587,1562,488,1509,2546
GRM6_3_529,1308,1143,2095,1758,3077,1623,2207,1086,2435,2079,2880,2464
GRPR_3_530,1614,3029,3656,4387,2277,7395,3859,3371,5317,2868,5083,1469
HCAR1_3_531,433,1066,807,1123,1006,892,893,777,725,446,262,557
HCAR2_3_532,999,1186,644,1268,441,1043,387,930,897,591,208,1138

HCAR3_3_533,3502,4887,3865,4473,4350,3585,4767,6322,2769,4763,2811,519
7
HCRTR1_3_534,1363,1346,732,1207,2979,1664,1276,1506,2154,1536,1083,210
6
HRH3_3_535,548,203,486,2162,645,2229,289,1222,639,2014,1862,1014
HTR1A_3_536,7531,7596,13168,12493,11567,8358,16051,12729,11655,10499,1
1405,10724
HTR1B_3_537,938,731,921,1237,36,2468,653,746,811,1124,1353,461
HTR1D_3_538,1941,1681,2188,3742,3458,1444,2632,2529,2699,3856,521,3525
HTR1E_3_539,158,392,423,750,1177,279,383,1093,526,1534,157,602
HTR1F_3_540,5422,5250,4847,5766,3719,5316,4944,5958,7631,5662,4188,584
0
HTR2B_3_541,931,2579,2220,2458,2099,2130,4939,1841,2934,1577,5938,2771
HTR2C_3_542,1393,1096,1250,1052,2504,649,4085,3039,3435,4495,795,1903
HTR5A_3_543,1613,2070,1561,1724,1848,699,3259,3357,1817,1370,1649,2357
HTR6_3_544,526,1278,721,1083,1834,622,555,1472,488,645,1073,440
KISS1R_3_545,208,905,1346,1373,999,1109,51,912,1372,1682,2351,1457
LGR4_3_546,911,1637,2321,2071,1284,1674,2341,1996,1928,1732,2465,1244
LGR5_3_547,1058,1619,1607,1867,990,2571,1935,3531,848,2558,1676,146
LHCGR_3_548,1238,2003,2386,1201,2151,1995,2185,1517,3,2525,6211,1841
LPAR2_3_549,286,580,943,585,1,1,71,2,41,1032,1522,1170
LPAR3_3_550,312,414,439,958,1562,1020,481,6,420,822,825,950
LPAR4_3_551,6218,8036,8161,11938,9282,11853,11007,16066,13893,13485,99
92,12726
LPHN2_3_552,3005,3470,3244,3258,2788,5122,3883,2264,2217,5756,1170,526
5
LPHN3_3_553,538,543,212,284,281,850,1090,857,108,160,470,693
MAS1_3_554,7702,10934,12349,14002,7516,20004,13395,15407,8697,13258,12
214,11002
MAS1L_3_555,878,1165,1633,1052,207,1715,851,2265,999,176,2890,988
MC1R_3_556,312,435,924,526,1166,519,333,1480,709,261,177,171
MC2R_3_557,1673,3165,1716,3610,982,3163,4640,2862,2035,710,3507,3131
MC3R_3_558,2274,2626,4253,2539,3542,1042,2596,3442,1323,1891,2968,4211
MC4R_3_559,1242,1645,3359,1883,2412,1357,2603,2071,947,167,459,1706
MC5R_3_560,1537,848,929,1543,1823,84,292,1633,494,4386,95,407
MCHR1_3_561,7314,8187,6948,9272,9501,6426,6257,6120,6633,12992,2675,99
07
MLNR_3_562,20,346,59,329,598,4,36,212,967,50,92,443
MRGPRD_3_563,732,2235,1886,1696,2393,966,2689,1012,1079,488,710,1593
MRGPRE_3_564,3733,7503,6009,6334,6465,8472,5824,3057,7555,7333,8392,59
30
MRGPRG_3_565,387,885,468,575,1115,2229,217,82,111,1149,250,560
MRGPRX1_3_566,706,37,48,56,18,122,0,178,2,163,0,1
MRGPRX2_3_567,696,1661,1621,1418,2124,345,1037,1684,1114,403,86,406
MRGPRX3_3_568,3510,3300,5020,7793,3210,6601,6128,4984,3780,8075,5231,5
168
MRGPRX4_3_569,446,1372,696,2185,942,1790,345,1265,1216,649,62,1160
MTNR1A_3_570,231,567,234,642,543,1944,531,3306,106,720,225,321
MTNR1B_3_571,448,250,1139,1233,4,799,117,215,505,1797,230,1233
NMBR_3_572,2149,2600,3380,3663,2392,4404,4174,4054,2842,2818,3537,4938

NMUR1_3_573,442,479,931,503,1565,599,188,456,450,1566,1414,1180
NMUR2_3_574,1944,1022,266,832,1482,1422,184,1724,1098,2097,427,1196
NPBWR1_3_575,133,627,305,543,304,234,899,184,217,1480,233,392
NPBWR2_3_576,1071,1485,626,930,11,557,874,1969,1192,573,2947,557
NPFFR1_3_577,284,184,497,535,1803,49,835,917,36,185,2121,91
NPY1R_3_578,1088,1740,1663,1599,1308,1181,1000,3828,790,2221,1975,2533
NPY2R_3_579,2883,3327,2551,2520,2332,3773,2640,2942,2937,3676,3635,125
7
NPY5R_3_580,5866,6956,8279,6875,4494,4531,5582,6024,4684,3271,9596,515
3
NTSR1_3_581,319,524,712,393,2182,394,1320,287,642,828,194,472
NTSR2_3_582,698,1396,1146,1654,2298,20,22,69,1340,590,186,1777
OMG_3_583,3161,4261,4853,5085,4368,3719,2979,6185,5338,9033,6885,1169
OPN1LW_3_584,917,803,1118,2130,1100,1188,943,1754,1431,653,1643,1399
OPN1MW2_3_585,1561,2969,3821,3677,3919,2730,2837,3243,3593,3421,6511,4
440
OPN1MW_3_586,1561,2969,3821,3677,3919,2730,2837,3243,3593,3421,6511,44
40
OPN1SW_3_587,745,1155,543,2056,447,439,1064,759,548,183,67,486
OPN3_3_588,996,577,650,537,423,224,1801,1322,1090,448,144,1167
OPN5_3_589,1272,918,1349,2364,1459,2553,2344,2972,2830,5361,4092,2097
OPRD1_3_590,210,451,331,636,1330,9,239,310,233,634,79,0
OPRK1_3_591,1014,2036,1436,4022,813,1362,2542,1524,904,3147,4504,243
OXER1_3_592,168,234,55,115,38,10,0,0,0,0,2939,634
OXGR1_3_593,1570,970,1409,1851,2217,2236,1690,1842,1294,891,4378,1596
OXTR_3_594,1841,2592,1799,2544,2947,2887,1967,3214,4888,2502,2464,5393
P2RY11_3_595,496,459,274,902,244,3,488,3616,531,1218,89,33
P2RY13_3_596,2260,3397,3100,4363,1740,1307,3578,2559,1894,2834,746,441
0
P2RY1_3_597,3003,2570,2074,2390,2129,2537,3429,3623,2496,4882,1397,223
5
P2RY4_3_598,377,701,1040,1028,1143,14,1094,493,636,1821,3590,512
P2RY8_3_599,201,168,55,128,102,205,30,530,47,2442,27,0
PPYR1_3_600,1664,2118,2785,1573,1625,2471,1759,2945,758,2576,4051,748
PRLHR_3_601,405,1582,566,2116,1844,1148,820,1303,889,268,970,305
PROKR1_3_602,1889,979,1466,2469,1574,3101,1380,1117,2197,1795,227,2174
PROKR2_3_603,9077,10582,9673,10713,6658,11584,12894,12284,13488,10557,
15057,12045
PTGDR_3_604,780,336,677,727,34,1981,1193,288,906,97,2356,2200
PTGER1_3_605,1458,1352,2062,1555,2342,5310,1689,2334,669,1448,3679,122
2
PTGER2_3_606,1140,591,851,540,1653,1358,172,1124,177,1068,4712,727
PTGER4_3_607,1246,1416,812,1446,1944,763,3167,2179,2250,1236,1585,2522
PTGIR_3_608,298,100,80,571,862,35,0,772,197,68,11,28
PTH2R_3_609,3402,4004,2511,3821,3444,2772,4031,3588,3816,3149,2228,436
0
QRFPR_3_610,11114,10118,11254,12617,9567,15508,13347,9718,8435,11927,8
426,11365
RHO_3_611,225,293,233,462,10,2,344,1,484,572,65,357
RRH_3_612,1826,2700,1624,2415,2602,2283,2341,4277,3491,2796,556,3110

RXFP1_3_613,1055,1779,1570,2925,3090,879,4522,1962,1016,1749,1816,6901
RXFP3_3_614,78,262,964,660,56,361,441,126,27,34,630,564
RXFP4_3_615,1827,2090,1799,966,2315,1774,1834,2379,2502,1404,2773,3243
S1PR1_3_616,263,622,303,144,9,269,33,119,89,723,156,825
S1PR2_3_617,591,533,289,623,80,720,1207,220,349,308,2,735
S1PR3_3_618,925,1138,1295,1768,686,3920,820,1109,201,1442,2084,896
S1PR4_3_619,733,53,485,1532,360,208,1662,2507,460,699,760,293
SCTR_3_620,5556,8176,6919,8471,5585,15013,8347,10786,6503,7249,8512,89
55
SSTR1_3_621,1055,1449,976,2433,1380,2168,1227,4618,1657,2239,6589,2364
SSTR2_3_622,3346,4347,4200,5683,3420,4931,5483,4025,5882,5461,1862,511
1
SSTR3_3_623,1251,894,1361,1157,1104,1576,1185,1303,1335,1181,765,631
SSTR4_3_624,713,843,1096,2277,500,1028,589,1301,301,1918,2377,3478
SUCNR1_3_625,3561,3398,3710,4541,4418,5253,3426,3608,7006,1831,4187,56
92
TAAR1_3_626,1616,2401,1759,1880,3506,536,2048,2428,1300,1033,2791,2499
TAAR5_3_627,3150,4079,4457,4552,3717,6117,3471,5724,4719,5012,4284,762
3
TAAR6_3_628,107,94,103,210,37,156,105,12,102,235,372,405
TAAR8_3_629,962,896,1357,1772,2946,494,488,814,1107,2115,7173,2506
TAAR9_3_630,18161,23275,22302,22627,25604,36423,30214,24762,20703,2115
8,28494,27265
TACR2_3_631,726,1296,1605,2465,3023,757,2109,2432,860,1193,2746,2009
TACR3_3_632,133,299,128,1058,29,244,255,1900,69,129,32,232
TAPT1_3_633,2360,3381,2946,4043,1862,5540,3131,3373,2724,4344,8039,335
0
TAS1R2_3_634,293,577,145,1056,580,24,134,59,618,698,73,20
TAS1R3_3_635,279,524,1295,686,503,80,3047,1748,334,30,1952,999
TAS2R10_3_636,150,680,156,572,202,909,803,38,337,11,1133,739
TAS2R13_3_637,2602,3429,1742,3055,5149,2006,2529,2894,1869,3308,3169,2
019
TAS2R14_3_638,5688,5923,6925,7560,4897,6044,8705,13372,3710,6708,7955,
9390
TAS2R16_3_639,7149,7655,8493,8101,7787,11396,9657,9752,8146,3330,10886
,4874
TAS2R1_3_640,6310,7062,8066,8870,7974,16288,9625,10531,6572,6523,5270,
5037
TAS2R20_3_641,4448,3984,4774,5913,4137,5127,4438,8418,6370,6061,10860,
10377
TAS2R31_3_642,1795,1813,1172,1533,1068,2142,2581,659,928,633,2256,327
TAS2R38_3_643,3875,6415,5100,9257,3667,6707,5577,4616,7004,8501,10071,
7035
TAS2R3_3_644,4974,5253,5858,8724,6148,6404,5536,14529,8471,3625,9832,4
210
TAS2R40_3_645,2233,2741,2984,4074,5146,2726,8885,4082,4864,2202,3446,3
245
TAS2R41_3_646,8811,9431,9367,8726,14306,17636,10845,15505,12357,15558,
10329,13369
TAS2R46_3_647,3253,3991,5348,4102,2885,6695,7846,4568,4909,2751,5613,7

726
TAS2R4_3_648,6505,7390,10271,6923,5999,7444,8555,7625,10230,9450,6935,5978
TAS2R5_3_649,1236,1048,2268,1398,297,932,2068,972,1221,3125,1137,1259
TAS2R60_3_650,2158,2199,2386,2867,2903,1202,1448,3505,1794,1347,4949,4548
TAS2R7_3_651,1618,1692,1801,2362,1433,3724,1381,2653,1637,2253,3448,3323
TAS2R8_3_652,1965,2338,2240,1495,2575,2711,1074,1649,1468,2165,1219,567
TAS2R9_3_653,3569,3951,3604,5294,5727,7216,2423,1898,5818,2663,2097,4579
TM2D1_3_654,3104,3785,4163,4062,7513,3279,6349,5090,2994,6495,3035,5895
TMEM11_3_655,151,552,314,1264,23,451,200,61,2,1132,189,80
TRHR_3_656,128,367,1126,642,508,239,4,2092,653,28,6,529
UTS2R_3_657,592,889,1189,916,1720,235,3108,1074,37,139,52,950
VIPR1_3_658,2429,2346,2298,1591,2385,3511,1371,3471,2360,2414,4820,936
VIPR2_3_659,1982,1107,909,1081,413,3198,1049,2046,1053,709,3516,1017
VN1R1_3_660,3099,3254,6152,5857,3653,5964,9910,1801,5179,3420,7827,5053
VN1R2_3_661,1776,3303,3365,3189,1363,3467,3406,2787,315,1721,2803,3614
VN1R4_3_662,2171,1982,1902,1767,2157,2817,1881,1988,3389,1779,1941,1846
ADCYAP1R1_3_663,719,1228,471,933,279,3529,1095,612,1124,997,148,204
ADORA1_3_664,202,124,703,31,1854,871,26,146,59,173,422,200
ADORA3_3_665,357,424,781,159,139,299,464,266,14,1217,1248,434
AGTR1_3_666,12432,14912,15519,21248,14931,25326,22861,25062,14503,23846,21953,17808
AGTRAP_3_667,1601,1909,1536,3280,2150,1799,1733,2078,3007,1666,2436,2702
AVPR2_3_668,15,0,38,110,139,0,127,0,0,0,168,687
CALCR_3_669,2915,3711,3901,3309,4223,4447,4474,4013,4861,2861,5424,3710
CASR_3_670,842,1709,1404,1122,2515,1082,1440,2308,1383,1561,3796,3655
CCR2_3_671,11760,13176,11937,13928,12731,20824,13255,15269,10627,13026,10660,9340
CCR3_3_672,7166,8187,10063,12521,5740,13283,12922,11966,11197,12818,8013,13128
CCR5_3_673,2680,3782,4134,4778,3519,7398,3975,5153,6169,2090,5977,3778
CCR6_3_674,1589,1577,1370,2759,127,2352,1278,945,1620,1738,1085,1113
CCRL2_3_675,3431,3558,5328,4626,4574,5901,4545,3746,4141,5566,3638,5514
CD97_3_676,14993,20421,22012,26256,16126,18341,23837,25345,16026,28839,28451,23246
CHRM2_3_677,1256,1953,716,1543,3259,4315,2285,3250,1629,195,5002,3290
CMKLR1_3_678,322,617,947,1539,377,1283,1961,151,1064,576,1683,2145
CRHR1_3_679,3235,4459,4192,5087,2973,6616,7139,7722,3492,2288,11992,9199
CRHR2_3_680,1674,3660,4121,4738,3836,3429,4257,2920,5093,4270,4843,532

4
CX3CR1_3_681,1357,1781,2081,2936,3532,2825,2405,3234,2610,2433,1151,21
95
CXCR2_3_682,4041,5161,2792,4945,5251,7867,7435,3995,3293,8700,4871,227
8
CXCR3_3_683,472,627,225,1340,1411,2083,2693,776,1099,380,2344,2141
CXCR4_3_684,560,363,648,188,86,7,906,0,2511,795,132,600
CXCR5_3_685,2485,3450,4118,4454,2385,1400,2560,2407,4328,1845,8677,551
1
DARC_3_686,553,974,1315,410,223,1306,273,904,1615,556,1085,567
DRD2_3_687,525,590,307,632,15,2117,550,816,667,1244,299,415
DRD3_3_688,594,1465,1511,2189,229,2147,1974,2351,2265,1302,1035,3718
EDNRB_3_689,3183,4385,3522,4462,5087,4248,5011,5076,2767,4714,9888,258
5
EMR2_3_690,662,1436,983,1408,2657,997,881,1294,5,1895,1757,841
FPR1_3_691,1431,2371,1685,2237,2956,1465,2269,1523,2144,1599,1709,2988
FSHR_3_692,1318,2153,2617,1090,795,338,2293,1348,1077,1233,2937,1043
FZD6_3_693,2542,4239,4230,4240,3132,2009,5118,3003,2481,2999,9124,6145
GABBR1_3_694,3615,2600,2716,4260,2573,3912,2876,4132,2486,1510,3518,20
89
GHSR_3_695,517,555,733,1476,690,1649,1108,594,335,13,1477,216
GNRHR_3_696,1628,1671,1680,2666,1974,1218,3967,3251,1966,2068,2246,174
5
GPBAR1_3_697,995,525,466,1192,1285,14,3556,530,390,228,215,2736
GPER_3_698,1067,680,374,581,227,2281,540,1021,512,232,2095,3034
GPR107_3_699,8075,7252,8776,13061,8443,6037,9011,7731,8187,6805,14531,
7363
GPR110_3_700,3896,5181,5441,5466,8223,8799,5978,10521,5569,9473,5775,4
546
GPR113_3_701,719,857,1776,2631,1985,761,254,1624,751,761,3117,1569
GPR116_3_702,1299,1339,2706,1034,500,834,1081,3306,436,441,2134,2157
GPR126_3_703,1648,2603,2272,5298,971,1859,3389,3852,1685,3608,470,3766
GPR155_3_704,1044,2018,1942,2397,542,460,2232,585,1295,1101,390,824
GPR156_3_705,1199,1864,1014,2107,111,2941,1573,1017,1895,4280,4874,132
1
GPR162_3_706,63,112,529,308,0,1784,996,1867,2,9,20,199
GPR17_3_707,679,857,349,890,831,454,1142,507,952,1920,56,972
GPR18_3_708,4564,6148,6394,5475,3952,7782,5356,7130,6494,12428,6505,44
51
GPR1_3_709,873,244,284,267,1447,61,360,697,186,143,262,250
GPR34_3_710,171,142,510,62,1677,279,21,13,720,6,157,115
GPR35_3_711,1409,1529,1034,3281,2243,4259,561,3838,853,1200,1348,3443
GPR56_3_712,595,343,734,675,257,259,1354,252,829,94,1406,624
GPR63_3_713,5493,5768,6168,4969,5642,12871,7014,7573,4934,7820,9740,72
29
GPR64_3_714,1959,1480,1729,2460,3319,1095,2207,2427,2372,2686,1973,280
3
GPR68_3_715,682,1176,431,1435,2031,386,352,469,838,1150,330,2049
GPR85_3_716,2588,3075,2383,2299,3414,3331,449,914,1184,3917,4813,3588
GPRC5C_3_717,13,247,326,23,7,42,29,197,47,244,1502,0

GRM1_3_718,301,480,1854,1572,366,624,976,1298,117,1202,1551,1167
GRM2_3_719,1567,2621,1016,1523,2693,243,750,1950,1117,186,786,1067
GRM5_3_720,2651,3602,2848,5018,2173,4254,5622,3657,3062,3264,5962,4557
GRM7_3_721,2491,1402,1929,2923,2441,4246,2379,2050,3615,990,2681,1077
GRM8_3_722,2073,2068,1443,2225,1391,1034,3217,2909,1998,2109,3484,641
HRH4_3_723,3964,4991,4984,5774,2984,4133,5842,9827,2057,4856,14283,336
9
HTR2A_3_724,1664,2360,2588,3091,839,6236,1524,4119,1643,2897,408,4293
HTR4_3_725,859,1086,1314,1769,1016,3200,1800,2254,1590,176,431,645
HTR7_3_726,5456,6035,7242,11241,7292,12807,5548,13346,8898,8941,5350,1
3784
LGR6_3_727,424,795,308,1411,269,6133,19,396,823,413,14,27
LPAR1_3_728,79,3,18,98,0,0,0,0,0,0,0,0
LPAR5_3_729,758,632,746,1119,551,454,929,471,735,149,1584,387
LPAR6_3_730,17145,20582,21101,22679,16046,21188,34268,27888,20982,3337
0,26423,21800
LPHN1_3_731,923,755,458,967,1313,1157,2862,2027,229,542,231,154
LTB4R2_3_732,572,459,605,832,1056,6,421,546,502,119,620,199
LTB4R_3_733,509,170,735,153,89,3,243,327,655,40,130,179
LYPD1_3_734,133,292,332,730,694,1,238,2,759,318,1011,89
MCHR2_3_735,6072,6402,4912,10269,5477,6207,7545,13343,6512,4638,3572,1
1944
MRGPRF_3_736,457,520,428,447,2050,1090,1219,784,1023,252,1210,1392
NPFFR2_3_737,3303,4659,3145,3250,2222,3535,4249,1382,2924,6278,4228,30
30
NPSR1_3_738,1282,2027,1501,1959,2725,1349,2252,2561,814,2555,2937,3171
O3FAR1_3_739,3289,3686,3155,4212,2729,5865,5419,9121,2289,3574,7214,52
19
OPN4_3_740,862,1071,989,704,2015,1791,3913,3257,1916,218,654,2327
OPRL1_3_741,1841,1697,1274,2094,1348,2436,1022,1302,571,3695,1841,1958
OPRM1_3_742,161,640,98,252,212,43,220,2,1227,692,257,114
P2RY10_3_743,2143,1788,2119,1671,851,2662,2979,1472,5688,4940,1157,178
7
P2RY12_3_744,10291,12556,12808,13148,10396,9302,16907,14539,7786,11780
,25092,18330
P2RY14_3_745,6863,5904,8275,8854,6625,9425,10155,9874,4923,8374,7972,7
615
P2RY2_3_746,136,695,1167,649,142,828,1372,77,740,90,424,80
P2RY6_3_747,1014,940,1370,1262,1200,83,951,3179,2004,1596,4262,1654
PTAFR_3_748,658,1423,1341,882,1800,2768,1225,1003,737,960,730,754
PTGER3_3_749,9239,10741,12865,16159,9359,14140,18584,19304,8810,14908,
13328,18414
PTGFR_3_750,4162,5338,4910,6333,5560,14307,3660,11521,4345,10521,5698,
5255
PTH1R_3_751,1181,2284,2817,2946,1279,1093,2786,4983,5678,2725,6408,327
8
RGR_3_752,846,1097,1881,1780,1500,198,1530,1637,606,1014,2148,437
RXFP2_3_753,5093,7208,8507,7456,4495,2048,7672,5922,6215,8967,5304,318
5
S1PR5_3_754,745,401,182,526,272,871,543,40,673,306,1726,521

SIGMAR1_3_755,651,300,450,522,622,30,252,360,1172,347,77,941
SSTR5_3_756,1492,2617,1414,3559,1780,1761,3401,4242,1692,2026,455,2676
TAAR2_3_757,3088,2710,3583,5809,5214,2801,5517,3046,4881,5602,6552,506
6
TACR1_3_758,1572,1729,1419,1536,2716,1473,2387,2761,1274,600,2333,3395
TAS1R1_3_759,1133,2568,3122,3433,3387,4294,2047,1615,2102,651,880,2610
TBXA2R_3_760,283,690,289,1723,821,801,2321,57,338,237,77,71
TPRA1_3_761,393,184,254,359,43,9,8,156,1173,139,71,7
TSHR_3_762,5726,4923,3161,6532,5436,7643,2439,5139,7091,7654,4996,2433
XCR1_3_763,1310,1963,2232,1858,871,2737,3335,3679,1378,4547,7426,2590
XPR1_3_764,6046,6327,7487,4281,6913,8194,7481,5545,4870,18297,7154,298
0
ADORA2A_3_765,2370,2250,2447,3294,2829,1591,1964,3767,1305,6046,3721,2
319
ADORA2B_3_766,1493,1390,2006,1674,3147,3197,3223,2532,1365,2112,4329,9
18
ADRA1B_3_767,1408,1557,1421,1796,669,694,216,1286,948,1456,408,2175
ADRA1D_3_768,377,172,495,1500,95,262,1361,181,258,140,920,303
ADRA2A_3_769,614,936,610,548,1702,195,641,119,1767,123,760,904
ADRA2B_3_770,2704,4007,3804,4690,4358,4556,5220,3365,5838,3906,1975,54
08
ADRA2C_3_771,231,795,1277,1137,1460,628,1379,1811,1301,210,147,755
ADRB1_3_772,491,922,358,1208,1101,1033,776,432,583,1152,411,1179
ADRB2_3_773,2391,3157,1964,3939,2333,3527,3609,4518,3289,6114,9023,180
5
ADRB3_3_774,640,1500,1715,2008,159,3361,239,732,661,3155,1576,566
AGTR2_3_775,5472,6102,4827,5432,5097,4512,6497,6474,7028,8117,4981,388
7
APLNR_3_776,239,352,728,105,21,122,437,22,961,205,1362,8
AVPR1A_3_777,2594,3570,3699,4429,6106,4202,3138,1892,5370,3861,2480,31
81
AVPR1B_3_778,3619,3435,2251,2478,4281,3225,3119,773,5127,4839,8638,200
3
BAI1_3_779,157,351,26,223,25,14,26,11,440,134,10,207
BAI2_3_780,787,416,755,1452,874,585,1342,1676,609,738,1886,1934
BAI3_3_781,6393,4547,6392,8781,6436,6377,7314,8456,9399,4896,3979,4672
BDKRB1_3_782,1083,779,797,527,466,888,1417,2919,1610,823,2400,1028
BDKRB2_3_783,1744,909,2152,2221,1776,417,1813,2829,855,2520,3785,2062
BRS3_3_784,7009,8669,8980,11008,5772,8320,9955,10148,4902,9009,8475,89
77
C3AR1_3_785,1156,2087,2697,2451,1752,535,1570,1710,3227,1096,2016,2107
C5AR1_3_786,275,526,1181,297,170,4278,188,411,180,882,6145,63
CALCRL_3_787,1755,1206,989,1627,895,238,1331,827,2822,197,421,2584
CCKAR_3_788,3600,3879,4044,5164,3879,5513,4420,5964,6412,6251,8232,421
4
CCKBR_3_789,1355,1271,1146,399,1657,65,3134,269,2156,1174,1928,1113
CCR1_3_790,2105,2365,3141,3716,3575,2061,5457,3953,3020,2180,4079,5377
CCR7_3_791,1373,1546,1714,3011,875,1255,2515,6840,1234,4743,1353,3817
CCR8_3_792,617,793,1072,1307,65,2073,760,652,373,1216,468,1483
CELSR1_3_793,696,493,744,580,559,1487,835,897,61,1385,137,933

CELSR2_3_794,3274,3484,2263,4223,2890,2919,3278,9044,4394,1801,4106,37
62
CELSR3_3_795,239,716,676,1189,149,1379,2977,967,90,84,884,595
CHRM1_3_796,693,922,600,1807,718,779,774,481,743,3233,3687,634
CHRM3_3_797,596,343,850,2008,59,342,699,756,47,1723,813,537
CHRM4_3_798,282,253,391,460,486,544,330,1435,463,25,41,482
CHRM5_3_799,3934,4113,4448,4985,3732,3510,7196,7319,8424,4333,5656,465
7
CXCR1_3_800,1494,2774,1382,2009,1788,2145,2240,2010,733,3892,743,322
CXCR6_3_801,1864,1761,1768,2417,1767,2560,3261,3708,2354,2400,2208,578
8
CXCR7_3_802,42,135,24,319,26,0,657,1188,608,0,1005,126
CYSLTR1_3_803,2585,2347,3090,3854,2718,4871,5427,3547,2398,2540,3420,5
645
CYSLTR2_3_804,7854,8102,6167,9379,6885,9335,4475,5938,11437,8995,6608,
5444
DRD1_3_805,1351,1735,2261,1762,5260,4043,2879,2317,856,2285,2244,2428
DRD4_3_806,119,19,877,379,0,12,224,270,2427,12,62,249
DRD5_3_807,464,484,784,1557,279,63,164,340,207,772,541,421
ELTD1_3_808,13068,15284,15097,14734,17112,20436,14455,17342,17323,1818
6,21362,18098
EMR1_3_809,935,898,718,960,2372,2308,1960,1047,877,901,3034,840
EMR3_3_810,1814,2059,2605,4302,1120,3114,2133,1920,505,4596,4477,3510
F2R_3_811,3243,4263,3939,5802,3402,2779,1874,7462,6673,5184,2794,4788
F2RL1_3_812,1466,1239,1881,2114,1823,230,3061,3986,1151,2229,630,2450
F2RL2_3_813,1551,2153,2687,1721,442,2410,2156,1445,3095,2274,2210,3154
F2RL3_3_814,905,1253,485,2113,1895,2186,844,2746,1924,2991,3772,911
FFAR1_3_815,233,171,134,231,1286,36,867,10,295,475,249,18
FFAR2_3_816,104,539,1009,465,23,352,798,753,162,462,541,370
FFAR3_3_817,1309,2274,3202,2687,2475,4983,1367,1843,5351,931,3337,1673
FZD10_3_818,150,991,426,444,433,1849,1261,10,162,702,5118,145
FZD1_3_819,1119,1944,1781,2489,2403,5113,6694,872,2333,1618,5370,2613
FZD2_3_820,6549,6341,6890,6988,4939,7255,9951,7718,6691,10024,7723,552
4
FZD4_3_821,895,988,662,608,879,1519,809,9,1022,219,68,687
FZD5_3_822,1511,3217,2486,3363,3742,6290,7670,3325,2499,2632,6857,2706
FZD7_3_823,4797,4668,5224,6978,2800,3996,5083,5538,6546,6292,8641,3409
FZD8_3_824,1624,2025,2504,3251,433,5448,2770,1295,1382,3284,3657,2854
FZD9_3_825,1359,597,999,1372,864,566,137,555,1940,157,1485,1672
GABBR2_3_826,1905,2192,1105,2275,1922,2357,3436,1239,3141,3619,5691,10
88
GALR1_3_827,9046,9005,9978,12667,10835,9073,12820,14733,7510,11171,891
9,13141
GALR2_3_828,1410,1916,1190,1911,1829,1452,2064,1160,1305,10287,2375,22
90
GALR3_3_829,382,86,185,396,52,308,265,452,242,1,53,145
GCGR_3_830,350,887,1042,628,66,549,106,273,358,277,193,494
GHRHR_3_831,1558,4251,2071,2711,2503,2502,2756,3037,2646,846,3983,1974
GIPR_3_832,125,431,170,396,183,76,1579,85,14,9,148,800
GLP1R_3_833,690,810,592,722,2794,598,1543,911,1198,861,3564,324

GLP2R_3_834,699,707,2088,1853,1155,1991,2168,1250,841,1321,3759,2835
GPR101_3_835,2663,3223,2505,2750,6036,9083,736,5696,4501,7431,2143,310
5
GPR108_3_836,704,1240,1471,1394,705,501,1203,749,1418,1883,2405,1602
GPR111_3_837,7673,8653,9880,10222,6175,8867,8593,9158,8887,9293,15538,
7350
GPR112_3_838,4544,4253,3941,5223,4932,10249,3472,5601,1609,2402,1099,2
816
GPR114_3_839,830,468,1007,1031,236,297,431,2994,493,1035,722,1843
GPR115_3_840,6118,5887,5361,6169,7615,7467,10214,4023,4624,5975,10626,
7505
GPR119_3_841,3296,4695,6389,5472,4141,5468,6655,6536,3708,2965,3303,76
69
GPR123_3_842,1313,1305,937,2400,2482,2360,1360,828,1183,1273,1138,3630
GPR124_3_843,1486,1507,1043,1744,151,2891,897,1302,741,2080,2391,1595
GPR125_3_844,7608,8529,6022,11308,10068,17836,10832,8067,7818,18209,94
76,8109
GPR128_3_845,5148,4285,6713,4852,3910,5670,9153,8604,5572,6394,9499,53
03
GPR12_3_846,800,746,659,1012,268,5,131,762,9,7,704,1784
GPR132_3_847,560,1128,172,825,496,276,1571,719,231,1710,269,349
GPR133_3_848,603,1434,404,1907,1295,1799,2416,2278,623,1521,2543,2191
GPR135_3_849,696,346,1540,1473,953,1023,896,707,1060,1507,1798,1171
GPR139_3_850,519,691,1774,1643,629,1229,613,441,975,305,705,659
GPR141_3_851,4785,7008,5530,9805,6758,8270,7887,5534,6460,4621,6759,55
90
GPR142_3_852,2768,3779,1994,4101,3339,4141,2657,2179,5174,4402,3980,39
00
GPR143_3_853,2187,1381,1766,1102,3294,1313,2583,1598,1184,1158,3069,10
60
GPR144_3_854,787,1439,751,1208,859,608,3029,2547,561,80,516,1362
GPR146_3_855,925,951,1350,849,385,3406,764,386,1183,995,874,1449
GPR148_3_856,2180,4928,3397,3823,3231,5717,3802,4086,2738,1251,5031,49
04
GPR149_3_857,6796,7846,8520,10441,4685,13676,14423,11241,9333,8024,929
1,11618
GPR150_3_858,1277,1121,738,2061,870,2761,611,119,799,2560,303,501
GPR151_3_859,1407,1506,1705,3645,1062,3854,3408,1433,812,2688,1251,510
2
GPR152_3_860,2204,3059,3367,2683,1105,1858,3291,3904,1317,1784,2991,19
70
GPR153_3_861,135,247,169,333,299,370,232,195,254,814,22,248
GPR157_3_862,4997,5156,4524,4601,5370,7321,4205,5606,6322,4611,8520,70
04
GPR158_3_863,1830,2780,3895,3027,1495,3441,5994,6319,2788,1448,2710,31
58
GPR15_3_864,14036,13959,16763,20563,11878,26480,19114,19279,18799,2281
9,24101,17408
GPR160_3_865,4477,3634,5488,4544,2776,5071,4743,3300,2287,2131,5754,38
90

GPR161_3_866,402,962,1530,1107,99,673,451,1470,831,917,74,263
GPR171_3_867,2652,5009,3029,5817,5288,6908,4062,6046,4749,4437,9854,28
97
GPR173_3_868,491,770,919,1795,174,2113,375,88,2388,425,469,849
GPR174_3_869,1225,1949,1856,1496,1211,3274,2384,1724,2324,1882,1866,36
96
GPR176_3_870,854,703,1508,2992,2263,2696,1117,1199,1147,1439,433,601
GPR179_3_871,487,1016,648,900,728,225,421,2655,1054,373,344,1484
GPR182_3_872,361,462,739,636,366,845,845,1393,1250,178,117,745
GPR183_3_873,1596,2536,2044,3231,2867,2992,3747,2319,3468,5670,6934,21
89
GPR19_3_874,6194,8737,10722,8002,6559,9129,8999,12656,6774,7886,15353,
6065
GPR20_3_875,2930,4684,4147,4730,1451,4073,4680,5547,2807,4212,1259,414
6
GPR21_3_876,1375,1595,1186,2200,1535,2803,1547,682,1146,1013,293,1102
GPR22_3_877,4775,4352,8047,7880,4492,4832,6998,8826,5922,4293,5662,666
6
GPR25_3_878,505,378,1165,629,152,186,1005,472,746,72,232,2
GPR27_3_879,385,543,239,1626,74,18,598,1,411,996,585,2
GPR31_3_880,596,943,780,884,1324,345,158,2419,423,2605,1278,236
GPR32_3_881,1445,1827,2102,2068,2744,681,1644,1053,2105,315,2944,1816
GPR37_3_882,9429,10462,7479,13591,8471,12743,15907,14940,9690,12667,10
589,10463
GPR37L1_3_883,499,520,619,399,1295,1157,1363,2218,496,1271,3629,1394
GPR39_3_884,299,390,196,75,25,93,126,286,20,51,1281,1222
GPR3_3_885,1126,1392,1395,1240,1483,742,1739,2335,2100,1957,795,3867
GPR45_3_886,1020,1441,2125,2142,1083,500,1564,937,569,3781,1010,2646
GPR4_3_887,1873,1712,2011,1733,5259,1344,1813,4037,2254,2871,233,5366
GPR50_3_888,1971,1050,2638,2879,2826,793,2493,2845,2584,2128,1816,1933
GPR52_3_889,6614,6082,5388,6707,4409,7221,5119,10504,8210,6583,5787,37
17
GPR55_3_890,1875,2796,2970,1744,1765,1366,697,3190,2077,992,3795,1773
GPR61_3_891,1235,1447,2622,1940,773,1585,3144,1570,897,2622,2552,893
GPR62_3_892,1787,1004,1148,1447,592,154,823,379,875,203,274,2962
GPR65_3_893,636,404,1264,2145,103,2329,1059,745,382,473,89,2013
GPR6_3_894,2122,2493,1638,1976,4149,2614,1300,4390,2766,4110,1593,1848
GPR75_3_895,1608,1906,2406,3580,1313,2225,1434,1095,2045,3301,994,2796
GPR77_3_896,1855,2094,2374,4298,5400,5063,1718,4623,1872,1187,692,853
GPR78_3_897,2632,3902,3170,4119,3661,3220,2382,5868,2954,3147,3293,396
9
GPR82_3_898,3305,3324,3360,3303,2167,1477,1914,4922,2879,3330,2259,384
5
GPR83_3_899,812,1291,1957,1281,1148,2858,2114,2757,1673,3899,99,729
GPR84_3_900,585,464,443,788,1583,13,360,1484,787,627,1349,3212
GPR87_3_901,2264,1812,2970,4978,4043,1602,4157,4471,5849,1832,576,3847
GPR88_3_902,577,58,141,1219,474,259,22,0,1313,1,352,5
GPR97_3_903,2791,2131,1795,2808,4387,1994,1685,1834,5421,3379,3428,376
3
GPR98_3_904,1741,1695,1568,4589,3243,2829,738,1608,3483,4261,587,3738

GPRC5A_3_905,204,476,308,177,1223,107,93,2922,738,403,601,208
GPRC5B_3_906,1275,974,444,805,1947,2418,1722,617,491,791,4713,1486
GPRC5D_3_907,2431,3329,2231,2527,5218,2719,3712,4244,3052,783,5268,577
6
GPRC6A_3_908,1695,1483,2289,2768,801,764,3775,1877,2559,2468,4134,2545
GRM3_3_909,3578,2726,3369,5149,8345,4623,6046,5436,5187,3951,1883,5142
GRM4_3_910,1630,1938,1262,1763,1837,139,1458,5003,2058,741,503,1745
GRM6_3_911,542,534,963,1096,198,3976,798,996,480,1410,1707,1562
GRPR_3_912,564,1305,1639,1272,1191,2956,1224,265,1407,252,3377,2310
HCAR1_3_913,4669,4316,4370,4671,5985,1770,4894,1513,7271,7208,7347,524
9
HCAR2_3_914,746,1463,1504,2164,721,2042,2539,1166,1840,1854,88,1493
HCAR3_3_915,746,1463,1504,2164,721,2042,2539,1166,1840,1854,88,1493
HCRTR1_3_916,3720,3285,4171,5237,4897,8322,4203,7601,5877,2659,3835,75
55
HRH3_3_917,143,658,42,228,612,0,36,813,17,1163,372,189
HTR1A_3_918,267,240,343,186,957,1318,734,246,56,3,11,76
HTR1B_3_919,783,1679,1535,1402,1325,484,254,998,1209,2177,2707,2776
HTR1D_3_920,851,1265,2131,2288,899,2008,569,2696,1987,1060,745,1422
HTR1E_3_921,4894,4574,4628,5314,3660,6289,4165,2541,4459,7526,6697,551
2
HTR1F_3_922,1428,2460,3110,2208,1276,2313,1485,2367,573,1212,5100,1633
HTR2B_3_923,2588,4263,3885,4694,3566,6119,3327,1722,4301,4561,7076,444
3
HTR2C_3_924,6082,10386,10232,10095,11276,14131,8522,4511,7394,7068,139
46,11350
HTR5A_3_925,6953,8406,9392,10196,4973,13055,11101,7751,10448,10119,197
18,9485
HTR6_3_926,209,177,435,439,122,35,550,105,4446,49,118,980
KISS1R_3_927,2407,4140,2368,2926,1891,1718,4140,4145,4691,3787,3639,23
67
LGR4_3_928,1336,2289,1975,2111,1461,469,2768,4397,239,5694,717,3337
LGR5_3_929,516,711,1251,1483,1323,4078,805,4207,568,83,258,1524
LHCGR_3_930,946,1315,512,737,415,1038,494,1225,97,847,49,685
LPAR2_3_931,1612,1617,2040,1034,1120,2384,2082,6583,2740,3079,4308,466
4
LPAR3_3_932,1229,926,1088,1119,1759,199,2793,1397,984,695,1193,1801
LPAR4_3_933,1414,1944,2947,2858,2692,2467,4707,5176,1517,1440,1777,239
2
LPHN2_3_934,1252,503,724,747,1168,937,1032,1300,1179,565,86,865
LPHN3_3_935,2354,2760,4569,6001,2921,3985,3964,3550,2945,8217,4370,687
5
MAS1_3_936,1615,1671,2022,1437,533,1471,459,854,598,1539,744,625
MAS1L_3_937,5666,6717,5954,9866,7547,7651,6924,7974,4396,9489,7559,120
66
MC1R_3_938,983,1189,1712,1056,554,1299,2234,246,2028,260,366,2918
MC2R_3_939,664,389,1165,1115,467,456,1825,601,1196,556,3194,689
MC3R_3_940,2063,2332,1387,3342,2274,1600,1752,2954,2576,1718,717,4149
MC4R_3_941,5072,7997,7412,8311,9600,6181,5359,5186,7338,4682,7321,6940
MC5R_3_942,720,927,603,1931,1437,793,1190,1251,356,24,47,637

MCHR1_3_943,1892,2391,2035,2585,3036,3847,1907,2704,2756,2252,2395,342
1
MLNR_3_944,690,233,674,1211,1597,216,12,889,3,798,117,273
MRGPRD_3_945,993,1517,1323,2592,1657,373,2438,641,704,761,587,2691
MRGPRE_3_946,497,920,831,403,87,261,986,1656,917,1170,2134,1101
MRGPRG_3_947,1563,806,879,2152,1604,630,1948,177,447,1977,1236,653
MRGPRX1_3_948,8451,10045,10115,12268,12558,12620,14983,12651,9963,1036
5,16539,18187
MRGPRX2_3_949,974,868,529,749,921,1133,636,1045,1069,689,1019,1082
MRGPRX3_3_950,1509,2528,2149,1410,1503,2267,2396,1903,2506,2999,1438,9
36
MRGPRX4_3_951,1464,1662,1569,2396,607,2264,806,1251,2979,2431,376,3927
MTNR1A_3_952,412,295,417,356,25,446,560,22,142,2896,18,264
MTNR1B_3_953,503,2865,1789,2182,2159,1807,1526,3525,1162,1154,1160,289
6
NMBR_3_954,7382,8379,7444,9773,10655,13144,7947,10546,8211,7758,9084,4
769
NMUR1_3_955,721,738,960,695,457,799,1186,1240,2223,1743,1048,284
NMUR2_3_956,5688,6910,8158,9630,6154,7343,12212,10386,8607,6265,10682,
11119
NPBWR1_3_957,220,627,546,516,315,278,1317,378,50,56,198,555
NPBWR2_3_958,2649,2846,2766,4410,3213,4915,1155,2522,2320,5359,4941,39
11
NPFFR1_3_959,1484,1462,1546,3534,2623,2287,1772,1790,2205,2471,3228,16
05
NPY1R_3_960,761,1516,1146,3036,261,523,1618,956,807,1155,1040,1901
NPY2R_3_961,1645,1745,1286,1306,2441,3776,1758,4164,866,3055,2199,618
NPY5R_3_962,316,274,915,121,327,4,1171,711,364,948,160,3
NTSR1_3_963,277,385,263,264,91,1,807,39,41,149,28,33
NTSR2_3_964,429,810,1282,2990,77,10,1936,1073,747,149,490,778
OMG_3_965,2606,2450,2400,2024,2278,2212,2462,2057,1528,833,2644,4309
OPN1LW_3_966,704,1559,1102,1654,575,1439,670,2184,433,716,1058,244
OPN1MW2_3_967,704,1559,1102,1654,575,1439,670,2184,433,716,1058,244
OPN1MW_3_968,704,1559,1102,1654,575,1439,670,2184,433,716,1058,244
OPN1SW_3_969,1478,1567,1568,828,877,952,76,260,2772,341,109,907
OPN3_3_970,317,363,882,387,307,814,210,1448,505,1954,1459,1883
OPN5_3_971,1975,2479,3924,2386,4597,6518,2708,2033,3589,2331,2351,5444
OPRD1_3_972,790,795,1161,1474,1772,1788,2017,1542,644,958,982,2434
OPRK1_3_973,5865,8067,7504,5633,4249,4847,4273,9975,13255,10828,6470,5
004
OXER1_3_974,259,827,240,709,240,405,222,139,213,438,101,251
OXGR1_3_975,1036,2004,2087,3684,1710,1511,4200,1237,2852,1169,2626,361
0
OXTR_3_976,715,1546,1706,1553,387,1835,2128,2501,621,2394,4066,2447
P2RY11_3_977,2079,2418,2395,3849,665,1287,4057,1536,3979,2106,2544,318
0
P2RY13_3_978,1177,1666,1120,2589,1390,2525,2604,2352,1730,2338,794,433
P2RY1_3_979,327,93,190,17,0,0,94,1,0,11,10,586
P2RY4_3_980,2565,3091,3910,3869,906,7127,2218,2142,1343,5189,4333,5626
P2RY8_3_981,2324,2388,2724,3134,989,1837,4334,3161,2375,4779,4974,1821

PPYR1_3_982,1045,1170,1157,1852,1154,2435,705,1175,303,1265,1674,1201
PRLHR_3_983,1716,1499,1420,1725,100,1108,1203,38,506,1769,307,2141
PROKR1_3_984,229,1644,343,299,1319,547,324,13,768,683,286,147
PROKR2_3_985,1408,1722,1814,1078,712,1658,3351,871,600,327,1049,298
PTGDR_3_986,574,1555,1775,1034,1812,1721,3155,2125,2448,1393,1584,3763
PTGER1_3_987,1490,1393,2071,1297,2747,5311,1691,2740,751,1452,3646,242
1
PTGER2_3_988,1814,3175,3600,3682,3590,4960,3015,3137,3692,4366,5309,26
22
PTGER4_3_989,1274,1825,1937,1848,380,1709,1980,5395,1169,1257,3461,145
4
PTGIR_3_990,657,596,1750,266,208,36,184,297,575,286,188,581
PTH2R_3_991,577,962,512,372,344,2319,1848,1779,780,1178,64,611
QRFPR_3_992,2618,3749,3604,4598,920,6038,2604,2783,3085,3505,6036,3413
RHO_3_993,1187,1423,1524,681,157,1072,93,254,1793,252,98,881
RRH_3_994,2490,2383,2763,3080,3005,2365,1546,5787,2667,3940,6114,1502
RXFP1_3_995,2174,2265,2679,3198,2171,2062,2856,2724,2832,4519,2484,183
3
RXFP3_3_996,1300,722,1374,791,583,4354,2649,216,1937,1762,1905,5421
RXFP4_3_997,888,1426,1284,1185,595,887,1346,2101,674,632,826,331
S1PR1_3_998,968,936,609,2382,918,2660,580,1167,917,1321,1817,523
S1PR2_3_999,316,796,1649,1187,870,964,523,1728,1398,3314,2967,910
S1PR3_3_1000,3435,5768,5641,6607,7083,6258,6859,6244,7505,7870,9718,95
00
S1PR4_3_1001,359,632,1371,1270,8,1969,211,4195,8,252,217,0
SCTR_3_1002,1722,1262,896,1360,1277,1215,809,2436,1768,1465,1209,2352
SSTR1_3_1003,1750,2970,3913,4274,2106,3854,3055,4364,2025,3057,3588,42
16
SSTR2_3_1004,1552,2764,1695,4719,1698,2094,3344,3823,1617,1214,874,190
5
SSTR3_3_1005,565,369,451,1030,576,466,1204,148,3201,114,50,1669
SSTR4_3_1006,529,197,336,280,399,986,39,392,329,666,164,443
SUCNR1_3_1007,3236,4107,3514,5051,2819,3193,4913,2954,4736,8130,11103,
4293
TAAR1_3_1008,6689,6542,7399,7521,7875,9331,12944,7223,11688,7225,15506
,10906
TAAR5_3_1009,839,2395,2521,2820,1211,1659,3536,2914,2179,1065,4909,152
0
TAAR6_3_1010,2553,2658,4197,3647,3441,1759,6758,3580,4571,2475,3582,48
66
TAAR8_3_1011,3127,3039,4634,4488,3898,1747,6724,3634,4860,1945,3879,47
98
TAAR9_3_1012,8675,8610,8400,13478,10462,11098,13292,16340,10225,17709,
9262,10013
TACR2_3_1013,3930,4782,3853,5381,4574,3152,4006,6458,4955,2817,3050,47
07
TACR3_3_1014,862,510,2419,1350,1645,786,2830,434,1221,4116,829,815
TAPT1_3_1015,3890,4282,7410,3459,4024,6712,5827,4700,6089,9315,2204,48
56
TAS1R2_3_1016,985,768,752,614,1178,197,714,737,844,68,1679,355

TAS1R3_3_1017,724,1318,746,1475,1262,831,567,5073,1525,3036,1546,1618
TAS2R10_3_1018,6442,6401,8125,9757,10163,11569,12507,4709,7774,8422,71
68,4793
TAS2R13_3_1019,7616,9707,12225,12926,10698,14642,14325,11812,6427,1137
9,10861,12022
TAS2R14_3_1020,2708,2832,3756,3472,2601,4299,2918,3661,3796,4924,3443,
3524
TAS2R16_3_1021,2097,2778,3626,2761,2445,3439,3838,2637,3726,2951,6626,
3370
TAS2R1_3_1022,7141,8306,7587,12538,7480,9775,8135,7681,7170,8939,12043
,11197
TAS2R20_3_1023,12388,12930,15414,14270,18386,18639,20605,22505,15274,1
8228,15609,16349
TAS2R31_3_1024,5026,6786,4612,6169,3354,7132,5646,7441,4260,6146,5206,
6781
TAS2R38_3_1025,904,1709,938,660,2264,1361,125,1491,1996,879,38,637
TAS2R3_3_1026,2130,2677,2907,3822,4082,2449,6587,5587,3907,3858,3865,4
305
TAS2R40_3_1027,528,525,290,595,1020,1310,77,275,862,100,313,282
TAS2R41_3_1028,1338,1456,1497,1318,569,1453,1641,1727,1413,3397,265,29
54
TAS2R46_3_1029,5026,6786,4612,6169,3354,7132,5646,7441,4260,6146,5206,
6781
TAS2R4_3_1030,6725,6012,10025,10545,8487,7908,12461,5812,7634,10909,17
973,9420
TAS2R5_3_1031,3782,3831,2585,3377,2507,3694,2975,3316,4360,5999,5136,4
382
TAS2R60_3_1032,2284,2008,2950,3144,2198,2248,3983,3844,1656,2593,2560,
1234
TAS2R7_3_1033,1721,2595,2250,3797,2325,5721,4086,2539,3294,1702,3336,3
237
TAS2R8_3_1034,7536,7967,7500,12254,9869,8123,12045,10708,6997,11093,15
204,12419
TAS2R9_3_1035,21974,27237,29963,35106,27858,36839,33658,35482,24075,36
153,31784,36671
TM2D1_3_1036,2920,3431,3638,4080,6355,2572,5679,3316,3883,4974,3408,43
67
TMEM11_3_1037,273,470,145,533,1574,1090,931,468,13,1660,165,157
TRHR_3_1038,2906,4080,2094,2752,5217,4589,1813,7838,5066,3756,1405,475
7
UTS2R_3_1039,324,367,90,398,576,28,106,1144,42,179,102,825
VIPR1_3_1040,1305,417,1168,655,657,1057,593,230,797,2197,900,1389
VIPR2_3_1041,2421,3596,3014,3767,4723,3538,3043,3106,8784,1665,2835,56
19
VN1R1_3_1042,10341,11272,13404,15338,12566,20515,9042,24654,13096,1118
3,15502,13315
VN1R2_3_1043,1927,3175,3424,4402,4314,3818,7981,4266,1901,6852,4360,56
87
VN1R4_3_1044,4441,4790,4683,5273,3700,9842,5669,4656,2945,3676,7405,72
94

ADCYAP1R1_3_1045,1039,2663,1845,2567,1180,3384,2566,3647,1381,3099,124
0,3276
ADORA1_3_1046,2051,2528,2709,3351,1220,1885,2541,4501,2593,912,906,157
8
ADORA3_3_1047,1327,1587,1640,1829,2561,2529,1084,1344,3955,657,2755,19
90
AGTR1_3_1048,10595,14622,16791,13782,16311,14160,17997,12504,15399,134
05,9952,16244
AGTRAP_3_1049,168,251,95,672,328,821,2,35,493,1426,26,44
AVPR2_3_1050,521,600,335,1559,1154,1007,287,1566,918,768,563,1222
CALCR_3_1051,871,1570,1726,2571,1390,1448,659,3374,1994,5275,3152,836
CASR_3_1052,3108,4181,3798,4128,2418,4043,2174,4270,2464,4832,6246,721
7
CCR2_3_1053,2826,3364,3209,5152,6774,2898,6199,5988,2043,3764,3387,251
7
CCR3_3_1054,1878,2030,1648,1473,2836,2657,4424,1825,772,4940,2033,2085
CCR5_3_1055,1829,2172,3656,2472,1675,4015,5440,948,1507,1012,2970,1934
CCR6_3_1056,501,1045,1737,632,448,1557,1766,1422,586,129,604,521
CCRL2_3_1057,3740,3172,2851,4302,4904,1753,6924,5006,5480,4144,6367,52
24
CD97_3_1058,1932,2041,1541,1402,2640,2624,1007,809,776,2603,891,2380
CHRM2_3_1059,862,333,1113,875,773,156,228,1986,141,1372,991,723
CMKLR1_3_1060,2153,3190,2802,3662,2388,7416,6052,1730,3924,3394,2150,3
262
CRHR1_3_1061,2101,2115,3712,2730,4030,1912,3485,1413,4131,1635,2473,59
00
CRHR2_3_1062,1887,932,1669,1303,1173,4329,35,1077,813,891,146,504
CX3CR1_3_1063,2826,4302,4576,4726,4214,4028,5846,4423,5659,5129,3399,5
737
CXCR2_3_1064,95,86,75,173,55,348,281,29,943,4,13,244
CXCR3_3_1065,160,640,358,961,110,1193,145,932,785,1049,177,977
CXCR4_3_1066,4335,4025,4625,9455,5321,8628,7292,6474,4088,4211,2453,45
40
CXCR5_3_1067,757,1035,362,1007,553,1172,368,2942,497,428,555,2270
DARC_3_1068,1307,1580,1611,1663,973,824,984,3559,723,2499,499,1867
DRD2_3_1069,3097,2651,4545,2709,3863,1320,5943,2312,2631,5910,4108,303
3
DRD3_3_1070,1117,1138,1035,1963,2049,4474,1785,31,594,1617,3666,2384
EDNRB_3_1071,2708,2669,2250,3356,3420,1737,2596,2977,2725,3761,2977,40
67
EMR2_3_1072,1099,1158,1379,1336,1223,486,567,1259,1719,2587,383,545
FPR1_3_1073,1061,1259,1555,1084,3082,3078,435,2174,2204,797,2269,739
FSHR_3_1074,3621,5694,4515,6872,3526,7600,9881,7158,7868,9303,5524,588
3
FZD6_3_1075,1962,1882,2514,2520,1040,1065,2989,3033,1838,679,821,2021
GABBR1_3_1076,994,1414,1013,1475,1138,1327,976,2103,1319,762,436,3006
GHSR_3_1077,246,290,238,149,8,477,16,54,2,185,450,159
GNRHR_3_1078,3495,4178,3588,4301,3218,6118,5140,2736,3181,2522,4502,32
36
GPBAR1_3_1079,63,442,221,373,31,345,78,27,233,228,8,895

GPER_3_1080,288,254,204,979,159,76,286,1140,2,44,319,188
GPR107_3_1081,7173,8463,8272,8234,8548,10692,14042,10681,7885,10213,97
47,8410
GPR110_3_1082,1460,2252,2300,3020,1685,2278,2583,1620,549,1306,2315,29
16
GPR113_3_1083,1438,945,1400,1224,907,144,826,784,1499,4326,327,1609
GPR116_3_1084,788,536,1004,830,28,729,2049,3389,238,81,3320,1683
GPR126_3_1085,2684,4009,4926,6035,3071,5679,3267,3656,1292,2749,11258,
2262
GPR155_3_1086,2386,1725,3459,3559,3554,4475,3766,4315,3164,2326,3555,4
886
GPR156_3_1087,2761,2800,2442,3616,1777,1227,1459,4030,4086,2029,3370,2
561
GPR162_3_1088,7618,9884,10339,9434,9252,12043,12870,10961,10907,14383,
7097,13453
GPR17_3_1089,579,699,471,301,60,604,276,59,24,22,194,2
GPR18_3_1090,1279,1523,2428,3314,1774,3676,4531,5396,141,679,1109,7399
GPR1_3_1091,3767,4351,3930,5878,4054,5169,6492,7792,3750,6951,9170,704
3
GPR34_3_1092,2229,3859,4200,5851,1716,4654,7493,5641,2866,4567,1875,21
88
GPR35_3_1093,508,346,91,29,6,16,1349,1,0,1426,253,890
GPR56_3_1094,1348,1648,1361,2461,591,620,1993,1639,356,1933,355,1086
GPR63_3_1095,888,634,419,550,1779,1100,222,2969,1038,46,337,79
GPR64_3_1096,800,719,589,1080,858,1122,629,1966,29,2,404,3464
GPR68_3_1097,174,106,743,148,322,22,1006,620,93,69,779,963
GPR85_3_1098,1061,982,1543,1654,981,487,1700,2102,1643,1646,2241,3125
GPRC5C_3_1099,1509,2980,2503,2141,2042,949,4402,2856,1232,1716,1872,38
55
GRM1_3_1100,1731,1984,2251,2575,2718,1243,2454,4119,2924,260,682,980
GRM2_3_1101,1427,2609,934,1513,2687,178,181,1487,931,179,776,1069
GRM5_3_1102,3254,5105,4125,5133,3115,6080,3378,3144,4739,5137,4408,788
1
GRM7_3_1103,864,1348,1225,1367,960,1092,1059,1421,830,4510,3556,2594
GRM8_3_1104,1338,1416,2322,3065,604,2578,717,3386,1776,1725,4126,4026
HRH4_3_1105,1988,1868,3997,3141,3808,3413,4764,5968,1626,2890,2463,106
4
HTR2A_3_1106,1103,1038,362,730,383,2979,422,1187,766,2199,2323,1216
HTR4_3_1107,919,888,724,970,972,1402,262,1034,1101,1247,2935,285
HTR7_3_1108,1742,2837,2120,3008,3170,1869,1716,2561,5101,1236,3491,415
6
LGR6_3_1109,1572,2619,1949,1606,1286,2348,3673,3674,4318,411,1299,1886
LPAR1_3_1110,8958,9892,12406,11131,12354,14215,11444,11604,14185,15340
,14229,11491
LPAR5_3_1111,1821,2759,3359,3941,3487,2681,2932,2376,2869,5709,3397,64
19
LPAR6_3_1112,315,1010,2425,2373,1096,381,1104,624,613,1226,2099,1772
LPHN1_3_1113,156,428,575,325,227,926,565,117,37,47,137,2787
LTB4R2_3_1114,939,1224,1218,1256,1112,124,716,1557,967,1130,79,789
LTB4R_3_1115,1203,2746,3149,3298,413,3389,3362,4511,177,2613,3350,3562

LYPD1_3_1116,2683,4806,5303,5975,3652,3363,4198,8079,5576,7223,5790,35
42
MCHR2_3_1117,15201,15807,18999,17903,22610,21935,27058,26186,19996,178
84,20375,17585
MRGPRF_3_1118,302,340,336,780,293,129,822,250,656,1149,11,55
NPFFR2_3_1119,4080,2266,4406,7337,2453,2859,4971,11159,7057,2597,2572,
2636
NPSR1_3_1120,2539,2057,2527,3085,2981,3787,4037,5791,4710,4471,7064,60
47
O3FAR1_3_1121,50,147,353,599,412,77,121,263,5,656,219,143
OPN4_3_1122,2159,2283,2271,2278,1551,1087,4882,3111,1398,2311,1723,298
9
OPRL1_3_1123,2313,3100,3088,2458,1143,1814,1147,912,2548,2725,1119,311
8
OPRM1_3_1124,1572,2077,1875,4254,3337,2515,1118,3433,1786,4384,7913,25
88
P2RY10_3_1125,1432,1626,1571,3071,1679,1719,1749,2068,1638,1264,2043,1
260
P2RY12_3_1126,2794,3843,3052,5899,4003,4945,6947,8577,5266,3770,5881,4
066
P2RY14_3_1127,1550,2015,2276,1304,3416,2143,2860,1068,1339,5119,2649,1
303
P2RY2_3_1128,2197,3701,1981,4563,4571,5404,4109,2677,1660,1764,1793,33
68
P2RY6_3_1129,30,46,287,81,52,530,8,296,294,783,99,128
PTAFR_3_1130,1091,1155,1230,1808,504,1112,863,935,984,685,786,957
PTGER3_3_1131,564,550,1743,554,118,315,1130,188,345,149,300,326
PTGFR_3_1132,15968,19012,19300,26849,19023,26078,27644,25544,14823,145
18,20748,25097
PTH1R_3_1133,1456,2988,3544,3034,4802,6323,4115,1697,2882,4049,3622,28
42
RGR_3_1134,914,1658,1426,1844,1112,2714,2562,1269,2188,470,4426,1275
RXFP2_3_1135,1433,1122,1525,866,511,3346,2698,1839,3068,1701,257,1416
S1PR5_3_1136,407,436,1144,572,903,455,1497,1065,657,59,325,671
SIGMAR1_3_1137,2082,2325,3385,2128,1341,2841,2409,2296,3135,2914,3457,
3841
SSTR5_3_1138,313,320,860,1672,255,32,401,942,1494,11,64,77
TAAR2_3_1139,7384,6132,6303,7259,6569,10045,9377,6267,5263,6320,5725,9
971
TACR1_3_1140,506,272,1381,1994,1255,605,272,314,2504,547,3626,3826
TAS1R1_3_1141,861,1294,1488,2051,1482,1693,2408,1304,918,2974,672,919
TBXA2R_3_1142,237,662,814,511,173,2971,741,60,114,87,1044,453
TPRA1_3_1143,216,283,463,755,2,284,2,1009,194,18,1187,975
TSHR_3_1144,6476,8749,9279,9598,7388,9810,17819,8899,9870,9561,5772,88
67
XCR1_3_1145,3661,6283,6617,8035,2182,5814,5511,9064,2572,9396,2827,677
1
XPR1_3_1146,4296,3556,6479,6630,5422,3535,3108,3803,6223,4184,8115,564
6
ADORA2A_3_1147,1199,1492,1198,1402,931,2996,1572,1466,1080,1407,2995,3

690
ADORA2B_3_1148,10674,12433,13108,13112,8547,19551,16455,16163,16190,12
325,19741,13095
ADRA1B_3_1149,4877,5752,7836,10270,5350,8268,8305,9963,6871,5266,13326
,6367
ADRA1D_3_1150,304,1281,346,1391,358,792,914,17,1344,148,91,640
ADRA2A_3_1151,2566,2160,2358,3533,5192,2192,2404,3037,3178,436,1792,45
51
ADRA2B_3_1152,106,730,704,790,786,129,380,1982,109,75,304,1066
ADRA2C_3_1153,62,43,18,30,302,0,0,55,498,14,0,384
ADRB1_3_1154,1007,1461,1664,1230,402,2428,2924,625,492,1590,3057,860
ADRB2_3_1155,860,1088,1335,1252,1171,1682,979,2132,3304,828,461,174
ADRB3_3_1156,134,325,81,125,451,1,41,433,106,300,823,0
AGTR2_3_1157,1449,1932,2808,1804,1684,2589,1979,2721,1065,1747,1034,43
7
APLNR_3_1158,714,1485,1363,1471,273,442,4088,70,2741,53,848,1133
AVPR1A_3_1159,196,608,298,352,25,74,1702,368,61,2259,66,410
AVPR1B_3_1160,675,256,967,748,186,1461,1397,62,1255,154,2590,1692
BAI1_3_1161,1255,3064,2097,2615,1003,2358,4545,2610,3406,6562,2747,121
2
BAI2_3_1162,658,1116,905,643,10,148,576,315,481,259,68,298
BAI3_3_1163,1810,940,749,1425,857,389,2910,673,2382,524,986,978
BDKRB1_3_1164,1312,1072,1394,777,1984,1734,1976,1845,1044,2421,1574,14
51
BDKRB2_3_1165,4847,5015,8176,8603,4717,10003,10081,7334,6485,12681,100
90,7998
BRS3_3_1166,6157,7436,7188,10663,6063,9050,6479,7780,7006,9031,7104,57
60
C3AR1_3_1167,1071,872,2086,2469,1827,185,665,1745,1252,3194,565,940
C5AR1_3_1168,2295,4401,3056,3796,3262,4190,2486,3679,5008,8947,6274,58
68
CALCRL_3_1169,7961,7659,7668,11642,17339,7441,8011,8325,8267,8340,7674
,9349
CCKAR_3_1170,2169,2362,3050,3593,2007,6981,4020,3541,2590,1368,3056,26
80
CCKBR_3_1171,567,973,538,904,704,582,1541,1158,1582,906,692,1542
CCR1_3_1172,2538,1507,1771,1812,1307,1254,2987,4607,2197,2424,2540,122
7
CCR7_3_1173,1179,1503,836,646,513,3111,1318,58,652,349,256,7
CCR8_3_1174,1352,2756,2417,2980,629,1674,4709,4963,3151,958,2799,2325
CELSR1_3_1175,427,866,508,259,306,29,388,1215,689,1111,210,198
CELSR2_3_1176,4544,6765,8141,9838,7179,9238,8286,6201,3999,6953,6168,7
215
CELSR3_3_1177,1479,1667,3229,2645,1362,1748,1897,128,2659,2232,2463,36
24
CHRM1_3_1178,4707,8504,6229,9575,11542,10037,10894,4686,5364,6596,8980
,5375
CHRM3_3_1179,635,691,809,233,57,258,303,269,799,239,1177,98
CHRM4_3_1180,436,359,718,1091,94,84,386,44,397,661,27,65
CHRM5_3_1181,376,552,984,279,1039,281,2484,1991,669,594,1408,728

CXCR1_3_1182,1822,2404,2078,2726,2743,2698,2626,3031,2049,2354,5470,18
93
CXCR6_3_1183,2976,2403,2334,4597,3019,2478,2511,5498,5104,6797,3465,25
78
CXCR7_3_1184,3502,2692,3603,3384,3478,4984,5979,3346,5318,2151,7622,32
81
CYSLTR1_3_1185,1167,620,1554,1484,888,697,2714,2038,1457,533,2264,1487
CYSLTR2_3_1186,555,1014,1038,1028,189,253,1319,2093,850,1060,144,702
DRD1_3_1187,464,67,265,321,663,2343,168,1,351,2,6,0
DRD4_3_1188,1011,1111,1880,1470,1341,1079,816,1931,2680,683,266,2233
DRD5_3_1189,1088,384,2603,869,1248,10,1271,303,2396,3741,198,1517
ELTD1_3_1190,4669,4751,4765,7155,5057,4345,6402,8158,3670,4565,7035,46
94
EMR1_3_1191,965,1809,1232,1918,1050,3817,3315,1850,1008,2181,4177,1178
EMR3_3_1192,6517,10277,9222,9689,6743,8274,8696,11316,9342,12452,12950
,10378
F2R_3_1193,174,595,591,497,213,153,1075,156,388,35,637,1090
F2RL1_3_1194,1311,1699,978,936,1786,2483,1289,589,1086,309,189,2127
F2RL2_3_1195,756,539,314,686,192,12,324,44,268,739,4,140
F2RL3_3_1196,960,3081,1513,2257,1075,4100,1016,4349,1577,1727,1875,189
7
FFAR1_3_1197,571,1782,702,1167,773,957,339,1422,2741,969,349,1001
FFAR2_3_1198,3993,4938,5048,4716,3825,3788,5627,5182,6641,4994,6310,46
83
FFAR3_3_1199,2665,2469,3997,5597,5314,7906,3845,3759,7129,5774,4228,44
77
FZD10_3_1200,1893,1851,1894,2770,739,1949,1381,478,3039,4388,199,1297
FZD1_3_1201,139,443,540,602,13,415,410,215,74,25,456,148
FZD2_3_1202,1243,1747,2690,2345,1500,3056,1925,2468,2766,3181,3101,267
7
FZD4_3_1203,18,257,11,484,0,1,24,1738,430,1,4,0
FZD5_3_1204,585,346,453,213,1487,321,932,1376,576,21,64,72
FZD7_3_1205,1206,3001,2035,2642,2217,4457,3390,3030,1537,2617,6493,100
9
FZD8_3_1206,6166,8305,7551,13117,5965,10167,13037,7687,11273,11941,695
0,13453
FZD9_3_1207,2884,3502,3549,3077,3745,4170,6036,2439,4731,3724,3634,256
6
GABBR2_3_1208,1741,2570,3519,2414,3269,2774,842,2012,1637,1974,6675,16
47
GALR1_3_1209,2004,1823,1426,2837,812,5045,2117,648,371,810,1424,438
GALR2_3_1210,758,1256,1016,1827,1519,369,1088,2077,1446,786,266,2616
GALR3_3_1211,367,198,598,490,13,14,2085,1931,599,667,332,2267
GCGR_3_1212,618,574,721,1622,422,1305,554,1215,1123,111,1329,924
GHRHR_3_1213,732,1002,435,864,625,814,1042,595,714,960,817,623
GIPR_3_1214,1486,3532,2010,2193,2369,3559,875,7076,3064,109,2836,2805
GLP1R_3_1215,1934,1889,2494,1931,1310,1954,2809,3929,2911,949,560,4174
GLP2R_3_1216,3339,3738,3749,4846,2792,6758,3696,3491,7361,7363,1782,53
24
GPR101_3_1217,300,441,662,1031,96,130,0,1207,141,90,229,326

GPR108_3_1218,4048,3646,3845,4806,1612,2418,5026,2605,3264,3420,4786,2
016
GPR111_3_1219,1188,1979,1578,1209,1452,2057,1288,57,2391,1889,357,2254
GPR112_3_1220,1044,2121,1682,1908,2914,2740,1225,2753,1211,2820,1175,1
260
GPR114_3_1221,589,728,428,1054,435,1469,1239,682,850,900,616,1858
GPR115_3_1222,5143,4266,5843,6081,7408,7266,7364,10499,5617,8994,5024,
8246
GPR119_3_1223,1195,435,1234,1148,2847,66,2780,94,311,1065,492,1294
GPR123_3_1224,782,1058,748,2090,445,1771,1432,1411,356,1055,228,775
GPR124_3_1225,500,285,640,368,167,380,844,276,641,29,1156,1532
GPR125_3_1226,3884,4497,4240,6360,2484,8323,5458,5694,6144,1853,2370,6
863
GPR128_3_1227,345,873,702,762,551,919,935,660,387,3267,3682,2198
GPR12_3_1228,12003,13113,13288,19991,18157,17903,10984,10736,15283,146
02,17276,10159
GPR132_3_1229,478,294,708,1014,417,2286,2208,691,2294,59,525,605
GPR133_3_1230,2111,3285,4440,7176,1327,2636,3100,7671,5678,1631,1948,5
138
GPR135_3_1231,1408,1952,2161,1468,1744,763,1839,1704,2019,49,2500,2143
GPR139_3_1232,596,510,552,331,429,523,685,96,674,2006,398,1535
GPR141_3_1233,1248,2297,2938,3896,2399,1643,3751,2606,3281,4012,2382,1
371
GPR142_3_1234,12209,15019,17327,18647,12629,15159,19558,16757,12461,21
477,14723,18676
GPR143_3_1235,2246,1947,3162,3213,3788,1529,1860,1562,3766,3008,2206,4
531
GPR144_3_1236,1593,2992,3985,2102,543,4726,3309,3022,959,3227,3312,124
7
GPR146_3_1237,1119,1409,956,1610,1028,690,1082,2108,676,564,1020,1755
GPR148_3_1238,831,951,1113,614,775,2516,1524,421,688,536,1505,748
GPR149_3_1239,70,652,150,5,43,335,198,131,1141,39,6,1
GPR150_3_1240,1145,273,958,705,1042,570,316,692,476,1251,3423,416
GPR151_3_1241,3081,2059,2813,3558,2334,4151,6031,3299,5162,2357,3199,4
139
GPR152_3_1242,1432,456,822,802,1099,1298,1369,1114,1313,863,3697,1930
GPR153_3_1243,1756,1145,1919,2081,378,1582,3569,4332,1356,1916,1273,23
25
GPR157_3_1244,497,611,225,1442,542,264,1143,44,1045,443,1928,561
GPR158_3_1245,1236,1386,697,1823,1012,1555,1288,732,895,823,1578,822
GPR15_3_1246,5258,4887,6407,6123,5066,7543,5566,2864,5750,8209,4785,57
51
GPR160_3_1247,4223,3769,3628,5764,3882,6324,4240,5040,6154,6321,2801,3
962
GPR161_3_1248,992,1356,813,1377,1201,5140,1479,873,1427,572,2830,1513
GPR171_3_1249,2688,2098,3766,3329,2055,1898,2593,3854,4140,3235,884,47
31
GPR173_3_1250,1954,1769,1953,2272,1710,3717,1209,5126,1989,1017,1329,3
057
GPR174_3_1251,897,604,654,669,515,275,1242,711,856,322,103,2365

GPR176_3_1252,1006,586,1218,932,426,1655,1293,605,1739,1920,4094,712
GPR179_3_1253,677,1514,1429,1339,1216,1520,1654,2151,1888,1518,1709,428
GPR182_3_1254,370,558,485,1092,2204,3658,1873,2008,2484,26,7572,212
GPR183_3_1255,2036,1026,2004,1924,1602,2495,3356,1954,2748,1457,1882,2372
GPR19_3_1256,1653,3100,3712,2783,2241,2014,4893,3846,2780,4182,1539,1899
GPR20_3_1257,1119,623,1739,1220,723,1454,1420,4159,1781,44,2196,479
GPR21_3_1258,396,1306,901,1527,402,1063,1188,424,403,122,125,2471
GPR22_3_1259,8966,7064,9901,13049,11468,12068,11329,19636,11553,9019,11848,16392
GPR25_3_1260,492,953,1060,722,3006,2454,647,842,634,190,1370,537
GPR27_3_1261,2512,2663,2858,4312,1974,3972,5032,4623,3064,2147,1999,1982
GPR31_3_1262,308,330,309,445,468,1429,592,1158,672,1004,260,1896
GPR32_3_1263,666,1011,643,876,1254,1533,1111,135,981,657,256,383
GPR37_3_1264,697,1059,637,883,110,3415,648,544,3073,2474,1333,1789
GPR37L1_3_1265,710,693,1038,842,530,1005,378,566,2317,1016,1445,160
GPR39_3_1266,3088,3347,3988,4202,560,2000,4096,3854,1195,1454,3811,5245
GPR3_3_1267,1306,1076,1170,1091,647,1055,1986,715,617,3558,6440,1153
GPR45_3_1268,575,940,979,1120,448,2117,75,2,255,146,39,99
GPR4_3_1269,1070,600,554,2006,608,2613,872,657,270,563,614,1472
GPR50_3_1270,693,725,1127,1147,595,1980,839,922,141,1866,268,1015
GPR52_3_1271,1164,976,2154,1287,897,1689,970,2611,1065,1400,3752,4328
GPR55_3_1272,478,1170,517,1492,38,3398,598,403,1644,1501,1377,891
GPR61_3_1273,1841,1602,1493,1825,1871,1267,1129,2708,3903,4951,205,2694
GPR62_3_1274,849,861,692,885,543,989,811,1776,958,9,90,831
GPR65_3_1275,12902,14728,13372,16749,18291,21490,15287,13200,16340,17327,20945,17878
GPR6_3_1276,907,607,701,1363,600,292,722,235,736,1254,149,1104
GPR75_3_1277,76,23,1,4,0,77,4,0,0,0,2,66
GPR77_3_1278,850,394,755,738,1486,3748,1363,438,2258,369,665,440
GPR78_3_1279,13,365,85,255,11,367,44,333,1380,1,652,885
GPR82_3_1280,664,1543,523,264,1,1952,2049,72,801,2437,887,426
GPR83_3_1281,1152,1326,1695,3179,957,2785,3672,2725,376,1022,1956,2693
GPR84_3_1282,422,338,386,580,128,38,59,225,171,1264,4559,621
GPR87_3_1283,1160,1080,1120,1661,985,501,1970,4116,1843,2543,1505,880
GPR88_3_1284,18,43,439,532,24,0,16,825,1,930,0,45
GPR97_3_1285,9858,15505,16421,17448,11681,22057,14846,11404,17908,17269,22265,25073
GPR98_3_1286,5234,4786,6535,7122,6624,9220,7244,8422,7287,3782,10910,11331
GPRC5A_3_1287,2372,1788,1641,3159,3548,1092,1479,1759,2773,887,1053,5217
GPRC5B_3_1288,903,1980,1368,1463,850,2879,2187,921,1247,2007,2792,2519
GPRC5D_3_1289,925,1379,1181,741,2242,1890,3126,3882,1231,1042,1301,1385

GPRC6A_3_1290,13005,13331,19388,19012,14853,16030,14195,15145,13503,10
127,26556,20531
GRM3_3_1291,1014,1336,830,98,1263,930,689,128,5,97,161,506
GRM4_3_1292,463,591,727,2552,531,122,20,154,46,2152,163,515
GRM6_3_1293,298,418,329,743,325,489,230,627,483,106,737,166
GRPR_3_1294,2862,4157,2983,4677,3704,4823,5013,4892,2890,4148,1975,479
2
HCAR1_3_1295,3413,3902,3607,5323,3572,4842,4170,8210,5164,4865,2087,43
65
HCAR2_3_1296,1382,2351,3736,1649,547,4646,1264,2395,2781,1900,4207,243
3
HCAR3_3_1297,1382,2351,3736,1649,547,4646,1264,2395,2781,1900,4207,243
3
HCRTR1_3_1298,8659,10557,8353,12038,11280,15687,11274,15132,10733,1109
9,14761,7748
HRH3_3_1299,712,259,685,1692,2513,2200,1719,620,1423,620,397,1857
HTR1A_3_1300,10314,11615,12582,16449,19295,19404,11569,15352,16606,175
05,15604,18870
HTR1B_3_1301,1330,1127,1116,1724,1006,206,1164,1250,673,1020,550,733
HTR1D_3_1302,1497,1950,655,2940,1785,3019,2095,425,2327,833,3332,2695
HTR1E_3_1303,2710,2815,2755,1038,2081,2262,2699,2269,2331,1629,6023,22
85
HTR1F_3_1304,3996,4077,4553,4297,2878,3414,2192,5594,5585,2106,3231,46
33
HTR2B_3_1305,1235,1938,907,1329,2402,1812,1769,1391,1703,4037,601,596
HTR2C_3_1306,4446,4326,7318,7025,13159,7750,9025,4979,4643,7927,5412,1
4707
HTR5A_3_1307,1008,1164,1161,1684,2029,3874,730,1367,2413,2435,643,1359
HTR6_3_1308,1573,3497,2704,2527,1532,5000,3831,3095,3838,5990,6197,127
1
KISS1R_3_1309,133,447,358,48,0,16,0,160,143,852,2058,73
LGR4_3_1310,2711,3752,2363,4447,2816,3631,4420,4610,3831,2348,5213,343
0
LGR5_3_1311,471,935,157,201,599,1748,1833,3,26,382,70,54
LHCGR_3_1312,4998,6620,6543,8453,10299,6894,6674,11266,6942,4750,4674,
7672
LPAR2_3_1313,1462,4077,3313,3679,1520,5634,4432,2163,2773,4658,3867,47
39
LPAR3_3_1314,2329,2286,2812,4712,105,728,2731,3909,2138,2177,10381,353
9
LPAR4_3_1315,2447,2113,3007,3453,2548,2478,3518,2742,1960,1778,882,312
1
LPHN2_3_1316,1093,1479,1879,1398,592,1718,2501,2722,1949,1417,5359,242
8
LPHN3_3_1317,798,802,983,1680,198,1149,1247,1003,1242,1605,295,399
MAS1_3_1318,821,887,1324,2084,1009,504,981,1432,40,27,454,422
MAS1L_3_1319,2209,2004,1494,3569,1092,4986,388,4601,7227,1948,906,2555
MC1R_3_1320,6688,4116,8441,8010,6767,9026,12039,9682,6985,6923,4428,86
34
MC2R_3_1321,830,872,1067,929,1251,1006,3005,128,328,4256,1751,91

MC3R_3_1322,1608,1833,2909,2127,2829,860,2052,2239,811,1655,1979,3576
MC4R_3_1323,5850,5656,9301,8505,7029,5614,14950,8653,4881,5715,4517,87
08
MC5R_3_1324,1186,799,640,1616,2095,1059,827,1679,2556,1390,225,871
MCHR1_3_1325,2106,2716,3364,2390,4350,3466,5626,1960,3756,497,527,4043
MLNR_3_1326,728,1077,608,1016,465,1158,1322,626,937,57,85,413
MRGPRD_3_1327,230,60,1,159,2740,0,27,0,0,4,2,0
MRGPRE_3_1328,1969,967,1951,2098,1104,3926,3924,1763,1693,2704,1206,17
63
MRGPRG_3_1329,2307,2730,3002,4198,3145,4420,3073,5078,2920,738,490,298
8
MRGPRX1_3_1330,3944,4744,3997,4536,5826,6559,3270,4811,6139,4616,3246,
2861
MRGPRX2_3_1331,5795,6829,7492,7216,8451,7155,6046,5587,9745,11776,1138
2,8465
MRGPRX3_3_1332,992,2968,2291,2804,1921,5636,1796,2350,4869,4359,1581,4
231
MRGPRX4_3_1333,699,799,749,1562,1287,2034,125,1449,927,773,351,1521
MTNR1A_3_1334,419,81,1321,405,1289,1093,1169,1510,463,2392,269,28
MTNR1B_3_1335,106,228,410,818,8,248,0,385,0,0,108,0
NMBR_3_1336,553,509,88,412,132,27,35,189,570,985,232,349
NMUR1_3_1337,205,736,1269,1130,113,112,0,1666,1357,473,181,217
NMUR2_3_1338,1953,1303,2009,1594,3847,2181,2931,2829,4012,2346,5860,40
02
NPBWR1_3_1339,3115,3123,2700,4101,1470,5198,2692,3568,1460,4296,1691,2
320
NPBWR2_3_1340,93,787,184,317,225,1272,1469,1569,2,72,51,571
NPFFR1_3_1341,975,1548,1883,1440,1193,2211,1623,1398,744,4094,1272,901
NPY1R_3_1342,1543,2544,2368,4903,2722,3170,2912,2891,2281,2082,4502,30
63
NPY2R_3_1343,7759,10290,11302,12735,6348,14067,9291,8860,9046,10604,23
132,7967
NPY5R_3_1344,1952,2513,2562,3685,3183,2380,5391,4831,4384,3632,1216,20
73
NTSR1_3_1345,605,239,1104,581,1042,1015,688,886,962,633,2244,607
NTSR2_3_1346,427,794,1280,2990,77,10,1881,287,742,148,486,769
OMG_3_1347,2940,3263,817,2290,2049,2564,181,1332,368,1818,3934,3208
OPN1LW_3_1348,574,2431,1219,2464,1347,1177,1970,3124,2453,2115,2271,23
72
OPN1MW2_3_1349,2694,3543,4192,2506,5542,2621,2412,1807,1774,3157,6257,
2901
OPN1MW_3_1350,2694,3543,4192,2506,5542,2621,2412,1807,1774,3157,6257,2
901
OPN1SW_3_1351,14795,19203,18261,20292,15968,19930,18414,22124,21578,26
031,15087,19704
OPN3_3_1352,938,895,829,2705,1207,886,1135,1194,880,2417,691,2185
OPN5_3_1353,2205,3031,3129,2366,1364,2327,2917,5045,918,1874,2129,3834
OPRD1_3_1354,2991,3601,3448,3862,2554,3652,2824,3967,3190,3180,2005,48
01
OPRK1_3_1355,721,765,416,905,623,539,1378,946,280,826,329,88

OXER1_3_1356,2860,2856,3713,2216,1755,2154,3440,2006,3234,1847,2083,12
67
OXGR1_3_1357,1248,926,1828,1740,1444,3068,1669,4407,2544,2490,2048,346
4
OXTR_3_1358,1068,1660,2285,2509,533,1884,2518,2227,616,3477,4905,625
P2RY11_3_1359,1022,1150,1115,522,364,846,604,310,1388,1552,2533,348
P2RY13_3_1360,2923,2144,2461,3003,1981,1328,2555,2102,3317,2603,3977,3
267
P2RY1_3_1361,3791,5767,4949,5294,4208,5616,6624,9326,7659,6790,5583,59
30
P2RY4_3_1362,837,1627,695,1630,855,3789,767,3096,1315,1272,396,985
P2RY8_3_1363,712,992,811,674,127,558,1814,1470,300,503,1446,997
PPYR1_3_1364,4421,6764,7982,8530,6380,6683,8187,8319,9380,10951,13104,
5222
PRLHR_3_1365,454,574,395,440,637,554,25,1082,257,6,6782,2582
PROKR1_3_1366,9137,9117,10237,10571,7657,10165,10776,9136,7139,7696,16
418,6450
PROKR2_3_1367,9752,9634,9355,10406,12481,16893,12472,7241,6210,19319,6
916,12128
PTGDR_3_1368,6492,6997,6826,7341,5527,5852,3533,7772,7272,3383,6883,65
60
PTGER1_3_1369,1072,319,865,219,1001,202,500,1085,83,370,1723,537
PTGER2_3_1370,1845,3318,3507,4169,3276,4955,3014,3845,3697,4733,5366,2
661
PTGER4_3_1371,3521,4690,6464,4150,4860,5109,8098,8696,3970,5764,3129,3
722
PTGIR_3_1372,1072,817,2093,699,284,420,1266,4579,1093,956,1203,3455
PTH2R_3_1373,468,511,1298,1729,840,2062,206,667,606,1912,3277,575
QRFPR_3_1374,749,1106,2057,2131,724,2819,1607,1421,1029,1529,2089,428
RHO_3_1375,1748,778,1381,306,2511,446,1676,1235,38,1130,113,1563
RRH_3_1376,917,945,1171,558,345,892,3129,429,2959,1795,1099,776
RXFP1_3_1377,3192,3148,3606,5454,2856,7250,6036,4773,1566,3525,712,168
2
RXFP3_3_1378,436,874,628,1549,291,1396,216,1224,1712,478,749,1396
RXFP4_3_1379,740,817,407,517,1714,255,1216,1184,959,572,100,617
S1PR1_3_1380,8,9,99,147,118,0,1063,186,4,7,1751,326
S1PR2_3_1381,540,309,609,325,434,730,981,164,1634,2070,174,356
S1PR3_3_1382,2818,4048,3763,3834,4068,4061,3369,3267,6499,3669,6982,51
45
S1PR4_3_1383,39,277,428,240,9,0,64,0,441,1,25,252
SCTR_3_1384,2218,2933,3056,4282,3477,4032,6945,4262,3780,5767,3876,253
0
SSTR1_3_1385,5860,8409,9807,11945,7248,9626,9350,13583,7755,10036,1399
0,10000
SSTR2_3_1386,2484,3146,5039,4963,3737,2306,3443,6156,3888,1257,3690,48
17
SSTR3_3_1387,842,1088,533,94,6,32,1183,907,179,29,11,1387
SSTR4_3_1388,777,1724,1292,1058,2026,848,465,1219,803,2275,1564,524
SUCNR1_3_1389,4009,3067,4762,3751,4507,6252,4351,1837,3782,5279,4855,3
702

TAAR1_3_1390,360,749,359,535,174,2626,252,812,612,515,130,371
TAAR5_3_1391,653,648,685,1508,43,2358,94,2,838,2895,1533,493
TAAR6_3_1392,4700,5379,4158,5824,3234,6204,4433,3553,6863,2082,3199,40
84
TAAR8_3_1393,4463,5182,4168,5677,3236,4855,4424,3469,6835,2093,4278,49
56
TAAR9_3_1394,154,57,68,15,24,25,50,1,1010,1052,139,0
TACR2_3_1395,2887,3333,4339,5646,1037,3897,4291,2945,3378,4083,4999,36
73
TACR3_3_1396,3935,4869,4161,5729,4586,2814,4009,8147,3859,4425,3070,43
71
TAPT1_3_1397,1332,2740,3088,3276,4861,3011,309,3150,3578,4723,2216,396
2
TAS1R2_3_1398,3654,5958,4311,6515,4758,7057,3280,12206,6754,5809,5505,
6567
TAS1R3_3_1399,4107,4463,7467,4309,2879,5443,6744,4153,4345,7169,3394,4
926
TAS2R10_3_1400,2668,2552,1691,2817,1973,2447,2405,974,1510,1110,749,16
45
TAS2R13_3_1401,2539,4315,3238,5610,1520,7522,4156,5002,5020,1845,9273,
4976
TAS2R14_3_1402,1935,2082,1797,1275,1820,1932,428,4090,2526,2338,5403,1
903
TAS2R16_3_1403,709,1239,1629,3194,572,5862,1399,2889,6,2879,563,2983
TAS2R1_3_1404,4278,4816,4816,3469,2768,3257,4889,4434,2764,2911,3869,2
875
TAS2R20_3_1405,4405,4526,5982,6886,5900,4267,8563,5045,3478,7660,9587,
2509
TAS2R31_3_1406,2700,3950,3145,6471,4199,7137,3555,3825,1937,6050,1254,
3109
TAS2R38_3_1407,9106,7847,8685,10160,11724,8170,7891,8846,8753,10468,10
468,7378
TAS2R3_3_1408,829,1927,3002,2576,1195,1996,592,1554,347,3523,2750,3838
TAS2R40_3_1409,10518,9575,8734,10835,7583,9771,6831,11351,10399,14552,
11763,13429
TAS2R41_3_1410,262,775,1146,831,2469,3242,18,679,554,1260,721,475
TAS2R46_3_1411,995,354,1054,2949,1329,626,2169,4676,2059,1509,3840,128
8
TAS2R4_3_1412,9776,11956,9268,11684,9943,16427,10242,8661,14700,9683,1
2542,16458
TAS2R5_3_1413,1977,1778,1550,2388,724,4010,2571,2847,1149,1368,1410,19
96
TAS2R60_3_1414,5929,5055,4320,8057,10130,5257,5805,7386,4516,7403,1032
0,5420
TAS2R7_3_1415,7503,7072,5509,10415,8748,9798,5893,8856,12087,7120,9271
,13367
TAS2R8_3_1416,2077,4430,4667,2598,2894,5058,5278,3816,4346,3705,7644,1
722
TAS2R9_3_1417,9980,8030,12120,8926,7704,17112,15367,13987,16737,11358,
13677,13058

TM2D1_3_1418,1066,345,1576,1889,1102,1194,2669,580,2374,425,1591,654
TMEM11_3_1419,238,908,469,150,1312,1035,291,178,1100,239,8,3598
TRHR_3_1420,1961,2627,4152,4435,2155,2909,3409,2213,2002,6259,7095,402
8
UTS2R_3_1421,209,396,543,421,1345,1074,35,646,316,95,90,666
VIPR1_3_1422,1809,1323,1993,1587,866,761,1849,809,2645,2312,823,1540
VIPR2_3_1423,1938,3326,4445,3697,1145,4171,3947,8600,3453,4675,3876,52
80
VN1R1_3_1424,7780,8174,10756,10536,9339,18480,12386,7151,12549,11369,1
7512,9499
VN1R2_3_1425,1123,865,1962,1899,1919,361,1607,2363,1540,1119,381,1830
VN1R4_3_1426,2140,3801,1753,2465,3266,5763,3039,2798,2718,3184,6873,29
65
ADCYAP1R1_3_1427,925,874,700,2608,721,1229,1641,3082,771,1097,620,632
ADORA1_3_1428,272,747,1264,378,425,788,2187,1000,127,722,149,1423
ADORA3_3_1429,1809,2674,2376,1972,704,2294,1262,936,2788,2529,8522,257
6
AGTR1_3_1430,1346,1370,1482,3415,3306,1277,1707,457,1949,976,2013,2223
AGTRAP_3_1431,2215,2062,2108,3144,1241,3273,727,601,1782,2547,3265,237
7
AVPR2_3_1432,1367,929,1351,1051,1137,187,1862,883,112,778,2666,1484
CALCR_3_1433,1374,982,1277,1581,1813,1371,784,2500,1273,1582,2434,965
CASR_3_1434,1143,1131,967,1737,561,732,1458,1365,831,169,472,1355
CCR2_3_1435,2318,1799,1744,3617,4091,5875,3389,3380,5496,7063,1562,214
5
CCR3_3_1436,6014,6195,8175,6787,7085,5522,7616,5512,7618,8897,9633,729
3
CCR5_3_1437,1105,2851,1404,2388,1726,2264,1748,2194,1243,1492,2463,235
4
CCR6_3_1438,6916,7046,7102,9771,7713,8575,8756,6953,10750,9235,2045,73
54
CCRL2_3_1439,3688,4942,5692,6421,4869,5233,5387,4662,4778,6433,4725,73
71
CD97_3_1440,14959,20622,23023,26434,14851,18283,23833,25321,16043,2881
4,30757,21723
CHRM2_3_1441,1903,1376,2031,2059,1505,3167,2102,423,1164,2989,3281,461
3
CMKLR1_3_1442,1583,1990,2566,2656,2368,3166,2240,5446,1840,2727,1694,1
786
CRHR1_3_1443,492,337,392,150,698,343,485,264,975,481,1135,202
CRHR2_3_1444,499,98,265,689,82,1050,33,51,2,6,25,372
CX3CR1_3_1445,2959,3564,3089,6078,4653,2695,4505,4588,3069,4885,4525,6
909
CXCR2_3_1446,317,548,411,167,333,91,351,10,679,1338,512,44
CXCR3_3_1447,615,660,972,705,309,3445,2486,1931,867,1905,1595,797
CXCR4_3_1448,148,948,744,998,30,7,1432,4180,1312,2463,2253,93
CXCR5_3_1449,676,537,795,998,1055,1460,2192,642,867,467,128,2863
DARC_3_1450,2027,1310,1465,2956,1885,1093,1923,2677,2324,2417,2532,310
0
DRD2_3_1451,1318,890,903,621,1359,2086,1454,240,1544,759,2944,1260

DRD3_3_1452,1271,696,725,2138,754,705,406,583,2206,115,801,1836
EDNRB_3_1453,756,782,831,1481,934,676,145,1121,1334,22,874,414
EMR2_3_1454,14959,20622,23023,26434,14851,18283,23833,25321,16043,2881
4,30757,21723
FPR1_3_1455,425,467,1728,364,405,166,1137,696,835,641,1309,836
FSHR_3_1456,662,816,655,1500,350,3279,3044,505,420,694,202,1527
FZD6_3_1457,1503,1790,564,1048,3185,588,1032,1765,2189,2346,727,2523
GABBR1_3_1458,324,197,1007,179,150,194,3175,1741,550,532,397,273
GHSR_3_1459,2242,4361,2446,3466,1816,1900,3674,4115,4754,4065,3686,239
0
GNRHR_3_1460,3978,7022,6998,7879,8685,8110,6198,10473,8178,6279,9295,7
560
GPBAR1_3_1461,48,280,331,64,618,584,58,2531,831,0,2,0
GPER_3_1462,1044,784,1359,1573,999,1767,275,1100,1193,1381,627,608
GPR107_3_1463,2418,2867,3229,6119,4169,3290,1830,8381,2224,3967,1152,9
298
GPR110_3_1464,638,692,452,870,740,1648,282,578,568,1133,941,2191
GPR113_3_1465,6290,6804,7632,12281,7337,8778,11957,9415,5592,9851,5780
,12558
GPR116_3_1466,4989,5725,5857,7738,5310,9140,5328,7815,8480,12318,8952,
7810
GPR126_3_1467,1351,1748,3051,475,643,1290,1594,563,381,1874,557,2124
GPR155_3_1468,1012,1192,890,2039,535,3330,1406,2335,1001,3583,1448,342
GPR156_3_1469,4041,3995,3443,3857,4206,5066,2901,4029,6246,6813,7017,4
435
GPR162_3_1470,1884,2070,1676,3030,2530,2693,2003,1712,1572,824,2060,27
34
GPR17_3_1471,561,1026,1190,1040,375,1660,396,555,149,1214,2292,1387
GPR18_3_1472,927,2602,2900,3043,1945,2523,4092,3503,6015,1390,2854,218
8
GPR1_3_1473,2190,3175,1599,1187,1168,2538,3478,1073,1534,1204,2121,716
GPR34_3_1474,3209,4921,7403,4794,6215,7125,7433,3280,5481,6048,2841,51
53
GPR35_3_1475,3536,5693,7438,7339,3463,4054,5630,7755,3975,5846,2270,75
78
GPR56_3_1476,405,480,551,874,71,171,2711,1013,201,495,27,1235
GPR63_3_1477,5045,6595,5590,7055,7790,10789,5166,5379,4173,7911,8624,6
715
GPR64_3_1478,1125,1949,2210,1945,1037,374,1091,2710,3159,2871,7509,279
0
GPR68_3_1479,0,0,1,112,0,0,0,0,0,0,0,464
GPR85_3_1480,1712,1433,2169,3204,1011,726,4854,1972,2235,675,2792,4203
GPRC5C_3_1481,1543,1879,2099,1623,2399,1872,2538,2150,1882,3254,1147,8
60
GRM1_3_1482,806,673,261,194,203,16,239,520,310,51,12,320
GRM2_3_1483,1487,1374,2068,783,365,234,1284,347,518,1474,3514,1553
GRM5_3_1484,4733,3882,5402,4561,4807,4527,6406,3936,4170,4162,3246,450
7
GRM7_3_1485,982,1562,509,1366,262,352,361,119,531,1003,438,401
GRM8_3_1486,3537,3093,3472,4769,5566,4914,4983,4727,5084,3497,2645,464

1
HRH4_3_1487,3253,3980,4356,6239,4501,5160,8532,3631,7677,5504,14949,30
36
HTR2A_3_1488,680,293,871,1288,320,728,2318,1728,1415,63,262,1607
HTR4_3_1489,3068,3135,4627,5545,5064,5797,3577,4985,3597,4222,2887,452
0
HTR7_3_1490,515,548,882,1394,2247,134,139,337,756,2082,160,1433
LGR6_3_1491,749,839,800,1264,915,3551,925,1016,507,1837,46,4
LPAR1_3_1492,6905,5495,7661,7943,5768,6541,6786,5952,7568,6185,6430,49
02
LPAR5_3_1493,1557,1574,2372,2008,1669,2669,4114,1414,1792,1737,3039,25
65
LPAR6_3_1494,2443,3797,4951,2287,773,988,3341,1467,2286,2873,5403,2865
LPHN1_3_1495,808,843,1405,566,1770,953,339,37,303,1292,1413,2619
LTB4R2_3_1496,718,767,1316,1214,213,1301,2267,251,2417,650,163,2006
LTB4R_3_1497,242,806,589,914,612,1482,929,2004,715,2320,1338,101
LYPD1_3_1498,1937,2422,2970,2572,3152,3780,2111,2226,2183,4419,1019,24
77
MCHR2_3_1499,12979,13318,18589,20857,21281,23317,20736,21691,17113,212
32,30733,14104
MRGPRF_3_1500,101,228,522,594,15,42,190,3,122,24,208,399
NPFFR2_3_1501,8058,9869,9044,11613,9836,12115,11064,13209,10633,12672,
12277,8355
NPSR1_3_1502,5970,5706,7275,5120,4883,5542,10336,4904,5716,8105,4824,1
2360
O3FAR1_3_1503,2944,2633,3886,2417,2862,921,2615,922,3060,2577,1549,263
8
OPN4_3_1504,0,14,0,0,0,0,0,0,0,0,0,0
OPRL1_3_1505,647,742,348,599,249,419,247,154,353,1064,510,239
OPRM1_3_1506,215,631,226,1460,453,739,42,58,73,591,844,1437
P2RY10_3_1507,1077,1708,1972,2646,1916,1657,1303,950,2649,2195,606,168
9
P2RY12_3_1508,6867,7014,7781,9454,8424,10844,8631,8509,5568,13026,8905
,8849
P2RY14_3_1509,724,1038,1333,615,1356,1871,1368,1248,2197,800,2603,1601
P2RY2_3_1510,559,859,221,291,1228,147,21,651,418,1134,68,78
P2RY6_3_1511,694,2148,1630,1763,515,2196,4319,1491,891,812,4256,2021
PTAFR_3_1512,148,909,664,2248,16,323,52,1744,0,1000,3313,2021
PTGER3_3_1513,1674,1674,2118,2087,2197,3092,2140,5375,2294,614,908,405
7
PTGFR_3_1514,678,1373,856,1281,1287,3273,1621,585,360,1352,1405,1433
PTH1R_3_1515,3187,2387,4616,6266,7604,5624,2984,4851,3566,5131,1143,34
24
RGR_3_1516,6960,8308,8565,8637,8973,11392,10501,10382,6988,13287,11503
,10708
RXFP2_3_1517,1101,1009,562,1492,1467,1464,1426,988,1678,186,886,87
S1PR5_3_1518,532,554,781,742,591,578,924,979,541,99,959,1002
SIGMAR1_3_1519,1082,750,1990,1462,1028,807,612,1330,957,2754,3207,2508
SSTR5_3_1520,323,276,424,875,5,633,294,82,155,40,617,549
TAAR2_3_1521,1708,1206,1051,1937,1138,4013,540,5840,1637,5272,237,787

TACR1_3_1522,2087,2239,2749,1276,1639,3422,3924,1936,1103,658,2241,895
TAS1R1_3_1523,8513,14163,11873,19869,9120,20047,12942,21386,17216,2086
4,13465,15258
TBXA2R_3_1524,596,120,269,728,312,72,28,343,1,38,41,300
TPRA1_3_1525,992,845,757,1382,27,473,988,938,1947,993,1215,48
TSHR_3_1526,14283,18054,18795,23309,15492,18629,21717,20620,18233,1393
4,17532,17409
XCR1_3_1527,759,707,590,771,1544,39,3351,1544,173,399,2676,44
XPR1_3_1528,3688,5707,5850,6510,5774,8012,8890,11232,7264,3158,8146,10
059
ADORA2A_3_1529,8171,6042,7722,11232,7565,4513,4133,5934,6631,4731,8953
,11833
ADORA2B_3_1530,904,180,770,541,53,1243,2861,87,761,1672,71,1252
ADRA1B_3_1531,2354,3806,2072,6061,2163,3959,3381,7460,5169,3264,2163,4
147
ADRA1D_3_1532,1551,1478,3174,2232,1185,832,2006,1461,2013,2018,1425,26
99
ADRA2A_3_1533,5685,6176,6090,7153,2852,12410,7551,6400,4170,9716,10392
,5214
ADRA2B_3_1534,304,825,664,1084,3197,315,302,1459,705,1614,1233,1749
ADRA2C_3_1535,398,1491,388,832,139,771,1097,545,1804,682,1036,263
ADRB1_3_1536,476,779,852,917,1176,1849,1309,223,1140,2176,4492,806
ADRB2_3_1537,1130,757,1383,966,2157,2252,975,3170,1499,1711,1226,1045
ADRB3_3_1538,580,856,631,649,741,1505,672,604,128,29,870,883
AGTR2_3_1539,6164,7585,7083,8142,10361,7099,5908,12724,9646,5245,8612,
9549
APLNR_3_1540,520,2026,1040,3726,201,1919,480,2864,1008,2847,421,5426
AVPR1A_3_1541,5077,6526,6297,10390,7135,7057,11229,9318,4695,6840,7451
,9690
AVPR1B_3_1542,3331,5149,4661,5806,2838,4287,3702,2807,4241,8152,4377,7
924
BAI1_3_1543,1090,925,1314,1230,506,594,733,602,762,847,390,290
BAI2_3_1544,3777,3668,6373,6757,4195,5276,5473,5830,5120,4793,5304,498
5
BAI3_3_1545,3558,4070,4038,6408,4132,3723,2298,6547,2639,6055,2740,356
6
BDKRB1_3_1546,3009,4855,2773,5057,3313,2981,4342,2408,2668,4166,4529,8
629
BDKRB2_3_1547,306,584,620,854,324,170,41,382,123,0,150,794
BRS3_3_1548,11203,11142,13388,11748,13700,17225,13517,10703,11610,1586
0,14351,15354
C3AR1_3_1549,6412,7398,6146,8881,4349,9594,6302,9945,4882,11086,2888,8
329
C5AR1_3_1550,5938,6590,9360,8386,7273,7911,10968,5062,7634,8495,7679,8
217
CALCRL_3_1551,10226,9637,12807,15701,12376,10482,14091,17622,15973,141
85,26375,8286
CCKAR_3_1552,2568,3518,3135,5695,2442,6046,5277,4419,6307,7245,9608,34
00
CCKBR_3_1553,1372,2343,3406,3321,1358,690,1287,1605,2022,5577,840,1707

CCR1_3_1554,761,689,762,2281,635,1262,606,2497,789,519,271,551
CCR7_3_1555,1394,1426,1585,1519,1598,1536,4442,536,1403,912,363,1435
CCR8_3_1556,1718,3120,2395,5382,2981,3469,1990,2169,4441,4116,4386,500
1
CELSR1_3_1557,1231,1167,2959,725,1127,3257,978,1100,459,81,693,257
CELSR2_3_1558,954,751,718,576,1110,517,1211,614,16,2559,1154,781
CELSR3_3_1559,196,287,687,464,21,939,134,39,314,104,2636,455
CHRM1_3_1560,665,562,359,1184,455,1589,1227,1219,490,1829,124,1607
CHRM3_3_1561,3618,4792,2812,5728,5086,6762,5391,6876,4108,6108,4485,74
46
CHRM4_3_1562,2793,2175,5088,3066,2752,2125,3058,685,5990,1299,1304,259
5
CHRM5_3_1563,1840,2790,1909,2290,2152,1644,4019,1242,1581,1536,1398,72
3
CXCR1_3_1564,1417,3835,2786,3232,4192,2814,2585,4052,2178,2944,6405,47
09
CXCR6_3_1565,627,1820,1128,375,224,780,2059,457,303,1636,2480,1001
CXCR7_3_1566,599,427,482,616,409,30,574,25,543,33,1248,1185
CYSLTR1_3_1567,1662,1622,2617,2913,1308,495,1511,3871,1593,478,1669,27
26
CYSLTR2_3_1568,2886,3766,4103,6988,3244,10807,3374,6028,4057,5429,5274
,4004
DRD1_3_1569,1332,1350,1469,2350,1695,4026,167,849,3631,2914,456,3557
DRD4_3_1570,689,513,1466,473,3,195,1288,389,22,443,356,56
DRD5_3_1571,230,794,628,1355,555,124,58,70,177,72,142,51
ELTD1_3_1572,1321,948,1385,1410,203,3885,1899,26,1321,4037,5087,2229
EMR1_3_1573,1144,920,703,1101,222,627,220,1117,93,1007,1368,1802
EMR3_3_1574,4456,6374,5448,7194,8235,6045,7612,5735,7511,3588,11263,71
58
F2R_3_1575,490,822,1999,1112,1675,34,3315,3127,164,1412,1117,1211
F2RL1_3_1576,4541,5600,5844,4051,6277,4390,9392,5443,4728,4078,6405,48
85
F2RL2_3_1577,823,1091,1587,1530,952,1093,225,3056,1883,680,3034,1757
F2RL3_3_1578,993,3212,1612,2311,1090,4175,1013,4470,1714,1749,1895,191
5
FFAR1_3_1579,388,300,542,800,1564,704,608,697,1148,410,2445,489
FFAR2_3_1580,247,526,396,108,1306,387,8,324,27,1061,161,575
FFAR3_3_1581,0,150,0,0,0,0,0,0,0,795,0,0
FZD10_3_1582,199,497,336,382,47,38,15,1738,941,875,150,656
FZD1_3_1583,2642,3220,4559,5047,3448,2776,5782,3865,2626,8730,3098,645
7
FZD2_3_1584,1511,3217,2486,3363,3742,6290,7670,3325,2499,2632,6857,270
6
FZD4_3_1585,964,1614,882,964,476,790,3488,340,779,732,116,886
FZD5_3_1586,6166,8305,7551,13117,5965,10167,13037,7687,11273,11941,695
0,13453
FZD7_3_1587,32,323,169,583,221,63,64,48,5,303,1948,248
FZD8_3_1588,1141,1381,2038,1884,2417,2439,1909,1243,1155,2788,2123,188
2
FZD9_3_1589,410,771,1487,2123,0,13,837,45,247,22,2883,2071

GABBR2_3_1590,2143,2114,2664,1893,2492,1149,1950,2262,3250,3257,312,11
74
GALR1_3_1591,63,175,231,117,116,245,275,38,275,0,7,440
GALR2_3_1592,1488,1899,1759,3674,836,3175,1506,3368,1063,6206,1348,202
9
GALR3_3_1593,250,340,323,287,210,16,194,27,1574,388,723,809
GCGR_3_1594,427,432,439,528,230,23,788,1861,619,701,377,990
GHRHR_3_1595,7061,6991,6879,7448,6913,10240,6649,2943,5615,7366,9353,1
1960
GIPR_3_1596,1965,1451,1018,1900,4322,561,659,4325,2455,1360,1158,1043
GLP1R_3_1597,171,1228,565,970,80,2115,402,37,22,231,764,1841
GLP2R_3_1598,1292,444,301,989,862,611,281,571,1490,733,201,1416
GPR101_3_1599,369,381,147,523,4,253,640,1016,57,0,65,6
GPR108_3_1600,478,558,490,336,190,1166,47,375,1288,303,275,1760
GPR111_3_1601,723,1118,1501,1253,1378,2077,1291,1801,1345,474,1264,114
0
GPR112_3_1602,3275,2608,4321,5483,5828,10225,2890,3498,2410,1644,7255,
4460
GPR114_3_1603,593,1390,768,856,1991,1959,906,1850,3943,1444,472,498
GPR115_3_1604,2879,3632,3470,3485,2190,8470,4355,5779,2180,2265,4810,3
363
GPR119_3_1605,1252,658,670,1429,4064,1192,237,581,648,276,2770,685
GPR123_3_1606,531,203,157,312,76,473,371,281,922,1647,887,279
GPR124_3_1607,1528,1329,1270,1621,2447,1961,1825,2102,1543,584,3374,37
54
GPR125_3_1608,6380,5391,6653,9527,6472,6939,5946,6493,12026,5887,14261
,8705
GPR128_3_1609,2547,2109,1948,3094,3263,7545,2591,1644,6773,7838,1853,2
880
GPR12_3_1610,1757,2708,2258,4136,2493,1246,2011,3267,2342,2981,1487,24
12
GPR132_3_1611,958,266,669,1952,46,1059,220,3685,26,2078,210,209
GPR133_3_1612,1982,1248,873,2294,144,2082,3864,1790,3146,2562,1543,211
9
GPR135_3_1613,92,224,77,248,228,906,485,47,210,65,283,583
GPR139_3_1614,4407,7060,6051,8226,3273,7500,7494,8523,8244,8479,6319,4
455
GPR141_3_1615,6256,6337,4616,5600,6243,5743,6171,5860,6484,8090,5334,5
592
GPR142_3_1616,4932,5498,4856,7144,4062,3030,5149,11282,3940,8349,3771,
6655
GPR143_3_1617,934,775,1412,2101,1043,1175,752,538,261,40,339,1073
GPR144_3_1618,200,263,652,401,894,15,379,500,231,2,19,667
GPR146_3_1619,5858,4808,4960,6402,4569,7944,5882,11370,7724,5840,12396
,5233
GPR148_3_1620,915,1495,1681,1012,1843,2371,2003,840,1149,1071,1299,933
GPR149_3_1621,1962,2838,3123,2172,2714,5132,4233,2865,2068,2918,939,21
11
GPR150_3_1622,728,1946,1949,863,920,3881,471,468,876,405,1009,1514
GPR151_3_1623,1433,2087,1592,717,2816,2463,3408,1695,2474,2989,4153,14

65
GPR152_3_1624,3050,4041,3357,3379,3903,4361,3334,3432,2236,2583,8189,5
096
GPR153_3_1625,586,381,372,751,135,925,2028,510,131,2,468,370
GPR157_3_1626,669,543,936,761,724,775,618,1160,316,447,307,2861
GPR158_3_1627,4423,3528,4929,6414,7004,6862,4539,3450,4184,6689,4986,6
602
GPR15_3_1628,513,1463,1950,1582,187,1536,4000,1858,274,2056,2899,646
GPR160_3_1629,3379,2716,4197,3692,3016,3529,3658,1875,2937,2756,1604,2
154
GPR161_3_1630,1339,1760,1104,2885,608,402,756,2690,1981,531,2435,4016
GPR171_3_1631,445,580,657,470,375,511,779,961,120,254,454,331
GPR173_3_1632,752,469,386,918,894,785,338,808,154,991,754,627
GPR174_3_1633,1969,2482,2536,2716,1317,2453,2779,4593,2336,2188,3064,1
425
GPR176_3_1634,533,567,590,306,1627,5,1331,901,228,647,1089,1161
GPR179_3_1635,136,433,1051,677,2168,456,156,2324,151,57,59,1277
GPR182_3_1636,876,738,918,1493,875,897,1337,610,991,89,763,749
GPR183_3_1637,879,884,550,2023,938,1560,643,2807,1581,2290,1894,898
GPR19_3_1638,897,308,1099,850,1756,2889,1387,402,511,960,189,1618
GPR20_3_1639,641,993,1482,1663,1920,1139,2453,307,332,1301,3491,885
GPR21_3_1640,12093,15037,15034,14571,15683,23808,15151,17855,11405,197
93,18880,15252
GPR22_3_1641,1538,2611,2231,2491,1636,4085,2642,3678,2593,2066,3865,28
09
GPR25_3_1642,83,433,92,339,339,611,1530,13,317,606,117,121
GPR27_3_1643,228,291,1323,1341,453,1506,2216,1719,735,75,495,2418
GPR31_3_1644,503,648,1345,393,1044,1742,1384,2029,117,189,133,858
GPR32_3_1645,1378,1552,1745,1477,387,1809,2897,2589,1544,2590,828,452
GPR37_3_1646,3048,4549,4599,4902,4458,5592,4446,3653,7286,3437,6975,56
12
GPR37L1_3_1647,767,885,1197,1016,835,1013,1159,726,2471,2513,3672,177
GPR39_3_1648,1922,2278,3340,2810,2243,3198,2867,3945,1311,2258,6319,51
79
GPR3_3_1649,4672,5445,6945,8225,9012,8013,5810,8757,6667,5043,13690,84
34
GPR45_3_1650,816,870,885,1092,2992,341,1225,1797,1138,248,1804,596
GPR4_3_1651,908,652,669,1401,2795,503,927,2972,1692,2923,2318,2757
GPR50_3_1652,1025,2161,1916,3437,2258,3283,2689,3219,611,1270,4655,164
8
GPR52_3_1653,2147,2135,1869,2423,1578,3838,3063,2599,4886,1717,2123,17
14
GPR55_3_1654,303,1150,193,891,1934,466,31,1630,1164,313,174,2066
GPR61_3_1655,369,184,52,40,1,942,0,0,231,11,1,0
GPR62_3_1656,1063,3195,1498,1200,922,3023,3079,1433,1799,2896,1341,111
8
GPR65_3_1657,4379,3996,4200,4535,3139,4840,2290,3645,3471,1751,2570,61
83
GPR6_3_1658,1049,622,749,919,967,187,354,4175,459,336,387,927
GPR75_3_1659,832,1458,706,1502,1727,1253,3018,2646,686,800,196,1388

GPR77_3_1660,456,1417,431,1506,1582,885,746,2851,609,1758,126,2665
GPR78_3_1661,573,933,721,1563,644,1489,1199,307,617,1901,1524,2936
GPR82_3_1662,2211,2277,4193,4757,2128,3532,3223,5467,3070,2554,5979,25
64
GPR83_3_1663,391,408,1105,1332,475,2019,637,1705,1423,1460,708,1398
GPR84_3_1664,1195,1142,1242,728,1142,1868,1717,1628,2170,339,1156,900
GPR87_3_1665,461,400,1186,1607,1425,3801,3318,3207,1218,36,2230,501
GPR88_3_1666,188,390,1426,795,409,475,329,350,434,605,348,459
GPR97_3_1667,662,679,1009,256,1072,32,17,2761,788,192,633,932
GPR98_3_1668,5887,8792,7588,8362,9162,6362,7969,10413,10578,11865,6756
,10911
GPRC5A_3_1669,1498,2101,1067,1515,841,2652,533,1324,757,1552,1359,764
GPRC5B_3_1670,1686,3379,2658,4562,1674,2125,2263,2645,2475,6565,1242,5
435
GPRC5D_3_1671,540,1577,1160,966,1069,1402,1257,184,3030,453,1327,537
GPRC6A_3_1672,1606,2245,3443,3489,1314,5776,1716,5260,4296,8885,6414,7
336
GRM3_3_1673,660,1596,1159,1016,61,267,1933,1044,1474,440,460,36
GRM4_3_1674,127,1252,529,562,682,1161,321,1990,467,2728,259,598
GRM6_3_1675,776,1418,990,1465,1864,996,1283,2453,3050,2468,3208,925
GRPR_3_1676,1068,235,681,889,419,417,251,2848,492,606,172,459
HCAR1_3_1677,704,1426,722,866,69,2974,852,2262,0,1977,1184,0
HCAR2_3_1678,2542,3372,2938,5583,2217,4618,3554,5736,2661,2715,4031,72
84
HCAR3_3_1679,2542,3372,2938,5583,2217,4618,3554,5736,2661,2715,4031,72
84
HCRTR1_3_1680,959,670,583,806,380,339,985,406,641,1073,1763,527
HRH3_3_1681,2316,2871,3018,5747,2715,2886,4617,2544,2841,2086,4304,553
5
HTR1A_3_1682,7340,7995,13178,13020,11132,9873,16103,12767,11087,11383,
11477,11044
HTR1B_3_1683,676,1153,936,1200,1481,2349,708,133,219,1033,209,1684
HTR1D_3_1684,535,498,1160,1330,473,1123,755,321,146,1790,600,862
HTR1E_3_1685,3587,4561,3700,5194,3763,3065,5149,4858,4312,7086,5739,30
81
HTR1F_3_1686,1021,658,1021,2645,1474,1470,1483,1127,1020,2158,3097,200
1
HTR2B_3_1687,1151,2280,2219,4191,3593,2781,1002,1604,546,425,3383,4544
HTR2C_3_1688,1027,1287,551,545,627,585,1636,211,380,1345,340,1110
HTR5A_3_1689,1494,2385,2552,1803,1049,2163,2187,1150,3246,2209,3103,23
52
HTR6_3_1690,410,736,462,915,1026,327,2015,947,724,954,1351,774
KISS1R_3_1691,547,129,301,665,41,38,657,397,277,166,179,32
LGR4_3_1692,11242,13319,11592,12652,12471,14575,17585,23614,17782,1311
9,12255,21614
LGR5_3_1693,1316,1589,1372,2861,2991,4402,1244,2583,1689,826,227,2109
LHCGR_3_1694,5817,6290,5579,7588,7541,4848,5920,6646,11401,5549,19428,
6995
LPAR2_3_1695,533,704,313,323,1062,2173,1861,3167,694,327,294,788
LPAR3_3_1696,1157,825,1727,1798,3336,1069,2375,3949,968,1595,2736,991

LPAR4_3_1697,397,1181,1428,1966,1714,1038,2288,2170,2174,1374,2349,119
2
LPHN2_3_1698,983,1050,667,772,929,1329,1254,3425,1268,2635,518,3136
LPHN3_3_1699,4214,3534,5753,5104,5405,13676,3109,5209,8522,3461,3562,5
113
MAS1_3_1700,1867,2888,1963,5810,2036,3467,1565,1803,1791,508,4854,2423
MAS1L_3_1701,6532,8896,8510,11666,9405,7776,7980,9239,4064,6601,9681,8
687
MC1R_3_1702,8518,7973,8132,13456,10135,9510,11818,10713,10448,11836,12
702,6523
MC2R_3_1703,3334,3943,2903,6085,5977,4003,1850,5402,3958,3016,2221,579
6
MC3R_3_1704,779,1422,1448,980,1676,1803,169,1329,2545,2144,2395,952
MC4R_3_1705,1637,2448,2979,3530,2716,1701,3918,4946,2790,51,4015,3158
MC5R_3_1706,2907,4815,4091,5578,3409,3775,6752,2836,3964,4081,2542,489
7
MCHR1_3_1707,475,1349,938,1742,444,1937,412,1120,686,845,1942,1189
MLNR_3_1708,4943,4710,6645,6620,5113,3585,4633,6603,5792,4083,1274,831
3
MRGPRD_3_1709,1688,3580,2782,3102,803,5431,3839,3769,1471,2084,3480,56
30
MRGPRE_3_1710,3575,3686,3359,4631,3477,8456,4953,4320,4163,4358,5373,4
742
MRGPRG_3_1711,3413,3621,1897,2448,3583,1784,681,1078,5740,4585,3219,50
82
MRGPRX1_3_1712,1509,2528,2149,1410,1503,2267,2396,1903,2506,2999,1438,
936
MRGPRX2_3_1713,4323,3240,5218,6456,4527,6064,5160,2874,4244,4095,7017,
6972
MRGPRX3_3_1714,83,117,209,376,50,68,42,0,177,2,14,593
MRGPRX4_3_1715,841,2052,2056,1322,1450,2223,2838,1902,2464,3186,1432,1
366
MTNR1A_3_1716,4695,7585,9038,6769,8257,4903,8258,11278,6858,6695,4252,
6864
MTNR1B_3_1717,1182,806,1020,1299,1761,264,1751,3041,249,1017,965,2852
NMBR_3_1718,895,2025,1610,1555,92,1900,2262,2040,1776,1912,1045,2191
NMUR1_3_1719,438,690,1007,760,355,2075,30,2524,251,860,91,1150
NMUR2_3_1720,3134,3207,3183,4612,3435,4669,3619,7805,4367,2284,5895,49
84
NPBWR1_3_1721,1011,160,591,1112,1268,1868,2898,1090,84,45,65,145
NPBWR2_3_1722,343,815,979,1784,384,831,1356,501,1572,2167,5412,1731
NPFFR1_3_1723,965,1014,1251,1089,348,2247,745,2433,628,1646,1224,1413
NPY1R_3_1724,2091,2735,2230,2975,4096,5406,2580,2652,3162,2443,6488,49
81
NPY2R_3_1725,6068,8031,8250,10030,6396,4119,9805,11354,6263,6784,6143,
13761
NPY5R_3_1726,1071,2272,1275,2334,2798,5167,1634,1723,2021,324,64,694
NTSR1_3_1727,4930,5582,3371,4509,6317,6813,6014,4457,5461,5398,4596,31
89
NTSR2_3_1728,1480,1396,1577,1075,3237,1064,1520,1603,344,1270,1893,103

OMG_3_1729,7920,7975,9057,11062,8162,14068,15698,11426,14167,12063,109
07,10212
OPN1LW_3_1730,2694,3543,4192,2506,5542,2621,2412,1807,1774,3157,6257,2
901
OPN1MW2_3_1731,4853,5115,4732,4064,2322,6788,3582,4104,5349,2388,7309,
4691
OPN1MW_3_1732,4853,5115,4732,4064,2322,6788,3582,4104,5349,2388,7309,4
691
OPN1SW_3_1733,1693,2374,1181,2715,2336,1950,1902,5350,1336,2137,1755,9
59
OPN3_3_1734,570,646,514,846,126,50,403,844,155,194,515,7
OPN5_3_1735,806,1408,1429,1304,908,179,3043,2166,1004,2067,489,802
OPRD1_3_1736,7694,11635,10167,10265,9441,13198,12176,8710,5307,8189,10
513,14434
OPRK1_3_1737,1010,1085,866,1877,927,763,2036,499,1729,890,2325,599
OXER1_3_1738,451,269,838,204,351,1191,78,244,508,377,126,242
OXGR1_3_1739,1202,1488,1579,2871,2218,1348,2530,1116,1091,2406,2605,41
03
OXTR_3_1740,1457,4111,1877,5109,3202,2286,2559,2787,3588,2455,9340,645
4
P2RY11_3_1741,846,1623,2115,1908,1395,3055,2105,2030,2316,1660,3530,28
35
P2RY13_3_1742,1008,1726,1193,1521,3407,2598,1127,1439,866,920,968,943
P2RY1_3_1743,4556,3969,5191,4336,4782,7697,4662,2909,3438,7267,4122,58
77
P2RY4_3_1744,793,779,827,820,627,295,1742,347,988,1206,2081,359
P2RY8_3_1745,487,1098,1411,1259,1380,415,1874,402,22,873,5397,56
PPYR1_3_1746,1615,1410,2883,3231,998,2863,1809,3048,1246,3300,3419,313
2
PRLHR_3_1747,720,351,373,430,319,195,623,1464,473,11,48,413
PROKR1_3_1748,3367,4724,3146,4427,4557,6276,4290,3645,2485,2396,9065,2
875
PROKR2_3_1749,1510,2226,1310,1747,2918,2491,3945,1777,1296,1930,2125,1
716
PTGDR_3_1750,1478,1587,2581,1666,2486,1575,606,2493,865,1274,3217,2979
PTGER1_3_1751,149,690,640,423,90,1366,266,664,188,118,7,143
PTGER2_3_1752,265,549,572,574,330,957,1195,18,250,87,332,1646
PTGER4_3_1753,3509,3523,4571,3900,4010,3247,3867,2038,2498,2723,3700,6
080
PTGIR_3_1754,90,214,64,339,250,0,216,0,122,192,0,25
PTH2R_3_1755,1752,2487,2043,2754,4732,2144,1788,2396,2396,1264,2086,14
76
QRFPR_3_1756,4114,3811,5433,7859,5098,3821,4656,10704,2678,3992,9412,4
946
RHO_3_1757,1304,602,462,737,2159,2184,693,1705,634,2099,385,1409
RRH_3_1758,1773,2578,2799,2864,1745,3314,2802,7119,1319,4552,2858,2734
RXFP1_3_1759,2686,2548,4484,3078,2330,4557,1020,5390,3199,6733,4556,38
75
RXFP3_3_1760,342,221,348,293,101,170,174,92,12,18,2377,274
RXFP4_3_1761,1276,1020,759,1150,1709,1370,217,1653,408,1114,3733,1755

S1PR1_3_1762,2242,3895,2939,5609,4560,3218,5114,3396,2082,2509,348,4525
S1PR2_3_1763,165,473,131,427,4,2076,1564,774,37,0,104,1134
S1PR3_3_1764,3289,3153,3485,2395,4245,2777,4167,5274,4527,857,5134,4067
S1PR4_3_1765,984,604,1300,773,618,857,1656,1736,969,469,10130,1577
SCTR_3_1766,1394,1236,1766,1396,1855,1495,1345,1753,2856,1456,444,972
SSTR1_3_1767,2235,2644,3601,2480,2226,2854,5687,4000,4055,2215,3278,3225
SSTR2_3_1768,644,713,535,123,2324,33,37,401,1142,481,2246,522
SSTR3_3_1769,695,591,468,854,577,248,379,1129,1239,103,59,95
SSTR4_3_1770,345,581,366,150,2573,1374,1296,243,40,566,1290,155
SUCNR1_3_1771,3302,4417,4022,4594,3584,3192,5296,3657,4281,8319,10957,4322
TAAR1_3_1772,7025,4436,8248,9208,6683,12074,9150,9359,6791,8084,4224,6057
TAAR5_3_1773,1560,2203,1998,1059,659,3874,1861,745,764,2784,1310,4835
TAAR6_3_1774,1244,1086,2356,2311,1999,1988,2172,3065,3059,2371,2039,3462
TAAR8_3_1775,4303,4768,6084,6453,6336,10293,9620,8233,5706,5775,5693,10710
TAAR9_3_1776,2307,2839,2609,2317,2118,2048,3147,4727,3247,2494,2987,2271
TACR2_3_1777,1885,2313,2411,1950,1598,2539,1901,1349,2233,1841,927,3033
TACR3_3_1778,5322,8916,7229,11747,6774,11027,10441,15340,9775,6532,5351,10672
TAPT1_3_1779,1145,1707,1763,1109,849,427,1478,921,1599,763,250,1702
TAS1R2_3_1780,879,772,1129,1625,624,687,1444,3041,977,639,2333,506
TAS1R3_3_1781,1977,1370,1715,3241,4541,4944,1388,5902,1707,1804,4057,2206
TAS2R10_3_1782,11961,14071,16375,17408,16105,21524,15125,19280,18352,13960,20624,13991
TAS2R13_3_1783,1761,3111,1537,3036,565,2417,3124,2334,487,1641,1356,6420
TAS2R14_3_1784,5266,7128,6459,7615,5155,8811,7022,7621,12918,7440,7379,10105
TAS2R16_3_1785,1247,2633,1776,3815,1969,3028,2983,4055,1345,2539,1028,2365
TAS2R1_3_1786,2409,1496,2388,3074,1661,3049,2948,2439,2058,2301,2528,1808
TAS2R20_3_1787,10052,7784,10105,11657,8367,10810,13464,12858,7463,11970,12849,10438
TAS2R31_3_1788,577,1001,1190,1414,812,4723,499,2973,455,2259,102,105
TAS2R38_3_1789,8267,11379,14485,12200,9601,14038,11979,7699,16348,12500,12758,9095
TAS2R3_3_1790,2247,1912,2934,3502,750,6385,4552,2795,2430,4227,3653,1913
TAS2R40_3_1791,4011,6520,5814,5432,4531,7756,3788,4960,9461,8060,7608,8978

TAS2R41_3_1792,5400,6384,6054,7464,7029,8158,6483,4659,3523,4418,10261
,6040
TAS2R46_3_1793,4107,5291,6665,5692,6123,6429,7787,2830,5884,9591,8742,
6233
TAS2R4_3_1794,2204,1909,3923,3935,1675,2671,2127,4835,1893,4649,5335,3
845
TAS2R5_3_1795,672,1024,1096,1099,1324,437,1283,767,146,1063,4820,1273
TAS2R60_3_1796,1241,633,1376,1979,1799,616,2766,3048,919,2272,959,294
TAS2R7_3_1797,4252,5375,9016,7156,4754,5499,6752,7441,9488,7189,8124,8
995
TAS2R8_3_1798,4611,6465,8129,8584,6692,5091,6338,7013,6414,12686,5650,
3583
TAS2R9_3_1799,6091,5259,5517,4369,3733,6644,6384,5238,5657,7779,4150,4
551
TM2D1_3_1800,4307,3271,6335,5363,6349,3782,7629,7105,6158,3811,7445,36
01
TMEM11_3_1801,1794,3093,2899,2537,1001,62,3715,2623,1075,1815,1743,110
7
TRHR_3_1802,7655,8711,9696,10081,10273,10690,7659,16241,7307,14439,118
90,11190
UTS2R_3_1803,44,100,106,88,80,0,42,19,698,53,25,91
VIPR1_3_1804,1964,1496,997,1218,1576,3072,1398,1043,1654,1714,6661,950
VIPR2_3_1805,2223,3778,2388,3238,1496,3441,959,2924,1754,5146,2773,168
3
VN1R1_3_1806,11375,11995,13307,16241,13654,16561,16435,11622,16779,119
79,12946,18694
VN1R2_3_1807,174,983,907,895,851,211,844,340,80,1547,1438,956
VN1R4_3_1808,2290,2101,2418,1966,1447,1448,3210,1172,4724,2331,1657,22
65
ADCYAP1R1_3_1809,465,466,103,505,503,293,295,95,436,1099,2191,144
ADORA1_3_1810,5837,7000,7504,9347,6308,6959,16114,10558,4873,11385,110
79,8434
ADORA3_3_1811,92,285,104,1119,126,1,209,1061,395,353,113,2253
AGTR1_3_1812,748,892,620,410,461,1037,936,129,1023,2221,1697,395
AGTRAP_3_1813,827,587,923,481,339,661,29,45,588,507,111,1444
AVPR2_3_1814,363,228,70,243,874,297,2539,5,1867,3,1,4
CALCR_3_1815,2214,4028,3401,2579,2815,4094,5652,3715,1393,2483,2125,22
47
CASR_3_1816,1268,1161,1042,1370,803,536,1932,753,946,931,216,2322
CCR2_3_1817,2275,2615,2149,3167,2534,1267,3420,3803,2366,2328,4144,176
0
CCR3_3_1818,562,1634,987,2181,544,853,1141,3113,2091,1992,1490,974
CCR5_3_1819,1688,3145,3176,2856,1823,3963,1244,3669,5006,1127,2745,393
4
CCR6_3_1820,6346,5798,4811,7156,5538,5606,5970,11734,5316,7733,9092,93
88
CCRL2_3_1821,2926,4272,3848,7131,2997,4367,5131,7964,4712,3965,1904,60
13
CD97_3_1822,5195,6815,6574,4328,4709,4147,5538,7570,3040,7976,13715,41
72

CHRM2_3_1823,2461,3369,4560,3587,2494,6287,5649,3644,2115,4997,3875,27
93
CMKLR1_3_1824,1048,2017,1630,2110,813,1936,911,841,2053,3834,2315,2694
CRHR1_3_1825,807,1553,1195,1175,1174,421,1601,547,682,207,732,571
CRHR2_3_1826,351,496,453,697,359,846,147,1134,175,339,2145,67
CX3CR1_3_1827,3980,6876,4978,5380,2909,8880,9306,6401,4032,8069,6402,4
511
CXCR2_3_1828,11546,13272,15061,17311,16108,15213,16424,22544,17415,121
80,8208,25064
CXCR3_3_1829,266,238,1193,1047,2,453,199,758,858,6,42,1609
CXCR4_3_1830,637,627,1739,1621,1906,1431,1746,875,1478,1896,965,475
CXCR5_3_1831,2257,2742,1744,2018,2200,1830,3484,3896,1444,2808,1075,54
02
DARC_3_1832,467,324,1405,598,1961,1617,948,308,163,64,2343,552
DRD2_3_1833,1847,2625,2381,3628,1770,3620,5664,1718,2689,2286,1880,152
0
DRD3_3_1834,1846,1752,1045,3065,1622,1481,3345,518,2754,3959,195,1351
EDNRB_3_1835,2649,1833,2403,3993,3110,1461,2454,3155,2576,4079,4296,30
44
EMR2_3_1836,1006,550,478,802,249,1525,564,1155,978,939,12,261
FPR1_3_1837,37,78,175,123,846,144,0,121,226,2,4,273
FSHR_3_1838,6530,10502,8363,10726,7398,12192,10788,11911,8653,8363,107
55,12377
FZD6_3_1839,2342,3076,3281,4341,4500,4531,4339,7075,3304,2971,2722,224
6
GABBR1_3_1840,1325,2253,2214,3674,2737,1137,3680,2666,2116,1898,1852,4
888
GHSR_3_1841,333,1312,1224,1257,816,877,817,90,1267,141,919,597
GNRHR_3_1842,2255,3773,3309,2356,6666,2654,4095,4211,1472,8106,5556,35
46
GPBAR1_3_1843,1143,885,106,456,1367,2345,131,644,897,1091,1172,1214
GPER_3_1844,2247,2128,3143,3134,3281,1327,3905,3602,4776,3808,1663,179
5
GPR107_3_1845,2457,3833,4804,4301,1278,7978,5859,5147,6037,7564,3843,2
434
GPR110_3_1846,2569,1955,2466,1457,3092,2702,3672,3000,3957,2081,5390,3
855
GPR113_3_1847,1334,2014,2050,3514,2223,6119,2861,4339,2829,1284,577,20
95
GPR116_3_1848,1375,821,2590,2578,1464,709,2833,1367,219,1249,147,937
GPR126_3_1849,2423,2546,3339,3344,1411,4396,3187,2504,2991,2053,4834,2
010
GPR155_3_1850,1976,2151,2723,3351,3228,6107,2107,970,3031,3590,1948,28
37
GPR156_3_1851,324,696,285,181,782,115,68,417,183,235,30,655
GPR162_3_1852,2037,2237,1883,2857,1841,1108,4579,2715,1974,5681,788,21
97
GPR17_3_1853,3430,5520,6762,5525,6569,7180,8602,6450,5668,2368,7934,38
41
GPR18_3_1854,1717,1215,810,953,2764,4285,873,1736,1868,1396,483,1406

GPR1_3_1855,122,80,123,60,343,1051,199,3,193,278,25,183
GPR34_3_1856,11374,13751,13118,18084,15615,22803,19220,12148,11716,186
95,18158,17310
GPR35_3_1857,873,354,1329,730,1048,785,1799,730,1209,59,123,1348
GPR56_3_1858,3185,3269,2921,6299,926,8516,5799,3981,2545,2884,2038,477
4
GPR63_3_1859,2779,2728,2364,3284,2341,3608,1226,3479,3481,3160,3996,86
3
GPR64_3_1860,6771,7250,9530,10604,12477,15927,14520,6540,10120,7707,92
94,6728
GPR68_3_1861,591,830,1502,871,1435,815,2386,2162,492,1322,805,877
GPR85_3_1862,5003,5309,6034,5034,6960,7172,4768,5857,2761,4560,9971,61
91
GPRC5C_3_1863,1425,3392,2676,5025,1688,2120,2267,2684,2469,6587,1250,5
442
GRM1_3_1864,6084,7540,7362,10035,6259,6602,9607,12061,4795,13562,13292
,6347
GRM2_3_1865,344,571,166,710,71,1801,34,6,164,58,2127,1080
GRM5_3_1866,1060,1674,1407,2856,649,3117,1460,1527,2660,3500,1703,2400
GRM7_3_1867,1583,1258,1157,2235,629,789,1081,3022,1140,2401,1687,2217
GRM8_3_1868,0,0,0,0,0,0,0,0,0,0,0,0
HRH4_3_1869,1884,3253,3009,4534,6266,3706,8422,9867,4146,3081,10049,30
18
HTR2A_3_1870,4801,3360,4617,4614,5133,4596,3954,5242,5826,4671,3489,34
30
HTR4_3_1871,681,674,491,602,2150,2834,701,233,114,419,1784,467
HTR7_3_1872,1236,838,1823,2445,877,397,975,1711,897,3255,4784,1097
LGR6_3_1873,1865,3630,2924,5672,2678,2808,6411,3283,1487,3649,7279,462
9
LPAR1_3_1874,2710,3839,4920,5532,3588,5607,3189,1871,2364,5132,1916,23
49
LPAR5_3_1875,3837,3385,3207,3647,2832,7001,2453,3169,5312,2938,7527,24
50
LPAR6_3_1876,1823,2389,2575,1495,3305,2908,434,4522,2173,5530,4293,181
0
LPHN1_3_1877,2821,2877,2942,3143,1957,6457,2717,3090,1460,3795,3737,33
58
LTB4R2_3_1878,129,165,77,402,0,459,62,484,68,3847,164,12
LTB4R_3_1879,2755,3420,1513,2182,2490,2287,4535,3319,1422,1833,4077,35
67
LYPD1_3_1880,676,475,646,977,575,2408,105,109,1259,1161,41,887
MCHR2_3_1881,974,1038,1476,1703,1851,1713,521,256,803,1811,590,941
MRGPRF_3_1882,328,985,461,642,1092,8,628,950,363,424,736,1667
NPFFR2_3_1883,7812,9196,9014,9040,7164,12854,8725,12363,15691,14993,11
231,7714
NPSR1_3_1884,2077,2611,1615,3577,3189,3607,2645,5304,3136,2104,3301,49
37
O3FAR1_3_1885,764,1656,1184,351,937,1911,1275,211,1092,839,1821,1356
OPN4_3_1886,2376,2572,2171,3880,2226,2400,1816,3547,1448,4358,3493,383
8

OPRL1_3_1887,3668,4457,2665,4237,4674,1983,6126,2319,3694,5982,2485,44
24
OPRM1_3_1888,1127,860,1038,1263,507,649,938,1174,413,1166,451,1255
P2RY10_3_1889,682,761,511,506,575,4146,363,1474,877,1098,474,341
P2RY12_3_1890,4782,5935,6416,6542,3828,4557,8275,11254,8308,5984,11449
,9303
P2RY14_3_1891,10219,10905,11091,15345,10348,15268,16877,10415,6282,900
6,8770,7995
P2RY2_3_1892,1522,3948,2741,4909,2839,7503,5831,2819,2313,2377,1231,19
86
P2RY6_3_1893,2853,3240,3801,5920,3436,4158,5107,2879,3471,9909,4403,52
74
PTAFR_3_1894,2024,1489,1510,2110,1391,3249,3865,1258,2245,1860,1768,17
82
PTGER3_3_1895,4143,5668,6930,6588,3068,5445,7315,4578,4851,10934,4951,
4444
PTGFR_3_1896,2984,3954,3997,5544,5313,3374,4544,2604,2664,2562,2702,69
93
PTH1R_3_1897,1018,1284,2288,1337,1828,771,1998,1571,1819,434,1809,2292
RGR_3_1898,825,901,896,960,1061,472,1768,341,410,356,294,374
RXFP2_3_1899,4610,6026,4625,5505,5149,7000,2068,5253,9379,6591,1339,88
54
S1PR5_3_1900,3411,2603,3026,2541,3470,1958,2400,2674,5449,3520,6257,33
03
SIGMAR1_3_1901,779,1347,1698,1926,2557,1260,1657,3387,831,1256,1837,23
52
SSTR5_3_1902,234,187,196,141,149,805,1087,86,19,248,420,2
TAAR2_3_1903,2126,2452,2173,3882,1032,3086,2471,3360,3204,2027,2182,39
00
TACR1_3_1904,2001,2253,3680,3063,3635,4455,3772,4484,3672,4798,1425,18
60
TAS1R1_3_1905,1569,1339,2059,2036,1349,1281,3052,2809,2394,441,1009,56
7
TBXA2R_3_1906,26,253,147,333,115,243,100,8,67,1,14,942
TPRA1_3_1907,48,135,104,922,110,1065,57,768,120,27,51,851
TSHR_3_1908,478,960,1577,584,24,794,1194,2099,549,1002,702,341
XCR1_3_1909,1028,756,1070,927,584,161,328,2266,2339,2617,1771,1503
XPR1_3_1910,3098,4653,5204,6109,9153,8603,3477,3358,4172,7367,5924,429
6
ADORA2A_3_1911,338,705,188,953,1191,290,588,15,488,433,161,963
ADORA2B_3_1912,353,937,497,548,18,1355,26,267,402,631,333,1671
ADRA1B_3_1913,720,1542,2283,2725,1616,1424,2546,1268,1306,777,81,1359
ADRA1D_3_1914,739,576,429,851,1478,712,1619,1701,664,1658,2505,1048
ADRA2A_3_1915,3956,5463,5875,8499,4766,5205,3190,7210,6195,6996,4869,5
355
ADRA2B_3_1916,5527,7188,7163,7160,3874,11277,9845,7786,4481,10681,1436
6,4400
ADRA2C_3_1917,48,845,608,735,9,650,500,229,322,384,34,65
ADRB1_3_1918,1204,1789,1174,1900,1042,668,1534,3373,1239,5007,1988,252
9

ADRB2_3_1919,2367,2383,2578,2921,1068,3631,3256,2914,3781,2374,1693,24
66
ADRB3_3_1920,1647,3630,2694,4051,3623,6963,6988,1687,3924,2408,2046,51
69
AGTR2_3_1921,456,586,274,2004,24,912,382,553,2996,105,110,904
APLNR_3_1922,1147,312,643,1157,1270,1981,3474,1606,778,58,574,291
AVPR1A_3_1923,2214,2798,2789,3485,3468,1347,1583,4748,3460,4959,2680,2
970
AVPR1B_3_1924,171,539,458,135,279,2067,1026,1483,158,380,433,271
BAI1_3_1925,406,395,538,867,788,398,299,890,35,410,429,700
BAI2_3_1926,400,247,706,422,235,136,50,994,150,65,812,945
BAI3_3_1927,1518,3349,3590,3610,1937,1793,4568,3634,5280,3556,3603,268
9
BDKRB1_3_1928,683,849,807,1723,1991,2274,820,3180,1256,2596,1383,1272
BDKRB2_3_1929,545,474,317,1284,875,667,102,2263,533,248,9,550
BRS3_3_1930,5392,5597,6232,6232,5030,8950,5668,7794,2756,3703,9649,355
8
C3AR1_3_1931,4937,8178,4802,6440,5570,6594,6205,6153,3942,3790,6212,57
46
C5AR1_3_1932,1063,1074,1014,793,532,1978,172,730,2641,947,273,1389
CALCRL_3_1933,3081,3219,4718,5088,2583,5307,6149,4245,7515,2041,2802,6
097
CCKAR_3_1934,18008,18616,17650,21271,19871,27741,22528,17825,16456,214
90,24774,27857
CCKBR_3_1935,1132,1165,1162,2115,80,3334,307,2016,530,2297,1698,4456
CCR1_3_1936,3363,3589,3458,3754,2105,3291,5220,4233,3993,3208,7683,253
5
CCR7_3_1937,274,412,201,965,7,7,4,576,17,3,21,647
CCR8_3_1938,1110,1265,3069,4545,3434,1201,3918,2547,2720,2684,980,2079
CELSR1_3_1939,600,1111,1477,2018,750,2140,1495,3599,261,476,1610,602
CELSR2_3_1940,429,616,201,506,336,308,66,340,698,660,24,1091
CELSR3_3_1941,214,1052,767,378,763,3641,108,1791,438,2104,273,666
CHRM1_3_1942,1445,1204,1362,1819,1742,797,2666,1786,2243,652,1397,525
CHRM3_3_1943,1252,1926,2623,2514,1491,2822,1884,3348,2355,2185,1924,14
07
CHRM4_3_1944,785,1745,738,1509,1055,3813,897,501,1632,1201,858,2841
CHRM5_3_1945,212,621,602,694,167,492,183,989,398,7,157,854
CXCR1_3_1946,3359,2089,2902,2688,2894,3309,7471,1699,6524,3686,7342,21
92
CXCR6_3_1947,2363,4324,2244,3947,3386,6084,4117,3057,1380,3828,10773,2
967
CXCR7_3_1948,1504,1317,1644,2600,343,679,2919,3624,186,2979,2855,2641
CYSLTR1_3_1949,4389,5241,3844,7218,4228,5825,5473,4530,9739,6999,8459,
9719
CYSLTR2_3_1950,10149,10620,12993,15367,14942,14982,19005,15487,10396,1
1875,17894,11702
DRD1_3_1951,559,329,215,511,778,427,503,145,205,116,372,265
DRD4_3_1952,594,496,56,103,433,153,631,723,2,80,2182,234
DRD5_3_1953,606,700,812,523,1047,1598,541,328,1910,439,490,1315
ELTD1_3_1954,7054,8141,9455,8307,9246,11101,11037,4393,8919,9187,8132,

9434
EMR1_3_1955,3202,4494,3656,2954,3543,2016,4194,3673,3668,5408,2124,338
1
EMR3_3_1956,158,460,115,946,57,8,629,1372,1800,5,410,1536
F2R_3_1957,6637,4275,3561,4224,2998,8254,4162,6688,5221,4901,5992,6473
F2RL1_3_1958,1576,1639,2045,3308,837,2201,2130,2445,6484,1345,1805,160
3
F2RL2_3_1959,1160,2281,3242,2244,2276,1120,4014,3779,871,1037,3931,150
9
F2RL3_3_1960,3034,4516,3012,3387,2938,6480,5033,1983,3286,3683,5840,55
49
FFAR1_3_1961,1074,376,1682,812,612,1088,2155,651,1955,115,115,683
FFAR2_3_1962,2551,2369,4340,3625,4030,5159,5373,807,4462,4890,4775,437
8
FFAR3_3_1963,6326,6532,6253,8416,7068,10000,9078,5939,4813,5852,9186,1
1174
FZD10_3_1964,2332,2535,1757,2243,1758,3447,1274,2162,4062,1476,7580,23
01
FZD1_3_1965,1447,1459,1797,1773,1362,1097,4240,2234,596,1634,2909,2147
FZD2_3_1966,1499,1985,2504,1408,2784,4232,6858,1535,436,5286,2245,423
FZD4_3_1967,2935,3298,4894,2729,3009,5753,3786,10539,7597,6723,1958,73
29
FZD5_3_1968,1146,804,1386,582,1150,1533,447,1517,1102,219,299,212
FZD7_3_1969,458,372,654,513,834,100,2184,487,696,217,4174,924
FZD8_3_1970,373,413,105,282,862,949,606,76,67,18,182,171
FZD9_3_1971,41,294,53,398,0,504,164,2059,0,6,508,482
GABBR2_3_1972,176,408,420,362,334,185,260,318,1063,28,254,64
GALR1_3_1973,2115,3031,3933,6006,3120,2421,1958,3163,1788,5491,2022,52
45
GALR2_3_1974,567,574,749,1609,1977,135,1737,350,558,2791,1683,2255
GALR3_3_1975,2352,3206,4121,6098,2300,4680,2414,5467,4210,7345,2091,30
43
GCGR_3_1976,1031,2471,1942,2714,1387,1816,2203,1800,1184,1210,1939,451
6
GHRHR_3_1977,904,1461,1754,1184,391,2736,921,1744,2288,149,2136,3931
GIPR_3_1978,891,1709,1905,2049,131,3129,1315,1785,1572,1235,546,2550
GLP1R_3_1979,44,487,42,125,0,0,0,128,1124,2,44,3
GLP2R_3_1980,750,1059,490,454,1000,66,418,319,303,533,116,677
GPR101_3_1981,1319,1833,1511,2162,1914,2659,2303,4047,1031,3317,1088,3
272
GPR108_3_1982,122,218,720,345,858,13,593,340,213,221,678,6
GPR111_3_1983,4617,6688,5862,10447,5488,6490,5942,7234,6037,7912,8535,
12150
GPR112_3_1984,1597,1583,1536,2596,1722,694,563,786,1007,1778,3548,897
GPR114_3_1985,5049,4346,5731,7580,4801,5162,6120,9201,6096,7911,7379,9
883
GPR115_3_1986,1284,1274,1797,4116,1161,4906,2134,3867,3206,2951,2331,1
775
GPR119_3_1987,2435,3866,2802,3254,1523,6230,4174,4193,3696,4349,2027,2
731

GPR123_3_1988,470,615,537,573,691,197,0,1,2107,19,14,39
GPR124_3_1989,919,726,235,764,1170,1267,594,1186,10,11,3989,57
GPR125_3_1990,1795,1325,3418,2917,1875,2083,5298,1783,3334,645,2222,27
75
GPR128_3_1991,438,308,1292,789,1448,1250,762,1796,997,1002,1344,456
GPR12_3_1992,734,910,1614,1246,2447,2530,1689,1443,457,2917,390,366
GPR132_3_1993,1042,2013,1990,2192,2302,2266,5448,115,1549,367,903,1972
GPR133_3_1994,2110,2747,2275,2668,1878,4435,2807,2686,4010,5146,2524,3
361
GPR135_3_1995,2403,3040,3081,3429,3184,1374,2502,1006,2496,3432,5361,1
612
GPR139_3_1996,2164,2433,1886,2372,2528,2709,2264,3627,3669,3324,2076,4
539
GPR141_3_1997,5651,6494,5380,8403,4165,12289,6687,8814,7893,10143,5957
,5405
GPR142_3_1998,12968,15810,17169,18831,12147,15200,19580,16731,11891,23
669,14761,18283
GPR143_3_1999,512,1324,706,752,1800,1586,655,2083,865,1379,2536,884
GPR144_3_2000,647,1361,1136,486,287,743,1715,1593,1234,890,4862,1617
GPR146_3_2001,3206,4657,5215,7619,6199,4527,5802,8760,4054,4770,2723,1
0050
GPR148_3_2002,1825,2009,1377,1657,302,2352,1047,1433,930,1623,449,4475
GPR149_3_2003,1435,1566,848,1396,372,895,1015,916,815,2503,659,955
GPR150_3_2004,388,525,547,989,333,294,920,1064,377,871,89,317
GPR151_3_2005,41,310,5,96,191,63,94,0,0,4,517,1
GPR152_3_2006,605,659,1413,1891,1762,1599,968,752,313,387,289,769
GPR153_3_2007,839,1839,1291,1249,2308,334,582,2951,1556,91,748,1187
GPR157_3_2008,5812,6091,5600,6084,8309,9594,5380,7766,7387,5358,10758,
7465
GPR158_3_2009,1226,1481,1226,1232,1775,593,1465,6001,1619,1488,796,147
4
GPR15_3_2010,2284,2424,3562,2738,1963,2627,3274,4312,2048,1799,3219,26
82
GPR160_3_2011,11874,13565,14510,14211,10718,17844,16339,13039,13480,19
753,26529,14909
GPR161_3_2012,1636,2046,1050,1025,1199,2317,2782,610,1335,706,1773,197
5
GPR171_3_2013,1467,1259,1324,1882,828,1245,1327,569,1439,603,48,59
GPR173_3_2014,103,479,146,223,675,1274,721,201,512,30,267,99
GPR174_3_2015,12824,16726,20021,22502,14605,16338,17912,18576,19480,21
434,16055,17452
GPR176_3_2016,5807,3956,6276,6809,6293,8840,6690,5050,7316,6489,5378,5
393
GPR179_3_2017,1287,2174,1367,2692,1270,4006,252,1690,1456,1231,1054,64
8
GPR182_3_2018,6121,9437,7410,8444,6695,3920,5924,7766,5182,9408,17496,
11084
GPR183_3_2019,7217,7740,8291,9303,7354,8701,12092,7867,8956,10712,1044
7,8352
GPR19_3_2020,23115,27317,25912,33088,26983,34947,25739,35159,28385,296

50,40371,29117
GPR20_3_2021,4558,2154,4310,4322,6438,1991,5037,5698,3643,2676,3364,4338
GPR21_3_2022,2301,3123,4197,5166,3426,7507,4056,4095,4758,756,3541,3460
GPR22_3_2023,1436,3284,1600,5544,813,1646,2360,3502,3028,2497,2608,4211
GPR25_3_2024,2675,2142,2600,3520,2238,2371,2905,2744,1328,5180,4930,2240
GPR27_3_2025,886,738,357,1320,2558,678,373,449,456,203,511,625
GPR31_3_2026,2553,4172,5517,4470,2695,7941,3735,1931,2540,2891,4607,2662
GPR32_3_2027,289,381,44,44,203,1017,34,133,443,213,2,839
GPR37_3_2028,2998,5005,2694,4168,1484,1351,1908,4121,1712,6293,2590,3964
GPR37L1_3_2029,3248,4928,3353,2603,3374,3274,3448,6468,1166,2532,507,4523
GPR39_3_2030,1348,1536,1343,374,754,2636,773,41,143,450,373,929
GPR3_3_2031,208,960,689,1032,527,415,1388,1451,1460,47,2531,2130
GPR45_3_2032,795,1254,1893,893,219,1815,1793,1290,363,924,3783,1153
GPR4_3_2033,769,1336,610,1575,280,1320,812,120,580,1175,4507,1559
GPR50_3_2034,19013,21952,19394,29000,15550,24031,27637,35414,32079,26410,23471,26911
GPR52_3_2035,3248,3694,3969,4577,3405,4439,5620,2342,2843,4789,2941,3909
GPR55_3_2036,3065,3281,2811,3657,3636,2732,4764,4835,3038,2575,5973,3328
GPR61_3_2037,1461,921,998,1715,1649,1725,3698,1620,1106,1663,4209,3140
GPR62_3_2038,1331,1903,1177,2500,1959,1352,769,2491,2302,567,1142,2832
GPR65_3_2039,985,941,811,948,789,553,697,520,1227,1208,35,672
GPR6_3_2040,696,999,980,1232,1220,607,1071,1637,658,1012,701,1073
GPR75_3_2041,6409,9434,9160,8728,4866,10010,8566,6314,10003,15277,8467,9235
GPR77_3_2042,1062,368,316,401,583,81,398,1443,1051,1308,82,2348
GPR78_3_2043,123,148,305,106,13,983,0,355,1809,32,21,74
GPR82_3_2044,825,237,147,109,171,39,16,1156,3,46,310,1334
GPR83_3_2045,18,239,277,161,13,479,176,8,1,316,99,6
GPR84_3_2046,722,701,474,820,826,1482,673,896,1168,698,225,107
GPR87_3_2047,559,1000,1514,913,1432,228,152,32,1526,701,50,762
GPR88_3_2048,187,145,139,424,8,253,2,3,196,0,1,93
GPR97_3_2049,9781,13961,14702,15236,9241,15507,17383,17674,15864,15736,20312,17935
GPR98_3_2050,465,828,457,729,3146,1031,561,4121,1582,202,1918,2699
GPRC5A_3_2051,3872,4922,5308,6787,3357,4399,2956,2735,4701,2223,3123,3197
GPRC5B_3_2052,822,1623,843,772,469,156,815,732,989,753,391,756
GPRC5D_3_2053,2191,1743,2135,3538,810,3433,3664,2537,2085,3325,594,3847
GPRC6A_3_2054,907,1442,1102,1428,1665,1204,1949,2286,1104,3854,2803,3218

GRM3_3_2055,2137,1868,2013,3490,827,5118,3453,6138,1727,1505,3116,2171
GRM4_3_2056,3981,6022,4778,5540,4453,3127,5718,7295,5703,2006,6879,523
1
GRM6_3_2057,1547,3182,3124,2276,916,1133,4041,1723,1565,2562,788,857
GRPR_3_2058,6499,10088,12233,10477,9120,9347,18282,13322,7187,4778,765
8,12398
HCAR1_3_2059,293,311,429,335,756,5,100,72,391,215,165,682
HCAR2_3_2060,2739,3428,3163,5460,2321,4259,4269,6265,2805,3651,3773,81
15
HCAR3_3_2061,2739,3428,3163,5460,2321,4259,4269,6265,2805,3651,3773,81
15
HCRTR1_3_2062,1955,2005,2619,3482,2765,6206,5582,4553,4756,5389,1310,5
660
HRH3_3_2063,2453,2197,2802,4385,2562,3506,5262,983,2915,2039,2788,4240
HTR1A_3_2064,631,416,512,723,575,0,1,65,27,623,1752,2
HTR1B_3_2065,1026,1176,467,2604,152,622,1484,417,969,641,225,2704
HTR1D_3_2066,326,103,99,507,0,2212,164,0,608,954,396,0
HTR1E_3_2067,1649,2771,2039,3163,1716,3242,1349,2189,2254,3910,2455,34
75
HTR1F_3_2068,964,251,468,1284,285,38,91,1210,1631,1993,305,148
HTR2B_3_2069,6276,4727,6273,11604,2826,5177,5982,8531,7613,7610,13544,
3472
HTR2C_3_2070,3983,4954,6158,3956,5891,7234,4751,7911,4027,3317,5412,39
49
HTR5A_3_2071,186,269,299,301,273,229,338,51,12,1518,1789,527
HTR6_3_2072,307,1290,746,511,80,3200,1143,1138,1899,208,239,1310
KISS1R_3_2073,723,488,631,831,618,668,507,998,744,628,127,128
LGR4_3_2074,801,906,815,787,1073,1581,2114,1807,323,365,1196,670
LGR5_3_2075,2953,2982,5739,2462,4259,5895,5056,1912,1181,1025,4891,399
6
LHCGR_3_2076,287,812,364,522,27,2299,830,3142,714,862,484,3188
LPAR2_3_2077,1965,1652,936,1616,104,60,720,2886,1584,1621,3197,1947
LPAR3_3_2078,2728,2518,3964,2318,2479,2443,3808,4662,2041,4101,5798,21
23
LPAR4_3_2079,2890,2473,2391,2161,1639,1853,4859,1195,2935,1203,3806,27
72
LPHN2_3_2080,5890,5407,7390,7679,3501,4483,9222,6493,3228,6206,3442,82
97
LPHN3_3_2081,6863,8754,9577,8946,7585,6323,6171,7495,8940,8190,8955,83
22
MAS1_3_2082,10083,12665,15040,17463,16813,29933,22800,11097,14558,1493
5,13680,19468
MAS1L_3_2083,1935,2512,1525,2156,2991,1956,5907,3968,2268,2277,3858,41
79
MC1R_3_2084,2385,1531,2287,3892,3262,3977,1842,1802,2010,4605,1288,280
5
MC2R_3_2085,1122,1401,1853,1912,97,822,2059,3073,933,337,3475,2196
MC3R_3_2086,2358,3392,1555,2880,3080,1854,2593,844,2559,2909,2800,1525
MC4R_3_2087,7472,10909,14363,14166,11761,11577,15880,12193,14037,11741
,12994,13153

MC5R_3_2088,1974,2091,2856,4202,2144,3173,1964,3478,3400,3687,6207,449
1
MCHR1_3_2089,330,498,582,601,943,82,405,420,245,328,257,237
MLNR_3_2090,522,161,347,287,153,80,1537,42,207,35,29,240
MRGPRD_3_2091,5505,5135,3475,5676,4918,4347,11193,5730,4906,3026,3781,
7318
MRGPRE_3_2092,481,571,1092,258,2896,71,3132,889,28,422,2352,53
MRGPRG_3_2093,960,1260,799,1038,64,2297,1180,664,260,580,1603,905
MRGPRX1_3_2094,2064,1998,1747,2584,4846,3816,4154,5893,3674,1155,1959,
4196
MRGPRX2_3_2095,924,1901,1277,1407,1260,1805,2264,1774,716,3961,1084,86
1
MRGPRX3_3_2096,10499,13395,15366,16110,13899,32880,22469,13363,14161,1
4828,11709,14963
MRGPRX4_3_2097,3143,2806,3228,2061,2719,3706,2286,4014,7550,5293,3229,
3140
MTNR1A_3_2098,5862,6507,6573,7592,7931,3818,5024,10499,9171,6184,5032,
9508
MTNR1B_3_2099,213,643,120,783,21,1271,401,106,179,18,119,140
NMBR_3_2100,2831,5424,3586,4180,3394,4242,2107,4759,2335,7499,2056,345
8
NMUR1_3_2101,918,457,581,1382,1315,1079,685,16,606,19,510,1424
NMUR2_3_2102,1065,801,801,960,89,56,891,719,1692,336,854,2830
NPBWR1_3_2103,330,54,161,150,21,1007,1,2129,0,0,0,0
NPBWR2_3_2104,493,95,437,123,34,798,41,131,90,318,463,214
NPFFR1_3_2105,2367,3742,5717,6009,2347,4519,3274,4981,4627,4000,3191,3
223
NPY1R_3_2106,1909,2589,3104,2952,1147,1480,3322,5094,1072,2633,5011,43
12
NPY2R_3_2107,1470,1352,3336,2859,3475,655,1834,358,426,2653,2962,3198
NPY5R_3_2108,2679,3320,4615,5223,3195,7179,3292,6909,4377,7439,5157,64
36
NTSR1_3_2109,1568,2605,1942,3109,4666,2054,1762,2458,1359,3057,6802,34
52
NTSR2_3_2110,858,1395,1439,923,1719,783,340,818,1418,1427,1956,1955
OMG_3_2111,2262,2349,2738,2546,2734,3603,4682,3079,2496,3095,2938,2024
OPN1LW_3_2112,4853,5115,4732,4064,2322,6788,3582,4104,5349,2388,7309,4
691
OPN1MW2_3_2113,330,793,1074,333,47,335,187,1104,538,1593,101,37
OPN1MW_3_2114,330,793,1074,333,47,335,187,1104,538,1593,101,37
OPN1SW_3_2115,4239,3196,3195,3989,6389,8678,2870,4609,4878,3015,5091,3
186
OPN3_3_2116,2626,2313,4157,3778,2376,3780,4371,4090,2581,3806,6128,579
3
OPN5_3_2117,1473,1561,1182,3089,1091,1678,980,1060,957,329,1871,3973
OPRD1_3_2118,1095,1063,665,661,458,4093,113,487,428,31,201,409
OPRK1_3_2119,223,514,201,542,71,170,37,101,112,65,701,0
OXER1_3_2120,556,239,832,362,96,600,384,1253,951,1384,536,34
OXGR1_3_2121,2643,3691,3355,4193,1892,3319,4812,3220,4256,3039,4695,54
56

OXTR_3_2122,676,328,254,360,171,1925,810,381,479,34,5,25
P2RY11_3_2123,150,31,219,532,2491,169,681,456,108,1,0,5
P2RY13_3_2124,1350,1175,2255,1889,3966,1324,1467,1132,2319,870,3939,14
16
P2RY1_3_2125,4800,5724,7819,7237,5337,9011,5909,11788,7115,4892,3178,5
803
P2RY4_3_2126,602,573,112,749,22,259,865,845,19,1682,127,94
P2RY8_3_2127,2308,2336,3342,3115,3290,5198,4059,1569,2932,2240,1313,71
30
PPYR1_3_2128,395,982,861,587,395,428,1379,595,689,861,68,432
PRLHR_3_2129,1517,1738,1247,1606,364,1057,1636,170,864,1896,408,1843
PROKR1_3_2130,145,717,169,506,164,600,50,556,15,274,664,734
PROKR2_3_2131,2087,2871,4171,3468,5730,808,5208,1561,4890,1564,2355,45
83
PTGDR_3_2132,1902,957,1959,3789,1555,2436,4016,1642,581,1282,2530,3545
PTGER1_3_2133,357,567,87,571,29,1262,861,2356,564,1077,82,6
PTGER2_3_2134,370,1260,374,505,602,1527,273,695,630,1737,246,299
PTGER4_3_2135,1565,1202,1607,1552,1219,3616,1390,1974,2057,2591,2093,1
626
PTGIR_3_2136,905,813,1156,703,102,857,1792,1130,1500,494,421,1844
PTH2R_3_2137,4714,4784,5522,5052,2569,9278,9902,5241,5599,4126,7136,59
32
QRFPR_3_2138,1060,834,860,2680,65,1687,3773,253,674,563,3350,2159
RHO_3_2139,446,312,446,722,1104,537,16,452,406,108,962,137
RRH_3_2140,2807,5510,5542,6694,5372,4503,10358,9670,4622,7406,11200,71
17
RXFP1_3_2141,1137,1699,1377,3506,1451,1605,1188,1682,887,1463,1811,353
5
RXFP3_3_2142,2312,2737,2046,2423,1138,3042,2498,2796,1513,2397,2904,53
28
RXFP4_3_2143,1954,2189,2357,2064,910,2478,3534,1826,2590,1145,1212,106
4
S1PR1_3_2144,157,705,55,591,396,493,314,64,704,1,1135,1093
S1PR2_3_2145,1263,3061,1927,2135,997,8149,1645,1345,2909,1342,1298,305
1
S1PR3_3_2146,2040,2448,3294,4829,780,2251,2910,3315,4045,1824,2613,401
8
S1PR4_3_2147,67,708,217,623,371,218,452,153,68,2029,741,790
SCTR_3_2148,1077,1487,2082,1633,782,1370,1388,1737,2517,1875,787,1510
SSTR1_3_2149,1712,3057,3922,4676,2105,4247,3587,4360,1926,3065,5965,43
44
SSTR2_3_2150,1790,2790,3651,3087,2144,3146,3625,4827,2384,5246,1813,25
62
SSTR3_3_2151,986,1189,984,2868,811,2783,1032,1439,3865,1076,883,1797
SSTR4_3_2152,443,637,708,892,194,938,476,601,580,34,493,290
SUCNR1_3_2153,926,1742,2272,1667,1094,2299,2378,485,1467,1388,1377,942
TAAR1_3_2154,21027,22059,25794,27282,22825,17021,20198,43153,18339,283
01,19800,33503
TAAR5_3_2155,7758,6928,6381,9615,12329,11911,10727,8103,11328,12239,12
562,9030

TAAR6_3_2156,1872,2312,1802,2578,3425,3421,2604,3340,1565,1258,5282,76
03
TAAR8_3_2157,1926,2086,2141,2238,2261,835,1864,1474,1715,7214,1665,995
TAAR9_3_2158,4780,6811,6878,6994,8354,8530,4354,6439,8910,6810,7814,68
56
TACR2_3_2159,555,550,697,1495,1530,75,479,376,550,321,1194,531
TACR3_3_2160,25,176,284,121,0,153,1,44,30,124,613,0
TAPT1_3_2161,6356,7054,7284,8574,10125,6631,6605,10814,6868,10747,5066
,8598
TAS1R2_3_2162,2614,4759,5451,5364,2967,6266,7591,7593,4719,5300,5273,7
165
TAS1R3_3_2163,1152,684,1067,1262,1598,1131,1051,1629,1194,2377,1306,17
12
TAS2R10_3_2164,1744,1486,2583,1882,1959,186,1960,2479,3050,317,2867,71
0
TAS2R13_3_2165,10610,10744,10734,10420,14298,17039,23372,17588,11278,1
1886,20548,10771
TAS2R14_3_2166,1929,3318,3900,4275,2601,4549,6923,7789,2384,2721,6570,
4005
TAS2R16_3_2167,3192,2488,4141,3516,2844,4409,5945,4761,3079,3187,2168,
6159
TAS2R1_3_2168,121,528,42,229,8,799,0,132,0,5,9,77
TAS2R20_3_2169,894,511,1472,1587,2200,1969,4075,1039,851,891,505,294
TAS2R31_3_2170,2004,3016,2247,3765,2629,2319,674,5999,1416,300,631,575
9
TAS2R38_3_2171,956,677,1408,1413,1237,759,1356,1292,164,474,600,2331
TAS2R3_3_2172,1259,1256,2015,1276,2333,1791,3886,2153,1040,2308,2962,2
516
TAS2R40_3_2173,4652,5850,5902,8420,5779,8487,4357,3552,3200,3749,7795,
9869
TAS2R41_3_2174,1870,2195,3292,2141,885,1955,3937,268,5801,869,2991,113
9
TAS2R46_3_2175,11365,12096,18109,15199,11015,22465,21121,18792,14052,9
336,13601,13714
TAS2R4_3_2176,2024,2204,2241,2974,906,2577,3448,5279,1602,1866,1819,27
79
TAS2R5_3_2177,1544,2241,1053,1603,936,2347,1359,724,1391,2737,1145,436
7
TAS2R60_3_2178,1020,1356,1268,2562,1049,826,2108,196,1742,1381,1735,25
84
TAS2R7_3_2179,2553,2814,2485,4628,2829,5432,2010,1781,4425,3992,2389,2
771
TAS2R8_3_2180,2699,2865,3235,2826,1154,4193,3876,1917,1939,6087,5420,2
248
TAS2R9_3_2181,3870,3904,4408,3014,9622,5561,6675,7357,2678,5783,9674,5
347
TM2D1_3_2182,1229,1589,838,852,185,920,466,1337,1232,608,135,1597
TMEM11_3_2183,246,168,317,439,221,164,161,0,720,983,88,19
TRHR_3_2184,1681,1226,2925,3000,1713,3175,1165,2067,959,3163,5172,3190
UTS2R_3_2185,507,587,1233,154,532,550,1099,480,107,89,457,543

VIPR1_3_2186,2667,2930,4066,6765,3028,5321,4414,7331,4161,4345,3070,70
82
VIPR2_3_2187,1734,3543,2786,3571,2543,2860,5028,3122,1585,2526,2024,24
70
VN1R1_3_2188,679,900,921,848,59,117,1996,1161,1044,753,208,245
VN1R2_3_2189,1506,1315,1010,1129,377,2768,2242,1867,2344,2287,2889,830
VN1R4_3_2190,1078,809,1277,1519,1131,424,703,254,553,1393,286,1353
ADCYAP1R1_3_2191,3940,4753,3547,5751,2767,4733,7270,2556,8532,7409,693
7,2864
ADORA1_3_2192,1840,1993,1868,5442,4508,2821,4063,5899,2630,1360,4942,1
922
ADORA3_3_2193,1517,1795,1494,2239,2572,3039,1645,5019,2466,1451,2086,2
334
AGTR1_3_2194,1270,1914,2056,1506,2295,1945,1147,1143,2343,1421,4513,34
44
AGTRAP_3_2195,1377,2079,2306,1972,2526,1465,4538,5564,1673,3448,825,12
01
AVPR2_3_2196,40,303,316,28,100,0,34,646,27,161,170,181
CALCR_3_2197,1645,2130,2879,2555,2973,3779,2442,3600,3471,528,2853,286
4
CASR_3_2198,1291,1926,1644,2612,1394,3012,1872,1174,4975,901,1125,1235
CCR2_3_2199,798,1396,1643,2275,1372,4928,1890,1916,878,2549,6336,1288
CCR3_3_2200,1369,760,632,1660,1218,1830,879,4299,4346,1195,1755,2065
CCR5_3_2201,4625,4525,2784,4238,4390,3728,9359,13234,4822,5614,5457,11
760
CCR6_3_2202,4877,6276,5735,6593,10217,9643,7793,3888,8892,4721,5330,58
88
CCRL2_3_2203,7016,7576,7385,11345,7683,9197,10404,10455,9382,6500,1851
7,6114
CD97_3_2204,1,1,1,0,1,0,1,1,0,0,4,0
CHRM2_3_2205,890,1310,840,2081,615,1063,3051,1124,135,1662,316,127
CMKLR1_3_2206,781,859,225,1127,1268,1514,631,859,236,825,107,1546
CRHR1_3_2207,1282,1878,1850,1730,2990,1084,1938,4458,3341,1080,2368,14
74
CRHR2_3_2208,1286,1883,2161,1959,3850,1079,1942,5462,3345,1080,2367,19
55
CX3CR1_3_2209,5688,6543,8423,8488,5986,5393,9804,7835,7819,6592,7650,6
066
CXCR2_3_2210,1698,2050,2905,3111,2141,5083,2917,265,1095,2375,3907,255
3
CXCR3_3_2211,1881,1737,1681,3552,3558,1895,2376,1407,4103,2084,2772,38
33
CXCR4_3_2212,1089,2967,2146,2962,1562,4406,4384,3303,4075,3874,641,140
6
CXCR5_3_2213,1503,1432,2578,3795,1466,2475,1477,3647,1270,1584,4537,13
55
DARC_3_2214,332,527,697,1726,637,512,2194,919,641,66,161,613
DRD2_3_2215,131,858,873,593,183,1345,601,355,125,653,2904,829
DRD3_3_2216,925,670,910,334,482,371,363,19,518,784,1011,2346
EDNRB_3_2217,533,1074,950,1571,1289,1044,1045,443,457,247,1156,2228

EMR2_3_2218,9312,12183,13274,15718,7365,21103,12337,17505,9946,13384,1
4236,9925
FPR1_3_2219,1574,1803,802,1378,525,2093,993,350,2782,717,852,608
FSHR_3_2220,3536,4165,4258,7102,2911,5722,5141,14040,2799,3599,5315,45
68
FZD6_3_2221,3430,3030,4197,4539,3478,3241,2688,4353,3509,8760,5534,410
3
GABBR1_3_2222,3315,4094,4095,6777,5779,7193,4357,10155,5008,5662,8352,
4601
GHSR_3_2223,2080,2073,3254,3414,1427,3766,4281,5840,5380,2135,6031,374
8
GNRHR_3_2224,319,539,784,1070,479,1312,919,643,134,1161,387,1340
GPBAR1_3_2225,38,46,408,351,596,205,685,12,2,49,234,29
GPER_3_2226,331,948,388,446,628,922,265,386,410,453,751,45
GPR107_3_2227,594,574,318,540,1271,175,1402,1206,554,1287,801,1379
GPR110_3_2228,8621,9631,10603,11169,10108,10666,15672,14169,11594,8101
,12378,11244
GPR113_3_2229,1915,2098,4264,3277,2992,4836,2159,2390,1178,1723,3754,2
061
GPR116_3_2230,2407,3619,3181,6278,2136,5587,4942,3380,4979,2221,10456,
2612
GPR126_3_2231,2195,1352,1502,2712,1993,1759,2172,4544,1232,4083,3349,9
67
GPR155_3_2232,2777,2159,2976,4197,2870,5060,3836,5526,1344,3453,2196,2
903
GPR156_3_2233,1397,713,1185,1579,102,765,1293,401,2163,903,1219,2119
GPR162_3_2234,1985,3590,3414,3311,3814,3526,3300,3766,2307,7070,1710,3
843
GPR17_3_2235,511,557,324,483,3,177,1213,20,799,41,291,153
GPR18_3_2236,2772,2769,2105,5172,4131,4217,3142,4901,2594,2441,2881,32
71
GPR1_3_2237,5231,7815,8152,8293,4190,2530,7755,4429,5444,4246,5667,615
8
GPR34_3_2238,4881,4062,8086,7977,2872,9347,6508,5473,4850,6143,6138,80
62
GPR35_3_2239,1272,990,142,1481,951,929,616,2012,95,1590,62,641
GPR56_3_2240,238,395,722,216,1312,2839,229,229,3080,295,50,243
GPR63_3_2241,11145,15345,16985,19891,17419,19006,18361,13814,17378,206
64,26973,22717
GPR64_3_2242,867,842,599,1344,1052,76,908,590,852,854,3750,867
GPR68_3_2243,8191,7692,8820,10036,6592,13247,10112,13535,9830,13092,65
39,14690
GPR85_3_2244,2270,2744,2713,3625,3269,3229,2100,3349,2193,1908,1918,35
45
GPRC5C_3_2245,1206,444,789,320,163,14,537,669,65,227,87,1129
GRM1_3_2246,1361,1376,1311,1591,544,1659,927,884,1162,2536,4429,647
GRM2_3_2247,194,234,657,906,302,1064,61,160,427,277,331,506
GRM5_3_2248,1052,1115,1070,786,2260,2345,272,934,2483,29,356,1816
GRM7_3_2249,2611,3258,4101,4437,6298,2996,1365,4524,2281,2572,3150,520
2

GRM8_3_2250,4469,5706,6584,5097,5400,7643,6073,9265,4061,7011,9118,298
9
HRH4_3_2251,4621,5829,5406,6163,6205,5377,6533,8486,3904,9158,6321,686
1
HTR2A_3_2252,232,232,774,139,135,53,1262,0,2,320,1182,29
HTR4_3_2253,1245,1476,1644,1280,1511,2355,3292,3081,1010,715,3213,1527
HTR7_3_2254,1399,1950,2427,2262,1102,1282,2055,824,1393,1228,4431,1431
LGR6_3_2255,1181,1533,1999,2238,2016,1189,3311,3149,2475,4561,3032,141
8
LPAR1_3_2256,1067,1234,1597,1274,3134,141,2358,17,1766,296,2780,365
LPAR5_3_2257,286,380,1405,645,858,69,1068,843,89,1407,212,1373
LPAR6_3_2258,2437,4806,3378,4759,4919,4015,6079,5591,4614,5885,5449,49
47
LPHN1_3_2259,441,198,282,119,26,1077,4,905,520,33,88,232
LTB4R2_3_2260,247,156,438,900,125,420,125,847,569,17,1,46
LTB4R_3_2261,576,850,1224,1326,754,1535,627,1164,1945,751,477,444
LYPD1_3_2262,412,236,1777,1586,452,1448,971,220,284,1036,139,118
MCHR2_3_2263,5287,6492,5067,4638,7059,3431,6298,3063,7907,3193,6403,48
52
MRGPRF_3_2264,1010,2163,1414,1908,2514,551,180,1058,2016,2434,173,2746
NPFFR2_3_2265,1953,1627,2148,1757,3797,4135,1095,3473,2853,2266,1479,1
586
NPSR1_3_2266,1416,1663,3329,3100,1760,5506,3855,7715,4169,3759,2222,13
36
O3FAR1_3_2267,3771,5788,3546,8285,4536,7761,7662,4346,3226,7338,4893,7
834
OPN4_3_2268,1394,1413,2197,3247,3152,1967,2710,2755,1667,2352,1741,331
3
OPRL1_3_2269,2659,3405,4511,4224,7385,3188,5227,7093,1394,4681,1879,36
94
OPRM1_3_2270,5315,7394,7605,7307,4940,9536,9759,8283,9578,7040,10980,1
2309
P2RY10_3_2271,9127,7694,12410,15726,12060,13309,14204,11165,12123,7257
,20017,10844
P2RY12_3_2272,5910,5899,6476,5806,5374,11839,6245,5331,4921,5916,6320,
5726
P2RY14_3_2273,2055,2588,3948,4460,810,6849,7220,5008,3232,3755,4848,29
62
P2RY2_3_2274,2377,2406,3490,4639,2496,4164,1415,2561,4026,6126,4341,35
20
P2RY6_3_2275,653,1160,858,2346,764,2586,1819,1086,244,941,1822,626
PTAFR_3_2276,249,1054,1656,2260,218,400,1188,1558,254,161,2504,1798
PTGER3_3_2277,157,248,122,209,255,1006,265,235,136,54,360,137
PTGFR_3_2278,3122,2762,5021,5234,4033,3033,7900,5446,3401,6944,4467,43
06
PTH1R_3_2279,1244,1120,1481,1542,593,813,1049,2674,2533,386,2951,1334
RGR_3_2280,862,1269,1579,2095,1100,158,2604,1238,2101,325,2549,1308
RXFP2_3_2281,1055,1442,1940,810,428,2933,1150,1338,1143,1885,4349,884
S1PR5_3_2282,269,262,22,461,359,84,410,339,210,6,2,1
SIGMAR1_3_2283,408,604,431,541,314,139,42,770,284,687,26,1142

SSTR5_3_2284,558,310,846,999,1274,1053,354,2231,1498,501,580,1398
TAAR2_3_2285,5047,5657,6510,8965,3974,7693,8033,6563,3475,8449,5922,55
42
TACR1_3_2286,1010,1448,1946,1447,290,401,918,1617,1685,481,205,459
TAS1R1_3_2287,1644,1743,2234,2226,2419,2745,1725,1072,1950,1743,2344,2
012
TBXA2R_3_2288,312,240,591,661,363,126,493,502,1032,73,14,1
TPRA1_3_2289,560,794,980,640,415,108,832,327,609,1151,60,1279
TSHR_3_2290,970,216,596,841,668,77,1025,256,337,1053,174,749
XCR1_3_2291,727,1211,901,1999,1706,2233,2510,949,636,536,3578,996
XPR1_3_2292,2687,4200,4838,4505,3638,6995,4367,6448,5353,7285,4860,305
5
ADORA2A_3_2293,449,200,303,559,1909,112,515,1,195,27,83,308
ADORA2B_3_2294,40,325,474,722,153,289,408,2,39,1158,66,448
ADRA1B_3_2295,969,1245,1170,1352,375,2411,1362,1982,227,2930,2254,2036
ADRA1D_3_2296,579,1446,979,1472,367,922,3774,207,1393,24,336,3127
ADRA2A_3_2297,325,898,372,785,585,177,867,262,717,690,972,237
ADRA2B_3_2298,10391,8837,13691,12251,10827,15082,10848,14084,13245,117
94,13846,11656
ADRA2C_3_2299,1964,2234,2224,2200,1185,2012,4335,3034,3302,4404,3966,1
897
ADRB1_3_2300,1019,1916,1423,1397,686,941,1578,904,730,4317,1575,1294
ADRB2_3_2301,422,307,144,376,95,436,212,904,142,210,15,22
ADRB3_3_2302,1075,1577,1310,2113,1574,1038,2117,3861,990,2821,1935,126
6
AGTR2_3_2303,2435,4313,2703,3694,3112,6612,4451,7265,1579,4021,2374,16
36
APLNR_3_2304,1709,2413,2144,2421,968,2434,1943,1867,1757,441,3218,3208
AVPR1A_3_2305,1232,1197,886,1573,3595,1472,2563,2086,891,1736,1702,157
8
AVPR1B_3_2306,52,209,219,708,671,2,118,269,881,1062,128,307
BAI1_3_2307,365,128,348,138,7,17,65,28,151,17,5,0
BAI2_3_2308,1162,1222,1830,1038,359,1040,1291,2810,980,404,699,1646
BAI3_3_2309,6284,6233,5515,7142,5045,11558,8901,3764,5838,6800,8081,82
15
BDKRB1_3_2310,734,1076,409,1718,1141,1058,223,1124,2035,390,504,4095
BDKRB2_3_2311,479,1151,279,840,994,359,1077,281,1165,406,945,3635
BRS3_3_2312,7059,9277,10645,12782,10515,5822,7545,10992,11851,13371,10
126,12887
C3AR1_3_2313,1580,2673,2175,4369,1506,1930,2194,3764,3470,1934,3555,16
99
C5AR1_3_2314,699,1551,1145,877,3332,368,695,1295,1267,1972,1080,557
CALCRL_3_2315,522,1576,1294,2289,523,2543,2676,1286,296,2163,1862,1442
CCKAR_3_2316,2916,4417,4409,3079,3469,6213,6228,3803,3205,1888,3754,37
40
CCKBR_3_2317,1851,2298,1360,2670,1845,2998,1915,3743,788,5699,4536,211
9
CCR1_3_2318,755,893,1084,543,538,1301,880,1251,693,1128,2130,172
CCR7_3_2319,2372,1861,2332,3506,1491,1274,3806,6868,1438,5102,4189,424
7

CCR8_3_2320,1188,1367,2294,1644,1101,1998,5108,1864,1470,1161,2388,127
8
CELSR1_3_2321,292,739,1273,621,1872,2722,76,1084,1316,1065,52,1330
CELSR2_3_2322,903,386,276,984,1449,62,493,1133,1594,806,2484,1246
CELSR3_3_2323,2063,2210,1797,3708,1857,3293,3355,1623,1674,2538,139,28
57
CHRM1_3_2324,246,445,871,821,223,119,1998,179,1134,641,1253,1589
CHRM3_3_2325,4165,5161,5769,6323,5752,9129,5684,8278,7403,8838,7763,80
66
CHRM4_3_2326,1845,2984,4157,8849,3210,2764,5675,6050,7450,5703,5059,72
04
CHRM5_3_2327,1444,1908,1955,2134,1868,1612,2058,3357,1198,1283,1327,23
32
CXCR1_3_2328,2512,2938,3246,3191,4252,2885,3321,2735,2053,1220,1501,29
57
CXCR6_3_2329,3853,4546,7879,8585,6678,2818,6196,7352,7864,6401,14608,5
058
CXCR7_3_2330,1043,1323,1785,1338,353,2069,1501,1176,1319,2066,4329,159
2
CYSLTR1_3_2331,2438,2023,3029,1635,1261,1895,2492,5131,3241,2576,2568,
2549
CYSLTR2_3_2332,1692,2115,873,1687,459,1966,535,3013,2468,1186,541,3224
DRD1_3_2333,327,340,586,77,12,177,480,42,199,45,83,157
DRD4_3_2334,67,22,22,51,14,0,16,0,0,2,17,0
DRD5_3_2335,1755,1133,1716,1076,1812,669,1948,3007,2228,4207,2058,3116
ELTD1_3_2336,3686,3758,5975,5366,7249,7424,3617,3398,3505,9394,4257,22
89
EMR1_3_2337,1730,1390,1171,1222,870,760,572,1777,1832,478,1771,1901
EMR3_3_2338,1066,645,1245,1454,929,2137,905,1353,1249,999,3392,1381
F2R_3_2339,6217,4697,7909,7361,4394,6957,5954,10744,9780,5709,12971,75
03
F2RL1_3_2340,1430,290,426,601,350,1391,435,70,167,171,51,253
F2RL2_3_2341,491,684,1153,1428,490,321,598,818,905,994,182,1435
F2RL3_3_2342,72,48,257,212,134,7,738,878,11,1002,34,7
FFAR1_3_2343,439,1431,1025,2296,2117,751,2026,2236,1524,595,924,2602
FFAR2_3_2344,2505,2544,3294,4408,3088,1856,2600,3290,1490,6744,5630,43
35
FFAR3_3_2345,1219,1226,1337,1293,1601,1245,1107,812,1029,1324,448,762
FZD10_3_2346,1666,2281,2888,2777,4564,1080,2023,2398,2413,1189,436,130
9
FZD1_3_2347,398,425,708,579,871,507,2267,2913,922,217,4160,950
FZD2_3_2348,653,460,712,636,1033,509,2260,2916,773,217,4196,950
FZD4_3_2349,1465,3291,2773,2904,1753,728,5027,1149,2479,5127,1465,2028
FZD5_3_2350,744,127,318,655,0,426,566,160,54,0,36,3
FZD7_3_2351,396,395,642,438,247,4099,547,1712,1048,471,267,2578
FZD8_3_2352,658,1011,520,263,891,273,1014,3224,1259,510,33,841
FZD9_3_2353,528,1546,791,564,151,259,294,223,168,98,271,289
GABBR2_3_2354,1408,1950,1426,2193,433,3518,983,2454,953,1282,389,638
GALR1_3_2355,4457,3907,5675,7847,3324,2598,4261,5217,2746,2443,4661,52
66

GALR2_3_2356,59,79,40,218,1,0,33,2,0,8,1,0
GALR3_3_2357,843,447,556,512,949,1404,2,69,1118,58,1068,344
GCGR_3_2358,1061,1101,1069,1108,943,1175,8,456,287,486,2923,1920
GHRHR_3_2359,342,518,853,460,110,973,2642,1018,1584,715,2274,453
GIPR_3_2360,2395,4968,4556,5816,2675,4379,6782,5477,5063,6712,3639,557
3
GLP1R_3_2361,726,1261,949,1335,552,618,1485,2447,1091,221,1161,546
GLP2R_3_2362,4006,4704,5083,8659,4519,3703,4208,3841,3122,2055,1893,81
10
GPR101_3_2363,160,279,351,350,131,675,580,179,98,1985,61,688
GPR108_3_2364,931,1526,1968,4545,967,2015,746,1897,1284,358,403,2257
GPR111_3_2365,5749,5915,7138,11506,5741,8674,4226,10212,5088,4457,4642
,12037
GPR112_3_2366,1889,4296,4978,4049,3352,2806,2419,6086,2150,3953,9279,8
219
GPR114_3_2367,533,1432,1189,1326,577,2531,1421,431,693,113,532,225
GPR115_3_2368,999,1834,2027,1945,1809,371,1680,2652,2914,921,2529,1933
GPR119_3_2369,1746,2959,2078,5923,1783,689,1486,2428,1566,2594,5469,41
09
GPR123_3_2370,1094,1366,911,2383,2772,2362,1369,834,1188,1262,870,3764
GPR124_3_2371,81,291,408,661,1471,0,1305,1169,100,92,675,500
GPR125_3_2372,3843,3694,4136,3228,2795,3279,5749,3513,6392,3606,4599,4
053
GPR128_3_2373,2036,2060,1484,2092,3045,3177,3547,1005,3830,5663,4431,5
42
GPR12_3_2374,73,71,734,681,148,486,243,126,205,1155,107,773
GPR132_3_2375,428,950,509,609,668,223,1282,42,471,7,120,1034
GPR133_3_2376,1225,1567,1697,2605,787,1721,2580,3121,2272,1264,1883,76
9
GPR135_3_2377,625,1387,1518,2074,1262,2849,1242,374,1927,1649,761,1436
GPR139_3_2378,12186,13695,13818,18698,12959,17889,21764,14424,14714,20
669,13420,19239
GPR141_3_2379,1644,872,1732,3007,1561,1833,1925,1824,468,1146,3326,158
2
GPR142_3_2380,3844,4700,3846,4082,5058,6519,3685,3944,4379,5102,3241,3
480
GPR143_3_2381,462,621,1573,588,1334,907,961,3009,493,583,4172,1335
GPR144_3_2382,1576,2962,3588,2359,756,4727,3546,1976,1313,3224,4897,12
35
GPR146_3_2383,1812,1393,2082,2297,2076,2571,1030,951,3266,1780,3398,32
34
GPR148_3_2384,185,329,498,776,69,318,916,1528,100,420,1107,555
GPR149_3_2385,2690,4671,3348,3395,4400,5438,4414,3233,1396,2746,4521,2
999
GPR150_3_2386,1045,1027,1544,1692,2061,1714,2691,1197,1276,1867,2516,1
463
GPR151_3_2387,555,1994,970,1238,276,3555,2728,35,201,1166,2709,831
GPR152_3_2388,1052,1024,563,955,1200,232,70,59,1309,312,97,320
GPR153_3_2389,1350,1234,1351,2668,1794,291,1241,2041,1844,2864,1404,11
50

GPR157_3_2390,499,124,318,1686,664,1546,1657,2374,865,2799,716,1215
GPR158_3_2391,1150,1932,2333,2862,2569,4946,2081,1403,5829,1171,3702,9
38
GPR15_3_2392,7210,9195,14356,11780,7214,9759,9938,16163,5736,5703,8054
,14410
GPR160_3_2393,1460,4012,3517,3134,708,4120,116,3295,4776,2509,4881,332
8
GPR161_3_2394,416,478,1052,292,2645,1060,16,836,868,73,283,683
GPR171_3_2395,1944,2466,3257,3124,4511,3199,2728,1129,2208,2954,3775,4
516
GPR173_3_2396,5333,5218,4297,6358,4664,3443,2594,4797,3712,8809,4986,6
670
GPR174_3_2397,766,1182,1509,842,593,1807,1370,610,1949,486,1127,2728
GPR176_3_2398,5619,4297,6047,5748,6011,8757,6388,4997,7650,6351,5424,5
286
GPR179_3_2399,1128,495,634,1615,509,1572,1264,1417,796,849,1475,145
GPR182_3_2400,965,964,526,1549,3523,849,626,960,730,1051,1324,1210
GPR183_3_2401,12318,14368,13986,16207,15462,14276,18597,20776,11093,16
611,13274,16220
GPR19_3_2402,1254,2836,3989,2737,2444,1192,4525,3575,2531,3325,2389,16
55
GPR20_3_2403,803,1165,1070,1041,1598,1061,1281,1220,413,3939,8506,1991
GPR21_3_2404,980,1602,1212,1505,950,1571,4134,609,726,2224,170,1563
GPR22_3_2405,185,227,134,284,30,221,55,548,141,7,276,126
GPR25_3_2406,14,164,438,290,180,10,395,25,58,1418,54,0
GPR27_3_2407,1150,1048,654,1451,653,813,483,1551,1038,1585,2053,129
GPR31_3_2408,503,113,192,975,624,0,2,449,17,19,4,34
GPR32_3_2409,1819,1215,2005,2906,457,2835,1635,717,1130,1573,1058,5426
GPR37_3_2410,2929,4124,2752,3683,4888,4692,4738,7825,2045,5454,5614,25
56
GPR37L1_3_2411,138,90,1129,235,401,589,671,101,323,20,1850,1
GPR39_3_2412,247,216,414,1040,1659,115,165,2174,140,99,2665,911
GPR3_3_2413,0,0,0,195,0,0,0,0,625,0,0,0
GPR45_3_2414,5905,5087,4820,7404,7965,4785,4009,6271,6096,3731,7068,98
76
GPR4_3_2415,666,323,1290,426,706,243,3585,241,790,2091,192,1632
GPR50_3_2416,1398,673,452,513,1141,937,1437,2440,386,2318,843,1193
GPR52_3_2417,438,1110,551,1858,1063,1573,306,2724,1946,706,2355,1342
GPR55_3_2418,958,903,1444,1925,2067,1528,1343,1119,618,1535,2912,2466
GPR61_3_2419,2201,2119,2275,3194,2358,3830,4197,6922,2475,1056,520,235
7
GPR62_3_2420,125,334,487,612,58,974,339,1491,678,275,1060,1623
GPR65_3_2421,6370,5460,5230,6305,7590,3302,10068,11884,6996,11616,9586
,6920
GPR6_3_2422,369,404,554,537,442,88,519,570,423,21,86,1696
GPR75_3_2423,2678,3058,2185,2817,2920,5464,2218,3662,2702,1574,4671,29
44
GPR77_3_2424,7868,9855,6298,12865,8858,9440,13211,10825,6086,15201,134
49,10681
GPR78_3_2425,2530,3307,3947,2444,2373,2606,2317,4728,2050,2817,3137,51

28
GPR82_3_2426,4528,6248,6211,5756,6060,7056,6902,7135,6885,8723,4993,35
36
GPR83_3_2427,3347,2546,4680,3802,4195,5566,6544,3686,4646,4272,6886,27
06
GPR84_3_2428,567,1064,735,219,1807,543,835,52,420,64,610,2251
GPR87_3_2429,1741,1520,2519,2179,527,3159,3714,1748,1272,483,1982,1044
GPR88_3_2430,257,381,205,420,429,236,525,624,488,72,264,32
GPR97_3_2431,13404,20803,23742,24197,15547,27733,20968,19315,26149,234
97,27248,32137
GPR98_3_2432,2812,1227,2118,2027,2546,2264,3319,2409,1752,2843,2792,31
00
GPRC5A_3_2433,1081,2349,2002,2515,948,2361,2717,1528,2580,1730,1587,44
09
GPRC5B_3_2434,503,373,740,190,643,607,12,2,506,106,121,289
GPRC5D_3_2435,322,367,189,602,606,730,367,1017,215,1184,325,250
GPRC6A_3_2436,138,150,20,12,515,0,0,0,0,780,0,0
GRM3_3_2437,10582,8459,14746,14927,9741,6687,12829,13931,13161,12373,7
813,12604
GRM4_3_2438,465,248,488,733,73,129,1031,1303,253,544,138,78
GRM6_3_2439,1529,3215,2960,1948,1283,1135,4281,1016,1553,2562,793,854
GRPR_3_2440,5175,4172,5707,7946,4856,8464,4861,17223,6523,5612,10040,3
366
HCAR1_3_2441,464,408,945,989,563,675,0,517,106,33,53,1036
HCAR2_3_2442,2154,1431,1986,2167,1118,1111,1655,1815,817,5257,5276,768
HCAR3_3_2443,2521,3652,3009,2095,3200,2794,4644,4162,1307,1203,8572,18
72
HCRTR1_3_2444,661,885,1392,1367,1018,811,1277,435,879,1488,161,1549
HRH3_3_2445,2266,3148,2297,2051,2621,3215,2522,412,1317,3024,2509,1076
HTR1A_3_2446,792,587,599,752,1332,120,538,1275,115,2093,617,402
HTR1B_3_2447,846,410,849,2644,781,291,1253,4640,3673,947,805,2330
HTR1D_3_2448,2637,2236,2244,3703,4450,5144,2436,4484,1448,1011,1964,56
03
HTR1E_3_2449,0,0,0,0,0,0,0,0,0,0,0,0
HTR1F_3_2450,766,1276,2637,1217,1558,1258,205,1474,507,2811,1434,1660
HTR2B_3_2451,5437,5829,5424,8328,4892,9684,5081,8311,6149,4922,4900,11
901
HTR2C_3_2452,138,438,572,87,237,110,149,1356,89,15,25,2427
HTR5A_3_2453,1491,896,1388,2067,2440,1528,1893,2386,2080,1644,1184,254
7
HTR6_3_2454,2082,1729,2705,2241,3268,1232,5633,4368,2677,3591,4117,415
7
KISS1R_3_2455,130,225,387,446,4,5,29,71,647,970,278,537
LGR4_3_2456,3142,2454,3279,4451,3110,3393,4192,5029,4572,1163,3817,484
8
LGR5_3_2457,791,1242,767,1874,1326,1380,1526,1374,2038,355,3675,1715
LHCGR_3_2458,1025,1487,594,1530,1634,1971,332,3465,1967,933,1845,240
LPAR2_3_2459,512,692,274,399,150,1173,231,151,562,1086,2734,249
LPAR3_3_2460,4595,4614,3709,5381,5785,7134,5734,5381,5862,3472,8267,66
21

LPAR4_3_2461,825,1036,1215,1996,2359,2444,2628,1783,728,2537,1844,1239
LPHN2_3_2462,3470,2968,3852,4054,2719,4787,1838,6096,1782,2093,3476,44
36
LPHN3_3_2463,1071,544,909,1228,1750,154,951,1894,901,961,46,1670
MAS1_3_2464,2174,1755,1691,2522,1858,215,1697,3014,2131,3298,2163,4140
MAS1L_3_2465,161,1049,102,333,785,1557,478,1493,291,135,742,1
MC1R_3_2466,539,228,213,147,52,1167,614,11,540,245,193,370
MC2R_3_2467,5850,5656,9301,8505,7029,5614,14950,8653,4881,5715,4517,87
08
MC3R_3_2468,2572,2770,3692,3046,3974,2049,3099,2896,6767,4612,4205,354
9
MC4R_3_2469,4509,7864,5569,5942,6293,6135,6004,8454,8084,17353,5931,71
72
MC5R_3_2470,2178,1816,2476,4297,2519,3874,1945,1822,561,5655,1641,1177
MCHR1_3_2471,1497,984,846,1150,2280,1097,281,614,1500,1143,660,990
MLNR_3_2472,3719,3883,4225,5820,1947,3547,2327,1302,5396,6536,9027,565
6
MRGPRD_3_2473,1354,952,707,1494,667,745,2146,2159,2987,1861,125,2125
MRGPRE_3_2474,499,285,197,1258,14,315,50,498,209,792,1381,1508
MRGPRG_3_2475,3827,5925,4396,6429,2648,9140,3455,6308,6895,2524,7984,4
182
MRGPRX1_3_2476,442,769,726,221,1609,64,45,315,582,67,109,735
MRGPRX2_3_2477,376,880,99,825,110,991,58,429,306,877,73,1571
MRGPRX3_3_2478,922,247,753,1042,21,157,273,538,2215,451,3257,2820
MRGPRX4_3_2479,855,1163,447,654,854,723,1203,2312,563,1503,403,1281
MTNR1A_3_2480,97,387,298,396,113,982,243,24,836,138,1253,845
MTNR1B_3_2481,113,132,795,206,589,359,1053,1772,4,1134,3,360
NMBR_3_2482,8940,8636,9299,14301,10253,11480,14807,15832,11160,12267,1
1332,10284
NMUR1_3_2483,739,768,815,1256,993,3028,1327,748,2348,1653,1495,2117
NMUR2_3_2484,993,1124,422,2867,325,1275,495,1030,749,1073,174,1360
NPBWR1_3_2485,1914,1975,2005,3320,2458,2642,2772,2441,4042,3647,3975,3
650
NPBWR2_3_2486,1144,1378,1431,827,858,1754,2959,2051,1945,1285,1320,155
5
NPFFR1_3_2487,176,324,77,323,360,711,107,89,217,3,449,341
NPY1R_3_2488,3903,4509,4867,4853,3627,6451,3202,9544,3104,4388,3625,52
45
NPY2R_3_2489,2412,3327,4553,2893,3149,4038,2829,3188,3814,1926,2284,41
92
NPY5R_3_2490,520,1515,1428,1555,702,952,1263,719,1651,1794,1370,1846
NTSR1_3_2491,3,120,302,270,0,34,0,16,2,4,5,8
NTSR2_3_2492,1028,1453,1125,1329,594,1396,3780,3240,1571,2151,1825,126
0
OMG_3_2493,4177,3891,5346,6318,7171,5082,4257,4968,2467,7339,4020,6674
OPN1LW_3_2494,6444,8285,8605,7529,6331,9398,11047,7530,8891,9449,6455,
7797
OPN1MW2_3_2495,6444,8285,8605,7529,6331,9398,11047,7530,8891,9449,6455
,7797
OPN1MW_3_2496,6444,8285,8605,7529,6331,9398,11047,7530,8891,9449,6455,

7797
OPN1SW_3_2497,1083,446,1075,1077,1099,127,863,902,1482,177,190,186
OPN3_3_2498,2797,3358,3666,4723,1756,4289,2676,2628,5078,4889,3235,421
2
OPN5_3_2499,790,976,962,558,1184,1536,1264,454,898,1445,743,1153
OPRD1_3_2500,507,1466,830,1418,596,3257,1042,1084,1194,907,96,2209
OPRK1_3_2501,1005,970,936,1120,1109,556,168,3415,725,1815,917,601
OXER1_3_2502,1359,844,1887,1898,1333,2504,1550,299,2312,3341,582,1164
OXGR1_3_2503,1258,646,1744,785,1189,2454,656,590,703,527,1166,1329
OXTR_3_2504,371,795,479,661,196,130,1123,1001,254,1834,184,440
P2RY11_3_2505,1706,2256,2316,1204,419,1271,731,1001,2071,1668,2703,498
P2RY13_3_2506,1335,1168,2372,1979,3945,1497,1480,1134,2148,868,3929,14
15
P2RY1_3_2507,1898,3235,2436,2141,1131,3999,3148,2172,2855,1812,2188,15
10
P2RY4_3_2508,625,834,251,389,280,818,489,114,41,88,4994,204
P2RY8_3_2509,224,293,127,1018,87,2491,0,379,0,5,3,417
PPYR1_3_2510,5124,7058,7896,8462,6391,5545,9551,8803,9730,11352,11696,
6601
PRLHR_3_2511,49,86,873,83,0,0,0,304,2051,212,2,32
PROKR1_3_2512,1683,1368,2101,1112,497,4948,3308,2607,1473,3618,3792,21
34
PROKR2_3_2513,605,723,473,1073,1488,1092,1653,992,1037,268,2200,246
PTGDR_3_2514,2281,5327,4048,3793,2947,2850,5613,1904,2951,2609,4670,29
85
PTGER1_3_2515,75,604,4,45,244,0,0,0,244,3,4,0
PTGER2_3_2516,6247,8048,8337,10362,6554,8293,7032,8188,6516,8364,7471,
7664
PTGER4_3_2517,418,672,219,388,47,125,156,1106,618,1558,252,498
PTGIR_3_2518,358,318,240,214,56,395,579,728,192,161,536,516
PTH2R_3_2519,2387,4542,3412,5147,1359,8978,2584,5178,4285,7838,1701,36
65
QRFPR_3_2520,983,2013,1871,2231,1336,513,767,195,953,640,613,3134
RHO_3_2521,2463,2911,7370,4262,4556,679,7120,3325,4742,4465,6247,2943
RRH_3_2522,5423,3792,3048,4120,6988,4481,3399,4605,7592,4909,540,3575
RXFP1_3_2523,3813,6158,6089,6597,8164,8298,5887,8896,6641,17126,9872,8
916
RXFP3_3_2524,1884,2615,1943,3648,2285,3795,1743,3737,2944,7176,4453,33
25
RXFP4_3_2525,52,70,64,22,0,0,21,2,131,1,7,957
S1PR1_3_2526,2285,3648,4531,6612,3346,6210,5351,4357,1796,5397,1340,42
06
S1PR2_3_2527,706,1376,886,670,1017,304,698,1089,1889,110,2860,1435
S1PR3_3_2528,44,97,61,45,25,0,249,2,0,4,720,2
S1PR4_3_2529,1172,792,609,900,1368,478,1352,46,440,2910,56,238
SCTR_3_2530,1499,2991,3550,3202,4797,6323,4055,1657,2676,4032,3603,282
4
SSTR1_3_2531,2555,2660,2700,4121,2409,2043,3069,5719,2890,3126,238,269
6
SSTR2_3_2532,3733,3652,4154,5763,2269,4923,5611,4539,5267,5441,1820,47

36
SSTR3_3_2533,814,648,854,1226,2486,1859,1009,2235,1672,566,451,1666
SSTR4_3_2534,500,310,286,725,110,1330,1227,3103,357,1749,11,1101
SUCNR1_3_2535,10712,11055,13856,16199,13653,11867,16106,15211,16246,15844,16004,18207
TAAR1_3_2536,10569,13645,15031,20045,13232,10969,15010,21855,8858,15579,24222,15417
TAAR5_3_2537,3524,4413,4518,4749,3857,6717,3209,5580,4666,5038,4308,7644
TAAR6_3_2538,12254,12352,16053,19593,16188,18774,13411,21777,15310,13870,18162,14002
TAAR8_3_2539,5599,6740,6965,8751,9770,11859,10035,11301,7140,8072,9912,3312
TAAR9_3_2540,4520,6596,6805,7110,7407,7955,3938,7143,7196,6282,9477,6766
TACR2_3_2541,812,1032,869,1388,684,556,696,2382,800,636,2648,1154
TACR3_3_2542,3588,4635,4133,6055,6886,2417,4948,4523,2474,7139,1836,3641
TAPT1_3_2543,3171,6208,5689,6629,4666,8057,7974,7155,9101,10680,12804,5116
TAS1R2_3_2544,768,751,1672,2544,154,2163,1749,457,1212,257,103,1386
TAS1R3_3_2545,1803,2821,1985,2009,2682,3620,6310,4352,4135,1547,2560,1874
TAS2R10_3_2546,2139,2177,5099,2693,987,4970,4148,7776,4816,4148,1633,4631
TAS2R13_3_2547,1477,1733,1508,2735,2490,2534,1773,1832,4946,1897,2751,2625
TAS2R14_3_2548,3226,3930,5273,3507,3092,911,4606,3295,3643,5472,3076,5334
TAS2R16_3_2549,1996,2177,2006,1572,3438,2417,2521,397,1433,1569,2543,4214
TAS2R1_3_2550,7855,7481,8862,9536,8560,12897,10068,7164,7312,11738,12545,14473
TAS2R20_3_2551,288,658,476,1531,1060,2674,1196,223,463,355,1289,1595
TAS2R31_3_2552,5767,8972,8018,10598,8647,12993,10569,4262,8943,14486,5619,11262
TAS2R38_3_2553,4957,3676,4783,6563,2488,8785,9398,6597,6086,12811,4863,6011
TAS2R3_3_2554,818,1910,1354,1142,1939,2875,967,1893,417,1340,254,561
TAS2R40_3_2555,2430,2204,1427,3674,1801,707,2461,4916,810,2565,5017,694
TAS2R41_3_2556,1358,1518,1110,2158,967,606,2581,651,1429,1392,2067,997
TAS2R46_3_2557,1039,1628,1600,2251,633,2462,1845,336,1219,1817,1046,753
TAS2R4_3_2558,2128,2618,2243,1753,2029,2088,1958,6601,1827,1911,5402,1279
TAS2R5_3_2559,2336,3276,3990,7455,3321,5415,2453,2171,2388,1087,2902,6473
TAS2R60_3_2560,2388,2163,2646,2741,2207,3066,2161,3343,2802,4084,1563,4032

TAS2R7_3_2561,4216,4320,8750,6249,6147,6802,3719,5608,4523,2299,3154,5
758
TAS2R8_3_2562,5230,5815,4631,4996,4184,12097,7100,6816,7567,4078,2755,
7863
TAS2R9_3_2563,3134,3423,3822,6373,4625,4684,8103,7124,2835,7673,7490,3
328
TM2D1_3_2564,4211,3822,4283,5218,4069,2110,1503,4254,4296,8065,8186,41
04
TMEM11_3_2565,9816,8952,9345,10557,6944,9449,7730,10590,11350,5392,120
93,5536
TRHR_3_2566,3468,5589,5228,7696,4192,12338,6654,7787,6959,3674,4780,93
05
UTS2R_3_2567,0,19,0,4,0,1,1,4269,2,10,122,1
VIPR1_3_2568,505,235,1412,1549,848,141,481,293,1125,99,672,740
VIPR2_3_2569,1770,2204,1271,2436,2448,2292,1508,2921,3788,567,696,2161
VN1R1_3_2570,12695,12500,13412,14300,14825,18917,14534,14765,11178,150
46,14677,14905
VN1R2_3_2571,1673,1662,792,1213,2353,209,2451,1093,1405,2289,3150,279
VN1R4_3_2572,733,1446,2154,1240,544,971,2567,2256,1941,2007,3624,337
ADCYAP1R1_3_2573,850,876,894,1246,787,6,612,1710,119,915,562,937
ADORA1_3_2574,422,176,769,322,2408,322,266,221,510,215,37,209
ADORA3_3_2575,1323,1494,2504,1604,1893,414,393,1641,2429,1369,472,949
AGTR1_3_2576,1343,850,1616,1439,1909,709,3212,2063,2884,1980,5769,2491
AGTRAP_3_2577,723,830,336,624,208,1380,48,1437,243,3765,127,1079
AVPR2_3_2578,85,52,52,291,27,8,4,291,145,8,136,102
CALCR_3_2579,1126,1431,1056,858,895,630,332,361,2357,1247,1545,368
CASR_3_2580,2112,1889,2287,3600,2209,2193,2237,3467,623,5468,2405,3783
CCR2_3_2581,1909,3159,3280,2864,1813,3946,1245,3651,4970,1135,5574,392
6
CCR3_3_2582,1954,844,1480,1025,960,212,1152,3233,2336,731,1994,1341
CCR5_3_2583,3385,4395,4184,6777,5173,4219,3772,5926,3633,5364,5560,584
8
CCR6_3_2584,4358,3833,5094,3433,3948,4066,5069,7873,5098,6049,5816,391
1
CCRL2_3_2585,1771,3106,2224,3837,2158,2457,495,5813,1874,4141,3866,238
0
CD97_3_2586,223,468,1269,398,1575,6,1147,2556,1750,412,562,2268
CHRM2_3_2587,4125,3811,4103,2509,5676,5565,3965,2620,4870,3559,406,365
8
CMKLR1_3_2588,581,1028,1660,1546,537,933,1939,3326,697,536,880,1473
CRHR1_3_2589,2441,3051,2340,4069,2578,1490,3004,5341,1821,2657,2963,22
16
CRHR2_3_2590,563,653,1579,320,410,309,200,33,664,2111,244,988
CX3CR1_3_2591,4936,5389,4642,6035,4285,10757,4099,4386,8174,8118,9324,
6946
CXCR2_3_2592,11985,13738,15873,18107,16185,19432,17956,23499,17884,157
60,10859,25122
CXCR3_3_2593,1477,1056,2714,2469,2456,415,646,2389,1666,641,4653,899
CXCR4_3_2594,2211,3316,1858,3107,3750,1826,2756,3466,583,3563,7216,257
2

CXCR5_3_2595,1942,1627,3196,4479,1927,2957,1981,4893,1865,1866,5543,14
96
DARC_3_2596,1234,1641,1320,1727,970,1278,911,3196,904,2502,498,1458
DRD2_3_2597,299,884,257,1100,26,63,200,0,0,883,180,1
DRD3_3_2598,2219,1966,2244,2652,1357,1870,1186,2254,2021,2029,2800,148
4
EDNRB_3_2599,8303,8382,7865,10875,7932,10895,14705,7496,7402,7916,1376
2,10578
EMR2_3_2600,2201,1412,2295,2578,275,2082,1459,2815,1767,2090,649,1736
FPR1_3_2601,19,83,101,157,11,34,574,1,0,3,51,0
FSHR_3_2602,430,1096,890,1809,262,2617,1326,1925,755,6849,251,1906
FZD6_3_2603,1169,2960,2292,3629,1025,3750,3709,8477,1717,837,4875,5179
GABBR1_3_2604,971,1904,1783,2035,809,1621,1118,2803,2074,1539,3854,318
2
GHSR_3_2605,988,964,1681,1783,2290,2186,2868,1979,2506,310,501,1409
GNRHR_3_2606,1094,1163,2150,1909,1524,885,2481,1147,504,4158,390,2491
GPBAR1_3_2607,101,25,529,16,1525,1026,52,0,0,4,48,489
GPER_3_2608,390,877,901,1567,220,579,31,2143,448,1620,175,1943
GPR107_3_2609,826,298,585,372,315,920,899,599,287,533,3620,151
GPR110_3_2610,1053,441,754,841,1203,94,841,981,1779,372,101,176
GPR113_3_2611,519,266,651,331,190,410,165,40,263,524,44,802
GPR116_3_2612,1227,1296,824,1923,1622,1778,783,2064,1027,1570,1496,125
8
GPR126_3_2613,4308,6191,6386,8224,5533,8561,5032,7299,3105,7434,5923,1
3871
GPR155_3_2614,1471,2722,2307,2837,2386,2914,3583,884,3114,280,1730,423
2
GPR156_3_2615,1388,1152,977,3981,984,3664,245,936,1770,807,452,26
GPR162_3_2616,426,1578,1028,1326,425,2501,258,1135,472,1996,818,413
GPR17_3_2617,411,901,1498,675,106,1505,394,58,3,968,2220,1045
GPR18_3_2618,5380,8808,7123,10899,7378,5146,9520,9420,6780,8269,11469,
11542
GPR1_3_2619,3710,3570,4003,4695,2866,5301,6297,4860,2976,3453,7934,466
4
GPR34_3_2620,4277,5964,4398,5760,4609,3732,4430,2754,4915,4854,4318,84
90
GPR35_3_2621,958,1100,528,556,516,66,1064,1479,488,2369,424,1473
GPR56_3_2622,13,161,101,77,26,213,55,65,95,70,173,488
GPR63_3_2623,10918,15278,16281,20009,17390,18229,18385,13796,17380,206
53,26005,22752
GPR64_3_2624,38,372,483,613,409,112,2,2590,8,2,51,51
GPR68_3_2625,857,1871,1175,598,1096,286,1746,789,1149,1263,3262,1044
GPR85_3_2626,415,257,277,660,1037,830,134,3316,678,348,506,1092
GPRC5C_3_2627,365,552,322,986,46,1566,534,2580,697,180,1590,1513
GRM1_3_2628,1577,1928,1351,1325,1773,6035,3051,2149,704,3700,874,2600
GRM2_3_2629,1358,1154,1422,1934,1109,2092,551,1765,2082,2010,902,2477
GRM5_3_2630,4722,5521,4438,4996,4609,4421,4615,5237,2537,5625,8018,782
4
GRM7_3_2631,1536,1876,1685,2014,909,2373,2852,786,2235,303,1766,789
GRM8_3_2632,5718,6208,7081,7760,6316,9929,4451,4817,3782,5501,7335,817

0
HRH4_3_2633,3739,4215,4872,5028,8609,4264,2875,3336,2944,6761,824,6601
HTR2A_3_2634,1050,2287,1682,1865,1613,543,1469,405,728,610,885,1218
HTR4_3_2635,2985,5603,4617,5083,3176,5682,4293,4055,4520,5080,3719,235
4
HTR7_3_2636,462,802,1009,1444,1444,189,1395,1686,731,2295,34,856
LGR6_3_2637,667,510,416,778,51,487,538,1825,764,410,631,445
LPAR1_3_2638,4065,3017,6468,4654,2823,3950,6351,4543,7136,1737,4442,62
26
LPAR5_3_2639,1390,2378,848,1125,945,1891,1462,2204,1357,1779,339,2436
LPAR6_3_2640,3769,5151,5707,7458,5978,4610,6844,5428,9151,5022,7961,88
57
LPHN1_3_2641,635,1377,1337,1825,1357,2544,2023,485,696,1595,1111,3144
LTB4R2_3_2642,0,1,85,19,25,0,179,0,0,0,3,250
LTB4R_3_2643,451,842,1679,1245,734,655,1467,3024,527,104,209,34
LYPD1_3_2644,573,1138,492,1164,1380,506,2295,84,128,2056,436,2184
MCHR2_3_2645,4973,5852,5116,4636,6768,3415,5984,3078,8366,3183,6354,49
69
MRGPRF_3_2646,255,144,601,566,156,194,1575,1698,149,61,385,73
NPFFR2_3_2647,2263,3834,2461,4688,2839,6193,5318,2001,4456,2521,4323,6
017
NPSR1_3_2648,4484,4461,4651,4315,4625,5256,5175,7174,4772,3756,4270,41
16
O3FAR1_3_2649,2531,2630,1522,2350,2416,4736,4650,951,3912,2153,2623,32
87
OPN4_3_2650,3001,3860,4544,5029,2839,5271,3654,3690,4282,3164,5726,683
5
OPRL1_3_2651,303,442,469,807,2235,0,610,4,0,16,219,2104
OPRM1_3_2652,1923,2381,1493,1572,1385,2761,2788,4339,2270,890,3695,584
3
P2RY10_3_2653,3720,5756,4552,5744,4493,10797,11233,11207,2787,3802,586
1,7187
P2RY12_3_2654,25009,26483,28921,36894,24511,35991,27471,31875,29379,36
182,31412,41858
P2RY14_3_2655,4122,5445,6697,5590,5485,6493,8072,5735,4485,7307,6202,8
491
P2RY2_3_2656,2853,3240,3801,5920,3436,4158,5107,2879,3471,9909,4403,52
74
P2RY6_3_2657,447,310,89,285,412,16,302,592,666,488,12,165
PTAFR_3_2658,512,405,517,740,232,104,3319,1700,845,55,3350,969
PTGER3_3_2659,8974,10991,12545,15434,9346,14179,17445,19210,8790,15429
,13256,17516
PTGFR_3_2660,3130,3157,5185,5359,4157,3946,9271,5558,4157,7005,4474,55
90
PTH1R_3_2661,2517,3222,4214,1866,3179,5632,2259,1462,5698,7457,3479,31
73
RGR_3_2662,3350,4150,4128,4077,3843,3853,4994,2883,5946,5175,1963,3437
RXFP2_3_2663,7121,6346,11303,14131,9921,17123,12049,15122,10004,14477,
8504,12791
S1PR5_3_2664,415,557,650,338,390,270,39,9,95,2122,7,676

SIGMAR1_3_2665,834,695,481,769,36,1353,702,505,221,1979,233,115
SSTR5_3_2666,258,48,272,123,662,300,0,70,0,1208,11,1
TAAR2_3_2667,1839,3493,4175,6306,4259,5393,1948,6201,4854,2666,2913,42
29
TACR1_3_2668,1751,2206,2499,3764,1634,3791,3167,5697,2017,1848,2186,98
5
TAS1R1_3_2669,807,1377,540,1559,861,2756,1438,1782,1061,741,286,1366
TBXA2R_3_2670,291,540,122,279,600,209,204,234,586,185,224,725
TPRA1_3_2671,38,1,160,234,26,466,2,7,172,1,0,147
TSHR_3_2672,2796,4399,5007,5997,3771,6324,4795,8853,1996,3717,3805,326
5
XCR1_3_2673,1683,1860,2874,2045,2710,3935,2971,934,4516,2190,3524,2181
XPR1_3_2674,9186,9262,8887,16760,8190,13522,8463,18257,10219,16270,184
89,14763
ADORA2A_3_2675,371,62,322,499,1087,99,284,51,153,240,36,1780
ADORA2B_3_2676,736,842,1443,719,66,858,1184,460,1123,151,354,919
ADRA1B_3_2677,2037,1820,1332,2065,2831,4197,2778,2524,1322,1747,1538,2
209
ADRA1D_3_2678,451,239,444,135,478,172,1908,404,350,52,12,271
ADRA2A_3_2679,257,390,699,652,482,847,2381,5610,7,21,849,1662
ADRA2B_3_2680,91,73,189,443,86,20,284,2067,255,12,480,97
ADRA2C_3_2681,1170,1185,1237,612,415,261,286,761,1000,301,2338,1173
ADRB1_3_2682,205,92,68,672,183,0,150,20,146,1075,32,160
ADRB2_3_2683,2801,2734,1986,2236,3325,2535,7168,2571,1836,1613,4812,31
29
ADRB3_3_2684,204,184,299,1230,2231,27,1462,160,656,337,207,860
AGTR2_3_2685,7104,6880,8234,8233,5558,9577,13121,14107,6943,10070,1043
8,9105
APLNR_3_2686,675,508,226,940,51,558,129,40,253,543,1073,1008
AVPR1A_3_2687,1647,3260,2197,4700,1448,2726,2846,1051,2578,6063,2055,6
486
AVPR1B_3_2688,1133,1050,251,370,2087,1780,341,357,4416,1014,2314,24
BAI1_3_2689,943,735,2004,1298,2427,1460,2375,873,4549,612,455,913
BAI2_3_2690,421,841,765,562,0,8,531,134,81,20,12,1394
BAI3_3_2691,2006,1527,2728,916,1948,3483,1408,2376,649,596,2319,3859
BDKRB1_3_2692,1175,1187,493,1554,318,535,2293,402,923,1625,375,1817
BDKRB2_3_2693,1978,1949,2789,3779,1977,2795,2304,1364,1860,6484,7070,2
361
BRS3_3_2694,7368,7646,7190,12123,4716,7546,6026,10008,5294,10488,9302,
7132
C3AR1_3_2695,3571,4621,6444,5101,5627,4473,7697,2501,2929,2967,7465,47
61
C5AR1_3_2696,529,208,398,123,335,106,162,331,385,345,10,35
CALCRL_3_2697,5207,7734,6414,7560,5788,5753,7316,9793,5662,7128,8339,1
0375
CCKAR_3_2698,4198,4771,6037,5159,3419,5522,7991,2106,4358,5656,7154,45
51
CCKBR_3_2699,880,1012,391,1128,111,1002,467,594,197,471,282,343
CCR1_3_2700,899,1226,1314,1895,1790,1013,472,593,2140,2419,2471,781
CCR7_3_2701,359,615,1148,277,949,734,103,419,7,6,4619,2574

CCR8_3_2702,1143,1006,899,1907,1043,3540,3110,1466,707,3821,2864,978
CELSR1_3_2703,1744,2208,2408,1771,1541,3240,1320,2046,1387,3479,2503,2
589
CELSR2_3_2704,1872,2845,2911,3564,5632,6308,1973,2861,2478,4492,1312,3
720
CELSR3_3_2705,285,1891,1376,794,601,4867,771,311,387,3424,215,1129
CHRM1_3_2706,2025,2142,3554,2701,1792,2875,4171,4022,1373,4094,3082,30
58
CHRM3_3_2707,861,798,1052,1800,2538,3071,1951,1937,3806,2761,266,1286
CHRM4_3_2708,2841,2597,2903,3932,2396,3723,6739,2185,1749,3533,6933,38
48
CHRM5_3_2709,1865,1605,973,4278,1289,2321,1441,1699,3743,3261,1240,110
0
CXCR1_3_2710,891,296,151,568,1319,246,671,1629,573,30,324,1130
CXCR6_3_2711,3041,3940,2871,4123,3137,8851,5803,2386,3410,5458,9195,44
75
CXCR7_3_2712,1364,1787,2717,3865,473,3374,4636,7833,3825,4182,1297,390
8
CYSLTR1_3_2713,10003,10337,10764,13922,14385,14700,13561,12994,12314,9
322,15952,11661
CYSLTR2_3_2714,3562,5793,4358,5752,3621,7048,3142,2736,3016,8684,7321,
3624
DRD1_3_2715,1431,1935,1344,1949,2517,1870,2467,1831,1160,2048,395,2123
DRD4_3_2716,510,485,238,941,67,282,1100,874,46,1013,39,2360
DRD5_3_2717,1508,1335,1618,3163,2862,1819,1649,1746,1006,1901,1359,197
4
ELTD1_3_2718,3199,3272,2422,3139,4912,4680,3655,7025,2605,3926,705,624
3
EMR1_3_2719,658,911,1449,979,1237,118,892,504,1982,330,634,528
EMR3_3_2720,9312,12183,13274,15718,7365,21103,12337,17505,9946,13384,1
4236,9925
F2R_3_2721,917,964,1011,846,90,160,348,1754,1283,529,3642,718
F2RL1_3_2722,1905,2175,3165,4571,1507,4904,2160,2919,2235,3875,475,481
7
F2RL2_3_2723,17192,18470,22176,21397,22009,38211,24117,25280,18599,118
62,38732,16925
F2RL3_3_2724,26,31,419,2,15,2,418,413,783,0,0,169
FFAR1_3_2725,2132,2211,2588,1700,1013,1152,1772,471,2558,3881,930,1692
FFAR2_3_2726,299,242,316,459,285,35,70,1003,191,12,28,75
FFAR3_3_2727,1148,2345,2475,3541,2024,1732,3397,1776,2062,2097,3257,19
09
FZD10_3_2728,1404,1781,1486,1509,2361,4190,1036,1363,3550,2416,1552,18
64
FZD1_3_2729,294,1436,855,456,866,31,1136,376,2221,169,172,202
FZD2_3_2730,6360,7918,8168,12486,9951,6875,9545,8830,9777,17373,17620,
8458
FZD4_3_2731,2783,3020,3530,3282,2723,7261,4093,2967,3403,1676,8673,292
0
FZD5_3_2732,1686,1514,2241,3351,547,4754,2954,1296,1853,3270,3486,2878
FZD7_3_2733,1819,2246,4043,2499,4735,3947,5841,2271,5107,2644,3784,253

8
FZD8_3_2734,351,582,507,546,548,375,45,497,660,1908,2408,3068
FZD9_3_2735,4414,5910,5135,7674,4814,10598,4530,8075,7017,7562,9671,42
56
GABBR2_3_2736,4766,7230,5542,7951,3022,11581,7880,7812,6719,6709,7325,
6907
GALR1_3_2737,2688,2683,2941,3557,328,5541,4361,3697,3804,2121,4071,440
2
GALR2_3_2738,744,701,341,409,198,768,1524,1826,1583,565,112,603
GALR3_3_2739,7781,8700,6539,10660,8075,4001,9920,9482,7041,10632,15032
,9968
GCGR_3_2740,1137,747,1194,1642,2321,705,3514,1144,3874,1823,2523,1043
GHRHR_3_2741,4904,4068,4963,5978,3580,5638,5564,5806,5596,6486,7991,59
39
GIPR_3_2742,2660,2469,4078,3757,3902,2428,3775,2538,2353,2014,3783,275
3
GLP1R_3_2743,377,492,424,832,519,488,359,1658,1445,1950,129,650
GLP2R_3_2744,4479,3464,6371,6025,8204,1153,6813,10151,2993,335,9478,36
70
GPR101_3_2745,584,658,501,818,309,1156,306,215,583,133,864,759
GPR108_3_2746,1340,1288,2425,2445,870,514,3169,2058,1499,1853,3787,312
0
GPR111_3_2747,2855,3913,3249,5783,3259,5788,3495,5806,4218,5614,4875,5
899
GPR112_3_2748,3043,3641,5039,7259,2099,7933,10146,7623,1577,3355,4073,
6264
GPR114_3_2749,1628,1857,1041,1700,760,276,1973,3754,2090,1376,301,3040
GPR115_3_2750,2776,3288,3549,5272,3975,4611,2765,6659,5272,7961,6049,6
399
GPR119_3_2751,1059,399,1021,1698,662,1332,1946,820,1116,1130,229,1593
GPR123_3_2752,279,245,757,1005,958,303,1450,82,241,121,1491,245
GPR124_3_2753,1767,1783,1469,1489,3085,837,262,423,1054,1764,108,465
GPR125_3_2754,4139,5849,4773,5447,5952,3046,7942,10535,5535,5606,3703,
6415
GPR128_3_2755,1535,1540,2258,2605,2862,1758,4530,539,1151,1815,914,304
0
GPR12_3_2756,2126,2909,2864,2123,2030,2780,4070,4983,4169,2923,5976,17
08
GPR132_3_2757,1117,670,577,634,0,62,1714,167,497,682,134,485
GPR133_3_2758,6404,7545,8308,10339,10601,7801,13863,11405,6687,12565,1
6182,12608
GPR135_3_2759,232,742,723,813,725,2581,589,597,635,2940,1742,1707
GPR139_3_2760,161,277,119,50,7,162,0,408,89,118,1368,213
GPR141_3_2761,1950,2568,2105,1520,2438,2038,2599,6623,1064,514,492,216
9
GPR142_3_2762,566,871,1376,2257,1125,401,1740,1273,899,561,923,1351
GPR143_3_2763,512,1141,1871,1372,497,1064,2074,2799,2871,520,5911,118
GPR144_3_2764,1312,933,1275,2275,1908,3698,1525,1030,539,3339,3736,189
9
GPR146_3_2765,931,1046,744,748,2169,744,2933,536,659,926,297,1134

GPR148_3_2766,599,535,892,694,39,322,1332,1025,161,668,1577,2460
GPR149_3_2767,9051,10422,10839,12804,12521,15576,12444,20221,8836,1083
6,5389,14206
GPR150_3_2768,466,471,113,368,546,187,138,16,439,286,29,8
GPR151_3_2769,950,1007,1507,1043,340,1424,446,562,882,1606,1656,1915
GPR152_3_2770,560,1062,673,564,340,267,2832,1768,546,5,423,1238
GPR153_3_2771,29,194,330,87,320,1511,1179,24,26,337,19,0
GPR157_3_2772,949,695,688,1080,1397,3396,70,18,1213,1774,923,33
GPR158_3_2773,1388,2522,1171,1076,995,2014,1919,733,481,3081,2690,3002
GPR15_3_2774,13946,13839,16280,20127,12403,25760,19160,19319,18774,211
33,23177,16488
GPR160_3_2775,8101,7715,10552,8391,7029,9319,8685,11120,5755,10846,113
97,7072
GPR161_3_2776,1093,1816,895,2534,3779,2253,2371,1083,907,3000,911,3000
GPR171_3_2777,17775,18779,20945,21686,22397,26743,27384,21816,21285,17
841,16371,28561
GPR173_3_2778,978,1973,1621,1113,320,3410,1393,167,668,2472,213,850
GPR174_3_2779,1303,1835,1679,1854,1499,2065,4561,1981,3051,1312,1764,1
850
GPR176_3_2780,670,645,611,1236,2261,88,1383,1251,2670,560,681,858
GPR179_3_2781,452,1099,547,1433,812,1010,330,75,137,1382,51,899
GPR182_3_2782,899,1477,602,1367,602,1226,2108,1979,514,2432,1017,1155
GPR183_3_2783,1027,1393,1521,1265,161,7144,891,947,1312,2759,4034,597
GPR19_3_2784,7398,9523,11110,12289,9370,13266,10511,15323,9350,9961,83
58,9379
GPR20_3_2785,920,1583,1050,1132,1591,56,1263,2310,313,333,486,622
GPR21_3_2786,666,839,906,407,1483,60,1336,119,1752,122,69,592
GPR22_3_2787,1182,1060,1478,1293,223,2777,464,3668,990,1021,3939,3287
GPR25_3_2788,846,884,410,1340,1469,1110,1159,637,615,242,560,761
GPR27_3_2789,189,2,204,18,27,0,385,4,1,21,3,467
GPR31_3_2790,429,489,200,470,58,5,239,6,50,5,32,377
GPR32_3_2791,1547,1147,1535,1125,1383,3897,473,1784,603,2424,1005,724
GPR37_3_2792,871,963,262,682,587,478,1309,322,130,47,2079,626
GPR37L1_3_2793,532,478,503,210,478,728,119,393,787,858,66,245
GPR39_3_2794,2886,2678,2196,3176,401,4843,2468,1472,3339,1747,3818,314
7
GPR3_3_2795,3134,2829,2937,3816,2691,3235,2450,1664,2356,5815,2799,183
2
GPR45_3_2796,938,2904,1869,3001,2131,4037,3183,2923,2481,570,2008,2380
GPR4_3_2797,673,632,693,1205,2768,407,909,3324,1645,2393,250,2270
GPR50_3_2798,1174,2769,1659,3147,1777,1074,1132,781,2096,2015,4065,962
GPR52_3_2799,219,138,302,142,189,164,92,116,256,98,21,490
GPR55_3_2800,245,279,79,468,0,0,25,70,255,0,22,145
GPR61_3_2801,1197,1199,1088,2197,1863,1471,2061,2661,325,3299,415,2217
GPR62_3_2802,564,396,328,285,32,1282,300,734,1199,5,544,115
GPR65_3_2803,1008,532,671,1000,444,790,188,301,823,76,113,2164
GPR6_3_2804,2129,2668,1722,2294,4098,2591,1292,4387,2817,4965,1607,208
9
GPR75_3_2805,754,1527,2034,2684,791,1491,1544,1217,1001,2437,2438,2826
GPR77_3_2806,292,481,574,1100,251,911,221,896,2,299,8,1247

GPR78_3_2807,698,1098,263,1326,832,199,354,59,142,541,2405,1445
GPR82_3_2808,995,883,1739,693,479,1646,474,742,836,1504,2772,2390
GPR83_3_2809,1968,1179,1661,3799,2477,728,2163,2204,745,1189,3158,1448
GPR84_3_2810,93,215,27,27,76,1,55,47,416,321,24,89
GPR87_3_2811,1139,2157,2625,3187,2280,3281,941,1260,1141,3891,1027,135
8
GPR88_3_2812,146,602,434,88,76,34,387,1,1029,415,126,191
GPR97_3_2813,655,940,1384,763,64,1976,1401,1225,2545,61,2116,2594
GPR98_3_2814,6914,5614,7549,5450,7832,7904,12875,8405,9707,10810,9665,
9956
GPRC5A_3_2815,5253,4970,7931,7035,4878,9990,7196,11016,5521,4337,8241,
4291
GPRC5B_3_2816,265,495,214,1741,275,196,603,1906,117,414,39,351
GPRC5D_3_2817,701,1295,272,973,1216,799,843,1955,747,1660,1322,1857
GPRC6A_3_2818,5221,4831,5698,6539,3208,5516,6659,3802,9234,6358,9029,8
840
GRM3_3_2819,1409,1912,1048,1522,1125,3170,2297,3237,2362,955,2520,3128
GRM4_3_2820,25,170,0,269,10,69,10,13,58,1,6,20
GRM6_3_2821,700,766,1296,1269,385,2442,555,965,1498,2562,169,852
GRPR_3_2822,307,782,382,413,1495,1922,36,971,484,8,69,781
HCAR1_3_2823,811,1344,1404,1776,694,4496,1950,2342,1622,903,2763,3709
HCAR2_3_2824,1880,2280,2529,2127,733,4331,2955,3396,2541,2068,2700,322
4
HCAR3_3_2825,877,873,1123,4204,402,764,2000,675,232,1432,935,1421
HCRTR1_3_2826,185,532,1261,1533,14,347,1027,1465,208,564,1398,809
HRH3_3_2827,459,782,488,649,503,3,2904,542,980,498,1239,1699
HTR1A_3_2828,664,1391,1187,1163,1658,1646,1023,47,2243,1496,140,130
HTR1B_3_2829,550,699,901,1184,659,5,219,12,440,426,628,1616
HTR1D_3_2830,818,2092,1400,2851,272,5784,3477,668,3087,4626,1060,1075
HTR1E_3_2831,869,1073,1446,1446,906,2078,732,985,1027,3090,1236,795
HTR1F_3_2832,1013,920,1249,2448,877,1183,2321,204,1045,1116,3376,2468
HTR2B_3_2833,583,508,2038,1246,3001,612,2861,401,456,707,649,2688
HTR2C_3_2834,783,1293,1705,1244,1461,1454,4175,625,864,1092,2867,1560
HTR5A_3_2835,509,286,235,519,428,501,561,344,430,364,630,706
HTR6_3_2836,480,768,723,1084,1442,858,127,893,2099,80,474,873
KISS1R_3_2837,600,465,1095,3165,566,1252,142,1191,608,467,799,948
LGR4_3_2838,4053,5249,5681,7953,5268,3596,5661,6225,5812,6373,4973,553
1
LGR5_3_2839,7293,7626,9051,9225,9169,8663,5666,8195,8147,7724,12024,98
77
LHCGR_3_2840,1696,2767,2339,3340,1486,3540,2934,4470,1937,3835,2305,31
15
LPAR2_3_2841,295,106,276,693,0,2,204,2266,142,472,1447,305
LPAR3_3_2842,4447,3986,3040,4746,5791,7124,4713,5419,5367,3442,8017,66
61
LPAR4_3_2843,5798,9191,7392,12509,8291,16884,14130,8056,5841,8261,4863
,17691
LPHN2_3_2844,462,150,428,598,516,741,524,8,1295,5,20,1010
LPHN3_3_2845,1915,2280,4744,2883,3240,3243,2515,4215,2607,4172,2325,20
97

MAS1_3_2846,1396,2102,2918,2268,1721,2383,2662,2962,823,2647,4963,2926
MAS1L_3_2847,2221,3179,2170,2191,2560,3972,2406,2967,2709,4865,6165,13
72
MC1R_3_2848,461,784,1611,1437,601,1643,764,2890,990,431,549,293
MC2R_3_2849,7541,8543,9170,10546,5910,16298,13116,7803,6507,10863,1369
2,8829
MC3R_3_2850,670,1028,224,968,43,4106,2045,35,4,1240,200,310
MC4R_3_2851,890,1504,1425,1596,234,1246,1206,255,222,636,303,1207
MC5R_3_2852,812,276,353,1067,1087,1943,334,391,367,36,2270,105
MCHR1_3_2853,959,873,1219,2161,1851,1211,1734,148,1724,1547,231,1549
MLNR_3_2854,816,392,148,594,358,26,125,307,139,850,39,395
MRGPRD_3_2855,3138,3624,2179,3142,3333,5374,2515,5209,5080,2937,530,26
42
MRGPRE_3_2856,779,512,215,480,762,574,1079,385,1179,792,1049,2036
MRGPRG_3_2857,771,610,1018,425,332,467,869,151,630,82,119,68
MRGPRX1_3_2858,1653,1612,1026,2637,1689,2056,2527,2640,479,286,395,175
1
MRGPRX2_3_2859,3902,4149,3901,5263,3515,5153,7783,6670,6269,3255,8141,
3498
MRGPRX3_3_2860,3143,2806,3228,2061,2719,3706,2286,4014,7550,5293,3229,
3140
MRGPRX4_3_2861,294,215,426,671,22,196,585,1307,69,33,1401,112
MTNR1A_3_2862,242,659,926,840,219,827,1269,1038,149,484,1818,32
MTNR1B_3_2863,372,1252,425,804,1372,2835,1913,1011,1415,622,2850,440
NMBR_3_2864,1244,1676,271,1699,2797,810,513,998,817,131,1028,1791
NMUR1_3_2865,650,850,533,1578,203,537,575,303,621,998,347,1346
NMUR2_3_2866,2677,1989,2699,5609,2603,1528,5992,6399,6643,2031,4735,47
53
NPBWR1_3_2867,399,111,527,1505,9,20,804,6,90,1,2321,249
NPBWR2_3_2868,2079,850,2168,2982,2868,764,3769,4384,1709,3558,744,3113
NPFFR1_3_2869,175,209,315,66,154,1,263,239,99,23,908,154
NPY1R_3_2870,507,376,438,1830,2087,1065,773,66,1803,89,455,5706
NPY2R_3_2871,706,1480,1393,1387,611,717,342,1145,2474,2137,366,1080
NPY5R_3_2872,1166,1315,2169,1115,1477,1375,3485,1396,616,2835,186,1526
NTSR1_3_2873,3332,2066,5236,4704,2185,2206,6523,3699,2267,2363,1427,12
45
NTSR2_3_2874,67,417,313,77,125,24,457,575,311,1,128,93
OMG_3_2875,934,1077,1385,1489,1143,1728,3217,2547,154,3508,2054,1749
OPN1LW_3_2876,1184,1733,1657,4611,1439,2720,988,5226,3068,276,1175,201
1
OPN1MW2_3_2877,6343,7540,8490,8043,6313,7219,13349,7544,7492,9422,6943
,7514
OPN1MW_3_2878,6343,7540,8490,8043,6313,7219,13349,7544,7492,9422,6943,
7514
OPN1SW_3_2879,2452,2558,2416,3210,1718,3023,2660,1802,1315,1999,3852,3
126
OPN3_3_2880,1641,2390,2741,2342,934,2387,3345,877,2031,3432,1878,1543
OPN5_3_2881,5207,6780,7031,6606,8885,7167,7119,5273,6493,2939,8590,464
5
OPRD1_3_2882,368,1352,753,1062,238,1758,2993,273,1169,59,211,3517

OPRK1_3_2883,2616,3543,4085,5632,3879,4172,4460,2085,4326,7176,1635,68
37
OXER1_3_2884,1127,1274,1786,828,707,177,2905,181,2691,1447,345,1996
OXGR1_3_2885,4603,6796,7008,9237,5326,10033,15480,10819,4082,13235,168
52,7122
OXTR_3_2886,339,390,406,92,608,1600,1635,96,135,12,46,81
P2RY11_3_2887,27,277,764,474,135,1398,2053,1,2,764,1278,1
P2RY13_3_2888,2193,2858,3201,1997,1037,1498,3749,2175,1901,4025,3830,2
050
P2RY1_3_2889,439,397,493,410,1866,1427,169,783,109,735,13,921
P2RY4_3_2890,571,136,309,781,583,233,929,2367,177,1054,113,615
P2RY8_3_2891,2,27,39,4,178,33,0,0,8,0,708,0
PPYR1_3_2892,3702,3771,4423,5109,1527,4501,2484,5357,4024,5917,3525,74
86
PRLHR_3_2893,13,338,9,346,18,1223,878,0,395,104,610,1663
PROKR1_3_2894,122,479,169,642,3,114,1177,393,280,27,317,353
PROKR2_3_2895,729,1124,1205,756,196,160,2344,40,586,463,3654,374
PTGDR_3_2896,362,1030,1988,1240,1284,249,2226,1498,188,521,2788,474
PTGER1_3_2897,580,1036,1021,663,42,981,2176,1730,700,1623,436,897
PTGER2_3_2898,431,294,231,935,799,1736,31,571,1,75,3194,1586
PTGER4_3_2899,554,247,411,1225,1192,1104,253,186,24,83,2295,1547
PTGIR_3_2900,200,564,866,765,327,342,2847,49,863,2969,2538,238
PTH2R_3_2901,1354,1359,1707,3015,2092,3236,2366,2200,1889,771,2764,304
1
QRFPR_3_2902,2201,1814,2252,4120,2086,1881,1426,4613,3543,2415,3476,16
73
RHO_3_2903,2484,2620,4630,4834,4248,4724,1574,1021,2596,2340,1621,2349
RRH_3_2904,5293,5483,7704,11263,8087,6340,7935,10529,8267,6448,8596,97
78
RXFP1_3_2905,2470,2026,1605,2267,2967,2595,1666,5296,1076,5262,3898,18
56
RXFP3_3_2906,834,863,821,808,706,669,482,1172,328,1356,617,586
RXFP4_3_2907,337,427,426,458,1860,49,142,167,25,62,2519,510
S1PR1_3_2908,2886,3349,4492,5111,3480,5129,3039,2381,2360,7722,7454,32
60
S1PR2_3_2909,2962,3188,3215,3239,1506,4101,1595,4087,3819,3774,1334,26
99
S1PR3_3_2910,1230,1352,1074,2056,1509,257,593,1042,1472,759,1413,570
S1PR4_3_2911,275,104,220,741,932,861,3898,147,1228,126,3086,217
SCTR_3_2912,218,553,523,603,641,197,546,79,10,336,143,5
SSTR1_3_2913,998,1670,1654,3106,858,1480,1595,1923,1019,897,763,2523
SSTR2_3_2914,2461,1735,1855,2748,3997,3926,1460,1645,3951,2739,5640,17
21
SSTR3_3_2915,526,662,942,535,224,13,1470,2389,297,1345,154,4
SSTR4_3_2916,78,1261,273,390,819,552,157,165,543,1972,220,41
SUCNR1_3_2917,3185,3828,2059,2827,1811,5344,4250,3515,3448,3499,5069,2
904
TAAR1_3_2918,6831,7842,8959,7974,11169,12886,12223,7806,9833,10126,105
04,9120
TAAR5_3_2919,1334,2331,2437,4625,1018,1136,3618,2441,1676,2755,5453,44

27
TAAR6_3_2920,5452,6935,4712,9505,4761,6563,8447,7370,4796,6618,12465,6
465
TAAR8_3_2921,651,1439,1825,1469,1163,1714,1879,2933,973,688,412,1230
TAAR9_3_2922,16679,15423,18542,22380,15473,17006,23229,24084,13673,195
88,28850,28843
TACR2_3_2923,1517,2688,2567,4156,2917,3522,2552,7214,2411,2704,805,246
8
TACR3_3_2924,6419,11483,11784,12315,10006,19209,11978,9732,6276,16098,
10374,13507
TAPT1_3_2925,4155,3544,2930,4510,4728,2338,5518,1707,5853,3283,1377,30
66
TAS1R2_3_2926,156,197,123,216,39,2731,1044,606,240,58,168,289
TAS1R3_3_2927,393,1057,1454,758,1174,2177,109,1370,1674,833,173,1498
TAS2R10_3_2928,2162,2383,2543,2369,2338,5990,1474,2891,1950,763,2183,1
718
TAS2R13_3_2929,3301,4358,5373,5172,4508,3686,5062,5515,1885,5569,1978,
4863
TAS2R14_3_2930,950,1640,1451,2369,1838,5041,751,4599,2316,5559,2089,48
19
TAS2R16_3_2931,7534,10181,8985,10401,10976,15830,11762,7268,11275,9986
,14169,11170
TAS2R1_3_2932,767,1245,908,2167,1374,405,723,1581,898,1109,2494,904
TAS2R20_3_2933,6800,8025,8403,11352,4937,8054,8808,7456,8391,12960,748
1,11192
TAS2R31_3_2934,2677,2586,3199,6216,3143,3233,3338,4593,3433,3895,3993,
10610
TAS2R38_3_2935,5484,7812,9161,5780,10389,9118,7727,11432,7796,10206,13
383,11304
TAS2R3_3_2936,1440,1904,1974,2280,2809,741,3407,1476,3291,493,3305,140
9
TAS2R40_3_2937,3892,4083,6343,5144,4629,5640,4006,6097,5323,6245,11298
,2567
TAS2R41_3_2938,2029,1755,3445,2503,2520,1711,3227,2737,2010,2520,4994,
2714
TAS2R46_3_2939,5767,8972,8018,10598,8647,12993,10569,4262,8943,14486,5
619,11262
TAS2R4_3_2940,648,1467,2148,3190,829,5347,2728,2504,1833,412,4450,2657
TAS2R5_3_2941,694,1488,1414,1265,1819,1831,791,3781,640,200,778,1826
TAS2R60_3_2942,5370,6545,6993,8988,4079,6172,5632,7584,6521,3237,7849,
5697
TAS2R7_3_2943,1407,1879,1661,3614,2177,1881,2575,1393,3506,376,565,215
9
TAS2R8_3_2944,6249,7962,6115,8364,4842,9473,7623,8982,5081,10129,7288,
8557
TAS2R9_3_2945,10700,10281,12548,13918,9769,14508,15958,15482,14669,948
9,9056,7377
TM2D1_3_2946,2489,2101,3414,2544,1688,1560,1683,970,1884,1495,1260,345
2
TMEM11_3_2947,484,778,496,764,1611,1799,392,182,973,577,1715,530

TRHR_3_2948,238,918,691,1553,967,620,940,396,443,250,306,1692
UTS2R_3_2949,124,429,544,500,70,201,252,351,48,7,3,502
VIPR1_3_2950,605,1584,1200,2187,827,844,373,499,1242,489,1743,1105
VIPR2_3_2951,808,2113,1431,371,1922,30,860,131,573,530,52,901
VN1R1_3_2952,916,633,504,528,98,1720,349,1659,474,2418,322,364
VN1R2_3_2953,6776,7557,9445,9709,6815,14507,9900,15900,6999,7325,8652,
10340
VN1R4_3_2954,1571,1133,1116,3020,2452,1775,2770,1986,907,2335,450,1479
ADCYAP1R1_3_2955,1202,336,645,672,786,50,378,473,1117,550,49,407
ADORA1_3_2956,1523,1825,3441,2917,1828,622,1005,3200,675,3106,3420,183
0
ADORA3_3_2957,923,1408,624,709,2911,1527,1347,298,708,957,3207,872
AGTR1_3_2958,6165,7572,7110,7456,3709,9871,6029,9146,9181,11463,10118,
10821
AGTRAP_3_2959,1413,2050,1981,1934,2346,1460,4466,4794,1681,3488,744,85
9
AVPR2_3_2960,1028,956,522,1812,78,755,813,1482,1402,290,1483,1590
CALCR_3_2961,1149,1808,2292,2493,2899,3130,2974,2958,1229,1203,2440,21
13
CASR_3_2962,973,638,1096,2267,955,10,1470,15,1778,1512,3060,1264
CCR2_3_2963,5366,7589,8691,10744,8846,14220,9155,15947,7467,8613,6272,
11921
CCR3_3_2964,504,353,318,750,838,736,209,979,504,407,1646,2585
CCR5_3_2965,8481,7523,9840,15067,3923,12133,12733,13952,6728,9613,1344
6,12852
CCR6_3_2966,4379,3774,6188,5369,4385,5529,7507,8194,7404,6226,4362,424
5
CCRL2_3_2967,2409,2894,2349,3421,1553,1904,4673,4032,1122,4306,1228,16
57
CD97_3_2968,2201,1412,2295,2578,275,2082,1459,2815,1767,2090,649,1736
CHRM2_3_2969,103,520,410,681,167,25,313,5,8,62,249,109
CMKLR1_3_2970,891,1515,1980,1475,598,3183,3114,1210,1308,889,6891,264
CRHR1_3_2971,4670,6379,5784,7147,5535,3926,5313,8417,4703,2934,6119,53
19
CRHR2_3_2972,4221,5258,4395,8018,7369,3970,9348,6792,6005,12559,1272,8
193
CX3CR1_3_2973,2724,2066,2831,3368,2711,4208,2729,3160,3322,3983,1603,4
201
CXCR2_3_2974,771,2049,1511,3210,622,2229,1231,587,1971,4153,4135,2715
CXCR3_3_2975,3038,2826,3124,4338,3855,4513,2326,2066,6165,3172,4130,45
12
CXCR4_3_2976,2064,2340,2653,3902,3424,7383,1916,3786,3476,3429,2370,40
60
CXCR5_3_2977,2953,1925,2240,4731,2823,2095,6287,1616,3343,4181,5633,15
17
DARC_3_2978,390,325,401,443,18,295,17,47,252,383,2310,0
DRD2_3_2979,123,914,195,665,584,6051,727,36,80,29,87,40
DRD3_3_2980,1208,724,1495,1132,1922,1280,1123,3603,242,327,2396,2042
EDNRB_3_2981,3831,4753,6114,7079,5429,5743,6337,4734,7094,6953,6373,38
77

EMR2_3_2982,421,980,1189,460,262,1790,94,1126,25,206,320,1094
FPR1_3_2983,791,472,1638,229,130,9,308,1145,851,784,2381,55
FSHR_3_2984,4528,5845,6575,9482,3842,5291,8590,7558,6884,2949,4820,792
6
FZD6_3_2985,5654,3033,4823,5633,4716,2820,8315,1770,4871,3224,611,4548
GABBR1_3_2986,2177,3090,2958,2344,2170,1629,3509,2378,463,5199,2393,35
19
GHSR_3_2987,2299,2820,2858,3809,4356,3891,2788,2929,3011,3423,2194,385
7
GNRHR_3_2988,5503,4182,5018,5280,3816,6224,7041,7512,8111,4669,4654,43
05
GPBAR1_3_2989,173,930,58,516,30,831,141,115,1525,7,103,532
GPER_3_2990,1641,2108,2524,2750,2597,1207,3118,3580,3288,2998,1623,178
5
GPR107_3_2991,223,820,427,556,1007,0,0,248,148,1920,8,44
GPR110_3_2992,2652,2214,3563,4141,3294,2796,3360,2238,1686,4116,3827,2
078
GPR113_3_2993,1365,2259,1532,2940,1487,1593,2575,331,3004,5586,2339,25
00
GPR116_3_2994,3647,2993,3518,3114,3978,3088,3564,3485,2608,2300,2450,2
683
GPR126_3_2995,1095,914,1899,1589,1363,1733,1497,2756,1825,1041,801,157
8
GPR155_3_2996,1631,1485,1435,2067,1688,4009,1174,2159,168,1009,3673,35
89
GPR156_3_2997,4205,2995,3268,5252,1829,6177,4802,5728,3022,4706,8776,7
099
GPR162_3_2998,94,128,590,79,0,613,98,2293,0,4,23,493
GPR17_3_2999,99,458,119,591,757,157,533,1636,541,1674,18,259
GPR18_3_3000,7241,8070,10351,8069,5120,10712,7283,11633,8342,10704,869
9,13184
GPR1_3_3001,3340,3672,3888,4586,2885,5285,6214,4844,2964,3791,7863,462
9
GPR34_3_3002,1348,2259,3930,3237,1440,2677,2990,4319,2151,1310,2708,24
58
GPR35_3_3003,824,1047,755,1272,866,2100,1728,3387,78,1228,2299,1484
GPR56_3_3004,829,1033,1409,1131,225,1671,1030,1790,479,2308,688,687
GPR63_3_3005,1196,759,834,900,202,775,1559,2249,2195,724,982,868
GPR64_3_3006,3135,5491,3653,6794,3802,3895,3577,6362,3042,5253,5642,67
56
GPR68_3_3007,1357,989,1804,2040,423,944,619,847,1824,45,578,1502
GPR85_3_3008,976,561,926,669,1239,348,2135,611,4459,444,53,2721
GPRC5C_3_3009,1085,687,1520,4373,833,2486,993,1332,611,749,469,2873
GRM1_3_3010,4032,6151,5831,10199,3323,14270,11869,6700,6474,4402,6923,
7467
GRM2_3_3011,443,823,840,440,10,2581,106,783,381,103,177,528
GRM5_3_3012,5371,8450,7586,10377,5389,17883,10593,8155,7282,10083,7287
,8981
GRM7_3_3013,2801,4628,4518,5838,4862,5822,8234,7310,3039,5785,4154,622
6

GRM8_3_3014,1873,2152,2192,4042,2234,4783,4325,2157,1358,4537,1025,347
4
HRH4_3_3015,3990,5907,6908,9061,4588,5666,5655,6157,9405,10638,2639,65
11
HTR2A_3_3016,2550,3055,2578,3125,1397,5071,2323,1227,2815,5142,4987,18
39
HTR4_3_3017,712,1221,1374,275,798,2556,1206,52,912,270,4430,828
HTR7_3_3018,2009,703,984,1197,148,301,461,389,1449,1044,173,2249
LGR6_3_3019,790,686,1311,508,245,672,827,191,1443,1860,568,470
LPAR1_3_3020,1826,2750,2704,3561,2321,2748,2388,2091,1685,1383,3883,10
91
LPAR5_3_3021,211,623,729,1164,37,103,69,1129,31,260,39,240
LPAR6_3_3022,2025,3239,1837,4011,3487,2038,2991,5460,2772,1524,910,341
7
LPHN1_3_3023,3562,3697,5875,6392,4679,9630,4387,8132,4864,1495,7243,78
79
LTB4R2_3_3024,527,140,645,44,2905,95,489,240,48,64,33,70
LTB4R_3_3025,674,363,525,1787,859,2904,269,283,54,31,19,2147
LYPD1_3_3026,313,189,158,693,35,394,42,337,53,4,9,180
MCHR2_3_3027,822,1212,1907,917,1002,2660,1064,1158,316,1413,741,4870
MRGPRF_3_3028,979,2228,2423,1518,956,2563,786,1013,3162,2132,2783,1865
NPFFR2_3_3029,4884,6214,4758,6563,2724,9707,5444,3783,5641,4816,5895,3
129
NPSR1_3_3030,8129,7458,8200,8420,4566,6893,8702,9754,7829,7913,5363,72
32
O3FAR1_3_3031,1015,1117,598,2039,1095,1061,759,2081,914,1779,707,1575
OPN4_3_3032,1764,2148,2433,998,5668,2257,3211,660,2414,830,388,1609
OPRL1_3_3033,1606,1049,1230,2886,666,2611,2316,2191,1214,974,865,1290
OPRM1_3_3034,4026,3318,4046,3837,2081,5232,4169,6631,3943,3398,11909,5
504
P2RY10_3_3035,2151,1617,2368,3571,1903,4506,1856,2813,1824,3798,1416,1
924
P2RY12_3_3036,7965,12782,13723,15053,11474,17420,13693,15639,10957,117
07,11248,13698
P2RY14_3_3037,475,1083,743,929,1822,2291,710,348,1848,746,605,161
P2RY2_3_3038,274,1087,3424,1257,1172,989,2734,164,1006,821,2316,868
P2RY6_3_3039,1196,1114,263,739,59,130,1672,737,2261,474,465,124
PTAFR_3_3040,1328,2466,1263,2775,611,1620,657,534,675,2466,1230,2809
PTGER3_3_3041,1766,2685,2362,2680,4078,2802,1978,3986,2832,7376,1763,2
631
PTGFR_3_3042,1400,2036,1537,1695,2478,6194,1819,1136,1241,3254,1435,49
9
PTH1R_3_3043,951,771,1306,2142,2231,3628,1451,1381,518,62,567,1821
RGR_3_3044,6840,8305,8697,8661,9029,11421,9970,10407,6962,13280,11560,
10336
RXFP2_3_3045,3024,2200,2091,6108,2381,2376,1501,3353,3558,3895,4119,46
37
S1PR5_3_3046,3333,2601,3042,2492,3573,2081,2398,2668,5468,3508,6158,32
90
SIGMAR1_3_3047,611,113,99,321,46,729,498,626,148,39,122,1846

SSTR5_3_3048,207,377,938,583,1254,869,1679,67,1369,576,528,152
TAAR2_3_3049,3968,4662,5014,7927,5341,6304,1091,10317,4658,8173,6521,7469
TACR1_3_3050,1140,1344,1175,1385,494,58,672,2518,698,556,1426,2165
TAS1R1_3_3051,2518,1933,2478,1518,2234,1859,2319,2152,4437,1155,3993,1276
TBXA2R_3_3052,300,145,280,156,26,32,2074,144,126,10,938,820
TPRA1_3_3053,709,1365,240,379,3296,597,666,103,191,790,742,606
TSHR_3_3054,1726,3013,2294,3937,2094,4775,2339,4528,6592,4775,349,3290
XCR1_3_3055,815,1470,1383,2648,88,1473,2738,773,456,19,1612,686
XPR1_3_3056,9263,9514,8949,17153,8220,14450,8520,17395,10284,16387,18637,14851
ADORA2A_3_3057,1102,415,944,1928,291,2677,976,1022,167,241,758,339
ADORA2B_3_3058,4095,5380,6491,9642,6553,6319,5384,6224,5321,6971,3870,11212
ADRA1B_3_3059,1141,871,537,711,1057,274,933,1996,766,180,688,1244
ADRA1D_3_3060,156,51,33,13,0,0,0,0,3,3,1,0
ADRA2A_3_3061,605,515,1057,802,786,461,1072,987,1161,195,2859,670
ADRA2B_3_3062,9027,8851,8669,9527,9480,4906,10970,9423,6301,9436,9657,9176
ADRA2C_3_3063,389,365,309,391,42,514,195,9,171,234,127,250
ADRB1_3_3064,604,1107,630,1686,130,2178,413,2171,703,1731,2404,1581
ADRB2_3_3065,2056,2402,805,2310,3287,2496,1207,3950,3211,2075,609,1962
ADRB3_3_3066,947,1987,818,3242,1245,4404,4936,1165,1755,2178,1827,2523
AGTR2_3_3067,5228,5134,5309,7229,5898,5501,5720,5804,7802,4459,2926,2536
APLNR_3_3068,473,596,620,1458,631,1985,121,31,200,46,125,2175
AVPR1A_3_3069,4141,4817,5251,6329,3856,4481,6139,2534,6941,7893,2628,6912
AVPR1B_3_3070,229,753,973,593,1534,2400,812,419,201,769,378,771
BAI1_3_3071,479,371,197,1513,95,54,301,1195,0,7,98,1342
BAI2_3_3072,596,99,339,1087,3,305,55,547,6,72,1164,28
BAI3_3_3073,306,495,775,604,101,258,777,1742,533,70,1184,478
BDKRB1_3_3074,4328,4075,6897,5729,3723,5092,5698,4374,5425,3694,3682,4375
BDKRB2_3_3075,1325,1281,2878,1231,2410,2791,4554,1826,2419,4242,6527,926
BRS3_3_3076,6562,7217,5346,10208,3104,4713,8781,7594,7695,8651,10816,10169
C3AR1_3_3077,3346,3829,2386,4255,5257,1320,3715,2148,1346,6411,3509,3520
C5AR1_3_3078,81,195,457,174,187,911,57,318,496,9,333,56
CALCRL_3_3079,1267,1323,1263,1451,109,2727,912,1382,484,827,3034,914
CCKAR_3_3080,1224,2086,1741,1623,1777,3469,3500,1497,1338,1391,3859,3284
CCKBR_3_3081,347,112,362,78,215,1888,154,972,82,191,36,220
CCR1_3_3082,365,500,644,607,320,507,626,30,770,868,410,998
CCR7_3_3083,203,298,58,66,149,2718,21,2425,794,87,159,402
CCR8_3_3084,1147,1011,910,2007,1046,3510,3472,1469,712,3820,2876,974
CELSR1_3_3085,678,781,2247,1045,1393,1378,947,2230,557,569,559,1387

CELSR2_3_3086,1498,1696,1088,1880,2026,3052,1577,1721,3488,2304,2787,1
900
CELSR3_3_3087,3558,4381,2417,4087,2186,5502,6229,2907,3237,2000,8137,3
376
CHRM1_3_3088,212,287,53,397,1261,225,800,971,335,516,809,1109
CHRM3_3_3089,7921,7777,8185,8547,11211,8910,8377,8072,9992,12276,6204,
9382
CHRM4_3_3090,231,580,956,1051,11,19,319,2174,343,79,1675,460
CHRM5_3_3091,1914,2085,2053,1993,2617,2765,1614,755,1703,2846,4513,161
3
CXCR1_3_3092,864,640,1413,689,394,2081,300,239,1709,95,367,382
CXCR6_3_3093,1069,2152,3078,1684,365,2343,432,3317,1907,3189,3765,2961
CXCR7_3_3094,3525,2780,3471,3454,3483,4968,5937,3310,5327,2141,7552,32
86
CYSLTR1_3_3095,706,2740,1748,2340,2622,2684,2940,1244,665,2962,1753,29
99
CYSLTR2_3_3096,895,1082,2116,975,746,2929,1567,2774,2620,1114,3016,595
DRD1_3_3097,6,143,81,265,0,6,1205,0,0,511,249,133
DRD4_3_3098,3040,3164,3865,5042,2104,4210,5512,4244,3939,8576,2314,422
9
DRD5_3_3099,586,269,268,414,758,612,755,778,943,432,957,155
ELTD1_3_3100,280,634,427,315,475,99,843,758,213,112,134,963
EMR1_3_3101,588,571,1048,1317,957,1569,1151,2575,1356,341,2030,1353
EMR3_3_3102,539,1345,1354,2229,439,1686,1445,414,1423,1797,2307,377
F2R_3_3103,1980,2367,2044,2880,1439,1800,1349,4951,561,2585,2057,5376
F2RL1_3_3104,2480,2426,2595,4467,1101,5742,2781,2891,3340,6932,2703,41
83
F2RL2_3_3105,2620,2217,1524,1645,2837,2949,2841,5423,1599,2311,762,237
3
F2RL3_3_3106,297,69,183,560,3,487,24,1109,144,30,2523,310
FFAR1_3_3107,1214,1015,2014,2653,626,1766,2120,1413,1374,1924,2520,125
7
FFAR2_3_3108,4481,5457,6047,4969,4682,4881,5879,6724,8460,5649,6423,49
39
FFAR3_3_3109,673,715,286,341,83,1273,803,545,302,158,1351,1027
FZD10_3_3110,44,80,90,501,5,3,535,70,94,2681,6,13
FZD1_3_3111,3260,3515,2723,3875,5205,6195,4299,1857,3855,4302,7121,627
3
FZD2_3_3112,5993,6128,6683,6677,5137,6388,9962,7764,6679,9405,7715,554
8
FZD4_3_3113,2533,3704,1967,3361,1412,2745,2949,4379,2019,322,1953,2784
FZD5_3_3114,449,1039,1081,1361,307,1020,2301,1880,1202,990,3911,787
FZD7_3_3115,34,377,666,124,3,980,254,18,545,755,1,633
FZD8_3_3116,170,238,229,169,633,34,41,165,12,0,1767,215
FZD9_3_3117,20,834,106,22,52,31,744,422,1,0,2,740
GABBR2_3_3118,2370,2076,1644,2764,2912,2567,2032,3898,2414,907,3069,35
3
GALR1_3_3119,610,930,876,1363,498,1305,1702,586,512,349,1717,606
GALR2_3_3120,519,295,68,387,23,307,112,5,274,141,177,1274
GALR3_3_3121,147,335,838,358,853,451,0,1117,227,72,151,266

GCGR_3_3122,595,886,286,352,360,1021,247,151,203,1347,1458,1267
GHRHR_3_3123,3431,5321,5107,5189,2090,4513,4712,3601,5386,7693,3616,4480
GIPR_3_3124,6047,4981,6125,5477,8261,3201,5911,5463,3440,7856,4432,6586
GLP1R_3_3125,893,367,671,956,141,76,210,582,1309,2751,1825,1866
GLP2R_3_3126,2357,4716,3102,6108,4440,8137,2499,4324,2971,6009,5491,3912
GPR101_3_3127,4998,7081,8501,8700,7261,6656,8306,8395,7831,7260,3971,8947
GPR108_3_3128,364,726,332,1094,42,959,558,1397,518,1164,2555,411
GPR111_3_3129,1549,1459,397,1695,864,4327,2393,1938,1187,1707,587,494
GPR112_3_3130,3954,4604,6318,5292,5008,10908,5108,7195,5092,8688,1780,3207
GPR114_3_3131,770,1308,894,581,2300,971,2514,974,1909,1877,949,77
GPR115_3_3132,7508,7802,9721,10119,6124,16876,10077,11356,9901,11152,10952,6992
GPR119_3_3133,731,525,195,1101,391,950,1582,1198,774,485,170,1377
GPR123_3_3134,1868,1661,1415,2736,1522,697,1735,1251,593,339,2331,2118
GPR124_3_3135,2190,3179,4105,6625,3525,4278,2500,4359,3750,4589,5171,4176
GPR125_3_3136,3645,3004,5080,5133,3639,3057,4388,5024,6116,4906,7987,7174
GPR128_3_3137,8660,11619,12981,15504,6091,13133,9057,12806,11363,18451,16340,22133
GPR12_3_3138,41,220,395,605,0,0,1,0,0,336,9,674
GPR132_3_3139,7683,9315,8577,10015,9195,8934,10206,7361,9943,7738,13024,12079
GPR133_3_3140,2180,3200,2549,3091,3949,4910,1617,6797,5538,6429,5884,3474
GPR135_3_3141,637,200,1127,921,18,76,299,446,168,951,418,554
GPR139_3_3142,854,1467,1326,1897,1733,1144,1849,5481,832,1833,181,2667
GPR141_3_3143,575,433,829,694,822,3494,983,1713,802,1842,713,16
GPR142_3_3144,1133,1733,1809,2074,1049,3890,2700,1459,1481,404,320,2979
GPR143_3_3145,880,1845,1081,912,2622,1630,1466,1715,2501,863,444,480
GPR144_3_3146,1354,680,568,977,946,470,549,1788,768,44,146,72
GPR146_3_3147,3007,2332,2402,5172,4430,4141,4246,1152,4403,2086,1326,2930
GPR148_3_3148,865,941,910,2444,2973,1007,1353,2453,3333,1115,2591,562
GPR149_3_3149,3697,5544,4864,4993,3162,6915,5092,5719,3745,6808,7167,6372
GPR150_3_3150,279,671,1036,405,276,199,139,185,18,8,947,9
GPR151_3_3151,8108,8276,11430,9621,6992,9369,10481,15861,15726,9355,10210,9195
GPR152_3_3152,18,77,120,339,512,23,0,47,67,765,13,40
GPR153_3_3153,841,1399,1541,1912,443,1467,814,344,2661,635,1729,1445
GPR157_3_3154,457,655,519,115,257,2375,98,177,501,9,1284,599
GPR158_3_3155,1198,1591,2493,3613,1356,1350,1789,3591,2231,4422,2291,2308

GPR15_3_3156,1009,729,1242,536,1652,1731,691,609,1123,344,240,1650
GPR160_3_3157,7370,7319,5417,10926,7012,10723,6972,14082,8831,4710,822
8,7250
GPR161_3_3158,1116,937,1234,1724,1408,2862,1616,1730,2791,2046,1632,14
49
GPR171_3_3159,7976,5985,9250,10759,11542,10084,5532,13696,11261,10629,
6171,8931
GPR173_3_3160,295,1443,563,378,649,1741,1782,613,3192,552,50,755
GPR174_3_3161,3145,3850,3396,5988,4052,4661,2460,4154,3955,3678,5230,6
122
GPR176_3_3162,2148,1823,3089,1756,2832,5759,6341,4418,2464,4542,4152,4
719
GPR179_3_3163,3191,3637,3386,2542,4125,1943,3785,1691,1467,1770,691,39
83
GPR182_3_3164,4496,6166,4987,5589,4480,5647,4782,7696,6299,6598,3671,4
752
GPR183_3_3165,3715,6468,5730,8500,4677,5178,5095,11162,2766,4722,6592,
6049
GPR19_3_3166,4962,5006,3794,3856,7252,6579,8979,4187,8533,4336,7340,63
20
GPR20_3_3167,1644,1152,1329,1309,1128,4151,297,2490,1119,4378,178,531
GPR21_3_3168,11411,12500,16846,17410,14193,12819,24597,18583,14815,119
70,19906,18011
GPR22_3_3169,3774,3943,5879,6502,10064,10129,8782,4732,5140,3046,3902,
3899
GPR25_3_3170,354,165,180,42,23,288,173,0,45,3,59,0
GPR27_3_3171,152,28,503,121,5,847,147,0,4,335,153,8
GPR31_3_3172,182,249,404,39,488,1454,130,150,296,61,129,71
GPR32_3_3173,4152,8087,7061,7990,7227,3941,12676,3997,5620,4649,4983,4
488
GPR37_3_3174,3867,3458,3676,3175,2711,2620,2093,4412,3678,2814,4000,32
63
GPR37L1_3_3175,1662,1335,2225,3004,1474,3267,333,4580,4032,3144,2668,1
843
GPR39_3_3176,406,441,36,683,14,12,354,527,0,8,8,1610
GPR3_3_3177,984,504,841,1244,1529,2536,1122,2688,597,56,406,1707
GPR45_3_3178,1319,1610,1404,2253,1022,1570,1699,1187,1305,4101,4914,18
46
GPR4_3_3179,412,209,69,47,42,82,39,51,408,57,173,1707
GPR50_3_3180,1992,2696,1799,2622,4451,4450,1752,1229,980,1073,3852,128
2
GPR52_3_3181,2172,2420,3323,4700,1684,1645,4509,7967,1878,4248,3016,43
13
GPR55_3_3182,4844,6010,5784,6901,6257,4175,5787,8039,4021,3764,7060,81
62
GPR61_3_3183,1357,923,1178,1478,1179,1557,1645,1390,1049,3753,2106,270
2
GPR62_3_3184,1355,3277,1629,1210,924,3021,3049,1435,1812,2864,1345,112
8
GPR65_3_3185,2363,2689,3376,2791,1676,2196,3868,2396,1786,3497,3392,22

54
GPR6_3_3186,660,514,486,725,1920,1414,1107,969,913,993,837,222
GPR75_3_3187,175,593,418,1137,1443,514,756,0,324,8,676,52
GPR77_3_3188,611,942,1307,229,176,2167,168,716,62,558,2006,1490
GPR78_3_3189,131,588,974,996,1386,648,754,112,414,456,1507,1016
GPR82_3_3190,322,718,638,1057,1487,4,114,1218,75,427,938,1381
GPR83_3_3191,381,922,883,1352,1529,3809,784,2897,271,181,2086,141
GPR84_3_3192,1911,2728,3426,3843,3610,2655,3646,2902,1830,1996,4284,31
31
GPR87_3_3193,3519,4544,3941,5203,4103,6076,3845,3928,2491,3411,7021,49
13
GPR88_3_3194,212,243,156,516,743,71,277,805,60,606,3494,36
GPR97_3_3195,179,641,469,410,465,952,531,741,406,2,463,382
GPR98_3_3196,2111,1317,2324,2872,1445,1434,3896,5156,3079,984,2587,474
3
GPRC5A_3_3197,3420,4952,3696,5269,5958,3762,7803,9542,6332,3059,5628,9
805
GPRC5B_3_3198,745,1074,1368,1708,406,914,2913,1891,757,1715,363,1632
GPRC5D_3_3199,1257,1513,1933,1610,1490,985,1713,1603,2610,991,370,1512
GPRC6A_3_3200,3374,4180,6430,5981,1579,7074,4093,6687,2267,4573,5222,8
715
GRM3_3_3201,2684,3199,2931,4538,3905,2941,3046,6402,1440,2905,8846,662
8
GRM4_3_3202,435,975,1347,1310,16,68,2297,263,484,1058,3253,2613
GRM6_3_3203,721,1115,2193,677,480,1109,1628,1730,677,1338,57,4271
GRPR_3_3204,8565,9235,9040,16627,12115,9903,8986,15035,12929,11595,832
3,10336
HCAR1_3_3205,242,328,340,541,425,358,44,837,157,20,388,181
HCAR2_3_3206,3502,4887,3865,4473,4350,3585,4767,6322,2769,4763,2811,51
97
HCAR3_3_3207,1880,2280,2529,2127,733,4331,2955,3396,2541,2068,2700,322
4
HCRTR1_3_3208,8162,9026,9214,12452,10905,10972,10004,13968,9323,12460,
14412,11940
HRH3_3_3209,1914,1037,1321,2781,2015,1313,2556,5411,584,2584,1264,1583
HTR1A_3_3210,200,52,656,2,0,0,142,0,2753,2282,0,1
HTR1B_3_3211,1126,958,2012,2102,482,1020,1295,969,1394,1282,1168,1874
HTR1D_3_3212,1209,2245,1485,3225,272,5766,4078,661,3080,4616,1086,1075
HTR1E_3_3213,0,0,2,0,0,0,0,0,0,0,0,0
HTR1F_3_3214,116,181,362,4,293,21,2822,1,11,4,393,0
HTR2B_3_3215,7466,7753,8345,9718,5336,11293,8924,5225,10661,4607,6599,
6847
HTR2C_3_3216,1749,1429,2437,2392,1437,1534,2947,1500,2042,3532,1514,38
80
HTR5A_3_3217,567,154,484,471,44,130,93,50,1030,8,121,53
HTR6_3_3218,49,260,116,166,74,519,158,1283,448,5,2034,2625
KISS1R_3_3219,1913,2235,3067,4200,4617,4277,1942,5438,2631,2617,3075,4
122
LGR4_3_3220,4672,3968,4581,7366,3571,4026,4845,5420,4513,4728,4362,656
5

LGR5_3_3221,1283,2197,2334,3545,4064,2672,4436,1882,4031,4856,6207,189
0
LHCGR_3_3222,6429,7843,9397,9308,9140,6617,10015,10214,7282,6751,9563,
11280
LPAR2_3_3223,857,758,387,443,707,2110,1804,2190,403,270,913,1213
LPAR3_3_3224,3273,3744,3400,3192,4771,4225,3482,1203,3328,2594,3859,38
17
LPAR4_3_3225,326,282,566,325,756,667,356,71,166,24,93,41
LPHN2_3_3226,2003,1422,1444,1971,1990,498,703,1186,1089,1692,1820,3594
LPHN3_3_3227,1412,3749,2691,4169,619,2397,2390,2582,1947,2851,4507,817
9
MAS1_3_3228,1985,2565,2204,1958,2765,3520,3847,4258,1321,1191,1221,102
6
MAS1L_3_3229,2469,3070,4676,4877,2904,6441,4089,5060,1456,2003,2042,23
14
MC1R_3_3230,8878,6539,8383,13343,9884,7442,10413,12273,8395,12380,1027
0,4710
MC2R_3_3231,1090,2189,2066,5147,3993,889,2849,1011,5121,1331,7765,2053
MC3R_3_3232,3178,3405,4126,3820,6289,2428,3755,3606,7002,5115,5246,508
0
MC4R_3_3233,379,234,511,325,2194,7,1936,1406,773,262,379,628
MC5R_3_3234,2954,3085,4928,5698,2118,2646,3323,4792,4131,2870,2285,356
1
MCHR1_3_3235,1322,1942,2048,2094,548,2333,2192,588,424,746,2032,3588
MLNR_3_3236,661,591,361,843,755,2751,775,468,729,671,37,407
MRGPRD_3_3237,699,341,381,263,1158,1865,312,722,810,1228,2550,962
MRGPRE_3_3238,586,645,933,797,1585,320,1168,4465,212,1537,737,1087
MRGPRG_3_3239,2304,2738,3002,4248,3137,4401,3075,5965,2942,733,493,298
9
MRGPRX1_3_3240,1287,1885,685,2453,1756,3675,2021,3060,721,1280,441,724
MRGPRX2_3_3241,767,719,1561,369,1581,3400,1154,664,549,4090,408,387
MRGPRX3_3_3242,1178,1028,1215,2153,877,3112,1337,538,1349,286,1141,971
MRGPRX4_3_3243,885,605,55,1534,354,946,137,1820,194,27,263,25
MTNR1A_3_3244,1572,2506,886,1101,3244,3913,162,658,1190,2629,5286,212
MTNR1B_3_3245,916,1168,438,385,94,757,847,1649,1611,1810,175,1939
NMBR_3_3246,2550,4264,3294,4477,2392,2159,1562,4099,4427,1607,3052,361
7
NMUR1_3_3247,511,853,288,486,109,858,80,547,243,794,106,49
NMUR2_3_3248,2153,2275,2641,1352,2706,2758,2419,2048,1896,3352,2149,10
28
NPBWR1_3_3249,2827,3712,5064,6507,2974,4917,2428,5661,5375,7530,3204,3
616
NPBWR2_3_3250,438,799,312,322,88,116,92,140,0,283,44,77
NPFFR1_3_3251,852,2269,2016,1812,2123,562,2060,4118,1843,835,1226,537
NPY1R_3_3252,2617,2346,2168,2849,1235,5513,3693,1473,2509,5482,3644,26
18
NPY2R_3_3253,851,1544,2227,2268,1928,3206,3104,2076,3491,1513,135,1707
NPY5R_3_3254,5055,6054,5519,7277,5976,5771,5529,7147,7426,8296,2504,58
15
NTSR1_3_3255,1462,1605,3555,3230,1938,1473,3319,2873,459,3587,1914,135

3
NTSR2_3_3256,805,794,728,847,324,322,1580,619,447,441,1502,204
OMG_3_3257,3706,5830,4438,6488,2621,8886,2853,3934,5289,3382,2378,6162
OPN1LW_3_3258,6343,7540,8490,8043,6313,7219,13349,7544,7492,9422,6943,
7514
OPN1MW2_3_3259,1186,1000,1382,2253,1822,1194,951,1760,1438,672,1651,16
15
OPN1MW_3_3260,1186,1000,1382,2253,1822,1194,951,1760,1438,672,1651,161
5
OPN1SW_3_3261,419,791,1444,1254,1132,1910,2308,2023,909,1883,855,570
OPN3_3_3262,1254,2460,2558,449,1447,4041,1071,2904,803,1247,2048,2626
OPN5_3_3263,1292,2263,1405,912,1065,470,1554,907,2513,644,859,1850
OPRD1_3_3264,1272,775,844,1717,429,190,760,689,789,327,2841,1793
OPRK1_3_3265,494,968,803,1132,735,580,531,3231,1724,720,1490,2452
OXER1_3_3266,871,1211,1094,730,847,675,1553,658,816,997,87,1236
OXGR1_3_3267,2235,3225,3527,3694,1986,6402,3236,3894,2316,2903,4026,50
38
OXTR_3_3268,701,842,388,984,580,2117,691,462,1375,9,240,301
P2RY11_3_3269,1031,1650,1917,958,870,3292,1657,842,1625,3282,680,1248
P2RY13_3_3270,1466,1767,2011,2552,611,2240,1163,2281,1802,617,1608,207
8
P2RY1_3_3271,4894,4147,5850,3546,3139,6131,4184,6123,4976,5362,5465,52
92
P2RY4_3_3272,1575,2258,1506,1350,172,3940,2117,2785,1449,909,484,2369
P2RY8_3_3273,99,416,502,490,188,612,296,317,643,41,60,141
PPYR1_3_3274,269,465,361,1299,247,477,938,2203,863,2208,2190,843
PRLHR_3_3275,661,214,4,879,0,1,9,0,455,301,0,22
PROKR1_3_3276,5410,4571,5294,5995,7033,6602,4401,4926,6738,5262,5052,4
316
PROKR2_3_3277,2507,2977,2145,2825,6992,8194,1795,2313,3276,3127,3829,2
478
PTGDR_3_3278,2120,2304,2952,1366,2536,3225,1483,3243,4348,2201,1259,33
42
PTGER1_3_3279,350,910,156,191,541,41,810,1,800,2849,4,367
PTGER2_3_3280,1717,1580,1280,2007,2352,1759,2170,4539,3796,1070,1812,3
023
PTGER4_3_3281,3422,5655,4639,6501,4661,1658,2134,2671,4733,10890,7408,
5015
PTGIR_3_3282,23,233,253,246,11,1068,37,194,0,4,206,36
PTH2R_3_3283,1910,2387,3119,4206,5496,9044,2849,5220,3126,1026,4161,25
83
QRFPR_3_3284,460,275,374,564,1568,263,397,385,780,453,855,209
RHO_3_3285,70,183,43,313,794,401,333,280,994,130,30,173
RRH_3_3286,1661,1958,1675,2037,2435,2248,1396,4296,1398,1100,1729,2418
RXFP1_3_3287,943,803,898,1452,1732,215,998,256,1130,2944,1075,171
RXFP3_3_3288,574,787,985,982,1375,969,2372,1057,1418,597,932,896
RXFP4_3_3289,104,366,320,906,135,3,98,724,58,511,214,397
S1PR1_3_3290,1937,3408,3335,3907,744,2039,3649,2193,2990,5775,4791,151
7
S1PR2_3_3291,531,501,575,487,295,6,665,559,444,551,1660,2309

S1PR3_3_3292,384,859,1196,294,5,3583,1085,16,909,62,1006,9
S1PR4_3_3293,240,786,700,497,1207,237,54,1120,1410,1693,381,623
SCTR_3_3294,1038,1085,1345,997,913,552,1058,297,1257,441,1981,616
SSTR1_3_3295,977,631,720,1091,1441,7,1840,648,315,1792,477,501
SSTR2_3_3296,606,1055,1650,1474,857,1474,2408,3741,1342,2952,1755,3192
SSTR3_3_3297,888,1257,1616,1754,1816,911,408,1394,2412,380,952,452
SSTR4_3_3298,609,538,545,824,961,548,1425,1003,364,216,21,202
SUCNR1_3_3299,11467,12429,18027,17099,10667,22350,17325,20836,12384,19
009,16144,20607
TAAR1_3_3300,17249,17758,20091,24753,16108,18590,17324,21283,18615,261
38,19633,24868
TAAR5_3_3301,1192,1594,985,2077,1492,3093,1568,6563,4405,1190,1348,209
4
TAAR6_3_3302,882,1482,865,782,1082,1090,984,686,930,1725,2133,1147
TAAR8_3_3303,6572,8136,7537,10870,6488,10210,8241,9776,4918,7449,7215,
12754
TAAR9_3_3304,4281,4908,5706,6859,2743,5786,8281,5872,4723,4573,3599,49
42
TACR2_3_3305,483,364,292,450,243,170,195,147,987,802,253,301
TACR3_3_3306,1754,2167,2052,1700,1377,2699,1615,871,2509,2335,899,2900
TAPT1_3_3307,15039,20943,23018,26773,14063,25454,35149,25490,17754,312
56,24172,33432
TAS1R2_3_3308,1070,1380,2730,3354,1528,4766,2169,4502,919,2065,2525,31
05
TAS1R3_3_3309,1826,1974,1609,3629,1887,2103,1773,2897,2517,1055,1259,2
987
TAS2R10_3_3310,1484,2866,1871,1397,2583,2084,2569,1326,502,1396,579,61
0
TAS2R13_3_3311,3533,5080,5877,5920,4935,5875,7002,1712,4182,7461,6847,
4990
TAS2R14_3_3312,1063,1374,4148,3437,830,1168,3579,1245,1628,3164,523,15
39
TAS2R16_3_3313,7187,7208,8192,10913,11849,14237,9270,8860,10922,9295,8
493,12887
TAS2R1_3_3314,1061,823,1737,1412,1563,3059,557,3149,632,1208,1071,1033
TAS2R20_3_3315,39279,47240,48835,47953,45143,51861,49449,68738,41888,4
9776,50323,43368
TAS2R31_3_3316,42049,49997,51638,51132,46981,55001,51074,68472,43474,5
0674,54618,45816
TAS2R38_3_3317,1962,854,1185,2370,1205,2909,1293,2555,695,1046,327,242
2
TAS2R3_3_3318,2673,1589,3500,2398,1912,730,2969,2588,303,5964,1709,241
0
TAS2R40_3_3319,14281,15533,16976,19368,17136,20779,14766,13573,15567,2
0378,21633,18621
TAS2R41_3_3320,1075,1574,779,1495,2814,1125,1178,2607,1560,1018,123,80
1
TAS2R46_3_3321,42049,49997,51638,51132,46981,55001,51074,68472,43474,5
0674,54618,45816
TAS2R4_3_3322,4979,3695,3056,5416,3242,3726,8185,4494,5902,5569,7585,6

732
TAS2R5_3_3323,3415,4395,4553,6741,2997,3704,6579,7967,5076,2772,7425,2804
TAS2R60_3_3324,408,781,469,351,30,622,626,217,817,669,1071,27
TAS2R7_3_3325,2888,3952,4461,4241,2390,4719,8419,6363,3788,5008,6876,5994
TAS2R8_3_3326,4271,5727,5909,4462,6030,8289,6562,4512,6911,7089,3461,5615
TAS2R9_3_3327,626,308,804,740,687,1095,153,2334,982,721,472,118
TM2D1_3_3328,3950,6168,8629,6374,4537,6792,10304,12611,9213,2478,8685,4874
TMEM11_3_3329,641,1303,1346,1636,372,2776,1160,459,1510,569,1435,246
TRHR_3_3330,35,0,32,202,0,0,0,25,5,212,12,0
UTS2R_3_3331,92,190,419,115,29,0,641,60,107,1028,91,1031
VIPR1_3_3332,3551,4635,4186,7181,5160,7603,5380,5700,5539,9887,4315,8015
VIPR2_3_3333,1530,860,2121,2194,2127,5087,828,4053,1499,2138,3703,3419
VN1R1_3_3334,3914,2964,3447,4086,3311,8583,5006,2095,4700,7548,8894,4646
VN1R2_3_3335,1146,963,1120,2191,1506,1582,2579,885,652,1215,472,1848
VN1R4_3_3336,91,42,116,48,0,0,0,233,4,12,104,22
ADCYAP1R1_3_3337,144,26,9,0,0,0,0,0,2,0,0,0
ADORA1_3_3338,460,458,494,1767,1634,1119,1917,159,898,151,66,1035
ADORA3_3_3339,10055,9468,11315,15122,12013,7155,5220,19804,11514,15045,12730,17533
AGTR1_3_3340,4598,6706,8779,6550,8847,10241,5424,4167,7936,4570,5207,10760
AGTRAP_3_3341,883,2086,1804,1404,705,963,3559,1944,3227,1411,746,711
AVPR2_3_3342,1885,854,1806,1836,1273,1878,1452,1713,1756,624,571,1282
CALCR_3_3343,688,396,1106,1583,1876,942,716,945,615,858,891,1138
CASR_3_3344,3526,6295,3655,5565,3572,7420,5114,5985,3796,6024,7936,5884
CCR2_3_3345,1324,1884,1358,2096,2189,1308,3396,3619,2656,113,587,975
CCR3_3_3346,1009,1270,1165,3425,1389,471,1077,1100,1478,861,671,991
CCR5_3_3347,424,903,1161,853,441,1276,590,903,714,26,1547,342
CCR6_3_3348,4442,4419,4049,5903,1594,4336,4810,5363,8060,3870,3377,2117
CCRL2_3_3349,1232,2861,1355,2051,2242,3490,1134,782,2913,1482,882,1629
CD97_3_3350,1013,1512,1025,1922,2658,1001,859,806,5,2208,490,848
CHRM2_3_3351,3318,2380,2917,4308,3865,2729,2452,4128,3586,6326,2140,2559
CMKLR1_3_3352,360,54,285,77,0,12,844,559,15,3,15,247
CRHR1_3_3353,517,1101,418,350,690,0,603,294,1475,0,3,6
CRHR2_3_3354,4488,5303,6247,6374,6448,4457,5273,9716,4988,2855,6677,7549
CX3CR1_3_3355,689,784,248,896,238,989,30,328,610,1026,133,300
CXCR2_3_3356,838,611,723,1097,1260,444,568,1093,180,63,999,945
CXCR3_3_3357,1740,1979,3475,2995,1486,4506,1724,3339,2203,2950,6023,3409
CXCR4_3_3358,851,1094,1265,1790,1872,446,1233,4825,788,3005,241,2204

CXCR5_3_3359,373,1788,1236,2920,521,2170,733,1986,3169,1233,2248,857
DARC_3_3360,152,206,17,652,624,184,1,1344,644,958,22,110
DRD2_3_3361,68,195,492,149,29,74,72,144,506,566,30,35
DRD3_3_3362,321,176,410,386,27,32,453,16,210,374,186,10
EDNRB_3_3363,4104,4603,6319,6051,2901,6071,7557,6193,2702,6221,4600,52
20
EMR2_3_3364,350,210,956,759,376,73,457,133,323,42,429,1305
FPR1_3_3365,425,739,323,665,837,603,1804,40,430,134,737,169
FSHR_3_3366,1929,2107,2010,3129,2193,1738,2472,7576,2522,1330,2504,274
8
FZD6_3_3367,2153,2162,2076,1493,1140,2501,1379,701,2619,363,1564,1108
GABBR1_3_3368,1791,1977,1172,2593,1918,703,1833,2158,2775,2881,986,250
4
GHSR_3_3369,288,194,756,144,10,37,1,1,0,3,533,843
GNRHR_3_3370,1280,809,1687,1097,157,577,3979,2299,434,441,4219,402
GPBAR1_3_3371,729,1037,440,1339,586,2187,228,1953,744,1327,1913,106
GPER_3_3372,1203,2452,981,757,846,2136,982,3362,1409,725,1336,2035
GPR107_3_3373,5023,7909,7583,8422,5244,7431,5124,6523,9563,10913,13735
,5448
GPR110_3_3374,3809,5406,5032,6135,4427,9330,5929,5007,5719,5985,10384,
9443
GPR113_3_3375,2663,3337,3041,4039,3952,1471,3606,6002,3196,4177,4019,3
749
GPR116_3_3376,2244,1905,2802,2789,5471,4745,4646,2540,2171,2981,3549,2
192
GPR126_3_3377,1566,1896,1999,2684,1917,1170,2354,5105,3374,4790,3155,1
687
GPR155_3_3378,1843,2203,2894,4007,4038,1100,2770,1517,1297,1702,1641,3
061
GPR156_3_3379,1834,1712,847,1411,1371,3056,1015,4305,848,1879,413,1548
GPR162_3_3380,7073,9205,9899,8591,7894,9733,10152,7539,10730,10868,674
3,13087
GPR17_3_3381,703,735,141,409,365,210,827,748,438,525,60,95
GPR18_3_3382,192,1727,857,1467,1480,3223,463,358,989,2260,215,45
GPR1_3_3383,2062,1513,1607,3020,1895,1498,2727,3446,2435,2346,1258,226
2
GPR34_3_3384,12397,13933,15559,19221,12720,20078,19256,12787,12258,212
11,10300,15013
GPR35_3_3385,511,2132,1218,917,580,1555,551,1503,903,895,219,1428
GPR56_3_3386,249,740,449,1956,311,406,484,805,1050,3322,275,902
GPR63_3_3387,807,624,932,1228,239,1529,312,895,586,147,705,1154
GPR64_3_3388,2382,3512,4626,4905,4771,10204,5630,2772,3604,3321,7839,1
1262
GPR68_3_3389,855,1985,1177,812,1096,285,1744,840,1042,1277,3363,1330
GPR85_3_3390,2961,3092,3675,6101,3593,5627,2344,3651,1691,5640,6700,47
95
GPRC5C_3_3391,1379,1628,2103,1474,2085,2792,1649,1857,1691,2883,933,68
1
GRM1_3_3392,4425,3698,4853,5380,4599,7000,7471,5926,2754,7177,7233,507
2

GRM2_3_3393,2374,2307,3175,2668,2784,5364,2707,2962,5213,1150,5199,235
4
GRM5_3_3394,2391,2556,3733,2873,2860,1617,4809,4932,1716,3628,6445,325
3
GRM7_3_3395,797,989,1230,1012,544,837,581,1467,1034,2272,1037,2495
GRM8_3_3396,5456,7415,8341,12739,10144,12658,8497,11598,7735,9910,1337
0,10582
HRH4_3_3397,685,1343,675,1788,2997,1101,1772,1380,804,1529,1231,850
HTR2A_3_3398,8728,9870,6323,11009,10637,18350,11147,7586,6098,12085,16
380,16725
HTR4_3_3399,4917,6994,7878,11045,5077,8433,7103,6483,5405,8508,7247,92
62
HTR7_3_3400,212,696,870,1032,596,1171,126,901,890,6,2718,148
LGR6_3_3401,3611,2307,2258,4285,1910,3156,4154,3569,1994,5172,5391,200
4
LPAR1_3_3402,5289,7828,7210,7472,7999,14703,7575,11145,7201,9141,11299
,6554
LPAR5_3_3403,1657,633,323,1338,940,1014,1586,515,487,391,1607,584
LPAR6_3_3404,3624,4166,3132,5228,4894,1963,5565,8831,3410,3113,6216,68
76
LPHN1_3_3405,1126,1007,1859,1711,1265,739,1579,2243,1007,350,478,1820
LTB4R2_3_3406,362,618,652,78,39,1242,55,22,1450,548,134,511
LTB4R_3_3407,1886,3753,3165,2881,3920,8418,5399,3244,2296,3620,4067,31
19
LYPD1_3_3408,733,643,809,1498,2556,1816,280,579,1191,761,855,1425
MCHR2_3_3409,1726,1635,2231,1382,2250,3756,1511,2070,336,1957,3259,593
5
MRGPRF_3_3410,1020,527,1666,1159,1479,89,1075,602,2134,2438,1759,668
NPFFR2_3_3411,8865,10596,7274,11504,8318,13637,13643,10555,10209,3412,
18198,14698
NPSR1_3_3412,8235,7486,9610,9350,5726,7801,9805,11188,8567,8302,6262,7
832
O3FAR1_3_3413,441,952,1079,632,875,386,45,1079,763,939,1261,3587
OPN4_3_3414,2260,2941,2878,5534,1859,2044,4247,6848,6083,4142,2045,334
6
OPRL1_3_3415,4408,3846,2887,4728,4053,5396,8976,3165,3864,2726,7061,73
51
OPRM1_3_3416,922,590,318,846,461,2324,611,1621,1680,588,1549,551
P2RY10_3_3417,2446,3922,1837,3213,930,53,3856,2413,3946,6178,2225,3697
P2RY12_3_3418,8001,12911,13757,15058,11478,17947,13736,15646,10975,135
12,13329,13706
P2RY14_3_3419,4909,5778,7757,8008,8090,5206,6771,6215,6694,6777,7709,9
672
P2RY2_3_3420,1608,1840,2586,2068,4407,1885,2651,5705,1997,4696,8752,20
74
P2RY6_3_3421,678,730,1053,1294,196,446,1084,1877,1248,117,3135,649
PTAFR_3_3422,694,910,1347,772,406,464,2082,262,1487,1855,740,92
PTGER3_3_3423,480,187,293,738,13,296,610,146,1403,463,580,1202
PTGFR_3_3424,3486,4732,5286,8991,7040,8366,4471,6073,5658,3903,7213,54
79

PTH1R_3_3425,3594,4948,4121,5185,4832,3783,3487,5425,6016,5598,9676,59
34
RGR_3_3426,1339,1660,3037,1435,4252,1175,815,1647,1360,2186,1249,2236
RXFP2_3_3427,7158,9034,10006,10753,5000,10402,5310,9769,8456,11020,193
97,11234
S1PR5_3_3428,269,371,323,159,341,76,511,920,817,687,37,1970
SIGMAR1_3_3429,1400,1995,1999,1918,1182,2188,1025,3769,3050,2822,4889,
4260
SSTR5_3_3430,699,364,412,351,292,241,262,106,745,69,24,230
TAAR2_3_3431,10000,8473,11023,17613,10335,13494,10917,18250,15692,1862
3,18596,11108
TACR1_3_3432,658,1928,1404,537,1138,455,1576,914,694,1548,1436,1250
TAS1R1_3_3433,2403,3252,3702,4633,2651,3594,2149,2910,3119,4586,3562,5
407
TBXA2R_3_3434,828,2918,3288,4094,3245,1899,3228,1369,3978,2092,2622,19
72
TPRA1_3_3435,2043,2663,3213,5977,3459,2852,4655,3580,2298,3807,6594,50
39
TSHR_3_3436,1974,3028,2514,3707,1192,4005,2436,3917,3721,1442,3233,567
8
XCR1_3_3437,573,350,127,1697,1,27,124,1432,60,289,58,599
XPR1_3_3438,884,962,1306,2119,2152,479,1064,1404,233,275,213,1593
ADORA2A_3_3439,5274,6918,5820,8389,6703,7574,12422,7074,4035,7807,9466
,4458
ADORA2B_3_3440,12195,13691,13939,14218,8989,19766,17526,16098,18002,12
145,22087,13197
ADRA1B_3_3441,499,804,65,621,718,423,307,1,40,43,49,20
ADRA1D_3_3442,233,210,254,916,154,2410,1012,940,142,578,178,0
ADRA2A_3_3443,115,68,56,655,0,0,774,1827,299,0,1,546
ADRA2B_3_3444,2067,1473,3107,1590,2098,319,4910,2852,3053,2324,2967,31
12
ADRA2C_3_3445,8568,11441,8723,11212,8320,5763,14783,12972,7340,11147,9
039,8636
ADRB1_3_3446,494,660,513,841,1640,2055,327,55,113,686,1146,537
ADRB2_3_3447,877,1844,2074,2031,726,656,1530,868,676,608,1709,1913
ADRB3_3_3448,843,1195,524,1573,496,525,119,1287,323,358,788,450
AGTR2_3_3449,4000,4091,4832,6724,3570,3100,3797,2807,4973,6388,8355,16
67
APLNR_3_3450,458,1073,1111,2172,293,1243,519,600,363,737,1112,1786
AVPR1A_3_3451,2884,3527,4463,3557,2794,6595,6209,2286,2534,3992,6394,1
945
AVPR1B_3_3452,7936,7768,8494,12880,13227,7258,10217,11067,9692,8907,11
546,14283
BAI1_3_3453,239,390,335,651,30,28,60,307,1910,205,328,129
BAI2_3_3454,1400,3040,1991,3849,2016,4012,417,2607,2931,2667,5849,3367
BAI3_3_3455,228,526,1159,808,154,258,334,1571,601,553,493,1204
BDKRB1_3_3456,914,1204,920,1925,577,3339,727,468,1804,802,854,790
BDKRB2_3_3457,2137,2124,1766,2117,2661,3215,2371,899,1092,4152,839,911
BRS3_3_3458,421,426,973,663,734,427,265,221,1250,49,3341,175
C3AR1_3_3459,1998,1652,3064,2334,1839,3298,3796,1638,3671,3895,3665,48

61
C5AR1_3_3460,36,111,211,32,0,0,285,0,1,375,233,5
CALCRL_3_3461,780,434,866,433,301,372,55,13,265,55,629,184
CCKAR_3_3462,1894,3110,2338,2926,1601,5711,4938,1435,800,1818,7766,422
5
CCKBR_3_3463,986,1232,827,925,110,995,1204,925,933,939,404,1150
CCR1_3_3464,2095,2620,2073,4616,2700,5054,2034,2554,2528,1773,2751,124
5
CCR7_3_3465,5944,6419,8329,6545,5642,5179,7253,5775,6247,9050,10978,48
29
CCR8_3_3466,779,802,1512,1669,408,1284,1078,1111,1957,240,2605,1625
CELSR1_3_3467,716,1784,1313,1933,1629,1413,2192,2258,948,3251,72,2177
CELSR2_3_3468,264,1152,317,92,1,1402,1535,62,1398,389,422,67
CELSR3_3_3469,627,2276,1721,846,1382,5130,1025,314,1563,2202,526,137
CHRM1_3_3470,34,5,558,37,122,1,53,275,0,11,15,456
CHRM3_3_3471,159,611,601,234,0,213,264,312,16,357,6,111
CHRM4_3_3472,3069,3798,4254,4921,2150,2795,3432,2139,5094,4598,4394,22
71
CHRM5_3_3473,1221,1293,1101,1242,617,2619,1925,461,1143,972,3264,1002
CXCR1_3_3474,256,192,36,184,551,1402,1,20,1,2,21,421
CXCR6_3_3475,9608,8734,11337,14214,9422,13674,10674,10105,15545,6590,1
8687,17756
CXCR7_3_3476,345,338,538,1174,1387,1029,212,695,763,1127,1110,62
CYSLTR1_3_3477,1656,3766,3049,4815,2845,6083,3037,3992,2972,1584,7484,
4061
CYSLTR2_3_3478,368,736,420,1546,81,321,379,1895,14,49,795,838
DRD1_3_3479,2290,3360,2094,3198,2524,2995,2849,4299,4088,3342,2763,570
9
DRD4_3_3480,273,262,630,219,8,1,1,3,244,23,46,571
DRD5_3_3481,1452,1300,1214,3189,2849,1823,1447,1641,1006,1892,1355,197
7
ELTD1_3_3482,3313,2273,2164,3923,2347,299,5413,3881,2182,3724,1701,830
6
EMR1_3_3483,1053,1604,1560,1670,336,1866,2389,1294,1132,318,1636,578
EMR3_3_3484,1548,2083,1885,2622,1453,799,2255,1293,1092,665,507,3819
F2R_3_3485,614,598,1768,1041,698,195,979,1862,455,1504,396,780
F2RL1_3_3486,480,778,787,691,602,106,347,313,1627,463,556,1258
F2RL2_3_3487,8137,7785,10072,11543,11615,6445,9553,7558,17370,10486,11
580,8436
F2RL3_3_3488,40,90,105,368,2,73,94,47,11,212,14,538
FFAR1_3_3489,457,450,218,427,1310,13,163,1393,53,391,7,0
FFAR2_3_3490,905,672,842,734,1269,654,12,126,46,2,57,90
FFAR3_3_3491,135,71,20,45,6,111,3,3049,14,68,274,142
FZD10_3_3492,9506,11544,12109,13928,10756,11287,10954,6803,12033,16169
,18566,7639
FZD1_3_3493,6360,7918,8168,12486,9951,6875,9545,8830,9777,17373,17620,
8458
FZD2_3_3494,379,521,405,878,1925,173,470,52,247,515,468,2082
FZD4_3_3495,389,269,118,289,38,304,467,1552,687,1024,180,331
FZD5_3_3496,530,802,803,520,889,1796,519,276,233,1005,714,234

FZD7_3_3497,520,585,935,1102,1490,290,963,517,55,1125,1882,1971
FZD8_3_3498,1971,1621,3357,2344,3504,1685,2769,3031,299,5149,1905,2310
FZD9_3_3499,558,637,1572,1546,363,845,140,1887,111,64,3193,611
GABBR2_3_3500,1557,1498,1117,2684,2041,2169,1509,1700,1331,537,1569,18
61
GALR1_3_3501,1447,1442,884,1668,1235,144,2573,1321,1789,2177,4334,1240
GALR2_3_3502,1258,1811,1597,2080,1823,1830,2089,585,1296,10742,3036,22
43
GALR3_3_3503,71,30,0,27,52,936,0,78,186,0,6,11
GCGR_3_3504,1060,1795,2839,2335,551,1412,2025,1871,604,1190,792,4058
GHRHR_3_3505,1261,944,1431,2329,1839,1077,2508,2443,1060,664,1057,3285
GIPR_3_3506,345,853,677,234,52,85,332,772,54,64,1582,216
GLP1R_3_3507,3768,3800,3879,6716,5378,5807,3854,6256,5172,5292,4731,68
36
GLP2R_3_3508,2083,2488,2770,2289,1816,3020,1521,1356,4491,2675,2854,43
9
GPR101_3_3509,1111,1497,977,2141,1488,3703,1004,4162,1656,1613,439,174
3
GPR108_3_3510,1391,1838,1935,1841,3140,2118,2020,1525,4530,1288,1926,2
701
GPR111_3_3511,443,460,507,410,625,1463,1037,798,403,984,155,343
GPR112_3_3512,4030,5586,6389,5049,5141,4296,7757,5336,9041,5867,2377,1
2552
GPR114_3_3513,1215,2123,1593,1861,1975,1506,3000,4598,1728,3931,2419,1
743
GPR115_3_3514,1275,1445,1347,1833,1289,1698,582,2821,889,2916,2098,128
9
GPR119_3_3515,958,855,494,512,755,1045,734,877,134,945,47,19
GPR123_3_3516,118,166,143,302,76,482,372,282,922,1649,17,279
GPR124_3_3517,5002,5447,4173,7381,4179,7877,5725,8190,5435,3295,6193,7
434
GPR125_3_3518,270,116,757,233,35,789,171,41,342,207,292,877
GPR128_3_3519,1434,2483,4374,1257,1825,961,4821,1139,1597,1856,352,143
2
GPR12_3_3520,416,700,1776,1275,253,1523,1353,3458,1207,1729,1865,2217
GPR132_3_3521,7401,9459,7834,9376,9204,8539,10032,6876,8989,7706,12951
,12057
GPR133_3_3522,676,558,379,1015,75,30,1407,1207,270,991,1290,248
GPR135_3_3523,1202,1795,1301,1599,1384,1890,2947,1345,1970,1640,333,25
57
GPR139_3_3524,11976,14390,14416,18625,13390,19006,22391,16300,14686,24
255,13404,19293
GPR141_3_3525,696,2163,1123,1722,1662,1187,2191,981,1398,1269,1389,645
GPR142_3_3526,1206,1046,1098,912,2000,1066,959,1135,346,1953,2547,1102
GPR143_3_3527,5601,5440,6826,5667,4397,4299,5307,4401,5681,8065,8966,4
841
GPR144_3_3528,1670,2029,1348,1455,479,1230,960,1180,113,190,663,718
GPR146_3_3529,413,477,158,846,1731,971,930,1046,746,616,32,2699
GPR148_3_3530,2365,2588,3357,3771,3543,998,2150,1956,3702,3676,458,343
0

GPR149_3_3531,6986,7653,8368,11304,6856,11799,14241,13376,6188,10800,10128,14492
GPR150_3_3532,158,250,1911,21,604,158,2651,0,1,6,2,25
GPR151_3_3533,1036,984,844,1273,294,702,41,1160,952,2730,1249,538
GPR152_3_3534,90,282,36,531,738,25,0,0,56,0,9,1065
GPR153_3_3535,356,1003,767,1221,415,203,1052,587,715,2525,700,847
GPR157_3_3536,1426,1586,1399,2745,1476,2573,3394,1105,2320,1829,3133,822
GPR158_3_3537,572,434,685,389,287,43,741,906,141,196,171,744
GPR15_3_3538,1120,1044,1481,2198,3213,767,1250,1146,622,1407,2724,2472
GPR160_3_3539,2645,2464,3150,2372,3552,2135,4484,2046,5207,3344,2989,4657
GPR161_3_3540,706,825,1285,1520,454,734,1496,4835,858,1129,3022,292
GPR171_3_3541,7936,9427,11889,9703,8439,13009,10061,5565,4759,7407,11084,5876
GPR173_3_3542,365,459,1172,1166,1134,2795,3382,2341,1695,74,1156,528
GPR174_3_3543,1268,1583,602,2719,1438,2053,2263,5434,1468,1699,1891,1163
GPR176_3_3544,1120,1090,1009,888,659,302,671,3064,2885,483,1816,456
GPR179_3_3545,642,1274,465,1057,503,880,1315,999,1269,341,232,1401
GPR182_3_3546,835,1139,968,1168,3379,2537,1226,2081,2066,571,479,1340
GPR183_3_3547,4250,5978,5239,6020,5454,9013,5234,5369,5157,6664,7483,7589
GPR19_3_3548,1566,2914,3126,3783,1787,3997,2404,5766,2649,3765,4588,3762
GPR20_3_3549,943,13,126,3,274,0,2,4,915,3,0,0
GPR21_3_3550,5127,5228,5548,6384,7027,6574,5820,6137,6850,5756,6512,4969
GPR22_3_3551,1787,1945,2712,3015,3111,3947,1932,1533,3255,3588,2040,3751
GPR25_3_3552,524,1043,718,991,349,892,3743,209,1255,22,336,829
GPR27_3_3553,524,459,185,430,2156,490,134,117,63,562,153,1119
GPR31_3_3554,1389,851,985,1784,4336,736,2332,3729,1857,1325,3221,470
GPR32_3_3555,1344,2494,826,4147,2192,3349,496,2103,3439,338,617,394
GPR37_3_3556,842,469,1203,1768,139,852,1350,2204,2959,63,929,606
GPR37L1_3_3557,966,1184,2292,2014,2829,2308,169,1949,3065,1325,2999,2585
GPR39_3_3558,365,373,594,963,17,490,65,46,401,146,106,200
GPR3_3_3559,698,1292,145,1711,143,35,35,13,1268,1212,87,113
GPR45_3_3560,410,75,379,360,325,138,29,571,654,32,117,456
GPR4_3_3561,695,961,106,613,140,2562,22,1140,123,1169,271,637
GPR50_3_3562,5226,7538,6123,10192,5524,4680,7887,8200,5043,5511,11497,11394
GPR52_3_3563,3578,4311,4943,5591,4102,6112,4687,6463,5009,2776,12474,6278
GPR55_3_3564,1833,2500,2022,3299,603,3834,2099,2995,1890,6633,2757,1171
GPR61_3_3565,617,1304,1371,1512,1193,443,1305,470,1824,583,2278,1397
GPR62_3_3566,576,470,42,25,264,1,25,0,1051,30,4,179
GPR65_3_3567,1069,1048,1803,2178,1505,1534,1363,1492,1158,1516,2151,12

59
GPR6_3_3568,1634,2429,1138,2235,443,1495,1657,5237,1462,1739,1233,2891
GPR75_3_3569,4555,4569,3922,6503,4606,7796,3198,8036,4815,5141,6364,66
72
GPR77_3_3570,958,1631,1303,1479,986,1541,1916,2732,2090,4766,4161,1879
GPR78_3_3571,747,402,823,194,743,1367,378,1235,399,126,87,327
GPR82_3_3572,2628,2008,2136,3676,1479,5238,3088,4580,4350,1411,5396,75
79
GPR83_3_3573,1695,1202,1916,874,3881,3711,3943,702,3814,1706,1001,1459
GPR84_3_3574,1089,756,395,638,615,667,16,425,294,58,1144,2390
GPR87_3_3575,11252,13254,11466,10874,13184,18110,15295,11044,12300,119
65,14185,15969
GPR88_3_3576,393,524,1057,706,166,167,408,4454,499,29,2823,176
GPR97_3_3577,818,282,301,760,230,535,210,846,1339,468,1064,778
GPR98_3_3578,0,0,0,0,0,0,0,673,0,0,0,0
GPRC5A_3_3579,912,1030,1926,1132,801,1149,1284,541,758,456,2653,1015
GPRC5B_3_3580,251,317,1612,665,86,275,2682,387,588,66,1170,954
GPRC5D_3_3581,1001,1836,2183,1449,3415,1166,2224,2386,341,1768,2738,16
01
GPRC6A_3_3582,4506,5710,4158,6199,5292,1773,6650,5257,4251,9038,8484,3
909
GRM3_3_3583,2648,4914,4548,3379,4069,4825,4546,3714,2907,5006,6981,505
9
GRM4_3_3584,594,378,109,584,273,1008,1289,2073,62,402,33,734
GRM6_3_3585,669,556,61,450,1571,44,496,1049,299,491,56,1370
GRPR_3_3586,1897,1308,2024,1449,2312,3337,2871,2192,2021,2883,826,2623
HCAR1_3_3587,482,963,619,824,554,176,168,616,809,1544,41,370
HCAR2_3_3588,2962,4036,3668,4413,1622,9877,3136,3467,2126,2236,1101,50
95
HCAR3_3_3589,2224,1885,2229,2205,1123,1114,1664,1814,1566,6019,5406,78
0
HCRTR1_3_3590,610,229,355,183,167,111,0,3,473,352,16,106
HRH3_3_3591,1274,1283,1286,1849,1497,1894,1186,2278,1551,919,1738,2299
HTR1A_3_3592,963,510,326,1047,1248,3124,467,1030,1035,529,597,1348
HTR1B_3_3593,331,1438,1448,1794,484,1505,2465,3014,1521,660,1095,2192
HTR1D_3_3594,898,1692,1480,2128,1404,1491,1854,2141,4055,2844,2001,403
8
HTR1E_3_3595,291,581,588,851,820,672,489,3915,554,86,200,515
HTR1F_3_3596,313,422,884,663,275,737,22,43,67,454,954,3
HTR2B_3_3597,207,365,336,349,9,1115,151,54,121,549,676,1142
HTR2C_3_3598,1730,2176,1638,2882,3610,1552,3020,1245,678,687,1484,1878
HTR5A_3_3599,455,1147,927,1170,443,1928,1743,2098,1102,253,590,368
HTR6_3_3600,24,132,79,83,0,1,0,215,2,938,7,6
KISS1R_3_3601,783,1738,1523,1490,921,2463,1191,4831,1236,1119,1208,185
5
LGR4_3_3602,3171,3551,4510,3532,2998,8170,6309,5388,2157,5281,4414,271
2
LGR5_3_3603,2319,3553,3052,2503,1360,5283,3103,873,2262,2357,1943,3053
LHCGR_3_3604,4676,3998,3838,7233,5173,3671,5455,4611,6919,3778,4071,89
06

LPAR2_3_3605,2319,2332,2567,4069,1072,3275,2526,1460,2227,6359,3214,16
49
LPAR3_3_3606,337,654,942,1335,1419,650,444,1355,735,433,2352,819
LPAR4_3_3607,1810,2929,1581,1570,2446,574,4451,2513,2495,406,3211,3470
LPHN2_3_3608,21968,25074,26545,31397,25195,30109,30797,32052,25102,254
59,25584,31413
LPHN3_3_3609,2297,2040,822,1751,2051,1061,4183,204,430,2714,3642,646
MAS1_3_3610,892,1285,1126,1565,1402,3523,1904,3194,956,997,239,316
MAS1L_3_3611,558,1585,1112,530,288,2051,499,1814,585,2457,609,512
MC1R_3_3612,1045,1456,1497,2203,596,105,935,355,790,6179,2570,1173
MC2R_3_3613,437,383,1350,1256,2372,2010,764,1844,82,1299,257,3119
MC3R_3_3614,1872,2277,1683,2558,2138,3490,4785,3054,3047,2955,673,2545
MC4R_3_3615,839,1341,695,1229,490,1171,566,411,1633,4102,263,1308
MC5R_3_3616,1457,2136,1878,2952,2335,1913,2955,1152,513,1371,5323,1264
MCHR1_3_3617,537,277,524,1071,292,1235,994,129,1915,24,59,166
MLNR_3_3618,922,1118,1295,1722,865,1467,1731,1872,2205,2340,910,632
MRGPRD_3_3619,625,213,371,383,147,898,670,209,443,179,5,22
MRGPRE_3_3620,3949,3011,3015,4862,3852,5708,4278,5468,4629,4109,5162,4
848
MRGPRG_3_3621,2558,3733,1811,2686,2492,1197,861,1010,4289,4543,885,428
8
MRGPRX1_3_3622,253,336,588,263,29,56,77,20,937,278,181,14
MRGPRX2_3_3623,3718,5008,6708,9152,4081,9896,5758,5368,7810,7004,2950,
8260
MRGPRX3_3_3624,885,605,55,1534,354,946,137,1820,194,27,263,25
MRGPRX4_3_3625,3630,4767,4988,5205,5025,7774,6426,5927,5590,6092,3473,
3696
MTNR1A_3_3626,1919,4058,2855,4666,2340,4606,4808,3935,2421,3992,2186,2
012
MTNR1B_3_3627,1422,1105,1575,2755,1483,1903,406,267,444,1315,3336,1154
NMBR_3_3628,2968,5880,4572,4753,5777,5049,7077,2628,4235,2927,9782,587
0
NMUR1_3_3629,139,147,264,594,945,291,117,834,11,1529,17,216
NMUR2_3_3630,1792,1855,2268,343,992,490,2241,1401,282,484,1617,2518
NPBWR1_3_3631,3055,3373,3418,4886,3160,6119,1829,3257,2240,6659,5476,3
534
NPBWR2_3_3632,2632,4470,2896,4127,2047,2501,3659,4219,2433,3445,2495,4
518
NPFFR1_3_3633,3425,4098,3640,5627,3841,4194,3062,5516,4464,4923,12525,
5743
NPY1R_3_3634,1036,1269,1254,2648,1178,1145,1129,2342,2032,3840,2137,42
97
NPY2R_3_3635,1534,1157,2194,1270,2540,1108,2538,1204,1991,104,134,1949
NPY5R_3_3636,343,8,109,392,0,0,0,0,0,5,51,0
NTSR1_3_3637,1150,427,343,1192,474,959,519,839,662,320,651,1607
NTSR2_3_3638,3715,3274,5181,7481,6650,4701,6016,4196,5339,4174,4463,69
61
OMG_3_3639,3711,2471,2670,3098,3150,4640,2167,5439,1139,3007,7690,3381
OPN1LW_3_3640,5522,5145,3879,4633,7392,4124,2225,7807,3860,2677,3128,3
562

OPN1MW2_3_3641,5522,5145,3879,4633,7392,4124,2225,7807,3860,2677,3128,
3562
OPN1MW_3_3642,5522,5145,3879,4633,7392,4124,2225,7807,3860,2677,3128,3
562
OPN1SW_3_3643,1740,2045,2361,3749,2233,3077,3635,3541,3655,3611,1931,3
731
OPN3_3_3644,658,736,623,1597,412,2948,127,1112,714,243,1116,2451
OPN5_3_3645,30,215,16,2,2,0,0,263,0,618,0,0
OPRD1_3_3646,3185,2736,4982,5264,3686,3176,6235,4284,1443,2750,5478,65
32
OPRK1_3_3647,1239,3260,2391,1354,2709,1592,2918,872,1343,1371,5235,143
5
OXER1_3_3648,243,283,152,97,169,50,73,524,140,95,24,64
OXGR1_3_3649,1155,1854,2162,1799,1352,3094,2191,1848,779,2225,2568,160
5
OXTR_3_3650,1071,1647,2579,2504,537,1889,2403,2231,624,3471,4886,1321
P2RY11_3_3651,494,294,216,649,1601,0,29,209,14,391,782,93
P2RY13_3_3652,1287,1408,838,1369,2578,3216,1562,1601,482,369,520,2477
P2RY1_3_3653,4533,5742,7793,7191,4826,8216,6340,11758,7790,4867,3106,5
819
P2RY4_3_3654,1779,3622,2901,2568,716,3024,4946,3094,5015,2648,1533,539
6
P2RY8_3_3655,0,0,0,0,0,0,0,0,0,0,0,0
PPYR1_3_3656,885,1761,2015,2584,529,160,843,2343,1909,2796,2333,3279
PRLHR_3_3657,1138,1568,2115,2960,1461,1552,1606,2659,1212,1589,1158,10
43
PROKR1_3_3658,362,509,261,488,305,3177,146,742,330,7,3,144
PROKR2_3_3659,1697,1733,1910,1903,1271,1152,761,2573,3531,2092,1718,27
57
PTGDR_3_3660,325,136,415,93,211,1288,310,128,135,122,56,405
PTGER1_3_3661,1309,1311,733,893,1559,413,516,787,1851,828,146,745
PTGER2_3_3662,1401,1996,2431,2428,2443,4528,5528,1244,985,1995,1742,21
12
PTGER4_3_3663,1552,2569,2213,2480,2568,3396,2708,3640,2036,3377,1075,1
787
PTGIR_3_3664,464,663,453,878,85,436,351,507,890,177,310,346
PTH2R_3_3665,422,568,1393,1032,1970,48,1851,847,58,2077,2348,2090
QRFPR_3_3666,2006,1652,1907,2095,1589,1298,2078,1105,3492,3576,2433,11
49
RHO_3_3667,710,530,1252,1283,1200,1295,446,1927,1689,1684,280,2983
RRH_3_3668,4729,3787,6110,6425,2099,7788,5536,8742,4645,4702,9711,5583
RXFP1_3_3669,3108,2206,3104,4739,4512,3179,6661,3332,4091,1677,3763,57
31
RXFP3_3_3670,1126,1967,1228,2547,1776,1083,2110,1411,251,3936,678,3881
RXFP4_3_3671,870,562,1888,1071,1353,1569,1027,2701,113,264,280,650
S1PR1_3_3672,2475,3344,4709,6975,9508,6294,4866,1542,3520,4080,10055,4
758
S1PR2_3_3673,251,168,88,171,325,28,107,122,457,4,456,1058
S1PR3_3_3674,908,2049,1086,882,864,596,1003,108,2416,3551,1040,2012
S1PR4_3_3675,119,68,212,512,379,0,239,8,25,0,14,440

SCTR_3_3676,2417,3964,4080,3500,2889,3172,5919,2899,7184,2788,2153,284
0
SSTR1_3_3677,1073,460,484,404,1290,105,218,2760,1468,569,736,1319
SSTR2_3_3678,1201,1676,1019,1900,1188,1860,2818,1013,885,1347,2088,363
6
SSTR3_3_3679,677,273,548,407,1516,502,29,358,1331,106,88,827
SSTR4_3_3680,10,28,241,296,8,28,0,37,0,1,0,25
SUCNR1_3_3681,1873,2329,1823,2734,1296,4085,2964,2165,3126,6656,3443,2
194
TAAR1_3_3682,1574,2569,2210,2333,1059,2527,2161,1118,1266,2450,2407,13
41
TAAR5_3_3683,1449,2265,1245,2645,3431,892,1303,3606,2266,3205,1812,187
6
TAAR6_3_3684,2175,2355,3307,2670,1618,1905,1839,4023,2647,2748,6773,24
42
TAAR8_3_3685,1351,2340,2314,2664,1798,5856,6800,4328,2614,279,2204,334
7
TAAR9_3_3686,2335,1905,2234,2599,1259,2704,2513,6953,4430,1883,2048,23
41
TACR2_3_3687,884,890,876,976,550,1551,1310,456,2204,2979,297,987
TACR3_3_3688,3654,4095,4639,4576,3680,4457,3879,5597,5925,6965,6403,25
48
TAPT1_3_3689,577,1135,1030,174,1969,166,986,159,1116,3438,1688,1098
TAS1R2_3_3690,3489,5334,4370,6752,5495,4492,7729,4561,6174,4112,6971,8
227
TAS1R3_3_3691,859,1495,1011,2245,2604,1174,2824,1053,2612,1547,9449,18
74
TAS2R10_3_3692,16357,21574,23553,25841,25950,37829,32437,32575,25009,2
4159,34600,27575
TAS2R13_3_3693,3507,4166,7101,5975,4928,8917,9129,7586,6542,5752,5253,
8882
TAS2R14_3_3694,2257,4707,4248,3627,3033,2086,6035,6010,1612,4989,6666,
4044
TAS2R16_3_3695,2081,3761,2695,3292,2263,2676,2846,7464,719,3205,1723,5
366
TAS2R1_3_3696,3853,3562,5372,3520,4572,2751,3522,3879,8142,4933,12461,
4359
TAS2R20_3_3697,4107,5291,6665,5692,6123,6429,7787,2830,5884,9591,8742,
6233
TAS2R31_3_3698,2657,2617,2683,3662,3257,3287,5770,2207,3589,2768,3145,
1669
TAS2R38_3_3699,450,1337,1139,2345,537,1237,2575,1112,502,380,1213,359
TAS2R3_3_3700,1163,1472,1083,1169,2472,1006,2811,302,3108,678,3886,163
5
TAS2R40_3_3701,4010,5083,5378,5101,4533,3276,6528,10077,4805,4425,4048
,5069
TAS2R41_3_3702,613,1352,1306,1035,1695,503,1211,1232,1283,1419,766,477
4
TAS2R46_3_3703,2866,2677,2903,4020,3618,3404,3150,3422,4101,3442,4217,
3231

TAS2R4_3_3704,2173,2362,4099,2481,2846,2330,2379,4109,1696,475,5564,39
81
TAS2R5_3_3705,1775,1681,1109,2893,2067,2057,2886,2833,957,3343,3349,24
04
TAS2R60_3_3706,526,1030,544,579,984,905,1146,937,369,902,472,742
TAS2R7_3_3707,1363,2501,1972,2331,4364,3680,2052,1082,1319,446,1997,24
26
TAS2R8_3_3708,5501,5251,8827,6089,3718,11809,8128,6986,5434,4583,5888,
7833
TAS2R9_3_3709,882,1226,1732,1596,617,4026,800,2101,417,1100,236,656
TM2D1_3_3710,678,1479,800,1482,1034,4501,1194,3341,1233,58,526,3436
TMEM11_3_3711,194,439,235,494,0,1117,2423,1208,182,11,1473,3041
TRHR_3_3712,1595,1841,2034,1476,1259,4255,2117,5608,2074,2175,2521,329
5
UTS2R_3_3713,839,909,251,989,1503,666,813,721,2398,1166,956,1188
VIPR1_3_3714,1039,1209,1491,1049,912,1734,1063,298,1269,440,1300,612
VIPR2_3_3715,457,491,505,421,1665,77,423,1985,750,386,421,42
VN1R1_3_3716,4067,6117,5278,7049,5735,3230,5750,10822,2794,4546,4305,4
372
VN1R2_3_3717,1670,2771,4298,5727,3152,2384,3112,4150,2175,2306,3565,71
01
VN1R4_3_3718,4649,6528,5724,5719,5932,3888,8653,6365,3051,8067,7998,41
83
ADCYAP1R1_3_3719,1816,1819,1039,2159,1119,1144,948,4905,3785,2828,2198
,2817
ADORA1_3_3720,210,446,484,480,69,58,73,527,196,13,177,55
ADORA3_3_3721,3419,2799,2779,4250,2312,4333,3689,5740,1767,3336,2898,5
860
AGTR1_3_3722,7338,9065,10316,10850,9648,13479,12152,10560,10201,11675,
10045,18741
AGTRAP_3_3723,589,378,943,913,901,194,362,1045,27,2329,6461,445
AVPR2_3_3724,315,645,346,806,474,1926,1565,98,945,54,2366,2273
CALCR_3_3725,2838,3700,3599,4883,3534,6617,2282,2552,3216,3499,1091,40
90
CASR_3_3726,355,677,869,575,77,1,1000,237,1193,285,226,746
CCR2_3_3727,338,347,586,821,897,1578,911,1899,881,938,2215,296
CCR3_3_3728,7041,6437,7472,6757,6786,8616,10330,5450,10451,10899,4235,
5981
CCR5_3_3729,1481,1198,458,2649,2327,2979,2031,3422,875,80,202,1242
CCR6_3_3730,68,346,41,391,52,9,227,280,39,13,18,1
CCRL2_3_3731,587,820,689,1487,1743,223,721,780,1103,30,507,114
CD97_3_3732,315,1054,675,493,349,10,2744,931,104,836,2952,498
CHRM2_3_3733,1564,1084,2272,3721,1203,2313,2810,1532,879,1828,391,2487
CMKLR1_3_3734,261,600,404,380,293,72,741,719,368,83,22,594
CRHR1_3_3735,1416,1169,2296,1318,2010,65,6001,2811,1511,2040,573,776
CRHR2_3_3736,723,1148,1046,802,107,163,374,2335,297,954,44,1257
CX3CR1_3_3737,8738,8913,10780,11496,11120,14715,13422,10588,6391,14839
,12927,11134
CXCR2_3_3738,864,640,1413,689,394,2081,300,239,1709,95,367,382
CXCR3_3_3739,1224,531,1477,1643,768,1217,2817,1624,1203,2668,1161,2549

CXCR4_3_3740,706,1499,1499,524,623,1107,1453,187,673,230,383,1209
CXCR5_3_3741,1283,1360,2261,3000,3555,3228,1898,6744,3675,829,2349,377
3
DARC_3_3742,883,1285,860,469,455,2315,948,272,694,1164,810,2019
DRD2_3_3743,1587,1020,1903,1631,1314,1527,2900,1042,610,1632,1566,497
DRD3_3_3744,969,1711,1785,2624,2658,3246,1999,2085,2401,2446,4183,2233
EDNRB_3_3745,3308,4184,3085,6254,3566,3994,2812,4312,2864,6610,3766,39
28
EMR2_3_3746,14993,20421,22012,26256,16126,18341,23837,25345,16026,2883
9,28451,23246
FPR1_3_3747,548,774,409,1005,69,497,1415,941,272,974,351,478
FSHR_3_3748,2789,3741,2288,5392,2211,1894,4338,4149,1821,4075,3633,301
7
FZD6_3_3749,2753,4075,4012,5867,3049,5349,4177,3786,3333,3188,6854,702
1
GABBR1_3_3750,89,867,531,1164,89,131,567,853,243,315,12,1789
GHSR_3_3751,123,333,24,21,0,0,1,79,21,8,88,0
GNRHR_3_3752,3398,2253,3846,5590,2019,3188,4513,5288,4494,4478,8051,31
39
GPBAR1_3_3753,826,1294,949,1328,191,1340,2203,1831,1855,1126,319,1786
GPER_3_3754,2697,3245,3599,7197,2206,4607,3875,3682,4985,5175,5359,527
4
GPR107_3_3755,4719,4932,3310,6657,4895,1262,6489,4754,7486,3523,7264,3
956
GPR110_3_3756,816,313,585,752,985,696,224,870,420,2334,3689,1054
GPR113_3_3757,363,606,585,927,63,337,2,1210,717,1520,160,980
GPR116_3_3758,5934,10063,6383,9005,7170,10740,7216,7814,8286,7969,5802
,4393
GPR126_3_3759,3737,3695,3588,3182,2190,5459,3659,3791,1148,2300,2697,4
229
GPR155_3_3760,1147,2512,1685,3475,2467,1616,1647,314,2219,4053,2344,34
31
GPR156_3_3761,2566,1877,2373,2837,590,4689,719,1048,330,2036,1696,1777
GPR162_3_3762,350,381,692,936,903,537,1421,1217,176,458,404,102
GPR17_3_3763,350,1684,1107,2675,2050,1152,3041,2149,456,1746,2322,343
GPR18_3_3764,1973,2254,1482,2583,2666,804,2464,1269,1502,2790,1169,282
6
GPR1_3_3765,1277,689,2078,2484,1337,1169,2163,2180,2068,1108,1769,1908
GPR34_3_3766,15610,18113,15143,25379,19245,41899,22064,30321,23719,215
46,34613,20692
GPR35_3_3767,818,1325,952,1292,825,708,1433,2394,1277,1710,447,2743
GPR56_3_3768,1024,1420,1043,1149,1373,827,301,623,784,930,660,1296
GPR63_3_3769,4721,5771,8323,7627,3393,11045,7562,10925,5796,6724,5295,
7505
GPR64_3_3770,525,817,558,1949,444,1057,1358,1484,670,1830,1115,2513
GPR68_3_3771,976,682,563,1549,851,323,1107,216,993,2277,1525,2317
GPR85_3_3772,4072,5604,5081,6198,8292,7035,5186,1617,5248,5541,9228,54
70
GPRC5C_3_3773,3560,4971,4638,6018,3774,5290,5091,4496,6015,4021,6215,7
344

GRM1_3_3774,1587,1820,1997,2671,2393,854,1918,2800,4643,2661,5900,4539
GRM2_3_3775,1016,380,2213,1143,1934,2798,1217,1789,874,4966,1795,854
GRM5_3_3776,833,618,560,295,1062,662,125,3539,443,1990,644,1076
GRM7_3_3777,2717,2678,5270,3479,2542,2627,2159,4212,3646,2922,4149,337
9
GRM8_3_3778,956,1607,2147,1513,2038,1952,1469,4055,1701,183,601,738
HRH4_3_3779,257,1015,212,760,924,326,560,249,234,90,147,432
HTR2A_3_3780,1296,870,210,511,641,950,101,1188,1095,853,650,1161
HTR4_3_3781,980,549,907,3047,116,1485,212,2042,100,1795,3413,949
HTR7_3_3782,3257,4838,3665,6461,3499,5020,3085,5980,5911,5300,3651,453
7
LGR6_3_3783,1580,1349,1687,1039,3580,3253,2938,2189,3134,1057,3543,151
7
LPAR1_3_3784,683,1736,1020,2145,1231,2095,1948,940,1182,4316,2404,1459
LPAR5_3_3785,3078,2715,3145,3839,2989,3420,2172,889,1021,2349,2695,257
8
LPAR6_3_3786,19138,20149,19318,22796,18549,25057,28725,22422,26879,244
66,26410,26194
LPHN1_3_3787,1183,698,1162,1896,200,172,319,152,848,2389,2214,2630
LTB4R2_3_3788,234,652,391,92,1052,1793,258,1,398,2,2,844
LTB4R_3_3789,853,1076,1475,897,476,2489,2161,2021,3006,1160,1219,1798
LYPD1_3_3790,3775,5576,6200,6712,4326,4773,6502,8184,5319,8651,9538,30
71
MCHR2_3_3791,2233,2147,1443,2497,2043,3221,3871,2199,3922,2045,5082,38
80
MRGPRF_3_3792,113,723,143,334,266,1460,114,32,107,317,3591,120
NPFFR2_3_3793,622,679,1212,1241,216,1479,562,272,1155,1430,1339,1632
NPSR1_3_3794,341,589,604,1159,252,159,323,934,433,332,335,380
O3FAR1_3_3795,3799,5863,3498,8355,4557,7793,7696,4100,3242,7411,4911,7
885
OPN4_3_3796,514,417,710,1060,446,573,620,1741,424,528,393,997
OPRL1_3_3797,448,960,157,881,119,1218,741,650,982,55,283,1527
OPRM1_3_3798,124,0,0,310,0,0,0,0,0,0,0,350
P2RY10_3_3799,380,746,762,655,1240,261,2584,160,1018,14,341,1071
P2RY12_3_3800,5891,6103,8871,7673,7847,14281,17008,10453,12387,4242,62
83,8232
P2RY14_3_3801,2941,3573,3141,4157,3455,6268,1260,5460,1630,5285,10721,
4142
P2RY2_3_3802,908,576,1126,1558,1344,3166,575,134,986,1781,670,2732
P2RY6_3_3803,2728,3688,5211,4041,4971,4901,2554,3531,1162,2023,7479,38
80
PTAFR_3_3804,1525,1936,1528,2481,1720,2489,1821,1050,1704,901,5922,318
6
PTGER3_3_3805,515,1690,924,1705,822,304,405,594,1756,127,299,3093
PTGFR_3_3806,2100,3486,2182,4036,1774,4228,3648,2975,5596,4087,1185,38
43
PTH1R_3_3807,2065,2983,3467,3992,3691,2638,2116,4924,4383,3953,1464,52
08
RGR_3_3808,490,435,469,237,224,1473,36,2297,360,864,39,105
RXFP2_3_3809,919,1106,2250,2826,2187,1253,1520,643,2270,3118,4366,3105

S1PR5_3_3810,51,116,214,450,4,11,1,220,1204,9,22,4
SIGMAR1_3_3811,795,413,175,467,358,77,257,332,314,795,1257,63
SSTR5_3_3812,1206,503,844,1647,3833,2584,1308,2,2497,208,175,262
TAAR2_3_3813,7485,6913,6913,8951,4889,7236,10754,5090,9573,7920,7914,1
0390
TACR1_3_3814,1741,1444,2490,1854,2079,2159,1305,2726,3434,3213,4125,14
93
TAS1R1_3_3815,2093,3121,974,2304,1689,1583,4113,2805,1442,7354,1534,17
12
TBXA2R_3_3816,187,256,152,568,23,270,727,8,223,23,941,1
TPRA1_3_3817,351,796,718,937,691,1217,1931,793,622,1403,45,441
TSHR_3_3818,1580,1326,1575,1824,1056,4940,2278,1136,2086,1659,2567,853
XCR1_3_3819,651,130,281,702,273,13,517,180,1912,11,435,197
XPR1_3_3820,4320,6753,5575,9662,11523,9743,3739,5724,6832,12775,5996,6
975
ADRA1D_3_3821,185,131,114,834,230,1695,8,52,0,1299,17,22
ADRA1D_3_3822,4206,5823,5273,7321,8236,11271,6122,9758,2067,8392,5046,
8798
ADRA1D_3_3823,283,744,660,837,916,5,629,256,906,158,2312,213
ADRA1D_3_3824,1104,1467,1401,1979,322,4364,2328,2109,2539,3872,1692,26
9
ADRA1D_3_3825,317,773,567,524,1528,761,1679,400,57,935,193,690
ADRA1D_3_3826,1796,2377,1224,3329,1141,5916,2584,2666,1166,1437,1296,4
417
ADRA1D_3_3827,196,109,156,331,16,0,91,18,13,32,25,66
ADRA1D_3_3828,1288,1409,2716,1108,635,478,2261,1235,1166,1315,2518,303
3
ADRA1D_3_3829,2875,2772,2225,4219,2353,1857,2915,4502,1953,7884,6851,5
366
ADRA1D_3_3830,1177,1355,1517,1635,1884,3886,1366,2179,810,460,4241,187
3
CCRL1_3_3831,2486,2847,3801,3753,2845,4884,4792,4049,5712,5399,5223,53
46
CCRL1_3_3832,2262,2226,2704,1307,3069,1212,808,2387,616,2093,1839,2885
CCRL1_3_3833,647,377,264,1070,2087,229,2702,4343,1041,1838,787,1683
CCRL1_3_3834,22,281,0,0,0,370,0,0,0,0,0,0
CCRL1_3_3835,1173,1236,1362,1605,1097,1320,2013,1036,3579,3482,1367,27
79
CCRL1_3_3836,3608,4593,4459,4514,4440,5752,5224,6494,4531,5551,9028,62
85
CCRL1_3_3837,1149,2033,1257,586,403,2898,3247,2028,897,1539,95,3035
CCRL1_3_3838,1657,2186,1279,2289,1623,1250,3230,2052,3167,2284,613,252
8
CCRL1_3_3839,684,957,1966,794,2417,3040,253,697,1878,1034,462,702
CCRL1_3_3840,1483,874,1422,1490,1373,1214,1771,479,827,1397,600,1442
CELSR1_3_3841,1753,1386,1728,1791,1291,2196,2669,972,661,3239,1285,352
1
CELSR1_3_3842,5012,4664,5521,6854,5773,8360,4819,6448,6365,10300,3396,
8647
CELSR1_3_3843,414,648,535,971,2462,1172,461,912,276,411,124,1351

CELSR1_3_3844,520,1157,1091,1724,2092,717,1667,865,2009,1271,298,3614
CELSR1_3_3845,2915,3088,3773,7208,4577,3977,5962,8554,2709,4357,7978,5374
CELSR1_3_3846,3836,4283,3864,6615,3414,6966,3212,4589,6710,7209,5682,3772
CELSR1_3_3847,6650,8841,8552,8146,4488,8717,3871,8252,6886,7419,15447,7092
CELSR1_3_3848,3657,5234,6537,7161,2817,7294,3312,7715,7584,8536,6780,6336
CELSR1_3_3849,3025,5539,6622,4669,2499,4918,3976,5815,5273,6084,2992,5265
CELSR1_3_3850,1598,2491,4584,3034,5316,5834,3719,4917,3489,2424,3245,3659
CX3CR1_3_3851,8065,11005,11764,10697,9114,10718,15610,16180,9731,13346,9802,12402
CX3CR1_3_3852,1893,3562,3014,2957,2252,2369,3370,4060,2058,6798,5529,1613
CX3CR1_3_3853,3150,3608,3606,3564,1093,4138,1586,1207,3694,1789,7722,5187
CX3CR1_3_3854,2906,5023,4734,4279,3478,6680,5680,3793,4233,3852,4354,6673
CX3CR1_3_3855,4102,6524,5615,7176,6860,7123,6522,9224,8562,9015,2790,8567
CX3CR1_3_3856,2432,1580,2696,3533,1497,2379,2480,1749,2233,2482,1224,3120
CX3CR1_3_3857,4183,4908,7943,8770,4767,10254,10449,5855,3872,7483,9344,9162
CX3CR1_3_3858,3336,4072,3685,4597,3755,2023,4000,5393,4761,6573,6345,4876
CX3CR1_3_3859,3319,3078,4237,5179,2709,6289,3365,4946,3499,4753,3924,6755
CX3CR1_3_3860,1159,2675,2234,2996,1205,6316,3371,2425,1098,3758,5318,3207
FZD9_3_3861,1683,991,1764,1247,1039,429,2126,466,961,457,333,274
FZD9_3_3862,1794,1950,2541,5497,4297,1572,3079,1110,1153,2705,2949,3983
FZD9_3_3863,5957,6710,6076,4462,5881,13032,5235,4343,6609,4722,6671,5105
FZD9_3_3864,5791,7036,7183,11862,9319,11965,8110,12243,7604,14304,13879,6657
FZD9_3_3865,5399,3969,4616,6764,2951,4826,3110,8590,4908,3583,3888,6552
FZD9_3_3866,1412,666,1083,1403,841,667,949,1657,717,81,1424,735
FZD9_3_3867,4625,7547,8225,7906,3163,13024,8398,8802,5494,9043,11148,6035
FZD9_3_3868,499,1345,1016,1536,639,444,553,935,514,1982,1256,1211
FZD9_3_3869,3286,5830,5498,5630,5218,5701,4152,6626,6020,4380,4256,4750
FZD9_3_3870,1508,3339,1697,3919,825,1730,2056,4650,2067,2483,4746,1560
LPAR1_3_3871,252,345,432,370,280,490,109,112,357,1862,287,422

LPAR1_3_3872,762,1354,2946,1589,545,734,1069,2714,3609,2413,1257,1571
LPAR1_3_3873,4699,5165,5242,6842,6600,7354,9082,4459,4518,4519,7840,69
20
LPAR1_3_3874,377,159,70,91,308,80,344,421,316,935,18,115
LPAR1_3_3875,416,456,629,524,1,0,856,151,1335,410,917,37
LPAR1_3_3876,232,287,175,352,1703,76,1874,554,122,884,672,1525
LPAR1_3_3877,192,739,201,191,11,189,471,114,61,745,394,1430
LPAR1_3_3878,1189,1465,1389,1095,868,84,4000,8492,1236,1912,1452,1025
LPAR1_3_3879,391,516,1581,671,1658,1058,882,1048,1449,266,25,28
LPAR1_3_3880,61,638,1533,923,530,551,810,73,42,120,177,4
PTGDR2_3_3881,1174,1002,1476,1626,1467,1927,236,2872,2301,1332,259,751
PTGDR2_3_3882,340,204,160,849,80,96,203,359,143,305,501,249
PTGDR2_3_3883,55,86,57,221,111,223,21,0,205,324,81,211
PTGDR2_3_3884,3847,4699,4688,8225,4942,8345,5011,5564,4159,8007,8216,9
855
PTGDR2_3_3885,137,192,204,465,414,1125,96,1300,56,11,434,46
PTGDR2_3_3886,656,679,522,2043,1092,378,1847,854,1620,1247,2741,1442
PTGDR2_3_3887,1500,1039,1520,1624,3496,1733,371,2860,2798,744,260,1152
PTGDR2_3_3888,834,1904,1795,1129,2670,395,1569,1575,930,1622,4062,1698
PTGDR2_3_3889,265,185,900,767,676,28,304,1302,218,28,1277,9
PTGDR2_3_3890,2419,2036,1923,2033,2620,2791,3283,2173,2547,2286,2180,1
363
TACR1_3_3891,417,1001,659,1698,140,1220,1331,2640,1143,1413,1737,2039
TACR1_3_3892,184,324,1396,1835,492,18,416,2157,1324,132,356,602
TACR1_3_3893,1186,1417,1074,1754,1485,879,1481,1237,2355,1643,1355,548
TACR1_3_3894,512,394,852,752,702,124,685,405,48,494,22,255
TACR1_3_3895,578,487,503,1599,337,913,565,401,1683,1157,460,249
TACR1_3_3896,1429,2667,2729,2716,1189,3087,2735,1576,2176,6993,2661,25
37
TACR1_3_3897,1453,1331,1519,2124,946,4114,2406,2207,1471,1844,2616,164
6
TACR1_3_3898,507,946,1373,2381,831,709,789,1356,847,3406,2090,1987
TACR1_3_3899,402,534,1368,1285,1284,1293,1159,160,1108,1029,1066,1268
TACR1_3_3900,699,1016,526,1888,1118,3704,344,1181,1312,3504,521,2199
