# Supplementary material for: Cross-species genetic screens identify transglutaminase 5 as a regulator of polyglutamine-expanded ataxin-1
Source: J Clin Invest. 2022 May 2;132(9):e156616. doi: 10.1172/JCI156616 (PMC9057624; doi:10.1172/JCI156616)
Supplement: Supplemental data set 4 [file jci-132-156616-s049.pdf]

```
sRNA,B1,B2,B3,B4,H1,H2,H3,H4,L1,L2,L3,L4
ABCC8_4_1,903,1045,1333,1021,583,744,1299,855,831,1485,658,413
AMFR_4_2,2991,2739,2600,2953,2547,1887,2570,3165,2137,1244,1649,2219
ANO1_4_3,1058,942,965,1123,1604,526,2584,1093,1314,969,1814,2224
ANO2_4_4,2054,2366,2445,2203,1853,2925,1912,1013,2896,2434,2874,1634
ANXA9_4_5,401,541,810,520,314,1150,843,66,376,1521,1668,796
AQP10_4_6,450,522,454,743,182,414,109,515,191,687,1399,497
AQP11_4_7,1534,1224,1455,923,876,2398,635,1814,946,1820,1531,2201
AQP12A_4_8,258,241,144,330,217,221,54,446,247,78,85,192
AQP2_4_9,707,885,932,655,671,1070,878,766,978,760,1076,477
AQP3_4_10,484,345,297,233,510,664,309,237,22,268,854,352
AQP5_4_11,1000,1239,1386,1479,975,2449,566,664,1302,2381,1535,1187
AQP6_4_12,122,141,11,116,4,8,0,1,0,0,130,50
AQP7_4_13,182,138,140,297,60,626,151,135,218,41,90,174
AQP8_4_14,328,405,293,395,342,122,97,350,69,733,509,433
B2M_4_15,229,461,465,547,407,975,1467,246,96,122,435,812
BEST2_4_16,1079,1082,1200,1490,903,1612,1623,1488,1845,2524,786,489
BEST4_4_17,93,40,112,298,0,1,1,0,0,0,486,1046
BSND_4_18,1989,1541,1610,1889,1416,1685,2341,1500,902,585,2318,2169
BTBD10_4_19,1381,880,1365,931,1611,1632,1089,1911,535,1515,2571,1041
BTNL2_4_20,2666,3044,3020,2290,3035,3484,1291,1864,2816,1718,2914,1909
C5orf62_4_21,1672,1920,1480,1323,1363,1165,2715,2060,1860,1837,1376,39
08
CACNA1B_4_22,790,993,664,700,560,310,93,202,361,266,948,465
CACNA1F_4_23,719,743,843,850,951,421,533,243,1364,421,1116,640
CACNA1S_4_24,1691,2396,2074,2272,2311,2467,2387,2103,1427,650,3668,211
6
CACNA2D1_4_25,1289,1433,1290,1261,1795,1139,1126,191,899,468,1234,985
CACNA2D3_4_26,304,228,175,199,2,0,195,499,13,1144,151,150
CACNA2D4_4_27,1583,1416,1449,1843,1802,1216,981,2217,1073,1208,2366,12
67
CACNG1_4_28,267,316,179,341,8,0,0,121,0,176,7,359
CACNG2_4_29,970,919,825,1124,1133,1209,209,383,284,769,865,1129
CACNG3_4_30,2029,1826,2447,2684,2873,2911,4203,3925,1216,1652,2679,254
1
CACNG4_4_31,906,915,838,784,586,547,44,1443,55,2190,1197,1994
CACNG5_4_32,1155,937,760,781,1035,935,463,1734,458,499,1222,1261
CACNG7_4_33,59,374,582,386,615,198,61,34,304,318,259,5
CACNG8_4_34,569,860,948,835,879,391,379,710,859,357,1098,506
CATSPER1_4_35,490,691,635,581,181,1391,291,207,841,76,784,1479
CATSPER3_4_36,659,688,750,640,494,474,646,599,543,1275,988,1050
CATSPER4_4_37,731,639,574,258,18,773,147,259,540,988,849,6
CCT8L2_4_38,408,294,171,375,415,113,475,446,186,362,31,228
CD160_4_39,98,109,267,154,211,126,0,145,66,0,3,10
CD163L1_4_40,408,620,389,498,1023,758,434,289,667,491,847,451
CD27_4_41,218,243,198,32,43,320,308,0,0,0,0,774
CD2_4_42,3188,2685,3019,2873,2763,2622,3487,2652,3270,2197,4567,3739
CD300C_4_43,269,246,146,175,196,175,201,5,434,166,462,66
CD3E_4_44,999,525,779,596,1155,745,1148,618,310,1776,1190,1217
CD3G_4_45,469,567,662,463,933,397,1115,310,381,309,419,919
```

CD5_4_46,64,126,112,192,514,13,124,154,204,0,268,348
CD5L_4_47,492,343,676,197,166,120,555,4,376,57,104,242
CD69_4_48,1074,1197,1076,1036,137,1366,977,769,873,743,1253,457
CD6_4_49,484,424,503,494,336,482,1417,431,272,1262,672,393
CD72_4_50,948,718,1059,781,1070,1523,1680,239,384,2819,1152,597
CD80_4_51,2265,2236,3033,2180,2682,1222,1701,2033,2087,2663,3936,2809
CFTR_4_52,1305,1221,1593,970,2031,2346,919,2947,969,867,1982,1092
CHRNA10_4_53,537,370,295,398,227,24,241,230,645,21,701,121
CHRNA2_4_54,117,186,272,77,166,17,2,19,202,46,772,122
CHRNA4_4_55,357,823,488,399,389,854,1333,458,301,12,326,688
CHRNA5_4_56,2078,1920,1632,1870,1505,1484,3362,2121,1930,2145,1789,244
2
CHRNA9_4_57,722,1155,984,1108,1017,439,828,328,1156,393,426,633
CHRNB1_4_58,1100,691,1339,1300,684,704,485,1818,776,2472,1560,925
CHRNB2_4_59,246,462,330,427,376,71,122,383,199,517,330,373
CHRNB3_4_60,376,397,372,462,1225,113,244,106,252,949,502,241
CHRNB4_4_61,836,838,989,954,585,845,1810,641,393,1228,1120,939
CHRND_4_62,458,566,433,334,207,527,436,429,511,46,967,221
CHRNE_4_63,770,550,416,722,384,654,158,904,228,1863,958,454
CHRNG_4_64,126,98,237,146,1,0,1,705,38,631,18,64
CLCA1_4_65,402,274,288,246,638,55,805,557,643,97,221,533
CLCA2_4_66,564,538,651,976,481,635,190,1314,658,1181,369,913
CLCA4_4_67,1439,1482,1343,1116,1439,426,928,1663,540,542,626,1480
CLCN1_4_68,209,320,145,476,746,559,100,135,344,993,60,668
CLCN4_4_69,599,711,752,799,674,842,1749,376,83,316,736,350
CLDN3_4_70,202,313,121,91,225,222,70,155,17,0,1,80
CLDN4_4_71,209,230,198,379,426,0,51,746,449,0,214,53
CLEC1A_4_72,793,692,771,813,305,574,450,948,677,202,1639,1013
CLIC1_4_73,176,231,192,226,6,611,232,10,309,24,110,347
CLIC2_4_74,0,0,0,0,0,0,0,0,0,0,0,0
CLIC3_4_75,356,81,174,85,687,4,3,255,73,0,270,231
CLIC4_4_76,200,120,71,127,0,0,0,184,49,7,61,11
CLIC6_4_77,1671,1620,2371,1656,2169,2149,899,1303,1707,2502,2533,2627
CLNS1A_4_78,807,1292,1222,866,1456,1705,573,1409,593,625,326,1484
CNGA2_4_79,1145,771,1335,895,782,1486,1442,1765,549,142,1493,1489
CNGA4_4_80,848,647,633,438,787,718,606,230,55,205,770,722
CNGB3_4_81,745,968,1101,863,348,1122,797,1132,627,1183,1413,2078
COLEC12_4_82,2037,1646,2367,1720,1447,1331,2485,2180,1881,1102,1547,16
44
CSF2RB_4_83,239,286,174,251,179,236,264,378,3,224,128,45
CUBN_4_84,1040,740,796,571,559,1054,2474,983,1142,202,987,1083
DCC_4_85,820,914,1039,879,574,860,707,1342,443,1822,720,697
DNER_4_86,810,910,837,1073,443,1296,1818,322,714,154,1193,807
EDAR_4_87,522,224,260,158,575,17,384,19,304,310,14,160
EPOR_4_88,539,550,973,696,77,627,1572,366,525,722,2536,915
FCER1A_4_89,690,387,762,885,892,483,687,533,164,935,747,937
FCER1G_4_90,312,347,351,717,2011,608,126,263,1616,497,163,656
FCGR1A_4_91,826,809,751,766,308,1031,801,475,454,905,936,643
FCGR2C_4_92,634,727,904,767,191,1853,1391,1120,156,344,727,762
FCGR3B_4_93,2459,2095,2564,2039,2788,1058,2753,2639,2018,2732,2575,192

2
FRRS1_4_94,879,959,1308,1245,1067,1635,1721,1636,993,1537,1179,1293
FXYD7_4_95,1169,1278,1339,1114,620,290,1116,2037,372,1159,1232,1620
GABRA3_4_96,534,482,304,356,164,2076,113,424,861,1604,341,268
GABRA6_4_97,2342,1988,2499,2322,2945,2449,2037,2896,1181,3616,3183,252
9
GABRB1_4_98,3436,3588,3972,3448,3010,4320,3379,5060,2859,5462,2994,389
3
GABRD_4_99,138,342,225,247,197,55,147,112,189,200,193,233
GABRE_4_100,580,409,423,440,533,4,10,127,649,752,226,481
GABRG1_4_101,589,601,781,778,729,1561,1060,255,402,883,1074,936
GABRG3_4_102,1755,1650,1948,1641,1620,2253,3048,1189,2322,382,1487,163
3
GABRP_4_103,193,300,406,494,616,211,349,221,267,2046,962,403
GABRQ_4_104,2909,2786,3000,3084,812,3604,4999,3820,1550,2509,4608,3153
GABRR1_4_105,227,336,357,317,3,139,239,314,648,1,733,245
GABRR2_4_106,1754,1245,1436,1118,2324,1315,849,1838,1316,1141,880,1189
GABRR3_4_107,581,794,1226,1216,1765,1092,1365,1881,656,1744,1557,312
GFRA3_4_108,330,319,308,500,184,90,82,654,1,1205,4,124
GPC1_4_109,310,360,373,289,191,859,555,264,27,41,520,167
GPC4_4_110,1248,939,980,1012,691,1746,1129,814,1453,223,826,1145
GPC6_4_111,302,386,617,311,483,430,152,209,81,99,142,407
GPR89B_4_112,744,1181,1106,1201,1443,1109,21,569,113,927,484,901
GRID1_4_113,26,142,171,152,75,12,413,104,535,31,49,188
GRID2_4_114,765,1318,902,725,1341,652,1493,615,842,790,843,655
GRIK3_4_115,440,347,632,485,1486,1825,1412,983,549,944,926,185
GRIK4_4_116,207,211,306,279,509,560,258,16,505,1332,194,508
GRIK5_4_117,342,319,292,374,241,611,62,543,153,141,236,248
GRIN2B_4_118,601,788,726,633,942,857,594,1452,1008,152,1038,1634
GRIN2C_4_119,536,290,581,390,1209,587,937,44,349,65,536,1238
GRIN2D_4_120,1225,1031,1677,1373,666,842,1479,636,1354,886,1178,367
GRIN3A_4_121,1997,2746,2945,3100,3776,3250,3264,1775,2358,2586,3220,40
47
GRIN3B_4_122,169,243,526,344,464,280,946,321,133,30,387,642
HAVCR2_4_123,344,889,1087,444,299,1153,972,869,381,958,591,361
HCN1_4_124,780,718,1034,693,1206,1138,855,837,539,436,1310,579
HCN2_4_125,320,229,322,948,479,83,68,723,99,5,208,389
HCN3_4_126,1257,2092,1514,1322,2760,2907,3232,1742,1854,2521,1653,1842
HCN4_4_127,688,673,677,602,647,529,990,259,680,1207,943,688
HLA-B_4_128,981,936,669,563,1266,704,875,1282,284,2677,369,1286
HLA-DOA_4_129,362,532,228,363,206,644,930,327,340,1254,521,279
HLA-DOB_4_130,976,1091,832,1365,1207,373,3120,1513,623,911,1369,1422
HLA-DPB1_4_131,254,182,242,381,340,141,99,672,469,0,220,286
HLA-
DQA1_4_132,6337,6691,7733,7157,9353,8542,10937,5964,6503,9365,9803,748
5
HLA-DQA2_4_133,161,172,283,276,415,427,0,87,410,14,294,385
HLA-DQB2_4_134,366,431,569,303,247,769,50,333,284,140,671,652
HLA-DRA_4_135,647,671,1136,717,856,560,495,1673,670,1718,827,915
HLA-DRB1_4_136,669,497,776,919,637,364,541,747,189,373,1719,754

HLA-DRB5_4_137,1131,1317,1270,1517,1278,892,2323,917,764,913,1823,1860
HLA-G_4_138,470,420,774,670,596,593,194,421,782,686,912,253
HTR3B_4_139,208,188,175,528,98,494,132,443,100,13,229,304
HTR3C_4_140,3637,3286,3685,3358,2316,3627,3319,4211,3493,3231,4206,404
4
HTR3E_4_141,603,369,921,680,151,679,260,449,335,2155,482,897
ICAM1_4_142,504,433,277,476,670,366,332,508,302,587,326,897
IFNAR1_4_143,854,1063,584,797,1588,936,626,607,1260,1025,765,923
IFNGR1_4_144,810,767,918,1111,383,661,471,534,551,222,1090,856
IFNGR2_4_145,219,342,466,287,16,226,322,245,79,1423,431,392
IGF1R_4_146,281,463,470,352,1125,223,507,262,170,169,171,704
IGF2R_4_147,383,384,444,637,496,248,59,239,53,32,768,364
IL10RA_4_148,1598,1669,1409,1843,1003,1395,539,1057,1585,556,2310,1794
IL10RB_4_149,0,0,0,0,0,0,0,0,0,0,0,0
IL12RB2_4_150,559,734,511,746,102,1123,396,828,603,383,1310,722
IL13RA1_4_151,1608,1276,1757,1876,705,1092,2277,1074,486,2109,2705,140
0
IL13RA2_4_152,566,471,539,490,398,157,175,484,582,281,309,484
IL17RA_4_153,861,962,585,925,618,299,234,1297,545,324,1341,367
IL17RB_4_154,1467,1144,1059,1679,1491,715,289,3000,1127,1089,2847,1669
IL18R1_4_155,2525,2841,3390,2315,3701,1338,3296,3528,2366,2506,2840,42
76
IL18RAP_4_156,740,578,977,757,908,565,1842,921,1657,1473,792,588
IL1R1_4_157,1432,1242,1830,1452,872,1250,1337,1501,1166,3498,771,800
IL1RAPL1_4_158,758,775,465,436,100,582,1200,558,363,509,366,502
IL1RAPL2_4_159,510,337,486,149,838,570,1,454,285,358,71,184
IL1RL2_4_160,349,123,190,298,60,137,525,701,491,1,170,76
IL20RA_4_161,2157,2087,2085,2422,2450,2673,2544,1667,1808,1795,1525,23
13
IL22RA1_4_162,128,130,164,152,462,9,508,81,239,678,440,25
IL23R_4_163,2854,3898,3894,3635,3722,2249,4555,5199,4095,3151,4699,298
8
IL27RA_4_164,405,740,445,437,322,1042,1390,1289,534,7,262,360
IL2RA_4_165,58,129,108,92,230,175,20,159,0,0,47,151
IL2RB_4_166,808,1569,1430,1472,930,2009,1234,1308,1114,1013,1038,671
IL2RG_4_167,224,218,339,256,1138,1027,173,339,264,42,63,405
IL3RA_4_168,872,920,1475,871,1561,1794,1337,525,1113,1001,638,1796
IL7R_4_169,4177,3566,3776,3228,4742,4526,3002,6307,3116,2472,4131,3160
IL9R_4_170,170,184,288,167,119,64,118,406,526,0,1133,220
ITFG1_4_171,263,218,218,275,282,392,251,0,0,64,0,250
ITGA2B_4_172,761,473,482,776,947,488,747,298,775,1076,367,491
ITGB3_4_173,686,973,904,1042,1377,893,303,667,580,1029,540,1056
ITGB7_4_174,697,735,930,829,447,821,866,1671,439,1714,1237,388
ITPR2_4_175,3812,3289,3693,3606,4192,5422,4012,4722,4162,2135,4921,487
2
ITPR3_4_176,567,674,1187,612,686,826,825,581,156,1212,111,1232
JPH3_4_177,320,347,318,260,660,618,339,354,557,36,721,46
KCMF1_4_178,924,1276,1119,959,1171,1870,894,183,1578,382,1251,1765
KCNA10_4_179,886,1059,1278,1247,747,1867,1077,1309,188,2935,506,1269
KCNA1_4_180,1116,755,984,768,511,579,1065,572,1119,562,980,1029

KCNA3_4_181,2282,2936,3290,2704,2403,2969,6086,3268,1493,3602,2803,419
9
KCNA4_4_182,3126,3002,3257,2638,2350,4003,2206,2078,2181,3229,3403,201
3
KCNA5_4_183,711,362,674,347,8,16,291,337,391,582,550,13
KCNA6_4_184,619,804,787,558,348,1311,298,1045,259,754,405,646
KCNA7_4_185,1182,1260,1396,1084,748,1177,2060,1026,1409,451,1152,503
KCNAB3_4_186,47,213,149,71,96,0,3,29,248,0,94,118
KCNB1_4_187,678,538,1014,1023,857,1655,242,719,560,926,829,1296
KCNB2_4_188,2344,2524,2984,2563,3879,568,998,3658,1554,2805,1844,3635
KCNC3_4_189,747,606,1044,638,1165,1838,1621,1077,259,30,1291,637
KCND1_4_190,656,388,451,275,67,116,535,900,500,480,71,413
KCND2_4_191,281,330,332,427,477,50,726,437,451,1176,32,76
KCNE1L_4_192,2378,2300,2171,2237,2021,2853,4199,2742,1551,625,1844,164
5
KCNE2_4_193,2863,2607,2746,2971,2502,3876,626,3383,2381,777,2499,2348
KCNE3_4_194,955,420,691,391,646,430,1060,235,1272,553,1490,623
KCNE4_4_195,538,406,656,860,597,1828,1604,855,469,445,569,825
KCNF1_4_196,89,375,127,309,33,479,1591,34,29,1284,48,221
KCNG1_4_197,321,218,354,280,674,1292,1120,66,205,649,803,227
KCNG2_4_198,55,0,0,73,0,0,0,0,260,0,0,132
KCNG4_4_199,35,155,193,64,0,419,3,2,108,0,5,162
KCNH3_4_200,2644,2699,3323,2810,1469,3045,1764,2186,2956,3228,2949,254
1
KCNH4_4_201,783,1095,735,955,558,285,1395,431,1949,596,1010,648
KCNH8_4_202,991,991,913,805,726,2211,611,163,991,1504,1285,653
KCNJ10_4_203,3640,3030,3643,3834,4157,4680,3215,4513,4614,4183,5145,35
17
KCNJ12_4_204,664,932,753,891,1147,1372,584,831,240,1189,300,292
KCNJ2_4_205,747,740,817,669,623,619,1769,1095,732,737,438,1131
KCNJ3_4_206,4285,3859,3539,4409,3326,4718,6866,4604,2366,5658,4188,475
0
KCNJ5_4_207,613,584,521,641,1097,325,1280,1191,800,376,400,386
KCNJ6_4_208,140,85,146,94,2,266,132,125,173,480,137,52
KCNJ8_4_209,1561,1062,1456,1516,161,690,1222,974,1308,2682,1927,1889
KCNJ9_4_210,507,523,615,703,481,284,296,397,622,370,1167,453
KCNK12_4_211,451,168,129,395,49,50,1252,140,337,0,114,262
KCNK13_4_212,650,774,682,563,733,907,682,330,171,2161,610,825
KCNK15_4_213,288,114,403,179,171,220,22,407,495,18,1012,166
KCNK18_4_214,1752,1511,2183,1634,1519,1437,881,1585,1004,739,3277,1965
KCNK1_4_215,98,82,47,70,60,66,584,195,28,27,12,9
KCNK3_4_216,48,82,8,134,4,14,74,301,1,175,0,85
KCNK4_4_217,633,304,241,541,135,1,13,48,271,229,225,152
KCNK5_4_218,286,412,597,259,494,267,333,530,318,1102,1108,409
KCNK6_4_219,2732,3220,3293,3573,2709,3282,717,4313,2132,1631,2168,3333
KCNK9_4_220,839,508,563,692,1204,537,402,913,461,688,2108,800
KCNMB1_4_221,246,209,387,512,269,198,257,49,227,67,429,263
KCNMB4_4_222,749,1214,716,946,706,1259,395,1090,1598,920,1107,331
KCNN1_4_223,492,340,410,281,26,357,282,1022,126,59,227,750
KCNN4_4_224,3465,3774,4624,4321,5071,5933,5691,4275,3623,3389,4774,412

2
KCNS1_4_225,458,616,720,485,138,1251,556,806,674,352,859,509
KCNS2_4_226,459,537,701,715,389,1073,1174,781,320,575,1159,785
KCNS3_4_227,600,564,766,468,496,1088,430,353,265,80,772,1275
KCNT1_4_228,361,491,703,288,577,747,675,338,148,2434,465,319
KCNT2_4_229,1222,1150,1433,872,1484,1503,1274,1165,1372,2217,926,1655
KCNU1_4_230,1659,1566,2284,1914,1263,2030,966,1536,863,1685,2754,2259
KCNV1_4_231,1761,1768,2398,1764,1580,3284,1887,1715,1347,1570,1331,138
9
KCNV2_4_232,702,508,682,632,666,1473,2535,1212,51,1567,1164,835
KCTD10_4_233,2857,1605,3015,2220,3116,2328,5034,2887,1393,2785,1916,32
28
KCTD11_4_234,279,271,434,327,135,184,519,692,261,688,744,737
KCTD12_4_235,487,359,283,89,81,775,126,456,584,0,163,365
KCTD13_4_236,380,512,266,311,406,193,81,784,78,261,106,387
KCTD14_4_237,404,671,685,414,271,872,524,339,296,159,178,953
KCTD16_4_238,373,264,271,242,326,198,653,595,1136,224,854,858
KCTD17_4_239,333,246,113,520,71,217,104,119,29,173,349,18
KCTD18_4_240,997,936,521,617,214,34,991,1091,59,1314,1187,1021
KCTD19_4_241,4051,3952,5065,3283,3774,3903,2800,2662,3709,2776,3485,43
73
KCTD20_4_242,864,1207,1531,964,954,1699,1096,1626,970,2170,1009,375
KCTD2_4_243,425,505,573,301,150,52,704,656,224,708,711,614
KCTD3_4_244,871,941,692,1195,1292,1109,520,3059,567,1469,849,1083
KCTD4_4_245,1885,2365,1990,1835,1776,654,2515,2146,1791,2894,3045,2038
KCTD5_4_246,283,244,86,520,70,217,103,133,22,173,349,18
KCTD8_4_247,2129,2376,2706,1860,1131,1102,1355,3827,1018,1015,1916,188
9
KCTD9_4_248,1080,1046,1237,811,657,709,1200,1967,500,524,619,1413
KIR3DL1_4_249,144,103,359,211,170,334,145,281,2,99,696,173
KLRB1_4_250,2315,3170,2849,2338,1811,1875,4224,1748,2325,902,3245,3622
LGALS3BP_4_251,251,332,407,243,787,696,0,80,1,171,767,369
LRP10_4_252,592,892,647,672,1359,945,751,1387,838,2376,368,540
LRP1B_4_253,1337,633,499,652,869,1493,485,1820,302,125,529,1076
LRP3_4_254,204,244,421,384,46,316,76,586,457,0,143,501
LRP5_4_255,666,691,809,465,1775,1065,752,205,985,146,324,920
LRP6_4_256,1111,1279,1956,759,2129,1421,1940,1113,479,317,1141,696
LRPAP1_4_257,213,246,231,173,53,132,256,154,23,663,273,92
LTBR_4_258,3527,4063,4559,3077,3533,3316,4820,4554,4071,5350,3766,3644
MARCO_4_259,418,151,414,321,38,562,148,230,11,449,433,568
MCOLN1_4_260,232,264,476,476,7,243,29,333,19,47,659,226
MCOLN2_4_261,1173,1286,1057,1497,950,754,1567,1415,1276,303,1883,1889
MCOLN3_4_262,658,1175,844,758,535,1378,966,343,941,3603,722,298
MCU_4_263,468,309,149,179,0,0,0,354,264,257,154,986
MFRP_4_264,520,617,806,696,693,675,995,566,443,299,883,735
MICB_4_265,436,601,633,877,533,521,38,957,876,771,583,665
MPL_4_266,69,191,251,207,32,485,0,110,30,273,237,196
MRC1_4_267,396,406,416,302,92,157,493,111,220,129,231,426
MRC2_4_268,314,95,319,433,309,983,99,276,60,1,582,298
NALCN_4_269,2912,2868,3490,2832,956,3640,1669,1746,3886,2111,2306,3076

NGFR_4_270,91,148,151,81,35,54,704,281,0,0,70,270
NISCH_4_271,88,189,216,180,75,254,3,170,72,193,102,188
NPTXR_4_272,734,558,757,679,886,1415,1327,905,66,357,780,1132
ORAI1_4_273,515,588,665,394,441,283,152,618,222,2189,501,512
P2RX1_4_274,435,314,644,339,329,204,893,253,22,503,390,378
P2RX3_4_275,233,414,442,376,39,381,225,464,675,224,666,511
P2RX4_4_276,678,1001,865,733,1030,734,861,818,570,329,450,1413
P2RX7_4_277,458,360,467,443,244,388,361,505,943,212,124,376
PEX5L_4_278,242,453,804,358,795,544,564,681,282,394,312,71
PGLYRP1_4_279,295,283,432,241,1066,454,1392,137,1009,0,236,554
PGLYRP2_4_280,1062,1090,1596,1571,1071,445,1025,919,1207,1407,2911,129
2
PGLYRP3_4_281,488,403,262,417,390,959,402,91,7,59,323,151
PGLYRP4_4_282,232,280,196,230,130,32,1,0,522,0,89,271
PGRMC1_4_283,1386,1711,1586,1361,2746,972,4829,1985,387,1395,1677,2477
PIP_4_284,737,613,772,602,303,489,1594,610,305,112,402,698
PKD1L1_4_285,1336,1077,1106,1432,804,247,652,1498,860,544,989,1921
PKD1L3_4_286,930,1276,872,1099,528,336,1537,1583,793,150,268,1157
PKD2L1_4_287,776,898,640,627,243,490,110,821,116,685,636,509
PKD2L2_4_288,2287,1628,2093,1752,1121,1762,3719,1722,1180,1164,4452,18
11
PKDREJ_4_289,2247,2463,2556,2559,814,1896,3662,1140,3193,2032,4211,307
3
PLXNA1_4_290,178,205,105,28,177,140,413,42,85,54,162,60
PLXNA2_4_291,2242,1952,1977,1745,1922,1986,2101,1952,1505,2899,2717,19
38
PLXNA3_4_292,478,507,335,342,144,680,606,74,324,46,675,488
PLXND1_4_293,2062,1844,1958,1712,1814,1983,2056,1693,1202,2898,2709,19
34
PRPH2_4_294,420,392,449,296,830,67,887,245,145,730,188,830
RARRES2_4_295,908,395,605,621,122,1601,2,14,643,1751,335,859
RASA3_4_296,289,463,317,361,517,258,327,237,8,15,718,652
RYR2_4_297,3438,3506,3226,3608,3533,2163,4877,2138,1513,1371,3213,3431
RYR3_4_298,1283,1328,1732,1216,1331,1452,1774,2065,2066,1083,2857,759
SARM1_4_299,574,756,857,900,950,653,2212,612,468,312,341,973
SCN10A_4_300,736,554,853,673,209,265,446,284,803,932,380,312
SCN11A_4_301,149,375,211,164,78,357,667,643,123,122,1101,213
SCN2B_4_302,556,445,437,394,417,417,632,105,329,374,1458,726
SCN4A_4_303,173,196,262,195,122,188,113,131,2,171,433,68
SCN7A_4_304,669,881,828,570,1285,1574,695,1791,376,536,1310,1483
SCN9A_4_305,1019,1124,1722,752,593,605,486,1030,1101,314,712,1306
SCNN1B_4_306,470,474,390,292,697,1209,909,440,293,493,787,293
SCNN1D_4_307,386,311,218,287,15,277,446,1222,295,497,232,29
SCNN1G_4_308,242,88,251,312,59,151,194,779,699,0,415,31
SCUBE1_4_309,2237,2061,2865,2091,2098,2261,4432,1486,2565,3045,1355,47
65
SELE_4_310,1391,1484,1457,1233,1212,2762,892,670,732,1215,1659,1963
SEMA5A_4_311,394,466,536,454,76,452,94,376,146,426,925,613
SETD1A_4_312,932,973,887,968,1332,1154,903,999,471,928,685,1773
SFRP1_4_313,467,243,148,111,11,114,253,192,190,99,336,289

SFRP4_4_314,638,747,543,708,405,713,1704,929,600,797,1002,362
SFRP5_4_315,2784,3781,3863,3112,3719,1948,3519,4261,1000,1266,4862,271
4
SHKBP1_4_316,49,66,49,42,0,0,59,97,8,1,265,5
SHROOM2_4_317,106,114,217,188,215,4,0,182,151,805,74,256
SIGLEC8_4_318,379,201,310,103,736,290,45,166,25,711,368,312
SLAMF1_4_319,906,1152,1579,1309,491,1057,711,651,407,1333,1640,1388
SLC6A18_4_320,237,410,548,363,104,282,9,844,6,221,811,268
SLC6A19_4_321,680,883,683,750,723,340,551,670,454,885,1348,718
SLC6A1_4_322,196,230,137,250,95,87,25,147,479,0,111,110
SLC6A3_4_323,875,929,1271,1172,551,822,1836,1125,247,322,1014,1335
SLC6A4_4_324,565,598,933,484,297,538,147,252,585,223,970,390
SLC9A1_4_325,444,387,444,312,1352,472,1018,1459,0,82,252,536
SLC9A3_4_326,91,136,40,169,0,62,642,0,0,183,18,0
SRCRB4D_4_327,121,61,37,13,7,53,126,45,0,204,9,3
STAB2_4_328,1092,1209,1480,1245,1192,1053,3020,1344,1167,306,734,2678
STX1B_4_329,935,490,693,407,487,849,96,570,620,570,905,507
THBD_4_330,743,754,983,589,1179,80,410,580,307,247,1107,669
TLR1_4_331,1871,2248,2141,2150,1769,3732,2423,1699,2069,2983,2253,1969
TLR2_4_332,3903,4344,3588,3575,5060,1539,8426,3467,2782,3577,3705,3647
TLR3_4_333,1174,1333,1313,1080,821,886,2112,1679,729,1962,706,1165
TLR4_4_334,666,899,541,766,164,889,390,181,278,2343,1613,428
TLR5_4_335,1373,1122,1159,996,1105,1888,1666,1351,473,3894,1428,1173
TLR6_4_336,1172,1391,1217,1382,1144,794,1143,617,843,1762,3820,937
TLR7_4_337,352,223,442,563,313,594,32,158,189,725,525,549
TLR8_4_338,1156,580,1006,936,546,1044,1305,1654,369,1079,980,592
TLR9_4_339,601,476,1206,512,852,84,1803,425,34,1454,373,570
TMEM37_4_340,275,406,491,301,779,634,1,49,456,433,339,189
TMEM38A_4_341,2089,2340,2184,2287,999,3306,2098,2432,2164,1675,2701,26
64
TMEM38B_4_342,1312,1208,1325,1006,2045,1553,1924,827,1409,393,2030,173
7
TNFAIP1_4_343,301,235,577,313,157,450,210,75,19,321,97,377
TNFRSF10A_4_344,396,605,788,445,779,799,1019,283,366,597,1320,874
TNFRSF10C_4_345,1032,1300,1473,1229,1151,2353,1165,2189,1038,855,730,9
67
TNFRSF10D_4_346,215,369,140,176,47,0,0,46,506,20,1695,282
TNFRSF11A_4_347,315,368,477,376,317,665,249,323,320,430,865,428
TNFRSF13B_4_348,381,404,481,235,199,266,860,50,312,1593,1240,460
TNFRSF13C_4_349,2606,2207,3376,2682,2754,2872,1893,2013,2407,1108,3817
,2353
TNFRSF14_4_350,717,345,689,431,78,242,812,959,311,825,703,340
TNFRSF1A_4_351,3335,2830,3499,3393,2981,1764,4675,3025,2319,3632,3429,
3118
TNFRSF1B_4_352,43,66,3,4,0,6,59,143,0,305,7,3
TNFRSF4_4_353,92,183,158,124,198,36,187,40,15,0,174,312
TNFRSF6B_4_354,436,526,670,186,873,550,544,341,445,208,911,331
TPCN2_4_355,586,811,957,818,407,677,551,967,135,868,1554,1200
TREM2_4_356,436,470,756,514,796,972,211,1137,56,412,572,252
TRPA1_4_357,3115,3062,3031,3390,4298,3651,3091,2881,2652,1883,5554,389

1
TRPC1_4_358,2315,2514,3306,2128,3967,2118,3913,2427,2786,1754,1584,312
8
TRPC5_4_359,1386,2032,1704,1794,1702,1590,1831,1714,1745,3040,1286,181
9
TRPC6_4_360,363,275,649,506,782,450,1196,410,152,31,241,311
TRPM1_4_361,548,627,855,615,747,392,80,29,218,28,937,178
TRPM2_4_362,204,348,619,346,60,469,279,363,6,334,524,87
TRPM5_4_363,352,440,328,251,175,230,521,165,199,26,86,266
TRPM7_4_364,325,394,745,389,148,869,820,1183,170,6,389,661
TRPM8_4_365,330,457,438,486,220,503,998,240,201,82,384,751
TRPV2_4_366,178,159,368,170,400,74,1044,359,401,3,241,230
TRPV3_4_367,2612,2212,2621,1884,2005,3247,2275,2567,1305,1521,2867,182
0
TRPV5_4_368,931,531,785,557,466,38,1068,1143,1776,612,223,1219
TRPV6_4_369,230,294,176,151,146,137,20,333,421,1151,113,264
TTYH3_4_370,306,261,385,442,820,338,289,233,18,17,527,931
ULBP1_4_371,643,292,541,867,168,232,359,428,989,461,1238,993
ULBP2_4_372,542,663,627,707,52,864,227,350,657,1668,510,486
ULBP3_4_373,643,294,542,780,168,229,254,428,986,462,1234,994
UNC5A_4_374,457,822,807,866,771,1063,208,607,450,438,1100,542
UNC5B_4_375,827,774,808,923,683,1619,992,1379,1412,1129,974,671
UNC5C_4_376,308,466,352,336,172,423,761,758,134,313,544,865
UTRN_4_377,1543,1402,1651,1887,1635,794,1689,1081,2573,1972,2834,1461
VDAC1_4_378,664,440,370,260,1164,0,151,463,582,32,43,62
ZACN_4_379,22,49,49,195,0,0,2,30,2,0,5,35
ABCC4_4_380,1261,1275,1682,1271,519,1116,1666,1510,1189,557,1191,1626
ABCC9_4_381,5473,5052,5478,5348,4578,2919,5776,5925,3592,5153,4866,561
2
ADIPOR1_4_382,752,895,789,769,987,1126,294,1287,1466,278,1356,1166
AGER_4_383,173,288,146,172,371,82,29,208,2,16,42,98
ANO6_4_384,376,198,262,499,14,160,656,42,522,411,307,94
ANTXR1_4_385,1235,1220,1399,1276,455,2656,1623,91,1586,4731,2087,2600
ANXA7_4_386,131,98,85,130,0,28,0,21,1,0,1,1
AQP4_4_387,623,214,186,281,1720,280,66,1193,1124,175,1519,273
ASGR1_4_388,330,411,495,278,143,270,942,499,691,1490,643,583
ASGR2_4_389,578,564,913,630,828,112,821,653,235,264,392,778
BEST1_4_390,4269,3349,3719,2658,3621,3107,2999,4570,3815,2456,3635,298
0
BEST3_4_391,601,860,851,429,564,397,614,635,505,850,715,1005
CACNA1A_4_392,738,769,907,930,1200,661,1301,811,559,443,427,598
CACNA1C_4_393,337,462,286,601,325,290,964,920,257,1079,385,743
CACNA1D_4_394,1952,2765,2693,1749,2531,1875,3824,2287,2034,2722,2483,1
916
CACNA1E_4_395,1111,1040,1475,1137,971,915,2007,664,1083,2106,1560,1808
CACNA1G_4_396,2428,2206,2029,2415,2355,1600,3677,1557,1348,2885,3302,1
292
CACNA1H_4_397,110,28,203,136,0,33,1,81,401,0,43,31
CACNA1I_4_398,663,586,864,446,454,1276,1481,736,1223,783,834,931
CACNA2D2_4_399,1814,1839,1971,1961,1215,1790,2881,1648,1949,583,1516,1

318
CACNB1_4_400,365,438,305,183,38,482,92,76,439,37,58,337
CACNB2_4_401,825,1207,1115,959,828,808,1026,824,631,2419,711,687
CACNB3_4_402,854,749,372,894,654,1282,193,359,743,11,513,332
CACNB4_4_403,439,645,707,526,964,83,497,1135,312,27,167,692
CACNG6_4_404,330,161,714,827,182,632,430,215,462,1240,268,744
CATSPER2_4_405,7013,7266,7108,6702,8738,9507,8465,4789,6480,7904,10100
,8112
CD14_4_406,187,357,135,354,32,4,52,13,330,659,746,22
CD163_4_407,231,190,45,255,51,78,55,124,284,8,3,67
CD247_4_408,1034,861,980,703,692,1768,86,587,464,1447,1168,872
CD302_4_409,1346,971,1035,960,1380,563,971,298,611,2754,1276,1046
CD36_4_410,1812,2008,1789,2038,1489,2492,1365,446,2058,3380,1879,2269
CD3D_4_411,918,608,1002,1042,1336,910,426,895,702,1022,1856,236
CD40_4_412,708,521,855,622,1310,540,1106,878,344,23,306,339
CD4_4_413,213,272,171,197,93,109,234,453,420,0,326,13
CD74_4_414,144,193,96,209,154,0,1,251,79,2,6,550
CD79A_4_415,149,140,104,27,1,36,29,327,287,48,90,0
CD79B_4_416,337,334,440,291,30,477,1,907,264,362,398,64
CD86_4_417,2755,2713,3403,3065,3566,2412,3913,3001,2540,1371,3045,2563
CEACAM1_4_418,809,1123,1224,866,918,1695,1763,879,608,2521,1029,935
CEACAM21_4_419,62,96,70,229,411,35,204,102,196,49,24,352
CHRFAM7A_4_420,252,365,176,287,532,851,107,385,135,22,260,182
CHRNA1_4_421,0,17,3,20,32,2,0,8,0,0,12,6
CHRNA3_4_422,1595,1915,1961,1411,2147,2666,1669,957,1369,3939,1180,217
0
CHRNA6_4_423,2478,2446,3276,2779,2138,3952,1914,2901,2162,3369,2725,13
27
CHRNA7_4_424,1897,1980,1427,1568,1503,3786,1328,1048,4165,1677,1765,10
05
CLCC1_4_425,589,590,890,695,546,726,237,475,68,594,876,842
CLCN2_4_426,501,709,606,582,154,32,1107,189,859,5,1014,691
CLCN3_4_427,886,1020,1525,1245,304,1555,2452,1147,566,701,2620,977
CLCN5_4_428,832,775,953,665,1103,455,642,64,1637,988,770,1238
CLCN6_4_429,364,317,273,359,245,199,344,6,37,209,327,131
CLCN7_4_430,209,227,195,50,387,181,7,124,95,47,4,156
CLCNKA_4_431,662,647,599,637,361,367,57,610,722,835,197,201
CLCNKB_4_432,662,647,599,637,361,367,57,610,722,835,197,201
CLEC1B_4_433,186,166,289,351,140,140,12,509,905,51,4,8
CLEC4A_4_434,348,358,520,328,796,436,293,337,1233,297,513,161
CNGA1_4_435,1026,924,1099,650,682,647,1665,1013,699,892,1102,558
CNGA3_4_436,2678,2256,3043,2456,1052,1798,1977,1923,1414,2402,2336,264
8
CNGB1_4_437,567,470,431,693,64,99,381,1050,20,634,181,217
CNTFR_4_438,316,224,370,364,179,241,303,394,190,9,303,180
CR2_4_439,282,382,274,197,196,221,142,308,3,1388,552,255
CSF2RA_4_440,695,863,439,855,419,725,1587,293,915,501,535,910
CSF3R_4_441,280,405,269,378,42,1316,396,178,59,74,44,593
CXADR_4_442,601,334,444,308,282,718,7,548,193,217,195,908
DAG1_4_443,2975,3594,3820,3572,6450,3494,3621,4011,2961,6614,3108,5326

DMBT1_4_444,235,339,441,381,65,610,314,109,69,108,31,615
EDA2R_4_445,113,235,195,129,318,291,160,998,10,391,235,357
EDNRA_4_446,366,644,560,680,399,512,1225,693,337,522,63,449
ENG_4_447,319,214,342,352,150,712,653,118,95,156,646,251
EVI2A_4_448,1028,1145,1044,893,1689,1248,2006,1514,950,1271,1791,1129
FCAMR_4_449,278,600,574,293,328,356,0,302,3,1057,486,37
FCGR1B_4_450,826,809,751,766,308,1031,801,475,454,905,936,643
FCGR2A_4_451,1215,931,989,973,1323,1048,1184,1418,863,912,2232,966
FCGR2B_4_452,2289,1921,2595,2315,2010,4392,3297,3570,1049,989,2189,146
1
FCGR3A_4_453,2459,2095,2564,2039,2788,1058,2753,2639,2018,2732,2575,19
22
FCGRT_4_454,154,164,363,442,173,1013,131,437,1,167,58,527
FGFRL1_4_455,303,328,288,168,107,341,305,98,119,530,218,284
FLT4_4_456,254,312,299,317,450,149,649,352,161,14,380,40
FXYD1_4_457,72,40,96,143,40,88,2,87,29,9,163,23
FXYD2_4_458,442,307,334,581,93,547,39,432,818,392,1490,224
FXYD4_4_459,1533,1741,1592,1698,1581,1765,2720,2070,3026,2094,2485,107
4
FXYD5_4_460,303,293,427,227,77,685,608,243,596,967,177,6
FXYD6_4_461,533,693,845,539,928,587,216,800,113,859,561,1321
GABRA1_4_462,191,124,268,147,144,450,170,289,48,132,244,879
GABRA2_4_463,5197,5174,5330,4035,5128,4858,4783,2319,3562,6489,7468,44
16
GABRA4_4_464,438,297,250,128,95,398,61,357,22,0,626,493
GABRA5_4_465,1337,1502,1514,1338,1154,1119,1194,1239,977,436,2101,633
GABRB2_4_466,1044,975,1428,1708,606,2759,522,98,1448,672,2033,1804
GABRB3_4_467,1758,2015,1836,1283,2099,1625,2889,1906,1230,1039,1887,18
94
GABRG2_4_468,1400,1475,1462,1733,2373,2083,1206,1714,2549,813,1906,109
5
GFRA1_4_469,621,323,435,529,204,518,30,74,469,204,958,1107
GFRA2_4_470,282,386,426,232,798,153,324,48,739,0,42,879
GFRA4_4_471,157,488,258,146,247,1133,1103,169,272,599,826,161
GHR_4_472,1415,1399,1863,1179,766,602,1213,829,993,633,1687,1055
GIPC1_4_473,980,729,614,677,1528,454,1275,382,223,191,912,1393
GLRA1_4_474,914,671,919,585,864,746,577,713,193,14,985,863
GLRA2_4_475,824,1206,1215,726,681,687,911,464,434,384,1389,582
GLRA3_4_476,1717,1500,1602,1692,1527,554,1611,1368,1723,861,1463,1862
GLRA4_4_477,779,685,926,852,374,975,501,754,588,829,1284,696
GLRB_4_478,1417,1372,1832,1759,1201,2048,387,1786,792,1529,1770,2204
GP6_4_479,470,340,454,533,526,440,244,451,405,98,513,801
GPM6A_4_480,327,660,899,508,387,1604,74,635,352,817,1378,285
GPR89A_4_481,744,1181,1106,1201,1443,1109,21,569,113,927,484,901
GRIA1_4_482,2454,2428,3182,3363,1939,2159,1914,3261,3528,5411,2528,292
6
GRIA2_4_483,371,318,270,245,52,312,36,62,299,199,949,166
GRIA3_4_484,2115,1969,1854,1521,3001,2267,2398,3272,1752,1199,1827,177
3
GRIA4_4_485,1355,1704,1674,1627,2052,1667,843,3035,1843,1578,3384,1731

GRIK1_4_486,2814,2081,2657,2380,1804,4153,2147,1703,1883,773,3426,2697
GRIN2A_4_487,1022,1479,921,1308,1777,335,1413,1337,874,2589,1431,2079
GRINA_4_488,115,111,93,89,184,1,383,0,72,0,23,0
HLA-DPA1_4_489,563,251,344,268,58,181,241,99,180,505,1373,160
HTR3A_4_490,956,1106,1106,1390,1046,544,953,671,671,591,1541,1851
HTR3D_4_491,961,1001,1092,1033,1406,1335,1995,1372,853,1332,1175,1230
HVCN1_4_492,326,187,556,357,95,335,251,32,35,1234,751,112
IFNAR2_4_493,443,474,682,596,621,382,486,567,902,264,362,508
IL11RA_4_494,315,375,500,654,244,480,1038,1368,212,106,149,817
IL12RB1_4_495,526,480,317,541,273,157,820,219,18,27,753,723
IL15RA_4_496,109,148,294,164,218,15,775,52,252,1044,156,14
IL1R2_4_497,571,849,675,627,802,223,400,565,692,638,448,731
IL1RAP_4_498,1079,709,1851,1167,636,1367,1739,1213,1050,1375,2168,1176
IL1RL1_4_499,669,509,708,226,441,1086,1296,506,963,204,559,379
IL21R_4_500,242,425,444,472,408,23,792,236,564,1242,411,229
IL22RA2_4_501,1384,1375,1602,1723,2249,999,4129,1316,1539,1667,2360,17
64
IL28RA_4_502,344,343,447,222,399,344,253,228,308,928,417,388
IL31RA_4_503,1042,758,1205,923,1581,436,837,1632,1089,1891,1231,972
IL4R_4_504,1704,2000,2576,2295,1586,2638,1100,1258,1816,1441,2685,1990
IL5RA_4_505,2231,2216,2771,2334,3822,1888,2641,2492,1517,1381,2323,242
5
IL6R_4_506,476,179,248,530,427,211,869,1198,86,5,1098,470
IL6ST_4_507,2095,2314,2425,2290,2817,1545,2868,2699,1188,2449,1414,257
2
ILDR1_4_508,140,197,316,282,534,291,143,512,907,34,820,293
ITGB1_4_509,1331,1124,1437,1350,621,1099,1065,1035,594,511,1888,1459
ITGB4_4_510,218,199,140,227,123,397,178,95,139,764,29,273
ITPR1_4_511,1492,1216,1284,1385,1667,1571,1836,623,1334,470,953,2014
KCNA2_4_512,742,864,1259,959,1161,759,725,854,281,572,329,1413
KCNAB1_4_513,360,231,171,207,33,228,117,10,37,358,323,284
KCNAB2_4_514,285,123,209,235,363,71,108,367,36,32,466,670
KCNC1_4_515,1608,1688,1569,1681,2031,2001,1543,2234,1305,2130,1957,400
KCNC2_4_516,743,724,776,652,368,993,300,1375,250,2468,227,1447
KCNC4_4_517,634,756,318,501,185,371,1443,751,51,952,153,375
KCND3_4_518,22,71,104,103,8,0,469,222,39,1,15,404
KCNE1_4_519,84,339,262,220,133,8,830,30,80,628,89,415
KCNG3_4_520,84,172,41,143,18,0,4,90,14,0,111,2
KCNH1_4_521,1457,1108,1385,1468,366,1459,2534,442,975,2302,2492,1492
KCNH2_4_522,718,341,350,264,425,376,417,682,699,0,1182,1007
KCNH5_4_523,87,339,90,109,0,7,22,189,10,289,0,1
KCNH6_4_524,90,136,115,122,4,90,45,85,272,17,33,822
KCNH7_4_525,1465,1607,2575,1943,1563,3480,2085,1287,2017,2535,2616,160
6
KCNIP1_4_526,279,593,398,394,134,925,248,276,265,11,844,287
KCNIP2_4_527,724,743,1029,1434,648,1263,2516,686,93,113,1122,523
KCNIP4_4_528,2041,1897,1623,1546,1775,2489,1864,765,814,1361,1674,2645
KCNJ11_4_529,0,0,0,0,0,0,0,0,0,0,0,0
KCNJ13_4_530,905,710,638,564,1270,1510,775,911,93,1720,654,673
KCNJ14_4_531,373,166,363,213,83,650,863,260,237,779,263,605

KCNJ15_4_532,578,661,962,612,875,1377,582,587,14,650,231,926
KCNJ16_4_533,1477,1009,1268,870,1250,1344,1359,832,564,416,804,1430
KCNJ1_4_534,2352,2550,2913,2795,1899,2133,3284,2334,1868,2104,2881,305
5
KCNJ4_4_535,12,28,41,3,0,17,0,0,3,0,5,0
KCNK10_4_536,990,676,1231,783,363,2379,1094,599,584,45,1902,870
KCNK16_4_537,276,218,355,260,118,146,565,190,46,353,282,52
KCNK17_4_538,548,571,515,528,136,636,2272,687,577,2222,1796,855
KCNK2_4_539,320,402,528,231,15,489,455,72,232,442,1024,279
KCNK7_4_540,364,357,251,341,495,199,114,921,7,884,681,255
KCNMA1_4_541,517,743,1207,655,179,522,754,1498,769,439,1139,397
KCNMB3_4_542,1053,1083,1011,842,932,1262,1683,871,1118,61,1112,1126
KCNN2_4_543,3177,3818,3655,3954,1809,3615,3926,4234,4078,6632,6098,430
7
KCNN3_4_544,971,1083,997,1172,831,860,733,1489,644,1309,1204,854
KCNQ1_4_545,529,330,553,554,1319,471,116,563,118,8,492,204
KCNQ2_4_546,1963,2358,1979,1342,2335,3420,1302,1533,1288,1558,1768,212
5
KCNQ3_4_547,2528,2007,2368,2561,3695,2449,4249,2483,2381,1280,2288,170
8
KCNQ4_4_548,520,465,727,502,742,358,505,1244,278,49,262,1236
KCNQ5_4_549,2117,1554,2061,1741,749,1584,1827,2419,1538,2748,2486,1730
KCNRG_4_550,992,891,1520,1060,776,1842,1790,747,1594,709,1643,1607
KCTD15_4_551,122,77,206,137,138,77,8,6,72,193,63,320
KCTD1_4_552,7554,6302,6654,6805,4960,7369,11664,8288,4703,5762,4474,70
06
KCTD6_4_553,351,486,626,573,309,562,365,777,656,762,655,835
KCTD7_4_554,410,540,594,615,603,204,461,293,662,933,510,875
KLRC1_4_555,1716,2583,2384,1865,852,919,2558,632,2492,2416,1791,3294
LEPR_4_556,18,69,0,0,0,315,0,7,0,0,323,183
LIFR_4_557,1828,2000,1702,2007,2735,2894,3024,2921,776,3260,3204,2384
LILRB1_4_558,1343,693,768,764,1156,894,782,933,1410,636,2168,672
LILRB3_4_559,1343,693,768,764,1156,894,782,933,1410,636,2168,672
LRP12_4_560,806,1162,1385,1504,336,2425,2518,1094,700,195,1700,1613
LRP8_4_561,391,645,593,342,223,955,201,511,938,0,325,168
MR1_4_562,639,733,903,787,263,217,214,345,232,1193,1544,1141
MSR1_4_563,343,468,567,248,451,679,1071,351,421,396,65,238
NCR2_4_564,2083,1588,2358,1998,1717,1739,1488,2373,734,3149,3344,1948
NCR3_4_565,389,213,167,238,508,35,852,300,88,74,18,482
NOX1_4_566,2219,2174,2414,2634,1517,2194,2252,2684,2502,1392,3417,2483
NOX5_4_567,455,322,487,421,985,136,635,316,110,2,622,829
NRP1_4_568,688,1098,930,690,812,184,1130,861,485,733,368,714
NUDT9_4_569,747,903,745,1139,684,803,1187,1129,470,1252,1542,808
OLR1_4_570,338,852,757,405,515,53,477,367,717,2093,705,945
OPCML_4_571,1781,2310,1555,1636,2084,1355,3365,2890,1255,61,2120,1690
OSMR_4_572,1275,1418,1552,1562,2261,1793,908,2653,1340,383,1791,1784
P2RX2_4_573,689,842,621,985,938,119,617,776,357,34,211,567
P2RX5_4_574,597,846,789,1039,522,821,531,960,517,2180,468,719
P2RX6_4_575,372,495,462,351,288,222,358,496,657,1679,789,441
PEX5_4_576,419,332,318,276,207,87,16,435,7,234,1092,365

PKD1_4_577,669,789,918,1152,761,1254,502,786,892,260,902,738
PKD1L2_4_578,100,146,64,168,102,10,59,52,2,0,156,95
PLA2R1_4_579,1271,754,671,1219,1250,1649,551,1114,651,179,942,1308
PLAUR_4_580,448,695,806,734,1018,419,621,877,540,29,417,897
PLXNB1_4_581,177,246,127,306,267,370,281,276,72,1439,178,280
PRKD2_4_582,58,21,13,156,23,51,5,350,0,589,34,42
PRLR_4_583,639,794,904,759,713,200,5,229,227,678,803,490
PTCH1_4_584,417,471,540,502,489,104,477,484,813,15,120,610
PTCH2_4_585,802,703,584,883,280,883,457,436,444,266,502,791
ROBO1_4_586,1500,1540,2337,1921,1192,2070,1293,1651,727,1540,1356,1177
ROBO2_4_587,1960,1792,2689,2591,3132,2604,2412,2304,2468,1412,2371,225
4
RYR1_4_588,1039,1035,1231,1469,394,1790,225,400,1453,610,1215,1643
SCARA3_4_589,1464,1103,1415,1377,1461,1678,785,1885,971,2537,1388,1086
SCARF1_4_590,381,186,99,202,425,307,480,281,73,121,296,120
SCARF2_4_591,263,380,318,566,440,323,452,515,171,472,715,139
SCN1A_4_592,2527,2299,2363,2579,2199,3735,2601,2391,1634,2110,3173,206
8
SCN2A_4_593,2084,2331,2173,1892,1350,2260,2767,1283,1121,731,2837,1818
SCN3A_4_594,4651,5221,5153,4721,5105,4989,7159,4276,2688,5114,6158,609
4
SCN3B_4_595,619,1262,959,861,267,960,1205,615,776,1620,1122,820
SCN4B_4_596,914,994,718,602,1091,1238,1306,1101,1086,435,1291,672
SCN5A_4_597,698,957,1012,1214,1374,1066,1274,764,1531,490,503,672
SCN8A_4_598,2202,2785,2994,3640,1767,3574,2750,2822,3109,1920,3058,320
8
SCNM1_4_599,156,63,196,264,58,510,429,219,48,37,242,253
SCNN1A_4_600,332,219,277,113,1,222,351,143,190,54,274,190
SLAMF6_4_601,731,1295,1433,1140,1650,807,2038,1134,1052,1162,407,1443
SLC6A2_4_602,811,516,740,694,356,999,460,572,144,507,881,658
TLR10_4_603,2575,2901,2698,2364,1838,2851,3646,2385,3341,2696,3705,261
8
TNFRSF10B_4_604,1054,583,1088,758,915,1398,554,1241,498,624,1283,1218
TNFRSF18_4_605,29,118,128,294,197,30,49,151,184,1,231,99
TNFRSF19_4_606,243,83,136,189,2,0,131,454,283,356,536,312
TNFRSF25_4_607,731,875,1010,629,751,1144,503,744,696,408,1444,1188
TNPO1_4_608,4004,4177,4473,4594,4508,6722,7799,4424,2608,3594,5739,339
4
TOMM40_4_609,66,105,121,91,32,3,0,277,10,4,5,92
TP53INP1_4_610,275,215,176,396,141,1373,18,493,144,114,1,318
TPCN1_4_611,769,641,710,482,564,1263,482,446,846,999,969,798
TRPC3_4_612,652,761,897,935,991,484,1248,1622,124,61,1415,515
TRPC4_4_613,384,381,565,555,317,846,548,535,6,316,259,627
TRPM3_4_614,1317,1626,1533,1416,1541,2347,989,1333,1818,1651,1261,889
TRPM4_4_615,1084,692,485,678,499,1308,626,767,745,1438,1520,1371
TRPM6_4_616,1058,1062,1266,799,1200,827,347,1210,519,537,771,436
TRPV1_4_617,354,511,99,179,635,249,283,292,0,223,254,52
TRPV4_4_618,184,180,94,245,160,26,139,135,78,3,18,561
TTYH1_4_619,384,491,755,512,632,410,1468,450,476,411,1254,401
TTYH2_4_620,451,742,661,529,447,434,227,1054,221,362,273,1112

VDAC2_4_621,412,837,821,595,270,573,3017,361,656,76,424,464
ANO7_4_622,810,770,664,904,463,484,937,1580,1237,887,724,1111
AQP1_4_623,641,894,1228,642,1588,865,644,291,313,2411,1191,1173
CLEC2D_4_624,177,272,315,267,758,149,666,9,225,609,118,214
CLEC7A_4_625,2916,2940,3519,3152,3020,1878,5665,3077,2717,2791,4852,29
19
CRLF2_4_626,1831,2504,2049,2596,1863,4281,2718,3190,2319,3158,2918,327
5
FXYD3_4_627,3024,2792,3068,2526,4028,2412,4599,2037,4235,2176,4849,413
7
HFE_4_628,871,1191,673,1246,541,668,835,840,988,752,1137,678
SCN1B_4_629,2423,2867,2947,3108,3080,2530,3274,2818,2736,2133,3545,215
0
TNFRSF8_4_630,1155,920,667,1367,597,1138,360,1113,234,972,938,772
TSPO_4_631,1118,1609,1360,1515,1002,1233,2288,1879,568,1167,2180,1688
ABCC8_4_632,171,110,196,236,35,37,68,235,234,10,3,126
AMFR_4_633,876,1544,1107,1435,1057,718,547,1015,710,815,1486,936
ANO1_4_634,1,121,27,82,4,98,161,67,1,14,11,2
ANO2_4_635,229,210,470,406,1195,206,255,255,105,528,531,1301
ANXA9_4_636,271,173,122,289,985,242,155,545,84,169,398,28
AQP10_4_637,953,1793,1307,1567,1146,1870,491,968,1608,1078,1564,1983
AQP11_4_638,162,364,41,190,0,274,272,220,172,1849,131,571
AQP12A_4_639,416,403,388,655,473,343,458,755,537,9,244,303
AQP2_4_640,380,332,218,275,47,374,592,27,448,137,88,405
AQP3_4_641,146,451,255,411,379,513,77,1,6,30,153,689
AQP5_4_642,231,297,126,278,316,687,554,137,145,24,19,403
AQP6_4_643,844,687,783,573,1142,861,1611,629,90,74,688,987
AQP7_4_644,717,1247,1167,1209,463,1458,1473,1575,985,1660,1138,1281
AQP8_4_645,34,61,44,85,37,9,97,20,1,0,5,18
B2M_4_646,642,928,1103,703,1365,860,1016,1166,183,482,475,473
BEST2_4_647,1170,1507,1451,1049,799,1942,471,1021,975,398,420,1311
BEST4_4_648,979,609,1141,852,2628,830,1302,634,1811,1414,1431,1389
BSND_4_649,48,158,142,115,250,95,11,0,0,16,77,112
BTBD10_4_650,878,628,726,455,884,317,178,191,638,1822,933,324
BTNL2_4_651,560,757,686,1014,189,1082,186,1064,331,761,1542,713
C5orf62_4_652,106,120,112,158,73,490,5,38,411,0,339,293
CACNA1B_4_653,75,72,274,254,146,10,0,429,21,0,0,38
CACNA1F_4_654,287,209,500,501,653,227,338,274,173,655,1180,552
CACNA1S_4_655,332,298,518,309,779,266,134,132,352,185,56,437
CACNA2D1_4_656,422,1269,1248,833,862,336,706,683,1125,937,921,920
CACNA2D3_4_657,735,408,396,652,1055,82,329,338,628,111,546,560
CACNA2D4_4_658,0,0,0,0,0,0,0,0,0,0,0,0
CACNG1_4_659,148,153,299,341,63,27,33,542,0,590,105,581
CACNG2_4_660,592,826,1078,841,1107,2298,645,306,174,2247,776,8
CACNG3_4_661,166,220,216,131,241,11,601,32,106,22,91,475
CACNG4_4_662,32,203,113,20,74,722,1,11,32,406,1118,1
CACNG5_4_663,1968,1774,1930,1775,1083,3246,1861,1294,1082,3399,1851,20
95
CACNG7_4_664,290,364,199,220,634,724,25,126,4,665,428,242
CACNG8_4_665,171,320,266,188,660,66,781,85,760,47,392,1446

CATSPER1_4_666,72,22,46,61,171,382,0,0,3,0,0,215
CATSPER3_4_667,0,6,9,0,0,0,0,0,0,0,0,0
CATSPER4_4_668,1869,2344,2784,3388,1052,2710,2566,3975,1314,1157,2621,
3138
CCT8L2_4_669,179,319,338,79,230,28,217,99,176,518,466,53
CD160_4_670,237,276,346,217,105,73,336,70,34,7,53,112
CD163L1_4_671,63,67,127,102,4,59,12,20,269,7,38,8
CD27_4_672,417,458,473,625,458,811,798,715,50,214,771,384
CD2_4_673,343,479,583,380,1506,1805,597,367,589,513,157,611
CD300C_4_674,409,332,468,279,336,649,177,183,50,84,74,129
CD3E_4_675,250,306,420,186,91,27,69,710,53,1245,826,651
CD3G_4_676,331,633,216,668,303,468,21,907,90,1,388,391
CD5_4_677,177,62,199,236,144,478,9,17,213,0,280,30
CD5L_4_678,797,1029,898,807,665,1991,1182,972,930,202,569,1104
CD69_4_679,995,1528,1238,1182,855,607,1727,715,1102,1483,1338,1882
CD6_4_680,119,36,73,101,0,1,10,10,39,2,29,66
CD72_4_681,33,125,53,22,11,3,553,2,4,1292,92,345
CD80_4_682,536,394,495,326,363,505,1357,277,138,101,128,530
CFTR_4_683,831,684,1245,800,1539,550,242,1269,942,1709,669,1826
CHRNA10_4_684,15,80,119,35,40,775,0,0,148,213,0,87
CHRNA2_4_685,418,351,668,632,729,998,1383,1194,54,1504,628,1308
CHRNA4_4_686,844,818,598,619,532,92,1066,628,281,214,722,463
CHRNA5_4_687,702,635,810,698,1193,660,692,307,841,81,501,1641
CHRNA9_4_688,1139,1984,1142,1562,1284,1267,1812,2038,1879,2781,1973,21
04
CHRNB1_4_689,1731,1556,1305,1726,1238,2327,2781,1103,2971,3499,2271,20
73
CHRNB2_4_690,639,731,952,839,1208,612,438,972,355,235,1964,820
CHRNB3_4_691,773,584,693,705,1193,226,711,690,422,136,779,1026
CHRNB4_4_692,435,496,252,184,726,345,412,513,232,2,287,640
CHRND_4_693,401,510,300,367,1060,406,439,658,749,266,591,356
CHRNE_4_694,1998,1690,1931,2027,1901,1043,1935,2377,2513,994,2767,1820
CHRNG_4_695,203,257,278,83,44,356,21,0,3,45,213,594
CLCA1_4_696,661,636,638,680,970,1189,145,590,758,374,353,1515
CLCA2_4_697,83,440,551,367,37,619,331,14,840,979,331,701
CLCA4_4_698,641,626,1062,822,235,863,563,1254,337,1235,1920,424
CLCN1_4_699,124,152,291,220,14,363,52,20,327,1472,242,3
CLCN4_4_700,296,323,372,145,289,476,530,93,409,102,460,239
CLDN3_4_701,125,51,80,35,39,1,19,15,0,0,2,0
CLDN4_4_702,1389,1210,1887,1298,989,436,1367,1085,897,1353,1162,1528
CLEC1A_4_703,1153,1683,1301,1204,1152,1510,2151,2283,956,1876,1827,189
0
CLIC1_4_704,269,227,524,504,96,482,718,445,34,1111,1430,549
CLIC2_4_705,705,573,765,705,1236,793,1440,976,1512,329,641,974
CLIC3_4_706,184,178,330,177,2,524,733,331,77,179,421,361
CLIC4_4_707,494,582,474,480,552,348,396,102,966,53,174,41
CLIC6_4_708,2519,2756,3263,3117,2138,1931,3636,2796,3050,2839,1635,185
8
CLNS1A_4_709,178,141,296,195,456,388,75,80,71,152,247,46
CNGA2_4_710,296,296,243,118,64,0,14,93,23,646,375,6

CNGA4_4_711,1437,1432,1717,1564,1384,2231,704,1206,1784,1609,1362,1085
CNGB3_4_712,853,1086,917,546,1094,271,723,1328,40,734,459,927
COLEC12_4_713,1054,1372,1341,1568,896,1902,229,844,1669,4073,863,1258
CSF2RB_4_714,228,472,420,375,611,256,37,551,91,520,1182,438
CUBN_4_715,1002,508,1270,695,629,772,950,800,681,458,443,307
DCC_4_716,2235,2380,2805,2213,1588,1827,2673,2991,1181,2671,2063,2248
DNER_4_717,102,563,1007,391,568,440,614,335,1026,369,1050,414
EDAR_4_718,466,591,655,650,900,713,668,839,525,610,572,792
EPOR_4_719,47,165,77,133,0,20,47,172,28,11,155,6
FCER1A_4_720,1579,1722,1744,1926,1220,2469,1871,635,1216,2376,1527,205
0
FCER1G_4_721,697,879,771,937,658,345,909,807,665,1522,1008,573
FCGR1A_4_722,3546,3325,3634,3164,2754,2919,4263,3481,2739,1447,4393,34
02
FCGR2C_4_723,1109,931,875,921,1257,1052,1437,1414,860,949,2224,987
FCGR3B_4_724,1092,1528,1211,1508,1432,2958,1500,2906,1562,201,1154,171
5
FRRS1_4_725,276,204,284,547,317,62,1191,0,1142,71,459,133
FXYD7_4_726,971,1085,880,1216,556,592,762,1682,901,971,626,1243
GABRA3_4_727,707,591,723,711,334,758,543,1263,449,428,1179,837
GABRA6_4_728,340,148,161,195,0,0,164,0,388,0,323,149
GABRB1_4_729,265,68,136,72,474,0,0,0,42,8,101,690
GABRD_4_730,346,390,339,271,658,793,129,536,167,1527,534,95
GABRE_4_731,237,579,349,295,0,147,931,81,229,161,457,0
GABRG1_4_732,1831,1426,1643,2249,1216,1845,1805,2216,1484,2051,3189,30
37
GABRG3_4_733,765,625,552,517,276,823,949,772,1511,498,1496,1244
GABRP_4_734,76,162,54,73,57,186,4,7,45,25,9,69
GABRQ_4_735,1596,1639,899,1686,2082,2538,969,1764,1055,1624,1959,1479
GABRR1_4_736,867,1259,1353,1589,1982,1097,775,1761,488,1340,1271,1571
GABRR2_4_737,217,244,341,266,203,650,1320,108,448,909,784,577
GABRR3_4_738,1007,655,967,1243,1387,1653,1418,974,1093,210,1383,1586
GFRA3_4_739,354,283,237,212,641,273,146,26,257,570,538,221
GPC1_4_740,228,577,348,362,233,1143,1313,183,124,283,898,248
GPC4_4_741,1346,1164,1419,817,974,785,498,1741,1248,1955,459,1746
GPC6_4_742,802,422,412,349,1038,126,423,467,161,566,558,329
GPR89B_4_743,739,1161,1119,1166,1519,1877,806,1119,463,3,1133,2065
GRID1_4_744,256,448,340,384,540,1038,89,456,80,241,475,916
GRID2_4_745,878,1261,1328,1565,1469,527,828,1247,1676,1056,1937,1193
GRIK3_4_746,701,878,988,884,783,718,1008,528,738,880,1133,712
GRIK4_4_747,285,573,414,635,337,520,151,344,208,750,552,530
GRIK5_4_748,155,381,269,240,60,46,76,115,0,951,838,289
GRIN2B_4_749,197,371,237,304,401,186,627,690,567,159,233,627
GRIN2C_4_750,372,336,447,391,6,650,1172,1,103,19,204,44
GRIN2D_4_751,513,176,482,513,22,851,377,816,162,0,383,170
GRIN3A_4_752,121,192,272,112,55,222,564,5,287,253,263,83
GRIN3B_4_753,1704,1716,2178,1586,2403,1680,2366,2661,2068,444,2347,298
1
HAVCR2_4_754,480,487,603,399,893,101,774,605,290,592,902,603
HCN1_4_755,218,128,258,122,136,645,122,69,519,4,86,74

HCN2_4_756,79,44,52,69,2,3,254,21,0,255,1,149
HCN3_4_757,1143,1972,1801,1561,1058,1854,2086,1714,1519,1359,3283,1651
HCN4_4_758,515,419,434,107,0,422,0,270,668,703,319,0
HLA-B_4_759,257,119,130,201,296,0,42,328,44,0,1,604
HLA-DOA_4_760,534,270,554,420,156,762,1175,186,93,1269,746,85
HLA-DOB_4_761,712,390,424,571,137,360,483,663,562,510,982,535
HLA-DPB1_4_762,195,97,151,306,24,295,200,322,152,346,149,109
HLA-DQA1_4_763,288,342,332,143,320,405,226,10,198,0,632,363
HLA-DQA2_4_764,357,175,191,375,1,202,12,540,752,269,519,880
HLA-DQB2_4_765,110,181,239,208,40,20,17,114,415,1125,259,44
HLA-DRA_4_766,239,662,369,652,877,146,875,131,25,87,564,351
HLA-DRB1_4_767,1052,901,851,779,570,672,1019,75,983,1695,578,522
HLA-DRB5_4_768,410,702,701,562,363,248,851,878,494,134,628,472
HLA-G_4_769,579,347,400,330,1098,64,481,289,137,1206,521,774
HTR3B_4_770,282,300,368,322,289,496,1867,190,235,1,123,99
HTR3C_4_771,2105,2540,2839,2068,1882,4149,4529,1547,1786,1056,2216,347
0
HTR3E_4_772,45,81,52,102,22,1,126,81,7,31,209,157
ICAM1_4_773,244,255,249,102,173,349,591,921,448,6,363,62
IFNAR1_4_774,3897,3643,4407,3593,4620,4598,6319,3954,3026,3071,4793,42
42
IFNGR1_4_775,585,485,350,350,544,127,318,622,519,656,413,151
IFNGR2_4_776,322,263,184,254,231,164,0,134,178,192,391,280
IGF1R_4_777,346,517,703,532,527,1071,714,751,155,599,634,273
IGF2R_4_778,640,516,635,414,329,405,142,511,518,72,231,524
IL10RA_4_779,1728,1209,1227,795,1315,1016,1081,946,1572,1649,1249,867
IL10RB_4_780,580,534,1089,1086,908,1662,1603,1448,781,1716,1116,1193
IL12RB2_4_781,1824,2577,2193,2464,2090,1979,1552,2284,1620,2080,2602,1
624
IL13RA1_4_782,3058,2710,3222,2667,2254,5216,3853,1656,1983,2165,2702,2
711
IL13RA2_4_783,241,585,538,304,570,144,88,530,216,60,1160,577
IL17RA_4_784,278,112,334,628,88,77,24,872,8,0,761,536
IL17RB_4_785,940,1469,1613,1377,1645,1215,644,1587,1086,781,1436,1605
IL18R1_4_786,4220,4059,4608,5045,2829,5915,5007,5441,2597,5719,2916,51
47
IL18RAP_4_787,235,231,528,164,125,134,27,240,587,262,102,256
IL1R1_4_788,281,331,230,268,211,179,15,252,181,140,230,281
IL1RAPL1_4_789,1078,1081,1262,1542,461,1565,924,884,229,680,2629,925
IL1RAPL2_4_790,403,795,577,384,243,877,767,383,160,210,757,721
IL1RL2_4_791,491,660,564,390,249,1147,648,579,320,213,213,245
IL20RA_4_792,152,98,333,217,65,128,397,46,229,378,13,82
IL22RA1_4_793,941,1178,1320,1562,781,1908,1545,976,974,1713,1202,2058
IL23R_4_794,1514,1700,2281,1908,2361,1497,2592,2803,731,2238,2571,1390
IL27RA_4_795,168,269,142,261,82,68,459,30,501,23,167,61
IL2RA_4_796,186,207,207,251,1,718,400,100,147,0,151,150
IL2RB_4_797,660,429,228,529,816,213,74,231,800,212,262,464
IL2RG_4_798,1033,817,813,744,988,1365,1667,1185,142,4864,309,629
IL3RA_4_799,279,350,551,266,397,156,250,161,684,205,1164,372
IL7R_4_800,204,229,316,542,262,584,490,490,15,240,844,316

IL9R_4_801,212,197,184,110,23,90,365,71,41,1042,472,109
ITFG1_4_802,156,147,126,92,51,53,111,0,65,0,372,546
ITGA2B_4_803,132,220,357,319,813,565,17,681,224,0,411,82
ITGB3_4_804,452,439,224,530,183,240,179,943,312,202,238,271
ITGB7_4_805,177,281,343,266,31,145,470,39,60,337,1253,158
ITPR2_4_806,1218,1430,2351,1373,2047,1094,1609,2294,1228,550,1862,1035
ITPR3_4_807,3394,3602,3671,3102,2919,4785,5540,3349,3023,4642,3071,344
7
JPH3_4_808,459,344,510,504,44,334,1140,1080,171,28,57,649
KCMF1_4_809,98,210,168,226,214,137,602,141,31,189,491,120
KCNA10_4_810,3148,3546,3752,3869,3162,3428,3169,5359,3361,2620,3805,35
49
KCNA1_4_811,1010,1013,1283,1498,1279,2360,1371,623,342,1179,2044,1437
KCNA3_4_812,137,128,135,178,28,231,883,505,369,0,0,296
KCNA4_4_813,145,74,197,123,261,2,0,205,1167,1665,22,621
KCNA5_4_814,191,242,401,399,413,652,426,287,357,65,466,532
KCNA6_4_815,2872,2834,2989,2953,1891,2609,2495,1667,2207,6013,3049,394
6
KCNA7_4_816,481,251,464,368,429,392,17,202,922,506,486,372
KCNAB3_4_817,274,352,312,255,317,326,285,178,36,198,118,129
KCNB1_4_818,1661,1816,1914,1671,1308,2078,3966,2044,2138,1456,2236,178
6
KCNB2_4_819,386,297,201,449,397,1,210,210,646,25,234,399
KCNC3_4_820,107,240,184,223,84,1007,132,6,520,338,872,115
KCND1_4_821,1101,1104,1119,1083,341,2003,410,1280,566,2820,1507,1262
KCND2_4_822,293,476,587,306,263,546,723,478,480,387,213,263
KCNE1L_4_823,1887,2024,2103,2124,1885,1059,1885,3484,1473,2690,3737,20
71
KCNE2_4_824,2059,1764,3318,2063,1407,2235,2169,2644,1515,3331,2445,318
9
KCNE3_4_825,884,1030,1135,864,619,852,1352,409,484,591,1187,581
KCNE4_4_826,0,36,49,48,49,0,0,0,0,0,263,1
KCNF1_4_827,159,311,247,132,289,81,374,209,339,0,302,77
KCNG1_4_828,93,219,325,237,1,289,402,0,128,4,14,139
KCNG2_4_829,720,495,603,480,1221,1202,817,842,658,410,1051,838
KCNG4_4_830,226,194,275,300,100,90,157,496,7,180,120,224
KCNH3_4_831,801,514,586,651,470,1168,265,634,239,466,874,1114
KCNH4_4_832,248,119,330,271,56,170,70,244,112,0,677,439
KCNH8_4_833,97,64,39,71,237,8,6,42,2,0,9,26
KCNJ10_4_834,240,400,312,175,1166,668,43,69,13,0,347,430
KCNJ12_4_835,402,398,608,691,895,418,231,194,491,607,560,728
KCNJ2_4_836,454,271,537,285,345,0,765,368,685,617,1157,589
KCNJ3_4_837,534,721,900,769,1071,1714,1874,521,365,85,604,610
KCNJ5_4_838,41,120,146,155,22,309,122,91,301,8,422,139
KCNJ6_4_839,1214,1515,1370,1464,1055,2008,1113,1834,452,1687,2081,877
KCNJ8_4_840,926,1071,1516,683,1011,355,1698,566,1092,547,1096,657
KCNJ9_4_841,427,527,455,493,216,1025,942,58,191,25,546,841
KCNK12_4_842,359,30,235,403,296,19,313,248,148,15,353,194
KCNK13_4_843,1942,1872,2131,2377,2547,1759,1153,2221,1543,1467,2067,29
05

KCNK15_4_844,366,511,358,265,305,674,115,208,403,314,179,220
KCNK18_4_845,105,249,245,273,236,107,188,392,133,8,352,526
KCNK1_4_846,332,213,174,438,319,142,8,90,318,37,42,81
KCNK3_4_847,129,79,136,41,45,7,329,8,58,218,372,21
KCNK4_4_848,141,86,138,209,280,251,16,32,0,6,50,98
KCNK5_4_849,475,458,187,291,750,289,90,664,122,116,355,713
KCNK6_4_850,181,60,113,41,767,1,50,31,138,181,127,119
KCNK9_4_851,177,163,257,261,239,95,70,98,64,297,54,9
KCNMB1_4_852,685,779,990,1000,251,234,1230,1401,511,79,1007,436
KCNMB4_4_853,388,550,530,418,156,128,372,1148,389,459,210,422
KCNN1_4_854,483,414,579,423,531,330,964,250,71,974,1431,660
KCNN4_4_855,286,416,361,137,590,18,96,118,223,0,200,159
KCNS1_4_856,29,118,59,143,81,0,431,2,0,1,2,191
KCNS2_4_857,2280,2067,2648,2331,3966,3166,2353,1904,1774,1917,1477,343
3
KCNS3_4_858,122,77,76,38,7,16,0,200,199,16,34,119
KCNT1_4_859,591,690,602,571,593,746,1205,1360,645,182,511,624
KCNT2_4_860,485,528,574,654,382,183,342,864,157,103,229,670
KCNU1_4_861,1430,1381,1509,1951,1955,1653,1381,989,1913,1911,1845,2755
KCNV1_4_862,23,15,25,21,33,0,0,0,0,0,0,0
KCNV2_4_863,85,148,270,280,118,31,863,435,87,306,65,238
KCTD10_4_864,233,106,210,326,286,269,23,4,121,2,120,77
KCTD11_4_865,574,292,520,307,221,541,1716,80,104,119,140,374
KCTD12_4_866,429,185,450,230,824,960,344,87,870,97,279,29
KCTD13_4_867,0,1,0,0,0,0,0,0,0,0,31,17
KCTD14_4_868,0,0,0,0,0,0,0,0,0,0,0,0
KCTD16_4_869,242,231,26,36,0,286,0,15,236,0,13,0
KCTD17_4_870,100,85,235,103,41,58,0,31,3,1,21,5
KCTD18_4_871,1207,1550,1584,1604,1059,2317,991,1371,903,1137,2287,1402
KCTD19_4_872,1724,1937,1831,1311,1365,1037,2650,1756,1274,770,2368,185
4
KCTD20_4_873,1377,1372,1401,1336,1643,1882,4074,965,1363,1021,2412,979
KCTD2_4_874,340,208,130,250,28,67,388,320,6,0,382,0
KCTD3_4_875,620,822,938,601,318,532,270,1030,409,1034,769,701
KCTD4_4_876,462,237,283,430,16,343,1387,660,18,632,151,437
KCTD5_4_877,65,32,106,211,28,0,13,0,41,0,33,0
KCTD8_4_878,592,517,749,368,430,573,896,587,138,2040,1331,1328
KCTD9_4_879,1149,1135,814,1224,2081,616,394,1054,1385,2076,890,1665
KIR3DL1_4_880,35,16,56,117,0,0,8,0,7,0,0,66
KLRB1_4_881,110,353,379,274,525,280,706,372,7,153,6,378
LGALS3BP_4_882,0,11,10,19,0,0,0,0,0,0,44,0
LRP10_4_883,265,99,617,133,999,107,66,704,4,1050,1083,64
LRP1B_4_884,1183,1144,2003,1327,1734,900,3768,879,1181,2131,1903,2098
LRP3_4_885,20,19,27,138,0,106,0,213,8,88,0,32
LRP5_4_886,0,0,0,0,0,0,0,0,0,0,0,0
LRP6_4_887,561,858,679,856,1426,363,2018,1711,815,960,816,766
LRPAP1_4_888,530,493,642,413,310,365,547,308,738,54,1201,792
LTBR_4_889,4551,4973,5297,4273,3664,4057,5951,5010,5281,6876,4776,4555
MARCO_4_890,133,157,306,234,55,176,853,163,626,0,27,86
MCOLN1_4_891,197,302,379,187,1184,1573,1,17,0,0,281,310

MCOLN2_4_892,794,689,1162,1074,290,914,1486,1085,402,779,740,1367
MCOLN3_4_893,3058,3194,3681,3542,2728,2910,6478,2658,2348,4336,5862,28
11
MCU_4_894,1678,1596,1377,1128,2339,1178,1891,629,481,621,1212,1447
MFRP_4_895,48,67,70,127,0,46,0,0,0,6,29,111
MICB_4_896,656,831,833,930,690,1235,236,1344,1082,853,528,1644
MPL_4_897,1229,966,1334,834,487,1401,3018,731,1219,610,1204,211
MRC1_4_898,327,291,346,235,557,418,545,300,520,138,438,203
MRC2_4_899,296,367,107,298,109,541,0,248,37,89,186,846
NALCN_4_900,1412,1320,1219,1207,2593,2447,718,992,1305,1916,1518,1929
NGFR_4_901,272,511,135,372,436,871,364,15,3,770,460,0
NISCH_4_902,63,177,193,387,386,615,38,156,237,120,0,332
NPTXR_4_903,181,85,376,53,305,0,790,28,0,613,203,73
ORAI1_4_904,470,660,510,561,375,661,1117,42,47,451,1136,354
P2RX1_4_905,634,806,643,605,378,1261,318,228,271,286,363,938
P2RX3_4_906,579,813,522,655,437,1161,253,998,190,1128,881,899
P2RX4_4_907,314,332,409,239,735,87,1969,488,241,82,510,499
P2RX7_4_908,2211,1601,2000,1470,648,1034,1595,2949,481,1327,2098,3048
PEX5L_4_909,1497,2063,1392,1518,1814,3415,1233,1590,1442,925,1675,1110
PGLYRP1_4_910,456,358,319,234,57,326,757,448,327,51,140,66
PGLYRP2_4_911,187,170,345,137,303,102,21,65,158,2,114,139
PGLYRP3_4_912,206,390,90,370,18,82,298,396,3,360,654,209
PGLYRP4_4_913,267,302,393,389,442,740,248,34,2,1533,309,152
PGRMC1_4_914,43,148,71,31,0,213,33,5,140,3753,572,389
PIP_4_915,2097,1731,1834,1740,1846,1466,2555,2052,1323,3611,1555,2146
PKD1L1_4_916,876,1243,1229,851,932,1177,1349,1436,1021,931,830,598
PKD1L3_4_917,886,448,625,617,246,722,777,740,691,152,749,1248
PKD2L1_4_918,712,752,1025,624,651,787,604,1224,274,99,867,475
PKD2L2_4_919,242,598,369,275,85,294,304,262,52,237,424,607
PKDREJ_4_920,3371,3876,4099,4486,3176,3796,7485,3039,3112,2890,5172,63
60
PLXNA1_4_921,735,477,659,506,570,274,1125,595,374,2594,457,840
PLXNA2_4_922,55,153,76,84,136,6,93,39,198,0,125,151
PLXNA3_4_923,58,321,239,142,279,0,650,0,0,47,5,420
PLXND1_4_924,283,103,246,174,70,660,39,282,440,0,132,465
PRPH2_4_925,1120,1147,1494,1458,532,1487,1736,966,832,139,2301,408
RARRES2_4_926,301,222,240,209,199,1610,7,325,554,391,1154,326
RASA3_4_927,509,479,429,500,410,894,30,1405,134,224,772,409
RYR2_4_928,0,0,0,0,0,0,0,0,0,0,0,0
RYR3_4_929,453,959,705,648,1124,1398,961,605,144,340,342,584
SARM1_4_930,57,123,70,257,0,5,2,0,430,0,16,101
SCN10A_4_931,1007,1317,823,816,864,2602,564,1487,2187,2498,1145,1013
SCN11A_4_932,184,169,123,179,430,50,72,183,175,338,512,130
SCN2B_4_933,262,42,166,149,99,451,0,211,361,9,425,8
SCN4A_4_934,103,176,203,132,64,122,168,96,32,0,55,5
SCN7A_4_935,4048,4703,3353,3769,3122,3746,5515,3832,3529,3826,4631,438
0
SCN9A_4_936,477,513,471,424,322,603,175,607,688,80,366,745
SCNN1B_4_937,650,372,735,953,139,1703,1217,559,443,427,776,1059
SCNN1D_4_938,741,624,979,620,1368,250,307,373,155,652,799,596

SCNN1G_4_939,358,50,314,173,567,19,235,200,625,149,16,183
SCUBE1_4_940,1904,1944,2932,2512,1533,2902,3323,3550,1856,3074,3013,25
68
SELE_4_941,177,84,168,71,71,509,128,16,2,1373,180,0
SEMA5A_4_942,643,617,584,776,866,614,227,581,106,1160,1704,1255
SETD1A_4_943,1476,1367,1415,925,1281,677,2354,1857,1200,1032,847,366
SFRP1_4_944,48,130,106,96,0,174,116,5,7,2,132,73
SFRP4_4_945,1567,2353,2555,1518,1547,3156,1820,1968,2014,860,1416,1885
SFRP5_4_946,72,138,86,203,42,261,23,37,10,9,8,23
SHKBP1_4_947,1766,1636,2086,1905,1412,1756,2138,3203,2173,3054,2227,36
11
SHROOM2_4_948,193,134,15,155,207,12,88,9,4,188,511,338
SIGLEC8_4_949,229,262,376,112,81,70,527,283,271,155,121,778
SLAMF1_4_950,485,465,852,644,160,611,1062,238,864,379,1982,451
SLC6A18_4_951,783,595,536,787,521,469,277,622,587,1223,490,254
SLC6A19_4_952,923,1468,1270,1553,1474,1397,1267,1427,459,3268,1522,205
0
SLC6A1_4_953,497,510,396,476,721,388,20,123,242,495,612,925
SLC6A3_4_954,2362,2433,2316,2792,1624,2454,4642,2050,1154,2111,1735,23
22
SLC6A4_4_955,391,324,482,159,148,448,256,324,302,72,83,333
SLC9A1_4_956,734,465,775,740,699,247,2575,269,571,401,1064,307
SLC9A3_4_957,86,144,143,173,422,441,54,95,4,139,33,30
SRCRB4D_4_958,275,222,250,194,8,101,462,301,226,810,49,512
STAB2_4_959,792,776,594,674,624,147,514,404,16,901,500,494
STX1B_4_960,202,227,143,109,252,370,35,241,177,471,253,111
THBD_4_961,594,799,876,997,1189,235,766,450,163,714,404,1632
TLR1_4_962,1838,2288,1948,1790,1061,1313,3806,745,701,2699,2313,1919
TLR2_4_963,1011,851,1114,746,689,1079,511,590,860,519,1772,1290
TLR3_4_964,849,991,1208,989,956,936,323,868,1501,185,1102,771
TLR4_4_965,2482,1839,2743,1657,2375,3368,2176,1299,3274,2867,3430,1932
TLR5_4_966,815,855,898,913,522,620,389,1196,446,2958,1815,1463
TLR6_4_967,0,0,0,0,0,0,0,0,0,0,0,0
TLR7_4_968,423,527,731,611,342,253,1016,825,525,591,729,1155
TLR8_4_969,1878,1856,1614,1240,1768,1923,1347,1994,1198,1493,620,1527
TLR9_4_970,321,349,150,265,103,138,185,95,546,66,174,122
TMEM37_4_971,82,0,61,0,0,0,0,0,0,0,0,0
TMEM38A_4_972,680,902,915,949,1344,1216,1173,909,964,81,1680,1257
TMEM38B_4_973,116,90,274,199,90,39,41,122,69,95,304,20
TNFAIP1_4_974,312,610,1023,968,233,842,879,825,629,487,801,1452
TNFRSF10A_4_975,409,176,275,341,672,272,299,399,387,292,149,159
TNFRSF10C_4_976,742,761,682,805,528,1075,1073,710,311,1609,926,1041
TNFRSF10D_4_977,234,407,236,267,275,118,238,607,272,178,236,314
TNFRSF11A_4_978,99,203,161,357,807,3,157,30,32,1021,332,749
TNFRSF13B_4_979,123,130,192,213,54,44,504,434,124,0,0,182
TNFRSF13C_4_980,2019,2008,2800,2446,1857,1728,1266,1576,1944,2903,3755
,1738
TNFRSF14_4_981,106,240,87,119,27,14,1,24,68,0,365,25
TNFRSF1A_4_982,88,75,97,6,9,105,1,323,190,0,11,4
TNFRSF1B_4_983,317,341,506,591,309,38,502,364,664,1259,197,155

TNFRSF4_4_984,328,603,808,545,207,343,402,946,548,1583,1490,532
TNFRSF6B_4_985,249,292,198,241,32,6,25,7,772,0,13,0
TPCN2_4_986,1750,2146,2003,2402,1866,2247,1328,1876,3152,1931,1455,242
8
TREM2_4_987,412,279,200,223,15,335,6,53,156,0,232,725
TRPA1_4_988,1032,602,939,499,543,941,435,902,813,750,1041,671
TRPC1_4_989,48,58,42,52,191,20,1,1,19,325,27,20
TRPC5_4_990,1902,2318,2116,1668,2801,2884,1545,2161,1512,1608,1741,270
9
TRPC6_4_991,252,423,278,433,444,643,1089,48,753,228,129,1043
TRPM1_4_992,1279,1428,1483,1465,3076,812,273,1619,760,1520,1688,1846
TRPM2_4_993,2329,3015,2574,3639,1765,792,2618,2560,2124,1490,4374,3727
TRPM5_4_994,449,468,370,628,230,237,891,611,162,2,280,535
TRPM7_4_995,623,442,698,530,192,1044,899,902,758,15,639,705
TRPM8_4_996,626,826,645,384,877,232,317,807,406,1295,390,471
TRPV2_4_997,630,661,591,630,473,275,775,256,1235,3,1334,572
TRPV3_4_998,1451,1333,1823,1351,1453,791,726,1566,1012,2602,1624,1913
TRPV5_4_999,1358,1802,1349,1601,1843,1219,1632,1427,1591,3145,1648,262
3
TRPV6_4_1000,163,167,161,271,1,419,393,638,0,989,169,127
TTYH3_4_1001,167,142,280,65,129,600,156,0,1,52,0,2
ULBP1_4_1002,342,400,593,350,400,635,557,281,19,899,568,358
ULBP2_4_1003,475,587,616,383,844,443,2021,880,956,31,121,534
ULBP3_4_1004,751,624,798,706,78,697,603,531,1053,339,1308,1415
UNC5A_4_1005,151,168,218,141,213,0,283,0,257,0,281,147
UNC5B_4_1006,1896,2199,2548,1757,2124,2331,2849,2039,1316,2510,1686,30
62
UNC5C_4_1007,151,208,212,241,332,9,531,137,74,422,90,1
UTRN_4_1008,323,297,440,325,296,364,759,450,536,28,1048,321
VDAC1_4_1009,552,710,570,549,968,678,362,1137,151,713,846,609
ZACN_4_1010,132,156,360,259,61,249,156,124,78,341,343,93
ABCC4_4_1011,1051,586,1186,1013,485,120,441,947,313,152,957,1798
ABCC9_4_1012,507,751,724,731,1309,450,261,564,635,604,1191,883
ADIPOR1_4_1013,741,1042,750,939,677,476,2132,897,518,2063,1024,709
AGER_4_1014,2776,2839,3008,2670,3596,1378,2201,2039,3024,5336,4687,186
0
ANO6_4_1015,242,180,163,167,357,56,146,272,58,338,43,79
ANTXR1_4_1016,492,632,571,569,1036,935,423,592,690,802,417,372
ANXA7_4_1017,142,102,83,98,119,360,504,120,100,51,45,102
AQP4_4_1018,271,373,280,671,230,209,549,381,1010,142,225,973
ASGR1_4_1019,62,9,16,68,0,24,0,0,0,1197,0,128
ASGR2_4_1020,807,927,916,1140,1285,1462,603,1322,772,435,1355,1033
BEST1_4_1021,781,695,956,846,754,529,301,612,106,1219,1616,1084
BEST3_4_1022,3677,3571,3617,3638,2790,4126,5381,3114,3358,2879,2318,44
59
CACNA1A_4_1023,408,453,473,507,233,671,453,1144,988,690,168,596
CACNA1C_4_1024,1181,1135,1497,1443,1399,1116,1053,2208,1187,297,1960,6
24
CACNA1D_4_1025,2013,2210,1960,2237,2990,3123,2048,2648,2468,1882,2217,
2248

CACNA1E_4_1026,270,156,371,198,273,36,55,98,0,128,80,339
CACNA1G_4_1027,414,273,412,355,53,132,519,320,137,181,251,359
CACNA1H_4_1028,363,446,432,499,313,1365,400,270,633,467,828,1167
CACNA1I_4_1029,1247,1408,1457,1183,1012,1757,1951,708,507,985,3208,118
8
CACNA2D2_4_1030,171,418,218,290,643,666,419,105,368,59,1245,416
CACNB1_4_1031,412,312,196,116,195,1021,185,584,40,78,431,177
CACNB2_4_1032,1350,1406,1833,1509,1491,1859,1216,1444,930,2695,1675,15
41
CACNB3_4_1033,87,242,120,216,5,47,107,1405,44,2227,0,6
CACNB4_4_1034,1174,998,1806,1061,1217,1691,1369,1766,357,34,750,987
CACNG6_4_1035,177,351,361,292,65,1,304,383,635,15,43,364
CATSPER2_4_1036,1297,1198,1602,1036,2824,2221,2195,1596,1070,1312,1697
,1097
CD14_4_1037,544,481,342,884,390,586,919,230,296,268,732,625
CD163_4_1038,174,231,226,216,78,279,0,6,0,238,64,207
CD247_4_1039,1185,1094,1740,1386,1230,1105,1464,1193,1221,999,2048,153
4
CD302_4_1040,455,733,704,435,949,308,327,210,607,586,457,693
CD36_4_1041,928,1221,950,931,1656,666,1162,1069,1191,285,798,1739
CD3D_4_1042,1110,850,952,1609,2299,1215,2021,1108,278,2419,1157,798
CD40_4_1043,580,424,343,619,985,637,1449,586,226,247,605,488
CD4_4_1044,574,539,654,633,997,829,972,520,712,791,404,950
CD74_4_1045,734,495,824,535,469,280,28,215,850,490,1343,801
CD79A_4_1046,3578,3283,3817,3520,4139,6545,5239,4011,1720,6002,3559,35
40
CD79B_4_1047,95,73,33,165,1,0,0,76,3,0,15,76
CD86_4_1048,51,291,300,243,430,438,373,688,105,162,771,371
CEACAM1_4_1049,1293,1575,1433,1816,1411,1888,498,573,1280,2811,1856,14
79
CEACAM21_4_1050,1273,1489,1889,1633,1363,2185,1673,2271,653,583,1930,1
359
CHRFAM7A_4_1051,1968,2119,2120,2727,2368,2860,1978,1491,1427,1492,3086
,1853
CHRNA1_4_1052,10,157,246,232,47,116,355,88,227,41,26,0
CHRNA3_4_1053,77,32,113,81,0,171,64,34,4,0,27,0
CHRNA6_4_1054,317,138,58,232,8,110,543,71,252,82,205,123
CHRNA7_4_1055,2058,2159,3064,1832,2611,1287,2882,2692,2851,1511,2020,2
014
CLCC1_4_1056,205,179,74,437,232,522,687,0,19,0,70,387
CLCN2_4_1057,947,1151,1172,1263,770,1158,1771,1242,931,574,1599,945
CLCN3_4_1058,566,862,1203,783,2328,775,1053,756,1065,773,1466,669
CLCN5_4_1059,1180,1676,1183,1413,1569,1802,472,2465,1084,2457,1992,145
4
CLCN6_4_1060,398,469,387,301,596,674,43,415,569,93,1175,594
CLCN7_4_1061,46,123,6,39,31,650,66,4,0,0,185,0
CLCNKA_4_1062,1224,1246,1665,1198,2061,1171,2563,1459,1719,1010,1909,1
231
CLCNKB_4_1063,403,412,698,363,794,319,259,266,235,224,787,414
CLEC1B_4_1064,1517,1568,1999,1645,1659,1504,2158,1514,1269,1115,1377,1

698
CLEC4A_4_1065,491,541,570,447,700,824,2062,1057,600,1375,1084,164
CNGA1_4_1066,296,197,403,139,296,485,110,193,89,67,98,98
CNGA3_4_1067,168,100,225,118,38,99,9,696,53,0,8,556
CNGB1_4_1068,201,173,113,503,582,566,37,1,311,72,392,419
CNTFR_4_1069,27,38,100,90,117,0,512,11,57,0,34,44
CR2_4_1070,694,496,603,729,379,449,114,819,412,754,1250,1083
CSF2RA_4_1071,402,268,493,369,237,87,39,201,208,948,906,572
CSF3R_4_1072,247,164,200,293,11,106,32,55,62,28,182,681
CXADR_4_1073,1694,2344,2519,2217,2364,2762,3500,2745,2662,1713,2857,20
19
DAG1_4_1074,1128,643,913,1203,622,1758,1625,1212,933,2870,1042,578
DMBT1_4_1075,359,302,297,241,229,154,331,286,925,738,1,122
EDA2R_4_1076,1025,757,639,401,909,1136,543,752,1221,626,786,808
EDNRA_4_1077,215,135,204,147,18,191,49,228,384,130,4,68
ENG_4_1078,75,251,145,81,0,2,975,546,1,25,83,217
EVI2A_4_1079,620,619,606,689,458,852,828,248,360,283,355,871
FCAMR_4_1080,888,932,1289,979,537,1676,1036,577,827,596,1255,1136
FCGR1B_4_1081,763,628,882,584,477,617,891,224,69,1012,1493,499
FCGR2A_4_1082,285,268,392,595,63,259,691,52,418,553,235,597
FCGR2B_4_1083,230,344,403,205,125,389,0,174,5,7,186,295
FCGR3A_4_1084,56,120,74,93,0,101,114,4,130,2,590,5
FCGRT_4_1085,1683,1286,1449,1541,1300,2106,777,2131,874,784,1006,1035
FGFRL1_4_1086,708,770,970,681,729,1551,1729,731,599,1653,301,1076
FLT4_4_1087,1208,1937,1620,2004,2839,3810,1883,902,757,1403,2320,1872
FXYD1_4_1088,233,267,218,137,40,71,97,141,280,1,806,652
FXYD2_4_1089,799,376,1068,659,351,478,631,171,1049,1690,719,656
FXYD4_4_1090,206,314,139,179,194,300,49,1,61,1326,264,578
FXYD5_4_1091,742,813,964,780,406,774,1615,441,890,1006,313,1248
FXYD6_4_1092,78,58,104,102,76,0,156,48,57,257,3,137
GABRA1_4_1093,283,414,497,520,826,454,633,437,86,226,207,1254
GABRA2_4_1094,1252,1221,1318,1426,2095,1442,1888,1661,667,3508,1747,18
16
GABRA4_4_1095,1043,852,1305,1187,738,1165,760,912,986,609,1275,1381
GABRA5_4_1096,202,123,159,173,27,3,49,66,0,6,1,107
GABRB2_4_1097,321,306,332,443,34,75,351,740,169,0,285,42
GABRB3_4_1098,211,137,18,129,0,17,22,445,216,206,4,78
GABRG2_4_1099,2625,2789,3017,2694,2354,3051,2300,3604,3883,3626,1390,2
413
GFRA1_4_1100,410,487,722,443,419,730,411,330,318,819,992,419
GFRA2_4_1101,802,624,660,573,727,230,699,432,613,44,232,987
GFRA4_4_1102,80,120,125,194,341,38,271,67,5,461,447,40
GHR_4_1103,1640,1475,2007,2201,1523,1593,1047,484,1619,1125,1721,2405
GIPC1_4_1104,293,380,189,358,154,29,11,152,52,0,119,75
GLRA1_4_1105,505,628,1153,811,825,990,472,981,1036,445,610,1014
GLRA2_4_1106,687,1507,1551,1168,895,514,2202,2706,2030,2446,1945,2568
GLRA3_4_1107,812,842,950,1050,772,771,689,828,766,1611,1140,919
GLRA4_4_1108,81,220,457,452,28,63,135,241,42,281,58,145
GLRB_4_1109,191,213,344,208,343,259,144,42,5,0,152,95
GP6_4_1110,1334,1297,1250,1115,1283,1925,1209,1503,1797,876,776,1223

GPM6A_4_1111,2061,1687,2108,1467,2179,2686,994,1171,911,2650,2165,1488
GPR89A_4_1112,739,1161,1119,1166,1519,1877,806,1119,463,3,1133,2065
GRIA1_4_1113,579,987,791,897,1041,994,353,591,447,136,1406,875
GRIA2_4_1114,809,457,703,449,1177,279,484,1760,113,1453,439,800
GRIA3_4_1115,350,244,445,629,331,275,1328,718,6,436,834,747
GRIA4_4_1116,679,421,709,823,460,332,568,465,456,124,1749,1160
GRIK1_4_1117,340,186,283,400,222,671,937,482,406,409,1001,747
GRIN2A_4_1118,979,1382,1041,1349,1441,831,1052,1368,712,942,1468,1026
GRINA_4_1119,338,459,458,392,394,295,388,202,189,537,819,169
HLA-DPA1_4_1120,17,10,28,56,5,23,22,0,0,0,0,5
HTR3A_4_1121,1291,1630,1739,1290,1592,1638,1421,729,962,1763,2064,1303
HTR3D_4_1122,806,788,944,632,551,584,1047,1036,1066,21,898,1322
HVCN1_4_1123,1141,920,1158,759,1403,1350,689,174,969,72,1106,740
IFNAR2_4_1124,453,220,373,177,864,382,60,114,129,549,154,234
IL11RA_4_1125,2024,2422,2061,1999,1098,2666,2193,874,4052,1208,1510,27
50
IL12RB1_4_1126,281,537,272,297,200,49,285,214,509,17,214,241
IL15RA_4_1127,216,242,170,151,21,1003,252,440,0,1,85,625
IL1R2_4_1128,2045,2096,2721,1975,1599,2442,2522,2128,1280,4155,1903,20
12
IL1RAP_4_1129,636,511,775,467,388,203,566,356,73,498,654,252
IL1RL1_4_1130,594,881,1161,800,843,2049,1018,782,1704,1646,1289,836
IL21R_4_1131,457,161,121,87,22,489,571,136,0,0,878,167
IL22RA2_4_1132,1512,1825,2212,1911,2498,1866,2358,2372,1734,1512,2396,
2209
IL28RA_4_1133,0,66,4,7,7,1,0,290,22,0,0,0
IL31RA_4_1134,497,648,563,655,1127,368,625,1233,481,137,787,739
IL4R_4_1135,61,36,139,13,342,0,149,71,0,105,22,165
IL5RA_4_1136,375,759,676,217,146,963,34,829,540,1663,953,113
IL6R_4_1137,2336,1930,1884,1844,1328,1963,2898,1619,998,2760,2458,1743
IL6ST_4_1138,1251,1034,1464,1217,1720,1445,2380,966,472,225,1667,729
ILDR1_4_1139,340,488,458,605,863,169,176,371,518,19,433,1013
ITGB1_4_1140,730,702,516,691,934,928,948,890,158,86,279,594
ITGB4_4_1141,133,74,25,24,262,9,45,18,34,0,0,0
ITPR1_4_1142,389,242,304,323,491,487,31,293,303,692,377,344
KCNA2_4_1143,921,902,1162,1028,599,600,1138,1373,678,603,1478,950
KCNAB1_4_1144,583,771,1101,903,516,441,815,634,211,360,1005,658
KCNAB2_4_1145,83,67,108,53,545,101,852,0,90,0,365,0
KCNC1_4_1146,634,780,1063,682,642,1082,924,507,277,700,1750,622
KCNC2_4_1147,748,548,484,408,467,362,489,871,357,1754,692,965
KCNC4_4_1148,413,673,717,476,315,8,264,358,172,221,486,996
KCND3_4_1149,491,337,331,294,225,432,12,289,1391,1,37,231
KCNE1_4_1150,168,226,261,115,357,28,1004,196,6,1799,210,87
KCNG3_4_1151,834,1234,1532,1194,1101,2156,719,1107,1020,150,1476,1057
KCNH1_4_1152,1497,1974,2195,1914,1741,2080,1758,1464,628,2195,1325,177
5
KCNH2_4_1153,830,1274,864,1415,448,1665,532,765,1475,1394,1173,1325
KCNH5_4_1154,1308,1240,1447,1424,868,2428,2482,1857,674,1543,1105,1666
KCNH6_4_1155,888,710,755,905,739,731,1288,1148,277,1162,705,737
KCNH7_4_1156,3321,3067,3799,3660,2931,4073,4333,2345,2792,1009,4679,35

31
KCNIP1_4_1157,65,9,59,127,49,120,137,1,0,0,2,123
KCNIP2_4_1158,493,555,511,648,914,723,1693,222,423,749,796,595
KCNIP4_4_1159,1019,1692,1842,1837,1663,3423,3056,2972,1635,1958,3963,2
884
KCNJ11_4_1160,335,307,284,454,221,334,1642,148,673,24,288,212
KCNJ13_4_1161,1086,1251,1115,924,482,963,1520,963,1179,1494,1186,2773
KCNJ14_4_1162,1,6,33,90,0,116,603,23,9,0,1,1
KCNJ15_4_1163,984,634,513,875,1440,1245,1710,442,832,1166,1545,783
KCNJ16_4_1164,390,195,334,218,27,0,996,131,4,1635,226,490
KCNJ1_4_1165,2848,2838,3135,3131,2868,3803,3824,2951,3483,3837,4318,21
79
KCNJ4_4_1166,298,216,273,89,19,467,23,382,489,0,320,112
KCNK10_4_1167,1046,1407,1028,1188,1133,1319,1391,853,955,377,1374,1159
KCNK16_4_1168,528,409,753,708,262,724,449,718,1783,710,248,284
KCNK17_4_1169,428,482,448,655,219,974,508,980,1030,860,830,214
KCNK2_4_1170,599,693,727,661,867,830,620,385,1431,703,479,397
KCNK7_4_1171,69,196,326,193,80,6,3,267,21,0,35,224
KCNMA1_4_1172,1539,1323,1167,1126,1275,593,143,776,356,137,842,359
KCNMB3_4_1173,459,461,682,383,588,534,64,1246,690,157,1487,722
KCNN2_4_1174,379,441,964,613,541,770,812,313,394,11,1043,472
KCNN3_4_1175,285,143,216,237,0,77,217,448,284,0,97,17
KCNQ1_4_1176,37,96,206,56,293,141,15,87,19,27,51,75
KCNQ2_4_1177,540,733,482,656,125,269,42,372,37,50,1007,890
KCNQ3_4_1178,572,671,351,295,394,296,825,81,41,1015,932,395
KCNQ4_4_1179,201,281,138,254,282,158,164,200,75,7,216,142
KCNQ5_4_1180,1639,1684,2576,1817,1788,2478,1005,1310,2386,2396,1752,18
34
KCNRG_4_1181,162,202,68,127,3,0,40,72,238,327,23,29
KCTD15_4_1182,120,318,260,284,21,232,657,246,17,205,189,248
KCTD1_4_1183,1511,2154,2666,1982,2857,3269,3303,1675,1934,3544,2352,26
61
KCTD6_4_1184,1785,2235,1908,1586,1371,1240,2407,2060,2076,692,1721,206
7
KCTD7_4_1185,248,689,622,474,417,820,365,420,88,3016,1011,463
KLRC1_4_1186,3200,2548,3845,2859,3193,4198,5339,4979,4497,1823,4790,34
33
LEPR_4_1187,3047,2835,3490,3466,3112,2671,4299,3072,2471,5565,3680,290
4
LIFR_4_1188,1029,1148,936,1195,709,2206,2767,1801,693,417,1484,1608
LILRB1_4_1189,63,80,99,157,1,98,61,256,5,119,126,53
LILRB3_4_1190,267,404,632,704,644,416,599,200,701,415,235,545
LRP12_4_1191,5550,5481,5834,4350,6797,6171,6376,4108,6681,5871,8695,45
08
LRP8_4_1192,384,184,305,270,267,50,147,499,119,92,392,396
MR1_4_1193,4855,3783,4232,3207,4315,2961,5053,2435,3342,3375,3148,5385
MSR1_4_1194,583,676,814,560,440,145,765,78,544,154,511,938
NCR2_4_1195,222,326,540,285,213,831,4,648,177,416,80,189
NCR3_4_1196,967,1180,1474,1369,974,1093,1421,1511,723,313,1331,1914
NOX1_4_1197,842,1324,1089,1278,1064,701,1353,1118,1906,562,1857,1097

NOX5_4_1198,301,273,293,220,277,539,769,157,452,93,581,217
NRP1_4_1199,610,912,743,584,603,590,14,486,671,460,522,836
NUDT9_4_1200,294,267,146,241,423,89,81,209,240,609,482,81
OLR1_4_1201,477,254,289,460,1221,199,32,144,569,16,617,568
OPCML_4_1202,842,584,1078,886,1114,534,608,1450,726,633,165,1468
OSMR_4_1203,637,820,513,797,512,559,1071,423,332,125,469,662
P2RX2_4_1204,306,226,318,172,209,112,438,506,181,0,410,522
P2RX5_4_1205,759,610,659,613,740,55,348,295,677,3364,273,457
P2RX6_4_1206,1,64,33,62,7,32,96,0,249,0,0,12
PEX5_4_1207,251,308,216,178,896,700,129,51,228,189,79,240
PKD1_4_1208,140,75,32,69,0,0,6,81,0,0,187,215
PKD1L2_4_1209,866,892,1518,1087,489,1651,1114,291,145,853,1612,366
PLA2R1_4_1210,1622,1585,1920,1714,1399,2448,2886,1949,1961,4204,1943,2
349
PLAUR_4_1211,297,112,143,396,118,70,164,276,247,0,43,20
PLXNB1_4_1212,1421,752,848,881,627,142,564,582,2036,1,848,958
PRKD2_4_1213,410,372,767,602,916,823,704,381,737,627,544,526
PRLR_4_1214,338,383,498,335,646,145,78,343,14,597,181,890
PTCH1_4_1215,395,410,758,800,782,678,915,659,254,63,173,910
PTCH2_4_1216,73,177,156,168,13,209,6,452,3,1430,189,34
ROBO1_4_1217,3893,3526,4640,3847,3854,4084,4637,2629,2867,4076,6389,54
53
ROBO2_4_1218,1203,1197,1311,1237,1577,540,4607,438,163,644,2131,1171
RYR1_4_1219,527,524,779,713,923,362,988,385,319,129,1256,860
SCARA3_4_1220,290,251,291,283,626,451,187,21,516,458,967,977
SCARF1_4_1221,185,450,291,65,433,0,80,892,295,1272,40,181
SCARF2_4_1222,439,232,250,130,502,150,267,580,13,170,702,50
SCN1A_4_1223,289,565,575,515,211,268,1852,509,619,1360,198,675
SCN2A_4_1224,3428,3412,3048,3068,3264,3652,4100,2318,1811,2215,5991,31
38
SCN3A_4_1225,1099,864,1000,1289,1055,850,464,705,1447,1350,868,1891
SCN3B_4_1226,105,108,174,22,233,119,64,50,531,439,388,86
SCN4B_4_1227,389,470,549,433,467,440,717,197,331,783,317,513
SCN5A_4_1228,2763,3118,3037,2880,2731,2701,3178,4880,2243,3623,4473,27
22
SCN8A_4_1229,971,1419,1340,1529,1277,560,1249,543,2416,3096,1131,2702
SCNM1_4_1230,105,265,217,152,201,131,862,47,79,0,378,344
SCNN1A_4_1231,981,1204,1013,1115,1032,3088,2031,621,1370,376,750,689
SLAMF6_4_1232,214,435,240,505,7,63,11,2,199,485,32,563
SLC6A2_4_1233,1041,740,492,491,445,116,41,833,893,848,337,2091
TLR10_4_1234,1821,1716,2020,2175,1660,2890,2041,1385,886,3014,1658,222
2
TNFRSF10B_4_1235,150,44,114,97,68,0,45,176,673,0,22,76
TNFRSF18_4_1236,169,219,420,160,8,85,858,326,108,45,1528,160
TNFRSF19_4_1237,3820,5379,5230,4168,6401,5207,6227,5381,5062,4561,4083
,4328
TNFRSF25_4_1238,564,393,430,204,444,972,64,746,66,823,113,560
TNPO1_4_1239,1032,1362,1464,1793,1036,687,1868,1663,1492,810,2092,1028
TOMM40_4_1240,39,87,94,135,1,726,9,19,0,0,450,46
TP53INP1_4_1241,328,328,233,237,141,290,495,393,59,336,524,302

TPCN1_4_1242,622,640,663,681,761,236,836,494,423,873,961,280
TRPC3_4_1243,181,202,274,229,17,39,157,229,51,33,15,178
TRPC4_4_1244,608,1006,699,749,1234,1097,1080,1495,185,1279,816,860
TRPM3_4_1245,399,372,334,234,149,73,524,7,513,42,145,362
TRPM4_4_1246,438,361,509,241,859,404,790,254,12,1043,277,978
TRPM6_4_1247,504,279,417,234,291,166,182,325,305,0,145,673
TRPV1_4_1248,225,119,329,388,182,49,0,400,1,1,443,52
TRPV4_4_1249,139,265,279,178,36,468,1,175,22,296,13,70
TTYH1_4_1250,400,596,468,436,619,909,693,237,356,179,1298,1175
TTYH2_4_1251,168,200,194,227,64,211,25,339,0,319,391,135
VDAC2_4_1252,2616,2636,2545,2741,2643,2367,3633,2721,2470,3475,2705,24
00
ANO7_4_1253,293,258,354,281,766,1040,72,394,1,656,65,103
AQP1_4_1254,300,535,871,478,1451,634,800,33,281,1719,693,1063
CLEC2D_4_1255,679,667,886,505,443,338,1977,416,754,676,109,526
CLEC7A_4_1256,301,320,376,301,65,1256,434,282,621,912,181,766
CRLF2_4_1257,950,1160,1223,1141,682,2593,2284,874,683,243,740,812
FXYD3_4_1258,2026,2469,2880,2344,2952,2503,2256,1992,2285,3541,3451,20
90
HFE_4_1259,399,260,578,344,292,225,585,357,77,305,570,232
SCN1B_4_1260,517,496,758,748,197,431,2335,953,1256,9,496,643
TNFRSF8_4_1261,97,412,51,202,111,268,28,396,617,687,221,91
TSPO_4_1262,4,0,32,50,0,220,0,0,378,0,0,0
ABCC8_4_1263,321,392,325,360,127,233,368,67,340,133,369,390
AMFR_4_1264,604,570,657,940,1086,886,486,707,842,710,810,1237
ANO1_4_1265,640,598,440,738,48,362,562,818,235,127,1067,483
ANO2_4_1266,2830,2778,2191,2379,1309,2885,1990,1893,1042,5174,4015,269
9
ANXA9_4_1267,132,245,154,273,31,70,759,49,107,14,170,66
AQP10_4_1268,240,153,269,193,86,1142,26,287,2,0,256,370
AQP11_4_1269,574,630,582,606,359,1101,1153,865,396,25,494,609
AQP12A_4_1270,336,386,371,383,92,850,4,133,78,600,531,279
AQP2_4_1271,145,133,187,73,77,78,652,60,126,0,32,24
AQP3_4_1272,353,330,372,512,1898,468,202,474,2,204,672,677
AQP5_4_1273,308,539,209,613,799,289,801,28,231,713,188,570
AQP6_4_1274,230,389,296,217,209,63,769,153,160,188,581,472
AQP7_4_1275,621,652,644,634,586,558,332,1440,647,316,975,877
AQP8_4_1276,31,97,29,68,15,20,0,1,5,17,592,0
B2M_4_1277,3868,3914,4591,4078,4807,6057,5929,3011,3008,1740,3649,3412
BEST2_4_1278,231,379,464,802,116,294,484,139,270,33,362,371
BEST4_4_1279,174,245,236,138,88,959,9,19,1,538,12,10
BSND_4_1280,146,67,130,117,12,201,145,312,598,0,90,112
BTBD10_4_1281,111,477,469,214,296,237,180,324,1500,31,1470,801
BTNL2_4_1282,385,595,510,585,989,117,852,360,214,543,345,929
C5orf62_4_1283,250,404,466,293,775,1280,277,101,581,251,37,635
CACNA1B_4_1284,1006,551,1049,845,1404,317,854,177,316,2043,1071,905
CACNA1F_4_1285,364,341,379,281,338,319,1321,96,364,6,963,109
CACNA1S_4_1286,5286,5210,5710,5516,4108,4921,5977,4867,3290,2856,4104,
4436
CACNA2D1_4_1287,2327,2973,3086,2015,1707,3774,3486,2776,1543,3933,1904

,2291
CACNA2D3_4_1288,287,378,304,289,112,299,296,269,405,14,180,934
CACNA2D4_4_1289,431,489,625,551,78,1267,556,661,339,102,836,249
CACNG1_4_1290,1319,1577,1554,1000,1021,2013,2345,1866,676,1647,2482,94
9
CACNG2_4_1291,573,588,642,476,547,110,1428,840,315,855,1245,875
CACNG3_4_1292,94,54,209,128,0,226,428,0,1,0,127,122
CACNG4_4_1293,526,423,681,374,971,576,421,329,437,228,293,430
CACNG5_4_1294,345,353,639,398,88,300,322,254,14,501,933,311
CACNG7_4_1295,258,275,213,194,566,33,378,30,452,186,81,1031
CACNG8_4_1296,153,177,171,225,170,98,565,98,242,90,143,224
CATSPER1_4_1297,688,711,674,779,557,912,565,1436,78,1785,620,1100
CATSPER3_4_1298,1570,1523,2260,1525,1169,1454,3752,2055,1261,477,1672,
2236
CATSPER4_4_1299,179,281,137,136,31,36,108,43,0,1710,123,55
CCT8L2_4_1300,241,403,383,532,323,72,686,174,233,20,128,423
CD160_4_1301,1296,2173,2022,1693,2677,1769,1032,1886,1440,1166,3161,14
59
CD163L1_4_1302,337,377,324,392,315,1141,514,181,147,49,134,495
CD27_4_1303,381,674,373,704,77,84,228,319,285,41,161,679
CD2_4_1304,370,259,513,453,340,431,964,679,212,83,122,906
CD300C_4_1305,169,195,617,406,312,401,107,42,20,604,381,88
CD3E_4_1306,101,158,152,135,155,178,90,56,54,14,481,204
CD3G_4_1307,323,173,365,278,679,252,504,17,1312,83,206,158
CD5_4_1308,353,579,517,568,489,401,833,721,56,720,1006,322
CD5L_4_1309,1021,1304,1574,1178,284,1461,1929,1597,1332,1383,1722,1279
CD69_4_1310,2909,3254,3048,2828,934,2388,2072,3029,2699,1136,2911,2616
CD6_4_1311,551,839,685,386,929,171,1271,1675,1069,486,1478,883
CD72_4_1312,1354,1459,1391,1649,769,920,2737,1393,585,1562,1483,1012
CD80_4_1313,628,216,432,488,201,580,650,683,555,19,495,387
CFTR_4_1314,310,492,551,320,100,171,671,0,4,138,338,136
CHRNA10_4_1315,138,332,147,249,134,144,104,219,152,335,50,297
CHRNA2_4_1316,345,826,509,399,394,884,1377,458,444,151,325,690
CHRNA4_4_1317,1341,1591,2117,1835,1504,1117,2416,2342,1252,2470,2153,2
408
CHRNA5_4_1318,1331,1521,1685,1408,2345,1785,4408,2673,1243,2277,2588,2
485
CHRNA9_4_1319,312,353,695,643,450,219,734,133,35,421,84,685
CHRNB1_4_1320,310,207,335,197,39,47,5,182,90,0,478,824
CHRNB2_4_1321,129,65,60,108,172,89,2,3,252,0,89,7
CHRNB3_4_1322,2664,3187,2280,3370,2627,2816,2584,2399,2264,3247,2942,4
074
CHRNB4_4_1323,674,661,742,488,53,344,407,631,669,919,801,1205
CHRND_4_1324,331,312,206,86,125,536,366,38,5,1,343,357
CHRNE_4_1325,2037,2115,1834,1611,2210,1926,1584,2125,2075,3242,1484,15
77
CHRNG_4_1326,311,447,671,392,910,592,33,105,315,15,295,116
CLCA1_4_1327,716,930,698,520,997,1617,191,480,1039,119,1517,321
CLCA2_4_1328,653,687,467,566,950,831,83,350,312,4714,731,524
CLCA4_4_1329,1616,1336,1977,1380,2728,1118,1831,1498,620,1978,2067,925

CLCN1_4_1330,2316,1416,2330,2205,2813,3137,1904,2750,1909,969,1983,288
0
CLCN4_4_1331,398,599,517,652,636,677,305,707,136,11,1224,507
CLDN3_4_1332,222,292,374,498,350,880,347,53,349,1,38,1255
CLDN4_4_1333,458,410,351,221,541,150,158,845,119,24,331,309
CLEC1A_4_1334,91,167,75,166,333,9,63,3,146,118,1,1
CLIC1_4_1335,47,22,157,157,0,0,170,1,0,0,44,69
CLIC2_4_1336,1808,1615,2230,2055,1718,2309,2397,1514,1715,1446,1335,14
05
CLIC3_4_1337,159,463,200,116,435,9,806,124,28,2297,1360,450
CLIC4_4_1338,1154,1606,1980,1318,2055,2146,950,658,1008,2043,3578,2020
CLIC6_4_1339,269,527,525,553,344,1615,318,696,597,232,558,285
CLNS1A_4_1340,211,122,188,198,16,456,281,230,532,42,3,166
CNGA2_4_1341,937,1273,1217,1122,1540,1393,1572,1685,808,1495,1416,2533
CNGA4_4_1342,570,496,733,545,239,240,1032,275,217,1330,350,362
CNGB3_4_1343,641,970,882,843,171,1909,630,295,498,38,635,586
COLEC12_4_1344,323,506,780,448,192,494,577,176,150,561,986,316
CSF2RB_4_1345,870,415,546,532,1051,493,1095,512,1349,115,172,782
CUBN_4_1346,3490,3614,4324,3758,2279,2412,4179,3994,3057,2247,4378,553
0
DCC_4_1347,1237,985,1535,1537,1377,1336,2017,1147,1241,1901,2383,1915
DNER_4_1348,419,350,224,359,537,215,226,218,254,148,323,209
EDAR_4_1349,255,340,361,359,714,68,38,347,123,62,633,434
EPOR_4_1350,193,341,237,200,8,171,26,178,61,24,320,118
FCER1A_4_1351,949,504,429,336,250,335,725,406,266,1,741,1141
FCER1G_4_1352,549,408,355,383,339,299,1180,433,18,145,779,488
FCGR1A_4_1353,3037,3748,3786,3695,4605,4759,5583,3056,2327,3457,4204,3
977
FCGR2C_4_1354,468,1043,476,269,978,793,556,598,240,1622,1013,649
FCGR3B_4_1355,1156,1702,1243,1545,1429,2903,1482,2965,1562,190,1633,18
35
FRRS1_4_1356,2204,2614,3015,1718,2362,3435,3368,2369,2419,3104,3123,25
70
FXYD7_4_1357,592,631,718,652,556,453,1722,1193,822,21,832,925
GABRA3_4_1358,1373,1354,1108,1192,1545,160,1032,1081,355,719,725,869
GABRA6_4_1359,2409,1969,2495,2292,2943,2443,2034,2882,1183,3618,3176,2
939
GABRB1_4_1360,432,285,543,484,214,1172,589,443,82,0,816,812
GABRD_4_1361,237,301,371,241,96,334,451,359,72,21,1116,478
GABRE_4_1362,138,191,446,402,283,9,384,306,34,55,705,341
GABRG1_4_1363,5128,3989,4304,4515,3398,4573,4795,4509,3346,2565,6157,3
677
GABRG3_4_1364,563,621,1058,735,67,1274,374,204,184,1703,684,746
GABRP_4_1365,237,383,391,229,119,426,15,96,118,888,355,289
GABRQ_4_1366,367,258,344,422,292,188,36,119,9,74,16,483
GABRR1_4_1367,554,428,657,334,412,880,371,821,40,12,395,372
GABRR2_4_1368,7053,6213,6750,5341,6738,7169,4088,6308,3368,5413,7319,7
117
GABRR3_4_1369,700,629,473,553,380,133,995,1193,766,1881,183,310
GFRA3_4_1370,822,655,657,772,686,825,485,707,368,1591,467,388

GPC1_4_1371,371,143,331,329,1857,128,28,207,413,13,59,147
GPC4_4_1372,889,1051,1163,1026,724,414,519,1072,543,1006,601,1475
GPC6_4_1373,3210,2319,3595,3407,2306,2700,1580,1431,1786,2273,3834,355
3
GPR89B_4_1374,362,528,883,598,442,349,828,690,469,695,1059,741
GRID1_4_1375,126,98,87,280,36,131,19,31,494,245,101,373
GRID2_4_1376,1717,1832,1912,1906,3297,1819,1072,2029,964,734,2348,1778
GRIK3_4_1377,946,667,563,755,439,18,605,885,326,2408,469,863
GRIK4_4_1378,1124,924,731,749,631,826,1346,839,630,413,1116,623
GRIK5_4_1379,93,136,98,126,411,38,226,188,206,101,715,81
GRIN2B_4_1380,457,375,441,465,419,970,530,362,675,364,884,521
GRIN2C_4_1381,240,147,289,264,145,246,548,48,671,1004,189,424
GRIN2D_4_1382,85,28,14,44,71,0,3,36,14,0,7,17
GRIN3A_4_1383,478,413,811,525,234,150,907,1382,727,335,150,22
GRIN3B_4_1384,1350,1316,1429,1269,1673,1661,2004,2442,1513,354,1825,22
70
HAVCR2_4_1385,213,40,98,42,0,0,0,0,0,0,78,2
HCN1_4_1386,454,613,409,602,711,378,584,137,679,56,680,249
HCN2_4_1387,239,162,72,140,0,0,15,5,0,139,0,12
HCN3_4_1388,212,211,254,257,180,1,112,91,55,34,193,104
HCN4_4_1389,1429,1254,1701,1005,1574,323,2401,1284,2073,1426,1440,875
HLA-B_4_1390,101,205,103,144,372,2,115,262,17,0,209,213
HLA-
DOA_4_1391,1559,2256,1794,1688,1948,1954,1028,1574,1025,1686,2223,1208
HLA-DOB_4_1392,656,284,518,450,368,414,342,314,794,232,358,231
HLA-DPB1_4_1393,61,103,129,233,72,5,107,244,101,278,156,479
HLA-
DQA1_4_1394,6322,6528,6448,5950,5102,10232,4851,5379,5306,3994,7253,46
44
HLA-
DQA2_4_1395,6337,6691,7733,7157,9353,8542,10937,5964,6503,9365,9803,74
85
HLA-
DQB2_4_1396,1769,1646,2884,1564,911,2021,1552,2048,3129,2412,1860,1734
HLA-DRA_4_1397,167,117,272,178,116,495,67,29,110,7,103,18
HLA-DRB1_4_1398,42,53,19,85,90,0,0,1,212,20,0,69
HLA-DRB5_4_1399,1130,1443,1471,1228,643,828,1591,1193,708,689,2150,915
HLA-G_4_1400,1262,1417,1360,992,2850,1737,1271,2259,649,1642,757,1792
HTR3B_4_1401,67,165,140,115,7,2,48,3,408,0,669,299
HTR3C_4_1402,399,237,264,272,5,2,0,239,381,3,364,45
HTR3E_4_1403,543,432,742,540,234,381,1387,867,544,283,1459,487
ICAM1_4_1404,923,728,961,778,1116,282,312,591,1849,216,505,979
IFNAR1_4_1405,1189,1992,1635,1175,1255,839,2960,1211,2196,3074,1196,12
22
IFNGR1_4_1406,1041,1107,1306,934,859,366,818,1547,596,338,620,1267
IFNGR2_4_1407,218,123,301,183,84,18,443,160,1,154,157,92
IGF1R_4_1408,234,239,320,200,346,2,49,54,0,75,935,837
IGF2R_4_1409,2441,1953,1899,2203,2594,2510,3352,3015,1616,857,1592,272
6
IL10RA_4_1410,136,309,279,157,217,188,497,26,0,7,252,46

IL10RB_4_1411,555,340,588,446,762,925,415,245,525,329,622,481
IL12RB2_4_1412,1492,1676,2024,2109,1461,3408,1674,1229,1254,2551,1797,
2426
IL13RA1_4_1413,4055,3728,3409,3643,3717,4198,4620,5299,2433,2284,3850,
1818
IL13RA2_4_1414,746,1128,967,727,914,904,1203,1319,621,1822,918,938
IL17RA_4_1415,188,194,476,209,288,605,10,182,439,61,393,271
IL17RB_4_1416,1369,1534,1348,1396,1323,1160,1520,1916,1302,848,1353,17
03
IL18R1_4_1417,1266,1200,1524,1592,898,1995,312,1009,980,2170,3051,1185
IL18RAP_4_1418,1917,1640,1812,2041,2567,1592,2630,1539,2317,966,2363,1
538
IL1R1_4_1419,428,735,371,585,182,1594,856,344,706,532,609,876
IL1RAPL1_4_1420,1578,1194,1459,1628,3139,839,961,1788,1193,804,959,219
5
IL1RAPL2_4_1421,116,139,320,137,0,222,112,937,0,134,170,158
IL1RL2_4_1422,621,579,498,530,450,197,1226,837,704,241,955,937
IL20RA_4_1423,1719,1265,1793,1341,1039,1659,2423,835,1818,1422,2092,13
27
IL22RA1_4_1424,772,782,1152,729,981,671,1559,916,329,959,366,1395
IL23R_4_1425,1696,2067,2027,2022,2294,1436,2527,1751,1306,923,4243,141
9
IL27RA_4_1426,480,698,582,365,542,1240,77,552,849,497,667,1005
IL2RA_4_1427,1257,1166,1069,1401,470,994,1353,1400,1571,1740,486,1364
IL2RB_4_1428,1407,1410,1213,678,853,1080,1868,644,955,696,1435,405
IL2RG_4_1429,502,623,746,588,1043,1055,773,1478,223,73,863,457
IL3RA_4_1430,198,341,284,309,378,199,18,13,525,99,426,72
IL7R_4_1431,2160,1954,2058,1886,1989,1242,2422,2922,2274,1503,3730,239
8
IL9R_4_1432,858,774,671,786,1104,32,243,363,1867,207,407,875
ITFG1_4_1433,2067,1759,2092,2450,1660,1570,1761,1847,1972,1573,1935,17
21
ITGA2B_4_1434,180,175,406,141,307,13,1607,174,4,41,140,26
ITGB3_4_1435,705,809,975,775,1072,908,1084,1329,132,559,831,966
ITGB7_4_1436,161,453,309,246,87,608,109,473,366,0,263,718
ITPR2_4_1437,2601,4073,3379,3005,3307,2706,3040,3343,3377,3822,4591,33
31
ITPR3_4_1438,250,621,515,450,912,301,627,432,415,77,268,611
JPH3_4_1439,280,570,286,546,415,793,120,1061,455,87,693,306
KCMF1_4_1440,1200,534,808,900,904,541,671,760,1462,265,533,859
KCNA10_4_1441,552,703,897,882,356,86,1196,697,681,130,1375,525
KCNA1_4_1442,1451,1773,1720,2313,1900,2127,1440,1193,1732,2214,976,148
5
KCNA3_4_1443,94,85,56,119,541,263,0,0,625,0,400,40
KCNA4_4_1444,1521,1223,1488,1814,975,1020,778,1611,1614,762,1354,1279
KCNA5_4_1445,139,31,113,42,10,3,709,464,0,1,298,352
KCNA6_4_1446,277,212,139,156,56,45,36,15,3,152,201,383
KCNA7_4_1447,109,35,220,193,0,25,110,173,513,240,31,151
KCNAB3_4_1448,319,141,310,183,640,460,317,123,164,1,258,94
KCNB1_4_1449,231,275,202,291,121,368,502,553,1,403,79,27

KCNB2_4_1450,1019,1022,898,1143,863,594,1651,965,1386,14,1737,847
KCNC3_4_1451,113,40,77,76,0,1,60,10,62,0,142,11
KCND1_4_1452,182,344,386,343,287,541,3,306,129,76,190,39
KCND2_4_1453,205,261,374,357,134,549,291,319,484,565,224,279
KCNE1L_4_1454,1454,991,1888,1535,2470,1612,750,1467,1810,149,3392,1475
KCNE2_4_1455,404,528,535,742,1033,956,658,464,543,1262,303,457
KCNE3_4_1456,179,121,120,82,146,11,12,155,0,737,73,25
KCNE4_4_1457,232,325,395,309,107,780,1073,116,409,540,211,875
KCNF1_4_1458,239,94,40,61,422,72,179,79,43,0,19,607
KCNG1_4_1459,243,284,122,196,200,256,56,540,554,1,50,361
KCNG2_4_1460,935,841,1393,904,376,1171,1082,820,1366,837,913,1667
KCNG4_4_1461,907,1010,761,905,735,1448,1621,616,560,736,1686,1563
KCNH3_4_1462,1111,1196,2131,1150,1614,1298,1571,1769,1781,452,2032,650
KCNH4_4_1463,66,19,76,228,47,475,347,77,57,1,123,234
KCNH8_4_1464,1434,1691,1498,1255,2338,1155,899,836,1407,625,1729,1456
KCNJ10_4_1465,157,179,103,121,12,0,149,562,0,84,119,205
KCNJ12_4_1466,569,767,570,924,608,1382,649,1111,240,1245,349,152
KCNJ2_4_1467,1329,1078,1357,1286,500,1889,962,938,472,1680,1635,1498
KCNJ3_4_1468,815,1048,774,1204,282,1187,1591,502,484,1430,1176,1200
KCNJ5_4_1469,347,429,210,429,79,122,0,31,72,271,24,156
KCNJ6_4_1470,2024,1602,1605,1808,2631,2157,2300,1243,1230,727,3165,186
7
KCNJ8_4_1471,417,514,780,678,465,131,396,529,228,22,577,818
KCNJ9_4_1472,692,781,600,771,569,680,544,670,444,1068,755,1155
KCNK12_4_1473,354,388,643,466,163,865,1114,283,106,249,628,590
KCNK13_4_1474,769,834,1137,657,604,1398,15,1245,470,447,910,1594
KCNK15_4_1475,8092,7979,8536,7894,6751,7283,10037,6370,5635,7286,8077,
8196
KCNK18_4_1476,465,500,702,331,216,239,470,633,203,219,535,707
KCNK1_4_1477,286,264,81,92,0,414,65,718,266,17,58,76
KCNK3_4_1478,263,255,251,220,71,737,0,352,0,222,2,245
KCNK4_4_1479,770,948,966,702,821,437,2272,453,343,123,877,922
KCNK5_4_1480,487,327,135,235,229,39,154,322,232,601,22,290
KCNK6_4_1481,323,122,450,71,69,82,253,165,210,0,32,454
KCNK9_4_1482,843,463,498,498,1034,364,401,905,459,380,2109,802
KCNMB1_4_1483,404,767,511,398,301,876,411,580,116,1913,1214,704
KCNMB4_4_1484,344,335,686,440,78,502,405,158,559,393,317,341
KCNN1_4_1485,486,563,961,579,113,610,974,870,446,39,1704,1000
KCNN4_4_1486,455,238,48,166,753,982,647,2,0,77,56,202
KCNS1_4_1487,349,461,330,416,227,643,1221,481,266,801,240,400
KCNS2_4_1488,240,201,273,267,0,7,0,6,342,4,0,1254
KCNS3_4_1489,1947,2597,2747,2463,1802,1908,3275,3128,2894,2975,2719,25
30
KCNT1_4_1490,273,105,177,115,353,147,15,40,162,0,7,353
KCNT2_4_1491,45,112,104,74,63,1,357,71,30,298,32,76
KCNU1_4_1492,3738,3909,4363,4010,4465,4380,5615,2353,3119,1985,7595,37
20
KCNV1_4_1493,1536,1750,1466,1874,885,1994,1097,876,1594,2431,1744,2184
KCNV2_4_1494,224,238,376,200,237,228,204,963,466,471,8,450
KCTD10_4_1495,1686,2023,2268,1844,1190,1399,1243,855,968,373,1695,2590

KCTD11_4_1496,612,330,625,398,160,984,318,82,1335,105,582,607
KCTD12_4_1497,65,149,115,144,2,170,45,0,0,152,246,251
KCTD13_4_1498,488,430,377,378,209,746,1024,258,464,205,150,321
KCTD14_4_1499,1167,967,1303,1512,1893,1182,1716,1839,1610,1176,1878,15
55
KCTD16_4_1500,1352,1060,1392,1317,1305,1525,1474,872,969,397,901,1223
KCTD17_4_1501,397,357,337,320,153,273,336,219,181,317,109,21
KCTD18_4_1502,669,524,654,384,270,491,213,661,773,578,425,870
KCTD19_4_1503,366,535,591,427,189,76,62,184,316,696,366,561
KCTD20_4_1504,198,262,263,186,188,45,534,42,244,142,151,169
KCTD2_4_1505,689,510,504,898,1257,507,894,987,325,709,573,132
KCTD3_4_1506,4212,3848,4193,3986,3667,3527,4379,4212,2741,6542,6032,39
97
KCTD4_4_1507,5558,6014,6470,6301,6373,10026,5948,6382,5799,7559,8146,4
258
KCTD5_4_1508,170,364,541,341,805,528,509,759,279,434,221,532
KCTD8_4_1509,964,523,765,828,564,34,156,653,527,1082,849,709
KCTD9_4_1510,2443,2393,2977,2145,1274,2741,3714,1518,1374,1628,4860,21
63
KIR3DL1_4_1511,873,754,1039,824,405,816,853,793,482,341,619,2254
KLRB1_4_1512,596,712,614,564,1099,768,758,1456,881,1558,668,211
LGALS3BP_4_1513,144,181,151,231,212,7,19,513,20,231,4,86
LRP10_4_1514,63,178,232,44,209,192,400,175,12,0,35,69
LRP1B_4_1515,1639,1894,1825,1872,803,1008,3289,2151,781,29,2210,2768
LRP3_4_1516,676,530,793,782,413,558,462,491,772,358,781,768
LRP5_4_1517,856,551,632,430,514,483,767,663,942,310,1129,633
LRP6_4_1518,1209,1028,1115,1484,1228,1027,612,1046,1210,1044,1596,986
LRPAP1_4_1519,260,180,108,313,197,478,191,134,255,472,64,459
LTBR_4_1520,1056,882,1089,1407,1129,799,1167,1206,290,1337,906,1984
MARCO_4_1521,649,553,496,517,621,291,1042,71,688,59,194,864
MCOLN1_4_1522,46,168,83,255,7,210,248,68,531,1,111,521
MCOLN2_4_1523,1058,1080,772,1410,529,1307,1727,628,392,811,1917,1027
MCOLN3_4_1524,2277,2270,3235,2890,2069,889,3081,1725,1883,3172,3587,13
93
MCU_4_1525,915,935,780,887,1039,1146,1517,917,823,862,1247,817
MFRP_4_1526,1128,773,727,1276,434,1856,1180,602,379,960,896,1265
MICB_4_1527,112,206,129,98,210,258,15,4,1,779,0,61
MPL_4_1528,389,353,453,285,325,421,195,257,344,62,377,320
MRC1_4_1529,728,589,664,912,769,455,480,781,199,1004,369,953
MRC2_4_1530,466,310,596,575,54,50,1068,831,614,40,1691,339
NALCN_4_1531,1550,1421,1379,1364,2820,2098,3378,1635,1130,620,1801,134
6
NGFR_4_1532,658,996,1045,825,811,420,913,450,681,66,699,679
NISCH_4_1533,678,621,692,684,343,1388,558,623,1063,136,590,628
NPTXR_4_1534,95,160,318,196,4,272,195,679,305,186,64,327
ORAI1_4_1535,75,171,89,29,1,580,37,0,161,0,0,6
P2RX1_4_1536,555,634,860,633,603,388,169,806,133,176,734,557
P2RX3_4_1537,499,285,289,371,409,376,407,385,205,441,382,307
P2RX4_4_1538,3374,2573,2960,3066,2235,4412,3635,2229,2946,1810,4401,34
35

P2RX7_4_1539,492,475,549,813,606,1181,357,320,648,18,570,677
PEX5L_4_1540,2760,2559,3302,2909,2474,2935,2830,2625,1829,2815,4193,16
55
PGLYRP1_4_1541,177,176,58,98,338,302,483,254,14,33,48,61
PGLYRP2_4_1542,493,462,279,276,763,539,112,429,895,434,527,376
PGLYRP3_4_1543,538,559,718,597,355,280,1148,570,445,516,1283,1326
PGLYRP4_4_1544,785,1049,971,880,578,317,974,379,1536,753,1400,680
PGRMC1_4_1545,604,526,420,469,459,193,643,345,587,6,462,504
PIP_4_1546,319,312,160,192,155,1065,253,423,115,236,63,233
PKD1L1_4_1547,1724,1422,2031,1200,681,2168,2329,2471,1841,552,2234,165
3
PKD1L3_4_1548,805,539,1018,690,1368,434,336,819,807,1741,781,1104
PKD2L1_4_1549,515,416,517,610,458,1023,995,510,392,1009,985,602
PKD2L2_4_1550,1638,1743,2370,1912,1739,1287,1241,2086,805,1236,1947,19
19
PKDREJ_4_1551,1084,926,1088,963,1458,1685,663,993,1723,902,1626,296
PLXNA1_4_1552,104,95,120,148,86,29,3,0,51,0,416,143
PLXNA2_4_1553,173,190,109,79,91,27,99,362,14,836,12,318
PLXNA3_4_1554,189,306,194,72,530,97,440,142,157,0,288,452
PLXND1_4_1555,415,465,545,569,766,611,606,275,1320,42,1070,132
PRPH2_4_1556,847,618,939,724,356,612,486,933,316,98,971,929
RARRES2_4_1557,251,281,139,190,292,274,180,482,15,7,133,232
RASA3_4_1558,278,391,639,211,546,1138,79,606,741,1049,805,911
RYR2_4_1559,1545,2361,1308,1890,1637,1933,821,1477,1739,2655,2337,1823
RYR3_4_1560,799,961,945,1471,1354,468,1151,1056,2050,1153,2492,1287
SARM1_4_1561,175,231,224,335,759,193,299,274,41,0,361,205
SCN10A_4_1562,1660,1425,1570,1704,960,530,1334,2028,1277,770,946,2249
SCN11A_4_1563,8327,8034,9870,8467,7873,8040,10248,9773,5850,8186,11706
,8454
SCN2B_4_1564,380,352,455,501,515,86,858,565,796,516,457,327
SCN4A_4_1565,536,797,886,667,753,221,920,1404,1096,558,482,623
SCN7A_4_1566,1181,1441,1932,1128,1492,1491,1154,1248,1232,749,1618,151
4
SCN9A_4_1567,51,73,1,93,412,0,0,0,0,0,0,0
SCNN1B_4_1568,162,109,198,206,175,178,723,4,311,28,562,0
SCNN1D_4_1569,143,321,163,70,206,431,8,163,14,445,14,522
SCNN1G_4_1570,233,308,338,146,334,86,828,21,248,61,686,291
SCUBE1_4_1571,13,185,130,130,115,186,5,0,549,0,342,113
SELE_4_1572,111,183,216,79,151,170,26,266,17,16,12,178
SEMA5A_4_1573,408,456,587,572,299,77,1142,182,441,151,1182,587
SETD1A_4_1574,304,353,275,213,121,244,81,139,247,32,256,330
SFRP1_4_1575,183,222,269,264,38,28,297,741,52,386,200,97
SFRP4_4_1576,2236,2944,2333,2838,2114,2931,3912,1714,2690,940,2111,302
7
SFRP5_4_1577,761,772,662,583,148,1745,926,142,602,91,504,144
SHKBP1_4_1578,241,188,263,143,45,272,38,2,304,1,1093,160
SHROOM2_4_1579,244,534,442,657,377,215,265,274,468,222,443,340
SIGLEC8_4_1580,475,469,438,440,763,797,281,211,70,103,405,292
SLAMF1_4_1581,723,920,1343,957,772,493,284,636,1146,137,1131,962
SLC6A18_4_1582,337,279,186,83,87,156,413,64,254,501,2,9

SLC6A19_4_1583,820,628,734,672,486,504,800,374,900,1113,561,286
SLC6A1_4_1584,255,443,572,535,477,255,979,278,243,208,794,507
SLC6A3_4_1585,110,206,133,137,2,110,299,177,823,73,430,600
SLC6A4_4_1586,1085,1373,1242,1192,232,2697,1447,622,1276,224,1759,712
SLC9A1_4_1587,549,137,297,157,32,559,19,44,15,178,310,91
SLC9A3_4_1588,1031,740,1241,812,411,449,538,1495,490,469,1210,518
SRCRB4D_4_1589,164,299,369,221,1735,446,514,632,429,14,292,324
STAB2_4_1590,2002,1267,1677,1338,2038,2454,3676,569,2089,2048,1251,107
2
STX1B_4_1591,3077,2884,3298,3548,3318,3928,1414,2404,1837,7505,3371,39
09
THBD_4_1592,181,165,116,123,37,0,47,108,104,0,15,220
TLR1_4_1593,1571,1760,1672,1878,1427,1407,1335,882,1772,1427,2215,2108
TLR2_4_1594,2611,3087,2852,2760,2969,3357,3724,3067,2298,3816,3351,301
4
TLR3_4_1595,821,909,1173,622,1105,843,586,1168,1319,1335,2405,839
TLR4_4_1596,3328,2944,4125,3032,3194,1847,3184,3710,2279,2679,3833,181
7
TLR5_4_1597,359,296,352,362,553,290,154,283,316,29,319,361
TLR6_4_1598,2082,2276,2303,1984,1347,2488,2957,1760,1669,1378,1682,260
4
TLR7_4_1599,1672,1408,1646,1504,1367,2104,1476,1054,897,1328,2116,932
TLR8_4_1600,176,292,575,397,342,116,74,224,203,430,170,143
TLR9_4_1601,400,435,574,596,141,370,477,524,237,56,763,392
TMEM37_4_1602,209,185,139,218,24,29,292,83,97,818,44,77
TMEM38A_4_1603,1476,1932,2265,1995,1412,1885,2062,2803,1937,1525,2711,
1945
TMEM38B_4_1604,487,876,673,766,779,1769,1431,372,685,537,1656,797
TNFAIP1_4_1605,628,384,567,562,261,1570,1,729,13,55,265,192
TNFRSF10A_4_1606,1562,1797,1890,1722,1928,2051,1920,2398,1412,2072,106
3,1000
TNFRSF10C_4_1607,2136,2224,1933,1831,1907,3156,2998,1105,1487,1715,233
5,2145
TNFRSF10D_4_1608,249,160,125,374,736,131,621,280,16,346,531,42
TNFRSF11A_4_1609,384,330,297,301,801,662,953,312,432,883,399,275
TNFRSF13B_4_1610,204,222,274,293,217,322,930,1631,472,1,1805,431
TNFRSF13C_4_1611,131,21,59,40,9,13,79,27,10,266,0,172
TNFRSF14_4_1612,500,475,506,378,587,311,1176,580,142,607,79,443
TNFRSF1A_4_1613,2385,2758,3412,2807,3000,4844,3615,3703,1945,5433,3445
,3851
TNFRSF1B_4_1614,563,667,385,404,726,2275,203,316,375,523,570,495
TNFRSF4_4_1615,151,51,55,111,0,6,16,2,0,0,13,69
TNFRSF6B_4_1616,191,240,293,157,578,3,460,21,438,7,17,279
TPCN2_4_1617,428,336,527,444,281,676,505,274,285,13,238,227
TREM2_4_1618,108,233,368,62,322,81,130,457,44,1,129,216
TRPA1_4_1619,1116,978,1039,1265,1090,1615,1404,952,1691,673,855,1191
TRPC1_4_1620,1621,1889,1675,1576,1523,1959,1596,1838,1862,2584,3044,14
51
TRPC5_4_1621,108,197,481,198,373,190,116,273,5,112,801,60
TRPC6_4_1622,3549,4554,4358,3914,4019,3225,4424,4542,4020,4992,5452,40

57
TRPM1_4_1623,606,652,998,586,452,1703,264,1394,1021,180,676,562
TRPM2_4_1624,139,188,92,258,123,1824,703,212,702,291,101,300
TRPM5_4_1625,443,322,430,496,415,32,108,524,407,933,1145,751
TRPM7_4_1626,1953,2364,2331,2424,2851,3798,5195,1838,3055,3671,2651,26
93
TRPM8_4_1627,385,414,256,353,96,411,294,270,0,288,435,108
TRPV2_4_1628,318,620,175,143,532,109,325,543,117,964,180,419
TRPV3_4_1629,5628,5389,5085,4525,4679,4246,6707,3875,5004,5274,5637,44
40
TRPV5_4_1630,933,524,678,822,771,477,1400,1332,325,170,701,673
TRPV6_4_1631,139,276,337,245,493,591,720,165,515,90,251,268
TTYH3_4_1632,152,208,260,129,7,488,124,312,802,88,20,486
ULBP1_4_1633,1521,1885,1687,1740,1190,1186,1989,1441,1565,1723,1105,11
13
ULBP2_4_1634,282,499,576,537,144,441,181,267,459,838,1067,549
ULBP3_4_1635,206,245,349,291,164,95,2,12,84,217,420,0
UNC5A_4_1636,947,995,1038,1278,649,1129,1620,669,1342,1484,2002,1535
UNC5B_4_1637,1122,1088,1404,1005,1464,710,2631,1263,1032,615,942,842
UNC5C_4_1638,702,768,633,903,653,1025,716,486,1237,1600,1065,1314
UTRN_4_1639,1027,667,883,914,427,1792,682,1190,909,1402,784,1900
VDAC1_4_1640,191,178,165,166,288,81,14,132,165,9,45,15
ZACN_4_1641,303,313,750,525,878,426,162,393,437,1274,935,269
ABCC4_4_1642,376,333,606,711,236,237,480,304,162,378,192,471
ABCC9_4_1643,299,495,262,446,375,558,750,742,295,117,47,382
ADIPOR1_4_1644,611,664,869,378,463,735,354,683,433,112,1963,531
AGER_4_1645,1465,1437,1607,1112,2462,1043,1373,1264,2182,1657,764,1785
ANO6_4_1646,2258,2144,2591,2329,2623,3674,1788,1394,2266,1765,2703,220
8
ANTXR1_4_1647,5138,5001,5088,4888,5787,6330,9233,4804,4408,4017,4618,6
448
ANXA7_4_1648,1177,1105,1311,1525,2766,1613,1493,624,135,715,968,3150
AQP4_4_1649,62,75,83,121,2,0,74,174,29,0,22,144
ASGR1_4_1650,378,172,582,288,407,184,376,791,444,302,607,52
ASGR2_4_1651,941,1187,1219,1143,1265,605,2448,1920,1079,2143,2043,1654
BEST1_4_1652,249,159,312,319,294,199,143,19,41,2,407,587
BEST3_4_1653,232,535,680,689,82,936,638,1445,832,356,322,856
CACNA1A_4_1654,829,902,1092,810,587,913,1740,660,651,1763,1323,1186
CACNA1C_4_1655,33,48,90,138,1,5,123,2,13,383,250,0
CACNA1D_4_1656,1851,1891,2015,1867,1669,2807,1527,1765,1260,551,1811,1
865
CACNA1E_4_1657,867,846,1205,878,333,776,684,1003,370,187,889,822
CACNA1G_4_1658,287,310,255,434,527,797,823,765,155,11,105,247
CACNA1H_4_1659,756,890,717,1283,437,904,1260,251,977,501,1470,537
CACNA1I_4_1660,16,12,77,84,966,13,55,11,0,0,0,12
CACNA2D2_4_1661,99,56,168,162,228,403,116,159,0,192,98,26
CACNB1_4_1662,1598,1408,2243,1112,1332,2473,1778,1203,1743,966,995,222
4
CACNB2_4_1663,427,652,722,861,860,510,506,1445,405,432,1142,1806
CACNB3_4_1664,1148,951,1360,1252,1007,1062,1120,191,460,217,883,1103

CACNB4_4_1665,231,719,520,301,329,732,202,154,606,571,284,441
CACNG6_4_1666,803,659,851,953,2450,2155,256,1205,602,283,1507,187
CATSPER2_4_1667,1657,2109,2257,1696,1762,2053,1704,3266,2723,2039,3292
,2621
CD14_4_1668,398,240,404,401,210,263,598,463,16,138,6,329
CD163_4_1669,305,430,475,418,197,603,442,334,395,250,978,1142
CD247_4_1670,514,782,331,283,517,198,36,770,116,490,87,552
CD302_4_1671,2495,2253,2883,2428,2222,2947,2545,1586,907,1876,2792,191
7
CD36_4_1672,2015,2019,2284,2114,954,3331,3403,1361,1622,933,3386,2650
CD3D_4_1673,9232,9318,10627,8979,8416,9241,11821,8958,8026,8277,13451,
10590
CD40_4_1674,1504,1860,1932,1860,1811,1351,1261,1815,1193,1317,1990,202
4
CD4_4_1675,710,1078,1242,1089,817,1192,1413,1477,852,224,1786,873
CD74_4_1676,211,244,247,149,44,15,206,149,94,724,138,32
CD79A_4_1677,331,366,439,485,676,213,173,35,384,111,429,1276
CD79B_4_1678,484,329,226,128,59,52,615,50,89,91,1283,82
CD86_4_1679,419,631,716,435,181,745,417,1086,598,306,168,269
CEACAM1_4_1680,1296,1349,1196,1551,799,1364,2063,1055,2381,2442,2348,9
19
CEACAM21_4_1681,2937,2267,2518,2567,2599,2072,4220,1956,3622,3528,4542
,1348
CHRFAM7A_4_1682,3382,3804,3659,3754,3218,4636,5101,3199,1964,3701,3542
,2958
CHRNA1_4_1683,687,665,538,430,419,1077,440,657,392,280,89,1308
CHRNA3_4_1684,1689,1780,2027,1859,1945,2837,1474,868,1762,4574,1333,25
68
CHRNA6_4_1685,581,341,211,442,630,107,32,609,777,691,181,979
CHRNA7_4_1686,659,727,746,779,496,356,1017,891,1010,2519,1072,780
CLCC1_4_1687,2089,1708,2089,1849,1365,1102,1981,1282,1030,547,2842,263
9
CLCN2_4_1688,297,549,809,713,1106,517,824,384,845,1897,1470,569
CLCN3_4_1689,80,98,38,106,153,0,533,73,6,358,1,26
CLCN5_4_1690,717,601,618,494,2613,155,775,1320,988,2,1161,894
CLCN6_4_1691,195,12,203,372,298,181,62,351,3,197,107,2
CLCN7_4_1692,308,180,76,313,6,130,333,165,72,110,111,58
CLCNKA_4_1693,1535,1850,2213,2161,1756,2785,2470,1357,575,3611,2079,23
17
CLCNKB_4_1694,1535,1850,2213,2161,1756,2785,2470,1357,575,3611,2079,23
17
CLEC1B_4_1695,777,572,314,217,842,745,430,1326,978,722,429,399
CLEC4A_4_1696,1113,711,909,904,1041,270,1813,544,1133,394,644,2331
CNGA1_4_1697,410,508,440,509,515,141,573,411,301,228,547,329
CNGA3_4_1698,712,1190,954,1181,994,2429,670,863,810,1253,1515,1631
CNGB1_4_1699,158,254,259,211,962,2246,1201,125,0,1338,0,66
CNTFR_4_1700,117,84,4,106,0,0,0,15,3,1,129,0
CR2_4_1701,1643,1677,2106,1429,536,1064,2331,2009,2695,242,1828,1604
CSF2RA_4_1702,628,639,669,757,567,340,2678,301,909,904,1336,993
CSF3R_4_1703,1679,2136,1860,1677,1945,1054,1922,2882,1945,1602,1426,19

34
CXADR_4_1704,307,398,263,525,182,374,133,668,129,657,209,302
DAG1_4_1705,120,86,49,163,0,27,2,0,11,6,324,75
EDA2R_4_1706,2564,2962,2744,2242,2449,3917,5287,1742,2703,2683,3484,36
93
EDNRA_4_1707,656,672,857,971,1406,690,187,740,495,652,234,623
ENG_4_1708,273,535,383,485,333,263,46,589,946,1417,244,734
EVI2A_4_1709,1057,1276,1420,1626,1184,3403,1179,881,505,1749,698,755
FCAMR_4_1710,563,517,216,402,817,370,250,295,338,71,263,351
FCGR1B_4_1711,3037,3748,3786,3695,4605,4759,5583,3056,2327,3457,4204,3
977
FCGR2A_4_1712,1302,1467,1421,1391,1969,1309,1975,699,1524,669,2391,828
FCGR2B_4_1713,1513,1171,1542,1114,1807,784,1757,1216,1946,900,2855,206
9
FCGR3A_4_1714,633,628,784,648,911,1502,780,270,184,153,261,866
FCGRT_4_1715,167,427,341,348,128,198,72,99,271,182,1262,108
FGFRL1_4_1716,707,765,758,714,702,1175,468,415,473,300,1224,688
FLT4_4_1717,78,96,199,121,181,2603,369,163,91,95,21,372
FXYD1_4_1718,44,82,149,118,21,2,94,518,17,0,15,6
FXYD2_4_1719,431,283,157,367,1681,111,67,41,986,310,594,77
FXYD4_4_1720,1663,1818,1554,1631,1572,1763,2713,2071,3023,2116,2485,89
8
FXYD5_4_1721,233,153,238,140,7,39,91,158,210,0,42,144
FXYD6_4_1722,56,97,199,32,3,279,14,12,4,14,156,83
GABRA1_4_1723,717,498,776,510,225,215,27,212,846,330,1860,395
GABRA2_4_1724,2535,2333,2535,2126,2745,2938,3802,3179,2757,1568,3135,2
645
GABRA4_4_1725,553,262,479,473,1098,922,4,251,7,448,104,179
GABRA5_4_1726,1648,1455,1857,1263,2137,2050,4187,1547,2498,2051,1271,1
372
GABRB2_4_1727,863,1050,1654,996,1704,1228,1189,1155,375,1058,855,1326
GABRB3_4_1728,536,616,566,786,307,1339,823,290,82,552,1537,1464
GABRG2_4_1729,2611,2495,2993,2711,2593,2631,2883,2595,1906,1536,2353,3
074
GFRA1_4_1730,876,1004,767,833,1148,490,416,888,916,1350,1119,1201
GFRA2_4_1731,838,1142,776,1193,641,1145,1389,607,836,1142,871,1072
GFRA4_4_1732,443,187,151,369,717,828,0,41,2,64,134,18
GHR_4_1733,2457,2570,2501,3137,2409,2909,2453,4364,2551,3047,4148,3534
GIPC1_4_1734,263,225,416,317,167,683,384,580,174,14,227,429
GLRA1_4_1735,1852,1711,1880,1791,2513,1146,1741,739,1679,890,2420,1151
GLRA2_4_1736,1440,1290,1392,1422,1185,572,1765,1088,1381,653,1585,1555
GLRA3_4_1737,758,830,875,813,339,1007,1352,999,749,429,538,832
GLRA4_4_1738,1745,1678,2542,1611,1179,2589,1233,784,1520,2855,2803,155
9
GLRB_4_1739,303,509,621,381,414,209,0,500,371,524,488,328
GP6_4_1740,385,381,489,651,893,752,25,989,404,210,609,835
GPM6A_4_1741,1528,1213,1312,934,166,713,1333,898,1544,936,2603,763
GPR89A_4_1742,362,528,883,598,442,349,828,690,469,695,1059,741
GRIA1_4_1743,967,777,1039,716,1605,449,1768,733,411,1100,2180,1066
GRIA2_4_1744,4945,5516,6040,5874,6085,6260,6780,5711,3094,4626,4879,52

42
GRIA3_4_1745,237,179,85,130,194,43,2,561,291,0,133,97
GRIA4_4_1746,427,423,626,211,713,223,1381,467,16,923,57,366
GRIK1_4_1747,3386,3803,3688,2951,2233,3613,4267,3105,4712,2729,5139,41
03
GRIN2A_4_1748,560,881,723,944,456,1058,339,969,1340,1256,651,652
GRINA_4_1749,756,658,603,606,404,238,1546,870,1339,822,424,484
HLA-DPA1_4_1750,173,89,117,143,52,1,202,169,145,0,521,146
HTR3A_4_1751,435,363,286,531,383,876,353,56,387,62,50,441
HTR3D_4_1752,656,565,896,907,275,348,1211,305,1851,86,1464,832
HVCN1_4_1753,5624,5247,4718,5789,7191,8272,4408,7233,4689,3681,6656,65
63
IFNAR2_4_1754,2495,2503,2124,2129,1954,2724,4196,1722,1497,1508,2034,2
411
IL11RA_4_1755,254,187,148,86,131,354,245,0,333,0,185,274
IL12RB1_4_1756,523,380,470,262,460,1367,1285,191,760,110,331,537
IL15RA_4_1757,81,99,393,164,31,264,0,0,99,633,13,768
IL1R2_4_1758,119,184,294,141,448,175,219,53,40,10,368,330
IL1RAP_4_1759,2068,2189,1824,1785,1793,1546,2444,1584,2988,2049,2599,1
753
IL1RL1_4_1760,377,507,801,706,278,1342,510,134,454,18,697,319
IL21R_4_1761,532,553,481,785,394,281,280,489,229,877,1493,383
IL22RA2_4_1762,800,859,1157,638,491,1028,435,2654,134,921,1146,1280
IL28RA_4_1763,543,1045,952,326,682,340,1014,146,835,340,205,480
IL31RA_4_1764,1825,1528,2101,1972,1362,1161,2281,1022,1903,1432,1932,2
903
IL4R_4_1765,474,598,534,409,389,1015,941,1072,534,580,186,439
IL5RA_4_1766,790,795,788,456,533,617,963,620,1736,659,672,871
IL6R_4_1767,633,361,503,173,116,109,396,57,75,108,302,965
IL6ST_4_1768,6927,6599,10470,7218,5543,9270,8838,7057,5606,6137,12428,
8483
ILDR1_4_1769,3963,4181,3953,3241,3563,5640,4197,3666,3986,3573,6383,62
82
ITGB1_4_1770,163,279,203,176,276,238,471,61,104,131,224,397
ITGB4_4_1771,53,199,181,211,6,149,319,657,133,0,227,7
ITPR1_4_1772,1031,1262,901,1579,725,1169,1426,1385,767,1718,1582,1221
KCNA2_4_1773,1479,1343,1870,1228,1728,1290,2644,1414,940,316,2176,1872
KCNAB1_4_1774,2433,2283,2381,1781,2689,2199,2810,1259,1723,3318,2258,2
411
KCNAB2_4_1775,1318,1300,1310,1816,1324,1353,1048,617,283,491,1596,923
KCNC1_4_1776,157,419,124,253,600,340,0,542,76,598,512,197
KCNC2_4_1777,403,588,453,304,562,195,643,745,462,99,550,172
KCNC4_4_1778,124,101,540,84,30,29,578,251,285,219,378,297
KCND3_4_1779,497,453,443,549,608,260,618,267,258,145,767,1209
KCNE1_4_1780,241,273,154,310,190,203,15,4,261,29,844,455
KCNG3_4_1781,1117,1541,1078,1395,1907,761,1932,1372,1709,3305,2166,183
8
KCNH1_4_1782,205,169,62,178,251,0,216,668,2,293,119,215
KCNH2_4_1783,2460,2457,2955,2610,1453,2928,1606,2178,2608,2604,2888,25
81

KCNH5_4_1784,228,220,281,352,1126,155,95,195,71,0,252,559
KCNH6_4_1785,97,94,221,73,122,143,40,297,163,400,228,89
KCNH7_4_1786,621,897,1236,887,1092,1306,938,1015,683,244,1145,720
KCNIP1_4_1787,549,638,744,754,588,170,601,964,1517,1296,1296,401
KCNIP2_4_1788,522,399,772,879,220,279,438,676,34,709,210,323
KCNIP4_4_1789,312,224,210,353,610,912,246,14,151,165,444,439
KCNJ11_4_1790,862,1118,1387,773,723,725,1467,860,60,540,291,626
KCNJ13_4_1791,1826,1444,1224,1384,1971,1586,562,1288,1292,540,1462,157
8
KCNJ14_4_1792,249,387,284,552,575,790,125,73,41,11,222,263
KCNJ15_4_1793,98,304,55,130,122,142,238,0,344,72,348,61
KCNJ16_4_1794,1540,1481,1611,1649,1549,2415,2212,941,949,764,3027,1917
KCNJ1_4_1795,694,575,829,517,835,901,531,654,397,4,735,847
KCNJ4_4_1796,141,164,137,249,0,54,112,132,489,2,230,255
KCNK10_4_1797,150,212,343,523,491,106,106,173,15,45,113,106
KCNK16_4_1798,940,958,1263,944,768,787,1346,945,1265,577,1971,1343
KCNK17_4_1799,770,730,788,632,344,137,863,1162,353,239,524,978
KCNK2_4_1800,329,218,163,307,71,218,42,298,717,286,125,214
KCNK7_4_1801,191,80,152,72,1,523,242,58,105,561,145,464
KCNMA1_4_1802,1018,1535,1301,1074,1864,1145,1606,997,289,1113,3196,110
3
KCNMB3_4_1803,991,1086,896,737,1747,594,294,1452,1367,410,1639,492
KCNN2_4_1804,3237,3489,4268,4115,3919,5286,4627,4657,4072,4466,3413,31
49
KCNN3_4_1805,132,130,243,261,561,378,169,45,22,0,494,87
KCNQ1_4_1806,199,311,257,244,84,363,3,134,47,6,222,263
KCNQ2_4_1807,1911,2275,2025,1337,2334,3414,1326,1536,1293,1511,1815,24
62
KCNQ3_4_1808,442,284,235,302,51,332,23,98,0,99,279,721
KCNQ4_4_1809,529,329,550,555,1316,471,116,562,117,8,489,204
KCNQ5_4_1810,1548,1503,1879,1441,1185,1135,775,1915,1300,2705,1879,186
6
KCNRG_4_1811,852,1034,1093,1676,431,555,1023,1211,910,1899,760,851
KCTD15_4_1812,1181,1254,1521,1641,1790,857,1331,1351,1740,1926,1349,20
51
KCTD1_4_1813,2204,2675,3173,2166,3471,2287,3799,3026,1579,475,3047,178
6
KCTD6_4_1814,3027,2244,2155,2240,2925,3029,3757,1926,3206,1761,2178,25
54
KCTD7_4_1815,696,353,584,566,213,219,259,1099,144,244,1397,151
KLRC1_4_1816,1075,1096,1185,1388,1051,1076,2013,2025,359,1328,1285,178
5
LEPR_4_1817,586,779,639,667,186,432,1320,293,615,4,1499,360
LIFR_4_1818,1664,1699,1845,1680,2376,2040,2281,2711,674,1050,2473,2026
LILRB1_4_1819,1243,1299,1064,1140,1550,1346,1442,525,754,465,987,581
LILRB3_4_1820,344,278,351,462,280,101,282,117,827,25,395,247
LRP12_4_1821,3086,3512,3793,3683,4246,5671,6536,3219,2837,2851,5038,29
70
LRP8_4_1822,486,348,452,426,359,224,135,827,128,19,333,452
MR1_4_1823,1184,1307,1229,1059,610,1970,831,1311,334,697,752,1995

MSR1_4_1824,1521,1638,1478,1381,1595,1423,1910,1230,890,1332,1455,1245
NCR2_4_1825,870,1053,846,680,1008,1317,389,258,849,2476,1531,1083
NCR3_4_1826,682,720,643,530,1380,754,410,772,578,73,444,961
NOX1_4_1827,470,733,630,590,452,312,323,458,327,20,678,253
NOX5_4_1828,437,305,400,568,42,213,272,149,590,251,554,884
NRP1_4_1829,2061,2475,2830,1601,1445,3185,2173,2206,1643,967,2407,2288
NUDT9_4_1830,4,74,254,99,0,19,286,180,21,0,105,0
OLR1_4_1831,4354,4862,4157,5016,5341,5079,5823,3569,3072,3920,6961,424
9
OPCML_4_1832,312,266,303,329,755,191,460,188,335,734,422,418
OSMR_4_1833,1558,1629,1807,1919,614,3280,1413,1686,2690,1595,1654,1917
P2RX2_4_1834,1077,885,1153,825,770,1046,537,603,645,851,678,1302
P2RX5_4_1835,95,220,149,85,206,148,228,191,46,0,111,12
P2RX6_4_1836,383,366,231,207,185,12,472,705,314,8,619,672
PEX5_4_1837,242,239,382,303,295,233,491,338,72,798,1111,222
PKD1_4_1838,300,202,292,222,736,1113,1265,989,67,15,265,266
PKD1L2_4_1839,335,99,286,130,365,0,817,41,11,25,34,11
PLA2R1_4_1840,2180,2546,2875,2561,2700,1913,2652,3206,2188,1452,3160,3
887
PLAUR_4_1841,600,582,412,578,184,589,165,1028,386,521,394,479
PLXNB1_4_1842,342,431,471,390,480,592,765,441,116,44,134,849
PRKD2_4_1843,282,271,174,263,113,252,494,148,109,0,25,74
PRLR_4_1844,248,337,242,220,433,515,517,666,112,221,78,291
PTCH1_4_1845,1810,1931,2388,1735,752,1298,3176,1938,981,1002,2207,1723
PTCH2_4_1846,359,392,149,457,160,486,0,39,0,753,2,497
ROBO1_4_1847,722,616,585,782,744,1787,681,896,602,87,1027,1860
ROBO2_4_1848,583,318,439,592,298,874,90,669,576,7,406,318
RYR1_4_1849,1715,1193,2057,1494,2478,1958,1707,1960,696,1219,1738,3252
SCARA3_4_1850,250,282,92,108,340,19,147,284,85,0,501,30
SCARF1_4_1851,748,724,688,554,77,57,420,1343,187,471,533,134
SCARF2_4_1852,399,591,1002,663,150,398,809,916,614,95,666,1085
SCN1A_4_1853,4370,5132,5257,6529,3006,6443,5604,4497,4570,2696,7091,61
27
SCN2A_4_1854,389,560,389,461,1463,914,395,244,713,324,564,23
SCN3A_4_1855,2527,2299,2363,2579,2199,3735,2601,2391,1634,2110,3173,20
68
SCN3B_4_1856,56,318,173,150,89,177,60,1,29,682,160,112
SCN4B_4_1857,196,209,217,252,207,1053,91,194,270,151,835,71
SCN5A_4_1858,546,434,366,449,633,838,615,366,197,221,762,481
SCN8A_4_1859,1168,884,1011,804,803,202,1021,1228,364,96,871,951
SCNM1_4_1860,422,567,394,599,210,1177,146,890,955,969,847,1372
SCNN1A_4_1861,280,192,433,468,627,272,354,132,321,490,620,75
SLAMF6_4_1862,844,495,603,1463,346,847,611,1298,581,714,1067,714
SLC6A2_4_1863,189,103,240,270,74,35,3,98,0,4,230,86
TLR10_4_1864,610,469,407,242,432,93,681,444,726,42,652,502
TNFRSF10B_4_1865,1987,2181,1806,1837,2388,2267,1885,1656,2005,576,2203
,2053
TNFRSF18_4_1866,52,101,129,46,0,15,0,0,0,0,0,0
TNFRSF19_4_1867,347,428,269,237,623,81,83,890,181,229,79,346
TNFRSF25_4_1868,282,281,253,137,79,98,237,19,146,9,83,414

TNPO1_4_1869,1671,1153,1649,897,1619,2055,2475,1471,1774,660,609,1400
TOMM40_4_1870,1096,755,1054,1044,442,933,153,879,1088,1263,1625,1580
TP53INP1_4_1871,867,917,1180,1052,994,1436,1228,302,1340,368,1766,850
TPCN1_4_1872,208,584,383,263,281,342,681,165,34,474,363,128
TRPC3_4_1873,2032,2320,2506,2450,1650,1589,1139,2502,2391,2399,2309,14
98
TRPC4_4_1874,217,349,302,242,532,49,129,279,778,228,699,265
TRPM3_4_1875,481,363,251,388,56,202,28,523,2,734,59,456
TRPM4_4_1876,540,601,930,685,643,522,535,1235,509,269,899,786
TRPM6_4_1877,1617,1099,1695,1224,1581,626,756,867,744,216,1400,2042
TRPV1_4_1878,98,233,352,84,16,334,487,170,668,59,527,143
TRPV4_4_1879,81,79,47,87,2,44,0,0,118,3,0,37
TTYH1_4_1880,419,352,590,448,938,437,613,236,5,17,663,1230
TTYH2_4_1881,1257,1342,1663,1383,1951,1614,1421,1031,1028,1049,1391,17
41
VDAC2_4_1882,249,439,543,287,267,285,158,142,238,1080,977,1021
ANO7_4_1883,176,257,265,218,138,242,49,443,23,1,259,404
AQP1_4_1884,710,464,793,674,572,168,766,769,291,954,241,1026
CLEC2D_4_1885,260,380,277,364,912,118,1038,410,1174,93,160,296
CLEC7A_4_1886,4327,4342,5904,4418,5574,5448,8101,4904,4288,2808,6251,5
526
CRLF2_4_1887,911,816,924,786,1086,618,838,1025,411,622,1081,1057
FXYD3_4_1888,2968,2658,2960,2900,3582,1889,4598,1827,3843,2177,4319,38
72
HFE_4_1889,779,682,599,709,1405,605,386,1121,750,1516,904,1117
SCN1B_4_1890,1287,1504,970,1466,1149,841,1358,1561,709,701,1272,1719
TNFRSF8_4_1891,414,429,266,314,453,322,94,64,1,228,75,50
TSPO_4_1892,29,84,94,381,118,483,313,129,616,9,194,328
ABCC8_4_1893,32,134,49,53,112,15,471,42,0,0,164,248
AMFR_4_1894,200,426,208,401,322,19,64,89,133,1,17,192
ANO1_4_1895,302,269,231,76,5,464,193,227,5,1488,343,161
ANO2_4_1896,178,176,88,186,1,288,77,63,0,124,10,111
ANXA9_4_1897,632,599,351,360,53,760,1,60,245,1184,4,1381
AQP10_4_1898,155,89,66,59,177,11,66,229,6,0,42,275
AQP11_4_1899,406,340,286,263,537,176,601,83,162,43,28,285
AQP12A_4_1900,505,356,502,425,346,727,629,211,155,119,215,587
AQP2_4_1901,114,184,133,247,41,933,0,0,10,0,0,30
AQP3_4_1902,216,395,225,252,447,0,0,0,4,0,1171,0
AQP5_4_1903,418,240,439,351,191,398,344,435,233,506,585,256
AQP6_4_1904,160,71,74,81,12,78,439,196,0,0,87,149
AQP7_4_1905,313,259,187,238,180,833,185,42,128,16,125,50
AQP8_4_1906,551,717,585,614,535,377,402,439,258,665,244,530
B2M_4_1907,925,1022,1953,883,1872,1588,498,1129,1408,498,2295,1688
BEST2_4_1908,33,51,79,53,3,192,1,163,17,0,10,126
BEST4_4_1909,74,58,27,51,2,0,0,18,18,0,32,6
BSND_4_1910,966,299,427,362,154,1137,150,674,199,396,1222,369
BTBD10_4_1911,1098,963,1225,1125,1087,479,1009,821,405,250,1752,890
BTNL2_4_1912,104,34,37,39,230,185,300,16,28,0,16,72
C5orf62_4_1913,69,86,195,64,47,797,42,13,1,290,27,158
CACNA1B_4_1914,725,345,658,511,125,486,498,213,1555,1546,353,1243

CACNA1F_4_1915,41,18,44,88,3,155,8,0,0,0,5,156
CACNA1S_4_1916,578,658,505,525,456,321,150,994,587,371,602,327
CACNA2D1_4_1917,2246,1912,2795,2394,1955,3163,2268,2257,4382,4024,2860
,3711
CACNA2D3_4_1918,561,578,623,456,965,799,586,406,649,959,706,496
CACNA2D4_4_1919,363,637,390,471,117,109,2028,96,39,1170,597,679
CACNG1_4_1920,163,262,244,283,0,37,98,270,72,144,180,317
CACNG2_4_1921,201,306,418,305,327,369,438,190,1059,349,754,527
CACNG3_4_1922,3445,3491,3264,3293,2902,3060,5336,3214,2566,4294,4867,3
455
CACNG4_4_1923,3393,3776,4287,3454,3019,3041,3493,3391,3760,2853,3919,4
218
CACNG5_4_1924,392,131,128,348,136,107,0,474,236,2,714,392
CACNG7_4_1925,818,1082,1101,755,489,712,2331,728,1072,263,1038,1074
CACNG8_4_1926,150,95,48,35,4,9,0,0,197,0,36,7
CATSPER1_4_1927,47,26,80,21,115,55,18,80,469,207,29,52
CATSPER3_4_1928,774,1167,1152,884,828,1385,513,580,919,1421,1204,726
CATSPER4_4_1929,210,491,371,223,645,429,343,404,58,42,372,107
CCT8L2_4_1930,131,289,362,257,737,324,296,145,635,3,279,21
CD160_4_1931,407,353,212,287,318,439,631,297,168,30,313,439
CD163L1_4_1932,675,368,353,359,409,1255,1469,406,258,85,834,438
CD27_4_1933,984,1467,984,1332,861,945,590,965,722,876,221,1201
CD2_4_1934,1146,854,829,1114,1073,779,1872,843,419,535,816,944
CD300C_4_1935,254,300,150,294,551,101,334,304,112,0,153,206
CD3E_4_1936,463,678,606,555,790,1496,645,342,262,523,322,556
CD3G_4_1937,1253,1360,1582,1431,1346,616,2665,1713,1558,1033,1747,575
CD5_4_1938,6,58,61,38,170,0,0,254,53,0,0,397
CD5L_4_1939,769,865,1083,806,276,734,1497,420,508,346,1082,1450
CD69_4_1940,794,874,834,996,99,794,987,596,694,640,1535,272
CD6_4_1941,881,576,616,954,1057,560,101,591,525,1100,446,687
CD72_4_1942,2582,3094,2439,1972,3137,2871,2340,1503,1499,1249,3650,186
8
CD80_4_1943,610,425,385,223,535,136,94,144,639,496,964,245
CFTR_4_1944,985,914,1330,832,775,449,1353,1218,133,2431,1220,435
CHRNA10_4_1945,908,741,1247,819,995,1188,237,1155,107,427,1213,677
CHRNA2_4_1946,1341,1591,2117,1835,1504,1117,2416,2342,1252,2470,2153,2
408
CHRNA4_4_1947,384,506,399,657,366,976,537,677,696,353,714,417
CHRNA5_4_1948,610,569,613,685,89,387,1238,1010,234,612,1028,1387
CHRNA9_4_1949,1352,1869,1438,1158,826,1122,2935,1103,1662,964,461,953
CHRNB1_4_1950,38,184,109,69,0,0,100,2,0,0,465,82
CHRNB2_4_1951,300,288,160,164,291,21,5,422,269,13,304,462
CHRNB3_4_1952,781,843,1084,439,469,176,308,324,709,543,490,875
CHRNB4_4_1953,219,445,269,418,459,352,202,490,192,0,543,708
CHRND_4_1954,62,21,51,125,265,17,187,212,113,3,24,7
CHRNE_4_1955,546,890,744,804,339,996,1255,1469,774,185,600,436
CHRNG_4_1956,61,184,247,326,387,234,1,871,43,1,375,24
CLCA1_4_1957,834,743,929,813,909,395,679,715,667,78,1195,1000
CLCA2_4_1958,413,447,420,300,252,222,1102,274,810,1108,180,722
CLCA4_4_1959,1212,626,908,945,182,847,438,1059,870,488,742,1322

CLCN1_4_1960,602,604,749,453,442,917,312,425,303,1048,752,1062
CLCN4_4_1961,409,250,407,429,681,1192,449,521,32,270,350,529
CLDN3_4_1962,300,389,454,309,72,445,73,278,680,354,196,441
CLDN4_4_1963,83,32,40,207,82,0,0,31,3,557,567,0
CLEC1A_4_1964,741,387,484,346,91,251,646,515,237,619,743,210
CLIC1_4_1965,1174,1187,1265,1329,1113,974,3794,697,233,2471,2084,1074
CLIC2_4_1966,157,65,68,63,1,14,82,0,28,0,2,243
CLIC3_4_1967,293,340,480,586,584,357,275,404,639,222,972,698
CLIC4_4_1968,1417,1339,1702,1678,989,2793,2838,828,1516,3856,1970,1621
CLIC6_4_1969,305,469,413,244,397,1078,615,215,830,0,365,271
CLNS1A_4_1970,312,205,484,588,614,271,411,1024,397,109,209,84
CNGA2_4_1971,980,855,1071,785,1831,1582,1348,1718,1615,1780,2249,8
CNGA4_4_1972,412,465,464,357,250,524,633,370,364,257,455,207
CNGB3_4_1973,1814,2567,2551,2612,3659,1812,1904,1579,1716,2696,2728,23
24
COLEC12_4_1974,212,209,193,224,59,111,286,817,202,1011,143,458
CSF2RB_4_1975,175,132,248,221,20,587,120,450,86,0,756,332
CUBN_4_1976,2324,2395,2630,2165,2497,1902,2938,2037,1363,2717,2513,261
2
DCC_4_1977,232,413,213,321,482,277,1164,901,560,60,488,24
DNER_4_1978,555,592,662,566,431,29,699,523,223,166,1192,743
EDAR_4_1979,745,526,1029,871,1201,288,705,666,1412,2324,513,1673
EPOR_4_1980,230,218,107,393,108,51,390,347,72,51,217,174
FCER1A_4_1981,902,548,854,459,1186,132,223,461,307,805,1175,304
FCER1G_4_1982,1165,1043,1186,946,945,831,980,1397,1546,341,626,1279
FCGR1A_4_1983,1312,1033,1602,1587,2619,1154,655,1331,604,522,754,396
FCGR2C_4_1984,429,535,749,593,729,731,770,443,190,2709,807,879
FCGR3B_4_1985,1071,899,986,989,1260,653,674,890,1589,1268,1170,991
FRRS1_4_1986,142,224,320,352,58,55,5,222,129,130,50,210
FXYD7_4_1987,642,941,1039,637,613,340,845,1390,276,861,2010,141
GABRA3_4_1988,182,844,478,654,234,502,417,520,315,68,208,269
GABRA6_4_1989,0,74,23,57,0,0,0,0,0,0,0,64
GABRB1_4_1990,734,557,506,451,700,573,320,467,228,485,572,778
GABRD_4_1991,2276,2225,2078,1425,1502,2233,1223,3481,1468,2670,2926,19
19
GABRE_4_1992,17,56,127,14,4,32,125,27,0,0,2,21
GABRG1_4_1993,1043,1559,1113,1029,825,941,2205,1546,1308,1462,883,993
GABRG3_4_1994,279,317,462,370,220,935,222,396,176,33,635,399
GABRP_4_1995,4806,4538,4870,4488,6388,6104,7536,5750,3137,4468,5624,34
50
GABRQ_4_1996,184,246,161,188,624,204,188,44,221,24,695,112
GABRR1_4_1997,826,898,872,646,1102,340,1156,528,613,163,1165,660
GABRR2_4_1998,108,198,101,119,198,101,141,151,111,0,12,128
GABRR3_4_1999,323,467,395,411,964,302,217,420,104,35,203,224
GFRA3_4_2000,419,334,366,504,39,366,311,687,552,538,533,301
GPC1_4_2001,0,0,6,0,0,0,0,0,0,0,0,0
GPC4_4_2002,2264,1531,2124,1957,1781,1005,1701,2252,933,1279,2593,1445
GPC6_4_2003,950,1200,1093,970,703,593,2931,1266,710,1068,891,725
GPR89B_4_2004,6136,4835,5235,5713,5534,6316,7129,6233,2771,2409,7812,5
038

GRID1_4_2005,101,120,114,97,42,0,598,136,12,7,137,98
GRID2_4_2006,340,402,369,643,179,376,160,666,101,328,106,102
GRIK3_4_2007,406,409,425,493,481,356,400,322,187,1299,612,1014
GRIK4_4_2008,221,303,247,423,80,27,162,271,105,731,55,301
GRIK5_4_2009,457,300,399,319,739,375,953,13,239,588,308,497
GRIN2B_4_2010,1245,1467,1470,1924,1887,1100,832,1790,1171,188,3967,207
0
GRIN2C_4_2011,298,158,261,182,18,2,282,169,25,51,43,154
GRIN2D_4_2012,475,700,580,438,99,1123,1317,165,640,203,69,216
GRIN3A_4_2013,482,694,986,897,632,353,164,1711,154,2,1568,806
GRIN3B_4_2014,197,306,435,408,115,588,846,165,642,713,380,32
HAVCR2_4_2015,376,621,785,500,750,457,108,852,669,15,738,172
HCN1_4_2016,601,697,735,596,216,558,924,366,692,520,722,858
HCN2_4_2017,262,259,446,368,543,188,119,309,632,805,407,646
HCN3_4_2018,34,40,23,60,39,77,210,60,6,0,34,52
HCN4_4_2019,202,346,272,161,313,76,224,487,178,12,222,597
HLA-B_4_2020,473,335,355,387,630,797,725,658,618,123,1144,329
HLA-DOA_4_2021,176,43,321,46,37,115,109,36,80,86,63,293
HLA-DOB_4_2022,646,514,879,702,540,1008,1145,640,356,294,668,558
HLA-DPB1_4_2023,528,216,402,619,1029,804,135,210,270,1124,358,505
HLA-DQA1_4_2024,273,358,398,307,422,329,113,293,624,79,651,175
HLA-DQA2_4_2025,143,191,175,131,620,194,400,5,0,192,112,50
HLA-DQB2_4_2026,11,43,75,119,425,0,30,16,0,0,0,10
HLA-
DRA_4_2027,1190,993,1188,1011,1439,807,1621,2157,1457,3323,2763,496
HLA-DRB1_4_2028,434,680,417,444,313,558,1418,578,65,502,333,36
HLA-
DRB5_4_2029,1398,1724,1471,1932,1072,751,2565,1841,727,2760,2529,2419
HLA-G_4_2030,370,425,345,267,331,38,1034,767,407,101,378,334
HTR3B_4_2031,91,232,175,141,786,1076,2,253,171,0,51,793
HTR3C_4_2032,238,245,226,332,105,188,77,722,55,191,203,105
HTR3E_4_2033,1186,1641,2122,1826,1984,2559,2477,1315,907,1255,1533,181
4
ICAM1_4_2034,605,462,451,575,658,332,327,725,268,596,575,833
IFNAR1_4_2035,1303,1226,1640,1170,3043,970,1513,821,1608,1258,2007,111
3
IFNGR1_4_2036,754,415,676,868,438,647,955,1469,923,330,1021,1232
IFNGR2_4_2037,1208,582,1097,1046,1948,546,1545,1435,851,714,1497,989
IGF1R_4_2038,1840,2315,2976,2563,2986,2303,4939,1501,1253,2056,3686,21
73
IGF2R_4_2039,435,304,446,589,403,160,802,216,726,231,981,507
IL10RA_4_2040,426,499,215,362,356,274,11,221,250,766,1358,417
IL10RB_4_2041,2917,1867,2524,1676,2816,1568,2424,1019,921,1698,1276,14
95
IL12RB2_4_2042,1250,1413,1341,1709,1661,1213,972,1920,723,1110,2108,93
5
IL13RA1_4_2043,644,878,867,568,330,1089,2914,1406,679,1534,1427,884
IL13RA2_4_2044,763,879,805,819,668,1242,368,532,435,1032,1255,1285
IL17RA_4_2045,509,465,518,455,525,378,296,195,299,379,444,637
IL17RB_4_2046,1029,851,1227,1051,929,635,1708,867,298,704,1191,1158

IL18R1_4_2047,1710,945,1805,1131,927,1449,1140,2180,279,107,1969,3076
IL18RAP_4_2048,794,766,631,491,138,120,1535,108,346,14,343,424
IL1R1_4_2049,1647,2148,2274,1990,1941,2160,1065,2912,1882,2417,2159,35
19
IL1RAPL1_4_2050,1997,2565,1728,1985,924,3476,2959,1334,1588,3450,1274,
2875
IL1RAPL2_4_2051,808,544,451,700,1010,2157,51,302,773,104,786,507
IL1RL2_4_2052,397,299,855,352,268,450,888,943,413,403,1134,736
IL20RA_4_2053,1603,1786,1289,1514,1646,2269,2673,1923,1618,4267,1462,3
501
IL22RA1_4_2054,582,463,435,550,280,770,929,861,600,696,141,641
IL23R_4_2055,1235,1076,1624,1373,971,707,2660,1666,627,1099,2276,1220
IL27RA_4_2056,423,294,546,398,161,291,197,310,769,94,462,529
IL2RA_4_2057,321,286,270,498,611,19,418,4,131,5,909,254
IL2RB_4_2058,650,474,197,527,816,214,74,374,762,446,262,466
IL2RG_4_2059,984,926,618,1109,251,936,891,884,1608,825,954,1040
IL3RA_4_2060,1084,1235,1447,1503,785,2194,797,1788,786,992,1749,1919
IL7R_4_2061,463,643,976,645,248,1762,691,404,52,652,746,469
IL9R_4_2062,1107,973,1148,1034,665,900,1319,1488,779,1181,1526,759
ITFG1_4_2063,3022,2878,3751,2623,3245,3972,1961,4255,5584,2046,3255,40
01
ITGA2B_4_2064,649,703,931,1269,1321,896,1024,1298,217,4987,1222,2002
ITGB3_4_2065,388,412,763,375,604,201,613,805,146,282,419,529
ITGB7_4_2066,352,354,359,289,277,86,733,919,418,484,432,558
ITPR2_4_2067,1225,836,616,1015,1263,224,234,1068,777,596,887,568
ITPR3_4_2068,926,1161,821,1721,1079,1255,1532,1118,710,1811,1126,1410
JPH3_4_2069,10,139,143,11,24,0,661,1,201,0,2,1
KCMF1_4_2070,490,717,751,568,1225,872,233,792,156,303,503,299
KCNA10_4_2071,702,358,664,322,1,16,291,536,391,578,550,34
KCNA1_4_2072,1337,1977,1869,2139,1431,2433,1785,1656,3363,2824,2531,19
15
KCNA3_4_2073,1217,1000,1095,1243,1042,933,359,671,1833,588,572,1163
KCNA4_4_2074,283,418,610,381,407,232,1095,200,184,120,1267,606
KCNA5_4_2075,1476,1477,1871,1673,1299,2472,3267,2005,806,637,2047,1346
KCNA6_4_2076,1202,971,1384,1270,1958,1535,3016,734,1035,1042,1760,1352
KCNA7_4_2077,1416,1223,1873,1648,1267,2757,107,1374,1827,674,2644,1956
KCNAB3_4_2078,643,555,969,934,387,662,636,565,272,616,903,751
KCNB1_4_2079,455,380,239,298,545,394,379,92,268,35,368,191
KCNB2_4_2080,1259,1537,1220,1460,2181,3299,2651,1884,1101,2462,1268,13
71
KCNC3_4_2081,189,106,301,111,92,284,112,162,60,0,384,122
KCND1_4_2082,140,63,119,210,113,204,555,551,56,301,44,0
KCND2_4_2083,3074,2715,3137,3452,3121,2832,4170,2253,3460,3638,3467,24
77
KCNE1L_4_2084,2378,2442,2225,2369,2019,3456,4202,2740,1553,670,2027,18
25
KCNE2_4_2085,1244,1006,1219,1110,1706,1028,1537,1183,1208,435,773,1659
KCNE3_4_2086,4287,5046,5370,4280,4976,5207,7487,6361,3835,5510,4526,43
75
KCNE4_4_2087,3439,3403,3610,3289,2931,3594,6897,2691,4189,1730,2779,34

44
KCNF1_4_2088,343,330,186,162,497,71,66,15,2,319,226,826
KCNG1_4_2089,330,269,382,380,58,1664,345,183,261,39,232,346
KCNG2_4_2090,117,29,17,145,53,0,44,4,8,0,4,543
KCNG4_4_2091,510,612,725,599,944,278,472,978,432,1136,635,1153
KCNH3_4_2092,438,582,193,411,322,377,164,15,115,493,354,546
KCNH4_4_2093,267,226,306,300,358,3,431,42,25,0,146,170
KCNH8_4_2094,2816,3472,3509,3161,4001,1778,2114,3971,2332,5641,4331,41
63
KCNJ10_4_2095,118,238,115,97,348,42,634,46,39,8,72,372
KCNJ12_4_2096,205,166,203,197,331,256,88,37,64,120,306,295
KCNJ2_4_2097,252,457,192,232,56,379,7,519,661,64,1,104
KCNJ3_4_2098,106,52,108,68,0,86,0,0,0,871,0,286
KCNJ5_4_2099,340,484,274,583,68,121,0,116,70,260,60,161
KCNJ6_4_2100,49,110,211,81,22,175,14,0,182,0,676,95
KCNJ8_4_2101,729,934,1367,1382,889,1051,1653,1127,630,2089,935,907
KCNJ9_4_2102,62,155,129,53,25,533,42,256,9,132,161,38
KCNK12_4_2103,1315,1612,1691,1436,1155,1213,2741,2417,1815,1112,2156,1
566
KCNK13_4_2104,715,594,627,695,746,1156,655,1004,391,28,841,482
KCNK15_4_2105,351,377,469,303,573,146,1791,434,557,489,13,179
KCNK18_4_2106,460,706,604,612,745,1763,461,638,100,130,1511,1623
KCNK1_4_2107,573,533,510,638,241,225,1207,319,778,550,856,209
KCNK3_4_2108,697,819,780,855,1016,282,482,743,575,929,938,989
KCNK4_4_2109,336,93,198,70,0,583,69,27,282,3,182,450
KCNK5_4_2110,621,834,655,651,660,734,234,324,446,337,418,1077
KCNK6_4_2111,357,305,368,318,427,19,3,1146,512,32,75,589
KCNK9_4_2112,460,376,180,257,57,139,292,340,64,12,391,206
KCNMB1_4_2113,88,86,92,135,6,199,565,17,25,0,487,27
KCNMB4_4_2114,431,608,560,332,507,261,6,427,153,1465,959,526
KCNN1_4_2115,831,802,1110,1240,404,464,1205,964,903,1715,1033,796
KCNN4_4_2116,492,194,338,312,769,1516,1522,422,263,60,337,276
KCNS1_4_2117,448,387,401,361,393,944,974,516,411,1584,888,470
KCNS2_4_2118,332,527,543,715,439,720,142,851,229,1187,119,334
KCNS3_4_2119,383,204,192,203,7,139,7,76,84,0,49,334
KCNT1_4_2120,233,96,93,78,169,102,863,50,183,0,398,6
KCNT2_4_2121,917,1044,1260,1297,1273,321,888,919,208,783,1625,1371
KCNU1_4_2122,2153,1845,2805,1717,881,2670,4313,1548,1411,761,2114,1514
KCNV1_4_2123,1501,1783,1487,1905,769,1962,1097,876,1574,2431,1743,2184
KCNV2_4_2124,2437,2708,2598,2923,1165,4285,2753,2378,2109,2907,4444,36
69
KCTD10_4_2125,793,1015,1572,1297,1160,98,1032,1116,101,219,1325,882
KCTD11_4_2126,733,856,866,657,580,1353,223,420,647,553,726,753
KCTD12_4_2127,281,313,367,447,882,573,607,25,391,1417,323,11
KCTD13_4_2128,727,845,1038,736,1434,311,488,798,569,311,1405,1299
KCTD14_4_2129,431,231,481,279,518,141,1111,340,60,201,48,491
KCTD16_4_2130,355,518,518,65,33,489,745,23,1,0,787,8
KCTD17_4_2131,26,13,104,76,23,269,0,153,3,421,2,52
KCTD18_4_2132,786,869,1130,726,325,623,1666,1308,567,546,995,1379
KCTD19_4_2133,189,308,419,213,221,162,163,469,328,43,978,223

KCTD20_4_2134,1378,1373,1399,1337,1648,1884,4072,964,1367,1024,2412,97
9
KCTD2_4_2135,686,273,547,682,940,1466,405,705,487,845,367,114
KCTD3_4_2136,241,284,186,459,405,10,222,62,64,519,301,380
KCTD4_4_2137,1661,1898,1533,1319,849,2336,1338,1267,2308,2560,938,733
KCTD5_4_2138,68,182,87,178,80,322,664,96,663,0,219,5
KCTD8_4_2139,66,244,176,63,74,642,489,327,21,1047,197,37
KCTD9_4_2140,2854,3105,3321,2508,2886,4464,3649,3895,3106,771,3935,243
2
KIR3DL1_4_2141,116,221,300,276,378,501,1020,49,105,609,409,329
KLRB1_4_2142,1930,1802,2010,1956,928,2578,1286,2346,1994,2422,3933,185
7
LGALS3BP_4_2143,520,279,349,149,259,1104,606,461,719,36,119,169
LRP10_4_2144,748,722,542,589,113,557,215,488,621,2181,1239,1519
LRP1B_4_2145,318,291,540,428,233,596,261,188,335,120,321,636
LRP3_4_2146,493,390,428,658,131,556,318,84,881,191,1259,739
LRP5_4_2147,539,450,562,278,1043,466,367,2,358,4,675,256
LRP6_4_2148,1065,975,786,622,1060,2673,1855,1006,546,680,721,926
LRPAP1_4_2149,14,30,101,373,0,30,81,70,10,2237,220,104
LTBR_4_2150,41,109,195,134,34,0,574,0,261,511,477,0
MARCO_4_2151,159,343,126,190,34,61,9,357,152,451,526,121
MCOLN1_4_2152,535,998,1455,634,218,1159,132,1013,715,1042,803,682
MCOLN2_4_2153,547,356,368,272,73,316,0,393,0,229,740,295
MCOLN3_4_2154,707,689,1029,742,1071,1909,886,709,566,1466,1900,1351
MCU_4_2155,555,447,722,807,428,556,340,597,834,334,568,483
MFRP_4_2156,34,4,42,85,0,0,14,0,24,0,11,525
MICB_4_2157,178,107,165,148,374,17,29,195,956,0,164,129
MPL_4_2158,198,75,214,146,304,0,736,205,6,12,762,95
MRC1_4_2159,3578,4145,4070,5082,4380,3610,4139,4614,2529,3019,4908,406
7
MRC2_4_2160,277,363,340,496,63,553,422,281,368,111,860,1562
NALCN_4_2161,384,240,428,153,287,25,340,83,375,262,943,64
NGFR_4_2162,740,953,908,872,760,349,1381,526,752,16,707,669
NISCH_4_2163,691,549,811,640,689,554,1607,393,1037,217,1921,282
NPTXR_4_2164,1134,1300,1091,1521,815,647,2548,1297,1912,648,1308,1654
ORAI1_4_2165,10,188,103,126,0,1,0,1,0,0,603,1050
P2RX1_4_2166,316,301,440,532,606,328,99,269,251,207,1035,594
P2RX3_4_2167,377,550,296,377,105,80,0,512,63,250,773,157
P2RX4_4_2168,642,398,337,244,161,33,560,124,197,77,541,183
P2RX7_4_2169,272,245,164,190,398,500,618,235,72,1329,194,325
PEX5L_4_2170,535,360,335,314,693,880,122,626,351,716,342,879
PGLYRP1_4_2171,442,522,622,720,108,233,394,423,265,134,1054,518
PGLYRP2_4_2172,175,277,320,311,165,280,300,713,292,93,405,428
PGLYRP3_4_2173,932,1106,1037,1064,1509,694,1190,1725,611,858,833,481
PGLYRP4_4_2174,2383,2143,2654,2297,2325,3225,3588,1660,1594,1097,4093,
3237
PGRMC1_4_2175,1783,1751,1789,2160,2327,1903,1942,2070,2251,1154,3670,2
710
PIP_4_2176,263,319,222,499,139,36,36,76,81,1683,168,126
PKD1L1_4_2177,923,694,1013,611,473,623,1130,412,1080,1161,792,948

PKD1L3_4_2178,998,718,697,736,695,701,637,432,562,718,1167,800
PKD2L1_4_2179,1286,1767,1600,1292,958,2591,1835,1168,509,2149,1318,133
4
PKD2L2_4_2180,637,633,610,554,415,577,310,590,164,20,553,552
PKDREJ_4_2181,767,696,1058,730,1919,1021,1094,1155,278,2289,932,1004
PLXNA1_4_2182,744,891,691,606,493,598,289,189,313,42,293,911
PLXNA2_4_2183,784,1065,867,1377,958,1237,1092,1007,911,315,1134,508
PLXNA3_4_2184,21,121,47,25,3,62,0,319,0,0,0,2
PLXND1_4_2185,249,228,106,243,650,311,102,104,6,424,98,455
PRPH2_4_2186,765,1008,861,766,308,1647,882,807,828,1105,1682,1624
RARRES2_4_2187,766,403,449,556,226,1708,61,386,638,247,399,536
RASA3_4_2188,856,592,855,651,908,814,254,519,188,344,559,934
RYR2_4_2189,2440,2405,2174,2585,2518,2216,3841,2229,2638,2752,2361,187
1
RYR3_4_2190,1084,1494,2043,1234,1552,1289,1719,1934,2033,296,2511,720
SARM1_4_2191,162,153,86,104,149,46,190,114,18,237,68,28
SCN10A_4_2192,534,391,489,578,772,273,829,288,636,606,497,986
SCN11A_4_2193,1267,1408,1204,941,935,348,957,581,718,217,1430,1646
SCN2B_4_2194,544,447,448,532,201,433,1416,547,1093,367,523,174
SCN4A_4_2195,6330,6238,6791,7362,3897,7982,6683,4776,5136,5326,8394,71
79
SCN7A_4_2196,2208,2003,2009,1822,2993,2786,2498,2364,2149,1933,1854,36
30
SCN9A_4_2197,1123,797,842,1118,1418,1243,1486,1233,587,1012,1645,1180
SCNN1B_4_2198,555,784,892,854,608,1041,96,1169,1043,578,1016,569
SCNN1D_4_2199,58,0,54,129,5,162,6,0,3,0,235,0
SCNN1G_4_2200,260,343,443,519,37,73,321,32,3,292,731,608
SCUBE1_4_2201,408,760,871,780,373,1249,665,560,456,494,442,423
SELE_4_2202,338,441,287,281,241,125,254,612,407,194,25,330
SEMA5A_4_2203,2231,2416,2369,2147,2434,989,2494,1248,1611,2563,3030,37
91
SETD1A_4_2204,124,145,183,80,0,233,71,64,31,0,751,26
SFRP1_4_2205,38,75,320,181,0,313,7,357,324,414,269,115
SFRP4_4_2206,51,1,0,0,0,0,0,0,0,0,0,0
SFRP5_4_2207,399,475,372,470,820,756,1198,477,437,280,1403,172
SHKBP1_4_2208,126,131,129,267,490,516,195,20,1,0,204,187
SHROOM2_4_2209,703,705,723,778,1904,887,1036,974,1112,390,612,866
SIGLEC8_4_2210,268,285,205,189,13,241,82,226,111,0,72,33
SLAMF1_4_2211,1944,2088,1879,2089,1339,1345,1815,1346,1235,3047,1040,2
153
SLC6A18_4_2212,1260,1007,1001,1213,874,2853,2070,544,1300,517,1285,677
SLC6A19_4_2213,3857,3400,3307,2699,3746,2802,3283,3328,3915,1027,3502,
2640
SLC6A1_4_2214,462,675,474,306,127,1072,219,219,280,0,350,224
SLC6A3_4_2215,325,339,161,206,215,243,1,14,543,127,178,632
SLC6A4_4_2216,840,722,1094,739,831,1468,1572,572,261,1367,1285,1289
SLC9A1_4_2217,59,33,80,114,6,32,13,0,229,109,18,409
SLC9A3_4_2218,717,685,834,670,714,736,573,728,529,152,893,917
SRCRB4D_4_2219,254,178,109,116,418,1,8,67,453,0,114,536
STAB2_4_2220,1454,1485,1148,1938,1709,1905,1345,1391,615,1589,1544,117

5
STX1B_4_2221,490,519,248,159,118,95,0,143,577,1,787,126
THBD_4_2222,505,842,1075,680,1075,57,758,238,348,21,356,1838
TLR1_4_2223,339,318,615,437,432,281,616,515,990,899,576,529
TLR2_4_2224,268,240,346,304,38,272,334,151,262,449,386,200
TLR3_4_2225,419,225,417,336,253,60,1156,486,251,49,339,572
TLR4_4_2226,2487,1997,2469,2085,3290,1706,2200,1949,2067,629,2572,2712
TLR5_4_2227,1212,1387,1241,1450,906,1349,1038,2444,851,140,1617,1950
TLR6_4_2228,1042,646,754,508,934,1290,822,390,721,1156,988,795
TLR7_4_2229,1674,2149,2169,1059,2231,1958,4181,3200,1970,434,2066,2609
TLR8_4_2230,2229,1961,2232,2204,1880,2024,1488,3355,2371,2681,3761,272
1
TLR9_4_2231,565,403,757,619,438,368,1003,448,637,723,800,556
TMEM37_4_2232,1281,1508,1246,1351,1019,2099,1186,2331,1469,794,1208,84
8
TMEM38A_4_2233,858,989,1146,973,486,1064,1085,685,387,1058,945,1837
TMEM38B_4_2234,2189,2163,2446,2552,3848,1038,2951,2300,1810,263,2153,2
271
TNFAIP1_4_2235,724,719,698,657,841,745,1,1008,705,77,408,1063
TNFRSF10A_4_2236,1410,1462,1923,1156,1708,2191,1785,1064,2792,1696,107
6,872
TNFRSF10C_4_2237,372,556,473,316,395,572,504,909,919,103,74,1208
TNFRSF10D_4_2238,1032,1300,1473,1229,1151,2353,1165,2189,1038,855,730,
967
TNFRSF11A_4_2239,646,510,878,754,158,980,121,556,89,208,596,216
TNFRSF13B_4_2240,249,76,77,103,0,130,0,220,209,0,0,80
TNFRSF13C_4_2241,897,1152,1209,1357,373,630,177,685,767,835,3218,1314
TNFRSF14_4_2242,3233,3352,4387,3173,4357,4008,3410,2746,4102,4468,3108
,3625
TNFRSF1A_4_2243,677,635,848,748,599,374,945,791,359,544,449,647
TNFRSF1B_4_2244,592,391,565,221,527,862,64,455,1383,1950,313,452
TNFRSF4_4_2245,87,174,63,228,158,983,0,0,78,39,69,207
TNFRSF6B_4_2246,382,355,265,414,14,148,318,353,187,1267,1103,1046
TPCN2_4_2247,273,131,192,360,341,0,345,25,449,27,265,861
TREM2_4_2248,949,733,895,788,1418,1062,217,836,455,492,1074,603
TRPA1_4_2249,675,195,122,224,8,77,136,360,342,620,467,275
TRPC1_4_2250,892,741,635,550,1526,280,920,819,306,695,852,804
TRPC5_4_2251,1322,849,1135,828,1488,528,694,1085,1834,1118,1670,612
TRPC6_4_2252,881,870,1072,950,1026,1058,576,800,600,915,1126,719
TRPM1_4_2253,392,339,437,359,281,506,312,947,97,12,234,360
TRPM2_4_2254,36,25,55,36,0,0,0,122,13,0,2,91
TRPM5_4_2255,517,714,496,638,378,624,517,605,568,1839,492,426
TRPM7_4_2256,130,153,149,278,47,45,141,477,272,287,280,3
TRPM8_4_2257,570,870,1020,1014,691,494,1067,2069,984,355,728,669
TRPV2_4_2258,360,320,833,502,464,658,1480,231,199,1,1791,1054
TRPV3_4_2259,250,358,534,581,106,660,1042,226,174,1743,480,960
TRPV5_4_2260,398,541,880,313,72,536,308,591,1532,1,1139,352
TRPV6_4_2261,101,172,155,17,20,1,26,0,219,0,391,28
TTYH3_4_2262,328,278,513,344,215,178,117,562,167,158,304,98
ULBP1_4_2263,2139,2530,3405,2714,3386,2070,2146,2248,2031,2505,1899,18

89
ULBP2_4_2264,407,344,265,267,71,138,305,181,119,6,529,97
ULBP3_4_2265,782,662,942,721,778,479,541,766,461,207,3009,370
UNC5A_4_2266,203,227,58,206,0,1,70,12,130,9,323,109
UNC5B_4_2267,741,585,493,595,651,301,768,744,826,586,830,289
UNC5C_4_2268,4136,3418,3977,4249,3953,4295,4702,2979,1785,3161,3921,48
76
UTRN_4_2269,786,545,809,825,270,971,717,427,903,1401,774,713
VDAC1_4_2270,349,299,224,562,233,4,293,288,320,348,646,529
ZACN_4_2271,1441,1342,1849,1848,2348,1149,1281,1988,633,2365,3818,1814
ABCC4_4_2272,2624,2951,2384,2583,1978,3230,2356,2569,2099,2538,3035,27
93
ABCC9_4_2273,551,525,600,463,644,753,212,762,620,333,1288,513
ADIPOR1_4_2274,780,898,788,875,991,1128,293,1286,1463,501,1360,1399
AGER_4_2275,31,0,12,12,0,0,0,1,3,0,0,0
ANO6_4_2276,2217,1967,2311,2229,1400,3782,1627,2002,1993,3420,2052,239
9
ANTXR1_4_2277,350,783,781,651,827,635,1223,735,688,2805,1044,597
ANXA7_4_2278,918,542,606,751,402,1833,402,560,402,32,822,852
AQP4_4_2279,555,286,840,820,951,228,618,451,237,5,635,805
ASGR1_4_2280,28,141,168,329,30,6,0,542,54,0,0,443
ASGR2_4_2281,1925,1615,1890,1625,2710,2956,2217,3050,718,2831,2750,240
5
BEST1_4_2282,566,686,475,646,881,62,1943,906,626,754,827,444
BEST3_4_2283,251,553,406,298,597,387,530,479,256,1045,492,94
CACNA1A_4_2284,905,501,987,842,1403,317,606,179,316,908,1244,907
CACNA1C_4_2285,6767,7168,7596,6928,5536,6580,7606,5900,5023,4566,7223,
6367
CACNA1D_4_2286,1322,1440,1677,1722,930,1675,1323,1759,672,3589,2269,20
31
CACNA1E_4_2287,722,716,959,857,275,1067,1365,2194,265,686,1180,474
CACNA1G_4_2288,1189,1419,1210,1313,1124,868,1222,1322,741,707,1368,157
4
CACNA1H_4_2289,255,411,690,422,109,51,926,292,674,1,1092,761
CACNA1I_4_2290,742,1212,1043,679,1528,475,693,823,500,281,825,655
CACNA2D2_4_2291,373,716,606,947,659,1999,432,138,267,877,897,943
CACNB1_4_2292,672,901,755,880,486,1186,655,548,1200,1789,1499,950
CACNB2_4_2293,1454,1346,2052,1274,1006,1770,1463,1694,1628,1110,673,19
60
CACNB3_4_2294,61,282,204,166,1261,779,15,38,189,50,45,363
CACNB4_4_2295,318,510,265,863,851,826,590,640,821,17,884,789
CACNG6_4_2296,379,650,520,506,782,371,1030,211,576,4,386,892
CATSPER2_4_2297,2868,3144,3570,2888,3326,3355,3045,3134,2132,4894,2801
,1806
CD14_4_2298,91,48,377,54,321,241,0,54,15,34,11,210
CD163_4_2299,641,750,799,922,344,356,1142,727,590,970,817,447
CD247_4_2300,595,1038,797,551,164,922,1253,810,440,86,586,610
CD302_4_2301,3207,3492,4128,3529,3262,2414,3364,2422,3021,3413,3266,44
47
CD36_4_2302,904,1062,1356,974,810,1223,731,1233,985,145,902,662

CD3D_4_2303,8741,9036,10261,8309,9956,9039,12359,9138,6359,8569,12470,
11155
CD40_4_2304,221,189,355,119,87,28,125,260,38,313,41,54
CD4_4_2305,116,246,123,124,105,389,99,254,329,0,232,69
CD74_4_2306,886,1092,782,1024,1074,426,756,719,769,1030,1851,588
CD79A_4_2307,48,25,32,45,29,328,0,604,0,0,0,141
CD79B_4_2308,91,63,166,65,4,8,788,8,64,0,215,75
CD86_4_2309,3114,3817,3458,3610,4884,6320,5748,3157,2091,1955,3584,253
0
CEACAM1_4_2310,397,306,434,343,146,219,430,137,982,431,972,755
CEACAM21_4_2311,2302,2139,2680,2399,2450,2842,3117,3033,1459,1137,2400
,2952
CHRFAM7A_4_2312,2058,2159,3064,1832,2611,1287,2882,2692,2851,1511,2020
,2014
CHRNA1_4_2313,609,271,725,575,501,8,694,3,272,1338,402,708
CHRNA3_4_2314,304,304,215,211,296,932,35,555,23,65,65,99
CHRNA6_4_2315,2490,2089,2694,2294,3167,2601,3600,2910,2368,2105,3185,1
864
CHRNA7_4_2316,294,372,403,391,1322,300,59,622,308,308,1127,598
CLCC1_4_2317,481,888,806,813,848,302,979,845,1231,926,1055,1468
CLCN2_4_2318,590,841,855,649,408,349,465,560,823,798,965,1348
CLCN3_4_2319,747,835,863,1031,456,1141,1316,600,1792,1276,1413,1189
CLCN5_4_2320,1567,1633,1956,1900,1086,2585,1987,2069,889,3198,2949,265
0
CLCN6_4_2321,1080,1223,1406,891,512,1049,2894,598,1817,2408,1653,1579
CLCN7_4_2322,91,289,276,208,118,12,90,371,200,3,254,192
CLCNKA_4_2323,559,669,477,541,315,964,329,693,310,298,551,333
CLCNKB_4_2324,1858,1974,2634,1497,735,1023,2731,1797,1476,2018,1673,15
82
CLEC1B_4_2325,845,1090,1116,537,458,982,631,230,1743,3849,894,562
CLEC4A_4_2326,410,407,468,395,1007,571,53,612,531,1903,686,165
CNGA1_4_2327,2717,2366,3266,2237,1055,1788,3290,1848,1314,2962,3150,22
51
CNGA3_4_2328,1038,1098,1233,1100,1305,2207,1817,1927,995,1405,1246,171
2
CNGB1_4_2329,580,467,431,695,64,99,381,1057,28,641,181,217
CNTFR_4_2330,533,448,244,560,1,614,453,546,332,24,779,176
CR2_4_2331,1345,1183,1795,1023,983,1404,733,1866,1517,459,1140,467
CSF2RA_4_2332,1828,2111,2024,1897,3366,2361,2353,2693,3957,1714,2018,2
785
CSF3R_4_2333,345,492,167,385,113,489,34,33,572,2139,46,289
CXADR_4_2334,684,742,681,797,283,715,1546,1242,560,174,1540,317
DAG1_4_2335,798,570,698,823,1975,153,1255,707,526,76,446,491
EDA2R_4_2336,798,757,828,758,848,664,336,1244,2185,1144,813,927
EDNRA_4_2337,446,646,663,570,308,722,358,1240,79,266,584,312
ENG_4_2338,112,239,85,263,18,310,367,7,18,685,28,343
EVI2A_4_2339,679,678,877,795,111,1685,1163,1408,1079,1366,1203,1828
FCAMR_4_2340,248,506,295,412,235,1352,318,431,81,361,1039,470
FCGR1B_4_2341,1312,1033,1602,1587,2619,1154,655,1331,604,522,754,396
FCGR2A_4_2342,2289,1921,2595,2315,2010,4392,3297,3570,1049,989,2189,14

61
FCGR2B_4_2343,69,415,336,229,73,449,2,653,173,463,19,338
FCGR3A_4_2344,1071,899,986,989,1260,653,674,890,1589,1268,1170,991
FCGRT_4_2345,577,554,822,611,500,290,1271,296,263,16,895,284
FGFRL1_4_2346,665,1075,923,951,546,1744,969,626,868,466,1466,1030
FLT4_4_2347,388,182,415,369,440,912,540,454,342,648,26,700
FXYD1_4_2348,1287,1281,1329,1081,1862,1061,1514,1076,1171,3808,1862,14
09
FXYD2_4_2349,882,950,738,1068,1435,891,973,668,438,569,1187,1333
FXYD4_4_2350,111,577,278,251,652,231,138,364,430,232,431,27
FXYD5_4_2351,205,491,518,625,561,578,1057,438,394,447,463,449
FXYD6_4_2352,647,483,405,437,534,516,1260,434,627,1365,835,564
GABRA1_4_2353,509,361,312,418,31,228,434,3,539,547,527,157
GABRA2_4_2354,1411,1471,1594,1812,1044,867,2419,1165,1500,1182,1223,39
10
GABRA4_4_2355,2158,1911,1997,2462,1743,2207,2148,1591,1713,757,1741,30
02
GABRA5_4_2356,202,74,132,149,45,521,0,146,0,0,0,84
GABRB2_4_2357,1817,1758,1812,1755,1241,2248,1557,1046,457,1640,1641,31
17
GABRB3_4_2358,806,724,996,980,619,1648,1203,730,576,1017,572,1121
GABRG2_4_2359,1502,1190,1376,1288,856,1192,742,1307,786,1975,1881,1966
GFRA1_4_2360,1964,1795,1837,1669,1771,734,1029,1801,151,3002,1611,1115
GFRA2_4_2361,1227,1255,1876,995,1525,2306,1562,737,1365,565,2170,957
GFRA4_4_2362,125,221,207,71,10,4,537,34,106,44,123,248
GHR_4_2363,239,255,244,344,679,95,850,208,0,85,766,276
GIPC1_4_2364,221,463,625,412,443,81,123,594,163,12,705,242
GLRA1_4_2365,347,329,420,224,643,569,339,508,150,726,514,326
GLRA2_4_2366,616,552,693,762,386,504,566,321,177,813,244,473
GLRA3_4_2367,148,376,336,365,205,972,141,280,700,236,194,270
GLRA4_4_2368,10,65,7,19,226,24,310,0,1,94,0,2
GLRB_4_2369,2010,1950,1910,1806,1643,2465,1361,1372,1559,3551,3136,284
6
GP6_4_2370,317,447,397,321,536,327,1703,778,667,348,509,447
GPM6A_4_2371,308,269,225,193,34,312,22,91,371,747,290,273
GPR89A_4_2372,6136,4835,5235,5713,5534,6316,7129,6233,2771,2409,7812,5
038
GRIA1_4_2373,639,621,1041,640,1243,1472,739,529,260,2126,970,898
GRIA2_4_2374,1607,1235,1487,1521,1433,2096,1635,1150,1463,726,1542,220
9
GRIA3_4_2375,1090,1308,1473,1487,1124,2036,995,2568,789,358,1583,1805
GRIA4_4_2376,2448,2727,1981,2312,1511,3677,3575,2720,1730,1857,1138,25
72
GRIK1_4_2377,701,876,991,887,785,724,1012,528,738,881,1139,713
GRIN2A_4_2378,539,438,798,520,1484,964,1110,710,1489,287,411,469
GRINA_4_2379,1819,2513,2464,2253,1676,1407,2849,1134,1259,1888,1435,23
16
HLA-DPA1_4_2380,811,871,1008,440,395,1146,2466,1026,1306,868,341,1722
HTR3A_4_2381,576,676,372,647,539,49,1603,605,678,737,1229,326
HTR3D_4_2382,592,899,863,732,1000,1344,645,756,718,1528,763,237

HVCN1_4_2383,218,234,337,389,28,31,1209,189,410,186,101,363
IFNAR2_4_2384,678,790,940,567,516,527,561,820,897,100,1107,544
IL11RA_4_2385,597,688,931,849,650,937,1052,318,422,110,593,782
IL12RB1_4_2386,1401,1230,1445,1458,2122,764,1348,1393,1238,390,2911,21
83
IL15RA_4_2387,279,285,354,269,371,32,1121,1047,452,203,236,558
IL1R2_4_2388,270,512,357,305,277,569,76,134,25,1061,168,288
IL1RAP_4_2389,746,719,794,685,1030,1997,1164,584,1734,773,1750,1106
IL1RL1_4_2390,475,498,1093,631,130,263,914,1016,385,387,1172,795
IL21R_4_2391,363,745,520,824,551,216,1534,1022,88,630,1491,94
IL22RA2_4_2392,226,237,221,243,261,379,125,369,125,187,144,147
IL28RA_4_2393,522,616,527,285,823,892,1479,197,480,539,1034,314
IL31RA_4_2394,348,253,480,439,93,286,323,198,94,521,1274,906
IL4R_4_2395,582,454,766,630,810,1630,1873,1024,323,1033,555,460
IL5RA_4_2396,353,684,629,727,492,642,1187,349,33,925,806,1133
IL6R_4_2397,475,179,248,531,427,208,869,1197,86,5,1101,471
IL6ST_4_2398,622,664,834,607,360,729,677,1439,793,189,1219,1257
ILDR1_4_2399,739,437,515,406,320,2885,539,867,257,1539,553,845
ITGB1_4_2400,590,651,662,622,1045,1302,339,1402,860,1128,1528,1441
ITGB4_4_2401,114,86,117,58,57,41,411,0,180,3,5,0
ITPR1_4_2402,1517,1432,2330,1414,2731,714,2381,1331,580,554,840,1700
KCNA2_4_2403,1,0,0,0,0,0,0,0,0,0,0,0
KCNAB1_4_2404,661,826,519,859,353,698,1443,655,788,550,359,1317
KCNAB2_4_2405,2166,1977,2026,2176,3205,2568,4127,1557,2311,2795,2726,3
222
KCNC1_4_2406,1210,1376,1676,1166,721,1301,1466,1121,2031,1605,1451,216
1
KCNC2_4_2407,3466,2345,4063,3218,2378,4664,4043,3053,4019,2410,3855,37
44
KCNC4_4_2408,1520,1942,1495,1965,2328,1177,2673,1275,1835,1382,2027,16
42
KCND3_4_2409,131,85,99,85,2,273,0,430,0,2,804,260
KCNE1_4_2410,754,963,585,585,677,1023,1130,931,615,558,1141,1290
KCNG3_4_2411,229,110,293,126,22,82,262,686,7,118,205,600
KCNH1_4_2412,510,556,209,171,155,199,28,436,960,684,14,580
KCNH2_4_2413,130,235,298,197,115,79,516,327,3,0,158,562
KCNH5_4_2414,1928,1591,1461,1885,3439,1544,314,651,1834,1967,1895,1878
KCNH6_4_2415,271,209,285,171,91,114,227,12,56,23,264,135
KCNH7_4_2416,759,998,766,594,1097,1032,547,620,337,477,1328,827
KCNIP1_4_2417,254,329,568,226,144,376,306,318,964,475,785,187
KCNIP2_4_2418,426,484,356,479,795,224,57,10,635,1,663,327
KCNIP4_4_2419,503,468,918,346,450,1011,638,726,38,335,1097,337
KCNJ11_4_2420,325,639,278,206,159,46,385,83,65,0,105,922
KCNJ13_4_2421,1347,2506,2507,1629,1891,3060,2719,1644,1803,1377,3563,1
716
KCNJ14_4_2422,764,811,1216,956,600,708,1007,625,403,497,1640,772
KCNJ15_4_2423,613,848,811,884,548,303,1343,389,1205,2250,1825,896
KCNJ16_4_2424,414,499,623,384,374,296,451,895,303,110,200,632
KCNJ1_4_2425,865,1271,1700,1146,1381,1542,2192,952,134,121,2077,1272
KCNJ4_4_2426,220,157,134,225,475,31,110,322,1,11,0,10

KCNK10_4_2427,457,567,374,413,1732,0,274,416,3,1,640,223
KCNK16_4_2428,209,161,55,232,670,0,781,530,9,199,30,294
KCNK17_4_2429,299,238,237,192,410,1100,140,362,339,253,154,165
KCNK2_4_2430,4115,3873,3490,3076,4195,5292,3234,3872,3818,2945,5521,55
23
KCNK7_4_2431,180,89,92,35,21,203,0,156,5,11,0,406
KCNMA1_4_2432,195,132,269,289,109,270,627,65,41,35,113,251
KCNMB3_4_2433,1707,1891,1787,1672,2288,3019,1109,1975,1263,811,2470,93
6
KCNN2_4_2434,313,239,476,367,71,427,828,348,35,162,120,355
KCNN3_4_2435,130,116,198,267,229,10,71,20,20,111,525,105
KCNQ1_4_2436,571,425,678,627,387,937,210,1033,197,9,240,745
KCNQ2_4_2437,710,849,722,634,505,279,449,519,465,1034,610,1285
KCNQ3_4_2438,68,84,76,99,324,28,0,11,0,0,142,13
KCNQ4_4_2439,641,733,486,355,641,1909,220,246,801,511,530,690
KCNQ5_4_2440,2565,2763,2562,3163,1601,2034,2036,2426,1125,1711,2932,25
34
KCNRG_4_2441,4236,4261,3790,3368,4817,4016,3748,3199,1912,3684,6916,41
62
KCTD15_4_2442,129,151,230,176,305,503,38,331,119,402,405,266
KCTD1_4_2443,347,667,556,371,335,241,1030,303,95,1206,663,761
KCTD6_4_2444,476,392,794,939,1041,548,728,183,1034,9,125,1215
KCTD7_4_2445,171,548,54,170,52,410,172,835,69,399,0,12
KLRC1_4_2446,1246,1181,1361,1016,1673,1055,2676,911,540,2250,1948,2174
LEPR_4_2447,1116,1353,1854,1576,2249,431,1011,2303,649,2171,1607,1820
LIFR_4_2448,479,459,221,225,203,180,116,181,7,0,215,620
LILRB1_4_2449,795,720,554,387,1053,1269,683,427,460,103,980,723
LILRB3_4_2450,251,84,124,114,41,131,0,52,161,4,276,78
LRP12_4_2451,1062,1352,1198,901,2090,1576,1806,2606,1309,523,2379,1942
LRP8_4_2452,269,235,162,230,56,20,192,703,250,711,82,798
MR1_4_2453,931,1088,1197,968,898,1790,871,1062,582,1406,637,1829
MSR1_4_2454,972,1249,901,1125,740,444,1241,1373,896,691,1277,1401
NCR2_4_2455,785,721,1210,547,61,1142,280,1359,567,63,777,1204
NCR3_4_2456,160,131,213,56,536,242,376,144,277,0,18,192
NOX1_4_2457,1902,2440,2567,2079,2959,852,1526,3294,2677,3267,2849,2598
NOX5_4_2458,1522,1664,1558,1493,956,1716,1811,840,1199,1805,1896,1238
NRP1_4_2459,406,308,275,323,114,460,440,355,37,1192,154,132
NUDT9_4_2460,1816,1620,2168,2017,843,764,319,904,1684,1796,2643,1839
OLR1_4_2461,754,614,713,761,636,1157,1064,315,447,549,932,1481
OPCML_4_2462,99,83,278,254,201,772,38,49,144,0,886,297
OSMR_4_2463,986,1181,1441,944,542,2556,876,758,257,1135,978,761
P2RX2_4_2464,420,377,542,542,754,374,270,77,657,566,192,81
P2RX5_4_2465,2421,2791,2539,2659,1167,2917,2098,2402,3019,2538,2483,20
87
P2RX6_4_2466,684,495,668,899,781,1600,228,429,120,38,78,1554
PEX5_4_2467,257,362,247,490,190,410,770,136,72,193,307,174
PKD1_4_2468,371,133,237,211,185,419,716,303,0,669,93,234
PKD1L2_4_2469,113,199,120,62,72,220,398,149,0,20,13,29
PLA2R1_4_2470,3229,2708,3321,2929,3433,3194,4794,3060,3396,1502,5029,2
204

PLAUR_4_2471,1183,909,1131,1191,1552,2223,494,1985,690,1378,829,1165
PLXNB1_4_2472,35,106,44,264,28,305,8,451,2,1,12,517
PRKD2_4_2473,209,347,149,476,367,767,18,11,144,0,127,8
PRLR_4_2474,197,224,65,340,20,549,106,48,340,0,454,244
PTCH1_4_2475,363,398,750,720,587,680,915,643,253,63,172,910
PTCH2_4_2476,307,346,315,291,689,178,0,10,81,0,437,210
ROBO1_4_2477,684,486,593,665,551,877,663,433,652,773,1005,465
ROBO2_4_2478,800,521,1359,777,551,377,235,174,397,2340,991,904
RYR1_4_2479,137,275,140,262,12,275,53,0,594,259,208,178
SCARA3_4_2480,629,298,494,718,364,246,484,18,476,104,462,1513
SCARF1_4_2481,538,409,594,227,762,750,497,536,534,774,1293,1105
SCARF2_4_2482,175,200,141,186,309,467,197,130,198,535,185,270
SCN1A_4_2483,8327,8034,9870,8467,7873,8040,10248,9773,5850,8186,11706,
8454
SCN2A_4_2484,4370,5132,5257,6529,3006,6443,5604,4497,4570,2696,7091,61
27
SCN3A_4_2485,586,483,372,300,573,0,154,1210,106,481,297,440
SCN3B_4_2486,646,1314,1009,982,270,959,1204,616,786,2022,1300,828
SCN4B_4_2487,801,933,1018,718,318,539,1272,828,441,702,1820,913
SCN5A_4_2488,122,75,166,102,28,0,236,115,0,0,60,0
SCN8A_4_2489,931,686,1014,1233,353,779,1657,690,294,855,1082,944
SCNM1_4_2490,80,150,168,92,83,8,0,37,203,2,0,28
SCNN1A_4_2491,252,233,104,192,1153,149,62,10,0,37,62,565
SLAMF6_4_2492,614,1059,701,634,971,605,738,482,875,242,656,327
SLC6A2_4_2493,831,885,994,1038,1020,752,1103,468,533,362,1939,878
TLR10_4_2494,408,565,622,951,247,461,2386,930,229,814,1075,543
TNFRSF10B_4_2495,391,199,293,278,731,200,208,410,257,153,471,370
TNFRSF18_4_2496,185,247,442,284,44,860,313,147,24,1,200,2
TNFRSF19_4_2497,525,645,709,750,1648,867,275,1638,494,297,406,738
TNFRSF25_4_2498,484,636,619,434,451,414,710,267,845,635,466,796
TNPO1_4_2499,1199,1749,1451,1360,1771,1863,2231,1571,1324,1690,691,135
9
TOMM40_4_2500,611,579,437,424,268,311,1658,1490,295,1482,1568,913
TP53INP1_4_2501,537,301,600,743,542,198,1028,2242,142,725,539,695
TPCN1_4_2502,1131,619,1016,1074,1521,1445,484,454,1261,294,2130,590
TRPC3_4_2503,3626,3770,3870,4843,2888,5169,2856,4735,2801,1404,3508,71
51
TRPC4_4_2504,609,439,521,705,396,723,208,752,123,683,611,697
TRPM3_4_2505,112,182,231,211,153,14,726,5,31,1,241,168
TRPM4_4_2506,397,258,382,471,412,32,108,524,407,852,1013,809
TRPM6_4_2507,1766,1175,1721,1474,938,1113,4439,1023,3111,1450,1542,877
TRPV1_4_2508,529,770,685,871,1079,1635,1620,709,755,1529,546,1166
TRPV4_4_2509,638,972,617,1147,1237,966,567,364,2578,2040,1650,497
TTYH1_4_2510,2004,1985,2518,2237,2298,1653,3829,1887,1161,1424,3470,37
49
TTYH2_4_2511,633,781,861,831,294,719,194,701,450,279,1138,558
VDAC2_4_2512,589,465,229,611,842,225,578,145,82,48,482,1121
ANO7_4_2513,97,133,184,72,11,5,74,89,16,3,646,144
AQP1_4_2514,299,321,374,405,320,1385,731,283,916,1058,216,643
CLEC2D_4_2515,264,162,139,151,322,78,20,303,118,74,8,34

CLEC7A_4_2516,791,668,635,1086,279,1452,1916,1024,407,300,783,417
CRLF2_4_2517,599,295,529,437,540,606,61,701,37,502,2504,585
FXYD3_4_2518,460,267,397,635,1235,752,477,834,166,42,720,445
HFE_4_2519,293,170,180,271,202,670,79,202,290,16,121,7
SCN1B_4_2520,194,197,120,282,0,601,61,351,223,0,35,221
TNFRSF8_4_2521,270,93,216,223,137,1173,0,46,126,22,82,458
TSPO_4_2522,1024,1092,1295,833,1857,825,1130,476,1385,951,1813,984
ABCC8_4_2523,902,702,1335,785,1105,1026,1573,822,412,871,1426,1365
AMFR_4_2524,124,221,304,377,452,128,45,377,545,33,238,176
ANO1_4_2525,258,420,628,82,61,487,598,923,109,820,430,133
ANO2_4_2526,2653,1957,2687,2678,1699,2425,4378,3124,2534,1192,5038,185
3
ANXA9_4_2527,503,378,453,441,91,155,67,232,976,540,0,524
AQP10_4_2528,848,1148,1294,1045,1192,2154,454,950,1000,317,1327,583
AQP11_4_2529,1394,1599,1630,1208,2091,1782,719,1458,1023,2347,1590,231
9
AQP12A_4_2530,1534,1364,1825,1513,1807,2294,1883,2094,1582,2097,2401,1
036
AQP2_4_2531,334,298,637,522,8,1117,791,30,26,381,1073,38
AQP3_4_2532,456,529,319,555,816,672,575,348,523,1645,770,665
AQP5_4_2533,256,100,215,292,1,21,1140,561,419,789,393,483
AQP6_4_2534,824,756,886,1026,817,674,217,577,1318,550,932,1797
AQP7_4_2535,577,269,291,437,623,289,320,175,1161,770,593,247
AQP8_4_2536,257,491,749,321,147,91,35,190,62,893,178,1243
B2M_4_2537,1637,2043,1848,1765,1742,1769,3449,2501,1973,4112,2562,1845
BEST2_4_2538,475,455,268,304,239,521,187,369,456,973,330,784
BEST4_4_2539,204,172,353,247,347,43,72,27,187,344,54,104
BSND_4_2540,1142,700,412,363,965,241,584,778,718,405,514,566
BTBD10_4_2541,531,574,670,578,344,734,835,1095,390,683,313,809
BTNL2_4_2542,138,301,105,172,17,45,0,0,0,0,110,305
C5orf62_4_2543,320,243,269,134,15,371,123,307,225,50,112,385
CACNA1B_4_2544,516,485,475,306,673,606,675,78,299,1254,1188,440
CACNA1F_4_2545,133,225,330,382,195,358,304,28,521,104,516,593
CACNA1S_4_2546,991,1186,1057,789,440,930,786,1295,755,654,1017,1580
CACNA2D1_4_2547,981,846,1046,770,2024,1689,1283,488,383,1950,1415,1020
CACNA2D3_4_2548,3237,3490,4211,3919,3597,4814,5546,3224,2880,4660,5079
,2569
CACNA2D4_4_2549,475,288,247,513,519,1074,306,284,1010,78,327,734
CACNG1_4_2550,0,0,0,0,0,0,0,0,0,0,0,0
CACNG2_4_2551,167,65,66,11,418,0,59,0,0,51,74,456
CACNG3_4_2552,149,239,107,224,105,0,453,118,7,698,47,359
CACNG4_4_2553,803,776,553,398,227,1006,248,412,469,543,938,1246
CACNG5_4_2554,1054,1230,1594,937,943,833,2340,719,1089,431,1246,1207
CACNG7_4_2555,608,574,411,446,224,556,877,863,364,119,802,808
CACNG8_4_2556,671,473,975,790,752,1260,1060,1562,670,1420,1243,668
CATSPER1_4_2557,315,327,259,345,404,203,207,84,203,209,128,553
CATSPER3_4_2558,189,309,261,356,185,461,0,248,25,0,142,409
CATSPER4_4_2559,218,190,423,597,210,1,329,361,319,2002,303,234
CCT8L2_4_2560,109,126,210,170,11,5,5,101,93,321,290,142
CD160_4_2561,690,832,638,860,310,1212,896,1139,1407,1434,1103,1077

CD163L1_4_2562,604,888,966,806,889,603,517,381,363,1597,1071,178
CD27_4_2563,616,737,659,646,1420,438,378,584,445,923,603,1637
CD2_4_2564,947,730,776,910,891,864,929,236,1136,703,1199,853
CD300C_4_2565,926,628,786,601,880,326,704,363,690,694,1158,528
CD3E_4_2566,1028,587,733,653,1026,28,817,705,293,146,843,597
CD3G_4_2567,1284,1287,1158,1169,1915,850,1085,1593,1386,908,2017,1026
CD5_4_2568,864,909,887,491,346,1073,192,1013,923,2666,722,1487
CD5L_4_2569,191,181,290,94,489,21,0,18,9,106,1354,175
CD69_4_2570,759,881,845,775,1193,2006,827,952,1145,126,905,992
CD6_4_2571,11,59,45,70,6,8,7,6,121,0,71,31
CD72_4_2572,650,396,511,663,766,283,178,427,956,28,753,985
CD80_4_2573,1336,1319,1642,1659,1763,1324,1500,1698,648,498,2123,1591
CFTR_4_2574,1007,1234,1432,1125,591,742,1990,410,1093,2138,1646,1377
CHRNA10_4_2575,545,667,1199,501,255,583,1149,535,251,2029,1514,1049
CHRNA2_4_2576,404,569,827,639,654,551,559,570,352,310,493,553
CHRNA4_4_2577,56,17,321,131,1180,0,51,30,69,0,106,126
CHRNA5_4_2578,1568,1657,2107,1798,1809,2627,1526,1120,1139,480,3305,21
34
CHRNA9_4_2579,1624,1274,1883,1379,2415,1256,2392,2334,1089,631,2281,12
29
CHRNB1_4_2580,38,24,51,36,13,1,0,5,1,0,0,45
CHRNB2_4_2581,232,263,366,218,587,138,159,109,47,690,47,258
CHRNB3_4_2582,139,305,219,187,94,530,8,97,40,939,113,814
CHRNB4_4_2583,1291,1347,1825,1105,1168,2282,2900,1262,745,632,1354,153
2
CHRND_4_2584,167,353,86,62,0,497,38,3,667,9,14,250
CHRNE_4_2585,532,273,579,553,318,693,235,581,132,818,794,342
CHRNG_4_2586,482,957,550,640,140,200,1155,1129,88,55,756,694
CLCA1_4_2587,437,465,564,387,331,709,407,696,759,161,90,456
CLCA2_4_2588,839,884,960,826,1141,1123,252,1012,710,358,1190,610
CLCA4_4_2589,2759,2168,2714,2984,2831,2318,3755,4056,2988,4045,4888,28
70
CLCN1_4_2590,69,130,195,21,0,353,1,495,2,166,26,72
CLCN4_4_2591,817,873,1074,858,477,777,1254,842,786,530,1676,715
CLDN3_4_2592,87,132,299,198,156,473,107,0,37,329,37,135
CLDN4_4_2593,657,584,460,566,692,304,1858,726,409,652,771,444
CLEC1A_4_2594,271,117,257,199,223,129,539,174,22,286,460,213
CLIC1_4_2595,473,247,548,136,100,348,309,323,148,797,447,354
CLIC2_4_2596,1682,1831,2373,2260,1219,1725,1404,2343,1814,1089,1160,22
87
CLIC3_4_2597,377,490,566,788,1641,547,1077,369,760,293,365,798
CLIC4_4_2598,1099,1025,1125,815,551,246,1197,1205,668,66,1193,749
CLIC6_4_2599,2148,1725,2151,2290,1401,1702,3620,1833,525,2212,1199,304
2
CLNS1A_4_2600,1001,831,1223,1226,1428,400,623,1312,909,2197,1667,1074
CNGA2_4_2601,232,159,141,160,116,533,28,102,51,22,1124,138
CNGA4_4_2602,646,870,1361,949,120,981,969,1785,686,909,876,947
CNGB3_4_2603,1404,702,1256,751,710,1735,534,489,1041,184,1375,497
COLEC12_4_2604,923,1148,1388,903,1199,1039,1327,1395,451,2341,1189,130
3

CSF2RB_4_2605,237,225,361,368,242,173,1047,309,647,280,847,63
CUBN_4_2606,541,317,516,600,242,858,637,368,247,553,788,1176
DCC_4_2607,398,405,501,362,87,256,794,745,190,260,689,246
DNER_4_2608,146,154,332,190,244,342,265,133,72,450,141,114
EDAR_4_2609,215,298,161,251,30,588,76,307,2,682,165,374
EPOR_4_2610,443,396,551,552,32,581,988,408,438,402,2021,821
FCER1A_4_2611,1206,1696,1859,1521,868,2794,1801,1320,1237,2696,2466,10
25
FCER1G_4_2612,705,852,781,965,654,431,911,806,664,1518,904,573
FCGR1A_4_2613,1387,1660,1906,1787,1140,2084,4200,1227,762,2287,1936,18
05
FCGR2C_4_2614,2289,1921,2595,2315,2010,4392,3297,3570,1049,989,2189,14
61
FCGR3B_4_2615,1334,1667,1466,1183,3192,671,1251,771,950,1712,2643,1662
FRRS1_4_2616,2028,2400,2963,1717,2362,3425,3150,1948,2151,3094,3118,27
41
FXYD7_4_2617,1329,1667,1574,1570,1126,646,1297,2625,1084,1503,2366,132
7
GABRA3_4_2618,625,663,985,786,576,787,1362,1415,418,865,750,814
GABRA6_4_2619,226,404,284,288,989,488,212,529,454,0,263,174
GABRB1_4_2620,1515,1629,1868,1483,1469,2683,1195,1162,978,496,1955,130
0
GABRD_4_2621,261,299,392,641,412,752,348,451,501,395,371,1228
GABRE_4_2622,79,104,144,100,0,52,124,162,79,12,39,265
GABRG1_4_2623,550,758,528,660,977,1102,10,837,955,218,861,1172
GABRG3_4_2624,1148,910,747,978,334,1671,683,1469,354,328,370,1127
GABRP_4_2625,5061,4678,4519,4481,6170,6101,7536,5759,2707,4809,4491,34
23
GABRQ_4_2626,243,273,338,281,4,1374,295,518,0,470,407,236
GABRR1_4_2627,628,333,214,222,11,4,32,177,0,537,47,226
GABRR2_4_2628,452,413,414,183,1884,880,921,956,201,1170,571,506
GABRR3_4_2629,944,1074,1033,1104,659,2118,2057,1224,456,189,932,976
GFRA3_4_2630,103,253,335,312,335,820,478,291,37,6,438,55
GPC1_4_2631,445,425,265,245,457,664,240,475,229,22,113,241
GPC4_4_2632,256,264,244,284,160,123,382,286,230,196,33,215
GPC6_4_2633,892,1226,755,672,1357,1982,1190,644,1148,951,1606,1173
GPR89B_4_2634,3099,3750,3636,3383,2323,4994,5203,2589,1795,3021,4683,1
961
GRID1_4_2635,168,225,171,496,869,26,41,522,108,44,383,477
GRID2_4_2636,2405,1689,2312,2017,3256,1656,3732,1361,1805,1717,2381,14
03
GRIK3_4_2637,134,176,193,210,47,30,4,25,2,39,195,30
GRIK4_4_2638,121,319,240,240,321,203,3,96,30,51,231,143
GRIK5_4_2639,905,839,1109,958,1737,2027,1525,786,1723,423,2357,1635
GRIN2B_4_2640,185,166,291,216,109,416,62,36,70,0,45,174
GRIN2C_4_2641,345,459,285,281,58,143,129,526,204,3,371,70
GRIN2D_4_2642,343,311,503,364,130,666,1172,1,103,19,200,132
GRIN3A_4_2643,490,507,545,769,1828,150,145,1598,1533,746,233,1029
GRIN3B_4_2644,243,335,204,156,103,252,168,183,340,277,345,114
HAVCR2_4_2645,217,167,250,168,159,739,3,41,638,17,342,187

HCN1_4_2646,1883,2271,2369,2556,1736,2295,1101,2154,1815,1153,2395,403
4
HCN2_4_2647,1122,1662,1241,969,2757,1695,1724,1731,1668,2128,1654,1591
HCN3_4_2648,337,319,369,262,264,446,1107,437,140,191,1106,143
HCN4_4_2649,108,204,226,298,104,27,69,9,1,71,79,262
HLA-B_4_2650,80,109,71,56,0,14,0,0,2,0,0,132
HLA-
DOA_4_2651,1635,1918,1886,1332,2373,1733,1766,1848,1229,2139,1669,928
HLA-DOB_4_2652,121,389,208,173,185,350,442,318,766,1,115,285
HLA-
DPB1_4_2653,2261,1940,2106,2250,1749,729,2301,3286,1739,2593,2902,2804
HLA-DQA1_4_2654,185,289,248,45,58,8,115,266,4,0,142,26
HLA-DQA2_4_2655,160,173,283,191,416,427,0,87,408,15,294,383
HLA-DQB2_4_2656,42,156,280,299,416,894,26,17,133,2,169,401
HLA-DRA_4_2657,413,613,391,233,267,769,10,326,134,2,218,204
HLA-DRB1_4_2658,933,1176,1075,1096,948,484,1130,1112,655,194,1771,891
HLA-DRB5_4_2659,933,1176,1075,1096,948,484,1130,1112,655,194,1771,891
HLA-G_4_2660,22,59,110,31,3,16,23,0,0,0,26,111
HTR3B_4_2661,1759,1777,1997,1865,872,2554,2237,1686,830,564,1619,1531
HTR3C_4_2662,206,256,238,109,52,57,694,200,289,549,236,823
HTR3E_4_2663,240,307,370,192,358,364,47,498,134,11,937,130
ICAM1_4_2664,229,262,318,152,275,93,75,162,78,304,156,1072
IFNAR1_4_2665,3197,2283,2727,2739,2793,3475,1900,2367,1595,2094,3964,3
413
IFNGR1_4_2666,587,898,586,613,821,468,995,628,403,407,741,700
IFNGR2_4_2667,381,552,639,353,3,829,1070,148,300,770,507,1110
IGF1R_4_2668,480,349,485,478,372,506,8,361,321,44,12,812
IGF2R_4_2669,1866,1116,1741,1329,2280,830,2134,1339,1996,2764,1573,190
9
IL10RA_4_2670,96,155,113,132,74,85,0,37,4,9,67,346
IL10RB_4_2671,906,784,952,1090,936,1838,198,232,315,1729,1955,947
IL12RB2_4_2672,599,932,493,703,1516,60,1666,981,341,880,952,789
IL13RA1_4_2673,829,1042,765,1020,305,2664,1297,1696,435,2543,699,965
IL13RA2_4_2674,1375,1033,1442,1610,418,2915,1665,1136,1567,1638,1191,2
267
IL17RA_4_2675,837,692,653,588,560,541,815,903,454,647,325,101
IL17RB_4_2676,147,96,139,206,278,71,141,12,485,0,167,599
IL18R1_4_2677,535,458,542,627,564,197,1381,497,92,917,1505,266
IL18RAP_4_2678,706,460,380,565,430,794,946,425,359,285,403,527
IL1R1_4_2679,3973,3262,3358,3910,3623,4634,3512,4058,2870,2000,5463,40
46
IL1RAPL1_4_2680,802,1027,908,558,781,743,508,366,390,1861,1772,1089
IL1RAPL2_4_2681,1750,1897,1489,2350,899,1553,795,2000,1501,1532,2940,2
747
IL1RL2_4_2682,111,139,350,65,576,567,569,3,12,15,159,63
IL20RA_4_2683,2116,2033,2032,2366,2448,2668,2428,1663,1804,1787,1522,2
310
IL22RA1_4_2684,1451,1220,1291,1048,602,863,808,1702,1467,423,883,2372
IL23R_4_2685,451,661,663,472,270,618,85,556,300,399,1251,499
IL27RA_4_2686,246,252,492,343,98,283,602,21,149,30,390,644

IL2RA_4_2687,206,199,530,282,128,586,57,68,47,78,719,671
IL2RB_4_2688,193,170,359,396,257,520,0,275,39,21,92,470
IL2RG_4_2689,1581,1744,1550,1413,1038,2392,2246,2507,2360,1503,1517,13
80
IL3RA_4_2690,418,285,563,426,270,767,233,550,653,103,105,461
IL7R_4_2691,206,100,158,172,49,76,694,19,37,41,73,189
IL9R_4_2692,461,176,353,185,276,155,301,13,180,0,140,525
ITFG1_4_2693,982,1634,1020,1201,781,1286,4240,2029,234,826,532,966
ITGA2B_4_2694,142,148,161,129,93,448,106,112,64,0,19,216
ITGB3_4_2695,1486,1438,1787,1664,1881,2672,2388,1162,1244,51,2074,1346
ITGB7_4_2696,591,684,633,770,263,871,1150,336,1508,197,1306,901
ITPR2_4_2697,641,788,1300,1015,405,214,662,1038,345,265,493,702
ITPR3_4_2698,586,336,482,256,564,414,957,343,929,155,368,200
JPH3_4_2699,272,311,300,290,15,107,797,332,70,31,785,8
KCMF1_4_2700,1297,602,994,653,211,629,1433,424,1490,51,1757,971
KCNA10_4_2701,708,717,1222,866,762,755,1298,374,148,773,741,1133
KCNA1_4_2702,2872,2834,2989,2953,1891,2609,2495,1667,2207,6013,3049,39
46
KCNA3_4_2703,467,526,369,406,945,179,306,709,148,491,0,140
KCNA4_4_2704,813,540,1137,1234,363,828,935,691,800,817,1777,1230
KCNA5_4_2705,2867,3271,3027,3010,2475,3470,2863,3979,3452,1952,3193,32
75
KCNA6_4_2706,711,362,674,347,8,16,291,337,391,582,550,13
KCNA7_4_2707,1311,966,1653,1347,2134,1837,1883,945,2507,1795,1608,1347
KCNAB3_4_2708,1211,1361,1757,1341,2218,633,1968,1704,274,1322,1257,170
2
KCNB1_4_2709,106,336,83,64,95,249,107,110,272,1,0,119
KCNB2_4_2710,1298,1671,1245,1406,1693,2658,2861,1885,954,2169,1269,147
2
KCNC3_4_2711,1711,1905,1662,2078,2660,982,2677,1276,1836,1384,2037,163
8
KCND1_4_2712,385,196,291,151,366,317,54,1,204,380,56,137
KCND2_4_2713,477,509,677,618,283,65,256,568,606,1279,382,745
KCNE1L_4_2714,2071,1719,2848,2481,2777,2416,2461,2287,1054,452,4554,28
74
KCNE2_4_2715,299,597,355,305,16,392,35,220,825,162,445,784
KCNE3_4_2716,1511,1206,1668,2152,2194,1027,748,3027,1014,368,653,1915
KCNE4_4_2717,725,997,1182,988,1230,471,301,569,472,286,568,1283
KCNF1_4_2718,830,1009,982,1183,1235,1514,1735,619,348,363,1242,1035
KCNG1_4_2719,573,843,1120,953,604,434,397,1062,1149,253,858,978
KCNG2_4_2720,49,191,253,251,311,17,0,565,21,432,140,130
KCNG4_4_2721,970,1055,845,1129,1526,1146,2108,1168,1267,1032,1740,1607
KCNH3_4_2722,0,22,26,41,0,27,17,21,42,0,34,74
KCNH4_4_2723,288,318,492,588,628,323,126,303,23,195,56,458
KCNH8_4_2724,163,139,152,117,0,17,74,373,1,844,34,87
KCNJ10_4_2725,286,412,333,242,134,48,1437,103,167,288,432,577
KCNJ12_4_2726,662,639,532,677,288,795,56,1031,513,447,949,812
KCNJ2_4_2727,700,903,609,654,656,2479,336,1649,278,2532,1268,1064
KCNJ3_4_2728,836,1115,764,684,800,986,1454,1127,767,108,1389,797
KCNJ5_4_2729,1763,1826,1996,1542,2305,2644,1596,1665,1460,879,2039,182

1
KCNJ6_4_2730,1226,1679,1444,1606,1053,2208,1534,1834,452,1688,2078,122
4
KCNJ8_4_2731,373,482,974,530,53,883,913,699,1353,1960,670,338
KCNJ9_4_2732,36,37,86,96,23,62,2,24,31,3,4,32
KCNK12_4_2733,521,666,611,924,329,1388,497,624,776,577,1144,1045
KCNK13_4_2734,1969,1808,2224,2393,2265,1854,1154,2154,1543,1467,2073,3
463
KCNK15_4_2735,1639,1978,1727,2218,1496,1801,2099,1915,894,1861,1587,21
60
KCNK18_4_2736,274,262,191,85,427,1,14,1218,43,1,3,249
KCNK1_4_2737,701,1083,773,618,913,1472,448,466,399,145,1314,370
KCNK3_4_2738,27,20,82,110,0,0,0,0,0,141,0,544
KCNK4_4_2739,1361,1468,1153,1460,984,1470,976,514,630,1304,1707,1394
KCNK5_4_2740,298,191,144,164,99,162,92,70,0,8,12,297
KCNK6_4_2741,436,320,387,299,1808,698,615,409,80,890,442,262
KCNK9_4_2742,72,52,94,262,140,0,356,44,14,2212,18,17
KCNMB1_4_2743,280,388,466,398,268,565,100,917,477,10,114,91
KCNMB4_4_2744,449,700,509,534,503,609,778,552,135,976,1139,980
KCNN1_4_2745,879,1181,1265,1197,1375,770,467,1377,1763,22,1637,920
KCNN4_4_2746,3774,4138,5255,4856,5416,6014,5838,4871,4384,4139,4927,41
57
KCNS1_4_2747,107,217,195,123,185,108,63,159,191,2097,447,126
KCNS2_4_2748,502,523,499,785,439,947,1077,528,387,341,1246,759
KCNS3_4_2749,172,276,53,261,68,0,43,124,0,29,793,3
KCNT1_4_2750,289,496,386,369,293,378,259,582,966,18,341,307
KCNT2_4_2751,1303,1671,1797,1277,1480,1750,1478,2583,425,2361,2080,994
KCNU1_4_2752,1026,1018,1004,988,1292,1254,1195,2016,798,651,429,919
KCNV1_4_2753,1668,1666,1216,1364,2257,3054,2119,2634,1063,828,1247,103
2
KCNV2_4_2754,607,681,1102,1012,1163,956,1509,892,941,454,897,694
KCTD10_4_2755,2266,2453,2929,2702,1144,3783,3257,1479,1591,1002,4462,2
543
KCTD11_4_2756,135,208,92,167,0,0,29,227,456,163,221,185
KCTD12_4_2757,98,242,274,283,76,146,98,253,176,28,123,222
KCTD13_4_2758,136,473,475,219,83,459,892,870,205,735,272,213
KCTD14_4_2759,3939,3652,3651,3176,3851,5360,5667,2266,2843,4607,5108,3
152
KCTD16_4_2760,2533,2793,2811,1898,4059,1290,1828,1414,2296,2923,1394,2
252
KCTD17_4_2761,105,71,71,283,4,7,48,36,25,942,125,0
KCTD18_4_2762,2199,2907,3043,3161,3198,4379,2456,3338,2750,5808,3558,4
580
KCTD19_4_2763,1776,1554,1485,1554,2566,959,1735,1534,1635,1848,1436,25
86
KCTD20_4_2764,1086,805,800,684,1994,819,1368,104,427,1734,914,1328
KCTD2_4_2765,1954,1582,1908,1749,2264,1208,2334,1936,1531,2608,2141,15
17
KCTD3_4_2766,1274,1289,1323,1535,1371,704,913,2230,1146,1243,2214,977
KCTD4_4_2767,378,591,509,532,569,521,232,582,507,36,149,853

KCTD5_4_2768,671,584,538,834,643,865,698,1179,360,430,629,1498
KCTD8_4_2769,3061,2883,3190,3051,3988,2120,3663,2977,2973,2729,3364,38
57
KCTD9_4_2770,1174,967,772,951,632,1385,292,1581,446,1361,1473,845
KIR3DL1_4_2771,766,434,396,477,785,956,120,200,178,666,407,746
KLRB1_4_2772,122,284,120,329,250,418,91,102,22,411,521,174
LGALS3BP_4_2773,876,774,505,637,244,1512,458,21,1230,811,549,435
LRP10_4_2774,657,1294,1055,854,705,913,883,1691,1039,1184,1623,1799
LRP1B_4_2775,935,1047,1202,1337,861,785,1145,488,1784,1349,500,1376
LRP3_4_2776,62,75,50,127,116,120,7,2,17,0,2,68
LRP5_4_2777,217,89,340,219,410,158,112,100,979,117,156,102
LRP6_4_2778,1564,1874,2040,1386,1640,1974,1232,1209,949,1197,1812,2337
LRPAP1_4_2779,418,448,307,711,650,573,796,258,1028,103,225,264
LTBR_4_2780,675,310,573,940,148,82,687,401,1126,15,656,479
MARCO_4_2781,172,6,244,321,1,0,5,0,0,0,0,0
MCOLN1_4_2782,1049,1117,1338,1367,355,1046,1397,1129,960,364,1035,1357
MCOLN2_4_2783,612,720,574,699,857,972,2025,529,123,625,1019,1378
MCOLN3_4_2784,486,628,486,360,32,325,718,272,280,609,268,329
MCU_4_2785,699,818,1091,1068,954,311,2346,254,700,827,625,1493
MFRP_4_2786,452,433,438,382,264,1961,889,543,414,38,159,387
MICB_4_2787,219,283,166,158,203,459,2,172,56,0,91,83
MPL_4_2788,391,311,376,321,339,790,403,251,51,616,283,284
MRC1_4_2789,472,443,509,350,314,476,315,170,534,641,859,926
MRC2_4_2790,620,569,757,634,389,216,474,1434,767,956,1623,1212
NALCN_4_2791,764,847,914,1144,1304,1301,1392,1218,1252,835,150,1586
NGFR_4_2792,483,389,493,284,138,125,169,483,117,52,167,191
NISCH_4_2793,782,537,445,392,1022,1640,87,593,699,310,1055,676
NPTXR_4_2794,535,666,1049,718,854,421,1930,489,404,283,350,401
ORAI1_4_2795,148,0,146,60,0,0,0,213,0,0,63,0
P2RX1_4_2796,769,847,1012,1002,742,1783,854,647,139,643,1055,814
P2RX3_4_2797,343,567,480,324,255,257,999,322,19,2,702,368
P2RX4_4_2798,1310,1451,1614,1696,1150,1521,1609,1914,1376,1697,1341,17
87
P2RX7_4_2799,312,218,317,363,608,305,35,883,28,599,290,199
PEX5L_4_2800,478,689,341,533,696,626,687,411,219,169,602,632
PGLYRP1_4_2801,77,159,132,103,159,191,180,95,138,0,0,19
PGLYRP2_4_2802,121,82,43,132,236,32,38,22,7,0,102,1
PGLYRP3_4_2803,1545,1685,2121,1938,959,1575,1423,1952,1491,1472,2348,2
135
PGLYRP4_4_2804,262,84,342,278,358,17,386,22,89,1,571,222
PGRMC1_4_2805,1376,1275,825,1094,1588,675,705,1653,373,988,1449,1213
PIP_4_2806,432,331,384,469,263,207,600,260,463,153,49,930
PKD1L1_4_2807,848,605,972,851,2079,590,564,256,718,1400,703,640
PKD1L3_4_2808,1584,1288,1529,1342,992,802,718,813,1397,2151,1978,1862
PKD2L1_4_2809,402,438,380,525,131,210,683,513,768,545,356,720
PKD2L2_4_2810,1177,940,793,1168,1673,738,372,226,1224,1108,557,1193
PKDREJ_4_2811,1533,2276,2357,1937,1613,1423,1662,1636,2389,1164,2022,2
055
PLXNA1_4_2812,608,827,803,1187,961,940,1125,1125,901,314,1122,439
PLXNA2_4_2813,284,381,420,243,270,110,601,498,85,155,314,219

PLXNA3_4_2814,525,601,552,932,100,724,720,578,114,920,401,494
PLXND1_4_2815,60,62,135,96,10,184,109,226,101,31,71,0
PRPH2_4_2816,71,188,84,40,448,0,3,0,336,0,136,337
RARRES2_4_2817,106,176,195,116,41,313,456,249,16,329,0,186
RASA3_4_2818,5494,4746,4925,4739,5327,5740,7300,2605,3082,8064,6538,51
95
RYR2_4_2819,975,1288,1325,1323,1094,1081,1721,875,1771,579,1222,1453
RYR3_4_2820,746,922,747,1071,567,727,942,754,1355,650,1430,1329
SARM1_4_2821,748,820,580,908,1536,395,2323,508,666,252,1759,1245
SCN10A_4_2822,833,728,736,523,275,545,280,931,105,318,670,600
SCN11A_4_2823,717,1164,956,745,851,924,1312,484,599,2297,1626,252
SCN2B_4_2824,419,531,474,512,171,134,944,472,893,67,1047,420
SCN4A_4_2825,3043,3020,2727,3188,3301,3661,3482,2550,1597,2105,5280,31
48
SCN7A_4_2826,2266,2441,2487,2582,1858,3030,1220,2115,1010,2696,4417,16
62
SCN9A_4_2827,4370,5132,5257,6529,3006,6443,5604,4497,4570,2696,7091,61
27
SCNN1B_4_2828,141,372,479,68,1,0,0,218,43,1053,172,194
SCNN1D_4_2829,140,94,110,150,318,7,344,74,419,3,101,174
SCNN1G_4_2830,502,649,844,767,248,559,874,78,639,657,241,1195
SCUBE1_4_2831,88,54,77,190,305,116,148,301,16,607,61,370
SELE_4_2832,783,692,765,1021,892,715,297,532,188,878,1676,389
SEMA5A_4_2833,232,111,89,212,168,0,227,198,16,0,135,35
SETD1A_4_2834,359,454,302,403,214,750,240,138,289,96,535,189
SFRP1_4_2835,535,547,339,565,374,379,419,863,178,256,564,2028
SFRP4_4_2836,290,170,379,272,52,128,760,321,509,78,171,536
SFRP5_4_2837,2775,2803,3970,3169,3401,3933,3464,2525,2719,2538,1723,38
82
SHKBP1_4_2838,182,185,417,175,7,243,92,292,104,97,923,536
SHROOM2_4_2839,326,288,419,518,106,985,479,173,395,80,699,727
SIGLEC8_4_2840,586,705,996,754,356,726,836,749,1370,232,1304,977
SLAMF1_4_2841,929,723,646,855,1138,558,633,1891,375,437,548,1058
SLC6A18_4_2842,1392,820,1110,1167,877,351,1624,909,459,921,2160,749
SLC6A19_4_2843,480,766,526,568,338,1649,650,427,446,2002,1229,1251
SLC6A1_4_2844,93,208,241,168,129,144,39,194,95,237,361,402
SLC6A3_4_2845,70,48,83,268,0,226,574,0,40,0,141,329
SLC6A4_4_2846,123,78,113,1,0,527,0,0,661,1182,0,0
SLC9A1_4_2847,255,261,271,225,43,34,285,333,224,17,404,230
SLC9A3_4_2848,120,142,102,205,33,60,101,214,0,0,11,109
SRCRB4D_4_2849,422,654,386,580,293,130,0,444,1139,1608,1243,252
STAB2_4_2850,2048,1591,2018,2219,3298,2072,2542,1222,1156,1507,2339,16
69
STX1B_4_2851,958,984,1033,1026,1216,1512,875,1221,1119,805,1189,1364
THBD_4_2852,473,742,847,674,434,174,285,359,41,1530,782,694
TLR1_4_2853,2457,2646,2894,2454,2316,3614,4666,3325,3178,1589,3911,240
8
TLR2_4_2854,156,152,374,334,220,514,925,84,172,19,1665,700
TLR3_4_2855,987,840,612,931,942,127,405,1401,523,186,681,1201
TLR4_4_2856,986,714,1324,855,700,582,2693,762,985,893,1190,771

TLR5_4_2857,1762,2407,2740,1515,2014,3030,2742,2506,1604,2420,1643,251
1
TLR6_4_2858,705,1016,1187,923,181,940,1817,467,965,2393,941,1714
TLR7_4_2859,330,499,356,283,270,393,303,200,425,458,473,213
TLR8_4_2860,1127,867,1017,1074,540,1658,1669,856,1204,470,1635,1945
TLR9_4_2861,291,501,288,248,545,175,754,56,441,1161,205,253
TMEM37_4_2862,256,395,481,517,971,1911,87,307,480,190,456,264
TMEM38A_4_2863,890,964,811,901,528,2568,1344,414,1047,35,778,1005
TMEM38B_4_2864,1151,1751,2034,1201,1098,2298,2635,1100,555,3476,2413,1
528
TNFAIP1_4_2865,181,134,135,225,48,5,61,77,600,70,561,244
TNFRSF10A_4_2866,329,385,399,207,18,171,245,269,1,2,263,128
TNFRSF10C_4_2867,324,49,207,105,112,7,8,337,551,335,72,68
TNFRSF10D_4_2868,364,233,432,142,441,1097,6,195,696,778,129,637
TNFRSF11A_4_2869,1055,1106,1071,1151,865,863,1029,1155,933,706,430,199
7
TNFRSF13B_4_2870,115,105,157,121,6,24,7,266,9,1032,1,449
TNFRSF13C_4_2871,39,43,81,125,0,722,0,917,25,604,9,0
TNFRSF14_4_2872,116,337,292,128,335,137,1774,221,217,14,94,470
TNFRSF1A_4_2873,1548,1563,1621,1843,2926,1325,788,2435,1125,3971,1874,
1552
TNFRSF1B_4_2874,352,314,562,276,342,648,1088,154,106,5,27,152
TNFRSF4_4_2875,77,197,66,130,106,204,0,140,37,1215,138,138
TNFRSF6B_4_2876,34,89,187,63,332,125,434,286,26,793,66,26
TPCN2_4_2877,580,320,428,535,359,275,276,296,299,502,502,277
TREM2_4_2878,288,407,309,249,668,512,442,423,1,239,494,108
TRPA1_4_2879,522,717,453,598,467,787,1008,274,1120,1045,205,364
TRPC1_4_2880,636,583,782,734,593,182,411,264,391,111,157,429
TRPC5_4_2881,1037,542,884,947,1015,779,213,274,1464,347,471,1069
TRPC6_4_2882,183,177,385,69,8,691,714,40,184,0,121,329
TRPM1_4_2883,920,884,1097,1081,417,1248,1376,1841,1769,982,1905,1173
TRPM2_4_2884,135,206,195,189,1,0,0,1,621,0,111,227
TRPM5_4_2885,349,175,213,350,77,341,291,112,36,239,586,430
TRPM7_4_2886,4481,3436,4429,3951,4835,2864,3147,5357,5865,3167,5958,32
21
TRPM8_4_2887,211,319,480,336,694,292,334,664,239,1611,483,307
TRPV2_4_2888,0,0,0,0,0,0,0,0,0,0,0,0
TRPV3_4_2889,120,96,69,143,91,63,53,425,0,0,211,105
TRPV5_4_2890,627,916,804,1045,597,745,1174,1249,152,1116,1310,1369
TRPV6_4_2891,646,609,953,870,1268,317,990,480,918,351,1027,614
TTYH3_4_2892,331,115,118,118,5,0,0,292,7,18,1,366
ULBP1_4_2893,831,638,828,794,1017,763,1549,769,642,890,1088,398
ULBP2_4_2894,706,857,794,571,851,489,1941,980,1199,194,240,720
ULBP3_4_2895,406,266,354,570,869,349,17,520,47,1036,196,322
UNC5A_4_2896,100,124,152,35,41,4,1,0,32,0,6,323
UNC5B_4_2897,955,1005,1085,1112,1634,131,950,1271,515,853,1254,1199
UNC5C_4_2898,1060,1368,1164,1228,1985,832,2179,1485,1562,1683,1077,897
UTRN_4_2899,1836,1305,1346,1117,1193,754,1887,908,1390,1225,1316,952
VDAC1_4_2900,860,1022,1172,573,682,742,1499,350,122,415,1542,654
ZACN_4_2901,247,233,131,256,390,121,10,29,267,692,14,24

ABCC4_4_2902,199,201,157,210,158,156,972,192,23,56,374,136
ABCC9_4_2903,562,410,419,665,891,369,909,625,279,970,207,359
ADIPOR1_4_2904,4399,4547,5318,4610,4122,5069,4284,4320,5592,4519,9369,
3875
AGER_4_2905,104,120,30,4,0,76,10,5,0,0,2,11
ANO6_4_2906,241,138,192,364,363,182,366,110,366,62,421,424
ANTXR1_4_2907,2587,2548,3414,2645,3171,1580,1472,2620,1660,1992,1676,3
436
ANXA7_4_2908,320,475,581,577,334,788,473,758,332,141,618,310
AQP4_4_2909,1750,1620,1621,1579,2227,936,1770,814,2257,2093,1476,2534
ASGR1_4_2910,289,130,174,165,222,6,290,20,709,19,202,339
ASGR2_4_2911,755,637,689,577,522,724,126,1234,954,1241,1480,557
BEST1_4_2912,448,506,1027,578,399,1645,145,736,390,810,518,648
BEST3_4_2913,974,780,1292,1013,1368,1825,997,1430,690,316,1139,1079
CACNA1A_4_2914,282,433,315,310,347,409,66,287,406,20,445,605
CACNA1C_4_2915,837,1062,708,1022,1136,601,733,408,739,1263,520,894
CACNA1D_4_2916,2048,2253,1965,2182,2996,3131,2662,2650,2821,1556,2238,
2282
CACNA1E_4_2917,201,242,188,197,268,108,36,314,5,0,115,70
CACNA1G_4_2918,348,391,121,185,40,215,473,28,323,549,218,52
CACNA1H_4_2919,125,168,364,164,455,113,109,257,371,31,64,459
CACNA1I_4_2920,626,451,446,409,668,424,486,608,319,865,572,1212
CACNA2D2_4_2921,739,1343,1147,1157,1749,357,1558,668,1728,478,1895,172
5
CACNB1_4_2922,1734,1893,2143,1585,2508,2160,1844,2479,1514,2417,2229,1
237
CACNB2_4_2923,725,279,388,696,451,92,202,115,1,484,372,88
CACNB3_4_2924,285,107,402,422,199,242,246,353,619,270,923,353
CACNB4_4_2925,2424,2152,2575,2014,1897,1758,2511,1466,1349,633,2903,16
58
CACNG6_4_2926,146,197,270,201,103,143,6,312,0,0,19,184
CATSPER2_4_2927,758,669,772,808,482,154,1103,46,1104,357,645,1367
CD14_4_2928,115,48,140,293,878,283,116,194,83,7,15,36
CD163_4_2929,18,256,184,100,35,5,1,8,93,3,70,455
CD247_4_2930,121,138,173,143,73,754,0,35,2,0,251,704
CD302_4_2931,1618,1434,1909,1548,1557,2394,1172,3011,871,646,2597,2031
CD36_4_2932,1208,1165,1257,938,1510,1077,2638,1426,1360,854,1095,1368
CD3D_4_2933,172,94,130,108,74,81,63,342,96,0,35,9
CD40_4_2934,0,0,30,0,0,0,0,0,0,0,0,0
CD4_4_2935,222,201,565,291,224,448,965,14,459,0,102,355
CD74_4_2936,108,354,138,174,39,409,0,50,0,2,570,229
CD79A_4_2937,472,225,426,445,837,252,170,725,174,479,1166,1660
CD79B_4_2938,68,225,232,125,1,23,369,1,0,0,326,303
CD86_4_2939,1416,1180,1198,1569,1766,1295,231,880,534,677,918,1270
CEACAM1_4_2940,2816,2440,2285,2747,2684,4285,3269,3063,1805,1390,3052,
2533
CEACAM21_4_2941,1179,1494,1867,1566,1361,2186,1676,2267,656,588,1931,1
362
CHRFAM7A_4_2942,1858,2055,2010,2630,2366,2851,1469,1193,1423,1499,3243
,1852

CHRNA1_4_2943,1493,1895,1529,1782,1267,2313,1675,1959,2191,2665,1451,1
062
CHRNA3_4_2944,729,573,496,460,315,432,325,332,1398,401,1009,1280
CHRNA6_4_2945,1169,1210,946,815,1313,1383,1852,1203,693,502,1192,908
CHRNA7_4_2946,252,365,176,287,532,851,107,385,135,22,260,182
CLCC1_4_2947,1459,1268,1192,1161,1491,347,1565,1010,888,1798,2092,1339
CLCN2_4_2948,490,459,556,503,464,417,1187,596,767,117,319,390
CLCN3_4_2949,1822,1860,1969,2303,1430,1116,1009,1835,2801,1333,2222,26
14
CLCN5_4_2950,179,250,393,368,40,421,145,239,422,487,468,474
CLCN6_4_2951,844,1007,976,1020,793,517,1174,1917,679,685,992,1051
CLCN7_4_2952,95,282,280,225,118,12,89,371,91,3,255,192
CLCNKA_4_2953,1962,2465,2370,1978,2725,1458,4381,2699,2191,1391,4188,2
755
CLCNKB_4_2954,187,63,18,53,436,0,0,5,0,31,0,3
CLEC1B_4_2955,1785,2247,2444,1947,1520,3543,2192,1694,1462,1293,2338,1
608
CLEC4A_4_2956,1348,1504,1857,1306,651,1358,1896,1240,2475,936,1582,187
3
CNGA1_4_2957,944,948,939,886,730,1198,674,1360,156,2919,1035,838
CNGA3_4_2958,294,399,279,499,6,115,6,106,88,909,480,349
CNGB1_4_2959,25,106,183,140,880,143,649,163,17,2,64,56
CNTFR_4_2960,119,175,77,111,14,623,515,1,357,1180,88,89
CR2_4_2961,250,273,331,378,10,368,21,0,92,31,17,98
CSF2RA_4_2962,453,345,467,370,141,105,618,775,787,396,538,477
CSF3R_4_2963,1260,1811,1723,1658,1966,1373,2305,3076,1839,1466,1176,15
62
CXADR_4_2964,1822,1812,2329,1557,1374,1432,2136,1902,1709,1858,1975,15
21
DAG1_4_2965,1094,1197,1730,1140,501,1203,2076,1857,2014,498,2334,2541
EDA2R_4_2966,711,444,504,530,452,979,841,321,203,534,363,233
EDNRA_4_2967,305,231,403,298,411,127,518,887,160,177,559,729
ENG_4_2968,243,180,162,201,576,119,504,55,605,287,364,380
EVI2A_4_2969,1448,1947,1815,1813,1274,1080,1260,484,1144,1181,3153,244
1
FCAMR_4_2970,1596,1343,1636,2100,3401,863,3657,2399,726,884,1491,1800
FCGR1B_4_2971,360,482,600,339,114,848,849,838,94,736,403,454
FCGR2A_4_2972,635,792,958,813,257,1861,1388,1141,156,344,722,765
FCGR2B_4_2973,700,825,727,1075,243,413,503,1003,594,1605,173,1476
FCGR3A_4_2974,283,297,644,619,356,802,619,640,246,105,1187,761
FCGRT_4_2975,380,289,280,82,428,71,1708,986,696,6,45,26
FGFRL1_4_2976,658,579,751,561,596,522,601,727,227,1377,467,922
FLT4_4_2977,99,121,149,79,0,0,3,237,1,4,412,253
FXYD1_4_2978,712,940,1080,606,603,527,497,705,523,1441,793,938
FXYD2_4_2979,1012,891,850,1022,641,1540,306,814,718,1472,513,1471
FXYD4_4_2980,382,401,524,613,359,778,734,327,59,725,248,346
FXYD5_4_2981,481,358,328,144,89,126,950,230,189,191,855,109
FXYD6_4_2982,27,208,308,135,2,416,2,309,58,510,11,4
GABRA1_4_2983,2086,1792,1896,2258,761,2200,2381,2161,3527,394,3170,343
6

GABRA2_4_2984,606,423,552,818,568,778,1167,988,378,124,387,471
GABRA4_4_2985,891,1247,858,1128,696,1069,673,1126,856,2265,2454,1401
GABRA5_4_2986,97,195,259,68,5,6,33,0,131,36,37,58
GABRB2_4_2987,516,633,850,838,978,581,350,212,533,234,1058,518
GABRB3_4_2988,210,287,100,181,873,1,40,60,0,152,161,367
GABRG2_4_2989,198,347,212,265,266,288,178,120,137,1004,401,434
GFRA1_4_2990,1818,1634,1397,1649,1692,1789,1468,1944,1273,1937,1922,20
58
GFRA2_4_2991,181,425,251,503,359,385,354,258,4,599,351,479
GFRA4_4_2992,128,164,146,242,340,358,789,425,387,24,0,5
GHR_4_2993,249,301,331,203,73,750,0,218,373,393,838,0
GIPC1_4_2994,229,264,91,312,72,12,13,24,152,0,113,49
GLRA1_4_2995,959,989,779,824,1343,2048,1067,1178,82,1622,1077,1529
GLRA2_4_2996,601,567,644,554,1050,906,1706,782,369,488,1451,835
GLRA3_4_2997,1293,603,1222,780,738,686,550,658,898,211,1360,2117
GLRA4_4_2998,112,375,429,234,175,379,82,31,903,0,166,245
GLRB_4_2999,440,712,472,470,543,379,975,514,81,34,1165,984
GP6_4_3000,133,330,352,291,627,447,48,205,370,325,488,887
GPM6A_4_3001,638,494,882,435,1075,493,1584,1386,834,4,743,751
GPR89A_4_3002,3099,3750,3636,3383,2323,4994,5203,2589,1795,3021,4683,1
961
GRIA1_4_3003,772,621,563,679,992,1233,1355,749,507,612,297,575
GRIA2_4_3004,2166,1979,2557,2184,2114,1880,1839,2606,2294,869,2506,254
5
GRIA3_4_3005,483,622,750,449,688,140,368,1029,133,436,754,661
GRIA4_4_3006,1772,1576,1986,1241,2389,2702,3044,1889,1817,1558,1163,18
51
GRIK1_4_3007,36,74,235,110,62,46,134,6,0,32,52,2
GRIN2A_4_3008,171,291,631,244,254,38,902,89,30,527,30,344
GRINA_4_3009,755,668,604,607,404,238,1547,866,1335,822,422,483
HLA-DPA1_4_3010,333,121,519,192,201,168,178,288,32,698,336,188
HTR3A_4_3011,148,580,241,220,367,175,292,212,412,512,177,356
HTR3D_4_3012,1559,1654,1819,1835,1902,1288,775,1785,1328,545,2727,1570
HVCN1_4_3013,737,285,346,259,1211,922,243,82,873,3,232,331
IFNAR2_4_3014,1673,1867,1735,1567,1222,3172,2537,2537,1233,2039,2925,1
960
IL11RA_4_3015,176,232,156,226,256,9,12,2,1,0,541,0
IL12RB1_4_3016,561,644,784,491,332,822,301,240,395,43,297,615
IL15RA_4_3017,333,445,538,232,239,184,1326,67,264,22,1048,237
IL1R2_4_3018,173,197,319,183,597,2,261,166,0,200,106,179
IL1RAP_4_3019,900,640,947,815,1453,479,734,45,640,121,268,768
IL1RL1_4_3020,1252,1433,1533,1254,1237,2075,989,657,1235,1182,1074,126
4
IL21R_4_3021,1568,1729,1929,1731,1008,2328,904,1373,1434,2029,1722,186
7
IL22RA2_4_3022,565,401,379,494,68,360,0,12,839,240,78,755
IL28RA_4_3023,817,1108,1063,588,222,600,761,576,618,2005,828,485
IL31RA_4_3024,1503,1357,1611,1750,2573,907,3115,1780,1860,1132,999,116
9
IL4R_4_3025,2053,1546,2186,2288,3301,731,1165,1840,801,1628,1853,1596

IL5RA_4_3026,1115,845,1007,1006,811,200,616,609,545,158,1174,1311
IL6R_4_3027,545,633,577,491,187,218,812,53,766,1664,1062,603
IL6ST_4_3028,2020,1834,2350,2974,2709,3914,2943,1734,3100,3617,2505,20
97
ILDR1_4_3029,1010,1448,934,1476,921,1108,383,1496,1537,784,425,823
ITGB1_4_3030,961,888,1243,816,752,330,2094,1329,1013,744,2114,522
ITGB4_4_3031,207,399,538,229,35,508,835,108,505,460,346,172
ITPR1_4_3032,608,1014,1151,740,1153,356,895,1613,487,1283,224,978
KCNA2_4_3033,658,350,637,666,1193,695,219,554,835,0,1130,285
KCNAB1_4_3034,1257,930,846,1190,1104,1906,1333,1979,471,699,1438,673
KCNAB2_4_3035,169,317,262,275,23,287,22,148,3,91,247,144
KCNC1_4_3036,5116,4344,4808,4861,5460,5303,7045,4194,3758,5307,5776,57
02
KCNC2_4_3037,11,262,94,206,0,1,31,32,52,0,0,1
KCNC4_4_3038,170,209,364,316,203,56,127,67,100,8,1039,553
KCND3_4_3039,402,191,545,214,441,11,217,662,95,15,367,338
KCNE1_4_3040,117,298,263,157,77,831,228,48,121,1,431,368
KCNG3_4_3041,884,843,970,802,199,771,948,261,764,1588,1442,618
KCNH1_4_3042,61,180,419,230,62,369,26,420,274,152,257,505
KCNH2_4_3043,266,354,368,419,197,331,71,262,1005,180,255,316
KCNH5_4_3044,1836,1380,1597,1390,1561,1496,2764,1660,2748,920,2478,225
0
KCNH6_4_3045,729,283,354,262,415,377,409,690,701,1,1196,1020
KCNH7_4_3046,192,351,265,204,449,107,165,86,60,979,193,109
KCNIP1_4_3047,409,298,299,352,607,498,821,386,883,1644,1195,377
KCNIP2_4_3048,1098,1206,1558,972,609,2051,953,1584,346,715,1358,1164
KCNIP4_4_3049,1469,1551,1772,1524,1414,1899,3270,1796,1207,1037,3259,1
861
KCNJ11_4_3050,383,402,141,490,712,51,595,514,415,1142,944,469
KCNJ13_4_3051,1611,1910,1552,1821,1811,1448,2718,1015,750,723,565,1981
KCNJ14_4_3052,1042,1027,1439,1119,1288,840,1929,1001,550,1376,2129,788
KCNJ15_4_3053,1057,1156,1483,1171,1198,753,1895,1916,908,278,1210,1760
KCNJ16_4_3054,327,518,492,411,361,190,562,5,614,181,1781,282
KCNJ1_4_3055,1272,1468,1730,1542,1350,1073,2177,1479,2383,2088,3227,21
78
KCNJ4_4_3056,299,493,292,389,389,591,579,151,108,356,245,908
KCNK10_4_3057,850,892,1574,1047,2090,861,597,734,1502,1887,972,1403
KCNK16_4_3058,34,24,106,72,40,52,0,12,171,0,21,57
KCNK17_4_3059,1745,1933,1767,1616,2141,1127,1387,1345,1757,902,2094,79
8
KCNK2_4_3060,537,362,554,508,21,219,91,303,546,636,237,735
KCNK7_4_3061,105,65,100,50,147,376,222,199,1,0,157,13
KCNMA1_4_3062,1321,1106,1412,1006,825,1639,931,736,1096,714,825,1125
KCNMB3_4_3063,2304,2935,3245,2381,2638,1329,2601,3048,1979,2061,1963,3
223
KCNN2_4_3064,1695,1865,2388,1980,931,1581,1042,1727,1554,3318,1939,142
5
KCNN3_4_3065,376,584,673,426,1243,601,282,144,419,683,726,269
KCNQ1_4_3066,58,224,103,114,41,126,323,398,127,0,0,598
KCNQ2_4_3067,196,298,392,258,518,713,77,98,443,12,146,362

KCNQ3_4_3068,876,514,769,833,1217,288,120,703,1793,1698,622,855
KCNQ4_4_3069,30,81,10,189,39,0,99,137,29,0,7,6
KCNQ5_4_3070,342,563,455,700,52,1079,1187,503,485,915,731,973
KCNRG_4_3071,1429,1219,1285,1128,1628,1114,2075,1361,2462,483,1632,811
KCTD15_4_3072,511,637,517,158,661,209,1217,678,65,803,843,1513
KCTD1_4_3073,662,1184,916,508,335,222,1711,967,393,374,1144,980
KCTD6_4_3074,1633,2053,2662,2353,2643,2198,2977,3111,1580,2352,2937,21
93
KCTD7_4_3075,168,209,123,70,0,8,1190,134,520,0,42,925
KLRC1_4_3076,3435,3086,3488,2902,4266,2829,3179,2760,2397,1636,2708,33
14
LEPR_4_3077,2306,2338,2842,2414,1731,2076,2187,2109,2698,2347,2222,172
7
LIFR_4_3078,1522,2092,1614,1407,1558,1672,1271,893,927,984,611,2461
LILRB1_4_3079,1244,1307,987,1161,1547,1341,1442,527,754,463,984,582
LILRB3_4_3080,575,879,927,806,1764,1301,478,645,1087,494,1051,1070
LRP12_4_3081,1820,1563,2077,1661,1342,2592,2271,1691,2121,3904,2742,19
93
LRP8_4_3082,432,528,709,474,606,480,778,374,223,1224,186,319
MR1_4_3083,237,249,269,199,46,84,296,49,175,393,211,248
MSR1_4_3084,704,682,808,1087,727,822,1053,1702,789,2040,1024,1785
NCR2_4_3085,909,897,1080,1304,421,1164,932,359,511,714,544,1079
NCR3_4_3086,21,20,34,111,2,70,88,446,160,133,2,38
NOX1_4_3087,494,430,597,173,761,32,68,618,1015,552,675,448
NOX5_4_3088,413,679,530,487,345,1527,779,376,460,983,1283,142
NRP1_4_3089,1004,1109,1469,1170,572,886,1902,1607,724,101,1082,440
NUDT9_4_3090,642,725,358,398,369,1522,250,122,140,1254,420,353
OLR1_4_3091,206,317,197,113,98,320,158,1,138,18,46,299
OPCML_4_3092,1754,1542,2344,1877,922,785,1178,1007,1048,874,1722,1768
OSMR_4_3093,1040,1225,1516,1157,1753,1214,2334,918,3221,328,2779,999
P2RX2_4_3094,540,229,541,399,537,137,1017,120,145,99,394,740
P2RX5_4_3095,882,997,1073,922,395,926,1094,658,834,1286,1343,967
P2RX6_4_3096,629,709,488,652,357,397,633,82,402,1429,994,300
PEX5_4_3097,220,304,463,364,236,264,651,71,0,12,625,598
PKD1_4_3098,167,395,204,260,50,0,232,246,4,132,113,34
PKD1L2_4_3099,378,439,604,314,668,179,206,31,246,425,322,284
PLA2R1_4_3100,528,684,924,762,1300,807,256,1656,149,110,710,661
PLAUR_4_3101,752,288,476,461,735,252,145,118,242,27,219,374
PLXNB1_4_3102,200,212,345,315,55,46,11,170,431,519,326,246
PRKD2_4_3103,755,636,531,618,263,842,94,174,1409,822,882,632
PRLR_4_3104,1629,1497,1697,1759,1734,2399,2381,1394,968,512,1040,2188
PTCH1_4_3105,3361,4344,3941,3745,3733,1885,2763,3719,3132,5211,4432,24
37
PTCH2_4_3106,116,382,332,432,0,3,450,346,1,706,166,15
ROBO1_4_3107,148,87,138,117,57,34,0,41,110,3,182,217
ROBO2_4_3108,569,628,465,814,740,1129,2330,267,770,1270,2,129
RYR1_4_3109,525,384,588,516,326,145,544,1058,82,688,1158,684
SCARA3_4_3110,71,60,64,160,86,211,8,177,10,7,32,414
SCARF1_4_3111,422,787,939,469,1299,294,300,418,628,165,1263,832
SCARF2_4_3112,39,201,319,125,0,156,18,722,0,0,112,67

SCN1A_4_3113,1390,1618,2163,1760,2422,2065,1474,1144,505,1358,1712,231
3
SCN2A_4_3114,282,247,454,283,380,27,744,1264,1320,1101,372,825
SCN3A_4_3115,1978,1793,1851,1746,2689,2164,2443,2523,1787,589,2780,213
6
SCN3B_4_3116,1251,1347,1356,1262,1325,1429,1036,1895,404,507,2049,1797
SCN4B_4_3117,57,177,163,138,0,5,88,354,1,40,943,287
SCN5A_4_3118,622,673,872,836,113,415,25,1141,885,2089,706,653
SCN8A_4_3119,1417,1496,1576,891,1546,2265,1115,736,2267,3241,2266,1526
SCNM1_4_3120,1052,1490,1289,1331,1156,1818,682,1990,1420,2115,1375,108
9
SCNN1A_4_3121,830,966,685,570,524,1522,1063,579,388,207,964,1058
SLAMF6_4_3122,279,256,310,306,362,408,11,954,140,83,862,478
SLC6A2_4_3123,106,61,47,48,1,76,1,387,122,2,2,111
TLR10_4_3124,504,780,809,536,691,808,182,252,525,1001,433,399
TNFRSF10B_4_3125,980,892,922,591,643,1379,1195,432,323,2615,1119,898
TNFRSF18_4_3126,271,101,211,157,104,594,316,528,788,21,39,217
TNFRSF19_4_3127,1238,1533,1766,1506,1613,4125,2004,1815,1163,2095,1454
,1904
TNFRSF25_4_3128,121,87,118,47,224,347,220,217,58,679,113,0
TNPO1_4_3129,672,725,719,957,651,1263,764,1283,255,616,754,277
TOMM40_4_3130,137,77,124,116,81,256,13,8,7,282,58,197
TP53INP1_4_3131,952,1314,1060,919,1562,1528,1040,695,820,1355,1045,153
3
TPCN1_4_3132,226,107,52,261,85,449,218,76,3,0,51,93
TRPC3_4_3133,1288,1163,1509,1038,2267,1479,787,1671,363,903,610,939
TRPC4_4_3134,412,614,506,541,752,1046,160,34,185,81,151,393
TRPM3_4_3135,545,762,648,816,546,469,652,915,678,776,1079,544
TRPM4_4_3136,573,829,1197,784,814,427,453,1020,639,127,995,942
TRPM6_4_3137,99,379,203,170,228,616,320,127,1,319,196,4
TRPV1_4_3138,400,399,568,547,487,685,1565,225,198,26,1438,540
TRPV4_4_3139,290,131,34,37,1,0,0,110,0,0,83,242
TTYH1_4_3140,820,920,705,850,631,990,1176,879,435,2108,1106,453
TTYH2_4_3141,892,706,639,902,554,1082,1290,460,829,1259,648,702
VDAC2_4_3142,539,728,441,533,726,412,212,1330,228,1035,550,517
ANO7_4_3143,430,352,539,271,1106,424,532,737,455,902,1504,135
AQP1_4_3144,844,512,400,1147,800,498,1050,682,949,1503,1779,1001
CLEC2D_4_3145,446,301,343,746,838,206,472,594,708,340,1718,639
CLEC7A_4_3146,2364,2483,2394,2457,1237,2396,2693,4159,2852,1782,3123,2
113
CRLF2_4_3147,9315,9827,9539,8840,6006,7436,11916,6779,8711,8339,11549,
11157
FXYD3_4_3148,1171,1234,1101,1158,689,2420,1450,939,1681,2196,1691,930
HFE_4_3149,495,590,771,556,359,908,721,583,169,1243,402,574
SCN1B_4_3150,2431,2883,2896,3169,2937,2066,2956,3111,2735,2122,3539,21
46
TNFRSF8_4_3151,196,390,298,285,30,530,158,263,701,475,584,96
TSPO_4_3152,172,403,164,95,352,233,375,3,189,385,319,16
ABCC8_4_3153,890,701,1290,785,1129,1028,1572,821,410,878,1425,1363
AMFR_4_3154,494,493,298,314,120,335,96,246,151,209,107,74

ANO1_4_3155,523,534,636,380,463,437,990,439,1128,253,496,447
ANO2_4_3156,426,247,401,234,692,575,384,412,180,6,296,334
ANXA9_4_3157,231,482,154,436,215,253,119,372,182,754,479,953
AQP10_4_3158,342,361,553,250,192,123,72,664,650,45,66,358
AQP11_4_3159,4544,4751,5345,3210,2962,5719,8468,4669,5240,4410,5315,41
67
AQP12A_4_3160,1555,1413,1838,1503,1706,2289,2517,2094,1120,2097,2396,1
034
AQP2_4_3161,222,203,384,236,529,79,165,24,49,472,1674,379
AQP3_4_3162,822,584,573,462,1209,803,433,535,782,1096,431,792
AQP5_4_3163,208,454,268,250,646,108,2,9,1226,0,4,0
AQP6_4_3164,193,145,154,163,28,0,0,2,2,141,339,85
AQP7_4_3165,735,1281,1173,1251,466,1465,1427,1483,984,1686,1137,1285
AQP8_4_3166,511,602,503,412,726,865,545,1109,163,322,293,616
B2M_4_3167,138,142,217,489,0,96,235,153,658,5,289,480
BEST2_4_3168,220,231,211,275,187,167,403,129,0,7,259,118
BEST4_4_3169,575,1082,1080,587,589,1791,1396,776,756,105,1125,1039
BSND_4_3170,56,121,162,115,130,255,2,9,68,1516,24,307
BTBD10_4_3171,3109,2726,3274,3162,1931,3321,2986,2539,2915,2870,3770,2
112
BTNL2_4_3172,410,106,182,151,0,38,5,22,106,3,21,5
C5orf62_4_3173,144,453,286,153,70,83,317,218,119,1520,608,255
CACNA1B_4_3174,618,1009,683,717,1149,821,1802,247,425,1394,430,1291
CACNA1F_4_3175,319,178,263,134,55,537,940,297,286,52,696,398
CACNA1S_4_3176,869,1026,740,1070,1140,371,794,407,741,1263,515,1238
CACNA2D1_4_3177,2977,2641,2432,2871,1957,3498,2200,2258,4104,1783,2893
,3564
CACNA2D3_4_3178,2758,3085,3504,2888,2243,3481,5017,4265,2858,1186,3964
,2525
CACNA2D4_4_3179,44,59,31,82,0,1,0,3,12,36,315,327
CACNG1_4_3180,927,522,613,1023,321,784,1665,2666,255,1276,553,1211
CACNG2_4_3181,253,393,155,118,62,105,18,458,55,195,67,299
CACNG3_4_3182,404,356,569,115,346,470,474,292,721,1619,737,97
CACNG4_4_3183,205,133,9,170,0,338,12,0,2,0,4,453
CACNG5_4_3184,245,380,344,335,249,283,117,185,296,171,49,263
CACNG7_4_3185,2024,1756,1767,1838,1440,3729,1864,1147,808,3950,2094,19
00
CACNG8_4_3186,81,79,90,162,101,4,55,301,175,0,65,35
CATSPER1_4_3187,1572,1313,1414,1601,1723,733,1750,646,1074,1250,1436,1
325
CATSPER3_4_3188,623,1224,1004,1356,1147,1381,810,2100,348,1007,1336,76
3
CATSPER4_4_3189,346,209,259,351,302,1158,562,1149,120,170,59,228
CCT8L2_4_3190,757,871,578,653,1263,1132,890,477,198,350,697,996
CD160_4_3191,3933,3667,3934,3768,3345,3786,5435,3115,3611,3398,3332,51
92
CD163L1_4_3192,272,209,450,233,42,282,22,68,1,1479,18,201
CD27_4_3193,1020,996,1486,969,1254,670,961,1566,286,1273,1484,728
CD2_4_3194,0,29,22,0,36,237,0,0,37,15,0,0
CD300C_4_3195,909,627,843,592,838,327,698,364,692,697,1163,526

CD3E_4_3196,3266,3489,3809,3149,3191,5013,3669,2019,3902,3275,5057,350
5
CD3G_4_3197,900,823,558,468,383,715,1130,759,141,744,1243,751
CD5_4_3198,42,0,4,74,50,0,64,1,0,138,5,0
CD5L_4_3199,530,775,861,568,246,1508,801,767,1316,424,871,1166
CD69_4_3200,2190,2310,2300,1923,1813,2461,1969,1797,1367,1818,1300,205
2
CD6_4_3201,192,375,220,255,15,0,31,469,389,151,157,450
CD72_4_3202,526,569,398,657,1061,1023,1406,1007,1180,1245,169,778
CD80_4_3203,745,1086,1060,796,756,356,1788,1105,1055,598,867,1005
CFTR_4_3204,100,61,83,126,0,52,121,0,41,2,298,5
CHRNA10_4_3205,422,631,356,318,433,1072,981,217,888,1649,907,328
CHRNA2_4_3206,991,830,479,508,228,108,655,978,618,803,385,488
CHRNA4_4_3207,134,261,222,138,104,49,210,128,5,118,552,142
CHRNA5_4_3208,1780,1195,1659,1524,1348,1545,1585,767,1691,854,1829,161
1
CHRNA9_4_3209,1016,1270,1229,1075,506,2804,2041,1153,1449,894,1692,122
2
CHRNB1_4_3210,290,362,851,517,286,558,724,185,334,2646,641,672
CHRNB2_4_3211,2117,1805,2323,1794,1620,1233,1346,2274,1355,1570,1544,1
861
CHRNB3_4_3212,136,165,344,140,4,119,62,71,374,0,34,98
CHRNB4_4_3213,614,809,537,450,261,234,245,1886,89,5,620,289
CHRND_4_3214,1603,1256,1958,1230,1423,1080,657,762,756,1029,2092,1535
CHRNE_4_3215,180,469,336,332,305,121,301,234,107,0,772,517
CHRNG_4_3216,537,801,435,531,922,293,1000,1223,30,580,301,387
CLCA1_4_3217,467,359,411,171,344,904,604,255,535,233,14,265
CLCA2_4_3218,1245,1269,1640,1796,100,543,866,1123,583,573,739,865
CLCA4_4_3219,444,809,824,340,795,800,860,193,558,700,310,447
CLCN1_4_3220,303,866,674,570,172,224,66,716,96,1254,451,358
CLCN4_4_3221,511,488,401,622,160,1101,400,104,161,247,2252,566
CLDN3_4_3222,496,511,663,645,475,463,640,344,164,282,510,918
CLDN4_4_3223,139,95,143,167,339,867,18,230,18,32,6,503
CLEC1A_4_3224,582,930,833,927,778,406,519,1955,1070,278,1379,572
CLIC1_4_3225,397,216,335,43,610,5,0,16,191,808,98,7
CLIC2_4_3226,1710,2056,2539,1885,1671,2505,3274,1977,1784,1981,2036,23
89
CLIC3_4_3227,119,116,144,169,31,23,164,210,179,0,1088,63
CLIC4_4_3228,1152,1529,1407,1029,1308,831,523,1021,648,1141,1214,1465
CLIC6_4_3229,1402,1012,1052,1221,823,1216,1804,951,713,1239,788,1267
CLNS1A_4_3230,200,284,224,303,28,127,677,469,50,12,519,322
CNGA2_4_3231,78,123,86,100,5,146,209,25,5,0,326,915
CNGA4_4_3232,335,267,290,288,34,202,21,42,74,2,10,717
CNGB3_4_3233,135,15,118,110,556,503,0,131,0,526,79,79
COLEC12_4_3234,1734,2001,1915,1786,1059,2891,834,2764,2536,1039,1248,1
568
CSF2RB_4_3235,259,79,236,158,13,187,110,689,20,1,236,177
CUBN_4_3236,264,282,442,118,293,20,131,490,201,879,56,191
DCC_4_3237,396,478,414,641,1148,169,246,103,762,869,425,267
DNER_4_3238,400,435,466,438,556,481,792,487,251,75,1142,352

EDAR_4_3239,219,227,243,482,284,832,380,127,158,2,129,631
EPOR_4_3240,458,302,401,346,57,179,322,445,112,16,539,559
FCER1A_4_3241,977,600,963,678,1327,329,450,776,70,1410,747,357
FCER1G_4_3242,1114,934,1153,982,500,734,1121,1513,1559,340,566,995
FCGR1A_4_3243,97,48,23,98,0,553,0,55,0,682,13,25
FCGR2C_4_3244,650,761,1062,879,898,293,750,1324,1204,108,2215,1331
FCGR3B_4_3245,283,297,644,619,356,802,619,640,246,105,1187,761
FRRS1_4_3246,2274,2566,2779,2990,2257,2758,2186,2879,2889,2616,2448,33
39
FXYD7_4_3247,116,7,90,19,617,1190,1,0,127,0,727,131
GABRA3_4_3248,482,883,500,510,376,705,243,1044,704,31,631,696
GABRA6_4_3249,8656,8880,8924,7624,8589,9608,9369,6447,5596,9397,7508,7
824
GABRB1_4_3250,3301,3520,3961,3455,3020,3895,3379,5054,2858,5460,2996,3
894
GABRD_4_3251,2401,2144,2124,1622,1867,2235,1229,3068,1738,2673,3197,27
61
GABRE_4_3252,651,557,772,478,735,938,843,349,219,429,654,143
GABRG1_4_3253,1450,1857,1849,1382,1076,1357,1866,1315,1469,1543,1992,1
234
GABRG3_4_3254,312,286,379,407,272,405,399,563,458,320,806,559
GABRP_4_3255,1456,923,1015,1054,911,859,990,96,952,594,999,1744
GABRQ_4_3256,248,277,164,295,262,2201,133,23,84,729,123,57
GABRR1_4_3257,3936,4210,4775,4570,5644,5088,6171,2784,4604,4548,5648,3
855
GABRR2_4_3258,291,319,164,151,24,300,253,127,76,446,0,74
GABRR3_4_3259,1757,1848,2526,1126,2363,3135,1093,1851,2992,1242,3077,1
560
GFRA3_4_3260,144,135,68,162,135,0,115,90,29,426,21,287
GPC1_4_3261,174,202,606,265,384,117,444,551,264,367,1,236
GPC4_4_3262,459,388,335,362,316,7,159,978,600,69,586,401
GPC6_4_3263,551,597,928,507,663,413,2603,669,383,2475,1661,591
GPR89B_4_3264,2807,2722,2603,2634,1576,3398,2793,1539,1784,1579,6168,1
952
GRID1_4_3265,715,730,894,842,567,1197,180,1452,825,1251,823,542
GRID2_4_3266,512,631,506,416,936,265,262,493,855,47,358,663
GRIK3_4_3267,398,468,381,701,788,722,185,338,211,755,192,552
GRIK4_4_3268,248,211,426,232,84,434,202,37,137,1726,534,698
GRIK5_4_3269,481,802,579,337,777,511,634,232,25,1237,49,218
GRIN2B_4_3270,668,815,717,677,927,935,634,1148,416,462,407,588
GRIN2C_4_3271,269,131,412,368,428,494,591,245,32,39,136,164
GRIN2D_4_3272,950,708,1364,1095,657,835,970,618,1030,869,895,208
GRIN3A_4_3273,1026,990,1289,818,752,441,258,778,646,2032,805,877
GRIN3B_4_3274,26,256,241,351,68,118,709,8,0,59,216,248
HAVCR2_4_3275,330,668,430,393,341,545,58,434,250,357,168,44
HCN1_4_3276,655,801,666,876,544,1042,1323,1172,475,13,482,1137
HCN2_4_3277,989,900,844,961,1717,732,614,371,646,1922,444,915
HCN3_4_3278,447,456,420,984,665,86,947,568,45,319,95,296
HCN4_4_3279,347,476,500,342,379,410,83,3,129,34,62,602
HLA-B_4_3280,543,486,613,639,1099,546,640,411,105,334,655,975

HLA-DOA_4_3281,1047,777,1431,757,795,1305,622,770,907,1324,1480,1041
HLA-DOB_4_3282,12,98,176,185,136,1,416,1,135,1,504,0
HLA-DPB1_4_3283,816,718,973,702,505,942,950,332,488,464,809,896
HLA-DQA1_4_3284,11,31,65,151,0,1,14,4,125,0,264,22
HLA-DQA2_4_3285,0,0,0,0,0,0,0,0,0,0,0,0
HLA-DQB2_4_3286,339,627,741,675,250,406,1081,637,243,494,900,941
HLA-DRA_4_3287,47,65,116,364,1012,302,9,46,72,998,238,145
HLA-
DRB1_4_3288,1031,1266,1119,1226,1171,484,1107,1098,656,195,1886,1007
HLA-DRB5_4_3289,68,34,60,178,512,85,3,6,29,29,34,358
HLA-G_4_3290,343,341,257,327,291,10,396,730,229,1,320,104
HTR3B_4_3291,1184,1418,1539,1281,1117,993,41,1566,479,401,1428,978
HTR3C_4_3292,684,845,1022,631,456,275,1063,1164,685,1549,1193,649
HTR3E_4_3293,2817,2803,3004,2881,3101,3043,3948,3325,2864,4131,4028,35
53
ICAM1_4_3294,424,258,369,502,615,694,72,1226,641,15,592,273
IFNAR1_4_3295,419,786,597,522,335,215,1078,350,646,896,171,771
IFNGR1_4_3296,956,1276,1292,1072,141,982,933,1140,990,868,938,758
IFNGR2_4_3297,555,795,642,512,67,1258,1415,26,437,734,611,1130
IGF1R_4_3298,1267,1674,1922,1944,1427,2049,3870,1807,1012,1937,3246,27
93
IGF2R_4_3299,340,462,548,395,347,371,661,70,857,80,32,373
IL10RA_4_3300,573,456,408,395,553,465,60,36,405,581,1489,529
IL10RB_4_3301,2154,2106,2917,1802,1095,2522,2431,2033,1454,2716,2007,1
776
IL12RB2_4_3302,177,304,301,258,274,544,30,67,21,457,119,84
IL13RA1_4_3303,597,678,602,736,742,211,1,682,621,974,309,562
IL13RA2_4_3304,843,822,743,713,1329,860,685,514,752,1056,323,1178
IL17RA_4_3305,89,251,243,87,313,286,9,583,281,0,28,22
IL17RB_4_3306,2983,3217,4265,3466,2796,4462,3101,4151,2934,3682,4839,4
456
IL18R1_4_3307,2452,2369,3054,1531,2343,2406,2471,3356,2420,4084,2225,2
824
IL18RAP_4_3308,1286,1178,1099,1035,1944,1525,1325,716,1096,692,868,165
3
IL1R1_4_3309,952,978,860,932,955,1203,876,983,492,206,1235,614
IL1RAPL1_4_3310,884,863,1170,715,632,218,1077,310,745,589,828,1711
IL1RAPL2_4_3311,755,903,423,459,487,644,541,607,620,589,143,280
IL1RL2_4_3312,116,611,344,381,30,658,147,16,12,39,612,30
IL20RA_4_3313,303,424,401,421,309,586,1588,434,337,791,1175,571
IL22RA1_4_3314,121,102,197,332,27,441,67,11,0,447,1,3
IL23R_4_3315,1874,2204,2530,2382,1644,2147,1732,2856,965,3367,2412,172
2
IL27RA_4_3316,753,996,1314,874,1151,977,1622,1086,982,1013,1849,1764
IL2RA_4_3317,1634,1408,1963,1519,3349,1445,2401,964,2527,1188,1307,255
1
IL2RB_4_3318,225,451,203,267,70,35,402,696,84,0,154,278
IL2RG_4_3319,2437,1907,2295,2624,2334,1398,1561,1977,2106,1014,2041,22
10
IL3RA_4_3320,551,641,535,613,955,315,149,807,572,597,183,756

IL7R_4_3321,213,388,350,309,236,108,375,715,156,0,313,639
IL9R_4_3322,601,568,670,714,1558,784,894,263,271,584,382,701
ITFG1_4_3323,453,328,597,206,120,54,5,351,711,566,115,466
ITGA2B_4_3324,442,611,320,405,188,680,447,291,253,836,72,894
ITGB3_4_3325,639,478,866,610,1041,393,960,375,662,1917,1166,265
ITGB7_4_3326,634,672,982,693,1485,1871,513,856,409,1079,452,974
ITPR2_4_3327,356,519,367,643,384,362,605,334,702,137,1026,289
ITPR3_4_3328,626,876,1009,566,206,821,536,817,1415,44,823,805
JPH3_4_3329,1020,1110,1361,1281,1607,685,1308,1569,588,505,1266,1261
KCMF1_4_3330,1306,1679,1149,1363,1822,1910,1468,826,877,1075,2099,1441
KCNA10_4_3331,420,410,666,332,834,292,983,657,582,64,224,588
KCNA1_4_3332,1300,2024,1839,1946,1236,1980,1423,1914,3070,2126,2160,17
00
KCNA3_4_3333,2799,2645,2935,2916,1885,2471,2489,1663,2640,6001,3046,37
54
KCNA4_4_3334,902,859,706,774,396,753,342,605,863,287,1351,1028
KCNA5_4_3335,1061,794,1429,1080,1192,1345,1472,317,2098,1757,1458,1043
KCNA6_4_3336,615,637,634,558,194,583,1282,801,197,563,524,359
KCNA7_4_3337,1144,837,1239,1049,2047,1257,1480,886,2468,1701,1233,1607
KCNAB3_4_3338,317,381,422,226,3,338,186,39,113,101,937,291
KCNB1_4_3339,196,326,322,214,287,11,21,147,118,38,135,240
KCNB2_4_3340,617,366,703,580,224,36,556,142,207,732,700,477
KCNC3_4_3341,198,121,609,81,54,29,583,293,285,925,383,348
KCND1_4_3342,699,733,663,562,1309,985,1746,787,932,922,639,291
KCND2_4_3343,179,231,165,336,192,459,410,17,305,68,12,67
KCNE1L_4_3344,33,118,45,59,27,10,0,15,0,379,171,48
KCNE2_4_3345,1004,1032,1410,951,421,1051,1052,88,145,2139,783,1500
KCNE3_4_3346,4482,5077,5528,4439,4800,3563,7293,6435,3600,4026,3621,53
91
KCNE4_4_3347,438,816,716,497,191,898,1744,504,295,252,600,332
KCNF1_4_3348,51,41,49,19,0,339,43,2,66,3,241,0
KCNG1_4_3349,155,76,79,69,0,0,0,0,0,0,0,62
KCNG2_4_3350,810,580,683,339,1001,547,712,552,510,193,1367,826
KCNG4_4_3351,688,345,291,242,584,1062,290,1272,485,462,877,806
KCNH3_4_3352,146,170,167,167,55,148,483,400,159,0,204,254
KCNH4_4_3353,830,1297,1016,1210,1060,613,1586,1131,1164,1374,727,837
KCNH8_4_3354,1025,1093,1113,785,1702,1215,946,1026,238,238,814,1536
KCNJ10_4_3355,474,638,417,488,225,1983,1067,209,255,543,945,310
KCNJ12_4_3356,593,474,524,607,258,313,2166,263,1008,88,882,771
KCNJ2_4_3357,412,531,359,222,1018,830,286,327,239,1462,119,67
KCNJ3_4_3358,1029,1091,886,867,1047,1088,1350,1531,941,65,716,833
KCNJ5_4_3359,197,267,416,379,847,351,1129,1182,4,9,453,248
KCNJ6_4_3360,394,512,383,492,244,203,106,473,888,316,1243,411
KCNJ8_4_3361,780,812,636,967,890,359,826,800,250,299,1711,833
KCNJ9_4_3362,322,144,204,267,81,1088,13,496,875,172,1027,649
KCNK12_4_3363,629,574,736,486,484,73,1128,241,216,1304,470,527
KCNK13_4_3364,906,869,662,574,1334,114,340,376,1136,1469,1127,621
KCNK15_4_3365,895,817,969,872,1699,1073,1441,707,606,1114,1530,720
KCNK18_4_3366,1177,1191,1242,1573,1879,1201,234,1522,125,1809,1497,120
4

KCNK1_4_3367,143,257,488,160,22,1071,1506,277,258,31,965,351
KCNK3_4_3368,8092,7979,8536,7894,6751,7283,10037,6370,5635,7286,8077,8
196
KCNK4_4_3369,132,94,108,127,34,116,0,244,259,0,24,36
KCNK5_4_3370,279,315,526,644,981,2393,153,812,91,6,352,318
KCNK6_4_3371,618,724,458,543,813,290,625,235,469,2098,868,695
KCNK9_4_3372,1007,1027,886,975,777,1019,1273,579,792,207,533,1086
KCNMB1_4_3373,252,451,449,249,26,111,675,314,311,0,242,490
KCNMB4_4_3374,689,758,987,484,519,299,784,577,727,839,397,514
KCNN1_4_3375,901,812,921,770,984,670,386,1111,846,838,1411,1553
KCNN4_4_3376,625,761,1326,861,368,506,2402,660,968,733,968,925
KCNS1_4_3377,9,10,111,62,186,1,2,0,28,0,92,269
KCNS2_4_3378,43,64,3,108,35,0,0,285,0,0,27,0
KCNS3_4_3379,733,446,354,583,250,553,292,224,1395,0,783,165
KCNT1_4_3380,310,253,297,143,41,33,100,65,248,33,48,411
KCNT2_4_3381,114,354,337,338,229,15,242,603,403,1,54,245
KCNU1_4_3382,1134,1244,1443,1227,1187,1122,1590,2033,585,610,1191,973
KCNV1_4_3383,299,468,427,264,57,931,335,340,210,219,554,378
KCNV2_4_3384,263,184,305,423,94,617,244,298,473,177,308,127
KCTD10_4_3385,199,87,366,111,17,684,121,498,22,17,612,107
KCTD11_4_3386,288,281,433,326,130,184,520,694,441,688,743,733
KCTD12_4_3387,75,94,139,298,82,0,56,5,6,0,255,107
KCTD13_4_3388,933,1147,1066,884,912,1498,794,762,1027,1060,939,846
KCTD14_4_3389,357,420,302,403,422,20,1114,418,355,332,239,537
KCTD16_4_3390,224,603,581,378,325,297,1577,433,70,552,452,188
KCTD17_4_3391,157,26,285,180,371,276,131,8,2,3,2,0
KCTD18_4_3392,2052,2111,2622,1644,2874,1869,1590,3246,1524,570,944,228
0
KCTD19_4_3393,575,694,612,440,934,56,770,720,569,308,685,219
KCTD20_4_3394,1313,1451,1770,1325,577,902,3408,589,1016,1138,1749,939
KCTD2_4_3395,2161,1869,1935,2395,2984,2334,1599,1881,1876,1438,2858,26
92
KCTD3_4_3396,616,358,735,356,272,141,171,702,322,649,267,1249
KCTD4_4_3397,642,501,668,313,477,223,358,523,410,43,1550,592
KCTD5_4_3398,694,570,514,723,643,852,698,1182,359,430,628,1492
KCTD8_4_3399,145,254,292,175,2,108,198,177,182,56,304,256
KCTD9_4_3400,737,488,585,433,1407,101,393,467,508,1064,1140,126
KIR3DL1_4_3401,526,621,696,755,859,692,829,526,376,11,1007,708
KLRB1_4_3402,778,725,485,739,1861,1446,783,450,905,273,344,1011
LGALS3BP_4_3403,186,492,374,372,509,291,0,462,258,401,569,433
LRP10_4_3404,443,350,280,282,6,82,239,405,342,178,80,345
LRP1B_4_3405,2549,2422,1712,2189,4186,1669,3243,2288,1847,1745,2918,29
48
LRP3_4_3406,770,602,817,1017,351,618,491,750,874,318,1292,851
LRP5_4_3407,489,487,651,783,216,104,144,1016,44,54,1060,770
LRP6_4_3408,2306,1787,2250,1644,1840,2168,4704,2108,1698,1585,2338,331
3
LRPAP1_4_3409,337,288,141,224,338,57,4,1424,12,248,929,327
LTBR_4_3410,0,6,0,0,0,0,0,0,0,0,0,0
MARCO_4_3411,173,252,115,211,1,113,213,983,102,194,202,37

MCOLN1_4_3412,32,65,127,104,89,23,0,88,0,365,45,3
MCOLN2_4_3413,676,560,774,881,1518,919,801,1152,1182,552,442,721
MCOLN3_4_3414,1601,1425,1938,1624,1434,1713,2731,1533,1939,2004,2540,1
678
MCU_4_3415,1352,1911,2308,2159,1757,1477,2232,667,2048,1640,2079,1661
MFRP_4_3416,418,657,644,746,620,329,235,1418,408,733,275,1091
MICB_4_3417,145,97,131,119,0,8,0,2,55,325,381,184
MPL_4_3418,44,174,159,181,154,124,67,75,75,88,264,224
MRC1_4_3419,1654,1645,2467,2401,2504,2353,3061,1979,1686,347,1187,2552
MRC2_4_3420,1162,749,865,862,525,1326,696,1135,1229,1101,1173,952
NALCN_4_3421,997,1445,1288,1482,1482,2144,343,1248,736,1145,2380,904
NGFR_4_3422,1202,922,1396,986,931,1348,1423,1266,271,670,1292,1789
NISCH_4_3423,934,670,877,1150,276,282,1720,1756,858,633,1043,1769
NPTXR_4_3424,273,147,546,545,543,22,5,1012,450,1215,1074,781
ORAI1_4_3425,95,111,295,146,187,19,0,0,951,0,422,80
P2RX1_4_3426,174,191,74,272,93,141,2,83,19,1,10,89
P2RX3_4_3427,489,532,455,493,323,508,536,850,199,461,236,144
P2RX4_4_3428,382,208,113,106,252,0,7,129,455,0,141,176
P2RX7_4_3429,694,515,792,641,662,537,429,562,1606,917,713,1054
PEX5L_4_3430,130,321,272,89,0,0,114,4,0,18,72,133
PGLYRP1_4_3431,335,191,225,152,138,539,227,261,0,186,465,0
PGLYRP2_4_3432,415,476,514,341,779,12,339,16,526,704,125,642
PGLYRP3_4_3433,1194,906,1719,1154,589,2110,2613,524,1021,716,2511,1049
PGLYRP4_4_3434,202,5,257,197,1,21,379,228,32,0,294,201
PGRMC1_4_3435,742,1006,878,1160,944,852,1412,768,732,34,908,1862
PIP_4_3436,2111,2371,1921,3194,1519,2787,2696,1891,825,2311,2392,1747
PKD1L1_4_3437,400,868,658,945,1039,11,1108,1479,985,580,1342,1034
PKD1L3_4_3438,418,562,491,262,357,441,483,116,271,11,643,473
PKD2L1_4_3439,1398,1618,1691,1425,1570,2254,2324,1533,1558,757,1938,19
95
PKD2L2_4_3440,131,484,158,382,35,448,346,801,64,83,424,58
PKDREJ_4_3441,1077,989,1219,1246,1085,173,931,1192,549,722,1033,1138
PLXNA1_4_3442,223,348,197,382,502,31,853,162,388,502,99,848
PLXNA2_4_3443,891,728,814,903,1052,844,1164,780,538,290,693,732
PLXNA3_4_3444,733,447,654,504,563,274,1122,589,372,2584,455,838
PLXND1_4_3445,272,132,157,286,309,0,128,36,68,0,348,101
PRPH2_4_3446,51,115,128,91,214,89,435,129,5,210,61,38
RARRES2_4_3447,474,420,749,329,212,125,269,201,106,19,498,283
RASA3_4_3448,513,537,552,711,855,659,1233,304,580,728,178,659
RYR2_4_3449,537,278,275,388,122,439,132,992,187,1368,724,169
RYR3_4_3450,2106,1848,2241,2361,2391,1978,1966,1651,1071,1307,2401,240
0
SARM1_4_3451,130,159,170,224,21,105,250,647,457,0,500,191
SCN10A_4_3452,416,525,449,493,783,955,465,1956,278,471,651,656
SCN11A_4_3453,3725,3181,3577,3199,3300,5599,3128,2394,2009,3601,4252,3
177
SCN2B_4_3454,602,626,1043,635,317,438,1785,250,574,2543,975,418
SCN4A_4_3455,325,753,1171,684,467,1321,1192,1002,313,348,2044,1046
SCN7A_4_3456,3045,2912,3082,2979,3144,3662,4097,2611,4039,2463,2843,49
92

SCN9A_4_3457,8327,8034,9870,8467,7873,8040,10248,9773,5850,8186,11706,
8454
SCNN1B_4_3458,836,855,1137,852,1053,1110,647,591,1445,329,681,517
SCNN1D_4_3459,444,395,715,467,138,1002,94,788,95,52,831,592
SCNN1G_4_3460,129,115,235,36,677,42,436,0,197,133,37,462
SCUBE1_4_3461,477,472,214,608,0,346,947,437,346,0,86,222
SELE_4_3462,382,657,590,533,720,573,465,778,504,1109,535,613
SEMA5A_4_3463,1433,1660,1361,1149,2207,1765,985,861,1233,1276,1691,138
8
SETD1A_4_3464,160,118,86,77,198,384,24,1,21,989,307,220
SFRP1_4_3465,355,212,249,341,359,309,216,524,571,2,38,311
SFRP4_4_3466,582,525,291,518,188,579,1135,259,18,1365,87,780
SFRP5_4_3467,2880,2854,3965,3108,3691,2873,3459,2523,2714,2501,1718,38
75
SHKBP1_4_3468,185,364,148,244,187,37,119,163,375,2,23,48
SHROOM2_4_3469,315,364,431,253,479,192,270,774,122,110,262,209
SIGLEC8_4_3470,698,607,550,524,1075,510,1123,521,1328,121,819,563
SLAMF1_4_3471,971,1165,1130,953,2261,1256,1574,900,685,99,1919,1065
SLC6A18_4_3472,567,680,650,693,680,1092,485,187,591,99,599,918
SLC6A19_4_3473,1390,1566,1553,1534,2590,1299,1641,1811,913,2571,1159,1
209
SLC6A1_4_3474,42,80,242,229,58,149,319,33,78,183,79,83
SLC6A3_4_3475,61,49,258,268,156,3,87,49,7,0,100,306
SLC6A4_4_3476,942,1261,999,910,856,903,489,953,239,361,1077,346
SLC9A1_4_3477,717,1183,1342,1428,1141,938,1481,1298,1259,1821,1892,882
SLC9A3_4_3478,304,203,352,206,758,178,166,348,5,0,211,584
SRCRB4D_4_3479,155,24,80,188,4,202,11,78,53,0,221,13
STAB2_4_3480,308,216,233,253,146,756,203,19,21,1,15,9
STX1B_4_3481,300,292,470,458,333,787,231,256,274,516,78,306
THBD_4_3482,369,192,223,157,136,379,213,80,74,70,509,224
TLR1_4_3483,1571,1316,1334,1411,1242,1720,1156,721,1111,428,1702,1377
TLR2_4_3484,1010,954,1225,1021,846,1221,2189,1190,430,968,1470,1346
TLR3_4_3485,256,344,448,333,85,46,391,334,282,13,203,360
TLR4_4_3486,853,804,1174,1193,1029,1531,655,1279,617,1427,808,802
TLR5_4_3487,225,371,182,199,365,168,286,408,40,550,122,160
TLR6_4_3488,1022,1042,1612,1037,1589,745,1275,508,561,1791,467,1587
TLR7_4_3489,991,824,916,652,293,517,669,1409,162,737,1544,227
TLR8_4_3490,207,296,211,231,526,675,161,989,557,50,280,115
TLR9_4_3491,362,161,234,482,82,565,486,420,22,1,1017,431
TMEM37_4_3492,1242,1689,1374,1475,999,2097,1185,1893,1475,792,1631,828
TMEM38A_4_3493,393,422,451,511,417,497,8,87,396,1,607,637
TMEM38B_4_3494,640,354,533,487,119,9,658,301,244,2129,795,833
TNFAIP1_4_3495,941,884,672,858,1391,466,1463,1579,505,321,778,876
TNFRSF10A_4_3496,202,318,384,103,98,734,183,508,201,0,208,505
TNFRSF10C_4_3497,1354,1656,1458,1423,1469,1343,778,1833,1338,1177,1281
,2234
TNFRSF10D_4_3498,330,593,565,599,582,1033,671,973,418,532,593,596
TNFRSF11A_4_3499,671,1009,875,967,730,1074,561,1231,1258,1654,1498,125
1
TNFRSF13B_4_3500,321,341,304,443,740,50,76,93,204,159,596,69

TNFRSF13C_4_3501,508,445,211,458,831,61,493,246,127,5,476,145
TNFRSF14_4_3502,426,223,463,250,51,233,399,684,25,387,136,646
TNFRSF1A_4_3503,2420,2546,2383,3018,1933,1775,2132,1115,2220,4504,4187
,2470
TNFRSF1B_4_3504,346,321,398,252,32,320,837,229,348,238,240,178
TNFRSF4_4_3505,101,315,407,238,653,86,146,142,28,805,50,24
TNFRSF6B_4_3506,117,205,200,112,47,412,68,56,438,0,192,134
TPCN2_4_3507,1082,1083,1344,1236,1475,661,1230,1317,1786,1140,591,1235
TREM2_4_3508,132,160,218,173,78,143,28,80,62,0,38,39
TRPA1_4_3509,124,396,488,577,335,20,744,873,111,81,187,401
TRPC1_4_3510,225,386,336,506,85,1088,342,461,505,1067,697,1738
TRPC5_4_3511,61,230,217,80,693,336,266,419,41,0,265,382
TRPC6_4_3512,268,514,432,756,866,406,893,460,891,1083,750,1093
TRPM1_4_3513,589,485,1032,269,400,622,1206,162,221,1736,743,856
TRPM2_4_3514,25,2,4,18,44,520,783,1,0,0,2,0
TRPM5_4_3515,105,257,62,140,0,787,50,202,170,7,124,104
TRPM7_4_3516,2044,1694,1774,2146,2122,2725,1932,1936,1012,1066,2420,19
09
TRPM8_4_3517,2153,2144,2075,2039,1845,2309,3400,1606,1264,2532,1681,16
48
TRPV2_4_3518,2104,1661,2227,1798,1256,1317,2286,1270,2373,1523,3301,68
6
TRPV3_4_3519,220,413,267,490,215,280,189,617,448,96,238,873
TRPV5_4_3520,3261,3858,3977,2992,3232,6019,5456,2586,3653,9121,5682,39
76
TRPV6_4_3521,1796,2162,2611,2581,1280,2764,1616,1742,1916,849,3857,269
1
TTYH3_4_3522,38,74,91,236,58,0,2,307,0,0,3,151
ULBP1_4_3523,643,523,473,856,2169,871,301,660,182,75,412,492
ULBP2_4_3524,347,165,312,309,561,164,993,587,327,55,672,188
ULBP3_4_3525,1643,1795,2072,1650,2332,2374,2713,1834,1286,2569,976,290
6
UNC5A_4_3526,138,89,85,232,802,51,484,0,155,355,0,18
UNC5B_4_3527,127,232,41,109,98,178,27,76,0,37,50,433
UNC5C_4_3528,653,614,469,742,1005,1953,287,744,820,94,1679,308
UTRN_4_3529,142,257,186,160,390,281,246,381,77,1,209,361
VDAC1_4_3530,482,346,278,567,914,464,1142,217,807,44,169,204
ZACN_4_3531,590,738,777,566,814,781,273,598,794,58,896,892
ABCC4_4_3532,2221,2135,2306,2246,859,1946,956,2849,2464,4597,2910,1203
ABCC9_4_3533,404,486,608,492,191,815,1762,124,947,436,1122,582
ADIPOR1_4_3534,2708,2376,2845,2903,3732,2930,3716,1530,1390,4349,2031,
2365
AGER_4_3535,220,286,416,355,278,228,683,84,142,38,365,764
ANO6_4_3536,575,663,460,323,892,278,1611,525,544,1260,634,478
ANTXR1_4_3537,1473,1562,1688,1784,1127,938,1333,2041,1493,325,1271,179
2
ANXA7_4_3538,215,243,262,223,855,249,72,167,73,154,513,955
AQP4_4_3539,285,591,925,430,537,612,581,640,1057,1060,83,164
ASGR1_4_3540,168,307,397,392,222,244,819,133,234,91,811,380
ASGR2_4_3541,411,444,544,368,292,505,988,864,189,1599,576,731

BEST1_4_3542,487,312,388,290,231,375,111,145,231,20,57,275
BEST3_4_3543,865,491,764,695,537,959,693,254,442,0,765,646
CACNA1A_4_3544,1338,1026,1639,1146,1488,512,1685,1217,1088,1294,1250,7
88
CACNA1C_4_3545,3242,2910,3759,3181,3257,4763,3798,2786,2028,2905,2752,
4385
CACNA1D_4_3546,675,687,901,1049,264,2089,1492,756,419,187,1118,1841
CACNA1E_4_3547,27,6,19,1,0,1,0,1,7,7,0,14
CACNA1G_4_3548,668,464,470,507,171,207,557,130,1245,491,598,524
CACNA1H_4_3549,186,92,144,150,343,395,80,376,283,567,27,68
CACNA1I_4_3550,2927,2768,2600,2575,4119,3369,3917,2204,2216,2892,3457,
2086
CACNA2D2_4_3551,181,148,172,186,51,40,324,366,161,144,44,230
CACNB1_4_3552,329,198,263,172,148,177,948,123,225,80,237,595
CACNB2_4_3553,282,313,231,288,119,145,724,121,50,110,332,533
CACNB3_4_3554,755,467,1622,813,607,886,140,85,887,1714,961,452
CACNB4_4_3555,132,41,99,57,0,4,263,14,40,0,18,16
CACNG6_4_3556,128,79,284,187,1180,173,880,80,3,198,358,346
CATSPER2_4_3557,1017,936,630,681,1444,707,2280,1765,392,832,238,685
CD14_4_3558,814,470,388,420,571,689,849,95,27,192,241,795
CD163_4_3559,234,208,443,234,42,279,22,68,1,1483,18,201
CD247_4_3560,384,439,333,325,842,33,784,73,955,114,10,24
CD302_4_3561,515,700,645,318,265,119,140,584,54,38,672,581
CD36_4_3562,982,1008,1357,909,809,1222,730,1080,1643,145,901,659
CD3D_4_3563,1317,1569,1178,1266,1498,777,1150,1239,716,782,2698,1375
CD40_4_3564,232,247,373,216,85,314,34,490,303,141,244,42
CD4_4_3565,407,706,399,681,421,289,1101,858,98,221,787,459
CD74_4_3566,105,347,125,276,66,24,1,211,12,390,361,155
CD79A_4_3567,874,595,663,547,993,990,378,999,775,1561,446,923
CD79B_4_3568,164,305,164,253,153,496,27,10,19,85,227,500
CD86_4_3569,2704,2512,2841,2609,2195,3605,2691,1807,423,2161,3078,2941
CEACAM1_4_3570,618,615,589,613,1081,839,1199,540,72,1123,411,650
CEACAM21_4_3571,718,865,890,584,779,1285,1974,522,284,45,1851,534
CHRFAM7A_4_3572,1,30,154,34,28,0,0,0,0,0,148,6
CHRNA1_4_3573,285,168,361,267,7,66,655,53,195,154,45,202
CHRNA3_4_3574,600,566,994,757,477,191,672,770,111,47,423,620
CHRNA6_4_3575,327,232,288,324,312,158,1177,239,307,1110,217,194
CHRNA7_4_3576,99,54,23,48,69,31,0,0,385,23,0,12
CLCC1_4_3577,43,81,29,35,6,17,0,569,0,0,105,6
CLCN2_4_3578,659,435,462,230,760,192,257,15,575,1364,532,312
CLCN3_4_3579,248,142,277,218,283,35,843,138,230,0,98,173
CLCN5_4_3580,696,1250,533,785,437,1480,894,1018,447,1058,1062,1165
CLCN6_4_3581,539,571,732,785,917,754,712,423,118,17,629,780
CLCN7_4_3582,164,258,183,227,535,340,858,64,0,543,313,80
CLCNKA_4_3583,3041,2820,2368,2557,3179,2393,3223,1273,1143,2422,3373,2
471
CLCNKB_4_3584,1962,2465,2370,1978,2725,1458,4381,2699,2191,1391,4188,2
755
CLEC1B_4_3585,349,790,693,706,305,672,458,1213,363,31,282,385
CLEC4A_4_3586,1737,1912,2376,1779,1595,1008,2251,1963,3323,525,1729,27

06
CNGA1_4_3587,1016,1008,1082,1224,1564,942,1243,1993,553,406,1550,886
CNGA3_4_3588,132,219,276,128,92,3,280,326,327,0,151,37
CNGB1_4_3589,79,87,78,212,32,57,45,15,272,28,24,68
CNTFR_4_3590,298,486,511,262,21,23,31,26,10,245,600,139
CR2_4_3591,235,340,461,373,49,32,1502,364,147,345,206,72
CSF2RA_4_3592,1779,1755,2132,1439,1594,3634,2843,1293,1550,3468,2543,1
175
CSF3R_4_3593,986,1008,1138,1002,1589,709,1516,1446,538,1840,1186,1029
CXADR_4_3594,639,651,879,968,343,1066,2192,703,889,283,1058,287
DAG1_4_3595,3006,3475,3872,3444,6461,3536,3622,3519,2966,6617,3448,457
1
EDA2R_4_3596,153,186,192,156,315,569,12,24,33,11,42,10
EDNRA_4_3597,4932,4748,5625,5350,6687,4940,5243,4930,2827,4682,5227,38
67
ENG_4_3598,134,106,109,91,44,203,201,251,56,388,616,73
EVI2A_4_3599,1694,1846,2024,1382,1551,2746,1995,2732,1117,1208,2120,23
61
FCAMR_4_3600,262,328,379,127,106,126,274,48,118,99,349,492
FCGR1B_4_3601,904,1031,717,840,504,1614,1496,439,330,1274,1415,1172
FCGR2A_4_3602,650,761,1062,879,898,293,750,1324,1204,108,2215,1331
FCGR2B_4_3603,1109,931,875,921,1257,1052,1437,1414,860,949,2224,987
FCGR3A_4_3604,304,210,490,356,32,142,201,454,2,700,440,333
FCGRT_4_3605,228,165,295,115,0,12,152,329,25,0,159,35
FGFRL1_4_3606,569,862,1125,589,401,922,1857,247,466,739,457,905
FLT4_4_3607,8,51,79,72,0,160,0,3,14,455,5,9
FXYD1_4_3608,60,421,198,176,171,68,0,1,45,0,0,482
FXYD2_4_3609,823,376,1069,683,351,479,633,171,1050,1691,720,657
FXYD4_4_3610,517,808,498,397,919,190,1291,470,312,2657,194,703
FXYD5_4_3611,389,188,351,138,268,12,528,261,255,9,128,187
FXYD6_4_3612,241,268,260,283,3,748,188,166,4,230,252,174
GABRA1_4_3613,1831,1970,1678,1811,1757,2409,2275,1313,619,1660,1283,70
5
GABRA2_4_3614,391,360,295,353,266,343,406,117,612,109,272,708
GABRA4_4_3615,1269,1302,1506,1479,1130,2255,1736,1567,1283,826,1738,11
10
GABRA5_4_3616,125,291,303,290,8,195,51,4,61,64,176,1048
GABRB2_4_3617,107,110,131,158,4,134,217,29,218,37,162,51
GABRB3_4_3618,1607,1629,1513,1548,2021,1604,2830,977,1413,1728,1818,16
24
GABRG2_4_3619,394,725,534,627,1078,438,573,289,933,73,1186,227
GFRA1_4_3620,588,585,670,300,557,1415,335,74,272,253,932,663
GFRA2_4_3621,163,104,92,283,74,70,145,129,83,1,161,46
GFRA4_4_3622,34,58,187,87,2,0,11,0,774,267,346,435
GHR_4_3623,656,636,716,691,157,1225,1382,849,105,3,340,508
GIPC1_4_3624,325,237,343,220,664,710,868,300,534,711,174,155
GLRA1_4_3625,526,523,595,985,970,1371,444,502,1429,2164,529,342
GLRA2_4_3626,1317,1705,1968,1721,2109,2176,3188,1364,763,698,2206,1592
GLRA3_4_3627,2054,1766,2401,2085,2003,862,975,1511,1809,2346,4328,2555
GLRA4_4_3628,758,1175,845,999,1327,1225,2055,390,1531,1164,1506,1091

GLRB_4_3629,1156,1244,1220,1388,1330,1293,1816,2340,1759,1727,1169,123
2
GP6_4_3630,196,312,316,226,153,30,7,389,23,0,68,536
GPM6A_4_3631,2108,2388,3044,2602,4338,1953,2777,2358,2215,2368,1454,23
40
GPR89A_4_3632,2807,2722,2603,2634,1576,3398,2793,1539,1784,1579,6168,1
952
GRIA1_4_3633,203,392,444,367,383,413,124,704,37,119,399,400
GRIA2_4_3634,0,22,0,0,0,0,0,0,0,0,0,0
GRIA3_4_3635,967,984,809,624,574,629,976,606,2112,757,2223,525
GRIA4_4_3636,647,736,1176,605,866,547,218,1283,208,1324,1628,1313
GRIK1_4_3637,654,552,796,641,1508,721,655,444,331,582,853,169
GRIN2A_4_3638,69,141,105,75,261,0,0,280,0,0,149,464
GRINA_4_3639,1795,2178,2036,1891,3274,1779,2525,2026,1904,2145,2351,36
80
HLA-DPA1_4_3640,360,441,727,678,680,351,221,146,167,314,815,424
HTR3A_4_3641,413,390,497,482,335,433,690,37,213,105,499,166
HTR3D_4_3642,134,162,399,164,76,19,163,120,665,13,69,500
HVCN1_4_3643,200,122,85,253,72,878,13,129,398,9,28,49
IFNAR2_4_3644,1096,831,1359,1083,2236,1250,1170,842,630,52,1471,1525
IL11RA_4_3645,602,725,1028,929,580,1104,1421,792,396,179,469,1702
IL12RB1_4_3646,966,732,809,814,959,550,746,1040,795,576,1193,751
IL15RA_4_3647,1402,1612,2353,1456,1215,1840,1992,1292,676,886,2241,222
9
IL1R2_4_3648,2005,1958,2450,1872,1762,1986,1774,2093,1142,1662,2971,16
64
IL1RAP_4_3649,762,855,981,566,1682,718,736,1202,499,1259,549,538
IL1RL1_4_3650,2251,2291,2931,3128,4211,3102,2565,2328,2997,2998,2682,2
567
IL21R_4_3651,1584,1700,1964,1765,1008,2458,295,1494,1809,2178,1887,186
7
IL22RA2_4_3652,1994,3236,2612,2320,1090,3770,3334,1668,1872,1986,2783,
2218
IL28RA_4_3653,0,0,0,0,0,0,0,0,0,0,0,0
IL31RA_4_3654,2609,2549,3299,2523,4205,3699,4000,1922,1517,610,4064,24
98
IL4R_4_3655,722,507,460,414,647,128,68,593,353,97,185,365
IL5RA_4_3656,6934,5500,5700,5398,5788,5436,6928,5962,4501,4332,8453,48
16
IL6R_4_3657,1033,1239,1537,1056,1747,960,2027,887,1092,5454,1449,1079
IL6ST_4_3658,421,339,780,535,155,430,624,381,715,542,1051,622
ILDR1_4_3659,519,641,818,853,672,1852,1361,21,572,665,1219,558
ITGB1_4_3660,702,747,964,599,898,452,816,1144,388,329,2020,632
ITGB4_4_3661,660,455,842,680,697,774,758,817,532,356,248,516
ITPR1_4_3662,774,588,662,876,413,924,648,1317,951,502,936,626
KCNA2_4_3663,473,561,830,807,732,280,2736,1006,146,1291,743,764
KCNAB1_4_3664,1043,963,908,720,2272,604,1029,2159,793,74,1210,2113
KCNAB2_4_3665,841,1017,1379,1122,883,839,1692,1001,807,478,2238,1238
KCNC1_4_3666,2593,2126,3053,2455,2945,3088,1582,3778,2294,2478,1975,25
66

KCNC2_4_3667,2116,1837,1948,1527,2533,1560,1953,1720,265,941,2440,1726
KCNC4_4_3668,156,383,161,382,55,568,40,118,0,129,344,130
KCND3_4_3669,466,306,494,463,269,114,914,165,290,560,413,489
KCNE1_4_3670,312,402,675,442,138,947,978,935,291,134,272,82
KCNG3_4_3671,1066,1245,1573,1176,763,1092,1491,1844,1458,1925,1725,234
3
KCNH1_4_3672,256,468,359,228,1,876,27,349,107,0,437,117
KCNH2_4_3673,293,381,664,244,0,1219,99,544,323,657,446,919
KCNH5_4_3674,463,495,603,540,524,856,166,922,254,43,437,636
KCNH6_4_3675,74,57,61,26,150,5,269,253,385,0,92,22
KCNH7_4_3676,1016,975,1224,931,953,1062,1778,1086,1216,2586,2108,1206
KCNIP1_4_3677,1495,1665,1865,1570,1186,1493,1793,1722,2872,722,1543,16
59
KCNIP2_4_3678,457,338,591,654,98,396,945,665,884,1533,856,355
KCNIP4_4_3679,1489,2092,2420,1956,2217,1665,2696,1616,1545,2147,2901,3
318
KCNJ11_4_3680,480,306,530,319,110,566,795,191,24,926,0,143
KCNJ13_4_3681,615,752,623,502,119,346,50,774,418,1335,620,1133
KCNJ14_4_3682,284,191,219,62,107,14,0,63,0,251,149,85
KCNJ15_4_3683,577,427,596,338,49,812,593,644,1057,823,756,618
KCNJ16_4_3684,645,441,593,343,352,1044,375,167,647,881,526,502
KCNJ1_4_3685,746,549,637,537,1047,233,485,359,401,1459,252,700
KCNJ4_4_3686,179,162,341,274,56,354,207,157,23,5,327,138
KCNK10_4_3687,6656,6831,8017,6588,5996,7572,7752,8673,5364,9267,8546,8
745
KCNK16_4_3688,108,53,24,261,0,15,46,10,45,48,143,104
KCNK17_4_3689,15,32,76,77,2,0,0,0,67,0,4,413
KCNK2_4_3690,548,364,385,456,253,345,273,590,40,488,737,704
KCNK7_4_3691,65,218,178,223,235,222,139,70,26,21,1,38
KCNMA1_4_3692,298,204,795,341,96,26,262,48,21,4,747,48
KCNMB3_4_3693,784,860,651,546,673,809,864,34,1361,360,867,953
KCNN2_4_3694,196,80,448,439,0,39,658,83,336,27,852,838
KCNN3_4_3695,509,556,431,431,989,278,247,310,510,725,455,235
KCNQ1_4_3696,322,124,464,138,321,9,227,767,92,0,788,243
KCNQ2_4_3697,536,279,402,344,941,466,105,412,89,3,25,210
KCNQ3_4_3698,226,422,266,404,337,348,575,265,678,12,180,688
KCNQ4_4_3699,126,93,171,112,37,264,330,13,286,251,69,391
KCNQ5_4_3700,2233,2231,2630,2705,2553,2777,4189,3697,965,1995,1899,324
9
KCNRG_4_3701,1032,917,1015,821,853,1742,1730,836,542,284,1584,1051
KCTD15_4_3702,1202,1186,1481,1624,1876,858,1335,1459,1826,1925,1348,15
98
KCTD1_4_3703,52,144,108,57,36,16,12,114,113,8,44,13
KCTD6_4_3704,1941,2918,2039,2321,1771,1668,1426,1575,2124,2267,3679,17
52
KCTD7_4_3705,57,144,121,135,2,10,346,547,290,0,459,67
KLRC1_4_3706,916,720,621,698,16,543,1178,602,704,613,321,665
LEPR_4_3707,0,0,0,0,0,0,0,0,0,0,0,0
LIFR_4_3708,3095,2816,3692,2855,2767,3697,5686,2772,1956,1131,3475,387
1

LILRB1_4_3709,276,186,334,460,31,138,273,407,542,76,119,282
LILRB3_4_3710,579,988,1137,899,1906,1534,429,670,1595,203,1206,1397
LRP12_4_3711,613,673,569,986,1129,39,772,1973,586,269,1181,703
LRP8_4_3712,528,592,393,582,500,1191,721,133,462,993,242,186
MR1_4_3713,709,633,812,1067,933,1838,358,730,1209,1998,681,338
MSR1_4_3714,348,490,431,481,121,551,551,733,134,732,955,1001
NCR2_4_3715,718,437,750,1001,931,1049,752,401,636,466,905,308
NCR3_4_3716,67,231,192,143,228,49,42,46,8,426,148,81
NOX1_4_3717,1470,1057,948,1101,1169,535,869,1327,1254,400,1530,903
NOX5_4_3718,1040,846,615,593,221,836,223,619,124,559,132,797
NRP1_4_3719,2040,2125,1891,1800,2002,483,1776,1555,1152,3366,2552,1699
NUDT9_4_3720,795,872,957,870,649,1362,380,1300,248,392,1081,935
OLR1_4_3721,628,534,790,896,492,1105,1701,373,625,298,1345,1133
OPCML_4_3722,1413,1589,2235,1353,1968,2039,2395,2157,1131,439,1378,110
3
OSMR_4_3723,236,278,288,146,23,71,1,102,14,694,195,164
P2RX2_4_3724,123,112,392,142,59,1,497,17,0,736,23,1
P2RX5_4_3725,483,435,672,406,8,684,698,412,691,1175,698,681
P2RX6_4_3726,282,321,377,262,292,309,439,138,280,458,205,376
PEX5_4_3727,656,1071,987,453,426,264,858,559,1342,746,947,1440
PKD1_4_3728,1736,1534,1835,1552,975,831,2323,1076,1007,1397,1804,1609
PKD1L2_4_3729,629,606,472,301,224,1387,66,998,24,1253,644,529
PLA2R1_4_3730,365,382,460,524,695,223,32,261,1807,147,1023,828
PLAUR_4_3731,80,80,100,43,94,54,121,11,38,0,294,59
PLXNB1_4_3732,2,87,79,169,33,12,0,7,244,0,0,0
PRKD2_4_3733,29,182,108,83,15,0,104,7,211,679,70,357
PRLR_4_3734,1640,1959,2037,1866,1321,2101,1710,1559,1381,2663,1731,258
7
PTCH1_4_3735,91,201,181,104,64,41,157,184,161,0,120,35
PTCH2_4_3736,1440,1311,1419,1496,1105,3484,2056,1634,1621,2430,2425,17
81
ROBO1_4_3737,2151,1699,2717,1563,1818,2820,2927,2231,1328,3318,2643,23
01
ROBO2_4_3738,578,790,637,834,281,1191,293,1067,632,157,865,815
RYR1_4_3739,710,807,714,793,517,1282,859,1098,755,510,1126,1293
SCARA3_4_3740,1217,1220,1843,1294,2010,1212,4028,1497,2133,1236,2876,2
285
SCARF1_4_3741,760,600,504,727,2320,648,503,1151,1112,3,842,1080
SCARF2_4_3742,494,294,422,314,570,566,541,755,47,973,1365,67
SCN1A_4_3743,2310,1849,1926,1761,1095,2346,3209,1963,1672,2125,2130,21
27
SCN2A_4_3744,2442,2565,3324,2645,1952,2063,3336,2274,1535,4017,4316,29
41
SCN3A_4_3745,1806,2129,2593,3021,1877,3512,2058,3217,2810,3792,4373,41
74
SCN3B_4_3746,213,111,419,282,80,36,314,64,310,192,598,264
SCN4B_4_3747,412,647,760,805,316,178,611,533,616,13,655,346
SCN5A_4_3748,341,534,520,311,454,158,892,1521,364,137,1010,226
SCN8A_4_3749,686,447,753,407,973,1049,945,544,247,773,529,535
SCNM1_4_3750,612,859,691,651,726,626,261,384,209,138,261,725

SCNN1A_4_3751,933,876,1159,969,175,210,945,227,247,73,560,1482
SLAMF6_4_3752,1009,1216,1191,1497,403,1846,1698,1301,999,687,1992,1438
SLC6A2_4_3753,948,594,1174,998,591,826,1030,871,573,1946,1015,1017
TLR10_4_3754,1196,1136,653,712,1303,2887,1100,1326,481,758,1189,1377
TNFRSF10B_4_3755,891,624,654,893,1859,1077,1544,1385,1259,494,312,1399
TNFRSF18_4_3756,397,289,393,340,364,405,208,151,52,51,998,642
TNFRSF19_4_3757,273,342,571,344,192,412,0,386,165,6,172,358
TNFRSF25_4_3758,120,121,136,139,58,17,163,607,19,1,120,123
TNPO1_4_3759,727,1129,1493,1303,714,453,2238,2834,948,666,855,2113
TOMM40_4_3760,595,260,421,296,207,126,1225,1486,772,430,1162,299
TP53INP1_4_3761,137,188,170,170,17,122,452,465,148,61,11,79
TPCN1_4_3762,489,756,781,1032,481,1818,466,1243,297,1370,2387,1035
TRPC3_4_3763,152,319,344,228,606,300,126,390,454,5,306,263
TRPC4_4_3764,1672,1560,1455,1364,630,1214,952,1761,1398,2010,1155,2116
TRPM3_4_3765,636,432,895,702,519,943,897,626,579,921,271,498
TRPM4_4_3766,11,112,69,48,348,37,89,8,1148,0,134,68
TRPM6_4_3767,1237,1383,1886,1046,809,1582,1443,569,730,498,831,1073
TRPV1_4_3768,66,284,239,128,87,25,10,46,69,0,160,188
TRPV4_4_3769,99,41,138,112,0,116,0,130,27,0,20,31
TTYH1_4_3770,152,21,137,68,86,361,0,110,123,0,163,141
TTYH2_4_3771,491,329,295,336,330,109,668,279,130,299,408,689
VDAC2_4_3772,854,981,673,742,594,1232,656,561,407,951,607,826
ANO7_4_3773,181,505,516,453,539,178,187,468,175,82,181,134
AQP1_4_3774,415,529,726,465,421,425,634,260,63,705,1083,590
CLEC2D_4_3775,288,134,415,267,215,15,363,299,52,131,371,75
CLEC7A_4_3776,1688,1016,1705,1500,1302,2034,2049,2624,1520,1949,1864,2
567
CRLF2_4_3777,3049,3096,3597,2984,2848,3787,4268,3149,3351,2519,5493,42
69
FXYD3_4_3778,219,210,297,54,735,397,463,0,0,69,235,70
HFE_4_3779,407,426,270,305,674,1360,394,330,798,280,978,44
SCN1B_4_3780,307,249,315,317,31,866,0,33,307,5,256,61
TNFRSF8_4_3781,479,394,551,431,331,206,255,361,155,229,214,523
TSPO_4_3782,1117,1548,1359,1480,998,1227,2295,1757,569,1164,1801,1691
ABCC8_4_3783,547,757,1487,727,1026,1221,1303,826,141,63,686,1103
AMFR_4_3784,447,591,570,433,344,395,994,98,632,103,1420,613
ANO1_4_3785,150,270,176,257,481,339,432,378,262,106,282,662
ANO2_4_3786,676,603,581,845,841,824,2254,712,656,542,28,622
ANXA9_4_3787,2037,1542,1849,1085,821,1413,1265,1422,911,2460,1414,2501
AQP10_4_3788,457,500,515,471,547,328,88,538,134,251,241,675
AQP11_4_3789,2140,1748,2182,1796,2276,3942,2354,2489,1282,3617,2184,23
08
AQP12A_4_3790,270,133,207,220,180,3,541,306,535,0,229,78
AQP2_4_3791,433,456,380,237,313,714,890,224,141,258,76,467
AQP3_4_3792,240,256,114,254,643,589,691,184,26,97,112,453
AQP5_4_3793,62,201,77,356,770,35,631,320,73,30,116,615
AQP6_4_3794,324,699,728,465,895,695,346,334,1141,932,969,542
AQP7_4_3795,1277,842,1304,1346,1223,928,468,1492,300,1636,1608,1728
AQP8_4_3796,69,302,196,236,5,57,684,297,85,1298,5,42
B2M_4_3797,657,358,847,376,1049,46,243,829,708,96,502,379

BEST2_4_3798,562,414,574,591,614,473,535,618,288,217,363,767
BEST4_4_3799,264,256,169,200,122,175,171,311,323,1,195,334
BSND_4_3800,732,517,680,632,391,288,553,370,564,78,866,349
BTBD10_4_3801,1118,1156,1371,1759,949,1408,2411,1358,1015,614,1665,162
6
BTNL2_4_3802,411,454,591,339,28,1826,617,434,1079,1,1303,126
C5orf62_4_3803,224,461,281,191,94,544,142,756,523,682,176,148
CACNA1B_4_3804,220,398,278,355,334,1078,348,278,140,336,129,245
CACNA1F_4_3805,91,131,28,48,9,31,0,4,4,14,185,52
CACNA1S_4_3806,425,504,506,327,870,723,514,603,103,0,227,450
CACNA2D1_4_3807,2418,2294,2649,2113,2140,1740,2934,941,2616,2295,3031,
1393
CACNA2D3_4_3808,544,334,420,677,222,89,44,164,342,366,700,934
CACNA2D4_4_3809,845,738,704,698,565,433,592,1137,717,659,888,210
CACNG1_4_3810,959,518,609,1013,598,784,1494,2681,254,1284,553,1214
CACNG2_4_3811,692,905,968,701,815,329,164,1279,53,1999,1818,1814
CACNG3_4_3812,704,359,283,408,179,354,588,536,673,710,286,58
CACNG4_4_3813,3402,3869,4260,3421,3017,3045,3494,3546,3758,2854,3908,4
314
CACNG5_4_3814,344,278,618,454,168,1046,588,166,10,0,803,218
CACNG7_4_3815,214,299,608,423,131,1944,490,598,170,55,868,125
CACNG8_4_3816,930,1071,1107,1037,1953,679,1534,875,1022,310,1221,805
CATSPER1_4_3817,902,845,988,399,458,968,541,551,1346,427,1654,694
CATSPER3_4_3818,558,1193,932,1211,1144,1341,434,2097,345,526,1435,755
CATSPER4_4_3819,667,994,720,621,664,1069,1188,809,650,217,862,1557
CCT8L2_4_3820,386,405,275,336,0,0,381,1218,192,311,510,394
CD160_4_3821,3757,4388,3948,2632,2911,3622,5879,4182,2531,3458,5315,44
73
CD163L1_4_3822,190,201,205,167,1174,1119,0,174,2,0,569,97
CD27_4_3823,949,568,747,604,1289,804,105,820,31,686,1114,935
CD2_4_3824,366,443,280,282,386,197,75,500,14,0,264,71
CD300C_4_3825,114,30,82,62,59,0,27,0,0,0,74,53
CD3E_4_3826,2342,2593,2966,2391,2936,3059,2768,2028,2827,2360,4390,324
8
CD3G_4_3827,170,410,452,282,3,558,346,314,856,400,237,392
CD5_4_3828,267,453,504,414,87,571,416,260,284,105,285,178
CD5L_4_3829,139,76,85,187,37,149,20,40,117,0,873,392
CD69_4_3830,979,960,1014,943,1144,1717,776,326,791,672,620,1363
CD6_4_3831,1407,1402,1609,1096,2358,1997,1780,2094,649,578,955,1303
CD72_4_3832,311,815,426,886,327,73,0,572,257,555,149,221
CD80_4_3833,862,1591,1142,1264,625,1485,810,734,871,527,243,902
CFTR_4_3834,456,482,455,404,307,773,755,65,322,218,359,392
CHRNA10_4_3835,1269,1247,1907,1035,1967,1834,1568,628,1125,881,2190,29
4
CHRNA2_4_3836,816,428,489,445,708,150,2121,484,778,318,1246,1037
CHRNA4_4_3837,839,643,631,767,618,786,646,1069,415,35,934,334
CHRNA5_4_3838,74,219,404,242,17,0,39,14,90,185,131,18
CHRNA9_4_3839,552,553,973,556,912,590,861,333,245,1108,779,491
CHRNB1_4_3840,1046,1017,1083,932,1401,1255,386,401,977,1230,1135,1011
CHRNB2_4_3841,350,385,439,325,423,420,234,712,437,802,110,336

CHRNB3_4_3842,1062,564,733,529,1242,1602,1314,636,849,2299,978,831
CHRNB4_4_3843,435,419,298,509,218,10,1261,1181,148,11,810,352
CHRND_4_3844,176,288,234,198,188,627,221,52,46,164,448,216
CHRNE_4_3845,252,97,246,140,3,1,414,10,78,137,40,33
CHRNG_4_3846,106,162,295,194,66,103,14,67,129,78,89,174
CLCA1_4_3847,316,264,395,299,253,41,267,426,457,29,91,845
CLCA2_4_3848,987,1492,1261,1088,1770,1469,1040,1225,771,763,1472,1267
CLCA4_4_3849,5785,6064,9178,5636,5806,3858,9057,8722,4866,6095,8735,63
80
CLCN1_4_3850,230,147,70,79,740,290,443,257,0,3,192,142
CLCN4_4_3851,736,689,645,551,442,928,679,49,613,420,1330,366
CLDN3_4_3852,271,87,365,261,611,286,28,259,128,523,40,99
CLDN4_4_3853,87,132,299,198,156,473,107,0,37,329,37,135
CLEC1A_4_3854,59,390,150,100,49,0,1543,189,1,945,101,77
CLIC1_4_3855,972,956,1313,885,733,952,1520,743,1451,334,577,1133
CLIC2_4_3856,246,483,749,355,481,538,1303,400,513,193,425,1139
CLIC3_4_3857,88,189,187,251,19,219,36,142,139,539,173,63
CLIC4_4_3858,411,395,264,260,484,2,52,372,234,277,261,311
CLIC6_4_3859,2644,2734,3933,3532,2951,3871,5355,3010,1890,2607,5202,43
87
CLNS1A_4_3860,530,563,377,375,501,923,1904,152,756,418,886,308
CNGA2_4_3861,31,35,117,80,21,0,0,0,0,0,0,12
CNGA4_4_3862,1086,1033,873,840,2575,603,828,1026,765,47,1072,879
CNGB3_4_3863,552,623,638,429,461,237,853,736,398,98,849,453
COLEC12_4_3864,479,698,642,965,235,248,1313,313,736,1222,535,854
CSF2RB_4_3865,132,365,185,515,123,197,194,278,1,495,2469,443
CUBN_4_3866,936,1106,933,985,892,1208,1157,731,318,414,2021,891
DCC_4_3867,419,218,377,463,218,255,186,547,211,274,506,294
DNER_4_3868,927,1258,890,795,1036,126,555,1384,52,1321,2105,1148
EDAR_4_3869,261,498,307,182,501,570,147,1105,507,51,918,83
EPOR_4_3870,339,100,73,169,14,361,696,786,328,645,459,453
FCER1A_4_3871,526,602,570,598,421,182,274,159,877,406,68,1069
FCER1G_4_3872,254,306,276,467,182,386,93,590,210,503,69,142
FCGR1A_4_3873,904,1031,717,840,504,1614,1496,439,330,1274,1415,1172
FCGR2C_4_3874,1513,1171,1542,1114,1807,784,1757,1216,1946,900,2855,206
9
FCGR3B_4_3875,304,210,490,356,32,142,201,454,2,700,440,333
FRRS1_4_3876,998,1197,1598,1377,1779,2724,844,1427,3076,1172,2348,3195
FXYD7_4_3877,938,491,899,575,1004,538,725,2284,536,10,997,429
GABRA3_4_3878,818,1042,710,438,561,1071,554,1206,301,831,288,1024
GABRA6_4_3879,2619,2704,2679,2816,2628,2983,1719,1526,2948,2605,2776,3
546
GABRB1_4_3880,1623,2002,2033,1952,1606,2734,1007,2122,2316,2822,1934,2
755
GABRD_4_3881,363,280,343,384,182,41,89,1060,580,46,254,638
GABRE_4_3882,689,786,649,536,929,126,784,1225,144,1155,914,578
GABRG1_4_3883,995,850,1111,1513,1277,600,859,1081,177,201,748,272
GABRG3_4_3884,513,553,758,505,215,234,422,362,243,217,971,242
GABRP_4_3885,1623,1478,2024,1896,1170,3142,1004,567,1265,2285,2620,134
7

GABRQ_4_3886,476,573,364,433,844,1300,129,380,170,1019,248,1016
GABRR1_4_3887,319,523,526,443,65,72,517,663,428,27,740,302
GABRR2_4_3888,1391,1134,1408,2070,608,2161,2720,1262,2012,2025,2101,16
51
GABRR3_4_3889,156,417,635,444,9,64,305,442,431,897,288,7
GFRA3_4_3890,644,755,604,689,1220,751,402,308,288,555,536,501
GPC1_4_3891,300,289,264,313,258,557,269,94,40,27,41,46
GPC4_4_3892,616,948,858,1035,410,455,954,1115,373,1060,1029,1267
GPC6_4_3893,2105,2262,2629,2111,2789,2103,2933,1766,2926,1471,2305,159
3
GPR89B_4_3894,1854,1751,1688,1211,2752,1821,1471,2319,494,733,1482,163
0
GRID1_4_3895,1662,1639,1926,1687,2694,970,1319,1347,1341,1388,1148,892
GRID2_4_3896,1578,1444,1500,1347,1316,2194,1720,863,986,1775,1523,1442
GRIK3_4_3897,195,209,765,486,49,363,235,642,117,288,105,527
GRIK4_4_3898,725,610,579,473,1046,218,1816,352,1228,229,381,138
GRIK5_4_3899,70,302,141,178,313,278,471,105,118,77,386,347
GRIN2B_4_3900,714,904,357,320,1040,529,523,692,43,229,161,869
GRIN2C_4_3901,161,268,206,247,55,74,233,244,14,664,156,243
GRIN2D_4_3902,234,349,301,89,202,1184,26,388,5,144,321,205
GRIN3A_4_3903,82,156,149,264,9,230,8,7,304,24,433,124
GRIN3B_4_3904,630,849,583,388,87,45,1185,58,407,12,563,440
HAVCR2_4_3905,238,142,75,26,2,0,52,25,15,0,0,1053
HCN1_4_3906,1438,1477,1756,1530,1567,2224,3023,949,1438,2642,2187,1149
HCN2_4_3907,365,264,351,426,498,654,1002,765,24,1067,620,577
HCN3_4_3908,1029,943,867,950,1713,798,613,371,648,1925,444,881
HCN4_4_3909,341,501,452,619,559,925,107,832,257,266,822,891
HLA-B_4_3910,381,511,342,280,330,38,1029,765,541,127,378,300
HLA-DOA_4_3911,147,161,193,122,0,0,16,191,20,285,173,275
HLA-DOB_4_3912,817,826,751,707,339,934,659,934,1437,825,426,942
HLA-DPB1_4_3913,263,482,416,354,262,68,752,485,451,122,452,1261
HLA-DQA1_4_3914,443,532,407,544,411,172,1167,200,193,339,690,595
HLA-DQA2_4_3915,0,0,0,0,0,0,0,0,0,0,0,0
HLA-DQB2_4_3916,152,38,108,30,0,583,0,6,0,0,20,4
HLA-DRA_4_3917,84,315,73,298,173,80,675,52,298,0,452,147
HLA-DRB1_4_3918,375,306,453,292,251,651,67,12,121,494,948,117
HLA-DRB5_4_3919,267,341,359,216,892,170,308,395,17,231,156,159
HLA-G_4_3920,234,82,101,132,229,14,428,103,1,515,68,85
HTR3B_4_3921,3583,4315,4696,4067,2920,4912,6338,3467,3205,5386,3903,46
85
HTR3C_4_3922,683,388,626,531,652,276,1535,471,207,262,200,441
HTR3E_4_3923,139,172,400,168,76,20,161,120,668,13,69,619
ICAM1_4_3924,523,548,458,442,251,78,422,82,664,268,133,656
IFNAR1_4_3925,2009,1632,1746,1366,3374,2403,2558,1730,2622,2314,2034,2
107
IFNGR1_4_3926,926,553,864,1009,226,736,782,361,1190,429,855,1345
IFNGR2_4_3927,289,687,676,406,132,610,545,269,0,218,1092,752
IGF1R_4_3928,5772,5937,7066,6081,6563,5123,10617,3817,4879,6630,8321,6
327
IGF2R_4_3929,184,42,63,34,11,112,433,26,151,3,13,22

IL10RA_4_3930,374,457,469,479,212,237,62,450,114,210,260,270
IL10RB_4_3931,1354,1348,1053,1248,979,2557,0,1009,973,1814,2343,816
IL12RB2_4_3932,1310,855,1098,768,778,918,927,690,250,592,1286,703
IL13RA1_4_3933,937,472,934,525,854,475,1104,1724,139,401,58,490
IL13RA2_4_3934,470,656,883,853,488,2428,1346,306,394,751,655,1748
IL17RA_4_3935,771,1175,819,1144,1166,326,1439,1759,1205,815,978,921
IL17RB_4_3936,846,982,797,1158,1624,160,476,561,767,62,1997,1297
IL18R1_4_3937,972,1037,752,735,1559,970,1272,513,1237,914,607,1284
IL18RAP_4_3938,547,452,712,594,895,848,505,905,840,124,475,564
IL1R1_4_3939,3314,2887,3078,3873,2653,3429,3784,3880,2790,1541,5208,37
21
IL1RAPL1_4_3940,1135,703,908,1155,608,588,545,1387,628,739,413,1115
IL1RAPL2_4_3941,817,962,934,892,554,1034,575,1463,806,1035,1007,823
IL1RL2_4_3942,202,117,140,229,0,18,696,65,241,1178,240,115
IL20RA_4_3943,1301,1472,1401,1195,1265,2584,1315,1499,864,550,515,1558
IL22RA1_4_3944,1078,814,1033,896,2059,2131,1272,1087,352,110,1214,1604
IL23R_4_3945,1586,1640,1691,1217,2329,679,1258,1850,801,1068,1001,1630
IL27RA_4_3946,1120,1349,1553,1098,1506,1687,1006,1086,1615,2094,1805,1
528
IL2RA_4_3947,299,69,216,214,289,81,393,508,13,197,138,399
IL2RB_4_3948,32,65,152,136,468,68,16,60,15,858,3,513
IL2RG_4_3949,2092,1709,1902,1851,759,1818,1865,2311,2104,358,1673,2091
IL3RA_4_3950,712,348,238,634,418,760,118,453,770,237,352,449
IL7R_4_3951,125,264,135,84,1042,365,348,1,33,23,196,481
IL9R_4_3952,76,172,218,110,535,286,22,195,119,573,63,92
ITFG1_4_3953,1880,1390,1707,1288,2001,1607,1232,2229,813,2084,2930,145
4
ITGA2B_4_3954,10,40,131,214,70,14,60,858,203,0,2,87
ITGB3_4_3955,413,378,500,486,474,283,220,440,584,69,1580,127
ITGB7_4_3956,227,351,345,394,307,77,393,417,15,759,69,390
ITPR2_4_3957,659,626,585,989,460,772,166,588,364,797,1198,930
ITPR3_4_3958,646,576,1062,627,779,734,1524,1164,124,1249,1411,706
JPH3_4_3959,601,424,637,319,726,345,348,139,253,205,360,234
KCMF1_4_3960,357,194,590,245,43,214,396,1452,1114,196,228,117
KCNA10_4_3961,1173,1002,1607,1133,1244,1200,1017,1575,1676,1639,1869,1
751
KCNA1_4_3962,1476,1477,1871,1673,1299,2472,3267,2005,806,637,2047,1346
KCNA3_4_3963,77,454,271,337,169,337,926,42,71,369,30,666
KCNA4_4_3964,476,500,459,375,164,717,1055,553,297,100,84,318
KCNA5_4_3965,273,235,222,94,100,5,211,400,275,357,400,538
KCNA6_4_3966,342,369,413,184,68,39,875,446,48,488,199,125
KCNA7_4_3967,447,266,320,140,276,528,430,28,282,14,138,442
KCNAB3_4_3968,445,315,426,362,186,1414,169,189,188,699,527,276
KCNB1_4_3969,104,194,267,105,174,313,205,51,125,0,139,762
KCNB2_4_3970,152,217,360,243,122,97,522,59,54,464,364,851
KCNC3_4_3971,267,272,301,251,120,229,75,71,34,148,173,210
KCND1_4_3972,1129,1090,1062,1012,342,2153,412,1177,208,2963,1500,1104
KCND2_4_3973,266,158,161,233,33,446,52,81,2,1,429,41
KCNE1L_4_3974,2021,2049,2189,2282,1890,1053,1891,3489,3055,2691,3725,2
378

KCNE2_4_3975,1100,1253,1567,1507,602,2138,285,1330,766,1091,1822,684
KCNE3_4_3976,529,293,508,203,268,0,1391,330,42,63,168,211
KCNE4_4_3977,3392,3193,3637,3614,2939,4286,5950,2747,3517,1383,3009,32
83
KCNF1_4_3978,128,89,385,120,117,0,54,2,3,0,27,360
KCNG1_4_3979,184,320,440,674,377,146,915,178,478,825,102,294
KCNG2_4_3980,27,210,51,38,224,252,0,0,1,0,72,1
KCNG4_4_3981,307,325,448,306,543,22,940,872,661,1057,179,243
KCNH3_4_3982,325,379,555,658,26,402,8,411,552,41,573,417
KCNH4_4_3983,337,384,343,695,187,591,467,182,480,342,969,909
KCNH8_4_3984,1758,1102,1671,845,1254,865,1306,649,620,897,1327,951
KCNJ10_4_3985,135,405,389,257,51,109,51,110,281,4,28,769
KCNJ12_4_3986,305,307,404,392,486,893,343,935,550,141,430,479
KCNJ2_4_3987,1866,1774,2300,1835,1739,925,2700,1914,2119,1079,2775,203
0
KCNJ3_4_3988,256,287,282,388,160,32,488,121,104,242,464,454
KCNJ5_4_3989,437,581,461,610,1359,312,68,118,870,185,462,662
KCNJ6_4_3990,209,408,296,489,72,327,1110,918,951,273,556,982
KCNJ8_4_3991,2873,2956,3031,2747,3216,3922,3208,3373,4362,3570,3428,19
69
KCNJ9_4_3992,55,62,49,83,98,0,9,6,0,182,163,36
KCNK12_4_3993,144,389,406,195,34,78,289,515,66,61,312,52
KCNK13_4_3994,580,611,798,531,734,679,682,405,169,2131,610,823
KCNK15_4_3995,355,161,280,98,379,18,74,229,198,0,260,318
KCNK18_4_3996,412,320,533,279,125,1,103,37,473,1046,358,149
KCNK1_4_3997,281,350,277,374,190,837,520,244,386,0,182,93
KCNK3_4_3998,95,112,190,55,1,49,346,3,1,128,33,9
KCNK4_4_3999,528,462,526,438,155,806,1028,358,5,1220,603,696
KCNK5_4_4000,216,203,334,352,97,436,2,589,518,3,89,909
KCNK6_4_4001,427,384,697,299,379,378,247,2,293,12,516,735
KCNK9_4_4002,8092,7979,8536,7894,6751,7283,10037,6370,5635,7286,8077,8
196
KCNMB1_4_4003,160,254,236,117,44,16,0,64,399,330,558,109
KCNMB4_4_4004,1397,1343,1077,1102,919,1529,614,2205,509,507,1340,915
KCNN1_4_4005,422,291,518,440,640,357,460,591,127,0,504,612
KCNN4_4_4006,273,538,463,434,298,963,243,118,20,784,222,1290
KCNS1_4_4007,93,398,248,319,103,60,265,172,13,6,1039,428
KCNS2_4_4008,655,588,1060,911,502,813,1005,715,312,307,704,1275
KCNS3_4_4009,52,125,75,316,412,0,21,569,134,0,0,205
KCNT1_4_4010,2656,2301,2311,2153,2413,1410,3462,3181,2266,945,2289,316
4
KCNT2_4_4011,1005,775,706,1201,990,644,313,875,563,480,848,1133
KCNU1_4_4012,583,635,586,191,287,199,1308,806,771,230,513,145
KCNV1_4_4013,636,328,608,472,6,246,487,599,931,629,574,303
KCNV2_4_4014,401,447,440,419,945,301,99,670,10,2072,94,350
KCTD10_4_4015,2929,1671,2927,2172,3301,2324,5029,2905,1395,2911,1919,3
237
KCTD11_4_4016,223,141,116,123,107,105,52,692,1,1858,547,78
KCTD12_4_4017,244,36,82,6,0,1,0,329,73,302,186,0
KCTD13_4_4018,1064,2242,1161,1320,2419,1213,638,2416,1139,2484,3213,99

1
KCTD14_4_4019,390,231,500,226,521,142,974,221,60,199,48,536
KCTD16_4_4020,0,0,0,0,0,0,0,0,0,0,0,0
KCTD17_4_4021,1775,1445,1719,1793,2136,1557,2334,1687,1371,2142,1782,1
263
KCTD18_4_4022,464,731,733,663,699,502,1797,458,266,420,621,469
KCTD19_4_4023,806,946,621,791,288,884,281,440,417,2157,1399,1919
KCTD20_4_4024,868,583,652,473,299,202,1437,621,628,104,1083,1594
KCTD2_4_4025,183,314,472,379,185,732,374,235,85,225,301,143
KCTD3_4_4026,1197,1239,1175,1437,1375,703,874,2902,749,1542,2210,952
KCTD4_4_4027,1128,1153,804,1109,1103,2099,493,1232,590,552,1627,1899
KCTD5_4_4028,384,315,581,589,669,106,2045,463,174,915,1303,379
KCTD8_4_4029,495,184,668,622,203,232,41,128,9,102,49,923
KCTD9_4_4030,1910,1977,1678,2042,1483,1936,1254,1497,1282,3146,2060,14
74
KIR3DL1_4_4031,759,642,594,335,541,118,278,278,987,398,1057,323
KLRB1_4_4032,1242,827,1081,1099,1317,2511,1700,1006,1482,846,2767,1809
LGALS3BP_4_4033,402,401,370,165,813,72,8,353,703,691,9,676
LRP10_4_4034,516,512,696,517,552,892,117,1472,502,52,814,288
LRP1B_4_4035,3357,3232,2941,2683,1902,2806,2854,1572,2311,3842,4737,39
20
LRP3_4_4036,426,337,626,573,167,152,1,635,200,668,988,141
LRP5_4_4037,433,240,271,281,586,835,194,323,264,230,127,617
LRP6_4_4038,1714,1730,2232,1790,1483,2785,1393,2675,902,1377,2955,2028
LRPAP1_4_4039,123,172,156,185,293,687,280,34,0,121,249,574
LTBR_4_4040,217,351,623,652,281,661,529,310,279,202,345,695
MARCO_4_4041,593,758,826,941,512,165,686,770,654,718,1117,961
MCOLN1_4_4042,1408,1369,1227,1170,887,1413,1811,898,992,1673,982,2273
MCOLN2_4_4043,1594,2114,2093,1853,2154,1672,2959,2478,463,701,1826,248
7
MCOLN3_4_4044,1025,919,1493,941,1703,1422,827,2404,742,1075,1411,2161
MCU_4_4045,1140,1051,1102,1234,767,371,624,1467,403,1329,1361,1765
MFRP_4_4046,461,708,741,666,614,172,337,1087,405,231,895,769
MICB_4_4047,685,1132,665,1038,1708,697,623,1634,1190,1364,518,1045
MPL_4_4048,445,826,573,579,812,413,244,703,102,333,455,241
MRC1_4_4049,2286,2586,2614,2806,1981,3203,2159,4096,2010,4426,4191,274
9
MRC2_4_4050,1137,1148,1394,1067,1316,673,982,804,929,816,1566,1240
NALCN_4_4051,201,11,132,247,0,332,516,2,3,34,34,37
NGFR_4_4052,570,529,561,741,456,826,801,622,527,1125,588,1722
NISCH_4_4053,244,96,70,104,634,2,0,0,439,0,130,210
NPTXR_4_4054,418,722,663,493,279,307,238,216,196,19,1581,284
ORAI1_4_4055,61,33,169,98,0,41,236,0,215,5,4,17
P2RX1_4_4056,2354,2179,1780,2361,881,2192,2615,1151,2556,2953,3164,127
7
P2RX3_4_4057,459,543,607,701,188,231,1845,469,887,943,559,718
P2RX4_4_4058,76,347,353,383,280,35,172,619,367,0,162,32
P2RX7_4_4059,617,416,468,354,127,10,99,59,74,5,569,350
PEX5L_4_4060,779,695,601,617,169,1141,877,947,514,392,185,533
PGLYRP1_4_4061,52,57,74,10,85,2,0,0,23,0,24,85

PGLYRP2_4_4062,518,707,480,509,241,280,638,325,439,69,449,1309
PGLYRP3_4_4063,118,110,72,113,24,2,6,20,91,0,15,23
PGLYRP4_4_4064,489,247,182,537,5,526,142,374,197,1,1163,312
PGRMC1_4_4065,306,599,714,600,285,873,1374,450,862,607,205,1112
PIP_4_4066,459,287,182,102,202,5,99,519,399,0,763,328
PKD1L1_4_4067,378,418,395,554,65,167,637,783,137,603,286,145
PKD1L3_4_4068,524,749,493,511,730,712,973,486,380,14,725,554
PKD2L1_4_4069,1057,984,955,1136,434,538,597,1161,1362,417,935,1034
PKD2L2_4_4070,2104,2720,3181,2128,828,2114,3473,3380,1522,1438,3911,23
43
PKDREJ_4_4071,346,331,321,381,724,2,169,365,265,547,952,164
PLXNA1_4_4072,992,886,886,752,1065,375,1090,871,605,478,644,888
PLXNA2_4_4073,789,767,573,870,232,762,550,608,480,816,521,1000
PLXNA3_4_4074,109,125,141,224,608,8,1116,1,3,2,169,137
PLXND1_4_4075,204,51,118,70,61,31,8,413,0,29,32,87
PRPH2_4_4076,930,1076,1749,1014,455,792,1163,845,432,2761,2606,794
RARRES2_4_4077,677,695,683,572,193,202,830,219,138,377,882,620
RASA3_4_4078,732,800,895,892,353,1221,847,731,795,235,1596,1678
RYR2_4_4079,880,641,1019,1029,1163,94,429,932,389,303,977,1327
RYR3_4_4080,2343,3238,2960,2943,2198,2892,3704,3096,1751,3651,2041,183
5
SARM1_4_4081,955,1079,960,868,1281,1738,671,471,251,753,798,1009
SCN10A_4_4082,403,314,355,256,87,1144,345,68,95,713,476,170
SCN11A_4_4083,156,108,225,43,205,0,251,334,532,18,0,268
SCN2B_4_4084,124,236,208,193,337,465,662,98,223,365,119,219
SCN4A_4_4085,175,260,384,270,72,412,59,77,148,789,211,27
SCN7A_4_4086,0,0,0,0,0,0,0,0,0,0,0,0
SCN9A_4_4087,267,496,329,337,171,763,665,900,84,38,635,233
SCNN1B_4_4088,734,480,440,650,272,163,870,178,358,773,796,724
SCNN1D_4_4089,131,58,130,365,26,14,8,379,304,190,103,438
SCNN1G_4_4090,213,314,369,269,240,135,271,261,61,700,195,241
SCUBE1_4_4091,412,171,190,254,92,426,75,141,431,6,85,721
SELE_4_4092,180,435,251,300,85,663,126,156,345,1,102,324
SEMA5A_4_4093,447,229,536,752,19,533,642,273,706,165,149,200
SETD1A_4_4094,577,569,454,684,900,500,22,155,1454,89,1069,297
SFRP1_4_4095,201,276,257,192,25,244,41,102,21,56,89,4
SFRP4_4_4096,98,322,141,185,17,90,281,89,700,56,794,152
SFRP5_4_4097,467,425,219,333,234,2,282,340,332,265,149,512
SHKBP1_4_4098,255,421,657,376,105,168,817,455,427,1248,276,212
SHROOM2_4_4099,141,397,104,159,260,0,469,1,465,384,42,235
SIGLEC8_4_4100,531,769,713,527,85,222,438,343,241,751,1549,491
SLAMF1_4_4101,354,222,576,296,395,323,327,548,339,12,352,321
SLC6A18_4_4102,42,172,112,14,0,174,90,26,48,217,5,0
SLC6A19_4_4103,1316,1511,1518,1515,2587,1298,1648,1808,917,2379,1161,9
95
SLC6A1_4_4104,527,447,404,551,533,77,374,135,208,182,621,599
SLC6A3_4_4105,377,576,293,397,252,629,241,386,63,31,487,728
SLC6A4_4_4106,1212,1130,1170,1109,865,965,1110,1065,920,1080,1250,1364
SLC9A1_4_4107,893,928,872,779,708,1715,1005,976,179,46,792,1018
SLC9A3_4_4108,684,719,834,797,714,738,577,926,936,153,1048,918

SRCRB4D_4_4109,519,580,447,385,510,920,315,348,276,16,631,167
STAB2_4_4110,336,686,581,723,25,1359,541,517,302,1333,476,987
STX1B_4_4111,454,897,1003,976,1078,199,1591,985,406,718,611,673
THBD_4_4112,230,229,437,322,501,848,517,481,203,201,241,63
TLR1_4_4113,1172,1391,1217,1382,1144,794,1143,617,843,1762,3820,937
TLR2_4_4114,0,23,0,0,0,0,0,0,0,0,0,0
TLR3_4_4115,508,326,348,378,1236,475,1544,177,448,33,315,77
TLR4_4_4116,426,403,469,539,178,456,235,374,262,172,1121,287
TLR5_4_4117,2649,2023,2674,3092,3332,3129,2934,2026,2647,3598,1936,275
7
TLR6_4_4118,1838,2288,1948,1790,1061,1313,3806,745,701,2699,2313,1919
TLR7_4_4119,0,0,6,0,0,0,0,0,0,0,0,0
TLR8_4_4120,3026,3100,2671,2952,1593,2208,4867,1883,3588,2896,3517,347
1
TLR9_4_4121,291,87,90,106,11,143,212,223,447,0,405,62
TMEM37_4_4122,128,10,203,69,8,24,52,0,0,0,20,156
TMEM38A_4_4123,562,335,312,314,282,53,1057,283,285,808,1087,356
TMEM38B_4_4124,323,204,154,148,397,455,267,105,766,11,786,30
TNFAIP1_4_4125,567,622,613,619,190,656,831,388,728,273,1297,996
TNFRSF10A_4_4126,1765,2032,2520,1463,2760,2867,2812,1149,3766,5359,242
0,1008
TNFRSF10C_4_4127,1596,1282,2263,1668,2181,1284,3508,1455,2352,988,1072
,1936
TNFRSF10D_4_4128,167,185,230,550,30,406,63,351,147,375,292,619
TNFRSF11A_4_4129,556,358,539,275,678,322,180,497,603,20,297,319
TNFRSF13B_4_4130,453,498,414,286,379,549,115,437,370,14,416,479
TNFRSF13C_4_4131,227,372,302,503,68,334,0,151,64,750,142,386
TNFRSF14_4_4132,493,360,387,240,175,194,193,637,1125,65,330,469
TNFRSF1A_4_4133,139,50,133,78,59,197,244,672,17,633,320,143
TNFRSF1B_4_4134,412,507,588,516,1,161,177,1286,314,546,426,366
TNFRSF4_4_4135,342,650,822,547,209,344,403,946,548,1585,1736,536
TNFRSF6B_4_4136,39,9,45,2,1,18,11,0,0,9,111,299
TPCN2_4_4137,758,850,1296,597,907,573,1318,376,579,778,509,448
TREM2_4_4138,615,375,311,216,6,596,6,136,431,0,280,727
TRPA1_4_4139,506,1069,911,849,616,587,1677,1424,516,1136,758,795
TRPC1_4_4140,761,989,940,631,905,476,1330,1373,275,1436,297,917
TRPC5_4_4141,1177,515,668,882,1057,1288,148,282,1180,317,661,598
TRPC6_4_4142,566,893,608,887,863,952,833,960,995,386,1791,1321
TRPM1_4_4143,1956,2148,1551,2015,952,2537,1289,735,849,1457,2997,2726
TRPM2_4_4144,87,16,61,251,0,1,7,395,26,0,373,3
TRPM5_4_4145,2487,3188,2713,3811,1770,1148,2625,2787,2131,1635,4383,37
44
TRPM7_4_4146,1463,1066,1427,1284,1004,1313,2183,1084,867,757,3672,589
TRPM8_4_4147,713,906,737,440,627,39,1576,1098,170,80,943,1067
TRPV2_4_4148,1716,1557,1760,1600,745,1546,581,1437,1226,518,1660,1356
TRPV3_4_4149,776,577,622,994,137,942,514,323,587,1870,718,520
TRPV5_4_4150,266,186,505,284,159,136,198,165,198,0,236,619
TRPV6_4_4151,1159,1052,1545,1228,1391,1279,1036,718,877,1529,1184,1462
TTYH3_4_4152,175,181,258,179,200,8,327,398,254,50,75,37
ULBP1_4_4153,843,389,449,628,856,771,32,337,484,98,649,413

ULBP2_4_4154,365,985,465,656,539,1059,280,1494,745,1006,748,878
ULBP3_4_4155,1554,1627,2111,1667,2294,2055,2712,2354,1217,2123,774,303
9
UNC5A_4_4156,2286,1767,2750,2002,2623,1815,4083,2071,1071,2217,1567,28
26
UNC5B_4_4157,1089,1254,928,1343,1620,1267,873,905,935,3325,1766,1664
UNC5C_4_4158,115,11,36,63,0,0,1,1,10,883,134,102
UTRN_4_4159,0,0,1,66,0,0,0,0,0,0,0,0
VDAC1_4_4160,458,662,615,653,1235,2103,603,700,448,364,1684,1354
ZACN_4_4161,1076,1426,1571,1121,1328,569,885,707,294,2001,1080,975
ABCC4_4_4162,911,672,1013,585,500,1268,1282,988,500,133,852,378
ABCC9_4_4163,1391,1202,1249,1240,2574,1739,1289,1234,1569,1007,1975,12
47
ADIPOR1_4_4164,203,222,188,161,981,200,1,216,150,1,18,76
AGER_4_4165,165,193,232,181,8,695,11,8,167,154,185,612
ANO6_4_4166,861,729,748,1022,592,883,1976,646,589,331,298,594
ANTXR1_4_4167,2,21,1,0,13,0,0,0,0,0,2,0
ANXA7_4_4168,134,147,356,83,148,140,16,106,2,880,116,370
AQP4_4_4169,231,632,916,505,771,463,580,644,1186,1179,144,343
ASGR1_4_4170,641,647,804,609,1102,218,895,297,914,1061,5,262
ASGR2_4_4171,140,149,284,211,608,1044,214,44,0,10,492,1
BEST1_4_4172,859,1394,769,1128,895,1446,2667,458,240,430,1818,1338
BEST3_4_4173,1366,1455,1210,925,2122,2267,690,2616,1446,375,867,1018
CACNA1A_4_4174,518,537,386,389,1243,171,288,62,48,67,1216,577
CACNA1C_4_4175,31,256,84,30,21,54,182,271,68,342,255,1
CACNA1D_4_4176,741,445,491,763,928,14,99,440,822,289,408,718
CACNA1E_4_4177,1019,510,1143,790,1392,236,774,57,98,2050,943,895
CACNA1G_4_4178,1008,957,1233,1147,1337,990,1518,1048,1009,1418,1029,16
17
CACNA1H_4_4179,1194,1225,1185,875,1478,2124,1270,1932,923,3034,1357,85
3
CACNA1I_4_4180,156,269,68,234,623,0,496,7,41,0,5,1
CACNA2D2_4_4181,495,752,452,741,501,345,993,1089,123,843,1705,452
CACNB1_4_4182,1323,1269,967,1300,1037,1926,355,1078,748,438,803,538
CACNB2_4_4183,440,246,528,245,366,100,244,374,27,274,185,405
CACNB3_4_4184,235,158,334,200,118,557,1,67,17,30,109,204
CACNB4_4_4185,316,218,410,327,398,36,38,51,20,391,1607,197
CACNG6_4_4186,702,578,915,854,52,421,1921,1721,82,1981,433,894
CATSPER2_4_4187,2038,1784,2147,2274,1383,2226,2360,3088,1979,2124,2108
,1704
CD14_4_4188,115,123,138,145,409,309,94,151,52,396,72,15
CD163_4_4189,199,128,354,264,461,824,538,91,360,0,169,55
CD247_4_4190,845,1212,1376,1132,1239,2053,1367,1850,804,811,917,1020
CD302_4_4191,398,298,347,399,112,22,64,167,14,39,344,427
CD36_4_4192,195,99,136,188,18,8,33,318,50,242,6,302
CD3D_4_4193,422,781,577,381,827,742,610,141,925,89,36,402
CD40_4_4194,225,197,219,441,482,39,120,694,287,417,190,370
CD4_4_4195,1279,823,1139,1285,1091,1123,1979,1371,896,1979,1823,968
CD74_4_4196,231,237,374,181,37,0,34,0,54,2293,1676,57
CD79A_4_4197,2923,3429,3218,2947,2813,3131,3671,2173,2864,3865,4276,40

83
CD79B_4_4198,167,141,95,205,87,2,70,241,699,725,376,52
CD86_4_4199,738,483,766,637,567,600,267,765,195,161,677,395
CEACAM1_4_4200,1584,1535,1642,1665,1151,1674,380,2037,1257,1372,1137,8
74
CEACAM21_4_4201,2780,2430,2409,2526,2343,2492,3357,2071,2572,3125,4418
,1568
CHRFAM7A_4_4202,425,358,400,407,124,84,595,188,288,1844,962,90
CHRNA1_4_4203,3143,4679,3882,4128,2773,3320,6403,4303,3039,3950,4087,3
729
CHRNA3_4_4204,1215,1263,1760,1614,1483,1063,1788,2382,827,2422,1990,23
06
CHRNA6_4_4205,1282,1637,1883,1786,1480,1121,2264,2342,1249,2471,2537,2
399
CHRNA7_4_4206,3382,3804,3659,3754,3218,4636,5101,3199,1964,3701,3542,2
958
CLCC1_4_4207,1199,1243,1609,1420,941,1930,892,1434,886,3182,929,798
CLCN2_4_4208,108,331,314,145,15,525,73,0,242,1,350,93
CLCN3_4_4209,404,441,640,399,262,344,1012,590,164,1529,898,664
CLCN5_4_4210,396,431,690,418,674,420,268,538,547,182,857,314
CLCN6_4_4211,836,986,1238,984,755,870,928,1490,1462,288,1581,1308
CLCN7_4_4212,329,220,281,404,730,80,539,309,415,75,1374,373
CLCNKA_4_4213,469,593,424,529,195,1302,849,307,93,860,385,339
CLCNKB_4_4214,3041,2820,2368,2557,3179,2393,3223,1273,1143,2422,3373,2
471
CLEC1B_4_4215,157,120,147,122,157,0,207,0,77,3,171,223
CLEC4A_4_4216,1973,2306,2369,2920,2278,3820,2336,2124,1551,2026,2139,2
530
CNGA1_4_4217,3784,4358,3877,3210,3882,4549,4756,3122,2577,2949,3682,28
70
CNGA3_4_4218,511,601,1075,959,614,1302,1249,1369,467,2901,1573,869
CNGB1_4_4219,39,7,36,53,12,1,2,6,25,6,8,164
CNTFR_4_4220,635,420,556,378,277,347,85,782,109,1410,205,213
CR2_4_4221,1102,1339,1281,1127,2213,2065,1251,1058,1009,1570,2785,748
CSF2RA_4_4222,189,289,292,165,244,171,874,80,283,778,345,375
CSF3R_4_4223,42,31,77,81,97,112,19,3,154,11,59,89
CXADR_4_4224,395,508,517,454,771,444,185,437,391,123,702,77
DAG1_4_4225,168,93,22,167,14,727,454,19,0,0,375,6
EDA2R_4_4226,267,227,327,327,225,123,335,411,214,88,327,813
EDNRA_4_4227,1193,1363,2152,1321,3271,1956,1436,950,1563,2493,740,1612
ENG_4_4228,595,919,738,578,670,748,629,1001,215,724,808,915
EVI2A_4_4229,1248,971,1180,1252,513,2208,694,1622,1324,1540,2743,1040
FCAMR_4_4230,1022,1142,1478,1231,1657,1530,627,1614,935,651,1180,900
FCGR1B_4_4231,3546,3325,3634,3164,2754,2919,4263,3481,2739,1447,4393,3
402
FCGR2A_4_4232,1513,1171,1542,1114,1807,784,1757,1216,1946,900,2855,206
9
FCGR2B_4_4233,641,249,378,401,164,1032,258,266,45,1558,335,115
FCGR3A_4_4234,1629,1515,1974,1546,1333,2799,1524,2011,1283,1589,1609,2
044

FCGRT_4_4235,190,101,106,50,0,368,31,0,0,0,37,92
FGFRL1_4_4236,641,936,1233,536,1266,980,1618,248,612,816,340,904
FLT4_4_4237,423,478,608,414,676,283,480,49,1011,1431,532,776
FXYD1_4_4238,1287,1253,1434,1271,2410,1044,1766,1033,1024,3023,1835,11
65
FXYD2_4_4239,363,291,341,623,493,247,245,280,942,736,580,660
FXYD4_4_4240,231,160,382,158,26,82,0,5,112,191,201,24
FXYD5_4_4241,384,313,285,337,536,569,293,259,358,38,551,1047
FXYD6_4_4242,135,45,99,89,0,179,62,136,18,55,18,10
GABRA1_4_4243,1172,979,1389,1552,1632,1655,1958,842,1718,434,1231,1197
GABRA2_4_4244,1150,1125,1422,1288,1642,1674,1626,1027,595,1413,1964,26
15
GABRA4_4_4245,50,93,238,143,10,37,33,10,293,690,68,251
GABRA5_4_4246,708,356,499,364,1568,977,446,531,978,418,869,119
GABRB2_4_4247,1980,1487,2269,1955,1781,3192,2576,1043,2371,3746,1455,1
832
GABRB3_4_4248,4317,4526,4626,4423,2936,7653,3162,3712,2958,5965,4315,4
991
GABRG2_4_4249,91,112,305,56,259,48,123,132,177,59,75,41
GFRA1_4_4250,853,406,769,678,692,680,867,431,157,271,1084,539
GFRA2_4_4251,889,776,709,863,317,180,748,225,914,1115,226,363
GFRA4_4_4252,133,118,339,209,13,212,149,297,314,630,101,0
GHR_4_4253,283,337,326,269,120,476,0,415,273,12,1,324
GIPC1_4_4254,301,371,137,253,165,17,9,152,3,0,101,49
GLRA1_4_4255,632,767,1189,912,1660,506,1337,877,352,392,721,740
GLRA2_4_4256,649,615,472,637,128,695,1066,984,469,661,119,278
GLRA3_4_4257,278,140,476,437,315,51,74,562,1463,263,143,141
GLRA4_4_4258,230,177,74,137,111,155,128,102,403,0,53,292
GLRB_4_4259,1111,1278,890,1593,915,653,1616,2090,675,411,2403,2016
GP6_4_4260,735,955,928,779,557,1063,1163,1448,1464,385,1016,1164
GPM6A_4_4261,827,600,541,860,811,58,479,691,1317,495,1448,1313
GPR89A_4_4262,1854,1751,1688,1211,2752,1821,1471,2319,494,733,1482,163
0
GRIA1_4_4263,2709,2395,2661,2906,2957,3174,2008,1500,1056,940,4600,191
8
GRIA2_4_4264,411,454,526,380,830,1490,127,755,144,34,663,130
GRIA3_4_4265,1040,1587,1382,1477,1095,2170,947,1518,990,982,1901,1437
GRIA4_4_4266,8078,6974,8440,8214,5006,8193,8338,9256,5903,9215,10323,7
146
GRIK1_4_4267,2048,2613,2599,2104,1566,3023,2591,2509,1282,1721,5039,28
16
GRIN2A_4_4268,386,572,480,384,843,1169,525,168,25,2115,313,842
GRINA_4_4269,1327,1145,1841,1053,827,642,1431,889,624,1428,1808,1191
HLA-DPA1_4_4270,162,143,47,42,0,0,124,163,209,116,6,154
HTR3A_4_4271,2509,2547,2588,2585,2118,2106,2593,3079,2679,2041,3370,32
65
HTR3D_4_4272,2105,2540,2839,2068,1882,4149,4529,1547,1786,1056,2216,34
70
HVCN1_4_4273,189,261,226,274,137,239,374,89,32,156,236,679
IFNAR2_4_4274,784,937,1078,899,835,715,877,280,1651,182,1664,1137

IL11RA_4_4275,471,298,663,197,850,699,123,1180,11,444,890,50
IL12RB1_4_4276,402,557,674,705,163,549,401,559,71,541,481,268
IL15RA_4_4277,410,359,616,304,153,574,905,105,249,672,593,326
IL1R2_4_4278,65,104,65,123,38,131,260,172,0,155,118,45
IL1RAP_4_4279,920,1016,796,789,352,583,1077,523,142,1411,412,677
IL1RL1_4_4280,49,87,89,225,4,1,312,3,436,27,5,6
IL21R_4_4281,178,80,24,156,0,148,22,52,1070,37,0,319
IL22RA2_4_4282,466,251,576,342,1722,215,523,655,532,4,320,65
IL28RA_4_4283,194,228,219,270,239,28,219,295,50,1,58,310
IL31RA_4_4284,894,843,1351,867,652,843,2533,445,683,42,934,769
IL4R_4_4285,1069,1098,1458,939,1287,399,1692,1053,769,265,1894,651
IL5RA_4_4286,977,1204,915,1205,888,1397,2137,965,529,874,1014,1175
IL6R_4_4287,910,1146,1301,1242,537,943,811,306,387,883,1288,1366
IL6ST_4_4288,236,300,296,165,122,0,0,61,276,37,481,197
ILDR1_4_4289,1516,1572,1156,1493,2210,2178,3058,1219,901,2098,2788,172
3
ITGB1_4_4290,660,846,594,735,899,193,722,336,87,472,432,372
ITGB4_4_4291,393,613,643,416,775,261,14,444,583,221,933,277
ITPR1_4_4292,695,674,757,743,245,874,612,845,755,91,245,1625
KCNA2_4_4293,3148,3546,3752,3869,3162,3428,3169,5359,3361,2620,3805,35
49
KCNAB1_4_4294,777,527,812,583,401,468,793,405,66,1740,969,1413
KCNAB2_4_4295,450,394,577,601,194,392,930,1396,717,642,212,505
KCNC1_4_4296,198,121,609,81,54,29,583,293,285,925,383,348
KCNC2_4_4297,2149,2456,2311,2678,2661,3363,2576,4149,2230,3294,3347,19
64
KCNC4_4_4298,337,243,286,202,21,381,82,55,261,357,665,513
KCND3_4_4299,252,39,290,50,81,1,72,0,620,0,41,305
KCNE1_4_4300,349,436,196,265,472,418,671,87,0,887,134,231
KCNG3_4_4301,673,801,997,938,714,1447,1190,861,356,480,561,351
KCNH1_4_4302,344,166,296,350,118,43,16,476,58,1255,89,175
KCNH2_4_4303,153,65,170,197,13,2,0,442,417,0,576,64
KCNH5_4_4304,743,614,772,740,715,1488,571,420,164,386,452,459
KCNH6_4_4305,105,224,98,115,103,257,106,177,84,1369,88,325
KCNH7_4_4306,817,803,1559,1055,446,548,1446,46,686,347,2386,1543
KCNIP1_4_4307,267,169,201,170,415,107,42,209,27,13,382,524
KCNIP2_4_4308,1184,1421,1487,1617,1845,1186,4893,1241,1936,978,1832,10
02
KCNIP4_4_4309,1143,908,1174,1059,1448,733,617,1355,764,2089,1879,1234
KCNJ11_4_4310,340,201,293,290,616,95,142,259,109,381,1055,567
KCNJ13_4_4311,3296,3237,3823,3442,3186,4167,3137,2754,4197,4107,3983,4
078
KCNJ14_4_4312,421,125,305,194,183,0,77,19,68,0,11,23
KCNJ15_4_4313,825,477,878,711,693,361,81,233,90,429,113,705
KCNJ16_4_4314,831,1157,1615,977,797,736,943,688,744,735,1375,1226
KCNJ1_4_4315,2676,2859,2851,2125,1898,3128,3590,2331,2601,2088,3573,32
58
KCNJ4_4_4316,808,365,949,462,1214,312,37,223,852,18,253,575
KCNK10_4_4317,203,634,589,508,4,32,547,20,277,128,549,485
KCNK16_4_4318,554,328,259,171,222,852,538,163,10,0,192,936

KCNK17_4_4319,129,243,59,143,21,5,5,103,0,79,0,244
KCNK2_4_4320,704,559,350,496,853,110,360,117,654,1,753,126
KCNK7_4_4321,8,13,4,79,0,0,0,0,0,0,146,0
KCNMA1_4_4322,863,1089,1310,1087,542,264,808,1483,718,1865,554,866
KCNMB3_4_4323,1539,1602,1396,1816,1419,1878,899,635,1103,4709,1831,109
8
KCNN2_4_4324,293,637,551,589,228,551,1678,778,57,1139,593,263
KCNN3_4_4325,937,616,756,824,198,291,870,739,377,298,900,948
KCNQ1_4_4326,211,308,261,245,9,0,0,594,35,362,858,30
KCNQ2_4_4327,593,700,794,448,498,422,741,195,341,675,571,1249
KCNQ3_4_4328,2907,2343,2834,3202,4284,2940,4237,2258,2316,1301,2983,17
83
KCNQ4_4_4329,144,198,110,89,41,907,52,219,118,15,203,65
KCNQ5_4_4330,448,452,601,770,69,1301,578,525,233,70,641,748
KCNRG_4_4331,927,675,519,265,150,804,741,658,134,17,770,1057
KCTD15_4_4332,4540,5528,5556,6768,6712,5464,4303,6129,5901,5722,4412,6
476
KCTD1_4_4333,1443,1556,2045,1707,2958,1287,1871,899,597,909,2007,1328
KCTD6_4_4334,679,1008,1276,924,2781,662,1784,1161,743,521,1931,1851
KCTD7_4_4335,398,135,114,120,594,13,178,3,456,88,58,170
KLRC1_4_4336,983,862,922,1081,931,1640,1058,1798,1099,501,803,604
LEPR_4_4337,3639,3886,3952,4037,2775,4004,4851,4671,3329,2461,4938,574
6
LIFR_4_4338,1901,1278,1863,1575,1059,1218,1973,2162,1354,926,1114,576
LILRB1_4_4339,1189,1316,1460,1604,1072,2152,1408,1207,703,968,1282,886
LILRB3_4_4340,1026,934,1061,651,790,409,1883,516,668,433,849,641
LRP12_4_4341,1805,1761,2384,1843,1726,1589,3277,2039,1938,1044,2870,19
34
LRP8_4_4342,246,369,229,270,173,318,12,331,0,138,302,525
MR1_4_4343,414,561,497,330,41,446,1311,1106,899,47,79,466
MSR1_4_4344,873,784,1164,1374,1526,1346,1469,1356,526,1568,1871,1213
NCR2_4_4345,224,186,380,160,94,45,300,58,105,130,206,163
NCR3_4_4346,988,744,570,504,975,656,1173,407,610,347,91,339
NOX1_4_4347,2216,2297,2447,2584,1511,2201,2689,2682,2502,1505,3414,259
8
NOX5_4_4348,437,439,472,387,343,437,580,436,50,1148,795,226
NRP1_4_4349,3687,3611,4276,3333,4158,3381,3942,2403,3987,3384,4477,352
9
NUDT9_4_4350,701,979,914,896,1093,1886,315,1370,765,774,1077,1230
OLR1_4_4351,269,280,414,272,56,421,38,276,454,1731,866,1181
OPCML_4_4352,1447,1621,2367,1301,1557,1621,3288,2558,1424,440,1378,159
7
OSMR_4_4353,1090,859,722,1025,168,1192,376,1384,755,978,820,2203
P2RX2_4_4354,584,522,358,212,306,456,461,111,165,16,481,576
P2RX5_4_4355,1033,1355,1241,1386,1560,658,1512,897,1170,1382,2328,2328
P2RX6_4_4356,710,837,822,595,36,395,1070,687,98,282,963,214
PEX5_4_4357,354,256,631,465,641,142,795,295,229,20,293,246
PKD1_4_4358,13,36,8,25,32,0,0,0,315,55,6,0
PKD1L2_4_4359,115,178,192,182,140,71,563,180,28,24,328,54
PLA2R1_4_4360,601,669,559,383,263,571,270,520,112,835,870,1129

PLAUR_4_4361,537,522,364,431,182,441,95,475,238,346,449,335
PLXNB1_4_4362,285,234,309,210,182,52,143,107,32,38,188,222
PRKD2_4_4363,787,981,1041,847,960,483,951,1803,226,277,1070,768
PRLR_4_4364,1960,2619,2744,2091,2419,2886,2077,2866,1195,3307,2388,218
3
PTCH1_4_4365,317,546,487,430,263,261,44,688,87,78,370,887
PTCH2_4_4366,208,588,463,463,572,477,233,610,92,2525,453,312
ROBO1_4_4367,295,283,239,272,453,15,721,237,17,1616,160,127
ROBO2_4_4368,1679,1991,2697,2291,1353,2176,2673,1550,2503,986,4609,276
6
RYR1_4_4369,550,385,559,433,240,529,283,788,1209,139,525,817
SCARA3_4_4370,1486,1119,1155,1453,2596,1301,1161,1510,1298,2030,1978,1
171
SCARF1_4_4371,531,670,839,923,580,927,1242,361,865,756,422,949
SCARF2_4_4372,161,184,90,406,155,359,222,351,66,10,342,93
SCN1A_4_4373,2468,2202,2829,2993,1949,2216,2608,3230,1226,854,2310,349
2
SCN2A_4_4374,5721,5788,6366,6288,5054,6927,4665,5868,3450,8139,7798,61
38
SCN3A_4_4375,1123,797,842,1118,1418,1243,1486,1233,587,1012,1645,1180
SCN3B_4_4376,505,463,525,468,316,728,680,711,250,177,678,489
SCN4B_4_4377,53,6,45,39,0,0,0,0,0,0,0,0
SCN5A_4_4378,912,1008,525,858,462,592,330,1683,525,1341,594,1364
SCN8A_4_4379,307,252,135,181,8,0,142,178,99,1,690,236
SCNM1_4_4380,230,516,559,248,138,728,186,594,374,401,139,475
SCNN1A_4_4381,800,933,741,1016,613,117,1791,824,671,1432,963,504
SLAMF6_4_4382,2989,2731,3911,3029,3147,2504,4695,2885,2802,3372,4941,3
171
SLC6A2_4_4383,368,464,411,483,407,721,946,727,502,1212,494,723
TLR10_4_4384,6727,6561,8031,6598,9232,6103,7757,5199,5179,6859,7274,53
95
TNFRSF10B_4_4385,232,336,499,297,174,381,325,650,65,401,309,128
TNFRSF18_4_4386,881,664,617,562,1189,336,1550,442,387,1429,280,776
TNFRSF19_4_4387,1638,1814,1676,1788,2588,1198,2346,616,1227,3157,1811,
1181
TNFRSF25_4_4388,634,630,882,525,428,1105,929,757,463,313,930,712
TNPO1_4_4389,234,331,193,295,368,433,149,580,26,754,15,218
TOMM40_4_4390,604,549,676,852,883,294,1525,471,2096,509,406,677
TP53INP1_4_4391,65,337,272,432,203,263,26,398,441,169,26,45
TPCN1_4_4392,302,430,329,474,254,297,365,35,555,286,1163,540
TRPC3_4_4393,1244,1089,1030,1084,556,746,854,1034,864,928,1444,983
TRPC4_4_4394,439,526,606,383,392,1173,5,351,175,607,613,137
TRPM3_4_4395,106,99,152,164,110,540,240,34,1,534,94,1
TRPM4_4_4396,939,647,734,671,813,1390,1613,1017,642,1783,1327,1119
TRPM6_4_4397,2453,1974,2736,2328,2921,3133,3165,3544,2132,2748,3180,31
32
TRPV1_4_4398,1247,1104,1126,1404,1459,1260,1049,1241,831,1367,1521,291
TRPV4_4_4399,689,790,776,783,878,1152,499,580,86,319,923,1345
TTYH1_4_4400,225,262,611,494,490,419,262,243,178,55,310,168
TTYH2_4_4401,1603,1447,1745,1865,1670,3484,1595,3242,675,2328,2780,119

7
VDAC2_4_4402,2405,2076,2461,2694,1522,3524,2679,2783,2236,917,2457,276
0
ANO7_4_4403,462,328,533,470,1718,24,212,400,182,90,1201,191
AQP1_4_4404,367,231,227,370,263,392,103,295,197,455,107,159
CLEC2D_4_4405,411,744,698,842,1062,677,301,736,383,725,1051,909
CLEC7A_4_4406,2680,2503,3235,2845,1966,862,4322,2138,1933,1419,2972,22
70
CRLF2_4_4407,524,655,925,512,1421,771,1270,316,305,191,837,426
FXYD3_4_4408,1781,2487,2504,2567,1738,1928,3096,1886,1443,3306,1286,30
07
HFE_4_4409,1263,1639,1549,1323,2029,2211,1017,1570,1661,3189,1485,1211
SCN1B_4_4410,177,372,240,370,354,31,541,124,115,14,394,250
TNFRSF8_4_4411,676,919,703,604,419,409,554,280,359,459,909,517
TSPO_4_4412,147,294,408,278,5,407,258,448,366,30,110,267
ABCC8_4_4413,35,28,43,18,3,27,5,1,525,87,29,0
AMFR_4_4414,461,563,602,437,571,932,581,912,352,848,311,647
ANO1_4_4415,781,663,1022,892,1216,668,1662,626,581,1757,412,840
ANO2_4_4416,317,447,200,186,48,257,356,2,506,4,162,275
ANXA9_4_4417,26,49,35,119,284,0,0,0,78,1002,0,277
AQP10_4_4418,209,304,117,219,1,0,440,406,5,167,155,95
AQP11_4_4419,270,259,118,110,129,7,351,414,324,2,56,141
AQP12A_4_4420,774,782,486,660,468,170,352,1772,553,247,357,1127
AQP2_4_4421,196,163,383,326,1,4,31,181,0,0,123,111
AQP3_4_4422,1292,1393,1571,1327,1118,1187,1204,1196,1789,1881,1391,227
5
AQP5_4_4423,619,686,305,725,188,2565,404,600,344,1318,495,866
AQP6_4_4424,322,150,312,33,388,82,510,433,409,0,27,344
AQP7_4_4425,520,525,461,437,262,810,186,626,183,678,192,347
AQP8_4_4426,143,126,146,105,18,79,258,155,91,0,18,7
B2M_4_4427,620,489,923,573,1039,273,471,389,895,672,519,737
BEST2_4_4428,516,517,644,449,1044,402,612,515,222,1099,94,939
BEST4_4_4429,393,376,332,464,234,426,482,194,173,1525,215,1492
BSND_4_4430,351,344,262,415,341,1171,775,230,95,96,839,141
BTBD10_4_4431,396,775,648,925,283,834,693,712,440,34,1141,492
BTNL2_4_4432,662,862,825,656,974,686,923,349,611,836,952,508
C5orf62_4_4433,793,793,1026,706,377,459,528,1297,1418,962,795,1926
CACNA1B_4_4434,140,86,193,159,26,30,483,0,229,0,28,104
CACNA1F_4_4435,1795,1302,1260,1388,1601,1100,1679,2201,240,1478,1511,1
551
CACNA1S_4_4436,105,193,355,153,161,178,494,60,121,21,394,141
CACNA2D1_4_4437,405,472,628,469,1398,130,480,288,80,0,746,379
CACNA2D3_4_4438,657,297,515,264,827,414,303,240,1,626,705,731
CACNA2D4_4_4439,748,720,809,868,226,576,1275,1117,218,944,1003,596
CACNG1_4_4440,280,221,192,243,154,42,19,40,764,6,180,415
CACNG2_4_4441,2379,2584,3230,2650,1490,2932,3153,2644,2327,2546,2291,1
983
CACNG3_4_4442,546,474,647,516,423,500,914,684,149,21,924,618
CACNG4_4_4443,421,722,769,596,742,1748,592,1647,808,504,956,729
CACNG5_4_4444,94,58,157,84,40,17,62,17,41,123,138,19

CACNG7_4_4445,451,419,460,814,770,289,124,399,329,58,879,460
CACNG8_4_4446,273,295,251,103,757,74,492,80,77,163,1321,378
CATSPER1_4_4447,1089,1010,1200,1047,699,863,2641,1542,596,1058,1413,11
06
CATSPER3_4_4448,381,556,428,524,565,989,1602,405,34,118,789,1170
CATSPER4_4_4449,102,66,104,309,0,3,97,101,1,76,37,885
CCT8L2_4_4450,790,520,827,619,232,726,546,1031,415,51,1412,371
CD160_4_4451,2355,3025,3101,2339,2257,3840,2765,2910,2143,1693,3721,26
66
CD163L1_4_4452,1447,1265,1393,1338,945,2419,3270,1170,2467,1260,2060,1
381
CD27_4_4453,521,548,651,480,505,268,543,302,116,27,486,667
CD2_4_4454,2358,2442,2773,2581,2603,3806,3461,1867,1407,2709,4876,3208
CD300C_4_4455,446,381,243,400,48,275,141,296,180,609,175,908
CD3E_4_4456,147,91,146,252,78,95,76,112,36,0,5,33
CD3G_4_4457,1332,1322,1570,1529,666,2764,2222,1059,1410,1903,662,1512
CD5_4_4458,708,606,666,579,1470,488,622,1207,489,129,553,1050
CD5L_4_4459,1538,1314,1517,1097,357,1363,2334,1192,1048,409,1045,1758
CD69_4_4460,1168,1258,1364,1228,1258,2199,907,1235,1071,676,976,1372
CD6_4_4461,45,117,67,176,6,25,35,65,576,24,0,21
CD72_4_4462,688,606,672,549,297,473,438,1261,1175,1977,861,461
CD80_4_4463,230,518,682,317,291,506,936,517,625,118,1435,421
CFTR_4_4464,1279,959,997,1713,365,1801,499,1312,1965,2495,1819,1937
CHRNA10_4_4465,347,359,678,353,551,1517,164,291,5,400,849,556
CHRNA2_4_4466,383,465,459,558,371,1120,590,726,803,377,927,446
CHRNA4_4_4467,418,351,668,632,729,998,1383,1194,54,1504,628,1308
CHRNA5_4_4468,1050,1318,847,1075,1128,2131,863,1826,1016,644,972,1563
CHRNA9_4_4469,1502,1116,1243,997,1176,1664,684,1128,1638,1214,607,1681
CHRNB1_4_4470,227,234,214,206,52,150,11,334,34,16,253,53
CHRNB2_4_4471,435,496,252,184,726,345,412,513,232,2,287,640
CHRNB3_4_4472,1464,1744,1662,941,2852,864,1347,1312,2777,1392,1546,121
3
CHRNB4_4_4473,472,698,853,517,16,1533,517,356,433,1965,70,27
CHRND_4_4474,482,596,290,549,1084,562,256,621,648,1109,2026,437
CHRNE_4_4475,70,250,315,270,116,266,265,64,25,19,116,158
CHRNG_4_4476,396,374,161,168,559,201,246,59,261,26,755,739
CLCA1_4_4477,1449,1451,1918,1308,1653,1693,734,1948,350,1881,2391,1228
CLCA2_4_4478,600,362,463,344,891,977,135,837,555,48,42,347
CLCA4_4_4479,560,677,1025,538,266,1391,500,457,951,542,881,1201
CLCN1_4_4480,205,202,201,286,862,11,230,920,0,2660,311,13
CLCN4_4_4481,2380,2666,2280,1782,1945,1751,2325,1583,2296,3201,2520,22
63
CLDN3_4_4482,1011,938,995,1041,270,984,609,1238,534,321,609,974
CLDN4_4_4483,499,215,296,181,552,578,329,249,53,2,705,106
CLEC1A_4_4484,2209,2092,2406,2276,2609,2643,2992,3241,2247,2186,3204,3
192
CLIC1_4_4485,486,62,359,264,34,461,14,1143,379,655,508,2
CLIC2_4_4486,1352,1036,1124,1382,2874,728,1199,1490,1103,1287,1641,764
CLIC3_4_4487,383,554,586,781,1659,547,1077,369,748,293,365,800
CLIC4_4_4488,3,3,0,1,0,2,0,1,2,0,0,86

CLIC6_4_4489,1524,1686,2248,1694,2171,2534,900,1302,1889,2790,2531,320
9
CLNS1A_4_4490,2976,3038,2816,2858,3711,2569,6211,1545,3313,2199,4406,2
288
CNGA2_4_4491,610,595,455,422,568,661,377,41,412,972,148,268
CNGA4_4_4492,1170,1298,1304,1109,2781,604,951,1002,639,90,1656,1020
CNGB3_4_4493,773,893,799,1040,1174,831,1062,2071,1183,721,949,1117
COLEC12_4_4494,559,351,890,526,659,845,496,1040,415,910,1375,657
CSF2RB_4_4495,100,112,219,248,228,371,448,314,221,1,31,228
CUBN_4_4496,615,904,715,804,59,1423,769,949,515,1218,2291,1918
DCC_4_4497,480,514,541,529,256,320,638,704,143,139,308,838
DNER_4_4498,111,232,125,106,0,11,1,179,23,181,226,91
EDAR_4_4499,287,261,287,122,641,383,200,743,77,39,362,13
EPOR_4_4500,390,326,343,255,915,252,91,119,547,484,320,746
FCER1A_4_4501,523,416,565,471,1246,291,417,519,48,386,584,236
FCER1G_4_4502,1498,2194,2079,1795,1744,808,783,1698,1535,3179,2497,198
4
FCGR1A_4_4503,143,146,299,282,263,21,402,34,488,39,244,271
FCGR2C_4_4504,700,825,727,1075,243,413,503,1003,594,1605,173,1476
FCGR3B_4_4505,1629,1515,1974,1546,1333,2799,1524,2011,1283,1589,1609,2
044
FRRS1_4_4506,224,217,221,172,318,112,641,378,233,31,309,372
FXYD7_4_4507,843,875,805,1016,879,1000,506,999,438,462,673,350
GABRA3_4_4508,1049,1322,1526,1490,1442,1612,1792,1039,1233,338,3307,22
19
GABRA6_4_4509,2484,2553,2736,2148,2584,2098,4219,1393,1091,3033,3409,2
688
GABRB1_4_4510,1174,1985,1439,1951,1856,1708,1416,998,1785,1177,2768,58
3
GABRD_4_4511,32,133,202,414,110,68,1,46,180,0,128,10
GABRE_4_4512,1548,1638,1331,1692,964,2241,2264,2314,1485,1505,1730,291
6
GABRG1_4_4513,620,797,847,876,866,498,1455,729,860,1453,807,529
GABRG3_4_4514,163,403,397,164,630,440,24,366,1,644,80,647
GABRP_4_4515,125,265,79,172,129,0,0,598,37,1428,170,409
GABRQ_4_4516,415,841,773,769,369,287,1072,604,107,164,1325,433
GABRR1_4_4517,1103,1002,1664,1198,1120,1332,1086,1298,857,772,1509,161
7
GABRR2_4_4518,128,149,246,278,257,61,145,190,88,1855,689,476
GABRR3_4_4519,1449,2078,1467,1626,2044,2204,1831,1873,2646,3640,1562,1
220
GFRA3_4_4520,629,608,846,670,758,552,510,856,109,777,777,540
GPC1_4_4521,0,0,0,0,0,0,0,0,0,0,0,0
GPC4_4_4522,595,360,569,483,243,54,333,110,91,1513,842,60
GPC6_4_4523,1912,1925,1989,1621,1908,1382,1603,1343,1347,2112,1361,218
0
GPR89B_4_4524,318,528,388,666,242,162,507,522,142,659,1995,547
GRID1_4_4525,514,912,725,791,539,1603,796,539,592,359,1025,547
GRID2_4_4526,1132,917,903,858,1189,113,641,767,2310,372,1129,1098
GRIK3_4_4527,372,173,493,546,338,472,281,521,228,235,363,576

GRIK4_4_4528,382,779,578,413,222,224,450,551,256,0,571,301
GRIK5_4_4529,119,163,42,105,63,31,13,57,23,0,35,85
GRIN2B_4_4530,131,180,67,62,168,33,0,42,282,34,5,157
GRIN2C_4_4531,1418,1021,1587,1283,655,866,1483,782,1348,739,1169,384
GRIN2D_4_4532,350,143,283,344,292,78,90,58,318,365,73,293
GRIN3A_4_4533,51,158,89,229,178,9,54,148,6,29,181,319
GRIN3B_4_4534,247,242,152,113,564,26,3,207,509,22,108,131
HAVCR2_4_4535,2068,2271,2848,2222,1642,1845,3872,3260,1332,1580,3786,3
458
HCN1_4_4536,145,89,210,207,27,43,5,1116,98,0,190,97
HCN2_4_4537,263,267,414,235,335,462,79,86,11,2,108,228
HCN3_4_4538,293,218,198,305,231,13,147,125,201,0,81,458
HCN4_4_4539,343,442,459,592,289,574,57,648,431,516,79,1076
HLA-B_4_4540,251,156,194,262,220,111,89,348,1,1,158,176
HLA-DOA_4_4541,99,72,139,198,191,122,4,160,521,439,311,299
HLA-DOB_4_4542,405,428,436,331,145,1721,350,505,487,1256,89,359
HLA-DPB1_4_4543,218,595,478,568,438,54,442,765,61,185,762,501
HLA-DQA1_4_4544,589,703,801,744,1050,641,533,960,498,975,1099,206
HLA-DQA2_4_4545,397,494,389,254,222,922,820,353,61,114,539,1052
HLA-DQB2_4_4546,585,260,160,124,646,974,59,113,605,486,500,37
HLA-DRA_4_4547,21,21,51,94,137,0,13,0,59,0,0,7
HLA-DRB1_4_4548,433,614,597,471,345,945,1376,320,219,460,1010,281
HLA-
DRB5_4_4549,1031,1266,1119,1226,1171,484,1107,1098,656,195,1886,1007
HLA-G_4_4550,556,425,780,719,597,597,194,504,788,686,932,255
HTR3B_4_4551,304,406,233,214,491,410,34,29,281,209,270,226
HTR3C_4_4552,4063,3581,3625,3720,2736,4367,4050,4643,2153,3020,4072,43
23
HTR3E_4_4553,807,787,954,634,558,585,1049,1040,1069,21,899,1323
ICAM1_4_4554,370,324,219,325,81,302,5,599,623,1477,125,191
IFNAR1_4_4555,3104,3343,3002,3075,2992,3277,3058,2927,3107,1323,3992,3
644
IFNGR1_4_4556,127,102,23,33,95,0,0,42,18,7,153,69
IFNGR2_4_4557,940,1035,1309,1159,1452,2111,1365,1706,534,1127,292,1083
IGF1R_4_4558,131,185,221,67,461,29,0,120,15,4,466,127
IGF2R_4_4559,887,884,963,1291,422,1471,2387,628,632,202,674,1278
IL10RA_4_4560,1045,1006,1178,1107,609,738,494,1421,514,1737,1331,1481
IL10RB_4_4561,1097,1069,957,1369,2478,345,934,1795,339,609,1301,1063
IL12RB2_4_4562,250,102,154,48,4,2,0,213,11,386,0,143
IL13RA1_4_4563,489,663,404,518,486,833,1192,674,1055,414,731,473
IL13RA2_4_4564,179,202,205,176,216,355,97,128,179,223,146,161
IL17RA_4_4565,271,178,354,269,203,93,39,256,821,180,312,922
IL17RB_4_4566,645,335,362,416,45,589,38,161,247,0,1295,296
IL18R1_4_4567,1027,1213,1919,1267,1531,2484,1776,1284,680,808,1718,170
7
IL18RAP_4_4568,434,377,459,386,212,765,155,375,451,597,1252,890
IL1R1_4_4569,1209,1011,1090,1541,1545,1304,1738,1312,1163,398,1256,229
8
IL1RAPL1_4_4570,1321,1873,1503,1537,1844,815,797,1515,1058,1023,1534,1
229

IL1RAPL2_4_4571,2171,2287,2507,2952,2141,2076,4852,2861,3159,1972,1879
,2643
IL1RL2_4_4572,1792,1240,2673,1797,1273,1304,1333,1445,841,1544,2371,15
92
IL20RA_4_4573,253,666,423,244,817,667,70,808,43,46,189,213
IL22RA1_4_4574,72,46,166,87,58,23,274,129,1,16,49,148
IL23R_4_4575,1972,2731,1992,3457,3587,2251,1828,2636,2402,2348,3086,37
08
IL27RA_4_4576,1086,1079,1223,1106,1041,2338,1040,434,1040,1465,2305,95
3
IL2RA_4_4577,367,611,439,371,142,797,274,155,332,774,43,766
IL2RB_4_4578,184,128,299,92,29,3,0,828,16,0,119,95
IL2RG_4_4579,491,320,284,360,947,89,311,170,537,964,765,387
IL3RA_4_4580,1097,1088,966,1116,410,1313,1160,1240,754,994,837,1061
IL7R_4_4581,640,820,760,642,874,840,1325,2006,634,266,610,723
IL9R_4_4582,163,174,258,410,1127,495,235,148,799,1178,392,447
ITFG1_4_4583,1982,2422,2047,2119,1702,1441,3049,1475,1513,2294,2357,19
85
ITGA2B_4_4584,103,240,220,176,16,12,0,48,0,0,0,187
ITGB3_4_4585,37,59,15,58,53,374,37,0,60,0,4,25
ITGB7_4_4586,248,94,205,238,47,338,103,299,39,720,59,73
ITPR2_4_4587,1183,1398,1080,1253,2993,2973,2136,1255,436,459,1700,705
ITPR3_4_4588,157,70,77,166,148,51,428,173,272,81,195,305
JPH3_4_4589,394,448,451,590,523,285,804,538,428,400,751,491
KCMF1_4_4590,659,494,577,615,1108,310,1029,945,1142,3768,548,2037
KCNA10_4_4591,267,283,382,188,264,38,137,310,69,1,116,128
KCNA1_4_4592,174,243,116,143,0,353,21,196,3,1,29,24
KCNA3_4_4593,623,671,689,809,1477,1778,164,700,648,403,840,1461
KCNA4_4_4594,907,527,1168,1186,365,832,929,911,808,817,1780,1431
KCNA5_4_4595,329,161,134,374,303,8,23,426,1116,1407,59,370
KCNA6_4_4596,395,360,791,266,1173,985,795,64,1121,150,536,495
KCNA7_4_4597,485,192,377,358,42,58,76,382,211,267,481,384
KCNAB3_4_4598,561,532,1077,692,331,1073,867,2128,635,929,581,205
KCNB1_4_4599,504,384,511,358,87,430,292,184,68,226,56,617
KCNB2_4_4600,952,781,954,1029,816,727,1297,1172,902,365,711,1196
KCNC3_4_4601,506,710,912,677,111,512,473,368,540,812,190,704
KCND1_4_4602,104,400,194,179,406,193,320,743,15,6,933,458
KCND2_4_4603,464,403,372,399,916,161,293,608,99,489,0,89
KCNE1L_4_4604,1817,1531,2487,2224,1906,2393,2152,1882,896,452,4458,245
3
KCNE2_4_4605,1311,1309,1488,1429,1779,1056,1805,1058,1169,1145,1063,20
92
KCNE3_4_4606,1908,1780,1839,2217,1773,2535,3372,1849,1794,3781,1366,19
31
KCNE4_4_4607,282,275,359,275,108,779,1071,116,409,423,292,799
KCNF1_4_4608,169,28,112,253,126,0,0,163,36,1,175,172
KCNG1_4_4609,556,373,398,208,1130,509,567,445,316,193,365,780
KCNG2_4_4610,195,334,520,356,151,45,1722,203,232,7,93,305
KCNG4_4_4611,203,147,169,155,41,490,125,480,4,94,86,32
KCNH3_4_4612,52,129,113,65,0,171,10,7,41,245,230,32

KCNH4_4_4613,442,596,621,739,399,903,683,911,160,254,615,564
KCNH8_4_4614,511,452,519,258,346,98,563,215,6,532,264,215
KCNJ10_4_4615,167,166,134,52,16,138,69,88,0,0,203,7
KCNJ12_4_4616,781,936,1358,989,973,592,902,947,637,663,1318,592
KCNJ2_4_4617,384,259,511,522,233,299,201,249,358,72,499,865
KCNJ3_4_4618,2247,2239,3014,2207,2209,4210,6446,3048,3744,1570,3445,23
55
KCNJ5_4_4619,223,471,515,220,664,11,1036,102,123,8,430,248
KCNJ6_4_4620,814,436,743,808,256,262,409,957,1002,99,581,579
KCNJ8_4_4621,3345,3671,3629,3239,3226,4183,3441,3374,4370,4264,3629,25
96
KCNJ9_4_4622,533,615,614,703,448,270,298,397,623,372,1167,452
KCNK12_4_4623,161,140,303,197,68,120,14,101,8,93,48,296
KCNK13_4_4624,49,16,27,26,2,0,0,5,0,0,14,28
KCNK15_4_4625,325,380,453,334,237,19,309,253,965,256,1401,701
KCNK18_4_4626,327,286,347,206,717,2,68,403,130,313,154,1389
KCNK1_4_4627,883,782,1021,958,917,212,1627,787,1155,1336,673,520
KCNK3_4_4628,706,776,868,615,666,280,483,352,825,924,932,715
KCNK4_4_4629,124,178,305,329,366,328,1377,592,348,0,447,479
KCNK5_4_4630,310,327,325,318,133,522,179,43,399,69,550,442
KCNK6_4_4631,1048,1330,799,889,1016,3034,1876,1245,572,396,1328,1061
KCNK9_4_4632,150,111,82,179,642,52,0,34,38,169,190,31
KCNMB1_4_4633,463,484,777,472,318,44,260,586,74,787,709,360
KCNMB4_4_4634,117,78,79,20,0,0,0,58,0,131,0,0
KCNN1_4_4635,100,188,39,97,1,11,133,69,142,0,89,52
KCNN4_4_4636,519,954,574,749,633,1420,1050,197,719,374,516,863
KCNS1_4_4637,152,348,499,123,196,493,236,74,455,0,345,838
KCNS2_4_4638,2349,3309,3641,2444,1663,1830,6674,1542,2324,2010,3810,28
91
KCNS3_4_4639,655,588,1060,911,502,813,1005,715,312,307,704,1275
KCNT1_4_4640,338,412,459,340,882,731,780,1022,558,0,166,491
KCNT2_4_4641,360,412,335,414,180,88,203,283,1,486,718,101
KCNU1_4_4642,35,42,120,285,195,8,10,396,14,670,7,255
KCNV1_4_4643,1514,1454,1372,1767,732,1043,658,1650,1229,1174,1540,1493
KCNV2_4_4644,187,190,339,599,690,528,278,0,120,561,601,228
KCTD10_4_4645,1203,887,1334,1146,829,1122,2399,1097,856,53,1979,764
KCTD11_4_4646,248,190,255,218,381,178,15,328,167,581,372,55
KCTD12_4_4647,376,189,276,126,146,807,40,212,20,3,322,221
KCTD13_4_4648,1140,1092,903,1438,1163,594,1455,662,1035,508,761,1041
KCTD14_4_4649,401,276,244,232,265,9,328,488,311,358,75,494
KCTD16_4_4650,888,666,928,829,714,1504,458,649,330,430,970,954
KCTD17_4_4651,267,355,591,114,722,520,69,229,324,108,230,280
KCTD18_4_4652,148,170,154,137,650,46,112,466,9,0,317,20
KCTD19_4_4653,512,595,562,672,154,949,979,713,132,248,1467,174
KCTD20_4_4654,194,329,343,192,288,342,1355,1,364,509,420,649
KCTD2_4_4655,57,38,71,26,0,0,0,0,80,1,1,27
KCTD3_4_4656,875,741,844,898,609,143,551,1122,1131,87,1210,1627
KCTD4_4_4657,201,178,311,223,303,371,22,165,214,0,257,72
KCTD5_4_4658,232,407,157,186,843,249,810,786,231,6,113,649
KCTD8_4_4659,2292,1989,2248,2737,1862,2184,2751,2740,1704,3424,4655,17

19
KCTD9_4_4660,1433,1587,783,1103,1120,722,2171,1087,2499,1289,1366,1813
KIR3DL1_4_4661,161,72,136,66,0,454,138,0,0,0,35,0
KLRB1_4_4662,301,522,581,245,252,540,453,497,879,1453,103,950
LGALS3BP_4_4663,3059,3498,3531,3874,1214,3864,3510,2137,3995,2942,5689
,3090
LRP10_4_4664,74,51,193,129,0,0,1,5,82,0,24,190
LRP1B_4_4665,247,201,180,283,481,286,331,121,448,50,199,927
LRP3_4_4666,29,71,98,216,2,0,6,190,1068,14,18,340
LRP5_4_4667,708,170,327,401,767,456,23,349,464,1291,700,863
LRP6_4_4668,2470,2173,1910,2459,2765,1970,1580,3249,1434,3359,2576,175
9
LRPAP1_4_4669,578,706,773,440,527,359,774,434,772,124,839,719
LTBR_4_4670,471,214,389,452,406,391,1089,209,95,4,460,94
MARCO_4_4671,51,146,125,73,0,223,224,82,25,1,68,299
MCOLN1_4_4672,256,189,196,100,395,143,146,524,601,996,349,100
MCOLN2_4_4673,555,388,681,393,308,426,843,1139,13,3,473,625
MCOLN3_4_4674,910,1030,857,995,1284,947,249,175,467,1738,1565,1763
MCU_4_4675,5387,5206,5017,5882,6079,7603,9368,6086,5453,8224,6717,6462
MFRP_4_4676,621,593,455,424,688,1589,648,242,246,402,479,330
MICB_4_4677,844,944,952,1175,56,1764,1590,1060,185,502,425,543
MPL_4_4678,381,681,693,494,410,1525,245,254,581,1401,732,829
MRC1_4_4679,1189,1000,1055,1147,1400,1455,1691,1198,1293,1192,378,614
MRC2_4_4680,2,110,20,86,3,0,127,1,8,34,32,179
NALCN_4_4681,9113,8824,8910,8066,11779,7088,14908,7765,6929,6467,11264
,10444
NGFR_4_4682,1268,1181,1461,1210,1246,1358,2013,1103,715,706,1815,1677
NISCH_4_4683,445,646,627,436,186,375,1331,445,247,1255,8,628
NPTXR_4_4684,1424,1393,1658,1273,1423,2094,568,1325,1356,1294,1760,168
3
ORAI1_4_4685,111,139,10,9,0,0,0,26,3,0,0,0
P2RX1_4_4686,492,264,316,560,180,251,318,282,462,87,246,580
P2RX3_4_4687,992,1367,1060,756,1348,570,388,2063,1341,690,326,1306
P2RX4_4_4688,316,314,159,260,45,15,77,8,42,114,461,292
P2RX7_4_4689,603,404,679,270,482,5,308,689,22,336,875,939
PEX5L_4_4690,810,969,1149,942,833,885,998,702,943,296,1459,817
PGLYRP1_4_4691,365,255,666,577,1173,197,844,837,267,998,545,900
PGLYRP2_4_4692,79,79,55,35,100,888,0,16,372,0,1,6
PGLYRP3_4_4693,1736,1898,1678,1454,1189,1527,1752,1181,441,2536,1288,1
438
PGLYRP4_4_4694,576,475,498,419,265,451,267,811,205,116,233,449
PGRMC1_4_4695,176,241,361,132,5,3,5,0,276,623,396,11
PIP_4_4696,857,1013,846,1018,1019,1054,2568,380,313,21,366,1905
PKD1L1_4_4697,1517,1399,1867,1431,3026,727,1717,975,1674,584,2003,2733
PKD1L3_4_4698,1450,1088,1394,1276,924,733,1735,853,2147,1182,1877,1218
PKD2L1_4_4699,75,6,2,42,0,0,67,0,0,0,0,0
PKD2L2_4_4700,300,316,336,244,187,654,81,582,687,1354,232,921
PKDREJ_4_4701,197,106,256,101,44,153,137,80,192,0,392,137
PLXNA1_4_4702,1166,833,806,1408,2045,877,558,1235,817,2244,472,1071
PLXNA2_4_4703,255,259,307,303,513,364,269,255,321,770,238,535

PLXNA3_4_4704,134,47,149,185,2,11,136,34,90,160,135,71
PLXND1_4_4705,668,897,918,1023,218,479,207,772,417,2305,490,782
PRPH2_4_4706,863,871,823,872,1064,545,1706,353,157,561,824,1754
RARRES2_4_4707,230,227,400,417,421,75,722,390,276,477,416,708
RASA3_4_4708,357,485,457,299,239,344,478,545,126,357,319,871
RYR2_4_4709,518,278,452,348,1043,101,183,634,144,18,477,556
RYR3_4_4710,87,200,145,213,162,29,54,60,336,0,446,612
SARM1_4_4711,150,187,127,61,428,163,469,473,176,2,216,161
SCN10A_4_4712,919,477,810,742,364,923,680,527,826,889,650,658
SCN11A_4_4713,2029,1607,1618,1948,3841,2455,1843,1146,1857,2647,2847,2
585
SCN2B_4_4714,40,50,167,44,18,36,53,10,19,2,62,97
SCN4A_4_4715,626,538,509,696,341,775,801,132,424,1033,526,1367
SCN7A_4_4716,1231,1362,1423,1744,2026,860,2203,667,1642,674,662,919
SCN9A_4_4717,2550,3404,3928,2881,2877,3880,1370,2364,3100,3049,1175,36
59
SCNN1B_4_4718,623,432,758,975,127,1700,1208,559,443,427,959,1058
SCNN1D_4_4719,3672,3407,3853,2696,4241,5042,3342,2970,2076,5069,2947,3
670
SCNN1G_4_4720,329,222,405,164,343,92,1387,923,26,535,153,393
SCUBE1_4_4721,381,289,305,187,1805,221,773,150,8,1223,727,280
SELE_4_4722,481,635,673,340,256,359,328,355,151,335,857,485
SEMA5A_4_4723,56,159,133,178,213,222,152,169,25,13,69,31
SETD1A_4_4724,114,205,339,57,26,43,4,37,1200,0,61,560
SFRP1_4_4725,2021,2422,2659,2393,2569,2090,3010,2689,1557,2962,1564,32
20
SFRP4_4_4726,1769,1845,1652,1912,3359,1194,1426,2006,990,1652,1634,217
2
SFRP5_4_4727,2199,1605,1547,2247,3092,2665,2167,2586,1179,2330,1231,30
84
SHKBP1_4_4728,1209,1549,1383,2002,928,923,2573,1312,1586,776,2274,1158
SHROOM2_4_4729,334,303,336,180,121,81,232,724,107,353,208,47
SIGLEC8_4_4730,1715,1733,1885,1850,1717,1901,2868,1224,341,457,1946,77
4
SLAMF1_4_4731,703,1042,1112,1086,2274,1117,1338,1020,254,1225,1396,446
SLC6A18_4_4732,866,1032,573,854,827,1143,1625,935,288,2028,516,1316
SLC6A19_4_4733,547,306,750,822,211,102,1166,1182,16,443,1249,591
SLC6A1_4_4734,841,726,678,404,749,781,645,451,550,374,918,292
SLC6A3_4_4735,166,117,193,37,1,242,333,66,76,0,425,157
SLC6A4_4_4736,300,347,373,258,1076,0,44,94,187,312,176,205
SLC9A1_4_4737,337,485,812,388,414,271,208,589,206,976,766,336
SLC9A3_4_4738,88,122,31,204,258,0,447,0,28,1,666,441
SRCRB4D_4_4739,358,504,563,509,998,261,302,245,173,576,265,847
STAB2_4_4740,94,198,100,131,535,315,126,305,14,342,80,231
STX1B_4_4741,738,603,648,578,606,286,539,1237,987,534,383,579
THBD_4_4742,88,358,227,324,103,245,741,246,84,0,321,76
TLR1_4_4743,2082,2276,2303,1984,1347,2488,2957,1760,1669,1378,1682,260
4
TLR2_4_4744,232,381,665,294,652,837,29,362,464,1225,455,317
TLR3_4_4745,2033,1844,2290,2009,874,3371,3174,1585,1593,1338,1854,2530

TLR4_4_4746,520,468,640,589,218,343,36,1022,934,55,223,506
TLR5_4_4747,8367,8682,9478,9928,6939,8536,10648,11190,8600,7741,12144,
9737
TLR6_4_4748,621,815,643,646,1073,1899,435,869,931,190,570,389
TLR7_4_4749,127,76,296,98,490,1153,0,690,481,0,167,116
TLR8_4_4750,851,1337,1128,1047,778,1592,1193,1402,638,444,1219,801
TLR9_4_4751,463,278,478,427,484,36,458,40,797,383,382,343
TMEM37_4_4752,472,606,599,251,584,190,68,402,7,694,334,131
TMEM38A_4_4753,16,7,22,58,0,64,0,4,0,0,66,0
TMEM38B_4_4754,550,544,770,781,383,857,719,840,1002,487,1019,523
TNFAIP1_4_4755,141,218,273,295,1467,37,107,525,15,948,205,661
TNFRSF10A_4_4756,578,387,598,467,540,215,210,455,57,623,507,646
TNFRSF10C_4_4757,668,647,772,676,820,762,134,627,233,291,549,662
TNFRSF10D_4_4758,1364,1676,1635,1906,1365,726,1000,1471,1351,1381,3974
,1236
TNFRSF11A_4_4759,282,238,170,273,194,131,132,321,153,114,450,252
TNFRSF13B_4_4760,146,71,275,313,337,13,344,574,50,5,120,271
TNFRSF13C_4_4761,543,257,235,366,208,281,53,179,131,0,380,320
TNFRSF14_4_4762,192,367,332,321,25,11,30,102,20,3,590,229
TNFRSF1A_4_4763,156,102,48,34,255,0,131,314,0,3,39,2
TNFRSF1B_4_4764,281,306,320,106,209,206,420,36,115,13,839,562
TNFRSF4_4_4765,98,2,42,128,0,36,2,0,5,0,0,3
TNFRSF6B_4_4766,488,748,613,182,864,1097,545,340,444,208,773,331
TPCN2_4_4767,742,703,634,801,96,185,304,153,165,1344,621,391
TREM2_4_4768,3220,2723,2634,2797,2918,3809,4898,3016,3137,2117,958,315
0
TRPA1_4_4769,2679,2739,3571,3007,3245,3233,3775,2536,1965,3625,6160,31
75
TRPC1_4_4770,2322,1763,2892,2208,1223,2038,1123,2115,2025,2649,3929,26
71
TRPC5_4_4771,549,547,399,389,549,421,375,148,104,256,674,475
TRPC6_4_4772,238,204,173,249,35,246,81,219,4,125,481,76
TRPM1_4_4773,206,187,249,562,602,65,118,274,1,185,955,403
TRPM2_4_4774,129,214,250,133,603,43,585,102,11,541,531,69
TRPM5_4_4775,357,200,284,446,411,11,108,331,297,234,630,744
TRPM7_4_4776,1884,1434,1877,1335,2620,989,2197,873,1562,1876,942,2132
TRPM8_4_4777,758,1009,1118,895,380,395,2494,782,201,515,658,709
TRPV2_4_4778,284,337,185,254,384,691,197,608,254,88,221,122
TRPV3_4_4779,654,1246,1042,764,1059,484,1287,1356,1709,2339,1544,1038
TRPV5_4_4780,320,278,484,458,774,214,48,137,18,265,536,266
TRPV6_4_4781,266,186,505,284,159,136,198,165,198,0,236,619
TTYH3_4_4782,162,161,611,251,132,248,494,369,478,779,1493,722
ULBP1_4_4783,268,413,305,324,77,128,299,296,217,150,611,948
ULBP2_4_4784,425,382,426,435,87,495,141,179,598,832,617,269
ULBP3_4_4785,364,162,437,221,4,140,518,93,55,28,539,124
UNC5A_4_4786,169,116,176,240,1,393,161,95,594,0,3,184
UNC5B_4_4787,735,287,376,510,10,457,775,855,0,791,625,236
UNC5C_4_4788,121,426,375,289,20,1038,1181,535,325,2,251,50
UTRN_4_4789,1194,1153,1180,717,820,1497,1510,708,911,1365,701,1344
VDAC1_4_4790,2027,1746,2123,2512,1399,2740,2858,1282,1591,963,2561,223

5
ZACN_4_4791,453,343,322,294,4,293,853,789,548,11,307,100
ABCC4_4_4792,829,1063,972,920,1153,1140,470,489,313,1269,551,1444
ABCC9_4_4793,2936,3064,3750,3120,4274,1754,2933,2470,4103,4908,6426,34
97
ADIPOR1_4_4794,1043,1070,1371,1113,862,1190,609,213,542,180,1840,1086
AGER_4_4795,255,529,237,261,64,1260,13,748,101,804,156,228
ANO6_4_4796,1177,1686,1716,1480,1091,538,1417,1849,829,1607,964,734
ANTXR1_4_4797,161,191,221,464,151,335,3,391,823,256,57,169
ANXA7_4_4798,1480,1662,1964,1500,1891,2208,1350,1604,1733,775,1734,211
0
AQP4_4_4799,506,322,748,391,390,791,65,976,261,476,434,542
ASGR1_4_4800,101,334,46,46,14,620,152,48,0,0,2,82
ASGR2_4_4801,1333,1251,1661,1414,2551,1299,1390,1338,991,1688,2795,124
9
BEST1_4_4802,151,378,188,266,586,341,366,246,59,267,578,906
BEST3_4_4803,2219,1969,1465,2198,1595,2338,1201,1917,1273,1015,1517,24
87
CACNA1A_4_4804,428,548,443,750,577,642,418,432,898,397,962,727
CACNA1C_4_4805,109,137,32,351,274,497,6,213,0,314,121,647
CACNA1D_4_4806,3242,2910,3759,3181,3257,4763,3798,2786,2028,2905,2752,
4385
CACNA1E_4_4807,779,978,837,630,769,475,444,584,463,947,1335,530
CACNA1G_4_4808,195,126,380,40,26,63,574,356,1,1,676,68
CACNA1H_4_4809,1138,1408,1252,1247,1125,865,1219,1320,744,702,1368,170
5
CACNA1I_4_4810,718,231,642,509,553,725,12,93,57,510,918,48
CACNA2D2_4_4811,48,68,133,262,28,78,0,38,254,32,0,9
CACNB1_4_4812,586,454,814,599,504,843,171,413,496,36,561,834
CACNB2_4_4813,276,164,119,63,5,0,16,19,16,0,7,160
CACNB3_4_4814,407,554,628,642,795,809,14,659,273,254,411,793
CACNB4_4_4815,180,434,345,359,337,210,855,651,3,1,134,434
CACNG6_4_4816,251,235,164,239,231,9,0,294,532,16,86,430
CATSPER2_4_4817,372,772,578,545,526,128,448,592,454,555,1099,860
CD14_4_4818,352,348,258,243,799,618,266,216,8,2,1041,328
CD163_4_4819,797,604,659,423,476,576,1586,112,378,992,189,425
CD247_4_4820,23,293,235,81,169,0,560,148,0,11,795,44
CD302_4_4821,842,674,1073,1014,506,1007,665,1661,481,267,1563,850
CD36_4_4822,195,329,284,165,34,170,1132,21,0,1201,450,316
CD3D_4_4823,148,49,198,328,573,0,0,197,1102,8,333,17
CD40_4_4824,200,446,529,208,295,1022,38,53,13,2176,601,341
CD4_4_4825,426,450,463,653,475,522,1309,542,176,1173,324,373
CD74_4_4826,999,1164,1001,1080,1084,577,1708,1548,1487,204,548,1621
CD79A_4_4827,98,17,122,95,219,0,106,9,2,32,699,81
CD79B_4_4828,481,248,310,256,401,532,200,780,155,0,304,510
CD86_4_4829,1195,1110,1738,1441,1110,1641,1448,1833,496,1835,784,2739
CEACAM1_4_4830,386,452,571,540,4,829,332,411,464,42,988,382
CEACAM21_4_4831,481,328,570,178,86,312,840,681,473,441,269,406
CHRFAM7A_4_4832,8,35,47,34,1,40,71,0,0,0,1,0
CHRNA1_4_4833,991,1359,938,1269,824,1450,126,1880,1472,393,607,998

CHRNA3_4_4834,514,526,467,921,417,1239,1132,1164,73,529,961,475
CHRNA6_4_4835,651,564,417,660,296,103,259,678,127,426,1057,609
CHRNA7_4_4836,65,124,57,130,236,436,27,104,0,0,21,28
CLCC1_4_4837,49,24,11,18,83,8,40,24,81,0,14,0
CLCN2_4_4838,409,319,630,429,532,32,461,699,54,511,317,86
CLCN3_4_4839,470,376,871,447,580,174,844,824,221,214,1447,780
CLCN5_4_4840,818,880,965,766,391,774,249,1165,418,1262,1567,722
CLCN6_4_4841,585,606,366,284,300,88,916,103,111,1557,371,642
CLCN7_4_4842,539,484,846,465,459,308,801,618,469,129,548,392
CLCNKA_4_4843,262,495,367,538,906,51,1353,790,553,990,473,94
CLCNKB_4_4844,262,495,367,538,906,51,1353,790,553,990,473,94
CLEC1B_4_4845,1002,1012,1161,773,878,451,2172,1404,1095,1293,1224,1151
CLEC4A_4_4846,777,841,985,782,333,917,330,482,656,281,963,609
CNGA1_4_4847,2493,1887,2356,2373,648,2004,1283,1462,1933,5265,3226,288
5
CNGA3_4_4848,434,316,361,434,428,56,309,409,112,263,469,786
CNGB1_4_4849,123,300,134,47,453,855,125,287,390,100,1,587
CNTFR_4_4850,209,133,107,228,0,113,78,251,139,36,101,704
CR2_4_4851,761,817,1192,793,648,488,2614,1090,428,1523,1576,645
CSF2RA_4_4852,3232,2799,3208,1993,2476,3388,3404,2048,2558,4172,2412,2
607
CSF3R_4_4853,427,266,735,449,326,215,338,72,261,50,1052,151
CXADR_4_4854,2497,2979,3242,2684,3055,2319,2281,2593,3219,1891,5192,27
01
DAG1_4_4855,167,169,325,202,494,76,283,372,252,0,408,17
EDA2R_4_4856,2233,2801,2504,2325,2746,3732,4098,2004,2287,2633,2744,35
33
EDNRA_4_4857,569,489,953,478,1092,479,1448,396,126,139,758,1036
ENG_4_4858,292,286,226,604,758,155,328,460,792,0,166,513
EVI2A_4_4859,1175,1138,875,899,379,1209,242,558,1087,1892,1295,877
FCAMR_4_4860,587,1024,902,984,260,1809,1994,2217,709,1179,503,733
FCGR1B_4_4861,641,1622,1093,1042,1259,898,561,900,950,196,554,1177
FCGR2A_4_4862,700,825,727,1075,243,413,503,1003,594,1605,173,1476
FCGR2B_4_4863,325,185,290,319,11,281,36,241,326,0,56,194
FCGR3A_4_4864,1456,1932,2082,1610,536,1195,2423,981,653,1092,2336,1956
FCGRT_4_4865,75,27,30,172,3,210,245,15,39,0,0,0
FGFRL1_4_4866,874,926,1217,836,832,1709,1900,885,629,1881,567,1275
FLT4_4_4867,393,406,417,241,513,234,2249,336,108,235,146,232
FXYD1_4_4868,733,1075,1159,801,956,689,770,796,605,1716,1091,873
FXYD2_4_4869,434,352,408,803,166,674,167,785,1159,396,1541,269
FXYD4_4_4870,259,372,376,216,160,748,142,554,279,5,120,107
FXYD5_4_4871,670,697,437,591,811,1169,342,508,601,244,143,824
FXYD6_4_4872,383,198,338,200,72,298,10,122,997,675,167,115
GABRA1_4_4873,1491,1162,1646,1141,1533,745,2490,1100,1594,339,2873,167
7
GABRA2_4_4874,683,772,884,735,593,630,2098,576,973,1120,1021,362
GABRA4_4_4875,1342,1592,1736,1385,1513,1548,1668,1640,1435,1568,1959,1
023
GABRA5_4_4876,548,690,647,661,162,594,257,901,411,607,180,388
GABRB2_4_4877,1427,1126,1230,1136,1173,1825,2023,402,585,339,1563,1640

GABRB3_4_4878,258,218,405,374,265,83,858,65,274,399,529,379
GABRG2_4_4879,725,634,1663,719,285,2065,1200,1031,273,317,911,939
GFRA1_4_4880,1808,2095,2022,2189,1740,1780,2458,1903,2037,1491,2148,20
21
GFRA2_4_4881,87,95,226,145,2,23,45,282,0,1128,123,70
GFRA4_4_4882,267,409,500,410,434,236,379,512,38,116,646,342
GHR_4_4883,445,237,220,504,256,142,29,307,840,438,299,224
GIPC1_4_4884,237,379,132,146,616,144,2,762,426,280,185,582
GLRA1_4_4885,1334,1142,1122,1197,822,2359,1514,1194,536,1281,1041,1362
GLRA2_4_4886,77,241,219,134,73,411,0,102,337,559,83,181
GLRA3_4_4887,203,290,495,211,31,278,2,210,260,467,408,415
GLRA4_4_4888,150,232,193,194,203,246,214,59,27,559,140,53
GLRB_4_4889,498,432,456,373,775,677,509,531,146,977,252,758
GP6_4_4890,146,100,396,200,456,196,376,748,18,338,436,110
GPM6A_4_4891,1708,1330,2111,1842,2196,573,1864,1486,1822,3040,2884,199
1
GPR89A_4_4892,318,528,388,666,242,162,507,522,142,659,1995,547
GRIA1_4_4893,1117,1158,1433,1378,1905,1261,530,797,1037,1494,1123,694
GRIA2_4_4894,925,937,1179,881,1854,922,332,878,759,768,1259,1175
GRIA3_4_4895,2286,2131,2441,3068,2419,858,3097,4477,2293,1852,3826,231
5
GRIA4_4_4896,192,132,327,78,458,0,228,182,13,0,16,244
GRIK1_4_4897,1040,1183,1383,1124,499,1522,650,1684,1064,793,1378,1020
GRIN2A_4_4898,143,370,121,116,0,125,89,445,0,71,260,43
GRINA_4_4899,43,120,119,63,301,1012,743,42,37,0,0,55
HLA-DPA1_4_4900,234,401,303,272,605,30,585,398,6,111,307,79
HTR3A_4_4901,82,48,96,239,23,16,7,142,29,7,42,133
HTR3D_4_4902,304,353,603,349,201,938,4,233,157,104,776,659
HVCN1_4_4903,3230,3664,4724,2992,4404,3548,6035,2969,3709,5376,4954,41
65
IFNAR2_4_4904,3702,3884,4806,3870,4192,4064,4094,3716,3063,5887,6581,4
590
IL11RA_4_4905,303,411,360,354,25,86,436,247,349,701,258,600
IL12RB1_4_4906,265,192,268,213,391,136,99,51,602,723,590,17
IL15RA_4_4907,53,90,231,13,0,248,471,6,7,791,0,346
IL1R2_4_4908,951,1357,1250,1085,1030,2011,1592,1379,1639,817,737,391
IL1RAP_4_4909,2343,1924,2665,2357,1817,2835,3343,3262,1594,1097,2070,2
814
IL1RL1_4_4910,4451,4325,4182,3283,4101,4069,4647,4036,4337,4758,5843,3
037
IL21R_4_4911,737,722,701,717,572,141,1044,482,1093,255,988,925
IL22RA2_4_4912,142,188,96,153,0,95,1,145,8,0,191,358
IL28RA_4_4913,66,45,117,76,0,0,0,0,0,4,17,0
IL31RA_4_4914,400,353,293,481,57,1169,12,27,120,1107,801,887
IL4R_4_4915,887,764,1154,761,2406,779,1642,614,1439,2249,501,1121
IL5RA_4_4916,1361,1333,1456,1165,1284,955,1207,928,1091,781,1503,599
IL6R_4_4917,498,501,815,616,551,1911,478,1039,107,58,693,1614
IL6ST_4_4918,753,730,911,711,673,314,1567,374,324,565,1027,690
ILDR1_4_4919,281,501,497,685,824,288,1090,659,381,593,826,635
ITGB1_4_4920,452,487,509,632,515,248,496,725,315,413,379,616

ITGB4_4_4921,113,112,131,57,22,37,0,29,402,1,441,9
ITPR1_4_4922,156,188,115,133,808,294,776,19,15,1085,553,103
KCNA2_4_4923,2113,2388,2284,2294,2894,1468,1989,2020,1435,2789,2970,28
58
KCNAB1_4_4924,203,406,451,231,127,7,37,548,83,4,337,826
KCNAB2_4_4925,341,336,228,260,345,334,472,51,661,892,276,415
KCNC1_4_4926,1575,1809,1589,1697,1596,2268,1505,1982,1306,2768,1955,39
9
KCNC2_4_4927,1141,654,875,655,1287,650,134,504,729,1225,519,1016
KCNC4_4_4928,60,70,13,48,0,0,1,8,0,0,7,3
KCND3_4_4929,659,1381,737,781,1752,1282,1001,1298,1225,540,1432,545
KCNE1_4_4930,45,94,115,321,12,129,38,248,196,1,0,128
KCNG3_4_4931,854,793,1015,840,2849,868,1906,954,381,2921,461,670
KCNH1_4_4932,1146,781,996,687,919,1491,614,265,1293,759,1213,1019
KCNH2_4_4933,77,219,180,150,68,61,70,165,0,219,141,22
KCNH5_4_4934,674,814,1080,700,1426,536,699,956,844,689,570,697
KCNH6_4_4935,2272,2402,2889,2333,1467,3017,1484,2149,2436,1788,1921,23
69
KCNH7_4_4936,577,769,634,506,417,751,556,383,186,1027,980,548
KCNIP1_4_4937,173,175,211,366,145,716,619,617,949,273,1117,31
KCNIP2_4_4938,566,677,1226,645,251,425,1740,526,489,633,1038,613
KCNIP4_4_4939,490,536,472,406,415,414,376,455,127,625,754,548
KCNJ11_4_4940,611,529,682,934,155,821,834,1191,201,4,1560,1131
KCNJ13_4_4941,1509,1693,1607,1062,1241,2667,1905,830,1247,604,1674,172
0
KCNJ14_4_4942,899,703,1028,637,955,631,1640,814,539,772,1721,236
KCNJ15_4_4943,279,474,261,490,547,370,254,184,218,122,952,70
KCNJ16_4_4944,1217,728,1270,990,1183,677,97,338,1400,404,900,1004
KCNJ1_4_4945,2671,2789,2802,2159,1889,3124,3581,2328,2591,2075,3557,32
56
KCNJ4_4_4946,1201,1215,1215,1477,2412,2921,3191,1393,874,4643,1371,183
9
KCNK10_4_4947,150,401,489,537,375,77,553,26,188,5,838,489
KCNK16_4_4948,525,287,240,467,454,97,237,701,510,415,336,731
KCNK17_4_4949,33,69,35,26,49,0,0,61,0,185,0,1
KCNK2_4_4950,545,546,713,285,356,781,1270,312,59,1668,449,94
KCNK7_4_4951,187,249,155,9,188,8,168,577,199,193,7,141
KCNMA1_4_4952,479,764,425,429,163,819,399,911,416,513,237,852
KCNMB3_4_4953,2439,3182,3277,2223,2799,1327,2604,3351,1967,2069,1961,3
236
KCNN2_4_4954,775,790,1096,876,1006,1052,2332,1104,846,381,1289,1170
KCNN3_4_4955,943,725,695,777,834,687,3198,267,1430,118,2340,888
KCNQ1_4_4956,83,50,81,45,283,25,73,8,233,0,52,143
KCNQ2_4_4957,647,599,653,562,734,264,1551,186,31,1578,1104,496
KCNQ3_4_4958,193,197,212,298,16,0,507,22,34,0,0,61
KCNQ4_4_4959,343,456,424,711,385,971,422,1263,309,33,315,569
KCNQ5_4_4960,24,58,0,40,0,0,0,0,0,0,0,219
KCNRG_4_4961,1600,1906,2396,1847,1891,1860,1479,2094,1302,1308,2132,16
27
KCTD15_4_4962,261,169,215,109,494,158,176,273,181,94,290,51

KCTD1_4_4963,649,534,660,532,83,220,583,629,748,1,401,352
KCTD6_4_4964,1725,1563,1211,1345,2310,1185,3628,1680,1359,2090,244,225
3
KCTD7_4_4965,644,319,489,608,642,517,689,215,423,606,703,917
KLRC1_4_4966,4239,4015,5006,3915,2615,5420,4898,3940,4494,3633,2656,43
14
LEPR_4_4967,946,565,799,988,223,70,913,1562,601,175,1887,1412
LIFR_4_4968,2190,2096,1823,2030,2154,2496,2163,1752,1110,849,2124,2188
LILRB1_4_4969,278,238,213,333,13,464,175,639,2,1618,981,82
LILRB3_4_4970,398,421,666,626,803,2240,886,56,465,805,1325,622
LRP12_4_4971,1161,940,973,803,453,174,966,411,1733,202,343,741
LRP8_4_4972,72,52,158,112,204,64,1,43,161,0,245,41
MR1_4_4973,994,1092,1240,967,901,1797,873,1061,586,1409,684,1934
MSR1_4_4974,197,232,369,233,0,192,167,151,277,298,3,176
NCR2_4_4975,296,254,288,349,121,191,87,163,56,195,242,502
NCR3_4_4976,248,482,474,304,268,656,36,518,543,57,469,498
NOX1_4_4977,960,940,1171,1107,981,1525,408,258,1193,2623,1288,772
NOX5_4_4978,813,721,998,1036,847,902,920,346,609,1655,796,1044
NRP1_4_4979,658,1093,917,693,812,184,1128,862,485,731,369,716
NUDT9_4_4980,800,496,569,564,185,361,583,1206,744,367,224,1065
OLR1_4_4981,545,681,1024,740,692,544,228,718,402,502,923,886
OPCML_4_4982,535,481,623,667,424,2017,750,604,400,681,900,52
OSMR_4_4983,5230,5870,5932,5863,6490,4749,7599,6318,4687,5700,8236,671
7
P2RX2_4_4984,268,197,212,355,663,125,212,319,898,82,0,219
P2RX5_4_4985,404,768,965,613,616,260,719,2005,129,1156,512,196
P2RX6_4_4986,583,686,693,694,685,853,1362,1911,960,403,1151,508
PEX5_4_4987,255,154,80,157,80,80,2,10,122,0,373,121
PKD1_4_4988,108,143,283,103,73,379,405,37,0,0,9,0
PKD1L2_4_4989,399,198,352,451,436,859,90,262,465,1598,413,394
PLA2R1_4_4990,59,30,79,159,0,0,0,0,0,0,43,2
PLAUR_4_4991,251,239,99,254,15,486,3,40,36,0,0,199
PLXNB1_4_4992,261,219,324,260,729,784,132,316,414,324,347,239
PRKD2_4_4993,696,531,676,650,345,680,614,9,375,2565,419,287
PRLR_4_4994,2527,2931,3040,2671,1587,2983,2933,2205,1968,4007,3714,265
3
PTCH1_4_4995,745,695,1052,797,906,588,1120,1171,889,806,980,1007
PTCH2_4_4996,222,252,384,633,579,197,64,693,85,204,39,152
ROBO1_4_4997,341,491,701,768,435,358,1810,775,412,943,1330,1146
ROBO2_4_4998,169,277,302,148,86,153,46,721,130,0,124,4
RYR1_4_4999,999,627,604,624,1443,449,408,993,556,503,1311,794
SCARA3_4_5000,421,573,399,413,155,382,1409,225,235,558,146,354
SCARF1_4_5001,50,41,79,26,0,0,0,528,65,0,0,0
SCARF2_4_5002,253,587,296,385,384,1277,142,318,72,35,308,112
SCN1A_4_5003,1146,999,1294,1223,868,986,1680,1472,627,366,1670,1097
SCN2A_4_5004,668,861,532,636,1073,502,1007,911,546,116,331,446
SCN3A_4_5005,4370,5132,5257,6529,3006,6443,5604,4497,4570,2696,7091,61
27
SCN3B_4_5006,1354,1256,2117,1534,837,996,1652,1036,1696,1504,2304,1537
SCN4B_4_5007,1557,1593,2143,1465,2181,3228,2512,2575,1261,1267,2469,13

80
SCN5A_4_5008,1065,884,937,1077,1620,1066,2020,1070,1114,1896,572,1102
SCN8A_4_5009,816,1230,1437,1018,515,1371,930,610,526,619,786,1149
SCNM1_4_5010,425,633,714,572,532,122,1055,954,314,38,700,394
SCNN1A_4_5011,77,227,325,177,147,0,714,33,76,0,114,107
SLAMF6_4_5012,2845,2281,3417,2904,3528,3156,3804,2247,3445,1942,4325,3
604
SLC6A2_4_5013,455,307,545,240,223,172,124,557,278,18,331,158
TLR10_4_5014,635,817,1158,861,690,131,1102,777,603,1056,1454,988
TNFRSF10B_4_5015,881,880,983,647,629,1497,1363,949,347,3004,1341,1411
TNFRSF18_4_5016,0,0,0,0,0,0,0,0,0,0,0,0
TNFRSF19_4_5017,2053,2109,2430,2488,2574,3217,1596,2373,1768,1857,3560
,2349
TNFRSF25_4_5018,2,80,35,34,4,104,1,184,87,8,0,70
TNPO1_4_5019,1127,1299,1130,1349,1603,689,2595,1894,1033,1178,2306,119
7
TOMM40_4_5020,189,248,275,381,351,527,160,721,372,795,568,1087
TP53INP1_4_5021,431,436,452,575,285,1174,917,295,163,1052,109,653
TPCN1_4_5022,167,270,140,210,57,10,213,386,48,164,123,354
TRPC3_4_5023,1151,1605,1207,952,659,1148,3560,1443,526,276,1587,1078
TRPC4_4_5024,882,968,738,822,1795,1392,782,685,1148,1372,1961,418
TRPM3_4_5025,536,544,462,620,432,137,1109,452,39,528,1151,775
TRPM4_4_5026,383,423,215,220,222,8,4,1,190,292,549,171
TRPM6_4_5027,100,46,42,57,257,0,80,5,87,1,0,548
TRPV1_4_5028,79,170,15,127,178,96,13,102,43,8,225,136
TRPV4_4_5029,47,150,150,94,6,0,0,580,0,4,40,64
TTYH1_4_5030,2053,1800,2069,1986,2942,1751,1686,1738,2520,1614,2686,29
76
TTYH2_4_5031,131,48,211,62,71,0,5,75,133,0,173,172
VDAC2_4_5032,579,747,481,560,459,934,195,539,331,966,489,492
ANO7_4_5033,654,449,588,780,391,554,422,863,254,370,1053,471
AQP1_4_5034,256,153,374,211,144,179,82,159,93,49,704,1047
CLEC2D_4_5035,861,842,538,738,1059,646,869,570,60,654,540,1250
CLEC7A_4_5036,2683,3129,2345,2763,2466,1509,736,1411,960,3212,3463,242
6
CRLF2_4_5037,9244,9695,9405,8784,6215,6939,11746,6970,8165,7781,11561,
10624
FXYD3_4_5038,1956,1536,2291,2120,1683,2755,2931,739,2320,1516,2335,212
6
HFE_4_5039,303,493,449,290,77,1011,366,110,75,10,117,709
SCN1B_4_5040,151,334,240,343,354,31,541,125,115,13,394,250
TNFRSF8_4_5041,400,140,78,395,48,190,33,269,471,3,63,137
TSPO_4_5042,123,125,184,130,1,46,25,327,2,0,0,52
ABCC8_4_5043,631,512,451,672,1656,636,720,479,846,34,270,347
AMFR_4_5044,4725,5211,5029,4373,2177,6018,5039,2616,3032,6717,7494,313
4
ANO1_4_5045,2224,2282,2185,2032,2926,2601,1811,1436,2503,2065,2550,129
8
ANO2_4_5046,1133,797,1253,1279,914,1565,1408,456,1313,234,867,1212
ANXA9_4_5047,308,409,740,503,411,336,731,97,314,281,349,222

AQP10_4_5048,755,1308,1200,1283,808,1714,751,520,1090,947,1527,1341
AQP11_4_5049,87,49,62,107,15,1,5,36,3,132,26,40
AQP12A_4_5050,330,337,448,419,232,54,4,238,15,366,617,119
AQP2_4_5051,1017,1349,1210,1174,1405,935,980,1056,616,222,1090,2192
AQP3_4_5052,2,13,86,12,56,0,0,102,72,1,23,13
AQP5_4_5053,200,355,395,355,345,246,24,200,665,204,337,726
AQP6_4_5054,38,95,245,84,40,2,406,1,3,0,853,180
AQP7_4_5055,379,544,500,532,178,793,538,676,607,1392,406,497
AQP8_4_5056,642,475,1002,682,574,686,1264,254,677,148,1721,620
B2M_4_5057,820,882,1045,1056,934,84,682,1220,740,878,958,1722
BEST2_4_5058,319,640,456,229,355,18,137,601,256,1,583,476
BEST4_4_5059,615,425,378,425,701,183,673,451,46,1292,295,426
BSND_4_5060,1659,1548,1504,1893,1414,1683,2338,1503,901,575,2155,2153
BTBD10_4_5061,1693,1934,1459,1493,1474,1572,1904,1137,2000,472,2658,23
56
BTNL2_4_5062,60,112,244,72,85,2,3,1,30,19,31,90
C5orf62_4_5063,355,499,387,481,330,257,959,152,363,200,431,465
CACNA1B_4_5064,450,483,453,748,576,641,418,429,894,535,958,725
CACNA1F_4_5065,172,501,641,196,40,185,946,320,523,437,357,547
CACNA1S_4_5066,166,133,199,185,190,419,157,4,134,0,154,79
CACNA2D1_4_5067,417,520,544,447,753,1388,33,499,485,317,124,366
CACNA2D3_4_5068,182,359,183,258,88,585,296,140,23,1,408,517
CACNA2D4_4_5069,430,206,438,83,450,13,3,179,618,0,36,805
CACNG1_4_5070,207,355,222,330,382,75,530,933,169,74,358,50
CACNG2_4_5071,63,103,21,148,6,13,7,215,0,0,0,566
CACNG3_4_5072,2359,2141,1571,1713,1388,1769,861,1445,1961,694,2277,229
8
CACNG4_4_5073,65,51,71,396,3,32,3,24,43,0,37,183
CACNG5_4_5074,182,346,155,276,540,386,244,8,36,47,17,829
CACNG7_4_5075,565,590,615,760,792,270,286,613,657,310,896,1116
CACNG8_4_5076,585,523,666,520,1816,655,1360,794,571,18,619,422
CATSPER1_4_5077,635,365,468,636,521,119,238,491,435,535,368,426
CATSPER3_4_5078,431,556,512,840,39,794,1755,741,213,695,1276,257
CATSPER4_4_5079,355,654,652,415,148,403,463,601,11,783,477,691
CCT8L2_4_5080,342,333,640,204,314,21,472,186,441,492,360,322
CD160_4_5081,782,1079,969,847,350,772,971,1088,726,1199,740,1011
CD163L1_4_5082,696,596,372,321,312,330,1559,455,390,600,21,364
CD27_4_5083,141,235,250,183,72,348,103,519,5,53,577,322
CD2_4_5084,0,0,92,0,0,0,0,0,0,0,0,0
CD300C_4_5085,191,305,182,111,386,260,190,1,69,563,223,316
CD3E_4_5086,784,900,899,667,1210,471,1618,933,1904,1513,1498,930
CD3G_4_5087,192,190,265,309,25,533,505,605,314,514,90,111
CD5_4_5088,150,75,110,104,171,201,203,520,56,90,85,234
CD5L_4_5089,74,23,112,88,1,26,439,0,1,7,828,212
CD69_4_5090,633,590,663,361,1251,204,861,704,194,53,773,1040
CD6_4_5091,308,328,260,290,281,146,1216,151,5,145,215,482
CD72_4_5092,752,740,568,449,377,1015,324,812,511,517,138,913
CD80_4_5093,338,339,704,357,210,347,1,388,169,44,569,183
CFTR_4_5094,1760,1786,1577,1594,1637,1874,2105,999,2160,980,1917,2032
CHRNA10_4_5095,430,118,278,248,357,627,627,19,343,0,72,115

CHRNA2_4_5096,412,436,394,706,432,508,219,545,213,4,96,788
CHRNA4_4_5097,610,1011,810,1051,434,1689,1155,1599,611,1387,706,1636
CHRNA5_4_5098,1577,1345,1435,1295,2712,734,1408,1083,496,3465,426,1216
CHRNA9_4_5099,2920,2452,3089,3092,2844,4085,3988,2334,3239,2705,2265,3
664
CHRNB1_4_5100,75,247,329,22,180,0,1191,43,4,0,208,100
CHRNB2_4_5101,53,118,89,209,354,211,0,1017,1,373,1,103
CHRNB3_4_5102,175,155,50,89,296,40,4,320,16,140,273,100
CHRNB4_4_5103,129,131,199,162,56,1,0,302,69,0,83,1508
CHRND_4_5104,389,986,1006,1286,350,1125,1880,598,17,349,616,662
CHRNE_4_5105,159,90,225,150,2,285,583,66,95,49,296,387
CHRNG_4_5106,191,492,368,282,272,545,918,400,7,3,148,244
CLCA1_4_5107,517,677,794,562,1655,686,574,907,283,172,1027,1513
CLCA2_4_5108,1083,1090,1209,1584,605,1584,1285,242,711,1930,442,1178
CLCA4_4_5109,304,314,309,143,295,573,75,90,257,5,329,10
CLCN1_4_5110,1358,1177,1264,1066,616,832,479,927,388,1008,1247,2153
CLCN4_4_5111,324,312,339,252,578,414,473,94,202,1555,27,154
CLDN3_4_5112,254,215,131,168,109,449,15,209,137,561,94,306
CLDN4_4_5113,572,639,731,567,163,837,180,1059,369,638,388,607
CLEC1A_4_5114,229,216,259,274,20,42,10,104,10,0,58,38
CLIC1_4_5115,139,68,79,191,175,0,348,34,109,8,446,85
CLIC2_4_5116,1233,864,1163,953,938,1176,751,1765,822,815,2178,1023
CLIC3_4_5117,3,3,1,19,2,121,0,0,0,0,89,0
CLIC4_4_5118,385,479,286,515,85,437,268,62,141,577,474,480
CLIC6_4_5119,431,273,198,326,954,255,537,833,269,196,790,516
CLNS1A_4_5120,1129,989,1429,909,1079,651,1378,665,662,774,775,726
CNGA2_4_5121,453,454,624,547,1058,146,859,624,378,966,539,653
CNGA4_4_5122,604,307,504,362,284,401,92,387,215,678,496,608
CNGB3_4_5123,2699,2387,3055,2580,2694,3881,2144,1646,2824,1223,3338,30
17
COLEC12_4_5124,556,215,449,187,195,1,180,368,42,116,65,271
CSF2RB_4_5125,177,101,121,122,75,0,58,17,35,2227,377,7
CUBN_4_5126,1022,740,816,1116,271,893,1514,399,694,2069,1263,635
DCC_4_5127,1035,1337,1892,1294,1875,859,1102,958,1216,406,849,1858
DNER_4_5128,1586,1637,1958,1547,2152,1470,2450,3298,1478,1542,1880,107
5
EDAR_4_5129,87,192,133,157,139,0,624,75,49,722,31,209
EPOR_4_5130,86,105,58,103,27,20,3,39,19,1,28,21
FCER1A_4_5131,761,460,299,308,765,224,235,6,72,357,629,215
FCER1G_4_5132,1035,799,758,534,547,417,1450,274,623,264,967,1058
FCGR1A_4_5133,641,373,373,381,522,1346,1058,1166,195,1591,76,14
FCGR2C_4_5134,3302,3153,3209,3746,3030,2444,2770,2324,2895,3142,5141,3
613
FCGR3B_4_5135,1456,1932,2082,1610,536,1195,2423,981,653,1092,2336,1956
FRRS1_4_5136,380,286,484,389,281,84,414,265,98,234,670,304
FXYD7_4_5137,475,445,763,546,475,1227,1413,1356,775,1580,100,592
GABRA3_4_5138,246,632,357,496,331,352,208,405,37,784,629,507
GABRA6_4_5139,781,683,747,828,989,300,1366,603,565,1113,1287,496
GABRB1_4_5140,687,957,1247,986,610,731,316,198,473,305,874,613
GABRD_4_5141,136,269,77,49,38,19,29,19,285,0,1,18

GABRE_4_5142,1811,1405,1806,1560,622,2641,2168,2719,1597,1150,3553,189
5
GABRG1_4_5143,365,582,473,364,507,605,1087,196,465,915,593,561
GABRG3_4_5144,193,221,200,135,88,483,129,10,7,18,156,145
GABRP_4_5145,939,1135,1401,1055,2438,1242,1555,1871,676,855,1721,807
GABRQ_4_5146,1089,862,1050,1056,745,1924,1978,916,1062,1227,1624,872
GABRR1_4_5147,476,540,470,420,814,1044,360,321,271,928,515,542
GABRR2_4_5148,1556,1359,1911,1459,1676,1411,3717,1830,1281,2394,2398,1
383
GABRR3_4_5149,1204,717,1647,1267,759,1518,1621,1269,380,757,912,1250
GFRA3_4_5150,162,22,26,8,1,0,1,0,34,0,12,69
GPC1_4_5151,259,408,575,574,316,204,814,311,183,424,849,341
GPC4_4_5152,504,393,867,444,450,171,1322,398,123,406,615,768
GPC6_4_5153,0,0,0,0,0,0,0,0,0,0,0,0
GPR89B_4_5154,289,206,268,335,213,391,565,221,578,254,718,80
GRID1_4_5155,479,567,606,523,536,2575,680,852,532,351,220,295
GRID2_4_5156,1379,1025,1014,1142,1142,1776,1090,1205,1059,962,1307,104
4
GRIK3_4_5157,105,160,130,250,107,250,32,86,157,164,10,147
GRIK4_4_5158,786,737,824,694,583,1202,756,1187,828,806,456,1377
GRIK5_4_5159,93,15,85,153,367,0,273,1,207,1,572,47
GRIN2B_4_5160,400,510,291,244,542,721,159,686,467,28,196,310
GRIN2C_4_5161,444,106,131,136,0,491,206,0,3,12,2,0
GRIN2D_4_5162,154,181,131,364,326,594,233,2,0,0,272,11
GRIN3A_4_5163,465,513,540,666,400,1294,604,312,303,504,520,423
GRIN3B_4_5164,270,493,493,365,277,851,421,190,786,1139,408,703
HAVCR2_4_5165,389,238,148,506,267,1983,108,0,42,509,783,19
HCN1_4_5166,1172,1174,1372,1301,822,1278,2656,1384,763,3104,521,905
HCN2_4_5167,262,249,444,83,18,434,753,255,0,12,0,15
HCN3_4_5168,1469,1735,1926,1356,468,1142,3399,2331,923,1191,747,972
HCN4_4_5169,1029,943,867,950,1713,798,613,371,648,1925,444,881
HLA-B_4_5170,109,196,82,183,677,10,130,11,1,12,96,496
HLA-DOA_4_5171,211,249,342,368,197,63,977,558,37,716,1538,284
HLA-DOB_4_5172,460,326,432,355,81,1430,652,254,154,178,497,544
HLA-DPB1_4_5173,403,282,246,262,38,129,84,26,82,410,326,143
HLA-
DQA1_4_5174,6129,6592,7475,6579,9333,8193,10845,5298,6106,9390,10272,7
266
HLA-
DQA2_4_5175,6322,6528,6448,5950,5102,10232,4851,5379,5306,3994,7253,46
44
HLA-DQB2_4_5176,215,73,268,106,339,428,193,198,111,25,153,61
HLA-DRA_4_5177,504,524,332,499,284,344,668,123,543,285,432,159
HLA-DRB1_4_5178,746,747,1285,532,797,354,1020,345,437,3395,680,1787
HLA-
DRB5_4_5179,1377,1427,1782,792,2276,1372,1192,1590,1746,1077,1781,949
HLA-G_4_5180,518,616,624,467,246,698,998,406,886,1747,173,1378
HTR3B_4_5181,694,1217,883,954,654,1860,1019,1125,1200,251,650,1685
HTR3C_4_5182,807,787,954,634,558,585,1049,1040,1069,21,899,1323
HTR3E_4_5183,1570,1664,1945,2093,1421,618,4072,1644,1935,381,2344,1309

ICAM1_4_5184,796,564,556,778,794,486,1275,457,1058,182,971,491
IFNAR1_4_5185,1236,1017,1594,1132,1616,2061,2005,1651,1413,852,2001,93
0
IFNGR1_4_5186,2958,2750,2769,2209,3359,1519,2815,2262,2331,4842,3896,2
638
IFNGR2_4_5187,1019,1339,1500,1325,1340,1602,3380,1502,1316,511,2136,12
48
IGF1R_4_5188,246,462,614,800,291,204,570,98,360,3,1159,340
IGF2R_4_5189,713,643,643,659,853,628,2,36,672,472,300,513
IL10RA_4_5190,351,472,525,578,367,194,143,324,345,56,1341,663
IL10RB_4_5191,863,881,1118,914,489,215,1361,975,490,1454,1241,1558
IL12RB2_4_5192,1262,1341,1521,1149,1315,1582,806,1579,861,618,1819,163
7
IL13RA1_4_5193,296,448,489,327,124,235,609,45,451,0,598,298
IL13RA2_4_5194,500,876,1023,523,1221,328,608,342,227,394,493,811
IL17RA_4_5195,901,762,776,680,518,1589,971,2018,395,382,766,1547
IL17RB_4_5196,1563,1685,1868,1252,1211,1133,3161,2991,993,2190,1627,12
53
IL18R1_4_5197,728,748,1229,1079,166,98,490,2157,401,1199,357,762
IL18RAP_4_5198,1302,1166,1479,1099,384,501,2365,351,1074,52,1368,1622
IL1R1_4_5199,4039,3768,3336,3568,2909,3937,1063,4364,3096,5798,4893,31
19
IL1RAPL1_4_5200,703,888,567,486,329,1452,782,344,912,570,570,620
IL1RAPL2_4_5201,561,574,684,717,789,1061,328,639,275,1141,831,375
IL1RL2_4_5202,1947,1448,1632,2152,2170,2066,1233,1014,1918,84,887,1406
IL20RA_4_5203,154,140,89,283,136,399,487,12,28,2,617,239
IL22RA1_4_5204,1097,970,879,761,1179,1333,815,1303,726,356,1062,1281
IL23R_4_5205,257,267,308,173,20,359,287,172,100,52,405,124
IL27RA_4_5206,118,118,436,46,1,9,526,124,575,312,176,133
IL2RA_4_5207,209,288,501,442,6,22,2654,306,9,283,604,577
IL2RB_4_5208,95,49,253,32,0,94,398,2,320,21,274,424
IL2RG_4_5209,1384,1558,1542,1446,612,1881,1240,1428,1790,505,3564,1053
IL3RA_4_5210,710,1119,1093,451,623,1413,1207,188,467,410,830,558
IL7R_4_5211,1427,1492,1247,977,1302,1173,1754,897,761,1148,1827,892
IL9R_4_5212,317,277,481,205,15,354,482,502,294,1157,217,527
ITFG1_4_5213,1588,1525,1623,1824,1853,1179,1437,846,1584,1292,2103,539
ITGA2B_4_5214,376,384,312,244,44,1246,12,77,66,127,283,226
ITGB3_4_5215,531,807,761,882,295,1191,779,234,688,719,898,2128
ITGB7_4_5216,247,458,417,211,200,729,615,158,351,1197,696,107
ITPR2_4_5217,873,731,690,670,395,1025,651,641,1210,36,1230,1442
ITPR3_4_5218,427,607,533,781,57,1106,585,1098,431,791,627,1007
JPH3_4_5219,508,395,825,404,778,656,174,709,1104,657,1092,331
KCMF1_4_5220,648,437,435,491,1134,371,19,381,405,47,272,273
KCNA10_4_5221,353,125,197,365,428,8,23,160,1109,1338,38,353
KCNA1_4_5222,326,189,146,144,69,83,536,40,1,0,883,124
KCNA3_4_5223,24,50,48,36,137,339,3,43,165,0,90,338
KCNA4_4_5224,1174,1310,1524,1081,1681,1157,2587,1139,736,321,1584,2036
KCNA5_4_5225,124,419,197,188,406,193,321,744,19,22,933,458
KCNA6_4_5226,374,259,419,315,374,714,1130,221,305,35,1066,604
KCNA7_4_5227,423,596,376,216,356,519,433,481,784,282,179,120

KCNAB3_4_5228,329,419,700,524,171,243,1074,616,374,126,861,252
KCNB1_4_5229,268,367,342,330,394,498,26,507,418,36,367,365
KCNB2_4_5230,363,298,236,231,255,113,504,237,108,175,336,564
KCNC3_4_5231,2549,2980,2804,2889,2823,1956,3812,2370,2028,1858,5158,19
60
KCND1_4_5232,26,25,224,62,37,82,360,15,0,0,88,25
KCND2_4_5233,493,453,707,343,377,154,314,425,637,917,520,673
KCNE1L_4_5234,16,71,31,4,15,57,477,86,0,5,17,52
KCNE2_4_5235,480,437,557,298,360,125,699,307,1224,1,612,685
KCNE3_4_5236,597,692,548,605,322,182,827,819,1062,464,1558,692
KCNE4_4_5237,760,972,882,934,547,1232,1009,1183,1119,1438,314,1239
KCNF1_4_5238,689,1111,1094,1161,513,1038,1088,525,831,347,1117,1189
KCNG1_4_5239,287,248,453,416,95,1642,345,147,512,62,17,164
KCNG2_4_5240,337,505,480,857,264,303,140,966,70,1008,97,668
KCNG4_4_5241,27,69,177,49,0,410,0,10,109,0,5,35
KCNH3_4_5242,325,314,249,362,325,623,216,284,177,698,334,394
KCNH4_4_5243,2486,2506,3008,2680,1453,3285,2354,2178,2615,2598,2893,24
11
KCNH8_4_5244,4149,4248,4730,4420,3701,5079,3943,4112,3760,4822,4424,52
63
KCNJ10_4_5245,460,587,738,620,691,605,614,449,167,887,565,873
KCNJ12_4_5246,335,99,166,109,415,280,573,347,0,3,3,391
KCNJ2_4_5247,163,390,377,592,325,202,659,182,246,330,139,477
KCNJ3_4_5248,815,1027,807,1213,288,1160,1593,500,481,1132,1162,1204
KCNJ5_4_5249,487,522,315,548,196,569,336,367,706,715,78,666
KCNJ6_4_5250,317,339,206,349,57,414,196,122,62,745,138,382
KCNJ8_4_5251,832,1060,1165,889,699,988,1014,628,798,1313,1134,1117
KCNJ9_4_5252,796,1039,1360,891,1054,595,2022,1237,477,715,2716,1069
KCNK12_4_5253,541,580,609,786,220,1345,700,527,509,542,1105,1025
KCNK13_4_5254,444,401,319,613,595,367,286,625,251,113,770,169
KCNK15_4_5255,3258,3806,3594,3136,4454,1520,4505,3303,2825,4273,4374,4
884
KCNK18_4_5256,761,443,732,644,95,1592,1010,527,379,295,1104,569
KCNK1_4_5257,1854,2193,2693,1772,1914,1727,1706,1563,1425,467,2474,279
6
KCNK3_4_5258,165,110,63,115,34,8,27,0,113,0,19,98
KCNK4_4_5259,636,322,232,520,177,278,116,47,272,226,141,95
KCNK5_4_5260,466,140,198,517,2,220,136,281,254,742,1023,749
KCNK6_4_5261,557,301,477,318,147,265,847,148,256,130,395,706
KCNK9_4_5262,82,57,35,17,12,328,38,49,5,545,17,3
KCNMB1_4_5263,618,1199,768,927,1590,238,869,1226,2465,252,87,790
KCNMB4_4_5264,411,469,451,405,424,400,259,471,110,431,652,1006
KCNN1_4_5265,162,321,309,214,187,107,1374,120,61,46,24,17
KCNN4_4_5266,923,1237,1371,1281,1128,811,1554,792,989,957,598,1346
KCNS1_4_5267,44,106,51,38,64,0,0,162,218,0,8,101
KCNS2_4_5268,809,1678,1307,1079,1035,1887,844,2323,1004,134,1288,1167
KCNS3_4_5269,191,219,103,266,422,398,356,64,12,23,309,272
KCNT1_4_5270,177,356,184,188,11,1,35,7,106,0,1,195
KCNT2_4_5271,3272,4239,5056,4600,3609,3606,5609,3732,3260,3553,4516,52
55

KCNU1_4_5272,437,798,1004,1045,1458,290,145,547,1062,1353,2080,1370
KCNV1_4_5273,2013,1510,1819,2219,849,2917,2557,2302,2143,2330,1749,222
5
KCNV2_4_5274,411,453,513,444,947,299,897,663,10,2071,94,351
KCTD10_4_5275,235,403,374,640,180,1278,1,423,391,62,62,248
KCTD11_4_5276,960,644,945,676,457,1295,1603,540,181,138,1439,875
KCTD12_4_5277,409,141,396,208,830,926,146,85,600,0,285,28
KCTD13_4_5278,149,231,319,298,1523,51,165,598,15,953,214,679
KCTD14_4_5279,838,1148,1039,780,603,287,1342,1201,679,437,800,962
KCTD16_4_5280,678,583,780,449,1047,1042,435,188,177,72,182,497
KCTD17_4_5281,49,107,70,77,403,30,4,201,149,0,12,722
KCTD18_4_5282,281,488,636,511,514,340,687,226,467,2253,141,145
KCTD19_4_5283,1718,2060,1860,1451,1581,932,3080,1754,1272,771,2339,184
8
KCTD20_4_5284,197,279,172,215,71,79,36,234,23,611,685,0
KCTD2_4_5285,86,98,70,24,90,137,0,12,250,106,13,158
KCTD3_4_5286,1312,1630,1637,1449,1234,1496,2136,1352,854,2014,2282,191
9
KCTD4_4_5287,476,249,284,413,85,289,929,652,18,628,151,434
KCTD5_4_5288,9,11,29,96,0,1,2,0,0,0,0,746
KCTD8_4_5289,566,354,669,302,181,147,399,659,936,17,910,109
KCTD9_4_5290,648,731,814,702,958,236,358,1443,1032,624,1110,504
KIR3DL1_4_5291,267,453,122,395,26,901,381,882,3,176,291,402
KLRB1_4_5292,1532,1465,1548,1827,1212,516,722,1265,1712,1789,1838,1840
LGALS3BP_4_5293,317,187,183,207,409,23,354,197,40,510,424,522
LRP10_4_5294,588,258,644,576,636,48,369,51,339,1327,475,164
LRP1B_4_5295,645,488,749,687,576,100,1229,453,357,707,952,1171
LRP3_4_5296,538,839,1065,614,451,516,547,548,924,1991,290,1215
LRP5_4_5297,1018,803,748,772,630,684,2011,894,736,521,558,1321
LRP6_4_5298,438,398,621,511,224,427,368,350,389,99,802,475
LRPAP1_4_5299,1619,1537,1777,1843,2409,2420,662,2402,1312,2357,1701,12
87
LTBR_4_5300,1157,641,795,1127,2448,1709,1092,572,752,1167,492,950
MARCO_4_5301,108,92,118,190,64,18,846,3,1,0,880,70
MCOLN1_4_5302,509,575,637,425,809,955,549,152,183,381,824,440
MCOLN2_4_5303,1452,1038,1598,1065,1506,874,1814,2677,1874,778,1695,196
1
MCOLN3_4_5304,1852,1786,2734,2360,451,1378,2197,971,1247,2413,2092,261
3
MCU_4_5305,1681,1953,1933,1700,3186,1524,1549,2393,1263,2431,2776,2029
MFRP_4_5306,66,71,75,101,4,2,2,110,135,587,161,60
MICB_4_5307,176,193,288,398,394,811,0,137,37,232,340,157
MPL_4_5308,191,304,195,170,388,101,100,301,26,129,285,8
MRC1_4_5309,558,396,824,437,439,641,1703,347,333,1003,1133,548
MRC2_4_5310,255,319,294,469,81,107,649,317,216,175,260,321
NALCN_4_5311,784,938,1036,683,622,374,778,217,747,127,796,560
NGFR_4_5312,549,290,376,240,69,292,320,494,73,154,622,1041
NISCH_4_5313,438,238,436,365,391,440,177,214,141,172,515,524
NPTXR_4_5314,578,693,984,819,847,362,2181,654,406,284,328,448
ORAI1_4_5315,128,308,162,284,210,419,1,0,454,0,220,2

P2RX1_4_5316,239,212,278,209,2,571,235,320,156,118,204,106
P2RX3_4_5317,413,298,393,190,197,868,37,1267,188,121,289,108
P2RX4_4_5318,273,79,183,193,100,48,38,98,498,0,55,111
P2RX7_4_5319,395,349,410,359,552,1077,5,456,1121,998,503,257
PEX5L_4_5320,1409,1306,1702,1608,1528,4034,832,363,1208,1288,1372,2063
PGLYRP1_4_5321,206,298,258,202,198,200,665,211,124,526,149,330
PGLYRP2_4_5322,66,141,171,116,80,871,0,10,41,6,5,259
PGLYRP3_4_5323,522,444,757,450,318,130,640,1027,606,488,402,864
PGLYRP4_4_5324,128,199,443,368,387,63,16,37,108,20,39,207
PGRMC1_4_5325,53,12,61,29,48,16,100,2,3,0,0,149
PIP_4_5326,5894,4526,4867,4961,4118,4271,7693,5138,5497,3407,4978,7305
PKD1L1_4_5327,298,322,513,388,262,105,470,571,117,6,125,323
PKD1L3_4_5328,499,178,252,317,876,48,262,215,147,2,250,395
PKD2L1_4_5329,1627,1757,1943,1458,1986,2267,2322,1817,1737,757,2024,24
22
PKD2L2_4_5330,1040,918,1201,1278,1389,215,1980,1139,757,646,1314,1334
PKDREJ_4_5331,595,555,494,728,1159,260,99,1585,118,714,341,678
PLXNA1_4_5332,312,317,289,329,36,731,332,59,207,255,345,321
PLXNA2_4_5333,736,804,803,1020,540,759,1754,793,896,165,731,307
PLXNA3_4_5334,436,206,544,355,6,57,137,50,417,127,466,462
PLXND1_4_5335,452,421,471,315,751,1186,348,438,197,2305,342,913
PRPH2_4_5336,251,549,593,581,226,598,1595,410,147,264,266,412
RARRES2_4_5337,257,167,356,309,612,310,119,79,139,1,295,170
RASA3_4_5338,201,231,290,355,939,157,132,37,156,11,884,131
RYR2_4_5339,480,540,586,542,545,316,229,589,323,951,458,403
RYR3_4_5340,403,560,518,224,263,605,460,63,100,475,370,164
SARM1_4_5341,418,400,405,322,950,309,98,9,307,649,276,491
SCN10A_4_5342,1065,884,937,1077,1620,1066,2020,1070,1114,1896,572,1102
SCN11A_4_5343,292,95,722,244,582,86,129,435,573,753,506,30
SCN2B_4_5344,228,176,229,270,266,26,348,424,65,2,532,41
SCN4A_4_5345,383,388,392,196,337,136,90,130,76,454,629,103
SCN7A_4_5346,1206,672,590,965,485,630,1486,945,675,405,639,1668
SCN9A_4_5347,1172,1233,1353,964,1413,971,1519,623,1083,2271,2157,1923
SCNN1B_4_5348,266,170,214,142,17,27,571,8,224,810,123,313
SCNN1D_4_5349,104,155,290,170,537,536,0,450,55,61,709,80
SCNN1G_4_5350,339,165,388,196,231,361,311,186,372,2,340,174
SCUBE1_4_5351,111,19,51,26,1,22,0,216,7,139,17,101
SELE_4_5352,1282,1008,1502,1364,1019,2242,2623,1650,1120,1212,1303,553
SEMA5A_4_5353,323,443,357,317,195,653,459,24,177,222,307,469
SETD1A_4_5354,246,192,252,250,16,368,35,50,231,15,602,241
SFRP1_4_5355,400,154,230,211,992,0,5,134,3,126,102,251
SFRP4_4_5356,6222,6242,6254,7268,6087,6866,6173,6162,7063,6758,5589,59
23
SFRP5_4_5357,100,59,251,166,0,82,34,189,0,444,136,425
SHKBP1_4_5358,17,19,24,45,182,0,32,2,0,0,23,613
SHROOM2_4_5359,459,693,635,403,310,51,274,918,2000,436,261,633
SIGLEC8_4_5360,236,74,116,200,131,114,125,19,8,0,22,30
SLAMF1_4_5361,630,641,1092,653,140,509,367,847,767,320,694,572
SLC6A18_4_5362,232,379,357,366,39,173,1813,222,7,249,287,387
SLC6A19_4_5363,9,2,97,74,52,0,0,0,35,0,0,0

SLC6A1_4_5364,1233,2078,1902,1530,1148,790,1154,2801,972,2785,3184,207
0
SLC6A3_4_5365,244,177,241,194,100,39,123,98,241,1141,355,355
SLC6A4_4_5366,268,256,180,229,675,312,105,928,582,34,179,188
SLC9A1_4_5367,29,4,22,7,8,0,0,22,0,0,0,0
SLC9A3_4_5368,722,434,669,626,914,374,1299,701,1058,225,998,439
SRCRB4D_4_5369,341,207,598,274,368,4,24,318,6,1,155,154
STAB2_4_5370,377,127,129,164,0,175,82,1,16,206,149,122
STX1B_4_5371,850,998,1204,1048,312,659,1124,257,672,1022,652,516
THBD_4_5372,8,1,81,0,7,0,0,0,4,0,45,1
TLR1_4_5373,1332,1212,937,941,260,1704,1529,942,616,1671,858,1159
TLR2_4_5374,6980,7758,8373,6902,6573,7197,9140,6259,6996,7441,11462,76
93
TLR3_4_5375,1527,1799,1747,1364,1561,997,2003,1236,546,856,1962,2294
TLR4_4_5376,3140,3902,3904,3808,3742,3516,4893,2015,5051,7515,4704,516
3
TLR5_4_5377,492,460,362,632,683,1172,264,310,500,62,322,618
TLR6_4_5378,855,974,949,1044,1319,874,442,834,550,1235,1645,1388
TLR7_4_5379,618,613,709,641,751,272,1107,367,296,1124,1743,698
TLR8_4_5380,1043,956,1273,1109,1218,1397,1166,762,1098,542,837,1251
TLR9_4_5381,396,512,433,593,207,548,547,294,649,13,810,741
TMEM37_4_5382,3,10,93,5,0,0,0,0,0,0,140,0
TMEM38A_4_5383,600,342,716,458,337,582,104,779,251,261,707,610
TMEM38B_4_5384,2068,2430,2297,2311,1799,1694,5029,2416,1429,1168,2506,
2350
TNFAIP1_4_5385,42,340,185,139,238,83,507,170,156,351,51,30
TNFRSF10A_4_5386,901,614,1073,907,532,1086,2209,740,301,348,2308,641
TNFRSF10C_4_5387,710,910,900,919,1326,30,455,545,452,692,1015,743
TNFRSF10D_4_5388,685,796,534,419,580,377,1081,284,237,220,934,108
TNFRSF11A_4_5389,474,386,859,771,649,109,484,1374,769,1174,972,916
TNFRSF13B_4_5390,387,177,535,183,23,597,767,678,67,250,658,18
TNFRSF13C_4_5391,2593,2532,3337,2812,2558,3086,2255,1974,2300,1202,390
4,3100
TNFRSF14_4_5392,302,248,144,288,333,0,290,498,38,164,474,199
TNFRSF1A_4_5393,119,225,162,109,4,173,94,935,541,901,107,928
TNFRSF1B_4_5394,455,247,551,250,541,56,37,1055,4,428,329,381
TNFRSF4_4_5395,70,22,24,157,20,12,0,5,0,0,36,8
TNFRSF6B_4_5396,251,219,351,373,180,330,414,1042,71,43,79,494
TPCN2_4_5397,887,607,1126,1049,445,1378,764,1585,631,613,888,977
TREM2_4_5398,55,33,59,9,2,7,0,0,0,0,0,36
TRPA1_4_5399,2674,2617,2826,2344,3041,4868,3693,1476,1632,3812,2992,20
55
TRPC1_4_5400,529,1060,1299,950,1041,1174,1581,897,1108,518,1201,1262
TRPC5_4_5401,815,916,751,1086,1690,429,1433,868,1476,531,1570,625
TRPC6_4_5402,414,828,765,402,464,0,172,360,116,2772,850,382
TRPM1_4_5403,102,119,116,169,22,617,189,593,7,886,834,217
TRPM2_4_5404,146,201,227,224,99,226,31,471,144,19,316,48
TRPM5_4_5405,2329,3015,2574,3639,1765,792,2618,2560,2124,1490,4374,372
7
TRPM7_4_5406,2213,2187,1958,1909,1931,2939,3843,1991,1210,1056,2424,14

33
TRPM8_4_5407,753,631,944,899,462,77,708,208,186,409,1225,240
TRPV2_4_5408,1708,1531,1793,1599,743,1547,580,1433,1224,517,1661,1649
TRPV3_4_5409,369,287,532,895,92,743,33,355,676,1319,443,463
TRPV5_4_5410,430,244,298,285,151,17,614,316,117,24,477,608
TRPV6_4_5411,370,424,368,384,642,200,533,440,226,139,526,447
TTYH3_4_5412,279,185,55,86,1090,205,293,59,6,1,536,83
ULBP1_4_5413,2059,2464,3411,2759,3387,2050,2141,1943,1825,2501,1399,18
93
ULBP2_4_5414,853,803,761,803,285,684,485,238,464,1027,737,1206
ULBP3_4_5415,554,579,755,513,676,358,56,974,121,14,2625,255
UNC5A_4_5416,444,821,842,838,772,1062,208,607,450,437,1101,464
UNC5B_4_5417,79,75,11,91,0,6,0,20,0,1329,17,114
UNC5C_4_5418,1316,1175,1179,1253,1726,1951,1326,879,1073,1886,1705,113
3
UTRN_4_5419,280,430,448,295,44,361,1,325,647,764,478,269
VDAC1_4_5420,603,490,538,613,592,154,1115,361,395,552,1010,838
ZACN_4_5421,379,312,669,660,458,799,1298,196,198,466,563,584
ABCC4_4_5422,1004,1280,1599,1674,1716,1072,2891,749,1819,935,1880,1816
ABCC9_4_5423,1344,1199,2067,1540,1643,2256,648,1713,2265,2642,982,1258
ADIPOR1_4_5424,1135,844,1240,1248,1571,635,1475,1364,396,99,1045,1238
AGER_4_5425,1087,1434,1333,1274,2483,1415,1010,1023,2505,1411,1058,144
3
ANO6_4_5426,1430,1186,1167,1451,1443,1082,839,425,421,206,1412,1659
ANTXR1_4_5427,496,766,647,489,810,302,652,227,63,7,1041,324
ANXA7_4_5428,5433,5309,5831,5146,5142,5017,4807,4320,2393,4162,4930,52
50
AQP4_4_5429,0,0,0,0,0,0,0,0,0,67,0,0
ASGR1_4_5430,439,400,295,258,65,67,299,77,200,1,79,228
ASGR2_4_5431,407,621,488,337,626,1295,346,190,349,0,97,558
BEST1_4_5432,110,108,49,188,4,119,31,359,5,575,133,48
BEST3_4_5433,293,347,262,182,95,0,606,913,65,27,149,257
CACNA1A_4_5434,484,503,710,638,226,101,1225,94,333,774,382,511
CACNA1C_4_5435,725,428,776,617,943,820,976,175,806,88,906,816
CACNA1D_4_5436,2060,2921,2902,2310,3458,3239,3832,2812,2048,3605,3381,
2148
CACNA1E_4_5437,1220,1104,1280,1702,2266,577,1423,1101,559,1693,2173,50
7
CACNA1G_4_5438,258,373,368,392,283,1064,149,554,196,59,132,227
CACNA1H_4_5439,174,248,234,366,726,291,642,269,133,663,159,510
CACNA1I_4_5440,746,645,581,760,422,712,1094,1086,55,662,561,788
CACNA2D2_4_5441,424,312,336,297,97,701,812,843,506,830,542,1125
CACNB1_4_5442,1024,1127,801,1021,376,1739,195,852,249,436,792,393
CACNB2_4_5443,288,503,491,630,741,828,453,479,110,484,665,862
CACNB3_4_5444,1080,1319,1312,1578,1137,2257,2331,1610,1602,1128,1602,6
26
CACNB4_4_5445,773,701,722,905,193,555,2105,357,323,727,533,382
CACNG6_4_5446,773,711,846,918,2656,2159,256,1448,725,284,1506,187
CATSPER2_4_5447,2615,2664,3189,2370,2009,2076,3942,2092,1686,2329,3733
,3208

CD14_4_5448,514,477,339,874,388,581,917,230,295,268,731,571
CD163_4_5449,235,238,309,456,489,251,931,544,116,545,64,179
CD247_4_5450,761,1200,1096,748,877,1264,1922,1014,679,303,389,939
CD302_4_5451,465,350,821,595,120,465,545,716,332,146,860,501
CD36_4_5452,3398,4807,3642,4217,2859,4349,5636,3633,4678,3591,5825,416
6
CD3D_4_5453,216,148,453,405,556,434,107,948,208,1179,207,236
CD40_4_5454,740,847,475,527,1031,1402,719,652,465,285,262,561
CD4_4_5455,228,400,369,295,49,1,220,328,136,3,220,174
CD74_4_5456,130,204,139,206,53,182,169,320,135,738,13,138
CD79A_4_5457,90,142,315,104,22,133,0,0,24,1555,358,2
CD79B_4_5458,184,353,325,295,256,208,229,227,130,10,19,62
CD86_4_5459,405,339,604,316,257,200,854,207,170,0,766,291
CEACAM1_4_5460,471,820,811,864,812,1128,138,1099,543,2049,885,1486
CEACAM21_4_5461,173,88,123,180,129,0,9,23,0,142,17,113
CHRFAM7A_4_5462,375,307,176,253,533,98,162,103,219,325,233,124
CHRNA1_4_5463,368,293,401,331,45,20,202,382,690,242,34,603
CHRNA3_4_5464,298,306,207,215,291,932,35,507,23,65,65,68
CHRNA6_4_5465,2160,2105,1973,2038,2196,2760,2007,1562,2413,1661,2458,1
539
CHRNA7_4_5466,160,109,153,379,508,53,15,713,222,513,482,2
CLCC1_4_5467,118,261,224,306,525,2,255,15,62,0,25,457
CLCN2_4_5468,282,232,277,81,373,357,661,231,43,585,584,452
CLCN3_4_5469,363,252,393,333,715,1092,451,521,32,268,345,533
CLCN5_4_5470,399,250,650,718,313,497,76,319,119,1152,768,1140
CLCN6_4_5471,1411,1537,1892,1480,1111,2544,3393,2483,938,1380,1778,100
8
CLCN7_4_5472,244,332,674,508,113,22,700,260,73,1406,158,309
CLCNKA_4_5473,29,25,41,124,2,43,108,0,33,0,243,1
CLCNKB_4_5474,2180,2907,2647,2581,2050,2985,2609,2502,2220,1580,4945,3
491
CLEC1B_4_5475,355,183,331,258,511,374,229,147,214,0,564,534
CLEC4A_4_5476,779,1045,1097,1122,890,924,1099,364,149,1082,675,1334
CNGA1_4_5477,528,367,677,318,790,148,911,378,443,515,139,1140
CNGA3_4_5478,1075,870,1027,669,788,646,1663,849,700,891,1104,320
CNGB1_4_5479,275,178,330,323,394,198,1221,74,341,743,483,469
CNTFR_4_5480,121,209,287,129,392,44,160,157,291,4,276,27
CR2_4_5481,652,611,658,409,713,397,950,880,1177,179,141,1003
CSF2RA_4_5482,652,366,774,807,716,1499,541,992,459,1613,705,636
CSF3R_4_5483,319,323,259,265,148,753,709,286,63,975,239,133
CXADR_4_5484,604,756,750,717,891,1045,1272,1021,430,887,740,352
DAG1_4_5485,821,1176,1167,1182,1499,1986,539,1061,469,951,1089,828
EDA2R_4_5486,531,377,879,672,770,42,667,1113,313,476,626,720
EDNRA_4_5487,4974,4956,5923,5360,7041,5282,6392,5301,3055,5069,5513,44
44
ENG_4_5488,219,146,114,115,162,128,53,139,127,0,408,42
EVI2A_4_5489,398,306,592,495,344,356,699,348,744,1240,1511,1063
FCAMR_4_5490,396,732,1023,760,724,1658,1432,511,692,214,2232,1178
FCGR1B_4_5491,1316,1053,1467,1482,759,1857,2218,2159,978,1066,1687,134
0

FCGR2A_4_5492,3302,3153,3209,3746,3030,2444,2770,2324,2895,3142,5141,3
613
FCGR2B_4_5493,223,76,201,69,399,39,77,81,11,170,111,189
FCGR3A_4_5494,1096,1103,1323,855,1343,663,1454,749,1005,2239,788,1406
FCGRT_4_5495,307,160,205,161,98,188,0,15,33,0,40,185
FGFRL1_4_5496,43,59,69,83,569,162,0,110,38,0,29,31
FLT4_4_5497,743,669,457,691,587,1162,479,639,435,440,771,968
FXYD1_4_5498,345,313,212,327,275,296,1064,149,301,94,274,174
FXYD2_4_5499,1535,1221,2064,1530,1808,1114,2609,909,2360,2656,1445,141
3
FXYD4_4_5500,195,166,246,127,115,379,586,150,236,0,14,351
FXYD5_4_5501,203,85,128,69,124,40,9,135,567,316,564,285
FXYD6_4_5502,629,477,406,467,639,470,1595,862,489,1395,1009,428
GABRA1_4_5503,2679,3401,3130,3374,2369,2084,2340,2190,2365,1758,3118,3
711
GABRA2_4_5504,1026,906,888,974,872,1411,1582,2259,732,1793,921,786
GABRA4_4_5505,807,393,897,535,321,47,957,626,91,1076,617,449
GABRA5_4_5506,180,268,326,231,172,80,37,95,197,202,343,60
GABRB2_4_5507,238,294,234,516,125,87,119,500,2,17,1198,322
GABRB3_4_5508,417,433,579,408,686,165,347,638,302,343,250,287
GABRG2_4_5509,132,321,247,69,109,525,63,313,128,482,269,117
GFRA1_4_5510,211,149,231,142,798,51,86,101,52,550,141,363
GFRA2_4_5511,1358,1421,1868,1325,2313,1535,3091,2857,1349,1573,1452,22
57
GFRA4_4_5512,469,332,1534,455,301,632,527,223,479,1838,1380,269
GHR_4_5513,1190,1558,846,1462,2175,2434,2069,1143,1662,459,2102,1325
GIPC1_4_5514,1441,1204,906,887,1726,784,1116,597,536,1350,1776,1502
GLRA1_4_5515,986,1058,1005,1033,1727,457,339,1617,868,103,687,975
GLRA2_4_5516,707,905,988,889,614,554,624,1251,1249,716,1133,143
GLRA3_4_5517,286,419,630,610,2,1006,451,935,756,106,724,187
GLRA4_4_5518,655,545,637,451,134,540,1111,698,124,556,453,480
GLRB_4_5519,1037,1036,1213,899,1712,880,1845,730,686,1122,1494,255
GP6_4_5520,13,0,12,9,0,0,0,0,0,0,0,0
GPM6A_4_5521,521,498,284,671,56,483,1175,774,267,0,273,616
GPR89A_4_5522,289,206,268,335,213,391,565,221,578,254,718,80
GRIA1_4_5523,429,314,388,656,412,593,1162,173,374,359,558,349
GRIA2_4_5524,813,457,716,450,1180,279,484,1131,112,1453,441,799
GRIA3_4_5525,1110,1306,1013,851,536,965,245,770,744,520,1554,617
GRIA4_4_5526,347,353,464,418,77,281,175,682,29,610,167,169
GRIK1_4_5527,2771,2275,1985,2484,1384,2605,2294,3284,3134,703,3743,179
2
GRIN2A_4_5528,570,744,897,640,1145,641,347,309,185,209,1038,858
GRINA_4_5529,35,188,41,52,15,110,227,0,0,0,62,2
HLA-DPA1_4_5530,687,730,501,413,555,627,114,276,215,1266,728,255
HTR3A_4_5531,67,40,161,94,12,295,4,2,10,2,1,225
HTR3D_4_5532,908,895,627,906,554,383,1418,1266,1035,400,835,1435
HVCN1_4_5533,747,851,780,813,739,568,424,1182,1237,1004,1460,1309
IFNAR2_4_5534,676,367,613,947,159,455,79,131,947,67,20,133
IL11RA_4_5535,2136,2571,2063,2123,1098,3018,2199,874,4050,815,1509,308
8

IL12RB1_4_5536,506,790,723,557,287,551,209,255,94,43,229,578
IL15RA_4_5537,558,823,835,779,1588,1476,1270,93,873,383,1284,1887
IL1R2_4_5538,3583,3637,3703,3404,2865,2701,4195,2909,4556,3962,5806,35
62
IL1RAP_4_5539,2422,2720,3388,2639,1944,3149,2933,2913,2754,1369,2701,3
638
IL1RL1_4_5540,349,288,706,392,460,185,544,319,198,0,134,113
IL21R_4_5541,457,568,697,331,417,461,207,17,375,690,984,468
IL22RA2_4_5542,2453,2666,2652,2283,2206,2659,1554,2502,1936,4155,3027,
3101
IL28RA_4_5543,301,239,367,402,130,825,129,373,76,153,270,176
IL31RA_4_5544,1931,1689,1909,1814,2053,1355,1002,1535,2906,1681,4878,1
537
IL4R_4_5545,200,280,140,202,68,80,191,182,77,12,379,215
IL5RA_4_5546,1525,1399,2059,2009,1627,1972,3404,1147,1215,670,2638,103
6
IL6R_4_5547,457,821,545,589,40,198,353,206,846,0,302,1228
IL6ST_4_5548,1146,1107,1572,780,763,1974,1831,1732,1761,1283,873,988
ILDR1_4_5549,326,195,334,353,909,75,178,362,177,71,239,230
ITGB1_4_5550,1076,1281,1248,1702,1989,2840,2446,1898,868,447,1394,1517
ITGB4_4_5551,168,282,262,202,155,21,887,184,180,334,94,349
ITPR1_4_5552,692,706,652,442,853,234,1149,618,599,395,601,902
KCNA2_4_5553,2349,2372,2468,2226,3188,1472,1989,2016,1718,3546,3175,28
62
KCNAB1_4_5554,433,280,606,319,36,414,68,274,278,338,813,493
KCNAB2_4_5555,502,549,1087,764,1281,244,1562,208,512,308,592,573
KCNC1_4_5556,249,336,291,80,751,207,1497,637,118,35,144,421
KCNC2_4_5557,542,363,503,324,568,75,647,519,501,282,306,198
KCNC4_4_5558,1389,1505,1052,1336,1414,2184,716,867,1465,2070,904,1411
KCND3_4_5559,123,418,448,497,689,43,25,236,153,185,371,524
KCNE1_4_5560,79,117,152,81,224,13,18,181,42,31,145,84
KCNG3_4_5561,208,91,180,306,88,2,253,50,238,4,1819,72
KCNH1_4_5562,703,748,674,598,279,612,1304,1724,144,639,933,279
KCNH2_4_5563,665,430,462,525,896,114,532,667,380,678,333,363
KCNH5_4_5564,637,687,826,640,467,943,1163,913,525,255,509,432
KCNH6_4_5565,1114,764,981,972,556,1031,1267,872,617,364,1228,1383
KCNH7_4_5566,1152,1826,1440,1291,1438,2272,1651,1421,620,125,1326,964
KCNIP1_4_5567,2034,2188,2511,2210,2967,2622,2467,1605,1518,1323,2187,2
406
KCNIP2_4_5568,557,869,738,702,1841,1521,337,73,455,66,1332,1443
KCNIP4_4_5569,1994,1669,2503,1891,1500,3348,5023,1338,1630,1485,2423,2
482
KCNJ11_4_5570,404,182,341,322,385,359,234,52,4,663,397,339
KCNJ13_4_5571,368,451,451,458,598,100,307,530,823,162,395,566
KCNJ14_4_5572,199,236,144,69,0,102,1077,1,136,1241,76,4
KCNJ15_4_5573,182,222,397,197,446,226,654,64,160,333,322,12
KCNJ16_4_5574,313,453,304,290,314,110,6,263,259,345,291,346
KCNJ1_4_5575,2380,2570,2424,2410,2021,2667,2910,2100,585,1919,2628,287
8
KCNJ4_4_5576,1654,1805,1656,1902,1102,673,796,2466,3070,1322,2899,1604

KCNK10_4_5577,568,565,710,646,538,233,1716,1034,221,1,1462,977
KCNK16_4_5578,128,350,270,249,167,35,69,54,209,1,416,114
KCNK17_4_5579,720,989,832,668,1164,496,729,548,845,1127,1404,1580
KCNK2_4_5580,295,233,541,493,213,189,676,360,213,194,147,1086
KCNK7_4_5581,123,345,397,281,77,252,277,0,130,12,368,277
KCNMA1_4_5582,2695,2552,2347,2296,2218,1785,1818,2194,2832,1591,1831,3
502
KCNMB3_4_5583,655,659,691,925,1176,826,870,444,594,1200,1055,444
KCNN2_4_5584,6304,6537,7554,7025,5023,6558,6960,4670,5862,8925,6918,52
74
KCNN3_4_5585,113,121,106,180,38,65,195,0,1,12,104,223
KCNQ1_4_5586,458,511,567,673,409,464,1154,412,398,677,540,531
KCNQ2_4_5587,662,561,637,523,795,264,1134,78,33,1580,1103,509
KCNQ3_4_5588,316,252,281,180,470,95,201,574,218,32,616,537
KCNQ4_4_5589,115,87,70,197,261,206,486,0,571,579,22,219
KCNQ5_4_5590,270,165,301,243,463,23,37,7,9,0,97,208
KCNRG_4_5591,1761,2060,2314,1770,1328,2793,2376,1510,927,280,1978,712
KCTD15_4_5592,4706,5619,5954,6827,5735,4849,4315,5945,5724,6160,4419,6
725
KCTD1_4_5593,2636,2506,3369,3184,2765,2453,3524,4392,2491,3648,1264,30
41
KCTD6_4_5594,417,298,391,839,741,348,25,154,944,1359,378,601
KCTD7_4_5595,53,86,69,195,8,106,160,115,33,645,5,21
KLRC1_4_5596,415,440,576,1026,877,698,917,720,571,1026,260,733
LEPR_4_5597,927,1184,1255,1259,722,479,383,541,955,1592,987,680
LIFR_4_5598,187,153,172,129,112,382,75,7,134,0,31,491
LILRB1_4_5599,939,1187,1448,1271,1556,2270,1744,1196,898,2574,1384,135
4
LILRB3_4_5600,87,289,55,284,0,0,0,745,5,2,369,652
LRP12_4_5601,1039,1068,1455,1339,647,695,730,1990,1176,1137,1623,1045
LRP8_4_5602,279,247,76,98,806,32,268,342,16,0,227,550
MR1_4_5603,417,583,576,638,178,379,373,525,421,724,423,234
MSR1_4_5604,564,772,409,588,304,817,595,596,837,1908,971,621
NCR2_4_5605,49,53,95,29,0,100,7,20,9,0,184,38
NCR3_4_5606,343,420,519,547,1078,43,9,170,640,31,101,13
NOX1_4_5607,996,679,1314,899,866,858,752,487,184,1281,532,653
NOX5_4_5608,432,704,488,668,349,1637,260,581,486,296,871,171
NRP1_4_5609,1979,2149,1861,1779,1971,431,1773,1551,1153,3391,2547,1697
NUDT9_4_5610,472,285,661,468,1000,551,1570,549,334,287,312,788
OLR1_4_5611,982,911,818,773,1818,460,900,709,412,407,318,1003
OPCML_4_5612,211,516,567,255,510,35,734,1353,312,14,48,344
OSMR_4_5613,1187,1342,1759,1260,1585,1882,2580,1724,803,1901,1729,2446
P2RX2_4_5614,340,80,141,118,0,224,221,65,134,20,74,720
P2RX5_4_5615,896,921,1301,863,709,1923,1974,589,415,283,800,614
P2RX6_4_5616,1596,2332,2677,2033,2147,3359,2052,2925,2650,1653,1813,17
00
PEX5_4_5617,0,0,0,0,0,0,0,0,0,0,0,0
PKD1_4_5618,226,227,420,299,270,258,373,40,15,428,112,338
PKD1L2_4_5619,324,710,915,575,922,450,1341,574,940,582,676,1178
PLA2R1_4_5620,820,644,718,595,954,451,863,549,231,1228,1280,1054

PLAUR_4_5621,200,225,510,464,217,72,852,393,55,1165,829,150
PLXNB1_4_5622,2821,2422,2750,3257,2325,516,4529,2705,969,1672,3878,305
4
PRKD2_4_5623,395,536,279,469,387,452,257,58,2,134,253,295
PRLR_4_5624,826,1430,1086,882,1567,768,721,1352,1393,381,1440,1131
PTCH1_4_5625,1891,1482,1986,1673,2012,2349,2157,2255,971,1522,695,1284
PTCH2_4_5626,64,89,117,175,359,230,34,1,18,2,283,44
ROBO1_4_5627,1331,1567,1957,1669,226,2305,2177,1574,1645,911,2627,1635
ROBO2_4_5628,377,282,171,468,238,265,373,372,123,537,179,32
RYR1_4_5629,572,721,602,708,291,824,1168,408,235,55,306,407
SCARA3_4_5630,745,978,895,761,843,611,631,896,598,1609,246,561
SCARF1_4_5631,670,755,630,432,1993,1401,1879,430,1349,516,1093,1130
SCARF2_4_5632,57,42,57,59,18,0,2,99,41,0,34,39
SCN1A_4_5633,3772,2961,3883,3960,5664,5050,7491,2985,3702,1955,2176,38
91
SCN2A_4_5634,422,621,762,513,652,120,516,610,271,38,614,756
SCN3A_4_5635,5721,5788,6366,6288,5054,6927,4665,5868,3450,8139,7798,61
38
SCN3B_4_5636,619,1139,878,864,265,690,1136,397,783,1887,1247,635
SCN4B_4_5637,1145,1289,1125,661,1352,809,1190,614,806,257,2054,821
SCN5A_4_5638,160,253,202,134,212,608,133,331,46,74,174,178
SCN8A_4_5639,362,765,447,365,822,711,525,1085,727,160,382,660
SCNM1_4_5640,388,401,371,303,85,464,46,680,219,0,178,393
SCNN1A_4_5641,118,270,263,155,386,254,120,76,90,2157,45,26
SLAMF6_4_5642,243,507,656,705,1074,436,608,352,1405,871,1093,587
SLC6A2_4_5643,252,197,310,242,722,3,53,32,616,1289,3,357
TLR10_4_5644,1514,1281,2473,1343,2268,1898,1670,884,2071,552,1375,1589
TNFRSF10B_4_5645,124,246,131,220,499,367,9,18,0,342,635,110
TNFRSF18_4_5646,38,25,124,37,0,0,0,0,11,0,0,0
TNFRSF19_4_5647,25,10,102,73,39,5,780,372,233,0,9,1
TNFRSF25_4_5648,330,322,345,161,329,99,5,147,475,1146,354,163
TNPO1_4_5649,798,589,1296,800,1522,581,636,1690,510,471,286,894
TOMM40_4_5650,439,132,328,239,148,129,891,605,57,800,325,221
TP53INP1_4_5651,2256,2060,3154,2798,3636,2775,3676,2863,1261,2662,4701
,2865
TPCN1_4_5652,2945,3145,3290,3245,3318,3316,3256,3688,2955,2551,4083,26
44
TRPC3_4_5653,89,44,52,0,0,2,0,0,0,0,0,0
TRPC4_4_5654,1145,1040,1210,542,333,1659,889,430,566,1364,1882,764
TRPM3_4_5655,66,62,130,80,0,0,0,90,5,123,82,1
TRPM4_4_5656,240,568,380,302,700,545,573,84,153,103,687,335
TRPM6_4_5657,467,381,217,332,155,208,142,465,166,419,642,585
TRPV1_4_5658,2035,1817,2110,1817,2497,1890,2340,2136,2418,2055,3015,19
21
TRPV4_4_5659,885,955,672,848,878,591,339,1554,465,811,762,911
TTYH1_4_5660,823,813,865,986,2435,739,1265,306,1311,6,627,602
TTYH2_4_5661,758,891,1238,905,1322,304,771,930,917,1028,1228,1220
VDAC2_4_5662,617,626,811,495,634,945,983,449,120,363,351,628
ANO7_4_5663,406,569,422,382,228,653,716,114,561,134,193,72
AQP1_4_5664,64,125,64,29,9,49,0,1,0,4,936,240

CLEC2D_4_5665,1198,1209,958,1543,473,871,1181,1522,1465,870,1086,1389
CLEC7A_4_5666,934,1155,1142,1047,1527,794,1597,1684,582,421,807,939
CRLF2_4_5667,1765,2197,2080,1696,1185,2463,1901,1513,1243,1696,1677,17
34
FXYD3_4_5668,462,277,404,626,1237,760,477,833,166,43,788,358
HFE_4_5669,347,296,674,439,350,471,159,473,192,29,483,501
SCN1B_4_5670,214,230,32,157,145,0,50,211,0,0,10,19
TNFRSF8_4_5671,1289,1360,1031,1072,1134,1602,2018,1281,588,1494,2154,1
892
TSPO_4_5672,982,1062,1340,808,1898,995,1249,438,1100,958,1184,985
ABCC8_4_5673,1585,1400,1718,1649,866,3538,1590,1365,1266,1950,1291,211
8
AMFR_4_5674,3255,2883,3058,3090,3023,2674,2573,3081,1819,2550,2244,258
5
ANO1_4_5675,481,671,560,668,1298,191,230,64,24,518,738,269
ANO2_4_5676,436,445,256,494,1207,780,611,68,445,1664,258,796
ANXA9_4_5677,414,285,380,241,251,831,185,67,94,22,883,70
AQP10_4_5678,317,355,294,234,316,318,147,55,136,104,702,10
AQP11_4_5679,292,196,72,218,455,19,188,23,0,6,38,121
AQP12A_4_5680,374,266,292,268,216,509,781,506,172,209,534,195
AQP2_4_5681,641,961,709,675,403,1034,662,932,964,1076,556,1141
AQP3_4_5682,25,49,61,30,1,9,45,0,12,22,6,554
AQP5_4_5683,273,203,140,162,283,86,375,375,499,725,402,270
AQP6_4_5684,9,102,155,8,145,0,843,2,1,194,7,217
AQP7_4_5685,336,318,745,523,769,187,101,293,440,363,420,170
AQP8_4_5686,141,96,48,218,49,13,13,91,102,0,185,219
B2M_4_5687,3751,3835,4832,3972,5030,6086,6386,2509,2807,927,3652,3271
BEST2_4_5688,748,951,1067,887,477,733,671,617,728,262,678,1287
BEST4_4_5689,522,560,502,721,464,505,628,604,474,58,35,258
BSND_4_5690,310,399,207,407,1243,251,55,91,87,494,222,507
BTBD10_4_5691,829,513,613,494,383,1049,447,77,124,99,36,458
BTNL2_4_5692,589,621,790,806,361,682,544,674,218,440,563,622
C5orf62_4_5693,915,829,1126,552,374,1027,529,1570,1410,1017,796,1797
CACNA1B_4_5694,277,259,176,243,44,674,34,175,419,0,68,135
CACNA1F_4_5695,389,702,751,824,268,506,1025,333,353,334,249,414
CACNA1S_4_5696,245,254,326,480,157,590,0,82,205,264,83,582
CACNA2D1_4_5697,444,361,606,517,632,262,1024,549,215,40,594,1115
CACNA2D3_4_5698,1082,940,1028,1288,781,856,1086,1894,670,1301,926,968
CACNA2D4_4_5699,200,107,246,208,223,1480,52,336,183,0,154,391
CACNG1_4_5700,2039,1997,2629,2106,1435,1617,3546,2398,1467,3509,3177,2
260
CACNG2_4_5701,607,438,628,610,395,636,346,49,338,190,546,570
CACNG3_4_5702,887,978,1026,1183,652,79,1422,1243,650,294,1516,931
CACNG4_4_5703,906,798,601,482,1144,1102,204,434,360,562,458,876
CACNG5_4_5704,243,305,419,302,221,77,214,392,79,493,283,321
CACNG7_4_5705,171,186,283,439,143,175,80,301,324,845,65,155
CACNG8_4_5706,211,202,84,247,0,148,0,36,388,0,5,422
CATSPER1_4_5707,598,851,869,604,590,1157,634,362,767,81,1477,951
CATSPER3_4_5708,1029,554,844,615,1617,983,568,279,290,811,2058,361
CATSPER4_4_5709,438,550,496,470,527,703,804,702,163,976,297,888

CCT8L2_4_5710,402,491,549,412,140,8,886,664,337,100,324,492
CD160_4_5711,2459,3220,2809,2314,3438,2699,4288,3191,1488,3127,3880,27
17
CD163L1_4_5712,157,119,197,166,76,36,83,1084,523,63,152,443
CD27_4_5713,752,971,920,671,997,556,419,553,31,1014,1362,1027
CD2_4_5714,1126,1893,2187,2092,1298,2149,2049,1456,1057,1519,3363,2032
CD300C_4_5715,0,77,167,204,0,352,0,133,0,0,0,6
CD3E_4_5716,39,125,105,94,16,482,20,124,93,140,0,214
CD3G_4_5717,256,231,285,224,115,162,419,184,162,97,206,176
CD5_4_5718,663,524,1238,710,802,375,1197,2205,316,1997,759,1298
CD5L_4_5719,2930,3374,2621,3478,3328,3402,3616,3608,2440,5609,4634,398
0
CD69_4_5720,894,611,498,484,820,504,788,1255,1028,691,707,719
CD6_4_5721,223,422,385,266,238,108,639,470,942,1082,40,476
CD72_4_5722,1469,1523,1389,1589,770,750,2726,1395,584,1568,1482,903
CD80_4_5723,1700,1672,2169,1345,1523,2174,2187,2362,1930,1197,2009,203
9
CFTR_4_5724,2065,1875,1783,2347,1574,2227,1136,1185,1099,1292,2109,147
8
CHRNA10_4_5725,445,88,262,305,792,278,68,22,168,1029,216,268
CHRNA2_4_5726,243,108,12,114,2,300,0,152,0,0,50,0
CHRNA4_4_5727,554,290,357,349,151,14,1542,125,115,147,248,256
CHRNA5_4_5728,1961,2095,1843,1928,1594,2112,1372,2089,1807,1796,2407,1
797
CHRNA9_4_5729,1200,1549,1213,917,652,571,1441,1201,1313,1303,1631,1378
CHRNB1_4_5730,453,318,507,179,56,110,723,208,141,78,789,68
CHRNB2_4_5731,402,396,199,402,882,82,162,270,1148,0,137,1014
CHRNB3_4_5732,3362,3417,4448,3082,2263,3250,4352,2923,2538,3602,3289,2
347
CHRNB4_4_5733,1874,1802,1557,1793,2050,2645,4152,1373,1188,2749,2435,1
254
CHRND_4_5734,790,835,667,928,489,1506,483,301,1387,208,406,616
CHRNE_4_5735,345,215,413,390,225,756,20,330,635,1079,209,152
CHRNG_4_5736,537,697,694,594,1019,492,1542,315,315,177,205,781
CLCA1_4_5737,324,581,288,712,551,846,201,318,572,215,350,536
CLCA2_4_5738,1410,1289,986,1139,1518,792,654,517,901,634,454,1507
CLCA4_4_5739,1164,1546,1505,1458,1207,1542,1221,936,751,1392,2446,915
CLCN1_4_5740,323,291,225,523,1394,158,585,272,372,654,314,395
CLCN4_4_5741,39,59,58,31,121,251,40,135,4,0,53,66
CLDN3_4_5742,290,220,185,150,545,188,132,212,29,17,219,12
CLDN4_4_5743,786,632,1104,501,697,937,932,1526,399,251,479,887
CLEC1A_4_5744,1378,1293,1873,941,1219,2111,952,912,1171,2977,1473,1176
CLIC1_4_5745,752,342,709,730,94,815,429,486,297,98,1192,449
CLIC2_4_5746,1419,998,1766,1168,916,1427,2641,1129,1038,803,1627,1094
CLIC3_4_5747,147,246,190,266,168,67,250,84,22,292,165,71
CLIC4_4_5748,769,1006,1013,1031,931,739,1000,720,717,790,948,565
CLIC6_4_5749,4433,4081,4162,4282,4572,4518,3081,4056,3181,3520,4191,46
90
CLNS1A_4_5750,334,398,238,393,105,732,43,500,147,454,173,361
CNGA2_4_5751,2028,2019,1754,1350,1874,2280,1050,1444,1551,1873,1553,17

67
CNGA4_4_5752,666,730,570,613,1674,438,162,792,519,258,1367,640
CNGB3_4_5753,2820,2300,2450,2520,2813,1587,2080,3590,3014,2749,5446,28
63
COLEC12_4_5754,895,924,1303,1271,1753,672,407,1232,225,1216,260,1048
CSF2RB_4_5755,31,50,71,84,97,1,0,7,109,0,11,157
CUBN_4_5756,1651,1557,1893,1674,2061,2564,1030,1156,882,1277,1910,942
DCC_4_5757,1544,1270,1970,1361,940,1034,3164,1202,1345,1250,478,820
DNER_4_5758,6845,7403,6370,7299,7348,10498,9180,7515,6843,3425,9124,70
17
EDAR_4_5759,272,390,457,149,496,67,352,475,896,33,17,985
EPOR_4_5760,767,685,1384,788,642,1058,1720,332,634,612,1206,1338
FCER1A_4_5761,883,925,1107,808,994,437,958,834,1166,426,377,932
FCER1G_4_5762,776,943,831,923,618,2144,860,1049,1004,355,906,932
FCGR1A_4_5763,230,355,417,253,159,721,325,90,212,3,435,305
FCGR2C_4_5764,5841,7020,7329,6690,4982,9609,8168,7349,5714,4853,6586,6
043
FCGR3B_4_5765,1096,1103,1323,855,1343,663,1454,749,1005,2239,788,1406
FRRS1_4_5766,805,1155,1180,825,467,438,1515,823,614,762,762,285
FXYD7_4_5767,359,450,478,205,118,688,72,355,620,57,604,527
GABRA3_4_5768,932,1011,1156,1211,732,1359,137,329,567,993,712,1618
GABRA6_4_5769,95,100,128,33,76,2,191,11,0,0,0,472
GABRB1_4_5770,1942,2405,2734,1977,1616,3754,1075,2333,2998,2859,2696,3
720
GABRD_4_5771,570,266,673,607,874,603,476,843,46,496,871,148
GABRE_4_5772,805,867,782,861,430,528,336,603,605,175,678,150
GABRG1_4_5773,503,713,1362,882,1157,174,1671,926,989,575,886,1152
GABRG3_4_5774,1011,1438,1251,1000,499,986,764,1752,467,536,1534,826
GABRP_4_5775,1030,877,946,702,274,87,368,124,994,446,787,649
GABRQ_4_5776,71,183,97,155,0,0,350,217,15,283,0,19
GABRR1_4_5777,128,149,255,274,255,61,145,191,88,1857,687,476
GABRR2_4_5778,308,401,371,441,413,269,928,327,73,479,357,741
GABRR3_4_5779,1511,1990,1747,2205,1860,2165,2679,921,935,1993,2283,191
7
GFRA3_4_5780,750,729,740,678,826,342,925,680,832,833,454,74
GPC1_4_5781,600,773,781,765,636,1101,334,922,522,841,692,388
GPC4_4_5782,250,144,171,253,108,26,98,52,124,74,59,243
GPC6_4_5783,1438,2163,1401,1390,858,1946,1686,1443,668,1502,2277,1663
GPR89B_4_5784,1756,1331,1382,1242,1472,122,910,1366,1549,4137,821,3186
GRID1_4_5785,236,162,298,237,187,24,660,168,398,106,641,373
GRID2_4_5786,193,421,123,236,206,80,274,387,741,18,1128,298
GRIK3_4_5787,369,324,292,300,248,659,99,380,221,120,563,447
GRIK4_4_5788,173,10,136,48,0,0,681,128,44,234,382,425
GRIK5_4_5789,212,348,359,220,192,52,284,347,17,0,268,280
GRIN2B_4_5790,396,257,302,734,9,0,52,582,314,755,244,145
GRIN2C_4_5791,53,157,97,140,0,210,69,91,126,971,0,354
GRIN2D_4_5792,170,177,186,56,324,1,220,3,4,247,16,587
GRIN3A_4_5793,957,1150,1242,941,1017,1012,1478,667,813,2224,1522,2356
GRIN3B_4_5794,446,687,512,593,662,490,474,988,920,787,1465,602
HAVCR2_4_5795,303,428,284,424,292,438,613,472,379,13,279,690

HCN1_4_5796,11477,10941,11919,10870,9009,13216,15672,12017,8860,13663,
13530,10166
HCN2_4_5797,1629,2024,2128,1642,413,2071,2238,1521,768,1773,3053,1906
HCN3_4_5798,305,706,609,433,662,259,289,143,671,91,214,490
HCN4_4_5799,812,1030,1257,792,252,1560,1634,1039,652,330,791,1094
HLA-B_4_5800,60,35,56,20,0,17,0,289,0,0,252,46
HLA-DOA_4_5801,233,372,335,443,346,126,2291,267,518,83,585,490
HLA-DOB_4_5802,417,352,453,562,772,142,125,196,0,3218,857,299
HLA-DPB1_4_5803,181,193,220,208,57,86,102,5,91,126,224,129
HLA-DQA1_4_5804,790,1143,1331,864,1067,845,745,321,1080,874,267,1752
HLA-DQA2_4_5805,273,358,398,307,422,329,113,293,624,79,651,175
HLA-
DQB2_4_5806,1774,1575,1891,1597,2380,2369,2951,1493,1662,588,2745,2717
HLA-DRA_4_5807,220,97,41,168,115,263,3,15,2,0,744,663
HLA-DRB1_4_5808,410,702,701,562,363,248,851,878,494,134,628,472
HLA-DRB5_4_5809,433,614,597,471,345,945,1376,320,219,460,1010,281
HLA-G_4_5810,455,342,384,442,846,791,721,654,616,123,1145,330
HTR3B_4_5811,305,437,309,581,467,408,147,277,458,109,2239,361
HTR3C_4_5812,3435,3373,3243,2843,3300,3850,3460,3595,3199,3885,4509,30
53
HTR3E_4_5813,126,262,193,155,19,255,6,191,224,24,14,0
ICAM1_4_5814,230,222,382,744,39,170,36,355,24,6,338,0
IFNAR1_4_5815,471,1246,695,826,741,403,2240,233,1141,599,1005,300
IFNGR1_4_5816,1591,1253,1578,2348,893,1005,1977,3208,1364,3360,2596,20
54
IFNGR2_4_5817,1202,2016,1685,1747,776,1362,1552,1396,979,1674,3258,185
6
IGF1R_4_5818,517,933,975,632,908,1040,1219,485,249,44,1439,1037
IGF2R_4_5819,496,674,886,660,933,158,1845,1003,926,348,1350,515
IL10RA_4_5820,348,257,163,242,81,74,700,227,455,100,366,176
IL10RB_4_5821,396,721,523,414,510,340,292,584,237,190,274,127
IL12RB2_4_5822,1039,1065,948,792,1132,1112,1402,771,171,1915,366,577
IL13RA1_4_5823,3980,3674,3617,3575,3706,4185,4509,5290,2438,2276,3839,
1855
IL13RA2_4_5824,723,500,628,801,949,764,385,623,415,590,950,164
IL17RA_4_5825,105,101,164,111,177,4,0,4,0,0,498,46
IL17RB_4_5826,502,649,585,402,217,975,153,791,522,390,616,512
IL18R1_4_5827,189,484,292,274,90,83,371,75,116,5,158,86
IL18RAP_4_5828,93,104,294,486,150,475,474,312,0,913,318,386
IL1R1_4_5829,101,98,255,441,159,385,401,42,183,12,976,501
IL1RAPL1_4_5830,728,653,689,728,1184,710,335,232,26,485,247,231
IL1RAPL2_4_5831,322,360,710,376,144,229,30,70,82,4,404,809
IL1RL2_4_5832,690,523,930,670,1094,488,812,1250,420,472,1091,778
IL20RA_4_5833,2453,2585,2652,2530,2491,5605,3060,3223,2026,1794,2025,1
953
IL22RA1_4_5834,68,171,185,182,322,308,6,5,157,309,121,17
IL23R_4_5835,623,903,1033,944,715,2716,1541,338,490,449,751,701
IL27RA_4_5836,543,436,511,367,1310,574,1191,675,432,0,404,380
IL2RA_4_5837,172,279,256,330,154,256,331,261,205,196,11,1081
IL2RB_4_5838,349,296,245,286,165,429,37,118,54,1572,379,1036

IL2RG_4_5839,92,103,150,86,0,824,235,175,7,80,34,41
IL3RA_4_5840,10,57,60,88,169,10,239,398,52,226,248,118
IL7R_4_5841,1152,872,1451,557,735,368,1315,1377,1167,1542,1065,1161
IL9R_4_5842,626,633,781,752,1517,785,1091,261,272,586,362,703
ITFG1_4_5843,519,609,668,633,719,791,1163,801,582,794,237,288
ITGA2B_4_5844,194,100,51,312,116,417,56,80,9,732,29,336
ITGB3_4_5845,960,950,776,1115,996,903,694,882,1509,806,1712,1031
ITGB7_4_5846,130,331,530,323,11,333,866,26,249,797,401,387
ITPR2_4_5847,630,763,925,959,792,906,824,746,404,1475,432,558
ITPR3_4_5848,154,230,265,297,4,733,383,364,34,237,9,256
JPH3_4_5849,156,202,435,199,590,592,139,511,575,252,0,397
KCMF1_4_5850,1285,1117,1595,1853,788,1668,759,1279,1091,1911,1453,1451
KCNA10_4_5851,934,997,677,894,2263,390,1497,522,1153,165,1260,1035
KCNA1_4_5852,421,515,276,489,458,401,16,249,94,56,168,690
KCNA3_4_5853,426,226,495,188,299,458,261,469,346,28,234,46
KCNA4_4_5854,3148,3546,3752,3869,3162,3428,3169,5359,3361,2620,3805,35
49
KCNA5_4_5855,183,298,182,239,300,240,473,0,374,410,150,17
KCNA6_4_5856,203,231,439,130,299,551,0,39,831,0,455,228
KCNA7_4_5857,898,707,538,779,390,2371,555,1258,1547,175,927,797
KCNAB3_4_5858,156,335,319,189,669,384,93,601,53,70,1109,194
KCNB1_4_5859,449,554,528,656,419,1243,424,372,108,487,528,849
KCNB2_4_5860,802,503,821,667,134,413,1020,582,378,34,728,750
KCNC3_4_5861,580,300,236,365,55,831,774,221,1199,56,488,572
KCND1_4_5862,1546,1731,2114,1806,1963,1401,2288,964,1121,3263,1237,132
2
KCND2_4_5863,491,337,331,294,225,432,12,289,1391,1,37,231
KCNE1L_4_5864,8,1,20,2,6,61,0,2,0,0,0,4
KCNE2_4_5865,600,591,510,709,586,1094,456,766,304,464,883,864
KCNE3_4_5866,4053,2895,4105,4130,4189,3028,2725,2366,2570,3840,5423,32
67
KCNE4_4_5867,117,147,160,192,320,123,156,59,1,38,9,287
KCNF1_4_5868,254,251,202,267,157,104,63,133,101,653,1171,418
KCNG1_4_5869,887,952,1132,704,765,628,739,795,819,2314,1053,757
KCNG2_4_5870,331,389,423,662,588,121,89,674,73,994,81,612
KCNG4_4_5871,29,93,95,146,0,1213,223,170,4,0,36,197
KCNH3_4_5872,241,201,407,486,38,856,674,417,233,355,2,71
KCNH4_4_5873,391,359,389,481,1058,456,675,144,882,42,76,162
KCNH8_4_5874,529,831,895,364,907,567,640,521,886,466,549,200
KCNJ10_4_5875,73,131,177,90,140,109,517,206,60,6,307,21
KCNJ12_4_5876,548,687,536,879,599,1376,385,1112,245,378,328,205
KCNJ2_4_5877,212,282,357,341,30,611,77,390,204,106,409,224
KCNJ3_4_5878,596,354,364,605,738,200,506,860,486,255,731,175
KCNJ5_4_5879,110,99,121,156,285,6,0,1077,901,3,255,54
KCNJ6_4_5880,573,676,652,427,470,94,751,825,548,411,467,1339
KCNJ8_4_5881,452,287,309,203,217,1,1243,95,381,1346,452,266
KCNJ9_4_5882,56,116,56,81,98,0,9,16,0,80,163,36
KCNK12_4_5883,97,55,11,81,70,65,0,1,0,0,0,0
KCNK13_4_5884,633,523,1176,636,1170,1225,2705,950,135,1932,441,585
KCNK15_4_5885,7858,8572,9165,7754,8346,6498,8891,7382,5688,9189,8628,1

0584
KCNK18_4_5886,130,171,266,284,8,706,136,907,124,349,366,112
KCNK1_4_5887,71,8,119,157,46,1,190,106,25,0,116,127
KCNK3_4_5888,202,114,249,174,43,220,10,262,358,19,1013,166
KCNK4_4_5889,1387,1487,1319,1647,685,972,960,1014,1289,1368,2176,1266
KCNK5_4_5890,413,491,743,411,277,186,208,1465,599,8,210,887
KCNK6_4_5891,906,1465,1171,629,1017,575,1168,1380,931,1055,1409,640
KCNK9_4_5892,572,602,595,536,367,979,416,188,468,691,843,491
KCNMB1_4_5893,796,660,798,516,414,491,0,456,594,1264,581,121
KCNMB4_4_5894,485,733,669,703,248,1102,748,347,245,448,1127,561
KCNN1_4_5895,526,541,267,486,354,921,765,647,334,648,919,520
KCNN4_4_5896,371,280,546,436,319,206,722,329,117,75,780,329
KCNS1_4_5897,396,247,373,460,371,267,1159,485,15,1,89,316
KCNS2_4_5898,386,460,972,823,250,190,872,915,1534,19,206,754
KCNS3_4_5899,79,78,333,124,96,2,0,861,0,0,54,544
KCNT1_4_5900,975,718,836,997,422,501,840,1209,851,1169,366,1046
KCNT2_4_5901,497,468,401,574,370,406,289,383,836,1317,11,766
KCNU1_4_5902,1373,1493,1898,1527,964,1395,1272,2711,1270,2591,3023,111
0
KCNV1_4_5903,417,547,498,563,784,266,339,740,558,307,108,374
KCNV2_4_5904,804,530,781,716,777,652,528,1303,407,160,253,712
KCTD10_4_5905,765,605,1021,631,617,404,664,598,566,131,1061,1131
KCTD11_4_5906,1009,1048,1028,709,458,830,497,1304,779,695,927,1732
KCTD12_4_5907,199,256,287,230,152,376,93,134,170,14,53,298
KCTD13_4_5908,270,163,347,244,594,453,130,502,0,34,13,73
KCTD14_4_5909,1353,1622,1642,1341,1887,1310,3074,1175,1819,1922,3132,1
124
KCTD16_4_5910,1727,1981,1604,1665,1195,1752,2513,2335,934,1708,812,183
4
KCTD17_4_5911,247,170,195,51,36,11,566,0,114,1,3,19
KCTD18_4_5912,2441,2084,2335,2104,1947,2720,1844,1213,2232,2133,1646,2
432
KCTD19_4_5913,512,492,519,468,432,367,711,1344,426,82,707,1436
KCTD20_4_5914,451,465,409,503,209,961,274,156,740,155,1060,696
KCTD2_4_5915,251,471,324,860,681,396,125,468,629,0,831,555
KCTD3_4_5916,49,34,166,59,22,27,1,15,171,954,1,91
KCTD4_4_5917,0,9,19,0,0,0,0,0,28,0,0,0
KCTD5_4_5918,201,565,248,242,733,806,833,688,277,1,113,443
KCTD8_4_5919,336,503,581,370,566,435,508,851,766,885,412,1265
KCTD9_4_5920,142,114,512,243,85,99,71,408,75,0,306,16
KIR3DL1_4_5921,954,938,1119,1115,1125,607,1489,818,858,1117,1019,1451
KLRB1_4_5922,600,897,740,1091,416,579,506,636,587,299,1651,1058
LGALS3BP_4_5923,86,168,69,166,3,1,31,0,0,0,55,17
LRP10_4_5924,694,896,1008,1236,340,2224,1185,1630,972,806,2146,658
LRP1B_4_5925,900,904,1063,1030,1699,1502,1441,964,842,1409,1332,800
LRP3_4_5926,1186,1421,1303,1487,1508,1374,1254,881,833,2705,1656,747
LRP5_4_5927,123,187,170,121,264,298,51,0,277,0,241,32
LRP6_4_5928,2521,2135,2260,2433,3618,3186,1535,1686,2097,1534,1794,148
5
LRPAP1_4_5929,42,87,75,89,1,18,7,80,0,0,74,74

LTBR_4_5930,967,924,614,1069,867,840,1364,831,708,199,834,1508
MARCO_4_5931,231,367,228,256,89,62,57,269,274,5,1077,237
MCOLN1_4_5932,71,298,118,112,65,140,393,138,186,224,2,223
MCOLN2_4_5933,1745,1529,1378,1546,1101,2185,1484,1527,1463,1761,2047,1
414
MCOLN3_4_5934,1019,974,1027,871,1232,694,1958,2302,967,576,1973,951
MCU_4_5935,190,61,118,208,292,356,2,27,114,0,60,138
MFRP_4_5936,181,325,259,226,108,54,536,408,92,150,149,568
MICB_4_5937,1104,1624,1514,1544,3788,1785,852,832,1048,3839,758,1747
MPL_4_5938,330,433,628,587,707,721,321,7,352,1044,98,562
MRC1_4_5939,156,207,214,393,600,99,45,17,147,0,975,36
MRC2_4_5940,332,332,482,472,420,950,970,309,243,6,693,699
NALCN_4_5941,1068,1057,1735,1049,614,184,874,1453,1094,519,2121,1344
NGFR_4_5942,153,198,36,17,112,6,5,386,601,529,1,267
NISCH_4_5943,148,142,366,199,1,216,96,1,148,194,342,421
NPTXR_4_5944,300,370,118,207,79,378,571,47,0,411,90,891
ORAI1_4_5945,469,690,498,560,377,661,1106,41,47,456,462,354
P2RX1_4_5946,641,510,815,576,566,309,1857,253,975,844,998,1195
P2RX3_4_5947,209,237,240,440,398,68,83,193,26,0,312,291
P2RX4_4_5948,1122,1483,1780,1021,830,1416,2416,702,1418,1013,801,1021
P2RX7_4_5949,331,569,318,577,259,221,251,644,293,308,1089,591
PEX5L_4_5950,236,378,450,381,743,91,1123,85,349,4,503,801
PGLYRP1_4_5951,338,549,824,462,622,385,484,384,384,483,225,797
PGLYRP2_4_5952,160,125,133,107,86,57,9,4,552,53,14,62
PGLYRP3_4_5953,1063,1129,1490,1050,2893,350,1081,1014,1500,588,1217,99
2
PGLYRP4_4_5954,1404,1837,2220,1563,1514,2523,2381,2285,1008,3694,2179,
2522
PGRMC1_4_5955,2978,2240,3595,2775,1559,2328,4333,3447,1840,2748,3247,2
303
PIP_4_5956,948,792,875,513,939,1106,1023,48,589,744,838,1374
PKD1L1_4_5957,352,340,281,290,407,190,551,72,697,340,50,313
PKD1L3_4_5958,125,93,158,340,80,219,25,350,233,3,260,581
PKD2L1_4_5959,603,617,616,728,1402,1259,2082,279,1608,105,1148,613
PKD2L2_4_5960,5576,5783,7043,5149,5779,4665,9461,4421,6880,3998,7219,6
051
PKDREJ_4_5961,724,1005,555,864,752,1885,371,1638,332,634,1317,306
PLXNA1_4_5962,108,125,141,224,609,8,1116,1,3,2,168,137
PLXNA2_4_5963,93,305,294,284,231,31,125,57,190,8,796,151
PLXNA3_4_5964,236,342,456,431,108,183,392,761,538,448,1149,453
PLXND1_4_5965,18,123,193,43,0,53,0,100,3,31,0,21
PRPH2_4_5966,107,174,440,61,105,308,39,280,427,19,118,38
RARRES2_4_5967,507,526,618,682,1180,288,742,477,592,747,848,916
RASA3_4_5968,452,299,599,449,225,537,511,432,651,26,477,385
RYR2_4_5969,2304,2089,2175,2164,1449,1632,1415,846,1364,4088,1521,2616
RYR3_4_5970,565,602,761,590,176,999,467,286,997,351,439,459
SARM1_4_5971,156,103,197,121,190,1,856,672,24,2844,117,658
SCN10A_4_5972,224,337,275,439,140,297,233,475,18,9,320,289
SCN11A_4_5973,582,871,1025,1447,366,1524,383,1627,273,433,1062,1180
SCN2B_4_5974,262,519,519,648,426,179,1071,291,86,851,223,212

SCN4A_4_5975,1133,1412,830,934,1082,2161,446,1411,2972,2510,1151,1493
SCN7A_4_5976,625,430,430,491,451,737,377,567,242,54,760,813
SCN9A_4_5977,297,580,577,514,780,956,1851,507,620,1534,198,675
SCNN1B_4_5978,644,512,616,443,381,651,646,306,360,274,708,556
SCNN1D_4_5979,247,444,149,264,305,16,8,41,99,13,211,82
SCNN1G_4_5980,475,620,537,664,1206,199,226,253,46,371,209,535
SCUBE1_4_5981,364,306,162,248,259,70,389,247,567,302,119,397
SELE_4_5982,246,417,220,194,470,129,139,64,444,705,362,91
SEMA5A_4_5983,87,122,107,189,48,57,106,99,17,395,127,167
SETD1A_4_5984,465,434,564,373,452,774,671,547,37,837,282,380
SFRP1_4_5985,305,487,218,466,948,138,43,226,160,42,72,836
SFRP4_4_5986,969,1133,1227,1216,1594,705,273,1248,541,842,1616,626
SFRP5_4_5987,73,77,21,43,193,1894,1983,4,1,145,106,138
SHKBP1_4_5988,154,440,205,192,303,801,246,0,1,0,735,363
SHROOM2_4_5989,48,328,66,42,2,532,306,0,7,0,0,1
SIGLEC8_4_5990,391,582,715,447,144,278,547,1028,76,49,512,580
SLAMF1_4_5991,493,337,622,640,714,314,149,504,164,332,450,871
SLC6A18_4_5992,314,321,435,215,187,124,309,158,89,456,543,242
SLC6A19_4_5993,1391,1766,1583,1565,1389,931,1337,1934,1104,1394,2530,2
317
SLC6A1_4_5994,182,177,130,236,155,39,57,68,11,60,212,224
SLC6A3_4_5995,440,645,508,544,647,364,399,171,151,12,707,332
SLC6A4_4_5996,1545,1215,1251,1734,1238,1099,1792,1910,832,2276,2038,10
53
SLC9A1_4_5997,227,261,208,122,84,431,495,231,65,36,121,26
SLC9A3_4_5998,372,151,267,289,89,5,631,150,65,0,109,188
SRCRB4D_4_5999,63,94,169,90,25,263,451,369,156,1,9,256
STAB2_4_6000,616,789,860,622,1798,711,1088,1018,719,943,1059,831
STX1B_4_6001,325,420,429,372,5,67,2045,13,541,826,819,560
THBD_4_6002,42,14,28,19,14,93,7,0,9,21,0,54
TLR1_4_6003,666,590,730,818,969,925,681,865,539,1139,1009,1355
TLR2_4_6004,2223,1804,2682,1924,1126,1853,2450,2649,1254,1754,2965,176
9
TLR3_4_6005,890,1274,958,1366,385,220,671,813,800,1039,797,1503
TLR4_4_6006,252,238,298,154,304,163,35,14,0,0,318,328
TLR5_4_6007,409,290,531,448,514,876,445,502,394,675,634,377
TLR6_4_6008,1620,856,852,1073,968,157,345,708,260,610,1908,1077
TLR7_4_6009,896,1356,1284,1290,499,625,1523,1949,908,3154,2762,996
TLR8_4_6010,1263,1389,1033,612,779,656,466,1147,1182,1027,2113,1006
TLR9_4_6011,76,122,260,206,14,396,285,9,435,14,141,14
TMEM37_4_6012,118,206,185,47,61,217,135,3,242,265,58,184
TMEM38A_4_6013,507,1069,902,540,59,1176,1259,20,740,1093,551,1131
TMEM38B_4_6014,1836,1446,2245,2003,1097,2093,2707,798,1400,3074,1765,3
177
TNFAIP1_4_6015,636,285,447,375,350,413,614,408,260,356,807,366
TNFRSF10A_4_6016,1216,1558,1809,1159,1636,1158,2966,2726,1573,1702,548
,1505
TNFRSF10C_4_6017,160,152,170,111,118,95,314,226,103,0,165,80
TNFRSF10D_4_6018,1284,1489,1252,981,1545,957,2670,1005,1759,3127,3165,
1580

TNFRSF11A_4_6019,1007,924,942,1196,1133,1765,362,799,666,945,905,1532
TNFRSF13B_4_6020,920,1134,1162,1673,2149,504,1495,1390,1272,811,338,93
9
TNFRSF13C_4_6021,1877,1734,2311,1919,2302,1840,2735,1929,1335,1051,170
6,2092
TNFRSF14_4_6022,163,335,299,271,9,35,29,86,18,2,577,168
TNFRSF1A_4_6023,1585,1628,1416,1627,2059,2292,2559,1930,1806,355,1788,
1789
TNFRSF1B_4_6024,191,28,193,102,27,0,264,93,4,0,28,1
TNFRSF4_4_6025,64,10,71,44,96,4,64,13,0,11,675,0
TNFRSF6B_4_6026,560,440,439,467,116,479,770,337,806,558,1150,189
TPCN2_4_6027,322,265,300,428,626,88,13,56,63,535,395,385
TREM2_4_6028,1594,1039,1175,1066,424,578,1461,1647,1499,620,974,1320
TRPA1_4_6029,318,328,490,665,326,570,445,171,526,0,375,347
TRPC1_4_6030,1034,1101,915,1663,1812,1075,1893,1013,961,1505,888,651
TRPC5_4_6031,255,306,199,153,539,382,543,33,56,76,116,343
TRPC6_4_6032,3427,4360,4431,3652,3466,3227,4075,4556,3934,5236,4398,40
72
TRPM1_4_6033,617,804,980,751,279,2320,537,510,876,1765,1462,747
TRPM2_4_6034,2487,3188,2713,3811,1770,1148,2625,2787,2131,1635,4383,37
44
TRPM5_4_6035,150,46,123,87,15,19,222,0,462,5,111,213
TRPM7_4_6036,530,1146,894,1003,894,1376,465,751,310,953,488,534
TRPM8_4_6037,355,673,607,460,272,633,57,666,171,860,28,194
TRPV2_4_6038,830,757,907,725,460,95,653,1038,857,517,2911,1049
TRPV3_4_6039,154,357,398,419,526,1129,696,50,318,66,750,549
TRPV5_4_6040,2005,2175,2255,2417,1402,2761,1126,1013,2022,887,3919,291
0
TRPV6_4_6041,92,20,15,36,207,115,198,277,12,0,48,237
TTYH3_4_6042,328,354,588,273,96,148,218,312,460,2,400,369
ULBP1_4_6043,1065,952,1206,1485,372,86,1170,990,663,2460,824,355
ULBP2_4_6044,189,461,291,247,414,67,259,123,47,645,507,126
ULBP3_4_6045,1488,1496,1652,1679,563,1303,1216,1660,1691,422,789,1401
UNC5A_4_6046,1068,1245,1102,1375,1523,1267,1463,1199,934,3324,1812,166
9
UNC5B_4_6047,3433,3546,2995,3718,4293,4518,3865,2677,3356,3026,5090,48
19
UNC5C_4_6048,66,63,128,59,377,0,0,36,16,0,65,27
UTRN_4_6049,84,243,247,204,290,10,1241,54,171,1,176,732
VDAC1_4_6050,208,132,209,149,247,36,150,280,83,35,95,339
ZACN_4_6051,1095,1384,1547,1080,1303,636,1079,540,105,1732,1072,936
ABCC4_4_6052,394,453,326,203,116,278,824,10,160,354,32,254
ABCC9_4_6053,1320,1212,1150,1298,1317,1578,1462,1930,532,1042,1115,123
1
ADIPOR1_4_6054,533,600,565,536,661,425,677,263,541,345,553,719
AGER_4_6055,203,116,167,333,350,377,56,453,2,53,972,463
ANO6_4_6056,766,385,468,163,971,598,221,68,447,375,1101,343
ANTXR1_4_6057,1150,936,1286,1251,1983,1773,1945,816,448,1430,919,717
ANXA7_4_6058,669,662,534,808,775,195,961,1035,22,24,1544,509
AQP4_4_6059,595,392,694,481,335,289,1290,1044,291,314,831,500

ASGR1_4_6060,370,207,332,427,135,90,72,65,312,1959,9,118
ASGR2_4_6061,1847,1513,1895,1592,2452,1620,2214,3049,718,1915,2747,244
5
BEST1_4_6062,2837,2668,3027,2891,890,3089,4314,3237,2957,1868,1500,138
3
BEST3_4_6063,443,587,297,350,473,616,65,150,99,873,202,290
CACNA1A_4_6064,701,604,791,913,779,909,1207,721,363,302,467,1006
CACNA1C_4_6065,318,218,270,163,491,207,93,29,150,1,137,296
CACNA1D_4_6066,2351,3242,3173,2459,3874,3552,3833,3044,2053,3773,3611,
2248
CACNA1E_4_6067,2080,1889,3418,2324,2574,1514,2835,2544,2516,432,628,22
58
CACNA1G_4_6068,228,336,236,400,100,57,36,241,75,64,20,254
CACNA1H_4_6069,718,231,642,509,553,725,12,93,57,510,918,48
CACNA1I_4_6070,190,153,117,155,173,93,268,54,528,122,263,61
CACNA2D2_4_6071,807,697,782,338,751,1396,881,520,223,1854,741,360
CACNB1_4_6072,439,292,584,590,983,62,139,669,373,187,305,88
CACNB2_4_6073,3948,3264,4064,4788,2865,4048,3747,4400,4793,1974,3705,4
563
CACNB3_4_6074,194,156,285,147,249,73,201,69,227,70,546,661
CACNB4_4_6075,2125,2449,2541,2326,1933,3057,3085,1988,2736,4220,3330,2
773
CACNG6_4_6076,4443,4298,4791,4029,3118,4438,4820,3840,4531,4258,4897,4
714
CATSPER2_4_6077,539,578,727,566,310,422,854,475,356,186,1019,846
CD14_4_6078,128,92,98,98,7,133,84,30,3,215,240,635
CD163_4_6079,937,820,792,1003,614,789,618,439,1001,773,938,684
CD247_4_6080,81,229,127,237,218,136,142,565,77,26,110,194
CD302_4_6081,169,328,304,210,174,144,90,549,41,206,1326,924
CD36_4_6082,934,590,889,986,1056,454,1947,688,1533,247,349,905
CD3D_4_6083,723,949,716,672,248,539,630,599,542,40,607,793
CD40_4_6084,441,540,558,433,256,539,256,406,183,282,370,336
CD4_4_6085,615,1206,1029,1105,782,1806,1406,1380,554,199,1571,1199
CD74_4_6086,194,319,121,243,75,303,0,71,86,348,0,286
CD79A_4_6087,871,467,820,545,240,1098,1176,945,846,804,915,832
CD79B_4_6088,222,237,115,175,250,78,15,109,334,0,360,29
CD86_4_6089,462,289,503,522,418,204,385,162,883,17,708,233
CEACAM1_4_6090,678,444,766,745,625,656,1531,527,443,30,347,333
CEACAM21_4_6091,213,303,335,344,242,66,270,307,273,111,100,165
CHRFAM7A_4_6092,608,363,264,450,3,1144,112,216,369,1369,340,791
CHRNA1_4_6093,1311,1186,1961,1715,1833,1803,1642,1336,1719,1197,2289,1
193
CHRNA3_4_6094,2856,2146,3065,2753,2798,3206,3450,4112,2697,1932,2707,1
623
CHRNA6_4_6095,553,406,369,615,18,820,1499,229,55,329,1126,406
CHRNA7_4_6096,145,324,166,199,250,289,103,185,4,764,387,197
CLCC1_4_6097,1622,1454,1631,1320,472,2057,2111,2799,695,1669,841,1714
CLCN2_4_6098,203,109,474,144,185,131,610,31,48,796,62,235
CLCN3_4_6099,1465,1268,1666,1599,1407,2277,2274,1412,1112,328,1980,151
6

CLCN5_4_6100,420,136,273,406,230,195,417,107,22,18,554,227
CLCN6_4_6101,7,79,20,77,0,281,0,6,3,0,31,0
CLCN7_4_6102,434,362,83,447,241,290,60,139,332,5,109,218
CLCNKA_4_6103,2180,2907,2647,2581,2050,2985,2609,2502,2220,1580,4945,3
491
CLCNKB_4_6104,246,298,431,334,51,234,334,107,335,63,648,398
CLEC1B_4_6105,5150,4707,4867,5293,5210,4804,4482,6475,3588,3275,6132,4
345
CLEC4A_4_6106,2303,2152,2316,2417,1110,1735,1890,1317,1135,1560,2621,3
113
CNGA1_4_6107,626,634,1103,687,233,1130,734,947,310,447,1131,1012
CNGA3_4_6108,110,173,189,249,732,186,576,156,583,0,196,59
CNGB1_4_6109,256,278,256,189,455,7,938,217,914,3,107,9
CNTFR_4_6110,2288,1981,2435,2571,1285,2058,2332,1544,2230,833,2366,193
4
CR2_4_6111,1308,1064,1311,1268,327,785,877,1549,254,1251,2401,1675
CSF2RA_4_6112,1900,2293,2844,1883,1559,2274,2334,1522,1769,3272,2823,2
723
CSF3R_4_6113,903,840,913,816,1658,677,1494,1408,630,630,974,941
CXADR_4_6114,353,276,212,152,616,332,55,323,263,4,522,440
DAG1_4_6115,188,146,216,239,39,196,134,408,278,246,556,160
EDA2R_4_6116,111,217,151,164,4,4,443,10,146,416,135,374
EDNRA_4_6117,23,95,40,117,10,29,256,64,12,2,103,155
ENG_4_6118,563,475,608,386,621,1240,611,109,307,792,556,793
EVI2A_4_6119,644,550,601,518,331,792,398,477,282,1062,339,800
FCAMR_4_6120,542,1063,958,952,260,1922,1992,2216,672,1180,504,731
FCGR1B_4_6121,387,501,798,562,80,148,797,35,210,443,1338,494
FCGR2A_4_6122,5841,7020,7329,6690,4982,9609,8168,7349,5714,4853,6586,6
043
FCGR2B_4_6123,1,14,3,34,0,0,0,112,1,0,14,7
FCGR3A_4_6124,8,0,0,0,0,0,0,0,0,0,0,0
FCGRT_4_6125,290,92,199,278,688,18,204,187,4,0,1098,1
FGFRL1_4_6126,135,224,129,48,62,4,165,1,172,30,0,228
FLT4_4_6127,260,153,136,225,109,0,513,74,136,39,162,48
FXYD1_4_6128,850,1166,1110,892,961,1812,1306,598,186,554,2537,656
FXYD2_4_6129,161,53,80,107,0,0,0,17,1,18,49,8
FXYD4_4_6130,94,238,19,131,73,21,256,11,220,4,2,11
FXYD5_4_6131,441,207,626,311,630,299,343,192,138,1,366,1264
FXYD6_4_6132,519,490,395,326,561,465,1279,748,493,1351,502,268
GABRA1_4_6133,330,163,344,279,329,262,349,17,235,813,239,35
GABRA2_4_6134,142,274,424,308,186,153,215,31,0,391,38,769
GABRA4_4_6135,2226,2360,2367,2756,2768,987,2291,3203,1177,1856,4138,14
25
GABRA5_4_6136,2025,1675,1837,1521,1869,1732,2273,1352,1049,3285,1841,2
446
GABRB2_4_6137,1171,1198,1396,1253,1122,1514,952,1299,1175,218,2017,190
5
GABRB3_4_6138,0,0,0,0,0,0,0,0,0,0,0,0
GABRG2_4_6139,1178,1415,1464,1082,473,2540,1582,2183,1114,1046,2396,84
3

GFRA1_4_6140,650,880,880,716,608,942,1595,753,494,1391,743,703
GFRA2_4_6141,1031,757,812,893,545,457,816,225,834,1196,364,339
GFRA4_4_6142,312,213,257,170,12,410,692,623,131,746,10,23
GHR_4_6143,710,908,911,585,1004,1151,3027,978,192,1570,1218,519
GIPC1_4_6144,366,432,557,375,355,419,1022,981,27,229,8,466
GLRA1_4_6145,85,250,219,131,73,468,0,102,337,557,24,181
GLRA2_4_6146,2065,2503,2169,2131,1848,1736,3670,1265,1570,2918,1854,27
64
GLRA3_4_6147,1129,867,1810,942,518,1240,521,466,898,126,2719,1476
GLRA4_4_6148,82,186,201,186,27,94,412,1431,135,187,247,421
GLRB_4_6149,612,746,634,507,1372,635,1106,996,1110,200,523,643
GP6_4_6150,701,993,1025,903,836,1273,535,1230,703,523,932,1619
GPM6A_4_6151,922,1010,1218,1016,679,1533,1142,1799,279,1117,794,1774
GPR89A_4_6152,1756,1331,1382,1242,1472,122,910,1366,1549,4137,821,3186
GRIA1_4_6153,995,1417,972,1207,492,2890,367,673,1016,1304,976,932
GRIA2_4_6154,189,208,264,495,110,158,196,593,2,163,83,99
GRIA3_4_6155,167,103,100,18,243,60,0,341,2,0,2,116
GRIA4_4_6156,2320,2534,3281,2279,3759,4523,2231,2271,1523,2270,3166,27
82
GRIK1_4_6157,561,610,736,737,561,1566,442,327,546,319,522,498
GRIN2A_4_6158,542,529,448,377,148,65,178,71,415,71,537,65
GRINA_4_6159,1570,1614,1804,2160,2046,1182,1256,1237,1039,609,1490,148
8
HLA-DPA1_4_6160,323,400,326,433,69,485,375,169,567,1056,516,230
HTR3A_4_6161,374,212,180,114,389,205,0,409,74,0,323,477
HTR3D_4_6162,1011,947,1505,1350,718,1479,904,975,2946,1053,957,1729
HVCN1_4_6163,5516,5141,4749,5408,7004,8254,4409,7022,4261,3670,6769,58
20
IFNAR2_4_6164,911,949,1050,1126,179,838,2077,465,682,651,1497,1398
IL11RA_4_6165,222,121,87,144,104,37,397,191,9,54,73,153
IL12RB1_4_6166,579,594,571,371,441,461,283,1031,375,1273,5,290
IL15RA_4_6167,203,183,311,398,711,272,155,11,87,170,140,214
IL1R2_4_6168,1029,1045,654,813,522,756,561,1600,192,680,1206,1685
IL1RAP_4_6169,693,829,856,766,1513,925,1053,738,1048,272,275,961
IL1RL1_4_6170,1567,1578,1482,1398,1849,1505,1798,1704,1299,2302,1943,9
99
IL21R_4_6171,381,424,381,340,120,773,704,660,70,69,146,93
IL22RA2_4_6172,683,919,986,880,1383,1495,1433,585,439,413,690,720
IL28RA_4_6173,656,557,631,529,451,762,184,436,552,230,931,2053
IL31RA_4_6174,397,682,535,548,277,43,178,249,145,182,535,615
IL4R_4_6175,1831,1135,1804,1901,2473,460,1159,1245,926,1231,1839,2077
IL5RA_4_6176,2053,2031,2016,2110,3741,1966,2020,2728,1501,1494,1636,19
23
IL6R_4_6177,69,43,84,171,18,12,0,53,2,0,122,163
IL6ST_4_6178,592,685,1103,784,303,1513,520,667,74,895,1469,959
ILDR1_4_6179,570,1177,675,663,1076,1338,802,877,371,173,1449,166
ITGB1_4_6180,2180,1998,2347,1432,1586,1653,1090,1107,1393,741,2566,107
9
ITGB4_4_6181,568,682,478,663,238,1232,1376,404,173,455,410,416
ITPR1_4_6182,432,427,450,344,655,268,562,681,295,516,687,435

KCNA2_4_6183,265,352,340,168,711,290,205,125,102,121,268,419
KCNAB1_4_6184,149,120,251,183,32,359,0,16,164,49,230,571
KCNAB2_4_6185,187,178,149,120,0,116,588,271,362,189,206,326
KCNC1_4_6186,353,484,343,212,751,309,728,639,264,322,484,258
KCNC2_4_6187,1376,1360,1250,1663,2687,2315,1343,329,1051,1652,2638,142
1
KCNC4_4_6188,813,934,857,790,1081,1376,1920,525,1434,575,997,723
KCND3_4_6189,156,136,287,242,253,72,18,106,3,31,309,159
KCNE1_4_6190,108,201,252,189,528,268,51,99,673,692,530,223
KCNG3_4_6191,114,185,188,194,176,6,731,3,112,2,213,407
KCNH1_4_6192,1205,1309,1501,1136,1057,1381,2126,2840,1433,1122,1679,14
97
KCNH2_4_6193,477,279,430,618,258,476,922,784,197,482,47,480
KCNH5_4_6194,334,297,208,314,1130,5,32,35,925,164,854,318
KCNH6_4_6195,101,340,148,282,364,1272,492,0,22,221,221,6
KCNH7_4_6196,623,875,1147,710,806,1245,959,565,635,316,660,550
KCNIP1_4_6197,317,407,678,393,892,878,594,174,317,805,296,262
KCNIP2_4_6198,1415,2257,1624,1809,1699,2103,2088,983,669,966,2124,2518
KCNIP4_4_6199,1500,1378,1327,1600,822,1809,1462,1224,1180,1141,1527,10
38
KCNJ11_4_6200,683,587,865,772,901,483,784,552,491,1101,591,499
KCNJ13_4_6201,918,874,760,987,710,1480,1060,785,344,144,2121,869
KCNJ14_4_6202,591,469,639,474,52,542,696,154,21,19,487,490
KCNJ15_4_6203,222,177,398,178,705,371,867,108,183,449,394,204
KCNJ16_4_6204,1374,1690,1134,1742,1386,2943,1015,1507,1467,1555,1375,1
936
KCNJ1_4_6205,87,153,196,115,40,0,62,99,2,2,299,30
KCNJ4_4_6206,20,180,64,75,7,0,41,64,0,0,17,219
KCNK10_4_6207,606,488,530,539,364,512,324,298,1103,1911,808,641
KCNK16_4_6208,1989,2082,1933,1804,2071,2221,1504,1286,2156,875,2364,79
1
KCNK17_4_6209,641,877,706,623,1164,565,729,548,848,337,1018,1585
KCNK2_4_6210,1210,1274,1679,1094,1026,2371,1473,1647,748,1551,1562,177
3
KCNK7_4_6211,829,795,1311,1157,723,1168,855,423,814,137,1713,732
KCNMA1_4_6212,375,363,176,244,12,6,798,102,1,0,1376,531
KCNMB3_4_6213,1528,1761,1459,1983,1920,1887,634,989,2562,4616,1110,205
6
KCNN2_4_6214,433,743,830,665,23,357,1136,548,397,209,642,861
KCNN3_4_6215,1726,2000,2015,2236,1543,2139,1477,1931,1631,1630,3200,19
65
KCNQ1_4_6216,151,111,134,103,18,78,7,63,164,2,266,0
KCNQ2_4_6217,370,457,554,457,595,137,416,118,344,1079,110,172
KCNQ3_4_6218,330,278,131,281,23,43,913,279,283,1979,488,324
KCNQ4_4_6219,50,369,162,302,122,11,1112,24,143,11,39,139
KCNQ5_4_6220,768,871,882,824,801,1313,853,1131,288,891,964,1304
KCNRG_4_6221,1079,942,1294,1024,1838,1690,2027,2566,254,1586,1317,758
KCTD15_4_6222,385,368,207,335,339,906,147,87,38,129,165,42
KCTD1_4_6223,50,79,106,36,18,72,1,40,10,0,124,0
KCTD6_4_6224,1747,2224,1895,1603,1366,1236,2401,1796,2070,690,1717,199

1
KCTD7_4_6225,396,167,164,207,5,382,700,59,8,2,148,34
KLRC1_4_6226,528,766,664,437,1200,815,3075,172,170,104,527,239
LEPR_4_6227,1434,1687,1870,1298,1014,1890,1960,2617,844,3071,1026,2150
LIFR_4_6228,2545,2297,3010,2809,1615,3261,2847,1710,1919,1971,3003,212
3
LILRB1_4_6229,234,123,218,162,614,83,251,62,5,48,518,792
LILRB3_4_6230,404,573,495,453,920,122,481,114,0,99,1140,396
LRP12_4_6231,2940,3790,2920,2991,1966,4242,4311,3303,2184,2010,3620,41
47
LRP8_4_6232,791,697,915,718,2593,668,1308,12,717,1690,139,363
MR1_4_6233,1626,2253,2566,2242,1953,1701,3842,1585,1777,3596,2620,1358
MSR1_4_6234,3917,3151,3474,3877,4209,3296,6352,4658,6623,3513,4486,357
6
NCR2_4_6235,251,658,795,677,484,715,508,753,623,636,1042,1067
NCR3_4_6236,900,927,1190,1204,989,1210,952,1160,547,317,1763,1745
NOX1_4_6237,1849,1892,2499,1718,2599,2950,2637,887,1878,2019,2643,1569
NOX5_4_6238,534,511,189,181,140,48,78,749,51,70,25,363
NRP1_4_6239,708,947,873,608,762,857,1101,1107,112,1840,607,577
NUDT9_4_6240,260,518,525,363,525,274,1160,281,385,447,73,236
OLR1_4_6241,4271,5009,4145,4861,5113,5108,5844,3351,3030,4092,6555,432
0
OPCML_4_6242,698,957,950,888,1196,1768,1418,753,303,1394,1065,1119
OSMR_4_6243,2577,2252,3369,2431,2410,2441,2369,3130,2907,2085,2758,337
8
P2RX2_4_6244,298,255,406,386,312,108,99,166,87,19,1356,29
P2RX5_4_6245,312,453,447,341,622,280,1878,717,560,594,738,436
P2RX6_4_6246,589,532,550,441,931,24,222,37,254,929,1020,353
PEX5_4_6247,152,234,134,97,43,173,137,196,221,368,363,334
PKD1_4_6248,819,1193,952,1467,910,1880,1236,329,1031,1293,1126,1523
PKD1L2_4_6249,360,500,557,594,129,332,972,1106,183,194,568,324
PLA2R1_4_6250,764,783,1194,1167,1057,1018,1828,1762,1107,446,652,653
PLAUR_4_6251,489,402,351,313,32,474,139,779,196,242,254,350
PLXNB1_4_6252,180,331,300,530,97,51,38,187,563,29,1079,402
PRKD2_4_6253,333,420,309,360,363,682,155,218,78,287,259,731
PRLR_4_6254,2422,2778,2350,2027,2232,3573,2966,3745,1885,1572,1591,317
0
PTCH1_4_6255,556,678,963,865,765,293,1217,475,564,255,227,966
PTCH2_4_6256,475,478,403,304,663,223,681,523,123,217,600,1
ROBO1_4_6257,1396,1902,2299,1630,1334,3704,1825,1634,1469,2674,2102,27
26
ROBO2_4_6258,585,702,735,498,1240,1092,1162,940,819,16,810,586
RYR1_4_6259,274,165,276,293,0,340,243,868,228,20,208,238
SCARA3_4_6260,566,865,789,641,716,977,666,868,1125,671,888,372
SCARF1_4_6261,154,24,246,38,0,178,2,0,0,0,32,261
SCARF2_4_6262,256,366,162,170,540,2,249,84,196,28,99,25
SCN1A_4_6263,3428,3412,3048,3068,3264,3652,4100,2318,1811,2215,5991,31
38
SCN2A_4_6264,2468,2202,2829,2993,1949,2216,2608,3230,1226,854,2310,349
2

SCN3A_4_6265,284,333,173,263,36,339,457,86,260,0,265,153
SCN3B_4_6266,563,318,295,695,176,312,593,624,138,183,514,134
SCN4B_4_6267,2268,1473,1914,1774,2095,1632,3862,2415,1182,738,2378,212
5
SCN5A_4_6268,798,1179,688,652,848,825,495,1363,1098,708,1099,910
SCN8A_4_6269,3293,3375,2997,3106,3760,3783,4099,2504,1815,2217,5985,31
37
SCNM1_4_6270,1436,1662,2820,1359,1554,1973,3602,1736,2686,1161,5086,17
27
SCNN1A_4_6271,1262,1284,1195,1400,1107,640,648,321,973,958,1255,1052
SLAMF6_4_6272,1832,1328,2084,2331,753,1685,2324,2010,2337,514,2014,168
6
SLC6A2_4_6273,1096,1147,1308,1192,899,198,737,766,1338,1499,1829,2684
TLR10_4_6274,1750,1519,1687,1410,2325,2353,1264,1543,1337,782,1738,989
TNFRSF10B_4_6275,1366,1591,1535,1212,1221,1297,1036,1222,870,4024,1272
,1300
TNFRSF18_4_6276,459,554,605,492,607,305,1582,522,488,946,949,836
TNFRSF19_4_6277,1417,925,1300,1264,1180,1449,1195,803,509,184,1095,115
0
TNFRSF25_4_6278,296,601,649,142,585,143,276,570,322,443,0,709
TNPO1_4_6279,947,945,1112,1255,1273,1342,2412,747,831,193,1752,1061
TOMM40_4_6280,156,53,119,89,593,1030,3,3,7,69,141,35
TP53INP1_4_6281,1254,1943,2077,2162,2527,3193,2219,1802,1510,3290,1157
,1598
TPCN1_4_6282,341,529,554,260,79,188,1158,489,506,261,417,526
TRPC3_4_6283,445,252,501,458,529,221,765,603,393,259,198,841
TRPC4_4_6284,1897,2159,2217,1680,1470,1296,1959,1485,2500,2167,1495,20
86
TRPM3_4_6285,185,463,284,258,28,304,253,219,2,705,114,139
TRPM4_4_6286,0,0,0,0,0,0,0,0,0,0,0,0
TRPM6_4_6287,224,258,278,181,51,250,239,48,451,115,237,163
TRPV1_4_6288,181,342,407,260,98,261,461,300,0,2215,113,206
TRPV4_4_6289,0,0,0,0,0,0,0,0,0,0,0,0
TTYH1_4_6290,424,625,390,761,602,897,172,955,562,120,123,886
TTYH2_4_6291,1112,767,1104,1078,513,913,2163,445,1038,210,568,1070
VDAC2_4_6292,985,1223,1325,1694,1089,1372,279,1923,534,1178,1282,897
ANO7_4_6293,79,152,183,352,28,482,160,65,0,25,107,218
AQP1_4_6294,1262,1565,1998,1427,961,1454,2137,1531,1937,1579,891,1395
CLEC2D_4_6295,366,372,320,358,744,1555,431,4,183,65,460,474
CLEC7A_4_6296,941,1360,913,1056,1269,2052,1559,995,434,432,804,951
CRLF2_4_6297,447,598,497,659,281,36,1314,392,1373,98,1015,306
FXYD3_4_6298,211,198,273,255,65,15,363,308,128,436,22,94
HFE_4_6299,343,335,668,469,120,850,1240,117,36,835,957,189
SCN1B_4_6300,373,321,257,235,263,127,217,442,166,242,441,42
TNFRSF8_4_6301,0,0,0,0,0,0,0,0,0,0,0,0
TSPO_4_6302,105,230,156,277,350,20,0,255,0,4,234,242
AGER_4_6303,1512,1627,1469,1682,1807,751,384,1640,1073,1475,1358,1606
AGER_4_6304,1040,1026,1556,858,660,1755,1682,665,833,581,1571,1752
AGER_4_6305,3489,4051,4358,4257,3491,2563,3830,3991,2987,3149,4382,351
6

AGER_4_6306,139,117,126,159,110,418,226,147,69,152,204,61
AGER_4_6307,964,1133,1477,890,835,1751,954,1484,526,590,1369,1044
AGER_4_6308,332,331,243,270,8,497,615,699,105,50,1112,443
AGER_4_6309,574,804,921,1039,613,634,741,1522,1443,83,932,1089
AGER_4_6310,2151,3114,3009,2291,1529,4499,2442,2872,3433,1498,2294,280
7
AGER_4_6311,3441,3850,4143,4229,3480,3117,3412,3984,3241,2943,4387,391
5
AGER_4_6312,3074,3126,3813,3522,3501,3096,3914,4031,2868,1987,4674,439
8
ANTXR1_4_6313,810,777,892,729,295,572,1670,476,1005,342,545,281
ANTXR1_4_6314,287,101,457,304,363,308,149,83,0,1730,788,743
ANTXR1_4_6315,848,791,986,707,588,905,1205,482,944,692,1912,867
ANTXR1_4_6316,1319,1747,2084,1535,3432,1742,1353,1356,2753,2000,2028,1
132
ANTXR1_4_6317,248,369,376,297,665,375,876,324,44,0,800,302
ANTXR1_4_6318,1266,1458,1095,1381,1280,763,1267,1531,344,406,909,1892
ANTXR1_4_6319,1961,1354,1743,1177,1277,1203,2091,685,801,681,2144,1154
ANTXR1_4_6320,579,664,710,621,739,659,590,330,235,42,132,587
ANTXR1_4_6321,3291,3237,2951,3182,2285,3483,4022,2206,2224,2642,1659,2
663
ANTXR1_4_6322,945,950,555,1110,1209,528,1011,802,214,48,98,987
AQP7_4_6323,2004,3008,2883,2248,1301,2298,3399,1460,709,1191,3363,1530
AQP7_4_6324,1802,2576,3371,1622,2068,3432,1971,2162,2199,1956,3722,344
9
AQP7_4_6325,455,419,725,551,231,706,342,210,33,1546,171,508
AQP7_4_6326,855,684,1024,947,703,690,749,361,183,30,1117,204
AQP7_4_6327,1163,1123,1803,1490,572,1441,1491,1656,1924,977,1786,1421
AQP7_4_6328,414,600,694,458,170,750,1507,1170,479,1052,744,584
AQP7_4_6329,406,423,365,289,57,664,182,122,44,378,114,286
AQP7_4_6330,405,1013,758,916,215,222,50,782,6,1233,29,494
AQP7_4_6331,4305,4309,5863,4972,4547,2856,6984,3233,3928,2782,4739,575
4
AQP7_4_6332,807,1151,1050,913,948,1876,1378,395,1199,786,195,626
ASIC1_4_6333,1008,1222,1562,789,890,1297,1523,760,955,1791,670,617
ASIC1_4_6334,564,478,794,863,450,1145,339,1774,388,674,1609,554
ASIC1_4_6335,166,241,248,335,14,258,74,147,29,98,208,307
ASIC1_4_6336,36,71,121,33,23,334,0,75,94,0,4,75
ASIC1_4_6337,448,361,979,410,666,517,774,623,260,87,201,485
ASIC1_4_6338,1107,883,1053,867,295,691,190,1591,1255,778,680,1070
ASIC1_4_6339,96,8,235,131,67,0,120,102,0,1,62,181
ASIC1_4_6340,722,410,524,628,768,30,1037,766,469,120,29,373
ASIC1_4_6341,398,380,408,649,197,954,325,406,102,793,292,940
ASIC1_4_6342,98,42,66,52,0,293,68,309,0,0,363,158
ASIC2_4_6343,2004,1875,3436,3034,3867,2070,1615,2884,2496,1994,4542,21
04
ASIC2_4_6344,380,392,620,646,1409,671,1218,455,513,8,59,195
ASIC2_4_6345,242,308,398,470,650,261,566,89,666,55,92,18
ASIC2_4_6346,220,419,371,312,31,914,4,240,110,1360,11,381
ASIC2_4_6347,4348,3951,4362,4426,5725,4058,3840,4557,4409,5158,4135,41

09
ASIC2_4_6348,572,365,520,465,801,159,116,94,287,578,479,162
ASIC2_4_6349,4722,4722,4895,4727,5494,5115,3731,4885,4680,5581,5011,54
05
ASIC2_4_6350,93,15,17,139,0,0,12,20,0,1,0,3
ASIC2_4_6351,2851,2830,3872,3673,5230,3693,2875,3966,3235,3121,5332,29
57
ASIC2_4_6352,2520,2073,2165,2545,2567,2700,2434,2711,1500,2753,1723,23
48
ASIC2_4_6353,626,692,891,611,2283,496,894,701,481,1789,248,1282
ASIC2_4_6354,2640,2091,2281,2207,2806,4042,1486,1079,2774,1033,2470,24
68
ASIC2_4_6355,1139,711,761,1055,373,621,666,920,482,1503,603,853
ASIC2_4_6356,120,201,269,232,6,96,669,46,0,92,9,166
ASIC2_4_6357,78,136,132,215,0,0,70,329,3,749,17,7
ASIC2_4_6358,1095,1148,1456,1474,1482,1512,1553,1976,830,480,675,2131
ASIC2_4_6359,277,476,426,451,90,438,279,62,213,137,1013,776
ASIC2_4_6360,789,462,605,536,725,273,473,437,51,138,948,336
ASIC2_4_6361,291,265,222,144,634,183,0,258,222,31,47,260
ASIC2_4_6362,1604,1731,886,1413,1513,2247,2578,1579,1454,2437,1427,610
ASIC3_4_6363,226,168,142,286,83,34,853,705,28,619,1337,383
ASIC3_4_6364,791,1343,1286,1013,655,613,300,2002,471,625,1603,1313
ASIC3_4_6365,304,171,235,60,76,432,695,625,191,129,807,43
ASIC3_4_6366,165,247,280,402,12,69,40,261,270,0,405,57
ASIC3_4_6367,286,578,480,357,39,2585,645,350,6,40,628,635
ASIC3_4_6368,46,89,135,25,258,321,0,20,26,1,304,318
ASIC3_4_6369,787,761,1009,386,961,397,1885,668,410,439,1495,779
ASIC3_4_6370,327,281,315,148,232,157,403,435,126,101,795,38
ASIC3_4_6371,1135,805,1023,922,1308,525,358,394,413,855,706,829
ASIC3_4_6372,346,524,412,373,2270,769,274,178,301,96,92,961
ASIC4_4_6373,196,142,125,179,670,322,0,68,49,0,228,791
ASIC4_4_6374,815,1240,1184,951,1034,390,353,994,406,273,1201,1758
ASIC4_4_6375,1496,1932,1584,2058,3168,1561,1860,2148,2160,1654,2119,22
16
ASIC4_4_6376,311,175,261,59,112,428,704,616,195,339,558,51
ASIC4_4_6377,963,1015,888,1031,934,468,1604,412,551,8,577,888
ASIC4_4_6378,317,92,84,322,139,20,55,521,946,61,0,83
ASIC4_4_6379,120,250,105,129,158,80,507,104,32,910,41,4
ASIC4_4_6380,1105,1355,1376,1048,2302,1311,1251,891,855,661,1209,1583
ASIC4_4_6381,380,96,111,102,37,5,69,192,38,0,7,79
ASIC4_4_6382,146,226,251,412,68,143,144,0,219,1,533,209
ASIC5_4_6383,2050,1629,1573,1887,1298,1365,1670,1990,2003,553,2051,158
3
ASIC5_4_6384,643,884,848,822,272,289,313,1607,704,336,2910,1299
ASIC5_4_6385,264,211,122,287,160,506,131,324,43,195,45,4
ASIC5_4_6386,1455,1362,1716,1396,710,274,906,1296,264,1268,1854,666
ASIC5_4_6387,1382,1436,1319,1112,1700,996,1221,2589,1664,1852,967,1370
ASIC5_4_6388,38,147,153,416,23,285,225,245,439,218,31,436
ASIC5_4_6389,1376,1310,1499,1800,1973,2109,1273,1767,1002,2091,2043,20
12

ASIC5_4_6390,4128,4799,5006,4811,5940,3690,5659,4092,4715,5749,4692,46
72
ASIC5_4_6391,1495,1356,1545,1319,919,1619,2349,770,638,2620,2500,912
ASIC5_4_6392,469,500,1067,665,715,824,699,531,887,187,934,426
CACNA1A_4_6393,211,250,145,304,193,954,39,206,622,0,428,529
CACNA1A_4_6394,715,976,1266,723,919,906,329,922,615,740,1838,1208
CACNA1A_4_6395,224,115,259,73,2,13,246,98,253,521,22,155
CACNA1A_4_6396,68,38,98,125,129,25,45,0,4,0,2,286
CACNA1A_4_6397,431,461,761,660,357,844,1336,592,814,320,371,591
CACNA1A_4_6398,441,405,663,454,460,440,874,935,256,8,1130,890
CACNA1A_4_6399,119,295,256,245,637,1444,555,382,674,1893,0,84
CACNA1A_4_6400,1078,817,844,957,691,1752,200,1216,1814,544,1400,397
CACNA1A_4_6401,397,429,481,581,453,551,386,1043,313,220,177,1357
CACNA1A_4_6402,237,100,17,131,732,886,240,14,347,0,238,13
CLIC6_4_6403,386,345,392,228,616,286,695,465,162,602,470,370
CLIC6_4_6404,2024,2028,2019,2097,1305,2147,2323,930,1332,1024,2061,194
9
CLIC6_4_6405,527,345,364,488,478,775,320,740,448,701,555,302
CLIC6_4_6406,1584,1632,1543,1290,907,843,4583,1034,1505,2632,2579,1681
CLIC6_4_6407,427,419,547,544,47,208,540,680,466,1,86,686
CLIC6_4_6408,1948,1787,2172,2027,1270,3109,2337,1024,1464,1022,2145,21
31
CLIC6_4_6409,1427,1520,1317,1175,905,841,3677,1038,1501,1930,2429,1779
CLIC6_4_6410,104,203,170,137,7,2,28,241,0,0,185,7
CLIC6_4_6411,388,184,301,170,3,417,228,439,25,67,190,171
CLIC6_4_6412,409,356,438,337,14,728,101,150,501,530,568,581
CNGB1_4_6413,680,668,441,606,893,257,210,950,16,779,884,941
CNGB1_4_6414,277,412,701,356,474,51,591,901,948,57,521,316
CNGB1_4_6415,1587,2528,3057,2055,1426,2490,2951,3342,1208,2300,2518,20
34
CNGB1_4_6416,1109,696,546,710,1261,1105,291,752,307,719,692,633
CNGB1_4_6417,3413,2775,3483,3225,4773,5330,6382,4797,1957,2371,2802,41
71
CNGB1_4_6418,1435,1042,893,1261,1305,1220,1147,607,1504,868,1639,1336
CNGB1_4_6419,99,180,232,129,120,50,48,170,1,123,808,229
CNGB1_4_6420,3521,2700,3699,3216,4939,5645,6250,4807,1961,2241,3995,41
76
CNGB1_4_6421,202,346,227,341,140,205,196,314,380,51,535,123
CNGB1_4_6422,102,397,264,241,1,902,912,440,0,0,344,185
SLC6A2_4_6423,2897,3778,3972,3849,2888,3627,4310,3171,4207,3531,4919,4
334
SLC6A2_4_6424,1493,1360,1897,2673,1989,2003,816,2127,1089,1928,1928,23
95
SLC6A2_4_6425,1816,1114,2395,1555,2031,1963,1238,1687,651,1171,1334,23
34
SLC6A2_4_6426,1690,1923,2367,2401,1827,1201,947,1996,1623,2627,2221,24
12
SLC6A2_4_6427,867,1140,1071,899,682,740,242,1197,453,555,1389,381
SLC6A2_4_6428,506,475,726,420,626,2310,931,870,78,284,1120,525
SLC6A2_4_6429,442,441,1274,438,534,798,868,237,91,901,553,766

SLC6A2_4_6430,2818,2969,3704,3305,2726,2731,3394,1839,3508,1262,3516,2
795
SLC6A2_4_6431,862,1438,1120,1280,759,740,1809,1366,881,195,3192,1290
SLC6A2_4_6432,2670,2770,3418,3267,2728,2746,3391,1642,3509,842,3878,27
89
GRIK5_4_6433,652,594,788,697,532,815,601,1902,1249,487,326,814
GRIK5_4_6434,597,333,445,360,148,119,236,442,563,2043,651,174
GRIK5_4_6435,510,462,384,531,253,0,416,697,85,22,444,907
GRIK5_4_6436,642,361,587,565,192,574,1211,337,175,566,665,426
GRIK5_4_6437,294,278,156,196,604,345,157,162,45,32,131,83
GRIK5_4_6438,181,459,424,455,150,0,99,68,859,103,0,0
GRIK5_4_6439,1199,1236,1493,1024,1615,1750,1749,1517,405,1270,1625,182
7
GRIK5_4_6440,1412,2262,1452,1421,1836,2097,3050,1181,1809,1175,1354,13
74
GRIK5_4_6441,605,412,838,592,1664,488,197,474,463,573,705,231
GRIK5_4_6442,311,185,189,132,230,203,157,50,449,0,1,574
KCNA4_4_6443,524,1130,829,844,859,469,577,1255,1121,2398,843,645
KCNA4_4_6444,751,816,871,1250,387,1427,199,885,221,207,900,1144
KCNA4_4_6445,255,235,639,345,491,989,771,238,157,1,148,251
KCNA4_4_6446,698,1079,920,566,1824,2198,776,855,179,1697,624,1233
KCNA4_4_6447,457,635,887,488,667,205,941,449,940,993,569,1127
KCNA4_4_6448,698,924,913,759,771,1122,197,637,572,230,489,503
KCNA4_4_6449,2013,1320,1981,1818,445,423,1319,1170,1383,1804,2551,1858
KCNA4_4_6450,755,515,568,595,61,1163,587,615,583,1265,822,541
KCNA4_4_6451,128,136,152,163,3,28,0,14,138,63,3,21
KCNA4_4_6452,143,152,136,117,26,2,319,659,244,0,14,169
KCNA5_4_6453,524,1130,829,844,859,469,577,1255,1121,2398,843,645
KCNA5_4_6454,652,689,717,740,795,1198,55,631,479,177,523,313
KCNA5_4_6455,536,192,200,65,60,124,11,35,63,25,95,111
KCNA5_4_6456,306,357,496,250,375,0,213,773,157,279,189,272
KCNA5_4_6457,56,99,105,117,0,29,0,404,19,0,39,1
KCNA5_4_6458,1375,1720,1450,1351,1033,1031,1815,1295,1445,168,1706,163
6
KCNA5_4_6459,253,337,183,51,121,283,8,38,303,7,1,529
KCNA5_4_6460,2425,2736,2144,2351,1714,4081,2540,2858,1604,762,3802,153
4
KCNA5_4_6461,664,724,815,690,332,798,123,1253,652,525,777,999
KCNA5_4_6462,1130,1251,1064,1071,505,1151,1293,1297,992,1,722,1914
KCNMB3_4_6463,6661,6839,5808,5728,5596,5566,5443,4412,4955,4108,7549,9
043
KCNMB3_4_6464,185,222,110,132,111,71,3,165,406,1,100,591
KCNMB3_4_6465,6462,6829,5385,5888,5591,6399,5340,5618,4912,3910,7192,9
086
KCNMB3_4_6466,1269,1502,1511,1316,924,1543,287,840,1072,672,1848,1388
KCNMB3_4_6467,2208,2039,2547,2913,2173,3192,1934,3446,2760,1649,3005,3
801
KCNMB3_4_6468,65,129,193,242,132,54,359,105,250,397,349,67
KCNMB3_4_6469,2110,2665,3315,3479,2383,3459,3073,2125,1621,1356,3964,3
137

KCNMB3_4_6470,112,135,191,154,2,23,0,38,314,142,0,184
KCNMB3_4_6471,86,205,263,308,274,74,18,356,195,73,505,15
KCNMB3_4_6472,714,968,730,1136,777,929,1292,1034,160,903,1410,1254
KCTD11_4_6473,144,128,157,193,0,302,0,299,4,119,47,0
KCTD11_4_6474,1594,1394,1216,1257,1869,2652,1778,1524,448,1356,1819,78
5
KCTD11_4_6475,634,749,832,707,545,1069,808,310,971,540,979,1219
KCTD11_4_6476,761,392,675,576,1940,213,1143,339,182,224,977,932
KCTD11_4_6477,487,839,1128,559,582,560,1028,813,529,1259,870,382
KCTD11_4_6478,1827,1893,2393,1687,1133,2932,2245,1753,1652,1826,2180,2
517
KCTD11_4_6479,311,266,362,264,868,6,2,268,502,341,13,419
KCTD11_4_6480,223,247,354,519,274,347,177,312,0,0,151,267
KCTD11_4_6481,256,321,295,362,105,61,32,173,1380,115,270,161
KCTD11_4_6482,445,528,480,457,104,241,105,817,505,97,790,168
LRP5_4_6483,372,434,363,440,624,377,581,140,16,235,649,516
LRP5_4_6484,435,676,633,325,643,398,390,247,55,234,615,709
LRP5_4_6485,24,10,112,82,0,0,39,124,0,0,4,28
LRP5_4_6486,105,187,126,29,389,0,334,333,91,251,29,0
LRP5_4_6487,513,265,556,284,340,259,580,89,220,78,293,252
LRP5_4_6488,8,23,315,56,0,37,313,55,2,0,28,246
LRP5_4_6489,477,585,454,595,795,624,650,571,75,281,685,716
LRP5_4_6490,226,255,208,287,1,122,17,110,13,61,678,254
LRP5_4_6491,349,747,478,510,656,432,395,320,156,234,757,739
LRP5_4_6492,1152,848,672,653,969,98,1135,445,1159,1609,1399,661
LRP12_4_6493,1407,1271,2300,1843,1388,2435,1237,1468,2048,2381,2112,12
84
LRP12_4_6494,246,458,505,522,11,207,285,733,349,176,218,10
LRP12_4_6495,1497,1200,1436,1089,597,789,1070,1043,667,1393,1047,1215
LRP12_4_6496,465,493,450,446,485,914,298,281,165,82,351,290
LRP12_4_6497,462,351,575,224,469,37,175,46,131,148,21,238
LRP12_4_6498,335,291,520,391,158,461,44,666,5,865,828,174
LRP12_4_6499,1742,2160,1621,1745,1050,1541,1304,2211,1987,2051,1212,20
58
LRP12_4_6500,214,275,506,254,555,185,101,337,502,50,241,623
LRP12_4_6501,589,578,660,655,15,1720,29,642,313,937,572,422
LRP12_4_6502,215,163,175,143,144,582,0,8,24,657,16,29
TNPO1_4_6503,7,114,183,83,0,3,55,115,4,1,3,157
TNPO1_4_6504,1195,1373,1185,1606,821,2061,1576,1563,1334,489,1310,1695
TNPO1_4_6505,415,409,425,485,23,373,962,203,129,473,445,667
TNPO1_4_6506,438,376,451,733,154,344,1533,534,1004,1662,335,451
TNPO1_4_6507,758,947,1240,1061,718,780,1765,1106,550,1904,973,1229
TNPO1_4_6508,44,122,16,28,58,5,0,4,0,49,126,30
TNPO1_4_6509,575,578,227,512,678,359,229,24,62,731,198,521
TNPO1_4_6510,1283,1383,1195,1582,1020,1775,1572,1564,1336,490,1310,169
2
TNPO1_4_6511,804,933,1126,1069,718,584,1766,928,584,1907,658,1388
TNPO1_4_6512,1493,1283,1664,1568,1783,2266,1968,1225,629,1240,1174,270
7
MFRP_4_6513,667,732,593,654,283,595,555,711,539,24,833,263

MFRP_4_6514,418,263,576,479,615,205,574,441,90,923,196,110
MFRP_4_6515,202,199,166,176,56,16,315,121,278,210,390,44
MFRP_4_6516,120,105,226,132,30,1,241,111,35,0,201,403
MFRP_4_6517,32,162,110,168,11,0,0,296,122,165,179,318
MFRP_4_6518,141,168,207,276,31,8,15,94,7,0,37,67
MFRP_4_6519,43,11,6,30,0,160,0,26,2,0,0,6
MFRP_4_6520,2181,2626,2695,2433,1958,5337,2369,1382,2255,932,1852,2036
MFRP_4_6521,202,219,292,119,170,305,454,777,142,49,155,99
MFRP_4_6522,927,601,767,906,1685,814,726,1253,172,764,515,719
MS4A2_4_6523,571,714,247,393,687,1194,388,282,306,510,457,675
MS4A2_4_6524,414,696,589,395,608,703,1262,306,626,57,478,462
MS4A2_4_6525,145,80,14,75,52,19,58,0,2,138,102,1
MS4A2_4_6526,707,410,691,498,201,1369,1940,573,517,1254,97,434
MS4A2_4_6527,176,285,372,340,23,1078,206,574,95,31,41,42
MS4A2_4_6528,3920,3672,4638,4387,3556,3093,4134,4319,3998,4227,3343,39
63
MS4A2_4_6529,134,104,136,370,193,197,362,338,615,1171,197,39
MS4A2_4_6530,536,243,344,113,71,286,484,708,438,305,484,812
MS4A2_4_6531,3934,3569,4640,4576,3864,3090,3710,4322,3683,3642,3828,39
62
MS4A2_4_6532,792,535,991,351,366,126,2085,347,234,441,1187,538
PLXNA1_4_6533,677,554,655,453,258,1005,460,347,798,80,309,246
PLXNA1_4_6534,463,580,602,771,674,438,508,305,687,443,785,224
PLXNA1_4_6535,469,317,510,317,29,1081,1092,981,490,391,405,236
PLXNA1_4_6536,153,347,245,318,122,743,1035,196,107,9,14,780
PLXNA1_4_6537,917,1097,963,941,614,549,1131,1263,1457,1032,362,1441
PLXNA1_4_6538,196,185,383,415,141,259,0,128,130,624,92,166
PLXNA1_4_6539,469,457,742,526,337,371,617,1526,280,1182,809,339
PLXNA1_4_6540,194,286,185,176,8,581,174,438,37,1,364,1032
PLXNA1_4_6541,213,304,238,208,59,701,202,441,37,1,366,1039
PLXNA1_4_6542,216,272,243,315,8,574,202,438,144,1,367,1025
PRKD2_4_6543,162,212,86,134,55,7,7,30,190,227,223,337
PRKD2_4_6544,1043,1068,1037,907,1348,1706,2384,651,1277,1413,1358,1520
PRKD2_4_6545,1089,819,964,738,523,205,454,518,256,442,609,386
PRKD2_4_6546,356,523,596,782,629,763,1002,701,558,285,522,281
PRKD2_4_6547,301,509,623,629,173,258,1550,892,644,93,346,1089
PRKD2_4_6548,1301,712,1087,1140,638,1233,1250,839,604,1202,379,1085
PRKD2_4_6549,913,1083,1071,1099,716,1213,1563,1610,995,455,1638,853
PRKD2_4_6550,1155,834,921,940,1090,858,1546,1060,2173,1027,1399,493
PRKD2_4_6551,460,523,403,583,424,168,130,679,132,1115,482,736
PRKD2_4_6552,1156,1084,1489,1326,1735,1060,2822,1975,1147,644,1302,131
7
SLC6A2_4_6553,233,236,201,382,256,21,170,362,835,1,145,883
SLC6A2_4_6554,141,163,195,303,16,25,193,41,116,2,441,78
SLC6A2_4_6555,493,702,773,364,239,313,422,21,163,780,564,239
SLC6A2_4_6556,539,558,601,681,962,202,353,757,892,566,823,479
SLC6A2_4_6557,1301,1426,1424,1738,1295,2286,2109,1592,1669,1066,955,24
72
SLC6A2_4_6558,1752,1715,1886,1288,1325,2123,2986,1985,1354,1249,1168,1
384

SLC6A2_4_6559,751,601,640,454,766,505,617,220,886,606,125,603
SLC6A2_4_6560,486,371,333,361,714,369,2077,417,197,406,982,571
SLC6A2_4_6561,1125,782,1109,794,975,385,1677,1096,335,1385,848,1785
SLC6A2_4_6562,2185,1530,2067,1889,2312,2142,2255,1269,1913,1140,2198,1
324
SLC6A2_4_6563,783,706,1330,1265,1183,1249,2082,1334,1224,529,1162,977
SLC6A2_4_6564,423,572,850,332,857,349,1376,295,386,51,379,718
SLC6A2_4_6565,1195,617,1117,655,1430,1447,681,581,1059,643,1297,1241
SLC6A2_4_6566,661,954,1123,835,835,369,346,481,307,241,1248,994
SLC6A2_4_6567,2042,1529,2022,1893,1410,1549,1218,2641,1071,599,2262,12
96
SLC6A2_4_6568,696,708,809,535,604,520,435,964,914,84,1366,498
SLC6A2_4_6569,348,251,621,398,34,17,8,668,204,578,431,14
SLC6A2_4_6570,131,246,268,244,0,600,690,4,209,72,393,113
SLC6A2_4_6571,576,539,488,319,833,223,132,1924,676,26,214,674
SLC6A2_4_6572,376,435,493,463,398,210,304,574,110,481,207,752
SLC6A4_4_6573,3111,2959,3212,2936,2353,1491,2350,3161,2544,1404,2370,2
483
SLC6A4_4_6574,2681,3889,3247,3343,2354,2688,2832,3235,2143,2448,2370,2
551
SLC6A4_4_6575,1210,1026,1716,894,734,722,1850,1722,911,916,2310,874
SLC6A4_4_6576,1301,1893,1581,1859,1580,1459,1711,2143,2169,3106,1440,1
469
SLC6A4_4_6577,628,732,326,660,666,480,603,687,275,99,112,639
SLC6A4_4_6578,2998,2925,3233,2813,1943,1491,2348,2957,2323,1403,2301,2
490
SLC6A4_4_6579,173,283,347,376,10,0,3,299,0,12,0,279
SLC6A4_4_6580,505,475,411,481,531,440,585,1435,442,263,744,901
SLC6A4_4_6581,470,552,862,505,528,617,1189,352,747,688,498,582
SLC6A4_4_6582,503,627,733,418,441,140,1258,705,379,29,452,262
SLC9C1_4_6583,704,463,466,601,866,8,735,265,161,823,332,1115
SLC9C1_4_6584,2479,2264,2923,2100,1807,3950,2656,3414,1943,2211,3132,2
833
SLC9C1_4_6585,226,103,261,81,739,1,264,2,100,225,689,29
SLC9C1_4_6586,1829,1854,1830,1646,959,2614,1349,2350,2753,617,1239,219
7
SLC9C1_4_6587,2618,2767,3116,3011,4826,2857,2845,3439,987,3672,3882,25
49
SLC9C1_4_6588,2240,3066,3520,2501,2185,1502,3373,3361,2135,1397,2700,2
533
SLC9C1_4_6589,1839,1967,2221,2169,2942,3279,2574,1756,1520,4209,1629,2
248
SLC9C1_4_6590,1612,1696,3155,1916,3243,1956,3999,2018,2025,2326,2469,2
538
SLC9C1_4_6591,2773,3018,3259,2686,3162,2399,4957,2193,5492,3557,2641,3
922
SLC9C1_4_6592,2064,1855,2061,1795,1573,2611,2850,1958,1750,2080,1720,2
646
SLC9C2_4_6593,636,704,1271,988,637,365,2647,866,310,662,1008,747
SLC9C2_4_6594,1406,1539,1841,1552,825,713,3424,1689,1999,484,1262,1174

SLC9C2_4_6595,651,388,866,544,42,159,1124,260,508,140,993,526
SLC9C2_4_6596,1786,2150,2316,2104,3407,2173,2715,1195,965,3042,2966,3155
SLC9C2_4_6597,665,789,873,566,298,1411,1196,1189,381,285,499,467
SLC9C2_4_6598,2657,2094,3085,2298,2535,2940,1937,1360,3014,2693,3847,904
SLC9C2_4_6599,2277,2164,2004,2112,4532,1422,1408,659,1625,1877,3082,1872
SLC9C2_4_6600,582,430,506,466,122,4,874,187,508,132,821,1186
SLC9C2_4_6601,375,353,341,183,185,158,54,78,404,233,701,1258
SLC9C2_4_6602,832,1292,915,1040,543,1274,864,549,1242,238,1175,963
TRPM2_4_6603,1386,892,1298,1428,1472,2279,1159,1566,1125,1432,2763,2321
TRPM2_4_6604,679,794,722,457,1384,1590,367,95,550,521,997,737
TRPM2_4_6605,39,180,46,109,2,122,178,237,214,0,5,6
TRPM2_4_6606,894,1297,1372,1550,2419,700,3223,1764,139,698,574,2467
TRPM2_4_6607,2137,1495,2145,1388,2094,1624,4504,1216,1573,2387,2919,1399
TRPM2_4_6608,74,181,86,221,193,244,500,721,17,272,137,614
TRPM2_4_6609,856,545,772,1048,338,1716,3463,876,819,831,2685,1186
TRPM2_4_6610,856,988,911,1072,462,1209,327,252,911,211,1487,1345
TRPM2_4_6611,3102,3614,3379,3062,1863,2781,3584,2110,1557,1675,4034,3219
TRPM2_4_6612,81,110,103,200,162,15,195,272,106,599,3,431
TSPO_4_6613,527,797,1025,400,965,1222,1432,603,1026,71,1309,1186
TSPO_4_6614,1090,1262,1132,1151,969,2099,798,1859,1230,2542,750,716
TSPO_4_6615,508,376,438,325,166,775,409,200,360,2,240,253
TSPO_4_6616,1773,2248,2284,2416,3015,3445,4101,756,530,2251,3081,2596
TSPO_4_6617,2449,2811,3012,2483,2088,2033,2862,2593,2368,2618,3042,2274
TSPO_4_6618,989,1334,1285,969,1593,1253,1129,1251,103,407,595,982
TSPO_4_6619,87,232,179,124,0,624,0,0,4,317,0,3
TSPO_4_6620,1319,1425,1572,1105,867,1414,1587,1339,267,1129,794,1412
TSPO_4_6621,822,764,574,686,1005,658,176,1103,603,5,1119,223
TSPO_4_6622,1614,1984,2168,1967,2729,3439,3256,755,365,2248,3087,2601
