# Supplementary material for: Cross-species genetic screens identify transglutaminase 5 as a regulator of polyglutamine-expanded ataxin-1
Source: J Clin Invest. 2022 May 2;132(9):e156616. doi: 10.1172/JCI156616 (PMC9057624; doi:10.1172/JCI156616)
Supplement: Supplemental data set 5 [file jci-132-156616-s050.pdf]

shRNA,B1,B2,B3,H1,H2,H3,L1,L2,L3
AAK1_5_1,1904,1797,1893,607,1612,671,934,1691,2237
ACP6_5_2,5489,5265,5216,4570,6022,5406,6263,2287,1474
ACPT_5_3,1194,1023,1139,1454,1933,1897,655,1017,1883
ACVR2A_5_4,3082,2693,3814,5348,3570,3269,4517,4469,3218
ACVR2B_5_5,1064,591,852,757,1021,63,335,553,1205
ADCK2_5_6,198,201,311,362,44,210,76,69,20
ADCK3_5_7,845,492,370,560,400,47,227,591,152
ADCK5_5_8,2580,3738,2530,1184,2732,2442,1234,2941,1786
ADPGK_5_9,1294,783,1343,582,2457,2638,999,1039,382
ADRBK1_5_10,500,846,739,989,2039,507,512,816,493
ADRBK2_5_11,831,962,900,727,1785,1406,580,1057,2350
AGK_5_12,964,1135,1155,393,2074,914,1079,1545,766
AK1_5_13,532,588,567,707,6,93,181,164,1039
AK7_5_14,278,363,292,152,210,725,512,414,0
AK8_5_15,238,551,582,91,758,392,413,313,147
ALK_5_16,1530,1242,972,2141,865,211,1197,777,1058
ALPI_5_17,63,299,863,605,1471,182,77,1100,631
ALPK2_5_18,814,797,1793,1390,621,1367,1176,921,1962
ALPK3_5_19,185,346,894,529,102,645,297,169,446
ALPP_5_20,1640,1501,1238,2551,1159,850,1824,802,1313
ALPPL2_5_21,491,162,300,708,227,103,176,55,141
ANKK1_5_22,489,775,1431,977,885,552,1360,154,751
ARAF_5_23,150,134,251,342,36,77,17,13,8
ATM_5_24,1860,2590,3287,3018,3407,4412,2281,2466,4066
AURKB_5_25,905,506,1183,380,1214,1268,585,456,491
BLK_5_26,1280,417,2314,1047,1820,1597,1459,1114,2573
BMPR1A_5_27,2375,2083,1650,1264,1410,621,2660,1444,2282
BMPR1B_5_28,2875,2341,3422,3107,1777,3740,1527,1942,854
BMPR2_5_29,712,757,1258,364,1319,1620,888,915,734
BPNT1_5_30,2213,2967,3442,873,2611,3069,2173,3340,5999
BRAF_5_31,1735,1924,2406,1048,1613,3573,2239,2259,3042
BRD3_5_32,1108,1095,1711,582,2174,1334,1346,100,203
BRSK1_5_33,3419,3121,3353,3802,4448,3616,3780,3783,9114
BRSK2_5_34,1637,1050,1742,676,1939,605,996,1084,2671
BTK_5_35,913,502,920,254,865,331,381,1168,580
BUB1B_5_36,3125,2547,3196,1770,5481,833,2910,3472,2495
BUB1_5_37,1176,408,354,115,99,899,545,420,1610
C20orf111_5_38,1152,1959,1821,676,2890,721,1516,2037,1057
C9orf96_5_39,423,737,840,1294,1252,1039,645,230,1616
CALM1_5_40,189,383,437,6,580,388,497,53,573
CALM3_5_41,347,848,502,566,2453,140,769,767,859
CAMK1_5_42,1183,1916,1689,2097,3337,1945,2240,1609,2119
CAMK1G_5_43,1018,474,1123,935,1041,179,649,242,1084
CAMK2N1_5_44,325,464,302,59,439,78,955,281,49
CAMK4_5_45,1500,952,2152,858,1312,1393,1808,585,2156
CAMKV_5_46,1626,1998,1030,2143,793,653,1387,522,3412
CARD11_5_47,307,872,959,235,1630,467,844,2059,402
CCL2_5_48,1175,1414,1787,2492,838,3049,1797,1247,795
CDC42BPB_5_49,801,1492,921,1726,2337,1497,615,1646,2212

CDC42BPG_5_50,89,148,173,1,51,404,166,196,1
CDK14_5_51,1588,1944,1449,782,1808,662,1588,2640,840
CDK15_5_52,101,501,483,136,451,210,0,651,35
CDK19_5_53,14,0,0,0,0,0,0,10,0
CDK3_5_54,24,11,65,18,35,33,1,36,0
CDK4_5_55,331,629,667,1008,166,1804,431,133,2472
CDK5R1_5_56,122,38,207,90,305,195,300,50,105
CDK7_5_57,3802,4668,4651,3284,2501,3105,3457,4083,6238
CDK8_5_58,2467,2485,3082,2045,2926,3942,3020,1266,4376
CDK9_5_59,1488,861,391,616,890,923,1010,1381,1162
CDKL1_5_60,2753,2932,2834,2941,1987,3393,1777,1200,4186
CDKL2_5_61,392,557,773,1675,801,307,1139,516,487
CDKL4_5_62,307,431,347,194,1705,623,988,42,1011
CDKN1B_5_63,2613,3609,4013,4639,2632,2959,1100,1673,3638
CERK_5_64,1488,1584,2087,2026,3832,2129,1522,2291,2029
CHKB_5_65,2945,1284,2391,1679,2187,1159,1728,780,3500
CHUK_5_66,598,608,872,1061,604,532,881,738,234
CIB2_5_67,768,477,1541,943,932,1496,427,620,938
CILP_5_68,2613,2394,2965,2230,2154,4270,2080,3015,1875
CKB_5_69,2412,2419,2846,3684,2353,2822,2975,3337,5029
CKM_5_70,69,153,353,80,86,44,776,34,1177
CKMT1A_5_71,1250,768,1976,286,2439,939,240,550,753
CKMT1B_5_72,1250,768,1976,286,2439,939,240,550,753
CKS1B_5_73,265,482,870,1222,120,1265,142,739,1081
CKS2_5_74,917,269,505,577,515,14,218,201,241
CLK2_5_75,3021,1626,2932,3514,4368,1664,3632,2068,2688
CLK4_5_76,1476,1883,1732,838,618,1474,1646,1165,1428
CMPK2_5_77,1014,727,976,632,636,277,791,675,966
CPNE3_5_78,390,149,235,241,222,4,50,466,93
CRIM1_5_79,556,365,796,216,713,2638,405,735,927
CRKL_5_80,1493,1282,2254,1489,1780,1132,1265,717,1781
CSF1R_5_81,136,45,308,1,0,2,124,0,0
CSNK1A1L_5_82,90,25,17,8,6,2,310,198,1406
CSNK1G1_5_83,603,957,900,2021,900,2027,621,650,459
CSNK1G2_5_84,134,139,621,181,260,1,30,162,138
CSNK2A2_5_85,1232,1642,1197,112,2342,1155,1881,492,735
CSNK2B_5_86,207,178,37,0,9,659,12,270,618
CTDSP2_5_87,185,412,626,1351,142,1600,200,147,870
DAPK1_5_88,326,676,481,133,1055,13,295,367,91
DAPK2_5_89,651,866,1575,570,1657,812,18,64,539
DAPK3_5_90,241,197,360,398,72,99,336,795,271
DBF4_5_91,1590,2167,1776,102,5381,532,1470,2413,3473
DCK_5_92,1831,1886,1347,1047,1229,2016,1542,1481,1882
DCLK3_5_93,474,474,787,204,103,674,839,754,1268
DGKE_5_94,1583,1650,2132,2443,2811,4515,1929,1819,1795
DGKI_5_95,1068,1436,1854,1412,2590,2210,1622,910,2371
DGKK_5_96,236,501,603,2962,294,660,1559,1184,880
DGKQ_5_97,145,81,151,170,533,53,839,46,10
DOLK_5_98,2081,713,1150,1060,2460,3726,844,807,933
DOT1L_5_99,60,328,235,192,11,459,53,149,1733

DUSP11_5_100,1500,1900,2568,891,1091,3241,1831,1476,3617
DUSP12_5_101,578,623,706,475,1056,2178,671,169,1191
DUSP14_5_102,118,123,339,108,20,682,389,149,0
DUSP16_5_103,669,1280,664,2507,612,607,808,249,731
DUSP18_5_104,914,619,1323,769,2184,548,265,513,4532
DUSP21_5_105,952,1027,971,370,1527,768,799,1575,1035
DUSP22_5_106,347,479,239,26,251,291,976,437,11
DUSP2_5_107,741,863,480,269,238,303,353,707,1214
DUSP3_5_108,144,260,158,145,257,6,3,1,3
DUSP5_5_109,190,235,409,73,110,469,64,177,651
DUSP7_5_110,1550,1479,921,847,787,1300,1514,1757,991
DUSP8_5_111,1384,1265,2215,653,1756,2693,1290,952,1008
DUSP9_5_112,426,369,1030,207,386,64,656,857,376
DYRK4_5_113,2519,2752,3402,1166,3050,2326,2626,4502,1936
EEF2K_5_114,1397,1829,1886,1246,740,3308,1883,1399,2089
EFNA2_5_115,3962,4644,5207,4227,3949,6388,3794,4358,5389
EFNA3_5_116,545,424,386,91,901,276,194,212,503
EFNA5_5_117,606,746,1064,1486,893,1123,648,699,905
EFNB3_5_118,1654,1666,1563,1640,755,537,2252,1015,1558
EIF2AK3_5_119,1803,2583,2288,891,5429,749,806,1700,1108
EIF2AK4_5_120,2247,3508,3632,1063,1777,1859,2921,3757,2396
EPHA1_5_121,846,1376,1433,643,894,2696,833,730,723
EPHA2_5_122,507,631,606,529,226,648,466,789,188
EPHA4_5_123,711,912,732,853,1364,242,442,817,130
EPHA7_5_124,890,618,688,675,295,283,741,1117,2338
EPHB1_5_125,502,454,418,1334,1427,74,479,451,844
EPHB3_5_126,1506,783,482,1669,2088,325,1347,1126,2305
EPHB4_5_127,227,287,298,576,309,21,380,3,0
EPHB6_5_128,1295,996,2475,159,1573,2101,1174,753,1361
ERN1_5_129,717,588,751,1443,2274,706,216,939,1420
ERN2_5_130,395,553,545,1,41,854,371,98,35
ETNK2_5_131,264,252,456,213,134,336,253,68,110
EYA3_5_132,161,83,595,144,606,999,74,68,494
FER_5_133,463,237,138,879,330,1,432,942,75
FLT3_5_134,340,659,514,64,923,458,626,349,642
FN3K_5_135,139,3,0,207,6,177,60,0,1055
FN3KRP_5_136,563,817,1012,658,1428,418,496,419,2442
FRK_5_137,2132,2699,1846,1146,2302,1586,2446,2190,2212
FUK_5_138,442,397,537,9,1796,504,179,211,0
G6PC3_5_139,41,55,72,227,28,0,307,94,211
G6PC_5_140,403,318,575,993,422,393,229,517,55
GAK_5_141,116,89,290,951,701,395,108,15,532
GALK1_5_142,2682,2438,1913,2354,3401,2760,2207,1342,1433
GDPD4_5_143,5634,4699,5117,4762,4772,6477,4044,4434,4675
GOLGA5_5_144,1194,1281,801,903,773,4250,512,1144,2569
GRK1_5_145,890,1101,826,810,669,880,1966,1000,1737
GRK5_5_146,714,1117,1383,506,1922,1675,648,1053,1298
GRK7_5_147,3546,3128,4070,4622,2997,4711,2449,3111,4303
GSG2_5_148,958,1091,555,1508,783,451,1105,375,2573
GSK3A_5_149,385,460,277,303,587,169,692,168,92

GUCY2C_5_150,1157,1077,665,1205,657,983,161,817,243
GUCY2D_5_151,3861,3808,5107,3214,2444,3671,4345,5207,4865
GUCY2F_5_152,2513,2868,3504,3236,3323,4325,2382,2351,5646
HIPK4_5_153,300,240,684,303,277,641,425,229,743
HKDC1_5_154,384,151,415,34,289,693,229,438,254
HOOK3_5_155,1155,1668,1501,841,965,1753,1742,1164,2430
HSPB8_5_156,2120,2066,2919,2626,2898,3429,2618,913,4874
HUNK_5_157,6066,7533,9018,6468,7797,7649,6212,3283,5992
HUS1_5_158,9062,8889,12519,11001,11084,11277,8414,11696,12384
IGBP1_5_159,2974,2241,2683,1543,2932,2977,2170,2310,4183
IGF1R_5_160,756,1003,651,348,1091,1492,452,547,856
IGF2R_5_161,454,629,985,823,911,913,561,177,31
ILKAP_5_162,861,989,1530,643,1618,1109,1492,599,986
IMPA2_5_163,38,124,280,5,53,7,206,240,406
INPP5A_5_164,594,644,528,258,382,922,577,592,793
INPP5B_5_165,1331,918,1285,1352,585,1186,854,297,1756
INPP5J_5_166,563,630,671,236,742,1376,625,647,984
INPPL1_5_167,612,414,1007,1741,1329,672,344,843,822
INSRR_5_168,689,702,325,98,790,115,547,774,880
IPMK_5_169,1196,1406,2249,907,1895,1290,1886,636,412
IPPK_5_170,276,516,527,0,1266,1633,467,62,53
IRAK2_5_171,60,634,65,167,154,373,38,104,12
ITK_5_172,1489,1370,2337,1778,590,1186,2119,860,1766
ITPKA_5_173,1079,623,810,539,1326,303,1563,335,1384
ITPKB_5_174,2595,3101,3488,973,3105,2671,2352,3529,2229
ITPKC_5_175,1323,1809,1609,1738,2889,780,907,1322,1965
JAK1_5_176,956,605,638,681,96,658,853,596,2124
JAK2_5_177,2134,2397,3242,1396,1490,2278,982,2657,3787
JAK3_5_178,2026,2221,2689,2748,588,4439,2639,767,2919
KDR_5_179,502,530,609,377,1064,1255,250,1369,793
KSR1_5_180,577,395,751,857,229,677,1175,431,1608
LATS1_5_181,2807,2609,1854,2114,2074,1994,2478,2059,729
LATS2_5_182,1745,891,1661,897,835,1152,1599,635,385
LLGL1_5_183,707,446,406,338,920,687,770,994,297
LMTK2_5_184,1137,976,939,676,571,571,349,791,527
LMTK3_5_185,62,254,117,132,68,145,4,38,526
LRRK1_5_186,652,691,568,394,195,953,56,158,37
LRRK2_5_187,7179,10038,8736,8690,10446,7369,7569,6581,9181
MAGI2_5_188,243,198,223,2,1,55,102,521,218
MAP2K1_5_189,342,617,480,996,64,468,632,1060,660
MAP2K2_5_190,171,140,748,98,32,1266,198,275,363
MAP2K4_5_191,162,306,249,370,26,141,11,43,1
MAP2K6_5_192,988,620,511,872,684,1142,1625,230,1320
MAP2K7_5_193,1008,440,1001,992,668,1825,2754,796,1741
MAP3K10_5_194,339,606,405,4,600,629,313,105,830
MAP3K11_5_195,418,624,434,356,149,1061,513,248,1652
MAP3K14_5_196,34,378,57,119,991,0,146,2,3
MAP3K15_5_197,1308,1594,2435,1490,1686,1869,1316,2104,457
MAP3K1_5_198,255,163,360,205,358,168,74,1,37
MAP3K2_5_199,1255,1254,2006,1247,631,334,841,1110,1831

MAP3K5_5_200,2103,2710,2420,2279,2130,4267,2333,1104,3053
MAP3K6_5_201,1167,772,925,610,1196,1302,1216,528,425
MAP3K8_5_202,105,143,38,81,1,2,126,35,6
MAP3K9_5_203,781,716,763,444,1488,330,855,653,3879
MAP4K2_5_204,962,60,423,25,548,296,456,96,72
MAP4K3_5_205,1008,813,1848,1643,1927,2035,1417,2207,3284
MAPK11_5_206,116,239,345,21,114,185,205,193,0
MAPK12_5_207,71,381,192,1,197,1299,30,4,3
MAPK13_5_208,884,1383,960,1510,1660,521,841,1128,570
MAPK15_5_209,148,80,0,0,0,0,0,0,0
MAPK4_5_210,324,374,868,148,374,539,56,82,48
MAPK6_5_211,3697,4841,4071,3288,4174,4076,4159,3587,2250
MAPKAPK3_5_212,677,861,1167,525,967,1016,533,564,1074
MARK1_5_213,32429,37331,41387,26706,31446,33995,30953,31143,49134
MAST2_5_214,1650,1806,2349,2355,2188,634,845,3116,905
MAST3_5_215,258,344,588,337,112,253,385,613,1596
MELK_5_216,2174,2562,3865,2014,3266,3196,2148,3657,3611
MERTK_5_217,2663,3070,3389,2136,2152,5840,2073,1721,3122
MEX3B_5_218,20,32,11,0,9,592,214,19,6
MPP3_5_219,266,307,153,336,237,42,262,66,17
MPP5_5_220,884,448,730,270,1214,972,1152,560,781
MST1R_5_221,194,71,210,0,177,86,115,68,0
MTM1_5_222,2593,1658,1757,1105,1447,2612,2560,1301,1170
MTMR1_5_223,1079,762,780,488,259,194,1281,544,656
MTMR4_5_224,338,343,480,333,85,659,115,238,67
MTMR6_5_225,456,528,666,666,1033,230,500,431,557
MTMR7_5_226,2776,3556,4389,2929,1311,4355,3470,3414,4478
MTMR9_5_227,1997,3132,2683,897,2924,1842,4066,3355,1773
MTOR_5_228,664,733,616,344,135,968,1691,416,62
MYLK2_5_229,820,1025,1574,576,1198,2295,316,1028,1845
MYLK3_5_230,2471,3938,4386,5533,3061,4121,2669,5530,3644
MYLK4_5_231,581,390,740,708,472,59,1089,520,584
MYO3A_5_232,2347,1914,1839,2135,4419,1385,1553,1964,1613
N4BP2_5_233,2816,3173,3882,1935,7556,3239,2462,2656,6482
NAGK_5_234,81,105,305,213,47,259,143,60,216
NAGS_5_235,22,52,119,93,39,115,70,198,0
NEK10_5_236,806,1348,1486,1087,879,1729,1329,2822,722
NEK5_5_237,412,799,1220,656,1891,534,1225,135,1266
NEK7_5_238,10242,13447,13939,11044,11557,18391,11102,12519,17422
NEK8_5_239,858,555,1129,1148,366,2988,92,535,567
NEK9_5_240,795,259,448,320,289,1066,274,594,12
NME3_5_241,940,408,383,1014,31,282,751,684,1368
NME4_5_242,349,525,563,942,135,999,615,610,206
NME5_5_243,2762,1736,2280,4288,2538,3571,3328,2990,3597
NME6_5_244,908,931,1121,963,507,2542,645,194,1631
NPR1_5_245,2049,1836,2688,1449,2918,1941,1835,1939,3212
NPRL2_5_246,448,784,407,83,292,19,298,241,169
NRBP1_5_247,1787,2308,2278,2943,2300,3684,1535,2505,2669
NRBP2_5_248,356,90,322,131,0,164,222,41,1366
NRK_5_249,1613,1400,1495,522,2192,1660,1459,2010,1411

NT5C1A_5_250,189,7,449,1,16,202,9,555,1
NT5C_5_251,776,1439,1163,130,714,1262,1348,1116,1151
NT5M_5_252,955,1336,1087,1215,1214,243,623,953,264
NUAK1_5_253,235,132,287,768,0,291,91,98,288
NUAK2_5_254,1808,3756,3593,1179,664,1228,1872,1831,1966
NUCKS1_5_255,2473,2076,2588,2316,2065,1842,1754,1868,4288
OXSR1_5_256,1297,1113,1497,533,1040,3349,1626,71,2795
PAK2_5_257,1475,1978,2129,812,1901,1890,2097,2103,385
PANK3_5_258,19,242,120,62,220,109,36,136,71
PANK4_5_259,844,270,655,4,544,644,605,362,595
PASK_5_260,1503,1676,1807,511,2185,1535,2562,1111,808
PBK_5_261,1116,1724,1666,2635,2435,1483,1392,1625,545
PCK1_5_262,4184,4353,6692,2059,3595,4735,4382,5004,9755
PDCD1_5_263,98,441,334,0,17,510,3,19,977
PDGFRA_5_264,948,905,1849,876,91,876,319,2152,646
PDGFRB_5_265,3350,2951,3576,3301,2588,3189,3832,1693,6630
PDGFRL_5_266,1696,1921,2251,2557,1731,3201,2711,1495,1262
PDIK1L_5_267,2448,2105,3895,1573,3104,2631,2242,2464,2627
PDK4_5_268,2881,3730,3088,2546,4220,4733,3278,2709,1795
PDP2_5_269,505,386,858,1508,348,1145,227,338,1768
PDXK_5_270,9,274,66,643,17,279,246,288,510
PFKFB1_5_271,909,1793,837,509,865,1194,939,1381,1087
PFKFB4_5_272,793,739,1021,110,1222,897,1020,726,1854
PFKL_5_273,1945,1911,2176,3573,1798,1620,1445,1725,1756
PGAM2_5_274,221,160,542,426,295,52,70,432,4
PGK1_5_275,204,492,71,33,474,13,187,280,132
PGK2_5_276,2725,2223,2632,2563,1135,2359,2489,2461,6128
PHKA2_5_277,389,333,540,32,698,394,294,406,801
PHKG1_5_278,743,691,1168,383,202,355,506,663,437
PI4K2A_5_279,649,1451,1141,1123,1995,2090,483,921,2951
PI4K2B_5_280,527,284,793,209,214,343,480,472,758
PIK3C2A_5_281,4747,3436,4741,5012,3478,3409,5812,4393,7079
PIK3C2B_5_282,80,197,52,243,245,275,27,15,98
PIK3C2G_5_283,97,431,226,525,343,331,137,940,68
PIK3C3_5_284,876,757,1377,633,402,1143,826,531,1369
PIK3CA_5_285,2861,3879,4383,2422,3908,4711,4756,3571,3507
PIK3CB_5_286,307,270,486,346,841,648,388,260,47
PIK3CD_5_287,563,702,511,370,279,875,587,749,1859
PIK3CG_5_288,2976,2961,3646,4186,1742,2950,2526,3064,1900
PIK3R2_5_289,2155,2439,2573,1333,2533,1365,2542,1503,5148
PIK3R4_5_290,1613,1704,1639,1536,2047,1999,2131,2097,4243
PIK3R6_5_291,2832,2608,1974,1799,1796,1996,2231,3830,4649
PIM2_5_292,5655,5068,8396,5472,10234,4978,7265,7486,7030
PIM3_5_293,930,1675,1167,1040,3567,2310,994,1625,3590
PINK1_5_294,717,390,927,912,1935,1054,667,990,486
PIP4K2A_5_295,611,343,737,726,442,896,818,173,749
PIP4K2B_5_296,2918,2730,3546,1414,2931,2685,2283,3751,2652
PIP5K1B_5_297,2890,3675,3627,2937,3362,1900,4347,4707,3560
PKDCC_5_298,688,634,884,2,894,117,201,1578,1939
PKN2_5_299,4485,4524,5057,3355,6642,7086,3696,2321,7323

PKN3_5_300,1268,1521,2180,513,1730,4796,867,1164,1496
PLK1_5_301,414,318,688,447,336,341,266,1634,522
PLK2_5_302,0,92,29,0,129,0,0,0,0
PLK3_5_303,540,397,406,667,1158,1455,315,749,684
PMVK_5_304,534,714,1143,373,565,1391,897,983,182
PNKP_5_305,0,16,97,0,0,109,131,0,31
POLD1_5_306,150,367,245,20,27,10,179,279,208
PON1_5_307,2431,4136,4342,3276,5057,1879,5767,2909,4279
PPAP2B_5_308,991,251,748,91,500,393,1169,646,23
PPEF2_5_309,2778,2322,3229,1649,3104,2938,2810,2300,5040
PPFIA3_5_310,379,269,593,36,140,18,282,22,353
PPFIA4_5_311,702,377,400,312,842,260,341,81,360
PPM1D_5_312,1318,1386,1125,808,1325,793,1249,1037,1073
PPM1E_5_313,1463,931,1119,900,1234,1517,599,1020,741
PPM1F_5_314,366,732,1086,420,709,800,726,623,363
PPM1G_5_315,129,107,298,142,1196,104,1,78,1
PPM1K_5_316,487,962,1180,839,2220,218,407,1377,131
PPM1L_5_317,1854,2399,2631,2132,2460,1516,2075,1739,1407
PPP1CC_5_318,7273,8253,10842,8275,11375,8900,7919,6774,12975
PPP1R12C_5_319,495,407,941,1234,328,882,643,878,769
PPP1R13B_5_320,3917,5683,5113,3297,4823,6380,2569,3646,7421
PPP1R14B_5_321,63,595,15,8,47,289,5,635,134
PPP1R15B_5_322,2063,2737,2586,2730,4599,1400,2419,2657,4452
PPP1R1A_5_323,519,876,704,960,57,725,259,660,166
PPP1R1C_5_324,941,935,1048,610,209,1800,571,890,4188
PPP1R2_5_325,1614,1470,2712,2604,1101,2467,2248,2862,1964
PPP1R3A_5_326,1223,1005,1322,924,336,1235,871,201,1559
PPP1R3C_5_327,441,459,567,17,416,795,754,528,1321
PPP1R3D_5_328,2,19,30,0,26,4,0,0,0
PPP1R7_5_329,352,215,234,768,45,341,22,33,1440
PPP2CA_5_330,1016,1822,1494,1670,3795,2675,1565,1356,198
PPP2CB_5_331,1099,253,1399,547,77,449,361,991,0
PPP2R1A_5_332,344,494,559,13,115,248,951,251,82
PPP2R2D_5_333,1381,1895,1613,1647,2706,1338,1198,1805,2117
PPP2R3B_5_334,1257,904,2118,697,345,448,544,906,902
PPP2R5B_5_335,1149,327,896,175,376,1550,566,988,35
PPP2R5E_5_336,2137,1793,2537,3572,4149,1566,2448,2038,1368
PPP3CC_5_337,1895,3017,3882,3468,4694,5666,2407,2737,8135
PPP3R1_5_338,441,810,1272,2,58,958,114,258,0
PPP3R2_5_339,107,233,378,90,177,366,459,29,29
PPP4C_5_340,86,0,83,0,0,0,1,0,14
PPTC7_5_341,1108,734,855,804,1164,172,567,1262,1236
PRKAA2_5_342,592,561,1035,610,782,794,955,577,250
PRKAB1_5_343,464,605,964,9,126,1634,966,115,823
PRKAB2_5_344,2858,2881,1711,961,1322,3417,1696,3427,2762
PRKACG_5_345,1498,1556,1357,777,774,788,1498,1228,2241
PRKAG3_5_346,146,362,225,88,43,852,347,40,1177
PRKAR2A_5_347,5265,5729,7572,3735,7677,6346,3575,4240,6201
PRKAR2B_5_348,1022,942,1397,1488,449,644,472,1452,358
PRKCA_5_349,589,214,739,611,327,0,728,530,83

PRKCE_5_350,1043,1639,1510,1822,3536,2993,1214,1342,2832
PRKCG_5_351,557,175,558,835,914,1005,917,759,92
PRKCH_5_352,1470,1745,2315,1386,2632,1976,1085,1568,1389
PRKCI_5_353,307,852,368,720,1065,769,42,361,603
PRKD1_5_354,1135,1540,1881,160,883,1769,1108,1451,760
PRKD3_5_355,3764,3186,5750,2034,7910,4652,2338,3101,3059
PRKG2_5_356,1214,2558,2935,1565,575,2865,2101,1259,4052
PRKX_5_357,484,307,93,2,31,7,45,63,393
PRPF4B_5_358,399,321,511,1123,50,133,480,273,714
PRPS1L1_5_359,1589,668,2337,1498,762,1813,1306,1721,2772
PSKH1_5_360,1471,1859,1635,3437,2419,857,1815,3228,1030
PSKH2_5_361,2015,2532,3236,2257,1001,1036,1735,2384,1335
PSPH_5_362,233,286,226,84,28,632,43,657,253
PSTK_5_363,2484,2415,2773,1455,2421,2312,2486,1971,3883
PTEN_5_364,1748,2313,1726,2251,1063,1553,3050,1724,3956
PTK6_5_365,256,213,157,197,52,286,381,111,1
PTP4A1_5_366,6508,8542,9154,7920,8426,10076,7615,7933,6433
PTPLA_5_367,2370,2915,2891,1368,2575,2523,3476,4258,4471
PTPLB_5_368,808,459,1322,745,317,806,595,965,2428
PTPN11_5_369,3141,2445,3164,1729,1524,3674,3991,2213,2951
PTPN14_5_370,487,553,525,828,1104,115,777,541,73
PTPN1_5_371,1369,1075,1369,830,2537,911,1395,984,2219
PTPN21_5_372,1545,2181,2607,96,1131,989,1862,1236,5405
PTPN23_5_373,1382,1652,2128,1628,1630,1190,1836,1471,3475
PTPN4_5_374,1189,1262,1192,2808,474,675,530,1170,1330
PTPN9_5_375,572,794,1536,850,326,1272,724,282,737
PTPRG_5_376,3128,3034,3191,1350,4408,1901,4198,2574,3131
PXK_5_377,1460,2156,2474,519,148,1252,1643,1360,3123
RAF1_5_378,1802,2810,2924,1917,2490,1853,1375,3715,2921
RBKS_5_379,1182,2123,1175,520,923,530,772,950,377
RFK_5_380,1508,1331,1742,1356,2447,2525,1915,1231,691
RIOK3_5_381,1522,1697,1696,1691,2805,1244,1846,1665,1994
RIPK1_5_382,392,642,429,836,428,237,1182,213,236
RIPK2_5_383,1775,1221,911,910,1612,747,648,1343,1348
RIPK3_5_384,722,708,879,576,1424,1547,588,1187,785
RIPK4_5_385,296,443,733,1175,9,203,159,24,420
RNASEL_5_386,1227,908,1416,897,707,1942,1318,1204,2597
RNGTT_5_387,896,1313,1454,943,429,818,531,527,2916
ROCK1_5_388,393,616,1392,0,198,756,121,656,2239
ROCK2_5_389,2178,2259,2926,1524,2470,2937,1437,1949,3126
ROS1_5_390,5824,5691,7238,4569,4430,5675,6528,4268,4305
RPS6KA3_5_391,1530,1888,2152,1897,2867,982,1191,1318,3492
RPS6KA6_5_392,2058,2075,2990,2434,3163,1410,3495,2082,6040
RPS6KB1_5_393,1769,1050,2209,2742,904,1912,2551,3107,4247
RPS6KB2_5_394,470,662,1161,848,2503,565,273,162,2813
RPS6KL1_5_395,1287,1608,1160,970,525,1201,735,889,1016
RSPO3_5_396,1984,2498,1772,3222,1994,1683,1823,2210,1267
SACM1L_5_397,665,712,1313,523,333,852,919,1104,2455
SBF1_5_398,32,226,333,0,52,6,143,349,203
SBK1_5_399,2865,2784,4224,2501,2836,4288,2472,1757,5452

SBK2_5_400,438,727,828,808,253,218,361,324,675
SEPHS2_5_401,3099,3028,2470,2558,2580,2823,2243,1528,3327
SGPP1_5_402,5603,6356,5849,5098,5033,5587,4520,6839,8733
SHPK_5_403,1869,1569,1265,1170,1279,3643,2055,2588,2240
SIK1_5_404,886,933,1269,1393,2091,709,2554,2054,1557
SIK2_5_405,428,599,1303,431,412,358,666,497,2264
SIK3_5_406,1692,2221,1833,4,1676,1255,1443,1015,901
SKAP2_5_407,1355,1461,2232,1964,1616,2409,908,1910,2075
SMG1_5_408,1551,3645,2417,1704,1792,1229,2105,2609,3150
SRMS_5_409,396,389,383,459,82,57,150,29,1002
SRP72_5_410,1101,1827,3000,1798,1390,2471,2232,2560,1716
SRPK1_5_411,1728,1658,2011,2273,673,2319,1444,4449,1107
SSH2_5_412,1939,1867,3044,3018,1260,3263,3174,2642,2468
SSH3_5_413,2545,2683,2915,974,1771,2636,792,1706,4367
STC1_5_414,1891,2024,2082,915,3517,1767,1360,3209,4275
STK10_5_415,1089,1124,1564,795,1698,2860,1577,851,65
STK11_5_416,1000,1120,1419,2344,2345,2391,1083,434,3375
STK16_5_417,471,398,862,414,656,1584,580,151,1446
STK17A_5_418,2474,1818,3793,1329,3985,3313,3076,2864,3040
STK17B_5_419,920,817,1325,563,829,508,794,749,655
STK25_5_420,14,60,146,4,0,0,10,11,10
STK32B_5_421,95,64,294,840,81,408,278,108,520
STK32C_5_422,21,0,28,4,1,5,3,76,2
STK33_5_423,189,71,62,104,673,61,53,37,1
STK35_5_424,126,78,19,0,0,0,94,77,5
STK38_5_425,687,1294,825,891,957,795,570,1098,983
STK38L_5_426,1864,2095,2360,3655,1878,1339,1757,601,910
STK39_5_427,357,584,869,517,1306,786,442,690,869
STK40_5_428,174,452,620,28,40,557,5,87,1737
STK4_5_429,2376,1129,1708,887,2509,2470,2314,825,2239
STYK1_5_430,845,1047,1305,366,406,1031,544,618,493
STYXL1_5_431,149,184,310,476,191,656,18,49,757
TAF1L_5_432,1598,1971,1406,863,800,1077,1781,2309,2263
TAOK3_5_433,4003,3181,4425,6263,4303,3288,3256,1508,4837
TBK1_5_434,1011,1036,898,1521,578,744,866,644,1549
TEC_5_435,1470,1935,2156,1267,1370,2949,2152,2092,1665
TEK_5_436,883,571,955,694,957,1513,753,777,677
TESK1_5_437,801,1201,865,2,588,1230,635,933,1301
TESK2_5_438,293,896,653,1046,1233,88,248,1402,289
THNSL1_5_439,417,495,455,1,515,968,285,91,203
TIE1_5_440,258,783,515,36,233,154,81,170,468
TK1_5_441,217,288,327,49,1265,399,262,426,652
TNK1_5_442,1764,1575,2164,1815,2697,787,1695,849,2245
TNNI3K_5_443,3928,4264,5417,3077,5161,4315,2700,3782,5290
TNS3_5_444,590,644,588,294,1168,812,261,1444,830
TP53RK_5_445,2027,1891,1814,254,1061,1442,3316,1333,1391
TRAT1_5_446,2305,2102,989,2915,2588,683,1490,1790,1134
TRIB1_5_447,1789,2156,2388,1095,2690,996,1435,2970,573
TRIB2_5_448,2284,1839,1877,2041,862,2387,1427,1601,2033
TRIB3_5_449,1002,1152,1106,1168,284,1034,802,1076,1815

TRIM27_5_450,40,10,415,0,59,32,1,0,0
TRIM28_5_451,181,549,559,391,645,772,721,770,1741
TRIO_5_452,1707,1604,1721,1259,1381,1444,836,1818,1360
TRPM7_5_453,907,696,1215,1376,1246,747,1885,432,729
TRRAP_5_454,1608,650,1368,1085,1313,306,505,440,1076
TSKS_5_455,1232,1011,1424,1163,802,1736,687,1364,489
TSSK1B_5_456,438,615,465,0,783,49,286,75,385
TSSK2_5_457,245,410,519,185,116,261,362,218,71
TSSK3_5_458,5699,4860,4808,5343,4290,5555,7512,5697,5299
TSSK6_5_459,565,740,1524,172,278,1660,213,728,343
TTBK1_5_460,1432,881,1564,2351,1084,1736,534,890,3165
TTBK2_5_461,570,421,782,44,299,351,218,281,671
TWF2_5_462,839,740,1275,554,432,1629,1301,1349,868
TXK_5_463,402,1465,600,520,1435,12,402,569,420
TYK2_5_464,560,1574,1053,1228,413,856,431,1190,747
TYRO3_5_465,10,50,135,7,1,1,141,3,14
UBLCP1_5_466,2460,1799,3145,2766,2882,1985,2373,3160,2170
UCK2_5_467,6728,8784,8189,4904,8976,7639,7138,8820,8518
ULK1_5_468,178,202,180,38,190,59,331,425,147
ULK3_5_469,549,725,1461,2081,1600,1283,1116,1208,581
ULK4_5_470,3114,2656,2802,3112,3233,2214,2487,3859,3992
VRK1_5_471,1109,791,1122,680,802,587,670,895,1072
WEE2_5_472,4874,4417,3698,5057,2814,4340,3305,3916,3503
WNK2_5_473,571,512,601,163,893,996,425,514,204
WNK4_5_474,341,275,287,251,30,335,337,395,557
XRCC6BP1_5_475,109,151,37,1,0,15,248,14,0
XYLB_5_476,1093,1288,2584,1002,570,2340,1326,766,4144
YES1_5_477,1995,1326,2161,1009,2634,1932,1476,1355,5045
AATK_5_478,194,405,934,251,69,72,317,437,169
ABL1_5_479,211,417,956,51,76,400,79,220,689
ABL2_5_480,492,409,675,136,623,322,319,137,518
ACP1_5_481,381,448,670,545,2712,743,254,250,10
ACP5_5_482,404,797,398,518,1297,439,124,101,26
ACPL2_5_483,212,282,215,16,518,67,72,82,2
ACPP_5_484,448,418,492,146,304,212,378,276,11
ACVR1B_5_485,778,1823,2477,1610,1269,1433,602,1543,3634
ACVR1C_5_486,1339,1697,1210,209,1948,970,2501,1825,3587
ACVR1_5_487,829,1409,937,1078,485,1001,666,1121,1752
ACVRL1_5_488,1176,1012,614,454,836,1079,707,65,649
ADCK1_5_489,688,788,1021,184,439,1911,614,14,469
ADCK4_5_490,462,93,245,1,87,0,349,221,10
ADK_5_491,1527,2009,3141,810,2454,2869,1910,2435,1153
AK2_5_492,1331,1505,2898,797,1168,1924,915,2430,1011
AK4_5_493,3310,4353,5397,2955,2681,4824,5304,4398,3933
AK5_5_494,1521,1946,1486,541,1288,2788,2004,1713,984
AKT1_5_495,480,895,383,456,273,162,754,519,607
AKT2_5_496,66,404,279,131,38,172,180,350,159
AKT3_5_497,1126,758,1402,547,2650,1648,1021,455,2005
ALDH18A1_5_498,1571,1393,2127,1246,1131,1396,2364,559,1247
ALPK1_5_499,20,133,51,21,7,25,20,168,63

ALPL_5_500,385,420,490,268,848,136,932,123,734
AMHR2_5_501,196,501,301,50,40,19,413,257,719
ANKHD1_5_502,474,685,857,1317,398,1016,221,855,1
APTX_5_503,1080,2360,2626,1624,1781,2899,1346,808,1760
ATRIP_5_504,4603,3940,5526,5563,4770,5528,3667,3472,4128
AURKA_5_505,462,624,567,33,568,274,408,388,500
AURKC_5_506,891,1200,1464,1052,541,1153,1374,1264,1730
AXL_5_507,1635,1800,1477,1563,1959,709,2830,995,1880
BAIAP2_5_508,29,170,297,72,9,161,174,106,1154
BCKDK_5_509,763,243,1531,2226,211,1799,811,846,818
BCR_5_510,1089,1077,1635,322,233,470,668,1188,326
BMP2K_5_511,750,1126,1141,185,1807,3173,256,1167,914
BMX_5_512,131,218,201,803,395,876,557,1441,0
BPGM_5_513,1273,1397,2495,953,1138,1125,1339,2275,2151
BRD2_5_514,226,268,165,494,643,479,553,631,820
BRD4_5_515,1284,1008,1645,1454,2159,648,1344,46,202
BRDT_5_516,990,1124,1650,1123,1484,1155,1316,2323,240
CAB39L_5_517,1347,2065,2349,4457,1451,2646,1552,1456,2232
CAMK1D_5_518,1034,1599,2121,640,993,1287,182,138,2708
CAMK2A_5_519,896,1368,1348,1163,1436,797,1217,1131,2102
CAMK2B_5_520,476,543,775,681,253,323,309,547,303
CAMK2D_5_521,589,474,466,155,193,89,1042,188,354
CAMK2G_5_522,987,998,1819,1435,682,892,641,642,1632
CAMKK1_5_523,96,101,23,250,69,1045,135,21,83
CAMKK2_5_524,4238,5633,5371,4505,4498,3786,3647,5974,8241
CASK_5_525,5917,5933,7880,5668,6482,4323,5686,7390,6082
CCT2_5_526,438,372,223,452,589,113,208,366,10
CDADC1_5_527,1812,2439,2847,3437,1669,3010,2710,2934,2955
CDC14A_5_528,1317,1361,1823,2691,2948,1090,1431,1406,1241
CDC14B_5_529,4447,5202,7345,4252,6634,6321,2804,6865,6476
CDC25A_5_530,1197,1195,1003,1397,603,3052,1003,769,643
CDC25B_5_531,299,157,767,5,285,428,537,41,1846
CDC25C_5_532,341,245,343,129,307,324,229,63,80
CDC42BPA_5_533,777,1247,1351,2819,1697,828,938,961,760
CDC7_5_534,375,614,930,680,587,2015,813,543,1611
CDK10_5_535,336,40,77,0,72,36,1403,13,309
CDK11A_5_536,1511,1144,1805,448,691,694,970,1041,1021
CDK11B_5_537,1511,1144,1805,448,691,694,970,1041,1021
CDK12_5_538,243,62,126,6,10,21,101,0,44
CDK13_5_539,126,59,334,1483,128,220,45,225,224
CDK16_5_540,1040,759,593,759,582,1116,419,76,156
CDK17_5_541,167,221,566,178,373,1201,327,17,232
CDK18_5_542,121,473,113,327,228,49,10,3,0
CDK20_5_543,327,559,524,738,100,1000,619,548,2646
CDK2_5_544,726,559,933,458,982,406,260,82,816
CDK5_5_545,607,1222,804,1003,687,96,571,1015,50
CDK6_5_546,2413,2527,2963,2801,1451,1780,2663,2986,2561
CDKL3_5_547,2523,3231,3957,2262,1190,3090,2742,4181,2516
CDKL5_5_548,3292,2824,4661,2438,2609,1417,2538,667,5396
CDKN1A_5_549,541,339,704,420,683,324,684,285,593

CDKN3_5_550,2533,3360,2787,3564,1765,1930,2030,3130,4196
CHEK1_5_551,3483,4165,4501,3544,3958,4278,1879,1988,2611
CHEK2_5_552,2855,3128,3684,2401,2664,1937,4273,2927,2630
CHKA_5_553,422,399,712,1051,103,2460,332,952,578
CIT_5_554,728,769,645,610,221,417,704,354,1911
CKMT2_5_555,225,200,316,312,35,772,786,325,642
CLK1_5_556,907,725,1499,965,1249,1077,596,1157,660
CLK3_5_557,154,441,411,44,203,853,671,256,53
CMPK1_5_558,445,1052,624,57,303,548,967,195,5
COASY_5_559,902,761,1099,2027,1221,624,540,487,517
COL4A3BP_5_560,1351,1166,2225,1431,1825,1246,1389,540,2227
CSK_5_561,704,678,777,640,649,843,1477,670,792
CSNK1A1_5_562,1463,2196,1331,1399,2362,1647,2362,2722,1402
CSNK1D_5_563,945,1097,616,2093,1290,266,538,417,748
CSNK1E_5_564,1022,1022,569,2043,809,1423,1142,308,725
CSNK1G3_5_565,1412,1915,1924,1445,1300,1391,987,2058,1124
CTDP1_5_566,1306,2411,1823,1703,2571,5199,1285,2759,1090
CTDSP1_5_567,1424,2304,2548,1779,1601,1971,2763,3114,2516
DCLK1_5_568,1019,1014,934,1058,857,808,1111,978,3039
DCLK2_5_569,708,631,567,448,1711,1049,643,126,717
DDR1_5_570,1285,803,1660,831,1960,1242,2023,1010,1777
DDR2_5_571,524,619,515,286,551,458,470,508,942
DGKA_5_572,3798,4655,5243,2801,5585,3611,2703,4401,3476
DGKB_5_573,529,933,630,121,667,279,742,195,846
DGKD_5_574,712,513,751,925,1424,1393,352,273,102
DGKG_5_575,166,437,340,248,30,324,165,62,1
DGKH_5_576,1447,2788,1838,999,2115,2670,1252,1184,765
DGKZ_5_577,355,603,435,607,19,197,133,23,21
DGUOK_5_578,3995,4112,3562,2720,3790,5780,5221,4206,3646
DLGAP5_5_579,252,482,489,272,561,966,539,330,137
DMPK_5_580,0,0,0,0,0,0,0,0,0
DSTYK_5_581,1233,1234,1663,1093,1708,2801,973,1745,2062
DTYMK_5_582,137,9,712,293,102,0,9,17,694
DUSP13_5_583,446,920,524,37,1407,101,903,1348,459
DUSP19_5_584,1730,2548,3071,1781,3275,3230,1557,809,1505
DUSP4_5_585,1316,1952,1531,2410,1000,1970,3198,1305,3004
DUSP6_5_586,226,149,205,298,62,172,484,325,0
DYRK1A_5_587,223,425,517,1163,21,1421,127,155,96
DYRK1B_5_588,1450,1329,1243,1169,2747,968,1442,1948,552
DYRK2_5_589,1513,791,1629,926,1741,1337,1862,1667,2520
DYRK3_5_590,1402,2154,2164,2063,1328,2661,2144,1514,2906
EFNA4_5_591,869,1395,1487,170,375,2545,847,256,625
EGFR_5_592,4576,5455,4033,4915,5348,3358,5618,4865,3807
EIF2AK2_5_593,0,0,0,0,0,0,0,0,0
EPHA10_5_594,382,170,462,9,388,267,349,5,640
EPHA3_5_595,345,172,327,1,77,275,113,253,1666
EPHA5_5_596,459,547,333,304,355,678,735,260,667
EPHA6_5_597,257,4,363,16,297,88,124,3,549
EPHA8_5_598,72,127,40,13,73,488,16,34,0
EPHB2_5_599,1519,846,2136,1186,2822,1868,1417,2615,1317

EPM2A_5_600,654,481,720,1591,5,1105,703,271,54
ERBB2_5_601,599,1160,715,687,1629,2467,1127,1073,245
ERBB4_5_602,1422,1752,2364,2379,1597,2794,1844,1670,707
ETNK1_5_603,3997,4313,6457,4025,4977,5031,5205,4759,3479
EXOSC10_5_604,522,499,276,18,1209,1543,250,387,2140
EYA1_5_605,228,141,401,537,55,26,8,112,24
EYA2_5_606,777,561,1169,499,771,1518,1621,1206,2394
EYA4_5_607,1562,2723,2135,1863,2230,1100,1801,2358,3283
FASTK_5_608,1660,2241,1546,1612,1850,576,1312,1345,1485
FBP1_5_609,519,922,740,166,397,691,742,130,1375
FES_5_610,970,1375,688,727,276,390,347,622,743
FGFR1_5_611,2600,2194,2923,3031,4592,2319,3417,3224,2782
FGFR2_5_612,1192,1865,2002,1944,2219,968,1866,1967,1932
FGFR3_5_613,1287,1921,2075,1904,2301,933,1940,1904,1945
FGFR4_5_614,931,851,1743,978,989,561,938,2333,1219
FGFRL1_5_615,580,608,689,409,99,6,681,69,7
FGR_5_616,1440,1504,1310,1892,3513,2363,2619,1332,1503
FLT1_5_617,2298,1431,1258,2676,2074,1689,1105,2931,1392
FLT4_5_618,898,675,2192,140,581,1164,711,484,696
FXN_5_619,757,289,955,664,693,1573,651,663,722
FYN_5_620,1156,811,1479,459,913,1019,1045,621,2697
FZR1_5_621,946,815,595,776,876,327,782,527,271
GALK2_5_622,532,1258,714,482,895,1628,831,822,40
GK_5_623,4952,3946,4962,3000,4328,6835,4006,3789,5183
GLYCTK_5_624,890,704,1083,23,169,652,145,906,1197
GNE_5_625,548,551,797,298,94,1464,304,219,435
GRK4_5_626,3326,4557,3773,4431,4913,3555,4383,3393,4966
GRK6_5_627,552,968,678,644,629,290,288,827,1124
GSK3B_5_628,1563,2023,1528,3679,2458,2617,2484,1394,2976
GUK1_5_629,318,203,416,1,2,220,225,369,0
HCK_5_630,475,527,481,751,224,284,53,666,116
HIPK1_5_631,184,245,75,231,241,316,366,207,217
HIPK2_5_632,974,1029,1366,865,717,1467,574,1053,2153
HIPK3_5_633,6422,5120,6402,6188,4099,6284,7435,6512,5396
ICK_5_634,2179,2335,3113,2600,4403,3223,1636,1974,5288
IKBKB_5_635,5725,5990,5285,4660,9268,4642,4412,4724,5383
IKBKE_5_636,637,604,599,228,1183,345,111,625,495
IKBKG_5_637,460,330,1014,239,98,565,188,407,1136
ILK_5_638,2096,1261,1870,562,2201,2115,2483,1453,2124
IMPA1_5_639,804,1203,149,1011,530,484,257,435,85
INPP1_5_640,1203,423,672,2352,903,1133,1172,620,1506
INPP4A_5_641,513,290,474,995,512,298,460,436,360
INPP4B_5_642,452,630,886,1047,832,729,772,442,853
INPP5D_5_643,184,291,304,9,2308,159,37,49,104
INSR_5_644,436,194,331,552,326,812,252,190,854
IP6K1_5_645,974,982,783,682,1395,914,275,838,205
IP6K3_5_646,183,1576,776,665,1408,996,625,219,985
IRAK1_5_647,123,63,264,54,0,273,161,37,16
IRAK3_5_648,1318,1595,1864,1083,3409,1199,1287,994,2457
IRAK4_5_649,892,604,978,336,632,678,599,783,1036

ITPK1_5_650,538,332,353,158,1216,471,151,106,196
KALRN_5_651,842,1040,1800,478,90,1118,840,582,476
KHK_5_652,97,254,88,84,53,101,391,88,303
KIF2A_5_653,283,316,421,1192,830,338,283,291,571
KIT_5_654,1167,694,1147,1225,1822,1933,1731,1369,272
LCK_5_655,1303,420,1400,1608,607,549,628,339,139
LHPP_5_656,2271,1273,4245,2218,2634,5587,2008,2029,2090
LIMK1_5_657,383,482,1104,348,368,411,216,98,2118
LIMK2_5_658,343,430,926,1756,82,831,250,311,1640
LTK_5_659,257,59,165,116,22,50,8,0,21
LYN_5_660,175,378,597,20,745,1246,366,128,726
MAGI3_5_661,206,827,249,74,1228,60,180,25,156
MAP2K3_5_662,158,25,220,90,5,43,101,42,443
MAP2K5_5_663,802,738,1070,1308,686,372,407,548,482
MAP3K12_5_664,188,61,281,332,0,1923,47,139,192
MAP3K13_5_665,157,258,626,1254,82,765,357,501,1159
MAP3K3_5_666,769,392,585,59,494,436,657,370,678
MAP3K4_5_667,883,627,700,1118,324,1686,911,781,1238
MAP3K7_5_668,1312,1323,881,1831,173,811,203,875,1263
MAP4K1_5_669,1356,1338,1338,2549,964,1262,1413,1598,1465
MAP4K4_5_670,1206,1236,1874,698,1317,2756,2195,1195,2219
MAP4K5_5_671,4010,2740,4166,1486,2698,3749,3627,2073,4888
MAPK10_5_672,899,345,1110,1535,825,338,1728,343,3870
MAPK14_5_673,5006,4580,6175,6551,9119,8403,3560,4387,6736
MAPK1_5_674,744,263,671,372,46,778,270,1058,1
MAPK3_5_675,976,1125,1075,149,1192,1082,622,712,636
MAPK7_5_676,180,58,94,124,3,1,11,101,74
MAPK8_5_677,968,1185,1724,202,872,990,268,1029,1032
MAPK9_5_678,608,338,989,1066,489,343,560,571,2530
MAPKAPK2_5_679,1376,1896,1528,1098,1731,1397,2066,2579,1580
MAPKAPK5_5_680,2109,1982,2092,1183,2405,2219,1831,1438,1544
MARK2_5_681,107,149,244,1052,43,224,103,48,30
MARK3_5_682,2487,1994,2090,2691,2088,2839,2712,3481,698
MARK4_5_683,844,937,1161,743,390,1015,844,1451,2744
MAST4_5_684,271,170,362,56,127,681,759,338,1
MASTL_5_685,1305,1698,2317,1495,1810,1874,1059,944,2226
MATK_5_686,299,431,742,164,693,121,701,111,148
MET_5_687,983,753,942,444,378,1594,608,1442,569
MINK1_5_688,553,773,986,224,197,69,550,135,19
MINPP1_5_689,10277,13895,15641,11396,11178,14148,13146,11652,11680
MKNK1_5_690,1113,797,997,738,905,601,1301,2032,2445
MKNK2_5_691,667,365,690,224,388,281,1438,107,1172
MLKL_5_692,1501,2064,3113,1163,2126,1934,1321,2406,3684
MTMR14_5_693,145,105,317,353,930,1,494,705,220
MTMR2_5_694,1013,1193,680,1594,648,393,1049,388,4
MTMR3_5_695,130,640,86,0,0,0,0,184,1
MUSK_5_696,1860,2122,1845,2691,1141,2288,1326,1087,5154
MVK_5_697,2090,2229,2643,3335,1588,2527,1900,1024,6735
MYO3B_5_698,767,1151,2804,520,1704,1570,895,591,2198
NADK_5_699,455,158,385,163,357,202,630,599,409

NCK1_5_700,1471,2019,1898,1649,2003,2309,1697,2351,2305
NCK2_5_701,236,334,485,263,517,978,473,717,783
NDRG1_5_702,1195,1209,1209,1161,741,1357,1225,668,3330
NEK11_5_703,7023,7639,8627,9221,11984,8574,7335,7319,9532
NEK1_5_704,1093,779,1729,1028,1012,3580,1411,908,2873
NEK2_5_705,1248,1180,1277,1325,3019,1000,1087,705,610
NEK3_5_706,394,569,926,2,1525,1565,495,25,52
NEK4_5_707,2129,1835,4397,2808,2315,2409,2486,2438,4292
NEK6_5_708,2242,2264,2155,2365,2022,2409,1725,1561,2238
NME2_5_709,1804,1967,1690,1710,1234,1360,1916,1337,1517
NME7_5_710,2531,1753,3003,2147,1582,1593,2806,1706,2780
NT5C1B_5_711,2231,2698,1314,822,3378,3229,1404,2396,2499
NT5C2_5_712,3886,3630,5003,4080,3135,4772,2762,3234,2473
NT5C3_5_713,3877,3792,3820,2916,5410,2709,4929,5292,2327
NT5E_5_714,462,545,1284,760,279,1041,433,718,76
NTRK1_5_715,583,375,769,42,236,788,556,304,40
NTRK2_5_716,2759,3999,5086,6222,3273,3220,3524,5904,3578
NTRK3_5_717,2076,1909,2729,2584,2507,4348,1698,2423,2868
NUDT4_5_718,2346,2272,2890,2305,3336,3347,3124,2258,1691
NUDT9_5_719,1340,1656,1365,2467,1738,1667,1639,1661,3120
OBSCN_5_720,1133,472,1637,1114,203,1610,811,792,1029
OCRL_5_721,2350,2415,2354,731,2725,1228,1381,1209,823
OXSM_5_722,756,951,1119,221,1866,927,210,272,1152
PACSIN1_5_723,481,367,341,16,316,110,505,1225,559
PAK4_5_724,1381,1621,2267,543,1200,1807,1046,734,1499
PAK6_5_725,656,797,670,285,73,1444,396,364,1622
PAK7_5_726,302,209,308,212,254,250,213,428,1
PANK1_5_727,313,369,444,455,239,209,396,27,620
PANK2_5_728,463,616,721,618,1017,1098,615,198,111
PCK2_5_729,1079,514,1100,331,377,625,1103,1178,2498
PDK2_5_730,200,313,387,2,230,820,145,134,727
PDK3_5_731,1230,1278,1693,1004,2234,1263,678,920,4897
PDPK1_5_732,846,750,917,772,692,757,741,788,1359
PEG3_5_733,339,155,296,661,1003,108,204,441,22
PFKFB2_5_734,1041,1742,1641,1836,2205,1100,1413,1680,1505
PFKFB3_5_735,474,1342,925,993,745,1257,1965,536,397
PFKM_5_736,914,1025,591,1554,251,1285,1095,232,713
PFKP_5_737,61,304,351,102,8,191,25,313,0
PHKA1_5_738,1420,2289,2647,2696,3549,1060,1116,1198,2071
PHKB_5_739,2056,3559,2818,1628,2136,1618,1710,3175,4359
PHKG2_5_740,536,335,261,1127,211,762,274,356,144
PHPT1_5_741,1478,1260,1796,398,1495,3312,2094,2192,2710
PI4KA_5_742,1006,1110,1083,891,1458,2021,1329,739,1406
PI4KB_5_743,8739,10957,11475,8748,11359,8227,8822,7990,12165
PIK3R1_5_744,137,4,512,307,9,1,680,0,1
PIK3R5_5_745,478,431,477,1315,627,331,616,553,12
PIKFYVE_5_746,1664,1633,2133,705,2538,1259,1069,1878,3046
PIP4K2C_5_747,2077,1383,1870,526,2646,3188,2577,1879,1687
PIP5K1A_5_748,658,463,1575,475,1472,1597,1254,800,1298
PIP5K1C_5_749,1630,1170,1428,1501,1135,1030,920,1594,1811

PIP5KL1_5_750,210,242,35,16,1,1027,5,91,10
PKLR_5_751,703,458,639,447,172,329,1081,312,480
PKMYT1_5_752,210,323,548,1,170,257,50,300,62
PKN1_5_753,501,270,1283,313,774,836,44,867,19
PLK4_5_754,1298,824,1071,2579,1714,1349,971,2187,1773
PNCK_5_755,213,375,728,1062,199,483,640,501,72
PODXL_5_756,382,562,199,0,825,140,138,2,0
PPAP2A_5_757,824,838,1715,81,808,380,756,1002,314
PPAP2C_5_758,122,94,118,282,86,16,58,38,65
PPEF1_5_759,404,412,846,623,1146,513,690,887,710
PPFIA1_5_760,1797,1145,1507,3873,466,1481,2027,1863,2029
PPFIA2_5_761,1830,2043,1784,2002,981,2368,1780,1777,2710
PPM1A_5_762,965,1682,679,971,807,439,1019,789,79
PPM1B_5_763,1314,2450,1827,599,3040,2148,1822,737,1756
PPM1M_5_764,871,705,1273,1105,358,734,1219,1062,766
PPP1CA_5_765,321,589,342,427,289,291,490,888,1165
PPP1CB_5_766,5583,4319,6215,6100,2902,6284,3603,3095,9991
PPP1R12A_5_767,807,1228,720,171,1422,368,1065,929,1096
PPP1R16B_5_768,139,353,584,244,170,44,980,719,17
PPP1R1B_5_769,171,20,195,25,0,459,26,206,0
PPP1R8_5_770,306,694,864,22,734,55,1353,1213,1301
PPP2R1B_5_771,3733,3219,5180,3034,3249,2269,2408,3810,2719
PPP2R2A_5_772,389,737,573,436,341,445,720,289,698
PPP2R2B_5_773,6928,7717,9239,8800,7674,9202,8267,5721,7645
PPP2R2C_5_774,874,562,656,1673,541,886,801,725,1143
PPP2R3A_5_775,3050,3371,3802,1242,1512,1760,2736,3684,7465
PPP2R4_5_776,368,825,343,264,279,430,907,205,212
PPP2R5A_5_777,1905,2065,2401,2520,2609,2165,1458,2387,3951
PPP2R5D_5_778,154,404,329,67,409,533,225,4,23
PPP3CA_5_779,3523,2891,4035,2634,3886,4146,3426,2473,6756
PPP3CB_5_780,1570,1856,1989,271,1776,2297,1991,1886,2378
PPP4R1_5_781,433,523,469,824,751,312,354,393,201
PPP5C_5_782,179,84,71,180,29,512,1,0,158
PPP6C_5_783,1162,822,1186,443,2955,363,1120,1517,680
PRKAA1_5_784,1244,2318,1827,1550,1791,4009,1958,1369,1099
PRKACA_5_785,289,255,379,104,10,244,115,299,65
PRKACB_5_786,1926,2712,1847,1323,2713,813,2347,2090,2395
PRKAG1_5_787,1283,742,1364,685,2255,2079,1547,666,4313
PRKAG2_5_788,8764,9724,10510,6502,10272,11192,7689,7768,16974
PRKAR1A_5_789,452,645,994,317,960,503,753,470,1138
PRKAR1B_5_790,1054,769,463,718,319,728,787,1763,1046
PRKCB_5_791,987,275,427,539,671,1412,1448,588,991
PRKCD_5_792,814,869,721,1140,635,1206,1004,1036,1413
PRKCQ_5_793,353,111,165,2,19,105,639,34,898
PRKCZ_5_794,826,528,1043,316,1047,762,395,466,773
PRKD2_5_795,220,56,286,178,2,40,53,2,44
PRKDC_5_796,1460,1855,2149,1926,1796,2829,1706,1376,2452
PRKG1_5_797,1544,1181,1209,405,1352,912,1499,1636,1320
PRPS2_5_798,5370,4437,8602,6026,6663,6597,5150,6101,8915
PTK2B_5_799,1054,773,1161,385,826,998,2012,622,781

PTK2_5_800,1536,1938,3012,2257,2591,2363,2692,2414,4430
PTK7_5_801,14,78,95,99,11,102,15,75,61
PTP4A2_5_802,3746,3054,4070,4306,4564,1759,2202,4811,2747
PTP4A3_5_803,226,448,155,47,464,1,54,28,467
PTPDC1_5_804,4749,5777,5592,5517,4895,5001,5959,5553,5289
PTPN12_5_805,1179,1373,1279,437,1728,829,1071,2334,956
PTPN13_5_806,966,1212,1412,285,1011,2121,1551,1283,2242
PTPN18_5_807,2000,1119,1733,864,1790,1882,1249,1490,905
PTPN22_5_808,3169,3573,5035,3886,5774,7532,3450,1388,5170
PTPN2_5_809,1135,830,931,733,727,761,925,146,1159
PTPN3_5_810,1742,1680,1037,811,2265,571,1398,1307,1273
PTPN5_5_811,165,104,147,420,40,0,575,25,47
PTPN6_5_812,433,354,427,985,457,431,425,138,1173
PTPN7_5_813,538,545,707,393,1599,538,1103,405,624
PTPRA_5_814,1304,1042,1188,2259,1908,1423,1161,777,774
PTPRB_5_815,1446,1541,1220,462,1278,1146,1443,37,1797
PTPRC_5_816,2276,3160,3455,3133,4267,2144,3644,4776,3276
PTPRD_5_817,2126,2371,2838,3029,643,2449,1770,1467,2533
PTPRE_5_818,719,939,1045,601,1905,1424,1458,1013,2006
PTPRF_5_819,92,194,84,20,51,18,40,61,1
PTPRH_5_820,2084,1439,940,1274,1046,1288,867,1740,711
PTPRJ_5_821,3221,2558,2649,3032,2966,1230,2848,2162,3285
PTPRK_5_822,498,0,370,28,503,432,343,28,0
PTPRM_5_823,247,230,229,260,172,279,985,518,80
PTPRN2_5_824,877,907,1395,694,22,3112,1080,925,3631
PTPRN_5_825,879,718,665,1358,348,237,477,1369,31
PTPRR_5_826,179,273,322,2,1,10,200,41,575
PTPRS_5_827,984,706,1188,169,755,771,823,727,567
PTPRT_5_828,451,161,101,253,84,129,189,154,125
PTPRU_5_829,642,565,1147,320,766,1602,1338,337,463
PTPRZ1_5_830,225,455,286,626,195,1162,595,642,612
RET_5_831,3590,3859,4531,2619,3974,6339,4324,3046,4247
RIOK1_5_832,1198,1383,2156,985,758,324,1119,1274,2072
RIOK2_5_833,4145,4900,5715,4778,3995,5708,3481,4731,4238
ROPN1L_5_834,931,308,834,497,966,1157,538,1012,553
RPS6KA1_5_835,911,632,1543,3,500,826,784,171,2109
RPS6KA2_5_836,1715,1318,2308,2644,1817,339,269,720,242
RPS6KA4_5_837,469,396,250,36,45,58,70,417,302
RPS6KA5_5_838,2448,2383,2751,1660,2075,2364,2162,2196,3620
RPS6KC1_5_839,3088,4093,3541,1792,1378,4081,2077,3080,3923
RYK_5_840,2314,2597,2693,1536,593,3786,1828,2828,3386
SET_5_841,2673,1944,2752,3349,2720,3066,1837,2380,2524
SGK1_5_842,665,883,630,1029,317,486,567,777,1011
SGK3_5_843,178,204,523,131,365,111,213,842,53
SIRPA_5_844,1595,1854,1551,2898,974,2144,1198,984,3087
SKAP1_5_845,3025,3105,3470,1520,3431,4080,2498,1699,3980
SNRK_5_846,6626,7829,8095,5148,6030,6167,6682,7459,5059
SPHK1_5_847,662,1041,1100,1750,1231,139,1199,1097,1244
SPHK2_5_848,227,264,117,14,489,70,571,73,268
SRC_5_849,264,47,337,1236,11,217,86,50,0

SRPK2_5_850,561,573,898,252,157,1521,570,776,321
SRPK3_5_851,301,246,204,722,67,169,355,70,714
SSH1_5_852,364,211,79,135,904,148,13,3,1817
STK19_5_853,893,1233,1124,287,1058,509,915,68,45
STK24_5_854,1293,1293,1598,914,2689,1030,763,978,1636
STK31_5_855,834,693,855,353,397,443,691,1050,462
STK36_5_856,81,101,23,33,14,60,134,148,5
STRADA_5_857,102,51,117,28,38,171,7,111,1396
STRADB_5_858,144,680,160,57,808,2,75,408,8
STYX_5_859,1139,780,1521,1179,378,645,1734,751,3283
SYK_5_860,1506,1010,1437,1678,756,1207,1947,891,945
SYNJ1_5_861,581,963,371,335,606,193,782,1227,212
SYNJ2_5_862,427,1052,611,1094,1197,1258,800,916,710
TAF1_5_863,1598,1971,1406,863,800,1077,1781,2309,2263
TAOK1_5_864,957,249,1111,478,948,911,567,580,218
TAOK2_5_865,240,458,249,5,178,1443,96,316,155
TBCK_5_866,1294,2083,1885,2956,1700,2338,2286,1418,3925
TEX14_5_867,843,1902,1234,823,243,1289,1219,907,1434
TGFBR1_5_868,167,770,520,893,590,420,78,661,0
TGFBR2_5_869,1657,1264,2250,1831,566,3087,989,908,1080
TGFBR3_5_870,809,1820,2131,1127,583,897,1149,604,2753
THTPA_5_871,267,323,529,30,934,320,557,191,650
TJP2_5_872,3558,4720,6541,3123,3257,4383,4029,4872,6138
TK2_5_873,1294,2214,3638,1595,2425,2417,2013,3954,3680
TLK1_5_874,1884,2567,2402,1325,2891,900,3170,1295,1097
TLK2_5_875,2250,2161,1944,2017,1538,1313,1520,2665,2804
TMEM134_5_876,1172,831,2679,952,1424,813,1645,1054,1607
TNIK_5_877,2397,2291,2912,1487,2053,1390,977,1826,2442
TNK2_5_878,1206,1123,1320,1040,781,2034,1020,2309,1075
TPK1_5_879,891,805,1174,1115,903,855,1458,335,381
TPTE2_5_880,6289,7129,13930,7376,6764,12744,9777,4655,11483
TPTE_5_881,452,252,511,173,759,841,1359,701,825
TRIM24_5_882,961,1520,1889,3445,1809,1170,273,1478,1254
TRIM33_5_883,1347,1578,1602,1973,1169,2306,2818,1424,1595
TRMT2A_5_884,46,47,40,225,323,0,18,0,257
TRPM6_5_885,1226,1419,1412,897,2774,598,1267,1487,1815
TSSK4_5_886,149,605,338,607,368,380,190,432,514
TTK_5_887,4150,4596,5255,2203,2779,5795,3307,3474,7973
TTN_5_888,344,1424,1401,71,2073,875,680,1291,1020
TWF1_5_889,2156,2248,2189,1600,1361,1950,1865,3828,3453
UCK1_5_890,831,1047,1236,957,1685,2719,828,606,76
UCKL1_5_891,482,390,813,656,28,153,554,174,531
UHMK1_5_892,1474,1849,2010,2431,2878,973,1546,665,2217
ULK2_5_893,4815,5392,5846,4819,5563,7922,3778,3613,6838
VRK2_5_894,987,1697,2503,2133,1698,1029,1416,1106,3154
VRK3_5_895,523,501,1324,924,252,1598,1056,469,657
WEE1_5_896,1836,2164,2404,2683,2832,2924,2211,2419,1906
WNK1_5_897,3006,2902,2547,1816,4076,2741,3146,5273,1556
WNK3_5_898,686,599,618,373,155,705,708,538,286
YSK4_5_899,1078,970,1897,583,1696,1977,1248,1013,1345

ZAP70_5_900,353,124,200,115,246,83,138,111,22
ACP2_5_901,352,468,203,1006,529,420,409,349,490
CDK1_5_902,6872,5802,7529,5411,6769,10406,7818,4747,6126
DUSP10_5_903,251,18,125,575,197,849,183,0,19
DUSP15_5_904,706,1028,1382,64,1067,66,303,980,352
ERBB3_5_905,803,848,973,845,649,845,1237,1131,1244
G6PC2_5_906,2706,2428,4117,2230,1402,3577,2399,3013,3253
IP6K2_5_907,1804,2365,2511,1250,1761,1888,1009,1107,3773
MYLK_5_908,498,573,873,781,1056,580,991,464,112
PRPS1_5_909,1831,905,2533,1632,1820,2620,2031,1936,2850
PTPMT1_5_910,76,155,813,442,285,470,5,60,336
PTPN20A_5_911,6458,5765,7687,6140,4592,6902,5299,7153,9121
PTPN20B_5_912,6458,5765,7687,6140,4592,6902,5299,7153,9121
SPEG_5_913,109,139,421,0,1,65,1386,7,890
STK32A_5_914,1396,1189,1809,1170,1251,1560,947,549,1079
PPP1R12B_5_915,592,626,557,92,322,1018,434,301,1218
PPP1R12B_5_916,2435,2557,2477,2753,3215,1794,1550,1383,4038
PPP1R12B_5_917,339,474,459,127,117,104,252,116,756
AAK1_5_918,74,24,37,52,33,65,26,59,9
ACP6_5_919,320,376,567,629,347,381,96,631,255
ACPT_5_920,564,1035,369,26,2463,616,649,380,142
ACVR2A_5_921,721,712,604,797,452,848,1036,885,421
ACVR2B_5_922,341,209,545,81,1399,608,134,492,780
ADCK2_5_923,2267,3265,2813,2353,2318,3198,2556,2205,2499
ADCK3_5_924,862,846,643,1025,993,647,847,354,300
ADCK5_5_925,16,127,349,175,34,562,130,615,11
ADPGK_5_926,1490,514,597,546,1027,685,69,466,6
ADRBK1_5_927,285,236,381,393,56,249,279,177,106
ADRBK2_5_928,524,564,851,738,85,1187,286,334,9
AGK_5_929,697,749,954,18,331,1454,456,1166,47
AK1_5_930,261,1175,244,72,1049,575,478,162,42
AK7_5_931,606,390,402,377,28,1140,645,24,0
AK8_5_932,52,2,146,0,2,0,1,0,176
ALK_5_933,709,636,810,374,802,1086,382,731,120
ALPI_5_934,36,73,153,35,0,138,285,201,11
ALPK2_5_935,606,779,392,16,531,421,280,833,933
ALPK3_5_936,931,368,1306,182,216,1808,1184,160,626
ALPP_5_937,1310,2648,1899,595,2808,1349,1546,1328,1863
ALPPL2_5_938,1310,2648,1899,595,2808,1349,1546,1328,1863
ANKK1_5_939,384,40,308,7,0,0,173,34,65
ARAF_5_940,455,342,399,840,1,589,565,1205,19
ATM_5_941,1457,1563,1402,1912,1116,1322,1495,1506,1637
AURKB_5_942,632,435,576,418,571,957,650,335,26
BLK_5_943,1913,1510,2538,1653,1668,2376,965,1788,6795
BMPR1A_5_944,667,628,427,282,899,620,1195,583,1728
BMPR1B_5_945,297,432,355,159,50,135,875,401,188
BMPR2_5_946,389,486,842,524,300,538,518,318,669
BPNT1_5_947,1060,1497,1915,1793,1754,672,1465,1295,2040
BRAF_5_948,1905,1873,2599,1691,1772,2613,723,2142,1775
BRD3_5_949,477,489,1082,593,1059,766,253,1018,217

BRSK1_5_950,1356,1180,1318,2137,725,802,1085,1451,674
BRSK2_5_951,994,1267,2105,462,1626,2129,742,5626,1263
BTK_5_952,218,393,336,1154,158,980,355,430,821
BUB1B_5_953,179,245,483,280,671,0,133,368,267
BUB1_5_954,369,194,302,129,434,838,117,838,839
C20orf111_5_955,517,82,159,1,260,804,516,311,1
C9orf96_5_956,77,272,332,107,0,49,0,3,0
CALM1_5_957,1160,1402,1107,878,26,1563,1463,1346,735
CALM3_5_958,664,558,787,740,597,662,670,695,1325
CAMK1_5_959,87,50,10,0,0,1,0,90,691
CAMK1G_5_960,1218,1342,1339,1466,439,790,1621,1276,557
CAMK2N1_5_961,333,455,302,46,440,83,963,289,50
CAMK4_5_962,462,776,955,25,3150,524,586,1135,1705
CAMKV_5_963,2737,3713,4913,3427,1721,4341,854,3308,6518
CARD11_5_964,1734,1496,2027,3067,2392,2023,2208,1990,4163
CCL2_5_965,461,583,465,758,152,1346,342,1772,78
CDC42BPB_5_966,1448,1043,1086,842,104,452,959,1499,2000
CDC42BPG_5_967,1044,1420,1659,360,656,2336,1278,990,2274
CDK14_5_968,2038,1720,3063,2026,3191,4315,1732,1291,3290
CDK15_5_969,2302,2611,2399,859,1185,796,1441,1151,2383
CDK19_5_970,467,230,247,0,99,443,302,10,133
CDK3_5_971,1371,733,2713,715,1375,1490,1407,1013,2480
CDK4_5_972,294,232,610,18,248,121,687,217,426
CDK5R1_5_973,762,879,806,1130,56,298,315,838,310
CDK7_5_974,4251,3859,4842,3734,9875,6505,2754,4185,7989
CDK8_5_975,1910,2025,3134,993,2020,2787,1647,947,3197
CDK9_5_976,1308,1368,2577,689,1893,2985,1778,1163,1239
CDKL1_5_977,782,1671,1399,1033,912,909,1100,1358,3202
CDKL2_5_978,1152,604,869,450,677,1256,692,350,1321
CDKL4_5_979,6823,8036,10856,5431,9250,8734,9296,8191,10578
CDKN1B_5_980,38,226,494,76,642,16,35,534,850
CERK_5_981,487,298,254,1155,695,879,942,921,13
CHKB_5_982,154,182,144,590,22,136,47,28,318
CHUK_5_983,2507,2855,3670,3545,3667,2677,2856,1643,2792
CIB2_5_984,1484,1349,1209,1703,1131,1266,1317,984,1174
CILP_5_985,518,214,417,254,717,189,1021,409,262
CKB_5_986,72,3,16,0,3,50,6,83,0
CKM_5_987,2214,2234,2404,2533,1908,2234,3390,3119,5154
CKMT1A_5_988,3679,4641,6615,2059,4290,5022,2934,4489,6312
CKMT1B_5_989,3679,4641,6615,2059,4290,5022,2934,4489,6312
CKS1B_5_990,13663,17571,19336,11593,14109,15932,14153,16478,26485
CKS2_5_991,664,834,1215,1516,467,1102,691,1370,1141
CLK2_5_992,254,410,704,135,649,1385,612,440,40
CLK4_5_993,1615,1717,1802,509,2300,927,1328,1385,693
CMPK2_5_994,2434,2852,4105,511,2396,5548,4489,2262,4385
CPNE3_5_995,404,840,540,23,557,640,1143,1185,222
CRIM1_5_996,281,511,956,106,311,128,462,1407,486
CRKL_5_997,34,136,415,27,0,12,16,281,15
CSF1R_5_998,478,1045,759,1437,1401,513,894,1005,1115
CSNK1A1L_5_999,1680,1987,2225,767,2016,2448,2074,2030,1769

CSNK1G1_5_1000,225,489,410,566,1569,1277,208,29,638
CSNK1G2_5_1001,248,315,313,537,713,85,96,442,414
CSNK2A2_5_1002,2655,3561,3778,2110,4557,2981,2554,1751,3848
CSNK2B_5_1003,506,98,289,1,900,0,0,201,0
CTDSP2_5_1004,274,682,997,767,141,518,480,351,2140
DAPK1_5_1005,1374,1750,1750,1698,1866,1411,2196,1598,1912
DAPK2_5_1006,384,704,537,229,1021,243,235,140,241
DAPK3_5_1007,337,434,312,283,17,20,385,53,0
DBF4_5_1008,1102,574,768,1302,1172,1230,914,717,462
DCK_5_1009,623,454,1071,500,943,602,451,338,1177
DCLK3_5_1010,650,797,1003,2464,1246,981,1333,311,1172
DGKE_5_1011,1246,1478,1751,2141,2544,3167,1750,1672,3178
DGKI_5_1012,566,631,1245,148,648,1409,866,491,790
DGKK_5_1013,234,608,773,515,670,112,798,235,383
DGKQ_5_1014,646,992,421,167,239,306,489,569,469
DOLK_5_1015,1211,1249,2332,892,693,1858,2519,813,3363
DOT1L_5_1016,1102,1349,1156,333,1657,1077,912,2736,1519
DUSP11_5_1017,1227,1447,1375,2691,984,1159,1223,2323,1941
DUSP12_5_1018,649,817,980,1052,441,274,826,1054,2415
DUSP14_5_1019,1678,1331,1796,2767,3507,3614,1245,926,2629
DUSP16_5_1020,3036,3485,3758,3478,4219,4269,2651,3860,2168
DUSP18_5_1021,2438,1778,2038,2839,1541,3039,1674,1758,1228
DUSP21_5_1022,1123,660,688,279,623,171,505,426,662
DUSP22_5_1023,802,1169,717,1734,174,1982,741,569,1618
DUSP2_5_1024,1061,930,975,642,585,365,1246,148,87
DUSP3_5_1025,102,1,122,87,115,5,126,106,119
DUSP5_5_1026,701,221,763,102,7,1,724,0,1231
DUSP7_5_1027,412,481,125,551,13,12,477,1,20
DUSP8_5_1028,3072,1328,2277,1998,771,4073,2444,2014,1177
DUSP9_5_1029,1830,1807,2117,1143,2065,2892,2311,1924,1624
DYRK4_5_1030,248,494,621,121,255,102,410,191,305
EEF2K_5_1031,194,237,290,72,0,121,63,212,111
EFNA2_5_1032,1015,1051,878,51,1223,726,1147,729,436
EFNA3_5_1033,444,649,364,283,810,229,1226,123,170
EFNA5_5_1034,4140,3813,5174,3616,2915,3443,3476,2154,2181
EFNB3_5_1035,1450,2213,2070,1328,2058,1185,2371,1906,1865
EIF2AK3_5_1036,371,279,350,448,1156,249,996,1046,771
EIF2AK4_5_1037,105,320,923,216,249,338,687,184,110
EPHA1_5_1038,514,658,480,330,321,994,116,82,89
EPHA2_5_1039,275,59,245,63,465,323,126,156,563
EPHA4_5_1040,1196,1718,1046,1781,2367,1084,561,2832,1195
EPHA7_5_1041,546,234,608,574,352,891,896,89,1481
EPHB1_5_1042,3613,3325,3675,3618,3636,4176,3301,3116,2416
EPHB3_5_1043,590,1485,1113,326,803,1045,784,445,951
EPHB4_5_1044,167,31,73,23,2,45,0,8,20
EPHB6_5_1045,888,975,1063,116,1920,633,1191,549,1103
ERN1_5_1046,3249,3148,3868,2088,3562,3886,2263,3464,2901
ERN2_5_1047,230,107,388,238,499,0,172,369,66
ETNK2_5_1048,466,358,955,382,75,201,956,751,26
EYA3_5_1049,535,476,771,334,151,4364,88,399,553

FER_5_1050,710,969,1184,1243,1083,435,1286,625,1500
FLT3_5_1051,731,72,718,1515,163,887,326,114,640
FN3K_5_1052,1686,3024,3643,920,2770,3718,2206,3763,2214
FN3KRP_5_1053,859,688,583,618,1895,3265,1242,554,324
FRK_5_1054,4079,5232,5737,4812,5343,5508,6083,3298,4487
FUK_5_1055,268,446,412,301,120,433,425,250,278
G6PC3_5_1056,418,395,598,31,248,142,1406,525,160
G6PC_5_1057,999,731,943,3306,113,536,588,1080,1322
GAK_5_1058,1264,1479,1513,1583,2733,514,923,1871,1235
GALK1_5_1059,2679,1913,1578,2543,3644,3157,1976,1289,1291
GDPD4_5_1060,5064,4262,6954,5945,4675,5736,5411,2752,2804
GOLGA5_5_1061,1081,1654,1678,1962,803,2149,798,1590,1649
GRK1_5_1062,6751,4994,5964,5866,4553,5877,6326,5755,5277
GRK5_5_1063,2,99,26,0,113,0,6,1,0
GRK7_5_1064,551,1288,1113,209,1190,2352,1135,669,1608
GSG2_5_1065,743,529,813,812,328,675,301,860,1458
GSK3A_5_1066,1793,1775,2717,1280,2580,3421,2460,1865,2976
GUCY2C_5_1067,1007,182,647,399,371,271,1328,507,181
GUCY2D_5_1068,2507,2995,4133,2859,2171,1868,2295,2905,1504
GUCY2F_5_1069,901,1518,1499,1061,1683,2554,656,750,674
HIPK4_5_1070,194,368,590,823,214,1041,141,389,8
HKDC1_5_1071,1250,1073,1072,124,321,1114,1004,1621,4066
HOOK3_5_1072,1605,1183,2008,478,610,3174,1093,1355,2239
HSPB8_5_1073,123,4,49,0,3,7,196,80,160
HUNK_5_1074,471,494,565,484,413,1196,525,518,1420
HUS1_5_1075,666,1157,1106,1238,1422,439,580,810,1482
IGBP1_5_1076,990,558,303,249,471,852,703,426,97
IGF1R_5_1077,1114,1302,1396,1255,346,574,2359,1333,1429
IGF2R_5_1078,154,363,460,244,54,560,211,236,395
ILKAP_5_1079,616,256,154,1719,833,639,148,127,112
IMPA2_5_1080,582,464,361,132,945,549,387,1110,105
INPP5A_5_1081,3994,4932,5374,3994,7277,9923,5103,4410,1819
INPP5B_5_1082,344,404,948,93,433,571,245,12,390
INPP5J_5_1083,874,1251,1233,2252,827,2416,1729,1276,1020
INPPL1_5_1084,171,169,246,444,210,33,117,18,363
INSRR_5_1085,1526,1380,1709,854,2345,1324,457,1190,1370
IPMK_5_1086,1426,2070,1832,1502,3787,1137,2168,631,1589
IPPK_5_1087,17,0,6,0,339,0,0,74,0
IRAK2_5_1088,286,317,430,11,106,841,14,56,41
ITK_5_1089,428,575,272,596,171,587,728,355,245
ITPKA_5_1090,887,214,639,226,610,1037,907,619,1665
ITPKB_5_1091,937,1010,1171,1750,1838,1540,1137,1734,913
ITPKC_5_1092,136,596,486,201,845,622,35,259,321
JAK1_5_1093,901,601,722,1215,642,2052,884,391,192
JAK2_5_1094,674,953,1127,271,757,1739,413,859,767
JAK3_5_1095,552,402,961,574,736,398,150,832,1294
KDR_5_1096,1324,1322,2125,1574,1037,1221,1010,1768,2286
KSR1_5_1097,387,103,95,36,382,231,15,25,95
LATS1_5_1098,963,518,484,194,1305,166,561,470,59
LATS2_5_1099,189,87,278,44,74,1021,666,2,0

LLGL1_5_1100,929,697,1747,916,2249,884,811,1566,1397
LMTK2_5_1101,1321,1432,1299,1241,595,2359,1240,1913,1626
LMTK3_5_1102,426,674,210,7,751,24,687,234,274
LRRK1_5_1103,1649,1368,2072,1085,2034,1383,1253,1208,503
LRRK2_5_1104,139,589,446,147,270,754,234,161,1
MAGI2_5_1105,732,717,916,189,575,587,375,623,1309
MAP2K1_5_1106,970,456,1172,736,92,910,1244,203,821
MAP2K2_5_1107,432,224,305,27,0,6,285,264,90
MAP2K4_5_1108,3513,3515,3832,4276,3469,5842,2931,3583,4453
MAP2K6_5_1109,1495,2203,1845,1659,3403,1983,1806,1409,2286
MAP2K7_5_1110,630,973,1581,1187,2687,814,388,1498,1243
MAP3K10_5_1111,445,530,998,131,4238,575,505,894,2238
MAP3K11_5_1112,138,101,213,0,0,24,0,365,0
MAP3K14_5_1113,298,291,702,681,116,504,140,975,437
MAP3K15_5_1114,557,488,774,792,420,23,1240,910,155
MAP3K1_5_1115,6678,6568,8243,4799,7548,7338,5935,8656,6600
MAP3K2_5_1116,2615,2203,3201,1085,2873,1907,2684,3131,4085
MAP3K5_5_1117,750,1203,966,263,660,959,968,503,1585
MAP3K6_5_1118,338,471,461,129,283,6,47,777,147
MAP3K8_5_1119,3810,2928,4610,3449,3134,2285,4625,3786,5008
MAP3K9_5_1120,359,701,399,142,426,675,710,627,684
MAP4K2_5_1121,515,1304,695,26,1463,133,502,523,659
MAP4K3_5_1122,2752,2518,3741,1075,1058,3169,999,2259,2984
MAPK11_5_1123,138,28,274,200,336,132,0,6,313
MAPK12_5_1124,655,381,976,7,361,600,57,484,1162
MAPK13_5_1125,716,1393,786,2514,1045,567,841,875,797
MAPK15_5_1126,0,0,0,0,0,0,0,0,0
MAPK4_5_1127,384,350,460,37,360,972,318,393,54
MAPK6_5_1128,426,76,116,4,0,25,446,0,0
MAPKAPK3_5_1129,2067,1159,1248,738,968,142,2483,1340,1094
MARK1_5_1130,224,349,983,207,271,398,130,30,5
MAST2_5_1131,254,227,352,143,86,286,37,10,11
MAST3_5_1132,135,123,92,64,38,133,530,423,0
MELK_5_1133,1612,1398,1373,1023,1098,702,1670,2394,3485
MERTK_5_1134,7826,10299,10363,6426,10521,7704,8529,7866,8892
MEX3B_5_1135,939,828,1243,927,1168,941,475,325,1343
MPP3_5_1136,876,1411,1428,111,549,421,427,835,419
MPP5_5_1137,697,352,1166,263,120,155,827,457,1662
MST1R_5_1138,327,318,361,540,19,0,791,840,234
MTM1_5_1139,7531,8553,9178,10063,7645,12287,8551,10134,9248
MTMR1_5_1140,2201,2738,2654,1812,1441,1779,1817,2660,2765
MTMR4_5_1141,797,800,804,57,435,544,321,214,722
MTMR6_5_1142,1034,941,443,348,675,411,1873,1269,363
MTMR7_5_1143,3323,3727,3399,3000,2375,1999,2767,2114,1138
MTMR9_5_1144,2095,1813,2431,524,1257,1834,1332,1493,2183
MTOR_5_1145,1557,1680,1932,955,1257,654,1146,2558,1524
MYLK2_5_1146,278,861,440,519,210,799,539,558,138
MYLK3_5_1147,52,117,94,0,11,14,32,341,127
MYLK4_5_1148,116,227,181,136,172,269,38,107,832
MYO3A_5_1149,104,99,122,37,257,263,77,4,9

N4BP2_5_1150,1932,1709,2181,1114,1429,2739,2157,1501,420
NAGK_5_1151,694,465,655,678,571,367,287,256,22
NAGS_5_1152,705,1243,799,666,342,307,989,951,1685
NEK10_5_1153,1251,1371,1428,1642,1289,468,1005,802,249
NEK5_5_1154,1163,827,1717,1067,654,820,587,793,2091
NEK7_5_1155,3875,5149,8222,5097,4291,4580,6255,4758,9187
NEK8_5_1156,156,160,169,292,0,58,169,153,0
NEK9_5_1157,509,303,215,211,133,450,801,299,21
NME3_5_1158,228,187,232,0,97,99,142,252,69
NME4_5_1159,214,158,251,0,185,16,1,1,15
NME5_5_1160,3045,3178,3279,3006,2524,1410,2862,3400,2964
NME6_5_1161,149,210,658,135,484,391,448,426,601
NPR1_5_1162,1272,1628,1281,1787,2011,456,1596,2042,1502
NPRL2_5_1163,176,52,243,0,207,584,263,549,463
NRBP1_5_1164,1381,1455,1984,1007,2014,3158,1890,780,3237
NRBP2_5_1165,679,604,721,1306,789,495,859,857,1008
NRK_5_1166,2278,2657,2996,2373,5580,2515,2489,1638,2028
NT5C1A_5_1167,280,636,548,246,264,275,344,1349,461
NT5C_5_1168,724,1223,1148,400,2313,664,847,1080,481
NT5M_5_1169,2,16,266,0,1,993,0,213,606
NUAK1_5_1170,521,199,264,4,0,638,208,8,0
NUAK2_5_1171,501,514,640,68,1064,446,226,636,1496
NUCKS1_5_1172,1019,923,1392,815,890,853,1302,1370,503
OXSR1_5_1173,1320,1140,1289,174,1237,934,1108,332,494
PAK2_5_1174,4048,4019,4947,2285,7945,3923,4217,3740,2972
PANK3_5_1175,3195,3214,3418,3380,1877,5615,2818,4024,3312
PANK4_5_1176,1928,2551,1673,1369,2498,3558,2423,1518,1958
PASK_5_1177,533,917,1018,198,589,1260,1482,277,546
PBK_5_1178,1314,1047,634,29,974,1028,543,965,892
PCK1_5_1179,566,594,704,1066,641,887,1366,463,763
PDCD1_5_1180,210,300,334,2,5,37,281,29,788
PDGFRA_5_1181,1373,1571,1706,1248,1735,1486,1036,1377,1726
PDGFRB_5_1182,495,460,699,1036,916,1661,640,368,769
PDGFRL_5_1183,8447,8506,10122,9147,7668,10285,7823,7393,7996
PDIK1L_5_1184,584,373,619,204,2127,1323,964,217,567
PDK4_5_1185,168,487,52,0,15,592,84,1,12
PDP2_5_1186,276,139,67,0,0,128,675,0,1
PDXK_5_1187,148,425,179,240,187,126,538,110,884
PFKFB1_5_1188,114,68,166,2132,70,502,582,139,302
PFKFB4_5_1189,48,173,319,482,31,15,18,58,486
PFKL_5_1190,1943,1900,2033,542,2850,554,1389,1792,2452
PGAM2_5_1191,497,659,514,384,2137,33,247,421,343
PGK1_5_1192,1015,2253,2265,2972,618,1430,1413,2180,2223
PGK2_5_1193,897,982,1058,575,1299,1410,569,94,752
PHKA2_5_1194,5058,6738,6349,6249,5698,6103,3430,5091,5622
PHKG1_5_1195,531,646,576,3795,319,174,240,661,932
PI4K2A_5_1196,632,717,924,1156,1079,1290,896,790,1344
PI4K2B_5_1197,1641,1936,2362,2305,2269,2694,2654,1389,2557
PIK3C2A_5_1198,1012,1003,1180,941,486,1237,1415,869,1162
PIK3C2B_5_1199,29,138,74,6,4,2,200,231,0

PIK3C2G_5_1200,3222,2304,2818,585,1293,3198,879,947,2034
PIK3C3_5_1201,1416,860,1188,669,674,591,993,1105,1466
PIK3CA_5_1202,296,523,352,400,71,734,1072,1877,103
PIK3CB_5_1203,1922,2227,1499,856,1198,1331,1353,2009,2600
PIK3CD_5_1204,117,203,441,35,558,41,191,52,7
PIK3CG_5_1205,1550,1396,1953,2168,1079,1102,1417,1299,1483
PIK3R2_5_1206,4514,4644,5334,3090,2055,4949,3465,3963,6019
PIK3R4_5_1207,871,588,402,767,951,1689,828,591,1396
PIK3R6_5_1208,557,306,1193,332,1465,755,655,1018,276
PIM2_5_1209,542,404,617,81,736,719,1104,231,1297
PIM3_5_1210,619,395,1146,2091,288,804,365,56,31
PINK1_5_1211,534,469,754,996,1260,559,1075,387,363
PIP4K2A_5_1212,457,584,1344,1216,801,568,844,2257,629
PIP4K2B_5_1213,133,422,397,47,7,541,295,237,11
PIP5K1B_5_1214,4026,3712,4035,2835,3773,3032,5038,3386,4812
PKDCC_5_1215,127,142,367,134,499,112,214,369,31
PKN2_5_1216,4104,3976,4814,2640,2216,3073,6306,2485,4195
PKN3_5_1217,305,416,178,499,4,1,461,354,444
PLK1_5_1218,564,561,1558,987,211,1288,1125,621,1854
PLK2_5_1219,390,467,312,795,103,1076,1218,344,858
PLK3_5_1220,661,857,928,3,868,1428,535,492,769
PMVK_5_1221,15,117,135,1,562,416,0,148,47
PNKP_5_1222,24,558,784,7,277,2,826,335,1265
POLD1_5_1223,920,449,782,89,629,197,139,33,835
PON1_5_1224,542,535,651,367,487,844,353,646,46
PPAP2B_5_1225,464,761,429,601,1471,493,474,735,278
PPEF2_5_1226,2441,2107,3182,1368,3096,2926,2812,2211,4112
PPFIA3_5_1227,301,332,533,28,110,137,242,235,6
PPFIA4_5_1228,132,43,260,33,0,690,127,365,30
PPM1D_5_1229,334,567,785,371,699,1177,122,592,37
PPM1E_5_1230,1613,1804,2121,1908,1527,2860,2493,1294,2453
PPM1F_5_1231,1166,386,1180,1492,229,892,446,137,1560
PPM1G_5_1232,111,482,94,763,360,1415,455,151,83
PPM1K_5_1233,771,568,786,1635,137,56,1105,723,749
PPM1L_5_1234,704,757,786,599,348,643,352,1101,680
PPP1CC_5_1235,3420,2306,3337,3853,4067,2666,2766,2975,3768
PPP1R12C_5_1236,202,20,261,2,565,424,46,169,0
PPP1R13B_5_1237,2808,2805,3133,2019,2695,4111,1562,2288,5415
PPP1R14B_5_1238,612,314,1394,2,594,515,708,257,6
PPP1R15B_5_1239,972,2690,2177,1883,2065,1381,1552,2041,1066
PPP1R1A_5_1240,267,388,341,11,265,297,279,216,742
PPP1R1C_5_1241,426,744,691,80,855,557,683,430,225
PPP1R2_5_1242,666,1074,943,237,1229,1779,925,654,1777
PPP1R3A_5_1243,1347,634,1766,1189,2126,1647,1697,583,1370
PPP1R3C_5_1244,3183,2737,3324,3467,2770,3223,3441,3226,2298
PPP1R3D_5_1245,332,566,615,677,1123,361,319,465,1517
PPP1R7_5_1246,293,873,425,0,60,5,573,31,11
PPP2CA_5_1247,360,267,384,187,233,520,583,344,180
PPP2CB_5_1248,508,1059,1048,669,489,478,1373,1315,679
PPP2R1A_5_1249,520,948,831,262,419,1233,359,154,796

PPP2R2D_5_1250,1279,1632,1719,805,1447,738,1539,1873,330
PPP2R3B_5_1251,1938,1612,2180,599,470,3719,1423,2007,757
PPP2R5B_5_1252,336,276,338,83,51,126,149,951,37
PPP2R5E_5_1253,4211,3746,3928,3316,2247,5642,2020,4365,3191
PPP3CC_5_1254,657,556,525,528,874,1271,668,332,159
PPP3R1_5_1255,417,773,554,215,235,622,271,189,288
PPP3R2_5_1256,74,125,121,0,931,287,344,0,0
PPP4C_5_1257,1062,1816,1770,812,1717,1824,459,1253,1777
PPTC7_5_1258,1338,1583,1583,1291,1957,3178,1763,662,2801
PRKAA2_5_1259,693,268,554,1077,569,1378,694,244,391
PRKAB1_5_1260,1078,761,1501,245,366,1000,662,556,496
PRKAB2_5_1261,1917,1269,1557,444,2992,1259,1165,1220,1311
PRKACG_5_1262,46,180,165,1,606,0,18,63,1052
PRKAG3_5_1263,239,569,103,0,0,1,16,334,41
PRKAR2A_5_1264,2856,4020,3105,1872,4191,5207,2357,4928,3100
PRKAR2B_5_1265,4044,3716,5347,4747,6164,3604,4956,4207,7799
PRKCA_5_1266,1149,1817,1215,1397,1881,505,1282,995,2312
PRKCE_5_1267,157,133,612,29,79,0,5,179,559
PRKCG_5_1268,985,1869,2316,896,860,4791,1886,2034,3638
PRKCH_5_1269,1189,1735,1406,447,969,1873,1317,1597,598
PRKCI_5_1270,2924,3918,3423,2242,466,6117,1916,3555,1750
PRKD1_5_1271,906,912,1213,1097,310,762,389,615,1408
PRKD3_5_1272,533,232,1103,5,34,1501,484,168,1
PRKG2_5_1273,801,816,821,68,664,1637,1083,564,501
PRKX_5_1274,904,662,931,158,215,162,64,415,0
PRPF4B_5_1275,342,359,173,32,910,168,376,1019,200
PRPS1L1_5_1276,3657,3294,3810,3779,3628,3256,4079,3016,8722
PSKH1_5_1277,143,592,535,417,5,16,19,178,441
PSKH2_5_1278,72,228,236,0,123,331,404,110,892
PSPH_5_1279,374,942,972,184,681,599,1017,1405,933
PSTK_5_1280,946,452,895,143,648,500,785,565,208
PTEN_5_1281,626,711,940,333,663,1105,145,991,1243
PTK6_5_1282,295,1072,496,23,622,184,555,471,483
PTP4A1_5_1283,207,523,478,903,430,305,281,153,790
PTPLA_5_1284,1005,827,1797,664,3630,1601,1163,1252,1096
PTPLB_5_1285,921,693,835,109,451,671,1285,1303,396
PTPN11_5_1286,153,109,284,926,65,2,635,36,107
PTPN14_5_1287,30,176,172,71,350,127,0,38,7
PTPN1_5_1288,752,933,1275,211,441,1068,1576,477,324
PTPN21_5_1289,2076,1553,1550,910,1642,942,1733,2712,1249
PTPN23_5_1290,1518,1056,1585,2147,2249,645,1463,1292,2622
PTPN4_5_1291,1838,2521,2290,2595,1228,2077,2023,1983,3195
PTPN9_5_1292,235,262,166,54,15,563,398,14,13
PTPRG_5_1293,0,0,0,0,0,0,0,0,0
PXK_5_1294,2324,2553,3207,2171,1841,2465,2515,1855,2939
RAF1_5_1295,221,315,623,347,172,1620,415,46,1384
RBKS_5_1296,465,1225,1374,1217,284,717,54,949,314
RFK_5_1297,2245,2777,3146,1672,3741,1493,1508,1351,2968
RIOK3_5_1298,3246,1816,2874,2601,1721,1887,2087,1627,2459
RIPK1_5_1299,867,425,1203,334,2194,635,928,617,1862

RIPK2_5_1300,434,656,631,1229,105,21,588,586,475
RIPK3_5_1301,3054,3169,2869,3377,4765,2199,2727,2687,1917
RIPK4_5_1302,931,544,490,499,992,808,294,880,2478
RNASEL_5_1303,1384,1438,1416,1592,1148,1826,1558,961,1391
RNGTT_5_1304,2376,4924,2923,3400,5573,1033,2165,2855,1630
ROCK1_5_1305,2475,3241,3326,2640,4082,3701,3034,2826,2172
ROCK2_5_1306,774,1005,745,704,606,1733,201,526,602
ROS1_5_1307,2643,2076,2304,1311,2795,2753,2687,1158,1363
RPS6KA3_5_1308,3976,3364,3374,3012,2082,3126,3197,3867,1484
RPS6KA6_5_1309,2261,1771,1584,144,1041,1297,2733,1786,670
RPS6KB1_5_1310,3454,3530,2664,3999,3956,1754,1831,2355,3633
RPS6KB2_5_1311,1903,1806,3153,4466,1351,1625,1041,3820,2015
RPS6KL1_5_1312,1107,1052,1164,1493,1712,538,483,351,2269
RSPO3_5_1313,1963,2520,1729,3287,2003,1692,1628,2217,1046
SACM1L_5_1314,1392,1755,2094,945,1067,738,1829,1860,496
SBF1_5_1315,809,989,784,717,514,1244,1021,1108,212
SBK1_5_1316,2765,2663,3802,2507,2258,3874,2458,1860,3861
SBK2_5_1317,204,332,245,0,344,554,21,270,0
SEPHS2_5_1318,794,1121,1008,1213,1073,901,153,1324,3290
SGPP1_5_1319,21527,23983,27223,23572,24176,21927,22065,19357,28447
SHPK_5_1320,355,461,497,342,654,858,346,298,433
SIK1_5_1321,449,402,276,1058,101,1037,489,151,735
SIK2_5_1322,447,729,1348,1251,197,1239,521,356,1009
SIK3_5_1323,350,579,374,53,360,913,229,828,826
SKAP2_5_1324,1213,1874,2689,266,1803,711,1238,1475,1556
SMG1_5_1325,685,524,761,368,3,501,131,734,174
SRMS_5_1326,530,390,1127,229,729,1183,608,1519,122
SRP72_5_1327,1866,1802,2819,1778,1870,2303,1010,1883,1469
SRPK1_5_1328,2343,2637,3581,3779,2798,2475,1957,1707,4279
SSH2_5_1329,891,549,1017,86,818,312,516,531,697
SSH3_5_1330,137,15,46,0,0,660,1,2,510
STC1_5_1331,219,151,213,412,137,99,126,67,334
STK10_5_1332,222,239,160,41,204,0,226,31,92
STK11_5_1333,329,252,217,39,623,499,289,1,0
STK16_5_1334,1188,2365,2599,1141,3874,1860,1285,2636,3421
STK17A_5_1335,4032,3225,4095,3129,3579,5389,4283,3254,8557
STK17B_5_1336,3731,4439,4991,3142,1212,3502,4054,3759,4337
STK25_5_1337,44,224,40,0,34,5,2,7,0
STK32B_5_1338,58,73,259,387,533,47,42,302,14
STK32C_5_1339,483,440,266,334,479,451,499,37,3
STK33_5_1340,287,441,434,572,375,1732,939,335,895
STK35_5_1341,292,29,142,107,1,291,457,156,581
STK38_5_1342,384,450,249,11,237,81,281,175,529
STK38L_5_1343,2473,2073,2750,3091,2781,1317,2331,2311,2313
STK39_5_1344,2011,2837,2628,4085,2144,4309,3348,1623,2368
STK40_5_1345,566,143,609,14,338,174,109,508,85
STK4_5_1346,1493,1908,2075,928,2998,1155,824,1970,711
STYK1_5_1347,100,149,93,0,354,1,38,33,2
STYXL1_5_1348,204,271,66,327,140,244,344,266,188
TAF1L_5_1349,4104,3872,4470,3863,4854,5556,4054,4247,5505

TAOK3_5_1350,1864,1705,1371,1917,2641,1367,1169,1380,3964
TBK1_5_1351,2485,1900,3336,1921,987,1091,3060,2393,5217
TEC_5_1352,7029,5878,6460,6135,6832,6680,7719,7056,5200
TEK_5_1353,542,516,713,200,776,889,799,653,1558
TESK1_5_1354,50,86,132,0,274,0,395,44,3
TESK2_5_1355,217,362,684,100,1293,5,24,1,302
THNSL1_5_1356,441,502,428,27,216,231,138,561,84
TIE1_5_1357,499,572,396,310,141,184,59,59,6
TK1_5_1358,31,407,154,7,17,540,121,25,245
TNK1_5_1359,185,406,511,184,584,910,235,156,14
TNNI3K_5_1360,796,645,442,252,795,130,1412,879,611
TNS3_5_1361,451,163,617,472,91,558,146,319,528
TP53RK_5_1362,617,672,740,150,464,356,193,405,2466
TRAT1_5_1363,4411,4744,3933,4506,4966,3640,4514,3292,3840
TRIB1_5_1364,61,133,19,2,17,100,47,0,17
TRIB2_5_1365,453,198,315,534,597,332,435,345,573
TRIB3_5_1366,98,16,154,592,1,1,0,542,0
TRIM27_5_1367,1687,2336,3075,2031,1959,2189,3231,3540,3843
TRIM28_5_1368,572,1059,1382,250,855,397,696,209,439
TRIO_5_1369,1856,1945,1599,1966,1699,2574,1785,2824,2184
TRPM7_5_1370,395,431,380,89,763,372,186,44,157
TRRAP_5_1371,1142,945,1627,811,1562,989,882,1188,914
TSKS_5_1372,1038,1306,1556,1167,1510,972,855,1079,2922
TSSK1B_5_1373,801,541,616,51,944,960,432,458,322
TSSK2_5_1374,1922,3530,2918,1911,4574,2698,1883,2027,1929
TSSK3_5_1375,826,1119,934,416,86,1371,585,2547,251
TSSK6_5_1376,1285,958,524,818,1520,959,1111,187,430
TTBK1_5_1377,662,700,320,2739,478,87,1144,241,9
TTBK2_5_1378,451,302,409,130,780,537,615,214,29
TWF2_5_1379,876,827,1194,170,1278,191,1053,509,477
TXK_5_1380,904,1163,1043,974,1713,1040,886,1787,391
TYK2_5_1381,131,130,337,0,46,113,410,69,835
TYRO3_5_1382,486,804,1029,1085,1699,766,227,73,1339
UBLCP1_5_1383,3851,3371,3480,2738,3821,3341,1511,2054,2981
UCK2_5_1384,342,832,1494,963,1767,2297,1003,491,734
ULK1_5_1385,386,112,164,98,11,15,896,41,9
ULK3_5_1386,304,391,314,214,176,802,246,505,307
ULK4_5_1387,737,989,1337,579,1667,359,444,1200,531
VRK1_5_1388,782,451,948,270,1614,941,531,290,1234
WEE2_5_1389,442,405,1026,50,730,1081,1009,172,285
WNK2_5_1390,258,227,530,192,59,406,631,304,2
WNK4_5_1391,350,438,214,1,4,115,4,0,0
XRCC6BP1_5_1392,2176,3441,3376,1306,4463,2961,2075,1810,2098
XYLB_5_1393,334,356,682,919,444,102,1301,278,890
YES1_5_1394,943,915,1160,1129,1587,261,1362,184,1618
AATK_5_1395,274,189,181,154,197,96,6,204,131
ABL1_5_1396,214,453,855,432,602,781,207,483,18
ABL2_5_1397,361,601,1014,1,136,1102,794,668,17
ACP1_5_1398,556,412,485,283,1581,1497,1407,914,309
ACP5_5_1399,1573,2635,2622,1849,1099,3208,996,2780,5150

ACPL2_5_1400,192,17,154,14,8,446,58,43,73
ACPP_5_1401,329,118,212,1,260,43,400,142,339
ACVR1B_5_1402,101,145,170,407,527,395,29,74,132
ACVR1C_5_1403,768,551,665,812,444,654,457,439,2592
ACVR1_5_1404,407,754,500,27,652,1648,520,500,695
ACVRL1_5_1405,28,18,125,6,0,0,40,0,2
ADCK1_5_1406,114,486,1041,57,40,330,272,120,117
ADCK4_5_1407,39,412,241,305,402,239,4,458,128
ADK_5_1408,387,510,973,885,360,1914,431,332,982
AK2_5_1409,18,1,6,371,0,33,17,11,0
AK4_5_1410,2005,1294,2214,1648,2776,1199,2095,839,628
AK5_5_1411,4323,3876,5032,4110,3206,4800,3257,4235,3282
AKT1_5_1412,216,49,96,0,39,196,16,0,0
AKT2_5_1413,528,1060,1154,2164,1021,972,1028,651,44
AKT3_5_1414,1466,1977,1551,730,2382,2443,1037,823,542
ALDH18A1_5_1415,508,1230,1076,901,553,524,1397,2545,669
ALPK1_5_1416,2828,3587,3197,1937,1919,4347,3385,4502,4562
ALPL_5_1417,1050,251,797,613,191,561,1270,782,415
AMHR2_5_1418,244,129,331,130,88,141,154,33,243
ANKHD1_5_1419,1334,1421,1730,565,1030,977,3225,1231,1039
APTX_5_1420,6180,8294,8665,5735,5520,4886,6892,6112,4816
ATRIP_5_1421,604,570,522,622,134,543,594,314,80
AURKA_5_1422,1689,1813,1848,1901,1909,2448,1516,2868,1356
AURKC_5_1423,99,893,331,0,3,49,50,677,699
AXL_5_1424,2224,1709,4343,441,3407,3047,1576,2482,2666
BAIAP2_5_1425,687,554,834,39,8,714,813,593,2020
BCKDK_5_1426,1547,1573,2087,1352,1200,2942,1029,1221,2279
BCR_5_1427,127,243,336,1167,0,457,14,1,2
BMP2K_5_1428,516,997,1147,1632,1330,595,433,1259,339
BMX_5_1429,367,963,644,1037,361,1267,175,126,480
BPGM_5_1430,4324,5241,5588,5774,4688,4304,3653,1468,4810
BRD2_5_1431,476,610,367,288,554,588,443,224,256
BRD4_5_1432,1878,2129,1834,636,2333,1918,1569,1631,651
BRDT_5_1433,789,715,777,1038,939,1403,995,1007,1193
CAB39L_5_1434,1938,2797,3039,5091,1899,2727,2778,2235,2507
CAMK1D_5_1435,731,682,383,689,360,297,402,435,173
CAMK2A_5_1436,2954,3354,4484,2413,2594,6236,3257,2522,5240
CAMK2B_5_1437,699,1408,1166,486,1245,976,1229,1882,1746
CAMK2D_5_1438,727,610,2376,2075,473,948,935,722,1204
CAMK2G_5_1439,466,868,582,1632,520,1264,732,1050,71
CAMKK1_5_1440,1601,1436,2283,2094,994,1450,3360,1423,240
CAMKK2_5_1441,2060,2269,2145,1683,1170,1260,1925,2369,2973
CASK_5_1442,1774,1020,1613,1277,1356,3039,2303,1154,2420
CCT2_5_1443,861,806,1384,618,552,1538,423,916,638
CDADC1_5_1444,628,1348,1935,424,146,483,387,448,167
CDC14A_5_1445,2027,1995,2569,1592,549,1603,1771,1821,3110
CDC14B_5_1446,34,405,116,235,48,7,15,6,9
CDC25A_5_1447,993,1230,1668,333,1071,1078,2233,663,1959
CDC25B_5_1448,985,1606,816,484,714,872,94,983,2415
CDC25C_5_1449,1383,1267,2571,1853,1121,1616,927,1950,1931

CDC42BPA_5_1450,1713,2506,1639,2915,3316,649,1637,1001,4361
CDC7_5_1451,4485,4714,5119,2318,4122,4313,4168,5953,3038
CDK10_5_1452,99,160,183,12,145,18,260,175,10
CDK11A_5_1453,837,574,807,1100,175,1025,880,622,2741
CDK11B_5_1454,519,386,745,162,743,1157,286,993,8
CDK12_5_1455,1463,1266,1704,909,864,530,1617,1081,274
CDK13_5_1456,474,488,762,194,666,967,514,1218,479
CDK16_5_1457,1347,1040,1260,1664,1910,2044,1031,757,384
CDK17_5_1458,727,623,795,153,377,419,735,1704,69
CDK18_5_1459,2499,3137,2616,3624,2188,3783,3584,2569,7783
CDK20_5_1460,43,152,474,22,55,1250,13,142,46
CDK2_5_1461,82,92,24,0,5,454,1,1,0
CDK5_5_1462,579,674,842,5,2,141,419,889,263
CDK6_5_1463,359,331,464,143,231,463,478,559,170
CDKL3_5_1464,415,635,442,534,813,745,234,470,1392
CDKL5_5_1465,741,793,473,223,1060,1044,1449,736,428
CDKN1A_5_1466,993,1029,1442,554,183,895,1650,688,2197
CDKN3_5_1467,1227,302,569,830,2767,1057,228,193,1097
CHEK1_5_1468,556,326,676,1548,1226,968,160,967,1019
CHEK2_5_1469,171,210,490,527,4,1082,167,220,343
CHKA_5_1470,2897,2925,3178,4422,2057,3619,4661,2711,3949
CIT_5_1471,719,894,1541,1339,1497,1206,493,1943,373
CKMT2_5_1472,848,781,1229,309,963,2037,952,963,1455
CLK1_5_1473,703,790,754,8,333,6,463,683,222
CLK3_5_1474,131,222,172,38,4,254,117,23,4
CMPK1_5_1475,557,654,747,610,562,385,592,278,77
COASY_5_1476,551,898,781,601,1005,905,632,431,1201
COL4A3BP_5_1477,1660,1853,2885,1687,2281,2129,1073,921,2092
CSK_5_1478,908,444,1274,56,86,62,507,494,601
CSNK1A1_5_1479,826,674,791,569,490,1544,92,1076,352
CSNK1D_5_1480,350,421,317,75,703,69,240,374,827
CSNK1E_5_1481,1,17,1,114,0,81,212,0,0
CSNK1G3_5_1482,378,522,779,161,574,1073,706,612,273
CTDP1_5_1483,604,590,480,447,45,410,332,1161,732
CTDSP1_5_1484,678,547,371,96,182,516,153,932,61
DCLK1_5_1485,2745,2683,4064,1657,3488,3223,3525,2396,5173
DCLK2_5_1486,844,741,977,1041,1573,688,496,277,974
DDR1_5_1487,374,895,716,2003,1137,256,273,407,1218
DDR2_5_1488,1543,877,1652,1165,1941,1619,2387,2620,1772
DGKA_5_1489,863,804,1271,577,941,1345,1208,1625,1513
DGKB_5_1490,741,1490,1490,883,2610,897,653,2142,226
DGKD_5_1491,1336,2741,2025,2893,2536,4678,3658,2528,4182
DGKG_5_1492,327,393,556,18,854,1093,80,600,125
DGKH_5_1493,476,254,722,513,55,336,871,120,857
DGKZ_5_1494,139,193,408,109,125,280,171,261,0
DGUOK_5_1495,4099,4285,3632,2736,3789,5829,5219,4429,4521
DLGAP5_5_1496,0,82,0,0,0,0,298,0,0
DMPK_5_1497,2215,1359,2333,864,2191,1997,2559,1447,2322
DSTYK_5_1498,344,83,415,195,5,358,153,370,269
DTYMK_5_1499,870,1980,1590,1538,3464,2132,1253,1514,1148

DUSP13_5_1500,615,794,696,320,1198,146,263,1195,194
DUSP19_5_1501,313,553,802,53,654,250,395,788,357
DUSP4_5_1502,503,878,1601,290,642,807,725,340,410
DUSP6_5_1503,339,647,585,1312,971,83,354,404,326
DYRK1A_5_1504,964,484,595,293,187,756,912,832,1350
DYRK1B_5_1505,109,0,0,0,37,1,0,0,0
DYRK2_5_1506,1481,2569,3142,363,3117,1938,3157,2018,3215
DYRK3_5_1507,292,636,438,254,98,1052,227,459,2
EFNA4_5_1508,605,382,372,23,934,325,461,60,1884
EGFR_5_1509,453,484,814,1088,532,811,483,436,2002
EIF2AK2_5_1510,2296,1717,1762,2075,1319,3416,1952,1316,2958
EPHA10_5_1511,16,3,71,2,7,106,51,196,100
EPHA3_5_1512,214,542,240,351,1134,514,251,36,851
EPHA5_5_1513,680,360,486,879,385,16,166,291,30
EPHA6_5_1514,847,1731,1420,386,939,389,1005,353,2
EPHA8_5_1515,627,350,189,655,1099,54,120,534,0
EPHB2_5_1516,436,73,367,250,257,1546,364,145,1253
EPM2A_5_1517,395,640,527,598,297,56,630,1037,259
ERBB2_5_1518,1296,1706,2494,1730,459,831,1828,942,664
ERBB4_5_1519,419,188,78,419,863,254,303,122,50
ETNK1_5_1520,2943,3766,4094,1240,5101,2541,2730,2018,3806
EXOSC10_5_1521,1193,1208,1553,2127,703,1135,976,966,155
EYA1_5_1522,194,410,444,74,463,420,851,61,983
EYA2_5_1523,684,808,403,536,79,460,354,321,88
EYA4_5_1524,1449,1316,962,2202,2261,1706,1430,516,2252
FASTK_5_1525,796,489,627,98,489,322,378,144,2535
FBP1_5_1526,798,669,529,284,489,563,352,406,136
FES_5_1527,485,895,1057,625,471,1394,849,189,1373
FGFR1_5_1528,430,901,909,995,259,761,414,219,1472
FGFR2_5_1529,280,319,716,344,239,1264,606,589,318
FGFR3_5_1530,770,453,1626,362,1486,1314,820,41,1488
FGFR4_5_1531,836,487,535,305,510,49,301,374,1948
FGFRL1_5_1532,884,373,1259,758,944,765,378,414,2080
FGR_5_1533,125,58,169,286,1,206,224,77,127
FLT1_5_1534,977,717,1357,1160,44,1877,1437,1274,1114
FLT4_5_1535,3692,2250,3019,1988,1214,2327,4021,3322,1426
FXN_5_1536,2210,2369,2492,2030,2099,3973,2393,2277,1377
FYN_5_1537,614,1432,2037,545,1571,2461,2232,1270,311
FZR1_5_1538,467,387,451,72,126,182,2,759,136
GALK2_5_1539,1273,962,855,711,2257,186,724,1640,1311
GK_5_1540,736,1029,1461,285,1793,1039,1222,1693,2533
GLYCTK_5_1541,294,151,296,18,426,373,20,136,65
GNE_5_1542,2143,2194,2865,805,1602,3009,1086,2249,2274
GRK4_5_1543,331,568,619,118,506,207,956,332,480
GRK6_5_1544,545,571,1094,953,653,1071,960,1184,1574
GSK3B_5_1545,673,466,317,108,0,838,326,31,0
GUK1_5_1546,225,110,393,14,0,0,1,43,493
HCK_5_1547,196,306,151,2,79,0,125,5,38
HIPK1_5_1548,421,74,334,193,177,976,895,475,230
HIPK2_5_1549,584,460,646,67,119,695,245,496,187

HIPK3_5_1550,0,0,0,0,0,0,0,0,0
ICK_5_1551,170,91,147,466,3,0,120,73,25
IKBKB_5_1552,288,195,489,2,609,0,70,1042,0
IKBKE_5_1553,1657,3070,1721,1727,3296,1791,887,1876,3063
IKBKG_5_1554,129,68,196,174,8,178,414,348,484
ILK_5_1555,3127,2918,2933,1391,2094,4002,2211,4674,4006
IMPA1_5_1556,2773,1886,2143,1985,2996,4196,2826,2300,2739
INPP1_5_1557,231,440,370,294,200,1172,154,6,302
INPP4A_5_1558,613,1445,1399,1997,184,316,944,1515,9
INPP4B_5_1559,539,512,477,121,82,666,312,146,45
INPP5D_5_1560,126,141,152,720,0,178,40,66,0
INSR_5_1561,315,62,150,2,129,0,1,158,37
IP6K1_5_1562,637,684,640,365,467,851,205,997,169
IP6K3_5_1563,868,1495,1473,632,2332,837,838,1969,72
IRAK1_5_1564,896,1305,1683,1881,565,1418,813,243,1297
IRAK3_5_1565,2389,2441,3002,2955,2270,2384,2613,2515,1838
IRAK4_5_1566,358,166,269,0,42,666,178,1636,729
ITPK1_5_1567,357,1149,885,12,1111,40,0,295,2
KALRN_5_1568,551,602,382,99,1041,996,111,1513,295
KHK_5_1569,459,452,928,22,649,1053,214,497,33
KIF2A_5_1570,169,155,299,414,586,264,58,76,1
KIT_5_1571,812,1057,1421,1791,1838,386,1132,1281,1361
LCK_5_1572,1136,789,864,1767,1744,811,1357,870,1851
LHPP_5_1573,791,271,1102,104,1414,330,734,227,259
LIMK1_5_1574,318,454,493,17,414,1004,358,344,850
LIMK2_5_1575,546,118,224,1435,347,588,1379,15,160
LTK_5_1576,246,164,155,1115,31,227,31,213,83
LYN_5_1577,501,449,635,515,91,84,746,1218,1525
MAGI3_5_1578,641,662,485,1812,954,398,1361,737,221
MAP2K3_5_1579,378,531,672,19,607,1488,379,749,349
MAP2K5_5_1580,3509,3332,3893,2318,4336,2550,3442,2223,4125
MAP3K12_5_1581,373,550,632,202,220,1054,582,864,207
MAP3K13_5_1582,278,226,633,192,1240,471,155,587,158
MAP3K3_5_1583,1848,2301,3218,3082,3372,1997,4302,2126,1449
MAP3K4_5_1584,4077,4469,6014,4282,3389,3176,4094,3215,7320
MAP3K7_5_1585,1595,1538,1694,1079,1781,1745,1021,270,1974
MAP4K1_5_1586,1337,1229,1436,1234,1165,1140,1610,877,1088
MAP4K4_5_1587,923,1151,642,2420,1005,1126,394,277,642
MAP4K5_5_1588,261,906,1399,166,307,266,655,478,57
MAPK10_5_1589,276,508,381,302,238,766,1299,238,1111
MAPK14_5_1590,1658,2101,1800,1657,2253,2064,1713,1899,1487
MAPK1_5_1591,906,1633,1621,1040,2582,1959,588,490,1357
MAPK3_5_1592,1381,1387,1274,88,3093,2632,1637,236,2236
MAPK7_5_1593,416,819,370,721,885,462,640,583,670
MAPK8_5_1594,960,1384,1298,337,625,785,1265,811,1735
MAPK9_5_1595,1497,1505,1381,880,1476,724,1277,579,2637
MAPKAPK2_5_1596,236,393,607,327,97,318,460,946,168
MAPKAPK5_5_1597,40,155,45,0,308,140,27,279,1
MARK2_5_1598,0,75,84,0,13,762,4,41,0
MARK3_5_1599,3688,4366,7542,3508,4802,3951,5202,5381,7593

MARK4_5_1600,596,432,754,1643,203,21,517,0,143
MAST4_5_1601,1582,1398,1533,172,1049,1448,694,2028,891
MASTL_5_1602,918,1243,1273,1464,1376,860,1074,1382,210
MATK_5_1603,101,145,295,352,152,238,42,292,17
MET_5_1604,1595,1426,1685,699,927,2622,1673,726,770
MINK1_5_1605,88,253,77,9,24,25,74,96,92
MINPP1_5_1606,1204,1704,1876,1906,2004,1487,3301,2743,1126
MKNK1_5_1607,380,961,792,44,189,175,1328,1208,430
MKNK2_5_1608,738,383,486,946,483,597,364,288,451
MLKL_5_1609,2048,2517,2202,1072,1833,3714,2096,1912,2646
MTMR14_5_1610,2610,2344,2967,2908,1509,1770,1918,1722,2012
MTMR2_5_1611,178,10,134,83,1,5,34,4,0
MTMR3_5_1612,828,1608,1300,702,315,1878,1310,1530,2124
MUSK_5_1613,1828,2061,1764,2684,1147,2292,1322,1081,5196
MVK_5_1614,321,215,371,42,55,67,81,552,10
MYO3B_5_1615,5140,4547,6145,3287,6326,7846,6655,4225,5013
NADK_5_1616,196,233,275,90,767,37,62,347,26
NCK1_5_1617,1643,1461,2275,908,904,1373,1535,1771,831
NCK2_5_1618,1411,1487,2475,2260,3871,815,1282,1542,2249
NDRG1_5_1619,124,179,92,3,147,1481,285,41,5
NEK11_5_1620,695,779,412,0,635,10,1201,391,381
NEK1_5_1621,1888,1772,2182,918,1089,2109,2107,1253,1440
NEK2_5_1622,3581,4569,4351,4269,4140,4135,4417,3252,7088
NEK3_5_1623,745,732,1176,290,554,3140,1268,1199,27
NEK4_5_1624,1,0,0,0,0,1,0,0,0
NEK6_5_1625,593,744,1487,467,466,1924,127,941,1764
NME2_5_1626,373,282,500,392,281,563,804,323,1114
NME7_5_1627,1581,1098,1662,1540,1199,754,2045,808,1359
NT5C1B_5_1628,1234,1393,1074,965,943,798,606,1694,1477
NT5C2_5_1629,635,309,1040,669,1674,2073,659,798,554
NT5C3_5_1630,1218,1659,2585,848,1641,1276,729,1163,224
NT5E_5_1631,21,85,125,35,318,120,215,4,1333
NTRK1_5_1632,89,286,92,649,345,62,199,190,303
NTRK2_5_1633,693,718,1217,1151,201,32,1213,821,874
NTRK3_5_1634,443,634,416,132,422,722,604,351,897
NUDT4_5_1635,141,0,127,0,0,211,275,0,1
NUDT9_5_1636,423,292,526,271,268,663,663,1417,312
OBSCN_5_1637,341,466,489,884,1894,0,285,893,1367
OCRL_5_1638,1907,2183,2767,1058,1978,2505,1695,1516,2948
OXSM_5_1639,1011,1093,647,151,2168,1182,1097,652,963
PACSIN1_5_1640,401,281,227,15,3,25,32,1023,488
PAK4_5_1641,365,502,567,805,499,138,569,657,419
PAK6_5_1642,296,385,458,271,64,1526,708,474,288
PAK7_5_1643,196,390,535,191,687,132,1296,181,49
PANK1_5_1644,1199,2538,2067,670,1627,1071,2295,1655,1832
PANK2_5_1645,3855,4418,3314,3524,4819,3373,5720,2811,2757
PCK2_5_1646,314,785,524,373,148,798,708,316,73
PDK2_5_1647,185,346,267,481,150,3,709,239,0
PDK3_5_1648,1784,1916,1867,2576,1051,1097,1706,1686,668
PDPK1_5_1649,1512,2029,2321,2072,966,2157,1058,2511,2774

PEG3_5_1650,1338,843,1117,1665,1775,934,555,2197,4066
PFKFB2_5_1651,3,2,1,0,0,0,0,7,0
PFKFB3_5_1652,1598,1586,1204,1480,2011,1742,1321,1333,405
PFKM_5_1653,462,235,444,158,252,302,179,725,1506
PFKP_5_1654,567,1000,722,393,1094,1024,1081,298,1352
PHKA1_5_1655,1468,2978,2599,1885,1767,2688,2870,2816,2912
PHKB_5_1656,1007,504,1302,808,1916,1923,1254,929,803
PHKG2_5_1657,83,88,319,0,64,220,148,80,520
PHPT1_5_1658,31,29,234,0,204,71,518,20,196
PI4KA_5_1659,368,238,538,559,357,506,244,88,1186
PI4KB_5_1660,373,270,507,289,4,520,531,299,695
PIK3R1_5_1661,9483,10193,11123,10709,10916,8156,11338,10710,12275
PIK3R5_5_1662,600,783,748,566,700,446,673,1516,389
PIKFYVE_5_1663,842,476,832,414,2534,999,532,660,756
PIP4K2C_5_1664,10,191,98,1,1,104,522,192,0
PIP5K1A_5_1665,571,496,1243,151,496,751,1707,231,905
PIP5K1C_5_1666,90,159,328,65,1262,819,4,56,2
PIP5KL1_5_1667,124,86,224,302,152,362,197,989,49
PKLR_5_1668,6,18,64,0,12,12,12,17,2
PKMYT1_5_1669,331,137,541,177,981,1080,208,548,54
PKN1_5_1670,603,280,391,524,91,723,38,0,0
PLK4_5_1671,846,1160,926,419,1669,35,2194,311,1992
PNCK_5_1672,71,156,7,0,318,0,9,753,1
PODXL_5_1673,306,545,232,313,903,588,83,181,550
PPAP2A_5_1674,191,341,157,85,505,25,38,223,0
PPAP2C_5_1675,637,744,947,730,1515,679,538,435,1214
PPEF1_5_1676,417,401,985,348,2150,832,389,779,136
PPFIA1_5_1677,457,510,341,588,1083,308,478,66,6
PPFIA2_5_1678,3196,2661,3362,1623,3406,5917,2078,2160,3389
PPM1A_5_1679,1606,3216,2045,1222,2930,2966,2774,2698,1699
PPM1B_5_1680,466,302,719,372,254,237,465,23,753
PPM1M_5_1681,768,165,277,82,50,1,453,297,19
PPP1CA_5_1682,699,939,634,1277,2446,786,175,811,1208
PPP1CB_5_1683,2178,2117,1985,849,2708,718,2174,3230,1993
PPP1R12A_5_1684,2330,1499,2180,2633,2992,2910,2204,2139,1924
PPP1R16B_5_1685,1796,2120,3196,2467,2358,2880,1736,1548,1608
PPP1R1B_5_1686,399,223,326,75,68,322,124,269,66
PPP1R8_5_1687,279,483,839,24,480,1024,139,272,1
PPP2R1B_5_1688,1150,631,1506,524,167,3084,655,252,1429
PPP2R2A_5_1689,1704,2357,2568,2783,3238,4746,2015,2759,1106
PPP2R2B_5_1690,1583,982,1709,765,267,193,2149,630,791
PPP2R2C_5_1691,250,186,306,0,85,38,781,55,106
PPP2R3A_5_1692,1247,2016,2126,1949,2743,2693,968,677,2655
PPP2R4_5_1693,261,692,346,78,201,1386,448,601,116
PPP2R5A_5_1694,478,936,340,714,1444,647,30,755,154
PPP2R5D_5_1695,956,728,738,108,343,485,70,514,1673
PPP3CA_5_1696,4230,4337,5879,2013,3816,3850,3988,4100,3780
PPP3CB_5_1697,2281,2131,2432,2267,1789,738,1996,1423,3445
PPP4R1_5_1698,562,763,1157,731,1193,402,728,956,124
PPP5C_5_1699,236,330,387,1108,1185,115,487,666,8

PPP6C_5_1700,738,628,768,240,481,962,67,743,105
PRKAA1_5_1701,1343,1376,2312,2552,4002,1711,1615,2474,1482
PRKACA_5_1702,1478,1114,954,1077,908,2015,1567,294,2920
PRKACB_5_1703,263,342,243,280,90,405,441,241,532
PRKAG1_5_1704,550,1308,943,471,788,1539,466,346,4225
PRKAG2_5_1705,50,300,284,360,205,80,122,93,3
PRKAR1A_5_1706,788,1314,2137,940,1251,2628,652,2287,4003
PRKAR1B_5_1707,515,574,1061,8,375,415,659,302,664
PRKCB_5_1708,579,1168,1190,1842,380,1595,570,1194,609
PRKCD_5_1709,821,674,735,436,554,877,1422,594,747
PRKCQ_5_1710,686,1188,1269,343,721,913,821,1238,464
PRKCZ_5_1711,943,887,1086,498,453,526,819,640,982
PRKD2_5_1712,150,307,505,295,239,964,272,740,736
PRKDC_5_1713,146,352,215,32,147,581,95,149,2
PRKG1_5_1714,407,546,384,236,1244,975,120,129,121
PRPS2_5_1715,22,139,302,0,303,374,0,1,0
PTK2B_5_1716,489,1772,677,434,823,931,150,452,1
PTK2_5_1717,3189,3262,4512,3283,4412,2854,4030,2959,4918
PTK7_5_1718,560,431,339,705,817,417,82,104,77
PTP4A2_5_1719,6609,6700,7958,3808,9106,6807,5148,6663,5871
PTP4A3_5_1720,375,460,336,21,59,6,553,3,0
PTPDC1_5_1721,922,1595,674,1020,645,2199,1117,431,578
PTPN12_5_1722,538,1604,844,46,496,1732,1136,536,467
PTPN13_5_1723,963,783,944,182,1711,1707,772,664,309
PTPN18_5_1724,13,228,145,55,10,0,2,76,25
PTPN22_5_1725,4950,5307,6957,4550,3192,5550,4149,3370,2568
PTPN2_5_1726,1249,1736,2501,2633,3588,1305,1645,1412,3733
PTPN3_5_1727,441,240,520,317,1410,125,664,393,79
PTPN5_5_1728,567,588,995,3,573,74,89,619,704
PTPN6_5_1729,322,60,237,12,5,15,42,93,487
PTPN7_5_1730,241,250,230,335,402,2,201,99,1
PTPRA_5_1731,521,217,971,5,68,178,611,478,757
PTPRB_5_1732,1597,2052,2132,2822,2050,2189,1884,1874,2395
PTPRC_5_1733,5379,6264,6781,5960,4252,6282,6799,4221,6745
PTPRD_5_1734,4506,5831,6462,2632,5720,7419,4299,4477,6586
PTPRE_5_1735,213,386,522,163,2493,148,379,736,1
PTPRF_5_1736,216,1215,1199,75,2439,948,1515,857,3068
PTPRH_5_1737,515,412,328,88,1112,1,524,8,3
PTPRJ_5_1738,266,268,164,3,84,15,483,338,450
PTPRK_5_1739,2145,2014,3321,5050,2924,3288,1743,1186,4821
PTPRM_5_1740,294,294,80,599,0,0,73,55,0
PTPRN2_5_1741,361,631,236,620,597,511,724,63,7
PTPRN_5_1742,755,289,774,785,614,260,573,892,1959
PTPRR_5_1743,771,736,710,1531,540,31,868,1144,1036
PTPRS_5_1744,162,149,385,0,236,198,146,134,213
PTPRT_5_1745,2414,2959,3690,723,3989,756,1188,1198,2211
PTPRU_5_1746,147,512,556,57,318,17,836,28,62
PTPRZ1_5_1747,2006,1805,1771,1901,1479,1372,2703,2021,4859
RET_5_1748,4771,5363,6370,3414,4305,5256,3913,5297,6221
RIOK1_5_1749,38,89,411,0,135,34,0,183,0

RIOK2_5_1750,1476,947,960,1084,1686,124,571,1140,839
ROPN1L_5_1751,491,578,798,19,366,699,122,255,2346
RPS6KA1_5_1752,1658,1295,1234,1021,2375,1469,799,1022,660
RPS6KA2_5_1753,544,348,588,269,910,639,426,829,102
RPS6KA4_5_1754,271,136,331,534,499,46,513,57,9
RPS6KA5_5_1755,228,230,526,684,635,0,517,148,119
RPS6KC1_5_1756,132,105,899,372,70,49,30,806,715
RYK_5_1757,2023,2377,2601,1787,2931,3032,2415,1259,3357
SET_5_1758,1295,1200,938,1462,1589,808,1674,2028,353
SGK1_5_1759,974,903,568,32,424,1072,871,402,1105
SGK3_5_1760,1182,2625,1728,1674,2909,2839,1131,2194,833
SIRPA_5_1761,1185,695,1527,827,1116,1033,908,1779,562
SKAP1_5_1762,3620,4806,4501,6576,7012,2996,5303,5240,5244
SNRK_5_1763,1603,1778,1707,1100,624,656,2195,1104,1809
SPHK1_5_1764,218,95,282,200,69,34,2,118,64
SPHK2_5_1765,570,1228,1490,718,418,1010,62,208,585
SRC_5_1766,566,263,404,533,76,151,49,476,111
SRPK2_5_1767,1899,2088,2278,2430,2130,1975,1507,1652,2507
SRPK3_5_1768,947,480,693,181,199,882,643,552,1087
SSH1_5_1769,1078,491,973,596,269,49,1824,858,1931
STK19_5_1770,611,1012,1084,920,1099,1771,1484,971,790
STK24_5_1771,7091,9353,9008,8071,10727,8468,8449,7315,8711
STK31_5_1772,1248,1342,1572,2236,904,719,205,1829,2257
STK36_5_1773,524,429,797,451,235,464,445,1286,1004
STRADA_5_1774,2726,4333,5209,2920,2532,5237,3868,3631,6746
STRADB_5_1775,1006,1092,1231,1576,1066,1443,348,1559,1294
STYX_5_1776,2009,2693,2511,2885,3717,2930,2338,3039,3125
SYK_5_1777,130,98,231,2,0,0,70,218,0
SYNJ1_5_1778,517,169,693,8,451,10,10,85,1136
SYNJ2_5_1779,2017,2350,2447,2065,1025,909,1944,1642,2482
TAF1_5_1780,44,185,47,169,135,60,337,468,790
TAOK1_5_1781,1363,2235,2878,2123,650,1368,2142,1748,3344
TAOK2_5_1782,357,319,560,22,39,742,626,188,32
TBCK_5_1783,1117,1828,2224,1939,1251,1804,2483,2372,935
TEX14_5_1784,1156,1157,756,198,1966,652,815,813,398
TGFBR1_5_1785,1086,560,1169,1075,100,1664,495,1302,951
TGFBR2_5_1786,3490,3672,4540,3878,2772,4624,3878,2903,4673
TGFBR3_5_1787,1361,1725,2009,2278,2744,1507,374,546,3177
THTPA_5_1788,50,12,0,434,2,59,2,27,0
TJP2_5_1789,2533,3401,2087,1298,2092,670,2679,1804,3163
TK2_5_1790,535,1069,1862,88,2018,2555,787,2349,1891
TLK1_5_1791,668,1389,1348,2693,607,2034,624,441,447
TLK2_5_1792,941,1175,757,492,1422,261,1288,416,1382
TMEM134_5_1793,535,146,156,10,377,579,41,183,598
TNIK_5_1794,1984,2600,1933,1247,862,1151,1327,1172,1662
TNK2_5_1795,636,588,487,98,6,95,683,1111,484
TPK1_5_1796,197,476,561,206,1096,132,407,496,92
TPTE2_5_1797,1672,3058,1554,2041,3392,3134,1008,3340,499
TPTE_5_1798,3084,2689,4259,3379,3737,3855,2391,2571,3543
TRIM24_5_1799,0,0,0,0,0,0,0,0,0

TRIM33_5_1800,923,976,722,1566,1608,168,1070,503,523
TRMT2A_5_1801,104,259,716,650,357,313,861,174,145
TRPM6_5_1802,392,286,281,722,299,119,84,296,259
TSSK4_5_1803,65,19,180,108,2,5,0,12,0
TTK_5_1804,2910,3053,3949,1835,2946,5843,3701,2686,2347
TTN_5_1805,2095,2191,3234,3375,3140,3457,3736,3965,2928
TWF1_5_1806,800,1105,1295,738,862,1861,1367,81,2535
UCK1_5_1807,1872,2071,2532,2082,1265,2293,550,2574,275
UCKL1_5_1808,1849,2452,2491,1923,1303,2725,1272,2340,1461
UHMK1_5_1809,2247,2462,2080,3827,1633,2554,1843,1775,3246
ULK2_5_1810,338,398,66,290,438,206,209,217,246
VRK2_5_1811,2687,3182,2953,3817,2551,3523,3032,4293,5965
VRK3_5_1812,373,677,632,1105,1067,767,205,30,1533
WEE1_5_1813,980,687,1532,1397,1179,1096,1854,1276,1088
WNK1_5_1814,4264,4553,5983,3485,5879,4720,4097,3185,6937
WNK3_5_1815,570,1677,1067,532,434,930,286,744,1258
YSK4_5_1816,336,566,510,265,1519,1042,640,117,259
ZAP70_5_1817,975,146,404,663,889,39,569,997,68
ACP2_5_1818,248,118,357,35,727,41,337,191,1
CDK1_5_1819,545,331,655,937,411,521,56,41,437
DUSP10_5_1820,295,556,525,1462,55,442,409,219,212
DUSP15_5_1821,15,62,251,228,36,0,19,8,11
ERBB3_5_1822,5,1,19,0,0,0,0,0,143
G6PC2_5_1823,2185,1612,1410,547,2297,626,1025,1779,1882
IP6K2_5_1824,85,427,142,135,42,296,38,361,676
MYLK_5_1825,246,1480,863,62,598,774,437,174,727
PRPS1_5_1826,3657,3294,3810,3779,3628,3256,4079,3016,8722
PTPMT1_5_1827,3559,5116,5951,1504,3345,4278,4579,4028,7632
PTPN20A_5_1828,314,492,522,197,652,109,245,563,419
PTPN20B_5_1829,314,492,522,197,652,109,245,563,419
SPEG_5_1830,300,486,200,538,230,100,345,491,976
STK32A_5_1831,919,1472,1566,1177,1980,1457,1406,1897,783
PPP1R12B_5_1832,134,135,472,13,50,568,211,292,150
PPP1R12B_5_1833,630,1203,902,590,288,611,384,803,360
PPP1R12B_5_1834,1862,2597,2459,2390,1699,2323,2390,2137,2740
AAK1_5_1835,585,312,500,207,0,284,690,320,1674
ACP6_5_1836,678,941,1246,652,463,1416,2584,1623,2336
ACPT_5_1837,960,740,1074,761,1068,697,1152,782,1113
ACVR2A_5_1838,550,689,327,693,1221,1436,1210,31,3
ACVR2B_5_1839,442,535,241,108,228,431,348,21,478
ADCK2_5_1840,720,1397,713,834,739,646,312,747,1570
ADCK3_5_1841,272,689,161,992,64,1003,810,1192,293
ADCK5_5_1842,28,138,38,0,0,0,0,0,0
ADPGK_5_1843,441,538,695,887,651,604,392,456,1477
ADRBK1_5_1844,128,15,9,811,2,0,2,2,3
ADRBK2_5_1845,903,512,786,1664,1159,1718,698,1066,3503
AGK_5_1846,591,507,966,1287,1109,1652,877,252,1232
AK1_5_1847,695,1238,958,1564,3290,888,1579,590,831
AK7_5_1848,347,352,514,43,321,166,288,80,30
AK8_5_1849,318,209,305,118,611,94,19,234,382

ALK_5_1850,245,526,641,1807,527,973,1028,171,365
ALPI_5_1851,3069,2177,2018,943,2744,2280,1908,1983,2014
ALPK2_5_1852,156,74,247,224,21,125,220,245,235
ALPK3_5_1853,870,302,522,155,694,618,757,453,421
ALPP_5_1854,1997,2305,2282,2656,2576,3487,1520,2017,1269
ALPPL2_5_1855,1640,1501,1238,2551,1159,850,1824,802,1313
ANKK1_5_1856,131,467,375,15,414,15,3,461,0
ARAF_5_1857,276,659,492,705,2489,538,353,1084,517
ATM_5_1858,1267,3517,1957,2164,1936,1496,930,2560,1470
AURKB_5_1859,406,207,467,124,162,114,363,216,957
BLK_5_1860,199,215,65,100,425,117,31,13,226
BMPR1A_5_1861,1097,1029,1242,10,1116,221,524,294,2273
BMPR1B_5_1862,4570,7743,5304,3546,5370,5154,4550,3506,4472
BMPR2_5_1863,4006,5148,5009,3427,4355,3995,4548,2900,4169
BPNT1_5_1864,665,638,1165,459,592,956,754,192,892
BRAF_5_1865,162,232,262,455,38,8,522,64,97
BRD3_5_1866,535,841,1489,1007,578,924,271,538,2355
BRSK1_5_1867,5909,6086,8840,7746,5761,6351,5915,7216,8043
BRSK2_5_1868,648,663,893,2055,1233,536,622,1081,700
BTK_5_1869,1427,1542,1196,2082,1799,801,1301,493,1450
BUB1B_5_1870,2073,1489,1894,3607,652,306,2530,1303,1205
BUB1_5_1871,1226,1152,1239,1413,2318,1866,478,2356,1695
C20orf111_5_1872,516,551,458,1040,633,664,617,249,9
C9orf96_5_1873,401,390,534,567,191,1483,791,385,212
CALM1_5_1874,269,153,883,500,438,197,599,36,10
CALM3_5_1875,662,846,741,1313,2221,865,573,548,94
CAMK1_5_1876,3312,3574,4744,2736,4550,3532,1790,2840,4348
CAMK1G_5_1877,1452,1427,1595,1001,1776,482,1633,2116,1494
CAMK2N1_5_1878,1450,1387,1848,587,1609,2264,1685,1437,934
CAMK4_5_1879,1578,1763,2005,258,2039,2865,2012,2165,6051
CAMKV_5_1880,3774,5079,5882,3812,4266,6373,3648,4072,3914
CARD11_5_1881,380,835,732,792,467,193,652,476,1115
CCL2_5_1882,1213,1235,2366,1337,2725,2294,1671,954,2921
CDC42BPB_5_1883,248,149,253,116,217,358,321,712,239
CDC42BPG_5_1884,441,277,464,139,82,634,704,172,163
CDK14_5_1885,480,1052,1197,353,486,2216,815,734,1726
CDK15_5_1886,657,625,1385,821,1159,678,298,207,576
CDK19_5_1887,426,852,195,712,500,412,757,905,55
CDK3_5_1888,899,941,409,79,1913,635,290,819,462
CDK4_5_1889,1391,1215,1029,341,803,1436,718,2427,2695
CDK5R1_5_1890,295,566,287,297,1102,303,613,541,4
CDK7_5_1891,562,438,656,664,477,714,609,883,539
CDK8_5_1892,2417,2936,3183,1448,5171,1969,2147,2640,5109
CDK9_5_1893,1314,1167,1537,895,958,1891,1089,904,3238
CDKL1_5_1894,879,1043,1124,858,912,1418,982,1005,3530
CDKL2_5_1895,916,714,938,285,1598,341,602,956,4013
CDKL4_5_1896,2531,1957,2099,2138,1793,4634,3120,1814,440
CDKN1B_5_1897,252,203,72,658,2,8,114,206,309
CERK_5_1898,1965,1749,2288,2854,4634,2153,1279,2523,1344
CHKB_5_1899,307,379,361,8,1,228,649,321,791

CHUK_5_1900,1552,962,1773,1014,1006,716,984,1124,1928
CIB2_5_1901,353,586,795,794,273,103,1095,731,980
CILP_5_1902,301,87,441,544,1,14,683,191,339
CKB_5_1903,4,239,163,1,104,86,22,237,60
CKM_5_1904,956,641,1847,943,2272,1682,425,90,1328
CKMT1A_5_1905,805,533,1216,907,1162,1424,569,1204,1649
CKMT1B_5_1906,805,533,1216,907,1162,1424,569,1204,1649
CKS1B_5_1907,2459,2435,2739,2159,1682,2969,3231,1939,2166
CKS2_5_1908,4029,4853,4805,4145,4492,3781,3090,3476,7644
CLK2_5_1909,649,811,634,498,511,916,384,1182,1853
CLK4_5_1910,950,1386,1154,945,775,635,1199,254,382
CMPK2_5_1911,800,356,1025,518,311,159,576,716,61
CPNE3_5_1912,0,0,2,0,0,0,0,0,0
CRIM1_5_1913,1316,1669,2047,477,1050,2308,1643,1623,2227
CRKL_5_1914,819,1283,1622,935,2096,1353,1181,1373,1792
CSF1R_5_1915,997,500,286,184,140,690,228,1014,16
CSNK1A1L_5_1916,121,232,190,8,158,312,7,502,25
CSNK1G1_5_1917,2001,1766,3230,3182,2461,314,2909,1714,3115
CSNK1G2_5_1918,4805,5562,5370,7541,3423,6757,4618,4170,9996
CSNK2A2_5_1919,2456,2039,2760,2106,1809,2475,1241,1626,2518
CSNK2B_5_1920,392,264,477,120,220,463,605,151,213
CTDSP2_5_1921,1093,496,541,343,475,14,261,401,10
DAPK1_5_1922,911,1330,1691,918,1025,2599,1413,1164,1861
DAPK2_5_1923,1462,1406,1724,820,2384,1280,660,1167,2263
DAPK3_5_1924,654,864,1532,570,1755,814,19,64,536
DBF4_5_1925,4090,3978,3884,3707,4116,4943,3698,5064,3403
DCK_5_1926,638,600,1335,380,541,372,638,852,262
DCLK3_5_1927,1561,2628,2270,693,3672,1067,2071,719,4652
DGKE_5_1928,774,689,1354,721,1153,1134,1298,1273,372
DGKI_5_1929,2021,2882,4672,3257,2848,3919,2593,2067,2800
DGKK_5_1930,314,169,469,1121,59,263,237,80,109
DGKQ_5_1931,1612,1275,1440,1584,1948,1805,1635,1379,1196
DOLK_5_1932,1408,1615,1796,1042,2899,1949,1536,2259,2655
DOT1L_5_1933,5366,5651,7279,7792,9288,7439,4018,6809,4314
DUSP11_5_1934,55,198,649,692,96,265,370,953,1507
DUSP12_5_1935,3165,2396,3786,3414,1365,3946,3546,2079,5700
DUSP14_5_1936,336,1636,1009,843,517,850,561,738,221
DUSP16_5_1937,1660,1745,1437,669,656,1853,1469,1177,2701
DUSP18_5_1938,1476,1598,2858,1479,1985,2552,1121,1857,980
DUSP21_5_1939,404,614,954,602,194,1016,398,242,2106
DUSP22_5_1940,2856,2090,2184,1568,1775,1849,2538,1448,3068
DUSP2_5_1941,2132,2750,1605,955,1622,2235,1511,1269,175
DUSP3_5_1942,894,510,766,532,593,157,750,555,523
DUSP5_5_1943,4300,4600,4355,3619,4550,5016,2956,4566,4273
DUSP7_5_1944,421,370,1035,206,383,58,643,863,381
DUSP8_5_1945,145,293,452,3,378,487,8,14,1
DUSP9_5_1946,705,724,596,678,489,1089,462,145,109
DYRK4_5_1947,1045,1464,1580,569,1763,942,855,792,857
EEF2K_5_1948,3609,3450,3028,2688,4107,4385,3621,2283,5619
EFNA2_5_1949,18,3,165,0,11,3,37,16,0

EFNA3_5_1950,44,120,23,1,1,2,31,278,57
EFNA5_5_1951,570,259,953,53,284,71,488,71,1823
EFNB3_5_1952,31,92,629,22,110,860,2,407,2
EIF2AK3_5_1953,1549,1276,1273,590,688,493,1254,980,3643
EIF2AK4_5_1954,735,555,853,1370,791,74,643,709,1294
EPHA1_5_1955,232,673,298,202,63,105,317,90,2
EPHA2_5_1956,658,639,154,145,72,161,311,807,134
EPHA4_5_1957,504,335,651,109,618,964,360,232,1808
EPHA7_5_1958,1042,1462,2404,2171,1733,718,2139,1788,601
EPHB1_5_1959,344,302,898,232,1088,360,738,328,690
EPHB3_5_1960,3609,4129,5233,5575,3579,3030,2627,2748,4452
EPHB4_5_1961,922,217,383,125,388,1345,637,516,44
EPHB6_5_1962,1373,1349,1940,1605,2347,1731,1184,1078,1134
ERN1_5_1963,891,1523,1767,2382,1778,397,1421,1691,1646
ERN2_5_1964,894,704,747,1329,145,298,1065,800,2163
ETNK2_5_1965,318,615,429,482,335,277,479,739,13
EYA3_5_1966,698,592,827,655,29,932,304,606,2
FER_5_1967,698,1072,1040,448,172,1154,521,536,705
FLT3_5_1968,5268,7581,7986,4158,5981,7550,5320,5599,6523
FN3K_5_1969,37,238,14,13,1278,1,17,29,13
FN3KRP_5_1970,1285,1129,1340,595,1751,1028,1068,1343,2429
FRK_5_1971,1826,1887,2826,1993,2097,3871,1319,2651,3052
FUK_5_1972,291,218,418,0,61,189,31,13,319
G6PC3_5_1973,671,810,1981,742,67,1545,1072,1636,1311
G6PC_5_1974,3450,2330,3551,2615,3259,2342,2995,1305,2577
GAK_5_1975,477,237,668,755,916,746,853,2379,302
GALK1_5_1976,136,437,385,158,347,1099,106,295,981
GDPD4_5_1977,2266,3320,3521,38,3153,3755,2757,1525,2521
GOLGA5_5_1978,1014,1474,1554,1339,2061,613,703,2105,1059
GRK1_5_1979,183,78,142,125,134,776,39,234,0
GRK5_5_1980,1055,205,599,800,385,514,427,281,1757
GRK7_5_1981,1383,865,1781,136,590,864,293,1414,2154
GSG2_5_1982,331,351,258,108,56,68,283,879,1227
GSK3A_5_1983,536,764,997,215,1118,1272,1344,880,280
GUCY2C_5_1984,0,245,204,1,105,280,22,170,871
GUCY2D_5_1985,356,204,483,1217,428,162,551,194,19
GUCY2F_5_1986,618,613,361,374,411,247,302,462,1143
HIPK4_5_1987,207,559,948,26,1275,775,183,856,840
HKDC1_5_1988,492,575,959,423,577,1099,572,454,1008
HOOK3_5_1989,4213,2924,3001,2170,3992,2996,3385,1495,4902
HSPB8_5_1990,1264,2177,1919,250,2180,2263,1204,1354,396
HUNK_5_1991,195,587,463,21,240,27,639,428,335
HUS1_5_1992,2914,4060,4776,695,6527,5955,4270,4949,4948
IGBP1_5_1993,270,153,121,0,56,0,576,42,4
IGF1R_5_1994,627,495,832,297,187,39,473,1047,48
IGF2R_5_1995,1356,2605,3201,2254,2310,2016,1218,1017,1035
ILKAP_5_1996,331,660,355,57,329,454,600,613,0
IMPA2_5_1997,83,256,667,770,746,75,118,312,2484
INPP5A_5_1998,510,1117,759,1077,1011,961,785,545,945
INPP5B_5_1999,786,1086,2222,533,2237,2113,1457,2189,500

INPP5J_5_2000,890,1405,1041,633,680,1911,1634,1108,612
INPPL1_5_2001,243,275,548,843,768,18,326,5,1558
INSRR_5_2002,583,239,340,172,200,297,437,481,1
IPMK_5_2003,0,4,58,51,0,383,69,0,98
IPPK_5_2004,271,1125,706,450,88,965,460,249,401
IRAK2_5_2005,185,114,324,4,0,47,1,4,274
ITK_5_2006,683,437,396,58,241,1321,205,259,311
ITPKA_5_2007,200,0,331,0,0,0,120,1,7
ITPKB_5_2008,76,214,150,67,39,154,222,1,34
ITPKC_5_2009,9,50,16,99,1,0,0,0,2
JAK1_5_2010,1822,1438,1855,1297,820,2717,1406,538,1807
JAK2_5_2011,2216,2547,3017,2100,3843,1764,2872,2467,2917
JAK3_5_2012,1721,1165,2289,726,1024,4954,1698,1070,3157
KDR_5_2013,880,946,930,758,1349,825,775,665,1007
KSR1_5_2014,516,201,210,180,189,223,288,582,349
LATS1_5_2015,1662,1612,1405,2642,1020,1357,984,855,1137
LATS2_5_2016,2202,1773,2396,1769,4356,1265,2593,2948,2252
LLGL1_5_2017,115,159,106,24,670,0,0,19,1
LMTK2_5_2018,141,56,199,29,0,16,48,96,7
LMTK3_5_2019,223,259,205,443,435,154,562,154,356
LRRK1_5_2020,180,288,533,288,149,161,639,459,87
LRRK2_5_2021,6387,7043,9719,6832,9044,10129,7869,7475,9456
MAGI2_5_2022,6861,9601,10453,6779,7872,11424,6425,7115,7424
MAP2K1_5_2023,60,19,81,5,235,72,321,9,81
MAP2K2_5_2024,443,243,650,102,33,410,588,167,1776
MAP2K4_5_2025,1309,452,1236,894,363,1596,1065,1035,1212
MAP2K6_5_2026,267,289,21,256,117,206,245,100,23
MAP2K7_5_2027,645,123,253,1006,125,21,29,5,109
MAP3K10_5_2028,697,1118,1367,1286,1823,356,204,985,2198
MAP3K11_5_2029,487,169,1,0,0,10,428,421,0
MAP3K14_5_2030,28,192,615,354,36,19,10,520,635
MAP3K15_5_2031,671,1280,1047,972,1810,822,896,482,1217
MAP3K1_5_2032,646,1093,705,690,753,625,1306,533,1241
MAP3K2_5_2033,3871,4772,5439,3512,4174,4708,4570,2791,6524
MAP3K5_5_2034,2114,1708,1936,3444,2955,3251,2995,929,1726
MAP3K6_5_2035,2178,2852,2769,2175,1129,2403,2891,2537,3819
MAP3K8_5_2036,785,306,779,2380,190,1240,836,674,1875
MAP3K9_5_2037,1590,1156,1835,1375,748,1092,1129,1037,1305
MAP4K2_5_2038,2686,2745,3846,2143,3995,3603,3023,2326,2469
MAP4K3_5_2039,1319,1373,2650,1378,1085,1601,800,3226,2642
MAPK11_5_2040,210,150,498,84,42,1,0,8,7
MAPK12_5_2041,3503,3210,4448,1803,4406,3373,3438,4073,2922
MAPK13_5_2042,1078,1568,1074,173,258,1647,891,707,1997
MAPK15_5_2043,917,928,595,2291,461,76,107,405,12
MAPK4_5_2044,515,775,754,1010,1851,6,444,88,1707
MAPK6_5_2045,1923,1747,2925,1860,3239,5485,3084,2072,1401
MAPKAPK3_5_2046,896,664,1031,1039,953,1357,1432,713,1377
MARK1_5_2047,649,902,1187,960,385,839,538,288,419
MAST2_5_2048,141,350,204,15,446,316,94,130,381
MAST3_5_2049,387,213,292,743,43,506,572,586,333

MELK_5_2050,1560,1231,1803,1161,1216,1902,1291,2298,2034
MERTK_5_2051,1506,837,2945,1630,2941,2818,1447,1513,765
MEX3B_5_2052,37,191,193,221,33,14,481,1,142
MPP3_5_2053,688,1423,858,108,1626,682,547,829,2970
MPP5_5_2054,24,19,18,8,13,230,28,271,0
MST1R_5_2055,3,165,232,146,0,170,94,1,1428
MTM1_5_2056,411,463,840,180,456,868,1056,158,748
MTMR1_5_2057,2197,1316,924,1294,1526,1809,1334,1177,2097
MTMR4_5_2058,211,146,778,46,690,939,185,193,507
MTMR6_5_2059,1523,1153,2204,577,1275,2631,1465,372,506
MTMR7_5_2060,360,289,846,428,396,791,1207,504,1063
MTMR9_5_2061,1833,1692,1671,3575,2259,2524,3631,1548,2863
MTOR_5_2062,45,10,45,30,0,0,255,256,71
MYLK2_5_2063,0,0,0,0,0,0,0,0,0
MYLK3_5_2064,141,194,194,114,218,44,14,270,71
MYLK4_5_2065,723,1490,1862,1906,838,2749,492,760,504
MYO3A_5_2066,3244,3346,3571,4508,1750,4273,3635,1244,4400
N4BP2_5_2067,607,1295,1258,1220,839,1904,1117,1166,1545
NAGK_5_2068,3131,1070,2129,2378,1841,1815,1605,543,2772
NAGS_5_2069,495,653,785,1342,543,372,862,993,501
NEK10_5_2070,135,209,405,6,44,896,312,229,5
NEK5_5_2071,2955,2146,3153,3286,2951,4955,3365,2237,4779
NEK7_5_2072,9444,13132,13765,11003,10788,18399,10410,12529,16708
NEK8_5_2073,742,803,714,500,1198,332,867,153,648
NEK9_5_2074,2267,2682,2705,2879,3258,1544,1210,2710,2324
NME3_5_2075,383,399,301,446,53,70,174,108,146
NME4_5_2076,417,613,690,527,18,360,341,257,2860
NME5_5_2077,772,1389,1571,1051,713,1538,1374,1185,1405
NME6_5_2078,1975,2228,2215,3322,4479,1524,2189,1064,3239
NPR1_5_2079,205,3,163,0,0,3,16,10,499
NPRL2_5_2080,308,221,326,214,253,230,185,409,97
NRBP1_5_2081,7,110,104,119,0,222,21,17,16
NRBP2_5_2082,134,31,87,221,125,22,1,112,868
NRK_5_2083,949,899,1176,528,1369,376,1289,866,4146
NT5C1A_5_2084,62,143,117,1,51,254,37,350,409
NT5C_5_2085,726,710,885,125,114,1256,1134,825,952
NT5M_5_2086,142,254,403,61,494,196,265,241,6
NUAK1_5_2087,1192,973,1095,381,1131,2399,1282,989,1519
NUAK2_5_2088,69,43,8,199,0,14,1,10,0
NUCKS1_5_2089,2351,2826,3335,1738,1232,3379,965,3602,2358
OXSR1_5_2090,1113,820,1279,1361,140,1193,952,922,1160
PAK2_5_2091,909,572,1582,486,486,584,671,694,2700
PANK3_5_2092,904,603,598,1174,1138,52,889,239,1461
PANK4_5_2093,1489,2095,1660,3259,1128,1119,1534,2471,1609
PASK_5_2094,587,395,299,0,6,375,4,135,41
PBK_5_2095,5120,3976,6475,6472,6214,6755,4343,3932,7291
PCK1_5_2096,944,919,1050,1447,483,1864,1330,129,390
PDCD1_5_2097,183,74,565,0,64,1,14,7,1527
PDGFRA_5_2098,385,185,578,9,81,78,425,687,149
PDGFRB_5_2099,689,386,974,900,1040,1751,1051,1512,1565

PDGFRL_5_2100,277,181,144,115,105,555,184,46,97
PDIK1L_5_2101,582,512,649,2217,273,1273,474,220,129
PDK4_5_2102,2382,3398,2454,2897,5037,2979,2729,1688,1832
PDP2_5_2103,309,152,150,48,495,132,292,92,277
PDXK_5_2104,2092,1477,892,1741,1724,646,2047,1813,701
PFKFB1_5_2105,1758,832,1910,1077,1650,1430,1305,1097,697
PFKFB4_5_2106,417,145,141,122,75,491,194,100,952
PFKL_5_2107,501,197,453,300,357,258,174,625,1535
PGAM2_5_2108,460,213,551,0,127,24,1196,156,588
PGK1_5_2109,13,30,2,0,19,0,0,0,70
PGK2_5_2110,214,220,452,46,50,222,168,78,235
PHKA2_5_2111,5350,6673,7305,3644,8148,4119,5067,4629,6046
PHKG1_5_2112,1368,992,1453,28,1947,1593,1482,577,1731
PI4K2A_5_2113,2375,2229,2863,1938,890,4146,1261,1439,2602
PI4K2B_5_2114,1503,1487,1317,1267,1704,819,1166,1249,1440
PIK3C2A_5_2115,2726,3395,3940,1776,2589,4204,3738,3890,2765
PIK3C2B_5_2116,1631,1530,2383,521,1817,2190,1779,835,1980
PIK3C2G_5_2117,186,181,490,569,47,324,68,17,0
PIK3C3_5_2118,2492,2513,3758,3181,2409,2868,2091,4044,3967
PIK3CA_5_2119,3956,4545,4888,3819,5557,5210,3658,3610,6089
PIK3CB_5_2120,644,287,598,460,743,668,25,303,678
PIK3CD_5_2121,238,62,171,0,0,44,13,100,38
PIK3CG_5_2122,448,497,430,28,1245,36,82,1084,1712
PIK3R2_5_2123,1730,1146,2251,1189,2325,2101,1729,3376,1335
PIK3R4_5_2124,510,559,845,388,1246,827,402,398,356
PIK3R6_5_2125,730,397,926,621,246,545,458,501,706
PIM2_5_2126,904,688,1232,62,596,2267,706,486,1055
PIM3_5_2127,664,649,975,169,376,2028,546,399,1348
PINK1_5_2128,832,886,722,351,1344,1052,784,632,637
PIP4K2A_5_2129,1162,1594,1664,2365,1952,1578,1100,1546,1954
PIP4K2B_5_2130,1974,1144,1211,1457,3057,1749,1752,1880,1493
PIP5K1B_5_2131,673,630,1525,717,585,1263,515,809,2508
PKDCC_5_2132,808,1126,1014,520,3735,1810,1387,409,441
PKN2_5_2133,1981,2411,2690,4822,598,3172,1995,1569,2931
PKN3_5_2134,123,527,638,119,840,850,136,364,103
PLK1_5_2135,240,132,705,281,66,138,333,3,1
PLK2_5_2136,259,710,861,22,1005,805,148,257,7
PLK3_5_2137,263,692,394,116,1069,458,82,222,340
PMVK_5_2138,574,1303,1489,604,755,1255,820,3962,3311
PNKP_5_2139,1512,1033,2431,1261,664,2924,1995,302,1050
POLD1_5_2140,289,98,259,1686,29,133,178,43,35
PON1_5_2141,1071,1300,1791,1707,421,3746,845,1777,1949
PPAP2B_5_2142,810,700,738,807,146,1798,752,932,1211
PPEF2_5_2143,1317,1122,1419,563,743,1353,1311,1304,622
PPFIA3_5_2144,970,327,725,507,123,795,1827,1034,1007
PPFIA4_5_2145,261,660,327,393,498,119,218,273,91
PPM1D_5_2146,1402,1796,2081,2040,1617,1624,779,1450,2771
PPM1E_5_2147,951,670,983,732,880,497,1114,1008,602
PPM1F_5_2148,1415,1883,1205,903,1625,947,1096,759,2422
PPM1G_5_2149,1218,1995,1773,2182,2419,1537,1326,2695,3080

PPM1K_5_2150,2005,2156,2400,1456,2735,1929,1147,2488,1513
PPM1L_5_2151,2614,2254,3750,2815,1637,2612,4026,1604,3768
PPP1CC_5_2152,452,1142,829,748,1705,724,150,602,1502
PPP1R12C_5_2153,215,379,579,307,1018,158,208,289,1194
PPP1R13B_5_2154,3913,5265,4790,3270,4116,6393,2577,3624,6023
PPP1R14B_5_2155,14,74,145,14,1542,6,0,0,27
PPP1R15B_5_2156,2399,2752,3211,2947,3373,3220,2165,2817,5041
PPP1R1A_5_2157,615,626,508,1163,1114,1548,931,549,95
PPP1R1C_5_2158,522,510,720,531,227,781,1026,532,1169
PPP1R2_5_2159,254,349,1376,2,26,604,437,52,1763
PPP1R3A_5_2160,1029,1347,1871,1598,1137,3099,1872,1956,2843
PPP1R3C_5_2161,3732,3233,4510,3646,2999,4217,4521,3461,3010
PPP1R3D_5_2162,518,122,150,0,1274,4,300,560,1343
PPP1R7_5_2163,790,803,1122,943,1446,1546,1308,332,2840
PPP2CA_5_2164,3107,3156,3969,2618,1866,4122,1675,2424,4251
PPP2CB_5_2165,390,253,685,608,908,24,27,130,7
PPP2R1A_5_2166,356,24,435,123,110,10,343,199,0
PPP2R2D_5_2167,1767,1022,1678,509,517,1406,1919,798,828
PPP2R3B_5_2168,820,606,321,810,429,252,280,569,266
PPP2R5B_5_2169,639,411,722,876,615,1579,379,189,2973
PPP2R5E_5_2170,1187,1570,1620,1320,2553,1160,542,1335,1944
PPP3CC_5_2171,308,887,420,699,820,1430,351,1666,851
PPP3R1_5_2172,1620,2319,3522,2187,1994,2384,2767,1525,2497
PPP3R2_5_2173,634,474,247,305,1467,634,349,373,533
PPP4C_5_2174,397,69,536,280,326,11,368,292,0
PPTC7_5_2175,707,462,650,2405,1224,334,870,1432,730
PRKAA2_5_2176,278,32,89,51,7,191,321,73,294
PRKAB1_5_2177,368,327,604,541,339,332,470,1807,363
PRKAB2_5_2178,1358,1676,2418,1146,2370,1668,1660,1436,1755
PRKACG_5_2179,687,631,377,487,910,938,262,675,326
PRKAG3_5_2180,2865,2705,3254,2916,2906,1813,2540,1622,1816
PRKAR2A_5_2181,696,1280,637,747,980,747,524,1644,754
PRKAR2B_5_2182,3020,4012,3973,3041,2748,2258,3253,3873,2073
PRKCA_5_2183,3352,2282,3318,3584,2542,4564,2091,2079,4433
PRKCE_5_2184,497,935,975,533,285,217,947,1062,1655
PRKCG_5_2185,391,445,154,99,1520,117,469,785,6
PRKCH_5_2186,1962,2516,3332,2341,2060,1450,2383,1948,1847
PRKCI_5_2187,3930,4692,6294,1700,3816,6262,4009,5891,6531
PRKD1_5_2188,5697,4486,5403,3511,5090,7033,6153,4079,5688
PRKD3_5_2189,3246,3133,3881,4335,4416,2683,2714,2698,4940
PRKG2_5_2190,1588,2065,2110,2402,2930,1325,1905,2359,458
PRKX_5_2191,1533,1681,1990,3857,2152,1534,666,344,2462
PRPF4B_5_2192,3287,4285,4195,3337,4747,4336,3444,2864,2644
PRPS1L1_5_2193,654,689,444,55,352,858,23,69,0
PSKH1_5_2194,1146,872,834,468,648,1459,1597,1987,704
PSKH2_5_2195,397,454,427,379,484,413,224,95,596
PSPH_5_2196,123,182,102,49,160,492,311,59,7
PSTK_5_2197,499,310,332,386,795,106,410,5,1239
PTEN_5_2198,4471,4976,6408,3107,3718,8000,3672,4530,6098
PTK6_5_2199,453,959,487,16,96,96,438,19,684

PTP4A1_5_2200,48,1,60,0,0,1,111,13,0
PTPLA_5_2201,560,1337,639,283,1575,177,1125,1003,2489
PTPLB_5_2202,808,546,471,560,803,755,939,865,99
PTPN11_5_2203,883,1192,1203,1470,429,1202,478,498,752
PTPN14_5_2204,566,1067,786,166,1381,1352,1302,633,735
PTPN1_5_2205,276,733,1158,451,195,0,81,559,987
PTPN21_5_2206,6328,5411,6760,5215,4653,4966,4852,7069,8996
PTPN23_5_2207,248,292,36,31,25,0,848,1117,0
PTPN4_5_2208,202,238,305,0,0,0,0,755,0
PTPN9_5_2209,354,426,395,352,129,368,292,91,303
PTPRG_5_2210,696,520,1091,461,795,345,1157,583,391
PXK_5_2211,3,30,140,26,7,17,16,121,0
RAF1_5_2212,1475,3074,2780,988,3119,3854,1152,1257,1686
RBKS_5_2213,2142,3827,2812,4229,3047,3670,2005,2217,5728
RFK_5_2214,1822,1663,2453,2974,2223,2187,3135,374,3158
RIOK3_5_2215,2006,2058,1858,2275,3940,2694,595,1664,1014
RIPK1_5_2216,637,577,895,742,744,1246,1084,418,688
RIPK2_5_2217,2757,1484,2449,4400,1029,2137,1766,1713,3182
RIPK3_5_2218,611,620,786,758,1140,1087,79,124,512
RIPK4_5_2219,585,42,322,63,146,356,12,286,8
RNASEL_5_2220,457,1151,286,695,1110,697,390,466,8
RNGTT_5_2221,629,888,607,233,1983,150,836,182,1483
ROCK1_5_2222,4334,4571,5192,6888,3423,6936,4334,5813,5536
ROCK2_5_2223,509,616,823,309,584,663,342,603,363
ROS1_5_2224,3994,5562,5081,2316,4654,6842,5778,2902,6079
RPS6KA3_5_2225,1832,1185,1785,3597,3003,2303,1883,1467,1005
RPS6KA6_5_2226,2823,1904,2661,1725,3007,804,919,1759,2245
RPS6KB1_5_2227,453,489,815,626,104,540,348,759,482
RPS6KB2_5_2228,2160,1909,2777,2269,1074,2941,2294,2667,4189
RPS6KL1_5_2229,311,164,173,281,960,897,885,71,854
RSPO3_5_2230,233,322,1194,41,489,682,1119,864,829
SACM1L_5_2231,2199,3144,4090,1958,2223,1910,2436,3513,3677
SBF1_5_2232,769,1066,901,556,558,1354,575,430,94
SBK1_5_2233,577,832,701,431,271,1190,280,280,118
SBK2_5_2234,870,1012,1696,382,1340,1525,367,485,1134
SEPHS2_5_2235,820,960,1581,1121,1181,870,534,500,166
SGPP1_5_2236,616,505,573,394,176,115,449,601,540
SHPK_5_2237,207,438,375,964,337,99,263,78,14
SIK1_5_2238,316,73,199,1,46,317,621,611,241
SIK2_5_2239,868,524,664,416,1268,793,716,531,147
SIK3_5_2240,148,348,430,325,723,29,659,160,270
SKAP2_5_2241,389,622,724,428,2,94,839,382,318
SMG1_5_2242,2093,1564,1906,827,997,2001,1475,2173,1632
SRMS_5_2243,212,250,349,485,74,8,306,24,49
SRP72_5_2244,256,477,177,1303,273,40,216,1,132
SRPK1_5_2245,1107,954,1574,672,2471,1496,987,1293,994
SSH2_5_2246,1182,1434,1672,741,859,715,1105,1746,2693
SSH3_5_2247,2537,2655,2905,972,1757,2638,790,1702,4326
STC1_5_2248,398,309,129,589,477,76,20,707,11
STK10_5_2249,93,174,467,0,23,54,6,321,74

STK11_5_2250,582,690,972,444,1859,187,774,422,374
STK16_5_2251,253,247,413,956,118,61,174,946,806
STK17A_5_2252,584,596,1389,427,547,693,1205,438,1108
STK17B_5_2253,2604,3301,3014,1755,2617,2788,3145,2629,4087
STK25_5_2254,534,795,968,452,142,307,1198,806,532
STK32B_5_2255,1952,2336,3868,4360,1788,2886,4250,1100,3572
STK32C_5_2256,938,944,1056,619,189,1311,768,690,674
STK33_5_2257,842,1115,968,323,622,1755,680,338,471
STK35_5_2258,212,293,97,780,178,66,249,344,474
STK38_5_2259,817,904,716,338,865,588,224,550,1
STK38L_5_2260,4005,3668,5894,3768,3147,3550,2957,2668,4510
STK39_5_2261,4180,3193,3245,2641,1995,6014,5134,3157,3030
STK40_5_2262,249,155,341,534,337,276,323,57,208
STK4_5_2263,4777,5130,5791,6684,8855,4465,6210,4705,7523
STYK1_5_2264,1403,1046,1143,1265,878,1419,799,1008,1800
STYXL1_5_2265,960,930,798,1302,932,1340,854,716,856
TAF1L_5_2266,534,599,942,1608,925,1159,935,91,1110
TAOK3_5_2267,2962,1968,4525,4686,4425,4163,4738,2682,3121
TBK1_5_2268,959,194,792,2334,239,82,671,434,795
TEC_5_2269,3242,4930,6436,4921,4735,4277,2876,5073,6269
TEK_5_2270,1369,1111,1784,2135,1236,1264,753,1209,3142
TESK1_5_2271,472,317,890,560,806,294,907,365,569
TESK2_5_2272,377,776,543,555,2482,959,915,963,316
THNSL1_5_2273,1514,1642,1556,988,377,2623,2493,659,5293
TIE1_5_2274,982,540,1084,410,896,1027,973,917,400
TK1_5_2275,28,89,60,4,3,7,184,13,0
TNK1_5_2276,946,390,1548,944,730,388,1116,811,20
TNNI3K_5_2277,853,1072,523,146,1096,470,768,141,258
TNS3_5_2278,413,340,538,229,348,1248,713,900,766
TP53RK_5_2279,509,36,239,206,7,26,1161,123,19
TRAT1_5_2280,514,987,171,199,364,594,140,140,2481
TRIB1_5_2281,3846,4790,5935,4389,3314,8010,5855,3210,6997
TRIB2_5_2282,44,424,24,183,69,30,0,278,422
TRIB3_5_2283,98,277,251,39,129,23,170,238,333
TRIM27_5_2284,1229,643,1000,133,290,605,979,722,79
TRIM28_5_2285,445,886,927,2306,528,522,914,348,154
TRIO_5_2286,1374,1720,2567,1881,1959,1467,1599,1446,1053
TRPM7_5_2287,2509,2883,5331,3304,1654,1486,3650,4061,1731
TRRAP_5_2288,1368,1466,1719,1481,3081,1541,629,2061,2881
TSKS_5_2289,160,437,366,261,6,145,549,204,453
TSSK1B_5_2290,3254,3256,5541,2160,2966,3368,3831,3308,2843
TSSK2_5_2291,64,24,445,372,150,125,112,0,85
TSSK3_5_2292,500,501,396,458,1183,744,1129,310,884
TSSK6_5_2293,520,418,357,303,667,161,778,696,493
TTBK1_5_2294,532,384,640,1727,1343,477,1049,484,349
TTBK2_5_2295,1629,1783,1261,1817,1206,1896,1730,1528,474
TWF2_5_2296,1374,892,1157,1601,441,1614,1300,1336,1052
TXK_5_2297,287,221,266,4,942,127,218,117,0
TYK2_5_2298,29,81,257,107,1,81,176,526,3
TYRO3_5_2299,438,144,247,121,32,460,81,51,20

UBLCP1_5_2300,429,544,770,459,273,785,470,651,0
UCK2_5_2301,616,284,579,710,557,895,196,204,1773
ULK1_5_2302,2302,2901,3532,2077,3864,3303,2381,2491,3663
ULK3_5_2303,591,518,334,50,526,803,248,505,307
ULK4_5_2304,5236,5917,7799,6338,5916,5344,6240,6638,4445
VRK1_5_2305,557,1248,1770,197,481,1531,798,1064,19
WEE2_5_2306,156,387,726,259,278,397,346,418,514
WNK2_5_2307,284,259,551,551,1032,42,74,581,255
WNK4_5_2308,204,283,197,151,44,1137,182,587,507
XRCC6BP1_5_2309,4033,4413,4723,3246,4128,5123,4780,3446,4619
XYLB_5_2310,3813,3649,3790,3444,5218,5819,2685,5619,4958
YES1_5_2311,512,741,703,994,885,326,633,528,240
AATK_5_2312,183,315,318,211,10,6,463,1,302
ABL1_5_2313,255,832,294,456,584,863,71,1419,1325
ABL2_5_2314,569,633,661,53,670,116,544,513,12
ACP1_5_2315,108,351,321,152,0,0,0,123,174
ACP5_5_2316,565,704,1145,960,1004,678,571,835,1443
ACPL2_5_2317,5,31,32,265,10,0,24,115,383
ACPP_5_2318,1191,1197,1654,1021,1462,1141,1733,1286,5121
ACVR1B_5_2319,320,520,527,431,591,547,141,1237,1240
ACVR1C_5_2320,1408,1333,1547,1140,1548,2224,1213,473,1238
ACVR1_5_2321,826,1047,1407,932,1298,2368,687,669,1968
ACVRL1_5_2322,522,95,477,378,647,126,175,659,630
ADCK1_5_2323,460,341,521,760,28,236,1917,297,665
ADCK4_5_2324,658,764,926,277,1046,196,336,1543,695
ADK_5_2325,1228,948,1333,900,2640,801,799,1309,2052
AK2_5_2326,613,761,605,768,628,488,232,627,447
AK4_5_2327,2432,2969,3337,2898,4199,3702,2885,1784,5277
AK5_5_2328,253,161,115,3,0,0,29,1,16
AKT1_5_2329,153,303,501,1092,1626,238,697,132,249
AKT2_5_2330,175,23,4,41,142,48,6,0,3
AKT3_5_2331,3142,1842,3005,1977,2166,2892,2124,1871,2099
ALDH18A1_5_2332,1051,1466,1211,2219,891,1435,517,591,1485
ALPK1_5_2333,533,1150,857,228,763,817,749,552,1264
ALPL_5_2334,69,166,405,1,30,150,289,317,0
AMHR2_5_2335,614,2034,1338,457,2192,1014,659,1457,2597
ANKHD1_5_2336,1184,548,863,2454,94,765,1002,961,895
APTX_5_2337,521,943,960,671,856,1635,1048,536,591
ATRIP_5_2338,260,414,1099,364,1054,12,668,1351,1512
AURKA_5_2339,3652,3492,2946,2550,3993,3913,3316,2380,4894
AURKC_5_2340,3290,2949,2827,3501,3261,3388,4784,1194,1712
AXL_5_2341,497,655,215,378,116,362,1307,905,1450
BAIAP2_5_2342,340,136,291,939,310,0,46,1074,0
BCKDK_5_2343,432,571,507,53,374,719,678,174,540
BCR_5_2344,161,318,608,146,120,167,392,14,2
BMP2K_5_2345,722,1134,1180,227,1628,1852,1462,849,1783
BMX_5_2346,883,623,987,965,452,1288,272,630,719
BPGM_5_2347,448,115,384,495,204,969,452,47,98
BRD2_5_2348,402,725,498,740,988,330,324,394,683
BRD4_5_2349,1200,637,1202,1857,1650,1533,528,1168,565

BRDT_5_2350,1659,1729,1663,2274,1906,1767,703,2579,1607
CAB39L_5_2351,1839,2384,2991,5343,1644,3794,1379,2322,1593
CAMK1D_5_2352,318,508,114,76,263,565,150,448,46
CAMK2A_5_2353,890,584,1159,563,652,1551,72,608,188
CAMK2B_5_2354,15,24,37,14,389,26,5,4,11
CAMK2D_5_2355,1513,1505,1822,1148,1456,1772,1785,1895,2990
CAMK2G_5_2356,1036,738,632,20,1505,820,1128,464,186
CAMKK1_5_2357,2425,3394,2851,2440,5538,2742,3024,4083,1773
CAMKK2_5_2358,265,575,245,101,232,69,403,857,345
CASK_5_2359,654,247,1125,131,185,63,471,362,3788
CCT2_5_2360,773,1375,1302,328,1110,1004,784,2323,3067
CDADC1_5_2361,675,553,1349,551,587,2304,268,1040,526
CDC14A_5_2362,3297,3681,4039,1528,3075,5312,3529,3458,1854
CDC14B_5_2363,3344,3152,3624,654,3021,2917,2580,2633,3298
CDC25A_5_2364,1359,814,1854,1933,325,1351,793,1577,1600
CDC25B_5_2365,468,423,416,257,2395,357,845,556,928
CDC25C_5_2366,2985,3097,4010,2873,1297,4903,1837,3664,2525
CDC42BPA_5_2367,971,898,1349,580,535,801,477,2085,3105
CDC7_5_2368,236,603,1137,494,673,278,242,1143,1236
CDK10_5_2369,72,450,778,3,165,532,205,1200,926
CDK11A_5_2370,1003,1246,1558,777,1891,645,763,1190,157
CDK11B_5_2371,1003,1246,1558,777,1891,645,763,1190,157
CDK12_5_2372,89,0,48,0,0,0,0,0,0
CDK13_5_2373,1085,717,1136,616,300,1316,501,481,2011
CDK16_5_2374,343,257,385,460,24,412,97,40,1
CDK17_5_2375,482,339,734,108,338,7,292,134,1320
CDK18_5_2376,360,479,486,48,883,604,86,315,1257
CDK20_5_2377,1333,2350,2124,1287,2748,2173,1696,1497,1224
CDK2_5_2378,305,176,878,1103,358,1214,290,311,368
CDK5_5_2379,256,273,1235,570,368,795,310,213,131
CDK6_5_2380,107,2039,1088,336,114,920,28,1002,1337
CDKL3_5_2381,9374,9633,14436,7656,7956,11903,7244,5016,10098
CDKL5_5_2382,1366,1880,2426,3177,2816,2202,928,2421,1870
CDKN1A_5_2383,745,1029,996,899,390,1711,570,349,1229
CDKN3_5_2384,703,406,511,239,1083,2756,284,534,597
CHEK1_5_2385,4132,6182,4816,4582,7487,5476,4311,6291,6999
CHEK2_5_2386,824,1047,2377,1421,1233,1840,1797,251,1687
CHKA_5_2387,2453,2905,2259,2859,3202,4297,2425,3308,2086
CIT_5_2388,116,727,502,329,2022,1671,37,726,595
CKMT2_5_2389,2260,2062,3591,2697,2092,1360,1948,2834,5461
CLK1_5_2390,2941,3152,3613,2266,2623,2554,2854,4310,3313
CLK3_5_2391,559,528,1078,109,201,371,507,980,524
CMPK1_5_2392,1045,605,1020,409,896,334,837,653,880
COASY_5_2393,639,826,594,407,354,769,383,636,1060
COL4A3BP_5_2394,596,123,677,205,112,439,892,106,526
CSK_5_2395,1820,2310,2374,2300,1257,2624,2195,2677,460
CSNK1A1_5_2396,1291,1455,2794,1194,1594,2848,1220,912,3353
CSNK1D_5_2397,396,430,191,240,7,256,10,5,51
CSNK1E_5_2398,230,383,933,409,319,376,319,426,0
CSNK1G3_5_2399,7853,8545,6835,8158,6229,9450,8651,6469,10205

CTDP1_5_2400,1239,2723,2135,1006,2187,1743,1075,2331,1657
CTDSP1_5_2401,329,1390,1143,64,1080,596,916,833,1572
DCLK1_5_2402,631,885,944,453,926,391,744,609,134
DCLK2_5_2403,2703,2152,2062,2666,2763,3120,1403,1769,3833
DDR1_5_2404,207,44,330,101,292,3,100,291,34
DDR2_5_2405,45,109,323,214,10,129,302,124,74
DGKA_5_2406,2566,3437,3314,4194,7302,2877,2799,2209,2909
DGKB_5_2407,2648,3790,2638,2257,2097,1835,1153,2197,2707
DGKD_5_2408,283,371,1125,583,10,765,291,123,773
DGKG_5_2409,943,1608,2789,486,1263,1970,1245,1929,856
DGKH_5_2410,1073,1197,1419,3222,1935,1017,917,1605,428
DGKZ_5_2411,199,360,780,130,48,160,320,594,66
DGUOK_5_2412,3608,3773,4087,3473,5213,3287,2276,3335,7502
DLGAP5_5_2413,2237,1910,2725,1117,1298,2825,1300,1970,1986
DMPK_5_2414,671,301,558,13,117,677,342,259,1118
DSTYK_5_2415,126,96,236,380,9,914,42,475,32
DTYMK_5_2416,2122,2765,2315,3279,5394,3717,1987,1763,3004
DUSP13_5_2417,1016,544,1234,957,475,826,2000,845,1770
DUSP19_5_2418,1313,743,1822,516,94,849,827,547,1165
DUSP4_5_2419,263,460,304,318,324,373,434,223,567
DUSP6_5_2420,94,365,22,288,125,51,3,154,55
DYRK1A_5_2421,1855,1553,1684,1090,3148,1228,1930,1757,554
DYRK1B_5_2422,3812,4529,6306,6059,3908,3861,5774,5527,3467
DYRK2_5_2423,1706,1474,2013,794,1306,1231,1382,750,1010
DYRK3_5_2424,561,531,1094,312,386,1927,296,771,686
EFNA4_5_2425,938,1593,2677,2933,2094,1267,3029,1307,4004
EGFR_5_2426,521,306,982,444,705,544,382,585,308
EIF2AK2_5_2427,5739,6287,7037,5077,7361,4957,5804,5648,9883
EPHA10_5_2428,930,1595,1552,2300,2022,1445,1544,755,933
EPHA3_5_2429,2032,789,2029,2317,1349,559,1988,691,3195
EPHA5_5_2430,1444,721,936,824,922,782,2023,1859,1214
EPHA6_5_2431,1874,1510,2146,2875,1716,1929,2345,1230,1218
EPHA8_5_2432,382,250,499,158,49,946,271,394,824
EPHB2_5_2433,295,282,539,0,0,713,117,559,268
EPM2A_5_2434,8992,10145,12987,10019,5694,11839,9176,8792,8779
ERBB2_5_2435,152,28,248,2055,29,33,76,19,0
ERBB4_5_2436,304,207,432,74,182,928,411,156,953
ETNK1_5_2437,465,691,429,42,238,546,542,193,1830
EXOSC10_5_2438,3288,3632,3650,1201,3873,2800,3264,5102,4184
EYA1_5_2439,726,1213,764,312,1437,634,963,657,2457
EYA2_5_2440,306,634,694,878,504,729,293,21,2352
EYA4_5_2441,317,396,1519,174,362,95,784,296,161
FASTK_5_2442,986,867,1837,1688,3480,1169,943,612,1942
FBP1_5_2443,5985,5734,5545,3774,6804,5558,5496,5018,5112
FES_5_2444,442,705,462,472,800,393,387,734,17
FGFR1_5_2445,3636,3643,5273,1637,3337,4143,3541,4778,4368
FGFR2_5_2446,101,358,299,382,1344,470,390,10,577
FGFR3_5_2447,201,196,755,93,190,558,302,395,484
FGFR4_5_2448,116,678,316,78,29,258,224,333,7
FGFRL1_5_2449,790,764,707,611,1520,288,284,1080,774

FGR_5_2450,760,448,970,669,331,834,897,319,1513
FLT1_5_2451,4251,3470,5547,2602,2983,4445,3300,3435,11565
FLT4_5_2452,397,371,551,475,387,511,6,1,402
FXN_5_2453,2202,2349,2265,2045,2107,3385,2412,2792,1678
FYN_5_2454,1444,1336,810,1097,1575,1623,1262,886,1713
FZR1_5_2455,878,766,491,959,136,192,704,470,188
GALK2_5_2456,676,943,674,499,1024,586,768,1135,756
GK_5_2457,1061,1261,790,2011,754,997,609,1500,1670
GLYCTK_5_2458,332,459,884,1,739,1227,497,79,763
GNE_5_2459,7033,7830,10712,7290,12020,9723,8513,6728,9249
GRK4_5_2460,3267,4561,3758,4434,4906,3546,4384,3394,4949
GRK6_5_2461,1623,1740,1123,1040,1018,540,1278,2086,1991
GSK3B_5_2462,570,470,554,461,292,489,272,459,271
GUK1_5_2463,211,95,96,8,57,149,672,133,43
HCK_5_2464,288,456,426,279,221,741,140,890,773
HIPK1_5_2465,442,1205,1670,628,187,1600,759,914,489
HIPK2_5_2466,566,1179,1135,274,879,695,519,1030,928
HIPK3_5_2467,854,571,1097,360,76,1456,384,491,1322
ICK_5_2468,333,523,387,28,79,166,1184,106,2180
IKBKB_5_2469,416,469,793,1028,858,120,121,156,371
IKBKE_5_2470,150,251,276,59,95,47,241,78,124
IKBKG_5_2471,807,831,1402,841,656,255,928,931,2408
ILK_5_2472,645,1070,892,674,1724,855,956,1223,1740
IMPA1_5_2473,678,353,892,1017,425,765,743,887,782
INPP1_5_2474,112,60,284,518,219,14,103,288,1
INPP4A_5_2475,2193,2136,3004,2913,1548,1010,2308,1951,1799
INPP4B_5_2476,390,93,403,327,452,116,387,608,0
INPP5D_5_2477,214,35,607,31,1413,175,588,1241,321
INSR_5_2478,97,0,0,0,0,0,404,0,0
IP6K1_5_2479,450,457,857,53,140,454,451,445,180
IP6K3_5_2480,706,895,1133,595,386,887,617,434,895
IRAK1_5_2481,333,272,336,2,232,489,236,56,285
IRAK3_5_2482,396,524,587,859,63,345,663,471,470
IRAK4_5_2483,1396,1136,1680,805,2224,881,1763,1738,1394
ITPK1_5_2484,497,647,223,590,58,1898,1523,507,85
KALRN_5_2485,1140,1243,1910,1444,808,1717,963,921,3232
KHK_5_2486,21,325,419,0,0,958,40,1,1017
KIF2A_5_2487,2809,3050,3802,3542,3516,5813,2277,2330,3693
KIT_5_2488,1551,2072,3501,2836,829,3474,3316,1438,2188
LCK_5_2489,514,877,1112,2004,1880,1676,653,1187,512
LHPP_5_2490,276,472,257,356,149,420,676,190,37
LIMK1_5_2491,208,232,737,18,522,1629,626,361,36
LIMK2_5_2492,1257,1027,1004,1253,338,735,406,1722,1519
LTK_5_2493,225,424,960,54,1380,71,144,12,33
LYN_5_2494,1004,352,927,1487,416,2320,757,1020,365
MAGI3_5_2495,423,564,766,336,920,969,362,330,1079
MAP2K3_5_2496,663,1141,1011,718,444,1172,1681,911,1506
MAP2K5_5_2497,600,1064,869,1632,872,504,423,1472,472
MAP3K12_5_2498,454,492,468,745,884,1648,415,465,225
MAP3K13_5_2499,280,457,251,383,109,839,1332,404,4

MAP3K3_5_2500,411,105,478,248,4,290,318,75,0
MAP3K4_5_2501,231,357,235,293,124,2198,1428,37,1281
MAP3K7_5_2502,905,841,1200,898,685,940,696,624,1535
MAP4K1_5_2503,996,712,1240,324,1291,1632,2310,912,822
MAP4K4_5_2504,155,68,472,1168,212,115,143,74,44
MAP4K5_5_2505,3118,3224,4274,2381,5305,3936,3754,2762,2951
MAPK10_5_2506,776,1451,1179,1205,849,141,2084,839,852
MAPK14_5_2507,1831,1903,1661,2317,886,2129,1682,1115,3465
MAPK1_5_2508,1697,985,2028,463,935,588,1803,1086,2532
MAPK3_5_2509,1117,726,1325,1452,1151,722,1605,655,737
MAPK7_5_2510,455,399,1043,323,384,1158,706,506,290
MAPK8_5_2511,123,143,204,56,57,265,92,176,110
MAPK9_5_2512,3879,4012,4097,3105,4354,5850,4070,4598,3388
MAPKAPK2_5_2513,334,282,845,613,448,124,584,370,143
MAPKAPK5_5_2514,510,325,189,239,33,186,100,827,228
MARK2_5_2515,610,1062,998,269,634,497,706,625,518
MARK3_5_2516,2105,3002,3090,1080,2614,2176,2660,1058,3136
MARK4_5_2517,261,581,123,397,161,5,38,513,230
MAST4_5_2518,941,536,885,3,58,337,504,1007,630
MASTL_5_2519,7673,10796,11031,8267,9113,12737,6414,10409,9495
MATK_5_2520,327,546,426,2,430,652,674,672,540
MET_5_2521,1756,2554,3050,953,2223,1737,1697,967,3336
MINK1_5_2522,2105,3304,4066,2844,2301,2659,3415,4455,5673
MINPP1_5_2523,581,785,1185,1597,1448,586,516,333,1203
MKNK1_5_2524,237,710,375,173,207,1079,171,488,976
MKNK2_5_2525,87,327,359,277,165,14,18,446,606
MLKL_5_2526,108,415,315,86,7,130,207,25,5
MTMR14_5_2527,11,18,40,40,141,11,126,35,0
MTMR2_5_2528,206,447,438,150,622,160,557,286,141
MTMR3_5_2529,326,166,25,20,0,3,314,39,119
MUSK_5_2530,3349,4141,4200,2742,4733,5734,4325,4003,5302
MVK_5_2531,73,113,237,597,107,697,50,49,18
MYO3B_5_2532,6992,9312,7519,5624,10931,7738,8384,6804,9855
NADK_5_2533,550,263,455,264,82,190,152,99,279
NCK1_5_2534,1053,1665,2600,841,1263,2153,1942,3643,1524
NCK2_5_2535,236,141,475,43,96,657,270,395,91
NDRG1_5_2536,71,304,661,209,0,288,825,209,1059
NEK11_5_2537,1315,859,1020,538,1332,1076,624,1188,1391
NEK1_5_2538,0,0,0,0,0,0,0,0,0
NEK2_5_2539,1784,1369,2162,2422,1367,2804,1910,2134,1357
NEK3_5_2540,60,71,216,41,333,212,143,21,0
NEK4_5_2541,2803,3484,3883,1975,6803,2245,2623,2836,4299
NEK6_5_2542,1780,1599,2893,735,1252,3769,1541,1420,1679
NME2_5_2543,1765,1942,1587,1723,1236,1370,1922,1336,1526
NME7_5_2544,0,199,190,0,0,1,0,275,1
NT5C1B_5_2545,184,53,94,331,119,282,227,0,59
NT5C2_5_2546,3895,3948,5433,2982,2475,3358,3965,2910,4869
NT5C3_5_2547,28,58,30,0,0,1,0,0,0
NT5E_5_2548,842,876,1046,2000,675,1286,407,1254,40
NTRK1_5_2549,768,677,1437,1547,491,1215,684,981,1198

NTRK2_5_2550,2606,2724,3869,2499,947,3724,3275,3689,2692
NTRK3_5_2551,918,852,708,454,0,210,135,731,13
NUDT4_5_2552,989,996,2166,158,502,1484,1491,1227,2343
NUDT9_5_2553,146,211,60,0,78,628,268,102,400
OBSCN_5_2554,194,37,389,791,728,60,598,11,340
OCRL_5_2555,3122,2932,4629,3234,3643,2346,3953,3369,3734
OXSM_5_2556,2252,2354,2241,1792,4169,1321,887,1285,4031
PACSIN1_5_2557,1751,1165,1742,309,58,557,952,596,1762
PAK4_5_2558,306,350,738,282,307,1049,135,499,1525
PAK6_5_2559,1675,2170,1791,1884,1104,4769,1925,1927,2226
PAK7_5_2560,1838,2122,1941,960,1895,2246,918,1809,1966
PANK1_5_2561,244,87,24,1,729,31,64,25,1212
PANK2_5_2562,96,63,207,92,3,126,103,37,577
PCK2_5_2563,253,12,769,136,153,2,30,4,1401
PDK2_5_2564,313,390,368,285,276,946,364,586,400
PDK3_5_2565,2431,2643,2802,3564,4030,2438,2064,1817,5362
PDPK1_5_2566,280,260,353,13,1623,471,216,517,945
PEG3_5_2567,276,547,1029,117,12,134,418,349,412
PFKFB2_5_2568,2121,2636,3664,3481,2935,2875,1368,4208,3764
PFKFB3_5_2569,561,570,270,410,739,423,674,488,593
PFKM_5_2570,178,23,52,264,19,266,69,3,0
PFKP_5_2571,397,623,677,429,162,359,1344,521,505
PHKA1_5_2572,2899,3224,2978,3873,2675,3586,2802,2602,3749
PHKB_5_2573,645,420,814,953,588,1138,505,348,626
PHKG2_5_2574,727,823,1374,1853,1590,575,1418,386,14
PHPT1_5_2575,99,401,193,0,135,11,226,356,55
PI4KA_5_2576,245,45,12,173,0,17,186,273,2
PI4KB_5_2577,1006,1110,1083,891,1458,2021,1329,739,1406
PIK3R1_5_2578,411,600,1067,479,779,1498,1374,132,1830
PIK3R5_5_2579,605,817,708,259,399,1335,251,1605,398
PIKFYVE_5_2580,540,755,1307,583,1107,1282,926,1034,2114
PIP4K2C_5_2581,414,400,672,461,121,889,859,334,725
PIP5K1A_5_2582,288,92,268,554,188,0,0,401,0
PIP5K1C_5_2583,158,324,124,49,389,222,111,166,123
PIP5KL1_5_2584,3,2,77,36,0,47,93,126,0
PKLR_5_2585,301,165,116,740,1520,301,137,0,491
PKMYT1_5_2586,146,481,85,8,639,209,357,305,42
PKN1_5_2587,1808,708,1621,642,488,1412,1137,1400,999
PLK4_5_2588,1576,1459,1513,1605,301,1353,556,1384,0
PNCK_5_2589,137,239,91,17,548,6,234,2,33
PODXL_5_2590,178,415,192,253,2,36,374,658,0
PPAP2A_5_2591,1634,964,2176,525,975,829,1425,1760,2074
PPAP2C_5_2592,324,424,450,1183,96,258,156,151,116
PPEF1_5_2593,1489,2014,2729,1407,781,2354,2733,2734,3783
PPFIA1_5_2594,738,1628,1314,474,1470,1178,1743,755,1647
PPFIA2_5_2595,29,64,155,278,133,920,501,4,4
PPM1A_5_2596,631,700,908,421,610,874,747,208,952
PPM1B_5_2597,550,809,720,1272,226,499,300,590,1723
PPM1M_5_2598,304,575,806,693,177,990,177,370,275
PPP1CA_5_2599,1841,3658,2085,1767,2617,2362,1885,2360,1306

PPP1CB_5_2600,7580,8449,10899,8262,11393,8641,7930,7050,13853
PPP1R12A_5_2601,1558,1238,1546,677,1255,1657,1693,2016,886
PPP1R16B_5_2602,629,449,1157,583,1160,177,1159,423,641
PPP1R1B_5_2603,368,605,828,648,141,573,541,857,758
PPP1R8_5_2604,5,108,2,0,3,251,0,0,0
PPP2R1B_5_2605,388,260,353,239,403,220,602,1700,106
PPP2R2A_5_2606,1584,1518,2773,1326,2547,1025,2256,1614,3947
PPP2R2B_5_2607,4105,4828,4773,3442,3886,3499,5834,3528,5987
PPP2R2C_5_2608,776,708,946,1493,827,1009,1267,566,670
PPP2R3A_5_2609,6746,8196,6476,5372,6747,10596,9020,6086,4341
PPP2R4_5_2610,172,153,150,0,0,571,342,66,421
PPP2R5A_5_2611,1990,1195,918,1366,1834,973,1011,738,536
PPP2R5D_5_2612,522,645,803,371,578,124,243,581,309
PPP3CA_5_2613,3156,3679,4312,1719,2801,1344,2253,2444,4600
PPP3CB_5_2614,1591,1243,1857,194,889,1502,1486,1006,3119
PPP4R1_5_2615,945,821,818,591,655,1014,439,1083,664
PPP5C_5_2616,205,381,969,238,69,341,886,641,486
PPP6C_5_2617,6017,9774,9568,6175,4549,11346,11030,8915,9866
PRKAA1_5_2618,403,781,117,381,482,487,187,386,307
PRKACA_5_2619,534,917,738,1766,1870,583,944,453,1139
PRKACB_5_2620,2370,3665,2714,3017,2592,3624,3606,3612,2028
PRKAG1_5_2621,1248,1282,1255,650,1071,1473,1716,1292,3267
PRKAG2_5_2622,13,0,51,0,0,0,12,0,60
PRKAR1A_5_2623,1824,2171,2688,2733,3335,6886,1338,2778,2445
PRKAR1B_5_2624,497,312,329,127,587,329,761,162,250
PRKCB_5_2625,684,518,1233,1351,392,575,293,432,4
PRKCD_5_2626,502,369,956,372,1071,859,319,288,265
PRKCQ_5_2627,838,797,863,926,1566,1017,2030,690,1593
PRKCZ_5_2628,478,740,551,351,844,197,790,421,639
PRKD2_5_2629,368,252,579,498,172,199,674,865,154
PRKDC_5_2630,1563,2478,1744,856,1369,1956,1606,704,2391
PRKG1_5_2631,2295,2015,2691,1295,2043,3673,2308,2408,4254
PRPS2_5_2632,1466,1123,1900,288,1797,1147,1513,735,2316
PTK2B_5_2633,586,625,1206,441,466,1346,934,371,928
PTK2_5_2634,9424,9054,9845,11550,6441,10355,7511,8510,5324
PTK7_5_2635,980,667,869,191,742,1030,477,2933,534
PTP4A2_5_2636,355,236,427,349,563,410,321,195,37
PTP4A3_5_2637,33,99,80,1,258,0,14,505,16
PTPDC1_5_2638,192,157,341,86,59,16,598,184,227
PTPN12_5_2639,2325,2613,2929,1229,2243,5180,1939,3213,1224
PTPN13_5_2640,2586,1903,2889,1249,2523,3468,3097,3703,1084
PTPN18_5_2641,3018,3724,4354,1632,4574,4939,2393,2800,4139
PTPN22_5_2642,3207,3692,5083,4369,5279,8861,2965,3432,3650
PTPN2_5_2643,667,858,1174,1178,1541,1505,1265,1295,30
PTPN3_5_2644,1789,1959,1855,1031,1099,3049,1877,2126,2386
PTPN5_5_2645,34,329,375,0,268,37,7,91,493
PTPN6_5_2646,193,406,697,1009,524,163,258,383,10
PTPN7_5_2647,183,961,448,85,1905,86,683,525,1010
PTPRA_5_2648,551,379,977,719,602,1044,1404,498,440
PTPRB_5_2649,751,1353,1363,24,2640,342,293,1582,101

PTPRC_5_2650,735,1034,1055,73,1353,1578,955,1260,1806
PTPRD_5_2651,443,955,823,69,1298,572,433,1271,2674
PTPRE_5_2652,566,831,903,2069,2085,526,218,8,417
PTPRF_5_2653,225,317,298,0,33,247,77,118,48
PTPRH_5_2654,182,16,46,0,490,2,2,1,0
PTPRJ_5_2655,443,198,472,1102,123,575,127,271,962
PTPRK_5_2656,2214,1834,2987,1351,2750,1808,1293,2339,2524
PTPRM_5_2657,922,786,922,344,1284,963,1006,1879,1331
PTPRN2_5_2658,1734,1586,1842,1662,2348,2455,1599,3055,1572
PTPRN_5_2659,340,662,206,611,600,506,732,55,4
PTPRR_5_2660,183,106,81,187,409,20,76,138,33
PTPRS_5_2661,148,246,231,620,873,1368,18,407,30
PTPRT_5_2662,460,179,363,278,583,1320,391,96,268
PTPRU_5_2663,226,262,64,55,739,6,141,0,593
PTPRZ1_5_2664,582,768,1024,431,1183,2836,137,430,851
RET_5_2665,313,312,266,384,390,8,430,64,0
RIOK1_5_2666,1576,1998,1230,1244,2250,2576,1340,1310,880
RIOK2_5_2667,11606,13119,12687,7857,14998,10220,12297,9602,15689
ROPN1L_5_2668,3175,2561,3103,3281,5029,3451,2731,1956,3712
RPS6KA1_5_2669,425,618,579,521,223,116,456,456,8
RPS6KA2_5_2670,928,1156,698,59,214,914,1255,505,522
RPS6KA4_5_2671,682,429,726,222,243,89,1455,196,1147
RPS6KA5_5_2672,906,1086,1786,1711,1720,1750,1332,351,330
RPS6KC1_5_2673,1087,1590,1698,1648,444,1591,1374,452,1585
RYK_5_2674,711,920,1217,656,968,850,1917,1162,1116
SET_5_2675,3331,3281,5159,4533,3430,5692,2801,3395,4887
SGK1_5_2676,800,1024,835,440,697,740,726,214,731
SGK3_5_2677,1984,2563,2266,1868,3854,698,846,988,1246
SIRPA_5_2678,1666,792,1379,2947,1388,1211,1666,864,622
SKAP1_5_2679,1273,2017,1238,733,1470,682,753,939,3292
SNRK_5_2680,349,661,937,288,638,598,318,849,1627
SPHK1_5_2681,144,194,196,212,708,6,376,218,37
SPHK2_5_2682,188,210,391,397,820,4,235,69,23
SRC_5_2683,153,214,143,310,480,101,24,674,43
SRPK2_5_2684,2074,3231,2628,1903,4214,3604,1069,1296,2534
SRPK3_5_2685,580,265,603,985,489,207,619,319,744
SSH1_5_2686,638,1025,1030,584,643,771,372,1334,681
STK19_5_2687,894,461,774,651,44,933,54,360,280
STK24_5_2688,1055,1718,1290,1659,760,2047,873,1355,3110
STK31_5_2689,141,396,381,980,403,84,1198,302,168
STK36_5_2690,1047,1575,1873,399,882,1097,853,1451,1005
STRADA_5_2691,1162,1072,1224,775,1231,1661,1599,1592,1811
STRADB_5_2692,1723,1039,1827,923,1649,1480,1104,1488,3239
STYX_5_2693,3799,4030,5827,4829,6349,7749,3067,4313,7953
SYK_5_2694,1179,1902,1997,1301,2531,1621,784,1436,1824
SYNJ1_5_2695,11,284,401,24,37,12,609,347,11
SYNJ2_5_2696,3899,4065,5238,5182,3807,6385,3162,5562,3810
TAF1_5_2697,1318,1206,1672,1841,2198,1579,1748,2061,2039
TAOK1_5_2698,1372,2293,3028,2584,1123,1615,2941,1217,3355
TAOK2_5_2699,332,243,781,1005,144,893,467,77,976

TBCK_5_2700,3130,2460,3187,1798,1915,4062,1475,2806,3161
TEX14_5_2701,607,814,1518,945,966,1787,546,1166,315
TGFBR1_5_2702,1771,1587,1807,641,2344,3022,1958,1650,930
TGFBR2_5_2703,964,1049,976,1588,1673,1093,1242,686,111
TGFBR3_5_2704,1372,2505,3452,1878,2311,4196,1890,2050,2120
THTPA_5_2705,2720,2577,4181,3559,3786,2649,4337,2100,2540
TJP2_5_2706,62,42,364,19,22,129,13,0,14
TK2_5_2707,2646,5026,3966,4896,2749,3102,3875,3630,2657
TLK1_5_2708,1002,1291,1218,1235,1327,1505,1434,531,1470
TLK2_5_2709,320,324,60,983,396,320,400,524,3
TMEM134_5_2710,687,494,894,516,364,1705,968,518,1694
TNIK_5_2711,1451,874,2470,1416,1026,1550,631,1077,1999
TNK2_5_2712,1718,1400,1553,391,1810,380,1978,884,2045
TPK1_5_2713,531,924,980,105,657,1070,561,540,26
TPTE2_5_2714,3081,3103,4094,2363,3367,5554,2265,2516,2945
TPTE_5_2715,6289,7129,13930,7376,6764,12744,9777,4655,11483
TRIM24_5_2716,2262,3757,3964,1386,2668,2590,2896,4428,6265
TRIM33_5_2717,882,837,1952,1023,933,2486,1261,750,926
TRMT2A_5_2718,714,1159,933,837,867,490,584,1984,2203
TRPM6_5_2719,2234,2918,3027,917,3694,3689,2747,2629,2994
TSSK4_5_2720,448,284,555,111,542,2422,783,395,726
TTK_5_2721,760,971,1363,690,943,1033,255,551,944
TTN_5_2722,4039,3304,5017,3371,5044,8823,5463,3865,3630
TWF1_5_2723,2769,3300,2235,3974,3061,2626,2682,1826,998
UCK1_5_2724,485,661,553,240,170,106,329,292,2
UCKL1_5_2725,233,509,265,337,1183,172,835,363,107
UHMK1_5_2726,679,1058,1458,1243,2171,697,1387,1005,880
ULK2_5_2727,897,903,1101,610,1222,1941,584,737,2369
VRK2_5_2728,202,418,65,986,194,449,1279,1373,324
VRK3_5_2729,111,560,428,378,96,405,2,96,15
WEE1_5_2730,1348,1004,1196,1848,3386,2119,1535,1528,1972
WNK1_5_2731,1239,1574,2841,1367,2480,2453,2585,2250,1627
WNK3_5_2732,177,86,103,0,8,1,142,0,169
YSK4_5_2733,310,248,443,39,244,294,80,541,48
ZAP70_5_2734,516,811,1132,152,1165,700,523,1265,3206
ACP2_5_2735,851,663,991,220,875,480,627,878,1265
CDK1_5_2736,313,792,508,257,13,113,456,280,209
DUSP10_5_2737,3644,3666,4766,3355,5751,3429,3636,2153,5215
DUSP15_5_2738,3392,3445,4257,2479,2941,3379,3946,2289,4951
ERBB3_5_2739,1183,936,1169,16,453,1704,2876,1088,994
G6PC2_5_2740,424,469,517,80,724,203,601,424,438
IP6K2_5_2741,325,191,753,860,96,898,475,548,94
MYLK_5_2742,4644,3497,3077,3755,1844,2789,3872,1941,2320
PRPS1_5_2743,3974,5385,5167,2210,6135,6032,3831,2164,6425
PTPMT1_5_2744,123,282,113,183,224,277,167,115,1205
PTPN20A_5_2745,2053,2650,3806,1780,3694,2171,3480,2789,5955
PTPN20B_5_2746,2053,2650,3806,1780,3694,2171,3480,2789,5955
SPEG_5_2747,348,368,308,1232,940,152,36,1,1426
STK32A_5_2748,733,562,694,1060,1407,1219,58,322,630
PPP1R12B_5_2749,580,701,1024,915,283,228,348,881,125

PPP1R12B_5_2750,631,648,803,200,1139,725,288,541,277
PPP1R12B_5_2751,4169,5244,4213,3207,3919,5087,3599,3073,3817
AAK1_5_2752,2611,4004,2793,2005,3659,1566,2723,3495,3466
ACP6_5_2753,923,851,835,1059,479,822,1254,388,1859
ACPT_5_2754,111,156,180,27,222,0,518,304,905
ACVR2A_5_2755,6657,6728,9659,6406,11174,5774,4943,6292,6293
ACVR2B_5_2756,274,344,171,107,160,42,208,69,798
ADCK2_5_2757,750,837,1323,886,879,1374,597,891,573
ADCK3_5_2758,2188,1346,2023,255,1604,3599,3472,1687,2459
ADCK5_5_2759,322,680,1102,904,290,335,15,162,1247
ADPGK_5_2760,2402,2167,3588,1395,1233,3598,1800,2418,1079
ADRBK1_5_2761,1707,1772,1815,1582,1303,482,1687,2599,3049
ADRBK2_5_2762,1523,1802,2000,333,3333,1269,1536,1863,2629
AGK_5_2763,2796,2944,4308,2593,1028,1857,3061,3783,5924
AK1_5_2764,395,368,310,1,434,0,457,717,0
AK7_5_2765,1673,1599,1135,945,2837,1633,1312,2120,1017
AK8_5_2766,783,238,243,135,559,5,79,34,221
ALK_5_2767,167,278,406,196,237,46,333,304,125
ALPI_5_2768,1767,1716,1918,3189,482,923,1369,350,255
ALPK2_5_2769,123,85,304,0,561,1160,98,28,378
ALPK3_5_2770,336,168,182,207,171,302,296,146,183
ALPP_5_2771,2996,2237,2237,1060,2811,2048,2060,2092,2049
ALPPL2_5_2772,1997,2305,2282,2656,2576,3487,1520,2017,1269
ANKK1_5_2773,66,304,111,119,2,3,67,480,1
ARAF_5_2774,903,618,1019,552,952,646,1858,427,1000
ATM_5_2775,1329,1711,1401,2144,1733,2610,873,1155,2660
AURKB_5_2776,3290,2949,2827,3501,3261,3388,4784,1194,1712
BLK_5_2777,345,254,482,609,301,248,274,1237,278
BMPR1A_5_2778,2962,4303,4271,672,6648,2202,5608,5643,3438
BMPR1B_5_2779,802,1264,1788,1172,1117,2257,766,1498,1259
BMPR2_5_2780,2994,5387,5405,2198,2440,6200,2860,6752,3791
BPNT1_5_2781,1769,1715,1602,2835,206,1880,1352,1460,472
BRAF_5_2782,929,1430,1440,2447,1855,1471,949,720,2917
BRD3_5_2783,951,943,694,740,591,738,426,1229,3643
BRSK1_5_2784,2226,1643,1902,1890,1241,1122,2863,1415,1619
BRSK2_5_2785,3167,2415,3093,3368,3272,2661,3280,3050,7245
BTK_5_2786,2272,1823,2137,1884,1543,1167,2464,1895,343
BUB1B_5_2787,1311,1063,1853,1442,784,2375,1543,1428,2974
BUB1_5_2788,1155,1074,1420,1312,1390,897,2375,347,1425
C20orf111_5_2789,738,1012,883,422,1026,1048,1289,1066,1595
C9orf96_5_2790,21,202,43,439,0,653,113,334,866
CALM1_5_2791,3367,2697,3445,1946,2524,2478,4487,2479,2497
CALM3_5_2792,216,239,882,1205,223,716,656,306,577
CAMK1_5_2793,296,432,339,570,581,743,507,620,22
CAMK1G_5_2794,94,67,21,0,0,78,0,120,126
CAMK2N1_5_2795,259,515,232,2,45,201,91,478,1
CAMK4_5_2796,1581,1767,2013,257,2063,2880,2022,2156,6091
CAMKV_5_2797,362,300,456,12,177,280,302,164,533
CARD11_5_2798,30,89,719,112,536,30,24,12,456
CCL2_5_2799,579,961,1060,304,1303,715,1237,504,670

CDC42BPB_5_2800,379,479,814,455,196,161,529,975,252
CDC42BPG_5_2801,1840,1701,2276,1915,3404,2819,2863,1802,1015
CDK14_5_2802,4326,3907,5637,4039,5040,2345,3230,4281,5578
CDK15_5_2803,2971,3011,4113,2705,3131,2731,2543,4340,3764
CDK19_5_2804,294,218,320,494,1021,27,46,421,319
CDK3_5_2805,1694,1109,775,926,1578,986,1138,1156,461
CDK4_5_2806,1047,1022,1577,657,872,173,749,1130,735
CDK5R1_5_2807,824,1349,969,242,1503,123,685,355,1760
CDK7_5_2808,1667,1745,1653,1500,1404,2018,1318,1798,255
CDK8_5_2809,1162,926,1336,337,1589,907,1899,397,1902
CDK9_5_2810,1447,974,564,578,938,993,707,1683,2080
CDKL1_5_2811,984,470,662,849,487,575,703,109,622
CDKL2_5_2812,274,490,286,362,288,544,368,1636,92
CDKL4_5_2813,1907,2282,1330,1538,1714,584,2379,1806,1077
CDKN1B_5_2814,210,193,516,241,643,598,389,91,2269
CERK_5_2815,1838,2060,3725,2614,1122,3423,1840,105,2496
CHKB_5_2816,441,230,80,229,93,0,36,7,1
CHUK_5_2817,936,1589,640,617,2306,1468,766,594,853
CIB2_5_2818,621,451,753,2111,294,385,371,491,973
CILP_5_2819,143,520,1693,799,257,1421,1047,779,241
CKB_5_2820,1768,2575,1745,1767,1516,1538,1106,1920,2467
CKM_5_2821,2157,1304,2417,677,2337,2964,1583,937,2920
CKMT1A_5_2822,4251,4390,3532,3536,6440,4462,5104,4764,2503
CKMT1B_5_2823,4251,4390,3532,3536,6440,4462,5104,4764,2503
CKS1B_5_2824,13288,16536,19881,14644,13169,17909,14561,15168,24703
CKS2_5_2825,4213,3572,5671,2175,3521,3554,4036,6157,3200
CLK2_5_2826,373,465,234,417,391,450,236,577,227
CLK4_5_2827,4090,3521,4782,4130,6409,4873,4621,5136,3305
CMPK2_5_2828,1386,1909,3057,1851,2166,1289,1219,605,924
CPNE3_5_2829,508,530,323,0,795,339,116,597,136
CRIM1_5_2830,248,449,556,104,163,666,739,601,325
CRKL_5_2831,376,512,128,1407,698,29,65,567,1091
CSF1R_5_2832,408,328,218,218,231,208,635,324,380
CSNK1A1L_5_2833,5672,4857,4767,3176,4053,4629,3121,4556,4954
CSNK1G1_5_2834,6773,7580,8380,6052,7597,8206,8060,6174,5924
CSNK1G2_5_2835,522,121,706,530,202,278,270,319,1
CSNK2A2_5_2836,488,936,734,365,997,231,102,580,283
CSNK2B_5_2837,140,154,537,46,7,15,439,137,161
CTDSP2_5_2838,343,1154,988,115,670,2339,91,1166,1148
DAPK1_5_2839,96,180,232,126,22,185,299,38,127
DAPK2_5_2840,560,194,426,704,407,3,457,216,0
DAPK3_5_2841,152,227,137,731,698,69,149,751,130
DBF4_5_2842,193,573,418,696,569,284,486,1366,268
DCK_5_2843,2452,2417,2741,3026,1398,3544,1662,589,2972
DCLK3_5_2844,854,765,1065,1263,555,883,624,910,1247
DGKE_5_2845,4076,3081,3852,1686,3652,3397,2561,2526,1330
DGKI_5_2846,1392,582,1001,867,890,1507,535,1011,457
DGKK_5_2847,890,685,1002,1098,786,616,1108,1010,131
DGKQ_5_2848,176,339,344,19,21,850,274,246,449
DOLK_5_2849,2356,1981,1663,1349,1559,1816,1301,1927,1349

DOT1L_5_2850,528,650,671,110,1942,442,1060,259,63
DUSP11_5_2851,1699,1013,845,751,315,1945,743,1309,411
DUSP12_5_2852,573,1224,1202,524,1530,148,1535,652,547
DUSP14_5_2853,1549,2148,3145,1336,3621,3722,728,4338,1105
DUSP16_5_2854,365,403,760,900,125,703,937,630,241
DUSP18_5_2855,330,752,413,75,774,277,294,499,165
DUSP21_5_2856,49,1052,584,263,11,295,468,320,315
DUSP22_5_2857,2203,3268,3177,3531,2601,1949,2711,3136,1635
DUSP2_5_2858,301,80,153,721,2,1062,4,742,413
DUSP3_5_2859,759,522,924,478,596,731,1053,512,539
DUSP5_5_2860,2167,2882,1924,1369,2575,4296,1606,1556,1013
DUSP7_5_2861,790,660,596,739,157,1089,404,147,162
DUSP8_5_2862,48,16,46,16,0,16,10,41,7
DUSP9_5_2863,708,643,550,671,266,1089,301,145,109
DYRK4_5_2864,1693,1246,1580,1873,2399,2361,1980,1031,1964
EEF2K_5_2865,353,440,852,589,1138,508,277,401,1111
EFNA2_5_2866,138,411,305,12,960,12,192,38,715
EFNA3_5_2867,1023,1160,1012,59,1232,1644,1275,1194,457
EFNA5_5_2868,327,823,964,131,312,203,469,169,49
EFNB3_5_2869,64,396,98,0,40,304,78,570,0
EIF2AK3_5_2870,1055,1434,1395,2904,1808,1202,2368,3543,2642
EIF2AK4_5_2871,369,480,606,169,68,1462,231,133,626
EPHA1_5_2872,428,352,482,330,276,495,290,637,213
EPHA2_5_2873,195,175,155,322,324,20,167,429,1189
EPHA4_5_2874,546,1155,783,1130,946,1708,169,1397,567
EPHA7_5_2875,4722,4366,5883,3514,6214,5763,4730,3911,4339
EPHB1_5_2876,273,1404,279,113,712,247,847,577,926
EPHB3_5_2877,351,506,73,331,893,123,22,34,3
EPHB4_5_2878,426,129,265,29,14,430,792,202,545
EPHB6_5_2879,1277,1482,1918,1592,2339,1721,1176,1074,1139
ERN1_5_2880,835,919,1208,928,930,1105,952,1231,1088
ERN2_5_2881,260,65,117,0,19,185,230,0,493
ETNK2_5_2882,211,323,143,351,37,154,55,436,510
EYA3_5_2883,600,1099,461,147,1002,311,561,149,257
FER_5_2884,696,763,1682,471,827,1333,321,699,753
FLT3_5_2885,2628,1933,3326,2408,3995,2526,2026,3189,2269
FN3K_5_2886,587,733,263,897,82,187,1,853,1
FN3KRP_5_2887,1809,1991,1853,670,2407,2091,2169,1704,1932
FRK_5_2888,1122,665,867,1501,248,317,375,577,45
FUK_5_2889,366,237,598,1498,601,100,18,126,833
G6PC3_5_2890,696,505,1025,289,778,801,402,688,1012
G6PC_5_2891,71,419,120,1,276,0,18,506,0
GAK_5_2892,301,294,351,251,80,1335,24,215,949
GALK1_5_2893,2927,2576,2047,3258,3813,3334,2364,1411,1323
GDPD4_5_2894,2332,1873,2005,4081,2266,2094,2623,1540,2580
GOLGA5_5_2895,1222,1939,1955,410,710,1036,913,1160,590
GRK1_5_2896,243,1073,314,103,221,483,51,1017,1
GRK5_5_2897,776,1582,1288,1118,2010,306,1152,613,2631
GRK7_5_2898,3354,3510,4603,1958,2663,3503,3604,2232,3677
GSG2_5_2899,378,304,507,658,232,85,333,295,2

GSK3A_5_2900,849,1375,1567,2098,1729,257,2088,1242,1176
GUCY2C_5_2901,2537,2556,3373,1471,2872,2399,1132,2088,2896
GUCY2D_5_2902,2941,3395,3539,2667,2338,4047,2268,2164,3675
GUCY2F_5_2903,556,625,1143,516,678,780,818,313,925
HIPK4_5_2904,227,281,168,207,209,254,122,27,76
HKDC1_5_2905,175,25,307,97,888,458,463,20,242
HOOK3_5_2906,490,1774,1139,669,1389,680,1022,842,2483
HSPB8_5_2907,220,391,275,400,325,38,2,252,183
HUNK_5_2908,0,0,0,0,0,0,0,1,0
HUS1_5_2909,1104,551,1090,907,730,1792,920,810,1101
IGBP1_5_2910,488,328,329,74,1300,325,127,414,1221
IGF1R_5_2911,875,532,966,122,1030,660,1362,1105,506
IGF2R_5_2912,324,310,349,219,348,355,350,747,44
ILKAP_5_2913,1375,1863,2235,2692,3977,2290,1259,1737,552
IMPA2_5_2914,342,149,620,410,265,1647,337,388,1418
INPP5A_5_2915,2469,2728,3131,1923,3738,5058,1917,3539,4407
INPP5B_5_2916,54,196,229,87,692,1111,519,122,20
INPP5J_5_2917,362,463,562,40,399,3,358,404,103
INPPL1_5_2918,1961,1809,1799,2551,3056,2682,3016,801,1690
INSRR_5_2919,47,658,114,571,1267,447,336,790,355
IPMK_5_2920,461,178,437,155,577,26,586,433,53
IPPK_5_2921,1752,1153,1324,1420,1644,434,1387,1335,876
IRAK2_5_2922,413,130,743,358,29,66,183,449,65
ITK_5_2923,281,344,500,202,332,115,277,1356,0
ITPKA_5_2924,57,156,39,479,335,0,256,3,0
ITPKB_5_2925,155,79,155,0,5,0,170,18,41
ITPKC_5_2926,484,638,1004,716,287,1731,704,610,2328
JAK1_5_2927,219,330,473,266,394,1030,18,731,552
JAK2_5_2928,1618,1912,2923,1476,1122,2522,1580,1364,3356
JAK3_5_2929,469,225,263,697,363,486,157,86,753
KDR_5_2930,519,719,996,735,1142,1154,1165,858,1172
KSR1_5_2931,659,704,468,637,960,632,548,872,230
LATS1_5_2932,4018,5560,4896,3909,5462,6906,5896,4711,8002
LATS2_5_2933,188,331,269,195,1058,41,593,72,0
LLGL1_5_2934,221,687,192,115,381,163,650,331,1249
LMTK2_5_2935,116,46,345,105,259,125,143,19,80
LMTK3_5_2936,1063,1887,1391,1311,2134,1062,1575,1489,1248
LRRK1_5_2937,983,361,607,1007,1148,567,358,316,224
LRRK2_5_2938,2380,3478,3304,3634,4125,2800,2296,3106,1216
MAGI2_5_2939,1641,1125,682,1810,1511,380,1193,1902,1475
MAP2K1_5_2940,1409,1347,1474,681,460,1466,1053,1602,487
MAP2K2_5_2941,2315,2088,3282,932,2743,1643,1677,2162,1390
MAP2K4_5_2942,2000,2416,2201,413,3005,2765,2341,606,835
MAP2K6_5_2943,1341,1376,2109,1822,895,2607,1519,1548,2027
MAP2K7_5_2944,705,1093,406,1115,1922,638,15,879,13
MAP3K10_5_2945,666,980,1074,1046,1043,1216,758,304,543
MAP3K11_5_2946,11,216,208,294,47,0,69,382,11
MAP3K14_5_2947,16,0,206,0,24,1,0,9,0
MAP3K15_5_2948,6327,5860,8988,1875,8245,6180,7274,5301,17456
MAP3K1_5_2949,1546,1265,2153,2064,128,533,1944,469,753

MAP3K2_5_2950,902,1071,1577,201,514,1339,855,1149,480
MAP3K5_5_2951,1275,1932,2530,868,1657,2273,1807,1325,1256
MAP3K6_5_2952,151,421,671,308,374,140,923,565,938
MAP3K8_5_2953,1198,1041,1851,1232,1915,866,902,1080,3114
MAP3K9_5_2954,189,219,258,92,472,448,233,452,393
MAP4K2_5_2955,1852,2559,3257,1441,2987,3869,1752,1741,3899
MAP4K3_5_2956,1501,1593,1915,1749,1687,1990,1202,1729,1506
MAPK11_5_2957,726,602,958,461,22,1440,501,888,3565
MAPK12_5_2958,20,282,763,0,454,581,55,0,391
MAPK13_5_2959,775,1282,1287,454,1169,1106,740,330,737
MAPK15_5_2960,537,471,1027,529,29,692,663,130,843
MAPK4_5_2961,381,320,516,228,174,890,273,182,266
MAPK6_5_2962,582,394,591,633,883,667,419,434,0
MAPKAPK3_5_2963,141,168,250,8,39,5,46,21,0
MARK1_5_2964,516,857,349,525,309,89,646,206,495
MAST2_5_2965,167,338,236,18,230,325,258,36,52
MAST3_5_2966,361,327,559,27,538,444,3,459,524
MELK_5_2967,919,975,953,788,95,201,698,2043,1654
MERTK_5_2968,429,1088,584,675,1436,92,484,256,1537
MEX3B_5_2969,35,584,172,1,383,647,304,48,2
MPP3_5_2970,629,1211,807,109,2533,866,728,409,2642
MPP5_5_2971,743,1561,1820,1676,2002,1461,507,1042,1351
MST1R_5_2972,31,239,207,6,15,16,73,183,271
MTM1_5_2973,0,0,0,0,0,0,0,1,0
MTMR1_5_2974,3296,2392,4458,2267,4225,2527,2672,1688,6108
MTMR4_5_2975,3709,4324,4281,1861,3243,2732,4207,3028,4067
MTMR6_5_2976,1785,2499,2909,1935,1717,3307,2191,2055,1298
MTMR7_5_2977,1366,578,1409,270,693,607,723,632,734
MTMR9_5_2978,423,1131,1352,1313,249,720,22,10,1767
MTOR_5_2979,127,380,90,0,6,0,444,520,44
MYLK2_5_2980,453,301,904,629,506,1298,620,1411,782
MYLK3_5_2981,2052,3184,3862,5429,2870,3396,2551,4581,3615
MYLK4_5_2982,3573,5725,4811,2960,4165,4620,4075,3833,3859
MYO3A_5_2983,3388,4945,4791,4738,7692,6367,6946,3357,5398
N4BP2_5_2984,2074,2447,2417,687,1554,4167,1273,2632,3859
NAGK_5_2985,1190,874,871,1454,2049,1183,1567,978,1469
NAGS_5_2986,1542,2229,2539,2745,2444,330,1864,1786,4072
NEK10_5_2987,2554,2182,2974,2338,2417,2010,2052,1272,1836
NEK5_5_2988,322,190,632,559,26,392,495,359,183
NEK7_5_2989,2896,2873,3162,1900,1513,562,2301,2560,4074
NEK8_5_2990,376,679,688,964,2,519,467,67,12
NEK9_5_2991,669,229,135,2,106,450,419,249,49
NME3_5_2992,185,23,41,199,0,197,147,0,95
NME4_5_2993,0,45,15,28,36,26,0,0,336
NME5_5_2994,1343,1796,1192,1385,3275,1163,1789,1193,1986
NME6_5_2995,1331,829,741,907,778,622,958,287,287
NPR1_5_2996,560,724,376,804,2395,282,630,371,328
NPRL2_5_2997,244,73,434,98,1665,223,145,282,0
NRBP1_5_2998,290,556,394,615,461,351,95,354,124
NRBP2_5_2999,265,155,182,25,5,318,667,230,380

NRK_5_3000,137,81,288,85,119,394,175,253,172
NT5C1A_5_3001,1343,1074,1094,527,313,691,1597,736,890
NT5C_5_3002,272,665,623,363,1410,635,73,286,114
NT5M_5_3003,386,969,232,25,410,2,25,261,1602
NUAK1_5_3004,794,744,1502,14,659,479,932,679,423
NUAK2_5_3005,103,218,246,2,175,2,206,3,2
NUCKS1_5_3006,1968,1532,2626,972,3397,3859,3158,2391,1299
OXSR1_5_3007,442,426,492,220,212,898,512,174,161
PAK2_5_3008,1452,938,1776,941,3168,3317,956,1209,1006
PANK3_5_3009,4353,5600,6265,1968,5138,7512,4601,4667,4228
PANK4_5_3010,94,106,21,0,304,23,228,79,0
PASK_5_3011,347,630,641,1401,1025,208,234,1080,825
PBK_5_3012,452,485,411,507,368,1017,247,1070,450
PCK1_5_3013,416,1194,804,205,661,866,1195,377,911
PDCD1_5_3014,569,203,655,22,832,176,268,638,2312
PDGFRA_5_3015,952,906,1721,873,175,947,373,2182,648
PDGFRB_5_3016,49,101,115,80,2,174,1,250,0
PDGFRL_5_3017,152,141,191,147,58,189,113,112,305
PDIK1L_5_3018,585,540,649,2281,355,1266,473,220,128
PDK4_5_3019,7731,10090,12803,9866,9011,11868,5503,7396,4647
PDP2_5_3020,1714,2417,2246,1160,1179,1168,1592,2203,797
PDXK_5_3021,375,625,1345,156,91,1013,625,491,1194
PFKFB1_5_3022,2369,1834,2382,3604,1890,1356,1944,780,1446
PFKFB4_5_3023,1171,567,1007,56,1210,1037,632,1641,3018
PFKL_5_3024,210,166,159,41,69,1190,16,40,30
PGAM2_5_3025,578,605,731,2047,450,1036,722,862,1221
PGK1_5_3026,1365,971,1538,670,493,1879,1201,1370,1568
PGK2_5_3027,1148,1082,1231,2316,1193,2704,2023,1552,1080
PHKA2_5_3028,4112,6853,6268,5297,6661,4554,5997,9703,7857
PHKG1_5_3029,762,854,713,372,1492,1781,623,508,275
PI4K2A_5_3030,638,749,975,1163,1075,1292,895,791,1356
PI4K2B_5_3031,6787,6900,6475,11086,5055,4655,5946,7454,6529
PIK3C2A_5_3032,3639,3883,4517,2320,3995,3852,4037,3332,6569
PIK3C2B_5_3033,873,617,1226,1442,742,1473,1217,623,4347
PIK3C2G_5_3034,1999,936,1519,2975,1462,1057,2482,435,1278
PIK3C3_5_3035,342,16,281,597,285,101,427,15,939
PIK3CA_5_3036,3282,3156,3172,3333,3284,8065,2552,3432,7506
PIK3CB_5_3037,2654,3342,2777,4752,3250,5603,2334,4511,2066
PIK3CD_5_3038,65,11,671,2,200,370,446,390,94
PIK3CG_5_3039,3340,4238,5801,4445,2479,5856,6283,4624,3900
PIK3R2_5_3040,1775,1199,2282,1255,2324,2103,1730,3337,1451
PIK3R4_5_3041,584,468,786,151,257,942,569,1327,164
PIK3R6_5_3042,1,2,4,0,0,0,4,0,0
PIM2_5_3043,30,80,5,4,1107,2,34,11,1
PIM3_5_3044,892,1878,1148,1057,3317,2148,955,1492,3592
PINK1_5_3045,607,694,418,154,162,1099,244,1064,135
PIP4K2A_5_3046,1033,847,1052,1094,992,592,1563,944,886
PIP4K2B_5_3047,589,551,418,1695,243,970,416,213,536
PIP5K1B_5_3048,2645,2357,3199,2249,2977,2409,3015,3026,3386
PKDCC_5_3049,4351,3909,4961,3753,6064,4402,5006,5683,3536

PKN2_5_3050,1781,2196,1306,2310,2727,511,1787,2696,2673
PKN3_5_3051,116,83,290,1304,466,139,213,255,140
PLK1_5_3052,141,205,54,45,19,386,55,0,146
PLK2_5_3053,3863,3979,4387,5404,4205,4712,3928,2882,3914
PLK3_5_3054,471,476,228,1624,253,2,23,96,753
PMVK_5_3055,662,790,1145,607,562,1399,879,994,185
PNKP_5_3056,1045,708,424,512,1100,553,562,834,1339
POLD1_5_3057,54,158,179,119,197,313,2,7,90
PON1_5_3058,3476,4740,5898,6532,3903,6698,4675,4542,8999
PPAP2B_5_3059,1363,713,1042,1002,1519,1194,1359,1646,433
PPEF2_5_3060,262,384,406,312,542,1281,27,304,566
PPFIA3_5_3061,773,363,425,308,903,395,344,8,774
PPFIA4_5_3062,461,1028,682,1551,447,1891,579,981,1259
PPM1D_5_3063,988,1618,1028,80,2509,838,449,1030,1445
PPM1E_5_3064,405,406,217,216,194,138,8,982,376
PPM1F_5_3065,772,479,838,1028,444,47,677,680,138
PPM1G_5_3066,72,255,277,608,61,838,103,311,1231
PPM1K_5_3067,1971,1329,2698,1825,1411,2320,1887,920,1872
PPM1L_5_3068,578,528,785,154,1216,302,615,1046,1171
PPP1CC_5_3069,196,116,220,14,594,116,1,104,283
PPP1R12C_5_3070,258,1134,730,12,570,329,1,366,588
PPP1R13B_5_3071,599,741,540,1336,339,533,467,718,246
PPP1R14B_5_3072,434,346,692,80,557,186,380,788,1029
PPP1R15B_5_3073,1604,2655,2052,1320,2079,1108,1411,1917,2329
PPP1R1A_5_3074,460,242,271,734,0,0,61,0,1
PPP1R1C_5_3075,670,416,674,485,1536,473,575,112,471
PPP1R2_5_3076,1726,1825,2037,3265,3078,2125,1369,1879,3756
PPP1R3A_5_3077,3494,3754,4219,4815,1673,3568,2987,3616,6225
PPP1R3C_5_3078,1490,1760,2268,2235,966,2077,1902,1748,2488
PPP1R3D_5_3079,308,221,21,1,36,67,315,351,389
PPP1R7_5_3080,418,176,322,71,378,1816,342,176,888
PPP2CA_5_3081,4425,6546,5952,4724,6567,6892,5791,9813,6318
PPP2CB_5_3082,1459,1735,1783,1956,3168,962,924,1647,2833
PPP2R1A_5_3083,330,393,594,144,950,619,592,278,843
PPP2R2D_5_3084,1228,1139,1164,270,1789,1160,1459,218,2876
PPP2R3B_5_3085,1461,1566,1192,1418,1182,3824,1215,1992,3120
PPP2R5B_5_3086,639,676,810,252,1648,1601,780,867,290
PPP2R5E_5_3087,726,1099,1816,346,2304,1408,699,1105,1609
PPP3CC_5_3088,1747,1599,3088,733,1887,2955,1011,2220,1421
PPP3R1_5_3089,1355,1962,1567,1266,734,1652,1611,2157,1740
PPP3R2_5_3090,1349,1017,1116,632,1726,814,2184,851,2178
PPP4C_5_3091,102,93,271,0,500,311,29,41,15
PPTC7_5_3092,885,1134,1217,779,1818,364,1095,723,1842
PRKAA2_5_3093,35,108,10,0,985,129,97,455,74
PRKAB1_5_3094,2007,2873,2658,701,3756,2491,1579,2143,2165
PRKAB2_5_3095,1173,1440,935,1463,705,290,1114,1280,100
PRKACG_5_3096,125,118,345,163,131,240,328,224,52
PRKAG3_5_3097,350,477,559,314,1060,18,849,386,25
PRKAR2A_5_3098,827,1873,1057,394,262,1094,1155,188,627
PRKAR2B_5_3099,308,708,715,124,809,138,998,1649,7

PRKCA_5_3100,2584,3521,2388,2920,1154,1208,2942,2347,2281
PRKCE_5_3101,678,889,513,296,642,8,834,96,1130
PRKCG_5_3102,321,800,544,802,289,861,325,905,88
PRKCH_5_3103,979,1450,1088,975,483,1728,1642,916,2310
PRKCI_5_3104,1109,1748,2638,1204,367,1225,627,307,2313
PRKD1_5_3105,498,670,1628,1998,296,165,947,648,472
PRKD3_5_3106,2142,2420,3065,1469,2661,3905,1664,2000,2294
PRKG2_5_3107,1716,2117,2266,2977,3783,816,773,2243,1359
PRKX_5_3108,198,198,567,602,200,123,393,758,214
PRPF4B_5_3109,676,1033,1238,435,457,1099,274,188,2461
PRPS1L1_5_3110,1227,1139,1448,2700,1685,1659,2676,1008,548
PSKH1_5_3111,1851,1349,1283,1936,954,358,2349,306,2278
PSKH2_5_3112,153,217,236,20,128,108,151,291,146
PSPH_5_3113,490,852,877,1047,237,727,277,850,789
PSTK_5_3114,2717,2417,3392,1444,2428,2407,2490,1751,4336
PTEN_5_3115,3711,2283,3978,2836,2663,2129,2160,4287,1930
PTK6_5_3116,1625,2224,2212,2377,2430,770,1343,996,3039
PTP4A1_5_3117,1995,2064,1455,1508,1904,1571,1511,1599,1125
PTPLA_5_3118,7633,9541,11536,8482,8821,6713,7195,7960,9440
PTPLB_5_3119,2068,1802,2531,2435,1559,4954,1720,1684,2577
PTPN11_5_3120,847,681,1350,295,463,296,1535,1362,2062
PTPN14_5_3121,876,1027,1073,552,481,2043,479,628,1518
PTPN1_5_3122,107,705,30,472,1981,416,728,285,47
PTPN21_5_3123,2076,2499,2245,1602,2110,3735,2858,3016,4120
PTPN23_5_3124,267,486,616,133,3,270,75,451,277
PTPN4_5_3125,722,965,1076,334,618,826,98,545,384
PTPN9_5_3126,509,351,873,1018,380,479,952,125,338
PTPRG_5_3127,385,771,882,92,95,624,425,7,514
PXK_5_3128,2492,2215,1720,1458,3074,3349,1867,3273,1089
RAF1_5_3129,292,359,556,460,133,835,315,608,776
RBKS_5_3130,462,118,443,94,1253,71,248,288,1000
RFK_5_3131,1288,484,1049,701,1177,942,1444,1508,410
RIOK3_5_3132,385,658,1264,133,9,875,143,715,1
RIPK1_5_3133,1342,1626,1472,1187,1632,1492,1599,1094,1124
RIPK2_5_3134,8744,8540,10904,6980,6857,9521,9010,8930,8207
RIPK3_5_3135,830,1204,863,487,1522,1172,632,1434,105
RIPK4_5_3136,218,441,68,160,92,15,170,232,46
RNASEL_5_3137,284,354,115,256,603,73,10,171,1080
RNGTT_5_3138,384,594,591,7,324,211,61,168,86
ROCK1_5_3139,732,454,399,285,114,452,595,209,19
ROCK2_5_3140,3485,4049,3879,3649,3686,3715,4684,4060,3218
ROS1_5_3141,978,2008,2184,1324,1530,2600,1637,930,1796
RPS6KA3_5_3142,1741,1471,2327,2647,1791,336,269,727,242
RPS6KA6_5_3143,1402,1608,2195,857,1270,1276,1263,1648,1721
RPS6KB1_5_3144,3678,3678,3840,3154,2950,1415,1902,3938,1571
RPS6KB2_5_3145,769,444,916,354,463,108,270,0,112
RPS6KL1_5_3146,629,652,539,683,770,392,377,212,179
RSPO3_5_3147,360,266,468,139,52,19,296,127,701
SACM1L_5_3148,840,1779,1346,682,2361,1184,1948,312,1725
SBF1_5_3149,1743,2043,1623,3666,2005,1000,1471,2246,2787

SBK1_5_3150,135,316,36,368,33,24,121,3,6
SBK2_5_3151,595,511,641,409,819,745,172,104,1755
SEPHS2_5_3152,618,298,289,342,897,395,777,790,1371
SGPP1_5_3153,1902,2357,2636,1322,4224,3986,2139,2506,4188
SHPK_5_3154,1313,991,1401,829,1088,1273,2546,1297,495
SIK1_5_3155,1115,978,1187,440,2013,879,644,107,1234
SIK2_5_3156,139,148,344,230,1419,397,13,129,494
SIK3_5_3157,529,704,660,289,1291,553,263,1223,134
SKAP2_5_3158,963,1072,752,2476,813,341,1286,657,210
SMG1_5_3159,174,225,202,13,604,847,92,461,115
SRMS_5_3160,836,692,1157,702,1936,1012,989,735,509
SRP72_5_3161,467,759,1174,0,1417,647,458,140,180
SRPK1_5_3162,1988,2612,3662,879,2032,3294,2268,2959,2945
SSH2_5_3163,1379,1650,2390,2230,1460,1153,2073,1474,1236
SSH3_5_3164,493,343,579,373,430,518,351,214,1122
STC1_5_3165,2580,3709,3433,1463,5655,3025,1904,2499,2614
STK10_5_3166,112,241,577,374,46,1003,297,211,424
STK11_5_3167,306,311,475,1205,20,1,350,360,12
STK16_5_3168,523,442,661,217,189,167,384,626,98
STK17A_5_3169,226,406,698,21,491,738,621,889,940
STK17B_5_3170,629,1596,1363,361,1645,1449,1139,1361,2668
STK25_5_3171,860,798,1387,1051,899,1056,541,893,1102
STK32B_5_3172,770,572,1211,191,524,1583,914,793,1863
STK32C_5_3173,106,191,26,1,10,183,303,1,196
STK33_5_3174,1400,2619,1965,1177,2350,2953,1019,1472,648
STK35_5_3175,466,650,517,186,11,383,533,497,632
STK38_5_3176,1423,657,2072,1049,1199,679,1209,967,2109
STK38L_5_3177,505,990,725,527,1499,2502,579,1141,832
STK39_5_3178,199,168,266,879,388,1187,675,204,21
STK40_5_3179,640,711,471,803,125,1030,703,228,1027
STK4_5_3180,2925,1800,3242,1306,1616,3227,2245,3340,3875
STYK1_5_3181,807,870,684,114,188,794,1182,268,3968
STYXL1_5_3182,84,119,264,193,697,19,318,395,110
TAF1L_5_3183,1883,2127,2203,1825,2024,3042,2095,2507,2191
TAOK3_5_3184,4506,4200,4131,5451,2973,4337,2102,3164,3501
TBK1_5_3185,10213,11113,13004,8162,10097,10539,11513,15610,12087
TEC_5_3186,3169,4026,4137,3698,3846,2083,3593,3221,6149
TEK_5_3187,1137,1760,2772,1012,977,1846,806,1655,1054
TESK1_5_3188,3250,3240,3889,5702,3623,4353,3681,4551,5504
TESK2_5_3189,1276,1682,2594,1568,2864,530,958,1830,973
THNSL1_5_3190,737,747,1407,469,1105,472,806,800,368
TIE1_5_3191,359,366,156,35,701,123,230,377,490
TK1_5_3192,354,347,395,0,55,291,51,29,1891
TNK1_5_3193,915,1056,1222,552,1473,329,1518,444,2268
TNNI3K_5_3194,1802,1860,1740,1472,735,858,1516,1520,1053
TNS3_5_3195,469,264,194,114,29,253,552,1,1895
TP53RK_5_3196,885,256,626,420,254,1738,1537,636,997
TRAT1_5_3197,654,544,1256,769,106,1971,415,130,1528
TRIB1_5_3198,988,1494,1163,1157,804,636,1169,1008,1543
TRIB2_5_3199,2272,1583,2346,2981,5599,1646,2523,951,3202

TRIB3_5_3200,512,338,1082,1127,1293,802,302,43,7
TRIM27_5_3201,601,523,779,1453,177,43,188,6,732
TRIM28_5_3202,271,236,81,137,384,13,321,289,2
TRIO_5_3203,1479,1271,1635,1553,1128,1316,1625,1773,1607
TRPM7_5_3204,521,583,438,516,312,191,263,302,0
TRRAP_5_3205,3131,3912,3317,2586,1685,4848,1198,3068,1718
TSKS_5_3206,997,559,730,127,1026,1428,134,1307,339
TSSK1B_5_3207,451,162,618,9,49,15,165,90,10
TSSK2_5_3208,398,581,422,945,125,613,349,531,284
TSSK3_5_3209,5978,5420,5533,6129,5473,6272,8612,5991,5331
TSSK6_5_3210,94,56,39,0,0,0,12,13,1
TTBK1_5_3211,462,781,251,1,230,1324,224,307,194
TTBK2_5_3212,760,680,656,1166,1172,508,400,131,958
TWF2_5_3213,475,594,635,713,542,518,1348,128,976
TXK_5_3214,3587,3530,4330,1197,2240,6061,2752,2180,7712
TYK2_5_3215,955,814,1005,1155,1347,313,408,610,888
TYRO3_5_3216,887,393,1669,358,427,813,834,1430,623
UBLCP1_5_3217,339,262,728,470,76,516,25,537,370
UCK2_5_3218,1438,1882,1455,582,3906,1618,1228,1598,5465
ULK1_5_3219,2606,2455,2782,3138,2058,4221,2894,1591,2053
ULK3_5_3220,1494,1348,2408,1804,3099,3756,1457,1367,2233
ULK4_5_3221,2006,1556,1822,943,2440,1466,1908,1064,1063
VRK1_5_3222,384,1071,936,331,589,1425,1018,703,527
WEE2_5_3223,1177,2068,2101,2610,1698,2604,2165,2301,4924
WNK2_5_3224,533,191,670,285,837,344,204,450,613
WNK4_5_3225,1035,1021,1090,24,725,1987,937,514,2697
XRCC6BP1_5_3226,456,300,650,867,117,555,324,724,1543
XYLB_5_3227,450,1126,772,125,856,634,873,247,713
YES1_5_3228,0,0,0,0,0,0,0,0,0
AATK_5_3229,125,202,84,351,11,56,97,12,1
ABL1_5_3230,887,927,717,846,634,534,747,240,558
ABL2_5_3231,466,329,659,255,255,1148,466,246,96
ACP1_5_3232,852,696,1015,738,730,1266,347,714,1149
ACP5_5_3233,1659,1197,2314,1198,1393,1915,2954,1564,1719
ACPL2_5_3234,510,507,681,131,1022,1311,296,421,508
ACPP_5_3235,2356,2511,3677,3762,3280,2750,2769,2457,3640
ACVR1B_5_3236,402,562,364,391,2620,422,235,358,112
ACVR1C_5_3237,1255,1137,1324,1264,1038,1491,1271,1089,1081
ACVR1_5_3238,222,641,797,951,420,142,333,536,750
ACVRL1_5_3239,217,105,115,30,413,2,127,40,25
ADCK1_5_3240,2568,2710,3187,3380,5174,4254,2826,3656,3873
ADCK4_5_3241,288,727,691,1187,992,1447,321,594,478
ADK_5_3242,2215,865,2438,1261,599,5341,1198,1606,2701
AK2_5_3243,11,259,30,258,0,622,0,0,0
AK4_5_3244,359,440,339,210,30,1170,522,60,14
AK5_5_3245,567,740,542,599,1309,446,366,377,787
AKT1_5_3246,568,225,750,1271,857,1646,268,132,1036
AKT2_5_3247,28,134,113,5,151,134,27,1,12
AKT3_5_3248,587,328,1081,0,1563,1410,692,78,2
ALDH18A1_5_3249,534,171,687,0,86,740,10,188,10

ALPK1_5_3250,10423,10487,12242,8529,8380,9743,7515,5799,11734
ALPL_5_3251,159,252,64,0,2001,17,122,399,0
AMHR2_5_3252,690,904,858,778,364,1469,1362,528,1982
ANKHD1_5_3253,108,117,219,46,23,756,41,5,92
APTX_5_3254,4705,4068,5658,2383,3495,3658,2152,4548,4750
ATRIP_5_3255,1456,1138,763,1670,840,1733,1127,759,823
AURKA_5_3256,88,380,524,18,162,0,110,286,32
AURKC_5_3257,1214,1384,2477,1972,2917,1504,2146,1670,1742
AXL_5_3258,1062,1606,1325,1124,1031,1101,1847,2494,984
BAIAP2_5_3259,1041,1071,676,70,437,332,1417,1981,469
BCKDK_5_3260,2035,1139,2198,2147,886,2888,1184,1811,1857
BCR_5_3261,923,592,1633,2048,167,1022,946,782,92
BMP2K_5_3262,448,447,493,319,722,105,356,223,459
BMX_5_3263,353,634,413,1142,295,579,361,432,228
BPGM_5_3264,1831,2061,3445,2587,1615,4085,2336,1835,2644
BRD2_5_3265,753,598,800,617,1154,1662,673,1090,656
BRD4_5_3266,531,1062,1230,62,31,239,1170,1139,854
BRDT_5_3267,399,645,363,476,149,148,252,232,559
CAB39L_5_3268,3781,4456,6609,4451,3552,2973,4975,4026,8586
CAMK1D_5_3269,356,285,405,28,275,135,394,516,558
CAMK2A_5_3270,589,708,806,700,252,325,544,546,444
CAMK2B_5_3271,122,7,11,104,9,0,54,42,0
CAMK2D_5_3272,391,383,294,239,71,619,1164,646,103
CAMK2G_5_3273,588,921,582,763,1853,561,471,411,92
CAMKK1_5_3274,99,293,247,121,53,3,259,250,856
CAMKK2_5_3275,217,227,572,16,90,291,647,79,136
CASK_5_3276,4693,4036,5262,3831,4416,5744,4731,2999,5050
CCT2_5_3277,332,371,561,448,70,543,766,207,94
CDADC1_5_3278,588,790,1100,682,104,538,493,135,374
CDC14A_5_3279,2467,3149,2906,3264,4731,3725,2343,2961,1638
CDC14B_5_3280,273,377,578,382,220,1424,645,417,1084
CDC25A_5_3281,447,168,181,5,22,786,491,88,456
CDC25B_5_3282,1044,739,820,2025,542,1503,517,749,923
CDC25C_5_3283,1140,1250,1052,1102,186,872,313,145,1009
CDC42BPA_5_3284,74,2,8,0,0,45,0,1,0
CDC7_5_3285,1621,1797,1428,2523,1239,1293,1748,1123,1377
CDK10_5_3286,439,315,396,443,52,761,70,227,1769
CDK11A_5_3287,3452,3132,4738,2637,4857,3536,4195,4051,3915
CDK11B_5_3288,3452,3132,4738,2637,4857,3536,4195,4051,3915
CDK12_5_3289,442,609,1036,224,317,347,177,596,2192
CDK13_5_3290,1269,1531,666,853,914,703,920,494,737
CDK16_5_3291,521,609,623,455,693,1069,503,246,394
CDK17_5_3292,178,326,303,1,40,269,2,360,0
CDK18_5_3293,3077,2846,2951,2569,1857,3192,2740,1882,2311
CDK20_5_3294,3275,4566,3773,5308,4475,4314,2796,2418,6164
CDK2_5_3295,408,197,460,386,687,114,17,178,1348
CDK5_5_3296,761,944,1297,907,419,1187,1013,381,1885
CDK6_5_3297,2395,2476,2543,2484,1322,1782,2664,2999,2559
CDKL3_5_3298,99,408,311,6,43,646,269,380,558
CDKL5_5_3299,1529,2333,2838,2278,3244,3504,2943,3248,4181

CDKN1A_5_3300,714,628,782,265,920,1232,968,507,903
CDKN3_5_3301,368,807,768,41,549,950,1707,521,226
CHEK1_5_3302,2755,3381,4166,3440,2862,3594,2494,2935,2437
CHEK2_5_3303,3570,4079,5144,5794,3677,4592,5138,3997,5137
CHKA_5_3304,2182,1530,2287,2699,1894,3050,2071,1256,4068
CIT_5_3305,781,844,1781,383,2842,1537,1490,1247,1377
CKMT2_5_3306,1888,946,2046,981,1762,2941,1389,946,2297
CLK1_5_3307,1179,1655,1913,1490,1722,1007,1557,1528,1820
CLK3_5_3308,134,383,443,495,1836,87,422,294,288
CMPK1_5_3309,5345,4880,5194,6189,7664,2363,6013,4511,7138
COASY_5_3310,343,418,539,7,512,736,487,350,1127
COL4A3BP_5_3311,962,1746,1028,509,2916,841,729,1454,748
CSK_5_3312,287,368,504,1019,1223,1071,493,344,330
CSNK1A1_5_3313,1724,1121,2008,2233,1289,1191,1742,1570,2681
CSNK1D_5_3314,378,176,242,282,21,156,112,73,13
CSNK1E_5_3315,386,75,544,47,225,46,270,271,0
CSNK1G3_5_3316,6574,5611,6247,5675,4492,6892,5530,5946,5253
CTDP1_5_3317,92,30,448,506,36,645,436,9,1340
CTDSP1_5_3318,725,1104,880,11,530,367,565,610,1043
DCLK1_5_3319,1678,1211,1954,2249,1661,1855,2277,1454,3881
DCLK2_5_3320,1099,2092,1472,807,2855,1287,1375,2219,1433
DDR1_5_3321,2338,1520,1597,599,1522,2930,837,1395,800
DDR2_5_3322,895,1092,965,1243,1208,1637,287,1936,862
DGKA_5_3323,1774,2501,2208,1439,2899,1798,3277,3961,2017
DGKB_5_3324,1491,2466,2171,1214,2624,3175,1123,2446,2848
DGKD_5_3325,799,1299,1135,1370,707,3211,733,1308,535
DGKG_5_3326,213,68,114,63,424,223,241,0,0
DGKH_5_3327,1934,1426,1373,1076,1881,850,2652,1214,2225
DGKZ_5_3328,99,11,155,0,1,0,327,19,71
DGUOK_5_3329,762,873,964,448,442,807,223,2280,1358
DLGAP5_5_3330,2183,2454,2327,987,1467,3067,2769,2145,2860
DMPK_5_3331,314,1007,711,14,169,464,1090,796,1394
DSTYK_5_3332,748,660,634,1417,483,21,1703,676,488
DTYMK_5_3333,1512,1895,1747,2291,3010,2534,1488,705,3192
DUSP13_5_3334,219,765,258,683,699,777,288,311,17
DUSP19_5_3335,319,493,913,551,262,26,592,199,1932
DUSP4_5_3336,1372,1498,1210,1389,116,913,805,1200,739
DUSP6_5_3337,619,1118,899,197,48,1414,367,465,605
DYRK1A_5_3338,11314,13378,14856,9356,22189,17789,12252,13002,12004
DYRK1B_5_3339,594,706,709,131,421,860,659,482,198
DYRK2_5_3340,567,603,848,192,859,930,746,301,735
DYRK3_5_3341,5170,5000,5210,6137,2729,4750,3261,4728,7094
EFNA4_5_3342,281,540,646,1184,224,408,512,372,76
EGFR_5_3343,1564,2170,1504,2181,2286,1223,1063,1993,652
EIF2AK2_5_3344,2875,2077,2413,1464,3093,1728,4185,2361,3303
EPHA10_5_3345,427,324,442,4,109,7,1450,2,26
EPHA3_5_3346,747,919,986,857,602,322,1068,1089,984
EPHA5_5_3347,4285,5260,6352,4623,3701,5886,3787,4204,5874
EPHA6_5_3348,312,348,337,335,711,159,751,730,223
EPHA8_5_3349,1030,750,768,862,1259,671,798,326,954

EPHB2_5_3350,897,550,1341,111,506,1265,778,911,1629
EPM2A_5_3351,8418,8618,9179,5789,10763,8866,8565,7294,6789
ERBB2_5_3352,458,418,414,37,28,269,165,159,85
ERBB4_5_3353,577,369,567,877,638,1120,1049,715,792
ETNK1_5_3354,951,1446,1292,658,635,2049,854,584,1771
EXOSC10_5_3355,380,409,657,429,388,680,189,1102,723
EYA1_5_3356,185,21,114,1,21,40,907,41,0
EYA2_5_3357,81,255,250,159,650,2,333,32,1
EYA4_5_3358,3723,3333,4330,4252,2528,5019,3954,4817,3578
FASTK_5_3359,506,787,531,1461,591,1516,535,527,511
FBP1_5_3360,683,1025,772,355,416,1016,1339,810,521
FES_5_3361,138,244,264,173,145,2,171,229,33
FGFR1_5_3362,709,630,767,1494,959,491,1597,583,76
FGFR2_5_3363,1570,1105,676,208,1175,661,542,492,1597
FGFR3_5_3364,817,789,922,391,717,1119,847,1199,710
FGFR4_5_3365,218,226,409,93,339,694,203,261,301
FGFRL1_5_3366,991,902,689,1146,765,698,794,1098,2177
FGR_5_3367,281,403,735,6,92,260,143,85,235
FLT1_5_3368,1925,3512,3091,2643,4378,3739,2653,3004,2960
FLT4_5_3369,190,131,388,524,807,458,252,30,258
FXN_5_3370,901,774,580,559,251,1080,99,744,256
FYN_5_3371,143,601,127,7,1179,22,312,5,24
FZR1_5_3372,11,83,11,0,3,1,319,17,3
GALK2_5_3373,222,179,146,14,3,137,183,103,1
GK_5_3374,1026,1353,1208,1907,576,911,742,493,1514
GLYCTK_5_3375,858,313,394,186,727,251,490,75,615
GNE_5_3376,1283,1352,1310,514,1893,3330,1788,827,363
GRK4_5_3377,597,900,441,60,855,1386,309,201,5
GRK6_5_3378,1021,860,1285,1450,906,1331,617,823,946
GSK3B_5_3379,239,842,478,36,250,234,637,293,2301
GUK1_5_3380,742,2060,1249,421,447,1599,765,367,750
HCK_5_3381,1088,621,1295,1602,1047,1177,1315,460,1719
HIPK1_5_3382,885,823,726,444,376,598,1240,74,1168
HIPK2_5_3383,533,164,487,822,8,293,1059,424,451
HIPK3_5_3384,973,1287,1467,497,1379,625,600,1433,951
ICK_5_3385,722,271,407,300,185,487,141,180,277
IKBKB_5_3386,1128,1389,1579,1279,735,1487,975,526,1384
IKBKE_5_3387,1237,1740,1434,1447,926,1433,1388,1551,877
IKBKG_5_3388,458,215,1244,130,115,741,349,258,470
ILK_5_3389,1832,2545,2890,1023,1792,2859,1773,1294,2640
IMPA1_5_3390,646,1158,782,103,304,1391,758,1105,640
INPP1_5_3391,150,27,19,0,1,1079,94,57,10
INPP4A_5_3392,255,453,261,130,1355,433,680,442,332
INPP4B_5_3393,1613,829,1659,2583,2248,2639,1742,1112,1244
INPP5D_5_3394,61,250,121,40,574,8,132,712,987
INSR_5_3395,207,389,390,14,174,543,312,129,2178
IP6K1_5_3396,185,476,599,27,117,593,151,309,15
IP6K3_5_3397,30,20,172,15,70,28,662,102,815
IRAK1_5_3398,171,644,193,0,596,0,45,19,21
IRAK3_5_3399,1789,1502,1622,299,1852,1003,2391,1376,463

IRAK4_5_3400,2010,2740,2424,1826,3922,3963,3209,2932,1640
ITPK1_5_3401,125,109,421,13,141,375,306,456,81
KALRN_5_3402,218,554,442,594,87,375,920,289,11
KHK_5_3403,558,458,559,286,516,20,225,404,43
KIF2A_5_3404,347,525,360,44,428,395,336,207,805
KIT_5_3405,800,345,475,1388,232,558,157,89,558
LCK_5_3406,996,667,653,45,137,552,494,910,3433
LHPP_5_3407,621,931,1168,1229,150,820,773,98,500
LIMK1_5_3408,146,105,147,730,384,6,37,63,15
LIMK2_5_3409,1829,2867,3409,2149,6833,5939,3018,1228,384
LTK_5_3410,39,380,89,518,962,13,0,35,6
LYN_5_3411,1445,1414,1156,869,835,803,147,558,3717
MAGI3_5_3412,1364,2040,2142,1108,1565,1053,1249,1741,2152
MAP2K3_5_3413,244,372,531,332,802,982,70,278,39
MAP2K5_5_3414,90,487,16,10,0,122,91,801,0
MAP3K12_5_3415,452,233,236,498,3,11,205,134,40
MAP3K13_5_3416,380,361,1372,441,106,713,585,114,2381
MAP3K3_5_3417,161,225,528,411,150,29,34,115,1359
MAP3K4_5_3418,917,1328,1262,464,922,704,481,375,868
MAP3K7_5_3419,425,306,333,51,97,398,685,201,0
MAP4K1_5_3420,999,712,561,706,1394,727,910,1261,311
MAP4K4_5_3421,6193,5253,8200,5228,6162,9543,5934,5027,14368
MAP4K5_5_3422,1646,2272,3404,3522,2211,2854,3460,1945,1545
MAPK10_5_3423,247,259,293,2,2,279,0,81,29
MAPK14_5_3424,3452,2722,4111,3100,2436,3922,3818,1363,4178
MAPK1_5_3425,1938,1849,1827,942,1314,994,942,893,2144
MAPK3_5_3426,39,101,326,0,0,0,16,0,0
MAPK7_5_3427,1256,1728,1196,1172,1415,2039,1494,474,1188
MAPK8_5_3428,974,1222,1026,1055,540,323,287,626,193
MAPK9_5_3429,434,497,809,13,753,758,200,547,1319
MAPKAPK2_5_3430,1535,1079,1820,773,1037,2385,1390,953,959
MAPKAPK5_5_3431,1456,1198,2573,962,1538,3599,919,706,2822
MARK2_5_3432,2250,2096,2746,2217,1101,1937,1770,1558,465
MARK3_5_3433,1412,1072,897,1295,1243,1240,1081,1426,649
MARK4_5_3434,412,506,508,69,34,788,59,218,86
MAST4_5_3435,425,608,540,382,32,694,462,437,1647
MASTL_5_3436,257,330,494,242,295,649,193,319,5
MATK_5_3437,282,247,502,70,601,751,598,132,558
MET_5_3438,1793,2245,2581,3038,3811,3165,1921,2064,3795
MINK1_5_3439,749,852,657,539,295,82,450,626,78
MINPP1_5_3440,421,219,933,1,220,730,513,1214,472
MKNK1_5_3441,1606,1653,1414,736,1238,887,1256,1224,1234
MKNK2_5_3442,323,593,497,3,134,426,298,1059,124
MLKL_5_3443,47,529,252,438,340,104,1235,74,2303
MTMR14_5_3444,2785,2155,3151,1454,3057,6009,2200,2148,1447
MTMR2_5_3445,4238,5368,6930,6203,5300,4796,5943,4869,9036
MTMR3_5_3446,1148,1406,1818,1829,1269,2207,958,1289,1480
MUSK_5_3447,81,208,103,0,168,263,16,183,0
MVK_5_3448,580,675,574,259,79,182,255,63,796
MYO3B_5_3449,1594,1605,1714,1501,2673,260,1314,2850,2386

NADK_5_3450,932,764,479,465,279,83,119,647,623
NCK1_5_3451,1280,1477,1829,433,1581,1526,1013,481,103
NCK2_5_3452,373,141,705,786,684,1369,954,281,64
NDRG1_5_3453,942,607,550,840,1339,459,656,332,503
NEK11_5_3454,1978,1948,1890,2102,2062,975,1687,3095,2603
NEK1_5_3455,540,606,1003,826,1172,2179,1071,895,14
NEK2_5_3456,135,467,92,48,334,200,17,125,83
NEK3_5_3457,3720,3374,3416,3260,2522,3112,5269,4259,4429
NEK4_5_3458,211,12,18,0,256,267,532,0,1
NEK6_5_3459,1018,591,997,546,523,1657,1566,658,593
NME2_5_3460,238,289,504,734,1210,983,517,227,797
NME7_5_3461,1072,1118,1588,696,1689,318,1496,1240,411
NT5C1B_5_3462,469,110,809,155,184,1179,424,1089,26
NT5C2_5_3463,420,536,569,548,782,1488,2027,204,8
NT5C3_5_3464,1165,1267,1566,879,931,2216,1643,1407,1465
NT5E_5_3465,5865,6941,9655,6486,7408,10828,4893,4305,12936
NTRK1_5_3466,431,469,331,658,421,1057,308,1054,327
NTRK2_5_3467,890,719,1246,1728,242,974,306,359,2319
NTRK3_5_3468,879,683,967,848,764,1297,825,1326,1171
NUDT4_5_3469,2049,1871,2065,2071,59,69,1365,1283,895
NUDT9_5_3470,2560,3771,2695,1593,3561,1340,1668,2047,5936
OBSCN_5_3471,104,11,69,271,242,66,599,0,0
OCRL_5_3472,1252,1493,1121,2683,684,1058,720,777,4838
OXSM_5_3473,4792,5141,5068,3792,4615,5464,5391,4347,5668
PACSIN1_5_3474,1295,1225,1365,1232,1097,2464,1736,1915,1166
PAK4_5_3475,840,1032,2061,1460,1469,619,2111,1131,1282
PAK6_5_3476,429,450,747,403,258,843,225,463,91
PAK7_5_3477,2920,5149,4699,3858,4477,4202,5190,3048,7589
PANK1_5_3478,936,703,1782,1185,1644,30,518,2265,1842
PANK2_5_3479,1249,1099,876,1987,1378,527,1668,1001,1370
PCK2_5_3480,599,303,861,38,138,1004,96,359,1
PDK2_5_3481,334,415,626,134,447,401,262,458,207
PDK3_5_3482,420,151,238,157,147,78,134,849,234
PDPK1_5_3483,180,331,384,33,975,232,162,28,265
PEG3_5_3484,507,483,426,16,69,187,572,376,2118
PFKFB2_5_3485,1526,1432,2080,2894,1090,1521,1152,2189,3931
PFKFB3_5_3486,793,739,1021,110,1222,897,1020,726,1854
PFKM_5_3487,1073,1213,1433,852,1056,855,1219,961,484
PFKP_5_3488,322,409,462,586,127,22,101,142,214
PHKA1_5_3489,738,1367,761,141,3416,652,2211,1476,258
PHKB_5_3490,2270,2211,2249,2682,698,1747,3461,1754,6458
PHKG2_5_3491,366,393,536,495,341,232,288,409,769
PHPT1_5_3492,285,176,479,192,690,34,152,2,1
PI4KA_5_3493,1459,1316,2063,588,1044,1347,923,576,428
PI4KB_5_3494,1683,1697,2725,1729,2102,1501,2362,1508,2621
PIK3R1_5_3495,1461,1680,1832,1686,1519,2070,1791,851,1980
PIK3R5_5_3496,655,544,382,766,951,417,463,31,60
PIKFYVE_5_3497,405,392,903,786,696,59,471,150,571
PIP4K2C_5_3498,651,466,925,1096,757,366,84,256,1109
PIP5K1A_5_3499,2552,1925,1907,3018,3170,1049,2912,961,1494

PIP5K1C_5_3500,352,122,530,463,640,90,37,268,506
PIP5KL1_5_3501,221,1103,391,0,13,79,296,24,31
PKLR_5_3502,1501,1058,1821,1324,1735,2610,2601,1035,1195
PKMYT1_5_3503,854,958,1348,1548,1061,2727,414,671,1136
PKN1_5_3504,336,132,186,2,105,8,266,478,95
PLK4_5_3505,337,691,703,609,469,679,1450,339,274
PNCK_5_3506,273,341,80,157,0,109,222,211,0
PODXL_5_3507,259,175,398,309,52,641,23,59,1480
PPAP2A_5_3508,1121,1053,1338,419,756,1287,1147,1255,1808
PPAP2C_5_3509,255,205,454,438,535,10,70,253,122
PPEF1_5_3510,435,475,613,849,855,1608,298,1504,787
PPFIA1_5_3511,343,424,786,815,34,385,281,186,1532
PPFIA2_5_3512,2776,3240,2865,2914,5045,1325,1628,4630,1599
PPM1A_5_3513,2973,3321,2525,3747,2399,1593,3783,3939,2317
PPM1B_5_3514,744,732,1510,876,67,1769,445,565,657
PPM1M_5_3515,588,1079,1546,79,1670,642,771,65,583
PPP1CA_5_3516,340,35,672,16,184,853,123,114,434
PPP1CB_5_3517,388,379,368,1057,322,607,519,820,432
PPP1R12A_5_3518,705,855,1186,120,1721,107,1169,421,325
PPP1R16B_5_3519,1704,1771,3244,2052,2358,3499,1750,1567,1620
PPP1R1B_5_3520,566,557,392,56,377,37,120,694,115
PPP1R8_5_3521,1008,1219,765,994,799,553,616,1458,266
PPP2R1B_5_3522,1169,1457,1668,1394,2510,2125,1600,938,2964
PPP2R2A_5_3523,678,943,1479,290,264,2186,751,926,394
PPP2R2B_5_3524,1085,977,1679,581,653,1455,1388,793,1414
PPP2R2C_5_3525,1045,1138,817,919,3096,192,340,1304,1856
PPP2R3A_5_3526,1357,1398,1950,1554,2235,1028,1662,2186,3611
PPP2R4_5_3527,186,218,237,728,82,178,260,96,2687
PPP2R5A_5_3528,1105,1559,1273,812,2215,940,914,1416,765
PPP2R5D_5_3529,798,1193,1034,173,965,297,381,728,930
PPP3CA_5_3530,128,619,730,204,302,55,144,587,2585
PPP3CB_5_3531,809,1111,1452,326,2910,607,1255,1174,968
PPP4R1_5_3532,1563,1386,1900,1991,1160,1919,1538,527,1250
PPP5C_5_3533,258,539,416,132,112,208,202,245,89
PPP6C_5_3534,2916,2219,4628,4091,2056,4235,2252,3008,7298
PRKAA1_5_3535,4347,3636,5897,2449,5284,3391,2527,4941,5161
PRKACA_5_3536,0,0,0,0,0,0,0,0,0
PRKACB_5_3537,2012,1124,2151,2710,1513,1346,2544,1951,1316
PRKAG1_5_3538,2040,2090,1952,3441,2551,2170,2380,2055,2272
PRKAG2_5_3539,502,231,258,296,293,1079,158,137,511
PRKAR1A_5_3540,1424,1729,1675,1402,1077,640,773,1055,814
PRKAR1B_5_3541,797,1167,1436,239,960,1660,1671,341,2536
PRKCB_5_3542,270,330,597,777,428,28,385,512,519
PRKCD_5_3543,244,479,578,926,118,733,61,125,212
PRKCQ_5_3544,1627,1077,2167,1467,1435,2242,1390,1274,1653
PRKCZ_5_3545,159,185,428,24,27,1166,461,147,10
PRKD2_5_3546,831,179,1031,424,9,794,7,103,538
PRKDC_5_3547,998,1132,1622,76,572,2597,763,1407,679
PRKG1_5_3548,706,1247,1043,714,615,504,742,745,1053
PRPS2_5_3549,1144,1179,1477,2705,1686,1937,2975,1007,1128

PTK2B_5_3550,1153,1597,1608,1095,2090,1448,1353,624,1462
PTK2_5_3551,165,325,290,115,497,98,38,176,60
PTK7_5_3552,302,389,242,156,56,907,693,1322,22
PTP4A2_5_3553,1542,1983,1750,754,1622,2391,2646,2496,1383
PTP4A3_5_3554,169,228,35,1,348,204,365,350,217
PTPDC1_5_3555,520,937,848,20,550,431,917,441,1528
PTPN12_5_3556,1828,2235,1474,3001,2106,2419,2061,3630,2216
PTPN13_5_3557,1323,2098,1704,1398,2706,2154,2206,1904,2558
PTPN18_5_3558,431,541,826,314,303,1271,1,129,968
PTPN22_5_3559,2730,1686,2449,2574,3075,4262,1490,2769,405
PTPN2_5_3560,987,806,1102,229,862,2769,1060,453,2727
PTPN3_5_3561,200,97,406,105,133,21,109,12,234
PTPN5_5_3562,715,331,107,209,145,282,3,178,399
PTPN6_5_3563,214,181,329,73,26,18,399,109,78
PTPN7_5_3564,165,438,521,603,507,786,415,313,10
PTPRA_5_3565,722,918,1218,227,1568,680,922,392,954
PTPRB_5_3566,395,516,532,550,204,519,389,774,30
PTPRC_5_3567,798,1655,1259,1036,3104,770,713,1434,947
PTPRD_5_3568,1767,1195,1463,684,190,2267,2066,1301,1785
PTPRE_5_3569,4186,4687,5326,5971,5877,4890,4975,6380,4915
PTPRF_5_3570,177,422,569,224,153,880,636,159,440
PTPRH_5_3571,243,211,443,1072,248,38,545,189,345
PTPRJ_5_3572,1378,1694,2157,801,2353,1020,845,1291,915
PTPRK_5_3573,1091,1495,1797,531,789,3308,1178,958,1042
PTPRM_5_3574,85,499,268,174,7,147,105,1213,1269
PTPRN2_5_3575,1198,586,930,1012,479,676,665,658,1933
PTPRN_5_3576,374,560,687,833,213,598,491,1704,235
PTPRR_5_3577,3412,3664,3464,3702,4810,3811,4754,2356,3375
PTPRS_5_3578,419,369,273,370,103,9,390,160,234
PTPRT_5_3579,226,217,322,19,544,857,554,404,369
PTPRU_5_3580,488,714,751,10,24,1040,599,4,0
PTPRZ1_5_3581,3758,4229,4258,2108,6679,3987,2404,3096,4877
RET_5_3582,2510,2483,3473,1211,2922,3994,2656,2420,3899
RIOK1_5_3583,152,73,418,423,29,145,205,303,85
RIOK2_5_3584,43,316,227,204,69,1624,192,85,512
ROPN1L_5_3585,2570,3012,4511,3528,1980,1921,3376,2524,1861
RPS6KA1_5_3586,953,544,1494,4,282,816,784,164,2108
RPS6KA2_5_3587,1302,1139,1997,753,1481,221,1109,1141,1130
RPS6KA4_5_3588,30,10,13,3,564,8,22,11,1
RPS6KA5_5_3589,712,807,1184,738,968,1909,554,535,264
RPS6KC1_5_3590,3462,3537,3879,5140,6186,4365,3194,3383,2018
RYK_5_3591,3718,6099,5004,3021,8022,3152,4031,6427,5064
SET_5_3592,892,385,987,1775,565,170,618,1006,974
SGK1_5_3593,843,912,1616,396,824,1837,671,1080,2471
SGK3_5_3594,2578,2200,2341,2016,2262,1473,1679,1134,3045
SIRPA_5_3595,329,33,125,112,2,125,104,2,14
SKAP1_5_3596,674,941,933,459,780,1800,956,1413,227
SNRK_5_3597,2574,2386,4160,3209,2580,687,3252,2601,898
SPHK1_5_3598,359,377,587,82,894,1394,233,672,582
SPHK2_5_3599,1332,1723,1795,707,2457,3143,522,751,1635

SRC_5_3600,540,743,1028,521,375,183,126,69,160
SRPK2_5_3601,919,1088,882,1512,2394,548,629,1459,1739
SRPK3_5_3602,433,179,416,1031,138,92,196,362,184
SSH1_5_3603,1858,1160,1307,1039,1421,2359,863,920,621
STK19_5_3604,427,139,648,473,175,193,728,109,293
STK24_5_3605,674,668,699,34,966,1254,585,609,1877
STK31_5_3606,1028,1545,1185,876,831,1722,933,1077,2825
STK36_5_3607,2014,2223,1841,665,1386,1913,1335,1079,3433
STRADA_5_3608,649,406,353,233,297,706,373,712,172
STRADB_5_3609,3569,3791,3718,4248,3223,3744,4383,4438,3639
STYX_5_3610,789,1160,819,631,467,531,1362,1534,1605
SYK_5_3611,3811,2847,3468,1897,2262,2702,1940,4104,5516
SYNJ1_5_3612,2794,5509,5467,6647,7015,2961,4690,3360,6344
SYNJ2_5_3613,672,813,1124,1622,1515,524,824,1240,1011
TAF1_5_3614,1883,2127,2203,1825,2024,3042,2095,2507,2191
TAOK1_5_3615,506,831,748,126,674,758,913,977,41
TAOK2_5_3616,944,1232,1091,640,2153,1294,1371,1194,875
TBCK_5_3617,490,545,1082,100,273,227,546,230,1320
TEX14_5_3618,96,81,171,0,2,0,3,0,98
TGFBR1_5_3619,2677,3430,3828,2209,2532,3186,2249,3607,3971
TGFBR2_5_3620,762,206,305,268,165,205,171,27,704
TGFBR3_5_3621,636,427,1271,384,963,1604,1052,1137,41
THTPA_5_3622,331,236,162,14,145,191,240,153,13
TJP2_5_3623,1810,2596,2555,1051,2737,2274,2321,2635,3133
TK2_5_3624,249,354,494,33,466,350,591,256,1111
TLK1_5_3625,1879,2907,2918,2137,2411,2685,1999,1115,2321
TLK2_5_3626,2348,2381,2068,3275,2739,2430,2011,2400,4864
TMEM134_5_3627,161,226,613,1,49,5,34,402,627
TNIK_5_3628,37,129,111,4,259,245,93,327,2
TNK2_5_3629,611,432,504,463,623,514,436,1109,238
TPK1_5_3630,220,52,301,220,266,22,14,468,13
TPTE2_5_3631,1272,1288,3095,1956,1764,2536,1323,548,2780
TPTE_5_3632,1281,1945,2175,2092,1192,460,1028,1287,1586
TRIM24_5_3633,1921,2310,1649,1195,1383,1892,2018,1204,2439
TRIM33_5_3634,2038,2106,2537,4046,2244,1855,2508,1243,5596
TRMT2A_5_3635,797,544,542,402,630,1500,430,116,978
TRPM6_5_3636,1454,1909,1872,1786,2081,3342,1728,912,3591
TSSK4_5_3637,0,0,0,0,0,0,0,0,0
TTK_5_3638,3237,4045,4906,5783,4699,8004,4749,4579,6333
TTN_5_3639,3383,4913,3788,3338,3563,3802,3390,2099,4020
TWF1_5_3640,694,857,1016,801,1052,2791,828,481,243
UCK1_5_3641,1426,2035,1631,1414,1573,2368,1482,1525,1002
UCKL1_5_3642,20,1,36,0,395,99,1,39,45
UHMK1_5_3643,2582,2295,3426,5098,2533,2805,2371,2437,2584
ULK2_5_3644,458,450,328,67,150,654,324,556,226
VRK2_5_3645,2438,2463,3028,2842,2788,2110,2273,1430,4292
VRK3_5_3646,474,456,1541,1962,448,1039,1276,1458,815
WEE1_5_3647,1731,2368,2272,2987,2664,942,810,2423,1571
WNK1_5_3648,1314,2027,1321,624,2797,1148,1070,1986,1234
WNK3_5_3649,858,1532,1993,173,1943,1338,901,68,2201

YSK4_5_3650,424,743,744,115,1359,390,274,722,1141
ZAP70_5_3651,911,581,313,534,257,462,760,268,2073
ACP2_5_3652,73,139,268,921,196,198,491,62,348
CDK1_5_3653,2424,3089,3584,880,2183,2532,1528,2697,4530
DUSP10_5_3654,3986,3909,5331,3385,5241,4425,4170,2176,7882
DUSP15_5_3655,1817,2213,1897,3613,1027,2475,1204,2140,2639
ERBB3_5_3656,3543,4032,4588,4227,5767,3886,3995,5637,5326
G6PC2_5_3657,635,621,538,382,1744,1018,476,260,1622
IP6K2_5_3658,566,504,423,381,556,538,482,141,103
MYLK_5_3659,436,594,1829,645,1183,1576,494,344,3162
PRPS1_5_3660,517,482,375,176,130,435,320,180,520
PTPMT1_5_3661,6,126,30,27,11,0,13,0,0
PTPN20A_5_3662,12408,13928,14155,18899,21841,20258,13601,18494,15980
PTPN20B_5_3663,12408,13928,14155,18899,21841,20258,13601,18494,15980
SPEG_5_3664,468,879,905,154,79,136,48,388,909
STK32A_5_3665,947,1389,2483,3098,1433,1559,1489,1953,1371
PPP1R12B_5_3666,1291,2263,1874,2457,2059,3105,1829,2979,4031
PPP1R12B_5_3667,333,412,451,525,137,646,337,85,644
PPP1R12B_5_3668,1067,1658,1144,697,1014,2664,375,510,1231
AAK1_5_3669,1303,1100,1657,1621,805,793,1486,860,4279
ACP6_5_3670,410,391,1185,293,1460,347,847,428,1151
ACPT_5_3671,195,136,412,265,57,3,581,25,209
ACVR2A_5_3672,563,976,1083,1097,316,948,212,1031,54
ACVR2B_5_3673,430,54,170,53,121,146,200,85,578
ADCK2_5_3674,2410,1872,2446,2051,1091,1536,2589,2399,4570
ADCK3_5_3675,1190,898,1274,1661,2669,384,1880,956,2044
ADCK5_5_3676,241,181,225,69,0,1,388,361,112
ADPGK_5_3677,2231,2437,2262,1218,1132,2713,1458,2728,1544
ADRBK1_5_3678,208,113,54,38,1628,223,383,237,1405
ADRBK2_5_3679,1355,618,2050,1274,814,1154,1190,1368,1103
AGK_5_3680,31,324,153,152,0,191,0,0,0
AK1_5_3681,527,557,326,721,6,94,180,165,1050
AK7_5_3682,0,0,0,0,0,0,0,0,0
AK8_5_3683,542,689,1455,1533,869,2170,520,128,1181
ALK_5_3684,3810,4965,5903,6113,4687,5301,5981,5429,6924
ALPI_5_3685,490,407,453,278,44,163,219,68,508
ALPK2_5_3686,201,73,32,6,2,48,54,42,107
ALPK3_5_3687,109,218,624,14,0,448,188,14,191
ALPP_5_3688,903,752,1107,956,1675,820,286,995,917
ALPPL2_5_3689,3240,2268,2298,1159,2833,2316,2106,2233,2102
ANKK1_5_3690,2655,1755,3074,1043,2751,2862,3220,2459,3647
ARAF_5_3691,1685,1204,1750,2971,1516,1209,1590,785,3910
ATM_5_3692,1554,857,1346,419,2000,823,1439,812,374
AURKB_5_3693,2740,3005,2361,1285,2949,2322,2718,3166,3535
BLK_5_3694,931,291,1090,1283,445,338,529,183,131
BMPR1A_5_3695,3377,3277,3617,1082,3758,5462,3950,3819,5936
BMPR1B_5_3696,1377,1702,1892,1799,2561,1621,1725,1900,2135
BMPR2_5_3697,2991,3170,4208,2839,2164,6311,3154,4075,5867
BPNT1_5_3698,2153,2044,1537,2202,2340,1899,901,1826,1580
BRAF_5_3699,3586,3899,4737,2423,5109,1929,4129,3196,4879

BRD3_5_3700,3997,3618,3004,5423,3996,2235,2730,1558,4789
BRSK1_5_3701,620,736,1493,1279,1334,534,1194,768,1708
BRSK2_5_3702,251,78,376,304,0,244,402,353,54
BTK_5_3703,329,91,402,594,0,15,102,1,401
BUB1B_5_3704,1916,1598,2041,3555,2145,2734,2413,952,1641
BUB1_5_3705,1329,1050,1950,1753,774,1733,411,1192,1862
C20orf111_5_3706,639,894,733,480,1529,1812,1064,543,649
C9orf96_5_3707,1499,1519,1462,595,1852,2731,2006,2554,1502
CALM1_5_3708,251,241,533,507,1603,1151,661,162,518
CALM3_5_3709,799,292,331,888,252,526,774,753,42
CAMK1_5_3710,1303,932,885,1841,806,1729,1629,1127,364
CAMK1G_5_3711,396,333,214,232,127,39,237,283,4
CAMK2N1_5_3712,600,959,865,304,493,1076,53,838,4724
CAMK4_5_3713,2552,3736,3761,4534,1872,2650,1973,4010,3086
CAMKV_5_3714,2791,2531,3558,1507,1627,2204,941,2476,3639
CARD11_5_3715,292,227,605,142,109,141,281,397,4
CCL2_5_3716,384,512,353,720,526,111,120,699,332
CDC42BPB_5_3717,650,294,500,162,246,769,698,158,692
CDC42BPG_5_3718,222,778,499,72,38,232,250,1060,150
CDK14_5_3719,855,986,1423,920,242,1033,1019,763,2942
CDK15_5_3720,182,300,1131,933,625,1670,391,183,2609
CDK19_5_3721,200,160,210,52,157,115,92,29,141
CDK3_5_3722,239,190,526,113,146,57,354,108,195
CDK4_5_3723,1524,2370,2133,1881,612,1764,1231,2682,559
CDK5R1_5_3724,1250,1563,1827,1231,1485,2554,1002,1320,1101
CDK7_5_3725,67,103,27,313,459,344,75,67,2
CDK8_5_3726,1721,2630,3619,2165,2870,2152,2384,2625,3302
CDK9_5_3727,189,261,486,3,316,177,316,498,66
CDKL1_5_3728,1951,2416,2324,1768,1642,2826,2309,1850,4121
CDKL2_5_3729,581,788,780,63,248,233,1308,206,2182
CDKL4_5_3730,820,529,536,1159,521,363,935,1057,2336
CDKN1B_5_3731,925,706,1002,447,1611,1726,455,611,2185
CERK_5_3732,598,274,638,1,174,426,372,216,48
CHKB_5_3733,1497,1137,1223,1041,1545,1724,670,1496,1900
CHUK_5_3734,14592,16147,19637,11335,15324,19503,14419,15039,25571
CIB2_5_3735,591,741,922,981,2606,1034,662,345,2240
CILP_5_3736,1281,1058,1187,753,1018,981,329,246,2554
CKB_5_3737,1190,1469,2590,2855,618,3156,1729,888,2422
CKM_5_3738,122,160,332,836,460,87,488,258,28
CKMT1A_5_3739,842,654,1442,780,1539,528,370,930,417
CKMT1B_5_3740,842,654,1442,780,1539,528,370,930,417
CKS1B_5_3741,2006,1941,2208,2317,968,1929,1715,848,2096
CKS2_5_3742,4026,4126,4709,3839,4410,3886,2987,3469,5746
CLK2_5_3743,675,509,834,226,437,1443,669,1429,1508
CLK4_5_3744,616,221,412,885,111,505,703,167,1397
CMPK2_5_3745,802,357,1043,515,312,209,578,716,61
CPNE3_5_3746,1609,1097,1555,632,2529,1266,1334,847,2401
CRIM1_5_3747,1780,1841,2047,1021,707,2013,1252,1290,1176
CRKL_5_3748,1194,1411,1177,414,1210,1643,2615,1110,2085
CSF1R_5_3749,402,505,857,0,867,49,384,1422,1914

CSNK1A1L_5_3750,1309,1394,2842,1197,1418,3114,1236,921,3587
CSNK1G1_5_3751,300,50,449,1261,623,309,205,142,329
CSNK1G2_5_3752,92,97,515,528,203,257,89,250,0
CSNK2A2_5_3753,1031,1120,1726,297,740,1899,1021,878,204
CSNK2B_5_3754,856,717,964,304,252,1511,331,591,813
CTDSP2_5_3755,291,226,427,476,755,735,571,93,353
DAPK1_5_3756,3917,5447,5443,2693,8237,5315,3265,4510,5692
DAPK2_5_3757,2011,1825,2085,2584,2756,2755,2025,1896,5351
DAPK3_5_3758,375,702,654,401,341,837,592,650,1032
DBF4_5_3759,1296,1037,1834,936,1364,2434,969,3028,680
DCK_5_3760,2517,2284,2763,3018,1385,3562,1761,585,2965
DCLK3_5_3761,550,544,417,1555,135,660,429,401,581
DGKE_5_3762,1493,1472,2188,2745,2618,3737,2101,1771,3214
DGKI_5_3763,1996,2444,2900,2292,2301,4040,3666,978,2666
DGKK_5_3764,698,992,618,1053,1197,689,518,1177,218
DGKQ_5_3765,989,943,1075,1148,1548,2028,1389,1612,2248
DOLK_5_3766,1817,2576,1642,213,1615,2051,1224,989,2168
DOT1L_5_3767,850,1403,1187,3086,1480,1103,852,1150,1757
DUSP11_5_3768,1490,1772,1847,2025,844,2226,1247,1976,1654
DUSP12_5_3769,2185,2414,2643,2501,2194,1985,1052,2277,2118
DUSP14_5_3770,829,420,1147,134,1034,1040,1066,818,899
DUSP16_5_3771,1016,524,1076,1063,1534,1745,383,398,1155
DUSP18_5_3772,1369,1660,2783,1496,1991,2825,1130,2046,948
DUSP21_5_3773,1441,1099,2496,1103,1492,2165,527,1224,840
DUSP22_5_3774,412,543,517,590,604,145,753,511,191
DUSP2_5_3775,956,981,956,1361,1339,919,185,212,2459
DUSP3_5_3776,458,904,803,59,1100,672,617,268,1578
DUSP5_5_3777,378,352,305,25,196,96,311,99,1369
DUSP7_5_3778,58,44,75,61,39,1,15,9,0
DUSP8_5_3779,971,1085,602,391,268,142,240,238,10
DUSP9_5_3780,1337,2152,2216,1664,2523,3374,1674,2823,1798
DYRK4_5_3781,1249,804,1606,1719,294,784,957,504,1925
EEF2K_5_3782,88,318,365,365,789,353,113,137,928
EFNA2_5_3783,800,973,1354,452,142,1755,1159,615,352
EFNA3_5_3784,719,1087,827,532,406,621,855,506,2024
EFNA5_5_3785,655,831,1138,58,1205,541,539,697,979
EFNB3_5_3786,110,271,253,0,0,517,7,0,6
EIF2AK3_5_3787,5328,5672,6107,5031,3160,5908,4999,4228,7519
EIF2AK4_5_3788,285,1003,806,172,1219,482,220,1069,2297
EPHA1_5_3789,65,83,18,38,3,3,2,7,22
EPHA2_5_3790,522,262,758,15,283,77,540,516,739
EPHA4_5_3791,1304,1420,1332,2924,1042,642,1350,911,1046
EPHA7_5_3792,1896,1408,1655,1291,2604,1398,1739,2582,1117
EPHB1_5_3793,467,235,95,33,669,421,451,244,129
EPHB3_5_3794,3774,4241,5670,5572,4539,3644,2524,3385,4420
EPHB4_5_3795,319,376,332,395,247,455,30,939,2331
EPHB6_5_3796,598,786,535,213,344,1208,79,262,397
ERN1_5_3797,1030,1892,2004,345,1911,1284,921,2777,113
ERN2_5_3798,220,376,444,567,160,425,646,503,635
ETNK2_5_3799,1641,1404,2368,1036,1796,1171,508,453,1028

EYA3_5_3800,726,426,208,408,29,832,388,283,900
FER_5_3801,1211,580,1163,304,1062,248,996,718,414
FLT3_5_3802,1804,2098,1662,1405,3491,2940,1756,1698,2998
FN3K_5_3803,166,290,295,1022,2070,267,621,275,99
FN3KRP_5_3804,1352,1639,911,1152,834,1850,2000,1472,391
FRK_5_3805,212,50,143,0,148,380,20,358,1145
FUK_5_3806,1701,1631,2056,1375,2113,1174,1449,1651,3950
G6PC3_5_3807,1354,1561,2223,917,1275,707,980,1161,2154
G6PC_5_3808,45,66,124,0,157,15,40,122,0
GAK_5_3809,2046,1633,1950,2485,2343,2153,1844,1237,1707
GALK1_5_3810,29,18,32,1,0,0,47,0,0
GDPD4_5_3811,320,795,429,114,369,368,318,925,0
GOLGA5_5_3812,914,1375,2027,1228,1617,2633,1342,1738,1735
GRK1_5_3813,6771,4977,5792,5415,4554,6704,6353,5569,4300
GRK5_5_3814,1195,885,1150,442,797,1664,1719,869,593
GRK7_5_3815,3414,3361,2818,4157,4421,5990,3196,4582,3764
GSG2_5_3816,1206,793,1569,2510,1115,2197,1037,1757,2182
GSK3A_5_3817,1759,1900,2726,1279,2095,3784,2309,1862,3201
GUCY2C_5_3818,1262,1491,1486,1056,1522,1958,711,931,859
GUCY2D_5_3819,769,322,686,900,1277,113,228,367,81
GUCY2F_5_3820,2062,1738,1751,572,1532,1443,1284,1317,1795
HIPK4_5_3821,301,1054,710,74,86,37,484,1034,450
HKDC1_5_3822,1678,2223,1625,1217,994,1683,2210,889,2597
HOOK3_5_3823,1130,623,1349,1253,448,533,921,281,1164
HSPB8_5_3824,2157,2649,2794,1695,2706,4802,2056,3329,2293
HUNK_5_3825,1626,1890,2656,3290,543,1271,1223,1650,2125
HUS1_5_3826,8331,8840,11323,10452,10266,10835,7545,10485,12748
IGBP1_5_3827,1171,1115,738,2631,1710,806,471,1701,1510
IGF1R_5_3828,521,467,1345,124,247,494,575,392,419
IGF2R_5_3829,1853,2092,1797,1730,2729,982,1340,2807,3893
ILKAP_5_3830,768,796,287,224,360,2195,52,775,547
IMPA2_5_3831,599,876,1086,1684,283,1398,452,273,1005
INPP5A_5_3832,1164,1207,764,953,2189,1007,1253,855,1137
INPP5B_5_3833,549,468,770,1743,524,1809,491,74,2838
INPP5J_5_3834,880,1181,778,530,2477,1216,640,1629,300
INPPL1_5_3835,1851,1771,1769,2545,2974,2660,2999,788,1689
INSRR_5_3836,252,110,293,0,21,67,10,337,331
IPMK_5_3837,918,890,1580,1269,1663,1709,1142,346,2265
IPPK_5_3838,247,676,219,80,236,1525,198,349,0
IRAK2_5_3839,760,348,861,381,1116,816,152,593,809
ITK_5_3840,434,766,1406,369,1249,1927,404,584,87
ITPKA_5_3841,322,429,539,190,450,238,628,582,607
ITPKB_5_3842,2060,2379,3988,3639,2510,3529,2115,2236,3952
ITPKC_5_3843,298,304,422,0,654,4,411,760,0
JAK1_5_3844,702,798,986,1301,1317,2439,733,2160,1895
JAK2_5_3845,1201,1289,948,1546,751,1384,584,428,409
JAK3_5_3846,542,300,654,8,524,1419,922,191,840
KDR_5_3847,299,1129,1076,315,424,475,989,1202,857
KSR1_5_3848,186,387,54,21,1,0,0,31,281
LATS1_5_3849,636,886,1470,128,1644,2356,680,987,183

LATS2_5_3850,1725,888,1665,888,842,1138,1598,630,382
LLGL1_5_3851,1571,1952,1945,468,1114,1846,878,2009,1260
LMTK2_5_3852,779,745,842,68,1050,387,665,592,192
LMTK3_5_3853,2,490,250,8,489,52,9,0,294
LRRK1_5_3854,344,558,472,2,393,34,373,6,726
LRRK2_5_3855,1824,2906,2627,2660,2549,4171,1867,3549,3832
MAGI2_5_3856,2712,2058,3041,2319,1842,4216,1611,2370,5446
MAP2K1_5_3857,100,421,401,95,141,176,15,79,313
MAP2K2_5_3858,282,25,16,0,1,654,25,189,0
MAP2K4_5_3859,513,402,570,1,786,2,662,391,82
MAP2K6_5_3860,1567,1595,1535,1649,2215,2002,1796,1414,2292
MAP2K7_5_3861,636,700,775,954,1011,732,325,818,857
MAP3K10_5_3862,247,157,535,730,119,35,680,95,112
MAP3K11_5_3863,271,41,73,15,88,257,146,153,1001
MAP3K14_5_3864,115,45,325,0,15,4,230,35,259
MAP3K15_5_3865,1993,2378,2158,1922,1570,2489,1664,1941,2939
MAP3K1_5_3866,484,295,181,291,23,243,269,771,655
MAP3K2_5_3867,377,514,767,420,91,620,326,555,663
MAP3K5_5_3868,264,82,229,237,33,230,451,271,438
MAP3K6_5_3869,443,449,408,46,2731,581,112,241,31
MAP3K8_5_3870,182,227,146,849,0,376,72,186,3
MAP3K9_5_3871,1391,1081,1508,1040,1884,2215,1106,1233,1613
MAP4K2_5_3872,193,171,116,152,348,2,79,0,30
MAP4K3_5_3873,425,180,252,18,481,10,179,4,75
MAPK11_5_3874,3389,3063,3236,2286,3157,3816,3446,2176,6757
MAPK12_5_3875,645,876,886,516,911,389,396,107,1843
MAPK13_5_3876,1469,2904,3491,2159,3630,901,1972,3290,2038
MAPK15_5_3877,575,1177,956,354,1173,792,618,563,324
MAPK4_5_3878,439,148,582,0,3,0,54,0,0
MAPK6_5_3879,1141,1145,737,1081,1084,672,397,1490,725
MAPKAPK3_5_3880,1105,1772,2273,945,2223,1572,1755,1052,2095
MARK1_5_3881,381,294,502,216,557,775,100,226,20
MAST2_5_3882,613,622,469,270,268,754,247,337,76
MAST3_5_3883,3506,3766,4630,2868,4119,4657,2818,3449,1567
MELK_5_3884,2162,2107,3746,835,3060,6054,3909,2864,5484
MERTK_5_3885,965,1231,1762,1144,2293,3331,1240,1450,876
MEX3B_5_3886,1071,2005,1549,1147,1286,631,1174,2051,2908
MPP3_5_3887,1698,2325,3448,693,5254,3244,2324,3126,4564
MPP5_5_3888,304,201,178,0,75,0,10,84,176
MST1R_5_3889,810,185,578,207,247,13,792,325,19
MTM1_5_3890,13094,12858,13744,12159,11291,14456,10173,10326,13412
MTMR1_5_3891,719,1056,1038,977,1417,736,1175,1668,945
MTMR4_5_3892,1616,1173,2065,721,1134,1294,1048,2365,927
MTMR6_5_3893,738,556,972,421,106,489,227,834,80
MTMR7_5_3894,2615,3062,2466,1101,6329,3562,3571,2411,2851
MTMR9_5_3895,5907,6972,8009,6642,6000,7386,6559,7426,5927
MTOR_5_3896,108,125,95,0,44,413,255,22,1046
MYLK2_5_3897,2166,3732,3192,2727,1857,1869,2241,2803,2783
MYLK3_5_3898,715,635,965,1138,1490,886,422,859,419
MYLK4_5_3899,823,1162,2097,335,608,2029,1217,1660,2051

MYO3A_5_3900,1087,1043,2101,271,1214,1193,788,1013,1478
N4BP2_5_3901,1236,1610,2407,1466,2266,1240,2561,1926,1661
NAGK_5_3902,2217,2270,3969,3501,3850,3649,1856,2818,968
NAGS_5_3903,1875,1988,1558,1067,2674,2130,1781,1645,1875
NEK10_5_3904,723,3067,1098,120,1674,2139,713,396,990
NEK5_5_3905,1112,1248,969,459,318,204,698,844,2016
NEK7_5_3906,661,658,768,364,571,607,344,328,24
NEK8_5_3907,136,257,444,0,0,0,247,0,966
NEK9_5_3908,647,867,1316,1015,294,829,59,320,1599
NME3_5_3909,384,109,261,59,16,84,17,0,0
NME4_5_3910,22,47,20,0,0,21,0,0,5
NME5_5_3911,2394,2356,2890,2111,2426,1478,1210,1310,1840
NME6_5_3912,198,274,296,92,49,950,4,6,564
NPR1_5_3913,1479,1236,1376,1463,783,2202,2025,1853,2594
NPRL2_5_3914,1014,619,1201,620,466,1874,844,507,1211
NRBP1_5_3915,1926,1761,2995,2303,568,1503,3143,1093,3407
NRBP2_5_3916,405,104,125,0,347,108,336,51,2
NRK_5_3917,545,552,336,2077,390,758,346,284,2341
NT5C1A_5_3918,17,259,166,411,0,11,256,2,0
NT5C_5_3919,712,965,366,565,28,66,400,356,1122
NT5M_5_3920,2173,2420,2594,1685,3237,2141,1539,1042,5811
NUAK1_5_3921,64,137,266,12,33,10,2,35,19
NUAK2_5_3922,1116,1328,1671,1586,797,1284,947,636,1746
NUCKS1_5_3923,2187,1493,2170,2082,2293,1118,1607,1303,777
OXSR1_5_3924,746,414,656,311,22,797,130,89,1522
PAK2_5_3925,166,211,552,391,145,282,65,517,50
PANK3_5_3926,722,408,1174,166,186,1896,913,505,2892
PANK4_5_3927,934,899,877,664,373,749,784,700,610
PASK_5_3928,867,1650,1647,661,1226,1782,1385,1206,3262
PBK_5_3929,1517,1922,2082,2631,2975,2346,1889,829,3073
PCK1_5_3930,280,541,590,433,58,5,42,308,36
PDCD1_5_3931,806,1543,1192,1112,601,516,806,576,1114
PDGFRA_5_3932,216,251,133,1,7,122,0,374,203
PDGFRB_5_3933,374,108,252,406,144,38,140,134,179
PDGFRL_5_3934,2683,3772,3720,3339,3953,2590,2333,4049,3606
PDIK1L_5_3935,313,355,548,669,127,143,384,264,242
PDK4_5_3936,866,1036,1744,1667,1972,2134,1213,1079,1628
PDP2_5_3937,759,476,731,1284,184,591,757,680,1745
PDXK_5_3938,161,72,43,103,1,2,523,159,455
PFKFB1_5_3939,1587,649,1327,2105,2118,544,1846,1606,1176
PFKFB4_5_3940,420,42,531,414,31,1550,41,1381,863
PFKL_5_3941,361,259,651,333,54,529,379,158,334
PGAM2_5_3942,791,1265,948,401,871,530,1023,1266,3543
PGK1_5_3943,2130,2983,2133,3167,4217,1400,2830,2405,3017
PGK2_5_3944,111,222,512,99,208,137,178,37,464
PHKA2_5_3945,441,269,582,289,157,412,157,129,141
PHKG1_5_3946,2558,2095,1944,747,1572,2250,3001,2931,2853
PI4K2A_5_3947,3523,2024,2887,2568,2242,3417,892,2650,3168
PI4K2B_5_3948,1338,1350,1803,1559,1332,2708,1366,2971,3279
PIK3C2A_5_3949,1264,1791,1471,1345,827,1922,769,647,3581

PIK3C2B_5_3950,989,1232,1102,950,162,561,520,677,377
PIK3C2G_5_3951,732,326,753,126,1319,1054,515,719,324
PIK3C3_5_3952,1682,1431,1105,1169,1808,891,809,1267,2184
PIK3CA_5_3953,2432,2141,2900,1147,2171,3041,2637,2677,1704
PIK3CB_5_3954,907,1234,705,441,596,776,1324,1261,2739
PIK3CD_5_3955,288,369,485,442,256,1367,290,163,57
PIK3CG_5_3956,354,49,141,471,167,341,255,9,5
PIK3R2_5_3957,1303,1328,1844,763,746,1244,855,1452,1338
PIK3R4_5_3958,4769,3678,5004,7093,1610,5412,3096,2018,4792
PIK3R6_5_3959,1994,932,2092,2119,1615,2051,2446,1047,911
PIM2_5_3960,524,102,310,34,203,250,953,30,1387
PIM3_5_3961,802,437,934,937,781,1713,448,1319,627
PINK1_5_3962,342,345,475,169,100,467,307,531,343
PIP4K2A_5_3963,3496,4113,3799,2948,4077,2024,2068,2532,5725
PIP4K2B_5_3964,305,264,598,587,1415,54,647,120,154
PIP5K1B_5_3965,2302,3121,3025,2851,3059,4473,3454,2313,2868
PKDCC_5_3966,541,689,874,404,95,124,214,401,431
PKN2_5_3967,3432,4317,5069,3899,6118,6448,3409,2769,5554
PKN3_5_3968,0,120,23,0,146,7,275,1,0
PLK1_5_3969,443,417,158,372,52,409,883,916,1
PLK2_5_3970,2648,1958,2501,1582,3720,2358,861,2138,1748
PLK3_5_3971,232,208,311,2,56,20,316,125,121
PMVK_5_3972,1053,409,1209,22,2247,1144,1316,967,3352
PNKP_5_3973,377,94,151,157,2,763,72,71,1
POLD1_5_3974,32,166,196,14,625,273,20,16,351
PON1_5_3975,1514,1783,2644,1916,1364,2088,1265,2605,2642
PPAP2B_5_3976,121,199,593,333,11,163,401,4,243
PPEF2_5_3977,269,598,357,375,480,390,411,457,1548
PPFIA3_5_3978,334,204,84,3,1,744,1,79,9
PPFIA4_5_3979,0,0,0,0,0,0,0,0,0
PPM1D_5_3980,333,569,808,371,706,1171,121,594,38
PPM1E_5_3981,390,232,224,148,157,408,77,83,196
PPM1F_5_3982,75,8,31,0,1,6,0,15,11
PPM1G_5_3983,2220,2967,4604,4171,2715,4264,2139,2708,3956
PPM1K_5_3984,2804,2702,3012,2745,885,2446,3718,1317,3005
PPM1L_5_3985,364,159,234,382,25,0,1,26,755
PPP1CC_5_3986,147,394,455,83,515,105,63,166,35
PPP1R12C_5_3987,414,132,469,206,3,0,281,1,877
PPP1R13B_5_3988,2910,2735,3136,1627,2975,3730,1579,2299,5419
PPP1R14B_5_3989,1581,837,1483,329,1420,952,1997,615,321
PPP1R15B_5_3990,1160,1240,1590,819,1658,965,1985,2091,1554
PPP1R1A_5_3991,787,519,1446,649,1266,382,604,1015,388
PPP1R1C_5_3992,44,83,38,152,176,248,99,6,25
PPP1R2_5_3993,353,585,922,447,3,893,597,926,66
PPP1R3A_5_3994,465,375,548,348,557,275,612,141,295
PPP1R3C_5_3995,1196,1321,1866,2221,1830,3564,1621,703,1733
PPP1R3D_5_3996,265,227,489,275,480,9,160,478,95
PPP1R7_5_3997,915,1066,948,798,1039,1284,565,445,1053
PPP2CA_5_3998,284,274,407,892,155,478,66,189,5
PPP2CB_5_3999,3528,3853,4609,1089,1975,5353,4776,2624,8062

PPP2R1A_5_4000,172,337,1085,1085,201,128,472,47,198
PPP2R2D_5_4001,605,777,1305,797,274,1290,623,591,3239
PPP2R3B_5_4002,1647,1195,2850,1043,512,881,1095,1239,2302
PPP2R5B_5_4003,517,109,967,57,115,1047,275,143,572
PPP2R5E_5_4004,2115,2107,3111,1458,2112,2672,1079,4380,1361
PPP3CC_5_4005,870,1364,1423,380,2833,1416,1592,1298,907
PPP3R1_5_4006,1500,1023,1074,622,1551,812,1689,1198,2108
PPP3R2_5_4007,726,437,1017,167,593,622,507,432,449
PPP4C_5_4008,993,852,1007,651,873,1114,787,691,910
PPTC7_5_4009,409,519,1155,86,66,100,694,701,249
PRKAA2_5_4010,612,817,970,270,241,420,627,680,314
PRKAB1_5_4011,925,793,747,583,1849,1529,2008,882,196
PRKAB2_5_4012,2952,3777,4915,3270,3416,2869,2834,3209,2916
PRKACG_5_4013,359,332,508,106,242,543,432,106,930
PRKAG3_5_4014,1289,1650,1083,141,344,120,2392,723,448
PRKAR2A_5_4015,85,397,821,583,1924,866,372,38,199
PRKAR2B_5_4016,580,651,1418,535,885,964,1273,1300,696
PRKCA_5_4017,445,843,365,703,41,910,1288,444,56
PRKCE_5_4018,112,146,16,143,78,76,456,114,11
PRKCG_5_4019,449,173,217,3,329,159,1014,96,168
PRKCH_5_4020,1566,1433,1626,1063,1874,1610,1879,2238,1316
PRKCI_5_4021,1682,1510,2332,2601,981,404,1542,192,503
PRKD1_5_4022,9041,9112,10249,9305,10111,10832,11096,10082,12700
PRKD3_5_4023,814,879,1130,2837,1251,1439,658,805,826
PRKG2_5_4024,837,699,1096,860,817,1345,598,1078,1578
PRKX_5_4025,1031,767,834,156,867,1777,415,1284,1492
PRPF4B_5_4026,656,919,736,478,377,1594,676,345,227
PRPS1L1_5_4027,3885,5310,5119,1318,6136,5758,3444,2214,6404
PSKH1_5_4028,541,697,630,186,663,751,1172,651,1141
PSKH2_5_4029,848,838,455,2667,957,59,334,135,1010
PSPH_5_4030,345,515,535,718,0,885,174,12,590
PSTK_5_4031,98,136,185,87,36,94,6,461,36
PTEN_5_4032,1203,1860,1945,2713,1561,2475,1109,1571,255
PTK6_5_4033,402,350,565,1793,324,40,283,111,394
PTP4A1_5_4034,722,1033,1176,1268,928,1590,605,2057,156
PTPLA_5_4035,188,113,378,0,0,63,0,240,5
PTPLB_5_4036,600,244,623,79,68,836,478,127,1499
PTPN11_5_4037,1850,1658,2708,2403,843,1723,2334,982,2997
PTPN14_5_4038,821,984,944,923,2079,2336,1122,681,92
PTPN1_5_4039,3188,2859,4768,2558,6156,5210,2888,2386,3952
PTPN21_5_4040,2170,2468,2251,2409,3548,3144,1544,1792,1756
PTPN23_5_4041,3562,4409,4295,3057,2127,5072,3397,4742,3209
PTPN4_5_4042,1387,1061,1660,1430,2138,612,642,1537,1174
PTPN9_5_4043,1123,904,1691,1440,1652,1216,464,1295,109
PTPRG_5_4044,667,317,602,697,356,510,151,115,152
PXK_5_4045,2404,2615,4568,2148,1571,3581,3051,1648,4758
RAF1_5_4046,183,182,340,72,163,388,3,897,11
RBKS_5_4047,982,1071,1132,420,235,617,1410,656,5928
RFK_5_4048,3644,4220,5113,5420,4078,5917,4062,3025,5034
RIOK3_5_4049,1704,1478,1490,2300,2707,393,1541,1758,1913

RIPK1_5_4050,2109,2497,3877,1814,2393,2237,2113,2005,2618
RIPK2_5_4051,197,751,501,32,317,187,482,193,89
RIPK3_5_4052,379,1257,491,846,985,1090,1565,859,555
RIPK4_5_4053,298,655,646,19,749,667,493,21,507
RNASEL_5_4054,812,1085,1176,749,606,1067,1071,353,2416
RNGTT_5_4055,0,0,0,0,0,0,0,0,0
ROCK1_5_4056,1318,1588,1605,2483,2455,3158,1905,1953,2872
ROCK2_5_4057,663,415,663,114,524,417,685,138,836
ROS1_5_4058,3519,3469,4773,3551,1861,5489,3207,2307,9827
RPS6KA3_5_4059,229,457,486,0,105,1345,0,0,437
RPS6KA6_5_4060,785,394,1071,662,799,1287,1419,410,677
RPS6KB1_5_4061,515,354,400,117,215,228,535,59,229
RPS6KB2_5_4062,727,447,1249,824,486,50,309,1203,854
RPS6KL1_5_4063,905,880,1188,949,437,909,1184,540,265
RSPO3_5_4064,1709,2294,2602,1698,2910,2317,2490,1947,4627
SACM1L_5_4065,667,1418,1562,1424,1939,2011,777,1384,1792
SBF1_5_4066,51,368,408,91,213,250,60,613,4
SBK1_5_4067,584,469,296,533,1262,284,766,624,1791
SBK2_5_4068,1,28,127,0,0,278,25,13,16
SEPHS2_5_4069,464,699,417,195,529,1,0,325,0
SGPP1_5_4070,502,738,1729,592,239,1100,715,347,2601
SHPK_5_4071,315,459,712,117,655,1640,593,384,1006
SIK1_5_4072,1878,2447,2782,788,1811,642,2056,1410,818
SIK2_5_4073,1344,1048,1074,2059,528,2069,1099,1288,3429
SIK3_5_4074,254,748,1146,672,75,723,681,748,1045
SKAP2_5_4075,2200,2619,1827,1773,1734,2680,2058,1994,1151
SMG1_5_4076,606,814,688,6,1959,1426,193,404,410
SRMS_5_4077,564,379,381,340,1229,294,1350,243,192
SRP72_5_4078,774,762,1043,244,610,417,800,943,188
SRPK1_5_4079,914,852,1379,641,1088,964,624,1104,1312
SSH2_5_4080,1533,1971,2189,728,1230,1539,1989,1524,2087
SSH3_5_4081,621,801,889,1244,983,975,299,823,678
STC1_5_4082,480,1464,1516,206,386,982,260,978,493
STK10_5_4083,355,564,663,75,760,371,726,1153,1107
STK11_5_4084,1713,2431,2998,1469,1682,1461,1718,1545,2843
STK16_5_4085,815,1153,1665,434,1158,747,553,792,299
STK17A_5_4086,2718,2552,3057,2518,4051,2747,989,1009,2002
STK17B_5_4087,1260,970,971,308,1391,345,546,280,1279
STK25_5_4088,650,1089,590,502,775,2103,362,224,507
STK32B_5_4089,581,466,663,126,1700,524,788,555,453
STK32C_5_4090,2556,3397,2946,2085,2944,2875,2019,3461,3332
STK33_5_4091,5605,5538,5548,4983,5961,5831,5917,3564,6232
STK35_5_4092,1707,2615,1860,2695,2373,2165,2017,1673,2742
STK38_5_4093,460,369,237,839,777,512,734,305,496
STK38L_5_4094,946,1917,1599,741,1357,1697,1141,580,356
STK39_5_4095,1361,2834,2535,1938,2232,2805,1212,2294,2122
STK40_5_4096,436,640,874,1142,415,1035,169,286,577
STK4_5_4097,2457,1873,2771,2496,1787,2092,2124,734,6101
STYK1_5_4098,584,864,679,134,1367,229,806,976,429
STYXL1_5_4099,563,728,414,67,205,84,304,90,59

TAF1L_5_4100,620,842,881,137,856,1237,731,1540,12
TAOK3_5_4101,1345,1114,2067,837,1670,2225,1693,241,885
TBK1_5_4102,1465,1633,2221,453,2431,576,2236,3761,3382
TEC_5_4103,4456,3683,3842,4372,3574,3119,2052,2382,4126
TEK_5_4104,3659,3297,4294,3482,3447,3402,3635,3921,2303
TESK1_5_4105,341,417,718,411,58,593,415,231,186
TESK2_5_4106,1472,1878,2209,1391,1535,2834,552,2309,3753
THNSL1_5_4107,1509,2560,2814,2105,1650,2251,1260,3941,4322
TIE1_5_4108,57,144,230,397,239,57,181,20,214
TK1_5_4109,58,143,115,0,127,0,1,38,18
TNK1_5_4110,141,82,370,92,162,278,32,545,61
TNNI3K_5_4111,958,1108,779,152,2957,413,1075,2834,1899
TNS3_5_4112,248,453,166,99,375,439,325,306,1179
TP53RK_5_4113,6048,4019,4505,3760,6237,4610,2558,3927,3681
TRAT1_5_4114,1480,1034,1153,444,672,1378,1319,703,2466
TRIB1_5_4115,754,631,657,233,1535,421,80,189,722
TRIB2_5_4116,79,23,60,17,69,652,218,25,0
TRIB3_5_4117,739,841,1345,234,511,1686,69,425,130
TRIM27_5_4118,603,763,439,529,980,379,539,1445,0
TRIM28_5_4119,486,593,473,1512,68,1074,1545,395,1483
TRIO_5_4120,453,743,541,324,1723,742,648,759,403
TRPM7_5_4121,5301,6799,7087,7281,8225,4794,4268,7447,10117
TRRAP_5_4122,232,316,600,868,326,25,352,316,228
TSKS_5_4123,41,8,255,0,0,0,0,80,0
TSSK1B_5_4124,738,853,1014,1102,321,1708,890,260,1638
TSSK2_5_4125,316,799,499,239,36,929,123,823,20
TSSK3_5_4126,189,135,122,34,186,478,142,363,8
TSSK6_5_4127,24,178,503,65,123,370,3,6,775
TTBK1_5_4128,76,314,79,430,30,102,103,198,2
TTBK2_5_4129,350,581,223,142,423,183,46,758,17
TWF2_5_4130,824,975,962,368,818,667,1477,1157,345
TXK_5_4131,3708,3078,4083,3063,1865,3701,2407,3155,4118
TYK2_5_4132,251,809,418,44,604,777,317,87,469
TYRO3_5_4133,148,26,52,206,211,0,15,156,1
UBLCP1_5_4134,729,878,799,210,860,218,1205,322,25
UCK2_5_4135,1043,1182,1376,1061,2827,1700,975,1605,1376
ULK1_5_4136,740,1217,1162,203,372,17,262,389,1786
ULK3_5_4137,875,1201,993,5,328,496,588,482,211
ULK4_5_4138,1508,1577,1360,2673,901,1177,700,1275,612
VRK1_5_4139,626,914,214,127,196,73,151,867,895
WEE2_5_4140,1422,2146,1840,1886,565,2598,1111,1818,587
WNK2_5_4141,1004,891,1808,2178,943,388,657,1543,1904
WNK4_5_4142,938,1217,1134,43,998,731,908,643,922
XRCC6BP1_5_4143,1804,2684,2643,1516,3120,1575,731,1084,2316
XYLB_5_4144,2783,2681,2701,4648,3561,3808,1722,3219,2411
YES1_5_4145,499,511,551,296,716,369,383,462,257
AATK_5_4146,761,894,732,300,590,834,1393,389,1611
ABL1_5_4147,147,250,656,216,1120,1201,508,250,1355
ABL2_5_4148,514,1526,783,455,3038,1567,1558,793,1067
ACP1_5_4149,250,384,188,48,858,555,118,231,12

ACP5_5_4150,373,661,604,191,220,330,22,322,385
ACPL2_5_4151,2161,2571,2303,843,2936,2298,1968,1718,1249
ACPP_5_4152,1850,1655,2676,1571,1380,3054,1354,2569,3827
ACVR1B_5_4153,309,66,343,251,822,864,165,276,113
ACVR1C_5_4154,2364,2250,3460,3711,4306,1176,2066,1896,2700
ACVR1_5_4155,572,487,553,190,329,1425,157,529,125
ACVRL1_5_4156,930,1392,907,753,400,860,1669,1401,837
ADCK1_5_4157,528,826,932,1288,390,1004,376,331,190
ADCK4_5_4158,53,215,115,1,4,555,123,221,52
ADK_5_4159,1470,1799,912,782,2595,955,849,911,1355
AK2_5_4160,318,729,1248,1080,1989,765,852,344,869
AK4_5_4161,3132,3545,4928,2949,1349,4219,5270,4242,3458
AK5_5_4162,1469,1352,1009,347,1001,1364,731,1330,536
AKT1_5_4163,130,53,72,76,12,0,2,0,0
AKT2_5_4164,705,331,1103,563,837,382,78,1136,692
AKT3_5_4165,4511,4727,4480,4964,5317,6252,5815,2630,7877
ALDH18A1_5_4166,805,591,697,1854,1818,651,36,1191,1567
ALPK1_5_4167,915,1418,942,1344,1374,1341,1708,280,2433
ALPL_5_4168,38,284,281,0,351,1216,32,456,53
AMHR2_5_4169,1881,2135,3623,608,4551,2949,756,1817,4420
ANKHD1_5_4170,1371,1285,1875,1209,919,342,1592,661,1027
APTX_5_4171,16,353,82,1,362,44,484,572,148
ATRIP_5_4172,1683,2350,1725,749,1543,1885,2983,2428,2346
AURKA_5_4173,730,837,772,544,473,342,857,1036,192
AURKC_5_4174,381,65,687,88,15,0,496,287,3
AXL_5_4175,774,251,557,243,130,991,978,921,5
BAIAP2_5_4176,410,450,153,117,264,100,584,0,545
BCKDK_5_4177,1269,838,1503,533,777,2308,1343,1136,2415
BCR_5_4178,1619,2150,2399,1304,1247,1465,2743,1217,3719
BMP2K_5_4179,1888,1801,2580,1617,1208,1683,1271,898,2427
BMX_5_4180,791,755,1075,956,1088,757,568,710,824
BPGM_5_4181,1521,2004,2070,1803,1152,1094,846,3174,4178
BRD2_5_4182,102,243,588,809,412,601,300,193,22
BRD4_5_4183,876,917,727,647,790,988,500,1208,3285
BRDT_5_4184,1550,2438,2090,2902,3534,3620,1910,1739,3145
CAB39L_5_4185,2275,3212,3556,5594,1793,4276,2442,2668,2501
CAMK1D_5_4186,2041,2548,2346,1845,2760,4236,2255,2202,2786
CAMK2A_5_4187,620,577,663,548,104,27,1148,59,819
CAMK2B_5_4188,527,221,490,468,482,153,331,1516,183
CAMK2D_5_4189,1335,904,946,1083,998,181,1621,301,1077
CAMK2G_5_4190,15159,17554,21006,11716,18079,15374,17583,13786,18215
CAMKK1_5_4191,679,616,973,333,540,1012,912,652,415
CAMKK2_5_4192,450,235,214,14,60,518,569,295,163
CASK_5_4193,1274,1444,2018,2049,1190,1090,930,1603,2611
CCT2_5_4194,1758,1547,2051,1710,1255,891,1048,824,800
CDADC1_5_4195,2565,1959,2540,2193,2025,1904,1640,1099,881
CDC14A_5_4196,171,208,178,0,53,51,397,73,283
CDC14B_5_4197,619,990,934,1095,1628,216,1078,673,1079
CDC25A_5_4198,763,1003,401,345,1210,768,437,614,132
CDC25B_5_4199,416,371,688,271,775,155,132,566,69

CDC25C_5_4200,1172,1238,2526,1218,2366,3082,1703,1493,2146
CDC42BPA_5_4201,252,1093,382,190,408,523,619,531,245
CDC7_5_4202,2221,3094,2654,835,2936,1171,3418,3628,1216
CDK10_5_4203,94,168,455,53,52,1818,24,201,303
CDK11A_5_4204,2047,1535,1586,607,1170,1615,1710,1154,701
CDK11B_5_4205,2047,1535,1586,607,1170,1615,1710,1154,701
CDK12_5_4206,1520,2162,1723,2600,649,1005,1368,1661,1887
CDK13_5_4207,1099,2362,731,1067,1293,1717,998,1492,742
CDK16_5_4208,1213,1292,1943,863,489,1059,1856,1690,886
CDK17_5_4209,387,230,268,312,286,232,305,125,434
CDK18_5_4210,1171,1138,1522,9,137,1438,864,499,2378
CDK20_5_4211,445,1254,373,253,32,384,853,911,274
CDK2_5_4212,365,1369,1017,366,1137,910,649,1535,1039
CDK5_5_4213,2430,2046,2928,3660,4412,2190,1694,2366,2477
CDK6_5_4214,578,268,410,98,82,479,158,491,130
CDKL3_5_4215,485,526,748,263,600,1128,160,542,41
CDKL5_5_4216,461,104,382,203,319,776,2,69,877
CDKN1A_5_4217,291,236,371,309,84,53,45,355,297
CDKN3_5_4218,1236,1284,1861,1737,822,2069,1095,1965,1544
CHEK1_5_4219,683,789,771,740,202,778,738,942,507
CHEK2_5_4220,1096,1870,1917,604,794,1929,1386,1384,891
CHKA_5_4221,1768,1557,2713,1005,4142,2669,892,2546,1494
CIT_5_4222,654,541,817,2139,596,674,1100,419,407
CKMT2_5_4223,43,234,150,245,171,355,62,51,229
CLK1_5_4224,443,380,291,405,323,840,651,440,440
CLK3_5_4225,372,68,398,400,0,3,29,81,0
CMPK1_5_4226,179,334,317,3,355,574,0,0,0
COASY_5_4227,357,347,336,569,329,610,35,515,233
COL4A3BP_5_4228,1247,511,909,815,269,1359,1126,582,977
CSK_5_4229,39,162,162,2,41,387,38,220,762
CSNK1A1_5_4230,170,1060,579,1,539,128,66,329,2
CSNK1D_5_4231,1685,2170,1881,488,734,3224,1222,2234,1224
CSNK1E_5_4232,176,235,654,50,261,587,213,176,235
CSNK1G3_5_4233,70,50,192,57,73,162,110,242,36
CTDP1_5_4234,1042,761,1235,206,264,1611,1263,1080,1116
CTDSP1_5_4235,217,139,119,422,10,178,159,38,668
DCLK1_5_4236,1470,1025,1637,1819,126,848,1105,1227,1349
DCLK2_5_4237,3625,3516,2873,1805,3816,4043,3651,1567,2677
DDR1_5_4238,1562,1569,1190,778,1799,1017,821,1016,880
DDR2_5_4239,53,45,362,304,586,182,499,77,207
DGKA_5_4240,1925,1938,2732,2091,1405,2376,2267,1432,1575
DGKB_5_4241,2118,2816,2703,2019,2947,2210,1815,2356,4407
DGKD_5_4242,81,66,129,171,120,88,26,81,3
DGKG_5_4243,339,381,719,381,79,272,691,414,125
DGKH_5_4244,450,946,706,160,1813,1116,1299,690,527
DGKZ_5_4245,835,1130,1483,774,587,1446,664,1473,1516
DGUOK_5_4246,1508,1577,1702,3549,1267,1332,1951,1526,1408
DLGAP5_5_4247,459,932,584,855,171,508,576,950,89
DMPK_5_4248,666,584,707,365,765,169,1761,150,35
DSTYK_5_4249,1066,663,944,1604,1033,3106,1950,1329,1878

DTYMK_5_4250,718,843,753,73,1186,330,570,804,2573
DUSP13_5_4251,110,215,137,13,420,194,80,78,478
DUSP19_5_4252,840,695,1585,1393,2456,1357,1318,594,1235
DUSP4_5_4253,507,364,642,95,870,371,533,254,1169
DUSP6_5_4254,207,348,787,1125,64,113,305,162,982
DYRK1A_5_4255,3787,5914,5593,1879,5105,2472,3264,3286,2221
DYRK1B_5_4256,172,592,1172,1738,5,49,16,204,567
DYRK2_5_4257,73,204,307,9,2,0,213,20,42
DYRK3_5_4258,308,462,654,437,259,547,231,343,562
EFNA4_5_4259,351,351,913,337,600,21,506,599,523
EGFR_5_4260,1175,1702,1684,1008,418,600,2016,202,2209
EIF2AK2_5_4261,1226,1180,1571,508,1425,870,690,751,2799
EPHA10_5_4262,1298,848,493,10,1134,455,191,530,604
EPHA3_5_4263,6233,8586,7937,8065,8253,6171,7972,8092,8155
EPHA5_5_4264,408,212,420,550,119,152,45,35,353
EPHA6_5_4265,4881,4894,4462,4150,4858,4430,6181,3601,4825
EPHA8_5_4266,1682,2179,3074,1525,1053,2227,870,1820,2667
EPHB2_5_4267,362,983,821,448,1633,27,1373,267,2083
EPM2A_5_4268,8110,10202,12385,9992,5604,11161,9206,10100,8745
ERBB2_5_4269,539,448,415,57,210,658,377,232,16
ERBB4_5_4270,777,834,839,1889,1353,964,525,742,3002
ETNK1_5_4271,136,399,471,55,1615,185,293,301,773
EXOSC10_5_4272,606,571,766,139,1318,361,505,184,291
EYA1_5_4273,457,524,513,575,423,743,349,335,535
EYA2_5_4274,1155,1161,1700,184,1573,574,1700,175,782
EYA4_5_4275,744,325,463,97,973,154,257,280,667
FASTK_5_4276,827,448,1170,1225,1072,1899,937,473,673
FBP1_5_4277,2012,2259,3042,3712,1491,2474,1994,3227,3933
FES_5_4278,649,891,1169,694,585,500,858,877,1792
FGFR1_5_4279,636,991,1376,955,1791,1293,1517,968,289
FGFR2_5_4280,146,108,218,169,80,604,491,135,171
FGFR3_5_4281,212,286,248,49,0,28,120,922,532
FGFR4_5_4282,68,116,97,0,0,0,0,0,0
FGFRL1_5_4283,686,570,843,726,1246,546,225,329,1659
FGR_5_4284,345,121,311,1301,744,712,89,373,6
FLT1_5_4285,2847,2605,4519,2507,1943,2244,1946,2624,3235
FLT4_5_4286,211,511,135,99,803,460,300,473,917
FXN_5_4287,2321,1627,1802,2405,1568,508,2227,1594,1995
FYN_5_4288,532,622,1339,602,238,1235,733,2809,1572
FZR1_5_4289,99,74,8,0,0,110,10,307,66
GALK2_5_4290,1735,1668,2514,1929,1795,2216,1395,1636,3355
GK_5_4291,1501,1208,1282,2397,1185,112,453,3125,2605
GLYCTK_5_4292,2762,2472,4560,3938,5468,4120,3922,2895,4019
GNE_5_4293,1495,1048,1546,540,1386,1671,916,1001,1445
GRK4_5_4294,4004,4483,4494,2674,3439,3863,4231,5234,4073
GRK6_5_4295,2100,2288,2544,2380,5609,2273,857,1972,632
GSK3B_5_4296,3275,2362,4346,3256,966,4492,933,1039,2798
GUK1_5_4297,225,41,44,136,13,228,31,2,16
HCK_5_4298,456,385,10,344,227,273,468,52,2
HIPK1_5_4299,3397,3442,5357,3603,2945,2471,3762,2833,2855

HIPK2_5_4300,361,505,859,141,112,1791,1605,432,347
HIPK3_5_4301,747,1123,1833,728,759,2647,1217,1189,805
ICK_5_4302,1,0,128,0,0,0,3,0,0
IKBKB_5_4303,1746,587,1753,1103,3278,1231,1034,547,2202
IKBKE_5_4304,3320,2842,2980,2180,2641,2568,4028,4322,3507
IKBKG_5_4305,2449,1330,1855,2273,1642,1189,841,1118,2006
ILK_5_4306,3232,3103,3147,3021,2089,3994,2350,4926,3978
IMPA1_5_4307,555,629,568,242,954,1302,421,408,1480
INPP1_5_4308,933,654,754,307,921,349,616,184,936
INPP4A_5_4309,794,254,194,1473,2,1236,297,104,62
INPP4B_5_4310,1099,602,1456,327,796,49,1477,105,1183
INPP5D_5_4311,270,536,468,1396,288,585,611,372,949
INSR_5_4312,286,425,61,0,130,999,958,16,0
IP6K1_5_4313,631,896,956,208,685,695,1210,359,3555
IP6K3_5_4314,2461,2535,2730,1868,4770,2493,986,1149,3400
IRAK1_5_4315,833,978,1043,393,57,727,341,1408,432
IRAK3_5_4316,601,631,1428,399,730,1137,2547,493,1044
IRAK4_5_4317,819,977,963,364,349,1735,775,612,1727
ITPK1_5_4318,731,876,927,878,215,849,477,641,16
KALRN_5_4319,764,1131,551,312,1187,1096,988,908,1410
KHK_5_4320,279,1062,635,156,724,1008,103,2384,565
KIF2A_5_4321,1149,857,768,316,263,717,530,158,54
KIT_5_4322,477,349,635,129,367,263,143,83,1296
LCK_5_4323,546,346,279,75,1001,315,211,800,312
LHPP_5_4324,593,508,507,354,306,489,500,390,1124
LIMK1_5_4325,287,844,547,60,606,472,526,457,960
LIMK2_5_4326,1273,1051,1112,1247,340,735,595,1752,1512
LTK_5_4327,623,790,644,528,138,429,551,1990,1733
LYN_5_4328,179,224,395,329,454,100,274,150,61
MAGI3_5_4329,643,1116,1795,785,1148,932,1029,1177,761
MAP2K3_5_4330,890,730,1448,578,303,1364,975,836,169
MAP2K5_5_4331,713,1377,1764,1243,552,990,1745,1152,1663
MAP3K12_5_4332,245,714,767,138,301,1,934,342,334
MAP3K13_5_4333,359,133,499,941,697,104,432,222,0
MAP3K3_5_4334,185,208,367,293,1957,577,15,529,314
MAP3K4_5_4335,1250,1109,1813,782,938,1296,458,3326,447
MAP3K7_5_4336,2034,2682,2342,1494,1896,1569,2170,3326,3524
MAP4K1_5_4337,2223,2435,3367,1419,2413,4742,2701,1570,2767
MAP4K4_5_4338,1799,2458,2093,1967,2284,1690,3403,1284,2731
MAP4K5_5_4339,1099,1348,1465,391,841,1561,1068,431,2201
MAPK10_5_4340,772,163,1135,558,366,1417,137,503,395
MAPK14_5_4341,203,353,421,242,1857,951,82,199,0
MAPK1_5_4342,927,877,1194,1333,558,302,237,1490,1301
MAPK3_5_4343,996,1447,1221,1018,584,249,601,503,1255
MAPK7_5_4344,589,1086,691,197,842,1923,1280,847,355
MAPK8_5_4345,4333,5469,4680,5285,7504,6092,4208,6247,3965
MAPK9_5_4346,1635,2064,2426,977,1821,4146,2513,964,3080
MAPKAPK2_5_4347,386,434,589,808,121,791,1236,661,519
MAPKAPK5_5_4348,189,178,569,132,232,388,300,12,119
MARK2_5_4349,97,156,499,179,54,249,255,168,245

MARK3_5_4350,261,809,366,516,790,170,640,52,1
MARK4_5_4351,589,1351,721,359,86,767,288,998,27
MAST4_5_4352,1752,1786,1741,637,2158,2081,1193,2138,555
MASTL_5_4353,242,260,471,193,1076,921,675,620,6
MATK_5_4354,140,588,660,674,165,24,319,171,96
MET_5_4355,5690,8546,11263,3839,10911,13662,10510,7834,10616
MINK1_5_4356,844,636,685,962,1784,539,289,309,1008
MINPP1_5_4357,1158,812,2006,1014,1388,1226,517,1722,1774
MKNK1_5_4358,498,673,795,1369,1040,446,981,322,171
MKNK2_5_4359,241,212,213,159,385,296,230,193,279
MLKL_5_4360,1415,1827,1206,670,472,875,1680,1677,343
MTMR14_5_4361,221,0,46,57,0,39,14,0,51
MTMR2_5_4362,2723,2128,3480,1835,2290,3195,1184,2141,2820
MTMR3_5_4363,126,214,369,387,0,542,525,737,72
MUSK_5_4364,4236,4346,4632,2512,4576,6656,4914,3483,7821
MVK_5_4365,858,750,1150,871,136,1755,1306,778,328
MYO3B_5_4366,1214,2205,1664,1780,2120,782,1227,1293,1979
NADK_5_4367,2822,1593,2335,1649,728,2239,2015,1946,4193
NCK1_5_4368,1994,2407,1765,500,927,1650,2443,2088,2058
NCK2_5_4369,1130,969,1403,2234,2267,1247,673,1260,2243
NDRG1_5_4370,1227,1031,1470,1057,1815,1922,1322,1383,2308
NEK11_5_4371,276,375,503,299,137,54,478,67,520
NEK1_5_4372,1072,1817,2056,2711,2248,1413,1595,1254,1905
NEK2_5_4373,1132,761,1136,1175,2355,1101,735,426,456
NEK3_5_4374,4060,3740,3954,2831,2281,2680,2200,3487,5987
NEK4_5_4375,2058,1539,1964,2130,580,1730,2690,1973,1571
NEK6_5_4376,397,78,329,249,328,508,99,112,567
NME2_5_4377,1483,800,2101,1815,1551,1353,854,605,3501
NME7_5_4378,250,408,347,102,159,11,284,22,682
NT5C1B_5_4379,6003,6708,8040,6278,6062,8831,5805,9314,9513
NT5C2_5_4380,2443,2373,2441,3430,2880,1193,2013,1931,2504
NT5C3_5_4381,3269,3763,3272,1265,3945,2350,1968,1216,4157
NT5E_5_4382,2279,1661,1799,1378,689,2912,1972,1563,1543
NTRK1_5_4383,241,583,1087,348,86,1508,495,26,1292
NTRK2_5_4384,581,511,485,92,405,1781,357,462,77
NTRK3_5_4385,319,147,121,21,299,263,232,57,72
NUDT4_5_4386,2190,2118,3082,3277,3189,3069,2801,2984,1801
NUDT9_5_4387,696,980,754,760,1510,843,131,567,214
OBSCN_5_4388,453,829,478,1073,30,902,386,488,441
OCRL_5_4389,351,672,708,676,546,159,591,1071,107
OXSM_5_4390,2103,2022,2400,2457,2848,1723,1116,2611,3090
PACSIN1_5_4391,198,361,322,5,330,421,482,391,401
PAK4_5_4392,1263,1362,2223,1022,1160,1656,1165,519,1324
PAK6_5_4393,537,486,1039,308,1138,109,372,24,138
PAK7_5_4394,249,508,435,595,617,430,245,1101,486
PANK1_5_4395,2061,1339,3470,1128,2381,3123,716,1509,2822
PANK2_5_4396,522,833,1023,41,1723,114,824,497,1656
PCK2_5_4397,4220,6632,4798,2927,7316,4097,5301,3855,4412
PDK2_5_4398,39,109,231,233,1554,12,367,22,2266
PDK3_5_4399,963,788,993,1113,2501,1165,835,1046,135

PDPK1_5_4400,0,0,0,0,0,0,0,0,0
PEG3_5_4401,227,109,525,4,174,1021,1460,792,568
PFKFB2_5_4402,98,258,88,466,622,340,47,36,25
PFKFB3_5_4403,514,416,428,1309,475,355,418,140,775
PFKM_5_4404,57,201,104,279,0,34,78,0,3
PFKP_5_4405,685,617,748,517,365,477,111,172,76
PHKA1_5_4406,1301,1595,1317,1464,1275,712,956,1014,358
PHKB_5_4407,1128,2328,1188,1323,2113,659,1709,1729,421
PHKG2_5_4408,729,854,863,1564,1123,767,1041,1181,1029
PHPT1_5_4409,679,610,848,1139,1227,1012,1341,556,1367
PI4KA_5_4410,2927,2536,3557,2169,3717,2377,2971,2382,2363
PI4KB_5_4411,1111,631,575,625,371,481,253,1295,913
PIK3R1_5_4412,279,358,231,607,126,364,36,500,0
PIK3R5_5_4413,157,271,90,80,93,11,275,951,95
PIKFYVE_5_4414,1739,1356,1635,4354,1573,1898,934,2743,2624
PIP4K2C_5_4415,291,242,420,277,690,306,1196,534,45
PIP5K1A_5_4416,2166,1417,2562,3097,2226,1810,721,1540,1553
PIP5K1C_5_4417,389,27,55,196,740,292,53,3,101
PIP5KL1_5_4418,182,185,660,1783,293,575,213,50,440
PKLR_5_4419,234,358,449,157,19,1,14,450,70
PKMYT1_5_4420,37,148,247,90,322,93,266,12,0
PKN1_5_4421,77,54,182,393,2,0,8,4,0
PLK4_5_4422,995,707,1307,955,593,206,599,336,1734
PNCK_5_4423,459,211,115,48,161,0,43,426,0
PODXL_5_4424,123,128,197,303,210,247,40,118,28
PPAP2A_5_4425,458,273,334,14,653,220,106,9,2
PPAP2C_5_4426,294,1518,758,422,806,369,325,782,509
PPEF1_5_4427,3181,3139,4038,2362,3959,2310,2811,3318,3123
PPFIA1_5_4428,1399,1847,1774,2049,1862,1026,2255,1040,322
PPFIA2_5_4429,394,439,414,291,530,27,1187,766,889
PPM1A_5_4430,2199,1640,1502,2693,435,2541,1356,2148,3677
PPM1B_5_4431,848,1068,1126,1328,836,1310,2178,756,180
PPM1M_5_4432,1153,934,866,0,144,20,174,1174,944
PPP1CA_5_4433,329,503,1050,686,1293,503,977,467,267
PPP1CB_5_4434,1974,1502,1650,2263,1863,1356,859,1229,806
PPP1R12A_5_4435,2516,2474,4719,3840,4348,5289,4340,2952,2108
PPP1R16B_5_4436,922,788,1342,209,446,1404,820,646,1205
PPP1R1B_5_4437,1679,1514,2029,645,520,2294,2931,3083,1663
PPP1R8_5_4438,1269,1685,2042,1081,413,1237,1313,588,2162
PPP2R1B_5_4439,511,716,1208,196,634,1106,954,617,748
PPP2R2A_5_4440,2875,2541,2460,3190,2191,3110,1396,2144,5647
PPP2R2B_5_4441,786,1125,1599,477,1524,970,1211,1319,3074
PPP2R2C_5_4442,557,108,1068,2,141,1233,234,211,796
PPP2R3A_5_4443,1303,1635,1704,2014,1531,1742,1916,1986,2004
PPP2R4_5_4444,1544,1401,1739,1106,1511,1847,723,399,2498
PPP2R5A_5_4445,233,215,272,13,52,47,539,541,92
PPP2R5D_5_4446,1210,865,1459,1265,924,302,892,925,443
PPP3CA_5_4447,1762,2117,1966,2593,5045,1222,1237,1504,2521
PPP3CB_5_4448,1931,1972,1883,1238,1977,2401,2447,897,1327
PPP4R1_5_4449,944,468,428,416,284,1008,820,828,727

PPP5C_5_4450,645,303,668,1167,542,1,241,1529,580
PPP6C_5_4451,886,613,191,1242,549,570,1090,756,406
PRKAA1_5_4452,2224,2756,3124,930,4080,3212,2315,2353,1534
PRKACA_5_4453,293,52,425,508,246,222,343,537,55
PRKACB_5_4454,894,1171,946,536,1612,1376,705,1730,262
PRKAG1_5_4455,947,1730,997,2648,2223,1792,1066,1434,1508
PRKAG2_5_4456,742,428,520,1280,2881,1156,912,792,1985
PRKAR1A_5_4457,801,1579,554,1222,2338,436,591,1816,275
PRKAR1B_5_4458,841,1071,1984,673,2596,1650,1118,1705,1268
PRKCB_5_4459,873,1074,1017,638,121,1128,476,498,381
PRKCD_5_4460,1126,291,915,1040,339,967,403,1271,302
PRKCQ_5_4461,183,301,297,19,73,101,200,480,102
PRKCZ_5_4462,0,6,282,1,129,1,364,18,0
PRKD2_5_4463,823,585,1004,1743,496,502,487,1267,89
PRKDC_5_4464,5882,5187,8260,5052,9325,5425,5485,5737,4896
PRKG1_5_4465,417,282,262,60,664,210,246,116,512
PRPS2_5_4466,1284,765,914,973,760,1557,2048,794,1476
PTK2B_5_4467,546,1017,683,1000,405,811,398,609,571
PTK2_5_4468,4918,6970,7505,3007,5011,4047,3329,5147,4332
PTK7_5_4469,1409,1516,1498,813,376,3216,1078,696,2753
PTP4A2_5_4470,994,2895,2043,1861,2222,2040,1967,2422,1759
PTP4A3_5_4471,397,30,61,304,608,794,327,230,164
PTPDC1_5_4472,595,1412,742,1569,973,538,1189,908,1203
PTPN12_5_4473,843,1005,1254,813,1775,1069,1980,1186,1831
PTPN13_5_4474,1417,1214,1320,2017,530,1299,1166,1797,511
PTPN18_5_4475,500,378,587,31,25,68,310,580,163
PTPN22_5_4476,2210,2276,3796,910,2122,1925,2098,3444,3863
PTPN2_5_4477,1757,3004,3066,1395,2759,2387,1892,2389,4102
PTPN3_5_4478,127,198,144,45,112,0,324,3,168
PTPN5_5_4479,323,155,636,421,331,469,52,7,753
PTPN6_5_4480,839,1669,1279,470,1197,3197,615,924,259
PTPN7_5_4481,230,58,458,309,270,604,1174,430,1
PTPRA_5_4482,158,461,323,292,456,864,104,459,1392
PTPRB_5_4483,5354,4354,6939,6382,4992,9325,2660,4633,5432
PTPRC_5_4484,4552,5108,4865,9837,6965,7524,3329,4458,6686
PTPRD_5_4485,4174,4308,5000,3576,2820,2896,3525,2058,6702
PTPRE_5_4486,34,177,26,21,0,138,139,26,15
PTPRF_5_4487,332,265,536,67,1096,1515,358,275,421
PTPRH_5_4488,1079,941,1251,934,769,1349,549,802,2089
PTPRJ_5_4489,745,1216,1199,812,1174,61,348,120,827
PTPRK_5_4490,630,809,728,170,973,195,475,889,61
PTPRM_5_4491,775,1647,1044,1261,2430,1186,968,463,848
PTPRN2_5_4492,1991,1336,2164,404,1464,1133,1163,846,590
PTPRN_5_4493,547,445,878,73,259,1484,23,3,54
PTPRR_5_4494,1185,1391,1724,483,1369,1039,1799,1984,1741
PTPRS_5_4495,182,128,140,83,53,1,467,0,13
PTPRT_5_4496,748,431,1266,3888,2469,1121,1153,781,3173
PTPRU_5_4497,525,269,532,1894,245,1525,711,429,685
PTPRZ1_5_4498,1251,2388,1279,1385,833,2402,1777,594,2049
RET_5_4499,4674,5479,6091,3421,3852,5670,4131,4927,6269

RIOK1_5_4500,1894,1344,3599,2039,481,1843,1482,1624,2581
RIOK2_5_4501,1695,1551,1536,2385,1100,1831,1636,2232,915
ROPN1L_5_4502,51,276,1,12,0,0,1,0,5
RPS6KA1_5_4503,1356,1849,2051,1192,1537,2343,1828,2066,3006
RPS6KA2_5_4504,607,371,840,1,529,1658,145,36,408
RPS6KA4_5_4505,155,4,10,9,1,5,10,1,0
RPS6KA5_5_4506,2108,2551,3452,2103,5123,2149,1243,787,4180
RPS6KC1_5_4507,504,674,615,489,621,2829,394,462,100
RYK_5_4508,608,826,974,491,382,623,458,648,2589
SET_5_4509,1321,1058,1789,2448,2110,2248,1877,1069,745
SGK1_5_4510,98,736,883,2,24,164,16,408,62
SGK3_5_4511,17209,18430,16083,16805,17734,11626,14679,14086,18755
SIRPA_5_4512,1199,1039,1392,1889,2429,1129,2263,663,246
SKAP1_5_4513,1529,992,1204,1966,2316,962,842,526,885
SNRK_5_4514,1145,489,532,18,167,1317,759,779,864
SPHK1_5_4515,550,846,1444,509,813,462,1562,578,544
SPHK2_5_4516,21,14,40,113,10,16,1,2,3
SRC_5_4517,400,317,155,141,112,459,83,142,72
SRPK2_5_4518,253,437,84,17,203,63,62,221,45
SRPK3_5_4519,161,107,96,2,439,2,428,48,0
SSH1_5_4520,598,1199,1170,1202,1808,1872,1126,353,1194
STK19_5_4521,652,785,596,2314,105,743,874,746,144
STK24_5_4522,729,834,1134,1656,396,583,610,1403,909
STK31_5_4523,3431,6215,6115,1859,5554,5453,3922,5484,4617
STK36_5_4524,150,121,461,4,18,480,95,27,292
STRADA_5_4525,997,969,1609,1718,1371,2082,1388,851,3089
STRADB_5_4526,467,188,635,403,106,440,305,98,379
STYX_5_4527,877,514,486,1256,618,493,1085,552,205
SYK_5_4528,1805,1686,2794,1231,1879,1390,1441,2101,2703
SYNJ1_5_4529,12346,12630,14215,16076,11914,11315,12538,13428,16813
SYNJ2_5_4530,245,296,327,1203,74,317,106,306,149
TAF1_5_4531,3388,3359,4368,3937,3175,6121,4825,3050,5815
TAOK1_5_4532,1238,1316,1757,1075,909,1912,898,722,2108
TAOK2_5_4533,465,253,409,36,313,336,858,174,5
TBCK_5_4534,1520,1231,1941,1426,2191,1864,1331,912,1967
TEX14_5_4535,2604,4535,3019,2648,2791,4279,2120,2161,4253
TGFBR1_5_4536,853,498,738,332,1229,254,645,1183,2082
TGFBR2_5_4537,696,840,1040,1292,1100,968,168,287,402
TGFBR3_5_4538,44,206,67,317,23,0,1,29,4
THTPA_5_4539,696,708,1301,672,1788,1483,881,1213,1295
TJP2_5_4540,594,227,862,328,1213,53,428,751,248
TK2_5_4541,433,254,184,5,33,708,191,175,174
TLK1_5_4542,1298,1093,1156,267,1669,719,1225,1691,3026
TLK2_5_4543,939,1231,1485,944,1290,1101,1918,645,3532
TMEM134_5_4544,635,499,1104,513,365,1611,980,521,1727
TNIK_5_4545,492,531,629,335,513,308,1078,295,84
TNK2_5_4546,250,602,87,27,102,214,4,76,277
TPK1_5_4547,2107,3851,2414,2917,3441,1659,3125,2349,1276
TPTE2_5_4548,12650,18911,15020,11017,15394,14393,12197,12854,17863
TPTE_5_4549,5708,4343,6139,3701,3290,7111,4801,3559,7154

TRIM24_5_4550,5122,4344,6506,4629,5904,5629,4249,5502,4172
TRIM33_5_4551,579,328,424,303,96,636,521,92,1090
TRMT2A_5_4552,1214,2236,1839,916,3430,1987,1189,789,1571
TRPM6_5_4553,558,489,313,171,959,32,70,255,544
TSSK4_5_4554,1532,1730,1245,3500,1790,406,1215,2429,1754
TTK_5_4555,5322,5986,6044,2462,7258,3965,6932,4869,8363
TTN_5_4556,3814,4193,3932,1532,2994,4134,3255,873,4534
TWF1_5_4557,876,656,497,788,647,678,447,1661,480
UCK1_5_4558,99,72,359,145,769,37,430,55,449
UCKL1_5_4559,409,499,494,188,1388,367,657,906,342
UHMK1_5_4560,452,187,575,38,3,45,50,658,551
ULK2_5_4561,1221,2038,1854,2620,765,3575,2083,1887,2083
VRK2_5_4562,271,124,247,511,94,394,883,176,219
VRK3_5_4563,837,734,671,338,5,1711,100,625,550
WEE1_5_4564,9862,9561,11950,5007,11531,11932,7740,8060,9402
WNK1_5_4565,592,1809,1280,271,768,651,636,1610,133
WNK3_5_4566,2299,2663,3634,3164,2478,3653,2496,2626,2489
YSK4_5_4567,264,291,915,150,504,137,54,264,31
ZAP70_5_4568,711,604,704,724,0,205,452,757,38
ACP2_5_4569,430,912,822,205,890,1070,510,842,142
CDK1_5_4570,2510,2529,2923,4499,3735,1838,2923,1199,4461
DUSP10_5_4571,1501,1698,1948,1282,1754,2638,1021,2162,1394
DUSP15_5_4572,704,1009,1382,63,981,66,301,978,349
ERBB3_5_4573,2543,2826,3231,3206,1246,3103,2271,3095,2960
G6PC2_5_4574,665,893,1203,361,937,2105,534,1074,87
IP6K2_5_4575,765,577,482,1568,1457,106,761,582,1402
MYLK_5_4576,811,667,774,131,1836,1305,500,555,109
PRPS1_5_4577,1483,2262,2565,1684,1471,3101,2156,2737,2162
PTPMT1_5_4578,219,248,544,273,302,394,159,140,1014
PTPN20A_5_4579,5124,4130,7289,4159,6882,5089,3222,5922,4705
PTPN20B_5_4580,5124,4130,7289,4159,6882,5089,3222,5922,4705
SPEG_5_4581,828,1160,1546,139,222,1620,1075,630,433
STK32A_5_4582,2133,3756,3493,2571,3658,978,1984,2462,3301
PPP1R12B_5_4583,5390,6354,6614,7338,3538,8539,4802,4638,8638
PPP1R12B_5_4584,536,928,724,187,1016,517,732,200,1602
PPP1R12B_5_4585,498,855,702,154,400,1214,556,437,927
AAK1_5_4586,213,140,111,0,413,32,224,1,0
ACP6_5_4587,1387,1538,2447,985,1662,1959,918,1401,4100
ACPT_5_4588,1190,1015,1117,1294,1939,1897,657,1266,1661
ACVR2A_5_4589,1524,1000,1852,957,892,1957,1098,1571,876
ACVR2B_5_4590,2173,3158,3672,1188,4725,4712,2458,2593,4043
ADCK2_5_4591,320,241,708,365,651,224,206,171,101
ADCK3_5_4592,331,452,536,877,286,640,80,62,383
ADCK5_5_4593,293,109,194,29,0,266,322,7,461
ADPGK_5_4594,743,1125,782,633,1410,787,250,782,743
ADRBK1_5_4595,599,1166,1279,885,875,630,1810,325,1122
ADRBK2_5_4596,2721,3111,3269,3793,2874,3868,2831,1246,3559
AGK_5_4597,1258,838,1094,1018,522,759,1020,1463,708
AK1_5_4598,4459,4602,4409,2156,2135,6164,3274,3887,5759
AK7_5_4599,184,227,169,91,440,144,450,210,1052

AK8_5_4600,2378,2881,4760,2922,2088,2807,2425,3321,5551
ALK_5_4601,366,814,513,0,20,466,510,32,683
ALPI_5_4602,2111,2270,2437,2633,2574,3521,1538,1822,1315
ALPK2_5_4603,2016,2245,2041,1225,1755,2617,1856,1330,3778
ALPK3_5_4604,561,298,354,5,536,26,742,310,966
ALPP_5_4605,549,292,706,1243,833,645,543,1578,185
ALPPL2_5_4606,903,752,1107,956,1675,820,286,995,917
ANKK1_5_4607,1099,1028,1476,1344,454,1196,1430,1063,1089
ARAF_5_4608,1024,1009,1840,1054,676,1545,1176,17,2454
ATM_5_4609,1567,1599,2622,724,1939,1906,1885,1467,2879
AURKB_5_4610,144,143,531,18,224,668,6,104,8
BLK_5_4611,97,141,166,345,215,195,302,224,25
BMPR1A_5_4612,1462,1501,1710,2732,658,900,1047,2203,306
BMPR1B_5_4613,2235,3127,3629,3094,1916,1175,2088,2531,1674
BMPR2_5_4614,849,1042,1156,169,1508,765,410,98,69
BPNT1_5_4615,547,1181,1199,102,943,1156,743,1834,1156
BRAF_5_4616,18,158,172,155,511,190,1,16,7
BRD3_5_4617,3670,2577,3092,2175,2863,4050,2595,3643,5342
BRSK1_5_4618,559,1091,307,151,0,15,165,992,818
BRSK2_5_4619,1990,1483,1826,1966,1237,1110,2187,1393,2079
BTK_5_4620,1113,1120,967,1108,556,719,1785,713,2035
BUB1B_5_4621,1256,959,1411,360,2079,460,1640,1344,655
BUB1_5_4622,517,546,744,415,336,705,1317,351,395
C20orf111_5_4623,127,135,289,360,29,113,41,1256,65
C9orf96_5_4624,110,125,265,14,612,651,216,403,443
CALM1_5_4625,1338,1356,1367,314,562,3867,973,875,355
CALM3_5_4626,1213,812,809,261,207,2466,542,126,747
CAMK1_5_4627,404,559,756,76,723,238,397,194,241
CAMK1G_5_4628,503,448,532,276,609,217,670,918,244
CAMK2N1_5_4629,7,20,209,0,219,25,162,337,1
CAMK4_5_4630,477,603,718,1029,2611,11,1697,923,2985
CAMKV_5_4631,1539,2269,2433,2140,2732,1323,2217,2169,2328
CARD11_5_4632,390,241,245,411,411,167,281,133,57
CCL2_5_4633,1612,1387,2400,504,2870,1962,389,791,262
CDC42BPB_5_4634,600,251,393,877,197,687,236,149,433
CDC42BPG_5_4635,358,443,734,1,963,360,409,384,921
CDK14_5_4636,1522,2650,2995,1377,1230,2392,3129,2576,3346
CDK15_5_4637,1074,969,1303,1248,1375,677,1275,1424,1037
CDK19_5_4638,2288,2522,2348,2099,1076,3162,1659,1987,2320
CDK3_5_4639,124,173,67,1,0,330,191,678,4
CDK4_5_4640,561,278,1661,478,954,938,771,881,1681
CDK5R1_5_4641,367,423,457,20,773,563,259,61,1
CDK7_5_4642,1327,1036,1711,1359,689,1152,704,583,3103
CDK8_5_4643,2059,1383,1907,1082,1560,3436,983,2707,1873
CDK9_5_4644,1118,1869,959,397,769,548,1427,341,1256
CDKL1_5_4645,936,1698,1676,689,2295,3410,1800,1363,1766
CDKL2_5_4646,2915,4154,4008,3242,2650,4293,3120,6017,3252
CDKL4_5_4647,15940,19252,18754,11747,16140,13797,15031,18109,22212
CDKN1B_5_4648,604,637,1198,383,459,334,872,1041,1285
CERK_5_4649,584,370,507,10,212,216,163,366,293

CHKB_5_4650,1062,831,1823,453,874,1207,1275,588,1478
CHUK_5_4651,7147,8440,8016,7478,6580,6270,7677,5574,9763
CIB2_5_4652,162,22,224,282,102,366,810,25,180
CILP_5_4653,1018,410,949,3137,667,186,355,444,653
CKB_5_4654,601,72,392,372,848,7,716,58,101
CKM_5_4655,105,200,53,117,62,18,147,31,56
CKMT1A_5_4656,1295,1693,1430,1488,3758,635,654,1534,2935
CKMT1B_5_4657,1295,1693,1430,1488,3758,635,654,1534,2935
CKS1B_5_4658,2279,1991,1972,2460,1788,2526,2044,3721,2484
CKS2_5_4659,4202,4563,5871,6217,4172,5443,4254,5353,4834
CLK2_5_4660,220,239,179,2,363,0,42,602,483
CLK4_5_4661,1209,1499,2302,1312,2265,1249,547,1391,1699
CMPK2_5_4662,576,407,1187,8,355,412,562,441,1089
CPNE3_5_4663,911,1137,1677,1570,386,1084,690,959,800
CRIM1_5_4664,150,26,502,0,23,1236,16,18,2
CRKL_5_4665,809,950,1113,279,957,909,1823,339,1387
CSF1R_5_4666,745,462,538,53,718,921,1199,465,480
CSNK1A1L_5_4667,740,438,352,186,1070,522,557,796,80
CSNK1G1_5_4668,347,290,383,603,222,261,745,96,35
CSNK1G2_5_4669,331,466,279,876,42,0,1,403,0
CSNK2A2_5_4670,192,298,808,1,125,11,0,307,303
CSNK2B_5_4671,1556,1036,936,750,518,465,1540,1232,2047
CTDSP2_5_4672,117,139,197,571,28,94,21,192,22
DAPK1_5_4673,1082,1567,1531,1131,987,605,1920,1095,1659
DAPK2_5_4674,746,782,714,477,1547,504,562,559,611
DAPK3_5_4675,463,664,510,1093,667,791,594,431,58
DBF4_5_4676,523,1210,984,234,963,1038,635,1972,1911
DCK_5_4677,1521,2880,2511,851,1696,1385,1849,3317,2123
DCLK3_5_4678,340,514,45,16,598,208,188,404,1584
DGKE_5_4679,752,568,1254,186,107,222,482,357,954
DGKI_5_4680,1167,643,1097,1205,1298,2339,1507,938,676
DGKK_5_4681,281,148,590,8,102,0,0,0,571
DGKQ_5_4682,20,7,38,4,0,32,4,0,2
DOLK_5_4683,663,1714,1566,1153,717,1827,1009,1569,1815
DOT1L_5_4684,4571,4716,5723,4399,6607,4682,4872,5323,5021
DUSP11_5_4685,1412,1138,616,425,1576,1017,1119,1325,133
DUSP12_5_4686,1672,1781,1643,14,2921,1922,930,1144,118
DUSP14_5_4687,1340,709,1301,1012,1967,542,975,525,1842
DUSP16_5_4688,218,132,241,513,495,0,59,330,353
DUSP18_5_4689,662,753,736,545,642,1587,413,564,281
DUSP21_5_4690,432,382,476,122,399,189,595,23,1487
DUSP22_5_4691,2016,1883,3188,1206,1943,3173,2950,2467,2448
DUSP2_5_4692,955,976,950,1356,1338,920,187,213,2451
DUSP3_5_4693,512,716,1073,707,1168,316,615,698,140
DUSP5_5_4694,476,456,476,1220,470,410,254,925,1
DUSP7_5_4695,299,141,302,35,147,252,473,152,25
DUSP8_5_4696,2414,2421,2015,2459,2143,3780,1503,4250,1529
DUSP9_5_4697,218,502,509,260,1487,425,356,437,541
DYRK4_5_4698,755,766,758,286,982,824,303,982,686
EEF2K_5_4699,407,162,275,46,589,877,260,292,520

EFNA2_5_4700,290,621,454,107,1958,646,209,34,1410
EFNA3_5_4701,2439,3487,3774,2396,3514,4520,2706,2807,4474
EFNA5_5_4702,439,421,718,1174,142,571,718,309,65
EFNB3_5_4703,833,962,1079,290,415,892,1503,635,1550
EIF2AK3_5_4704,831,1454,1512,804,1606,1269,571,1149,1070
EIF2AK4_5_4705,917,955,875,417,702,651,869,921,557
EPHA1_5_4706,839,1115,1171,129,1166,1059,1748,851,784
EPHA2_5_4707,39,119,30,0,0,278,254,532,129
EPHA4_5_4708,650,727,788,2957,619,155,174,1650,1229
EPHA7_5_4709,792,647,710,662,293,283,730,1114,1481
EPHB1_5_4710,331,311,679,611,375,567,746,179,1037
EPHB3_5_4711,694,441,671,1662,134,608,342,244,1184
EPHB4_5_4712,271,335,107,113,9,195,721,296,18
EPHB6_5_4713,2156,2034,2645,1540,2596,3613,1606,1683,2211
ERN1_5_4714,231,525,683,119,902,250,18,361,73
ERN2_5_4715,137,237,156,26,645,33,48,344,4
ETNK2_5_4716,400,335,333,998,148,692,362,690,158
EYA3_5_4717,210,292,475,0,197,161,34,121,0
FER_5_4718,240,343,492,58,152,11,421,185,10
FLT3_5_4719,2604,2902,3567,1747,2576,2695,2703,730,1897
FN3K_5_4720,83,97,260,352,42,198,3,7,11
FN3KRP_5_4721,948,918,931,814,1322,572,623,1481,721
FRK_5_4722,5025,5737,7693,5081,6687,6851,5498,7463,5775
FUK_5_4723,339,412,295,105,299,1124,183,206,446
G6PC3_5_4724,1213,1315,1437,3328,2059,379,1175,1803,2836
G6PC_5_4725,1118,1572,854,1001,589,705,533,1147,435
GAK_5_4726,351,441,675,245,147,908,446,17,181
GALK1_5_4727,414,852,246,825,233,326,832,166,13
GDPD4_5_4728,1464,2005,1856,1525,1642,1387,604,245,3719
GOLGA5_5_4729,293,631,232,82,454,309,521,420,0
GRK1_5_4730,361,885,267,124,428,648,335,80,594
GRK5_5_4731,422,692,746,354,2609,1219,399,1032,306
GRK7_5_4732,802,383,380,0,1,373,294,885,804
GSG2_5_4733,105,334,409,207,237,430,247,41,660
GSK3A_5_4734,994,337,956,731,354,2343,559,914,1022
GUCY2C_5_4735,224,350,250,211,252,63,324,233,305
GUCY2D_5_4736,2019,1834,1808,1470,1305,642,672,1322,1823
GUCY2F_5_4737,18089,19841,20009,18646,18384,25286,16943,15749,21522
HIPK4_5_4738,1041,1154,2036,223,645,753,135,1285,1058
HKDC1_5_4739,810,836,1403,81,0,872,483,419,4
HOOK3_5_4740,3755,3327,3266,4134,6942,2041,3132,4092,3258
HSPB8_5_4741,55,418,797,37,1,1416,193,503,15
HUNK_5_4742,204,423,164,265,0,59,240,65,0
HUS1_5_4743,2190,1738,2470,1681,2388,3057,1577,1538,2626
IGBP1_5_4744,1096,1530,1109,609,1146,889,566,685,1659
IGF1R_5_4745,1272,1404,2658,2264,1280,2586,3891,1186,5197
IGF2R_5_4746,266,621,899,709,424,859,1096,266,72
ILKAP_5_4747,315,724,873,355,253,263,320,572,1286
IMPA2_5_4748,645,312,455,9,1282,364,698,403,158
INPP5A_5_4749,429,677,546,757,1764,290,652,953,504

INPP5B_5_4750,1636,1426,1624,983,1237,2552,1237,845,2223
INPP5J_5_4751,137,309,385,582,908,327,625,342,12
INPPL1_5_4752,364,37,554,5,113,80,27,11,0
INSRR_5_4753,777,681,544,972,1285,1367,928,733,526
IPMK_5_4754,3527,6432,6306,5491,5575,6182,5107,5500,3689
IPPK_5_4755,92,162,146,21,14,25,4,124,17
IRAK2_5_4756,222,87,242,41,76,29,9,140,0
ITK_5_4757,892,1029,1135,1869,442,1208,1944,796,1367
ITPKA_5_4758,41,119,114,277,10,109,32,73,0
ITPKB_5_4759,775,1055,880,498,914,1505,395,574,968
ITPKC_5_4760,93,92,81,55,392,317,30,15,185
JAK1_5_4761,354,348,669,348,204,863,751,717,435
JAK2_5_4762,1519,1392,1177,1589,1654,3056,1169,1483,1331
JAK3_5_4763,86,186,194,0,139,605,446,27,46
KDR_5_4764,2073,2560,3324,3392,2726,2494,1615,1643,2790
KSR1_5_4765,1557,2188,2335,1831,3131,2076,1878,1350,1864
LATS1_5_4766,1850,1612,1544,2897,2105,1487,1781,1865,1297
LATS2_5_4767,1223,1333,889,1490,1760,1316,1317,1803,2072
LLGL1_5_4768,918,835,1708,279,2147,384,688,368,2181
LMTK2_5_4769,449,214,256,0,27,0,355,0,0
LMTK3_5_4770,961,250,330,853,416,100,595,234,209
LRRK1_5_4771,581,998,1101,1031,565,1057,679,290,657
LRRK2_5_4772,1397,356,1659,1048,802,786,554,781,990
MAGI2_5_4773,649,1061,609,665,818,594,150,50,467
MAP2K1_5_4774,197,151,794,203,187,145,169,164,269
MAP2K2_5_4775,133,17,16,1,0,2,198,0,5
MAP2K4_5_4776,225,187,435,23,178,595,67,223,34
MAP2K6_5_4777,155,463,655,308,54,799,129,239,149
MAP2K7_5_4778,2225,1802,1878,1302,2666,1189,1266,2721,675
MAP3K10_5_4779,574,527,274,12,379,599,962,482,722
MAP3K11_5_4780,0,127,62,34,0,0,79,91,0
MAP3K14_5_4781,515,647,579,693,497,1379,450,1276,733
MAP3K15_5_4782,1550,3206,1773,1320,1373,1498,1591,1580,1931
MAP3K1_5_4783,688,305,442,596,329,196,828,216,544
MAP3K2_5_4784,1825,1516,2291,642,1585,2318,2259,2372,1019
MAP3K5_5_4785,620,731,1245,923,1219,1219,1395,1049,1766
MAP3K6_5_4786,447,392,813,979,232,216,347,661,15
MAP3K8_5_4787,540,502,641,1003,829,457,1414,111,3
MAP3K9_5_4788,135,35,191,175,3,10,57,0,437
MAP4K2_5_4789,1226,983,2234,2582,378,2262,1134,102,1834
MAP4K3_5_4790,2951,2151,3619,1517,2217,3609,1332,3368,4220
MAPK11_5_4791,150,247,158,303,84,0,101,11,1427
MAPK12_5_4792,50,37,98,167,17,101,39,209,379
MAPK13_5_4793,310,187,579,126,2,2,140,56,36
MAPK15_5_4794,571,124,156,318,12,257,221,3,4
MAPK4_5_4795,363,220,453,75,508,364,65,1,494
MAPK6_5_4796,1436,1787,2019,607,2405,1131,1326,2318,1799
MAPKAPK3_5_4797,2248,2321,3024,2848,2073,1863,2931,1868,2619
MARK1_5_4798,1604,1761,1758,726,1799,2901,1824,2018,1949
MAST2_5_4799,1930,633,1391,609,501,397,303,95,2481

MAST3_5_4800,2279,2410,2275,1867,3310,1320,711,3767,1182
MELK_5_4801,2595,3862,4186,1208,5016,3845,2854,3109,5324
MERTK_5_4802,12435,13501,20706,15024,19717,19145,12665,13586,15720
MEX3B_5_4803,67,47,247,134,0,65,389,305,631
MPP3_5_4804,775,895,983,2148,1876,627,97,931,312
MPP5_5_4805,137,69,116,157,79,191,100,17,460
MST1R_5_4806,443,632,856,922,230,836,757,429,2230
MTM1_5_4807,1082,1918,1187,1030,2133,1070,880,393,2626
MTMR1_5_4808,2453,2866,4259,2711,2642,3180,1847,3167,5434
MTMR4_5_4809,1433,815,920,432,1756,1256,1103,2417,839
MTMR6_5_4810,484,1085,364,375,488,317,877,742,5
MTMR7_5_4811,553,780,639,124,1151,546,361,125,56
MTMR9_5_4812,48,157,57,0,16,48,40,49,28
MTOR_5_4813,434,449,553,116,304,577,488,259,236
MYLK2_5_4814,460,144,345,133,442,58,292,184,271
MYLK3_5_4815,511,583,474,192,516,454,918,407,1806
MYLK4_5_4816,420,253,443,113,34,0,467,83,224
MYO3A_5_4817,1769,2228,2281,2139,2170,2909,1575,1601,2671
N4BP2_5_4818,39,117,326,444,179,169,400,191,182
NAGK_5_4819,1410,1310,1981,347,1369,1782,553,393,2424
NAGS_5_4820,347,131,359,1,0,326,13,17,0
NEK10_5_4821,273,240,275,814,26,59,129,197,584
NEK5_5_4822,137,105,349,0,360,27,140,226,27
NEK7_5_4823,227,132,98,718,17,147,1,102,58
NEK8_5_4824,1680,1092,2200,701,1694,3069,2462,683,1099
NEK9_5_4825,2099,2514,2695,2988,2769,1160,1919,3211,3629
NME3_5_4826,300,214,1279,191,2442,12,317,253,1488
NME4_5_4827,53,549,172,561,539,30,0,28,0
NME5_5_4828,594,753,590,259,73,166,173,138,3
NME6_5_4829,134,111,145,0,291,8,0,6,0
NPR1_5_4830,582,529,729,1095,816,576,914,158,345
NPRL2_5_4831,1961,1977,2115,934,1471,2486,2620,1169,966
NRBP1_5_4832,1405,916,701,1216,710,277,361,568,829
NRBP2_5_4833,1098,820,1412,595,1589,935,939,801,769
NRK_5_4834,1061,363,518,1338,267,796,576,217,371
NT5C1A_5_4835,543,496,285,6,26,1166,35,120,0
NT5C_5_4836,542,531,485,604,619,200,46,535,606
NT5M_5_4837,600,704,500,177,284,223,1040,462,304
NUAK1_5_4838,4098,4009,4304,3958,4466,5244,2922,4237,4216
NUAK2_5_4839,1247,1325,1138,886,1396,1471,1702,1144,284
NUCKS1_5_4840,497,881,564,404,800,660,354,631,1201
OXSR1_5_4841,329,420,580,102,706,107,553,163,845
PAK2_5_4842,898,644,1395,488,1388,743,334,1239,237
PANK3_5_4843,620,352,887,648,314,396,682,372,1634
PANK4_5_4844,322,110,143,1,423,183,43,140,14
PASK_5_4845,393,774,344,224,107,595,752,729,229
PBK_5_4846,1180,633,888,1122,999,1796,1425,94,947
PCK1_5_4847,634,546,659,73,1789,1579,961,1268,2058
PDCD1_5_4848,0,312,320,11,548,720,200,10,0
PDGFRA_5_4849,276,293,413,581,244,121,238,160,186

PDGFRB_5_4850,1818,1896,2431,532,920,3408,1018,2050,1848
PDGFRL_5_4851,443,652,476,359,164,12,459,629,548
PDIK1L_5_4852,729,774,556,848,580,1167,765,529,709
PDK4_5_4853,1858,2445,2300,1326,2507,3951,1971,2938,701
PDP2_5_4854,1548,1899,2083,1247,2126,2757,1091,1991,2821
PDXK_5_4855,617,957,2041,126,1470,187,423,1409,506
PFKFB1_5_4856,2023,1207,1971,1798,1802,1433,1771,1193,579
PFKFB4_5_4857,41,175,202,100,119,218,57,93,8
PFKL_5_4858,1596,1099,2598,2344,2443,544,1679,2606,2531
PGAM2_5_4859,499,660,604,384,2136,213,248,424,344
PGK1_5_4860,170,496,282,629,155,483,517,38,266
PGK2_5_4861,426,581,576,1102,1726,587,173,507,889
PHKA2_5_4862,601,816,514,116,361,436,323,1153,506
PHKG1_5_4863,528,679,772,1018,371,1025,440,579,1505
PI4K2A_5_4864,503,891,1066,336,771,1911,641,835,1063
PI4K2B_5_4865,302,671,340,568,1305,1047,377,242,1220
PIK3C2A_5_4866,1100,983,1182,1359,1702,1467,1631,923,3016
PIK3C2B_5_4867,391,332,119,63,212,403,164,528,2
PIK3C2G_5_4868,68,571,512,169,17,17,322,562,132
PIK3C3_5_4869,1376,890,1016,1229,2651,107,975,1809,1925
PIK3CA_5_4870,1217,716,1205,696,1419,419,1664,1236,160
PIK3CB_5_4871,878,951,995,866,246,1121,715,1283,360
PIK3CD_5_4872,277,58,74,0,18,2,260,93,46
PIK3CG_5_4873,699,1035,828,1073,2207,1251,835,1489,86
PIK3R2_5_4874,1697,1362,1472,1654,854,1701,2098,630,522
PIK3R4_5_4875,295,193,343,106,72,219,136,386,6
PIK3R6_5_4876,1274,1892,2143,439,730,1410,1186,1419,215
PIM2_5_4877,415,531,1242,297,483,479,203,1019,570
PIM3_5_4878,612,664,1058,1401,166,723,759,13,179
PINK1_5_4879,147,68,93,0,33,5,0,0,126
PIP4K2A_5_4880,722,951,1536,920,1316,213,1301,981,3324
PIP4K2B_5_4881,194,311,191,302,36,19,85,29,30
PIP5K1B_5_4882,1427,1508,2526,3334,1313,2675,1844,1072,1518
PKDCC_5_4883,787,584,726,1436,484,842,549,298,1067
PKN2_5_4884,1015,978,574,394,467,5,970,736,154
PKN3_5_4885,569,760,470,802,924,403,567,215,722
PLK1_5_4886,661,197,621,1346,228,521,1138,243,17
PLK2_5_4887,503,604,714,500,719,260,857,1120,826
PLK3_5_4888,414,45,344,5,448,280,155,683,438
PMVK_5_4889,146,632,390,76,32,933,392,407,815
PNKP_5_4890,400,150,371,1271,110,16,294,26,1
POLD1_5_4891,123,220,100,24,259,104,643,117,83
PON1_5_4892,2932,3110,3053,1627,3064,3358,4775,2966,2926
PPAP2B_5_4893,1130,741,1964,1142,146,1574,1227,506,1275
PPEF2_5_4894,451,502,361,37,194,2,618,567,297
PPFIA3_5_4895,388,1072,974,212,458,1450,328,856,1090
PPFIA4_5_4896,103,9,17,1,1,23,33,3,571
PPM1D_5_4897,566,837,837,364,415,1306,484,369,1171
PPM1E_5_4898,2410,3886,3925,3330,1958,2919,2620,2571,7311
PPM1F_5_4899,363,15,0,0,0,417,599,0,0

PPM1G_5_4900,2226,2913,4690,4196,2713,4261,2135,1801,4799
PPM1K_5_4901,887,1975,1508,331,2236,977,1868,947,378
PPM1L_5_4902,389,327,623,428,95,531,776,444,441
PPP1CC_5_4903,2070,1940,3070,2419,1447,3786,2184,2671,3400
PPP1R12C_5_4904,201,51,49,106,17,10,122,86,1
PPP1R13B_5_4905,913,1210,1363,758,500,1767,822,1356,2874
PPP1R14B_5_4906,559,475,741,861,217,1075,383,120,205
PPP1R15B_5_4907,0,0,0,0,0,0,0,0,0
PPP1R1A_5_4908,231,340,475,162,116,420,306,879,519
PPP1R1C_5_4909,520,1574,1500,878,1661,829,572,701,1140
PPP1R2_5_4910,807,1681,1058,390,949,2884,1205,1166,2203
PPP1R3A_5_4911,1697,1993,2243,1744,1439,997,2128,2207,3398
PPP1R3C_5_4912,1176,1357,1722,2297,1831,3216,1310,611,1727
PPP1R3D_5_4913,176,544,20,17,85,1032,294,118,11
PPP1R7_5_4914,257,391,388,496,290,0,0,1,0
PPP2CA_5_4915,231,723,372,345,454,668,167,319,525
PPP2CB_5_4916,3196,3042,4508,2901,4403,2150,3109,2170,3875
PPP2R1A_5_4917,515,472,361,305,277,455,600,306,79
PPP2R2D_5_4918,5474,5686,6276,4548,5111,3355,4633,4246,8029
PPP2R3B_5_4919,1668,1499,1650,1823,1478,2573,1833,1971,1937
PPP2R5B_5_4920,157,196,546,181,68,125,131,282,0
PPP2R5E_5_4921,4408,4486,5655,3415,4488,4063,5458,4073,9530
PPP3CC_5_4922,1561,826,1979,768,2037,1896,868,1067,1614
PPP3R1_5_4923,1153,1629,1305,1426,722,1415,1018,1301,4305
PPP3R2_5_4924,893,311,430,227,3338,368,331,88,258
PPP4C_5_4925,458,804,1599,59,1663,1503,1173,253,1264
PPTC7_5_4926,316,888,1096,712,535,963,363,308,109
PRKAA2_5_4927,966,771,603,2018,959,141,534,1733,264
PRKAB1_5_4928,1406,737,2161,1291,749,1713,1323,962,1519
PRKAB2_5_4929,1015,644,962,816,497,680,1342,1951,758
PRKACG_5_4930,484,814,572,656,561,169,277,806,976
PRKAG3_5_4931,438,515,619,315,1064,18,857,584,26
PRKAR2A_5_4932,1491,1969,2996,524,1137,2502,1469,2499,1749
PRKAR2B_5_4933,184,117,81,694,33,0,25,20,56
PRKCA_5_4934,318,451,392,453,365,124,392,596,446
PRKCE_5_4935,892,1062,874,246,775,1143,881,513,1412
PRKCG_5_4936,886,411,514,549,87,333,251,335,569
PRKCH_5_4937,250,135,105,3,60,96,328,17,105
PRKCI_5_4938,224,734,391,757,24,857,952,227,787
PRKD1_5_4939,1093,1327,1995,2732,865,2263,3959,1916,3094
PRKD3_5_4940,1649,1715,1449,1630,3050,1880,2576,1431,2193
PRKG2_5_4941,331,163,442,108,298,1093,471,730,354
PRKX_5_4942,1335,1415,1127,1108,878,1434,611,1812,1805
PRPF4B_5_4943,1337,1231,1914,936,1372,407,1149,652,1487
PRPS1L1_5_4944,404,807,1555,521,1761,396,695,236,201
PSKH1_5_4945,416,270,445,221,891,333,525,832,16
PSKH2_5_4946,355,591,1476,338,442,1362,264,610,597
PSPH_5_4947,332,453,419,128,82,24,533,782,283
PSTK_5_4948,989,1414,1771,1040,3765,877,1754,1032,1283
PTEN_5_4949,1555,1110,1112,368,3083,1228,1334,378,2471

PTK6_5_4950,1244,1582,1390,1651,1814,2075,1270,1209,985
PTP4A1_5_4951,1494,2851,2300,1227,938,2624,1825,2359,3649
PTPLA_5_4952,2058,1786,1928,596,1451,2092,1109,2158,1216
PTPLB_5_4953,2533,3330,4692,1650,1531,4324,2398,4217,2919
PTPN11_5_4954,1297,741,1575,526,1052,568,1040,510,605
PTPN14_5_4955,2764,1711,2654,1090,3985,2538,1944,2139,5813
PTPN1_5_4956,131,109,299,6,7,34,7,383,149
PTPN21_5_4957,484,257,412,512,309,1012,151,177,161
PTPN23_5_4958,1192,417,1030,863,1042,792,895,289,843
PTPN4_5_4959,2341,1704,2629,2160,554,1814,1988,1112,2170
PTPN9_5_4960,283,678,578,73,18,302,817,341,633
PTPRG_5_4961,707,844,750,606,648,88,1795,370,959
PXK_5_4962,1434,2359,3001,640,1349,2600,1436,890,4876
RAF1_5_4963,694,536,825,667,434,381,74,331,1061
RBKS_5_4964,2019,3369,2299,4237,3038,2405,2013,2217,5726
RFK_5_4965,443,377,424,648,450,348,406,573,433
RIOK3_5_4966,5638,8067,6627,5071,4219,5858,5431,5573,2397
RIPK1_5_4967,3533,4354,4389,3219,6165,3530,4165,4386,3407
RIPK2_5_4968,7573,9704,9479,6874,13001,6644,10548,4780,8080
RIPK3_5_4969,486,607,840,150,254,529,854,418,463
RIPK4_5_4970,806,1350,1490,1915,706,1610,1288,834,1479
RNASEL_5_4971,823,559,1011,679,769,501,616,538,107
RNGTT_5_4972,1228,1411,1550,651,880,813,1036,815,3269
ROCK1_5_4973,5072,7710,8435,4717,6971,7778,5455,6343,9454
ROCK2_5_4974,4337,4302,3999,4812,1711,7117,5132,2772,3545
ROS1_5_4975,1604,1263,2466,661,178,54,1505,1628,834
RPS6KA3_5_4976,708,576,460,8,82,147,90,354,969
RPS6KA6_5_4977,1891,2493,3372,2281,2728,861,2203,2978,4359
RPS6KB1_5_4978,1380,1433,1434,555,764,1326,1164,754,465
RPS6KB2_5_4979,722,1721,1148,428,448,1785,790,879,437
RPS6KL1_5_4980,4869,4914,6621,6645,3682,7605,5647,5558,6359
RSPO3_5_4981,829,615,781,422,391,1024,307,1689,1243
SACM1L_5_4982,238,240,660,265,41,33,484,1373,0
SBF1_5_4983,221,123,264,14,52,135,109,79,775
SBK1_5_4984,170,46,169,0,2,453,0,321,228
SBK2_5_4985,490,478,370,405,386,658,151,86,830
SEPHS2_5_4986,143,843,586,766,672,1016,258,346,71
SGPP1_5_4987,1854,1958,1942,2257,3104,1830,1479,2738,3359
SHPK_5_4988,431,107,692,499,4,176,697,0,276
SIK1_5_4989,46,111,537,229,809,882,153,69,20
SIK2_5_4990,191,13,250,0,15,517,0,63,1079
SIK3_5_4991,249,326,637,217,828,436,403,254,311
SKAP2_5_4992,1740,1475,1778,1298,732,2100,367,828,1556
SMG1_5_4993,885,1187,1959,1254,1657,1236,1022,1048,1413
SRMS_5_4994,0,17,238,1,78,0,109,26,1
SRP72_5_4995,1020,325,526,909,343,546,600,976,4013
SRPK1_5_4996,741,1537,1847,661,1723,1769,1165,563,2522
SSH2_5_4997,1532,1625,3271,1503,1635,1820,1452,2057,2160
SSH3_5_4998,206,362,488,705,451,256,691,304,396
STC1_5_4999,993,668,548,695,132,174,32,1305,2118

STK10_5_5000,375,237,343,60,1,395,8,831,29
STK11_5_5001,195,775,463,491,10,729,20,568,13
STK16_5_5002,513,563,886,1140,652,229,954,992,897
STK17A_5_5003,1406,1387,2243,1107,1254,1483,1430,1110,447
STK17B_5_5004,443,41,278,8,244,0,22,153,1419
STK25_5_5005,557,970,565,585,421,461,674,695,552
STK32B_5_5006,1018,750,1223,594,1202,1283,885,1046,1018
STK32C_5_5007,65,154,466,46,145,90,88,343,360
STK33_5_5008,406,501,878,49,768,2014,443,337,681
STK35_5_5009,1427,1813,1334,993,815,1181,399,358,3058
STK38_5_5010,2171,2876,3123,1382,5007,3450,3418,1683,2878
STK38L_5_5011,1771,1398,2468,2066,2177,1047,2630,719,4244
STK39_5_5012,3328,2271,3593,1421,737,3218,2576,2231,4046
STK40_5_5013,420,789,752,398,1542,171,1173,289,585
STK4_5_5014,736,1423,1628,516,979,1506,1534,1312,1000
STYK1_5_5015,130,271,402,410,430,799,113,297,288
STYXL1_5_5016,512,581,680,1075,714,768,354,1223,332
TAF1L_5_5017,3388,3359,4368,3937,3175,6121,4825,3050,5815
TAOK3_5_5018,359,111,184,674,27,97,178,223,92
TBK1_5_5019,1277,1499,1769,695,856,2927,1190,972,1738
TEC_5_5020,1553,2012,2556,3000,4200,2325,1341,1981,3461
TEK_5_5021,3825,3798,5902,2184,3483,3450,4414,5087,5286
TESK1_5_5022,142,55,92,604,93,347,131,95,73
TESK2_5_5023,1121,1258,1909,1257,2060,2572,2609,1271,1837
THNSL1_5_5024,728,763,1014,822,1003,269,183,551,2526
TIE1_5_5025,91,157,207,49,78,16,89,32,30
TK1_5_5026,283,349,459,845,528,329,378,1014,1016
TNK1_5_5027,553,661,303,566,185,618,63,220,159
TNNI3K_5_5028,4491,5514,5192,3087,3467,5994,5645,5549,2856
TNS3_5_5029,211,250,447,180,17,456,61,277,16
TP53RK_5_5030,201,387,711,216,960,161,1042,531,36
TRAT1_5_5031,1352,1502,2324,1183,1469,1765,1268,1094,608
TRIB1_5_5032,137,162,227,16,60,88,426,375,29
TRIB2_5_5033,574,388,492,329,255,2,293,769,684
TRIB3_5_5034,224,228,90,40,1435,348,191,441,6
TRIM27_5_5035,635,598,302,50,60,1513,1244,715,110
TRIM28_5_5036,189,497,328,1,125,373,353,89,39
TRIO_5_5037,764,1131,551,312,1187,1096,988,908,1410
TRPM7_5_5038,1897,2630,3511,2356,529,2536,2432,1974,3820
TRRAP_5_5039,1637,1389,1999,1140,1840,3022,1923,2576,1632
TSKS_5_5040,2264,3374,3586,2779,3388,2941,2591,2572,1672
TSSK1B_5_5041,1078,1107,1394,1794,169,126,448,388,1042
TSSK2_5_5042,844,380,831,678,660,887,1239,152,751
TSSK3_5_5043,512,197,356,208,338,28,202,569,941
TSSK6_5_5044,617,435,8,966,854,514,893,660,1
TTBK1_5_5045,506,400,535,341,14,102,839,31,0
TTBK2_5_5046,1163,476,564,211,9,190,748,384,221
TWF2_5_5047,186,387,301,11,47,379,225,26,554
TXK_5_5048,1056,670,688,1328,455,630,521,586,2331
TYK2_5_5049,485,622,154,529,33,119,52,195,38

TYRO3_5_5050,636,169,841,235,60,654,542,662,500
UBLCP1_5_5051,2601,2130,3215,888,1453,1720,2702,2990,7786
UCK2_5_5052,489,190,355,21,50,384,27,38,0
ULK1_5_5053,212,291,353,6,342,526,441,211,570
ULK3_5_5054,209,181,619,96,77,898,34,753,584
ULK4_5_5055,2801,3608,4126,2041,2190,3280,3837,3311,4483
VRK1_5_5056,1329,1371,2191,2172,963,1981,1273,2501,614
WEE2_5_5057,6357,5416,6378,4779,6439,6883,4952,6178,7269
WNK2_5_5058,1256,1588,1048,867,3579,398,1456,1600,1433
WNK4_5_5059,820,159,389,419,9,1118,209,184,1474
XRCC6BP1_5_5060,3095,3740,4080,2895,4222,4923,3183,2907,6657
XYLB_5_5061,643,911,1105,824,1208,1737,856,1444,608
YES1_5_5062,2644,1858,2442,2115,2552,3119,1841,1896,3406
AATK_5_5063,1757,1782,2258,873,3727,2327,657,1764,1997
ABL1_5_5064,904,779,1961,532,2340,1803,2150,575,2092
ABL2_5_5065,1501,882,970,480,1235,804,2144,1025,647
ACP1_5_5066,651,726,377,44,453,80,562,417,45
ACP5_5_5067,474,736,27,1649,21,137,571,957,0
ACPL2_5_5068,796,704,547,362,1022,430,608,35,803
ACPP_5_5069,1493,1823,2017,2863,1761,992,1788,1968,1263
ACVR1B_5_5070,1604,1501,1622,163,803,1452,1817,1166,1418
ACVR1C_5_5071,3172,3758,4263,3349,4387,3071,3169,6422,5910
ACVR1_5_5072,818,408,775,143,1649,768,721,1231,880
ACVRL1_5_5073,786,1062,1103,2342,3742,858,316,572,526
ADCK1_5_5074,286,581,637,195,818,648,53,7,984
ADCK4_5_5075,319,286,1052,218,632,210,552,392,390
ADK_5_5076,1857,1273,2141,1555,1929,1348,647,1107,1885
AK2_5_5077,144,358,256,175,733,292,334,313,48
AK4_5_5078,351,352,539,322,782,696,865,293,829
AK5_5_5079,39,9,64,7,0,32,64,0,12
AKT1_5_5080,431,441,387,937,818,99,919,271,149
AKT2_5_5081,80,144,482,625,109,331,322,536,0
AKT3_5_5082,1913,2584,1785,374,2690,2776,2151,1288,1687
ALDH18A1_5_5083,862,929,1450,1752,1133,1529,1642,1156,1083
ALPK1_5_5084,793,1133,1331,1663,1310,2333,176,612,2292
ALPL_5_5085,706,367,290,641,1397,468,305,1226,1018
AMHR2_5_5086,440,1725,1156,120,77,707,1086,319,211
ANKHD1_5_5087,971,1080,625,1224,848,31,1109,646,472
APTX_5_5088,1017,933,1691,184,1101,1042,1363,704,1218
ATRIP_5_5089,68,348,834,1036,146,321,500,17,2225
AURKA_5_5090,517,603,894,282,300,398,525,526,2159
AURKC_5_5091,3293,2907,3087,3516,2824,3649,4805,1686,1844
AXL_5_5092,958,1162,736,747,60,2139,299,1070,1083
BAIAP2_5_5093,1938,2034,2003,2239,2462,1142,1725,2060,1244
BCKDK_5_5094,570,471,418,638,4,151,750,242,977
BCR_5_5095,83,70,388,4,13,0,21,90,169
BMP2K_5_5096,532,181,848,100,242,268,278,105,127
BMX_5_5097,304,358,402,0,307,64,502,136,1486
BPGM_5_5098,1992,2141,2127,2430,3126,1657,2469,2469,1950
BRD2_5_5099,153,184,401,44,136,720,141,97,118

BRD4_5_5100,461,1170,891,646,1479,614,508,549,7
BRDT_5_5101,1033,773,1300,502,270,903,114,1127,662
CAB39L_5_5102,1144,973,1402,324,1036,1221,922,2141,436
CAMK1D_5_5103,816,1022,1110,304,731,1415,952,398,2035
CAMK2A_5_5104,15,25,38,14,386,26,5,4,11
CAMK2B_5_5105,88,343,513,763,316,271,86,265,134
CAMK2D_5_5106,450,826,1073,1198,586,591,321,1673,4471
CAMK2G_5_5107,659,604,556,351,321,639,1073,264,16
CAMKK1_5_5108,1822,2721,2845,2137,4481,2976,3490,3921,1770
CAMKK2_5_5109,1308,1210,1226,1086,1025,621,1787,649,3105
CASK_5_5110,61,102,84,0,0,0,358,0,0
CCT2_5_5111,2499,1980,3361,2573,2053,2135,1663,1589,3632
CDADC1_5_5112,935,1182,1540,1690,871,2723,366,221,1323
CDC14A_5_5113,257,81,215,105,20,88,153,29,0
CDC14B_5_5114,929,999,1332,2186,985,849,1133,487,296
CDC25A_5_5115,198,224,324,1,49,567,270,180,204
CDC25B_5_5116,810,1444,1458,1284,2337,339,1510,501,322
CDC25C_5_5117,3325,4942,4637,4466,4391,4341,3955,5593,2863
CDC42BPA_5_5118,134,314,148,54,518,403,121,480,21
CDC7_5_5119,2237,1705,2409,1571,1038,1971,1806,1339,5469
CDK10_5_5120,260,726,194,420,61,534,143,119,78
CDK11A_5_5121,7693,7720,7837,5190,8167,7700,6098,6478,8164
CDK11B_5_5122,141,230,1126,134,291,47,751,502,320
CDK12_5_5123,2800,3145,4407,2422,2277,2330,1882,2938,4199
CDK13_5_5124,732,1236,790,449,629,64,1117,837,977
CDK16_5_5125,636,582,436,7,583,1120,418,76,153
CDK17_5_5126,4084,4049,4687,4748,4817,5554,5184,2144,2486
CDK18_5_5127,903,236,1016,1601,318,588,663,134,401
CDK20_5_5128,3770,4760,4289,4123,5784,4786,3141,3078,5186
CDK2_5_5129,397,367,455,1387,1344,532,106,42,551
CDK5_5_5130,1143,587,972,712,960,361,1253,885,858
CDK6_5_5131,654,208,290,1,249,203,15,29,67
CDKL3_5_5132,512,929,598,43,371,260,629,245,63
CDKL5_5_5133,174,476,175,4,268,9,356,94,157
CDKN1A_5_5134,744,768,993,298,160,471,1206,684,1295
CDKN3_5_5135,709,375,522,436,317,75,932,599,1242
CHEK1_5_5136,16904,17143,18315,15470,20234,17291,15297,13912,19167
CHEK2_5_5137,1951,1759,1809,1448,1000,1985,1406,1474,1162
CHKA_5_5138,301,108,464,521,0,1268,272,0,280
CIT_5_5139,272,482,468,123,601,238,1019,568,1357
CKMT2_5_5140,4156,4283,3729,3546,6439,4474,5098,4788,3121
CLK1_5_5141,1461,1886,2409,1454,731,1424,1166,2925,2400
CLK3_5_5142,1513,1529,2596,1442,1426,1683,947,1821,3520
CMPK1_5_5143,989,1394,1894,999,1374,2592,1112,2261,452
COASY_5_5144,1653,1851,2452,492,1591,413,752,782,2601
COL4A3BP_5_5145,2732,2907,3505,2540,1132,3728,2750,1829,2087
CSK_5_5146,52,14,86,0,9,0,43,220,1
CSNK1A1_5_5147,5672,4857,4767,3176,4053,4629,3121,4556,4954
CSNK1D_5_5148,1044,1598,2204,2122,1148,1710,685,1346,5132
CSNK1E_5_5149,101,0,53,3,0,306,3,22,0

CSNK1G3_5_5150,5292,4347,6275,3841,8945,3235,6150,4750,7896
CTDP1_5_5151,832,1766,2157,1996,3890,2087,611,1302,1390
CTDSP1_5_5152,1308,1193,1115,1659,697,802,1614,1944,1720
DCLK1_5_5153,2104,1449,1649,1275,1435,3345,1924,867,2808
DCLK2_5_5154,3021,2127,3115,2826,2867,4412,1712,1637,5125
DDR1_5_5155,1459,1468,1366,582,3125,1113,1704,1481,2737
DDR2_5_5156,520,169,586,194,61,1041,374,123,29
DGKA_5_5157,143,187,357,90,187,30,6,6,0
DGKB_5_5158,811,294,1567,1117,44,3113,436,613,69
DGKD_5_5159,3091,2770,3913,2967,4196,4192,4685,3735,2343
DGKG_5_5160,493,508,1062,112,806,406,533,793,571
DGKH_5_5161,44,493,742,109,170,455,196,187,2
DGKZ_5_5162,398,518,671,300,706,362,749,840,2650
DGUOK_5_5163,2527,2806,2792,1088,1968,2409,1150,1428,2175
DLGAP5_5_5164,1385,1284,1279,2126,530,189,663,1279,319
DMPK_5_5165,222,431,69,0,0,4,1,546,312
DSTYK_5_5166,1586,1743,2081,675,1572,490,1112,716,2425
DTYMK_5_5167,764,665,1070,1006,32,467,759,115,1721
DUSP13_5_5168,444,375,252,358,149,6,384,130,453
DUSP19_5_5169,1724,1837,2295,1173,3590,1538,966,1724,3978
DUSP4_5_5170,234,157,458,1,125,552,0,657,1100
DUSP6_5_5171,339,627,559,1307,972,85,374,256,333
DYRK1A_5_5172,1053,659,976,1371,241,1253,525,414,981
DYRK1B_5_5173,636,425,1067,1100,124,911,543,153,1635
DYRK2_5_5174,182,832,455,49,817,732,1,958,19
DYRK3_5_5175,786,1141,753,843,1670,1653,1558,396,1990
EFNA4_5_5176,64,159,305,744,587,889,76,803,39
EGFR_5_5177,567,960,985,1147,1663,1089,1016,1609,552
EIF2AK2_5_5178,1447,2337,2074,2885,598,3079,1872,3398,2209
EPHA10_5_5179,318,147,196,194,0,73,4,177,538
EPHA3_5_5180,316,514,346,717,219,83,93,772,187
EPHA5_5_5181,67,216,384,39,58,1019,787,181,1192
EPHA6_5_5182,1023,2087,1648,1921,1224,560,795,1993,1666
EPHA8_5_5183,411,324,701,631,665,452,508,199,546
EPHB2_5_5184,1090,801,1321,3400,2089,1669,672,2107,3425
EPM2A_5_5185,632,536,662,887,1657,47,867,395,908
ERBB2_5_5186,1209,1255,795,1030,627,1014,568,692,650
ERBB4_5_5187,1637,2054,1383,571,2447,2442,1871,822,1473
ETNK1_5_5188,805,1066,1091,867,649,1387,309,121,299
EXOSC10_5_5189,0,0,0,0,0,0,0,0,0
EYA1_5_5190,706,620,1024,1511,875,287,1104,435,1140
EYA2_5_5191,1998,2305,2898,2765,3288,2559,1280,1386,2204
EYA4_5_5192,690,1471,865,539,656,862,467,1426,1838
FASTK_5_5193,835,644,1287,1666,3377,1168,625,616,1684
FBP1_5_5194,627,909,698,126,694,588,816,130,1402
FES_5_5195,412,429,203,611,888,300,739,137,1090
FGFR1_5_5196,427,451,520,306,287,1160,1295,382,344
FGFR2_5_5197,878,1072,746,1360,907,1032,1553,877,168
FGFR3_5_5198,449,322,484,6,20,558,196,665,0
FGFR4_5_5199,1533,1347,1289,1784,1548,1586,1623,1166,2263

FGFRL1_5_5200,499,360,1058,202,477,403,687,67,1615
FGR_5_5201,390,600,821,221,2,295,421,1,0
FLT1_5_5202,252,202,279,2038,2,2265,124,20,1
FLT4_5_5203,148,1,31,3,7,112,33,206,10
FXN_5_5204,1173,705,1754,925,1167,995,1783,1274,1107
FYN_5_5205,655,622,1043,1302,589,1338,717,803,239
FZR1_5_5206,298,490,208,0,0,429,50,1,4
GALK2_5_5207,267,310,738,199,76,339,291,210,65
GK_5_5208,1234,2092,2432,1489,1927,1509,1030,1684,3516
GLYCTK_5_5209,1,0,6,1,34,1,38,0,4
GNE_5_5210,2188,2800,3264,3942,2739,2613,1922,2131,3095
GRK4_5_5211,336,747,409,506,215,1249,70,654,673
GRK6_5_5212,1129,683,1195,1350,806,1324,627,579,668
GSK3B_5_5213,10,5,225,0,47,0,257,101,36
GUK1_5_5214,116,40,350,319,95,6,16,137,68
HCK_5_5215,1035,718,1188,848,409,2288,141,187,685
HIPK1_5_5216,1876,972,1065,1209,1207,1099,921,1370,693
HIPK2_5_5217,22,704,764,137,176,15,41,28,544
HIPK3_5_5218,1440,1792,1825,420,1041,3014,1947,1097,1322
ICK_5_5219,486,218,472,937,526,717,829,130,7
IKBKB_5_5220,2189,2270,2587,1379,3916,2770,3312,2254,4781
IKBKE_5_5221,272,87,221,17,30,330,155,188,116
IKBKG_5_5222,638,1196,1203,251,615,1639,1346,729,1139
ILK_5_5223,1014,826,1021,2954,588,631,615,869,649
IMPA1_5_5224,5712,6792,6473,5907,6604,7793,7202,3991,6793
INPP1_5_5225,559,642,389,755,1064,1187,934,215,421
INPP4A_5_5226,266,374,190,21,96,264,54,16,18
INPP4B_5_5227,276,395,99,262,86,14,102,41,215
INPP5D_5_5228,723,1944,1121,1237,1545,629,955,512,2549
INSR_5_5229,1351,1134,1665,998,1080,709,664,788,3449
IP6K1_5_5230,442,686,572,479,594,1651,649,1382,1059
IP6K3_5_5231,481,281,632,810,294,331,508,682,905
IRAK1_5_5232,1188,731,1420,608,921,1673,1358,524,2810
IRAK3_5_5233,2062,1626,2282,1507,1923,1811,3465,1549,1494
IRAK4_5_5234,444,191,245,1,767,283,297,275,14
ITPK1_5_5235,157,159,5,518,955,0,219,2,0
KALRN_5_5236,28,181,64,37,112,85,6,216,143
KHK_5_5237,254,186,256,129,66,404,641,898,1320
KIF2A_5_5238,2471,2768,4808,3033,4144,3201,2496,1595,4819
KIT_5_5239,5078,5379,4303,3508,7876,5569,3836,4675,6584
LCK_5_5240,3102,3361,2834,3496,2281,3876,2989,2591,3243
LHPP_5_5241,1840,3051,2435,2478,1666,2906,1914,2951,4146
LIMK1_5_5242,717,770,554,266,274,225,628,101,489
LIMK2_5_5243,522,449,655,21,42,423,1161,17,1
LTK_5_5244,407,447,33,33,0,3,125,1,534
LYN_5_5245,504,1105,610,542,366,93,1082,379,178
MAGI3_5_5246,84,179,421,7,9,14,13,21,9
MAP2K3_5_5247,576,471,598,1217,25,1026,549,1072,31
MAP2K5_5_5248,1573,1108,817,1386,863,330,1237,840,1174
MAP3K12_5_5249,884,1148,2020,1685,453,919,1290,602,661

MAP3K13_5_5250,94,36,170,0,54,357,173,1,236
MAP3K3_5_5251,1209,867,1324,1627,1391,675,1202,702,1178
MAP3K4_5_5252,954,749,1184,1269,914,814,820,594,729
MAP3K7_5_5253,188,287,510,237,246,381,775,840,235
MAP4K1_5_5254,466,710,702,505,174,246,182,184,539
MAP4K4_5_5255,1511,1492,1187,1742,2454,2022,1641,2680,1098
MAP4K5_5_5256,374,60,486,1084,385,29,807,149,178
MAPK10_5_5257,1263,1365,1439,410,1222,939,881,921,2193
MAPK14_5_5258,0,184,1,900,0,1,0,0,1
MAPK1_5_5259,1527,1627,1772,1302,2082,1308,2130,1160,4267
MAPK3_5_5260,784,1357,726,542,1177,1331,1503,392,1791
MAPK7_5_5261,397,171,789,600,58,220,71,426,1591
MAPK8_5_5262,1525,1253,1368,361,1775,2626,1357,973,1706
MAPK9_5_5263,1670,1135,1289,1251,933,322,1523,1290,1961
MAPKAPK2_5_5264,701,367,675,464,143,4513,384,871,1106
MAPKAPK5_5_5265,119,27,102,90,791,89,824,335,37
MARK2_5_5266,579,541,831,608,743,254,509,382,1246
MARK3_5_5267,523,1582,1523,2213,1071,1057,944,1070,1126
MARK4_5_5268,326,97,154,140,214,92,52,211,0
MAST4_5_5269,603,142,520,500,151,130,336,172,5
MASTL_5_5270,3541,4031,4372,3479,2257,5197,2354,4286,4854
MATK_5_5271,839,298,833,215,629,19,1408,1881,680
MET_5_5272,2394,1843,3582,1211,2902,3348,2110,1323,3464
MINK1_5_5273,1218,2423,1787,903,2817,1938,1368,2561,1714
MINPP1_5_5274,495,265,232,83,8,1,635,242,37
MKNK1_5_5275,4465,5600,4480,2467,4675,4046,4062,5793,7416
MKNK2_5_5276,2360,1979,3744,2390,1312,4364,2707,1561,1166
MLKL_5_5277,3472,3581,4266,4829,3605,5069,3481,4259,4806
MTMR14_5_5278,4688,6434,5843,2626,7278,4994,6354,5784,8228
MTMR2_5_5279,2997,3653,4068,5145,3164,3654,3295,2177,4359
MTMR3_5_5280,134,537,795,100,782,251,257,370,163
MUSK_5_5281,1301,1258,1472,338,1387,985,448,1277,2545
MVK_5_5282,370,445,495,320,352,1725,639,73,525
MYO3B_5_5283,1531,1452,1614,1881,1371,1944,1283,1105,2538
NADK_5_5284,495,201,388,18,0,948,421,621,0
NCK1_5_5285,595,267,463,1646,556,728,955,708,49
NCK2_5_5286,29,657,587,18,1913,323,510,270,6
NDRG1_5_5287,302,182,822,331,535,388,51,294,52
NEK11_5_5288,386,676,650,78,642,773,295,844,213
NEK1_5_5289,517,598,788,763,306,184,636,620,397
NEK2_5_5290,805,406,765,761,458,1034,728,780,2218
NEK3_5_5291,2181,2748,4214,4362,2804,2967,2233,2282,5221
NEK4_5_5292,2831,3375,4352,1350,6530,3230,3701,2522,4336
NEK6_5_5293,72,0,39,89,2,1,20,0,363
NME2_5_5294,668,853,954,1676,931,1099,1036,356,1133
NME7_5_5295,2295,1338,1473,1350,1644,1295,878,1847,3504
NT5C1B_5_5296,1967,1190,1644,1812,985,958,1404,761,767
NT5C2_5_5297,2076,2077,3356,2839,2970,3435,3240,1963,2976
NT5C3_5_5298,750,1085,1316,653,911,313,595,474,538
NT5E_5_5299,182,504,179,0,261,33,5,0,5

NTRK1_5_5300,416,375,589,604,294,483,253,182,71
NTRK2_5_5301,471,420,374,1,24,983,117,2,616
NTRK3_5_5302,119,314,257,439,0,0,0,442,1
NUDT4_5_5303,4500,3499,4986,4344,1628,5172,3085,3312,2523
NUDT9_5_5304,1077,1260,2006,984,1140,3143,999,1142,2423
OBSCN_5_5305,537,1178,662,529,1746,211,407,1735,702
OCRL_5_5306,2503,2137,3219,2871,2986,1753,3331,1861,2066
OXSM_5_5307,1403,1424,2147,1115,2111,2663,1347,736,3143
PACSIN1_5_5308,1768,1239,1619,138,357,576,1073,647,1714
PAK4_5_5309,104,271,281,0,30,405,205,95,104
PAK6_5_5310,331,907,530,545,1161,218,199,110,174
PAK7_5_5311,274,180,233,1004,326,479,575,226,90
PANK1_5_5312,1552,1914,2278,1402,2227,1380,1381,2162,1565
PANK2_5_5313,1458,1420,1735,2247,3307,2497,1750,1604,1943
PCK2_5_5314,112,371,593,133,447,386,134,177,659
PDK2_5_5315,915,905,587,552,183,581,1068,530,1085
PDK3_5_5316,1206,1414,2551,1701,3767,2379,2116,1108,2495
PDPK1_5_5317,1446,960,1630,1665,318,3349,927,643,1042
PEG3_5_5318,743,1672,1683,1085,1170,1129,1205,244,734
PFKFB2_5_5319,3237,2157,3457,2645,1873,3145,1328,2261,3682
PFKFB3_5_5320,89,81,297,0,0,159,4,1,104
PFKM_5_5321,473,442,689,777,901,184,183,362,662
PFKP_5_5322,432,919,621,2,1132,156,30,220,40
PHKA1_5_5323,5032,4278,4945,3651,4646,4410,4615,4168,6845
PHKB_5_5324,602,983,1027,754,1585,788,1091,267,315
PHKG2_5_5325,1714,2478,2683,1284,2382,2630,1043,4818,1451
PHPT1_5_5326,510,1303,1714,2076,831,749,1600,1352,342
PI4KA_5_5327,1173,543,942,1601,1190,1262,1606,668,451
PI4KB_5_5328,8391,9848,11030,8748,10598,9601,9485,8330,11733
PIK3R1_5_5329,1047,3061,1586,1492,4723,1809,2402,1674,2612
PIK3R5_5_5330,1231,1151,2011,70,2352,3050,765,1145,768
PIKFYVE_5_5331,824,725,743,201,771,665,728,1272,1288
PIP4K2C_5_5332,151,74,136,4,48,145,270,0,181
PIP5K1A_5_5333,877,849,884,936,696,810,810,247,318
PIP5K1C_5_5334,307,140,407,447,39,1093,235,152,816
PIP5KL1_5_5335,21,204,6,232,11,52,8,7,0
PKLR_5_5336,149,235,70,9,128,98,6,306,1
PKMYT1_5_5337,15,106,14,3,24,2,1,50,29
PKN1_5_5338,408,156,301,371,10,495,201,414,161
PLK4_5_5339,767,1409,698,679,218,802,721,972,184
PNCK_5_5340,137,9,368,278,457,0,0,639,194
PODXL_5_5341,1609,2240,2409,961,1917,2822,1691,1345,1870
PPAP2A_5_5342,4044,4130,3737,1789,2958,2966,4013,2945,3077
PPAP2C_5_5343,1704,1877,2129,902,1026,706,1335,1211,1371
PPEF1_5_5344,5069,5413,4861,4187,3602,3262,4374,3931,5485
PPFIA1_5_5345,917,693,1000,642,709,729,1056,415,98
PPFIA2_5_5346,808,314,1249,36,1059,508,337,138,448
PPM1A_5_5347,166,217,351,154,0,0,2,230,535
PPM1B_5_5348,284,52,159,643,448,450,76,152,311
PPM1M_5_5349,1144,698,2156,615,768,2584,643,1640,1837

PPP1CA_5_5350,638,803,702,756,690,208,9,710,97
PPP1CB_5_5351,2033,1942,2981,3785,2255,3172,2598,2125,3157
PPP1R12A_5_5352,1234,870,1003,761,1791,1656,1247,987,1264
PPP1R16B_5_5353,1395,661,616,1163,381,673,823,739,961
PPP1R1B_5_5354,61,140,266,443,195,340,9,13,6
PPP1R8_5_5355,1086,270,632,1866,71,349,802,447,201
PPP2R1B_5_5356,2057,1629,3294,836,1506,928,2396,1874,594
PPP2R2A_5_5357,353,117,218,179,227,289,440,355,876
PPP2R2B_5_5358,782,1127,1606,479,1529,974,1203,1323,3052
PPP2R2C_5_5359,46,171,14,1317,2,6,22,134,1
PPP2R3A_5_5360,2557,2652,3053,1878,2416,3055,2705,3325,3598
PPP2R4_5_5361,863,1156,967,768,914,1880,752,1082,1485
PPP2R5A_5_5362,1587,1231,2090,2846,1150,1411,979,1559,390
PPP2R5D_5_5363,454,614,590,218,466,1685,1095,216,231
PPP3CA_5_5364,1537,1833,1922,863,3385,1386,1649,1299,3980
PPP3CB_5_5365,2992,3187,2723,1043,2875,3404,3291,1240,1766
PPP4R1_5_5366,2059,1260,1666,774,1287,652,965,884,1153
PPP5C_5_5367,320,71,274,0,9,5,64,3,358
PPP6C_5_5368,3285,4151,5260,4987,4743,6669,4327,3711,7856
PRKAA1_5_5369,1323,745,1504,1691,1078,1999,1765,1475,1653
PRKACA_5_5370,2217,2926,3290,4151,1935,3404,2283,1727,3509
PRKACB_5_5371,37,2,341,62,0,37,43,0,0
PRKAG1_5_5372,255,235,188,189,86,37,28,5,705
PRKAG2_5_5373,5751,7830,6955,9620,9223,7664,7878,6949,7047
PRKAR1A_5_5374,1538,2345,2194,265,1116,3719,1580,2295,3494
PRKAR1B_5_5375,646,739,1662,1570,357,558,1570,687,1911
PRKCB_5_5376,2299,858,2216,3074,2193,2075,682,1680,3658
PRKCD_5_5377,2534,2098,2886,1719,1044,1589,4287,2498,1733
PRKCQ_5_5378,905,795,859,927,1572,1017,2185,688,1585
PRKCZ_5_5379,1690,865,1285,740,1486,893,1511,820,1795
PRKD2_5_5380,97,283,277,0,186,445,159,428,130
PRKDC_5_5381,2190,3484,3211,4656,1873,5690,1902,3033,1994
PRKG1_5_5382,2128,1732,2162,1102,2161,3511,1092,1369,3079
PRPS2_5_5383,3213,3968,3384,2826,2606,2672,3303,4147,1934
PTK2B_5_5384,382,191,952,162,901,137,392,812,564
PTK2_5_5385,841,681,785,479,278,1104,805,416,343
PTK7_5_5386,472,426,871,463,15,339,257,664,73
PTP4A2_5_5387,4718,4049,6129,3511,4034,4123,4307,6184,4844
PTP4A3_5_5388,1854,1922,1883,1645,1396,2399,1231,1900,2035
PTPDC1_5_5389,905,768,1867,723,1528,1481,871,1004,2256
PTPN12_5_5390,4150,3599,5196,5116,2911,4888,2654,3444,4474
PTPN13_5_5391,2767,2596,3600,2082,5197,2046,1730,1978,5055
PTPN18_5_5392,1107,872,1030,539,2099,1875,851,836,1031
PTPN22_5_5393,165,285,234,521,208,19,2,305,274
PTPN2_5_5394,4360,4068,4102,896,4274,3087,2375,2135,3905
PTPN3_5_5395,594,774,1414,247,2117,900,681,1458,1866
PTPN5_5_5396,628,506,1046,234,193,892,1005,1047,185
PTPN6_5_5397,425,420,1107,476,149,108,1587,72,556
PTPN7_5_5398,406,385,481,18,838,982,260,311,145
PTPRA_5_5399,780,490,837,139,57,2264,242,1110,1529

PTPRB_5_5400,1536,1847,2125,879,2840,3082,1075,768,750
PTPRC_5_5401,2002,1817,3250,1503,3570,2622,1374,2837,427
PTPRD_5_5402,6585,7411,6875,4128,7416,6474,5710,7513,9975
PTPRE_5_5403,2969,2991,3206,2489,2895,2585,1492,2330,1891
PTPRF_5_5404,427,230,98,0,392,0,319,218,847
PTPRH_5_5405,853,594,1015,2098,840,65,227,1757,2399
PTPRJ_5_5406,2048,3054,2925,3503,3927,5422,1492,3433,1569
PTPRK_5_5407,2483,1838,2604,2366,2291,3254,3323,2979,4239
PTPRM_5_5408,284,544,434,1654,219,272,73,910,408
PTPRN2_5_5409,378,93,210,188,0,4,51,25,551
PTPRN_5_5410,107,1,116,0,26,392,1,14,0
PTPRR_5_5411,410,720,1298,903,560,373,861,1138,569
PTPRS_5_5412,1755,2278,2885,701,1883,2073,1866,3101,3530
PTPRT_5_5413,829,902,319,626,673,50,828,1187,839
PTPRU_5_5414,172,227,190,15,417,0,60,136,294
PTPRZ1_5_5415,545,1688,617,612,609,72,1393,657,927
RET_5_5416,423,460,474,685,0,759,231,479,1067
RIOK1_5_5417,430,268,375,309,396,133,223,133,41
RIOK2_5_5418,1629,793,1167,1371,1894,450,885,328,485
ROPN1L_5_5419,741,458,912,737,575,600,99,356,889
RPS6KA1_5_5420,1,95,4,0,881,160,0,38,5
RPS6KA2_5_5421,554,808,864,530,1057,4,66,209,3066
RPS6KA4_5_5422,309,425,456,987,29,1857,243,674,972
RPS6KA5_5_5423,515,930,634,267,1133,1395,589,562,11
RPS6KC1_5_5424,402,271,387,348,0,292,223,215,295
RYK_5_5425,7347,7173,8199,4529,4849,5691,6755,6184,7450
SET_5_5426,4185,4139,5482,3240,3592,5843,3325,3156,7303
SGK1_5_5427,1742,2502,2383,1906,1968,2558,3453,2423,2537
SGK3_5_5428,3609,3387,3391,3616,2429,4819,2647,2614,5282
SIRPA_5_5429,3843,4539,4472,3769,2027,6495,4598,3143,6674
SKAP1_5_5430,1496,1908,1868,1194,1702,4464,638,1156,3020
SNRK_5_5431,1062,447,903,166,1000,1652,678,759,644
SPHK1_5_5432,1156,1634,1773,346,1441,1413,1361,1094,2697
SPHK2_5_5433,205,563,606,3,17,454,371,339,0
SRC_5_5434,572,1004,1002,1970,558,59,745,997,475
SRPK2_5_5435,5183,6840,6628,5355,6028,7803,6171,6065,9464
SRPK3_5_5436,627,220,213,10,79,30,786,555,94
SSH1_5_5437,473,248,273,481,3,1185,743,475,317
STK19_5_5438,488,520,794,42,676,1308,431,66,839
STK24_5_5439,206,700,317,535,26,253,135,8,1525
STK31_5_5440,1218,1484,1516,1601,580,1146,764,1459,1497
STK36_5_5441,788,787,976,1310,1874,1378,120,1816,1830
STRADA_5_5442,247,173,259,435,88,423,207,31,863
STRADB_5_5443,1083,944,1447,1150,2905,834,1660,2237,1440
STYX_5_5444,1007,1393,809,1112,862,976,1272,1372,1612
SYK_5_5445,394,547,831,466,358,507,519,347,271
SYNJ1_5_5446,375,647,822,707,1370,31,13,504,208
SYNJ2_5_5447,321,405,308,232,1084,73,193,79,152
TAF1_5_5448,326,167,161,45,469,2,3,19,137
TAOK1_5_5449,2477,1255,3454,878,1233,2801,2756,3285,5076

TAOK2_5_5450,752,1025,1223,1209,447,1387,984,1254,1719
TBCK_5_5451,49,198,340,207,307,101,90,463,666
TEX14_5_5452,2324,3756,3344,3652,3813,4916,2408,930,2060
TGFBR1_5_5453,892,351,784,826,615,1160,618,233,1256
TGFBR2_5_5454,1573,1789,1592,842,444,1537,1493,759,332
TGFBR3_5_5455,1071,943,2386,1062,1275,2028,579,1930,2132
THTPA_5_5456,485,727,497,432,1096,301,717,661,166
TJP2_5_5457,4052,3669,3864,3185,3580,4293,6461,2201,3456
TK2_5_5458,1326,2358,1721,1602,1587,1650,847,1483,1427
TLK1_5_5459,1439,916,1457,1028,2558,1236,2173,1426,902
TLK2_5_5460,6518,6262,7497,4396,8411,5497,5565,4612,6997
TMEM134_5_5461,2288,3396,3078,2980,4238,2648,2747,2484,3364
TNIK_5_5462,652,674,539,2436,598,1193,760,1107,1067
TNK2_5_5463,646,455,240,547,198,625,390,689,1167
TPK1_5_5464,72,125,615,182,82,462,124,360,57
TPTE2_5_5465,5708,4343,6139,3701,3290,7111,4801,3559,7154
TPTE_5_5466,4523,4147,5052,4588,4080,5783,3645,3452,2820
TRIM24_5_5467,615,1497,1423,202,883,295,967,407,1779
TRIM33_5_5468,3312,4156,4911,4096,5621,4679,3306,3440,6635
TRMT2A_5_5469,357,876,652,562,699,144,417,1592,2152
TRPM6_5_5470,2917,2697,2307,1301,2351,3285,3564,3468,4740
TSSK4_5_5471,504,689,870,1236,486,1812,684,618,874
TTK_5_5472,31,37,5,3,0,2,107,4,0
TTN_5_5473,240,230,322,52,669,69,425,292,877
TWF1_5_5474,108,714,385,0,144,949,695,587,119
UCK1_5_5475,1674,1763,2151,1299,2287,993,1211,1430,1356
UCKL1_5_5476,123,585,625,7,8,29,5,177,409
UHMK1_5_5477,2097,2104,3364,2895,3867,2144,2644,2358,2938
ULK2_5_5478,479,362,859,255,1643,353,1297,730,882
VRK2_5_5479,585,532,852,924,177,1246,640,561,1338
VRK3_5_5480,777,300,292,1270,291,173,349,155,325
WEE1_5_5481,2802,1608,2456,1582,4385,1677,1665,3035,1755
WNK1_5_5482,180,125,105,297,397,6,298,247,381
WNK3_5_5483,4780,6472,5723,4227,10021,5763,5948,5992,1770
YSK4_5_5484,1631,1901,1346,2222,991,2028,816,1812,2094
ZAP70_5_5485,147,19,102,6,2,0,799,43,14
ACP2_5_5486,1108,962,831,1175,1297,1321,1042,967,1327
CDK1_5_5487,2323,1832,1963,1038,2042,3054,1715,600,1528
DUSP10_5_5488,295,212,935,10,394,302,1414,161,1882
DUSP15_5_5489,1679,1425,2011,935,1125,1482,707,1027,2685
ERBB3_5_5490,434,225,677,1,148,113,222,514,963
G6PC2_5_5491,345,216,202,142,49,333,34,575,792
IP6K2_5_5492,928,998,1363,1625,1209,2318,1051,644,1127
MYLK_5_5493,644,1029,275,867,550,1022,61,1192,268
PRPS1_5_5494,732,1596,1217,775,282,1358,1183,1521,621
PTPMT1_5_5495,426,28,207,338,0,566,40,42,39
PTPN20A_5_5496,1382,2169,2271,625,1032,1416,1409,1835,1280
PTPN20B_5_5497,1382,2169,2271,625,1032,1416,1409,1835,1280
SPEG_5_5498,722,1151,1188,781,21,882,61,190,53
STK32A_5_5499,2403,1596,2574,779,1585,978,2452,1811,1688

PPP1R12B_5_5500,2757,3044,3224,3642,2371,4804,2906,3554,5814
PPP1R12B_5_5501,683,1208,904,589,290,614,647,811,373
PPP1R12B_5_5502,59,47,8,3,91,84,9,0,0
AAK1_5_5503,1902,1840,3180,2702,2346,3775,2524,3133,3547
ACP6_5_5504,1860,2280,2462,2223,3313,2246,2489,2292,801
ACPT_5_5505,1366,1289,2742,1295,943,1490,4269,1981,1995
ACVR2A_5_5506,1069,756,839,1020,776,994,778,382,2072
ACVR2B_5_5507,310,268,330,1,53,3,618,133,88
ADCK2_5_5508,920,1462,2046,2422,1441,2032,1365,706,1958
ADCK3_5_5509,118,170,123,39,668,134,897,56,1
ADCK5_5_5510,415,51,168,1059,3,157,102,240,141
ADPGK_5_5511,1169,600,1112,661,1954,2243,434,150,473
ADRBK1_5_5512,316,301,365,701,451,1121,670,607,151
ADRBK2_5_5513,1345,1574,1831,607,1761,1481,2623,988,2020
AGK_5_5514,477,403,954,158,713,358,327,467,123
AK1_5_5515,4496,4664,4749,1460,2140,5804,3290,4001,5790
AK7_5_5516,2811,3139,5153,2219,1877,3288,4454,1700,4466
AK8_5_5517,223,460,911,146,763,105,108,2181,1164
ALK_5_5518,906,1223,1113,210,798,427,1533,1450,177
ALPI_5_5519,688,738,832,60,45,956,214,1524,913
ALPK2_5_5520,145,282,305,183,476,79,1000,644,21
ALPK3_5_5521,228,647,676,124,0,16,404,330,338
ALPP_5_5522,3899,3419,3641,3539,1071,2401,4710,4437,7179
ALPPL2_5_5523,3899,3419,3641,3539,1071,2401,4710,4437,7179
ANKK1_5_5524,402,475,857,627,516,1200,118,1024,320
ARAF_5_5525,3399,1983,2837,967,2385,4054,4081,2071,3974
ATM_5_5526,1930,1422,1759,874,414,985,1241,775,2098
AURKB_5_5527,3293,2907,3087,3516,2824,3649,4805,1686,1844
BLK_5_5528,813,907,1042,531,1072,355,684,598,716
BMPR1A_5_5529,6585,7424,8196,5405,8078,6227,7661,5504,9156
BMPR1B_5_5530,3941,4763,4718,5091,4069,5313,2803,4053,7754
BMPR2_5_5531,3115,3233,4559,3013,3748,6292,3081,3412,6782
BPNT1_5_5532,728,773,1013,965,1031,891,351,1461,1268
BRAF_5_5533,3771,2661,3204,4642,2259,4361,2859,2743,4164
BRD3_5_5534,472,179,271,753,5,23,320,4,171
BRSK1_5_5535,273,119,544,1660,367,93,262,110,372
BRSK2_5_5536,509,486,902,20,957,551,970,831,1676
BTK_5_5537,208,200,816,528,947,499,193,140,235
BUB1B_5_5538,1167,1627,1940,1016,2315,2048,1342,1063,476
BUB1_5_5539,1150,1068,1400,804,536,1423,1547,1244,1415
C20orf111_5_5540,2801,1600,3065,1442,2045,4704,2503,2571,4084
C9orf96_5_5541,382,423,514,109,128,1081,361,863,1108
CALM1_5_5542,2277,2118,1784,864,2111,3272,2595,1035,1425
CALM3_5_5543,768,357,462,61,730,376,563,590,232
CAMK1_5_5544,2100,2252,2821,1658,4095,2941,1718,3817,2514
CAMK1G_5_5545,440,1040,228,519,559,137,267,28,1
CAMK2N1_5_5546,333,468,305,60,442,83,981,281,49
CAMK4_5_5547,280,407,192,447,100,450,539,436,25
CAMKV_5_5548,1849,1913,1496,791,267,2508,1729,1664,1224
CARD11_5_5549,1000,953,850,2653,330,240,1313,715,666

CCL2_5_5550,361,450,361,1352,882,762,251,701,619
CDC42BPB_5_5551,708,552,736,163,245,761,758,142,697
CDC42BPG_5_5552,572,727,637,464,760,516,438,874,1474
CDK14_5_5553,1608,1280,1604,988,545,836,651,1176,2891
CDK15_5_5554,2597,2439,3690,1966,2353,2108,2333,2809,2453
CDK19_5_5555,1912,2490,1477,2875,2986,1296,1583,2218,520
CDK3_5_5556,433,522,481,261,31,528,330,356,93
CDK4_5_5557,1989,2504,3444,2630,3222,2608,2512,2739,3349
CDK5R1_5_5558,193,282,125,140,59,118,246,122,192
CDK7_5_5559,343,433,252,26,417,739,172,806,159
CDK8_5_5560,1375,1021,1654,409,663,747,843,1018,207
CDK9_5_5561,778,645,1065,116,939,1128,853,621,327
CDKL1_5_5562,1716,2193,2013,1503,2399,3105,1632,3147,2101
CDKL2_5_5563,1143,870,1343,805,1478,1004,1096,716,1299
CDKL4_5_5564,3169,2640,3010,1987,3094,3654,2371,1668,2315
CDKN1B_5_5565,2121,2288,2801,2735,3269,2928,3018,1255,2365
CERK_5_5566,423,333,689,132,858,189,399,474,528
CHKB_5_5567,1205,1244,1573,1102,907,2969,668,1537,1071
CHUK_5_5568,382,256,495,138,29,406,221,163,1152
CIB2_5_5569,399,163,252,239,22,242,351,268,23
CILP_5_5570,150,428,536,244,39,351,269,102,39
CKB_5_5571,1747,2130,2722,970,2751,1645,1155,2243,1632
CKM_5_5572,858,928,1405,354,157,2650,1078,724,826
CKMT1A_5_5573,537,802,777,936,1668,887,736,1622,2653
CKMT1B_5_5574,537,802,777,936,1668,887,736,1622,2653
CKS1B_5_5575,1879,1856,3200,1639,2179,2546,3677,1806,2005
CKS2_5_5576,259,545,465,182,212,285,223,156,80
CLK2_5_5577,87,30,83,0,2,0,235,140,0
CLK4_5_5578,241,461,542,248,2,283,1204,221,575
CMPK2_5_5579,1850,2554,1945,634,1292,699,1298,2013,3707
CPNE3_5_5580,3163,4045,5367,4140,2168,4136,1951,3717,4505
CRIM1_5_5581,327,693,668,331,634,289,118,911,553
CRKL_5_5582,1029,1094,974,679,771,1502,1230,934,2045
CSF1R_5_5583,211,115,220,41,13,168,310,79,698
CSNK1A1L_5_5584,3859,3384,4683,1827,2995,4404,3111,3207,4682
CSNK1G1_5_5585,559,338,376,1518,941,819,1219,172,1094
CSNK1G2_5_5586,82,3,110,381,259,26,56,0,0
CSNK2A2_5_5587,53,17,28,20,61,359,280,11,186
CSNK2B_5_5588,646,213,521,36,65,612,249,115,21
CTDSP2_5_5589,407,625,572,34,799,997,84,1049,1343
DAPK1_5_5590,602,358,195,524,3,1722,480,188,871
DAPK2_5_5591,577,949,1054,328,1485,1285,1061,619,3123
DAPK3_5_5592,1797,1564,1770,1575,1389,741,1903,889,2658
DBF4_5_5593,3688,4902,5348,3685,4174,7785,4021,5706,4953
DCK_5_5594,5416,5888,5651,4904,4498,8233,4587,5927,5838
DCLK3_5_5595,1182,1039,857,136,447,1048,558,466,1352
DGKE_5_5596,3758,3316,3481,2004,4123,3885,3354,2648,3710
DGKI_5_5597,501,387,571,494,133,358,164,81,395
DGKK_5_5598,296,416,1104,432,99,606,183,346,260
DGKQ_5_5599,931,930,1036,1138,1536,2014,1213,1616,2223

DOLK_5_5600,443,567,497,136,778,796,820,188,269
DOT1L_5_5601,1578,1442,2466,1298,1300,3175,1562,1158,537
DUSP11_5_5602,1712,1605,1872,1071,1640,2770,2380,2136,1119
DUSP12_5_5603,2194,2841,2718,1200,2059,1397,2503,1211,1229
DUSP14_5_5604,298,398,255,118,1,35,114,26,0
DUSP16_5_5605,1876,2075,3198,314,2073,3991,1701,1169,2205
DUSP18_5_5606,2395,1740,2046,3095,1559,3043,1211,1316,1223
DUSP21_5_5607,447,636,814,32,70,17,408,603,261
DUSP22_5_5608,1261,1093,1604,2182,3560,4094,1904,1139,1347
DUSP2_5_5609,383,599,851,598,806,582,630,591,243
DUSP3_5_5610,16,195,179,0,393,12,113,32,15
DUSP5_5_5611,362,120,391,37,38,240,132,65,2
DUSP7_5_5612,1933,2050,2136,1965,3583,1805,2469,1110,2854
DUSP8_5_5613,412,243,533,440,285,650,233,321,270
DUSP9_5_5614,271,297,135,46,510,0,52,7,1150
DYRK4_5_5615,1994,2382,2124,810,3283,2307,4154,3778,4151
EEF2K_5_5616,688,324,284,179,165,827,420,350,211
EFNA2_5_5617,616,1014,888,810,398,816,1267,1003,1165
EFNA3_5_5618,1100,1994,1866,621,2552,2053,1466,1158,267
EFNA5_5_5619,705,1101,1168,391,2669,1295,699,261,117
EFNB3_5_5620,305,238,516,164,281,1713,275,392,1496
EIF2AK3_5_5621,2225,1470,3350,2243,2510,5525,2472,2217,3779
EIF2AK4_5_5622,4796,4118,5808,4637,3966,7756,4747,4739,6685
EPHA1_5_5623,242,167,579,201,1251,381,42,562,100
EPHA2_5_5624,307,320,732,848,1140,804,367,782,719
EPHA4_5_5625,418,87,304,81,187,208,517,70,65
EPHA7_5_5626,910,1386,1066,1549,764,1421,1176,1047,519
EPHB1_5_5627,591,719,1102,709,1072,1425,191,218,540
EPHB3_5_5628,194,48,424,646,1058,913,387,514,21
EPHB4_5_5629,267,210,437,0,0,561,21,10,261
EPHB6_5_5630,436,430,490,168,1621,1296,471,1061,50
ERN1_5_5631,360,930,911,69,382,747,589,788,124
ERN2_5_5632,361,259,237,35,178,185,1,1484,4
ETNK2_5_5633,494,764,701,460,710,860,735,1301,601
EYA3_5_5634,609,872,789,252,693,410,525,424,1038
FER_5_5635,1923,1202,1561,3147,2480,1895,1933,1364,937
FLT3_5_5636,620,548,351,368,275,545,392,436,13
FN3K_5_5637,755,1034,1883,819,426,2073,609,1914,2198
FN3KRP_5_5638,718,725,1415,727,1357,511,630,722,2476
FRK_5_5639,1322,1484,1225,2577,1411,1969,1202,1214,4997
FUK_5_5640,597,858,726,190,1047,906,653,1766,347
G6PC3_5_5641,727,589,235,605,47,379,303,407,133
G6PC_5_5642,421,396,315,1059,684,259,138,841,652
GAK_5_5643,19,211,727,62,88,1141,125,401,30
GALK1_5_5644,298,395,643,270,680,5,181,494,915
GDPD4_5_5645,761,439,751,333,94,443,405,489,858
GOLGA5_5_5646,3659,3540,3755,3024,1713,2867,2816,2939,4402
GRK1_5_5647,393,1153,496,582,1685,1435,445,544,854
GRK5_5_5648,476,350,942,195,1434,55,540,88,47
GRK7_5_5649,315,639,384,25,365,135,544,232,10

GSG2_5_5650,1334,1419,1726,1000,1750,1910,2354,1095,2199
GSK3A_5_5651,4781,3926,4205,2636,4234,3561,2385,3208,2589
GUCY2C_5_5652,2405,1983,2259,2950,4514,1050,1489,2389,6778
GUCY2D_5_5653,394,450,436,8,500,268,389,66,205
GUCY2F_5_5654,437,585,884,1116,1021,275,495,1151,615
HIPK4_5_5655,993,963,796,450,3,712,311,119,732
HKDC1_5_5656,2102,2587,2331,1431,2864,3789,2546,2646,3570
HOOK3_5_5657,145,318,896,497,429,453,62,524,0
HSPB8_5_5658,238,54,102,9,81,60,3,2,5
HUNK_5_5659,3559,5551,6211,2887,6673,6121,5540,6840,3554
HUS1_5_5660,1130,1230,1016,1725,608,565,726,398,1420
IGBP1_5_5661,37,367,65,334,0,0,227,169,651
IGF1R_5_5662,4005,5302,5711,2562,5551,4041,3176,3371,3252
IGF2R_5_5663,557,1075,994,84,458,1228,1448,1071,4485
ILKAP_5_5664,297,511,1028,673,2239,423,119,551,129
IMPA2_5_5665,718,1140,1737,1962,1693,2445,1864,1785,3366
INPP5A_5_5666,723,2083,1939,1566,724,1926,1133,1203,904
INPP5B_5_5667,2010,1245,2110,1544,952,2041,1095,1339,1157
INPP5J_5_5668,88,298,551,371,262,1044,941,320,25
INPPL1_5_5669,787,778,773,1650,951,1245,760,783,883
INSRR_5_5670,75,303,269,665,1609,628,685,762,146
IPMK_5_5671,2783,3148,3654,2620,4696,4011,3416,2975,5407
IPPK_5_5672,262,245,224,450,785,279,871,127,1322
IRAK2_5_5673,63,117,354,134,11,259,303,757,587
ITK_5_5674,838,941,876,1033,3925,1137,563,1186,1266
ITPKA_5_5675,268,35,69,282,0,568,94,0,2
ITPKB_5_5676,17,122,3,34,7,99,247,55,206
ITPKC_5_5677,491,513,797,44,33,3,245,1047,2363
JAK1_5_5678,4422,4501,3734,4575,7372,4704,5427,5929,3512
JAK2_5_5679,2199,2443,3075,2185,3727,3468,2489,2969,6782
JAK3_5_5680,550,889,702,1297,605,1099,264,1545,286
KDR_5_5681,653,826,771,52,355,689,912,1540,331
KSR1_5_5682,404,594,476,278,35,627,274,289,228
LATS1_5_5683,1185,996,1000,1147,219,826,2219,542,783
LATS2_5_5684,21,14,169,4,436,14,85,2,3
LLGL1_5_5685,1250,620,939,616,1212,1370,378,596,1084
LMTK2_5_5686,521,371,497,248,1280,334,327,192,6
LMTK3_5_5687,128,100,679,254,668,273,307,544,1279
LRRK1_5_5688,220,163,314,2,0,1035,311,4,468
LRRK2_5_5689,5081,6554,6884,7145,7731,7894,3777,5113,10881
MAGI2_5_5690,1469,1362,960,462,1220,1647,1236,514,661
MAP2K1_5_5691,1005,1123,1490,928,280,2245,992,2209,1254
MAP2K2_5_5692,2649,2142,3666,949,2840,1803,1426,1875,1795
MAP2K4_5_5693,702,272,390,92,412,617,1405,463,31
MAP2K6_5_5694,196,294,606,95,463,169,310,268,2
MAP2K7_5_5695,411,991,1398,464,220,294,493,442,1732
MAP3K10_5_5696,193,194,478,551,110,334,369,46,638
MAP3K11_5_5697,704,278,854,290,64,856,1547,712,229
MAP3K14_5_5698,106,185,136,850,681,496,203,408,3
MAP3K15_5_5699,898,825,1199,414,1236,2650,596,1103,939

MAP3K1_5_5700,742,1430,1106,2078,271,1436,136,767,88
MAP3K2_5_5701,1112,928,1571,1100,2512,2291,1210,832,1814
MAP3K5_5_5702,986,1819,1537,542,843,372,868,1223,709
MAP3K6_5_5703,2156,2842,2750,2170,1114,2401,2880,2551,3797
MAP3K8_5_5704,573,1047,643,166,192,791,368,715,827
MAP3K9_5_5705,124,279,354,88,535,458,472,532,49
MAP4K2_5_5706,332,633,731,2245,687,590,58,635,967
MAP4K3_5_5707,1355,1204,1086,2008,963,1370,457,2091,1372
MAPK11_5_5708,646,524,463,1210,565,645,252,507,100
MAPK12_5_5709,1779,1365,2190,1623,573,1185,2403,2237,1482
MAPK13_5_5710,793,1245,2157,2068,1359,1436,1605,630,4288
MAPK15_5_5711,3481,3309,5296,3644,5068,2884,4296,2499,2735
MAPK4_5_5712,245,494,436,510,135,16,361,773,379
MAPK6_5_5713,422,404,218,13,142,337,3,121,511
MAPKAPK3_5_5714,722,706,622,544,1567,551,256,616,1583
MARK1_5_5715,51,250,282,273,19,229,404,18,63
MAST2_5_5716,1376,1407,1978,2413,1485,829,458,2688,134
MAST3_5_5717,2722,2833,3438,2858,2992,4359,1711,1081,1577
MELK_5_5718,5563,5295,5955,4285,2415,7399,5330,6091,7494
MERTK_5_5719,4626,4950,5331,4755,5291,4619,6105,4029,6024
MEX3B_5_5720,598,543,502,274,345,329,140,648,855
MPP3_5_5721,677,616,1039,26,1598,634,404,308,173
MPP5_5_5722,1705,2443,2350,1301,4500,1466,2583,1377,4678
MST1R_5_5723,4,174,328,30,104,350,0,18,733
MTM1_5_5724,1140,1296,1695,249,733,1873,1233,1780,1557
MTMR1_5_5725,3019,2149,3143,1843,3872,1460,2675,1056,4299
MTMR4_5_5726,297,525,1097,136,1235,691,333,586,438
MTMR6_5_5727,0,0,0,0,0,0,0,0,0
MTMR7_5_5728,2711,3836,4360,1275,3968,3460,2576,4129,6410
MTMR9_5_5729,623,719,599,2517,1336,1190,111,598,75
MTOR_5_5730,303,196,195,40,79,65,354,212,183
MYLK2_5_5731,507,205,17,155,16,411,247,43,0
MYLK3_5_5732,621,180,795,17,730,100,500,242,1101
MYLK4_5_5733,817,2028,2282,2130,1217,3053,525,759,2535
MYO3A_5_5734,858,1259,1249,32,1194,549,349,414,988
N4BP2_5_5735,1126,1263,1103,2350,104,375,716,1276,578
NAGK_5_5736,456,900,382,51,290,945,505,577,857
NAGS_5_5737,709,1242,754,581,334,433,987,1089,1903
NEK10_5_5738,888,1568,1625,1153,1397,903,1434,673,2527
NEK5_5_5739,1042,785,879,18,258,1020,381,863,377
NEK7_5_5740,603,705,357,55,321,507,456,515,982
NEK8_5_5741,845,605,972,1549,649,511,1182,627,1230
NEK9_5_5742,568,419,802,566,323,1208,432,595,15
NME3_5_5743,2720,2620,1969,1903,1051,1911,1885,2366,1586
NME4_5_5744,60,234,70,0,332,2,9,184,477
NME5_5_5745,2887,2789,2449,2992,1758,1182,2498,3387,4012
NME6_5_5746,518,175,560,511,1193,14,392,150,14
NPR1_5_5747,456,953,843,845,321,1590,552,1268,141
NPRL2_5_5748,534,313,566,219,366,446,339,297,1329
NRBP1_5_5749,976,1283,747,128,1234,615,349,1113,98

NRBP2_5_5750,50,448,260,0,44,92,15,52,977
NRK_5_5751,157,502,294,0,818,0,55,18,0
NT5C1A_5_5752,148,113,176,13,326,135,7,15,0
NT5C_5_5753,92,113,501,2,26,605,151,9,1206
NT5M_5_5754,3700,2872,5804,3031,2525,5371,3801,4244,5068
NUAK1_5_5755,0,204,195,0,1235,0,0,0,0
NUAK2_5_5756,905,597,396,234,2763,216,564,146,94
NUCKS1_5_5757,2759,2464,3297,2703,2959,1306,3852,1358,1562
OXSR1_5_5758,666,843,1142,264,1570,646,1394,405,1690
PAK2_5_5759,695,582,616,2297,2816,2436,1104,630,772
PANK3_5_5760,380,207,368,59,381,146,164,670,364
PANK4_5_5761,608,535,1443,2522,1172,465,278,1728,94
PASK_5_5762,1400,1048,2629,1324,1601,2144,1160,1139,1476
PBK_5_5763,2877,2306,2842,1789,1989,2662,2984,3201,2671
PCK1_5_5764,1363,2373,3282,1658,4541,2801,1398,880,1278
PDCD1_5_5765,606,874,976,1810,365,651,480,1045,777
PDGFRA_5_5766,755,807,993,1892,1176,1369,160,1052,708
PDGFRB_5_5767,516,401,748,102,28,177,223,1304,2192
PDGFRL_5_5768,468,932,615,1823,1164,48,420,524,218
PDIK1L_5_5769,292,500,393,423,105,193,155,697,135
PDK4_5_5770,712,899,1387,1184,962,1611,1276,709,4503
PDP2_5_5771,432,1020,366,57,13,162,411,554,367
PDXK_5_5772,383,628,1278,155,89,1028,718,562,1178
PFKFB1_5_5773,795,1042,1161,2301,800,1351,681,911,517
PFKFB4_5_5774,343,151,458,464,766,627,113,62,483
PFKL_5_5775,263,238,296,137,303,201,218,686,475
PGAM2_5_5776,228,208,651,316,221,371,273,6,74
PGK1_5_5777,5130,6771,7026,5125,10678,7469,6809,7224,4427
PGK2_5_5778,1903,2185,2115,1282,1159,4332,1122,1847,952
PHKA2_5_5779,1666,1823,1539,1459,1439,2251,1464,2632,1434
PHKG1_5_5780,1062,466,936,181,754,1135,801,678,143
PI4K2A_5_5781,146,232,300,119,35,0,15,2,0
PI4K2B_5_5782,106,449,337,685,319,118,278,69,116
PIK3C2A_5_5783,1613,2273,3934,1664,2841,2209,1450,4124,718
PIK3C2B_5_5784,131,205,144,325,92,408,6,43,305
PIK3C2G_5_5785,595,661,335,988,2046,118,338,1413,62
PIK3C3_5_5786,1694,1089,1846,2541,1611,478,2127,1762,2366
PIK3CA_5_5787,73,52,0,0,0,0,353,1,0
PIK3CB_5_5788,109,404,96,300,1210,1,433,353,3
PIK3CD_5_5789,549,913,1666,69,2222,4232,441,1450,691
PIK3CG_5_5790,219,660,767,260,521,17,5,80,180
PIK3R2_5_5791,668,993,1053,229,3,181,712,298,1
PIK3R4_5_5792,657,292,1020,421,660,534,275,416,3274
PIK3R6_5_5793,1804,2196,2871,2355,961,1592,1632,2483,1021
PIM2_5_5794,1769,1884,2372,1694,962,904,881,1155,196
PIM3_5_5795,154,179,10,173,10,31,120,0,0
PINK1_5_5796,1705,2103,3291,1302,1535,2050,2985,3633,3056
PIP4K2A_5_5797,613,1125,1350,524,1368,1782,1272,1256,4284
PIP4K2B_5_5798,1648,1147,1147,1461,3045,1789,1434,1761,923
PIP5K1B_5_5799,314,480,610,875,138,253,10,292,160

PKDCC_5_5800,691,680,914,2,961,183,231,1587,1942
PKN2_5_5801,3590,4451,5212,2124,1742,2456,5116,2923,3517
PKN3_5_5802,880,841,1283,620,468,1189,1251,471,1878
PLK1_5_5803,1,36,69,75,0,0,0,97,0
PLK2_5_5804,1544,2027,1774,636,1348,2626,658,1027,930
PLK3_5_5805,595,530,615,47,829,638,581,672,358
PMVK_5_5806,118,490,274,623,88,622,36,603,843
PNKP_5_5807,1027,1747,1368,679,883,1162,1136,1077,1148
POLD1_5_5808,114,239,100,302,811,7,0,87,1
PON1_5_5809,538,454,457,170,256,27,413,557,1826
PPAP2B_5_5810,1476,983,1439,725,1228,3180,1079,1213,1199
PPEF2_5_5811,363,710,1202,87,1208,224,553,538,857
PPFIA3_5_5812,58,781,322,0,43,987,379,969,4
PPFIA4_5_5813,361,564,338,652,127,515,83,489,201
PPM1D_5_5814,402,400,766,35,330,1087,461,312,484
PPM1E_5_5815,668,230,760,226,162,130,679,118,388
PPM1F_5_5816,72,86,31,0,0,0,0,229,0
PPM1G_5_5817,142,173,506,110,152,867,288,49,472
PPM1K_5_5818,643,319,456,5,0,62,1,58,1572
PPM1L_5_5819,4347,5612,6679,3006,4509,3774,4305,2725,10806
PPP1CC_5_5820,1235,913,1046,1578,1200,156,870,631,659
PPP1R12C_5_5821,54,111,154,36,34,259,47,148,0
PPP1R13B_5_5822,1371,567,949,823,778,1878,1846,1225,511
PPP1R14B_5_5823,29,124,437,8,231,15,49,79,1108
PPP1R15B_5_5824,549,770,929,1039,1221,1232,1259,958,1428
PPP1R1A_5_5825,379,372,357,393,25,211,351,610,94
PPP1R1C_5_5826,336,395,507,60,832,578,350,158,370
PPP1R2_5_5827,6073,5442,4880,4550,4455,4265,5120,4711,5308
PPP1R3A_5_5828,681,1001,923,1822,1368,363,168,359,323
PPP1R3C_5_5829,742,617,778,802,251,70,662,331,1990
PPP1R3D_5_5830,389,474,619,1387,640,210,465,152,268
PPP1R7_5_5831,1211,1143,1654,491,326,1054,220,750,860
PPP2CA_5_5832,3554,4713,6663,3097,4007,5468,4729,2962,7723
PPP2CB_5_5833,175,397,441,107,248,667,142,245,61
PPP2R1A_5_5834,10,92,395,3,152,10,0,373,22
PPP2R2D_5_5835,2348,2843,3136,1857,3860,1697,3147,2726,2098
PPP2R3B_5_5836,761,543,1073,370,297,1576,601,115,1458
PPP2R5B_5_5837,72,161,337,352,59,247,357,283,202
PPP2R5E_5_5838,1035,1365,1417,1162,1025,1253,1344,432,1434
PPP3CC_5_5839,299,516,410,202,223,733,310,1447,100
PPP3R1_5_5840,386,398,334,0,0,214,0,200,0
PPP3R2_5_5841,94,49,68,391,1,2,156,34,68
PPP4C_5_5842,321,100,251,11,39,142,346,433,6
PPTC7_5_5843,0,0,0,0,0,0,0,0,0
PRKAA2_5_5844,359,384,323,6,2,187,1306,210,486
PRKAB1_5_5845,1435,1737,2097,607,1867,1796,1201,1486,1224
PRKAB2_5_5846,531,1075,1244,242,726,687,1122,1543,894
PRKACG_5_5847,2213,2985,3295,4148,1939,3425,2281,1729,3526
PRKAG3_5_5848,1154,1520,1775,1591,617,2987,1728,1835,3665
PRKAR2A_5_5849,392,202,368,68,166,80,44,277,370

PRKAR2B_5_5850,578,608,739,279,349,1065,1299,927,525
PRKCA_5_5851,735,170,981,703,827,162,1425,219,971
PRKCE_5_5852,1466,794,1746,2067,1678,1726,1963,737,774
PRKCG_5_5853,543,1658,1109,461,863,1046,1339,699,705
PRKCH_5_5854,324,365,362,5,940,491,352,40,336
PRKCI_5_5855,1627,1525,1482,451,1418,1793,628,732,3110
PRKD1_5_5856,259,250,301,84,636,0,804,370,5
PRKD3_5_5857,1533,2251,2762,1088,1472,1645,2158,1392,6364
PRKG2_5_5858,2903,2618,3567,2037,3628,4915,3312,1562,8175
PRKX_5_5859,1203,702,1319,1969,3458,871,853,253,1437
PRPF4B_5_5860,1288,2205,1413,1289,2413,1265,1827,2993,1243
PRPS1L1_5_5861,964,950,1104,3192,1060,1397,379,859,392
PSKH1_5_5862,1731,1988,2470,1392,3857,3463,2641,2812,3159
PSKH2_5_5863,1747,1549,2679,2154,796,1625,1918,2437,1914
PSPH_5_5864,305,400,642,818,674,285,559,666,1936
PSTK_5_5865,379,229,601,65,76,0,8,765,0
PTEN_5_5866,783,907,1232,1012,952,856,562,2023,691
PTK6_5_5867,522,368,227,1069,144,37,728,146,0
PTP4A1_5_5868,360,876,217,111,788,269,338,492,207
PTPLA_5_5869,1762,2132,2189,1599,2357,4113,2817,2114,1589
PTPLB_5_5870,3038,3224,4990,2106,4410,3005,5577,3748,7893
PTPN11_5_5871,1705,1148,2387,1469,1895,2362,1307,2571,1404
PTPN14_5_5872,605,921,850,898,2229,1661,660,540,177
PTPN1_5_5873,9189,7815,9590,10669,7333,9426,7299,6800,7824
PTPN21_5_5874,762,1103,1194,350,1738,885,1316,785,710
PTPN23_5_5875,3926,3178,3468,4144,4101,2700,4861,1699,3351
PTPN4_5_5876,39,231,172,0,0,0,0,743,0
PTPN9_5_5877,431,1301,962,291,2234,538,390,974,1752
PTPRG_5_5878,5124,5138,5717,3635,2355,6245,3425,4868,6929
PXK_5_5879,721,712,1233,176,1046,849,592,2155,492
RAF1_5_5880,384,965,898,235,799,487,559,164,28
RBKS_5_5881,624,352,319,139,686,360,417,534,137
RFK_5_5882,2349,2867,3333,1438,3830,1569,2580,857,2770
RIOK3_5_5883,3670,2390,3994,1698,619,3588,2136,2083,3519
RIPK1_5_5884,211,146,272,180,193,10,127,69,140
RIPK2_5_5885,925,990,1696,1003,2161,1229,1060,461,1369
RIPK3_5_5886,46,140,209,138,60,721,92,39,41
RIPK4_5_5887,26,91,102,161,9,16,70,7,199
RNASEL_5_5888,846,326,673,48,215,46,1000,33,9
RNGTT_5_5889,260,896,817,1,792,1722,729,1108,1508
ROCK1_5_5890,663,478,654,791,409,297,214,141,410
ROCK2_5_5891,3221,4916,4775,1604,3980,2928,4624,4210,4586
ROS1_5_5892,256,341,732,130,8,1558,215,602,1709
RPS6KA3_5_5893,2902,2244,3453,4369,4612,3855,3272,2431,3150
RPS6KA6_5_5894,532,437,607,204,980,1974,437,375,537
RPS6KB1_5_5895,345,132,344,196,292,490,317,398,55
RPS6KB2_5_5896,3195,2422,3960,3418,5246,4722,2392,2342,4418
RPS6KL1_5_5897,267,371,638,197,1104,572,41,394,89
RSPO3_5_5898,2397,3226,3479,1424,3416,2860,2679,2880,3961
SACM1L_5_5899,139,732,933,304,96,933,291,18,892

SBF1_5_5900,593,224,1318,522,252,991,491,619,820
SBK1_5_5901,205,364,85,283,125,152,378,107,427
SBK2_5_5902,326,178,488,17,855,684,161,26,1707
SEPHS2_5_5903,1031,2373,1226,620,1561,953,1034,724,2069
SGPP1_5_5904,1543,1713,2192,1245,630,3873,1529,2155,2242
SHPK_5_5905,834,285,900,164,711,732,924,42,1974
SIK1_5_5906,531,159,654,148,227,718,239,200,8
SIK2_5_5907,386,147,338,621,194,119,387,292,4
SIK3_5_5908,1734,2267,1569,1612,2979,2015,1576,1810,1268
SKAP2_5_5909,960,1180,794,84,1388,410,284,290,1206
SMG1_5_5910,1637,1805,2813,687,522,1510,275,783,2674
SRMS_5_5911,364,279,298,84,83,9,107,273,91
SRP72_5_5912,3182,2878,3985,1244,3036,1517,2519,3521,4556
SRPK1_5_5913,653,1280,1604,695,980,833,1396,861,856
SSH2_5_5914,614,354,385,86,3,690,0,0,0
SSH3_5_5915,833,960,646,309,311,2477,220,115,3373
STC1_5_5916,258,787,676,0,1516,618,364,1291,8
STK10_5_5917,1094,929,1870,1180,689,1000,239,423,3378
STK11_5_5918,189,738,405,492,10,534,20,474,0
STK16_5_5919,586,332,919,562,531,290,906,852,100
STK17A_5_5920,598,740,791,913,697,624,156,432,399
STK17B_5_5921,1146,1598,1424,634,1897,833,781,904,1733
STK25_5_5922,362,147,99,8,565,0,922,56,5
STK32B_5_5923,632,992,727,833,485,732,183,407,929
STK32C_5_5924,483,710,584,204,708,1219,606,1250,1616
STK33_5_5925,390,644,491,416,1003,882,299,93,128
STK35_5_5926,545,491,553,1774,12,581,22,56,708
STK38_5_5927,446,1239,1248,503,299,541,683,628,282
STK38L_5_5928,4305,2918,4280,2875,2923,3865,3896,2061,6986
STK39_5_5929,627,577,516,558,406,426,1367,653,485
STK40_5_5930,2121,2019,2148,1095,2620,4511,1712,789,2758
STK4_5_5931,747,597,956,1200,307,1581,433,440,1002
STYK1_5_5932,579,970,1159,408,6,1714,658,1797,737
STYXL1_5_5933,1004,560,782,956,186,1317,726,1734,1757
TAF1L_5_5934,9416,8775,10507,9096,7517,12517,7950,6890,8431
TAOK3_5_5935,8872,8933,9405,6506,12143,6000,6993,5093,10285
TBK1_5_5936,1098,1319,2349,1420,1941,2672,1051,2558,1950
TEC_5_5937,826,799,1096,166,1268,290,636,132,374
TEK_5_5938,1474,832,1363,1041,1720,1749,959,802,813
TESK1_5_5939,385,518,466,11,651,755,0,887,680
TESK2_5_5940,76,18,193,158,30,30,375,99,27
THNSL1_5_5941,876,1073,1175,870,1508,1815,1910,1434,1072
TIE1_5_5942,3,67,3,0,0,0,0,0,0
TK1_5_5943,997,1058,848,1112,1866,1147,1054,1017,1201
TNK1_5_5944,358,1132,1109,205,1016,1176,539,1628,304
TNNI3K_5_5945,1234,1200,1045,1723,3169,980,1163,1707,1389
TNS3_5_5946,1169,400,630,867,591,454,751,889,314
TP53RK_5_5947,798,1456,2087,510,1225,1629,800,938,906
TRAT1_5_5948,494,400,418,333,460,844,665,232,1004
TRIB1_5_5949,2079,1090,3303,602,3348,954,1089,585,2540

TRIB2_5_5950,688,587,959,2494,758,180,721,1159,517
TRIB3_5_5951,1046,819,1037,1879,2429,2544,1039,1387,975
TRIM27_5_5952,1144,1182,635,1502,1166,51,759,796,529
TRIM28_5_5953,225,448,50,0,139,14,19,330,479
TRIO_5_5954,1780,1919,1898,1164,1248,2114,1564,1501,1046
TRPM7_5_5955,1570,1284,1391,2696,1531,1367,1388,871,1269
TRRAP_5_5956,275,508,711,67,1174,286,261,344,1123
TSKS_5_5957,618,492,393,9,597,182,1382,954,92
TSSK1B_5_5958,274,614,504,898,194,582,292,547,124
TSSK2_5_5959,661,615,1803,739,2371,1114,506,1677,3429
TSSK3_5_5960,622,488,306,821,351,686,410,629,97
TSSK6_5_5961,237,163,247,572,236,60,83,376,320
TTBK1_5_5962,407,126,131,4,18,932,5,10,362
TTBK2_5_5963,1039,769,1401,921,1430,358,1257,1152,1078
TWF2_5_5964,2504,2065,2293,1062,2188,2247,1954,746,2860
TXK_5_5965,6549,8425,9850,10188,6753,5044,7876,8570,5690
TYK2_5_5966,333,311,889,560,786,1582,555,169,488
TYRO3_5_5967,623,712,775,722,480,817,603,493,1187
UBLCP1_5_5968,398,414,707,48,2162,173,317,1332,477
UCK2_5_5969,1136,1709,1849,668,1755,144,1034,2009,1427
ULK1_5_5970,329,296,468,0,1227,441,255,259,509
ULK3_5_5971,687,805,761,157,1375,219,841,340,32
ULK4_5_5972,917,521,692,223,189,492,491,760,215
VRK1_5_5973,730,409,386,656,985,120,504,304,736
WEE2_5_5974,0,0,0,0,0,0,0,0,0
WNK2_5_5975,217,330,291,560,411,20,305,117,28
WNK4_5_5976,1433,1511,1254,906,874,1878,2122,1446,3542
XRCC6BP1_5_5977,1814,1992,2445,2382,1093,612,1473,1764,5471
XYLB_5_5978,722,420,1365,413,491,133,710,69,104
YES1_5_5979,1544,1656,1649,1811,2508,1387,1030,1634,967
AATK_5_5980,217,689,568,99,39,24,638,302,876
ABL1_5_5981,1041,1192,1237,633,2400,439,815,1562,674
ABL2_5_5982,610,741,1305,304,1210,2087,960,1019,571
ACP1_5_5983,4141,5195,6337,3289,4524,4963,3203,2987,4647
ACP5_5_5984,330,877,814,472,355,741,210,176,1363
ACPL2_5_5985,1866,3210,2587,2142,4056,3686,693,2507,2382
ACPP_5_5986,1477,932,1003,1289,531,1945,958,928,1866
ACVR1B_5_5987,573,977,793,1437,301,259,417,550,634
ACVR1C_5_5988,286,239,319,158,71,16,119,32,0
ACVR1_5_5989,772,932,603,1849,792,1195,427,1023,474
ACVRL1_5_5990,398,485,472,0,1,811,327,0,874
ADCK1_5_5991,343,681,883,8,459,1803,707,110,13
ADCK4_5_5992,1586,1497,2231,1304,2544,821,1732,1558,2221
ADK_5_5993,1495,1927,1067,805,2609,1083,939,1256,2034
AK2_5_5994,1098,910,1298,424,493,1068,986,1134,1356
AK4_5_5995,1991,1760,1853,675,1873,2145,2510,1031,1739
AK5_5_5996,1959,1281,2064,2247,2829,1148,1132,2209,2424
AKT1_5_5997,467,898,381,453,273,163,754,451,604
AKT2_5_5998,3983,4608,5472,4723,4531,4669,3556,3770,4789
AKT3_5_5999,449,183,187,602,143,386,500,121,765

ALDH18A1_5_6000,518,189,717,175,207,305,492,563,69
ALPK1_5_6001,132,133,333,11,29,26,3,14,0
ALPL_5_6002,495,302,587,221,146,893,428,147,246
AMHR2_5_6003,376,502,275,433,482,327,179,97,1295
ANKHD1_5_6004,2184,1997,2241,2218,947,1119,2783,1265,1662
APTX_5_6005,3550,4832,4588,2249,3275,4883,2641,2895,7784
ATRIP_5_6006,116,111,305,0,6,37,17,40,21
AURKA_5_6007,2674,3032,4103,1005,1630,3090,2538,2827,4443
AURKC_5_6008,534,759,728,953,1369,409,722,273,395
AXL_5_6009,68,88,78,4,0,25,43,0,0
BAIAP2_5_6010,42,100,332,68,51,177,131,100,1469
BCKDK_5_6011,426,926,616,1285,1138,89,671,244,1089
BCR_5_6012,161,341,275,185,67,199,228,679,336
BMP2K_5_6013,981,866,642,1189,617,47,219,594,141
BMX_5_6014,1786,1404,2887,1336,1901,2173,2036,1726,4847
BPGM_5_6015,1475,2064,1779,1814,1158,1088,852,3186,4135
BRD2_5_6016,174,246,153,1,0,80,8,203,7
BRD4_5_6017,804,616,844,1131,1348,1755,1073,399,163
BRDT_5_6018,1262,1022,1723,1008,3086,2360,1126,2011,1559
CAB39L_5_6019,1532,1082,2336,967,631,2009,725,1048,1414
CAMK1D_5_6020,23,418,32,38,124,2,4,13,705
CAMK2A_5_6021,527,482,1662,67,122,790,269,283,364
CAMK2B_5_6022,1192,1032,2777,574,1638,2891,1662,1306,4866
CAMK2D_5_6023,593,465,759,119,1885,1564,185,506,409
CAMK2G_5_6024,323,820,600,102,512,1151,95,641,514
CAMKK1_5_6025,312,276,623,962,152,8,594,57,0
CAMKK2_5_6026,3702,4760,6228,4716,4262,5392,4519,4204,5583
CASK_5_6027,658,452,696,156,493,632,931,820,242
CCT2_5_6028,462,853,1035,272,189,475,377,392,1251
CDADC1_5_6029,430,154,334,85,160,91,188,305,142
CDC14A_5_6030,799,936,999,545,1268,1098,1340,519,1278
CDC14B_5_6031,815,1048,480,510,209,25,1891,398,952
CDC25A_5_6032,102,115,54,0,16,188,246,18,3
CDC25B_5_6033,1332,1634,1006,634,723,1276,586,1332,1783
CDC25C_5_6034,986,1193,1558,615,1132,1813,1066,1442,365
CDC42BPA_5_6035,1013,1447,1580,2368,897,1645,1105,1003,3374
CDC7_5_6036,94,366,195,118,918,164,191,330,34
CDK10_5_6037,2864,2808,3893,2052,1398,4092,1894,1600,2534
CDK11A_5_6038,195,337,128,54,18,237,170,82,20
CDK11B_5_6039,7693,7720,7837,5190,8167,7700,6098,6478,8164
CDK12_5_6040,2852,3092,4062,3490,2926,2112,2844,2008,4154
CDK13_5_6041,5760,5473,5691,5578,6894,6829,6793,5448,7596
CDK16_5_6042,159,689,693,1004,1662,264,239,663,182
CDK17_5_6043,2282,2935,3128,4046,3314,2195,2218,2731,2512
CDK18_5_6044,973,598,1189,809,986,2023,1251,297,610
CDK20_5_6045,495,824,1463,483,947,871,668,814,1069
CDK2_5_6046,970,746,1660,1332,394,1777,453,1304,1206
CDK5_5_6047,723,1130,804,993,683,767,569,1011,50
CDK6_5_6048,1177,2241,1123,836,1047,823,791,309,513
CDKL3_5_6049,2162,2368,3524,1588,485,2790,2542,3726,2487

CDKL5_5_6050,1589,1136,1699,582,1098,390,1886,896,3409
CDKN1A_5_6051,408,545,344,906,128,711,282,847,1910
CDKN3_5_6052,521,895,952,151,381,1229,1272,683,34
CHEK1_5_6053,2810,3324,4166,2092,5133,4325,2584,2780,4720
CHEK2_5_6054,1408,1192,1570,196,2542,1494,894,2132,1503
CHKA_5_6055,504,1224,864,503,500,1103,724,1276,900
CIT_5_6056,146,582,308,128,80,44,270,80,5
CKMT2_5_6057,386,320,393,181,74,207,620,410,577
CLK1_5_6058,1029,682,917,1129,1532,1046,395,867,307
CLK3_5_6059,496,333,525,128,240,505,373,741,2671
CMPK1_5_6060,1928,1487,1955,1462,1355,4881,1459,1388,2910
COASY_5_6061,1096,1282,1611,835,2583,1962,1611,1863,1618
COL4A3BP_5_6062,500,87,659,1129,15,184,298,349,375
CSK_5_6063,2298,2101,1767,789,1318,2767,1522,1706,1730
CSNK1A1_5_6064,4708,5743,6314,5890,6045,4593,3041,4329,6448
CSNK1D_5_6065,280,345,646,85,181,1510,411,412,22
CSNK1E_5_6066,471,601,493,203,701,299,259,462,119
CSNK1G3_5_6067,1604,1242,1952,630,1292,2120,924,1421,3394
CTDP1_5_6068,1263,1158,1057,2061,633,1609,2061,953,1829
CTDSP1_5_6069,400,454,972,650,569,93,411,330,48
DCLK1_5_6070,580,721,1042,756,1424,694,503,557,99
DCLK2_5_6071,919,1177,1133,656,1208,446,686,1000,1405
DDR1_5_6072,303,68,176,148,466,366,86,83,514
DDR2_5_6073,305,355,699,127,266,1368,277,1,114
DGKA_5_6074,515,807,420,683,561,468,1040,61,255
DGKB_5_6075,815,1485,1691,1105,1508,1187,758,2008,1555
DGKD_5_6076,1069,1378,1120,309,1702,1022,639,1773,1550
DGKG_5_6077,2466,2448,3591,1501,2500,1854,1880,1985,1023
DGKH_5_6078,222,230,392,322,836,106,177,96,1247
DGKZ_5_6079,622,872,784,279,569,1265,877,624,1142
DGUOK_5_6080,217,542,38,2,0,0,51,24,368
DLGAP5_5_6081,4239,5592,6672,5019,7205,4601,7010,4749,8221
DMPK_5_6082,132,213,184,46,296,104,2,119,13
DSTYK_5_6083,3339,3331,3423,3076,2201,2433,2228,3423,786
DTYMK_5_6084,1487,1276,1387,1386,1737,1953,1501,1260,1653
DUSP13_5_6085,104,412,141,227,87,244,11,243,0
DUSP19_5_6086,215,81,597,655,223,637,598,347,62
DUSP4_5_6087,644,455,1106,941,1573,3071,409,939,1570
DUSP6_5_6088,97,365,22,284,129,2,4,170,55
DYRK1A_5_6089,4136,5040,5860,5835,5118,3639,6087,5317,2425
DYRK1B_5_6090,19,48,211,64,51,128,9,192,2
DYRK2_5_6091,1016,1804,1216,1206,59,2123,929,1348,3253
DYRK3_5_6092,371,139,546,27,512,629,447,56,95
EFNA4_5_6093,191,632,543,49,974,588,288,995,523
EGFR_5_6094,3831,2962,4710,2316,3067,3279,3388,5940,4524
EIF2AK2_5_6095,2830,3478,3842,1564,2752,1759,1959,2253,4617
EPHA10_5_6096,453,132,360,42,0,355,118,207,36
EPHA3_5_6097,1962,2592,3396,3807,1372,2139,783,3161,2692
EPHA5_5_6098,433,1097,982,503,63,943,1691,156,827
EPHA6_5_6099,201,121,408,0,8,96,142,32,620

EPHA8_5_6100,2641,1993,2452,3384,2221,2205,1827,1495,1597
EPHB2_5_6101,539,1032,947,54,1569,72,1147,148,263
EPM2A_5_6102,1270,557,901,997,2078,544,1387,1052,2397
ERBB2_5_6103,70,0,65,0,5,0,3,335,62
ERBB4_5_6104,1697,1894,1894,2336,2224,2220,2053,1647,1281
ETNK1_5_6105,1652,3010,2672,2061,2860,3887,2204,1907,1557
EXOSC10_5_6106,1639,2887,2399,1844,1232,1586,1807,1198,513
EYA1_5_6107,461,323,444,167,165,885,538,137,348
EYA2_5_6108,716,943,943,866,2802,844,1195,551,1209
EYA4_5_6109,693,118,431,1005,168,357,140,351,149
FASTK_5_6110,238,325,871,367,592,147,531,144,2
FBP1_5_6111,796,692,758,1475,1883,2029,1274,1028,1104
FES_5_6112,191,174,92,322,137,280,23,392,204
FGFR1_5_6113,790,791,1062,526,323,1795,1131,546,1960
FGFR2_5_6114,501,1007,1115,529,1554,690,656,212,143
FGFR3_5_6115,396,163,437,157,99,1,78,29,885
FGFR4_5_6116,41,384,527,0,369,214,158,219,8
FGFRL1_5_6117,551,566,1119,203,477,613,698,103,1648
FGR_5_6118,346,469,440,3,1408,5,9,339,215
FLT1_5_6119,1528,827,1242,313,1072,937,1474,475,525
FLT4_5_6120,537,488,682,947,1378,327,483,535,277
FXN_5_6121,437,258,930,728,342,513,186,565,192
FYN_5_6122,142,721,606,0,7,729,125,260,603
FZR1_5_6123,374,376,420,963,904,1699,250,204,41
GALK2_5_6124,349,815,585,1569,743,935,442,513,693
GK_5_6125,1966,935,1069,752,688,1416,976,616,3710
GLYCTK_5_6126,478,1268,876,736,1867,634,1244,1541,1158
GNE_5_6127,235,420,482,274,722,241,253,309,181
GRK4_5_6128,3400,4131,5641,3618,4535,4573,2412,4121,4667
GRK6_5_6129,435,762,382,501,668,3439,95,508,3385
GSK3B_5_6130,1563,1764,2241,1072,1156,2169,1174,977,1537
GUK1_5_6131,6379,7463,7966,6581,7646,7381,5184,4180,11025
HCK_5_6132,510,152,368,948,368,107,533,123,404
HIPK1_5_6133,761,350,517,951,1897,163,842,700,581
HIPK2_5_6134,971,551,872,743,13,50,208,179,201
HIPK3_5_6135,2658,2963,3263,2172,3605,1237,2850,2661,1995
ICK_5_6136,1317,1179,1715,349,586,2595,1030,373,420
IKBKB_5_6137,245,297,248,102,84,1196,263,9,4
IKBKE_5_6138,2855,2177,2853,383,5506,3454,999,4017,1327
IKBKG_5_6139,313,67,259,532,162,533,107,79,0
ILK_5_6140,33,82,496,71,30,52,17,2,153
IMPA1_5_6141,3317,3652,6051,5645,6306,3438,2643,4838,7113
INPP1_5_6142,134,205,242,155,135,14,270,258,777
INPP4A_5_6143,620,1318,724,585,804,1084,1283,1210,28
INPP4B_5_6144,1435,1518,2566,1219,1034,1642,362,1314,3488
INPP5D_5_6145,185,113,431,1,87,86,12,1,35
INSR_5_6146,380,539,618,15,2038,932,306,750,238
IP6K1_5_6147,653,246,320,687,213,114,218,625,154
IP6K3_5_6148,124,226,96,31,5,0,264,114,36
IRAK1_5_6149,1199,1071,1925,799,931,2357,1684,972,2641

IRAK3_5_6150,779,698,1077,443,557,916,1170,457,411
IRAK4_5_6151,2646,2789,3865,2889,3767,3664,2888,2723,418
ITPK1_5_6152,196,10,116,24,1449,651,170,114,65
KALRN_5_6153,453,176,384,393,74,494,372,578,104
KHK_5_6154,684,992,1569,978,300,1254,420,733,2464
KIF2A_5_6155,2183,2360,2951,2698,2351,2896,3161,1533,1738
KIT_5_6156,865,801,1105,147,483,433,810,1221,120
LCK_5_6157,1655,2970,2150,933,2621,2364,1118,2579,3066
LHPP_5_6158,56,193,87,19,21,179,142,239,5
LIMK1_5_6159,498,517,466,673,1481,709,195,363,193
LIMK2_5_6160,19,9,28,0,5,8,0,4,2
LTK_5_6161,185,25,411,663,38,1187,281,456,903
LYN_5_6162,1244,2823,1824,632,1586,1415,1928,1340,1648
MAGI3_5_6163,2340,2179,3416,4437,3717,3407,2588,3680,4177
MAP2K3_5_6164,237,498,255,55,134,211,346,216,256
MAP2K5_5_6165,225,334,272,180,101,79,120,560,506
MAP3K12_5_6166,687,1534,1968,1087,1909,1242,1488,1635,1836
MAP3K13_5_6167,42,201,409,132,0,582,0,41,576
MAP3K3_5_6168,107,256,364,328,1774,87,278,699,860
MAP3K4_5_6169,3460,3794,3787,4439,2202,4711,3084,2040,3791
MAP3K7_5_6170,1719,1632,2148,1196,1650,805,1500,1519,1982
MAP4K1_5_6171,403,251,192,393,29,673,727,185,431
MAP4K4_5_6172,1112,1390,1654,708,2387,1402,1989,1230,892
MAP4K5_5_6173,195,568,317,58,296,1,437,15,590
MAPK10_5_6174,1387,1483,1545,413,1235,954,881,923,2227
MAPK14_5_6175,1104,1090,747,1007,684,749,800,526,1577
MAPK1_5_6176,1052,424,774,959,1074,552,564,625,2012
MAPK3_5_6177,348,610,395,1453,596,107,97,470,41
MAPK7_5_6178,656,1228,1532,611,938,525,593,522,581
MAPK8_5_6179,458,362,759,8,321,382,214,486,181
MAPK9_5_6180,597,973,614,171,708,313,1509,460,276
MAPKAPK2_5_6181,260,594,805,861,170,731,354,296,1770
MAPKAPK5_5_6182,1942,1893,1829,1575,1757,2458,2553,1975,1301
MARK2_5_6183,467,476,778,579,694,180,489,348,994
MARK3_5_6184,1833,1798,1948,1258,1010,1697,2736,2752,2459
MARK4_5_6185,2996,3305,4608,3158,4051,4655,3254,3024,4719
MAST4_5_6186,835,873,951,513,1418,1043,928,1377,238
MASTL_5_6187,6056,6879,6529,5591,7178,5999,6040,6675,11719
MATK_5_6188,106,401,268,22,138,1054,250,302,76
MET_5_6189,1421,1280,1200,2308,1789,551,464,1186,1213
MINK1_5_6190,683,2464,1505,1784,2931,1404,1140,1653,2678
MINPP1_5_6191,480,771,580,258,927,213,1667,1396,353
MKNK1_5_6192,64,284,150,146,9,295,630,359,56
MKNK2_5_6193,495,593,622,439,1083,15,364,741,19
MLKL_5_6194,710,893,922,992,691,703,857,635,547
MTMR14_5_6195,586,514,867,1620,863,1508,831,1538,1399
MTMR2_5_6196,417,137,422,289,7,10,105,63,0
MTMR3_5_6197,2088,1548,2410,1149,896,2794,2199,795,2211
MUSK_5_6198,706,515,126,481,181,318,192,43,963
MVK_5_6199,27,71,40,0,0,24,88,17,350

MYO3B_5_6200,590,571,503,86,457,258,689,195,528
NADK_5_6201,1068,1439,1700,1479,2372,1256,900,785,1476
NCK1_5_6202,3202,5320,4267,2178,3281,3605,3291,3731,6802
NCK2_5_6203,687,874,1712,608,2734,571,659,855,44
NDRG1_5_6204,2422,2993,2378,1313,1683,2897,1253,3088,2995
NEK11_5_6205,508,850,625,276,2169,146,421,914,360
NEK1_5_6206,3222,3810,3514,2886,4463,3212,2454,3873,5442
NEK2_5_6207,273,360,514,2,0,905,22,130,1796
NEK3_5_6208,1168,913,1399,1340,478,1983,956,1560,548
NEK4_5_6209,2686,2123,3270,3065,1705,2449,2470,1562,5126
NEK6_5_6210,1803,1903,2436,2595,1798,3336,1500,2561,1471
NME2_5_6211,141,391,211,305,1595,113,21,1398,976
NME7_5_6212,1858,2041,1856,292,4208,2258,2201,1419,1775
NT5C1B_5_6213,715,803,711,421,867,1374,855,785,946
NT5C2_5_6214,1307,1526,898,1508,1304,1475,711,1276,1283
NT5C3_5_6215,1686,977,1487,2431,1780,2465,1393,1199,1408
NT5E_5_6216,317,191,305,18,321,755,979,457,890
NTRK1_5_6217,137,321,269,419,816,1,79,380,1
NTRK2_5_6218,419,542,264,1555,1351,172,458,513,150
NTRK3_5_6219,141,315,675,25,125,573,945,372,808
NUDT4_5_6220,5109,5687,6182,6415,3231,6834,4739,5207,5494
NUDT9_5_6221,1940,1263,1768,1054,2048,2468,2344,993,2695
OBSCN_5_6222,62,130,128,120,911,45,15,29,1
OCRL_5_6223,2018,1608,2455,1758,1810,1950,2755,2930,1820
OXSM_5_6224,1452,1487,2009,1537,1915,1753,1243,754,3132
PACSIN1_5_6225,282,538,507,229,773,227,450,532,1637
PAK4_5_6226,178,148,174,6,43,912,387,88,0
PAK6_5_6227,550,140,303,672,65,320,149,200,89
PAK7_5_6228,585,903,1026,1274,2303,686,1328,1177,2091
PANK1_5_6229,801,1482,1208,987,1928,2327,1748,276,819
PANK2_5_6230,571,863,515,344,2032,248,392,494,514
PCK2_5_6231,117,178,86,20,8,197,51,297,0
PDK2_5_6232,1052,861,1183,579,1403,1370,1049,760,1178
PDK3_5_6233,2846,3023,3416,2969,4828,2768,2940,804,2789
PDPK1_5_6234,538,649,974,1659,101,240,132,486,973
PEG3_5_6235,2017,2428,3665,723,2197,1795,1011,2007,1944
PFKFB2_5_6236,212,437,333,37,443,390,99,27,210
PFKFB3_5_6237,1039,1474,1896,1896,1638,1188,1427,525,1244
PFKM_5_6238,994,1079,1157,1194,798,292,923,1529,1373
PFKP_5_6239,203,76,69,7,0,107,6,40,305
PHKA1_5_6240,2973,4149,4170,3386,5086,8189,3282,2903,1932
PHKB_5_6241,994,826,670,745,281,233,850,892,1840
PHKG2_5_6242,960,1354,1012,2002,83,1487,1754,1986,1853
PHPT1_5_6243,256,215,544,5,122,222,125,592,540
PI4KA_5_6244,1678,1352,1790,806,2099,2603,1855,2023,1984
PI4KB_5_6245,261,381,338,160,111,453,489,179,505
PIK3R1_5_6246,466,644,832,305,775,91,405,447,352
PIK3R5_5_6247,14,201,42,98,1,130,52,5,0
PIKFYVE_5_6248,539,971,1133,1596,1234,633,722,322,365
PIP4K2C_5_6249,476,764,601,947,739,824,1075,596,450

PIP5K1A_5_6250,509,799,1434,867,803,335,1218,595,1449
PIP5K1C_5_6251,118,24,136,121,0,753,1,44,116
PIP5KL1_5_6252,469,260,744,136,245,497,1060,549,1698
PKLR_5_6253,192,240,105,0,0,867,51,140,141
PKMYT1_5_6254,30,128,0,4,0,38,54,1,1
PKN1_5_6255,1098,1065,1352,97,387,1384,859,2509,280
PLK4_5_6256,833,717,851,1099,847,669,519,137,349
PNCK_5_6257,325,413,488,498,441,1595,915,331,85
PODXL_5_6258,845,463,1016,986,1228,646,1395,987,336
PPAP2A_5_6259,370,372,96,731,372,1680,83,298,154
PPAP2C_5_6260,242,887,356,380,1356,844,479,271,341
PPEF1_5_6261,983,932,905,298,885,1019,1091,549,444
PPFIA1_5_6262,1150,2268,1259,1059,1174,849,920,787,1926
PPFIA2_5_6263,1849,1308,1634,1129,1646,2337,2126,1170,4557
PPM1A_5_6264,1077,599,576,75,1137,268,407,584,20
PPM1B_5_6265,2740,2325,3580,2987,1849,3159,2102,2608,3044
PPM1M_5_6266,363,787,510,51,95,135,120,411,1109
PPP1CA_5_6267,123,444,113,0,43,0,65,260,1078
PPP1CB_5_6268,1092,1279,1536,711,1491,740,722,703,1088
PPP1R12A_5_6269,678,641,681,731,1183,632,685,951,194
PPP1R16B_5_6270,78,202,411,6,38,66,206,58,79
PPP1R1B_5_6271,806,475,542,657,1131,3160,1552,576,507
PPP1R8_5_6272,923,1268,910,1329,1502,2133,1536,2065,426
PPP2R1B_5_6273,1203,1586,2196,259,1105,1890,1484,300,849
PPP2R2A_5_6274,5968,6292,6355,4638,3322,4646,7775,3739,5887
PPP2R2B_5_6275,231,302,376,93,163,120,1133,281,179
PPP2R2C_5_6276,283,312,732,310,95,192,226,142,130
PPP2R3A_5_6277,4046,4733,4604,1703,5138,1874,3249,3518,2065
PPP2R4_5_6278,417,578,359,94,26,1485,251,14,0
PPP2R5A_5_6279,2606,2074,3891,1149,3646,2757,2905,2112,3554
PPP2R5D_5_6280,1089,1020,738,62,344,1874,938,472,629
PPP3CA_5_6281,496,451,327,171,512,539,302,735,783
PPP3CB_5_6282,354,630,466,202,219,1003,509,1722,61
PPP4R1_5_6283,5632,5064,7640,2672,6187,4285,6873,4579,7375
PPP5C_5_6284,298,49,260,302,277,384,247,39,12
PPP6C_5_6285,399,170,156,13,77,80,399,140,245
PRKAA1_5_6286,700,304,353,909,296,128,236,681,121
PRKACA_5_6287,850,1331,1194,591,1154,365,855,1772,403
PRKACB_5_6288,2175,2230,4377,2824,1053,1221,2492,1986,543
PRKAG1_5_6289,66,675,234,317,0,273,32,691,2
PRKAG2_5_6290,6515,7418,6654,8945,8784,5569,7504,6899,5503
PRKAR1A_5_6291,283,300,385,25,366,1110,105,724,376
PRKAR1B_5_6292,255,376,535,223,128,606,307,254,39
PRKCB_5_6293,1028,1346,1751,1164,3227,1189,1065,1127,1337
PRKCD_5_6294,1555,2131,2127,2242,1694,2282,2227,1828,1623
PRKCQ_5_6295,4403,5972,5937,2358,5168,4324,5375,3770,6553
PRKCZ_5_6296,263,229,352,558,151,235,415,38,781
PRKD2_5_6297,181,594,286,140,176,494,324,449,622
PRKDC_5_6298,1084,998,2248,810,1802,376,734,1355,1699
PRKG1_5_6299,4925,4189,5481,5864,5604,2676,4757,3318,4415

PRPS2_5_6300,5759,4782,9017,6053,6747,6633,6609,6215,9718
PTK2B_5_6301,200,539,261,147,21,374,484,592,545
PTK2_5_6302,1631,1043,875,810,846,710,692,1493,253
PTK7_5_6303,465,514,1263,275,770,1286,325,893,984
PTP4A2_5_6304,1001,717,948,1334,1145,584,375,406,2079
PTP4A3_5_6305,120,30,856,470,5,225,253,27,797
PTPDC1_5_6306,1602,1325,1647,124,391,1191,405,255,2642
PTPN12_5_6307,696,828,1064,638,1027,397,576,1152,103
PTPN13_5_6308,2106,1596,3315,2760,1845,720,2086,1452,1638
PTPN18_5_6309,548,617,850,115,66,541,944,426,389
PTPN22_5_6310,468,660,742,1020,861,557,1034,1033,506
PTPN2_5_6311,1873,3319,3168,1396,2947,2866,1891,2962,4109
PTPN3_5_6312,1330,1657,1493,1359,1747,1805,1412,848,1718
PTPN5_5_6313,2383,1766,2635,1562,3213,2272,1812,3130,2727
PTPN6_5_6314,131,22,335,4,107,530,68,461,78
PTPN7_5_6315,305,72,70,13,109,164,222,264,194
PTPRA_5_6316,4586,5266,5206,4757,3621,5344,4213,5379,4991
PTPRB_5_6317,1416,1705,2280,1257,2467,3659,1306,755,3978
PTPRC_5_6318,1106,686,809,1936,1129,290,1089,448,183
PTPRD_5_6319,324,293,652,455,127,906,124,143,235
PTPRE_5_6320,2229,1446,2727,1172,916,2909,1613,1954,4406
PTPRF_5_6321,187,262,254,167,86,104,237,404,442
PTPRH_5_6322,426,401,190,292,1950,31,292,515,1250
PTPRJ_5_6323,8826,8080,9716,5931,11437,10615,8101,9261,5884
PTPRK_5_6324,1063,170,554,189,647,682,364,822,1502
PTPRM_5_6325,178,355,473,883,34,148,286,618,895
PTPRN2_5_6326,389,884,1257,983,795,606,1566,15,2667
PTPRN_5_6327,1111,696,1495,551,1089,986,995,1374,1602
PTPRR_5_6328,804,940,1006,1204,147,2744,88,221,1542
PTPRS_5_6329,1764,2291,2978,705,1893,2080,1866,3128,3549
PTPRT_5_6330,301,227,290,18,2,467,570,65,58
PTPRU_5_6331,44,74,321,374,169,11,94,152,4
PTPRZ1_5_6332,3402,6065,6097,3829,6508,6276,3961,5917,3556
RET_5_6333,392,670,593,173,1305,1370,295,241,538
RIOK1_5_6334,358,32,349,121,4,254,206,134,151
RIOK2_5_6335,144,218,81,622,0,0,112,496,0
ROPN1L_5_6336,1016,1724,1573,1562,2411,887,1962,1313,330
RPS6KA1_5_6337,439,1292,657,348,853,573,1494,357,70
RPS6KA2_5_6338,1147,1001,1417,637,1668,1298,1013,743,862
RPS6KA4_5_6339,368,485,854,40,1461,133,453,177,470
RPS6KA5_5_6340,340,134,0,0,0,0,0,0,583
RPS6KC1_5_6341,0,0,0,0,0,0,0,0,0
RYK_5_6342,545,851,432,1218,648,1267,1388,1340,793
SET_5_6343,2240,2418,3420,4188,2348,5862,2017,2329,6523
SGK1_5_6344,650,638,839,2119,454,786,1048,1503,765
SGK3_5_6345,1075,1695,1482,1304,1090,1149,1396,2186,157
SIRPA_5_6346,309,441,476,1301,24,645,467,52,814
SKAP1_5_6347,568,557,969,674,646,364,363,79,1218
SNRK_5_6348,594,530,1085,2,1134,185,136,432,210
SPHK1_5_6349,1419,522,1374,473,1279,633,1047,767,2238

SPHK2_5_6350,34,253,41,0,108,2,0,23,1035
SRC_5_6351,0,0,33,0,0,0,0,0,0
SRPK2_5_6352,2896,2735,2365,3003,1946,2406,1989,2317,2293
SRPK3_5_6353,592,695,459,89,824,302,103,183,1193
SSH1_5_6354,611,269,586,527,31,1863,1343,495,309
STK19_5_6355,52,174,374,1111,130,27,178,208,1101
STK24_5_6356,3317,3933,4562,3253,2521,2137,3772,3670,5597
STK31_5_6357,2120,1649,1710,2069,2033,1732,1924,1101,975
STK36_5_6358,999,1091,917,1221,262,1843,1514,1164,2471
STRADA_5_6359,936,868,545,150,568,614,497,378,615
STRADB_5_6360,2150,2019,1582,2542,1445,1685,2347,1957,1525
STYX_5_6361,883,683,296,1273,160,1667,562,1045,432
SYK_5_6362,767,1428,720,1240,1734,1162,1167,775,618
SYNJ1_5_6363,1345,1449,2243,2060,2454,1371,1229,1850,2790
SYNJ2_5_6364,235,295,821,14,500,455,180,211,23
TAF1_5_6365,2264,1416,2362,1267,1126,3113,2646,2280,843
TAOK1_5_6366,3810,3632,4792,5778,2921,5228,5366,5956,4810
TAOK2_5_6367,429,140,393,1,38,468,320,471,79
TBCK_5_6368,698,3171,2155,414,1680,1506,872,2120,2091
TEX14_5_6369,79,236,335,21,1003,909,140,343,1231
TGFBR1_5_6370,4370,3679,4707,3691,2844,5206,4759,5125,4898
TGFBR2_5_6371,293,411,288,0,362,0,8,829,692
TGFBR3_5_6372,1025,889,2380,1055,1274,2029,569,1928,2128
THTPA_5_6373,1261,1459,1441,159,1319,1346,722,979,2013
TJP2_5_6374,348,140,242,159,751,4,88,57,860
TK2_5_6375,127,445,556,7,4,194,49,189,0
TLK1_5_6376,521,583,492,490,162,468,749,829,770
TLK2_5_6377,458,692,871,151,223,906,273,637,677
TMEM134_5_6378,584,691,756,606,460,285,474,611,1234
TNIK_5_6379,219,318,360,313,215,30,296,19,2071
TNK2_5_6380,329,267,265,691,110,95,257,631,480
TPK1_5_6381,752,682,1056,840,574,810,979,342,133
TPTE2_5_6382,4523,4147,5052,4588,4080,5783,3645,3452,2820
TPTE_5_6383,3275,3039,4111,2705,2279,3964,2997,2183,4113
TRIM24_5_6384,1341,664,792,583,1898,2237,1221,516,3670
TRIM33_5_6385,1181,1901,1878,1928,1524,957,1126,1910,499
TRMT2A_5_6386,614,785,1214,173,1285,1072,470,848,983
TRPM6_5_6387,2324,2269,2974,902,1719,3240,1981,2011,2167
TSSK4_5_6388,1002,745,1256,474,1225,398,690,334,873
TTK_5_6389,422,252,219,126,18,107,584,97,0
TTN_5_6390,2873,2930,3863,3685,4093,3820,4777,2266,2521
TWF1_5_6391,2067,1882,2225,2970,2512,1810,1489,723,1111
UCK1_5_6392,359,433,456,189,1152,57,226,374,716
UCKL1_5_6393,282,411,450,228,296,251,172,526,180
UHMK1_5_6394,67,208,572,125,269,381,95,602,196
ULK2_5_6395,4931,5147,5970,4897,3640,4889,4002,3722,5585
VRK2_5_6396,6391,6964,6322,6911,6872,7509,6480,5440,6942
VRK3_5_6397,423,138,359,3,58,566,28,6,38
WEE1_5_6398,1041,697,790,137,42,1026,687,383,79
WNK1_5_6399,395,483,335,198,207,68,745,755,823

WNK3_5_6400,1830,1234,2308,3167,1695,2145,1130,2003,527
YSK4_5_6401,3156,4574,3303,3420,3943,564,2628,2099,2018
ZAP70_5_6402,452,389,312,72,305,242,481,365,6
ACP2_5_6403,373,470,427,7,1038,925,728,483,982
CDK1_5_6404,806,779,1490,1236,978,549,760,613,2082
DUSP10_5_6405,1746,1612,2129,1555,1650,1479,938,1634,2606
DUSP15_5_6406,245,93,151,76,144,122,76,179,319
ERBB3_5_6407,710,280,677,35,151,71,758,54,1
G6PC2_5_6408,572,302,382,27,311,834,142,204,1088
IP6K2_5_6409,5431,6185,4416,2785,6951,4890,6552,5852,4240
MYLK_5_6410,1115,1120,2172,2917,868,2840,1619,915,878
PRPS1_5_6411,1258,1123,2008,1169,1434,3923,985,967,1411
PTPMT1_5_6412,391,590,1035,1034,1917,659,446,1351,403
PTPN20A_5_6413,1226,1429,2056,1311,579,1074,1817,1802,2564
PTPN20B_5_6414,1226,1429,2056,1311,579,1074,1817,1802,2564
SPEG_5_6415,314,132,295,35,124,419,37,105,223
STK32A_5_6416,89,27,199,4,0,0,113,43,80
PPP1R12B_5_6417,2770,2605,2631,2366,3124,2561,1917,946,2578
PPP1R12B_5_6418,2756,3089,2546,3232,4251,1812,2067,1643,4353
PPP1R12B_5_6419,2072,2611,2633,2398,1709,2314,2398,2143,2722
AAK1_5_6420,719,794,591,655,729,571,858,669,1889
ACP6_5_6421,666,1096,1354,676,586,606,363,566,782
ACPT_5_6422,248,248,277,145,0,341,30,522,288
ACVR2A_5_6423,532,435,1066,593,383,861,453,334,1857
ACVR2B_5_6424,195,190,343,295,9,186,35,19,828
ADCK2_5_6425,543,853,661,110,993,598,453,412,14
ADCK3_5_6426,206,352,221,171,890,1,85,11,0
ADCK5_5_6427,128,136,319,4,0,7,15,9,145
ADPGK_5_6428,1167,1446,1556,15,1865,649,701,747,2564
ADRBK1_5_6429,548,343,267,708,774,65,1059,219,39
ADRBK2_5_6430,933,470,986,1540,136,514,814,263,770
AGK_5_6431,624,597,854,1289,1109,1243,879,255,1227
AK1_5_6432,304,414,593,1324,858,42,713,293,360
AK7_5_6433,1035,720,1158,486,2307,716,915,2762,90
AK8_5_6434,324,986,429,6,1463,285,493,438,464
ALK_5_6435,1561,1466,726,322,956,600,1992,502,2447
ALPI_5_6436,553,156,1248,1005,224,351,235,76,400
ALPK2_5_6437,298,176,336,153,35,309,66,76,0
ALPK3_5_6438,945,1532,1356,1566,782,2236,929,2167,845
ALPP_5_6439,582,639,894,417,861,63,1008,992,166
ALPPL2_5_6440,582,639,894,417,861,63,1008,992,166
ANKK1_5_6441,1637,1694,2219,552,1184,2510,2508,2369,2780
ARAF_5_6442,810,1581,1415,1318,1689,1511,1333,1042,837
ATM_5_6443,5901,6808,7562,7301,7901,5444,6011,6816,6628
AURKB_5_6444,96,287,205,248,1,509,265,0,12
BLK_5_6445,36,219,273,3,913,763,28,0,432
BMPR1A_5_6446,4,127,185,106,141,0,47,204,0
BMPR1B_5_6447,776,1210,1010,1043,190,967,197,676,471
BMPR2_5_6448,130,139,260,13,326,501,210,46,14
BPNT1_5_6449,488,1022,911,44,424,385,906,633,1509

BRAF_5_6450,1899,1831,1725,1135,2142,1079,1702,2898,1238
BRD3_5_6451,406,618,672,421,18,243,783,824,605
BRSK1_5_6452,2535,2144,2165,3816,3855,2235,3307,2271,4228
BRSK2_5_6453,158,55,177,22,573,298,72,64,391
BTK_5_6454,1005,955,1782,1561,972,1635,1417,1448,469
BUB1B_5_6455,1030,1456,1519,1085,583,390,1539,1248,1082
BUB1_5_6456,666,747,978,445,447,164,494,759,80
C20orf111_5_6457,179,387,326,0,7,453,239,272,4
C9orf96_5_6458,229,183,363,695,382,673,286,80,11
CALM1_5_6459,1246,832,886,132,967,558,858,421,403
CALM3_5_6460,585,630,389,131,347,228,584,381,0
CAMK1_5_6461,2514,3184,3178,2283,1196,3007,2947,2674,6422
CAMK1G_5_6462,9,151,534,970,12,514,24,0,650
CAMK2N1_5_6463,213,269,226,46,1676,191,758,820,1707
CAMK4_5_6464,492,781,615,1410,643,1104,171,425,1185
CAMKV_5_6465,1932,1727,2241,1571,1732,1697,922,1609,652
CARD11_5_6466,583,484,743,589,845,968,995,413,34
CCL2_5_6467,106,19,23,249,0,36,35,36,0
CDC42BPB_5_6468,2647,4355,3097,2514,4074,2956,2232,3818,3507
CDC42BPG_5_6469,842,1057,1830,1843,243,347,484,816,1739
CDK14_5_6470,285,1018,998,501,636,870,462,716,40
CDK15_5_6471,861,405,463,702,422,448,740,63,550
CDK19_5_6472,1091,1948,1773,731,648,2291,1574,704,1161
CDK3_5_6473,636,617,852,503,382,1220,904,494,91
CDK4_5_6474,79,629,238,1125,3,0,107,528,1764
CDK5R1_5_6475,877,1687,1746,2781,1613,1234,1709,1702,1940
CDK7_5_6476,999,1396,1173,1406,3033,910,718,2405,760
CDK8_5_6477,1707,1456,2829,1861,3179,1749,2252,1065,2547
CDK9_5_6478,235,571,186,6,131,366,1405,477,181
CDKL1_5_6479,1771,2380,2994,512,2360,1732,1827,3550,2044
CDKL2_5_6480,609,1246,861,32,652,1208,1026,1001,262
CDKL4_5_6481,208,199,307,599,116,323,428,134,13
CDKN1B_5_6482,301,306,363,361,103,371,341,727,157
CERK_5_6483,700,806,761,245,1927,689,173,925,763
CHKB_5_6484,948,771,1202,207,958,172,1500,119,1332
CHUK_5_6485,527,542,349,748,150,147,56,1275,27
CIB2_5_6486,1309,1118,1121,1176,991,1138,1190,861,869
CILP_5_6487,321,520,822,209,20,1095,295,77,1860
CKB_5_6488,83,263,289,841,805,549,793,7,384
CKM_5_6489,662,1057,1391,206,789,2781,1194,379,1742
CKMT1A_5_6490,1544,1498,1323,2393,556,1969,1499,1815,1546
CKMT1B_5_6491,1544,1498,1323,2393,556,1969,1499,1815,1546
CKS1B_5_6492,2370,2039,2019,2824,1790,2524,1985,3709,2499
CKS2_5_6493,3754,4342,4330,3432,3605,3512,2872,3017,6327
CLK2_5_6494,2385,1935,3145,2299,2209,2601,2240,1110,1352
CLK4_5_6495,1038,1542,1308,464,2255,583,1730,1291,524
CMPK2_5_6496,1639,1106,1451,1097,2097,883,1388,413,408
CPNE3_5_6497,148,555,135,22,1010,343,394,322,413
CRIM1_5_6498,808,1276,772,870,3160,343,616,521,2268
CRKL_5_6499,358,513,249,44,50,847,72,814,1163

CSF1R_5_6500,358,310,373,293,217,329,466,415,547
CSNK1A1L_5_6501,740,699,926,687,694,150,424,830,891
CSNK1G1_5_6502,3202,2788,2952,3548,2936,3051,2237,3383,4044
CSNK1G2_5_6503,776,26,323,816,474,0,313,0,2
CSNK2A2_5_6504,1004,805,1297,360,884,1434,281,469,933
CSNK2B_5_6505,577,192,353,55,25,855,238,201,190
CTDSP2_5_6506,1367,535,841,782,1128,904,654,276,166
DAPK1_5_6507,4420,3151,4683,3976,2688,2472,3492,2836,2946
DAPK2_5_6508,36,9,4,1,2,4,285,5,3
DAPK3_5_6509,31,47,13,0,2,71,53,0,2
DBF4_5_6510,102,126,33,0,340,0,46,0,0
DCK_5_6511,2863,2263,2753,2084,3876,3599,1589,1817,4718
DCLK3_5_6512,682,436,712,22,779,409,1086,886,372
DGKE_5_6513,1201,823,1407,1355,2405,2237,1326,961,2349
DGKI_5_6514,267,126,257,41,447,52,214,332,82
DGKK_5_6515,809,985,1311,460,2656,1683,591,140,323
DGKQ_5_6516,208,412,207,686,25,209,409,183,3
DOLK_5_6517,168,9,259,0,29,373,13,179,0
DOT1L_5_6518,877,971,1304,634,1502,386,332,2618,20
DUSP11_5_6519,5451,5380,7005,6511,2212,5477,5222,3705,8516
DUSP12_5_6520,1423,928,2726,1685,981,1266,866,183,2034
DUSP14_5_6521,4264,4531,5625,3600,4323,4898,3714,3049,4870
DUSP16_5_6522,342,596,631,19,1911,884,665,481,52
DUSP18_5_6523,495,658,540,417,420,142,267,86,28
DUSP21_5_6524,1454,1126,942,338,2652,1514,1935,1692,1682
DUSP22_5_6525,833,648,1195,132,554,153,914,392,27
DUSP2_5_6526,891,767,985,113,1293,1643,559,763,382
DUSP3_5_6527,258,67,87,126,54,30,208,57,3
DUSP5_5_6528,96,69,227,692,29,232,301,27,1178
DUSP7_5_6529,323,386,328,119,395,215,37,512,500
DUSP8_5_6530,30,305,1,3,0,0,38,1,0
DUSP9_5_6531,281,258,624,26,569,446,197,606,1119
DYRK4_5_6532,1315,1338,1805,88,2294,2371,1714,628,3099
EEF2K_5_6533,318,867,453,892,55,573,410,231,738
EFNA2_5_6534,675,893,832,245,1015,1183,290,805,80
EFNA3_5_6535,132,36,221,1563,45,200,269,28,900
EFNA5_5_6536,421,316,557,150,814,206,351,471,35
EFNB3_5_6537,175,190,381,650,0,156,23,201,518
EIF2AK3_5_6538,1259,499,1119,727,926,999,1745,856,697
EIF2AK4_5_6539,378,450,750,19,37,26,329,482,48
EPHA1_5_6540,768,485,788,1249,1699,1287,257,180,349
EPHA2_5_6541,226,122,364,317,3,167,136,759,19
EPHA4_5_6542,1790,2086,1848,1542,2227,1674,2604,892,817
EPHA7_5_6543,879,1194,1189,1561,1124,514,909,1006,1905
EPHB1_5_6544,442,976,1189,55,1227,385,638,766,621
EPHB3_5_6545,777,539,1607,236,537,940,615,410,206
EPHB4_5_6546,550,332,370,768,274,888,930,613,553
EPHB6_5_6547,113,457,284,463,7,603,28,252,1852
ERN1_5_6548,978,714,519,866,1890,813,804,545,2190
ERN2_5_6549,312,107,469,173,138,437,205,28,202

ETNK2_5_6550,347,277,384,291,1091,537,822,320,21
EYA3_5_6551,322,189,316,4,511,639,0,0,4
FER_5_6552,4243,4658,6119,3719,5882,2860,4038,5911,4096
FLT3_5_6553,357,330,1043,108,759,619,724,768,139
FN3K_5_6554,758,1034,1875,819,809,2091,608,1906,2173
FN3KRP_5_6555,214,432,712,10,296,367,31,172,127
FRK_5_6556,837,1279,1134,534,1896,2180,1280,1306,1427
FUK_5_6557,137,33,253,0,0,227,137,1,13
G6PC3_5_6558,113,72,600,0,572,482,328,0,292
G6PC_5_6559,91,66,224,80,148,221,115,47,96
GAK_5_6560,76,171,303,634,3,234,96,1,197
GALK1_5_6561,821,1311,1014,229,884,515,1208,1457,1009
GDPD4_5_6562,5117,5047,5787,4530,6421,5638,6880,6760,8640
GOLGA5_5_6563,994,1456,1267,1331,2038,610,698,2082,1046
GRK1_5_6564,199,32,92,718,49,216,300,0,485
GRK5_5_6565,2007,1754,2133,3343,1460,795,1720,708,26
GRK7_5_6566,753,1070,1240,656,1729,899,315,373,922
GSG2_5_6567,531,513,387,219,479,1294,134,363,698
GSK3A_5_6568,1491,1798,1720,2269,596,1311,1742,1007,1405
GUCY2C_5_6569,829,617,375,464,1144,1274,379,623,17
GUCY2D_5_6570,3616,5238,4910,3875,4340,5243,2770,2952,5096
GUCY2F_5_6571,578,940,1074,1344,278,1258,953,889,199
HIPK4_5_6572,214,276,676,78,22,10,289,1,0
HKDC1_5_6573,444,406,652,568,254,1441,258,211,914
HOOK3_5_6574,918,1316,1612,315,2791,778,853,722,1271
HSPB8_5_6575,606,392,934,562,70,208,905,186,477
HUNK_5_6576,267,272,718,13,1673,653,444,444,1619
HUS1_5_6577,711,268,887,988,632,1147,1129,334,490
IGBP1_5_6578,0,0,0,0,0,0,0,0,0
IGF1R_5_6579,239,770,739,43,111,786,453,1063,189
IGF2R_5_6580,1208,1694,1613,326,1933,471,1054,875,1199
ILKAP_5_6581,1637,1869,1693,758,2292,2095,2657,773,2180
IMPA2_5_6582,1286,1365,1706,141,953,667,1180,922,1966
INPP5A_5_6583,628,624,979,207,396,1115,336,50,393
INPP5B_5_6584,2881,4463,4250,2866,1952,2550,2182,3157,3482
INPP5J_5_6585,293,174,257,84,102,558,158,346,853
INPPL1_5_6586,1433,290,584,1768,350,1407,1417,269,1293
INSRR_5_6587,1444,774,1480,267,861,2075,1320,725,491
IPMK_5_6588,559,708,619,1026,250,408,663,326,1131
IPPK_5_6589,445,340,384,1549,378,195,293,343,326
IRAK2_5_6590,747,522,533,925,206,1037,323,1777,645
ITK_5_6591,1022,1396,1674,1527,1170,2636,2408,984,1364
ITPKA_5_6592,2242,1134,2777,1207,1478,3724,2010,2144,2704
ITPKB_5_6593,147,206,63,222,349,239,152,442,272
ITPKC_5_6594,571,418,622,306,179,640,614,265,1995
JAK1_5_6595,1053,863,748,1991,1163,2005,971,1672,304
JAK2_5_6596,1072,936,1150,1235,2707,1144,1177,1611,1369
JAK3_5_6597,663,950,955,1876,213,786,582,226,14
KDR_5_6598,984,997,781,673,1150,603,1226,180,747
KSR1_5_6599,7,2,1,1,4,507,10,4,1

LATS1_5_6600,1239,668,1125,67,596,1065,1907,672,1740
LATS2_5_6601,506,1333,208,436,116,72,221,1912,909
LLGL1_5_6602,1660,2010,1949,1589,757,1661,1402,1049,3618
LMTK2_5_6603,401,619,734,219,308,269,688,965,939
LMTK3_5_6604,1,68,41,0,900,120,2,22,115
LRRK1_5_6605,193,160,261,318,658,64,281,53,377
LRRK2_5_6606,1051,852,1246,507,784,1456,350,627,1240
MAGI2_5_6607,2532,2274,2365,1107,3256,1656,2636,1638,4363
MAP2K1_5_6608,2254,1871,3237,1104,2808,1722,1258,2321,1588
MAP2K2_5_6609,155,146,459,37,210,438,118,200,7
MAP2K4_5_6610,1667,2627,1186,1422,2045,479,2388,2973,3666
MAP2K6_5_6611,1056,730,660,1343,699,582,678,346,986
MAP2K7_5_6612,158,244,291,340,465,701,930,323,140
MAP3K10_5_6613,561,288,456,241,2,293,330,435,443
MAP3K11_5_6614,196,173,350,14,8,15,410,15,0
MAP3K14_5_6615,898,1638,1136,1161,954,905,1281,1566,156
MAP3K15_5_6616,143,65,183,5,22,158,66,17,263
MAP3K1_5_6617,901,572,840,731,792,62,161,402,15
MAP3K2_5_6618,2476,2279,2108,1284,2000,1151,2316,1813,569
MAP3K5_5_6619,2322,2148,1850,1800,3974,3503,2657,1439,1410
MAP3K6_5_6620,617,267,365,546,20,1338,417,521,93
MAP3K8_5_6621,17,56,42,0,173,162,69,42,43
MAP3K9_5_6622,613,794,413,429,73,1377,872,456,782
MAP4K2_5_6623,128,153,217,3,852,714,382,292,383
MAP4K3_5_6624,3268,2671,2933,2693,3321,1835,1831,3653,1260
MAPK11_5_6625,716,852,925,1955,250,1239,1152,575,1622
MAPK12_5_6626,742,156,430,65,155,475,413,131,434
MAPK13_5_6627,170,16,206,356,0,139,186,147,1185
MAPK15_5_6628,265,176,364,413,48,174,342,52,1787
MAPK4_5_6629,215,116,653,810,90,258,335,173,98
MAPK6_5_6630,1407,1307,1383,1340,1968,1260,1497,1536,3131
MAPKAPK3_5_6631,62,50,284,244,137,645,121,95,2
MARK1_5_6632,764,962,1026,226,1815,1512,579,694,147
MAST2_5_6633,1932,2236,3624,3634,3067,1582,2511,2972,4937
MAST3_5_6634,829,690,712,606,348,1600,877,400,1361
MELK_5_6635,1283,1947,1975,777,2523,1900,1338,1705,1666
MERTK_5_6636,2542,1866,2901,2268,2102,4480,764,1711,3271
MEX3B_5_6637,448,479,180,783,954,500,163,2,343
MPP3_5_6638,55,78,79,14,14,0,1,6,1099
MPP5_5_6639,1966,1491,2344,2857,1310,2401,2701,1948,3729
MST1R_5_6640,1261,1305,1299,4837,1958,2841,607,2695,1446
MTM1_5_6641,1174,716,1230,172,1695,2224,749,319,1843
MTMR1_5_6642,1212,1008,1788,322,1177,2502,1078,1273,1172
MTMR4_5_6643,2144,2813,2257,3046,3251,2988,2794,2557,1362
MTMR6_5_6644,942,704,672,63,873,974,628,57,720
MTMR7_5_6645,1180,891,1650,974,1178,1073,907,931,1219
MTMR9_5_6646,435,659,348,1,203,21,1121,635,174
MTOR_5_6647,238,230,124,383,0,308,966,197,48
MYLK2_5_6648,1977,2594,3042,1397,2261,2087,2154,2198,2221
MYLK3_5_6649,194,734,835,69,766,189,220,98,195

MYLK4_5_6650,1833,1463,1770,1184,524,1511,830,387,1454
MYO3A_5_6651,760,1328,1119,787,472,986,1095,1832,1445
N4BP2_5_6652,900,1090,1093,1371,377,1692,1236,1080,621
NAGK_5_6653,149,245,284,183,143,91,103,179,176
NAGS_5_6654,789,1236,1026,668,714,504,1022,1241,1922
NEK10_5_6655,84,112,127,85,160,221,15,387,27
NEK5_5_6656,4,0,11,0,0,0,0,0,0
NEK7_5_6657,809,150,496,18,557,490,653,206,322
NEK8_5_6658,141,158,181,1,169,3,273,49,39
NEK9_5_6659,1293,1975,1723,978,1554,1979,1192,1508,2304
NME3_5_6660,2400,2507,1455,1772,1044,1895,1349,2163,697
NME4_5_6661,274,301,201,0,22,15,414,277,0
NME5_5_6662,526,1000,1190,159,1537,1340,979,424,2193
NME6_5_6663,189,156,187,659,129,136,13,585,39
NPR1_5_6664,616,1435,754,155,1017,772,1353,111,950
NPRL2_5_6665,405,232,472,467,3,878,168,484,846
NRBP1_5_6666,726,359,232,490,190,214,262,143,17
NRBP2_5_6667,611,698,1016,1195,816,1721,832,683,147
NRK_5_6668,505,1798,1539,246,1351,497,390,1287,1721
NT5C1A_5_6669,53,822,248,12,1381,46,17,409,818
NT5C_5_6670,347,126,588,45,0,195,431,43,392
NT5M_5_6671,1388,1556,2601,910,3108,1523,1643,2945,2032
NUAK1_5_6672,298,372,466,556,1511,897,115,382,27
NUAK2_5_6673,469,748,742,461,317,1079,670,719,487
NUCKS1_5_6674,1801,1824,2463,2205,1966,621,3241,1081,1365
OXSR1_5_6675,294,271,243,459,170,310,46,114,273
PAK2_5_6676,87,362,142,166,297,992,111,239,207
PANK3_5_6677,142,137,638,190,165,513,429,51,181
PANK4_5_6678,339,318,708,97,133,31,411,26,391
PASK_5_6679,642,162,748,555,519,523,486,578,247
PBK_5_6680,2697,2574,3085,1013,2671,2717,2424,2211,2474
PCK1_5_6681,315,342,544,1,350,999,612,197,4436
PDCD1_5_6682,762,832,780,30,1068,1491,618,416,2
PDGFRA_5_6683,997,886,1037,690,1295,597,1796,699,2873
PDGFRB_5_6684,911,544,1103,35,236,22,899,737,5583
PDGFRL_5_6685,1361,1629,1519,1176,1565,1127,523,1100,1017
PDIK1L_5_6686,1357,2474,2100,747,2583,1441,750,3394,3980
PDK4_5_6687,974,802,1254,809,1128,205,527,1364,389
PDP2_5_6688,489,720,668,310,53,240,452,297,225
PDXK_5_6689,348,612,1233,153,5,1015,820,562,1165
PFKFB1_5_6690,93,307,636,72,13,154,344,241,1284
PFKFB4_5_6691,1865,3307,3342,3866,2629,3134,2074,2080,3349
PFKL_5_6692,2678,3402,2639,1255,4363,941,1721,3141,2763
PGAM2_5_6693,314,533,239,5,77,30,276,2,703
PGK1_5_6694,15,75,162,7,18,0,1,368,0
PGK2_5_6695,5135,6587,7209,5265,10722,6992,6816,6847,4489
PHKA2_5_6696,1562,1877,3362,564,1957,1750,1717,1450,1965
PHKG1_5_6697,1884,2237,1828,2432,1248,3175,1696,1299,1702
PI4K2A_5_6698,239,754,601,67,929,651,649,188,1185
PI4K2B_5_6699,1359,1844,1679,1072,1992,640,1803,1743,2031

PIK3C2A_5_6700,3023,2787,4281,3965,1526,4974,1936,2273,3454
PIK3C2B_5_6701,818,331,276,814,2670,65,261,157,743
PIK3C2G_5_6702,1560,1537,2145,1921,941,1526,1989,924,2078
PIK3C3_5_6703,333,380,403,37,207,1555,263,327,418
PIK3CA_5_6704,699,979,1140,839,157,794,456,533,979
PIK3CB_5_6705,1064,1123,1374,1498,2429,1798,1097,1129,273
PIK3CD_5_6706,842,1032,1794,821,913,1058,2177,852,707
PIK3CG_5_6707,893,495,863,774,1321,607,1094,711,516
PIK3R2_5_6708,6,108,6,0,0,13,0,0,330
PIK3R4_5_6709,783,1464,1120,1369,1286,835,1314,261,1242
PIK3R6_5_6710,70,216,177,415,1,6,576,235,217
PIM2_5_6711,536,683,534,301,914,1019,499,787,1394
PIM3_5_6712,786,1407,865,846,3157,1936,1040,1230,3101
PINK1_5_6713,265,711,166,439,146,146,424,90,105
PIP4K2A_5_6714,6059,6510,6868,6551,3501,9524,5099,10958,7417
PIP4K2B_5_6715,277,97,503,540,161,510,539,181,1
PIP5K1B_5_6716,4387,3487,5179,1400,3837,4482,5240,5029,4651
PKDCC_5_6717,121,32,24,2,0,343,2,0,0
PKN2_5_6718,2368,2173,3135,1962,3170,2309,4258,3570,2974
PKN3_5_6719,136,43,236,522,26,16,175,89,25
PLK1_5_6720,154,312,220,133,86,144,113,47,0
PLK2_5_6721,659,582,1012,289,86,629,308,1230,595
PLK3_5_6722,110,428,882,330,1093,6,629,22,138
PMVK_5_6723,123,210,352,228,234,152,88,257,304
PNKP_5_6724,670,211,353,0,803,27,95,650,7
POLD1_5_6725,338,269,333,637,234,241,865,751,12
PON1_5_6726,801,1062,1010,1034,1617,1136,733,660,1599
PPAP2B_5_6727,529,329,757,289,579,282,388,872,285
PPEF2_5_6728,2494,2753,3256,2233,2637,1499,2822,2253,837
PPFIA3_5_6729,1743,1830,1199,1301,771,550,1751,943,4438
PPFIA4_5_6730,181,84,70,48,1113,58,306,57,76
PPM1D_5_6731,749,792,286,336,236,1005,286,223,2701
PPM1E_5_6732,143,315,385,345,249,39,794,49,738
PPM1F_5_6733,51,119,151,1,0,35,686,155,599
PPM1G_5_6734,1118,1132,1043,1458,1164,1495,856,1272,1334
PPM1K_5_6735,406,279,230,89,128,21,280,107,0
PPM1L_5_6736,1127,832,845,1342,2700,869,523,863,464
PPP1CC_5_6737,2378,3247,2611,1872,3065,2977,3793,3234,4214
PPP1R12C_5_6738,36,31,245,79,3,0,185,67,0
PPP1R13B_5_6739,747,352,750,1318,318,577,416,378,164
PPP1R14B_5_6740,191,607,355,208,896,839,497,778,790
PPP1R15B_5_6741,1006,500,572,113,1026,920,1077,282,124
PPP1R1A_5_6742,1048,1085,1117,2070,555,1631,784,446,982
PPP1R1C_5_6743,560,537,553,1138,725,1960,518,340,1113
PPP1R2_5_6744,1826,1041,2031,2705,1145,1237,708,949,3504
PPP1R3A_5_6745,2027,1422,1253,1498,1128,603,2940,1087,202
PPP1R3C_5_6746,4876,4974,7193,3900,6107,8551,5215,3092,4916
PPP1R3D_5_6747,137,223,217,15,181,214,22,107,1
PPP1R7_5_6748,1790,1453,2126,1515,878,2861,1783,2181,753
PPP2CA_5_6749,4903,5694,6812,4252,6718,5793,6381,5070,6461

PPP2CB_5_6750,165,128,159,173,118,300,439,128,46
PPP2R1A_5_6751,81,89,182,0,0,0,174,1,0
PPP2R2D_5_6752,1357,426,1366,580,267,227,1500,168,752
PPP2R3B_5_6753,1929,1706,2080,1048,1088,1021,1139,1234,1838
PPP2R5B_5_6754,487,676,1045,704,1439,1724,1097,667,792
PPP2R5E_5_6755,6,11,42,0,0,0,0,6,0
PPP3CC_5_6756,1665,1790,1855,1191,1200,1279,749,2377,814
PPP3R1_5_6757,0,0,0,0,0,0,0,0,0
PPP3R2_5_6758,257,284,464,450,227,984,244,1104,965
PPP4C_5_6759,694,1417,1722,603,1949,1449,904,946,703
PPTC7_5_6760,204,153,669,71,72,1371,354,378,397
PRKAA2_5_6761,1726,1656,1409,1405,1637,652,1263,1610,741
PRKAB1_5_6762,336,649,677,1111,365,1410,703,50,627
PRKAB2_5_6763,63,413,368,249,1388,799,109,221,2
PRKACG_5_6764,698,710,891,379,445,330,1709,832,649
PRKAG3_5_6765,441,105,307,208,497,170,338,662,466
PRKAR2A_5_6766,236,271,737,365,9,690,764,47,370
PRKAR2B_5_6767,555,242,548,1,759,1298,846,402,13
PRKCA_5_6768,661,550,713,468,763,714,245,658,983
PRKCE_5_6769,1577,1292,1331,1142,1740,1459,1577,906,2125
PRKCG_5_6770,765,550,404,30,134,1149,382,161,1666
PRKCH_5_6771,604,922,552,2,1529,21,461,417,207
PRKCI_5_6772,1408,966,2064,2121,1566,2166,1056,1507,731
PRKD1_5_6773,980,1459,1182,1272,1078,595,1197,801,1026
PRKD3_5_6774,411,631,839,1101,185,1207,1123,1066,1249
PRKG2_5_6775,219,232,92,51,200,3,197,457,30
PRKX_5_6776,404,642,442,230,1123,190,434,397,281
PRPF4B_5_6777,3434,3880,4290,3644,3604,5783,2573,3308,8031
PRPS1L1_5_6778,437,660,761,63,242,144,376,174,394
PSKH1_5_6779,475,635,723,1495,675,632,261,697,3788
PSKH2_5_6780,248,298,391,157,85,206,71,405,1
PSPH_5_6781,100,172,503,1452,2,251,349,120,1614
PSTK_5_6782,2763,2218,2430,1216,1113,3722,1803,1371,4342
PTEN_5_6783,1888,2457,3233,1495,2042,2171,1908,1667,2404
PTK6_5_6784,12,61,1,19,0,1,0,0,365
PTP4A1_5_6785,8409,9067,13368,8321,12461,9062,10144,9852,12940
PTPLA_5_6786,1934,2182,2249,2101,2356,4096,2825,2102,1592
PTPLB_5_6787,303,459,1051,881,46,359,564,1442,1142
PTPN11_5_6788,2378,2265,2514,3878,2225,1856,2918,3107,4675
PTPN14_5_6789,234,52,77,2,131,0,6,2,9
PTPN1_5_6790,1883,3065,2746,2924,2217,5684,2936,2076,2706
PTPN21_5_6791,438,254,607,300,164,611,682,830,742
PTPN23_5_6792,574,348,923,67,458,369,278,190,1530
PTPN4_5_6793,1017,1879,1177,164,1251,736,296,625,4038
PTPN9_5_6794,2263,2265,2660,2031,2811,1999,2665,1464,2210
PTPRG_5_6795,384,599,322,1328,155,1694,461,660,988
PXK_5_6796,1580,2277,2132,1708,2400,2585,1288,2181,3320
RAF1_5_6797,203,189,466,0,392,67,469,1,0
RBKS_5_6798,231,322,685,937,863,1490,279,1111,922
RFK_5_6799,339,959,242,596,2171,165,1061,240,163

RIOK3_5_6800,522,1350,1077,201,1997,1164,562,764,602
RIPK1_5_6801,379,327,931,244,167,91,205,502,495
RIPK2_5_6802,240,143,202,15,494,137,39,507,59
RIPK3_5_6803,787,513,616,495,936,413,1632,137,1091
RIPK4_5_6804,453,259,515,281,81,701,819,481,655
RNASEL_5_6805,4500,4622,4408,2531,2970,4335,3823,6731,2870
RNGTT_5_6806,993,1476,1908,1114,1349,1135,1508,1492,1451
ROCK1_5_6807,1223,1737,1399,1470,1310,1041,865,1002,813
ROCK2_5_6808,416,270,80,674,723,111,448,8,328
ROS1_5_6809,414,616,390,828,207,730,240,194,8
RPS6KA3_5_6810,2829,3789,3803,2883,2646,2360,4235,3073,3321
RPS6KA6_5_6811,2546,2751,3346,3315,2822,2940,1059,1921,2058
RPS6KB1_5_6812,1903,2837,3231,1309,3736,2533,2561,1849,3136
RPS6KB2_5_6813,1773,1667,3176,4596,1160,1648,838,3872,2483
RPS6KL1_5_6814,242,148,423,295,57,362,287,479,839
RSPO3_5_6815,117,204,482,133,189,241,511,22,1
SACM1L_5_6816,506,758,713,683,1509,1922,1369,435,754
SBF1_5_6817,364,559,559,12,146,396,934,605,10
SBK1_5_6818,256,163,743,192,1188,17,206,367,1193
SBK2_5_6819,168,349,244,26,164,51,256,0,34
SEPHS2_5_6820,3108,3109,2599,2581,2600,2838,2264,1539,3361
SGPP1_5_6821,748,543,887,501,52,496,773,259,431
SHPK_5_6822,224,776,484,321,1085,381,67,226,1
SIK1_5_6823,305,451,273,32,1,265,232,1,1205
SIK2_5_6824,2374,2214,2013,2365,1733,2317,3053,2034,3386
SIK3_5_6825,146,329,583,493,10,145,48,184,35
SKAP2_5_6826,1013,604,1425,467,1522,1534,1084,836,692
SMG1_5_6827,921,731,1256,956,939,1439,650,930,941
SRMS_5_6828,289,277,12,20,11,0,0,276,8
SRP72_5_6829,1188,1320,927,960,397,574,978,735,1005
SRPK1_5_6830,423,288,739,735,671,344,983,350,486
SSH2_5_6831,2291,1837,2531,1219,2288,3942,1396,1516,3259
SSH3_5_6832,485,286,288,691,33,369,18,264,193
STC1_5_6833,4043,4957,6287,3028,3797,4880,3485,3496,3147
STK10_5_6834,970,934,949,120,1085,342,1428,764,674
STK11_5_6835,576,1190,822,2379,215,292,690,1329,1539
STK16_5_6836,480,389,472,100,313,41,239,429,556
STK17A_5_6837,1286,1409,1381,865,1380,629,599,2041,1390
STK17B_5_6838,3404,3988,5284,3615,4057,4864,2314,3234,8230
STK25_5_6839,215,79,119,136,112,325,97,371,1408
STK32B_5_6840,892,697,913,692,2492,1831,850,338,1664
STK32C_5_6841,467,110,525,459,81,6,695,0,7
STK33_5_6842,6470,6113,6513,3191,7233,7474,7606,4579,6065
STK35_5_6843,149,89,153,42,513,272,64,40,0
STK38_5_6844,271,465,830,8,513,1430,225,175,776
STK38L_5_6845,4514,2588,4781,2252,3958,3484,3110,2901,2688
STK39_5_6846,553,426,1204,1186,833,1055,419,373,1551
STK40_5_6847,1504,1449,1620,275,675,327,1013,1322,1870
STK4_5_6848,574,1033,1300,797,1680,890,439,205,1433
STYK1_5_6849,414,324,846,653,127,751,494,930,298

STYXL1_5_6850,2998,2646,3371,922,5122,3703,2467,1748,2665
TAF1L_5_6851,480,358,481,346,691,808,81,97,559
TAOK3_5_6852,413,290,598,335,678,1363,1004,1112,141
TBK1_5_6853,668,2463,2116,1065,2604,799,674,1640,2402
TEC_5_6854,432,514,354,761,229,516,953,246,80
TEK_5_6855,1299,2876,2043,2115,3350,5281,1078,1529,1836
TESK1_5_6856,92,223,74,75,12,0,600,195,790
TESK2_5_6857,1240,778,1004,357,921,1416,984,1720,390
THNSL1_5_6858,156,746,225,47,596,8,161,1417,3
TIE1_5_6859,404,852,486,49,139,24,90,95,395
TK1_5_6860,1487,1134,1755,1785,406,357,1667,1778,1364
TNK1_5_6861,203,813,642,202,568,852,478,231,109
TNNI3K_5_6862,2692,1840,2786,2927,2936,2838,1502,1737,2765
TNS3_5_6863,3433,3806,3679,1208,4015,4295,2266,2458,4605
TP53RK_5_6864,175,429,431,29,162,144,298,83,3
TRAT1_5_6865,2211,1693,2441,2210,1543,3587,3459,3357,3186
TRIB1_5_6866,239,161,160,238,487,38,32,245,9
TRIB2_5_6867,2896,2285,3097,1611,2607,2218,3180,2196,5487
TRIB3_5_6868,126,571,238,0,436,5,216,353,1074
TRIM27_5_6869,1728,1094,2143,923,2196,2270,1406,2118,4282
TRIM28_5_6870,179,92,194,172,51,294,48,26,85
TRIO_5_6871,383,312,642,902,784,545,496,738,426
TRPM7_5_6872,1319,1306,2604,2255,1047,1589,2279,978,3442
TRRAP_5_6873,427,280,489,616,498,349,194,139,199
TSKS_5_6874,157,142,125,69,18,160,17,11,44
TSSK1B_5_6875,1957,3587,3041,1905,4563,2727,1865,2370,1917
TSSK2_5_6876,1108,1142,1347,1790,167,126,496,412,1046
TSSK3_5_6877,225,91,126,532,50,639,221,66,1
TSSK6_5_6878,1307,1035,570,817,1518,962,1370,203,431
TTBK1_5_6879,625,156,244,80,146,349,296,51,105
TTBK2_5_6880,1072,1534,1061,1696,2392,447,686,1143,1526
TWF2_5_6881,309,510,667,719,186,1043,421,116,521
TXK_5_6882,1483,1194,1891,2521,1604,1554,718,3469,4086
TYK2_5_6883,465,858,894,21,726,1760,82,312,1092
TYRO3_5_6884,142,251,125,320,414,601,62,16,708
UBLCP1_5_6885,946,448,1897,1145,582,1618,1040,103,479
UCK2_5_6886,619,262,574,449,696,645,197,375,1762
ULK1_5_6887,3283,3328,4105,3363,3801,4718,2513,3385,4195
ULK3_5_6888,1826,1293,2117,1963,1713,2322,2496,2021,474
ULK4_5_6889,456,409,615,637,590,1134,403,928,1283
VRK1_5_6890,1452,1023,1192,588,696,2072,1799,1106,1623
WEE2_5_6891,388,415,528,275,758,979,916,455,596
WNK2_5_6892,278,312,503,1009,2,292,316,283,188
WNK4_5_6893,290,317,328,6,54,790,540,717,0
XRCC6BP1_5_6894,2412,1522,2010,727,2360,802,872,1186,1014
XYLB_5_6895,687,911,293,1090,246,2,221,785,926
YES1_5_6896,524,650,814,533,1192,690,1388,980,1091
AATK_5_6897,67,154,218,95,14,18,166,392,231
ABL1_5_6898,34,145,241,1,0,0,744,468,147
ABL2_5_6899,664,658,453,1676,720,725,226,1180,697

ACP1_5_6900,141,209,348,330,7,65,144,429,368
ACP5_5_6901,269,299,224,0,228,462,63,29,33
ACPL2_5_6902,720,540,725,2163,184,559,599,899,1604
ACPP_5_6903,422,720,336,132,191,287,206,645,45
ACVR1B_5_6904,609,690,843,509,0,749,154,182,1411
ACVR1C_5_6905,397,546,523,509,1124,130,722,227,196
ACVR1_5_6906,270,335,329,551,24,59,476,220,188
ACVRL1_5_6907,338,525,471,250,91,2057,635,53,256
ADCK1_5_6908,930,446,302,159,1648,81,182,829,213
ADCK4_5_6909,184,97,250,160,130,253,604,229,214
ADK_5_6910,934,1238,2191,1527,1860,1882,632,271,2005
AK2_5_6911,0,102,68,0,0,0,242,0,0
AK4_5_6912,1131,1255,1401,975,523,719,1659,1934,2046
AK5_5_6913,497,1667,803,3,924,1995,764,944,164
AKT1_5_6914,49,418,137,1,63,985,655,0,0
AKT2_5_6915,895,1214,1547,415,1220,496,1016,1973,650
AKT3_5_6916,1485,1682,1597,1397,2055,1583,1031,1082,1701
ALDH18A1_5_6917,343,194,351,107,241,71,342,332,163
ALPK1_5_6918,1392,1517,2002,1172,2788,2288,2889,1515,4989
ALPL_5_6919,650,431,1283,207,24,212,250,161,1816
AMHR2_5_6920,168,74,256,27,174,14,269,343,293
ANKHD1_5_6921,541,1195,1019,1257,1972,256,1029,1133,2517
APTX_5_6922,342,739,730,256,406,618,770,1268,366
ATRIP_5_6923,1210,369,1016,300,834,1962,1132,403,846
AURKA_5_6924,946,1298,2006,1508,1563,969,1377,2918,1656
AURKC_5_6925,1492,1198,1321,973,1151,1718,442,1062,461
AXL_5_6926,1067,760,997,489,1518,822,730,1161,2264
BAIAP2_5_6927,120,306,274,6,45,633,124,5,886
BCKDK_5_6928,464,748,444,1458,352,93,438,547,482
BCR_5_6929,498,562,1176,442,383,7,451,705,575
BMP2K_5_6930,2014,1774,3133,2214,2933,785,1505,2154,1113
BMX_5_6931,1708,1178,1510,1121,1886,2124,956,1312,3184
BPGM_5_6932,4117,3151,2775,2736,4833,4482,2218,2272,2262
BRD2_5_6933,668,930,435,61,295,244,406,310,1363
BRD4_5_6934,321,439,723,613,684,781,512,168,1082
BRDT_5_6935,127,98,158,0,0,371,203,509,783
CAB39L_5_6936,6441,6479,7150,6113,9768,8723,4773,7157,8399
CAMK1D_5_6937,317,411,305,5,970,577,239,99,68
CAMK2A_5_6938,282,88,218,0,584,371,109,50,97
CAMK2B_5_6939,1160,1281,1065,831,728,1522,1837,981,3189
CAMK2D_5_6940,108,237,384,56,35,76,108,489,85
CAMK2G_5_6941,5359,4148,6613,3566,6546,6722,3393,4691,8880
CAMKK1_5_6942,203,269,591,75,19,655,60,168,6
CAMKK2_5_6943,326,1028,809,866,1345,52,487,570,544
CASK_5_6944,730,978,1508,1398,56,382,1203,1030,161
CCT2_5_6945,789,770,918,1693,908,1564,853,57,565
CDADC1_5_6946,4199,3882,6264,4313,3977,5534,5956,4387,6534
CDC14A_5_6947,758,1001,1268,1117,2302,1467,664,1859,1724
CDC14B_5_6948,2182,2004,3227,697,1108,3477,1365,1818,2053
CDC25A_5_6949,1183,829,1742,612,91,1260,724,1457,919

CDC25B_5_6950,243,147,161,134,61,161,574,9,20
CDC25C_5_6951,888,1220,1761,2289,904,1666,1454,1569,3418
CDC42BPA_5_6952,855,1464,1338,1250,286,999,1771,637,1159
CDC7_5_6953,772,333,408,697,3,331,470,340,484
CDK10_5_6954,263,385,252,339,286,738,361,1119,41
CDK11A_5_6955,939,2118,2279,2133,2621,1646,1238,988,1017
CDK11B_5_6956,939,2118,2279,2133,2621,1646,1238,988,1017
CDK12_5_6957,852,1080,998,498,369,1249,571,1042,1039
CDK13_5_6958,4923,4070,3555,2277,971,3721,2956,2046,2119
CDK16_5_6959,361,402,322,685,545,568,446,357,899
CDK17_5_6960,898,896,834,282,766,1601,718,389,528
CDK18_5_6961,2028,3197,2952,3504,2039,3400,3780,2133,7366
CDK20_5_6962,187,854,410,311,1083,837,343,1,0
CDK2_5_6963,121,285,528,615,448,1087,155,398,908
CDK5_5_6964,560,345,797,31,504,688,264,409,156
CDK6_5_6965,1511,1532,2612,3286,3991,2128,1436,3209,1104
CDKL3_5_6966,400,374,419,202,1312,517,708,369,13
CDKL5_5_6967,1336,1818,1413,1241,520,3373,2076,1373,830
CDKN1A_5_6968,578,453,272,8,1952,309,607,200,171
CDKN3_5_6969,105,143,520,129,302,220,213,193,459
CHEK1_5_6970,1871,1689,1681,393,624,834,1863,184,2672
CHEK2_5_6971,187,329,101,97,546,146,83,32,3
CHKA_5_6972,1452,2224,2557,2867,2926,1410,1431,1730,1780
CIT_5_6973,174,27,91,415,1024,48,197,46,17
CKMT2_5_6974,109,1,17,0,1,3,184,3,16
CLK1_5_6975,2585,2862,3112,3277,3290,3656,3287,4817,2749
CLK3_5_6976,404,622,664,426,1434,2038,632,1371,969
CMPK1_5_6977,3399,3842,4959,4627,5074,5393,3715,3082,3925
COASY_5_6978,557,718,705,1,362,163,706,406,96
COL4A3BP_5_6979,1266,1795,2779,2281,458,1446,1965,1336,1323
CSK_5_6980,260,501,349,625,1832,691,792,465,937
CSNK1A1_5_6981,3859,3384,4683,1827,2995,4404,3111,3207,4682
CSNK1D_5_6982,1590,1898,1764,1277,2316,743,1600,2140,364
CSNK1E_5_6983,1681,2172,1919,488,735,3223,1237,2237,1228
CSNK1G3_5_6984,3569,2075,3094,2208,5393,3433,3543,1734,5397
CTDP1_5_6985,83,5,17,4,0,30,84,0,113
CTDSP1_5_6986,1721,2356,2988,1524,1630,2249,3257,3126,2596
DCLK1_5_6987,808,1003,1198,419,1625,1001,703,590,671
DCLK2_5_6988,1198,1033,1408,698,1462,796,544,1847,3119
DDR1_5_6989,189,157,351,83,218,459,700,244,2
DDR2_5_6990,107,341,282,25,106,185,80,3,30
DGKA_5_6991,0,0,0,0,0,0,0,0,0
DGKB_5_6992,1464,2004,2250,1575,2271,1267,1464,1122,2207
DGKD_5_6993,1364,1206,1465,222,2411,575,2075,1060,2232
DGKG_5_6994,2947,3219,3962,2082,3560,6713,3387,4586,6123
DGKH_5_6995,526,1063,1173,426,2338,890,972,1126,267
DGKZ_5_6996,1525,1036,1040,1205,305,1585,1105,1443,1038
DGUOK_5_6997,266,592,575,424,1595,812,446,120,1060
DLGAP5_5_6998,540,220,490,379,239,399,782,771,345
DMPK_5_6999,210,197,513,38,127,438,318,12,1252

DSTYK_5_7000,2626,2997,2093,1851,3219,1153,1585,2032,4718
DTYMK_5_7001,1124,846,1908,1090,1522,1584,1043,1108,1059
DUSP13_5_7002,1391,783,1528,1011,1315,1074,2647,953,1257
DUSP19_5_7003,169,170,453,180,368,14,376,132,1015
DUSP4_5_7004,0,0,0,0,0,0,0,0,0
DUSP6_5_7005,970,1908,1170,1987,1363,2378,627,893,2902
DYRK1A_5_7006,469,279,794,527,345,531,776,65,612
DYRK1B_5_7007,678,1207,962,1039,1125,798,532,181,441
DYRK2_5_7008,56,754,789,6,2188,541,564,1152,346
DYRK3_5_7009,1462,1360,2044,629,1955,551,1039,1432,1958
EFNA4_5_7010,279,465,422,211,579,1171,275,119,689
EGFR_5_7011,458,835,222,415,802,112,189,567,1298
EIF2AK2_5_7012,2108,3312,3104,2529,1569,3523,766,1384,3123
EPHA10_5_7013,1420,940,539,93,1462,645,617,815,124
EPHA3_5_7014,509,468,811,554,8,1047,848,47,956
EPHA5_5_7015,21,158,371,24,54,499,81,105,230
EPHA6_5_7016,2194,1778,2823,556,2595,1313,962,1310,330
EPHA8_5_7017,2565,2107,3038,2132,1878,2428,1520,1596,3313
EPHB2_5_7018,2,99,288,0,534,0,0,0,165
EPM2A_5_7019,100,138,18,0,39,16,0,9,0
ERBB2_5_7020,2046,1943,2379,2035,1677,2835,3318,1409,1950
ERBB4_5_7021,1246,1410,1540,1559,1011,2969,1116,1032,1074
ETNK1_5_7022,673,1425,951,726,50,1285,584,293,298
EXOSC10_5_7023,3593,4362,6871,4786,1707,5391,5060,2956,4838
EYA1_5_7024,461,239,517,302,267,276,101,146,47
EYA2_5_7025,166,246,283,1,30,474,460,301,0
EYA4_5_7026,1569,1893,1857,2810,1868,491,610,1280,368
FASTK_5_7027,797,895,1433,408,1471,1357,617,157,200
FBP1_5_7028,384,252,980,433,2053,202,570,200,611
FES_5_7029,495,190,89,3,0,145,0,243,0
FGFR1_5_7030,537,200,781,247,153,750,569,541,223
FGFR2_5_7031,717,418,1074,420,1732,796,778,751,368
FGFR3_5_7032,673,937,967,654,220,1836,658,1149,690
FGFR4_5_7033,338,212,294,830,12,0,90,160,558
FGFRL1_5_7034,1072,808,1529,841,1315,1015,456,485,2550
FGR_5_7035,0,0,243,0,2,3,0,19,0
FLT1_5_7036,2047,1899,2923,1852,1992,4450,1997,2021,1183
FLT4_5_7037,901,802,666,91,850,121,1095,679,436
FXN_5_7038,1439,1513,1458,1344,1064,507,525,656,2316
FYN_5_7039,253,283,158,728,607,684,20,7,150
FZR1_5_7040,886,527,574,159,189,270,726,565,877
GALK2_5_7041,1523,2173,2057,3259,1823,2790,1395,1324,2139
GK_5_7042,887,2562,1823,587,1552,3048,1712,1964,1159
GLYCTK_5_7043,348,201,299,17,431,379,38,132,65
GNE_5_7044,5117,5200,7453,4166,7485,6588,4669,3451,6707
GRK4_5_7045,657,545,1563,280,903,1236,1064,343,993
GRK6_5_7046,230,359,495,823,0,149,359,0,1
GSK3B_5_7047,1514,2000,2039,2843,4468,2672,2928,1116,1751
GUK1_5_7048,654,1897,909,425,405,686,819,297,739
HCK_5_7049,430,364,592,246,452,451,372,213,374

HIPK1_5_7050,1559,3010,2610,1101,2330,3554,2130,2749,2844
HIPK2_5_7051,501,480,546,394,363,461,232,736,1511
HIPK3_5_7052,1945,2051,2253,1152,1183,900,2340,2133,2739
ICK_5_7053,255,354,149,432,241,88,198,247,8
IKBKB_5_7054,409,481,223,2,22,0,17,1,984
IKBKE_5_7055,53,406,299,3,117,124,168,9,1030
IKBKG_5_7056,565,1203,897,687,865,2176,1269,549,1089
ILK_5_7057,313,30,112,11,71,153,66,24,28
IMPA1_5_7058,1206,1249,2016,435,964,1736,889,386,2171
INPP1_5_7059,807,450,811,392,1786,117,85,481,262
INPP4A_5_7060,1322,2066,2426,643,866,3186,1002,2509,3538
INPP4B_5_7061,734,496,566,1019,663,702,899,531,771
INPP5D_5_7062,497,393,731,23,315,0,689,720,123
INSR_5_7063,340,459,160,151,266,220,390,461,86
IP6K1_5_7064,449,220,272,188,134,208,164,39,5
IP6K3_5_7065,213,304,343,406,451,85,389,266,217
IRAK1_5_7066,146,104,427,3,36,70,256,37,476
IRAK3_5_7067,1481,2660,2220,1161,3569,2478,1666,2424,3841
IRAK4_5_7068,2164,2834,2448,2641,3984,3984,3198,2947,1649
ITPK1_5_7069,546,757,711,176,555,432,1302,211,700
KALRN_5_7070,474,943,1672,482,316,977,538,639,441
KHK_5_7071,409,267,345,289,25,718,161,8,90
KIF2A_5_7072,1066,1594,1913,1465,445,1882,2506,1340,1312
KIT_5_7073,170,212,98,154,337,1673,165,839,38
LCK_5_7074,607,570,1569,1216,588,686,515,826,662
LHPP_5_7075,457,375,604,160,959,31,232,246,504
LIMK1_5_7076,737,604,660,1029,913,649,206,724,1386
LIMK2_5_7077,316,435,547,60,334,302,110,312,238
LTK_5_7078,46,327,321,2,438,124,37,250,4
LYN_5_7079,2,1,17,102,0,261,0,2,25
MAGI3_5_7080,740,987,2155,1854,2453,2531,1545,475,757
MAP2K3_5_7081,378,653,1145,928,155,573,295,1082,408
MAP2K5_5_7082,1846,1928,2460,770,3579,825,1851,2572,2616
MAP3K12_5_7083,978,1067,692,154,304,1573,996,960,848
MAP3K13_5_7084,285,288,289,345,24,292,63,65,705
MAP3K3_5_7085,554,356,717,1021,593,1565,42,350,69
MAP3K4_5_7086,209,75,205,664,139,560,0,140,14
MAP3K7_5_7087,665,1462,1305,1349,1977,1113,715,1320,381
MAP4K1_5_7088,1322,1349,1274,2225,956,1193,1416,2132,1470
MAP4K4_5_7089,1055,1056,895,350,1,2269,340,1235,1286
MAP4K5_5_7090,638,165,476,1109,121,1503,227,62,73
MAPK10_5_7091,10,42,32,0,47,0,2,47,0
MAPK14_5_7092,2265,2748,3019,2307,3184,2228,2626,1565,1602
MAPK1_5_7093,585,1512,865,39,838,506,1167,1335,1232
MAPK3_5_7094,1968,1082,1673,1822,1153,1684,1343,594,1254
MAPK7_5_7095,236,228,295,374,40,620,154,13,241
MAPK8_5_7096,240,405,382,232,0,493,676,661,290
MAPK9_5_7097,1131,1116,1342,1574,1339,483,1318,798,806
MAPKAPK2_5_7098,1613,1121,980,1272,146,1091,1010,389,2603
MAPKAPK5_5_7099,37,188,188,1,0,18,211,5,270

MARK2_5_7100,124,319,133,409,87,317,337,326,9
MARK3_5_7101,223,160,181,18,75,568,850,139,300
MARK4_5_7102,322,404,684,32,1022,816,349,749,211
MAST4_5_7103,543,633,560,382,32,1218,462,437,1657
MASTL_5_7104,1634,1527,1921,844,3329,3025,2968,1718,1853
MATK_5_7105,131,77,214,13,14,364,0,10,190
MET_5_7106,493,575,1015,165,517,1071,972,71,926
MINK1_5_7107,577,676,1040,42,1199,1483,640,1405,346
MINPP1_5_7108,1067,978,1765,2649,1499,1641,1376,1781,2795
MKNK1_5_7109,2548,2592,3251,1670,2692,3985,2621,1537,2864
MKNK2_5_7110,632,1629,1776,400,722,868,206,653,1468
MLKL_5_7111,17,93,369,203,56,137,452,4,233
MTMR14_5_7112,471,156,1183,634,300,302,610,808,1140
MTMR2_5_7113,2164,2194,2163,961,1787,2951,1140,2150,1560
MTMR3_5_7114,190,573,527,898,2,1612,393,257,869
MUSK_5_7115,610,1050,810,107,2211,87,1154,445,703
MVK_5_7116,92,431,73,61,90,9,1556,367,0
MYO3B_5_7117,556,639,601,25,893,772,391,383,498
NADK_5_7118,368,156,483,101,222,523,119,499,203
NCK1_5_7119,586,509,412,165,91,191,346,131,497
NCK2_5_7120,567,272,515,505,235,12,342,674,228
NDRG1_5_7121,3,1,64,0,1,1,171,0,327
NEK11_5_7122,3500,4695,3985,3646,5996,6100,4335,3754,7805
NEK1_5_7123,5165,6989,5820,5384,7445,6725,6099,4907,6585
NEK2_5_7124,1321,713,916,1275,907,817,1000,1158,718
NEK3_5_7125,1914,1969,2164,1554,3041,2715,873,477,2365
NEK4_5_7126,587,640,555,1187,321,2,251,473,834
NEK6_5_7127,377,180,324,153,467,187,343,135,22
NME2_5_7128,236,170,199,2,14,335,40,627,1
NME7_5_7129,673,1004,1877,359,1350,1590,1186,1735,1772
NT5C1B_5_7130,5562,6304,7170,6910,4288,8828,5530,6983,9174
NT5C2_5_7131,2510,2725,2642,1210,3235,3110,1567,2245,3663
NT5C3_5_7132,711,1015,1366,1230,918,970,625,700,527
NT5E_5_7133,332,685,691,1170,423,1581,821,77,298
NTRK1_5_7134,1994,1935,1709,1184,2127,2198,756,1403,3522
NTRK2_5_7135,265,177,887,773,0,696,469,2,1593
NTRK3_5_7136,101,240,236,82,73,20,226,695,140
NUDT4_5_7137,2067,1710,2081,2134,627,2632,1705,2330,1142
NUDT9_5_7138,1565,2411,2596,1792,3141,2680,1490,1775,3956
OBSCN_5_7139,357,382,403,849,608,498,131,256,1172
OCRL_5_7140,1469,1432,2103,646,1091,2538,964,1757,1684
OXSM_5_7141,1810,2046,1866,999,1145,2458,1452,1120,1751
PACSIN1_5_7142,1362,1649,2266,1006,1458,1293,1702,2111,1232
PAK4_5_7143,418,479,435,288,62,425,155,130,466
PAK6_5_7144,202,50,248,24,116,331,147,512,240
PAK7_5_7145,3088,2331,2275,1261,4524,3261,1581,2911,1649
PANK1_5_7146,358,565,590,523,1190,394,665,396,1009
PANK2_5_7147,3853,2845,4352,3754,2812,2579,2711,2449,2587
PCK2_5_7148,67,63,244,62,180,17,49,504,54
PDK2_5_7149,1056,871,1189,582,1737,1370,1059,764,1188

PDK3_5_7150,416,477,634,225,351,418,608,676,2331
PDPK1_5_7151,716,649,1069,1100,2005,1167,561,512,266
PEG3_5_7152,1512,1740,2666,1984,1929,2920,1375,1462,3970
PFKFB2_5_7153,1212,698,786,118,663,1157,864,948,1213
PFKFB3_5_7154,871,545,636,305,438,666,776,250,683
PFKM_5_7155,555,403,666,616,1414,1663,869,554,114
PFKP_5_7156,462,444,834,634,106,1606,1025,213,880
PHKA1_5_7157,1080,993,1062,549,2394,1004,1909,1492,1211
PHKB_5_7158,826,1172,1608,1065,1101,2309,981,547,1897
PHKG2_5_7159,398,294,170,756,172,1061,544,1003,146
PHPT1_5_7160,486,419,760,1106,743,516,1101,462,820
PI4KA_5_7161,2451,2911,3133,3230,3662,3432,1685,2550,1716
PI4KB_5_7162,491,186,576,1031,112,51,75,109,349
PIK3R1_5_7163,51,169,376,186,97,390,347,271,6
PIK3R5_5_7164,2488,2546,2439,3018,5292,2139,3421,1830,2346
PIKFYVE_5_7165,1746,1610,1807,4331,1575,1903,950,3024,2616
PIP4K2C_5_7166,600,596,765,304,1668,727,366,573,251
PIP5K1A_5_7167,2140,1929,2840,2277,2575,2203,2017,2315,2808
PIP5K1C_5_7168,134,269,172,242,9,41,15,376,717
PIP5KL1_5_7169,181,463,59,4,10,273,287,10,6
PKLR_5_7170,211,299,238,291,23,417,66,100,271
PKMYT1_5_7171,156,214,9,592,118,11,66,0,0
PKN1_5_7172,605,158,424,234,944,463,697,260,3
PLK4_5_7173,1953,3270,1933,3283,1340,1465,880,3033,1950
PNCK_5_7174,523,727,786,27,337,128,1488,28,331
PODXL_5_7175,164,623,356,414,264,403,366,550,100
PPAP2A_5_7176,167,210,22,0,0,0,149,27,0
PPAP2C_5_7177,56,27,87,0,90,74,0,410,253
PPEF1_5_7178,6448,7751,8422,8395,8644,6310,6822,5821,6108
PPFIA1_5_7179,581,381,589,28,225,1485,512,216,1625
PPFIA2_5_7180,7525,8302,8303,7471,7651,12800,6243,7762,9487
PPM1A_5_7181,211,318,186,141,458,44,586,164,724
PPM1B_5_7182,993,1573,1264,234,849,772,1535,1061,1405
PPM1M_5_7183,494,533,415,655,804,246,53,561,32
PPP1CA_5_7184,1144,1201,753,1202,121,276,153,593,1065
PPP1CB_5_7185,1208,847,791,1631,1186,264,1140,699,1158
PPP1R12A_5_7186,1966,2272,3551,2481,1012,1470,2011,1062,3003
PPP1R16B_5_7187,3344,3528,3578,2722,3616,2887,3014,2770,1830
PPP1R1B_5_7188,189,402,426,223,1265,375,371,446,1426
PPP1R8_5_7189,144,117,201,11,117,257,24,0,0
PPP2R1B_5_7190,292,46,323,352,205,716,255,226,5
PPP2R2A_5_7191,16331,14046,20521,12533,17774,14986,20569,15001,24076
PPP2R2B_5_7192,210,112,273,696,189,1200,172,596,136
PPP2R2C_5_7193,479,644,1372,666,610,1422,965,505,1785
PPP2R3A_5_7194,1194,1786,1410,813,910,1254,1149,3742,985
PPP2R4_5_7195,2287,3468,2859,479,4009,5453,3189,3422,4497
PPP2R5A_5_7196,1248,1255,1745,589,991,1703,1053,1513,2376
PPP2R5D_5_7197,607,671,945,474,368,817,755,615,187
PPP3CA_5_7198,2886,3246,3080,2105,2732,2076,2214,5122,5180
PPP3CB_5_7199,3471,3303,3863,3428,1818,2837,3675,2278,6334

PPP4R1_5_7200,379,327,720,1253,2035,554,506,417,76
PPP5C_5_7201,795,462,815,574,611,217,97,345,317
PPP6C_5_7202,366,188,484,918,116,151,364,446,876
PRKAA1_5_7203,3032,4857,5085,2309,1636,3960,4431,6007,4223
PRKACA_5_7204,1081,2621,2184,2129,2491,2630,1202,3346,2322
PRKACB_5_7205,1378,1499,2676,3358,1465,1945,2719,2703,3116
PRKAG1_5_7206,839,1051,1224,733,1741,354,1229,369,714
PRKAG2_5_7207,299,393,492,297,278,287,492,236,351
PRKAR1A_5_7208,1428,1001,1132,3171,2190,628,893,1187,1830
PRKAR1B_5_7209,1867,1700,1588,1120,2248,1836,626,1243,2708
PRKCB_5_7210,677,1178,532,396,452,1673,278,213,2581
PRKCD_5_7211,416,708,339,170,1107,457,865,705,623
PRKCQ_5_7212,1713,1198,2399,1063,1436,2372,1646,1114,1667
PRKCZ_5_7213,701,631,896,565,263,1385,1146,808,1997
PRKD2_5_7214,328,494,139,10,201,24,474,215,151
PRKDC_5_7215,586,412,711,654,307,480,160,186,48
PRKG1_5_7216,214,287,370,0,5,320,23,641,18
PRPS2_5_7217,988,1050,921,394,2622,433,1807,307,3643
PTK2B_5_7218,219,422,323,178,107,225,58,34,9
PTK2_5_7219,1476,1930,2142,1772,1677,2427,1056,1211,2587
PTK7_5_7220,393,141,241,925,0,77,281,51,130
PTP4A2_5_7221,258,813,432,1252,21,53,365,61,1119
PTP4A3_5_7222,521,1009,805,1077,963,386,624,608,1589
PTPDC1_5_7223,804,383,270,38,412,871,319,864,100
PTPN12_5_7224,880,2092,1025,2773,373,856,502,2929,395
PTPN13_5_7225,682,722,904,323,1163,2124,1288,1112,529
PTPN18_5_7226,495,214,142,25,100,847,141,103,13
PTPN22_5_7227,2107,2617,2619,2115,3480,3087,3629,1462,4303
PTPN2_5_7228,2686,3949,4223,1847,6208,4636,1806,2429,6820
PTPN3_5_7229,922,568,653,201,1131,727,1329,952,276
PTPN5_5_7230,184,142,218,225,125,714,957,184,1365
PTPN6_5_7231,591,611,655,153,362,549,454,399,489
PTPN7_5_7232,640,472,908,249,695,251,866,3527,802
PTPRA_5_7233,2995,3023,3596,2504,2924,2608,1509,2348,1916
PTPRB_5_7234,9217,8141,9785,5130,10083,9173,6665,7647,8097
PTPRC_5_7235,1167,1340,1262,1965,1292,1385,1333,758,1813
PTPRD_5_7236,2037,1677,1350,763,2764,1053,1422,1952,1946
PTPRE_5_7237,1705,2214,1524,1010,1525,2721,1629,1002,1197
PTPRF_5_7238,255,90,482,228,3,362,156,171,115
PTPRH_5_7239,1725,1565,1585,992,1920,2234,1616,860,1296
PTPRJ_5_7240,711,841,869,461,1684,504,333,995,1026
PTPRK_5_7241,0,7,18,0,0,0,0,12,0
PTPRM_5_7242,4801,4048,4828,4913,3255,6152,4979,4325,4281
PTPRN2_5_7243,439,113,223,4,15,28,536,11,19
PTPRN_5_7244,994,1684,1826,573,2266,2014,1196,834,250
PTPRR_5_7245,173,163,164,877,15,714,6,2,280
PTPRS_5_7246,952,777,584,1366,242,2162,391,95,894
PTPRT_5_7247,639,629,1636,876,699,620,46,827,2352
PTPRU_5_7248,143,271,29,13,454,26,151,83,131
PTPRZ1_5_7249,9482,9472,10451,6415,12285,11106,10269,7908,12055

RET_5_7250,12,40,94,27,0,0,1,0,0
RIOK1_5_7251,2146,4359,3053,1557,3593,2991,1905,3310,2844
RIOK2_5_7252,3049,3306,4596,4178,2988,2273,1224,3344,7599
ROPN1L_5_7253,435,561,346,722,760,307,358,158,775
RPS6KA1_5_7254,1015,956,1226,734,1942,795,902,450,1244
RPS6KA2_5_7255,151,389,173,0,465,19,277,114,0
RPS6KA4_5_7256,204,5,85,5,7,0,363,22,874
RPS6KA5_5_7257,1089,1077,2489,2240,1624,1891,3087,830,3838
RPS6KC1_5_7258,421,577,1107,1188,38,1042,404,1043,24
RYK_5_7259,2537,4028,4028,1812,4298,2656,2724,2354,4675
SET_5_7260,262,274,193,17,206,366,53,18,138
SGK1_5_7261,758,1458,1327,866,1282,899,1175,665,1490
SGK3_5_7262,2120,2101,3129,2338,5400,2468,1999,4421,7336
SIRPA_5_7263,677,1094,491,275,1054,611,741,186,488
SKAP1_5_7264,873,322,647,791,754,724,219,993,447
SNRK_5_7265,142,695,427,0,824,1,26,338,895
SPHK1_5_7266,235,723,244,58,824,294,291,1374,17
SPHK2_5_7267,199,58,124,762,1,4,75,138,0
SRC_5_7268,36,12,36,0,221,0,7,135,0
SRPK2_5_7269,116,331,239,163,141,313,543,104,52
SRPK3_5_7270,71,187,144,216,165,830,195,224,788
SSH1_5_7271,680,962,717,163,824,101,1215,663,277
STK19_5_7272,654,496,862,53,454,1063,690,426,2261
STK24_5_7273,1827,2346,3008,2529,3191,2520,1652,2697,857
STK31_5_7274,229,169,472,346,434,1112,637,8,2180
STK36_5_7275,227,7,115,2,46,66,0,321,7
STRADA_5_7276,487,947,1061,268,914,1682,1393,1531,750
STRADB_5_7277,316,475,431,54,350,583,443,832,355
STYX_5_7278,1638,2914,2317,3155,2468,4708,2655,1759,3083
SYK_5_7279,1488,1648,1854,1421,2082,1738,1705,2940,2443
SYNJ1_5_7280,6100,9442,9550,5135,10718,8765,6658,7982,10442
SYNJ2_5_7281,2569,3945,2976,2482,2823,2972,2385,3383,3263
TAF1_5_7282,1142,574,1207,976,1825,2229,1878,990,2969
TAOK1_5_7283,758,704,1122,607,2873,832,504,999,1836
TAOK2_5_7284,107,276,61,0,34,325,35,254,7
TBCK_5_7285,737,1118,1126,755,594,939,530,1438,1103
TEX14_5_7286,339,377,763,112,88,494,535,422,939
TGFBR1_5_7287,565,302,412,80,77,434,169,401,130
TGFBR2_5_7288,510,378,360,121,507,149,76,197,121
TGFBR3_5_7289,321,397,425,825,139,209,50,928,441
THTPA_5_7290,2601,2131,3802,3609,3067,2583,3201,2103,2079
TJP2_5_7291,779,899,1074,908,191,419,1002,685,788
TK2_5_7292,4727,4154,5157,4092,3566,6262,5138,3815,5204
TLK1_5_7293,2412,3188,2132,1039,2153,3401,1449,2157,2792
TLK2_5_7294,10816,13789,13483,5757,11526,12401,8836,8380,13562
TMEM134_5_7295,1257,803,2802,1031,1428,815,1646,1139,1618
TNIK_5_7296,1866,2227,1435,367,1324,955,1434,1669,928
TNK2_5_7297,304,491,1035,386,706,851,521,167,1168
TPK1_5_7298,210,148,482,551,42,9,577,734,23
TPTE2_5_7299,3275,3039,4111,2705,2279,3964,2997,2183,4113

TPTE_5_7300,12732,19058,14984,10998,15396,14378,12172,12821,17829
TRIM24_5_7301,1043,1183,2691,400,1246,705,621,983,3690
TRIM33_5_7302,1596,2664,3731,1411,3298,4504,2229,2069,1037
TRMT2A_5_7303,92,664,841,205,34,450,690,1147,401
TRPM6_5_7304,63,74,90,136,148,338,18,52,209
TSSK4_5_7305,1184,1534,1292,1086,483,772,1607,2491,1086
TTK_5_7306,3571,3210,4322,3000,5214,4930,3205,1968,2720
TTN_5_7307,812,742,1816,566,2423,1608,200,869,3192
TWF1_5_7308,265,210,38,559,98,93,439,93,785
UCK1_5_7309,702,486,489,990,704,534,197,187,79
UCKL1_5_7310,283,543,775,3020,707,527,1803,111,207
UHMK1_5_7311,323,459,851,457,765,872,136,506,743
ULK2_5_7312,1831,2503,1702,710,1399,1189,1969,732,1025
VRK2_5_7313,646,553,1280,608,1452,1241,1026,721,827
VRK3_5_7314,1315,1386,903,1138,3234,872,1646,1113,1680
WEE1_5_7315,360,435,807,615,1487,551,340,1109,370
WNK1_5_7316,471,383,1015,96,250,1225,465,699,637
WNK3_5_7317,4762,4759,6088,6298,4569,4839,3559,5246,5938
YSK4_5_7318,907,1170,994,979,833,761,812,650,1859
ZAP70_5_7319,169,15,180,96,66,0,74,38,2
ACP2_5_7320,83,781,513,162,223,1193,256,252,544
CDK1_5_7321,801,1825,1487,2099,123,989,2245,691,2723
DUSP10_5_7322,1218,1037,1230,1177,911,1429,430,261,1925
DUSP15_5_7323,3006,2782,3269,3947,4040,2943,2341,2090,5130
ERBB3_5_7324,211,318,377,15,882,1057,104,15,479
G6PC2_5_7325,913,407,853,811,371,1116,312,1379,1022
IP6K2_5_7326,1736,2341,2493,1233,1735,1882,1006,1102,3761
MYLK_5_7327,1370,1344,1620,1691,1309,1153,310,690,1667
PRPS1_5_7328,1964,2583,2789,1393,2406,3364,1678,2000,2069
PTPMT1_5_7329,222,270,550,276,303,394,193,142,1019
PTPN20A_5_7330,5379,6038,7598,5196,6575,7808,4633,5288,11832
PTPN20B_5_7331,5379,6038,7598,5196,6575,7808,4633,5288,11832
SPEG_5_7332,261,270,711,905,9,121,394,11,266
STK32A_5_7333,2736,4092,2869,2184,3673,3245,2621,4397,2006
PPP1R12B_5_7334,280,119,692,171,90,685,157,27,791
PPP1R12B_5_7335,782,582,919,477,175,696,551,333,302
PPP1R12B_5_7336,903,725,974,749,653,939,942,347,322
AAK1_5_7337,1095,604,1708,1390,2632,1514,2705,1723,1685
ACP6_5_7338,739,590,1764,525,1634,897,907,762,3384
ACPT_5_7339,205,137,134,0,0,718,18,43,0
ACVR2A_5_7340,477,733,271,343,695,307,840,0,369
ACVR2B_5_7341,1,24,7,32,162,12,22,0,0
ADCK2_5_7342,489,986,868,130,1272,592,449,748,14
ADCK3_5_7343,235,148,172,14,25,86,42,176,6
ADCK5_5_7344,602,443,677,557,38,533,305,48,674
ADPGK_5_7345,408,921,740,363,676,260,400,121,556
ADRBK1_5_7346,242,176,91,93,11,89,356,261,3
ADRBK2_5_7347,535,530,529,799,1883,648,1088,429,41
AGK_5_7348,706,787,1200,1048,225,378,1288,878,1100
AK1_5_7349,1504,1090,1695,476,3572,1580,855,765,600

AK7_5_7350,645,497,611,275,42,133,73,525,348
AK8_5_7351,1266,666,1806,1208,288,2333,1263,1792,1102
ALK_5_7352,73,114,504,176,125,13,104,220,0
ALPI_5_7353,1677,2475,2770,2237,1604,794,2254,1206,4286
ALPK2_5_7354,291,452,486,554,1348,535,421,290,150
ALPK3_5_7355,181,429,1,0,25,53,0,108,25
ALPP_5_7356,814,809,1165,1487,2037,843,288,984,725
ALPPL2_5_7357,814,809,1165,1487,2037,843,288,984,725
ANKK1_5_7358,1156,1302,846,466,549,830,1058,1100,1499
ARAF_5_7359,2202,2011,2453,732,1374,3701,1390,2937,3722
ATM_5_7360,223,383,259,981,442,111,1435,398,57
AURKB_5_7361,448,861,421,299,359,10,446,1161,1250
BLK_5_7362,1685,480,1218,117,1282,749,1364,591,2073
BMPR1A_5_7363,452,429,345,305,243,1022,1147,412,443
BMPR1B_5_7364,1841,2004,2267,2155,2936,2215,673,1912,4006
BMPR2_5_7365,381,170,153,202,589,190,708,293,869
BPNT1_5_7366,2419,2914,3487,648,2971,2572,2336,3151,5575
BRAF_5_7367,4143,5263,6138,2553,8409,5558,5600,3828,5360
BRD3_5_7368,514,1294,1037,318,3782,496,818,1599,775
BRSK1_5_7369,623,646,598,57,989,302,723,464,38
BRSK2_5_7370,25,183,385,0,92,1803,193,28,299
BTK_5_7371,595,448,2060,1050,636,1400,237,496,1641
BUB1B_5_7372,613,457,872,1040,126,673,1211,175,65
BUB1_5_7373,11093,12182,14521,7864,11054,14669,9350,10447,9762
C20orf111_5_7374,510,645,890,755,298,655,584,1990,847
C9orf96_5_7375,483,483,1332,79,1216,2027,943,738,1861
CALM1_5_7376,832,615,728,267,548,1359,1720,1139,839
CALM3_5_7377,146,303,278,106,1000,601,168,381,465
CAMK1_5_7378,2794,2700,3634,1749,1994,2762,1370,1120,3284
CAMK1G_5_7379,409,690,520,91,799,27,162,115,1428
CAMK2N1_5_7380,574,813,1013,538,1746,1137,514,1232,930
CAMK4_5_7381,7,135,100,0,465,0,44,217,2
CAMKV_5_7382,941,1049,1437,792,696,563,277,1421,581
CARD11_5_7383,223,253,477,492,396,392,59,245,232
CCL2_5_7384,1267,1734,2208,2995,2150,3040,2000,1065,796
CDC42BPB_5_7385,292,6,678,292,35,293,135,1,689
CDC42BPG_5_7386,178,431,95,174,155,255,389,289,0
CDK14_5_7387,349,600,442,577,114,0,294,259,1817
CDK15_5_7388,2453,1541,2390,2148,3737,2264,2401,1484,2291
CDK19_5_7389,356,941,443,352,825,1346,139,625,224
CDK3_5_7390,1196,982,1932,2152,885,1795,485,1400,1280
CDK4_5_7391,235,65,264,317,13,658,40,220,398
CDK5R1_5_7392,225,204,469,88,291,748,88,493,333
CDK7_5_7393,1899,1353,2127,2697,1461,2049,1154,1429,1525
CDK8_5_7394,1455,2022,1929,421,4030,2105,1467,1346,604
CDK9_5_7395,935,447,1162,1441,810,578,569,829,125
CDKL1_5_7396,261,119,346,240,33,3,222,291,45
CDKL2_5_7397,937,1520,1501,635,2084,1671,2035,886,619
CDKL4_5_7398,914,1041,1936,1162,1573,2052,542,1365,1659
CDKN1B_5_7399,426,426,349,202,0,259,871,374,39

CERK_5_7400,206,193,549,0,288,701,557,328,0
CHKB_5_7401,3520,2373,2670,3038,1845,2814,2847,1859,2032
CHUK_5_7402,372,209,970,374,587,476,145,558,21
CIB2_5_7403,170,350,507,225,240,13,21,248,1925
CILP_5_7404,947,412,1036,858,2726,1331,1262,618,703
CKB_5_7405,1,113,548,19,0,0,26,938,298
CKM_5_7406,669,1108,1420,206,927,2793,1198,730,1750
CKMT1A_5_7407,240,232,270,26,196,146,9,1461,289
CKMT1B_5_7408,240,232,270,26,196,146,9,1461,289
CKS1B_5_7409,14608,18755,22761,13431,15624,19222,15261,16972,30559
CKS2_5_7410,1485,1564,2206,1066,1149,1041,2015,1606,940
CLK2_5_7411,913,924,958,269,573,59,340,316,507
CLK4_5_7412,137,419,637,319,66,144,568,1151,278
CMPK2_5_7413,718,321,753,297,388,91,260,50,94
CPNE3_5_7414,677,938,672,1784,981,80,234,891,1229
CRIM1_5_7415,681,672,804,1046,778,868,1307,933,1210
CRKL_5_7416,805,957,1923,160,16,806,1223,391,104
CSF1R_5_7417,1097,2470,2461,447,841,1382,2290,2451,704
CSNK1A1L_5_7418,2699,3700,3347,1291,2921,2932,1337,4873,2150
CSNK1G1_5_7419,1101,2060,1158,1047,1035,1027,639,1646,1591
CSNK1G2_5_7420,812,411,715,278,332,966,402,757,351
CSNK2A2_5_7421,1877,2016,2599,3564,1958,1056,3373,2013,1506
CSNK2B_5_7422,431,435,320,26,233,136,263,350,2365
CTDSP2_5_7423,255,25,459,565,88,136,46,159,1339
DAPK1_5_7424,185,169,422,236,591,422,542,222,187
DAPK2_5_7425,4086,4162,3782,3021,2673,3887,4574,4778,6834
DAPK3_5_7426,5,2,10,123,0,11,0,0,0
DBF4_5_7427,508,427,579,353,242,186,909,650,1108
DCK_5_7428,942,1017,1438,1162,1294,1071,1124,967,770
DCLK3_5_7429,1049,733,1208,567,1283,750,1596,583,1318
DGKE_5_7430,217,171,192,66,99,392,11,117,643
DGKI_5_7431,1741,1585,1661,741,720,1976,1184,681,966
DGKK_5_7432,1048,2048,1714,780,2041,1252,1295,1268,1962
DGKQ_5_7433,1599,1286,1415,1577,1928,1525,1615,1374,1190
DOLK_5_7434,272,186,885,34,46,710,339,432,484
DOT1L_5_7435,295,909,671,15,591,812,331,1065,464
DUSP11_5_7436,121,219,218,201,89,1161,404,517,7
DUSP12_5_7437,818,647,1010,506,223,419,254,548,1182
DUSP14_5_7438,369,150,603,598,285,713,183,295,1256
DUSP16_5_7439,198,767,807,387,1793,487,384,998,333
DUSP18_5_7440,521,409,1059,201,389,329,336,2033,1997
DUSP21_5_7441,338,399,425,498,132,530,57,175,971
DUSP22_5_7442,212,303,1027,604,456,600,210,1123,1111
DUSP2_5_7443,327,448,601,568,30,119,117,360,8
DUSP3_5_7444,539,301,362,705,0,50,104,4,0
DUSP5_5_7445,294,383,358,224,767,241,83,382,1081
DUSP7_5_7446,276,398,793,1128,62,115,201,226,992
DUSP8_5_7447,52,120,102,138,54,174,93,15,0
DUSP9_5_7448,562,491,622,267,88,761,350,771,312
DYRK4_5_7449,1695,1890,1486,740,697,1744,539,1144,1781

EEF2K_5_7450,479,940,975,743,421,1504,902,852,329
EFNA2_5_7451,780,1384,1833,731,1976,1993,747,890,333
EFNA3_5_7452,1,249,150,0,142,0,390,12,1
EFNA5_5_7453,275,311,457,632,144,385,672,441,54
EFNB3_5_7454,892,636,1270,969,429,1034,873,606,82
EIF2AK3_5_7455,629,990,986,856,203,1172,1579,1456,483
EIF2AK4_5_7456,1320,657,1873,1781,2431,786,333,835,2849
EPHA1_5_7457,1652,2166,3067,1512,1022,2226,871,1806,2667
EPHA2_5_7458,1520,2079,3316,1513,980,2084,769,1964,2626
EPHA4_5_7459,143,573,202,106,202,500,143,71,285
EPHA7_5_7460,539,594,567,205,293,899,193,876,245
EPHB1_5_7461,1375,2007,1317,1697,1415,1289,1021,704,1096
EPHB3_5_7462,370,1097,713,93,1208,211,373,418,1150
EPHB4_5_7463,3749,5025,6346,4276,5774,6301,4969,4836,9956
EPHB6_5_7464,519,1066,1351,721,1318,1861,185,1197,1768
ERN1_5_7465,396,530,713,100,731,377,63,591,607
ERN2_5_7466,798,433,421,433,678,460,363,481,13
ETNK2_5_7467,728,521,839,863,1202,20,282,1747,2307
EYA3_5_7468,872,1004,917,1496,451,368,892,1294,938
FER_5_7469,193,405,190,7,90,37,346,918,1561
FLT3_5_7470,1510,2527,2364,1518,4628,1840,2268,3693,2098
FN3K_5_7471,911,1063,1369,842,750,504,1100,1109,2106
FN3KRP_5_7472,346,172,344,92,148,328,111,415,380
FRK_5_7473,0,0,8,0,0,0,0,0,0
FUK_5_7474,330,49,385,86,47,4,562,1,9
G6PC3_5_7475,152,355,397,144,126,599,114,644,147
G6PC_5_7476,212,514,442,200,971,76,68,21,548
GAK_5_7477,96,166,377,197,679,33,313,373,2
GALK1_5_7478,747,512,646,390,106,356,452,1389,388
GDPD4_5_7479,992,848,1894,958,693,1670,993,1514,1083
GOLGA5_5_7480,1378,1673,1409,984,1670,1803,1051,1087,441
GRK1_5_7481,406,230,321,553,825,788,361,58,34
GRK5_5_7482,921,1791,2314,2191,1361,1813,1680,1135,1149
GRK7_5_7483,749,977,1224,418,1401,1003,720,1218,772
GSG2_5_7484,103,50,84,2,0,0,0,42,0
GSK3A_5_7485,1032,1651,1197,149,1775,1730,1571,2081,259
GUCY2C_5_7486,1139,1420,2125,1580,1634,1146,3142,884,2110
GUCY2D_5_7487,1157,1068,1404,193,782,540,1050,594,1894
GUCY2F_5_7488,2140,573,1787,916,1290,1256,752,869,267
HIPK4_5_7489,287,188,52,44,28,8,81,0,43
HKDC1_5_7490,1191,1053,939,1496,925,1330,1605,742,2837
HOOK3_5_7491,899,1093,1289,1020,897,1251,507,960,1199
HSPB8_5_7492,1345,1266,1329,2068,1050,1011,581,591,1828
HUNK_5_7493,120,254,234,18,1120,0,637,825,2327
HUS1_5_7494,559,715,371,206,734,680,546,494,141
IGBP1_5_7495,981,760,2153,805,164,1126,720,755,618
IGF1R_5_7496,265,216,291,235,296,165,238,106,81
IGF2R_5_7497,266,114,182,57,90,708,157,254,0
ILKAP_5_7498,0,0,0,0,0,0,0,0,0
IMPA2_5_7499,215,610,706,166,315,119,455,111,843

INPP5A_5_7500,499,1241,1050,36,1889,1057,532,1265,152
INPP5B_5_7501,856,1110,2171,526,2251,2141,1495,2372,610
INPP5J_5_7502,322,591,472,529,586,627,246,741,925
INPPL1_5_7503,1137,904,971,185,985,1695,766,760,1585
INSRR_5_7504,330,582,126,783,32,572,200,222,703
IPMK_5_7505,1884,993,2214,2407,1442,625,2601,1145,2146
IPPK_5_7506,183,194,222,83,59,420,113,147,199
IRAK2_5_7507,386,391,479,19,361,482,376,735,167
ITK_5_7508,314,494,206,513,1135,393,239,120,205
ITPKA_5_7509,85,834,118,334,74,624,266,1506,2
ITPKB_5_7510,392,222,492,643,395,656,318,36,990
ITPKC_5_7511,1668,1978,1451,1297,2568,1942,334,2752,479
JAK1_5_7512,335,81,692,1245,849,770,484,393,1306
JAK2_5_7513,1597,2047,2745,2749,1185,2816,744,2371,3821
JAK3_5_7514,2268,1519,2947,3235,1714,1883,2177,1070,3003
KDR_5_7515,5720,6089,6564,4635,7270,5397,3640,5858,6096
KSR1_5_7516,462,191,272,87,493,79,212,179,2
LATS1_5_7517,1355,1132,1225,521,1517,1348,1265,2492,2747
LATS2_5_7518,162,255,318,0,172,102,28,142,1
LLGL1_5_7519,751,345,722,763,854,713,287,484,1058
LMTK2_5_7520,1718,1323,2204,2006,1403,2219,1111,1332,35
LMTK3_5_7521,442,273,614,3,321,376,688,11,481
LRRK1_5_7522,454,833,965,90,981,882,1529,310,2223
LRRK2_5_7523,2349,2277,3021,562,4072,1993,3012,2414,6311
MAGI2_5_7524,567,653,1306,0,52,642,690,439,61
MAP2K1_5_7525,470,408,352,62,423,152,546,183,1425
MAP2K2_5_7526,223,579,1040,244,971,1959,91,107,160
MAP2K4_5_7527,2002,2086,1964,2264,2520,1958,2430,2041,3020
MAP2K6_5_7528,518,326,974,94,161,1530,9,323,2199
MAP2K7_5_7529,250,658,842,185,44,120,249,1101,2172
MAP3K10_5_7530,261,159,91,26,0,102,180,301,60
MAP3K11_5_7531,142,2,7,0,0,135,2,0,0
MAP3K14_5_7532,16,2,81,0,29,35,5,557,21
MAP3K15_5_7533,37,281,63,0,1,0,84,327,45
MAP3K1_5_7534,1114,525,1438,820,482,1457,799,545,263
MAP3K2_5_7535,0,95,123,0,0,0,0,16,0
MAP3K5_5_7536,718,220,824,388,1153,958,112,295,2255
MAP3K6_5_7537,430,151,61,7,593,3,18,2,10
MAP3K8_5_7538,670,535,635,844,713,1872,1085,457,854
MAP3K9_5_7539,317,197,243,441,296,14,689,14,7
MAP4K2_5_7540,697,56,169,883,361,612,515,153,45
MAP4K3_5_7541,284,117,200,74,1244,194,88,291,1210
MAPK11_5_7542,810,1022,1290,314,2102,857,544,965,2263
MAPK12_5_7543,3838,3390,4507,1812,4420,3389,3894,4087,2950
MAPK13_5_7544,21,113,89,45,4,0,4,10,2
MAPK15_5_7545,1380,545,639,978,142,104,1305,668,1421
MAPK4_5_7546,626,253,1003,890,348,505,646,570,194
MAPK6_5_7547,287,136,194,381,0,0,369,493,100
MAPKAPK3_5_7548,510,971,602,2060,2313,68,982,989,700
MARK1_5_7549,938,480,915,1559,649,632,552,567,1003

MAST2_5_7550,658,198,528,56,87,1297,298,15,1116
MAST3_5_7551,295,550,281,7,242,31,266,142,91
MELK_5_7552,2698,2223,2676,2824,2678,992,1805,1993,5245
MERTK_5_7553,1271,1602,2578,1118,1015,2277,2431,1868,161
MEX3B_5_7554,278,412,329,306,228,631,246,175,20
MPP3_5_7555,245,179,364,230,155,539,388,141,92
MPP5_5_7556,2485,2128,2866,3442,1161,1568,1807,1842,3465
MST1R_5_7557,335,106,383,457,140,1096,382,41,129
MTM1_5_7558,632,879,1543,680,615,685,711,654,267
MTMR1_5_7559,647,440,1401,467,1198,2677,766,856,650
MTMR4_5_7560,253,620,732,73,884,581,7,599,772
MTMR6_5_7561,1246,1199,1194,723,2561,1045,1635,1082,597
MTMR7_5_7562,48,42,311,63,152,300,40,50,9
MTMR9_5_7563,323,175,202,1,86,394,3,252,37
MTOR_5_7564,143,254,132,106,123,1053,184,44,19
MYLK2_5_7565,49,290,86,12,1381,1,58,114,146
MYLK3_5_7566,671,829,1390,120,1869,962,267,95,2294
MYLK4_5_7567,1493,1159,1390,1289,1583,610,1731,1000,1742
MYO3A_5_7568,1019,1447,2148,2140,1672,1167,2739,931,2043
N4BP2_5_7569,744,716,544,119,833,756,670,445,1048
NAGK_5_7570,1462,598,1562,505,339,257,738,1239,1762
NAGS_5_7571,741,446,478,515,75,871,842,500,1012
NEK10_5_7572,691,499,586,1490,515,311,819,320,208
NEK5_5_7573,1378,2130,1882,711,1902,1595,1560,1705,962
NEK7_5_7574,4987,4377,5113,4839,4217,3749,5704,5178,4923
NEK8_5_7575,1254,1598,2099,1963,2611,2360,1340,2579,1369
NEK9_5_7576,422,1058,705,548,1612,747,366,756,1861
NME3_5_7577,2430,2348,1941,1891,1046,1958,1835,2326,1901
NME4_5_7578,576,444,1314,166,486,228,516,225,2605
NME5_5_7579,3641,3881,6145,4203,2368,5397,4115,2289,1943
NME6_5_7580,162,161,153,9,302,489,89,15,419
NPR1_5_7581,470,288,243,419,320,621,622,73,139
NPRL2_5_7582,453,54,163,457,306,994,75,2,316
NRBP1_5_7583,51,121,179,315,113,234,47,4,0
NRBP2_5_7584,374,172,284,285,590,250,243,79,0
NRK_5_7585,694,1036,885,740,807,655,611,398,562
NT5C1A_5_7586,216,321,703,2,807,648,1351,672,705
NT5C_5_7587,2,16,259,0,0,1022,78,217,626
NT5M_5_7588,1099,983,1093,644,1520,1464,1387,1109,3088
NUAK1_5_7589,1419,1151,2135,2153,1687,3906,2034,2819,1985
NUAK2_5_7590,528,307,455,151,1051,88,219,232,14
NUCKS1_5_7591,519,588,442,13,518,141,1270,1511,5
OXSR1_5_7592,528,336,724,1,347,40,653,0,694
PAK2_5_7593,14,18,98,10,21,14,3,1,143
PANK3_5_7594,418,383,483,406,1444,350,233,557,1405
PANK4_5_7595,410,697,611,32,311,809,991,1268,291
PASK_5_7596,542,724,1076,1,0,994,178,467,0
PBK_5_7597,1815,2224,2367,2717,4421,2631,2356,958,3079
PCK1_5_7598,99,179,394,485,195,236,236,134,11
PDCD1_5_7599,119,230,297,180,479,28,117,277,48

PDGFRA_5_7600,596,437,963,2069,913,416,1342,789,826
PDGFRB_5_7601,733,501,1128,708,1854,952,804,643,1083
PDGFRL_5_7602,1855,2292,2399,3495,1752,3212,3029,1506,1271
PDIK1L_5_7603,1176,1125,1356,1519,2038,1559,922,3034,1004
PDK4_5_7604,19893,19799,26430,21353,24550,27082,19735,18167,25485
PDP2_5_7605,691,780,1460,905,223,236,933,1221,1052
PDXK_5_7606,225,12,439,7,0,578,297,20,143
PFKFB1_5_7607,201,244,206,346,384,68,187,498,0
PFKFB4_5_7608,1865,2043,2241,1186,3372,1690,2902,1705,1276
PFKL_5_7609,359,362,157,17,1,981,595,270,1108
PGAM2_5_7610,402,722,329,0,5,0,0,106,1076
PGK1_5_7611,417,264,277,9,98,104,80,171,41
PGK2_5_7612,554,803,478,391,669,732,684,196,293
PHKA2_5_7613,381,294,526,763,342,1834,403,369,102
PHKG1_5_7614,1727,1792,1983,2540,2420,2950,1847,735,1575
PI4K2A_5_7615,700,515,1397,560,732,1996,728,710,2613
PI4K2B_5_7616,1427,1133,1602,2152,1912,918,792,1798,3505
PIK3C2A_5_7617,1702,1070,1021,582,458,1198,572,1776,646
PIK3C2B_5_7618,1509,2833,2567,2201,2627,2872,1502,2754,2288
PIK3C2G_5_7619,385,948,1271,647,594,1572,406,915,1448
PIK3C3_5_7620,503,145,622,20,946,383,235,351,50
PIK3CA_5_7621,1348,754,1089,1934,2283,1934,944,693,2460
PIK3CB_5_7622,875,894,1152,316,2586,975,1326,655,1728
PIK3CD_5_7623,949,606,647,873,388,458,586,812,1230
PIK3CG_5_7624,3249,2615,2982,4455,3304,1229,3157,1841,1692
PIK3R2_5_7625,1606,1288,1424,1642,766,1668,2088,629,525
PIK3R4_5_7626,87,57,641,1,57,17,445,9,79
PIK3R6_5_7627,2032,2956,2649,2915,3088,1700,1465,2427,3887
PIM2_5_7628,1266,1447,1876,2734,394,936,2069,743,2035
PIM3_5_7629,280,1013,535,245,22,334,458,75,656
PINK1_5_7630,410,94,850,42,818,1485,215,1021,347
PIP4K2A_5_7631,723,931,1109,2298,1098,748,1641,1371,88
PIP4K2B_5_7632,1975,2374,2706,2888,2258,2329,1346,2109,3901
PIP5K1B_5_7633,1350,1515,1049,643,982,1544,1744,2234,477
PKDCC_5_7634,696,603,470,792,1491,472,388,943,24
PKN2_5_7635,0,0,0,0,0,0,0,0,0
PKN3_5_7636,85,54,48,66,136,43,30,95,2
PLK1_5_7637,243,353,427,42,0,1020,281,158,4
PLK2_5_7638,2139,1102,1681,511,1407,930,1031,547,3279
PLK3_5_7639,1258,1023,837,1053,1281,1346,1115,1522,782
PMVK_5_7640,448,1123,768,486,179,576,641,511,1350
PNKP_5_7641,2679,2180,2435,2838,2828,2077,1839,2085,3725
POLD1_5_7642,297,315,309,333,489,184,343,77,246
PON1_5_7643,3360,5830,4172,8286,4465,7383,4398,4376,6254
PPAP2B_5_7644,1511,1472,1746,2271,2121,438,1675,1059,2291
PPEF2_5_7645,185,0,591,3,22,1570,55,0,0
PPFIA3_5_7646,88,147,338,4,23,695,10,510,375
PPFIA4_5_7647,498,588,730,307,1120,73,383,313,47
PPM1D_5_7648,1575,1133,1565,2457,180,1397,1173,377,1245
PPM1E_5_7649,1102,574,1142,316,488,459,614,231,1231

PPM1F_5_7650,725,1022,959,653,1486,1179,1007,1156,518
PPM1G_5_7651,274,317,179,79,141,27,582,95,313
PPM1K_5_7652,8223,7460,9604,5959,9567,11282,9401,7348,11307
PPM1L_5_7653,925,1555,2002,1040,3279,1930,1228,3474,1633
PPP1CC_5_7654,4360,2881,4714,5339,4804,4122,4770,2200,4541
PPP1R12C_5_7655,262,193,371,96,321,255,359,54,412
PPP1R13B_5_7656,1551,1090,2043,1417,1799,1943,911,2109,1890
PPP1R14B_5_7657,296,511,338,323,212,458,211,372,26
PPP1R15B_5_7658,1438,752,1399,1115,915,1039,1189,1532,2773
PPP1R1A_5_7659,736,630,1008,1100,803,680,1732,630,690
PPP1R1C_5_7660,833,528,711,400,1581,910,580,191,617
PPP1R2_5_7661,1645,2513,3989,1924,5037,1598,2612,2010,3426
PPP1R3A_5_7662,10120,8688,11847,10826,7470,10478,10383,9170,9062
PPP1R3C_5_7663,650,139,258,1,98,366,355,1,28
PPP1R3D_5_7664,228,115,332,155,1214,16,592,5,14
PPP1R7_5_7665,456,90,132,141,327,101,1599,267,18
PPP2CA_5_7666,94,67,188,27,0,25,48,383,4
PPP2CB_5_7667,136,268,454,221,1208,778,189,19,1
PPP2R1A_5_7668,422,407,503,933,73,984,902,328,159
PPP2R2D_5_7669,1041,868,1474,1124,1043,522,1044,1972,829
PPP2R3B_5_7670,274,375,484,0,17,0,644,231,212
PPP2R5B_5_7671,298,322,214,373,553,183,169,289,5
PPP2R5E_5_7672,306,143,609,1185,577,68,810,171,43
PPP3CC_5_7673,1183,1783,1699,619,2843,1300,2371,2746,3503
PPP3R1_5_7674,1154,1630,1311,1440,723,1418,1017,1297,4343
PPP3R2_5_7675,151,184,85,1,0,405,9,6,0
PPP4C_5_7676,1833,729,1227,715,988,521,2346,274,595
PPTC7_5_7677,743,1864,1420,3222,1704,982,1186,1237,1689
PRKAA2_5_7678,3293,3250,3415,3188,3098,2305,1893,2386,5416
PRKAB1_5_7679,347,469,685,1493,23,301,679,435,490
PRKAB2_5_7680,1001,1209,2388,608,1001,1252,1688,1131,347
PRKACG_5_7681,2653,2064,2254,4532,907,2389,1330,2871,1832
PRKAG3_5_7682,665,747,622,844,1084,821,1319,369,1186
PRKAR2A_5_7683,2105,2840,2518,811,1531,4012,2934,2390,2426
PRKAR2B_5_7684,1082,1486,1155,167,2161,637,1020,1310,78
PRKCA_5_7685,1709,918,1685,1228,2891,1135,2022,2540,1184
PRKCE_5_7686,85,89,121,56,8,61,22,20,533
PRKCG_5_7687,449,375,872,1431,272,14,595,162,1
PRKCH_5_7688,1080,1331,1453,503,1662,2051,416,114,647
PRKCI_5_7689,190,100,160,797,68,7,104,271,206
PRKD1_5_7690,309,130,594,27,0,51,470,185,181
PRKD3_5_7691,486,537,366,728,729,505,131,616,201
PRKG2_5_7692,2477,3125,3287,2324,2638,1339,3715,2746,6567
PRKX_5_7693,335,350,586,532,1069,692,291,553,84
PRPF4B_5_7694,61,98,80,0,135,0,0,288,1
PRPS1L1_5_7695,632,614,1199,686,352,1432,436,494,1400
PSKH1_5_7696,392,291,466,942,751,397,424,90,51
PSKH2_5_7697,139,148,118,0,14,56,87,118,0
PSPH_5_7698,1068,803,1462,645,423,755,2100,951,332
PSTK_5_7699,703,509,1410,462,207,1404,1195,992,275

PTEN_5_7700,123,0,11,4,1,0,0,1,3
PTK6_5_7701,61,15,121,0,3,85,377,0,665
PTP4A1_5_7702,5226,6085,6539,5209,9163,5906,7462,5561,7760
PTPLA_5_7703,2748,3255,3518,2613,1551,2332,2352,2669,1212
PTPLB_5_7704,1388,967,1342,1351,238,1663,1754,282,1085
PTPN11_5_7705,1664,1107,1632,1274,607,2122,2414,1385,2637
PTPN14_5_7706,613,646,605,1043,63,1159,584,373,980
PTPN1_5_7707,419,306,411,832,104,128,689,306,117
PTPN21_5_7708,520,104,306,586,7,7,79,0,0
PTPN23_5_7709,472,185,331,69,372,409,254,136,9
PTPN4_5_7710,1253,1605,1333,701,2579,1777,833,1577,1885
PTPN9_5_7711,480,694,1046,95,982,229,1268,327,2063
PTPRG_5_7712,1474,1034,809,122,802,348,959,1898,2111
PXK_5_7713,3,0,201,0,77,0,0,0,0
RAF1_5_7714,1180,1428,1451,1954,1611,1461,753,1758,2461
RBKS_5_7715,745,618,552,760,135,545,1054,363,1108
RFK_5_7716,923,2118,1995,1281,1855,1947,703,1082,1436
RIOK3_5_7717,1589,1173,2291,957,1820,1101,980,1164,859
RIPK1_5_7718,1606,2422,2440,2433,2496,3928,3151,2276,4983
RIPK2_5_7719,1605,1448,1912,559,3365,1155,1144,2166,2826
RIPK3_5_7720,1366,1697,2233,922,1308,2126,879,1716,1233
RIPK4_5_7721,1121,1219,1946,1713,1694,1918,1408,1512,1555
RNASEL_5_7722,915,1821,1414,204,1343,715,1354,2476,1188
RNGTT_5_7723,119,261,242,0,1,9,204,33,0
ROCK1_5_7724,2038,1352,2628,1253,2245,2694,1399,1403,855
ROCK2_5_7725,1362,1035,1231,969,1080,1528,821,437,1053
ROS1_5_7726,151,51,257,0,0,3,113,109,0
RPS6KA3_5_7727,384,219,943,1428,156,90,84,141,2723
RPS6KA6_5_7728,593,1182,1182,918,1339,734,1163,1151,1151
RPS6KB1_5_7729,283,493,726,642,513,588,594,733,251
RPS6KB2_5_7730,309,99,612,135,167,309,327,222,89
RPS6KL1_5_7731,194,55,31,21,192,62,174,90,0
RSPO3_5_7732,1487,871,1085,455,1582,518,955,308,1382
SACM1L_5_7733,570,963,498,493,474,41,548,739,75
SBF1_5_7734,148,228,126,7,210,271,167,135,166
SBK1_5_7735,372,108,200,762,22,0,29,0,271
SBK2_5_7736,419,460,522,60,1412,610,1665,337,1303
SEPHS2_5_7737,153,368,1189,180,976,3,203,227,995
SGPP1_5_7738,1517,1490,2382,1541,1045,2298,1042,2154,2951
SHPK_5_7739,409,464,615,140,149,299,591,119,782
SIK1_5_7740,872,1160,810,747,489,863,531,333,1371
SIK2_5_7741,1410,1206,1044,1462,642,648,1142,968,188
SIK3_5_7742,286,130,178,1335,23,127,3,93,45
SKAP2_5_7743,205,327,291,25,130,7,292,154,516
SMG1_5_7744,1513,2529,2413,1440,2174,2234,2325,2785,1707
SRMS_5_7745,207,9,532,838,326,681,0,150,99
SRP72_5_7746,1559,1296,1944,1164,913,2051,1994,1466,1663
SRPK1_5_7747,0,0,0,0,0,0,0,0,0
SSH2_5_7748,727,1068,1017,1116,730,1010,1348,1096,1181
SSH3_5_7749,354,405,531,690,512,37,1050,2188,492

STC1_5_7750,423,1170,463,833,991,586,395,573,1028
STK10_5_7751,684,1048,870,353,1684,688,147,927,1742
STK11_5_7752,312,188,231,0,20,50,158,5,959
STK16_5_7753,333,468,221,2,3,218,390,93,35
STK17A_5_7754,348,394,370,129,130,653,130,250,141
STK17B_5_7755,3636,4283,4668,2668,4881,4145,2480,3812,2784
STK25_5_7756,903,795,847,367,215,1031,911,1128,1417
STK32B_5_7757,1773,1272,2164,831,633,879,2001,1264,3803
STK32C_5_7758,0,51,157,119,3,65,222,90,0
STK33_5_7759,2161,2221,2443,1445,2440,1554,2008,1891,624
STK35_5_7760,1046,1188,1339,1129,749,1513,482,1619,1530
STK38_5_7761,2078,2459,2731,2176,1862,4115,1971,1168,2908
STK38L_5_7762,504,442,366,253,293,3,589,993,663
STK39_5_7763,1074,1012,707,1114,125,328,340,1761,368
STK40_5_7764,1969,2396,2182,1593,2199,2169,2086,1959,754
STK4_5_7765,1246,2117,2042,2516,1185,1657,1726,1810,4296
STYK1_5_7766,323,855,362,8,729,1189,501,786,22
STYXL1_5_7767,1591,1455,1234,522,1101,2301,881,882,1362
TAF1L_5_7768,2294,1367,2349,1381,1126,3124,2672,2274,841
TAOK3_5_7769,2293,2994,2858,1341,3063,2006,2885,2660,3706
TBK1_5_7770,2079,2568,3055,5765,2102,2268,883,1510,5676
TEC_5_7771,1069,1075,995,221,189,525,644,2169,413
TEK_5_7772,1269,1735,1456,984,1494,1447,1433,472,2332
TESK1_5_7773,112,536,230,141,459,229,657,160,61
TESK2_5_7774,592,822,1031,519,603,1186,627,864,1438
THNSL1_5_7775,665,394,489,32,250,682,239,1291,1740
TIE1_5_7776,509,698,1272,0,338,439,34,1299,1259
TK1_5_7777,715,410,988,715,420,391,670,2079,406
TNK1_5_7778,122,452,191,47,0,294,164,113,137
TNNI3K_5_7779,1008,1132,1677,1231,1007,331,1545,1791,1032
TNS3_5_7780,26,866,503,962,26,402,58,345,410
TP53RK_5_7781,670,278,461,312,420,793,1217,347,424
TRAT1_5_7782,5188,5257,4428,5439,5435,3388,5165,4324,4106
TRIB1_5_7783,2157,2600,3128,3726,899,2513,1360,3026,1239
TRIB2_5_7784,565,640,328,191,320,92,98,168,2665
TRIB3_5_7785,1298,1816,2297,1743,2347,1625,594,1346,433
TRIM27_5_7786,337,255,568,1229,828,1118,874,1235,827
TRIM28_5_7787,854,1062,607,482,2056,192,947,779,909
TRIO_5_7788,1658,1471,1992,1971,2586,1127,1386,1475,2490
TRPM7_5_7789,4586,3957,4771,4037,4690,1863,3836,3909,7226
TRRAP_5_7790,340,216,848,591,173,660,337,497,11
TSKS_5_7791,504,501,355,282,112,942,387,223,813
TSSK1B_5_7792,561,572,117,501,711,539,536,512,835
TSSK2_5_7793,449,1153,1540,268,878,2430,1086,491,2229
TSSK3_5_7794,362,642,320,1104,264,4,729,2226,1042
TSSK6_5_7795,299,368,517,346,163,132,197,140,24
TTBK1_5_7796,22,260,210,13,209,160,184,3,17
TTBK2_5_7797,2190,1708,2285,2176,1284,1967,427,1867,2820
TWF2_5_7798,229,282,121,562,45,534,449,227,0
TXK_5_7799,291,249,278,99,398,423,389,290,3

TYK2_5_7800,714,320,469,213,141,494,1125,483,2859
TYRO3_5_7801,870,955,1375,1509,1246,2234,837,1676,693
UBLCP1_5_7802,1513,1049,2133,1405,1681,409,1480,2749,1261
UCK2_5_7803,1418,1444,1826,1106,432,745,1298,756,179
ULK1_5_7804,270,509,908,544,71,808,612,179,628
ULK3_5_7805,1404,1080,1422,551,3184,1695,2313,1504,2453
ULK4_5_7806,2033,2154,2107,1333,3850,1516,2470,2726,1467
VRK1_5_7807,113,116,271,235,442,331,97,31,95
WEE2_5_7808,1474,2643,2523,1468,1558,2760,1974,2864,1835
WNK2_5_7809,1,4,33,0,1,0,0,0,6
WNK4_5_7810,458,613,1121,467,339,1062,436,164,210
XRCC6BP1_5_7811,1490,1034,1504,2607,726,2039,981,1243,1963
XYLB_5_7812,1832,1064,1452,3186,442,1146,1301,928,1579
YES1_5_7813,1645,1830,2072,1379,2196,2889,1710,1053,2328
AATK_5_7814,368,339,177,1477,39,297,319,50,73
ABL1_5_7815,429,138,114,11,86,755,633,404,1232
ABL2_5_7816,1220,973,1360,641,2542,902,812,1371,856
ACP1_5_7817,3310,2209,3128,2163,2519,4614,3211,4226,1848
ACP5_5_7818,163,611,176,795,729,611,224,789,370
ACPL2_5_7819,81,279,153,0,380,110,257,61,34
ACPP_5_7820,829,838,1180,736,840,784,701,852,1603
ACVR1B_5_7821,179,58,514,536,31,0,124,362,801
ACVR1C_5_7822,2972,3065,4295,3075,795,2794,4234,2511,4212
ACVR1_5_7823,868,529,699,270,1153,196,356,1000,1039
ACVRL1_5_7824,49,35,105,0,83,14,0,73,13
ADCK1_5_7825,4086,3974,4616,4373,3829,3974,3099,3269,1080
ADCK4_5_7826,455,563,794,75,709,1303,4,272,493
ADK_5_7827,6600,9155,7519,6112,6785,6042,6009,5535,8736
AK2_5_7828,1632,1978,2246,730,1239,3551,1574,1416,969
AK4_5_7829,429,785,849,1088,873,53,658,257,1876
AK5_5_7830,5551,5171,7227,4618,4655,3378,4049,5970,10206
AKT1_5_7831,1195,1686,1777,1725,1891,1753,1367,1417,1114
AKT2_5_7832,806,555,1369,333,734,1392,584,370,651
AKT3_5_7833,1251,1115,1311,1499,386,345,566,704,158
ALDH18A1_5_7834,750,487,937,473,229,1386,664,19,3254
ALPK1_5_7835,763,816,1121,379,584,1898,505,920,397
ALPL_5_7836,31,68,109,528,0,19,1,0,30
AMHR2_5_7837,261,53,65,845,0,0,9,15,2
ANKHD1_5_7838,1753,1259,1507,1365,1065,1016,1584,1256,2615
APTX_5_7839,4632,3974,5568,2391,3483,3415,2140,4539,4745
ATRIP_5_7840,102,313,328,151,1069,271,286,315,119
AURKA_5_7841,1742,1085,1821,1327,2233,4151,1209,771,344
AURKC_5_7842,1513,1645,1014,1721,552,868,507,1555,2577
AXL_5_7843,45,17,55,60,3,35,160,88,475
BAIAP2_5_7844,396,330,148,263,762,755,59,545,173
BCKDK_5_7845,845,1094,1674,721,352,559,2168,505,2522
BCR_5_7846,285,572,707,229,241,1567,448,1218,399
BMP2K_5_7847,7,136,212,20,4,378,191,253,16
BMX_5_7848,3562,3141,3987,2403,3370,4127,3616,3069,5576
BPGM_5_7849,258,86,172,22,393,11,97,102,47

BRD2_5_7850,2197,2424,2484,1256,2928,1693,1369,3030,3707
BRD4_5_7851,1355,962,1369,584,728,2452,3310,309,1393
BRDT_5_7852,64,108,153,160,203,40,10,107,810
CAB39L_5_7853,1146,1593,1144,2489,1944,701,1163,854,845
CAMK1D_5_7854,131,219,223,141,443,155,147,179,2
CAMK2A_5_7855,169,281,220,0,69,49,138,10,134
CAMK2B_5_7856,252,57,482,264,1,15,147,360,37
CAMK2D_5_7857,72,289,179,55,201,63,51,110,145
CAMK2G_5_7858,447,783,607,596,901,890,969,149,1204
CAMKK1_5_7859,131,328,141,543,92,8,33,178,87
CAMKK2_5_7860,963,730,1181,608,1757,625,613,905,61
CASK_5_7861,1849,2755,2809,3209,6221,1178,2500,844,706
CCT2_5_7862,309,123,327,346,350,594,65,101,867
CDADC1_5_7863,2506,4359,4248,2509,5951,6289,2247,4206,7541
CDC14A_5_7864,1181,2241,1649,1640,660,1841,433,689,128
CDC14B_5_7865,207,265,69,54,448,13,301,85,706
CDC25A_5_7866,839,1306,1126,1,1347,1352,567,553,1431
CDC25B_5_7867,294,398,645,53,1383,206,499,30,1086
CDC25C_5_7868,4138,5651,4980,2233,8146,4671,3215,4459,8143
CDC42BPA_5_7869,1787,2883,2006,1661,1506,1820,1941,2968,1846
CDC7_5_7870,76,126,480,521,226,386,216,139,82
CDK10_5_7871,32,38,35,0,1,113,54,35,5
CDK11A_5_7872,1432,1460,2193,1233,3569,2062,2465,1993,3341
CDK11B_5_7873,1432,1460,2193,1233,3569,2062,2465,1993,3341
CDK12_5_7874,2876,2635,4689,1766,2805,3326,3550,2505,1401
CDK13_5_7875,1033,1016,455,131,761,1929,1645,1459,648
CDK16_5_7876,69,10,194,1,1,20,37,933,0
CDK17_5_7877,323,1178,824,288,1007,532,474,392,659
CDK18_5_7878,70,235,361,18,228,878,267,34,1
CDK20_5_7879,352,702,224,761,24,649,242,389,1203
CDK2_5_7880,1657,2236,2179,3258,2749,3460,2455,1025,3313
CDK5_5_7881,404,443,1050,106,211,35,620,1040,141
CDK6_5_7882,2897,2653,3449,1500,3069,3032,2623,1726,1088
CDKL3_5_7883,1764,2412,3546,4804,740,2590,4197,1885,5256
CDKL5_5_7884,2020,3307,3260,1016,1159,1540,2529,2260,3671
CDKN1A_5_7885,343,343,283,1,1495,41,359,218,164
CDKN3_5_7886,2206,3508,3646,1130,3121,3145,1991,3150,2703
CHEK1_5_7887,1611,1755,1494,919,584,2202,2767,1407,466
CHKA_5_7888,2083,2723,3597,1170,3026,2912,1840,2805,2686
CIT_5_7889,406,753,465,128,1275,890,1655,521,1321
CKMT2_5_7890,4298,4415,3517,3724,4861,2367,4755,3659,3487
CLK1_5_7891,1303,1452,1289,1612,1075,1100,964,1798,1219
CLK3_5_7892,869,688,669,1405,1387,761,783,525,2576
CMPK1_5_7893,3395,4229,4968,4646,5377,5391,3744,3099,3955
COASY_5_7894,189,1052,298,35,22,790,41,1404,948
COL4A3BP_5_7895,3239,3957,4019,3953,5649,3632,1798,2942,4210
CSK_5_7896,420,1296,670,468,1,659,257,1228,84
CSNK1A1_5_7897,2870,3725,3400,1441,2906,2927,1328,4864,2177
CSNK1D_5_7898,653,529,641,1851,312,368,225,482,1313
CSNK1E_5_7899,322,230,510,90,744,347,269,293,787

CSNK1G3_5_7900,4408,5342,5400,3731,6276,4238,4560,3908,3458
CTDP1_5_7901,333,252,316,7,86,164,61,96,47
CTDSP1_5_7902,273,580,272,2,179,792,40,1010,1
DCLK1_5_7903,2662,4684,3743,1294,3585,1585,3242,2252,5404
DCLK2_5_7904,110,290,220,237,161,618,26,365,1
DDR1_5_7905,782,1534,930,407,472,529,1731,968,175
DDR2_5_7906,4865,6906,7576,4289,4774,6162,3442,4865,10031
DGKA_5_7907,643,862,737,577,548,814,528,1152,1092
DGKB_5_7908,464,533,448,1189,739,445,800,351,444
DGKD_5_7909,766,474,251,8,104,86,126,370,81
DGKG_5_7910,60,213,295,0,586,389,266,1946,158
DGKH_5_7911,4764,5184,5936,3456,6972,6421,4411,4649,5298
DGKZ_5_7912,103,63,167,375,90,0,139,426,9
DGUOK_5_7913,748,810,817,319,2138,1008,499,804,1183
DLGAP5_5_7914,932,747,237,109,913,1072,1227,1239,984
DMPK_5_7915,101,259,207,284,8,4,412,19,65
DSTYK_5_7916,615,260,1362,995,410,1127,347,458,1351
DTYMK_5_7917,863,1043,1149,1888,1313,852,718,1818,868
DUSP13_5_7918,353,569,785,672,407,1447,905,422,708
DUSP19_5_7919,1475,2020,2079,1079,2844,1125,967,722,636
DUSP4_5_7920,2809,1645,2767,1813,2337,1240,2287,1967,3600
DUSP6_5_7921,1017,1768,1400,192,467,2684,535,1136,637
DYRK1A_5_7922,759,956,1075,49,840,799,705,52,297
DYRK1B_5_7923,86,0,27,0,0,3,378,68,0
DYRK2_5_7924,494,292,642,896,1062,418,426,290,489
DYRK3_5_7925,1220,1411,2752,1424,1267,3232,965,1225,2837
EFNA4_5_7926,57,25,87,0,1,28,0,0,9
EGFR_5_7927,6700,7833,7955,7155,9472,11298,6295,5579,6882
EIF2AK2_5_7928,339,594,341,202,545,894,215,1200,84
EPHA10_5_7929,305,431,272,566,1204,2,452,61,600
EPHA3_5_7930,323,131,336,62,455,582,209,47,2
EPHA5_5_7931,696,936,1304,628,288,48,1014,1378,955
EPHA6_5_7932,935,907,1085,409,1816,471,1781,1134,1635
EPHA8_5_7933,474,263,833,738,56,961,17,341,1342
EPHB2_5_7934,362,1247,883,344,554,1164,547,456,826
EPM2A_5_7935,312,888,354,2,0,0,14,7,0
ERBB2_5_7936,232,515,472,402,237,640,98,357,111
ERBB4_5_7937,887,1348,1085,1731,1571,566,1106,721,685
ETNK1_5_7938,1627,2903,2625,2062,3259,3637,2534,1907,1557
EXOSC10_5_7939,1007,1326,1190,465,454,811,350,600,439
EYA1_5_7940,1197,976,1155,1562,1763,166,1112,1270,690
EYA2_5_7941,204,350,109,2,395,613,516,115,864
EYA4_5_7942,589,913,916,383,924,906,650,960,542
FASTK_5_7943,21,61,12,0,24,18,199,97,0
FBP1_5_7944,518,1346,1122,596,766,1023,1271,488,1156
FES_5_7945,1014,772,38,640,715,341,211,514,720
FGFR1_5_7946,778,821,488,586,387,322,397,549,1157
FGFR2_5_7947,548,555,282,71,26,664,73,312,22
FGFR3_5_7948,438,589,480,285,365,436,174,458,185
FGFR4_5_7949,200,194,222,12,14,37,151,94,0

FGFRL1_5_7950,112,45,297,0,8,175,2,2,0
FGR_5_7951,472,462,124,99,680,114,708,63,441
FLT1_5_7952,773,729,702,1716,442,1557,1369,779,1006
FLT4_5_7953,498,301,576,1449,924,388,37,359,42
FXN_5_7954,807,738,1180,1704,968,1753,1218,307,321
FYN_5_7955,1148,843,1363,1048,449,1756,992,325,1959
FZR1_5_7956,138,147,139,0,486,214,24,47,104
GALK2_5_7957,3828,3695,4673,3972,1831,3937,4086,2250,3725
GK_5_7958,1183,458,778,1428,479,1121,507,1032,1510
GLYCTK_5_7959,580,1557,999,1289,2064,636,1242,1431,1165
GNE_5_7960,1367,1820,2730,726,2773,2594,2270,1323,937
GRK4_5_7961,751,1256,652,308,981,782,716,291,673
GRK6_5_7962,0,0,0,0,0,0,0,0,0
GSK3B_5_7963,1816,2232,2934,1791,2575,4525,1823,1947,2393
GUK1_5_7964,81,76,440,22,86,2,16,98,1026
HCK_5_7965,143,26,90,11,369,154,7,19,5
HIPK1_5_7966,2123,2958,2355,4262,1617,2192,4505,2484,2276
HIPK2_5_7967,730,1551,1669,707,742,1119,1189,1528,1763
HIPK3_5_7968,669,193,444,230,859,277,72,1046,530
ICK_5_7969,939,740,840,86,306,277,1011,723,926
IKBKB_5_7970,2224,2335,2764,1379,3904,2759,3267,2905,4756
IKBKE_5_7971,1042,535,282,373,57,496,1290,379,195
IKBKG_5_7972,221,196,364,832,404,1154,194,172,32
ILK_5_7973,1037,860,1058,3190,575,629,772,852,648
IMPA1_5_7974,133,523,231,496,747,127,51,234,250
INPP1_5_7975,507,1041,516,100,1255,423,103,23,1747
INPP4A_5_7976,308,287,391,95,1604,186,204,144,9
INPP4B_5_7977,3509,4505,3887,2792,4804,4585,3845,3485,7011
INPP5D_5_7978,546,400,57,71,816,346,151,197,749
INSR_5_7979,203,400,341,21,41,275,24,236,1042
IP6K1_5_7980,291,775,999,281,534,423,331,1163,20
IP6K3_5_7981,533,418,655,448,254,569,435,326,492
IRAK1_5_7982,186,62,86,0,0,0,0,0,424
IRAK3_5_7983,130,444,1390,48,789,373,513,354,426
IRAK4_5_7984,962,986,1462,2753,1579,1429,876,660,1778
ITPK1_5_7985,2183,1701,2184,1274,2714,2021,1859,1973,3268
KALRN_5_7986,493,1176,703,922,551,319,1170,1009,2476
KHK_5_7987,4,27,90,4,3,401,186,22,0
KIF2A_5_7988,5296,4867,6890,3888,5092,6861,6691,4990,4355
KIT_5_7989,130,78,291,268,138,364,122,60,18
LCK_5_7990,239,10,125,64,13,1,0,20,0
LHPP_5_7991,541,786,443,42,599,237,146,213,50
LIMK1_5_7992,164,183,120,4,0,371,196,0,289
LIMK2_5_7993,448,279,78,46,97,408,0,543,0
LTK_5_7994,356,243,406,424,14,288,353,53,824
LYN_5_7995,229,580,319,176,392,183,310,732,581
MAGI3_5_7996,338,440,625,110,294,397,456,31,4
MAP2K3_5_7997,513,1323,1201,738,72,291,341,460,1406
MAP2K5_5_7998,634,702,854,786,156,869,1335,257,1761
MAP3K12_5_7999,813,898,834,358,822,655,70,559,590

MAP3K13_5_8000,280,371,316,124,46,136,112,245,695
MAP3K3_5_8001,594,474,699,567,387,1414,271,245,239
MAP3K4_5_8002,433,587,410,544,137,537,338,59,1160
MAP3K7_5_8003,316,436,324,74,46,193,504,332,48
MAP4K1_5_8004,641,462,1079,357,849,539,900,538,1011
MAP4K4_5_8005,531,777,835,305,1951,125,578,1127,1223
MAP4K5_5_8006,2220,1106,2098,2002,1671,2478,1410,622,2088
MAPK10_5_8007,1962,822,1473,996,1522,1254,2460,1640,1079
MAPK14_5_8008,454,326,477,296,301,1710,235,312,1680
MAPK1_5_8009,363,373,400,8,459,37,31,66,3413
MAPK3_5_8010,339,100,59,2,524,196,228,227,89
MAPK7_5_8011,179,598,625,1337,133,467,0,87,0
MAPK8_5_8012,2570,1576,2860,3421,2204,1337,1198,1318,1801
MAPK9_5_8013,2095,2484,2788,1065,1865,2348,3332,2607,1588
MAPKAPK2_5_8014,837,432,308,10,914,186,1226,126,1198
MAPKAPK5_5_8015,1742,2012,1754,3260,4301,3548,1656,1872,2984
MARK2_5_8016,716,930,1126,604,1313,53,1773,538,1239
MARK3_5_8017,948,1551,694,569,351,496,2150,1386,977
MARK4_5_8018,1091,737,581,1289,507,1969,1243,1364,745
MAST4_5_8019,1528,1755,1803,765,1563,2782,833,1710,3975
MASTL_5_8020,1297,1623,2193,1479,1239,1905,1092,938,2202
MATK_5_8021,314,227,500,10,776,42,335,171,84
MET_5_8022,780,713,526,555,747,324,289,211,1643
MINK1_5_8023,143,269,325,29,54,290,972,583,5
MINPP1_5_8024,176,169,35,384,87,108,106,60,52
MKNK1_5_8025,50,165,529,4,266,1,486,122,900
MKNK2_5_8026,950,507,616,706,1825,114,426,344,1162
MLKL_5_8027,1510,1444,2241,622,2114,2954,752,1325,3205
MTMR14_5_8028,1489,2412,3447,1978,789,3712,1071,1444,2479
MTMR2_5_8029,512,728,445,263,183,1317,458,478,1240
MTMR3_5_8030,2090,874,1602,1153,1852,1605,1663,1777,967
MUSK_5_8031,600,153,475,7,117,30,250,122,34
MVK_5_8032,0,395,17,0,0,16,17,107,0
MYO3B_5_8033,1722,1781,1876,868,2423,2994,1634,2001,1603
NADK_5_8034,2058,2417,1703,3263,1437,1147,2001,1233,2404
NCK1_5_8035,1255,1190,2149,1383,1589,1508,1316,2293,2874
NCK2_5_8036,22,60,319,3,103,78,122,4,0
NDRG1_5_8037,344,536,767,18,710,1013,112,367,75
NEK11_5_8038,73,493,192,23,878,16,84,989,696
NEK1_5_8039,1347,1113,1278,2048,946,1734,1855,1424,981
NEK2_5_8040,59,25,6,0,0,126,1,28,0
NEK3_5_8041,1240,1266,1400,1389,353,1440,1754,531,2486
NEK4_5_8042,451,361,1136,123,94,598,815,698,183
NEK6_5_8043,1840,2409,2051,2380,2030,2402,1745,1572,2262
NME2_5_8044,1386,1053,709,896,1569,701,1870,1405,657
NME7_5_8045,902,1373,1212,477,217,961,526,2913,236
NT5C1B_5_8046,204,226,54,0,1414,1,40,454,442
NT5C2_5_8047,2640,2725,2343,2082,431,1915,1480,2284,1401
NT5C3_5_8048,1612,1215,1966,1324,2272,1403,1207,1365,1408
NT5E_5_8049,3005,2094,2749,3388,4976,1681,2950,865,1844

NTRK1_5_8050,38,453,209,753,27,39,0,67,42
NTRK2_5_8051,825,806,1394,890,1535,1503,517,940,1830
NTRK3_5_8052,486,775,493,315,712,697,685,199,346
NUDT4_5_8053,3177,3037,3735,3718,3556,5442,3550,5532,2809
NUDT9_5_8054,852,1588,1012,521,1541,1722,791,591,1261
OBSCN_5_8055,60,49,33,50,10,54,0,99,1
OCRL_5_8056,2673,2750,3310,434,1410,1953,2362,2175,4026
OXSM_5_8057,220,43,142,71,35,702,19,178,1
PACSIN1_5_8058,17,443,120,0,412,68,0,157,3
PAK4_5_8059,33,377,151,93,141,709,94,205,1115
PAK6_5_8060,1110,961,1298,293,57,1897,273,425,933
PAK7_5_8061,659,297,1020,536,754,64,384,37,44
PANK1_5_8062,1350,2245,1469,816,1355,2068,1935,1287,2490
PANK2_5_8063,2390,1739,2635,3181,3273,1681,1550,2521,1832
PCK2_5_8064,1027,652,931,328,827,412,1081,724,2505
PDK2_5_8065,714,809,651,616,422,844,798,888,649
PDK3_5_8066,995,1167,864,772,699,897,1298,715,117
PDPK1_5_8067,3327,3707,3899,3142,2048,4183,2724,2101,4220
PEG3_5_8068,1474,1668,2594,1766,1920,2766,1325,1461,3975
PFKFB2_5_8069,571,703,997,652,748,928,483,681,3254
PFKFB3_5_8070,1485,2111,1783,2970,2329,2031,1498,1017,2520
PFKM_5_8071,1618,1315,2456,2583,358,1060,842,655,876
PFKP_5_8072,834,445,1934,179,858,759,34,677,1467
PHKA1_5_8073,1415,1586,1194,2344,1704,2179,1721,902,2879
PHKB_5_8074,2391,3309,3295,1007,2975,3938,2686,2431,5441
PHKG2_5_8075,2247,2651,2923,1111,3840,1833,1583,2088,3670
PHPT1_5_8076,174,287,341,336,203,427,473,944,193
PI4KA_5_8077,926,678,881,508,1042,1141,579,905,634
PI4KB_5_8078,1510,1001,1289,478,1918,1782,1310,846,514
PIK3R1_5_8079,2056,1728,1809,1411,941,1532,1311,1196,1839
PIK3R5_5_8080,2833,2957,4470,1605,1503,2511,3164,3654,2725
PIKFYVE_5_8081,1478,1034,1293,399,629,1485,628,1135,567
PIP4K2C_5_8082,4363,4485,4355,4890,1168,5734,4401,3668,4574
PIP5K1A_5_8083,2167,4041,5057,1052,5340,5550,2117,2935,5351
PIP5K1C_5_8084,270,620,772,159,582,23,440,209,1841
PIP5KL1_5_8085,382,314,278,39,753,1932,842,539,716
PKLR_5_8086,262,552,64,7,1,0,2,4,46
PKMYT1_5_8087,335,183,355,437,92,370,35,88,1332
PKN1_5_8088,1822,1635,3039,2170,2245,1681,2424,1460,4384
PLK4_5_8089,3813,4211,4533,2482,3198,3913,4184,4790,4849
PNCK_5_8090,526,557,975,166,802,6,666,88,1088
PODXL_5_8091,78,217,340,64,138,314,79,563,1441
PPAP2A_5_8092,2388,1221,1959,2387,993,2688,3209,778,3883
PPAP2C_5_8093,333,276,346,602,4,1416,515,101,50
PPEF1_5_8094,856,861,489,269,257,285,515,856,361
PPFIA1_5_8095,773,363,383,309,883,265,16,8,351
PPFIA2_5_8096,976,722,1026,185,962,401,1274,230,297
PPM1A_5_8097,134,18,726,22,59,377,154,40,35
PPM1B_5_8098,1570,2201,2530,1902,3870,2411,421,1715,4504
PPM1M_5_8099,1090,760,840,478,344,348,1129,485,1796

PPP1CA_5_8100,1151,1031,864,667,390,979,320,1309,2253
PPP1CB_5_8101,145,524,534,366,1165,519,740,496,396
PPP1R12A_5_8102,795,1607,1418,743,1475,17,1118,1204,1532
PPP1R16B_5_8103,368,391,257,588,770,448,349,647,52
PPP1R1B_5_8104,957,1205,623,744,297,1712,242,709,125
PPP1R8_5_8105,192,95,137,17,92,14,405,522,1017
PPP2R1B_5_8106,1069,1308,1640,85,1900,1385,757,817,2289
PPP2R2A_5_8107,1014,927,2132,575,1525,1749,1367,1727,1702
PPP2R2B_5_8108,58,159,115,0,13,169,30,150,370
PPP2R2C_5_8109,430,160,164,0,83,631,58,130,194
PPP2R3A_5_8110,694,662,1143,537,226,1150,2341,517,3997
PPP2R4_5_8111,4068,7134,6001,1680,4184,7142,4080,3888,4361
PPP2R5A_5_8112,2684,3156,3183,2972,3923,1679,1317,1599,2901
PPP2R5D_5_8113,946,875,835,1161,1203,699,948,99,320
PPP3CA_5_8114,3430,4713,4363,4448,3771,5014,2523,3258,3870
PPP3CB_5_8115,5095,6886,7203,4458,3754,9616,4007,3715,3987
PPP4R1_5_8116,652,335,716,428,48,144,173,384,3147
PPP5C_5_8117,785,899,365,496,758,603,1527,987,843
PPP6C_5_8118,391,417,838,614,2552,345,93,282,1559
PRKAA1_5_8119,826,933,1175,898,1432,526,1175,606,1120
PRKACA_5_8120,993,1021,870,1039,423,837,795,2404,1027
PRKACB_5_8121,7802,7335,9440,6535,6340,4500,6715,8008,17739
PRKAG1_5_8122,1493,3018,3235,912,3830,4667,3874,2055,3670
PRKAG2_5_8123,1170,1477,1307,1042,1710,2734,1694,1876,2634
PRKAR1A_5_8124,750,1560,794,945,1171,585,729,818,1017
PRKAR1B_5_8125,1448,1431,1700,896,2290,437,3525,2181,1716
PRKCB_5_8126,2593,1985,1685,1084,2490,3057,1509,1605,1666
PRKCD_5_8127,68,417,337,8,1,34,343,126,2
PRKCQ_5_8128,62,1,0,753,0,0,102,0,1
PRKCZ_5_8129,228,578,422,951,0,1,313,203,614
PRKD2_5_8130,197,336,99,71,578,34,107,526,1220
PRKDC_5_8131,1485,2041,1451,549,1584,1999,1203,1483,3134
PRKG1_5_8132,60,178,97,38,40,319,221,20,308
PRPS2_5_8133,560,503,698,466,1428,205,434,205,846
PTK2B_5_8134,379,310,642,566,805,851,756,426,349
PTK2_5_8135,922,1308,898,204,1488,126,1778,1938,2433
PTK7_5_8136,2550,2964,3017,1908,4249,3333,3390,2448,2602
PTP4A2_5_8137,847,270,477,34,332,642,270,277,1795
PTP4A3_5_8138,1576,1897,2130,1661,1904,2386,1222,1905,2032
PTPDC1_5_8139,1552,1372,1678,1849,2023,1672,553,1543,1747
PTPN12_5_8140,864,498,691,547,856,1295,881,272,668
PTPN13_5_8141,8,72,107,6,54,14,52,30,6
PTPN18_5_8142,1877,1970,2362,1432,4147,3204,2272,2623,4066
PTPN22_5_8143,2443,2782,3142,1760,2032,3835,2194,3281,1866
PTPN2_5_8144,358,768,507,302,762,2293,311,562,846
PTPN3_5_8145,51,137,120,0,70,1,128,527,182
PTPN5_5_8146,323,757,316,7,164,1042,272,441,1051
PTPN6_5_8147,365,378,488,1440,852,27,120,727,50
PTPN7_5_8148,0,0,0,0,0,1791,0,787,0
PTPRA_5_8149,1072,818,1282,860,1700,968,1614,941,1782

PTPRB_5_8150,47,120,129,2,85,102,583,28,16
PTPRC_5_8151,595,1037,1576,1003,439,909,1327,460,1086
PTPRD_5_8152,3862,3004,5348,2863,3228,6072,4557,3706,1955
PTPRE_5_8153,965,1025,1821,1056,1265,813,322,1200,306
PTPRF_5_8154,262,424,845,337,357,241,240,133,3174
PTPRH_5_8155,552,302,182,1276,151,897,256,304,978
PTPRJ_5_8156,3225,1932,3343,3004,3720,4079,3924,2218,3758
PTPRK_5_8157,2815,2894,3317,2821,2061,2563,1601,4241,2366
PTPRM_5_8158,666,373,1320,325,121,100,559,829,215
PTPRN2_5_8159,784,454,1279,279,863,2005,338,1047,895
PTPRN_5_8160,3029,3860,3996,2466,7562,2952,3269,4388,2690
PTPRR_5_8161,1450,2882,3499,2973,3824,2320,2073,1989,3940
PTPRS_5_8162,963,788,718,1350,241,2735,394,182,946
PTPRT_5_8163,3068,4430,5045,3942,6117,3374,3502,2077,1492
PTPRU_5_8164,1164,935,1333,438,1125,880,1777,657,550
PTPRZ1_5_8165,3413,4057,3936,3530,4485,4966,2755,2339,3577
RET_5_8166,797,977,881,1833,705,905,802,665,645
RIOK1_5_8167,402,424,1133,54,630,387,168,646,1685
RIOK2_5_8168,1759,1663,1764,710,2087,824,1516,1689,2246
ROPN1L_5_8169,431,209,575,342,1215,330,663,90,71
RPS6KA1_5_8170,257,515,1000,91,508,1167,168,214,1469
RPS6KA2_5_8171,381,1007,428,271,450,37,298,546,51
RPS6KA4_5_8172,582,1061,911,759,4512,473,191,1882,302
RPS6KA5_5_8173,3808,4378,6294,4042,3328,4110,2422,4293,2700
RPS6KC1_5_8174,63,4,642,10,0,57,59,11,15
RYK_5_8175,1152,943,1969,1397,1034,1671,1323,1521,1167
SET_5_8176,1845,1938,2376,2379,3363,857,2473,1680,1402
SGK1_5_8177,419,700,1028,46,425,488,248,560,1187
SGK3_5_8178,841,848,1568,772,874,779,814,92,2800
SIRPA_5_8179,272,735,572,6,1065,5,322,263,559
SKAP1_5_8180,2341,1884,2069,1236,3716,2798,2621,852,1369
SNRK_5_8181,599,369,604,981,791,1092,549,237,1199
SPHK1_5_8182,627,546,728,960,552,2200,1194,190,365
SPHK2_5_8183,77,39,123,19,157,83,108,55,284
SRC_5_8184,1048,785,833,657,2317,366,791,765,1353
SRPK2_5_8185,664,720,564,79,183,484,196,564,1585
SRPK3_5_8186,354,180,475,117,0,100,71,388,41
SSH1_5_8187,710,355,842,0,119,465,591,95,269
STK19_5_8188,504,826,953,1438,905,813,803,953,2551
STK24_5_8189,25,29,9,135,0,0,9,225,10
STK31_5_8190,249,217,472,367,2,841,373,168,1
STK36_5_8191,862,1258,1225,1230,259,1891,1401,1761,2066
STRADA_5_8192,908,666,701,513,1556,536,900,1,84
STRADB_5_8193,583,719,568,1019,0,591,96,340,2594
STYX_5_8194,2488,1899,3023,1115,1612,3822,1640,3354,5148
SYK_5_8195,1458,898,899,2342,837,1761,1487,917,1893
SYNJ1_5_8196,210,110,202,25,16,19,87,327,0
SYNJ2_5_8197,439,938,795,769,420,400,254,1035,502
TAF1_5_8198,4083,4054,4691,3865,4860,5516,4032,4228,5492
TAOK1_5_8199,675,707,989,660,2663,137,440,478,1358

TAOK2_5_8200,2074,2757,2844,1989,2994,582,1657,3062,1358
TBCK_5_8201,9163,12745,11993,7091,9999,8581,8966,10970,13618
TEX14_5_8202,350,481,645,120,267,278,246,476,764
TGFBR1_5_8203,2184,1857,2390,2110,1787,1567,1838,616,4834
TGFBR2_5_8204,499,328,347,229,282,6,153,514,1217
TGFBR3_5_8205,799,870,1338,287,679,369,91,305,1391
THTPA_5_8206,310,565,565,225,683,924,626,1898,66
TJP2_5_8207,880,1153,717,1144,1650,702,452,934,755
TK2_5_8208,386,123,760,93,474,155,133,270,21
TLK1_5_8209,2217,1716,3206,1566,871,1836,3002,2245,1748
TLK2_5_8210,1482,2428,1786,1645,979,2435,2388,2475,3576
TMEM134_5_8211,2306,3587,3008,2957,4203,2649,2767,2495,3356
TNIK_5_8212,212,99,606,165,55,4,353,103,229
TNK2_5_8213,20,81,264,0,165,399,16,230,2
TPK1_5_8214,568,719,760,716,1432,770,745,277,1601
TPTE2_5_8215,1369,1597,2054,590,1178,1128,1366,1559,2358
TPTE_5_8216,1258,1018,904,1543,648,491,1228,610,307
TRIM24_5_8217,1624,1600,1833,2019,1415,2083,1129,1436,265
TRIM33_5_8218,1393,1508,2008,790,1943,512,1600,670,3604
TRMT2A_5_8219,104,23,40,0,72,16,266,6,73
TRPM6_5_8220,580,417,225,191,3,184,54,350,105
TSSK4_5_8221,299,356,287,273,394,156,538,168,169
TTK_5_8222,1525,1612,1939,766,3168,1884,773,820,4059
TTN_5_8223,569,546,1229,1275,181,779,1505,320,884
TWF1_5_8224,2677,3679,3005,982,1754,2911,1556,4230,3541
UCK1_5_8225,121,184,358,240,201,36,460,217,499
UCKL1_5_8226,41,221,173,325,284,206,25,681,73
UHMK1_5_8227,1736,1695,2450,1961,3054,3486,2185,1519,1542
ULK2_5_8228,1162,1356,1038,456,718,1132,851,1248,478
VRK2_5_8229,855,1286,1605,569,757,3852,1431,920,3603
VRK3_5_8230,4073,4366,4895,4555,4065,6850,2663,4618,7229
WEE1_5_8231,1309,1643,2099,734,1237,1535,965,1216,1278
WNK1_5_8232,1362,1640,1901,1986,604,2687,1271,507,740
WNK3_5_8233,1130,1700,1731,224,2666,1359,1020,958,911
YSK4_5_8234,645,603,1142,232,1063,2447,333,453,390
ZAP70_5_8235,542,707,1009,78,200,2178,920,429,734
ACP2_5_8236,875,703,1156,2352,439,1261,586,1063,302
CDK1_5_8237,893,668,1391,1794,593,1134,610,376,1389
DUSP10_5_8238,1525,1257,1902,1081,891,681,1389,583,4302
DUSP15_5_8239,501,462,356,591,412,597,372,500,421
ERBB3_5_8240,2041,2047,1885,2123,1849,2048,3284,1863,1925
G6PC2_5_8241,186,201,132,0,6,134,216,14,0
IP6K2_5_8242,87,103,210,41,365,587,9,28,69
MYLK_5_8243,95,110,440,490,321,13,617,5,298
PRPS1_5_8244,942,941,1012,3158,983,1375,387,849,383
PTPMT1_5_8245,3231,1933,3359,2299,1903,3142,3436,2514,1381
PTPN20A_5_8246,681,1168,684,11,385,560,302,506,96
PTPN20B_5_8247,681,1168,684,11,385,560,302,506,96
SPEG_5_8248,22,15,794,0,1236,21,161,0,733
STK32A_5_8249,278,170,578,0,0,941,270,104,340

PPP1R12B_5_8250,606,621,1012,1119,279,383,571,880,122
PPP1R12B_5_8251,1740,2241,2272,2125,2268,1867,1434,1336,3323
PPP1R12B_5_8252,1089,1428,1565,586,1299,1413,1609,392,1727
AAK1_5_8253,844,1545,1369,911,399,654,1138,773,1241
ACP6_5_8254,205,748,417,62,20,835,333,414,336
ACPT_5_8255,471,84,143,409,98,563,209,3,14
ACVR2A_5_8256,1677,1306,2328,301,2286,1326,1524,1551,2658
ACVR2B_5_8257,0,1,0,0,0,0,0,0,0
ADCK2_5_8258,2097,1861,1744,148,606,1537,2112,2003,2160
ADCK3_5_8259,49,105,57,48,12,22,137,320,2
ADCK5_5_8260,241,1177,1058,338,3152,111,137,740,94
ADPGK_5_8261,385,548,840,596,363,1223,295,827,550
ADRBK1_5_8262,799,710,664,660,374,1070,559,564,1261
ADRBK2_5_8263,508,396,1027,1262,418,528,536,453,1675
AGK_5_8264,954,955,933,741,543,1536,1433,1039,478
AK1_5_8265,156,139,133,197,11,66,14,0,0
AK7_5_8266,1436,2164,2196,1981,3096,2387,348,2433,1390
AK8_5_8267,103,271,435,354,879,626,231,124,239
ALK_5_8268,313,280,274,0,626,205,11,433,329
ALPI_5_8269,367,785,605,971,446,471,373,1196,43
ALPK2_5_8270,400,400,399,677,513,60,53,59,135
ALPK3_5_8271,866,677,638,792,324,1132,292,574,978
ALPP_5_8272,1702,2464,2808,2365,1683,798,1865,1231,4292
ALPPL2_5_8273,1702,2464,2808,2365,1683,798,1865,1231,4292
ANKK1_5_8274,362,583,646,444,903,1109,529,135,280
ARAF_5_8275,278,224,569,4,226,886,734,37,795
ATM_5_8276,6227,7259,6946,6566,10395,6865,7002,8021,11831
AURKB_5_8277,396,1468,1012,1085,607,1155,249,1181,610
BLK_5_8278,716,970,1403,2068,970,1580,969,598,609
BMPR1A_5_8279,164,238,194,4,147,111,258,579,2
BMPR1B_5_8280,565,1228,737,939,1727,884,418,440,966
BMPR2_5_8281,2576,2151,3018,2794,4119,4851,2808,2211,4660
BPNT1_5_8282,229,408,523,210,267,353,284,301,1267
BRAF_5_8283,2713,2462,3490,2683,3702,4108,2853,2779,6123
BRD3_5_8284,5131,3026,4335,2421,3356,5846,4648,5354,6560
BRSK1_5_8285,338,246,182,203,364,1151,40,26,180
BRSK2_5_8286,720,971,889,687,80,1731,1114,211,1390
BTK_5_8287,1767,2326,2075,1136,3820,2859,1417,2236,2722
BUB1B_5_8288,6644,10044,12228,8041,13832,7196,7743,11094,12281
BUB1_5_8289,258,1013,563,22,622,330,138,193,280
C20orf111_5_8290,1434,1461,920,885,404,1612,740,472,1121
C9orf96_5_8291,363,1175,193,626,1086,110,348,689,401
CALM1_5_8292,622,371,449,699,270,549,87,418,25
CALM3_5_8293,43,69,214,5,379,775,150,0,8
CAMK1_5_8294,2380,3023,3077,2930,4282,2952,3231,3068,2484
CAMK1G_5_8295,2765,1870,2359,3229,2632,1275,2920,2761,5025
CAMK2N1_5_8296,953,1217,1185,529,1417,1614,1075,782,246
CAMK4_5_8297,891,581,1765,821,1888,1562,188,1598,85
CAMKV_5_8298,544,988,758,193,262,1040,716,576,193
CARD11_5_8299,144,144,395,9,44,440,133,143,651

CCL2_5_8300,2144,1915,3164,2574,4424,3378,1739,1423,3086
CDC42BPB_5_8301,805,1316,1234,1010,2376,805,1176,978,899
CDC42BPG_5_8302,85,155,252,3,57,304,652,514,987
CDK14_5_8303,173,7,203,656,83,188,239,204,29
CDK15_5_8304,4563,5675,5471,4216,3420,4592,2689,2446,2404
CDK19_5_8305,1040,1518,814,545,1602,1559,517,681,917
CDK3_5_8306,744,1896,1443,517,674,1359,800,568,741
CDK4_5_8307,395,528,620,1287,187,371,535,507,290
CDK5R1_5_8308,374,392,755,1292,2608,1014,1130,9,18
CDK7_5_8309,3718,5290,4862,807,1750,4088,5309,3815,2322
CDK8_5_8310,1025,1192,1483,656,489,1902,1351,447,1365
CDK9_5_8311,1412,1005,1020,747,652,317,1038,1423,1982
CDKL1_5_8312,2438,1972,2462,1977,434,2716,3308,1951,2832
CDKL2_5_8313,890,908,576,853,1347,317,428,1046,623
CDKL4_5_8314,13044,9358,13960,10880,12082,14077,9229,9406,10096
CDKN1B_5_8315,995,793,995,709,1589,1718,598,646,2186
CERK_5_8316,158,216,216,337,353,510,1399,992,18
CHKB_5_8317,470,1471,1127,989,537,1869,439,1667,1020
CHUK_5_8318,167,503,276,765,229,497,633,191,516
CIB2_5_8319,1736,1801,2040,1757,1176,1343,2700,1611,1613
CILP_5_8320,597,1183,719,1567,376,1275,196,697,404
CKB_5_8321,375,143,506,988,186,377,117,181,358
CKM_5_8322,1805,2406,1533,1147,1850,2092,1323,1688,2299
CKMT1A_5_8323,2746,2098,3206,2749,3129,3577,1964,2534,2785
CKMT1B_5_8324,2746,2098,3206,2749,3129,3577,1964,2534,2785
CKS1B_5_8325,2663,2941,2840,2089,1468,2862,2983,1926,1786
CKS2_5_8326,1449,1797,2106,2421,3248,1125,823,1650,2852
CLK2_5_8327,235,140,537,818,1068,4,630,377,187
CLK4_5_8328,1627,1738,1989,515,2317,936,1338,1406,700
CMPK2_5_8329,319,134,99,46,5,50,225,174,57
CPNE3_5_8330,566,778,815,167,845,693,671,663,1757
CRIM1_5_8331,1351,1949,2048,668,1388,1117,1820,1412,1641
CRKL_5_8332,156,417,257,288,179,297,90,290,461
CSF1R_5_8333,154,316,158,564,0,46,131,279,0
CSNK1A1L_5_8334,100,226,79,4,346,100,37,95,45
CSNK1G1_5_8335,720,837,1240,1288,318,621,1156,1073,875
CSNK1G2_5_8336,230,326,406,256,232,296,550,14,978
CSNK2A2_5_8337,2195,1867,2665,2080,2322,2919,2062,1606,2395
CSNK2B_5_8338,101,80,297,33,134,44,331,351,846
CTDSP2_5_8339,3313,4636,4612,3926,4408,6743,4697,2125,2971
DAPK1_5_8340,1240,1413,1543,254,772,1306,1388,1292,622
DAPK2_5_8341,779,1949,1494,323,836,1666,1059,1316,867
DAPK3_5_8342,394,287,706,86,1184,2468,362,1556,768
DBF4_5_8343,1155,960,1272,127,2091,1265,467,466,3077
DCK_5_8344,345,693,369,171,760,344,185,549,925
DCLK3_5_8345,1135,602,718,102,1334,2651,1014,913,668
DGKE_5_8346,921,1195,1713,537,934,3868,1361,2031,1586
DGKI_5_8347,5,59,86,1,110,0,144,96,150
DGKK_5_8348,633,914,547,183,1084,386,131,1010,20
DGKQ_5_8349,330,161,292,24,42,481,125,48,16

DOLK_5_8350,103,448,227,6,770,138,63,115,119
DOT1L_5_8351,2609,3294,2884,2972,3992,1994,2492,3617,3820
DUSP11_5_8352,3103,2660,3820,2542,2191,3497,3338,2040,3069
DUSP12_5_8353,635,379,849,235,586,774,187,1011,2067
DUSP14_5_8354,115,118,65,0,1505,686,9,9,5
DUSP16_5_8355,1516,779,1569,554,1937,1229,669,3227,1010
DUSP18_5_8356,883,972,1596,248,2479,1573,1243,1923,1498
DUSP21_5_8357,1292,1546,1350,1732,1210,338,1084,2865,499
DUSP22_5_8358,2634,1973,2172,1558,1752,1582,2515,1441,3029
DUSP2_5_8359,2224,2782,1768,962,1784,3367,1205,1283,175
DUSP3_5_8360,198,986,740,333,1000,793,274,924,878
DUSP5_5_8361,281,227,523,115,11,1384,85,221,78
DUSP7_5_8362,910,1199,1549,251,901,1527,1054,1453,3028
DUSP8_5_8363,3054,2661,3089,3329,2256,5440,1540,4775,1651
DUSP9_5_8364,811,1118,862,357,658,834,1170,586,390
DYRK4_5_8365,1157,914,1422,16,546,1049,549,1642,932
EEF2K_5_8366,135,86,409,0,34,0,32,132,208
EFNA2_5_8367,338,381,802,143,506,2040,401,882,778
EFNA3_5_8368,59,136,144,284,7,8,168,6,604
EFNA5_5_8369,626,694,1145,727,1007,1578,932,347,1383
EFNB3_5_8370,1598,1210,1571,2296,727,2045,666,1629,292
EIF2AK3_5_8371,776,1072,699,347,988,170,1512,947,617
EIF2AK4_5_8372,136,138,44,0,499,22,0,201,0
EPHA1_5_8373,1574,1299,1664,853,2023,2600,2601,945,832
EPHA2_5_8374,500,480,408,73,278,641,404,172,158
EPHA4_5_8375,2274,2510,2145,821,1776,2699,1568,2499,2336
EPHA7_5_8376,196,182,414,621,385,306,475,224,507
EPHB1_5_8377,185,118,109,195,343,438,109,190,225
EPHB3_5_8378,1638,1749,2028,504,833,1248,913,354,2743
EPHB4_5_8379,301,257,106,212,7,15,610,2,263
EPHB6_5_8380,1442,1129,976,554,539,1469,1562,1251,1564
ERN1_5_8381,401,651,402,673,1223,224,58,304,732
ERN2_5_8382,1808,1704,2946,1764,989,957,1789,851,1208
ETNK2_5_8383,372,284,582,3,467,274,951,670,81
EYA3_5_8384,1727,1897,1429,3082,1892,2897,1818,1066,1291
FER_5_8385,4435,5040,4812,4624,3387,2647,4645,3623,1232
FLT3_5_8386,653,695,691,495,1098,574,1169,551,881
FN3K_5_8387,1028,1779,1453,1026,261,1447,1160,866,2039
FN3KRP_5_8388,259,418,196,17,624,9,336,928,58
FRK_5_8389,621,267,338,0,4,20,489,600,1
FUK_5_8390,496,167,1054,8,737,126,199,864,1269
G6PC3_5_8391,31,547,399,28,395,708,786,110,0
G6PC_5_8392,2302,2720,2470,2847,1337,1794,2577,953,1838
GAK_5_8393,932,1987,1268,1885,1475,526,364,941,2534
GALK1_5_8394,1796,2061,2524,1740,1727,1978,1700,998,2528
GDPD4_5_8395,1162,1585,1720,477,671,1040,618,1769,1228
GOLGA5_5_8396,1811,3215,3308,737,2684,1567,2365,543,3377
GRK1_5_8397,3092,2809,2794,2754,2604,3684,1624,3158,1323
GRK5_5_8398,355,403,319,1,95,828,264,218,1412
GRK7_5_8399,214,84,78,118,3,10,146,161,1177

GSG2_5_8400,1199,1088,1048,651,996,1812,681,649,1462
GSK3A_5_8401,1102,340,961,769,377,2355,560,1126,1030
GUCY2C_5_8402,873,93,446,397,53,1411,464,323,388
GUCY2D_5_8403,470,342,1132,24,563,564,38,439,621
GUCY2F_5_8404,375,364,396,397,340,97,554,440,28
HIPK4_5_8405,55,520,422,0,177,362,372,13,489
HKDC1_5_8406,846,408,816,64,1999,736,680,690,2213
HOOK3_5_8407,1043,936,799,583,1562,1541,1756,1899,1773
HSPB8_5_8408,6440,6251,6756,4681,6775,5430,5348,5307,3820
HUNK_5_8409,253,317,172,582,808,552,367,488,1
HUS1_5_8410,1781,2221,2297,662,3042,1265,1848,2763,1445
IGBP1_5_8411,456,509,995,1756,1570,1002,385,464,861
IGF1R_5_8412,1616,1678,1778,1004,2374,3495,459,1110,1084
IGF2R_5_8413,1431,577,1211,621,808,198,810,1121,1954
ILKAP_5_8414,478,707,412,782,137,53,706,785,180
IMPA2_5_8415,1548,1733,2212,937,1192,3400,1752,2419,905
INPP5A_5_8416,2658,2990,2472,2036,4691,4915,1952,3573,4455
INPP5B_5_8417,1355,1496,2085,335,2602,2860,864,949,7
INPP5J_5_8418,553,1141,1524,1774,1264,1531,368,790,1229
INPPL1_5_8419,1332,1036,1898,413,272,1295,505,1672,1675
INSRR_5_8420,472,271,621,12,405,530,170,21,981
IPMK_5_8421,994,1007,2042,3588,762,3986,736,2384,4885
IPPK_5_8422,3633,3565,6354,3194,4068,4151,4194,3191,8064
IRAK2_5_8423,261,107,95,368,0,57,72,498,241
ITK_5_8424,531,1168,920,795,534,1049,678,1550,278
ITPKA_5_8425,129,615,232,0,1397,113,227,742,1
ITPKB_5_8426,575,1664,1087,904,527,297,1036,931,1764
ITPKC_5_8427,63,84,126,59,3,1,232,76,17
JAK1_5_8428,2425,2594,3198,2342,3019,3690,1745,2142,2733
JAK2_5_8429,3427,3483,6194,5345,7068,4783,3942,5474,3980
JAK3_5_8430,1779,2079,2070,2424,2457,1452,642,1764,396
KDR_5_8431,501,903,820,19,163,511,56,1360,824
KSR1_5_8432,156,311,452,262,726,364,326,250,752
LATS1_5_8433,59,48,52,279,366,110,28,201,374
LATS2_5_8434,808,638,887,277,285,1450,94,9,1750
LLGL1_5_8435,104,199,477,69,202,72,369,59,322
LMTK2_5_8436,593,361,885,581,1381,761,744,427,933
LMTK3_5_8437,367,369,311,268,261,26,1456,123,315
LRRK1_5_8438,423,488,742,349,347,2193,405,535,549
LRRK2_5_8439,3487,4498,4110,3417,3378,3757,3041,5291,5177
MAGI2_5_8440,736,1551,1089,1786,717,890,2701,1383,1880
MAP2K1_5_8441,702,1394,1631,172,943,640,1542,2024,822
MAP2K2_5_8442,61,333,13,0,0,73,0,590,32
MAP2K4_5_8443,679,1048,1155,1143,1359,453,700,1051,623
MAP2K6_5_8444,439,942,1677,798,420,903,370,297,905
MAP2K7_5_8445,332,512,549,169,596,1844,679,115,121
MAP3K10_5_8446,172,471,310,1426,60,1253,576,282,386
MAP3K11_5_8447,345,334,686,1192,256,743,464,107,473
MAP3K14_5_8448,672,1617,593,787,1136,657,521,1460,537
MAP3K15_5_8449,1326,1425,1114,367,1776,266,651,1183,1508

MAP3K1_5_8450,296,643,642,495,693,388,552,920,1449
MAP3K2_5_8451,824,627,718,1995,1341,1084,232,610,1289
MAP3K5_5_8452,331,416,648,37,340,366,377,294,886
MAP3K6_5_8453,643,547,442,533,23,1049,310,255,978
MAP3K8_5_8454,1514,1663,2138,2506,2270,4782,2033,1907,2704
MAP3K9_5_8455,280,114,216,545,125,95,117,638,1091
MAP4K2_5_8456,607,851,854,197,67,515,212,152,208
MAP4K3_5_8457,460,582,458,503,1416,1394,1089,1080,551
MAPK11_5_8458,220,369,403,780,60,295,276,86,1037
MAPK12_5_8459,2455,2619,2101,2220,1711,2609,1172,1331,746
MAPK13_5_8460,2362,2227,2780,3099,3929,5630,1473,3579,2520
MAPK15_5_8461,235,400,225,1583,385,747,370,70,140
MAPK4_5_8462,339,49,58,116,18,0,2,154,0
MAPK6_5_8463,337,464,592,190,318,1100,213,308,569
MAPKAPK3_5_8464,534,738,1292,1578,747,1566,1629,707,696
MARK1_5_8465,2405,3729,3202,1807,6917,2960,3888,5094,6565
MAST2_5_8466,166,472,848,652,294,321,290,440,1
MAST3_5_8467,378,200,341,45,8,1083,588,267,8
MELK_5_8468,899,549,813,1669,612,246,181,50,347
MERTK_5_8469,3288,5409,4050,3631,4152,5463,3890,4990,5107
MEX3B_5_8470,6,24,53,138,0,0,3,12,3
MPP3_5_8471,186,98,418,53,6,608,119,74,3169
MPP5_5_8472,623,401,778,409,217,214,823,718,135
MST1R_5_8473,109,79,79,480,5,8,955,11,16
MTM1_5_8474,515,608,223,202,2033,448,368,297,319
MTMR1_5_8475,484,890,899,1112,2024,436,1238,764,1260
MTMR4_5_8476,1045,722,825,584,609,104,878,430,351
MTMR6_5_8477,886,830,1226,367,415,1532,1540,215,361
MTMR7_5_8478,505,466,328,0,673,392,459,537,1087
MTMR9_5_8479,3011,4008,3858,1613,6748,4078,2506,1342,5259
MTOR_5_8480,1148,1016,1756,1969,480,1140,250,1528,2989
MYLK2_5_8481,1875,1298,1523,802,984,1311,1709,914,2425
MYLK3_5_8482,50,472,253,767,92,260,2,163,58
MYLK4_5_8483,255,317,350,4,11,50,7,608,117
MYO3A_5_8484,2127,1728,2802,2155,1771,2361,2090,2193,1667
N4BP2_5_8485,1303,1491,1966,2006,1221,2697,1354,1585,2463
NAGK_5_8486,58,226,245,104,11,0,309,83,198
NAGS_5_8487,57,310,470,8,85,199,26,251,15
NEK10_5_8488,972,496,1206,837,1396,476,1142,55,828
NEK5_5_8489,1,0,0,0,0,54,1,13,0
NEK7_5_8490,327,309,647,7,448,1289,292,278,2
NEK8_5_8491,541,389,839,232,130,443,487,140,29
NEK9_5_8492,597,986,344,398,484,467,1151,1027,1046
NME3_5_8493,1204,693,1827,482,1769,1640,2337,753,1243
NME4_5_8494,421,866,264,167,10,129,20,133,0
NME5_5_8495,111,140,459,192,245,835,53,210,19
NME6_5_8496,1415,2074,1945,1081,3578,496,1686,1679,1286
NPR1_5_8497,68,16,123,0,90,314,0,33,0
NPRL2_5_8498,368,445,368,157,46,97,366,489,9
NRBP1_5_8499,297,583,661,502,694,66,333,660,566

NRBP2_5_8500,23,533,24,0,482,390,4,198,52
NRK_5_8501,1074,1273,1758,668,1418,1161,993,635,1848
NT5C1A_5_8502,351,614,1102,1116,718,1333,828,1136,529
NT5C_5_8503,241,429,678,232,137,336,278,676,51
NT5M_5_8504,71,123,328,134,831,368,44,42,56
NUAK1_5_8505,215,255,350,0,1059,699,20,224,240
NUAK2_5_8506,642,1148,570,143,722,815,332,1348,1
NUCKS1_5_8507,3357,4049,4453,3130,4723,3025,3160,3962,4911
OXSR1_5_8508,684,955,623,716,136,193,387,758,922
PAK2_5_8509,494,1197,965,571,1826,302,1198,830,147
PANK3_5_8510,722,437,1203,166,185,1888,911,462,2879
PANK4_5_8511,71,203,183,133,8,70,188,191,169
PASK_5_8512,13525,15689,14798,11374,15066,14331,13514,13251,15279
PBK_5_8513,1553,1342,1604,1425,1944,1554,1355,1684,1863
PCK1_5_8514,775,798,1358,862,3001,1088,1266,399,691
PDCD1_5_8515,1672,2104,1925,1166,1192,2329,1427,1880,1888
PDGFRA_5_8516,1082,1258,1095,312,1395,2179,1110,401,1913
PDGFRB_5_8517,1689,1647,2039,543,4868,1273,2392,1723,2635
PDGFRL_5_8518,719,1032,1187,1010,1693,950,1281,899,1956
PDIK1L_5_8519,3976,3286,2611,4957,1125,1825,2743,2235,683
PDK4_5_8520,151,154,291,544,51,730,443,3,663
PDP2_5_8521,862,1231,1265,758,626,1052,690,1070,1642
PDXK_5_8522,398,225,273,17,66,399,280,45,83
PFKFB1_5_8523,16,31,18,0,1,0,0,0,0
PFKFB4_5_8524,454,301,333,217,851,192,115,94,0
PFKL_5_8525,5,7,82,1,9,0,23,0,0
PGAM2_5_8526,1478,1353,2487,546,663,1285,835,992,2160
PGK1_5_8527,341,301,619,243,676,1064,119,149,360
PGK2_5_8528,572,562,1095,637,1305,2102,498,1662,1491
PHKA2_5_8529,2144,2032,2537,3041,2888,775,1538,2473,1717
PHKG1_5_8530,44,33,5,0,0,1432,1,37,0
PI4K2A_5_8531,135,180,203,1,781,137,84,98,1031
PI4K2B_5_8532,1315,2985,2623,1002,3810,1940,3269,2057,907
PIK3C2A_5_8533,3397,4469,3352,1398,7473,5061,3395,4221,5330
PIK3C2B_5_8534,1983,1339,1863,1474,1992,3516,1545,2297,4042
PIK3C2G_5_8535,1523,1292,1644,391,987,1903,230,2555,202
PIK3C3_5_8536,811,2699,2126,833,1262,2869,922,2038,2110
PIK3CA_5_8537,4297,4689,4397,4422,4789,9356,2757,4885,8588
PIK3CB_5_8538,1192,1729,1292,346,1482,1788,1055,1105,2199
PIK3CD_5_8539,993,742,1107,677,1017,600,324,1016,847
PIK3CG_5_8540,1077,868,1548,868,43,3019,537,1102,2620
PIK3R2_5_8541,760,1083,1031,687,204,971,1090,590,2606
PIK3R4_5_8542,109,217,366,240,311,7,326,745,124
PIK3R6_5_8543,1442,1541,2750,1624,1149,1592,961,2211,1046
PIM2_5_8544,1445,924,602,1374,991,217,571,337,280
PIM3_5_8545,284,1037,566,244,22,332,458,74,1114
PINK1_5_8546,347,875,602,522,0,910,748,60,138
PIP4K2A_5_8547,912,794,1441,1129,410,393,334,652,3357
PIP4K2B_5_8548,749,121,735,24,632,170,664,353,2916
PIP5K1B_5_8549,530,614,1234,1578,1125,331,662,556,2232

PKDCC_5_8550,1295,1932,2172,616,1494,1485,881,1853,1928
PKN2_5_8551,4731,6143,6271,3509,6864,7596,4719,4251,3026
PKN3_5_8552,4211,3289,5296,3052,3343,3765,4952,2060,6007
PLK1_5_8553,116,534,277,275,534,214,435,393,392
PLK2_5_8554,2178,1481,2499,999,2424,3667,1898,2111,1227
PLK3_5_8555,1250,1708,1448,1049,460,967,2202,1615,382
PMVK_5_8556,1053,1055,1189,964,1571,1106,1186,921,650
PNKP_5_8557,63,238,230,597,10,241,217,91,158
POLD1_5_8558,51,27,261,8,188,104,132,251,241
PON1_5_8559,428,1075,842,1121,1802,237,551,284,871
PPAP2B_5_8560,1016,255,798,146,504,388,1179,649,28
PPEF2_5_8561,471,824,1037,659,516,239,349,808,380
PPFIA3_5_8562,781,396,637,582,266,659,294,330,79
PPFIA4_5_8563,103,232,113,184,0,20,0,4,857
PPM1D_5_8564,189,290,127,324,90,1,206,611,95
PPM1E_5_8565,326,329,177,1068,152,203,213,292,303
PPM1F_5_8566,111,166,6,6,57,0,2,0,703
PPM1G_5_8567,356,559,392,420,203,1621,408,173,186
PPM1K_5_8568,82,194,257,1,471,507,209,254,323
PPM1L_5_8569,1084,744,1307,766,850,1115,1092,746,73
PPP1CC_5_8570,1329,698,2104,1072,464,489,1249,452,697
PPP1R12C_5_8571,128,180,133,140,15,0,10,83,694
PPP1R13B_5_8572,718,876,1005,471,2257,1406,688,1080,194
PPP1R14B_5_8573,878,478,888,7,357,1944,76,688,99
PPP1R15B_5_8574,0,0,0,0,0,0,0,0,0
PPP1R1A_5_8575,1055,1112,1118,2073,561,1632,789,445,985
PPP1R1C_5_8576,957,1881,1017,1534,1629,1677,1835,1719,788
PPP1R2_5_8577,1628,2313,3774,2309,4558,1894,2344,2027,3573
PPP1R3A_5_8578,916,1002,548,581,494,815,362,220,777
PPP1R3C_5_8579,1186,1305,1738,2221,1818,2878,1344,701,1721
PPP1R3D_5_8580,321,320,606,282,68,1182,582,23,957
PPP1R7_5_8581,492,420,357,131,739,443,259,284,280
PPP2CA_5_8582,1314,1340,2071,1897,3292,2048,1935,1586,1985
PPP2CB_5_8583,788,1599,2257,2601,1546,1547,1448,1389,1853
PPP2R1A_5_8584,311,274,393,1,307,757,394,44,723
PPP2R2D_5_8585,492,324,252,672,52,34,61,25,84
PPP2R3B_5_8586,927,643,1534,955,1135,2158,819,622,2972
PPP2R5B_5_8587,1169,2174,2169,2593,4217,1010,1500,2099,1919
PPP2R5E_5_8588,5139,4949,5250,9633,5256,5665,4353,3599,4060
PPP3CC_5_8589,861,1124,1894,592,729,494,1869,1465,2515
PPP3R1_5_8590,933,1388,1239,535,1636,343,2100,549,2921
PPP3R2_5_8591,4117,3626,4677,2574,5868,5269,3236,5951,2797
PPP4C_5_8592,991,240,822,1259,341,422,916,191,37
PPTC7_5_8593,32,108,275,66,0,708,315,59,2
PRKAA2_5_8594,176,668,698,540,707,1081,1270,776,793
PRKAB1_5_8595,3872,3820,4987,2667,4651,4040,3051,2935,3302
PRKAB2_5_8596,1756,1913,1775,1625,2165,874,1837,1717,1083
PRKACG_5_8597,527,384,633,33,15,912,228,120,7
PRKAG3_5_8598,620,969,1255,909,789,1415,1222,935,738
PRKAR2A_5_8599,194,21,1,3,12,291,41,49,91

PRKAR2B_5_8600,1931,3079,2922,2250,1355,1862,1550,1422,3336
PRKCA_5_8601,1986,1347,892,1797,1159,1839,1657,1339,1285
PRKCE_5_8602,941,1473,1343,438,444,479,508,353,941
PRKCG_5_8603,3983,4608,5472,4723,4531,4669,3556,3770,4789
PRKCH_5_8604,916,1140,1021,1824,583,704,1797,941,2064
PRKCI_5_8605,1569,2245,1321,768,2392,2889,1050,2865,929
PRKD1_5_8606,639,500,972,833,448,121,105,537,2360
PRKD3_5_8607,10612,11011,12455,11434,11979,11875,12292,11923,13012
PRKG2_5_8608,1618,2051,1952,1264,2291,2974,2128,1796,906
PRKX_5_8609,294,478,375,658,166,105,741,385,21
PRPF4B_5_8610,2427,1992,2888,3609,1025,2248,2802,2964,4225
PRPS1L1_5_8611,3169,3065,3038,2821,2076,2269,3295,3899,1924
PSKH1_5_8612,1660,1852,2315,2252,2441,2579,1047,1397,2610
PSKH2_5_8613,368,363,478,365,207,92,314,219,923
PSPH_5_8614,1645,1063,1398,450,1878,2153,547,852,740
PSTK_5_8615,795,509,768,1594,347,900,1188,1213,452
PTEN_5_8616,5,227,421,31,567,92,24,120,2
PTK6_5_8617,361,129,44,151,278,0,121,8,0
PTP4A1_5_8618,386,293,285,896,217,305,166,641,305
PTPLA_5_8619,1080,998,1525,1043,1998,152,1167,2729,9
PTPLB_5_8620,2404,3921,5288,2944,2154,2981,3876,2270,4620
PTPN11_5_8621,1382,998,2433,697,2195,1663,3380,1106,992
PTPN14_5_8622,855,931,1408,558,660,565,1460,1152,1269
PTPN1_5_8623,257,214,216,28,1758,342,354,390,112
PTPN21_5_8624,1131,1961,1226,1375,1530,948,1246,842,2289
PTPN23_5_8625,91,78,369,32,4,15,222,11,21
PTPN4_5_8626,273,295,1074,568,983,330,439,201,194
PTPN9_5_8627,477,179,1050,84,928,232,352,238,830
PTPRG_5_8628,1227,1695,1402,1358,343,1322,1563,2178,49
PXK_5_8629,1071,764,1221,1553,703,790,592,1023,755
RAF1_5_8630,284,216,273,204,320,281,196,155,3
RBKS_5_8631,1195,848,1665,1311,911,832,1168,642,1175
RFK_5_8632,528,568,475,1216,458,593,634,564,1026
RIOK3_5_8633,2041,2573,2570,1871,1640,2343,2251,705,2240
RIPK1_5_8634,945,426,1117,539,2171,632,927,608,1875
RIPK2_5_8635,332,321,1134,134,1317,288,668,156,1849
RIPK3_5_8636,373,364,604,529,327,692,159,125,1094
RIPK4_5_8637,274,66,228,277,3,152,0,14,885
RNASEL_5_8638,255,344,404,28,717,356,35,475,1821
RNGTT_5_8639,951,1176,1281,903,2280,1969,1456,751,1321
ROCK1_5_8640,4,4,33,31,0,0,1,78,52
ROCK2_5_8641,5926,5025,8117,4494,7434,7309,4134,6793,6505
ROS1_5_8642,2281,1884,2585,2026,3184,2845,3666,2658,4687
RPS6KA3_5_8643,1895,1313,1746,813,848,1344,1261,1347,1064
RPS6KA6_5_8644,1218,892,1538,1574,426,2236,467,1072,2714
RPS6KB1_5_8645,824,1624,1327,602,2045,1336,673,1448,1778
RPS6KB2_5_8646,749,423,725,477,95,453,641,423,393
RPS6KL1_5_8647,179,418,484,3039,317,794,297,106,768
RSPO3_5_8648,1596,1540,2913,1762,1666,2504,2407,1556,3758
SACM1L_5_8649,6624,7565,7973,3211,6926,10000,5995,6530,7424

SBF1_5_8650,71,28,136,7,2,0,99,857,0
SBK1_5_8651,439,76,36,0,864,149,105,156,1
SBK2_5_8652,133,6,269,0,0,146,566,100,16
SEPHS2_5_8653,569,382,186,292,6,964,73,214,112
SGPP1_5_8654,353,1141,837,240,344,197,936,1622,2668
SHPK_5_8655,14,13,32,213,0,0,5,1,26
SIK1_5_8656,2530,1771,2697,2983,2968,2362,2644,2069,3727
SIK2_5_8657,163,548,505,28,102,1154,241,438,193
SIK3_5_8658,303,461,500,385,386,962,798,438,1515
SKAP2_5_8659,561,373,485,12,724,21,280,197,483
SMG1_5_8660,1316,707,1178,1931,2280,1075,842,667,518
SRMS_5_8661,113,243,216,33,1757,165,0,34,34
SRP72_5_8662,908,675,1422,637,287,1303,758,1461,775
SRPK1_5_8663,5192,6841,6906,4831,6626,6648,5552,7229,9580
SSH2_5_8664,537,69,286,48,18,622,284,321,212
SSH3_5_8665,90,199,165,29,20,12,4,10,0
STC1_5_8666,555,129,699,450,460,24,4,264,1355
STK10_5_8667,677,871,860,349,1728,2147,1712,93,866
STK11_5_8668,1003,967,1324,2361,2561,2398,1089,434,3387
STK16_5_8669,254,256,552,136,1164,123,873,1100,1229
STK17A_5_8670,1164,1510,1369,419,777,1169,1158,1645,1872
STK17B_5_8671,3092,3769,3868,4583,4449,3547,3617,3966,10189
STK25_5_8672,2043,2387,2410,1129,2744,3567,1976,2457,3009
STK32B_5_8673,1161,669,1793,833,631,886,1471,1261,2285
STK32C_5_8674,2321,1602,2625,2479,5337,3609,2007,1531,1235
STK33_5_8675,663,928,1040,192,581,409,148,595,1308
STK35_5_8676,1816,1644,2054,2085,3095,2827,1863,652,1431
STK38_5_8677,1286,839,2086,1792,837,1547,2153,462,1690
STK38L_5_8678,524,1390,690,767,1384,1662,749,706,1999
STK39_5_8679,332,61,402,2,0,108,33,0,0
STK40_5_8680,188,204,265,33,30,0,172,462,51
STK4_5_8681,2583,2461,3235,2466,5463,2643,1755,3690,1997
STYK1_5_8682,784,1493,2136,1031,357,2702,635,760,923
STYXL1_5_8683,460,431,576,1194,877,61,605,509,770
TAF1L_5_8684,351,349,403,147,499,486,99,857,2424
TAOK3_5_8685,657,773,1030,593,288,312,852,1011,368
TBK1_5_8686,1121,2311,1751,979,3727,644,1594,2160,1804
TEC_5_8687,794,336,1325,97,41,468,1303,1236,838
TEK_5_8688,148,178,13,0,13,5,12,41,0
TESK1_5_8689,3405,4100,4729,5300,3937,4806,4523,5437,5460
TESK2_5_8690,260,370,316,71,245,673,280,367,1002
THNSL1_5_8691,957,740,1194,1960,305,1049,628,416,752
TIE1_5_8692,1278,1091,1381,3228,717,1469,1582,1507,2578
TK1_5_8693,1002,572,1386,420,495,1090,899,649,100
TNK1_5_8694,213,412,200,9,1563,0,43,164,111
TNNI3K_5_8695,4530,6227,5280,3084,3444,6333,6204,6020,2852
TNS3_5_8696,1166,1243,1386,781,474,3080,1214,2241,632
TP53RK_5_8697,394,369,477,1099,427,409,830,259,108
TRAT1_5_8698,1396,912,1447,611,1921,3523,1386,2081,2921
TRIB1_5_8699,302,279,188,0,5,0,658,9,0

TRIB2_5_8700,659,733,1231,1491,93,1025,155,824,142
TRIB3_5_8701,784,573,331,218,412,467,851,329,179
TRIM27_5_8702,1796,2254,2662,2078,1546,3304,1352,814,3010
TRIM28_5_8703,605,1250,478,1012,694,224,65,515,146
TRIO_5_8704,250,127,209,134,24,21,26,111,1492
TRPM7_5_8705,3555,4284,3499,3554,4723,2520,3482,4283,3433
TRRAP_5_8706,249,522,541,524,436,361,971,233,696
TSKS_5_8707,274,174,426,103,381,219,551,167,677
TSSK1B_5_8708,3289,3405,5582,1391,3686,3440,3577,3261,2946
TSSK2_5_8709,517,284,89,413,251,149,436,184,107
TSSK3_5_8710,127,185,608,0,334,277,208,912,0
TSSK6_5_8711,109,276,151,139,51,1328,630,326,14
TTBK1_5_8712,748,622,1203,223,210,1969,1117,1087,2231
TTBK2_5_8713,4190,5261,5457,3014,5447,5240,2414,6936,6922
TWF2_5_8714,475,139,327,440,853,409,414,157,243
TXK_5_8715,650,615,812,1012,1210,775,1147,265,249
TYK2_5_8716,93,67,255,59,0,35,6,431,88
TYRO3_5_8717,1222,116,633,318,249,172,932,186,179
UBLCP1_5_8718,733,1722,1057,741,631,1008,1078,1349,2415
UCK2_5_8719,646,1038,840,116,265,335,1595,380,67
ULK1_5_8720,246,244,190,8,847,702,0,8,138
ULK3_5_8721,958,1419,1221,202,155,850,1539,1357,2322
ULK4_5_8722,750,984,945,438,1813,110,581,553,432
VRK1_5_8723,255,497,596,245,218,1679,180,173,74
WEE2_5_8724,1241,1040,1318,2037,1244,1067,1373,638,1126
WNK2_5_8725,211,246,733,11,3,58,146,244,289
WNK4_5_8726,462,374,696,0,57,100,74,11,1045
XRCC6BP1_5_8727,1032,1295,1033,555,956,1799,1108,961,1207
XYLB_5_8728,285,525,256,5,539,174,141,448,925
YES1_5_8729,323,326,707,20,243,502,563,157,293
AATK_5_8730,91,152,219,550,55,2,727,11,0
ABL1_5_8731,148,48,7,30,466,15,14,28,8
ABL2_5_8732,536,802,1452,1101,2003,614,656,1372,1007
ACP1_5_8733,1369,1241,1158,1346,2715,935,914,795,212
ACP5_5_8734,391,770,1257,699,322,937,644,170,750
ACPL2_5_8735,194,517,614,645,694,520,382,294,867
ACPP_5_8736,510,631,1061,857,161,1216,390,637,808
ACVR1B_5_8737,880,1608,1426,714,2035,1252,932,1183,478
ACVR1C_5_8738,58,171,158,199,220,67,176,119,0
ACVR1_5_8739,1015,806,1233,1096,667,1136,539,1658,2382
ACVRL1_5_8740,287,285,792,20,509,566,22,1825,1943
ADCK1_5_8741,2553,2929,3307,3390,5219,4280,2596,3486,3861
ADCK4_5_8742,372,259,475,1106,279,784,153,62,141
ADK_5_8743,1502,1481,1635,1294,3900,1908,1189,821,1866
AK2_5_8744,564,161,420,332,0,404,647,98,441
AK4_5_8745,5356,6651,7638,5017,7693,7954,4409,7903,6727
AK5_5_8746,1085,2092,1197,2687,901,2398,1368,742,1108
AKT1_5_8747,524,534,1013,411,923,353,279,566,1560
AKT2_5_8748,446,881,959,2172,411,648,1030,651,44
AKT3_5_8749,401,449,571,692,926,409,251,467,440

ALDH18A1_5_8750,643,1368,1233,1048,1457,1167,932,1033,875
ALPK1_5_8751,104,554,214,0,30,280,914,417,125
ALPL_5_8752,628,467,1309,205,56,157,207,160,1759
AMHR2_5_8753,1010,977,1095,777,366,1399,1362,529,2356
ANKHD1_5_8754,310,349,547,179,14,7,326,6,682
APTX_5_8755,334,236,364,443,687,61,129,199,160
ATRIP_5_8756,1019,929,1371,622,1261,1919,1044,1731,3604
AURKA_5_8757,653,490,1744,642,291,2322,319,469,1940
AURKC_5_8758,356,396,352,159,149,437,70,1284,255
AXL_5_8759,772,445,696,1169,1722,102,671,252,2771
BAIAP2_5_8760,458,351,682,48,0,84,62,577,1
BCKDK_5_8761,472,704,159,893,850,28,377,16,378
BCR_5_8762,281,203,548,513,45,362,248,57,376
BMP2K_5_8763,2567,2600,3716,2313,3280,3295,3043,4239,3298
BMX_5_8764,3171,3825,4267,2556,2945,3421,3248,3210,3060
BPGM_5_8765,924,640,791,891,863,769,555,727,1076
BRD2_5_8766,690,843,589,1147,1059,229,520,987,20
BRD4_5_8767,11,331,8,286,568,198,2,488,0
BRDT_5_8768,3252,2748,5081,2406,2152,4897,2726,1912,2971
CAB39L_5_8769,434,571,746,336,8,397,132,19,934
CAMK1D_5_8770,9,0,11,0,0,0,0,0,0
CAMK2A_5_8771,1149,1031,2616,574,1639,1535,1668,1317,4884
CAMK2B_5_8772,195,73,95,291,191,13,35,41,0
CAMK2D_5_8773,1141,829,856,1293,607,940,1111,671,135
CAMK2G_5_8774,663,340,698,175,389,702,126,115,894
CAMKK1_5_8775,750,35,230,90,708,75,463,13,246
CAMKK2_5_8776,4248,5806,5534,4522,4666,3815,3653,6178,8288
CASK_5_8777,4949,4817,6506,5158,5133,7160,6513,4336,7397
CCT2_5_8778,638,565,461,117,650,660,270,657,79
CDADC1_5_8779,1256,1633,1637,1705,2051,1091,386,2185,3271
CDC14A_5_8780,672,918,1397,468,596,1603,533,646,2025
CDC14B_5_8781,458,1347,1478,682,799,1016,473,430,700
CDC25A_5_8782,1193,842,1967,558,801,1240,746,1467,922
CDC25B_5_8783,1278,1519,2699,1493,1484,2412,2342,1406,488
CDC25C_5_8784,2070,2017,3159,4956,2855,2407,1140,2312,2145
CDC42BPA_5_8785,861,744,1396,384,617,848,795,821,581
CDC7_5_8786,819,664,982,1699,599,1137,949,1309,1119
CDK10_5_8787,654,327,447,408,194,787,725,204,1357
CDK11A_5_8788,5283,4717,5928,3571,5574,5249,3823,5851,5054
CDK11B_5_8789,5283,4717,5928,3571,5574,5249,3823,5851,5054
CDK12_5_8790,4294,5748,7330,4237,3283,6533,3745,5448,6510
CDK13_5_8791,325,181,61,433,60,270,393,240,731
CDK16_5_8792,151,334,234,498,8,203,66,433,5
CDK17_5_8793,822,926,853,514,1750,1246,1174,929,1055
CDK18_5_8794,206,104,191,59,9,0,7,1,0
CDK20_5_8795,152,205,192,191,415,286,544,75,157
CDK2_5_8796,150,177,133,22,1377,0,99,19,180
CDK5_5_8797,93,228,92,77,225,186,765,36,108
CDK6_5_8798,387,322,413,142,230,463,486,499,327
CDKL3_5_8799,1307,2064,1578,777,1796,550,1241,693,1681

CDKL5_5_8800,5612,6542,6423,4515,7477,5805,5076,5851,12248
CDKN1A_5_8801,261,398,349,972,169,137,24,541,428
CDKN3_5_8802,1478,1065,1824,1178,1335,1133,1667,1600,408
CHEK1_5_8803,1777,1772,2396,1389,3581,1884,2396,1337,2465
CHEK2_5_8804,2096,1884,1808,1015,1476,3073,1311,1451,1520
CHKA_5_8805,3135,3423,4367,2057,3457,2917,2743,2772,5646
CIT_5_8806,1488,1372,1481,1779,941,4153,862,493,2242
CKMT2_5_8807,1190,1449,1125,1016,765,3411,1336,809,873
CLK1_5_8808,5126,4622,6497,5149,3544,7543,5648,4631,7282
CLK3_5_8809,2332,2956,3350,1755,2293,3115,2403,2496,4060
CMPK1_5_8810,856,572,1031,408,709,268,874,722,1046
COASY_5_8811,6418,6383,5726,3332,6990,8348,3994,6097,10730
COL4A3BP_5_8812,399,813,1268,1014,2410,1719,556,574,167
CSK_5_8813,877,1037,1065,903,463,1466,660,546,957
CSNK1A1_5_8814,3119,2750,2196,2484,3377,3174,2928,5819,2880
CSNK1D_5_8815,240,510,707,16,1,153,0,331,375
CSNK1E_5_8816,484,726,872,352,2004,403,1026,347,716
CSNK1G3_5_8817,2641,3315,4401,5067,2027,3700,2302,2638,3334
CTDP1_5_8818,950,1344,1384,1350,1021,2200,600,1969,738
CTDSP1_5_8819,454,359,410,173,180,170,229,579,603
DCLK1_5_8820,185,511,631,355,68,608,55,310,1452
DCLK2_5_8821,316,669,829,609,300,557,456,556,683
DDR1_5_8822,70,3,32,0,0,9,9,89,0
DDR2_5_8823,5210,7604,6023,3314,3706,7715,6133,4834,6051
DGKA_5_8824,487,266,582,84,52,901,412,615,3059
DGKB_5_8825,1032,1380,2219,1291,463,953,1801,510,511
DGKD_5_8826,531,693,556,1480,1770,1354,1435,664,614
DGKG_5_8827,834,855,1899,707,1685,653,525,788,621
DGKH_5_8828,756,1174,2087,809,596,769,691,803,2021
DGKZ_5_8829,25,105,261,175,2,169,80,225,12
DGUOK_5_8830,195,434,623,126,479,329,404,256,332
DLGAP5_5_8831,1827,2322,1542,1041,1672,1594,745,973,5774
DMPK_5_8832,54,182,370,128,57,541,90,4,0
DSTYK_5_8833,947,905,1530,211,2347,1803,1938,553,1776
DTYMK_5_8834,125,294,225,1,568,23,93,48,515
DUSP13_5_8835,656,685,1145,1461,693,107,319,1978,2796
DUSP19_5_8836,717,1519,974,442,718,459,853,479,2378
DUSP4_5_8837,478,472,436,34,57,1042,270,970,81
DUSP6_5_8838,550,161,562,492,895,347,7,129,1665
DYRK1A_5_8839,1793,2337,1863,1131,3527,1919,1598,2011,1989
DYRK1B_5_8840,585,131,904,15,566,1578,937,124,3174
DYRK2_5_8841,330,627,315,535,1154,271,754,4,865
DYRK3_5_8842,829,992,1125,945,988,332,787,1193,1884
EFNA4_5_8843,2255,4351,5063,2058,5899,4719,4149,3668,3784
EGFR_5_8844,13,0,172,16,1,264,16,1,0
EIF2AK2_5_8845,394,371,530,514,374,462,1118,346,1585
EPHA10_5_8846,253,188,118,1362,1,117,0,705,0
EPHA3_5_8847,2814,2175,2961,6647,2527,2647,2231,2894,3550
EPHA5_5_8848,2708,1709,2099,2080,569,1893,1800,2203,1528
EPHA6_5_8849,431,329,336,318,923,495,216,113,34

EPHA8_5_8850,17,95,138,0,129,156,0,73,0
EPHB2_5_8851,2266,1932,2982,1854,3838,2995,2379,2892,1351
EPM2A_5_8852,1195,443,437,198,1746,237,513,80,416
ERBB2_5_8853,425,535,422,117,479,455,545,14,268
ERBB4_5_8854,328,120,838,1042,953,848,14,71,201
ETNK1_5_8855,665,670,817,617,345,475,777,256,707
EXOSC10_5_8856,442,314,463,47,1007,758,153,48,287
EYA1_5_8857,34,158,116,1,1,0,200,0,12
EYA2_5_8858,22,21,92,83,0,2,52,16,350
EYA4_5_8859,818,1111,936,281,1238,372,901,1283,3111
FASTK_5_8860,1747,2667,3185,2560,1806,1319,2997,2140,5144
FBP1_5_8861,869,940,941,177,807,707,819,130,1396
FES_5_8862,942,1280,687,465,277,390,333,618,741
FGFR1_5_8863,727,408,914,1039,421,1704,1195,608,1731
FGFR2_5_8864,18,109,183,1,207,180,56,58,57
FGFR3_5_8865,122,250,56,51,508,4,212,542,106
FGFR4_5_8866,725,909,605,341,223,696,811,175,146
FGFRL1_5_8867,471,158,547,236,488,288,627,135,229
FGR_5_8868,757,1599,975,2352,909,328,664,722,942
FLT1_5_8869,625,837,878,103,1156,417,489,521,414
FLT4_5_8870,414,557,511,449,208,236,131,417,647
FXN_5_8871,2055,1856,1580,2005,3194,616,1386,1370,4886
FYN_5_8872,334,184,284,237,13,345,166,220,43
FZR1_5_8873,260,246,400,1,348,285,262,618,473
GALK2_5_8874,446,436,597,231,131,399,521,1207,31
GK_5_8875,148,231,172,413,34,490,173,387,347
GLYCTK_5_8876,187,85,380,69,52,0,18,2,0
GNE_5_8877,208,307,608,208,313,705,222,435,384
GRK4_5_8878,280,279,670,239,1378,69,357,150,775
GRK6_5_8879,862,1013,970,174,1523,1283,683,966,2144
GSK3B_5_8880,2305,2015,2512,969,1746,2746,1246,2378,7225
GUK1_5_8881,3,152,90,290,626,1,48,706,270
HCK_5_8882,962,1057,672,1530,473,1648,728,829,2841
HIPK1_5_8883,388,248,322,473,1605,1,546,2,575
HIPK2_5_8884,2032,2084,1734,973,2422,2302,1950,1240,1854
HIPK3_5_8885,1418,1568,2469,853,1561,3972,1501,1236,713
ICK_5_8886,2801,1775,3255,2269,2068,1693,3811,1914,2233
IKBKB_5_8887,264,210,1159,314,766,166,490,6,2338
IKBKE_5_8888,249,243,313,1,63,281,495,0,143
IKBKG_5_8889,161,158,54,126,77,10,18,179,102
ILK_5_8890,67,119,198,3,56,45,555,21,179
IMPA1_5_8891,1103,910,1673,905,1410,1756,1516,686,1072
INPP1_5_8892,1348,715,900,1047,318,750,834,1407,2283
INPP4A_5_8893,1169,1420,2743,1871,1448,2647,1473,812,1617
INPP4B_5_8894,1223,670,1644,687,1061,1013,974,2902,1175
INPP5D_5_8895,757,533,413,523,79,1138,571,911,422
INSR_5_8896,2848,2697,2575,4156,2578,2370,1815,1465,4444
IP6K1_5_8897,268,735,1026,281,532,425,331,1159,20
IP6K3_5_8898,2269,1135,1874,718,1680,1566,1011,998,3828
IRAK1_5_8899,184,99,140,913,0,1,605,240,0

IRAK3_5_8900,854,735,1185,2994,1461,454,874,944,446
IRAK4_5_8901,2862,2537,4080,3554,3942,3658,3192,3191,1010
ITPK1_5_8902,92,249,376,133,242,0,42,236,158
KALRN_5_8903,173,87,205,14,384,32,514,44,0
KHK_5_8904,117,34,5,0,25,0,175,146,493
KIF2A_5_8905,1096,2182,1367,2377,1064,1505,904,606,2969
KIT_5_8906,1411,1404,1066,1438,891,2185,1133,842,892
LCK_5_8907,233,281,359,0,61,62,39,297,65
LHPP_5_8908,614,1243,1382,2512,3255,1018,418,1229,3140
LIMK1_5_8909,378,689,449,1025,1342,757,303,752,110
LIMK2_5_8910,641,262,729,247,610,553,395,211,446
LTK_5_8911,155,247,289,1,215,4,16,543,1
LYN_5_8912,221,382,890,1406,112,256,321,47,89
MAGI3_5_8913,559,1194,1040,1814,1336,186,411,338,151
MAP2K3_5_8914,904,1618,782,831,2000,284,317,1245,6
MAP2K5_5_8915,201,204,306,992,218,79,762,673,159
MAP3K12_5_8916,360,374,607,43,144,555,220,204,214
MAP3K13_5_8917,238,857,845,81,601,162,97,252,906
MAP3K3_5_8918,1020,624,1081,235,1011,1403,283,197,385
MAP3K4_5_8919,187,158,105,77,367,25,680,190,52
MAP3K7_5_8920,175,379,752,2,309,144,36,1129,9
MAP4K1_5_8921,2959,2581,3700,2519,2145,3482,2454,4725,2019
MAP4K4_5_8922,1054,1037,1521,2035,1076,1176,1368,124,921
MAP4K5_5_8923,2381,2763,2541,1255,1778,3323,3636,1300,2959
MAPK10_5_8924,737,350,727,285,352,61,604,266,936
MAPK14_5_8925,189,162,455,279,530,181,158,88,70
MAPK1_5_8926,668,377,550,296,1361,795,943,82,922
MAPK3_5_8927,932,838,815,288,1031,1277,1107,489,867
MAPK7_5_8928,93,385,325,232,49,313,153,12,271
MAPK8_5_8929,3084,2677,2886,1259,4128,4670,2331,3331,1858
MAPK9_5_8930,115,300,224,198,492,340,496,316,66
MAPKAPK2_5_8931,334,451,423,209,20,33,498,1578,63
MAPKAPK5_5_8932,554,423,454,904,428,685,608,109,673
MARK2_5_8933,1938,2056,1968,2587,1533,1840,2725,3832,485
MARK3_5_8934,232,611,466,83,400,275,410,159,531
MARK4_5_8935,1078,1331,1001,681,835,1442,166,458,2379
MAST4_5_8936,574,114,474,31,114,102,376,24,454
MASTL_5_8937,1239,1684,1663,412,1733,1886,1975,451,2400
MATK_5_8938,248,263,269,58,1179,504,28,412,549
MET_5_8939,110,397,357,0,754,750,66,173,2
MINK1_5_8940,1014,1163,1220,916,1639,358,802,1319,685
MINPP1_5_8941,1099,386,723,1028,487,821,407,48,502
MKNK1_5_8942,4293,4983,4392,2835,4329,3484,3766,4565,5623
MKNK2_5_8943,265,63,38,77,171,104,35,76,767
MLKL_5_8944,82,16,78,274,0,18,8,0,1
MTMR14_5_8945,962,1563,694,99,1387,1003,1257,1603,2845
MTMR2_5_8946,1477,1412,2202,1685,1898,1713,3480,1244,1129
MTMR3_5_8947,1302,1149,1706,2062,466,880,1701,1197,594
MUSK_5_8948,720,965,1438,545,601,802,1152,673,966
MVK_5_8949,306,144,511,476,1,1122,918,318,670

MYO3B_5_8950,4205,4876,4990,1285,3131,4145,3658,3377,10992
NADK_5_8951,2300,2210,2272,1658,2337,3049,2384,2293,1387
NCK1_5_8952,1111,1407,1640,782,2574,1815,1841,1328,2092
NCK2_5_8953,1471,1384,1169,1946,1242,849,1175,950,1328
NDRG1_5_8954,125,78,328,5,103,18,52,5,4
NEK11_5_8955,8118,8203,9165,7511,6825,7721,6446,6263,6230
NEK1_5_8956,267,451,688,387,253,629,585,640,434
NEK2_5_8957,699,629,1044,916,481,598,427,1209,627
NEK3_5_8958,1303,1445,2154,687,1483,3406,2135,1007,4043
NEK4_5_8959,493,1208,832,744,591,619,242,1125,1159
NEK6_5_8960,46,870,413,127,145,215,11,1337,46
NME2_5_8961,11,4,124,554,506,42,3,259,153
NME7_5_8962,3197,2159,2854,2087,1660,3821,1272,2554,1485
NT5C1B_5_8963,16,0,148,0,0,184,0,0,1
NT5C2_5_8964,1983,2697,2376,1809,1758,1161,1334,2236,3608
NT5C3_5_8965,3659,3892,4773,2431,3389,4253,2369,2590,5778
NT5E_5_8966,1877,2755,2691,1677,2942,2769,2193,2316,5034
NTRK1_5_8967,489,565,268,389,992,130,327,553,7
NTRK2_5_8968,613,663,383,552,45,756,639,299,262
NTRK3_5_8969,1463,1160,2062,683,1534,1118,615,1557,747
NUDT4_5_8970,393,362,168,547,355,146,367,295,509
NUDT9_5_8971,612,1503,1850,1013,380,1100,537,1278,1439
OBSCN_5_8972,87,90,105,18,86,2,116,21,10
OCRL_5_8973,935,994,1089,497,2486,1651,440,736,2609
OXSM_5_8974,400,356,357,996,494,245,256,85,229
PACSIN1_5_8975,445,446,158,30,744,266,33,83,9
PAK4_5_8976,89,341,276,160,48,46,22,4,85
PAK6_5_8977,213,154,449,69,1,36,229,907,915
PAK7_5_8978,1346,1652,1296,2563,1421,1154,1523,1131,1429
PANK1_5_8979,1256,1643,1838,1444,983,581,757,1510,3243
PANK2_5_8980,2894,2940,2864,3339,2349,2692,3612,2295,2517
PCK2_5_8981,166,378,391,0,604,11,434,5,1663
PDK2_5_8982,1133,1292,1942,1082,620,2769,1814,1739,1448
PDK3_5_8983,916,1521,1214,243,927,161,627,1252,3041
PDPK1_5_8984,3533,2312,3951,3866,2430,2542,3209,2885,2924
PEG3_5_8985,1050,319,1093,799,105,892,843,329,1349
PFKFB2_5_8986,274,351,874,20,0,176,166,579,73
PFKFB3_5_8987,1249,1902,2667,1466,3708,2984,796,1973,282
PFKM_5_8988,183,123,355,177,0,1,361,5,25
PFKP_5_8989,191,86,385,482,18,6,680,92,4
PHKA1_5_8990,2683,2736,2256,591,1329,833,2352,1709,2063
PHKB_5_8991,1793,1938,3006,2024,3751,1575,2235,2197,3440
PHKG2_5_8992,785,1645,1135,992,1546,938,1340,2108,366
PHPT1_5_8993,516,463,742,137,499,513,436,103,122
PI4KA_5_8994,670,434,637,1434,770,556,1147,293,1495
PI4KB_5_8995,222,302,500,56,459,1127,49,82,1379
PIK3R1_5_8996,2024,2362,2745,1990,3528,2134,2959,3530,3353
PIK3R5_5_8997,66,305,26,1,12,12,429,20,346
PIKFYVE_5_8998,1940,2820,2527,1697,1962,1299,2387,2230,5240
PIP4K2C_5_8999,1356,1073,2318,1432,2869,1927,2036,686,2065

PIP5K1A_5_9000,2050,1876,2360,564,4380,4189,1200,962,1765
PIP5K1C_5_9001,865,740,165,6,84,1051,76,31,612
PIP5KL1_5_9002,264,1033,237,0,17,598,499,595,206
PKLR_5_9003,668,432,317,630,881,169,594,404,715
PKMYT1_5_9004,292,358,415,133,1124,127,438,397,370
PKN1_5_9005,194,365,611,23,532,439,336,660,62
PLK4_5_9006,2400,2647,2312,2031,3136,3129,1245,3212,2664
PNCK_5_9007,29,241,316,1,64,0,12,618,172
PODXL_5_9008,326,180,717,20,0,19,285,262,1699
PPAP2A_5_9009,681,936,760,845,1058,142,471,293,1864
PPAP2C_5_9010,665,1046,1696,1630,1657,1402,1125,987,1476
PPEF1_5_9011,2575,3769,2431,2789,3117,3612,2479,2983,2900
PPFIA1_5_9012,1395,1795,1765,2055,2056,1028,2336,1039,320
PPFIA2_5_9013,3158,3647,2972,2069,3460,2249,3316,4217,2528
PPM1A_5_9014,1872,877,1690,1800,1249,1846,623,996,2200
PPM1B_5_9015,1015,1279,1053,1734,438,587,942,1359,1267
PPM1M_5_9016,5,6,18,43,0,95,91,1,81
PPP1CA_5_9017,598,585,655,187,1044,274,207,1153,13
PPP1CB_5_9018,7524,8186,10936,8286,11385,8787,7917,6777,13006
PPP1R12A_5_9019,180,366,265,484,0,175,1483,86,60
PPP1R16B_5_9020,458,746,657,681,52,703,272,182,374
PPP1R1B_5_9021,899,1691,2785,294,1534,2026,589,524,1188
PPP1R8_5_9022,921,1426,1858,1078,414,1235,754,1175,2157
PPP2R1B_5_9023,3805,3273,5616,3072,3121,2417,2875,3019,3320
PPP2R2A_5_9024,1329,1122,1937,723,1785,917,816,2320,3316
PPP2R2B_5_9025,493,420,1160,763,566,1259,303,672,2282
PPP2R2C_5_9026,417,578,420,1231,509,456,835,122,151
PPP2R3A_5_9027,1617,1253,2096,1266,2182,992,2261,904,3086
PPP2R4_5_9028,242,398,398,99,184,160,119,301,79
PPP2R5A_5_9029,2405,2154,1865,3376,1546,2427,2325,3038,1003
PPP2R5D_5_9030,800,1194,993,175,958,298,381,733,933
PPP3CA_5_9031,1204,491,1205,2356,620,2387,116,305,1413
PPP3CB_5_9032,3326,4994,5208,4560,5602,8757,3038,3384,6011
PPP4R1_5_9033,1215,1465,1896,762,1095,1777,593,2166,1922
PPP5C_5_9034,1114,830,1040,1626,542,2365,963,1115,1530
PPP6C_5_9035,2758,3417,3536,1562,6920,3918,2848,2753,3723
PRKAA1_5_9036,334,697,415,1684,75,226,90,1133,955
PRKACA_5_9037,2333,2543,2519,1712,2500,2343,980,2312,3323
PRKACB_5_9038,2068,2807,2088,1426,2592,1607,1765,2128,3873
PRKAG1_5_9039,2092,2681,2194,87,2508,1942,1673,1823,5468
PRKAG2_5_9040,174,251,175,604,121,270,11,87,11
PRKAR1A_5_9041,0,0,547,0,243,588,0,0,0
PRKAR1B_5_9042,249,79,187,87,0,88,102,93,2
PRKCB_5_9043,1242,2122,1419,1063,1270,1661,2015,1367,1609
PRKCD_5_9044,9,362,37,1,0,2,42,39,757
PRKCQ_5_9045,124,161,240,599,1,72,103,491,425
PRKCZ_5_9046,666,618,1116,3176,171,647,1161,610,1177
PRKD2_5_9047,499,653,1057,578,22,214,128,306,300
PRKDC_5_9048,1353,1844,1927,2520,610,2069,851,2301,1587
PRKG1_5_9049,1860,1067,1358,1394,677,1918,1566,1579,1688

PRPS2_5_9050,450,1400,1745,707,968,392,608,1042,2009
PTK2B_5_9051,213,260,7,1258,11,519,17,244,16
PTK2_5_9052,196,264,583,78,594,631,570,404,26
PTK7_5_9053,510,1131,1185,483,283,1085,643,380,2445
PTP4A2_5_9054,1519,1446,2179,1917,1916,2972,1391,1425,972
PTP4A3_5_9055,448,393,185,0,3,4,61,58,191
PTPDC1_5_9056,161,188,303,510,283,138,456,81,134
PTPN12_5_9057,2482,2748,3175,1702,1927,3838,2113,3270,1879
PTPN13_5_9058,416,325,105,0,266,4,898,373,367
PTPN18_5_9059,532,453,1063,168,400,873,310,455,622
PTPN22_5_9060,5418,6647,5592,5522,5676,6330,5151,4483,2797
PTPN2_5_9061,4111,4695,4407,5130,5016,2936,5428,3172,4653
PTPN3_5_9062,12,98,160,510,15,586,18,18,1
PTPN5_5_9063,0,53,294,0,287,0,0,0,0
PTPN6_5_9064,2,29,316,4,1,0,0,0,0
PTPN7_5_9065,143,373,427,29,415,35,224,12,276
PTPRA_5_9066,430,256,334,379,1206,257,450,466,1552
PTPRB_5_9067,630,870,1513,627,448,1926,1438,120,1401
PTPRC_5_9068,262,68,87,0,0,207,313,8,770
PTPRD_5_9069,1679,1972,2991,1463,2972,1551,1360,2301,2704
PTPRE_5_9070,1366,1361,2400,2310,768,1228,1850,2447,2610
PTPRF_5_9071,179,88,550,76,389,83,380,108,688
PTPRH_5_9072,1927,2176,2184,1457,1463,1547,1486,696,2921
PTPRJ_5_9073,1338,865,1050,1065,761,1384,925,756,1309
PTPRK_5_9074,742,890,1522,746,689,2135,910,1240,1674
PTPRM_5_9075,1282,782,919,1363,355,2541,1388,1309,736
PTPRN2_5_9076,1321,1366,1629,712,1579,1059,1138,858,1122
PTPRN_5_9077,650,437,502,117,553,1757,569,41,33
PTPRR_5_9078,1238,2377,3182,1068,5339,2550,1233,2364,7330
PTPRS_5_9079,150,565,284,237,547,123,849,527,1508
PTPRT_5_9080,566,423,348,26,449,217,433,705,17
PTPRU_5_9081,327,452,491,362,178,1362,526,612,1873
PTPRZ1_5_9082,2013,2877,1732,1615,2008,2253,1715,1206,2277
RET_5_9083,234,71,223,88,270,566,173,58,184
RIOK1_5_9084,39,142,345,128,388,32,37,24,2
RIOK2_5_9085,524,1201,749,259,444,107,596,589,364
ROPN1L_5_9086,425,450,1122,92,161,132,550,134,1924
RPS6KA1_5_9087,1604,1617,1277,882,788,1299,1230,338,37
RPS6KA2_5_9088,342,405,221,344,61,310,151,83,1485
RPS6KA4_5_9089,280,841,909,600,1050,299,388,389,1050
RPS6KA5_5_9090,214,552,252,102,185,3,307,285,318
RPS6KC1_5_9091,3924,3600,3418,4987,4771,2629,3682,2272,2285
RYK_5_9092,513,239,944,1150,82,1490,259,141,123
SET_5_9093,953,682,1554,2039,1661,1847,1242,605,903
SGK1_5_9094,508,613,452,250,118,260,42,854,1160
SGK3_5_9095,18747,18321,19313,18816,20801,18625,16115,15872,20940
SIRPA_5_9096,624,327,186,367,102,285,350,113,14
SKAP1_5_9097,434,648,290,1369,288,337,205,861,900
SNRK_5_9098,1731,1276,2512,1497,587,1473,2654,1437,2660
SPHK1_5_9099,614,215,669,646,36,7,210,443,118

SPHK2_5_9100,11,6,173,18,1,0,334,354,2
SRC_5_9101,517,65,534,103,87,131,79,50,0
SRPK2_5_9102,609,397,815,164,406,61,118,954,822
SRPK3_5_9103,955,969,844,1240,2249,966,373,1290,1136
SSH1_5_9104,1305,1143,996,844,1824,1833,389,890,1878
STK19_5_9105,125,158,122,0,147,33,2,437,22
STK24_5_9106,7312,7043,8341,7894,8800,9472,6692,8328,7690
STK31_5_9107,2753,4199,4345,3222,4363,2959,4139,5181,5080
STK36_5_9108,30,10,658,0,12,209,30,143,7
STRADA_5_9109,610,259,467,396,181,139,739,364,613
STRADB_5_9110,990,2040,1843,1353,1876,1185,1664,963,930
STYX_5_9111,17409,18746,20731,14096,20126,16270,17848,17535,20672
SYK_5_9112,3548,2789,3454,1901,2252,2709,1943,4094,5510
SYNJ1_5_9113,1225,1155,840,579,904,475,1703,281,1755
SYNJ2_5_9114,897,1013,623,419,1098,175,261,1648,1156
TAF1_5_9115,344,443,141,152,156,57,354,251,199
TAOK1_5_9116,1139,626,673,1318,376,2032,1885,516,707
TAOK2_5_9117,253,497,344,393,557,69,464,804,1
TBCK_5_9118,1141,3178,2181,1331,4437,1266,515,2162,310
TEX14_5_9119,650,119,142,0,225,1273,0,321,17
TGFBR1_5_9120,638,1618,921,1363,1622,1731,1250,842,1355
TGFBR2_5_9121,891,1734,1358,1361,2761,2028,1705,1452,1197
TGFBR3_5_9122,191,83,197,112,85,3,27,28,59
THTPA_5_9123,72,33,110,431,25,779,378,5,0
TJP2_5_9124,179,282,503,385,618,90,301,7,80
TK2_5_9125,67,307,317,297,33,291,174,225,572
TLK1_5_9126,1441,1381,1392,599,1016,1046,2012,583,1298
TLK2_5_9127,1016,1578,1681,2022,1755,907,1811,627,1925
TMEM134_5_9128,1829,2253,2041,602,1987,2310,1176,1387,3648
TNIK_5_9129,3775,4043,3898,4528,4210,3589,3530,3261,4820
TNK2_5_9130,190,2,228,0,0,0,0,11,321
TPK1_5_9131,1016,1560,1569,644,1312,1283,1739,758,898
TPTE2_5_9132,1068,1328,1624,99,1803,1741,1029,1326,2605
TPTE_5_9133,789,1189,846,922,2027,2988,1367,833,1784
TRIM24_5_9134,370,529,549,512,1912,181,124,58,651
TRIM33_5_9135,410,589,503,106,799,277,285,110,73
TRMT2A_5_9136,580,414,460,174,109,1194,95,100,320
TRPM6_5_9137,80,70,484,16,165,0,58,36,576
TSSK4_5_9138,364,342,122,73,5,339,98,53,295
TTK_5_9139,7609,7879,7863,7584,11643,7454,8410,8477,6162
TTN_5_9140,2500,2627,3429,2236,1429,4758,3294,2005,2496
TWF1_5_9141,2234,1952,2299,329,4750,2224,1931,1301,761
UCK1_5_9142,312,452,611,2164,125,102,582,53,559
UCKL1_5_9143,590,895,864,1301,2359,1850,1866,292,488
UHMK1_5_9144,241,122,506,106,254,357,328,248,9
ULK2_5_9145,10655,10498,11536,7640,11038,8928,8898,8181,7448
VRK2_5_9146,7931,8676,7964,8485,8945,9783,8411,6895,8974
VRK3_5_9147,121,114,5,42,291,38,157,131,56
WEE1_5_9148,3078,1761,2839,2280,4632,2979,1893,3517,2646
WNK1_5_9149,1139,516,1596,1893,244,1706,704,979,4122

WNK3_5_9150,2526,4368,3531,5515,4049,2993,4608,2734,2928
YSK4_5_9151,1732,1418,2734,1667,2216,1202,2242,1355,1458
ZAP70_5_9152,1117,1531,2430,2095,2502,1838,1274,1340,2588
ACP2_5_9153,98,386,449,560,456,11,1,549,368
CDK1_5_9154,890,1527,1446,1048,1113,1730,540,1585,880
DUSP10_5_9155,322,373,176,0,66,576,979,494,0
DUSP15_5_9156,3327,3017,3274,5188,4031,2955,2425,2551,5138
ERBB3_5_9157,892,544,830,249,999,232,463,1466,3139
G6PC2_5_9158,780,927,1423,701,979,432,1489,1008,2782
IP6K2_5_9159,698,615,922,97,1353,556,456,331,2795
MYLK_5_9160,263,277,411,57,1,330,63,74,315
PRPS1_5_9161,265,247,429,281,49,41,509,307,34
PTPMT1_5_9162,3513,4176,5275,1602,3449,4922,3399,4033,5860
PTPN20A_5_9163,3887,5154,4592,3519,4840,5971,2839,4469,5058
PTPN20B_5_9164,3887,5154,4592,3519,4840,5971,2839,4469,5058
SPEG_5_9165,400,759,879,70,1093,138,448,231,157
STK32A_5_9166,417,887,548,844,136,959,81,207,7
PPP1R12B_5_9167,680,824,841,75,529,1768,439,632,1352
PPP1R12B_5_9168,1719,2052,1692,1962,2560,1025,1101,514,2365
PPP1R12B_5_9169,770,857,698,154,399,1216,545,440,917
MOK_5_9170,1190,1819,2826,524,681,1139,1826,1854,3103
MOK_5_9171,1211,1440,1123,2318,572,2884,1911,1039,1585
MOK_5_9172,3767,4063,4321,4666,6551,3730,5335,4422,2247
MOK_5_9173,229,267,315,82,7,34,381,10,12
MOK_5_9174,1452,1043,1008,1130,386,762,1107,1192,685
MOK_5_9175,86,223,120,46,609,355,51,674,4
MOK_5_9176,897,1067,1848,2484,671,1503,1671,1158,430
MOK_5_9177,4365,4461,4236,3079,6674,4135,2897,1815,2980
MOK_5_9178,3541,4044,4022,3502,7748,3711,5180,4237,2218
MOK_5_9179,7053,9043,8071,7333,8656,9913,7124,4777,9276
AK4_5_9180,791,577,777,1776,1024,496,673,335,1495
AK4_5_9181,1754,1727,2618,1594,1930,727,2518,2036,1067
AK4_5_9182,291,514,657,453,1415,97,261,881,608
AK4_5_9183,1753,1728,2508,1585,1948,720,2515,2043,1062
AK4_5_9184,86,115,64,44,218,247,4,15,1
AK4_5_9185,719,599,1252,561,209,1960,543,840,118
AK4_5_9186,770,760,653,2070,558,1161,654,251,55
AK4_5_9187,485,309,1289,549,5,1420,230,27,1431
AK4_5_9188,2309,2134,2577,1652,2745,3569,1863,1555,3224
AK4_5_9189,2150,2049,2527,1649,2726,3565,1882,1426,3219
ALPK3_5_9190,1418,1754,1626,1837,1911,3260,1857,870,2630
ALPK3_5_9191,1180,677,1613,624,872,3063,1551,1025,2464
ALPK3_5_9192,1236,1816,1646,1695,2104,2879,1902,898,2716
ALPK3_5_9193,8483,10323,10183,6156,13227,6693,8426,8193,10141
ALPK3_5_9194,204,583,172,750,196,431,343,554,1280
ALPK3_5_9195,497,367,395,831,544,459,711,410,796
ALPK3_5_9196,621,664,1467,628,204,559,66,1595,194
ALPK3_5_9197,4992,4396,5537,2996,3068,5383,5030,6662,3401
ALPK3_5_9198,251,239,260,137,223,177,220,179,400
ALPK3_5_9199,324,822,208,1657,705,56,177,730,3018

CHEK2_5_9200,5001,6383,7342,6500,9276,5463,4173,4959,6827
CHEK2_5_9201,3611,3975,5649,4216,5903,3414,3770,2978,4920
CHEK2_5_9202,1253,715,1104,393,112,440,1593,109,1163
CHEK2_5_9203,1318,2609,2251,496,1434,4491,2047,2226,1096
CHEK2_5_9204,1342,2651,2821,1004,1250,2002,1663,1238,3780
CHEK2_5_9205,117,164,198,57,380,70,162,35,23
CHEK2_5_9206,194,313,687,894,85,852,425,119,0
CHEK2_5_9207,1709,1672,1545,1245,687,1365,1918,2003,1430
CHEK2_5_9208,2091,1863,2679,2954,3951,2634,3106,3792,3865
CHEK2_5_9209,5954,6462,6660,3465,5143,6699,7885,4126,12305
COASY_5_9210,1361,2150,1502,1709,692,941,2216,927,1615
COASY_5_9211,5897,6406,7120,6775,6074,7806,5401,8508,9668
COASY_5_9212,1687,3267,3754,1680,3124,1961,4412,1940,3006
COASY_5_9213,2146,2358,2536,1718,1615,3336,2573,1783,3731
COASY_5_9214,1653,1573,1479,4959,662,1911,2536,1116,1620
COASY_5_9215,446,404,448,100,70,806,320,231,895
COASY_5_9216,630,681,888,135,892,1393,90,540,197
COASY_5_9217,1730,1977,1931,1528,1773,2787,2039,2969,1687
COASY_5_9218,6314,6936,8954,7855,10405,6266,5585,9424,10736
COASY_5_9219,5598,6008,6460,5475,4666,7305,6945,7409,9492
CSNK2A2_5_9220,217,234,337,2,218,64,419,44,2356
CSNK2A2_5_9221,2160,2967,2783,794,1803,4711,1952,1936,1930
CSNK2A2_5_9222,404,863,1061,990,2326,1425,547,1040,1581
CSNK2A2_5_9223,918,1227,1755,860,477,1505,1128,713,1135
CSNK2A2_5_9224,2578,1523,1943,3679,2911,2996,2648,2005,2281
CSNK2A2_5_9225,5970,7560,7313,7912,5811,5809,6389,9649,8358
CSNK2A2_5_9226,138,87,184,0,0,0,0,0,0
CSNK2A2_5_9227,1639,4156,3526,2214,2201,3546,2309,1780,1544
CSNK2A2_5_9228,1488,1664,2659,1801,1366,1170,1013,2975,2332
CSNK2A2_5_9229,1830,1711,2239,1245,1731,2391,2924,2074,1612
CTDSP2_5_9230,1303,2096,1510,1253,1886,2914,1249,1412,4363
CTDSP2_5_9231,1237,1465,1510,992,769,479,2134,1497,2017
CTDSP2_5_9232,2250,2365,3437,2602,4413,2769,3136,3647,3028
CTDSP2_5_9233,2153,2066,1716,2208,2137,1401,1934,1722,2774
CTDSP2_5_9234,427,777,1349,618,817,956,469,394,78
CTDSP2_5_9235,5117,7283,6965,3850,8613,7890,5025,7120,7479
CTDSP2_5_9236,301,213,156,128,8,170,300,74,137
CTDSP2_5_9237,307,635,577,371,214,238,692,843,2130
CTDSP2_5_9238,337,693,235,1113,366,370,239,547,209
CTDSP2_5_9239,2772,2221,2636,1295,3623,3237,1531,2114,1383
DDR2_5_9240,574,224,648,328,372,994,699,674,117
DDR2_5_9241,351,90,225,602,4,41,177,496,8
DDR2_5_9242,267,368,305,925,686,20,33,804,1407
DDR2_5_9243,651,857,1292,842,638,1446,938,872,1424
DDR2_5_9244,462,234,604,86,633,525,239,474,661
DDR2_5_9245,230,594,274,2745,13,250,128,552,0
DDR2_5_9246,2881,3133,4280,3201,1761,3515,3149,2520,1452
DDR2_5_9247,910,1981,552,736,1948,829,1217,227,63
DDR2_5_9248,64,123,587,28,1016,113,213,47,127
DDR2_5_9249,264,591,288,323,36,507,150,0,300

DLGAP5_5_9250,2101,1319,1563,1676,1476,2198,1281,901,1052
DLGAP5_5_9251,3780,3703,4648,2674,3895,6174,3543,4709,4027
DLGAP5_5_9252,3042,3535,3610,2785,5505,3287,2520,3257,3470
DLGAP5_5_9253,612,809,568,727,860,1017,286,1237,1060
DLGAP5_5_9254,386,315,509,180,1258,41,499,646,236
DLGAP5_5_9255,2122,3038,1990,2694,2180,1694,2449,2183,1918
DLGAP5_5_9256,828,588,744,590,549,1259,1035,1006,1207
DLGAP5_5_9257,3967,5204,5145,5063,5871,2996,6695,3355,4287
DLGAP5_5_9258,1935,1460,1313,1128,2965,1334,1901,1748,1373
DLGAP5_5_9259,1585,1930,1760,2380,3216,1657,1963,2412,994
DUSP12_5_9260,559,474,690,481,899,1926,1340,483,1585
DUSP12_5_9261,1423,1396,1199,1600,159,841,973,1353,760
DUSP12_5_9262,240,786,403,255,396,712,250,346,265
DUSP12_5_9263,111,90,102,0,28,3,50,3,6
DUSP12_5_9264,1118,1086,1031,658,1073,409,1188,198,178
DUSP12_5_9265,569,328,736,854,392,430,534,550,2223
DUSP12_5_9266,238,471,592,69,149,1636,735,588,731
DUSP12_5_9267,538,440,798,530,698,1005,221,494,636
DUSP12_5_9268,1070,787,1117,356,1021,543,907,1373,1563
DUSP12_5_9269,563,925,499,557,832,99,1110,882,2000
EIF2AK2_5_9270,935,1053,1206,1267,527,2305,1293,735,757
EIF2AK2_5_9271,849,1087,1449,1043,1615,2265,851,1720,1779
EIF2AK2_5_9272,1691,2007,1907,782,735,2905,1590,2924,3009
EIF2AK2_5_9273,4056,5946,6384,3282,6243,4917,4965,6143,11187
EIF2AK2_5_9274,4299,4718,5180,6364,5486,7004,4094,4448,3540
EIF2AK2_5_9275,206,469,686,154,462,867,191,389,835
EIF2AK2_5_9276,921,786,885,760,618,1434,1352,1149,1608
EIF2AK2_5_9277,3128,3301,4554,2342,3257,5478,3084,3832,4034
EIF2AK2_5_9278,1683,2477,2677,1917,2811,3185,3297,2545,1799
EIF2AK2_5_9279,1467,2350,1460,1271,1403,1528,1637,874,2153
FGFR1_5_9280,929,521,1321,121,88,2041,260,1002,64
FGFR1_5_9281,726,803,1131,154,119,436,514,427,250
FGFR1_5_9282,4056,4607,4513,4471,4526,3187,5381,2298,4080
FGFR1_5_9283,350,381,391,461,706,534,700,349,1378
FGFR1_5_9284,910,895,1222,423,786,698,777,1353,387
FGFR1_5_9285,439,778,625,370,1127,604,422,718,708
FGFR1_5_9286,3994,4997,4479,4627,4896,2984,4903,2548,6025
FGFR1_5_9287,1008,878,442,188,1300,586,1694,638,791
FGFR1_5_9288,709,909,694,506,735,906,606,2059,895
FGFR1_5_9289,1280,2089,2345,1844,1478,2925,2699,1100,4199
FYN_5_9290,503,1013,1295,751,506,1120,878,1118,818
FYN_5_9291,791,945,787,1416,533,1335,847,747,3547
FYN_5_9292,3330,1886,3026,3269,4587,1717,3241,2191,3461
FYN_5_9293,214,121,624,127,511,388,45,1068,0
FYN_5_9294,2384,2750,3140,2398,2097,2443,2690,1870,2088
FYN_5_9295,493,934,1193,187,1154,892,808,978,2148
FYN_5_9296,1406,1075,1557,1199,1091,547,1168,1027,1342
FYN_5_9297,335,591,361,12,174,145,152,104,608
FYN_5_9298,1703,1253,1606,2020,1145,181,1139,1391,3472
FYN_5_9299,1299,1446,1496,898,793,2663,2279,1767,2190

FZR1_5_9300,756,574,1052,1079,469,429,1191,475,306
FZR1_5_9301,816,1330,1573,1797,1449,813,1304,71,1
FZR1_5_9302,833,542,739,1278,1309,254,554,484,301
FZR1_5_9303,121,252,458,398,296,13,316,175,21
FZR1_5_9304,588,313,453,678,411,655,625,425,719
FZR1_5_9305,3775,5054,3532,3127,3466,3125,3152,4445,3644
FZR1_5_9306,2330,2459,1492,1116,1521,1307,908,2184,2124
FZR1_5_9307,1682,1298,2008,1375,1174,1915,1555,1326,2426
FZR1_5_9308,807,1226,1055,1150,1157,691,1422,1032,627
FZR1_5_9309,760,891,1162,1119,842,376,783,278,1286
GALK2_5_9310,1061,1261,790,2011,754,997,609,1500,1670
GALK2_5_9311,451,227,529,451,427,186,272,58,58
GALK2_5_9312,702,209,641,505,294,241,513,344,147
GALK2_5_9313,4952,3946,4962,3000,4328,6835,4006,3789,5183
GALK2_5_9314,611,147,66,33,0,10,410,24,1472
GALK2_5_9315,808,705,1469,2324,1012,937,555,745,877
GALK2_5_9316,2125,2305,2172,1274,3128,1877,1461,2469,2137
GALK2_5_9317,1626,1266,1795,562,793,1210,398,1181,333
GALK2_5_9318,1846,2009,3143,2764,2327,4188,1954,2278,3645
GALK2_5_9319,3649,4861,5212,3143,4652,5435,3284,5046,6233
GUCY2C_5_9320,285,678,248,25,265,559,822,48,258
GUCY2C_5_9321,1832,2722,2974,1718,1104,1786,1777,2088,3835
GUCY2C_5_9322,484,1032,1165,1789,1023,1004,1076,583,1172
GUCY2C_5_9323,797,868,648,593,295,450,34,113,1088
GUCY2C_5_9324,611,1194,895,1489,1599,1769,308,768,97
GUCY2C_5_9325,2744,3455,3464,1527,3041,2435,3181,4444,2628
GUCY2C_5_9326,467,396,471,993,133,768,360,102,75
GUCY2C_5_9327,3875,5188,4810,5881,4081,3748,2767,6193,2836
GUCY2C_5_9328,464,465,426,410,189,45,299,431,193
GUCY2C_5_9329,3867,4762,4602,5839,3369,3726,2760,5753,2833
KIF2A_5_9330,527,1231,1026,339,986,841,273,1048,239
KIF2A_5_9331,386,1172,1089,1146,2421,407,459,1283,510
KIF2A_5_9332,93,293,150,450,96,771,83,19,452
KIF2A_5_9333,152,24,227,0,3,0,139,0,582
KIF2A_5_9334,168,267,351,34,0,195,761,90,816
KIF2A_5_9335,1217,1691,1952,1253,353,1284,1883,909,597
KIF2A_5_9336,113,719,525,0,521,73,349,1730,476
KIF2A_5_9337,3241,2638,3159,4188,2941,1806,3113,1980,4875
KIF2A_5_9338,193,340,353,751,1014,291,152,228,1355
KIF2A_5_9339,2181,1076,2579,3123,707,793,2370,1426,142
HOOK3_5_9340,1556,1249,1966,642,1120,1242,1401,1038,1957
HOOK3_5_9341,2209,1514,2839,525,1140,1790,2429,1602,1931
HOOK3_5_9342,1480,1222,2442,2350,1016,754,3048,913,1755
HOOK3_5_9343,208,224,381,1,32,22,257,212,29
HOOK3_5_9344,1076,626,813,861,2468,1916,865,679,1545
HOOK3_5_9345,201,336,75,271,77,86,127,265,1946
HOOK3_5_9346,163,324,130,3,2064,255,112,52,0
HOOK3_5_9347,775,1296,1667,1768,940,1488,784,379,558
HOOK3_5_9348,450,678,86,810,706,350,181,1674,264
HOOK3_5_9349,223,60,251,41,0,302,4,803,0

LLGL1_5_9350,195,61,189,357,38,166,3,179,4
LLGL1_5_9351,1817,2621,2861,569,2749,2482,2074,1656,3208
LLGL1_5_9352,216,186,523,58,2,191,228,253,1154
LLGL1_5_9353,429,168,39,0,9,1,272,371,6
LLGL1_5_9354,1269,1400,1426,2090,576,159,1359,318,489
LLGL1_5_9355,222,953,332,190,855,1420,767,640,1913
LLGL1_5_9356,17,138,135,6,10,5,140,0,144
LLGL1_5_9357,6,1,220,32,63,198,9,88,110
LLGL1_5_9358,503,511,977,196,691,210,229,256,452
LLGL1_5_9359,397,438,275,3,1200,932,85,320,321
MAGI1_5_9360,250,587,501,603,440,341,1184,346,391
MAGI1_5_9361,0,20,105,0,0,0,0,19,0
MAGI1_5_9362,2077,1604,1461,1578,1484,2343,2354,1942,1958
MAGI1_5_9363,246,256,353,113,116,401,141,269,919
MAGI1_5_9364,992,1160,1250,693,1207,2563,727,263,215
MAGI1_5_9365,2527,2374,2576,1115,3056,2235,1818,2386,1673
MAGI1_5_9366,1720,1119,1984,4539,1598,694,1572,664,2933
MAGI1_5_9367,244,480,587,692,233,277,129,212,939
MAGI1_5_9368,183,283,404,226,216,358,84,83,0
MAGI1_5_9369,799,1088,1212,789,634,1336,348,1147,1380
MAP4K2_5_9370,259,463,537,56,453,1,51,269,81
MAP4K2_5_9371,945,917,655,29,530,481,290,574,784
MAP4K2_5_9372,106,613,380,127,556,85,126,49,323
MAP4K2_5_9373,664,1147,1435,972,1804,945,763,1511,3506
MAP4K2_5_9374,311,772,972,520,174,624,21,975,624
MAP4K2_5_9375,358,714,595,601,15,263,74,211,918
MAP4K2_5_9376,358,264,301,243,453,634,367,319,643
MAP4K2_5_9377,318,477,39,512,41,214,112,127,50
MAP4K2_5_9378,744,1075,529,1084,626,614,630,687,1250
MAP4K2_5_9379,428,152,265,23,363,1035,292,373,25
MAP4K5_5_9380,56,98,14,21,336,71,189,51,12
MAP4K5_5_9381,21,891,562,111,67,89,112,977,651
MAP4K5_5_9382,551,127,421,1013,37,215,372,469,197
MAP4K5_5_9383,3868,4837,4304,2296,3335,5381,2739,5517,3118
MAP4K5_5_9384,237,329,366,23,206,134,69,274,4
MAP4K5_5_9385,113,78,393,216,190,510,14,52,1
MAP4K5_5_9386,246,773,846,5,34,835,58,250,1017
MAP4K5_5_9387,832,1328,1268,747,584,1383,1456,549,1792
MAP4K5_5_9388,2603,1507,3111,2788,4230,1613,2556,815,1572
MAP4K5_5_9389,674,543,392,24,840,1179,313,600,171
STK19_5_9390,2601,2487,4809,2095,752,5350,3318,3435,2156
STK19_5_9391,4648,5684,6340,3997,8544,2599,4555,6198,8337
STK19_5_9392,1560,2150,2440,2088,1495,1514,1737,1304,2609
STK19_5_9393,2160,3282,2667,1077,4212,1626,3467,2490,2116
STK19_5_9394,610,679,986,1992,1037,1345,1325,443,1737
STK19_5_9395,5995,5441,8118,6572,5448,9537,7560,7787,12239
STK19_5_9396,1356,1709,1852,2345,1093,1136,967,1744,2545
STK19_5_9397,3390,4644,3758,4608,4440,2700,2824,3276,5290
STK19_5_9398,2428,3559,2327,6228,4347,3214,2727,2377,3312
STK19_5_9399,2380,3595,2842,2643,3640,3783,2006,5178,3808

MPP2_5_9400,1428,1924,1142,214,377,722,1097,1404,1112
MPP2_5_9401,4241,3766,3689,5003,1553,3072,3446,2777,3758
MPP2_5_9402,1142,1340,1337,958,1403,1729,2191,3061,999
MPP2_5_9403,3546,3681,3382,2145,4858,6743,1433,2338,2668
MPP2_5_9404,932,1145,1587,792,2528,1527,1037,1581,1653
MPP2_5_9405,822,710,1042,398,972,682,470,656,185
MPP2_5_9406,525,388,320,877,449,510,1826,515,4
MPP2_5_9407,102,134,313,68,280,30,34,712,361
MPP2_5_9408,1808,1709,1362,891,1151,3281,2278,1836,1988
MPP2_5_9409,747,1270,1766,1711,561,1347,580,1232,1166
MPP3_5_9410,343,840,805,191,462,517,259,920,845
MPP3_5_9411,1091,589,861,876,180,533,1213,502,634
MPP3_5_9412,191,248,859,246,321,331,248,422,391
MPP3_5_9413,499,172,588,16,746,10,717,40,248
MPP3_5_9414,476,488,189,539,24,525,287,74,731
MPP3_5_9415,261,407,266,677,4,640,505,201,5
MPP3_5_9416,681,251,647,32,446,604,773,337,588
MPP3_5_9417,69,230,376,128,2,15,18,239,4675
MPP3_5_9418,299,351,523,42,863,64,32,114,966
MPP3_5_9419,1236,1704,1310,717,4024,498,2528,2074,923
MTMR9_5_9420,366,266,670,431,155,1020,706,295,928
MTMR9_5_9421,1982,2808,2049,2786,2137,2238,2295,2010,2250
MTMR9_5_9422,350,748,988,707,1504,404,822,727,1501
MTMR9_5_9423,1004,1132,1467,1467,2860,614,807,453,120
MTMR9_5_9424,535,167,339,567,442,73,640,312,173
MTMR9_5_9425,2033,1861,1971,2445,3001,2259,941,1843,1331
MTMR9_5_9426,145,592,207,423,126,378,489,246,16
MTMR9_5_9427,196,380,1186,1526,871,643,543,805,351
MTMR9_5_9428,1437,619,698,227,624,673,272,602,351
MTMR9_5_9429,1145,1245,1142,726,337,1323,1477,1303,582
NDRG1_5_9430,1390,1870,1596,1128,1485,2133,897,674,3133
NDRG1_5_9431,0,0,0,0,0,0,0,0,0
NDRG1_5_9432,2092,1890,2170,2891,1040,3803,1387,1656,2388
NDRG1_5_9433,837,1074,1241,64,970,499,389,180,1330
NDRG1_5_9434,1536,1475,1634,849,1888,2179,3213,1934,1749
NDRG1_5_9435,1858,1907,1896,3303,1685,1485,1558,1673,6113
NDRG1_5_9436,6377,7307,9520,4639,6271,10115,4595,7469,10620
NDRG1_5_9437,1763,2300,2159,1129,1774,1926,1757,652,2334
NDRG1_5_9438,211,390,515,353,111,9,129,2,2433
NDRG1_5_9439,1091,600,376,682,186,813,1305,702,864
NPRL2_5_9440,773,1710,841,503,1719,1472,457,1723,3574
NPRL2_5_9441,461,98,451,379,0,1027,127,6,0
NPRL2_5_9442,737,703,869,1132,1729,838,1051,619,583
NPRL2_5_9443,154,241,759,1,0,24,159,34,989
NPRL2_5_9444,3261,2472,4519,2953,2716,3295,2028,2095,6592
NPRL2_5_9445,305,585,741,415,259,1819,1,2176,6
NPRL2_5_9446,106,229,143,107,1276,221,259,28,544
NPRL2_5_9447,377,211,558,370,196,79,227,865,979
NPRL2_5_9448,2521,2551,2045,1129,2490,2892,2268,2120,767
NPRL2_5_9449,32,8,131,2,175,3,52,0,98

OXSR1_5_9450,214,285,200,131,662,48,200,226,0
OXSR1_5_9451,1262,1760,1725,801,872,1015,633,734,891
OXSR1_5_9452,586,442,329,319,355,725,111,1163,104
OXSR1_5_9453,570,368,879,512,839,1588,738,164,3636
OXSR1_5_9454,0,68,8,0,0,0,0,0,402
OXSR1_5_9455,539,553,719,1648,77,210,143,656,127
OXSR1_5_9456,445,141,332,85,1318,141,121,105,620
OXSR1_5_9457,484,271,393,6,6,625,382,820,535
OXSR1_5_9458,362,343,508,12,820,337,191,327,1529
OXSR1_5_9459,51,161,451,85,107,370,85,121,0
PDPK1_5_9460,2054,2162,2180,1034,1823,830,1352,1895,3669
PDPK1_5_9461,1471,1055,2234,2556,1750,1089,1667,862,692
PDPK1_5_9462,553,648,917,315,328,2105,13,27,306
PDPK1_5_9463,2801,4091,3257,1871,4358,3842,3071,3662,2945
PDPK1_5_9464,191,13,16,1,104,3,39,542,0
PDPK1_5_9465,2355,1823,2555,1913,4336,3244,1227,2184,3025
PDPK1_5_9466,191,511,789,632,2224,1054,108,280,1561
PDPK1_5_9467,1426,1393,985,1717,849,722,1381,830,39
PDPK1_5_9468,2646,2682,4228,2196,2524,2050,2586,4003,3489
PDPK1_5_9469,1694,2589,3672,4770,2639,4143,3780,1866,3081
PKMYT1_5_9470,391,1074,1269,103,976,423,672,403,355
PKMYT1_5_9471,300,679,334,62,789,660,484,7,542
PKMYT1_5_9472,149,148,522,8,655,351,207,2,1518
PKMYT1_5_9473,838,676,1617,282,495,255,532,935,2785
PKMYT1_5_9474,145,143,187,913,1383,42,1,505,1
PKMYT1_5_9475,909,512,860,973,573,1762,2554,727,2268
PKMYT1_5_9476,105,21,245,55,363,75,312,6,102
PKMYT1_5_9477,1535,2308,2062,2522,2498,2967,1438,2258,3347
PKMYT1_5_9478,1047,1105,684,102,674,728,934,1596,762
PKMYT1_5_9479,1050,444,939,577,328,841,1069,669,420
PKN1_5_9480,965,1376,2019,1339,145,2023,1226,1513,2413
PKN1_5_9481,546,527,713,1027,457,406,1528,287,1017
PKN1_5_9482,301,525,680,5,330,214,687,276,928
PKN1_5_9483,172,69,201,30,0,14,63,55,25
PKN1_5_9484,2520,3513,3313,948,3179,5158,1355,1106,2789
PKN1_5_9485,1581,2483,2132,1626,992,1498,2821,2746,1875
PKN1_5_9486,646,774,774,546,1533,453,1178,240,774
PKN1_5_9487,2084,1891,2316,1856,2048,2602,1271,1764,3830
PKN1_5_9488,5485,5415,6895,4578,5474,5719,5124,7805,5197
PKN1_5_9489,262,183,132,584,1253,178,471,564,3
PODXL_5_9490,139,1287,1288,0,985,4,210,531,809
PODXL_5_9491,1487,1409,1180,437,839,3347,936,1079,175
PODXL_5_9492,1080,825,1467,645,322,1049,814,260,671
PODXL_5_9493,522,851,909,773,126,2257,140,672,272
PODXL_5_9494,376,278,455,119,234,325,15,51,464
PODXL_5_9495,427,183,827,424,641,1217,286,425,811
PODXL_5_9496,335,565,431,1306,107,1309,505,139,214
PODXL_5_9497,1428,1959,1430,966,1501,1207,926,1173,2805
PODXL_5_9498,329,612,583,1284,104,1141,572,142,213
PODXL_5_9499,347,199,244,91,2,0,4,165,13

PRKD2_5_9500,67,719,87,1743,20,2,226,440,137
PRKD2_5_9501,970,1105,2109,1311,3720,2151,688,1258,958
PRKD2_5_9502,733,647,976,2363,696,1588,1085,536,544
PRKD2_5_9503,1307,1319,1044,1334,1092,397,1511,988,3021
PRKD2_5_9504,432,668,297,634,120,237,1357,349,820
PRKD2_5_9505,677,1547,964,711,2039,1163,1057,1002,87
PRKD2_5_9506,2443,1593,1719,3226,1679,1567,3289,2707,6270
PRKD2_5_9507,578,1191,2045,182,1482,1042,450,810,1775
PRKD2_5_9508,348,347,119,240,569,170,354,133,57
PRKD2_5_9509,1916,3026,2682,2184,1121,3977,1981,2504,5885
PTEN_5_9510,2032,3046,2043,1116,1073,4875,2255,1305,3041
PTEN_5_9511,365,88,33,0,1,62,233,374,0
PTEN_5_9512,604,547,507,591,248,586,211,378,43
PTEN_5_9513,838,883,1573,1648,1388,1427,222,319,2272
PTEN_5_9514,1433,848,1664,254,2748,3890,951,639,1402
PTEN_5_9515,258,5,238,6,10,0,63,181,0
PTEN_5_9516,32,5,29,0,0,95,3,0,13
PTEN_5_9517,435,307,858,753,84,268,170,56,252
PTEN_5_9518,685,798,1294,1069,135,969,266,742,518
PTEN_5_9519,499,314,90,780,216,0,176,11,1
PTPMT1_5_9520,660,301,626,431,316,264,305,512,292
PTPMT1_5_9521,736,514,695,341,1012,989,416,1221,3
PTPMT1_5_9522,103,39,553,0,631,39,0,0,0
PTPMT1_5_9523,64,96,134,2,0,36,0,0,0
PTPMT1_5_9524,3,28,32,23,39,0,0,120,0
PTPMT1_5_9525,454,574,697,263,1852,124,1212,703,474
PTPMT1_5_9526,36,105,68,235,583,2,260,0,147
PTPMT1_5_9527,456,593,738,262,1862,421,1219,715,475
PTPMT1_5_9528,298,577,834,9,406,65,303,178,192
PTPMT1_5_9529,10,30,145,1,385,315,3,47,34
PTPN18_5_9530,140,272,139,12,104,40,71,128,17
PTPN18_5_9531,4186,4205,4596,4447,5748,6369,4232,4339,6438
PTPN18_5_9532,2906,3326,2829,4276,4701,3472,1776,3584,3819
PTPN18_5_9533,478,1264,578,1309,2149,1311,1333,1198,617
PTPN18_5_9534,537,1199,702,974,162,271,104,694,466
PTPN18_5_9535,544,1371,1742,698,481,1077,357,553,1370
PTPN18_5_9536,1836,1708,1917,1537,1316,1607,1385,1943,1512
PTPN18_5_9537,1206,1386,1739,1223,1143,2175,1647,992,1752
PTPN18_5_9538,1211,2694,3234,2978,1648,2859,2438,794,2467
PTPN18_5_9539,401,639,516,31,1062,930,291,395,1611
PTPRR_5_9540,2338,2129,2830,2334,641,4190,2486,1918,2840
PTPRR_5_9541,783,879,1465,1186,1072,2029,966,833,1531
PTPRR_5_9542,257,364,541,3,642,627,101,378,1
PTPRR_5_9543,1035,1524,965,48,240,1675,596,3043,1669
PTPRR_5_9544,1578,2911,3696,2225,2200,3360,2430,3992,2638
PTPRR_5_9545,2439,2380,2506,983,1143,3075,1790,3316,2040
PTPRR_5_9546,117,343,264,170,1646,249,264,557,2
PTPRR_5_9547,749,753,875,799,1573,135,1238,597,1282
PTPRR_5_9548,814,765,975,654,1972,480,1208,1417,1430
PTPRR_5_9549,2002,1306,2055,420,1091,605,681,904,2242

PTPRU_5_9550,132,38,9,34,133,5,91,0,0
PTPRU_5_9551,1300,1416,1056,1613,451,862,926,740,641
PTPRU_5_9552,1743,1403,2015,3710,2800,1716,1907,823,2188
PTPRU_5_9553,503,586,877,166,927,944,767,339,639
PTPRU_5_9554,536,828,724,605,802,515,202,835,358
PTPRU_5_9555,361,566,572,420,283,2188,489,388,31
PTPRU_5_9556,4386,2957,3958,3845,2492,1936,3561,2238,2191
PTPRU_5_9557,3645,3995,3126,4636,5010,2861,3339,3254,4496
PTPRU_5_9558,704,622,1116,611,801,2144,343,408,462
PTPRU_5_9559,1302,1598,1688,1071,1732,2703,1107,1001,895
SGK3_5_9560,1093,662,739,732,460,364,618,1164,1177
SGK3_5_9561,1856,1537,2422,320,2363,2053,1457,880,1654
SGK3_5_9562,170,125,499,1283,12,30,697,126,445
SGK3_5_9563,127,100,122,293,3,3,135,58,7
SGK3_5_9564,280,155,150,364,96,28,242,159,27
SGK3_5_9565,750,1002,684,61,1970,3548,1464,666,889
SGK3_5_9566,444,559,510,190,188,502,433,586,162
SGK3_5_9567,1835,3981,2457,2017,2854,2057,2995,3480,2757
SGK3_5_9568,561,527,1074,931,514,896,142,1871,1238
SGK3_5_9569,150,217,305,39,745,503,23,103,187
SRMS_5_9570,1129,732,1122,1729,878,666,991,553,1231
SRMS_5_9571,643,942,691,686,2073,1471,423,67,1681
SRMS_5_9572,249,17,983,146,20,598,284,6,566
SRMS_5_9573,517,305,956,22,733,770,966,1144,350
SRMS_5_9574,23,258,24,21,0,0,0,0,0
SRMS_5_9575,45,618,384,91,231,112,28,663,0
SRMS_5_9576,367,147,741,96,306,525,798,603,268
SRMS_5_9577,665,257,1313,117,908,827,1532,1535,522
SRMS_5_9578,648,571,938,1086,620,1471,79,589,137
SRMS_5_9579,639,618,884,2018,1255,768,594,420,349
STK4_5_9580,259,272,487,526,0,26,48,17,295
STK4_5_9581,71,245,0,0,64,90,74,5,881
STK4_5_9582,400,350,574,602,260,222,203,204,61
STK4_5_9583,295,104,478,75,9,295,146,14,545
STK4_5_9584,199,291,259,939,139,670,303,37,245
STK4_5_9585,253,220,683,105,660,208,587,171,1191
STK4_5_9586,616,328,377,596,335,105,91,513,564
STK4_5_9587,225,63,412,34,2,108,156,0,805
STK4_5_9588,394,788,439,461,468,229,470,410,0
STK4_5_9589,620,859,1113,566,954,2942,660,1885,1284
STK24_5_9590,4667,3550,7286,6800,5763,8094,4123,3341,4694
STK24_5_9591,3327,4984,4203,2638,3834,3045,3350,5600,8740
STK24_5_9592,285,67,166,33,574,360,115,123,145
STK24_5_9593,185,347,304,439,342,173,155,374,516
STK24_5_9594,858,587,889,1253,1426,1059,395,163,2
STK24_5_9595,251,306,533,748,637,622,677,415,385
STK24_5_9596,1450,1843,2449,2238,1670,1868,1689,3126,4316
STK24_5_9597,1352,1819,2462,2207,1668,1866,1553,3112,4325
STK24_5_9598,1676,1235,2042,2407,1790,1792,2577,2644,3016
STK24_5_9599,960,1543,1740,1635,2229,1269,1521,1660,1154
