# Supplementary material for: Cross-species genetic screens identify transglutaminase 5 as a regulator of polyglutamine-expanded ataxin-1
Source: J Clin Invest. 2022 May 2;132(9):e156616. doi: 10.1172/JCI156616 (PMC9057624; doi:10.1172/JCI156616)
Supplement: Supplemental data set 6 [file jci-132-156616-s051.pdf]

shRNA,B1,B2,B3,B4,H1,H2,H3,H4,L1,L2,L3,L4
A2M_8_1,228,332,191,155,0,1240,0,0,1,570,163,2636
ACE2_8_2,2310,2497,1884,1865,2441,2490,3915,3045,3734,2236,3333,1262
ACTR6_8_3,1834,1973,1100,1563,1077,2239,2778,1852,1296,2227,2167,1172
ADAM11_8_4,847,1020,502,646,258,1418,1125,632,515,285,309,1269
ADAM19_8_5,941,923,1378,782,1800,1360,1989,1515,2109,1057,3455,848
ADAM20_8_6,4224,3171,3497,3473,2930,1504,1708,2983,3539,3082,3355,5303
ADAM21_8_7,3231,3426,2829,2775,1469,3854,2257,1787,1624,3421,1869,4910
ADAM2_8_8,4200,4649,3727,3747,4808,4893,3181,3709,5086,5230,4690,6667
ADAM30_8_9,3683,4268,2723,3252,4072,3796,5176,4496,2097,2766,4266,4857
ADAM7_8_10,3713,3278,3613,4668,3930,3949,4215,5190,2282,5285,3487,7750
ADAM9_8_11,1922,1503,1191,1253,1763,1661,360,1188,2573,1341,1885,2181
ADAMTS10_8_12,1004,1078,723,564,433,575,1944,1157,929,584,1488,932
ADAMTS12_8_13,2800,2027,2167,2435,3137,2037,1925,1429,1911,4838,2703,2
502
ADAMTS15_8_14,2206,1878,2106,1941,2813,2242,3443,2493,1365,1653,1620,1
851
ADAMTS18_8_15,4387,3227,4340,5215,3646,5122,3813,3621,4574,4537,4177,6
084
ADAMTS19_8_16,2171,1760,1460,1783,1427,2310,641,780,1483,2575,2184,148
7
ADAMTS1_8_17,4691,3640,2957,4180,3762,5695,5273,1608,3563,3733,3767,47
60
ADAMTS20_8_18,7194,7807,6944,7491,8463,8341,7275,8134,7885,6072,9047,9
256
ADAMTS3_8_19,8122,6967,7213,7551,6161,6491,8341,7664,8771,7216,8841,83
89
ADAMTS5_8_20,213,275,499,236,103,553,34,64,14,303,206,472
ADAMTS6_8_21,3271,2898,2968,2682,3155,5301,1885,4096,3278,3608,4796,37
88
ADAMTS7_8_22,1015,960,1191,1651,1158,1172,1535,1890,551,1295,2164,1024
ADAMTS8_8_23,330,278,450,428,102,48,94,626,190,993,340,1253
AEBP1_8_24,327,268,256,353,335,1005,1056,184,2,460,594,372
AFG3L2_8_25,2858,3317,2711,2451,2515,3405,4650,4159,3899,2059,3708,327
9
AFM_8_26,3525,3859,3680,3424,4188,5700,3356,1653,2561,7259,6768,3748
AFP_8_27,143,202,24,190,168,0,15,508,79,162,113,44
AGTPBP1_8_28,1308,2121,1302,1862,1149,1894,1886,1823,1143,1932,1418,37
37
ALG10B_8_29,30,66,2,26,582,0,0,0,190,0,0,0
AMBP_8_30,246,621,675,223,0,92,0,108,2,172,0,1467
ANGPT4_8_31,181,160,145,178,6,210,767,101,97,55,19,98
ANGPTL1_8_32,5711,5735,4200,4648,5455,6571,5476,6578,4074,9889,6080,71
46
ANGPTL2_8_33,490,290,321,460,316,300,274,181,678,152,466,362
ANGPTL3_8_34,1532,1871,1246,1967,1829,1981,2065,3187,2802,2016,1552,23
58
ANGPTL7_8_35,1045,779,822,715,1164,2158,1585,147,565,1936,856,801
ANKH_8_36,291,141,269,247,46,96,285,260,137,179,192,541
AP1G2_8_37,666,1108,453,675,1736,1600,535,266,1443,1107,1737,638

AP1M2_8_38,1152,763,639,980,1022,1143,1790,806,873,1586,649,1237
AP1S1_8_39,9,0,2,3,242,10,1,0,92,34,107,0
AP1S2_8_40,33228,36881,31009,32220,32921,37980,38885,34302,32780,35590
,40147,40077
AP1S3_8_41,1607,1202,1146,1708,2192,867,1778,1777,1198,1540,1288,1370
AP3B1_8_42,417,370,284,628,0,238,211,778,447,365,901,1698
AP3B2_8_43,1465,1363,801,1018,1287,830,2448,1490,809,1064,1441,1504
AP3S1_8_44,3104,3148,2277,2771,1581,3356,2463,3337,3031,4389,2687,2898
AP3S2_8_45,466,535,752,366,569,638,370,331,332,434,392,507
AP4B1_8_46,1994,2875,2275,2209,2062,3139,599,2660,3539,4461,2471,3163
AP4M1_8_47,50,67,129,126,0,0,0,425,0,299,17,348
APBA1_8_48,3527,3452,2857,3210,1748,2572,3631,2164,1904,4937,3197,3772
APBA3_8_49,582,461,547,790,142,889,142,1273,126,144,283,397
APOA1_8_50,329,367,166,392,80,2,556,465,31,699,235,256
APOA2_8_51,712,753,813,829,466,1110,1057,1261,911,540,885,804
APOA4_8_52,146,182,493,292,87,218,846,401,428,15,4,1198
APOB_8_53,1773,2229,2081,1900,2172,1271,2665,2509,1800,2553,3721,4033
APOC1_8_54,1531,1702,1469,1793,2986,3063,2106,1453,621,1632,3505,1680
APOC2_8_55,381,364,121,452,250,109,285,559,1750,682,0,618
APOC3_8_56,1848,1230,1077,1637,326,2210,746,1734,1182,905,694,1649
APOC4_8_57,2493,3048,2512,1946,5320,1967,1381,1635,2554,2307,3199,4978
APOD_8_58,565,537,605,564,300,90,659,404,106,685,638,896
APOE_8_59,879,549,612,922,603,690,1692,532,460,1027,332,1499
APOF_8_60,557,590,445,517,831,636,76,403,69,1450,1039,265
APOH_8_61,3881,3300,4522,4544,3672,5107,2988,4466,2517,5010,2477,4387
APOL6_8_62,562,384,624,284,848,261,921,1335,347,349,161,233
APOM_8_63,510,272,361,475,735,468,132,411,480,293,612,847
AQP12B_8_64,391,675,616,871,82,548,178,2563,78,1138,673,843
ARF5_8_65,235,170,232,199,518,291,35,139,517,87,161,463
ARF6_8_66,474,497,485,403,980,407,370,349,139,905,347,748
ARPP19_8_67,2038,2507,2206,1865,2209,2219,2692,2383,1337,2943,3358,251
2
ASTL_8_68,2487,2797,3405,3019,3146,2832,3387,839,3715,3663,2316,2600
ATOX1_8_69,664,616,649,500,1446,1487,1584,610,289,1109,115,64
ATP13A1_8_70,317,466,411,629,497,495,138,523,258,355,835,387
ATP13A3_8_71,2672,2526,2904,2031,2764,3494,2663,1740,1363,3541,2269,25
01
ATP13A4_8_72,1637,2433,2273,1685,1184,3119,4135,1726,1213,2374,5024,23
14
ATP13A5_8_73,1715,1969,2060,2418,1549,1985,2012,2877,2093,1965,3349,45
73
AZGP1_8_74,1820,2037,1773,1795,1235,2240,2420,1383,1401,1600,1708,1105
AZU1_8_75,570,579,440,322,184,511,1153,616,461,172,1251,353
BCL2L10_8_76,1435,1566,1577,1156,1783,1342,1204,1623,677,753,2985,694
BET1_8_77,1132,1526,986,1370,992,1117,1207,680,1829,2383,412,4724
BGLAP_8_78,711,798,827,740,1190,1553,972,1172,481,410,1905,690
BOC_8_79,1391,973,705,1246,999,784,583,2197,991,514,1322,1226
BPI_8_80,4481,4907,4447,3698,5597,4007,6007,2212,4134,4495,5890,3621
BPIFC_8_81,791,1119,795,1022,2128,41,1535,215,2355,1327,1174,2698
C16orf7_8_82,433,207,200,383,105,112,23,347,69,368,47,951

C1orf162_8_83,1300,1292,1676,987,378,1387,953,1260,1578,1618,1645,1701
C1QA_8_84,206,241,168,109,258,21,141,40,64,141,241,853
C1QB_8_85,1537,2008,1268,970,1914,2136,366,702,1685,1611,1904,2195
C1RL_8_86,2462,2161,1779,2147,2330,2201,617,1534,3816,2603,3238,2029
C20orf141_8_87,262,268,218,129,0,379,64,571,419,71,2,136
C3_8_88,1069,1519,1165,1254,2348,1507,3600,1626,1767,1610,1620,2107
C4A_8_89,164,46,197,101,382,0,54,14,4,89,12,0
C5_8_90,4156,3956,4002,3929,3715,4960,6744,3538,3953,3023,5315,4821
C7orf31_8_91,2500,2596,2362,2739,3159,3702,6583,3710,2395,4151,4229,23
25
C8G_8_92,1158,1153,1215,1089,948,273,1055,921,1090,1688,1747,1352
CALM2_8_93,871,345,840,1098,954,580,671,1435,819,605,1680,848
CALY_8_94,147,110,33,189,263,1,40,917,447,153,1,750
CAMLG_8_95,1494,2206,1856,1743,2391,3641,1281,1638,2147,2949,1303,1116
CAPN11_8_96,876,1038,1302,768,486,492,1145,1143,811,1479,614,511
CAPN5_8_97,1024,1386,1021,938,821,1264,83,2184,347,1382,773,1937
CAPN6_8_98,926,615,565,667,567,818,396,496,668,557,1179,654
CARTPT_8_99,580,127,344,468,149,92,242,158,835,539,1115,318
CCL13_8_100,585,529,593,624,1246,1764,1158,50,321,671,906,1401
CCND1_8_101,2280,2548,2064,2049,1567,3200,1276,2267,2494,1402,1881,179
9
CD1A_8_102,721,693,727,831,281,1683,405,1231,923,1562,771,1112
CD52_8_103,656,504,492,648,900,1691,112,337,488,418,1549,923
CDCP2_8_104,1330,678,875,1049,1284,1954,884,55,2142,1380,808,1086
CDH5_8_105,617,670,723,606,191,1253,379,263,355,769,379,475
CHMP7_8_106,77,0,40,32,0,39,206,1,130,11,6,1
CLDN16_8_107,6727,7209,6797,6711,9584,7606,6189,4007,7382,8670,9080,87
32
CLEC3B_8_108,627,425,332,354,124,994,280,939,3,437,816,319
CLSTN2_8_109,2059,1928,1642,2186,1780,1491,1777,1997,1905,2632,578,244
6
CLVS2_8_110,2652,1936,1434,2170,2785,1857,2458,2985,2296,3148,3321,348
5
CNIH3_8_111,2019,2348,1513,1839,1453,4002,2155,1896,2850,2183,3216,234
1
CNOT6_8_112,3240,2233,2683,3081,3808,3192,3188,3975,2300,3098,2348,339
9
CNTNAP1_8_113,502,393,415,666,1005,550,748,235,210,271,819,815
COG1_8_114,291,526,202,257,138,729,88,250,132,97,439,421
COG3_8_115,3254,3738,2404,2635,2695,3002,2765,2048,2454,5469,3273,4306
COG7_8_116,377,257,161,341,4,110,76,362,1214,101,60,1152
COG8_8_117,165,66,14,30,0,163,308,2,5,241,266,212
COL10A1_8_118,363,317,350,263,144,591,814,475,128,468,160,2349
COL14A1_8_119,2266,2362,2523,2919,923,2338,2337,3339,3073,3789,2652,30
23
COL15A1_8_120,799,744,717,907,466,865,829,1336,781,1486,635,1480
COL16A1_8_121,946,620,768,881,437,1540,1353,297,964,216,1237,613
COL17A1_8_122,3660,4368,3641,3253,3511,4576,3281,4434,4095,2523,4023,4
120
COL1A1_8_123,664,620,475,743,569,837,746,911,318,273,870,393

COL1A2_8_124,2079,1870,1665,2448,1051,2133,2277,2461,1777,1555,2779,35
59
COL21A1_8_125,1910,1980,1883,1596,1159,4256,1477,1796,2377,2127,960,76
3
COL22A1_8_126,1467,1458,1379,1235,2177,1548,1962,1494,1928,943,849,158
3
COL23A1_8_127,181,280,385,229,682,241,37,23,217,487,210,329
COL24A1_8_128,5156,5247,5188,5153,6050,6152,5162,6062,5564,6098,10173,
8948
COL27A1_8_129,1850,1829,1548,1735,1116,1811,2624,868,2852,2387,2215,12
75
COL3A1_8_130,253,565,311,347,58,1611,124,168,71,136,122,1410
COL4A1_8_131,1545,2015,1375,1550,1142,2103,1794,2203,775,1589,839,1825
COL4A2_8_132,2050,1605,1352,1620,927,802,1098,2041,2746,2966,1278,2294
COL4A3_8_133,1391,1282,1306,1677,61,286,2551,1106,1439,1210,214,2011
COL4A4_8_134,650,558,319,490,320,148,329,841,1218,447,521,344
COL5A1_8_135,1665,1798,737,966,1692,3109,1352,1023,1094,1647,926,555
COL5A2_8_136,10074,12974,8363,9829,11125,13066,9123,10099,9698,12806,1
0063,11919
COL5A3_8_137,238,750,358,509,228,999,1031,113,419,1300,1414,974
COL6A1_8_138,1248,1639,1257,1103,3015,1412,2733,685,639,1978,494,1140
COL7A1_8_139,507,861,531,340,692,58,541,1113,96,156,154,674
COL8A2_8_140,752,562,172,389,0,40,569,244,381,36,46,18
COL9A2_8_141,45,183,79,75,631,281,23,9,0,1,6,31
COL9A3_8_142,784,863,400,595,352,1678,1026,1765,2,82,130,1431
COMMD1_8_143,4011,3746,3778,3572,2262,2317,4666,4966,3624,3977,6963,33
75
COPB2_8_144,2132,2908,3038,3307,2981,2526,2248,2498,2116,4424,2678,239
6
COPG2_8_145,308,251,420,206,315,381,862,666,396,459,249,700
COPZ1_8_146,370,430,218,560,118,811,139,77,1049,514,581,1806
COPZ2_8_147,977,1207,691,1050,1355,994,1318,766,806,2481,870,304
CORIN_8_148,2567,1855,1620,2036,3342,3336,1830,2276,4261,2407,2457,170
5
COX18_8_149,1783,1454,1885,1974,2437,1386,3978,1562,1685,3067,2032,176
9
CPLX1_8_150,354,312,390,660,186,557,363,633,556,666,105,742
CPLX3_8_151,481,274,336,476,835,1009,1143,431,480,195,554,509
CPNE6_8_152,627,883,896,346,1188,2204,417,342,601,1738,1184,219
CPXM2_8_153,1401,1132,918,1083,1405,687,648,1076,895,615,2167,1467
CRABP1_8_154,1223,1968,1176,1521,875,1382,1916,624,1224,1825,822,967
CRH_8_155,1153,1007,1168,1177,789,269,902,1531,649,1311,1976,477
CSE1L_8_156,1813,1355,1646,1673,342,2083,3155,2154,1657,2361,1561,2834
CTSW_8_157,2353,2393,2564,2891,1698,2525,4026,2866,1226,1259,1608,2760
CXCL10_8_158,1875,2260,1729,2331,1375,3965,2712,2461,2236,3646,3696,88
7
CYGB_8_159,46,0,0,73,0,0,0,0,0,0,260,0
CYTH3_8_160,506,678,421,604,449,1226,237,436,1259,912,358,739
DDI2_8_161,1245,1506,1779,1888,2009,2925,1506,902,691,1585,4213,1706
DIRC2_8_162,649,914,480,690,1352,447,598,1468,1109,597,602,1426

DISP1_8_163,1841,1530,1452,1208,1733,1550,2930,576,1954,2004,1459,787
DLL4_8_164,1184,1332,931,916,509,1970,2007,711,1529,393,1267,1016
DNAJC5B_8_165,900,792,639,1039,1232,942,921,1176,487,1312,1323,1155
DNAJC6_8_166,1283,1449,1322,1016,1051,997,841,402,1681,1226,1713,639
DOC2A_8_167,786,824,881,669,306,670,1123,765,882,816,1764,940
DOC2B_8_168,551,476,212,646,938,377,90,1576,171,318,362,975
DSCAML1_8_169,213,116,71,58,65,436,158,77,94,413,585,774
ECEL1_8_170,1164,760,754,674,1125,803,3317,2024,2031,1901,1160,426
EID2_8_171,1764,1673,1943,1825,1078,3566,1103,938,1561,2422,2558,2698
EPCAM_8_172,1718,1513,1085,1130,1336,1322,1451,429,384,1319,1762,808
EXOC2_8_173,3775,4892,3485,3275,1537,6915,4563,2976,4325,4817,5717,472
6
EXOC3_8_174,1385,2057,1017,1281,1145,732,1628,686,3324,715,1675,846
F11R_8_175,372,298,238,243,112,699,311,55,40,328,233,23
FABP1_8_176,1113,844,1162,1052,2629,2087,3352,1048,563,1056,556,1826
FABP2_8_177,1301,1127,914,1594,2547,2082,1423,1650,1599,531,953,2012
FABP3_8_178,598,625,289,386,344,797,85,1092,692,500,521,107
FABP4_8_179,994,897,618,746,1587,801,477,1608,882,525,1142,583
FABP7_8_180,5846,5683,4801,5713,8304,7757,7719,4440,6213,5886,6630,531
4
FABP9_8_181,6503,6532,4933,5445,6502,4583,3071,7892,4300,6929,5958,717
3
FAM101A_8_182,209,428,510,359,142,598,0,674,281,188,2,1697
FAM117A_8_183,263,518,510,246,833,109,62,722,697,56,1011,22
FAM57A_8_184,1200,1374,1285,887,1044,1479,1894,2660,1593,2274,1314,157
0
FAP_8_185,1409,2058,1272,939,1446,1583,1189,807,1393,1793,1776,1055
FBF1_8_186,1693,1179,1229,1050,706,825,1739,3806,1173,2125,1085,1776
FCN1_8_187,1979,2283,1915,2220,465,2122,1433,2937,2760,1502,1798,2368
FDX1_8_188,1243,938,900,1433,1618,2497,1444,2271,212,229,802,2239
FDX1L_8_189,719,509,377,516,20,1040,101,795,250,388,807,592
FGF4_8_190,679,328,838,729,416,693,437,199,1220,1245,911,879
FGL2_8_191,558,1308,438,527,677,1433,430,1429,625,1202,838,733
FOLR3_8_192,1222,1293,1192,1399,329,706,1623,1887,602,842,361,2037
FOLR4_8_193,721,905,545,695,1491,1270,805,1839,174,447,719,889
FRG1_8_194,1643,1550,1504,1586,2028,2727,1338,1946,1546,814,1397,1715
FTL_8_195,3579,2953,2907,3410,3009,4421,3363,2842,3768,3347,4155,3539
FXC1_8_196,229,213,162,350,144,38,8,19,804,53,93,61
GABARAP_8_197,426,427,335,582,336,57,761,210,164,131,81,1133
GGA2_8_198,674,836,849,753,1187,1380,267,986,436,532,781,176
GJA1_8_199,326,121,295,66,391,87,24,1313,91,361,217,149
GJA3_8_200,141,170,29,178,0,59,1,0,15,169,19,0
GJA4_8_201,1093,277,303,989,221,559,182,770,590,319,252,1175
GJA8_8_202,233,446,676,339,42,98,99,568,42,229,199,265
GJB2_8_203,883,999,1136,1233,1181,813,1328,322,1337,1642,1287,333
GJB4_8_204,1209,1061,1194,1373,600,1845,1313,2618,1414,932,567,1881
GJB5_8_205,2110,3022,2434,2445,2689,5217,3499,1547,3076,4152,4596,2458
GJC2_8_206,260,120,189,216,60,11,364,618,431,643,265,1
GJC3_8_207,1169,1481,1409,1277,537,1047,402,1726,644,1194,2535,660
GJD2_8_208,4514,4139,3330,2983,3025,4591,4341,3611,3363,5237,3990,4313

GJD3_8_209,512,1148,509,465,6,798,232,322,1038,1031,680,1385
GJD4_8_210,60,30,75,298,166,491,73,85,122,0,14,8
GKN1_8_211,53,123,116,40,106,26,77,5,0,267,8,35
GLCCI1_8_212,2491,2051,1346,1774,2687,1039,2739,1021,2145,3228,1712,20
08
GLTP_8_213,380,63,287,154,68,70,569,386,388,1,718,303
GLYATL2_8_214,299,446,516,320,218,265,374,377,0,1131,128,309
GP9_8_215,303,346,173,119,235,708,21,81,52,126,129,0
GPIHBP1_8_216,974,1006,1261,1276,1021,1710,779,716,1338,410,1392,1491
GPR180_8_217,7548,7757,6644,6814,7568,9468,5577,5775,9450,8468,9212,62
97
GRN_8_218,565,676,418,617,541,765,604,546,207,1428,140,1188
GZMH_8_219,3964,4494,4238,3893,3719,4271,3433,3449,6017,4700,6029,5694
GZMK_8_220,3928,3067,3755,2692,4264,2663,2987,2435,4767,2581,4270,4182
GZMM_8_221,187,55,244,126,5,83,21,8,36,28,23,47
HBA1_8_222,896,720,807,1210,179,1047,2746,2240,1412,1572,1175,962
HBA2_8_223,896,720,807,1210,179,1047,2746,2240,1412,1572,1175,962
HBE1_8_224,331,387,425,379,285,319,1101,304,1,681,386,957
HBZ_8_225,405,810,551,427,309,340,28,812,168,1201,315,879
HECA_8_226,292,281,350,395,549,601,213,247,315,606,98,343
HGFAC_8_227,206,194,290,70,6,111,523,0,298,135,68,119
HIAT1_8_228,1851,1867,2273,2180,1413,3149,1173,2753,766,2211,2418,2682
HLA-DQB1_8_229,638,689,659,660,333,1211,581,2075,476,1689,578,494
HMCN1_8_230,4068,4546,4509,4858,4151,5325,6592,3432,2943,5549,5930,609
3
HMHA1_8_231,881,1062,549,1193,1851,702,450,1076,1636,1107,1017,668
HPCAL4_8_232,558,416,367,341,1642,129,1439,183,205,557,22,966
HPR_8_233,2139,2063,2024,2592,2568,2255,2211,4413,829,2101,1947,4142
HPX_8_234,4450,5849,5144,6084,6222,9435,6451,3933,4875,6372,6671,7527
HSP90B1_8_235,2167,2167,2161,2907,2094,1871,2309,1747,2529,3319,2997,3
842
HTRA1_8_236,2397,1859,1861,2322,4038,3313,2351,2025,2502,2286,2257,397
8
HTRA4_8_237,2262,1938,1908,2234,1248,1638,1445,2612,1145,2801,2745,255
7
IFNG_8_238,1222,1437,832,1104,1139,796,342,1054,1433,1055,1915,1165
IGFBP7_8_239,1274,1407,1353,1492,2358,1489,838,2180,2331,784,1410,426
IL12B_8_240,1268,1247,1112,1021,698,1446,1337,517,2289,1629,753,2589
IL13_8_241,171,145,210,75,380,1,124,8,376,59,218,150
IL17A_8_242,213,495,632,564,70,652,185,3,330,469,686,917
IL1A_8_243,739,1098,699,699,128,1679,626,735,884,590,771,1156
IL1B_8_244,2896,3249,3336,3590,3074,3164,2144,3343,3557,4627,5358,2841
IL3_8_245,1510,1488,1578,1277,914,2914,1293,564,1100,1979,2609,1720
IL5_8_246,1845,1671,1351,1669,3312,1285,1339,1375,1543,1112,1653,1470
INSL3_8_247,159,218,322,370,14,743,99,83,207,787,311,135
IPO13_8_248,378,752,483,359,78,127,421,743,340,517,186,76
IPO4_8_249,1435,1912,748,1227,1128,2174,1162,1425,1461,1476,1617,565
IPO5_8_250,1583,1183,1380,1683,667,836,1231,964,1165,1594,1134,3203
IPO7_8_251,2033,1879,1842,1995,2058,2780,2909,3407,1112,2263,947,3124
IPO9_8_252,781,424,469,416,221,577,839,344,671,164,346,206

ITGA10_8_253,127,396,158,182,8,223,25,141,8,50,429,817
ITGA11_8_254,2539,2613,2126,2492,2419,2014,3439,2686,3068,2421,2537,2233
ITGA2_8_255,1535,1432,2018,1765,2466,1277,1597,1833,3244,1677,1215,1678
ITGA4_8_256,1739,1858,1033,1502,1104,2297,1894,2113,1572,1645,2099,2192
ITGA5_8_257,744,1103,1296,562,712,646,817,909,305,1656,569,2484
ITGA8_8_258,1184,1083,1395,1329,598,1595,1755,2154,2144,1683,814,734
ITGAX_8_259,1105,1328,840,1271,602,682,628,2054,432,777,1503,3786
ITGB5_8_260,2558,3169,2313,2711,3358,3597,3310,2444,3010,1697,5023,1495
ITGB6_8_261,613,522,657,1010,1065,1239,1146,451,729,1461,1119,2123
ITGB8_8_262,7363,7321,5798,7320,7077,6497,10518,10536,8857,10355,6529,7981
ITLN1_8_263,890,937,485,573,1891,497,534,706,808,497,321,435
KDELR1_8_264,507,641,807,764,93,192,2108,355,1580,475,642,3062
KEL_8_265,367,125,130,233,1082,558,1367,154,10,45,1925,108
KIF20A_8_266,1146,1194,588,558,387,1356,1976,1635,1685,1388,363,1767
KIF3B_8_267,673,613,738,558,516,1274,98,910,1097,552,647,1634
KIF5A_8_268,1020,710,657,750,642,784,1453,467,1446,862,845,658
KLK13_8_269,1049,922,1090,869,1082,236,1081,768,365,985,1240,15
KLK14_8_270,1001,938,949,733,96,1415,1442,48,1602,1469,518,859
KLK4_8_271,414,482,87,483,60,721,37,47,64,232,950,231
KLK9_8_272,1720,1805,1319,2419,1643,2515,723,1637,923,3210,2528,829
KPNA1_8_273,1201,1343,1339,1354,1032,547,1741,1113,1602,638,999,2226
KPNA2_8_274,1436,2214,993,1882,2133,1684,1289,2702,1456,1692,2323,1915
KPNA3_8_275,2530,2260,1716,2480,4049,2782,1564,1977,3730,3229,2759,1764
KPNA4_8_276,429,770,770,786,590,558,90,124,109,694,87,614
KPNA6_8_277,3495,2842,2853,3446,2044,4541,5522,2100,3576,3286,5020,3964
KPNB1_8_278,1165,1374,1223,1476,211,2705,1869,699,1306,669,576,1063
KRT12_8_279,931,681,501,422,2232,415,638,885,103,614,1398,459
KRT7_8_280,346,362,540,567,158,797,736,909,476,805,599,1018
KRT8_8_281,1992,2575,1481,2623,1398,2699,1352,2775,538,2160,2360,4167
KRTAP5-4_8_282,960,811,660,528,620,912,1009,502,1004,108,64,145
LASP1_8_283,308,64,73,106,57,1,223,90,45,29,119,52
LBP_8_284,3344,3395,3088,3069,1820,4614,3936,3496,4866,4620,4328,5020
LCN12_8_285,3170,2725,3470,3559,2849,1762,3081,3508,1466,2815,4457,2078
LCN1_8_286,3148,3335,3466,3776,4345,2389,4668,3857,3703,4019,4478,4437
LCN2_8_287,972,1638,1706,1856,1332,1027,1189,1961,791,2652,1271,624
LCN8_8_288,348,148,628,812,745,1357,4,502,392,68,56,1205
LCN9_8_289,1774,2054,1292,1390,1175,3262,1170,870,1541,2626,1672,2771
LDLRAD2_8_290,364,311,395,284,4,551,308,0,209,768,487,9
LDLRAP1_8_291,495,856,965,853,263,233,434,278,152,798,1334,644
LMAN1_8_292,1684,1492,1574,1902,2165,3219,1318,1309,1439,1863,2558,764
LMAN2_8_293,495,639,831,570,379,920,903,579,107,183,163,509
LPA_8_294,1106,1092,718,1038,1600,1295,321,1270,863,427,2214,977

LRIG1_8_295,591,395,740,558,218,578,1365,694,340,325,292,382
LRP2_8_296,4178,3239,4244,3342,5551,5633,5839,2754,3825,3944,6853,4005
LRRC4B_8_297,0,0,19,5,0,0,0,0,0,6,5,1
LRRCC1_8_298,3541,4138,3217,3169,5595,2121,8834,2546,2390,2562,3820,34
54
LYST_8_299,1396,1258,745,790,1401,1616,479,1638,1026,405,1660,599
MAL2_8_300,1205,1598,1294,1738,1138,3118,384,1266,1592,1332,1580,1597
MATN3_8_301,708,678,647,591,538,795,76,366,428,331,1084,919
MFSD8_8_302,2964,3181,2677,3897,4932,4363,3391,3444,3512,3561,3068,211
7
MFSD9_8_303,942,748,1178,879,1297,837,339,250,1970,731,2453,564
MMAA_8_304,372,363,324,344,1462,340,244,173,223,108,569,319
MMACHC_8_305,795,591,819,583,2124,1450,895,1606,681,299,1084,620
MMGT1_8_306,2565,2089,1597,2245,1321,4044,2358,2936,1988,1651,2448,441
6
MMP11_8_307,313,136,233,158,40,1130,24,266,37,209,72,165
MMP13_8_308,1573,1470,1262,1909,1385,2695,1854,1399,861,884,3293,1109
MMP15_8_309,457,1129,638,649,422,814,2854,760,1376,661,102,95
MMP16_8_310,3538,4188,3011,3378,4145,5667,3720,3166,2743,3621,4040,358
9
MMP17_8_311,86,313,39,181,49,450,12,12,6,0,6,26
MMP19_8_312,1088,1248,1233,1144,2790,1729,455,2415,1771,1446,518,1239
MMP24_8_313,484,699,601,655,177,30,1207,883,535,577,1576,786
MMP25_8_314,651,499,382,659,175,112,123,384,97,147,279,83
MMP26_8_315,716,740,409,762,1000,760,143,821,342,1266,169,297
MMP27_8_316,5895,5987,4850,4947,4717,5562,6407,7143,4196,7191,6935,726
4
MRS2_8_317,2074,1954,2179,2463,3372,2634,3485,2278,2307,3538,4150,2104
MSTN_8_318,1117,1185,1144,767,1119,1022,1329,724,1049,775,730,1184
MTX2_8_319,1470,1275,1002,1008,600,1302,1875,1101,1366,2504,665,912
MUC2_8_320,510,421,198,505,214,337,534,96,706,487,117,78
NAPG_8_321,2355,2848,1818,2526,1788,2489,1799,1837,2622,3307,4929,1867
NAPSA_8_322,930,943,393,416,191,198,137,296,382,774,998,1887
NCOA5_8_323,2772,2274,2683,3068,3004,2182,3215,4053,3499,3088,4760,285
2
NGF_8_324,165,119,157,185,157,284,566,13,137,33,164,95
NID1_8_325,1275,798,924,641,477,2001,902,896,1128,421,1076,1127
NPC1_8_326,715,746,800,925,915,695,551,867,603,519,469,1150
NPEPPS_8_327,543,727,663,725,1174,1941,786,553,903,940,212,1815
NPPB_8_328,48,3,41,51,3,0,1,0,1,270,10,3
NPY_8_329,695,898,567,651,765,109,572,956,464,896,1504,344
NSMCE1_8_330,323,232,609,513,358,221,60,968,102,1198,24,79
NUP107_8_331,160,136,57,207,181,5,5,86,151,162,281,222
NUP133_8_332,284,461,248,247,651,913,99,221,13,403,287,390
NUP153_8_333,509,386,342,341,860,205,564,541,524,729,149,644
NUP160_8_334,451,597,432,574,195,1772,464,675,49,321,223,346
NUP210_8_335,1678,1370,1465,603,1749,1335,2090,903,1521,2153,3933,396
NUP214_8_336,3243,2634,3010,2861,3793,3212,2335,2497,2016,2690,4135,61
75
NUP35_8_337,1120,1169,1521,1221,472,1631,923,1704,794,1206,1222,693

NUP37_8_338,2407,2400,2262,2225,2053,2619,2871,1981,2308,2256,3376,294
5
NUP54_8_339,135,666,194,431,96,872,232,147,177,28,4,6
NUP88_8_340,877,432,577,1009,913,466,342,793,718,878,289,690
NUPL2_8_341,2599,3204,1772,1877,2907,2716,3372,1815,3256,3036,2911,193
2
NUTF2_8_342,1274,1183,1174,1454,1966,1616,1481,1328,1181,1347,909,2074
NXF2B_8_343,1520,1606,1266,1426,734,2771,4899,803,1744,1473,1369,1929
NXF2_8_344,1520,1606,1266,1426,734,2771,4899,803,1744,1473,1369,1929
NXF3_8_345,190,291,259,369,447,441,333,422,423,104,74,502
NXT1_8_346,1613,1936,2376,1631,730,2481,1831,1605,1425,1065,3043,2718
OAZ2_8_347,149,128,188,144,785,71,494,40,14,110,66,67
OBP2A_8_348,1612,1580,1290,1309,1589,933,983,1006,1491,2277,1541,871
OBP2B_8_349,1612,1580,1290,1309,1589,933,983,1006,1491,2277,1541,871
OCA2_8_350,791,1005,365,891,1100,815,713,530,175,672,991,1271
OGFOD1_8_351,1442,1720,906,1243,1558,1943,1909,1228,1416,440,1570,1465
OGFOD2_8_352,220,105,161,134,361,79,64,181,216,68,0,199
OGFR_8_353,429,572,517,180,515,0,99,320,320,174,1371,528
OVCH1_8_354,423,279,405,708,132,331,562,605,444,661,927,14
OVCH2_8_355,773,988,1038,1010,330,315,1339,248,578,1334,30,1287
OXNAD1_8_356,1895,1926,1214,1870,1794,2329,1590,2388,2041,2462,2201,23
68
OXT_8_357,153,27,103,275,136,66,324,181,8,7,369,16
PANX1_8_358,1119,1080,711,750,351,597,1605,694,424,1094,874,406
PAQR7_8_359,783,800,637,807,383,1359,1001,895,1396,639,1349,996
PCDHB11_8_360,289,635,239,380,684,455,416,656,154,179,873,36
PCDHB16_8_361,3122,2952,3131,3196,2764,2315,4264,4288,4918,3686,3350,5
802
PCSK4_8_362,134,289,126,210,329,439,171,335,123,416,325,88
PCSK7_8_363,4897,4715,4229,3628,7245,4660,6626,4292,2738,4526,5860,670
7
PEA15_8_364,1044,1129,1311,1086,383,855,1497,868,831,1282,1620,1521
PET112_8_365,340,251,178,214,1441,141,0,292,40,216,191,102
PEX13_8_366,2479,2866,2818,2791,3793,4440,3923,4478,2138,3635,4192,292
1
PEX7_8_367,1852,1305,1331,1671,3219,1277,1215,2395,3098,814,3465,2196
PF4_8_368,911,942,450,479,417,517,879,167,645,488,381,899
PFN3_8_369,1325,924,1121,877,1676,1495,970,1141,307,1039,1284,1603
PHEX_8_370,5492,4095,5924,4129,4773,8363,6813,2479,5448,5058,8001,6394
PIGR_8_371,444,512,215,407,801,118,346,334,90,67,529,242
PITPNA_8_372,1209,1254,775,1093,584,1256,2035,508,816,1984,978,1192
PITPNB_8_373,685,1163,582,685,1952,736,2304,347,908,412,1409,705
PLLP_8_374,640,375,517,455,960,53,498,96,1327,181,980,377
PLP2_8_375,142,265,330,522,299,620,35,293,45,697,334,84
PLXNB2_8_376,718,775,788,1312,195,1122,42,1884,1122,438,1130,1471
PNMA2_8_377,397,372,582,475,544,1118,549,304,347,801,607,647
PPP1R14A_8_378,611,501,516,503,1423,73,93,60,191,597,30,547
PPP1R14C_8_379,2740,3289,2583,3425,2883,2034,4247,4005,2176,1999,2058,
3616
PPP1R15A_8_380,753,681,671,500,559,1017,1374,897,250,1068,891,688

PPRC1_8_381,121,279,91,155,286,178,2,29,7,15,1145,14
PPY_8_382,1220,956,1095,1189,475,3041,1323,819,1354,488,951,1699
PRB3_8_383,186,216,145,174,225,110,40,6,79,296,244,335
PROCR_8_384,510,332,465,577,557,119,373,91,365,330,1362,1058
PROS1_8_385,1809,1582,1529,1506,2287,1369,2073,616,1293,1864,1647,2226
PROZ_8_386,4431,3581,3984,4552,6448,3466,2947,5006,4340,4028,4249,5096
PRPF18_8_387,1090,1113,1118,1047,472,734,1835,1027,1629,1681,1879,1271
PRSS12_8_388,3637,3826,3295,4253,3742,2757,4813,3679,3108,4700,3447,55
97
PRSS22_8_389,699,789,616,711,719,404,294,950,1016,575,2034,2254
PRSS27_8_390,115,114,156,96,282,23,466,0,30,819,3,103
PRSS33_8_391,396,308,233,532,623,792,237,91,383,276,236,590
PRSS36_8_392,891,519,1107,484,614,580,336,663,230,478,1666,1559
PRSS8_8_393,128,113,159,303,78,276,184,153,306,148,145,422
PSCA_8_394,821,248,417,329,611,1079,482,38,669,360,199,1274
PTOV1_8_395,269,149,218,471,140,184,266,173,304,82,271,7
RABIF_8_396,7457,6667,7056,7391,7578,9385,5928,7991,5707,7110,7798,137
41
RAMP1_8_397,73,27,21,5,4,0,0,70,7,0,1,5
RAMP2_8_398,1469,1794,1083,1843,1165,2049,4492,1474,2197,2768,1072,195
0
RAMP3_8_399,2114,1772,1632,1559,1860,1004,1379,1431,1420,3421,1428,255
4
RANBP17_8_400,3756,2901,2733,2549,3551,2648,4624,4336,3848,2663,4746,5
249
RASSF9_8_401,3547,4909,3550,3895,2558,6458,6562,5114,4773,3825,4934,49
10
REEP5_8_402,131,171,143,297,0,0,142,0,0,0,0,0
RHAG_8_403,208,343,277,230,199,135,50,149,378,421,304,780
RLBP1_8_404,631,705,535,684,796,2107,509,739,576,530,709,1477
RPL15_8_405,10,82,117,7,0,1,301,0,2,0,5,42
S100A12_8_406,1626,845,822,1595,1678,801,890,2148,1041,1117,3421,2263
S100A1_8_407,1010,1425,1122,1021,822,2374,2666,759,1616,1769,1299,912
S100A2_8_408,965,880,573,669,1135,1042,826,2232,175,1480,803,850
S100A3_8_409,1383,373,401,1118,865,541,235,644,2201,723,846,303
S100A6_8_410,866,1185,1000,1613,950,349,615,1313,687,2479,1451,722
S100B_8_411,484,187,214,441,554,485,391,1072,300,296,776,178
S100P_8_412,3000,3304,3599,2971,3094,5159,3709,2440,4188,3999,3015,440
5
SAA4_8_413,247,315,285,426,57,245,464,299,565,391,25,483
SCAMP1_8_414,1993,2387,1140,1810,1234,1414,3057,975,3696,1676,1943,176
1
SCAMP2_8_415,830,223,296,517,396,1149,307,793,130,148,656,1701
SCFD2_8_416,1246,1260,532,995,1512,856,538,629,1149,726,1219,601
SCLT1_8_417,5731,7349,5055,5400,3579,7066,8638,5175,6867,6534,3495,578
2
SCPEP1_8_418,717,612,617,600,1193,857,293,934,1759,296,373,1028
SDC2_8_419,1029,937,1119,1455,491,932,1787,1074,1126,1014,1407,1182
SEC14L3_8_420,129,96,211,133,53,8,36,3,147,0,27,313
SEC22A_8_421,1111,1322,918,953,775,1511,1369,478,390,1479,1713,1491

SEC23A_8_422,4140,2992,2885,4170,3515,4923,3011,5481,3463,4632,5510,66
03
SEC24A_8_423,12678,11924,13047,12170,11741,14209,12729,10065,14916,163
58,21389,15964
SEC24D_8_424,2328,2871,2256,2554,2833,3061,2733,2938,2078,3523,1418,28
02
SEC61A1_8_425,585,331,601,587,534,185,70,380,571,471,229,589
SEC61B_8_426,541,697,527,598,721,0,13,281,542,46,176,823
SEC62_8_427,608,732,465,382,787,747,409,330,615,567,300,426
SEC63_8_428,2421,3386,3051,3246,1129,4983,3107,1586,2370,3529,1536,272
8
SELP_8_429,765,1213,807,955,2677,595,1383,1501,1185,625,669,2158
SERINC1_8_430,4114,5656,4491,4072,5196,6632,5692,5715,4756,5803,6625,6
213
SERPINA11_8_431,983,1148,818,1405,658,1287,1114,664,426,989,989,951
SERPINA12_8_432,251,433,381,408,333,934,332,723,214,573,1715,309
SERPINA3_8_433,1216,1428,1122,1323,1089,984,1175,529,328,652,1901,659
SERPINA4_8_434,645,702,527,751,1117,589,711,356,555,750,769,1190
SERPINA5_8_435,438,533,430,1019,555,604,1078,702,819,409,55,1233
SERPINA7_8_436,157,311,494,91,322,85,1370,0,38,66,381,1
SERPINB10_8_437,135,123,91,124,0,271,0,65,210,78,236,272
SERPINB12_8_438,664,959,649,498,1900,1126,2248,713,1159,113,97,942
SERPINB13_8_439,725,1363,616,527,933,1433,840,297,421,531,1186,704
SERPINB1_8_440,1122,1261,1406,1155,2205,416,1387,958,1027,1819,1305,26
34
SERPINB4_8_441,1039,1094,827,1387,2047,1336,1257,1126,629,1200,817,906
SERPINB9_8_442,5275,4809,4190,4819,5208,4323,6650,4396,7112,7064,7565,
5568
SERPINC1_8_443,709,760,442,562,746,2290,554,615,915,1864,1024,1524
SERPIND1_8_444,2872,2835,2418,2881,3436,1919,2283,2030,5309,3778,2375,
3096
SERPINF1_8_445,524,273,432,275,719,512,628,409,737,183,106,478
SFXN1_8_446,1490,1833,975,1286,1370,2187,2881,2310,978,3530,1892,2393
SFXN2_8_447,1642,1560,904,2747,1571,2120,1529,2540,2035,1125,872,1127
SFXN3_8_448,123,172,360,266,7,17,4,118,1,76,164,40
SFXN4_8_449,2837,2953,2915,2572,3609,2896,4091,1617,3046,3957,2637,283
3
SFXN5_8_450,1069,1260,1130,918,458,1157,1856,473,892,2414,1709,802
SLC15A5_8_451,3982,3789,4342,3657,1873,5088,2362,2763,3053,3675,7648,5
719
SLC16A13_8_452,460,416,442,294,174,1009,565,502,612,10,74,459
SLC16A14_8_453,2532,2357,2767,1840,1435,3893,2245,1675,1971,4139,1430,
2271
SLC16A9_8_454,2353,1989,2064,2244,1806,1948,1883,1672,3487,1826,3265,3
043
SLC17A9_8_455,287,261,81,203,3,205,576,1,25,198,923,544
SLC22A20_8_456,589,548,723,1172,833,476,1078,867,1063,439,664,426
SLC22A24_8_457,846,1092,962,865,1162,923,616,544,1463,1275,1053,1405
SLC22A25_8_458,1110,580,947,697,1893,2666,1025,624,1905,1193,1686,630
SLC25A23_8_459,2265,2226,2534,2423,2126,2660,1754,2816,2745,3967,5268,

1668
SLC25A29_8_460,748,650,686,569,128,148,280,1021,635,630,1999,737
SLC25A31_8_461,152,20,299,191,204,11,0,44,586,50,67,0
SLC25A38_8_462,679,765,222,677,105,493,572,279,481,843,3,919
SLC25A42_8_463,474,635,826,398,5,89,584,660,2404,317,7,1083
SLC25A46_8_464,1569,1213,1586,1530,1222,452,681,3142,1691,2448,2785,18
82
SLC25A48_8_465,713,529,464,892,161,570,1604,282,1443,1931,679,611
SLC38A7_8_466,576,653,768,762,332,261,909,1759,151,314,1125,190
SLC38A9_8_467,3332,3383,3385,3525,3265,4179,4283,2548,4740,3947,3971,5
346
SLC41A2_8_468,2086,2110,2158,2713,2487,2821,3307,2412,2228,1677,2726,7
90
SLC45A1_8_469,635,876,738,802,577,704,885,610,642,175,1382,1303
SLC45A3_8_470,1443,1073,1501,919,2764,588,3444,1233,911,586,1789,1383
SLC47A1_8_471,17938,18287,16222,18231,21153,19395,20386,16464,15798,20
178,19528,22558
SLC48A1_8_472,601,702,186,359,169,315,503,505,95,19,806,182
SLC5A12_8_473,3657,3800,2782,3696,3899,4067,3044,4322,2964,5046,4302,4
106
SLC6A17_8_474,488,490,667,431,911,683,386,175,141,318,802,138
SLC7A14_8_475,3085,3165,3953,2163,2097,3795,1693,3042,3207,2819,2911,2
663
SLIT2_8_476,4950,4471,4185,4874,4150,5166,4286,4548,5055,3965,4654,616
2
SNAP29_8_477,4032,3916,2861,4584,3080,3289,3784,6141,4775,5506,4386,27
42
SNX12_8_478,2218,2039,2325,1770,1802,3268,2194,1402,1838,1817,2703,291
1
SNX13_8_479,2117,2370,2317,2001,3899,1509,1580,1435,2282,2366,3200,370
9
SNX17_8_480,1329,1211,1087,1402,1721,1616,911,1644,581,857,1542,1333
SNX19_8_481,3890,4195,2947,3387,2452,3946,2364,2486,4279,4407,5492,223
5
SNX22_8_482,134,84,66,71,244,0,0,178,211,76,0,30
SNX24_8_483,392,520,414,812,454,451,6,758,383,116,768,465
SNX2_8_484,316,178,422,542,364,505,243,46,329,116,109,357
SNX4_8_485,2257,2380,2504,2151,2306,3832,3098,1827,1925,2949,4974,2534
SNX8_8_486,235,668,187,312,77,956,97,1065,679,2047,269,287
SNX9_8_487,474,826,298,237,413,260,304,289,847,399,249,745
SORCS2_8_488,364,475,608,508,590,352,671,1135,238,541,401,609
SORCS3_8_489,2080,1920,1630,2223,1738,4161,2816,2014,2795,3202,2083,24
08
SORL1_8_490,3008,3809,2825,3098,2235,3222,5400,2153,5339,4214,4191,248
5
SOS1_8_491,4290,3558,3631,4163,2655,3146,3081,4548,4818,4380,4988,5245
SST_8_492,232,357,363,582,0,125,508,236,645,879,361,814
ST13_8_493,1573,2234,1199,1744,2117,2954,2327,1153,1841,3225,1428,1717
STAB1_8_494,454,472,163,221,663,212,950,286,395,325,386,300
STARD4_8_495,3209,3652,2021,3600,2584,3816,4225,2949,5252,2345,4244,22

62
STARD5_8_496,798,817,1205,945,418,951,744,565,517,624,1343,2814
STARD6_8_497,1414,2230,1296,1386,1773,2484,2346,2063,3402,2734,1115,35
69
STEAP1_8_498,3692,2996,3129,3899,4341,4749,3815,2901,3760,2856,4388,41
02
STMN4_8_499,877,566,684,761,942,345,743,345,740,1039,850,836
STX11_8_500,228,390,257,221,136,464,4,472,530,150,457,394
STX18_8_501,765,927,990,387,539,1631,1164,258,178,1305,250,1399
STX4_8_502,280,488,395,276,434,442,576,1475,471,797,122,15
STX5_8_503,496,728,420,559,706,82,827,723,184,53,1196,0
STX6_8_504,166,292,167,373,142,134,467,714,686,362,917,586
STX7_8_505,1495,1273,1433,1150,2408,775,1508,1640,2377,1825,1394,2812
STXBP3_8_506,516,731,403,385,199,922,759,787,89,124,349,957
SVOP_8_507,327,300,269,284,263,284,160,137,1613,617,137,178
SYP_8_508,1776,1658,1771,2092,1372,1159,1149,1949,1917,1386,1244,1263
SYT10_8_509,5745,6035,5181,6694,7470,6084,5061,6030,3037,8715,5033,495
7
SYT11_8_510,148,98,112,147,0,53,11,331,1,0,572,144
SYT13_8_511,381,600,390,246,248,67,37,805,204,116,146,369
SYT16_8_512,956,1075,532,726,602,1177,935,929,924,616,770,778
SYT4_8_513,529,1010,585,864,475,1431,537,364,709,1146,992,997
SYT5_8_514,682,336,439,310,412,196,195,92,390,587,700,371
SYT6_8_515,1342,813,1279,1293,2886,2598,1625,1955,2130,1011,905,1254
SYT7_8_516,589,378,585,604,3157,295,537,378,369,305,95,1581
SYT8_8_517,553,557,570,773,209,1647,436,1299,391,1798,112,1685
SYT9_8_518,396,820,374,273,305,42,1225,356,869,1086,774,336
TAS2R42_8_519,4582,4566,4264,4800,4418,4771,3367,3842,3581,4393,3918,6
419
TBC1D9_8_520,2742,3067,2636,2914,2550,1433,884,2192,2417,4218,2868,430
9
TCN1_8_521,1805,2547,1340,1877,1634,2589,1928,2254,3123,957,1468,2307
TEKT4_8_522,566,659,629,745,461,388,1588,927,365,952,518,910
TF_8_523,2048,1957,1250,1940,1752,1810,2665,847,1245,2641,2865,1460
TG_8_524,997,778,645,1403,842,743,282,575,546,787,1202,189
TGFB1_8_525,286,222,241,34,0,469,4,105,138,0,87,97
TIMM10_8_526,885,286,333,531,325,549,652,1128,136,182,383,626
TIMM13_8_527,596,602,578,703,830,453,891,1733,1143,540,352,541
TIMM17A_8_528,1902,1629,1849,1439,1438,2050,823,2450,1606,2325,1691,27
84
TIMM22_8_529,1482,1042,1131,1442,1846,1129,794,589,1879,2139,1171,1249
TIMM23_8_530,1821,1206,1437,2140,1332,1671,755,1612,390,959,1645,513
TIMM44_8_531,161,26,140,184,24,249,30,44,500,757,35,514
TIMM8B_8_532,703,339,553,355,689,545,575,488,1384,100,407,822
TIMM9_8_533,6230,7101,6102,6974,5046,9120,6103,6272,5323,6613,8506,102
15
TLL2_8_534,1,1,10,2,11,0,546,0,0,1,157,0
TM9SF2_8_535,1792,1684,2091,1132,2911,1489,1362,1394,604,971,2008,1259
TM9SF3_8_536,1928,1804,1626,1673,1804,1421,1347,2009,1639,1576,2623,20
93

TM9SF4_8_537,2418,2053,2461,1470,1504,3581,5463,1230,1414,1128,2597,91
2
TMCO3_8_538,1188,1553,881,1341,2553,1183,333,1497,488,1657,1537,879
TMED10_8_539,5469,4117,4478,4401,3395,3706,2147,4307,2591,3467,4595,31
32
TMED1_8_540,373,226,90,241,287,147,6,357,199,107,239,949
TMED2_8_541,4152,3939,3636,3602,2487,5595,5512,4152,3588,5704,4591,456
9
TMED3_8_542,3244,3367,2981,2904,2432,1490,3143,2178,1331,1784,3759,326
0
TMED4_8_543,102,183,8,62,210,17,65,312,7,537,115,109
TMED7_8_544,724,690,520,835,538,469,645,270,533,473,929,596
TMED9_8_545,652,487,351,521,683,542,316,128,274,652,44,147
TMEM104_8_546,378,249,302,299,101,259,1042,727,162,152,645,377
TMPRSS11B_8_547,447,387,409,204,161,1284,352,671,693,27,58,40
TMPRSS11D_8_548,3303,2243,2475,2685,2761,1865,2473,2215,2329,3256,3734
,3959
TMPRSS11E_8_549,1055,1655,872,959,666,1701,1131,750,1000,1421,909,2607
TMPRSS11F_8_550,2397,1950,2610,1951,1869,1650,779,1332,4642,1876,1696,
2190
TMPRSS12_8_551,907,895,698,715,238,1189,398,2790,550,1124,534,706
TMPRSS5_8_552,293,353,158,272,560,432,0,1690,21,358,275,320
TMPRSS6_8_553,224,420,618,393,147,922,1407,184,204,373,179,293
TMPRSS7_8_554,2393,2754,3253,2275,1827,4883,2863,1084,2238,3171,2814,2
891
TMPRSS9_8_555,25,60,19,33,2,397,0,381,171,67,87,545
TNC_8_556,2318,1732,1416,1594,2938,2386,1749,1670,2328,2400,1545,2410
TNF_8_557,1301,1639,884,1734,2469,1251,2194,1222,1057,578,2491,931
TNFRSF9_8_558,1153,1110,764,1254,1599,988,1301,1002,726,812,2413,897
TNNC2_8_559,1604,1672,1833,1718,2556,1939,1749,1751,2721,2228,1448,174
6
TNN_8_560,457,393,664,608,346,996,772,328,450,695,846,1369
TNNI3_8_561,495,676,675,450,815,892,311,238,149,398,1325,347
TNR_8_562,2097,1363,1764,1802,1375,1422,1428,1346,1704,2588,2871,1798
TOMM20_8_563,1857,1706,1583,1537,2095,1103,2411,3056,2107,1655,2697,17
94
TOMM22_8_564,3205,3571,3153,3298,1389,4471,4632,3626,3814,3248,5887,37
41
TOMM70A_8_565,522,374,684,602,1772,643,723,682,293,150,1398,460
TOMM7_8_566,169,322,53,256,603,433,512,7,21,332,195,0
TPSG1_8_567,571,1186,485,295,828,864,159,185,403,553,14,1195
TRAK2_8_568,0,0,0,0,0,0,0,0,0,0,0,0
TRAPPC10_8_569,3344,4348,2973,3531,4606,3214,3821,5710,2612,2398,4509,
3400
TRAPPC8_8_570,2103,2630,2061,2491,2225,3250,2595,2664,679,2527,1340,13
09
TSNAX_8_571,1789,1632,1770,1768,1291,1059,301,378,1816,1689,3875,2503
TTPA_8_572,1429,966,891,1072,1182,1284,1142,2108,608,1469,784,1594
TTR_8_573,73,174,209,72,486,15,121,30,9,83,0,61
TUBA1A_8_574,2991,3191,1803,3294,2119,3066,4109,2376,1949,3302,4320,28

06
TUBA1C_8_575,2991,3191,1803,3294,2119,3066,4109,2376,1949,3302,4320,28
06
TUBA3D_8_576,531,280,136,176,1277,226,43,870,520,1045,36,202
TUBA3E_8_577,2424,2707,1574,2505,1643,3023,2704,2813,1996,2628,3941,45
23
TUBA4A_8_578,993,975,1093,1113,1613,1454,948,1474,1646,1251,1736,1006
TUBB1_8_579,1,1,1,1,0,0,1,0,0,1,2,1
TUBB2A_8_580,654,612,616,789,376,303,1110,892,361,188,2132,1463
TUBB2B_8_581,654,612,616,789,376,303,1110,892,361,188,2132,1463
TUBB6_8_582,221,317,589,208,818,41,77,42,17,620,589,541
TUBB8_8_583,166,575,321,195,0,452,810,27,383,2,430,712
TUBE1_8_584,1550,1495,1500,1071,666,1730,3234,710,1753,1100,2445,2049
TUBG1_8_585,1515,1792,1558,1084,1489,2511,2117,1340,653,1570,1309,1300
TUBG2_8_586,1515,1792,1558,1084,1489,2511,2117,1340,653,1570,1309,1300
TXNDC8_8_587,1181,2035,1450,1461,1504,1847,1138,3134,1995,1827,2648,29
78
UCP2_8_588,737,526,627,591,303,622,450,1203,344,657,202,786
USO1_8_589,1287,1001,1558,1359,1109,1826,1588,908,1098,1099,1438,826
VAMP2_8_590,660,550,242,483,185,221,646,1,157,688,312,622
VAMP5_8_591,191,639,319,144,55,593,953,534,304,400,59,51
VKORC1L1_8_592,1407,1950,1252,1107,1455,1577,1707,1917,1441,1807,1556,
1734
VPS18_8_593,1251,1863,1449,1445,2853,727,2228,774,1619,1238,2086,839
VPS26B_8_594,1148,1039,697,1225,423,1087,967,1178,1446,1432,1189,1226
VPS33A_8_595,3160,3291,3598,3140,3885,4110,2102,4667,3568,3310,4709,23
24
VPS33B_8_596,299,275,378,494,392,1936,425,10,4,487,40,308
VPS35_8_597,2393,2651,2046,1856,2299,5029,2320,1776,1843,1926,2511,139
6
VPS39_8_598,4273,4084,3952,3473,2668,4664,3457,1388,3067,3965,4764,211
7
VPS45_8_599,555,619,385,454,169,1572,608,636,757,687,1002,847
VPS4B_8_600,2080,2289,2186,1419,1824,2665,998,1648,2327,1155,2103,2830
VSIG2_8_601,2323,2089,1405,2318,4116,4139,989,2229,891,2175,396,2448
VTI1A_8_602,1124,1241,965,1059,1534,493,1153,324,1057,1537,354,660
VTI1B_8_603,1165,966,819,624,803,317,430,1240,686,531,836,237
VTN_8_604,1346,773,511,1150,1658,417,651,2388,1591,375,478,1409
VWF_8_605,1109,970,1556,1165,812,616,1323,824,836,1204,1669,1049
XK_8_606,420,642,216,156,449,1194,136,66,221,884,333,250
XPO1_8_607,996,721,1202,1331,361,738,1251,1468,58,651,520,3514
XPO4_8_608,3230,4295,3409,2980,4546,3035,3684,2998,4555,4819,5333,4934
XPO5_8_609,669,288,423,1009,380,253,1546,979,665,927,1768,3002
XPO6_8_610,178,308,164,216,471,0,392,120,19,181,132,176
XPO7_8_611,2710,1896,2586,2174,2523,4411,3238,3015,3906,1635,3884,2292
ACTL6A_8_612,1045,910,776,1208,991,1250,730,2152,320,1182,1422,1370
ADAM12_8_613,2545,2044,2430,2508,2784,1971,3748,1982,2565,2102,7221,41
00
ADAM15_8_614,2390,3485,2334,2748,2103,4248,1924,3048,3148,3305,1798,48
84

ADAM18_8_615,3064,3097,2636,3292,3241,3896,4402,2604,1843,1827,2994,42
70
ADAM22_8_616,897,937,829,976,700,1265,2445,1368,718,1124,165,1761
ADAM29_8_617,763,996,530,949,1841,845,600,1264,326,1466,403,646
ADAM33_8_618,454,667,581,542,324,198,463,397,463,1120,341,456
ADAM8_8_619,252,364,501,395,349,564,10,452,498,82,939,289
ADAMTS13_8_620,750,759,1106,642,162,1004,865,1653,467,506,219,755
ADAMTS14_8_621,620,509,477,311,656,652,128,355,878,280,720,561
AKAP12_8_622,207,323,301,240,331,521,84,42,265,94,392,531
ANGPT1_8_623,464,285,474,290,373,410,164,755,668,91,531,159
ANGPTL4_8_624,2784,2764,2445,2269,3804,2809,3527,975,2819,3325,1936,33
65
AP1B1_8_625,779,406,440,508,1667,385,803,631,609,859,870,714
AP1G1_8_626,14,16,0,54,0,0,0,0,0,0,132,0
AP1M1_8_627,179,205,112,151,765,746,246,94,6,276,8,14
AP2A1_8_628,1298,812,868,1079,2203,1010,999,608,1325,883,1259,891
AP2A2_8_629,353,205,319,169,104,927,315,45,60,357,366,611
AP2B1_8_630,299,447,127,390,274,1267,16,90,431,98,58,252
AP2M1_8_631,1078,638,1019,919,2624,661,728,973,572,2366,2022,653
AP2S1_8_632,292,213,298,410,54,65,624,694,272,61,48,122
AP3D1_8_633,79,76,177,140,72,3,326,50,42,31,246,489
AP3M1_8_634,6795,6647,6093,6502,8204,7675,10541,7602,7902,5615,10710,8
315
AP3M2_8_635,169,120,300,244,20,17,43,368,17,232,440,301
AP4S1_8_636,830,407,305,319,785,320,584,413,960,493,782,149
APAF1_8_637,4932,3469,3571,3661,5573,3007,8209,4029,6012,5053,5956,560
9
APBA2_8_638,484,992,674,359,1036,1722,910,401,753,281,336,1009
APOL1_8_639,2593,3067,2445,2930,1849,2552,2695,4224,1761,1979,1191,429
2
APOL3_8_640,196,11,46,111,0,0,647,0,16,58,1,106
APP_8_641,1939,1545,1768,2002,1465,2023,2859,4284,1711,1968,1745,2565
ARFGAP3_8_642,445,559,576,750,658,237,251,188,569,293,968,1164
ARHGAP33_8_643,261,232,362,190,417,22,436,110,154,100,614,541
ARL6_8_644,0,0,0,0,0,0,0,0,0,0,0,0
ATP13A2_8_645,459,335,606,385,1201,536,1591,415,105,655,816,319
BAX_8_646,1512,1281,1497,943,2212,1570,1753,2566,434,1024,1634,1154
BCAP29_8_647,5470,4152,4571,5002,6084,4305,5634,4833,4823,3629,5714,44
94
BCAP31_8_648,668,387,391,530,23,1207,1011,821,634,899,311,428
BCL2_8_649,5599,5452,5222,4935,2379,5005,5564,2811,4911,7083,5665,7215
BCL2L2_8_650,964,713,656,439,396,788,298,356,492,558,229,57
BID_8_651,672,779,665,1150,167,1442,499,878,1842,844,579,524
BSG_8_652,610,677,466,579,1205,988,1137,112,739,778,1108,563
C1QC_8_653,3467,4473,3132,3327,2762,6287,4435,1881,3202,3822,8170,3648
CANX_8_654,260,188,202,233,1,126,37,68,163,166,65,200
CAPN9_8_655,454,631,344,365,1145,927,273,485,1205,701,127,937
CAPNS1_8_656,398,802,653,563,990,1963,303,326,426,945,909,324
CCT6B_8_657,2304,2196,1816,2615,730,3748,3846,2327,3348,979,2645,2869
CD19_8_658,666,800,632,753,655,51,279,1434,213,97,498,1040

CD22_8_659,1979,2040,1575,1757,1215,1269,1764,1966,3176,1961,1229,1984
CD33_8_660,824,820,756,703,1079,149,593,953,441,814,1184,629
CD44_8_661,511,408,523,613,714,176,164,534,260,155,495,901
CD55_8_662,941,1242,830,494,1425,704,393,787,2268,540,2006,713
CDH17_8_663,2641,3370,2455,2688,2344,3051,5114,3420,1968,2586,4974,115
2
CFHR4_8_664,4563,3864,3003,3906,4407,3610,5548,4017,4366,3978,4277,205
0
CIZ1_8_665,145,70,87,168,233,68,0,550,175,273,425,376
COG2_8_666,2277,2405,2506,2257,2625,1847,1331,1938,993,2894,2619,4072
COG4_8_667,136,453,91,269,460,96,6,28,6,486,0,4
COG5_8_668,4591,5448,4721,6785,2966,8074,4882,5383,3980,6493,6986,8998
COG6_8_669,410,335,214,505,54,94,219,156,367,191,989,1695
COL11A1_8_670,10452,10755,9518,9817,10902,11710,14034,11577,9458,13132
,10947,8679
COL11A2_8_671,1343,1291,1057,1086,1065,901,2580,1165,1032,524,681,881
COL12A1_8_672,2609,3577,2946,3249,3369,4862,3094,4726,2924,1563,1863,4
234
COL13A1_8_673,1369,958,869,1928,2669,1222,2871,1204,1720,1252,1241,131
9
COL18A1_8_674,440,606,497,547,281,1137,794,1047,186,1363,579,1428
COL25A1_8_675,1233,1455,1343,1109,872,2854,903,798,1205,1295,1289,3932
COL2A1_8_676,1226,842,949,1010,551,1109,2493,1314,885,1770,1294,1269
COL4A5_8_677,1071,773,1008,1325,192,1556,851,1775,1176,931,794,2386
COL4A6_8_678,1629,1469,1347,1457,1934,3410,1658,1985,1508,1517,2258,13
47
COL6A2_8_679,470,1559,742,540,1052,1906,132,738,107,537,1798,1470
COL6A3_8_680,697,642,546,973,1028,1006,1128,678,1277,581,231,296
COL8A1_8_681,218,250,84,137,521,146,758,1211,6,508,75,0
COL9A1_8_682,396,311,286,308,251,107,603,201,358,235,1023,174
COPA_8_683,224,228,288,147,6,3,1402,449,348,233,273,321
COPB1_8_684,884,1137,1154,920,1945,1633,830,231,658,460,1150,666
COPE_8_685,588,498,228,436,261,180,1011,418,525,218,1097,1136
CPA4_8_686,340,320,216,385,6,460,1399,290,468,57,342,458
CPNE1_8_687,724,529,791,1043,1435,1585,858,182,195,773,704,865
CPNE7_8_688,1409,1810,1697,1375,1673,1571,1567,2217,1906,1505,1894,275
7
CPXM1_8_689,468,693,309,417,0,323,1131,123,0,80,346,113
CPZ_8_690,1565,1692,1016,1279,2628,1415,730,756,1407,540,1861,1367
CRABP2_8_691,1343,1910,1425,1039,1656,1454,1917,2315,1827,1603,1721,19
88
CTLA4_8_692,6078,5552,5476,4934,5302,6581,3945,5016,5036,6875,8177,689
9
CTNS_8_693,100,122,86,75,6,290,143,13,231,93,332,32
CXCL12_8_694,720,781,658,802,195,1408,1457,488,1131,405,652,368
DPP10_8_695,5742,4882,4929,6091,4487,4654,6210,7487,5707,5115,8773,577
3
DPP6_8_696,1574,2081,1488,1702,1483,1881,2627,2199,2423,3406,1490,1418
ECM1_8_697,1012,1090,1085,688,1227,669,1356,256,1103,473,1053,369
EGF_8_698,501,556,362,592,680,1648,329,447,654,662,544,1239

EIF2C2_8_699,265,288,179,261,394,131,445,272,169,345,21,437
EIF2D_8_700,447,346,380,550,526,32,99,649,445,349,260,950
ENSA_8_701,591,298,211,909,158,244,300,629,1474,691,849,1423
EPB41L4B_8_702,2663,2211,2649,1958,2511,3654,3115,4176,2354,5071,2555,
2592
EPB42_8_703,656,301,291,558,130,2328,946,160,233,299,1049,785
ETFA_8_704,7747,7688,5986,7679,4982,7711,6684,8816,9198,6690,7455,5376
ETFB_8_705,390,339,397,390,34,358,34,347,79,104,1317,522
EXOC1_8_706,2871,2722,3105,2270,1951,5418,4114,1946,2316,4164,3603,584
8
EXOC4_8_707,1005,658,1068,1081,661,1367,1760,1452,880,788,594,1795
EXOC6_8_708,3325,3014,2553,2990,5222,4397,2035,3695,2861,4554,3241,270
2
EXOC7_8_709,597,1019,1140,424,481,2787,2332,219,235,1522,650,764
F8_8_710,3299,4100,3289,4125,2860,5437,5453,2829,1939,3879,3331,5354
FABP6_8_711,965,815,673,1134,1581,238,899,16,898,1123,1111,1429
FAM131A_8_712,96,179,281,138,12,235,0,53,209,406,281,53
FAM63B_8_713,1235,899,936,1348,1634,575,733,1174,749,452,1933,1504
FANCA_8_714,3082,2921,3038,3497,4596,2466,3954,4943,4997,3663,4392,274
5
FCN3_8_715,2281,2354,2165,2538,2816,3324,2349,3539,2546,4188,1153,3154
FGA_8_716,303,389,328,407,73,16,739,145,871,120,233,554
FGB_8_717,324,492,1060,549,608,240,2080,53,442,616,464,1030
FGF13_8_718,329,470,579,599,754,490,563,42,131,214,327,83
FGG_8_719,614,778,1296,854,1155,643,724,1140,836,718,1072,1622
FIBCD1_8_720,1344,927,986,983,1737,1974,1727,1570,1158,1119,340,1692
FLVCR2_8_721,755,1195,893,1044,567,1345,436,1596,441,1684,746,755
FOLR1_8_722,718,668,663,537,285,2149,628,828,1411,1576,528,1421
FOLR2_8_723,338,496,151,321,137,663,112,546,853,106,151,1296
GGA1_8_724,3907,2786,2792,3226,6853,3449,3579,2967,6261,4037,4269,3916
GGA3_8_725,1315,1272,995,1519,988,2280,1701,1689,977,1908,1543,2333
GJA5_8_726,1139,1297,1163,1056,1853,1633,1026,1110,214,506,3443,1555
GJB1_8_727,1713,2587,1004,1789,1710,1849,2761,676,1086,1506,1031,2364
GJB3_8_728,316,316,269,585,197,566,2000,152,1004,164,558,653
GJB6_8_729,3138,4232,3605,3428,2092,4436,3622,5075,3151,4972,3364,6913
GLYATL1_8_730,1587,706,1016,1228,953,1156,987,474,978,2176,2754,661
GNRH1_8_731,635,597,458,529,604,901,555,157,145,986,26,217
GOLGA3_8_732,184,142,133,187,112,300,58,68,272,766,9,18
GOPC_8_733,2235,1818,2037,2402,1305,3441,3829,2324,3208,3308,3098,2552
GOSR1_8_734,2925,3588,2655,3137,2581,2847,2458,1347,2771,3279,4696,234
6
GOSR2_8_735,1039,683,485,427,578,1333,995,1179,1564,471,751,759
GPRASP1_8_736,457,840,570,389,1432,568,1260,274,362,773,474,404
GRB2_8_737,1453,1406,719,1155,3159,3401,809,957,322,1110,1943,2136
HABP2_8_738,1171,1632,1126,1152,1946,1640,2132,1688,1520,1708,2368,129
5
HDLBP_8_739,3399,3405,2950,3135,2222,3429,3530,2733,4177,3788,4710,276
0
HEPH_8_740,128,167,186,119,8,35,142,23,268,65,287,60
HNRNPU_8_741,658,603,527,458,416,1175,345,441,192,341,1502,382

HOMER2_8_742,1679,1388,1183,1470,1516,440,2227,1985,2135,1074,3864,215
4
HPN_8_743,1447,1544,1459,1457,2118,2295,1993,1615,2227,1539,1034,2328
HSDL2_8_744,2875,3149,2659,3054,3763,4331,2497,2654,3430,814,4438,3524
IGF1_8_745,213,251,328,433,429,55,574,89,235,271,107,428
IGFBP3_8_746,611,617,423,586,587,1115,551,417,229,611,297,943
IPO11_8_747,2420,2696,1999,2213,2211,3013,2371,3939,1753,2889,1941,237
8
IPO8_8_748,3683,3589,3789,3443,3280,2570,3120,5329,4664,3153,4745,3432
ITGAL_8_749,3945,4515,3027,4194,4147,2615,3652,4131,2894,5637,4726,282
2
ITGAM_8_750,1796,1578,1433,1457,2387,2518,2345,1389,329,1375,949,1429
ITGAV_8_751,1513,1275,1447,2299,1927,1146,2199,1094,1538,1775,1111,183
2
ITGB2_8_752,736,643,546,300,337,233,143,459,1505,767,964,756
KDELR2_8_753,1475,1781,917,1510,1767,1816,2646,1028,1485,1972,1851,159
0
KDELR3_8_754,2253,3477,2389,2626,3919,3182,2112,3666,1127,2588,1421,15
97
KIF13A_8_755,887,1097,701,698,1183,1542,537,464,1950,1635,906,285
KIF17_8_756,1235,1741,1267,1644,1212,1837,800,1898,663,865,2970,1633
KIF1B_8_757,308,337,185,565,142,392,884,979,501,350,1395,936
KLK10_8_758,1896,2327,1852,2612,3417,2456,1680,2899,1333,3621,3569,150
9
KLK11_8_759,171,127,164,405,115,255,117,189,149,4,34,825
KLK12_8_760,436,348,319,119,1237,101,116,826,33,768,276,388
KLK15_8_761,574,665,415,733,433,335,1934,614,426,382,229,1303
KLK5_8_762,336,550,201,161,276,472,51,116,195,232,537,513
KLK6_8_763,2102,1812,2082,2010,2393,1995,2813,3491,3677,2036,2725,2578
LAMB3_8_764,112,141,218,253,0,191,341,784,135,336,5,316
LDB3_8_765,507,776,395,659,395,910,507,616,851,226,564,573
LDLR_8_766,125,278,134,110,338,1527,16,6,16,231,6,1
LTA_8_767,3691,3864,3913,3671,4224,3318,4340,3935,4315,4482,5481,5731
LTF_8_768,2799,3093,2011,2331,4068,1682,3445,2559,1257,2447,2389,2505
M6PR_8_769,300,529,519,529,78,1405,53,554,789,1240,741,823
MASP1_8_770,1612,1957,1806,2549,1570,2390,2798,1550,1150,2637,1581,162
1
MB_8_771,350,443,362,222,8,21,18,133,455,854,383,725
MCFD2_8_772,6294,6430,3993,6031,8338,4994,7162,6539,6029,7638,9245,510
6
MCL1_8_773,0,0,0,0,0,0,0,0,0,0,0,0
MEFV_8_774,740,180,784,349,327,339,332,403,62,322,158,573
MFAP4_8_775,222,118,209,156,292,515,1,65,39,194,189,10
MFSD10_8_776,189,108,211,352,1,663,206,64,513,225,39,860
MFSD1_8_777,704,789,512,1177,732,398,603,1176,492,340,137,1388
MFSD5_8_778,680,564,472,359,72,907,486,913,257,1171,1284,184
MLC1_8_779,2323,1852,1664,2052,1125,2051,1949,659,2090,3156,2746,1786
MSLN_8_780,1015,606,626,741,470,1022,1792,413,608,445,702,176
MTX1_8_781,399,352,231,602,967,390,383,469,154,1222,520,87
MUC1_8_782,388,256,219,189,380,139,242,287,989,92,178,847

NCAM1_8_783,0,0,0,0,0,0,0,0,0,0,0,0
NNAT_8_784,675,824,1006,679,1516,1042,886,973,1420,735,1556,998
NOXO1_8_785,116,146,132,160,170,0,63,439,0,1033,199,0
NPC1L1_8_786,514,410,654,389,263,1490,899,626,886,316,353,372
NPRL3_8_787,962,915,623,369,3130,1322,1408,866,1046,875,99,169
NRXN1_8_788,887,828,1052,1258,698,1246,463,1088,1016,1230,375,800
NRXN2_8_789,1548,1542,1174,1174,2027,483,733,1184,730,625,2972,2205
NRXN3_8_790,3916,4242,4245,4159,4770,4093,5328,3114,4827,3369,4623,315
1
NUP155_8_791,1121,1229,1032,1322,1057,1089,4788,1415,1110,1270,1186,10
90
NUP50_8_792,4647,3429,5021,3576,5304,5972,4333,3010,3467,5863,7274,432
0
NUP62_8_793,989,814,796,1695,1435,2209,1700,817,1262,1903,1797,2054
NUP98_8_794,3398,3086,2535,2907,2330,3261,2660,4014,4223,3883,3971,218
6
NUPL1_8_795,362,371,666,370,2193,258,0,653,1423,1650,76,28
NXF1_8_796,538,520,680,450,510,305,865,692,536,823,471,1182
NXNL2_8_797,712,1117,679,1295,1086,858,648,1040,575,562,1381,945
NXT2_8_798,1216,1206,1104,1369,860,1916,1505,2342,1268,1060,1851,917
OAZ3_8_799,248,295,153,298,147,22,72,4,9,108,4,135
PACSIN2_8_800,345,69,390,92,0,120,53,0,0,671,0,0
PANX2_8_801,1529,631,669,1110,799,668,683,756,1092,1206,1650,2135
PCDHA6_8_802,519,1125,536,562,721,630,836,1343,351,1047,1103,1531
PCDHGA5_8_803,4223,3086,3775,3485,3868,5096,3413,2523,2937,1971,5024,3
164
PCLO_8_804,6003,5644,4434,6824,8280,6421,7046,6525,6336,6589,5074,6291
PCSK5_8_805,3012,4288,3391,3127,4656,7643,1716,3903,3734,3981,3076,567
7
PCSK6_8_806,193,110,299,245,765,150,121,129,4,711,1437,1156
PCTP_8_807,3604,3217,3781,3126,3595,2505,4867,3833,6085,4474,6243,5151
PDYN_8_808,1309,1170,1105,1142,560,2323,1952,750,1711,901,1924,400
PDZD3_8_809,3988,4163,3278,3789,4915,3358,4630,4996,2821,6104,3289,503
3
PDZK1_8_810,270,395,299,271,666,842,434,384,288,343,161,61
PGAP2_8_811,1160,1138,1012,908,564,1543,967,982,882,1032,1175,1548
PGF_8_812,174,320,274,117,2,1119,0,350,226,700,91,56
PIK3R3_8_813,445,315,36,262,95,327,295,28,1390,30,39,116
PITPNC1_8_814,406,513,306,724,171,243,26,579,477,752,246,715
PITPNM1_8_815,447,703,467,870,142,1256,952,464,1503,897,1303,749
PITPNM3_8_816,1516,1866,1876,1455,427,1862,351,1458,1714,1534,1326,131
5
PLEC_8_817,1643,2113,1716,2718,185,2073,2151,3399,2083,2279,2458,3168
PLIN3_8_818,214,275,218,260,787,1,291,312,102,440,279,36
PLTP_8_819,243,60,93,77,3,12,145,0,77,82,250,159
PNKD_8_820,2856,2665,3137,2944,2629,3638,5056,3567,3170,3219,4952,4828
POMC_8_821,179,116,312,184,16,854,0,31,59,7,237,178
PORCN_8_822,130,59,303,141,132,595,8,234,200,5,497,218
PREPL_8_823,366,286,393,471,1108,84,1,1756,324,5,121,3
PRNP_8_824,666,658,342,628,194,936,363,468,761,366,136,488

PRSS21_8_825,1257,2366,1510,1412,1261,2325,892,1284,1414,2000,2417,157
6
PRSS35_8_826,988,1008,672,967,1070,235,893,678,1081,1035,1387,263
PSEN1_8_827,2205,1839,1316,1578,2400,1873,2290,1982,2128,1405,1806,525
4
PSEN2_8_828,416,215,176,319,13,78,195,512,59,774,573,139
RABEP1_8_829,2847,3196,2917,2772,2744,4403,3228,2724,2217,2333,2424,28
47
RACGAP1_8_830,1410,1500,1296,1639,1163,3040,554,1252,1001,2826,1231,13
68
RARRES1_8_831,811,624,798,681,614,693,811,632,1359,1357,924,111
RASA1_8_832,2303,2713,2091,2486,2471,3234,2760,2588,2692,4207,2098,109
5
RELN_8_833,285,240,359,588,159,58,527,242,75,77,358,320
RHCE_8_834,248,330,179,448,12,191,7,201,518,104,280,8
RHD_8_835,428,613,569,773,225,1021,146,541,991,634,283,690
RIMS2_8_836,553,380,336,472,100,1110,485,412,76,98,84,149
RRBP1_8_837,729,517,628,784,1199,413,1030,561,1302,598,1271,479
RUFY1_8_838,4158,5115,4132,4640,2983,4403,7189,4691,4924,5186,3528,729
2
S100A13_8_839,594,594,778,606,505,252,689,631,519,877,670,470
S100A4_8_840,1229,1087,748,1119,1009,1970,503,325,1371,1824,110,1255
SAA1_8_841,1856,1866,1379,1029,1421,1673,2557,1614,1265,1916,1338,1560
SCAMP3_8_842,4026,3136,3648,3093,2948,2562,5563,3347,3326,4956,3911,42
67
SCARB1_8_843,2253,1820,2366,2020,2345,1259,951,1242,2089,3100,1079,117
3
SCFD1_8_844,4608,5872,4977,4948,6128,6763,5829,4363,5917,8246,10978,55
62
SEC13_8_845,182,333,341,442,641,388,177,823,642,198,135,26
SEC14L1_8_846,5985,6302,5638,6231,5696,6169,4894,7149,8648,5631,10615,
9265
SEC14L2_8_847,317,319,348,262,117,42,271,231,105,818,356,674
SEC14L4_8_848,329,198,664,446,98,50,404,502,1291,1018,717,271
SEC23B_8_849,761,1033,790,1040,612,1989,2028,712,204,672,1228,1201
SEC24B_8_850,980,943,978,1093,1244,1292,224,1723,456,986,2423,625
SEC24C_8_851,2461,1950,2176,2350,1068,2345,2324,3255,2274,1599,3246,40
02
SEC61A2_8_852,1396,1496,1276,1440,2529,1223,1367,902,390,829,1468,2915
SEC61G_8_853,1251,1507,1175,1221,1995,1697,1882,1217,1394,2486,1282,24
07
SEH1L_8_854,1403,1209,619,1102,476,2131,917,1101,840,1485,387,862
SERINC2_8_855,211,305,215,270,21,13,345,556,671,166,382,8
SERINC3_8_856,1278,889,510,955,1954,624,1083,786,451,1002,1542,1029
SERPINA10_8_857,3377,4134,4166,4483,3954,5394,7460,4403,3876,4088,6232
,5762
SERPINA1_8_858,3270,3660,2220,2712,3046,3888,4281,3852,3013,2333,4762,
3913
SERPINB2_8_859,352,417,127,254,202,580,586,74,209,44,681,302
SERPINB6_8_860,1321,956,795,1007,668,1005,1078,1019,2827,1621,1756,122

9
SERPINB8_8_861,5061,4710,4156,4801,5190,4181,6144,4396,6771,7063,7540,
5566
SERPINE1_8_862,505,794,688,516,1422,616,495,642,542,205,612,548
SERPINF2_8_863,129,296,259,238,53,1,130,0,89,242,3,10
SERPING1_8_864,5911,5938,5496,6351,5521,8805,6594,4254,5154,8016,5270,
6448
SERPINH1_8_865,591,559,291,837,261,1094,1020,151,11,2313,138,448
SERPINI1_8_866,1641,1615,1676,2000,1334,2295,1657,969,3228,871,2852,29
74
SFI1_8_867,194,169,267,108,114,14,397,218,9,362,1089,49
SFTPA1_8_868,565,238,388,335,320,1265,673,2,81,492,797,301
SH3D19_8_869,884,668,585,530,716,980,1420,469,832,1030,612,935
SIL1_8_870,530,373,382,495,509,290,50,507,108,1660,437,755
SLC25A25_8_871,2245,1839,2356,1606,2746,1738,1878,1050,2421,2509,1450,
2174
SLC25A36_8_872,1470,1684,941,1413,1986,2549,822,676,1486,704,912,524
SLC25A45_8_873,125,107,56,127,646,95,1,13,8,323,30,196
SLC38A10_8_874,706,801,520,334,1199,420,138,1168,134,1204,591,1647
SLC41A3_8_875,537,403,584,575,215,1166,412,129,554,237,593,4
SLC43A3_8_876,431,813,667,950,452,719,1719,1196,145,1050,809,114
SLC44A2_8_877,1284,1357,1097,1845,3603,613,258,2025,1183,1168,937,2101
SLC44A4_8_878,653,583,777,576,304,926,1161,300,20,797,956,923
SLC44A5_8_879,323,419,215,315,1552,620,82,16,443,206,149,36
SLC46A1_8_880,477,525,265,314,429,627,45,634,1,365,616,367
SLC47A2_8_881,526,246,633,557,979,596,1430,518,304,435,393,359
SLC50A1_8_882,353,398,327,494,0,635,1924,1377,342,467,1237,200
SLC6A20_8_883,1630,1201,1006,2303,1665,476,1526,1592,1155,2108,1808,20
67
SNAP23_8_884,198,288,255,541,139,285,318,504,88,7,91,1006
SNAP25_8_885,2867,3120,2184,2868,2644,3939,5017,3642,2306,6174,4655,43
36
SNX10_8_886,985,772,561,1162,743,219,1103,1090,246,518,1253,1980
SNX11_8_887,1179,2360,1718,813,1062,2367,1232,644,416,2183,1500,592
SNX14_8_888,17,78,15,85,0,64,0,0,139,0,6,75
SNX15_8_889,355,281,288,317,484,283,272,225,549,336,606,385
SNX16_8_890,1925,2624,2320,1656,2652,2871,3707,1879,1465,2725,1212,244
7
SNX18_8_891,173,494,306,297,0,14,46,513,476,122,12,2024
SNX1_8_892,567,409,521,531,222,1021,287,540,895,287,667,483
SNX3_8_893,1365,929,1187,1031,923,853,785,1280,1612,870,866,2355
SNX5_8_894,1924,1659,1631,1317,2302,1914,2761,1614,1308,2375,2254,1575
SNX6_8_895,1610,1759,1616,2190,1546,2058,1911,974,1838,2193,2103,1155
SNX7_8_896,2282,1745,2021,2105,2300,2521,1071,3373,2118,3048,3046,3832
SORCS1_8_897,1278,1314,919,1677,1099,799,1683,1266,1582,718,1402,2657
SORT1_8_898,673,674,470,961,291,535,1517,1368,146,640,508,1051
SPNS1_8_899,483,487,289,218,572,477,285,427,193,711,291,50
SRI_8_900,2784,2229,2570,2964,1105,1311,3790,3692,2412,961,2906,3809
STARD3_8_901,577,584,992,720,355,754,1307,196,291,569,750,336
STAU1_8_902,2391,2223,1946,2195,3931,3215,2994,3028,3562,2924,3249,305

5
STEAP2_8_903,3400,3284,2518,3201,3815,4293,2807,2994,6133,5577,2816,3477
STEAP3_8_904,386,626,584,412,937,887,750,170,1288,409,504,136
STIM2_8_905,1563,1435,1194,1536,2617,2988,3052,1490,1342,1174,532,2428
STX16_8_906,1654,1149,827,1064,303,279,1054,899,1075,429,1046,1616
STX1A_8_907,590,361,392,258,89,573,1085,72,531,285,1318,672
STX2_8_908,1896,1174,1283,1537,2673,1587,1726,1288,1970,907,2550,2030
STX3_8_909,32,76,30,138,0,15,51,35,17,17,5,388
STXBP1_8_910,243,261,173,263,64,284,447,595,122,670,65,181
STXBP2_8_911,2861,3425,2382,2760,1220,3710,4329,3956,3853,2775,1727,2084
SV2B_8_912,345,440,553,422,132,449,796,584,277,511,572,152
SYN1_8_913,405,820,472,193,157,2016,808,348,212,1332,229,626
SYNGR1_8_914,204,131,197,142,1,38,52,247,0,50,0,256
SYNPR_8_915,2357,1582,1255,2677,2962,1801,2775,4520,1356,1914,2375,2217
SYPL1_8_916,1887,1956,1294,1886,2865,2595,2851,1776,1017,1644,1226,2162
SYT12_8_917,77,150,292,118,23,97,1092,133,21,159,103,7
SYT14_8_918,1759,2098,1472,2449,1149,1687,2967,1624,2626,3557,3319,2136
SYT15_8_919,162,295,131,265,184,44,42,97,66,16,104,0
SYT1_8_920,38,84,17,0,0,1,0,0,0,2,4,107
SYT2_8_921,578,527,450,501,808,941,1263,209,282,1493,338,1022
SYT3_8_922,1145,1078,820,1255,2001,2270,2080,969,1038,1320,894,808
TAPBP_8_923,489,415,513,760,1166,481,14,449,34,394,542,677
TC2N_8_924,303,281,393,305,370,401,159,590,220,532,422,250
TCN2_8_925,175,195,467,174,393,625,116,758,162,194,196,965
TCOF1_8_926,350,323,111,473,444,1279,2,4,221,489,289,358
TFPI_8_927,1381,1388,1167,1338,1111,1263,688,667,1195,881,1589,635
TFR2_8_928,513,496,532,175,228,21,378,21,460,277,680,31
TFRC_8_929,973,773,886,981,1116,880,297,1639,979,649,420,1063
TGFB2_8_930,213,109,112,164,143,263,32,159,0,2,39,29
TIMM17B_8_931,205,81,152,164,0,0,974,35,1,149,217,127
TINAGL1_8_932,410,322,224,282,571,142,62,0,465,872,572,343
TLL1_8_933,1191,1123,1329,1526,2440,1701,2195,1589,1233,2968,3022,1291
TM9SF1_8_934,2827,2183,1996,1803,3081,2165,1994,3615,1614,1915,2156,1769
TMC6_8_935,2734,1682,2207,2237,2332,3825,2603,2076,2560,1250,2869,1682
TMPRSS11A_8_936,655,725,691,749,469,1987,1799,720,741,2064,1041,2246
TMPRSS13_8_937,294,428,465,185,93,653,98,158,386,23,759,325
TMPRSS2_8_938,345,340,667,452,39,612,476,684,393,1277,467,903
TMPRSS4_8_939,812,779,440,673,394,965,1515,1147,449,1034,348,1195
TNFSF11_8_940,963,1029,919,1071,824,1296,1346,2055,1594,1364,842,296
TNFSF13B_8_941,1432,698,854,1468,1341,2839,1371,1131,706,1435,1313,984
TNPO2_8_942,921,1274,787,924,742,1002,1643,1453,635,1063,1906,982
TOM1_8_943,1767,1747,1408,1299,1998,1191,437,1675,2391,478,1650,1625
TOM1L2_8_944,950,1082,869,1247,538,448,2327,951,1404,834,589,318
TSC1_8_945,69,185,129,173,315,454,45,30,118,309,169,345

TSC2_8_946,151,302,243,297,14,197,130,37,82,682,238,430
TUBA8_8_947,691,719,965,427,212,160,427,2571,184,261,1886,55
TUBB3_8_948,221,317,589,208,818,41,77,42,17,620,589,541
TUBD1_8_949,2772,2253,2670,2823,1955,1804,3406,2742,1620,2215,3250,484
1
UCP3_8_950,480,698,388,866,260,1762,237,738,314,939,157,299
UPF3A_8_951,1641,2320,1919,1593,1063,1703,1216,1704,1621,2115,1735,207
3
UPF3B_8_952,1391,813,1233,828,79,1478,2342,519,1791,409,425,2061
VAMP1_8_953,428,270,87,273,19,33,121,85,270,206,18,746
VAMP7_8_954,1992,1320,1540,1603,2110,1980,3494,1482,1297,1518,1757,270
4
VCAM1_8_955,4717,3566,4036,4461,3862,5046,5020,4618,5183,4275,6640,668
7
VLDLR_8_956,2731,2620,1871,2277,2529,1487,5837,3173,2405,2314,3529,411
6
VPS13A_8_957,943,802,873,774,1726,1113,200,429,922,1418,1480,837
VPS13B_8_958,2849,3057,2421,2412,2878,3738,3168,1331,1622,2739,4549,14
99
VPS16_8_959,1540,1736,1618,1341,2208,1809,1546,1412,853,2224,770,1156
VPS26A_8_960,7483,7194,7295,7160,5143,8523,8157,6528,8591,7852,11042,6
325
VPS28_8_961,352,415,389,315,483,305,119,72,49,646,40,670
ZFYVE16_8_962,3073,3980,2275,2389,3207,4239,791,3534,2746,3411,2822,57
49
ZNF160_8_963,348,242,99,191,248,72,617,418,529,394,16,231
ZP3_8_964,418,727,423,626,119,778,255,923,226,335,1154,420
BET1L_8_965,335,340,407,238,145,215,14,215,343,308,8,983
C2orf83_8_966,1231,1309,426,1219,1122,563,872,550,1086,1782,452,890
ERP29_8_967,554,1116,1032,859,333,921,172,411,1829,580,631,1290
FGF1_8_968,1255,1606,1118,1479,757,1582,876,1073,497,1456,1623,399
LYNX1_8_969,1013,1057,636,767,997,1251,431,201,587,761,1345,666
MMP28_8_970,351,145,233,292,72,178,15,55,348,241,318,247
PDPN_8_971,455,581,304,342,245,633,786,1286,834,414,1610,575
SNX21_8_972,766,938,595,388,1363,1528,947,395,8,272,1732,2329
TIMM8A_8_973,298,361,246,304,97,429,77,42,96,503,227,311
VEGFA_8_974,1026,994,697,836,534,1215,2814,665,789,929,1001,1147
CDH23_8_975,557,228,651,721,1197,1977,767,468,1582,254,1260,306
CDH23_8_976,2548,2018,2015,2376,3066,2077,2471,2223,2635,2500,541,1612
CDH23_8_977,293,336,203,174,522,80,101,338,277,236,65,35
CDH23_8_978,268,326,31,271,0,391,11,629,10,249,11,499
CDH23_8_979,3086,2932,2124,2878,5453,2448,3772,1826,1646,2581,3358,219
3
TNXB_8_980,433,473,517,607,1009,896,837,35,57,501,883,832
TNXB_8_981,899,893,686,1000,206,985,865,416,880,965,1417,1895
A2M_8_982,807,735,507,531,464,1598,1492,542,612,1124,773,939
ACE2_8_983,771,624,469,625,2114,486,437,1664,1511,1278,946,1261
ACTR6_8_984,1912,2192,1671,1457,1683,2536,429,1759,2057,1708,1338,1988
ADAM11_8_985,221,109,73,229,931,123,42,255,17,260,477,2
ADAM19_8_986,2357,2325,2060,2666,3656,2245,1131,3459,2300,2438,2935,22

57
ADAM20_8_987,2560,2498,2529,2203,2070,2562,2726,2328,2958,2257,2867,30
08
ADAM21_8_988,3154,4214,3490,5014,5711,3506,5047,3998,3107,5116,4512,35
29
ADAM2_8_989,834,1139,1684,1100,1604,1222,1345,1673,1268,1104,1112,1652
ADAM30_8_990,869,818,972,945,1091,1074,2054,1421,497,1055,520,641
ADAM7_8_991,1214,1143,1090,1313,1324,616,1842,586,2871,1565,2161,1860
ADAM9_8_992,1082,1119,641,1219,1216,1141,854,1067,1510,2030,2312,441
ADAMTS10_8_993,560,310,775,584,1126,167,613,290,131,303,814,981
ADAMTS12_8_994,1254,1940,1475,1011,396,1345,2755,1939,2123,2259,1350,1
273
ADAMTS15_8_995,710,963,874,779,15,1126,1072,995,1082,970,1072,2150
ADAMTS18_8_996,558,605,915,400,666,1379,198,1060,1586,1401,630,542
ADAMTS19_8_997,1288,1164,632,936,1282,1064,1932,1695,732,1388,488,1294
ADAMTS1_8_998,1662,1344,1495,2398,2466,1722,2711,2325,1852,2550,2303,2
480
ADAMTS20_8_999,186,215,192,111,358,222,546,76,156,222,515,404
ADAMTS3_8_1000,2802,2647,2203,2625,4416,2771,2619,1914,3959,2178,1988,
2221
ADAMTS5_8_1001,1029,965,998,983,849,1121,1266,275,972,1301,899,1463
ADAMTS6_8_1002,427,855,349,470,51,1245,1184,276,927,873,263,669
ADAMTS7_8_1003,525,464,261,383,160,18,408,1422,143,150,572,957
ADAMTS8_8_1004,1498,1274,1117,1592,1122,780,2098,1694,1188,1393,787,90
9
AEBP1_8_1005,581,448,401,420,448,713,891,2462,202,721,750,361
AFG3L2_8_1006,2139,2139,1990,2062,2394,2481,1555,2597,1672,1770,2489,1
725
AFM_8_1007,2442,2317,2009,1875,3895,871,4059,1273,1422,1877,1553,2251
AFP_8_1008,317,130,113,204,196,262,166,100,724,758,40,113
AGTPBP1_8_1009,1662,1928,1077,1939,3891,754,603,1448,2373,1291,1832,26
55
ALG10B_8_1010,428,392,230,257,626,436,895,554,472,53,401,291
AMBP_8_1011,1600,1949,2305,1329,1713,1750,2571,1406,1640,1756,2171,296
1
ANGPT4_8_1012,195,55,115,141,17,2,24,1,657,54,273,89
ANGPTL1_8_1013,237,299,96,260,451,477,1056,1364,410,126,32,329
ANGPTL2_8_1014,1106,1128,841,1434,903,2147,3820,615,309,829,1115,2025
ANGPTL3_8_1015,3305,3367,3091,4090,5360,5534,3805,2682,5299,4675,3941,
4249
ANGPTL7_8_1016,621,681,537,266,172,594,664,1045,619,273,1998,1318
ANKH_8_1017,1523,1959,1486,2482,1069,3110,1628,1765,2141,3493,2965,117
4
AP1G2_8_1018,2378,2982,2514,2558,2196,4159,2180,2556,2535,3726,2907,42
00
AP1M2_8_1019,767,596,803,570,1349,1469,526,254,1524,773,317,299
AP1S1_8_1020,1127,1429,1266,1930,1901,3322,2497,1370,1019,2203,1173,16
93
AP1S2_8_1021,247,395,313,253,10,1601,42,185,93,664,514,369
AP1S3_8_1022,1075,1177,1058,942,896,1132,1616,916,1051,1282,1593,1692

AP3B1_8_1023,1007,697,1233,665,122,1195,37,623,611,962,1666,1495
AP3B2_8_1024,753,668,426,560,736,2442,258,348,175,3,228,67
AP3S1_8_1025,810,752,328,691,295,939,43,1475,19,1184,843,1402
AP3S2_8_1026,876,729,430,435,732,1023,457,877,430,820,746,31
AP4B1_8_1027,775,832,789,812,431,1127,270,1699,803,844,1089,866
AP4M1_8_1028,1524,1991,1598,1698,1798,2116,2527,1380,1305,2228,2669,23
33
APBA1_8_1029,221,356,119,362,17,244,597,53,54,652,668,0
APBA3_8_1030,1080,1044,752,402,2020,1064,826,346,1118,880,938,404
APOA1_8_1031,100,131,139,200,311,3,6,108,74,356,24,908
APOA2_8_1032,285,313,245,360,40,392,254,78,102,1018,496,309
APOA4_8_1033,612,625,158,245,1140,1265,956,614,137,129,120,1027
APOB_8_1034,1281,1451,1277,1482,1336,1973,1130,625,2145,3077,2341,2262
APOC1_8_1035,638,174,193,292,23,335,52,187,802,663,112,50
APOC2_8_1036,2660,2936,1731,2665,3319,2273,2585,2474,2696,4781,4231,34
47
APOC3_8_1037,248,264,119,238,78,16,163,168,460,177,99,40
APOC4_8_1038,2208,1745,1364,1945,4030,1498,1287,1393,1920,1435,1392,33
22
APOD_8_1039,1843,1599,1572,2004,2401,1993,1462,1986,2303,1555,3488,241
9
APOE_8_1040,91,204,215,289,70,701,4,633,17,233,64,642
APOF_8_1041,2442,2484,1898,2105,3250,4912,3021,3193,3387,2045,2023,450
4
APOH_8_1042,1847,1515,1699,1704,2389,2148,1640,2511,1305,1342,1162,149
8
APOL6_8_1043,434,327,260,267,91,1221,537,0,356,359,105,29
APOM_8_1044,5448,5521,4266,5337,3832,8194,6076,6540,3146,5077,6539,610
6
AQP12B_8_1045,837,631,897,818,2246,845,935,1171,646,917,623,274
ARF5_8_1046,4264,4153,3629,3885,4426,5724,6131,4239,4353,4983,3676,416
6
ARF6_8_1047,119,9,126,160,0,0,0,65,0,92,101,635
ARPP19_8_1048,2516,2665,2627,2405,3180,1915,5927,3730,2055,3092,1903,1
561
ASTL_8_1049,1967,1764,1912,1675,2589,795,1658,1678,1158,2205,1636,1750
ATOX1_8_1050,382,239,46,255,40,101,101,75,1080,155,45,711
ATP13A1_8_1051,995,1256,540,845,614,433,734,432,208,729,863,1512
ATP13A3_8_1052,3533,2412,2391,2622,676,1943,1616,2445,2849,2987,2134,2
915
ATP13A4_8_1053,4507,4033,3643,4208,3359,5079,3968,3479,3060,6385,5857,
4022
ATP13A5_8_1054,1679,1974,661,1625,314,881,1423,1363,1609,1331,3350,115
0
AZGP1_8_1055,1062,1082,437,1108,499,775,2024,940,1479,1258,810,522
AZU1_8_1056,61,107,74,4,0,149,19,1,40,495,197,517
BCL2L10_8_1057,67,64,82,179,19,332,96,0,45,183,8,66
BET1_8_1058,1791,1663,1374,1365,2804,1260,1404,774,2070,1399,1963,866
BGLAP_8_1059,530,513,369,703,246,562,290,25,16,470,2043,170
BOC_8_1060,966,998,869,1124,1188,484,1216,311,1212,1435,2175,783

BPI_8_1061,107,233,63,228,49,204,4,291,0,438,51,400
BPIFC_8_1062,1054,1115,605,982,374,814,1724,1259,1013,1131,1258,1181
C16orf7_8_1063,326,276,492,398,170,960,37,139,337,148,367,886
C1orf162_8_1064,8560,7413,7718,9353,6149,8805,8410,12344,6468,7102,139
36,11192
C1QA_8_1065,448,389,425,357,38,2031,260,92,1052,901,376,83
C1QB_8_1066,0,0,0,0,0,0,0,0,0,0,0,0
C1RL_8_1067,687,579,353,515,128,511,467,980,647,381,33,244
C20orf141_8_1068,386,303,377,279,459,842,1,18,267,585,269,43
C3_8_1069,1026,604,752,859,652,1255,814,390,520,955,751,418
C4A_8_1070,246,364,285,89,935,560,26,159,1,332,94,109
C5_8_1071,3040,3137,2516,2676,1488,1597,3719,3988,2511,2914,3657,4481
C7orf31_8_1072,1398,1313,1273,963,1357,884,2849,1622,531,1601,2685,243
7
C8G_8_1073,1446,1147,1021,1400,1054,2426,764,1556,1244,899,1056,2777
CALM2_8_1074,944,1062,693,1246,1127,1324,250,430,2137,2331,1298,603
CALY_8_1075,609,230,288,229,9,58,584,949,87,41,20,128
CAMLG_8_1076,5915,6696,5425,5471,6700,6274,8642,8794,6861,7613,6787,10
361
CAPN11_8_1077,177,212,30,94,31,0,0,71,0,0,482,0
CAPN5_8_1078,1766,2236,2336,1909,2381,2235,3653,2784,1259,2364,1688,14
76
CAPN6_8_1079,1357,1137,1181,1027,377,967,1840,1776,1151,1914,923,2382
CARTPT_8_1080,2715,1929,1651,2142,1739,1674,3502,2271,3066,2695,1353,1
827
CCL13_8_1081,1603,1530,1583,1181,1284,1816,1200,2181,652,1722,1226,165
8
CCND1_8_1082,652,651,749,746,1332,1220,886,663,292,705,273,73
CD1A_8_1083,1411,1928,1575,1409,1491,4193,2255,2260,1621,1160,1993,172
2
CD52_8_1084,35,107,182,65,1,0,0,15,93,55,3,4
CDCP2_8_1085,719,1099,945,645,84,823,680,18,419,715,288,473
CDH5_8_1086,231,589,648,381,84,114,1176,600,763,547,157,527
CHMP7_8_1087,2116,1725,1839,2026,2899,2142,1642,991,1358,3107,3348,236
4
CLDN16_8_1088,1748,1309,1695,1504,1212,1557,1768,1281,1767,1437,1414,3
085
CLEC3B_8_1089,1011,1010,631,996,972,1073,1008,764,198,442,602,965
CLSTN2_8_1090,132,45,57,6,40,30,55,202,95,441,14,13
CLVS2_8_1091,578,572,404,622,626,1092,931,272,987,604,600,201
CNIH3_8_1092,2573,2216,2466,2713,2893,3525,1184,1268,2429,2046,2644,25
29
CNOT6_8_1093,3002,1957,2256,2860,3428,3529,2353,3060,4020,3209,3122,30
36
CNTNAP1_8_1094,907,714,441,888,257,749,379,393,394,388,473,780
COG1_8_1095,4464,4672,2970,4268,4787,5994,3628,3870,3503,4057,4490,622
8
COG3_8_1096,3613,3572,3412,3621,4006,3836,3558,3560,3023,4020,4183,420
9
COG7_8_1097,13,99,0,68,0,0,0,0,0,496,0,0

COG8_8_1098,478,515,914,720,126,513,417,452,1390,722,1315,414
COL10A1_8_1099,665,849,465,475,304,473,824,108,411,593,466,842
COL14A1_8_1100,1834,1960,1899,2172,2392,1930,1830,1330,1175,2902,3708,
2076
COL15A1_8_1101,482,446,239,473,376,308,308,446,74,137,95,474
COL16A1_8_1102,158,234,373,517,84,117,138,83,449,180,507,176
COL17A1_8_1103,776,1094,480,753,598,1861,1808,370,1024,1980,2742,832
COL1A1_8_1104,644,621,476,760,573,831,744,911,170,274,871,212
COL1A2_8_1105,638,1175,727,870,2895,685,299,1025,1171,1359,473,2843
COL21A1_8_1106,1577,736,1251,1057,924,1584,491,748,1219,1146,667,1940
COL22A1_8_1107,2284,2983,2527,2410,4242,3786,1463,2158,2957,1616,2340,
2199
COL23A1_8_1108,533,512,793,841,33,701,215,529,23,579,983,146
COL24A1_8_1109,236,369,194,289,176,263,11,272,172,8,7,5
COL27A1_8_1110,572,775,641,744,1113,1119,1468,236,850,406,1592,52
COL3A1_8_1111,140,213,168,134,0,83,3,17,84,343,178,5
COL4A1_8_1112,922,405,458,737,1827,914,386,1024,947,253,1027,561
COL4A2_8_1113,1700,1193,1592,1650,1462,1680,740,1137,793,1138,2337,135
5
COL4A3_8_1114,309,433,395,189,863,602,399,853,753,182,275,402
COL4A4_8_1115,612,661,1054,863,764,1069,1153,1010,673,1047,1600,368
COL5A1_8_1116,2988,3374,2672,3103,1763,2713,3713,3543,3533,2026,3644,1
267
COL5A2_8_1117,1065,1075,1210,1184,1467,1353,1696,2639,1067,239,2203,14
64
COL5A3_8_1118,455,584,602,296,341,474,918,85,718,419,988,1023
COL6A1_8_1119,559,667,942,696,163,1255,394,871,1172,654,1160,482
COL7A1_8_1120,170,283,297,422,0,1170,72,720,0,665,48,33
COL8A2_8_1121,626,257,139,347,246,406,83,210,128,177,16,257
COL9A2_8_1122,559,1040,454,600,153,384,1098,911,574,1054,691,546
COL9A3_8_1123,250,242,558,579,1289,277,996,112,176,66,0,831
COMMD1_8_1124,2852,2529,1905,1754,2005,3251,1639,2090,1746,2729,1737,1
890
COPB2_8_1125,640,971,786,872,212,135,1629,643,102,832,1142,535
COPG2_8_1126,5927,6661,5893,6457,7808,6170,4662,5096,7793,6366,8838,51
73
COPZ1_8_1127,1874,1435,1307,1979,1643,2001,693,1211,2522,2375,3237,159
7
COPZ2_8_1128,5354,5261,6069,5550,5756,3956,6503,4254,7047,5257,7873,77
75
CORIN_8_1129,953,896,774,836,899,1072,1574,98,317,661,354,893
COX18_8_1130,1881,1603,1486,2179,2641,1148,1469,2374,1493,2598,1988,30
34
CPLX1_8_1131,672,332,380,766,451,1049,410,1174,105,680,380,153
CPLX3_8_1132,2743,3053,2460,3031,3471,3842,1817,4507,2970,2590,3726,29
76
CPNE6_8_1133,3384,3314,2762,2285,3112,4240,2579,2010,3175,4086,3457,32
25
CPXM2_8_1134,1380,774,968,1065,811,1173,2019,758,604,521,1311,1808
CRABP1_8_1135,1805,2507,1936,1848,679,4708,2754,2936,1011,1650,3206,19

48
CRH_8_1136,639,539,317,419,864,119,88,60,663,445,739,485
CSE1L_8_1137,5581,5636,6440,4701,4812,6143,6289,7916,5040,4831,5054,44
64
CTSW_8_1138,390,511,248,449,41,1528,94,102,231,312,318,708
CXCL10_8_1139,565,701,327,374,240,490,422,804,90,380,205,816
CYGB_8_1140,102,109,181,196,90,165,71,70,0,276,317,846
CYTH3_8_1141,6761,7164,5688,6446,5632,5950,6252,6918,7364,7913,6981,99
07
DDI2_8_1142,812,676,775,563,1066,963,718,1104,12,705,1688,226
DIRC2_8_1143,881,743,699,490,490,532,553,788,1198,951,1520,825
DISP1_8_1144,573,620,744,392,123,227,292,674,145,668,196,587
DLL4_8_1145,998,975,671,741,1387,1722,2918,362,1404,1378,537,1118
DNAJC5B_8_1146,190,420,246,482,150,541,198,28,207,290,1195,291
DNAJC6_8_1147,1386,1430,1021,965,1603,1594,654,1079,1582,1360,396,898
DOC2A_8_1148,517,734,517,427,144,1602,94,789,245,1022,258,1385
DOC2B_8_1149,337,118,73,80,99,14,193,432,55,155,3,13
DSCAML1_8_1150,174,192,44,71,272,1,87,28,26,72,194,7
ECEL1_8_1151,400,401,156,134,267,973,433,111,221,423,113,667
EID2_8_1152,297,272,311,342,76,418,670,533,606,103,201,867
EPCAM_8_1153,340,320,152,141,463,81,142,272,253,318,11,187
EXOC2_8_1154,367,226,222,323,89,355,224,161,430,463,311,245
EXOC3_8_1155,1527,1394,946,1462,551,1731,2919,960,2615,1321,671,934
F11R_8_1156,0,0,0,0,0,0,0,0,0,0,0,0
FABP1_8_1157,334,396,291,408,1033,149,279,1181,325,202,55,548
FABP2_8_1158,8119,9110,7687,8631,6800,7562,5787,5900,7444,6972,9577,94
26
FABP3_8_1159,1932,1970,2245,1552,3315,2569,3272,2074,1988,2306,2514,17
15
FABP4_8_1160,647,644,788,319,812,629,898,209,295,771,305,8
FABP7_8_1161,557,595,370,619,269,300,808,305,379,415,480,1286
FABP9_8_1162,1849,1976,2083,2460,1926,1604,2312,2689,1872,1495,1706,31
95
FAM101A_8_1163,552,593,415,507,844,195,530,574,230,13,240,1377
FAM117A_8_1164,125,380,337,195,264,73,635,33,87,89,2,144
FAM57A_8_1165,340,357,248,376,675,149,13,558,807,201,31,487
FAP_8_1166,2571,2961,2705,2500,1647,5831,2371,3377,3511,3856,3000,3175
FBF1_8_1167,338,344,161,256,251,359,109,295,93,148,20,272
FCN1_8_1168,1959,2250,1915,2198,464,2117,1431,2880,2750,1361,1788,2154
FDX1_8_1169,383,150,371,200,339,340,327,267,432,250,99,414
FDX1L_8_1170,297,328,502,615,429,297,0,1409,319,178,119,118
FGF4_8_1171,229,153,394,482,21,303,931,703,184,141,338,369
FGL2_8_1172,3066,2540,2916,2117,3998,3157,3626,2654,2907,2158,3919,227
2
FOLR3_8_1173,406,145,216,367,26,850,220,31,246,400,162,31
FOLR4_8_1174,683,540,332,608,330,554,1636,1189,354,399,209,717
FRG1_8_1175,2326,2046,2088,1826,1995,1379,1744,1707,2750,1789,2523,253
6
FTL_8_1176,1191,1371,1047,1461,691,1133,611,1505,1161,1091,626,464
FXC1_8_1177,870,1081,717,1253,562,1154,1092,1849,1575,459,1858,1542

GABARAP_8_1178,245,49,124,205,22,161,117,67,4,4,0,2
GGA2_8_1179,435,557,463,576,737,753,876,264,273,673,1643,485
GJA1_8_1180,571,631,931,741,387,735,1646,880,16,1368,53,1520
GJA3_8_1181,952,678,521,787,799,370,1662,176,300,665,405,356
GJA4_8_1182,194,204,210,249,0,36,118,180,361,180,69,11
GJA8_8_1183,2032,2220,1801,1723,451,3102,2160,1718,3057,3638,2630,2663
GJB2_8_1184,1846,1818,995,1281,1710,1644,2396,890,1657,1922,1906,2438
GJB4_8_1185,1954,1721,1775,1911,3767,1534,1675,932,1162,1243,864,4020
GJB5_8_1186,666,616,738,976,432,971,1499,334,221,707,1289,1327
GJC2_8_1187,298,125,78,341,379,30,386,9,59,18,0,415
GJC3_8_1188,1314,1350,913,1438,1463,740,814,879,1146,3167,1132,1213
GJD2_8_1189,3955,3614,2567,2458,3548,3646,4711,3558,3316,4137,4038,296
2
GJD3_8_1190,256,305,259,391,0,1135,3,0,434,186,70,1614
GJD4_8_1191,86,136,104,114,0,608,1,15,78,573,24,0
GKN1_8_1192,655,1278,748,569,624,320,595,265,346,1018,638,743
GLCCI1_8_1193,242,190,190,712,771,438,129,282,285,174,269,810
GLTP_8_1194,798,736,1132,727,1185,294,1135,375,1026,1959,729,1517
GLYATL2_8_1195,3165,3738,2988,3714,2320,3832,2575,1428,2032,2302,3215,
3773
GP9_8_1196,57,108,28,134,95,518,634,203,13,589,28,0
GPIHBP1_8_1197,523,441,326,232,47,917,2072,200,5,267,1254,48
GPR180_8_1198,3058,2167,2363,2712,2576,3034,3774,3453,3354,2214,2479,2
172
GRN_8_1199,510,413,361,326,6,925,96,470,646,46,920,809
GZMH_8_1200,597,460,656,546,1356,20,917,623,494,551,1878,657
GZMK_8_1201,1560,1910,1297,1224,426,1274,1359,1527,361,1379,2104,393
GZMM_8_1202,55,138,14,16,2,2,12,91,10,19,0,355
HBA1_8_1203,290,212,237,269,25,380,989,213,492,1098,538,6
HBA2_8_1204,290,212,237,269,25,380,989,213,492,1098,538,6
HBE1_8_1205,580,615,597,306,620,317,470,193,338,851,437,22
HBZ_8_1206,1084,756,1425,1033,2542,687,695,1325,46,1255,1044,251
HECA_8_1207,263,592,385,519,389,715,935,423,617,1052,547,516
HGFAC_8_1208,1406,1574,675,1259,581,1855,2609,1028,239,953,357,309
HIAT1_8_1209,398,328,447,516,739,470,942,903,577,250,607,125
HLA-DQB1_8_1210,400,571,506,827,290,1029,1200,674,501,774,432,1239
HMCN1_8_1211,1705,2298,1300,1829,2316,1578,1961,1559,3357,1485,869,160
1
HMHA1_8_1212,147,251,370,396,144,153,22,828,45,78,194,47
HPCAL4_8_1213,340,622,583,529,193,642,321,906,147,229,539,26
HPR_8_1214,1323,1320,644,710,789,1033,2396,1131,1601,2244,544,623
HPX_8_1215,162,269,134,394,80,360,74,193,63,55,10,612
HSP90B1_8_1216,3051,2477,3436,3337,4706,4838,1692,2474,3120,2143,3675,
2751
HTRA1_8_1217,2364,2558,1338,1740,2733,896,2479,1355,3174,3563,2335,252
1
HTRA4_8_1218,374,508,347,357,815,151,1399,290,382,1758,658,600
IFNG_8_1219,277,78,266,184,121,23,132,137,515,34,425,363
IGFBP7_8_1220,2135,2675,2132,2575,3056,4417,3299,2820,1091,2679,2811,3
159

IL12B_8_1221,1450,1124,713,1582,333,1273,332,1475,1690,1854,2920,3467
IL13_8_1222,916,1416,1556,1636,562,3056,1100,3499,2197,2169,1084,924
IL17A_8_1223,272,474,199,178,48,153,1341,161,196,421,91,895
IL1A_8_1224,1704,1974,1474,1991,1312,2651,4859,687,2270,2265,1959,1286
IL1B_8_1225,715,349,682,569,478,150,622,517,417,156,894,330
IL3_8_1226,1728,1456,1475,1562,1071,670,2459,665,2569,629,2089,1723
IL5_8_1227,797,806,676,995,613,1324,373,402,547,752,108,2239
INSL3_8_1228,1263,820,1117,1226,896,1884,2001,1661,1471,1664,400,2175
IPO13_8_1229,682,471,337,515,1166,1404,263,1437,992,63,318,536
IPO4_8_1230,146,153,277,110,115,79,205,28,338,44,73,360
IPO5_8_1231,1189,1116,929,1452,1552,783,1720,877,1143,766,1154,406
IPO7_8_1232,1508,1288,1764,1615,847,1677,826,1091,1886,1861,2641,1396
IPO9_8_1233,536,401,595,291,273,154,443,79,161,466,318,28
ITGA10_8_1234,1629,1553,1229,1128,552,2122,1307,1969,2163,1272,3267,14
75
ITGA11_8_1235,3120,3262,3288,2447,2266,2788,2591,3908,2248,3384,2776,3
630
ITGA2_8_1236,10601,11191,8964,10597,6966,9085,19004,14102,9342,11859,1
4706,14395
ITGA4_8_1237,2529,3215,2679,2455,2943,2873,2407,2542,2065,3023,2678,30
66
ITGA5_8_1238,622,666,423,768,614,120,359,1472,446,427,778,190
ITGA8_8_1239,5531,6442,5142,5758,5603,4998,4009,4516,7505,6356,5932,69
81
ITGAX_8_1240,548,686,666,330,368,276,222,1410,1165,1317,298,345
ITGB5_8_1241,2102,2738,1957,2276,2786,3313,1943,1459,1922,1451,3838,12
66
ITGB6_8_1242,1612,1819,1209,1429,542,1987,982,2206,2012,1462,809,1831
ITGB8_8_1243,559,906,652,953,166,884,1119,641,940,778,667,847
ITLN1_8_1244,664,592,691,406,757,228,68,6,823,310,569,731
KDELR1_8_1245,685,480,495,513,291,142,0,100,788,106,445,778
KEL_8_1246,1493,1979,1375,1788,867,1737,2568,2989,1631,1828,1516,1965
KIF20A_8_1247,2224,2485,1809,2838,3586,2106,1496,2534,2501,2741,3795,2
068
KIF3B_8_1248,1440,1855,1333,1290,1475,754,1950,993,1592,925,1945,1437
KIF5A_8_1249,1559,966,671,1481,584,694,391,1968,969,1076,957,904
KLK13_8_1250,334,302,335,239,297,312,465,435,120,17,600,63
KLK14_8_1251,369,129,13,140,279,0,0,0,0,99,45,1
KLK4_8_1252,300,673,394,401,359,456,788,5,281,1052,789,101
KLK9_8_1253,11,0,0,0,0,0,0,0,0,0,0,0
KPNA1_8_1254,1799,1563,1687,2078,703,1001,1465,2887,1902,1981,168,3463
KPNA2_8_1255,506,367,350,394,980,341,51,474,276,500,511,181
KPNA3_8_1256,2426,2419,2606,2419,2609,1955,1738,2145,2464,3539,2624,14
75
KPNA4_8_1257,327,178,202,183,327,181,78,116,71,78,330,246
KPNA6_8_1258,676,639,975,521,729,1477,92,52,300,572,548,610
KPNB1_8_1259,636,730,704,613,415,528,802,875,436,641,517,240
KRT12_8_1260,1785,2273,1301,2276,1855,1562,2433,191,3637,2590,2161,336
5
KRT7_8_1261,1026,601,1023,631,401,393,498,603,1510,798,1932,498

KRT8_8_1262,271,402,494,406,413,320,457,1214,148,333,325,438
KRTAP5-4_8_1263,108,69,206,76,413,205,542,24,514,42,0,819
LASP1_8_1264,2487,2763,2094,2725,2403,867,3228,2435,1371,1402,3287,319
7
LBP_8_1265,174,196,224,312,1,572,42,496,272,178,899,220
LCN12_8_1266,1901,1979,2231,2415,2959,2235,1274,2604,1677,2600,2961,13
68
LCN1_8_1267,106,295,179,312,436,182,632,104,858,597,97,1
LCN2_8_1268,295,236,317,429,309,12,82,9,649,471,509,0
LCN8_8_1269,452,179,438,350,90,232,505,379,1103,20,204,1352
LCN9_8_1270,334,252,214,551,101,361,4,535,618,226,35,223
LDLRAD2_8_1271,455,812,631,1081,217,445,290,995,276,1123,740,1059
LDLRAP1_8_1272,831,945,903,1163,576,535,1885,1547,318,604,759,1010
LMAN1_8_1273,1638,986,1076,1770,2959,694,887,1201,1330,2328,3208,1416
LMAN2_8_1274,9607,9336,9306,8479,10070,9964,10328,7097,9564,8020,11855
,9266
LPA_8_1275,486,768,813,460,1214,1034,408,1305,411,451,14,1292
LRIG1_8_1276,250,843,322,294,16,339,2672,246,338,530,74,3
LRP2_8_1277,17087,18915,14018,15721,15621,20539,23925,16882,18590,1913
7,19010,18791
LRRC4B_8_1278,307,249,108,34,88,278,5,26,0,1,347,3
LRRCC1_8_1279,4161,4867,5243,4140,3031,7166,4921,3585,5010,3962,3449,2
847
LYST_8_1280,1609,1306,1064,955,2312,1103,1342,1125,1607,1623,2475,1526
MAL2_8_1281,440,483,346,464,732,218,1100,956,524,366,295,255
MATN3_8_1282,609,404,491,88,494,140,103,157,209,164,642,445
MFSD8_8_1283,849,1041,1103,805,75,1128,2952,347,706,732,1063,1986
MFSD9_8_1284,748,759,619,464,473,361,1069,1137,693,407,1115,761
MMAA_8_1285,2094,1905,1640,2045,1885,3623,1348,1595,3402,1425,2495,420
6
MMACHC_8_1286,933,1240,1502,832,352,982,914,2037,351,2027,659,1009
MMGT1_8_1287,3588,3300,2694,3189,4211,4417,3639,5181,2432,4115,4168,36
89
MMP11_8_1288,463,696,680,686,313,1088,929,150,732,405,598,178
MMP13_8_1289,1036,894,903,1281,494,990,862,1087,2060,2181,897,1415
MMP15_8_1290,506,660,600,387,398,294,846,727,932,590,901,582
MMP16_8_1291,1720,1601,1247,1670,1260,2089,2209,1187,2407,807,3133,228
6
MMP17_8_1292,2724,2409,2224,2654,2927,3891,2427,2363,1998,4107,2157,43
18
MMP19_8_1293,358,284,801,369,547,106,36,225,593,127,92,928
MMP24_8_1294,1223,1121,865,974,400,1780,1970,386,612,1174,432,1523
MMP25_8_1295,1866,2196,1811,1900,3309,1360,3078,1394,2399,2297,3618,17
85
MMP26_8_1296,355,504,406,458,360,16,147,241,1014,330,257,1679
MMP27_8_1297,701,1095,814,440,478,974,825,2134,1175,209,2248,1043
MRS2_8_1298,1907,2358,2447,2230,2195,3388,4492,1630,1081,1576,4538,256
7
MSTN_8_1299,1686,1436,1882,2314,1478,2956,2101,2033,4039,2741,3452,344
1

MTX2_8_1300,2528,2782,2470,2501,2723,1238,3404,3004,2244,1819,2853,374
5
MUC2_8_1301,426,430,282,362,468,318,87,0,1,733,3,47
NAPG_8_1302,382,293,295,735,236,649,188,33,442,100,675,273
NAPSA_8_1303,609,369,236,462,1212,398,293,791,812,501,47,2217
NCOA5_8_1304,935,1024,640,1075,2469,2106,766,518,2360,849,1250,566
NGF_8_1305,538,664,465,512,604,100,446,318,286,1323,43,1361
NID1_8_1306,1516,2182,1742,1468,2360,1367,821,3263,244,2279,1757,1282
NPC1_8_1307,1803,2327,1451,1493,1852,2724,821,2514,2085,889,3716,2750
NPEPPS_8_1308,1619,1921,1419,1420,2673,1703,1939,835,1060,1240,1130,15
57
NPPB_8_1309,169,66,109,219,0,227,295,25,0,12,0,0
NPY_8_1310,1262,1394,1604,1272,2035,1126,752,1309,722,1152,5019,1275
NSMCE1_8_1311,1556,1150,958,1130,1885,1550,635,886,1253,1923,1913,1223
NUP107_8_1312,9898,9973,8692,9946,14308,10113,13152,11429,10220,10275,
12230,10308
NUP133_8_1313,1794,1715,1251,1817,2038,1277,553,3065,2144,1366,2356,17
08
NUP153_8_1314,2710,2965,2786,2098,4505,4861,4728,2894,2086,2756,3748,3
380
NUP160_8_1315,1179,1275,1144,1351,1588,1761,2061,1137,698,1236,1951,13
26
NUP210_8_1316,345,1035,316,461,646,418,453,35,152,653,688,1232
NUP214_8_1317,2567,2095,1813,2049,1466,2330,2680,1744,1656,3363,1176,2
360
NUP35_8_1318,1639,1490,1190,1334,2090,1293,1756,643,2703,904,2257,1807
NUP37_8_1319,926,740,719,524,2652,415,1401,710,533,614,293,230
NUP54_8_1320,334,159,338,314,1321,281,169,927,216,203,505,25
NUP88_8_1321,1744,2355,1764,1741,818,2332,503,1209,2042,2162,1626,2389
NUPL2_8_1322,2662,3773,2007,3103,4899,4753,4762,2999,3127,1763,2835,59
91
NUTF2_8_1323,904,937,772,841,921,673,1093,600,2017,506,342,3525
NXF2B_8_1324,756,570,662,668,50,446,93,2685,1276,238,644,2017
NXF2_8_1325,756,570,662,668,50,446,93,2685,1276,238,644,2017
NXF3_8_1326,981,1197,629,784,1290,649,28,1501,206,629,1553,850
NXT1_8_1327,835,553,667,461,780,684,1007,662,533,72,1410,348
OAZ2_8_1328,1092,1113,847,1017,1877,1181,1463,1774,799,1871,401,1461
OBP2A_8_1329,698,627,919,783,1207,228,738,63,357,1279,2392,1113
OBP2B_8_1330,301,115,166,69,404,25,285,137,52,472,1,313
OCA2_8_1331,1078,841,1252,1149,813,2186,1963,459,1186,749,1227,1461
OGFOD1_8_1332,1350,1263,1498,1518,2379,2540,3203,2415,2178,1373,2343,1
567
OGFOD2_8_1333,84,399,111,150,39,402,0,359,0,407,1,42
OGFR_8_1334,1092,1148,1074,896,929,1050,1621,477,510,871,752,2116
OVCH1_8_1335,583,691,964,608,316,1645,225,699,114,637,1777,267
OVCH2_8_1336,546,1198,742,1063,926,2494,716,362,587,1002,299,1372
OXNAD1_8_1337,1179,1400,900,1170,357,1419,546,272,1113,672,3408,2524
OXT_8_1338,509,528,654,1133,87,680,1439,981,361,52,980,505
PANX1_8_1339,1221,849,1443,1222,836,1236,1051,776,1041,880,661,1155
PAQR7_8_1340,537,635,560,499,269,369,1752,429,281,627,1121,316

PCDHB11_8_1341,337,521,161,238,5,854,751,349,938,361,853,105
PCDHB16_8_1342,647,466,560,554,108,257,655,43,482,294,637,1294
PCSK4_8_1343,722,889,448,533,1178,1448,1999,114,375,1954,299,989
PCSK7_8_1344,201,222,162,194,87,332,263,522,89,849,399,0
PEA15_8_1345,167,154,285,155,4,24,284,1,435,5,0,31
PET112_8_1346,822,935,654,637,833,585,2750,2217,539,635,2339,1448
PEX13_8_1347,1563,1362,1424,1401,1887,3369,2935,2245,1925,1398,4609,13
30
PEX7_8_1348,779,1331,724,830,342,1146,1025,478,583,1756,714,2675
PF4_8_1349,1477,1020,1164,1335,2418,1714,876,1489,2139,893,2363,1094
PFN3_8_1350,2111,2026,2053,1716,1666,2839,2586,3179,2118,2768,3483,374
6
PHEX_8_1351,7946,6410,5836,6782,8732,8020,8447,10440,10408,6971,6219,6
774
PIGR_8_1352,269,186,181,206,2,103,23,461,560,134,265,1181
PITPNA_8_1353,4937,3971,4124,4243,5394,2946,2324,3964,5309,7108,5914,3
728
PITPNB_8_1354,2891,2355,1755,3153,4669,1754,3644,3710,2705,2646,3795,3
936
PLLP_8_1355,249,237,377,228,1534,765,2148,1299,105,488,381,654
PLP2_8_1356,592,832,482,407,833,510,1601,581,837,519,695,316
PLXNB2_8_1357,109,95,134,243,76,35,364,274,23,2,0,1
PNMA2_8_1358,846,709,941,897,580,1098,230,568,1371,515,1258,1678
PPP1R14A_8_1359,1087,870,407,858,786,1458,12,1240,2382,262,1592,514
PPP1R14C_8_1360,2052,2459,1961,2299,1773,2936,1011,2032,1085,2798,3062
,2472
PPP1R15A_8_1361,199,188,192,244,632,433,211,38,8,44,324,273
PPRC1_8_1362,1057,1640,1142,833,650,2004,226,1677,790,2013,917,599
PPY_8_1363,1270,1004,1152,1198,474,3131,1654,929,1357,516,759,1870
PRB3_8_1364,605,830,911,519,543,680,658,414,232,617,1063,805
PROCR_8_1365,711,1330,594,474,348,2125,777,527,756,798,1314,528
PROS1_8_1366,2439,2088,1677,2275,2993,3054,2208,2950,2525,3820,3412,22
38
PROZ_8_1367,4418,3692,3940,4607,6483,3487,2957,4635,4326,4324,4274,514
3
PRPF18_8_1368,820,984,601,899,894,1145,317,323,366,1363,733,367
PRSS12_8_1369,467,711,667,345,990,604,532,1113,362,428,188,88
PRSS22_8_1370,1151,899,979,1139,1340,516,1499,296,716,1150,450,1414
PRSS27_8_1371,238,284,171,369,77,0,0,0,0,112,5,946
PRSS33_8_1372,215,223,143,215,0,1,493,5,38,206,0,301
PRSS36_8_1373,293,420,213,165,161,206,300,159,844,323,301,77
PRSS8_8_1374,652,414,381,472,473,715,367,101,266,179,278,620
PSCA_8_1375,317,142,143,108,1139,143,16,13,429,82,27,200
PTOV1_8_1376,952,1375,1117,1154,944,1766,1325,840,1237,951,838,1289
RABIF_8_1377,312,276,134,220,18,14,492,0,543,1,0,19
RAMP1_8_1378,183,335,72,246,146,9,5,12,283,183,31,968
RAMP2_8_1379,1173,1575,913,1168,2203,438,385,940,1773,444,1279,822
RAMP3_8_1380,241,628,217,72,678,422,772,423,48,339,115,1003
RANBP17_8_1381,378,159,372,305,571,744,304,0,281,315,116,307
RASSF9_8_1382,1149,1330,971,1462,1390,2242,91,2232,213,1220,2213,1209

REEP5_8_1383,499,402,330,597,618,287,304,354,302,807,569,486
RHAG_8_1384,1852,2388,1850,1242,1539,2702,1374,2054,2172,1546,3701,132
7
RLBP1_8_1385,543,463,390,756,367,808,1191,1338,884,432,563,474
RPL15_8_1386,1021,720,1795,1374,400,569,2909,2402,880,1266,2908,1550
S100A12_8_1387,3259,2662,2320,2603,4164,2536,3901,3429,1512,3751,3617,
1996
S100A1_8_1388,146,118,157,120,48,248,862,0,0,15,361,760
S100A2_8_1389,1083,652,1090,443,1314,636,721,213,52,1331,2614,150
S100A3_8_1390,3682,3443,3474,3215,3537,3080,7352,3843,4390,3185,5321,5
022
S100A6_8_1391,901,310,431,509,82,73,842,1078,138,478,490,2349
S100B_8_1392,3634,2571,3094,3631,3261,3804,3254,4566,2797,4321,3396,47
25
S100P_8_1393,474,642,735,586,292,863,590,43,520,362,260,547
SAA4_8_1394,928,669,576,286,539,1518,642,455,1313,1781,180,1674
SCAMP1_8_1395,337,340,170,223,90,76,119,335,77,406,81,632
SCAMP2_8_1396,998,836,694,588,2842,799,816,878,1232,359,536,385
SCFD2_8_1397,2172,2463,1895,2076,1356,2074,1212,2278,2582,2746,2074,20
09
SCLT1_8_1398,1238,1632,989,1321,713,676,318,1116,829,1207,975,1308
SCPEP1_8_1399,407,345,421,296,605,993,570,556,384,656,649,758
SDC2_8_1400,2500,2232,2142,2838,2516,2670,3225,1434,2020,3876,2575,239
3
SEC14L3_8_1401,550,591,162,179,150,352,15,573,300,564,199,181
SEC22A_8_1402,362,229,322,263,25,64,47,49,152,627,72,491
SEC23A_8_1403,3464,4031,3412,2837,3731,3583,3906,3174,2847,2687,3520,6
148
SEC24A_8_1404,7850,6919,6624,6569,6832,4716,7419,6984,3937,4968,9574,8
313
SEC24D_8_1405,1321,1320,1029,947,837,745,1521,278,910,1285,859,1361
SEC61A1_8_1406,2217,1730,1677,2544,1901,2470,3551,1989,2368,2196,3627,
1964
SEC61B_8_1407,1503,1117,1340,1603,3060,1373,787,1271,1565,1063,1652,31
06
SEC62_8_1408,1255,1328,641,882,1066,1312,573,140,1479,822,1252,1105
SEC63_8_1409,1045,1150,993,959,1970,2232,792,1348,1029,1177,563,1064
SELP_8_1410,4908,4826,5753,4655,6543,4048,7528,4819,4057,5693,5830,643
9
SERINC1_8_1411,1494,1449,1564,1645,774,2286,1530,1550,2101,662,3322,20
38
SERPINA11_8_1412,1477,1379,1137,1696,1981,2597,1864,1733,1680,1316,179
7,1385
SERPINA12_8_1413,765,553,389,656,1037,83,701,497,909,847,305,537
SERPINA3_8_1414,852,1210,1110,1160,1221,619,462,929,1490,1266,908,1934
SERPINA4_8_1415,870,952,551,1080,127,1138,792,945,300,645,796,822
SERPINA5_8_1416,3919,2187,2376,3283,4426,2960,1283,5588,3137,3626,3786
,1706
SERPINA7_8_1417,4608,4890,3927,4744,3089,9035,6274,3994,4061,3880,3912
,4283

SERPINB10_8_1418,814,243,557,663,1319,883,642,1377,324,93,851,1774
SERPINB12_8_1419,2938,2923,2187,2016,780,1844,2199,2147,2663,2433,2227,2924
SERPINB13_8_1420,493,453,676,367,1288,164,292,847,280,730,1060,29
SERPINB1_8_1421,712,847,613,506,293,554,57,1060,718,243,103,701
SERPINB4_8_1422,1961,1949,2047,1831,4119,2623,1846,1077,2528,1186,1252,4033
SERPINB9_8_1423,426,224,494,155,45,154,1828,141,447,101,355,228
SERPINC1_8_1424,103,156,176,169,0,330,74,155,47,73,187,72
SERPIND1_8_1425,567,493,400,533,608,348,951,1826,2128,706,248,341
SERPINF1_8_1426,215,45,268,192,0,853,0,0,1,65,6,181
SFXN1_8_1427,782,631,474,712,878,156,1017,558,1201,732,560,73
SFXN2_8_1428,1511,1293,1358,1886,1337,2480,512,2126,772,392,2858,899
SFXN3_8_1429,488,299,476,506,744,624,1757,832,50,188,1415,353
SFXN4_8_1430,909,1200,573,933,362,2104,948,536,1097,1641,1507,947
SFXN5_8_1431,2039,2005,2041,1545,1693,1377,1090,694,1052,3198,3035,2565
SLC15A5_8_1432,5720,5730,4672,4551,3771,6634,6058,2952,4229,4721,6990,3528
SLC16A13_8_1433,5988,5158,4538,5069,5878,3637,6044,5795,4469,4263,3313,7501
SLC16A14_8_1434,576,1407,564,632,997,1029,1129,1797,205,215,1155,1081
SLC16A9_8_1435,1111,886,912,980,303,1140,624,1237,1467,1463,1566,601
SLC17A9_8_1436,899,993,740,854,222,1214,3325,972,1095,413,320,1029
SLC22A20_8_1437,174,97,108,97,0,1,4,2,100,53,20,820
SLC22A24_8_1438,3576,3699,3114,2127,3143,3335,4366,3220,1899,5423,2065,2345
SLC22A25_8_1439,1223,1030,1354,1262,1102,3442,1696,1884,659,1371,1724,2636
SLC25A23_8_1440,251,92,200,140,126,331,169,131,619,15,23,77
SLC25A29_8_1441,206,285,231,188,15,18,2,394,177,276,17,216
SLC25A31_8_1442,1123,1232,926,1239,1631,1922,1066,830,1156,1113,1563,2846
SLC25A38_8_1443,478,643,397,469,801,211,371,452,63,158,141,1127
SLC25A42_8_1444,535,716,431,601,1243,999,594,194,1187,373,486,75
SLC25A46_8_1445,814,1025,765,1149,1080,1149,122,450,0,435,662,1451
SLC25A48_8_1446,280,667,712,590,7,843,152,923,605,1102,613,1027
SLC38A7_8_1447,421,519,564,336,322,989,868,590,327,520,408,1723
SLC38A9_8_1448,3552,3226,2422,2871,3313,4037,4978,2045,3690,4051,3728,4295
SLC41A2_8_1449,1093,963,661,638,1339,1339,993,1996,539,1061,627,1309
SLC45A1_8_1450,1106,733,1282,736,1289,154,114,772,1484,158,878,1048
SLC45A3_8_1451,318,504,390,353,29,22,1,33,1,0,601,88
SLC47A1_8_1452,1865,1366,1521,1621,2992,1275,3197,1592,1390,2342,2456,1696
SLC48A1_8_1453,351,1233,521,416,487,1259,58,465,986,1222,566,839
SLC5A12_8_1454,1324,1127,1203,921,784,916,392,1033,1100,1254,1599,1664
SLC6A17_8_1455,328,322,186,193,508,665,357,319,1104,1021,469,377
SLC7A14_8_1456,1006,1099,678,700,1116,1046,364,902,1366,1776,1700,2837
SLIT2_8_1457,6161,6961,5496,6598,7108,8413,10022,8849,5791,7779,6037,8

413
SNAP29_8_1458,3123,3029,3587,3110,2044,2787,1246,5698,2027,1998,3500,6
406
SNX12_8_1459,774,1252,715,513,573,623,195,435,88,915,700,1280
SNX13_8_1460,786,653,710,820,1161,401,180,67,247,745,244,1162
SNX17_8_1461,647,704,1080,447,314,365,994,1257,524,742,575,619
SNX19_8_1462,3192,3424,3136,3602,6611,5124,3529,3162,3925,3771,2277,33
39
SNX22_8_1463,150,126,72,425,64,359,53,15,56,40,231,1312
SNX24_8_1464,601,456,413,223,817,0,1047,417,0,337,671,0
SNX2_8_1465,199,243,154,359,321,285,717,550,523,494,386,8
SNX4_8_1466,1602,1567,1049,1869,307,2085,1812,901,1134,909,2253,1508
SNX8_8_1467,1475,1429,1245,1494,2778,1039,1377,1077,2192,1512,1087,138
7
SNX9_8_1468,833,926,539,977,1288,460,450,59,269,330,355,342
SORCS2_8_1469,731,444,388,801,297,907,185,917,632,457,778,278
SORCS3_8_1470,279,267,270,250,400,394,1128,265,93,692,624,1179
SORL1_8_1471,2353,3316,2752,2669,3501,2030,3635,3190,3146,3172,3286,47
21
SOS1_8_1472,432,381,311,432,375,294,671,520,1029,453,356,386
SST_8_1473,2882,2300,2663,2606,4112,1956,4588,2005,2461,2379,3258,2506
ST13_8_1474,366,524,464,447,640,189,11,55,204,919,41,2
STAB1_8_1475,2395,1566,1420,2445,1590,1999,3673,817,1538,1912,1588,262
2
STARD4_8_1476,660,1003,328,616,864,1427,2669,1848,491,818,4,120
STARD5_8_1477,1057,924,1092,619,863,2055,1125,600,1478,431,1397,286
STARD6_8_1478,6453,6324,6008,7584,6929,7691,8316,7303,7199,7878,9688,1
0134
STEAP1_8_1479,2885,3242,2592,2750,2848,4918,3449,2615,3215,3427,6646,5
066
STMN4_8_1480,507,204,565,220,29,21,833,138,267,456,54,360
STX11_8_1481,450,397,257,502,48,293,82,154,335,545,192,530
STX18_8_1482,2599,1869,2375,2384,2077,1677,1523,1878,3606,2775,2248,11
79
STX4_8_1483,542,530,868,790,948,234,638,179,971,587,2055,614
STX5_8_1484,186,497,141,146,113,100,335,164,0,84,20,275
STX6_8_1485,2644,1999,1740,2102,2210,3829,2588,1365,2490,1134,2209,372
9
STX7_8_1486,487,343,346,432,1182,503,255,539,434,352,195,1025
STXBP3_8_1487,856,1028,720,964,625,2176,616,24,863,458,1312,1656
SVOP_8_1488,73,98,103,282,238,44,344,7,97,300,831,320
SYP_8_1489,308,561,630,440,260,455,1284,193,251,760,340,233
SYT10_8_1490,854,760,464,646,488,929,238,2116,515,1158,568,274
SYT11_8_1491,860,821,614,1350,389,638,514,1215,639,525,1012,1097
SYT13_8_1492,1720,1210,1471,1325,2707,1546,1963,711,366,698,2316,2646
SYT16_8_1493,353,388,275,497,265,577,171,15,1275,795,1196,487
SYT4_8_1494,761,799,632,618,158,861,1463,719,940,440,579,944
SYT5_8_1495,526,292,295,454,358,154,786,1063,564,525,140,208
SYT6_8_1496,55,258,258,183,0,2291,17,140,12,821,100,339
SYT7_8_1497,1629,1636,1780,1304,2547,898,2423,2357,408,1940,1412,1145

SYT8_8_1498,154,272,302,237,179,293,293,53,0,164,333,661
SYT9_8_1499,1074,462,1053,744,1169,719,847,717,1669,1455,422,1181
TAS2R42_8_1500,688,313,505,604,474,272,351,529,370,964,513,516
TBC1D9_8_1501,1435,2093,1951,1676,1229,2214,1047,2442,1061,1524,1403,1
120
TCN1_8_1502,1740,2131,1856,1849,1299,2237,2430,1486,1658,2122,1779,524
0
TEKT4_8_1503,448,140,224,346,63,0,737,2,3,1216,499,816
TF_8_1504,1319,810,1328,1339,306,807,1424,2925,958,1460,3724,1280
TG_8_1505,868,970,649,946,1107,718,1025,249,1173,892,617,960
TGFB1_8_1506,9598,10373,8553,9689,14714,7514,12763,11908,9370,10732,10
222,11650
TIMM10_8_1507,481,662,277,653,1028,102,87,68,588,824,292,802
TIMM13_8_1508,122,166,79,232,0,1027,0,0,304,141,923,4
TIMM17A_8_1509,3324,3797,2657,3602,3104,3391,3440,2441,3506,4474,5407,
3624
TIMM22_8_1510,720,535,509,532,255,395,1455,1348,342,914,1146,566
TIMM23_8_1511,3198,3762,3603,3671,4231,3353,3204,2086,3346,4265,6064,4
048
TIMM44_8_1512,494,554,560,638,764,408,534,900,607,344,616,979
TIMM8B_8_1513,143,203,149,107,425,135,262,700,870,634,206,242
TIMM9_8_1514,2987,3312,2859,3450,1350,5387,2600,3664,2807,2402,1053,29
21
TLL2_8_1515,414,173,344,387,1406,247,949,336,0,945,1078,557
TM9SF2_8_1516,6075,7648,6386,6787,4040,6570,6687,7879,5229,7280,7858,1
0548
TM9SF3_8_1517,1376,1220,1703,1653,1172,1975,1354,329,1430,2920,1076,23
64
TM9SF4_8_1518,991,1546,1175,1045,2286,1118,1904,1674,3407,980,1306,157
7
TMCO3_8_1519,725,811,775,776,269,598,1423,1679,437,1290,1193,334
TMED10_8_1520,2970,3750,2143,3759,2585,2822,5542,3271,3431,2512,3804,3
772
TMED1_8_1521,449,259,521,381,29,393,97,1084,161,126,2362,636
TMED2_8_1522,418,193,202,460,38,0,745,0,1747,134,505,0
TMED3_8_1523,1004,882,1067,959,602,1385,546,984,678,1252,740,330
TMED4_8_1524,309,602,438,262,378,84,350,1242,79,270,619,243
TMED7_8_1525,1738,2389,1897,1682,1604,1694,3539,1280,1438,1759,4742,13
27
TMED9_8_1526,4625,5331,4336,4197,3626,6319,5651,4503,6054,5983,3576,47
52
TMEM104_8_1527,708,554,525,871,757,505,363,1253,924,595,852,1541
TMPRSS11B_8_1528,1824,1735,1744,1370,2414,2609,1569,2091,1504,1463,111
8,1745
TMPRSS11D_8_1529,543,319,313,782,24,685,776,336,37,923,109,2237
TMPRSS11E_8_1530,2066,2010,1815,1785,1091,1345,1813,2731,3153,3016,290
8,1831
TMPRSS11F_8_1531,4325,5827,3677,5149,4401,4754,4616,4860,4922,4444,405
4,4517
TMPRSS12_8_1532,303,803,452,477,10,420,1181,0,943,1562,369,850

TMPRSS5_8_1533,2077,2435,1527,1910,1107,2724,2669,611,4345,2392,2786,2
487
TMPRSS6_8_1534,252,224,11,386,569,118,65,0,7,22,32,81
TMPRSS7_8_1535,2603,2462,2539,2449,2682,3167,2293,3625,954,764,2891,28
63
TMPRSS9_8_1536,121,116,159,125,106,47,194,119,154,143,158,11
TNC_8_1537,1664,2313,1433,1904,2422,1124,2545,1745,2395,2369,1457,1572
TNF_8_1538,121,278,59,79,11,74,0,209,17,456,3,133
TNFRSF9_8_1539,2471,2681,2548,2445,2492,3452,2162,1247,2159,2890,2880,
1781
TNNC2_8_1540,1536,1649,1777,1609,1978,1593,1749,1389,2263,1967,2478,17
36
TNN_8_1541,362,134,349,219,1476,22,905,149,134,74,180,450
TNNI3_8_1542,1081,665,657,693,1449,1046,441,237,1030,1415,492,692
TNR_8_1543,1055,901,964,860,755,1247,715,443,440,874,424,2572
TOMM20_8_1544,1466,1131,946,745,1517,1629,991,1132,1335,607,646,1344
TOMM22_8_1545,468,380,315,400,105,762,771,302,341,460,127,210
TOMM70A_8_1546,2510,1927,1816,2041,1849,2796,2124,1563,3289,2698,3159,
1330
TOMM7_8_1547,1149,1731,745,1000,1921,2144,1937,337,1483,1330,2568,644
TPSG1_8_1548,187,228,140,83,9,8,22,21,56,448,610,82
TRAK2_8_1549,353,711,460,656,436,228,127,198,899,262,227,375
TRAPPC10_8_1550,905,886,835,695,550,2081,884,913,837,819,1153,1501
TRAPPC8_8_1551,1269,1417,1090,1636,3135,2744,2174,813,1016,2240,832,21
91
TSNAX_8_1552,476,562,431,716,1403,872,1137,170,172,436,599,843
TTPA_8_1553,864,920,650,948,1039,1707,737,821,199,1720,1679,1507
TTR_8_1554,587,391,857,669,299,464,412,937,352,2094,749,329
TUBA1A_8_1555,1423,1537,1235,1166,1098,1791,1988,242,2212,837,593,1585
TUBA1C_8_1556,1105,1239,1236,880,1018,553,1380,452,575,1602,1022,2033
TUBA3D_8_1557,1496,1861,1284,1987,1788,1357,561,1264,1607,1852,1568,17
28
TUBA3E_8_1558,568,1166,507,699,509,1697,160,628,297,614,1021,1087
TUBA4A_8_1559,724,936,481,593,818,833,935,219,677,474,417,564
TUBB1_8_1560,248,600,251,124,204,704,289,208,768,110,365,234
TUBB2A_8_1561,1395,1211,947,650,1509,949,1925,1190,1906,534,1394,755
TUBB2B_8_1562,2152,1691,1738,2329,2355,1421,2989,1792,2827,3800,1106,1
772
TUBB6_8_1563,2194,2105,1750,2571,3585,1794,2790,1615,2068,1988,2719,25
28
TUBB8_8_1564,650,653,530,519,781,1334,517,535,1016,789,1347,284
TUBE1_8_1565,1954,2629,1934,2151,3081,3462,2873,1895,1689,3339,2308,37
47
TUBG1_8_1566,710,830,810,539,421,231,94,1031,373,790,672,1406
TUBG2_8_1567,367,612,243,217,176,149,30,266,305,425,354,1711
TXNDC8_8_1568,1463,1373,1300,1269,2723,2489,2462,2229,1517,1389,1731,1
929
UCP2_8_1569,396,362,377,439,551,120,231,174,51,304,713,658
USO1_8_1570,2965,3045,2115,3157,3067,3001,3299,1891,2840,4186,3411,184
1

VAMP2_8_1571,8049,9073,6509,7447,9843,10981,10681,8061,7127,7317,8277,
8766
VAMP5_8_1572,888,1154,952,819,442,1712,592,834,824,1027,1064,596
VKORC1L1_8_1573,470,474,285,311,351,829,83,381,229,563,4,256
VPS18_8_1574,766,401,325,387,273,88,39,28,232,36,497,1992
VPS26B_8_1575,1403,1475,1207,1453,2480,2143,2033,1137,1394,1149,2219,1
829
VPS33A_8_1576,5561,5766,4765,5402,5161,5146,6090,4304,6777,5669,4984,7
029
VPS33B_8_1577,165,347,79,243,305,17,158,147,95,488,142,81
VPS35_8_1578,2380,3112,2150,3360,3850,4740,1307,4116,3869,3139,2927,56
79
VPS39_8_1579,2118,2623,1740,2552,1843,2771,2513,1426,2076,1543,6817,44
47
VPS45_8_1580,2433,2601,1622,2255,3453,1553,1484,2251,2384,4159,3816,15
49
VPS4B_8_1581,2242,2680,2242,2110,2894,2517,5270,1588,1808,4143,4481,37
19
VSIG2_8_1582,509,526,256,740,283,621,234,951,1048,154,287,249
VTI1A_8_1583,1980,1276,1959,1512,1492,1504,1379,943,1834,2893,1030,227
2
VTI1B_8_1584,1544,1459,1818,1244,2500,2327,3277,638,1832,2174,2455,364
8
VTN_8_1585,168,83,218,221,153,0,323,19,560,37,319,248
VWF_8_1586,796,1452,617,785,361,640,1522,824,2173,352,938,580
XK_8_1587,112,532,37,174,123,202,372,25,0,290,794,1
XPO1_8_1588,1482,1690,1303,1244,2643,1889,1375,1352,1867,847,2504,1373
XPO4_8_1589,2100,1997,1392,1662,1602,920,1300,1536,1266,3193,1802,1807
XPO5_8_1590,1533,1495,1568,1008,3158,1372,739,872,1333,1844,2619,2090
XPO6_8_1591,743,988,712,496,308,54,681,360,10,686,112,124
XPO7_8_1592,1286,1384,1170,1039,941,995,2824,1148,850,1317,2102,926
ACTL6A_8_1593,1456,1041,1347,998,1096,1682,1169,929,1296,2097,1875,262
6
ADAM12_8_1594,178,252,103,231,259,416,36,75,193,116,293,153
ADAM15_8_1595,193,292,391,305,0,267,0,0,0,0,1057,11
ADAM18_8_1596,1066,1028,1138,2066,1584,2230,361,611,292,369,1793,2630
ADAM22_8_1597,943,935,728,763,789,1707,1198,1032,523,1711,273,1771
ADAM29_8_1598,429,213,239,400,441,828,37,256,217,509,256,250
ADAM33_8_1599,245,193,256,82,3,277,118,599,1135,194,137,752
ADAM8_8_1600,816,578,398,421,79,800,957,72,532,1122,1748,377
ADAMTS13_8_1601,536,406,629,820,861,325,295,830,48,741,843,654
ADAMTS14_8_1602,143,460,33,196,8,375,640,69,42,1220,27,42
AKAP12_8_1603,1330,841,1146,1243,503,692,443,249,1945,2154,1293,1230
ANGPT1_8_1604,1419,1846,1242,1363,1677,1765,844,769,2777,2995,1707,170
6
ANGPTL4_8_1605,0,0,0,0,0,0,0,0,0,0,0,0
AP1B1_8_1606,4770,4256,3867,4596,5302,5530,5553,3139,5836,7581,7562,47
04
AP1G1_8_1607,3078,3150,3024,2964,6164,3074,2941,3846,3551,3919,3388,34
07

AP1M1_8_1608,261,219,144,60,268,168,60,5,349,78,482,46
AP2A1_8_1609,432,452,120,705,32,1061,81,563,83,59,263,2478
AP2A2_8_1610,254,374,229,205,1,233,111,174,550,253,74,59
AP2B1_8_1611,1742,2142,1109,2210,997,893,2088,1316,1530,2419,3001,1316
AP2M1_8_1612,742,1067,467,206,172,14,145,31,532,537,159,794
AP2S1_8_1613,94,27,150,2,0,0,0,0,0,635,525,614
AP3D1_8_1614,321,249,182,551,687,285,0,158,108,255,261,576
AP3M1_8_1615,395,368,230,457,154,945,491,1127,25,121,1,351
AP3M2_8_1616,369,273,187,475,47,194,234,546,178,511,592,102
AP4S1_8_1617,574,848,1021,599,315,570,1,949,433,110,262,954
APAF1_8_1618,627,496,612,437,989,937,1716,881,1235,1002,933,1361
APBA2_8_1619,1599,1422,1096,1280,3491,676,1195,991,1166,1220,855,2161
APOL1_8_1620,759,1254,858,1306,487,1842,638,1797,124,1039,527,1150
APOL3_8_1621,1604,1690,1266,1752,3447,1618,1691,3204,2094,2121,2624,44
73
APP_8_1622,60,165,23,191,19,448,5,14,81,77,70,8
ARFGAP3_8_1623,3065,2879,2711,2205,2475,2865,2448,2166,2528,3812,2511,
2832
ARHGAP33_8_1624,375,317,257,463,143,166,1129,1470,364,619,570,56
ARL6_8_1625,2875,2336,2054,2770,3402,2440,3323,2639,2463,2518,3531,429
8
ATP13A2_8_1626,551,437,472,793,263,496,420,436,353,277,426,1116
BAX_8_1627,718,366,368,329,505,212,1,1516,643,144,1748,568
BCAP29_8_1628,3487,3118,3351,4166,5934,4236,5203,3155,3032,3053,4753,3
587
BCAP31_8_1629,144,157,122,88,3,266,20,20,672,8,89,8
BCL2_8_1630,912,771,841,819,1297,487,1476,1271,441,1011,1708,694
BCL2L2_8_1631,41,110,132,117,13,337,2,1,373,24,0,0
BID_8_1632,816,555,444,706,244,632,640,212,1365,238,940,2602
BSG_8_1633,48,138,66,37,14,2,76,116,4,336,873,1
C1QC_8_1634,2751,3490,2495,2665,1793,6447,4190,1131,2489,1679,6376,284
7
CANX_8_1635,1937,1697,1210,1403,2967,2985,1070,1753,2483,2615,2222,240
3
CAPN9_8_1636,5902,5973,4536,5219,6391,5390,3486,4565,4731,7390,6291,77
60
CAPNS1_8_1637,961,566,777,720,1898,241,697,1328,1458,999,147,1741
CCT6B_8_1638,2140,2095,1589,1785,1218,1695,688,1414,2620,1398,2959,139
5
CD19_8_1639,1020,1056,1002,1476,767,1713,576,351,639,805,838,1535
CD22_8_1640,993,1332,1371,1617,1359,365,691,1060,1148,1896,1737,1929
CD33_8_1641,3192,3283,3115,3692,4936,4159,3387,2590,2869,1078,3186,332
3
CD44_8_1642,437,593,532,230,493,101,96,88,764,247,576,229
CD55_8_1643,1300,1208,1017,903,883,2778,949,1345,129,2057,1408,626
CDH17_8_1644,232,165,81,143,5,277,27,192,153,364,67,615
CFHR4_8_1645,1709,2078,1986,1557,1080,1801,1342,1556,818,2126,1792,220
8
CIZ1_8_1646,640,547,371,685,543,602,293,263,180,652,59,202
COG2_8_1647,963,653,758,991,549,556,800,617,1028,1102,1456,1349

COG4_8_1648,1359,1312,849,1220,1373,1821,774,880,1413,1268,2206,857
COG5_8_1649,317,356,438,553,86,803,4,1189,21,41,890,225
COG6_8_1650,1710,1502,2051,1666,768,2310,1973,1161,2613,2572,2694,3048
COL11A1_8_1651,436,409,303,602,256,925,264,191,1220,1682,118,861
COL11A2_8_1652,570,462,264,330,0,1458,30,99,407,44,466,517
COL12A1_8_1653,259,551,107,497,1,380,95,477,573,59,3,94
COL13A1_8_1654,1703,1247,1152,2047,2604,1413,3388,1478,1823,1193,1981,
1165
COL18A1_8_1655,146,234,156,239,27,1,4,184,42,225,43,52
COL25A1_8_1656,873,854,643,1397,426,2090,1145,2116,2052,1359,508,1038
COL2A1_8_1657,214,296,158,153,13,89,7,8,276,37,243,10
COL4A5_8_1658,1066,945,1006,1062,926,748,431,888,400,1513,1833,755
COL4A6_8_1659,495,472,404,427,243,705,89,1081,82,816,1332,8
COL6A2_8_1660,519,275,242,105,158,971,20,416,17,726,129,475
COL6A3_8_1661,1691,1498,1828,1065,1235,2542,2303,1422,1666,1807,2670,2
409
COL8A1_8_1662,1345,1085,1164,1203,432,1769,1144,629,1606,1938,2801,233
9
COL9A1_8_1663,443,460,390,393,240,2,300,428,1383,1067,584,79
COPA_8_1664,2422,2710,2662,2171,1812,2974,2803,1952,2911,2218,3115,311
4
COPB1_8_1665,348,334,325,401,180,433,138,730,224,790,627,291
COPE_8_1666,213,380,46,180,3,578,35,12,36,39,29,205
CPA4_8_1667,696,785,708,678,974,610,198,901,707,228,581,575
CPNE1_8_1668,1368,1205,963,1251,2419,1752,1571,943,694,1777,2722,1674
CPNE7_8_1669,0,0,0,0,0,0,0,0,0,0,0,0
CPXM1_8_1670,187,373,67,217,284,405,0,1,189,361,6,567
CPZ_8_1671,1283,2193,1017,830,2323,3486,1679,1220,1404,1643,2372,1001
CRABP2_8_1672,645,1182,786,1096,818,646,146,687,651,679,1891,566
CTLA4_8_1673,32,26,105,145,97,31,0,125,23,57,51,79
CTNS_8_1674,591,494,633,632,103,219,808,1099,150,1130,206,111
CXCL12_8_1675,441,352,434,267,1327,551,607,934,398,50,120,616
DPP10_8_1676,2199,2749,1766,2233,2126,3359,2599,1261,3625,2259,1899,45
70
DPP6_8_1677,807,616,594,794,595,223,186,656,247,770,525,1042
ECM1_8_1678,1288,1556,898,1174,1973,473,1025,822,975,1177,1778,1146
EGF_8_1679,1851,2819,2044,2060,2214,1418,1989,1504,2568,2685,3368,4575
EIF2C2_8_1680,769,1275,1424,1556,2022,1794,1166,1179,507,1542,1601,102
7
EIF2D_8_1681,843,1202,1090,1330,683,1035,896,940,922,945,463,2278
ENSA_8_1682,1581,1180,1547,1389,1032,1358,546,3461,1308,3518,721,369
EPB41L4B_8_1683,586,398,478,505,818,358,283,456,819,41,226,439
EPB42_8_1684,151,310,110,197,1,77,0,1294,360,222,403,45
ETFA_8_1685,2945,3435,3354,3092,3079,2240,3948,2181,2228,6228,2528,445
4
ETFB_8_1686,414,243,396,413,4,669,23,506,141,59,869,818
EXOC1_8_1687,942,949,788,600,1920,2397,1367,2524,415,1029,1767,566
EXOC4_8_1688,362,380,248,151,304,311,313,527,152,360,309,320
EXOC6_8_1689,1412,780,921,1479,1650,754,1422,995,750,1323,1062,978
EXOC7_8_1690,660,868,628,597,286,1567,306,106,481,994,757,1090

F8_8_1691,1093,1070,731,778,2036,1148,2346,742,2479,644,562,1532
FABP6_8_1692,743,568,199,181,1298,111,412,1893,194,642,408,272
FAM131A_8_1693,557,157,218,410,799,294,271,51,1774,107,59,284
FAM63B_8_1694,953,910,891,991,727,683,274,608,704,673,1096,1173
FANCA_8_1695,1598,1472,1359,1110,1194,1537,4632,1187,2186,1887,931,199
5
FCN3_8_1696,923,876,522,983,971,631,898,771,936,1276,876,1435
FGA_8_1697,576,1110,1125,643,388,989,818,2198,240,921,478,676
FGB_8_1698,993,1125,1173,881,644,919,2518,433,476,1510,1286,653
FGF13_8_1699,1114,1238,1079,1612,2706,1427,1990,1195,910,515,907,2026
FGG_8_1700,411,438,264,192,634,50,42,26,499,265,9,220
FIBCD1_8_1701,585,1154,645,760,1383,1025,946,387,681,464,673,344
FLVCR2_8_1702,652,663,944,441,1229,1409,117,347,173,61,797,522
FOLR1_8_1703,416,518,363,496,707,21,1054,508,254,517,82,859
FOLR2_8_1704,1038,627,974,894,1,1196,862,545,1008,1306,798,2548
GGA1_8_1705,259,425,233,144,13,1104,397,560,42,892,8,34
GGA3_8_1706,390,293,201,342,287,493,41,248,1040,1254,279,589
GJA5_8_1707,510,483,462,673,592,256,267,693,274,263,244,508
GJB1_8_1708,672,442,618,864,997,800,454,645,295,686,744,170
GJB3_8_1709,1818,2039,1756,2369,2606,1761,2311,1374,1037,1238,1477,169
2
GJB6_8_1710,719,777,738,684,699,720,296,636,950,710,715,62
GLYATL1_8_1711,3009,2748,2984,2257,3152,3155,862,2611,4492,3121,1141,3
338
GNRH1_8_1712,289,57,210,296,2,174,0,0,238,59,300,57
GOLGA3_8_1713,883,1033,824,968,391,785,705,390,1562,1094,742,1902
GOPC_8_1714,256,301,204,307,113,150,204,626,35,689,114,203
GOSR1_8_1715,9,0,36,14,0,0,9,0,0,0,0,0
GOSR2_8_1716,530,247,483,459,448,340,59,757,140,1198,416,220
GPRASP1_8_1717,963,754,492,852,1171,625,759,576,376,820,479,680
GRB2_8_1718,1615,1444,1597,1535,1365,2179,836,250,1217,436,1845,543
HABP2_8_1719,2040,1602,2135,1916,1384,2981,3856,1168,942,1874,2566,242
0
HDLBP_8_1720,417,340,316,389,228,1026,306,700,635,322,599,477
HEPH_8_1721,380,533,653,530,1098,318,452,93,58,892,494,499
HNRNPU_8_1722,190,177,261,111,1,1,141,52,65,432,498,53
HOMER2_8_1723,578,419,669,490,252,492,566,341,1264,784,985,385
HPN_8_1724,874,461,406,740,396,1215,651,88,667,232,179,410
HSDL2_8_1725,208,391,722,377,29,697,290,247,965,901,47,227
IGF1_8_1726,881,1415,708,752,699,1671,1194,433,744,215,949,985
IGFBP3_8_1727,807,589,421,843,810,809,291,238,668,179,1577,76
IPO11_8_1728,647,683,969,1056,1095,513,867,422,537,980,478,653
IPO8_8_1729,6668,6068,6182,4894,5915,8679,7505,8232,5112,7056,6748,522
5
ITGAL_8_1730,1803,924,1522,1211,578,926,3118,1628,1885,980,3968,1795
ITGAM_8_1731,3280,3307,3169,3452,4989,4696,2770,2948,3421,3137,4339,37
60
ITGAV_8_1732,1075,670,1136,852,606,730,977,543,1041,728,1351,1204
ITGB2_8_1733,821,816,770,883,1348,342,1085,1123,894,454,1262,1044
KDELR2_8_1734,5647,5081,5385,5702,3811,9437,6372,4346,8118,8264,4896,8

434
KDELR3_8_1735,455,741,307,553,137,1926,328,2705,916,672,317,203
KIF13A_8_1736,1343,1388,1663,1443,1249,1549,1892,1342,2286,1345,1664,1
604
KIF17_8_1737,259,99,120,407,308,37,6,324,42,318,1,1270
KIF1B_8_1738,3497,3931,3530,4398,4133,5774,3223,2341,3175,2776,4233,24
65
KLK10_8_1739,513,571,539,607,358,233,483,543,558,326,48,144
KLK11_8_1740,132,184,67,181,255,396,37,1,5,20,1,96
KLK12_8_1741,444,509,489,358,309,354,348,716,813,724,905,186
KLK15_8_1742,128,138,153,232,765,0,58,25,263,128,183,506
KLK5_8_1743,455,406,431,358,704,227,157,914,214,790,155,27
KLK6_8_1744,598,594,824,756,666,602,943,411,386,1379,600,856
LAMB3_8_1745,1307,1833,1547,1837,1380,2515,1716,2051,2203,2081,829,336
5
LDB3_8_1746,422,385,97,221,137,0,847,108,47,249,29,200
LDLR_8_1747,403,238,525,586,697,212,400,219,486,463,635,810
LTA_8_1748,305,457,650,246,718,116,1150,413,248,174,1370,662
LTF_8_1749,586,651,860,965,736,1062,1807,1915,829,427,1320,365
M6PR_8_1750,715,1014,1110,842,2189,1494,2616,781,1596,861,1507,1750
MASP1_8_1751,1305,905,1181,1402,2091,1031,620,1514,594,711,2751,862
MB_8_1752,441,198,326,231,77,96,13,46,670,243,33,703
MCFD2_8_1753,1403,1454,1079,1669,1333,1175,2963,1253,2711,748,1207,214
8
MCL1_8_1754,1440,884,646,1705,671,1165,970,1917,969,2552,2222,1813
MEFV_8_1755,2128,1288,1372,1390,938,944,257,778,2108,1570,1563,1605
MFAP4_8_1756,590,567,750,554,184,644,1296,680,1296,1077,530,1461
MFSD10_8_1757,141,103,40,108,0,0,126,0,260,423,226,0
MFSD1_8_1758,1667,1747,1175,1601,793,2252,1692,1412,2074,1711,1983,207
5
MFSD5_8_1759,386,496,371,654,4,32,489,1051,940,382,888,126
MLC1_8_1760,479,209,289,267,60,421,461,469,198,204,1752,51
MSLN_8_1761,121,317,199,368,30,881,219,219,475,190,28,364
MTX1_8_1762,348,242,47,231,0,12,18,427,901,41,15,267
MUC1_8_1763,410,973,912,734,10,368,310,739,1889,479,199,1086
NCAM1_8_1764,695,780,963,580,1911,1678,435,1085,929,745,770,954
NNAT_8_1765,485,671,314,567,1431,226,259,509,661,789,95,199
NOXO1_8_1766,1,0,0,0,0,0,0,0,0,0,0,1
NPC1L1_8_1767,4396,4093,3815,4299,2161,4723,3870,3886,4727,3109,4505,5
772
NPRL3_8_1768,2407,2311,2599,1460,2167,3135,803,2560,1945,1558,4802,557
8
NRXN1_8_1769,671,607,568,678,2055,404,1202,570,808,490,1299,258
NRXN2_8_1770,2261,2048,2371,1920,2688,3399,2716,2631,2373,1566,3582,14
20
NRXN3_8_1771,1415,1556,1174,1058,2472,912,1120,658,616,1306,796,277
NUP155_8_1772,115,87,49,36,0,0,7,92,23,29,240,395
NUP50_8_1773,3056,3381,2838,3477,4060,2735,1319,2631,4731,3657,3162,42
15
NUP62_8_1774,1296,1383,881,1219,1805,810,1380,1280,679,1458,1774,1457

NUP98_8_1775,865,1302,623,1081,659,459,941,289,888,1589,735,1124
NUPL1_8_1776,893,806,578,540,440,2065,1142,1765,590,1304,563,1600
NXF1_8_1777,461,436,424,448,1469,514,155,406,806,1150,1320,524
NXNL2_8_1778,253,148,225,106,271,395,0,421,0,173,516,29
NXT2_8_1779,689,538,613,664,980,449,558,2459,239,361,1130,934
OAZ3_8_1780,366,478,151,356,630,36,0,27,403,415,196,820
PACSIN2_8_1781,375,419,439,553,675,427,317,841,24,118,1504,368
PANX2_8_1782,311,357,202,537,302,288,605,781,32,244,274,36
PCDHA6_8_1783,797,815,722,444,849,962,223,324,717,587,959,1322
PCDHGA5_8_1784,365,620,327,300,591,197,821,136,247,65,655,121
PCLO_8_1785,1177,830,1004,767,758,1981,548,863,1640,2205,536,1134
PCSK5_8_1786,877,635,894,1097,483,1051,1829,1531,330,857,1439,523
PCSK6_8_1787,142,44,82,60,2,17,20,223,122,4,188,7
PCTP_8_1788,837,891,1238,676,625,1687,401,410,540,583,1079,774
PDYN_8_1789,333,327,251,280,203,208,399,287,386,183,104,227
PDZD3_8_1790,785,740,617,591,675,1212,1397,647,810,475,248,1347
PDZK1_8_1791,966,881,1015,1291,884,1194,1467,1016,2047,623,567,2027
PGAP2_8_1792,4453,5204,4428,4110,5372,5695,5230,4631,3789,4448,6345,72
97
PGF_8_1793,151,527,349,408,271,354,33,243,33,1212,722,575
PIK3R3_8_1794,891,1387,830,1175,1969,774,456,990,2293,1036,1981,1085
PITPNC1_8_1795,917,1334,978,1415,2173,2365,1424,1086,703,689,2374,277
PITPNM1_8_1796,378,981,431,770,707,502,79,48,390,824,2006,251
PITPNM3_8_1797,974,967,951,1320,605,1248,1100,2073,1275,1705,556,1988
PLEC_8_1798,1798,1628,1586,1716,1297,1206,769,1820,1565,1613,2146,2770
PLIN3_8_1799,220,174,139,180,767,72,563,287,30,191,231,577
PLTP_8_1800,441,155,458,688,558,100,2194,104,579,152,73,315
PNKD_8_1801,226,150,683,193,0,16,966,6,7,1312,0,391
POMC_8_1802,384,226,130,252,544,6,212,133,127,7,292,152
PORCN_8_1803,572,735,596,677,766,211,209,412,207,273,183,237
PREPL_8_1804,1285,967,1098,1431,1206,374,2251,1407,804,387,702,4386
PRNP_8_1805,705,651,462,703,927,967,23,455,434,337,615,494
PRSS21_8_1806,93,124,330,214,9,47,1113,31,1,376,260,38
PRSS35_8_1807,2659,1999,2739,2104,1817,1744,4675,3271,1430,2452,4175,1
758
PSEN1_8_1808,702,979,802,450,1784,1441,378,1083,435,1385,916,983
PSEN2_8_1809,812,1253,1062,943,1993,1424,1796,922,1354,1296,2078,1142
RABEP1_8_1810,503,261,520,426,280,799,219,853,724,706,978,124
RACGAP1_8_1811,372,505,374,348,269,472,1076,396,660,890,974,473
RARRES1_8_1812,1887,1516,1529,1648,2913,1481,2346,1226,2352,1628,2228,
2164
RASA1_8_1813,3122,2741,2820,2810,4792,1327,3194,2413,4683,3253,1955,51
56
RELN_8_1814,1275,833,664,1184,1935,511,1441,1444,154,1023,1529,378
RHCE_8_1815,357,490,514,374,48,449,683,375,157,184,1220,834
RHD_8_1816,32,115,25,331,199,260,60,4,0,171,434,719
RIMS2_8_1817,512,650,572,669,1419,1175,475,640,624,345,989,563
RRBP1_8_1818,1486,1866,1612,1634,1301,2670,2699,937,1212,829,1861,1869
RUFY1_8_1819,1746,1103,1937,1169,2052,2182,872,1369,2271,937,1884,1116
S100A13_8_1820,956,652,435,1269,1088,562,281,1117,1754,787,560,2311

S100A4_8_1821,1597,1046,742,1263,1721,868,1311,1845,1221,1662,75,1439
SAA1_8_1822,1004,1116,1036,1008,1922,252,505,390,1669,1401,526,924
SCAMP3_8_1823,356,408,150,577,40,25,202,333,144,635,55,199
SCARB1_8_1824,464,868,595,591,528,301,974,1077,89,492,2891,706
SCFD1_8_1825,665,721,468,693,602,729,630,962,295,619,903,75
SEC13_8_1826,661,1117,589,373,492,183,1249,1037,1826,579,1632,1361
SEC14L1_8_1827,3244,2823,2151,3614,2705,3350,3463,3439,3583,3028,3548,
4174
SEC14L2_8_1828,1028,1164,769,947,1752,2095,1291,539,452,1019,1274,1199
SEC14L4_8_1829,1082,1081,806,847,2539,1814,710,2283,1057,1536,384,1346
SEC23B_8_1830,948,1200,896,1064,930,2066,260,1239,163,224,755,1243
SEC24B_8_1831,1523,1879,1758,1711,1910,2120,649,2211,1646,1041,2357,23
75
SEC24C_8_1832,648,631,579,750,3,2010,587,648,151,855,708,714
SEC61A2_8_1833,751,661,845,408,760,497,1129,1021,626,503,946,1680
SEC61G_8_1834,1699,1545,1347,1327,1745,980,790,2299,810,2010,2570,3017
SEH1L_8_1835,1938,1679,1176,1370,2256,588,2664,1643,1871,2545,3073,126
2
SERINC2_8_1836,193,103,371,365,17,34,342,615,319,262,14,523
SERINC3_8_1837,392,279,339,368,507,288,1093,723,562,600,296,539
SERPINA10_8_1838,2872,2680,2380,2495,4561,3022,4866,3914,1310,2757,486
2,3320
SERPINA1_8_1839,371,326,473,365,210,444,7,92,174,277,323,85
SERPINB2_8_1840,1204,1309,1173,1087,2346,1476,574,2429,906,2050,1958,1
187
SERPINB6_8_1841,5009,4430,3779,4443,5012,4832,6172,3910,6519,6243,7064
,5222
SERPINB8_8_1842,1005,902,670,986,783,421,465,1365,1017,980,411,1549
SERPINE1_8_1843,459,712,890,614,775,125,1863,1310,95,585,2560,927
SERPINF2_8_1844,127,68,276,83,23,123,2,72,608,93,577,38
SERPING1_8_1845,95,48,141,1,4,786,0,9,52,397,199,152
SERPINH1_8_1846,732,1143,933,979,1076,1440,1444,3055,190,454,183,535
SERPINI1_8_1847,1372,1774,1111,1605,1945,398,2260,692,1648,846,1668,20
03
SFI1_8_1848,676,666,230,377,1,204,72,503,546,714,695,1072
SFTPA1_8_1849,1240,1650,1133,1094,924,1691,993,768,1685,565,505,1069
SH3D19_8_1850,316,220,133,130,241,390,16,115,12,50,200,28
SIL1_8_1851,369,562,699,263,2,79,802,2232,78,327,652,835
SLC25A25_8_1852,411,413,270,415,382,1214,161,588,34,622,467,123
SLC25A36_8_1853,4015,3706,3567,3941,5615,4039,5441,2933,3019,4678,3705
,5630
SLC25A45_8_1854,518,657,426,700,945,647,2232,1891,648,670,721,376
SLC38A10_8_1855,923,439,734,577,1506,243,282,1353,649,1913,1523,849
SLC41A3_8_1856,523,823,501,545,356,553,2972,129,507,2309,252,1906
SLC43A3_8_1857,4288,3880,3649,3297,6241,2252,5217,2918,4063,4666,6187,
2908
SLC44A2_8_1858,658,956,834,689,628,848,1179,182,493,1013,863,472
SLC44A4_8_1859,1824,2391,1705,2151,1786,2073,2497,2968,2947,1948,2458,
2197
SLC44A5_8_1860,1211,1520,983,1143,238,711,259,1332,1239,1414,1855,1267

SLC46A1_8_1861,649,679,948,705,667,369,1426,327,1039,422,1431,2093
SLC47A2_8_1862,477,656,334,322,1560,506,461,644,242,939,605,555
SLC50A1_8_1863,304,228,72,193,152,466,602,577,154,89,8,238
SLC6A20_8_1864,300,213,156,362,33,357,23,693,153,674,32,669
SNAP23_8_1865,4500,4102,3738,3116,1000,5641,4747,2722,4108,4129,6050,5
861
SNAP25_8_1866,275,319,378,378,177,152,381,658,602,1041,101,231
SNX10_8_1867,3219,3733,1626,2650,1192,2156,2163,6580,2802,5295,2765,40
25
SNX11_8_1868,841,762,743,675,321,1168,1536,537,1643,1011,309,718
SNX14_8_1869,460,251,188,261,261,2239,81,178,278,167,100,436
SNX15_8_1870,83,83,0,96,0,1,0,0,0,0,890,0
SNX16_8_1871,1685,2123,2048,1572,2415,2490,1020,2509,1136,1518,3874,21
56
SNX18_8_1872,666,827,929,664,8,1946,1279,487,44,1099,1294,379
SNX1_8_1873,1159,1284,1124,1352,1503,2063,456,3598,1753,442,576,1309
SNX3_8_1874,4660,4131,3689,3780,2793,4399,4407,3692,5352,3737,6105,492
3
SNX5_8_1875,2088,1747,1328,2138,552,3171,809,1786,2448,1611,1371,1387
SNX6_8_1876,945,1173,980,1437,1313,1619,1383,2196,1207,845,1363,344
SNX7_8_1877,2049,1903,1582,2801,1273,615,2512,1565,2451,2716,2381,1247
SORCS1_8_1878,421,710,430,547,1000,1019,195,788,415,1184,9,1166
SORT1_8_1879,400,909,386,196,0,1665,514,836,46,1647,644,86
SPNS1_8_1880,2220,3349,2669,2400,1948,3493,3158,3609,2559,2876,3621,35
45
SRI_8_1881,4920,5854,4552,4280,7248,5145,5085,3566,3617,4836,6948,5717
STARD3_8_1882,1163,1218,874,1121,1066,404,1313,1802,2480,850,764,1121
STAU1_8_1883,1116,905,728,896,892,1893,523,1942,653,974,903,519
STEAP2_8_1884,1522,1589,1782,1152,3510,661,1451,2476,1069,1635,1372,18
95
STEAP3_8_1885,121,211,21,196,0,0,1,1,9,6,2,157
STIM2_8_1886,987,1196,1102,1628,1412,1305,730,1840,946,752,2792,639
STX16_8_1887,307,508,208,561,713,165,213,138,1128,1183,673,253
STX1A_8_1888,81,13,9,7,63,0,0,0,257,67,17,0
STX2_8_1889,3539,3898,3348,3211,6462,3470,2194,3189,4492,2696,6467,481
3
STX3_8_1890,911,780,554,209,293,857,470,462,649,21,301,834
STXBP1_8_1891,615,581,513,801,565,875,540,290,251,267,214,434
STXBP2_8_1892,88,118,137,55,9,296,305,170,73,131,263,201
SV2B_8_1893,4698,4460,3311,3844,4436,5769,5073,5691,3894,4723,6850,422
8
SYN1_8_1894,751,430,259,421,199,659,65,988,156,468,1427,1106
SYNGR1_8_1895,574,1018,556,706,1145,407,285,251,713,935,2209,647
SYNPR_8_1896,359,479,498,640,96,512,79,123,189,573,125,212
SYPL1_8_1897,1774,2235,1497,2113,1747,1132,2731,1766,400,2607,2412,237
9
SYT12_8_1898,646,683,322,607,798,499,935,976,338,803,1325,132
SYT14_8_1899,711,1103,1297,980,766,1937,1197,89,1111,840,548,1150
SYT15_8_1900,578,980,761,832,641,1205,47,1174,498,488,360,487
SYT1_8_1901,1431,2223,1562,2207,2487,1210,3435,3031,1400,1336,2831,190

2
SYT2_8_1902,898,851,1259,606,1121,307,1270,1033,731,571,1448,1091
SYT3_8_1903,160,258,19,162,323,5,311,12,388,12,158,187
TAPBP_8_1904,1191,1273,1139,1380,1504,869,1083,1951,1523,2169,642,2169
TC2N_8_1905,1328,1079,1275,1725,973,1692,1675,1969,1594,1530,1000,1048
TCN2_8_1906,335,347,253,398,1161,1361,0,274,126,338,87,290
TCOF1_8_1907,824,645,958,1255,702,263,847,1600,1323,827,1945,1941
TFPI_8_1908,3856,3354,2522,3278,3701,3621,3512,4340,1706,4782,5906,456
5
TFR2_8_1909,434,300,357,154,212,959,13,973,253,15,0,335
TFRC_8_1910,689,698,790,688,542,150,205,1327,1338,1654,946,435
TGFB2_8_1911,2638,2457,2066,3242,2315,4765,2511,1430,2444,3702,4315,29
66
TIMM17B_8_1912,792,1210,725,1190,1540,1275,581,1551,814,482,806,754
TINAGL1_8_1913,300,420,292,282,65,341,211,60,137,62,81,852
TLL1_8_1914,984,946,670,466,225,531,410,1788,1246,345,710,1333
TM9SF1_8_1915,1016,957,636,681,1315,709,435,1061,386,921,332,1408
TMC6_8_1916,109,116,216,192,26,135,102,318,1,25,291,1
TMPRSS11A_8_1917,4442,3933,3965,3840,2771,3822,6196,3565,4013,4368,306
4,4989
TMPRSS13_8_1918,570,261,652,650,787,624,867,119,735,899,1869,850
TMPRSS2_8_1919,252,376,229,451,1,78,444,0,86,101,366,54
TMPRSS4_8_1920,313,405,265,324,424,337,133,86,598,1635,339,38
TNFSF11_8_1921,686,1048,1139,661,715,521,885,624,456,1517,2333,985
TNFSF13B_8_1922,1874,1341,810,1491,1272,1325,1504,1574,1301,1234,1590,
1427
TNPO2_8_1923,122,141,85,72,3,387,0,3,37,36,26,68
TOM1_8_1924,183,469,107,117,495,1,0,9,0,110,207,306
TOM1L2_8_1925,1189,1106,981,1710,563,1409,949,1611,1976,1038,1396,1341
TSC1_8_1926,232,332,334,272,101,369,356,285,301,618,73,216
TSC2_8_1927,1017,1142,1049,958,231,909,278,798,1029,2803,1104,962
TUBA8_8_1928,251,442,375,365,3,251,33,318,38,0,427,734
TUBB3_8_1929,2098,1952,1758,2510,3503,1656,2543,1455,1964,2069,2420,25
29
TUBD1_8_1930,3399,3420,3429,3245,4793,3604,4189,3729,2503,3094,5855,33
61
UCP3_8_1931,108,41,29,115,0,0,0,19,0,101,0,2
UPF3A_8_1932,1253,1380,1052,1967,659,2358,1698,3105,1666,1202,2625,163
4
UPF3B_8_1933,400,243,228,151,1,598,563,4,18,228,75,551
VAMP1_8_1934,1055,857,539,1115,713,2461,4,1408,1428,848,792,1656
VAMP7_8_1935,1480,1423,1359,1366,1999,1317,2698,1003,1728,1470,1163,16
37
VCAM1_8_1936,1508,1444,1079,1123,1065,1314,3141,2625,755,1336,1976,493
VLDLR_8_1937,839,1761,894,838,2337,1149,691,840,590,1356,504,603
VPS13A_8_1938,1110,1188,1402,916,1364,1933,2482,1621,1005,1634,2375,42
77
VPS13B_8_1939,889,730,1452,730,948,103,2225,1258,958,928,425,1222
VPS16_8_1940,399,662,531,653,318,728,1708,356,623,320,216,1221
VPS26A_8_1941,436,363,416,430,604,303,433,559,329,552,289,154

VPS28_8_1942,138,190,79,122,5,27,39,28,264,395,925,0
ZFYVE16_8_1943,2464,3019,2015,2622,1884,5030,1600,746,2237,2208,3297,2
973
ZNF160_8_1944,2156,1584,1683,2163,1360,2186,2797,926,3245,1893,2527,18
92
ZP3_8_1945,1107,694,897,929,1136,419,542,1016,1452,370,847,911
BET1L_8_1946,1066,945,1138,685,498,1549,1025,1336,1112,1569,2259,668
C2orf83_8_1947,343,322,269,305,594,251,476,545,142,116,770,1263
ERP29_8_1948,2171,2945,1892,1435,2718,2180,1306,2772,1268,2835,2931,23
74
FGF1_8_1949,3718,3405,3232,4198,2524,1938,3626,3397,4922,1731,6345,544
9
LYNX1_8_1950,1008,633,669,740,1650,370,502,189,607,956,1265,308
MMP28_8_1951,4709,5196,3635,4773,5679,5446,4591,3613,8192,5423,4486,46
19
PDPN_8_1952,580,359,347,250,457,6,174,5,102,313,754,848
SNX21_8_1953,578,564,513,454,4,1199,1098,268,1147,93,689,826
TIMM8A_8_1954,321,261,314,304,379,550,454,259,248,457,351,528
VEGFA_8_1955,1,34,1,1,907,0,0,1,0,4,0,0
CDH23_8_1956,668,1103,639,1022,456,1165,770,1004,1596,688,1100,915
CDH23_8_1957,855,1051,733,833,1268,690,1186,1941,800,1207,927,1453
CDH23_8_1958,906,994,402,837,1072,333,1797,1237,954,370,776,1796
CDH23_8_1959,129,336,422,48,8,366,557,452,7,20,194,494
CDH23_8_1960,0,0,0,0,0,0,0,0,0,0,0,0
TNXB_8_1961,434,218,171,242,569,390,228,96,610,804,223,262
TNXB_8_1962,1211,777,995,737,828,542,1302,782,1419,982,1641,637
A2M_8_1963,1007,1149,1287,1000,1284,1230,990,1642,1058,834,519,907
ACE2_8_1964,2764,2395,1952,2913,1601,3850,2368,1499,3416,1950,1945,328
0
ACTR6_8_1965,3029,3250,3839,4854,3759,2020,2805,2520,2554,3398,3165,43
70
ADAM11_8_1966,478,540,482,569,703,395,1242,437,841,388,881,956
ADAM19_8_1967,1926,2048,1578,1619,1538,1131,1688,3467,1522,3248,1593,1
514
ADAM20_8_1968,1279,1573,1217,1050,2215,964,853,2320,1371,1117,853,1602
ADAM21_8_1969,818,942,892,1335,464,1058,632,1356,1198,834,2100,339
ADAM2_8_1970,2659,3055,2193,3009,2697,1945,5336,2914,1354,3890,4718,18
30
ADAM30_8_1971,4703,4234,3325,4090,3362,3818,3155,5413,3976,3137,3499,4
819
ADAM7_8_1972,572,1014,583,502,305,306,1145,762,305,993,831,1587
ADAM9_8_1973,3895,3719,3923,4342,1902,3080,4615,3028,4039,4177,6758,43
70
ADAMTS10_8_1974,543,174,349,270,246,306,86,162,331,330,618,763
ADAMTS12_8_1975,1182,1303,1157,1220,1376,1742,1118,731,366,1958,827,12
65
ADAMTS15_8_1976,1988,2076,2271,1829,1319,1109,2185,1576,3030,1764,2360
,3026
ADAMTS18_8_1977,861,730,507,966,1707,255,1075,1017,1320,1117,1443,1862
ADAMTS19_8_1978,1971,1673,1306,1750,3578,2605,1398,1958,1431,901,3102,

1139
ADAMTS1_8_1979,473,673,616,379,513,309,1403,363,291,578,305,1294
ADAMTS20_8_1980,1244,999,1651,1260,179,1758,1481,1692,367,3540,651,260
3
ADAMTS3_8_1981,550,731,424,418,298,275,878,598,494,1095,237,417
ADAMTS5_8_1982,386,572,392,443,227,1864,197,1246,773,192,128,703
ADAMTS6_8_1983,478,417,584,448,147,278,1039,423,51,73,2566,543
ADAMTS7_8_1984,4201,3230,3401,3206,3001,2495,2621,4331,3634,5770,5405,
3489
ADAMTS8_8_1985,1197,1481,919,1109,659,859,1074,2993,1390,978,2606,1458
AEBP1_8_1986,1112,1252,683,494,1980,713,1113,1384,271,196,1673,1724
AFG3L2_8_1987,1860,2095,955,1174,2051,2822,829,679,1699,2186,2997,2736
AFM_8_1988,2436,2673,1891,2565,2537,3861,2281,3388,1264,2730,3818,3420
AFP_8_1989,314,601,419,417,833,565,582,153,395,379,1800,161
AGTPBP1_8_1990,3514,4218,2820,3642,4459,5612,2846,3917,3749,3490,4434,
3524
ALG10B_8_1991,3074,2670,2809,3784,1363,4152,2717,6162,2485,3467,3746,6
332
AMBP_8_1992,416,344,129,274,351,256,15,507,210,45,71,124
ANGPT4_8_1993,161,74,43,110,1253,23,19,462,147,12,0,0
ANGPTL1_8_1994,1261,1019,990,827,939,1309,1178,1008,981,743,953,1853
ANGPTL2_8_1995,996,1006,710,1085,786,1138,693,1112,2396,1648,1415,1299
ANGPTL3_8_1996,1078,938,1456,1411,1100,2739,1670,1915,978,677,1585,853
ANGPTL7_8_1997,348,442,269,466,8,2064,721,409,183,413,493,271
ANKH_8_1998,673,660,892,1307,97,470,205,324,92,1208,1630,1074
AP1G2_8_1999,235,118,126,276,188,269,486,413,435,13,150,510
AP1M2_8_2000,590,504,329,469,337,182,229,749,922,42,766,555
AP1S1_8_2001,262,228,163,312,8,8,119,1,107,249,552,156
AP1S2_8_2002,373,337,124,288,44,345,490,1581,50,339,54,162
AP1S3_8_2003,35648,37743,32075,33681,34710,41553,40611,37375,34238,363
41,41044,39956
AP3B1_8_2004,1719,1251,973,1069,2016,1377,992,1660,391,1623,1399,1529
AP3B2_8_2005,243,275,282,174,48,433,206,659,187,178,316,380
AP3S1_8_2006,608,616,497,793,914,613,1197,614,273,132,861,624
AP3S2_8_2007,2672,3811,3096,3629,2743,3354,4723,3607,3148,4940,3723,37
71
AP4B1_8_2008,922,733,723,815,1231,404,987,643,562,441,875,612
AP4M1_8_2009,454,200,658,417,124,67,138,4,291,388,406,866
APBA1_8_2010,2938,2367,2297,2255,1924,3661,3133,1737,1745,2961,3331,24
03
APBA3_8_2011,323,137,216,362,14,220,522,1089,1087,38,627,35
APOA1_8_2012,2286,2328,1399,1734,3090,1003,2033,1968,2538,2164,2523,23
05
APOA2_8_2013,286,188,405,398,461,110,313,37,745,126,1014,79
APOA4_8_2014,262,491,153,429,104,976,20,418,304,89,402,593
APOB_8_2015,592,865,708,634,426,1193,31,726,886,342,142,177
APOC1_8_2016,2280,1675,2029,2552,1580,2510,1950,1523,1916,1226,2154,27
69
APOC2_8_2017,266,182,400,79,714,587,313,618,758,26,140,48
APOC3_8_2018,1546,982,1090,1062,1185,548,1246,788,380,464,2010,1245

APOC4_8_2019,235,295,444,430,14,500,299,736,351,139,636,139
APOD_8_2020,2880,2634,2053,2325,3083,2523,1873,1471,3461,3218,3665,249
5
APOE_8_2021,1015,877,1287,734,867,2343,1132,2083,1207,527,1244,189
APOF_8_2022,197,107,305,66,138,88,201,65,348,232,13,655
APOH_8_2023,1892,2273,1491,1555,1958,2150,1187,1137,2043,1799,2026,365
5
APOL6_8_2024,672,847,490,593,872,1239,674,355,1210,324,767,1061
APOM_8_2025,861,1034,786,1123,2105,1987,2200,3031,2521,1369,2160,194
AQP12B_8_2026,670,611,515,547,507,152,942,618,1204,758,108,366
ARF5_8_2027,3415,3650,3737,3174,2515,2975,2858,3278,4203,1651,4456,553
7
ARF6_8_2028,89,76,263,337,280,20,23,86,0,97,138,45
ARPP19_8_2029,4597,4617,4121,4711,3383,7133,5401,6822,3715,4475,3360,5
411
ASTL_8_2030,744,835,548,601,233,192,676,158,268,1096,354,1443
ATOX1_8_2031,1100,2056,1579,1330,1478,2147,1053,613,1340,995,617,1226
ATP13A1_8_2032,0,7,0,0,40,0,0,0,0,0,0,0
ATP13A3_8_2033,1492,1912,1617,1887,1582,1153,2149,1471,2095,1334,2229,
2166
ATP13A4_8_2034,2763,2987,2814,2604,1860,3131,3517,2578,2349,2472,4086,
4170
ATP13A5_8_2035,2053,2140,1392,1485,1018,1252,2178,3096,1209,2574,488,4
95
AZGP1_8_2036,3189,2205,3178,3140,2724,2919,1015,4351,2557,1645,2989,11
21
AZU1_8_2037,791,893,824,1113,762,558,114,966,867,804,25,291
BCL2L10_8_2038,2079,1504,1038,2071,2486,1984,959,2715,2106,1011,2434,2
599
BET1_8_2039,1031,1746,743,1112,1196,579,1426,736,2099,607,252,1865
BGLAP_8_2040,140,121,82,36,23,69,37,15,83,440,34,27
BOC_8_2041,473,409,560,366,81,272,1169,299,504,725,592,956
BPI_8_2042,780,887,838,709,744,1051,1622,646,202,1086,782,1704
BPIFC_8_2043,114,49,116,96,0,0,36,3,2,1040,73,1
C16orf7_8_2044,319,273,87,282,9,394,1,442,1,128,15,28
C1orf162_8_2045,732,1002,536,981,1741,1096,600,277,1314,702,1067,283
C1QA_8_2046,279,124,83,250,101,35,336,7,124,179,206,1000
C1QB_8_2047,355,349,216,343,570,202,106,268,335,143,69,1329
C1RL_8_2048,393,600,470,265,261,1106,538,372,162,973,261,1142
C20orf141_8_2049,478,94,232,271,1262,523,689,620,1492,44,726,43
C3_8_2050,666,694,1373,995,1987,2285,1874,1262,560,720,780,2128
C4A_8_2051,1813,1945,1143,2023,21,1294,1459,569,2966,1047,1883,2843
C5_8_2052,1231,1717,1231,1491,1077,511,2959,1470,1511,997,2155,1263
C7orf31_8_2053,673,651,393,342,1093,374,1207,515,463,230,730,1432
C8G_8_2054,1156,1155,1191,1096,951,273,1063,923,997,1698,1737,1356
CALM2_8_2055,1738,1595,1988,2416,2084,976,2422,1821,2065,943,2370,1932
CALY_8_2056,166,77,203,122,138,46,41,5,16,70,87,33
CAMLG_8_2057,340,471,416,509,419,538,498,569,385,160,264,871
CAPN11_8_2058,1243,1186,1139,1171,1703,1684,969,1996,1739,420,1434,239
2

CAPN5_8_2059,632,744,1071,941,16,1357,683,325,379,1179,956,784
CAPN6_8_2060,993,1222,669,1730,698,1838,976,1853,698,1139,2457,1666
CARTPT_8_2061,677,804,613,358,828,209,986,618,825,1096,972,832
CCL13_8_2062,7448,6971,5766,8069,7881,9090,8492,6553,6567,8178,10615,8
056
CCND1_8_2063,156,185,128,124,30,0,1,0,0,125,51,1249
CD1A_8_2064,1340,1517,1248,1502,2046,194,1636,1879,1171,949,952,2154
CD52_8_2065,1813,1591,1981,1729,2096,2144,1266,2272,2330,1851,804,3040
CDCP2_8_2066,261,229,147,70,1,87,558,9,17,514,17,12
CDH5_8_2067,953,1173,790,1197,273,2510,2418,1568,799,2253,372,1664
CHMP7_8_2068,1383,1064,1254,1394,1507,1057,637,413,1062,989,1235,1752
CLDN16_8_2069,3557,3128,2793,2672,3937,4630,4609,4001,3334,2996,2428,1
719
CLEC3B_8_2070,1314,1738,484,703,1776,1127,784,1749,931,464,368,1283
CLSTN2_8_2071,470,540,424,956,397,266,917,328,413,869,640,398
CLVS2_8_2072,1131,1038,998,699,522,970,2033,1143,710,1063,943,1147
CNIH3_8_2073,201,148,91,59,303,608,0,51,97,142,1007,5
CNOT6_8_2074,304,546,173,381,296,117,140,466,69,15,272,276
CNTNAP1_8_2075,630,675,879,624,509,491,817,755,522,1222,1215,1192
COG1_8_2076,518,697,637,367,83,447,144,1183,73,578,178,254
COG3_8_2077,347,575,226,361,0,545,213,205,864,643,177,194
COG7_8_2078,613,1254,1102,1017,1264,2581,976,2268,1196,687,2795,729
COG8_8_2079,3966,4515,3588,5026,2645,5733,6448,7099,4039,4635,5990,638
3
COL10A1_8_2080,894,1010,636,1031,1215,387,718,391,1380,1395,1631,1816
COL14A1_8_2081,5058,6126,4378,5772,6289,4334,6180,7129,4762,6956,4449,
8221
COL15A1_8_2082,913,1310,935,1206,1335,768,121,690,571,491,409,1894
COL16A1_8_2083,1301,834,688,737,698,900,1212,1725,1065,1362,681,847
COL17A1_8_2084,1435,1388,1414,1675,434,779,1695,1234,1510,1077,1928,16
58
COL1A1_8_2085,706,836,524,732,708,1124,2683,161,1024,706,311,728
COL1A2_8_2086,1104,1125,1362,798,98,810,1820,1317,612,366,608,932
COL21A1_8_2087,2026,938,1257,1713,1766,463,772,1503,3358,1623,2494,160
3
COL22A1_8_2088,2315,3236,2533,2530,4247,4638,1468,2171,2973,1946,2346,
3121
COL23A1_8_2089,529,820,780,764,1866,475,954,664,895,761,428,186
COL24A1_8_2090,1955,1506,2206,1735,1646,1985,3544,2191,1576,1981,2708,
3149
COL27A1_8_2091,663,608,268,318,62,313,213,61,910,122,857,200
COL3A1_8_2092,1086,1106,1372,877,1504,754,1585,361,1194,1103,1879,1189
COL4A1_8_2093,570,1002,259,248,37,335,652,829,220,858,1015,168
COL4A2_8_2094,521,736,668,524,427,1139,1886,515,153,350,693,1854
COL4A3_8_2095,921,877,1028,682,2248,465,467,1106,847,561,860,426
COL4A4_8_2096,1051,930,1249,1114,451,983,4109,489,4165,2080,1210,1067
COL5A1_8_2097,644,591,960,923,735,645,1075,525,781,2,1049,490
COL5A2_8_2098,16,4,93,44,85,62,129,10,1,1,70,0
COL5A3_8_2099,1295,940,800,583,1163,2138,635,1260,583,578,2682,1510
COL6A1_8_2100,503,904,566,756,493,1401,328,1550,141,735,479,345

COL7A1_8_2101,783,1062,798,693,167,450,1955,700,1850,962,1058,2112
COL8A2_8_2102,474,686,664,362,1375,808,332,891,589,415,994,102
COL9A2_8_2103,489,778,591,332,632,856,741,379,420,413,967,368
COL9A3_8_2104,685,576,888,1203,142,1105,548,185,447,775,94,1680
COMMD1_8_2105,765,646,502,967,1516,588,703,866,417,867,1008,652
COPB2_8_2106,1149,664,646,684,2351,1032,1000,1184,1988,2130,1324,865
COPG2_8_2107,1786,1757,1180,1471,2383,1771,1122,1484,2644,1068,2117,81
6
COPZ1_8_2108,424,517,346,551,725,927,1338,818,311,232,910,1031
COPZ2_8_2109,1246,635,461,899,667,1813,1463,125,724,613,1486,899
CORIN_8_2110,284,223,211,315,147,289,147,11,586,637,7,92
COX18_8_2111,2053,1683,1447,1684,453,1589,1318,2404,1343,1340,1240,222
7
CPLX1_8_2112,221,470,499,228,14,34,37,480,293,476,28,226
CPLX3_8_2113,582,513,235,68,141,784,981,1,2,459,619,151
CPNE6_8_2114,1477,2142,1431,1308,1437,2529,799,1532,948,2452,2816,1394
CPXM2_8_2115,1457,1429,1228,1601,3024,1425,1835,1243,1340,723,2541,164
1
CRABP1_8_2116,1075,1093,1138,745,1987,1081,1139,227,1879,675,1763,520
CRH_8_2117,886,376,914,732,165,298,22,520,618,426,480,1885
CSE1L_8_2118,11647,12676,10706,11773,13462,12465,12176,13424,13644,145
46,12979,11902
CTSW_8_2119,244,676,296,364,2,23,47,445,109,119,785,1031
CXCL10_8_2120,730,523,558,473,333,272,218,266,414,910,1167,447
CYGB_8_2121,255,429,859,188,1070,63,598,472,9,209,174,0
CYTH3_8_2122,929,850,488,497,1673,1090,694,1008,575,582,452,1512
DDI2_8_2123,5186,5135,4257,5009,4881,4002,6131,5289,5689,5885,8020,635
1
DIRC2_8_2124,382,494,538,658,383,311,178,897,904,1166,1636,162
DISP1_8_2125,4132,3225,3400,4096,5096,4708,5373,3365,4162,3774,6320,52
74
DLL4_8_2126,1631,1464,1417,1513,1210,1210,760,1125,1836,1397,732,1529
DNAJC5B_8_2127,1824,1956,1356,1614,1713,1801,2527,2685,386,1061,3340,3
161
DNAJC6_8_2128,3408,4105,3761,3699,2716,3667,4279,3162,3077,2625,4710,2
542
DOC2A_8_2129,556,611,824,129,24,299,621,895,1113,1046,1152,2520
DOC2B_8_2130,596,381,327,404,231,84,437,1330,422,696,214,569
DSCAML1_8_2131,839,967,711,873,1032,358,1488,106,1265,524,938,870
ECEL1_8_2132,300,164,113,186,1312,462,7,24,24,512,129,115
EID2_8_2133,2635,3327,2760,3068,2377,2442,4088,5514,2270,4079,4921,673
0
EPCAM_8_2134,3754,3187,3953,3917,5065,6459,3191,3781,3007,3580,4792,72
33
EXOC2_8_2135,2882,3585,2282,3069,4582,2239,3080,3828,5151,3720,2026,30
81
EXOC3_8_2136,1123,1044,847,948,964,1010,2954,374,1427,282,2639,600
F11R_8_2137,471,207,466,534,209,395,606,932,363,221,575,1141
FABP1_8_2138,225,336,206,97,75,700,31,81,855,475,71,101
FABP2_8_2139,1412,1391,672,1086,3179,1290,1516,1735,1170,1115,1646,110

2
FABP3_8_2140,796,772,209,363,499,25,1042,320,414,543,348,487
FABP4_8_2141,1143,1465,936,1070,684,737,1606,267,1271,1853,1016,1326
FABP7_8_2142,288,350,182,182,2,181,3,53,123,0,0,2
FABP9_8_2143,493,771,458,644,1167,1108,829,738,640,382,164,118
FAM101A_8_2144,1111,895,1101,1494,2641,1614,1593,816,772,565,1270,474
FAM117A_8_2145,2519,2424,2437,3183,1784,2465,2776,4447,1895,1459,3687,
2934
FAM57A_8_2146,2134,1983,1873,1701,1400,1873,2086,2454,1004,1797,873,24
11
FAP_8_2147,135,586,328,266,1207,317,200,445,35,780,0,13
FBF1_8_2148,132,77,74,227,46,169,668,209,45,86,75,189
FCN1_8_2149,3645,3971,3359,3428,5436,4883,4330,3109,3221,3898,3829,674
4
FDX1_8_2150,622,335,404,410,8,690,46,493,662,1235,194,404
FDX1L_8_2151,250,623,277,432,114,119,503,791,5,153,65,121
FGF4_8_2152,221,288,9,136,9,41,7,0,0,169,0,595
FGL2_8_2153,170,338,105,253,578,93,97,31,43,17,81,602
FOLR3_8_2154,165,281,133,273,19,49,742,17,120,123,168,8
FOLR4_8_2155,989,876,685,1159,1261,1410,2168,1583,960,1088,2601,713
FRG1_8_2156,367,693,323,396,266,297,158,1536,30,672,431,1470
FTL_8_2157,3534,2963,2812,3316,3019,4425,3373,2862,3770,3018,3628,3542
FXC1_8_2158,523,784,725,561,245,843,1436,292,558,581,529,430
GABARAP_8_2159,4880,3837,4152,4131,6042,4128,4217,4574,4213,5151,5617,
3669
GGA2_8_2160,407,319,145,222,792,320,276,361,455,229,700,557
GJA1_8_2161,2181,1766,1952,2152,2925,1204,2959,1766,2918,1691,1558,355
2
GJA3_8_2162,1574,1048,1677,1035,1125,1087,1890,1970,1606,1072,1923,150
1
GJA4_8_2163,1134,1648,779,1313,752,391,777,591,957,1177,704,1329
GJA8_8_2164,2037,2261,1894,1754,452,3412,2169,1716,3228,3659,2700,2660
GJB2_8_2165,1204,1289,1664,1080,1508,2039,2952,15,1370,1188,2201,1818
GJB4_8_2166,260,459,241,252,541,340,196,521,488,482,390,874
GJB5_8_2167,649,451,529,733,1651,910,694,1606,492,1399,1062,190
GJC2_8_2168,701,390,549,426,1340,155,1140,2,198,756,854,208
GJC3_8_2169,623,369,884,928,778,1082,1557,1846,647,540,578,2205
GJD2_8_2170,3374,2808,3621,3538,2960,3748,3618,2581,3334,4009,5423,257
6
GJD3_8_2171,731,1287,654,555,66,909,273,378,1104,1142,719,1375
GJD4_8_2172,4470,4525,4842,4377,5802,4191,3393,2560,6367,5450,7271,307
8
GKN1_8_2173,2961,2904,2747,2854,2534,5000,4804,2512,4408,3136,1812,243
6
GLCCI1_8_2174,897,1188,728,1039,631,1573,1291,1142,864,1252,1409,1070
GLTP_8_2175,417,689,626,308,447,844,609,44,356,62,376,1045
GLYATL2_8_2176,462,968,391,364,946,694,635,1600,270,492,342,434
GP9_8_2177,171,124,168,43,34,144,27,164,35,164,1,41
GPIHBP1_8_2178,476,571,551,475,565,771,310,890,219,696,1728,965
GPR180_8_2179,1570,1576,1684,1845,1508,1776,1701,2339,2924,1925,1652,2

219
GRN_8_2180,266,392,286,300,157,568,63,127,573,344,206,354
GZMH_8_2181,1164,1636,770,1098,2248,1574,1481,1636,793,878,609,1367
GZMK_8_2182,3023,2827,2948,2008,4063,2054,2628,1216,4211,2137,3738,396
1
GZMM_8_2183,247,422,273,202,7,166,14,128,395,151,777,94
HBA1_8_2184,438,554,489,741,640,88,586,1199,1264,241,370,1812
HBA2_8_2185,438,554,489,741,640,88,586,1199,1264,241,370,1812
HBE1_8_2186,1883,2202,2033,2354,2254,2306,1422,1829,1426,959,2832,979
HBZ_8_2187,336,171,220,153,155,86,291,7,711,117,439,165
HECA_8_2188,3357,2863,2226,2976,3449,2749,3124,4837,2689,3344,1866,138
4
HGFAC_8_2189,306,505,263,92,555,135,686,5,178,555,323,160
HIAT1_8_2190,2095,2677,1471,1602,1595,2429,1531,1552,1308,2144,963,151
5
HLA-DQB1_8_2191,290,475,127,232,80,407,230,203,234,349,220,200
HMCN1_8_2192,1113,1261,615,796,827,1649,1335,1194,404,551,1816,696
HMHA1_8_2193,640,700,361,637,702,594,8,251,760,1477,409,2136
HPCAL4_8_2194,287,407,680,340,112,941,83,441,162,126,1036,20
HPR_8_2195,1239,1236,1468,1326,2228,1573,1624,1290,3041,910,2203,1626
HPX_8_2196,175,130,45,30,4,119,166,0,496,0,241,5
HSP90B1_8_2197,4220,4449,4085,4596,5506,6375,6854,3704,4857,5944,5032,
2799
HTRA1_8_2198,629,842,831,768,755,540,1010,723,594,1001,1055,387
HTRA4_8_2199,1536,1692,1416,1405,2066,2131,2929,1433,1398,2290,1820,23
43
IFNG_8_2200,364,573,561,177,555,739,1110,256,71,390,1925,921
IGFBP7_8_2201,4077,4215,3587,4247,2809,3623,3391,2933,3246,5178,4400,2
450
IL12B_8_2202,129,133,524,53,861,41,140,112,25,116,543,27
IL13_8_2203,1193,1018,1530,1119,1513,1233,667,1132,1864,1693,866,1725
IL17A_8_2204,1450,2088,2053,2101,659,2974,3485,2207,2049,1687,2296,780
IL1A_8_2205,1644,1203,1216,1244,1933,1598,1651,648,295,1460,1129,1111
IL1B_8_2206,1128,982,1249,1101,492,1018,1983,2118,1406,1536,1786,1694
IL3_8_2207,1517,1493,1632,1322,909,2925,1292,561,1104,1974,2615,1731
IL5_8_2208,315,336,238,181,165,328,641,32,356,258,138,543
INSL3_8_2209,1079,1132,690,1283,1406,860,811,1398,871,969,2222,1999
IPO13_8_2210,373,315,309,195,872,65,0,751,342,142,102,115
IPO4_8_2211,355,448,295,471,41,785,855,6,457,387,454,83
IPO5_8_2212,2241,2599,1729,2349,2333,984,3132,1848,1682,2255,1747,3433
IPO7_8_2213,2937,3740,2847,3595,2410,2046,3215,3407,1314,3574,3904,437
3
IPO9_8_2214,1371,816,825,955,1804,666,274,441,2009,1451,680,769
ITGA10_8_2215,775,437,548,856,708,392,872,873,480,397,942,1405
ITGA11_8_2216,912,619,801,1195,1451,779,1077,607,392,601,942,630
ITGA2_8_2217,474,321,233,265,832,104,15,248,981,290,1,4
ITGA4_8_2218,3098,3726,2773,2415,3041,2348,3158,4008,2483,2473,4578,30
28
ITGA5_8_2219,279,367,178,378,44,100,283,745,624,287,1565,412
ITGA8_8_2220,1163,915,454,806,1170,1785,530,378,1521,837,790,980

ITGAX_8_2221,352,403,679,90,105,21,535,385,540,221,163,209
ITGB5_8_2222,454,658,444,337,381,1231,177,1626,280,955,585,804
ITGB6_8_2223,674,959,942,644,162,635,1760,981,1011,1103,807,1894
ITGB8_8_2224,6281,5510,5392,6087,4043,8173,8947,7600,5432,8791,8668,47
74
ITLN1_8_2225,418,687,409,428,64,870,282,589,71,29,773,281
KDELR1_8_2226,560,495,693,612,381,45,1283,707,518,211,83,1065
KEL_8_2227,1745,1940,1721,1983,2597,2498,560,1452,698,2275,2303,1371
KIF20A_8_2228,831,987,698,1128,790,847,1921,699,482,870,980,1315
KIF3B_8_2229,57,69,188,71,28,0,530,1,29,27,1,2
KIF5A_8_2230,2069,1653,2031,2066,1218,1467,2739,224,404,1476,2915,1877
KLK13_8_2231,242,343,83,104,71,0,15,0,267,49,349,375
KLK14_8_2232,5340,5856,4932,4977,4653,6161,3485,3554,2899,5616,6172,78
61
KLK4_8_2233,913,883,997,795,1168,994,1303,720,953,1190,856,925
KLK9_8_2234,2012,2179,1579,2503,1631,2477,987,1784,1330,3759,2535,1242
KPNA1_8_2235,1714,1250,1537,1082,2816,2192,2234,2653,1792,475,3511,174
3
KPNA2_8_2236,1786,2164,1581,1346,689,1819,842,1630,1824,1840,460,1329
KPNA3_8_2237,555,392,416,571,928,760,45,1140,991,950,279,1986
KPNA4_8_2238,2252,2098,2392,1832,2967,1890,3072,1057,2139,2930,2187,18
30
KPNA6_8_2239,683,1111,677,1237,811,997,1501,1250,158,747,855,1836
KPNB1_8_2240,312,421,609,601,235,250,642,574,205,403,409,377
KRT12_8_2241,276,317,302,232,160,191,317,307,393,372,606,117
KRT7_8_2242,382,650,269,601,50,106,214,4,307,218,684,1796
KRT8_8_2243,190,218,149,200,58,883,997,286,134,114,307,663
KRTAP5-4_8_2244,359,481,847,292,54,177,185,473,29,706,31,58
LASP1_8_2245,1155,1326,1091,1516,1463,3268,1177,1092,1310,1545,1781,12
85
LBP_8_2246,560,535,577,615,806,372,0,742,584,1002,115,178
LCN12_8_2247,259,243,399,326,237,329,645,112,101,11,0,129
LCN1_8_2248,3487,4850,2908,3228,2585,3689,6546,3716,4466,4722,7595,249
1
LCN2_8_2249,475,434,499,606,350,410,1535,832,339,428,549,718
LCN8_8_2250,559,328,400,386,1246,314,421,237,1194,603,56,624
LCN9_8_2251,5983,6043,5763,4940,6256,4809,4296,3407,5371,8623,8099,767
3
LDLRAD2_8_2252,63,35,9,96,0,4,843,9,511,15,44,1
LDLRAP1_8_2253,252,607,146,385,184,274,447,153,473,637,277,708
LMAN1_8_2254,4200,4803,3841,3833,7957,3648,4348,5507,3960,6170,4713,52
62
LMAN2_8_2255,2580,3200,1961,1722,1836,998,2802,1056,1874,2208,3726,334
4
LPA_8_2256,2009,1760,1704,1389,1011,1754,2311,2812,2253,2685,2103,2098
LRIG1_8_2257,537,339,209,402,232,1005,340,1148,657,930,225,1334
LRP2_8_2258,2660,2408,2564,2483,2896,2903,4029,3474,1585,3135,2497,229
0
LRRC4B_8_2259,585,668,405,647,966,323,9,580,90,1114,74,219
LRRCC1_8_2260,1411,863,1681,1389,1531,1387,306,1124,1636,927,2135,1638

LYST_8_2261,2343,2630,2302,2656,3458,3691,1743,3760,2863,3508,2198,238
9
MAL2_8_2262,7600,7798,8131,7324,9356,11313,6199,6784,10335,10267,7223,
8959
MATN3_8_2263,1534,2308,1791,1481,896,823,764,1029,1565,1442,1356,2355
MFSD8_8_2264,3084,2358,1901,2621,5738,1373,4705,2923,3379,2622,2876,31
60
MFSD9_8_2265,1071,1181,1267,1282,1247,664,522,1143,508,1011,1048,580
MMAA_8_2266,147,429,155,318,1076,266,199,411,549,626,322,94
MMACHC_8_2267,102,23,80,0,4,1,0,226,51,7,42,118
MMGT1_8_2268,1589,2500,1115,940,2156,816,798,1018,1815,1411,910,1457
MMP11_8_2269,837,1232,659,1007,1218,709,1594,1178,1147,2259,812,1325
MMP13_8_2270,2777,2031,1567,1860,1825,1792,1002,2004,4153,2205,1695,28
79
MMP15_8_2271,317,129,132,341,1,568,143,522,12,124,1198,812
MMP16_8_2272,3749,4255,2553,3270,3696,3553,1884,2235,2986,6179,5336,51
83
MMP17_8_2273,485,519,271,390,122,657,776,719,682,294,1229,433
MMP19_8_2274,2048,2020,1901,2203,2138,1785,1586,2639,2079,1818,2847,22
07
MMP24_8_2275,222,142,175,244,874,158,163,107,0,214,99,55
MMP25_8_2276,556,894,541,566,455,1125,1401,1037,79,469,271,1095
MMP26_8_2277,959,977,927,962,835,1524,807,836,841,1731,815,1576
MMP27_8_2278,2386,3296,1765,1814,4532,3736,1769,3090,889,2707,1119,326
7
MRS2_8_2279,1169,1177,564,988,1539,785,279,1415,636,1218,899,1393
MSTN_8_2280,2372,2592,1646,2596,2086,1596,4051,2247,2919,4409,2687,335
5
MTX2_8_2281,1440,1648,1360,942,1249,1487,849,1900,1092,1907,2572,915
MUC2_8_2282,856,991,688,677,261,1208,463,1086,1860,1510,1639,580
NAPG_8_2283,265,370,478,291,405,520,15,489,332,252,45,644
NAPSA_8_2284,441,467,844,276,6,274,29,18,861,17,32,416
NCOA5_8_2285,1728,2136,1334,1568,1278,1455,1969,1018,2345,2375,2079,13
22
NGF_8_2286,7024,7757,6268,7082,3478,7195,6682,6660,6020,7257,7169,8765
NID1_8_2287,1349,1044,1612,1729,1292,1083,2282,2219,1344,1203,1997,232
8
NPC1_8_2288,1335,1893,1745,1858,1920,2294,3357,1521,1441,2537,2078,305
6
NPEPPS_8_2289,372,221,156,228,2,291,246,10,114,187,328,2
NPPB_8_2290,446,363,495,246,639,259,335,116,223,51,30,622
NPY_8_2291,1097,1162,1025,1247,2425,948,1188,1227,1105,1819,1698,2379
NSMCE1_8_2292,654,394,477,674,226,482,373,273,1203,986,1323,408
NUP107_8_2293,2134,1856,1589,2252,2084,1775,1562,4469,2138,1844,1930,1
656
NUP133_8_2294,5166,4989,4650,3983,6777,4369,6216,6744,5628,6489,6217,6
266
NUP153_8_2295,1342,1719,1507,1578,1085,2815,1724,2024,1270,865,2683,25
77
NUP160_8_2296,1109,985,800,700,1297,2106,741,669,1075,1477,1156,536

NUP210_8_2297,301,229,129,362,649,213,535,1010,196,41,114,621
NUP214_8_2298,1230,1100,1456,889,404,357,879,72,2723,1723,1526,1496
NUP35_8_2299,1065,768,775,620,846,1032,668,920,1177,1133,200,580
NUP37_8_2300,1472,1351,870,1416,1968,1870,574,541,1002,2917,1206,3030
NUP54_8_2301,1633,2342,1530,1199,1658,1824,369,919,1485,3571,1804,1098
NUP88_8_2302,284,169,153,106,192,160,217,965,357,33,121,39
NUPL2_8_2303,533,480,854,842,745,301,1401,1331,1048,787,459,325
NUTF2_8_2304,1483,1250,1084,1634,2550,2301,2740,1559,1688,1873,2138,36
17
NXF2B_8_2305,6249,6624,6384,6955,10530,7285,7089,6636,6185,5670,7983,7
528
NXF2_8_2306,6249,6624,6384,6955,10530,7285,7089,6636,6185,5670,7983,75
28
NXF3_8_2307,354,569,566,554,489,1257,74,564,907,603,517,16
NXT1_8_2308,883,854,587,751,235,757,3213,824,242,199,2122,3086
OAZ2_8_2309,851,688,449,766,503,2311,534,670,1023,605,1272,1237
OBP2A_8_2310,1225,1092,846,1268,1226,2102,851,796,1845,1169,2613,1673
OBP2B_8_2311,3329,3218,3752,3865,4228,1906,5859,3382,3858,3897,5220,38
42
OCA2_8_2312,304,147,297,250,1212,483,230,128,306,310,713,893
OGFOD1_8_2313,4365,4729,4314,3547,5075,2161,4176,5455,2459,4180,6543,5
428
OGFOD2_8_2314,918,689,610,671,1482,764,573,581,957,327,115,288
OGFR_8_2315,682,429,295,329,971,677,700,282,801,503,779,432
OVCH1_8_2316,1590,1786,2108,1599,3824,1902,1042,1756,1114,828,1748,177
6
OVCH2_8_2317,3101,3633,2480,2542,4075,3033,4449,1315,1572,4815,3832,26
73
OXNAD1_8_2318,1535,1781,1022,941,3118,2304,1873,1767,594,1304,870,1248
OXT_8_2319,467,452,796,742,1402,872,467,976,436,327,909,583
PANX1_8_2320,2404,1812,1990,1938,2591,2199,879,588,1550,1924,2025,1154
PAQR7_8_2321,1408,1704,1252,1532,1852,2567,907,2804,1155,875,1082,1530
PCDHB11_8_2322,1010,786,890,719,1385,361,2186,855,611,1506,1668,651
PCDHB16_8_2323,790,890,1111,759,1161,1048,2265,281,952,765,1641,1614
PCSK4_8_2324,704,1286,473,894,1181,1222,1570,151,6,1040,1227,62
PCSK7_8_2325,1327,760,854,1075,1132,1398,861,1076,271,1007,1236,3287
PEA15_8_2326,1334,1654,1536,1200,1093,1967,2268,1256,2244,615,1356,306
6
PET112_8_2327,460,138,252,286,14,28,536,21,166,70,188,90
PEX13_8_2328,3051,2024,2662,3048,3342,2565,7008,2544,3416,2982,2773,49
03
PEX7_8_2329,573,432,212,901,118,1141,1110,257,710,715,538,799
PF4_8_2330,136,6,75,171,551,39,270,5,0,0,209,125
PFN3_8_2331,139,52,49,56,348,120,11,107,59,185,4,73
PHEX_8_2332,14,2,0,0,0,0,0,0,0,0,0,0
PIGR_8_2333,101,27,16,154,673,10,125,20,370,252,0,594
PITPNA_8_2334,917,961,1309,1275,209,1064,553,627,859,1252,1394,1134
PITPNB_8_2335,5608,4924,5196,4653,3806,4494,5446,8931,4450,5243,8876,6
101
PLLP_8_2336,668,722,350,524,311,527,35,233,2013,438,454,986

PLP2_8_2337,1161,1462,1486,1322,784,546,1174,1742,1188,1412,1170,1952
PLXNB2_8_2338,827,714,1118,678,1480,564,1176,907,853,1464,1138,775
PNMA2_8_2339,1001,712,793,700,779,1445,202,437,719,538,546,1705
PPP1R14A_8_2340,2550,2553,2522,2084,2906,2721,2352,2139,3308,2702,2372
,3020
PPP1R14C_8_2341,681,850,806,617,818,575,1561,688,1194,991,2781,390
PPP1R15A_8_2342,315,243,457,287,15,501,266,43,763,478,684,223
PPRC1_8_2343,195,195,362,345,55,25,918,5,108,181,1780,79
PPY_8_2344,1001,612,722,943,1682,1102,517,1644,1654,1339,1557,1008
PRB3_8_2345,386,319,209,575,632,396,9,226,93,856,337,781
PROCR_8_2346,134,258,92,170,196,82,129,1,105,119,27,242
PROS1_8_2347,1856,2541,2561,2286,3252,3405,2002,1639,3035,2766,4988,32
39
PROZ_8_2348,102,147,238,115,2,265,16,173,0,465,157,64
PRPF18_8_2349,742,908,791,899,1192,593,1726,910,388,1219,696,258
PRSS12_8_2350,6096,6168,5243,4550,5110,6652,8612,5147,6123,3495,5298,3
633
PRSS22_8_2351,576,533,239,519,217,348,141,623,1014,330,505,395
PRSS27_8_2352,1125,813,958,1286,824,2228,397,1092,654,719,356,700
PRSS33_8_2353,186,145,160,153,350,133,409,748,765,416,149,0
PRSS36_8_2354,309,343,417,97,163,1161,1,67,828,66,1410,41
PRSS8_8_2355,576,669,330,522,2067,194,130,831,65,924,93,218
PSCA_8_2356,697,749,755,663,794,333,2066,464,652,906,756,399
PTOV1_8_2357,923,1036,677,818,1829,809,416,1438,1177,781,576,1245
RABIF_8_2358,7794,7429,7584,8004,7661,9694,8397,9445,5815,7673,8423,15
872
RAMP1_8_2359,137,468,324,181,6,226,318,566,98,251,235,315
RAMP2_8_2360,539,502,623,587,673,490,269,317,836,1519,904,1017
RAMP3_8_2361,300,278,190,339,35,37,411,348,43,189,256,35
RANBP17_8_2362,576,771,447,754,202,1252,188,1451,970,568,1014,666
RASSF9_8_2363,642,775,521,771,767,1148,356,1160,1311,616,885,934
REEP5_8_2364,1525,1212,1062,1904,1406,1102,1832,128,2372,1204,3054,821
RHAG_8_2365,3703,3002,2900,2493,4423,4183,2578,3235,4072,3469,1763,545
7
RLBP1_8_2366,347,758,629,670,1474,1118,1616,2175,293,469,760,808
RPL15_8_2367,672,587,619,241,277,691,1067,448,444,397,604,877
S100A12_8_2368,2507,2501,2525,2074,2698,1288,1150,2345,2345,2469,1993,
1702
S100A1_8_2369,928,1217,1200,1225,680,1279,533,3353,712,599,669,1861
S100A2_8_2370,1990,2432,1318,1844,1053,2620,2333,3586,1374,2755,4136,3
346
S100A3_8_2371,394,36,136,539,1409,284,0,967,434,304,263,39
S100A6_8_2372,436,866,302,714,20,55,930,1805,473,149,92,50
S100B_8_2373,771,1176,823,834,537,1074,752,188,371,1121,1391,505
S100P_8_2374,221,265,248,141,682,392,176,1413,28,3,2,0
SAA4_8_2375,1614,1899,1847,1293,1267,1479,680,2467,769,2890,4690,2715
SCAMP1_8_2376,9396,9192,8401,10487,11377,10706,10174,8963,10146,12459,
13635,10490
SCAMP2_8_2377,409,195,458,477,751,175,35,411,532,482,969,570
SCFD2_8_2378,531,774,507,744,955,730,233,187,102,389,1348,553

SCLT1_8_2379,1338,850,1262,1133,1110,2364,1151,1048,320,993,1198,1318
SCPEP1_8_2380,788,921,833,419,250,41,670,464,632,559,345,413
SDC2_8_2381,838,561,748,901,1065,522,599,1209,1006,364,1924,413
SEC14L3_8_2382,3774,4027,4210,3994,2789,3608,4383,5433,3241,5422,4526,
2947
SEC22A_8_2383,1939,1245,1085,1467,1480,2418,2311,2216,1802,2036,1406,1
632
SEC23A_8_2384,1422,1677,1553,1961,1827,1616,2372,1050,1758,1969,2138,1
578
SEC24A_8_2385,2273,2068,2300,2213,3756,3111,3759,1656,984,1944,2848,41
35
SEC24D_8_2386,2429,2075,1682,1302,1910,2885,1574,2014,1384,2481,1437,9
47
SEC61A1_8_2387,291,363,103,358,16,321,122,229,25,1061,408,256
SEC61B_8_2388,904,813,1135,925,1269,335,1518,1666,2138,1235,1483,2694
SEC62_8_2389,1027,1873,1099,1125,1333,1117,739,662,1184,1323,2367,2307
SEC63_8_2390,204,306,233,452,38,268,49,719,101,1148,370,78
SELP_8_2391,0,0,0,0,0,0,0,0,0,0,0,0
SERINC1_8_2392,751,1412,997,867,598,1999,784,251,1126,906,2430,701
SERPINA11_8_2393,1774,2602,1866,1461,2388,3163,4707,2057,4420,2048,273
5,2904
SERPINA12_8_2394,4074,3974,4219,3645,1790,5021,2171,4624,4393,4064,516
7,4433
SERPINA3_8_2395,702,602,413,885,2042,1486,1502,436,845,447,248,723
SERPINA4_8_2396,338,161,269,229,122,254,409,1,2,829,47,180
SERPINA5_8_2397,659,702,1007,961,1890,2618,1144,817,1317,788,1167,946
SERPINA7_8_2398,1207,912,1285,1253,2261,998,1403,651,1928,1114,2095,85
7
SERPINB10_8_2399,538,866,465,492,184,1090,16,587,620,454,212,1266
SERPINB12_8_2400,1059,532,771,1050,3377,950,1013,1924,2177,1505,2058,7
39
SERPINB13_8_2401,2222,2113,1684,2290,1344,3902,3469,1300,1500,1703,237
5,2920
SERPINB1_8_2402,990,858,1184,1235,596,980,2291,2005,2659,1306,2550,674
SERPINB4_8_2403,943,888,603,1113,273,1362,884,1347,1223,1969,275,584
SERPINB9_8_2404,1087,748,742,1017,1678,739,1726,478,202,1494,494,664
SERPINC1_8_2405,1560,1133,1217,1202,1841,1114,1860,2431,1469,1212,1324
,777
SERPIND1_8_2406,161,55,176,108,0,0,3,365,68,463,1007,855
SERPINF1_8_2407,718,589,673,690,368,597,380,931,652,624,397,883
SFXN1_8_2408,2652,3777,3271,3196,1705,4208,3996,2811,4295,3442,4389,25
20
SFXN2_8_2409,1173,1027,1024,1199,1240,1067,1423,175,837,660,1001,1334
SFXN3_8_2410,3677,3596,2820,3007,2599,3233,4598,3417,3413,4945,5114,58
11
SFXN4_8_2411,2492,3415,2559,2022,2008,3816,2572,3800,3757,3048,2834,14
49
SFXN5_8_2412,905,777,1090,794,2446,477,564,374,1329,809,822,622
SLC15A5_8_2413,2943,2508,2347,2222,3056,2664,2213,1908,3005,4485,2172,
4196

SLC16A13_8_2414,779,471,338,424,670,951,15,57,629,696,1510,259
SLC16A14_8_2415,1307,1683,967,1333,528,837,1374,652,529,879,1478,1118
SLC16A9_8_2416,1034,1363,1297,1041,641,597,471,511,617,1956,1758,392
SLC17A9_8_2417,1589,1535,1712,1503,1227,1255,2396,1878,1730,937,2020,9
03
SLC22A20_8_2418,371,387,258,898,3,627,88,122,1,271,560,536
SLC22A24_8_2419,678,664,531,640,1064,810,590,766,354,453,867,73
SLC22A25_8_2420,766,854,410,635,786,258,285,392,527,989,417,1121
SLC25A23_8_2421,4773,5352,3137,4442,4997,5220,5832,5495,2889,4368,7051
,4995
SLC25A29_8_2422,333,149,115,174,13,197,0,6,0,659,244,0
SLC25A31_8_2423,2153,1610,1124,1497,1644,3366,2921,4022,2146,2138,2171
,1860
SLC25A38_8_2424,9379,8901,7698,9172,8754,9446,9300,10383,11577,10034,1
0894,8507
SLC25A42_8_2425,160,254,267,87,141,73,0,291,0,1,302,195
SLC25A46_8_2426,4511,3469,3589,4287,2837,4428,4111,4517,2043,5093,4233
,2757
SLC25A48_8_2427,300,306,122,277,295,434,105,467,520,534,719,539
SLC38A7_8_2428,867,862,856,533,898,1532,257,854,1355,1759,657,511
SLC38A9_8_2429,2837,2248,2441,2413,6773,3435,3414,2514,1751,3195,1782,
4326
SLC41A2_8_2430,740,710,831,648,249,221,466,853,814,539,317,1413
SLC45A1_8_2431,739,644,490,666,1583,939,411,108,309,1382,478,769
SLC45A3_8_2432,115,62,71,17,4,0,38,750,0,579,542,324
SLC47A1_8_2433,860,781,775,633,210,1468,60,863,563,412,722,400
SLC48A1_8_2434,357,118,123,103,343,29,327,181,84,859,0,241
SLC5A12_8_2435,1031,1412,1252,1457,3204,2709,2314,2355,1038,1658,938,1
872
SLC6A17_8_2436,3690,3672,2465,2704,4158,3439,3362,4458,3567,4149,4946,
3652
SLC7A14_8_2437,1425,1587,1396,1351,337,2591,2569,2325,1087,1738,2194,1
680
SLIT2_8_2438,537,349,524,331,608,18,1123,68,51,408,267,123
SNAP29_8_2439,1783,2044,1434,1667,2480,1490,2227,2757,874,2129,463,259
3
SNX12_8_2440,2558,2676,2594,2430,2477,3188,1828,2921,1741,2030,2820,25
56
SNX13_8_2441,2366,2292,1559,2663,4303,2458,2860,1413,2591,2073,3001,17
63
SNX17_8_2442,1805,1841,1277,2053,1287,1497,2584,996,3648,2888,2513,250
0
SNX19_8_2443,2092,1882,2258,2049,1552,3408,2440,2143,2314,1429,3714,24
30
SNX22_8_2444,510,350,783,551,681,1228,1938,429,358,287,135,1108
SNX24_8_2445,4003,3316,2966,2671,4693,4500,4217,2050,3335,2312,2874,41
01
SNX2_8_2446,799,991,1009,635,1997,543,2673,570,686,961,2852,738
SNX4_8_2447,521,479,782,662,230,963,957,247,174,1109,894,732
SNX8_8_2448,219,177,288,232,32,24,483,444,158,839,364,986

SNX9_8_2449,472,243,99,309,80,1162,13,1934,746,289,13,53
SORCS2_8_2450,2278,2036,1551,1863,2372,3003,2850,1875,2980,2580,1517,3
067
SORCS3_8_2451,561,959,718,919,415,1114,645,936,452,501,643,1021
SORL1_8_2452,375,386,295,383,783,162,301,555,54,636,105,517
SOS1_8_2453,2742,2709,2832,1984,2587,5588,3688,3225,3670,3287,4655,495
1
SST_8_2454,756,739,376,924,151,362,1144,904,855,323,1028,779
ST13_8_2455,3130,3549,3137,2723,4998,7022,2870,2843,2494,2677,5623,232
6
STAB1_8_2456,1300,1459,919,1337,1765,1885,2220,814,2263,2485,1183,2918
STARD4_8_2457,652,559,554,500,943,63,604,421,1612,29,140,205
STARD5_8_2458,677,704,914,632,1292,614,867,1028,929,542,1982,1344
STARD6_8_2459,2759,2893,2836,2988,4289,3087,4513,3439,2951,2264,3901,4
500
STEAP1_8_2460,3332,2936,2835,3492,4328,4738,3154,2357,3647,2556,3961,4
095
STMN4_8_2461,254,199,152,361,316,46,148,265,532,321,480,534
STX11_8_2462,25,135,191,39,2,128,116,0,0,0,97,405
STX18_8_2463,929,1167,819,632,24,624,1199,407,514,277,2536,584
STX4_8_2464,379,283,367,324,290,139,47,508,474,434,1097,382
STX5_8_2465,73,284,70,144,1019,64,299,296,99,542,238,147
STX6_8_2466,901,931,1156,658,936,1427,2064,667,302,1819,1717,621
STX7_8_2467,3446,2923,3166,2704,3704,5884,3062,4370,3867,4234,4042,464
5
STXBP3_8_2468,249,368,145,170,810,2,3,1,648,459,596,934
SVOP_8_2469,1426,1111,1109,1204,997,1614,1084,1937,751,1839,2755,2174
SYP_8_2470,485,967,816,640,1348,289,1092,833,285,1609,2454,1241
SYT10_8_2471,931,678,1143,548,638,389,158,19,1045,1140,1440,140
SYT11_8_2472,204,171,203,223,1112,116,361,290,28,302,264,174
SYT13_8_2473,440,435,294,489,276,65,374,199,331,0,454,564
SYT16_8_2474,1537,1610,723,1419,1432,913,1157,1671,770,1726,1210,3265
SYT4_8_2475,1083,1133,654,1073,1468,307,1064,1618,692,559,1305,1524
SYT5_8_2476,2712,3383,2692,3426,3523,1563,3704,2352,2685,2652,3102,131
0
SYT6_8_2477,491,315,470,231,595,485,0,181,452,697,2034,1726
SYT7_8_2478,675,678,452,615,1527,619,38,468,381,398,367,393
SYT8_8_2479,998,793,794,1040,667,2170,1406,1826,1489,522,1381,983
SYT9_8_2480,1503,1006,1376,1114,650,2159,864,652,976,1751,2899,965
TAS2R42_8_2481,8111,7956,7184,7547,7506,9319,7968,11003,8402,7914,1240
1,8442
TBC1D9_8_2482,1751,2325,1403,1707,1588,1189,1352,1696,2098,1338,2034,2
234
TCN1_8_2483,1519,1544,919,1493,1117,2517,1740,1809,2360,1170,1295,715
TEKT4_8_2484,28,45,85,101,0,146,37,261,0,65,214,38
TF_8_2485,1622,1719,1719,1819,1644,1163,1827,729,1457,2415,2327,3186
TG_8_2486,309,82,356,326,41,0,62,254,54,646,27,281
TGFB1_8_2487,1352,1302,797,1038,1385,2233,2419,1461,748,766,1207,856
TIMM10_8_2488,1643,2138,1371,1567,505,1585,1231,1602,1418,2106,1359,22
80

TIMM13_8_2489,74,138,201,406,9,30,157,102,0,229,1184,97
TIMM17A_8_2490,739,1217,1236,1030,1408,753,1562,196,1347,1779,1491,557
TIMM22_8_2491,1057,815,1054,926,331,637,1339,37,439,1270,327,2421
TIMM23_8_2492,677,687,919,866,1534,503,1998,2033,727,530,884,790
TIMM44_8_2493,2513,2082,1741,1670,930,1328,1854,1611,1440,2094,3192,21
80
TIMM8B_8_2494,273,102,84,367,55,17,565,958,74,216,25,732
TIMM9_8_2495,6291,6404,6980,7111,7880,6476,6067,8579,7033,8021,6527,93
39
TLL2_8_2496,3372,3155,2878,3429,4958,2741,2873,3474,1499,3604,2493,337
5
TM9SF2_8_2497,3929,3275,2455,3458,3336,4687,4460,719,3738,4530,4145,46
79
TM9SF3_8_2498,5938,5495,5744,6198,4929,7954,7338,4345,7844,7043,5251,6
958
TM9SF4_8_2499,894,745,663,854,316,930,1559,266,809,743,578,1895
TMCO3_8_2500,999,1029,1223,861,441,320,1317,544,590,2043,1424,663
TMED10_8_2501,827,1007,631,714,654,647,703,562,664,1174,1421,234
TMED1_8_2502,1022,1137,964,1513,1183,1838,1505,1176,701,977,2258,1950
TMED2_8_2503,1768,1626,1620,2317,1933,1104,3106,2497,3357,2513,1987,87
1
TMED3_8_2504,3127,2521,2009,3076,2678,3505,4084,2802,3069,3941,4368,47
08
TMED4_8_2505,732,741,479,446,127,1007,282,701,224,1428,341,417
TMED7_8_2506,2010,2709,2126,2453,2631,2738,4475,2817,3023,2949,2975,25
13
TMED9_8_2507,616,787,1091,559,586,1123,845,2393,552,1871,1208,302
TMEM104_8_2508,113,116,12,97,37,42,485,14,0,200,197,4
TMPRSS11B_8_2509,415,291,410,186,77,406,17,443,586,366,312,195
TMPRSS11D_8_2510,10466,11795,11016,12080,9381,12230,13724,11700,9293,1
6120,11925,12591
TMPRSS11E_8_2511,2240,1708,1546,1928,1369,2182,2643,2106,1083,1815,300
0,1486
TMPRSS11F_8_2512,1221,813,982,925,1466,1465,1502,1442,1086,1346,1632,9
58
TMPRSS12_8_2513,4932,5182,4402,4946,4417,4760,4937,4765,5855,4949,6392
,2574
TMPRSS5_8_2514,619,564,674,774,66,412,1026,76,929,1458,958,795
TMPRSS6_8_2515,548,327,711,454,1145,1904,286,75,675,1764,440,130
TMPRSS7_8_2516,358,187,521,466,645,32,268,727,182,453,152,1013
TMPRSS9_8_2517,232,217,354,296,283,269,161,2,903,4,741,118
TNC_8_2518,463,299,537,638,473,1059,1456,110,1148,1148,893,922
TNF_8_2519,280,528,149,344,208,175,9,29,6,122,53,27
TNFRSF9_8_2520,375,697,236,362,51,576,207,387,170,533,493,1013
TNNC2_8_2521,1599,1235,1785,1169,1204,1606,847,1076,559,861,592,1266
TNN_8_2522,1010,1292,790,816,1071,1517,877,629,1126,1040,597,1585
TNNI3_8_2523,225,400,435,237,993,100,202,502,145,420,901,219
TNR_8_2524,1428,1335,1224,1430,1559,2053,1693,1988,808,1879,1048,1324
TOMM20_8_2525,1994,2233,2074,2527,2658,2116,4761,2205,2660,2054,3881,1
843

TOMM22_8_2526,425,398,269,139,125,173,160,282,96,361,241,463
TOMM70A_8_2527,1111,1392,1010,914,1070,1882,338,1508,1346,1670,976,184
4
TOMM7_8_2528,464,827,393,355,561,841,946,606,306,594,444,193
TPSG1_8_2529,317,232,398,541,892,317,20,1256,0,307,222,1107
TRAK2_8_2530,826,842,665,1140,526,1046,392,597,1173,611,520,928
TRAPPC10_8_2531,8250,9165,8400,8673,9485,10969,9618,7530,8743,10001,12
762,10262
TRAPPC8_8_2532,4350,4504,3523,3206,3985,3587,2298,3607,2989,4090,2981,
3831
TSNAX_8_2533,1668,1901,1778,979,1181,694,2738,1599,1695,2026,765,1004
TTPA_8_2534,459,295,573,402,94,158,449,10,928,176,484,599
TTR_8_2535,826,835,566,973,752,485,1142,177,1149,1270,1411,1144
TUBA1A_8_2536,783,522,556,312,1019,866,1252,162,1,335,207,1415
TUBA1C_8_2537,2834,2515,1804,2725,2919,4087,2855,2094,1321,2104,2261,2
310
TUBA3D_8_2538,1032,572,352,560,1068,1874,1067,629,2392,457,1637,77
TUBA3E_8_2539,1554,1296,1693,1604,1913,1238,3354,940,1786,2365,929,821
TUBA4A_8_2540,388,569,194,347,12,232,819,303,708,57,775,58
TUBB1_8_2541,601,516,322,422,664,504,1841,1194,200,507,891,1403
TUBB2A_8_2542,1184,1054,1304,1034,567,1637,703,632,1274,2571,1362,1614
TUBB2B_8_2543,1184,1054,1304,1034,567,1637,703,632,1274,2571,1362,1614
TUBB6_8_2544,1377,2521,1192,1601,1046,2456,1444,1537,911,1801,2008,287
4
TUBB8_8_2545,895,722,1009,1129,1080,964,732,601,546,583,355,424
TUBE1_8_2546,2070,1964,1163,1862,956,2637,3646,829,1454,2123,1060,1293
TUBG1_8_2547,2189,2451,1621,1905,2340,1257,1883,1719,1617,679,4378,174
6
TUBG2_8_2548,565,313,333,642,207,70,178,383,101,175,470,201
TXNDC8_8_2549,3306,3367,3962,3434,2596,3404,3623,3260,3516,4240,6909,3
642
UCP2_8_2550,476,473,356,557,616,814,216,1938,83,822,203,533
USO1_8_2551,1798,2130,2028,2159,1843,2666,2574,2433,3117,1550,3474,295
2
VAMP2_8_2552,1233,1394,1827,1462,724,1049,1764,1211,1296,862,1564,1257
VAMP5_8_2553,924,1670,1338,752,843,1541,795,1550,1050,1941,620,458
VKORC1L1_8_2554,591,419,538,760,135,557,643,379,240,1133,478,86
VPS18_8_2555,1010,1220,1399,808,1755,2083,874,547,547,1418,3256,1490
VPS26B_8_2556,45,128,26,181,0,382,3,33,10,271,94,7
VPS33A_8_2557,4991,4431,5337,5160,6275,5810,6549,6314,4739,8569,7922,4
942
VPS33B_8_2558,791,1194,903,587,682,801,647,171,552,751,1436,1717
VPS35_8_2559,1707,1291,1333,835,1565,978,904,1555,1576,1598,1339,1497
VPS39_8_2560,396,689,610,939,1014,1333,706,101,818,811,330,1048
VPS45_8_2561,987,865,634,859,941,1443,1253,461,850,959,705,644
VPS4B_8_2562,1683,2004,1033,2132,826,1116,1883,2088,1957,1111,1563,205
1
VSIG2_8_2563,141,227,105,148,374,145,285,305,388,234,682,239
VTI1A_8_2564,2221,1465,1581,2048,2295,768,1056,3493,1594,1697,1670,224
4

VTI1B_8_2565,841,1180,1059,1171,933,1289,690,969,506,1110,1110,586
VTN_8_2566,402,328,282,617,943,547,492,156,704,120,267,539
VWF_8_2567,836,736,754,546,2338,390,78,739,1033,152,111,1359
XK_8_2568,3357,3696,1757,2746,3149,2182,2283,1474,2305,5270,4282,4734
XPO1_8_2569,1971,2584,2081,2898,2828,3835,4121,2682,2632,1809,1810,170
4
XPO4_8_2570,747,294,443,612,1399,213,1954,44,651,267,816,927
XPO5_8_2571,340,400,432,519,1540,1596,560,388,402,1330,1009,261
XPO6_8_2572,1426,1101,926,1471,1424,1235,1322,1862,743,882,655,2138
XPO7_8_2573,581,828,792,739,243,839,151,217,464,712,1173,1016
ACTL6A_8_2574,1090,1006,1009,744,1118,960,1112,1454,1009,2155,2000,206
9
ADAM12_8_2575,675,453,666,428,122,316,324,542,368,518,972,702
ADAM15_8_2576,138,189,11,307,1,138,457,402,215,164,2,1
ADAM18_8_2577,685,625,471,798,375,276,376,784,449,592,823,973
ADAM22_8_2578,347,418,388,454,50,391,251,475,95,663,457,163
ADAM29_8_2579,2319,1562,2081,2130,2629,2548,3892,2402,1463,2002,1663,2
955
ADAM33_8_2580,1268,2109,1475,1772,3108,1751,1976,1431,1580,2652,2667,1
869
ADAM8_8_2581,338,569,222,690,71,499,376,468,11,116,941,321
ADAMTS13_8_2582,65,450,467,75,84,45,963,88,52,18,29,623
ADAMTS14_8_2583,3178,3437,2800,3105,4623,4132,5719,3010,2770,2407,3270
,2363
AKAP12_8_2584,1424,1486,1261,1501,1020,1335,505,1726,2169,1781,1853,98
4
ANGPT1_8_2585,719,959,510,453,968,666,2745,322,809,227,230,511
ANGPTL4_8_2586,432,410,595,242,40,400,703,70,237,41,1007,345
AP1B1_8_2587,1468,1779,1233,1252,1598,872,148,2257,572,1836,765,2632
AP1G1_8_2588,966,667,394,599,974,586,1836,573,1215,509,1545,1129
AP1M1_8_2589,1395,1238,1220,1060,1441,1214,545,2743,1160,1475,1868,201
8
AP2A1_8_2590,2657,2976,2419,2945,2680,3008,4393,3136,2133,2334,1905,39
26
AP2A2_8_2591,781,588,664,601,416,1232,419,291,1280,410,456,1231
AP2B1_8_2592,1724,1240,997,927,544,759,556,197,810,628,938,2672
AP2M1_8_2593,228,435,331,246,95,204,367,548,1555,111,99,93
AP2S1_8_2594,2702,2469,2003,2197,2117,2523,3427,2456,3572,2050,2619,30
20
AP3D1_8_2595,1506,1353,1528,1683,1441,1966,1164,2016,1308,2499,1823,22
83
AP3M1_8_2596,601,911,581,525,519,125,587,1998,853,902,990,1667
AP3M2_8_2597,327,140,188,281,289,637,201,68,346,117,453,240
AP4S1_8_2598,5123,4836,4232,4923,8257,3497,6091,3947,3922,4291,4016,58
90
APAF1_8_2599,1230,1261,1175,1673,446,1675,1110,1510,1658,964,1948,259
APBA2_8_2600,552,619,772,627,411,1335,6,848,95,1791,512,1360
APOL1_8_2601,389,130,589,402,1320,5,458,473,1545,729,256,687
APOL3_8_2602,8262,9174,7865,8745,7186,10996,9106,8211,11005,10986,8268
,10111

APP_8_2603,1326,1205,1669,1245,903,2741,1737,1398,1219,629,4303,609
ARFGAP3_8_2604,1511,1420,1106,863,1561,1295,1254,2263,2113,1664,1373,1
338
ARHGAP33_8_2605,437,366,470,252,80,924,478,944,150,260,372,736
ARL6_8_2606,829,859,610,621,1650,711,1517,560,1630,555,1323,704
ATP13A2_8_2607,538,367,447,822,259,430,380,476,298,218,419,1118
BAX_8_2608,1525,1071,1080,970,2038,1312,1313,1414,1461,2766,1693,1114
BCAP29_8_2609,1158,966,1081,983,1533,741,592,1407,1693,908,1066,1418
BCAP31_8_2610,1036,1155,536,712,1653,1017,659,783,926,1842,612,662
BCL2_8_2611,365,706,262,498,96,120,666,317,122,736,90,930
BCL2L2_8_2612,2645,2632,2295,2558,2806,2218,2031,4168,2079,2505,2374,3
836
BID_8_2613,6375,6269,6040,6310,5886,5768,7594,5398,6244,8025,7458,6075
BSG_8_2614,579,570,733,715,1019,1184,618,1663,466,753,1609,794
C1QC_8_2615,1524,1733,1979,1626,1388,2273,3086,1891,2237,1720,2515,186
3
CANX_8_2616,836,512,485,457,8,807,413,1032,1332,608,859,527
CAPN9_8_2617,1594,1667,1225,1576,2166,3036,441,1180,1892,1249,1323,174
6
CAPNS1_8_2618,0,0,0,0,0,0,0,0,0,0,0,0
CCT6B_8_2619,2181,2210,1699,1522,1633,3370,3472,1771,1983,1331,2570,23
43
CD19_8_2620,1622,1723,1126,1552,1501,2017,948,1022,2004,1170,2096,1171
CD22_8_2621,1526,967,702,788,619,754,4103,112,605,759,628,2505
CD33_8_2622,570,668,446,522,774,309,1166,193,506,333,296,663
CD44_8_2623,155,168,139,297,30,231,63,3,2,59,491,409
CD55_8_2624,184,363,516,419,135,846,107,240,248,775,644,863
CDH17_8_2625,476,456,289,214,41,631,21,0,71,606,10,1278
CFHR4_8_2626,4349,3663,3323,3854,2511,4220,6042,4256,3878,3500,3989,29
82
CIZ1_8_2627,916,522,500,1068,311,610,569,481,460,286,43,1029
COG2_8_2628,907,827,708,901,1140,994,699,1900,2433,827,422,1774
COG4_8_2629,717,689,929,699,2236,388,882,510,2474,907,334,1917
COG5_8_2630,2048,1849,1184,2110,1440,2382,1511,2079,1967,1615,1096,294
8
COG6_8_2631,3296,3012,2219,2782,2112,3612,3296,3546,4817,3154,4198,272
8
COL11A1_8_2632,721,569,841,561,906,572,572,1908,1380,675,879,1711
COL11A2_8_2633,750,708,822,806,1064,1140,2099,699,904,555,410,563
COL12A1_8_2634,262,403,327,317,469,221,1024,58,280,765,355,253
COL13A1_8_2635,584,643,452,402,1239,880,551,306,137,679,449,579
COL18A1_8_2636,437,811,314,603,319,307,456,609,835,680,436,579
COL25A1_8_2637,204,346,411,364,626,431,203,564,81,342,468,373
COL2A1_8_2638,574,621,504,615,383,696,729,263,383,1593,1012,515
COL4A5_8_2639,784,798,995,694,363,604,838,165,952,1170,1376,849
COL4A6_8_2640,4555,4186,2890,4148,4185,3841,2472,2947,5530,4492,3944,4
633
COL6A2_8_2641,1052,1020,899,1255,1027,1505,777,858,1154,1839,734,627
COL6A3_8_2642,795,489,566,491,716,134,845,535,161,1413,813,515
COL8A1_8_2643,1265,1050,1056,1040,646,1901,1134,627,1222,1739,2615,225

2
COL9A1_8_2644,871,1077,863,695,1240,1426,436,723,1381,840,1515,2143
COPA_8_2645,1672,1350,1547,1305,3871,1879,1806,508,1209,1523,1396,422
COPB1_8_2646,1384,1077,1163,1909,1099,1866,640,2499,977,2032,2274,274
COPE_8_2647,144,209,104,201,8,0,551,142,428,419,8,52
CPA4_8_2648,392,686,308,585,1021,819,793,158,437,1793,433,416
CPNE1_8_2649,665,900,960,417,1270,819,1516,1065,264,848,737,1100
CPNE7_8_2650,941,401,603,565,879,557,814,186,1853,158,297,932
CPXM1_8_2651,616,841,723,532,3435,1076,1084,567,860,1285,264,1655
CPZ_8_2652,853,968,666,853,405,1869,881,915,1542,555,851,970
CRABP2_8_2653,2964,2386,2430,3079,2675,3276,1912,3892,1831,3859,3410,1
826
CTLA4_8_2654,1728,1947,1422,1321,1318,2134,2475,2536,2110,2470,827,338
8
CTNS_8_2655,676,556,509,494,444,467,35,442,271,757,235,33
CXCL12_8_2656,1945,1453,1256,1488,2161,1850,637,2865,2174,2553,1482,30
97
DPP10_8_2657,2767,2672,3103,3425,3034,4541,3022,1351,1616,3363,3369,45
72
DPP6_8_2658,641,605,430,457,25,757,1172,283,540,680,1197,595
ECM1_8_2659,362,333,212,182,573,993,588,177,209,311,465,335
EGF_8_2660,951,1176,779,1142,999,1102,858,1128,891,1334,2706,1242
EIF2C2_8_2661,746,774,603,500,919,710,1224,821,983,1392,852,353
EIF2D_8_2662,805,689,262,523,22,474,364,33,26,936,499,27
ENSA_8_2663,1335,1619,1725,1661,1563,1568,1706,383,1348,973,1659,999
EPB41L4B_8_2664,2263,2496,2023,1952,2028,1782,2703,1822,2299,3178,2236
,2166
EPB42_8_2665,2118,2469,2540,1772,2435,1466,659,2404,3977,2032,2636,230
5
ETFA_8_2666,403,447,205,298,530,332,309,158,978,376,336,264
ETFB_8_2667,1706,1729,1773,1877,2037,1359,1420,2233,3934,1784,2735,217
1
EXOC1_8_2668,3925,4546,3738,3510,4314,4456,5222,4419,4455,4054,6373,34
85
EXOC4_8_2669,414,443,536,486,512,455,232,243,241,705,646,302
EXOC6_8_2670,1770,1365,1639,905,970,902,813,1027,2321,1609,1589,1056
EXOC7_8_2671,740,1050,491,369,343,481,197,440,124,109,220,205
F8_8_2672,3316,4233,3437,4142,2887,5457,5483,2850,1759,3492,3358,5458
FABP6_8_2673,963,841,1085,1014,1017,901,891,708,622,991,769,1243
FAM131A_8_2674,90,12,42,161,88,75,57,0,7,13,6,14
FAM63B_8_2675,2302,1722,1439,1835,1572,2533,1406,2500,2661,2838,3434,2
141
FANCA_8_2676,137,191,141,85,21,278,565,893,306,79,42,282
FCN3_8_2677,506,178,661,539,332,506,1444,273,931,1429,358,118
FGA_8_2678,1989,3205,1923,1910,1705,2285,2548,1436,1647,3200,2481,2481
FGB_8_2679,484,313,231,266,711,633,146,297,624,480,149,283
FGF13_8_2680,4557,4878,4372,5216,5096,5409,4963,6832,4796,5109,4530,58
05
FGG_8_2681,1029,690,865,914,1587,387,2687,1418,1261,1137,0,1143
FIBCD1_8_2682,382,487,394,305,335,957,686,590,517,203,350,1002

FLVCR2_8_2683,601,790,475,582,454,838,338,729,605,395,79,464
FOLR1_8_2684,1560,1485,1394,1489,1417,1593,3565,2023,2377,1268,1832,11
14
FOLR2_8_2685,427,443,443,407,662,1406,345,1597,123,178,600,908
GGA1_8_2686,204,229,12,72,76,386,0,143,50,691,21,0
GGA3_8_2687,729,348,650,690,29,1027,336,891,1496,669,6,262
GJA5_8_2688,656,754,820,578,1372,1024,796,876,739,514,834,712
GJB1_8_2689,1017,900,749,1075,1082,805,1322,1649,524,1121,695,919
GJB3_8_2690,415,594,369,556,165,436,575,321,161,696,717,1488
GJB6_8_2691,1692,1942,1005,1198,2032,810,2257,1342,4263,1924,3099,1237
GLYATL1_8_2692,2049,2166,2170,1889,2492,2167,3076,2598,3349,1858,3558,
3289
GNRH1_8_2693,1327,1483,1783,1346,1324,1241,1413,2420,2264,2183,2473,27
37
GOLGA3_8_2694,125,64,61,149,188,96,20,15,81,213,40,437
GOPC_8_2695,4069,3745,3576,4982,4647,5780,4599,4842,4487,4620,5563,638
9
GOSR1_8_2696,1643,1441,1583,1536,2381,1415,2189,720,2987,1464,3342,129
3
GOSR2_8_2697,2217,1603,2756,1387,2462,1831,1140,1111,4084,1739,1499,30
75
GPRASP1_8_2698,1531,1402,1168,1653,363,1122,1441,1498,2012,2124,1844,2
557
GRB2_8_2699,667,508,350,374,669,7,514,1606,1331,344,487,40
HABP2_8_2700,492,588,391,403,102,374,317,624,596,985,671,71
HDLBP_8_2701,1678,1458,1202,1254,1315,1265,766,1199,1910,675,454,1593
HEPH_8_2702,3158,3755,2417,4760,3624,3142,3144,3461,5099,3402,2072,676
9
HNRNPU_8_2703,968,526,682,1024,342,1282,769,1397,623,365,681,1316
HOMER2_8_2704,1454,1357,1646,1668,1140,1654,837,2186,945,1079,1910,976
HPN_8_2705,1,0,0,0,0,0,0,0,0,0,0,0
HSDL2_8_2706,270,318,59,189,1,504,226,3,157,1038,119,610
IGF1_8_2707,685,795,618,911,254,593,165,1914,196,203,376,1084
IGFBP3_8_2708,1374,1032,1055,1379,484,2515,2844,651,549,1917,910,1927
IPO11_8_2709,3118,2962,2811,3044,2841,2539,4260,4175,1937,3285,4930,43
07
IPO8_8_2710,2098,2334,2865,1620,1500,1899,3415,4114,3089,912,3527,3770
ITGAL_8_2711,316,322,698,327,920,136,1652,250,3,0,16,26
ITGAM_8_2712,318,528,436,550,102,1048,822,507,414,1794,76,1
ITGAV_8_2713,645,712,708,737,645,867,1322,930,906,1388,453,149
ITGB2_8_2714,207,374,323,49,322,429,1234,135,176,387,1700,72
KDELR2_8_2715,895,1192,1139,765,798,1551,667,321,1308,948,828,575
KDELR3_8_2716,251,300,254,44,670,270,17,5,37,181,484,14
KIF13A_8_2717,199,67,111,61,0,500,78,204,41,134,270,1415
KIF17_8_2718,513,663,334,449,550,1266,845,211,381,393,466,569
KIF1B_8_2719,444,924,590,636,589,932,279,1528,579,482,501,69
KLK10_8_2720,1060,1000,1146,1002,2064,1105,2538,891,834,1233,836,562
KLK11_8_2721,89,81,74,12,17,128,0,408,0,15,61,10
KLK12_8_2722,385,586,460,512,120,689,393,180,184,333,125,536
KLK15_8_2723,418,1060,477,508,628,102,321,382,273,502,517,177

KLK5_8_2724,1664,1557,1831,1495,1272,1028,529,3770,1044,1413,1814,911
KLK6_8_2725,1245,2029,1380,1915,664,337,3082,1464,1332,446,393,1847
LAMB3_8_2726,317,337,200,231,487,450,143,503,147,260,261,218
LDB3_8_2727,1505,1404,1458,1149,674,3372,1656,981,1087,1233,1139,1942
LDLR_8_2728,478,48,159,779,410,23,727,118,0,753,0,847
LTA_8_2729,133,191,287,458,2,461,205,0,35,66,984,8
LTF_8_2730,920,680,966,1054,1199,1548,877,132,486,1320,1944,510
M6PR_8_2731,693,817,453,672,525,979,294,326,733,717,1243,223
MASP1_8_2732,3904,4645,3916,3860,6226,5448,5001,2013,1973,4540,5089,52
82
MB_8_2733,497,352,444,445,612,54,612,442,603,611,157,147
MCFD2_8_2734,1672,2059,1826,2120,1866,1864,834,1357,854,2920,2254,1704
MCL1_8_2735,672,1062,599,755,147,281,555,97,564,476,621,1042
MEFV_8_2736,571,597,588,771,324,1193,1495,200,534,585,148,942
MFAP4_8_2737,407,211,528,355,5,941,201,120,217,335,262,314
MFSD10_8_2738,680,278,364,300,0,146,1284,52,829,249,254,5
MFSD1_8_2739,3416,2660,2566,3603,3714,2840,1864,2405,4178,2247,5645,37
03
MFSD5_8_2740,1860,1828,2177,1670,2372,1264,1135,1595,1530,2136,2144,14
55
MLC1_8_2741,1319,861,576,1066,1037,1301,1456,494,72,817,144,2449
MSLN_8_2742,255,249,175,358,1334,194,266,10,63,224,85,686
MTX1_8_2743,646,539,549,760,427,1303,231,803,605,291,1187,1451
MUC1_8_2744,302,224,509,452,83,538,262,331,205,352,352,631
NCAM1_8_2745,2682,2675,2323,2887,2841,4644,1611,1918,2885,4196,3234,31
45
NNAT_8_2746,109,33,94,62,97,0,147,0,1,22,0,50
NOXO1_8_2747,208,0,0,57,0,0,0,0,0,64,0,0
NPC1L1_8_2748,486,717,605,589,1341,556,8,129,245,714,304,932
NPRL3_8_2749,709,747,770,833,604,617,591,338,544,574,1605,971
NRXN1_8_2750,1735,2671,1905,2162,1060,2797,2307,1283,1509,2033,3145,32
41
NRXN2_8_2751,1011,797,713,575,458,1250,518,933,662,864,810,1057
NRXN3_8_2752,1577,2102,2139,1820,1307,2016,1803,3116,1671,1340,2004,16
38
NUP155_8_2753,374,627,452,480,581,25,624,771,353,611,117,952
NUP50_8_2754,0,0,0,0,0,0,0,0,0,0,0,0
NUP62_8_2755,331,353,805,987,1,523,552,192,35,615,331,435
NUP98_8_2756,1557,2021,1249,2056,1152,1403,721,2430,807,2629,3842,1411
NUPL1_8_2757,8141,7442,8759,7202,6800,11384,11550,5978,7512,10369,1190
1,12406
NXF1_8_2758,1630,1866,1832,1856,1001,1483,3447,560,2682,1648,2088,1708
NXNL2_8_2759,774,1242,696,1302,1099,863,647,1043,763,740,1385,945
NXT2_8_2760,934,526,849,798,969,1079,1621,1719,336,725,589,1273
OAZ3_8_2761,798,775,895,906,836,1319,1517,1546,445,760,740,1969
PACSIN2_8_2762,728,774,815,739,259,1092,127,678,1408,1191,36,223
PANX2_8_2763,643,687,821,805,1545,678,1404,399,585,708,1451,1194
PCDHA6_8_2764,362,97,273,63,1081,3,877,885,1,210,46,181
PCDHGA5_8_2765,1312,1146,1343,1706,1475,418,2679,2136,1344,896,1057,10
05

PCLO_8_2766,3109,3260,2387,2861,3421,5154,2852,3271,2776,4426,4587,656
5
PCSK5_8_2767,212,154,112,211,3,538,362,181,464,39,183,2
PCSK6_8_2768,1169,811,1141,1013,426,583,446,839,229,1847,1405,815
PCTP_8_2769,673,404,403,472,179,437,820,689,261,389,969,213
PDYN_8_2770,681,611,454,432,241,374,652,108,1691,1453,604,460
PDZD3_8_2771,144,177,254,237,28,8,0,2,534,132,24,288
PDZK1_8_2772,1732,1800,1637,925,1570,1696,1877,1186,2215,1535,1206,160
1
PGAP2_8_2773,628,744,447,622,832,945,278,636,612,511,514,916
PGF_8_2774,1150,424,567,795,403,818,199,175,761,83,104,89
PIK3R3_8_2775,1791,1768,1770,2026,1612,2837,2709,2638,2389,2234,1784,2
186
PITPNC1_8_2776,64,275,224,322,89,453,503,4,256,961,186,366
PITPNM1_8_2777,96,39,289,110,424,182,78,12,213,89,176,189
PITPNM3_8_2778,1456,1552,969,1125,2703,1137,2035,933,583,1230,1817,116
1
PLEC_8_2779,1989,2139,2375,2201,1463,2685,2349,1700,1136,2938,1702,226
5
PLIN3_8_2780,735,858,525,691,371,327,850,1316,903,1381,33,416
PLTP_8_2781,943,1058,1143,457,1086,244,1203,1361,1439,1235,821,26
PNKD_8_2782,2245,1981,2592,2483,2088,2568,4663,3230,2661,2476,3578,300
2
POMC_8_2783,216,147,244,373,272,87,52,35,183,193,109,237
PORCN_8_2784,223,135,295,158,857,292,1595,191,57,323,332,537
PREPL_8_2785,575,574,469,260,1417,661,180,963,342,961,1117,271
PRNP_8_2786,944,1072,1203,1024,578,890,394,444,1162,657,1690,1306
PRSS21_8_2787,2429,2412,2086,3150,1016,3363,2925,3364,1727,2088,2872,3
616
PRSS35_8_2788,2786,2641,2217,3057,2108,2161,1986,2240,4625,4579,2177,4
619
PSEN1_8_2789,1132,1066,615,952,1238,748,1876,1282,872,1497,1091,1377
PSEN2_8_2790,439,499,376,606,458,1030,119,42,66,102,332,896
RABEP1_8_2791,2007,1595,1865,2206,2752,1880,1689,513,2796,2907,2250,85
8
RACGAP1_8_2792,1469,1958,1079,1805,531,3862,1148,725,1102,2384,727,160
3
RARRES1_8_2793,1912,1739,1474,1729,1762,820,1705,1952,2989,3733,1967,1
515
RASA1_8_2794,2867,2721,1953,2024,1239,3812,3937,1658,843,2163,3019,257
4
RELN_8_2795,191,610,96,148,272,266,602,510,100,728,373,6
RHCE_8_2796,428,613,569,773,225,1021,146,541,991,634,283,690
RHD_8_2797,2354,2833,3144,1742,2726,3282,1843,1260,1594,3485,3097,2770
RIMS2_8_2798,1043,924,733,951,1839,777,429,1289,1147,1465,161,1225
RRBP1_8_2799,694,465,105,196,639,1106,813,799,132,246,117,101
RUFY1_8_2800,1194,1057,1085,1077,1758,760,1561,2820,1115,829,1964,747
S100A13_8_2801,942,644,432,1267,1082,520,832,1120,1748,787,556,2311
S100A4_8_2802,593,641,585,638,500,966,427,153,980,913,800,874
SAA1_8_2803,951,1088,1856,1089,1155,963,2514,1192,1199,888,3008,1652

SCAMP3_8_2804,4911,5081,5803,6046,4162,5000,4897,5675,6944,4874,6972,6
394
SCARB1_8_2805,823,1045,916,916,813,481,2100,712,1444,1226,1145,649
SCFD1_8_2806,1092,1238,1154,1157,2037,1348,999,1579,620,1045,2229,406
SEC13_8_2807,591,653,352,715,820,408,470,1404,652,677,323,602
SEC14L1_8_2808,1261,1197,1013,1242,2115,489,1147,2006,776,1467,1095,50
0
SEC14L2_8_2809,1827,1972,1927,2353,3542,1264,1734,2248,2991,2199,2568,
4118
SEC14L4_8_2810,372,707,709,742,603,151,296,20,442,414,377,961
SEC23B_8_2811,310,230,297,445,256,419,238,274,538,435,115,425
SEC24B_8_2812,2145,1854,2904,2262,1337,2451,3223,3546,2174,2278,2095,8
53
SEC24C_8_2813,503,293,487,185,410,250,875,894,340,360,22,290
SEC61A2_8_2814,1726,1392,1213,1474,1960,2697,2330,2192,1267,1802,1436,
4633
SEC61G_8_2815,1998,1947,1258,1506,798,3559,1399,2073,1161,1219,2666,27
60
SEH1L_8_2816,1131,1375,1246,1446,1348,1997,926,2500,896,2036,986,2405
SERINC2_8_2817,382,292,547,312,196,409,244,343,749,250,549,168
SERINC3_8_2818,10437,11248,9485,10872,10543,11859,11336,15320,9594,110
17,17728,11533
SERPINA10_8_2819,1665,1678,1812,1863,3283,1751,2093,920,508,2040,1958,
832
SERPINA1_8_2820,864,589,562,356,496,1091,1335,235,877,808,622,327
SERPINB2_8_2821,358,321,251,216,101,489,126,1097,184,489,135,1097
SERPINB6_8_2822,714,800,934,637,1285,1270,384,1204,504,1379,922,1577
SERPINB8_8_2823,2589,2865,2080,2571,3296,1646,970,2586,1643,3912,2864,
5221
SERPINE1_8_2824,708,802,988,637,1298,538,769,1009,702,848,989,314
SERPINF2_8_2825,1015,484,650,381,1724,820,648,671,1415,799,722,274
SERPING1_8_2826,2212,1693,1880,1736,1313,2766,752,809,1760,2036,1677,2
040
SERPINH1_8_2827,286,363,364,317,80,632,593,624,281,914,415,192
SERPINI1_8_2828,4552,5435,4556,5378,6070,7264,3594,5607,5315,7226,7815
,7628
SFI1_8_2829,3833,3925,2585,3149,2571,1788,5516,3496,5102,3261,4368,312
8
SFTPA1_8_2830,631,314,605,542,374,719,123,228,481,387,1098,823
SH3D19_8_2831,275,633,163,268,0,1105,2,165,4,158,198,201
SIL1_8_2832,3432,3059,2437,2481,2213,3746,2862,4890,1360,3001,5567,335
8
SLC25A25_8_2833,1126,1095,873,979,1722,1341,973,786,3062,955,646,3194
SLC25A36_8_2834,627,1200,595,1147,197,1857,1257,849,935,1553,99,1437
SLC25A45_8_2835,507,576,416,1214,1341,165,84,393,395,411,515,1755
SLC38A10_8_2836,1580,1298,968,1198,1851,1338,1051,848,1674,1202,1492,3
422
SLC41A3_8_2837,455,263,350,225,68,191,683,214,586,98,796,255
SLC43A3_8_2838,1461,1914,2207,2192,227,2695,2861,1488,1537,2204,1550,3
236

SLC44A2_8_2839,1779,1914,1338,1409,1408,2949,3912,1610,2450,1281,1851,
985
SLC44A4_8_2840,1865,1593,1316,1823,1504,2834,5303,787,2220,1291,2195,2
012
SLC44A5_8_2841,3036,2152,2052,2996,2931,2695,4566,2108,3282,3339,2466,
3061
SLC46A1_8_2842,583,654,456,800,1005,817,1099,837,1149,99,1111,766
SLC47A2_8_2843,590,790,1143,727,277,804,331,472,105,1198,847,751
SLC50A1_8_2844,46,310,128,51,8,98,0,10,14,49,0,123
SLC6A20_8_2845,593,763,371,354,328,1157,63,680,1111,716,618,882
SNAP23_8_2846,1016,1044,381,1124,535,310,691,1907,508,1389,844,419
SNAP25_8_2847,3690,4127,2841,3725,4722,5216,7580,5219,2780,7083,5213,4
753
SNX10_8_2848,6065,6079,5850,6230,5101,7260,8520,5680,4610,7149,7571,86
54
SNX11_8_2849,598,488,705,763,391,836,40,167,510,494,1289,1005
SNX14_8_2850,2176,1435,1505,2866,3197,2449,1447,1430,2030,945,1834,126
4
SNX15_8_2851,523,615,525,581,503,507,602,234,271,542,337,907
SNX16_8_2852,1896,1696,2550,2354,4245,2465,2420,3056,3641,3157,2397,47
84
SNX18_8_2853,252,276,249,135,241,304,410,283,855,352,559,164
SNX1_8_2854,901,1073,592,483,651,594,462,432,345,1701,515,869
SNX3_8_2855,1263,958,1129,1030,469,998,1322,1266,1403,750,900,2335
SNX5_8_2856,1392,1710,1477,1387,1336,2970,1927,1580,2110,1599,590,1084
SNX6_8_2857,1508,1273,1738,1381,460,902,2126,1315,1089,2144,2090,1284
SNX7_8_2858,1394,1952,1332,970,1215,1212,463,956,968,2564,1409,2338
SORCS1_8_2859,4500,4000,4685,3601,5581,2562,5131,4484,5462,2263,7033,4
268
SORT1_8_2860,672,384,615,378,250,240,80,342,135,404,830,660
SPNS1_8_2861,755,561,321,512,323,95,919,476,262,212,483,604
SRI_8_2862,0,0,0,0,0,0,0,0,0,0,0,0
STARD3_8_2863,1002,902,644,812,280,761,1197,447,282,740,868,2630
STAU1_8_2864,1618,1348,1320,1804,2155,676,1863,1136,3288,575,1716,3198
STEAP2_8_2865,1070,1402,891,1001,3241,988,464,611,867,1060,703,1399
STEAP3_8_2866,239,426,230,397,111,576,195,884,19,768,564,330
STIM2_8_2867,409,627,357,415,1246,109,226,702,819,517,331,353
STX16_8_2868,915,1236,835,1201,594,1508,516,1688,1180,895,654,1195
STX1A_8_2869,232,47,72,248,1474,4,0,17,3,0,73,540
STX2_8_2870,1013,1203,1158,1261,2854,177,864,902,395,2297,860,1268
STX3_8_2871,838,1162,875,954,1947,1336,646,583,1173,1014,838,1542
STXBP1_8_2872,367,304,238,460,1437,20,879,519,259,91,516,107
STXBP2_8_2873,465,446,133,601,564,82,26,163,0,154,509,364
SV2B_8_2874,2795,3016,2636,2556,3055,1986,3406,3525,2429,2402,3768,241
5
SYN1_8_2875,2365,3130,1623,1988,3311,2932,1123,2481,3109,2569,2752,211
8
SYNGR1_8_2876,821,794,860,998,959,1590,300,1100,2767,2198,489,452
SYNPR_8_2877,275,355,273,181,521,306,1,55,703,2,243,8
SYPL1_8_2878,1787,2073,1651,1877,1892,1129,2786,2234,2187,3155,2207,23

98
SYT12_8_2879,376,475,184,443,395,481,344,147,195,249,485,482
SYT14_8_2880,3763,4662,3341,5278,2815,3794,4518,6502,5017,5417,3432,40
81
SYT15_8_2881,370,652,420,543,25,241,446,294,393,172,189,1630
SYT1_8_2882,2044,3040,1891,2352,988,2347,3046,4714,2835,3515,1911,2547
SYT2_8_2883,1979,2117,1812,2095,2819,2498,886,1884,2261,1532,2365,1865
SYT3_8_2884,984,1250,781,934,1273,721,1385,996,1740,1501,336,222
TAPBP_8_2885,288,287,291,254,3,116,367,191,131,24,671,247
TC2N_8_2886,2664,3349,2584,2715,3944,3043,2559,2174,2145,3078,2835,290
6
TCN2_8_2887,251,348,406,225,109,510,150,606,351,170,708,713
TCOF1_8_2888,88,81,0,15,20,0,0,2,190,9,418,0
TFPI_8_2889,1446,872,1401,1585,1028,1158,518,1164,561,1872,1649,894
TFR2_8_2890,1439,1350,1247,1296,1765,579,934,996,528,4241,344,2434
TFRC_8_2891,1078,944,878,596,652,1007,158,660,667,1969,1264,1193
TGFB2_8_2892,2245,2367,2133,2206,1818,2499,2601,3294,2654,3110,3023,15
30
TIMM17B_8_2893,495,521,426,263,90,713,424,226,890,619,1213,187
TINAGL1_8_2894,1015,1551,1481,1131,2294,1331,1273,1452,1814,1889,1596,
2170
TLL1_8_2895,2553,3362,3111,2117,3956,3339,3801,3679,3420,3427,3115,318
7
TM9SF1_8_2896,25,6,10,88,0,0,0,183,0,0,0,0
TMC6_8_2897,1559,1898,1575,1469,1600,2390,2902,1013,1998,2903,3924,134
5
TMPRSS11A_8_2898,1769,2113,1679,1565,1799,735,1893,587,724,2512,2042,1
62
TMPRSS13_8_2899,706,731,321,578,175,687,401,1301,903,888,386,1032
TMPRSS2_8_2900,302,390,167,281,422,1079,202,776,703,586,726,205
TMPRSS4_8_2901,1195,1108,411,995,1293,2700,1681,1322,737,478,1225,1844
TNFSF11_8_2902,340,73,50,103,0,0,25,13,323,47,1,81
TNFSF13B_8_2903,2234,2210,1834,1811,1741,3127,2116,2124,1686,2023,982,
3662
TNPO2_8_2904,3174,4380,3207,3862,4599,5366,4062,4437,3340,3982,2874,55
07
TOM1_8_2905,1915,1793,1614,1538,884,5269,1120,642,2376,2380,4478,2108
TOM1L2_8_2906,1245,1075,1053,1617,530,1080,947,1514,1831,1119,1401,116
2
TSC1_8_2907,4104,4775,4170,4582,3576,5888,5086,5120,3590,6657,4648,277
0
TSC2_8_2908,505,564,187,467,51,289,177,289,95,228,1305,857
TUBA8_8_2909,307,569,103,204,299,433,74,436,70,468,20,251
TUBB3_8_2910,1305,2057,1148,1415,1562,2363,1032,1936,876,1368,1218,168
0
TUBD1_8_2911,3702,2696,4343,3408,3240,1966,3603,3508,3582,3258,6597,53
65
UCP3_8_2912,345,111,274,518,14,27,6,315,501,434,447,137
UPF3A_8_2913,1212,892,1188,1303,1281,1421,433,399,2042,1813,817,796
UPF3B_8_2914,585,751,858,1007,1327,880,1743,543,1811,833,1256,897

VAMP1_8_2915,130,262,449,317,49,123,881,963,216,121,35,925
VAMP7_8_2916,3328,3864,3930,3253,3656,4408,2137,2903,5840,3334,5243,49
05
VCAM1_8_2917,293,290,494,688,47,116,37,78,257,589,1264,13
VLDLR_8_2918,972,1889,1236,699,1074,1239,1022,691,1050,968,930,973
VPS13A_8_2919,748,491,803,478,510,467,1433,15,552,982,1522,20
VPS13B_8_2920,966,1127,760,1276,1026,884,2007,1436,1076,508,249,1748
VPS16_8_2921,1037,1335,1049,885,1326,885,755,364,444,1915,1556,1134
VPS26A_8_2922,1261,629,1248,1457,672,1025,1990,1041,987,650,2964,631
VPS28_8_2923,866,1129,837,1408,1240,664,2612,1451,427,2626,575,670
ZFYVE16_8_2924,780,969,960,932,1425,1161,1125,1935,565,1099,829,1907
ZNF160_8_2925,2330,2179,1899,2499,2653,2050,1800,2461,3361,3293,2560,2
694
ZP3_8_2926,748,774,810,606,1000,1244,897,1885,580,563,1073,962
BET1L_8_2927,84,21,50,34,82,276,0,13,1,318,0,83
C2orf83_8_2928,667,307,335,511,1415,56,613,306,329,184,89,1033
ERP29_8_2929,523,626,532,639,656,283,523,224,378,990,965,1516
FGF1_8_2930,2005,1887,2215,1774,4627,4371,4914,977,2438,2076,3137,3391
LYNX1_8_2931,297,263,246,183,527,515,197,6,36,372,486,21
MMP28_8_2932,534,125,42,139,1057,154,664,2,469,181,356,1218
PDPN_8_2933,1017,1001,740,691,766,2239,715,529,2152,1389,2190,832
SNX21_8_2934,431,626,481,663,199,708,191,569,350,71,1863,400
TIMM8A_8_2935,532,617,558,492,20,343,686,66,910,171,192,201
VEGFA_8_2936,815,717,790,974,519,535,934,225,1964,822,554,1739
CDH23_8_2937,630,1142,780,1082,903,875,1137,1006,993,684,860,1168
CDH23_8_2938,601,627,621,1025,1217,14,1179,310,590,602,1210,845
CDH23_8_2939,263,277,632,397,559,242,384,26,408,756,313,293
CDH23_8_2940,591,348,606,555,128,299,246,1658,445,1074,722,302
CDH23_8_2941,507,512,672,178,1347,318,434,38,1755,143,433,55
TNXB_8_2942,41,234,49,43,286,18,443,0,76,186,4,30
TNXB_8_2943,472,710,241,608,746,1262,952,1543,566,1003,493,425
A2M_8_2944,1981,2294,956,1883,2912,2478,1228,1944,2475,1892,2880,3346
ACE2_8_2945,4490,4076,3774,5218,4302,4248,4730,3218,3082,2393,4193,586
8
ACTR6_8_2946,11715,11109,9390,9618,10244,11830,9824,6279,9308,8771,130
28,11218
ADAM11_8_2947,381,689,387,378,1967,512,1779,729,32,954,293,420
ADAM19_8_2948,695,313,481,791,648,267,1118,1260,612,1305,773,671
ADAM20_8_2949,1200,1519,1299,1024,806,1591,343,1110,1627,1988,920,1482
ADAM21_8_2950,1300,2160,941,1080,884,1906,739,678,1200,1946,2029,2544
ADAM2_8_2951,213,274,374,490,0,458,78,203,475,123,202,438
ADAM30_8_2952,2924,2574,2545,1871,2531,2630,3805,1779,2139,1726,1872,3
410
ADAM7_8_2953,297,375,251,330,1169,1003,94,73,15,247,697,73
ADAM9_8_2954,2567,2253,2515,2486,2081,910,1847,2618,3207,3225,4075,328
4
ADAMTS10_8_2955,515,494,318,787,578,564,573,332,280,1167,543,686
ADAMTS12_8_2956,646,727,566,497,1319,575,306,923,1500,924,917,90
ADAMTS15_8_2957,590,301,340,495,453,253,610,227,540,458,257,558
ADAMTS18_8_2958,341,567,448,595,0,82,1102,793,532,455,1126,701

ADAMTS19_8_2959,2417,2167,2124,2511,3318,2977,2466,1903,2004,3818,2677
,2470
ADAMTS1_8_2960,993,979,545,1036,429,561,1377,1856,1308,925,549,1528
ADAMTS20_8_2961,1580,1824,852,1787,1279,1988,1998,2151,1038,1438,2154,
1921
ADAMTS3_8_2962,514,411,602,795,250,502,477,848,521,314,1566,872
ADAMTS5_8_2963,2474,1684,1995,2026,2158,1468,2510,1118,2491,3469,2912,
2827
ADAMTS6_8_2964,586,1069,647,832,1016,997,1591,61,294,692,1302,611
ADAMTS7_8_2965,1346,1320,1597,1006,304,490,2560,1910,748,1055,1532,137
0
ADAMTS8_8_2966,1319,1697,1282,1425,967,1700,1183,3445,1676,1776,2693,1
804
AEBP1_8_2967,522,768,665,785,347,223,2319,44,41,439,1100,598
AFG3L2_8_2968,585,418,778,827,125,905,1073,1254,481,953,343,1630
AFM_8_2969,1632,1972,1400,1392,1962,1358,2333,1408,1454,1375,1312,1648
AFP_8_2970,1512,1104,1326,1889,767,1527,1616,2115,2336,1238,567,1101
AGTPBP1_8_2971,2460,2621,2657,2409,3815,2733,3462,2079,4238,2994,3164,
4428
ALG10B_8_2972,2557,2638,2587,2413,4512,3198,3314,2090,2854,3490,2735,2
540
AMBP_8_2973,2132,2604,1827,1628,2175,3380,1402,2044,2886,2766,4766,234
8
ANGPT4_8_2974,304,117,244,71,14,4,949,0,0,194,45,918
ANGPTL1_8_2975,988,712,475,782,1062,682,326,1017,712,1094,185,921
ANGPTL2_8_2976,2548,3482,2594,2207,6049,2655,2171,2770,3196,4540,3673,
4106
ANGPTL3_8_2977,160,151,127,21,48,83,156,0,36,174,146,65
ANGPTL7_8_2978,1078,1165,1142,1209,713,149,721,1067,871,408,1819,1294
ANKH_8_2979,1479,854,1126,1060,2364,789,1451,1020,664,1188,1813,2427
AP1G2_8_2980,664,795,370,688,540,259,1186,150,818,358,660,772
AP1M2_8_2981,4172,4214,3719,3349,5574,1643,5190,3393,3269,4710,3815,67
25
AP1S1_8_2982,1158,1362,1321,1287,781,1404,1549,1219,1845,1321,1167,253
6
AP1S2_8_2983,1209,953,1073,1173,1223,947,1817,1643,1553,795,573,1689
AP1S3_8_2984,1915,2571,1879,2566,2247,3224,2014,4968,2255,2564,3409,21
01
AP3B1_8_2985,523,524,377,664,1124,767,1534,520,548,394,258,114
AP3B2_8_2986,725,978,908,585,390,649,863,518,568,1115,952,1112
AP3S1_8_2987,1084,1042,1284,632,1705,491,995,640,1798,1128,820,507
AP3S2_8_2988,943,1065,1452,1229,719,1525,3550,711,1264,676,930,513
AP4B1_8_2989,172,147,229,391,0,53,216,511,432,444,477,34
AP4M1_8_2990,431,232,290,367,166,107,104,267,145,322,622,47
APBA1_8_2991,652,560,597,581,748,292,17,2102,1121,432,1359,107
APBA3_8_2992,115,377,70,257,122,398,74,4,91,325,143,38
APOA1_8_2993,1318,1727,1238,930,1235,1898,877,1661,2616,2332,1052,279
APOA2_8_2994,395,367,476,324,725,839,520,46,905,442,1025,386
APOA4_8_2995,285,279,342,368,925,840,168,130,177,908,867,396
APOB_8_2996,2316,1895,1926,2062,1984,2305,1486,2375,669,2903,3065,3713

APOC1_8_2997,1158,1005,1003,1104,2192,912,1344,367,808,995,1475,478
APOC2_8_2998,920,933,976,1051,896,796,956,541,1344,1339,1028,1554
APOC3_8_2999,1820,1109,1037,1541,325,2211,782,1737,1176,814,694,1637
APOC4_8_3000,1316,1138,1019,1067,860,797,1440,1320,749,626,2815,309
APOD_8_3001,538,770,570,914,1252,887,1091,1623,296,1009,572,513
APOE_8_3002,967,802,800,912,382,1585,2009,244,626,1505,617,711
APOF_8_3003,118,105,287,437,33,1263,184,82,113,179,641,603
APOH_8_3004,4574,4549,3326,5183,3420,4316,2540,7477,5501,3372,3542,461
8
APOL6_8_3005,721,1022,906,1121,141,586,1477,407,889,926,1105,1260
APOM_8_3006,5557,5629,4378,5499,4055,8217,6116,6649,3155,5135,6565,613
4
AQP12B_8_3007,462,646,568,752,1268,936,1059,1184,880,250,1502,1147
ARF5_8_3008,280,51,91,134,191,11,140,114,282,0,91,443
ARF6_8_3009,830,983,1044,980,1653,1022,1254,533,690,1126,632,642
ARPP19_8_3010,1517,1196,2016,1263,1044,2234,450,893,2295,2479,2630,311
0
ASTL_8_3011,609,386,520,508,78,498,18,456,12,898,244,1391
ATOX1_8_3012,144,275,159,151,5,113,685,6,333,385,10,6
ATP13A1_8_3013,122,148,263,43,142,432,27,1,196,628,40,1
ATP13A3_8_3014,2824,3439,2421,2640,2080,4951,4087,1364,2566,1942,3544,
3489
ATP13A4_8_3015,642,913,1023,890,560,705,859,463,382,535,1007,1727
ATP13A5_8_3016,1217,1415,870,1311,416,2184,2155,2400,1975,1161,1074,18
94
AZGP1_8_3017,138,191,256,210,376,764,98,345,60,12,825,328
AZU1_8_3018,1327,1302,918,1352,204,964,89,2843,1217,1572,712,1531
BCL2L10_8_3019,542,642,902,370,104,610,363,307,352,760,789,878
BET1_8_3020,901,851,826,832,915,207,93,1110,888,444,671,1472
BGLAP_8_3021,546,399,306,434,411,426,403,321,617,556,256,188
BOC_8_3022,363,150,261,226,0,368,57,3,50,43,11,148
BPI_8_3023,474,428,333,454,9,880,69,562,293,465,241,1380
BPIFC_8_3024,945,724,796,924,2210,1814,431,1034,680,325,539,523
C16orf7_8_3025,327,12,10,92,7,5,154,0,376,36,55,1337
C1orf162_8_3026,355,321,330,482,15,15,28,521,357,205,64,259
C1QA_8_3027,1719,1328,1917,1115,1971,792,1848,1197,1383,1525,2937,2160
C1QB_8_3028,1354,1400,1001,1161,2449,1029,1026,1124,932,824,1205,831
C1RL_8_3029,229,300,112,259,18,1830,0,19,405,15,292,106
C20orf141_8_3030,307,202,294,346,229,354,14,249,316,501,395,72
C3_8_3031,996,508,541,1241,320,869,1246,1327,964,1547,905,771
C4A_8_3032,373,232,389,379,38,640,178,113,288,126,70,995
C5_8_3033,977,1030,516,900,989,1498,1349,1347,2430,1228,731,1860
C7orf31_8_3034,1641,1327,971,906,687,2578,740,1759,1346,1521,2203,2286
C8G_8_3035,310,388,65,145,25,24,93,156,122,89,166,4
CALM2_8_3036,595,980,939,1091,463,840,451,563,325,1142,831,955
CALY_8_3037,835,987,799,1427,1373,375,1156,2274,1250,553,1412,2197
CAMLG_8_3038,1871,1572,1220,938,874,1339,2535,642,1755,1904,553,1630
CAPN11_8_3039,807,585,534,650,899,908,934,883,724,413,504,1732
CAPN5_8_3040,1777,1033,1269,1078,992,1234,1574,1436,2016,1706,3466,397
0

CAPN6_8_3041,466,319,317,176,69,520,331,191,94,42,11,432
CARTPT_8_3042,265,168,181,128,64,56,3,181,97,91,309,330
CCL13_8_3043,1685,1632,1581,1175,1290,1809,1202,2186,657,1724,1220,165
4
CCND1_8_3044,511,565,391,415,119,373,272,131,354,82,840,72
CD1A_8_3045,133,273,185,171,164,0,1,34,850,360,0,320
CD52_8_3046,3388,3946,3470,3717,3425,3920,2729,3513,4890,2746,4706,359
1
CDCP2_8_3047,218,454,360,475,1403,12,214,149,208,568,438,1338
CDH5_8_3048,940,886,1057,976,1000,2432,1934,154,195,1714,817,2422
CHMP7_8_3049,336,295,355,565,456,169,836,1000,339,234,44,967
CLDN16_8_3050,707,612,466,748,731,774,542,472,1042,932,687,472
CLEC3B_8_3051,2469,2328,2543,2747,1736,3212,3216,1936,1565,4038,2847,2
923
CLSTN2_8_3052,738,567,569,568,400,86,1242,495,1210,629,266,369
CLVS2_8_3053,852,942,1022,1313,834,1609,1302,1421,1542,812,951,1127
CNIH3_8_3054,504,375,528,321,483,4,913,690,10,37,1778,1390
CNOT6_8_3055,3475,2974,2522,3055,4970,4337,3641,1329,3468,3388,4528,51
03
CNTNAP1_8_3056,706,917,504,752,551,1575,629,415,992,202,896,303
COG1_8_3057,1241,1491,1334,1427,372,2389,1138,753,1667,796,1965,585
COG3_8_3058,302,128,218,181,158,54,166,14,134,213,451,602
COG7_8_3059,2959,2477,1945,2844,1102,1555,2949,2365,4023,5190,2042,304
9
COG8_8_3060,2249,2609,2474,2518,2728,2597,2736,2555,2773,2689,2880,232
8
COL10A1_8_3061,660,1088,721,1009,424,1688,1817,1199,1744,624,851,471
COL14A1_8_3062,430,807,546,479,192,788,914,386,199,525,1562,488
COL15A1_8_3063,1428,1125,1020,834,1036,755,2023,1790,1247,2190,2199,16
85
COL16A1_8_3064,508,259,362,343,15,113,62,104,505,358,518,92
COL17A1_8_3065,480,656,518,660,752,328,1035,405,179,473,619,725
COL1A1_8_3066,482,222,554,378,196,868,397,436,222,545,168,193
COL1A2_8_3067,578,1030,438,360,2025,592,1006,831,490,430,476,544
COL21A1_8_3068,1287,1110,1331,1050,1143,344,1034,1249,1031,1367,1452,1
659
COL22A1_8_3069,1195,1621,1022,1281,1107,1356,423,827,1045,1136,800,193
8
COL23A1_8_3070,137,149,228,77,37,30,741,197,67,277,252,119
COL24A1_8_3071,1267,945,855,1038,1239,1163,619,22,1861,494,820,2102
COL27A1_8_3072,499,621,613,505,140,37,2402,326,564,585,493,482
COL3A1_8_3073,1243,1422,1335,1311,2189,1840,1064,956,1663,930,1429,126
5
COL4A1_8_3074,362,718,360,504,263,461,366,83,171,160,192,42
COL4A2_8_3075,968,1535,986,908,2640,1562,295,866,667,617,1291,1765
COL4A3_8_3076,0,0,0,0,0,0,0,0,0,0,0,0
COL4A4_8_3077,458,549,372,664,214,213,710,483,117,323,641,1787
COL5A1_8_3078,601,712,275,725,174,757,209,450,725,189,806,680
COL5A2_8_3079,439,451,343,483,366,380,466,688,480,582,734,715
COL5A3_8_3080,680,544,627,518,192,485,94,471,388,499,402,742

COL6A1_8_3081,1188,1728,1344,831,1907,1721,2333,1252,2838,2543,1083,23
17
COL7A1_8_3082,811,585,321,177,117,661,2576,102,1783,185,591,324
COL8A2_8_3083,647,305,505,791,475,610,903,222,1045,692,844,399
COL9A2_8_3084,211,377,189,135,948,549,173,19,147,50,56,494
COL9A3_8_3085,1714,1552,1492,2058,1351,2137,2643,1325,1386,1711,2484,1
579
COMMD1_8_3086,1091,703,968,1344,169,1221,1284,1216,1303,1340,1070,1596
COPB2_8_3087,1195,719,874,758,413,1124,1628,228,859,1634,2165,866
COPG2_8_3088,1671,1862,2093,1927,1914,330,3127,1810,1438,1461,1257,103
3
COPZ1_8_3089,2056,1357,1575,1995,1709,749,2949,1883,2479,1612,2995,198
2
COPZ2_8_3090,489,635,630,484,595,954,1937,873,803,216,638,712
CORIN_8_3091,479,73,193,333,214,298,83,325,1439,379,4,176
COX18_8_3092,2908,3523,3442,3225,1850,4273,2748,4831,2539,4679,3660,29
19
CPLX1_8_3093,195,278,63,151,717,29,150,346,50,24,484,51
CPLX3_8_3094,536,394,419,284,381,567,150,127,295,97,485,38
CPNE6_8_3095,1414,1783,1613,1231,1673,1469,1507,2220,1891,1512,1896,21
69
CPXM2_8_3096,7961,9758,10209,9300,9175,8857,10042,11602,12034,9049,100
58,11345
CRABP1_8_3097,1188,1363,1607,1790,1858,1495,2333,2253,1536,2500,1304,1
778
CRH_8_3098,334,258,183,414,50,87,403,503,19,153,266,199
CSE1L_8_3099,1300,1186,1431,995,559,955,188,411,649,733,2432,1701
CTSW_8_3100,658,716,433,343,1122,322,9,19,632,748,17,3191
CXCL10_8_3101,3227,2274,2232,2056,2103,2074,1748,3417,1686,2504,3440,3
465
CYGB_8_3102,693,560,296,442,592,110,924,277,409,136,803,313
CYTH3_8_3103,357,459,439,320,189,1155,356,46,117,1039,154,1029
DDI2_8_3104,768,1261,392,786,691,1142,1175,628,487,690,688,1384
DIRC2_8_3105,2635,2724,2644,2650,2967,2281,1843,2572,2501,3763,2453,24
05
DISP1_8_3106,412,383,393,440,573,443,2,606,196,193,4,471
DLL4_8_3107,1208,1294,767,1042,756,850,384,943,987,2452,1020,1310
DNAJC5B_8_3108,985,836,995,1209,322,2509,636,1632,797,904,1293,1500
DNAJC6_8_3109,1839,2033,1230,1682,1459,1428,2118,774,770,3049,1018,232
8
DOC2A_8_3110,3248,2716,2699,2722,2716,4020,2972,3527,3233,3578,4358,21
09
DOC2B_8_3111,984,930,688,687,2067,378,1230,356,628,900,1597,609
DSCAML1_8_3112,310,540,351,306,254,546,20,326,682,453,482,112
ECEL1_8_3113,1072,941,1260,1079,2962,989,281,2458,276,660,348,1969
EID2_8_3114,1017,974,1000,1045,1511,1149,769,741,1442,985,2366,854
EPCAM_8_3115,1040,978,618,773,1122,1595,1667,1731,662,1381,900,442
EXOC2_8_3116,569,497,290,432,382,272,750,226,301,790,458,903
EXOC3_8_3117,926,661,1376,612,665,656,704,1063,401,393,2378,1310
F11R_8_3118,753,1208,665,911,755,741,634,1023,1423,719,398,655

FABP1_8_3119,948,1063,1131,750,885,1342,1032,663,544,1399,45,879
FABP2_8_3120,1239,933,1040,1302,1985,2314,1072,1030,605,826,1199,2157
FABP3_8_3121,857,966,1073,761,364,2255,239,1001,931,417,497,869
FABP4_8_3122,687,919,996,1032,975,1828,1469,2310,484,1689,844,1746
FABP7_8_3123,3295,3255,2524,2890,3993,3738,5802,3606,5021,3367,5563,44
37
FABP9_8_3124,6610,6451,5010,5773,6487,4991,3072,7922,4275,6703,5943,75
06
FAM101A_8_3125,600,552,777,314,1112,411,842,264,394,184,1115,195
FAM117A_8_3126,236,158,106,243,94,1095,210,435,169,305,24,156
FAM57A_8_3127,1170,1005,1484,1446,153,1207,1757,1228,1318,1877,1932,11
77
FAP_8_3128,805,1194,1107,1025,1496,2842,484,540,1641,899,1243,718
FBF1_8_3129,495,649,401,716,861,43,1007,383,774,174,526,74
FCN1_8_3130,1546,1616,1235,1240,3523,1117,3479,2714,1142,2337,2384,141
9
FDX1_8_3131,520,251,703,643,638,165,603,40,357,542,612,279
FDX1L_8_3132,364,376,386,414,141,907,689,182,92,174,504,372
FGF4_8_3133,1029,1030,864,1037,473,605,1957,424,1101,1295,180,439
FGL2_8_3134,823,837,641,978,474,1205,1886,670,993,1866,383,541
FOLR3_8_3135,419,240,325,405,396,103,95,11,1287,541,131,86
FOLR4_8_3136,1162,760,533,1094,1171,383,980,675,1259,808,2238,631
FRG1_8_3137,1315,1295,1518,1461,813,2519,2808,2871,1353,1701,724,1593
FTL_8_3138,593,421,446,365,1372,743,1046,1510,719,1171,1054,294
FXC1_8_3139,246,94,118,136,126,7,0,384,100,88,267,40
GABARAP_8_3140,1031,1034,1194,1518,1290,460,556,1000,1140,1764,842,120
4
GGA2_8_3141,345,371,356,725,429,330,236,780,773,600,270,473
GJA1_8_3142,1028,1693,1004,1258,771,1263,637,620,1419,1186,2010,1668
GJA3_8_3143,1092,716,957,900,829,509,1755,192,314,931,1322,357
GJA4_8_3144,216,334,64,311,190,638,155,0,112,361,343,479
GJA8_8_3145,124,292,226,49,50,194,39,12,287,184,236,348
GJB2_8_3146,1085,1530,941,988,1770,1011,2793,1755,939,1850,2272,2573
GJB4_8_3147,2042,2043,2063,2529,2349,1996,1920,2839,1871,1696,1765,273
7
GJB5_8_3148,752,380,775,454,190,475,871,161,106,115,439,210
GJC2_8_3149,218,189,276,142,455,127,853,8,301,25,312,909
GJC3_8_3150,1159,1092,1219,1512,1533,1804,390,891,457,778,1480,1909
GJD2_8_3151,790,1132,1032,1001,2417,1089,883,615,1415,1106,1071,1337
GJD3_8_3152,528,674,481,725,590,338,23,2020,932,292,884,46
GJD4_8_3153,810,703,586,523,1412,1571,419,259,549,685,1133,509
GKN1_8_3154,360,534,510,253,941,314,792,213,114,1150,268,329
GLCCI1_8_3155,982,1463,977,1117,834,565,2493,1759,1095,2144,1403,2040
GLTP_8_3156,657,426,346,140,69,235,560,380,396,1,719,305
GLYATL2_8_3157,1074,1290,896,970,89,1329,1102,517,1672,2012,783,3553
GP9_8_3158,395,185,89,70,29,387,3,239,127,39,131,37
GPIHBP1_8_3159,1828,1530,1561,1713,1373,2427,2204,1171,2212,1635,1075,
2515
GPR180_8_3160,2388,2737,2498,2745,2420,4474,3181,2506,1938,2878,1997,2
041

GRN_8_3161,566,895,886,590,1852,367,1485,686,829,955,674,131
GZMH_8_3162,482,722,479,1091,784,141,655,368,392,155,661,136
GZMK_8_3163,220,154,244,267,283,41,64,1002,913,253,160,2
GZMM_8_3164,172,195,282,145,132,515,616,14,527,58,726,232
HBA1_8_3165,1991,1169,1312,1917,767,1030,217,2595,2436,1278,1472,1394
HBA2_8_3166,1991,1169,1312,1917,767,1030,217,2595,2436,1278,1472,1394
HBE1_8_3167,96,184,236,143,87,9,0,11,11,274,611,7
HBZ_8_3168,0,0,51,14,0,0,0,0,0,0,0,10
HECA_8_3169,891,948,802,1151,630,832,669,703,1605,1456,2190,1016
HGFAC_8_3170,2996,2842,3352,3321,2298,3432,1478,1860,2065,3782,2701,50
62
HIAT1_8_3171,765,1052,1509,1120,393,774,615,23,936,1287,2809,1628
HLA-DQB1_8_3172,619,642,397,268,8,807,176,1065,143,883,201,737
HMCN1_8_3173,1287,1296,1185,1256,1027,1997,626,1047,830,1882,1950,947
HMHA1_8_3174,383,368,530,203,105,469,26,0,335,134,154,48
HPCAL4_8_3175,387,186,360,244,277,538,450,205,610,333,435,325
HPR_8_3176,442,253,137,123,6,112,163,41,429,12,7,126
HPX_8_3177,4171,4477,4444,5573,5842,8407,6921,3200,4497,6098,4225,5147
HSP90B1_8_3178,1523,1917,1514,2234,1696,2754,3494,1982,2276,1806,1772,
1639
HTRA1_8_3179,1085,511,424,448,632,1786,1277,629,860,1374,1105,481
HTRA4_8_3180,1685,2160,1918,1982,1917,1177,1158,1596,1723,1635,4618,21
17
IFNG_8_3181,3729,3551,3009,3226,1500,2889,2354,3905,3145,3932,3195,335
6
IGFBP7_8_3182,1075,1609,1487,1484,516,717,1614,1355,1279,2472,1082,168
6
IL12B_8_3183,3254,2593,2537,2283,2223,1341,1639,3336,2590,3077,3102,20
59
IL13_8_3184,1265,1583,1310,1149,1063,4006,1365,1323,1770,1999,2444,185
4
IL17A_8_3185,590,802,685,783,1304,1757,495,836,1043,557,1178,958
IL1A_8_3186,781,1070,758,545,1436,928,457,488,341,412,1382,1781
IL1B_8_3187,339,275,200,289,328,676,460,100,157,1011,426,334
IL3_8_3188,1043,1219,1357,1134,1521,2000,287,2455,2193,1247,468,1462
IL5_8_3189,835,645,583,703,555,652,503,443,731,678,159,1142
INSL3_8_3190,314,181,79,370,133,85,48,69,125,191,140,145
IPO13_8_3191,535,421,683,783,1036,668,1147,707,421,1237,244,214
IPO4_8_3192,429,307,342,405,912,393,131,569,224,167,120,17
IPO5_8_3193,2537,1961,2095,1788,2530,1241,3677,961,1252,1617,4767,2396
IPO7_8_3194,2732,3434,2685,2591,2153,4632,6671,1942,1831,5973,2273,407
4
IPO9_8_3195,569,429,681,957,858,1132,1841,800,1156,653,248,560
ITGA10_8_3196,3560,4316,3321,3417,3299,3123,3314,3873,4281,6128,4805,3
461
ITGA11_8_3197,3148,3197,3315,2456,2270,2426,2600,3931,2254,3368,2785,3
618
ITGA2_8_3198,440,481,270,256,444,427,473,317,1703,266,1028,582
ITGA4_8_3199,389,245,102,287,186,119,15,603,107,232,551,747
ITGA5_8_3200,506,912,908,398,0,573,461,398,814,520,693,2106

ITGA8_8_3201,1957,1962,2185,1956,2239,2398,3090,2863,2715,1699,2191,21
60
ITGAX_8_3202,830,729,589,313,1374,803,542,956,1186,1338,358,594
ITGB5_8_3203,118,117,241,154,11,269,753,259,0,220,2,260
ITGB6_8_3204,620,275,175,733,729,59,837,974,1239,151,149,1460
ITGB8_8_3205,1175,874,1262,1133,1802,1520,1015,1883,1752,1699,2568,182
2
ITLN1_8_3206,568,447,635,523,1013,743,302,586,628,356,1607,1097
KDELR1_8_3207,1952,1777,2075,1931,2046,1524,1525,1755,1948,1956,1849,7
52
KEL_8_3208,1928,2546,1820,2091,1945,2339,3166,3306,2662,2712,2968,2296
KIF20A_8_3209,1266,996,1300,717,531,1186,2153,616,1909,550,1637,1087
KIF3B_8_3210,2201,1987,1537,1747,1657,2507,1622,2915,2456,1819,2200,25
65
KIF5A_8_3211,156,232,170,233,4,387,0,424,195,19,761,64
KLK13_8_3212,1144,1273,1269,1031,400,1475,1016,1593,1248,2828,2111,167
1
KLK14_8_3213,5271,5794,4824,4983,4663,5301,3501,3577,3546,5356,6046,70
20
KLK4_8_3214,411,471,135,508,56,672,129,47,34,235,1075,504
KLK9_8_3215,336,428,265,95,1042,380,202,1,154,594,102,23
KPNA1_8_3216,890,791,891,967,651,1507,1125,1695,255,462,986,1005
KPNA2_8_3217,1096,461,729,634,177,567,370,568,1001,81,1598,866
KPNA3_8_3218,2256,1622,2149,1991,1224,2396,1559,2108,4293,622,3045,433
2
KPNA4_8_3219,730,884,505,1009,780,748,414,1347,766,1273,887,768
KPNA6_8_3220,792,866,369,313,233,1498,832,235,348,609,1052,663
KPNB1_8_3221,2137,1767,2152,2095,1376,2236,1033,2441,1877,1900,1204,28
23
KRT12_8_3222,297,634,435,245,576,950,1197,576,143,406,415,42
KRT7_8_3223,650,795,776,631,807,793,2470,533,954,408,846,2090
KRT8_8_3224,1,106,10,27,0,7,0,0,0,6,1,4
KRTAP5-4_8_3225,2467,1829,1762,1529,3239,3094,2131,1127,3086,1895,1953
,2956
LASP1_8_3226,662,625,332,832,823,441,2653,625,296,1292,270,568
LBP_8_3227,522,723,507,416,694,822,916,1573,920,503,860,649
LCN12_8_3228,2846,2454,3014,3089,2520,1399,3572,3429,1291,2347,3597,18
43
LCN1_8_3229,798,681,829,708,1387,848,616,206,308,484,1364,33
LCN2_8_3230,1342,1137,1480,1412,1092,655,1867,2157,1039,1283,1180,1028
LCN8_8_3231,261,175,19,225,653,56,1841,654,65,36,300,8
LCN9_8_3232,2860,3296,2771,2932,4816,3947,3338,1685,2040,4148,3479,131
3
LDLRAD2_8_3233,290,403,317,144,182,398,236,248,186,750,596,171
LDLRAP1_8_3234,1647,1076,1331,1471,2576,2113,2250,1688,1760,790,2639,1
122
LMAN1_8_3235,3452,4146,3565,3456,5552,3077,4327,5157,3933,5761,4512,46
68
LMAN2_8_3236,312,276,311,320,467,411,186,792,455,283,330,142
LPA_8_3237,743,272,565,554,638,1056,178,790,1629,539,560,430

LRIG1_8_3238,661,835,577,664,411,332,2112,1391,1216,108,341,1056
LRP2_8_3239,1297,1336,1063,1444,1186,1959,1113,678,3251,1639,571,1512
LRRC4B_8_3240,182,162,125,302,259,651,112,889,66,199,553,105
LRRCC1_8_3241,1833,2233,1414,1777,1350,3181,758,1237,1859,1782,897,287
6
LYST_8_3242,2394,2930,3089,3461,3272,3098,2299,2958,1933,3543,3972,348
6
MAL2_8_3243,1087,1634,972,1285,563,2006,357,1131,736,1056,419,2374
MATN3_8_3244,701,739,413,703,896,1331,230,870,792,396,148,631
MFSD8_8_3245,4639,4393,4271,4349,3966,3900,2620,3640,5004,3748,4304,55
89
MFSD9_8_3246,2691,3260,3122,2232,3228,2506,3857,1835,2172,3272,2941,18
20
MMAA_8_3247,1123,1288,685,1248,1140,1132,234,1056,1931,902,970,915
MMACHC_8_3248,196,255,313,251,126,314,286,134,7,31,82,281
MMGT1_8_3249,594,587,807,713,222,549,1465,644,2113,721,397,1359
MMP11_8_3250,1447,1239,1500,1842,2871,1585,2932,2461,2002,1482,1517,87
4
MMP13_8_3251,253,257,173,188,10,0,474,46,604,59,79,42
MMP15_8_3252,947,687,603,556,1297,1190,551,77,318,1384,1080,35
MMP16_8_3253,1763,2274,1136,1919,1493,3899,2740,2653,1994,1394,1625,20
35
MMP17_8_3254,1861,1829,2486,2841,2104,1603,1339,4318,2309,1707,2234,29
96
MMP19_8_3255,3099,2022,2372,2734,3476,3256,4377,2362,2921,3019,2671,35
48
MMP24_8_3256,1033,825,610,568,543,1599,942,57,535,403,722,802
MMP25_8_3257,187,207,142,381,646,2,408,481,11,13,509,481
MMP26_8_3258,2903,2175,1880,1854,1406,2504,2386,4433,3278,1535,2014,26
12
MMP27_8_3259,149,77,224,145,1,225,18,0,168,1,24,155
MRS2_8_3260,541,707,395,374,594,163,293,279,397,1248,599,242
MSTN_8_3261,619,937,330,838,1840,265,109,2868,1473,900,208,601
MTX2_8_3262,1422,1428,981,1582,741,1096,2588,1608,2656,1778,2690,2909
MUC2_8_3263,108,47,64,148,0,10,91,30,532,82,127,712
NAPG_8_3264,774,480,300,297,301,615,532,175,460,706,851,766
NAPSA_8_3265,417,421,371,357,1360,74,621,112,1208,423,628,537
NCOA5_8_3266,289,205,101,492,36,79,7,1874,652,366,565,57
NGF_8_3267,5517,5142,4709,5415,4059,5649,8032,5239,5866,6392,7395,6939
NID1_8_3268,911,1236,912,1080,1451,1619,1530,1014,938,1448,1102,681
NPC1_8_3269,1120,1156,552,1340,1427,1276,526,577,1630,1533,1074,823
NPEPPS_8_3270,1459,1348,1152,1653,2607,1080,1441,2201,2318,2247,1562,1
912
NPPB_8_3271,130,348,64,220,17,245,3,412,5,479,16,0
NPY_8_3272,1391,1400,1775,1254,2320,1441,1207,1210,1082,1695,1576,2716
NSMCE1_8_3273,901,1404,1091,1069,1468,1373,2309,1190,322,1984,107,1502
NUP107_8_3274,2134,2221,1799,1564,2121,3516,809,1243,1661,2088,4263,12
38
NUP133_8_3275,1248,749,1118,1030,939,937,1331,716,1507,1499,1048,1067
NUP153_8_3276,18,196,40,250,0,44,1,0,71,93,32,33

NUP160_8_3277,2579,2965,2303,2497,3759,2691,2233,4199,3139,3563,3643,4
182
NUP210_8_3278,334,422,383,385,765,64,299,405,146,920,1057,40
NUP214_8_3279,315,365,343,406,258,168,54,379,182,796,75,513
NUP35_8_3280,299,526,370,352,51,1331,8,1964,104,870,389,325
NUP37_8_3281,140,293,433,312,861,188,592,525,141,64,872,156
NUP54_8_3282,1495,1330,1222,1349,1281,927,1835,735,1445,761,3489,942
NUP88_8_3283,549,357,371,856,243,982,108,598,451,854,416,1081
NUPL2_8_3284,2738,2737,2042,2769,3840,6256,3044,1803,3109,4207,4000,29
17
NUTF2_8_3285,2707,2420,2553,2825,961,2783,2713,1442,2322,5804,4164,388
0
NXF2B_8_3286,2606,1934,1913,2256,1419,3348,4208,2537,3423,1969,3850,25
32
NXF2_8_3287,2606,1934,1913,2256,1419,3348,4208,2537,3423,1969,3850,253
2
NXF3_8_3288,5194,4671,4057,4340,7498,5078,3558,5719,6018,5137,6243,667
5
NXT1_8_3289,525,292,378,421,701,450,249,13,232,1491,986,188
OAZ2_8_3290,426,594,433,421,251,1271,902,345,481,684,787,601
OBP2A_8_3291,553,767,551,486,588,1009,843,233,1262,320,921,186
OBP2B_8_3292,3570,4494,2743,2701,2633,2620,7232,3070,4383,5295,6704,28
68
OCA2_8_3293,531,363,278,601,79,326,40,236,553,64,163,235
OGFOD1_8_3294,1143,1004,337,914,470,1214,490,736,1229,1403,964,1012
OGFOD2_8_3295,210,371,475,348,563,808,138,55,95,351,425,762
OGFR_8_3296,555,724,501,943,60,2041,1007,461,1128,198,330,1100
OVCH1_8_3297,2767,2774,2778,3890,4176,2079,4015,3004,3008,4524,6907,29
53
OVCH2_8_3298,3013,2810,2367,3258,4011,2561,2503,1312,3076,3799,4247,30
20
OXNAD1_8_3299,2330,2479,2737,2389,1949,1943,4319,1784,2593,2282,7198,1
405
OXT_8_3300,285,278,137,241,392,175,120,304,21,599,1366,77
PANX1_8_3301,1977,814,1713,1333,1456,811,2527,873,1551,1865,1788,1632
PAQR7_8_3302,331,247,499,337,155,179,303,339,223,243,776,323
PCDHB11_8_3303,2381,3006,2011,2845,3043,2183,1647,3799,1665,3101,3955,
4093
PCDHB16_8_3304,1106,1525,1248,1346,875,1738,915,1226,308,1385,2354,162
4
PCSK4_8_3305,37,75,122,60,0,143,205,17,2,20,287,70
PCSK7_8_3306,944,627,608,578,937,554,235,837,376,314,587,850
PEA15_8_3307,1138,836,984,877,2558,968,1545,611,1085,1933,2145,749
PET112_8_3308,749,680,722,546,1493,409,1006,503,1189,900,961,453
PEX13_8_3309,4636,5312,5635,5355,4980,9162,5652,6288,4852,8955,5670,48
25
PEX7_8_3310,357,193,326,352,186,96,194,188,232,527,457,269
PF4_8_3311,5053,4977,4386,4222,5302,7148,6843,4682,4267,4038,4001,6130
PFN3_8_3312,209,130,260,70,288,54,8,99,297,178,466,52
PHEX_8_3313,875,1118,812,967,240,1010,466,793,1022,823,1812,323

PIGR_8_3314,398,424,184,333,244,65,131,150,184,1327,403,554
PITPNA_8_3315,505,816,759,497,687,850,897,545,492,411,692,972
PITPNB_8_3316,510,488,370,567,650,40,79,681,368,784,138,437
PLLP_8_3317,278,118,218,182,59,719,142,213,655,532,224,197
PLP2_8_3318,6557,8026,5986,6270,4024,6668,5897,8931,7441,6576,6014,722
2
PLXNB2_8_3319,1758,1994,1344,1431,3068,894,1446,1277,1397,2766,1225,24
61
PNMA2_8_3320,170,152,131,146,510,348,537,743,454,286,275,253
PPP1R14A_8_3321,476,510,588,777,1135,45,1214,252,191,1619,1671,814
PPP1R14C_8_3322,1457,1520,960,1531,2312,648,2520,2291,1773,1636,2186,3
546
PPP1R15A_8_3323,681,1325,767,1215,703,1699,1267,217,1417,1174,258,3027
PPRC1_8_3324,1295,917,585,329,477,2057,1400,1001,972,1184,719,2724
PPY_8_3325,1266,1131,1201,890,1829,952,352,1966,1213,1340,1439,1066
PRB3_8_3326,136,245,199,74,320,306,20,3,138,104,144,4
PROCR_8_3327,229,161,226,95,397,369,830,625,159,34,31,18
PROS1_8_3328,1782,1757,2062,1845,2206,698,1925,2915,2214,1447,2717,204
2
PROZ_8_3329,400,338,398,574,1069,578,478,2037,301,124,367,876
PRPF18_8_3330,4731,4889,4707,4685,4589,4249,5554,3602,2937,4706,6204,5
737
PRSS12_8_3331,1721,2078,1461,1847,2695,2911,2311,1231,2856,2774,1894,2
883
PRSS22_8_3332,1279,952,1029,1164,2379,194,1401,1023,921,1033,361,936
PRSS27_8_3333,281,195,281,60,14,54,843,50,9,236,768,2
PRSS33_8_3334,334,137,396,182,1,623,398,206,353,31,31,373
PRSS36_8_3335,296,286,72,666,1227,138,349,569,467,123,154,0
PRSS8_8_3336,175,66,178,197,144,151,10,286,15,30,59,300
PSCA_8_3337,397,451,796,541,1357,764,1487,1,45,922,863,0
PTOV1_8_3338,891,939,566,754,1494,569,390,221,559,864,1569,462
RABIF_8_3339,5483,5783,4438,5536,7459,8187,5434,4469,7373,4564,4345,60
06
RAMP1_8_3340,150,89,38,111,0,0,0,144,357,162,47,82
RAMP2_8_3341,1265,970,898,1228,1984,1464,1147,895,379,1475,248,1017
RAMP3_8_3342,493,346,337,206,15,639,395,1,375,513,594,352
RANBP17_8_3343,1429,2148,1691,1753,1461,1315,2007,2156,1679,2312,1580,
2483
RASSF9_8_3344,3687,3866,3001,3420,3289,4808,5490,2184,2086,4612,4497,2
750
REEP5_8_3345,445,902,662,518,396,231,263,552,470,840,486,845
RHAG_8_3346,533,626,367,769,468,747,96,45,253,1932,924,719
RLBP1_8_3347,1208,1737,1401,1311,1993,879,786,1681,816,1062,1067,1594
RPL15_8_3348,1311,1787,1872,1622,876,2138,2766,4590,1141,1313,2399,159
6
S100A12_8_3349,541,764,424,696,680,373,888,369,792,512,1045,1064
S100A1_8_3350,495,262,249,392,171,103,167,214,139,527,93,385
S100A2_8_3351,1853,2535,1209,1851,1049,2599,1878,3153,1141,2735,4118,3
700
S100A3_8_3352,312,535,289,520,597,1270,169,452,687,329,96,421

S100A6_8_3353,774,815,648,752,854,497,835,736,923,978,910,216
S100B_8_3354,1104,1220,1264,1376,1743,1018,1553,2685,527,2111,1872,658
S100P_8_3355,227,355,188,173,0,88,1,4,92,325,502,129
SAA4_8_3356,2113,1529,1538,1797,2179,1547,2270,1028,1464,1608,1081,295
7
SCAMP1_8_3357,2730,2570,2911,2143,2284,3727,2487,1327,2482,1756,3196,3
406
SCAMP2_8_3358,1736,2406,1890,1630,2001,2552,3069,1669,1270,1420,1675,8
87
SCFD2_8_3359,1487,2232,1300,1609,2304,1361,1902,956,2341,2130,1323,165
9
SCLT1_8_3360,1032,1341,814,1314,2387,850,320,131,695,110,1323,2476
SCPEP1_8_3361,396,15,17,155,226,416,0,438,1558,61,42,117
SDC2_8_3362,1184,1122,1171,869,618,1170,1769,894,979,1093,2459,1166
SEC14L3_8_3363,989,728,871,694,661,1870,1066,1231,1138,1994,1031,420
SEC22A_8_3364,1469,1766,1345,1539,2208,2910,1090,970,1551,697,2517,243
3
SEC23A_8_3365,548,484,267,610,20,345,182,514,439,663,711,555
SEC24A_8_3366,917,426,530,619,1216,597,1786,214,1505,895,188,1118
SEC24D_8_3367,1027,1579,1224,1526,1206,2654,1892,1341,1913,1311,1900,1
424
SEC61A1_8_3368,664,731,754,402,1577,490,76,882,451,390,1229,635
SEC61B_8_3369,804,811,611,660,558,1318,399,1482,740,553,1196,974
SEC62_8_3370,951,867,654,1053,281,722,1139,852,1383,598,1372,1585
SEC63_8_3371,2451,3582,2968,2199,4506,2426,4225,2407,2295,2473,4585,40
11
SELP_8_3372,730,634,363,493,421,247,77,1360,917,528,1721,773
SERINC1_8_3373,3553,3309,3302,3912,4982,5899,3177,4018,4166,3965,2881,
2786
SERPINA11_8_3374,276,85,413,119,177,356,48,35,331,184,430,712
SERPINA12_8_3375,464,481,360,614,55,43,1602,542,29,360,265,76
SERPINA3_8_3376,442,872,322,592,305,965,99,310,260,677,385,349
SERPINA4_8_3377,0,0,0,0,0,0,0,0,0,0,0,0
SERPINA5_8_3378,2320,2697,1924,2708,1709,2661,2629,2038,1683,4185,1998
,1003
SERPINA7_8_3379,1127,1122,1170,1267,2177,865,3215,1008,2107,594,1614,3
14
SERPINB10_8_3380,2732,2503,1805,2540,1656,2401,1570,2363,4047,2551,180
4,3783
SERPINB12_8_3381,400,307,337,528,1031,36,1084,1989,364,748,22,27
SERPINB13_8_3382,526,439,469,581,538,791,948,1032,840,949,489,592
SERPINB1_8_3383,420,474,152,230,609,380,13,553,255,351,272,35
SERPINB4_8_3384,3718,3690,3583,3558,3409,4936,3413,4671,2808,4173,4753
,4941
SERPINB9_8_3385,487,242,315,147,416,316,47,36,328,833,296,394
SERPINC1_8_3386,2357,2483,2703,2501,1521,4430,2405,2348,2228,3084,3063
,3169
SERPIND1_8_3387,734,860,1192,882,1294,908,791,1641,1462,1110,883,294
SERPINF1_8_3388,1417,889,1063,1336,962,1688,1465,1534,1220,2325,664,72
4

SFXN1_8_3389,641,742,872,810,1073,374,1373,1866,1062,1809,1130,439
SFXN2_8_3390,338,468,308,443,550,300,1706,761,28,1104,771,1418
SFXN3_8_3391,341,471,312,445,553,311,1713,763,28,1107,776,1427
SFXN4_8_3392,3707,3259,2196,2537,1259,1748,2705,3234,1976,1377,5564,48
95
SFXN5_8_3393,459,731,549,524,506,838,1469,173,279,618,900,770
SLC15A5_8_3394,1197,1256,1900,1316,1992,2431,1317,878,1113,1813,1523,1
904
SLC16A13_8_3395,1022,624,1061,899,480,1885,1519,694,658,1298,1086,1113
SLC16A14_8_3396,1751,1378,1684,1741,2583,2191,2302,2188,2083,3226,2224
,1740
SLC16A9_8_3397,1842,1751,1530,1508,1442,1013,1461,1682,1756,1733,1900,
1567
SLC17A9_8_3398,3007,3053,2768,3011,4951,3556,5835,2230,2505,4249,5895,
3992
SLC22A20_8_3399,3045,3694,2927,3680,3725,3620,2435,2675,2831,3622,4335
,3423
SLC22A24_8_3400,1083,797,802,800,1098,1128,1495,244,666,1018,466,649
SLC22A25_8_3401,443,197,143,265,55,124,116,1,205,1203,413,61
SLC25A23_8_3402,558,414,394,623,467,34,569,963,1076,191,924,335
SLC25A29_8_3403,25,70,443,38,0,415,511,0,0,95,0,0
SLC25A31_8_3404,1921,1931,1632,2177,528,1863,1053,710,1449,2052,2041,3
042
SLC25A38_8_3405,3968,4025,3448,4690,4256,2991,3413,4078,3213,3668,6183
,4426
SLC25A42_8_3406,4277,4890,3861,4694,3837,4033,5493,3725,5813,4923,5797
,5773
SLC25A46_8_3407,4401,3739,3186,4176,5074,5920,5686,3767,5485,6691,4239
,4273
SLC25A48_8_3408,245,514,398,331,909,607,1588,596,51,655,552,207
SLC38A7_8_3409,159,133,704,223,193,1231,661,1,1,47,5,298
SLC38A9_8_3410,1596,1695,1684,1348,2330,2527,2315,820,1108,1036,2295,1
638
SLC41A2_8_3411,259,191,386,369,54,56,759,25,29,107,2,2202
SLC45A1_8_3412,239,277,381,311,486,135,417,239,529,60,1171,120
SLC45A3_8_3413,952,1139,894,841,789,701,1814,653,817,1542,1227,2597
SLC47A1_8_3414,508,757,396,441,2758,1131,116,1767,1173,664,880,434
SLC48A1_8_3415,682,991,590,581,621,681,1964,409,330,599,685,176
SLC5A12_8_3416,242,129,327,224,1930,273,88,281,534,209,398,557
SLC6A17_8_3417,2188,1983,1773,2016,1623,2476,1467,3650,1309,2967,2744,
1871
SLC7A14_8_3418,901,638,762,724,1098,256,441,693,1709,616,1093,990
SLIT2_8_3419,219,81,31,51,4,8,1,28,0,44,0,138
SNAP29_8_3420,695,497,670,940,827,492,685,73,229,685,1704,196
SNX12_8_3421,1934,1968,1570,1782,2090,1337,1832,2339,3306,1904,1483,96
0
SNX13_8_3422,7180,8683,6383,6550,6192,6081,11630,6589,8155,6902,10161,
6967
SNX17_8_3423,1062,1154,909,1034,1600,1804,726,340,582,796,675,170
SNX19_8_3424,1060,1688,1432,1649,1387,1277,1658,1216,722,387,1831,2457

SNX22_8_3425,0,0,0,0,0,0,0,0,0,0,0,0
SNX24_8_3426,2260,2995,1961,1929,592,2900,3932,2710,1324,3032,2577,359
2
SNX2_8_3427,1188,811,748,647,396,873,275,345,1500,982,1235,3498
SNX4_8_3428,766,1179,450,1000,1215,1106,316,1020,1341,703,1947,566
SNX8_8_3429,232,178,345,96,43,49,650,0,579,36,22,1466
SNX9_8_3430,1517,1818,1134,1318,757,771,1686,1747,1031,1209,1672,1676
SORCS2_8_3431,395,332,310,251,1,787,1442,1847,315,257,630,263
SORCS3_8_3432,2103,2089,1634,2299,1978,4161,2815,2017,3132,3206,1769,2
403
SORL1_8_3433,2055,1933,1771,2396,2194,2787,400,841,2058,2775,6170,3766
SOS1_8_3434,3534,3179,3191,3486,3747,3012,2791,2111,3111,3029,2289,623
2
SST_8_3435,932,557,820,840,447,866,1164,1414,206,814,1992,486
ST13_8_3436,1056,1269,1727,1034,580,1158,1004,981,1276,1294,295,823
STAB1_8_3437,811,1127,988,950,975,1500,419,478,1381,469,502,2709
STARD4_8_3438,159,67,115,114,1018,0,42,0,420,5,179,22
STARD5_8_3439,1608,1495,1397,800,2578,364,956,2367,956,1145,1422,2224
STARD6_8_3440,311,536,463,310,165,369,1145,40,837,245,785,171
STEAP1_8_3441,609,706,933,605,570,756,870,300,51,482,1209,1171
STMN4_8_3442,2064,1350,1638,1808,1905,2397,1730,1096,3402,2266,1903,16
65
STX11_8_3443,584,607,331,498,1569,1118,22,560,155,424,98,1247
STX18_8_3444,571,314,131,184,103,303,127,46,189,235,342,838
STX4_8_3445,126,37,119,49,0,0,33,0,13,0,599,2
STX5_8_3446,1466,1533,1060,1641,1442,1002,1677,1309,869,3051,1963,1314
STX6_8_3447,676,612,496,749,931,1135,631,139,897,701,299,435
STX7_8_3448,5224,4878,6019,4889,6693,6782,6349,7385,6995,5731,8771,580
5
STXBP3_8_3449,999,700,693,859,743,1027,67,811,695,1029,779,1712
SVOP_8_3450,1506,1237,1252,938,41,1782,1770,194,2465,1716,754,1318
SYP_8_3451,440,362,124,261,0,0,0,0,912,25,461,694
SYT10_8_3452,694,623,719,782,45,948,134,1244,644,1867,1762,313
SYT11_8_3453,524,945,726,564,734,341,18,411,546,1173,894,1136
SYT13_8_3454,737,780,385,783,113,181,330,1637,1718,843,1150,222
SYT16_8_3455,986,812,799,752,561,181,607,636,328,465,1637,458
SYT4_8_3456,4571,5213,4466,3837,5342,4223,5065,6777,4483,3307,3770,656
4
SYT5_8_3457,342,488,314,598,420,510,437,300,1223,433,450,566
SYT6_8_3458,1716,856,1384,1530,522,726,1304,676,828,990,2916,1444
SYT7_8_3459,1726,1824,920,915,443,526,1549,337,840,1256,2422,1629
SYT8_8_3460,186,638,405,194,538,580,536,64,9,295,298,322
SYT9_8_3461,5204,5147,4932,5346,4718,3573,5865,5239,4492,4548,6409,540
3
TAS2R42_8_3462,4529,4563,4069,4190,4106,5863,6356,5640,4559,4841,4412,
3847
TBC1D9_8_3463,540,595,392,523,130,806,52,883,1213,547,308,473
TCN1_8_3464,1655,2381,1238,1325,1596,2279,1118,506,2757,717,3010,1682
TEKT4_8_3465,128,98,218,110,1,9,821,17,7,37,146,650
TF_8_3466,242,363,277,248,26,427,1,0,121,662,1,11

TG_8_3467,647,712,407,277,283,1060,654,321,906,804,499,492
TGFB1_8_3468,9589,10103,8535,9714,14685,7503,12749,11886,9349,10708,10
207,11655
TIMM10_8_3469,2001,1894,2044,2114,1655,1930,2492,2765,1923,702,1036,15
82
TIMM13_8_3470,642,659,750,749,829,608,1070,1378,1255,1013,107,594
TIMM17A_8_3471,908,1371,677,993,535,2208,650,1201,881,1367,1295,2133
TIMM22_8_3472,1642,2120,1730,1343,2435,1571,1829,2821,1818,1013,3396,1
540
TIMM23_8_3473,1413,1460,897,760,1650,297,789,1353,2291,1133,1838,568
TIMM44_8_3474,915,1292,971,1065,1114,1424,340,1358,693,1959,513,1325
TIMM8B_8_3475,386,486,108,311,147,1390,688,661,19,894,145,281
TIMM9_8_3476,124,150,100,27,0,253,0,844,7,346,11,18
TLL2_8_3477,2099,3068,2560,1971,3134,2713,3403,3414,3578,2267,2310,356
5
TM9SF2_8_3478,2480,2287,3092,2775,3954,1415,2098,2987,1594,2138,4016,2
826
TM9SF3_8_3479,3757,3982,3323,3212,4030,5084,3170,3388,2152,4291,5806,5
661
TM9SF4_8_3480,626,363,486,563,537,338,360,589,381,772,502,1161
TMCO3_8_3481,2864,2056,2294,2352,2787,3329,1699,3334,2667,2188,2757,32
07
TMED10_8_3482,3144,3410,3102,3226,2265,4323,4912,3007,2567,3929,4310,2
893
TMED1_8_3483,389,129,200,322,28,196,147,141,1050,461,186,184
TMED2_8_3484,5920,6049,5000,5190,6937,6667,6163,5832,6186,4245,9026,60
60
TMED3_8_3485,242,155,94,261,156,577,771,277,23,42,212,1024
TMED4_8_3486,672,979,382,1233,414,1343,389,49,330,737,565,1413
TMED7_8_3487,2498,2323,3073,1661,1376,1095,2231,1728,3158,3694,3338,31
67
TMED9_8_3488,4637,5320,4082,4236,3339,6323,5169,3684,6512,6302,3582,47
19
TMEM104_8_3489,582,608,475,765,288,615,958,471,364,1407,1129,588
TMPRSS11B_8_3490,1017,1223,1221,906,340,287,572,702,1566,328,490,875
TMPRSS11D_8_3491,439,528,228,456,690,373,289,244,154,476,867,483
TMPRSS11E_8_3492,3539,3216,2999,3494,3062,3547,4048,2483,4215,6350,433
3,4617
TMPRSS11F_8_3493,2581,1979,2448,2025,2083,2656,1200,1134,3961,2133,141
1,1007
TMPRSS12_8_3494,301,252,119,146,444,114,16,150,669,127,14,104
TMPRSS5_8_3495,1025,421,1115,680,1473,408,85,573,947,582,347,551
TMPRSS6_8_3496,997,1145,679,1351,830,904,1152,939,1449,1446,68,2422
TMPRSS7_8_3497,1124,1355,1220,1718,109,1693,1601,1596,1551,1433,810,14
42
TMPRSS9_8_3498,616,498,502,295,396,0,33,246,285,621,919,1410
TNC_8_3499,384,219,204,167,116,874,534,1788,4,0,228,254
TNF_8_3500,1155,1062,886,1274,1609,1001,1008,1029,674,610,1632,1499
TNFRSF9_8_3501,895,1199,1004,1060,825,275,1524,1323,901,786,1826,547
TNNC2_8_3502,610,220,244,299,1228,15,1030,327,375,651,959,1104

TNN_8_3503,842,1006,800,832,139,461,1192,799,48,575,389,2265
TNNI3_8_3504,587,605,525,488,88,287,647,303,262,307,290,314
TNR_8_3505,817,690,852,474,851,903,708,265,1111,1361,1198,691
TOMM20_8_3506,581,672,299,399,407,1450,582,196,215,329,333,427
TOMM22_8_3507,342,319,424,515,120,300,275,163,194,247,1662,171
TOMM70A_8_3508,977,919,891,1094,1680,498,1769,855,913,1914,359,1453
TOMM7_8_3509,261,501,341,318,196,1027,116,33,84,127,284,683
TPSG1_8_3510,478,265,506,353,599,532,209,235,746,333,199,1327
TRAK2_8_3511,1463,1102,997,1315,780,2801,1365,912,851,889,918,890
TRAPPC10_8_3512,801,977,949,840,1759,1837,785,952,888,584,902,2498
TRAPPC8_8_3513,1384,1429,1029,1515,761,2247,258,1425,2457,1110,1270,29
58
TSNAX_8_3514,1408,1489,1592,1667,797,899,1615,2132,1152,2500,4066,1595
TTPA_8_3515,2493,1863,1699,1945,2879,1284,2406,1819,836,3068,3195,2423
TTR_8_3516,172,25,59,200,8,0,6,103,93,564,29,0
TUBA1A_8_3517,2834,2515,1804,2725,2919,4087,2855,2094,1321,2104,2261,2
310
TUBA1C_8_3518,1019,1021,1198,1393,798,1365,495,1074,740,483,978,630
TUBA3D_8_3519,763,436,393,725,3188,956,1147,1395,530,123,557,1293
TUBA3E_8_3520,1023,647,302,633,1606,2039,178,24,185,630,698,329
TUBA4A_8_3521,2570,2260,1741,2226,2934,3415,2835,2082,1696,1686,2353,2
296
TUBB1_8_3522,551,685,447,458,25,874,102,8,623,661,80,8
TUBB2A_8_3523,3586,3376,3172,3311,3426,4224,3880,4416,3705,5170,2938,2
372
TUBB2B_8_3524,3586,3376,3172,3311,3426,4224,3880,4416,3705,5170,2938,2
372
TUBB6_8_3525,111,242,152,106,345,4,273,474,22,200,32,503
TUBB8_8_3526,1306,854,1353,1237,978,931,560,1193,2387,1149,838,1153
TUBE1_8_3527,3203,3067,1797,2645,2449,1408,3729,2110,5576,4086,1867,34
52
TUBG1_8_3528,1046,894,1024,896,654,945,1360,853,1132,988,574,2945
TUBG2_8_3529,1741,2096,1615,1750,1184,1147,1637,1614,978,522,4076,951
TXNDC8_8_3530,10560,10979,11442,11523,12879,13027,10215,10785,12996,12
794,17963,10497
UCP2_8_3531,907,965,1416,1108,1493,700,1101,1328,2006,528,2548,1558
USO1_8_3532,857,802,762,532,54,196,621,876,910,2000,1054,554
VAMP2_8_3533,1548,1299,1914,1597,1521,1223,707,2592,505,2051,2629,1744
VAMP5_8_3534,410,441,400,262,11,150,632,439,418,187,456,545
VKORC1L1_8_3535,1781,2010,2134,2523,1126,1986,1963,726,3158,1178,1817,
2456
VPS18_8_3536,1761,1694,1679,1648,1166,924,3072,1735,1473,1510,841,1460
VPS26B_8_3537,205,195,216,74,10,140,60,710,3,31,142,8
VPS33A_8_3538,763,772,964,831,1285,3032,359,1283,503,976,1868,270
VPS33B_8_3539,3192,3073,2349,2603,2643,3904,4033,4040,2486,3731,4502,2
748
VPS35_8_3540,505,318,546,406,442,46,182,77,53,69,115,253
VPS39_8_3541,2820,2309,2180,2225,3669,3874,1484,2116,2447,1523,4308,20
20
VPS45_8_3542,3982,3801,2623,3558,3500,3356,5133,3799,5782,4517,4817,49

97
VPS4B_8_3543,2327,2348,2452,1901,1384,2105,2408,1188,2899,1111,4521,37
28
VSIG2_8_3544,425,110,206,381,334,280,180,822,553,424,1892,725
VTI1A_8_3545,830,744,777,757,1338,2354,1148,1182,1120,1148,1019,1392
VTI1B_8_3546,1017,1280,1007,1553,2331,983,1983,661,2705,901,825,440
VTN_8_3547,308,526,472,324,273,850,1247,158,1308,729,246,692
VWF_8_3548,626,396,1044,449,277,240,1632,203,1053,433,551,364
XK_8_3549,1524,1957,1167,1866,2029,921,268,3064,1494,1585,1896,870
XPO1_8_3550,2148,1607,1996,2158,1599,3325,3335,2375,2455,2020,2486,382
5
XPO4_8_3551,883,813,428,1210,980,489,1307,2248,1062,209,119,1280
XPO5_8_3552,1380,1815,1820,1539,882,1124,826,1246,1190,1703,2309,1465
XPO6_8_3553,6845,5416,5727,6630,7672,6235,5571,5393,4405,6481,6799,657
7
XPO7_8_3554,689,816,527,364,403,1000,572,811,1598,1418,317,504
ACTL6A_8_3555,1641,1067,963,1770,312,732,790,1202,957,2385,1595,1710
ADAM12_8_3556,586,569,476,288,465,217,545,275,316,571,1358,233
ADAM15_8_3557,386,491,409,385,828,950,277,329,720,1113,1067,17
ADAM18_8_3558,2947,2109,2459,2730,3755,4893,3448,1718,2284,3568,4931,3
206
ADAM22_8_3559,535,571,690,285,61,174,132,83,782,223,436,904
ADAM29_8_3560,49,139,73,45,0,71,0,0,158,2,0,277
ADAM33_8_3561,16,27,60,92,0,75,0,5,1,92,66,0
ADAM8_8_3562,516,983,508,895,613,532,1137,1102,2091,528,4,701
ADAMTS13_8_3563,716,440,436,554,598,1346,87,97,433,369,549,750
ADAMTS14_8_3564,344,196,156,209,34,145,20,75,525,586,281,68
AKAP12_8_3565,860,828,655,437,518,149,1035,855,84,373,1320,406
ANGPT1_8_3566,2088,1955,1587,1743,3019,2547,1897,2216,1232,1595,3539,2
392
ANGPTL4_8_3567,496,303,299,331,103,491,110,827,219,180,559,365
AP1B1_8_3568,51,105,41,34,11,838,0,6,247,302,58,0
AP1G1_8_3569,337,260,214,261,230,399,76,357,169,806,392,417
AP1M1_8_3570,815,846,355,611,937,913,654,526,528,528,428,934
AP2A1_8_3571,1286,1419,971,1063,1305,1426,1598,1252,569,2360,524,1215
AP2A2_8_3572,333,151,216,384,0,0,27,520,1,3,39,88
AP2B1_8_3573,2487,2313,1970,3240,2626,1930,4041,3349,1689,4153,3257,29
88
AP2M1_8_3574,713,475,331,924,663,146,1057,799,371,303,168,731
AP2S1_8_3575,2314,2502,2422,2113,1552,2564,2653,854,1720,3123,3746,257
6
AP3D1_8_3576,1047,583,872,1162,673,852,1236,922,1216,773,1479,1621
AP3M1_8_3577,2578,2331,2713,2102,3121,4940,2589,1676,2013,3137,3948,32
86
AP3M2_8_3578,714,562,582,1208,782,671,1200,350,595,1229,433,1615
AP4S1_8_3579,1072,1111,671,1039,425,1186,813,1443,749,2006,339,1652
APAF1_8_3580,1781,1782,2189,1459,1478,1690,2244,1407,3397,4007,1256,22
86
APBA2_8_3581,1341,951,516,678,1360,2583,1241,1348,752,1043,665,2468
APOL1_8_3582,381,122,589,384,1322,4,458,475,1475,731,252,693

APOL3_8_3583,595,248,925,556,1801,174,3,2224,42,452,899,1966
APP_8_3584,412,422,252,487,1005,26,54,408,19,7,332,273
ARFGAP3_8_3585,506,297,448,573,220,1341,231,1484,681,355,362,272
ARHGAP33_8_3586,447,1034,385,590,577,1211,911,1680,96,915,1036,637
ARL6_8_3587,751,1065,625,1063,662,1548,264,269,267,509,305,1044
ATP13A2_8_3588,573,533,391,653,35,34,721,86,478,773,456,783
BAX_8_3589,782,615,552,649,240,1234,1966,605,2020,79,838,263
BCAP29_8_3590,1136,1522,1315,1367,2115,1430,2304,1215,1198,402,736,119
9
BCAP31_8_3591,257,232,215,421,47,63,1083,930,5,66,32,22
BCL2_8_3592,1148,1278,684,560,1614,1848,1390,1440,1714,2459,1963,1832
BCL2L2_8_3593,1499,1709,1249,2354,2340,2856,1560,999,1457,2605,2346,25
12
BID_8_3594,101,275,153,175,328,266,262,1120,233,3,42,206
BSG_8_3595,430,328,213,296,903,923,28,450,797,251,649,272
C1QC_8_3596,1682,1630,1298,2123,1258,2753,989,851,2051,1306,3437,3853
CANX_8_3597,717,560,1113,605,368,2182,615,735,1032,2025,212,1165
CAPN9_8_3598,1186,1127,1120,928,295,1861,2790,1199,1597,1910,692,585
CAPNS1_8_3599,662,678,642,851,210,569,1560,657,512,1814,1491,834
CCT6B_8_3600,2845,3693,3462,3524,3319,5371,3074,3379,2110,5304,2873,30
67
CD19_8_3601,529,635,593,541,622,796,981,126,446,988,105,674
CD22_8_3602,885,585,803,788,448,869,932,1244,685,1866,909,967
CD33_8_3603,365,670,619,392,916,863,560,297,1049,949,557,806
CD44_8_3604,876,1032,708,985,818,185,1127,366,1794,1387,1585,1210
CD55_8_3605,1597,1704,1332,1621,1462,1969,2456,615,1807,1595,1264,1853
CDH17_8_3606,1082,1314,1023,1052,786,714,770,1041,393,1031,1538,932
CFHR4_8_3607,1567,1381,1106,1285,2920,3327,2256,1203,2372,1698,2226,14
75
CIZ1_8_3608,1296,684,1314,1192,2041,1764,2681,1107,415,1259,1552,888
COG2_8_3609,533,398,488,651,396,466,1698,95,677,355,105,211
COG4_8_3610,343,224,412,633,184,167,55,243,374,29,423,521
COG5_8_3611,1839,2181,1553,1670,1700,1306,1501,2766,1471,3474,2383,297
9
COG6_8_3612,1255,1510,1557,1408,690,1994,1865,1272,742,1371,1030,2017
COL11A1_8_3613,854,680,775,484,200,720,616,422,520,497,615,583
COL11A2_8_3614,299,205,270,534,758,8,13,469,5,67,0,609
COL12A1_8_3615,3037,3467,2206,2796,3185,5997,2810,3630,2939,4155,5056,
2409
COL13A1_8_3616,489,479,757,792,895,1970,9,1056,311,232,452,651
COL18A1_8_3617,200,5,16,33,0,1,22,0,813,240,0,406
COL25A1_8_3618,1150,1353,515,1106,1089,2729,737,635,1470,97,2290,813
COL2A1_8_3619,606,309,328,950,35,188,649,476,305,20,864,1538
COL4A5_8_3620,516,970,687,756,28,2624,357,737,508,1316,1389,910
COL4A6_8_3621,1485,801,1314,1281,1202,343,376,1258,1232,680,510,578
COL6A2_8_3622,128,384,168,198,9,269,25,415,16,230,11,271
COL6A3_8_3623,162,44,0,71,219,5,0,0,0,0,0,210
COL8A1_8_3624,323,456,768,913,149,1145,806,563,423,48,471,23
COL9A1_8_3625,980,1183,880,1065,303,1372,577,914,1377,1472,889,2144
COPA_8_3626,1037,1186,823,1292,467,2713,1171,2275,998,1481,1386,1949

COPB1_8_3627,628,926,458,716,110,874,383,671,403,1136,363,1643
COPE_8_3628,874,668,513,726,862,882,473,584,369,1524,4988,608
CPA4_8_3629,428,244,181,196,492,26,62,304,1221,4,940,872
CPNE1_8_3630,1639,1356,1073,1438,1313,4387,902,631,1691,577,51,2226
CPNE7_8_3631,188,163,167,320,34,0,754,1141,0,415,5,610
CPXM1_8_3632,357,332,371,442,195,603,721,499,168,138,197,303
CPZ_8_3633,577,187,285,780,220,134,95,56,216,3,828,56
CRABP2_8_3634,561,503,490,413,960,397,62,272,243,285,1765,822
CTLA4_8_3635,408,708,637,573,310,556,268,201,715,800,575,527
CTNS_8_3636,252,113,185,107,0,105,220,1,597,131,709,1
CXCL12_8_3637,387,158,157,324,507,70,38,866,70,1,163,0
DPP10_8_3638,450,894,581,646,0,0,0,195,462,1596,1385,1673
DPP6_8_3639,822,906,737,784,934,1066,876,1658,308,1231,1189,688
ECM1_8_3640,248,467,453,200,138,71,124,326,44,189,921,526
EGF_8_3641,2458,2339,2028,2360,1775,1300,1714,4301,3065,4111,3289,3134
EIF2C2_8_3642,380,242,311,452,133,565,56,187,84,576,407,364
EIF2D_8_3643,178,140,230,151,170,399,434,59,154,132,11,206
ENSA_8_3644,1950,2135,2122,2132,2045,2370,1952,522,1790,1490,2293,1192
EPB41L4B_8_3645,278,211,155,181,800,268,18,305,95,56,123,215
EPB42_8_3646,539,801,728,517,684,851,235,572,473,1143,872,628
ETFA_8_3647,420,291,396,214,67,155,25,4,42,384,32,635
ETFB_8_3648,1651,1458,1292,1297,943,1146,1764,2575,1069,2234,1346,1385
EXOC1_8_3649,1088,1089,1079,1207,1143,1465,1989,558,1276,779,1530,938
EXOC4_8_3650,949,518,654,574,220,954,570,1199,201,1225,2375,268
EXOC6_8_3651,2259,1914,1874,1940,1106,1735,1444,1256,1199,2544,2814,33
51
EXOC7_8_3652,2068,2306,1160,1824,1287,2836,788,1627,2883,2668,1774,243
6
F8_8_3653,291,76,165,624,0,30,892,538,1,166,1015,131
FABP6_8_3654,577,531,575,646,73,871,22,553,353,436,457,882
FAM131A_8_3655,821,663,618,765,750,317,1410,626,767,1298,868,33
FAM63B_8_3656,1893,2140,1778,1617,2453,2241,992,1450,1794,1996,2426,40
61
FANCA_8_3657,387,695,303,593,1852,1010,134,194,445,229,118,132
FCN3_8_3658,315,589,175,589,93,298,491,259,1031,436,392,842
FGA_8_3659,819,270,567,631,709,557,30,461,540,573,1376,448
FGB_8_3660,208,332,328,204,888,157,184,166,39,130,633,298
FGF13_8_3661,7504,7302,7207,8410,9131,8517,8405,8697,5219,8927,9315,12
182
FGG_8_3662,919,1148,842,750,896,471,606,741,874,1190,1762,1979
FIBCD1_8_3663,554,318,390,191,0,1095,0,0,1,52,256,446
FLVCR2_8_3664,918,809,821,436,302,1538,116,1407,637,748,878,1858
FOLR1_8_3665,708,739,594,891,362,1005,99,508,460,586,543,1161
FOLR2_8_3666,780,603,647,908,941,497,1372,1354,569,227,194,276
GGA1_8_3667,213,247,397,137,35,843,804,43,7,360,787,40
GGA3_8_3668,649,635,673,537,1888,626,441,1825,840,1145,1381,513
GJA5_8_3669,157,168,34,49,295,139,74,12,208,176,64,39
GJB1_8_3670,857,968,866,784,1678,984,353,1653,158,498,1761,877
GJB3_8_3671,807,972,741,920,426,300,133,2102,310,415,846,1601
GJB6_8_3672,3219,2646,2453,2937,2895,1641,2132,5459,2926,4183,3881,455

3
GLYATL1_8_3673,12,62,0,90,0,13,11,0,7,0,75,6
GNRH1_8_3674,1691,1477,1165,1349,940,1503,1581,1487,2359,1883,1352,132
1
GOLGA3_8_3675,1112,581,706,656,451,473,890,1177,241,475,922,2597
GOPC_8_3676,4516,3946,3142,3151,4040,5050,4117,1169,3764,6337,4698,489
9
GOSR1_8_3677,2892,3543,2656,3127,2581,2841,2453,1346,2014,3268,4707,18
53
GOSR2_8_3678,579,285,478,521,667,362,153,584,578,229,999,724
GPRASP1_8_3679,218,108,150,91,141,109,152,292,500,182,21,1006
GRB2_8_3680,82,298,69,284,0,856,34,335,78,335,353,188
HABP2_8_3681,597,562,611,968,286,249,1323,760,830,702,528,1488
HDLBP_8_3682,485,1000,687,611,135,211,1469,173,1173,311,464,101
HEPH_8_3683,841,1029,1364,932,839,727,904,1935,1970,351,1468,816
HNRNPU_8_3684,1918,1525,1589,1490,2320,2520,1365,1729,2953,2841,2766,2
049
HOMER2_8_3685,490,717,650,760,319,880,560,697,1788,33,343,988
HPN_8_3686,0,0,0,0,0,0,0,0,0,0,0,0
HSDL2_8_3687,601,873,608,640,589,1181,256,749,1042,1001,2055,134
IGF1_8_3688,350,301,249,206,420,693,81,381,411,105,1088,22
IGFBP3_8_3689,213,306,258,150,34,2,304,178,621,138,1672,247
IPO11_8_3690,1475,1334,850,1528,1450,1062,1173,850,919,988,1389,765
IPO8_8_3691,340,377,415,319,9,142,298,318,627,241,292,345
ITGAL_8_3692,393,502,83,268,65,449,827,1,177,64,774,41
ITGAM_8_3693,656,1224,279,715,728,805,1071,3,626,672,766,669
ITGAV_8_3694,1602,1509,1393,1640,2273,1513,2206,2189,976,2333,1755,204
9
ITGB2_8_3695,589,1274,239,526,78,7,164,776,1113,802,752,1179
KDELR2_8_3696,2201,2125,2538,2059,1662,2645,3426,2643,2887,2722,2501,1
813
KDELR3_8_3697,396,307,608,569,385,45,1287,704,312,210,83,961
KIF13A_8_3698,1311,1750,1485,1205,1644,1420,2930,711,962,1098,656,2436
KIF17_8_3699,194,280,157,154,642,117,460,422,298,31,119,346
KIF1B_8_3700,409,362,250,412,633,1094,313,190,148,96,690,810
KLK10_8_3701,544,628,308,660,183,863,209,878,239,1253,289,397
KLK11_8_3702,126,69,61,32,0,184,21,0,118,33,187,0
KLK12_8_3703,404,469,502,392,70,570,23,1520,983,190,686,2012
KLK15_8_3704,1327,1238,911,1245,418,362,1407,833,1461,928,1517,417
KLK5_8_3705,457,564,296,420,375,148,112,729,27,381,1271,523
KLK6_8_3706,892,1143,1122,1184,966,1671,1831,1883,1592,713,869,1403
LAMB3_8_3707,1295,1081,1367,1941,850,567,1680,1758,957,433,582,793
LDB3_8_3708,202,65,353,137,9,132,603,111,268,423,243,154
LDLR_8_3709,1157,1532,1045,1366,1338,1356,3128,1958,610,1276,437,1167
LTA_8_3710,608,503,253,252,1508,997,232,1,358,478,592,1718
LTF_8_3711,807,1063,766,1038,1179,1084,490,1335,544,759,705,646
M6PR_8_3712,1554,1482,1091,1450,619,1777,1431,852,413,1734,1262,728
MASP1_8_3713,961,1070,1010,747,576,1116,283,493,504,2301,513,515
MB_8_3714,246,178,129,136,120,362,0,0,498,257,157,0
MCFD2_8_3715,4904,5395,4263,5418,6695,5623,4257,4947,3851,4981,5595,57

15
MCL1_8_3716,1048,1074,1248,654,1472,382,1517,188,946,636,2502,472
MEFV_8_3717,1308,811,1014,817,1782,825,1650,421,532,558,1281,889
MFAP4_8_3718,734,1042,805,701,949,598,611,920,890,433,749,1812
MFSD10_8_3719,241,269,373,227,6,160,473,40,620,314,266,101
MFSD1_8_3720,314,364,225,210,896,174,530,33,753,241,361,321
MFSD5_8_3721,188,320,97,366,26,124,158,13,26,460,404,57
MLC1_8_3722,1932,2142,1538,2149,2207,3015,1437,719,2338,3173,1987,3387
MSLN_8_3723,1055,607,626,742,473,1023,1803,415,611,445,991,175
MTX1_8_3724,788,839,627,878,1327,1289,2024,1126,851,1693,813,612
MUC1_8_3725,3803,4223,3740,4237,4731,2792,2510,2593,3488,6831,5876,470
0
NCAM1_8_3726,214,389,369,198,362,705,740,1322,637,554,507,317
NNAT_8_3727,950,907,911,592,1300,619,1618,1223,374,1105,519,1169
NOXO1_8_3728,106,56,43,96,64,64,173,9,0,0,0,9
NPC1L1_8_3729,80,79,194,79,158,355,200,1,61,23,39,266
NPRL3_8_3730,202,303,193,285,222,402,93,198,851,225,5,116
NRXN1_8_3731,446,401,512,414,81,1460,407,664,637,494,609,1109
NRXN2_8_3732,1075,863,160,515,894,12,152,196,249,1374,1107,226
NRXN3_8_3733,454,505,365,500,675,1528,187,324,449,911,197,410
NUP155_8_3734,969,1164,634,988,597,1981,2069,2313,1042,875,1096,1368
NUP50_8_3735,126,245,462,124,9,8,525,0,93,41,572,58
NUP62_8_3736,186,424,159,417,74,244,22,490,85,33,138,555
NUP98_8_3737,693,1140,684,1187,1363,1586,1005,1029,1319,2383,970,954
NUPL1_8_3738,944,1365,1201,1250,2706,1770,596,300,647,1146,404,1167
NXF1_8_3739,215,422,252,142,65,131,785,20,45,368,50,782
NXNL2_8_3740,483,298,328,274,572,410,24,165,472,62,133,94
NXT2_8_3741,457,745,462,595,491,1692,94,1114,935,936,316,742
OAZ3_8_3742,224,216,107,231,214,246,1005,136,153,214,32,340
PACSIN2_8_3743,642,514,499,588,13,486,43,234,178,634,1110,545
PANX2_8_3744,553,297,295,168,253,61,12,55,1172,224,277,2
PCDHA6_8_3745,1440,1073,974,1012,1780,1210,980,1075,1597,1526,1902,138
8
PCDHGA5_8_3746,689,468,387,812,314,1127,1092,225,846,284,639,594
PCLO_8_3747,1887,1462,2383,1938,1679,2982,3671,2510,2309,2619,1557,194
8
PCSK5_8_3748,759,469,440,733,110,6,694,614,85,190,48,1426
PCSK6_8_3749,244,400,109,220,351,258,108,922,55,268,51,891
PCTP_8_3750,2472,2350,3188,2649,1494,4775,3252,4166,1906,3539,3486,358
9
PDYN_8_3751,671,775,452,462,1255,220,152,789,645,440,837,1107
PDZD3_8_3752,1493,1678,1464,1122,1257,2101,1633,565,2625,2169,1191,360
1
PDZK1_8_3753,1158,1102,1630,1796,458,2072,1381,718,1489,763,2770,1399
PGAP2_8_3754,3044,3125,1777,2663,3608,4946,2897,6007,3227,3122,2337,38
81
PGF_8_3755,936,995,840,1247,797,1569,1237,777,1561,823,1965,695
PIK3R3_8_3756,1222,1541,885,816,93,2543,1652,1788,1269,2317,2738,3099
PITPNC1_8_3757,2563,2393,2765,1850,1480,2458,3749,2012,2988,2452,2931,
2502

PITPNM1_8_3758,479,512,510,947,1771,686,1503,1967,165,881,290,339
PITPNM3_8_3759,2437,1974,2125,1775,1872,4561,2224,3031,2621,1635,2801,
1962
PLEC_8_3760,474,928,767,781,689,1597,2723,786,84,1640,73,861
PLIN3_8_3761,721,967,732,861,1216,1996,427,1481,969,1005,939,1295
PLTP_8_3762,574,1053,884,660,329,641,544,1108,796,825,748,324
PNKD_8_3763,283,179,410,403,116,450,509,345,597,65,583,177
POMC_8_3764,53,21,134,194,126,67,22,674,36,336,32,76
PORCN_8_3765,776,1014,873,875,821,256,271,704,517,950,1494,519
PREPL_8_3766,1920,2208,2548,2990,3819,3944,3140,3104,651,2558,3604,379
0
PRNP_8_3767,398,381,233,340,3,224,474,204,43,36,443,711
PRSS21_8_3768,258,315,236,169,789,755,138,239,585,475,239,22
PRSS35_8_3769,2806,2741,2211,2379,2913,2111,2476,3609,2545,4530,2553,2
548
PSEN1_8_3770,681,1325,767,1215,703,1699,1267,217,1417,1174,258,3027
PSEN2_8_3771,436,166,132,269,30,616,358,617,306,169,1524,112
RABEP1_8_3772,1178,1057,1108,815,217,725,1587,891,1520,1460,1384,525
RACGAP1_8_3773,1129,676,650,876,344,185,703,36,646,417,1115,1071
RARRES1_8_3774,2119,2374,1889,2576,2677,4831,2451,2305,2449,3495,3353,
3194
RASA1_8_3775,2638,2829,3129,3125,2936,3693,4302,4179,2336,2467,5297,25
52
RELN_8_3776,1152,1141,1191,1095,1954,2829,2265,2231,2383,1710,2291,178
8
RHCE_8_3777,2368,2855,3039,1724,2578,3259,1827,1260,1558,3448,3101,276
6
RHD_8_3778,185,381,264,487,26,465,317,243,32,922,288,606
RIMS2_8_3779,1272,1383,1572,1345,668,1325,1648,1273,1740,1166,1979,110
9
RRBP1_8_3780,1018,1038,717,498,990,1250,1246,832,1706,1451,505,59
RUFY1_8_3781,115,185,97,75,51,21,82,335,21,23,2,30
S100A13_8_3782,647,718,552,937,30,594,414,843,996,166,329,684
S100A4_8_3783,455,766,431,747,51,1344,531,0,802,493,719,33
SAA1_8_3784,2251,1754,1554,1743,1014,3503,1685,2457,2438,2120,2488,160
1
SCAMP3_8_3785,831,760,606,627,1400,742,544,596,1586,310,942,530
SCARB1_8_3786,701,884,506,783,1154,500,297,532,1572,804,865,540
SCFD1_8_3787,1763,1532,1503,1364,2733,1862,1586,945,1845,1657,2174,217
2
SEC13_8_3788,532,572,483,839,813,121,192,873,766,1129,218,1179
SEC14L1_8_3789,1445,1427,1187,1239,892,1106,343,732,1796,2747,1864,133
4
SEC14L2_8_3790,1086,534,1100,1514,581,1147,461,1776,1257,1430,1592,168
6
SEC14L4_8_3791,587,710,679,709,857,1053,953,376,523,1100,958,71
SEC23B_8_3792,4448,3579,3625,3887,4071,2311,6609,2475,3812,3672,3854,3
302
SEC24B_8_3793,1727,1851,1812,2033,1256,2633,2190,1070,2461,1446,2765,2
441

SEC24C_8_3794,1050,1469,1602,1244,2676,815,179,1607,689,1984,2049,1513
SEC61A2_8_3795,1246,1247,1800,1268,3463,598,1877,948,1789,1214,1376,26
34
SEC61G_8_3796,1049,923,705,1036,523,1358,1601,1230,1532,1308,464,574
SEH1L_8_3797,922,837,1198,1279,477,1450,1351,2534,1066,377,1119,1341
SERINC2_8_3798,815,687,574,479,62,25,509,411,488,450,536,549
SERINC3_8_3799,2826,3076,2690,3143,4364,2350,2589,2656,3007,4462,3546,
4486
SERPINA10_8_3800,1305,1625,1145,836,791,479,705,2886,994,1442,1456,261
SERPINA1_8_3801,212,51,65,274,5,329,1,47,32,103,22,16
SERPINB2_8_3802,961,921,724,558,319,1573,667,991,2455,187,326,1116
SERPINB6_8_3803,1749,2328,1461,1806,1621,2608,2728,1607,479,2375,1251,
2054
SERPINB8_8_3804,558,440,561,606,623,480,351,406,538,506,1218,291
SERPINE1_8_3805,748,219,465,489,187,511,471,455,1513,160,454,1189
SERPINF2_8_3806,1382,2545,1193,1794,1357,1383,2002,1930,1868,1135,2092
,2426
SERPING1_8_3807,1351,621,846,889,151,992,1621,941,535,942,1039,1155
SERPINH1_8_3808,1562,1112,1162,1081,1845,923,1269,1677,1635,1781,888,1
584
SERPINI1_8_3809,1831,2513,869,1232,1347,2749,1485,314,1153,1885,712,13
97
SFI1_8_3810,241,607,349,140,6,2,633,476,117,65,380,2646
SFTPA1_8_3811,830,674,400,743,1047,1150,622,988,1221,754,62,125
SH3D19_8_3812,2067,2532,2340,2090,2527,2200,641,4739,193,2939,3619,293
1
SIL1_8_3813,405,581,376,487,196,578,485,543,636,290,1073,452
SLC25A25_8_3814,1516,2141,1548,1985,1170,498,1344,2070,1086,2175,3365,
1309
SLC25A36_8_3815,2718,2987,2059,2670,3669,2727,3371,3674,3024,2847,3185
,1739
SLC25A45_8_3816,849,1406,930,1138,402,907,1048,1095,1189,2059,982,2300
SLC38A10_8_3817,712,605,351,629,315,448,2184,737,278,1051,411,448
SLC41A3_8_3818,222,246,429,349,12,108,88,349,92,163,90,514
SLC43A3_8_3819,313,248,353,281,55,621,82,403,20,649,1526,526
SLC44A2_8_3820,1051,876,1096,650,1233,947,2325,408,1761,1003,2883,246
SLC44A4_8_3821,1147,1129,1344,1981,1287,1583,2046,1933,1146,1589,1982,
2701
SLC44A5_8_3822,707,859,683,876,973,302,688,902,927,787,1653,799
SLC46A1_8_3823,3262,3078,3186,3825,2308,5985,5370,3374,2772,2510,4419,
3463
SLC47A2_8_3824,596,922,438,481,15,608,773,163,1726,329,1143,940
SLC50A1_8_3825,888,433,711,727,804,168,1384,1338,2151,477,1280,2117
SLC6A20_8_3826,1678,1222,871,2253,1708,128,1618,1214,1488,1874,1511,27
07
SNAP23_8_3827,2510,1448,2017,2216,1486,2998,1687,1570,2604,1283,4066,2
001
SNAP25_8_3828,1760,1992,1630,1783,1889,986,1186,1840,1711,1714,1492,88
3
SNX10_8_3829,627,624,597,484,1717,576,635,451,139,326,688,416

SNX11_8_3830,612,1384,749,826,25,2135,1512,982,187,981,580,2270
SNX14_8_3831,1409,1447,1071,923,1199,1430,1891,2286,918,1916,2298,1119
SNX15_8_3832,467,493,451,465,455,233,404,207,250,470,312,795
SNX16_8_3833,408,556,27,579,419,427,344,691,68,308,56,76
SNX18_8_3834,776,466,309,467,203,363,1247,812,757,231,500,1187
SNX1_8_3835,2745,2472,2234,2710,3812,2249,2343,4718,2982,3544,4460,503
7
SNX3_8_3836,443,268,835,596,652,357,512,905,632,133,110,190
SNX5_8_3837,1620,2105,2023,1874,3378,2926,1658,2587,2144,1860,2322,236
3
SNX6_8_3838,2335,2304,2281,1842,3012,1914,1260,1416,1573,1405,3148,142
9
SNX7_8_3839,2211,2124,2015,2800,2352,1749,2141,2047,3055,1971,2060,175
6
SORCS1_8_3840,1363,1580,1848,1341,1089,2106,1299,2370,1344,2544,1269,1
698
SORT1_8_3841,1286,1218,1023,1204,1724,1947,1774,1117,1236,971,393,512
SPNS1_8_3842,1207,1373,1617,1116,1168,1298,1668,1222,1567,717,2998,953
SRI_8_3843,1435,1020,1500,1455,1545,1010,2295,1755,969,879,1373,2693
STARD3_8_3844,1939,1643,1605,1885,1909,1781,1473,866,1177,2461,3872,36
22
STAU1_8_3845,614,848,652,550,263,1853,1476,719,458,737,348,879
STEAP2_8_3846,2572,3711,1873,2693,3531,3199,3195,2952,2218,1778,2135,5
140
STEAP3_8_3847,284,330,249,126,411,492,102,114,360,118,619,3
STIM2_8_3848,855,721,670,766,112,778,693,379,902,655,1934,1881
STX16_8_3849,1368,1366,1099,896,2588,946,1562,1216,1339,953,1687,1693
STX1A_8_3850,790,685,664,597,2281,2,1558,1068,830,303,2387,21
STX2_8_3851,761,676,646,654,515,539,197,1267,561,557,527,115
STX3_8_3852,272,527,429,407,77,91,1437,542,26,582,1151,992
STXBP1_8_3853,1327,1062,907,1568,2031,597,2019,1401,257,655,1673,1990
STXBP2_8_3854,587,832,555,801,50,307,318,58,1356,392,518,2105
SV2B_8_3855,51,164,38,304,169,472,431,0,569,1,17,465
SYN1_8_3856,1507,1490,1482,1584,203,955,432,1225,1874,1534,2092,2327
SYNGR1_8_3857,171,184,172,111,320,477,139,38,115,202,259,117
SYNPR_8_3858,1078,921,500,940,1003,1232,339,330,1295,1077,1124,1192
SYPL1_8_3859,2952,2032,2690,2398,3368,1569,1655,3370,2154,3112,4212,44
50
SYT12_8_3860,295,734,276,290,486,296,230,730,127,984,549,234
SYT14_8_3861,3131,4155,2158,2683,2414,2347,2216,2726,2408,3252,3691,41
77
SYT15_8_3862,1784,1731,1623,1507,866,3175,2413,2909,2095,1500,870,1962
SYT1_8_3863,1943,2042,1279,2495,973,2132,2314,1240,2454,1101,2732,1219
SYT2_8_3864,790,946,1301,1297,1289,1323,236,771,949,2098,2210,1157
SYT3_8_3865,961,834,618,1003,1502,1096,362,1491,560,1595,596,1071
TAPBP_8_3866,30,78,71,265,15,91,0,254,1,380,3,224
TC2N_8_3867,2081,1370,1253,2754,1974,964,3486,3318,1535,1312,1308,2093
TCN2_8_3868,224,175,297,72,67,232,0,358,38,900,0,0
TCOF1_8_3869,280,288,471,332,750,1014,1199,1044,376,220,1581,1223
TFPI_8_3870,4033,3530,3368,3503,1859,3731,3575,2132,3039,3388,2724,275

8
TFR2_8_3871,1069,1286,814,783,1257,445,881,2316,1012,980,1326,971
TFRC_8_3872,6429,6341,5426,6838,5768,5195,6959,9538,6567,6381,4466,791
6
TGFB2_8_3873,1575,1346,931,802,1531,2405,1436,1173,932,1086,1998,871
TIMM17B_8_3874,833,856,637,879,1534,587,1265,1726,994,696,1291,2409
TINAGL1_8_3875,369,350,123,575,305,0,428,413,37,203,612,1270
TLL1_8_3876,5244,5165,4948,4717,4694,7667,6166,5349,5965,5085,7915,509
2
TM9SF1_8_3877,2976,2187,2135,1638,3075,2677,2797,3523,1840,2431,2189,1
766
TMC6_8_3878,662,632,443,559,638,1183,629,883,491,630,1807,2270
TMPRSS11A_8_3879,478,687,357,524,912,1600,806,698,122,768,281,934
TMPRSS13_8_3880,1594,1735,1444,1870,2111,1069,1934,1043,1202,1432,2659
,1136
TMPRSS2_8_3881,1037,881,568,702,539,1152,1285,1299,764,1375,874,1528
TMPRSS4_8_3882,273,344,157,297,1002,72,10,677,27,278,254,562
TNFSF11_8_3883,548,594,534,844,1090,1222,355,1060,438,975,1735,1991
TNFSF13B_8_3884,1087,1228,1317,812,1220,1470,820,534,872,859,1949,1864
TNPO2_8_3885,463,492,159,334,544,384,620,221,901,341,112,57
TOM1_8_3886,404,410,183,205,21,84,73,37,89,180,518,217
TOM1L2_8_3887,4853,4727,4295,3069,6624,3476,4167,3341,4889,5307,6188,5
733
TSC1_8_3888,785,736,530,800,125,2441,2402,166,384,2370,561,1007
TSC2_8_3889,0,0,0,0,0,0,0,0,0,0,0,0
TUBA8_8_3890,1091,977,1110,1487,961,1585,1573,416,742,1052,2053,1616
TUBB3_8_3891,496,498,489,55,4,331,69,681,87,131,509,143
TUBD1_8_3892,429,561,769,486,922,565,1601,90,686,651,553,1434
UCP3_8_3893,2414,2285,1975,2291,1863,2184,2677,3123,1274,3958,2272,271
6
UPF3A_8_3894,499,512,472,473,1405,104,583,25,495,349,739,288
UPF3B_8_3895,149,159,103,118,342,331,35,27,55,122,249,78
VAMP1_8_3896,1128,947,778,930,609,1510,2421,310,957,1641,1180,735
VAMP7_8_3897,1055,1215,1040,1147,1634,1793,794,701,1389,3419,857,3152
VCAM1_8_3898,1230,1663,891,1716,114,2666,1394,1952,384,974,2478,1836
VLDLR_8_3899,814,735,622,665,377,1177,298,10,483,615,1357,436
VPS13A_8_3900,3441,3210,3259,3711,3831,3514,6065,5621,3648,3886,4051,2
517
VPS13B_8_3901,267,427,143,398,357,805,149,277,290,167,261,135
VPS16_8_3902,299,460,360,603,573,369,982,1613,267,1298,1706,647
VPS26A_8_3903,2423,1843,1858,2954,2216,2828,3747,3010,1796,1524,3259,5
378
VPS28_8_3904,777,1192,1061,983,894,922,1368,734,1348,1006,1137,583
ZFYVE16_8_3905,2273,3122,2067,2210,2691,2106,3604,3568,2430,4260,2685,
1858
ZNF160_8_3906,1596,1106,1248,1830,1185,1689,2307,2263,1530,1743,1462,1
590
ZP3_8_3907,543,627,416,797,624,319,427,1799,238,449,86,1753
BET1L_8_3908,398,236,457,380,187,639,61,254,570,144,396,270
C2orf83_8_3909,234,215,162,220,947,95,292,492,9,388,69,3

ERP29_8_3910,1474,1715,831,1339,622,1522,636,466,1758,1052,802,508
FGF1_8_3911,2458,2332,2868,1916,1600,2031,1906,1798,2441,3024,3018,152
0
LYNX1_8_3912,104,180,252,125,99,66,1,160,34,276,211,324
MMP28_8_3913,1156,622,1422,561,1237,617,973,1586,371,790,1916,1235
PDPN_8_3914,123,167,168,159,0,126,227,2,19,68,262,66
SNX21_8_3915,136,226,156,405,185,18,679,176,210,670,185,18
TIMM8A_8_3916,1515,1301,1146,1123,138,2055,1778,825,518,1263,681,550
VEGFA_8_3917,187,129,128,252,29,35,494,101,3,414,42,53
CDH23_8_3918,519,355,626,708,1216,1924,1231,262,1455,308,1258,282
CDH23_8_3919,448,475,630,565,740,14,1338,319,659,391,974,933
CDH23_8_3920,596,623,288,403,108,483,420,908,441,917,867,0
CDH23_8_3921,975,800,357,454,933,591,94,1149,310,264,521,476
CDH23_8_3922,351,247,84,341,76,530,1870,1183,683,120,275,216
TNXB_8_3923,844,551,434,805,818,320,547,567,1472,950,445,140
TNXB_8_3924,880,324,1015,682,455,221,706,344,369,463,648,839
A2M_8_3925,138,604,112,303,629,820,171,1545,47,10,356,830
ACE2_8_3926,3900,5020,4537,4464,4687,3927,2606,4253,5308,5994,4769,551
3
ACTR6_8_3927,2179,1473,2031,1727,1312,2128,1026,2803,1750,2658,1025,14
44
ADAM11_8_3928,1483,1824,1219,1251,2552,2666,1321,1139,979,2073,1900,13
84
ADAM19_8_3929,282,419,435,266,1025,441,291,159,690,696,1563,193
ADAM20_8_3930,279,301,107,375,66,138,115,143,344,341,91,807
ADAM21_8_3931,343,188,507,513,374,33,9,393,481,299,15,0
ADAM2_8_3932,1132,723,898,1138,732,349,474,458,343,721,1721,1969
ADAM30_8_3933,226,297,242,137,1282,211,55,1366,0,675,1,1415
ADAM7_8_3934,6696,5969,5124,7010,5161,6209,7138,6562,7390,5290,8761,66
79
ADAM9_8_3935,2762,2898,2373,3033,2591,2428,3491,2332,2621,3439,4117,44
60
ADAMTS10_8_3936,1072,786,549,164,502,611,0,256,1258,655,927,362
ADAMTS12_8_3937,202,481,498,358,187,856,1178,101,221,519,412,1347
ADAMTS15_8_3938,482,345,237,537,585,156,345,301,1005,377,668,387
ADAMTS18_8_3939,2549,2276,2743,2374,1746,1837,1439,5417,1519,2154,3089
,2644
ADAMTS19_8_3940,1079,1035,875,965,1223,1935,1581,1790,551,946,1563,236
2
ADAMTS1_8_3941,556,369,378,425,515,634,605,821,395,343,2,318
ADAMTS20_8_3942,261,228,256,102,16,511,18,46,220,16,697,73
ADAMTS3_8_3943,318,307,516,362,537,151,1487,753,129,284,797,1378
ADAMTS5_8_3944,2150,1538,1986,1846,3227,1023,2246,1458,3757,2497,2574,
1836
ADAMTS6_8_3945,4254,5737,4999,5260,2219,6113,5131,5856,5850,7125,4466,
6311
ADAMTS7_8_3946,199,253,486,268,101,111,51,40,39,636,393,7
ADAMTS8_8_3947,468,564,389,378,384,135,134,239,592,297,692,1080
AEBP1_8_3948,571,355,398,457,443,692,897,2534,270,823,748,322
AFG3L2_8_3949,844,582,598,893,652,1338,260,1061,673,740,1469,674

AFM_8_3950,1373,613,796,1146,1394,1217,531,1262,2015,800,506,673
AFP_8_3951,1445,1697,630,1371,1208,3243,2059,479,1253,970,1912,467
AGTPBP1_8_3952,2748,2514,3049,1980,1563,2805,5620,2800,2363,3743,2097,3287
ALG10B_8_3953,1022,716,814,1054,57,770,778,400,621,1054,984,1794
AMBP_8_3954,1252,1208,1322,1466,239,540,283,1163,332,883,1110,683
ANGPT4_8_3955,1784,1741,1959,1836,2401,2547,1485,2385,2333,1121,2700,2304
ANGPTL1_8_3956,1580,1176,695,1329,417,583,1399,1182,845,673,2068,1470
ANGPTL2_8_3957,2828,2472,1996,2418,2772,2224,2944,3268,1794,1783,2624,2731
ANGPTL3_8_3958,3264,2713,2686,3176,2949,4054,3008,2321,1520,3047,6015,3695
ANGPTL7_8_3959,107,144,217,98,6,3,1,2,40,156,57,246
ANKH_8_3960,1479,1489,1223,1358,1416,2567,446,320,1599,1638,1265,1250
AP1G2_8_3961,143,74,56,158,0,123,0,4,0,6,1,19
AP1M2_8_3962,761,304,557,279,214,652,491,608,1237,356,630,159
AP1S1_8_3963,163,248,240,153,28,145,127,108,339,32,486,38
AP1S2_8_3964,7041,5865,5842,6077,5374,4318,7912,6214,8600,4197,5831,6377
AP1S3_8_3965,958,1386,534,1037,627,1387,1637,610,1126,300,1054,1024
AP3B1_8_3966,615,774,418,1010,610,844,477,1631,749,630,259,1287
AP3B2_8_3967,1878,1356,1314,1886,1365,731,753,1030,1396,2177,2235,1269
AP3S1_8_3968,2239,2487,1692,2209,2015,3448,3876,1461,3866,2248,1079,1701
AP3S2_8_3969,2606,3825,3109,3585,2766,3354,4731,3617,3157,4947,3719,2596
AP4B1_8_3970,6031,6383,5792,6403,8208,8239,5966,6531,7492,7023,9664,7565
AP4M1_8_3971,1201,1411,981,1148,1242,1649,1985,359,775,1994,1973,1494
APBA1_8_3972,618,181,434,448,312,193,883,33,828,590,589,525
APBA3_8_3973,876,779,822,886,857,404,535,1199,1174,923,1327,610
APOA1_8_3974,376,551,290,163,115,119,205,546,93,225,950,12
APOA2_8_3975,460,179,351,281,280,237,247,93,707,203,396,427
APOA4_8_3976,1424,1162,1423,1293,364,1387,1273,1209,3048,1609,2734,1176
APOB_8_3977,2827,2749,2035,2189,4288,3508,3041,2319,839,3635,3835,2351
APOC1_8_3978,2530,2635,1929,2543,1179,5254,3374,3390,1793,2516,3001,2583
APOC2_8_3979,3201,3841,3025,3803,3430,3963,4050,3127,5337,3441,4015,4566
APOC3_8_3980,331,288,310,242,103,401,31,88,298,43,19,957
APOC4_8_3981,368,381,320,446,198,164,242,41,216,958,498,1303
APOD_8_3982,644,672,635,538,785,267,524,410,651,668,615,67
APOE_8_3983,850,644,627,866,541,1991,1107,476,347,658,114,834
APOF_8_3984,1271,1446,1151,1692,659,646,1489,1935,1408,1303,1999,2648
APOH_8_3985,669,742,590,823,2197,722,216,67,629,1067,995,652
APOL6_8_3986,1424,1549,1505,1380,2497,1927,1406,2254,3439,2292,2213,2153
APOM_8_3987,374,440,978,261,62,313,42,1030,244,25,341,235

AQP12B_8_3988,640,691,357,460,1100,645,465,355,911,703,480,861
ARF5_8_3989,250,37,124,219,1189,730,18,482,176,286,42,91
ARF6_8_3990,1183,874,787,901,464,1100,164,1355,1047,1814,283,545
ARPP19_8_3991,474,792,320,712,110,97,317,120,452,805,35,622
ASTL_8_3992,658,434,488,633,857,1057,433,103,325,13,244,86
ATOX1_8_3993,533,538,468,708,298,887,439,548,450,1183,1460,610
ATP13A1_8_3994,1264,1372,1120,1552,1940,1708,1281,1236,1070,2143,2975,
1566
ATP13A3_8_3995,1552,1355,1379,1293,2196,580,576,1061,768,2458,1600,181
0
ATP13A4_8_3996,1059,1145,388,921,2525,1102,305,201,1331,339,739,835
ATP13A5_8_3997,1023,1094,1453,1395,567,880,906,964,730,1649,1606,2561
AZGP1_8_3998,409,1014,698,750,217,878,407,688,281,412,109,612
AZU1_8_3999,255,664,751,409,2,1332,120,1383,1631,1035,283,131
BCL2L10_8_4000,328,305,413,247,324,316,11,25,605,297,340,87
BET1_8_4001,1190,997,1100,896,1633,2033,1510,1676,2409,1942,2250,908
BGLAP_8_4002,496,216,364,233,75,0,264,865,144,881,291,629
BOC_8_4003,158,228,229,65,200,995,108,373,110,398,473,230
BPI_8_4004,802,383,438,277,912,673,357,577,557,33,385,801
BPIFC_8_4005,11430,12175,11243,12047,10731,14647,9946,9463,10945,10985
,9664,10952
C16orf7_8_4006,1257,1957,1664,1417,823,619,1878,3061,1913,1441,2029,18
93
C1orf162_8_4007,8468,7646,7751,9175,6152,8780,8864,12641,6003,7506,133
22,11616
C1QA_8_4008,163,225,207,258,715,57,479,348,78,647,73,539
C1QB_8_4009,1318,880,847,1342,1012,871,1168,808,907,1078,841,2062
C1RL_8_4010,2457,2267,1830,2127,2308,2190,614,1532,3792,2602,2947,2027
C20orf141_8_4011,56,361,142,207,0,39,133,663,52,19,0,12
C3_8_4012,571,457,839,871,393,121,1113,956,604,658,617,802
C4A_8_4013,738,952,1190,1013,1266,1252,1515,1428,210,450,788,497
C5_8_4014,2347,2089,2402,2344,2338,3368,1308,1771,1645,1786,3342,4890
C7orf31_8_4015,2969,3534,4083,3624,3607,3716,2548,3089,3883,2625,5746,
3923
C8G_8_4016,135,94,144,74,8,391,245,3,785,394,224,51
CALM2_8_4017,2241,2946,1669,2143,1325,2339,3494,3184,2632,1696,2748,23
50
CALY_8_4018,167,56,217,204,271,0,656,0,77,0,281,370
CAMLG_8_4019,1295,1787,1059,1542,894,1354,1553,598,1576,1997,1237,1236
CAPN11_8_4020,976,1176,1052,1106,647,1964,1064,1778,2119,888,431,1155
CAPN5_8_4021,299,253,200,341,52,175,6,0,18,155,499,218
CAPN6_8_4022,2072,2156,1917,2585,2051,2231,1979,2252,3261,3380,4061,38
79
CARTPT_8_4023,38,97,130,159,9,168,6,35,153,114,49,6
CCL13_8_4024,278,680,180,494,638,699,197,715,26,417,558,142
CCND1_8_4025,371,702,476,871,387,74,384,648,254,195,737,249
CD1A_8_4026,134,201,17,259,485,0,83,0,130,9,102,123
CD52_8_4027,545,525,599,628,734,641,757,545,604,565,1563,535
CDCP2_8_4028,1126,1015,793,748,835,1656,1833,1378,1389,958,1477,1936
CDH5_8_4029,157,158,257,311,203,53,12,870,83,316,240,491

CHMP7_8_4030,443,547,876,230,212,726,385,33,174,319,672,960
CLDN16_8_4031,988,878,896,1059,1393,1910,1035,1076,805,596,1507,655
CLEC3B_8_4032,497,225,259,434,870,35,301,396,34,199,209,1390
CLSTN2_8_4033,586,541,494,514,136,349,329,360,92,880,822,477
CLVS2_8_4034,0,0,0,1,0,0,0,0,0,0,0,0
CNIH3_8_4035,185,659,580,151,23,209,37,132,170,863,868,52
CNOT6_8_4036,725,1051,711,1048,340,362,501,590,1171,604,676,121
CNTNAP1_8_4037,265,140,233,25,653,323,4,88,0,229,47,0
COG1_8_4038,2074,1572,915,1391,1595,2391,1150,814,1577,1794,1505,2163
COG3_8_4039,1869,1603,1419,1696,3066,1820,1830,1269,1209,2322,2564,198
4
COG7_8_4040,1123,639,683,1101,2719,701,1875,762,396,1779,523,1053
COG8_8_4041,538,368,198,405,623,787,117,92,182,48,574,918
COL10A1_8_4042,408,368,137,190,32,248,264,108,3,13,491,507
COL14A1_8_4043,4904,5659,4738,4952,6300,9228,3898,3280,2982,4574,7301,
6945
COL15A1_8_4044,820,1157,472,890,233,1535,1240,848,137,1369,897,376
COL16A1_8_4045,458,414,614,387,1410,298,1537,479,12,247,110,117
COL17A1_8_4046,401,179,373,429,408,144,76,125,459,399,213,370
COL1A1_8_4047,248,304,212,511,209,124,63,699,207,229,56,1006
COL1A2_8_4048,4882,3564,2892,4047,7116,3453,4764,6517,6764,5192,3131,7
369
COL21A1_8_4049,318,298,306,296,129,897,27,907,960,477,101,232
COL22A1_8_4050,0,0,0,14,0,0,0,0,0,0,0,0
COL23A1_8_4051,956,724,342,437,862,792,174,1280,835,241,278,1498
COL24A1_8_4052,732,455,654,611,714,691,333,775,611,538,2967,1561
COL27A1_8_4053,129,96,33,185,7,403,17,774,102,1074,33,320
COL3A1_8_4054,763,608,195,525,33,1806,1031,369,746,0,547,364
COL4A1_8_4055,1804,1829,1340,1973,1505,2977,1546,4898,1632,2010,3349,2
172
COL4A2_8_4056,1506,1707,1507,1750,1007,1484,1154,1226,1072,1549,1659,2
504
COL4A3_8_4057,1681,1502,1691,1534,1556,2515,748,1370,2217,691,3191,205
1
COL4A4_8_4058,2348,2464,1297,1783,3007,1986,3505,1660,1459,2368,2372,5
256
COL5A1_8_4059,778,1073,900,1143,1559,704,540,1251,156,993,1381,306
COL5A2_8_4060,1857,1638,1918,2239,1712,1792,2971,2970,3864,1721,1665,3
445
COL5A3_8_4061,1270,1176,1246,924,1406,1205,1299,2361,794,1005,1227,147
8
COL6A1_8_4062,1854,1686,1438,1588,1615,2698,1968,2280,2083,1592,1085,1
634
COL7A1_8_4063,372,378,478,791,1200,425,201,278,158,14,22,447
COL8A2_8_4064,2677,2774,1817,2706,2951,3530,1702,1915,1691,2637,3536,1
990
COL9A2_8_4065,300,653,257,197,295,381,686,36,546,24,416,341
COL9A3_8_4066,176,124,223,257,0,964,3,242,274,367,428,80
COMMD1_8_4067,2784,2812,2018,2151,2825,2096,1806,2607,1771,2327,1462,2
111

COPB2_8_4068,1262,1153,1116,958,1788,1289,885,615,479,1176,2835,1512
COPG2_8_4069,1202,1110,661,835,2496,1243,603,1860,902,583,1298,658
COPZ1_8_4070,708,647,634,489,309,150,1445,687,1225,82,29,1327
COPZ2_8_4071,926,1032,878,667,1146,1087,1698,1894,968,1396,1992,526
CORIN_8_4072,755,597,554,403,88,248,1211,850,280,1856,587,899
COX18_8_4073,540,728,522,614,1022,690,529,1324,1189,486,932,162
CPLX1_8_4074,211,154,160,250,1,3,3,0,0,292,317,1
CPLX3_8_4075,236,192,126,154,1,7,23,145,149,105,36,97
CPNE6_8_4076,1120,1266,1260,1372,345,347,2018,534,1508,1392,1076,1106
CPXM2_8_4077,343,401,464,533,87,608,273,472,160,27,256,98
CRABP1_8_4078,73,87,90,76,564,63,733,14,23,229,432,867
CRH_8_4079,334,369,116,97,0,0,0,15,0,236,16,734
CSE1L_8_4080,445,457,215,597,174,8,15,538,424,13,272,844
CTSW_8_4081,385,504,280,392,42,1524,94,102,284,266,314,660
CXCL10_8_4082,696,773,596,599,939,353,685,202,1124,490,292,622
CYGB_8_4083,484,194,158,120,7,1348,857,34,303,30,174,155
CYTH3_8_4084,654,499,708,655,1540,1675,581,706,687,576,2214,1147
DDI2_8_4085,863,972,703,998,792,1531,199,1122,1132,726,1010,2954
DIRC2_8_4086,2341,3519,1785,2667,3167,3006,2900,3195,2130,5172,1738,15
09
DISP1_8_4087,1285,1042,537,782,1252,1273,296,1441,445,1282,1412,1319
DLL4_8_4088,384,645,34,206,102,16,104,1576,733,746,525,6
DNAJC5B_8_4089,1698,2120,1467,1771,2120,1132,1441,1191,944,1270,713,15
20
DNAJC6_8_4090,1640,2071,1782,1607,2113,1568,3217,1487,1713,1406,1505,9
61
DOC2A_8_4091,665,879,755,339,265,261,300,240,395,680,992,516
DOC2B_8_4092,837,1164,373,1432,21,1184,772,266,273,1879,1481,729
DSCAML1_8_4093,1112,1101,1134,1483,772,937,1222,612,1567,1203,940,2692
ECEL1_8_4094,817,965,530,1033,1915,1190,1028,714,519,662,468,1406
EID2_8_4095,546,1038,450,405,355,660,328,442,1479,861,1695,264
EPCAM_8_4096,1222,750,596,822,2,485,818,1464,777,688,1788,265
EXOC2_8_4097,1098,1038,768,949,446,1552,1594,1288,1928,1508,1134,1649
EXOC3_8_4098,956,812,656,766,179,1067,940,1151,476,631,809,607
F11R_8_4099,304,161,269,596,313,74,937,190,293,273,609,566
FABP1_8_4100,638,579,756,724,449,718,646,1275,661,1184,953,381
FABP2_8_4101,2511,2340,2648,2453,1848,2328,3178,2824,2993,3027,3048,37
50
FABP3_8_4102,3450,4056,3110,3111,2675,3958,4109,4602,2059,1997,2555,24
54
FABP4_8_4103,232,59,351,300,17,33,658,70,303,133,241,71
FABP7_8_4104,1962,1723,2027,1621,2061,1888,1422,1113,810,1426,1709,110
6
FABP9_8_4105,2757,2025,1914,2169,740,1820,4149,3010,1416,2823,2239,261
4
FAM101A_8_4106,756,780,287,506,23,615,467,231,181,132,740,276
FAM117A_8_4107,2814,2686,2537,2506,2336,2556,5133,3277,2737,3462,4479,
4332
FAM57A_8_4108,136,284,173,242,925,416,433,69,52,121,315,562
FAP_8_4109,311,685,231,203,342,623,458,0,2,479,53,22

FBF1_8_4110,949,370,601,616,326,395,558,1118,1112,655,415,1804
FCN1_8_4111,367,1132,558,506,356,294,247,955,593,687,858,1162
FDX1_8_4112,2185,2734,2551,3046,3117,2374,1595,3137,1189,3653,3272,183
2
FDX1L_8_4113,381,326,323,232,51,163,997,10,148,117,22,26
FGF4_8_4114,1713,1277,1205,1485,1850,1497,1286,2496,1409,679,2095,1888
FGL2_8_4115,1358,1743,1248,1052,3131,2041,569,1973,903,1409,1148,1939
FOLR3_8_4116,207,504,411,231,35,65,78,96,661,196,114,1090
FOLR4_8_4117,287,102,107,91,182,235,96,353,93,822,222,53
FRG1_8_4118,1156,1662,1169,1156,502,1403,207,2149,713,827,1652,2179
FTL_8_4119,326,323,218,396,343,416,748,500,630,193,356,4
FXC1_8_4120,1172,907,576,1227,1540,786,1436,1083,1470,979,2703,1724
GABARAP_8_4121,1384,1392,1485,1302,1368,1113,936,381,974,1196,2286,228
1
GGA2_8_4122,1728,1397,1192,1548,994,2216,2354,803,1819,1896,1605,2196
GJA1_8_4123,4495,4805,4137,4188,6101,4796,3615,3948,4826,5502,2835,477
8
GJA3_8_4124,330,453,275,736,588,930,1187,420,114,337,181,771
GJA4_8_4125,1317,1360,1621,2010,2229,1462,587,1505,1166,1983,3566,3465
GJA8_8_4126,330,453,275,736,588,930,1187,420,114,337,181,771
GJB2_8_4127,413,470,429,211,808,477,604,724,517,534,695,1469
GJB4_8_4128,356,507,448,450,686,36,21,13,1067,387,490,152
GJB5_8_4129,1931,1949,1145,1612,2000,3175,2640,1526,1378,1708,1551,170
9
GJC2_8_4130,385,672,284,613,1,568,555,30,63,711,133,1831
GJC3_8_4131,858,858,883,1339,1765,933,689,3110,919,312,683,620
GJD2_8_4132,985,1205,873,890,765,1109,1539,1487,1546,1799,1223,2215
GJD3_8_4133,541,919,594,575,763,112,378,268,388,557,427,954
GJD4_8_4134,199,464,318,235,421,103,28,376,284,384,39,49
GKN1_8_4135,2241,2166,1784,1489,3399,1680,2464,1147,1724,2660,2836,182
9
GLCCI1_8_4136,2132,2420,1840,2195,2565,3625,4413,2135,2245,2475,2295,2
625
GLTP_8_4137,445,453,148,547,223,676,465,500,468,310,110,986
GLYATL2_8_4138,689,993,619,702,179,450,603,584,963,156,471,769
GP9_8_4139,201,87,69,112,618,596,0,577,498,96,582,888
GPIHBP1_8_4140,114,437,180,21,10,35,1,1,27,1200,0,15
GPR180_8_4141,844,652,602,686,213,3,135,127,1715,258,518,981
GRN_8_4142,387,328,119,278,449,529,550,844,487,173,561,162
GZMH_8_4143,624,1039,892,843,2075,805,333,530,1074,1108,84,673
GZMK_8_4144,2986,2945,3482,3345,3172,4337,3164,2401,3811,2439,4005,388
9
GZMM_8_4145,317,410,217,157,802,437,0,29,0,28,831,5
HBA1_8_4146,401,455,532,322,235,1331,667,324,46,76,1076,4
HBA2_8_4147,401,455,532,322,235,1331,667,324,46,76,1076,4
HBE1_8_4148,709,1218,343,833,149,1358,319,1272,583,1007,357,1212
HBZ_8_4149,531,339,424,327,478,209,132,32,516,237,910,178
HECA_8_4150,454,598,481,372,539,81,453,294,966,854,574,350
HGFAC_8_4151,207,119,47,49,17,7,3,4,909,226,435,15
HIAT1_8_4152,4242,4550,3485,4414,5148,3917,4846,3680,4560,3549,4461,44

44
HLA-DQB1_8_4153,367,342,385,243,147,592,1033,558,504,244,174,752
HMCN1_8_4154,10441,10112,7476,9331,8003,8701,10216,8000,10160,7238,133
53,9412
HMHA1_8_4155,66,110,101,209,0,15,10,63,151,329,1,428
HPCAL4_8_4156,461,740,412,843,862,88,1254,1353,74,1028,207,843
HPR_8_4157,277,475,434,351,899,482,1052,149,329,810,44,412
HPX_8_4158,571,463,598,542,231,512,28,1421,760,544,270,648
HSP90B1_8_4159,2967,3723,2537,2969,2676,5363,3165,4734,1918,3253,2899,
2305
HTRA1_8_4160,959,861,689,646,1185,830,2054,760,395,1235,354,657
HTRA4_8_4161,967,1103,727,1013,1577,940,886,1038,957,1065,825,1600
IFNG_8_4162,500,417,127,431,294,159,445,53,330,110,316,406
IGFBP7_8_4163,846,1045,806,952,510,912,601,2467,978,930,571,1989
IL12B_8_4164,873,593,531,780,204,145,527,3,469,916,274,704
IL13_8_4165,1476,1642,1345,1233,1061,3677,1372,1331,1776,2003,2452,174
7
IL17A_8_4166,928,1280,914,821,240,962,12,1,1069,1584,731,1028
IL1A_8_4167,1696,1270,1265,1066,872,644,2199,537,1703,776,922,1978
IL1B_8_4168,640,526,693,299,666,196,918,676,1593,39,721,455
IL3_8_4169,784,475,624,609,908,644,340,440,445,655,455,652
IL5_8_4170,1086,1581,1375,1186,401,2570,1336,2486,1509,2349,2190,1675
INSL3_8_4171,1169,792,1145,1230,883,1798,1973,1601,1449,1671,389,2095
IPO13_8_4172,1785,1658,1762,1661,1234,697,2972,1170,1162,820,2966,2168
IPO4_8_4173,712,466,536,227,283,834,416,83,760,795,1038,739
IPO5_8_4174,418,209,89,106,6,683,27,186,69,663,4,266
IPO7_8_4175,1791,1909,2092,1512,2851,1468,1384,1602,2033,1379,1992,145
8
IPO9_8_4176,699,346,251,544,1641,101,1236,734,310,62,1536,34
ITGA10_8_4177,362,331,298,430,0,456,2,239,390,980,41,47
ITGA11_8_4178,310,267,228,553,94,376,27,36,265,106,73,623
ITGA2_8_4179,1968,1691,1615,1915,1967,3236,2369,1735,1717,2637,2017,27
64
ITGA4_8_4180,1544,1800,1594,1398,1528,2510,847,2290,2259,1429,3101,202
0
ITGA5_8_4181,75,216,387,89,156,962,56,88,0,107,117,41
ITGA8_8_4182,2627,1729,1779,1804,3158,3432,2779,3297,1200,1401,2622,35
57
ITGAX_8_4183,1168,1063,968,1184,2446,1075,2819,775,1384,1384,1646,1150
ITGB5_8_4184,109,291,404,438,25,816,20,48,535,144,24,700
ITGB6_8_4185,1471,1017,1338,1270,1075,1696,935,1020,1418,626,3040,227
ITGB8_8_4186,130,112,65,90,7,68,17,12,488,775,14,132
ITLN1_8_4187,288,240,226,262,179,128,1254,734,715,208,1601,286
KDELR1_8_4188,953,1255,1051,1181,1882,1504,1602,1063,1773,1461,2828,12
79
KEL_8_4189,729,831,930,399,632,1511,748,586,1236,646,1151,601
KIF20A_8_4190,565,630,575,734,811,2159,729,480,900,1018,678,1715
KIF3B_8_4191,208,210,286,452,591,68,126,549,460,262,22,239
KIF5A_8_4192,504,162,171,417,1463,342,532,1197,327,695,979,2
KLK13_8_4193,2270,1992,1967,2041,2574,2000,2065,1802,3398,2347,2435,32

20
KLK14_8_4194,624,850,653,306,727,35,841,720,59,759,1140,1255
KLK4_8_4195,132,123,94,158,0,68,104,54,419,0,1,4
KLK9_8_4196,718,1105,741,1025,1233,1877,1280,1040,929,1430,742,418
KPNA1_8_4197,1505,943,945,1020,1110,593,757,1254,1133,327,223,2660
KPNA2_8_4198,4998,4870,4001,4608,7159,5609,4022,5686,5102,4555,5170,42
88
KPNA3_8_4199,805,686,481,382,369,527,88,454,112,470,202,276
KPNA4_8_4200,5728,4571,4645,4382,4424,4763,4256,3243,3534,5408,7320,94
65
KPNA6_8_4201,730,747,378,320,234,1515,832,235,349,613,1127,660
KPNB1_8_4202,4519,4052,4771,5268,4342,6804,5577,5386,3872,4805,3766,34
43
KRT12_8_4203,1511,1186,1089,961,1194,1696,1975,1054,1351,1347,1459,171
7
KRT7_8_4204,391,556,284,388,432,1211,667,107,226,972,559,1348
KRT8_8_4205,323,516,284,344,429,1204,665,107,225,965,557,1349
KRTAP5-4_8_4206,2728,1803,1984,1187,3541,3925,2450,1308,2672,1682,2198
,2343
LASP1_8_4207,2563,2768,2105,2764,2412,880,3241,2447,1375,1411,3300,319
5
LBP_8_4208,3355,3308,3090,3067,1821,4613,3936,3489,4874,4621,4325,5019
LCN12_8_4209,1327,1408,869,1371,2906,1559,641,775,764,1799,1522,481
LCN1_8_4210,3845,4062,4115,4667,4926,2658,6763,4161,4467,3836,6107,437
8
LCN2_8_4211,408,436,530,103,142,577,383,927,104,580,306,122
LCN8_8_4212,50,113,69,17,30,3,6,38,0,94,148,0
LCN9_8_4213,1370,1248,1240,1198,434,803,186,1692,639,1812,2213,1191
LDLRAD2_8_4214,383,592,412,85,4,1061,1066,0,461,648,518,484
LDLRAP1_8_4215,244,491,189,386,415,170,275,288,25,727,287,307
LMAN1_8_4216,3131,3975,3326,4171,3263,5365,4985,2492,4410,5063,3020,38
84
LMAN2_8_4217,1746,1802,1761,1511,1743,1733,1206,1656,1520,2641,975,162
9
LPA_8_4218,2121,2167,1904,1957,2141,1552,2740,2524,1503,2508,1673,2651
LRIG1_8_4219,692,540,285,468,1203,751,838,1138,562,985,340,2297
LRP2_8_4220,76,540,317,364,582,232,33,68,285,27,30,720
LRRC4B_8_4221,374,80,84,54,32,124,93,32,652,339,178,7
LRRCC1_8_4222,5411,5112,4238,5197,4684,3569,5359,4962,3977,4029,5688,5
745
LYST_8_4223,1979,2159,1422,1596,1480,1771,718,1027,1724,1822,2150,1302
MAL2_8_4224,653,530,656,465,296,514,896,354,1241,786,2061,1202
MATN3_8_4225,1057,2409,1488,1077,2036,1302,2526,944,979,1148,221,1296
MFSD8_8_4226,4098,4991,3393,3942,4191,4158,1860,4518,2515,3447,4805,54
49
MFSD9_8_4227,1125,1413,1210,922,1519,519,733,433,465,2465,1443,876
MMAA_8_4228,5404,5399,5735,5183,2246,4963,6589,4532,7013,5846,8022,823
9
MMACHC_8_4229,129,114,229,83,0,550,317,367,0,3,411,149
MMGT1_8_4230,4100,3686,3571,3163,5177,3979,6104,4707,2877,3814,5883,31

51
MMP11_8_4231,407,468,492,409,49,528,257,957,229,346,214,392
MMP13_8_4232,1970,1487,954,1264,2258,1444,1205,1881,1650,1400,2385,299
4
MMP15_8_4233,691,543,231,640,1016,440,153,769,358,762,427,452
MMP16_8_4234,506,780,582,332,380,344,1353,117,331,168,989,164
MMP17_8_4235,3570,3428,3373,2248,3730,4924,1468,2355,4037,2438,4862,22
26
MMP19_8_4236,317,534,226,615,383,126,711,375,519,514,189,653
MMP24_8_4237,88,53,44,35,4,316,25,131,2,81,35,114
MMP25_8_4238,2553,2164,2601,2135,2577,1938,2858,1381,3784,3303,3448,22
58
MMP26_8_4239,449,311,345,239,52,262,67,519,795,235,208,278
MMP27_8_4240,8165,8354,8598,9539,9071,7629,8623,8328,7452,11243,8585,7
671
MRS2_8_4241,6282,5940,4459,5771,8037,4530,8186,4538,7078,3334,7096,680
1
MSTN_8_4242,193,687,449,409,1469,907,172,217,347,889,173,89
MTX2_8_4243,1151,1170,800,1009,864,2216,308,634,890,1528,513,621
MUC2_8_4244,75,34,194,37,0,0,0,0,2,670,0,384
NAPG_8_4245,4720,4646,4347,5591,3313,3164,5229,5252,4585,6883,2938,796
6
NAPSA_8_4246,1000,731,650,1054,473,889,233,1403,1667,585,381,1155
NCOA5_8_4247,238,196,93,235,0,112,1364,50,119,1170,474,51
NGF_8_4248,940,1095,872,904,545,367,1642,30,5,1298,1210,1616
NID1_8_4249,1564,1740,839,2189,213,1305,1856,875,1207,1476,2175,1003
NPC1_8_4250,1076,1274,745,858,1309,1357,1263,928,729,1209,840,715
NPEPPS_8_4251,2156,1212,1023,1637,1594,479,1878,1375,1771,2037,2846,36
1
NPPB_8_4252,889,978,909,1318,1046,1370,2108,995,828,1209,1328,1379
NPY_8_4253,316,128,534,244,32,182,135,690,11,367,465,462
NSMCE1_8_4254,2110,1735,1482,1925,2698,2088,2030,3034,2446,1433,2123,1
073
NUP107_8_4255,961,1026,1057,733,1245,1320,1911,625,673,1187,428,483
NUP133_8_4256,2825,3762,3013,3869,3102,4807,2349,2471,2535,1511,3629,3
637
NUP153_8_4257,1288,1574,1926,1871,2618,3217,790,1428,853,2518,925,2069
NUP160_8_4258,1134,1543,1017,1617,1002,426,1023,1328,2099,735,588,2742
NUP210_8_4259,1833,1935,1622,1101,2269,2170,2594,2184,2383,1882,773,23
82
NUP214_8_4260,1318,1809,1124,1950,1442,2435,720,1829,2139,1855,3092,14
68
NUP35_8_4261,883,465,515,990,1101,896,940,1364,612,264,590,1174
NUP37_8_4262,3173,3308,2963,2512,5576,3725,3241,5106,2481,3454,4193,36
98
NUP54_8_4263,1942,1742,2101,1887,2433,1952,3066,1405,1181,2250,1990,12
60
NUP88_8_4264,2032,2215,2212,1933,1329,2055,1848,1589,2560,1853,2168,24
19
NUPL2_8_4265,1595,1877,1262,1937,973,2164,2223,1131,2121,3701,735,3288

NUTF2_8_4266,902,931,763,771,888,670,1108,576,2011,503,341,3505
NXF2B_8_4267,674,508,393,476,230,1537,609,919,963,1657,1587,1481
NXF2_8_4268,674,508,393,476,230,1537,609,919,963,1657,1587,1481
NXF3_8_4269,678,780,760,1315,678,1214,746,75,722,931,411,531
NXT1_8_4270,4075,4171,3542,3195,2172,7760,4442,4163,3991,4971,3965,341
7
OAZ2_8_4271,2282,1818,2074,1402,3971,2078,1095,2702,1832,2053,1441,266
7
OBP2A_8_4272,835,372,555,461,192,1445,286,1108,525,1151,186,464
OBP2B_8_4273,3742,3861,4176,4405,4739,1942,6774,4138,4465,4171,6303,42
91
OCA2_8_4274,659,819,879,787,334,1238,595,448,17,1712,631,1078
OGFOD1_8_4275,389,565,670,617,1034,1154,1,662,526,374,490,33
OGFOD2_8_4276,362,203,347,437,48,794,177,1011,37,438,72,227
OGFR_8_4277,521,530,285,356,879,425,563,645,587,201,1675,1242
OVCH1_8_4278,460,214,370,220,710,116,437,532,260,497,521,767
OVCH2_8_4279,665,569,598,973,1256,1017,269,506,807,619,1622,452
OXNAD1_8_4280,391,452,169,190,181,518,4,62,0,1074,16,41
OXT_8_4281,497,526,724,1116,284,680,1702,926,361,41,969,597
PANX1_8_4282,378,340,35,329,481,0,32,557,917,209,68,1027
PAQR7_8_4283,166,337,246,295,2,138,88,0,36,385,512,112
PCDHB11_8_4284,239,151,382,192,0,39,839,39,309,248,144,763
PCDHB16_8_4285,2372,2447,1600,1835,3342,3061,105,1724,1393,1778,1784,1
442
PCSK4_8_4286,1000,1576,1005,1355,1101,2699,955,149,1344,1273,1680,1520
PCSK7_8_4287,513,296,506,353,837,1344,684,195,661,852,123,602
PEA15_8_4288,578,606,396,392,1575,428,422,328,321,379,266,68
PET112_8_4289,2533,3141,2471,2751,949,4215,3607,2855,3462,3530,3302,46
44
PEX13_8_4290,452,410,407,826,230,291,1801,889,492,27,860,1338
PEX7_8_4291,378,458,404,735,149,1142,172,258,2,522,536,548
PF4_8_4292,573,360,425,284,240,589,22,31,607,545,134,220
PFN3_8_4293,1332,920,1120,877,1681,1553,974,1146,306,986,1282,1605
PHEX_8_4294,671,492,549,266,1005,1178,616,471,241,2180,1028,288
PIGR_8_4295,1491,1803,1117,1301,1194,1381,1411,2759,2804,2307,1569,181
5
PITPNA_8_4296,218,180,191,257,387,178,1,390,344,415,122,266
PITPNB_8_4297,1199,664,850,867,1630,793,1129,831,946,314,1157,1590
PLLP_8_4298,309,550,1008,418,702,596,967,488,761,1295,29,89
PLP2_8_4299,1450,2013,954,1604,1042,1466,1406,3447,2245,2887,1290,2875
PLXNB2_8_4300,978,1377,1141,1321,98,1009,1365,1787,948,1749,407,1945
PNMA2_8_4301,1018,740,1043,946,1789,1082,659,2037,969,1298,867,1146
PPP1R14A_8_4302,1345,1874,1308,972,1209,1034,1447,1152,1140,2059,2022,
1112
PPP1R14C_8_4303,3260,3032,2260,3226,3662,4822,2955,2240,3500,4487,2683
,5163
PPP1R15A_8_4304,728,921,594,703,303,1661,369,338,1091,662,580,883
PPRC1_8_4305,144,182,396,309,353,434,38,427,248,143,431,33
PPY_8_4306,1470,1058,1107,1224,1714,1220,547,2785,1269,1722,2261,1130
PRB3_8_4307,1759,1298,2012,1608,857,1718,1858,2041,2000,1991,3247,1270

PROCR_8_4308,848,1284,813,710,2550,1821,2390,702,1906,685,1635,853
PROS1_8_4309,860,632,834,1142,955,1427,326,1263,848,831,984,916
PROZ_8_4310,4349,4310,4818,3442,3254,6820,3770,2515,5432,5002,5247,631
2
PRPF18_8_4311,1473,1764,1415,1214,277,2281,1988,1562,1099,734,1757,284
0
PRSS12_8_4312,3851,3954,3355,4719,4445,2768,4819,3696,3378,4718,3631,5
623
PRSS22_8_4313,1827,1970,1965,1994,864,1726,4065,2313,1786,1227,2591,35
86
PRSS27_8_4314,143,512,41,161,403,699,156,0,0,84,56,458
PRSS33_8_4315,691,707,835,762,393,1586,734,427,26,782,869,246
PRSS36_8_4316,1585,1453,1521,1203,1892,2011,1958,1131,2143,1636,2202,1
116
PRSS8_8_4317,220,124,235,43,531,196,241,53,104,54,163,299
PSCA_8_4318,578,565,612,404,368,1076,301,385,439,189,661,321
PTOV1_8_4319,445,543,391,598,333,311,480,458,255,1380,181,1152
RABIF_8_4320,4973,5455,3997,4929,5065,4655,4374,6820,3740,5094,4023,61
73
RAMP1_8_4321,446,289,300,35,260,401,26,23,104,217,504,30
RAMP2_8_4322,943,975,1496,1263,532,1169,1055,1880,556,2075,2379,674
RAMP3_8_4323,512,535,148,245,124,834,1,1779,358,687,6,756
RANBP17_8_4324,956,936,291,1011,315,2159,797,869,863,648,378,459
RASSF9_8_4325,316,422,347,630,392,19,612,476,111,83,170,80
REEP5_8_4326,599,690,723,608,1315,993,141,44,1776,886,233,1009
RHAG_8_4327,4145,4369,3954,3575,3487,3584,2705,3203,3255,1476,5397,556
8
RLBP1_8_4328,914,1063,572,509,1263,1169,223,631,1351,918,2377,635
RPL15_8_4329,299,301,164,482,241,15,250,292,337,133,99,320
S100A12_8_4330,142,98,563,266,12,206,1178,1,516,9,783,474
S100A1_8_4331,516,693,380,948,674,964,532,1317,165,595,669,1358
S100A2_8_4332,474,339,262,337,1308,372,710,214,50,938,493,149
S100A3_8_4333,1118,1277,860,1186,311,637,817,1747,866,2158,1680,1434
S100A6_8_4334,58,15,61,77,25,8,197,0,64,2,0,627
S100B_8_4335,1295,1733,1444,1535,1735,1463,1211,2136,2201,993,1961,366
S100P_8_4336,1807,1552,1444,1652,2062,3619,2743,2204,1743,2284,1961,22
35
SAA4_8_4337,637,491,453,414,876,615,1009,15,741,140,1010,486
SCAMP1_8_4338,1323,924,786,1325,2385,1288,2499,1223,961,1189,849,1560
SCAMP2_8_4339,1342,841,1025,1135,1597,950,1176,1089,1781,1896,604,490
SCFD2_8_4340,299,266,385,305,87,449,34,688,608,589,168,332
SCLT1_8_4341,5851,7475,5177,5392,3772,7098,8375,5558,7326,6567,3882,61
27
SCPEP1_8_4342,2170,2292,2089,2992,744,2010,2714,1566,1566,2207,2364,19
61
SDC2_8_4343,1199,1205,1033,1139,188,373,1488,402,1192,425,1215,881
SEC14L3_8_4344,1274,852,1155,1004,566,1299,1114,780,845,1417,1877,1711
SEC22A_8_4345,9855,9764,7818,10336,8176,12972,11414,10367,8461,11235,1
0080,12620
SEC23A_8_4346,47,47,60,29,64,327,5,0,4,5,0,155

SEC24A_8_4347,82,180,122,62,6,241,43,12,10,506,146,273
SEC24D_8_4348,229,153,229,152,8,417,414,95,372,499,7,3
SEC61A1_8_4349,174,290,411,225,332,113,971,250,336,439,362,404
SEC61B_8_4350,280,539,461,558,998,111,118,257,487,338,186,175
SEC62_8_4351,2425,2680,2062,2167,3232,1850,3207,1794,2946,4234,3472,31
49
SEC63_8_4352,4583,4052,3979,3501,2080,3791,6058,4490,5220,3655,3440,37
24
SELP_8_4353,671,1197,1048,762,189,1786,1932,939,569,1412,622,1407
SERINC1_8_4354,2039,2899,2133,2598,1232,3611,1922,2540,1783,3498,5004,
1081
SERPINA11_8_4355,1983,2602,1673,2380,3066,3067,1717,2103,1215,1520,184
2,3677
SERPINA12_8_4356,1457,1387,1351,1542,1599,1754,1466,1812,1343,1016,126
1,662
SERPINA3_8_4357,894,1084,952,608,678,884,1177,867,784,540,880,711
SERPINA4_8_4358,2051,2190,1746,1500,2457,2367,1163,2495,433,1493,4281,
202
SERPINA5_8_4359,805,872,650,958,477,321,2293,1188,341,1053,1133,1013
SERPINA7_8_4360,623,679,556,618,967,484,438,665,1204,824,269,154
SERPINB10_8_4361,1583,1442,957,1947,2871,955,1665,2221,1161,1205,380,2
900
SERPINB12_8_4362,2761,2535,2293,2888,2141,3311,3796,2398,2420,1811,302
1,5543
SERPINB13_8_4363,352,547,566,670,1643,533,670,381,157,731,1078,151
SERPINB1_8_4364,802,753,850,877,735,1768,1712,592,1166,653,645,1046
SERPINB4_8_4365,2499,1809,1856,2214,1517,1566,2926,2698,2166,1933,2430
,1186
SERPINB9_8_4366,1642,2075,1219,1969,1399,2590,1711,983,1627,3184,2459,
2321
SERPINC1_8_4367,1077,402,760,984,794,283,1221,545,1282,557,1806,1683
SERPIND1_8_4368,161,69,147,62,527,110,200,398,82,73,357,447
SERPINF1_8_4369,1450,1409,1599,1123,2553,1673,1561,1610,1128,1652,3134
,2100
SFXN1_8_4370,580,445,372,927,277,504,166,677,1497,907,645,78
SFXN2_8_4371,1394,1134,1599,1531,1576,2920,682,2210,1162,1147,2110,107
4
SFXN3_8_4372,1147,932,1159,901,412,1126,355,1148,1844,576,2261,547
SFXN4_8_4373,483,675,428,502,520,1069,363,1316,352,313,316,271
SFXN5_8_4374,946,676,1135,1070,2858,730,490,412,2881,973,857,2297
SLC15A5_8_4375,702,959,778,582,219,901,596,865,2272,827,805,552
SLC16A13_8_4376,342,416,550,335,377,627,635,105,316,170,2,1472
SLC16A14_8_4377,1883,1264,1210,1192,1744,1840,1381,2168,1966,845,2002,
2219
SLC16A9_8_4378,441,293,554,354,223,249,1660,517,237,991,170,61
SLC17A9_8_4379,114,478,505,536,0,0,1,382,0,240,1483,46
SLC22A20_8_4380,157,93,132,30,190,493,24,90,148,49,300,386
SLC22A24_8_4381,1007,1019,1024,807,1100,1143,1500,245,241,814,977,652
SLC22A25_8_4382,402,369,140,406,233,9,38,816,221,507,432,1039
SLC25A23_8_4383,544,622,714,587,155,497,1651,342,1266,108,545,591

SLC25A29_8_4384,258,54,100,151,0,62,656,299,242,3,915,102
SLC25A31_8_4385,1834,2493,1762,1842,3239,1568,2341,2753,2132,1801,3517,3003
SLC25A38_8_4386,350,488,525,393,2,296,900,477,464,273,52,660
SLC25A42_8_4387,1214,1331,876,1615,845,417,1730,587,1342,1371,1333,1768
SLC25A46_8_4388,1282,1326,1432,1165,1145,1351,1944,1723,1508,1289,1198,2822
SLC25A48_8_4389,685,547,382,507,899,757,529,571,930,1385,1136,1226
SLC38A7_8_4390,743,623,530,566,1427,643,117,507,1438,427,1786,627
SLC38A9_8_4391,2565,1685,1655,1705,1695,1586,2432,2911,918,863,2436,1933
SLC41A2_8_4392,521,368,422,233,503,764,1172,961,976,1108,702,378
SLC45A1_8_4393,420,519,693,409,595,495,42,638,1690,1539,599,895
SLC45A3_8_4394,706,991,832,766,786,697,1601,653,810,1538,1145,2338
SLC47A1_8_4395,359,247,317,388,252,445,265,244,146,105,85,145
SLC48A1_8_4396,890,719,613,412,239,1530,929,654,248,238,788,285
SLC5A12_8_4397,1181,968,1503,1530,511,1142,2433,818,814,1465,2261,1452
SLC6A17_8_4398,203,246,257,116,2,358,464,116,122,1,647,62
SLC7A14_8_4399,1586,1315,1086,1271,1974,1577,1659,1701,1962,1900,1961,2900
SLIT2_8_4400,1291,806,875,953,1353,861,1124,1945,1717,492,918,285
SNAP29_8_4401,10587,9273,7739,8443,8150,7784,9050,6396,11273,6506,8342,8582
SNX12_8_4402,0,0,0,0,0,0,0,0,0,0,0,0
SNX13_8_4403,915,376,631,717,1755,904,2334,745,364,457,742,1536
SNX17_8_4404,726,543,490,813,789,562,658,391,792,513,619,1067
SNX19_8_4405,702,544,763,645,928,653,671,1856,358,823,1513,292
SNX22_8_4406,1100,917,976,1165,598,1285,1122,682,678,924,700,1547
SNX24_8_4407,73,306,12,184,28,0,0,161,69,133,155,0
SNX2_8_4408,398,322,386,267,295,305,32,703,703,449,577,155
SNX4_8_4409,365,353,468,279,645,406,56,301,375,288,662,576
SNX8_8_4410,0,0,0,0,0,0,0,0,0,1,0,0
SNX9_8_4411,1003,1274,913,1005,1785,903,195,1267,1074,1366,2209,1159
SORCS2_8_4412,1626,1609,1170,1192,823,1546,1647,2336,2031,1887,864,1562
SORCS3_8_4413,419,251,599,111,132,1,274,12,951,467,213,273
SORL1_8_4414,595,1028,313,860,914,1541,458,808,330,965,1029,771
SOS1_8_4415,3443,3250,3091,3407,3040,3779,2608,4646,2621,4042,6257,4074
SST_8_4416,1487,1802,1135,858,528,531,2216,1246,741,2450,1853,2178
ST13_8_4417,2118,1436,1211,1688,3156,2245,1832,3383,3154,1636,1736,1881
STAB1_8_4418,290,216,207,194,15,257,7,182,11,382,431,499
STARD4_8_4419,3464,6171,4467,4190,5554,7169,5061,3582,3200,5812,5128,3729
STARD5_8_4420,1353,1715,1767,1892,2186,2335,1286,1095,1758,1947,2279,3965
STARD6_8_4421,1806,2175,1664,2106,3179,1801,1010,1642,2171,2673,3015,1643

STEAP1_8_4422,2287,2690,2465,3136,2508,2692,3013,2095,2753,2278,4211,2
773
STMN4_8_4423,633,468,334,789,1445,396,209,314,380,486,1010,741
STX11_8_4424,374,176,298,108,477,0,1473,851,334,41,99,177
STX18_8_4425,742,854,819,660,1363,655,1066,423,720,266,466,943
STX4_8_4426,792,982,971,678,822,1217,474,313,694,498,691,669
STX5_8_4427,328,265,95,268,740,337,459,73,514,131,145,62
STX6_8_4428,2746,3101,2599,3415,3949,4788,2836,2061,2870,2917,3597,365
8
STX7_8_4429,1705,1800,1591,1631,1720,1382,2633,1824,2549,1587,1650,222
7
STXBP3_8_4430,1162,1541,701,1138,905,1080,1743,403,739,1228,547,1736
SVOP_8_4431,218,153,111,162,347,198,5,244,559,764,87,5
SYP_8_4432,141,186,122,235,651,194,472,0,144,38,25,72
SYT10_8_4433,1862,3085,1875,2734,1054,1023,4928,2261,1546,3253,3588,37
66
SYT11_8_4434,509,376,274,693,262,1228,159,282,546,353,75,1058
SYT13_8_4435,759,692,1320,573,33,992,1863,2572,44,714,970,464
SYT16_8_4436,2521,2072,2424,2105,765,1911,3904,1404,2792,2616,5169,406
9
SYT4_8_4437,958,926,1056,895,1340,1040,589,1069,121,1080,1105,332
SYT5_8_4438,816,300,814,655,668,342,98,1468,1239,203,348,3051
SYT6_8_4439,1219,1334,1362,665,1909,2200,1023,1366,1760,710,1039,665
SYT7_8_4440,69,100,62,0,1,198,0,3,0,0,367,0
SYT8_8_4441,158,163,379,642,186,15,31,76,119,1,0,147
SYT9_8_4442,1192,1056,930,1403,2239,1136,1526,1182,814,1253,1729,1477
TAS2R42_8_4443,1598,1256,1086,1356,759,1499,2358,1822,1906,2407,1505,2
307
TBC1D9_8_4444,637,1022,1334,643,479,581,295,738,650,1290,2853,714
TCN1_8_4445,1114,885,796,861,1300,1079,1598,490,1389,2465,1450,5872
TEKT4_8_4446,628,846,515,857,1048,424,396,410,968,1389,802,1299
TF_8_4447,1052,1039,890,791,190,1855,416,1667,831,715,1026,545
TG_8_4448,1524,755,950,1531,1704,101,119,903,3107,1887,717,407
TGFB1_8_4449,584,413,266,352,1198,2090,233,42,198,55,242,26
TIMM10_8_4450,1142,1628,1324,1206,3402,2384,1894,1185,2364,1858,1105,7
55
TIMM13_8_4451,710,811,1015,946,1383,1463,422,622,1160,2188,1195,503
TIMM17A_8_4452,3732,2705,2638,3027,1132,5047,4088,3368,3155,3586,4686,
4808
TIMM22_8_4453,112,389,426,77,44,15,64,132,12,518,152,925
TIMM23_8_4454,5884,4777,4700,4359,3247,5448,6527,3935,5604,4501,8371,6
387
TIMM44_8_4455,523,422,442,224,15,354,269,147,412,587,147,98
TIMM8B_8_4456,154,335,124,229,0,345,611,403,1,284,65,188
TIMM9_8_4457,5492,5234,6050,6171,4375,5546,6084,8274,5626,7439,5962,75
49
TLL2_8_4458,776,1114,618,1347,2274,64,1118,69,1029,798,78,384
TM9SF2_8_4459,838,1052,993,914,867,1082,1230,711,408,2076,1596,1367
TM9SF3_8_4460,893,485,380,528,625,320,482,1713,1827,756,665,1293
TM9SF4_8_4461,2514,1515,1845,2321,1640,3025,2709,1639,2160,2290,2884,2

576
TMCO3_8_4462,2592,2066,2390,3759,1543,1996,3690,2091,1450,3090,3346,46
58
TMED10_8_4463,832,768,722,301,690,1113,728,657,413,1129,581,759
TMED1_8_4464,1913,2013,1128,1404,930,1715,502,1233,1777,2926,1431,2403
TMED2_8_4465,383,773,283,407,259,462,707,488,55,426,215,763
TMED3_8_4466,813,522,712,671,1390,20,172,1394,508,52,709,96
TMED4_8_4467,884,834,1025,949,1857,1627,721,159,1058,1273,1416,1169
TMED7_8_4468,4346,4685,3599,3321,5221,4530,3385,3998,4603,5747,3404,43
65
TMED9_8_4469,966,1258,690,735,842,1948,1121,884,1111,186,468,2334
TMEM104_8_4470,184,173,142,308,83,285,2029,12,414,48,79,88
TMPRSS11B_8_4471,1895,2249,2461,1774,3228,2562,4386,2007,2230,2964,409
7,2650
TMPRSS11D_8_4472,432,277,484,710,571,1506,34,1332,100,496,420,971
TMPRSS11E_8_4473,1234,1075,1391,1303,1525,700,2711,506,2150,463,1501,1
408
TMPRSS11F_8_4474,4567,3365,2935,4381,6112,4258,4330,5005,5324,4638,436
1,6609
TMPRSS12_8_4475,1092,1138,892,863,2387,1224,1268,1386,867,1047,326,215
7
TMPRSS5_8_4476,577,608,391,487,512,716,184,323,46,503,1411,41
TMPRSS6_8_4477,1159,1415,1701,1306,2004,1416,1434,1084,2101,1226,2268,
1859
TMPRSS7_8_4478,650,449,348,406,984,1055,1021,179,413,228,549,663
TMPRSS9_8_4479,139,128,206,94,20,22,8,64,289,0,733,10
TNC_8_4480,468,605,441,516,792,648,503,940,782,942,1886,295
TNF_8_4481,1001,1110,1007,1051,470,1021,390,1320,896,1039,1011,762
TNFRSF9_8_4482,478,107,154,498,471,1,260,632,697,501,370,1590
TNNC2_8_4483,597,507,687,421,146,1009,722,551,277,605,418,615
TNN_8_4484,1146,1384,1074,1271,699,1211,1739,2574,1695,1142,1083,2599
TNNI3_8_4485,296,626,390,315,603,1008,560,140,1,7,801,498
TNR_8_4486,1532,1878,1585,1786,758,1476,2877,664,1795,2098,1946,1263
TOMM20_8_4487,447,820,639,753,240,2096,258,98,834,560,552,312
TOMM22_8_4488,3158,3574,2993,3023,1387,4239,4007,2867,2808,3064,5460,3
765
TOMM70A_8_4489,8776,8899,9059,8732,7989,12192,7453,9699,7894,11319,116
93,9840
TOMM7_8_4490,434,976,314,325,0,939,194,5,5,685,450,135
TPSG1_8_4491,1083,831,884,779,644,694,1018,1108,950,1077,1182,1049
TRAK2_8_4492,1184,1341,682,1392,902,1412,988,1418,755,1566,1262,1740
TRAPPC10_8_4493,686,634,502,683,74,283,544,712,285,1582,815,2437
TRAPPC8_8_4494,256,290,16,281,6,499,106,669,16,286,175,350
TSNAX_8_4495,242,150,199,287,41,257,363,11,434,671,226,968
TTPA_8_4496,4496,5001,4441,4263,3545,4701,4068,5564,6640,5445,4185,590
0
TTR_8_4497,296,204,262,131,825,135,274,26,754,145,167,397
TUBA1A_8_4498,1019,1021,1198,1393,798,1365,495,1074,740,483,978,630
TUBA1C_8_4499,2986,2541,2450,2386,3904,3369,3230,2478,1877,3028,4569,1
721

TUBA3D_8_4500,1554,1296,1693,1604,1913,1238,3354,940,1786,2365,929,821
TUBA3E_8_4501,1508,1441,1211,1201,1209,1409,3191,398,1574,1171,803,208
9
TUBA4A_8_4502,1843,2766,1312,1828,2567,2181,2436,1192,2214,3763,3375,1
496
TUBB1_8_4503,1440,1366,1726,1415,1680,1054,1970,1505,1393,1437,3100,14
77
TUBB2A_8_4504,671,443,892,870,1213,559,335,890,699,874,955,284
TUBB2B_8_4505,671,443,892,870,1213,559,335,890,699,874,955,284
TUBB6_8_4506,2172,2102,1846,2582,3567,1721,2779,1488,2079,2288,2804,25
39
TUBB8_8_4507,1634,1800,1404,1893,2165,1223,2830,2024,1139,818,2328,258
3
TUBE1_8_4508,2706,3175,2790,2593,2365,3132,2596,2721,3572,3500,3070,28
18
TUBG1_8_4509,594,690,463,847,202,311,4,1045,782,283,517,636
TUBG2_8_4510,3160,2601,2051,2015,3440,3189,2448,1758,4048,2394,1960,11
83
TXNDC8_8_4511,2088,2335,1680,2016,2087,3022,2581,3595,2069,2807,2721,2
793
UCP2_8_4512,348,310,267,317,932,392,272,258,316,348,53,279
USO1_8_4513,2211,1936,1609,1756,2523,839,1630,1722,1272,2281,1730,1563
VAMP2_8_4514,126,239,234,164,10,1369,303,462,0,88,746,114
VAMP5_8_4515,895,994,503,1401,495,907,1409,306,55,1471,87,1666
VKORC1L1_8_4516,1376,1988,1053,1991,2690,708,926,1022,2808,1486,1588,1
473
VPS18_8_4517,372,470,485,713,645,123,134,129,2044,292,747,143
VPS26B_8_4518,548,318,477,465,1712,951,429,250,1139,475,270,1227
VPS33A_8_4519,1276,1254,1667,1149,867,1857,1338,1952,1559,1531,1260,10
63
VPS33B_8_4520,2767,3352,2417,3597,3162,5000,3174,3277,3393,1701,2978,4
306
VPS35_8_4521,301,207,340,377,337,170,42,551,54,337,437,662
VPS39_8_4522,3456,4469,2944,4209,2598,4629,3030,4822,5055,2943,3416,35
23
VPS45_8_4523,718,729,1072,429,1418,1141,860,206,637,484,439,216
VPS4B_8_4524,620,486,783,926,241,537,13,528,217,676,348,336
VSIG2_8_4525,255,278,218,128,759,79,92,357,800,330,115,532
VTI1A_8_4526,994,1055,636,793,1909,1479,1327,477,986,1677,1705,2609
VTI1B_8_4527,323,296,217,449,196,208,142,305,279,661,703,148
VTN_8_4528,1378,782,568,1241,1672,418,659,2615,1604,421,748,1425
VWF_8_4529,1224,1256,920,929,1419,513,324,96,314,925,1007,916
XK_8_4530,170,74,219,6,13,304,0,1,20,331,1,712
XPO1_8_4531,3149,2232,2600,3023,2731,1491,2716,2038,4003,2338,2126,233
1
XPO4_8_4532,243,186,207,195,266,475,1062,318,184,170,210,671
XPO5_8_4533,238,273,247,203,11,84,57,346,272,274,122,25
XPO6_8_4534,145,360,280,226,8,493,436,1169,370,1047,1157,156
XPO7_8_4535,258,180,112,397,176,678,503,1198,104,92,943,1304
ACTL6A_8_4536,830,1039,667,1054,672,1379,311,965,1299,1835,1599,1605

ADAM12_8_4537,500,228,221,486,34,147,566,547,54,59,388,974
ADAM15_8_4538,334,417,316,428,191,45,167,359,414,200,60,494
ADAM18_8_4539,1325,1496,1217,1496,1488,1356,1669,1361,1834,675,1815,49
2
ADAM22_8_4540,576,683,542,933,36,37,284,711,968,281,510,2289
ADAM29_8_4541,749,940,632,801,580,1850,1301,769,1121,1415,1792,1412
ADAM33_8_4542,715,473,558,917,1999,180,341,478,228,904,1342,941
ADAM8_8_4543,211,105,148,401,364,276,1072,369,327,109,1002,551
ADAMTS13_8_4544,3602,3254,3157,2990,2834,1829,3737,1597,2702,5917,3524
,4790
ADAMTS14_8_4545,7650,6694,7437,6799,6895,7662,6142,7177,8391,7958,8723
,8167
AKAP12_8_4546,326,356,71,152,266,1014,24,435,0,237,63,262
ANGPT1_8_4547,1388,1348,948,1196,563,1518,2395,3271,1957,1070,1354,205
8
ANGPTL4_8_4548,324,468,143,456,276,428,521,142,343,136,747,233
AP1B1_8_4549,243,189,347,444,755,772,83,852,350,13,671,605
AP1G1_8_4550,1399,2131,1634,2035,583,1627,2666,1159,1158,1418,2712,219
6
AP1M1_8_4551,868,600,665,645,1761,873,379,722,614,564,800,312
AP2A1_8_4552,1849,2232,1741,1703,1956,2647,2323,1531,2300,4185,3317,17
11
AP2A2_8_4553,228,186,220,361,584,63,60,10,12,51,875,12
AP2B1_8_4554,3317,2980,2733,3886,3116,4122,4730,4631,2584,4914,4221,26
10
AP2M1_8_4555,2014,1492,1878,1531,3134,841,2763,814,1550,1018,2153,1864
AP2S1_8_4556,2299,2398,1703,1847,3199,2749,1997,1450,1265,2075,4007,44
00
AP3D1_8_4557,662,467,638,477,324,338,952,1,429,650,1271,4
AP3M1_8_4558,1098,1054,1061,1070,2118,928,609,341,1717,1093,868,808
AP3M2_8_4559,741,912,686,872,2043,1126,690,2226,1843,1318,570,791
AP4S1_8_4560,237,328,333,388,166,441,474,246,720,380,281,269
APAF1_8_4561,1485,1434,1219,1664,1889,525,3059,919,1946,1691,1796,1706
APBA2_8_4562,812,1096,329,733,441,1168,1656,1195,1254,833,362,325
APOL1_8_4563,774,580,532,562,1123,291,188,827,126,503,709,670
APOL3_8_4564,2076,1429,2166,2232,4164,3225,1182,3010,1124,1912,2847,37
12
APP_8_4565,987,462,401,694,790,564,18,591,1359,235,1500,133
ARFGAP3_8_4566,1875,1293,1743,1142,2896,1513,754,485,735,1076,921,986
ARHGAP33_8_4567,78,83,317,139,56,949,126,0,127,137,421,254
ARL6_8_4568,1004,1348,1707,381,1437,674,199,1376,330,221,814,229
ATP13A2_8_4569,3134,3090,2588,3747,3329,3124,3109,2854,4116,3270,4098,
3658
BAX_8_4570,810,1063,1067,1102,1219,831,1098,467,886,2308,2108,1157
BCAP29_8_4571,6045,6533,6447,5380,5659,6870,8099,7733,8698,5113,7319,8
461
BCAP31_8_4572,1779,2204,1650,1496,682,2107,1847,2930,772,1250,1202,129
8
BCL2_8_4573,1116,1191,669,551,1607,1788,1362,1437,1718,2419,1964,1504
BCL2L2_8_4574,1264,1530,1039,1221,863,1084,1180,596,1532,1184,1229,182

7
BID_8_4575,11278,10120,10432,10982,11534,10192,15390,12278,12564,14816
,11684,10700
BSG_8_4576,126,283,231,188,674,735,280,90,115,220,851,632
C1QC_8_4577,260,380,372,231,131,1795,1310,3,383,336,830,47
CANX_8_4578,5568,5384,4686,5459,5831,4701,6726,2745,5046,5279,5995,528
7
CAPN9_8_4579,983,742,728,1145,951,64,1566,91,875,1517,966,1232
CAPNS1_8_4580,1086,661,1056,814,486,346,1235,570,878,638,1127,513
CCT6B_8_4581,628,711,682,650,547,824,1132,625,210,635,441,171
CD19_8_4582,625,938,385,1003,0,274,432,1397,530,258,680,794
CD22_8_4583,557,696,570,359,542,202,1194,1317,873,1512,983,980
CD33_8_4584,3758,3480,2511,3656,4889,5408,2471,3071,3419,4464,4187,605
4
CD44_8_4585,2676,3077,3323,2058,1122,4569,1747,2779,2882,3462,3595,394
1
CD55_8_4586,1266,1216,1075,1192,884,2838,921,1592,204,2091,1514,680
CDH17_8_4587,1350,1227,1437,1228,1826,1378,1280,1405,768,2188,626,1402
CIZ1_8_4588,1378,1037,1111,1039,562,90,1944,857,2585,1142,620,1587
COG2_8_4589,701,719,861,471,903,538,1753,662,901,288,1264,304
COG4_8_4590,378,308,306,293,641,123,15,71,453,113,794,515
COG5_8_4591,969,987,983,776,1029,1058,2362,75,1067,522,1064,1193
COG6_8_4592,1877,1934,1446,1772,4100,1595,3787,1763,2119,1821,1982,190
8
COL11A1_8_4593,832,443,710,611,234,48,958,132,1003,713,1919,1105
COL11A2_8_4594,101,98,31,221,90,351,51,539,65,232,4,22
COL12A1_8_4595,1696,981,1123,1057,2096,1341,1931,768,1655,1089,2215,12
51
COL13A1_8_4596,281,191,167,195,392,259,453,531,287,276,32,125
COL18A1_8_4597,344,333,758,383,573,221,271,887,1101,347,474,628
COL25A1_8_4598,241,62,172,159,0,272,2,149,335,104,487,551
COL2A1_8_4599,76,56,53,249,60,7,0,484,81,93,453,107
COL4A5_8_4600,996,1417,970,1002,2415,987,776,784,963,1091,1250,1295
COL4A6_8_4601,543,376,199,168,110,6,453,11,1114,635,199,127
COL6A2_8_4602,476,365,511,261,669,418,399,363,730,412,655,375
COL6A3_8_4603,749,426,341,369,12,452,832,71,1042,211,1631,978
COL8A1_8_4604,118,82,15,224,28,2,441,9,240,137,0,0
COL9A1_8_4605,527,313,588,270,1041,580,537,28,629,138,811,315
COPA_8_4606,1177,1479,1351,1420,1111,588,2361,1670,978,1075,2297,1823
COPB1_8_4607,785,818,940,807,2891,173,1455,1093,1128,1020,1687,4845
COPE_8_4608,90,59,428,9,0,2,6,9,0,401,0,0
CPA4_8_4609,1083,681,671,553,578,995,1477,70,598,1451,2056,1020
CPNE1_8_4610,321,299,368,325,317,575,642,226,267,423,137,314
CPNE7_8_4611,1,0,0,0,0,0,0,0,0,0,0,0
CPXM1_8_4612,667,1339,686,487,714,1631,275,683,552,727,1284,893
CPZ_8_4613,384,244,187,230,13,59,447,43,2033,672,543,13
CRABP2_8_4614,3243,2711,2575,3065,1718,6411,4054,2229,1963,3240,2670,4
519
CTLA4_8_4615,475,344,469,865,475,856,268,1099,838,680,373,321
CTNS_8_4616,3344,3317,2946,3086,4494,4468,3739,5945,2973,3170,6710,537

2
CXCL12_8_4617,677,671,582,605,180,1200,458,92,446,361,764,668
DPP10_8_4618,1310,954,1592,1849,296,969,1015,1854,813,1249,1560,687
DPP6_8_4619,239,325,510,350,0,300,356,248,542,672,1326,1311
ECM1_8_4620,649,927,719,781,136,868,1990,1215,506,485,986,357
EGF_8_4621,910,814,694,840,431,510,853,277,203,462,1068,1178
EIF2C2_8_4622,971,1427,1530,1080,843,1543,868,1910,2048,850,2336,1125
EIF2D_8_4623,750,651,627,721,739,778,1080,1082,725,706,691,430
ENSA_8_4624,1478,1453,1222,1254,1553,968,653,875,589,3266,1946,300
EPB41L4B_8_4625,1862,2965,2067,1885,2429,4263,1842,1843,1994,1885,2284
,2629
EPB42_8_4626,331,462,198,378,254,37,14,155,18,260,212,615
ETFA_8_4627,1015,1277,1540,914,1704,667,682,83,665,1924,2879,445
ETFB_8_4628,95,133,173,64,27,89,97,26,923,11,3,207
EXOC1_8_4629,6187,5475,5807,5611,6912,8221,7286,8293,5410,5244,7618,54
05
EXOC4_8_4630,1654,2984,2104,2374,1454,4429,2604,2623,2614,1905,6047,27
84
EXOC6_8_4631,97,608,117,171,533,21,152,121,7,240,102,171
EXOC7_8_4632,544,331,274,651,791,115,90,668,787,324,583,221
F8_8_4633,1128,1452,844,1096,1157,1610,1881,847,638,1024,956,477
FABP6_8_4634,5442,4764,4237,5278,7854,5217,4349,5659,5770,5468,5788,80
88
FAM131A_8_4635,2376,3172,2402,2182,3039,3598,5575,1631,2613,3379,4176,
5026
FAM63B_8_4636,625,481,267,818,1179,303,53,859,752,739,135,1057
FANCA_8_4637,1592,1094,1622,1453,2882,1033,1628,1717,2539,1725,2079,23
38
FCN3_8_4638,868,1365,833,1358,536,1327,1203,2538,868,2401,1464,2587
FGA_8_4639,210,418,80,218,456,995,291,243,1094,93,180,197
FGB_8_4640,702,694,548,724,92,792,2071,927,40,396,1435,214
FGF13_8_4641,8316,7262,7501,7444,8145,8631,8062,8153,8797,7754,8483,88
04
FGG_8_4642,206,316,624,584,69,51,654,420,103,234,20,591
FIBCD1_8_4643,538,471,401,570,119,317,732,1338,1034,557,523,715
FLVCR2_8_4644,755,904,515,458,1029,751,61,1828,686,887,342,58
FOLR1_8_4645,1171,1479,1197,1236,433,420,1816,938,994,538,1028,2789
FOLR2_8_4646,2677,3279,2371,1964,1529,2273,2400,2422,2902,2987,1837,16
05
GGA1_8_4647,774,953,741,955,198,2150,600,1127,904,1215,792,410
GGA3_8_4648,1236,981,699,1157,2404,1579,226,466,2462,681,1591,398
GJA5_8_4649,1993,1730,2215,1489,2000,1863,1707,756,1972,2074,3724,2055
GJB1_8_4650,181,382,79,417,6,11,8,283,289,231,1,424
GJB3_8_4651,2686,1945,1623,1480,2423,1616,353,2582,1355,2724,2378,1206
GJB6_8_4652,397,45,37,291,23,27,359,501,55,394,73,287
GLYATL1_8_4653,10328,10121,9832,9202,9266,9537,11393,11027,12423,8904,
9628,10336
GNRH1_8_4654,1352,1578,1677,1526,2020,761,996,2793,2529,2139,2294,2257
GOLGA3_8_4655,429,183,176,306,21,260,115,872,1006,13,136,32
GOPC_8_4656,499,737,736,745,19,1680,111,1724,1393,1392,452,1838

GOSR1_8_4657,188,146,33,42,0,2,169,697,156,80,61,94
GOSR2_8_4658,444,641,362,604,524,563,964,660,867,592,1116,597
GPRASP1_8_4659,698,740,700,621,1190,485,869,170,475,611,788,1298
GRB2_8_4660,694,842,623,1084,783,1179,537,1105,633,1199,1043,1681
HABP2_8_4661,2860,3423,2606,3051,4897,6061,4463,2190,4685,4501,2682,41
26
HDLBP_8_4662,422,11,113,267,0,2,54,8,569,0,494,131
HEPH_8_4663,195,486,381,166,78,819,543,712,43,274,553,633
HNRNPU_8_4664,4422,4350,3841,4913,4666,7056,2181,4920,5987,4454,7652,5
295
HOMER2_8_4665,284,521,318,495,782,1655,154,89,162,307,939,608
HPN_8_4666,701,503,315,1211,696,406,1593,833,947,907,1413,1073
HSDL2_8_4667,800,1402,1157,1201,822,1291,517,303,864,1574,1810,1374
IGF1_8_4668,549,475,589,479,495,1244,1114,485,424,167,948,876
IGFBP3_8_4669,183,161,101,281,411,263,170,758,329,68,657,874
IPO11_8_4670,541,529,373,377,0,117,1064,563,1370,719,574,293
IPO8_8_4671,606,111,311,210,325,579,276,486,487,150,193,391
ITGAL_8_4672,621,432,641,432,777,976,815,771,1183,220,820,1367
ITGAM_8_4673,575,657,564,785,1194,292,1704,972,389,1122,1474,1677
ITGAV_8_4674,1812,1850,1614,1369,1455,2307,1354,1228,2641,1859,2506,17
87
ITGB2_8_4675,495,412,593,551,82,139,500,382,92,1118,272,162
KDELR2_8_4676,1638,1786,1470,1700,2329,3001,3895,1193,1599,1941,2107,2
389
KDELR3_8_4677,3230,4299,2709,3326,2796,4228,2346,4128,4443,4361,3401,4
482
KIF13A_8_4678,2295,2039,1757,1985,2579,2148,3829,864,2581,2277,2443,29
44
KIF17_8_4679,51,12,60,58,7,32,5,62,407,325,340,84
KIF1B_8_4680,2071,1900,2503,2012,1421,2462,2634,3846,2002,1648,2396,12
52
KLK10_8_4681,436,238,259,333,1,1113,26,731,596,25,297,1495
KLK11_8_4682,284,606,192,264,1140,386,41,125,32,235,260,429
KLK12_8_4683,65,30,31,139,0,74,0,0,365,0,9,127
KLK15_8_4684,286,240,224,434,1014,2,667,49,280,804,593,93
KLK5_8_4685,3107,3167,3453,3083,4150,4957,3928,5195,4798,4372,3778,319
6
KLK6_8_4686,872,823,683,359,543,399,687,358,845,1300,660,312
LAMB3_8_4687,1935,1608,1753,1824,964,1840,1280,2427,1062,1741,1638,172
0
LDB3_8_4688,210,203,120,207,81,125,127,620,75,340,1,76
LDLR_8_4689,743,649,843,712,451,891,1774,885,48,606,1428,891
LTA_8_4690,2885,2644,3099,3073,4531,1835,3736,2657,3021,3474,4714,3855
LTF_8_4691,2636,3002,1893,2151,3626,1278,2918,2562,1254,1796,2385,2513
M6PR_8_4692,2322,1865,2314,2917,1956,1819,2827,4959,2808,2756,2134,181
1
MASP1_8_4693,1541,1905,1414,1590,2413,2450,1001,1023,1754,1241,1792,29
03
MB_8_4694,996,826,1374,1152,1726,1890,1043,1340,413,983,1184,1147
MCFD2_8_4695,1534,1997,1621,2455,1349,3188,2254,2240,2233,511,2411,179

3
MCL1_8_4696,39,288,210,101,0,172,51,7,17,2,636,0
MEFV_8_4697,1839,2163,1546,1714,3416,1100,4144,703,1594,1602,1528,1809
MFAP4_8_4698,475,428,548,394,330,384,1108,361,361,248,141,189
MFSD10_8_4699,787,477,426,933,121,857,640,1142,520,918,219,1117
MFSD1_8_4700,410,269,381,630,83,149,347,811,1463,160,342,338
MFSD5_8_4701,2165,1648,2387,3004,2808,2243,2463,3539,2419,1599,3040,61
02
MLC1_8_4702,566,310,306,636,391,262,1152,567,135,297,48,753
MSLN_8_4703,339,395,599,418,145,480,845,94,145,198,94,684
MTX1_8_4704,142,312,107,103,879,323,36,61,17,44,67,20
MUC1_8_4705,494,364,234,413,179,204,65,13,274,191,180,808
NCAM1_8_4706,1181,1483,979,721,808,3240,106,60,985,2588,1151,771
NNAT_8_4707,644,660,409,744,1421,367,261,622,606,534,73,360
NOXO1_8_4708,1280,1417,1114,1621,959,2137,1962,2842,2293,1459,827,1212
NPC1L1_8_4709,543,570,647,514,1242,916,1667,113,1283,1012,509,164
NPRL3_8_4710,2413,2354,2502,1439,1994,2612,1064,1980,1863,1711,4719,53
65
NRXN1_8_4711,376,254,285,441,837,378,621,18,1135,410,533,507
NRXN2_8_4712,349,395,592,428,54,0,273,684,316,139,34,7
NRXN3_8_4713,380,617,382,297,601,98,113,224,88,143,1208,663
NUP155_8_4714,361,323,210,243,503,333,276,13,250,422,153,687
NUP50_8_4715,472,324,322,905,472,785,933,642,681,316,723,55
NUP62_8_4716,763,900,716,973,109,598,1308,626,313,684,1102,872
NUP98_8_4717,446,449,487,721,1413,885,575,604,409,343,924,744
NUPL1_8_4718,1718,2131,1559,1954,578,1901,1913,622,1145,1280,1513,2019
NXF1_8_4719,2955,3529,2800,3205,1985,3676,4724,3463,3107,4965,6301,635
6
NXNL2_8_4720,215,200,363,69,9,41,82,138,155,87,794,0
NXT2_8_4721,994,870,1152,1016,515,707,1363,745,230,1301,857,1016
OAZ3_8_4722,740,936,598,519,161,469,1819,197,269,902,355,984
PACSIN2_8_4723,617,430,311,265,147,712,1268,220,667,391,125,1272
PANX2_8_4724,336,146,152,266,586,1,481,14,997,181,254,0
PCDHA6_8_4725,1323,1419,1015,1058,1981,1119,1388,281,955,1938,772,1778
PCDHGA5_8_4726,617,934,251,282,206,776,314,170,237,224,223,47
PCLO_8_4727,2134,1292,2063,1514,2994,1726,1732,1675,2226,3669,2936,237
4
PCSK5_8_4728,540,500,454,429,1390,202,470,661,590,389,350,569
PCSK6_8_4729,1056,1149,1025,1726,810,1409,1523,465,889,1541,1567,1229
PCTP_8_4730,921,814,938,652,566,1140,569,390,550,897,945,483
PDYN_8_4731,549,532,610,692,1216,875,71,635,262,644,1099,713
PDZD3_8_4732,3606,3689,3305,3630,2528,4238,3830,2199,2924,4972,5265,45
24
PDZK1_8_4733,480,545,343,780,461,292,317,767,1052,570,1292,650
PGAP2_8_4734,954,546,730,1173,1170,341,988,436,574,596,318,164
PGF_8_4735,384,362,572,430,396,362,791,1427,305,459,664,23
PIK3R3_8_4736,3065,3425,2924,2977,2477,2141,6592,3643,2370,2791,6600,5
570
PITPNC1_8_4737,377,920,323,279,0,404,126,80,483,129,899,285
PITPNM1_8_4738,129,154,185,198,15,13,218,45,281,33,6,42

PITPNM3_8_4739,1434,1359,1182,1786,1438,846,1868,1890,1391,2084,2163,1
143
PLEC_8_4740,364,243,240,336,70,247,177,908,1087,125,354,165
PLIN3_8_4741,864,547,507,392,86,990,640,524,932,57,2044,815
PLTP_8_4742,107,61,21,127,1,4,10,141,0,227,63,0
PNKD_8_4743,298,96,375,174,21,64,547,235,155,98,216,12
POMC_8_4744,655,351,416,846,126,545,361,296,1151,557,1876,1872
PORCN_8_4745,518,354,632,502,354,752,528,166,259,715,1099,891
PREPL_8_4746,1006,796,961,1149,1638,829,249,552,401,1362,1347,520
PRNP_8_4747,281,437,442,407,0,1,0,78,199,222,290,180
PRSS21_8_4748,1858,2250,1687,2504,1126,2593,2424,757,521,968,1252,2885
PRSS35_8_4749,226,255,143,109,39,97,96,671,35,371,197,207
PSEN1_8_4750,740,564,423,877,339,345,691,824,1075,937,320,529
PSEN2_8_4751,294,344,159,205,498,185,81,127,484,1042,83,108
RABEP1_8_4752,1468,1323,1475,1792,1481,1983,751,2743,666,1775,1425,274
9
RACGAP1_8_4753,1899,2174,1781,1967,1743,3861,1810,820,2051,2595,2308,1
197
RARRES1_8_4754,1692,1600,1222,1184,3802,354,736,452,588,1678,1907,927
RASA1_8_4755,4039,4041,4362,4134,4397,3653,4066,2653,3758,4679,3548,47
90
RELN_8_4756,3739,3360,2420,3818,5934,3775,3496,5078,3356,5824,4760,326
9
RHCE_8_4757,89,41,119,138,121,318,168,23,384,1,160,10
RHD_8_4758,132,93,221,103,1,97,19,94,45,403,2,323
RIMS2_8_4759,0,0,0,0,0,0,0,0,0,0,0,0
RRBP1_8_4760,155,204,102,216,44,139,406,2,6,146,550,59
RUFY1_8_4761,1464,1864,1899,1105,1897,2338,939,2585,1045,1092,3018,103
3
S100A13_8_4762,2057,1790,1662,1504,1853,1758,1251,2178,1167,1415,3417,
676
S100A4_8_4763,1071,1429,811,1446,115,699,503,806,1386,2327,780,1168
SAA1_8_4764,1423,1400,1005,771,1326,1027,1493,1509,1117,1046,1938,1523
SCAMP3_8_4765,1187,1985,868,1112,3736,1414,1382,673,726,1964,797,1179
SCARB1_8_4766,117,151,317,53,12,1,384,0,374,330,27,0
SCFD1_8_4767,1843,1578,1815,1408,1021,2694,3975,722,1623,1806,3496,333
8
SEC13_8_4768,444,725,685,667,1078,144,38,281,276,629,573,1942
SEC14L1_8_4769,3405,3114,2668,3634,2712,4677,3464,3244,3889,3910,3545,
3556
SEC14L2_8_4770,556,779,686,786,1120,1253,708,1040,195,400,1212,1623
SEC14L4_8_4771,355,375,449,384,47,558,311,22,122,662,692,237
SEC23B_8_4772,250,357,242,399,414,35,704,1102,71,162,62,531
SEC24B_8_4773,819,731,925,1022,860,389,1039,1234,633,1155,1251,1771
SEC24C_8_4774,5204,5154,4329,5231,5301,8659,3145,3455,8591,4979,6891,5
016
SEC61A2_8_4775,631,813,376,541,181,978,182,184,463,202,943,593
SEC61G_8_4776,363,396,147,390,652,586,6,677,367,206,1268,279
SEH1L_8_4777,470,262,477,321,379,86,193,377,594,223,92,227
SERINC2_8_4778,521,578,627,657,356,368,897,292,534,497,108,312

SERINC3_8_4779,613,524,633,553,151,548,256,483,1607,470,1213,1294
SERPINA10_8_4780,540,581,505,891,748,2024,48,952,727,612,1194,565
SERPINA1_8_4781,253,133,105,266,784,6,209,155,9,160,166,93
SERPINB2_8_4782,237,191,146,378,1,90,163,286,419,167,348,641
SERPINB6_8_4783,475,713,811,560,1680,1020,316,1391,512,323,1190,1056
SERPINB8_8_4784,659,877,675,526,1311,173,898,3,414,643,472,392
SERPINE1_8_4785,838,872,388,654,1719,1863,1400,470,1250,558,522,1384
SERPINF2_8_4786,1482,1430,1476,1412,1494,1595,835,1795,913,1475,1884,2
158
SERPING1_8_4787,2497,2220,2353,1879,2950,1574,3159,3380,2651,2464,2783
,1774
SERPINH1_8_4788,1157,1301,1165,1424,2410,1499,1806,2100,334,1315,1732,
1031
SERPINI1_8_4789,705,459,686,746,257,1255,937,797,244,318,262,500
SFI1_8_4790,200,14,114,72,792,36,11,175,9,254,29,91
SFTPA1_8_4791,686,726,1014,998,5,1177,73,301,328,605,572,1449
SH3D19_8_4792,1199,1177,958,1029,974,2471,458,833,1377,791,241,887
SIL1_8_4793,911,814,821,585,1951,719,532,616,525,395,1643,234
SLC25A25_8_4794,2388,2151,2195,1805,2742,2885,1764,1327,2811,2311,1434
,2727
SLC25A36_8_4795,1135,650,745,472,476,1509,813,27,268,597,854,291
SLC25A45_8_4796,606,533,430,539,907,29,1600,275,168,868,63,487
SLC38A10_8_4797,641,993,793,784,630,1210,557,1416,221,1186,1112,897
SLC41A3_8_4798,94,230,352,134,0,37,1196,505,71,492,539,27
SLC43A3_8_4799,1289,1593,1427,1032,1400,1149,3109,312,737,2164,3435,14
68
SLC44A2_8_4800,2546,2699,1939,2540,3017,2058,3855,2773,1551,3294,3415,
3433
SLC44A4_8_4801,211,45,134,134,279,8,1,228,59,75,504,177
SLC44A5_8_4802,335,507,257,504,3,478,473,856,182,481,341,4
SLC46A1_8_4803,825,607,356,427,330,629,714,1356,1543,1610,859,1318
SLC47A2_8_4804,1102,1723,792,1838,774,1003,376,2211,1605,761,1961,1656
SLC50A1_8_4805,3247,2904,2809,3744,2064,4142,3345,4338,3743,1899,4410,
2889
SLC6A20_8_4806,487,897,415,635,231,890,323,199,1034,1470,473,1044
SNAP23_8_4807,1471,1136,1439,1211,2235,2001,1738,1474,1165,502,1450,87
0
SNAP25_8_4808,276,134,223,377,120,185,472,180,112,332,108,15
SNX10_8_4809,648,674,687,1202,512,1193,1198,694,1250,425,876,738
SNX11_8_4810,1836,1676,735,734,3942,2541,2722,320,1354,1946,1459,1660
SNX14_8_4811,6170,5509,6607,6253,9951,6379,9162,7371,6320,5434,9285,72
49
SNX15_8_4812,176,106,3,103,0,2,2,0,73,0,11,5
SNX16_8_4813,955,848,1027,1206,997,1638,1187,750,485,1063,620,352
SNX18_8_4814,265,380,219,312,379,200,2571,49,42,1072,229,501
SNX1_8_4815,1237,1144,1150,1078,970,1067,1451,1242,609,1081,1239,862
SNX3_8_4816,3442,2589,2747,3431,2626,2515,5908,3665,1501,1752,5233,589
4
SNX5_8_4817,840,635,670,1198,641,1263,852,1401,2039,551,314,697
SNX6_8_4818,4175,3725,3923,3541,3927,3572,4505,1878,3836,5639,4779,377

1
SNX7_8_4819,421,270,208,243,1240,315,534,387,465,540,978,706
SORCS1_8_4820,222,365,278,291,0,603,35,0,38,176,41,289
SORT1_8_4821,357,503,157,215,582,878,4,97,347,347,181,380
SPNS1_8_4822,759,565,322,516,327,96,920,475,262,212,485,607
SRI_8_4823,519,536,470,472,2070,541,338,262,511,593,16,294
STARD3_8_4824,1288,1185,611,848,1060,1357,776,1706,1341,988,1171,1076
STAU1_8_4825,1147,1203,1335,823,2196,983,2049,305,2776,1479,2385,1785
STEAP2_8_4826,818,831,1097,1309,179,247,943,2978,635,811,3068,3398
STEAP3_8_4827,884,727,532,674,1744,346,63,1891,610,1106,771,905
STIM2_8_4828,925,1190,943,1345,1318,2085,837,702,820,1005,368,1690
STX16_8_4829,940,1094,852,1016,566,1166,1116,1215,548,704,1866,847
STX1A_8_4830,655,1134,927,1060,452,736,1500,762,249,866,499,1075
STX2_8_4831,816,802,724,433,750,1216,109,1195,875,1254,678,845
STX3_8_4832,490,791,481,506,467,310,655,509,54,142,829,131
STXBP1_8_4833,1168,1268,835,1303,1375,838,2373,1904,3292,1967,970,587
STXBP2_8_4834,270,335,272,221,0,268,500,470,443,419,348,544
SV2B_8_4835,784,429,758,777,1299,201,1208,1478,669,785,793,1987
SYN1_8_4836,179,479,79,80,238,768,84,308,209,605,133,10
SYNGR1_8_4837,746,673,476,710,963,517,629,723,1124,487,770,1342
SYNPR_8_4838,5334,5413,3664,4883,3743,4792,5373,4058,6208,5602,4145,69
01
SYPL1_8_4839,1960,2078,2027,2022,4906,2205,2091,2351,1780,1436,2254,28
10
SYT12_8_4840,298,267,108,290,278,320,0,536,21,230,34,0
SYT14_8_4841,585,1210,661,637,1167,1029,287,140,486,904,2683,1339
SYT15_8_4842,1458,2017,968,1581,822,1024,1098,770,1727,1168,1498,2093
SYT1_8_4843,1193,1287,1157,1700,711,1178,1090,648,666,2177,1179,1419
SYT2_8_4844,257,301,205,151,208,541,384,385,68,2,456,40
SYT3_8_4845,162,315,614,282,37,287,524,585,453,593,752,61
TAPBP_8_4846,421,398,634,429,442,98,1343,577,993,64,498,460
TC2N_8_4847,1377,1081,736,846,264,844,1001,841,700,494,1903,1904
TCN2_8_4848,135,112,186,48,1,5,69,22,656,62,76,977
TCOF1_8_4849,454,769,681,582,160,1007,331,247,488,696,337,707
TFPI_8_4850,2063,2503,1723,2540,3557,1138,489,861,1677,3062,3555,4899
TFR2_8_4851,363,345,475,475,1,67,344,1185,264,14,505,649
TFRC_8_4852,3192,2940,2827,3174,2740,1711,1892,4472,3398,1700,4805,337
2
TGFB2_8_4853,656,958,1151,879,432,189,807,852,267,2470,839,1185
TIMM17B_8_4854,299,116,236,381,426,38,42,6,0,150,7,638
TINAGL1_8_4855,1516,1195,1066,1011,1316,533,870,1423,2773,1595,1016,35
39
TLL1_8_4856,2162,2216,2376,2180,2857,3269,1936,2614,2717,3842,5704,191
9
TM9SF1_8_4857,433,296,452,460,172,667,1724,677,688,117,648,474
TMC6_8_4858,1733,1678,1475,1703,1082,2137,1951,1441,1793,1941,2942,732
TMPRSS11A_8_4859,600,706,589,336,155,449,194,297,683,416,554,400
TMPRSS13_8_4860,4854,4475,3933,4543,1999,6080,4020,5692,4853,4950,5436
,2593
TMPRSS2_8_4861,437,509,455,436,576,221,1040,1406,472,474,575,308

TMPRSS4_8_4862,4329,4002,3776,4013,2717,4042,3445,3943,2457,6530,5508,
6463
TNFSF11_8_4863,0,0,0,0,0,0,0,0,0,0,0,0
TNFSF13B_8_4864,2059,1902,1657,1986,1446,3417,1710,1698,2594,2402,2022
,2693
TNPO2_8_4865,712,476,533,591,1075,828,1583,156,1024,553,961,1083
TOM1_8_4866,359,320,442,440,13,938,852,1220,1526,316,962,639
TOM1L2_8_4867,378,584,627,707,195,286,1030,1330,254,1228,369,648
TSC1_8_4868,3370,3138,1949,2418,3925,2936,3942,3919,4407,1990,2434,306
0
TSC2_8_4869,926,898,736,1185,1712,486,60,909,943,1030,530,1850
TUBA8_8_4870,3529,3578,2459,2720,4325,4092,2746,3752,3520,4014,2792,33
93
TUBB3_8_4871,644,627,328,762,396,1477,25,78,460,53,1247,406
TUBD1_8_4872,1732,1445,814,1461,2027,1977,1866,1882,1895,725,1185,882
UCP3_8_4873,1703,842,961,840,1113,1549,1270,809,167,1742,432,1502
UPF3A_8_4874,669,526,548,472,345,466,804,40,233,395,1047,1272
UPF3B_8_4875,797,788,488,872,577,2606,1209,840,1497,633,219,956
VAMP1_8_4876,1085,748,731,901,903,2448,1863,242,249,441,928,1254
VAMP7_8_4877,2589,2627,2891,3617,3002,2880,3202,3143,2562,4234,3492,30
55
VCAM1_8_4878,1165,1836,1036,1386,115,3054,1338,1176,230,1243,2611,1841
VLDLR_8_4879,1491,1554,2105,1186,1514,2290,1597,2130,395,1158,1524,209
7
VPS13A_8_4880,975,836,729,1045,1448,609,983,2567,1008,997,780,918
VPS13B_8_4881,276,249,276,676,5,137,55,370,252,24,1798,596
VPS16_8_4882,500,573,750,338,112,785,1011,496,624,828,423,335
VPS26A_8_4883,1475,980,779,845,2817,737,497,2214,1160,921,1453,1606
VPS28_8_4884,288,186,451,237,217,338,989,667,408,60,133,340
ZFYVE16_8_4885,3299,3682,2446,3457,2939,3055,3576,2291,3462,3229,3440,
3031
ZNF160_8_4886,115,135,105,193,1,518,698,294,255,155,44,123
ZP3_8_4887,412,314,248,246,1143,894,59,248,106,373,4,596
BET1L_8_4888,176,130,461,369,326,190,74,358,0,59,55,71
C2orf83_8_4889,690,313,693,379,265,346,241,441,194,380,563,434
ERP29_8_4890,1249,1099,639,967,2093,1607,1177,296,1044,1684,1953,881
FGF1_8_4891,1446,1573,1091,1730,1623,2109,1419,1179,1574,1404,2103,117
6
LYNX1_8_4892,175,79,24,64,0,0,2,40,0,14,8,9
MMP28_8_4893,1362,1453,1211,1330,797,1608,1386,1933,489,1709,1967,2376
PDPN_8_4894,1796,2179,1116,1988,754,1634,1728,2654,1044,2003,3425,763
SNX21_8_4895,596,767,894,456,3448,535,22,223,1005,1212,805,1051
TIMM8A_8_4896,1883,1245,2208,1366,1155,865,1971,1510,2036,1905,1557,22
54
VEGFA_8_4897,603,383,665,540,384,418,649,123,296,50,1217,600
CDH23_8_4898,225,173,334,39,1,17,605,26,598,6,0,234
CDH23_8_4899,616,674,720,1039,941,14,1348,318,663,674,1255,945
CDH23_8_4900,171,216,157,280,7,9,258,261,335,325,106,1146
CDH23_8_4901,247,427,437,297,159,800,13,69,58,197,54,200
CDH23_8_4902,586,529,344,588,2248,996,135,258,225,354,926,1261

TNXB_8_4903,663,513,957,661,1024,848,855,21,235,461,842,209
TNXB_8_4904,179,92,363,313,259,112,419,9,17,87,32,463
A2M_8_4905,429,351,516,682,1116,59,0,850,615,208,225,414
ACE2_8_4906,649,1070,681,259,871,1297,163,676,1094,260,276,409
ACTR6_8_4907,3075,3333,2677,2820,4049,3536,2313,3967,3967,2437,3189,25
12
ADAM11_8_4908,4368,4519,3495,3845,2994,4097,2884,2117,4416,3567,5966,2
673
ADAM19_8_4909,676,537,520,588,0,549,1462,856,1643,30,386,33
ADAM20_8_4910,2587,2996,2987,1471,3057,2680,1255,2385,2749,2744,2141,2
855
ADAM21_8_4911,3475,3545,2980,3857,4078,4361,2435,2361,2865,4860,2660,3
541
ADAM2_8_4912,3435,2916,1925,3263,3581,3211,2898,2989,1180,4790,3501,34
94
ADAM30_8_4913,3363,3162,2171,3215,2774,2649,3255,3728,4050,3624,2634,3
257
ADAM7_8_4914,1945,2709,1984,2310,3983,3733,2940,2426,2986,2937,3356,21
74
ADAM9_8_4915,592,374,502,438,458,388,260,409,367,447,857,461
ADAMTS10_8_4916,709,521,542,654,746,1603,1091,757,667,2678,128,327
ADAMTS12_8_4917,727,520,524,478,638,398,1063,813,1054,820,520,838
ADAMTS15_8_4918,855,923,734,995,721,439,341,1124,1935,1035,839,1542
ADAMTS18_8_4919,2573,3581,1937,2460,1716,2669,3076,2328,3896,3958,3299
,3911
ADAMTS19_8_4920,5024,5040,4651,4865,4739,6942,6262,3043,6068,4986,6774
,5635
ADAMTS1_8_4921,825,823,628,820,753,354,1346,1871,867,552,466,114
ADAMTS20_8_4922,8493,8473,9068,8470,9141,9056,11608,7246,9303,10562,10
463,8420
ADAMTS3_8_4923,405,359,400,749,0,871,784,466,0,1081,213,823
ADAMTS5_8_4924,1046,916,996,769,604,1127,550,1502,1083,1119,703,808
ADAMTS6_8_4925,1960,1866,1575,1504,898,979,2671,1083,1418,1906,2300,18
14
ADAMTS7_8_4926,287,425,421,406,15,173,101,633,498,239,17,810
ADAMTS8_8_4927,331,278,515,430,102,48,95,626,189,995,339,1255
AEBP1_8_4928,259,121,215,268,115,447,5,31,50,315,80,147
AFG3L2_8_4929,3158,2186,2155,1748,1688,1932,3530,1369,3438,3004,2159,3
724
AFM_8_4930,349,533,638,373,294,292,279,506,257,59,973,607
AFP_8_4931,619,637,463,782,870,1111,1837,449,472,635,1119,1113
AGTPBP1_8_4932,3860,2943,4348,3771,2745,3490,3624,3013,6625,4273,4960,
6454
ALG10B_8_4933,1152,768,839,654,783,1477,1179,637,736,984,1016,1248
AMBP_8_4934,648,469,409,436,263,313,100,143,137,952,269,461
ANGPT4_8_4935,774,870,859,989,587,1037,548,1743,983,818,1072,504
ANGPTL1_8_4936,1268,1328,849,1013,2914,1466,976,232,765,1157,997,1879
ANGPTL2_8_4937,760,417,586,827,612,113,541,980,565,1166,568,414
ANGPTL3_8_4938,4034,5008,4396,5256,3877,5998,5591,6228,4809,3982,5869,
6567

ANGPTL7_8_4939,600,585,513,309,207,119,386,698,532,230,238,358
ANKH_8_4940,909,885,818,1150,345,1753,397,1302,2111,658,2648,1761
AP1G2_8_4941,1230,988,1039,1303,1071,2073,1982,1719,705,895,1552,2157
AP1M2_8_4942,179,205,112,151,765,746,246,94,6,276,8,14
AP1S1_8_4943,1454,1247,1946,1418,1955,2574,1197,1179,726,1900,2981,680
AP1S2_8_4944,3688,3039,3530,3040,2140,4198,3138,2932,2932,4276,5102,49
91
AP1S3_8_4945,2763,3327,2779,3332,3754,4172,3772,5299,3164,2188,5548,22
25
AP3B1_8_4946,3354,3318,3270,2210,5049,1886,3068,3166,1983,4593,4418,31
47
AP3B2_8_4947,1467,1178,1343,1761,411,1721,1774,1796,916,1011,938,1820
AP3S1_8_4948,1307,1379,1089,1339,3385,866,1586,428,558,901,2334,3048
AP3S2_8_4949,2124,2264,2079,1811,1591,1843,1622,1684,3454,1873,1861,23
95
AP4B1_8_4950,433,660,232,508,170,106,3,741,371,465,74,110
AP4M1_8_4951,1328,1329,1619,1516,2160,1733,2513,1591,1522,313,416,2799
APBA1_8_4952,817,1145,430,677,657,1171,1588,1195,1033,837,585,325
APBA3_8_4953,810,916,904,972,831,761,475,312,914,1131,1289,1175
APOA1_8_4954,286,365,381,362,462,114,264,863,536,728,221,413
APOA2_8_4955,3164,3183,2443,2398,4999,3766,4057,1948,2526,2050,2800,20
94
APOA4_8_4956,219,415,301,376,275,63,873,1251,98,221,368,158
APOB_8_4957,880,689,895,1044,516,148,1133,686,1000,1076,1027,1177
APOC1_8_4958,2230,2369,1936,2196,1682,970,2147,2567,1757,2655,2130,350
2
APOC2_8_4959,3792,4147,3147,4403,3474,3468,4167,3547,6325,3780,4457,54
33
APOC3_8_4960,348,314,344,336,269,168,145,949,924,363,249,621
APOC4_8_4961,1110,812,955,872,1323,608,1356,333,1340,1359,1345,774
APOD_8_4962,6268,4981,5078,6563,6948,9486,4775,6602,7080,7352,9198,105
21
APOE_8_4963,971,656,685,875,1205,1498,519,687,1287,898,486,1388
APOF_8_4964,592,703,691,639,87,1140,1680,924,535,1029,872,1198
APOH_8_4965,1900,2279,1518,1796,2483,2171,1123,1104,2268,1801,1617,432
0
APOL6_8_4966,567,773,454,709,706,1153,417,522,1227,1091,322,777
APOM_8_4967,1084,826,927,823,1043,675,1139,1229,975,1495,710,688
AQP12B_8_4968,2180,2118,1838,2261,3496,1925,1274,2942,2583,2563,4024,2
118
ARF5_8_4969,391,413,451,208,0,161,994,167,673,782,78,433
ARF6_8_4970,828,576,741,472,566,322,58,1021,594,661,427,394
ARPP19_8_4971,698,590,726,881,34,921,798,678,1459,1076,863,1453
ASTL_8_4972,1656,2195,1912,1990,1280,1824,1732,1361,1843,2300,1602,860
ATOX1_8_4973,267,381,366,327,366,294,48,279,330,406,908,3
ATP13A1_8_4974,323,276,441,171,12,913,526,14,3,749,573,897
ATP13A3_8_4975,8914,7332,7370,8888,7907,6972,7369,8075,7831,8513,10892
,10547
ATP13A4_8_4976,3874,3897,3922,3631,4331,4235,4353,3966,2851,3626,6238,
8922

ATP13A5_8_4977,3703,4163,4034,3145,4009,2960,5378,2107,3407,3430,3290,
5403
AZGP1_8_4978,2399,1932,2350,2134,2531,2606,2505,3172,2145,3907,2064,23
03
AZU1_8_4979,257,509,551,674,535,300,195,531,74,697,155,303
BCL2L10_8_4980,1773,1674,1146,1223,4345,2143,1737,757,1459,980,1742,29
96
BET1_8_4981,1424,1977,1770,1715,1903,3374,2425,2241,672,1429,2172,2971
BGLAP_8_4982,293,238,170,516,33,110,519,596,152,19,294,21
BOC_8_4983,3004,3323,3102,3703,3219,4345,2867,5012,2508,5145,5616,2186
BPI_8_4984,658,486,548,434,771,1217,2081,527,806,545,612,1170
BPIFC_8_4985,1404,1444,1461,1680,1282,3475,1248,594,1548,1164,930,677
C16orf7_8_4986,479,165,261,267,452,226,393,308,793,234,1482,1055
C1orf162_8_4987,84,227,118,167,307,177,316,403,17,46,85,408
C1QA_8_4988,100,174,21,90,83,14,11,0,5,0,112,0
C1QB_8_4989,64,33,12,3,8,0,0,3,141,2,61,2
C1RL_8_4990,1465,1560,967,1528,2613,1586,2223,1018,1007,2108,1436,1916
C20orf141_8_4991,249,169,145,174,2,99,318,51,469,443,96,81
C3_8_4992,537,527,352,228,152,110,124,5,689,231,1046,686
C4A_8_4993,396,75,92,160,505,988,338,0,10,16,2,0
C5_8_4994,4275,2760,3057,3726,5435,3945,3388,6248,3617,3557,3336,2882
C7orf31_8_4995,680,424,707,770,1277,140,148,1336,884,131,727,1540
C8G_8_4996,2308,1781,1750,2267,1214,2813,1521,1846,2140,2193,1647,3378
CALM2_8_4997,2641,1909,1990,2375,1800,2542,1517,1079,3778,2963,2614,22
43
CALY_8_4998,852,1290,593,556,667,503,1810,737,573,1794,685,1086
CAMLG_8_4999,442,592,232,362,208,288,818,193,46,670,487,663
CAPN11_8_5000,2026,1236,1781,2042,3113,970,1647,2306,1164,1959,1492,25
27
CAPN5_8_5001,1526,1486,839,1431,1844,2541,1384,1601,1410,2326,1177,188
8
CAPN6_8_5002,1355,1438,739,922,2283,1166,2086,1111,2250,2887,1005,1425
CARTPT_8_5003,1628,1219,990,1663,2216,1190,2400,1672,2043,2251,1103,21
07
CCL13_8_5004,540,386,701,658,549,164,1967,184,996,514,57,1248
CCND1_8_5005,776,1258,941,513,159,1313,329,603,512,801,409,1111
CD1A_8_5006,1157,1116,1159,613,1111,2026,1058,1201,399,1096,764,316
CD52_8_5007,3075,3818,3357,3344,2957,4597,2249,3084,4917,2361,5312,359
7
CDCP2_8_5008,1632,1406,987,1236,1380,1117,655,1503,991,949,1933,1017
CDH5_8_5009,1223,1282,1152,1311,220,1568,231,1804,580,892,1881,820
CHMP7_8_5010,1078,946,783,904,375,528,240,834,1987,950,245,1908
CLDN16_8_5011,53,102,235,206,3,25,2,45,2,116,305,31
CLEC3B_8_5012,223,95,54,87,2,370,515,193,268,123,18,78
CLSTN2_8_5013,884,1102,1244,816,1135,1732,1761,556,666,931,1318,1279
CLVS2_8_5014,959,1001,1005,1030,1972,1606,1290,1017,1428,817,738,1256
CNIH3_8_5015,2007,1653,1491,1661,1906,2861,57,1241,2767,2450,2210,1219
CNOT6_8_5016,1064,1325,703,808,1279,340,452,1370,794,1916,1136,859
CNTNAP1_8_5017,700,595,1091,404,423,1915,4220,412,544,10,774,44
COG1_8_5018,1012,1235,660,1359,279,491,1215,1400,1274,2539,1261,1910

COG3_8_5019,3079,3516,2890,3068,3344,2466,4573,3070,1728,2697,4429,282
7
COG7_8_5020,658,565,570,352,163,877,617,521,412,101,857,193
COG8_8_5021,311,504,355,527,101,285,609,133,1147,1447,539,354
COL10A1_8_5022,628,359,183,213,338,367,431,1172,287,95,192,0
COL14A1_8_5023,602,575,326,664,865,394,794,393,58,908,939,1143
COL15A1_8_5024,805,409,589,691,1035,550,1407,35,277,416,103,119
COL16A1_8_5025,233,366,354,136,1378,155,126,327,410,58,388,714
COL17A1_8_5026,3488,3833,2667,2868,6186,3362,3552,2404,3203,4453,3535,
1950
COL1A1_8_5027,374,80,363,387,24,68,0,0,943,0,228,124
COL1A2_8_5028,702,643,854,1060,822,861,971,981,148,985,723,2088
COL21A1_8_5029,467,506,374,363,449,738,421,173,1075,386,865,420
COL22A1_8_5030,1578,2355,1233,1249,4113,1067,1049,1490,1233,1644,1591,
1295
COL23A1_8_5031,478,263,742,314,559,257,29,170,451,419,229,335
COL24A1_8_5032,109,149,151,145,1,231,47,4,219,103,60,51
COL27A1_8_5033,289,363,264,532,7,91,885,775,9,412,1,8
COL3A1_8_5034,809,946,1281,747,703,825,206,2121,459,1775,1014,885
COL4A1_8_5035,3652,3960,3175,4326,5591,4639,4683,4944,4115,6282,5414,6
535
COL4A2_8_5036,191,232,289,216,64,45,44,134,308,36,3,32
COL4A3_8_5037,4549,5940,5142,4605,7189,5760,3612,5539,4114,4507,6727,6
963
COL4A4_8_5038,1759,1624,1880,1573,3288,2872,1857,4292,837,1663,2179,24
06
COL5A1_8_5039,387,454,712,520,173,1081,432,1326,54,222,1383,1258
COL5A2_8_5040,209,318,178,301,114,162,36,26,28,150,491,119
COL5A3_8_5041,1031,1096,860,890,2615,662,751,704,1363,1510,1543,2206
COL6A1_8_5042,662,564,597,767,490,1660,386,1774,192,748,1495,1026
COL7A1_8_5043,543,864,493,301,159,138,1045,5,388,340,221,13
COL8A2_8_5044,1454,1148,1153,1433,2147,1007,1793,1173,2005,2752,963,25
25
COL9A2_8_5045,252,72,115,124,703,119,36,59,97,107,272,0
COL9A3_8_5046,642,518,600,592,683,392,513,366,615,228,1367,2070
COMMD1_8_5047,400,644,666,612,197,853,444,1073,2,922,631,105
COPB2_8_5048,1872,2073,1804,1852,2300,1412,1329,1864,1964,1987,1984,22
67
COPG2_8_5049,672,687,612,1038,579,2018,857,385,831,909,716,1809
COPZ1_8_5050,1437,1935,1111,1324,319,577,1164,977,1084,1502,2028,2003
COPZ2_8_5051,660,558,619,860,40,776,19,1672,796,221,81,1488
CORIN_8_5052,4247,2948,2545,3572,4457,3934,4572,2346,3080,3425,5094,72
34
COX18_8_5053,2237,2145,2181,1918,3835,2391,5102,2449,1357,3574,2392,22
82
CPLX1_8_5054,529,543,626,505,144,743,89,122,1176,801,16,475
CPLX3_8_5055,114,114,123,162,45,196,159,20,85,207,183,517
CPNE6_8_5056,193,180,188,362,189,930,268,254,554,675,348,469
CPXM2_8_5057,325,552,169,121,362,49,952,1276,405,192,10,827
CRABP1_8_5058,1103,1911,1123,1288,874,1501,1910,624,1228,1817,567,968

CRH_8_5059,644,471,458,502,708,190,1739,611,253,223,408,864
CSE1L_8_5060,56,260,75,105,570,1,119,36,0,254,260,101
CTSW_8_5061,704,524,619,517,1211,1462,444,621,1057,163,2862,981
CXCL10_8_5062,830,926,890,768,1284,1288,1741,1386,305,874,1712,811
CYGB_8_5063,539,328,298,361,728,9,250,233,373,780,780,329
CYTH3_8_5064,173,226,99,231,66,15,3,3,219,123,36,46
DDI2_8_5065,1928,2041,1948,2068,2248,1629,2102,2051,2157,1487,4101,330
4
DIRC2_8_5066,1024,1182,1173,1402,3019,1213,931,867,946,1453,1460,800
DISP1_8_5067,402,186,304,262,348,1042,1053,38,190,16,1116,498
DLL4_8_5068,381,261,444,455,971,281,349,249,245,372,141,288
DNAJC5B_8_5069,1407,1047,1026,1232,876,1321,1826,930,919,2051,1258,216
9
DNAJC6_8_5070,688,1315,679,425,1096,300,191,286,639,850,1629,333
DOC2A_8_5071,1835,1756,853,1742,866,1844,1014,2150,2097,2336,966,846
DOC2B_8_5072,133,0,217,128,70,0,1302,636,485,182,0,235
DSCAML1_8_5073,703,498,272,534,1443,683,60,916,114,16,439,751
ECEL1_8_5074,2749,2314,1960,1690,3457,1866,2736,2296,2508,1633,1866,19
68
EID2_8_5075,439,141,215,269,7,37,252,126,8,113,351,746
EPCAM_8_5076,0,0,0,0,0,0,0,0,0,0,0,0
EXOC2_8_5077,4849,7146,5814,6300,5159,6769,6802,4674,5042,4013,9065,60
75
EXOC3_8_5078,1219,1476,1381,1345,2864,1821,43,1049,1625,1177,1548,1184
F11R_8_5079,700,1036,701,1005,849,1222,448,2004,914,1761,351,1521
FABP1_8_5080,649,1167,586,914,1136,630,939,1014,1573,445,718,263
FABP2_8_5081,960,1032,671,791,267,1234,671,1158,962,877,1004,2909
FABP3_8_5082,446,604,556,576,833,501,547,263,251,494,409,601
FABP4_8_5083,1967,1350,1255,1751,2968,2136,1518,1787,2066,2661,1795,19
42
FABP7_8_5084,892,813,846,700,639,1592,3760,443,751,268,317,373
FABP9_8_5085,2216,1501,1492,1624,3200,1706,2014,556,3221,2909,967,1825
FAM101A_8_5086,777,465,425,433,122,174,48,681,1503,440,106,1725
FAM117A_8_5087,1881,1556,1833,1935,1123,2271,2363,2357,921,1353,1891,1
536
FAM57A_8_5088,197,193,117,79,4,660,2,65,17,292,41,274
FAP_8_5089,2962,2843,2570,3156,3189,2827,3252,3838,3222,3949,2692,4004
FBF1_8_5090,972,871,940,1266,825,726,1633,1469,1448,1952,620,582
FCN1_8_5091,492,506,422,349,247,380,1031,458,243,729,1011,152
FDX1_8_5092,852,1037,801,749,1097,1408,687,537,80,630,1280,163
FDX1L_8_5093,446,615,486,566,1661,1377,239,481,455,602,286,1295
FGF4_8_5094,1760,1309,1121,1507,1842,1626,1288,2484,1399,848,2485,1879
FGL2_8_5095,695,596,533,773,2279,903,874,752,165,100,1314,421
FOLR3_8_5096,3646,3423,3444,3189,3269,3754,2436,3024,2623,3926,6724,46
60
FOLR4_8_5097,664,451,418,746,662,96,384,1177,225,447,948,134
FRG1_8_5098,1960,2825,1797,1841,1504,1595,1738,2731,979,925,2737,3652
FTL_8_5099,2298,2371,2084,2697,3731,3358,4978,1566,1931,2582,970,4941
FXC1_8_5100,692,771,505,329,351,231,551,382,449,1264,708,451
GABARAP_8_5101,5448,4403,4317,4678,6561,4793,6504,5319,5624,5308,5809,

3684
GGA2_8_5102,767,402,522,538,239,420,253,779,1093,1164,653,478
GJA1_8_5103,4458,4640,4217,5615,4645,6355,5102,6183,1998,5535,7096,500
6
GJA3_8_5104,1317,1360,1621,2010,2229,1462,587,1505,1166,1983,3566,3465
GJA4_8_5105,2383,2412,2527,2291,4067,3416,1575,1336,2286,3583,2983,450
2
GJA8_8_5106,895,920,738,781,1182,817,732,900,1077,131,995,989
GJB2_8_5107,924,1136,941,1050,427,1763,582,1442,1351,1811,1058,1055
GJB4_8_5108,1395,1245,1202,1296,1497,756,1118,1226,435,2371,1176,1187
GJB5_8_5109,1442,1282,1335,1249,1588,2434,1152,1474,1137,1328,2869,148
GJC2_8_5110,1197,1300,1484,1146,524,1180,236,507,2190,1544,1405,920
GJC3_8_5111,85,23,0,19,0,32,0,326,1,0,0,14
GJD2_8_5112,3015,2622,3168,3380,2937,3714,2510,2569,3042,3630,4185,256
8
GJD3_8_5113,442,382,399,398,706,1054,404,596,601,311,1173,395
GJD4_8_5114,837,955,638,844,680,777,2281,1289,1162,2693,971,1012
GKN1_8_5115,813,926,1033,872,1029,813,685,1223,753,865,633,560
GLCCI1_8_5116,3159,3281,2043,2254,4034,2240,4969,2627,3708,3830,2872,2
525
GLTP_8_5117,3418,4042,2657,2341,2424,3614,4304,1724,2610,3259,3135,226
3
GLYATL2_8_5118,3401,3406,2403,2805,2953,4189,1404,1868,2770,2718,5809,
2405
GP9_8_5119,322,1132,444,222,561,349,578,0,188,1315,61,768
GPIHBP1_8_5120,632,539,686,924,682,848,1287,620,310,455,249,603
GPR180_8_5121,7974,7984,7665,7183,7670,10010,9343,6953,8234,10574,8294
,9052
GRN_8_5122,99,210,68,151,49,44,28,263,117,165,17,694
GZMH_8_5123,1274,1302,820,900,1907,860,1255,759,308,2917,742,1059
GZMK_8_5124,1243,745,1531,1057,1190,2254,2334,1119,2701,1725,2005,1116
GZMM_8_5125,545,453,565,231,274,1130,5,56,12,620,514,377
HBA1_8_5126,1238,1332,884,1239,842,763,1860,2312,758,1000,1013,1444
HBA2_8_5127,1238,1332,884,1239,842,763,1860,2312,758,1000,1013,1444
HBE1_8_5128,4554,5173,4111,3652,5615,5054,3719,4948,2994,4485,6340,676
6
HBZ_8_5129,385,344,524,448,228,138,155,2,1072,338,25,546
HECA_8_5130,851,639,1118,730,703,2011,253,145,1952,906,918,626
HGFAC_8_5131,31,398,269,252,304,442,0,60,212,6,17,336
HIAT1_8_5132,969,1184,499,899,533,1108,751,1223,66,220,72,87
HLA-DQB1_8_5133,167,160,131,134,43,441,128,69,167,32,67,799
HMCN1_8_5134,11859,12798,12269,12381,9735,11745,13989,15790,11475,1424
7,15549,12494
HMHA1_8_5135,1344,1550,792,1227,1369,869,475,1650,2134,1250,1065,637
HPCAL4_8_5136,455,502,337,457,1284,1742,158,38,160,103,66,947
HPR_8_5137,1414,1304,1193,1380,1659,1682,1065,1535,1123,817,1111,999
HPX_8_5138,644,581,284,570,717,1147,458,913,933,799,715,715
HSP90B1_8_5139,2818,2125,2599,2044,2813,2786,2873,2022,2576,3156,3122,
3964
HTRA1_8_5140,445,994,361,732,1477,890,75,1231,758,63,361,213

HTRA4_8_5141,2846,3530,2325,3070,3958,3998,2679,1946,4021,3445,3360,41
30
IFNG_8_5142,2219,2035,1717,2060,3670,1262,2115,2792,1452,2396,4836,399
6
IGFBP7_8_5143,95,335,515,177,111,368,78,39,9,303,488,250
IL12B_8_5144,734,289,274,669,11,82,1151,743,319,480,894,1009
IL13_8_5145,482,208,359,308,7,5,58,857,526,153,299,176
IL17A_8_5146,158,78,109,69,252,165,0,15,287,163,124,17
IL1A_8_5147,988,1189,817,943,1706,1526,581,2210,417,2348,1219,927
IL1B_8_5148,586,412,457,459,227,301,1662,338,422,429,833,905
IL3_8_5149,2201,2442,2145,2595,4385,2038,1039,2462,3503,2320,856,4394
IL5_8_5150,2141,2341,2405,2269,3748,1660,1948,2459,2478,1708,3279,2424
INSL3_8_5151,264,630,414,384,206,1028,181,217,595,728,264,187
IPO13_8_5152,2627,2243,2381,2343,1599,2139,2014,2289,2768,2807,3961,12
28
IPO4_8_5153,1570,1385,1025,1399,1258,1105,618,1824,1462,935,2052,2406
IPO5_8_5154,2000,2236,1984,2581,3417,4735,2026,1176,1847,3385,2428,139
1
IPO7_8_5155,0,0,0,0,0,0,0,0,0,0,0,0
IPO9_8_5156,1663,1840,1972,2068,1559,2279,2109,2392,946,2543,1889,2531
ITGA10_8_5157,929,1197,1343,516,1134,142,1509,728,1099,1245,858,1305
ITGA11_8_5158,1280,1603,1516,1764,1234,1873,1750,419,2645,2261,3117,16
25
ITGA2_8_5159,416,351,572,596,406,835,172,451,464,336,16,1029
ITGA4_8_5160,917,646,402,813,394,960,710,1097,909,165,1538,791
ITGA5_8_5161,1552,1027,1634,969,1921,215,1171,1471,2432,1337,2128,647
ITGA8_8_5162,1336,1184,1304,1557,953,1179,850,1214,459,720,1062,475
ITGAX_8_5163,486,657,403,429,320,391,643,699,435,646,533,400
ITGB5_8_5164,4044,4150,3869,4381,5675,4312,3525,1996,3469,2051,3207,72
74
ITGB6_8_5165,2861,2380,2580,2422,3803,1116,4149,2235,3247,3620,2783,30
01
ITGB8_8_5166,1181,1462,1286,820,1575,1424,967,1221,600,1369,1962,3483
ITLN1_8_5167,326,646,161,536,4,1,336,28,893,472,45,914
KDELR1_8_5168,266,339,302,142,3,222,257,552,316,290,779,1096
KEL_8_5169,631,579,334,721,245,821,261,119,403,857,1412,1077
KIF20A_8_5170,148,122,351,115,37,322,39,64,20,237,25,26
KIF3B_8_5171,1215,725,977,1196,579,1583,237,1058,436,402,1742,2817
KIF5A_8_5172,316,291,85,262,130,124,11,31,708,106,109,111
KLK13_8_5173,1913,1814,1965,1967,1390,2592,3097,1983,911,1586,4672,239
9
KLK14_8_5174,388,566,292,318,270,770,12,786,426,503,412,724
KLK4_8_5175,1709,1548,1502,1535,1973,2577,1359,3419,1467,941,2682,1148
KLK9_8_5176,994,913,532,935,1402,1418,2166,825,443,772,1874,1430
KPNA1_8_5177,234,205,188,292,280,153,183,110,64,148,78,200
KPNA2_8_5178,4917,4760,3909,4499,7113,5596,4016,5683,5405,4551,5171,42
56
KPNA3_8_5179,3047,2895,2735,2478,3137,2656,3285,2124,2122,3635,6182,32
00
KPNA4_8_5180,2252,1363,1752,1774,2132,3475,1024,2689,1784,929,3035,112

3
KPNA6_8_5181,605,926,484,836,248,787,1158,473,58,859,354,275
KPNB1_8_5182,1261,1832,2251,1156,1851,511,960,2189,769,2160,1813,1913
KRT12_8_5183,731,507,740,547,499,741,2067,446,875,545,1396,239
KRT7_8_5184,266,593,303,380,1154,128,79,0,75,465,323,92
KRT8_8_5185,752,651,665,488,191,681,782,194,328,563,370,69
KRTAP5-4_8_5186,518,239,169,264,448,1,441,19,807,789,222,173
LASP1_8_5187,516,651,494,477,686,696,214,1249,973,880,1075,436
LBP_8_5188,1330,1710,932,1149,903,849,425,1286,1610,1719,1794,1698
LCN12_8_5189,405,323,451,235,142,195,364,211,449,246,30,96
LCN1_8_5190,794,617,595,590,112,1422,131,757,279,440,1350,805
LCN2_8_5191,433,450,644,351,553,843,159,271,849,809,836,362
LCN8_8_5192,916,1050,814,765,744,1427,658,1210,351,1102,998,411
LCN9_8_5193,1057,1318,1418,1163,485,1277,824,1456,1824,2248,1156,359
LDLRAD2_8_5194,481,493,401,761,755,378,371,584,16,351,675,73
LDLRAP1_8_5195,298,212,319,159,1024,70,47,3,276,26,2,299
LMAN1_8_5196,444,173,334,359,19,333,53,1,514,125,518,101
LMAN2_8_5197,807,911,617,515,973,1541,894,938,46,619,756,984
LPA_8_5198,3778,2902,3622,3636,4071,1734,1999,3735,2942,4301,2768,5669
LRIG1_8_5199,1390,1815,968,2345,1103,2854,857,671,718,3247,1677,1621
LRP2_8_5200,73,22,53,30,71,0,0,11,0,4,0,27
LRRC4B_8_5201,175,328,108,185,421,204,15,201,20,12,0,1
LRRCC1_8_5202,1073,946,691,1056,1597,906,1054,1199,1042,1146,868,1579
LYST_8_5203,3161,2802,2321,2686,3415,2849,3451,1830,4580,3635,4823,227
0
MAL2_8_5204,885,1296,953,1142,708,2237,1055,760,1002,687,1828,438
MATN3_8_5205,893,815,827,865,818,1048,301,1301,439,1169,2020,1393
MFSD8_8_5206,1389,697,1234,964,1254,1818,134,806,1587,1247,2616,762
MFSD9_8_5207,1126,940,680,1450,1654,871,1478,883,1955,303,403,395
MMAA_8_5208,878,1005,626,711,1476,989,1434,1249,462,969,997,971
MMACHC_8_5209,708,911,495,593,1341,375,1273,500,525,693,1293,1004
MMGT1_8_5210,1126,481,637,780,442,422,193,523,1061,155,616,2664
MMP11_8_5211,748,572,779,674,467,606,541,217,804,1573,658,462
MMP13_8_5212,2015,1295,1165,1246,1074,705,828,874,779,1823,599,2157
MMP15_8_5213,927,1197,1112,750,209,2609,2114,1136,1455,1869,644,360
MMP16_8_5214,487,477,556,414,788,503,68,421,380,100,505,820
MMP17_8_5215,929,815,654,698,580,924,1201,1241,34,81,171,617
MMP19_8_5216,1312,1223,1201,798,873,1247,2058,1837,1063,1832,1610,424
MMP24_8_5217,492,318,455,265,122,0,93,618,175,172,138,93
MMP25_8_5218,414,302,65,347,430,67,1047,251,4,553,873,659
MMP26_8_5219,1046,653,800,687,686,693,590,902,2225,1352,691,585
MMP27_8_5220,3263,2539,2578,2915,4319,3711,4017,2913,4473,3841,2261,53
86
MRS2_8_5221,479,256,573,289,700,564,596,588,71,720,879,271
MSTN_8_5222,1189,1007,750,1069,2019,1645,1006,1553,1121,901,2403,1098
MTX2_8_5223,624,366,516,445,454,1085,448,326,673,641,28,694
MUC2_8_5224,538,599,633,334,1616,356,128,1723,492,152,547,494
NAPG_8_5225,1606,1177,639,1343,2017,532,734,2134,1888,1866,991,1455
NAPSA_8_5226,791,559,693,666,1078,1754,1771,936,332,1308,453,1517
NCOA5_8_5227,897,1317,1202,1218,359,793,1440,587,131,1222,318,828

NGF_8_5228,2815,3126,1405,2418,3306,3618,3731,1485,1728,3059,1667,3674
NID1_8_5229,3100,3340,3301,2612,3976,3667,1756,2151,2577,4691,3118,164
1
NPC1_8_5230,618,424,225,864,665,158,2,510,512,742,559,573
NPEPPS_8_5231,665,585,363,320,159,318,1067,647,290,917,840,272
NPPB_8_5232,193,232,112,224,32,34,1,31,584,378,40,17
NPY_8_5233,1870,2395,1422,1628,3308,3659,4224,2311,988,2386,2694,1715
NSMCE1_8_5234,1296,1948,1135,1397,1333,815,1247,1923,2372,1770,1489,16
15
NUP107_8_5235,4148,3686,4318,4227,3976,7257,5201,5885,4342,4968,4780,3
230
NUP133_8_5236,2041,2731,2090,2326,2026,3718,2417,1818,2564,1525,2510,1
421
NUP153_8_5237,1784,1522,1175,1186,2580,1350,2214,1453,2712,1330,1238,1
903
NUP160_8_5238,728,617,520,583,872,655,534,479,387,512,662,1445
NUP210_8_5239,678,987,663,634,343,1509,267,581,424,1059,707,809
NUP214_8_5240,1672,1638,1221,1215,2216,2113,1423,2776,1847,2666,1916,2
517
NUP35_8_5241,575,303,363,537,724,496,73,859,816,303,843,93
NUP37_8_5242,1352,1628,887,1237,849,2034,2160,1286,1431,1700,2295,1543
NUP54_8_5243,245,367,321,300,436,23,330,18,16,801,1852,1039
NUP88_8_5244,1176,1221,1385,999,2331,783,1522,1347,2188,2125,3023,1248
NUPL2_8_5245,6911,5761,4910,6317,6676,11483,6248,4694,7501,7425,6000,6
086
NUTF2_8_5246,532,239,313,488,77,1384,67,124,208,127,855,67
NXF2B_8_5247,6762,6128,5151,5937,6168,4529,7651,7394,6305,4734,6526,98
68
NXF2_8_5248,6762,6128,5151,5937,6168,4529,7651,7394,6305,4734,6526,986
8
NXF3_8_5249,368,439,426,208,259,287,507,89,121,164,691,667
NXT1_8_5250,2680,2539,2471,1767,2049,4967,2736,2976,2825,2995,3059,279
6
OAZ2_8_5251,896,1032,934,1123,534,1383,813,863,752,524,907,1164
OBP2A_8_5252,2025,1429,2062,2039,2473,1316,1932,1261,2635,2604,3732,24
68
OBP2B_8_5253,1170,1039,811,1146,949,1478,850,791,1838,681,2592,1615
OCA2_8_5254,357,99,506,395,75,323,671,177,96,203,526,342
OGFOD1_8_5255,502,645,416,703,292,115,101,375,319,130,643,1000
OGFOD2_8_5256,587,587,242,362,664,1018,322,434,152,103,198,314
OGFR_8_5257,3772,4996,2738,4168,3515,3961,2597,3335,3500,4856,3468,618
9
OVCH1_8_5258,3175,3580,2538,3299,3658,4409,2458,2338,3663,4297,3496,28
08
OVCH2_8_5259,1187,1264,880,1548,1756,1515,230,2627,1119,967,2039,1049
OXNAD1_8_5260,2218,1528,1909,2144,1439,4504,2305,1095,1677,1180,1778,6
03
OXT_8_5261,154,28,102,273,137,68,7,184,0,7,373,32
PANX1_8_5262,1971,1507,1799,1257,5192,1988,2253,923,942,1649,387,913
PAQR7_8_5263,2197,1638,2038,1486,1592,2458,2356,767,1531,1014,5598,299

3
PCDHB11_8_5264,261,87,136,32,16,457,397,2,274,88,6,380
PCDHB16_8_5265,4012,3066,2990,3263,2758,2320,4866,2007,4092,3556,3546,2235
PCSK4_8_5266,584,545,872,601,2062,585,1535,371,330,567,988,1786
PCSK7_8_5267,653,896,943,1144,403,1237,2370,139,736,1324,1168,704
PEA15_8_5268,1967,2169,1950,2058,557,1661,2766,2263,980,2435,2550,2801
PET112_8_5269,471,260,639,369,367,350,510,195,280,258,230,361
PEX13_8_5270,3387,3648,2699,3800,3165,5192,3320,3548,2727,4867,3706,2245
PEX7_8_5271,526,795,444,606,80,1047,695,1100,302,2490,634,364
PF4_8_5272,480,542,333,318,2440,369,1084,0,229,785,639,0
PFN3_8_5273,2096,2038,1902,1729,1668,2906,2585,3191,2111,2772,3598,3763
PHEX_8_5274,643,854,712,723,565,1355,360,1652,665,620,530,190
PIGR_8_5275,1375,1674,1122,1443,1021,1181,2559,2380,2849,2053,1704,2205
PITPNA_8_5276,1613,1966,1559,1814,2019,1328,1786,1541,1284,1925,2281,2753
PITPNB_8_5277,3602,3922,3019,3425,3784,4113,3836,2999,3951,4775,5337,3558
PLLP_8_5278,3321,3464,3035,2579,3994,5960,2992,3892,1955,4023,4235,4235
PLP2_8_5279,802,545,157,401,985,165,1287,404,142,1159,403,56
PLXNB2_8_5280,2469,2808,2103,2128,3102,2243,4999,2439,2498,2424,1510,2782
PNMA2_8_5281,1475,1072,1359,1066,504,1045,1601,1420,467,439,528,1139
PPP1R14A_8_5282,476,268,730,241,10,0,0,1,164,217,719,980
PPP1R14C_8_5283,142,225,60,124,242,541,278,464,102,39,124,250
PPP1R15A_8_5284,1153,1243,927,488,917,898,427,626,201,631,852,599
PPRC1_8_5285,463,431,678,772,699,222,1346,585,1089,833,598,997
PPY_8_5286,670,686,599,520,173,1194,785,91,864,107,861,875
PRB3_8_5287,575,1068,400,602,120,824,333,263,640,324,531,262
PROCR_8_5288,1137,1221,912,825,631,996,1168,2458,332,1185,1673,490
PROS1_8_5289,633,968,780,823,886,1577,1143,2147,233,1174,898,1845
PROZ_8_5290,390,370,194,226,326,149,439,164,268,242,873,387
PRPF18_8_5291,3004,3148,3187,2667,2400,3033,3391,3403,3047,4842,3590,2967
PRSS12_8_5292,1493,924,1376,1201,2045,1451,1860,2499,1451,1299,1151,889
PRSS22_8_5293,598,1182,963,667,314,557,145,36,75,1216,848,1011
PRSS27_8_5294,419,932,376,571,3238,853,813,351,246,182,933,539
PRSS33_8_5295,524,167,297,428,282,196,386,533,572,607,180,112
PRSS36_8_5296,1019,1023,864,955,1050,1772,1156,1904,749,1805,1454,1206
PRSS8_8_5297,458,284,364,266,312,49,1026,355,425,553,912,40
PSCA_8_5298,424,367,399,329,627,184,800,83,54,133,471,546
PTOV1_8_5299,862,1037,666,808,1500,795,415,1419,1172,783,576,874
RABIF_8_5300,469,953,357,552,1891,1713,946,777,34,165,703,4
RAMP1_8_5301,296,308,275,363,415,69,809,15,1611,964,801,11
RAMP2_8_5302,1053,1552,791,1149,2171,423,388,570,1785,331,1215,922

RAMP3_8_5303,602,915,827,850,229,498,224,1211,1413,2072,721,1193
RANBP17_8_5304,1281,780,865,1145,717,1458,271,1336,707,945,459,1071
RASSF9_8_5305,1422,1574,2030,1309,2073,2658,3446,703,1512,1333,1499,23
78
REEP5_8_5306,1514,1128,906,1228,1077,1547,2090,828,461,999,1472,424
RHAG_8_5307,99,255,54,21,2,0,0,0,27,320,0,40
RLBP1_8_5308,998,1059,1051,755,1466,653,1842,299,1122,1322,1688,1554
RPL15_8_5309,951,1013,806,1352,920,1773,1797,914,1085,1547,2894,1024
S100A12_8_5310,445,648,321,432,779,1369,975,842,240,429,670,95
S100A1_8_5311,1535,1406,1382,1615,2518,1698,454,2566,1156,1619,1188,35
20
S100A2_8_5312,771,607,763,755,152,906,628,684,193,309,312,958
S100A3_8_5313,11,27,106,38,104,19,7,0,3,0,7,0
S100A6_8_5314,674,1224,803,682,1429,2623,1283,1421,532,615,2015,1121
S100B_8_5315,165,390,259,442,52,606,626,13,165,2,117,253
S100P_8_5316,656,734,813,714,293,1553,88,120,479,197,279,512
SAA4_8_5317,847,808,760,1240,1421,1235,268,755,312,449,326,959
SCAMP1_8_5318,484,356,213,441,94,306,193,1138,829,381,170,392
SCAMP2_8_5319,4471,3922,4099,4515,3266,4120,4947,4039,3633,5586,6891,3
759
SCFD2_8_5320,832,1017,435,871,1117,1433,1886,1238,717,861,300,1129
SCLT1_8_5321,2378,2222,1671,2166,2817,1358,2074,2091,1632,2238,2829,28
12
SCPEP1_8_5322,439,688,536,950,41,1891,38,508,611,589,544,503
SDC2_8_5323,1016,1319,595,1354,1052,825,2184,1113,1063,2433,152,1487
SEC14L3_8_5324,457,338,337,485,157,540,774,2065,1006,326,132,320
SEC22A_8_5325,6362,7371,5297,5319,6109,4456,8317,5703,5341,6086,8435,4
838
SEC23A_8_5326,3553,4167,3535,3000,3767,4131,3893,3590,2832,3314,3494,5
869
SEC24A_8_5327,381,12,37,14,0,0,47,1,246,47,672,0
SEC24D_8_5328,698,624,617,637,2427,395,651,481,454,645,307,491
SEC61A1_8_5329,600,380,434,481,2738,854,87,282,1137,323,686,1138
SEC61B_8_5330,8449,9539,9326,9763,10814,10618,11094,9516,7658,7860,118
24,12752
SEC62_8_5331,145,158,27,76,125,24,0,92,87,25,359,104
SEC63_8_5332,1268,1345,1637,1545,488,1013,982,2848,1848,579,1308,1340
SELP_8_5333,446,696,333,424,441,532,381,476,945,628,512,572
SERINC1_8_5334,442,462,311,432,704,969,162,866,367,499,282,941
SERPINA11_8_5335,133,163,226,177,252,892,572,670,461,139,338,288
SERPINA12_8_5336,1489,731,899,1362,1030,1020,877,172,2107,2628,908,491
SERPINA3_8_5337,1798,1539,1512,1238,860,699,397,2255,3803,1228,1429,14
67
SERPINA4_8_5338,929,461,747,705,317,1032,318,112,1639,1004,636,691
SERPINA5_8_5339,246,253,499,194,771,6,514,28,347,734,147,143
SERPINA7_8_5340,390,268,192,417,161,678,49,840,462,671,185,84
SERPINB10_8_5341,5012,4554,3970,5100,4850,4979,4677,6049,3417,4727,682
6,5318
SERPINB12_8_5342,1601,1602,1193,2155,950,1136,2216,2688,2008,1897,2299
,2626

SERPINB13_8_5343,1137,1417,1222,982,1380,2079,277,1189,1011,1029,983,1
329
SERPINB1_8_5344,466,288,255,396,1354,403,16,261,832,399,111,306
SERPINB4_8_5345,218,58,122,251,26,79,29,3,529,76,611,46
SERPINB9_8_5346,500,793,403,306,102,73,571,452,12,454,402,839
SERPINC1_8_5347,400,301,608,435,287,145,644,254,741,186,382,738
SERPIND1_8_5348,1248,883,928,1039,412,1816,1001,997,1689,403,817,833
SERPINF1_8_5349,998,1459,870,1028,250,1729,1163,493,953,1573,1200,1315
SFXN1_8_5350,512,269,325,479,504,105,419,640,410,343,309,739
SFXN2_8_5351,581,459,479,504,834,571,291,400,416,882,995,607
SFXN3_8_5352,753,550,516,418,783,1391,474,752,1162,575,2778,630
SFXN4_8_5353,713,526,626,590,1247,364,1371,479,971,928,751,1303
SFXN5_8_5354,1047,1232,949,718,143,1728,2225,482,1825,978,944,514
SLC15A5_8_5355,3249,3100,2089,2755,3051,5172,3020,3591,3492,4019,4531,
4938
SLC16A13_8_5356,787,759,943,708,443,1007,1988,68,831,14,1365,1183
SLC16A14_8_5357,11869,11628,11015,12164,11687,15415,11966,14488,11950,
12517,13359,12125
SLC16A9_8_5358,1158,1430,620,929,638,866,1997,400,1020,1419,960,1164
SLC17A9_8_5359,1487,1725,1692,1896,653,3336,2619,507,1796,2129,1839,14
49
SLC22A20_8_5360,163,173,148,299,11,90,466,55,0,24,1,2
SLC22A24_8_5361,874,298,546,719,329,145,2287,183,1699,706,1874,514
SLC22A25_8_5362,0,0,0,0,0,0,0,0,0,0,0,0
SLC25A23_8_5363,536,893,282,746,89,58,129,472,836,974,1045,9
SLC25A29_8_5364,459,533,290,236,1261,444,465,226,487,231,689,364
SLC25A31_8_5365,1961,1477,1719,1888,1673,827,1389,1961,1920,1181,2497,
3614
SLC25A38_8_5366,1915,2343,2031,1905,2822,2343,2087,2308,1566,2611,2005
,1422
SLC25A42_8_5367,348,448,509,272,120,353,591,2,163,480,559,823
SLC25A46_8_5368,1096,1496,978,881,1201,1421,1732,710,870,1664,585,728
SLC25A48_8_5369,606,995,364,551,389,1068,845,60,369,630,352,933
SLC38A7_8_5370,264,477,321,213,201,534,2,0,3,346,795,271
SLC38A9_8_5371,4093,4533,2736,3123,5154,6129,3179,2177,3605,3308,4841,
2151
SLC41A2_8_5372,2414,2976,2612,2694,2099,3324,3620,2463,3837,2237,2565,
4503
SLC45A1_8_5373,260,259,355,197,19,152,1393,441,266,505,127,475
SLC45A3_8_5374,3595,4597,3198,3955,3704,4676,2991,3642,2791,4415,5020,
4924
SLC47A1_8_5375,2412,3063,2643,1936,2670,5700,2472,2166,3794,2075,3779,
1815
SLC48A1_8_5376,698,669,1056,1097,1365,71,418,678,153,470,536,824
SLC5A12_8_5377,4285,4004,4024,3944,4353,2139,5724,3791,3868,3147,3488,
3588
SLC6A17_8_5378,459,584,238,475,46,715,411,262,704,468,119,185
SLC7A14_8_5379,830,657,656,422,1611,1378,634,55,450,461,158,534
SLIT2_8_5380,2558,2681,1708,1824,2293,1908,2291,2876,2482,2843,1862,42
03

SNAP29_8_5381,4911,5271,4900,5181,6090,4773,4129,5714,5114,3724,7938,4
402
SNX12_8_5382,39,44,34,66,89,63,9,64,174,0,63,9
SNX13_8_5383,10241,11102,8472,8596,8381,10023,14661,8707,8005,13035,96
16,12436
SNX17_8_5384,2498,2861,2192,2291,1880,1878,901,1994,2913,2905,3679,384
7
SNX19_8_5385,750,683,1181,519,1681,1001,633,984,417,326,1286,383
SNX22_8_5386,541,755,789,572,966,760,875,360,475,1105,1004,1813
SNX24_8_5387,168,290,65,186,221,68,171,0,387,1194,100,381
SNX2_8_5388,829,777,896,828,759,744,2569,822,107,561,1885,1298
SNX4_8_5389,874,1023,1016,825,2650,343,2071,1175,1087,1286,2177,795
SNX8_8_5390,1000,971,281,585,145,724,19,508,889,2092,621,543
SNX9_8_5391,560,678,412,495,582,1348,315,298,1490,1015,827,172
SORCS2_8_5392,348,289,278,298,0,27,10,10,274,346,84,1473
SORCS3_8_5393,626,816,590,734,2250,860,369,701,1084,307,1030,463
SORL1_8_5394,892,853,796,667,464,58,1576,862,453,1569,857,1693
SOS1_8_5395,1135,902,740,1142,1441,897,598,834,473,1864,791,1395
SST_8_5396,2818,2146,2473,2719,3912,2160,3838,1978,2450,1090,2822,2641
ST13_8_5397,910,766,686,755,1036,1690,897,1116,1158,1495,491,1251
STAB1_8_5398,41,21,41,9,0,24,0,0,7,0,0,0
STARD4_8_5399,1990,1859,1732,1975,1579,2019,1575,2194,1890,2579,2236,2
807
STARD5_8_5400,325,142,72,102,8,174,11,127,58,299,37,83
STARD6_8_5401,2853,3208,2458,2740,4414,3436,4080,3091,3524,2586,4365,3
552
STEAP1_8_5402,1144,747,1417,733,1901,1556,2664,1561,963,2258,1046,1764
STMN4_8_5403,806,830,629,933,1198,551,457,1402,61,1184,608,1785
STX11_8_5404,280,366,380,55,316,197,388,99,30,303,791,559
STX18_8_5405,195,68,53,280,0,466,0,42,14,17,2,4
STX4_8_5406,1141,946,790,765,317,969,567,387,1104,1391,1412,816
STX5_8_5407,178,183,179,382,117,325,81,187,74,276,730,34
STX6_8_5408,2915,2841,2682,2834,2778,2775,3186,3184,2711,3522,3002,320
0
STX7_8_5409,5181,4263,3820,4526,3605,5074,4264,3104,4174,3094,3798,399
9
STXBP3_8_5410,971,700,604,546,350,13,1156,1959,423,190,2151,420
SVOP_8_5411,256,391,186,209,347,845,62,476,6,327,15,1441
SYP_8_5412,891,1008,1042,899,1413,1326,1160,1110,1004,2045,1289,893
SYT10_8_5413,136,206,145,417,53,35,327,212,34,107,69,2
SYT11_8_5414,1613,1551,1195,1804,1516,3202,1515,1617,2648,1204,1728,17
19
SYT13_8_5415,159,372,132,210,1986,389,104,7,500,150,17,17
SYT16_8_5416,1783,1503,1651,1909,712,2227,987,949,835,1584,2099,2394
SYT4_8_5417,4384,4663,4142,3772,5486,4214,5073,6735,4015,3308,3630,602
6
SYT5_8_5418,1744,1739,1174,1490,752,695,3286,1248,2319,1094,3477,732
SYT6_8_5419,1044,1277,1488,1500,448,399,871,1523,1674,1123,1891,2809
SYT7_8_5420,413,322,456,292,1571,756,617,513,487,16,13,325
SYT8_8_5421,781,1197,328,607,432,1584,190,2547,582,1041,628,274

SYT9_8_5422,136,251,237,210,29,53,46,424,368,163,411,205
TAS2R42_8_5423,1733,1760,1952,1667,1507,2708,1011,1406,1709,2704,2757,
1547
TBC1D9_8_5424,1139,927,1066,781,1501,1962,887,366,1888,471,1040,1880
TCN1_8_5425,1111,551,1222,791,929,1811,1527,1106,405,1550,1632,1353
TEKT4_8_5426,303,645,489,594,940,586,351,1763,463,649,260,900
TF_8_5427,929,812,774,737,667,669,695,956,1614,1540,1696,204
TG_8_5428,83,86,193,42,27,2,953,219,4,0,0,7
TGFB1_8_5429,1483,1317,1669,1481,1940,1051,832,1688,458,882,1850,2511
TIMM10_8_5430,402,365,395,358,26,309,875,1686,411,191,312,27
TIMM13_8_5431,846,790,777,579,201,1051,604,814,974,163,1734,719
TIMM17A_8_5432,545,693,421,743,1537,638,27,1047,392,817,731,898
TIMM22_8_5433,957,1519,883,2019,1618,554,1418,2775,1754,1602,1887,1558
TIMM23_8_5434,305,734,467,604,190,393,397,818,294,1040,477,92
TIMM44_8_5435,360,323,284,377,345,285,636,156,380,517,245,213
TIMM8B_8_5436,1560,1114,2133,878,2956,374,2057,1655,2199,1331,2454,900
TIMM9_8_5437,357,642,280,463,187,684,184,1590,1130,557,1040,881
TLL2_8_5438,127,234,152,86,675,50,53,594,0,51,29,23
TM9SF2_8_5439,2155,2488,2374,2502,2885,1997,3118,1467,2180,2725,2263,3
802
TM9SF3_8_5440,532,255,129,445,397,30,111,103,21,96,150,0
TM9SF4_8_5441,1283,868,893,1084,861,472,492,1360,1940,953,481,915
TMCO3_8_5442,111,180,90,122,1,66,63,30,4,359,0,84
TMED10_8_5443,666,791,999,727,1772,278,2312,2036,643,407,524,452
TMED1_8_5444,180,116,41,77,5,279,92,48,206,286,3,374
TMED2_8_5445,4160,3838,3635,3489,2652,5369,5898,4161,2943,5905,4576,49
03
TMED3_8_5446,517,309,406,464,7,361,645,129,743,53,1016,324
TMED4_8_5447,13,65,84,18,0,27,38,0,21,0,18,0
TMED7_8_5448,144,405,467,215,0,113,640,14,60,149,972,236
TMED9_8_5449,132,113,104,227,28,1,101,13,437,74,48,8
TMEM104_8_5450,565,826,388,604,1324,695,324,49,298,1553,427,959
TMPRSS11B_8_5451,2311,2073,2091,2586,3508,2766,2669,3317,2818,1556,160
9,2384
TMPRSS11D_8_5452,0,0,0,0,0,0,0,0,0,0,0,0
TMPRSS11E_8_5453,1126,956,1036,891,1206,947,957,1178,597,2030,1940,309
2
TMPRSS11F_8_5454,212,176,185,470,136,290,226,156,287,221,79,48
TMPRSS12_8_5455,660,470,535,462,1128,1238,160,147,480,819,1191,1068
TMPRSS5_8_5456,581,589,642,723,311,580,413,1549,1698,817,535,1615
TMPRSS6_8_5457,483,349,241,349,543,244,741,104,371,193,1159,473
TMPRSS7_8_5458,1362,1247,1094,1091,949,1591,1341,1659,982,666,330,1064
TMPRSS9_8_5459,284,315,70,160,99,155,648,136,15,302,59,35
TNC_8_5460,774,761,577,996,149,324,197,623,407,476,399,1890
TNF_8_5461,413,960,914,462,330,1057,771,307,225,48,569,509
TNFRSF9_8_5462,2113,1476,1883,1444,2897,1632,3014,51,1486,2030,3251,37
87
TNNC2_8_5463,1561,1630,1427,1276,865,1529,1466,2893,3490,2280,1123,239
9
TNN_8_5464,872,591,719,687,338,1247,1412,1034,1886,847,1489,507

TNNI3_8_5465,484,262,280,575,1034,56,86,1268,1205,344,487,352
TNR_8_5466,921,1901,847,968,1321,1238,1260,2223,634,2848,1426,1316
TOMM20_8_5467,557,441,283,345,174,560,859,760,274,0,964,131
TOMM22_8_5468,93,207,69,284,52,268,119,2,185,257,33,259
TOMM70A_8_5469,7642,6293,6017,7241,5015,7780,6024,8636,5329,5834,6985,
8434
TOMM7_8_5470,2457,2523,1464,1696,3049,2077,2913,2842,2606,2221,2483,15
94
TPSG1_8_5471,568,656,632,260,81,6,151,41,870,228,221,176
TRAK2_8_5472,872,857,752,513,1475,203,863,910,306,1603,1581,1552
TRAPPC10_8_5473,574,467,390,570,294,347,101,507,1150,157,1171,672
TRAPPC8_8_5474,1757,1541,2087,2143,3240,2117,1256,435,1272,1675,1989,2
891
TSNAX_8_5475,2233,2047,1158,1872,1302,2261,1353,2204,2351,1785,756,370
0
TTPA_8_5476,963,598,635,643,843,180,352,798,212,263,836,570
TTR_8_5477,5954,4639,4638,5183,5059,3270,5099,5868,6588,5290,5364,5140
TUBA1A_8_5478,2986,2541,2450,2386,3904,3369,3230,2478,1877,3028,4569,1
721
TUBA1C_8_5479,3031,2303,2104,2337,3618,2046,1447,2604,3143,3110,2375,3
704
TUBA3D_8_5480,564,1049,516,645,465,1610,160,596,291,516,327,491
TUBA3E_8_5481,1367,1201,1200,812,1469,2282,1397,1953,1360,1169,2361,97
0
TUBA4A_8_5482,0,0,0,0,0,0,0,0,0,0,0,0
TUBB1_8_5483,693,484,884,691,170,372,358,405,708,667,1058,1110
TUBB2A_8_5484,709,595,654,275,519,1865,396,1157,241,950,610,1735
TUBB2B_8_5485,709,595,654,275,519,1865,396,1157,241,950,610,1735
TUBB6_8_5486,688,623,354,759,1083,1471,25,78,459,52,1241,399
TUBB8_8_5487,3757,2694,3630,3200,3462,4241,5811,4555,2529,3809,7247,58
81
TUBE1_8_5488,1429,1506,758,1033,2300,1536,1534,1075,1360,1592,1024,173
0
TUBG1_8_5489,2912,3230,2269,2837,2985,2518,5632,1531,1978,3075,4539,40
95
TUBG2_8_5490,431,181,303,90,535,382,1174,715,252,248,738,606
TXNDC8_8_5491,770,544,565,814,1823,1381,366,616,236,455,196,854
UCP2_8_5492,1172,947,890,1012,2649,425,1720,1042,916,619,1206,1334
USO1_8_5493,1027,1402,1603,1616,452,2755,808,551,596,1605,1554,2758
VAMP2_8_5494,961,994,875,808,820,496,306,615,1270,1678,232,1293
VAMP5_8_5495,906,975,723,1299,708,1716,1103,299,1868,3008,1171,161
VKORC1L1_8_5496,1698,1279,771,1095,602,836,424,814,2890,1548,1492,1170
VPS18_8_5497,504,341,296,448,70,559,389,289,23,42,638,411
VPS26B_8_5498,1870,1549,1747,2347,2356,4050,1380,1160,1907,2405,2066,1
965
VPS33A_8_5499,2008,2520,2141,2029,2885,3482,5469,2434,2400,1527,3579,2
539
VPS33B_8_5500,866,748,921,610,1869,1129,360,697,802,837,1146,830
VPS35_8_5501,879,613,605,504,226,1033,249,840,1230,1126,963,1034
VPS39_8_5502,552,299,646,408,686,296,196,877,1430,183,791,811

VPS45_8_5503,239,488,216,309,525,79,1095,154,580,1188,248,159
VPS4B_8_5504,599,1049,516,548,533,238,483,639,1870,432,967,1038
VSIG2_8_5505,427,226,373,172,267,570,82,0,14,146,850,629
VTI1A_8_5506,5447,5729,5550,5648,6647,5704,5795,3640,3803,4774,4175,76
15
VTI1B_8_5507,2464,2407,3644,2381,3350,2084,5338,2428,3533,1737,3794,26
93
VTN_8_5508,346,429,497,864,1,716,351,904,36,77,170,1035
VWF_8_5509,334,129,310,207,0,16,849,15,68,0,0,9
XK_8_5510,1131,1155,1055,735,873,409,315,1383,1053,1203,1032,535
XPO1_8_5511,1580,1605,1402,1258,2216,2548,936,2219,2983,3056,1168,1190
XPO4_8_5512,809,649,878,1130,1935,299,676,482,511,392,630,419
XPO5_8_5513,899,784,412,824,1440,851,754,492,830,433,2278,423
XPO6_8_5514,1888,1490,1456,1790,3690,1948,1421,863,1450,1497,1572,1088
XPO7_8_5515,920,1158,1068,889,1636,307,479,3317,754,703,867,1168
ACTL6A_8_5516,481,637,291,309,95,6,151,467,151,2028,414,2621
ADAM12_8_5517,561,392,690,722,864,558,725,315,1144,337,876,361
ADAM15_8_5518,783,647,869,827,334,1675,2257,1989,1521,526,856,1319
ADAM18_8_5519,1422,1990,2001,1491,2103,2826,2491,1387,2500,1237,3712,4
386
ADAM22_8_5520,1397,955,1385,840,703,244,433,614,173,1121,1288,1134
ADAM29_8_5521,901,944,892,642,1018,954,1232,1594,1279,472,1299,573
ADAM33_8_5522,492,429,343,540,538,235,787,704,127,563,350,27
ADAM8_8_5523,564,570,310,759,22,187,597,190,228,716,542,817
ADAMTS13_8_5524,564,611,164,489,863,696,522,418,728,650,1280,743
ADAMTS14_8_5525,916,1256,910,750,260,105,557,551,403,1057,474,837
AKAP12_8_5526,1230,1024,752,701,874,838,1222,89,751,1260,1122,566
ANGPT1_8_5527,491,774,770,446,1408,618,3430,579,716,420,1571,432
ANGPTL4_8_5528,323,517,355,189,18,740,1,609,742,494,33,187
AP1B1_8_5529,970,1281,1300,1449,925,1970,786,313,910,617,854,990
AP1G1_8_5530,270,439,228,344,16,192,1124,262,435,1038,152,178
AP1M1_8_5531,775,1030,590,780,335,640,197,779,453,397,1782,444
AP2A1_8_5532,330,400,385,481,67,1161,0,3,429,1201,531,315
AP2A2_8_5533,668,555,624,460,5,1306,199,785,28,606,30,222
AP2B1_8_5534,2150,1904,2385,2266,5017,1790,2216,2195,2909,3396,3424,21
14
AP2M1_8_5535,2161,2730,1772,2535,2572,2939,1085,4126,660,2346,3715,202
7
AP2S1_8_5536,213,276,467,542,9,464,100,1116,206,7,274,188
AP3D1_8_5537,205,122,299,748,37,7,528,655,2,299,728,0
AP3M1_8_5538,1385,1014,840,1028,902,812,389,1271,940,1770,1476,335
AP3M2_8_5539,956,980,648,900,2109,529,599,1193,1082,1797,626,752
AP4S1_8_5540,1614,2503,2783,3040,1563,2253,2478,2953,2095,1705,2631,46
83
APAF1_8_5541,1228,1382,1486,1472,285,1110,2637,1213,1402,1668,1131,249
6
APBA2_8_5542,2100,1862,1557,1101,1390,2562,1277,720,1304,2396,1877,147
0
APOL1_8_5543,2372,2181,2879,2290,4184,2512,4695,1837,2025,2396,3924,36
25

APOL3_8_5544,778,403,576,422,1417,65,217,737,1008,596,303,158
APP_8_5545,829,627,362,980,1145,196,2296,1701,1073,584,764,308
ARFGAP3_8_5546,2989,2891,2925,2873,3783,2901,3498,5498,2845,3540,5364,
3446
ARHGAP33_8_5547,195,21,42,168,206,7,850,31,75,63,33,642
ARL6_8_5548,1650,1750,1988,1824,2040,2560,3629,1965,1471,2423,3830,109
6
ATP13A2_8_5549,2413,2197,2293,2314,3330,2792,2029,2387,2119,1860,2197,
3341
BAX_8_5550,1515,1279,1500,942,2212,1563,1754,2581,434,1019,1636,1153
BCAP29_8_5551,917,1059,1057,850,653,1356,917,475,220,1728,1486,1512
BCAP31_8_5552,1860,1711,1135,1106,2663,1665,969,1202,1434,1302,1301,53
5
BCL2_8_5553,44,290,120,117,1,34,19,42,1,6,11,697
BCL2L2_8_5554,437,664,441,683,378,682,75,232,416,884,345,316
BID_8_5555,1341,578,1027,998,1568,1537,1606,1305,544,1826,2006,1472
BSG_8_5556,902,856,990,1001,2088,1376,766,589,1489,825,281,502
C1QC_8_5557,1395,1560,1902,1341,1230,2262,2574,1679,2221,1744,2460,185
6
CANX_8_5558,1069,1078,989,1349,868,735,315,1023,607,1891,1885,1430
CAPN9_8_5559,949,702,778,990,1345,490,611,592,293,591,336,1764
CAPNS1_8_5560,387,378,387,269,273,679,129,440,1660,918,655,120
CCT6B_8_5561,278,117,413,317,24,614,352,927,332,210,53,1009
CD19_8_5562,246,399,320,117,814,5,1041,1562,1014,90,44,1761
CD22_8_5563,36,104,66,42,246,0,0,99,59,543,31,0
CD33_8_5564,279,230,246,131,74,130,234,103,61,453,1,11
CD44_8_5565,2630,2687,2292,2647,1851,4178,2953,2129,3208,2601,3404,167
4
CD55_8_5566,3086,2271,2786,3603,3844,4089,3051,5152,2874,3403,5114,437
9
CDH17_8_5567,405,367,144,431,0,315,0,16,363,746,59,513
CFHR4_8_5568,6058,5205,4108,5254,6405,5624,6484,6255,6492,5446,5926,36
94
CIZ1_8_5569,2945,3566,2772,2407,1907,5082,4076,2612,2748,3342,3085,239
1
COG2_8_5570,609,603,457,529,864,306,0,1408,992,144,304,40
COG4_8_5571,1524,2225,1315,1976,900,1563,64,634,1156,918,2020,2574
COG5_8_5572,871,989,928,678,1029,947,1654,58,1029,527,1059,1199
COG6_8_5573,422,582,290,288,821,486,447,227,250,206,593,119
COL11A1_8_5574,1966,1784,2027,1728,2359,2277,1986,2130,2022,3466,1507,
1636
COL11A2_8_5575,531,468,339,232,558,197,33,34,1255,1243,438,14
COL12A1_8_5576,2820,3658,3026,3298,3382,4858,3106,4710,2924,1562,1872,
4235
COL13A1_8_5577,750,911,704,590,1269,741,522,404,55,650,1190,769
COL18A1_8_5578,1246,1374,1257,1129,1939,1198,1172,798,1365,1056,2979,2
346
COL25A1_8_5579,37,209,186,246,1,33,1,1023,0,100,174,672
COL2A1_8_5580,292,628,367,248,547,154,1092,562,142,656,1147,685
COL4A5_8_5581,1755,1412,1555,1703,2294,2301,4305,1807,828,1220,3156,16

49
COL4A6_8_5582,197,428,303,231,269,573,247,360,4,386,687,780
COL6A2_8_5583,544,285,183,305,315,27,124,591,50,83,547,955
COL6A3_8_5584,888,942,697,1141,559,937,765,428,1021,509,773,97
COL8A1_8_5585,399,747,276,213,211,209,993,66,1080,725,236,1011
COL9A1_8_5586,260,243,283,223,334,77,829,598,145,227,424,77
COPA_8_5587,582,726,761,566,364,225,1206,171,750,2211,759,1007
COPB1_8_5588,574,745,573,616,48,569,778,632,555,353,256,204
COPE_8_5589,608,499,502,1083,64,46,46,850,1518,810,799,384
CPA4_8_5590,1015,978,820,1129,1315,2024,621,942,465,1157,1326,574
CPNE1_8_5591,2160,1205,1363,1409,1650,1086,872,3015,956,1971,889,1217
CPNE7_8_5592,180,130,109,129,70,128,2,1147,40,11,544,677
CPXM1_8_5593,487,214,335,484,0,862,196,2129,717,512,1028,182
CPZ_8_5594,338,517,624,468,797,258,61,112,573,911,447,331
CRABP2_8_5595,589,603,747,788,642,1644,766,681,352,1615,1386,123
CTLA4_8_5596,3453,3551,3339,3998,2669,3641,4321,5397,2675,4503,4697,59
50
CTNS_8_5597,244,151,465,320,8,323,317,161,1962,230,259,204
CXCL12_8_5598,1387,1516,1438,1692,281,1091,1053,4145,1238,3235,1282,23
03
DPP10_8_5599,1056,1082,1389,1606,1106,719,1413,2019,761,272,444,845
DPP6_8_5600,1658,1511,1244,1479,3077,673,1389,2211,657,346,778,2892
ECM1_8_5601,274,493,85,108,1,615,442,558,330,28,1588,673
EGF_8_5602,1405,1056,1541,1304,2501,451,1437,1545,929,1699,1035,1398
EIF2C2_8_5603,359,580,290,320,40,176,214,344,412,71,450,638
EIF2D_8_5604,2513,1558,2111,2154,1767,1814,1875,625,3198,2257,1767,271
4
ENSA_8_5605,2018,2317,2074,1398,1715,1682,4578,1874,1125,1331,3008,267
4
EPB41L4B_8_5606,3179,3536,3239,2686,3585,4663,2137,2591,2621,3371,4746
,2824
EPB42_8_5607,432,525,1238,766,791,41,778,568,169,108,1944,585
ETFA_8_5608,2588,2617,2319,2763,3473,2116,4863,2455,3334,4590,3783,338
8
ETFB_8_5609,204,398,258,490,447,155,1127,282,16,487,201,280
EXOC1_8_5610,547,343,737,823,61,131,630,112,1230,669,665,1061
EXOC4_8_5611,606,764,446,426,44,755,814,1455,590,852,23,1618
EXOC6_8_5612,2287,2444,2509,1961,1994,4028,990,1991,2523,2633,2950,328
3
EXOC7_8_5613,546,878,652,310,1038,773,677,219,365,968,825,366
F8_8_5614,1767,2136,1768,1852,1720,2460,2384,3325,4377,2003,1797,3109
FABP6_8_5615,552,755,487,647,68,70,304,1007,864,1204,1816,337
FAM131A_8_5616,114,130,235,127,1,99,917,9,38,18,0,242
FAM63B_8_5617,689,893,879,1042,763,1761,1086,2622,1441,215,358,1684
FANCA_8_5618,157,145,291,119,6,4,74,234,13,63,91,7
FCN3_8_5619,464,170,113,329,766,194,3,816,607,260,452,499
FGA_8_5620,269,213,275,185,677,347,681,0,1127,433,4,0
FGB_8_5621,1835,2302,1901,1936,3108,2063,3558,1012,2199,1268,2455,2396
FGF13_8_5622,7318,7412,7080,8457,9180,8556,8152,8744,5217,9043,9357,12
211

FGG_8_5623,7077,6907,6757,6840,6321,7405,7269,5406,4751,7065,7157,1033
8
FIBCD1_8_5624,1548,1365,1606,1531,1191,836,2353,2030,190,1552,1458,235
4
FLVCR2_8_5625,264,574,253,435,791,499,595,1815,252,183,297,175
FOLR1_8_5626,60,15,40,69,6,5,134,850,3,0,210,73
FOLR2_8_5627,256,334,86,283,176,444,185,0,123,356,513,13
GGA1_8_5628,205,237,156,141,387,598,254,70,1,864,124,165
GGA3_8_5629,233,271,353,439,101,348,526,255,28,206,712,79
GJA5_8_5630,459,704,479,441,1321,376,435,325,740,521,580,606
GJB1_8_5631,527,675,480,703,591,337,23,2007,795,294,885,46
GJB3_8_5632,1687,1716,1802,2353,2113,1707,1656,2288,1055,1274,1638,251
6
GJB6_8_5633,417,274,288,420,0,1028,301,73,351,304,115,302
GLYATL1_8_5634,559,891,761,504,261,462,2118,855,905,1886,524,1563
GNRH1_8_5635,371,108,392,278,22,56,189,73,407,96,476,623
GOLGA3_8_5636,983,995,990,1015,248,1487,1038,3485,709,1208,2414,1495
GOPC_8_5637,710,1122,662,893,1679,2033,1662,678,1075,1200,1580,1567
GOSR1_8_5638,385,840,842,286,1084,300,1092,32,162,793,1769,433
GOSR2_8_5639,1252,1752,1331,1224,939,2242,887,564,832,1200,705,1428
GPRASP1_8_5640,1632,2080,1965,1589,2299,2666,2132,1664,1952,3236,2898,
2041
GRB2_8_5641,2780,1832,2127,2309,945,1319,2897,2098,2862,4464,3276,3957
HABP2_8_5642,3028,3428,2484,2727,4536,6349,3691,2136,4793,4741,3124,35
77
HDLBP_8_5643,745,1281,947,1013,452,1371,497,1380,829,893,492,814
HEPH_8_5644,627,965,416,350,1629,673,233,84,819,1262,693,255
HNRNPU_8_5645,156,445,315,432,103,626,635,369,133,324,52,23
HOMER2_8_5646,547,416,333,350,895,203,471,546,1005,650,1229,213
HPN_8_5647,838,953,853,1012,728,1060,1191,1456,833,1112,595,2094
HSDL2_8_5648,1272,1045,1216,909,1345,1719,893,2013,33,1234,1042,874
IGF1_8_5649,72,76,10,160,25,0,0,115,55,185,6,40
IGFBP3_8_5650,850,1034,1090,1841,529,1470,1160,1487,756,1663,1223,2198
IPO11_8_5651,1383,1556,1617,1957,698,1238,1377,1640,650,1227,1237,2301
IPO8_8_5652,4106,5071,4412,3782,2923,5595,3615,3582,1947,6913,6665,513
8
ITGAL_8_5653,301,321,88,471,188,354,1463,60,72,546,356,485
ITGAM_8_5654,600,380,413,984,774,582,186,472,752,219,236,493
ITGAV_8_5655,249,89,209,188,166,103,25,918,78,495,454,207
ITGB2_8_5656,103,78,183,155,89,1,84,11,49,26,52,0
KDELR2_8_5657,1509,1988,1174,1357,761,3444,777,1476,1423,1406,2226,122
7
KDELR3_8_5658,776,870,506,533,2628,254,616,1623,886,1754,1530,740
KIF13A_8_5659,709,474,379,654,2084,396,548,267,1187,591,1077,1007
KIF17_8_5660,399,431,552,252,126,148,101,54,828,378,729,351
KIF1B_8_5661,1658,1398,979,1108,1122,2429,2044,1646,1889,825,1996,227
KLK10_8_5662,457,519,744,198,86,221,515,863,316,371,2151,152
KLK11_8_5663,517,558,434,465,935,473,448,1211,1223,362,583,230
KLK12_8_5664,319,385,211,528,116,146,214,213,996,624,507,118
KLK15_8_5665,132,187,58,376,287,146,3,81,8,214,8,393

KLK5_8_5666,244,368,219,273,0,959,16,387,191,756,461,15
KLK6_8_5667,250,42,33,22,4,401,107,61,5,247,6,2
LAMB3_8_5668,1550,1669,1540,2118,2399,2035,3141,2102,1915,3355,1563,11
25
LDB3_8_5669,350,368,267,140,473,222,0,12,498,371,54,7
LDLR_8_5670,199,503,287,304,239,536,182,92,21,677,67,108
LTA_8_5671,431,473,368,751,13,56,779,583,194,684,7,1117
LTF_8_5672,468,369,297,735,1031,440,119,480,64,599,479,666
M6PR_8_5673,1568,1041,1252,1022,2205,1179,1213,1132,646,1076,2463,2310
MASP1_8_5674,624,414,108,469,67,1457,156,661,203,66,630,632
MB_8_5675,573,586,341,590,323,95,410,303,572,355,1635,184
MCFD2_8_5676,588,870,602,899,1027,1366,1243,503,1257,637,146,1015
MCL1_8_5677,3839,2717,2551,2890,3003,3530,7733,2135,3084,3981,2110,517
7
MEFV_8_5678,569,459,564,205,2,238,322,1774,223,603,415,586
MFAP4_8_5679,564,651,333,889,678,456,382,344,268,1140,232,1661
MFSD10_8_5680,1150,414,448,646,2207,181,1281,1524,853,377,871,1204
MFSD1_8_5681,639,400,573,444,811,1169,1318,78,518,445,1079,547
MFSD5_8_5682,4910,5089,4099,5077,8928,5022,5729,6118,4630,4737,5427,48
42
MLC1_8_5683,272,382,79,93,0,259,3,73,0,775,363,580
MSLN_8_5684,637,1178,643,531,704,1523,635,345,1266,436,850,645
MTX1_8_5685,831,524,523,775,501,889,551,468,710,939,1028,750
MUC1_8_5686,521,764,1051,425,172,775,751,40,908,1189,125,1019
NCAM1_8_5687,2437,2400,2260,2496,2776,2607,3590,1665,4768,2950,3012,27
06
NNAT_8_5688,663,744,839,650,1511,855,122,915,1406,677,1561,986
NOXO1_8_5689,251,135,171,106,678,561,1120,0,8,3,55,835
NPC1L1_8_5690,1167,1442,1257,1431,885,1874,1607,2129,656,2086,1131,179
9
NPRL3_8_5691,785,528,791,1057,1400,1237,291,375,903,960,849,571
NRXN1_8_5692,734,1320,1583,847,796,1044,1380,506,190,583,726,1313
NRXN2_8_5693,380,313,232,428,227,295,469,251,502,605,142,1478
NRXN3_8_5694,143,159,178,127,52,31,221,2,372,14,716,27
NUP155_8_5695,1837,1933,1516,2508,2195,1456,3921,3173,1135,1748,2351,2
446
NUP50_8_5696,569,449,510,360,978,400,1228,201,199,714,538,384
NUP62_8_5697,663,467,472,586,617,537,363,458,782,2113,801,398
NUP98_8_5698,3875,4449,3879,3718,3414,3600,4879,3846,3051,5579,4228,40
33
NUPL1_8_5699,448,374,394,333,21,281,7,18,449,1203,0,664
NXF1_8_5700,587,292,138,381,80,30,197,82,395,112,4,87
NXNL2_8_5701,1310,1688,1074,1426,652,1217,1063,1058,1976,518,666,522
NXT2_8_5702,4280,3461,2990,3514,4941,4741,3514,4196,3852,3080,3849,534
6
OAZ3_8_5703,242,588,349,1120,497,1479,307,467,122,1307,3,319
PACSIN2_8_5704,758,333,267,911,927,94,79,240,64,156,863,418
PANX2_8_5705,72,14,76,44,216,0,0,36,1,15,1,2219
PCDHA6_8_5706,8015,6499,6685,6711,7306,9437,5467,6662,7493,7375,7878,8
671

PCDHGA5_8_5707,621,578,385,673,311,311,616,524,588,541,1470,400
PCLO_8_5708,1286,1663,1143,2096,875,1911,1435,1271,1193,1400,1564,2628
PCSK5_8_5709,872,721,613,793,831,548,1777,420,716,393,848,1033
PCSK6_8_5710,279,218,48,126,18,233,2432,5,1386,61,387,43
PCTP_8_5711,1333,1515,1523,2016,1423,1620,1604,1554,1072,1253,2148,159
7
PDYN_8_5712,425,484,260,373,1381,242,246,144,222,231,172,700
PDZD3_8_5713,1264,1203,829,1045,632,1064,1137,305,472,931,1350,1173
PDZK1_8_5714,1045,1354,1038,984,1089,1583,1657,1847,335,1490,1255,1070
PGAP2_8_5715,2184,2231,1489,2031,2978,3630,2187,3917,1832,2033,1233,40
86
PGF_8_5716,555,295,434,219,643,159,62,963,234,150,1065,76
PIK3R3_8_5717,668,595,348,543,205,801,458,407,794,320,396,294
PITPNC1_8_5718,3919,3715,3234,3311,4296,5419,5097,2666,2423,3392,3133,
1526
PITPNM1_8_5719,1449,1775,1015,1630,2169,1649,1215,1243,1103,3420,1590,
1637
PITPNM3_8_5720,424,624,646,349,1054,466,1132,862,841,945,95,32
PLEC_8_5721,241,214,182,224,205,587,45,3,0,167,448,827
PLIN3_8_5722,235,201,119,458,162,520,8,471,113,450,56,364
PLTP_8_5723,116,361,31,77,10,16,139,48,251,1376,3,218
PNKD_8_5724,951,845,411,445,1045,1894,892,717,858,200,111,117
POMC_8_5725,80,24,30,209,160,114,2,486,124,5,162,891
PORCN_8_5726,271,328,100,249,182,136,63,151,165,207,440,276
PREPL_8_5727,659,676,672,471,489,977,64,307,547,959,669,1694
PRNP_8_5728,515,487,311,402,488,696,37,223,882,535,621,896
PRSS21_8_5729,232,150,55,80,28,24,781,255,153,229,89,185
PRSS35_8_5730,615,438,438,613,429,1193,441,20,53,75,2,620
PSEN1_8_5731,3417,3098,3621,3856,3324,3424,2782,2157,4076,3825,4513,36
82
PSEN2_8_5732,158,70,175,172,68,22,17,127,34,666,9,27
RABEP1_8_5733,4920,5278,5045,4581,4624,6443,6812,5235,5951,4042,5594,5
590
RACGAP1_8_5734,1568,1781,1605,1559,1352,2998,1706,913,1624,2060,1392,1
083
RARRES1_8_5735,271,178,197,357,408,182,216,599,286,54,180,29
RASA1_8_5736,1642,1507,1182,1987,1056,2451,1430,1931,1714,1917,776,190
2
RELN_8_5737,1732,2223,1940,1777,1462,1332,548,910,1221,2191,2600,2096
RHCE_8_5738,853,905,822,906,1357,1509,636,1072,393,738,358,1161
RHD_8_5739,1812,1528,1163,1250,2417,1680,662,865,2725,1871,1324,2754
RIMS2_8_5740,2556,3026,3317,2560,2281,3756,4682,2922,1433,2211,3247,48
10
RRBP1_8_5741,1518,1923,1860,1880,239,2015,542,3357,2061,1578,761,1721
RUFY1_8_5742,807,1255,660,774,865,1561,1501,1720,1038,167,96,636
S100A13_8_5743,448,449,552,731,92,100,12,130,20,1056,788,443
S100A4_8_5744,2295,2386,3127,2250,1788,2986,2912,3543,2382,1263,5139,2
750
SAA1_8_5745,1240,1041,1302,1819,1283,1009,805,2220,399,1500,1759,1138
SCAMP3_8_5746,870,912,930,526,1563,876,625,1048,776,939,748,264

SCARB1_8_5747,1600,1556,1526,1317,1242,1838,2080,712,692,881,2656,2054
SCFD1_8_5748,737,99,556,512,139,173,572,462,549,97,530,516
SEC13_8_5749,389,320,529,516,798,386,4,1,926,514,363,0
SEC14L1_8_5750,1469,1192,1071,1135,199,1362,947,1529,659,1217,1280,187
9
SEC14L2_8_5751,330,345,269,297,148,758,605,120,965,323,446,168
SEC14L4_8_5752,305,243,113,130,556,849,146,288,440,410,368,303
SEC23B_8_5753,154,206,95,242,120,485,14,462,4,32,20,420
SEC24B_8_5754,912,1082,433,642,129,930,691,919,55,333,666,1511
SEC24C_8_5755,747,392,528,1029,1664,258,262,914,543,423,628,976
SEC61A2_8_5756,540,459,352,453,38,391,1092,443,384,94,413,993
SEC61G_8_5757,957,953,675,1079,551,1363,1226,587,1540,1395,378,648
SEH1L_8_5758,725,1098,693,916,504,697,2350,831,589,280,831,962
SERINC2_8_5759,3620,3433,3102,3045,4825,2450,2027,4440,2886,2437,5100,
5076
SERINC3_8_5760,778,1307,1097,372,624,1005,152,524,989,605,1826,880
SERPINA10_8_5761,593,326,265,531,1382,1182,112,466,538,287,771,1485
SERPINA1_8_5762,307,391,830,611,127,1874,231,211,860,337,1044,1616
SERPINB2_8_5763,113,99,154,232,260,465,14,117,117,126,54,19
SERPINB6_8_5764,300,293,190,289,106,1059,246,466,516,337,206,30
SERPINB8_8_5765,1769,1180,1522,1402,1410,2134,1457,607,1710,2929,1697,
2046
SERPINE1_8_5766,1240,1120,931,1481,690,1607,1523,928,1522,1236,1750,11
29
SERPINF2_8_5767,733,655,671,751,356,1122,495,702,139,940,1356,1567
SERPING1_8_5768,1309,902,942,869,1451,670,428,1197,1284,683,877,992
SERPINH1_8_5769,868,627,485,851,2124,1319,870,1264,274,999,1835,638
SERPINI1_8_5770,1232,1317,1575,1781,1680,1686,1752,630,1313,2608,1616,
928
SFI1_8_5771,1473,1292,1551,1784,1318,799,561,1378,2403,1554,1579,1418
SFTPA1_8_5772,799,471,533,735,2057,162,733,895,75,1048,49,315
SH3D19_8_5773,1303,1173,789,1318,367,1370,674,1407,903,777,1044,1232
SIL1_8_5774,433,493,634,304,1184,24,1816,1010,1891,534,1796,1986
SLC25A25_8_5775,650,499,407,825,878,582,470,364,978,667,848,237
SLC25A36_8_5776,1184,992,759,811,498,1643,458,974,1365,977,891,2182
SLC25A45_8_5777,783,368,707,717,851,1217,519,600,908,810,1620,161
SLC38A10_8_5778,850,463,221,538,2,83,35,5,442,165,668,105
SLC41A3_8_5779,1597,1548,1365,1632,869,919,2953,492,1906,1716,1185,155
3
SLC43A3_8_5780,535,403,390,428,475,596,483,340,1507,102,198,337
SLC44A2_8_5781,811,752,426,640,605,598,1240,425,21,561,3180,305
SLC44A4_8_5782,351,220,310,314,432,1187,1426,811,1140,76,459,439
SLC44A5_8_5783,794,1061,601,562,253,823,24,848,1648,1073,977,707
SLC46A1_8_5784,414,667,230,318,808,842,1051,467,100,137,78,360
SLC47A2_8_5785,1126,1078,1279,988,760,2702,1939,709,1186,925,1444,1152
SLC50A1_8_5786,739,636,730,445,1689,564,1,348,655,172,419,300
SLC6A20_8_5787,351,49,151,213,26,3,584,8,293,139,0,76
SNAP23_8_5788,1987,1090,1316,1425,1321,934,1456,1162,2375,1875,992,156
1
SNAP25_8_5789,2686,2688,1967,2468,1746,2563,5560,3175,1862,4675,3977,3

366
SNX10_8_5790,941,793,1103,1102,1399,797,591,973,1210,542,1870,752
SNX11_8_5791,532,407,328,491,1273,345,641,56,131,273,958,189
SNX14_8_5792,732,313,793,467,441,1451,585,627,210,280,258,261
SNX15_8_5793,18,65,65,11,0,0,0,200,0,556,1,2
SNX16_8_5794,376,309,339,519,1146,76,233,1018,65,757,791,1483
SNX18_8_5795,637,1323,627,696,948,2449,1133,75,1082,75,340,134
SNX1_8_5796,1072,1285,1103,1387,881,1031,1150,2572,1015,1267,1657,2076
SNX3_8_5797,366,466,505,193,852,514,104,154,440,199,111,71
SNX5_8_5798,1503,2391,1285,2024,1353,2406,2440,2522,1183,3070,1492,363
0
SNX6_8_5799,5331,5314,4246,5112,8722,7168,5590,7010,4466,4958,7631,598
1
SNX7_8_5800,1615,1353,1139,2014,1909,1986,1357,1613,2343,2087,2382,176
8
SORCS1_8_5801,1519,1186,1287,1181,592,1286,1555,767,1919,778,2188,2504
SORT1_8_5802,1161,1216,684,1158,2268,1398,728,443,689,682,1713,1077
SPNS1_8_5803,2392,3508,2946,2680,3137,3712,3129,3592,2538,3014,4101,35
54
SRI_8_5804,944,861,582,511,437,772,1836,1876,575,171,468,735
STARD3_8_5805,2172,1539,1545,1402,4039,1512,2315,2160,1775,1468,900,38
55
STAU1_8_5806,1471,1475,1779,2048,1965,786,942,1291,2664,1556,1581,839
STEAP2_8_5807,2523,1704,1867,2617,3449,2382,2745,2863,2776,2321,1877,3
649
STEAP3_8_5808,484,161,245,227,927,191,307,104,218,743,174,318
STIM2_8_5809,1340,1496,1303,1394,1753,2802,1003,1532,1018,4062,2109,14
90
STX16_8_5810,196,499,297,302,669,244,233,431,205,174,298,735
STX1A_8_5811,281,225,163,293,403,1068,187,401,517,441,7,941
STX2_8_5812,1102,1109,1006,1390,1098,1229,828,404,942,369,1892,1074
STX3_8_5813,1126,961,817,750,255,386,590,672,1058,419,921,2186
STXBP1_8_5814,1683,2071,1588,1702,1655,1595,2807,1433,1485,1143,1182,1
769
STXBP2_8_5815,201,237,187,150,41,636,841,210,239,471,24,275
SV2B_8_5816,277,199,179,349,532,262,728,317,327,99,451,55
SYN1_8_5817,1543,1484,1648,1329,2003,1005,963,1292,1270,1390,1095,953
SYNGR1_8_5818,54,61,178,157,280,139,224,197,156,343,291,147
SYNPR_8_5819,653,1068,602,632,941,880,1487,117,286,310,617,1398
SYPL1_8_5820,319,392,235,294,633,203,1,199,523,163,227,1347
SYT12_8_5821,213,62,349,288,271,315,96,3,293,121,90,509
SYT14_8_5822,3303,2784,2777,2829,2484,3424,3874,4065,5841,2131,3439,26
62
SYT15_8_5823,227,307,311,213,111,239,16,263,203,224,255,1661
SYT1_8_5824,3120,2683,3023,3490,3321,4883,4257,4523,2931,2634,4686,521
1
SYT2_8_5825,9,76,0,199,0,13,0,4,409,0,0,0
SYT3_8_5826,568,419,270,850,222,150,225,43,218,387,536,624
TAPBP_8_5827,406,166,239,639,909,64,860,2163,552,435,484,1
TC2N_8_5828,2126,1769,2264,3005,1356,1504,2348,2125,4206,2394,2276,872

TCN2_8_5829,452,356,229,132,38,293,196,318,213,1,183,565
TCOF1_8_5830,357,265,607,343,228,153,75,19,319,239,869,581
TFPI_8_5831,7718,8715,8303,8115,8662,12843,8599,8727,5752,10776,11137,
10976
TFR2_8_5832,1215,1147,907,1234,2380,1539,773,1101,1522,651,1098,2038
TFRC_8_5833,407,520,282,293,339,284,444,99,258,391,702,1114
TGFB2_8_5834,1969,2088,1670,2331,2688,3569,2359,5275,3178,2617,3195,16
92
TIMM17B_8_5835,200,169,120,360,27,17,556,395,63,358,207,196
TINAGL1_8_5836,615,549,610,725,887,290,976,403,235,961,1005,646
TLL1_8_5837,98,99,296,168,0,203,765,199,8,412,176,7
TM9SF1_8_5838,1546,1859,1297,1728,3882,1391,1445,1235,1943,2386,1446,2
429
TMC6_8_5839,137,39,289,91,10,115,236,641,58,156,562,110
TMPRSS11A_8_5840,819,833,1230,980,761,2020,1328,621,765,1808,1042,1681
TMPRSS13_8_5841,757,511,655,919,33,998,227,431,1017,821,635,2280
TMPRSS2_8_5842,393,695,790,328,210,300,35,678,109,501,874,948
TMPRSS4_8_5843,326,409,271,477,916,10,236,686,17,163,110,978
TNFSF11_8_5844,1274,1585,1020,1178,1254,1851,1898,863,1768,362,1032,14
26
TNFSF13B_8_5845,831,896,778,524,1187,965,782,820,1285,865,374,987
TNPO2_8_5846,263,52,38,31,513,96,3,27,50,22,1082,2
TOM1_8_5847,116,22,36,71,311,3,0,10,0,18,0,0
TOM1L2_8_5848,1536,1147,998,1201,1022,642,737,715,1309,1357,483,619
TSC1_8_5849,387,144,284,247,1154,211,377,666,65,76,446,228
TSC2_8_5850,277,150,98,151,498,427,147,258,0,561,305,1
TUBA8_8_5851,1993,2128,1581,2131,3759,3497,1029,1466,3028,2627,4999,98
8
TUBB3_8_5852,707,572,652,270,517,1867,370,1152,242,947,604,1732
TUBD1_8_5853,981,577,527,787,1169,2176,460,553,592,1904,813,1445
UCP3_8_5854,1033,675,738,585,889,1602,762,806,180,1032,432,1263
UPF3A_8_5855,1246,1369,1051,1961,661,2343,1698,3105,1650,1193,2626,163
3
UPF3B_8_5856,2117,2524,2482,2553,1668,3809,3974,2886,2779,2441,1448,18
30
VAMP1_8_5857,1243,668,590,617,1683,503,249,989,565,553,682,1038
VAMP7_8_5858,805,533,500,556,671,571,658,203,260,406,124,170
VCAM1_8_5859,1121,1283,768,1121,2600,1608,1884,1771,1677,820,458,833
VLDLR_8_5860,282,211,269,100,49,60,822,160,342,1082,31,228
VPS13A_8_5861,2060,1789,1237,1919,2902,1402,911,1668,1749,1230,1357,20
98
VPS13B_8_5862,2913,3254,2423,2779,3215,1389,2530,2208,3582,2845,2462,2
449
VPS16_8_5863,1296,1515,1494,1943,757,1430,925,1252,993,1944,2706,911
VPS26A_8_5864,1316,1251,1772,1382,785,481,931,1247,1791,1306,3881,1822
VPS28_8_5865,253,340,194,331,436,548,1546,150,966,216,253,411
ZFYVE16_8_5866,761,1365,832,1079,712,439,1196,2975,696,762,1243,420
ZNF160_8_5867,6134,6331,6636,6138,8648,5960,9343,3932,9568,6562,7849,6
219
ZP3_8_5868,736,1294,500,837,300,624,133,412,69,1693,403,552

BET1L_8_5869,689,602,652,810,2804,1563,39,884,1192,718,3118,468
C2orf83_8_5870,490,465,345,361,596,251,1249,547,929,116,1073,1266
ERP29_8_5871,1373,1437,829,1288,385,1516,632,467,1453,1052,798,506
FGF1_8_5872,279,346,207,282,420,2,188,678,257,54,928,181
LYNX1_8_5873,861,857,457,853,609,461,370,881,948,1638,538,898
MMP28_8_5874,2090,2055,1896,1668,3362,2621,2200,520,2476,2958,3796,264
6
PDPN_8_5875,876,893,622,944,853,1517,279,1046,452,885,1293,421
SNX21_8_5876,253,237,289,299,237,37,476,118,85,477,171,404
TIMM8A_8_5877,213,85,193,118,49,9,152,29,12,671,51,162
VEGFA_8_5878,112,88,124,230,107,37,5,305,444,15,49,176
CDH23_8_5879,355,323,337,746,184,1822,118,1020,334,401,231,558
CDH23_8_5880,426,474,415,913,1204,2,1125,7,509,502,987,841
CDH23_8_5881,1158,1226,635,1012,1118,1518,2520,1383,1342,1193,785,2461
CDH23_8_5882,385,121,150,256,1221,171,496,248,824,8,601,252
CDH23_8_5883,743,773,634,887,715,248,448,1669,968,411,1209,695
TNXB_8_5884,144,79,135,141,69,120,122,3,443,40,18,574
A2M_8_5885,282,129,94,102,265,68,120,5,38,283,373,187
ACE2_8_5886,2793,2918,1675,2293,3404,2708,2198,3216,2850,2499,2512,322
5
ACTR6_8_5887,5621,5909,5600,5740,5202,7230,5443,5730,6586,5862,7059,86
42
ADAM11_8_5888,2431,2207,1852,2212,3068,3340,2035,1904,2074,1656,1186,2
611
ADAM19_8_5889,1032,773,1071,657,2037,604,1584,1050,250,738,1259,402
ADAM20_8_5890,2548,1945,1684,2066,3668,2695,1929,2351,1642,1443,2035,2
529
ADAM21_8_5891,2313,2116,2190,1917,1965,1069,2751,1329,1231,890,3637,23
47
ADAM2_8_5892,18298,17438,15678,16282,16945,17257,20603,19604,21340,164
89,15611,25153
ADAM30_8_5893,2414,1403,1717,2885,1945,3588,2386,4482,2949,2744,2574,3
773
ADAM7_8_5894,519,1075,780,725,1519,846,1558,1148,510,708,1813,2225
ADAM9_8_5895,640,968,1217,549,527,641,810,797,827,332,1122,1156
ADAMTS10_8_5896,1325,1210,1479,1493,724,1334,2357,2240,2595,1517,2022,
2003
ADAMTS12_8_5897,119,77,113,93,39,62,151,62,7,19,4,60
ADAMTS15_8_5898,194,411,196,131,231,297,736,197,477,160,435,154
ADAMTS18_8_5899,866,773,824,1293,1282,1509,1297,820,515,1376,1869,1686
ADAMTS19_8_5900,4678,4522,4484,5407,2617,4988,6022,5808,4473,3440,8106
,4624
ADAMTS1_8_5901,1081,1057,1028,875,200,1850,825,1482,191,242,876,2707
ADAMTS20_8_5902,1565,1755,1279,1352,1519,2184,2819,1696,2386,2277,1462
,2209
ADAMTS3_8_5903,1232,959,1502,1261,1178,1379,1068,2029,1094,931,774,235
3
ADAMTS5_8_5904,466,448,452,389,295,745,380,220,849,573,334,402
ADAMTS6_8_5905,2215,1750,1274,1588,516,2310,2791,1346,3647,2485,3521,2
005

ADAMTS7_8_5906,1589,982,1289,939,1113,1014,1871,392,4419,1825,416,560
ADAMTS8_8_5907,1567,1490,1183,1001,1939,447,4905,2085,598,2846,1916,11
70
AEBP1_8_5908,468,392,318,386,437,712,763,2212,124,451,610,361
AFG3L2_8_5909,424,292,320,300,368,659,772,115,349,207,579,407
AFM_8_5910,195,294,144,47,95,348,695,126,12,67,100,18
AFP_8_5911,2632,2610,2549,3515,2872,3671,3958,2878,1207,2064,3583,2364
AGTPBP1_8_5912,518,588,307,298,169,788,1157,105,402,416,134,372
ALG10B_8_5913,9083,10079,10108,10453,9272,11240,9054,11254,10967,11356
,11063,12710
AMBP_8_5914,1917,1782,1196,1736,1455,1101,1663,708,3756,2443,2123,1317
ANGPT4_8_5915,970,1120,675,1135,1325,1828,2094,236,1607,1464,949,1000
ANGPTL1_8_5916,507,483,326,421,111,82,75,432,795,492,71,199
ANGPTL2_8_5917,2192,2358,2765,2431,3564,4186,2332,3257,1668,4197,2930,
2583
ANGPTL3_8_5918,3719,4300,3896,3787,3910,3394,4002,5660,3390,4609,4370,
4696
ANGPTL7_8_5919,644,951,652,619,661,1552,573,1816,1259,734,1238,537
ANKH_8_5920,1227,766,1081,988,1877,1582,2313,623,939,1023,1398,801
AP1G2_8_5921,0,0,0,0,0,0,0,0,0,0,0,0
AP1M2_8_5922,322,124,248,146,75,1010,9,1196,25,847,397,454
AP1S1_8_5923,3189,3054,3004,2776,3529,3667,6286,3565,4050,2859,2519,41
01
AP1S2_8_5924,1509,1407,1086,1902,1743,167,2351,1093,1686,1364,1969,784
AP1S3_8_5925,1935,2435,1600,1440,1399,1636,3627,1378,1065,2845,1055,12
42
AP3B1_8_5926,1028,1211,979,781,756,202,831,2292,278,774,1491,1865
AP3B2_8_5927,358,177,270,297,162,62,527,559,169,190,353,635
AP3S1_8_5928,1815,2288,1879,1916,722,3643,733,1887,1778,3033,3250,1390
AP3S2_8_5929,922,1407,1005,578,352,643,1301,395,234,1973,850,508
AP4B1_8_5930,963,628,1288,992,704,921,1278,408,1461,1039,1334,1370
AP4M1_8_5931,953,1018,648,1046,2565,760,219,821,292,551,341,1060
APBA1_8_5932,1373,1415,1122,1189,1521,1254,1139,1619,1462,1702,1898,14
84
APBA3_8_5933,1025,1496,654,1114,1244,1404,1553,401,1009,327,1780,1143
APOA1_8_5934,252,314,192,242,3,40,920,0,481,555,154,326
APOA2_8_5935,943,743,536,902,639,935,705,336,620,1353,1277,654
APOA4_8_5936,370,565,504,268,122,514,631,735,723,431,2212,182
APOB_8_5937,1032,810,782,919,710,1502,467,1371,320,1821,843,1766
APOC1_8_5938,1254,1903,1997,1408,1465,1273,1186,931,3527,1603,1236,114
7
APOC2_8_5939,81,144,76,133,118,176,0,108,0,198,7,58
APOC3_8_5940,148,205,185,454,359,19,1,1,0,0,80,452
APOC4_8_5941,687,902,252,869,631,685,655,650,1141,348,153,388
APOD_8_5942,1578,1960,1957,2288,873,3147,1862,2156,2549,2292,4027,4681
APOE_8_5943,1144,879,1241,548,1009,1848,2065,382,683,709,1329,2633
APOF_8_5944,230,605,145,425,160,280,104,113,542,300,810,455
APOH_8_5945,1309,1095,975,1158,1643,678,626,1181,1632,1492,1592,2409
APOL6_8_5946,670,261,814,274,169,244,1272,1419,718,470,862,435
APOM_8_5947,160,248,316,263,62,20,1791,6,273,399,745,217

AQP12B_8_5948,2701,2526,2236,2495,4089,3060,1498,2544,2964,2941,4483,2
111
ARF5_8_5949,1513,2174,913,1061,955,531,1128,1423,1065,1937,765,563
ARF6_8_5950,219,200,246,150,160,193,138,30,34,274,380,449
ARPP19_8_5951,404,448,287,385,583,672,107,348,293,524,227,1042
ASTL_8_5952,537,803,488,588,1362,1182,153,589,621,1242,587,1100
ATOX1_8_5953,184,126,21,70,0,45,467,36,925,116,25,7
ATP13A1_8_5954,362,514,487,305,196,718,297,276,35,274,275,1151
ATP13A3_8_5955,152,121,89,183,0,0,173,781,393,256,142,44
ATP13A4_8_5956,620,352,241,343,800,1523,66,2,1191,591,211,57
ATP13A5_8_5957,555,488,226,464,66,1214,345,1387,203,524,267,394
AZGP1_8_5958,4335,3202,3906,3859,5395,3367,3324,4231,4575,2609,6012,63
16
AZU1_8_5959,704,370,294,718,1304,207,124,1198,376,285,247,65
BCL2L10_8_5960,2231,1964,1361,1246,4976,3053,2460,701,2019,1266,1625,2
541
BET1_8_5961,934,812,1148,975,1195,445,35,406,1126,777,697,916
BGLAP_8_5962,296,363,268,232,48,99,480,108,1,467,400,281
BOC_8_5963,2319,2375,2175,2590,1629,2702,1338,2134,1428,1244,2392,2693
BPI_8_5964,2573,2540,2698,2756,3638,2111,3762,2896,1445,2255,4733,1635
BPIFC_8_5965,538,598,675,494,344,441,728,622,804,772,216,656
C16orf7_8_5966,59,143,174,222,90,227,1,23,144,177,271,450
C1orf162_8_5967,1158,1350,1193,1129,986,1531,1259,1404,134,967,1940,21
13
C1QA_8_5968,401,94,245,238,337,202,487,556,359,95,617,240
C1QB_8_5969,2169,2362,1816,1746,2224,2661,1558,876,2216,2215,2448,2711
C1RL_8_5970,49,256,58,4,0,0,11,40,0,0,5,93
C20orf141_8_5971,269,224,186,258,0,1069,452,308,750,183,549,102
C3_8_5972,315,154,317,14,1431,42,710,394,438,0,501,40
C4A_8_5973,1259,852,702,547,313,301,782,71,478,453,257,1705
C5_8_5974,1111,1391,648,1314,494,780,908,1334,760,1839,1543,529
C7orf31_8_5975,3431,2898,3242,2707,4438,2266,5279,3404,3857,3399,4489,
2769
C8G_8_5976,173,238,311,169,290,530,134,75,1,149,721,645
CALM2_8_5977,3373,3536,3435,2587,4316,4555,5019,2780,3858,2389,3524,28
46
CALY_8_5978,59,183,359,214,425,120,487,185,40,517,566,23
CAMLG_8_5979,7324,7753,5448,8096,8931,8531,5088,7634,9981,8307,6285,91
54
CAPN11_8_5980,242,450,192,70,518,6,212,33,53,291,346,223
CAPN5_8_5981,923,1007,825,713,786,936,694,472,870,579,2004,385
CAPN6_8_5982,337,734,522,559,1,772,429,295,103,415,249,468
CARTPT_8_5983,735,1202,563,718,1109,897,983,1126,1025,948,843,1528
CCL13_8_5984,1020,1251,1040,993,1160,1103,893,1149,531,2253,933,1792
CCND1_8_5985,1341,2025,1563,1029,1313,2318,2124,1278,152,1211,1275,302
2
CD1A_8_5986,900,1025,752,858,595,664,1075,803,1067,1193,935,338
CD52_8_5987,1645,1084,1103,1043,970,2473,2459,1017,1842,559,1874,1619
CDCP2_8_5988,261,266,362,366,340,0,135,141,0,132,0,633
CDH5_8_5989,1066,1148,1506,1165,1198,1517,550,53,634,1497,1690,1292

CHMP7_8_5990,553,729,489,547,522,289,305,1090,429,1170,341,618
CLDN16_8_5991,758,875,858,732,47,610,806,279,620,726,1558,1252
CLEC3B_8_5992,789,1261,1332,1119,1098,401,1150,776,1074,131,1406,522
CLSTN2_8_5993,59,21,29,74,0,20,134,0,1,5,306,124
CLVS2_8_5994,643,277,249,522,600,24,451,1198,494,595,652,501
CNIH3_8_5995,254,138,104,458,60,328,368,922,520,312,257,46
CNOT6_8_5996,625,945,520,712,462,713,62,1326,589,240,446,1011
CNTNAP1_8_5997,1919,1702,1654,1851,1157,1963,1082,2971,1525,2936,2533,
3228
COG1_8_5998,7595,9146,7118,8451,9667,9521,14560,7877,8751,8658,8614,10
585
COG3_8_5999,683,660,390,558,316,733,89,1555,190,1749,251,656
COG7_8_6000,1866,1535,1429,1134,3472,1541,928,2060,1664,2016,982,3315
COG8_8_6001,495,445,553,795,378,2344,1038,274,396,844,851,2295
COL10A1_8_6002,507,584,310,336,309,436,71,606,759,414,666,380
COL14A1_8_6003,659,762,772,992,1178,366,134,821,913,825,384,75
COL15A1_8_6004,1934,1480,1238,1334,1086,605,488,3033,4836,1633,513,814
COL16A1_8_6005,2454,2623,2236,1831,881,3692,3314,2076,836,1613,4727,22
69
COL17A1_8_6006,1995,1904,1132,1894,904,2226,1102,1671,2469,2252,2570,3
145
COL1A1_8_6007,253,127,301,257,52,973,345,45,11,217,195,103
COL1A2_8_6008,280,563,168,288,254,231,1142,784,894,816,917,213
COL21A1_8_6009,1599,1679,1677,1428,1836,2212,1137,2646,625,2552,669,14
16
COL22A1_8_6010,1054,486,417,814,923,230,1169,1548,805,985,128,1045
COL23A1_8_6011,1074,1077,695,893,622,766,308,760,950,1138,654,503
COL24A1_8_6012,1656,1531,1166,1645,358,2357,1105,954,959,1721,1471,105
7
COL27A1_8_6013,529,758,841,526,1713,0,36,423,4,919,112,748
COL3A1_8_6014,748,467,605,499,1766,723,738,9,722,476,1507,12
COL4A1_8_6015,584,378,583,782,771,440,136,228,291,748,697,349
COL4A2_8_6016,619,325,372,524,671,176,990,455,497,471,384,144
COL4A3_8_6017,2988,2802,2105,2457,3572,4341,1856,2679,3867,3315,2396,3
961
COL4A4_8_6018,1862,2149,2143,1502,1977,1921,1291,1727,4037,2526,1908,1
361
COL5A1_8_6019,873,807,612,913,17,1751,640,1530,578,570,2064,1531
COL5A2_8_6020,1335,817,1214,1020,2289,854,1835,647,1980,935,1430,915
COL5A3_8_6021,1056,729,466,785,618,958,1462,785,1037,1207,549,835
COL6A1_8_6022,736,526,378,505,960,814,277,905,614,468,384,319
COL7A1_8_6023,37,142,156,187,52,0,3,1,161,12,5,1311
COL8A2_8_6024,249,578,164,335,49,0,99,7,0,503,72,3
COL9A2_8_6025,514,958,425,516,125,383,1010,827,420,1083,686,535
COL9A3_8_6026,1645,1466,1293,2117,1719,2126,2579,1753,1534,1755,1641,1
593
COMMD1_8_6027,3197,3066,2339,3031,2830,2327,1803,2783,1771,2223,4093,2
993
COPB2_8_6028,12640,13697,12276,12697,13928,16757,12710,11871,13648,125
23,14120,17387

COPG2_8_6029,860,758,465,603,820,365,296,1308,474,1227,756,1424
COPZ1_8_6030,1285,1082,1082,781,777,864,379,1118,1095,1313,47,1672
COPZ2_8_6031,1912,1990,1447,1659,2858,898,2257,2346,1163,4118,2871,293
5
CORIN_8_6032,2057,2201,2034,1947,1302,1604,1767,1285,3235,2888,1776,15
15
COX18_8_6033,4148,2779,3626,3493,4226,3215,7409,4280,2616,3166,4365,44
34
CPLX1_8_6034,229,135,225,316,38,41,204,107,356,113,262,516
CPLX3_8_6035,501,203,345,422,593,510,174,109,889,116,446,630
CPNE6_8_6036,1026,1078,1309,1226,345,156,1977,475,1120,1359,1073,518
CPXM2_8_6037,947,1387,782,666,1831,1030,883,1259,985,2134,309,515
CRABP1_8_6038,2671,2713,1796,2579,1803,1751,2084,2688,3086,4224,3665,1
271
CRH_8_6039,557,382,373,419,1703,552,520,362,546,387,710,191
CSE1L_8_6040,1926,1477,1292,1785,3099,1824,2647,1130,2587,1145,1434,28
54
CTSW_8_6041,1083,1396,1132,724,1883,727,310,1444,1225,1184,1225,3328
CXCL10_8_6042,588,713,483,622,908,351,685,202,1120,488,293,618
CYGB_8_6043,363,354,211,310,258,360,26,98,239,756,239,103
CYTH3_8_6044,2082,2197,1695,2153,1457,1729,1104,1045,2031,2635,1788,21
89
DDI2_8_6045,1879,2034,1444,1981,2656,2524,1437,1778,2354,1578,2893,679
DIRC2_8_6046,4986,4125,4140,4333,3775,4038,6407,3849,6199,4885,4262,48
38
DISP1_8_6047,1506,1901,1090,1203,759,1607,242,1998,1405,1389,3050,1200
DLL4_8_6048,309,199,181,476,100,138,209,784,22,485,166,418
DNAJC5B_8_6049,572,416,829,484,437,1055,786,275,808,796,756,285
DNAJC6_8_6050,2694,2378,2573,2036,2238,1730,2406,1553,1475,1434,2441,3
303
DOC2A_8_6051,571,699,375,660,134,729,16,94,1440,372,202,1668
DOC2B_8_6052,1445,1056,1030,855,695,1160,1796,2186,772,601,1155,1300
DSCAML1_8_6053,555,386,347,345,1525,449,1584,618,417,344,702,773
ECEL1_8_6054,699,615,641,761,213,256,576,1168,509,1208,939,759
EID2_8_6055,620,992,677,580,1248,1328,1047,1097,500,702,1417,1225
EPCAM_8_6056,4751,4731,4503,4920,2817,6157,3822,4610,3764,7276,5365,67
83
EXOC2_8_6057,1566,1810,1246,1544,3028,2244,352,1943,1332,1473,870,1081
EXOC3_8_6058,707,391,459,838,1202,160,278,690,782,90,270,1272
F11R_8_6059,67,33,81,262,25,175,162,0,105,161,0,37
FABP1_8_6060,1303,1025,847,830,450,1213,1681,783,1418,1112,1269,489
FABP2_8_6061,1964,2752,1765,1923,883,4401,2212,2274,1510,2549,1986,168
9
FABP3_8_6062,2178,2880,2256,1836,1521,1962,1534,1840,1785,2494,4860,25
09
FABP4_8_6063,1150,1132,1111,810,867,629,1426,1030,1459,483,1130,1125
FABP7_8_6064,1922,1826,1779,1789,1783,2761,2017,1703,3658,1638,2143,21
59
FABP9_8_6065,698,771,829,622,1671,2389,219,1130,319,1161,802,1023
FAM101A_8_6066,700,796,267,668,87,567,502,76,212,57,731,296

FAM117A_8_6067,568,518,526,681,715,478,426,1939,759,1076,1593,475
FAM57A_8_6068,1363,1187,981,1503,1009,1652,540,823,2410,1231,736,2221
FAP_8_6069,5135,5006,5391,5185,5943,5686,7764,4811,7202,5131,8887,4568
FBF1_8_6070,65,182,399,414,276,1266,168,171,218,484,46,394
FCN1_8_6071,1823,1566,2516,2143,1636,1423,3088,2966,1906,2259,2937,162
3
FDX1_8_6072,1098,886,997,1263,1376,1619,285,1631,1000,1223,1669,511
FDX1L_8_6073,447,614,487,567,1662,1376,471,478,456,603,285,1291
FGF4_8_6074,279,189,143,316,695,313,607,24,272,0,0,0
FGL2_8_6075,1411,775,802,759,900,595,1060,850,1100,1469,1896,1550
FOLR3_8_6076,297,308,555,296,63,103,111,1418,487,7,1460,691
FOLR4_8_6077,391,758,268,397,239,940,620,303,703,598,801,701
FRG1_8_6078,5539,6767,5422,7515,6036,7680,4702,6461,6324,6506,6591,640
9
FTL_8_6079,1348,970,659,1467,389,1504,1245,909,1369,2283,484,1036
FXC1_8_6080,2099,2372,2177,2371,1481,3259,2655,3545,2159,2639,4307,436
0
GABARAP_8_6081,2377,2673,2090,2197,2724,4817,1457,1704,1757,1118,3524,
2754
GGA2_8_6082,714,523,549,485,79,224,1299,746,35,403,1,456
GJA1_8_6083,1007,574,693,485,161,725,984,236,786,1175,736,1711
GJA3_8_6084,458,102,249,558,1283,37,1027,13,28,84,361,554
GJA4_8_6085,203,126,136,84,5,158,93,185,54,38,272,0
GJA8_8_6086,2838,2838,2394,2661,2661,3708,3085,2857,5041,3893,5420,276
3
GJB2_8_6087,323,184,480,143,262,86,34,222,92,243,56,507
GJB4_8_6088,107,318,78,377,16,461,39,0,111,144,820,760
GJB5_8_6089,1994,1536,1082,1240,1342,1084,385,1713,1394,1413,2312,1445
GJC2_8_6090,131,328,137,34,989,223,6,36,0,355,35,0
GJC3_8_6091,0,0,0,46,0,0,0,0,0,0,0,0
GJD2_8_6092,1048,1923,713,909,1020,1007,2596,603,852,1332,1382,1724
GJD3_8_6093,359,275,239,271,765,392,1549,468,107,122,0,582
GJD4_8_6094,420,223,504,182,106,206,1351,203,343,1190,82,67
GKN1_8_6095,2489,2105,1531,2332,881,1309,2842,4056,2922,1568,2500,3625
GLCCI1_8_6096,908,1224,885,1148,611,903,28,558,832,2267,1607,493
GLTP_8_6097,592,700,747,624,655,477,397,656,660,554,1242,465
GLYATL2_8_6098,1002,1118,1107,1223,682,1359,1326,1555,757,1461,1316,57
5
GP9_8_6099,161,148,112,127,26,20,0,29,0,98,5,488
GPIHBP1_8_6100,2225,1770,1772,2081,1985,2727,2462,1646,2494,1743,1512,
3109
GPR180_8_6101,549,1122,735,814,919,1195,1062,1305,1014,1383,882,450
GRN_8_6102,158,99,57,169,0,43,55,5,280,93,32,25
GZMH_8_6103,412,381,239,447,2162,190,394,106,562,524,422,1112
GZMK_8_6104,1045,938,838,547,649,1000,411,497,980,939,1282,34
GZMM_8_6105,950,967,779,846,1178,703,1443,1235,727,289,471,62
HBA1_8_6106,669,724,504,388,697,504,662,1884,1646,1256,551,1185
HBA2_8_6107,669,724,504,388,697,504,662,1884,1646,1256,551,1185
HBE1_8_6108,389,422,335,206,356,807,536,14,15,356,1171,186
HBZ_8_6109,438,554,489,741,640,88,586,1199,1264,241,370,1812

HECA_8_6110,119,110,230,7,104,0,0,0,1,249,4,0
HGFAC_8_6111,1039,883,422,908,2279,917,728,709,1137,1393,383,671
HIAT1_8_6112,3376,2975,2994,4331,2085,4128,3291,1897,1682,2233,6842,27
92
HLA-DQB1_8_6113,721,806,706,376,272,689,716,1817,560,1055,278,961
HMCN1_8_6114,886,1327,901,1090,688,1103,589,1300,914,1282,746,1655
HMHA1_8_6115,1452,1553,884,1572,1778,1034,443,1809,2316,1431,1929,1112
HPCAL4_8_6116,655,136,546,285,28,263,2372,612,93,619,235,2695
HPR_8_6117,802,1152,618,1006,1796,1885,1199,2113,2253,1489,510,1587
HPX_8_6118,321,355,116,136,1201,174,66,19,474,60,68,338
HSP90B1_8_6119,538,369,463,538,243,340,126,763,525,202,218,1520
HTRA1_8_6120,629,797,830,740,765,391,998,729,594,912,1060,387
HTRA4_8_6121,796,1102,774,1424,110,269,1290,361,1303,845,253,1791
IFNG_8_6122,7137,8305,7073,6460,7764,8813,9068,7119,9827,4794,11209,82
06
IGFBP7_8_6123,509,724,533,228,536,110,58,169,401,1103,1345,709
IL12B_8_6124,1149,1547,1206,1433,499,1338,2331,916,622,2116,1743,1249
IL13_8_6125,417,349,326,891,1033,968,266,597,737,244,1013,182
IL17A_8_6126,535,457,651,313,794,1611,661,449,579,560,477,181
IL1A_8_6127,2717,3996,2472,3148,2048,1512,1353,3771,2490,2037,4505,420
0
IL1B_8_6128,2401,2727,2318,2669,1343,2852,1996,2614,2439,3392,1924,420
8
IL3_8_6129,552,616,409,784,451,81,719,594,313,804,275,990
IL5_8_6130,643,518,490,251,409,557,2127,910,441,211,1176,1341
INSL3_8_6131,1082,1396,765,1406,1005,1046,1175,1670,859,1390,2294,2029
IPO13_8_6132,3245,3155,2735,3066,2762,4116,3900,2843,2045,2986,2564,42
30
IPO4_8_6133,978,731,975,826,524,2702,387,2141,2171,740,366,588
IPO5_8_6134,2157,1670,1265,1547,2395,1705,1261,2319,961,1012,1568,1334
IPO7_8_6135,1100,1287,910,1360,56,855,1373,1665,1479,1374,299,1875
IPO9_8_6136,1096,920,628,818,349,1298,720,1256,834,33,1753,2261
ITGA10_8_6137,420,377,141,475,233,391,257,47,1020,1007,199,1188
ITGA11_8_6138,484,686,536,543,1007,1506,682,16,654,59,26,912
ITGA2_8_6139,2289,1978,1750,1831,2795,2228,3089,2499,3799,764,2059,175
0
ITGA4_8_6140,1560,1387,1410,2181,1106,2765,1983,2449,2515,1788,2890,23
12
ITGA5_8_6141,1779,1412,1573,1706,1789,1630,1031,1550,1186,595,2416,226
6
ITGA8_8_6142,237,477,427,334,504,967,136,197,127,149,1195,302
ITGAX_8_6143,812,764,375,557,1459,2726,525,1029,75,1015,861,918
ITGB5_8_6144,232,59,206,179,719,49,1029,314,1176,1,583,5
ITGB6_8_6145,1161,1399,731,859,1623,2695,1236,1261,991,1439,1034,1011
ITGB8_8_6146,1322,919,1913,1381,1765,1169,1641,2437,479,469,1107,2848
ITLN1_8_6147,2340,2471,1535,2778,2755,1546,2290,1884,2947,2471,3157,28
06
KDELR1_8_6148,154,45,101,113,170,17,11,3,485,31,133,144
KEL_8_6149,1440,1692,1614,2285,1324,2252,2734,1519,804,1450,2997,2370
KIF20A_8_6150,1143,450,861,777,758,1567,677,789,189,174,1631,1296

KIF3B_8_6151,186,88,169,100,10,51,191,228,125,62,5,17
KIF5A_8_6152,2633,2621,1376,1945,2998,3122,3744,2719,2812,2163,1745,30
35
KLK13_8_6153,4004,4180,4180,5750,3130,4691,4380,5782,4865,3699,4460,44
78
KLK14_8_6154,884,639,501,722,1128,235,1590,689,704,987,1680,1545
KLK4_8_6155,355,383,328,612,49,45,112,92,1455,28,1012,54
KLK9_8_6156,1194,1283,1008,624,1241,851,1665,919,1300,1404,1005,586
KPNA1_8_6157,1075,1224,1294,1259,1042,813,1318,2500,2071,2336,858,1636
KPNA2_8_6158,1073,1249,656,1480,1479,799,863,1194,1758,626,2050,268
KPNA3_8_6159,422,371,241,268,249,606,38,766,121,815,177,1030
KPNA4_8_6160,713,909,826,646,1454,583,1441,707,719,559,2247,671
KPNA6_8_6161,182,281,289,182,1,459,139,229,6,603,0,758
KPNB1_8_6162,2478,3412,2736,2755,2236,1804,2622,5608,3770,3195,4464,14
54
KRT12_8_6163,512,458,349,415,41,1317,516,144,103,887,586,10
KRT7_8_6164,250,637,419,128,312,595,1059,952,412,559,542,6
KRT8_8_6165,558,439,917,441,113,400,470,916,19,343,140,144
KRTAP5-4_8_6166,712,1212,736,510,738,1530,1135,651,867,694,678,770
LASP1_8_6167,105,47,169,42,1,1348,0,1090,43,0,66,5
LBP_8_6168,1061,1619,1265,937,205,697,563,1147,784,1064,788,2276
LCN12_8_6169,484,578,648,444,42,185,592,115,323,372,1363,561
LCN1_8_6170,453,1011,446,796,898,966,822,238,189,999,482,499
LCN2_8_6171,414,265,260,494,324,371,161,418,477,280,940,450
LCN8_8_6172,382,486,458,368,290,596,848,756,708,689,432,498
LCN9_8_6173,317,380,371,220,102,279,92,1463,624,486,279,608
LDLRAD2_8_6174,267,25,120,87,0,139,0,370,1,247,9,3
LDLRAP1_8_6175,621,644,278,985,10,432,1431,285,462,551,401,934
LMAN1_8_6176,3619,3449,2825,2651,2547,3143,3432,4844,1820,4712,4201,24
11
LMAN2_8_6177,9535,9406,9047,8564,10672,9386,10539,6413,9597,8020,11883
,9280
LPA_8_6178,430,315,1031,331,834,984,233,176,394,418,908,952
LRIG1_8_6179,329,194,149,451,640,5,17,349,80,145,1334,322
LRP2_8_6180,798,1444,855,976,847,2351,1118,643,649,657,1764,863
LRRC4B_8_6181,164,106,70,111,574,45,238,0,11,0,466,464
LRRCC1_8_6182,1541,1317,1104,1800,1177,809,1073,1085,2326,1247,523,156
9
LYST_8_6183,646,1112,222,580,151,610,1455,221,293,882,622,609
MAL2_8_6184,1162,1043,1263,877,1285,1953,2066,1478,1020,1023,1214,421
MATN3_8_6185,4861,4942,4820,3851,4199,6262,3316,4428,4174,6319,5513,26
03
MFSD8_8_6186,242,433,395,266,2,344,235,275,104,717,523,362
MFSD9_8_6187,3071,2501,2044,2370,3093,4967,1808,4826,4076,3080,3626,29
19
MMAA_8_6188,1543,1703,962,1503,1321,2552,1656,1796,1600,2221,1581,1523
MMACHC_8_6189,703,851,750,599,273,300,656,2162,69,774,625,80
MMGT1_8_6190,1140,1376,1388,1116,1496,1583,1196,1384,1315,1364,2155,39
29
MMP11_8_6191,205,284,141,72,2,46,11,935,0,130,2,179

MMP13_8_6192,671,927,475,816,595,1279,1031,2230,778,99,1302,1972
MMP15_8_6193,524,636,460,715,1015,1014,257,359,348,576,400,759
MMP16_8_6194,342,422,297,695,207,140,506,467,154,71,169,62
MMP17_8_6195,977,935,753,473,1504,501,244,572,61,558,417,1473
MMP19_8_6196,2897,2380,2433,3264,3108,5631,4374,1802,1505,2871,3181,42
01
MMP24_8_6197,288,236,225,60,310,110,9,254,3,90,33,568
MMP25_8_6198,1753,1025,1450,1868,1870,943,1408,1169,1098,731,1674,1884
MMP26_8_6199,546,526,680,601,2539,869,146,478,672,1026,772,1045
MMP27_8_6200,1234,1297,1268,1287,936,1351,778,964,1237,1418,1479,2291
MRS2_8_6201,1816,1835,1577,1747,3232,735,1351,1874,1601,502,1302,1850
MSTN_8_6202,796,350,672,286,56,740,297,81,1212,446,603,153
MTX2_8_6203,2414,1595,1883,1755,1957,3194,2080,3999,1166,381,1175,2625
MUC2_8_6204,1374,1207,936,978,401,2698,962,1275,1243,1365,1603,1141
NAPG_8_6205,2986,2512,2942,3254,4010,2169,5038,4450,3934,2030,3521,449
5
NAPSA_8_6206,440,307,542,350,598,21,5,638,440,307,349,25
NCOA5_8_6207,1145,1584,1073,1771,2800,1628,3710,587,1398,1452,2167,184
7
NGF_8_6208,5508,5314,4721,5412,4319,5035,5989,6833,5493,5766,6216,8478
NID1_8_6209,320,265,192,95,194,187,48,459,250,496,112,118
NPC1_8_6210,393,333,365,402,75,1116,531,532,253,844,339,599
NPEPPS_8_6211,206,99,376,260,497,926,133,192,681,16,1600,42
NPPB_8_6212,619,527,883,392,195,645,109,297,300,158,680,783
NPY_8_6213,1860,1557,2017,1247,1140,2872,1048,1319,1191,2320,700,2372
NSMCE1_8_6214,726,786,906,1166,777,919,904,519,261,511,1943,1331
NUP107_8_6215,1066,1344,931,1182,2167,985,832,521,1077,821,990,1146
NUP133_8_6216,3863,3943,3283,3460,3760,4236,4066,7368,3673,4760,5883,6
535
NUP153_8_6217,1070,1130,900,905,1514,935,1173,2722,1844,674,631,1602
NUP160_8_6218,1418,1236,1439,1417,1122,843,731,328,1525,791,2133,2107
NUP210_8_6219,1318,766,1000,875,2437,1667,1050,1851,1096,954,1163,755
NUP214_8_6220,3957,3892,4173,3303,4010,3928,4620,4794,4225,4443,4054,3
760
NUP35_8_6221,1916,1863,1246,1800,2274,2155,667,2503,1528,1773,1738,277
4
NUP37_8_6222,4518,4241,4581,4676,1199,3847,4288,5151,5651,5072,4479,50
37
NUP54_8_6223,911,1478,1499,763,774,1696,2209,778,1069,1389,945,1192
NUP88_8_6224,218,498,394,400,658,717,35,440,242,1388,484,0
NUPL2_8_6225,1650,2201,992,1178,2676,1398,2422,1760,1036,2181,2705,182
6
NUTF2_8_6226,2603,2247,2407,2812,950,2768,2693,1439,1973,5770,3931,386
8
NXF2B_8_6227,8068,8271,7934,8394,9215,12270,8043,9787,8196,11596,10353
,10642
NXF2_8_6228,8068,8271,7934,8394,9215,12270,8043,9787,8196,11596,10353,
10642
NXF3_8_6229,632,823,749,567,554,288,157,1638,482,635,647,2000
NXT1_8_6230,3410,3802,3003,3323,1609,6045,2913,4102,3520,3739,3738,224

6
OAZ2_8_6231,914,892,649,1100,649,385,2078,121,1067,715,1433,1210
OBP2A_8_6232,3115,2370,2389,3366,2007,4490,5100,5067,3591,2714,4244,20
60
OBP2B_8_6233,716,320,546,560,521,184,883,763,597,988,953,984
OCA2_8_6234,1293,1817,1884,1401,1061,1553,1516,1233,963,2209,3084,470
OGFOD1_8_6235,1047,1044,652,863,1022,1404,884,1841,886,553,446,1461
OGFOD2_8_6236,272,492,470,384,596,1014,409,56,105,342,413,625
OGFR_8_6237,218,380,227,252,758,1107,184,49,183,323,773,8
OVCH1_8_6238,816,690,345,1137,615,1413,777,270,1096,1538,744,1119
OVCH2_8_6239,6653,5648,5586,7023,5687,8240,6476,6505,4714,7056,7155,64
99
OXNAD1_8_6240,382,579,268,203,464,113,0,973,380,205,492,899
OXT_8_6241,469,528,798,747,1400,872,468,979,437,324,910,583
PANX1_8_6242,596,787,470,947,953,516,317,393,1134,792,1176,1635
PAQR7_8_6243,653,585,387,388,535,696,20,590,549,766,364,282
PCDHB11_8_6244,411,584,673,367,777,76,1962,840,258,187,554,316
PCDHB16_8_6245,337,521,161,238,5,854,751,349,938,361,853,105
PCSK4_8_6246,813,1161,1175,1538,511,2115,2579,1071,990,2162,1166,739
PCSK7_8_6247,539,280,356,371,467,542,150,66,117,1416,307,847
PEA15_8_6248,493,590,478,545,875,642,859,1447,261,539,593,1734
PET112_8_6249,1404,1078,1091,854,2080,940,1316,1305,1890,1416,1362,188
8
PEX13_8_6250,3770,3569,2897,3988,2279,3914,3055,4098,2779,4625,3136,42
66
PEX7_8_6251,1945,939,1802,1857,1741,2177,2306,1745,1408,2239,2144,1445
PF4_8_6252,540,485,692,442,31,779,2091,277,1020,648,904,18
PFN3_8_6253,94,683,42,355,415,311,5,0,482,14,0,588
PHEX_8_6254,2338,1902,2350,1356,1984,1836,2011,1060,2763,2354,1039,821
PIGR_8_6255,939,859,914,540,247,499,790,1356,795,1128,815,1861
PITPNA_8_6256,2698,2572,1792,2148,2117,1662,1312,2612,2779,3408,4502,3
847
PITPNB_8_6257,812,688,376,753,2441,1451,1079,1311,294,207,147,380
PLLP_8_6258,112,110,359,323,0,730,387,168,752,316,616,1
PLP2_8_6259,905,786,583,875,782,61,979,550,1978,895,1078,1459
PLXNB2_8_6260,429,546,675,738,598,1087,1463,272,226,363,615,242
PNMA2_8_6261,1365,978,1159,1121,1022,1127,1549,877,1817,1781,2319,2401
PPP1R14A_8_6262,673,204,525,246,1271,548,82,963,58,943,37,225
PPP1R14C_8_6263,615,957,516,389,94,731,334,68,1282,1211,1035,478
PPP1R15A_8_6264,981,1033,861,1478,773,1245,925,192,1763,1097,559,1555
PPRC1_8_6265,224,174,139,261,481,359,287,54,252,223,10,31
PPY_8_6266,854,793,935,1180,115,795,1407,1766,827,2772,560,246
PRB3_8_6267,1901,1616,1407,1656,872,773,1719,1012,1472,1091,1317,1160
PROCR_8_6268,5,0,98,68,1,0,0,0,0,0,0,0
PROS1_8_6269,206,415,153,483,808,349,0,364,0,321,631,682
PROZ_8_6270,586,550,346,854,217,718,0,456,804,404,673,1401
PRPF18_8_6271,831,820,654,728,659,727,1936,757,936,1146,1261,762
PRSS12_8_6272,2123,2718,2269,1937,3878,3183,2518,2572,3190,3272,1870,4
019
PRSS22_8_6273,1397,1726,1707,1771,1429,2205,3521,1608,1304,1117,2058,3

429
PRSS27_8_6274,1175,715,1421,1307,288,574,1258,2088,735,457,895,1809
PRSS33_8_6275,496,939,490,441,752,1410,195,631,485,936,358,174
PRSS36_8_6276,176,173,157,402,5,381,125,120,423,197,20,211
PRSS8_8_6277,757,840,803,962,1022,606,646,115,403,1288,492,1229
PSCA_8_6278,994,1469,881,1145,1066,1137,1230,620,362,1374,2231,1691
PTOV1_8_6279,133,287,315,181,11,505,297,9,574,1529,174,14
RABIF_8_6280,8770,8759,8709,9265,7604,11062,9744,12210,7091,8639,8415,
16724
RAMP1_8_6281,255,293,238,241,0,15,509,249,541,552,61,761
RAMP2_8_6282,993,842,1096,1146,1590,1207,1106,568,598,333,809,878
RAMP3_8_6283,1343,1759,1058,1474,797,2549,2930,2756,1685,1308,914,1643
RANBP17_8_6284,10177,9498,8643,9406,8525,7036,11186,8567,9833,9579,120
05,11313
RASSF9_8_6285,682,556,542,444,482,1161,644,299,457,520,886,1089
REEP5_8_6286,7193,7168,5189,7864,6784,5827,8683,10978,8071,9444,6171,1
0976
RHAG_8_6287,491,610,249,327,71,408,0,726,1049,282,309,1008
RLBP1_8_6288,1404,1705,1435,1016,1430,1992,2024,689,1362,2175,2026,179
3
RPL15_8_6289,1411,1632,1374,1784,1704,1409,2181,1408,1499,2015,3192,29
80
S100A12_8_6290,691,726,1052,905,708,856,1537,743,788,1438,1269,1681
S100A1_8_6291,668,597,650,958,2652,963,556,1029,336,472,706,3865
S100A2_8_6292,185,116,117,137,0,21,0,5,177,291,336,99
S100A3_8_6293,1310,1399,1105,1716,653,852,1467,1698,679,2133,2706,1193
S100A6_8_6294,767,1463,923,732,1434,2495,1271,1429,533,617,2041,1127
S100B_8_6295,3337,4413,3866,4107,3426,4442,2702,3326,4372,4010,4097,31
02
S100P_8_6296,441,101,161,270,192,19,0,65,50,399,134,207
SAA4_8_6297,250,361,285,373,5,440,59,0,574,174,143,1
SCAMP1_8_6298,277,518,468,654,1655,701,622,61,159,396,615,84
SCAMP2_8_6299,4588,4076,4368,4532,3273,3847,5272,4030,3643,5803,6416,4
328
SCFD2_8_6300,181,342,325,394,6,1006,260,666,127,42,319,1
SCLT1_8_6301,1315,1316,1414,642,1102,1309,1274,1531,798,849,591,749
SCPEP1_8_6302,884,516,1131,729,971,1302,1404,427,570,980,3,804
SDC2_8_6303,1934,1106,1713,1533,2894,1713,1339,3607,1597,464,2013,2883
SEC14L3_8_6304,474,826,695,689,132,1236,440,335,1118,268,701,1651
SEC22A_8_6305,617,636,698,581,874,1476,1589,1348,214,1475,282,191
SEC23A_8_6306,2341,2183,1696,2324,792,3404,3868,2445,2990,2258,3267,11
76
SEC24A_8_6307,223,391,127,267,382,173,678,654,28,287,697,608
SEC24D_8_6308,2897,3853,3004,3462,3407,2859,6870,5599,3187,3899,2796,4
232
SEC61A1_8_6309,1112,883,986,612,2248,892,990,740,1280,1453,1186,345
SEC61B_8_6310,191,132,180,112,24,435,1287,20,421,21,80,692
SEC62_8_6311,1122,1143,914,917,250,1294,2207,733,1566,1647,1486,3867
SEC63_8_6312,3606,4431,3360,4159,4095,4088,6716,4509,4623,6265,6294,38
25

SELP_8_6313,900,348,240,343,115,565,246,111,933,191,216,535
SERINC1_8_6314,840,1165,810,1062,674,1480,1942,671,809,1267,684,709
SERPINA11_8_6315,3544,3150,2328,3829,1694,3174,2507,1865,3373,2151,385
4,3659
SERPINA12_8_6316,872,451,607,961,1153,947,710,924,1151,1240,832,687
SERPINA3_8_6317,1178,1276,1118,1253,1089,959,1173,530,317,649,1814,663
SERPINA4_8_6318,173,55,325,28,346,187,20,36,151,33,4,566
SERPINA5_8_6319,577,943,1111,1044,461,1016,241,509,708,1001,763,1166
SERPINA7_8_6320,1864,1481,1558,1356,1151,1739,1323,1771,1330,2654,3875
,985
SERPINB10_8_6321,910,884,772,1252,1376,1741,760,883,783,1609,2114,709
SERPINB12_8_6322,913,334,715,911,1376,378,964,1718,1883,780,698,721
SERPINB13_8_6323,2146,1748,1761,2689,1226,3883,3157,969,1137,1264,2244
,3494
SERPINB1_8_6324,3408,3881,3396,4045,4116,5720,4324,3166,2932,4952,3351
,4275
SERPINB4_8_6325,1707,1565,1163,1695,4666,2909,1654,1023,1129,1514,1510
,671
SERPINB9_8_6326,523,398,395,433,450,479,455,89,1613,380,254,729
SERPINC1_8_6327,1219,1341,863,722,1501,848,3034,349,1980,1365,1297,976
SERPIND1_8_6328,1203,1466,1667,1667,1075,2251,670,1302,1845,1706,1501,
1230
SERPINF1_8_6329,713,540,458,198,61,605,37,812,712,1226,150,155
SFXN1_8_6330,5415,5731,6345,5898,6797,5397,5653,5804,3424,7511,5844,49
46
SFXN2_8_6331,785,1087,837,808,658,1246,183,551,338,1939,404,477
SFXN3_8_6332,1108,997,1071,1387,887,598,676,1125,747,1557,1943,1217
SFXN4_8_6333,1012,869,738,1010,624,1076,1179,583,1320,1270,1145,2580
SFXN5_8_6334,799,908,1028,884,1257,1161,1200,955,1200,1287,2098,1636
SLC15A5_8_6335,4194,3938,3509,3709,2963,5119,4237,1979,3356,3889,3458,
6070
SLC16A13_8_6336,980,1243,742,1443,1271,1107,2031,891,1232,1424,2142,21
25
SLC16A14_8_6337,73,114,299,373,23,207,61,1012,117,135,429,290
SLC16A9_8_6338,203,493,213,244,25,210,134,213,373,294,472,109
SLC17A9_8_6339,235,131,37,110,16,336,1,485,297,273,12,226
SLC22A20_8_6340,1509,1231,1531,1586,2145,1584,1599,2607,773,1752,2094,
1110
SLC22A24_8_6341,4397,4125,3385,3520,3934,3348,6391,2849,6498,3712,2932
,6194
SLC22A25_8_6342,941,1194,894,1368,1445,750,1606,642,1084,1514,963,1149
SLC25A23_8_6343,3334,3013,2532,3136,2343,4529,4239,3270,2689,2327,3313
,2763
SLC25A29_8_6344,197,218,619,107,399,749,282,2,269,33,834,4
SLC25A31_8_6345,4113,4939,4569,4377,7188,6232,5339,2977,3928,5454,5564
,5821
SLC25A38_8_6346,812,1083,1106,820,1325,1192,1478,1540,980,1067,1833,22
51
SLC25A42_8_6347,906,766,459,647,1093,917,1079,559,37,354,309,1112
SLC25A46_8_6348,3320,3320,2853,2829,4387,3824,3449,1649,3323,4274,3970

,3018
SLC25A48_8_6349,270,646,665,556,6,838,112,921,552,1100,615,1022
SLC38A7_8_6350,466,541,616,691,792,56,251,145,654,162,765,985
SLC38A9_8_6351,1410,1118,1214,994,2975,1975,1318,136,1398,1182,1306,25
81
SLC41A2_8_6352,5040,5428,5651,5072,4536,9326,6222,6712,6049,6650,3851,
3817
SLC45A1_8_6353,526,388,552,329,18,263,2138,0,643,1078,0,41
SLC45A3_8_6354,765,824,566,1224,1223,649,297,1440,2533,1415,830,2909
SLC47A1_8_6355,785,776,865,838,477,511,181,1135,892,734,1204,1201
SLC48A1_8_6356,1692,1172,2062,1573,3849,1325,1843,1877,1295,1396,1218,
1989
SLC5A12_8_6357,694,554,524,845,759,456,1492,230,2254,346,409,1019
SLC6A17_8_6358,1535,1654,1446,1474,1199,1903,1425,2129,2709,1381,2273,
2762
SLC7A14_8_6359,805,692,384,869,684,915,100,2,170,59,462,186
SLIT2_8_6360,966,1297,924,1091,1994,412,2776,472,1698,1402,2651,1459
SNAP29_8_6361,2012,1794,2085,2666,3214,1704,4113,1733,1390,2660,1698,3
601
SNX12_8_6362,901,628,1490,1040,827,219,307,443,803,260,1575,1252
SNX13_8_6363,4747,4751,4213,4729,5656,8424,6208,3905,4375,6164,4636,68
38
SNX17_8_6364,2598,3188,2209,2707,3655,5533,2137,1864,4360,2639,5388,24
79
SNX19_8_6365,1413,902,1075,1330,1552,3229,2193,1371,1051,1869,1252,239
3
SNX22_8_6366,820,535,904,990,677,999,686,708,385,1068,860,914
SNX24_8_6367,894,1510,1157,1189,113,1103,955,1838,1220,750,1208,1593
SNX2_8_6368,2223,2411,2309,1928,2563,3789,1488,299,1884,1973,3465,2515
SNX4_8_6369,993,690,431,644,581,37,929,736,957,656,52,220
SNX8_8_6370,3007,2074,2439,2254,2980,2940,1721,1161,1661,1618,3059,248
4
SNX9_8_6371,1587,1549,1661,1077,347,1638,1800,1817,1742,2511,3318,1545
SORCS2_8_6372,220,406,561,283,3,506,574,147,1505,671,34,85
SORCS3_8_6373,6056,5240,4015,6072,5180,6781,7039,7940,4641,6439,7664,7
006
SORL1_8_6374,356,437,501,502,231,164,477,541,441,877,128,262
SOS1_8_6375,227,316,153,119,226,86,5,40,1,28,393,2
SST_8_6376,314,663,174,471,45,517,26,896,54,1132,227,360
ST13_8_6377,3884,4184,4022,3956,3972,2783,2726,2322,3539,4354,5824,879
4
STAB1_8_6378,3150,3832,2506,4289,1338,3476,2658,4182,3477,6179,3818,40
21
STARD4_8_6379,2117,2502,2264,1907,1815,4262,2161,1966,1685,3266,1740,2
586
STARD5_8_6380,2397,1570,1991,2212,1371,3653,1692,1192,2428,2051,3561,2
908
STARD6_8_6381,4305,3705,3014,4744,3077,4294,4359,3401,4384,5116,4127,3
763
STEAP1_8_6382,1355,1825,1428,1536,1350,2000,1254,691,1171,892,652,1763

STMN4_8_6383,11,5,59,17,0,5,0,0,32,0,0,278
STX11_8_6384,320,118,100,451,421,0,0,61,390,24,166,12
STX18_8_6385,2244,2874,2216,2439,3331,2273,4114,1980,1512,5003,3777,33
63
STX4_8_6386,1270,1357,713,747,1657,843,1349,874,1155,868,1114,805
STX5_8_6387,1148,862,608,780,2025,529,1793,1333,959,1099,1284,2217
STX6_8_6388,478,451,441,168,284,1021,12,308,394,280,1289,249
STX7_8_6389,1307,1517,1263,1252,1178,1369,2191,1816,1330,1576,1705,191
8
STXBP3_8_6390,296,256,442,180,878,361,37,93,166,153,22,66
SVOP_8_6391,420,473,264,626,84,116,768,741,123,189,148,1180
SYP_8_6392,161,117,137,108,96,615,19,13,335,38,0,23
SYT10_8_6393,1,22,1,1,1,1,1,0,2,3,4,0
SYT11_8_6394,804,731,606,1317,387,638,515,1485,638,651,1006,869
SYT13_8_6395,1430,1273,1015,1248,124,731,1408,1057,1519,2141,2038,1110
SYT16_8_6396,458,587,463,597,507,844,600,383,589,1,935,86
SYT4_8_6397,472,550,574,556,227,1017,1092,506,327,386,613,236
SYT5_8_6398,680,328,238,502,254,701,132,1142,576,135,528,1266
SYT6_8_6399,1093,733,506,867,1242,1621,756,891,773,776,746,1929
SYT7_8_6400,1786,1790,922,846,568,525,2285,336,840,1257,2823,1094
SYT8_8_6401,261,250,228,193,312,37,96,315,620,147,254,40
SYT9_8_6402,1046,590,1169,1100,2094,2234,275,178,575,1168,1344,830
TAS2R42_8_6403,775,585,1159,848,1535,398,1538,1949,2789,934,1002,953
TBC1D9_8_6404,1336,1280,643,1080,2036,2053,985,1504,659,1030,2010,1877
TCN1_8_6405,1835,2132,1288,1932,1467,1318,1099,1157,3318,2615,829,1043
TEKT4_8_6406,982,518,684,780,192,1901,678,1715,1370,1544,1710,1565
TF_8_6407,121,174,297,37,470,457,98,616,300,190,10,226
TG_8_6408,848,696,615,770,771,317,590,43,888,1781,2159,786
TGFB1_8_6409,1156,988,691,1336,103,632,288,1570,577,1083,1384,2082
TIMM10_8_6410,256,343,152,529,737,312,471,319,141,298,139,254
TIMM13_8_6411,638,626,781,789,1315,1291,423,618,1149,2181,1193,502
TIMM17A_8_6412,3652,2641,2582,2993,1279,5061,4340,3219,2923,3639,4622,
4926
TIMM22_8_6413,620,619,397,488,1011,392,440,294,235,717,701,187
TIMM23_8_6414,514,779,430,177,764,84,394,910,653,216,463,1810
TIMM44_8_6415,291,320,93,123,7,509,155,93,413,10,30,555
TIMM8B_8_6416,139,267,277,143,5,1014,130,10,41,320,20,2
TIMM9_8_6417,2898,2908,2671,3269,1364,5347,2597,3652,2810,2397,1051,29
20
TLL2_8_6418,1159,1264,1023,1101,298,677,1258,951,1436,1715,1410,714
TM9SF2_8_6419,3495,3865,3441,3375,2447,2302,8285,3680,2074,3482,4677,4
209
TM9SF3_8_6420,1223,928,776,685,498,492,207,800,1575,933,823,1461
TM9SF4_8_6421,2062,1704,1567,1445,2387,1800,2596,2348,1783,1032,1791,4
23
TMCO3_8_6422,539,790,493,486,212,466,584,142,361,174,666,551
TMED10_8_6423,3627,3509,3105,3738,2829,4592,5486,3368,4734,3621,6043,2
866
TMED1_8_6424,474,497,243,372,116,377,228,203,1046,619,698,1300
TMED2_8_6425,270,151,311,29,182,40,395,505,28,353,311,250

TMED3_8_6426,767,561,1024,692,573,1172,802,575,1097,1584,219,2045
TMED4_8_6427,477,190,222,548,256,511,358,522,352,236,481,145
TMED7_8_6428,8260,8077,8555,7469,10564,8377,9999,10522,7419,5674,11031
,9326
TMED9_8_6429,266,310,332,277,268,187,179,423,185,575,194,304
TMEM104_8_6430,249,570,263,330,442,869,595,429,173,692,1455,570
TMPRSS11B_8_6431,926,894,739,619,1333,181,845,533,514,1002,195,0
TMPRSS11D_8_6432,9525,12262,8720,10054,4972,11732,9578,12447,8160,1194
0,10286,13782
TMPRSS11E_8_6433,1249,1135,1397,1317,1525,700,2638,503,2141,462,1500,1
410
TMPRSS11F_8_6434,526,869,365,556,265,183,733,1,359,202,17,155
TMPRSS12_8_6435,157,227,154,143,65,41,38,12,3,568,61,22
TMPRSS5_8_6436,3446,3125,3167,2972,4498,3435,3389,3710,3205,3298,3398,
4130
TMPRSS6_8_6437,539,847,149,256,149,701,747,642,0,361,670,1542
TMPRSS7_8_6438,950,881,878,788,238,578,1257,466,741,1266,399,2393
TMPRSS9_8_6439,243,362,155,198,191,150,461,89,503,327,0,169
TNC_8_6440,702,160,154,94,0,464,0,149,815,741,40,843
TNF_8_6441,597,181,286,489,1001,764,1355,314,109,188,48,778
TNFRSF9_8_6442,2068,2297,1656,2639,2049,2789,1266,1145,1983,2618,1838,
5070
TNNC2_8_6443,471,607,353,382,1182,777,379,918,877,535,253,7
TNN_8_6444,840,1012,970,954,550,757,510,934,204,2203,2574,383
TNNI3_8_6445,1133,1544,1329,1168,1097,1776,1948,1585,2018,1606,2400,13
48
TNR_8_6446,930,794,877,720,1188,1391,2966,913,1052,735,1754,2471
TOMM20_8_6447,1838,1690,1534,1516,2089,1101,2207,3054,2125,1646,2678,1
770
TOMM22_8_6448,488,559,415,561,29,1134,765,267,1062,557,335,1061
TOMM70A_8_6449,2830,2797,3585,3577,3239,2095,3845,1424,1610,2727,3078,
4203
TOMM7_8_6450,445,235,363,229,0,58,176,89,69,192,387,321
TPSG1_8_6451,205,460,182,182,256,1028,0,232,364,397,453,310
TRAK2_8_6452,1221,2408,1592,1520,530,4013,657,652,810,2491,3211,2908
TRAPPC10_8_6453,933,1362,900,611,220,1417,823,1189,1279,594,444,430
TRAPPC8_8_6454,1015,1113,1042,1258,1462,3177,2055,1237,2174,817,2319,1
90
TSNAX_8_6455,961,1485,1119,1066,1174,1759,233,654,157,1623,1430,2070
TTPA_8_6456,716,565,786,1179,637,373,218,610,1474,1016,115,4
TTR_8_6457,1001,1000,863,914,656,1824,512,847,1775,827,649,706
TUBA1A_8_6458,3031,2303,2104,2337,3618,2046,1447,2604,3143,3110,2375,3
704
TUBA1C_8_6459,2125,1664,1517,1715,1287,291,3240,2373,3058,2725,3928,21
41
TUBA3D_8_6460,1023,647,302,633,1606,2039,178,24,185,630,698,329
TUBA3E_8_6461,915,747,804,577,334,1192,276,1074,163,588,694,909
TUBA4A_8_6462,2559,2277,1694,2236,3389,1609,1397,3148,2519,2448,1707,3
764
TUBB1_8_6463,274,333,238,228,22,909,755,557,492,114,126,943

TUBB2A_8_6464,2166,2134,1749,2568,3571,1749,3087,1616,2045,1840,2716,2
528
TUBB2B_8_6465,2166,2134,1749,2568,3571,1749,3087,1616,2045,1840,2716,2
528
TUBB6_8_6466,395,145,166,36,1529,232,909,14,973,1,10,714
TUBB8_8_6467,253,290,363,516,381,281,571,220,139,349,372,256
TUBE1_8_6468,5792,4758,3371,4807,4647,7172,4039,3905,6025,4061,5099,71
11
TUBG1_8_6469,1161,1187,1739,1147,1374,2125,1947,488,1522,1133,605,724
TUBG2_8_6470,719,772,590,584,1055,840,423,235,178,733,389,216
TXNDC8_8_6471,9794,10386,10825,10629,11931,11792,9238,10025,12170,1104
1,17197,9951
UCP2_8_6472,1213,1217,816,1270,1242,2114,294,912,1010,163,716,1088
USO1_8_6473,775,965,590,712,470,953,675,708,1463,636,777,793
VAMP2_8_6474,598,579,476,326,582,726,581,695,643,1105,249,1440
VAMP5_8_6475,217,588,76,309,56,354,10,0,0,444,67,86
VKORC1L1_8_6476,4473,4499,4968,4442,5601,4791,5268,6000,4197,5652,7536
,5922
VPS18_8_6477,641,1133,595,791,516,700,685,1174,397,229,615,1013
VPS26B_8_6478,226,109,177,146,0,535,34,212,233,109,545,141
VPS33A_8_6479,911,710,638,631,299,1494,751,1098,114,725,925,1289
VPS33B_8_6480,626,520,465,532,811,728,1378,213,484,955,1087,628
VPS35_8_6481,383,601,85,197,41,135,15,725,886,602,202,253
VPS39_8_6482,2649,2893,2726,2107,2820,4554,1344,2163,5429,3792,3410,19
87
VPS45_8_6483,1516,1726,1275,1508,928,5303,4589,1294,1412,2380,2363,957
VPS4B_8_6484,2275,2675,2691,3179,1037,1473,2348,2530,1902,2488,5413,27
15
VSIG2_8_6485,2442,2809,1789,3097,2061,1934,1160,2485,2254,1576,2487,28
48
VTI1A_8_6486,1105,1331,1067,1135,2101,1841,1674,599,1679,1474,1390,805
VTI1B_8_6487,770,867,516,393,1641,832,711,1272,624,1367,1852,1291
VTN_8_6488,331,250,211,281,636,121,490,108,1065,291,296,788
VWF_8_6489,1034,791,391,611,1091,716,48,221,395,1168,303,682
XK_8_6490,2205,2319,1603,1664,1782,1512,1355,1299,2825,2212,1186,1821
XPO1_8_6491,1699,1391,1764,1705,463,1179,1522,471,2106,3001,2906,1752
XPO4_8_6492,507,244,524,261,310,283,540,10,645,334,1388,2810
XPO5_8_6493,1825,2218,1788,1861,1323,2075,2617,1346,1081,1938,2770,453
5
XPO6_8_6494,653,500,388,644,343,391,745,1394,428,617,1613,1132
XPO7_8_6495,2151,2002,1999,2138,2737,525,3694,1323,1561,1573,2198,935
ACTL6A_8_6496,1800,1997,1751,1667,2469,854,1669,885,1111,1703,2667,144
7
ADAM12_8_6497,2558,2881,2035,2550,2941,2942,2070,3770,3029,3411,4614,4
033
ADAM15_8_6498,1779,2158,1516,2174,858,1859,2208,1206,1767,3538,1815,18
15
ADAM18_8_6499,5223,4951,4380,5742,5889,3362,6279,4039,5342,4351,8372,6
673
ADAM22_8_6500,2040,1834,2111,1660,1573,2015,1592,3693,2396,673,2586,14

57
ADAM29_8_6501,514,578,533,543,755,115,442,74,1304,1084,596,1613
ADAM33_8_6502,838,325,369,387,18,344,85,31,197,448,1044,312
ADAM8_8_6503,351,472,651,474,1093,1009,2314,863,454,870,1081,14
ADAMTS13_8_6504,3187,3120,2759,2977,2479,3715,3306,3202,2029,3894,3140
,3291
ADAMTS14_8_6505,393,435,522,304,285,685,50,615,412,209,149,616
AKAP12_8_6506,967,1044,1320,803,430,929,2345,952,306,1547,1660,765
ANGPT1_8_6507,119,543,147,215,120,51,447,315,31,402,101,134
ANGPTL4_8_6508,1,0,0,3,0,39,0,0,0,5,0,0
AP1B1_8_6509,921,1063,841,602,1100,723,937,63,433,695,277,1374
AP1G1_8_6510,729,842,805,732,453,132,271,1017,875,690,542,1686
AP1M1_8_6511,167,78,77,66,1414,172,0,61,0,100,43,446
AP2A1_8_6512,918,737,1292,1049,1534,768,1571,2321,312,3059,2257,2523
AP2A2_8_6513,488,380,648,899,2005,361,1380,442,854,126,1325,93
AP2B1_8_6514,6494,6677,5579,6873,6766,8529,4782,5004,6948,8133,7660,71
65
AP2M1_8_6515,1509,1739,1048,1597,2027,1882,733,3204,701,2132,2416,1457
AP2S1_8_6516,1005,1188,504,777,702,2357,1895,347,1097,1285,1969,1663
AP3D1_8_6517,3080,3459,2786,3384,3266,3454,1987,4234,1345,2831,4509,19
96
AP3M1_8_6518,585,702,681,659,225,1392,1224,682,716,1303,623,664
AP3M2_8_6519,1672,2054,1715,1575,2116,2621,1314,1666,1185,2180,1798,34
04
AP4S1_8_6520,1114,1073,1143,955,272,2614,394,1420,1143,800,2472,136
APAF1_8_6521,2560,3165,2891,2228,3196,2316,1827,1997,1138,2874,4068,27
01
APBA2_8_6522,286,329,415,469,396,500,347,529,626,392,449,241
APOL1_8_6523,517,348,587,258,124,1484,75,645,298,791,1045,302
APOL3_8_6524,387,511,671,377,415,146,289,276,724,186,302,587
APP_8_6525,2133,1646,2093,2389,5815,198,1946,944,3189,1343,2266,2387
ARFGAP3_8_6526,1240,1234,1154,1679,1132,2665,400,734,2238,1895,887,150
0
ARHGAP33_8_6527,483,470,412,451,1076,1640,95,1009,553,234,92,273
ARL6_8_6528,4141,3871,3813,3800,7932,4778,5121,2970,2231,4846,2697,472
3
ATP13A2_8_6529,180,109,142,195,0,319,0,239,47,51,210,49
BAX_8_6530,235,429,95,166,282,407,207,442,612,385,488,188
BCAP29_8_6531,2636,2404,2268,1832,4581,3604,3655,3844,1997,1919,3162,2
745
BCAP31_8_6532,483,412,722,556,432,1187,1631,60,218,71,162,574
BCL2_8_6533,220,502,94,103,12,588,905,15,441,53,41,15
BCL2L2_8_6534,1542,2144,1900,1789,1480,2500,1967,1082,954,1948,1807,27
38
BID_8_6535,85,145,220,122,3,575,377,67,12,169,18,1745
BSG_8_6536,1942,1884,1976,2087,2266,1781,2393,1864,1802,2026,3584,3124
C1QC_8_6537,2210,2282,1470,1609,1861,3087,2077,927,2137,2175,3254,2457
CANX_8_6538,2579,1895,1876,2144,3912,2334,2881,1508,3751,2603,2280,447
0
CAPN9_8_6539,563,676,612,765,569,800,971,662,641,218,1106,1056

CAPNS1_8_6540,555,400,281,264,395,167,1584,219,1494,517,319,253
CCT6B_8_6541,3393,2297,2717,2362,3925,2282,2267,2288,2534,3575,2721,26
67
CD19_8_6542,910,749,562,555,974,680,519,547,65,641,1089,1284
CD22_8_6543,747,544,595,1039,447,1707,3,1010,492,1022,233,303
CD33_8_6544,3509,2859,2552,3190,4039,2147,2883,4833,2776,3376,2411,360
1
CD44_8_6545,510,375,438,348,313,63,222,435,463,411,1359,667
CD55_8_6546,1237,1572,1301,1528,2914,2012,1379,1381,616,1852,803,2460
CDH17_8_6547,28,53,7,179,12,0,744,234,3,32,261,4
CFHR4_8_6548,3004,3009,2509,3289,2111,1134,3057,2341,1559,4711,3116,31
20
CIZ1_8_6549,2471,2226,2765,2772,2911,4517,3726,5166,3090,2456,2345,376
1
COG2_8_6550,1109,1103,826,1329,723,1379,813,853,1882,1013,770,3264
COG4_8_6551,793,729,750,693,1018,2334,2280,467,428,977,252,1104
COG5_8_6552,621,1320,737,850,353,475,930,223,535,343,549,781
COG6_8_6553,1594,1335,1488,1283,986,2284,853,523,1663,1283,1662,1510
COL11A1_8_6554,2732,2038,2458,2556,2251,1840,1610,905,1744,3071,4277,5
633
COL11A2_8_6555,383,683,480,471,1206,488,614,142,64,995,500,463
COL12A1_8_6556,1083,1551,1177,1368,1926,1646,1284,1110,2514,1717,1738,
1618
COL13A1_8_6557,451,111,125,338,42,268,15,692,407,422,358,468
COL18A1_8_6558,484,283,260,256,314,971,226,213,92,43,75,45
COL25A1_8_6559,504,877,402,422,693,1011,456,648,675,164,220,1203
COL2A1_8_6560,336,162,200,341,405,139,527,255,120,71,595,182
COL4A5_8_6561,0,0,13,7,0,0,0,0,0,0,1,0
COL4A6_8_6562,299,358,572,269,398,143,567,137,122,271,1343,314
COL6A2_8_6563,460,340,261,352,1657,226,847,152,430,154,879,549
COL6A3_8_6564,252,265,391,235,12,28,26,49,21,178,254,95
COL8A1_8_6565,1167,924,680,1093,1147,1121,643,1702,589,1140,1251,1676
COL9A1_8_6566,1985,2203,2769,2050,842,912,1825,1967,1184,1706,3186,281
8
COPA_8_6567,617,703,532,610,788,530,1562,700,789,695,87,750
COPB1_8_6568,341,472,377,344,207,508,1035,558,675,718,401,1
COPE_8_6569,2473,2090,2981,2597,1967,2808,983,2621,1673,2265,2604,1119
CPA4_8_6570,565,352,403,364,1690,1145,450,1151,263,715,191,2556
CPNE1_8_6571,1081,702,907,761,1776,917,807,481,474,491,1024,636
CPNE7_8_6572,1408,1087,1415,1572,737,1993,1751,1113,1649,2577,2292,197
2
CPXM1_8_6573,301,299,180,232,124,209,243,284,310,476,514,377
CPZ_8_6574,781,450,618,760,1306,882,94,307,196,400,1075,641
CRABP2_8_6575,708,653,477,612,387,293,1310,802,319,216,663,360
CTLA4_8_6576,1128,1181,646,1095,432,830,8,980,1385,649,556,1404
CTNS_8_6577,1875,2275,1417,2051,1670,3187,1380,1265,2077,2563,1546,125
8
CXCL12_8_6578,759,1149,625,1015,1145,1023,621,1514,1087,2382,789,1749
DPP10_8_6579,367,306,517,399,0,584,250,688,8,210,323,531
DPP6_8_6580,3835,4446,3372,4049,3609,5350,4464,3551,4323,3233,2868,386

0
ECM1_8_6581,1141,869,532,1100,947,936,825,1531,1457,1227,625,466
EGF_8_6582,1061,892,657,1435,1282,589,490,680,1979,1082,803,511
EIF2C2_8_6583,1201,1396,1153,1476,2233,1204,359,1143,2717,904,1640,211
2
EIF2D_8_6584,4393,3976,3254,3131,5123,5172,3918,2486,4136,4206,3542,65
47
ENSA_8_6585,222,159,574,257,16,94,483,20,47,654,618,28
EPB41L4B_8_6586,772,650,1124,755,859,1003,2726,461,670,1339,553,423
EPB42_8_6587,517,595,580,532,1563,635,635,1193,604,305,518,344
ETFA_8_6588,340,429,221,216,1152,272,108,177,83,462,68,18
ETFB_8_6589,468,531,848,572,579,853,725,38,302,415,606,800
EXOC1_8_6590,1784,1548,1249,1537,1533,1201,1263,2875,1648,2451,1034,33
68
EXOC4_8_6591,366,562,650,544,1591,330,302,813,498,1317,527,1002
EXOC6_8_6592,1097,1658,966,867,776,1862,541,1590,1200,2486,1411,895
EXOC7_8_6593,1727,1647,1367,1879,1735,1729,1460,1651,830,1582,1233,301
2
F8_8_6594,937,1041,638,1284,928,1591,914,682,914,1589,1583,1153
FABP6_8_6595,663,312,515,943,254,275,550,483,700,802,753,1106
FAM131A_8_6596,1738,1355,1023,1856,1920,1240,809,2579,1723,1324,65,153
7
FAM63B_8_6597,347,462,455,270,14,95,16,322,7,327,1204,20
FANCA_8_6598,815,843,869,931,1463,1568,2084,1179,978,1341,1731,674
FCN3_8_6599,215,348,300,323,570,379,1504,321,175,409,509,313
FGA_8_6600,1063,595,1040,1090,781,1595,450,1002,1059,593,353,1071
FGB_8_6601,1891,1190,1378,955,2123,1735,1921,1437,2733,1135,3411,1776
FGF13_8_6602,1034,1007,454,656,60,87,442,10,1827,966,300,1222
FGG_8_6603,2352,2401,1753,1418,1898,1716,2402,2123,758,2487,3154,3718
FIBCD1_8_6604,424,406,202,489,189,194,329,205,252,267,357,777
FLVCR2_8_6605,446,221,545,235,183,296,330,395,50,550,348,226
FOLR1_8_6606,402,568,340,375,572,532,115,496,599,290,152,1293
FOLR2_8_6607,3348,2689,2369,2889,2181,1930,4742,1803,2094,3323,1550,67
31
GGA1_8_6608,588,220,341,280,178,81,2,332,942,413,228,845
GGA3_8_6609,291,363,222,290,34,65,0,113,0,432,120,86
GJA5_8_6610,408,292,306,261,6,405,70,648,249,635,923,37
GJB1_8_6611,1162,1165,492,776,421,518,991,1342,593,435,478,1806
GJB3_8_6612,256,194,226,219,11,112,0,375,652,25,452,120
GJB6_8_6613,846,832,940,720,700,1285,521,708,1074,1677,1066,327
GLYATL1_8_6614,730,836,334,791,506,537,403,690,413,1182,791,702
GNRH1_8_6615,323,688,383,910,468,560,289,658,178,719,1443,783
GOLGA3_8_6616,439,208,500,212,628,388,1021,67,364,564,521,244
GOPC_8_6617,513,274,455,732,0,927,1383,379,3,545,63,93
GOSR1_8_6618,2857,2973,2222,2571,3977,3231,3959,4602,2393,2541,2456,26
88
GOSR2_8_6619,2165,1581,2696,1346,2481,1849,1148,1117,4085,1743,1502,20
94
GPRASP1_8_6620,284,273,333,292,146,62,980,23,412,692,683,485
GRB2_8_6621,2166,1169,1029,1924,4286,1508,2491,2640,1339,1568,1320,974

HABP2_8_6622,3047,3261,2422,2507,2382,4069,2292,1451,3179,4984,1062,32
27
HDLBP_8_6623,772,1064,458,775,2699,1547,150,627,955,1290,660,799
HEPH_8_6624,2558,1733,2056,1742,2839,1956,3342,2713,3751,2588,2828,265
7
HNRNPU_8_6625,117,10,98,402,18,0,86,0,3,0,428,633
HOMER2_8_6626,546,534,262,345,791,1124,218,33,478,789,1403,1722
HPN_8_6627,145,316,198,216,2,166,147,843,220,137,314,123
HSDL2_8_6628,392,301,281,456,728,391,487,749,183,244,341,237
IGF1_8_6629,997,855,526,1026,557,993,1199,1233,1409,472,62,926
IGFBP3_8_6630,1054,966,697,925,949,927,940,301,441,654,1532,137
IPO11_8_6631,1484,1661,1371,1240,1780,1344,3145,3966,1504,1684,650,279
5
IPO8_8_6632,5312,4671,5625,5084,7303,7069,7843,3814,5198,4611,6425,561
1
ITGAL_8_6633,449,489,441,284,518,1872,360,463,812,197,1784,20
ITGAM_8_6634,351,359,326,402,97,456,82,172,238,691,634,785
ITGAV_8_6635,4721,4779,5099,4037,6476,4010,7612,6054,5131,6376,5204,53
54
ITGB2_8_6636,1732,1242,1710,1845,1224,911,2483,1551,1905,410,1039,1289
KDELR2_8_6637,822,1136,662,745,672,868,824,1063,1370,549,853,1565
KDELR3_8_6638,338,320,246,290,1021,121,518,173,13,366,868,25
KIF13A_8_6639,727,496,494,887,978,529,640,579,894,121,41,87
KIF17_8_6640,548,607,399,401,1128,1979,688,508,314,487,354,110
KIF1B_8_6641,837,1217,747,871,1002,864,2467,372,1122,1528,1062,199
KLK10_8_6642,191,66,466,65,3,521,7,7,15,0,0,0
KLK11_8_6643,175,150,126,197,91,95,236,464,131,231,942,320
KLK12_8_6644,280,377,266,151,401,304,126,2,133,147,46,26
KLK15_8_6645,184,632,386,582,78,330,1,18,56,1044,2,1161
KLK5_8_6646,387,226,188,143,95,58,470,161,864,436,517,83
KLK6_8_6647,447,653,533,914,616,390,654,337,592,375,857,824
LAMB3_8_6648,254,321,175,359,754,432,423,677,59,62,13,218
LDB3_8_6649,164,612,247,273,956,1,100,328,710,220,943,927
LDLR_8_6650,209,317,215,155,898,112,667,84,13,122,1529,4
LTA_8_6651,275,194,384,316,1143,115,377,740,48,461,276,523
LTF_8_6652,3316,2813,2798,2802,4293,3891,5253,1030,2494,3502,2974,5578
M6PR_8_6653,225,404,214,417,77,76,55,227,66,147,281,36
MASP1_8_6654,327,458,271,400,360,988,35,395,813,612,141,633
MB_8_6655,940,1103,815,802,234,764,1855,994,1373,1548,545,2343
MCFD2_8_6656,853,997,703,491,414,570,352,611,1549,870,759,691
MCL1_8_6657,557,565,414,283,320,520,364,529,0,1162,897,1767
MEFV_8_6658,1709,1961,1377,1465,2683,1104,2713,911,1863,1701,1848,893
MFAP4_8_6659,2602,2511,2102,2316,3989,2690,1697,3479,3021,2095,2089,60
75
MFSD10_8_6660,521,355,399,666,555,153,369,824,464,1315,773,392
MFSD1_8_6661,2947,2579,2324,2582,3285,1974,2276,3760,4523,4158,2806,56
58
MFSD5_8_6662,976,836,784,1230,980,1415,355,880,1465,1079,1644,1107
MLC1_8_6663,595,516,995,340,26,363,315,100,337,402,555,348
MSLN_8_6664,1194,1123,864,779,185,1082,640,1299,579,1013,900,2163

MTX1_8_6665,294,319,139,174,4,110,90,103,427,17,3,47
MUC1_8_6666,1663,1281,1472,1259,337,1493,1906,1050,777,2137,1231,1469
NCAM1_8_6667,2558,2515,2204,2724,2844,4659,1604,1924,2888,4176,3238,31
45
NNAT_8_6668,389,319,282,295,1261,183,120,370,764,443,64,245
NOXO1_8_6669,414,263,291,322,1334,672,623,9,1330,505,287,424
NPC1L1_8_6670,5757,6310,7049,5167,7517,6576,11188,7396,5894,7090,6551,
9124
NPRL3_8_6671,806,1311,881,1198,2781,1449,751,398,510,1170,378,568
NRXN1_8_6672,941,684,769,882,916,1008,398,1842,1733,939,1128,1397
NRXN2_8_6673,64,10,130,85,0,110,7,0,0,2,109,115
NRXN3_8_6674,1127,969,886,1207,1347,742,966,638,837,309,179,884
NUP155_8_6675,898,872,1238,1001,519,539,980,1247,875,795,1252,1436
NUP50_8_6676,726,569,954,848,1295,1437,1694,856,707,1118,496,1387
NUP62_8_6677,2228,2118,1875,1908,1037,1558,958,2355,3007,2905,2066,297
8
NUP98_8_6678,2067,2226,2322,2087,3384,1169,3867,2863,465,2699,3162,238
9
NUPL1_8_6679,2566,1928,1967,2772,2713,2575,3883,3004,1598,4390,3224,26
46
NXF1_8_6680,361,549,469,232,516,358,710,693,726,412,549,201
NXNL2_8_6681,1320,1696,1066,1439,644,1223,1037,1024,1987,519,667,523
NXT2_8_6682,1570,1993,1536,1264,1346,1360,644,2082,1021,1481,1528,2211
OAZ3_8_6683,888,1125,789,882,415,1263,989,231,648,677,1825,3112
PACSIN2_8_6684,1139,1105,937,1000,1686,1041,1595,1356,1485,856,1165,16
03
PANX2_8_6685,612,624,528,517,893,105,438,1732,746,662,385,1083
PCDHA6_8_6686,2992,3163,2577,2711,4678,5108,3096,4057,2328,3792,3826,3
463
PCDHGA5_8_6687,877,614,478,646,689,275,70,191,596,1046,949,611
PCLO_8_6688,3632,3711,2917,3895,2354,2362,4734,2652,5786,2981,5034,455
8
PCSK5_8_6689,866,1266,875,505,368,738,161,1816,707,1374,466,537
PCSK6_8_6690,563,716,605,559,1778,355,332,282,571,1048,599,731
PCTP_8_6691,1329,1211,1235,1319,1018,679,126,1017,739,1014,1111,2101
PDYN_8_6692,205,187,552,230,440,95,40,25,80,6,200,53
PDZD3_8_6693,307,82,408,456,2,269,179,66,54,92,69,84
PDZK1_8_6694,2580,2486,2191,2475,3268,1922,751,1242,1329,1184,1740,168
1
PGAP2_8_6695,1692,1807,1471,1675,1266,1917,1509,1508,1632,2755,697,314
4
PGF_8_6696,646,528,881,812,424,60,124,409,266,442,557,307
PIK3R3_8_6697,275,551,365,131,68,19,3,534,140,89,898,300
PITPNC1_8_6698,556,858,708,443,219,536,2749,458,395,1457,457,1435
PITPNM1_8_6699,23,0,8,2,0,0,1,0,0,0,0,0
PITPNM3_8_6700,1675,1884,815,1156,1450,4012,793,2123,1167,1605,1185,14
81
PLEC_8_6701,190,108,204,385,48,136,31,1,70,276,701,221
PLIN3_8_6702,1567,1643,1576,1950,2107,1603,3049,1996,1202,851,1732,276
7

PLTP_8_6703,291,575,225,461,322,272,549,396,317,763,1341,49
PNKD_8_6704,19,14,127,49,40,565,0,0,120,67,275,35
POMC_8_6705,75,51,74,40,3,360,0,69,100,80,129,51
PORCN_8_6706,810,668,548,559,773,488,521,664,1514,559,1156,386
PREPL_8_6707,1338,1595,1555,1609,666,970,1150,1398,1297,2079,1353,902
PRNP_8_6708,1107,1542,607,1118,2369,998,261,487,885,1090,947,637
PRSS21_8_6709,252,19,59,139,4,140,5,15,2,28,57,101
PRSS35_8_6710,799,1624,856,918,503,1021,2384,363,257,1099,2274,525
PSEN1_8_6711,663,1000,694,891,284,470,1001,92,857,667,1131,713
PSEN2_8_6712,171,170,165,114,238,2,34,20,3,573,650,396
RABEP1_8_6713,307,306,307,425,558,138,569,398,5,335,489,627
RACGAP1_8_6714,429,312,457,98,229,1708,799,619,51,355,759,182
RARRES1_8_6715,11,103,150,82,0,0,0,0,589,0,0,0
RASA1_8_6716,468,953,611,643,30,426,1124,1032,403,691,411,1751
RELN_8_6717,1630,1626,1784,1892,271,1483,2489,1463,441,1780,3031,1711
RHCE_8_6718,330,258,521,316,120,199,550,94,357,96,1083,242
RHD_8_6719,367,440,534,207,185,513,447,72,546,681,555,352
RIMS2_8_6720,856,1115,717,739,365,1076,1194,1060,1368,281,1087,580
RRBP1_8_6721,609,412,99,461,191,191,88,746,189,68,36,270
RUFY1_8_6722,1344,1089,1086,1229,1762,762,1559,3290,1119,1426,1962,763
S100A13_8_6723,590,635,435,458,86,671,141,142,343,178,350,573
S100A4_8_6724,1388,605,788,1196,728,371,276,2422,924,420,473,1247
SAA1_8_6725,1087,1053,839,1312,607,1885,1634,785,1616,703,1398,1562
SCAMP3_8_6726,1259,1322,1709,1178,1595,844,2767,2094,1523,1452,1911,20
44
SCARB1_8_6727,626,1413,742,585,407,809,796,195,1647,1534,750,1941
SCFD1_8_6728,1327,1184,726,1506,347,282,1411,543,626,1410,785,2494
SEC13_8_6729,259,145,123,146,2,424,405,2,508,35,82,2
SEC14L1_8_6730,2583,2296,2396,2060,1899,3140,2996,1968,2846,2472,2242,
1445
SEC14L2_8_6731,395,611,220,509,166,742,649,428,219,265,490,731
SEC14L4_8_6732,64,207,57,163,177,106,686,59,210,1218,30,20
SEC23B_8_6733,1440,1566,641,961,776,941,559,131,491,1686,2400,2413
SEC24B_8_6734,1824,2631,1507,1873,1439,1648,1990,1027,814,2405,2292,20
14
SEC24C_8_6735,1758,1681,1425,1405,1718,1112,2701,1051,871,1579,2388,18
06
SEC61A2_8_6736,1660,1045,614,857,1689,131,2154,1510,1652,1187,685,1824
SEC61G_8_6737,1525,1145,1385,1176,2410,876,1244,1146,2223,1442,1906,12
64
SEH1L_8_6738,2971,3032,2217,2956,3403,3048,1767,5664,1991,2492,3515,28
60
SERINC2_8_6739,898,868,871,836,270,448,680,571,719,1270,470,2094
SERINC3_8_6740,1896,2283,1981,1516,940,2290,1494,2223,1958,2282,3076,3
799
SERPINA10_8_6741,1906,2321,1978,2960,3966,1892,612,798,3835,2335,2594,
2496
SERPINA1_8_6742,141,244,105,293,93,50,240,277,232,77,446,152
SERPINB2_8_6743,649,766,673,600,593,1005,940,270,767,1120,483,392
SERPINB6_8_6744,7987,7989,9452,9258,10471,9039,9083,9278,7860,9729,140

23,10723
SERPINB8_8_6745,1173,1172,1426,1432,2441,1939,2619,561,1380,708,1886,1052
SERPINE1_8_6746,503,900,334,603,436,594,277,423,875,325,552,945
SERPINF2_8_6747,564,544,807,507,450,1808,908,1668,266,624,1564,1327
SERPING1_8_6748,833,1272,833,812,725,1614,2193,1159,175,900,2479,1988
SERPINH1_8_6749,99,229,283,54,121,49,4,165,107,148,80,615
SERPINI1_8_6750,992,1285,856,974,494,937,1866,2923,2569,2390,1649,1572
SFI1_8_6751,2303,1708,2122,2339,1261,3488,2468,1647,1630,2449,3183,1262
SFTPA1_8_6752,551,840,354,531,810,439,1693,7,234,703,426,766
SH3D19_8_6753,4173,4247,3877,3295,4059,4671,6065,5770,2776,4030,1907,4717
SIL1_8_6754,452,344,255,473,398,546,233,417,128,338,521,460
SLC25A25_8_6755,1412,2191,1956,1661,1892,1569,1786,2279,1306,1872,2945,1506
SLC25A36_8_6756,3366,3415,2568,2868,3365,2988,2677,945,3029,4167,1380,3824
SLC25A45_8_6757,406,591,257,256,188,229,1015,711,1027,581,45,590
SLC38A10_8_6758,1639,908,1115,994,2258,753,757,1516,1250,2262,1689,3772
SLC41A3_8_6759,488,415,405,474,387,1093,141,1419,8,512,392,1334
SLC43A3_8_6760,693,962,1145,831,1632,1488,625,633,1123,968,960,3491
SLC44A2_8_6761,215,224,289,313,695,123,20,78,69,187,215,298
SLC44A4_8_6762,203,238,407,318,453,6,319,411,129,188,217,157
SLC44A5_8_6763,916,782,825,866,184,375,1193,1137,1347,935,2718,130
SLC46A1_8_6764,1060,1458,717,1321,955,1908,1739,999,1301,710,705,1393
SLC47A2_8_6765,853,1162,1340,858,830,1119,3932,648,1818,1913,1679,650
SLC50A1_8_6766,1080,1020,531,639,539,676,1309,985,499,272,448,1556
SLC6A20_8_6767,3694,4436,3037,3490,4030,2591,3827,5640,1760,5281,4117,5898
SNAP23_8_6768,1360,1860,937,1117,2283,2181,3530,2231,1206,2791,1475,1552
SNAP25_8_6769,849,600,720,899,12,400,708,801,1003,371,2294,2385
SNX10_8_6770,914,497,839,975,424,790,452,694,969,945,392,999
SNX11_8_6771,1051,954,1201,1336,508,1333,1414,1435,971,1346,1833,3220
SNX14_8_6772,8047,8533,6378,7200,6881,9727,8145,6768,6341,11297,9471,9882
SNX15_8_6773,889,600,751,968,959,535,317,694,961,773,868,676
SNX16_8_6774,284,412,309,200,92,198,790,52,432,129,538,441
SNX18_8_6775,413,217,341,452,93,931,95,104,47,430,45,1337
SNX1_8_6776,2408,2141,2432,2506,1956,2529,1648,1665,1865,2204,3792,1357
SNX3_8_6777,1342,1365,1576,1608,1369,1457,1986,2955,2668,1828,2092,3849
SNX5_8_6778,2311,2059,2366,2620,2777,3023,2344,2181,1807,2984,5602,4220
SNX6_8_6779,1925,2059,2856,2263,1616,2696,2441,2152,1976,2933,3207,3315
SNX7_8_6780,2107,2178,1912,2097,2351,2691,1885,2718,2041,2569,1927,247

7
SORCS1_8_6781,1057,1174,500,892,1190,1130,1560,1317,1303,542,266,1520
SORT1_8_6782,636,668,696,784,645,306,370,507,208,636,606,1132
SPNS1_8_6783,194,188,157,266,199,145,526,1,343,366,39,221
SRI_8_6784,2869,3342,2410,2155,3223,4159,4540,2078,4946,4959,4706,4096
STARD3_8_6785,431,310,413,326,1398,220,58,363,97,88,1163,48
STAU1_8_6786,1536,1425,1364,1327,829,3383,1859,2415,446,1549,1506,407
STEAP2_8_6787,447,627,445,588,269,510,62,409,279,276,174,530
STEAP3_8_6788,239,241,105,320,611,49,488,662,377,150,305,329
STIM2_8_6789,1388,1722,1444,1072,492,887,457,1247,1002,2058,2424,1321
STX16_8_6790,570,716,664,534,1063,565,326,163,492,176,355,195
STX1A_8_6791,809,976,746,1045,1618,1452,1336,1494,553,657,508,421
STX2_8_6792,240,220,430,497,1107,0,303,825,632,174,425,207
STX3_8_6793,530,829,451,626,1353,417,1039,20,694,739,910,293
STXBP1_8_6794,238,480,94,199,65,357,0,916,12,671,320,288
STXBP2_8_6795,108,62,92,64,52,18,0,97,21,30,283,193
SV2B_8_6796,1076,994,1258,934,658,1296,1441,737,633,1283,1342,985
SYN1_8_6797,2567,2117,1822,2232,2879,2948,2527,1043,2303,2201,2360,154
6
SYNGR1_8_6798,374,614,232,342,710,108,103,115,1257,355,32,925
SYNPR_8_6799,1056,499,531,744,1321,2125,937,1942,571,286,542,629
SYPL1_8_6800,1912,1477,1142,1032,2460,2896,7918,2919,1527,1702,550,270
7
SYT12_8_6801,336,213,397,347,1,13,0,10,11,342,473,1477
SYT14_8_6802,315,204,273,240,185,228,569,1450,316,284,474,723
SYT15_8_6803,437,377,444,328,485,1515,471,470,60,823,745,1451
SYT1_8_6804,1081,887,1487,1541,701,1277,2121,324,1726,1121,2272,1230
SYT2_8_6805,877,992,1344,1216,1166,1220,370,665,1023,1960,1991,1128
SYT3_8_6806,413,313,253,422,1859,1012,113,38,27,820,369,1518
TAPBP_8_6807,494,745,285,381,664,11,276,593,71,460,569,698
TC2N_8_6808,2210,2238,2204,1922,2489,3610,2489,2200,2633,2020,2818,178
1
TCN2_8_6809,864,619,905,687,152,884,924,261,293,978,1086,315
TCOF1_8_6810,1025,1404,1032,1450,1118,2201,951,1636,1995,1008,1675,238
1
TFPI_8_6811,1500,2512,1553,1802,590,2523,3154,1196,919,1831,2221,1236
TFR2_8_6812,1041,1153,801,904,1480,769,887,2712,2080,657,757,1011
TFRC_8_6813,1961,1711,1835,1793,2444,2612,3605,3354,1132,2014,1891,228
6
TGFB2_8_6814,282,216,259,77,231,15,6,40,304,108,271,61
TIMM17B_8_6815,946,328,471,432,1196,732,918,529,964,783,1759,1717
TINAGL1_8_6816,943,932,991,694,1152,732,1555,838,521,1248,794,1115
TLL1_8_6817,1709,1103,1062,1781,862,823,1277,1536,853,1595,822,2807
TM9SF1_8_6818,944,1509,1101,828,47,741,527,528,653,723,2497,1567
TMC6_8_6819,821,1057,910,615,1088,656,838,1516,533,953,603,1644
TMPRSS11A_8_6820,1821,2642,1942,1954,760,1591,2605,3385,1667,1607,1415
,1931
TMPRSS13_8_6821,346,128,124,146,1200,325,385,157,45,69,88,353
TMPRSS2_8_6822,4026,4220,4730,4933,8182,3123,6441,3602,2447,2610,4846,
3950

TMPRSS4_8_6823,669,541,480,505,403,449,803,397,442,1475,926,801
TNFSF11_8_6824,1656,2081,2277,1565,2084,1629,2224,1429,1115,3191,2468,
2808
TNFSF13B_8_6825,385,717,333,327,373,698,137,1085,17,436,386,1160
TNPO2_8_6826,267,326,606,299,99,316,852,640,829,672,34,153
TOM1_8_6827,394,729,249,554,1134,991,1142,201,587,926,1355,759
TOM1L2_8_6828,1855,1365,1952,1557,746,1695,1279,654,2876,2302,1848,195
5
TSC1_8_6829,4604,4749,4702,4919,6034,5306,5806,5781,5284,6849,5947,422
1
TSC2_8_6830,207,176,233,300,8,65,2,150,89,344,12,35
TUBA8_8_6831,1412,1188,1033,1290,1371,1115,2537,586,1650,1050,477,2084
TUBB3_8_6832,553,598,433,486,929,764,760,226,162,117,93,867
TUBD1_8_6833,1311,1253,1324,1256,1442,1335,1972,1150,1635,1315,764,239
6
UCP3_8_6834,124,219,127,511,231,27,200,3,0,187,0,18
UPF3A_8_6835,2669,3018,2078,2925,1899,4999,2332,2960,2281,6466,3672,27
22
UPF3B_8_6836,557,813,542,575,529,769,977,472,765,1423,123,11
VAMP1_8_6837,1120,926,880,1077,1835,543,1377,2202,1827,829,1516,1573
VAMP7_8_6838,225,493,304,291,633,1077,26,563,152,405,12,584
VCAM1_8_6839,951,960,1021,1118,495,1729,743,827,964,761,1013,1018
VLDLR_8_6840,2443,1939,2043,1728,2137,1842,1969,2161,1569,1341,1653,31
87
VPS13A_8_6841,1265,1395,1928,1436,1934,2044,1819,2487,1213,1148,3057,2
889
VPS13B_8_6842,445,389,354,153,0,372,476,105,676,412,98,295
VPS16_8_6843,26,194,119,370,16,235,3,0,0,84,318,442
VPS26A_8_6844,3610,3552,3639,3494,4404,2898,7157,2443,3083,3758,6024,5
723
VPS28_8_6845,1263,1056,822,1140,1391,1745,330,527,1066,1404,1745,172
ZFYVE16_8_6846,4315,4742,4773,5856,5138,3893,6533,2336,4811,6270,4138,
5637
ZNF160_8_6847,6120,6522,6482,6040,8685,6490,8238,3945,9310,6345,7858,6
217
ZP3_8_6848,1357,1714,1773,1652,871,2840,2376,2638,1995,1043,2012,1537
BET1L_8_6849,928,687,679,491,933,353,302,672,378,887,307,248
C2orf83_8_6850,252,336,474,126,730,933,143,3,188,53,263,172
ERP29_8_6851,907,740,866,390,730,296,366,168,800,463,1290,51
FGF1_8_6852,1810,1851,1929,1686,2003,4093,4432,861,2523,2362,2023,2228
LYNX1_8_6853,1259,1281,865,1332,1030,1665,668,1314,1582,1370,2424,747
MMP28_8_6854,330,415,394,346,1,512,492,314,777,198,565,307
PDPN_8_6855,619,749,429,449,1769,102,397,455,583,1051,172,1182
SNX21_8_6856,510,656,615,249,3564,606,22,2,916,1066,597,1060
TIMM8A_8_6857,1024,1709,995,1502,535,1043,1058,2050,1732,1422,644,1178
VEGFA_8_6858,1486,1349,1112,830,1673,2175,699,1889,168,1128,838,1897
CDH23_8_6859,360,360,323,419,1313,422,489,745,238,435,419,953
CDH23_8_6860,2453,2079,1802,2207,2514,2094,2416,2643,1936,2450,1143,15
12
CDH23_8_6861,392,361,266,236,248,29,617,395,60,129,48,1119

CDH23_8_6862,387,121,150,261,1232,171,511,250,831,8,612,251
CDH23_8_6863,2865,2879,2082,2825,5459,2708,3760,1828,1687,2596,3096,25
08
TNXB_8_6864,1056,1116,665,1100,1472,983,158,829,793,519,353,1884
A2M_8_6865,1014,842,956,896,527,697,2186,675,1118,1161,836,1301
ACE2_8_6866,1378,1243,1306,1087,1038,1021,2159,1031,1613,632,1490,2159
ACTR6_8_6867,32071,29579,24326,30294,27860,24533,36151,26913,30636,274
20,36318,35054
ADAM11_8_6868,971,687,823,462,1173,1108,848,789,517,387,1131,383
ADAM19_8_6869,296,405,300,239,588,2,93,502,58,720,29,158
ADAM20_8_6870,2265,1926,2586,2021,3016,3956,2835,1469,2428,2273,4587,5
246
ADAM21_8_6871,1533,1601,1394,1797,1623,1236,1464,1885,1875,1845,2510,2
579
ADAM2_8_6872,790,785,717,520,728,75,289,551,1784,398,965,498
ADAM30_8_6873,2206,2389,2385,2371,1693,3061,1827,1460,2257,2362,2061,2
610
ADAM7_8_6874,165,319,165,295,1,30,14,567,488,261,220,242
ADAM9_8_6875,861,501,460,759,664,292,1025,125,986,661,1330,418
ADAMTS10_8_6876,551,822,554,668,233,1253,954,1143,376,869,857,183
ADAMTS12_8_6877,1046,1004,440,847,578,1571,1621,1103,376,443,632,944
ADAMTS15_8_6878,312,409,342,686,172,501,482,507,801,1232,340,198
ADAMTS18_8_6879,130,221,228,72,0,885,0,208,288,388,332,5
ADAMTS19_8_6880,693,1298,724,847,1318,796,853,360,1875,530,938,387
ADAMTS1_8_6881,114,429,359,236,90,527,179,704,72,103,109,694
ADAMTS20_8_6882,1392,1687,1637,1227,982,1015,2572,334,2025,1689,2237,2
156
ADAMTS3_8_6883,1778,2501,1360,2057,2127,2825,2385,1044,1430,3490,2213,
2364
ADAMTS5_8_6884,2557,2743,2843,2255,4509,1112,2651,2060,2639,2845,3725,
2708
ADAMTS6_8_6885,1468,1593,1738,1183,364,1808,844,619,1614,1652,1248,765
ADAMTS7_8_6886,826,725,720,850,776,1135,1868,1469,817,1501,936,1818
ADAMTS8_8_6887,579,755,419,628,1728,324,719,306,209,356,370,272
AEBP1_8_6888,295,553,161,240,300,1673,76,498,471,25,316,199
AFG3L2_8_6889,1672,1495,1093,1886,3045,1695,733,1398,1120,1062,3188,28
77
AFM_8_6890,2723,3425,2174,3043,1047,2398,2211,2610,1934,4106,2320,3055
AFP_8_6891,2406,2802,1827,2433,2894,2677,2025,2474,2465,1877,2730,3185
AGTPBP1_8_6892,559,440,764,738,496,210,473,699,44,421,400,520
ALG10B_8_6893,2135,2597,1727,2810,1177,2254,3342,3297,1949,2381,3429,2
079
AMBP_8_6894,875,987,868,700,594,539,911,889,643,1654,720,2463
ANGPT4_8_6895,412,403,514,474,394,307,278,929,522,210,428,222
ANGPTL1_8_6896,4165,5532,4240,4321,4042,6292,8444,5817,2957,4463,6962,
4427
ANGPTL2_8_6897,200,331,43,277,54,723,6,218,1,116,787,677
ANGPTL3_8_6898,691,574,258,442,907,496,1141,161,1214,1403,603,414
ANGPTL7_8_6899,760,1075,827,673,164,1546,2086,627,868,857,461,351
ANKH_8_6900,797,1032,771,1130,1220,945,1220,1308,421,1640,379,1033

AP1G2_8_6901,643,852,488,624,360,244,1176,167,807,211,635,771
AP1M2_8_6902,2286,1748,1771,1603,3146,2030,466,1284,1384,2391,2224,896
AP1S1_8_6903,250,88,67,302,8,22,136,412,715,277,21,145
AP1S2_8_6904,869,971,862,1099,1059,949,1508,787,1218,996,1348,1028
AP1S3_8_6905,4624,4705,4391,3901,4311,7457,2815,5438,4970,5788,7238,28
81
AP3B1_8_6906,671,678,872,724,800,375,1244,107,431,60,895,54
AP3B2_8_6907,767,929,932,989,232,1295,1066,830,633,2678,762,1381
AP3S1_8_6908,2760,2921,2019,2794,3735,3144,3885,1911,4115,2583,1140,32
89
AP3S2_8_6909,841,1171,969,568,732,620,1396,583,244,2069,884,788
AP4B1_8_6910,1022,868,854,623,691,1553,521,95,1503,554,727,806
AP4M1_8_6911,2267,1880,1884,1872,2301,2686,1336,1535,470,2716,1484,183
5
APBA1_8_6912,1068,1210,1117,1428,1449,432,651,394,1454,1032,1119,3868
APBA3_8_6913,1064,778,755,1193,692,1130,238,518,471,585,1227,2190
APOA1_8_6914,366,563,300,167,118,44,228,595,30,231,1048,16
APOA2_8_6915,623,687,405,750,382,175,51,6,1908,5,497,157
APOA4_8_6916,281,482,292,414,11,54,413,1090,281,174,61,56
APOB_8_6917,490,410,203,385,109,280,361,167,734,55,256,530
APOC1_8_6918,870,1292,1524,1131,1203,531,1040,1144,2843,1503,864,1107
APOC2_8_6919,3812,4093,3117,4544,3748,3429,4008,3919,7797,3470,4197,54
14
APOC3_8_6920,806,998,867,577,772,732,1634,180,592,559,1367,1227
APOC4_8_6921,694,1112,745,547,591,1500,1332,756,1509,349,1588,415
APOD_8_6922,382,304,374,311,228,681,686,343,505,701,312,442
APOE_8_6923,181,154,334,65,13,2,0,802,137,465,174,83
APOF_8_6924,907,947,636,984,1107,816,379,1592,1094,748,465,2359
APOH_8_6925,828,494,367,618,921,1402,500,1080,1313,574,152,189
APOL6_8_6926,477,836,544,731,491,661,443,962,273,338,116,2275
APOM_8_6927,381,305,60,434,525,266,1081,264,716,219,400,148
AQP12B_8_6928,49,13,16,9,1,4,0,0,0,0,0,0
ARF5_8_6929,256,101,35,149,59,342,341,478,219,1030,0,719
ARF6_8_6930,620,583,309,549,132,755,512,244,685,846,290,989
ARPP19_8_6931,1695,670,798,1395,1364,903,566,1404,479,622,2147,2198
ASTL_8_6932,2331,2339,2242,2949,780,2732,2730,1340,3268,5239,7423,3964
ATOX1_8_6933,198,180,166,154,9,0,302,518,3,568,401,608
ATP13A1_8_6934,658,681,250,318,481,109,84,169,25,14,496,438
ATP13A3_8_6935,455,571,354,481,785,631,380,486,973,337,219,1218
ATP13A4_8_6936,461,115,112,26,0,316,24,0,0,75,336,326
ATP13A5_8_6937,824,957,496,383,143,249,754,1103,55,695,1023,275
AZGP1_8_6938,350,197,246,151,2,378,336,600,1689,508,278,1495
AZU1_8_6939,397,353,319,150,156,50,19,428,309,51,457,350
BCL2L10_8_6940,2096,1530,1083,2047,1933,1735,956,1972,2466,1264,2419,2
472
BET1_8_6941,1668,1806,1468,1574,1377,2265,1128,2053,1838,1312,1494,333
2
BGLAP_8_6942,701,709,536,658,187,358,388,824,346,392,589,888
BOC_8_6943,1995,1817,2043,1640,2301,1421,2981,2122,3778,1783,2787,2733
BPI_8_6944,657,273,460,981,254,324,82,473,272,3,1293,94

BPIFC_8_6945,818,646,1117,780,229,698,2524,1166,947,670,914,850
C16orf7_8_6946,880,1069,578,866,108,954,1900,1085,115,1481,1702,845
C1orf162_8_6947,247,459,283,392,7,669,9,63,64,164,242,27
C1QA_8_6948,205,121,49,24,0,3,18,433,643,0,0,0
C1QB_8_6949,145,166,181,187,401,108,238,386,165,212,422,0
C1RL_8_6950,354,273,261,197,320,64,159,163,634,394,190,509
C20orf141_8_6951,1713,1156,1450,1702,2052,1552,2584,1925,1508,1743,152
8,2315
C3_8_6952,516,325,404,563,628,721,497,1120,970,28,389,1268
C4A_8_6953,284,420,545,284,73,916,1474,639,179,377,1498,770
C5_8_6954,258,445,164,237,8,12,1,114,320,33,635,694
C7orf31_8_6955,858,1279,882,1374,1819,1871,1428,1351,1211,641,284,2252
C8G_8_6956,2134,1471,1404,1750,496,1565,1060,876,1769,2096,1327,1817
CALM2_8_6957,1132,1113,927,582,534,1435,1038,1621,1627,1298,1996,1064
CALY_8_6958,328,175,146,388,0,11,3,614,445,68,968,265
CAMLG_8_6959,1613,2024,1259,1139,788,1807,2589,1732,1489,1421,928,770
CAPN11_8_6960,812,635,808,742,862,56,226,490,400,404,541,822
CAPN5_8_6961,151,78,18,161,250,22,323,95,5,57,45,44
CAPN6_8_6962,973,1308,1003,1025,1238,720,944,1123,902,901,1875,717
CARTPT_8_6963,601,139,345,465,157,91,240,158,864,535,1236,318
CCL13_8_6964,612,327,327,263,1041,286,365,89,318,110,398,488
CCND1_8_6965,402,237,314,586,457,21,435,611,508,267,104,453
CD1A_8_6966,405,382,583,532,1530,33,501,341,450,434,652,808
CD52_8_6967,325,286,454,428,561,140,1631,712,968,173,641,457
CDCP2_8_6968,161,211,348,169,133,420,523,250,84,105,133,117
CDH5_8_6969,807,770,783,969,953,343,1268,565,956,936,858,1199
CHMP7_8_6970,308,516,614,652,1,1457,1521,0,54,1021,40,520
CLDN16_8_6971,1192,1595,1632,1704,2702,3537,1562,1168,1571,2075,785,14
97
CLEC3B_8_6972,2173,2044,2409,2380,2714,3224,3228,1939,1145,2764,2862,2
498
CLSTN2_8_6973,317,245,279,414,195,209,44,93,97,393,201,489
CLVS2_8_6974,802,759,648,336,653,1023,1570,36,776,579,297,543
CNIH3_8_6975,4477,4496,4626,4917,5806,5139,5035,4787,7140,3827,5339,79
17
CNOT6_8_6976,1190,1359,1205,1442,1682,2053,1048,1872,1958,829,1864,127
0
CNTNAP1_8_6977,469,379,593,353,508,521,163,272,1109,793,624,345
COG1_8_6978,680,872,684,835,650,804,264,1328,804,937,1312,1577
COG3_8_6979,475,471,1051,706,1353,63,848,543,665,557,316,68
COG7_8_6980,1632,2659,1255,1812,1039,2180,1091,1994,2220,1707,2610,253
0
COG8_8_6981,741,1294,871,1350,321,2514,214,1227,509,2060,967,1601
COL10A1_8_6982,362,238,250,402,56,95,21,308,6,165,265,39
COL14A1_8_6983,3575,2699,2678,2533,3145,1927,3704,1482,4113,3799,5273,
3152
COL15A1_8_6984,822,587,895,733,279,802,198,649,688,484,1194,814
COL16A1_8_6985,114,225,51,152,0,8,0,1,0,5,136,38
COL17A1_8_6986,235,235,280,306,162,117,854,141,115,269,431,224
COL1A1_8_6987,293,451,278,108,297,71,8,21,29,14,118,647

COL1A2_8_6988,163,389,205,433,168,111,14,253,4,344,851,83
COL21A1_8_6989,1549,1258,877,1941,950,1876,683,2752,1791,1231,1100,240
7
COL22A1_8_6990,221,106,293,228,93,3,153,67,60,65,521,238
COL23A1_8_6991,834,438,594,883,718,1336,543,785,341,970,883,1167
COL24A1_8_6992,339,310,166,339,123,264,689,668,29,352,291,243
COL27A1_8_6993,62,106,137,97,4,1,0,60,3,5,23,0
COL3A1_8_6994,251,151,429,147,1078,115,105,693,208,575,433,42
COL4A1_8_6995,1215,1590,570,947,1164,880,597,1226,2282,2153,2289,1820
COL4A2_8_6996,320,421,381,383,1094,1394,2,0,1244,0,42,2
COL4A3_8_6997,914,697,1121,643,645,1198,1859,437,634,500,819,1059
COL4A4_8_6998,423,486,472,413,207,229,638,58,873,627,158,443
COL5A1_8_6999,262,193,187,510,52,80,52,1240,24,643,1163,54
COL5A2_8_7000,612,213,404,422,1101,639,103,913,476,268,631,887
COL5A3_8_7001,846,729,617,575,999,482,1440,900,1330,742,1487,103
COL6A1_8_7002,1418,922,1172,1126,1854,802,761,43,487,306,3234,1244
COL7A1_8_7003,232,305,453,93,289,804,218,205,665,173,617,498
COL8A2_8_7004,34,53,32,82,2,39,0,1,2,72,127,186
COL9A2_8_7005,77,26,12,90,0,1,416,3,103,3,258,0
COL9A3_8_7006,551,630,629,778,218,72,1145,799,1112,499,493,125
COMMD1_8_7007,1633,1549,1555,1873,1516,2083,1297,1671,2295,2415,2560,2
357
COPB2_8_7008,903,786,828,1060,1596,1319,1287,1049,546,923,1476,435
COPG2_8_7009,737,767,776,731,824,1271,0,946,677,547,445,740
COPZ1_8_7010,685,310,336,275,778,992,35,191,474,387,320,238
COPZ2_8_7011,2718,1902,1726,1768,1375,2133,2470,2290,3098,1506,1829,18
48
CORIN_8_7012,615,704,704,365,762,929,572,369,763,347,889,592
COX18_8_7013,1756,1477,1425,2293,1369,1559,1814,3117,2034,1316,2819,26
94
CPLX1_8_7014,955,721,1092,714,1028,388,695,425,404,423,831,1015
CPLX3_8_7015,583,431,338,539,404,571,67,704,520,541,236,259
CPNE6_8_7016,873,846,1152,1040,161,935,1376,625,1237,1804,707,2044
CPXM2_8_7017,2122,2898,1960,2164,966,3215,1300,2255,1082,2826,2531,336
1
CRABP1_8_7018,2751,2755,1761,2683,1798,1753,2083,3059,3115,4210,3638,1
271
CRH_8_7019,168,191,154,188,2,3,700,245,379,38,8,0
CSE1L_8_7020,3765,3937,2661,3249,3500,2237,3133,2522,4167,3681,4204,72
92
CTSW_8_7021,41,55,61,241,13,27,0,53,294,10,27,68
CXCL10_8_7022,2277,2358,1481,2082,2419,1713,2312,1724,2766,2197,1288,1
501
CYGB_8_7023,278,117,243,363,792,116,413,167,258,288,265,131
CYTH3_8_7024,1444,906,1363,1614,2302,1146,1002,1812,1407,2393,1532,477
DDI2_8_7025,2579,1769,1931,2106,1527,2374,2050,1631,2405,1516,3667,161
9
DIRC2_8_7026,147,126,206,182,12,128,10,0,31,92,6,0
DISP1_8_7027,2307,2429,1545,2218,3859,1645,1234,608,4238,1969,1314,330
6

DLL4_8_7028,662,885,710,667,1117,1723,1222,526,810,1138,482,507
DNAJC5B_8_7029,852,663,833,772,558,1131,9,768,238,494,1976,895
DNAJC6_8_7030,397,224,344,521,346,505,721,229,991,275,198,1238
DOC2A_8_7031,568,378,759,593,395,677,931,1555,1081,483,981,873
DOC2B_8_7032,449,499,284,428,408,96,473,1084,104,484,52,604
DSCAML1_8_7033,1077,677,753,951,415,822,164,155,422,550,891,974
ECEL1_8_7034,2703,2715,2719,2484,973,1982,2574,2077,5297,3665,2734,281
7
EID2_8_7035,656,358,713,613,634,171,701,1401,443,324,1666,877
EPCAM_8_7036,791,972,932,1423,491,914,900,227,517,696,268,1016
EXOC2_8_7037,1217,1283,794,1292,583,2216,1852,2518,418,628,843,261
EXOC3_8_7038,2171,1750,1393,1878,1713,3588,1535,1632,1731,1359,1669,29
28
F11R_8_7039,4473,5717,4934,4239,4871,3385,7063,5424,2517,6765,5655,580
1
FABP1_8_7040,697,528,512,468,1516,750,1179,889,1204,1060,816,1104
FABP2_8_7041,8206,9348,7932,8465,6808,8820,5769,6660,8292,7397,9562,98
28
FABP3_8_7042,1296,872,1215,1139,919,1790,891,1172,786,733,2914,1700
FABP4_8_7043,1194,1593,996,1189,690,741,1528,267,1281,1854,1021,1327
FABP7_8_7044,1960,2446,1812,2857,2350,3073,1699,2966,1345,3970,2836,48
35
FABP9_8_7045,1254,1858,1184,1268,1637,745,1960,1384,1177,1234,1489,234
7
FAM101A_8_7046,912,800,461,1108,1693,1735,560,314,852,977,1005,218
FAM117A_8_7047,247,151,333,402,82,122,449,238,77,210,553,564
FAM57A_8_7048,266,125,225,499,327,0,171,786,329,74,892,0
FAP_8_7049,496,416,203,319,512,564,157,985,900,128,9,115
FBF1_8_7050,447,569,528,963,555,818,1024,1645,112,1449,396,1493
FCN1_8_7051,3263,3558,3074,3217,4187,3299,4460,3094,2532,2798,3263,636
7
FDX1_8_7052,411,407,733,666,287,678,277,1397,358,73,229,1898
FDX1L_8_7053,645,571,603,584,271,947,519,650,254,454,1389,690
FGF4_8_7054,1051,1039,954,1124,481,607,1923,338,1240,1469,181,436
FGL2_8_7055,3085,2349,1960,2118,2546,3049,2584,1948,2427,1967,1910,169
6
FOLR3_8_7056,2257,1114,1474,2042,2560,1667,1182,1674,2300,2278,929,191
2
FOLR4_8_7057,257,658,610,481,177,462,0,62,7,389,28,296
FRG1_8_7058,1272,1285,781,1167,1285,753,1479,749,2836,901,1698,869
FTL_8_7059,694,698,1333,717,910,1650,277,1107,1345,540,1496,884
FXC1_8_7060,1186,870,668,1309,1535,785,1468,1389,1475,1004,3145,2007
GABARAP_8_7061,0,0,0,0,0,0,0,0,0,0,0,0
GGA2_8_7062,165,121,50,97,1,78,115,481,18,212,2,288
GJA1_8_7063,468,478,342,308,789,12,273,217,853,313,1659,858
GJA3_8_7064,400,329,179,351,363,527,75,563,115,92,85,889
GJA4_8_7065,124,110,102,306,13,12,62,29,42,124,397,0
GJA8_8_7066,902,1454,806,1180,12,1723,563,769,931,1843,187,1298
GJB2_8_7067,1835,1502,1630,1769,2306,783,1610,1256,1839,2392,2059,643
GJB4_8_7068,1071,1510,2032,1139,2651,2493,224,2469,1205,2492,1378,2463

GJB5_8_7069,1989,1957,1316,1625,2019,3177,2654,1837,1389,1714,1559,173
2
GJC2_8_7070,794,528,320,893,1122,674,367,1027,1468,62,1053,312
GJC3_8_7071,433,520,442,512,661,987,625,738,844,252,724,33
GJD2_8_7072,791,1020,798,1056,1433,586,776,721,461,2173,879,739
GJD3_8_7073,429,370,389,381,954,1051,399,590,599,319,753,388
GJD4_8_7074,325,253,316,249,166,493,219,334,394,377,190,262
GKN1_8_7075,399,294,349,539,992,1850,45,542,508,195,501,646
GLCCI1_8_7076,568,558,393,387,161,9,790,111,554,424,811,105
GLTP_8_7077,383,226,330,368,240,218,1015,185,409,612,166,29
GLYATL2_8_7078,986,1024,971,1485,3212,419,1011,1107,549,2137,192,594
GP9_8_7079,165,628,63,536,0,0,753,0,14,242,12,287
GPIHBP1_8_7080,1806,1608,1803,2040,1812,2559,1912,1623,2467,1018,1710,
2576
GPR180_8_7081,1318,868,1472,915,745,452,2571,901,1567,1276,2329,863
GRN_8_7082,363,137,284,266,1860,247,555,115,855,211,824,176
GZMH_8_7083,173,287,379,236,111,674,169,266,107,52,286,321
GZMK_8_7084,519,776,348,1026,281,1176,14,486,788,438,331,1270
GZMM_8_7085,337,294,559,579,958,446,501,1,674,660,340,342
HBA1_8_7086,1108,1016,597,861,914,1407,360,485,1131,205,148,499
HBA2_8_7087,1108,1016,597,861,914,1407,360,485,1131,205,148,499
HBE1_8_7088,518,492,554,859,437,490,743,84,1051,729,260,1023
HBZ_8_7089,1991,1169,1312,1917,767,1030,217,2595,2436,1278,1472,1394
HECA_8_7090,966,723,583,627,1169,986,812,885,2769,1206,1677,928
HGFAC_8_7091,444,464,504,729,384,589,199,411,649,470,1206,1697
HIAT1_8_7092,2073,1673,1409,1396,1162,1352,1929,856,1544,2354,2103,185
1
HLA-
DQB1_8_7093,1714,1585,1283,1660,1309,1141,1030,2230,2710,2285,1432,221
6
HMCN1_8_7094,1215,1265,1035,1203,600,2250,1591,1882,433,1217,1472,763
HMHA1_8_7095,169,78,70,175,0,5,9,266,28,84,802,662
HPCAL4_8_7096,635,694,822,506,1181,794,214,2,414,1373,1189,297
HPR_8_7097,938,1220,715,903,129,1643,905,843,900,1216,582,1229
HPX_8_7098,1875,1062,1423,1428,822,2182,1685,2931,864,1415,1358,1280
HSP90B1_8_7099,224,178,72,174,50,503,448,641,129,148,216,61
HTRA1_8_7100,1565,986,1132,1496,795,1453,421,1255,370,866,1224,1629
HTRA4_8_7101,1534,1907,1703,1438,3309,1421,1821,3031,293,2329,1948,163
4
IFNG_8_7102,1344,1615,953,1332,1134,1044,347,2361,1438,1458,2104,1172
IGFBP7_8_7103,546,152,658,438,671,302,219,230,246,569,258,144
IL12B_8_7104,889,826,707,593,2500,1072,582,517,1714,709,1974,1201
IL13_8_7105,275,349,358,770,1,478,286,49,109,684,291,243
IL17A_8_7106,18,19,112,44,0,26,0,4,0,10,0,9
IL1A_8_7107,1306,1033,773,1053,469,1018,652,231,2632,2518,1181,1580
IL1B_8_7108,4670,4248,4092,3472,4971,3549,2868,4183,5075,6674,7023,578
4
IL3_8_7109,1144,1307,1523,1533,1389,1897,1619,1518,729,2502,2061,2834
IL5_8_7110,833,1675,782,1555,621,1200,2059,1062,1461,1545,1050,1222
INSL3_8_7111,188,262,189,343,1442,4,196,453,487,1,0,11

IPO13_8_7112,618,355,225,343,222,408,0,184,438,214,989,736
IPO4_8_7113,476,177,616,292,821,310,50,19,125,114,745,352
IPO5_8_7114,1417,1335,1126,1341,655,1134,2651,793,2541,1507,2373,1375
IPO7_8_7115,699,794,1044,500,1555,429,768,886,748,682,178,764
IPO9_8_7116,307,278,468,297,455,406,258,4,704,706,77,2003
ITGA10_8_7117,292,366,308,368,246,539,178,428,119,270,1434,1507
ITGA11_8_7118,2177,2469,1907,1980,2015,2045,2369,2680,2661,2395,2118,2
214
ITGA2_8_7119,2664,2200,1757,1816,1165,1437,2285,1508,3036,1442,2385,17
30
ITGA4_8_7120,5539,4740,3665,5299,6450,3620,5804,7718,6018,4971,5201,42
22
ITGA5_8_7121,1835,2557,2309,2314,1076,1416,2048,3218,2767,2274,3289,54
35
ITGA8_8_7122,847,1396,822,1110,1227,250,32,1628,700,1113,880,1427
ITGAX_8_7123,1667,1760,1030,2017,1625,1592,1406,1572,517,2940,3439,202
0
ITGB5_8_7124,1640,1663,1631,1461,1692,1077,3483,1829,1805,1040,3007,14
00
ITGB6_8_7125,914,946,832,574,768,783,1141,958,646,236,1508,1409
ITGB8_8_7126,1153,1654,922,1119,884,3176,451,335,1148,2272,2163,1308
ITLN1_8_7127,827,543,595,839,692,829,782,350,881,537,1833,1424
KDELR1_8_7128,817,476,433,392,1121,2053,268,54,789,806,183,147
KEL_8_7129,2264,2170,2421,2271,2930,4643,3942,3700,1367,3782,3272,3485
KIF20A_8_7130,676,868,717,835,791,572,2609,1680,446,1127,632,497
KIF3B_8_7131,781,1220,938,1057,599,1081,1188,1392,4,838,1332,1126
KIF5A_8_7132,359,704,614,878,742,354,483,1308,162,385,953,1717
KLK13_8_7133,825,1505,1103,835,762,861,108,305,1563,565,1090,701
KLK14_8_7134,766,758,806,587,1459,926,236,1317,331,299,624,266
KLK4_8_7135,1027,877,1009,902,1165,986,1287,710,953,1172,851,924
KLK9_8_7136,920,1230,1067,1195,806,1244,702,497,3793,913,516,783
KPNA1_8_7137,2048,2639,2872,2128,3120,3193,1577,1769,2954,3269,3698,17
22
KPNA2_8_7138,728,1124,904,760,1673,809,564,534,787,790,1933,802
KPNA3_8_7139,3675,3182,2272,2787,2624,5225,3094,2159,3456,4111,3329,51
96
KPNA4_8_7140,1102,1051,1482,1142,790,1642,1384,1698,1377,1060,1670,233
3
KPNA6_8_7141,234,84,445,121,136,379,124,0,392,126,375,9
KPNB1_8_7142,1028,830,1099,758,443,563,1190,781,909,627,1593,676
KRT12_8_7143,1565,1394,1231,1381,1857,2391,2251,775,2008,1205,1469,169
5
KRT7_8_7144,466,174,149,485,110,457,102,164,66,186,489,492
KRT8_8_7145,1292,851,773,1091,1634,1369,2514,2004,807,1019,1498,547
KRTAP5-4_8_7146,2391,1653,2008,1163,3476,3212,1636,1277,2635,1735,3156
,1978
LASP1_8_7147,381,319,258,475,269,358,992,217,1949,351,32,1946
LBP_8_7148,1114,798,1076,1022,854,2021,1258,916,1691,662,1100,1130
LCN12_8_7149,639,691,577,779,672,828,308,976,298,835,737,1117
LCN1_8_7150,561,850,442,405,197,1920,73,210,361,454,599,776

LCN2_8_7151,610,453,266,847,1546,410,256,247,594,304,921,210
LCN8_8_7152,159,152,263,507,433,42,356,529,20,226,135,119
LCN9_8_7153,401,323,544,640,357,275,195,436,158,1567,460,758
LDLRAD2_8_7154,628,531,511,569,312,918,566,484,65,412,523,701
LDLRAP1_8_7155,581,899,601,431,1158,412,352,734,402,962,394,329
LMAN1_8_7156,346,965,399,380,436,1951,91,2177,1461,684,568,770
LMAN2_8_7157,349,612,714,321,345,847,2547,550,480,127,1198,569
LPA_8_7158,232,276,282,325,419,258,30,205,118,497,717,478
LRIG1_8_7159,2045,2006,1946,2639,2674,3087,990,2907,1562,3635,2123,229
3
LRP2_8_7160,237,164,255,500,67,31,11,292,452,94,12,476
LRRC4B_8_7161,542,262,567,421,253,28,1149,513,612,120,520,159
LRRCC1_8_7162,496,428,328,411,331,1506,546,1162,351,69,32,46
LYST_8_7163,1901,1677,1639,1473,1494,4328,2365,1674,1531,2488,2158,353
7
MAL2_8_7164,837,912,626,586,612,571,686,1576,1094,773,1558,378
MATN3_8_7165,1531,1103,1675,928,1892,1251,1013,497,1568,1166,1604,1212
MFSD8_8_7166,2212,2228,1693,1171,1715,1925,3813,2068,3046,3552,2140,80
7
MFSD9_8_7167,563,196,893,593,1846,475,1260,910,820,166,1445,585
MMAA_8_7168,319,606,195,481,8,226,131,585,451,280,163,706
MMACHC_8_7169,695,677,1117,905,567,998,916,690,718,411,2696,371
MMGT1_8_7170,1225,1245,871,714,1195,1103,760,880,338,737,1583,528
MMP11_8_7171,722,655,445,751,1822,833,486,1392,417,697,274,509
MMP13_8_7172,1291,1672,1028,1370,1610,1256,2288,169,1254,881,2307,1728
MMP15_8_7173,339,295,273,317,470,386,2,502,24,0,170,1
MMP16_8_7174,79,198,31,88,0,329,119,1,6,30,1143,116
MMP17_8_7175,1416,1018,1334,1775,1880,943,603,1161,840,721,1662,1870
MMP19_8_7176,8814,7585,7180,9582,8108,8928,8196,6954,10433,8910,12788,
11003
MMP24_8_7177,176,188,192,187,1365,1,17,64,0,25,959,349
MMP25_8_7178,401,355,207,460,440,514,291,195,130,526,1079,411
MMP26_8_7179,61,283,144,140,116,56,219,0,101,37,221,161
MMP27_8_7180,1728,1543,1652,1978,1740,1291,1813,2048,1787,2976,1687,17
05
MRS2_8_7181,121,135,103,92,174,161,17,0,0,7,46,937
MSTN_8_7182,969,1138,828,854,850,1395,1799,266,521,787,728,1469
MTX2_8_7183,3816,3669,2685,3500,5394,4645,2798,2954,3119,2509,3970,421
8
MUC2_8_7184,707,463,384,221,1052,357,1194,713,1313,461,1050,660
NAPG_8_7185,505,338,530,622,428,575,4,588,10,412,921,450
NAPSA_8_7186,1008,1079,684,757,608,130,1292,698,1178,1937,244,289
NCOA5_8_7187,112,268,224,45,47,144,35,31,176,0,1168,63
NGF_8_7188,1226,1439,1771,2013,1651,1892,1897,943,1255,564,1049,659
NID1_8_7189,941,1111,885,669,704,2170,1832,91,1048,413,1546,786
NPC1_8_7190,1487,1560,1457,1461,1894,1921,1393,1967,2061,2053,1244,192
3
NPEPPS_8_7191,1729,2524,1942,2700,2858,2751,3692,2059,1141,1906,2668,2
393
NPPB_8_7192,82,144,246,274,474,256,540,4,224,468,50,0

NPY_8_7193,1333,1516,1131,1625,1188,831,1122,1442,1521,1876,3464,2318
NSMCE1_8_7194,529,566,602,938,87,875,416,959,249,864,2128,1389
NUP107_8_7195,3547,3815,3607,3878,3472,2578,3073,2028,2943,3650,4777,5
089
NUP133_8_7196,1237,515,631,783,1462,1934,1837,893,349,498,413,1328
NUP153_8_7197,1112,935,972,714,1015,1424,1155,746,1563,352,185,1008
NUP160_8_7198,1455,1116,1442,1509,1703,1946,820,1595,1322,1017,2980,11
17
NUP210_8_7199,485,310,359,299,377,71,303,449,749,165,106,289
NUP214_8_7200,2454,2547,2079,2820,2498,4652,2030,3665,2489,4764,2170,4
219
NUP35_8_7201,2816,3154,1970,2428,2782,4003,3265,2160,2561,2233,1583,33
87
NUP37_8_7202,774,1033,781,975,388,872,1854,489,1444,656,1228,378
NUP54_8_7203,2508,2787,2571,2478,973,3953,2157,2688,4836,2828,2793,426
3
NUP88_8_7204,254,296,159,453,1,742,688,846,137,396,23,405
NUPL2_8_7205,5782,5561,6146,5777,7972,4999,8130,5274,4828,5299,8607,58
66
NUTF2_8_7206,1,0,1,75,0,0,0,1,0,0,0,243
NXF2B_8_7207,1665,1978,1336,2082,1731,2221,2541,2777,1881,2882,1767,16
08
NXF2_8_7208,1665,1978,1336,2082,1731,2221,2541,2777,1881,2882,1767,160
8
NXF3_8_7209,1519,2288,1656,1371,2203,2298,1491,1671,3706,1458,1607,618
NXT1_8_7210,3138,3305,2024,2455,3382,5411,2044,1305,4597,4170,1636,329
0
OAZ2_8_7211,458,684,460,655,546,1628,89,132,1494,935,585,1597
OBP2A_8_7212,597,868,611,564,577,1090,1038,302,1239,405,900,357
OBP2B_8_7213,194,260,225,316,252,176,2,2,52,396,0,491
OCA2_8_7214,1524,1369,1348,1377,1268,2684,2244,662,867,1039,1465,3012
OGFOD1_8_7215,3514,3354,2352,3615,3602,4592,3445,4154,3437,5060,4268,2
400
OGFOD2_8_7216,373,505,234,347,146,208,25,448,2233,571,1151,699
OGFR_8_7217,713,917,559,886,254,513,188,90,521,675,1288,2361
OVCH1_8_7218,0,0,0,0,0,0,0,0,0,0,0,0
OVCH2_8_7219,2211,2249,2152,2348,1607,1776,3360,2553,944,2946,3632,356
6
OXNAD1_8_7220,2296,2360,1898,2266,2141,2634,2907,2748,2000,2905,3336,2
563
OXT_8_7221,387,253,310,161,573,554,0,1,32,27,989,0
PANX1_8_7222,446,490,428,567,743,516,543,0,314,708,500,542
PAQR7_8_7223,377,238,308,728,801,131,28,789,1016,644,338,647
PCDHB11_8_7224,891,830,986,1121,1167,1785,1335,539,822,1042,704,551
PCDHB16_8_7225,2636,2570,1994,2027,1825,3351,2314,3726,2418,2254,2782,
2483
PCSK4_8_7226,595,1237,634,1099,783,321,544,412,198,804,465,93
PCSK7_8_7227,549,399,280,490,1265,197,270,68,365,413,77,154
PEA15_8_7228,179,151,28,151,0,25,147,1,1078,56,633,472
PET112_8_7229,708,562,385,556,442,493,232,744,155,928,1521,1711

PEX13_8_7230,625,406,862,873,1906,661,2394,1222,670,742,383,703
PEX7_8_7231,1125,1756,1185,1580,1406,1146,1298,1746,1110,2444,1329,143
9
PF4_8_7232,884,1147,879,935,874,318,788,679,919,393,1452,390
PFN3_8_7233,293,194,195,198,35,12,43,28,118,19,0,91
PHEX_8_7234,1126,1611,1397,1096,882,968,2717,2709,1238,1187,2278,1510
PIGR_8_7235,972,1016,675,753,198,641,847,195,293,392,636,1591
PITPNA_8_7236,322,745,667,615,1900,841,2201,1157,11,529,71,1760
PITPNB_8_7237,1170,1433,1066,1456,1320,223,2333,1116,1037,931,297,1942
PLLP_8_7238,873,759,679,619,467,800,1784,1109,1643,957,329,525
PLP2_8_7239,696,417,787,467,79,572,1208,188,2059,407,247,88
PLXNB2_8_7240,503,876,460,426,120,461,234,420,284,447,90,891
PNMA2_8_7241,876,1239,1181,873,1090,1381,1140,1458,1299,799,1187,1523
PPP1R14A_8_7242,373,376,327,291,914,444,101,13,58,787,476,129
PPP1R14C_8_7243,904,942,489,583,1364,1474,241,411,412,1176,1365,898
PPP1R15A_8_7244,155,402,122,189,661,435,13,5,0,10,573,20
PPRC1_8_7245,147,250,78,292,1740,241,21,2,66,0,11,259
PPY_8_7246,1009,967,968,1250,541,1063,1669,2148,1572,3105,818,635
PRB3_8_7247,365,264,373,455,1171,98,211,823,352,235,646,562
PROCR_8_7248,1185,686,585,1041,655,1040,67,1066,484,1169,655,828
PROS1_8_7249,2584,3160,3168,3193,4620,2525,2245,1814,2745,2860,3101,30
43
PROZ_8_7250,968,1632,953,1055,798,1048,667,864,772,969,505,1142
PRPF18_8_7251,2248,1926,929,1849,2409,1446,2677,1100,1822,2052,1541,13
03
PRSS12_8_7252,339,211,247,450,656,1,17,0,717,0,349,1419
PRSS22_8_7253,602,281,98,596,507,105,1,481,858,930,583,45
PRSS27_8_7254,865,1041,828,420,1314,2211,1727,602,1802,686,883,386
PRSS33_8_7255,129,246,60,149,0,38,0,7,1201,27,37,7
PRSS36_8_7256,271,321,90,65,0,186,358,51,179,83,1595,0
PRSS8_8_7257,141,106,332,251,150,25,243,243,1,42,169,37
PSCA_8_7258,635,403,258,376,793,354,131,47,32,650,602,389
PTOV1_8_7259,491,267,363,1026,12,30,224,24,665,301,648,53
RABIF_8_7260,421,367,353,231,174,471,990,179,857,535,1109,685
RAMP1_8_7261,388,385,521,377,796,6,558,413,597,440,100,454
RAMP2_8_7262,796,562,410,582,232,1001,481,852,492,1355,390,1329
RAMP3_8_7263,1106,1354,751,1338,833,1382,1361,1235,3627,1189,785,1202
RANBP17_8_7264,753,2149,1114,1088,1574,2935,190,1804,579,2956,2388,549
RASSF9_8_7265,2292,3232,1936,2106,2683,4157,4670,2206,4394,1813,3182,2
749
REEP5_8_7266,5486,5739,3805,5601,5242,4495,6959,6325,5731,6741,4254,66
05
RHAG_8_7267,1745,2102,1750,2142,2278,2958,2443,1152,1178,1974,2721,278
2
RLBP1_8_7268,2311,2365,1846,2042,1844,2463,1287,1393,4165,2467,2484,23
92
RPL15_8_7269,328,297,237,253,167,82,66,334,25,9,470,14
S100A12_8_7270,1780,2177,1469,2223,4018,1502,742,1591,2503,1680,2320,2
765
S100A1_8_7271,821,412,860,136,557,587,862,157,6,305,2556,132

S100A2_8_7272,601,421,891,658,498,116,324,727,1055,1028,707,215
S100A3_8_7273,509,498,343,862,605,216,963,1337,1403,476,739,704
S100A6_8_7274,180,218,445,138,54,163,229,468,296,88,966,19
S100B_8_7275,3369,4530,4065,4251,3399,4450,2706,3677,4371,5367,4641,31
07
S100P_8_7276,2064,1763,1513,1776,2303,3640,2741,2395,2615,2871,1974,22
15
SAA4_8_7277,2627,2931,2521,2423,3512,3140,4225,2318,1341,4242,3987,139
3
SCAMP1_8_7278,4220,2999,3553,3174,3981,4509,3923,3090,3783,3412,4489,5
066
SCAMP2_8_7279,2612,2620,2798,2677,2983,2715,4122,3903,847,2483,3909,33
97
SCFD2_8_7280,2552,2884,3859,2675,3882,4058,2786,3315,4452,3713,3889,22
50
SCLT1_8_7281,1835,1798,1385,1766,1225,2269,990,2048,2198,3266,3369,278
3
SCPEP1_8_7282,827,802,850,945,40,883,1891,328,2034,1214,2088,1631
SDC2_8_7283,614,801,466,706,23,417,87,834,582,371,309,154
SEC14L3_8_7284,328,338,159,202,97,496,105,12,235,242,399,303
SEC22A_8_7285,851,819,630,652,730,1142,412,1381,374,190,1120,691
SEC23A_8_7286,558,594,709,628,231,1243,93,290,428,1650,1343,457
SEC24A_8_7287,472,975,382,240,32,50,73,582,91,544,986,1051
SEC24D_8_7288,1139,1522,1350,1523,2849,768,439,509,1653,1094,1964,125
SEC61A1_8_7289,572,999,505,616,1197,1312,973,432,646,1003,185,1219
SEC61B_8_7290,5601,5566,4647,4487,2702,5482,7929,5928,3702,6412,4209,4
652
SEC62_8_7291,1334,1907,1655,1756,916,2161,2483,2121,2157,2824,1933,128
2
SEC63_8_7292,395,435,469,450,575,474,240,202,743,214,512,946
SELP_8_7293,285,344,381,662,593,265,542,142,305,570,258,1148
SERINC1_8_7294,47,95,86,45,177,32,3,14,6,14,286,36
SERPINA11_8_7295,3762,3329,2692,3848,2708,4000,3350,1863,3597,2962,385
2,4046
SERPINA12_8_7296,473,487,320,327,523,151,532,938,210,650,799,164
SERPINA3_8_7297,201,130,204,56,14,578,1335,0,52,372,15,0
SERPINA4_8_7298,285,181,161,357,270,380,696,238,11,321,295,539
SERPINA5_8_7299,1515,1543,1410,1667,2499,1057,1432,3295,1693,1392,1879
,2059
SERPINA7_8_7300,773,708,570,516,323,638,515,778,344,1355,441,782
SERPINB10_8_7301,2048,1331,1679,1778,3550,3209,2687,3566,1848,1322,313
8,1908
SERPINB12_8_7302,536,331,473,588,526,613,432,113,320,296,545,500
SERPINB13_8_7303,930,960,1190,1096,1204,720,1428,1044,1322,2100,2116,6
37
SERPINB1_8_7304,4301,4396,3802,3660,5379,5884,4246,4046,3401,3437,4750
,5865
SERPINB4_8_7305,597,612,411,670,854,793,718,777,1554,633,489,895
SERPINB9_8_7306,265,342,170,176,1057,3,74,267,133,1073,279,335
SERPINC1_8_7307,582,579,709,433,1095,49,400,918,873,334,607,217

SERPIND1_8_7308,952,1546,1161,1257,511,1408,1300,1098,1295,1239,888,15
76
SERPINF1_8_7309,315,382,175,226,79,248,371,278,389,181,804,517
SFXN1_8_7310,512,480,1037,486,110,490,1212,1040,154,333,1977,884
SFXN2_8_7311,285,250,378,242,253,44,252,1,274,213,514,253
SFXN3_8_7312,486,446,337,445,330,433,594,350,970,624,423,140
SFXN4_8_7313,3675,3232,2391,2858,1603,1855,2483,2903,1617,1859,6467,46
76
SFXN5_8_7314,494,229,330,230,1073,201,119,94,288,74,178,0
SLC15A5_8_7315,517,274,138,432,1925,351,816,578,49,330,487,596
SLC16A13_8_7316,502,672,609,736,473,16,1664,1084,452,1615,827,10
SLC16A14_8_7317,409,700,375,590,188,983,504,74,453,503,407,892
SLC16A9_8_7318,372,328,380,263,96,115,37,500,195,179,466,42
SLC17A9_8_7319,281,269,177,196,179,865,330,109,685,304,72,213
SLC22A20_8_7320,3043,3695,2883,3677,3713,3620,2454,2675,2825,3456,4328
,3427
SLC22A24_8_7321,462,481,919,498,436,405,1243,43,622,81,691,265
SLC22A25_8_7322,697,885,695,1535,1500,151,608,1133,788,1580,1105,407
SLC25A23_8_7323,3618,2579,2666,3212,2614,4701,3692,3280,2689,2882,2999
,2921
SLC25A29_8_7324,941,388,714,568,1216,26,59,440,937,113,862,770
SLC25A31_8_7325,1652,2441,1486,1850,3052,3520,1603,1552,1306,2358,1941
,1448
SLC25A38_8_7326,1644,1154,954,1817,2242,1492,2581,1355,1150,3283,1953,
3178
SLC25A42_8_7327,191,200,117,194,116,154,87,13,56,523,61,173
SLC25A46_8_7328,1398,1337,1470,1065,1228,1180,1586,863,2389,1484,1061,
3082
SLC25A48_8_7329,145,194,55,193,110,494,0,1,10,260,0,1258
SLC38A7_8_7330,526,750,320,700,462,726,995,265,904,854,971,1133
SLC38A9_8_7331,9466,9700,9272,9085,9818,7953,16753,7812,10797,9386,118
79,13527
SLC41A2_8_7332,236,56,104,132,7,59,26,460,37,289,32,0
SLC45A1_8_7333,2036,1696,1898,2000,1198,2522,1212,798,1668,2969,2723,2
155
SLC45A3_8_7334,375,403,171,140,106,173,185,272,672,533,806,114
SLC47A1_8_7335,2951,3555,2844,2458,2836,6745,2476,2169,5903,2710,4840,
2547
SLC48A1_8_7336,1882,1679,1631,1588,933,1499,825,2017,2165,2030,1155,16
98
SLC5A12_8_7337,1124,1025,473,755,877,946,1067,498,1119,844,1424,619
SLC6A17_8_7338,3261,3154,3041,3298,4523,3372,5189,1290,2447,4517,5960,
4651
SLC7A14_8_7339,3420,3365,4141,2078,2545,4055,2371,2966,4003,2735,2777,
2619
SLIT2_8_7340,2332,2685,2139,2147,2642,3441,1322,2438,3119,2988,1494,34
61
SNAP29_8_7341,433,413,262,516,1200,1170,62,399,202,1630,17,336
SNX12_8_7342,777,563,687,547,1068,1460,1315,258,758,1069,1033,538
SNX13_8_7343,5097,5120,4354,5584,5657,8839,6211,5269,4624,6414,6301,77

71
SNX17_8_7344,183,46,209,241,95,96,356,215,118,33,1006,18
SNX19_8_7345,2777,3080,1506,2146,3128,2394,2538,2177,2236,2668,2223,25
35
SNX22_8_7346,480,696,385,478,597,198,18,75,480,1159,336,1284
SNX24_8_7347,3561,2995,3175,2348,4972,3418,4021,4854,2743,3244,5215,37
59
SNX2_8_7348,1410,1825,1474,1757,1369,1573,2841,1636,1111,2791,1565,106
6
SNX4_8_7349,3692,3765,3142,3174,2093,1740,3938,2711,5180,4288,5116,288
5
SNX8_8_7350,1112,589,925,1081,493,703,573,1733,1508,1877,1135,2259
SNX9_8_7351,664,787,330,393,665,1925,307,170,236,0,445,246
SORCS2_8_7352,656,151,331,266,1631,363,490,189,68,527,83,49
SORCS3_8_7353,400,69,408,307,385,44,338,279,315,272,412,627
SORL1_8_7354,630,671,473,734,604,133,462,333,1266,593,156,954
SOS1_8_7355,361,359,442,830,129,480,642,1621,1104,380,492,712
SST_8_7356,344,168,195,417,583,484,1347,152,810,341,455,290
ST13_8_7357,373,342,280,284,808,5,791,242,288,673,366,171
STAB1_8_7358,71,91,77,91,61,69,170,195,9,538,25,20
STARD4_8_7359,493,406,585,280,654,1120,644,195,212,722,521,627
STARD5_8_7360,556,294,257,389,753,815,86,84,99,82,139,763
STARD6_8_7361,1751,2162,1658,2064,3175,1435,1007,1635,2168,2587,3013,1
637
STEAP1_8_7362,1254,1898,996,950,935,2028,2856,1481,1209,1634,2112,949
STMN4_8_7363,674,627,859,1022,676,2351,104,883,1151,378,420,1976
STX11_8_7364,136,195,90,141,187,5,13,65,45,153,400,518
STX18_8_7365,997,870,1087,1163,1393,1169,137,546,1433,1435,3074,1005
STX4_8_7366,68,32,79,87,6,0,525,163,2,1,564,78
STX5_8_7367,424,410,476,478,550,1622,1,599,579,651,589,109
STX6_8_7368,898,913,611,700,685,625,523,235,736,416,672,741
STX7_8_7369,442,771,499,929,761,556,78,578,545,414,170,618
STXBP3_8_7370,3739,4514,2757,3949,2151,5906,4444,2631,3272,3001,5511,4
198
SVOP_8_7371,844,1071,482,632,1273,883,517,449,1346,684,878,1187
SYP_8_7372,2695,2671,2683,2214,1645,3531,2305,3263,2619,2816,2883,4241
SYT10_8_7373,294,193,178,89,122,221,452,8,59,280,271,94
SYT11_8_7374,539,482,460,264,70,583,731,1038,190,330,108,771
SYT13_8_7375,249,297,337,292,338,239,31,379,42,212,189,377
SYT16_8_7376,45,73,30,103,0,57,0,1,1,1,41,244
SYT4_8_7377,897,1073,1150,1384,1258,1693,1146,1262,1107,1334,865,1687
SYT5_8_7378,800,429,246,392,633,710,110,611,1037,963,387,1904
SYT6_8_7379,404,436,209,435,18,967,504,247,29,578,17,0
SYT7_8_7380,373,603,219,286,911,375,452,58,16,140,55,276
SYT8_8_7381,266,258,40,245,350,2,453,722,4,1,1,405
SYT9_8_7382,861,753,1415,829,678,1439,1844,728,1436,642,629,657
TAS2R42_8_7383,9173,9254,8058,8049,9272,12255,7900,6269,8060,11509,113
19,9486
TBC1D9_8_7384,608,611,472,276,642,400,469,770,1189,467,354,490
TCN1_8_7385,1523,1734,1864,2064,1596,3615,2172,1391,979,1785,2045,3994

TEKT4_8_7386,1186,952,1094,953,1456,1303,1078,553,1405,1297,1843,800
TF_8_7387,942,989,1119,534,524,2086,1111,2459,512,981,818,743
TG_8_7388,372,744,396,259,98,243,1057,422,49,821,1680,883
TGFB1_8_7389,742,417,404,284,321,10,205,251,716,174,44,555
TIMM10_8_7390,1387,1671,1063,1081,921,1403,1479,1521,695,2397,2317,597
TIMM13_8_7391,387,334,359,146,568,0,1286,0,497,271,2,1184
TIMM17A_8_7392,558,291,390,361,103,425,790,453,703,421,1547,112
TIMM22_8_7393,1600,1689,1568,1242,2372,2650,1215,2515,1273,2608,3702,2
250
TIMM23_8_7394,3192,3605,3560,3652,4254,3368,3231,2101,3365,4078,6109,4
064
TIMM44_8_7395,1953,2101,942,1716,1917,1525,2003,1734,1308,2996,2857,17
45
TIMM8B_8_7396,2007,2281,1517,1526,1592,1708,2485,1593,948,810,2933,253
3
TIMM9_8_7397,1277,1253,1115,1968,1121,3590,1134,2730,1107,558,1549,946
TLL2_8_7398,56,40,81,30,0,0,107,0,67,0,6,114
TM9SF2_8_7399,2333,2379,2063,2326,2477,2344,2711,2019,2576,3994,3924,2
771
TM9SF3_8_7400,652,468,497,512,202,971,1025,688,993,550,114,1041
TM9SF4_8_7401,791,986,834,1104,243,2197,476,218,766,1185,759,1100
TMCO3_8_7402,3238,4285,3217,3336,2898,3791,2804,3805,4413,3719,5875,54
05
TMED10_8_7403,1110,1259,1138,977,826,1104,757,1735,1748,356,1136,1756
TMED1_8_7404,2056,2109,1208,1412,932,1705,499,1213,1779,2911,1032,2394
TMED2_8_7405,433,1086,900,1255,688,640,1428,926,310,1487,1721,2730
TMED3_8_7406,959,685,590,645,669,997,787,720,992,552,1118,280
TMED4_8_7407,413,964,414,358,342,1624,670,622,71,260,671,1318
TMED7_8_7408,1010,750,932,1064,1101,579,580,1235,1217,1800,1307,682
TMED9_8_7409,329,824,413,327,335,1473,1300,507,74,180,636,1273
TMEM104_8_7410,291,738,324,503,45,871,84,512,163,57,515,223
TMPRSS11B_8_7411,197,139,175,111,123,3,1,927,395,253,0,434
TMPRSS11D_8_7412,137,283,294,201,83,246,92,291,649,185,244,265
TMPRSS11E_8_7413,2070,3887,2832,2257,3301,4562,2344,2818,4677,2475,448
7,4887
TMPRSS11F_8_7414,1747,1905,1504,1794,2224,2226,3218,4104,1515,1569,448
0,2307
TMPRSS12_8_7415,2399,1754,1563,2316,2225,3146,1667,1915,2447,2199,3732
,1881
TMPRSS5_8_7416,1506,1439,1317,1420,1094,2433,1769,1234,1351,2593,2015,
1462
TMPRSS6_8_7417,75,88,49,320,0,67,20,1,149,401,328,61
TMPRSS7_8_7418,460,357,366,332,398,121,148,369,127,28,531,380
TMPRSS9_8_7419,1184,1131,1015,1406,415,1341,4030,1831,1650,688,1536,22
77
TNC_8_7420,242,422,441,264,218,928,14,374,113,102,1137,518
TNF_8_7421,415,1011,937,524,329,1129,773,308,227,199,1005,508
TNFRSF9_8_7422,749,716,701,962,1429,1547,381,1194,430,1026,881,570
TNNC2_8_7423,1163,1076,1246,1144,1012,1572,671,2773,2356,1930,1008,148
3

TNN_8_7424,1216,1287,1123,1517,778,1245,759,482,254,1236,665,686
TNNI3_8_7425,2480,2327,2468,2220,1647,1882,2261,3282,1794,2946,5281,57
05
TNR_8_7426,421,311,116,156,1097,676,98,679,662,441,760,28
TOMM20_8_7427,370,397,263,461,131,967,619,157,355,607,71,88
TOMM22_8_7428,1334,1360,1066,1414,497,1095,1728,1904,1367,1106,2400,23
73
TOMM70A_8_7429,1006,904,822,1059,2253,342,179,701,572,2053,929,702
TOMM7_8_7430,180,393,375,179,8,678,141,396,0,685,800,600
TPSG1_8_7431,188,327,118,222,188,237,108,18,378,311,154,612
TRAK2_8_7432,94,144,129,96,24,220,304,117,193,936,177,2
TRAPPC10_8_7433,3677,3092,2585,2432,1835,2713,3138,2931,4539,4114,3894
,4149
TRAPPC8_8_7434,3170,3396,2975,2547,4024,4422,4620,3065,3645,3962,4266,
4728
TSNAX_8_7435,0,0,0,0,0,0,0,0,0,0,0,0
TTPA_8_7436,916,900,581,632,1063,372,413,779,775,810,1693,1168
TTR_8_7437,474,653,813,676,166,463,1664,2,351,1149,1411,1467
TUBA1A_8_7438,2125,1664,1517,1715,1287,291,3240,2373,3058,2725,3928,21
41
TUBA1C_8_7439,1009,838,1599,1085,778,643,1967,933,690,2287,1032,1369
TUBA3D_8_7440,1508,1441,1211,1201,1209,1409,3191,398,1574,1171,803,208
9
TUBA3E_8_7441,1496,1861,1284,1987,1788,1357,561,1264,1607,1852,1568,17
28
TUBA4A_8_7442,1070,1176,1053,1084,1061,1126,1732,219,2083,731,536,573
TUBB1_8_7443,2037,1615,1534,1703,1282,283,3256,2156,3083,2714,3922,213
1
TUBB2A_8_7444,679,744,509,1089,452,1273,22,917,588,402,677,807
TUBB2B_8_7445,679,744,509,1089,452,1273,22,917,588,402,677,807
TUBB6_8_7446,416,303,115,328,686,1455,13,30,143,146,208,152
TUBB8_8_7447,931,800,742,553,937,767,1968,224,554,117,93,1082
TUBE1_8_7448,1938,2646,1479,2250,2261,4474,2242,1811,3520,2776,2423,25
37
TUBG1_8_7449,2510,2065,2387,1591,2410,758,1200,1589,3871,3740,2089,240
7
TUBG2_8_7450,576,365,732,233,55,592,370,642,695,478,1063,607
TXNDC8_8_7451,3688,4206,3992,3437,3485,4432,1771,3336,3018,3133,4407,3
101
UCP2_8_7452,3344,2864,2197,3444,1970,2200,3913,2161,2085,3583,6558,175
4
USO1_8_7453,1984,1647,1543,1577,1464,2687,3453,2713,1398,2790,2443,146
8
VAMP2_8_7454,5163,5610,4461,5354,5666,6141,4215,4366,6064,5838,3168,51
50
VAMP5_8_7455,1968,2139,1773,1800,1970,2208,2823,2142,1554,2498,1957,18
40
VKORC1L1_8_7456,696,626,989,732,441,502,1643,1126,603,683,1335,1739
VPS18_8_7457,692,711,442,667,109,510,228,899,624,1000,1006,1796
VPS26B_8_7458,650,1130,521,1044,1566,2427,1921,410,425,840,2011,393

VPS33A_8_7459,828,1067,911,2005,1751,1072,626,629,459,2607,261,1448
VPS33B_8_7460,971,829,995,704,742,718,1239,1118,856,627,1159,1970
VPS35_8_7461,489,500,363,228,366,312,365,755,879,283,747,1213
VPS39_8_7462,1344,1440,1335,1315,1684,3796,4283,1503,647,1279,1967,204
9
VPS45_8_7463,821,980,582,755,583,699,140,696,790,455,1597,2255
VPS4B_8_7464,2895,2653,2109,2296,5339,1490,3153,3041,2088,2223,3722,27
67
VSIG2_8_7465,248,161,309,166,674,99,5,57,12,657,20,315
VTI1A_8_7466,584,729,622,519,155,224,62,91,61,823,1426,429
VTI1B_8_7467,1160,1118,1183,891,643,1648,394,1056,1574,1322,713,1058
VTN_8_7468,636,960,774,705,1539,1445,816,96,49,1087,87,16
VWF_8_7469,182,72,100,112,68,430,961,32,4,35,389,3
XK_8_7470,431,525,558,329,801,911,554,855,566,150,344,757
XPO1_8_7471,4090,3914,3085,4289,3577,4016,7187,2947,2653,4737,5277,633
1
XPO4_8_7472,2633,2540,2890,2275,2833,3959,3067,2946,3960,3207,3193,280
7
XPO5_8_7473,7188,8144,7225,6977,4718,11423,7354,8632,4448,9622,8948,67
83
XPO6_8_7474,1636,1939,1783,1512,427,3040,2112,3350,918,1248,1409,645
XPO7_8_7475,2203,2523,1963,2313,1562,2092,2514,1989,1865,3098,1559,109
5
ACTL6A_8_7476,836,616,604,844,1559,487,690,1199,534,805,648,1682
ADAM12_8_7477,519,405,398,701,385,59,530,931,216,635,195,658
ADAM15_8_7478,812,307,568,520,153,2194,539,731,415,610,90,845
ADAM18_8_7479,3485,2094,2079,2912,1162,2441,3327,3092,2406,2216,5003,5
081
ADAM22_8_7480,720,414,381,439,277,78,853,333,435,197,338,589
ADAM29_8_7481,1707,1763,1048,1891,3096,1991,995,2379,1377,1654,2198,14
05
ADAM33_8_7482,1312,2154,1480,1932,3331,2138,1986,1195,1585,2603,2654,2
022
ADAM8_8_7483,1294,1060,1326,1575,666,886,666,698,1614,409,377,2573
ADAMTS13_8_7484,306,112,94,124,0,14,456,117,30,152,101,64
ADAMTS14_8_7485,394,149,203,160,757,824,905,31,788,72,158,179
AKAP12_8_7486,918,470,412,646,291,538,619,150,723,449,426,1645
ANGPT1_8_7487,1128,1471,1017,1023,1000,1635,2386,716,623,870,1217,1291
ANGPTL4_8_7488,2321,1761,1697,1719,2299,1463,577,2070,1679,2501,4714,2
984
AP1B1_8_7489,971,1068,839,1129,1899,1209,962,1244,683,1217,524,1053
AP1G1_8_7490,2130,2295,1867,1864,3187,3397,2563,1545,2313,2562,988,184
9
AP1M1_8_7491,698,805,407,495,368,1186,652,450,749,424,302,699
AP2A1_8_7492,527,1104,472,935,542,484,78,359,678,1237,902,278
AP2A2_8_7493,351,125,428,303,645,388,1701,42,26,247,248,349
AP2B1_8_7494,4674,6799,4176,4539,10567,7991,2957,4065,5325,7966,6482,7
045
AP2M1_8_7495,1802,1703,1408,1120,1241,2386,977,1245,1303,1808,1329,107
7

AP2S1_8_7496,554,734,475,643,293,647,621,1164,985,810,338,864
AP3D1_8_7497,586,324,460,478,3,44,232,166,567,391,45,26
AP3M1_8_7498,875,552,613,778,117,122,512,757,1315,846,850,1464
AP3M2_8_7499,1322,864,1161,1268,990,1454,517,754,921,995,1128,2056
AP4S1_8_7500,6302,5979,5112,5801,8566,6528,7805,3952,5316,6334,5421,73
76
APAF1_8_7501,764,643,525,402,1525,45,1624,564,594,339,979,1003
APBA2_8_7502,1014,1095,889,853,542,1107,664,168,747,1854,2562,2569
APOL1_8_7503,789,851,646,841,1202,979,1830,500,1615,1149,603,1022
APOL3_8_7504,409,436,673,339,450,184,666,203,686,124,300,366
APP_8_7505,2207,2306,1470,2689,3021,3674,2973,3148,1588,2187,1996,2536
ARFGAP3_8_7506,555,528,447,613,1570,319,303,624,824,436,824,81
ARHGAP33_8_7507,154,225,155,286,121,0,2,404,129,6,280,0
ARL6_8_7508,2423,3085,1453,1678,2500,3971,2176,3462,2661,3657,2495,361
2
ATP13A2_8_7509,672,969,1127,1006,892,1835,1750,346,672,346,144,1378
BAX_8_7510,196,304,417,153,504,480,628,20,552,406,226,1055
BCAP29_8_7511,1575,1233,686,939,612,1428,1200,1178,522,2231,1611,487
BCAP31_8_7512,238,24,236,373,7,41,3,41,252,79,127,67
BCL2_8_7513,5642,5510,5208,4965,2377,5209,5544,3084,4896,7262,5634,718
9
BCL2L2_8_7514,323,287,318,597,19,722,129,30,176,171,43,91
BID_8_7515,9218,7096,7639,8146,9577,6371,10767,10177,11963,10448,7871,
9364
BSG_8_7516,425,645,447,360,199,478,164,142,526,291,235,642
C1QC_8_7517,468,521,330,841,101,116,30,60,27,397,839,140
CANX_8_7518,2880,2462,2641,2316,3419,1845,1414,4709,3462,2122,4147,393
1
CAPN9_8_7519,509,131,334,490,25,483,0,378,236,130,322,324
CAPNS1_8_7520,281,160,245,385,74,6,1110,290,231,210,75,135
CCT6B_8_7521,755,1036,828,877,572,471,848,382,296,724,1222,1116
CD19_8_7522,529,463,268,520,1075,515,546,1319,1506,652,289,910
CD22_8_7523,513,566,599,500,633,1060,1812,1791,57,251,1031,410
CD33_8_7524,690,660,525,300,613,1586,1593,387,298,95,388,139
CD44_8_7525,398,431,327,132,632,1092,103,0,908,402,332,600
CD55_8_7526,1446,1458,1147,1481,984,897,1727,1822,1157,2211,2034,641
CDH17_8_7527,2694,3872,2656,3116,1402,4327,3535,2025,2409,3241,1700,59
48
CFHR4_8_7528,2982,3589,3005,2880,1843,3196,3153,2957,3266,3605,4121,48
89
CIZ1_8_7529,264,261,515,329,1279,73,0,50,939,332,311,649
COG2_8_7530,2047,2282,3213,1854,1597,2130,4142,805,1628,3694,1543,2231
COG4_8_7531,1227,988,1362,912,1186,2138,1982,1980,673,975,1412,1953
COG5_8_7532,1644,2004,1541,1790,2400,1341,2856,1567,3506,1800,2842,176
4
COG6_8_7533,2962,2904,2279,2304,2970,2654,3517,2735,1946,2677,2624,210
5
COL11A1_8_7534,2073,3272,1794,2489,2573,3123,3133,1391,2711,3224,2410,
2713
COL11A2_8_7535,357,635,191,531,844,738,62,758,1739,109,612,1056

COL12A1_8_7536,1678,1789,1311,2112,3243,1598,915,1439,2998,1936,2787,4
625
COL13A1_8_7537,522,626,383,708,928,135,315,297,179,917,856,413
COL18A1_8_7538,772,1042,979,1238,1135,800,938,1592,1269,1183,1502,1779
COL25A1_8_7539,126,250,58,180,496,36,8,1337,487,54,0,87
COL2A1_8_7540,376,383,603,448,465,541,6,1047,1395,905,1456,1078
COL4A5_8_7541,821,321,1165,592,547,520,1346,1634,675,770,760,109
COL4A6_8_7542,1272,1513,1689,1735,2606,1629,2533,2449,1752,1635,1436,2
179
COL6A2_8_7543,415,543,983,355,550,353,209,605,570,384,1040,388
COL6A3_8_7544,2233,2074,2187,1470,1027,1761,627,2293,2771,2506,1886,22
80
COL8A1_8_7545,1214,2256,1126,1100,557,1257,591,1413,1093,2116,2124,102
1
COL9A1_8_7546,310,332,82,258,247,79,84,7,167,239,264,435
COPA_8_7547,270,422,255,338,226,324,9,297,161,589,399,129
COPB1_8_7548,6604,7702,7561,7184,4544,7206,6984,4769,6185,5685,8993,58
12
COPE_8_7549,552,296,167,157,736,279,212,1361,1104,165,117,991
CPA4_8_7550,549,933,764,468,690,783,276,296,613,2,1151,438
CPNE1_8_7551,251,182,181,376,42,796,411,831,381,103,1404,9
CPNE7_8_7552,0,0,0,0,0,0,0,0,0,27,0,0
CPXM1_8_7553,81,66,72,110,0,68,383,211,21,28,19,94
CPZ_8_7554,188,280,244,163,281,2,148,109,139,298,270,873
CRABP2_8_7555,490,371,348,511,292,734,373,102,444,796,577,436
CTLA4_8_7556,1691,1939,1435,1258,999,2132,2480,2390,2127,2474,833,3419
CTNS_8_7557,11713,12565,11082,11775,13182,12152,14380,11672,13905,1315
8,14857,15353
CXCL12_8_7558,130,39,7,25,11,1,0,585,69,45,491,604
DPP10_8_7559,2015,1901,1955,2025,1099,1975,2922,872,1856,1674,2554,154
3
DPP6_8_7560,2733,2366,2341,2842,2929,2022,1768,2078,3889,3167,2193,495
7
ECM1_8_7561,106,173,169,74,3,201,63,29,414,471,59,152
EGF_8_7562,215,301,213,178,2,511,552,4,264,27,470,133
EIF2C2_8_7563,483,356,386,394,286,74,329,226,150,254,1101,385
EIF2D_8_7564,359,825,907,568,139,426,887,1139,86,451,78,497
ENSA_8_7565,2623,2779,2460,2352,3835,4239,3157,2212,2363,2836,3250,337
3
EPB41L4B_8_7566,604,699,643,1044,775,1372,745,208,1262,1995,289,1484
EPB42_8_7567,675,1023,578,832,161,646,418,675,816,791,216,358
ETFA_8_7568,861,855,855,1029,239,788,82,1683,895,737,1339,1455
ETFB_8_7569,1680,1737,1773,1837,2046,1357,1424,2234,3943,1794,2749,217
8
EXOC1_8_7570,784,482,472,571,224,855,1276,284,1292,702,668,906
EXOC4_8_7571,563,983,442,517,306,1133,310,160,1195,1069,765,1579
EXOC6_8_7572,1369,1332,1204,1300,1204,957,1051,1816,678,1591,2168,1542
EXOC7_8_7573,170,311,323,366,699,330,741,165,749,88,437,62
F8_8_7574,1113,1116,1299,1380,66,444,233,1070,1168,1153,787,2727
FABP6_8_7575,1537,1189,1703,1519,1067,302,1845,1625,1548,809,3781,2828

FAM131A_8_7576,2707,2004,2057,1998,1897,1742,1808,2789,2532,1761,2227,
2079
FAM63B_8_7577,365,299,473,763,49,323,45,228,667,964,55,556
FANCA_8_7578,1084,775,974,1376,638,2443,1175,1043,1973,1311,560,938
FCN3_8_7579,2255,2392,2144,2452,3041,3443,2402,3488,2537,4890,1151,319
1
FGA_8_7580,193,533,569,376,667,544,614,406,518,449,430,318
FGB_8_7581,834,915,711,1057,88,196,1716,1184,436,798,1699,1324
FGF13_8_7582,366,265,188,339,1290,598,29,128,561,155,415,112
FGG_8_7583,1522,1082,994,1510,2428,1534,1237,1158,2618,2276,905,2242
FIBCD1_8_7584,256,771,282,418,271,786,71,696,332,988,245,35
FLVCR2_8_7585,569,514,427,310,272,218,517,405,126,677,912,888
FOLR1_8_7586,493,769,600,880,215,936,1278,961,678,515,672,363
FOLR2_8_7587,419,240,325,405,396,103,95,11,1287,541,131,86
GGA1_8_7588,751,1263,1191,1088,859,387,731,216,469,661,682,1231
GGA3_8_7589,331,338,659,516,42,557,2,1342,1048,261,1117,1213
GJA5_8_7590,90,416,72,161,321,24,0,823,424,357,553,56
GJB1_8_7591,498,690,657,251,920,32,257,814,806,844,2123,81
GJB3_8_7592,451,581,512,1032,157,610,514,588,739,515,817,635
GJB6_8_7593,7576,7771,5971,8488,8314,5799,6871,7508,6399,11655,9062,10
912
GLYATL1_8_7594,1854,2560,1719,1451,2144,1856,1310,3845,2239,1326,2786,
3185
GNRH1_8_7595,1702,1441,1174,1327,1162,1507,1663,1483,2374,1893,1554,13
17
GOLGA3_8_7596,437,196,412,230,1093,178,195,626,87,273,148,87
GOPC_8_7597,711,615,877,948,978,780,1077,646,868,1060,1219,1424
GOSR1_8_7598,925,844,1201,999,1201,1875,661,874,2417,983,622,181
GOSR2_8_7599,434,447,388,342,265,563,412,389,483,411,782,17
GPRASP1_8_7600,959,902,758,694,938,546,1291,565,117,965,881,1033
GRB2_8_7601,564,896,627,860,1252,269,1473,631,167,729,403,2215
HABP2_8_7602,376,367,441,535,30,16,1098,1494,310,124,745,847
HDLBP_8_7603,673,605,683,716,776,277,888,790,1064,701,326,816
HEPH_8_7604,367,658,414,524,386,225,1145,615,200,583,248,1670
HNRNPU_8_7605,2480,2150,2365,2038,1698,3430,3724,2511,1741,3333,4355,3
986
HOMER2_8_7606,836,1193,927,1235,809,1342,988,911,1321,580,1293,1061
HPN_8_7607,95,453,128,34,566,23,29,29,187,115,0,1
HSDL2_8_7608,3726,4258,4017,3798,4688,4645,2079,3337,3989,5317,6211,63
04
IGF1_8_7609,312,199,203,386,349,726,125,663,278,39,381,635
IGFBP3_8_7610,9,62,5,6,0,556,0,54,330,0,163,142
IPO11_8_7611,2219,1713,1833,1531,2719,3734,1410,1790,2341,2500,2031,25
57
IPO8_8_7612,1341,1187,1545,1292,2131,1787,1699,964,1825,1013,1530,2688
ITGAL_8_7613,672,342,465,688,941,360,139,712,913,1141,451,949
ITGAM_8_7614,651,822,403,431,1069,288,890,66,950,458,360,913
ITGAV_8_7615,1230,1533,1751,1768,1171,1805,1578,1556,1972,1426,2151,54
2
ITGB2_8_7616,529,423,643,667,82,773,504,382,91,1210,273,383

KDELR2_8_7617,7624,8582,6843,7822,7213,9731,7795,9556,6844,6597,9812,1
0440
KDELR3_8_7618,589,659,690,322,293,398,161,1744,325,251,469,58
KIF13A_8_7619,616,563,1063,424,715,1626,2350,506,1965,1545,307,996
KIF17_8_7620,596,250,227,119,208,139,35,839,240,196,128,799
KIF1B_8_7621,920,1213,1303,952,1140,1091,840,1809,599,727,2101,665
KLK10_8_7622,1302,1070,959,901,859,1476,626,1421,1080,1084,709,1555
KLK11_8_7623,540,785,144,446,414,134,35,271,1641,980,334,1896
KLK12_8_7624,617,327,442,462,199,630,212,294,864,590,569,576
KLK15_8_7625,164,198,382,335,53,5,68,33,103,538,33,36
KLK5_8_7626,344,572,527,226,256,1,711,548,748,1442,168,27
KLK6_8_7627,522,250,1035,598,1322,78,1871,34,1218,428,1318,96
LAMB3_8_7628,2444,2358,2424,2351,1040,1797,4546,1840,1877,2390,3900,18
41
LDB3_8_7629,1368,1172,1388,1124,624,2748,1621,772,1004,671,877,1919
LDLR_8_7630,276,317,241,348,5,0,30,108,70,66,195,423
LTA_8_7631,381,237,335,356,65,112,1938,36,468,33,847,36
LTF_8_7632,570,650,477,690,630,202,1218,527,97,518,589,343
M6PR_8_7633,1848,1716,1356,1777,813,2109,1646,1167,731,2238,2062,745
MASP1_8_7634,1037,1602,1130,1087,1129,2015,943,750,809,1360,1326,1378
MB_8_7635,618,651,209,740,1014,748,1209,93,354,353,202,1180
MCFD2_8_7636,121,304,108,160,28,150,0,12,12,24,2,549
MCL1_8_7637,1630,1799,1601,1508,1705,2024,1798,1923,677,2188,535,1438
MEFV_8_7638,816,503,799,603,794,828,1731,241,878,644,2579,777
MFAP4_8_7639,259,119,107,89,515,43,115,395,144,217,328,1
MFSD10_8_7640,913,746,624,1294,1049,1505,274,1638,736,821,980,565
MFSD1_8_7641,322,398,414,292,320,0,527,0,106,341,127,895
MFSD5_8_7642,600,390,410,566,98,754,154,582,839,627,513,1008
MLC1_8_7643,444,508,315,327,343,912,1134,1603,447,512,315,559
MSLN_8_7644,312,665,465,521,1461,726,1687,880,437,676,1037,161
MTX1_8_7645,134,255,322,221,56,424,66,97,3,115,5,569
MUC1_8_7646,953,737,579,651,370,339,169,733,73,1459,482,508
NCAM1_8_7647,31,51,111,96,7,91,8,47,42,73,59,11
NNAT_8_7648,252,187,97,194,6,119,21,105,388,74,85,34
NOXO1_8_7649,259,327,280,361,375,25,44,81,0,240,20,55
NPC1L1_8_7650,378,412,372,372,883,499,4,934,171,226,81,201
NPRL3_8_7651,208,208,212,207,156,270,456,124,214,223,277,101
NRXN1_8_7652,251,211,226,185,0,142,3,184,341,105,368,85
NRXN2_8_7653,781,1025,724,855,58,733,656,1951,394,530,764,1289
NRXN3_8_7654,4044,3600,3458,3422,5063,6163,4409,2184,4755,3344,3816,41
34
NUP155_8_7655,15,8,128,91,27,0,0,2,333,46,0,19
NUP50_8_7656,213,353,601,327,33,19,887,777,321,78,1102,1774
NUP62_8_7657,37,166,92,253,8,77,72,28,77,187,97,210
NUP98_8_7658,374,467,472,349,471,678,168,1094,583,284,782,104
NUPL1_8_7659,769,850,583,694,182,1050,1592,1035,263,1415,1675,1221
NXF1_8_7660,392,272,322,347,69,154,38,268,21,545,205,650
NXNL2_8_7661,39,27,60,113,281,46,107,3,12,44,483,21
NXT2_8_7662,918,990,617,1093,1053,1387,1631,1710,419,2113,2632,255
OAZ3_8_7663,2323,2426,2378,2668,2250,2953,2620,1421,3063,2088,3083,188

9
PACSIN2_8_7664,1050,1429,1666,1070,1364,571,668,1324,1506,920,299,146
PANX2_8_7665,910,636,697,503,704,386,1326,648,940,783,1492,827
PCDHA6_8_7666,347,163,307,46,311,230,0,689,347,19,143,31
PCDHGA5_8_7667,402,710,510,423,153,133,256,812,241,734,319,450
PCLO_8_7668,1146,1325,1462,1045,1503,1761,836,1420,678,954,1559,1250
PCSK5_8_7669,1939,1882,2101,1728,1611,2211,1775,2269,1705,1441,1831,39
10
PCSK6_8_7670,78,281,180,265,278,79,602,439,949,72,4,2
PCTP_8_7671,67,140,50,257,27,436,174,0,122,15,10,445
PDYN_8_7672,469,419,319,382,523,88,545,33,82,988,908,456
PDZD3_8_7673,427,287,595,859,426,430,480,847,829,677,327,591
PDZK1_8_7674,2109,1823,1492,1195,2756,2253,3228,1406,1245,2093,2436,12
40
PGAP2_8_7675,623,609,1095,484,483,1089,1387,316,1009,1612,437,953
PGF_8_7676,217,277,347,453,0,125,385,538,544,398,271,330
PIK3R3_8_7677,2395,1758,1613,2399,2688,534,2347,1980,3114,2599,1813,13
83
PITPNC1_8_7678,4132,3990,3474,3463,4846,5930,5160,3055,2582,4739,3434,
1874
PITPNM1_8_7679,960,718,1081,1586,962,3091,2553,327,389,2004,1026,1044
PITPNM3_8_7680,1337,1205,1233,1359,426,2004,883,786,938,2365,1664,1385
PLEC_8_7681,675,824,873,836,11,993,1517,1704,278,62,358,1189
PLIN3_8_7682,73,201,50,70,127,1,304,1,36,88,21,589
PLTP_8_7683,1386,1884,1231,856,394,1786,1445,772,3091,1805,1302,2526
PNKD_8_7684,264,159,151,148,306,234,406,38,520,377,411,533
POMC_8_7685,180,150,318,22,0,0,0,0,307,1,634,9
PORCN_8_7686,188,309,431,344,281,553,423,73,371,1192,92,2082
PREPL_8_7687,1551,1822,1333,2089,2621,1252,1230,4047,2554,1432,2018,29
49
PRNP_8_7688,200,28,260,36,0,3,1119,92,28,161,890,35
PRSS21_8_7689,404,1017,430,577,961,835,571,1128,1027,598,82,338
PRSS35_8_7690,4413,4184,3505,4929,2533,4391,8309,4327,3272,5719,5464,5
244
PSEN1_8_7691,858,776,280,487,1088,53,431,643,673,699,779,493
PSEN2_8_7692,240,180,189,355,1,260,442,787,0,330,269,0
RABEP1_8_7693,656,511,453,590,1352,91,1701,405,675,548,525,1450
RACGAP1_8_7694,638,798,536,762,1873,535,1765,568,247,866,2049,1827
RARRES1_8_7695,1257,764,1159,898,942,1198,1474,1367,865,1597,598,1813
RASA1_8_7696,7704,8238,8232,8318,12135,10356,5459,10646,10356,9817,989
3,12586
RELN_8_7697,921,1279,1404,1688,265,1154,3289,1375,322,1606,1559,1948
RHCE_8_7698,421,471,364,266,61,27,39,146,162,186,572,209
RHD_8_7699,807,782,673,674,976,422,542,1050,755,324,1543,726
RIMS2_8_7700,564,435,159,397,155,420,19,1033,427,1502,300,77
RRBP1_8_7701,862,1383,1041,981,1434,1193,921,404,524,1061,221,479
RUFY1_8_7702,1251,945,1082,1104,1519,1029,868,1224,977,1300,27,478
S100A13_8_7703,771,703,422,869,1112,679,994,988,957,1181,962,117
S100A4_8_7704,1659,1538,2122,1692,539,2304,1982,2895,2189,1264,4408,20
33

SAA1_8_7705,1558,1564,1867,2133,1430,1370,3145,753,1444,1743,2559,1196
SCAMP3_8_7706,5156,5264,5895,6145,4013,5025,5170,5731,6962,5103,7031,6
428
SCARB1_8_7707,362,243,336,315,36,713,164,367,287,289,2213,366
SCFD1_8_7708,376,510,381,1021,625,1499,1138,2074,246,593,924,983
SEC13_8_7709,136,103,102,91,263,31,312,85,35,108,135,377
SEC14L1_8_7710,1910,2397,1445,1622,2171,3589,2454,964,2260,2920,2633,1
464
SEC14L2_8_7711,1967,1992,2158,2425,3979,1264,1740,2246,3300,2201,2561,
4132
SEC14L4_8_7712,153,262,229,336,7,37,0,0,434,207,69,251
SEC23B_8_7713,826,662,1071,592,188,445,829,226,1919,271,372,489
SEC24B_8_7714,4808,5391,4212,4265,3307,7186,5674,2636,2596,5090,5370,4
723
SEC24C_8_7715,3007,3369,3034,3155,5676,3589,3987,4689,3950,2420,2792,3
484
SEC61A2_8_7716,430,92,291,325,115,149,216,98,829,41,232,734
SEC61G_8_7717,275,235,361,330,599,235,82,334,17,301,196,408
SEH1L_8_7718,1049,806,728,716,550,2522,630,708,548,1294,352,450
SERINC2_8_7719,514,685,524,904,305,747,747,470,1395,538,1418,1045
SERINC3_8_7720,10453,11523,9440,11015,10889,11832,10903,16050,9831,111
69,17677,10821
SERPINA10_8_7721,510,816,417,474,207,1900,730,62,738,720,376,313
SERPINA1_8_7722,772,369,447,530,387,699,94,820,1932,546,470,860
SERPINB2_8_7723,5951,5144,4903,4861,6691,4644,6737,3569,5440,7463,5308
,4726
SERPINB6_8_7724,500,783,525,483,1140,1107,23,385,1520,466,6,1612
SERPINB8_8_7725,992,953,889,747,2222,1754,613,1734,864,946,656,1553
SERPINE1_8_7726,532,563,206,213,26,1389,204,5,16,656,98,235
SERPINF2_8_7727,192,276,179,71,316,285,49,308,573,8,15,2
SERPING1_8_7728,2550,2480,2031,2099,2273,2261,2555,1250,2050,1904,1223
,2367
SERPINH1_8_7729,220,40,22,88,4,875,0,0,263,391,0,0
SERPINI1_8_7730,1337,1043,1477,1167,735,1119,985,1270,2409,919,2899,21
36
SFI1_8_7731,1479,1348,1615,1772,1320,1194,558,1384,2757,1689,1580,1447
SFTPA1_8_7732,408,624,399,287,775,1165,247,74,789,1285,609,341
SH3D19_8_7733,503,337,448,382,569,752,225,471,782,1575,613,972
SIL1_8_7734,84,175,235,360,0,17,473,10,0,310,0,0
SLC25A25_8_7735,1863,2363,1896,2321,1809,880,1355,2114,1094,2251,3377,
2231
SLC25A36_8_7736,1157,952,1402,1768,1645,623,92,416,926,1359,1924,568
SLC25A45_8_7737,391,735,397,573,363,561,571,420,973,254,422,414
SLC38A10_8_7738,1545,1394,1627,1186,956,871,911,2220,930,1293,1126,184
6
SLC41A3_8_7739,514,628,336,635,118,640,91,545,116,22,396,2053
SLC43A3_8_7740,320,310,124,356,47,224,64,296,68,279,310,626
SLC44A2_8_7741,2572,2681,2672,2822,3035,2905,2964,2055,2206,5718,3204,
2773
SLC44A4_8_7742,926,946,853,757,2121,985,3166,1376,1304,1051,3017,1575

SLC44A5_8_7743,788,451,534,534,1372,644,796,54,1334,197,1316,288
SLC46A1_8_7744,2011,2130,2463,1925,3234,1136,2016,2081,3280,1585,1766,
2994
SLC47A2_8_7745,26,166,103,83,0,0,10,239,85,15,0,435
SLC50A1_8_7746,361,380,220,346,160,133,6,73,14,125,68,1109
SLC6A20_8_7747,965,1017,499,1011,1405,666,1034,1072,1188,843,532,1623
SNAP23_8_7748,3951,3835,3909,3007,1144,5284,5439,3253,4190,3724,5857,4
067
SNAP25_8_7749,873,622,317,666,69,700,175,532,350,298,570,1627
SNX10_8_7750,1201,459,858,542,600,305,2030,2574,1002,995,2151,7
SNX11_8_7751,199,485,157,787,212,52,211,92,137,639,390,854
SNX14_8_7752,2669,4067,2646,3637,1927,2487,2634,4704,2175,4292,4055,55
46
SNX15_8_7753,626,634,416,554,288,1221,220,218,331,431,106,412
SNX16_8_7754,2230,2305,2100,2387,1793,1691,2078,3243,3347,1457,3176,21
97
SNX18_8_7755,32,75,89,81,371,89,30,22,24,497,20,29
SNX1_8_7756,261,208,461,220,550,76,83,248,222,255,814,455
SNX3_8_7757,3461,3822,3031,3289,4260,5267,1997,817,3374,4470,2348,5853
SNX5_8_7758,4179,3810,3501,3817,3102,3844,5128,4823,2259,3649,5331,504
2
SNX6_8_7759,4570,4680,3324,4137,5931,8208,2649,6645,4048,5667,6835,293
0
SNX7_8_7760,861,803,782,905,1417,982,260,137,101,541,1728,1747
SORCS1_8_7761,367,76,384,146,0,8,26,0,0,1,1409,9
SORT1_8_7762,1519,1258,1451,1249,1523,1691,3023,1613,1986,1296,3473,11
17
SPNS1_8_7763,1144,787,1257,1027,1501,408,1378,784,399,1168,1526,1195
SRI_8_7764,4,14,10,0,0,8,0,0,0,0,0,0
STARD3_8_7765,1232,1455,873,1191,1321,405,1318,2017,2479,1034,1261,157
5
STAU1_8_7766,1318,903,863,1139,1699,2548,954,344,1876,720,705,503
STEAP2_8_7767,2938,2760,2662,2284,2918,2986,2756,3673,1617,4171,5766,3
331
STEAP3_8_7768,866,725,473,664,1739,340,62,1883,610,1119,766,903
STIM2_8_7769,1262,1146,688,1769,1268,951,1758,1463,391,428,271,1246
STX16_8_7770,876,1484,1042,601,165,1607,484,1729,1005,430,614,272
STX1A_8_7771,405,147,338,458,1925,1172,292,320,142,216,163,58
STX2_8_7772,186,406,322,66,0,1343,384,285,270,1,170,1207
STX3_8_7773,3372,3928,2985,3836,2159,1326,2760,2289,2757,2035,4583,578
2
STXBP1_8_7774,546,446,387,558,979,519,758,121,334,361,310,127
STXBP2_8_7775,1860,1190,1643,1860,3284,1227,1179,1260,1424,1196,1281,2
177
SV2B_8_7776,2383,1964,1330,1337,3041,1282,1987,1559,1155,1458,1768,249
9
SYN1_8_7777,0,0,0,0,0,0,0,0,0,0,0,0
SYNGR1_8_7778,308,25,105,220,72,30,0,18,667,1,8,144
SYNPR_8_7779,760,777,652,645,1524,315,154,584,470,463,1129,272
SYPL1_8_7780,13919,14716,12572,13847,19351,16076,16844,13090,11775,155

65,14094,13325
SYT12_8_7781,162,275,142,417,0,61,16,94,217,87,162,497
SYT14_8_7782,546,615,530,605,18,187,0,635,839,600,648,1073
SYT15_8_7783,1092,1365,1198,999,796,566,828,660,493,1794,3965,2509
SYT1_8_7784,1528,2291,1719,1783,2144,1370,2930,1140,2399,1474,1746,377
5
SYT2_8_7785,2702,3013,1989,2494,3757,3454,2893,5223,2302,2958,2839,268
0
SYT3_8_7786,326,379,366,394,457,750,738,494,257,499,210,209
TAPBP_8_7787,324,443,232,483,499,336,1,7,63,593,138,186
TC2N_8_7788,1274,1428,670,1243,897,1418,1998,1249,1082,1292,2046,1679
TCN2_8_7789,1588,1874,954,1220,1530,3037,738,1106,2216,934,859,2084
TCOF1_8_7790,3670,3832,3219,3708,4245,2449,2432,3217,4937,4476,6552,40
83
TFPI_8_7791,982,866,675,782,901,1288,729,447,639,646,1119,2221
TFR2_8_7792,1019,948,890,703,658,1060,356,483,731,2013,1445,1110
TFRC_8_7793,1042,750,1081,897,527,519,484,2882,1492,610,1003,1129
TGFB2_8_7794,1185,1140,1049,1010,5519,446,918,1174,1566,2029,1710,478
TIMM17B_8_7795,235,66,119,193,14,108,56,65,506,108,3,20
TINAGL1_8_7796,403,224,229,369,63,378,1169,237,279,131,384,282
TLL1_8_7797,1647,1986,1414,1441,2732,2033,2260,2439,2142,2049,1948,161
7
TM9SF1_8_7798,661,419,610,501,2296,739,32,503,13,352,133,476
TMC6_8_7799,825,1019,689,858,473,911,1785,477,1459,1278,402,1719
TMPRSS11A_8_7800,8284,7356,6840,7473,9656,10588,4912,8145,7646,8718,89
84,13731
TMPRSS13_8_7801,1396,1748,844,1337,2379,2161,1764,1429,722,1374,1210,9
39
TMPRSS2_8_7802,232,195,48,307,22,8,2,1,41,63,186,4
TMPRSS4_8_7803,750,710,407,365,103,992,1035,86,661,214,585,1207
TNFSF11_8_7804,1287,955,891,935,2243,1125,463,1714,249,1331,2466,1068
TNFSF13B_8_7805,671,680,917,1525,1122,1063,465,930,718,718,909,1442
TNPO2_8_7806,479,523,417,548,277,387,122,562,917,562,557,573
TOM1_8_7807,1294,2234,1462,953,1053,1493,2647,1857,2890,1508,1893,1625
TOM1L2_8_7808,4149,4394,3320,4833,4772,4869,4031,4686,4817,4792,3258,3
782
TSC1_8_7809,87,368,298,171,1,167,5,605,0,645,417,169
TSC2_8_7810,723,740,844,801,997,665,1629,1620,624,1323,1708,1585
TUBA8_8_7811,293,478,210,251,42,9,11,161,343,137,180,477
TUBB3_8_7812,2231,2117,1848,2696,3586,1750,2777,1631,2087,1981,2861,25
57
TUBD1_8_7813,1988,2171,2117,2326,3492,2505,3262,550,3380,2463,3456,179
4
UCP3_8_7814,129,115,125,143,88,7,0,8,2,28,246,72
UPF3A_8_7815,4032,3359,3729,3737,3393,2426,3928,2124,4120,4188,5384,38
29
UPF3B_8_7816,1793,1589,1486,1800,1564,1520,3346,2085,1276,2296,768,129
2
VAMP1_8_7817,281,302,121,299,101,591,507,68,292,108,314,10
VAMP7_8_7818,525,384,377,512,113,1146,25,286,404,594,338,597

VCAM1_8_7819,1096,1021,800,1156,784,2126,528,705,726,1210,1052,1072
VLDLR_8_7820,760,844,1170,841,544,1274,1047,1367,596,2698,599,1628
VPS13A_8_7821,1741,1803,1272,1623,1338,867,1974,2190,2050,1890,1225,12
76
VPS13B_8_7822,2619,2279,2069,2044,2910,2572,3383,1752,3201,3368,1497,2
837
VPS16_8_7823,318,304,238,450,88,619,279,318,52,310,26,255
VPS26A_8_7824,2737,3109,2667,3634,2185,1884,4739,3469,1418,2788,5225,4
283
VPS28_8_7825,0,0,0,0,0,0,0,0,0,0,0,0
ZFYVE16_8_7826,3243,4062,2655,3278,2873,3561,3002,3835,4246,2826,3276,
3722
ZNF160_8_7827,470,1113,753,974,53,1643,1138,360,329,751,776,551
ZP3_8_7828,255,233,60,225,680,15,128,306,126,138,1,223
BET1L_8_7829,0,0,0,1,0,1,1,0,0,0,0,0
C2orf83_8_7830,2142,2082,2017,1230,3972,1862,2143,2289,1130,2598,1810,
3329
ERP29_8_7831,499,652,662,591,266,1076,313,463,321,583,402,548
FGF1_8_7832,675,895,890,1360,765,724,95,790,633,586,815,699
LYNX1_8_7833,879,937,793,1125,388,632,665,1292,1427,962,2245,310
MMP28_8_7834,1163,1236,1492,966,1504,1135,2480,841,1304,1004,1259,1345
PDPN_8_7835,778,883,711,479,781,681,45,2687,443,514,150,468
SNX21_8_7836,377,527,532,447,1781,499,88,140,1029,1060,314,675
TIMM8A_8_7837,272,168,287,230,94,113,1291,26,74,95,4,397
VEGFA_8_7838,2395,2555,2700,2948,3148,3989,3181,2491,3233,3167,1791,33
40
CDH23_8_7839,1013,537,504,669,599,1847,468,1123,949,278,1829,384
CDH23_8_7840,843,1001,611,510,40,1760,1028,787,1517,843,901,350
CDH23_8_7841,212,82,138,325,27,47,0,0,0,0,43,0
CDH23_8_7842,232,171,334,265,563,597,161,686,32,255,522,658
CDH23_8_7843,1627,1272,1265,1508,1083,2465,1298,3666,2314,1377,1279,21
41
TNXB_8_7844,669,512,965,659,1027,848,857,22,236,461,842,209
TNXB_8_7845,530,760,995,696,626,218,599,1681,899,396,1787,1218
A2M_8_7846,749,736,635,797,309,285,773,1053,880,659,917,812
ACE2_8_7847,807,1450,1412,629,331,244,1555,1018,1036,377,767,155
ACTR6_8_7848,1053,1247,1224,1324,337,1147,1149,1664,1310,924,1993,3953
ADAM11_8_7849,219,74,179,75,116,2,567,87,60,0,67,24
ADAM19_8_7850,1329,1858,1040,865,1517,1226,1511,1055,1100,1091,1379,13
15
ADAM20_8_7851,3476,2707,2948,2700,2474,4932,3142,3125,1841,2626,5224,4
687
ADAM21_8_7852,988,1278,1225,1119,1196,704,1189,2590,1812,1875,682,1292
ADAM2_8_7853,3008,3381,2354,2611,2706,3686,2810,2348,3192,4784,2606,20
00
ADAM30_8_7854,3995,3677,3390,3446,3346,3505,4920,1724,3628,4008,2719,4
769
ADAM7_8_7855,938,1103,1241,757,1239,1103,2233,180,583,1047,1213,1313
ADAM9_8_7856,2633,2162,1843,2409,2014,2142,2782,2205,1823,1378,3357,33
59

ADAMTS10_8_7857,1201,1248,980,1142,1171,1210,1408,251,1346,1520,1650,7
89
ADAMTS12_8_7858,411,801,387,522,337,558,717,224,217,1685,418,430
ADAMTS15_8_7859,1600,1193,1513,1276,1655,1014,2083,434,1110,1225,1872,
1810
ADAMTS18_8_7860,1399,1359,1374,1520,328,2446,1061,2014,379,1655,2726,2
235
ADAMTS19_8_7861,1365,1384,654,984,1281,1133,1928,1785,722,1539,533,128
8
ADAMTS1_8_7862,4005,3668,4934,3810,2851,2842,4209,2128,2743,4837,6544,
8709
ADAMTS20_8_7863,258,122,234,489,13,58,19,137,155,121,297,87
ADAMTS3_8_7864,1475,867,1011,1071,371,2156,1167,683,632,570,1594,2314
ADAMTS5_8_7865,55,14,51,70,0,303,25,21,0,0,146,25
ADAMTS6_8_7866,934,1026,1121,868,1791,2226,1962,1395,722,616,909,1108
ADAMTS7_8_7867,1012,958,816,1320,1559,1599,664,813,1170,1118,1804,1351
ADAMTS8_8_7868,721,807,711,265,1374,634,638,231,1382,780,691,959
AEBP1_8_7869,638,756,683,796,768,2094,198,196,255,709,240,748
AFG3L2_8_7870,637,538,581,492,376,1282,373,35,909,182,990,235
AFM_8_7871,592,835,775,463,426,547,0,100,153,1035,433,453
AFP_8_7872,460,367,379,532,107,99,160,695,573,192,990,379
AGTPBP1_8_7873,1463,859,834,1027,1648,2516,630,965,698,1181,549,1054
ALG10B_8_7874,1338,1571,1337,1212,1486,1351,2719,1735,876,916,1338,253
8
AMBP_8_7875,922,678,748,547,2058,476,3004,649,682,593,495,2300
ANGPT4_8_7876,779,1104,857,998,2121,1154,2152,1181,1982,1119,1755,1112
ANGPTL1_8_7877,1189,311,1004,527,195,632,613,713,1005,1718,425,2166
ANGPTL2_8_7878,1062,725,1064,1168,472,373,784,567,1339,956,399,1214
ANGPTL3_8_7879,2206,2082,2053,1416,1060,2621,2073,2124,2666,1900,2407,
2115
ANGPTL7_8_7880,1701,2073,1716,1467,1241,1411,1715,980,1028,1254,2713,1
470
ANKH_8_7881,548,523,624,701,431,213,535,11,615,304,1103,923
AP1G2_8_7882,1335,1306,1032,1365,1209,2085,1860,1712,556,965,1623,2298
AP1M2_8_7883,1245,1369,1008,903,800,692,2108,498,395,1685,2331,1397
AP1S1_8_7884,3038,3192,2763,2898,3061,3226,4294,4999,3450,3441,2859,37
18
AP1S2_8_7885,560,412,466,545,54,413,508,1,621,343,405,73
AP1S3_8_7886,4447,4635,4391,3949,3900,7434,2799,5432,4388,5794,7254,28
91
AP3B1_8_7887,642,979,429,578,981,908,1126,648,671,844,424,534
AP3B2_8_7888,1541,1793,1109,1657,1408,1100,1761,1862,3187,817,565,1331
AP3S1_8_7889,2379,2584,1987,2470,2341,2447,3176,2596,2320,3143,4441,33
15
AP3S2_8_7890,385,524,384,297,169,338,391,142,178,487,525,307
AP4B1_8_7891,4250,4018,3770,4286,2918,3330,4386,3825,4469,5141,3110,51
01
AP4M1_8_7892,2504,1981,2137,1677,1299,3830,1831,3445,1673,1364,1474,15
87
APBA1_8_7893,463,179,382,430,330,78,864,106,843,634,331,529

APBA3_8_7894,433,436,482,357,349,352,1213,480,101,637,787,763
APOA1_8_7895,593,255,239,818,1389,152,44,1972,18,359,588,695
APOA2_8_7896,4586,5251,4069,4262,4178,3501,6104,3961,5079,4242,7575,52
93
APOA4_8_7897,1924,1671,1961,2127,2200,2758,2606,1379,2056,1576,3006,28
09
APOB_8_7898,1461,1673,1172,1302,910,1349,813,1636,1403,665,1516,1327
APOC1_8_7899,1639,1709,1665,2168,3116,3240,2543,1429,618,1751,3515,229
9
APOC2_8_7900,1048,1456,1021,1106,1183,1125,1356,757,746,1834,892,1486
APOC3_8_7901,1463,898,1097,993,1175,547,1244,794,377,439,1938,1242
APOC4_8_7902,671,799,309,1041,650,213,1032,855,226,576,222,1265
APOD_8_7903,1265,1052,811,783,1347,783,405,2218,515,1318,1964,656
APOE_8_7904,752,637,615,880,534,1986,1418,475,355,660,114,832
APOF_8_7905,429,495,212,209,1762,107,488,395,149,116,310,292
APOH_8_7906,688,813,630,728,994,1004,706,808,892,553,761,689
APOL6_8_7907,1177,747,520,920,939,1163,515,1314,201,1584,563,719
APOM_8_7908,862,1427,1422,1320,405,1010,733,813,1131,2316,71,467
AQP12B_8_7909,747,724,576,451,249,1297,1263,1245,813,301,1712,435
ARF5_8_7910,789,712,592,693,233,516,1364,264,135,715,912,875
ARF6_8_7911,426,570,313,352,934,475,86,863,178,414,779,142
ARPP19_8_7912,983,836,1035,907,1786,1334,480,1609,1308,856,1308,3078
ASTL_8_7913,669,799,552,724,62,3167,1621,2435,318,1359,2066,3271
ATOX1_8_7914,663,980,1360,471,496,505,994,855,806,736,1368,1196
ATP13A1_8_7915,535,289,279,350,391,115,231,315,679,627,215,141
ATP13A3_8_7916,3087,2377,2571,3184,3963,2129,1715,2019,2783,2406,3857,
2794
ATP13A4_8_7917,800,784,710,744,1043,988,891,2098,1550,307,1436,511
ATP13A5_8_7918,408,574,386,745,340,796,477,50,682,853,259,573
AZGP1_8_7919,781,582,346,755,1050,611,522,381,355,572,1017,2010
AZU1_8_7920,217,115,75,256,89,208,183,126,109,664,50,5
BCL2L10_8_7921,1434,1587,1469,1237,1808,1342,888,1671,674,766,2930,634
BET1_8_7922,254,499,273,202,0,250,1249,198,115,143,104,299
BGLAP_8_7923,353,198,127,484,9,128,365,31,657,914,593,143
BOC_8_7924,281,425,514,333,368,320,576,295,43,357,953,348
BPI_8_7925,481,389,416,576,481,983,970,478,24,1347,1946,451
BPIFC_8_7926,1777,2011,1826,1575,1136,888,1097,1360,1658,2526,3656,169
4
C16orf7_8_7927,2130,2115,2149,2767,2107,2486,1082,1720,3185,2456,2787,
1782
C1orf162_8_7928,1320,1194,966,968,1666,1984,692,1896,267,470,2408,1691
C1QA_8_7929,33,69,37,9,0,1,488,0,2,0,0,0
C1QB_8_7930,1305,1491,1491,2218,812,1075,1438,655,1670,2351,1973,1389
C1RL_8_7931,386,344,147,305,81,234,319,279,81,102,10,499
C20orf141_8_7932,811,694,600,781,862,1102,2079,848,430,443,709,498
C3_8_7933,114,254,127,328,0,285,4,232,0,36,0,311
C4A_8_7934,604,519,468,415,697,218,188,997,309,189,842,413
C5_8_7935,541,511,1038,508,36,2273,754,883,56,1801,791,597
C7orf31_8_7936,3553,3160,2805,2991,3880,3150,2076,1772,2914,3074,2750,
3671

C8G_8_7937,235,164,264,247,639,329,1052,132,130,168,145,931
CALM2_8_7938,4287,3570,3152,4017,5217,4060,3124,3025,4383,5019,4604,52
53
CALY_8_7939,1189,1561,1051,1157,949,1333,432,690,1547,2824,1273,1730
CAMLG_8_7940,1330,1177,991,841,233,1772,1797,1151,1864,795,786,317
CAPN11_8_7941,159,372,232,275,143,643,166,474,1233,53,12,85
CAPN5_8_7942,1973,1876,1658,2338,2303,2192,2098,795,1540,867,2126,3644
CAPN6_8_7943,388,625,457,461,660,444,167,223,207,1054,148,700
CARTPT_8_7944,192,300,101,61,0,2,0,0,82,0,315,0
CCL13_8_7945,514,926,455,862,708,1609,1508,253,599,1331,1255,658
CCND1_8_7946,1644,1350,1727,1170,2041,3167,1361,668,2037,1594,1811,214
7
CD1A_8_7947,988,1551,909,1010,1547,1489,1077,1646,375,1179,1566,241
CD52_8_7948,456,717,473,299,498,239,345,716,396,8,1262,1216
CDCP2_8_7949,163,292,165,151,324,595,2,480,208,91,850,15
CDH5_8_7950,1552,1584,1757,1888,1896,1849,1147,1765,1325,1296,1769,111
3
CHMP7_8_7951,244,319,132,311,302,114,209,12,68,609,79,4
CLDN16_8_7952,438,447,254,339,756,377,356,304,242,167,610,1229
CLEC3B_8_7953,1004,1391,1199,1443,1124,645,1893,1133,1216,488,1369,832
CLSTN2_8_7954,1080,1242,1198,1275,1121,369,1751,850,1696,509,1346,390
CLVS2_8_7955,489,910,183,181,104,410,976,391,1126,428,181,473
CNIH3_8_7956,508,358,582,659,223,283,447,162,758,396,616,397
CNOT6_8_7957,295,206,317,437,721,471,973,558,192,35,386,122
CNTNAP1_8_7958,1899,1492,1295,1205,2213,1710,1792,2369,2237,1767,1783,
470
COG1_8_7959,5886,6538,3830,5030,4157,6795,3115,5255,4277,6112,6455,459
3
COG3_8_7960,4065,4034,3303,3397,4798,4744,3261,4367,3010,3126,6831,531
9
COG7_8_7961,1214,986,953,1181,1866,2600,620,1437,1419,539,1288,1432
COG8_8_7962,107,106,289,47,69,53,414,89,1,56,0,302
COL10A1_8_7963,670,868,658,620,529,573,523,863,565,94,361,1016
COL14A1_8_7964,1011,1044,582,598,501,1599,323,620,1437,458,1371,898
COL15A1_8_7965,609,429,503,691,742,1073,620,499,615,1362,871,526
COL16A1_8_7966,43,27,85,81,1,105,76,0,0,0,1,1
COL17A1_8_7967,1522,1434,1142,963,628,893,1372,1750,2344,474,2070,859
COL1A1_8_7968,80,201,130,153,165,75,928,56,439,5,23,133
COL1A2_8_7969,2700,2398,2240,2531,4689,3357,2209,4486,1685,1968,1900,2
000
COL21A1_8_7970,1137,915,1129,945,1363,1967,1191,615,1615,541,1169,1357
COL22A1_8_7971,2142,1780,1870,2132,489,2266,582,2419,1979,2070,2095,14
40
COL23A1_8_7972,41,42,240,142,78,206,36,139,125,94,486,81
COL24A1_8_7973,1641,1165,1058,1036,156,1235,989,1695,2297,1293,3577,97
4
COL27A1_8_7974,87,185,267,247,4,719,2,53,0,20,344,10
COL3A1_8_7975,1088,1253,1346,1107,2096,1087,2402,1924,928,1018,944,817
COL4A1_8_7976,1678,1383,1606,1830,1489,1462,3495,2512,1017,1175,1400,2
529

COL4A2_8_7977,324,588,583,136,345,864,567,31,16,620,394,54
COL4A3_8_7978,922,1008,1013,665,809,678,536,424,757,1348,677,1274
COL4A4_8_7979,6,1,89,11,0,0,105,58,1,0,161,152
COL5A1_8_7980,641,684,457,928,517,636,1197,116,531,874,645,1723
COL5A2_8_7981,555,533,352,590,553,474,232,40,412,253,391,572
COL5A3_8_7982,316,437,251,116,502,31,306,57,59,949,186,95
COL6A1_8_7983,364,378,411,159,160,317,13,470,622,183,313,961
COL7A1_8_7984,401,477,255,482,70,478,632,3,104,138,950,977
COL8A2_8_7985,91,195,297,362,207,101,3,42,338,43,68,824
COL9A2_8_7986,305,256,33,154,24,0,253,38,99,0,0,5
COL9A3_8_7987,217,83,158,164,0,0,8,0,0,287,506,245
COMMD1_8_7988,1001,882,1121,799,298,685,812,1014,275,2200,980,2
COPB2_8_7989,2331,2136,1724,1516,1897,1285,1664,1779,2698,2966,3348,27
10
COPG2_8_7990,1585,1713,1172,1283,3098,2660,2325,2285,2187,1617,920,588
COPZ1_8_7991,464,422,608,454,330,780,287,898,860,695,489,2592
COPZ2_8_7992,1265,1120,1331,1673,2443,1389,1751,1371,1587,1879,1837,10
51
CORIN_8_7993,1043,1044,1546,1309,157,1241,455,548,610,927,631,504
COX18_8_7994,301,381,492,442,79,125,96,194,674,475,137,896
CPLX1_8_7995,545,236,560,462,419,403,16,229,679,738,648,1007
CPLX3_8_7996,604,412,738,696,850,54,6,747,153,7,1514,717
CPNE6_8_7997,4721,3355,3526,3619,5000,5154,8109,2461,2370,5091,4950,44
73
CPXM2_8_7998,3217,2439,3640,2638,6637,1849,4231,4235,1821,2367,1895,53
04
CRABP1_8_7999,604,314,274,572,417,171,527,986,194,1194,506,1116
CRH_8_8000,1006,1154,1001,1412,300,1505,222,879,899,620,2207,2793
CSE1L_8_8001,0,0,0,0,0,0,0,0,0,0,0,0
CTSW_8_8002,0,0,0,0,0,0,0,0,0,0,0,0
CXCL10_8_8003,0,0,0,0,0,0,0,0,0,0,0,0
CYGB_8_8004,0,0,0,0,0,0,0,0,0,0,0,0
CYTH3_8_8005,0,0,0,0,0,0,0,0,0,0,0,0
DDI2_8_8006,0,0,0,0,0,0,0,0,0,0,0,0
DIRC2_8_8007,0,0,0,0,0,0,0,0,0,0,0,0
DISP1_8_8008,0,0,0,0,0,0,0,1,0,0,0,0
DLL4_8_8009,0,0,0,0,0,0,0,0,0,0,0,0
DNAJC5B_8_8010,135,266,66,399,150,196,196,28,207,287,349,282
DNAJC6_8_8011,34,61,192,70,0,0,702,376,0,0,0,0
DOC2A_8_8012,0,0,0,0,0,0,0,0,0,0,0,0
DOC2B_8_8013,0,0,0,0,0,0,0,0,0,0,0,0
DSCAML1_8_8014,0,0,0,0,0,0,0,0,0,0,0,0
ECEL1_8_8015,0,0,0,0,0,0,0,0,0,0,0,0
EID2_8_8016,1535,1639,1486,1666,531,2826,1091,929,1551,2064,2548,2408
EPCAM_8_8017,0,0,0,0,0,0,0,0,0,0,0,0
EXOC2_8_8018,0,0,0,0,0,0,0,0,0,0,0,0
EXOC3_8_8019,0,0,0,0,0,0,0,0,0,0,0,0
F11R_8_8020,0,0,0,0,0,0,0,0,0,0,0,0
FABP1_8_8021,0,0,0,0,0,0,0,0,0,0,0,0
FABP2_8_8022,0,0,0,0,0,0,0,0,0,0,0,0

FABP3_8_8023,0,0,0,0,0,0,0,0,0,0,0,0
FABP4_8_8024,0,0,0,0,0,0,0,0,0,0,0,0
FABP7_8_8025,0,0,0,0,0,0,0,0,0,0,0,0
FABP9_8_8026,1,2,0,0,1,2,0,0,0,0,5,0
FAM101A_8_8027,256,159,71,149,138,1,9,0,4,0,33,11
FAM117A_8_8028,0,0,0,0,0,0,0,0,0,0,0,0
FAM57A_8_8029,0,0,0,0,0,0,0,0,0,0,0,0
FAP_8_8030,0,0,0,0,0,0,0,0,0,0,0,0
FBF1_8_8031,0,0,0,0,0,0,0,0,0,0,0,0
FCN1_8_8032,0,0,0,0,0,0,0,0,0,0,0,0
FDX1_8_8033,518,251,698,641,634,164,602,40,357,532,603,279
FDX1L_8_8034,0,0,0,0,0,0,0,0,0,0,0,0
FGF4_8_8035,0,0,0,0,0,0,0,0,0,0,0,0
FGL2_8_8036,0,0,0,0,0,0,0,0,0,0,0,0
FOLR3_8_8037,0,0,0,0,0,0,0,0,0,0,0,0
FOLR4_8_8038,0,0,0,0,0,0,0,0,0,0,0,0
FRG1_8_8039,0,0,0,0,0,0,0,0,0,0,0,0
FTL_8_8040,0,0,0,0,0,0,0,0,0,0,0,0
FXC1_8_8041,0,0,0,0,0,0,0,0,0,0,0,0
GABARAP_8_8042,0,0,0,0,0,0,0,0,0,0,0,0
GGA2_8_8043,0,0,0,0,0,0,0,0,0,0,0,0
GJA1_8_8044,0,0,0,0,0,0,0,0,0,0,0,0
GJA3_8_8045,2383,2412,2527,2291,4067,3416,1575,1336,2286,3583,2983,450
2
GJA4_8_8046,0,0,0,0,0,0,0,0,0,0,0,0
GJA8_8_8047,0,0,0,0,0,0,0,0,0,0,0,0
GJB2_8_8048,0,0,0,0,0,0,0,0,0,0,0,0
GJB4_8_8049,2457,1884,1418,1332,1439,1367,353,2560,1355,2703,2373,1195
GJB5_8_8050,0,0,0,0,0,0,0,0,0,0,0,0
GJC2_8_8051,258,117,187,213,60,11,355,615,430,639,264,1
GJC3_8_8052,0,0,0,0,0,0,0,0,0,0,0,0
GJD2_8_8053,0,0,0,0,0,0,0,0,0,0,0,0
GJD3_8_8054,0,0,0,0,0,0,0,0,0,0,0,0
GJD4_8_8055,0,0,0,0,0,0,0,0,0,0,0,0
GKN1_8_8056,0,0,0,0,0,0,0,0,0,0,0,0
GLCCI1_8_8057,0,0,0,0,0,0,0,0,0,0,0,0
GLTP_8_8058,0,0,0,0,0,0,0,0,0,0,0,0
GLYATL2_8_8059,0,0,0,0,0,0,0,0,0,0,0,0
GP9_8_8060,0,0,0,0,0,0,0,0,0,0,0,0
GPIHBP1_8_8061,0,0,0,0,0,0,0,0,0,0,0,0
GPR180_8_8062,0,0,0,0,0,0,0,0,0,0,0,0
GRN_8_8063,218,71,90,99,5,443,90,7,631,0,11,61
GZMH_8_8064,0,0,0,0,0,0,0,0,0,0,0,0
GZMK_8_8065,0,1,0,0,0,0,0,0,0,0,0,0
GZMM_8_8066,2,1,1,0,0,0,0,0,0,0,1,0
HBA1_8_8067,0,0,0,0,0,0,0,0,0,0,0,0
HBA2_8_8068,0,0,0,0,0,0,0,0,0,0,0,0
HBE1_8_8069,58,161,227,121,0,8,0,0,11,169,602,7
HBZ_8_8070,0,0,0,0,0,0,0,0,0,0,0,0
HECA_8_8071,0,0,0,0,0,0,0,0,0,0,0,0

HGFAC_8_8072,0,0,0,0,0,0,0,0,0,0,0,0
HIAT1_8_8073,0,0,0,0,0,0,0,0,0,0,0,0
HLA-DQB1_8_8074,0,0,0,0,0,0,0,0,0,0,0,0
HMCN1_8_8075,0,0,0,0,0,0,0,0,0,0,0,0
HMHA1_8_8076,0,0,0,0,0,0,0,0,0,0,0,0
HPCAL4_8_8077,0,0,0,0,0,0,0,0,0,0,0,0
HPR_8_8078,0,0,0,0,0,0,0,0,0,0,0,0
HPX_8_8079,9,2,3,5,0,1,5,10,2,1,5,5
HSP90B1_8_8080,0,0,0,0,0,0,0,0,0,0,0,0
HTRA1_8_8081,253,225,276,220,327,911,289,474,210,544,567,314
HTRA4_8_8082,3,1,2,3,0,3,2,1,0,9,2,1
IFNG_8_8083,0,0,0,0,0,0,0,0,0,0,0,0
IGFBP7_8_8084,0,0,0,0,0,0,0,0,0,0,0,0
IL12B_8_8085,0,0,0,0,0,0,0,0,0,0,0,0
IL13_8_8086,651,490,415,581,368,667,168,1417,912,350,374,348
IL17A_8_8087,0,0,0,0,0,0,0,0,0,0,0,0
IL1A_8_8088,0,0,0,0,0,0,0,0,0,0,0,0
IL1B_8_8089,0,0,0,0,0,0,0,0,0,0,0,0
IL3_8_8090,0,0,0,0,0,0,0,0,0,0,0,0
IL5_8_8091,0,0,0,0,0,0,0,0,0,0,0,0
INSL3_8_8092,0,0,0,0,0,0,0,0,0,0,0,0
IPO13_8_8093,0,0,0,0,0,0,0,0,0,0,0,0
IPO4_8_8094,0,0,0,0,0,0,0,0,0,0,0,0
IPO5_8_8095,0,0,0,0,0,0,0,0,0,0,0,0
IPO7_8_8096,0,0,0,0,0,0,0,0,0,0,0,0
IPO9_8_8097,0,0,0,0,0,0,0,0,0,0,0,0
ITGA10_8_8098,0,1,0,1,1,1,0,1,0,0,0,0
ITGA11_8_8099,0,0,0,0,0,0,0,0,0,0,0,0
ITGA2_8_8100,0,0,0,0,0,0,0,0,0,0,0,0
ITGA4_8_8101,0,0,0,0,0,0,0,0,0,0,0,0
ITGA5_8_8102,0,0,0,0,0,0,0,0,0,0,0,0
ITGA8_8_8103,0,0,0,0,0,0,0,0,0,0,0,0
ITGAX_8_8104,0,0,0,0,0,0,0,0,0,0,0,0
ITGB5_8_8105,0,0,0,0,0,0,0,0,0,0,0,0
ITGB6_8_8106,0,0,0,0,0,0,0,0,0,0,0,0
ITGB8_8_8107,0,0,0,0,0,0,0,0,0,0,0,0
ITLN1_8_8108,0,0,0,0,0,0,0,0,0,0,0,0
KDELR1_8_8109,0,0,0,0,0,0,0,0,0,0,0,0
KEL_8_8110,0,0,0,0,0,0,0,0,0,0,0,0
KIF20A_8_8111,0,0,0,0,0,0,0,0,0,0,0,0
KIF3B_8_8112,0,0,0,0,0,0,0,0,0,0,0,0
KIF5A_8_8113,0,0,0,0,0,0,0,0,0,0,0,0
KLK13_8_8114,0,0,0,0,0,0,0,0,0,0,0,0
KLK14_8_8115,0,0,0,0,0,0,0,0,0,0,0,0
KLK4_8_8116,0,0,0,0,0,0,0,0,0,0,0,0
KLK9_8_8117,0,0,0,0,0,0,0,0,0,0,0,0
KPNA1_8_8118,0,0,0,0,0,0,0,0,0,0,0,0
KPNA2_8_8119,0,0,0,0,0,0,0,0,0,0,0,0
KPNA3_8_8120,0,0,0,0,0,0,0,0,0,0,0,0
KPNA4_8_8121,0,0,0,0,0,0,0,0,0,0,0,0

KPNA6_8_8122,0,0,0,0,0,0,0,0,0,0,0,0
KPNB1_8_8123,0,0,0,0,0,0,0,0,0,0,0,0
KRT12_8_8124,0,0,0,0,0,0,0,0,0,0,0,0
KRT7_8_8125,0,0,0,0,0,0,0,0,0,0,0,0
KRT8_8_8126,0,0,0,0,0,0,0,0,0,0,0,0
KRTAP5-4_8_8127,0,0,0,0,0,0,0,0,0,0,0,0
LASP1_8_8128,306,63,73,104,57,1,221,90,44,29,118,52
LBP_8_8129,0,0,0,0,0,0,0,0,0,0,0,0
LCN12_8_8130,0,0,0,0,0,0,0,0,0,0,0,0
LCN1_8_8131,0,0,0,0,0,0,0,0,0,0,0,0
LCN2_8_8132,0,0,0,0,0,0,0,0,0,0,0,0
LCN8_8_8133,0,0,0,0,0,0,0,0,0,0,0,0
LCN9_8_8134,0,0,0,0,0,0,0,0,0,0,0,0
LDLRAD2_8_8135,0,0,0,0,0,0,0,0,0,0,0,0
LDLRAP1_8_8136,0,0,0,0,0,0,0,0,0,0,0,0
LMAN1_8_8137,1857,2043,1809,2288,3332,2223,2758,3628,989,1864,2723,139
5
LMAN2_8_8138,0,0,0,0,0,0,0,0,0,0,0,0
LPA_8_8139,0,0,0,0,0,0,0,0,0,0,0,0
LRIG1_8_8140,0,0,0,0,0,0,0,0,0,0,0,0
LRP2_8_8141,0,0,0,0,0,0,0,0,0,0,0,0
LRRC4B_8_8142,0,0,0,0,0,0,0,0,0,0,0,0
LRRCC1_8_8143,7,10,10,6,4,2,13,5,9,7,4,5
LYST_8_8144,0,0,0,0,0,0,0,0,0,0,0,0
MAL2_8_8145,0,0,0,0,0,0,0,0,0,0,0,0
MATN3_8_8146,0,0,0,0,0,0,0,0,0,0,0,0
MFSD8_8_8147,0,0,0,0,0,0,0,0,0,0,0,0
MFSD9_8_8148,0,0,0,0,0,0,0,0,0,0,0,0
MMAA_8_8149,0,0,0,0,0,0,0,0,0,0,0,0
MMACHC_8_8150,0,0,0,0,0,0,0,0,0,0,0,0
MMGT1_8_8151,0,0,0,0,0,0,0,0,0,0,0,0
MMP11_8_8152,1125,1221,822,1224,2519,750,680,2312,1479,2179,822,1345
MMP13_8_8153,0,0,0,0,0,0,0,0,0,0,0,0
MMP15_8_8154,1,6,4,2,0,0,16,1,0,0,0,0
MMP16_8_8155,0,0,0,0,0,0,0,0,0,0,0,0
MMP17_8_8156,0,0,0,0,0,0,0,0,0,0,0,0
MMP19_8_8157,0,0,0,0,0,0,0,0,0,0,0,0
MMP24_8_8158,0,0,0,0,0,0,0,0,0,0,0,0
MMP25_8_8159,0,0,0,0,0,0,0,0,0,0,0,0
MMP26_8_8160,0,0,0,0,0,0,0,0,0,0,0,0
MMP27_8_8161,0,0,0,0,0,0,0,0,0,0,0,0
MRS2_8_8162,0,0,0,0,0,0,0,0,0,0,0,0
MSTN_8_8163,0,0,0,0,0,0,0,0,0,0,0,0
MTX2_8_8164,0,0,0,0,0,0,0,0,0,0,0,0
MUC2_8_8165,0,0,0,0,0,0,0,0,0,0,0,0
NAPG_8_8166,0,0,0,0,0,0,0,0,0,0,0,0
NAPSA_8_8167,0,0,0,0,0,0,0,0,0,0,0,0
NCOA5_8_8168,0,0,0,0,0,0,0,0,0,0,0,0
NGF_8_8169,0,0,0,0,0,0,0,0,0,0,0,0
NID1_8_8170,0,0,0,0,0,0,0,0,0,0,0,0

NPC1_8_8171,0,0,0,0,0,0,0,0,0,0,0,0
NPEPPS_8_8172,0,0,0,0,0,0,0,0,0,0,0,0
NPPB_8_8173,312,359,406,234,566,256,330,113,179,39,29,618
NPY_8_8174,0,0,0,0,0,0,0,0,0,0,0,0
NSMCE1_8_8175,3,3,2,4,2,2,3,0,0,0,3,4
NUP107_8_8176,0,0,0,0,0,0,0,0,0,0,0,0
NUP133_8_8177,0,0,0,0,0,0,0,0,0,0,0,0
NUP153_8_8178,0,0,0,0,0,0,0,0,0,0,0,0
NUP160_8_8179,0,0,0,0,0,0,0,0,0,0,0,0
NUP210_8_8180,0,0,0,0,0,0,0,0,0,0,0,0
NUP214_8_8181,0,0,0,0,0,0,0,0,0,0,0,1
NUP35_8_8182,1003,1133,1495,1165,466,1597,917,1680,786,1188,1220,686
NUP37_8_8183,0,0,0,0,0,0,0,0,0,0,0,0
NUP54_8_8184,0,0,0,0,0,0,0,0,0,0,0,0
NUP88_8_8185,0,0,0,0,0,0,0,0,0,0,0,0
NUPL2_8_8186,0,0,0,0,0,0,0,0,0,0,0,0
NUTF2_8_8187,108,74,162,99,0,1008,0,122,0,15,557,41
NXF2B_8_8188,0,0,0,0,0,0,0,0,0,0,0,0
NXF2_8_8189,0,0,0,0,0,0,0,0,0,0,0,0
NXF3_8_8190,0,0,0,0,0,0,0,0,0,0,0,0
NXT1_8_8191,0,0,0,0,0,0,1,1,0,0,0,1
OAZ2_8_8192,0,0,0,0,0,0,0,0,0,0,0,0
OBP2A_8_8193,0,0,0,0,0,0,0,0,0,0,0,0
OBP2B_8_8194,835,372,555,461,192,1445,286,1108,525,1151,186,464
OCA2_8_8195,592,787,824,701,326,1201,480,420,12,1304,617,1053
OGFOD1_8_8196,0,0,0,0,0,0,0,0,0,0,0,0
OGFOD2_8_8197,363,396,266,792,257,147,950,940,894,426,529,579
OVCH1_8_8198,0,0,0,0,0,0,0,0,0,0,0,0
OVCH2_8_8199,0,0,0,0,0,0,0,0,0,0,0,0
OXNAD1_8_8200,0,0,0,0,0,0,0,0,0,0,0,0
OXT_8_8201,0,0,0,0,0,0,0,0,0,0,0,0
PANX1_8_8202,0,0,0,0,0,0,0,0,0,0,0,0
PAQR7_8_8203,0,0,0,0,0,0,0,0,0,0,0,0
PCDHB11_8_8204,0,0,0,0,0,0,0,0,0,0,0,0
PCDHB16_8_8205,0,0,0,0,0,0,0,0,0,0,0,0
PCSK4_8_8206,0,0,0,0,0,0,0,0,0,0,0,0
PCSK7_8_8207,0,0,0,0,0,0,0,0,0,0,0,0
PEA15_8_8208,0,0,0,0,0,0,0,0,0,0,0,0
PET112_8_8209,0,0,0,0,0,0,1,0,0,0,0,0
PEX13_8_8210,0,2,0,0,0,0,1,0,0,0,0,0
PEX7_8_8211,3,3,4,6,3,2,5,4,1,6,2,2
PF4_8_8212,4185,3999,3870,3598,5200,6980,5573,4551,3228,3419,3843,4859
PFN3_8_8213,92,22,19,42,341,60,3,29,1,159,0,2
PHEX_8_8214,1,0,0,0,0,0,0,0,0,0,0,0
PIGR_8_8215,0,0,0,0,0,0,0,0,0,0,0,0
PITPNA_8_8216,0,0,0,0,0,0,0,0,0,0,0,0
PITPNB_8_8217,0,0,0,0,0,0,0,0,0,0,0,0
PLLP_8_8218,0,0,0,0,0,0,0,0,0,0,0,0
PLP2_8_8219,0,0,0,0,0,0,0,0,0,0,0,0
PLXNB2_8_8220,0,0,0,0,0,0,0,0,0,0,0,0

PNMA2_8_8221,0,0,0,0,0,0,0,0,0,0,0,0
PPP1R14A_8_8222,0,0,0,0,0,0,0,0,0,0,0,0
PPP1R14C_8_8223,654,846,805,530,816,575,1543,684,1177,986,1945,389
PPP1R15A_8_8224,0,0,0,0,0,0,0,0,0,0,0,0
PPRC1_8_8225,0,0,0,0,0,0,0,0,0,0,0,0
PPY_8_8226,1150,969,947,683,1359,667,355,1963,812,1130,1264,675
PRB3_8_8227,0,0,0,0,0,0,0,0,0,0,0,0
PROCR_8_8228,0,0,0,0,0,0,0,0,0,0,0,0
PROS1_8_8229,0,0,0,0,0,0,0,0,0,0,0,1
PROZ_8_8230,0,0,0,0,0,0,0,0,0,0,0,0
PRPF18_8_8231,0,0,0,0,0,0,0,0,0,0,0,0
PRSS12_8_8232,0,0,0,0,0,0,0,0,0,0,0,0
PRSS22_8_8233,0,0,0,0,0,0,0,0,0,0,0,0
PRSS27_8_8234,0,0,0,0,0,0,0,0,0,0,0,0
PRSS33_8_8235,0,0,0,0,0,0,0,0,0,0,0,0
PRSS36_8_8236,0,0,0,0,0,0,0,0,0,0,0,0
PRSS8_8_8237,0,0,0,0,0,0,0,0,0,0,0,0
PSCA_8_8238,0,0,0,0,0,0,0,0,0,0,0,0
PTOV1_8_8239,0,0,0,0,0,0,0,0,0,0,0,0
RABIF_8_8240,0,0,0,0,0,0,0,0,0,0,0,0
RAMP1_8_8241,0,0,0,0,0,0,0,0,0,0,0,0
RAMP2_8_8242,0,0,0,0,0,0,0,0,0,0,0,0
RAMP3_8_8243,0,0,0,0,0,0,0,0,0,0,0,0
RANBP17_8_8244,0,0,0,0,0,0,0,0,0,0,0,0
RASSF9_8_8245,0,1,0,0,0,0,0,0,0,0,0,0
REEP5_8_8246,0,0,0,0,0,0,0,0,0,0,0,0
RHAG_8_8247,0,0,0,0,0,0,0,0,0,0,0,0
RLBP1_8_8248,0,0,0,0,0,0,0,0,0,0,0,0
RPL15_8_8249,1,2,1,3,2,5,2,3,4,1,9,1
S100A12_8_8250,404,712,370,475,225,367,459,366,781,507,1038,1049
S100A1_8_8251,147,157,299,173,1006,604,113,5,23,214,4,676
S100A2_8_8252,0,0,0,0,0,0,0,0,0,0,0,0
S100A3_8_8253,2839,2532,2444,2478,2639,1880,6104,3255,3461,1837,4173,3
400
S100A6_8_8254,0,0,0,0,0,0,0,0,0,0,0,0
S100B_8_8255,0,0,0,0,0,0,0,0,0,0,0,0
S100P_8_8256,1345,1118,1047,1366,1993,1831,2351,1766,1270,1577,1911,21
96
SAA4_8_8257,0,0,0,0,0,0,0,0,0,0,0,0
SCAMP1_8_8258,13,9,9,6,7,10,37,5,8,6,12,8
SCAMP2_8_8259,0,0,0,0,0,0,0,0,0,0,0,0
SCFD2_8_8260,8,6,3,4,4,4,1,4,4,6,5,2
SCLT1_8_8261,0,0,0,0,0,0,0,0,0,0,0,0
SCPEP1_8_8262,0,0,0,0,0,0,0,0,0,0,0,0
SDC2_8_8263,0,0,0,0,0,0,0,0,0,0,0,0
SEC14L3_8_8264,0,0,0,0,0,0,0,0,0,0,0,0
SEC22A_8_8265,0,0,0,0,0,0,0,0,0,0,0,0
SEC23A_8_8266,0,0,0,0,0,0,0,0,0,0,0,0
SEC24A_8_8267,0,0,0,0,0,0,0,0,0,0,0,0
SEC24D_8_8268,0,0,0,0,0,0,0,0,0,0,0,0

SEC61A1_8_8269,0,0,0,0,0,0,0,0,0,0,0,0
SEC61B_8_8270,0,0,0,0,0,0,0,0,0,0,0,0
SEC62_8_8271,0,0,0,0,0,0,0,0,0,0,0,0
SEC63_8_8272,0,0,0,0,0,0,0,0,0,0,0,0
SELP_8_8273,0,0,0,0,0,0,0,0,0,0,0,0
SERINC1_8_8274,0,0,0,0,0,0,0,0,0,0,0,0
SERPINA11_8_8275,4,5,6,10,4,8,7,3,10,1,10,8
SERPINA12_8_8276,0,0,0,0,0,0,0,0,0,0,0,0
SERPINA3_8_8277,656,742,408,647,183,236,1940,1169,306,913,607,489
SERPINA4_8_8278,0,0,0,0,0,0,0,0,0,0,0,0
SERPINA5_8_8279,0,0,0,0,0,0,0,0,0,0,0,0
SERPINA7_8_8280,1850,1471,1553,1329,1146,1726,1313,1762,1323,2632,3866
,967
SERPINB10_8_8281,0,0,0,0,0,0,0,0,0,0,0,0
SERPINB12_8_8282,0,0,0,0,0,0,0,0,0,0,0,0
SERPINB13_8_8283,0,0,0,0,0,0,0,0,0,0,0,0
SERPINB1_8_8284,0,0,0,0,0,0,0,0,0,0,0,0
SERPINB4_8_8285,0,0,0,0,0,0,0,0,0,0,0,0
SERPINB9_8_8286,0,0,0,0,0,0,0,0,0,0,0,0
SERPINC1_8_8287,0,0,0,0,0,0,0,0,0,0,0,0
SERPIND1_8_8288,0,0,0,0,0,0,0,0,0,0,0,0
SERPINF1_8_8289,0,0,0,0,0,0,0,0,0,0,0,0
SFXN1_8_8290,0,0,0,0,0,0,0,0,0,0,0,0
SFXN2_8_8291,0,0,0,0,0,0,0,0,0,0,0,0
SFXN3_8_8292,0,0,0,0,0,0,0,0,0,0,0,0
SFXN4_8_8293,0,0,0,0,0,0,0,0,0,0,0,0
SFXN5_8_8294,489,229,293,229,1064,201,102,94,288,74,44,0
SLC15A5_8_8295,0,0,0,0,0,0,0,0,0,0,0,0
SLC16A13_8_8296,215,234,268,220,18,316,527,492,151,10,6,448
SLC16A14_8_8297,0,0,0,0,0,0,0,0,0,0,0,0
SLC16A9_8_8298,0,0,0,0,0,0,0,0,0,0,0,0
SLC17A9_8_8299,0,0,0,0,0,0,0,0,0,0,0,0
SLC22A20_8_8300,0,0,0,0,0,0,0,0,0,0,0,0
SLC22A24_8_8301,0,0,0,0,0,0,0,0,0,0,0,0
SLC22A25_8_8302,0,0,0,0,0,0,0,0,0,0,0,0
SLC25A23_8_8303,0,0,0,0,0,0,0,0,0,0,0,0
SLC25A29_8_8304,0,0,0,0,0,0,0,0,0,0,0,0
SLC25A31_8_8305,0,0,0,0,0,0,0,0,0,0,0,0
SLC25A38_8_8306,0,0,0,0,0,0,0,0,0,0,0,0
SLC25A42_8_8307,0,0,0,0,0,0,0,0,0,0,0,0
SLC25A46_8_8308,0,0,0,1,3,0,0,0,0,0,1,0
SLC25A48_8_8309,426,463,325,418,748,636,430,386,900,1153,1117,1127
SLC38A7_8_8310,0,0,0,0,0,0,0,0,0,0,0,0
SLC38A9_8_8311,2535,2154,2244,2280,5886,2650,2836,1629,1742,2952,1763,
3846
SLC41A2_8_8312,0,0,0,0,0,0,0,0,0,0,0,0
SLC45A1_8_8313,0,0,0,0,0,0,0,0,0,0,0,0
SLC45A3_8_8314,0,0,0,0,0,0,0,0,0,0,0,0
SLC47A1_8_8315,0,0,0,0,0,0,0,0,0,0,0,0
SLC48A1_8_8316,1,3,0,0,3,1,0,2,2,0,1,1

SLC5A12_8_8317,0,0,0,0,0,0,0,0,0,0,0,0
SLC6A17_8_8318,0,0,0,0,0,0,0,0,0,0,0,0
SLC7A14_8_8319,0,0,0,0,0,0,0,0,0,0,0,0
SLIT2_8_8320,0,0,0,0,0,0,0,0,0,0,0,0
SNAP29_8_8321,0,0,0,0,0,0,0,0,0,0,0,0
SNX12_8_8322,0,0,0,0,0,0,0,0,0,0,0,0
SNX13_8_8323,0,0,0,0,0,0,0,0,0,0,0,0
SNX17_8_8324,0,0,0,0,0,0,0,0,0,0,0,0
SNX19_8_8325,0,0,0,0,0,0,0,0,0,0,0,0
SNX22_8_8326,0,0,0,0,0,0,0,0,0,0,0,0
SNX24_8_8327,0,0,0,0,0,0,0,0,0,0,0,0
SNX2_8_8328,0,0,0,0,0,0,0,0,0,0,0,0
SNX4_8_8329,0,0,0,0,0,0,0,0,0,0,0,0
SNX8_8_8330,0,0,0,0,0,0,0,0,0,0,0,0
SNX9_8_8331,0,0,0,0,0,0,324,0,0,0,0,0
SORCS2_8_8332,0,0,0,0,0,0,0,0,0,0,0,0
SORCS3_8_8333,0,0,0,0,0,1,0,0,0,1,1,0
SORL1_8_8334,0,0,0,0,0,0,0,0,0,0,0,0
SOS1_8_8335,0,0,0,0,0,0,0,0,0,0,0,0
SST_8_8336,700,676,326,873,151,359,732,901,714,321,328,717
ST13_8_8337,0,0,0,0,0,0,0,0,0,0,0,0
STAB1_8_8338,31,82,26,52,3,46,58,10,8,511,0,3
STARD4_8_8339,0,0,0,0,0,0,0,0,0,0,0,0
STARD5_8_8340,0,0,0,0,0,0,0,0,0,0,0,0
STARD6_8_8341,0,0,0,0,0,0,0,0,0,0,0,0
STEAP1_8_8342,0,0,0,0,0,0,0,0,0,0,0,0
STMN4_8_8343,0,0,0,0,0,0,0,0,0,0,0,0
STX11_8_8344,0,0,0,0,0,0,0,0,0,0,0,0
STX18_8_8345,0,0,0,0,0,0,0,0,0,0,0,0
STX4_8_8346,0,0,0,0,0,0,0,0,0,1,0,0
STX5_8_8347,0,0,0,0,0,0,0,0,0,0,0,0
STX6_8_8348,0,0,0,0,0,0,0,0,0,0,0,0
STX7_8_8349,0,0,0,0,0,0,0,0,0,0,0,0
STXBP3_8_8350,0,0,0,0,0,0,0,0,0,0,0,0
SVOP_8_8351,0,0,0,0,0,0,0,0,0,0,0,0
SYP_8_8352,0,0,0,0,0,0,0,0,0,0,0,0
SYT10_8_8353,0,0,0,0,0,0,0,0,0,0,0,0
SYT11_8_8354,0,0,0,0,0,0,0,0,0,0,0,0
SYT13_8_8355,957,902,563,1036,120,483,911,931,791,2081,1162,963
SYT16_8_8356,0,0,0,0,0,0,0,0,0,0,0,0
SYT4_8_8357,0,0,0,0,0,0,0,0,0,0,0,0
SYT5_8_8358,141,341,201,179,241,114,322,62,631,228,296,129
SYT6_8_8359,1339,462,810,1132,35,583,819,463,543,769,1483,527
SYT7_8_8360,0,0,0,0,0,0,1,0,0,0,0,0
SYT8_8_8361,221,234,11,168,347,2,440,710,2,0,0,374
SYT9_8_8362,1,1,0,0,1,3,2,1,0,0,1,0
TAS2R42_8_8363,0,0,0,0,0,0,0,0,0,0,0,0
TBC1D9_8_8364,0,0,0,0,0,0,0,0,0,0,0,0
TCN1_8_8365,0,0,0,0,0,0,0,0,0,0,0,0
TEKT4_8_8366,0,0,0,0,0,0,0,0,0,0,0,0

TF_8_8367,0,0,0,0,0,0,0,0,0,0,0,0
TG_8_8368,0,0,0,0,0,0,0,0,0,0,0,0
TGFB1_8_8369,0,0,0,0,0,0,0,0,0,0,0,0
TIMM10_8_8370,133,54,19,40,76,537,68,16,133,1,19,115
TIMM13_8_8371,0,0,0,0,0,0,0,0,0,0,0,0
TIMM17A_8_8372,0,0,0,0,0,0,0,0,0,0,0,0
TIMM22_8_8373,0,0,0,0,0,0,0,0,0,0,0,0
TIMM23_8_8374,0,0,0,0,0,0,0,0,0,0,0,0
TIMM44_8_8375,1,0,0,0,1,0,2,3,0,0,2,1
TIMM8B_8_8376,83,219,245,97,0,984,124,10,41,313,19,1
TIMM9_8_8377,0,0,0,0,0,0,0,0,0,0,0,0
TLL2_8_8378,0,0,0,0,0,0,0,0,0,0,0,0
TM9SF2_8_8379,0,0,0,0,0,0,0,0,0,0,0,0
TM9SF3_8_8380,2,0,0,0,0,0,0,0,0,0,0,0
TM9SF4_8_8381,0,0,0,0,0,0,0,0,0,0,0,0
TMCO3_8_8382,0,0,0,0,0,0,0,0,0,0,0,0
TMED10_8_8383,0,0,0,0,0,0,0,0,0,0,0,0
TMED1_8_8384,0,0,1,1,0,0,2,0,1,0,1,1
TMED2_8_8385,1559,1635,1479,1820,1375,2889,2028,907,1344,3108,1636,303
5
TMED3_8_8386,0,0,0,0,0,0,0,0,0,0,0,0
TMED4_8_8387,0,0,0,0,0,0,0,0,0,0,0,0
TMED7_8_8388,0,0,0,0,0,0,0,0,0,0,0,0
TMED9_8_8389,0,0,0,0,0,0,0,0,0,0,0,0
TMEM104_8_8390,546,485,378,652,630,262,299,876,486,517,789,1186
TMPRSS11B_8_8391,0,0,0,0,0,0,0,0,0,0,0,0
TMPRSS11D_8_8392,0,0,0,0,0,0,0,0,0,0,0,0
TMPRSS11E_8_8393,0,0,0,0,0,0,0,0,0,0,0,0
TMPRSS11F_8_8394,0,0,0,0,0,0,0,0,0,0,0,0
TMPRSS12_8_8395,0,0,0,0,0,0,0,0,0,0,0,0
TMPRSS5_8_8396,1090,1189,936,1152,701,2141,592,1126,601,1946,1639,1289
TMPRSS6_8_8397,0,0,0,0,0,0,0,0,0,0,0,0
TMPRSS7_8_8398,0,0,0,0,0,0,0,0,0,0,0,0
TMPRSS9_8_8399,0,0,0,0,0,0,0,0,0,0,0,0
TNC_8_8400,0,0,0,0,0,0,0,0,0,0,0,0
TNF_8_8401,736,786,733,723,354,1015,386,944,896,718,1004,327
TNFRSF9_8_8402,0,0,0,0,0,0,0,0,0,0,0,0
TNNC2_8_8403,0,0,0,0,0,0,0,0,0,0,0,0
TNN_8_8404,0,0,0,0,0,0,0,0,0,0,0,0
TNNI3_8_8405,0,0,0,0,0,0,0,0,0,0,0,0
TNR_8_8406,0,0,0,0,0,0,0,0,0,0,0,0
TOMM20_8_8407,0,0,0,0,0,0,0,0,0,0,0,0
TOMM22_8_8408,0,0,0,0,0,0,0,0,0,0,0,0
TOMM70A_8_8409,0,0,0,0,0,0,0,0,0,0,0,0
TOMM7_8_8410,118,209,247,66,8,0,107,293,0,674,413,591
TPSG1_8_8411,0,0,0,0,0,0,0,0,0,0,0,0
TRAK2_8_8412,0,0,0,0,0,0,0,0,0,0,0,0
TRAPPC10_8_8413,0,0,0,0,0,0,0,0,0,0,0,0
TRAPPC8_8_8414,0,0,0,0,1,0,0,0,0,0,0,0
TSNAX_8_8415,0,0,0,0,0,0,1,0,0,0,0,0

TTPA_8_8416,0,0,0,0,0,0,0,0,0,0,0,0
TTR_8_8417,0,0,0,0,0,0,0,0,0,0,0,0
TUBA1A_8_8418,1009,838,1599,1085,778,643,1967,933,690,2287,1032,1369
TUBA1C_8_8419,1930,2197,1226,2298,1231,1789,3621,2080,1717,2011,2627,2
073
TUBA3D_8_8420,1367,1201,1200,812,1469,2282,1397,1953,1360,1169,2361,97
0
TUBA3E_8_8421,0,0,0,0,0,0,0,0,0,0,0,0
TUBA4A_8_8422,509,989,444,581,397,1582,129,595,245,503,306,469
TUBB1_8_8423,0,0,0,0,0,0,0,0,0,0,0,0
TUBB2A_8_8424,875,1579,724,1187,654,1657,276,1327,329,965,726,990
TUBB2B_8_8425,0,0,0,0,0,0,0,0,0,0,0,0
TUBB6_8_8426,190,105,141,282,384,699,282,94,139,4,73,226
TUBB8_8_8427,0,0,0,0,0,0,0,0,0,0,0,0
TUBE1_8_8428,0,0,0,0,0,0,0,0,0,0,0,0
TUBG1_8_8429,3160,2601,2051,2015,3440,3189,2448,1758,4048,2394,1960,11
83
TUBG2_8_8430,0,0,0,0,0,0,0,0,0,0,0,0
TXNDC8_8_8431,0,0,0,0,0,0,0,0,0,0,0,0
UCP2_8_8432,0,0,0,0,0,0,0,0,0,0,0,0
USO1_8_8433,0,0,0,0,0,0,0,0,0,0,0,0
VAMP2_8_8434,0,0,0,0,0,0,0,0,0,0,0,0
VAMP5_8_8435,0,0,0,0,0,0,0,0,0,0,0,0
VKORC1L1_8_8436,1213,837,504,774,442,446,422,700,2227,1393,1103,539
VPS18_8_8437,0,0,0,0,0,0,0,0,0,0,0,0
VPS26B_8_8438,0,0,0,0,0,0,0,0,0,0,0,0
VPS33A_8_8439,0,0,0,0,0,0,0,0,0,0,0,0
VPS33B_8_8440,0,0,0,0,0,0,0,0,0,0,0,0
VPS35_8_8441,0,0,0,0,0,0,0,0,0,0,0,0
VPS39_8_8442,0,0,0,0,0,0,0,0,0,0,0,0
VPS45_8_8443,0,0,0,0,0,0,0,0,0,0,0,0
VPS4B_8_8444,0,0,0,0,0,0,0,0,0,0,0,0
VSIG2_8_8445,0,0,0,0,0,0,0,0,0,0,0,0
VTI1A_8_8446,0,0,0,0,0,0,0,0,0,0,0,0
VTI1B_8_8447,0,0,0,0,0,0,0,0,0,0,0,0
VTN_8_8448,0,0,0,0,0,0,0,0,0,0,0,0
VWF_8_8449,0,0,0,0,0,0,0,0,0,0,0,0
XK_8_8450,0,0,0,0,0,0,0,0,0,0,0,0
XPO1_8_8451,0,0,0,0,0,0,0,0,0,0,0,0
XPO4_8_8452,3180,4156,3314,2960,4517,3012,3149,2644,4514,4788,5117,489
3
XPO5_8_8453,0,0,0,8,0,0,0,0,0,0,0,0
XPO6_8_8454,0,10,2,0,0,0,0,0,0,0,0,0
XPO7_8_8455,0,0,0,0,0,0,0,0,0,0,0,0
ACTL6A_8_8456,0,0,0,0,0,0,0,0,0,0,0,0
ADAM12_8_8457,9,7,2,4,6,6,3,9,9,4,14,7
ADAM15_8_8458,0,0,1,0,0,0,0,0,0,0,0,0
ADAM18_8_8459,0,0,0,0,0,0,0,0,0,0,0,0
ADAM22_8_8460,0,0,0,0,0,0,0,0,0,0,0,0
ADAM29_8_8461,2,0,4,2,1,0,2,0,0,0,0,0

ADAM33_8_8462,0,0,0,0,0,0,0,0,0,0,0,0
ADAM8_8_8463,0,0,0,0,0,0,0,0,0,0,0,0
ADAMTS13_8_8464,0,0,0,0,0,0,0,0,0,0,0,0
ADAMTS14_8_8465,0,0,0,0,0,0,0,0,0,0,0,0
AKAP12_8_8466,0,0,0,0,0,0,0,0,0,0,0,0
ANGPT1_8_8467,0,0,0,0,0,0,0,0,0,0,0,0
ANGPTL4_8_8468,0,0,0,0,0,0,0,0,0,0,0,0
AP1B1_8_8469,0,0,0,0,0,0,0,0,0,0,0,0
AP1G1_8_8470,0,0,0,0,0,0,0,0,0,0,0,0
AP1M1_8_8471,0,0,0,0,0,0,0,0,0,0,0,0
AP2A1_8_8472,0,0,0,0,0,0,0,0,0,0,0,0
AP2A2_8_8473,0,1,0,0,0,0,0,0,0,0,0,0
AP2B1_8_8474,0,0,0,0,0,0,0,0,0,0,0,0
AP2M1_8_8475,0,0,0,0,0,0,0,0,0,0,0,0
AP2S1_8_8476,729,678,348,493,688,1263,956,80,589,923,1398,1561
AP3D1_8_8477,0,0,0,0,0,0,0,0,0,0,0,0
AP3M1_8_8478,0,0,0,0,0,0,0,0,0,0,0,0
AP3M2_8_8479,0,0,0,0,0,0,0,0,0,0,0,0
AP4S1_8_8480,1061,1105,666,1028,419,1177,809,1434,738,2002,337,1627
APAF1_8_8481,0,0,0,0,0,0,0,0,0,0,0,0
APBA2_8_8482,1,3,1,2,2,1,2,5,1,3,0,2
APOL1_8_8483,0,0,0,0,0,0,0,0,0,0,0,0
APOL3_8_8484,0,0,0,0,0,0,0,0,0,0,0,0
APP_8_8485,0,0,0,0,0,0,0,0,0,0,0,0
ARFGAP3_8_8486,0,0,0,0,0,0,0,0,0,0,0,0
ARHGAP33_8_8487,50,29,8,116,20,378,30,0,7,11,2,238
ARL6_8_8488,0,0,0,0,0,0,0,0,0,0,0,0
ATP13A2_8_8489,0,0,0,0,0,0,0,0,0,0,0,0
BAX_8_8490,0,0,0,0,0,0,0,0,0,0,0,0
BCAP29_8_8491,743,661,831,729,647,1103,909,475,218,1602,876,672
BCAP31_8_8492,0,0,0,0,0,0,0,0,0,0,0,0
BCL2_8_8493,0,0,0,0,0,0,0,0,0,0,0,0
BCL2L2_8_8494,0,0,0,0,0,0,0,0,0,0,0,0
BID_8_8495,4407,3400,4076,4315,5728,3699,6820,5595,6135,5949,3845,4586
BSG_8_8496,0,0,0,0,0,0,0,0,0,0,0,0
C1QC_8_8497,0,0,0,0,0,0,0,0,0,0,0,0
CANX_8_8498,0,0,0,0,0,0,0,0,0,0,0,0
CAPN9_8_8499,0,0,0,0,0,0,0,0,0,0,0,0
CAPNS1_8_8500,0,0,0,1,0,0,0,0,0,0,0,0
CCT6B_8_8501,0,0,0,0,0,0,0,0,0,0,0,0
CD19_8_8502,0,0,0,0,0,0,0,0,0,0,0,0
CD22_8_8503,0,0,0,0,0,0,0,0,0,0,0,0
CD33_8_8504,11,3,4,5,2,3,4,4,2,4,4,3
CD44_8_8505,0,0,0,0,0,0,0,0,0,0,0,0
CD55_8_8506,0,0,0,0,0,0,0,0,0,0,0,0
CDH17_8_8507,0,0,0,0,0,0,0,0,0,0,0,0
CFHR4_8_8508,0,0,0,0,0,0,0,0,0,0,0,0
CIZ1_8_8509,0,0,0,0,0,0,0,0,0,0,0,0
COG2_8_8510,0,0,0,0,0,0,0,0,0,0,0,0
COG4_8_8511,0,0,0,0,0,0,0,0,0,0,0,0

COG5_8_8512,0,0,0,0,0,0,0,0,0,0,0,0
COG6_8_8513,0,0,0,0,0,0,0,0,0,0,0,0
COL11A1_8_8514,0,0,0,0,0,0,0,0,0,0,0,0
COL11A2_8_8515,0,0,0,0,0,0,0,0,0,0,0,0
COL12A1_8_8516,0,0,0,0,0,0,0,0,0,0,0,0
COL13A1_8_8517,729,433,394,549,219,676,1141,448,1015,161,1003,63
COL18A1_8_8518,0,0,0,0,0,0,0,0,0,0,0,0
COL25A1_8_8519,0,0,0,0,0,0,0,0,0,0,0,0
COL2A1_8_8520,0,0,0,0,0,0,0,0,0,0,0,0
COL4A5_8_8521,0,0,0,0,0,0,0,0,0,0,0,0
COL4A6_8_8522,0,0,0,0,0,0,0,0,0,0,0,0
COL6A2_8_8523,0,0,0,0,0,0,0,0,0,0,0,0
COL6A3_8_8524,0,0,0,0,0,0,0,0,0,0,0,0
COL8A1_8_8525,0,0,0,0,0,0,0,0,0,0,0,0
COL9A1_8_8526,0,0,0,0,0,0,0,0,0,0,0,0
COPA_8_8527,0,0,0,0,0,0,0,0,0,0,0,0
COPB1_8_8528,0,0,0,0,0,0,0,0,0,0,0,0
COPE_8_8529,0,0,0,0,0,0,0,0,0,0,0,0
CPA4_8_8530,0,0,0,0,0,0,0,0,0,0,0,0
CPNE1_8_8531,0,0,0,0,0,0,0,0,0,0,0,0
CPNE7_8_8532,0,0,0,0,0,0,0,0,0,0,1,0
CPXM1_8_8533,0,0,0,0,0,0,0,0,0,0,0,0
CPZ_8_8534,1341,2006,1214,1106,1152,2869,1846,1702,1890,1523,1774,1172
CRABP2_8_8535,0,0,0,0,0,0,0,0,0,0,0,0
CTLA4_8_8536,0,0,0,0,0,0,0,0,0,0,0,0
CTNS_8_8537,0,0,0,0,0,0,0,0,0,0,0,0
CXCL12_8_8538,0,0,0,0,0,0,0,0,0,0,0,0
DPP10_8_8539,0,0,0,0,0,0,0,0,0,0,0,0
DPP6_8_8540,0,0,0,0,0,0,0,0,0,0,0,0
ECM1_8_8541,0,0,0,0,0,0,0,0,0,0,0,0
EGF_8_8542,0,0,0,0,0,0,0,0,0,0,0,0
EIF2C2_8_8543,0,0,0,0,0,0,0,0,0,0,0,0
EIF2D_8_8544,0,0,0,0,0,0,0,0,0,0,0,0
ENSA_8_8545,0,0,0,0,0,0,0,0,0,0,0,0
EPB41L4B_8_8546,1,0,1,0,0,2,0,0,2,0,0,1
EPB42_8_8547,0,0,0,0,0,0,0,0,0,0,0,0
ETFA_8_8548,0,0,0,0,0,0,0,0,0,0,0,0
ETFB_8_8549,0,0,0,0,0,0,0,0,0,0,0,0
EXOC1_8_8550,0,0,0,0,0,0,0,0,0,0,0,0
EXOC4_8_8551,0,0,0,0,0,0,0,0,0,0,0,0
EXOC6_8_8552,0,0,0,0,0,0,0,0,0,0,0,0
EXOC7_8_8553,1640,1494,1319,1748,1731,1716,1438,1646,825,1569,1229,300
0
F8_8_8554,1747,2075,1745,1824,1701,2447,2366,3312,4188,1995,1789,3090
FABP6_8_8555,0,0,0,0,0,0,0,0,0,0,0,0
FAM131A_8_8556,114,119,235,124,1,99,913,9,38,18,0,240
FAM63B_8_8557,0,0,0,0,0,0,0,0,0,0,0,0
FANCA_8_8558,0,0,0,0,0,0,0,0,0,0,0,0
FCN3_8_8559,0,0,0,0,0,0,0,0,0,0,0,0
FGA_8_8560,0,0,0,0,0,0,0,0,0,0,0,0

FGB_8_8561,0,0,0,0,0,0,0,0,0,0,0,0
FGF13_8_8562,0,0,0,0,0,0,0,0,0,0,0,0
FGG_8_8563,0,0,0,0,0,0,0,0,0,0,0,0
FIBCD1_8_8564,0,0,0,0,1,0,0,0,0,0,0,0
FLVCR2_8_8565,637,796,373,373,613,625,9,1786,618,540,337,56
FOLR1_8_8566,0,0,0,0,0,0,0,0,0,0,0,1
FOLR2_8_8567,0,0,0,0,0,0,0,0,0,0,0,0
GGA1_8_8568,0,0,0,0,0,0,0,0,0,0,0,0
GGA3_8_8569,0,0,0,0,0,0,0,0,0,0,0,0
GJA5_8_8570,0,0,0,0,0,0,0,0,0,0,0,0
GJB1_8_8571,0,3,1,1,2,0,0,3,0,0,1,1
GJB3_8_8572,0,0,0,0,0,0,0,0,0,0,0,0
GJB6_8_8573,0,0,0,0,0,0,0,0,0,0,0,0
GLYATL1_8_8574,0,0,0,0,0,0,0,0,0,0,0,0
GNRH1_8_8575,0,0,0,0,0,0,0,0,0,0,0,0
GOLGA3_8_8576,0,0,0,0,0,0,0,0,0,0,0,0
GOPC_8_8577,0,0,0,0,0,0,0,0,0,0,0,0
GOSR1_8_8578,0,0,0,0,0,0,0,0,0,0,0,0
GOSR2_8_8579,0,0,0,0,0,0,0,0,0,0,0,0
GPRASP1_8_8580,0,0,0,0,0,0,0,0,0,0,0,0
GRB2_8_8581,82,297,69,282,0,853,33,334,77,335,351,188
HABP2_8_8582,0,0,0,0,0,0,1,0,0,0,2,1
HDLBP_8_8583,0,0,0,0,0,0,0,0,0,0,0,0
HEPH_8_8584,1,0,0,1,3,1,0,2,0,2,2,0
HNRNPU_8_8585,0,0,0,0,0,0,0,0,0,0,0,0
HOMER2_8_8586,0,0,0,0,0,0,0,0,0,0,0,0
HPN_8_8587,0,0,0,0,0,0,0,0,0,0,0,0
HSDL2_8_8588,0,0,0,0,0,0,0,0,0,0,0,0
IGF1_8_8589,0,0,0,0,0,0,0,0,0,0,0,0
IGFBP3_8_8590,0,0,0,0,0,0,0,0,0,0,0,0
IPO11_8_8591,0,0,0,0,0,0,0,0,0,0,0,0
IPO8_8_8592,0,0,0,0,0,0,0,0,0,0,0,0
ITGAL_8_8593,0,0,0,0,0,0,0,0,0,0,0,0
ITGAM_8_8594,0,0,0,0,0,0,0,0,0,0,0,0
ITGAV_8_8595,0,0,0,0,0,0,0,0,0,0,0,0
ITGB2_8_8596,0,0,0,0,0,0,0,0,0,0,0,0
KDELR2_8_8597,0,0,0,0,0,0,0,0,0,0,0,0
KDELR3_8_8598,0,0,0,0,0,0,0,0,0,0,0,0
KIF13A_8_8599,0,0,0,0,0,0,0,0,0,0,0,0
KIF17_8_8600,2,0,0,1,0,0,0,0,0,0,0,0
KIF1B_8_8601,0,0,0,0,0,0,0,0,0,0,0,0
KLK10_8_8602,0,0,0,0,0,0,0,0,0,0,0,0
KLK11_8_8603,0,0,0,0,0,0,0,0,0,0,0,0
KLK12_8_8604,0,0,0,0,0,0,0,0,0,0,0,0
KLK15_8_8605,0,0,0,0,0,0,0,0,0,0,0,0
KLK5_8_8606,0,0,0,0,0,0,0,0,0,0,0,0
KLK6_8_8607,0,0,0,0,0,0,0,0,0,0,0,0
LAMB3_8_8608,0,0,0,0,0,0,0,0,0,0,0,0
LDB3_8_8609,0,0,0,0,0,0,0,0,0,0,0,0
LDLR_8_8610,0,0,0,0,0,0,0,0,0,0,0,0

LTA_8_8611,0,0,0,0,0,0,0,1,0,0,0,0
LTF_8_8612,0,0,0,0,0,0,0,0,0,0,0,0
M6PR_8_8613,0,0,0,0,0,0,0,0,0,0,0,0
MASP1_8_8614,0,0,0,0,0,0,0,0,0,0,0,0
MB_8_8615,0,0,0,0,0,0,0,0,0,0,0,0
MCFD2_8_8616,0,0,0,0,0,0,0,0,0,0,0,0
MCL1_8_8617,0,0,0,0,0,0,0,0,0,0,0,0
MEFV_8_8618,0,0,0,0,0,0,0,0,0,0,0,0
MFAP4_8_8619,0,0,0,0,0,0,0,0,0,0,0,0
MFSD10_8_8620,0,0,0,0,0,0,0,0,0,0,0,0
MFSD1_8_8621,0,0,0,0,0,0,0,0,0,0,0,0
MFSD5_8_8622,0,0,0,0,0,0,0,0,0,0,0,0
MLC1_8_8623,0,0,0,0,0,0,0,0,0,0,0,0
MSLN_8_8624,0,0,0,2,3,1,2,4,0,1,1,0
MTX1_8_8625,0,0,0,0,0,0,0,0,0,0,0,0
MUC1_8_8626,0,0,0,0,0,0,0,0,0,0,0,0
NCAM1_8_8627,0,0,0,0,0,0,0,0,0,0,0,0
NNAT_8_8628,0,0,0,0,0,0,0,0,0,0,0,0
NOXO1_8_8629,660,828,631,1284,732,1292,1148,2540,1254,1035,629,964
NPC1L1_8_8630,0,0,0,0,0,0,0,0,0,0,0,0
NPRL3_8_8631,0,0,0,0,0,0,0,0,0,0,0,0
NRXN1_8_8632,269,94,364,137,0,1,1,385,139,95,34,8
NRXN2_8_8633,0,0,0,0,0,0,0,0,0,0,0,0
NRXN3_8_8634,0,0,0,0,0,0,0,0,0,0,0,0
NUP155_8_8635,0,0,0,0,0,0,0,0,0,0,0,0
NUP50_8_8636,564,446,477,356,973,393,1214,201,198,706,533,382
NUP62_8_8637,0,0,0,0,0,0,0,0,0,0,0,0
NUP98_8_8638,0,0,0,0,0,0,0,0,0,0,0,0
NUPL1_8_8639,0,0,0,0,0,0,0,0,0,0,0,0
NXF1_8_8640,0,0,0,0,0,0,0,0,0,0,0,0
NXNL2_8_8641,0,0,0,0,0,0,0,0,0,0,0,0
NXT2_8_8642,0,0,0,0,0,0,0,0,0,0,0,0
OAZ3_8_8643,316,399,136,220,418,36,0,27,400,412,195,33
PACSIN2_8_8644,0,0,0,0,0,0,0,0,0,0,0,0
PANX2_8_8645,0,0,0,0,0,0,0,0,0,0,0,0
PCDHA6_8_8646,0,0,0,0,0,0,0,0,0,0,0,0
PCDHGA5_8_8647,0,0,0,0,0,0,0,0,0,0,0,0
PCLO_8_8648,0,0,0,0,0,0,0,0,0,0,0,0
PCSK5_8_8649,0,0,0,0,0,0,0,0,0,0,0,0
PCSK6_8_8650,0,0,0,0,0,0,0,0,0,0,0,0
PCTP_8_8651,0,0,0,0,0,0,0,0,0,0,0,0
PDYN_8_8652,0,0,0,0,0,0,0,0,0,0,0,0
PDZD3_8_8653,0,0,0,0,0,0,0,0,0,0,0,0
PDZK1_8_8654,0,0,0,0,0,0,0,0,0,0,0,0
PGAP2_8_8655,0,0,0,0,0,0,0,0,0,0,0,0
PGF_8_8656,0,0,0,0,0,0,0,0,0,0,0,0
PIK3R3_8_8657,0,0,0,0,0,0,0,0,0,0,0,0
PITPNC1_8_8658,0,1,0,0,0,0,0,0,0,0,0,0
PITPNM1_8_8659,0,0,0,0,0,0,0,0,0,0,0,0
PITPNM3_8_8660,0,0,0,0,0,0,0,0,0,0,0,0

PLEC_8_8661,0,0,0,0,0,0,0,0,0,0,0,0
PLIN3_8_8662,0,0,0,0,0,0,0,0,0,0,0,0
PLTP_8_8663,0,0,0,0,0,0,0,0,0,0,0,0
PNKD_8_8664,0,0,0,0,0,0,0,0,0,0,0,0
POMC_8_8665,0,0,0,0,0,0,0,0,0,0,0,0
PORCN_8_8666,0,0,0,0,0,0,0,0,0,0,0,0
PREPL_8_8667,0,0,0,0,0,0,0,0,0,0,0,0
PRNP_8_8668,41,0,0,0,0,0,0,0,0,0,0,0
PRSS21_8_8669,0,0,0,0,0,0,0,0,0,0,0,0
PRSS35_8_8670,0,0,0,0,0,0,0,0,0,0,0,0
PSEN1_8_8671,662,990,690,821,283,468,998,92,853,656,1124,710
PSEN2_8_8672,0,0,0,0,0,0,0,0,0,0,0,0
RABEP1_8_8673,0,0,0,0,0,0,0,0,0,0,0,0
RACGAP1_8_8674,0,0,0,0,0,0,0,0,0,0,0,0
RARRES1_8_8675,1248,754,1150,892,938,1195,1458,1351,855,1586,596,1795
RASA1_8_8676,0,0,0,0,0,0,0,0,0,0,0,0
RELN_8_8677,0,0,0,0,0,0,0,0,0,0,0,0
RHCE_8_8678,0,0,0,0,0,0,0,0,0,0,0,0
RHD_8_8679,0,0,0,0,0,0,0,0,0,0,0,0
RIMS2_8_8680,0,0,0,0,0,0,0,0,0,0,0,0
RRBP1_8_8681,0,0,0,0,0,0,0,0,0,0,0,0
RUFY1_8_8682,1458,1847,1888,1096,1880,2316,932,2565,1040,1085,2987,103
1
S100A13_8_8683,0,0,0,0,0,0,0,0,0,0,0,0
S100A4_8_8684,0,0,0,0,0,0,0,0,0,0,0,0
SAA1_8_8685,1176,1031,1294,1797,792,1000,802,2204,396,1492,1474,1127
SCAMP3_8_8686,0,0,0,0,0,0,0,0,0,0,0,0
SCARB1_8_8687,0,0,0,0,0,0,0,0,0,0,0,0
SCFD1_8_8688,0,0,0,0,0,0,0,0,0,0,0,0
SEC13_8_8689,0,0,0,0,0,0,0,0,0,0,0,0
SEC14L1_8_8690,0,0,0,0,0,0,0,0,0,0,0,0
SEC14L2_8_8691,0,0,0,0,0,0,0,0,0,0,0,0
SEC14L4_8_8692,0,0,0,0,0,0,0,0,0,0,0,0
SEC23B_8_8693,0,0,0,0,0,0,0,0,0,0,0,0
SEC24B_8_8694,0,0,0,0,0,0,0,0,0,0,0,0
SEC24C_8_8695,0,0,0,0,0,0,0,0,0,0,0,0
SEC61A2_8_8696,0,32,0,0,0,0,0,0,0,0,0,0
SEC61G_8_8697,2,2,1,1,5,4,3,0,4,5,1,2
SEH1L_8_8698,0,0,0,0,0,0,0,0,0,0,0,0
SERINC2_8_8699,0,0,0,0,0,0,0,0,0,0,0,0
SERINC3_8_8700,0,0,0,0,0,0,0,0,0,0,0,0
SERPINA10_8_8701,0,0,0,0,0,0,0,0,0,0,0,0
SERPINA1_8_8702,0,0,0,0,0,0,0,0,0,0,0,0
SERPINB2_8_8703,0,1,0,0,1,1,0,0,0,0,1,0
SERPINB6_8_8704,0,0,0,0,0,0,0,0,0,1,0,0
SERPINB8_8_8705,0,0,0,0,0,0,0,0,0,0,0,0
SERPINE1_8_8706,0,0,0,0,0,0,0,1,0,0,0,0
SERPINF2_8_8707,0,0,0,0,0,0,0,0,0,0,0,0
SERPING1_8_8708,0,0,0,0,0,0,0,0,0,0,0,0
SERPINH1_8_8709,0,0,0,0,0,0,0,0,0,0,0,0

SERPINI1_8_8710,0,0,0,0,0,0,0,0,0,0,0,0
SFI1_8_8711,0,0,0,0,0,0,0,0,0,0,0,0
SFTPA1_8_8712,0,0,0,0,0,0,0,0,0,0,0,0
SH3D19_8_8713,251,602,132,252,0,880,0,165,4,157,100,198
SIL1_8_8714,0,0,0,0,0,0,0,0,0,0,0,0
SLC25A25_8_8715,0,0,0,0,0,0,0,0,0,0,0,0
SLC25A36_8_8716,0,0,0,0,0,0,0,0,0,0,0,0
SLC25A45_8_8717,0,0,0,0,0,0,0,0,0,0,0,0
SLC38A10_8_8718,0,0,0,0,0,0,0,0,0,0,0,0
SLC41A3_8_8719,0,0,0,0,0,0,0,0,0,0,0,0
SLC43A3_8_8720,0,0,0,0,0,0,0,0,0,0,0,0
SLC44A2_8_8721,0,0,0,0,0,0,0,0,0,0,0,0
SLC44A4_8_8722,0,0,0,0,0,0,0,0,0,0,0,0
SLC44A5_8_8723,0,0,0,0,0,0,0,0,0,0,0,0
SLC46A1_8_8724,278,504,84,397,896,357,540,40,446,47,597,304
SLC47A2_8_8725,0,0,0,0,0,0,0,0,0,0,0,0
SLC50A1_8_8726,0,0,0,0,0,0,0,0,0,0,0,0
SLC6A20_8_8727,0,0,0,0,0,0,0,0,0,0,0,0
SNAP23_8_8728,0,0,0,0,0,0,0,0,0,0,0,0
SNAP25_8_8729,0,0,0,0,0,0,0,0,0,0,0,0
SNX10_8_8730,0,0,0,0,0,0,0,0,0,0,0,0
SNX11_8_8731,0,0,0,0,0,0,0,0,0,0,0,0
SNX14_8_8732,0,0,0,0,0,0,0,0,0,0,0,0
SNX15_8_8733,0,0,0,0,0,0,0,0,0,0,0,0
SNX16_8_8734,0,0,0,0,0,0,0,0,0,0,0,0
SNX18_8_8735,0,0,0,0,0,0,0,0,0,0,0,0
SNX1_8_8736,0,0,0,0,0,0,0,0,0,0,0,0
SNX3_8_8737,0,0,0,0,0,0,0,0,0,0,0,0
SNX5_8_8738,0,0,0,0,0,0,0,0,0,0,0,0
SNX6_8_8739,5130,5096,4123,4799,8228,7103,5112,6411,4268,4622,7219,542
6
SNX7_8_8740,0,0,0,0,0,0,0,0,0,0,0,0
SORCS1_8_8741,0,0,0,0,0,0,0,0,0,0,0,0
SORT1_8_8742,0,0,0,0,0,0,0,0,0,0,0,0
SPNS1_8_8743,0,0,0,0,0,0,0,0,0,0,0,0
SRI_8_8744,0,0,0,0,0,0,0,0,0,0,0,0
STARD3_8_8745,0,0,0,0,0,0,0,0,0,0,0,0
STAU1_8_8746,0,0,0,0,0,0,0,0,0,0,0,0
STEAP2_8_8747,0,0,0,0,0,0,0,0,0,0,0,0
STEAP3_8_8748,0,0,2,2,1,0,1,0,2,0,0,0
STIM2_8_8749,0,0,0,0,0,0,0,0,0,0,0,0
STX16_8_8750,0,0,0,0,0,0,0,0,0,0,0,0
STX1A_8_8751,0,0,0,0,0,0,0,0,0,0,0,0
STX2_8_8752,0,0,0,0,0,0,0,0,0,0,0,0
STX3_8_8753,0,0,0,0,0,0,0,0,0,0,0,0
STXBP1_8_8754,0,0,0,0,0,0,0,0,0,0,0,0
STXBP2_8_8755,0,0,0,0,0,0,0,0,0,0,0,0
SV2B_8_8756,0,0,0,0,0,0,0,0,0,0,0,0
SYN1_8_8757,0,0,0,0,0,0,0,0,0,0,0,0
SYNGR1_8_8758,0,0,0,0,0,0,0,0,0,0,0,0

SYNPR_8_8759,0,0,0,0,0,0,0,0,0,0,0,0
SYPL1_8_8760,0,0,0,0,0,0,0,0,0,0,0,0
SYT12_8_8761,0,0,0,0,0,0,0,0,0,0,0,0
SYT14_8_8762,3718,4464,3320,5234,2797,3768,3034,6015,4986,5087,3410,40
55
SYT15_8_8763,0,0,0,0,0,0,0,0,0,0,0,0
SYT1_8_8764,0,0,0,0,0,0,1,0,0,0,0,0
SYT2_8_8765,0,0,0,0,0,0,0,0,0,0,0,0
SYT3_8_8766,0,0,1,0,0,0,0,0,0,0,0,0
TAPBP_8_8767,0,0,0,0,0,0,0,0,0,0,0,0
TC2N_8_8768,0,0,0,0,0,0,0,0,0,0,0,0
TCN2_8_8769,0,0,0,0,0,0,0,0,0,0,0,0
TCOF1_8_8770,0,0,0,0,0,0,0,0,0,0,0,0
TFPI_8_8771,0,0,0,0,0,0,0,0,0,0,0,1
TFR2_8_8772,0,0,0,0,0,0,0,0,0,0,0,0
TFRC_8_8773,0,0,0,0,0,0,0,0,0,0,0,0
TGFB2_8_8774,556,620,1064,738,427,70,799,217,217,2130,832,958
TIMM17B_8_8775,0,0,0,0,0,0,0,0,0,0,0,0
TINAGL1_8_8776,0,0,0,0,0,0,0,0,0,0,0,0
TLL1_8_8777,0,0,0,0,0,0,0,0,0,0,0,0
TM9SF1_8_8778,0,0,0,0,0,0,0,0,0,0,0,0
TMC6_8_8779,0,0,0,0,0,0,0,0,0,0,0,0
TMPRSS11A_8_8780,0,0,0,0,0,0,0,0,0,0,0,0
TMPRSS13_8_8781,0,0,0,0,0,0,0,0,0,0,0,0
TMPRSS2_8_8782,0,0,0,0,0,0,0,0,0,0,0,0
TMPRSS4_8_8783,0,0,0,0,0,0,0,0,0,0,0,0
TNFSF11_8_8784,0,0,0,0,0,0,0,0,0,0,0,0
TNFSF13B_8_8785,354,680,325,325,170,651,132,1051,1,436,303,1022
TNPO2_8_8786,0,0,0,0,0,0,0,0,0,0,0,0
TOM1_8_8787,0,0,0,0,0,0,0,0,0,0,0,0
TOM1L2_8_8788,0,0,0,0,0,0,0,0,0,0,0,0
TSC1_8_8789,0,0,0,0,0,0,0,0,0,0,0,0
TSC2_8_8790,0,0,0,0,0,0,0,0,0,0,0,0
TUBA8_8_8791,0,0,0,0,0,0,0,0,0,0,0,0
TUBB3_8_8792,1245,1177,893,596,1331,945,1915,1183,1665,494,1387,752
TUBD1_8_8793,0,0,0,0,0,0,0,0,0,0,0,0
UCP3_8_8794,0,0,0,0,0,0,0,0,0,0,0,0
UPF3A_8_8795,0,0,0,0,0,0,0,0,0,0,0,0
UPF3B_8_8796,3,20,1,19,0,1,1,1,0,1,0,4
VAMP1_8_8797,0,0,0,0,0,0,0,0,0,0,0,0
VAMP7_8_8798,0,0,0,0,0,0,0,0,0,0,0,0
VCAM1_8_8799,0,0,0,0,0,0,0,0,0,0,0,0
VLDLR_8_8800,0,0,0,0,0,0,0,0,0,0,0,0
VPS13A_8_8801,0,0,0,0,0,0,0,0,0,0,0,0
VPS13B_8_8802,0,0,0,0,0,0,0,0,0,0,0,0
VPS16_8_8803,0,0,0,0,0,0,0,0,0,0,0,0
VPS26A_8_8804,0,0,0,0,0,0,0,0,0,0,0,0
VPS28_8_8805,576,1110,625,554,760,1184,2161,331,1820,462,764,482
ZFYVE16_8_8806,0,0,0,0,0,0,0,0,0,0,0,0
ZNF160_8_8807,0,0,0,0,0,0,0,0,0,0,0,0

ZP3_8_8808,0,0,0,0,0,0,0,0,0,0,0,0
BET1L_8_8809,0,0,0,0,0,0,0,0,0,0,0,0
C2orf83_8_8810,0,0,0,0,0,0,0,0,0,0,0,0
ERP29_8_8811,0,0,0,0,0,0,0,0,0,0,0,0
FGF1_8_8812,0,0,0,0,0,0,0,0,0,0,0,0
LYNX1_8_8813,0,0,0,0,1,0,0,0,0,1,1,89
MMP28_8_8814,0,0,0,0,0,0,0,0,0,0,0,0
PDPN_8_8815,0,0,0,0,0,0,0,0,0,0,0,0
SNX21_8_8816,267,401,729,206,1753,467,18,139,91,18,422,576
TIMM8A_8_8817,0,0,0,0,0,0,0,0,0,0,0,0
VEGFA_8_8818,0,0,0,0,0,0,0,0,0,0,0,0
CDH23_8_8819,317,273,310,392,1160,1742,1132,260,1361,195,810,252
CDH23_8_8820,0,0,0,0,0,0,0,0,0,0,0,0
CDH23_8_8821,0,0,0,0,0,0,0,0,0,0,0,0
CDH23_8_8822,232,156,257,257,563,595,158,590,32,255,518,658
CDH23_8_8823,0,0,0,0,0,0,0,0,0,0,0,0
TNXB_8_8824,0,0,0,0,0,0,0,0,0,0,0,0
TNXB_8_8825,0,0,0,0,0,0,0,0,0,0,0,0
A2M_8_8826,0,0,0,0,0,0,0,0,0,0,0,0
ACE2_8_8827,0,0,0,0,0,0,0,0,0,0,0,0
ACTR6_8_8828,0,0,0,0,0,0,0,0,0,0,0,0
ADAM11_8_8829,0,0,0,0,0,0,0,0,0,0,0,0
ADAM19_8_8830,0,0,0,0,0,0,0,0,0,0,0,0
ADAM20_8_8831,0,0,0,0,0,0,0,0,0,0,0,0
ADAM21_8_8832,0,0,0,0,0,0,0,0,0,0,0,0
ADAM2_8_8833,0,0,1,0,0,0,0,0,0,0,1,0
ADAM30_8_8834,0,0,0,0,0,0,0,0,0,0,0,0
ADAM7_8_8835,0,0,0,0,0,0,0,0,0,0,0,0
ADAM9_8_8836,0,0,0,0,0,0,0,0,0,0,0,0
ADAMTS10_8_8837,0,0,0,0,0,0,0,0,0,0,0,0
ADAMTS12_8_8838,0,0,0,0,0,0,0,0,0,0,0,0
ADAMTS15_8_8839,5,6,1,4,2,3,4,0,7,2,1,9
ADAMTS18_8_8840,0,0,0,0,0,0,0,0,0,0,0,0
ADAMTS19_8_8841,741,848,385,1007,836,736,718,431,1044,1459,1522,282
ADAMTS1_8_8842,0,0,0,0,0,0,0,0,0,0,0,0
ADAMTS20_8_8843,0,0,0,0,0,0,0,0,0,0,0,0
ADAMTS3_8_8844,0,1,0,0,0,0,0,0,0,0,0,0
ADAMTS5_8_8845,0,0,0,0,0,0,0,0,0,0,0,0
ADAMTS6_8_8846,0,0,0,0,0,0,0,0,0,0,0,0
ADAMTS7_8_8847,0,0,0,0,0,0,0,0,0,0,0,0
ADAMTS8_8_8848,0,1,0,0,0,0,1,0,0,0,1,0
AEBP1_8_8849,0,0,0,0,0,0,0,0,0,0,0,0
AFG3L2_8_8850,0,0,0,0,0,0,0,0,0,0,0,0
AFM_8_8851,0,0,0,0,0,0,0,0,0,0,0,0
AFP_8_8852,0,0,0,0,0,0,0,0,0,0,0,0
AGTPBP1_8_8853,0,0,1,0,0,0,0,0,1,0,1,2
ALG10B_8_8854,0,0,0,0,0,0,0,0,0,0,0,1
AMBP_8_8855,0,0,0,0,0,0,0,0,0,0,0,0
ANGPT4_8_8856,0,0,0,0,0,0,0,0,0,0,0,0
ANGPTL1_8_8857,0,0,0,0,0,0,0,0,0,0,0,0

ANGPTL2_8_8858,0,0,0,0,0,0,0,0,0,0,0,0
ANGPTL3_8_8859,0,0,0,0,0,0,0,0,0,0,0,0
ANGPTL7_8_8860,0,0,0,0,0,0,0,0,0,0,0,0
ANKH_8_8861,0,0,0,0,0,0,0,0,0,0,0,0
AP1G2_8_8862,0,0,0,0,0,0,0,0,0,0,0,0
AP1M2_8_8863,0,0,0,0,0,0,0,0,0,0,0,0
AP1S1_8_8864,181,157,89,225,0,8,0,0,86,243,540,153
AP1S2_8_8865,0,0,0,0,0,0,0,0,0,0,0,0
AP1S3_8_8866,0,0,0,0,0,0,0,0,0,0,0,0
AP3B1_8_8867,0,0,0,0,0,0,0,0,0,0,0,0
AP3B2_8_8868,0,0,0,0,0,0,0,0,0,0,0,0
AP3S1_8_8869,0,0,0,0,0,0,0,0,0,0,0,0
AP3S2_8_8870,0,0,0,0,0,0,0,0,0,0,0,0
AP4B1_8_8871,1,0,1,1,0,3,0,0,0,0,0,0
AP4M1_8_8872,0,0,0,0,0,0,0,0,0,0,0,0
APBA1_8_8873,763,559,572,314,1585,175,682,306,721,219,902,267
APBA3_8_8874,0,0,0,0,0,0,0,0,0,0,0,0
APOA1_8_8875,0,0,0,0,0,0,0,0,0,0,0,0
APOA2_8_8876,0,0,0,0,0,0,0,0,0,0,0,0
APOA4_8_8877,0,0,0,0,0,0,0,0,0,0,0,0
APOB_8_8878,0,0,0,0,0,0,0,0,0,0,0,0
APOC1_8_8879,0,0,0,0,0,0,0,0,0,0,0,0
APOC2_8_8880,0,0,0,0,0,0,0,0,0,0,0,0
APOC3_8_8881,0,0,0,0,0,0,0,0,0,0,0,0
APOC4_8_8882,2,0,0,1,11,0,4,1,5,3,5,0
APOD_8_8883,381,303,370,309,227,411,679,339,503,698,311,440
APOE_8_8884,0,0,0,0,0,0,0,0,0,0,0,0
APOF_8_8885,0,0,0,0,0,0,0,0,0,0,0,0
APOH_8_8886,0,0,0,0,0,0,0,0,0,0,0,0
APOL6_8_8887,0,0,0,0,0,0,0,0,0,0,0,0
APOM_8_8888,0,0,0,0,0,0,0,0,0,0,0,0
AQP12B_8_8889,0,0,0,0,0,0,0,0,0,0,0,0
ARF5_8_8890,0,0,0,0,0,0,0,0,0,0,0,0
ARF6_8_8891,322,462,147,515,53,608,28,96,505,806,255,899
ARPP19_8_8892,0,0,0,0,0,0,0,0,0,0,0,0
ASTL_8_8893,175,183,17,106,232,48,139,32,0,0,150,45
ATOX1_8_8894,0,0,0,0,0,0,0,0,0,0,0,0
ATP13A1_8_8895,0,0,0,0,0,0,0,0,0,0,0,0
ATP13A3_8_8896,0,0,0,0,0,0,0,0,0,0,0,0
ATP13A4_8_8897,2560,2123,2188,2334,2743,1711,2201,2662,2280,2634,3753,
1087
ATP13A5_8_8898,0,0,0,0,0,0,0,0,0,0,0,0
AZGP1_8_8899,0,0,0,0,0,0,0,0,1,0,0,0
AZU1_8_8900,0,0,0,0,0,0,0,0,0,0,0,0
BCL2L10_8_8901,0,0,0,0,0,0,0,0,0,0,0,0
BET1_8_8902,0,0,0,0,0,0,0,0,0,0,0,0
BGLAP_8_8903,0,0,0,0,0,0,0,0,0,0,0,0
BOC_8_8904,0,0,0,0,0,0,0,0,0,0,0,0
BPI_8_8905,0,0,0,0,0,0,0,0,0,0,0,0
BPIFC_8_8906,0,0,0,0,0,0,0,0,0,0,0,0

C16orf7_8_8907,0,0,0,0,0,0,0,0,0,0,0,0
C1orf162_8_8908,880,665,749,497,376,831,842,344,395,806,1282,924
C1QA_8_8909,0,0,0,0,0,0,0,0,0,0,0,0
C1QB_8_8910,0,0,0,0,0,0,0,0,0,0,0,1
C1RL_8_8911,0,0,0,0,0,0,0,0,0,0,0,0
C20orf141_8_8912,0,0,0,0,0,0,0,0,0,0,0,0
C3_8_8913,0,0,0,0,0,0,0,0,0,0,0,0
C4A_8_8914,0,0,0,0,0,0,0,0,0,0,0,0
C5_8_8915,0,0,0,0,0,0,0,0,0,0,0,0
C7orf31_8_8916,0,0,0,0,0,0,0,0,0,0,0,0
C8G_8_8917,0,0,4,1,1,0,0,0,1,2,3,0
CALM2_8_8918,0,0,0,0,0,0,0,0,0,0,0,0
CALY_8_8919,0,0,0,0,0,0,0,0,0,0,0,0
CAMLG_8_8920,0,0,0,0,0,0,0,0,0,0,0,0
CAPN11_8_8921,0,0,0,0,0,0,0,0,0,0,0,0
CAPN5_8_8922,0,0,0,0,0,0,0,0,0,0,0,0
CAPN6_8_8923,0,0,0,0,0,0,0,0,0,0,0,0
CARTPT_8_8924,0,0,0,0,0,0,0,0,0,0,0,0
CCL13_8_8925,237,621,142,479,558,695,195,709,25,410,552,142
CCND1_8_8926,0,0,0,0,0,0,0,0,0,0,0,0
CD1A_8_8927,0,0,0,0,0,0,0,0,0,0,0,0
CD52_8_8928,1147,1017,1301,1068,1496,946,248,886,1986,1442,665,2461
CDCP2_8_8929,131,198,290,111,133,410,494,95,81,99,72,39
CDH5_8_8930,0,0,0,0,0,0,0,0,0,0,0,0
CHMP7_8_8931,0,0,0,0,0,0,0,0,0,0,0,0
CLDN16_8_8932,0,0,0,0,0,0,0,0,0,0,0,0
CLEC3B_8_8933,0,0,0,0,0,0,0,0,0,0,0,0
CLSTN2_8_8934,0,0,0,0,0,0,0,0,0,0,0,0
CLVS2_8_8935,0,0,0,1,1,3,2,0,2,1,3,0
CNIH3_8_8936,0,0,0,0,0,0,0,0,0,0,0,0
CNOT6_8_8937,1124,1284,1069,1207,1449,1149,1042,1855,1620,824,1850,126
7
CNTNAP1_8_8938,0,0,0,0,0,0,0,0,0,0,0,0
COG1_8_8939,0,0,0,0,0,0,0,0,0,0,0,0
COG3_8_8940,0,0,0,0,0,0,0,0,0,0,0,0
COG7_8_8941,0,0,0,0,0,0,0,0,0,0,0,0
COG8_8_8942,0,0,0,0,0,0,0,0,0,0,0,0
COL10A1_8_8943,0,0,0,0,0,0,0,0,0,0,0,0
COL14A1_8_8944,0,0,0,0,0,0,0,0,0,0,0,0
COL15A1_8_8945,0,0,0,0,0,0,0,0,0,0,0,0
COL16A1_8_8946,0,0,0,0,0,0,0,0,0,0,0,0
COL17A1_8_8947,0,0,0,0,0,0,0,0,0,0,0,0
COL1A1_8_8948,3,2,1,1,1,1,3,0,1,0,2,2
COL1A2_8_8949,0,0,0,0,0,0,0,0,0,0,0,0
COL21A1_8_8950,0,0,0,0,0,0,0,0,0,0,0,0
COL22A1_8_8951,0,1,0,0,0,0,0,0,0,0,0,0
COL23A1_8_8952,0,0,0,0,0,0,0,0,0,0,0,0
COL24A1_8_8953,0,0,0,0,0,0,0,0,0,0,0,0
COL27A1_8_8954,0,0,0,0,0,0,0,0,0,0,0,0
COL3A1_8_8955,0,0,0,0,0,0,0,0,0,0,0,0

COL4A1_8_8956,1,164,1,66,1,434,1,0,507,260,2,1
COL4A2_8_8957,0,0,0,0,0,0,0,0,0,0,0,0
COL4A3_8_8958,0,0,0,0,0,0,0,0,0,0,0,0
COL4A4_8_8959,0,0,0,0,0,0,0,0,0,0,0,0
COL5A1_8_8960,175,65,85,461,50,46,51,875,23,40,896,53
COL5A2_8_8961,0,0,0,0,0,0,0,0,0,0,0,0
COL5A3_8_8962,0,0,0,0,0,0,0,0,0,0,0,0
COL6A1_8_8963,0,0,0,0,0,0,0,0,0,0,0,0
COL7A1_8_8964,0,0,0,0,0,0,0,0,0,0,0,0
COL8A2_8_8965,383,479,381,239,140,110,187,312,329,550,343,1052
COL9A2_8_8966,0,0,0,0,0,0,0,0,0,0,0,0
COL9A3_8_8967,0,0,0,0,1,0,0,0,0,0,1,0
COMMD1_8_8968,0,0,0,0,0,0,0,0,0,0,0,0
COPB2_8_8969,0,0,0,0,0,0,0,0,0,0,0,0
COPG2_8_8970,0,0,0,0,0,0,0,0,0,0,0,0
COPZ1_8_8971,0,0,0,0,0,0,0,0,0,0,0,0
COPZ2_8_8972,0,0,0,0,0,0,0,0,0,0,0,0
CORIN_8_8973,0,0,0,0,0,0,0,0,0,0,0,0
COX18_8_8974,1291,1192,1075,1459,980,1245,952,2139,1444,854,2160,1702
CPLX1_8_8975,224,130,224,313,38,40,43,107,354,63,261,517
CPLX3_8_8976,0,0,0,0,0,0,0,0,0,0,0,0
CPNE6_8_8977,0,0,0,0,0,0,0,0,0,0,0,0
CPXM2_8_8978,0,0,0,0,0,0,0,0,0,0,0,0
CRABP1_8_8979,0,0,0,0,0,0,0,0,0,0,0,0
CRH_8_8980,0,0,0,0,0,0,0,0,0,0,0,0
CSE1L_8_8981,0,0,0,0,0,0,0,0,0,0,0,0
CTSW_8_8982,0,0,0,0,0,0,0,0,0,0,0,0
CXCL10_8_8983,0,0,0,0,0,0,0,0,0,0,0,0
CYGB_8_8984,0,0,0,0,0,0,0,0,0,0,0,0
CYTH3_8_8985,0,0,0,0,0,0,0,0,0,0,0,0
DDI2_8_8986,0,0,0,0,0,0,0,0,0,0,0,0
DIRC2_8_8987,637,908,769,930,1413,1033,804,541,756,704,902,692
DISP1_8_8988,0,0,0,0,0,0,0,0,0,0,0,0
DLL4_8_8989,0,0,0,0,0,0,0,0,0,0,0,0
DNAJC5B_8_8990,0,0,0,0,0,0,0,0,0,0,0,0
DNAJC6_8_8991,0,0,0,0,0,0,0,0,0,0,0,0
DOC2A_8_8992,0,0,0,0,0,0,0,0,0,0,0,0
DOC2B_8_8993,0,0,0,0,0,0,0,0,0,0,0,0
DSCAML1_8_8994,0,0,0,0,0,0,0,0,0,0,0,0
ECEL1_8_8995,0,0,0,0,0,0,0,0,0,0,0,0
EID2_8_8996,0,0,0,0,0,0,0,0,0,0,0,0
EPCAM_8_8997,0,0,0,0,0,0,0,0,0,0,0,0
EXOC2_8_8998,0,0,0,0,0,0,0,0,0,0,0,0
EXOC3_8_8999,0,0,0,0,0,0,0,0,0,0,0,0
F11R_8_9000,0,0,0,0,0,0,0,0,0,0,0,0
FABP1_8_9001,0,0,0,0,0,0,0,0,0,0,0,0
FABP2_8_9002,0,0,0,0,0,0,0,0,0,0,0,0
FABP3_8_9003,2,3,0,1,1,0,0,0,1,2,0,0
FABP4_8_9004,0,0,1,0,2,3,2,7,0,3,1,7
FABP7_8_9005,0,0,0,0,0,0,0,0,0,0,0,0

FABP9_8_9006,0,0,0,0,0,0,0,0,0,0,0,0
FAM101A_8_9007,0,0,4,2,0,0,0,0,6,1,0,1
FAM117A_8_9008,0,0,0,0,0,0,0,0,0,0,0,0
FAM57A_8_9009,0,0,0,0,0,0,0,0,0,0,0,0
FAP_8_9010,0,0,0,0,0,0,0,0,0,0,0,0
FBF1_8_9011,0,0,0,0,0,0,0,0,0,0,0,0
FCN1_8_9012,0,0,0,0,0,0,0,0,0,0,0,0
FDX1_8_9013,0,0,0,0,0,0,0,0,0,0,0,0
FDX1L_8_9014,2,0,2,0,2,3,0,2,0,0,4,0
FGF4_8_9015,0,0,0,0,0,0,0,0,0,0,0,0
FGL2_8_9016,0,0,0,0,0,0,0,0,0,0,0,0
FOLR3_8_9017,0,0,0,1,0,0,0,0,1,0,0,1
FOLR4_8_9018,0,0,0,0,0,0,0,0,0,0,0,0
FRG1_8_9019,0,0,0,0,0,0,0,0,0,0,0,0
FTL_8_9020,1,2,2,1,0,3,0,1,2,2,3,4
FXC1_8_9021,668,877,705,1026,557,1146,665,1245,1044,374,1184,1533
GABARAP_8_9022,0,0,0,0,0,0,0,0,0,0,0,0
GGA2_8_9023,0,0,0,0,0,0,0,0,0,0,0,0
GJA1_8_9024,1205,1348,1613,1987,2218,1398,584,1490,1159,1972,2593,3445
GJA3_8_9025,156,142,141,253,7,31,854,163,7,232,2,328
GJA4_8_9026,0,0,0,0,0,0,0,0,0,0,0,0
GJA8_8_9027,0,0,0,0,0,0,0,0,0,0,0,0
GJB2_8_9028,0,0,0,2,0,0,0,1,0,0,0,0
GJB4_8_9029,781,856,726,779,1122,358,814,718,218,1189,716,1145
GJB5_8_9030,0,0,0,0,0,0,0,0,0,0,0,0
GJC2_8_9031,0,0,0,0,0,0,0,0,0,0,0,0
GJC3_8_9032,0,0,0,0,0,0,0,0,0,0,0,0
GJD2_8_9033,0,0,0,0,0,0,0,0,0,0,0,0
GJD3_8_9034,0,0,0,0,0,0,0,0,0,0,0,0
GJD4_8_9035,0,0,0,0,0,0,0,0,0,0,0,0
GKN1_8_9036,0,0,0,0,0,0,0,0,0,0,0,0
GLCCI1_8_9037,0,0,0,0,0,0,0,0,0,0,0,0
GLTP_8_9038,0,0,0,0,0,0,0,0,0,0,0,0
GLYATL2_8_9039,0,0,0,0,0,0,0,0,0,0,0,0
GP9_8_9040,0,0,0,0,0,0,0,0,0,0,0,0
GPIHBP1_8_9041,0,0,0,0,0,0,0,0,0,0,0,0
GPR180_8_9042,0,0,0,0,0,0,0,0,0,0,0,0
GRN_8_9043,0,0,0,0,0,0,0,0,0,0,0,0
GZMH_8_9044,0,0,0,0,0,0,0,0,0,0,0,0
GZMK_8_9045,0,0,0,0,0,0,0,0,0,0,0,0
GZMM_8_9046,0,0,0,0,0,0,0,0,0,0,0,0
HBA1_8_9047,0,0,0,0,0,0,0,0,0,0,0,0
HBA2_8_9048,0,0,0,0,0,0,0,0,0,0,0,0
HBE1_8_9049,0,0,0,0,0,0,0,0,0,0,0,0
HBZ_8_9050,0,0,0,0,0,0,0,0,0,0,0,0
HECA_8_9051,0,0,0,0,0,0,0,0,0,0,0,0
HGFAC_8_9052,0,0,0,0,0,0,0,0,0,0,0,0
HIAT1_8_9053,9,20,4,4,7,10,2,6,9,4,12,17
HLA-DQB1_8_9054,160,319,282,334,267,996,506,273,299,739,130,1088
HMCN1_8_9055,0,0,0,0,0,0,0,0,0,0,0,0

HMHA1_8_9056,59,69,88,201,0,5,10,55,148,325,1,86
HPCAL4_8_9057,0,0,0,0,0,0,0,0,0,0,0,0
HPR_8_9058,0,0,0,0,0,0,0,0,0,0,0,0
HPX_8_9059,3042,4481,3433,3886,4905,6214,4436,2542,3342,4263,4391,5095
HSP90B1_8_9060,5,2,3,3,6,8,1,3,1,6,1,7
HTRA1_8_9061,0,0,0,0,0,0,0,0,0,0,0,0
HTRA4_8_9062,0,0,0,0,0,0,0,0,0,0,0,0
IFNG_8_9063,0,0,0,0,0,0,0,0,0,0,0,0
IGFBP7_8_9064,0,0,0,0,0,0,0,0,0,0,0,0
IL12B_8_9065,0,0,0,0,0,0,0,0,0,0,0,0
IL13_8_9066,0,0,0,0,0,0,0,0,0,0,0,0
IL17A_8_9067,0,0,0,0,0,0,0,0,0,0,0,0
IL1A_8_9068,0,0,0,0,0,0,0,0,0,0,0,0
IL1B_8_9069,0,0,0,0,0,0,0,0,0,0,0,0
IL3_8_9070,0,0,0,0,0,0,0,0,0,0,0,0
IL5_8_9071,0,0,0,0,0,0,0,0,0,0,0,0
INSL3_8_9072,0,0,0,0,0,0,0,0,0,0,0,0
IPO13_8_9073,0,0,0,0,0,0,0,0,0,0,0,0
IPO4_8_9074,0,0,0,0,0,0,0,0,0,0,0,0
IPO5_8_9075,0,0,0,0,0,0,0,0,0,0,0,0
IPO7_8_9076,3,3,1,0,2,1,1,2,0,1,1,0
IPO9_8_9077,0,0,0,0,0,0,0,0,0,0,0,0
ITGA10_8_9078,0,0,0,0,0,0,0,0,0,0,0,0
ITGA11_8_9079,0,0,0,0,0,0,0,0,0,0,0,0
ITGA2_8_9080,0,0,0,0,0,0,0,0,0,0,0,0
ITGA4_8_9081,899,1184,793,609,1721,893,673,1379,684,688,585,942
ITGA5_8_9082,0,0,0,0,0,0,0,0,0,0,0,0
ITGA8_8_9083,0,0,0,0,0,0,0,0,0,0,0,0
ITGAX_8_9084,0,0,0,0,0,0,0,0,0,0,0,0
ITGB5_8_9085,274,568,307,144,250,1206,170,1530,44,853,363,693
ITGB6_8_9086,0,0,0,0,0,0,0,0,0,0,0,0
ITGB8_8_9087,0,0,0,0,0,0,0,0,0,0,0,0
ITLN1_8_9088,0,0,0,0,0,0,0,0,0,0,0,0
KDELR1_8_9089,0,0,0,0,0,0,0,0,0,0,0,0
KEL_8_9090,0,0,0,0,0,0,0,0,0,0,0,0
KIF20A_8_9091,0,0,0,0,0,0,0,0,0,0,0,0
KIF3B_8_9092,652,486,719,504,510,1089,94,906,830,246,645,1369
KIF5A_8_9093,0,0,0,0,0,0,0,0,0,0,0,0
KLK13_8_9094,0,0,0,0,0,0,0,0,0,0,0,0
KLK14_8_9095,0,0,0,0,0,0,0,0,0,0,0,0
KLK4_8_9096,0,0,4,1,0,0,1,2,3,3,3,1
KLK9_8_9097,0,0,0,0,0,0,0,0,0,0,0,0
KPNA1_8_9098,0,0,0,0,0,0,0,0,0,0,0,0
KPNA2_8_9099,0,0,0,0,0,0,0,0,0,0,0,0
KPNA3_8_9100,1,0,0,0,0,1,1,3,0,0,3,1
KPNA4_8_9101,0,0,0,0,0,0,0,0,0,0,0,0
KPNA6_8_9102,0,0,0,0,0,0,0,0,0,0,0,0
KPNB1_8_9103,0,0,0,0,0,0,0,0,0,0,0,0
KRT12_8_9104,0,0,0,0,0,0,0,0,0,0,0,0
KRT7_8_9105,0,0,0,0,0,0,0,0,0,0,0,0

KRT8_8_9106,0,0,0,0,0,0,0,0,0,0,0,0
LASP1_8_9107,0,0,0,0,0,0,0,0,0,0,0,0
LBP_8_9108,0,0,0,0,0,1,0,0,0,0,0,0
LCN12_8_9109,0,0,0,0,0,0,0,0,0,0,0,0
LCN1_8_9110,793,676,817,696,1379,845,613,186,308,483,1354,33
LCN2_8_9111,0,0,0,0,0,0,0,0,0,0,0,0
LCN8_8_9112,0,0,0,0,0,0,0,0,0,0,0,0
LCN9_8_9113,0,0,0,0,0,0,0,0,0,0,0,0
LDLRAD2_8_9114,381,591,409,85,4,1058,1065,0,458,644,516,483
LDLRAP1_8_9115,0,0,0,0,0,0,0,0,0,0,0,0
LMAN1_8_9116,1,3,0,1,0,1,0,4,5,0,2,0
LMAN2_8_9117,0,0,0,0,0,0,0,0,0,0,0,0
LPA_8_9118,0,0,0,0,0,0,0,0,0,0,0,0
LRIG1_8_9119,0,0,0,0,0,0,0,0,0,0,0,0
LRP2_8_9120,0,0,0,0,0,0,0,0,0,0,0,0
LRRC4B_8_9121,0,0,0,0,0,0,0,0,0,0,0,0
LRRCC1_8_9122,0,0,0,0,0,0,0,0,0,0,0,0
LYST_8_9123,0,0,0,0,0,0,0,0,0,0,0,0
MAL2_8_9124,663,540,693,596,257,650,1736,733,467,149,1206,416
MATN3_8_9125,0,0,0,0,0,0,0,0,0,0,0,0
MFSD8_8_9126,0,1,0,1,0,0,0,0,0,1,0,0
MFSD9_8_9127,0,0,0,0,0,0,0,0,0,0,0,0
MMAA_8_9128,2,0,0,0,1,1,1,0,0,0,0,0
MMACHC_8_9129,0,0,0,0,0,0,0,0,0,0,0,0
MMGT1_8_9130,0,0,0,0,0,0,0,0,0,0,0,0
MMP11_8_9131,0,0,0,0,0,0,0,0,0,0,0,0
MMP13_8_9132,0,0,0,0,0,0,0,0,1,0,0,0
MMP15_8_9133,0,0,0,0,0,0,0,0,0,0,0,0
MMP16_8_9134,0,0,0,0,0,0,0,0,0,0,0,0
MMP17_8_9135,968,1235,1454,1806,1226,828,912,2341,1113,462,750,1945
MMP19_8_9136,0,0,0,0,0,0,0,0,0,0,0,0
MMP24_8_9137,0,0,0,0,0,0,0,0,0,0,0,0
MMP25_8_9138,0,0,0,0,0,0,0,0,0,0,0,0
MMP26_8_9139,0,0,0,0,0,0,0,0,0,0,0,0
MMP27_8_9140,0,0,0,0,0,0,0,0,0,0,0,0
MRS2_8_9141,0,0,0,0,0,0,0,0,0,0,0,0
MSTN_8_9142,1109,1151,1142,764,1039,1018,1324,718,1043,773,728,1178
MTX2_8_9143,0,0,0,0,0,0,0,0,0,0,0,0
MUC2_8_9144,0,0,0,0,0,0,0,0,0,0,0,0
NAPG_8_9145,0,0,0,0,0,0,0,0,0,0,0,0
NAPSA_8_9146,0,0,0,0,0,0,0,0,0,0,0,0
NCOA5_8_9147,0,0,0,0,0,0,0,0,0,0,0,0
NGF_8_9148,0,0,0,0,0,0,0,0,0,0,0,0
NID1_8_9149,0,0,0,0,0,0,0,0,0,0,0,0
NPC1_8_9150,0,0,0,0,0,0,0,0,0,0,0,0
NPEPPS_8_9151,0,0,0,0,0,0,0,0,0,0,0,0
NPPB_8_9152,0,0,0,0,0,0,0,0,0,0,0,0
NPY_8_9153,0,0,0,0,0,0,0,0,0,0,0,0
NSMCE1_8_9154,0,0,0,0,0,0,0,0,0,0,0,0
NUP107_8_9155,0,0,0,0,0,0,0,0,0,0,0,0

NUP133_8_9156,0,0,0,0,0,0,0,0,0,0,0,0
NUP153_8_9157,0,0,0,0,0,0,0,0,0,0,0,0
NUP160_8_9158,0,0,0,0,0,0,0,0,0,0,0,0
NUP210_8_9159,0,0,0,0,0,0,0,0,0,0,0,0
NUP214_8_9160,1394,1466,928,1004,2205,1964,966,2075,1611,2649,1535,240
7
NUP35_8_9161,0,0,0,0,0,0,0,0,0,0,0,0
NUP37_8_9162,0,0,2,0,1,0,1,1,3,2,2,0
NUP54_8_9163,0,0,0,0,0,0,0,0,0,0,0,0
NUP88_8_9164,0,0,0,0,0,0,0,0,1,0,0,0
NUPL2_8_9165,2,1,5,2,1,1,1,0,1,4,1,4
NUTF2_8_9166,0,0,0,0,0,0,0,0,0,0,0,0
NXF2B_8_9167,0,0,0,0,0,0,0,0,0,0,0,0
NXF2_8_9168,0,0,0,0,0,0,0,0,0,0,0,0
NXF3_8_9169,0,0,0,0,0,0,0,0,0,0,0,0
NXT1_8_9170,1446,1679,2170,1388,728,2467,1823,1173,1228,1062,3026,2437
OAZ2_8_9171,0,0,0,0,0,0,0,0,0,0,0,0
OBP2A_8_9172,301,115,166,69,404,25,285,137,52,472,1,313
OBP2B_8_9173,0,0,0,0,0,0,0,0,0,0,0,0
OCA2_8_9174,0,0,0,0,0,0,0,0,0,0,0,0
OGFOD1_8_9175,0,0,0,0,0,0,0,0,0,0,0,0
OGFOD2_8_9176,0,0,0,0,0,0,0,0,0,0,0,0
OGFR_8_9177,0,0,0,0,0,0,0,0,0,0,0,0
OVCH1_8_9178,0,0,0,0,0,0,0,0,0,0,0,0
OVCH2_8_9179,0,0,0,0,0,0,0,0,0,0,0,0
OXNAD1_8_9180,0,0,0,0,0,0,0,0,0,0,0,0
OXT_8_9181,0,0,0,0,0,0,0,0,0,0,0,0
PANX1_8_9182,0,0,0,0,0,0,0,0,0,0,0,0
PAQR7_8_9183,2,1,0,2,1,4,2,2,1,3,11,1
PCDHB11_8_9184,0,0,0,0,0,0,0,0,0,0,0,0
PCDHB16_8_9185,0,0,0,0,0,0,0,0,0,0,0,0
PCSK4_8_9186,0,0,0,0,0,0,0,0,0,0,0,0
PCSK7_8_9187,0,0,0,0,0,0,0,0,0,0,0,0
PEA15_8_9188,0,0,0,0,0,0,0,0,0,0,0,0
PET112_8_9189,0,0,0,0,0,0,0,0,0,0,0,0
PEX13_8_9190,0,0,0,0,0,0,0,0,0,0,0,0
PEX7_8_9191,0,0,0,0,0,0,0,0,0,0,0,0
PF4_8_9192,0,0,0,0,0,0,0,0,0,0,0,0
PFN3_8_9193,704,560,516,468,203,915,1446,1534,202,1590,884,1467
PHEX_8_9194,0,0,0,0,0,0,0,0,0,0,0,0
PIGR_8_9195,0,0,0,0,0,0,0,0,0,0,0,0
PITPNA_8_9196,0,0,0,0,0,0,0,0,0,0,0,0
PITPNB_8_9197,0,0,0,0,0,0,0,0,0,0,0,0
PLLP_8_9198,0,0,0,0,0,0,0,0,0,0,0,0
PLP2_8_9199,0,0,0,0,0,0,0,0,0,0,0,0
PLXNB2_8_9200,0,0,0,0,0,0,0,1,0,0,0,0
PNMA2_8_9201,0,0,0,0,0,0,0,0,0,0,0,0
PPP1R14A_8_9202,0,0,0,0,0,0,0,0,0,0,0,0
PPP1R14C_8_9203,0,0,0,0,0,0,0,0,0,0,0,0
PPP1R15A_8_9204,0,2,2,0,0,0,0,0,0,1,0,2

PPRC1_8_9205,0,0,0,0,0,0,0,0,0,0,0,0
PPY_8_9206,0,0,0,0,0,0,0,0,0,0,0,0
PRB3_8_9207,0,0,0,0,0,0,0,0,0,0,0,0
PROCR_8_9208,0,0,0,0,0,0,0,0,0,0,0,0
PROS1_8_9209,0,0,0,0,0,0,0,0,0,0,0,0
PROZ_8_9210,0,0,0,0,0,0,0,0,0,0,0,0
PRPF18_8_9211,0,0,0,0,0,0,0,0,0,0,0,0
PRSS12_8_9212,0,0,0,0,0,0,0,0,0,0,0,0
PRSS22_8_9213,0,0,0,0,0,0,0,0,0,0,0,0
PRSS27_8_9214,0,0,0,0,0,0,0,0,0,0,0,0
PRSS33_8_9215,0,0,0,0,0,0,0,0,0,0,0,0
PRSS36_8_9216,0,0,0,0,0,0,0,0,0,0,0,0
PRSS8_8_9217,0,0,0,0,0,0,0,0,0,0,0,0
PSCA_8_9218,0,0,0,0,0,0,0,0,0,0,0,0
PTOV1_8_9219,0,0,0,0,0,0,0,0,0,0,0,0
RABIF_8_9220,0,0,0,0,0,0,0,0,0,0,0,0
RAMP1_8_9221,0,0,0,0,0,0,0,0,0,0,0,0
RAMP2_8_9222,0,0,0,0,0,0,0,0,0,0,0,0
RAMP3_8_9223,0,0,0,0,0,0,0,0,0,0,0,0
RANBP17_8_9224,0,0,0,0,0,0,0,0,0,0,0,0
RASSF9_8_9225,0,0,0,0,0,0,0,0,0,0,0,0
REEP5_8_9226,0,0,0,0,0,0,0,0,0,0,0,0
RHAG_8_9227,0,0,0,0,0,0,0,0,0,0,0,0
RLBP1_8_9228,0,0,0,0,0,0,0,0,0,0,0,0
RPL15_8_9229,0,0,0,0,0,0,0,0,0,0,0,0
S100A12_8_9230,0,0,0,0,0,0,0,0,0,0,0,0
S100A1_8_9231,0,0,0,0,0,0,0,0,0,0,0,0
S100A2_8_9232,0,1,34,0,0,1,2,0,0,1,0,0
S100A3_8_9233,0,0,0,0,0,0,0,0,0,0,0,0
S100A6_8_9234,419,634,466,809,498,297,173,500,558,1268,782,539
S100B_8_9235,0,0,0,1,0,1,0,0,0,0,0,0
S100P_8_9236,0,0,0,0,0,0,0,0,0,0,0,0
SAA4_8_9237,0,0,0,0,0,0,0,0,0,0,0,0
SCAMP1_8_9238,0,0,0,0,0,0,0,0,0,0,0,0
SCAMP2_8_9239,0,0,0,0,0,0,0,0,0,0,0,0
SCFD2_8_9240,0,0,0,0,0,0,0,0,0,0,0,0
SCLT1_8_9241,0,0,0,0,0,0,0,0,0,0,0,0
SCPEP1_8_9242,0,0,0,0,0,0,0,0,0,0,0,0
SDC2_8_9243,0,0,0,0,0,0,0,0,0,0,0,0
SEC14L3_8_9244,0,0,0,0,0,0,0,0,0,0,0,0
SEC22A_8_9245,0,0,0,0,0,0,0,0,0,0,0,0
SEC23A_8_9246,2025,2094,1602,2056,788,3130,3835,2425,2974,2130,2535,11
68
SEC24A_8_9247,0,0,0,0,0,0,0,0,0,0,0,0
SEC24D_8_9248,0,0,0,0,0,0,0,0,0,0,0,0
SEC61A1_8_9249,0,0,0,0,0,0,0,0,0,0,0,0
SEC61B_8_9250,1,0,1,1,3,8,2,0,2,2,3,5
SEC62_8_9251,0,0,0,0,0,0,0,0,0,0,0,0
SEC63_8_9252,10,0,0,0,2,1,0,0,2,1,0,2
SELP_8_9253,0,0,0,0,0,0,0,0,0,0,0,0

SERINC1_8_9254,0,0,0,0,0,0,0,0,0,0,0,0
SERPINA11_8_9255,0,0,0,0,0,0,0,0,0,0,0,0
SERPINA12_8_9256,0,0,0,0,0,0,0,0,0,0,0,0
SERPINA3_8_9257,409,843,320,589,305,964,98,307,193,673,227,347
SERPINA4_8_9258,0,0,0,0,0,0,0,0,0,0,0,0
SERPINA5_8_9259,0,0,1,2,0,1,0,0,2,0,1,2
SERPINA7_8_9260,0,0,0,0,0,0,0,0,0,0,0,0
SERPINB10_8_9261,0,0,0,0,0,0,0,2,0,0,0,2
SERPINB12_8_9262,0,0,0,0,0,0,0,0,0,0,0,0
SERPINB13_8_9263,0,0,0,0,0,0,0,0,0,0,0,0
SERPINB1_8_9264,0,0,0,0,0,0,0,0,0,1,0,0
SERPINB4_8_9265,0,0,0,0,0,0,0,0,0,0,0,0
SERPINB9_8_9266,1,0,0,0,0,0,0,0,0,0,0,0
SERPINC1_8_9267,0,0,0,0,0,0,0,0,0,0,0,0
SERPIND1_8_9268,0,0,0,0,0,0,0,0,0,0,0,0
SERPINF1_8_9269,0,0,2,1,0,0,0,0,0,0,0,0
SFXN1_8_9270,0,0,0,0,0,0,0,0,0,0,0,0
SFXN2_8_9271,0,0,0,0,0,0,0,0,0,0,0,0
SFXN3_8_9272,0,0,0,0,0,0,0,0,0,0,0,0
SFXN4_8_9273,876,913,553,654,189,388,333,507,311,366,1181,1216
SFXN5_8_9274,893,773,1077,747,2417,475,561,372,1320,804,815,618
SLC15A5_8_9275,0,0,0,0,0,0,0,0,0,0,0,0
SLC16A13_8_9276,0,0,0,0,0,0,0,0,0,0,0,0
SLC16A14_8_9277,0,0,0,0,0,0,0,0,0,0,0,0
SLC16A9_8_9278,0,0,0,0,0,0,0,0,0,0,0,0
SLC17A9_8_9279,0,0,0,0,0,0,0,0,0,0,0,0
SLC22A20_8_9280,0,0,0,0,0,0,0,0,0,0,0,0
SLC22A24_8_9281,0,0,0,0,0,0,0,0,0,0,0,0
SLC22A25_8_9282,0,0,0,0,0,0,0,0,0,0,0,0
SLC25A23_8_9283,0,0,0,0,0,0,0,0,0,0,0,0
SLC25A29_8_9284,0,0,0,0,0,0,0,0,0,0,0,0
SLC25A31_8_9285,0,0,0,0,0,0,0,0,0,0,0,0
SLC25A38_8_9286,0,0,0,0,0,0,0,0,0,0,0,0
SLC25A42_8_9287,816,598,352,559,1075,363,1061,485,35,238,291,949
SLC25A46_8_9288,0,0,0,0,0,0,0,0,0,0,0,0
SLC25A48_8_9289,0,0,0,0,0,0,0,0,0,0,0,0
SLC38A7_8_9290,0,0,0,0,0,0,0,0,0,0,2,0
SLC38A9_8_9291,0,0,0,0,0,0,0,0,0,0,0,0
SLC41A2_8_9292,0,0,0,0,0,0,0,0,0,0,0,0
SLC45A1_8_9293,0,0,0,0,0,0,0,0,0,0,0,0
SLC45A3_8_9294,0,0,0,0,0,0,0,0,0,0,0,0
SLC47A1_8_9295,0,0,0,0,0,0,0,0,0,0,0,0
SLC48A1_8_9296,0,0,0,0,0,0,0,0,0,0,0,0
SLC5A12_8_9297,0,0,0,0,0,0,0,0,0,0,0,0
SLC6A17_8_9298,0,0,0,0,0,0,0,0,0,0,0,0
SLC7A14_8_9299,0,0,0,0,0,0,0,0,0,0,0,0
SLIT2_8_9300,0,0,0,0,0,0,0,0,0,0,0,0
SNAP29_8_9301,0,0,0,0,0,0,0,0,0,0,0,0
SNX12_8_9302,38,43,34,64,87,63,9,63,174,0,63,9
SNX13_8_9303,0,0,0,0,0,0,0,0,0,0,0,0

SNX17_8_9304,0,0,0,0,0,0,0,0,0,0,0,0
SNX19_8_9305,0,0,0,0,0,0,0,0,0,0,0,0
SNX22_8_9306,0,0,0,0,0,0,0,0,0,0,0,0
SNX24_8_9307,0,0,0,0,0,0,0,0,0,0,0,0
SNX2_8_9308,0,0,0,0,0,0,0,0,0,0,0,0
SNX4_8_9309,0,0,0,0,0,0,0,0,0,0,0,0
SNX8_8_9310,0,0,0,0,0,0,0,0,0,0,0,0
SNX9_8_9311,2,0,2,4,4,0,0,0,1,0,0,0
SORCS2_8_9312,0,0,0,0,0,0,0,0,0,0,0,0
SORCS3_8_9313,0,0,0,0,0,0,0,0,0,0,0,0
SORL1_8_9314,0,0,0,0,0,0,0,0,0,0,0,0
SOS1_8_9315,0,0,0,0,0,0,0,0,0,0,0,0
SST_8_9316,1,0,0,0,0,0,0,0,0,0,0,0
ST13_8_9317,0,0,0,0,0,0,0,0,0,0,0,0
STAB1_8_9318,0,0,0,0,0,0,0,0,0,0,0,0
STARD4_8_9319,0,0,0,0,0,0,0,0,0,0,0,0
STARD5_8_9320,268,414,427,354,1260,488,454,739,380,498,1300,748
STARD6_8_9321,0,0,0,0,0,0,0,0,0,0,0,0
STEAP1_8_9322,0,0,0,0,0,0,0,0,0,0,0,0
STMN4_8_9323,0,0,0,0,0,0,0,0,0,0,0,0
STX11_8_9324,283,98,135,63,62,0,77,336,44,41,98,175
STX18_8_9325,0,0,0,0,0,0,0,0,0,0,0,0
STX4_8_9326,0,0,0,0,0,0,0,0,0,0,0,0
STX5_8_9327,0,0,0,0,0,0,0,0,0,0,0,0
STX6_8_9328,0,0,0,0,0,0,0,0,0,0,0,0
STX7_8_9329,0,0,0,0,0,0,0,0,0,0,0,0
STXBP3_8_9330,0,0,0,0,0,0,0,0,0,0,0,1
SVOP_8_9331,0,0,0,0,0,0,0,0,0,0,0,0
SYP_8_9332,0,0,0,0,0,0,0,0,0,0,0,0
SYT10_8_9333,0,0,0,0,0,0,0,0,0,0,0,0
SYT11_8_9334,0,0,0,0,0,0,0,0,0,0,0,0
SYT13_8_9335,2,2,1,3,0,2,6,5,3,6,3,5
SYT16_8_9336,0,0,0,0,0,0,0,0,0,0,0,0
SYT4_8_9337,0,0,0,0,0,0,0,0,0,0,0,0
SYT5_8_9338,9,73,0,196,0,12,0,4,401,0,0,0
SYT6_8_9339,0,0,0,0,0,0,0,0,0,0,0,0
SYT7_8_9340,0,0,0,0,0,0,0,0,0,0,0,0
SYT8_8_9341,0,0,0,0,0,0,0,0,0,0,0,0
SYT9_8_9342,0,0,0,0,0,0,0,0,0,0,0,0
TAS2R42_8_9343,0,0,0,0,0,0,0,0,0,0,0,0
TBC1D9_8_9344,0,0,0,0,0,0,0,0,0,0,0,0
TCN1_8_9345,0,0,0,0,0,0,0,0,0,0,0,0
TEKT4_8_9346,0,0,0,0,0,0,0,0,0,0,0,0
TF_8_9347,0,0,0,0,0,0,0,0,0,0,0,0
TG_8_9348,0,0,0,0,0,0,0,0,0,0,0,0
TGFB1_8_9349,806,660,382,828,103,313,89,917,562,743,997,1704
TIMM10_8_9350,0,0,0,1,0,0,0,0,0,0,0,0
TIMM13_8_9351,0,0,0,0,0,0,0,0,0,0,0,0
TIMM17A_8_9352,906,1361,674,986,529,2192,640,1191,878,1353,1277,2115
TIMM22_8_9353,0,0,0,0,0,0,0,0,0,0,0,0

TIMM23_8_9354,0,0,0,0,0,0,0,0,0,0,0,0
TIMM44_8_9355,0,0,0,0,0,0,0,0,0,0,0,0
TIMM8B_8_9356,0,0,0,0,0,0,0,0,0,0,0,0
TIMM9_8_9357,0,0,0,0,0,0,0,0,0,0,0,0
TLL2_8_9358,0,0,0,0,0,0,0,0,0,0,0,0
TM9SF2_8_9359,0,0,0,0,0,0,0,0,0,0,0,0
TM9SF3_8_9360,0,0,0,0,0,0,0,0,0,0,0,0
TM9SF4_8_9361,0,0,0,0,0,0,0,0,0,0,0,0
TMCO3_8_9362,0,0,0,0,0,0,0,0,0,0,0,0
TMED10_8_9363,0,0,0,0,0,0,0,0,0,0,0,0
TMED1_8_9364,0,0,0,0,0,0,0,0,0,0,0,0
TMED2_8_9365,0,0,0,0,0,0,0,0,0,0,0,0
TMED3_8_9366,0,0,0,0,0,0,0,0,0,0,0,0
TMED4_8_9367,0,0,0,0,0,0,0,0,0,0,0,0
TMED7_8_9368,0,0,0,0,0,0,0,0,0,0,0,0
TMED9_8_9369,0,0,0,0,0,0,0,0,0,0,0,0
TMEM104_8_9370,0,0,0,0,0,0,0,0,0,0,0,0
TMPRSS11B_8_9371,0,0,0,0,0,0,0,0,0,0,0,0
TMPRSS11D_8_9372,0,0,0,0,0,0,0,0,0,0,0,0
TMPRSS11E_8_9373,0,0,0,0,0,0,0,0,0,0,0,0
TMPRSS11F_8_9374,0,0,0,0,0,0,0,0,0,0,0,0
TMPRSS12_8_9375,0,0,0,0,0,0,0,0,0,0,0,0
TMPRSS5_8_9376,0,0,0,0,0,0,0,0,0,0,0,0
TMPRSS6_8_9377,0,0,0,0,0,0,0,0,0,0,0,0
TMPRSS7_8_9378,0,0,0,0,0,0,0,0,0,0,0,0
TMPRSS9_8_9379,0,0,0,0,0,0,0,0,0,0,0,0
TNC_8_9380,0,0,0,0,0,0,0,0,0,0,0,0
TNF_8_9381,84,211,2,40,10,76,1,204,17,0,3,132
TNFRSF9_8_9382,0,0,0,0,0,0,0,0,0,0,0,0
TNNC2_8_9383,645,593,531,495,516,994,471,2393,1277,1592,290,416
TNN_8_9384,834,891,959,942,550,751,506,932,203,2192,2471,377
TNNI3_8_9385,0,0,0,0,0,0,0,0,0,0,0,0
TNR_8_9386,0,0,0,0,0,0,0,0,0,0,0,0
TOMM20_8_9387,0,0,0,0,0,0,0,0,0,0,0,0
TOMM22_8_9388,386,161,137,111,124,172,148,280,61,360,234,463
TOMM70A_8_9389,0,0,0,0,0,0,0,0,0,0,0,0
TOMM7_8_9390,0,0,0,0,0,0,0,0,0,0,0,0
TPSG1_8_9391,275,218,336,499,473,311,6,1238,0,300,219,1091
TRAK2_8_9392,0,0,0,0,0,0,0,0,0,0,0,0
TRAPPC10_8_9393,0,0,0,0,0,0,0,0,0,0,0,0
TRAPPC8_8_9394,0,0,0,0,0,0,0,0,0,0,0,0
TSNAX_8_9395,0,0,0,0,0,0,0,0,0,0,0,0
TTPA_8_9396,0,0,0,0,0,0,0,0,0,0,0,0
TTR_8_9397,293,204,257,131,823,132,273,26,749,141,167,397
TUBA1A_8_9398,1930,2197,1226,2298,1231,1789,3621,2080,1717,2011,2627,2
073
TUBA1C_8_9399,1423,1537,1235,1166,1098,1791,1988,242,2212,837,593,1585
TUBA3D_8_9400,1649,1509,1793,1191,1103,1426,1626,1346,462,1438,1459,25
36
TUBA3E_8_9401,0,0,0,0,0,0,0,0,0,0,0,0

TUBA4A_8_9402,0,0,0,0,0,0,0,0,0,0,0,0
TUBB1_8_9403,0,0,0,0,0,0,0,0,0,0,0,0
TUBB2A_8_9404,2152,1691,1738,2329,2355,1421,2989,1792,2827,3800,1106,1
772
TUBB2B_8_9405,875,1579,724,1187,654,1657,276,1327,329,965,726,990
TUBB6_8_9406,0,0,0,0,0,0,0,0,0,0,0,0
TUBB8_8_9407,0,0,0,0,0,0,0,0,0,0,0,0
TUBE1_8_9408,0,0,0,0,0,0,0,0,0,0,0,0
TUBG1_8_9409,431,181,303,90,535,382,1174,715,252,248,738,606
TUBG2_8_9410,0,0,0,0,0,0,0,0,0,0,0,0
TXNDC8_8_9411,0,0,0,0,0,0,0,0,0,0,0,0
UCP2_8_9412,0,0,0,0,0,0,0,0,0,0,0,0
USO1_8_9413,0,0,0,0,0,0,0,0,0,0,0,0
VAMP2_8_9414,0,0,0,0,0,0,0,0,0,0,0,0
VAMP5_8_9415,0,0,0,0,0,0,0,0,0,0,0,0
VKORC1L1_8_9416,0,0,0,0,0,0,0,0,0,0,0,0
VPS18_8_9417,0,0,0,0,0,0,0,0,0,0,0,0
VPS26B_8_9418,0,0,0,0,0,0,0,0,0,0,0,0
VPS33A_8_9419,0,0,0,0,0,0,0,0,0,0,0,0
VPS33B_8_9420,0,0,0,0,0,0,0,0,0,0,0,0
VPS35_8_9421,0,0,0,0,0,0,0,0,0,0,0,0
VPS39_8_9422,0,0,0,0,0,0,0,0,0,0,0,0
VPS45_8_9423,0,0,0,0,0,0,0,0,0,0,0,0
VPS4B_8_9424,3,3,5,2,3,9,0,2,3,1,4,8
VSIG2_8_9425,0,0,0,0,0,0,0,0,0,0,0,0
VTI1A_8_9426,0,0,0,0,0,0,0,0,0,0,0,0
VTI1B_8_9427,1413,1559,1127,879,2155,1129,999,2162,1035,1741,2308,1388
VTN_8_9428,0,0,0,0,0,0,0,0,0,0,0,0
VWF_8_9429,0,0,0,0,0,0,0,0,0,0,0,0
XK_8_9430,0,0,0,0,0,0,0,0,0,0,0,0
XPO1_8_9431,0,0,0,0,0,0,0,0,0,0,0,0
XPO4_8_9432,0,0,0,0,0,0,0,0,0,0,0,0
XPO5_8_9433,0,0,0,0,0,0,0,0,0,0,0,0
XPO6_8_9434,0,0,0,0,0,0,0,0,0,0,0,0
XPO7_8_9435,0,0,0,0,0,0,0,0,0,0,0,0
ACTL6A_8_9436,0,0,0,0,0,0,0,0,0,0,0,0
ADAM12_8_9437,0,0,0,0,0,0,0,0,0,0,0,0
ADAM15_8_9438,0,0,0,0,0,0,0,0,0,0,0,0
ADAM18_8_9439,0,0,0,0,0,0,0,0,0,0,0,0
ADAM22_8_9440,0,0,0,0,0,0,0,0,0,0,0,0
ADAM29_8_9441,0,0,0,0,0,0,0,0,0,0,0,0
ADAM33_8_9442,0,0,0,0,0,0,0,0,0,0,0,0
ADAM8_8_9443,0,0,0,0,0,0,0,0,0,0,0,0
ADAMTS13_8_9444,0,0,0,0,0,0,0,0,0,0,0,0
ADAMTS14_8_9445,0,0,0,0,0,0,0,0,0,0,0,0
AKAP12_8_9446,0,0,0,0,0,0,0,0,0,0,0,0
ANGPT1_8_9447,0,0,0,0,0,0,0,0,0,0,0,0
ANGPTL4_8_9448,0,0,0,0,0,0,0,0,0,0,0,0
AP1B1_8_9449,0,0,0,0,0,0,0,0,0,0,0,0
AP1G1_8_9450,0,0,0,0,0,0,0,0,0,0,0,0

AP1M1_8_9451,664,977,509,746,332,635,196,658,449,186,1774,439
AP2A1_8_9452,0,0,0,0,0,0,0,0,0,0,0,0
AP2A2_8_9453,0,0,0,0,0,0,0,0,0,0,0,0
AP2B1_8_9454,0,0,0,0,0,0,0,0,0,0,0,0
AP2M1_8_9455,0,0,0,0,0,0,0,0,0,0,0,0
AP2S1_8_9456,0,0,0,0,0,0,0,0,0,0,0,0
AP3D1_8_9457,0,0,0,0,0,0,1,0,0,0,1,0
AP3M1_8_9458,0,0,0,0,0,0,0,0,0,0,0,0
AP3M2_8_9459,0,0,0,0,0,0,0,0,0,0,0,0
AP4S1_8_9460,1,2,4,8,1,1,1,6,4,5,3,2
APAF1_8_9461,0,0,0,0,0,0,0,0,0,0,0,0
APBA2_8_9462,0,0,0,0,0,0,0,0,0,0,0,0
APOL1_8_9463,0,0,0,0,0,0,0,0,0,0,0,0
APOL3_8_9464,0,0,0,0,0,0,0,0,0,0,0,0
APP_8_9465,0,0,0,0,0,0,0,0,0,0,0,0
ARFGAP3_8_9466,4,2,2,1,0,1,2,6,0,0,1,1
ARHGAP33_8_9467,0,0,0,0,0,0,0,0,0,0,0,0
ARL6_8_9468,0,0,0,0,0,0,0,0,0,0,0,0
ATP13A2_8_9469,0,0,0,0,0,0,0,0,0,0,0,0
BAX_8_9470,0,0,0,0,0,0,0,0,0,0,0,0
BCAP29_8_9471,0,0,0,0,0,0,0,0,0,0,0,0
BCAP31_8_9472,0,0,0,0,0,0,0,0,0,0,0,0
BCL2_8_9473,0,0,0,0,0,0,0,0,0,0,0,0
BCL2L2_8_9474,0,0,0,0,0,0,0,0,0,0,0,0
BID_8_9475,0,0,0,0,0,0,0,0,0,0,0,0
BSG_8_9476,0,0,0,0,0,0,0,0,0,0,0,0
C1QC_8_9477,0,0,0,0,0,0,0,0,0,0,0,0
CANX_8_9478,0,0,0,0,336,0,0,0,0,0,0,0
CAPN9_8_9479,0,0,0,0,0,0,0,0,0,0,0,0
CAPNS1_8_9480,0,0,0,0,0,0,0,0,0,0,0,0
CCT6B_8_9481,0,0,0,0,0,0,0,0,0,0,0,0
CD19_8_9482,866,640,945,1029,753,1267,573,350,357,795,829,846
CD22_8_9483,0,0,0,0,0,0,0,0,0,0,0,0
CD33_8_9484,0,0,0,0,0,0,0,0,0,0,0,0
CD44_8_9485,0,0,0,0,0,0,0,0,0,0,0,0
CD55_8_9486,0,0,0,0,0,0,0,0,0,0,0,0
CDH17_8_9487,0,0,0,0,0,0,0,0,0,0,0,0
CFHR4_8_9488,0,0,0,0,0,0,0,0,0,0,0,0
CIZ1_8_9489,0,0,0,0,0,0,0,0,0,0,0,0
COG2_8_9490,0,0,0,0,0,0,0,0,0,0,0,0
COG4_8_9491,0,0,0,0,0,0,0,0,0,0,0,0
COG5_8_9492,0,0,0,0,0,0,0,0,0,0,0,0
COG6_8_9493,0,0,0,0,0,0,0,0,0,0,0,0
COL11A1_8_9494,0,0,0,0,0,0,0,0,0,0,0,0
COL11A2_8_9495,0,0,0,0,0,0,0,0,0,0,0,0
COL12A1_8_9496,0,0,0,0,0,0,0,0,0,0,0,0
COL13A1_8_9497,0,0,0,0,0,0,0,0,0,0,0,0
COL18A1_8_9498,0,0,0,0,0,0,0,0,0,0,0,0
COL25A1_8_9499,0,0,0,0,0,0,0,0,0,0,0,0
COL2A1_8_9500,0,0,0,0,0,0,0,0,0,0,0,0

COL4A5_8_9501,0,0,0,0,0,0,0,0,0,0,0,0
COL4A6_8_9502,0,0,0,0,0,0,0,0,0,0,0,0
COL6A2_8_9503,0,0,0,0,0,0,0,0,0,0,0,0
COL6A3_8_9504,0,0,0,1,0,0,0,0,0,0,0,0
COL8A1_8_9505,0,0,0,0,0,0,0,0,0,0,0,0
COL9A1_8_9506,0,0,0,0,0,0,0,0,0,0,0,0
COPA_8_9507,0,0,0,0,0,0,0,0,0,0,0,0
COPB1_8_9508,0,0,0,0,0,0,0,0,0,0,0,0
COPE_8_9509,0,0,0,0,0,0,0,0,0,0,0,0
CPA4_8_9510,321,519,295,464,1014,814,595,153,434,1445,430,409
CPNE1_8_9511,0,0,0,0,0,0,0,0,0,0,0,0
CPNE7_8_9512,0,0,0,0,0,0,0,0,0,0,0,0
CPXM1_8_9513,0,0,0,0,0,0,0,0,0,0,0,0
CPZ_8_9514,0,1,1,0,0,0,0,0,0,1,0,0
CRABP2_8_9515,0,0,0,0,0,0,0,0,0,0,0,0
CTLA4_8_9516,0,0,0,0,0,0,0,0,0,0,0,0
CTNS_8_9517,0,0,0,0,0,0,0,0,0,0,0,0
CXCL12_8_9518,0,0,0,0,0,0,0,0,0,0,0,0
DPP10_8_9519,0,1,0,0,0,0,0,0,0,0,0,0
DPP6_8_9520,0,0,0,0,0,0,0,0,0,0,0,0
ECM1_8_9521,0,0,0,0,0,0,0,0,0,0,0,0
EGF_8_9522,0,0,0,0,0,0,0,0,0,0,0,0
EIF2C2_8_9523,692,1003,1111,1354,1753,1775,1159,797,500,1527,960,1018
EIF2D_8_9524,0,0,0,0,0,0,0,0,0,0,0,0
ENSA_8_9525,0,0,0,0,0,0,0,0,0,0,0,0
EPB41L4B_8_9526,0,0,0,0,0,0,0,0,0,0,0,0
EPB42_8_9527,0,0,0,0,0,0,0,0,0,0,0,0
ETFA_8_9528,0,0,0,0,0,0,0,0,0,0,0,0
ETFB_8_9529,0,0,0,0,0,0,0,0,0,0,0,0
EXOC1_8_9530,1,0,0,1,0,0,0,1,0,0,1,0
EXOC4_8_9531,0,0,0,0,0,0,0,0,0,0,0,0
EXOC6_8_9532,0,0,0,0,0,0,0,0,0,0,0,0
EXOC7_8_9533,0,0,0,0,0,0,0,0,0,0,0,0
F8_8_9534,0,0,0,0,0,0,0,0,0,0,0,0
FABP6_8_9535,685,749,615,990,1347,238,889,15,690,1118,1023,1427
FAM131A_8_9536,0,0,0,0,0,0,0,0,0,0,0,0
FAM63B_8_9537,0,0,0,0,0,0,0,0,0,0,0,0
FANCA_8_9538,0,0,0,0,0,0,0,0,0,0,0,0
FCN3_8_9539,0,0,0,0,0,0,0,0,0,0,0,0
FGA_8_9540,0,0,0,0,0,0,0,0,0,0,0,0
FGB_8_9541,0,0,0,0,0,0,0,0,0,0,0,0
FGF13_8_9542,0,0,0,0,0,0,0,0,0,0,0,0
FGG_8_9543,0,0,0,0,0,0,0,0,0,0,0,0
FIBCD1_8_9544,291,256,183,340,18,167,322,11,8,53,214,444
FLVCR2_8_9545,0,0,0,0,0,0,0,0,0,0,0,0
FOLR1_8_9546,419,240,325,405,396,103,95,11,1287,541,131,86
FOLR2_8_9547,0,0,0,0,0,0,0,0,0,0,0,0
GGA1_8_9548,0,0,0,0,0,0,0,0,0,0,0,0
GGA3_8_9549,158,114,258,206,118,116,0,131,2,296,123,248
GJA5_8_9550,0,0,0,0,0,0,0,0,0,0,0,0

GJB1_8_9551,0,0,0,0,0,0,0,0,0,0,0,0
GJB3_8_9552,3,1,0,3,2,1,0,1,1,1,1,4
GJB6_8_9553,7330,7728,5858,7962,7823,5751,6437,6558,6369,11604,9016,10
513
GLYATL1_8_9554,0,0,0,0,0,0,0,0,0,0,0,0
GNRH1_8_9555,0,0,0,0,0,0,0,0,0,0,0,0
GOLGA3_8_9556,0,0,0,0,0,0,0,0,0,0,0,0
GOPC_8_9557,0,1,0,0,0,0,0,0,1,0,0,0
GOSR1_8_9558,0,0,0,0,0,0,0,0,0,0,0,0
GOSR2_8_9559,567,354,294,200,270,662,979,305,778,260,440,189
GPRASP1_8_9560,0,0,0,2,0,0,0,0,1,1,0,2
GRB2_8_9561,0,0,0,0,0,0,0,0,0,0,0,0
HABP2_8_9562,0,0,0,0,0,0,0,0,0,0,0,0
HDLBP_8_9563,0,0,0,0,0,0,0,0,0,0,0,0
HEPH_8_9564,0,0,0,0,0,0,0,0,0,0,0,0
HNRNPU_8_9565,0,0,0,0,0,0,0,0,0,0,0,0
HOMER2_8_9566,0,0,0,0,0,0,0,0,0,0,0,0
HPN_8_9567,2,0,2,2,1,3,3,3,2,2,0,7
HSDL2_8_9568,0,0,0,0,0,0,0,0,0,0,0,0
IGF1_8_9569,913,1408,718,746,687,1668,1195,483,761,216,957,1004
IGFBP3_8_9570,0,0,0,0,0,0,0,0,0,0,0,0
IPO11_8_9571,0,0,0,0,0,0,0,0,0,0,1,0
IPO8_8_9572,0,0,0,0,0,0,0,0,0,0,0,0
ITGAL_8_9573,0,0,0,0,0,0,0,0,0,0,0,0
ITGAM_8_9574,0,0,0,0,0,0,0,0,0,0,0,0
ITGAV_8_9575,0,0,0,0,0,0,0,0,0,0,0,0
ITGB2_8_9576,0,0,0,0,0,0,0,0,0,0,0,0
KDELR2_8_9577,0,0,0,0,0,0,0,0,0,0,0,0
KDELR3_8_9578,0,0,0,0,0,0,0,0,0,0,0,0
KIF13A_8_9579,0,0,0,0,0,0,0,0,0,0,0,0
KIF17_8_9580,0,0,0,0,0,0,0,0,0,0,0,0
KIF1B_8_9581,0,0,0,0,0,0,0,0,0,0,0,0
KLK10_8_9582,0,0,0,0,0,0,0,0,0,0,0,0
KLK11_8_9583,0,0,0,0,0,0,0,0,0,0,0,0
KLK12_8_9584,64,14,21,115,0,8,0,0,359,0,9,127
KLK15_8_9585,0,0,0,0,0,0,0,0,0,0,0,0
KLK5_8_9586,0,0,0,0,0,0,0,0,0,0,0,0
KLK6_8_9587,0,0,0,0,0,0,0,0,0,0,0,0
LAMB3_8_9588,0,0,0,0,0,0,0,0,0,0,0,0
LDB3_8_9589,0,0,0,0,0,0,0,0,0,0,0,0
LDLR_8_9590,0,0,0,0,0,0,0,0,0,0,0,0
LTA_8_9591,0,0,0,0,0,0,0,0,0,0,0,0
LTF_8_9592,0,0,0,0,0,0,0,0,0,0,0,0
M6PR_8_9593,0,0,0,0,0,0,0,0,0,0,0,0
MASP1_8_9594,0,0,0,0,0,0,0,0,0,0,0,0
MB_8_9595,0,0,0,0,0,0,0,0,0,0,0,0
MCFD2_8_9596,2877,3284,2126,2946,4976,4649,2788,2340,1996,3144,1728,34
96
MCL1_8_9597,0,0,0,0,0,0,0,0,0,0,0,0
MEFV_8_9598,0,0,0,0,0,0,0,0,0,0,0,0

MFAP4_8_9599,0,0,0,0,0,0,0,0,0,0,0,0
MFSD10_8_9600,0,0,0,0,0,0,0,0,0,0,0,0
MFSD1_8_9601,0,0,0,0,0,0,0,0,0,0,0,0
MFSD5_8_9602,0,0,0,0,0,0,0,0,0,0,0,0
MLC1_8_9603,2,3,4,0,0,0,0,0,0,24,0,0
MSLN_8_9604,0,0,0,0,0,0,0,0,0,0,0,0
MTX1_8_9605,0,0,0,0,0,0,0,0,0,0,0,0
MUC1_8_9606,0,0,0,0,0,0,0,0,0,0,0,0
NCAM1_8_9607,0,0,0,0,0,0,0,0,0,0,0,0
NNAT_8_9608,0,0,0,0,0,0,0,0,0,0,0,0
NOXO1_8_9609,0,0,0,0,0,0,0,0,0,0,0,0
NPC1L1_8_9610,0,0,0,0,0,0,0,0,0,0,0,0
NPRL3_8_9611,2,0,1,2,5,1,1,2,0,1,0,0
NRXN1_8_9612,0,0,0,0,0,0,0,0,0,0,0,0
NRXN2_8_9613,0,0,0,0,0,0,0,0,0,0,0,0
NRXN3_8_9614,0,0,0,0,0,0,0,0,0,0,0,0
NUP155_8_9615,0,0,0,0,0,0,0,0,0,0,0,0
NUP50_8_9616,554,525,814,793,1290,1411,1677,852,701,987,495,1378
NUP62_8_9617,0,0,0,0,0,0,0,0,0,0,0,0
NUP98_8_9618,1814,2100,2062,1849,3361,1166,3841,2559,463,2687,3139,232
7
NUPL1_8_9619,0,0,0,0,0,0,0,0,0,0,0,0
NXF1_8_9620,1463,1442,1462,1601,433,1423,3410,555,2648,762,2060,1234
NXNL2_8_9621,0,0,0,0,1,0,2,0,0,0,0,0
NXT2_8_9622,0,0,0,0,0,0,0,0,0,0,0,0
OAZ3_8_9623,0,0,0,0,0,0,0,0,0,0,0,0
PACSIN2_8_9624,0,0,0,0,0,0,0,0,0,0,0,0
PANX2_8_9625,0,0,0,0,0,0,0,0,0,0,0,0
PCDHA6_8_9626,0,0,0,0,0,0,0,0,0,0,0,0
PCDHGA5_8_9627,0,1,0,0,0,0,1,0,0,0,0,0
PCLO_8_9628,0,0,0,0,0,0,0,0,0,0,0,0
PCSK5_8_9629,3,16,7,4,14,12,4,6,11,5,7,12
PCSK6_8_9630,0,0,0,0,0,1,0,0,0,0,0,0
PCTP_8_9631,0,0,0,0,0,0,0,0,0,0,0,0
PDYN_8_9632,0,0,0,0,0,0,0,0,0,0,0,0
PDZD3_8_9633,0,1,0,1,0,0,0,0,2,0,1,0
PDZK1_8_9634,0,0,0,0,0,0,0,0,0,0,0,0
PGAP2_8_9635,0,0,0,0,0,0,0,0,0,0,0,0
PGF_8_9636,60,310,235,234,168,278,29,214,1,1042,129,567
PIK3R3_8_9637,0,0,0,0,0,0,0,0,0,0,0,0
PITPNC1_8_9638,0,0,0,0,0,0,0,0,0,0,0,0
PITPNM1_8_9639,0,0,0,0,0,0,0,0,0,0,0,0
PITPNM3_8_9640,0,0,0,0,0,0,0,0,0,0,0,0
PLEC_8_9641,0,1,0,8,0,2,1,3,2,1,0,0
PLIN3_8_9642,0,0,0,0,0,0,0,0,0,0,0,0
PLTP_8_9643,0,0,0,0,0,0,0,0,0,0,0,0
PNKD_8_9644,0,0,0,0,0,0,0,0,0,0,0,0
POMC_8_9645,0,0,0,0,0,0,0,0,0,0,0,0
PORCN_8_9646,0,0,0,0,0,0,0,0,0,0,0,0
PREPL_8_9647,0,0,0,0,0,0,0,0,0,0,0,0

PRNP_8_9648,89,220,203,216,0,1,0,77,3,143,128,19
PRSS21_8_9649,0,0,0,0,0,0,0,0,0,0,0,0
PRSS35_8_9650,0,0,0,0,0,0,0,0,0,0,0,0
PSEN1_8_9651,0,0,0,0,0,0,0,0,0,0,0,0
PSEN2_8_9652,0,0,0,0,0,0,1,0,1,0,0,0
RABEP1_8_9653,0,0,0,0,0,0,0,0,0,0,0,0
RACGAP1_8_9654,0,0,0,0,0,0,0,0,0,0,0,0
RARRES1_8_9655,0,0,0,0,0,0,0,0,0,0,0,0
RASA1_8_9656,0,0,0,0,0,0,0,0,0,0,0,0
RELN_8_9657,0,0,0,0,0,0,0,0,0,0,0,0
RHCE_8_9658,32,115,25,331,199,260,60,4,0,171,434,719
RHD_8_9659,0,0,0,0,0,0,0,0,0,0,0,0
RIMS2_8_9660,0,0,0,0,0,0,0,0,0,0,0,0
RRBP1_8_9661,0,0,0,0,0,0,0,0,0,0,0,0
RUFY1_8_9662,0,0,0,0,0,0,0,0,0,0,0,0
S100A13_8_9663,0,0,0,0,0,0,0,0,0,0,0,0
S100A4_8_9664,153,75,83,39,544,322,642,152,1,160,462,131
SAA1_8_9665,0,0,0,0,0,0,0,0,0,0,0,0
SCAMP3_8_9666,0,0,0,0,0,0,0,0,0,0,0,0
SCARB1_8_9667,0,0,0,0,0,0,0,0,0,0,0,0
SCFD1_8_9668,0,0,0,0,1,0,0,0,0,0,1,0
SEC13_8_9669,0,0,0,0,0,0,0,0,0,0,0,0
SEC14L1_8_9670,0,0,0,0,0,0,0,0,0,0,0,0
SEC14L2_8_9671,0,0,0,0,0,0,0,0,0,0,0,0
SEC14L4_8_9672,0,0,0,0,0,0,0,0,0,0,0,0
SEC23B_8_9673,0,0,0,0,0,0,0,0,0,0,0,0
SEC24B_8_9674,0,0,0,0,0,0,0,0,0,0,0,0
SEC24C_8_9675,0,0,0,0,0,0,0,0,0,0,0,0
SEC61A2_8_9676,0,0,0,0,0,0,0,0,0,0,0,0
SEC61G_8_9677,0,0,0,0,0,0,0,0,0,0,0,0
SEH1L_8_9678,0,0,0,0,0,0,0,0,0,0,0,0
SERINC2_8_9679,0,0,0,0,0,0,0,0,0,0,0,0
SERINC3_8_9680,0,0,0,0,0,0,0,0,0,0,0,0
SERPINA10_8_9681,0,0,0,0,0,0,0,0,0,0,0,0
SERPINA1_8_9682,0,0,0,0,0,0,0,0,0,0,0,0
SERPINB2_8_9683,0,0,0,0,0,0,0,0,0,0,0,0
SERPINB6_8_9684,0,0,0,0,0,0,0,0,0,0,0,0
SERPINB8_8_9685,0,0,0,0,0,0,0,0,0,0,0,0
SERPINE1_8_9686,0,0,0,0,0,0,0,0,0,0,0,0
SERPINF2_8_9687,0,0,0,0,0,0,0,0,0,0,0,0
SERPING1_8_9688,0,0,0,0,0,0,0,0,0,0,0,0
SERPINH1_8_9689,0,0,0,0,0,0,0,0,0,0,0,0
SERPINI1_8_9690,7,4,3,44,5,14,11,5,5,8,10,5
SFI1_8_9691,0,0,0,0,0,0,0,0,0,0,0,0
SFTPA1_8_9692,0,0,0,0,0,0,0,0,0,0,0,0
SH3D19_8_9693,0,0,0,0,0,0,0,0,0,0,0,0
SIL1_8_9694,0,0,0,0,0,0,0,0,0,0,0,0
SLC25A25_8_9695,0,0,0,0,0,0,0,0,0,0,0,0
SLC25A36_8_9696,0,0,0,0,0,0,0,0,0,0,0,0
SLC25A45_8_9697,0,0,0,0,0,0,0,0,0,0,0,0

SLC38A10_8_9698,0,0,0,0,0,0,0,0,0,0,0,0
SLC41A3_8_9699,0,0,0,0,0,0,0,0,0,0,0,0
SLC43A3_8_9700,0,0,0,0,0,0,0,0,0,0,0,0
SLC44A2_8_9701,0,0,0,0,0,0,0,0,0,0,0,0
SLC44A4_8_9702,0,0,0,0,0,0,0,0,0,0,0,0
SLC44A5_8_9703,0,0,0,0,0,0,0,0,0,0,0,0
SLC46A1_8_9704,0,0,0,0,0,0,0,0,0,0,0,0
SLC47A2_8_9705,0,0,0,0,0,0,0,0,0,0,0,0
SLC50A1_8_9706,0,1,0,0,1,0,0,0,0,0,0,0
SLC6A20_8_9707,820,885,609,1011,818,432,998,693,1036,1056,772,828
SNAP23_8_9708,0,0,0,0,0,0,0,0,0,0,0,0
SNAP25_8_9709,3,2,3,2,0,4,2,6,2,0,1,0
SNX10_8_9710,0,0,0,0,0,0,0,0,0,0,0,0
SNX11_8_9711,0,0,0,0,0,0,0,0,0,0,0,0
SNX14_8_9712,0,0,0,0,0,0,0,0,0,0,0,0
SNX15_8_9713,0,0,0,0,0,0,0,0,0,0,0,0
SNX16_8_9714,0,0,0,0,0,0,0,0,0,0,0,0
SNX18_8_9715,0,0,0,0,0,0,0,0,0,0,0,0
SNX1_8_9716,0,0,0,0,0,0,0,0,0,0,0,0
SNX3_8_9717,0,0,0,0,0,0,0,0,0,0,0,0
SNX5_8_9718,0,0,0,0,0,0,0,0,0,0,0,0
SNX6_8_9719,1,1,2,0,0,0,0,0,0,0,0,0
SNX7_8_9720,0,0,0,0,0,0,0,0,0,0,0,0
SORCS1_8_9721,0,0,0,0,0,0,0,0,0,0,0,0
SORT1_8_9722,0,0,0,0,0,0,1,0,1,0,0,0
SPNS1_8_9723,0,0,0,0,0,0,0,0,0,0,0,0
SRI_8_9724,0,0,0,0,0,0,0,0,0,0,0,0
STARD3_8_9725,0,0,0,0,0,0,0,0,0,0,0,0
STAU1_8_9726,0,0,0,0,0,0,0,0,0,0,0,0
STEAP2_8_9727,0,0,0,0,2,0,1,0,0,0,0,1
STEAP3_8_9728,0,0,0,0,0,0,0,0,0,0,0,0
STIM2_8_9729,0,0,0,0,0,0,0,0,0,0,0,0
STX16_8_9730,0,0,0,0,0,0,0,0,0,0,0,0
STX1A_8_9731,0,0,0,0,0,0,0,0,0,0,0,0
STX2_8_9732,0,0,0,0,0,0,0,0,0,0,0,0
STX3_8_9733,0,0,0,0,0,0,0,0,0,0,0,0
STXBP1_8_9734,0,0,0,0,0,0,0,0,0,0,0,0
STXBP2_8_9735,0,0,0,0,0,0,0,0,0,0,0,0
SV2B_8_9736,0,1,0,0,0,0,0,0,0,0,0,0
SYN1_8_9737,0,0,0,0,0,0,0,0,0,0,0,0
SYNGR1_8_9738,0,0,0,0,0,0,0,0,0,0,0,0
SYNPR_8_9739,0,0,0,0,0,0,0,0,0,0,0,0
SYPL1_8_9740,10782,10905,9836,11131,14554,13192,12500,8980,9506,12876,
10821,9536
SYT12_8_9741,0,0,0,0,0,0,0,0,0,0,0,0
SYT14_8_9742,0,0,0,0,0,0,0,0,0,0,0,0
SYT15_8_9743,0,0,0,0,0,0,0,0,0,0,0,0
SYT1_8_9744,0,0,0,0,0,0,0,0,0,0,0,0
SYT2_8_9745,0,0,0,0,0,0,0,0,0,0,0,0
SYT3_8_9746,335,292,267,328,128,9,16,34,1021,198,1,1392

TAPBP_8_9747,393,163,239,634,906,64,794,2156,546,430,483,2
TC2N_8_9748,0,0,0,0,0,0,0,0,0,0,0,0
TCN2_8_9749,0,0,0,0,0,0,0,0,0,0,0,0
TCOF1_8_9750,0,0,0,0,0,0,0,0,0,0,0,0
TFPI_8_9751,0,0,0,0,0,0,0,0,0,0,0,0
TFR2_8_9752,316,327,300,260,612,603,120,261,286,1088,431,62
TFRC_8_9753,6313,6267,5174,6544,5495,5175,6921,9519,6548,6318,4441,753
5
TGFB2_8_9754,0,0,0,0,0,0,0,0,0,0,0,0
TIMM17B_8_9755,0,0,0,0,0,0,0,0,0,0,0,0
TINAGL1_8_9756,0,0,0,0,0,0,0,0,0,0,0,0
TLL1_8_9757,0,0,0,0,0,0,0,0,0,0,0,0
TM9SF1_8_9758,0,0,0,0,0,0,0,0,0,0,0,0
TMC6_8_9759,0,0,0,0,0,0,0,1,0,0,140,0
TMPRSS11A_8_9760,491,489,294,527,343,1555,502,615,505,1864,859,1650
TMPRSS13_8_9761,0,0,0,0,0,0,0,0,0,0,0,0
TMPRSS2_8_9762,0,0,0,0,0,0,0,0,0,0,0,0
TMPRSS4_8_9763,0,0,0,0,0,0,0,0,0,0,0,0
TNFSF11_8_9764,0,0,0,0,0,0,0,0,0,0,0,0
TNFSF13B_8_9765,0,0,0,0,0,0,0,0,0,0,0,0
TNPO2_8_9766,0,0,0,0,0,0,0,0,0,0,0,0
TOM1_8_9767,0,0,0,0,0,0,0,0,0,0,0,0
TOM1L2_8_9768,0,0,0,0,0,0,0,0,0,0,0,0
TSC1_8_9769,68,173,52,161,309,442,44,30,115,245,18,236
TSC2_8_9770,0,0,0,0,0,0,0,0,0,0,0,0
TUBA8_8_9771,540,553,902,556,946,55,1989,1770,550,589,2067,582
TUBB3_8_9772,0,0,0,0,0,0,0,0,0,0,0,0
TUBD1_8_9773,0,0,0,0,0,0,0,0,0,0,0,0
UCP3_8_9774,0,0,0,0,0,0,0,0,0,0,0,0
UPF3A_8_9775,0,0,0,0,0,0,0,0,0,0,0,0
UPF3B_8_9776,0,0,0,0,0,0,0,0,0,0,0,0
VAMP1_8_9777,0,0,0,0,0,0,0,0,0,0,0,0
VAMP7_8_9778,0,0,0,0,0,0,0,0,0,0,0,0
VCAM1_8_9779,3,1,1,1,0,0,1,1,0,6,0,0
VLDLR_8_9780,0,0,0,0,0,0,0,0,0,0,0,0
VPS13A_8_9781,0,0,0,0,0,0,0,0,0,0,0,0
VPS13B_8_9782,0,0,0,0,0,0,0,0,0,0,0,0
VPS16_8_9783,0,0,0,0,0,0,0,0,0,0,0,0
VPS26A_8_9784,0,0,0,0,0,0,0,0,0,0,0,0
VPS28_8_9785,0,0,0,0,0,0,0,0,0,0,0,0
ZFYVE16_8_9786,0,0,0,0,0,0,0,0,0,0,0,0
ZNF160_8_9787,0,0,0,0,0,0,0,0,0,0,0,0
ZP3_8_9788,0,0,0,0,0,0,0,0,0,0,0,0
BET1L_8_9789,3,65,14,0,0,0,0,0,1,104,0,1
C2orf83_8_9790,0,0,0,0,0,0,0,0,0,0,0,0
ERP29_8_9791,0,0,0,0,0,0,0,0,0,0,0,0
FGF1_8_9792,0,0,0,0,0,0,0,0,0,0,0,0
LYNX1_8_9793,0,0,0,0,0,0,0,0,0,0,0,0
MMP28_8_9794,0,0,1,0,0,0,2,0,1,1,1,0
PDPN_8_9795,415,212,281,172,443,6,93,5,74,197,538,733

SNX21_8_9796,0,0,0,0,0,0,0,0,0,0,0,0
TIMM8A_8_9797,0,0,0,0,0,0,0,0,0,0,0,0
VEGFA_8_9798,0,0,0,0,0,0,0,0,0,0,0,0
CDH23_8_9799,0,0,0,0,0,0,0,0,0,0,0,0
CDH23_8_9800,1481,1619,1084,1076,1189,1917,2095,2662,1933,1777,1563,11
12
CDH23_8_9801,117,160,393,206,553,231,351,26,0,736,0,0
CDH23_8_9802,264,326,30,271,0,387,11,623,10,227,9,495
CDH23_8_9803,0,0,0,0,0,0,0,0,0,0,1,0
TNXB_8_9804,0,0,0,0,0,0,0,0,0,0,0,0
TNXB_8_9805,0,0,0,0,0,0,0,0,0,0,0,0
SLC25A23_8_9806,0,0,0,0,0,0,0,0,0,0,0,0
SLC25A23_8_9807,0,0,0,0,0,0,0,0,0,0,0,0
SLC25A23_8_9808,0,0,0,0,0,0,0,0,0,0,0,0
SLC25A23_8_9809,0,0,0,0,0,0,0,0,0,0,0,0
SLC25A23_8_9810,0,0,0,0,0,0,0,0,0,0,0,0
SLC25A23_8_9811,0,0,0,0,0,0,0,0,0,0,0,0
SLC25A23_8_9812,0,0,0,0,0,0,0,0,0,0,0,0
SLC25A23_8_9813,0,0,0,0,0,0,0,0,0,0,0,0
SLC25A23_8_9814,0,0,0,0,0,0,0,0,0,0,0,0
SLC25A23_8_9815,0,0,0,0,0,0,0,0,0,0,0,0
SERPINA2_8_9816,0,0,0,0,0,0,0,0,0,0,0,0
SERPINA2_8_9817,0,0,0,0,0,0,0,0,0,0,0,0
SERPINA2_8_9818,0,0,0,0,0,0,0,0,0,0,0,0
SERPINA2_8_9819,0,0,0,0,0,0,0,0,0,0,0,0
SERPINA2_8_9820,0,0,0,0,0,0,0,0,0,0,0,0
SERPINA2_8_9821,0,0,0,0,0,0,0,0,0,0,0,0
SERPINA2_8_9822,0,0,0,0,0,0,0,0,0,0,0,0
SERPINA2_8_9823,0,0,0,0,0,0,0,0,0,0,0,0
SERPINA2_8_9824,0,0,0,0,0,0,0,0,0,0,0,0
SERPINA2_8_9825,0,0,0,0,0,0,0,0,1,0,0,0
ARFGAP3_8_9826,0,0,0,0,0,0,0,0,0,0,0,0
ARFGAP3_8_9827,0,0,0,0,0,0,0,0,0,0,0,0
ARFGAP3_8_9828,0,0,0,0,0,0,0,0,0,0,0,0
ARFGAP3_8_9829,0,0,0,0,0,0,0,0,0,0,0,0
ARFGAP3_8_9830,0,0,0,0,0,0,0,0,0,0,0,0
ARFGAP3_8_9831,0,0,0,0,0,0,0,0,0,0,0,0
ARFGAP3_8_9832,0,0,0,0,0,0,0,0,0,0,0,0
ARFGAP3_8_9833,0,0,0,0,0,0,0,0,0,0,0,0
ARFGAP3_8_9834,0,0,0,0,0,0,0,0,0,0,0,0
ARFGAP3_8_9835,0,0,0,0,0,0,0,0,0,0,0,0
CAPN5_8_9836,0,0,0,0,0,0,0,0,0,0,0,0
CAPN5_8_9837,0,0,0,0,0,0,0,0,0,0,0,0
CAPN5_8_9838,0,0,0,0,0,0,0,0,0,0,0,0
CAPN5_8_9839,0,0,0,0,0,0,0,0,0,0,0,0
CAPN5_8_9840,0,0,0,0,0,0,0,0,0,0,0,0
CAPN5_8_9841,0,0,0,0,0,0,0,0,0,0,0,0
CAPN5_8_9842,0,0,0,0,0,0,0,0,0,0,0,0
CAPN5_8_9843,0,0,0,0,0,0,0,0,0,0,0,0
CAPN5_8_9844,0,0,0,0,0,0,0,0,0,0,0,0

CAPN5_8_9845,0,0,0,0,0,0,1,0,0,0,0,0
CCL13_8_9846,0,0,0,0,0,0,0,0,0,0,0,0
CCL13_8_9847,0,0,0,0,0,0,0,0,0,0,0,0
CCL13_8_9848,0,0,0,0,0,0,0,0,0,0,0,0
CCL13_8_9849,0,0,0,0,0,0,0,0,0,0,0,0
CCL13_8_9850,0,0,0,0,0,0,0,0,0,0,0,0
CCL13_8_9851,0,0,0,0,0,0,0,0,0,0,0,0
CCL13_8_9852,0,0,0,0,0,0,0,0,0,0,0,0
CCL13_8_9853,0,0,0,0,0,0,0,0,0,0,0,0
CCL13_8_9854,0,0,0,0,0,0,0,0,0,0,0,0
CCL13_8_9855,0,0,0,0,0,0,0,0,0,0,0,0
CNOT6_8_9856,0,0,0,0,0,0,0,0,0,0,0,0
CNOT6_8_9857,0,0,0,0,0,0,0,0,0,0,0,0
CNOT6_8_9858,0,0,1,0,1,0,0,0,0,0,0,0
CNOT6_8_9859,0,0,0,0,0,0,0,0,0,0,0,0
CNOT6_8_9860,0,0,0,0,0,0,0,0,0,0,0,0
CNOT6_8_9861,0,0,0,0,0,0,0,0,0,0,0,0
CNOT6_8_9862,0,0,0,0,0,0,0,0,0,0,0,0
CNOT6_8_9863,0,0,0,0,0,0,0,0,0,0,0,0
CNOT6_8_9864,0,0,0,0,0,0,0,0,0,0,0,0
CNOT6_8_9865,0,0,0,0,0,0,0,0,0,0,0,0
PCDHB11_8_9866,0,0,0,0,0,0,0,0,0,0,0,0
PCDHB11_8_9867,0,0,0,0,0,0,0,0,0,0,0,0
PCDHB11_8_9868,0,0,0,0,0,0,0,0,0,0,0,0
PCDHB11_8_9869,0,0,0,0,0,0,0,0,0,0,0,0
PCDHB11_8_9870,0,0,0,0,0,0,0,0,0,0,0,0
PCDHB11_8_9871,0,0,0,0,0,0,0,0,0,0,0,0
PCDHB11_8_9872,0,0,0,0,0,0,0,0,0,0,0,0
PCDHB11_8_9873,0,0,0,0,0,0,0,0,0,0,0,0
PCDHB11_8_9874,0,0,0,0,0,0,0,0,0,0,0,0
PCDHB11_8_9875,0,0,0,0,0,0,0,0,0,0,0,1
CPA4_8_9876,0,0,0,0,0,0,0,0,0,0,0,0
CPA4_8_9877,0,0,0,0,0,0,0,0,0,0,0,0
CPA4_8_9878,0,0,0,0,0,0,0,0,0,0,0,0
CPA4_8_9879,0,0,0,0,0,0,0,0,0,0,1,0
CPA4_8_9880,0,0,0,0,0,0,0,0,0,0,0,0
CPA4_8_9881,0,0,0,0,0,0,0,0,0,0,0,0
CPA4_8_9882,0,0,0,0,0,0,0,0,0,0,0,0
CPA4_8_9883,0,0,0,0,0,0,0,0,0,0,0,0
CPA4_8_9884,0,0,0,0,0,0,0,0,0,0,0,0
CPA4_8_9885,0,0,0,0,0,0,0,0,0,0,0,0
CPNE1_8_9886,0,0,0,0,0,0,0,0,0,0,0,0
CPNE1_8_9887,0,0,0,0,0,0,0,0,0,0,0,0
CPNE1_8_9888,0,0,0,0,0,0,0,0,0,0,0,0
CPNE1_8_9889,0,0,0,0,0,0,0,0,0,0,0,0
CPNE1_8_9890,0,0,0,0,0,0,0,0,0,0,0,0
CPNE1_8_9891,0,0,0,0,0,0,0,0,0,0,0,0
CPNE1_8_9892,0,0,0,0,0,0,0,0,0,0,0,1
CPNE1_8_9893,0,0,0,0,0,0,0,0,0,0,0,0
CPNE1_8_9894,0,0,0,0,0,0,0,0,0,0,0,0

CPNE1_8_9895,0,0,0,0,0,0,0,0,0,0,0,0
CPQ_8_9896,0,0,0,0,0,0,0,0,0,0,0,0
CPQ_8_9897,0,0,0,0,0,0,0,0,0,0,0,0
CPQ_8_9898,0,0,0,0,0,0,0,0,0,0,0,0
CPQ_8_9899,0,0,0,0,0,0,0,0,0,0,0,0
CPQ_8_9900,0,0,0,0,0,0,0,0,0,0,0,0
CPQ_8_9901,0,0,0,0,0,0,0,0,0,0,0,0
CPQ_8_9902,0,0,0,0,0,0,0,0,0,0,0,0
CPQ_8_9903,0,0,0,0,0,0,0,0,0,0,0,0
CPQ_8_9904,0,0,0,0,0,0,0,0,0,0,0,0
CPQ_8_9905,0,0,0,0,0,0,0,0,0,0,0,0
CRABP1_8_9906,0,0,0,0,0,0,0,0,0,0,0,0
CRABP1_8_9907,0,0,0,0,0,0,0,0,0,0,0,0
CRABP1_8_9908,0,0,0,0,0,0,0,0,0,0,0,0
CRABP1_8_9909,0,0,0,0,0,0,0,0,0,0,0,0
CRABP1_8_9910,0,0,0,0,0,0,0,0,0,0,0,0
CRABP1_8_9911,0,0,0,0,0,0,0,0,0,0,0,0
CRABP1_8_9912,0,0,0,0,0,0,0,0,0,0,0,0
CRABP1_8_9913,0,0,0,0,0,0,0,0,0,0,0,0
CRABP1_8_9914,0,0,0,0,0,0,0,0,0,0,0,0
CRABP1_8_9915,0,0,0,0,0,0,0,0,0,0,0,0
CTLA4_8_9916,0,0,0,0,0,0,0,0,0,0,0,0
CTLA4_8_9917,0,0,0,0,0,0,0,0,0,0,0,0
CTLA4_8_9918,0,0,0,0,0,0,0,0,0,0,0,0
CTLA4_8_9919,0,0,0,0,0,0,0,0,0,0,0,0
CTLA4_8_9920,0,0,0,0,0,0,0,0,0,0,0,0
CTLA4_8_9921,0,0,0,0,0,0,0,0,0,0,0,0
CTLA4_8_9922,0,0,0,0,0,0,0,0,0,0,0,0
CTLA4_8_9923,0,0,0,0,0,0,0,0,0,0,0,0
CTLA4_8_9924,0,0,0,0,0,0,0,0,0,0,0,0
CTLA4_8_9925,0,0,0,0,0,0,0,0,0,0,0,0
CTLA4_8_9926,0,0,0,0,0,0,0,0,0,0,0,0
CTLA4_8_9927,0,0,0,0,0,0,0,0,0,0,0,0
CTLA4_8_9928,0,0,0,0,0,0,0,0,0,0,0,0
CTLA4_8_9929,0,0,0,0,0,0,0,0,0,0,0,0
CTLA4_8_9930,0,0,0,0,0,0,0,0,0,0,0,0
CTLA4_8_9931,0,0,0,0,0,0,0,0,0,0,0,0
CTLA4_8_9932,0,0,0,0,0,0,0,0,0,0,0,0
CTLA4_8_9933,0,0,0,0,0,0,0,0,0,0,0,0
CTLA4_8_9934,0,0,0,0,0,0,0,0,0,0,0,0
CTPS1_8_9935,0,0,0,0,0,1,0,0,0,0,0,0
CTPS1_8_9936,0,0,0,0,0,0,0,0,0,0,0,0
CTPS1_8_9937,0,0,0,0,0,0,0,0,0,0,0,0
CTPS1_8_9938,0,0,0,0,0,0,0,0,0,0,0,0
CTPS1_8_9939,0,0,0,0,0,0,0,0,0,0,0,0
CTPS1_8_9940,0,0,0,0,0,0,0,0,0,0,0,0
CTPS1_8_9941,0,0,0,0,0,0,0,0,0,0,0,0
CTPS1_8_9942,0,0,0,0,0,0,0,0,0,0,0,0
CTPS1_8_9943,0,0,0,0,0,0,0,0,0,0,0,0
CTPS1_8_9944,0,0,0,0,0,0,0,0,0,0,0,0

CXCL10_8_9945,0,0,0,0,0,0,0,0,0,0,0,0
CXCL10_8_9946,0,0,0,0,0,0,0,0,0,0,0,0
CXCL10_8_9947,0,0,0,0,0,0,0,0,0,0,0,0
CXCL10_8_9948,0,0,0,0,0,0,0,0,0,0,0,0
CXCL10_8_9949,0,0,0,0,0,0,0,0,0,0,0,0
CXCL10_8_9950,0,0,0,0,0,0,0,0,0,0,0,0
CXCL10_8_9951,0,0,0,0,0,0,0,0,0,0,0,0
CXCL10_8_9952,0,0,0,0,0,1,0,1,0,0,2,0
CXCL10_8_9953,0,0,0,0,0,0,0,0,0,0,0,0
CXCL10_8_9954,0,0,0,0,0,0,0,0,0,0,0,0
ETFA_8_9955,0,0,0,0,0,0,0,0,0,0,0,0
ETFA_8_9956,0,0,0,0,0,0,0,0,0,0,0,0
ETFA_8_9957,0,0,0,0,0,0,0,0,0,0,0,0
ETFA_8_9958,0,0,0,0,0,0,0,0,0,0,0,0
ETFA_8_9959,0,0,0,0,0,0,0,0,0,0,0,0
ETFA_8_9960,0,0,0,0,0,0,0,0,0,0,0,0
ETFA_8_9961,0,0,0,0,0,0,0,0,0,0,0,0
ETFA_8_9962,0,0,0,0,0,0,0,0,0,0,0,0
ETFA_8_9963,0,0,0,0,0,0,0,0,0,0,0,0
ETFA_8_9964,0,0,0,0,0,0,0,0,0,0,0,0
EXOC4_8_9965,0,0,0,0,0,0,0,0,0,0,0,0
EXOC4_8_9966,0,0,0,0,0,0,0,0,0,0,0,0
EXOC4_8_9967,0,0,0,0,0,0,0,0,0,0,0,0
EXOC4_8_9968,0,0,0,0,0,0,0,0,0,0,0,0
EXOC4_8_9969,0,0,0,0,0,0,0,0,0,0,0,0
EXOC4_8_9970,0,0,0,0,0,0,0,0,0,0,0,0
EXOC4_8_9971,0,0,0,0,0,0,0,0,0,0,0,0
EXOC4_8_9972,0,0,0,0,0,0,0,0,0,0,0,0
EXOC4_8_9973,0,0,0,0,0,0,1,0,0,0,0,0
EXOC4_8_9974,0,0,0,0,0,0,0,0,0,0,0,0
FANCA_8_9975,0,0,0,0,0,0,0,0,0,0,0,0
FANCA_8_9976,0,0,0,0,0,0,0,0,0,0,0,0
FANCA_8_9977,0,0,0,0,0,0,0,0,0,0,0,0
FANCA_8_9978,0,0,0,0,0,0,0,0,0,0,0,0
FANCA_8_9979,0,0,0,0,0,0,0,0,0,0,0,0
FANCA_8_9980,0,0,0,0,0,0,0,0,0,0,0,0
FANCA_8_9981,0,0,0,0,0,0,0,0,0,0,0,0
FANCA_8_9982,0,0,0,0,0,0,0,0,0,0,0,0
FANCA_8_9983,0,0,0,0,0,0,0,0,0,0,0,0
FANCA_8_9984,0,0,0,0,0,0,0,0,0,0,0,0
FBF1_8_9985,0,0,0,0,0,0,0,0,0,0,0,0
FBF1_8_9986,0,0,0,0,0,0,0,0,0,0,0,0
FBF1_8_9987,0,0,0,0,0,0,0,0,0,0,0,0
FBF1_8_9988,0,0,0,0,0,0,0,0,0,0,0,0
FBF1_8_9989,0,0,0,0,0,0,0,0,0,0,0,0
FBF1_8_9990,0,0,0,0,0,0,0,0,0,0,0,0
FBF1_8_9991,0,0,0,0,0,0,0,0,0,0,0,0
FBF1_8_9992,0,0,0,0,0,0,0,0,0,0,0,0
FBF1_8_9993,0,0,0,0,0,0,0,0,0,0,0,0
FBF1_8_9994,0,0,0,0,0,0,0,0,1,0,0,0

FGF13_8_9995,0,0,0,0,0,0,0,0,0,0,0,0
FGF13_8_9996,0,0,0,0,0,0,0,0,0,0,0,0
FGF13_8_9997,0,0,0,0,0,0,0,0,0,0,0,0
FGF13_8_9998,0,0,0,0,0,0,0,0,0,0,0,0
FGF13_8_9999,0,0,0,0,0,0,0,0,0,0,0,0
FGF13_8_10000,0,0,0,0,0,0,0,0,0,0,0,0
FGF13_8_10001,0,0,0,0,0,0,0,0,0,0,0,0
FGF13_8_10002,0,0,0,0,0,0,0,0,0,0,0,0
FGF13_8_10003,0,0,0,0,0,0,0,0,0,0,0,0
FGF13_8_10004,0,0,0,0,0,0,0,0,0,0,0,0
GJA9_8_10005,0,0,0,0,0,0,0,0,0,0,0,0
GJA9_8_10006,0,0,0,0,0,0,0,0,0,0,0,0
GJA9_8_10007,0,0,0,0,0,0,0,0,0,0,0,0
GJA9_8_10008,0,0,0,0,0,0,0,0,0,0,0,0
GJA9_8_10009,0,0,0,0,0,0,0,0,0,0,0,0
GJA9_8_10010,0,0,0,0,0,0,0,0,0,0,0,0
GJA9_8_10011,0,0,0,0,0,0,0,0,0,0,0,0
GJA9_8_10012,0,0,0,0,0,0,0,0,0,0,0,0
GJA9_8_10013,1,1,1,1,1,1,0,0,0,0,1,0
GJA9_8_10014,0,0,0,0,0,0,0,0,0,0,0,0
GJD2_8_10015,0,0,0,0,0,0,0,0,0,0,0,0
GJD2_8_10016,0,0,0,0,0,0,0,0,0,0,0,0
GJD2_8_10017,0,0,0,0,0,0,0,0,0,0,0,0
GJD2_8_10018,0,0,0,0,0,0,0,0,0,0,0,0
GJD2_8_10019,0,0,0,0,0,0,0,0,0,0,0,0
GJD2_8_10020,0,0,0,0,0,0,0,0,0,0,0,0
GJD2_8_10021,0,0,0,0,0,0,0,0,0,0,0,0
GJD2_8_10022,0,0,0,0,0,0,0,0,0,0,0,0
GJD2_8_10023,0,0,0,0,0,0,0,0,0,0,0,0
GJD2_8_10024,0,0,0,0,0,0,0,0,0,0,0,0
GJD3_8_10025,0,0,0,0,0,0,0,0,0,0,0,0
GJD3_8_10026,0,0,0,1,0,0,0,0,0,0,0,0
GJD3_8_10027,0,0,0,0,0,0,0,0,0,0,0,0
GJD3_8_10028,0,0,0,0,0,0,0,0,0,0,0,0
GJD3_8_10029,0,0,0,0,0,0,0,0,0,0,0,0
GJD3_8_10030,0,0,0,0,0,0,0,0,0,0,0,0
GJD3_8_10031,0,0,0,0,0,0,0,0,0,0,0,0
GJD3_8_10032,0,0,0,0,0,0,0,0,0,0,0,0
GJD3_8_10033,0,0,0,0,0,0,0,0,0,0,0,0
GJD3_8_10034,0,0,0,0,0,0,0,0,0,0,0,0
GKN1_8_10035,0,0,0,0,0,0,0,0,0,0,0,0
GKN1_8_10036,0,0,0,0,0,0,0,0,0,0,0,0
GKN1_8_10037,0,0,0,0,0,0,0,0,0,0,0,0
GKN1_8_10038,0,0,0,0,0,0,0,0,0,0,0,0
GKN1_8_10039,0,0,0,0,0,0,0,0,0,0,0,0
GKN1_8_10040,0,0,0,0,0,0,0,0,0,0,0,0
GKN1_8_10041,0,0,0,0,0,0,0,0,0,0,0,0
GKN1_8_10042,0,0,0,0,0,0,0,0,0,0,0,0
GKN1_8_10043,0,0,0,0,0,0,0,0,0,0,0,0
GKN1_8_10044,1,0,0,0,0,0,0,0,0,0,0,0

SH3D19_8_10045,0,0,0,0,0,0,0,0,0,0,0,0
SH3D19_8_10046,0,0,0,0,0,0,0,0,0,0,0,0
SH3D19_8_10047,0,0,0,0,0,0,0,0,0,0,0,0
SH3D19_8_10048,0,0,0,0,0,0,0,0,0,0,0,0
SH3D19_8_10049,0,0,0,0,0,0,0,0,0,0,0,0
SH3D19_8_10050,0,0,0,0,0,0,0,0,0,0,0,0
SH3D19_8_10051,0,0,0,0,0,0,0,0,0,0,0,0
SH3D19_8_10052,0,0,0,0,0,0,0,0,0,0,0,0
SH3D19_8_10053,0,0,0,0,0,0,0,0,0,0,0,0
SH3D19_8_10054,0,0,0,0,0,0,0,0,0,0,0,0
HBB_8_10055,0,0,0,0,0,0,0,0,0,0,0,0
HBB_8_10056,0,0,0,0,0,0,0,0,0,0,0,0
HBB_8_10057,0,0,0,0,0,0,0,0,0,0,0,0
HBB_8_10058,0,0,0,0,0,0,0,0,0,0,0,0
HBB_8_10059,0,0,0,0,0,0,0,0,0,0,0,0
HBB_8_10060,0,0,0,0,0,0,0,0,0,0,0,0
HBB_8_10061,0,0,0,0,0,0,0,0,0,0,0,0
HBB_8_10062,415,293,243,561,393,59,78,2,520,399,255,813
HBB_8_10063,0,0,0,0,0,0,0,0,0,0,0,0
HBB_8_10064,0,0,0,0,0,0,0,0,0,0,0,0
HECA_8_10065,0,0,0,0,0,0,0,0,0,0,0,0
HECA_8_10066,0,0,0,0,0,0,0,0,0,0,0,0
HECA_8_10067,0,0,0,1,0,0,0,0,0,0,0,0
HECA_8_10068,0,0,0,0,0,0,0,0,0,0,0,0
HECA_8_10069,0,0,0,0,0,0,0,0,0,0,0,0
HECA_8_10070,0,0,0,0,0,0,0,0,0,0,0,0
HECA_8_10071,0,0,0,0,0,0,0,0,0,0,0,0
HECA_8_10072,0,0,0,0,0,0,0,0,0,0,0,0
HECA_8_10073,0,0,0,0,0,0,0,0,0,0,0,0
HECA_8_10074,0,0,0,0,0,0,0,0,0,0,0,0
HOMER2_8_10075,0,0,0,0,0,0,0,0,0,0,0,0
HOMER2_8_10076,0,0,0,0,0,0,0,0,0,0,0,0
HOMER2_8_10077,0,0,0,0,0,0,0,0,0,0,0,0
HOMER2_8_10078,0,0,0,0,0,0,0,0,0,0,0,0
HOMER2_8_10079,0,0,0,0,0,0,0,0,0,0,0,0
HOMER2_8_10080,0,0,0,0,0,0,0,0,0,0,0,0
HOMER2_8_10081,0,0,0,0,0,0,0,0,0,0,0,0
HOMER2_8_10082,0,0,0,0,0,0,0,0,0,0,0,0
HOMER2_8_10083,0,0,0,0,0,0,0,0,0,0,0,0
HOMER2_8_10084,0,0,0,0,0,0,0,0,0,0,0,0
SOS1_8_10085,0,0,0,0,0,0,0,0,0,0,0,0
SOS1_8_10086,0,0,0,0,0,0,0,0,0,0,0,0
SOS1_8_10087,0,0,0,0,0,0,0,0,0,0,0,0
SOS1_8_10088,0,0,0,0,0,0,0,0,0,0,0,0
SOS1_8_10089,0,0,0,0,0,0,0,0,0,0,0,0
SOS1_8_10090,0,0,0,0,0,0,0,0,0,0,0,0
SOS1_8_10091,0,0,0,0,0,0,0,0,0,0,0,0
SOS1_8_10092,0,0,0,0,0,0,0,0,0,0,0,0
SOS1_8_10093,0,0,0,0,0,0,0,0,0,0,0,0
SOS1_8_10094,0,0,0,0,0,0,0,0,0,0,0,0

INSL3_8_10095,0,0,0,0,0,0,0,0,0,0,0,0
INSL3_8_10096,0,0,0,0,0,0,0,0,0,0,0,0
INSL3_8_10097,0,0,0,0,0,0,0,0,0,0,0,0
INSL3_8_10098,0,0,0,0,0,0,0,0,0,0,0,0
INSL3_8_10099,0,0,0,0,0,0,0,2,0,0,0,0
INSL3_8_10100,0,0,0,0,0,0,0,0,0,0,0,0
INSL3_8_10101,0,0,0,0,0,0,0,0,0,0,0,0
INSL3_8_10102,0,0,0,0,0,0,0,0,0,0,0,0
INSL3_8_10103,0,0,0,0,0,0,0,0,0,0,0,0
INSL3_8_10104,0,0,0,0,0,0,0,0,0,0,0,0
KPNA6_8_10105,0,0,0,0,0,0,0,0,0,0,0,0
KPNA6_8_10106,0,0,0,0,0,0,0,0,0,0,0,0
KPNA6_8_10107,0,0,0,0,0,0,0,0,0,0,0,0
KPNA6_8_10108,0,0,2,0,0,2,0,0,1,0,0,2
KPNA6_8_10109,0,0,0,0,0,0,0,0,0,0,0,0
KPNA6_8_10110,0,0,0,0,0,0,0,0,0,0,0,0
KPNA6_8_10111,0,0,0,0,0,0,0,0,0,0,0,0
KPNA6_8_10112,0,0,0,0,0,0,0,0,0,0,0,0
KPNA6_8_10113,0,0,0,0,0,0,0,0,0,0,0,0
KPNA6_8_10114,0,0,0,0,0,0,0,0,0,0,0,0
KRTAP5-2_8_10115,960,811,660,528,620,912,1009,502,1004,108,64,145
KRTAP5-2_8_10116,0,0,0,0,0,0,0,0,0,0,0,0
KRTAP5-2_8_10117,0,0,0,0,0,0,0,0,0,0,0,0
KRTAP5-2_8_10118,0,0,0,0,0,0,0,0,0,0,0,0
KRTAP5-2_8_10119,257,79,145,57,241,72,104,13,169,133,17,101
KRTAP5-2_8_10120,0,0,0,0,0,0,0,0,0,0,0,0
KRTAP5-2_8_10121,0,0,0,0,0,0,0,0,0,0,0,0
KRTAP5-2_8_10122,108,69,206,76,413,205,542,24,514,42,0,819
LRIG1_8_10123,0,0,0,0,0,0,0,0,0,0,0,0
LRIG1_8_10124,0,0,0,0,0,0,0,0,0,0,0,0
LRIG1_8_10125,0,0,0,0,0,0,0,0,0,0,0,0
LRIG1_8_10126,0,0,0,0,0,0,0,0,0,0,0,0
LRIG1_8_10127,0,0,0,0,0,0,0,0,0,0,0,0
LRIG1_8_10128,0,0,0,0,0,0,0,0,0,0,0,0
LRIG1_8_10129,0,0,0,0,0,0,0,0,0,0,0,0
LRIG1_8_10130,0,0,0,0,0,0,0,0,0,0,0,0
LRIG1_8_10131,0,0,0,0,0,0,0,0,0,0,0,0
LRIG1_8_10132,0,0,0,0,0,0,0,0,0,0,0,0
MB_8_10133,0,0,0,0,0,0,0,0,0,0,0,0
MB_8_10134,0,0,0,0,0,0,0,0,0,0,0,0
MB_8_10135,0,0,0,0,0,0,0,0,0,0,0,0
MB_8_10136,0,0,0,0,0,0,0,0,0,0,0,0
MB_8_10137,0,0,0,0,0,0,0,0,0,0,0,0
MB_8_10138,0,0,0,0,0,0,0,0,0,0,0,0
MB_8_10139,0,0,0,0,0,0,0,0,0,0,0,0
MB_8_10140,0,0,0,0,0,0,0,0,0,0,0,0
MB_8_10141,0,0,0,0,0,0,0,0,0,0,0,0
MB_8_10142,0,0,0,0,0,0,0,0,0,0,0,0
MMP25_8_10143,0,0,0,0,0,0,0,0,0,0,0,0
MMP25_8_10144,0,0,0,0,0,0,0,0,0,0,0,0

MMP25_8_10145,0,0,0,0,0,0,0,0,0,0,0,0
MMP25_8_10146,0,0,0,0,0,0,0,0,0,0,0,0
MMP25_8_10147,0,0,0,0,0,0,0,0,0,0,0,0
MMP25_8_10148,0,0,0,0,0,0,0,0,0,0,0,0
MMP25_8_10149,0,0,0,0,0,0,0,0,0,0,0,0
MMP25_8_10150,0,0,0,0,0,0,0,0,0,0,0,0
MMP25_8_10151,0,0,0,0,0,0,0,0,0,0,0,0
MMP25_8_10152,0,0,0,0,0,0,0,0,0,0,0,0
MSRB1_8_10153,0,0,0,0,0,0,0,0,0,0,0,0
MSRB1_8_10154,0,0,0,0,0,0,0,0,0,0,0,0
MSRB1_8_10155,0,0,0,0,0,0,0,0,0,0,0,0
MSRB1_8_10156,0,0,0,0,0,0,0,0,0,0,0,0
MSRB1_8_10157,0,0,0,0,0,0,0,0,0,0,0,0
MSRB1_8_10158,0,0,0,0,0,0,0,0,0,0,0,0
MSRB1_8_10159,0,0,0,0,0,0,0,0,0,0,0,0
MSRB1_8_10160,0,0,0,0,0,0,0,0,0,0,0,0
MSRB1_8_10161,0,0,0,0,0,0,0,0,0,0,0,0
MSRB1_8_10162,0,0,0,0,0,0,0,0,0,0,0,0
NAPSA_8_10163,0,0,0,0,0,0,0,0,0,0,0,0
NAPSA_8_10164,0,0,0,0,0,0,0,0,0,0,0,0
NAPSA_8_10165,0,0,0,0,0,0,0,0,0,0,0,0
NAPSA_8_10166,0,0,0,0,0,0,0,0,0,0,0,0
NAPSA_8_10167,0,0,0,0,0,0,0,0,0,0,0,0
NAPSA_8_10168,0,0,0,0,0,0,0,0,0,0,0,0
NAPSA_8_10169,0,0,0,0,0,0,0,0,0,0,0,0
NAPSA_8_10170,0,0,0,0,0,0,0,0,0,0,0,0
NAPSA_8_10171,0,0,0,0,0,0,0,0,0,0,0,0
NAPSA_8_10172,0,0,0,0,0,0,0,0,0,0,0,0
NPRL3_8_10173,0,0,0,0,0,0,0,0,0,0,0,0
NPRL3_8_10174,0,0,0,0,0,0,0,0,0,0,0,0
NPRL3_8_10175,0,0,0,0,0,0,0,0,0,0,0,0
NPRL3_8_10176,0,0,1,0,0,0,0,0,0,0,0,0
NPRL3_8_10177,0,0,0,0,0,0,0,0,0,0,0,0
NPRL3_8_10178,0,0,0,0,1,0,0,0,0,0,0,0
NPRL3_8_10179,0,0,0,0,0,0,0,0,0,0,0,0
NPRL3_8_10180,0,0,0,0,0,0,0,0,0,0,0,0
NPRL3_8_10181,0,0,0,0,0,0,0,0,0,0,0,0
NPRL3_8_10182,0,0,0,0,0,0,0,0,0,0,0,0
PCDHA@_8_10183,0,0,0,0,0,0,0,0,0,0,0,0
PCDHA@_8_10184,0,0,0,0,0,0,0,0,0,0,0,0
PCDHA@_8_10185,0,0,0,0,0,0,0,0,0,0,0,0
PCDHA@_8_10186,0,0,0,0,0,0,0,0,0,0,0,0
PCDHA@_8_10187,0,0,0,0,0,0,0,0,0,0,0,0
PCDHA@_8_10188,0,0,0,0,0,0,0,0,0,0,0,0
PCDHA@_8_10189,0,0,0,0,0,0,0,0,0,0,0,0
PCDHA@_8_10190,0,0,0,0,0,0,0,0,0,0,0,0
PCDHA@_8_10191,0,0,0,0,0,0,0,0,0,0,0,0
PCDHA@_8_10192,0,0,0,0,0,0,0,0,0,0,0,0
PCDHA6_8_10193,0,0,0,0,0,0,0,0,0,0,0,0
PCDHA6_8_10194,0,0,0,0,0,0,0,0,0,0,0,0

PCDHA6_8_10195,0,0,0,0,0,0,0,0,0,0,0,0
PCDHA6_8_10196,0,0,0,0,0,0,0,0,0,0,0,0
PCDHA6_8_10197,0,0,0,0,0,0,0,0,0,0,0,0
PCDHA6_8_10198,0,0,0,0,0,0,0,0,0,0,0,0
PCDHA6_8_10199,0,0,0,0,0,0,0,0,0,0,0,0
PCDHA6_8_10200,0,0,0,0,0,0,0,0,0,0,0,0
PCDHA6_8_10201,0,0,0,0,0,0,0,0,0,0,0,0
PCDHA6_8_10202,0,0,0,0,0,0,0,0,0,0,0,0
PCDHB16_8_10203,0,0,0,0,0,0,0,0,0,0,0,0
PCDHB16_8_10204,1,0,0,0,0,0,0,0,0,0,0,0
PCDHB16_8_10205,0,0,0,0,0,0,0,0,0,0,0,0
PCDHB16_8_10206,0,0,0,0,0,0,0,0,0,0,0,0
PCDHB16_8_10207,0,0,0,0,0,0,0,0,0,0,0,0
PCDHB16_8_10208,0,0,0,0,0,0,0,0,0,0,0,0
PCDHB16_8_10209,0,0,0,0,0,0,0,0,0,0,0,0
PCDHB16_8_10210,0,0,0,0,0,0,0,0,0,0,0,0
PCDHB16_8_10211,0,0,0,0,0,0,0,0,0,0,0,0
PCDHB16_8_10212,0,0,0,0,0,0,0,0,0,0,0,0
PCDHGA5_8_10213,0,0,0,0,0,0,0,0,0,0,0,0
PCDHGA5_8_10214,0,0,0,0,0,0,0,0,0,0,0,0
PCDHGA5_8_10215,0,0,0,0,0,0,0,0,0,1,0,0
PCDHGA5_8_10216,0,0,0,0,0,0,0,0,0,0,0,0
PCDHGA5_8_10217,0,0,0,0,0,0,0,0,0,0,0,0
PCDHGA5_8_10218,0,0,0,0,0,0,0,0,0,0,0,0
PCDHGA5_8_10219,0,0,0,0,0,0,0,0,0,0,0,0
PCDHGA5_8_10220,0,0,0,0,0,0,0,0,0,0,0,0
PCDHGA5_8_10221,0,0,0,0,0,0,0,0,0,0,0,0
PCDHGA5_8_10222,0,0,0,0,0,0,0,0,0,0,0,0
RPL10_8_10223,0,0,0,0,0,0,0,0,0,0,0,0
RPL10_8_10224,0,0,0,0,0,0,0,0,0,0,0,0
RPL10_8_10225,0,0,0,0,0,0,0,0,0,0,0,0
RPL10_8_10226,0,0,0,0,0,0,0,0,0,0,0,0
RPL10_8_10227,0,0,0,0,0,0,0,0,0,0,0,0
RPL10_8_10228,0,0,0,0,0,0,0,0,0,0,0,0
RPL10_8_10229,0,0,0,0,0,0,0,0,0,0,0,0
RPL10_8_10230,0,0,0,0,0,0,0,0,0,0,0,0
RPL10_8_10231,0,0,0,0,0,0,0,0,0,0,0,0
RPL10_8_10232,0,0,0,0,0,0,0,0,0,0,0,0
PNKD_8_10233,0,0,0,0,0,0,0,0,0,0,0,0
PNKD_8_10234,0,0,0,0,0,0,0,0,0,0,0,0
PNKD_8_10235,0,0,0,0,0,0,0,0,0,0,0,0
PNKD_8_10236,0,0,0,0,0,0,0,0,0,0,0,0
PNKD_8_10237,0,0,0,0,0,0,0,0,0,0,0,0
PNKD_8_10238,0,0,0,0,0,0,0,0,0,0,0,0
PNKD_8_10239,0,0,0,0,0,0,0,0,0,0,0,0
PNKD_8_10240,0,0,0,0,0,0,0,0,0,0,0,1
PNKD_8_10241,0,0,0,0,0,0,0,0,0,0,0,0
PNKD_8_10242,0,0,0,0,0,0,0,0,0,0,0,0
PPY_8_10243,0,0,0,0,0,0,0,0,0,0,0,0
PPY_8_10244,0,0,0,0,0,0,0,0,0,0,0,0

PPY_8_10245,0,0,0,0,0,0,0,0,0,0,0,0
PPY_8_10246,0,0,0,0,0,0,0,0,0,0,0,0
PPY_8_10247,0,0,0,0,0,0,0,0,0,0,0,0
PPY_8_10248,0,0,0,0,0,0,0,0,0,0,0,0
PPY_8_10249,0,0,0,0,0,0,0,0,0,0,0,0
PPY_8_10250,0,0,0,0,0,0,0,0,0,0,0,0
PPY_8_10251,0,0,0,0,0,0,0,0,0,0,0,0
PPY_8_10252,0,0,0,0,0,0,0,0,0,0,0,0
PRNP_8_10253,0,0,0,0,0,0,0,0,0,0,0,0
PRNP_8_10254,0,0,0,0,0,0,0,0,0,0,0,0
PRNP_8_10255,0,0,0,0,0,0,0,0,0,0,0,0
PRNP_8_10256,0,0,0,0,0,0,0,0,0,0,0,0
PRNP_8_10257,0,0,0,0,0,0,0,0,0,0,0,0
PRNP_8_10258,0,0,0,0,0,0,0,0,0,0,0,0
PRNP_8_10259,0,0,0,0,0,0,0,0,0,0,0,0
PRNP_8_10260,0,0,0,0,0,0,0,0,0,0,0,0
PRNP_8_10261,0,0,0,0,0,0,0,0,0,0,0,0
PRNP_8_10262,0,0,0,0,0,0,0,0,0,0,0,0
RPL15_8_10263,0,0,0,0,0,0,0,0,0,0,0,0
RPL15_8_10264,0,0,0,0,0,0,0,0,0,0,0,0
RPL15_8_10265,0,0,0,0,0,0,0,0,0,0,0,0
RPL15_8_10266,0,0,0,0,0,0,0,0,0,0,0,0
RPL15_8_10267,0,0,0,0,0,0,0,0,0,0,0,0
RPL15_8_10268,0,0,0,0,0,0,0,0,0,0,0,0
RPL15_8_10269,0,0,0,0,0,0,0,0,0,0,0,0
RPL15_8_10270,0,0,0,0,0,0,0,0,0,0,0,0
RPL15_8_10271,0,0,0,0,0,0,0,0,0,0,0,0
RPL15_8_10272,3,1,1,0,2,2,2,0,0,3,2,1
SEC14L3_8_10273,0,0,0,0,0,0,0,0,0,0,0,0
SEC14L3_8_10274,0,0,0,0,0,0,0,0,0,0,0,0
SEC14L3_8_10275,0,0,0,0,0,0,0,0,0,0,0,0
SEC14L3_8_10276,0,0,0,0,0,0,0,0,0,0,0,0
SEC14L3_8_10277,0,0,0,0,0,0,0,0,0,0,0,0
SEC14L3_8_10278,0,0,0,0,0,0,0,0,0,0,0,0
SEC14L3_8_10279,0,0,0,0,0,0,0,0,0,0,0,0
SEC14L3_8_10280,0,0,0,0,0,0,0,0,0,0,0,0
SEC14L3_8_10281,0,0,0,0,0,0,0,0,0,0,0,0
SEC14L3_8_10282,0,0,0,0,0,0,0,0,0,0,0,0
SERPINB8_8_10283,0,0,0,0,0,0,0,0,0,0,0,0
SERPINB8_8_10284,0,0,0,0,0,0,0,0,0,0,0,0
SERPINB8_8_10285,0,0,0,0,0,0,0,0,0,0,0,0
SERPINB8_8_10286,0,0,0,0,0,0,0,0,0,0,0,0
SERPINB8_8_10287,0,0,0,0,0,0,0,0,0,0,0,0
SERPINB8_8_10288,0,0,0,0,0,0,0,0,0,0,0,0
SERPINB8_8_10289,0,0,0,0,0,0,0,0,0,0,0,0
SERPINB8_8_10290,0,0,0,0,0,0,0,0,0,0,0,0
SERPINB8_8_10291,0,0,0,0,0,0,0,0,0,0,0,0
SERPINB8_8_10292,0,0,0,0,0,0,0,0,0,0,0,0
SFI1_8_10293,0,0,0,0,0,0,0,0,0,0,0,0
SFI1_8_10294,0,0,0,0,0,0,0,0,0,0,0,0

SFI1_8_10295,0,0,0,0,0,0,0,0,0,0,0,0
SFI1_8_10296,0,0,0,0,0,0,0,0,0,0,0,0
SFI1_8_10297,0,0,0,0,0,0,0,0,0,0,0,0
SFI1_8_10298,0,0,0,0,0,0,0,0,0,0,0,0
SFI1_8_10299,0,0,0,0,0,0,0,0,0,0,0,0
SFI1_8_10300,0,0,0,0,0,0,0,0,0,0,0,0
SFI1_8_10301,0,0,0,0,0,0,0,0,0,0,0,0
SFI1_8_10302,0,0,0,0,0,0,0,0,0,0,0,0
SLC23A1_8_10303,0,0,0,0,0,0,0,0,0,0,0,0
SLC23A1_8_10304,0,0,0,0,0,0,0,0,0,0,0,0
SLC23A1_8_10305,0,0,0,0,0,0,0,0,0,0,0,0
SLC23A1_8_10306,0,0,0,0,0,0,0,0,0,0,0,0
SLC23A1_8_10307,0,0,0,0,0,0,0,0,0,0,0,0
SLC23A1_8_10308,0,0,0,0,0,0,0,0,0,0,0,0
SLC23A1_8_10309,0,0,0,0,0,0,0,0,0,0,0,0
SLC23A1_8_10310,0,0,0,0,0,0,0,0,0,0,0,0
SLC23A1_8_10311,0,0,0,0,0,0,0,0,0,0,0,0
SLC23A1_8_10312,0,0,0,0,0,0,0,0,0,0,0,0
SPNS1_8_10313,0,0,0,0,0,0,0,0,0,0,0,0
SPNS1_8_10314,0,0,0,0,0,0,0,0,0,0,0,0
SPNS1_8_10315,0,0,0,0,0,0,0,0,0,0,0,0
SPNS1_8_10316,0,0,0,0,0,0,0,0,0,0,0,0
SPNS1_8_10317,0,0,0,0,0,0,0,0,0,0,0,0
SPNS1_8_10318,0,0,0,0,0,0,0,0,0,0,0,0
SPNS1_8_10319,0,0,0,0,0,0,0,0,0,0,0,0
SPNS1_8_10320,0,0,0,0,0,0,0,0,0,0,0,0
SPNS1_8_10321,0,0,0,0,0,0,0,0,0,0,0,0
SPNS1_8_10322,0,0,0,0,0,0,0,0,0,0,0,0
SPNS1_8_10323,0,0,0,0,0,0,0,0,0,0,0,0
SPNS1_8_10324,0,0,0,0,0,0,0,0,0,0,0,0
SPNS1_8_10325,0,0,0,0,0,0,0,0,0,0,0,0
SPNS1_8_10326,0,0,0,0,0,0,0,0,0,0,0,0
SPNS1_8_10327,0,0,0,0,0,0,0,0,0,0,0,0
SPNS1_8_10328,0,0,0,0,0,0,0,0,0,0,0,0
SPNS1_8_10329,0,0,0,0,0,0,0,0,0,0,0,0
SPNS1_8_10330,0,0,0,0,0,0,0,0,0,0,0,0
SPNS1_8_10331,0,0,0,0,0,0,0,0,0,0,0,0
SPNS1_8_10332,0,0,0,0,0,0,0,0,0,0,0,0
TINAGL1_8_10333,0,0,0,0,0,0,0,0,0,0,0,0
TINAGL1_8_10334,0,0,0,0,0,0,0,0,0,0,0,0
TINAGL1_8_10335,0,0,0,0,0,0,0,0,0,0,0,0
TINAGL1_8_10336,0,0,1,0,0,0,0,0,0,0,0,0
TINAGL1_8_10337,0,0,0,0,0,0,0,0,0,0,0,0
TINAGL1_8_10338,0,0,0,0,0,0,0,0,0,0,0,1
TINAGL1_8_10339,0,0,0,0,0,0,0,0,0,0,0,0
TINAGL1_8_10340,0,0,0,0,0,0,0,0,0,0,0,1
TINAGL1_8_10341,0,0,0,0,0,0,0,0,0,0,0,0
TINAGL1_8_10342,0,0,0,0,0,0,0,0,0,0,0,0
TMPRSS4_8_10343,0,0,0,0,0,0,0,0,0,0,0,0
TMPRSS4_8_10344,0,0,0,0,0,0,0,0,0,0,0,0

TMPRSS4_8_10345,0,0,0,0,0,0,0,0,0,0,0,0
TMPRSS4_8_10346,0,0,0,0,0,0,0,0,0,0,0,0
TMPRSS4_8_10347,0,0,0,0,0,0,0,0,0,0,0,0
TMPRSS4_8_10348,0,0,0,0,0,0,0,0,0,0,0,0
TMPRSS4_8_10349,0,0,0,0,0,0,0,0,0,0,0,0
TMPRSS4_8_10350,0,0,0,0,0,0,0,0,0,0,0,0
TMPRSS4_8_10351,0,0,0,0,0,0,0,0,0,0,0,0
TMPRSS4_8_10352,0,0,0,0,0,0,0,0,0,0,0,0
TNNI3_8_10353,1,0,0,0,0,0,0,0,0,0,0,0
TNNI3_8_10354,0,0,0,0,0,0,0,0,0,0,0,0
TNNI3_8_10355,0,0,0,0,0,0,0,0,0,0,0,0
TNNI3_8_10356,0,0,0,0,0,0,0,0,0,0,0,0
TNNI3_8_10357,0,0,0,0,0,0,0,0,0,0,0,0
TNNI3_8_10358,0,0,0,0,0,0,0,0,0,0,0,0
TNNI3_8_10359,0,0,0,0,0,0,0,0,0,0,0,0
TNNI3_8_10360,0,0,0,0,0,0,0,0,0,0,0,0
TNNI3_8_10361,0,0,0,0,0,0,0,0,0,0,0,0
TNNI3_8_10362,0,0,0,0,0,0,0,0,0,0,0,0
TRAPPC8_8_10363,0,0,0,0,0,0,0,0,0,0,0,0
TRAPPC8_8_10364,0,0,0,0,0,0,0,0,0,0,0,0
TRAPPC8_8_10365,0,0,0,0,0,0,0,0,0,0,0,0
TRAPPC8_8_10366,0,0,0,0,0,0,0,0,0,0,0,0
TRAPPC8_8_10367,0,0,0,0,0,0,0,0,0,0,0,0
TRAPPC8_8_10368,0,0,0,0,0,0,0,0,0,0,0,0
TRAPPC8_8_10369,0,0,0,0,0,0,0,0,0,0,0,0
TRAPPC8_8_10370,0,0,0,0,0,0,0,0,0,0,0,0
TRAPPC8_8_10371,0,0,0,0,0,0,0,0,0,0,0,0
TRAPPC8_8_10372,0,1,0,0,0,0,0,0,0,0,1,0
TUBB2A_8_10373,1244,1173,893,594,355,945,1913,1177,1663,494,1385,750
TUBB2A_8_10374,0,0,0,0,0,0,0,0,0,0,0,0
TUBB2A_8_10375,0,0,0,0,0,0,0,0,0,0,0,0
TUBB2A_8_10376,854,712,1059,680,320,1107,518,616,1103,1545,1326,625
TUBB2A_8_10377,682,617,317,740,1080,1453,23,78,450,52,1215,34
TUBB2A_8_10378,0,0,1,0,0,0,0,0,0,0,0,0
TUBB2A_8_10379,0,0,0,0,0,0,0,0,0,0,0,0
TUBB2A_8_10380,1,0,1,1,0,0,0,0,0,1,0,1
TUBB2A_8_10381,0,0,0,0,0,0,0,0,1,0,0,0
TUBB4A_8_10382,1305,2057,1148,1415,1562,2363,1032,1936,876,1368,1218,1
680
TUBB4A_8_10383,384,332,233,178,417,959,1476,551,835,102,11,1390
TUBB4A_8_10384,553,598,433,486,929,764,760,226,162,117,93,867
TUBB4A_8_10385,1245,1177,893,596,1331,945,1915,1183,1665,494,1387,752
TUBB4A_8_10386,1,1,0,1,0,1,1,0,2,1,0,0
TUBB4A_8_10387,0,0,0,0,0,0,0,0,0,0,0,0
TUBB4A_8_10388,0,0,0,0,0,0,0,0,0,0,0,0
TUBB4A_8_10389,644,627,328,762,396,1477,25,78,460,53,1247,406
TUBB4A_8_10390,0,0,0,0,0,0,0,0,0,0,0,0
TUBB4A_8_10391,2166,2134,1749,2568,3571,1749,3087,1616,2045,1840,2716,
2528
TUBB4B_8_10392,0,0,0,0,0,0,0,0,0,0,0,0

TUBB4B_8_10393,0,0,0,0,0,0,0,0,0,0,0,0
TUBB4B_8_10394,0,0,0,0,0,0,0,0,0,0,0,0
TUBB4B_8_10395,166,575,321,195,0,452,810,27,383,2,430,712
TUBB4B_8_10396,1067,948,1284,954,564,1623,526,628,1270,2558,1357,1269
TUBB4B_8_10397,888,717,1007,1122,1074,962,732,595,541,579,350,419
TUBB4B_8_10398,0,0,0,0,0,0,0,0,0,0,0,0
TUBB4B_8_10399,553,598,433,486,929,764,760,226,162,117,93,867
TUBB4B_8_10400,0,0,0,0,0,0,0,0,0,0,0,0
TUBB4B_8_10401,688,623,354,759,1083,1471,25,78,459,52,1241,399
VSIG2_8_10402,0,0,0,0,0,0,0,0,0,0,0,0
VSIG2_8_10403,0,0,0,0,0,0,0,0,0,0,0,0
VSIG2_8_10404,0,0,0,0,0,0,0,0,0,0,0,0
VSIG2_8_10405,0,0,0,0,0,0,0,0,0,0,0,0
VSIG2_8_10406,0,0,0,0,0,0,0,1,0,0,0,0
VSIG2_8_10407,0,0,0,0,0,0,0,0,0,0,0,0
VSIG2_8_10408,0,0,0,0,0,0,0,0,0,0,0,1
VSIG2_8_10409,0,0,0,0,0,0,0,0,0,0,0,0
VSIG2_8_10410,0,0,0,0,0,0,0,0,0,0,0,0
VSIG2_8_10411,0,0,0,0,0,0,0,0,0,0,0,0
ZNF160_8_10412,0,0,0,0,0,0,0,0,0,0,0,0
ZNF160_8_10413,0,0,0,0,0,0,0,0,0,0,0,0
ZNF160_8_10414,0,0,0,0,0,0,0,0,0,0,0,0
ZNF160_8_10415,0,0,0,0,0,0,0,0,0,0,0,0
ZNF160_8_10416,0,0,0,0,0,0,0,0,0,0,0,0
ZNF160_8_10417,0,0,0,0,0,0,0,0,0,0,0,0
ZNF160_8_10418,0,0,0,0,0,0,0,0,0,0,0,0
ZNF160_8_10419,0,0,0,0,0,0,0,0,0,0,0,0
ZNF160_8_10420,0,0,0,0,0,0,0,0,0,0,0,0
ZNF160_8_10421,0,0,0,0,0,0,0,0,0,0,0,0
