# Supplementary material for: Cross-species genetic screens identify transglutaminase 5 as a regulator of polyglutamine-expanded ataxin-1
Source: J Clin Invest. 2022 May 2;132(9):e156616. doi: 10.1172/JCI156616 (PMC9057624; doi:10.1172/JCI156616)
Supplement: Supplemental data set 7 [file jci-132-156616-s052.pdf]

shRNA,B1,B2,B3,B4,H1,H2,H3,H4,L1,L2,L3,L4
ABCA10_6_1,7513,6303,7940,6958,6288,9245,6856,8730,10059,3687,11390,68
57
ABCA13_6_2,4129,2774,4014,4185,3674,2991,4575,4499,2678,4198,4607,4517
ABCA1_6_3,3164,2495,4334,3993,3342,2766,4221,4689,2461,3892,4929,5326
ABCA3_6_4,1742,2232,2201,2908,1235,2458,1578,1152,2197,2810,4292,2265
ABCA4_6_5,13359,10785,13003,14362,13453,12074,12188,15902,11802,12078,
15116,10460
ABCA6_6_6,4177,4568,5336,5612,3075,6123,5659,4697,3661,5792,5030,5624
ABCA7_6_7,1439,1558,1611,1459,1665,1835,1730,2996,892,2202,2393,1735
ABCA8_6_8,501,716,830,709,939,251,1015,553,201,981,894,856
ABCA9_6_9,12865,12786,15688,14312,13331,19052,16972,15828,13400,16333,
18601,14382
ABCB10_6_10,689,725,1040,648,1292,962,335,416,137,610,1359,328
ABCB11_6_11,2015,1669,1703,1467,1101,309,1489,763,1123,3038,2222,2739
ABCB1_6_12,24666,22097,26521,27373,24048,25014,23235,24397,20999,32354
,30820,23392
ABCB6_6_13,3381,2677,3832,3388,3997,3281,2248,2501,854,4717,2781,4921
ABCB7_6_14,1532,2053,1686,1682,4170,1810,821,1349,996,1997,2655,1324
ABCB8_6_15,2261,1963,2319,2599,2080,1764,3018,1577,1654,2183,4711,1982
ABCC12_6_16,3047,2872,3435,2742,1340,1492,2871,2389,916,5781,2239,4222
ABCC2_6_17,658,598,926,963,1735,689,662,1304,385,1368,2026,282
ABCC8_6_18,4000,3806,4153,3854,3953,4465,4667,2205,4515,3072,4264,5352
ABCD1_6_19,523,134,440,456,21,1690,372,1398,653,1206,650,53
ABCD2_6_20,2135,1989,2615,2349,2803,2926,3725,1771,1771,1801,2776,2494
ABCD4_6_21,820,591,634,799,615,2630,201,503,1384,280,667,651
ABCF3_6_22,182,273,371,357,373,193,153,550,252,816,146,277
ABCG2_6_23,1744,1423,1719,1821,2238,1023,1733,1267,972,2026,2475,590
ABCG5_6_24,988,562,1240,1033,890,1538,2435,431,696,1212,3240,549
ABCG8_6_25,907,1360,990,685,1616,374,1127,766,1033,2536,1237,402
ANXA10_6_26,3901,3396,2946,3923,2396,6283,5486,3191,3163,4641,4191,463
8
ANXA1_6_27,1454,1744,1827,1907,4360,2733,1792,3457,759,1849,1944,2477
ANXA3_6_28,1216,1666,1914,1914,1908,2340,1637,2515,1047,1161,2572,3553
ANXA4_6_29,443,830,607,634,163,1220,99,1094,331,1984,1150,802
ANXA5_6_30,1297,1003,1711,1678,1464,1565,2070,1731,1328,802,1662,744
ANXA8_6_31,9235,8376,11560,10447,7346,10191,10071,10794,6621,8118,1149
6,10229
ANXA9_6_32,1070,572,868,1399,1508,1197,1126,436,896,92,539,479
ATP10A_6_33,1494,1416,2191,1606,730,2942,4576,1966,2411,3224,2078,2331
ATP10B_6_34,7332,6598,7383,7313,6830,8088,6363,6023,5676,10452,6735,68
35
ATP10D_6_35,6397,6426,7501,7850,5386,9413,8947,8086,7380,8269,9878,614
2
ATP11B_6_36,9373,7147,10002,9044,6859,9466,9366,11015,8353,10158,10584
,7261
ATP1A2_6_37,1386,1163,1170,1677,1640,460,1249,2267,1161,2536,2540,1371
ATP1A3_6_38,1170,1090,1022,1416,1401,119,1284,2508,1117,2246,2382,813
ATP1B1_6_39,1580,1228,1983,1642,1872,1596,3739,2199,1052,1651,1536,290
9

ATP1B2_6_40,4135,4970,6502,4874,2935,5309,3527,4407,5396,3457,5635,541
8
ATP1B3_6_41,1107,593,2128,1611,1075,680,1509,1203,1875,2505,752,2129
ATP4A_6_42,4632,4161,5188,5116,2936,4769,4154,7040,2656,6595,5293,4378
ATP4B_6_43,761,768,1122,695,691,923,954,945,803,1086,688,1375
ATP5B_6_44,2134,2485,3749,1910,1769,2500,3581,3448,1287,3262,5604,1204
ATP5F1_6_45,4270,3772,3726,3277,6158,2188,4826,4911,3565,7749,4354,381
0
ATP5I_6_46,1532,1509,1979,1573,872,2823,712,2907,1802,4138,2960,895
ATP5L2_6_47,5771,4160,6780,5778,4133,9054,6323,6152,4978,5464,8063,477
0
ATP5L_6_48,5771,4160,6780,5778,4133,9054,6323,6152,4978,5464,8063,4770
ATP5O_6_49,3119,3627,3431,3691,3290,4724,3169,5921,3317,4250,7241,4974
ATP6AP1_6_50,797,589,952,1165,360,639,780,1808,529,408,1874,922
ATP6AP2_6_51,3195,2406,3479,3068,2242,2413,1460,1964,1244,3706,1939,42
54
ATP6V0A2_6_52,1803,1738,1390,1540,820,1931,2036,2224,1558,2412,1512,98
7
ATP6V0D1_6_53,681,676,1114,557,616,507,1361,252,279,1099,272,754
ATP6V0D2_6_54,10058,9929,10326,10358,10088,10359,13759,14656,10026,147
53,10621,11244
ATP6V0E1_6_55,1544,1141,1714,1418,886,450,1827,1451,597,1799,869,2490
ATP6V1A_6_56,713,578,749,1055,1231,792,824,664,426,431,1326,1090
ATP6V1B1_6_57,1588,1620,1532,976,3648,2704,2299,1737,366,2849,2038,251
0
ATP6V1B2_6_58,13075,11571,14058,12344,11930,13768,12537,10757,11151,13
790,15442,13489
ATP6V1C1_6_59,3622,3187,3991,4368,3182,3462,4978,4323,3183,6022,5498,4
153
ATP6V1D_6_60,4640,4135,4256,4755,4657,4811,4351,5102,3434,4836,4955,33
08
ATP6V1E2_6_61,1673,2065,2259,2139,2969,449,2083,2630,808,1774,2782,266
1
ATP6V1G1_6_62,7274,5719,8529,8067,7950,5440,10711,6823,4413,6321,9949,
6164
ATP7A_6_63,2067,1029,1790,1279,1221,3293,1314,673,2335,1574,2174,1242
ATP8A2_6_64,4741,3537,4771,4036,4283,6188,3824,3929,3706,3951,5994,376
5
ATP8B1_6_65,322,419,464,626,1204,816,294,923,0,1,331,0
ATP8B4_6_66,2076,3232,3764,3227,590,1885,1628,3915,3128,1058,3567,2111
ATP9A_6_67,676,530,730,517,513,164,316,253,211,921,186,196
ATP9B_6_68,2190,1566,2009,1819,2357,881,1643,1568,2161,2433,2129,3019
ATPAF2_6_69,733,435,1213,1516,591,609,490,1629,1538,1238,1365,1531
FLVCR1_6_70,329,197,585,224,188,111,627,425,11,1074,565,384
RHBG_6_71,675,205,440,639,375,292,1292,1421,483,849,1203,971
RHCG_6_72,95,116,19,199,299,1,0,0,0,644,0,865
SLC10A1_6_73,1797,2202,2126,2179,2500,1796,2067,3253,2167,3220,3515,21
60
SLC10A2_6_74,6248,5882,7830,7124,4170,6354,9310,7086,7558,7152,9136,45
83

SLC10A4_6_75,5730,4783,5261,5807,2961,2075,9436,4563,4100,5453,7594,60
00
SLC10A5_6_76,7695,7928,7707,7842,10515,10672,12390,10875,5564,9573,877
5,7871
SLC10A6_6_77,1408,1091,1079,988,532,302,305,1496,320,1089,3454,2343
SLC11A1_6_78,1561,1480,1963,1916,1488,2746,911,924,1186,1565,1694,3638
SLC12A2_6_79,10081,9359,12591,12920,10411,9694,12481,10578,10393,13069
,12867,17000
SLC12A7_6_80,3452,3488,3892,3364,4159,4629,4391,1594,1951,3625,3536,34
27
SLC12A9_6_81,370,706,970,814,512,207,1539,1145,373,1503,796,2857
SLC13A1_6_82,3344,3738,3995,4049,3920,4119,3259,2643,3456,3919,3950,27
73
SLC13A4_6_83,2306,2146,3161,2520,1246,1805,2491,2940,2464,1424,4468,29
40
SLC15A1_6_84,10525,9820,12094,12367,10217,10181,10577,15311,9048,14426
,13465,16847
SLC15A3_6_85,1754,1235,1349,1212,2302,2536,2790,2018,694,2128,2354,824
SLC15A4_6_86,2352,1956,2771,2356,3125,2833,2363,1791,2129,2421,3869,14
19
SLC16A10_6_87,1427,1552,1839,1721,2280,627,3097,2936,820,1278,2078,266
0
SLC16A11_6_88,2105,1431,2359,1638,2055,788,2792,1479,1099,2021,1848,66
2
SLC16A12_6_89,6268,5905,6286,6253,7323,7636,7651,8702,6480,6209,8278,7
206
SLC16A2_6_90,2813,2695,2994,3026,2412,2985,3503,2150,1995,2046,4193,22
73
SLC16A5_6_91,1102,1180,1264,995,996,1288,1752,765,1071,1176,1919,434
SLC16A7_6_92,1700,1357,1037,1507,353,1701,3315,1322,424,2373,1062,964
SLC16A8_6_93,1794,1898,1802,1847,2004,1214,1809,1663,578,2840,2628,509
SLC17A1_6_94,1464,1756,1764,1823,1046,903,2180,3113,1089,1384,2001,154
6
SLC17A4_6_95,3150,3483,3445,3008,3334,4459,2389,2052,3470,3289,3600,28
41
SLC17A5_6_96,1064,1094,1445,1506,417,1680,1666,1049,303,64,1155,1423
SLC17A6_6_97,9643,9179,12832,9072,10540,13223,9816,18224,7736,10929,13
260,10418
SLC17A7_6_98,1023,967,1160,1336,420,645,393,231,1676,1762,1360,3048
SLC18A2_6_99,699,831,886,1032,290,295,780,1873,551,272,449,1415
SLC18A3_6_100,3326,3691,3068,3137,3528,4656,4197,2388,2288,3104,4773,2
721
SLC19A2_6_101,3554,3295,4124,3823,2755,5047,4509,7341,4046,4062,5247,2
543
SLC19A3_6_102,1767,1508,1758,1857,1212,436,988,2102,1136,2911,2856,170
6
SLC1A1_6_103,5833,6451,9426,8065,4302,9148,6972,8355,6864,8226,8920,59
80
SLC1A6_6_104,1389,1663,1507,1771,1082,925,2069,1825,756,474,1880,409
SLC1A7_6_105,6004,6630,9058,7902,4156,10673,6909,8184,6884,8405,8670,5

759
SLC20A1_6_106,4453,3418,5442,4832,5197,6730,6709,3831,5129,2870,8300,4
715
SLC20A2_6_107,1796,1076,1532,1136,1664,955,2131,1229,1877,4806,1539,18
02
SLC22A10_6_108,745,696,1027,1352,921,317,2620,1028,988,450,355,765
SLC22A11_6_109,621,971,674,838,231,2278,1393,564,345,1132,1419,667
SLC22A13_6_110,427,321,464,264,1430,334,48,353,111,58,780,245
SLC22A14_6_111,1284,1068,1809,1302,1172,1177,506,1133,1380,2426,1904,1
806
SLC22A15_6_112,3043,2585,3204,3798,1681,3641,2969,4984,1857,1190,4291,
3821
SLC22A16_6_113,5139,4076,5273,4607,5095,5343,4877,3590,2728,4901,7414,
7657
SLC22A18AS_6_114,1524,1050,1312,1549,2275,481,1412,1266,1142,1170,1822
,1360
SLC22A2_6_115,970,799,640,861,1261,213,482,392,477,1194,1285,1757
SLC22A3_6_116,568,520,1043,828,109,490,524,82,733,751,1099,453
SLC22A4_6_117,3364,3454,4277,4036,2785,6222,5255,2803,3310,3074,4821,2
332
SLC22A5_6_118,2787,2885,4049,2824,2174,4620,2269,3827,3397,3685,4884,4
949
SLC22A9_6_119,2138,1873,1776,2644,1168,1789,2741,1845,1795,2838,4154,1
156
SLC24A1_6_120,6137,4861,5169,4601,6861,6704,6488,6031,6595,8489,5802,3
189
SLC24A3_6_121,837,521,568,757,1343,1592,1338,574,385,420,1052,321
SLC24A5_6_122,856,1119,935,1159,942,629,2554,598,647,1005,1269,802
SLC24A6_6_123,3571,3829,4888,3495,4044,4882,3596,6782,2669,3012,5575,3
495
SLC25A10_6_124,2577,3421,3757,3436,3115,4727,2312,2588,1947,2085,5089,
2873
SLC25A12_6_125,945,1057,1515,1717,1542,1547,805,1913,493,1777,1319,144
4
SLC25A15_6_126,2434,1767,2756,2250,2146,2634,3268,6475,2375,1905,3467,
2756
SLC25A16_6_127,2814,3822,3963,3606,2745,2641,6428,2068,2850,2917,3544,
3544
SLC25A17_6_128,5955,4439,5825,6259,6132,6389,5995,4301,4046,7404,5938,
5296
SLC25A18_6_129,363,148,505,672,205,406,191,438,401,1064,314,126
SLC25A1_6_130,594,266,523,688,177,88,1262,80,587,1426,2086,892
SLC25A20_6_131,1245,1720,1653,1333,2255,665,1672,2204,1181,1919,1829,2
040
SLC25A28_6_132,810,1211,1643,1302,1501,584,1791,1712,710,957,1089,1267
SLC25A2_6_133,1486,1865,1886,2118,1985,940,1677,2864,1593,1239,2738,14
54
SLC25A32_6_134,6185,4959,5776,5862,5771,4944,6483,6559,5193,6489,5198,
3290
SLC25A37_6_135,3918,3452,4252,3576,2232,3259,4389,4053,2687,3106,3460,

2592
SLC25A43_6_136,1923,2061,2664,2407,2001,2363,1983,1596,2551,1145,3380,
3462
SLC25A44_6_137,887,1322,1322,1130,2831,2290,1455,1280,422,670,2617,644
SLC25A4_6_138,1275,1162,1003,1935,711,1899,2190,1266,761,3109,1057,130
5
SLC25A5_6_139,1050,923,1274,1192,1277,2820,1202,1955,976,331,1124,883
SLC25A6_6_140,1800,1476,2286,1668,1739,2713,2065,3150,2410,3549,2603,5
83
SLC26A10_6_141,1874,1662,2226,1903,1558,3543,1833,1647,1359,2402,2877,
2503
SLC26A2_6_142,2671,3038,3097,2895,3584,4887,2460,2704,2697,3490,5365,3
954
SLC26A3_6_143,1371,1228,1654,1195,681,899,684,2404,1044,3063,1522,1146
SLC26A4_6_144,6709,7337,9073,8028,6890,10777,6808,12574,5546,7346,1321
4,7946
SLC27A1_6_145,7720,4949,7466,8795,4815,7411,10069,8908,4857,10185,7290
,7520
SLC27A3_6_146,369,214,336,567,12,185,30,56,34,73,1042,1132
SLC27A4_6_147,3399,3298,4000,4082,6418,2665,2423,3944,3986,5625,4175,4
959
SLC27A5_6_148,638,1576,633,1108,213,2474,381,746,699,408,811,361
SLC28A2_6_149,1499,1735,2168,1446,1516,4911,1345,1435,1865,2301,2213,1
928
SLC29A2_6_150,1348,1284,2027,1637,2125,1990,1567,2460,622,734,1857,441
SLC2A10_6_151,1095,1412,2251,1823,996,1182,1999,1656,1482,2081,4785,95
0
SLC2A12_6_152,2482,2604,2849,2016,779,3221,772,1533,2439,1368,2588,214
9
SLC2A13_6_153,996,1116,1623,1099,1265,443,1707,1739,791,487,2277,1268
SLC2A14_6_154,4460,3063,4807,4907,3509,3917,2668,6932,2448,9298,5977,4
266
SLC2A1_6_155,630,526,589,484,162,127,174,657,2,448,219,372
SLC2A2_6_156,4586,4530,4612,4641,3629,4126,7204,7283,4367,4686,7032,45
49
SLC2A3_6_157,4460,3063,4807,4907,3509,3917,2668,6932,2448,9298,5977,42
66
SLC2A4_6_158,1255,1416,1120,1398,2775,2445,1075,581,148,3187,757,1765
SLC2A4RG_6_159,1560,1569,1582,1635,2909,2827,2404,2419,1181,2518,1178,
1004
SLC2A7_6_160,836,532,766,909,25,50,252,969,659,898,1286,575
SLC2A8_6_161,2021,2455,2466,2746,1400,3134,2073,3284,1985,4212,5133,25
32
SLC30A10_6_162,2479,1896,2299,2128,1155,3026,2987,3226,1497,2759,4809,
1790
SLC30A1_6_163,0,0,0,0,0,0,0,0,0,0,0,0
SLC30A3_6_164,2546,2715,3218,3221,2966,2355,2587,5034,1702,4114,3539,1
873
SLC30A4_6_165,3493,2746,3427,3397,3074,2827,4902,4299,2682,4489,2804,5
428

SLC30A9_6_166,1400,1333,1182,1497,1348,888,1736,686,807,1161,1548,1771
SLC31A1_6_167,813,872,1166,968,499,1235,1029,3548,891,2058,428,1446
SLC31A2_6_168,2379,2413,3202,2554,3337,1976,3297,2584,3021,1878,2585,1
976
SLC32A1_6_169,4491,3343,4911,3772,2789,8202,3727,3114,1911,3123,5964,6
371
SLC35A3_6_170,5524,4813,5786,5381,5267,4817,5241,6383,4749,4907,5170,5
199
SLC35A4_6_171,179,390,105,239,6,100,101,19,15,0,0,0
SLC35A5_6_172,938,958,1213,1517,1099,1564,1434,1742,942,1072,1886,2310
SLC35B1_6_173,5115,4252,3798,5607,3336,5294,6632,4025,5787,3707,6664,5
655
SLC35B2_6_174,3491,3634,4523,4921,4023,4780,3945,4698,2680,2692,5720,4
430
SLC35B4_6_175,1348,1041,1604,1292,1258,2778,1923,1470,857,3207,2565,10
53
SLC35D1_6_176,1123,1106,1780,1576,2022,1410,1242,837,68,2068,2084,2807
SLC35D2_6_177,1395,1530,1867,1331,1314,806,2301,1601,2440,5217,1055,91
8
SLC35D3_6_178,1112,392,927,755,652,56,805,1720,773,1695,1530,336
SLC35E1_6_179,555,370,562,773,187,872,841,38,268,718,649,408
SLC35E3_6_180,2751,1891,2891,2867,3818,3573,1536,2542,1059,2572,3775,3
198
SLC35E4_6_181,1656,1041,1866,1285,249,1019,1790,2656,1606,1229,1718,16
95
SLC35F1_6_182,1163,1333,1344,1004,1276,1571,435,1808,1069,2696,2687,72
6
SLC35F2_6_183,539,431,651,775,1983,353,151,281,128,989,1136,852
SLC35F3_6_184,4550,4114,5503,4473,5418,5841,4824,4512,1857,7506,5285,5
319
SLC35F4_6_185,4756,5248,4692,5821,4387,4542,8443,5954,3157,7177,5488,3
627
SLC35F5_6_186,4529,3433,4751,4880,3756,5150,6007,4979,3141,3360,5288,3
959
SLC36A1_6_187,1756,1162,1900,1662,2045,789,783,1335,959,1216,2363,1074
SLC36A2_6_188,3051,3360,3673,3734,4129,4262,3604,4331,2102,3599,4090,4
471
SLC36A4_6_189,2803,2381,3656,2892,797,4003,1388,2838,2023,4169,4899,26
32
SLC37A1_6_190,935,740,1423,830,1567,1054,444,1140,703,637,1639,1199
SLC38A2_6_191,12083,13477,12508,11814,12122,17753,12605,13768,10150,12
942,15988,15128
SLC38A3_6_192,2292,3089,3533,2928,3708,2572,1514,2273,1185,2219,2267,2
693
SLC38A5_6_193,2072,1769,2984,2979,1324,1722,2642,4019,1555,3052,2498,7
95
SLC39A1_6_194,2668,2806,2835,3343,1932,2758,3139,2812,2132,3398,5137,1
660
SLC39A2_6_195,1310,1321,2191,1268,1595,1660,884,2986,807,1020,1531,214
4

SLC39A9_6_196,3203,1905,2883,2921,2647,5525,3972,4665,2399,4796,4057,900
SLC3A1_6_197,3871,3609,4868,4020,5494,2686,4776,6579,3980,2496,5580,4694
SLC40A1_6_198,6193,7058,8669,8033,7920,7705,7125,7398,5924,6798,9193,7485
SLC41A1_6_199,793,615,722,700,446,2001,45,547,592,936,444,1336
SLC43A2_6_200,389,251,309,497,648,214,397,803,663,200,343,479
SLC44A1_6_201,1353,1574,1790,1676,708,1812,1856,1307,1325,1381,2824,3860
SLC46A2_6_202,1292,1504,1868,1851,801,680,1464,422,1090,2818,2694,1435
SLC4A1AP_6_203,6893,7757,7421,7821,8214,9890,10025,6357,8137,7856,8604,9091
SLC4A1_6_204,2385,2384,3532,3092,3351,1820,3666,1167,1654,3322,5471,1853
SLC4A7_6_205,15025,12101,16000,18352,15410,19411,13962,18411,11176,22687,19490,13616
SLC4A9_6_206,3410,2586,2731,2705,2913,4538,3608,2094,2131,3454,3535,2526
SLC5A11_6_207,1643,638,1371,1079,1063,1297,1688,1367,1814,504,615,2088
SLC5A1_6_208,1949,2658,3207,2555,2475,2646,2712,980,2160,2363,2156,2815
SLC5A2_6_209,10335,8803,10660,10817,12555,10160,8158,14066,7238,10562,12421,9948
SLC5A3_6_210,521,500,288,790,1285,1060,496,742,62,672,512,169
SLC5A4_6_211,516,708,862,682,82,954,690,367,158,1478,539,535
SLC5A5_6_212,2441,2404,3194,2896,2068,2055,2062,2775,2049,4065,5814,2425
SLC5A6_6_213,1473,960,1489,1000,400,107,919,1332,1095,1289,886,1373
SLC5A7_6_214,2371,1864,3192,2154,2721,1875,1141,4855,1828,3574,3616,1192
SLC5A8_6_215,4744,4653,5113,5235,4450,6992,4145,6518,2357,4288,5475,7121
SLC6A11_6_216,766,771,1577,1150,949,1731,370,374,349,966,289,904
SLC6A14_6_217,3953,2604,3598,4344,3279,2667,2515,3277,4616,6117,4424,3647
SLC6A16_6_218,2041,2369,3039,2442,2642,1459,1898,894,2648,3700,1085,3916
SLC6A19_6_219,1128,1019,1535,1471,2150,2140,434,1018,2026,2151,2158,3238
SLC6A1_6_220,632,633,853,929,962,490,1297,1005,1640,291,1173,475
SLC6A3_6_221,1085,1707,1671,1500,2316,1247,1934,755,470,389,2215,2297
SLC6A4_6_222,1082,1007,1107,1142,1142,414,614,479,935,2241,634,1484
SLC6A7_6_223,350,128,202,538,19,20,538,658,136,877,816,829
SLC7A10_6_224,1920,2138,1662,2006,1293,3619,3636,1321,2550,1639,1591,1407
SLC7A11_6_225,5662,5169,6103,6668,6122,5275,4298,5307,2399,2838,8466,5518
SLC7A13_6_226,2809,3163,2255,2848,2409,3655,3335,1618,2201,2767,3728,5008

SLC7A1_6_227,3502,3195,3395,3642,2183,3496,3361,3793,3909,3450,4264,36
60
SLC7A4_6_228,2347,1807,3108,2652,1371,1903,3668,1571,1580,5925,4045,20
85
SLC7A5_6_229,1477,2096,1881,1932,3018,3452,1841,1987,1185,3018,1838,18
44
SLC8A2_6_230,851,347,953,753,346,1454,727,199,420,322,606,921
SLC9A1_6_231,855,617,1103,776,251,287,178,508,48,501,264,181
SLC9A2_6_232,629,613,538,485,209,384,390,22,161,40,819,79
SLC9A3_6_233,521,466,812,309,244,303,262,208,545,133,1945,566
SLC9A3R1_6_234,360,571,459,715,553,483,562,1287,482,718,1166,483
SLC9A4_6_235,2657,2721,3252,2869,3965,4906,2135,3577,1154,5752,2574,19
58
SLC9A5_6_236,406,357,514,364,425,599,1179,257,89,1706,768,349
SLC9A7_6_237,2362,2090,2045,2180,2294,2617,1578,2674,1370,2416,1733,22
72
SLC9A8_6_238,1488,1447,1498,1143,981,1653,1889,2228,1106,2947,1523,170
8
SLC9A9_6_239,2749,2705,2900,3001,3738,2944,2356,3229,1736,4380,3079,25
90
SLCO1B1_6_240,2881,4084,3983,3932,3011,4847,5481,4592,4591,4013,3310,4
492
SLCO1B3_6_241,3296,2886,3347,3706,5015,3096,1936,2814,3374,5804,4045,5
813
SLCO2A1_6_242,959,531,954,861,1168,436,345,156,1084,47,1534,336
SLCO4A1_6_243,4165,4680,4610,4290,3826,2797,3481,5337,2493,2523,5319,3
618
SLCO4C1_6_244,5701,4821,6169,6421,8414,10119,4553,7303,3780,9767,6381,
5963
SLCO6A1_6_245,687,787,1059,1149,507,2075,1170,344,686,527,1846,2142
SV2A_6_246,3662,3196,3821,3761,2675,1218,2505,5415,4562,2354,4035,3017
TAP1_6_247,1885,1444,1129,2031,401,3173,2518,3337,1366,1886,2208,1787
UCP1_6_248,2525,2689,2461,2785,3280,4374,3940,2373,2781,4013,2523,2632
ABCA12_6_249,888,676,994,602,485,329,719,1026,341,321,1317,536
ABCA2_6_250,1181,1347,1309,1452,1197,2071,1789,1302,873,1097,2116,896
ABCA5_6_251,2403,3021,3436,2667,2550,2695,3712,3498,3127,3231,3339,234
3
ABCB4_6_252,661,1065,521,729,297,1172,1540,1608,1085,967,1475,279
ABCB9_6_253,2934,2281,3103,2987,4325,2890,1324,4033,2341,2170,5388,269
0
ABCC10_6_254,1089,744,1088,836,521,396,528,1917,1046,528,1127,751
ABCC11_6_255,4947,5486,5328,6534,4040,6531,8176,7165,4654,6516,8112,69
92
ABCC1_6_256,437,393,367,571,19,23,236,473,697,51,113,314
ABCC3_6_257,1946,2401,1876,2446,1795,940,2656,3126,1458,1373,2539,1633
ABCC4_6_258,3679,3132,4754,4727,4440,4167,3558,3829,3312,6449,6005,552
3
ABCC5_6_259,1367,1012,1377,1743,850,935,1009,2779,1294,1910,1560,933
ABCC9_6_260,378,310,167,427,249,527,123,523,267,202,360,4
ABCD3_6_261,2029,2097,2189,1624,2499,1446,910,1934,1786,1705,3516,1504

ABCE1_6_262,2939,2189,3073,3274,2576,4257,4194,2145,3688,4734,3759,381
8
ABCF1_6_263,861,1029,777,919,534,1537,117,326,444,2112,652,1493
ABCF2_6_264,2832,3006,3230,2914,907,3193,3461,4285,1884,3906,4245,2492
ABCG1_6_265,1385,1819,1156,1048,518,1145,57,978,1097,2069,1748,611
ABCG4_6_266,2321,2438,2862,2770,3050,2047,4114,3464,3179,4057,4362,192
9
ANXA11_6_267,1388,1142,1647,1311,2949,511,301,470,1183,667,855,1192
ANXA13_6_268,9276,9313,10617,9387,11298,12032,9614,10214,9343,18922,12
345,7549
ANXA2_6_269,2525,3002,2702,3307,3114,5688,3318,3235,1447,4422,3206,249
3
ANXA6_6_270,3945,3712,3509,3406,2919,5129,1546,4705,5289,4717,3703,474
0
ANXA7_6_271,945,728,1066,1106,1092,812,145,943,704,435,1261,2157
ATP11A_6_272,5505,4517,6599,5325,5904,4267,6671,7508,4424,5793,8066,52
74
ATP11C_6_273,1469,1644,2087,1942,1741,3481,808,2512,534,1680,3377,1418
ATP12A_6_274,1641,1556,2337,1848,1727,600,1902,424,1093,1540,2121,718
ATP1A1_6_275,751,889,1204,963,621,792,1621,1434,1360,307,865,1505
ATP1B4_6_276,11760,12761,15902,15561,12256,14225,11788,14618,11693,126
33,20929,16906
ATP2A1_6_277,3201,3870,4327,4030,4259,3634,5769,3501,2967,3553,4691,50
54
ATP2A2_6_278,1554,1488,1960,2056,884,4615,2447,664,4052,2116,2790,2649
ATP2A3_6_279,3222,3630,4264,4002,4246,3835,5292,3491,2971,3507,4432,46
76
ATP2B1_6_280,8400,7576,9971,8287,7045,6336,9047,7847,5305,7708,11837,1
0457
ATP2B3_6_281,664,314,596,291,14,2678,1620,248,107,1256,46,343
ATP2B4_6_282,2181,1779,2808,2413,2928,2256,1501,2299,1785,2132,3531,22
68
ATP2C1_6_283,625,681,788,856,259,163,329,954,145,145,95,895
ATP5A1_6_284,1038,680,803,733,745,265,432,213,1373,793,551,2378
ATP5C1_6_285,3518,4566,4529,4115,2600,3548,4825,3664,3419,4977,6914,32
85
ATP5D_6_286,6558,6605,6344,6079,7522,7417,6070,6812,5205,6187,6749,654
9
ATP5E_6_287,1853,1251,1596,1539,386,1034,1188,1400,939,3023,2364,1488
ATP5G1_6_288,1860,2587,1883,2108,1246,2724,1300,4476,1267,1160,772,146
2
ATP5G2_6_289,488,433,719,485,254,956,351,2321,513,280,687,236
ATP5G3_6_290,1718,1642,2362,1854,1721,1376,1873,1671,954,3159,1880,964
ATP5H_6_291,5384,4782,5077,5889,3239,6502,3597,7319,3227,6716,7807,417
6
ATP5J_6_292,3372,3044,4055,3620,3442,3254,5089,6386,2256,3953,4869,466
2
ATP6V0A1_6_293,1806,1513,1284,1300,2670,754,1002,1638,765,2939,1288,92
3
ATP6V0A4_6_294,3170,4021,3661,3568,2662,3832,2447,1641,2526,1954,3238,

2746
ATP6V0B_6_295,843,665,328,808,1163,1404,811,1285,996,1081,575,2033
ATP6V0C_6_296,1452,1583,1793,1456,842,1047,2584,2789,1949,1136,2706,14
15
ATP6V1C2_6_297,1039,825,1062,1133,540,2760,914,1112,2194,619,1629,762
ATP6V1E1_6_298,1547,1194,1930,1572,1224,721,2221,2007,1168,1759,2498,1
707
ATP6V1F_6_299,733,671,1149,1160,1388,1218,2357,2321,200,1138,331,529
ATP6V1G2_6_300,353,462,253,351,276,464,180,10,0,23,100,167
ATP6V1H_6_301,2963,2525,3052,2516,1828,3917,4065,3584,3321,5131,5160,4
715
ATP7B_6_302,936,1001,1237,1247,989,2133,2114,2297,1327,527,878,986
ATP8A1_6_303,2612,2087,2308,2670,2254,1564,3109,3440,3692,2426,3448,28
02
ATP8B2_6_304,1916,2471,2064,1898,2228,2147,885,1850,2968,1070,3608,309
3
ATP8B3_6_305,1577,1546,2153,1593,1528,3677,1444,3049,1349,338,1981,226
8
ATPAF1_6_306,4011,4205,4065,4752,2017,7159,3400,3759,4328,3392,3418,49
11
GC_6_307,7495,6397,8270,6451,6676,5560,6720,5695,6552,6230,8790,9040
SERINC5_6_308,13465,13573,16907,16150,11102,19149,13572,11028,11552,15
183,26656,17422
SLC10A3_6_309,410,340,467,515,326,238,364,21,187,583,1642,486
SLC11A2_6_310,1099,1067,1441,811,1402,350,2258,606,467,323,934,684
SLC12A1_6_311,6047,5438,5810,6782,6462,4164,8544,6701,3817,7065,11145,
5412
SLC12A3_6_312,1017,806,1237,991,516,498,767,192,618,1153,1920,843
SLC12A4_6_313,949,1055,896,781,1442,634,771,523,1248,585,2338,1926
SLC12A5_6_314,4503,4402,5935,5448,3464,3582,8287,5229,3255,6081,9063,7
353
SLC12A6_6_315,2502,1881,2603,2642,2685,3218,2562,2513,4348,1886,3153,3
060
SLC12A8_6_316,1503,1324,1709,1677,1545,1780,2274,3412,936,1052,2032,15
37
SLC13A2_6_317,4784,4814,4781,4965,4608,2911,6272,3275,3117,3507,6726,2
804
SLC13A3_6_318,5229,5069,4771,5132,2812,4426,4429,3608,3696,6575,6477,5
990
SLC13A5_6_319,585,452,779,717,123,137,460,552,648,759,1942,178
SLC14A1_6_320,1865,1880,2796,2259,2542,3024,2380,3284,1534,2431,2579,7
63
SLC14A2_6_321,1713,1681,2414,2341,1932,2584,1632,1323,1010,2598,3413,1
794
SLC15A2_6_322,2060,1944,2132,2095,1118,2399,3991,2235,1615,5599,1873,3
396
SLC16A1_6_323,4640,4389,3627,4307,5862,3568,3781,3891,5437,5750,4413,5
613
SLC16A3_6_324,1732,1803,2222,2047,1877,1873,3170,4292,3718,4036,1748,1
599

SLC16A4_6_325,6694,5482,7764,6582,9205,7935,5921,9547,4275,7015,8979,7
918
SLC16A6_6_326,6205,5383,6758,7094,5624,5804,6368,8600,4449,9166,6784,9
321
SLC17A3_6_327,3057,3129,3506,3533,2235,2607,2893,6825,2766,3767,6014,4
466
SLC17A8_6_328,2385,3027,2850,2408,3599,3858,3435,2943,792,1944,2660,13
54
SLC18A1_6_329,871,221,813,870,2157,191,60,1229,1197,29,2448,477
SLC19A1_6_330,10215,10512,13094,10884,11153,10739,12507,11816,12709,11
483,11711,11863
SLC1A2_6_331,2836,1871,2085,2796,2750,2369,1638,3642,2649,3240,4793,22
42
SLC1A3_6_332,7855,7407,8492,7986,7645,9581,10297,9596,6510,6213,9262,9
181
SLC1A4_6_333,599,574,517,471,92,1360,54,213,942,1481,1302,1149
SLC1A5_6_334,2031,2106,2262,2048,2033,1411,2206,1856,1135,2083,4384,27
77
SLC22A12_6_335,2259,2290,2633,2641,2123,4809,4349,3182,2325,3014,3432,
2568
SLC22A17_6_336,2885,2798,2253,2803,2533,2280,2319,3981,3821,2455,3020,
4488
SLC22A18_6_337,1597,2140,1585,1412,1035,528,1689,2556,2289,3371,2001,1
938
SLC22A1_6_338,6164,6061,6773,6292,6023,8903,6179,7282,5117,8321,10616,
8781
SLC22A23_6_339,2681,2991,2475,2665,1130,3591,3630,3223,3144,3035,2660,
2760
SLC22A6_6_340,911,1087,1244,1418,1972,2534,1741,2095,1559,1400,738,234
2
SLC22A7_6_341,232,225,664,501,24,440,422,2394,313,340,527,483
SLC22A8_6_342,427,421,548,450,475,377,890,467,550,18,618,363
SLC23A3_6_343,528,444,970,517,75,934,493,577,439,211,1261,271
SLC24A2_6_344,525,365,645,735,291,649,1897,700,5,698,788,34
SLC24A4_6_345,6686,5418,6740,6675,4146,7363,6132,7149,5475,4941,10341,
6209
SLC25A11_6_346,4486,4415,4728,4914,2400,6372,5250,4201,3872,7335,6461,
6155
SLC25A13_6_347,1139,876,1030,967,1223,2014,1132,1856,2970,546,612,300
SLC25A14_6_348,1347,1495,2142,1478,1148,2415,2216,1577,1942,2945,1920,
2447
SLC25A19_6_349,3755,2328,3879,4474,4222,5647,4839,4136,2113,4107,3783,
3345
SLC25A21_6_350,1663,1634,975,1708,1315,1812,1883,1611,1340,3053,736,27
67
SLC25A22_6_351,814,494,835,649,139,1190,648,430,1848,369,1399,1340
SLC25A26_6_352,660,1462,1736,1069,746,849,1323,861,1205,1294,1207,2015
SLC25A27_6_353,2281,2101,2217,1611,3184,4767,2287,1897,3515,2215,3101,
1718
SLC25A3_6_354,5590,5669,7896,6547,4730,5727,6132,5853,5603,5087,9126,6

263
SLC26A11_6_355,699,623,877,749,121,2533,2576,1222,405,1206,1084,747
SLC26A1_6_356,1445,1312,1485,1342,255,935,1742,981,1353,1320,2948,1104
SLC26A5_6_357,2831,3634,3368,3062,6007,5189,3617,2547,2777,2508,4958,4
176
SLC26A6_6_358,1635,1905,2427,1664,2193,1113,584,504,1976,1324,2788,120
5
SLC26A7_6_359,8971,7884,11597,10770,8722,12421,8789,9865,7676,11776,13
783,6439
SLC26A8_6_360,7214,6150,7712,8346,7708,6932,5055,9378,9569,6046,9908,1
1475
SLC26A9_6_361,4337,3913,5425,4955,3065,2176,7089,5637,5302,4773,5140,6
212
SLC27A2_6_362,1602,1449,1651,1689,1733,1305,3061,2173,1585,3539,2263,1
959
SLC27A6_6_363,2184,2131,2971,2586,3492,2732,2575,1708,1770,4433,3362,2
746
SLC28A3_6_364,2461,2203,1839,2299,3421,1722,2103,1656,1821,1390,2273,2
134
SLC29A1_6_365,514,746,828,603,2385,82,904,718,273,331,520,287
SLC29A3_6_366,930,748,1033,1096,414,172,37,1064,455,1542,1204,536
SLC29A4_6_367,519,472,691,746,441,367,1074,108,979,1270,1065,1277
SLC2A11_6_368,3735,2656,3794,3788,5330,3149,3091,3722,2832,3628,5272,2
827
SLC2A5_6_369,4192,5029,6263,5323,2148,6532,7434,5661,3705,5286,9337,35
88
SLC2A6_6_370,598,481,723,507,623,1083,1046,834,682,5,720,384
SLC2A9_6_371,2832,2161,2883,3438,1777,1193,2666,3408,2329,1752,4589,52
78
SLC30A2_6_372,1546,2102,1687,1467,1853,315,1407,1001,1209,2599,1500,96
5
SLC30A6_6_373,7145,8180,8795,9216,5366,9532,7508,6267,4585,11309,6062,
7252
SLC30A7_6_374,1338,1292,1437,1228,1698,1271,734,817,758,1306,3447,1603
SLC30A8_6_375,1526,1170,1462,1469,1330,1291,1335,2745,2225,2201,1399,1
722
SLC33A1_6_376,1846,1226,1848,1731,1716,1130,2596,902,611,1723,2102,151
7
SLC34A1_6_377,330,637,808,538,137,1813,1265,1036,246,108,376,218
SLC34A2_6_378,1750,2037,3064,2832,1164,467,1801,1393,2069,911,1285,344
4
SLC34A3_6_379,3819,3570,4048,3850,4256,2951,4890,4630,3652,3123,3831,3
329
SLC35A1_6_380,5253,4231,5383,5959,1527,5741,5278,8731,4107,6304,8204,5
214
SLC35A2_6_381,384,1037,511,661,0,164,271,300,260,372,1745,782
SLC35B3_6_382,390,301,190,322,218,0,152,499,315,202,643,158
SLC35C1_6_383,672,549,395,363,1311,502,358,411,1146,632,695,266
SLC35C2_6_384,1043,800,796,614,88,605,1409,500,9,2718,783,368
SLC35E2_6_385,1435,2271,1525,2383,2402,558,775,2070,3606,1904,1340,367

8
SLC36A3_6_386,6007,6591,8078,6994,7766,6040,5483,6113,4398,6673,9297,5
955
SLC37A2_6_387,1042,740,1003,850,686,578,1076,661,1375,960,1918,401
SLC37A3_6_388,2260,2043,2172,2251,1683,1424,3325,1603,1303,3188,1353,1
998
SLC37A4_6_389,659,247,795,484,216,1529,77,106,659,471,921,1003
SLC38A11_6_390,4314,4816,5304,4867,5249,4165,3376,3271,5334,3938,3191,
4575
SLC38A1_6_391,5861,6015,7167,6535,3883,8345,6906,6996,5707,7354,9483,7
151
SLC38A4_6_392,862,778,968,714,38,854,17,125,558,775,1070,744
SLC38A6_6_393,2451,2557,2930,2150,2417,2831,3259,3339,1911,2055,2048,2
843
SLC39A10_6_394,1002,838,1371,2179,919,737,1494,1395,1312,1044,1382,528
SLC39A11_6_395,786,741,947,1174,441,617,1586,362,1120,3245,688,431
SLC39A12_6_396,3955,3368,3623,3617,3646,4049,2618,2272,3284,4010,5292,
3065
SLC39A13_6_397,490,393,522,711,226,528,158,0,510,200,11,168
SLC39A14_6_398,12341,11224,12399,13654,16888,12190,13302,13344,10091,1
5931,17202,10230
SLC39A4_6_399,4562,4102,5615,5308,3483,4618,7615,6465,4600,6280,7083,6
961
SLC39A5_6_400,385,333,461,455,761,117,85,391,77,884,678,259
SLC39A6_6_401,4129,3144,4522,4107,4365,2065,5000,3908,3486,2732,2689,4
572
SLC39A7_6_402,562,672,784,1058,323,102,1844,363,419,685,470,780
SLC39A8_6_403,3254,2635,3556,3558,1907,8835,4226,2372,2550,3943,4122,2
455
SLC3A2_6_404,1034,462,956,1009,1810,819,474,1282,681,1126,782,1176
SLC43A1_6_405,577,1181,1106,906,794,1596,860,567,1024,2415,1149,871
SLC44A3_6_406,696,849,586,835,1612,2832,1328,758,912,243,883,676
SLC4A10_6_407,375,613,432,661,119,571,435,920,37,565,1923,948
SLC4A11_6_408,1020,1053,1143,1226,1787,695,377,2009,1033,2785,3585,120
6
SLC4A2_6_409,1156,908,538,740,966,419,678,1142,432,306,297,1298
SLC4A3_6_410,867,938,1723,1417,790,1440,2058,2023,897,2587,1462,1405
SLC4A4_6_411,3020,2479,3274,3346,2678,2949,3799,2438,1940,2956,4782,22
94
SLC4A8_6_412,405,558,760,746,58,456,2455,2129,518,280,2060,1467
SLC5A10_6_413,423,344,621,570,369,634,456,790,29,656,469,357
SLC5A9_6_414,335,949,473,645,913,14,325,747,414,10,1335,323
SLC6A12_6_415,4629,4036,6400,5591,4872,6144,7369,4168,4924,7684,8175,5
903
SLC6A13_6_416,1395,1124,1658,1422,1470,1571,638,1598,2109,592,1001,198
8
SLC6A15_6_417,2652,3128,3386,3849,4131,2266,4353,2657,4047,4296,3963,2
987
SLC6A2_6_418,1455,1784,2102,1582,876,638,2182,1561,2845,1123,1714,1973
SLC6A6_6_419,1727,1850,2349,2753,2705,556,1319,2959,1487,3427,2947,221

3
SLC6A8_6_420,1502,1947,1619,1782,2956,1887,1666,871,1336,3194,2167,147
5
SLC6A9_6_421,5083,4814,5842,5941,8197,6091,6940,5859,5159,5731,4219,55
44
SLC7A2_6_422,1711,1495,1441,1700,1158,569,1074,3031,1289,850,1890,2215
SLC7A3_6_423,10024,11047,11265,11738,7633,9714,13714,10772,7927,14658,
15409,11666
SLC7A6_6_424,4172,4020,3794,3532,2617,3754,3373,4221,4278,5014,5181,36
34
SLC7A7_6_425,4985,4888,6130,6341,5382,4400,4876,5847,4017,9403,8970,68
47
SLC7A8_6_426,1369,1082,1969,1432,2367,608,1012,3659,1182,757,1629,3193
SLC7A9_6_427,1725,1984,2133,1995,2145,3278,2127,1571,2688,2317,2628,23
23
SLC8A1_6_428,0,0,60,176,0,0,0,0,0,0,0,107
SLC8A3_6_429,405,685,660,468,866,649,737,496,444,322,33,405
SLC9A3R2_6_430,751,798,578,471,170,1518,1571,362,889,641,739,559
SLC9A6_6_431,3774,3720,4254,4217,3211,7079,4009,3709,3402,4539,6561,47
26
SLCO1A2_6_432,4150,3294,4170,4994,7346,2973,5627,4695,3060,5661,8154,3
592
SLCO1C1_6_433,2574,2344,2584,2067,1670,1639,2267,2305,1277,3135,2480,2
770
SLCO2B1_6_434,3949,2874,3284,3349,5357,2441,2386,3555,1711,4540,2668,3
359
SLCO3A1_6_435,972,1414,1377,1233,1226,1260,1047,1715,770,455,1896,1428
SLCO5A1_6_436,445,1018,1053,1046,800,626,1133,2444,776,2505,226,2027
ABCB5_6_437,2510,3403,3526,3293,3876,3985,2046,3057,1600,2869,3018,143
9
ABCC6_6_438,1503,841,1552,1552,960,2036,1678,390,1234,2037,661,1231
ATP1A4_6_439,1895,2677,2321,2207,2593,2428,1165,1441,1627,1859,3220,21
69
ATP5J2_6_440,484,381,569,1107,46,609,893,535,411,220,63,776
ATP5S_6_441,5718,5290,6544,6502,5376,4365,4486,5115,5117,5484,10599,42
98
ATP6V1G3_6_442,1580,1724,1730,2289,332,896,2567,1022,1064,3001,3528,24
58
ATPIF1_6_443,871,523,1257,976,745,471,1219,936,448,996,701,1265
SLC10A7_6_444,2781,2093,2892,3041,2287,3824,4005,2465,1646,3226,3313,3
129
SLC28A1_6_445,2957,3220,3856,3943,5195,4169,3374,4852,2987,3143,7348,7
046
SLC30A5_6_446,9450,10537,12057,10436,5606,9270,9211,6726,8780,13178,13
467,9851
SLC39A3_6_447,980,747,918,802,963,1075,1532,1508,541,810,733,1336
ABCA10_6_448,3365,2338,3388,2923,1524,2704,3387,3323,2537,4286,5436,16
11
ABCA13_6_449,6389,4991,6764,5922,4281,9981,3760,6993,7363,7648,6532,60
30

ABCA1_6_450,1285,1331,1296,1677,816,1231,2659,646,1898,359,3692,1699
ABCA3_6_451,469,238,271,426,423,139,69,1630,11,236,58,471
ABCA4_6_452,611,811,646,940,429,447,1160,935,603,514,1517,490
ABCA6_6_453,567,896,994,620,564,804,2227,354,424,2332,789,326
ABCA7_6_454,1121,1239,1751,1295,1977,1316,1551,1446,1831,1479,1954,257
8
ABCA8_6_455,3101,2168,3820,3663,972,2887,3093,4953,2130,2032,2479,4923
ABCA9_6_456,1518,1400,1942,2050,1145,1147,1439,2187,1719,1627,2178,712
ABCB10_6_457,1768,969,2205,1700,1522,1279,3056,2443,1239,1842,2972,100
7
ABCB11_6_458,1624,1677,1753,1280,983,917,1862,1607,1077,1430,2078,1387
ABCB1_6_459,5811,4686,5753,6888,5557,4503,8250,5756,4440,4624,8996,220
5
ABCB6_6_460,172,290,176,261,9,438,33,50,24,475,34,320
ABCB7_6_461,3424,3063,4409,2609,721,1732,3577,5792,2655,4805,3717,2806
ABCB8_6_462,1245,1355,1257,1462,1028,1497,1080,305,2445,1865,1624,2584
ABCC12_6_463,1156,1133,1311,1211,549,598,808,2767,689,2988,1455,734
ABCC2_6_464,4911,3607,6561,4637,2356,4138,3793,6917,4132,4079,6203,667
4
ABCC8_6_465,236,364,153,400,584,170,101,1350,481,453,793,128
ABCD1_6_466,342,198,529,477,138,890,152,145,129,846,332,643
ABCD2_6_467,2854,2134,3445,2822,2478,3450,4904,4053,2688,3249,2670,252
4
ABCD4_6_468,854,697,576,673,517,403,38,863,1116,492,846,282
ABCF3_6_469,1007,435,935,802,515,29,700,25,2446,366,1471,747
ABCG2_6_470,2550,3513,4191,3539,830,1750,2546,2634,2361,5551,4719,6571
ABCG5_6_471,2637,2152,2609,2759,1732,655,1670,5158,1670,2119,3955,1623
ABCG8_6_472,1872,2928,2388,2825,940,2573,2176,1918,2161,2931,3807,4386
ANXA10_6_473,1456,1507,1419,1910,1457,1269,2311,1307,1745,1186,1908,25
37
ANXA1_6_474,1041,1274,1445,1139,1105,2377,1677,296,589,401,1355,1336
ANXA3_6_475,2918,3058,4345,3789,1944,1629,4000,3825,4850,3432,4826,334
6
ANXA4_6_476,1776,1712,2180,2082,3064,1760,1847,1811,2015,2782,2096,245
4
ANXA5_6_477,1535,1783,2255,2078,2264,836,1719,1238,478,1192,3678,3419
ANXA8_6_478,3851,3634,5001,5235,4586,7329,5281,5143,4981,5360,5979,374
8
ANXA9_6_479,2516,3183,3772,3024,4815,2409,3966,2864,3032,4280,4275,181
8
ATP10A_6_480,2161,1733,2343,2353,1035,2957,2325,2659,1951,3127,2096,19
36
ATP10B_6_481,371,631,760,794,942,838,298,98,1225,191,398,680
ATP10D_6_482,743,704,846,696,648,588,1955,421,593,1386,681,86
ATP11B_6_483,291,351,532,606,358,36,89,395,412,6,299,3
ATP1A2_6_484,339,550,679,755,12,496,448,346,199,704,338,1025
ATP1A3_6_485,2263,2284,1932,2137,2750,2240,1975,2673,3111,1174,2259,17
37
ATP1B1_6_486,7379,6621,7985,7323,6781,4001,9257,6763,6589,9612,11329,8
453

ATP1B2_6_487,1057,1160,1412,1389,1040,1152,1056,543,348,924,2340,1154
ATP1B3_6_488,1922,1489,1964,2026,357,1297,1671,1689,1506,2720,2911,100
7
ATP4A_6_489,1577,1678,2222,1463,1720,2451,2550,1818,2371,989,2945,1742
ATP4B_6_490,1754,2001,1808,2382,1769,2058,943,1864,2544,3301,2951,1561
ATP5B_6_491,7647,5986,9131,7364,7364,7704,10130,6213,8857,8173,10925,9
448
ATP5F1_6_492,778,872,658,732,930,2267,1253,471,52,448,799,194
ATP5I_6_493,723,465,622,809,406,689,840,1829,818,470,1087,1099
ATP5L2_6_494,1212,1119,1985,1517,1020,2214,231,810,806,3451,1335,2687
ATP5L_6_495,1212,1119,1985,1517,1020,2214,231,810,806,3451,1335,2687
ATP5O_6_496,2078,2557,2625,2733,2474,3626,1874,4073,1394,1182,3052,322
4
ATP6AP1_6_497,408,92,277,267,485,345,54,147,862,487,711,83
ATP6AP2_6_498,3883,2740,2930,3147,634,2725,4030,4229,2331,5313,3235,23
29
ATP6V0A2_6_499,1029,1171,1042,1325,32,239,780,796,251,2833,1791,234
ATP6V0D1_6_500,664,462,877,806,97,693,1035,113,123,375,535,24
ATP6V0D2_6_501,695,1459,1592,1250,2480,519,1162,1173,1299,976,1037,776
ATP6V0E1_6_502,1849,1693,1994,2166,1600,2245,1954,3675,1243,5082,3767,
1968
ATP6V1A_6_503,1778,2310,1917,1408,2210,2213,1084,1524,2315,2420,2516,9
53
ATP6V1B1_6_504,594,376,383,689,580,590,1059,152,1324,217,811,374
ATP6V1B2_6_505,1568,1619,1706,1901,1946,1969,2831,2900,1314,4233,3395,
1645
ATP6V1C1_6_506,1663,837,1360,1599,271,1662,2395,1975,167,1997,4574,853
ATP6V1D_6_507,4147,3475,3356,3777,5029,3411,4272,4336,4985,7436,6706,5
037
ATP6V1E2_6_508,8099,7686,9249,10303,8113,9677,14948,7951,7982,7510,115
32,8694
ATP6V1G1_6_509,3259,3403,5250,3934,3749,2823,3341,4335,2483,2739,6332,
2850
ATP7A_6_510,2925,1882,3353,3114,4177,6314,1956,3866,2304,3448,4101,247
8
ATP8A2_6_511,114,251,415,304,718,53,14,656,45,14,516,167
ATP8B1_6_512,1431,1679,2068,1723,2481,1279,1545,1588,2046,1294,1219,21
59
ATP8B4_6_513,5865,3238,5644,4591,5951,5956,5041,6004,5281,4811,4353,63
05
ATP9A_6_514,3960,3365,4861,4289,2870,3601,6487,4283,3971,5127,4651,432
1
ATP9B_6_515,898,691,978,770,1526,671,960,1382,740,583,922,1453
ATPAF2_6_516,574,657,876,549,566,81,614,388,160,1329,796,745
FLVCR1_6_517,2198,1646,2228,1939,982,3882,1677,1563,2114,2493,3077,979
RHBG_6_518,3490,3593,4162,3752,2836,5124,4480,3157,2940,3751,5490,3246
RHCG_6_519,678,782,1309,864,208,807,2236,773,1226,1411,678,1056
SLC10A1_6_520,1716,1242,2641,2241,1993,2336,2126,2778,1787,1584,2799,2
566
SLC10A2_6_521,421,810,682,1072,1888,835,1758,347,272,1199,237,933

SLC10A4_6_522,886,889,1255,1945,1983,192,1463,3368,1155,1511,1819,711
SLC10A5_6_523,2745,3034,2900,2722,2935,5041,3096,2218,2112,3561,3360,2
993
SLC10A6_6_524,732,629,928,949,578,521,1379,1000,628,320,891,421
SLC11A1_6_525,1669,1490,2025,1987,1484,2759,916,972,1181,1772,2110,288
0
SLC12A2_6_526,1559,1418,1326,1285,1003,1658,1741,2245,1231,2475,913,19
89
SLC12A7_6_527,937,482,934,1046,118,883,797,308,815,2186,103,1137
SLC12A9_6_528,423,749,710,815,1360,861,1671,483,39,1044,823,470
SLC13A1_6_529,9138,8778,9275,10678,9663,5940,11146,11522,7908,10107,13
307,8449
SLC13A4_6_530,794,745,1104,881,827,1267,107,292,447,1617,411,161
SLC15A1_6_531,10796,9883,12167,12267,10234,10565,11456,15300,8705,1445
4,13474,17542
SLC15A3_6_532,1696,1288,1420,1008,1927,1656,2768,2387,307,2118,2151,10
68
SLC15A4_6_533,1997,1463,2387,1940,945,2006,3160,2045,1548,2111,3854,19
45
SLC16A10_6_534,1541,1316,1753,1174,3283,1608,1241,3619,880,1224,1597,1
192
SLC16A11_6_535,1001,910,1000,912,669,475,1471,873,945,701,2195,334
SLC16A12_6_536,3264,2792,3295,3049,3349,2239,2545,4191,1594,2398,3430,
3148
SLC16A2_6_537,300,403,253,588,1357,780,16,435,22,166,50,497
SLC16A5_6_538,422,668,696,665,713,349,959,537,418,896,358,810
SLC16A7_6_539,11512,10837,13724,12416,12442,11944,9665,16253,8595,1464
2,15393,12929
SLC16A8_6_540,519,504,215,241,233,0,1703,48,70,70,830,1577
SLC17A1_6_541,2016,2148,2003,2276,137,1323,2230,1602,3030,1863,2696,15
22
SLC17A4_6_542,1698,1653,3104,2816,1776,1331,4048,3704,3618,2939,4413,1
644
SLC17A5_6_543,2940,2795,2545,2994,3809,2238,2740,2520,1101,1303,2532,1
900
SLC17A6_6_544,3077,2110,3819,3046,1655,5468,5495,2729,3576,2711,2292,2
805
SLC17A7_6_545,1865,1438,1872,1918,1463,2534,1233,1422,985,1173,2718,40
42
SLC18A2_6_546,501,577,790,523,2955,1039,1601,731,433,923,1475,595
SLC18A3_6_547,8934,9324,8186,9434,9055,9285,7957,7397,5763,8666,10299,
8445
SLC19A2_6_548,3673,3336,4125,3990,2758,5059,4653,7794,4046,4663,5360,2
529
SLC19A3_6_549,3594,3394,4422,3550,2414,3318,2774,2820,4154,5515,3778,3
488
SLC1A1_6_550,1550,2026,1392,1401,1504,1221,1342,465,357,1122,862,586
SLC1A6_6_551,4586,4803,6365,5228,5083,4753,6664,4895,5929,5235,6961,38
53
SLC1A7_6_552,930,787,1396,1247,620,1196,1904,1420,768,1178,1199,1641

SLC20A1_6_553,653,660,686,1077,1519,1285,517,450,515,1538,898,1528
SLC20A2_6_554,2935,3060,3659,2469,3888,3466,3011,2422,1799,3888,4549,2
812
SLC22A10_6_555,1355,1849,1267,1334,3018,3498,1008,1694,1646,2341,1421,
1196
SLC22A11_6_556,662,570,1396,1008,426,669,920,1661,693,1682,1030,430
SLC22A13_6_557,1825,2067,2495,2428,922,1914,1898,3687,2574,1394,2284,1
607
SLC22A14_6_558,3636,3421,4067,3531,2929,5047,3732,3359,1276,2255,3735,
3709
SLC22A15_6_559,1900,1605,2713,2170,1064,763,1478,779,1170,2617,1644,11
39
SLC22A16_6_560,1286,1190,1082,1636,2167,1172,2076,379,799,662,1236,123
8
SLC22A18AS_6_561,2750,1958,2250,2105,2109,1902,3830,3839,1490,2082,335
5,1286
SLC22A2_6_562,5888,5325,7195,6470,3878,6765,6108,5457,3656,4473,10426,
5832
SLC22A3_6_563,519,346,687,669,2125,752,1760,1233,477,249,489,325
SLC22A4_6_564,1979,1461,2032,1877,4356,423,2394,2692,530,2289,1484,297
1
SLC22A5_6_565,2801,2387,4485,3261,2044,3952,3439,3369,3553,3296,6093,1
948
SLC22A9_6_566,2525,2719,3523,4338,1864,3644,3949,2182,2700,2359,4286,3
849
SLC24A1_6_567,806,353,915,690,1046,752,400,229,1049,617,243,1
SLC24A3_6_568,6157,5095,7568,7233,7717,7382,7692,11284,5507,6909,9316,
6051
SLC24A5_6_569,2912,2675,3373,2345,4013,2783,2266,3553,3368,2275,6011,3
403
SLC24A6_6_570,4283,3108,4285,5136,2289,4546,3454,4572,3609,4705,3568,4
352
SLC25A10_6_571,2473,2490,3239,2515,1865,2460,2586,2332,1091,1448,2571,
2172
SLC25A12_6_572,4355,4983,4952,5383,3090,6256,6710,4217,4863,3406,5648,
6753
SLC25A15_6_573,4233,5034,6530,5675,6274,7270,3383,6161,4283,6025,6688,
4773
SLC25A16_6_574,1254,1041,1163,1050,1187,825,1141,2010,590,341,2292,108
2
SLC25A17_6_575,3421,3083,3396,3460,3232,3705,2266,3177,2777,3595,4264,
3466
SLC25A18_6_576,1817,1804,1542,1820,2057,982,559,2058,819,2046,2072,139
6
SLC25A1_6_577,601,192,186,144,2,3,55,38,40,648,3,31
SLC25A20_6_578,213,107,474,475,210,147,913,1147,25,123,6,528
SLC25A28_6_579,1184,1361,2562,1821,1541,53,1194,2614,1210,1114,3144,15
20
SLC25A2_6_580,2460,2385,3047,2678,1816,2748,2361,3345,1495,2382,4059,1
534

SLC25A32_6_581,481,240,564,258,677,5,67,697,31,674,619,1036
SLC25A37_6_582,3848,3466,4378,4012,2866,3514,4308,5535,2203,3214,3576,
2510
SLC25A43_6_583,1902,2589,2818,3044,2400,855,2923,1845,1130,2403,3327,2
412
SLC25A44_6_584,1717,1550,2169,1886,2386,1294,1348,1176,982,2535,1902,3
059
SLC25A4_6_585,702,595,781,551,956,970,649,527,682,395,741,677
SLC25A5_6_586,810,1332,944,988,189,252,1512,1087,1346,1299,498,760
SLC25A6_6_587,328,174,327,280,407,383,318,98,180,358,217,62
SLC26A10_6_588,3396,3971,3831,3773,5395,4393,2694,4426,2965,2670,4665,
3889
SLC26A2_6_589,1131,1129,1491,1523,1176,994,1385,994,1426,1227,2365,870
SLC26A3_6_590,3288,2798,3154,4266,2615,5769,2712,3463,3168,2138,1790,4
375
SLC26A4_6_591,2115,1887,2958,2631,2868,2523,2316,3182,2348,499,4051,14
08
SLC27A1_6_592,2347,3058,2897,3092,3666,3191,2684,3212,1571,2783,4639,2
946
SLC27A3_6_593,2166,1611,2784,3227,3545,954,3081,2983,1611,3042,2723,16
39
SLC27A4_6_594,1309,1137,1444,1562,1456,397,1782,1583,1131,1682,2006,12
36
SLC27A5_6_595,752,1752,1231,895,1138,934,2085,492,1011,692,224,1046
SLC28A2_6_596,7113,6380,6145,7267,6143,7193,6500,7589,4007,5409,6669,4
347
SLC29A2_6_597,1347,1047,1426,1266,739,1160,782,858,1137,1149,1271,928
SLC2A10_6_598,918,504,796,1088,50,681,360,337,571,524,618,516
SLC2A12_6_599,1404,2543,2140,2030,2052,1412,1625,2269,392,1626,1405,17
20
SLC2A13_6_600,1196,944,1457,1039,825,3217,1522,594,795,2290,769,1382
SLC2A14_6_601,2353,2113,2951,3116,3212,1455,2095,3277,1951,4962,2554,3
602
SLC2A1_6_602,3370,3697,4564,3791,3787,3802,4469,3306,3457,3005,4988,44
48
SLC2A2_6_603,1504,1403,1956,1844,735,1726,2360,1537,951,1052,3792,3603
SLC2A3_6_604,205,184,478,264,34,2,653,361,112,1315,818,146
SLC2A4_6_605,413,542,1057,632,172,65,446,39,975,641,315,813
SLC2A4RG_6_606,1052,829,617,1234,846,1331,912,784,761,470,586,1576
SLC2A7_6_607,2769,1942,2590,2391,3655,4986,2571,2310,1184,1031,3293,32
36
SLC2A8_6_608,936,710,303,562,658,77,19,1070,296,328,102,281
SLC30A10_6_609,2248,2456,2952,2830,3613,2819,1609,3080,2188,2963,3049,
3033
SLC30A1_6_610,3877,2964,4237,4084,2950,1838,4709,4883,4581,4467,4287,2
680
SLC30A3_6_611,56,15,201,179,0,0,0,0,513,69,2,0
SLC30A4_6_612,763,590,632,1049,574,831,83,921,1081,386,1594,163
SLC30A9_6_613,1871,1240,2222,1964,1657,2492,2341,1424,265,597,2858,221
4

SLC31A1_6_614,467,576,913,867,143,265,1118,1572,798,407,1394,523
SLC31A2_6_615,1893,2267,2237,2275,1123,2531,1368,1028,804,1967,3645,23
47
SLC32A1_6_616,223,283,817,272,66,636,1560,668,5,125,100,36
SLC35A3_6_617,13728,12659,15595,13812,15653,13798,14295,16762,11242,12
030,15122,13335
SLC35A4_6_618,110,412,411,374,30,261,464,680,352,338,32,339
SLC35A5_6_619,3499,2962,3690,4330,1932,2304,5234,2307,3693,3399,5947,2
570
SLC35B1_6_620,6167,6248,8374,6593,4235,8381,4595,5950,5465,4324,9578,5
458
SLC35B2_6_621,702,424,511,767,217,2458,465,93,549,423,2028,363
SLC35B4_6_622,4316,3713,4884,4661,4219,5217,5227,3204,2793,3013,7948,4
765
SLC35D1_6_623,3622,4086,4694,4171,4390,5837,3588,6858,2789,4007,4791,4
222
SLC35D2_6_624,9884,11110,11728,10427,5868,9691,11190,11830,8230,14361,
11370,9005
SLC35D3_6_625,489,497,669,947,559,245,72,2073,204,274,985,99
SLC35E1_6_626,313,81,215,415,377,221,914,950,250,0,577,1035
SLC35E3_6_627,3321,2379,4634,3413,4245,5086,5015,5702,2865,3245,4712,2
486
SLC35E4_6_628,197,459,434,357,201,1118,18,63,191,720,49,485
SLC35F1_6_629,2599,3217,3775,3891,2550,3125,3017,2556,1268,2898,5063,2
513
SLC35F2_6_630,673,855,751,595,70,655,269,830,15,710,743,196
SLC35F3_6_631,760,570,1190,762,1331,722,408,1364,572,713,632,2208
SLC35F4_6_632,1982,1435,2231,2030,1420,2552,3225,5067,1075,2567,2536,2
426
SLC35F5_6_633,1190,1561,1884,2274,2925,1244,1262,2465,1133,1858,2633,1
726
SLC36A1_6_634,428,389,421,251,416,17,1727,0,10,85,207,299
SLC36A2_6_635,1810,2660,3150,2446,2772,3657,2093,3149,2914,3375,2648,2
566
SLC36A4_6_636,3610,2930,3489,3976,1980,3541,2815,5934,2648,4920,5851,3
401
SLC37A1_6_637,8444,7660,10135,9009,8560,10805,9940,7966,8634,11034,132
54,11574
SLC38A2_6_638,4561,4005,4788,5005,4400,4931,1700,3455,2570,5075,4415,4
108
SLC38A3_6_639,3604,4981,4749,5148,2856,3508,7416,4135,2686,3697,3595,6
095
SLC38A5_6_640,479,302,561,471,14,530,196,251,27,174,1116,772
SLC39A1_6_641,1629,901,1422,1841,823,1712,450,1654,455,403,3384,1909
SLC39A2_6_642,1122,510,837,963,228,307,275,137,1684,156,957,961
SLC39A9_6_643,580,517,472,686,43,681,340,315,497,58,140,439
SLC3A1_6_644,1496,1702,1001,1665,957,346,1122,1955,1633,1318,1551,1551
SLC40A1_6_645,1085,625,985,1141,1318,239,416,2797,910,1378,871,1425
SLC41A1_6_646,2061,1670,2224,2154,2045,1634,2067,3554,2116,847,2558,18
05

SLC43A2_6_647,498,179,323,569,333,38,163,72,182,639,538,77
SLC44A1_6_648,968,859,752,748,1483,1387,259,509,527,579,1966,1240
SLC46A2_6_649,1493,1480,1497,1844,3321,1160,1328,1189,879,1337,2515,24
16
SLC4A1AP_6_650,489,421,1041,539,137,600,491,673,194,77,1118,429
SLC4A1_6_651,1448,1853,1536,1798,662,1382,2101,2455,1827,1539,3996,217
1
SLC4A7_6_652,2556,1982,3195,2783,1702,3129,1670,1574,478,1736,4908,205
8
SLC4A9_6_653,1382,993,1059,1430,832,1632,2271,2922,696,1699,1859,920
SLC5A11_6_654,1475,1556,1904,1470,764,1325,1872,1608,1330,2082,1821,94
9
SLC5A1_6_655,1076,1284,1307,1606,2187,2384,2947,1682,963,3219,2689,179
2
SLC5A2_6_656,191,640,314,854,1478,524,598,406,132,968,2469,402
SLC5A3_6_657,3367,3691,3329,4226,5524,3751,5142,3229,2136,2570,3661,20
98
SLC5A4_6_658,1655,1312,1658,1537,1484,2512,1980,766,943,2894,1585,1745
SLC5A5_6_659,399,248,296,505,576,95,167,49,14,490,159,449
SLC5A6_6_660,112,10,147,324,420,0,0,0,108,0,0,1
SLC5A7_6_661,2952,3127,3313,3227,5073,2776,3138,1771,2613,1100,3405,24
98
SLC5A8_6_662,2780,3269,3762,2760,3415,2894,4537,2122,3338,3707,5590,47
34
SLC6A11_6_663,1348,1409,2050,2145,995,1121,1847,2128,1060,2085,2390,40
08
SLC6A14_6_664,10420,8013,9851,9965,10530,13076,9105,11611,8008,11528,1
2028,9397
SLC6A16_6_665,1359,958,1165,1338,211,930,982,1168,1502,598,1113,578
SLC6A19_6_666,2567,2389,3515,3448,1367,1563,1412,2630,2183,3277,4653,1
350
SLC6A1_6_667,1615,1479,1441,1585,849,1690,1087,1041,208,951,1348,1176
SLC6A3_6_668,3886,3159,3706,4255,3096,7627,6095,5589,3827,3180,5152,33
89
SLC6A4_6_669,340,421,261,239,241,771,381,5,662,266,213,19
SLC6A7_6_670,789,869,1040,778,199,805,419,817,255,335,812,815
SLC7A10_6_671,2946,2996,4248,4010,2839,4567,2140,1243,3334,3661,4874,2
407
SLC7A11_6_672,1608,1451,1952,1461,2369,851,3285,1666,1651,1954,2498,96
9
SLC7A13_6_673,1228,1941,1945,1815,1249,2992,2523,2185,1103,1410,1106,3
065
SLC7A1_6_674,1682,2740,3165,2810,1520,3528,2272,2767,1783,2850,4033,26
61
SLC7A4_6_675,648,879,1125,739,862,1524,1368,617,1275,1848,1456,813
SLC7A5_6_676,3501,3952,5428,4553,3280,5483,4892,4093,3644,5183,4074,53
68
SLC8A2_6_677,826,347,528,435,588,6,133,121,52,105,475,449
SLC9A1_6_678,1189,559,1655,1838,1410,706,1004,948,1399,3333,1100,1449
SLC9A2_6_679,3018,2152,2806,2681,3465,3717,2796,4628,3437,4513,3766,31

63
SLC9A3_6_680,1103,1163,1363,981,344,1206,1166,462,503,836,610,181
SLC9A3R1_6_681,908,983,1513,1287,3193,257,2096,1010,1765,637,783,354
SLC9A4_6_682,1557,1789,1847,1757,354,1472,1031,1219,3026,2312,2568,142
2
SLC9A5_6_683,816,877,634,559,996,1857,277,232,1428,54,1076,960
SLC9A7_6_684,2458,2666,2839,2560,2232,2072,5147,1920,1610,3718,1528,24
90
SLC9A8_6_685,2748,2434,2746,2884,1735,3361,3457,4506,2381,2936,3824,28
90
SLC9A9_6_686,2785,2464,3267,2458,1480,3782,3989,2015,3079,4525,2161,39
96
SLCO1B1_6_687,10202,8647,11639,10877,12957,12119,11348,8890,9141,13508
,12263,10464
SLCO1B3_6_688,3466,3167,3804,3266,2207,3526,2522,3080,4170,3913,4610,4
756
SLCO2A1_6_689,4540,4593,5074,5398,3249,6560,4930,5931,4733,5759,5073,6
745
SLCO4A1_6_690,1280,1082,816,1445,1167,881,1028,622,1289,2262,2354,2145
SLCO4C1_6_691,722,364,661,678,253,0,1156,507,207,342,683,340
SLCO6A1_6_692,3412,2235,3772,3785,3081,4268,3209,2996,2856,2927,5754,3
495
SV2A_6_693,1053,787,798,556,963,1990,391,1984,455,794,915,1247
TAP1_6_694,215,137,158,127,493,1184,152,14,805,0,19,812
UCP1_6_695,5223,5489,6771,4769,7190,3596,4677,6378,6934,10634,9953,515
7
ABCA12_6_696,1438,1689,2417,2385,1938,1765,1949,3045,1547,1524,3403,15
68
ABCA2_6_697,434,265,479,413,1914,646,19,1109,477,361,354,331
ABCA5_6_698,2654,1888,2479,2970,4326,2401,3045,3338,2411,4996,2795,271
4
ABCB4_6_699,2264,2582,3341,2230,3547,2815,1741,2561,791,2507,4776,2510
ABCB9_6_700,1711,1651,2361,1417,1025,1511,1141,2370,2463,534,1525,1245
ABCC10_6_701,3864,3771,3533,3990,4972,4399,5588,2809,4878,4283,3404,41
23
ABCC11_6_702,667,255,587,624,2,13,439,684,385,317,150,538
ABCC1_6_703,1459,1491,1174,1615,538,3421,1336,2521,358,1554,2872,1144
ABCC3_6_704,1126,1277,1447,1424,1100,2594,369,1276,1473,579,2417,1413
ABCC4_6_705,807,841,1593,1187,794,649,1935,1822,2185,2092,3127,1415
ABCC5_6_706,2501,2100,2373,2249,2153,1034,2055,2444,2938,1964,2921,122
2
ABCC9_6_707,1603,1108,2257,2180,1083,246,2513,3021,680,2379,2850,2206
ABCD3_6_708,2859,3723,3541,4178,3853,4257,3220,4734,1720,3465,3832,415
7
ABCE1_6_709,463,534,693,906,1228,1121,1914,1463,412,912,766,102
ABCF1_6_710,2754,2409,2977,2938,3271,2112,2915,1760,2356,2435,3592,108
0
ABCF2_6_711,1077,1114,1063,915,696,306,1236,893,321,872,2300,942
ABCG1_6_712,570,849,705,595,1360,540,661,1506,364,243,234,157
ABCG4_6_713,703,408,366,413,196,157,685,584,1128,56,890,799

ANXA11_6_714,567,1346,983,685,961,495,2045,2357,858,353,187,255
ANXA13_6_715,1803,1551,2212,1939,1214,2515,2614,1906,1512,3645,3234,32
36
ANXA2_6_716,2090,1409,1593,1843,1918,1370,2106,2302,2255,1726,1510,127
9
ANXA6_6_717,602,780,695,811,1096,8,1102,932,605,308,249,779
ANXA7_6_718,951,757,1415,1234,973,805,2046,1041,965,494,482,975
ATP11A_6_719,1237,931,1098,1643,252,654,2563,1519,850,383,1753,3160
ATP11C_6_720,2086,2314,2861,3285,2184,3620,3706,2327,2857,1924,3470,22
62
ATP12A_6_721,4115,3726,4673,4180,4831,4269,4844,3853,6026,6658,4973,52
65
ATP1A1_6_722,2863,2039,2505,2394,4562,2665,3517,1913,2723,2220,2872,31
66
ATP1B4_6_723,1753,1270,1453,2364,1358,1235,1303,1005,685,799,1551,1097
ATP2A1_6_724,681,345,984,698,498,1015,642,496,162,335,920,51
ATP2A2_6_725,2903,2879,3862,3002,5799,2814,3170,2216,1034,2451,3485,21
82
ATP2A3_6_726,1504,1443,1513,1726,2922,3597,2092,1246,1227,1650,2356,12
48
ATP2B1_6_727,6456,6706,7850,6736,5760,5403,8811,5536,5494,4921,9671,79
75
ATP2B3_6_728,290,391,714,280,841,854,9,689,3,893,583,1194
ATP2B4_6_729,2018,1811,2187,1651,1589,2542,3582,1938,1508,2805,3293,12
48
ATP2C1_6_730,603,393,494,305,624,522,690,420,775,961,601,515
ATP5A1_6_731,1242,1265,1440,1155,1516,1981,1263,996,701,873,1935,880
ATP5C1_6_732,855,1074,1287,669,567,484,368,579,710,1019,279,852
ATP5D_6_733,966,749,901,610,667,646,222,1331,932,211,1074,1393
ATP5E_6_734,0,102,0,2,383,0,0,0,0,0,0,0
ATP5G1_6_735,757,1255,672,1077,849,1912,810,1018,703,1223,2210,529
ATP5G2_6_736,1949,2100,1959,1358,1346,579,1191,1646,950,1682,2172,90
ATP5G3_6_737,1549,1710,999,1560,1469,917,890,411,1394,1879,1823,3443
ATP5H_6_738,783,1009,1372,1166,639,1199,453,1880,1095,1375,315,627
ATP5J_6_739,3250,3722,3624,3177,3272,3389,4096,3282,3591,3753,3963,505
5
ATP6V0A1_6_740,2486,2087,2186,2512,1496,3102,2190,3672,3100,2566,3716,
1740
ATP6V0A4_6_741,1628,1526,1862,1763,2841,717,4320,1538,3006,1787,1113,1
803
ATP6V0B_6_742,2961,3559,4038,4797,3654,4568,5010,6057,2348,4623,4239,3
594
ATP6V0C_6_743,1040,700,1410,1772,262,3211,2172,2167,430,2669,3116,2775
ATP6V1C2_6_744,342,76,354,260,562,175,358,161,44,188,0,1
ATP6V1E1_6_745,565,777,1179,1010,1441,648,766,858,167,1363,1179,731
ATP6V1F_6_746,211,217,323,486,11,317,499,163,111,2,1269,415
ATP6V1G2_6_747,618,226,731,343,153,789,556,1135,723,841,434,920
ATP6V1H_6_748,5302,6327,7408,6088,4095,4521,5857,9391,3417,6983,9792,3
709
ATP7B_6_749,701,846,845,558,549,107,323,534,485,1187,305,1548

ATP8A1_6_750,2426,1687,3114,3004,4538,2271,1636,3302,2561,3572,3927,37
11
ATP8B2_6_751,2425,2722,2886,2922,3064,3148,2902,2871,4237,2052,3806,21
47
ATP8B3_6_752,1712,1336,2201,1874,2008,1442,1920,3197,2104,2187,2363,37
46
ATPAF1_6_753,5089,3450,5499,4442,5816,5530,6742,5737,3431,5139,5708,44
99
GC_6_754,1615,1138,2492,1709,1194,680,2153,625,1087,1200,784,716
SERINC5_6_755,3538,3536,4610,3878,2675,3882,5178,2321,3035,4548,5778,4
544
SLC10A3_6_756,835,823,993,958,3342,544,1600,1774,333,882,1152,1251
SLC11A2_6_757,872,1057,1100,1660,1101,1430,1466,435,1860,1402,1081,111
0
SLC12A1_6_758,7822,7072,8202,8167,7670,11469,9324,7499,6351,9281,10333
,8050
SLC12A3_6_759,2466,3276,3179,3054,3285,3706,3495,3723,2051,3216,5096,2
066
SLC12A4_6_760,4259,3999,3900,4714,3007,5564,5125,4128,3918,4016,5200,4
214
SLC12A5_6_761,3376,2934,3668,3920,2803,4527,2806,3547,3008,3571,4749,2
213
SLC12A6_6_762,937,934,1260,997,2047,943,1218,2228,196,570,1811,762
SLC12A8_6_763,4715,5222,6084,4955,5231,3999,5340,5736,5346,3740,6581,5
310
SLC13A2_6_764,619,232,375,848,209,256,184,235,161,39,155,292
SLC13A3_6_765,1024,539,936,1407,916,2131,69,584,1383,1218,1080,105
SLC13A5_6_766,499,1295,1588,989,1265,714,1238,832,980,207,413,775
SLC14A1_6_767,3141,2379,2666,2345,3538,2788,1534,1625,2017,2601,3310,2
369
SLC14A2_6_768,1967,2703,3212,2261,1093,798,1967,4381,3316,2208,2559,30
08
SLC15A2_6_769,5963,5664,6561,6713,5894,5351,4351,6807,6209,3839,7694,5
367
SLC16A1_6_770,2012,1765,2486,2070,595,2176,2615,1867,1406,1154,2396,54
1
SLC16A3_6_771,2224,1945,2454,3204,3151,1216,3111,2008,2518,2903,2515,1
503
SLC16A4_6_772,3451,4119,4958,3874,3566,4947,6069,4115,2811,4955,6327,3
029
SLC16A6_6_773,5127,4404,4666,5013,4960,3681,4716,4377,3419,5658,8261,4
869
SLC17A3_6_774,3107,3459,4330,3754,4688,2205,6933,4408,2965,3455,3774,4
031
SLC17A8_6_775,1984,1193,2400,2484,1724,1561,2054,997,1885,3412,787,831
SLC18A1_6_776,574,638,1264,602,1152,6,480,781,622,258,1375,250
SLC19A1_6_777,603,796,514,784,345,172,882,777,107,2140,995,317
SLC1A2_6_778,208,150,339,151,79,3,38,130,146,5,937,509
SLC1A3_6_779,871,561,608,1251,1218,13,233,902,146,874,570,492
SLC1A4_6_780,1343,1234,624,734,540,858,603,160,484,396,1791,810

SLC1A5_6_781,2765,3078,3702,3275,2225,3448,3145,1711,2157,4010,2652,29
99
SLC22A12_6_782,772,652,514,425,809,740,677,89,687,674,507,230
SLC22A17_6_783,1385,1048,1618,1607,1139,1069,2863,1753,767,1614,2407,6
93
SLC22A18_6_784,1088,339,1078,975,837,1043,599,454,638,449,411,1244
SLC22A1_6_785,1242,1253,1383,1469,397,566,1889,734,697,728,1589,1091
SLC22A23_6_786,1846,1303,1788,1816,3487,2403,1495,1309,1975,1902,1407,
1734
SLC22A6_6_787,3462,2540,3206,3074,4324,3380,3694,3903,1607,2592,4402,5
939
SLC22A7_6_788,9,0,0,7,0,0,0,0,0,0,0,0
SLC22A8_6_789,836,838,1009,866,447,678,242,887,105,61,1546,529
SLC23A3_6_790,1070,541,881,1344,229,444,1536,1457,166,880,1054,798
SLC24A2_6_791,2301,2101,2453,2385,3268,2136,2330,2243,1536,1943,7527,2
222
SLC24A4_6_792,2127,1254,2882,2388,2475,711,1558,2046,2533,2073,2338,12
53
SLC25A11_6_793,2482,2760,2735,3315,1883,3116,3933,4407,3145,3425,2173,
3627
SLC25A13_6_794,4700,4600,4004,5273,2375,6742,5439,3748,4661,2852,4468,
7087
SLC25A14_6_795,5040,4940,6316,5764,5064,6640,5903,4544,5048,5391,7604,
7204
SLC25A19_6_796,290,644,908,758,414,254,223,375,156,195,110,947
SLC25A21_6_797,3186,2584,3355,3019,3776,3828,5003,3586,2693,2000,2489,
2448
SLC25A22_6_798,211,200,249,149,308,29,320,179,58,7,35,137
SLC25A26_6_799,407,268,314,577,61,767,117,326,704,1078,198,602
SLC25A27_6_800,6042,5908,6121,5971,5890,11695,6686,8925,7512,8587,4714
,7932
SLC25A3_6_801,869,737,834,606,232,1545,592,3054,654,4451,1643,3202
SLC26A11_6_802,3518,3112,3891,3614,3680,1782,3876,4743,4488,4389,6599,
3028
SLC26A1_6_803,888,950,883,1231,897,1286,1357,576,812,440,744,1191
SLC26A5_6_804,1323,931,1255,1257,1876,967,892,1822,472,1573,2989,463
SLC26A6_6_805,2933,2483,3979,3354,3576,1691,3994,1854,2971,4620,5569,3
290
SLC26A7_6_806,1384,1081,1772,918,1945,865,966,1240,577,1089,1223,586
SLC26A8_6_807,3707,3993,3887,4061,3306,5919,5394,3809,3908,4845,4368,2
951
SLC26A9_6_808,765,495,1133,667,143,302,1149,983,999,1333,514,598
SLC27A2_6_809,4438,4499,4970,5843,3000,3201,5578,5457,3103,6403,6662,7
924
SLC27A6_6_810,5188,5790,6259,5600,4056,4836,4522,5055,5176,7710,7695,5
817
SLC28A3_6_811,910,1025,1078,1140,2253,706,568,966,1128,826,1928,767
SLC29A1_6_812,1843,1971,2766,2204,1394,3326,1219,1550,2848,1760,2019,2
383
SLC29A3_6_813,1443,944,1508,1045,531,598,1315,2290,475,2157,1678,220

SLC29A4_6_814,1174,947,703,947,2292,528,1196,601,713,1311,535,771
SLC2A11_6_815,408,351,322,363,232,32,296,113,132,185,710,253
SLC2A5_6_816,987,592,483,636,1182,212,596,1409,338,1384,1202,972
SLC2A6_6_817,128,153,148,361,0,29,78,354,53,337,198,427
SLC2A9_6_818,2446,1195,1878,1826,2746,2012,2426,3218,2041,2261,1924,95
2
SLC30A2_6_819,253,300,333,792,67,655,774,1215,1363,1117,1052,106
SLC30A6_6_820,818,918,848,1306,1209,484,2027,1751,1113,432,1420,1206
SLC30A7_6_821,1758,2288,2732,1979,3790,1576,4783,5140,1209,1722,2206,1
212
SLC30A8_6_822,5658,5465,6889,6017,6776,7146,6800,3908,4696,7270,9369,6
332
SLC33A1_6_823,2565,1949,2864,3116,2772,1325,5497,2152,1895,1798,4626,2
298
SLC34A1_6_824,460,350,603,437,516,651,836,128,699,607,148,409
SLC34A2_6_825,1256,1176,2026,1578,2816,1851,785,1231,1368,2053,2062,18
39
SLC34A3_6_826,1593,1259,2019,2045,1898,1493,1873,1643,2277,1476,965,11
13
SLC35A1_6_827,4056,2563,4297,3534,4431,2860,4322,2754,3358,4855,4581,4
033
SLC35A2_6_828,1089,1546,1589,1582,1426,2128,769,1186,817,1802,2477,201
3
SLC35B3_6_829,1728,1436,2336,1979,818,2984,1918,2636,1021,1652,3721,27
90
SLC35C1_6_830,145,304,405,224,9,392,240,66,442,927,163,2
SLC35C2_6_831,2984,2510,2946,2553,3297,2881,2223,1395,3771,3476,4866,1
780
SLC35E2_6_832,507,457,893,917,390,577,377,191,130,336,356,576
SLC36A3_6_833,5019,4660,4865,5331,3791,4969,6594,6258,3861,5063,5116,5
564
SLC37A2_6_834,973,412,853,1068,1605,348,1123,725,90,1233,1193,1035
SLC37A3_6_835,1441,1170,2028,1333,450,1109,2008,3001,1072,987,1930,152
7
SLC37A4_6_836,1749,1391,1826,1922,911,2776,1062,778,1381,2044,3123,102
0
SLC38A11_6_837,5093,5091,6165,5493,3144,7046,6761,5365,3783,6794,7284,
5236
SLC38A1_6_838,1150,853,1721,1272,1959,2090,355,1950,1310,939,3373,1594
SLC38A4_6_839,3089,2475,3611,3737,4278,5426,2537,2257,2131,3572,8150,3
269
SLC38A6_6_840,899,356,1152,749,151,991,1173,335,402,1985,1124,1573
SLC39A10_6_841,3267,3974,3135,3896,3899,6251,4610,2793,3446,4060,4866,
4061
SLC39A11_6_842,261,257,93,404,144,1063,68,98,6,1058,992,453
SLC39A12_6_843,2807,3086,4437,3016,1780,2835,3417,3803,1860,4581,1911,
2780
SLC39A13_6_844,2980,2532,3369,3676,3163,2079,2793,1901,3268,3182,3073,
3649
SLC39A14_6_845,10358,9195,9913,10867,12722,8233,10353,10307,7914,13122

,14039,6737
SLC39A4_6_846,980,520,897,634,315,1634,1225,1035,1460,103,697,184
SLC39A5_6_847,664,708,798,836,1580,269,516,654,593,423,308,221
SLC39A6_6_848,3562,2968,3788,3328,3568,5225,2889,4526,1792,3052,6695,4
924
SLC39A7_6_849,1277,1413,1956,1718,1725,1505,453,1249,1320,2594,1951,17
09
SLC39A8_6_850,3615,4033,4673,4184,4025,4414,3097,3918,4152,2376,3588,1
435
SLC3A2_6_851,942,537,900,848,936,922,788,1652,1363,1847,2844,4056
SLC43A1_6_852,802,896,763,477,222,920,1028,809,997,419,584,1110
SLC44A3_6_853,2828,2320,2695,3037,2546,2643,2704,3631,1978,2195,3377,3
720
SLC4A10_6_854,2884,1974,3249,2439,3495,2817,2203,3335,3116,1509,2731,2
533
SLC4A11_6_855,291,153,275,311,792,192,409,304,977,357,27,330
SLC4A2_6_856,1728,1379,2860,2413,2789,1781,3452,1898,2411,2220,1679,22
19
SLC4A3_6_857,1638,1074,1591,1392,280,3129,1778,1173,1732,714,1157,1441
SLC4A4_6_858,994,724,886,1036,276,1465,1264,567,896,403,1520,2425
SLC4A8_6_859,2237,2126,2512,1960,1815,2056,2102,1842,985,1180,3759,320
7
SLC5A10_6_860,1146,1647,1289,2006,2095,3235,657,1642,1409,808,764,1069
SLC5A9_6_861,5495,4776,6748,5420,4915,4683,5796,5847,6325,8054,4468,56
44
SLC6A12_6_862,3971,2709,3380,4004,1477,4573,3018,5141,2277,4757,3787,4
973
SLC6A13_6_863,1445,1759,1808,1856,1827,2615,2672,952,882,1560,1760,125
1
SLC6A15_6_864,20803,17936,21107,20648,16439,24041,23445,19734,12982,27
579,26824,14382
SLC6A2_6_865,3080,2379,3488,3083,2997,3587,3692,1803,3200,1743,3834,16
99
SLC6A6_6_866,898,872,1266,1659,621,2454,430,1549,145,608,1793,2446
SLC6A8_6_867,2600,2009,1997,2130,1994,2428,2246,2338,1681,4710,2160,14
58
SLC6A9_6_868,920,657,658,628,869,2233,984,1157,756,1335,1865,663
SLC7A2_6_869,2780,2907,3112,2992,2684,6104,3260,4002,3870,4156,4724,25
45
SLC7A3_6_870,566,300,531,275,297,1823,0,1,402,7,10,771
SLC7A6_6_871,626,449,543,640,182,528,66,516,533,363,872,44
SLC7A7_6_872,850,1025,1017,1550,802,951,62,2052,1036,1352,2368,1426
SLC7A8_6_873,177,76,431,200,0,58,1426,134,4,43,395,634
SLC7A9_6_874,1731,1315,1580,1389,2254,519,2362,317,628,1574,1713,1284
SLC8A1_6_875,1574,1713,2487,2121,473,395,773,3745,1339,2765,1768,1376
SLC8A3_6_876,1416,1400,2346,1860,1450,1844,2181,2806,2882,1946,4110,23
95
SLC9A3R2_6_877,1266,812,1112,1237,786,1100,2647,918,377,623,1425,604
SLC9A6_6_878,441,360,457,624,1774,109,1313,840,127,6,97,357
SLCO1A2_6_879,3247,3020,3410,3059,3589,3611,3619,2514,2725,5890,5169,5

859
SLCO1C1_6_880,1427,1402,1999,1538,3092,1803,674,1744,1564,2563,1553,2813
SLCO2B1_6_881,10083,8980,11138,11183,11682,10354,10198,11936,12064,11004,14899,7490
SLCO3A1_6_882,649,182,445,409,710,60,654,494,97,1904,458,910
SLCO5A1_6_883,4891,4794,5108,5470,4262,4085,4521,7265,3456,7010,6702,5713
ABCB5_6_884,2335,2365,3224,1797,2086,2664,2412,3200,2286,1834,3370,2395
ABCC6_6_885,622,614,683,848,419,75,967,548,739,935,298,400
ATP1A4_6_886,2379,2018,2750,2881,1809,2205,4005,1086,1206,470,3830,5292
ATP5J2_6_887,958,695,845,699,376,453,865,467,736,1532,895,1295
ATP5S_6_888,6450,6078,8619,7659,6573,7659,8474,5771,7433,6741,10744,7381
ATP6V1G3_6_889,468,123,266,207,2365,170,34,642,0,110,68,94
ATPIF1_6_890,896,1179,642,1146,40,1154,832,3850,476,1341,510,925
SLC10A7_6_891,799,601,1177,657,844,672,284,999,615,494,1065,802
SLC28A1_6_892,586,618,714,702,1034,1383,1281,650,962,8,533,1015
SLC30A5_6_893,2669,2795,2512,2663,2159,3312,2407,2842,2916,2217,5238,1936
SLC39A3_6_894,4910,4888,5725,5279,4618,2312,7116,5712,3773,6037,4449,5601
ABCA10_6_895,1462,1166,1496,1152,1067,2343,608,1102,812,988,2675,1284
ABCA13_6_896,1235,1649,1873,2046,1292,1932,3637,1326,603,698,2059,3632
ABCA1_6_897,3576,3475,3929,3334,4504,3901,3422,3223,3318,3001,3910,3493
ABCA3_6_898,1125,1342,1450,1419,2216,1029,1040,2332,2342,1310,2123,1054
ABCA4_6_899,7519,7373,8286,9996,6684,9989,9543,9011,9067,10676,12345,6876
ABCA6_6_900,908,682,971,1090,81,542,1057,999,1277,1225,891,750
ABCA7_6_901,828,640,699,1053,316,227,1852,1501,338,241,1409,312
ABCA8_6_902,3917,3516,5693,5575,4169,4267,4972,4337,3025,5081,8252,5425
ABCA9_6_903,5306,5495,5541,6137,6760,7338,5115,3480,3175,5602,7519,5142
ABCB10_6_904,1626,1473,1785,2060,907,1043,2781,2304,1101,1357,1865,1339
ABCB11_6_905,3172,3030,3091,2992,3808,1967,3639,2411,2477,3198,5144,4301
ABCB1_6_906,807,1055,1124,1305,474,1550,1004,679,275,1391,1459,672
ABCB6_6_907,4092,3192,4940,4051,3843,5986,4115,3972,6116,7240,6018,3015
ABCB7_6_908,819,924,940,1152,729,522,1895,2375,399,1524,839,1509
ABCB8_6_909,187,864,503,574,704,855,942,155,1084,426,52,156
ABCC12_6_910,4502,5381,5905,5561,3349,6878,3718,4455,5233,5863,5347,3650
ABCC2_6_911,920,325,944,539,184,555,886,433,180,232,1281,925

ABCC8_6_912,2492,2179,2539,2091,2387,2151,2326,5058,2817,2774,2821,126
3
ABCD1_6_913,516,189,933,688,535,32,1280,99,1099,76,872,599
ABCD2_6_914,2196,2565,2847,2690,1301,3363,1903,2021,2323,4293,1109,167
6
ABCD4_6_915,740,967,860,870,253,139,1957,264,343,1427,1310,877
ABCF3_6_916,637,361,859,948,577,1297,556,1292,890,651,256,602
ABCG2_6_917,1202,1940,1634,1501,1766,2352,2863,1152,1405,1594,1650,167
1
ABCG5_6_918,645,413,473,402,301,985,436,835,390,1141,553,1424
ABCG8_6_919,975,1475,1961,1730,1464,3289,1330,2388,1853,1447,2039,1316
ANXA10_6_920,1199,1788,1848,1478,637,2050,1099,3562,603,3608,3094,2628
ANXA1_6_921,6090,4465,5508,5266,5406,2804,6191,5845,4529,7074,6183,591
2
ANXA3_6_922,2952,3017,3644,4069,3324,3816,4714,4318,2668,3245,3925,325
8
ANXA4_6_923,4082,3160,3964,4240,6911,3511,3167,3957,3868,4186,8434,436
0
ANXA5_6_924,705,956,1314,1038,651,2397,1201,1991,859,1014,1950,2093
ANXA8_6_925,2790,2453,2851,2416,3178,2492,2466,2935,1490,3954,2965,142
7
ANXA9_6_926,1456,1919,1661,1633,212,811,1114,1784,1110,1814,1707,1934
ATP10A_6_927,1131,1048,1000,1521,1852,778,1001,300,1480,288,676,1735
ATP10B_6_928,1636,956,1965,1724,785,1071,1552,2737,1241,580,2216,677
ATP10D_6_929,4022,4313,4495,5442,4182,4755,4419,7354,3486,7700,6184,53
52
ATP11B_6_930,1469,1184,1362,1425,2129,848,1229,1926,3318,2849,3702,107
2
ATP1A2_6_931,925,1021,1349,1129,517,565,1332,1577,936,1376,1572,463
ATP1A3_6_932,1514,1611,1919,2331,1639,2798,2182,1969,1476,1022,2099,28
50
ATP1B1_6_933,5701,5609,6333,6792,4205,7094,6499,5957,3639,5083,5173,56
96
ATP1B2_6_934,533,379,618,563,137,33,1318,1595,458,252,588,678
ATP1B3_6_935,1185,458,948,869,190,268,943,565,1066,369,1374,461
ATP4A_6_936,2870,3361,4160,3276,3637,2801,4300,1807,4074,5326,3777,290
0
ATP4B_6_937,233,247,590,578,35,78,482,2,732,422,423,271
ATP5B_6_938,440,386,585,473,21,928,200,621,495,792,98,1489
ATP5F1_6_939,0,0,0,0,0,0,0,0,0,0,0,0
ATP5I_6_940,414,218,534,397,621,1438,1084,346,173,259,67,354
ATP5L2_6_941,5976,4780,6758,6131,4327,10468,6602,7214,4700,6050,8306,5
063
ATP5L_6_942,5976,4780,6758,6131,4327,10468,6602,7214,4700,6050,8306,50
63
ATP5O_6_943,2230,1987,2470,2307,620,2767,1965,1711,1724,1130,2298,2619
ATP6AP1_6_944,884,940,636,1035,1209,582,247,1236,260,1095,2073,804
ATP6AP2_6_945,3089,3452,3064,2972,2282,1798,3830,5039,3316,2648,2858,1
862
ATP6V0A2_6_946,12879,11905,14777,13352,11818,16455,16241,16981,14396,1

5492,12989,13393
ATP6V0D1_6_947,573,498,561,775,719,2170,186,1169,906,425,968,80
ATP6V0D2_6_948,2621,2120,2355,2491,3160,6210,3144,2212,2209,2232,2973,
2349
ATP6V0E1_6_949,7109,6521,8634,7764,8580,8050,8142,8194,5477,10769,1001
9,7444
ATP6V1A_6_950,549,279,446,645,497,0,0,296,11,4,1036,804
ATP6V1B1_6_951,790,1092,1016,1152,2538,1067,285,1691,260,1763,1652,708
ATP6V1B2_6_952,395,1199,647,744,1120,502,935,724,627,1312,1210,614
ATP6V1C1_6_953,3626,3856,4819,3817,3140,3545,3827,3627,3388,2898,4271,
3297
ATP6V1D_6_954,2238,2242,3016,2450,3051,2801,2895,2640,647,1846,3356,28
13
ATP6V1E2_6_955,1298,917,1365,1393,1311,677,1403,2008,784,1488,771,1209
ATP6V1G1_6_956,887,348,542,382,62,502,426,102,285,981,473,650
ATP7A_6_957,3273,2176,2822,2439,4375,2140,2936,3382,2640,2448,4140,236
2
ATP8A2_6_958,9354,8434,12237,11197,13421,10457,10884,12053,9053,8447,1
5034,9016
ATP8B1_6_959,1138,1188,1175,1255,907,1995,933,1770,241,801,1461,795
ATP8B4_6_960,4565,5649,5194,4735,5744,5898,5528,6615,4936,8899,7334,42
50
ATP9A_6_961,1433,1491,1289,1142,3523,2116,918,1557,463,1454,1513,1097
ATP9B_6_962,1456,1838,1554,1538,831,1350,2950,2936,1630,2562,1871,1840
ATPAF2_6_963,918,688,627,763,1363,893,705,512,621,136,1960,952
FLVCR1_6_964,3005,3730,3114,4151,3156,2229,3225,4223,3190,5955,3465,31
63
RHBG_6_965,1053,1555,1874,1590,1634,941,3039,2015,2140,1820,1435,1893
RHCG_6_966,825,973,1601,1283,807,1993,799,237,574,3330,1937,1076
SLC10A1_6_967,1425,1817,1731,1862,1798,2931,3145,1140,497,3849,2185,30
75
SLC10A2_6_968,1322,1155,949,1546,405,1274,1438,1912,617,83,2586,1607
SLC10A4_6_969,4685,3622,4583,4892,5077,3574,5979,5643,4164,3309,5237,4
245
SLC10A5_6_970,3037,3554,3456,3151,2922,5163,2899,3057,1780,3490,4217,2
834
SLC10A6_6_971,1159,954,1684,1784,2106,1150,876,810,1622,1985,1158,672
SLC11A1_6_972,2751,2737,2878,3007,2087,3301,4020,3319,3033,3822,3514,3
215
SLC12A2_6_973,1050,977,1169,829,682,2510,520,1313,381,2531,3069,1331
SLC12A7_6_974,713,1285,1138,876,546,1863,1592,1024,1414,416,1252,1083
SLC12A9_6_975,135,186,175,61,2,0,0,459,3,0,0,0
SLC13A1_6_976,3047,3309,2911,3660,2705,4035,1826,1885,1130,3123,3713,1
061
SLC13A4_6_977,3180,2386,3160,2900,4247,4217,3410,2214,3569,2217,4368,2
899
SLC15A1_6_978,6602,7334,8873,9551,10614,8036,6768,10588,4611,10200,127
31,9466
SLC15A3_6_979,5988,5562,6854,6306,4909,5347,5595,5025,5609,5899,6897,4
774

SLC15A4_6_980,729,661,1106,896,740,470,841,1808,1155,1584,2624,1394
SLC16A10_6_981,6708,6663,6935,7259,7214,6802,3404,6614,6781,6680,7578,
5530
SLC16A11_6_982,365,303,364,759,586,54,620,881,314,529,512,37
SLC16A12_6_983,4141,3707,5452,4646,2532,5726,5147,7395,2746,5198,5795,
4426
SLC16A2_6_984,2322,1943,2183,1822,2062,2050,3094,1791,1323,5071,3724,1
968
SLC16A5_6_985,332,325,151,369,978,39,633,20,31,130,995,1714
SLC16A7_6_986,3071,2772,2627,2518,2168,2056,1884,1390,2735,4302,2423,5
233
SLC16A8_6_987,6512,6355,6356,6244,8757,5574,6911,4873,5636,5728,5720,5
226
SLC17A1_6_988,2814,2519,2584,3215,2091,2915,3451,2824,3023,1837,3643,2
175
SLC17A4_6_989,574,413,430,891,359,1742,1340,461,187,812,671,31
SLC17A5_6_990,26129,25820,31123,31702,26711,31545,29821,27778,29287,38
776,41237,36900
SLC17A6_6_991,7630,6802,9045,8800,7919,9908,8502,10298,5209,9193,12526
,7650
SLC17A7_6_992,331,694,460,726,27,942,236,664,69,304,742,45
SLC18A2_6_993,724,493,611,497,657,1565,1266,198,229,2697,959,818
SLC18A3_6_994,11010,9444,9860,11388,11539,10714,7016,8593,5936,10878,1
1643,9554
SLC19A2_6_995,2615,2413,2713,2620,1400,1568,2876,2180,2347,3799,3033,2
518
SLC19A3_6_996,2261,1711,2433,2966,2399,1191,884,2564,2395,2520,2657,37
50
SLC1A1_6_997,10547,12376,12899,11889,7956,16205,10993,12211,15099,1008
0,10722,11210
SLC1A6_6_998,180,87,414,471,303,308,1926,13,2,3,634,28
SLC1A7_6_999,945,949,1093,1308,783,889,653,552,1220,1357,961,744
SLC20A1_6_1000,882,792,1043,740,902,124,1303,1476,881,1199,2023,2248
SLC20A2_6_1001,2129,2474,2413,3299,2160,2910,449,3349,1925,2524,3300,2
851
SLC22A10_6_1002,6532,6442,7208,6585,5236,6724,8847,8102,4525,9023,9902
,7913
SLC22A11_6_1003,2831,3889,3893,2919,1368,3136,3685,2682,2730,3784,3149
,2915
SLC22A13_6_1004,204,387,339,509,154,358,1615,0,1,0,235,451
SLC22A14_6_1005,1149,1019,986,1149,2310,1614,1202,2263,743,1019,1094,6
41
SLC22A15_6_1006,13188,12010,15767,15006,20006,12078,13732,14058,9150,1
6471,16100,14670
SLC22A16_6_1007,2120,1935,2420,1727,3450,1662,1907,2273,1609,2509,2596
,3817
SLC22A18AS_6_1008,623,755,1354,993,974,1149,1368,1148,211,1475,714,192
4
SLC22A2_6_1009,3286,3392,3704,3700,3632,4570,5371,2866,3804,3204,5962,
2755

SLC22A3_6_1010,738,840,663,1369,1051,1394,601,483,619,695,1447,783
SLC22A4_6_1011,2787,2885,4049,2824,2174,4620,2269,3827,3397,3685,4884,
4949
SLC22A5_6_1012,2554,2107,3292,2936,3077,3056,3097,4645,2929,5526,2967,
2561
SLC22A9_6_1013,2135,1863,1771,2680,1159,1786,2722,1838,1792,2807,4156,
1156
SLC24A1_6_1014,555,240,736,279,0,153,237,1073,1163,47,577,119
SLC24A3_6_1015,10697,9138,11008,10684,7073,11830,7729,8845,7567,14389,
15750,12338
SLC24A5_6_1016,8405,8187,9005,8933,10675,10951,8844,11676,6800,13473,1
0551,9907
SLC24A6_6_1017,2198,2453,2190,2383,1501,4732,1944,3710,1014,2847,4183,
2514
SLC25A10_6_1018,260,182,407,243,577,225,132,46,475,1,90,69
SLC25A12_6_1019,1987,1501,2033,2042,2236,1079,1296,1859,2185,2294,3293
,1793
SLC25A15_6_1020,2308,2313,2442,2902,1792,2090,4142,4861,2520,3249,3798
,3582
SLC25A16_6_1021,1292,1643,1613,1877,1509,415,1833,1301,2355,769,2126,1
264
SLC25A17_6_1022,8098,7771,10806,9541,6446,6420,10517,9261,9814,10864,1
1054,10705
SLC25A18_6_1023,1041,874,1766,2287,520,497,2486,583,1559,3423,1539,108
0
SLC25A1_6_1024,787,841,867,1124,348,1559,1483,1594,503,657,720,1321
SLC25A20_6_1025,1039,1245,1172,1523,467,703,638,1139,544,892,3141,1109
SLC25A28_6_1026,1652,1648,1243,1352,1875,1246,987,3405,1307,2483,1874,
2377
SLC25A2_6_1027,2294,2375,2796,3329,3120,1484,2557,3618,2437,4163,2619,
2336
SLC25A32_6_1028,4164,4406,5420,4108,1982,4866,5293,6421,3092,4852,9413
,6939
SLC25A37_6_1029,367,960,781,539,155,237,499,690,466,36,77,369
SLC25A43_6_1030,965,1248,1143,973,1003,1671,1597,1117,575,161,923,2333
SLC25A44_6_1031,3207,2200,3645,2753,1203,2025,1560,3534,4934,3580,3329
,3366
SLC25A4_6_1032,2163,1843,2923,2163,1821,2111,2564,3820,962,1862,2193,3
231
SLC25A5_6_1033,15,2,0,0,0,0,0,0,0,0,0,0
SLC25A6_6_1034,2140,2042,1991,1862,1768,2682,3224,1748,2197,2571,2778,
1967
SLC26A10_6_1035,1437,2484,2073,2437,2221,1808,1810,1035,1203,3998,847,
3169
SLC26A2_6_1036,1813,1388,1950,2164,3717,2124,1767,2899,1293,1608,1671,
3058
SLC26A3_6_1037,8708,8160,9759,10903,6676,11504,8697,11409,5205,11386,1
5797,10477
SLC26A4_6_1038,1736,1261,2259,1779,2289,2677,2819,1326,2523,753,1119,1
271

SLC27A1_6_1039,187,197,394,344,80,633,773,173,534,325,865,480
SLC27A3_6_1040,1017,1194,1135,1705,363,1409,2018,1730,1237,1074,1120,9
38
SLC27A4_6_1041,2076,1590,1684,1324,1346,1156,1440,1485,2781,3030,1761,
1266
SLC27A5_6_1042,1235,970,1797,1454,824,938,928,3253,1969,3535,903,2040
SLC28A2_6_1043,1698,1627,2303,1693,2186,930,1886,2607,1746,2263,2125,7
90
SLC29A2_6_1044,2481,2115,3405,3012,2490,2941,4976,1380,2453,4393,3050,
1870
SLC2A10_6_1045,1306,1279,1136,1083,508,4089,2804,1666,453,2587,1828,67
0
SLC2A12_6_1046,2261,1761,2579,1911,1496,1229,4005,2391,922,1878,2330,2
364
SLC2A13_6_1047,629,641,566,500,123,422,731,242,327,1664,2175,128
SLC2A14_6_1048,3310,2991,3508,3716,2409,2582,3224,4634,2245,3268,6844,
4571
SLC2A1_6_1049,2125,1749,2688,1818,1298,2396,3202,3701,1603,1314,3609,2
790
SLC2A2_6_1050,4597,3621,3970,4299,3839,6091,5748,7161,4101,5059,5104,4
147
SLC2A3_6_1051,5949,6524,7966,6709,5279,10735,6909,7169,5196,8033,6311,
5634
SLC2A4_6_1052,803,816,1073,909,763,1728,654,534,147,1394,1182,1113
SLC2A4RG_6_1053,853,566,1385,591,331,341,2254,149,1381,722,477,343
SLC2A7_6_1054,2523,2167,3183,2839,1908,3429,1292,2470,2406,3755,4136,3
340
SLC2A8_6_1055,1607,907,1260,1969,908,1544,823,389,1065,736,943,2673
SLC30A10_6_1056,1322,1162,1201,1699,1392,1865,1840,2275,1333,401,1332,
2431
SLC30A1_6_1057,5309,5057,6182,5154,6244,3514,6794,3992,6233,6366,8707,
6659
SLC30A3_6_1058,253,338,310,476,105,212,278,264,31,323,866,457
SLC30A4_6_1059,2118,2045,3149,2364,4109,3034,2902,1682,1906,3849,3671,
1560
SLC30A9_6_1060,2234,1755,2901,3355,1150,2415,3111,2261,628,2678,3697,1
924
SLC31A1_6_1061,3981,3596,3603,4486,5475,3164,4194,5952,2556,4470,3703,
5070
SLC31A2_6_1062,7869,6997,7946,7592,5052,7542,8281,10955,5498,11155,115
01,8684
SLC32A1_6_1063,209,203,237,356,487,118,280,780,106,788,448,430
SLC35A3_6_1064,3782,3277,3803,3403,1824,2749,3922,3082,2562,2337,4690,
5202
SLC35A4_6_1065,6128,5747,6526,7070,6072,7924,6460,8299,4428,7098,8636,
5317
SLC35A5_6_1066,9187,9166,10889,9700,6995,13359,11688,12085,8213,8669,1
1361,15946
SLC35B1_6_1067,2847,2600,3328,3884,3827,2620,4769,7703,1203,2076,4406,
2362

SLC35B2_6_1068,1655,1879,1302,1586,1850,1587,2706,1169,797,1402,1435,1
311
SLC35B4_6_1069,2617,2588,3670,3469,3030,4230,2420,2745,1625,3288,3169,
4130
SLC35D1_6_1070,1160,1491,1252,1141,302,591,869,547,723,641,1354,1993
SLC35D2_6_1071,2514,2134,2002,2431,1538,3555,1931,758,3072,2749,2786,8
27
SLC35D3_6_1072,840,466,790,883,1430,2049,1063,1045,77,1097,569,173
SLC35E1_6_1073,64,34,224,298,11,8,87,66,11,343,359,44
SLC35E3_6_1074,2666,2103,3075,2977,2401,3741,1605,2529,2674,3603,5178,
2634
SLC35E4_6_1075,1230,558,1091,857,209,247,739,1020,1356,629,1499,848
SLC35F1_6_1076,2465,2186,3157,3207,2282,3350,3888,1621,2701,3375,2999,
3312
SLC35F2_6_1077,9427,9915,10465,9978,7643,13698,11021,7822,6273,9064,95
41,6007
SLC35F3_6_1078,1369,1330,1309,1435,212,1381,1053,1228,1113,2506,2484,2
552
SLC35F4_6_1079,4340,4210,5636,4901,3841,5460,5320,5532,5575,5188,6623,
4931
SLC35F5_6_1080,2580,2702,3027,3672,3453,3504,4397,2335,3863,3387,3810,
5441
SLC36A1_6_1081,1183,700,1412,1161,1194,209,460,691,1002,1055,2138,1911
SLC36A2_6_1082,1800,2772,1999,2412,945,1073,2034,1344,2409,2736,1663,3
311
SLC36A4_6_1083,2650,1867,2962,2844,2098,1635,1933,1657,2422,2991,4260,
2208
SLC37A1_6_1084,1271,991,1676,1433,1298,669,2248,3597,278,558,1746,1878
SLC38A2_6_1085,1228,1518,1761,1543,2309,1547,1066,2562,1427,1148,2941,
976
SLC38A3_6_1086,3949,4629,4867,4905,2203,2139,7026,4172,5196,5504,6244,
4948
SLC38A5_6_1087,3054,2497,3694,3889,3063,2843,3189,4641,3972,1822,4608,
2898
SLC39A1_6_1088,2787,2684,2911,3414,2595,2486,3136,2811,2275,3440,5140,
1662
SLC39A2_6_1089,1261,1218,1116,2226,1716,2106,1995,2025,876,540,1642,88
2
SLC39A9_6_1090,2253,1826,2356,2878,1229,1675,3495,1350,1670,3534,3175,
2457
SLC3A1_6_1091,1986,1554,1878,2096,2889,661,1477,3148,947,2130,1480,132
9
SLC40A1_6_1092,6044,6617,8502,8868,4802,6911,9479,10261,6094,10549,955
1,5737
SLC41A1_6_1093,504,297,459,729,1214,1299,597,137,712,354,533,596
SLC43A2_6_1094,507,1181,667,630,497,438,769,453,693,501,1162,272
SLC44A1_6_1095,1898,2149,2614,1435,2521,1476,4316,2258,2057,1629,3928,
2755
SLC46A2_6_1096,1199,1525,2138,1671,965,1229,2580,1287,849,874,2922,501
SLC4A1AP_6_1097,1451,951,1566,1559,698,1471,1171,1578,1947,1621,2067,1

969
SLC4A1_6_1098,668,515,729,959,439,894,431,704,248,2027,955,300
SLC4A7_6_1099,780,1437,1173,1253,329,1785,245,489,790,874,820,1660
SLC4A9_6_1100,3018,2746,3589,3396,4127,3922,4225,1759,2623,2656,5101,4
852
SLC5A11_6_1101,515,301,725,585,80,1850,987,2079,689,13,1226,1233
SLC5A1_6_1102,1975,2223,2460,2832,1038,4042,1897,1539,2072,2521,3669,6
157
SLC5A2_6_1103,347,701,494,417,922,133,23,389,346,570,1393,1
SLC5A3_6_1104,5507,4632,6018,6225,6758,5469,6483,4992,3594,8574,6606,5
691
SLC5A4_6_1105,4963,5098,6029,6186,7499,5176,5599,5514,3936,8272,5407,5
395
SLC5A5_6_1106,4981,5642,6608,5596,7029,8965,6749,7927,2617,10142,6863,
4934
SLC5A6_6_1107,936,907,664,1044,1265,146,352,521,427,840,350,330
SLC5A7_6_1108,1220,1398,1483,1575,1234,1101,1273,2008,1364,1658,816,94
7
SLC5A8_6_1109,2403,2696,3590,3492,1657,6456,2531,4308,3118,3114,6179,4
147
SLC6A11_6_1110,703,759,1214,890,551,509,2181,521,469,1149,581,880
SLC6A14_6_1111,4436,4620,5057,5585,3411,5895,7137,6349,5152,6290,5635,
5925
SLC6A16_6_1112,1963,1826,2856,2264,2060,1994,2802,3757,1347,3760,2633,
1616
SLC6A19_6_1113,781,1425,1236,715,737,1763,1738,2045,1663,137,1361,484
SLC6A1_6_1114,1101,1214,2019,2006,1550,2822,1427,1034,945,2067,2805,16
00
SLC6A3_6_1115,868,1113,1279,1574,1160,1723,1342,527,1142,711,1758,716
SLC6A4_6_1116,7344,6686,6813,6293,5851,7977,9001,6293,4484,7754,5465,9
420
SLC6A7_6_1117,202,236,316,423,130,10,182,59,61,285,21,308
SLC7A10_6_1118,2158,2008,1845,2309,2333,4997,2984,2216,1884,2116,1283,
1598
SLC7A11_6_1119,17532,17102,19440,18145,18903,19839,20488,20342,18080,2
0432,25698,19847
SLC7A13_6_1120,1821,1543,1461,2288,1398,2643,985,1012,1956,508,2322,23
04
SLC7A1_6_1121,8861,9534,9630,10164,7539,7258,7775,9519,6988,10125,1438
8,10951
SLC7A4_6_1122,243,167,633,588,17,897,5,1038,38,585,289,1077
SLC7A5_6_1123,2365,2324,2825,2303,3132,3202,2696,1740,2053,2047,2502,2
850
SLC8A2_6_1124,5577,5549,5904,6238,4381,5968,3507,7105,4731,4391,5503,2
530
SLC9A1_6_1125,594,774,1332,604,653,933,1828,229,775,1006,1802,44
SLC9A2_6_1126,351,330,678,597,919,375,473,702,536,0,1888,544
SLC9A3_6_1127,1746,1559,2365,1911,3901,973,2159,2067,81,1008,774,760
SLC9A3R1_6_1128,1121,630,1073,913,785,418,740,272,619,1159,573,1383
SLC9A4_6_1129,1015,1058,1321,1071,2143,1084,1394,1678,911,779,1062,539

SLC9A5_6_1130,854,1088,741,656,393,1097,348,1269,286,307,1662,827
SLC9A7_6_1131,2611,2256,1934,2536,3820,3588,3595,1673,2621,2208,2793,1
144
SLC9A8_6_1132,312,170,200,396,123,578,3,309,114,108,18,341
SLC9A9_6_1133,2394,2316,2593,2551,1254,4287,1949,1079,1291,4382,4305,2
960
SLCO1B1_6_1134,2088,1406,3045,3345,3636,2091,3380,3832,2019,1601,4191,
2128
SLCO1B3_6_1135,3178,2884,4167,3038,3482,2084,4009,2668,1985,1534,3223,
3749
SLCO2A1_6_1136,5643,6558,6350,6499,6589,6699,6167,8001,3813,3903,7680,
5023
SLCO4A1_6_1137,1141,790,1530,1307,2306,715,1663,15,888,1898,2643,1062
SLCO4C1_6_1138,2298,3357,3443,3419,2029,5799,2672,2688,1691,3174,5422,
2842
SLCO6A1_6_1139,8786,9034,10418,10095,9484,12202,9486,8252,7773,9455,13
682,9066
SV2A_6_1140,3494,3126,3674,3667,2055,1225,2042,5414,4574,2351,3756,303
3
TAP1_6_1141,4354,4299,5198,5674,4318,3614,3928,5955,3121,3490,10248,10
098
UCP1_6_1142,1871,2128,2666,2797,5626,3471,1230,2136,2818,3303,3361,383
3
ABCA12_6_1143,4387,4152,4871,4714,3661,4534,5462,3710,5154,3779,5891,5
965
ABCA2_6_1144,464,625,476,345,948,47,847,349,94,473,1724,834
ABCA5_6_1145,2083,2083,2245,1991,2410,2727,2902,4090,1320,1951,3141,17
84
ABCB4_6_1146,3899,4579,6067,4766,3519,3624,2248,4808,3647,4631,4942,66
85
ABCB9_6_1147,2481,2509,3263,2845,1070,4630,4206,2043,4206,3927,3620,21
71
ABCC10_6_1148,917,996,905,837,1383,960,1003,350,912,292,2024,1815
ABCC11_6_1149,1039,1501,1343,1492,1062,990,1636,2472,691,626,1253,1130
ABCC1_6_1150,433,439,797,605,859,519,985,19,192,789,885,385
ABCC3_6_1151,368,468,663,838,377,803,472,554,313,537,120,354
ABCC4_6_1152,751,1211,932,904,1073,1093,2314,578,801,857,958,2431
ABCC5_6_1153,1048,1173,1222,743,954,562,584,1296,432,1186,1593,1192
ABCC9_6_1154,1129,1039,912,967,784,2054,655,1166,1239,683,1710,1269
ABCD3_6_1155,5441,4917,5526,5213,8281,7302,5749,6952,5167,7481,9475,77
56
ABCE1_6_1156,1419,1397,2049,1716,2213,1303,2286,1960,1925,628,1800,220
5
ABCF1_6_1157,983,1180,879,871,1056,1988,359,703,782,2332,1781,1589
ABCF2_6_1158,1086,1186,1642,1493,158,1416,1389,1295,574,677,1922,1325
ABCG1_6_1159,6894,6966,7788,7441,6221,8878,6769,10910,5200,6408,4588,6
897
ABCG4_6_1160,1710,2255,2192,2024,1708,2428,2324,2077,1748,2728,3279,20
83
ANXA11_6_1161,278,290,528,209,1047,418,378,47,155,33,274,251

ANXA13_6_1162,2843,3419,3579,3721,3233,2595,2946,3097,2233,5160,5448,3
166
ANXA2_6_1163,761,803,1475,954,1780,1155,1249,687,1366,488,1306,339
ANXA6_6_1164,917,480,664,600,1702,1227,1988,857,239,116,405,199
ANXA7_6_1165,2643,2327,2703,2404,1902,2701,3594,2333,2115,4985,2943,30
06
ATP11A_6_1166,1560,874,1042,985,320,724,888,2926,1649,1013,757,1791
ATP11C_6_1167,539,711,659,751,913,313,554,404,616,428,990,1218
ATP12A_6_1168,746,905,512,656,2469,440,608,856,583,297,1108,774
ATP1A1_6_1169,792,523,587,591,858,978,181,91,245,30,637,421
ATP1B4_6_1170,2716,2615,3088,2251,1183,3154,3151,2318,2231,4971,3592,3
105
ATP2A1_6_1171,1087,961,786,1209,652,2784,927,1077,477,2221,2459,666
ATP2A2_6_1172,2217,3020,3313,3394,2702,4021,3916,2493,2383,1947,2048,4
225
ATP2A3_6_1173,440,424,625,722,1127,249,956,300,675,202,72,1063
ATP2B1_6_1174,933,930,1318,1108,623,475,1470,1751,276,1059,1351,1269
ATP2B3_6_1175,2883,4519,4010,2561,2971,4277,2781,3069,2998,2556,6725,3
707
ATP2B4_6_1176,1680,1328,1742,1126,1278,2899,1138,3212,1408,1010,2080,2
046
ATP2C1_6_1177,3858,4482,5293,4939,2472,4217,4585,6081,4580,4883,4965,4
504
ATP5A1_6_1178,3703,2490,3577,3747,2536,2765,3987,3963,4008,2845,3357,2
722
ATP5C1_6_1179,1121,1075,1159,1247,2023,1440,1247,907,441,1560,2080,952
ATP5D_6_1180,246,341,190,181,7,16,319,75,464,173,334,80
ATP5E_6_1181,129,0,0,0,0,0,0,150,0,0,0,0
ATP5G1_6_1182,990,1169,793,545,539,1180,739,1686,1562,725,1065,849
ATP5G2_6_1183,236,181,406,211,270,124,293,12,13,82,883,139
ATP5G3_6_1184,451,193,92,310,3,162,184,66,135,85,595,88
ATP5H_6_1185,4151,3114,2739,3512,1544,3979,4950,3953,1684,6244,4419,44
61
ATP5J_6_1186,1716,1543,2421,2099,4343,1618,1734,3236,2819,1862,2475,24
66
ATP6V0A1_6_1187,6323,6048,6472,7307,3138,11331,7891,8774,11154,4800,71
80,7346
ATP6V0A4_6_1188,7171,6613,7640,8269,10611,10603,10250,10158,6478,7799,
14924,5122
ATP6V0B_6_1189,955,780,827,786,810,920,1613,808,106,822,930,1384
ATP6V0C_6_1190,785,652,819,757,1021,446,784,108,534,1012,1852,1359
ATP6V1C2_6_1191,3833,2914,3452,3221,3111,5955,2514,2664,1642,5579,4594
,4921
ATP6V1E1_6_1192,3534,4150,4183,4015,3552,3242,5207,3800,3352,3636,6426
,6212
ATP6V1F_6_1193,45,57,184,178,19,3,8,0,0,492,248,525
ATP6V1G2_6_1194,137,538,178,122,5,25,8,199,0,2793,368,398
ATP6V1H_6_1195,766,473,789,895,389,476,1302,812,1034,1935,1021,410
ATP7B_6_1196,793,661,965,995,1535,237,1229,1429,1127,1694,950,337
ATP8A1_6_1197,1983,2242,1766,2015,1588,3021,2027,2053,654,1493,2805,21

51
ATP8B2_6_1198,586,640,810,639,1112,949,371,971,523,758,373,572
ATP8B3_6_1199,1992,1831,2962,2406,1851,2395,2924,2773,1484,1738,3033,2
581
ATPAF1_6_1200,225,472,534,528,238,1346,1294,151,106,162,270,852
GC_6_1201,2778,2402,3315,2741,2572,778,2903,3229,3251,3253,4578,4856
SERINC5_6_1202,2119,1923,2494,2675,3469,3131,1862,4385,3330,2917,2184,
2968
SLC10A3_6_1203,350,499,869,747,312,3753,243,505,326,562,63,142
SLC11A2_6_1204,1507,1547,2315,1656,2684,1475,3469,1496,295,4015,2770,2
422
SLC12A1_6_1205,7324,6044,7587,8268,7134,5264,10097,7943,4399,6513,1051
6,7049
SLC12A3_6_1206,560,520,862,875,60,485,227,305,438,733,2511,2148
SLC12A4_6_1207,3375,3582,4044,3470,4173,4640,4954,1585,1928,3629,3633,
3538
SLC12A5_6_1208,6062,7130,7479,7205,6242,8935,6004,8925,5654,7936,10276
,6157
SLC12A6_6_1209,5124,4195,7014,5518,4391,7762,5981,5629,5565,2568,6869,
6388
SLC12A8_6_1210,2229,1341,1967,1696,2277,2152,1543,1543,1757,4104,1639,
1128
SLC13A2_6_1211,4788,4846,4656,5079,4591,3307,6245,3245,3250,4163,6726,
3225
SLC13A3_6_1212,2299,2438,3175,3291,4001,3719,2767,2895,1670,2015,3538,
2379
SLC13A5_6_1213,636,430,963,1249,259,1442,1004,504,146,888,1937,952
SLC14A1_6_1214,2067,1862,1954,2606,968,2940,2117,4981,1313,2670,2637,1
995
SLC14A2_6_1215,729,1017,864,647,659,1358,898,226,2079,317,476,133
SLC15A2_6_1216,7389,6785,8380,8393,7915,8040,7190,8173,7957,7530,10293
,7886
SLC16A1_6_1217,2074,2253,2440,2243,2325,2086,1972,2654,1888,2705,5289,
1597
SLC16A3_6_1218,1794,1568,2469,2239,1044,1696,1745,1456,1185,5076,2556,
2782
SLC16A4_6_1219,4205,3933,4414,4997,4799,3982,7018,5252,2998,6730,7711,
6568
SLC16A6_6_1220,399,301,395,437,594,566,77,412,160,543,45,105
SLC17A3_6_1221,2988,2533,3900,3404,2014,3928,3869,3272,2917,4495,5162,
2844
SLC17A8_6_1222,0,0,0,0,0,0,0,0,0,0,0,0
SLC18A1_6_1223,615,267,543,383,139,1316,37,1080,559,2002,166,220
SLC19A1_6_1224,1449,1169,1730,1248,1995,1470,3087,1964,1881,866,3033,1
700
SLC1A2_6_1225,4255,3389,5269,4740,3429,3492,4651,5720,3142,4712,5348,5
493
SLC1A3_6_1226,3525,3168,3598,3140,4584,1658,2829,3799,2451,3586,3702,3
038
SLC1A4_6_1227,1681,1596,1786,1591,1624,2289,2638,1804,1091,3242,2746,3

368
SLC1A5_6_1228,549,440,182,386,19,624,67,772,346,586,779,434
SLC22A12_6_1229,1536,834,993,1224,2101,1654,1012,1126,1520,3433,744,12
60
SLC22A17_6_1230,1047,1513,2078,1655,3480,2420,1601,1435,1485,2194,3672
,1669
SLC22A18_6_1231,935,728,801,1142,652,1783,608,705,335,692,1373,510
SLC22A1_6_1232,493,326,500,312,64,349,97,111,512,378,1176,865
SLC22A23_6_1233,9952,9320,10793,12047,8490,13521,8980,12031,10218,1279
0,16327,9105
SLC22A6_6_1234,345,397,392,263,0,667,19,438,737,135,307,18
SLC22A7_6_1235,478,276,683,546,426,159,1321,2029,871,685,1457,204
SLC22A8_6_1236,1692,1310,2068,1868,896,1645,1954,299,1814,1695,1863,17
24
SLC23A3_6_1237,1289,1423,1388,1318,864,1318,3697,1289,1104,938,2274,59
3
SLC24A2_6_1238,9424,10124,11838,9813,9031,7218,9251,11315,4672,8519,10
835,8794
SLC24A4_6_1239,1480,1517,1417,2225,1137,1461,2317,1733,2386,815,1486,7
29
SLC25A11_6_1240,2247,1542,2464,2340,2866,659,1111,1104,1264,1466,2855,
1646
SLC25A13_6_1241,3856,2996,4836,4905,4781,6939,5657,5170,4878,5842,6355
,3752
SLC25A14_6_1242,1549,1526,2074,2408,1020,2255,4052,1939,1100,1331,3591
,2935
SLC25A19_6_1243,1195,964,1356,945,513,139,795,1243,873,176,1345,464
SLC25A21_6_1244,1295,896,1463,1510,2811,2595,959,1093,2152,2133,1510,6
07
SLC25A22_6_1245,1804,1965,2050,2072,2634,516,2588,1965,803,1326,3489,2
360
SLC25A26_6_1246,3366,2511,3277,4006,2903,4884,3040,3072,5080,4750,2199
,4813
SLC25A27_6_1247,5331,3870,5092,5044,5054,6759,5999,4755,4581,3959,6224
,6084
SLC25A3_6_1248,691,1183,848,929,879,178,1794,1246,863,248,770,516
SLC26A11_6_1249,1349,1318,1555,1663,824,1917,1819,2308,862,2302,2544,2
339
SLC26A1_6_1250,1435,1353,1664,1520,1703,1125,1741,1980,976,1841,684,20
21
SLC26A5_6_1251,2316,2490,3673,3184,1791,3122,2713,4531,2719,3941,2072,
2446
SLC26A6_6_1252,4017,3701,5539,5804,2023,6162,5584,8762,3005,3346,5447,
6636
SLC26A7_6_1253,1030,1386,1117,1352,955,59,1330,1023,1344,790,1326,713
SLC26A8_6_1254,286,655,517,435,83,26,5,351,19,49,74,204
SLC26A9_6_1255,646,987,1329,1112,557,638,3033,773,1212,799,3123,1169
SLC27A2_6_1256,983,924,2060,1862,1315,1280,1589,1448,1540,2823,1872,20
99
SLC27A6_6_1257,4241,4026,4435,5380,5075,3368,4316,5325,2498,4372,7695,

6720
SLC28A3_6_1258,3511,2759,3748,3555,3302,5646,3625,3684,2709,6894,5166,
4302
SLC29A1_6_1259,886,818,1056,947,1268,2154,844,1603,401,1312,1547,343
SLC29A3_6_1260,3307,3327,4391,4691,3278,7273,1797,4039,2859,3569,3292,
3227
SLC29A4_6_1261,3903,3797,5933,4246,7006,2723,4889,8010,3056,6991,3318,
4201
SLC2A11_6_1262,707,767,1469,1199,512,677,1088,468,1041,714,2198,745
SLC2A5_6_1263,1357,1457,1523,1674,926,1617,973,1523,1079,1004,2488,104
2
SLC2A6_6_1264,129,319,528,278,35,39,363,201,938,703,120,467
SLC2A9_6_1265,125,46,123,272,0,269,20,22,33,9,31,2
SLC30A2_6_1266,842,746,748,767,1070,1455,774,1661,111,1058,1199,1608
SLC30A6_6_1267,1781,1948,3114,2167,1326,1564,1630,1019,2119,1970,1902,
1634
SLC30A7_6_1268,9871,10069,11464,10364,12723,14648,11948,11418,10469,11
446,12781,10160
SLC30A8_6_1269,587,643,556,529,745,448,453,1162,489,1539,797,591
SLC33A1_6_1270,2116,2070,1880,2436,2726,3727,2716,1571,2717,3326,2848,
1606
SLC34A1_6_1271,1707,1579,1758,1979,2415,1894,1126,3996,1175,3345,1328,
1596
SLC34A2_6_1272,543,277,827,426,20,684,134,598,37,161,2250,740
SLC34A3_6_1273,3030,2742,3272,2993,3666,2577,4028,3688,3229,2367,2547,
3703
SLC35A1_6_1274,545,419,674,443,569,142,632,315,100,661,311,391
SLC35A2_6_1275,538,771,1007,816,393,806,1037,1443,588,196,1880,2072
SLC35B3_6_1276,9496,9553,11050,9018,5116,10956,6957,6375,6631,9947,111
21,6809
SLC35C1_6_1277,916,484,1190,1330,1391,244,979,936,1566,1529,3047,529
SLC35C2_6_1278,384,390,392,690,86,1178,1060,335,197,1007,93,201
SLC35E2_6_1279,481,553,1170,644,1013,539,430,741,445,2528,2175,614
SLC36A3_6_1280,1832,1296,2360,1755,1243,2625,1359,1837,1364,901,2602,1
544
SLC37A2_6_1281,1533,1423,1831,1893,1307,1295,1355,2614,2305,2513,907,2
599
SLC37A3_6_1282,1601,2041,2619,2961,1538,1974,1828,1638,2200,2993,1798,
2400
SLC37A4_6_1283,139,265,416,423,1,4,367,0,38,134,494,15
SLC38A11_6_1284,3598,3099,3969,3369,9144,3348,3095,3447,1794,3026,3345
,6702
SLC38A1_6_1285,14086,15545,15781,14138,13773,14636,15583,15328,14764,1
4523,17321,18053
SLC38A4_6_1286,11511,12011,10841,11375,10649,18125,11529,10923,11103,1
2988,18048,12663
SLC38A6_6_1287,6961,7253,8077,7468,4033,9117,7202,8517,3438,8100,10257
,10470
SLC39A10_6_1288,5426,5218,7115,7382,7154,10586,7981,7227,6878,7542,977
2,10639

SLC39A11_6_1289,2719,2951,3196,3083,2665,3066,1922,3780,2069,2562,4687
,3435
SLC39A12_6_1290,2413,2707,2331,2848,2572,3134,2536,2666,4115,3215,1385
,2700
SLC39A13_6_1291,3564,3019,3187,3238,8446,3300,4227,2919,2069,3752,3683
,5677
SLC39A14_6_1292,780,106,622,433,502,573,348,88,1125,854,157,342
SLC39A4_6_1293,1929,1778,2385,2274,2755,3851,2068,1917,696,3341,1480,2
661
SLC39A5_6_1294,187,72,173,162,846,170,6,3,279,282,18,479
SLC39A6_6_1295,3634,4081,5302,4458,3264,2728,4137,4862,3165,5831,4955,
3242
SLC39A7_6_1296,447,726,390,701,40,137,899,1080,367,381,881,1658
SLC39A8_6_1297,1872,1394,1207,1134,1143,1723,1645,2337,1873,1516,2345,
851
SLC3A2_6_1298,242,471,426,344,165,20,169,160,85,992,815,88
SLC43A1_6_1299,606,711,411,662,871,421,511,123,797,833,479,428
SLC44A3_6_1300,932,760,654,372,267,145,1625,293,609,563,701,727
SLC4A10_6_1301,1826,1724,2641,2126,916,1097,1849,809,2287,1497,2417,21
46
SLC4A11_6_1302,2640,2473,3044,3082,1491,3686,2821,2903,2208,5817,2806,
1825
SLC4A2_6_1303,267,320,307,339,656,12,111,150,154,145,1591,59
SLC4A3_6_1304,3340,1896,2683,2919,2315,2686,3302,3550,1986,2909,2767,2
276
SLC4A4_6_1305,3540,2853,4038,3101,4400,4925,2944,4137,3291,5120,6708,4
200
SLC4A8_6_1306,1500,1218,1402,1825,538,861,1080,1352,592,1242,3029,1483
SLC5A10_6_1307,1952,1357,1983,1171,1943,1889,2224,1831,726,1070,1926,3
81
SLC5A9_6_1308,1878,1318,1832,1120,1544,2509,2231,1938,798,1022,1933,37
4
SLC6A12_6_1309,5713,6401,7495,6935,6515,6626,6102,8211,5948,5855,7748,
5134
SLC6A13_6_1310,326,462,458,569,1,17,858,0,139,77,446,478
SLC6A15_6_1311,2981,3385,3869,4594,5360,6786,2927,2512,2533,3204,4503,
2188
SLC6A2_6_1312,975,1122,1367,1268,1048,1124,592,544,1650,1839,1265,1085
SLC6A6_6_1313,242,42,123,244,362,184,333,124,20,112,67,205
SLC6A8_6_1314,2096,2079,2854,2511,2542,1143,1446,3642,3218,981,3790,15
02
SLC6A9_6_1315,252,96,307,546,281,679,68,1378,126,207,679,379
SLC7A2_6_1316,2715,2602,3065,3153,2465,2112,2777,2849,1208,3552,3325,3
289
SLC7A3_6_1317,2105,1847,3181,1590,2163,1778,1919,1244,1610,4187,3756,2
080
SLC7A6_6_1318,466,35,900,150,68,558,118,639,528,3,659,1760
SLC7A7_6_1319,634,742,637,445,480,352,1380,1897,1113,1540,456,527
SLC7A8_6_1320,1493,1153,1601,1159,592,1002,1241,745,913,294,956,272
SLC7A9_6_1321,390,640,324,290,796,211,493,36,18,820,122,68

SLC8A1_6_1322,14307,13811,15573,15679,16243,17509,11867,14390,12884,15
823,18350,14151
SLC8A3_6_1323,366,197,697,551,221,579,347,162,325,571,430,146
SLC9A3R2_6_1324,1451,1405,1497,2231,517,1262,3210,2141,716,1624,979,14
08
SLC9A6_6_1325,516,934,758,802,818,598,1103,597,809,374,1786,622
SLCO1A2_6_1326,666,319,462,396,203,774,184,509,917,247,1325,96
SLCO1C1_6_1327,2660,2571,3153,3151,2495,4440,4384,3347,2761,6130,3049,
2441
SLCO2B1_6_1328,1364,1570,1360,1625,1454,1487,1596,3224,2559,1715,2512,
353
SLCO3A1_6_1329,82,96,42,186,104,32,184,34,3,359,319,68
SLCO5A1_6_1330,2068,2675,2564,1542,1665,1615,1202,1200,1611,2703,4729,
4112
ABCB5_6_1331,2334,2778,2708,2653,2584,1959,3383,1215,2585,1931,4303,20
21
ABCC6_6_1332,317,531,425,443,1,149,227,0,441,138,14,23
ATP1A4_6_1333,2045,2415,2388,2442,741,1767,1430,1865,1150,3160,3744,25
73
ATP5J2_6_1334,6999,5440,7764,7592,2706,4920,5881,6253,3526,9866,5971,8
079
ATP5S_6_1335,388,460,716,360,546,345,575,144,128,371,455,379
ATP6V1G3_6_1336,7733,8651,8580,9743,12797,11448,9963,8577,4301,9029,12
441,7966
ATPIF1_6_1337,1483,870,1232,1370,1936,2007,1339,1888,2690,1764,2243,14
60
SLC10A7_6_1338,3000,3934,3663,3798,4685,4625,3596,4752,3129,3391,4076,
4008
SLC28A1_6_1339,1477,1414,1547,2536,2523,3421,2549,896,1004,3092,2085,2
283
SLC30A5_6_1340,1994,2047,2430,1939,1533,1389,1826,2583,1905,2633,3501,
2986
SLC39A3_6_1341,4915,4858,5641,5383,4519,2322,7121,5723,3841,6086,4353,
5424
ABCA10_6_1342,5480,5824,6630,4988,8697,5739,7174,6712,5295,5262,6147,8
035
ABCA13_6_1343,5520,5084,6968,6325,7888,7693,8210,5031,3637,5379,9216,5
532
ABCA1_6_1344,4716,3612,5905,6096,4395,4952,5459,6160,3667,5015,7469,78
46
ABCA3_6_1345,1079,1102,1440,1682,1734,1111,1783,2013,871,3741,2769,680
ABCA4_6_1346,1268,1152,1463,1808,757,1110,861,1019,1564,1708,639,1785
ABCA6_6_1347,2061,1739,2469,1912,1275,1798,2360,3958,2677,2081,1716,20
28
ABCA7_6_1348,665,594,1041,514,111,758,269,209,326,1166,631,1024
ABCA8_6_1349,5768,5474,6246,6826,7210,8691,5912,4950,4670,8393,6628,46
01
ABCA9_6_1350,1847,2460,2535,2601,2100,2461,1812,2085,2815,2958,3630,32
14
ABCB10_6_1351,212,282,247,508,0,375,252,418,81,48,314,87

ABCB11_6_1352,3111,3112,3543,4003,3318,1583,4334,2307,2466,5552,4749,4
525
ABCB1_6_1353,2777,2791,3246,2632,3582,3384,1547,4000,2363,3368,2377,38
95
ABCB6_6_1354,856,531,920,1170,2816,1016,767,1213,122,696,1232,1740
ABCB7_6_1355,9971,9736,11982,10645,9979,8837,12764,8312,6203,9066,1254
6,11644
ABCB8_6_1356,3221,3481,3558,3609,2845,1152,3559,4203,2090,2909,5607,28
34
ABCC12_6_1357,1940,1250,1564,2359,864,3424,1156,1551,2281,1589,1475,12
32
ABCC2_6_1358,1187,830,1069,1192,337,835,1183,443,541,472,1170,1095
ABCC8_6_1359,667,1068,928,1202,848,733,251,634,1494,1264,1461,620
ABCD1_6_1360,3123,2865,4185,3775,4574,4887,4471,3889,4449,7497,5005,27
28
ABCD2_6_1361,727,1488,1344,1160,423,228,1806,1630,1164,1507,1634,1448
ABCD4_6_1362,2585,2557,2789,3818,1804,4479,3035,2651,2094,2233,3736,29
56
ABCF3_6_1363,1348,1850,1649,1972,837,2165,900,1600,1652,882,1856,1194
ABCG2_6_1364,1876,1707,3588,2675,2403,2250,4017,4233,2426,2308,3671,16
13
ABCG5_6_1365,815,813,1311,800,97,925,756,376,269,1729,434,695
ABCG8_6_1366,4100,3980,4588,5334,7973,5727,4955,3722,3898,4303,3699,36
89
ANXA10_6_1367,702,564,652,494,608,317,377,903,1756,1477,1286,512
ANXA1_6_1368,2088,1644,2386,1811,1193,1605,985,770,1964,870,4960,2225
ANXA3_6_1369,2290,1939,2366,2250,2769,2625,1009,2278,1163,1156,3321,30
22
ANXA4_6_1370,2219,1946,2315,2611,1614,3202,2722,2727,421,2177,3393,144
0
ANXA5_6_1371,945,690,701,1051,833,1493,471,198,460,337,897,1224
ANXA8_6_1372,810,426,642,732,608,461,82,1082,313,185,353,960
ANXA9_6_1373,1443,2032,1776,2038,611,588,1029,1723,2469,1874,1909,1547
ATP10A_6_1374,4468,5035,5435,4838,4577,3535,5214,4830,4299,2561,7016,6
120
ATP10B_6_1375,4281,3846,5641,5515,3327,4613,4070,3546,3790,3634,5610,2
918
ATP10D_6_1376,1471,1584,2004,1763,1753,3477,828,2046,534,1687,3169,141
8
ATP11B_6_1377,2661,1707,1999,2866,1917,2147,865,1754,1336,2302,3256,20
27
ATP1A2_6_1378,1734,1502,1121,1349,670,3357,1357,2624,1891,2059,2099,16
71
ATP1A3_6_1379,1031,1289,1139,1551,1533,1471,2484,1686,778,643,1151,197
0
ATP1B1_6_1380,1095,876,856,973,1095,1419,671,258,494,1329,1221,632
ATP1B2_6_1381,4049,5082,6500,4924,2915,5299,3032,4408,5381,3731,5645,5
373
ATP1B3_6_1382,573,770,842,645,710,744,1644,2231,58,917,984,1093
ATP4A_6_1383,585,1035,1019,875,1566,1206,1564,637,638,963,756,90

ATP4B_6_1384,544,317,423,918,257,365,272,789,737,1944,752,754
ATP5B_6_1385,423,439,712,686,1594,74,239,1217,1369,219,828,149
ATP5F1_6_1386,1795,2027,2447,2262,2093,400,2635,2794,1870,863,2452,206
5
ATP5I_6_1387,503,521,304,501,245,893,370,43,543,43,99,447
ATP5L2_6_1388,4298,3699,4187,4785,2816,4748,6150,3129,4657,5891,5473,3
430
ATP5L_6_1389,4298,3699,4187,4785,2816,4748,6150,3129,4657,5891,5473,34
30
ATP5O_6_1390,2228,2564,2334,2276,1576,2150,5362,3689,1443,2211,5638,15
88
ATP6AP1_6_1391,5751,4384,7419,6258,7224,9082,7177,7186,3551,4075,7983,
6888
ATP6AP2_6_1392,308,240,463,667,1293,141,305,419,1387,337,798,343
ATP6V0A2_6_1393,1467,1291,2353,1871,1899,2142,782,814,2077,1300,3977,1
141
ATP6V0D1_6_1394,5743,4551,6791,5917,5180,8239,4666,5140,4517,4737,8623
,8135
ATP6V0D2_6_1395,4308,3968,4451,4065,4501,3804,3103,7392,3556,2346,3699
,3777
ATP6V0E1_6_1396,841,688,1008,440,1466,93,621,568,1014,1377,1821,347
ATP6V1A_6_1397,2989,3136,3846,3152,3762,3552,6031,4128,1914,3522,5738,
2693
ATP6V1B1_6_1398,1345,1625,1452,1185,1440,2345,1646,2718,1065,596,1671,
1574
ATP6V1B2_6_1399,12894,11395,14053,12200,11841,13751,12989,10730,10981,
13774,15215,13014
ATP6V1C1_6_1400,1052,1104,1714,1084,2156,383,1243,1613,1490,963,1891,1
235
ATP6V1D_6_1401,1974,1305,1730,2079,1324,2522,1976,1396,1608,2161,3002,
2488
ATP6V1E2_6_1402,1545,1243,1672,1478,1014,2141,835,3242,856,423,1625,28
83
ATP6V1G1_6_1403,1895,1864,2331,2288,2193,1298,3836,1187,3018,2026,3764
,1030
ATP7A_6_1404,13084,11338,13983,14818,12339,13508,9723,16324,9520,19540
,16919,15100
ATP8A2_6_1405,5920,5306,7045,7758,6096,7908,8237,9064,8666,6636,10616,
7441
ATP8B1_6_1406,1358,1120,1353,880,1172,759,1329,1842,685,645,2130,1205
ATP8B4_6_1407,298,310,485,290,123,189,138,219,258,219,988,87
ATP9A_6_1408,1091,518,1417,1354,812,1621,1659,858,1079,1970,1339,1408
ATP9B_6_1409,3800,3068,3617,3719,2657,2863,3963,3239,2372,3302,4098,33
12
ATPAF2_6_1410,464,580,549,434,230,395,1009,721,475,1132,719,330
FLVCR1_6_1411,6306,5855,6772,6614,5030,5960,6838,6182,6135,7847,9352,5
065
RHBG_6_1412,743,1190,1414,1441,2146,1481,1443,978,1869,3016,1202,1598
RHCG_6_1413,785,1022,1076,1503,110,2929,839,506,1062,118,1121,721
SLC10A1_6_1414,2699,3198,3308,3327,2080,3799,3296,3358,2834,2557,3251,

3788
SLC10A2_6_1415,4314,3116,5461,5158,2235,6059,6913,5006,3958,3707,9857,
4752
SLC10A4_6_1416,94,78,197,123,1,59,30,9,70,422,0,21
SLC10A5_6_1417,3643,3453,3885,4451,1861,3853,3796,4729,4052,7113,4482,
3392
SLC10A6_6_1418,2791,3338,3178,3703,2979,7085,3018,3714,5044,3534,6519,
4194
SLC11A1_6_1419,2578,3236,4006,3515,3788,4285,4030,3941,3983,2125,3211,
3824
SLC12A2_6_1420,3467,3178,3655,4009,3125,1892,3818,3257,4287,2594,6767,
3307
SLC12A7_6_1421,961,1105,1511,1174,699,2259,2705,1176,1305,1560,1271,46
5
SLC12A9_6_1422,961,1179,1400,1070,1735,928,1648,1805,2454,2260,626,138
6
SLC13A1_6_1423,3092,2672,2512,2967,2035,1510,2595,4335,1904,1463,2354,
2832
SLC13A4_6_1424,1050,1130,1125,1222,351,1060,1850,1749,1970,1653,2022,1
593
SLC15A1_6_1425,4370,3225,4505,3739,2019,4077,4345,3506,2494,4489,6862,
4309
SLC15A3_6_1426,54,104,222,295,625,149,661,966,133,281,163,2
SLC15A4_6_1427,3078,2758,3711,3702,4047,2527,4281,2650,2802,2553,5982,
3606
SLC16A10_6_1428,3025,3257,3698,3816,7694,3830,1434,2089,4574,4220,3803
,2709
SLC16A11_6_1429,1841,1033,1777,1575,377,3036,2063,950,1368,2239,3179,1
559
SLC16A12_6_1430,2778,2470,3404,3084,3523,5564,4179,2421,3108,3428,6603
,2543
SLC16A2_6_1431,712,237,743,689,1027,1254,834,667,195,248,1807,337
SLC16A5_6_1432,516,335,451,378,87,453,119,226,229,2120,340,874
SLC16A7_6_1433,10578,10215,12214,13541,10228,15043,13296,12819,8336,13
618,14105,7462
SLC16A8_6_1434,473,294,869,726,313,205,637,1035,167,174,787,166
SLC17A1_6_1435,8239,8620,9863,8438,8501,7640,10451,12092,6086,9349,115
06,10749
SLC17A4_6_1436,1694,2400,1449,2042,2418,1159,1192,4058,1299,1774,1695,
2844
SLC17A5_6_1437,5090,4643,5730,5183,4842,7916,6002,4937,7431,7536,5868,
4454
SLC17A6_6_1438,8544,7799,8686,7457,4670,4386,8974,11814,9014,7275,1173
4,8619
SLC17A7_6_1439,2230,1843,2591,2797,2065,3752,2187,1357,2045,1116,1972,
1734
SLC18A2_6_1440,3427,2612,3021,4070,6168,5602,3785,3526,3546,1813,3986,
4665
SLC18A3_6_1441,521,762,701,741,135,88,363,271,516,666,116,976
SLC19A2_6_1442,323,513,448,782,181,6,810,1213,55,1399,584,453

SLC19A3_6_1443,4087,3073,4936,5018,3604,5476,3491,6676,2058,2883,6018,
4293
SLC1A1_6_1444,1512,1704,2466,1378,2976,1147,1127,1781,3567,1399,2909,1
571
SLC1A6_6_1445,3948,4506,6116,4428,4614,4532,6280,4747,3510,3795,5683,3
950
SLC1A7_6_1446,1108,1152,1238,1241,3009,2778,1105,1281,1137,2339,1491,5
95
SLC20A1_6_1447,2393,2860,2323,2754,2010,1403,2983,3487,586,2502,3569,2
234
SLC20A2_6_1448,1893,2073,2393,2682,4346,2417,1175,2539,2904,2085,3499,
2635
SLC22A10_6_1449,3731,3800,4437,4146,4825,3244,4666,3672,2161,4829,4615
,1496
SLC22A11_6_1450,3938,3185,4270,3970,3179,1821,2241,4306,3866,2582,5058
,5920
SLC22A13_6_1451,2874,2537,3865,2491,4246,4286,4389,2630,3800,3644,4615
,3316
SLC22A14_6_1452,2305,1754,1698,1650,1203,4247,1925,1654,2328,3107,3992
,1577
SLC22A15_6_1453,1074,937,1187,666,250,775,1642,722,744,1724,708,1172
SLC22A16_6_1454,9523,9914,11447,10819,7028,14195,9766,13242,8401,8706,
10206,9838
SLC22A18AS_6_1455,2000,1253,1905,2118,2178,1722,1921,2134,2154,2397,16
28,1021
SLC22A2_6_1456,2682,3272,3401,2903,3385,1435,1431,3176,1482,3296,2289,
3365
SLC22A3_6_1457,823,1279,953,959,1913,123,1100,332,434,1798,939,1958
SLC22A4_6_1458,2788,2921,3330,3210,3535,1503,2748,3733,1533,3490,3093,
2588
SLC22A5_6_1459,1527,550,1384,881,1380,431,1880,308,722,1972,1607,864
SLC22A9_6_1460,2496,3498,3479,2890,2015,2720,5134,3268,3103,6101,3595,
4109
SLC24A1_6_1461,1718,1700,1867,1725,748,2812,1308,2059,3581,1897,2850,2
246
SLC24A3_6_1462,706,962,502,607,266,12,424,446,729,1545,944,587
SLC24A5_6_1463,1415,1872,1122,1293,817,1240,1564,770,2667,914,2900,174
6
SLC24A6_6_1464,294,516,476,569,265,961,568,501,42,564,628,402
SLC25A10_6_1465,6224,5352,5381,4846,4134,6006,6028,4957,5135,5093,7367
,4568
SLC25A12_6_1466,1925,2204,1705,2654,2022,2049,1886,2126,2043,2353,3332
,1997
SLC25A15_6_1467,1886,1848,1752,1865,510,2476,3516,1508,1669,1683,2814,
1728
SLC25A16_6_1468,4625,4574,4444,4754,6869,5890,7612,4883,3665,2775,6073
,3988
SLC25A17_6_1469,3657,3918,4464,4448,2998,3812,3836,4659,2797,5514,6644
,4391
SLC25A18_6_1470,537,681,562,569,75,1488,468,525,123,39,2114,61

SLC25A1_6_1471,462,413,426,487,109,329,1458,58,161,133,486,441
SLC25A20_6_1472,1493,1448,894,898,1310,584,1042,538,1163,159,1272,683
SLC25A28_6_1473,389,1054,1263,803,1278,1641,518,582,420,1569,1340,1432
SLC25A2_6_1474,17694,16910,18927,18659,17509,21929,17928,18731,16783,18982,20131,19246
SLC25A32_6_1475,313,537,538,442,702,1066,1893,208,222,474,43,836
SLC25A37_6_1476,955,962,1046,934,232,272,1320,940,538,701,912,519
SLC25A43_6_1477,3037,2306,3411,3266,1857,5888,3368,3139,2028,2405,5533,2331
SLC25A44_6_1478,860,755,723,682,698,331,613,57,701,358,726,1026
SLC25A4_6_1479,157,527,761,775,218,3,439,416,730,677,1531,12
SLC25A5_6_1480,2641,2017,2656,2795,1560,1496,2799,853,3378,1443,3932,2884
SLC25A6_6_1481,5100,5761,7378,6166,5332,8490,6751,6355,6150,6476,6205,5209
SLC26A10_6_1482,663,422,866,790,486,420,1100,1030,925,428,817,825
SLC26A2_6_1483,1606,1644,1786,2022,1558,2332,1603,2474,1172,1434,3096,2393
SLC26A3_6_1484,1562,1381,1878,1614,1985,2171,1865,2752,2024,1452,1508,1268
SLC26A4_6_1485,6828,5464,7116,6844,3703,6469,5263,11048,6434,7305,6613,7461
SLC27A1_6_1486,7605,8052,9115,8498,7024,7068,6074,10845,8802,9602,10637,8646
SLC27A3_6_1487,1004,1198,1083,824,1142,2471,648,1365,392,238,1760,1135
SLC27A4_6_1488,571,578,1075,905,446,1126,497,1023,579,808,664,732
SLC27A5_6_1489,1210,754,1384,2001,518,1553,920,1177,2721,1663,1807,1110
SLC28A2_6_1490,3702,3916,5066,5774,4776,5611,5855,4494,2321,3836,4580,3032
SLC29A2_6_1491,252,345,56,207,308,155,7,213,711,2,357,423
SLC2A10_6_1492,392,475,586,690,32,599,127,576,248,244,1057,347
SLC2A12_6_1493,1651,1470,1639,1386,380,3253,822,1467,1157,3170,2603,2635
SLC2A13_6_1494,639,658,689,408,219,33,483,78,257,279,695,1581
SLC2A14_6_1495,1120,491,1448,1208,1123,1472,5,1012,681,1085,954,1
SLC2A1_6_1496,345,141,379,172,597,51,133,554,43,278,239,1033
SLC2A2_6_1497,11778,11356,14224,13122,14832,10557,14763,15267,10655,13962,17148,13113
SLC2A3_6_1498,708,956,841,1079,197,1763,1986,1197,598,1570,1099,723
SLC2A4_6_1499,4750,4108,4673,5002,4857,3527,3571,3453,2848,4122,3579,4988
SLC2A4RG_6_1500,1272,1226,1442,955,602,701,96,772,1168,1759,2652,3568
SLC2A7_6_1501,519,422,541,790,334,274,826,1322,499,198,487,1167
SLC2A8_6_1502,1917,1320,2390,1481,1521,737,1286,1155,1510,3598,2680,2014
SLC30A10_6_1503,3812,3401,4540,5654,4001,6037,4809,3225,3203,5546,6347,5321
SLC30A1_6_1504,2588,2946,3711,3615,2258,1770,3576,2282,2688,4536,3181,2467

SLC30A3_6_1505,529,356,1169,1110,169,33,867,1421,255,196,1299,192
SLC30A4_6_1506,1663,1999,2422,2462,873,3173,2145,1238,1057,1920,3159,9
87
SLC30A9_6_1507,1947,2023,1549,2652,3155,454,3864,2627,1626,2645,3071,2
229
SLC31A1_6_1508,2161,2176,3192,2746,2491,3664,5195,4462,1163,1993,1826,
2352
SLC31A2_6_1509,4208,4953,5774,5606,2645,7898,4369,5304,3164,4017,5665,
5411
SLC32A1_6_1510,636,426,722,881,91,545,849,1302,455,522,819,1
SLC35A3_6_1511,4714,4139,5874,6340,8150,3695,5319,7345,6163,7547,10121
,4072
SLC35A4_6_1512,731,442,584,579,509,2030,129,413,1792,2682,644,1479
SLC35A5_6_1513,886,879,825,816,430,221,582,533,313,786,1073,518
SLC35B1_6_1514,2370,2603,2934,2721,2955,3007,2080,2305,2314,3728,4619,
2922
SLC35B2_6_1515,524,461,1052,812,224,279,879,130,1882,808,530,481
SLC35B4_6_1516,5993,4494,6162,6022,3443,5314,6355,7653,5389,5362,5498,
6628
SLC35D1_6_1517,6222,7168,8398,8142,4261,10099,8664,4356,6998,6959,1045
3,8913
SLC35D2_6_1518,9701,11062,11572,10378,5855,8653,10940,12000,8148,13278
,11376,8974
SLC35D3_6_1519,585,638,672,164,1987,238,1743,1272,286,199,731,365
SLC35E1_6_1520,1839,2501,2700,2364,1999,2177,2479,2494,1979,2307,3196,
3152
SLC35E3_6_1521,149,262,267,273,292,517,587,465,313,570,406,934
SLC35E4_6_1522,4683,3587,4453,4588,2609,6881,4539,2482,3650,6420,3789,
1986
SLC35F1_6_1523,1605,2240,1461,2079,2222,1940,2415,1571,790,2306,1890,1
952
SLC35F2_6_1524,2213,2477,3167,1955,2362,3880,3140,3419,2012,1995,1875,
3630
SLC35F3_6_1525,4966,6626,6432,6630,6020,5757,5884,7188,3270,5643,10106
,6147
SLC35F4_6_1526,909,956,1107,1143,549,1058,2442,488,1624,962,851,1081
SLC35F5_6_1527,2105,2802,2862,3093,3186,2302,4527,2402,1770,2043,4208,
3785
SLC36A1_6_1528,4565,5239,5853,4928,3245,3816,4794,6464,3810,5787,6991,
4366
SLC36A2_6_1529,180,107,226,152,199,444,1,324,43,436,187,307
SLC36A4_6_1530,5365,5078,5939,5951,5042,7777,7846,5083,4345,8083,8893,
6232
SLC37A1_6_1531,380,530,749,1072,1449,1763,856,262,70,725,1059,252
SLC38A2_6_1532,7333,6548,8445,6975,8378,7022,7375,9743,4586,4411,6890,
4440
SLC38A3_6_1533,1754,1431,1701,1777,2290,2120,2185,3302,2470,1619,3780,
1667
SLC38A5_6_1534,1280,1365,1459,1416,2594,332,1064,1901,1492,1674,4185,6
95

SLC39A1_6_1535,746,597,729,997,1459,393,700,916,759,52,1010,206
SLC39A2_6_1536,4351,3610,3883,3790,2947,7022,6933,1195,2110,3508,5385,
6494
SLC39A9_6_1537,218,142,104,283,0,628,231,253,403,798,152,39
SLC3A1_6_1538,2582,1790,3142,2956,1678,2150,2820,1115,2051,2100,4079,2
494
SLC40A1_6_1539,4369,4542,4375,4311,2129,2641,5610,4152,2350,5496,4442,
4165
SLC41A1_6_1540,782,452,567,677,2119,828,825,108,411,1380,453,783
SLC43A2_6_1541,1816,1414,1985,2461,2020,2611,2478,1166,1703,2343,2568,
2257
SLC44A1_6_1542,1145,1179,1263,1413,958,1009,958,2857,1661,1499,1551,70
6
SLC46A2_6_1543,6756,6880,6408,6185,4390,5444,8787,11614,4644,8281,9132
,7846
SLC4A1AP_6_1544,2588,1767,2773,2749,3941,3357,3921,3776,1623,2504,5606
,2900
SLC4A1_6_1545,938,525,855,960,106,1383,172,1208,466,1522,573,1005
SLC4A7_6_1546,2809,3140,2559,2940,2337,4388,2290,2992,1916,3117,4189,2
764
SLC4A9_6_1547,808,725,787,637,1138,1475,364,1252,703,337,369,791
SLC5A11_6_1548,9790,8155,10341,11212,11326,16910,12234,10561,6482,7284
,11268,8355
SLC5A1_6_1549,3661,2243,2506,2216,3965,1104,1474,2634,1829,4473,1367,2
296
SLC5A2_6_1550,3918,3068,2817,2956,3276,2733,4174,2688,1688,4691,5429,3
953
SLC5A3_6_1551,1634,1583,1415,2067,1178,1207,2873,1243,1011,3884,2569,8
33
SLC5A4_6_1552,1268,1021,1635,1289,987,2742,1398,2067,672,2434,2392,329
6
SLC5A5_6_1553,993,751,1419,629,1453,294,1169,889,515,907,1250,1225
SLC5A6_6_1554,1973,1665,1664,2317,2179,1792,3656,1775,2460,1127,3501,4
719
SLC5A7_6_1555,4138,3819,4285,4901,4532,4630,4136,4713,4311,4074,5828,5
036
SLC5A8_6_1556,3158,3098,3319,3606,1475,1403,2528,1998,4353,3651,2183,2
256
SLC6A11_6_1557,4939,4948,5363,5331,5222,7588,5964,2675,3841,2986,7192,
3655
SLC6A14_6_1558,2542,2327,2645,2912,3021,5658,1521,1975,2417,5304,2851,
3232
SLC6A16_6_1559,5462,6000,6781,6067,6823,8481,7994,5283,7639,6786,8233,
4902
SLC6A19_6_1560,6324,6881,8242,7846,9225,6017,8334,5393,4842,11246,8906
,9294
SLC6A1_6_1561,1862,1774,2308,1786,424,1555,2401,1063,572,3245,1985,201
1
SLC6A3_6_1562,1823,1706,2001,1912,1049,2650,2538,674,1932,1784,2032,33
98

SLC6A4_6_1563,1314,903,1249,1448,3400,613,955,3006,1064,2322,2989,1677
SLC6A7_6_1564,1018,1289,1302,1352,727,1811,1908,1178,555,1677,2525,696
SLC7A10_6_1565,894,935,709,843,198,13,394,803,652,1173,611,342
SLC7A11_6_1566,3892,3710,3944,3971,4842,2999,5147,3611,2944,2927,7759,
4584
SLC7A13_6_1567,5861,4707,7724,6814,8133,10863,7296,5587,5778,6921,6581
,9732
SLC7A1_6_1568,1412,2382,1079,1917,785,1469,1430,946,1670,695,2069,1341
SLC7A4_6_1569,995,836,1165,1601,1009,683,2124,343,559,694,1340,2074
SLC7A5_6_1570,4642,3797,5143,4882,5691,5331,5034,5875,4131,4059,9380,3
643
SLC8A2_6_1571,658,456,547,480,651,121,512,209,56,7,231,144
SLC9A1_6_1572,237,154,185,210,673,493,521,1281,21,18,575,662
SLC9A2_6_1573,737,743,1027,1189,1341,1274,326,1441,14,2054,1600,1423
SLC9A3_6_1574,1762,1626,2102,2118,673,3256,3641,2222,1984,1389,2994,19
55
SLC9A3R1_6_1575,704,671,367,451,677,756,42,339,451,102,976,302
SLC9A4_6_1576,6051,5038,7104,7993,5737,5242,7660,10905,4109,4767,8755,
9976
SLC9A5_6_1577,672,461,430,656,1056,1022,290,51,259,234,1086,432
SLC9A7_6_1578,922,331,899,1069,292,996,1491,2368,633,2074,2282,1046
SLC9A8_6_1579,402,763,646,768,0,618,299,44,542,264,1128,87
SLC9A9_6_1580,752,1034,1325,877,1986,133,1246,2126,552,495,1155,1923
SLCO1B1_6_1581,8842,8086,9051,8263,10248,9245,8253,11226,8314,12504,11
256,6869
SLCO1B3_6_1582,6684,6725,8304,8663,4890,10647,9375,6127,5189,8910,7874
,9763
SLCO2A1_6_1583,4577,4633,5216,5375,4314,6548,4932,5948,4746,5758,5090,
6907
SLCO4A1_6_1584,947,833,1269,1288,1242,1055,517,1372,457,1790,2201,1625
SLCO4C1_6_1585,4221,4109,4881,4846,5278,4255,5671,5078,4091,5445,7408,
4580
SLCO6A1_6_1586,8420,7721,9140,9203,7747,6873,10064,7569,7309,7958,1255
6,9586
SV2A_6_1587,612,874,597,857,181,206,1197,1016,389,1764,867,818
TAP1_6_1588,9516,8230,11155,10363,9295,11982,7299,10661,6472,17520,130
19,7884
UCP1_6_1589,1939,1573,2329,1941,1403,1609,1836,3454,1005,1691,2054,386
4
ABCA12_6_1590,3082,2919,3755,3593,1823,2425,2853,5152,3080,5101,5358,4
040
ABCA2_6_1591,1454,811,1305,1238,226,1261,890,955,524,3393,251,1682
ABCA5_6_1592,7531,7707,8286,7500,8236,11116,11444,8390,6192,8371,8174,
6912
ABCB4_6_1593,4352,4569,4776,4411,3064,3856,3015,6308,2950,4373,5589,35
00
ABCB9_6_1594,958,945,795,1066,867,1633,556,146,1221,1992,2109,774
ABCC10_6_1595,746,768,1285,983,573,682,685,434,665,1145,598,464
ABCC11_6_1596,813,1566,1864,1650,1161,1395,1935,787,852,2443,1445,1199
ABCC1_6_1597,3363,3333,4033,3529,2681,6019,4932,1038,2714,5905,4073,47

74
ABCC3_6_1598,6069,6448,6196,6866,6272,9390,4211,7802,6306,7157,8923,70
25
ABCC4_6_1599,5095,4626,5827,5577,5470,4548,6043,6002,3762,4488,7218,45
05
ABCC5_6_1600,424,787,1268,1221,930,233,424,1054,421,1106,1169,1219
ABCC9_6_1601,1555,1494,2210,1875,2227,1492,3208,1158,1197,1513,1989,33
07
ABCD3_6_1602,1305,1605,1493,2056,2016,2335,1085,1886,935,1300,943,2542
ABCE1_6_1603,2165,2403,2555,2258,3463,3260,2346,1955,2615,1533,3748,45
8
ABCF1_6_1604,1445,2227,2709,2383,882,1864,3428,2143,1156,2424,2311,238
3
ABCF2_6_1605,467,1001,1067,400,337,324,786,451,433,2,660,31
ABCG1_6_1606,1302,875,1692,1559,2375,440,976,1772,1076,2486,3198,937
ABCG4_6_1607,391,512,633,870,424,747,2907,59,59,106,942,1343
ANXA11_6_1608,2222,3436,3669,3201,1703,2249,2610,3561,2911,3240,4006,3
612
ANXA13_6_1609,2387,1985,1603,1604,3939,1287,1367,2194,1097,690,1128,17
81
ANXA2_6_1610,3218,3194,3765,3570,2259,2870,3772,4647,1583,2936,3727,27
67
ANXA6_6_1611,2894,3173,3973,3321,2408,6316,4115,3293,3343,4612,3377,28
94
ANXA7_6_1612,1177,968,1128,1201,1797,327,574,2316,1104,1318,1157,1091
ATP11A_6_1613,1575,1067,1397,825,2551,1570,903,1426,1410,2304,1172,787
ATP11C_6_1614,3390,3939,4352,4474,3618,3364,3560,3952,3580,5463,3345,4
390
ATP12A_6_1615,4020,3269,4572,2928,4086,5521,2131,8155,3346,3486,6790,4
233
ATP1A1_6_1616,937,636,992,747,138,1120,403,775,1256,1015,1422,245
ATP1B4_6_1617,7865,7764,10008,8010,7178,9953,8473,12806,5723,7112,1125
5,10555
ATP2A1_6_1618,1025,1031,1217,1628,2646,878,1231,998,371,1500,2216,1350
ATP2A2_6_1619,1961,1741,2272,2455,2299,1187,3105,1919,2505,1706,3399,3
256
ATP2A3_6_1620,357,640,881,479,1014,335,318,93,207,148,219,281
ATP2B1_6_1621,755,776,810,935,320,1451,409,802,577,2189,1395,726
ATP2B3_6_1622,1900,1845,1655,1896,1736,1832,2101,1457,1110,1602,1022,2
001
ATP2B4_6_1623,1184,1232,1704,1254,1129,308,2126,1556,1244,1513,658,131
9
ATP2C1_6_1624,2332,2186,2089,1848,2208,1050,2230,1816,2478,2767,2708,1
532
ATP5A1_6_1625,4033,4130,5218,5443,5047,4774,4095,5452,3651,6014,7755,6
122
ATP5C1_6_1626,8798,6519,9080,9404,7925,6918,5916,8897,6721,6816,9789,6
955
ATP5D_6_1627,771,699,639,1038,1269,204,84,978,1006,439,1708,541
ATP5E_6_1628,1641,1420,2144,1956,1053,2877,860,2479,2013,2428,2617,174

2
ATP5G1_6_1629,722,574,573,850,251,719,990,1031,253,63,326,1074
ATP5G2_6_1630,1201,1231,1170,1250,43,142,890,494,1817,1746,908,494
ATP5G3_6_1631,1150,1745,1900,1612,616,3338,837,1354,744,2790,3733,3129
ATP5H_6_1632,4170,2892,4343,3827,3512,4931,3509,4808,3151,3495,4109,43
40
ATP5J_6_1633,975,917,1093,915,533,1273,1042,1170,377,662,1245,1473
ATP6V0A1_6_1634,1122,1266,1240,1289,591,309,1492,2555,1413,1845,2338,6
36
ATP6V0A4_6_1635,1733,602,1438,1058,350,1362,1119,627,2222,2095,1444,15
26
ATP6V0B_6_1636,2459,2162,2323,2631,2059,2942,663,1483,646,2682,3423,12
06
ATP6V0C_6_1637,2376,2662,2657,2868,2872,5336,2574,2391,2210,4080,2741,
2327
ATP6V1C2_6_1638,1814,1200,2234,1840,1348,2233,1388,2008,1729,1442,1860
,1459
ATP6V1E1_6_1639,2707,2445,3864,3530,2933,5561,5654,3017,3166,3975,4771
,3096
ATP6V1F_6_1640,214,452,579,955,1,277,224,1895,260,911,104,266
ATP6V1G2_6_1641,625,469,546,471,95,157,2,14,20,718,528,467
ATP6V1H_6_1642,3791,3325,4369,4268,3559,4282,5767,5094,2746,4916,5105,
5027
ATP7B_6_1643,2037,1894,1767,1566,755,1331,1313,4897,1455,2838,2530,163
4
ATP8A1_6_1644,1438,1276,1171,1131,1334,479,863,3165,882,665,2270,1782
ATP8B2_6_1645,326,397,333,362,3,34,558,506,738,375,154,220
ATP8B3_6_1646,958,1344,756,1131,1065,242,1917,129,498,1280,1105,555
ATPAF1_6_1647,2415,2314,3115,2734,1866,748,3097,1840,2436,2315,3368,26
09
GC_6_1648,2638,2933,3786,2786,4537,3476,5661,2655,2076,3472,4984,2659
SERINC5_6_1649,6632,4872,6817,6195,7881,6056,6246,9150,6979,7249,9048,
6975
SLC10A3_6_1650,470,509,398,353,353,54,1035,192,139,379,391,904
SLC11A2_6_1651,5475,4860,5589,6338,5014,5919,5604,7847,3806,4423,7354,
3930
SLC12A1_6_1652,2750,2790,3967,3473,2762,3310,2269,2379,1911,1696,6762,
4552
SLC12A3_6_1653,390,541,462,420,269,1049,363,644,154,0,237,13
SLC12A4_6_1654,124,339,189,386,0,1565,0,532,256,721,0,717
SLC12A5_6_1655,1402,1596,1790,1744,1747,1933,3648,1409,1412,2807,1237,
1189
SLC12A6_6_1656,3353,3917,4497,4163,2840,3891,1927,4197,2999,2376,4648,
2555
SLC12A8_6_1657,6855,7966,8461,7372,6902,9806,12052,10227,8196,7398,115
13,11933
SLC13A2_6_1658,967,523,883,1400,906,2268,69,469,1402,1350,939,137
SLC13A3_6_1659,3834,3558,4300,3880,3100,4214,4021,3598,2625,2500,5893,
3836
SLC13A5_6_1660,2007,2869,2846,1748,2509,1072,2353,1999,1808,3624,2883,

3026
SLC14A1_6_1661,2385,2557,3055,2949,3338,1309,2526,3642,2944,4503,4386,
4433
SLC14A2_6_1662,2374,2549,2670,2918,1216,1743,2106,3314,1705,1989,2794,
2967
SLC15A2_6_1663,2480,2381,2313,2696,3457,3145,1599,3500,1322,3600,2654,
2003
SLC16A1_6_1664,3106,3361,4977,4013,5262,4753,4437,3444,3859,6828,4921,
3262
SLC16A3_6_1665,485,559,843,911,1775,523,250,615,293,983,1074,520
SLC16A4_6_1666,3248,3471,3640,3224,1092,1504,2602,2174,2391,1727,6063,
6233
SLC16A6_6_1667,3008,4086,3537,3697,2037,1654,2629,3589,3081,4206,5139,
3211
SLC17A3_6_1668,1341,1382,883,1452,1701,365,1780,922,799,1708,1170,962
SLC17A8_6_1669,2218,2130,3136,3268,2796,2916,1725,2652,2800,2020,4863,
2667
SLC18A1_6_1670,5892,5243,6514,7157,6742,9482,5155,6659,5439,8143,8840,
4656
SLC19A1_6_1671,2864,2846,3365,3945,3928,3163,2432,2503,1936,2547,4480,
2030
SLC1A2_6_1672,1285,957,1015,1218,862,72,521,164,941,82,2092,1882
SLC1A3_6_1673,4324,4350,4812,4147,4249,6185,3060,4798,3393,2508,5535,5
353
SLC1A4_6_1674,1120,1572,959,1163,646,1298,3145,1680,624,1564,1944,1610
SLC1A5_6_1675,3314,3569,3933,3518,3296,3349,3346,5215,1590,3132,4076,4
132
SLC22A12_6_1676,2627,1799,3330,3189,2108,5261,2894,3446,3183,2729,4031
,3197
SLC22A17_6_1677,1031,1478,2044,1655,1820,1970,2004,1657,691,1031,2688,
841
SLC22A18_6_1678,591,584,922,794,672,1100,679,61,738,429,804,954
SLC22A1_6_1679,6205,6143,6773,6267,6006,8917,6941,7249,4834,7391,10063
,7692
SLC22A23_6_1680,1510,2056,2001,2060,1373,1573,2209,2682,1299,1758,3096
,2965
SLC22A6_6_1681,1018,1276,1314,1112,626,2984,1348,1738,690,1181,203,833
SLC22A7_6_1682,1757,1517,2068,2054,878,2171,1585,1879,1529,2616,2394,2
154
SLC22A8_6_1683,1625,1364,1606,1551,1060,1533,1535,2306,1416,1801,1978,
949
SLC23A3_6_1684,1426,1543,2264,1781,1333,1110,3064,1648,1181,4369,3474,
2770
SLC24A2_6_1685,789,1261,1388,1047,221,1066,875,1482,884,1218,688,2029
SLC24A4_6_1686,1690,1081,1612,1700,2479,2758,2583,1370,386,1580,1607,8
55
SLC25A11_6_1687,432,636,700,632,159,704,1491,718,405,190,1631,1014
SLC25A13_6_1688,2142,1925,2083,2482,2520,1144,2213,1346,1974,2119,1994
,1730
SLC25A14_6_1689,688,1123,1278,912,388,1069,806,773,285,259,1524,514

SLC25A19_6_1690,1511,1483,1423,1163,651,1179,3092,1880,1086,2132,1164,
2192
SLC25A21_6_1691,1730,1457,1689,1530,1861,1463,1047,608,3044,3417,1741,
1084
SLC25A22_6_1692,2410,1757,2517,2146,2727,1906,2788,2645,2292,2121,2386
,2124
SLC25A26_6_1693,1878,1546,1991,1994,925,1495,2397,1894,1641,1486,1921,
1465
SLC25A27_6_1694,1794,1116,1632,1540,2411,3455,871,1523,557,1277,3185,1
212
SLC25A3_6_1695,836,565,1113,810,829,634,839,1267,358,1480,535,97
SLC26A11_6_1696,330,182,265,419,1,775,73,1488,69,743,479,21
SLC26A1_6_1697,6526,4925,8660,6060,5799,5805,4503,6534,6666,10428,5969
,9921
SLC26A5_6_1698,2942,2879,4378,3771,5142,4182,6887,4259,3764,2834,6167,
2902
SLC26A6_6_1699,455,576,906,664,1242,1675,1793,1317,1440,280,1338,1477
SLC26A7_6_1700,1775,1529,2485,2608,2384,1196,2260,762,2432,1580,1710,1
341
SLC26A8_6_1701,7556,6998,9748,8503,9756,10806,7642,7372,5780,7558,9229
,8052
SLC26A9_6_1702,933,861,1696,1294,935,1383,1476,1467,40,2521,3368,1639
SLC27A2_6_1703,845,1213,974,805,700,1255,1096,1280,1255,1012,946,345
SLC27A6_6_1704,632,830,953,696,908,335,159,1115,472,208,1690,816
SLC28A3_6_1705,3375,2787,3703,3788,1601,2859,2479,4854,2478,4403,3171,
3954
SLC29A1_6_1706,1866,1956,2598,2058,1389,2909,906,1282,2100,1750,1892,1
924
SLC29A3_6_1707,8149,7494,7797,8553,10467,8907,6775,8702,8615,8792,8689
,7539
SLC29A4_6_1708,840,942,1362,1019,1416,758,2157,1697,712,893,1015,1413
SLC2A11_6_1709,2122,2115,1961,2221,2335,886,1593,2294,2677,2913,2180,1
982
SLC2A5_6_1710,4561,3637,4193,4223,4910,3633,3551,4331,3998,5644,5202,4
682
SLC2A6_6_1711,401,701,480,646,996,338,156,328,370,318,326,980
SLC2A9_6_1712,1859,1599,2359,2185,1047,1438,3211,1576,1343,2913,1444,3
164
SLC30A2_6_1713,901,756,872,755,951,1765,152,561,754,466,450,1605
SLC30A6_6_1714,8784,8491,9922,9572,7442,9791,10580,8717,8014,8831,1022
1,8215
SLC30A7_6_1715,2899,2761,3039,2825,1593,2268,3584,2989,1493,1998,3487,
4012
SLC30A8_6_1716,895,1239,1178,1462,870,1489,655,577,838,1827,593,1444
SLC33A1_6_1717,3670,2957,4422,3830,3733,3188,4761,6267,3496,4699,4242,
3086
SLC34A1_6_1718,866,732,892,648,3194,50,1302,152,663,590,1292,711
SLC34A2_6_1719,1831,1476,1778,1732,283,2226,1456,1259,1460,2337,2590,1
455
SLC34A3_6_1720,636,1061,829,792,303,767,601,425,398,2210,3080,429

SLC35A1_6_1721,2156,1832,2261,2341,2735,3633,1370,1614,1312,1312,3722,
2170
SLC35A2_6_1722,362,332,480,836,353,211,740,415,1187,672,381,231
SLC35B3_6_1723,1533,982,1409,1556,659,447,3153,1873,1625,1516,870,604
SLC35C1_6_1724,418,464,513,688,219,42,200,935,489,493,202,404
SLC35C2_6_1725,1131,641,1127,869,1433,738,1024,956,225,722,1282,1958
SLC35E2_6_1726,567,688,1523,1220,354,312,927,91,146,437,1345,1293
SLC36A3_6_1727,1821,2028,2291,2366,1915,438,2372,3782,1348,2046,2685,1
963
SLC37A2_6_1728,3372,3846,5095,4645,6146,2412,3386,3758,3776,3728,5401,
4900
SLC37A3_6_1729,3906,3582,3450,3896,2209,4884,5454,7907,4338,3307,8052,
5160
SLC37A4_6_1730,1275,1275,1982,1599,614,2236,907,1265,185,2973,2213,307
8
SLC38A11_6_1731,3599,3110,3805,4003,3284,6239,3709,8059,3211,5611,3918
,3807
SLC38A1_6_1732,4705,5017,6303,6494,5675,5581,7269,7361,4604,6898,6055,
5719
SLC38A4_6_1733,5123,4495,5904,5921,3328,5063,5564,6015,5462,5968,8548,
5049
SLC38A6_6_1734,3518,3991,4316,3435,2952,5021,4457,7908,3318,5336,3894,
3324
SLC39A10_6_1735,2479,2227,3321,3007,1852,2813,2947,1246,3164,1786,6134
,1833
SLC39A11_6_1736,1776,1350,2165,1273,1757,1634,2974,564,1903,2287,811,1
592
SLC39A12_6_1737,4740,4745,5779,5751,9481,6401,7279,4260,5573,6701,4812
,7930
SLC39A13_6_1738,3577,3085,3168,3304,8750,3012,4258,2854,2193,3769,4176
,5707
SLC39A14_6_1739,13243,11910,13086,14149,11965,17607,8504,10722,13340,1
6872,17625,11757
SLC39A4_6_1740,903,503,893,653,948,2635,966,1350,1142,136,866,1202
SLC39A5_6_1741,49,169,36,292,2,7,14,8,42,180,145,15
SLC39A6_6_1742,1334,495,1127,1142,215,1018,948,582,143,923,3138,409
SLC39A7_6_1743,805,789,1305,964,659,3038,2725,806,1539,1261,774,2179
SLC39A8_6_1744,2865,2810,2123,2727,3328,3214,1655,2231,2769,3471,3116,
2955
SLC3A2_6_1745,3450,3044,4452,3441,3603,7153,3430,3885,4506,3184,4478,1
696
SLC43A1_6_1746,1775,2492,2539,2124,2424,2454,1173,3687,1114,1738,2590,
2050
SLC44A3_6_1747,1380,1414,1488,1434,1668,1292,2224,985,670,1877,737,684
SLC4A10_6_1748,4022,4218,5033,5233,3758,8841,3945,4538,4342,6340,7610,
3772
SLC4A11_6_1749,879,680,928,980,1766,402,1555,1641,784,2109,1639,799
SLC4A2_6_1750,622,887,1022,1007,1271,934,458,446,903,1504,1861,436
SLC4A3_6_1751,1327,1284,1116,914,376,1406,867,1093,1003,1588,1242,1399
SLC4A4_6_1752,1221,1334,1320,1629,455,1500,1640,989,562,1434,942,1424

SLC4A8_6_1753,2558,2364,3157,2379,2917,2511,3053,3764,1519,4239,2472,7685
SLC5A10_6_1754,969,590,893,1398,1873,814,535,2173,889,934,1648,436
SLC5A9_6_1755,1529,1744,1623,2117,1427,1583,1528,2676,1010,2224,1993,2543
SLC6A12_6_1756,2663,1691,3067,2684,1517,4990,2593,2210,2177,4292,2863,1410
SLC6A13_6_1757,1710,1299,2486,1674,515,1055,1398,1872,1364,2269,3451,1392
SLC6A15_6_1758,4684,3846,4820,5118,3200,6608,3922,3645,4620,2710,2737,3464
SLC6A2_6_1759,1793,1401,2367,2642,1721,842,4317,3548,2220,2411,3737,2213
SLC6A6_6_1760,3535,4024,3728,3536,3989,5619,3850,3054,3887,4725,4562,3977
SLC6A8_6_1761,683,663,673,717,516,379,386,786,672,464,296,762
SLC6A9_6_1762,822,445,402,657,3,2007,1106,474,849,0,701,130
SLC7A2_6_1763,3137,3210,3063,3029,2451,3780,1535,3610,1872,1327,5705,3049
SLC7A3_6_1764,560,549,979,754,763,138,254,987,952,477,550,1201
SLC7A6_6_1765,2865,2230,2918,2852,3169,3274,2162,2884,2297,3837,2568,1743
SLC7A7_6_1766,6505,7083,9178,8190,8969,8057,11005,10811,10597,11700,12773,10098
SLC7A8_6_1767,1909,1019,1802,2080,599,2027,923,1840,2518,1088,2019,1665
SLC7A9_6_1768,2745,3559,3026,2935,1424,3215,5812,5038,3045,4332,2047,4267
SLC8A1_6_1769,675,570,779,791,561,2284,1125,987,303,406,1488,1219
SLC8A3_6_1770,408,261,516,557,802,746,944,148,221,1394,656,703
SLC9A3R2_6_1771,1302,1840,1555,1524,2103,829,1821,766,247,2965,1211,961
SLC9A6_6_1772,5420,5017,5270,6530,5149,6210,5862,3558,6754,4511,7077,5580
SLCO1A2_6_1773,1994,1656,1518,1367,1320,2077,2443,812,3491,1491,1467,1328
SLCO1C1_6_1774,3208,2872,4308,3737,4556,2685,7605,4237,3493,2590,4240,2363
SLCO2B1_6_1775,643,253,578,929,190,313,159,1319,305,780,724,935
SLCO3A1_6_1776,4241,3625,4204,4583,4651,2471,3716,2772,2796,6414,7052,5733
SLCO5A1_6_1777,5050,5835,7712,6882,7111,9779,7142,9356,7352,9908,6271,7724
ABCB5_6_1778,3012,2060,3159,3407,3151,6686,2639,4702,2307,2692,4154,3721
ABCC6_6_1779,90,40,81,270,0,56,5,705,0,626,348,40
ATP1A4_6_1780,975,710,941,1206,1828,1350,507,929,486,576,1123,1046
ATP5J2_6_1781,3538,4315,3979,4718,5650,4508,7617,4104,3978,3721,6726,3391
ATP6V1G3_6_1782,3831,3359,4300,4435,3858,6057,3542,5307,2837,3940,5585

,6669
ATPIF1_6_1783,2085,1806,2101,2284,1215,2052,2430,2528,1074,2038,3685,1
635
SLC10A7_6_1784,966,519,715,1155,743,1058,2480,1060,404,1000,903,686
SLC28A1_6_1785,607,374,454,460,98,170,79,549,226,596,462,150
SLC30A5_6_1786,1665,1363,2367,1933,2794,2264,1116,1196,2319,2159,2298,
3409
SLC39A3_6_1787,886,482,1081,930,1049,1352,1373,1448,1181,75,1968,765
ABCA10_6_1788,3458,2677,3321,4020,2496,4063,2468,3169,3504,2428,6626,2
782
ABCA13_6_1789,5958,5581,6512,7315,8856,11747,7192,9863,6409,13491,9843
,9041
ABCA1_6_1790,4490,4544,6655,5912,2136,3883,9271,8597,5261,4765,11659,2
655
ABCA3_6_1791,3571,2687,3382,2976,2495,4469,4436,1735,3191,2910,5883,20
63
ABCA4_6_1792,97,238,723,265,298,27,587,255,0,459,67,322
ABCA6_6_1793,1502,2302,1934,2476,764,272,1050,1780,450,2956,4310,2385
ABCA7_6_1794,346,603,666,478,554,602,122,121,490,737,557,2590
ABCA8_6_1795,3352,3692,4034,3335,1094,3355,4524,6524,3843,4764,4896,37
07
ABCA9_6_1796,907,1014,1630,1545,1327,495,1095,465,1735,1725,1448,841
ABCB10_6_1797,1753,1959,1774,2329,2849,2536,2079,3353,948,1849,4312,90
7
ABCB11_6_1798,5358,5415,6437,5728,5204,5073,4811,4006,3752,5463,7179,6
426
ABCB1_6_1799,385,722,817,693,391,1226,685,2043,897,425,305,1568
ABCB6_6_1800,854,1226,1090,1004,1581,1710,1408,1270,259,1692,166,421
ABCB7_6_1801,2054,2763,2826,2580,1930,3801,1100,2985,2310,1022,1941,89
9
ABCB8_6_1802,1260,1507,1515,1254,838,1076,698,70,1191,59,2792,599
ABCC12_6_1803,3399,3348,4074,4049,3309,5868,5299,4207,3328,2666,7706,4
053
ABCC2_6_1804,2,2,1,41,1,1,0,0,1,2,0,130
ABCC8_6_1805,3738,4145,3858,3648,4358,3988,2308,4815,2707,3932,5840,50
89
ABCD1_6_1806,1594,1371,1425,1401,1164,952,1298,953,1612,737,2524,1754
ABCD2_6_1807,11064,10866,13308,12517,6525,11216,11702,16414,7045,12106
,11298,9607
ABCD4_6_1808,830,785,1103,860,501,1301,720,241,137,813,1301,1382
ABCF3_6_1809,4408,4686,5517,5090,2449,5141,3585,3586,4995,4464,5754,45
17
ABCG2_6_1810,4477,4314,5348,5254,3830,3375,7315,5343,3598,3502,7283,35
63
ABCG5_6_1811,5302,5714,6292,5900,4622,3846,4013,6851,2853,4812,6881,44
28
ABCG8_6_1812,187,424,363,327,688,154,231,1048,665,329,507,313
ANXA10_6_1813,9389,9989,12881,9619,9898,13525,11382,13262,11301,10960,
10115,9302
ANXA1_6_1814,917,866,1142,983,1913,1055,1001,1073,1226,392,2387,954

ANXA3_6_1815,3299,3334,4128,3831,3993,6097,2349,3120,3322,4332,6709,26
94
ANXA4_6_1816,40,12,62,83,364,20,1,0,130,28,3,150
ANXA5_6_1817,1769,1571,2120,2114,2802,3473,3152,2435,1228,1294,2604,19
43
ANXA8_6_1818,2898,3669,3838,3326,5835,3225,3779,3403,3281,4059,4625,36
53
ANXA9_6_1819,1032,759,1201,1539,1788,2192,425,855,684,556,2381,904
ATP10A_6_1820,328,289,391,489,47,339,242,3,1018,170,54,86
ATP10B_6_1821,2590,3185,3421,2303,3153,2589,1900,2869,1195,2545,3903,2
487
ATP10D_6_1822,2130,3095,2403,2077,3733,3943,2995,2009,2787,1921,4412,3
005
ATP11B_6_1823,1961,2459,1615,1612,835,2100,1175,2028,1605,510,3121,441
6
ATP1A2_6_1824,2379,2018,2750,2881,1809,2205,4005,1086,1206,470,3830,52
92
ATP1A3_6_1825,1485,2027,1757,2000,968,1038,1218,1006,1077,1130,1126,13
73
ATP1B1_6_1826,2186,1694,3257,2464,1725,2670,3285,1512,2038,3258,2985,3
132
ATP1B2_6_1827,852,834,698,926,938,336,647,767,756,1003,375,568
ATP1B3_6_1828,5689,8227,8329,7092,4882,5342,10180,6991,4753,7545,7468,
8122
ATP4A_6_1829,3212,2238,3019,2869,2135,1989,1709,3694,3011,4339,4448,20
05
ATP4B_6_1830,127,280,243,320,277,0,21,223,5,824,282,232
ATP5B_6_1831,2810,2998,3656,3534,1401,2798,6189,3077,2258,3987,5791,33
19
ATP5F1_6_1832,3495,3136,3330,3081,2220,1908,5639,2952,3705,2663,4135,4
415
ATP5I_6_1833,890,465,1143,742,1159,276,1061,1026,1111,667,1302,1403
ATP5L2_6_1834,11653,10789,14128,11949,14147,14147,15808,17200,7862,134
80,10376,9692
ATP5L_6_1835,247,309,601,383,720,211,648,2112,90,346,305,358
ATP5O_6_1836,784,1015,770,627,236,376,562,564,163,273,685,233
ATP6AP1_6_1837,1031,781,867,1122,321,539,403,701,3036,365,1238,2776
ATP6AP2_6_1838,467,1019,1304,1221,879,1604,1965,1279,857,1061,698,162
ATP6V0A2_6_1839,5718,6328,8059,7250,8322,8577,7726,9162,7214,5283,7788
,6509
ATP6V0D1_6_1840,3280,2423,2839,3025,1386,4187,3160,4353,2841,3279,3062
,2021
ATP6V0D2_6_1841,1830,1328,1331,1933,1509,3445,1385,3044,1037,1594,2302
,2310
ATP6V0E1_6_1842,337,186,128,311,3,1,105,3,173,499,0,139
ATP6V1A_6_1843,2328,1286,2342,2235,1689,3107,2182,2356,2005,1242,3510,
2744
ATP6V1B1_6_1844,1642,1899,2105,2399,2763,519,2371,4069,1231,3367,2723,
3360
ATP6V1B2_6_1845,3239,2948,3688,3307,2986,3338,2391,3224,2445,4175,4232

,4716
ATP6V1C1_6_1846,0,0,0,0,0,0,0,1,0,0,0,0
ATP6V1D_6_1847,152,451,585,468,34,172,879,402,313,385,501,864
ATP6V1E2_6_1848,980,675,1093,1153,986,149,602,447,1096,1488,2425,2001
ATP6V1G1_6_1849,1854,1752,2315,2339,2200,1439,3801,1210,3009,2035,3727
,1023
ATP7A_6_1850,1436,1214,1944,1029,3859,1766,984,1735,1316,893,4049,3025
ATP8A2_6_1851,714,839,950,819,150,0,570,0,302,709,1966,346
ATP8B1_6_1852,1699,1892,2366,1485,979,2398,1056,2326,2137,3644,1867,96
2
ATP8B4_6_1853,4008,3579,3388,3789,5180,5090,3833,3131,3618,3352,5268,2
484
ATP9A_6_1854,11950,12304,14710,13741,13056,16427,15408,13857,12446,209
33,15400,11555
ATP9B_6_1855,689,848,811,930,159,1067,1268,140,523,2356,971,30
ATPAF2_6_1856,713,779,1021,1145,2005,357,1077,1602,453,601,1848,979
FLVCR1_6_1857,1557,1308,1944,1577,2047,506,1563,867,1254,2996,1345,109
9
RHBG_6_1858,293,418,373,769,224,414,868,174,363,11,259,21
RHCG_6_1859,569,495,486,525,974,485,548,597,363,454,153,70
SLC10A1_6_1860,3242,3793,4176,3619,3557,3941,3732,2913,2758,3258,4981,
4827
SLC10A2_6_1861,193,373,625,424,324,1,460,129,239,560,374,440
SLC10A4_6_1862,2001,1399,1841,1707,1460,3360,1278,1667,1826,1530,1824,
406
SLC10A5_6_1863,9170,9108,12072,11557,8084,12561,9824,8871,5394,9156,11
507,11577
SLC10A6_6_1864,2975,2590,2780,3992,2696,2862,2431,3335,1966,4305,4584,
2499
SLC11A1_6_1865,2266,2270,2493,2328,2325,2327,2665,2137,2562,3300,2619,
4038
SLC12A2_6_1866,8430,7767,9508,9389,9020,7549,11013,9125,8093,10004,996
1,9218
SLC12A7_6_1867,1420,1456,2105,1551,391,1846,1885,2524,1402,2987,1266,8
61
SLC12A9_6_1868,379,407,662,670,494,122,470,43,39,470,1713,109
SLC13A1_6_1869,13564,15037,16171,15202,14480,16136,17899,14821,13186,1
5670,19683,16595
SLC13A4_6_1870,2416,1836,2460,2124,1123,1322,1517,2364,2775,1415,4834,
2154
SLC15A1_6_1871,3744,3215,3935,4389,2219,3733,4045,4597,2877,5594,4366,
4693
SLC15A3_6_1872,906,558,1082,736,583,1221,255,959,379,150,1145,536
SLC15A4_6_1873,228,123,205,200,0,93,20,54,0,0,3,27
SLC16A10_6_1874,1286,1471,1586,1464,2284,619,3092,1798,762,1277,2084,2
280
SLC16A11_6_1875,1435,1672,1493,1506,2118,1837,276,524,533,2765,1173,10
27
SLC16A12_6_1876,1945,1631,1609,1565,2337,1544,2025,2254,1220,2677,1777
,1511

SLC16A2_6_1877,1484,1211,1834,1920,1051,783,1006,842,983,525,2275,779
SLC16A5_6_1878,385,676,732,459,441,214,450,973,54,991,691,1145
SLC16A7_6_1879,2278,3182,2621,3406,3709,5029,2365,2217,2514,1945,2635,
1851
SLC16A8_6_1880,1190,1229,1139,702,254,946,737,606,869,1183,305,561
SLC17A1_6_1881,2052,1884,2924,2715,1863,1425,2429,4145,2076,1908,3335,
747
SLC17A4_6_1882,1073,1218,1405,1410,283,1125,1982,1523,666,1003,4056,13
24
SLC17A5_6_1883,3900,2908,3638,3365,5276,2865,3473,3862,2694,4476,3759,
1333
SLC17A6_6_1884,8335,7847,10228,10419,8564,12088,8439,8769,9260,11097,1
0838,11058
SLC17A7_6_1885,1217,986,1128,1236,593,1114,717,1015,968,164,681,205
SLC18A2_6_1886,6735,5882,7667,7437,7190,7906,6007,6743,4294,7000,7186,
5631
SLC18A3_6_1887,1555,1510,1602,2246,2520,1419,2258,2377,778,1154,2465,3
046
SLC19A2_6_1888,13156,12685,14272,14538,15276,16612,16748,14678,10437,1
6667,13730,16328
SLC19A3_6_1889,4075,3677,3502,3721,3412,5107,3087,4386,2603,4518,4860,
2502
SLC1A1_6_1890,1380,1266,2036,1915,1577,3324,1869,2212,2242,1487,1964,2
604
SLC1A6_6_1891,3867,3691,4136,4473,4878,4034,5206,4919,3722,4958,7887,4
612
SLC1A7_6_1892,5392,4841,5124,4682,3914,4894,4608,5702,4701,3851,5980,6
856
SLC20A1_6_1893,667,694,670,921,479,762,113,981,892,609,824,661
SLC20A2_6_1894,1080,1301,1057,851,537,1429,456,2480,553,65,1469,768
SLC22A10_6_1895,2560,3207,3889,3509,2914,4160,2335,1511,2619,2290,6885
,1944
SLC22A11_6_1896,178,314,286,281,504,665,139,241,697,253,3,153
SLC22A13_6_1897,1289,1244,1201,1209,1114,870,1456,293,541,878,1388,108
2
SLC22A14_6_1898,9468,7405,11192,8290,9308,11363,12292,7800,8112,12393,
13338,9714
SLC22A15_6_1899,2223,1680,2730,2394,2199,1641,1782,4243,2824,2455,1929
,3445
SLC22A16_6_1900,2962,2952,3890,3335,1462,2819,2153,1906,1718,5938,3680
,5165
SLC22A18AS_6_1901,561,259,604,997,1052,114,214,42,149,537,146,862
SLC22A2_6_1902,2542,2042,2798,2618,3815,2632,1975,1804,3991,2728,3404,
1805
SLC22A3_6_1903,4033,3917,4650,4080,2325,2816,3043,3229,2128,5727,7480,
2974
SLC22A4_6_1904,2121,1228,2318,1731,2515,1387,1491,3426,1062,1348,1707,
1916
SLC22A5_6_1905,2397,1823,2226,2210,1244,1857,2026,3028,1537,2477,2908,
1474

SLC22A9_6_1906,1186,1388,2044,1255,1764,1332,2642,3714,525,3813,5023,1
778
SLC24A1_6_1907,4258,4697,5011,5840,4646,6080,5461,6632,2787,6081,7024,
5588
SLC24A3_6_1908,1244,1192,1599,1659,204,757,2735,947,1356,1296,2054,976
SLC24A5_6_1909,1517,1633,2059,1668,637,1153,1773,1793,1299,1560,1760,1
968
SLC24A6_6_1910,532,545,612,692,783,1126,102,398,102,2637,972,330
SLC25A10_6_1911,775,805,942,1173,749,330,938,630,313,2276,853,998
SLC25A12_6_1912,5030,3070,3417,4260,3952,3895,5387,2874,4332,4625,7343
,4458
SLC25A15_6_1913,5021,5053,6239,5833,7641,4855,5249,3225,3485,5818,6437
,6536
SLC25A16_6_1914,9050,8276,10612,10072,6205,8612,8917,12735,8452,13385,
12235,11345
SLC25A17_6_1915,527,889,705,663,159,371,163,174,553,344,879,644
SLC25A18_6_1916,4095,3902,4604,4247,5486,1248,2797,3243,2700,5358,5221
,6164
SLC25A1_6_1917,1181,911,1025,940,798,2224,2287,505,818,1200,1388,602
SLC25A20_6_1918,1140,1539,1819,1276,653,1387,1245,2548,1197,575,2829,9
74
SLC25A28_6_1919,263,245,450,357,235,172,601,452,196,579,524,384
SLC25A2_6_1920,1605,2381,2179,2124,1993,641,2189,2520,966,2697,2827,39
26
SLC25A32_6_1921,6441,6648,6929,7070,4916,12672,6793,9545,8121,6560,109
50,7783
SLC25A37_6_1922,1527,1197,1772,1672,833,1006,4275,1544,2582,1502,1804,
592
SLC25A43_6_1923,1693,1888,2044,2330,3264,2834,2383,2089,1614,2393,4309
,2440
SLC25A44_6_1924,892,1240,884,618,1008,926,769,775,624,385,1337,586
SLC25A4_6_1925,1265,1403,1446,1700,3195,2723,2442,2544,1334,1429,1092,
505
SLC25A5_6_1926,6858,7021,9676,8527,6761,9911,9837,9058,6900,9330,8245,
7788
SLC25A6_6_1927,892,488,474,681,208,44,605,4,1172,2672,192,1253
SLC26A10_6_1928,1311,1874,3052,1941,3817,2627,1377,3336,781,4622,1683,
811
SLC26A2_6_1929,6403,4708,7078,6471,3956,3900,7707,9130,4845,7808,11077
,5866
SLC26A3_6_1930,7428,6179,7939,7725,6184,7408,8066,6873,5983,7093,8620,
8472
SLC26A4_6_1931,2072,2251,2598,2687,5057,2620,2034,741,1804,2430,3358,2
603
SLC27A1_6_1932,228,320,270,459,858,1,152,794,225,856,504,169
SLC27A3_6_1933,249,1010,733,824,87,389,431,851,1128,48,752,2195
SLC27A4_6_1934,1831,824,1119,1225,817,906,2342,362,1079,472,949,1292
SLC27A5_6_1935,2275,2155,2517,3011,1171,2807,2194,2937,2114,1892,3823,
4444
SLC28A2_6_1936,2511,2235,2580,2995,2371,1593,2220,3038,2145,1918,4040,

607
SLC29A2_6_1937,593,595,615,490,1523,2429,80,895,451,301,683,1311
SLC2A10_6_1938,678,809,486,758,103,190,429,1086,168,729,977,1268
SLC2A12_6_1939,1055,1361,1578,1538,1087,806,927,1051,748,1035,1319,109
3
SLC2A13_6_1940,5409,5809,5547,6099,3137,9449,4683,6688,3705,6870,5512,
3613
SLC2A14_6_1941,5549,5599,7807,7224,6827,9159,6029,7462,4346,7611,9771,
6275
SLC2A1_6_1942,3678,3992,4450,4156,5026,4724,4544,3864,2043,1323,8860,5
860
SLC2A2_6_1943,994,1606,1495,1495,1837,740,2268,2485,1987,2324,1306,141
2
SLC2A3_6_1944,529,586,436,476,275,395,121,232,570,623,369,1141
SLC2A4_6_1945,2494,2562,2215,2753,3662,3652,1325,5506,1695,3314,4740,3
103
SLC2A4RG_6_1946,1920,1884,1972,1915,1298,1932,884,1360,2013,1012,1600,
1519
SLC2A7_6_1947,678,815,1276,1703,1717,1465,1916,325,1242,513,1376,1253
SLC2A8_6_1948,897,901,781,1111,1406,1222,725,736,1391,1647,676,856
SLC30A10_6_1949,2948,3984,3886,3438,2272,3397,4601,4057,2184,3131,4874
,3600
SLC30A1_6_1950,2724,2007,3029,2660,3316,3737,2712,3258,1451,3430,4055,
2038
SLC30A3_6_1951,870,744,950,870,234,445,1446,498,1147,1491,1920,345
SLC30A4_6_1952,5951,6371,6452,5745,3796,6760,6840,6716,4761,11845,7569
,4972
SLC30A9_6_1953,933,762,980,1188,2323,431,1019,1348,479,1171,1021,1677
SLC31A1_6_1954,345,185,188,582,1048,357,55,448,501,442,637,602
SLC31A2_6_1955,3105,3042,3731,3254,3522,2079,4542,3447,3685,2131,3047,
2161
SLC32A1_6_1956,2649,2525,3559,2479,2024,4022,2463,2507,3180,3072,4106,
3298
SLC35A3_6_1957,14867,14316,15272,15142,18460,17251,15043,15095,13266,1
2561,18214,14531
SLC35A4_6_1958,974,964,1948,1180,916,1444,1360,733,845,723,2313,775
SLC35A5_6_1959,6593,6652,8252,7615,5278,10903,7667,7686,6894,9251,8867
,8173
SLC35B1_6_1960,2702,2569,3197,2973,2603,3034,2498,2819,3496,3700,3688,
2480
SLC35B2_6_1961,1064,1043,821,1370,1575,785,887,1395,1098,1372,748,495
SLC35B4_6_1962,1047,782,735,1339,69,1895,318,1807,484,1059,2849,167
SLC35D1_6_1963,1603,2368,1850,2120,3778,1992,2417,1414,1115,2001,3961,
4253
SLC35D2_6_1964,395,914,691,830,721,436,1291,61,503,1195,985,97
SLC35D3_6_1965,1335,1838,1156,1695,1845,3042,1493,1759,1425,645,1878,1
371
SLC35E1_6_1966,864,1767,2177,1267,1039,2823,593,1135,1593,1571,1173,14
01
SLC35E3_6_1967,12854,12701,15709,15425,12492,15688,15759,14093,9872,18

793,19210,15056
SLC35E4_6_1968,4024,3180,3619,3650,2493,5791,1910,2354,2848,4110,4214,
2121
SLC35F1_6_1969,2953,1974,3133,3292,2713,2416,4627,2590,1233,1243,3115,
2900
SLC35F2_6_1970,5568,6180,8293,7256,6482,8633,6707,3620,6627,5907,9604,
10169
SLC35F3_6_1971,1906,2087,2961,2547,1681,3888,2694,1634,4159,2358,3266,
3589
SLC35F4_6_1972,5395,5519,6238,6351,4318,5118,5655,6145,6085,4540,9092,
6813
SLC35F5_6_1973,696,821,1501,858,0,1166,1015,683,243,1651,1045,777
SLC36A1_6_1974,488,596,586,807,410,1050,1466,1281,108,1176,1819,661
SLC36A2_6_1975,1153,1381,1721,1465,1681,1471,811,672,897,1496,3056,120
1
SLC36A4_6_1976,4021,2829,3530,3852,2730,3547,2844,6222,2744,4846,5823,
3415
SLC37A1_6_1977,1461,891,1697,1147,381,617,2100,1905,1230,2198,1220,210
5
SLC38A2_6_1978,1273,1443,2020,1636,2373,1141,1400,2800,775,2481,1189,2
125
SLC38A3_6_1979,793,1214,1237,1045,582,1098,904,1487,546,1406,596,830
SLC38A5_6_1980,3508,2704,3445,2510,2482,3564,3761,3626,3935,3497,3773,
4102
SLC39A1_6_1981,1126,994,1074,1369,1629,1524,158,608,1148,953,1418,1057
SLC39A2_6_1982,1307,1231,1280,2299,1714,2096,1999,2196,874,882,1641,13
96
SLC39A9_6_1983,4941,4625,5746,5843,3799,5121,5870,6565,5048,5047,7397,
3193
SLC3A1_6_1984,7875,6539,7615,8335,6488,14599,9039,4858,7225,7657,8513,
6826
SLC40A1_6_1985,7531,8400,9138,7453,5608,12289,9458,6509,7481,10916,122
01,6846
SLC41A1_6_1986,378,371,360,540,756,92,422,734,341,543,820,472
SLC43A2_6_1987,626,549,481,653,26,1062,218,203,588,64,948,818
SLC44A1_6_1988,6019,5477,7126,6816,4214,7858,6537,8733,3777,7013,9594,
7954
SLC46A2_6_1989,1477,1477,1491,1847,3325,1155,1318,1185,875,1334,2510,2
374
SLC4A1AP_6_1990,3740,4301,4618,4187,2438,4358,1203,2511,4320,4047,6429
,5974
SLC4A1_6_1991,1852,1702,1448,1672,1243,1208,1012,2334,1204,2138,1976,1
470
SLC4A7_6_1992,1372,1428,1317,1349,1316,967,1743,1274,1087,1732,1973,18
17
SLC4A9_6_1993,1388,991,1064,1421,831,1870,2267,2913,691,1844,1645,882
SLC5A11_6_1994,468,210,397,625,197,1792,90,50,571,467,111,204
SLC5A1_6_1995,963,646,1697,1217,1856,563,1080,1049,864,1581,813,807
SLC5A2_6_1996,1486,964,1550,875,1979,1190,1163,1731,709,1010,798,364
SLC5A3_6_1997,526,335,668,505,817,150,858,254,90,48,625,987

SLC5A4_6_1998,1398,1615,1132,1305,720,1116,1091,2770,789,2205,2067,131
0
SLC5A5_6_1999,469,376,470,317,779,1117,729,927,140,43,2086,478
SLC5A6_6_2000,1281,1098,1622,1834,1607,461,1846,1475,2067,1025,2125,12
07
SLC5A7_6_2001,1403,1075,1342,1352,2505,1740,1328,2454,436,1132,2274,16
24
SLC5A8_6_2002,2572,1897,2074,2677,2671,3919,2728,2284,1224,1231,4567,2
875
SLC6A11_6_2003,6472,6278,6212,6304,5890,6819,6631,5947,5174,7593,5747,
10610
SLC6A14_6_2004,2220,1676,2376,2105,942,3678,2305,3235,2511,2315,2643,2
351
SLC6A16_6_2005,2017,2395,2633,2301,1294,1729,3027,3529,1334,3833,2727,
1091
SLC6A19_6_2006,1510,1674,1451,1689,1441,1947,1523,1884,698,2380,787,18
45
SLC6A1_6_2007,796,1039,773,1045,1534,625,1351,1486,322,648,206,1298
SLC6A3_6_2008,615,727,476,544,362,1904,283,981,484,154,1021,608
SLC6A4_6_2009,0,0,0,0,0,0,0,0,0,0,0,0
SLC6A7_6_2010,7934,8928,9920,11044,13563,8928,13001,12419,8252,9265,12
831,9173
SLC7A10_6_2011,1470,2227,2373,2414,1782,3494,2392,1765,2579,1528,3284,
2539
SLC7A11_6_2012,1657,1920,1826,1333,1689,923,1100,1656,1781,927,1326,15
89
SLC7A13_6_2013,2486,2140,2462,2643,1742,3093,3398,3687,1412,2631,2156,
2068
SLC7A1_6_2014,1928,2361,2734,2151,2026,1827,3354,1806,1931,3098,2136,4
670
SLC7A4_6_2015,994,808,1103,1497,1004,679,2109,341,557,688,1324,1924
SLC7A5_6_2016,488,696,641,946,720,1682,1302,1399,398,598,758,446
SLC8A2_6_2017,1215,1138,1026,1169,93,1312,2226,1385,1064,2477,2669,797
SLC9A1_6_2018,586,557,661,663,800,935,515,182,815,484,994,1012
SLC9A2_6_2019,2043,1717,2329,1870,1482,1196,1127,2078,1052,2388,2148,2
161
SLC9A3_6_2020,401,355,139,476,0,0,2074,872,107,89,9,195
SLC9A3R1_6_2021,444,167,296,262,861,678,10,1498,360,97,166,622
SLC9A4_6_2022,6886,5980,9162,7130,6916,8145,8000,7685,9220,11137,10026
,7692
SLC9A5_6_2023,1761,1375,1707,1458,1888,1281,1804,1237,1821,2831,1847,3
165
SLC9A7_6_2024,1275,745,843,757,230,103,958,498,1233,1122,1961,146
SLC9A8_6_2025,2771,2993,3156,2971,3229,2225,2267,1516,3271,4451,3380,2
678
SLC9A9_6_2026,4385,4744,4848,5703,3537,3314,4376,4211,3356,4519,4918,4
278
SLCO1B1_6_2027,3199,3085,4429,3962,3802,3057,3562,4134,3681,3975,4854,
3433
SLCO1B3_6_2028,4291,3664,3267,4054,4212,3991,5499,4659,4381,5052,4272,

5172
SLCO2A1_6_2029,468,628,787,762,281,137,661,289,365,486,387,1177
SLCO4A1_6_2030,271,529,483,297,358,529,1242,917,395,1,140,337
SLCO4C1_6_2031,5529,5262,5829,6112,2628,4502,7787,7137,5325,6678,10167
,8948
SLCO6A1_6_2032,1999,2704,2373,3076,4828,947,2259,2506,901,3784,3005,34
67
SV2A_6_2033,2996,3769,3797,3219,1751,3489,4062,4534,3014,2402,4043,442
7
TAP1_6_2034,417,494,652,561,110,357,334,459,230,732,353,445
UCP1_6_2035,8299,8454,10935,9721,11247,10564,9584,9697,5965,7155,9483,
10225
ABCA12_6_2036,4015,4913,4964,4166,3868,2622,5618,3355,3154,4471,4162,3
465
ABCA2_6_2037,1709,2518,1492,1914,674,2203,2709,1568,1896,3544,2458,248
6
ABCA5_6_2038,6470,7064,6614,7029,4896,5902,4359,6026,4983,4857,8148,33
70
ABCB4_6_2039,14649,15942,17896,16461,14422,19976,21895,18624,14878,159
55,20822,16108
ABCB9_6_2040,544,748,326,560,83,1720,956,261,77,1128,464,346
ABCC10_6_2041,620,716,742,601,501,724,67,564,105,2837,1121,187
ABCC11_6_2042,1524,1377,1591,1709,1854,2766,972,1861,1096,2155,1975,26
52
ABCC1_6_2043,373,432,792,592,857,520,986,19,191,577,867,372
ABCC3_6_2044,1775,1706,2235,2407,3523,736,1504,1861,3004,2832,5772,107
5
ABCC4_6_2045,269,384,637,489,15,0,1224,679,37,1790,832,239
ABCC5_6_2046,957,617,626,830,405,685,1036,807,84,1349,1935,394
ABCC9_6_2047,884,818,1041,1506,1248,346,348,1066,892,1862,1045,2000
ABCD3_6_2048,5208,4443,5240,5175,3905,8498,4277,4789,4957,6736,6862,40
21
ABCE1_6_2049,9447,7563,9399,7826,6916,11901,17194,11595,5709,9939,1350
5,11089
ABCF1_6_2050,775,228,605,341,427,277,334,233,349,751,700,418
ABCF2_6_2051,2028,1580,2187,2549,2276,4514,2583,1843,1319,1549,3810,48
84
ABCG1_6_2052,441,709,596,352,371,1024,578,48,283,799,61,259
ABCG4_6_2053,224,140,263,400,617,375,42,328,15,328,20,0
ANXA11_6_2054,4085,3881,3967,5304,5508,3498,5063,4660,5175,5746,7071,4
882
ANXA13_6_2055,804,967,1085,1038,1395,636,1047,505,367,684,1295,791
ANXA2_6_2056,261,112,296,257,17,608,25,1979,172,450,13,271
ANXA6_6_2057,2324,2064,2267,2107,688,3126,1125,1675,1177,2051,2688,183
9
ANXA7_6_2058,1632,1097,881,1858,1622,2658,457,1458,1221,1075,754,1528
ATP11A_6_2059,1582,1926,2490,2565,1836,1575,1761,3881,2471,1491,4034,2
098
ATP11C_6_2060,1534,1946,2729,2255,1933,1745,2515,1398,1591,1791,1846,5
777

ATP12A_6_2061,3803,2664,4537,5274,2825,4045,3348,4098,3885,4309,4826,3
213
ATP1A1_6_2062,10514,8153,10916,10841,8993,11431,9861,10123,7887,8904,1
5765,10785
ATP1B4_6_2063,566,358,374,806,831,741,341,963,412,252,2099,81
ATP2A1_6_2064,646,427,1141,1166,315,352,469,133,197,2723,729,615
ATP2A2_6_2065,7480,6326,8808,9121,4352,6043,8941,7776,6029,11881,11213
,10470
ATP2A3_6_2066,3988,3561,5337,5304,5366,7285,4933,5340,3889,3854,7964,3
375
ATP2B1_6_2067,755,531,1443,864,348,628,548,493,259,961,1374,459
ATP2B3_6_2068,520,604,724,630,1685,778,1113,79,709,212,1138,881
ATP2B4_6_2069,851,501,651,1030,23,583,1086,306,91,1849,289,1690
ATP2C1_6_2070,3052,3046,4158,5169,2373,3122,3036,7428,3107,4502,6191,5
904
ATP5A1_6_2071,1635,1404,2465,1727,1982,2409,2523,4072,1902,683,2598,23
03
ATP5C1_6_2072,4856,5228,6422,5676,5279,5766,7085,5410,4212,7637,6698,6
829
ATP5D_6_2073,1072,714,1211,990,2096,185,694,829,339,1954,680,76
ATP5E_6_2074,1022,991,936,730,132,1688,175,1663,647,1542,96,1019
ATP5G1_6_2075,516,688,610,659,732,309,617,466,931,807,1105,884
ATP5G2_6_2076,1088,887,1730,1496,636,2085,2583,1686,1135,1467,1853,103
0
ATP5G3_6_2077,1432,1521,2310,2210,2378,1137,1798,1562,1342,3901,2923,2
889
ATP5H_6_2078,3982,3024,4261,3749,3506,4595,3526,4812,3154,3365,4110,33
12
ATP5J_6_2079,2371,2077,2426,1915,1245,3951,2053,2080,1209,3458,2354,26
34
ATP6V0A1_6_2080,8907,8178,9793,8846,13146,15618,12334,10024,9147,10882
,13908,10027
ATP6V0A4_6_2081,5220,5308,6117,5789,4202,7143,6671,7538,4130,5499,7246
,9457
ATP6V0B_6_2082,721,918,967,936,863,640,481,125,445,292,976,428
ATP6V0C_6_2083,1497,2296,2043,2428,1034,1679,4023,2171,996,727,4116,15
96
ATP6V1C2_6_2084,857,1081,1117,895,853,859,561,1271,140,1198,1122,731
ATP6V1E1_6_2085,1322,1501,1482,1495,1552,1795,2105,1699,811,2053,1895,
1576
ATP6V1F_6_2086,169,82,39,187,0,25,0,0,31,115,61,0
ATP6V1G2_6_2087,4,6,3,8,7,6,0,4,4,5,12,6
ATP6V1H_6_2088,2315,3086,2518,2641,1756,647,2069,2783,922,2539,4032,46
97
ATP7B_6_2089,968,1303,1017,1153,1044,2866,643,1892,697,841,960,1234
ATP8A1_6_2090,1905,1913,2616,2509,4097,1686,2139,5642,1888,3528,3039,7
21
ATP8B2_6_2091,7102,9081,9947,9130,8953,12576,8441,6739,7761,12053,1038
8,10984
ATP8B3_6_2092,355,684,718,678,445,892,326,494,127,1069,1013,71

ATPAF1_6_2093,18177,15469,18615,18499,12691,22102,15757,19715,14572,13
177,17017,20242
GC_6_2094,6228,5820,7476,7787,6732,4267,8723,8285,7280,8543,9654,7084
SERINC5_6_2095,365,638,283,607,415,1513,1077,523,174,233,998,452
SLC10A3_6_2096,399,485,409,378,932,16,458,1,40,106,725,1
SLC11A2_6_2097,2137,2288,2311,2185,3359,1442,1981,2149,2069,1983,1931,
1822
SLC12A1_6_2098,3845,3743,4674,4905,3496,3429,2859,3013,5434,3992,4499,
4149
SLC12A3_6_2099,575,484,480,610,53,1065,1839,1405,14,78,642,170
SLC12A4_6_2100,8861,10305,9655,10083,4548,10374,8327,13460,8508,10961,
13611,11155
SLC12A5_6_2101,585,833,920,485,463,114,566,196,465,1845,914,134
SLC12A6_6_2102,1375,840,1101,1316,163,1741,1417,1040,692,591,937,799
SLC12A8_6_2103,2605,2300,3677,2692,3131,4067,3016,3882,2246,3118,3749,
3240
SLC13A2_6_2104,1046,1339,774,1137,1650,1575,1809,642,375,864,443,631
SLC13A3_6_2105,667,628,909,657,600,359,770,906,1032,834,1365,477
SLC13A5_6_2106,1004,1205,1266,1218,2511,995,513,1644,837,351,2366,1401
SLC14A1_6_2107,2918,3810,4232,3830,2627,4029,4247,3275,2077,2807,5531,
3602
SLC14A2_6_2108,2470,2687,2423,2753,1723,3096,2032,1161,3637,2385,5527,
4027
SLC15A2_6_2109,1762,998,1644,1908,1282,1624,1688,1314,1009,1023,3017,1
300
SLC16A1_6_2110,12020,10562,12205,13147,6793,17391,14272,14073,9677,109
65,16941,8560
SLC16A3_6_2111,2647,2176,3090,2284,1367,1396,2455,3141,3182,2341,3814,
1234
SLC16A4_6_2112,3734,3194,4142,4112,5757,4089,3314,4166,4331,4143,5629,
3173
SLC16A6_6_2113,3279,2925,3850,2911,2233,1761,2780,4773,2438,2157,5082,
2745
SLC17A3_6_2114,231,343,249,196,13,5,179,218,659,116,265,1
SLC17A8_6_2115,3461,3022,3636,2874,1806,2986,2872,4478,3462,2638,4615,
3975
SLC18A1_6_2116,1960,1732,2230,3116,2796,3045,3775,1480,2481,1784,3514,
2645
SLC19A1_6_2117,687,683,945,1003,956,140,1721,817,1658,899,914,765
SLC1A2_6_2118,1931,2172,1882,2124,3394,3512,3773,1895,1260,327,2598,47
87
SLC1A3_6_2119,6443,5806,5864,5950,3860,4159,6769,6676,3167,6735,7418,8
217
SLC1A4_6_2120,1038,1187,1720,1206,95,448,1257,619,941,1872,1378,538
SLC1A5_6_2121,272,102,243,91,443,542,8,36,0,142,50,28
SLC22A12_6_2122,1661,1832,2362,2315,3306,820,4049,2182,1633,1808,1998,
1841
SLC22A17_6_2123,175,340,494,148,211,451,182,307,366,386,667,244
SLC22A18_6_2124,753,730,1541,1455,980,466,222,557,1356,585,1633,733
SLC22A1_6_2125,3917,3916,4712,4981,2867,5525,6133,5750,3445,3106,6148,

5158
SLC22A23_6_2126,899,285,843,687,146,713,339,385,1044,429,1317,1493
SLC22A6_6_2127,536,540,588,701,1503,774,264,724,246,240,2,149
SLC22A7_6_2128,321,333,293,410,1205,90,148,40,170,676,415,277
SLC22A8_6_2129,1114,537,1207,1338,2226,497,655,2601,362,303,474,1688
SLC23A3_6_2130,881,594,508,623,1504,639,307,363,1280,812,1326,1936
SLC24A2_6_2131,1477,1384,1290,1117,721,1750,1954,2022,966,3235,768,162
1
SLC24A4_6_2132,1164,596,1321,728,558,918,173,56,1559,992,188,967
SLC25A11_6_2133,2918,3433,4262,4219,4643,8736,3928,2456,3477,3176,3718
,5438
SLC25A13_6_2134,1680,1458,958,1174,1093,1303,353,3541,2519,195,1716,28
75
SLC25A14_6_2135,13033,11614,15871,15209,13074,16949,19380,13009,13700,
19647,19787,15804
SLC25A19_6_2136,325,645,550,462,266,127,85,489,21,213,36,1081
SLC25A21_6_2137,139,201,236,300,1115,0,1371,0,0,0,2,1
SLC25A22_6_2138,3006,2261,2777,2512,4229,2224,2909,2519,2776,2645,3113
,2152
SLC25A26_6_2139,7049,7978,8765,9188,10344,6611,6777,9471,5514,10441,90
45,10754
SLC25A27_6_2140,5624,5655,5755,5601,5703,11663,6392,7978,7743,8187,469
1,7231
SLC25A3_6_2141,5535,3920,5086,5651,7371,7326,2514,4768,3472,5854,8354,
5275
SLC26A11_6_2142,2199,2073,3372,2765,478,4458,3675,2108,2572,2468,2713,
1914
SLC26A1_6_2143,8769,6125,10750,7918,7312,6627,7256,9121,7837,11046,689
0,11558
SLC26A5_6_2144,3765,1371,3084,2592,4380,2169,5102,3638,5153,5906,3992,
2336
SLC26A6_6_2145,774,1093,700,868,1036,557,272,367,594,11,388,1572
SLC26A7_6_2146,4382,4322,4296,3477,3817,5133,4397,3628,4666,6983,5964,
6960
SLC26A8_6_2147,601,676,849,1052,1792,1189,1420,1088,281,861,839,377
SLC26A9_6_2148,2150,1448,1141,1492,567,2132,1074,1192,1755,1322,2062,2
077
SLC27A2_6_2149,520,308,518,362,115,194,764,1400,512,1630,817,313
SLC27A6_6_2150,72,63,30,92,2,163,0,0,5,0,31,0
SLC28A3_6_2151,2091,2861,1989,2184,1174,2445,1584,3393,1760,1605,2140,
1606
SLC29A1_6_2152,345,613,489,653,470,998,873,339,110,2363,548,473
SLC29A3_6_2153,6698,6130,6888,6706,8289,5651,6251,8461,3828,7262,8888,
4958
SLC29A4_6_2154,956,736,1023,648,1202,761,1045,1143,558,177,1816,1313
SLC2A11_6_2155,1089,1641,1431,2007,1448,789,1008,796,888,2525,2254,173
1
SLC2A5_6_2156,1101,1173,1092,1452,646,748,1443,768,1114,905,2090,701
SLC2A6_6_2157,175,766,234,357,24,67,712,1458,3,836,481,24
SLC2A9_6_2158,953,903,1324,1534,466,1421,1120,1508,975,2821,636,2024

SLC30A2_6_2159,577,325,527,643,20,13,716,681,151,1647,393,159
SLC30A6_6_2160,952,1086,1015,1497,2078,1245,205,459,1340,1550,1773,876
SLC30A7_6_2161,376,240,559,626,347,1299,125,568,388,442,525,354
SLC30A8_6_2162,1490,1494,1091,1512,765,3032,1263,582,2106,551,1093,157
4
SLC33A1_6_2163,1619,1286,1919,1717,1372,3193,3122,1969,1764,837,2286,1
134
SLC34A1_6_2164,496,746,605,709,564,1206,662,920,16,583,99,120
SLC34A2_6_2165,253,475,194,416,5,219,226,266,1067,262,811,184
SLC34A3_6_2166,5829,6668,6038,6877,5218,7269,9055,5739,4692,7403,10504
,6682
SLC35A1_6_2167,2293,3235,2362,2317,2893,3296,2551,2790,1509,3245,3609,
3856
SLC35A2_6_2168,384,116,142,580,1422,153,69,0,20,330,83,1056
SLC35B3_6_2169,451,527,502,809,154,450,425,913,86,1555,188,815
SLC35C1_6_2170,814,877,884,486,685,1333,989,1048,170,272,163,779
SLC35C2_6_2171,1685,2313,2785,2280,3250,1115,1259,2155,1470,2729,1804,
1748
SLC35E2_6_2172,966,779,1154,1088,687,2749,911,443,1357,2120,1444,706
SLC36A3_6_2173,734,743,919,1146,260,197,1207,1984,1072,612,1918,336
SLC37A2_6_2174,286,239,412,301,389,42,1010,917,214,1080,277,213
SLC37A3_6_2175,791,544,926,1082,28,2459,789,352,68,1519,2297,1119
SLC37A4_6_2176,4713,5008,5895,5241,3578,5473,7584,4003,2883,5707,5636,
3474
SLC38A11_6_2177,7017,6742,8155,8828,9035,8098,6031,9268,4502,9405,9560
,10902
SLC38A1_6_2178,2603,2607,2967,2897,3049,2426,4129,3848,1760,1623,2759,
3739
SLC38A4_6_2179,394,363,654,632,1312,652,905,335,46,47,161,378
SLC38A6_6_2180,6147,5609,6366,6679,3378,10542,7983,5303,6292,5725,8525
,6760
SLC39A10_6_2181,1691,1842,2402,3282,2040,644,4039,2257,3411,4057,4366,
2816
SLC39A11_6_2182,1935,1607,1806,2342,921,503,1808,810,1623,3000,2048,31
23
SLC39A12_6_2183,2765,2790,3806,3181,5017,3793,4732,5400,3361,3400,5173
,3426
SLC39A13_6_2184,590,307,312,498,444,1717,1,616,87,1537,291,467
SLC39A14_6_2185,18336,17135,18272,19956,23287,21049,19065,18181,17122,
22226,23762,16021
SLC39A4_6_2186,586,520,1045,939,460,4020,390,1523,626,892,530,438
SLC39A5_6_2187,219,201,231,586,24,235,1635,17,290,91,1390,987
SLC39A6_6_2188,1015,1267,939,1009,1021,132,396,626,762,1229,1736,230
SLC39A7_6_2189,3624,5414,4636,4370,2753,4755,3147,5252,4637,4629,5127,
4987
SLC39A8_6_2190,4450,4608,5390,5222,4850,6804,1906,5820,3343,4922,8258,
3899
SLC3A2_6_2191,752,995,367,860,278,132,492,346,375,1122,1561,1563
SLC43A1_6_2192,376,327,236,585,1403,373,11,925,302,610,423,853
SLC44A3_6_2193,4048,3712,4496,3479,2512,3311,4147,2978,4012,3533,4085,

3258
SLC4A10_6_2194,13803,13240,15745,14897,13400,15629,19106,17107,9358,17
199,18074,18390
SLC4A11_6_2195,700,1134,862,689,137,983,464,1367,1332,1000,1385,375
SLC4A2_6_2196,1627,1249,1822,1669,754,4136,2102,1452,981,1528,3290,167
0
SLC4A3_6_2197,879,1110,1122,1051,585,726,1248,2114,1891,2726,909,481
SLC4A4_6_2198,6817,6454,8705,8145,8829,8462,8127,6100,8941,5344,10383,
8454
SLC4A8_6_2199,1930,1786,1987,1762,687,1583,1563,1719,745,1366,2592,269
8
SLC5A10_6_2200,824,1091,700,561,0,1,341,1150,668,653,346,1218
SLC5A9_6_2201,440,377,359,305,1560,767,1091,330,368,212,347,1240
SLC6A12_6_2202,3598,3246,4937,4435,4351,5592,5995,4086,3842,4319,6689,
5369
SLC6A13_6_2203,5849,4893,6873,6514,5496,4398,8479,5942,4877,5090,10678
,5120
SLC6A15_6_2204,4667,4632,5091,6115,2993,4616,4000,5971,3915,7065,7234,
3633
SLC6A2_6_2205,714,882,820,502,78,188,605,1256,1848,1108,251,719
SLC6A6_6_2206,3444,3702,2966,3116,3874,4605,1877,3728,3905,4799,3587,4
179
SLC6A8_6_2207,2389,2130,3597,2794,2704,1785,5027,1573,3304,3054,3552,2
752
SLC6A9_6_2208,1938,1867,2082,1871,2104,1525,3760,2791,1702,3460,3547,2
785
SLC7A2_6_2209,3555,3621,3477,3873,2147,4342,3762,6018,3799,4040,5140,3
535
SLC7A3_6_2210,1253,1374,2461,1638,2026,909,1134,3189,2191,2561,2280,13
57
SLC7A6_6_2211,1499,1152,1544,1454,870,1656,708,1750,1116,1222,1996,277
7
SLC7A7_6_2212,5304,5055,6234,5673,2451,4537,8027,5117,4126,6158,5728,4
640
SLC7A8_6_2213,1513,1902,1163,2460,3805,2100,2893,1016,1840,599,1982,28
98
SLC7A9_6_2214,2263,1902,2777,3296,1974,1526,2279,2520,1781,1973,6880,6
10
SLC8A1_6_2215,5855,5219,6751,5652,4258,9740,3257,6435,6280,5080,5561,4
244
SLC8A3_6_2216,488,267,463,252,639,437,285,1543,393,264,168,398
SLC9A3R2_6_2217,1115,1287,932,1087,1425,2110,904,560,1069,921,2117,144
4
SLC9A6_6_2218,3780,3082,3871,3271,4125,6360,2579,3785,5720,4805,5712,4
573
SLCO1A2_6_2219,2346,1904,3303,2380,2152,5878,1634,2344,2225,1044,3186,
2063
SLCO1C1_6_2220,3539,3264,3604,3825,1147,3146,4221,3746,1146,3416,5578,
3047
SLCO2B1_6_2221,2851,3378,3722,3619,4890,3817,4755,5385,5039,4138,5743,

2899
SLCO3A1_6_2222,854,1307,1277,1533,742,952,1305,3301,669,709,1868,122
SLCO5A1_6_2223,1858,1453,2662,2899,3169,1621,1625,2874,1813,1365,3417,
2418
ABCB5_6_2224,2717,2755,3803,2174,2928,4057,2838,3797,3190,2244,4010,22
20
ABCC6_6_2225,4414,5075,5607,5124,3604,5646,8915,2907,5309,4651,4439,46
52
ATP1A4_6_2226,3916,3250,4233,4608,4682,5866,4669,3287,2293,6322,4655,3
691
ATP5J2_6_2227,2878,3524,4050,4055,2798,3601,2927,5619,2193,3844,4544,3
643
ATP5S_6_2228,8468,8465,10150,9856,6880,11846,6850,5243,6743,7979,11007
,8325
ATP6V1G3_6_2229,8506,8224,10291,10490,6984,7698,11607,9781,6588,7783,1
0721,13045
ATPIF1_6_2230,4627,3950,4774,5006,5421,2999,2918,4507,2256,5184,6452,3
247
SLC10A7_6_2231,2306,3233,3356,2611,1551,2429,3790,2430,2293,3654,3318,
3724
SLC28A1_6_2232,555,557,577,492,227,1721,273,348,251,209,712,342
SLC30A5_6_2233,5699,4007,5795,5422,2003,8596,7082,8547,5711,4175,4382,
5236
SLC39A3_6_2234,935,870,1064,958,939,1357,398,2368,1721,1264,2169,741
ABCA10_6_2235,7419,6399,9368,8416,8574,8054,8219,7423,8961,9506,10453,
6777
ABCA13_6_2236,1580,1842,1919,2510,1256,1118,2821,1767,2102,1717,3266,2
486
ABCA1_6_2237,3839,3459,4757,4869,3570,3625,4484,4775,4627,4397,7274,49
02
ABCA3_6_2238,526,798,1042,452,1142,132,565,729,1422,134,128,1455
ABCA4_6_2239,779,730,964,1141,863,1841,353,863,1454,637,1107,602
ABCA6_6_2240,10963,11126,12101,11775,12300,13586,12191,14154,13102,158
31,11277,11096
ABCA7_6_2241,202,298,235,889,23,1213,1015,1431,430,967,320,221
ABCA8_6_2242,1857,1716,2172,1482,990,3746,2572,709,449,2826,2890,833
ABCA9_6_2243,1433,822,1092,1291,739,1463,1437,1015,1140,1583,2584,258
ABCB10_6_2244,812,975,786,1018,769,1096,952,2354,555,593,343,1364
ABCB11_6_2245,2054,1333,1758,1977,1125,856,2488,2978,3318,115,2691,926
ABCB1_6_2246,3719,2977,4046,3070,3168,3074,4070,6479,1820,3094,7398,36
50
ABCB6_6_2247,3885,3668,5109,3357,1589,3726,4894,2626,2229,4311,5756,28
98
ABCB7_6_2248,1412,1028,1801,1633,469,2095,1591,2502,1232,1633,1982,136
4
ABCB8_6_2249,815,1089,1128,1263,1643,861,1580,1622,176,341,1374,1306
ABCC12_6_2250,1789,1115,1344,1822,3487,1227,1112,2334,1432,1477,727,18
52
ABCC2_6_2251,698,626,702,392,612,266,440,232,1053,56,1475,270
ABCC8_6_2252,3722,4130,3600,3682,4688,3992,2540,5150,2733,3869,6399,50

88
ABCD1_6_2253,2775,2724,2776,3041,572,4584,2660,4033,2882,5136,1781,213
2
ABCD2_6_2254,1855,1637,2039,2121,1796,2514,1158,1073,1454,3238,2167,17
46
ABCD4_6_2255,1729,1842,1999,2109,1204,2765,2350,3771,737,2723,2598,136
9
ABCF3_6_2256,2141,1419,2092,2095,1057,2628,2497,1490,808,3462,1952,277
6
ABCG2_6_2257,1871,1751,2669,1753,265,1001,1402,2433,3104,1204,3609,823
ABCG5_6_2258,1236,1142,1432,1299,591,1161,816,1934,870,1354,498,1192
ABCG8_6_2259,856,645,1227,933,1597,699,7,1640,1035,93,1441,83
ANXA10_6_2260,427,651,711,460,640,519,105,19,223,1510,253,1035
ANXA1_6_2261,5922,4611,5577,5345,6146,2798,6200,5866,4596,6929,6170,59
11
ANXA3_6_2262,3475,3465,3500,3135,2567,5378,6994,6278,2792,2424,3720,17
31
ANXA4_6_2263,2186,1841,2755,2621,1555,5976,1782,2432,1268,2772,1656,94
8
ANXA5_6_2264,869,516,997,693,539,58,1858,637,403,1675,1156,628
ANXA8_6_2265,9313,8420,11496,10623,6756,10184,10040,11013,7031,8055,11
365,10724
ANXA9_6_2266,1246,907,816,1055,1644,767,952,595,1329,328,2521,1110
ATP10A_6_2267,498,263,738,361,1043,264,852,1141,306,549,185,163
ATP10B_6_2268,1737,1390,1850,1651,2972,1152,1624,3033,1653,486,4048,12
93
ATP10D_6_2269,369,446,745,747,391,866,527,1384,727,672,2045,342
ATP11B_6_2270,9356,7934,9818,8416,9679,9149,8002,6571,8333,5853,12252,
9336
ATP1A2_6_2271,732,1153,754,854,44,2122,982,1116,854,1002,1286,153
ATP1A3_6_2272,3558,4109,5536,4083,4136,3876,5107,2383,5293,5610,6061,4
446
ATP1B1_6_2273,644,482,666,810,307,417,312,544,219,1366,1298,1314
ATP1B2_6_2274,490,815,749,857,579,623,130,518,301,253,32,262
ATP1B3_6_2275,742,528,1143,908,90,754,682,3313,1063,1794,1132,912
ATP4A_6_2276,0,14,0,0,0,57,0,0,0,0,0,0
ATP4B_6_2277,680,672,886,1085,798,124,1317,1125,461,359,1578,546
ATP5B_6_2278,7731,6884,9949,9289,9125,6400,8143,9754,6843,4577,10455,1
1145
ATP5F1_6_2279,37,107,149,116,0,512,0,0,0,0,0,0
ATP5I_6_2280,622,513,1260,1071,1859,1843,2254,436,1323,1621,671,967
ATP5L2_6_2281,2839,2440,2631,2486,4768,3259,2379,2712,2533,3752,2734,3
528
ATP5L_6_2282,11653,10789,14128,11949,14147,14147,15808,17200,7862,1348
0,10376,9692
ATP5O_6_2283,1638,1541,1635,1477,2270,1090,2242,2870,276,2538,3065,114
7
ATP6AP1_6_2284,597,755,953,424,631,358,226,639,684,1174,450,1133
ATP6AP2_6_2285,3131,3133,3040,3095,1747,2599,2194,2684,1890,2026,4106,
3300

ATP6V0A2_6_2286,743,661,1510,1143,1723,546,1697,1830,295,477,696,1044
ATP6V0D1_6_2287,487,479,883,852,540,855,209,280,823,2587,1450,81
ATP6V0D2_6_2288,2116,1654,1909,1925,1334,1205,2760,2231,1884,1274,1871
,2190
ATP6V0E1_6_2289,6263,4332,6777,5575,7878,5015,6786,5803,4322,5057,8460
,7230
ATP6V1A_6_2290,1287,1694,1339,1405,1259,1584,591,975,2455,1021,1620,64
3
ATP6V1B1_6_2291,752,803,903,906,1494,1463,1875,772,357,806,356,926
ATP6V1B2_6_2292,4350,3469,4686,4826,2672,5033,5168,7243,5165,3284,6364
,5286
ATP6V1C1_6_2293,4178,3468,3780,5713,4123,5316,6278,3360,3951,3980,4333
,4892
ATP6V1D_6_2294,1153,1378,1571,1432,2133,850,618,1644,3414,4465,1985,32
38
ATP6V1E2_6_2295,4711,4052,4137,4100,4378,4841,4880,5523,3511,4587,7325
,3398
ATP6V1G1_6_2296,1765,2071,2102,1692,2624,1130,3498,2182,1017,577,2451,
1214
ATP7A_6_2297,2947,2679,3384,3156,2500,1541,4548,3723,1000,3694,2933,39
43
ATP8A2_6_2298,5172,3848,5955,4954,3747,5470,3754,5983,3045,3711,8218,7
154
ATP8B1_6_2299,970,416,819,1168,533,529,2049,661,1752,971,2035,877
ATP8B4_6_2300,9186,8064,10246,11196,9292,14289,12557,10481,9560,9573,1
2892,9140
ATP9A_6_2301,1516,1764,2054,2111,2094,1917,1447,854,817,1025,2091,1049
ATP9B_6_2302,3567,2898,3709,4259,4103,5421,5223,4279,4227,2155,4230,53
10
ATPAF2_6_2303,3753,3002,3735,4129,3194,5463,4899,4626,2532,6488,3896,3
237
FLVCR1_6_2304,185,95,256,205,0,98,1,2002,227,55,57,383
RHBG_6_2305,492,499,579,485,1162,320,356,131,428,206,342,262
RHCG_6_2306,1914,1689,2292,1914,1575,3055,3117,2323,1418,2391,3246,198
1
SLC10A1_6_2307,523,296,598,426,72,877,256,466,194,98,1384,1187
SLC10A2_6_2308,1210,876,1420,920,822,675,1283,769,1272,1708,1629,1117
SLC10A4_6_2309,3468,2603,4307,4676,3121,6151,5192,3763,3028,2501,3770,
3757
SLC10A5_6_2310,3137,2897,2930,3065,1016,1636,2391,5711,1356,3024,4722,
3112
SLC10A6_6_2311,242,497,484,541,7,1039,979,609,676,210,439,433
SLC11A1_6_2312,831,835,1405,1334,70,1663,1318,1421,1908,1801,1844,520
SLC12A2_6_2313,1651,1080,1381,944,1473,1497,1134,1496,528,1008,1983,21
55
SLC12A7_6_2314,787,474,561,648,447,440,709,1362,510,1166,1206,442
SLC12A9_6_2315,1336,1007,1172,1080,738,1063,587,906,553,1099,1143,838
SLC13A1_6_2316,13695,13185,15700,14627,14439,14464,12336,13549,11992,1
7157,19793,18050
SLC13A4_6_2317,4172,4180,4129,4307,5687,2480,4477,5839,3463,5741,5642,

3662
SLC15A1_6_2318,2789,3375,3997,3727,4931,4508,2481,3152,3437,5719,4550,
3995
SLC15A3_6_2319,11215,9588,11388,11147,8815,12502,11409,9936,8936,11114
,14384,10225
SLC15A4_6_2320,2251,2569,3551,2702,2218,4424,4544,5070,1704,3165,4093,
3304
SLC16A10_6_2321,5079,4349,5981,6165,6585,5616,5358,4119,5187,7624,1014
7,6147
SLC16A11_6_2322,859,413,761,708,679,1308,212,555,1367,259,933,201
SLC16A12_6_2323,2782,2352,2701,2117,3312,4550,2959,1858,1252,1693,3085
,2685
SLC16A2_6_2324,1458,1260,1184,878,842,1751,1246,1704,452,987,482,1248
SLC16A5_6_2325,1169,1400,1253,1193,1286,2142,1229,1824,1206,1013,2667,
278
SLC16A7_6_2326,4665,5327,5436,4562,4183,6706,6175,6640,4674,6323,6854,
4388
SLC16A8_6_2327,789,1023,956,545,994,440,806,507,929,53,2029,1086
SLC17A1_6_2328,1461,1348,1824,1032,524,466,1988,1512,1677,1753,3612,27
27
SLC17A4_6_2329,1199,670,1196,1657,67,1038,3772,958,731,2962,1192,1927
SLC17A5_6_2330,25713,25630,31074,31252,26057,29192,29456,27269,29164,3
8380,39628,36881
SLC17A6_6_2331,1573,1377,2151,1328,3574,2079,1330,1613,912,1925,2485,6
72
SLC17A7_6_2332,1189,652,827,656,624,750,502,1400,247,1053,1269,248
SLC18A2_6_2333,4119,4174,5554,5695,2191,4402,4490,3628,4148,6330,6844,
2903
SLC18A3_6_2334,154,191,140,344,1417,1571,106,1,111,125,171,36
SLC19A2_6_2335,10187,10949,11188,10607,5752,16304,10579,12352,8560,971
9,11497,8649
SLC19A3_6_2336,626,432,659,558,137,1913,86,113,1178,535,99,363
SLC1A1_6_2337,559,415,1049,1040,805,1682,50,1237,345,161,1551,710
SLC1A6_6_2338,1272,646,1099,976,2207,2739,1634,842,202,951,1624,470
SLC1A7_6_2339,3618,3342,3463,3953,4734,3894,5035,4205,2820,4874,8089,3
119
SLC20A1_6_2340,4654,4533,5442,4699,1038,9517,5153,4658,3674,7574,7546,
3889
SLC20A2_6_2341,2344,3189,2774,3084,1505,3825,2017,4862,2028,3896,2885,
1268
SLC22A10_6_2342,2128,2461,2717,2155,2861,3615,2710,2968,1894,1929,2224
,3673
SLC22A11_6_2343,414,358,375,337,415,11,13,25,17,202,1037,402
SLC22A13_6_2344,582,508,863,247,78,419,869,1091,68,208,498,1247
SLC22A14_6_2345,1157,1100,1293,1102,605,1732,2274,349,880,572,1612,109
9
SLC22A15_6_2346,776,364,524,728,1004,679,230,1389,9,2240,921,1113
SLC22A16_6_2347,1025,983,1017,1308,575,935,779,514,1652,1004,1270,567
SLC22A18AS_6_2348,223,509,659,318,918,1109,55,181,217,28,692,163
SLC22A2_6_2349,7386,5858,7184,7296,7588,4866,9017,6776,5482,5576,9160,

4524
SLC22A3_6_2350,1707,2365,2118,1333,1435,1023,3663,1600,938,2216,2373,1
477
SLC22A4_6_2351,3028,1989,3249,2933,2506,2143,1299,2048,1703,2929,4115,
2241
SLC22A5_6_2352,2161,2369,2510,2665,1677,2840,3294,4001,1580,4093,2879,
3518
SLC22A9_6_2353,891,592,1055,790,372,875,1140,510,128,724,1617,338
SLC24A1_6_2354,1667,1444,1743,1534,1758,1369,1527,969,1427,1721,2679,8
51
SLC24A3_6_2355,567,290,311,475,3,800,2166,41,737,1800,784,33
SLC24A5_6_2356,14189,14337,16302,15433,14402,16137,20158,19195,12757,1
3661,18827,19143
SLC24A6_6_2357,2662,2814,3100,3940,972,4246,4700,2005,2265,3673,3828,1
905
SLC25A10_6_2358,342,48,142,97,55,470,18,0,319,166,173,75
SLC25A12_6_2359,8348,7699,9593,9724,8180,9563,9081,9544,7049,9783,1012
4,12355
SLC25A15_6_2360,7283,7091,8678,8268,9494,11047,10115,10181,7894,6490,7
213,7756
SLC25A16_6_2361,991,766,891,1038,272,725,521,1714,1230,713,1968,649
SLC25A17_6_2362,1353,1316,2033,1497,672,725,914,927,994,1825,1061,2081
SLC25A18_6_2363,1121,1339,1175,1500,1547,552,1525,305,931,1535,1554,71
3
SLC25A1_6_2364,1647,1793,1408,1094,192,968,1868,1332,571,1760,1773,180
7
SLC25A20_6_2365,810,1179,1101,1035,529,988,1513,1647,516,1416,1900,193
7
SLC25A28_6_2366,901,637,1310,365,1299,174,1127,1541,446,873,1597,2111
SLC25A2_6_2367,1815,1053,2588,2821,1204,2226,2330,3414,2664,1347,3019,
1732
SLC25A32_6_2368,3107,1901,2367,2968,2626,2380,4794,3697,2728,1199,4274
,4029
SLC25A37_6_2369,3207,3019,3712,3422,2054,6086,2724,4570,2886,3059,4507
,2418
SLC25A43_6_2370,1601,1611,2094,2263,2911,2936,2509,1673,1611,2232,3871
,1673
SLC25A44_6_2371,847,552,1335,535,493,1025,293,1282,405,1600,306,355
SLC25A4_6_2372,1454,824,2027,1570,2168,4100,2256,2059,1111,1979,1782,1
568
SLC25A5_6_2373,868,1016,676,482,1006,300,1658,671,782,3371,1523,271
SLC25A6_6_2374,1316,1269,1401,1268,1313,422,724,554,837,819,1990,2525
SLC26A10_6_2375,897,753,900,828,661,1501,1691,379,53,1682,544,2101
SLC26A2_6_2376,2420,1904,1649,1695,328,834,397,1153,647,2979,2350,2153
SLC26A3_6_2377,1880,1822,2699,3501,2807,3066,1962,2361,2632,2023,4440,
1912
SLC26A4_6_2378,1252,979,1326,1542,1181,981,1816,2534,1078,1269,2888,21
67
SLC27A1_6_2379,7872,4827,7732,8749,4548,7580,10439,8692,4845,9357,8351
,7758

SLC27A3_6_2380,479,743,1000,804,2989,2258,364,2022,732,1777,1888,528
SLC27A4_6_2381,530,537,485,487,199,138,501,644,400,606,226,397
SLC27A5_6_2382,926,929,940,1015,599,2147,633,2546,851,3196,1334,596
SLC28A2_6_2383,3061,3196,3838,3983,5208,4982,4129,4436,3135,3219,7476,7053
SLC29A2_6_2384,495,313,477,690,183,300,146,232,260,464,1374,525
SLC2A10_6_2385,497,485,799,593,237,610,83,780,786,490,228,569
SLC2A12_6_2386,597,689,1228,1263,1557,2106,1084,172,479,1058,147,1530
SLC2A13_6_2387,2803,1721,2658,3282,2456,2446,4810,3565,1987,2490,3155,2251
SLC2A14_6_2388,3259,2814,4246,3892,2335,2381,2730,4220,2949,3658,6947,3131
SLC2A1_6_2389,3252,3589,4371,3709,3782,3810,4752,3309,3661,2769,5010,4471
SLC2A2_6_2390,7007,7755,7278,7759,5033,10105,8238,8960,7678,11859,11760,6118
SLC2A3_6_2391,5549,5599,7807,7224,6827,9159,6029,7462,4346,7611,9771,6275
SLC2A4_6_2392,921,1079,670,943,811,711,1758,675,1704,1316,1790,1066
SLC2A4RG_6_2393,691,561,788,770,455,2955,168,406,515,831,1198,762
SLC2A7_6_2394,4910,4449,5199,4895,7351,8648,5455,5024,2209,5665,5735,6545
SLC2A8_6_2395,3481,3370,4305,4446,3464,4020,6033,3386,1100,2518,5919,4405
SLC30A10_6_2396,3442,3331,3413,3633,5563,3770,4812,4826,2347,3497,4615,4774
SLC30A1_6_2397,318,243,391,415,18,21,420,703,226,12,541,76
SLC30A3_6_2398,4514,3544,4065,4026,3115,5075,6406,3441,3270,5985,5970,3766
SLC30A4_6_2399,3390,2067,3354,3320,1648,3491,4118,4766,2453,3716,1878,2182
SLC30A9_6_2400,694,580,930,1175,285,366,876,1023,118,1126,1595,886
SLC31A1_6_2401,3106,2687,2849,3670,4269,2331,2537,3694,2062,3244,2626,3981
SLC31A2_6_2402,2993,2414,3095,3301,3067,4575,3262,7269,2226,1735,4429,2503
SLC32A1_6_2403,426,681,413,517,483,142,1198,474,113,49,177,785
SLC35A3_6_2404,10602,9346,10346,9606,11709,10290,8888,10566,8219,9604,11434,9794
SLC35A4_6_2405,1734,1648,1472,1653,3075,2073,2052,2652,2584,1957,3240,3465
SLC35A5_6_2406,14259,11358,12436,14108,11844,12479,12169,9448,14884,13995,16543,11639
SLC35B1_6_2407,2874,2394,3496,3389,2947,2147,5875,5265,1742,2120,3273,2273
SLC35B2_6_2408,566,514,369,557,291,420,96,446,379,80,277,1088
SLC35B4_6_2409,770,495,1282,1059,3038,258,832,557,346,1610,407,519
SLC35D1_6_2410,1384,1172,1604,1493,1423,1056,2872,1931,1188,2039,4138,1468
SLC35D2_6_2411,445,509,537,250,764,201,337,1181,627,853,775,344

SLC35D3_6_2412,840,649,436,542,1093,631,611,308,677,181,51,2019
SLC35E1_6_2413,526,319,223,516,455,401,926,339,199,66,708,333
SLC35E3_6_2414,444,891,940,1092,400,732,348,1401,269,422,1161,1554
SLC35E4_6_2415,403,296,496,277,8,1298,19,608,259,292,894,519
SLC35F1_6_2416,879,308,786,791,861,1084,891,1003,631,250,65,519
SLC35F2_6_2417,2293,2231,2659,2989,1417,2812,3344,2475,2812,2461,2360,
2441
SLC35F3_6_2418,1010,1042,1384,1219,375,866,721,1874,543,2519,961,1009
SLC35F4_6_2419,2647,2531,2876,2679,1822,3471,2894,3883,2109,3215,3559,
2144
SLC35F5_6_2420,13047,12819,15821,14196,12468,13261,16025,14396,14124,1
4356,18753,15258
SLC36A1_6_2421,4458,3896,5297,5430,6977,5752,3951,3489,4124,6273,6618,
6935
SLC36A2_6_2422,532,944,895,916,819,150,743,449,614,566,2037,806
SLC36A4_6_2423,2302,1361,2313,1860,1910,1056,2745,2437,1824,1159,1849,
1754
SLC37A1_6_2424,1071,1145,1863,1611,1153,3554,2439,3226,535,2483,1679,1
298
SLC38A2_6_2425,983,988,1469,1391,988,2288,2096,1311,1528,1366,1439,115
2
SLC38A3_6_2426,882,880,1161,1074,695,1125,362,465,877,1197,1351,1062
SLC38A5_6_2427,578,340,660,696,137,749,82,959,267,929,396,64
SLC39A1_6_2428,146,348,322,214,0,183,79,10,273,124,158,803
SLC39A2_6_2429,3647,3745,4275,4184,4598,2008,1679,4010,3772,4003,2703,
4916
SLC39A9_6_2430,636,1701,1590,1721,2258,123,2469,1894,1110,1188,1167,43
3
SLC3A1_6_2431,2845,3627,2666,3191,3173,3883,2756,3356,3117,3643,3915,2
074
SLC40A1_6_2432,1290,1768,2704,1597,1464,1156,1651,1707,2066,482,1909,1
184
SLC41A1_6_2433,1844,2557,2203,2512,1467,2220,1454,4246,3689,1754,3374,
2061
SLC43A2_6_2434,661,665,1140,964,128,998,1670,3012,161,866,1492,215
SLC44A1_6_2435,1833,2407,2121,1687,2493,2576,1895,3473,2106,2811,3108,
1043
SLC46A2_6_2436,505,207,536,543,620,935,630,452,302,11,152,84
SLC4A1AP_6_2437,1193,1456,1723,1301,2252,467,1884,2693,554,965,2959,11
45
SLC4A1_6_2438,305,586,377,477,113,213,408,1126,132,1068,57,248
SLC4A7_6_2439,989,854,1265,1504,1198,870,1000,1245,996,4088,682,572
SLC4A9_6_2440,2072,1198,2045,2818,3260,2803,1249,2599,2500,2581,3672,1
482
SLC5A11_6_2441,2497,3320,3496,2812,1900,4544,3803,3724,2241,3402,3680,
2543
SLC5A1_6_2442,1263,1082,1224,1505,537,851,1060,2210,601,949,1338,1960
SLC5A2_6_2443,10446,8942,10871,10935,12562,10634,7787,13812,7252,10563
,12146,9938
SLC5A3_6_2444,1899,1885,1857,2142,748,591,3102,2387,1212,2194,2577,786

SLC5A4_6_2445,4219,3858,3465,3436,3923,4375,5832,2059,2064,3478,5320,2
420
SLC5A5_6_2446,964,1321,1222,1158,1142,400,870,839,974,2620,2221,1121
SLC5A6_6_2447,1465,909,1068,1722,916,1624,1443,425,987,1266,5199,1425
SLC5A7_6_2448,1730,1554,2465,2279,1189,1497,1128,1489,1507,1556,3766,1
197
SLC5A8_6_2449,1670,1169,1918,1665,2675,2876,2552,3445,2333,579,1591,21
32
SLC6A11_6_2450,10,0,0,0,0,0,0,0,0,0,0,0
SLC6A14_6_2451,6896,5995,6377,6827,5320,6520,9102,6625,4304,6411,6240,
6324
SLC6A16_6_2452,835,744,714,855,418,1175,322,725,1560,1524,420,1228
SLC6A19_6_2453,4560,4636,5360,5271,4782,4461,4854,6974,3852,5754,8049,
5214
SLC6A1_6_2454,341,336,555,313,409,143,16,62,423,507,79,284
SLC6A3_6_2455,820,672,686,583,703,58,703,420,439,596,383,486
SLC6A4_6_2456,3218,2533,3078,3068,2387,1334,4979,1589,1642,1512,5180,1
831
SLC6A7_6_2457,610,577,838,911,685,141,217,1855,299,569,1162,542
SLC7A10_6_2458,1103,533,1147,912,1213,207,1276,615,353,1654,1620,1158
SLC7A11_6_2459,578,794,1124,1259,212,1966,1840,1626,610,1484,926,1238
SLC7A13_6_2460,3652,3764,3927,3491,3555,4790,4120,5379,2634,4048,3466,
4432
SLC7A1_6_2461,4358,4195,5165,4372,4876,4786,3903,5968,5018,2534,5157,6
200
SLC7A4_6_2462,712,666,619,1065,963,1409,622,1028,448,202,658,1045
SLC7A5_6_2463,773,487,478,1056,39,1143,783,81,1356,440,664,1668
SLC8A2_6_2464,110,321,355,261,66,529,686,14,260,433,1748,935
SLC9A1_6_2465,6923,6250,8298,7494,4237,9532,9332,5741,7852,6348,7293,5
284
SLC9A2_6_2466,6051,5038,7104,7993,5737,5242,7660,10905,4109,4767,8755,
9976
SLC9A3_6_2467,635,978,743,721,389,335,429,1354,451,906,626,494
SLC9A3R1_6_2468,2152,1633,1962,2363,1916,1698,3788,2967,4272,2536,3355
,1021
SLC9A4_6_2469,6865,6599,9128,8493,7003,8231,8968,10226,8302,5734,11705
,8249
SLC9A5_6_2470,939,647,918,1134,917,531,1638,1123,1203,3126,1930,1643
SLC9A7_6_2471,946,1137,2013,1332,545,552,1406,1041,1729,102,1759,273
SLC9A8_6_2472,5630,6075,6696,7184,7130,11950,7663,4291,6014,3564,8202,
10213
SLC9A9_6_2473,242,275,537,520,128,369,148,252,1296,221,681,858
SLCO1B1_6_2474,1722,1250,1721,1201,1409,1328,2007,2170,1360,3302,1413,
3268
SLCO1B3_6_2475,4081,5639,5669,5816,2597,4827,2959,3761,5370,3678,5475,
4073
SLCO2A1_6_2476,1447,1714,2031,1361,1064,2337,2031,3507,1891,1882,3548,
938
SLCO4A1_6_2477,448,937,1213,761,874,700,862,835,668,1576,924,1361
SLCO4C1_6_2478,933,1158,1648,1285,2008,623,1774,2066,1750,2286,2080,21

69
SLCO6A1_6_2479,2986,3735,4165,3718,3756,5952,2807,5090,2404,6159,4913,
2988
SV2A_6_2480,2389,1890,2124,2770,1871,2191,3298,2804,2223,568,3934,3430
TAP1_6_2481,2280,2378,3636,3006,1943,1771,2951,2852,1549,1639,2751,311
6
UCP1_6_2482,8674,7672,8990,7894,9992,7991,8342,8675,6653,10764,8876,10
331
ABCA12_6_2483,5398,5008,5779,5077,7067,6086,6060,6152,4711,5952,6001,7
188
ABCA2_6_2484,1574,2094,1326,1599,677,1865,3320,1576,1170,3547,2467,248
6
ABCA5_6_2485,2877,3109,3709,4173,5384,5214,3377,3176,4009,3941,2987,22
20
ABCB4_6_2486,4412,4737,3677,3986,4124,3665,5535,2985,4614,7762,5482,44
02
ABCB9_6_2487,727,843,1363,1439,760,360,704,4391,458,912,681,3122
ABCC10_6_2488,1851,2029,1967,2540,2732,1890,1841,1147,495,560,3063,220
8
ABCC11_6_2489,226,251,199,334,547,10,380,48,712,182,85,71
ABCC1_6_2490,2242,1337,2234,2191,1753,3938,1665,199,2200,2188,1883,214
7
ABCC3_6_2491,1440,991,1391,1258,1612,1061,1002,1411,1123,941,2105,1429
ABCC4_6_2492,8173,8130,9383,8494,5581,9965,9378,8939,6896,10030,7263,1
0003
ABCC5_6_2493,617,475,535,847,538,179,842,779,1943,898,788,1303
ABCC9_6_2494,996,1093,1184,1135,1550,1042,1696,1359,907,1289,1212,975
ABCD3_6_2495,3029,3204,3307,3570,2176,7128,4841,4766,2363,3106,4439,21
29
ABCE1_6_2496,1038,1668,1737,1430,2392,2961,2664,2155,1982,2541,1856,19
05
ABCF1_6_2497,1197,480,982,1376,1220,1673,865,136,877,1099,1113,696
ABCF2_6_2498,893,1250,793,1138,1222,1365,1003,736,146,973,1218,1157
ABCG1_6_2499,2885,3279,2976,2091,1836,3961,1885,3626,1463,2998,2257,10
92
ABCG4_6_2500,405,1079,942,1260,920,1054,1408,1421,1278,495,1406,1171
ANXA11_6_2501,4753,4747,5808,4285,8183,6884,3004,3527,3455,4221,6979,4
107
ANXA13_6_2502,1069,1097,1626,1476,1156,1349,3073,411,844,1864,2247,124
1
ANXA2_6_2503,680,613,553,832,413,262,70,854,1008,1017,575,399
ANXA6_6_2504,612,365,624,600,546,211,98,247,820,81,893,181
ANXA7_6_2505,1012,1337,1354,1270,1018,1830,542,837,1726,1213,1788,1967
ATP11A_6_2506,250,276,164,233,907,5,0,220,69,0,3,481
ATP11C_6_2507,1576,1544,2165,1813,1977,1638,2265,1135,1336,311,2182,13
60
ATP12A_6_2508,4006,3216,4209,3763,5559,3798,3749,6609,4402,1755,3395,4
494
ATP1A1_6_2509,5253,4437,5980,5252,5919,5852,5590,6062,4266,7343,8714,5
239

ATP1B4_6_2510,12844,13502,15286,15603,11743,16300,12715,16385,11404,12
060,21080,17061
ATP2A1_6_2511,778,601,888,766,83,988,567,605,734,635,1957,488
ATP2A2_6_2512,3400,2211,4011,4084,3339,3545,3278,5828,2861,2593,4170,2
129
ATP2A3_6_2513,2782,2886,3618,3596,2209,4680,3614,3676,1852,2455,3195,2
635
ATP2B1_6_2514,1919,1395,1539,2337,1898,1160,1041,1768,1379,2464,2843,9
53
ATP2B3_6_2515,2168,1953,1593,1306,2029,2126,231,1664,1276,2169,2763,10
46
ATP2B4_6_2516,2109,1910,2385,2324,3829,3683,1354,2032,2169,1000,2636,2
593
ATP2C1_6_2517,6409,6313,6250,7144,7099,8052,10048,7773,7475,7098,9394,
7093
ATP5A1_6_2518,4398,3694,4308,5135,3543,4162,5521,4080,4953,3197,4944,8
512
ATP5C1_6_2519,1102,1204,1310,954,1006,1482,1190,1272,1468,1595,1340,15
82
ATP5D_6_2520,6605,6521,6552,6326,6972,6397,5873,6778,5171,5938,7231,65
45
ATP5E_6_2521,834,723,808,1188,831,993,265,168,1813,1153,1946,1231
ATP5G1_6_2522,1897,1981,2371,2679,2990,535,3386,2686,1850,4285,3761,15
23
ATP5G2_6_2523,1238,623,1054,1203,789,1122,1180,367,743,1306,3251,1304
ATP5G3_6_2524,1008,1015,1811,1496,987,2202,1280,1620,658,1094,1597,127
6
ATP5H_6_2525,3136,3070,3067,2503,3475,1958,1720,2996,1928,1962,3440,11
41
ATP5J_6_2526,1921,1622,2527,1735,2126,2498,1691,2143,968,2548,3150,226
2
ATP6V0A1_6_2527,862,785,1099,1154,1911,1366,2068,2280,1352,329,2258,46
2
ATP6V0A4_6_2528,2875,3166,3153,3360,3709,3418,3169,3314,2455,3353,1873
,2956
ATP6V0B_6_2529,2402,2220,2067,2372,949,2168,2829,2941,1114,3014,2726,1
355
ATP6V0C_6_2530,237,259,477,292,54,207,197,1017,244,0,350,451
ATP6V1C2_6_2531,1331,1702,2321,2302,3173,1107,3707,2595,2165,2386,2865
,3418
ATP6V1E1_6_2532,2125,2344,2584,2350,2216,2410,2966,1754,2473,1631,4059
,4388
ATP6V1F_6_2533,1139,1494,1946,1646,835,884,2337,2292,482,1346,954,633
ATP6V1G2_6_2534,1190,1472,1008,1468,1901,1403,147,1157,1340,996,1490,1
869
ATP6V1H_6_2535,6018,6523,5915,6756,11287,5411,6271,9747,6667,7808,9703
,6207
ATP7B_6_2536,1693,1690,1306,2083,1672,658,1340,3446,1299,859,3116,2343
ATP8A1_6_2537,1854,2396,2489,1927,966,2681,1069,1914,1851,3314,2549,28
13

ATP8B2_6_2538,880,812,1031,383,541,189,1911,340,465,1239,332,933
ATP8B3_6_2539,547,362,558,313,298,1196,27,804,279,240,210,480
ATPAF1_6_2540,673,1069,1082,996,931,1507,126,535,590,1618,1243,712
GC_6_2541,901,1261,1142,961,1105,1451,1185,915,1260,479,2024,1006
SERINC5_6_2542,17149,16080,19360,18375,13570,19072,15990,17581,14104,2
0816,22919,17936
SLC10A3_6_2543,883,415,1086,1275,660,179,268,535,787,2467,1613,555
SLC11A2_6_2544,1090,976,1317,871,1100,1578,2056,1078,1482,439,1044,106
2
SLC12A1_6_2545,2784,2864,3315,3005,3198,3900,3400,1688,3505,4392,3366,
4830
SLC12A3_6_2546,849,854,1014,773,593,1530,1320,935,1099,1470,666,1490
SLC12A4_6_2547,1723,1868,1804,2214,3623,934,2073,1121,1789,2301,2937,4
89
SLC12A5_6_2548,429,293,805,403,3035,604,1023,115,200,128,728,151
SLC12A6_6_2549,1054,1294,1044,1196,1665,326,796,862,682,1830,2421,1711
SLC12A8_6_2550,5798,5228,7469,6259,4637,9735,7030,6409,4083,5438,7355,
5763
SLC13A2_6_2551,1991,2581,2897,2829,2364,1854,2033,3162,4419,3216,4692,
1398
SLC13A3_6_2552,2204,2329,2316,2642,1594,1615,2136,2982,2372,855,3582,2
518
SLC13A5_6_2553,1178,1370,1374,1434,520,2508,1149,1318,1027,1036,2083,2
868
SLC14A1_6_2554,771,651,1374,823,1004,494,944,289,603,215,1329,1429
SLC14A2_6_2555,577,491,626,469,530,1360,1496,1313,698,577,865,1255
SLC15A2_6_2556,496,360,542,692,502,1065,913,1690,271,305,1357,658
SLC16A1_6_2557,4449,4120,3416,4365,5738,3240,5106,4067,6580,5746,4132,
4461
SLC16A3_6_2558,1475,1612,2545,2395,1153,599,1801,1466,956,2904,1410,21
55
SLC16A4_6_2559,2425,1895,3227,2541,1825,2573,2223,3077,2430,3247,3137,
3372
SLC16A6_6_2560,1170,744,1023,1646,591,2007,1572,2581,242,730,1340,314
SLC17A3_6_2561,1992,1983,2332,2351,1683,339,1850,2415,2322,1557,2188,2
063
SLC17A8_6_2562,935,820,1363,1563,1701,995,1605,1448,715,452,1960,1771
SLC18A1_6_2563,5193,5156,6270,5373,6806,2367,7043,5380,4322,7450,8415,
4313
SLC19A1_6_2564,734,706,1087,1365,519,995,1285,465,1088,486,672,2201
SLC1A2_6_2565,891,606,924,1043,682,268,594,2130,1707,594,757,939
SLC1A3_6_2566,1428,1091,1765,1828,459,1477,1981,1161,1095,3011,2277,24
98
SLC1A4_6_2567,329,245,670,257,273,0,611,833,449,192,595,118
SLC1A5_6_2568,1479,1530,2188,2067,1640,2893,1596,841,1360,1396,2566,15
52
SLC22A12_6_2569,1196,1120,1726,1824,654,848,2295,3088,1447,2446,2925,1
897
SLC22A17_6_2570,1108,1176,1257,999,784,937,1256,1071,286,673,1184,1172
SLC22A18_6_2571,463,889,590,815,1619,1190,644,1174,675,2000,1180,1097

SLC22A1_6_2572,3362,3457,3351,2988,1388,5317,3429,3273,2675,3103,2800,
3033
SLC22A23_6_2573,3342,3378,3521,4094,2920,3849,5162,4308,2109,4065,5749
,2999
SLC22A6_6_2574,2493,2257,3257,2350,2527,1124,3171,2173,3435,3423,2300,
3368
SLC22A7_6_2575,269,345,251,260,8,1096,353,16,3,374,24,981
SLC22A8_6_2576,1212,1374,1388,1200,1480,2329,1071,1014,1038,944,1120,1
396
SLC23A3_6_2577,1465,1677,2022,1963,1108,2106,1999,1092,1265,1594,1963,
1064
SLC24A2_6_2578,4029,4382,5050,5263,5904,4305,6119,7498,4817,6876,6708,
3907
SLC24A4_6_2579,2141,2668,2645,2443,2777,2784,2733,2006,1134,2752,4960,
3074
SLC25A11_6_2580,1647,1481,2707,2156,2953,1026,527,2193,1468,1045,1442,
2599
SLC25A13_6_2581,667,981,1096,993,965,1824,752,2005,797,422,865,1594
SLC25A14_6_2582,4470,4767,5299,4514,4468,4082,4827,2501,2623,6493,6223
,5980
SLC25A19_6_2583,953,749,673,502,612,1985,635,337,365,1813,771,52
SLC25A21_6_2584,1840,2043,2464,2608,1316,1952,4263,1720,2127,2065,2896
,3254
SLC25A22_6_2585,255,217,386,398,0,669,12,251,148,16,789,713
SLC25A26_6_2586,9042,9105,10706,9858,10092,8629,9824,8828,9373,11293,1
0642,10229
SLC25A27_6_2587,5152,3856,5158,4954,4563,6919,5950,4711,4530,3925,6203
,6046
SLC25A3_6_2588,2299,3027,2624,2548,1007,5991,4773,1500,2482,3207,2670,
1554
SLC26A11_6_2589,1337,920,1321,1161,1149,944,633,1991,1939,995,1055,753
SLC26A1_6_2590,1514,1309,1473,1374,255,927,1738,977,1084,1322,2949,110
6
SLC26A5_6_2591,1011,569,733,1049,320,284,286,1014,1193,1086,1553,708
SLC26A6_6_2592,928,1561,1664,1194,1268,670,913,503,577,637,926,1804
SLC26A7_6_2593,727,917,900,1094,1313,723,985,901,210,668,934,1576
SLC26A8_6_2594,5208,6475,8170,7177,4827,5274,6774,7417,5733,6625,6845,
7703
SLC26A9_6_2595,763,483,680,824,97,596,1416,639,1082,592,482,91
SLC27A2_6_2596,1883,1695,3202,1808,1795,2258,2651,2195,1257,1589,3005,
3017
SLC27A6_6_2597,1828,2678,2091,2098,1206,4305,3821,2114,1900,4190,2380,
3658
SLC28A3_6_2598,3430,2306,3149,2099,2898,2943,1979,2454,3668,3007,2157,
1245
SLC29A1_6_2599,4818,4248,4758,4738,4269,3465,7545,3843,2999,4120,5634,
4628
SLC29A3_6_2600,8177,7043,7625,8817,9959,10647,7175,8662,8450,8502,8676
,8525
SLC29A4_6_2601,741,545,938,681,895,181,685,1070,347,427,1638,2035

SLC2A11_6_2602,1440,1232,1689,2063,1259,340,151,1049,840,1366,2184,512
2
SLC2A5_6_2603,2082,2252,1862,2156,2410,2574,1829,2799,1703,1224,3103,1
771
SLC2A6_6_2604,261,502,651,321,15,325,25,1261,296,1021,1509,1012
SLC2A9_6_2605,2644,1586,2643,2790,1593,2768,1666,3043,904,4215,2588,29
56
SLC30A2_6_2606,185,365,259,185,100,814,648,68,273,364,76,176
SLC30A6_6_2607,4955,5830,6275,6679,4656,8159,8349,5510,3101,4967,6372,
6221
SLC30A7_6_2608,10475,9398,11863,10681,10984,13022,10945,9080,9295,1507
7,12372,9089
SLC30A8_6_2609,1209,686,804,1182,922,262,662,567,903,1444,1500,1736
SLC33A1_6_2610,2757,1848,2351,3078,1768,5059,368,1775,1606,2704,4115,1
839
SLC34A1_6_2611,1011,1311,1967,1755,746,1703,1100,1284,1998,1795,2510,1
881
SLC34A2_6_2612,2635,2362,3198,3053,2730,4012,3019,3356,3107,2138,3150,
2731
SLC34A3_6_2613,1597,1759,1621,1399,673,1544,2044,1111,2517,2291,2319,1
028
SLC35A1_6_2614,1502,1136,1789,1769,1702,3290,1866,2408,2033,972,1491,1
023
SLC35A2_6_2615,2735,3095,3609,2764,4030,3627,1956,2872,1684,4615,6689,
3514
SLC35B3_6_2616,1751,1233,1325,1605,1116,1608,2697,2928,1658,1622,3510,
2273
SLC35C1_6_2617,291,232,639,544,224,419,14,97,1237,1340,781,12
SLC35C2_6_2618,2187,1390,2958,2184,2844,1434,1196,600,2356,3028,2967,9
59
SLC35E2_6_2619,2560,2930,3124,3293,1777,5750,4199,1472,2310,4879,5596,
2091
SLC36A3_6_2620,1571,1477,1400,1646,2537,1322,1760,876,939,1608,2034,82
9
SLC37A2_6_2621,2603,1409,2225,2099,2902,1631,1476,1122,1218,1947,2704,
1413
SLC37A3_6_2622,4331,3147,4212,4351,3807,2976,4159,4459,4612,2002,7726,
4584
SLC37A4_6_2623,803,1024,1259,1437,1986,208,401,2384,633,2451,1014,1082
SLC38A11_6_2624,4103,4294,4312,4539,2257,3935,3751,7530,3936,5691,8423
,2449
SLC38A1_6_2625,797,544,874,1127,890,841,2554,1242,573,435,1457,580
SLC38A4_6_2626,1450,1391,1647,1458,1972,962,551,1081,2059,1205,1652,12
17
SLC38A6_6_2627,1330,988,2389,1885,736,2845,1518,2032,1948,2154,2381,20
19
SLC39A10_6_2628,6318,4755,5955,5689,6746,6310,4151,5492,4483,5761,6352
,6256
SLC39A11_6_2629,250,129,338,203,69,0,112,2,408,687,67,0
SLC39A12_6_2630,619,822,957,656,1186,596,278,299,190,264,1449,1734

SLC39A13_6_2631,2019,2493,2826,2544,2134,2064,2129,3099,2455,2429,4221
,3925
SLC39A14_6_2632,1694,1583,2069,1853,1269,1870,727,3287,1301,770,1607,8
58
SLC39A4_6_2633,6376,6623,7036,7566,6940,6067,4817,6806,6012,9423,8478,
6986
SLC39A5_6_2634,602,393,344,651,982,23,896,9,10,1237,232,1331
SLC39A6_6_2635,1266,1928,1965,2571,2574,2548,2682,1130,1025,2049,1023,
1126
SLC39A7_6_2636,2193,2619,2719,2950,1865,1069,1743,3116,2025,3876,2191,
1661
SLC39A8_6_2637,534,517,759,1095,1354,574,385,461,98,685,763,1197
SLC3A2_6_2638,9497,8821,10785,10493,8779,8039,10737,12153,6962,7616,13
974,9527
SLC43A1_6_2639,494,833,507,1077,683,180,1078,1338,80,1675,1587,359
SLC44A3_6_2640,1973,2228,2222,2464,1885,3087,873,1898,1763,3320,3706,2
465
SLC4A10_6_2641,1646,2094,2006,2240,2057,1377,1644,2830,3592,3396,2983,
2339
SLC4A11_6_2642,393,690,524,602,254,299,763,144,127,527,725,845
SLC4A2_6_2643,632,346,481,700,21,568,551,447,137,1051,1123,383
SLC4A3_6_2644,1096,979,1331,1013,895,682,484,543,2373,225,1527,84
SLC4A4_6_2645,1164,888,1590,1267,1820,995,2413,1105,899,912,998,1276
SLC4A8_6_2646,1988,2396,2703,2962,768,1731,2032,2520,790,2851,3583,114
2
SLC5A10_6_2647,579,484,523,598,1423,694,563,156,293,284,1931,433
SLC5A9_6_2648,1336,1246,1529,1530,230,1996,3167,899,1588,581,2172,1454
SLC6A12_6_2649,6087,5282,6620,5959,4539,9125,6102,4410,3886,5561,8152,
6906
SLC6A13_6_2650,3074,3381,3176,3286,2609,2956,2558,4914,1375,4699,5820,
3806
SLC6A15_6_2651,2036,2149,2810,1925,1467,3936,1804,2040,1881,3410,1912,
2227
SLC6A2_6_2652,752,736,1018,1246,2235,1656,2486,1283,1020,938,1456,850
SLC6A6_6_2653,2552,2576,2576,3237,1159,2377,4548,2227,2125,1086,4036,3
764
SLC6A8_6_2654,922,1172,1278,1023,613,302,2324,857,1641,731,1467,587
SLC6A9_6_2655,6349,6201,8858,8539,5819,8226,10466,7189,5984,7278,12208
,8666
SLC7A2_6_2656,1959,1118,2120,2236,2115,1651,3825,2035,1980,515,2547,13
05
SLC7A3_6_2657,687,1201,1079,980,123,147,948,1571,627,1518,1041,891
SLC7A6_6_2658,1194,1523,1669,1510,655,1861,823,2447,699,804,2732,1070
SLC7A7_6_2659,2041,1852,2099,2082,2319,2031,2089,2102,1553,2276,2896,2
496
SLC7A8_6_2660,2736,2177,2286,2341,2119,2783,1754,2084,3294,1690,3333,3
115
SLC7A9_6_2661,2540,2745,3306,2749,1311,1998,2933,1463,2766,2712,2994,1
579
SLC8A1_6_2662,1275,1536,1198,1161,2032,1986,888,1637,577,1718,1351,153

1
SLC8A3_6_2663,245,210,286,463,362,127,28,2,17,207,968,486
SLC9A3R2_6_2664,235,100,414,677,72,12,272,504,75,302,1095,565
SLC9A6_6_2665,2322,2240,3428,3465,3781,1382,2244,3570,1723,3890,4490,2
242
SLCO1A2_6_2666,839,1287,1388,1874,2182,3101,1218,1860,368,2281,1202,15
58
SLCO1C1_6_2667,4488,4412,4629,4775,3715,8313,5163,4582,5303,5362,5817,
5288
SLCO2B1_6_2668,459,130,178,494,0,0,222,3,42,346,184,965
SLCO3A1_6_2669,513,275,370,245,25,361,916,55,0,556,234,584
SLCO5A1_6_2670,2019,2423,2634,2507,1968,3485,2451,2698,1278,4555,2889,
2239
ABCB5_6_2671,1103,1411,1817,1664,1828,876,2113,828,1627,1540,1129,2624
ABCC6_6_2672,1576,1911,1479,1319,1352,2642,1922,1398,2866,1796,1175,16
24
ATP1A4_6_2673,4782,4750,5887,5321,4403,4809,6009,3333,7442,8084,5601,4
927
ATP5J2_6_2674,868,934,887,1277,1222,1982,571,851,1160,302,1786,309
ATP5S_6_2675,4704,3528,5745,4992,5777,3856,7038,7418,2550,7769,5229,62
87
ATP6V1G3_6_2676,18065,19576,21636,21997,21539,26467,20048,20525,16300,
23835,28684,24106
ATPIF1_6_2677,475,190,789,348,895,1014,555,435,1333,611,840,199
SLC10A7_6_2678,4069,3344,4763,4125,5221,3240,6043,6574,4756,3281,4416,
3420
SLC28A1_6_2679,818,681,918,952,1,421,47,490,949,709,1689,1625
SLC30A5_6_2680,1837,1749,1823,1912,3279,2192,2731,1437,1190,1114,2229,
1900
SLC39A3_6_2681,5298,5794,6759,5722,3940,5878,6236,6098,4787,6336,11465
,6430
ABCA10_6_2682,4110,4437,3561,4303,4237,6106,2613,5572,3889,3782,6012,4
643
ABCA13_6_2683,11607,9493,11499,11097,10623,18377,9728,15093,7470,13712
,12898,11495
ABCA1_6_2684,632,356,1032,596,198,13,1342,272,945,783,1635,478
ABCA3_6_2685,488,360,786,468,204,1276,316,89,233,730,511,4
ABCA4_6_2686,3956,3642,4068,5560,3115,4123,4425,4801,3266,5162,5776,30
16
ABCA6_6_2687,958,1354,1127,1226,1610,1450,2464,2047,1588,264,1658,1480
ABCA7_6_2688,835,937,592,1012,1509,824,351,1656,718,2401,746,1202
ABCA8_6_2689,3005,2822,3532,3490,2990,1435,4242,2421,2070,3671,6422,41
33
ABCA9_6_2690,1091,1416,1633,1651,1880,3911,1633,2176,1351,1003,1820,36
1
ABCB10_6_2691,1050,1002,1865,1883,1432,1417,1489,2151,3232,452,1566,71
ABCB11_6_2692,393,307,440,351,71,2,0,345,46,31,185,73
ABCB1_6_2693,11967,10053,12591,12830,10441,12867,12784,15010,10137,123
97,12296,10939
ABCB6_6_2694,649,542,455,445,1889,157,648,1400,462,178,349,342

ABCB7_6_2695,1390,1414,1830,1656,400,1344,265,2495,1120,2059,3048,884
ABCB8_6_2696,1099,695,1195,899,1955,740,1435,1542,1028,2613,1796,507
ABCC12_6_2697,4142,4194,5997,5368,7867,6119,5532,5280,2914,3222,6351,4
521
ABCC2_6_2698,1183,908,1818,1209,724,331,2616,1172,1231,570,849,2586
ABCC8_6_2699,2917,2680,2698,3296,2666,3163,3307,2580,3806,4717,4238,22
11
ABCD1_6_2700,605,162,306,660,402,2485,24,434,221,155,434,794
ABCD2_6_2701,223,80,599,322,39,127,99,163,2,15,921,93
ABCD4_6_2702,212,596,357,316,161,353,1099,1,1248,29,232,133
ABCF3_6_2703,1739,1290,1003,1276,1049,1594,2312,825,1188,809,1704,576
ABCG2_6_2704,1290,765,1540,1970,579,108,1425,1339,636,2399,2140,1048
ABCG5_6_2705,1680,1661,1879,1752,1240,669,1804,1172,888,1203,4116,1868
ABCG8_6_2706,521,615,806,847,336,411,2080,397,346,66,106,240
ANXA10_6_2707,6765,5531,6202,5886,6453,7905,7654,9500,4572,8376,11392,
4841
ANXA1_6_2708,1719,1691,2107,2559,1790,2211,1951,789,1379,4009,2764,669
ANXA3_6_2709,7045,5488,8048,6849,7442,6755,8059,10346,6010,7727,10176,
5544
ANXA4_6_2710,1079,631,725,786,920,1,1252,246,1042,701,47,1337
ANXA5_6_2711,811,717,1238,1295,2281,4997,1647,676,1043,1397,1460,1876
ANXA8_6_2712,1379,2072,2142,2643,742,1302,5858,2904,1380,1536,3495,238
9
ANXA9_6_2713,3967,4115,4556,4291,4923,1859,4247,5773,5011,4978,4005,31
72
ATP10A_6_2714,5488,4381,5124,5956,4376,2893,4358,6837,4899,3679,7450,6
795
ATP10B_6_2715,376,433,529,635,167,156,259,454,321,42,208,48
ATP10D_6_2716,1021,987,784,840,830,1376,615,1688,1112,1378,1080,354
ATP11B_6_2717,33,48,53,25,607,41,0,195,88,0,0,0
ATP1A2_6_2718,814,535,997,929,1120,1659,446,1075,1353,256,1783,282
ATP1A3_6_2719,431,837,1036,1217,921,808,1679,1427,813,387,1492,706
ATP1B1_6_2720,1696,2189,1693,1717,1580,1044,1861,1218,876,933,1508,117
9
ATP1B2_6_2721,298,431,295,249,18,937,450,361,32,5,702,398
ATP1B3_6_2722,2827,2720,3273,2172,2287,2290,2472,2859,930,3681,3374,23
11
ATP4A_6_2723,1606,1309,1921,1906,1207,853,1839,1521,2806,3524,7086,343
7
ATP4B_6_2724,1095,888,1085,1059,2033,932,1467,2733,1595,1560,708,1169
ATP5B_6_2725,1837,2241,2003,1989,1245,2512,2323,1538,1494,2309,3541,48
5
ATP5F1_6_2726,3538,3241,3773,3944,2775,3033,2826,4491,4326,4712,4624,5
301
ATP5I_6_2727,258,417,116,258,853,13,472,194,95,871,51,28
ATP5L2_6_2728,557,476,591,756,1537,512,152,1335,265,412,357,436
ATP5L_6_2729,2158,2238,1838,2679,1559,827,1994,2119,1109,3112,2572,217
9
ATP5O_6_2730,1248,1016,1408,1442,2845,975,1345,941,595,436,1177,1088
ATP6AP1_6_2731,1117,587,936,600,803,1473,293,831,809,981,688,1832

ATP6AP2_6_2732,2628,2705,2988,3252,2969,2473,6005,2072,2703,985,1560,2
497
ATP6V0A2_6_2733,1033,470,769,474,696,187,550,305,576,442,1781,1381
ATP6V0D1_6_2734,1023,1124,1375,1816,110,1116,1534,725,2365,1432,2128,1
266
ATP6V0D2_6_2735,5971,6160,6551,5765,6794,5029,13115,5059,7591,3549,773
1,7693
ATP6V0E1_6_2736,2139,1878,2249,2141,1394,3002,2872,3126,1962,2147,2488
,3837
ATP6V1A_6_2737,1680,1823,1686,1950,923,1679,1593,2748,1588,2366,2036,1
889
ATP6V1B1_6_2738,1696,1385,2037,2106,940,2203,2008,960,1240,1334,3363,2
605
ATP6V1B2_6_2739,1892,1763,1828,1680,1666,4065,1533,4373,975,1588,1431,
1190
ATP6V1C1_6_2740,3938,3209,4608,3689,4031,4719,3893,6092,2928,6158,3679
,3639
ATP6V1D_6_2741,4702,3646,4718,4827,3922,3953,2476,5139,6223,5307,5977,
2884
ATP6V1E2_6_2742,673,580,805,720,542,1190,1301,298,105,409,1247,473
ATP6V1G1_6_2743,904,806,818,697,614,704,180,776,668,1325,1171,1172
ATP7A_6_2744,1835,1655,2133,2039,1710,1786,2068,773,1841,1183,1556,234
1
ATP8A2_6_2745,2004,2071,3039,2898,1131,4469,1534,2175,2275,3393,2922,2
474
ATP8B1_6_2746,642,846,1078,907,185,732,1237,277,1140,808,1031,2366
ATP8B4_6_2747,1047,662,808,879,1256,287,969,2053,688,1904,592,1668
ATP9A_6_2748,350,879,921,295,713,901,145,479,158,988,624,317
ATP9B_6_2749,817,402,878,683,1262,638,33,156,302,1335,1316,123
ATPAF2_6_2750,664,662,981,660,409,581,1135,634,213,2332,341,1375
FLVCR1_6_2751,372,325,395,346,1,147,606,277,641,352,236,484
RHBG_6_2752,1049,1326,641,898,1578,824,929,1128,1121,1847,1336,481
RHCG_6_2753,1746,2202,2093,1826,1939,745,5024,1725,1875,2681,2556,2138
SLC10A1_6_2754,1825,2237,2346,2246,2518,1813,2144,3268,2396,3240,3525,
1489
SLC10A2_6_2755,4236,4402,6365,5964,3341,4950,4057,5086,4759,5332,5876,
6541
SLC10A4_6_2756,2991,2163,2417,3243,784,2807,2136,2641,1417,3716,2966,4
874
SLC10A5_6_2757,2303,1448,2078,2199,2341,1430,995,2760,1256,3998,4744,1
599
SLC10A6_6_2758,2812,2466,2710,3721,2683,2848,2420,3863,1962,4355,4460,
2495
SLC11A1_6_2759,788,850,1287,1578,780,2389,1373,3612,583,1133,1891,1364
SLC12A2_6_2760,785,725,636,692,291,1454,758,1402,360,115,1448,317
SLC12A7_6_2761,1205,1032,1420,1383,54,688,779,2960,1713,1640,1532,754
SLC12A9_6_2762,1209,927,1161,1223,1216,1817,307,1410,1557,1097,733,861
SLC13A1_6_2763,10595,10404,11201,13225,11287,8361,13722,10131,8665,103
61,13729,12614
SLC13A4_6_2764,691,1011,1256,1192,475,1270,1507,888,827,502,1435,1774

SLC15A1_6_2765,2390,1686,1865,2182,2802,2214,2430,1522,1924,2209,2229,
2488
SLC15A3_6_2766,801,674,1094,819,1316,1388,1262,1111,415,533,984,664
SLC15A4_6_2767,809,1367,869,1160,411,1398,2615,63,79,84,1081,3015
SLC16A10_6_2768,6303,6241,8882,6345,5028,8099,8246,7745,5316,6775,9703
,5227
SLC16A11_6_2769,1485,1987,1445,2069,2109,1533,2518,1966,1746,3141,2033
,1689
SLC16A12_6_2770,1403,1096,1427,1276,1460,1220,686,606,1068,1629,271,18
75
SLC16A2_6_2771,840,883,887,1106,730,556,1709,806,712,1512,651,908
SLC16A5_6_2772,1124,1040,866,1464,2047,1076,171,1974,235,968,852,826
SLC16A7_6_2773,2604,2737,2878,3603,2720,3167,4709,1536,3482,2068,5379,
4352
SLC16A8_6_2774,4651,3923,5300,4792,2487,5966,8102,5695,5060,5868,8846,
4702
SLC17A1_6_2775,2350,2944,2868,2560,2205,2249,2203,3539,3787,3197,3123,
1955
SLC17A4_6_2776,4929,4940,5025,5097,8328,2039,5268,6774,3920,7825,6139,
4042
SLC17A5_6_2777,847,908,1493,1367,1965,491,2931,1536,1945,637,2890,883
SLC17A6_6_2778,2671,3140,2903,3127,2038,2225,2604,6393,2906,3814,5671,
2679
SLC17A7_6_2779,352,291,360,319,51,0,303,78,111,0,386,396
SLC18A2_6_2780,655,849,604,738,341,961,1245,1702,1319,1651,354,163
SLC18A3_6_2781,460,491,583,849,559,6,1699,200,849,1009,1513,1709
SLC19A2_6_2782,654,1455,944,1382,645,920,816,1077,655,1032,2711,791
SLC19A3_6_2783,826,893,1065,735,41,909,1300,938,140,157,925,489
SLC1A1_6_2784,1370,1558,1806,1783,1887,980,1075,1863,1648,1116,2485,12
48
SLC1A6_6_2785,3651,3347,3984,3208,3408,3676,5353,5211,3175,2375,3788,4
185
SLC1A7_6_2786,736,428,748,569,343,157,1132,204,1283,562,48,293
SLC20A1_6_2787,5418,4805,5151,6180,2399,4878,4232,4755,2942,6614,9735,
3799
SLC20A2_6_2788,2464,2337,2812,2070,2681,2526,2921,4010,1393,2144,4648,
1146
SLC22A10_6_2789,1614,1570,1453,1969,1710,1406,1846,1744,1018,2887,2715
,2218
SLC22A11_6_2790,3832,3265,4264,3993,3159,1817,2237,4306,3884,2569,5057
,6079
SLC22A13_6_2791,1074,930,1108,1111,941,1638,493,386,510,1551,2298,762
SLC22A14_6_2792,999,945,970,898,598,743,170,485,1364,417,1086,198
SLC22A15_6_2793,2229,2506,2857,2713,1855,3089,4570,995,1522,2276,3139,
2616
SLC22A16_6_2794,1565,947,1191,1300,326,193,333,814,585,522,1740,928
SLC22A18AS_6_2795,1739,1277,1339,1744,2311,1086,1720,633,1055,1175,154
5,1493
SLC22A2_6_2796,1367,1324,1401,1513,924,2120,1270,1308,703,849,1403,119
9

SLC22A3_6_2797,5058,5215,6788,6354,6263,7714,6454,6157,2838,7074,4689,
4234
SLC22A4_6_2798,1350,1183,1723,1300,1764,1138,2675,2902,880,2254,1838,1
803
SLC22A5_6_2799,942,912,1309,1572,1572,995,860,1461,816,2266,1403,431
SLC22A9_6_2800,1636,1639,2095,1579,866,1228,1478,3578,1472,1119,2069,1
105
SLC24A1_6_2801,2549,2634,3142,2985,3148,4014,2438,1721,2224,1977,4699,
1804
SLC24A3_6_2802,410,252,433,262,110,251,1220,1085,135,93,1841,219
SLC24A5_6_2803,2326,2097,2182,1884,2102,1383,1132,4343,2141,1697,3080,
2671
SLC24A6_6_2804,1831,1618,1440,1721,1584,1659,1804,3260,872,1311,1261,6
31
SLC25A10_6_2805,6467,5535,5939,5389,3535,5729,4868,6355,4895,6104,6453
,4802
SLC25A12_6_2806,8953,9037,11195,10084,10382,11703,9008,9174,9814,11253
,12085,6333
SLC25A15_6_2807,1323,1043,1520,1497,2583,254,1934,1619,1172,4164,3340,
2630
SLC25A16_6_2808,3528,3209,3808,3566,5642,4257,4348,2827,1406,3632,3820
,2316
SLC25A17_6_2809,7873,7773,9499,8709,5848,6171,7889,10671,7564,9673,116
44,7022
SLC25A18_6_2810,1605,1390,1500,1848,533,1278,2236,1328,709,1706,3086,1
640
SLC25A1_6_2811,1254,1412,1805,1955,1657,3356,1326,409,752,2722,2543,88
8
SLC25A20_6_2812,3770,4496,4194,4160,2851,4190,5310,4141,3330,3201,4515
,5085
SLC25A28_6_2813,341,306,569,660,380,1104,659,402,502,198,1035,133
SLC25A2_6_2814,2434,1767,2756,2250,2146,2634,3268,6475,2375,1905,3467,
2756
SLC25A32_6_2815,1678,1971,1742,1953,524,1959,2063,916,3178,3380,1926,1
613
SLC25A37_6_2816,843,320,650,834,492,208,509,318,239,248,244,1020
SLC25A43_6_2817,482,476,637,569,876,31,329,697,384,146,1129,264
SLC25A44_6_2818,400,862,546,630,1135,787,1249,359,10,1987,783,136
SLC25A4_6_2819,2032,2225,2187,1867,2204,4232,3967,1989,1657,4447,3368,
3079
SLC25A5_6_2820,1382,1042,1752,1224,766,2591,2643,502,109,1306,1288,197
9
SLC25A6_6_2821,487,672,695,747,590,673,1393,1159,1636,1027,713,40
SLC26A10_6_2822,1783,1431,1616,1066,200,1558,994,2650,2013,2215,3512,1
762
SLC26A2_6_2823,5035,5670,5824,5402,4509,5348,4313,6330,4816,8283,8027,
4584
SLC26A3_6_2824,438,466,369,428,70,63,1271,869,555,1704,658,199
SLC26A4_6_2825,2513,1818,3253,2703,4616,2748,1642,1826,2750,2945,5466,
2967

SLC27A1_6_2826,647,305,626,513,758,722,298,1,214,251,61,954
SLC27A3_6_2827,740,312,494,391,1038,1746,392,410,49,386,1295,1178
SLC27A4_6_2828,4820,5475,4514,5417,3438,6054,3867,3732,3720,5668,4498,
4837
SLC27A5_6_2829,2242,1933,2116,2587,3106,1246,2367,1192,1338,2229,3049,
2268
SLC28A2_6_2830,1111,1969,2130,1760,514,1386,3631,917,902,650,1764,1729
SLC29A2_6_2831,1881,1739,2182,2351,1756,2321,524,2701,2525,2202,5898,1
253
SLC2A10_6_2832,1219,1632,1498,1530,519,714,1684,2068,2141,2040,2121,57
8
SLC2A12_6_2833,655,549,1114,982,743,304,1635,1269,782,20,637,932
SLC2A13_6_2834,3261,3101,3163,3045,3182,3176,1043,2157,1756,1696,2293,
3362
SLC2A14_6_2835,530,584,433,486,280,397,118,237,576,627,371,1136
SLC2A1_6_2836,1980,1408,2126,2072,1705,2723,989,2292,338,1561,3121,127
8
SLC2A2_6_2837,549,366,437,540,212,615,974,110,111,1370,1026,94
SLC2A3_6_2838,4551,4251,6449,4715,4770,6546,5203,4433,4124,7006,5797,4
625
SLC2A4_6_2839,693,1026,928,714,1569,1256,1418,704,1054,437,744,318
SLC2A4RG_6_2840,519,113,283,370,13,102,34,63,2,114,471,161
SLC2A7_6_2841,333,545,486,369,941,558,134,172,314,4,631,61
SLC2A8_6_2842,2187,2459,3414,2879,2201,3621,3091,3388,1964,4091,5595,2
233
SLC30A10_6_2843,2521,2678,3091,3098,4512,3424,2903,3586,2850,3428,3992
,3873
SLC30A1_6_2844,1084,1216,1387,1390,332,429,276,413,1124,1520,2105,216
SLC30A3_6_2845,587,554,536,726,497,1111,1769,99,354,1441,636,791
SLC30A4_6_2846,5711,4803,8540,7439,7322,10290,7046,7112,6256,4312,8783
,6434
SLC30A9_6_2847,2921,2881,2293,3832,4097,1378,3975,3996,2476,3424,4206,
2988
SLC31A1_6_2848,981,1458,1462,1708,578,1316,740,718,879,573,1662,665
SLC31A2_6_2849,2758,2766,3289,3088,3512,1978,5266,2168,3733,2119,2599,
1955
SLC32A1_6_2850,843,622,1485,1235,1617,544,1852,974,370,1673,230,564
SLC35A3_6_2851,10030,10448,12194,10511,11189,11051,11391,12087,7630,99
94,12266,9162
SLC35A4_6_2852,2199,1931,3509,2756,522,3241,2078,3377,1315,3946,4169,1
054
SLC35A5_6_2853,5894,6123,7216,7301,7278,9727,7020,6169,6340,9793,6023,
5520
SLC35B1_6_2854,1197,1041,1068,1253,2138,409,672,797,322,1117,851,1495
SLC35B2_6_2855,1880,986,1760,1629,1539,1853,1617,658,1085,1650,2043,31
33
SLC35B4_6_2856,417,266,717,703,129,248,413,924,1100,175,1340,142
SLC35D1_6_2857,2318,2461,3249,3398,1904,1226,2930,4961,1766,2714,5225,
2261
SLC35D2_6_2858,3341,3170,3712,3713,3143,3397,5170,4103,2962,3765,5184,

3836
SLC35D3_6_2859,1778,1603,2353,2230,2491,1717,2632,986,1349,747,2446,28
34
SLC35E1_6_2860,274,701,597,543,11,163,481,340,155,75,615,204
SLC35E3_6_2861,6187,5637,7978,8963,6167,5683,8203,10803,6110,6161,1013
2,7292
SLC35E4_6_2862,1045,351,584,695,1700,1403,780,681,925,141,383,316
SLC35F1_6_2863,396,386,1155,737,669,1096,1192,1046,615,2141,578,358
SLC35F2_6_2864,1878,1706,2620,2655,1300,979,3479,1307,1197,2590,3260,3
060
SLC35F3_6_2865,2517,3082,3350,3881,2845,3380,3532,3437,2928,4451,3606,
2841
SLC35F4_6_2866,371,317,561,359,868,246,137,451,47,940,329,157
SLC35F5_6_2867,592,464,718,655,124,359,991,1136,1601,998,1076,1226
SLC36A1_6_2868,460,511,719,642,209,364,148,94,90,357,2102,247
SLC36A2_6_2869,4417,3324,4481,3471,3476,3195,3352,4195,3843,3333,5645,
2605
SLC36A4_6_2870,1717,1755,2204,2022,2133,3476,2142,1100,1905,1866,2437,
1731
SLC37A1_6_2871,8227,6251,8083,8266,10547,9447,12663,6998,10132,8911,12
678,6798
SLC38A2_6_2872,6860,5186,7051,4952,7527,7313,7311,9532,7335,5911,8235,
5382
SLC38A3_6_2873,268,368,507,425,1267,708,824,46,184,1090,381,1349
SLC38A5_6_2874,693,881,934,833,86,978,512,494,388,526,460,818
SLC39A1_6_2875,846,764,843,654,200,974,61,898,160,633,1127,1324
SLC39A2_6_2876,2357,1980,2568,2885,2437,2960,2718,4940,1808,2646,3686,
3903
SLC39A9_6_2877,1204,1233,1239,1348,483,1862,1374,1812,813,376,1353,401
SLC3A1_6_2878,1754,1277,1932,1948,1265,3135,2705,1835,1155,4880,3300,1
649
SLC40A1_6_2879,2976,2644,3481,3489,3491,5628,3198,4878,2719,2750,3357,
1845
SLC41A1_6_2880,1379,968,1569,1602,376,2397,384,2104,782,1457,2603,2122
SLC43A2_6_2881,947,811,838,967,1806,496,911,1394,1105,509,1095,884
SLC44A1_6_2882,11873,11474,13394,14300,8371,12456,15492,14847,8140,137
08,15537,16665
SLC46A2_6_2883,1388,1360,1881,2134,1282,627,2335,2080,1295,1919,3492,2
698
SLC4A1AP_6_2884,726,428,857,807,392,1070,1132,1897,252,880,549,293
SLC4A1_6_2885,7096,7566,7941,8084,10211,7050,6530,6835,4808,9054,9342,
7668
SLC4A7_6_2886,4941,5243,4947,6296,4807,12796,6094,5965,4797,6778,10040
,7580
SLC4A9_6_2887,1592,1132,1729,1682,2275,1617,2321,1392,1189,722,2477,90
2
SLC5A11_6_2888,4672,3727,5819,5462,4451,4326,5392,4501,5156,4430,8784,
3815
SLC5A1_6_2889,591,337,456,530,803,83,743,325,176,584,579,336
SLC5A2_6_2890,1590,1056,1549,1454,1079,1309,353,2910,1367,1290,925,213

1
SLC5A3_6_2891,6034,6937,7219,7699,8062,6015,8090,8082,8968,7134,10172,
6558
SLC5A4_6_2892,679,583,894,1158,65,36,366,3143,973,418,1685,444
SLC5A5_6_2893,2438,2296,2736,2225,1703,2327,4108,3582,1992,5033,5135,2
579
SLC5A6_6_2894,351,725,335,554,276,15,252,133,598,394,718,710
SLC5A7_6_2895,1133,1187,1446,1491,609,1004,2305,1094,1147,1707,1311,10
86
SLC5A8_6_2896,10147,9384,12197,12264,8960,13873,10069,8203,9433,11260,
15015,10013
SLC6A11_6_2897,4443,4169,5596,4599,4314,5876,4443,5231,4817,4140,7223,
5508
SLC6A14_6_2898,7063,7113,7645,8019,7096,10495,6311,5169,5040,4546,6773
,8162
SLC6A16_6_2899,2191,1932,2133,2613,2202,1174,4447,3137,1639,2454,1394,
1781
SLC6A19_6_2900,4823,4584,5651,5209,4756,4438,5485,5960,4195,5724,8025,
5350
SLC6A1_6_2901,1590,1632,2036,2180,4209,1082,2026,2506,1424,3393,1867,9
20
SLC6A3_6_2902,1222,944,1100,1129,1067,3226,1324,1257,346,1339,1297,308
SLC6A4_6_2903,2699,2265,3149,2678,3014,4097,2043,5043,2002,1773,2938,3
289
SLC6A7_6_2904,2149,2018,2796,2057,3295,4921,2549,3606,1909,1913,2628,2
756
SLC7A10_6_2905,3365,2569,2993,4072,3069,4515,2119,3071,1678,5305,4017,
3431
SLC7A11_6_2906,2983,2346,2687,2653,2184,4271,3205,2497,2388,2634,3932,
4227
SLC7A13_6_2907,5518,5802,6457,5811,4950,6702,6973,5352,6706,8029,6861,
5210
SLC7A1_6_2908,998,738,1061,1157,547,1248,657,1538,966,594,1548,1170
SLC7A4_6_2909,3365,3747,4497,4498,2605,3854,4898,5124,3089,2568,5076,6
332
SLC7A5_6_2910,5398,5408,7564,6230,5903,4753,5231,4729,6171,8764,7494,6
939
SLC8A2_6_2911,2378,2850,3033,2462,2244,1793,1904,1087,2435,1950,1197,8
66
SLC9A1_6_2912,1093,964,1890,1639,1654,1180,3206,1823,1264,934,2722,152
7
SLC9A2_6_2913,5127,5725,6112,6590,9284,5538,4322,3555,4450,8517,8738,3
086
SLC9A3_6_2914,2091,2052,2695,2434,675,3377,4008,2326,2589,2672,3785,22
53
SLC9A3R1_6_2915,2394,1768,1635,2342,1589,1881,2870,2526,3419,2187,3324
,967
SLC9A4_6_2916,2581,2984,2298,2695,3890,3163,2326,1609,1973,4567,3865,3
278
SLC9A5_6_2917,1049,1246,1247,1195,956,2693,1008,1241,1028,2683,2837,20

70
SLC9A7_6_2918,659,395,690,828,2924,335,1056,199,726,215,1938,247
SLC9A8_6_2919,799,1151,1287,1122,964,837,596,1236,1125,914,1797,575
SLC9A9_6_2920,4708,4069,4919,5122,4848,3904,7335,5684,3502,6080,9939,5
058
SLCO1B1_6_2921,6675,6908,8197,8549,4670,10941,9337,5689,5806,9416,7905
,9215
SLCO1B3_6_2922,7684,6640,8747,7543,8004,10921,6247,8264,10164,8322,139
70,8576
SLCO2A1_6_2923,622,697,599,701,74,467,311,116,620,453,744,37
SLCO4A1_6_2924,1281,1748,2077,1741,1418,1063,2134,2370,952,1046,1556,1
779
SLCO4C1_6_2925,5905,5037,6681,6618,8471,10232,4587,7350,3993,9831,6408
,6775
SLCO6A1_6_2926,8067,8018,8881,9046,6845,8782,7862,9667,5626,8216,8359,
8443
SV2A_6_2927,418,598,681,539,1086,484,849,1256,607,657,32,65
TAP1_6_2928,822,610,724,815,116,249,105,154,391,35,825,244
UCP1_6_2929,8362,7606,8770,7595,9983,7547,7475,9141,6652,10718,9295,79
26
ABCA12_6_2930,6102,6533,6454,6055,7190,5292,4702,8877,5811,5411,7041,6
823
ABCA2_6_2931,504,422,613,524,3,733,302,940,958,380,884,525
ABCA5_6_2932,7556,7310,10292,8773,7972,9852,10208,10804,6342,10509,119
00,10163
ABCB4_6_2933,3719,2977,4046,3070,3168,3074,4070,6479,1820,3094,7398,36
50
ABCB9_6_2934,1879,1749,1491,1994,2294,1613,1520,1312,2075,1883,2516,92
0
ABCC10_6_2935,992,1044,739,1295,417,1218,999,1361,485,1256,1405,891
ABCC11_6_2936,2395,2339,3002,2502,3530,2233,3774,2401,1597,2032,4187,2
362
ABCC1_6_2937,763,713,733,1320,1110,1394,1452,105,1074,946,317,829
ABCC3_6_2938,1531,1519,1735,1914,1127,2645,2134,1048,2045,769,3164,275
0
ABCC4_6_2939,2671,1788,3005,2430,2676,2999,1011,424,1860,2056,3191,306
6
ABCC5_6_2940,1248,966,1264,1458,660,815,1578,365,1049,1150,1661,601
ABCC9_6_2941,2572,2823,3659,3196,2484,2703,2256,2222,4284,5573,3067,43
77
ABCD3_6_2942,3760,3146,4159,3437,2559,3925,3429,2250,3070,2653,5258,35
18
ABCE1_6_2943,1621,2146,1944,1688,2272,3608,1610,3357,1424,1895,3195,86
1
ABCF1_6_2944,283,350,734,358,267,16,1012,1266,11,124,318,384
ABCF2_6_2945,2669,2965,3438,3648,2914,4092,3461,3585,2712,3585,4069,36
59
ABCG1_6_2946,1557,1309,1050,1250,1458,368,1748,400,470,1878,1296,1127
ABCG4_6_2947,210,504,517,778,1232,728,744,180,244,2,100,284
ANXA11_6_2948,1595,1404,1524,1429,1273,1399,394,902,567,2021,1715,1664

ANXA13_6_2949,2535,2277,3218,3095,2551,3209,4388,3276,2951,2981,2903,2
789
ANXA2_6_2950,3080,2295,3374,2856,2785,3969,1969,1282,3331,2715,4581,25
78
ANXA6_6_2951,657,337,590,640,20,884,1822,35,169,0,114,765
ANXA7_6_2952,381,594,697,737,168,580,264,26,1011,957,672,738
ATP11A_6_2953,1124,975,1281,976,1488,1081,3333,1085,1087,228,222,1435
ATP11C_6_2954,2403,1855,2011,1965,726,2177,1289,1886,1524,2038,3575,19
50
ATP12A_6_2955,620,594,874,741,639,723,255,1098,425,1687,498,1484
ATP1A1_6_2956,3190,3631,2643,3520,1803,3263,4104,4336,1504,2921,3614,3
105
ATP1B4_6_2957,1314,1181,1427,1541,793,1032,2921,1445,960,1207,2523,695
ATP2A1_6_2958,274,390,216,360,43,519,560,78,350,166,134,41
ATP2A2_6_2959,2794,1773,2601,3138,1788,2955,4061,2858,1688,2749,3149,2
724
ATP2A3_6_2960,1361,1120,1094,1123,1333,837,1500,2271,833,1300,1263,258
4
ATP2B1_6_2961,5546,5197,6481,5549,4204,8351,5951,8869,5923,5447,5570,4
513
ATP2B3_6_2962,563,456,454,684,81,1047,101,202,230,654,200,349
ATP2B4_6_2963,6866,6726,8349,7271,6304,7715,5912,7253,4474,6425,9231,5
733
ATP2C1_6_2964,2989,2579,3015,2779,3155,1971,3221,3047,2171,5416,4029,4
218
ATP5A1_6_2965,860,976,1079,1092,1110,1465,1533,449,788,897,1020,768
ATP5C1_6_2966,2530,2257,2418,2122,3899,1173,2978,2855,2536,4741,1763,3
333
ATP5D_6_2967,219,463,419,420,926,72,867,683,97,1,351,1123
ATP5E_6_2968,1728,1233,1589,1532,351,902,1185,1399,849,2594,2409,1375
ATP5G1_6_2969,118,87,58,124,0,0,98,0,0,0,449,0
ATP5G2_6_2970,1990,1867,2320,1587,1425,728,874,2738,181,1084,4996,2498
ATP5G3_6_2971,729,594,1153,676,1099,1786,289,1572,943,468,1125,473
ATP5H_6_2972,1630,1058,1495,1536,1311,591,2025,2867,2108,695,976,445
ATP5J_6_2973,1878,2235,2684,1910,792,3186,3000,4075,1628,2071,2462,313
1
ATP6V0A1_6_2974,2764,2591,2573,2752,3645,747,1651,2664,1444,2011,3465,
2675
ATP6V0A4_6_2975,3470,2582,3077,3138,5472,3993,4006,2727,2718,2941,4846
,3309
ATP6V0B_6_2976,398,354,498,306,85,27,13,898,738,389,443,160
ATP6V0C_6_2977,2935,2849,3278,3225,3482,3620,3516,3165,2906,3729,4194,
2526
ATP6V1C2_6_2978,2996,1919,3270,2677,1591,1687,3832,2600,1325,1827,4454
,2213
ATP6V1E1_6_2979,402,264,529,626,546,9,436,554,145,550,1170,111
ATP6V1F_6_2980,677,612,739,1094,638,831,1426,1676,727,1204,1026,1055
ATP6V1G2_6_2981,1965,1643,1803,1933,1539,949,2899,2114,1285,2648,3478,
2122
ATP6V1H_6_2982,728,898,1149,1047,358,1261,610,2064,332,2432,2456,389

ATP7B_6_2983,1491,1890,2645,1739,820,2328,1388,1902,1488,1962,2089,205
9
ATP8A1_6_2984,2128,1995,1945,1860,1542,2874,2148,518,1807,1001,2829,27
30
ATP8B2_6_2985,1018,1500,1615,1763,1777,468,1168,716,1335,950,1046,1002
ATP8B3_6_2986,714,853,906,705,708,805,1269,728,846,141,2037,936
ATPAF1_6_2987,2581,2498,2898,2629,258,2190,2649,2142,1251,2903,3737,36
22
GC_6_2988,2838,3417,3171,3438,3686,3727,1858,5821,1545,2354,2784,6756
SERINC5_6_2989,392,612,175,352,7,873,20,474,195,161,92,51
SLC10A3_6_2990,829,219,244,478,467,204,654,1,5,1495,519,57
SLC11A2_6_2991,436,339,482,882,312,188,1089,1136,114,1282,263,1663
SLC12A1_6_2992,2231,2274,2777,2944,2761,2731,2330,3915,2168,1730,3115,
2032
SLC12A3_6_2993,416,336,601,408,122,566,444,515,417,904,130,484
SLC12A4_6_2994,4004,3825,3913,4483,3023,5559,4987,4328,4284,3356,5372,
4230
SLC12A5_6_2995,1407,1415,1564,1511,2319,1362,485,1299,1588,1198,2301,1
545
SLC12A6_6_2996,1616,1002,1708,1077,624,1721,1586,1653,202,789,1907,823
SLC12A8_6_2997,3162,2868,4082,3562,3181,3414,4180,3125,3163,3559,4784,
3638
SLC13A2_6_2998,682,670,923,757,1022,416,1504,460,343,1837,1155,942
SLC13A3_6_2999,876,595,476,404,370,854,372,132,78,919,652,1680
SLC13A5_6_3000,5332,3929,6397,5943,4045,6536,5793,8721,4505,5362,4897,
5013
SLC14A1_6_3001,885,928,644,840,1092,168,290,1675,486,275,926,1082
SLC14A2_6_3002,1677,1348,1630,2094,2137,2099,1850,3465,560,2130,2498,2
258
SLC15A2_6_3003,1501,1605,2573,2146,1747,3285,2030,2349,1058,1276,3237,
481
SLC16A1_6_3004,1621,1788,2099,2181,3157,4173,1903,2099,1477,2597,2016,
2185
SLC16A3_6_3005,266,39,293,69,1429,108,39,678,17,2,480,187
SLC16A4_6_3006,790,1302,967,1075,869,1232,1457,880,437,687,1842,1342
SLC16A6_6_3007,3633,2805,4249,3369,2325,2971,2486,5946,3497,6286,5731,
3555
SLC17A3_6_3008,870,1233,1525,1107,967,1060,725,1544,476,1733,1607,1516
SLC17A8_6_3009,2492,2207,2063,2236,3264,1929,2283,2393,2578,1910,3848,
2309
SLC18A1_6_3010,3314,4206,3699,3647,3516,3953,3338,3679,3328,6904,2102,
4022
SLC19A1_6_3011,809,711,1055,1018,545,1457,543,1141,69,159,1613,123
SLC1A2_6_3012,1214,1486,1690,1709,2227,876,2137,2338,846,248,3449,1405
SLC1A3_6_3013,2400,2368,2237,2105,4250,2209,2186,3073,1602,1223,2007,1
136
SLC1A4_6_3014,1364,1807,1251,1370,1598,1247,847,829,1871,505,1974,1319
SLC1A5_6_3015,1921,2001,2052,2184,2052,3049,1377,3482,1856,1668,3657,2
601
SLC22A12_6_3016,2591,2135,3262,2420,2402,3124,3152,4557,1977,2480,3015

,1963
SLC22A17_6_3017,716,591,608,459,241,1053,932,1152,351,774,824,898
SLC22A18_6_3018,399,332,372,560,589,17,768,167,722,757,329,690
SLC22A1_6_3019,487,338,511,592,645,260,1071,570,819,766,921,571
SLC22A23_6_3020,3425,3557,4245,3184,5064,3343,3357,2004,4410,3440,3707
,2780
SLC22A6_6_3021,799,807,993,636,382,418,1821,482,526,282,1563,660
SLC22A7_6_3022,0,0,0,0,0,0,0,2,0,0,0,0
SLC22A8_6_3023,1075,1457,1149,1430,2663,2066,2348,772,279,1049,689,178
6
SLC23A3_6_3024,2727,2529,2986,2336,2424,3868,1341,3261,2090,2955,1935,
1980
SLC24A2_6_3025,4307,3713,4944,4644,5989,4869,4945,4464,2820,5230,4208,
7262
SLC24A4_6_3026,984,558,831,811,722,1305,1166,795,496,886,1241,1539
SLC25A11_6_3027,476,882,450,703,1195,1713,149,2470,140,371,833,47
SLC25A13_6_3028,1200,559,1023,1742,1722,691,791,1089,2325,1108,382,792
SLC25A14_6_3029,6160,5277,6272,6152,6153,6285,6248,5004,5640,7135,4837
,6979
SLC25A19_6_3030,4732,3611,5220,5348,5065,5375,3476,5517,6161,6088,7034
,4268
SLC25A21_6_3031,2397,2219,3116,2265,3418,3177,2620,3911,2179,3536,2293
,2415
SLC25A22_6_3032,1960,1612,1574,1594,3034,1178,2738,843,1865,1996,2744,
1332
SLC25A26_6_3033,1060,971,1006,940,555,718,1416,336,1053,1936,1943,1902
SLC25A27_6_3034,4842,5078,5904,5235,3455,5002,4892,3669,3770,8924,6571
,4184
SLC25A3_6_3035,3378,2638,3550,3544,3503,3014,3796,3098,2491,5115,5299,
2164
SLC26A11_6_3036,922,984,888,1083,58,156,909,315,1415,145,2176,677
SLC26A1_6_3037,4926,3499,5807,4751,2572,5430,4263,5560,2791,5215,3526,
5358
SLC26A5_6_3038,1704,1647,2322,2223,1173,1921,1468,1688,751,1687,2567,1
169
SLC26A6_6_3039,747,976,666,1083,560,182,497,1475,303,798,666,1201
SLC26A7_6_3040,2421,2478,3301,3147,2791,4404,5972,2600,3428,4194,5232,
3378
SLC26A8_6_3041,974,798,588,545,5,1940,308,1106,1227,1712,1958,1017
SLC26A9_6_3042,1048,686,448,556,303,959,151,410,842,422,1093,1670
SLC27A2_6_3043,769,426,875,506,5,1299,390,1337,969,998,531,1172
SLC27A6_6_3044,2495,2567,1993,2152,4000,3083,2490,2468,875,1285,2820,2
268
SLC28A3_6_3045,2153,1810,2734,2274,1888,2098,2395,2319,891,4095,3151,2
388
SLC29A1_6_3046,232,543,666,656,1469,860,245,1457,51,142,45,680
SLC29A3_6_3047,2822,2153,3611,2447,1076,3405,1454,3499,2874,2547,5613,
3664
SLC29A4_6_3048,1210,939,793,948,2295,522,993,675,1217,1315,541,776
SLC2A11_6_3049,2189,1911,2509,2210,2362,3893,1353,2875,1408,3077,3105,

1902
SLC2A5_6_3050,859,730,1101,1036,658,2754,1617,2177,1131,657,3034,1180
SLC2A6_6_3051,641,937,865,1031,37,3330,689,389,1886,1012,524,1163
SLC2A9_6_3052,882,400,910,1078,586,57,767,1030,1023,679,956,440
SLC30A2_6_3053,22280,24061,24285,28021,17891,31798,29405,25778,18050,2
7982,29524,23178
SLC30A6_6_3054,2056,1510,2172,2380,2851,460,1798,3135,3064,2048,2608,1
895
SLC30A7_6_3055,2280,1162,1045,1739,767,695,485,1391,1071,687,490,1073
SLC30A8_6_3056,1162,1276,999,1349,453,2320,1105,517,2083,205,503,806
SLC33A1_6_3057,5599,5696,6030,7303,9013,8201,6674,7774,4277,5792,10067
,3964
SLC34A1_6_3058,589,487,408,897,232,1807,972,895,227,126,1011,1521
SLC34A2_6_3059,596,513,793,357,413,1272,294,189,495,273,193,1043
SLC34A3_6_3060,782,667,768,916,880,1489,619,1344,1223,578,1174,613
SLC35A1_6_3061,3053,2492,2787,2449,2678,3220,1890,3274,3380,2314,2571,
2356
SLC35A2_6_3062,1054,946,1205,1229,1927,1768,1317,1306,1522,569,1575,10
61
SLC35B3_6_3063,1045,746,1912,1783,1013,717,512,1556,340,873,1198,1394
SLC35C1_6_3064,282,364,454,657,258,223,208,74,584,471,390,263
SLC35C2_6_3065,1378,905,720,1090,354,1934,912,2014,1926,626,738,1488
SLC35E2_6_3066,4049,4078,3920,4961,3475,2672,4153,3092,2358,4498,6708,
3279
SLC36A3_6_3067,1457,1901,1868,1549,2333,1952,2246,1737,840,2307,4063,9
08
SLC37A2_6_3068,157,64,145,301,53,20,99,29,410,323,119,0
SLC37A3_6_3069,2637,3201,2857,2301,1992,2824,2449,2764,2385,3149,1699,
1632
SLC37A4_6_3070,928,1087,811,839,753,151,1096,802,1540,1005,1088,446
SLC38A11_6_3071,3335,2583,4496,3055,2903,2912,5488,6238,4370,3256,4541
,4317
SLC38A1_6_3072,1141,1578,1218,1395,1792,1721,1942,834,537,1063,1525,92
2
SLC38A4_6_3073,2892,3822,4515,4897,4241,5920,4610,3263,5523,4917,3712,
2936
SLC38A6_6_3074,4637,4346,4912,5051,3568,4717,4309,6645,2699,5448,8555,
4218
SLC39A10_6_3075,5966,5715,5916,6834,5987,4685,5935,9052,4971,8295,4443
,4530
SLC39A11_6_3076,3256,3219,3259,3561,2446,2180,4093,4588,1895,4617,3772
,3146
SLC39A12_6_3077,1199,1651,1721,1271,522,2736,2500,1893,1478,2007,1587,
1480
SLC39A13_6_3078,351,532,840,1043,234,309,39,1494,97,83,586,1456
SLC39A14_6_3079,967,510,774,603,1395,476,640,345,829,1808,2580,1054
SLC39A4_6_3080,1188,1312,2482,2043,982,2559,1689,1565,872,1249,1017,19
97
SLC39A5_6_3081,1169,1479,1575,1414,588,1395,1249,2446,852,2788,1848,16
60

SLC39A6_6_3082,1584,2015,1992,1267,1574,2147,2829,1106,2900,3158,1236,
2855
SLC39A7_6_3083,3858,5356,5020,5262,3441,6006,4753,5025,4737,5413,5768,
5626
SLC39A8_6_3084,1144,759,2066,2004,1350,666,439,920,907,2236,2396,208
SLC3A2_6_3085,1003,1344,1171,1105,844,967,882,2154,1762,716,1054,2212
SLC43A1_6_3086,673,1527,893,741,780,3840,669,731,820,965,2234,1569
SLC44A3_6_3087,2661,2952,3542,3566,2406,1709,4459,4161,1795,5132,3671,
3654
SLC4A10_6_3088,2219,1981,2533,2786,2081,2506,2523,2998,2413,2689,3316,
2147
SLC4A11_6_3089,1886,1726,2619,2352,2355,3713,2562,2179,1073,1929,3485,
3094
SLC4A2_6_3090,262,415,377,649,184,333,26,1053,171,615,592,89
SLC4A3_6_3091,361,324,596,541,406,341,623,529,630,717,326,328
SLC4A4_6_3092,1361,1428,1707,1313,633,1529,1344,2377,1316,1600,2630,71
7
SLC4A8_6_3093,682,1069,1353,546,978,636,603,529,598,1453,1051,871
SLC5A10_6_3094,1776,1237,1356,1216,1271,2510,2524,2924,1515,1975,502,1
365
SLC5A9_6_3095,307,314,903,393,300,367,212,907,78,135,286,1204
SLC6A12_6_3096,1715,2054,1865,2001,3305,1761,1506,903,1690,2653,2422,1
729
SLC6A13_6_3097,5641,4850,6638,6206,5517,4366,8718,5949,4861,4828,10065
,4854
SLC6A15_6_3098,671,464,354,553,1282,36,247,792,1006,508,367,364
SLC6A2_6_3099,965,776,1451,1368,1865,2904,1159,1592,2693,1162,2976,622
SLC6A6_6_3100,2983,3507,3044,3416,2128,4757,2434,4254,2785,833,4219,28
51
SLC6A8_6_3101,214,168,40,135,311,576,61,218,0,0,2,232
SLC6A9_6_3102,531,479,347,444,145,1114,148,342,158,232,78,260
SLC7A2_6_3103,3420,2450,3668,3376,2486,3115,3714,5337,2467,5067,5700,2
894
SLC7A3_6_3104,1529,1687,1907,1839,1583,560,1975,1883,1270,2789,3352,14
01
SLC7A6_6_3105,1641,1361,1519,1558,1109,342,751,1744,517,1675,692,752
SLC7A7_6_3106,1898,2104,2090,2331,2601,1854,2735,1253,3232,1070,2786,2
826
SLC7A8_6_3107,72,12,73,97,0,127,0,543,0,48,61,78
SLC7A9_6_3108,7071,6811,8107,7133,5956,8979,6961,6864,3754,9396,12234,
5682
SLC8A1_6_3109,8060,8705,10375,9575,8236,11683,10670,10878,9615,7538,11
546,11336
SLC8A3_6_3110,2896,2913,4490,4632,2740,5457,3959,3203,3255,2412,4699,4
140
SLC9A3R2_6_3111,490,882,1136,929,973,541,602,329,80,542,948,739
SLC9A6_6_3112,4847,5573,5719,5639,2852,11290,5293,3460,3749,4265,6758,
4444
SLCO1A2_6_3113,393,388,487,803,490,669,106,13,284,152,1204,1741
SLCO1C1_6_3114,8374,8588,7863,9298,11433,6985,10312,9139,6574,8212,865

0,12385
SLCO2B1_6_3115,2367,1497,1680,2166,1368,2572,2573,646,2045,2465,3394,2
666
SLCO3A1_6_3116,222,510,443,569,37,246,200,53,903,251,342,185
SLCO5A1_6_3117,13616,13252,16541,15417,12262,18072,16787,15656,9567,15
119,19840,11173
ABCB5_6_3118,3895,4254,5431,3993,3802,6722,3537,4587,3786,3568,5051,25
06
ABCC6_6_3119,388,449,589,346,94,707,310,1668,653,1030,188,82
ATP1A4_6_3120,634,648,1117,713,1172,394,133,1768,873,2658,234,272
ATP5J2_6_3121,4115,3969,4062,4775,3997,5217,3559,4936,3374,8574,6268,5
676
ATP5S_6_3122,845,700,894,967,1322,277,1395,2506,790,942,1431,1313
ATP6V1G3_6_3123,11277,8581,11137,10812,7403,11201,11776,10141,8700,103
87,11960,11899
ATPIF1_6_3124,2102,2245,2404,2559,1677,662,2667,2621,2291,2206,1258,58
8
SLC10A7_6_3125,7699,6154,8881,7340,8011,7458,6332,8752,6173,8048,10376
,9404
SLC28A1_6_3126,969,550,1130,1390,466,1540,258,1589,312,1407,1011,1319
SLC30A5_6_3127,675,828,625,440,763,191,2333,1597,2240,116,1711,166
SLC39A3_6_3128,518,317,272,303,568,88,64,51,402,252,131,468
ABCA10_6_3129,3253,3207,4317,3895,3948,3470,2503,2052,3191,2305,6025,4
415
ABCA13_6_3130,9906,11570,12146,11295,11971,12470,14492,14173,9518,1445
1,16196,12078
ABCA1_6_3131,1746,1244,2110,1805,2074,2283,991,1816,828,1880,2180,742
ABCA3_6_3132,1423,971,1694,2008,1131,3573,2154,1457,1480,1148,1850,262
5
ABCA4_6_3133,2572,2432,2834,3123,3217,4176,2178,2196,2926,2559,2302,24
58
ABCA6_6_3134,5790,5406,6648,5021,5568,3539,6461,5555,3893,5452,5836,48
96
ABCA7_6_3135,1840,2426,2644,2035,630,2156,2297,2213,2090,1476,2875,211
4
ABCA8_6_3136,9264,9611,10128,8960,10780,6757,9237,12473,7766,14375,128
81,12039
ABCA9_6_3137,4666,5317,6197,5675,6940,8613,7693,2728,5670,4855,6405,34
54
ABCB10_6_3138,1696,1322,1835,777,1472,2361,3015,1450,2053,727,1288,100
5
ABCB11_6_3139,1004,612,1331,1165,585,261,450,1256,451,485,370,428
ABCB1_6_3140,553,876,805,742,693,305,563,100,1217,1168,1885,214
ABCB6_6_3141,4128,3088,4968,4042,4589,5084,4734,4704,6087,7465,5773,29
32
ABCB7_6_3142,3162,2814,5312,4219,2686,6288,2704,4326,4869,3160,4909,44
45
ABCB8_6_3143,912,1069,1408,1432,1267,757,457,301,1668,2406,1830,792
ABCC12_6_3144,3155,2265,3351,2975,3576,1481,2148,5084,2920,3355,3823,3
385

ABCC2_6_3145,1430,1785,1581,2018,1674,1599,1576,1508,1617,1134,3282,14
10
ABCC8_6_3146,299,171,279,209,191,0,589,97,0,864,0,274
ABCD1_6_3147,1540,1699,1646,1187,920,2288,660,447,486,1001,1873,1491
ABCD2_6_3148,1173,970,1189,1428,1068,883,408,852,1710,1976,1328,999
ABCD4_6_3149,649,489,761,551,510,1178,529,744,201,1133,1626,851
ABCF3_6_3150,2487,2464,2804,3149,1305,1400,4239,1642,3042,2522,4762,21
82
ABCG2_6_3151,1925,2452,3069,2453,1210,4186,2265,3094,1477,4523,3391,18
08
ABCG5_6_3152,1992,1521,2199,1805,2654,1156,680,2284,666,2096,1931,2377
ABCG8_6_3153,1861,1130,1170,1270,2097,1151,2016,1541,2798,1822,926,703
ANXA10_6_3154,6951,5608,6552,5643,7615,8363,7274,10038,5277,8394,10772
,4330
ANXA1_6_3155,3983,4742,4596,4644,3164,2391,4496,2668,3349,5852,4577,53
92
ANXA3_6_3156,4059,2717,3802,4526,3962,5195,2150,2602,3291,5201,4221,45
43
ANXA4_6_3157,1976,802,1352,1808,2167,2514,2654,2694,559,2219,1524,2161
ANXA5_6_3158,1414,1321,1017,1262,1797,1173,1565,1830,1562,1278,1693,76
7
ANXA8_6_3159,1482,1022,835,699,468,4225,304,422,318,985,1318,1117
ANXA9_6_3160,185,374,316,360,471,2,338,72,436,2665,135,1236
ATP10A_6_3161,3399,2562,4376,3563,3061,4276,3961,4201,2132,3259,3579,3
325
ATP10B_6_3162,2706,2883,3297,2927,2495,2802,2238,2622,3223,3969,2411,2
906
ATP10D_6_3163,1036,452,764,854,713,486,526,728,540,773,856,761
ATP11B_6_3164,342,401,1065,886,317,998,1971,1424,88,1427,385,364
ATP1A2_6_3165,10463,8383,11248,11013,9894,10575,9240,10224,9033,8006,1
3452,9605
ATP1A3_6_3166,10391,8090,10871,10832,9934,10141,9637,8350,9528,8013,13
443,9620
ATP1B1_6_3167,2284,1881,2495,2234,2628,905,2662,1816,1966,2138,1709,13
97
ATP1B2_6_3168,661,303,587,789,952,362,65,1584,1367,1143,166,268
ATP1B3_6_3169,1362,981,1445,1775,838,890,1673,3057,1061,3254,2569,480
ATP4A_6_3170,473,1003,914,1123,1307,572,226,815,160,942,234,363
ATP4B_6_3171,356,105,563,296,598,135,326,262,0,18,1028,12
ATP5B_6_3172,16051,16539,19439,17031,12166,21637,14936,17765,12958,159
91,21666,13707
ATP5F1_6_3173,243,267,299,328,278,1138,259,237,256,564,450,309
ATP5I_6_3174,902,575,928,659,976,451,488,771,2443,893,1701,938
ATP5L2_6_3175,4639,3466,4300,3775,3784,4908,4494,5467,1940,4494,5658,4
020
ATP5L_6_3176,1066,354,1238,1633,796,1292,1417,988,1116,1076,1569,357
ATP5O_6_3177,1253,1748,1717,1619,2832,793,1886,1416,1194,1073,1609,174
9
ATP6AP1_6_3178,1316,1210,1192,1046,3274,1120,1380,2092,1108,1706,3110,
2996

ATP6AP2_6_3179,1525,980,1447,891,246,839,876,730,1032,1297,2468,2986
ATP6V0A2_6_3180,1541,1149,1900,1572,1330,489,752,791,1338,1738,2038,20
22
ATP6V0D1_6_3181,1674,2323,2514,2105,1883,2048,2070,3194,2050,2417,1413
,1117
ATP6V0D2_6_3182,2823,1928,2606,3184,4644,2600,1729,2405,3099,3539,3473
,3101
ATP6V0E1_6_3183,3427,3329,3464,3775,2621,3252,3290,4801,3672,5185,6325
,4054
ATP6V1A_6_3184,2256,1894,2467,3302,2042,3206,4128,2145,2095,3816,3427,
2109
ATP6V1B1_6_3185,1296,981,1108,1293,2068,1655,912,295,692,783,465,1334
ATP6V1B2_6_3186,401,685,825,526,1791,86,267,418,392,656,832,708
ATP6V1C1_6_3187,741,1155,885,1330,1498,1078,1466,557,634,1148,522,713
ATP6V1D_6_3188,3831,3288,3236,3168,3803,3371,4397,3825,3449,6783,5216,
4758
ATP6V1E2_6_3189,2900,2524,2784,3352,2552,3709,6004,4320,4148,3564,3605
,3021
ATP6V1G1_6_3190,513,463,596,641,482,506,396,1221,960,392,2185,1225
ATP7A_6_3191,2010,2214,2732,1877,1725,1952,3182,1855,1286,3376,2673,36
50
ATP8A2_6_3192,984,992,645,922,302,1265,1751,840,1771,324,1648,1196
ATP8B1_6_3193,1078,741,1267,977,366,116,994,563,572,864,1901,429
ATP8B4_6_3194,1422,1469,1210,1422,331,1024,1363,2199,600,1128,1689,255
7
ATP9A_6_3195,2015,2474,2870,2344,1535,1222,1521,3577,1124,2534,1801,28
37
ATP9B_6_3196,1006,1358,1225,2240,537,1614,1789,1391,1558,2392,2143,143
2
ATPAF2_6_3197,404,537,743,567,349,156,224,288,98,195,1226,319
FLVCR1_6_3198,139,393,570,492,280,317,9,386,406,1163,787,944
RHBG_6_3199,343,220,493,509,69,27,20,44,52,172,703,171
RHCG_6_3200,2017,2173,2838,3288,923,1594,1366,2726,2381,4153,3149,3364
SLC10A1_6_3201,1654,1361,2283,2863,854,2691,2185,3114,3133,3758,2039,1
865
SLC10A2_6_3202,1244,543,898,857,659,781,809,2217,222,2239,360,708
SLC10A4_6_3203,3258,2116,4092,4304,3106,6097,5165,3731,2999,2490,3743,
3721
SLC10A5_6_3204,1002,1694,1445,1404,2166,1334,2557,2390,2386,2522,1846,
1802
SLC10A6_6_3205,2657,2513,3044,2850,2125,2988,2476,4017,2407,2058,1423,
2598
SLC11A1_6_3206,537,607,696,629,225,27,293,16,889,1197,402,132
SLC12A2_6_3207,7484,7412,7280,7776,5199,7268,9060,7509,8069,9333,11191
,6697
SLC12A7_6_3208,858,1118,1194,887,48,3463,1414,109,1716,2239,1222,1112
SLC12A9_6_3209,815,1361,887,857,1140,1425,2644,718,198,862,1764,226
SLC13A1_6_3210,8651,7935,10350,9401,5992,5777,6230,8830,6944,9198,1291
5,14636
SLC13A4_6_3211,1225,1499,762,1432,670,2099,4358,1379,1274,607,1961,454

SLC15A1_6_3212,9,22,0,0,0,0,0,0,0,0,319,0
SLC15A3_6_3213,1210,2061,1859,1939,1359,524,4651,2231,1297,3076,1654,2
223
SLC15A4_6_3214,503,201,352,458,163,26,107,43,51,299,879,0
SLC16A10_6_3215,5357,5797,6419,7365,3085,4336,5561,6288,5665,6684,8482
,8518
SLC16A11_6_3216,302,220,274,221,47,166,919,555,177,161,525,20
SLC16A12_6_3217,1449,1608,1694,2058,378,708,2248,2608,2030,706,2020,19
52
SLC16A2_6_3218,4170,3240,4485,5006,4602,4913,3357,3504,3241,6020,4512,
2550
SLC16A5_6_3219,498,206,454,396,228,48,764,333,342,96,545,870
SLC16A7_6_3220,1211,1377,1231,1791,1776,2087,738,3540,760,1914,1950,78
7
SLC16A8_6_3221,571,345,503,910,560,56,400,347,1102,140,1088,895
SLC17A1_6_3222,7099,4998,7570,7702,5470,6854,7738,11094,4901,6716,8960
,5554
SLC17A4_6_3223,977,924,655,925,810,2087,964,482,515,603,469,108
SLC17A5_6_3224,2538,2313,2568,3176,3165,1997,3697,1737,2548,4945,5146,
3140
SLC17A6_6_3225,2958,2510,2658,3832,1745,2551,2736,2905,2455,3334,2385,
2343
SLC17A7_6_3226,3192,2242,3211,2720,3114,1006,4109,4400,2374,6208,3120,
2082
SLC18A2_6_3227,2334,2267,2135,2556,2090,2760,1407,2328,3934,2712,2720,
2178
SLC18A3_6_3228,518,618,598,779,1468,1067,438,259,129,319,1997,466
SLC19A2_6_3229,988,1465,1667,1493,1093,1945,2392,1132,1225,1048,1584,1
107
SLC19A3_6_3230,2081,2890,2183,2529,2811,2095,2572,4197,2741,2278,5373,
2830
SLC1A1_6_3231,5027,6201,5770,6119,6302,4557,6371,4115,4803,7266,8934,8
913
SLC1A6_6_3232,681,681,551,772,547,1358,702,800,163,1072,1214,290
SLC1A7_6_3233,136,234,217,537,287,81,266,4,0,362,11,360
SLC20A1_6_3234,1144,1503,1588,1794,1744,1917,2416,1321,1418,1565,2561,
1798
SLC20A2_6_3235,576,522,623,1128,932,304,276,970,700,1038,821,57
SLC22A10_6_3236,7571,7676,8819,8558,9237,9191,9801,9619,4114,9062,1266
0,6003
SLC22A11_6_3237,297,283,361,437,193,99,248,136,463,729,654,495
SLC22A13_6_3238,2411,2414,2312,2397,2687,1796,1523,622,1524,3920,3203,
2384
SLC22A14_6_3239,2201,2238,2117,2117,1612,3223,1580,3449,2714,748,1002,
2473
SLC22A15_6_3240,1537,1662,1712,1387,1511,1283,1330,2500,2338,2503,3272
,3021
SLC22A16_6_3241,3602,3889,4675,3847,1549,5236,3650,3113,5190,4174,5977
,4899
SLC22A18AS_6_3242,2189,2590,2983,2741,2345,5539,3253,1510,2096,2775,38

13,2019
SLC22A2_6_3243,350,303,195,350,25,373,199,165,84,187,221,165
SLC22A3_6_3244,1400,1163,1792,1610,628,1602,1099,943,1053,1253,1946,42
8
SLC22A4_6_3245,2119,1951,2619,1787,1536,1034,1886,2418,2549,1288,4738,
2734
SLC22A5_6_3246,1879,1276,2110,2420,1911,1643,3243,1831,1653,3460,2537,
1826
SLC22A9_6_3247,620,164,885,445,457,425,558,130,180,540,869,5
SLC24A1_6_3248,1901,1532,2120,2236,3551,821,2549,810,963,2664,3043,360
0
SLC24A3_6_3249,1095,776,1408,1805,788,1576,1429,2742,1007,2073,2551,10
90
SLC24A5_6_3250,1804,1923,1514,1190,723,539,1127,1819,2128,615,1410,228
1
SLC24A6_6_3251,2676,3498,2670,2918,2456,5817,3213,1144,2797,3127,3698,
1766
SLC25A10_6_3252,227,118,303,308,9,316,144,22,10,22,54,14
SLC25A12_6_3253,725,540,577,465,674,1250,1361,513,973,987,1780,671
SLC25A15_6_3254,3488,3532,3797,3843,3945,4206,3652,3472,2812,2976,8596
,3881
SLC25A16_6_3255,1840,2056,2728,2528,1255,1705,3091,2372,1842,1739,1229
,1655
SLC25A17_6_3256,791,1030,819,684,100,47,651,691,519,1729,1656,765
SLC25A18_6_3257,851,1122,957,1521,1623,1013,670,1091,1200,3504,1151,11
90
SLC25A1_6_3258,2050,2117,2998,2386,1852,2545,1663,1051,1083,2064,2439,
3788
SLC25A20_6_3259,430,656,866,554,490,90,445,628,88,183,484,817
SLC25A28_6_3260,1156,1488,2555,1812,1543,52,1189,2999,1210,1081,3154,1
515
SLC25A2_6_3261,2683,2206,3102,3529,2735,1790,2041,1262,3111,2734,4542,
2836
SLC25A32_6_3262,6256,5428,7782,7157,4980,6083,6528,10229,5171,7025,766
5,7181
SLC25A37_6_3263,1051,772,949,981,1310,1240,491,361,1275,395,1941,911
SLC25A43_6_3264,3270,4050,4540,4193,3401,2638,2910,3852,2773,5615,3228
,2771
SLC25A44_6_3265,651,712,847,960,1813,1016,281,1149,297,1129,568,451
SLC25A4_6_3266,3944,3830,4875,4109,4810,4038,6542,4962,4930,5015,5508,
4722
SLC25A5_6_3267,586,953,839,831,679,767,2030,86,513,586,2003,1342
SLC25A6_6_3268,5820,5194,5203,5892,7340,3552,5740,4856,2112,5001,6116,
5526
SLC26A10_6_3269,427,221,252,105,98,236,109,129,107,128,489,13
SLC26A2_6_3270,2060,1674,2798,2028,2118,3085,1810,3230,1548,2196,3268,
3446
SLC26A3_6_3271,4570,4207,5725,4314,3784,4355,4846,2967,5093,3871,5663,
4158
SLC26A4_6_3272,1723,1684,2108,1866,2189,2053,2332,1974,2148,1668,2576,

1939
SLC27A1_6_3273,1137,1217,1132,1060,1184,643,904,1241,1385,1044,1413,23
06
SLC27A3_6_3274,2204,1546,2758,3254,3559,964,3103,2992,1427,3051,2742,1
544
SLC27A4_6_3275,2685,2050,2880,2050,1129,2966,1925,2268,997,1780,5219,3
001
SLC27A5_6_3276,2216,2428,2507,2674,915,2824,2437,2028,2600,3036,3118,3
432
SLC28A2_6_3277,879,699,581,619,813,528,206,433,527,309,555,492
SLC29A2_6_3278,1517,1193,1968,1467,925,2027,1060,2196,1154,1043,1914,1
477
SLC2A10_6_3279,245,541,723,644,56,727,675,849,1196,95,795,469
SLC2A12_6_3280,1568,2008,1753,1200,1455,1172,1728,1251,1015,1064,1361,
312
SLC2A13_6_3281,9278,7802,11734,10816,8790,11147,10313,13247,7373,9237,
13685,11140
SLC2A14_6_3282,3787,3474,4188,3715,4304,4022,5309,2689,1802,3407,5632,
3566
SLC2A1_6_3283,2122,2577,2735,2635,3144,2622,829,3205,2096,1172,3219,13
41
SLC2A2_6_3284,2247,1066,2580,2838,2377,2576,884,2006,2056,857,2788,340
6
SLC2A3_6_3285,3259,2814,4246,3892,2335,2381,2730,4220,2949,3658,6947,3
131
SLC2A4_6_3286,401,887,511,419,34,75,21,415,670,1878,341,439
SLC2A4RG_6_3287,578,999,555,733,1457,1519,210,655,77,796,1018,592
SLC2A7_6_3288,1454,2220,1667,1352,2332,2997,1967,3153,1223,1998,1362,1
561
SLC2A8_6_3289,2195,1570,2214,2207,1094,730,3365,2026,1799,2761,2214,16
19
SLC30A10_6_3290,2223,1633,2080,2297,776,2465,2652,4043,1453,4049,2039,
2246
SLC30A1_6_3291,5956,5841,6288,6131,5890,4890,6134,3421,6393,5558,7411,
5525
SLC30A3_6_3292,845,783,1172,1391,1062,968,678,2417,416,1289,1317,1472
SLC30A4_6_3293,1315,1616,1421,1446,436,1113,1315,1684,1733,386,1611,11
32
SLC30A9_6_3294,972,503,1354,1658,456,3011,935,1352,816,512,1213,687
SLC31A1_6_3295,1260,1008,1475,1255,204,552,1928,3201,502,708,852,1395
SLC31A2_6_3296,275,325,529,396,216,2,625,3,59,69,813,71
SLC32A1_6_3297,753,630,615,1169,1485,331,196,73,202,309,725,1689
SLC35A3_6_3298,14292,13781,18898,16472,11906,17858,17156,18904,11435,2
2074,18900,10366
SLC35A4_6_3299,1167,869,1026,1651,376,541,880,672,908,2501,2562,549
SLC35A5_6_3300,3973,3430,3996,3653,3363,3032,2958,3258,3345,5189,7060,
6706
SLC35B1_6_3301,4834,5448,4921,4785,5755,6999,3918,2997,4466,7302,7464,
3606
SLC35B2_6_3302,1062,853,1054,899,553,511,913,2878,440,335,1237,240

SLC35B4_6_3303,4785,4000,5528,4931,4470,7254,6621,7932,2628,4274,7789,
5522
SLC35D1_6_3304,4356,4566,4791,4452,3767,5029,4478,3674,5088,4275,5679,
4830
SLC35D2_6_3305,6330,5471,6461,6170,5145,7187,5732,6412,3870,7678,9345,
3863
SLC35D3_6_3306,239,100,394,393,3,366,0,302,0,347,28,786
SLC35E1_6_3307,1805,1855,1778,1810,2919,2761,2645,1566,2059,3437,2562,
2276
SLC35E3_6_3308,9583,8434,10060,10391,10098,8506,12294,9844,7681,9144,9
987,14290
SLC35E4_6_3309,1719,1108,1527,1990,1007,3627,3416,2428,1565,3908,2927,
412
SLC35F1_6_3310,2302,2325,2186,1683,3569,1643,1476,1513,1939,1645,3622,
2742
SLC35F2_6_3311,5165,4083,4533,5445,3817,5307,5696,5150,2975,6448,4450,
5582
SLC35F3_6_3312,2797,2705,3869,2944,2100,2460,3395,2588,3078,2777,3882,
4401
SLC35F4_6_3313,268,446,523,410,289,1676,50,96,12,504,278,0
SLC35F5_6_3314,804,1259,1173,1003,1817,423,11,1192,346,475,1967,1100
SLC36A1_6_3315,424,227,318,585,345,321,289,104,26,200,410,173
SLC36A2_6_3316,4565,5239,5853,4928,3245,3816,4794,6464,3810,5787,6991,
4366
SLC36A4_6_3317,2550,2562,2654,2434,879,5351,2532,3208,1573,2706,2448,1
315
SLC37A1_6_3318,1028,1045,861,1986,1310,1428,795,1153,168,1270,998,2494
SLC38A2_6_3319,1158,668,1343,1494,700,745,1071,875,1691,3458,1767,1359
SLC38A3_6_3320,855,731,1481,1206,1111,829,2255,1136,451,706,2304,1686
SLC38A5_6_3321,1204,1587,1760,1721,1144,1007,2117,1194,1262,3230,4506,
1239
SLC39A1_6_3322,535,857,563,985,1040,393,47,321,408,1844,1256,501
SLC39A2_6_3323,1112,1198,1485,1270,706,1787,295,2619,1795,1171,1878,12
32
SLC39A9_6_3324,3227,3292,4651,4140,2067,1894,3613,2884,5000,3187,5949,
2780
SLC3A1_6_3325,1494,1183,1953,1398,367,1356,2016,1741,2485,1910,3633,15
83
SLC40A1_6_3326,3930,4495,3229,4475,3389,5578,4891,3260,4856,3193,5480,
2399
SLC41A1_6_3327,1833,2007,1701,1243,2629,1419,2637,1066,1796,1540,2659,
1844
SLC43A2_6_3328,817,1025,932,941,1231,923,288,1083,1238,564,987,1718
SLC44A1_6_3329,3137,3016,3402,3464,2395,5295,5902,4285,1554,2388,3173,
4188
SLC46A2_6_3330,935,483,1358,888,862,1057,1370,478,439,1367,1358,1182
SLC4A1AP_6_3331,5806,5612,6640,5400,5707,5745,6301,5118,8815,7395,6618
,5003
SLC4A1_6_3332,901,859,1089,934,82,928,134,152,1176,731,1325,2342
SLC4A7_6_3333,4003,4375,4733,4482,3010,6122,7489,7283,5015,5415,6081,3

967
SLC4A9_6_3334,1678,1722,1378,1701,1829,1969,1725,3270,417,2149,2212,18
59
SLC5A11_6_3335,392,379,497,440,1708,284,136,404,663,270,735,850
SLC5A1_6_3336,524,1370,848,597,191,837,1046,533,471,445,861,657
SLC5A2_6_3337,1575,1644,2660,1875,3644,1477,2471,1346,645,2804,2829,71
4
SLC5A3_6_3338,7921,7590,8306,8482,5527,5988,6045,7048,7722,7820,12356,
7615
SLC5A4_6_3339,1372,1370,1941,2163,2425,987,1340,1895,1176,1081,1251,11
48
SLC5A5_6_3340,820,965,884,1204,464,1761,1808,674,38,1543,634,2186
SLC5A6_6_3341,1809,1848,1814,1847,2028,4062,2363,3008,1354,1941,2185,3
047
SLC5A7_6_3342,1426,1548,1440,1827,1135,917,1470,4181,759,4850,2858,222
8
SLC5A8_6_3343,2615,1937,2129,2958,2382,3661,3484,2353,1473,1668,3544,3
188
SLC6A11_6_3344,827,1073,968,883,937,1502,1130,1274,1235,1113,545,556
SLC6A14_6_3345,2702,1999,2618,2424,1304,2166,3078,2421,1686,1419,1733,
1594
SLC6A16_6_3346,1796,1388,1525,2053,800,644,2081,923,700,4449,2923,1265
SLC6A19_6_3347,1509,1090,1818,1349,1304,748,2332,2320,2254,927,2071,21
53
SLC6A1_6_3348,889,1041,1471,1482,1115,1011,748,309,1088,1004,838,935
SLC6A3_6_3349,80,39,220,6,6,1,195,7,1,113,25,414
SLC6A4_6_3350,533,386,610,492,221,1179,77,39,107,274,355,266
SLC6A7_6_3351,102,420,224,102,274,426,390,58,1,104,564,5
SLC7A10_6_3352,1834,1972,1621,1447,1276,2787,3557,2821,1116,1691,1391,
1394
SLC7A11_6_3353,7819,8993,10877,10356,8704,8809,8937,11565,9891,9943,14
157,6673
SLC7A13_6_3354,1435,1698,1691,1597,1920,589,1583,531,1406,1316,1450,11
5
SLC7A1_6_3355,937,1294,1091,1088,1045,838,2181,259,171,2486,2315,587
SLC7A4_6_3356,1822,1786,1236,2172,1160,1746,2140,1221,1191,2581,2015,1
312
SLC7A5_6_3357,5645,5295,8066,6413,6003,3446,5900,4594,7357,8733,6003,7
057
SLC8A2_6_3358,220,351,194,218,2,0,27,52,22,277,0,8
SLC9A1_6_3359,688,852,983,512,854,3,62,1850,677,687,541,1545
SLC9A2_6_3360,5574,5687,6264,5070,1365,9513,5279,6543,6419,6090,7570,3
018
SLC9A3_6_3361,2361,1950,2847,2102,1582,2202,3028,2269,2855,4209,3704,5
193
SLC9A3R1_6_3362,1824,1285,1962,1937,825,1818,967,3737,2191,2256,2892,1
986
SLC9A4_6_3363,1960,1071,1731,1475,2159,1596,864,1583,1753,1136,1807,19
04
SLC9A5_6_3364,1026,873,964,995,2329,830,1424,1364,360,803,338,312

SLC9A7_6_3365,54,326,99,144,450,0,164,135,233,1,9,255
SLC9A8_6_3366,1555,1021,1236,1363,3051,1862,1503,1400,677,675,4057,108
8
SLC9A9_6_3367,4188,2776,4310,4909,5671,4570,3134,5821,2852,5681,6555,7
766
SLCO1B1_6_3368,9801,8285,11301,10554,12934,11556,10325,8724,9136,12449
,11721,10456
SLCO1B3_6_3369,4574,3810,4680,4256,3051,5811,3713,3941,3230,2204,6806,
5117
SLCO2A1_6_3370,1738,1994,2297,2203,2178,1783,2508,2113,965,2407,3593,1
778
SLCO4A1_6_3371,1177,1485,1277,1248,1958,1466,1423,1238,1354,1587,1676,
998
SLCO4C1_6_3372,3331,3569,4291,4202,2437,2212,6161,2800,2904,4210,4891,
2928
SLCO6A1_6_3373,1383,865,1567,1779,305,2759,1738,1529,896,752,913,2054
SV2A_6_3374,638,291,1176,774,910,705,607,674,465,212,1116,355
TAP1_6_3375,1069,821,1370,1234,348,1123,1143,3036,1535,2309,591,1678
UCP1_6_3376,2109,2544,2098,2470,2391,3112,2125,4358,1508,4331,2986,331
2
ABCA12_6_3377,1729,812,1503,1294,1493,1585,979,1955,951,2005,1139,2564
ABCA2_6_3378,934,1066,1553,1261,2001,1340,1307,1557,848,918,2085,631
ABCA5_6_3379,11735,12299,15530,13183,11773,8778,16217,21920,12698,2096
2,20159,16332
ABCB4_6_3380,5178,5572,5932,6958,6814,4483,3191,6253,4078,7074,6240,82
38
ABCB9_6_3381,872,371,681,703,696,57,883,958,710,377,404,590
ABCC10_6_3382,1581,1127,1502,1661,1238,570,2221,191,692,2200,1139,233
ABCC11_6_3383,2160,1947,2711,2531,1967,644,1721,2920,1901,4285,3486,21
60
ABCC1_6_3384,1403,1167,598,1303,273,202,552,407,143,1350,1419,788
ABCC3_6_3385,1325,1137,1522,1579,1120,3085,2724,1500,1489,3410,2729,36
51
ABCC4_6_3386,2495,3096,3825,3284,2306,3330,3771,2845,2785,4337,3325,20
72
ABCC5_6_3387,1609,1502,1916,2136,2045,3086,2939,1142,2630,4321,1160,14
69
ABCC9_6_3388,15669,12502,16633,15676,13485,16524,19055,15588,15167,136
33,16567,13544
ABCD3_6_3389,5062,5570,6223,7087,3377,8386,7511,4963,5210,4073,6704,35
11
ABCE1_6_3390,1187,752,1076,1066,482,235,747,863,465,243,310,992
ABCF1_6_3391,707,852,996,995,160,1572,1123,517,163,1053,2315,338
ABCF2_6_3392,1448,1537,1902,1212,994,388,2784,504,1462,1478,1414,1393
ABCG1_6_3393,697,1141,1050,791,622,434,1562,988,93,3099,1675,619
ABCG4_6_3394,338,86,616,348,1,754,534,203,20,18,58,185
ANXA11_6_3395,1489,1588,1813,1660,2350,2791,2708,1490,1552,3609,3340,1
509
ANXA13_6_3396,1674,1358,1519,1530,1722,301,1287,1906,1810,940,1444,164
2

ANXA2_6_3397,1254,1036,987,1398,1756,1310,1320,1483,322,803,2896,867
ANXA6_6_3398,855,1011,971,1530,441,2869,1861,541,560,1890,2220,1226
ANXA7_6_3399,4902,4437,4971,5218,4265,6503,4010,6358,3789,3897,3788,55
80
ATP11A_6_3400,3226,4067,4650,4238,2670,3845,3511,4294,4090,5974,4138,3
489
ATP11C_6_3401,3140,3241,2460,2628,1523,3039,2865,3078,1641,2081,2458,2
691
ATP12A_6_3402,1943,1275,1868,2414,801,1251,2184,649,1088,1052,1970,911
ATP1A1_6_3403,855,1225,1021,1363,1009,686,1034,750,1051,1250,1627,1066
ATP1B4_6_3404,5315,4767,6854,6788,2036,3665,5104,4784,3494,5612,6489,4
157
ATP2A1_6_3405,879,400,821,870,1426,378,1114,720,273,151,2047,576
ATP2A2_6_3406,3199,2826,3688,4226,5291,1913,3482,3109,1849,2456,3300,2
858
ATP2A3_6_3407,2885,2892,3715,3737,2212,4675,3599,3457,1848,2632,3193,2
656
ATP2B1_6_3408,1127,1256,1404,1571,1463,3730,3717,444,956,3749,1459,150
1
ATP2B3_6_3409,771,1071,974,1260,1474,615,983,1120,1527,582,857,114
ATP2B4_6_3410,6199,5616,6656,6679,7356,4720,9682,5683,6795,5427,7509,7
544
ATP2C1_6_3411,1101,537,982,689,823,1688,1615,1085,623,746,1271,957
ATP5A1_6_3412,1799,2130,2555,2852,3119,1832,2757,1212,4153,2227,1833,1
899
ATP5C1_6_3413,4068,5166,6023,4762,4938,5964,6166,5336,4143,3497,7302,6
168
ATP5D_6_3414,992,1068,1280,1144,500,1590,647,1040,2884,737,773,1712
ATP5E_6_3415,1934,1995,2328,2407,2931,2822,1445,3125,2161,2594,3307,29
26
ATP5G1_6_3416,1668,1076,1962,1895,1498,2831,3605,2427,1309,1063,449,90
8
ATP5G2_6_3417,1964,1257,1907,1696,1303,2031,1940,2704,1623,1197,2439,9
28
ATP5G3_6_3418,3057,4145,3597,3485,3392,4214,3733,2175,3739,5096,3225,3
740
ATP5H_6_3419,19279,17568,22304,22939,19577,17869,26516,22650,13251,192
12,26096,18929
ATP5J_6_3420,3951,3965,3785,3872,2765,5590,4242,6384,2469,7579,5495,33
05
ATP6V0A1_6_3421,1438,658,1553,1204,3077,737,1392,461,1423,1555,1626,19
42
ATP6V0A4_6_3422,3110,3050,2691,3324,3419,1978,3396,3095,1829,2820,3737
,2495
ATP6V0B_6_3423,1342,1455,1105,1648,789,153,1005,788,746,2036,1408,992
ATP6V0C_6_3424,442,174,365,276,40,3,877,1285,534,287,818,1105
ATP6V1C2_6_3425,1874,2061,2127,2161,2179,1080,1699,1548,1601,2283,3918
,2095
ATP6V1E1_6_3426,3929,3201,4769,3977,4608,5041,4066,5005,2582,4105,5134
,3676

ATP6V1F_6_3427,468,339,865,576,80,1091,375,549,1350,733,386,651
ATP6V1G2_6_3428,1097,472,1379,1254,406,458,854,1576,1120,2899,1750,149
1
ATP6V1H_6_3429,1957,2187,1826,2303,1455,824,1409,690,1357,2233,1978,70
8
ATP7B_6_3430,1109,1533,1519,1427,1011,1265,1249,1812,385,1820,2362,181
4
ATP8A1_6_3431,5646,5818,6393,5943,5117,4744,4073,8921,3217,4564,5089,5
259
ATP8B2_6_3432,4327,4108,5233,5119,3431,3847,4308,5227,5922,3660,8627,2
149
ATP8B3_6_3433,1354,2139,2646,1825,821,2684,1946,1218,1500,656,4594,246
2
ATPAF1_6_3434,7910,7488,8830,8309,7871,11791,9317,11962,7987,6996,9861
,12029
GC_6_3435,3266,3053,3928,3793,2857,2137,4530,4759,3269,4527,5778,2670
SERINC5_6_3436,5094,5224,5433,5261,8062,6909,4681,4061,3754,7274,4844,
8426
SLC10A3_6_3437,629,587,876,826,609,117,874,804,290,705,1125,389
SLC11A2_6_3438,413,332,627,761,922,228,2407,660,971,1072,1082,1805
SLC12A1_6_3439,1716,1228,1534,1824,2018,2632,1381,857,831,693,2393,132
2
SLC12A3_6_3440,3840,3793,4430,4958,4641,3031,5139,3784,3908,4136,6671,
4658
SLC12A4_6_3441,1226,1026,1153,1452,555,3063,857,1416,2144,1457,1018,93
0
SLC12A5_6_3442,958,1010,1772,1712,1748,101,2309,1350,296,820,1316,1177
SLC12A6_6_3443,961,834,862,901,1046,1025,1548,1150,511,693,522,415
SLC12A8_6_3444,853,182,375,182,1003,690,463,10,1644,525,129,419
SLC13A2_6_3445,14390,16306,13742,14264,15116,21143,9242,14763,8846,137
63,20732,13980
SLC13A3_6_3446,211,198,334,403,62,1404,31,454,82,69,50,113
SLC13A5_6_3447,1337,1024,1385,1629,423,591,1348,1445,703,2471,2385,110
6
SLC14A1_6_3448,968,1142,1010,943,1800,686,1423,749,448,737,880,1668
SLC14A2_6_3449,927,688,1665,1287,2842,811,1003,437,586,500,1478,349
SLC15A2_6_3450,808,1215,936,1129,459,269,1175,246,406,1021,2111,604
SLC16A1_6_3451,1836,1692,1700,1458,1214,1145,2206,1878,2042,1874,3918,
3111
SLC16A3_6_3452,671,537,329,638,463,1461,371,158,1189,261,1136,711
SLC16A4_6_3453,3252,2844,4206,3726,4042,3224,4986,2736,4172,3104,3856,
3370
SLC16A6_6_3454,1540,1867,1810,2065,998,3604,1511,1276,1008,3538,1603,3
324
SLC17A3_6_3455,1527,1336,2265,1938,3785,3228,1323,2102,943,3220,1502,2
307
SLC17A8_6_3456,1659,1997,2455,2283,2762,1524,1954,4817,2365,3591,3838,
3486
SLC18A1_6_3457,3263,3622,3566,3251,3353,3437,3214,3668,3282,6918,2101,
4087

SLC19A1_6_3458,1161,1317,981,1404,1366,1433,1161,1959,1853,2652,387,22
47
SLC1A2_6_3459,1365,968,2003,1901,434,2357,1790,746,1460,1314,3809,1025
SLC1A3_6_3460,3086,1864,2785,2797,2563,2089,5971,4266,2306,3459,3101,2
744
SLC1A4_6_3461,2066,2082,2010,2549,3678,895,1982,1616,2455,1772,2562,15
92
SLC1A5_6_3462,848,715,942,812,1579,1015,896,661,72,699,2808,1975
SLC22A12_6_3463,1149,485,633,900,1765,1178,1042,1004,1403,3349,733,102
4
SLC22A17_6_3464,1180,957,1242,1286,622,518,2022,978,1593,335,700,914
SLC22A18_6_3465,508,392,307,383,249,522,712,519,234,641,129,136
SLC22A1_6_3466,666,465,689,567,605,466,402,1291,2001,784,2137,552
SLC22A23_6_3467,1033,1009,1138,1265,1218,560,1295,483,1683,1272,1015,1
17
SLC22A6_6_3468,831,603,658,790,447,1120,167,822,362,413,1750,174
SLC22A7_6_3469,285,457,819,697,1091,172,858,595,147,2034,1206,488
SLC22A8_6_3470,2065,1757,2096,1856,2297,1018,728,2944,1560,3913,1312,1
570
SLC23A3_6_3471,559,526,679,738,215,1063,676,34,909,314,848,640
SLC24A2_6_3472,4358,3550,4897,5664,2252,5484,5198,6007,3464,9162,9705,
5257
SLC24A4_6_3473,858,576,378,699,610,127,659,2408,216,901,690,453
SLC25A11_6_3474,972,585,890,699,332,211,572,344,1665,1162,1019,447
SLC25A13_6_3475,7362,6355,6696,6690,7032,7126,6295,4845,5444,10277,861
2,8060
SLC25A14_6_3476,237,146,540,307,23,0,98,54,331,10,390,91
SLC25A19_6_3477,4602,3320,5065,5193,5058,4484,3473,5524,5292,5518,7086
,4259
SLC25A21_6_3478,1907,2103,2553,2622,1317,2286,4389,1783,1721,2225,2897
,3366
SLC25A22_6_3479,1782,2027,2244,2163,2606,513,2590,1584,807,1844,3121,2
439
SLC25A26_6_3480,906,593,790,952,808,503,221,2085,944,933,402,156
SLC25A27_6_3481,2118,2225,2560,2106,3738,3735,2349,3890,2715,2241,2805
,1675
SLC25A3_6_3482,2624,2509,2977,3364,1534,1712,2451,2515,1881,2181,4049,
3881
SLC26A11_6_3483,1993,2202,2557,2127,1917,1374,3869,2205,1667,2675,4781
,1179
SLC26A1_6_3484,422,494,357,384,524,2265,267,1516,240,1038,740,645
SLC26A5_6_3485,2655,1985,2715,2412,4209,800,1384,2217,1986,2840,4030,2
933
SLC26A6_6_3486,366,203,369,484,142,726,375,2293,172,245,619,136
SLC26A7_6_3487,936,559,1234,877,863,438,2600,293,777,981,974,741
SLC26A8_6_3488,9905,9483,11167,11990,12215,11578,10303,10918,9734,8079
,10448,9244
SLC26A9_6_3489,1008,1187,1070,1253,911,1203,754,725,1210,2501,1882,190
0
SLC27A2_6_3490,675,552,1321,928,852,527,1775,561,3,453,1802,55

SLC27A6_6_3491,4170,4427,4009,3923,5186,3755,4252,4375,3363,4449,4393,
4005
SLC28A3_6_3492,4145,3727,5719,3952,4429,2599,5133,3442,4165,3344,6528,
3169
SLC29A1_6_3493,2558,2869,2189,3105,1648,1904,2788,3811,2361,1939,3321,
4566
SLC29A3_6_3494,1109,741,1055,1217,81,537,954,1137,769,167,449,1653
SLC29A4_6_3495,4240,4168,5612,4552,6736,3963,7079,8023,3346,7721,2950,
4259
SLC2A11_6_3496,169,190,575,414,530,323,276,275,425,841,673,0
SLC2A5_6_3497,1995,2330,2290,2443,2817,2526,1773,865,846,2568,4536,259
3
SLC2A6_6_3498,959,953,1597,1719,2035,857,963,746,486,1827,1712,1562
SLC2A9_6_3499,927,895,758,1264,1817,485,2006,817,1385,972,1655,1645
SLC30A2_6_3500,22681,24592,24535,28075,18456,33887,29804,25859,18253,2
8044,29888,23516
SLC30A6_6_3501,1340,1073,1576,1388,1481,1588,567,743,1336,1731,1916,13
79
SLC30A7_6_3502,1771,2313,2072,2364,988,3034,2273,2527,1793,2230,3310,1
279
SLC30A8_6_3503,2855,2365,2950,3044,3230,3532,3200,3013,1260,1271,3076,
2482
SLC33A1_6_3504,2184,2714,2484,2354,1816,2849,1921,1880,2662,3666,4385,
1699
SLC34A1_6_3505,398,917,275,441,504,135,34,401,481,25,590,226
SLC34A2_6_3506,6118,6120,7225,6906,7370,11293,8140,5756,5457,5065,6534
,6684
SLC34A3_6_3507,5342,5842,5810,6677,4701,7302,9284,5783,4585,6734,9823,
7392
SLC35A1_6_3508,3623,3535,3172,2972,3230,3133,1515,4286,4139,3129,2504,
2757
SLC35A2_6_3509,1550,1693,1818,2519,1892,2035,2271,1555,2293,4000,2914,
2992
SLC35B3_6_3510,815,921,1068,714,129,3866,584,295,636,87,839,460
SLC35C1_6_3511,617,356,243,405,898,302,1505,853,241,1234,182,680
SLC35C2_6_3512,663,587,579,626,1423,338,226,771,503,155,333,212
SLC35E2_6_3513,2157,1533,3007,2310,2527,1463,2012,2145,1860,2979,3969,
1388
SLC36A3_6_3514,1818,2093,1954,2133,2383,1721,2416,3451,2059,1096,2381,
1597
SLC37A2_6_3515,4049,3582,4005,4017,2281,6624,5248,2946,2960,4674,4988,
4512
SLC37A3_6_3516,996,591,744,1212,638,864,1351,737,1693,1248,804,408
SLC37A4_6_3517,800,1015,1254,1433,1973,208,633,2410,682,2444,1010,1084
SLC38A11_6_3518,2985,2945,2316,3055,2647,3322,2950,2894,2337,1983,3231
,1728
SLC38A1_6_3519,768,954,723,1394,562,1605,577,1008,439,2155,1877,3053
SLC38A4_6_3520,17834,17340,20662,19448,14986,15544,20703,15430,10235,1
8584,28904,13396
SLC38A6_6_3521,4975,4521,5176,5251,3887,2830,6292,5755,3660,6641,7275,

3794
SLC39A10_6_3522,977,901,935,708,252,591,1487,281,610,673,1236,503
SLC39A11_6_3523,569,93,870,626,446,265,590,1499,708,43,174,1284
SLC39A12_6_3524,295,494,472,477,109,92,247,42,154,612,365,864
SLC39A13_6_3525,424,857,908,643,437,152,322,364,598,650,1277,526
SLC39A14_6_3526,842,1031,901,1243,1249,1120,1111,1474,664,2103,1561,90
4
SLC39A4_6_3527,1049,368,502,559,823,1272,18,225,86,564,629,664
SLC39A5_6_3528,356,337,152,88,9,251,315,79,207,23,65,73
SLC39A6_6_3529,1265,1599,1716,1858,785,941,2099,954,2340,174,1153,1939
SLC39A7_6_3530,1845,1496,1657,1919,362,2072,1434,2030,2300,1259,3605,2
039
SLC39A8_6_3531,3722,3717,4924,4959,2933,3602,5264,6011,2664,4435,5452,
3786
SLC3A2_6_3532,709,790,740,1159,451,1654,2147,681,1146,2403,969,600
SLC43A1_6_3533,679,772,256,320,152,128,463,267,52,222,2219,830
SLC44A3_6_3534,623,711,422,1132,355,692,687,2273,483,284,612,620
SLC4A10_6_3535,1489,1614,1990,1940,2299,882,1378,1342,486,1832,2431,12
51
SLC4A11_6_3536,1554,1596,2099,2116,744,1010,1631,359,1591,1253,998,198
4
SLC4A2_6_3537,816,674,631,1134,951,2128,1216,621,119,1092,839,570
SLC4A3_6_3538,624,454,656,682,402,1043,470,701,427,396,370,1093
SLC4A4_6_3539,4850,4647,5283,6314,7062,6488,4404,5868,5428,9332,4443,7
667
SLC4A8_6_3540,2673,1943,3119,2664,3678,2810,1127,3640,3340,1828,2765,2
752
SLC5A10_6_3541,881,822,824,751,760,163,1313,301,417,695,1562,1379
SLC5A9_6_3542,1605,1080,818,856,1006,503,1002,892,1112,1009,1282,329
SLC6A12_6_3543,1113,766,927,1030,444,667,1460,297,168,1852,1563,129
SLC6A13_6_3544,2913,2655,3084,3502,3560,5074,4148,4003,618,3093,5007,4
415
SLC6A15_6_3545,4777,3522,6343,5881,3532,3378,3378,9095,6821,4278,6609,
4720
SLC6A2_6_3546,1243,917,1291,1068,219,404,574,1969,165,1009,1104,902
SLC6A6_6_3547,1749,1008,1459,2014,817,1500,2303,401,1314,3803,3507,246
1
SLC6A8_6_3548,1601,1159,1817,1583,1351,3348,1191,1089,472,817,2062,276
7
SLC6A9_6_3549,1047,1320,1404,1679,2007,3687,1072,1621,1217,2200,2911,8
65
SLC7A2_6_3550,1882,1779,1596,2479,2917,2021,2899,1853,1337,1378,3211,2
366
SLC7A3_6_3551,1077,1034,778,972,500,394,609,1495,320,722,2252,425
SLC7A6_6_3552,901,693,856,1053,1695,1467,1598,580,1030,1120,1229,644
SLC7A7_6_3553,410,767,1304,753,215,831,646,204,135,631,1070,2018
SLC7A8_6_3554,3068,3558,3969,3850,4559,4373,5050,3526,2281,3123,6516,2
119
SLC7A9_6_3555,730,1014,1031,1341,1423,901,666,883,356,1448,2496,1154
SLC8A1_6_3556,636,682,332,485,1037,438,740,647,1044,151,658,1096

SLC8A3_6_3557,1946,1905,2690,2496,1595,3381,2897,2236,1366,2720,3087,3
407
SLC9A3R2_6_3558,683,824,1003,640,13,6,580,578,252,1149,657,640
SLC9A6_6_3559,1361,1584,1240,1432,2046,2950,2442,1920,742,4297,1837,17
43
SLCO1A2_6_3560,2810,2500,3180,3493,2496,1261,4693,2365,2460,3586,4083,
3192
SLCO1C1_6_3561,3932,3713,4629,3517,5992,6031,4964,3255,2599,4449,6127,
5208
SLCO2B1_6_3562,9294,8128,10513,9943,10518,8748,8893,10525,10918,8421,1
3163,7700
SLCO3A1_6_3563,2039,2604,1849,1687,1154,3790,2163,2092,2385,3584,4636,
3399
SLCO5A1_6_3564,1606,1237,1431,1251,1521,1006,433,541,2914,637,2302,164
3
ABCB5_6_3565,1308,1356,1219,1760,1768,2016,2021,566,692,1302,3712,3153
ABCC6_6_3566,1109,1437,1911,1477,2107,792,1546,30,1129,488,2489,1771
ATP1A4_6_3567,2210,3136,2958,2952,2405,3348,2375,2377,1842,3651,2772,2
734
ATP5J2_6_3568,1590,1481,1275,1056,144,2630,1517,418,1765,2972,1611,172
7
ATP6V1G3_6_3569,8207,8986,8946,10098,12469,12535,9894,8592,4823,9516,1
3143,7636
ATPIF1_6_3570,3879,3803,4232,4511,6132,3578,3109,4890,3438,3588,6939,1
858
SLC10A7_6_3571,4310,5633,5794,5361,4363,6986,6601,4296,2236,5297,6079,
6367
SLC28A1_6_3572,1948,2639,3457,2728,1749,1757,2977,2504,1710,2205,3106,
5004
SLC30A5_6_3573,3385,3191,2877,3528,3566,3252,4281,2493,2578,3230,3457,
3169
SLC39A3_6_3574,600,326,494,579,830,2,639,418,406,65,150,275
ABCA10_6_3575,1347,1211,1548,1069,475,800,1169,734,1276,1986,744,1289
ABCA13_6_3576,1224,1187,1802,1775,1234,3750,1488,1096,1771,3474,673,88
8
ABCA1_6_3577,762,652,866,969,631,1691,1053,1515,932,546,1260,1632
ABCA3_6_3578,1041,1738,1746,1614,655,1542,3190,1334,411,1320,866,3119
ABCA4_6_3579,18285,17141,22916,19696,14205,22928,16933,18109,17861,170
55,22752,14661
ABCA6_6_3580,11515,12587,13514,12335,13655,11080,13723,12824,9880,9754
,16803,14088
ABCA7_6_3581,1451,253,864,958,0,3655,1519,910,628,4,590,24
ABCA8_6_3582,6529,5803,7831,6151,5367,8700,6652,10883,5399,7901,9271,7
016
ABCA9_6_3583,6529,5803,7831,6151,5367,8700,6652,10883,5399,7901,9271,7
016
ABCB10_6_3584,2064,1830,2159,2069,2207,3552,1021,1662,2894,2230,2390,2
935
ABCB11_6_3585,118,67,449,385,88,383,103,802,39,149,434,250
ABCB1_6_3586,3984,3439,4023,4028,5101,3283,4632,3766,4499,3677,4740,62

01
ABCB6_6_3587,491,462,519,316,54,17,243,490,393,328,131,421
ABCB7_6_3588,739,865,1065,1057,1267,1538,731,753,1324,658,796,492
ABCB8_6_3589,2231,2281,2847,2462,1775,1251,3250,3582,2890,2832,3500,22
75
ABCC12_6_3590,3109,2846,3623,4053,3371,2902,2937,3491,3078,3872,5050,2
324
ABCC2_6_3591,6589,7331,8194,8463,9473,8477,9800,7048,7012,6521,13014,8
690
ABCC8_6_3592,1743,2119,2803,2145,1458,3769,2357,1312,2645,5139,3226,34
72
ABCD1_6_3593,1416,1593,1495,940,915,2298,660,448,481,1004,1619,1490
ABCD2_6_3594,667,498,1154,1106,1413,626,314,819,970,523,1291,732
ABCD4_6_3595,1554,2106,1398,1571,1445,1140,960,1877,1418,3566,2490,237
2
ABCF3_6_3596,5583,5158,6059,5595,5607,5250,6135,4031,7313,4332,7453,74
36
ABCG2_6_3597,3021,3311,3550,3357,6970,2364,3104,3745,2335,2811,5346,32
78
ABCG5_6_3598,523,833,963,554,1018,703,1221,606,809,806,450,1312
ABCG8_6_3599,491,471,612,528,505,825,985,912,563,568,625,1379
ANXA10_6_3600,9648,9571,12901,9986,10148,12692,11500,13616,11290,10354
,10969,9204
ANXA1_6_3601,4931,4142,4621,3837,3321,4845,4957,2693,3389,5985,6923,53
38
ANXA3_6_3602,2726,2615,3885,3287,2722,2997,3567,2630,3039,3119,4533,47
39
ANXA4_6_3603,6688,5251,7463,5919,5118,6643,6294,6156,4046,10041,7020,1
0582
ANXA5_6_3604,809,683,1002,524,824,951,1686,1505,127,2378,1445,678
ANXA8_6_3605,3853,4050,4570,4599,3360,4496,5404,3344,3852,4530,6359,61
57
ANXA9_6_3606,452,307,349,461,139,9,473,1205,537,213,435,137
ATP10A_6_3607,1788,1579,2702,1610,2408,1148,2459,2890,2997,1250,4291,3
302
ATP10B_6_3608,441,515,834,716,75,1681,148,711,1240,1695,1157,615
ATP10D_6_3609,1060,798,1099,1342,945,2502,1572,1996,1528,1020,1499,171
0
ATP11B_6_3610,18328,17112,21397,20657,18469,20988,23613,19120,15704,23
026,25071,19786
ATP1A2_6_3611,1047,1807,1171,935,899,426,1222,627,604,1070,1839,1312
ATP1A3_6_3612,2379,2018,2750,2881,1809,2205,4005,1086,1206,470,3830,52
92
ATP1B1_6_3613,6954,6143,7511,7169,4219,4018,8413,6630,6747,8826,10442,
8362
ATP1B2_6_3614,610,519,723,430,80,466,1957,499,343,713,778,335
ATP1B3_6_3615,2687,2960,2728,2885,2529,3886,2474,2743,1561,800,3275,15
21
ATP4A_6_3616,3747,3522,4744,4225,4254,4807,3971,5122,5048,6724,6395,53
88

ATP4B_6_3617,669,737,710,856,765,121,428,304,1404,1445,1559,862
ATP5B_6_3618,1212,1412,1788,1640,1190,3148,3073,2616,1540,2671,1038,10
48
ATP5F1_6_3619,3750,3502,3999,4037,2795,4059,2682,4835,4302,4707,4688,5
400
ATP5I_6_3620,380,493,503,561,234,20,430,530,751,863,1418,332
ATP5L2_6_3621,3756,2884,3122,3526,2399,1071,2827,3467,1888,3747,4012,4
541
ATP5L_6_3622,4639,3466,4300,3775,3784,4908,4494,5467,1940,4494,5658,40
20
ATP5O_6_3623,1999,1989,2259,2140,4709,1573,2214,2112,916,2827,949,1152
ATP6AP1_6_3624,1249,1099,1388,1065,249,2561,1423,1828,1052,758,1051,12
20
ATP6AP2_6_3625,512,432,646,490,603,937,700,433,630,344,752,1068
ATP6V0A2_6_3626,1018,1586,1186,1578,728,1335,2969,602,1298,3149,2090,2
255
ATP6V0D1_6_3627,1259,827,1665,1190,732,1171,1599,1020,850,489,3666,188
2
ATP6V0D2_6_3628,2003,1550,2263,1744,465,1175,2136,1216,1616,1975,3302,
1552
ATP6V0E1_6_3629,755,1032,871,541,2,335,1255,1143,666,379,1054,451
ATP6V1A_6_3630,2126,1502,1643,2074,3553,2306,1391,3358,1318,2429,2090,
1696
ATP6V1B1_6_3631,1729,1118,1431,1941,3466,886,4224,876,430,1442,2457,10
91
ATP6V1B2_6_3632,1741,1918,2281,1612,1312,1639,1245,3560,3155,697,3158,
2319
ATP6V1C1_6_3633,2741,2386,3001,2132,2379,2026,3406,3965,3934,2530,4530
,2627
ATP6V1D_6_3634,4865,3591,4648,4847,4170,4411,3060,5152,6172,5935,6288,
3091
ATP6V1E2_6_3635,668,1063,829,949,785,382,897,355,601,1387,1557,754
ATP6V1G1_6_3636,1596,1634,1951,1918,1167,309,2095,2850,1698,1779,2875,
726
ATP7A_6_3637,2873,3710,4325,4045,5752,3190,2455,4298,3179,4433,3058,29
91
ATP8A2_6_3638,2853,2962,3111,2757,3154,2845,2268,2818,2571,2122,2992,2
830
ATP8B1_6_3639,408,145,430,538,1568,1,121,761,579,116,272,206
ATP8B4_6_3640,2644,3053,2863,2887,3342,4305,2545,1528,2307,2161,2925,3
093
ATP9A_6_3641,5286,5093,6312,5632,7398,4140,4667,6810,5192,6438,7770,66
63
ATP9B_6_3642,1196,1223,1679,1152,1058,359,543,2509,1590,591,992,3184
ATPAF2_6_3643,1600,2163,2494,2208,1673,1967,2371,2253,1238,2012,3828,1
863
FLVCR1_6_3644,992,895,1402,1142,785,973,663,858,924,223,1889,1209
RHBG_6_3645,1414,1459,2282,2130,907,1761,1963,2822,1748,1745,2276,762
RHCG_6_3646,115,0,47,168,0,0,0,25,0,0,0,0
SLC10A1_6_3647,2026,2239,2575,2966,609,1527,2719,4447,1900,3098,2705,1

949
SLC10A2_6_3648,932,1273,1412,1360,2394,2012,1132,1129,613,2491,610,129
9
SLC10A4_6_3649,2316,2488,2785,2506,2111,1461,2043,5937,3695,2749,3609,
2540
SLC10A5_6_3650,1860,2089,3251,2873,1538,3366,1139,3374,1931,2188,3642,
1079
SLC10A6_6_3651,3165,3741,3871,4333,3000,7625,3237,3711,5994,3767,7275,
4758
SLC11A1_6_3652,1911,1920,2185,1758,1095,1621,2659,2653,1434,972,2157,1
891
SLC12A2_6_3653,10437,9523,13227,11644,10056,9285,11413,12165,9665,9090
,13963,12628
SLC12A7_6_3654,511,792,457,802,104,35,585,953,616,966,1183,1113
SLC12A9_6_3655,681,775,920,935,287,2595,590,317,369,2156,1354,643
SLC13A1_6_3656,1112,1331,1092,1574,971,1347,1805,1265,233,627,1910,663
SLC13A4_6_3657,314,195,326,426,292,319,621,175,283,286,114,106
SLC15A1_6_3658,932,1172,1605,1083,2097,2058,1621,1579,1108,2468,3385,1
101
SLC15A3_6_3659,643,592,635,811,1036,391,111,61,850,68,107,1902
SLC15A4_6_3660,11153,10871,13553,12780,9539,12245,15301,16333,11094,83
34,16898,9645
SLC16A10_6_3661,4016,2588,4103,3971,3861,1839,2212,1900,3061,3246,3133
,3647
SLC16A11_6_3662,1773,2203,1725,2062,2092,718,2121,1697,3044,1810,2370,
2902
SLC16A12_6_3663,2542,2647,2596,2422,4148,4289,2981,2921,2539,2485,4923
,967
SLC16A2_6_3664,983,1121,1223,1075,680,1621,949,495,1282,220,1854,1164
SLC16A5_6_3665,1361,1693,1770,2548,1816,2050,1647,4707,1282,1078,3291,
2831
SLC16A7_6_3666,885,860,1041,896,880,1375,2125,1111,613,385,1204,1284
SLC16A8_6_3667,215,246,177,324,47,66,84,0,604,5,191,251
SLC17A1_6_3668,2497,2637,1972,2020,3049,1066,428,1325,1407,2951,2592,2
452
SLC17A4_6_3669,1574,1180,1776,1872,1417,1480,1294,2041,1223,2770,1486,
2908
SLC17A5_6_3670,2355,2540,2885,2624,2558,1555,3622,2295,2411,2031,3577,
1653
SLC17A6_6_3671,2662,2324,3028,2826,1536,4481,3516,3977,2121,3812,4487,
3439
SLC17A7_6_3672,755,372,537,432,133,905,833,911,281,191,216,287
SLC18A2_6_3673,821,852,829,729,417,516,1297,429,305,2148,966,809
SLC18A3_6_3674,448,362,693,654,438,1014,1910,1059,287,150,1475,2978
SLC19A2_6_3675,4289,3744,4394,4614,6422,2775,876,2924,1548,7261,4411,3
635
SLC19A3_6_3676,1650,1510,2000,1636,974,4621,1653,1425,1896,1260,3043,4
67
SLC1A1_6_3677,785,438,788,1027,2,1292,203,796,1039,788,414,523
SLC1A6_6_3678,3692,3338,3450,3889,4174,3344,3808,3945,2989,4739,6743,4

020
SLC1A7_6_3679,743,811,900,925,559,1558,804,1792,658,160,1259,513
SLC20A1_6_3680,3500,3216,3529,3175,2530,4162,3301,4037,1907,3610,1937,
3116
SLC20A2_6_3681,458,547,197,473,32,988,685,12,34,1503,245,1
SLC22A10_6_3682,2479,2881,3454,3268,2744,4227,2828,3971,3125,2860,2767
,2727
SLC22A11_6_3683,1423,1330,1163,903,409,780,2009,3142,537,92,1221,481
SLC22A13_6_3684,6881,7143,8828,8239,5281,10509,13614,7067,5629,9843,10
571,7072
SLC22A14_6_3685,5811,4555,6367,6307,4501,5469,7643,6302,5695,3967,6507
,2796
SLC22A15_6_3686,2443,1707,2524,2668,2371,3183,2165,1704,612,3081,2998,
4920
SLC22A16_6_3687,1347,2128,2701,1685,3024,2995,1033,1760,2689,1693,2874
,1095
SLC22A18AS_6_3688,1676,1188,1533,1475,1929,1058,1580,3726,862,485,1886
,2884
SLC22A2_6_3689,625,797,657,696,1241,3009,878,0,447,14,737,463
SLC22A3_6_3690,3825,3037,4139,3850,4718,2561,2536,3553,3772,3534,3335,
6692
SLC22A4_6_3691,1219,1294,1939,1635,1317,1254,1993,1722,1130,3422,3716,
1419
SLC22A5_6_3692,3150,2194,3159,2822,2661,2138,3719,2180,1418,2562,4356,
1796
SLC22A9_6_3693,2421,2712,3316,3387,1606,2668,3306,2363,1512,2364,5443,
3241
SLC24A1_6_3694,3241,2823,3692,3668,3435,4541,5074,4936,3062,3106,7694,
2260
SLC24A3_6_3695,960,689,736,1299,390,1840,212,1278,483,1291,1799,1976
SLC24A5_6_3696,2434,2591,3064,1910,1975,1807,4119,2164,1289,1676,2632,
1663
SLC24A6_6_3697,1511,1196,1555,1863,1789,1586,959,1732,1636,1328,1288,9
26
SLC25A10_6_3698,1081,1393,1860,1893,1071,1246,1962,2465,1066,1623,1553
,1809
SLC25A12_6_3699,863,618,832,881,920,780,757,643,810,1339,1556,1021
SLC25A15_6_3700,2015,1914,2410,2162,2786,1916,2800,2932,2019,2378,3464
,3289
SLC25A16_6_3701,0,0,8,4,0,0,37,0,0,0,0,25
SLC25A17_6_3702,1815,1946,2407,2613,1751,3370,3302,1718,1319,913,5142,
2841
SLC25A18_6_3703,468,475,617,262,1049,0,293,1126,1101,155,507,1476
SLC25A1_6_3704,645,760,1140,1473,943,774,739,994,1470,569,1481,958
SLC25A20_6_3705,1024,718,1294,849,1386,164,1755,786,870,385,1132,85
SLC25A28_6_3706,734,422,703,805,378,973,1384,285,107,438,1052,50
SLC25A2_6_3707,3060,2808,3161,2935,2545,1433,2830,3088,1674,2833,7014,
3555
SLC25A32_6_3708,6421,6354,7865,8114,10703,11882,6102,8449,7548,9341,10
385,8567

SLC25A37_6_3709,4095,3616,4931,4137,2740,3701,4760,5563,2267,3296,4112,2533
SLC25A43_6_3710,15092,13587,15344,17502,19061,20886,18309,15037,14049,22254,21751,14796
SLC25A44_6_3711,915,825,684,706,326,292,331,814,482,637,2508,1666
SLC25A4_6_3712,917,802,1149,1181,2540,1943,1586,1811,1278,496,1268,1525
SLC25A5_6_3713,4482,4240,4973,3807,5232,5279,7201,5321,2977,5080,3805,7425
SLC25A6_6_3714,836,846,2088,1309,1258,1309,2881,753,1087,875,1700,820
SLC26A10_6_3715,876,1071,1199,1293,614,946,1150,603,943,1008,1834,1423
SLC26A2_6_3716,1093,1134,1449,1096,1969,995,2778,1095,882,1880,2190,978
SLC26A3_6_3717,1762,1698,1784,1289,1164,1694,958,225,1165,1927,2231,1560
SLC26A4_6_3718,1655,1825,2778,2011,3387,3507,1887,845,2123,2340,3550,3404
SLC27A1_6_3719,421,194,285,355,328,319,562,26,388,25,393,137
SLC27A3_6_3720,1549,1289,2145,1550,1643,1631,1427,1503,2928,1828,1510,1674
SLC27A4_6_3721,547,294,547,535,353,1518,138,439,8,504,1412,38
SLC27A5_6_3722,1108,1600,1275,1210,492,762,1784,636,1340,1466,1711,984
SLC28A2_6_3723,3031,2895,3958,3625,6009,6560,3993,2138,1860,4080,4375,3245
SLC29A2_6_3724,337,403,446,367,301,680,181,279,98,1138,668,150
SLC2A10_6_3725,608,1073,687,672,620,154,278,192,1035,27,1874,803
SLC2A12_6_3726,379,697,1036,507,1284,663,415,43,109,424,220,1843
SLC2A13_6_3727,1353,1145,1633,1469,256,1827,278,996,2101,581,3069,3050
SLC2A14_6_3728,16889,15491,19132,18135,14320,17970,15938,15052,13072,18830,16250,19179
SLC2A1_6_3729,343,544,498,403,380,49,519,27,305,230,132,194
SLC2A2_6_3730,4177,3850,4577,5402,2011,4233,3781,3624,3333,7547,5443,4520
SLC2A3_6_3731,16889,15491,19132,18135,14320,17970,15938,15052,13072,18830,16250,19179
SLC2A4_6_3732,2293,2859,4405,3284,1857,2365,5019,2944,3298,2252,5145,3001
SLC2A4RG_6_3733,493,866,757,621,372,1530,400,2895,194,1350,880,2152
SLC2A7_6_3734,1639,924,1446,1042,607,2162,1053,1389,1073,1754,2377,1620
SLC2A8_6_3735,515,520,885,756,909,489,294,1685,682,419,1163,232
SLC30A10_6_3736,395,192,807,715,594,357,344,507,283,837,246,256
SLC30A1_6_3737,4084,2774,4036,4538,4413,2897,2825,3605,4387,4375,4537,2607
SLC30A3_6_3738,517,290,477,601,309,447,839,434,103,236,523,447
SLC30A4_6_3739,1267,1369,2060,1727,2125,2246,1924,2628,2693,1747,2615,2013
SLC30A9_6_3740,1785,2222,2985,1924,862,1091,2673,2763,1232,1407,1480,3406
SLC31A1_6_3741,396,537,1198,480,826,735,452,449,309,1147,579,1021

SLC31A2_6_3742,2858,2518,3108,3270,2810,5954,3278,7277,2473,1738,4457,
2706
SLC32A1_6_3743,672,752,353,368,1922,0,469,2302,550,496,379,1
SLC35A3_6_3744,10205,10291,11233,12432,12474,21853,16852,13065,14438,1
6694,13977,12061
SLC35A4_6_3745,1890,2434,2028,2104,3057,3368,2216,3271,1943,1776,3334,
2303
SLC35A5_6_3746,950,1036,844,1351,714,446,1571,499,276,2318,1581,1494
SLC35B1_6_3747,986,831,782,1054,710,84,1237,2562,1425,1328,2145,1022
SLC35B2_6_3748,1053,1152,2275,1560,1696,896,1282,826,613,108,3091,450
SLC35B4_6_3749,4127,3471,4650,4294,4150,5217,5116,3239,2522,3011,8061,
4636
SLC35D1_6_3750,997,801,1264,891,1291,857,727,264,1645,1782,342,967
SLC35D2_6_3751,3576,2815,2985,3707,2489,4702,3513,1860,1755,1514,3570,
3162
SLC35D3_6_3752,599,568,520,815,1043,1153,387,1379,249,476,1785,1031
SLC35E1_6_3753,678,1178,667,975,672,237,1163,542,324,957,724,1495
SLC35E3_6_3754,5287,4841,6854,7008,4384,7381,7563,8660,7315,6464,7820,
6062
SLC35E4_6_3755,269,363,200,498,2,473,717,508,85,333,733,1085
SLC35F1_6_3756,980,796,1015,1147,357,569,509,2246,186,2566,1106,486
SLC35F2_6_3757,12120,12440,13760,12987,10310,16844,12432,12579,7120,11
703,13451,8368
SLC35F3_6_3758,1480,1737,2684,2369,1769,2699,3462,1413,4021,2289,2175,
3318
SLC35F4_6_3759,684,817,694,900,214,872,521,915,253,749,715,165
SLC35F5_6_3760,947,1153,1911,1423,1484,5540,825,2877,1611,911,1867,133
4
SLC36A1_6_3761,4446,4008,5500,5443,6187,5754,3483,3280,3769,6283,6623,
6624
SLC36A2_6_3762,1794,2667,2102,2551,939,569,2043,1179,2415,2761,1700,30
11
SLC36A4_6_3763,5647,5355,6185,5427,4072,7550,8372,5935,4482,5464,7312,
6747
SLC37A1_6_3764,1028,1138,760,1981,1279,1421,796,1148,169,1277,993,2491
SLC38A2_6_3765,3173,3093,3782,3923,3250,4502,3508,1857,2448,3827,5524,
5321
SLC38A3_6_3766,701,612,603,777,771,1036,1151,498,82,278,834,346
SLC38A5_6_3767,540,489,366,444,1022,718,863,229,212,819,1478,156
SLC39A1_6_3768,151,230,328,117,0,49,0,43,0,6,119,2
SLC39A2_6_3769,2167,1930,2290,2709,2156,2951,2646,5037,1873,1831,3837,
3887
SLC39A9_6_3770,1217,970,1961,1371,1134,81,2060,2314,543,1782,1934,619
SLC3A1_6_3771,219,729,459,337,104,485,193,318,184,294,269,61
SLC40A1_6_3772,1224,1190,1537,1836,471,1349,1547,1870,1178,1264,2312,1
357
SLC41A1_6_3773,746,417,1033,651,205,348,353,1010,656,2836,647,1155
SLC43A2_6_3774,1395,682,1132,1134,423,623,1099,3302,992,412,1018,477
SLC44A1_6_3775,603,632,702,583,676,16,1008,109,614,50,668,1007
SLC46A2_6_3776,1460,1106,1882,1440,2018,2902,1592,972,350,2159,1316,32

89
SLC4A1AP_6_3777,3041,2925,3529,3868,3130,2617,3999,2549,1664,3895,5191
,2246
SLC4A1_6_3778,1717,1556,1730,1599,1733,2778,714,1379,698,906,2309,1506
SLC4A7_6_3779,2245,2928,3067,2519,1971,1220,3002,2734,2249,3750,3562,3
480
SLC4A9_6_3780,4905,4933,5074,5598,5003,5722,4521,6266,3975,6624,5485,5
505
SLC5A11_6_3781,648,471,565,488,776,911,322,62,157,632,973,219
SLC5A1_6_3782,586,989,865,1255,327,1362,991,629,789,868,1296,983
SLC5A2_6_3783,432,693,501,934,1045,740,652,1953,317,1739,326,744
SLC5A3_6_3784,1301,1130,1964,2760,1907,2239,1345,3245,1294,2599,2497,1
829
SLC5A4_6_3785,3117,2208,3748,3475,1868,3052,4015,3479,1292,1615,4755,2
740
SLC5A5_6_3786,195,213,259,243,21,58,1177,98,222,468,25,17
SLC5A6_6_3787,8727,7063,9238,9999,7250,9479,7021,10588,4879,8635,11567
,10205
SLC5A7_6_3788,5663,5266,7072,6248,6933,6335,7791,8382,3140,5346,7126,7
921
SLC5A8_6_3789,3662,3574,3897,3828,5818,2360,2923,5401,4300,3259,5163,5
183
SLC6A11_6_3790,5886,6085,6398,6256,4998,6337,6452,7871,5518,7896,5133,
9102
SLC6A14_6_3791,9154,7797,8955,9448,9371,9650,6988,10586,8527,8175,1127
6,10042
SLC6A16_6_3792,2650,1244,2927,1938,1537,641,778,1717,1158,2626,3106,38
09
SLC6A19_6_3793,154,416,425,425,136,1513,965,252,6,62,0,1276
SLC6A1_6_3794,2828,2372,3638,3662,3522,3177,3328,2619,1880,1622,3909,1
976
SLC6A3_6_3795,408,260,349,695,616,1143,1303,219,85,322,399,376
SLC6A4_6_3796,3936,5049,6165,5604,7164,4196,2484,5768,3634,4330,6413,4
788
SLC6A7_6_3797,458,521,932,917,2715,741,1613,474,1380,1175,1342,1837
SLC7A10_6_3798,930,353,787,615,423,302,488,609,168,324,1362,585
SLC7A11_6_3799,1927,2500,2419,2850,1015,5532,2115,3338,2364,3385,3107,
1888
SLC7A13_6_3800,581,452,801,452,20,509,91,120,1168,310,329,54
SLC7A1_6_3801,2432,3475,2994,3424,1819,3440,1009,5145,2292,4797,2406,3
344
SLC7A4_6_3802,546,785,876,1286,1570,1651,1061,915,152,457,730,288
SLC7A5_6_3803,408,725,498,696,539,652,169,552,1006,270,35,363
SLC8A2_6_3804,876,660,884,791,1850,1718,829,1056,1446,496,1170,111
SLC9A1_6_3805,578,426,479,565,459,690,641,124,514,254,944,356
SLC9A2_6_3806,0,0,0,0,0,0,0,0,0,0,0,0
SLC9A3_6_3807,1618,1023,1323,1626,2756,665,804,1356,869,506,663,2361
SLC9A3R1_6_3808,1555,1345,1344,1133,1525,4215,1165,679,1294,825,1929,3
60
SLC9A4_6_3809,3065,3450,3333,3157,2253,1715,3451,3336,5952,3103,4410,3

955
SLC9A5_6_3810,1956,1612,2477,1570,2426,1511,2398,4221,1418,1773,2985,1
441
SLC9A7_6_3811,5747,6364,7001,7175,4030,5121,4783,4772,4589,5717,7389,2
546
SLC9A8_6_3812,568,666,1388,1088,1109,1638,1154,2398,486,2039,1287,591
SLC9A9_6_3813,2943,2886,3308,3596,3538,3249,3567,2193,3226,3507,5641,1
725
SLCO1B1_6_3814,4571,4091,4946,4467,3081,5843,3726,3956,3297,2255,7181,
5539
SLCO1B3_6_3815,2788,2973,3238,3459,4576,4822,3259,2799,3022,3530,4369,
3423
SLCO2A1_6_3816,494,757,698,748,45,106,658,692,244,856,567,9
SLCO4A1_6_3817,1275,927,812,1508,1168,878,1040,624,1098,2255,2354,2142
SLCO4C1_6_3818,5877,5893,6192,6201,5977,4913,8644,6126,5171,6280,6638,
7801
SLCO6A1_6_3819,2658,2929,3094,3442,1739,3404,3590,3719,2627,2917,3291,
1429
SV2A_6_3820,1776,1283,2461,1700,1468,1708,1566,2578,486,1373,721,873
TAP1_6_3821,1649,1740,1893,2036,1093,3098,1188,2930,439,3390,2185,2165
UCP1_6_3822,1461,1046,1396,977,1406,636,1446,1291,1367,616,2132,1601
ABCA12_6_3823,4788,4546,5831,5084,9231,4061,4128,4110,4519,5591,6153,5
821
ABCA2_6_3824,717,557,544,949,243,1675,1811,731,158,1477,566,793
ABCA5_6_3825,7440,6755,9731,7661,8281,9990,11064,10237,7722,11226,1125
6,8492
ABCB4_6_3826,10365,10916,12116,11628,11082,16603,13691,12458,11402,122
49,13609,14248
ABCB9_6_3827,1137,639,951,1034,375,1513,229,447,738,341,909,546
ABCC10_6_3828,686,743,860,634,1058,919,423,290,1416,825,1699,800
ABCC11_6_3829,866,657,436,701,149,771,635,26,68,359,1114,581
ABCC1_6_3830,736,809,755,766,870,386,1342,516,979,164,905,344
ABCC3_6_3831,618,938,1514,1086,799,2129,1107,406,184,205,1064,1095
ABCC4_6_3832,4819,3742,5049,4862,5217,5325,4342,5789,4397,6508,6243,39
61
ABCC5_6_3833,609,165,496,277,136,129,188,142,859,221,23,768
ABCC9_6_3834,2788,2491,3728,3460,3279,4215,2444,3079,1961,2441,2428,33
84
ABCD3_6_3835,3263,3134,4913,3756,3558,5376,4773,3536,5922,4502,6097,53
68
ABCE1_6_3836,3240,2743,4144,4000,6289,7628,6920,6497,1973,5536,3637,38
23
ABCF1_6_3837,857,756,983,775,210,594,135,398,690,108,1690,593
ABCF2_6_3838,8145,8016,9882,10308,10349,11142,12363,8625,7386,13396,14
394,9064
ABCG1_6_3839,841,724,674,1486,367,1589,2036,148,843,1437,1891,836
ABCG4_6_3840,4722,4774,5033,3926,3502,5606,3679,6932,2737,4353,9676,75
65
ANXA11_6_3841,2304,2301,3108,2682,2550,4870,2325,2955,4175,2685,3883,3
699

ANXA13_6_3842,1080,754,1717,1199,1114,1156,718,911,1775,1234,1694,604
ANXA2_6_3843,3288,2890,3839,3686,2918,2688,4411,4189,3467,4487,5159,34
93
ANXA6_6_3844,923,1587,800,829,1677,1157,349,1475,1887,917,1544,853
ANXA7_6_3845,10356,10978,13204,12404,9329,16060,12265,7914,11051,16389
,18128,10868
ATP11A_6_3846,473,678,903,381,741,1672,210,272,472,472,324,199
ATP11C_6_3847,1697,1673,1969,1841,2540,3817,1973,3201,964,1818,1907,20
58
ATP12A_6_3848,2805,2418,3401,3747,2534,2713,3754,3934,1771,4673,3815,5
029
ATP1A1_6_3849,1380,1185,1486,1042,574,785,1412,1768,258,548,893,1612
ATP1B4_6_3850,96,63,48,122,0,0,110,1,168,0,105,1
ATP2A1_6_3851,406,853,887,784,596,733,565,744,225,1340,1854,385
ATP2A2_6_3852,2899,2446,3396,3440,1787,4007,2322,2560,1256,1582,4164,4
041
ATP2A3_6_3853,910,456,713,772,111,657,354,955,212,1027,177,131
ATP2B1_6_3854,2034,2201,2518,2498,2055,2249,1999,2307,1287,2217,1574,1
501
ATP2B3_6_3855,1704,1793,1860,673,692,1536,1490,2488,1706,1268,1328,142
7
ATP2B4_6_3856,1957,1864,2327,1731,1580,2521,3882,1946,1508,2874,3304,1
318
ATP2C1_6_3857,1082,700,1182,1060,473,947,1037,4393,948,2814,1745,2289
ATP5A1_6_3858,1336,1318,1221,1032,1247,733,2611,1128,894,944,823,1220
ATP5C1_6_3859,2140,1410,2015,2297,3332,2454,1650,3456,1624,2885,1572,1
035
ATP5D_6_3860,814,738,893,552,712,866,207,1338,920,208,852,981
ATP5E_6_3861,0,103,0,2,391,0,0,0,0,0,0,0
ATP5G1_6_3862,225,68,122,78,0,153,0,153,0,355,114,0
ATP5G2_6_3863,963,1105,726,521,541,1170,739,1674,1566,715,1064,811
ATP5G3_6_3864,1200,1633,1447,1247,1776,933,832,936,619,2457,2919,1469
ATP5H_6_3865,19256,17634,22198,22811,19478,19239,25832,23068,13239,191
64,25749,18798
ATP5J_6_3866,7794,7195,10760,9376,11548,6857,10769,7031,7058,8198,7485
,8433
ATP6V0A1_6_3867,1644,1435,1836,1939,1798,1116,2911,3811,1507,1364,3982
,1730
ATP6V0A4_6_3868,2251,2091,2568,2294,2623,3822,2140,1928,2603,2685,2676
,3147
ATP6V0B_6_3869,705,881,1372,935,944,637,2081,917,749,968,750,498
ATP6V0C_6_3870,492,476,386,611,1138,1109,248,1282,139,1574,966,456
ATP6V1C2_6_3871,1478,1249,1786,1535,2951,1086,2840,1752,1903,1533,2142
,905
ATP6V1E1_6_3872,2104,2046,2617,2824,1873,1594,4451,1409,846,3757,3833,
3142
ATP6V1F_6_3873,1561,1020,1700,1006,1118,1989,1285,639,902,1529,3422,65
4
ATP6V1G2_6_3874,1605,1418,1801,1825,786,1369,2268,2120,712,3389,2267,1
067

ATP6V1H_6_3875,1337,1357,1826,1318,1065,1807,2142,855,1283,3228,3479,1457
ATP7B_6_3876,735,916,1035,716,658,2216,1078,478,1502,422,1439,1105
ATP8A1_6_3877,1994,2825,2421,2792,1575,3133,2329,1850,2491,3370,2845,1685
ATP8B2_6_3878,45,140,21,144,0,0,18,5,31,0,813,4
ATP8B3_6_3879,582,529,681,424,218,109,980,68,334,708,497,768
ATPAF1_6_3880,3175,2222,3549,3182,3242,3266,4300,3486,2680,2720,4590,1907
GC_6_3881,0,0,0,0,0,0,0,0,0,0,0,0
SERINC5_6_3882,1670,1535,1652,1719,1624,1294,2453,1703,1520,3100,3275,1549
SLC10A3_6_3883,873,759,409,672,862,119,113,17,509,381,846,332
SLC11A2_6_3884,158,300,479,230,313,184,185,0,732,615,1083,62
SLC12A1_6_3885,2175,1755,2586,2395,1470,4719,815,737,1476,4002,3922,2136
SLC12A3_6_3886,1290,1169,877,1220,2263,560,1161,427,1272,760,1147,516
SLC12A4_6_3887,471,726,771,763,163,19,1041,190,574,1153,1296,413
SLC12A5_6_3888,1206,1977,1303,1452,3180,2061,1664,1334,1265,1226,3200,2003
SLC12A6_6_3889,1616,1862,2414,1977,1473,5117,3146,1730,2428,2177,1949,2589
SLC12A8_6_3890,643,636,574,543,467,194,150,1510,390,254,248,424
SLC13A2_6_3891,227,316,251,297,8,477,412,30,341,1226,548,61
SLC13A3_6_3892,647,889,1260,1195,276,54,893,1455,540,1028,1230,1107
SLC13A5_6_3893,962,813,1344,867,1622,555,963,1434,1123,1632,2739,1737
SLC14A1_6_3894,2562,2125,3058,2272,1621,1655,2129,5975,1909,2789,7010,1475
SLC14A2_6_3895,2327,2039,3560,2807,1384,1336,3130,1905,1042,2694,3373,3170
SLC15A2_6_3896,5672,4540,5661,5761,5323,7331,4248,5197,3816,9182,7570,5209
SLC16A1_6_3897,8127,6518,7181,7979,8339,5658,5262,6543,5547,7641,7033,3844
SLC16A3_6_3898,2817,3096,4605,4275,3049,4805,3354,2886,2093,4169,4531,4481
SLC16A4_6_3899,737,895,1475,975,421,1052,334,1792,517,1618,706,1041
SLC16A6_6_3900,5885,7011,7670,8007,6409,8583,8353,7060,5455,8115,10655,6940
SLC17A3_6_3901,2273,2890,2401,3586,2120,4106,2562,2832,1560,3586,2924,1163
SLC17A8_6_3902,1109,896,802,1158,917,1184,607,426,397,1596,2424,356
SLC18A1_6_3903,848,1049,1479,1062,1302,39,1859,1034,638,1243,1616,517
SLC19A1_6_3904,10381,10807,13476,11197,11846,11097,12501,12177,13376,11548,11729,11842
SLC1A2_6_3905,424,321,540,837,313,609,480,723,173,170,173,483
SLC1A3_6_3906,2171,1206,2257,2369,2481,2711,1793,1126,670,2229,3883,1190
SLC1A4_6_3907,655,729,957,911,1313,1300,1787,539,566,801,1207,755
SLC1A5_6_3908,2973,2875,2701,3104,2069,3276,2501,5042,2665,1436,4664,3

558
SLC22A12_6_3909,2022,2117,2215,2147,1787,2778,1721,2403,587,3292,1887,
1850
SLC22A17_6_3910,2432,1974,2707,2479,2354,2116,1366,683,1683,2002,3921,
1866
SLC22A18_6_3911,125,139,458,378,558,516,258,1139,2,743,410,354
SLC22A1_6_3912,755,1054,1683,845,1141,612,1560,551,974,339,77,860
SLC22A23_6_3913,3128,3363,3059,3101,1139,3779,3615,3439,3138,3407,2788
,3196
SLC22A6_6_3914,4294,3010,4623,4648,3776,3439,4912,5597,2896,3261,5650,
3625
SLC22A7_6_3915,573,1187,778,643,1779,121,1439,458,208,657,408,1019
SLC22A8_6_3916,750,573,585,632,99,197,179,797,851,188,359,1522
SLC23A3_6_3917,710,748,768,530,264,1411,892,166,1317,941,217,877
SLC24A2_6_3918,1543,1806,1548,1549,734,1863,1602,1729,836,1880,3230,13
46
SLC24A4_6_3919,5351,5407,6284,6163,6290,7754,4355,6396,6126,8951,8568,
5093
SLC25A11_6_3920,2643,2672,2733,3368,1367,3500,3639,5565,3013,2455,2175
,3888
SLC25A13_6_3921,9580,9618,11350,10918,11660,12564,9573,9173,9902,12481
,11590,6794
SLC25A14_6_3922,848,749,616,945,303,2335,862,833,472,524,3924,643
SLC25A19_6_3923,778,412,930,706,1904,501,125,848,542,1392,236,152
SLC25A21_6_3924,1162,1329,1275,1591,402,1562,225,1563,814,1781,1604,11
43
SLC25A22_6_3925,723,617,993,925,388,80,333,439,565,41,606,1694
SLC25A26_6_3926,488,292,542,382,1049,482,465,841,740,33,886,946
SLC25A27_6_3927,3121,4314,4486,4747,3910,2214,6057,4866,2478,3417,5136
,3346
SLC25A3_6_3928,965,698,1040,1384,433,1117,2087,363,965,1121,1912,1159
SLC26A11_6_3929,1110,1289,857,698,1055,1088,891,1382,660,166,285,263
SLC26A1_6_3930,407,365,525,894,878,45,119,29,779,806,1382,423
SLC26A5_6_3931,7453,6218,8665,8325,9828,9380,12122,8679,7322,7411,8939
,7477
SLC26A6_6_3932,907,1044,670,946,507,131,1413,834,1450,534,1207,1008
SLC26A7_6_3933,3251,3211,3510,3468,2269,3333,5228,4341,2790,5024,3313,
4010
SLC26A8_6_3934,7430,6903,9730,8354,9770,10782,7506,7464,5782,7257,9307
,8043
SLC26A9_6_3935,864,923,743,1071,359,416,1191,942,1018,512,481,765
SLC27A2_6_3936,4955,4943,4795,4625,4384,3264,7630,6180,3068,4109,5895,
2617
SLC27A6_6_3937,1939,1905,2285,1838,1698,3861,2154,4534,2337,4150,2746,
3080
SLC28A3_6_3938,1533,1051,2001,2258,1646,2245,2082,2152,2605,2103,3658,
1920
SLC29A1_6_3939,4243,4827,5949,5484,5575,5302,8808,4159,2713,5407,7312,
3834
SLC29A3_6_3940,1410,1306,1745,1511,1158,354,2136,541,1007,2561,2913,10

55
SLC29A4_6_3941,1579,1367,1561,1828,1144,1432,3838,1513,3063,1041,735,1
605
SLC2A11_6_3942,3588,4049,4362,3816,1647,4385,5922,3078,4282,4821,3788,
4615
SLC2A5_6_3943,823,885,788,568,132,87,442,1386,540,250,2233,1189
SLC2A6_6_3944,530,831,760,562,90,9,513,15,629,183,465,544
SLC2A9_6_3945,844,954,1573,1272,703,455,946,939,1427,1894,1293,1082
SLC30A2_6_3946,1281,1887,2028,1789,2927,1115,1392,1484,1922,2612,1777,
1772
SLC30A6_6_3947,1251,1223,1926,1268,1149,1277,2057,1618,2218,346,2120,2
483
SLC30A7_6_3948,465,389,714,790,267,1190,487,1199,147,210,1505,5
SLC30A8_6_3949,3210,3605,3671,3604,3332,5030,2365,2019,3297,2781,4855,
3540
SLC33A1_6_3950,1227,1457,1537,1561,2151,2598,1434,2525,1571,872,844,25
38
SLC34A1_6_3951,576,610,650,1078,589,943,133,573,731,277,933,1110
SLC34A2_6_3952,540,321,661,719,1249,540,1332,255,455,821,532,554
SLC34A3_6_3953,3952,3679,4332,4047,3887,2679,4942,4694,3765,3026,3117,
3776
SLC35A1_6_3954,1416,910,1692,1474,677,2059,945,1133,807,434,1835,1940
SLC35A2_6_3955,2120,1944,2051,2136,3566,1109,4642,2085,887,1745,4447,2
995
SLC35B3_6_3956,609,675,784,560,159,987,359,376,15,928,353,706
SLC35C1_6_3957,1902,2056,2587,2697,2493,2732,1746,2786,2509,2921,2250,
2256
SLC35C2_6_3958,1347,1864,2220,1831,1246,2917,2785,1392,621,1745,1464,7
63
SLC35E2_6_3959,721,729,1105,889,249,1041,875,537,290,1892,966,1316
SLC36A3_6_3960,823,843,2025,1697,933,1702,719,643,1593,3576,1361,1183
SLC37A2_6_3961,5,0,262,140,825,0,0,0,63,0,223,7
SLC37A3_6_3962,704,724,744,699,661,826,770,1277,1324,2132,826,1075
SLC37A4_6_3963,302,553,711,656,2925,614,163,576,1603,983,1190,88
SLC38A11_6_3964,6750,5662,7708,7249,7529,6561,6053,7323,5525,8017,9470
,7955
SLC38A1_6_3965,5123,5005,6060,5911,3453,4950,5515,9620,3387,5874,5804,
3098
SLC38A4_6_3966,6422,5617,5074,5309,7345,7339,7566,5749,3843,5595,5376,
6409
SLC38A6_6_3967,1775,1255,1872,1576,1073,1634,1644,1038,755,2228,3636,8
65
SLC39A10_6_3968,1678,2354,2536,2210,1658,3003,987,3117,2351,2828,1574,
2230
SLC39A11_6_3969,599,635,332,669,5,1145,843,16,81,507,794,1102
SLC39A12_6_3970,1288,1033,1182,1251,1738,1891,2936,1572,906,1985,3165,
554
SLC39A13_6_3971,381,298,475,306,546,20,702,83,25,30,735,159
SLC39A14_6_3972,2662,2082,3402,2877,2818,1959,4298,4861,3087,6066,2937
,2288

SLC39A4_6_3973,166,750,420,410,664,835,628,62,34,898,94,581
SLC39A5_6_3974,1191,1458,1756,1561,762,1642,1619,2455,849,2881,2210,22
69
SLC39A6_6_3975,2684,2690,2741,2739,2920,1830,2676,1885,897,3828,2209,3
958
SLC39A7_6_3976,2193,1792,1989,2402,1351,2382,916,2337,2264,2121,4484,2
119
SLC39A8_6_3977,3155,2518,3626,3820,2621,5362,3521,2145,3091,5298,2619,
5197
SLC3A2_6_3978,1469,1283,1337,1249,1106,1819,223,210,851,680,726,791
SLC43A1_6_3979,673,1037,1185,1305,2363,1281,1815,1503,403,1148,1392,21
26
SLC44A3_6_3980,4218,3725,4325,4042,4271,3278,5440,3825,2164,2832,6611,
5831
SLC4A10_6_3981,2356,1908,2237,2442,2920,2528,1099,2466,1532,1147,3289,
2817
SLC4A11_6_3982,1026,865,1156,1235,467,1036,789,912,560,2297,2028,1993
SLC4A2_6_3983,484,313,463,776,666,418,1021,585,146,212,353,99
SLC4A3_6_3984,1790,1674,1449,1675,1240,1202,1015,2338,1063,2157,1975,1
838
SLC4A4_6_3985,1372,1223,1779,1092,837,2114,748,2963,1037,1498,1059,221
4
SLC4A8_6_3986,723,810,1132,887,713,502,1389,761,1216,396,2930,1461
SLC5A10_6_3987,871,412,499,409,1,121,534,558,623,542,380,1744
SLC5A9_6_3988,1148,1090,1111,1177,1352,1885,1298,2064,941,2447,876,800
SLC6A12_6_3989,1171,1261,1151,1333,823,2059,1351,693,1173,1591,933,119
8
SLC6A13_6_3990,2385,2170,2562,2970,1531,2999,2673,3254,3692,2740,3858,
2688
SLC6A15_6_3991,5248,4909,5766,6463,4514,3874,6839,3274,5115,4413,8313,
8989
SLC6A2_6_3992,1221,789,1274,1185,1839,580,713,1410,1137,1788,1585,1594
SLC6A6_6_3993,2996,3593,3007,3438,2138,4786,2620,4269,2784,834,4238,28
66
SLC6A8_6_3994,390,303,318,456,0,22,112,1377,153,13,559,37
SLC6A9_6_3995,6663,7122,9143,8966,6576,7803,10536,7655,6032,9419,13036
,9057
SLC7A2_6_3996,1197,1268,1311,1327,144,2015,2811,112,232,1319,1187,1341
SLC7A3_6_3997,1608,1250,1313,1436,2085,782,941,3522,1786,633,1973,1649
SLC7A6_6_3998,1229,878,1355,1598,215,1414,1487,1919,315,2359,1796,1907
SLC7A7_6_3999,1759,2354,2485,2407,2797,3072,2358,1604,1045,839,3000,29
85
SLC7A8_6_4000,3059,3535,4021,3900,4565,4365,5082,3522,2287,3137,6559,2
130
SLC7A9_6_4001,2266,2283,2691,1994,2419,1857,1867,2836,1797,1895,3047,3
078
SLC8A1_6_4002,2501,2320,3850,2897,2266,3642,2182,1848,3153,5134,1423,1
218
SLC8A3_6_4003,1206,1164,1323,1249,991,570,986,314,1102,841,1020,499
SLC9A3R2_6_4004,224,47,131,204,1,0,1,202,421,92,409,0

SLC9A6_6_4005,7777,7507,8389,7738,7894,8354,8127,9271,7707,8621,9023,6
236
SLCO1A2_6_4006,1074,389,991,818,272,98,2614,457,1130,660,327,493
SLCO1C1_6_4007,3036,2688,4217,3143,3662,3807,5152,3325,2436,3901,4601,
4752
SLCO2B1_6_4008,590,556,952,630,1053,1196,262,4,194,2084,1367,504
SLCO3A1_6_4009,242,189,483,561,292,423,0,409,12,529,342,342
SLCO5A1_6_4010,1932,1556,1743,2123,2705,1428,2437,3506,2709,2485,3388,
1383
ABCB5_6_4011,3234,3270,4708,3648,5174,3928,4953,3573,3046,4945,5213,46
84
ABCC6_6_4012,611,310,583,500,1564,403,416,503,676,438,1366,443
ATP1A4_6_4013,2030,1311,2485,2064,2183,2591,2000,3932,952,1255,1262,18
68
ATP5J2_6_4014,2786,2824,3792,3567,2718,2381,2884,5597,1864,3739,3867,3
640
ATP5S_6_4015,6164,5792,8099,7541,6779,7831,8474,6162,7585,6723,10044,6
079
ATP6V1G3_6_4016,6422,5306,7092,7425,7418,5647,10029,6752,3865,5059,106
09,5921
ATPIF1_6_4017,2080,2144,2333,2460,1677,659,2033,2605,2146,2196,1260,58
3
SLC10A7_6_4018,1947,1205,2710,3001,1331,1278,1837,2894,1283,818,1877,3
295
SLC28A1_6_4019,434,443,207,539,55,947,48,83,396,931,893,371
SLC30A5_6_4020,2721,1629,2635,2959,2686,2068,3408,1721,2342,1845,4041,
2873
SLC39A3_6_4021,481,685,617,510,31,368,718,1009,534,672,632,324
ABCA10_6_4022,9417,9677,10776,9816,7810,16203,14389,11511,7359,12819,1
2389,15044
ABCA13_6_4023,5940,5918,7135,7475,6393,8885,7453,9978,7315,7212,10424,
5090
ABCA1_6_4024,3262,3551,3179,3153,6017,2601,2288,2925,2557,2062,4794,20
44
ABCA3_6_4025,333,390,360,390,302,149,465,210,528,114,100,776
ABCA4_6_4026,6104,6361,8716,8713,8276,9018,10795,6964,10308,9115,12397
,6056
ABCA6_6_4027,3045,2934,3756,3147,5760,4215,3547,3202,3019,5551,6525,26
71
ABCA7_6_4028,874,986,639,1101,1507,822,339,1662,716,2765,993,1565
ABCA8_6_4029,5485,6179,6679,6549,7733,9204,5617,8280,3857,7382,6907,60
09
ABCA9_6_4030,142,188,225,213,10,230,114,128,99,133,538,127
ABCB10_6_4031,1346,1505,1376,1223,462,959,1400,1000,1443,1113,1950,220
1
ABCB11_6_4032,0,0,0,0,0,0,0,0,284,0,333,0
ABCB1_6_4033,5337,6282,5810,5893,5567,6250,5394,6774,4414,8659,7738,76
21
ABCB6_6_4034,1431,1305,1252,1417,1697,451,1579,911,601,892,1103,1427
ABCB7_6_4035,616,591,578,894,486,865,185,447,737,293,1512,454

ABCB8_6_4036,2094,1872,2705,2340,1778,1254,3258,3597,2900,2194,3297,22
19
ABCC12_6_4037,2385,1544,2103,3130,4117,2677,1377,2707,2538,3591,4817,3
948
ABCC2_6_4038,620,478,683,548,1830,99,213,43,60,175,112,1766
ABCC8_6_4039,3823,3293,5428,5068,2120,4875,4345,5150,3474,3094,4069,51
02
ABCD1_6_4040,857,981,1088,929,2163,1550,566,701,1527,1933,1557,2211
ABCD2_6_4041,2071,1464,1755,1899,1723,1426,1804,3250,1535,1490,3130,15
80
ABCD4_6_4042,1438,1414,1866,1439,1299,1393,854,3253,235,715,1721,786
ABCF3_6_4043,1008,803,732,1059,634,567,1403,499,899,1616,1782,839
ABCG2_6_4044,1407,1834,1694,1469,1424,2094,1008,515,1528,1051,2028,185
6
ABCG5_6_4045,4268,4033,5814,4856,6913,4370,5381,4582,1239,5376,3838,50
48
ABCG8_6_4046,530,403,710,700,803,230,19,191,329,1199,88,800
ANXA10_6_4047,2914,2509,3031,4060,2443,2821,2518,3310,1703,4440,4586,1
578
ANXA1_6_4048,1182,1891,959,1446,326,1633,874,466,2043,1106,716,1457
ANXA3_6_4049,3718,2710,4061,3755,2769,5148,2906,3663,1938,1698,6393,15
41
ANXA4_6_4050,598,857,858,891,1175,819,674,283,391,1744,347,562
ANXA5_6_4051,2938,2142,3661,3026,4471,5063,4415,7551,2101,4067,5308,44
17
ANXA8_6_4052,868,742,1238,1000,1040,888,1058,1166,583,1399,1297,1312
ANXA9_6_4053,428,303,468,610,378,0,0,410,592,0,449,0
ATP10A_6_4054,2325,3368,3459,4086,3049,5553,3419,4782,1989,4541,6145,2
734
ATP10B_6_4055,1055,809,1082,1381,942,2510,1562,2324,1520,1018,1407,169
2
ATP10D_6_4056,3240,3371,4263,3395,2984,3904,2343,1991,2596,3448,3829,3
922
ATP11B_6_4057,7037,7074,8728,8540,7826,7462,7421,9773,5868,10164,12940
,6371
ATP1A2_6_4058,960,596,494,946,47,1120,1568,1632,2075,1665,1118,411
ATP1A3_6_4059,4122,4488,3235,4113,1972,5648,6030,5654,1616,4243,5342,3
372
ATP1B1_6_4060,1479,1347,1670,1722,2348,1977,1698,1027,1356,918,2152,27
01
ATP1B2_6_4061,1350,1506,1345,1086,1511,1033,1033,843,1440,913,1429,765
ATP1B3_6_4062,978,1427,1269,1005,979,1285,1218,875,1241,1073,2205,1856
ATP4A_6_4063,754,767,1014,760,456,1008,621,964,603,973,613,201
ATP4B_6_4064,227,280,332,341,154,279,255,33,249,557,1794,393
ATP5B_6_4065,387,637,787,797,1559,39,337,412,1092,540,2240,358
ATP5F1_6_4066,1040,943,1397,1066,163,1040,1820,1685,937,4312,1714,2583
ATP5I_6_4067,933,664,1161,437,1053,265,1497,1929,1177,225,1841,1116
ATP5L2_6_4068,3156,2347,2660,3148,2366,992,2186,3319,1652,3713,3352,37
30
ATP5L_6_4069,3188,3231,3121,3158,5228,2867,2215,2393,3172,2571,3469,48

65
ATP5O_6_4070,1494,1682,1677,1761,1097,2312,1600,2133,2191,1180,3442,15
78
ATP6AP1_6_4071,939,1033,1503,1087,2322,5342,1431,1018,1241,495,2528,57
3
ATP6AP2_6_4072,2963,3580,4130,4273,2299,4597,3951,4474,1472,5482,7004,
6030
ATP6V0A2_6_4073,2386,2916,3405,3012,1974,948,2077,2939,3490,4458,4771,
3804
ATP6V0D1_6_4074,643,412,846,713,236,535,925,2812,895,472,622,2185
ATP6V0D2_6_4075,3051,2830,3260,3038,1063,2718,3299,4323,2098,2622,4274
,3309
ATP6V0E1_6_4076,2058,1908,2195,2088,1398,2987,2881,2614,1753,2128,2471
,3882
ATP6V1A_6_4077,2605,3305,3776,3810,3821,5649,5388,4053,5036,4669,2582,
1986
ATP6V1B1_6_4078,1058,1139,1891,1846,1295,1684,1961,3422,2158,1305,2482
,1114
ATP6V1B2_6_4079,1253,755,629,906,706,441,2930,804,1141,1092,655,236
ATP6V1C1_6_4080,214,79,52,76,0,0,0,0,0,96,0,0
ATP6V1D_6_4081,321,207,395,278,257,113,8,498,25,176,476,42
ATP6V1E2_6_4082,1323,978,991,773,1158,826,1025,569,424,1649,2203,54
ATP6V1G1_6_4083,641,558,938,932,482,448,406,737,1154,419,2417,1592
ATP7A_6_4084,4602,4369,5774,4873,5493,5104,4989,6315,5388,8313,5815,56
67
ATP8A2_6_4085,2308,1868,2301,2630,1850,2629,2974,1822,3187,1852,4477,1
091
ATP8B1_6_4086,755,739,1108,1307,578,816,1421,891,725,1627,467,1060
ATP8B4_6_4087,1415,2491,1679,2023,2853,1852,873,3255,1878,3058,1056,27
12
ATP9A_6_4088,657,453,554,632,394,1310,1184,1413,230,610,1915,596
ATP9B_6_4089,6197,6008,6991,5953,3776,6696,5819,5992,3129,6340,6893,41
39
ATPAF2_6_4090,353,373,1009,603,691,460,207,287,150,443,1232,403
FLVCR1_6_4091,783,608,1030,1246,351,331,1491,1917,530,868,1520,1300
RHBG_6_4092,1007,399,706,495,255,288,1288,432,532,54,681,257
RHCG_6_4093,1911,1693,2335,1951,2028,3044,3113,2321,1423,2479,3249,198
5
SLC10A1_6_4094,606,375,919,419,1131,1427,19,270,1191,1858,430,30
SLC10A2_6_4095,456,229,202,778,291,782,105,50,0,165,531,181
SLC10A4_6_4096,1375,1188,1718,2149,635,1471,2086,2564,1679,1200,2160,1
695
SLC10A5_6_4097,3414,2811,4203,3678,4175,3252,3646,1767,3439,5311,4309,
4016
SLC10A6_6_4098,7707,7388,9114,9479,10472,11629,10416,12494,10291,8240,
13560,7992
SLC11A1_6_4099,5295,5164,6571,5007,3338,3377,3677,5918,3134,5042,5533,
7556
SLC12A2_6_4100,2024,1972,2187,1632,2599,1567,1863,2496,1888,2863,2515,
3224

SLC12A7_6_4101,1048,911,1483,1315,1499,1321,1678,1246,681,2066,1163,63
2
SLC12A9_6_4102,600,430,381,406,295,14,827,41,63,38,713,20
SLC13A1_6_4103,2688,2666,2484,2795,2492,3557,2098,1177,1584,1147,3469,
1748
SLC13A4_6_4104,894,634,509,563,283,326,250,1010,183,435,1318,525
SLC15A1_6_4105,3212,2517,2756,3143,2693,3983,3397,3636,2464,3110,2101,
2977
SLC15A3_6_4106,367,154,442,233,1,0,1039,454,0,1502,0,0
SLC15A4_6_4107,1219,1065,1297,1455,376,674,537,465,2495,1197,1123,966
SLC16A10_6_4108,1694,1802,1665,1711,2986,1254,1277,2252,3264,2920,2483
,1630
SLC16A11_6_4109,787,702,884,778,509,404,1055,403,85,1148,972,919
SLC16A12_6_4110,3151,3418,3828,4468,4618,4394,3300,4927,4152,4109,4640
,3203
SLC16A2_6_4111,664,799,509,1049,1819,1513,915,1123,948,139,1124,418
SLC16A5_6_4112,266,106,329,216,514,163,577,160,43,89,210,67
SLC16A7_6_4113,11165,11547,11335,12018,10884,12555,14978,13907,9753,91
57,13306,10596
SLC16A8_6_4114,497,759,640,656,391,692,681,672,258,312,467,965
SLC17A1_6_4115,2573,3154,3654,3313,3675,6198,3922,3325,3229,4962,4054,
1768
SLC17A4_6_4116,1086,565,1220,1220,694,2703,1297,1020,1596,1507,1333,15
17
SLC17A5_6_4117,1610,1185,1960,1571,2335,548,552,1206,776,2195,980,2239
SLC17A6_6_4118,5675,5747,6744,7783,4132,7349,3866,4212,4191,3829,8031,
6604
SLC17A7_6_4119,178,288,136,306,1276,1,21,62,308,259,85,15
SLC18A2_6_4120,1121,971,1359,1129,1722,865,1278,1222,1256,592,1776,194
6
SLC18A3_6_4121,6172,5396,6401,6670,5596,6147,4725,7150,4051,4637,7618,
6356
SLC19A2_6_4122,1338,1932,1229,1698,1035,994,1798,1400,861,1890,3487,11
57
SLC19A3_6_4123,1574,1571,2480,1903,1931,2932,3249,3956,1283,1518,2245,
2926
SLC1A1_6_4124,1640,1402,1755,1962,1085,1768,1420,2244,859,842,2956,107
8
SLC1A6_6_4125,477,591,489,484,770,16,59,698,106,622,967,1195
SLC1A7_6_4126,499,333,534,487,141,734,940,1815,365,197,555,28
SLC20A1_6_4127,4639,4013,3546,3763,4688,5323,5053,3928,3808,3769,2214,
3555
SLC20A2_6_4128,1191,912,959,1532,894,565,1253,2233,1164,430,732,1424
SLC22A10_6_4129,1331,1666,1159,1010,557,1416,1295,1600,850,815,3465,12
66
SLC22A11_6_4130,670,966,1574,1016,1242,1254,2701,1248,538,1267,642,203
2
SLC22A13_6_4131,739,747,949,1415,585,1974,2190,2327,829,373,1221,897
SLC22A14_6_4132,1121,770,1353,1477,1050,577,293,2407,2475,1441,1269,24
6

SLC22A15_6_4133,597,775,580,1277,414,772,133,1091,851,579,1056,292
SLC22A16_6_4134,1609,1077,915,1627,1100,2783,1555,1548,544,426,997,456
SLC22A18AS_6_4135,109,125,515,345,568,204,92,99,597,392,8,204
SLC22A2_6_4136,2897,3403,3311,3753,5265,1342,3158,6225,4028,4477,6079,
2118
SLC22A3_6_4137,3472,3699,4119,4732,5432,3195,3568,3008,3266,5432,4155,
5693
SLC22A4_6_4138,391,652,776,1098,1003,290,2247,170,218,938,731,893
SLC22A5_6_4139,316,306,724,528,22,223,582,880,440,605,134,264
SLC22A9_6_4140,1590,1747,2207,2786,1388,1012,2815,2402,1091,1610,1217,
1207
SLC24A1_6_4141,3331,3365,3415,3557,2491,1411,3550,6296,2357,2992,3805,
1962
SLC24A3_6_4142,1344,1465,1837,1367,1270,1842,1643,1949,1786,893,1264,2
349
SLC24A5_6_4143,2360,3104,3170,3047,4271,2423,5523,2860,3291,3220,4877,
3066
SLC24A6_6_4144,1248,914,1249,1275,531,227,1129,225,1353,2706,2801,1007
SLC25A10_6_4145,578,632,595,844,405,1377,1708,975,173,24,988,175
SLC25A12_6_4146,1002,1150,1610,1526,528,1883,1417,1143,521,4202,697,11
65
SLC25A15_6_4147,675,722,895,803,582,561,1136,1199,824,2278,1356,569
SLC25A16_6_4148,8220,6798,8940,8298,5341,10294,8512,10657,7129,11835,1
0928,10414
SLC25A17_6_4149,7183,7065,8509,7907,5471,4480,6996,9930,6868,9072,1019
4,5787
SLC25A18_6_4150,729,699,1008,1070,191,1854,1208,329,631,945,1200,447
SLC25A1_6_4151,2043,2394,2483,1889,3330,2100,1950,4247,1895,2151,1495,
3455
SLC25A20_6_4152,763,1374,768,1194,1300,714,1489,1755,838,1530,1905,705
SLC25A28_6_4153,770,549,1445,860,193,767,108,380,84,650,396,69
SLC25A2_6_4154,17894,16810,19254,18462,17679,21842,18063,18790,16655,1
8304,20089,18920
SLC25A32_6_4155,7450,6316,7156,6788,6722,7474,6616,7678,5967,7878,6358
,4388
SLC25A37_6_4156,6139,4600,6160,7448,11529,7737,4946,7904,5479,7529,961
7,4480
SLC25A43_6_4157,14821,13699,15467,17641,18136,21408,18404,15363,13869,
22492,22240,15713
SLC25A44_6_4158,5359,5485,5206,5243,6720,5797,5920,5315,4548,6932,8781
,6694
SLC25A4_6_4159,1394,1192,2128,1331,2797,3561,3487,1753,2298,2113,1237,
2155
SLC25A5_6_4160,5858,5203,5242,5899,7381,4041,5731,4856,2105,4989,6532,
5530
SLC25A6_6_4161,710,757,770,488,288,509,948,1131,1020,1345,1093,289
SLC26A10_6_4162,1706,1575,2057,2067,892,2268,2703,2849,2199,897,2380,2
529
SLC26A2_6_4163,2757,3628,3941,4237,1454,2517,3134,2027,4244,4395,5754,
3269

SLC26A3_6_4164,1100,1026,1065,769,342,453,1765,959,1036,1881,2765,425
SLC26A4_6_4165,2705,2349,1966,2529,2084,3920,2364,3394,1624,5176,3270,
2725
SLC27A1_6_4166,581,612,437,361,440,731,988,10,1020,408,1664,190
SLC27A3_6_4167,676,709,681,785,1576,141,1063,717,564,352,736,216
SLC27A4_6_4168,1505,2241,1688,2120,2136,1966,2501,2033,1114,2241,2334,
2472
SLC27A5_6_4169,367,867,788,771,975,691,1419,469,156,478,1567,168
SLC28A2_6_4170,1153,1089,1518,1680,702,1700,478,1041,1152,714,2932,101
4
SLC29A2_6_4171,632,517,856,687,1226,172,936,539,943,1179,812,645
SLC2A10_6_4172,393,300,334,397,1217,742,83,1,257,240,1068,112
SLC2A12_6_4173,1144,1360,1918,1955,1369,3350,842,872,1193,1758,2275,15
89
SLC2A13_6_4174,1333,1775,1616,1929,648,3484,1784,1823,963,1409,2146,19
78
SLC2A14_6_4175,2764,1969,3672,3443,2735,4549,2279,2249,3890,1090,5753,
3025
SLC2A1_6_4176,1172,759,1049,791,1383,816,1340,619,331,1029,1972,819
SLC2A2_6_4177,1517,1313,1284,1116,1344,1218,1391,644,924,749,407,1234
SLC2A3_6_4178,500,306,602,227,51,416,799,13,43,1344,346,66
SLC2A4_6_4179,1444,1456,1483,1907,3216,3004,1617,684,931,3302,925,3355
SLC2A4RG_6_4180,381,408,471,428,12,647,1185,477,19,865,218,145
SLC2A7_6_4181,1458,1527,1724,1882,776,2628,1382,1781,551,3745,3916,102
7
SLC2A8_6_4182,269,268,495,181,52,821,17,314,83,320,463,148
SLC30A10_6_4183,2541,2978,3521,3723,2248,2046,3599,2295,2770,4662,3394
,2468
SLC30A1_6_4184,3835,2922,4090,3722,2953,1848,4174,3539,4581,4499,3788,
2670
SLC30A3_6_4185,516,469,862,453,283,1460,2519,491,88,128,74,336
SLC30A4_6_4186,1070,1365,1291,1588,1952,444,3299,1192,1360,976,1428,10
90
SLC30A9_6_4187,1418,2106,1962,1743,357,1928,1627,1664,2342,2518,2004,1
779
SLC31A1_6_4188,1306,1097,1792,1765,2072,1195,2424,1809,600,2141,2840,3
333
SLC31A2_6_4189,3270,3276,4350,3533,3876,2721,3923,5244,3584,5432,4655,
5049
SLC32A1_6_4190,1161,1025,914,1231,1082,3105,865,2402,2518,1346,1390,13
14
SLC35A3_6_4191,588,341,761,528,566,167,3,0,187,1117,807,475
SLC35A4_6_4192,2385,1943,3548,2796,529,3227,2072,3368,1448,3954,4474,1
058
SLC35A5_6_4193,9343,9959,11787,11175,10286,14887,9701,12516,9909,13578
,14283,7881
SLC35B1_6_4194,1053,1593,1938,1949,2434,1225,2248,2709,1450,781,3188,1
719
SLC35B2_6_4195,1055,1224,1312,1277,1948,659,925,1176,950,789,1291,642
SLC35B4_6_4196,1040,1349,972,949,26,1138,1430,735,831,1061,280,585

SLC35D1_6_4197,947,888,1275,1188,3022,543,315,550,1023,855,1190,1216
SLC35D2_6_4198,998,984,604,872,334,1350,1328,1170,523,1361,1268,905
SLC35D3_6_4199,608,751,730,558,1283,25,1138,496,126,169,2776,289
SLC35E1_6_4200,693,482,910,826,2340,602,590,961,414,442,1103,1117
SLC35E3_6_4201,652,699,498,894,639,2369,485,1029,1038,796,262,978
SLC35E4_6_4202,5813,5402,6467,4899,5521,4281,8015,7075,3717,4271,6791,
5289
SLC35F1_6_4203,4726,4100,5029,6105,4161,3633,6404,6380,4160,6794,4668,
6154
SLC35F2_6_4204,1756,1272,1547,1580,1363,1872,2697,4122,1023,3941,3302,
871
SLC35F3_6_4205,742,711,688,540,326,296,1097,358,988,1403,458,747
SLC35F4_6_4206,1087,1424,1621,1279,502,279,637,634,616,2003,894,359
SLC35F5_6_4207,2694,1972,2322,2628,2211,2887,3949,4298,1833,1092,2928,
1489
SLC36A1_6_4208,2353,1614,2214,2433,2408,494,2616,4053,2038,4003,2378,1
826
SLC36A2_6_4209,5856,6278,7804,7311,7320,5891,8543,5428,4579,6439,8757,
4916
SLC36A4_6_4210,4172,4512,4339,4684,3846,4831,6981,8755,2860,4212,4120,
4354
SLC37A1_6_4211,8393,7779,9936,8649,8117,10718,9475,7941,8495,11474,136
00,10660
SLC38A2_6_4212,967,574,1087,950,97,724,1190,1132,1532,219,1956,1477
SLC38A3_6_4213,1463,1243,1979,1739,1016,2021,1325,4117,869,788,2046,20
10
SLC38A5_6_4214,3440,3069,4691,3433,4578,4277,3417,2941,2465,3541,8058,
2547
SLC39A1_6_4215,915,833,1813,1343,515,663,2105,318,643,618,1962,407
SLC39A2_6_4216,2997,2037,2593,2262,1243,4783,5384,1954,1898,4069,4117,
3172
SLC39A9_6_4217,1708,1804,2296,2216,1068,1169,2240,1823,796,912,2812,12
36
SLC3A1_6_4218,16248,15555,18230,17263,23739,19152,17566,21974,17206,18
956,24124,16804
SLC40A1_6_4219,4232,4361,4062,4032,2127,2514,5588,3493,2357,5533,4457,
3479
SLC41A1_6_4220,686,609,534,623,1150,921,931,18,376,727,1549,425
SLC43A2_6_4221,912,638,545,743,713,580,867,519,573,251,611,1684
SLC44A1_6_4222,94,95,87,0,0,0,0,0,0,1380,0,564
SLC46A2_6_4223,393,530,740,431,744,236,1392,129,363,407,671,475
SLC4A1AP_6_4224,3197,2679,3798,3942,3736,4367,3003,4170,3348,2645,4091
,4925
SLC4A1_6_4225,138,119,182,77,392,5,596,0,0,0,2,569
SLC4A7_6_4226,5048,5629,6924,6751,6082,5996,4766,5156,7536,6278,8692,7
285
SLC4A9_6_4227,1140,1110,1013,944,1788,834,699,1265,2187,381,1256,1319
SLC5A11_6_4228,3172,3341,4910,4122,5439,4252,4057,4833,2483,7379,6335,
4225
SLC5A1_6_4229,2047,1888,2015,2355,2185,1306,2586,931,2877,1665,1302,14

02
SLC5A2_6_4230,51,293,119,128,231,0,125,54,15,8,821,77
SLC5A3_6_4231,1234,622,811,375,187,70,586,814,864,1181,1347,867
SLC5A4_6_4232,2554,2853,4022,3493,3037,2805,3658,5658,2456,4619,5668,6
318
SLC5A5_6_4233,5149,5465,6716,5410,7520,9279,8206,7950,3112,10427,6885,
4932
SLC5A6_6_4234,1278,1045,1131,1062,1268,325,2047,1713,313,756,1168,1050
SLC5A7_6_4235,4009,3914,4225,4515,3713,3844,5474,3593,2655,3692,3400,4
326
SLC5A8_6_4236,7250,6546,7763,9808,5055,10377,5707,4845,4711,4033,12863
,6697
SLC6A11_6_4237,1109,938,532,906,1282,1746,1287,467,1242,2258,973,575
SLC6A14_6_4238,3125,2859,3178,3189,3474,5219,2818,2245,3696,920,3338,4
004
SLC6A16_6_4239,92,173,326,203,303,88,29,105,33,177,395,100
SLC6A19_6_4240,5281,4765,4762,4970,4325,9857,3124,6041,3455,4785,6754,
4071
SLC6A1_6_4241,950,527,1478,1208,1180,386,669,1741,372,269,807,1160
SLC6A3_6_4242,1567,1399,1685,1820,2171,758,1145,954,1910,2190,1747,170
6
SLC6A4_6_4243,5611,5844,5121,5313,3414,6015,6142,6350,2575,7756,7602,6
112
SLC6A7_6_4244,589,444,527,444,193,734,226,469,127,681,559,498
SLC7A10_6_4245,1071,742,760,882,2450,696,669,1970,339,839,1276,1698
SLC7A11_6_4246,3540,4073,3698,4053,8018,3402,3610,5509,3592,3556,2338,
5675
SLC7A13_6_4247,3025,2676,3601,4003,1734,4441,3548,1993,2632,3270,3877,
3196
SLC7A1_6_4248,694,514,427,497,61,773,291,32,316,46,1062,754
SLC7A4_6_4249,2424,2337,2570,2164,2020,2156,1593,1430,1439,3723,3620,1
547
SLC7A5_6_4250,739,195,404,440,11,233,132,17,1502,248,1473,783
SLC8A2_6_4251,1156,1244,1001,1254,534,1394,3158,1274,935,2525,3296,524
SLC9A1_6_4252,697,724,1054,1159,226,1962,1451,542,1211,899,2603,185
SLC9A2_6_4253,1114,857,1249,969,415,906,1135,926,623,323,1646,1372
SLC9A3_6_4254,552,421,669,551,476,266,151,518,776,737,382,169
SLC9A3R1_6_4255,1497,1363,2224,1547,892,967,2076,1140,1527,1523,2136,1
600
SLC9A4_6_4256,1257,529,1162,730,282,18,1606,1171,483,1382,923,396
SLC9A5_6_4257,341,560,740,597,16,114,534,172,410,340,1520,100
SLC9A7_6_4258,1910,1641,1547,1988,3253,3117,3035,887,1815,1822,2246,40
2
SLC9A8_6_4259,4368,4309,3454,4443,3248,4661,3589,2659,1842,6276,3901,3
661
SLC9A9_6_4260,2072,1960,2295,1817,1433,1164,2032,1056,1591,1038,2165,1
006
SLCO1B1_6_4261,2788,2973,3238,3459,4576,4822,3259,2799,3022,3530,4369,
3423
SLCO1B3_6_4262,411,520,537,692,1381,960,949,602,96,396,406,107

SLCO2A1_6_4263,1082,1215,2334,1359,1848,1899,961,1881,2486,3382,2149,4
071
SLCO4A1_6_4264,841,703,947,1076,152,544,1586,178,30,146,867,835
SLCO4C1_6_4265,1452,1437,1774,1275,432,703,2648,894,1414,553,2425,972
SLCO6A1_6_4266,1510,2019,2488,2267,1657,2871,3916,2309,1045,1193,4143,
2217
SV2A_6_4267,2101,2243,2209,2441,1028,2587,873,2188,1354,1057,1707,1604
TAP1_6_4268,1928,2227,2332,2058,2494,4010,2433,2388,1461,1291,2620,211
6
UCP1_6_4269,1238,710,766,1368,453,1491,1175,801,2168,1829,1227,803
ABCA12_6_4270,1463,1203,1140,887,1401,3080,1014,2621,1119,1517,1520,14
15
ABCA2_6_4271,1067,1707,1262,1423,445,237,1473,995,591,1317,2424,1443
ABCA5_6_4272,1046,1239,443,747,1030,752,1113,1044,1064,688,1452,69
ABCB4_6_4273,1950,2331,2543,3039,1810,3350,1873,3181,1439,2068,3364,23
62
ABCB9_6_4274,1728,1318,1788,2258,1297,2033,2380,223,1461,1085,1775,491
ABCC10_6_4275,166,104,383,225,218,8,24,7,122,57,90,72
ABCC11_6_4276,1320,1818,2139,1713,1019,1440,1101,2293,3231,1804,1286,1
780
ABCC1_6_4277,1462,958,1622,1343,750,135,2540,1406,1524,1695,2796,619
ABCC3_6_4278,745,668,886,1332,1755,252,1517,674,561,872,913,863
ABCC4_6_4279,606,935,594,750,1,674,28,469,25,33,892,150
ABCC5_6_4280,1112,888,1606,1112,628,1829,2083,2386,2214,712,504,1519
ABCC9_6_4281,3134,2982,4042,3893,2912,4736,4091,4573,4304,3101,4733,30
80
ABCD3_6_4282,2186,1896,2136,2300,4165,1737,3682,3405,2293,1911,2331,16
76
ABCE1_6_4283,2291,1997,2862,2548,3779,3763,5284,3502,2141,3265,4868,25
62
ABCF1_6_4284,99,118,181,150,91,6,548,334,15,216,78,213
ABCF2_6_4285,2101,1933,2844,2067,1482,3783,2690,2285,1402,1884,4246,82
1
ABCG1_6_4286,1282,1427,1629,1357,1421,2701,2035,1021,1363,551,2971,347
2
ABCG4_6_4287,2484,2665,2781,3688,2677,1275,1855,3329,3268,2132,2101,43
46
ANXA11_6_4288,2971,3873,3315,3096,3917,3390,2691,2894,5167,4796,3705,3
091
ANXA13_6_4289,1265,1587,1865,1595,2702,745,1181,1074,585,1100,1382,183
4
ANXA2_6_4290,2287,1799,3477,2895,1798,2252,2210,3852,1234,1580,2701,31
54
ANXA6_6_4291,809,892,788,781,1110,608,1035,903,1634,418,1255,1079
ANXA7_6_4292,1210,1283,1303,783,786,2516,733,841,29,1706,1229,3144
ATP11A_6_4293,1071,1069,1087,1725,841,926,547,982,849,1505,953,902
ATP11C_6_4294,2505,3168,3690,2390,2355,2352,3024,2826,1415,2510,3571,3
853
ATP12A_6_4295,1660,2007,1948,1719,2782,2377,635,1690,1852,1825,2299,13
91

ATP1A1_6_4296,2303,1927,1996,2650,1957,3350,3105,3364,2532,3065,2587,3
840
ATP1B4_6_4297,7829,8101,10288,8043,7227,9978,8515,12924,5790,7028,1130
6,10613
ATP2A1_6_4298,314,203,408,353,76,299,845,446,63,884,44,588
ATP2A2_6_4299,1886,1725,2369,1798,2338,1274,1758,2803,761,1775,844,938
ATP2A3_6_4300,2430,2037,2215,1888,4459,4350,2467,1360,2956,3169,4518,1
124
ATP2B1_6_4301,1375,1142,1456,1390,2455,1860,1304,1698,1115,1675,1563,2
875
ATP2B3_6_4302,931,973,1500,1055,1112,1194,1898,620,1638,1126,1136,1113
ATP2B4_6_4303,1531,1856,2687,1479,3357,2890,1761,1968,2643,1633,2578,1
924
ATP2C1_6_4304,1567,1630,2191,2412,1293,1066,1008,1435,2206,1186,3076,2
307
ATP5A1_6_4305,1656,2096,2008,1998,1249,1232,1211,1533,1355,1444,3235,1
148
ATP5C1_6_4306,3629,4095,6146,5350,4555,3385,3275,3343,2329,2490,4936,5
741
ATP5D_6_4307,346,211,408,624,503,552,607,38,1046,555,438,29
ATP5E_6_4308,0,0,0,0,0,0,0,0,0,0,0,1
ATP5G1_6_4309,453,407,594,572,2335,287,169,145,209,748,730,683
ATP5G2_6_4310,3229,3270,3784,3479,2190,4915,2345,2987,2243,3990,3708,3
657
ATP5G3_6_4311,1878,1510,1490,1677,1478,2206,827,1570,917,1850,1724,132
0
ATP5H_6_4312,1721,1885,1537,1501,2174,1764,283,1037,666,1523,595,480
ATP5J_6_4313,6540,6213,6227,6903,5793,3845,4528,8616,6288,10011,10603,
7370
ATP6V0A1_6_4314,512,636,362,373,284,569,147,271,3,980,754,527
ATP6V0A4_6_4315,2263,2133,1945,2914,3316,1511,1953,2300,1726,1977,4389
,2313
ATP6V0B_6_4316,519,937,735,446,904,446,598,497,0,644,506,1621
ATP6V0C_6_4317,476,543,593,936,1126,510,914,1806,19,273,528,727
ATP6V1C2_6_4318,2386,2345,3354,2546,1757,3476,3155,1146,2951,3511,2362
,2335
ATP6V1E1_6_4319,2913,2430,3737,3344,3236,5024,6171,2283,3011,3151,4333
,2698
ATP6V1F_6_4320,1144,882,1640,1670,1210,2191,1566,1065,542,1630,1308,21
68
ATP6V1G2_6_4321,1609,1643,1929,2028,792,1378,2279,2136,965,3419,2268,1
078
ATP6V1H_6_4322,1688,2386,1979,1518,48,2884,2262,2208,969,604,2875,2122
ATP7B_6_4323,4064,3392,4473,3977,2771,5919,9813,5981,2915,4480,5695,23
09
ATP8A1_6_4324,1371,1579,1465,1700,1285,1738,1207,637,2604,2501,2462,46
0
ATP8B2_6_4325,2646,2783,3861,3494,4919,2892,2827,2903,2838,2704,5066,3
817
ATP8B3_6_4326,1564,1392,1165,1733,849,995,2080,1020,1128,654,2023,1398

ATPAF1_6_4327,3394,2872,4612,3156,3973,4351,1922,4266,2996,4314,4785,2
812
GC_6_4328,3522,2870,3195,4154,2777,3493,3028,4721,2602,3350,4544,3129
SERINC5_6_4329,4658,3802,5377,4185,6703,2984,4255,6884,3146,7715,6517,
5699
SLC10A3_6_4330,1362,1031,1293,1442,503,395,524,330,739,464,1977,1132
SLC11A2_6_4331,1212,2147,1182,1764,768,2073,1272,226,1017,1513,2908,14
22
SLC12A1_6_4332,1752,1656,2387,2330,2853,2328,3984,566,2204,2100,2880,2
892
SLC12A3_6_4333,1926,1684,1930,1864,862,1674,1921,3220,2347,1320,2788,3
542
SLC12A4_6_4334,1543,1259,2159,1802,728,1709,3838,1011,2064,2034,1672,3
240
SLC12A5_6_4335,1105,1178,1641,1460,1434,2156,1514,3069,805,395,1573,37
1
SLC12A6_6_4336,1625,1687,2159,2113,1870,1242,1530,1607,3030,1335,2058,
2152
SLC12A8_6_4337,1579,1853,2190,1979,1192,2736,1941,2024,993,1513,2650,2
489
SLC13A2_6_4338,409,695,1246,770,607,151,70,668,421,383,1557,1101
SLC13A3_6_4339,843,580,439,471,2365,734,9,1254,124,1072,737,180
SLC13A5_6_4340,1405,1114,1191,1356,3146,803,817,1527,838,2359,2835,794
SLC14A1_6_4341,2513,2321,3377,3107,2702,2928,3881,3395,2843,4004,5201,
5056
SLC14A2_6_4342,523,515,245,292,522,29,129,315,1112,0,556,1261
SLC15A2_6_4343,5330,5369,7092,6563,5533,3798,4700,4753,6013,8407,10365
,8391
SLC16A1_6_4344,412,706,530,466,222,497,807,1199,671,1033,978,853
SLC16A3_6_4345,561,218,79,257,255,237,30,43,1098,787,207,191
SLC16A4_6_4346,4811,4229,4717,4934,2541,2796,6010,5082,4376,4045,5716,
3950
SLC16A6_6_4347,6531,6315,8186,8477,7242,6595,9675,7601,8932,6033,10526
,8119
SLC17A3_6_4348,2484,1841,2743,2239,2376,3367,1742,2001,1608,2730,2935,
1303
SLC17A8_6_4349,3359,1811,3187,2995,2624,2118,2656,3835,1646,3575,4894,
1925
SLC18A1_6_4350,4948,3953,5417,5272,8571,4827,6432,7060,5758,5041,8743,
3098
SLC19A1_6_4351,242,377,278,434,57,66,430,389,53,216,61,435
SLC1A2_6_4352,4457,4862,5005,6334,7546,7302,6786,5615,7182,6181,8536,5
346
SLC1A3_6_4353,3473,3629,4927,3987,4001,4556,3441,5134,3026,4405,6596,3
083
SLC1A4_6_4354,2106,1890,2850,2168,2386,3012,1919,5462,1309,1926,2601,1
645
SLC1A5_6_4355,975,943,1042,1165,979,1482,1885,1935,1457,1114,1389,946
SLC22A12_6_4356,1717,1579,1628,1575,2688,2226,1344,529,1715,1196,2440,
2526

SLC22A17_6_4357,1242,1414,1847,1922,1169,2156,1062,1070,776,2172,2155,
865
SLC22A18_6_4358,261,205,1015,759,555,56,366,3,401,464,945,618
SLC22A1_6_4359,7031,6627,6245,7506,6948,6846,6709,10464,9438,9727,1060
0,6785
SLC22A23_6_4360,6346,6002,7220,7788,5707,6603,6244,8479,7495,10013,994
2,5731
SLC22A6_6_4361,835,1370,1267,1292,589,702,1684,1461,881,406,969,689
SLC22A7_6_4362,816,1402,917,1168,3384,676,257,907,966,2542,2295,1360
SLC22A8_6_4363,630,945,1001,790,484,1950,565,1555,120,58,877,380
SLC23A3_6_4364,586,347,739,1109,544,4,760,1930,222,379,1165,452
SLC24A2_6_4365,7499,6974,7453,8697,4097,6559,7508,8774,2021,6142,8102,
5053
SLC24A4_6_4366,2716,3124,2946,2519,2418,2394,3759,2033,1820,3568,1587,
2324
SLC25A11_6_4367,839,1500,1578,1087,736,600,1029,1455,286,1122,996,759
SLC25A13_6_4368,10443,11358,14246,12679,11844,12364,16433,17483,11485,
13249,17593,12477
SLC25A14_6_4369,2174,2304,2106,2682,1142,2116,654,4081,2625,1644,2767,
1784
SLC25A19_6_4370,263,482,211,381,339,186,140,568,53,1393,493,15
SLC25A21_6_4371,2012,1310,1422,1606,2174,683,3879,1017,406,1277,1638,8
49
SLC25A22_6_4372,544,448,602,437,671,1509,490,386,887,3183,244,411
SLC25A26_6_4373,988,1613,1976,1596,1928,2001,1967,1149,908,1300,2596,2
914
SLC25A27_6_4374,1571,1360,1115,1327,1247,1102,1866,676,533,1760,2104,1
569
SLC25A3_6_4375,3387,3210,3902,4536,1894,4904,4339,1295,4188,6018,2820,
2541
SLC26A11_6_4376,531,560,348,352,118,964,134,1213,111,611,380,202
SLC26A1_6_4377,211,324,307,203,110,0,2,1077,0,0,730,274
SLC26A5_6_4378,565,810,920,722,1720,617,293,1579,276,827,1546,1768
SLC26A6_6_4379,511,279,637,569,10,1120,814,476,834,1084,1010,833
SLC26A7_6_4380,6541,6954,6891,7448,6085,7866,9420,8208,5568,5410,9713,
6375
SLC26A8_6_4381,5655,7261,8813,8449,9820,7035,5703,6466,3780,8231,9448,
6480
SLC26A9_6_4382,954,958,957,993,1362,2036,1018,349,949,464,608,349
SLC27A2_6_4383,5009,5114,6770,5978,9565,10329,5071,3046,2844,5342,6171
,7314
SLC27A6_6_4384,1870,1527,1507,1350,1305,2685,2776,858,1005,2044,2029,1
270
SLC28A3_6_4385,902,1010,1537,1323,91,1001,318,875,940,1017,524,692
SLC29A1_6_4386,451,444,468,214,307,41,1303,74,43,94,160,287
SLC29A3_6_4387,4430,4558,5496,5962,5207,9829,2234,5792,3541,5373,4487,
4101
SLC29A4_6_4388,3120,3394,4100,3336,2363,3361,2630,4189,3006,3242,1762,
3346
SLC2A11_6_4389,1268,1267,1223,1226,199,1515,1117,649,386,821,1369,1053

SLC2A5_6_4390,1141,1310,1510,1602,1070,741,3312,2133,398,1226,2803,114
4
SLC2A6_6_4391,629,589,708,848,111,130,609,513,297,231,115,296
SLC2A9_6_4392,1458,1558,1908,1822,3576,3390,3073,355,1828,2123,3560,21
83
SLC30A2_6_4393,1215,1598,1735,1256,2338,1602,1821,1529,807,950,1902,72
7
SLC30A6_6_4394,2039,1414,2319,1850,2629,630,1415,1605,1158,1230,1915,1
521
SLC30A7_6_4395,2117,1439,2510,1920,4499,3235,2984,4568,1627,3384,2622,
1320
SLC30A8_6_4396,487,479,724,515,322,797,446,968,298,594,1648,1261
SLC33A1_6_4397,2000,1803,2534,2418,955,2207,2835,1181,1820,1474,1195,3
026
SLC34A1_6_4398,300,180,745,279,109,112,327,438,582,1005,781,0
SLC34A2_6_4399,912,1143,1227,1105,1358,1311,634,2005,969,1200,3182,538
SLC34A3_6_4400,969,650,546,884,530,266,910,794,191,82,2077,204
SLC35A1_6_4401,448,438,498,453,347,961,192,1164,569,877,2147,496
SLC35A2_6_4402,422,363,286,461,237,936,92,1786,219,1414,668,76
SLC35B3_6_4403,7954,8206,8959,10094,5105,11222,11103,8761,4982,8882,79
70,13049
SLC35C1_6_4404,1191,1145,1279,1706,1195,651,1375,1105,799,982,1833,155
9
SLC35C2_6_4405,908,443,385,553,611,133,746,638,278,514,559,308
SLC35E2_6_4406,824,1103,990,1245,531,526,991,1111,442,1086,677,2474
SLC36A3_6_4407,1203,1281,1629,1605,1556,203,1642,1747,807,437,1428,893
SLC37A2_6_4408,3021,2316,2871,2943,4255,4158,2334,2892,1511,3606,2734,
1850
SLC37A3_6_4409,1692,1464,1752,2067,600,454,1779,3086,1075,632,2269,321
1
SLC37A4_6_4410,438,890,671,597,554,123,1239,1440,351,1199,351,620
SLC38A11_6_4411,7943,7283,9032,9882,10374,9283,7365,10378,5375,9877,11
992,11995
SLC38A1_6_4412,3081,3080,3067,2800,3077,3278,2834,3080,3589,5495,6752,
5513
SLC38A4_6_4413,126,274,1,61,0,289,6,0,3,0,1,0
SLC38A6_6_4414,1815,1579,1888,1511,1521,889,2087,2068,557,2278,884,896
SLC39A10_6_4415,2512,2677,3479,3202,1860,2806,2034,2068,1440,3313,3356
,3622
SLC39A11_6_4416,3931,3565,4569,4040,1404,3624,4454,3794,3283,3197,6372
,4062
SLC39A12_6_4417,4315,3980,4120,4724,3496,6960,5761,3734,3084,4717,4956
,4686
SLC39A13_6_4418,762,620,1201,830,748,876,468,345,38,2512,743,1152
SLC39A14_6_4419,1175,1427,1421,1813,1108,4159,174,1252,1892,1167,1197,
2044
SLC39A4_6_4420,476,626,706,646,747,383,180,1715,361,349,937,948
SLC39A5_6_4421,1168,844,1080,1183,1133,995,2308,791,1583,51,1366,1353
SLC39A6_6_4422,1275,1439,1686,1598,3363,582,3004,1840,1679,3023,2907,1
467

SLC39A7_6_4423,1055,1417,1070,1322,846,98,979,3350,2073,972,2435,1419
SLC39A8_6_4424,7304,7057,7725,8091,8326,6093,8832,8162,5853,11272,6581
,7520
SLC3A2_6_4425,4070,3579,3718,3243,4103,3307,3592,4406,2033,4851,3418,6
064
SLC43A1_6_4426,605,562,457,284,588,476,133,743,3,432,162,60
SLC44A3_6_4427,2577,2668,3149,3525,1376,3252,1683,2470,3710,2778,2612,
3473
SLC4A10_6_4428,2846,2193,2668,3237,3238,3527,4076,2029,3072,1826,4944,
1974
SLC4A11_6_4429,815,574,789,1018,1077,224,330,1124,343,1152,1648,1060
SLC4A2_6_4430,247,239,852,552,120,176,847,865,252,62,271,194
SLC4A3_6_4431,1107,1316,761,1314,911,2558,1131,1899,1890,1629,1145,876
SLC4A4_6_4432,525,904,911,799,433,486,1021,153,519,25,454,1452
SLC4A8_6_4433,462,602,987,883,546,495,879,1327,536,87,818,542
SLC5A10_6_4434,5105,5208,7134,6511,7799,4584,6184,7299,4773,6868,10530
,6965
SLC5A9_6_4435,568,495,542,584,352,1671,44,136,0,914,270,153
SLC6A12_6_4436,151,486,650,332,188,605,965,958,17,55,618,733
SLC6A13_6_4437,3429,2988,3741,4156,2779,5509,5789,5521,1861,4721,5533,
5789
SLC6A15_6_4438,1215,1327,728,1090,1380,1013,1776,1311,953,2097,2298,21
25
SLC6A2_6_4439,3132,2903,3889,4316,5384,2040,3185,5497,3842,3588,3064,5
004
SLC6A6_6_4440,5694,5419,7331,6588,5173,7557,5728,6584,2949,6821,7719,6
482
SLC6A8_6_4441,1027,1716,1093,1013,1576,447,666,285,1210,603,518,1702
SLC6A9_6_4442,6080,6482,7756,8066,5817,7741,9799,6995,5509,9126,10841,
8316
SLC7A2_6_4443,5393,5509,5175,4300,4641,4141,5595,3391,7305,6852,6890,4
697
SLC7A3_6_4444,2924,2013,3092,3382,2438,2606,2776,2969,2958,3376,4320,5
660
SLC7A6_6_4445,655,972,1968,1066,1191,1170,1832,1666,398,550,955,852
SLC7A7_6_4446,3295,3811,3426,3390,4227,4493,2739,4626,3211,3839,4649,2
823
SLC7A8_6_4447,4126,3832,4089,4311,5265,5772,4354,6880,3325,3160,4245,3
195
SLC7A9_6_4448,2730,3138,3416,3246,4628,2740,4450,3368,1728,4727,2392,3
905
SLC8A1_6_4449,3101,2705,4141,4010,2167,1469,3874,4604,3693,1912,2970,3
825
SLC8A3_6_4450,2291,2947,2390,3466,1629,3002,1698,3485,2522,1093,3080,7
52
SLC9A3R2_6_4451,452,790,637,990,863,475,122,577,373,935,644,536
SLC9A6_6_4452,1117,560,1060,749,1887,100,562,1234,203,630,1190,2336
SLCO1A2_6_4453,3500,2886,4750,3933,3669,6913,4948,2445,3909,3306,4996,
3983
SLCO1C1_6_4454,3391,2976,3742,3774,2509,3880,4711,4957,3323,1828,6687,

3588
SLCO2B1_6_4455,896,665,1257,1097,653,475,2135,1255,1054,702,1678,1454
SLCO3A1_6_4456,2430,2597,2506,1897,544,4259,3107,1850,2298,3224,3443,1
756
SLCO5A1_6_4457,1101,925,1301,1524,647,1320,967,1953,1931,1678,1886,649
ABCB5_6_4458,2667,2635,3049,2224,1614,3811,2998,2151,1799,4066,2912,32
27
ABCC6_6_4459,597,563,931,1585,610,598,1403,2161,826,687,883,613
ATP1A4_6_4460,1124,1204,1424,919,1197,1017,2370,766,1090,557,1707,1156
ATP5J2_6_4461,5375,4861,7189,6234,2166,4507,5434,4169,4124,7534,5696,8
049
ATP5S_6_4462,1596,1241,1427,1814,1117,2501,1578,2738,828,2450,1949,324
0
ATP6V1G3_6_4463,354,581,398,274,431,149,430,70,363,1878,1486,687
ATPIF1_6_4464,1433,1203,1198,1076,1302,1433,903,1246,1121,1070,1870,79
9
SLC10A7_6_4465,2770,3052,3890,4331,1830,2405,2743,4730,2173,3515,5780,
3875
SLC28A1_6_4466,613,337,620,800,824,1174,905,1876,234,839,930,854
SLC30A5_6_4467,3106,3331,3536,3762,3288,2735,2081,4114,3563,4649,5861,
3819
SLC39A3_6_4468,781,971,1137,944,2585,2692,2419,514,141,1169,729,352
ABCD1_6_4469,2755,2990,2914,3177,4277,2335,2212,2156,1971,3832,3865,26
61
ABCD1_6_4470,1040,441,858,533,722,129,292,1832,578,875,532,629
ABCD1_6_4471,252,41,54,266,31,29,0,61,0,5,33,305
ABCD1_6_4472,450,85,388,90,7,210,618,1837,2,1562,3,322
ABCD1_6_4473,479,399,1105,1125,80,906,388,816,377,685,352,874
ABCD1_6_4474,2773,3061,2915,3230,4303,2321,2223,2151,2264,3781,3852,26
40
ABCD1_6_4475,472,364,379,474,450,135,42,1468,446,869,0,1472
ABCD1_6_4476,838,237,991,1042,919,566,47,528,185,708,903,749
ABCD1_6_4477,223,242,429,102,89,1495,51,1046,328,72,325,477
ABCD1_6_4478,222,168,285,212,501,91,25,1003,132,20,25,219
ATP2B4_6_4479,850,563,932,771,1608,559,1800,499,707,909,834,307
ATP2B4_6_4480,2304,2254,2772,1246,1090,2763,1979,3559,2415,1597,2710,2
465
ATP2B4_6_4481,710,364,612,329,35,2918,1672,188,60,1032,3,747
ATP2B4_6_4482,1574,1860,2469,2198,1322,3304,1959,938,783,2411,2062,141
7
ATP2B4_6_4483,581,745,529,638,544,208,842,84,442,691,709,2206
ATP2B4_6_4484,6941,6515,7588,7477,7122,4754,10726,6768,7663,5593,7005,
6921
ATP2B4_6_4485,3504,3603,4578,3888,3791,4048,4485,4626,4290,2731,6639,4
213
ATP2B4_6_4486,1137,1202,997,1171,645,343,864,1499,885,3161,1141,2866
ATP2B4_6_4487,749,879,1331,1030,367,520,1032,848,986,1236,1390,380
ATP2B4_6_4488,2225,2869,3634,3262,3390,3702,3234,2827,2379,4964,1461,3
764
SLC6A2_6_4489,14997,14318,18019,16715,17803,11996,20964,17251,15311,16

294,21596,16844
SLC6A2_6_4490,5717,5966,7361,6197,3557,5132,7110,5426,4608,9057,5399,8
440
SLC6A2_6_4491,2845,4023,3665,2948,1769,1912,2961,3954,2988,2778,5620,1
529
SLC6A2_6_4492,3155,3739,4065,4060,3285,3368,3086,4697,2321,2550,6169,2
194
SLC6A2_6_4493,1821,1942,2597,2066,1700,1583,3078,1854,518,1543,4150,15
08
SLC6A2_6_4494,567,541,730,715,904,639,1167,136,1,17,449,986
SLC6A2_6_4495,1914,1564,2244,2321,1798,4762,2633,2938,2212,1542,3342,1
242
SLC6A2_6_4496,11225,11388,13667,11984,11428,14352,11524,13216,10944,15
740,18678,12188
SLC6A2_6_4497,3407,3972,4759,3964,3369,4197,4044,4745,2288,7375,9459,5
979
SLC6A2_6_4498,10701,10799,12961,11449,11269,13181,11015,12734,10579,15
525,17326,11686
GC_6_4499,1361,2146,2091,1648,1189,817,989,1783,1175,1081,2034,414
GC_6_4500,2178,1951,3267,2507,2287,2345,2619,1480,3580,3158,2978,2279
GC_6_4501,7877,8296,9739,7922,12873,14161,9303,9988,8485,7562,10151,75
78
GC_6_4502,91,137,383,342,1949,128,2016,426,84,44,289,341
GC_6_4503,170,304,380,295,1178,424,27,849,1014,116,521,372
GC_6_4504,6644,7017,8102,6430,10270,10691,9757,9389,7884,6770,8461,600
5
GC_6_4505,814,512,816,619,55,368,51,519,187,0,289,5
GC_6_4506,412,283,197,99,618,0,207,420,4,1,1381,536
GC_6_4507,1367,2161,2139,1617,1189,823,993,1749,983,1088,2008,274
GC_6_4508,8258,8658,9934,8259,11326,16224,10521,10888,9004,8550,10111,
8289
SLC6A5_6_4509,596,820,1300,1338,341,490,1069,1309,1218,275,304,1265
SLC6A5_6_4510,2679,1791,1687,2038,1807,1524,2809,1108,1209,728,2309,60
9
SLC6A5_6_4511,2079,2742,2321,2314,2882,3699,2399,2862,1631,2093,2637,2
632
SLC6A5_6_4512,2884,2303,2923,3209,2166,4951,2008,3445,1955,3811,3938,2
582
SLC6A5_6_4513,7285,6092,8817,8049,7278,8187,7842,7131,7264,11223,10602
,7407
SLC6A5_6_4514,11459,11335,12721,11177,11220,13274,14644,15158,9318,160
83,13658,11018
SLC6A5_6_4515,1384,1983,1993,2333,810,1176,2119,4578,2328,2624,3008,30
65
SLC6A5_6_4516,3374,3091,2651,3718,2791,4204,6947,2546,2733,2925,3865,4
240
SLC6A5_6_4517,4307,4663,5267,5436,3020,4934,5881,6267,4178,7003,8619,6
661
SLC6A5_6_4518,10152,8784,11150,10669,14320,12529,15684,10956,9059,1113
3,13684,11537

SLC6A2_6_4519,596,820,1300,1338,341,490,1069,1309,1218,275,304,1265
SLC6A2_6_4520,2679,1791,1687,2038,1807,1524,2809,1108,1209,728,2309,60
9
SLC6A2_6_4521,2079,2742,2321,2314,2882,3699,2399,2862,1631,2093,2637,2
632
SLC6A2_6_4522,2884,2303,2923,3209,2166,4951,2008,3445,1955,3811,3938,2
582
SLC6A2_6_4523,7285,6092,8817,8049,7278,8187,7842,7131,7264,11223,10602
,7407
SLC6A2_6_4524,11459,11335,12721,11177,11220,13274,14644,15158,9318,160
83,13658,11018
SLC6A2_6_4525,1384,1983,1993,2333,810,1176,2119,4578,2328,2624,3008,30
65
SLC6A2_6_4526,3374,3091,2651,3718,2791,4204,6947,2546,2733,2925,3865,4
240
SLC6A2_6_4527,4307,4663,5267,5436,3020,4934,5881,6267,4178,7003,8619,6
661
SLC6A2_6_4528,10152,8784,11150,10669,14320,12529,15684,10956,9059,1113
3,13684,11537
SLC4A4_6_4529,1847,2315,1975,2393,784,3877,2033,1580,2267,1544,4191,31
84
SLC4A4_6_4530,2239,1742,2739,1966,1218,2080,1611,1559,1873,2887,3987,3
186
SLC4A4_6_4531,2232,2053,2674,2700,1681,3422,2697,3446,3145,3079,1708,4
692
SLC4A4_6_4532,2242,2578,2943,3096,636,2723,1557,2816,2499,2581,1976,28
79
SLC4A4_6_4533,2352,2051,2897,2299,1340,2039,1557,1487,1767,2651,3331,3
528
SLC4A4_6_4534,1177,574,1132,1521,106,697,3881,2414,815,2303,1016,651
SLC4A4_6_4535,1156,776,1392,1053,1459,1514,581,1243,1089,1588,2191,949
SLC4A4_6_4536,2818,3037,2668,3219,1481,1702,3081,3803,1832,5717,3135,2
433
SLC4A4_6_4537,782,320,711,811,192,153,256,203,1925,579,1428,77
SLC4A4_6_4538,3800,3690,4696,4227,5837,3211,5298,3152,2996,3068,3989,3
128
SLC6A2_6_4539,2394,2613,1890,2629,1003,2105,2580,3708,1333,4506,4180,2
830
SLC6A2_6_4540,891,693,1168,1229,652,921,103,13,1366,1170,875,477
SLC6A2_6_4541,1877,2936,2077,2224,1378,2124,3283,2530,1937,636,3835,15
07
SLC6A2_6_4542,3673,3797,4440,3427,3298,4029,3882,3072,3943,2755,3892,3
627
SLC6A2_6_4543,3937,3166,5061,4432,2240,5845,5274,6630,4600,4290,4466,2
914
SLC6A2_6_4544,2038,1671,2824,1907,3315,1796,2158,1521,2310,2209,1904,1
763
SLC6A2_6_4545,712,985,868,983,530,325,1101,1411,118,497,1377,368
SLC6A2_6_4546,751,708,339,710,35,716,705,789,672,510,948,1045
SLC6A2_6_4547,1815,784,1160,1413,1202,514,2167,1157,1057,1682,2217,109

4
SLC6A2_6_4548,803,1078,1443,1257,2415,251,580,1512,514,1726,1886,1318
SLC6A4_6_4549,7164,5996,7545,7106,11335,5313,6318,6484,7008,8727,6559,
7925
SLC6A4_6_4550,11537,11425,15005,12415,11166,12101,13839,10203,9860,168
81,13010,10797
SLC6A4_6_4551,2153,1555,2003,2003,1698,1842,1951,2045,2892,1497,1546,1
931
SLC6A4_6_4552,1630,1563,1980,2460,890,1407,1283,2294,1632,2163,1945,18
00
SLC6A4_6_4553,2136,1724,2486,2282,952,839,2141,906,2326,2303,1831,2161
SLC6A4_6_4554,7169,5972,7716,7247,11416,5340,6317,6824,6747,8903,6584,
7977
SLC6A4_6_4555,1196,1389,1266,1203,971,501,201,947,190,1304,631,164
SLC6A4_6_4556,1274,953,1187,1198,678,2628,1137,538,600,1175,2517,1374
SLC6A4_6_4557,1537,1467,2198,2887,616,1082,2983,3789,1877,2940,2631,21
62
SLC6A4_6_4558,1505,1489,2181,2407,1445,1378,1557,1961,1384,1435,2319,1
165
SLC7A8_6_4559,634,480,698,362,372,168,638,331,246,384,721,555
SLC7A8_6_4560,432,516,1005,837,543,423,1266,282,188,332,936,268
SLC7A8_6_4561,3824,3338,4312,5301,4924,5430,3283,5870,3327,2850,9469,4
573
SLC7A8_6_4562,3763,3404,4173,5187,4100,5064,3833,5870,3341,2848,9585,4
059
SLC7A8_6_4563,912,1072,1249,1355,166,387,1578,1689,402,805,1232,1694
SLC7A8_6_4564,1677,1196,2246,1825,1189,717,1640,2773,1872,2971,2772,13
05
SLC7A8_6_4565,4977,4960,5805,5135,6060,3748,7366,6928,3510,5146,7499,3
506
SLC7A8_6_4566,903,590,1233,946,393,919,1104,601,13,441,1878,431
SLC7A8_6_4567,872,783,1298,1165,24,1289,2467,1251,496,1475,1471,1097
SLC7A8_6_4568,5092,4955,5784,4959,6063,3751,7423,6916,4105,5976,6650,3
409
SLC18A1_6_4569,2773,2559,3053,2865,2483,3025,3798,3926,1824,3325,4687,
2270
SLC18A1_6_4570,498,515,483,573,317,1146,1334,393,489,613,794,398
SLC18A1_6_4571,3023,2677,3013,3715,4925,3782,3621,3233,4364,5172,4915,
3410
SLC18A1_6_4572,326,287,552,425,641,459,745,583,52,829,352,371
SLC18A1_6_4573,433,286,545,333,127,186,619,120,38,1262,1169,873
SLC18A1_6_4574,3170,2807,3096,3661,4956,3784,3633,3244,5002,6268,4933,
3425
SLC18A1_6_4575,8940,8760,9855,9925,9316,6045,10576,8492,8062,10523,137
67,8291
SLC18A1_6_4576,262,564,463,888,232,517,962,244,502,734,1438,491
SLC18A1_6_4577,507,408,572,668,26,2762,1544,372,779,610,1275,216
SLC18A1_6_4578,1267,1220,1244,1278,1339,789,823,509,1109,1282,1156,969
SLC22A5_6_4579,477,561,718,736,124,565,243,1831,885,1382,570,1050
SLC22A5_6_4580,1674,1602,1947,1991,1201,1995,2279,1309,1281,1087,4554,

2522
SLC22A5_6_4581,637,639,492,887,357,228,463,1670,632,529,1179,314
SLC22A5_6_4582,601,326,326,561,1202,1444,210,78,54,38,29,2066
SLC22A5_6_4583,2517,1815,2782,2373,6037,1965,2811,2993,2443,2849,3751,
2763
SLC22A5_6_4584,16773,18013,19693,19360,18329,21285,17116,21374,19310,1
9516,20674,19435
SLC22A5_6_4585,2115,1732,1593,2532,1317,2547,2602,3146,1100,2457,1919,
1781
SLC22A5_6_4586,2714,2418,2908,3235,3119,5430,3089,2581,2413,2554,3677,
2093
SLC22A5_6_4587,1912,1359,2335,2345,1722,1823,2939,2960,1643,1831,2058,
2895
SLC22A5_6_4588,1147,697,928,1223,283,604,1400,1911,894,779,1523,1048
SLC23A2_6_4589,928,544,1452,1025,393,1676,867,1346,305,802,1639,579
SLC23A2_6_4590,1367,1603,1737,1603,379,538,2347,993,815,1391,3916,2586
SLC23A2_6_4591,3429,3363,3169,3652,3482,3835,3656,2724,2190,5465,3327,
2118
SLC23A2_6_4592,1219,595,1204,1650,523,1621,141,1557,2436,1367,2356,598
SLC23A2_6_4593,1732,2016,2279,1971,731,2141,1895,2668,1662,3125,3393,1
640
SLC23A2_6_4594,2321,1978,2858,2215,3580,2619,3309,2888,1808,817,4412,2
671
SLC23A2_6_4595,1354,1437,1111,1076,2911,828,707,1267,779,2756,1370,124
9
SLC23A2_6_4596,997,1251,1433,1245,603,631,1606,1083,563,1141,1160,1356
SLC23A2_6_4597,2137,2307,2247,2626,3891,3398,2634,4417,3780,4136,4524,
2477
SLC23A2_6_4598,1695,695,1507,1579,139,1616,1595,2341,1054,1948,2300,47
8
SLC25A15_6_4599,0,0,9,72,0,0,295,0,0,0,0,248
SLC25A15_6_4600,1816,2120,2863,2632,2066,3634,2696,2769,2798,1310,3627
,1488
SLC25A15_6_4601,1253,973,1179,788,599,469,204,1192,1699,2356,1576,752
SLC25A15_6_4602,10,15,0,72,0,0,0,0,0,0,0,0
SLC25A15_6_4603,303,184,425,667,396,100,274,17,178,521,825,492
SLC25A15_6_4604,1366,786,958,1089,206,2047,1494,2462,223,583,1579,1580
SLC25A15_6_4605,1245,1650,984,1885,1287,2613,1816,1032,1068,1790,1349,
2642
SLC25A15_6_4606,3485,3250,4468,3526,4689,3595,3840,4000,1458,4446,7658
,3117
SLC25A15_6_4607,3791,3343,4611,3491,2092,3626,3589,2989,2761,3035,4815
,3389
SLC25A15_6_4608,958,1582,1520,1603,180,854,1871,2005,2124,2887,1564,13
39
SLC25A16_6_4609,2585,2749,3963,2932,2811,3698,2328,3327,2202,2219,4060
,3085
SLC25A16_6_4610,2357,2159,3204,2850,2261,3220,2376,2792,1777,2856,4777
,3540
SLC25A16_6_4611,1132,824,1645,1247,1162,1102,1489,1545,1144,1235,1105,

677
SLC25A16_6_4612,848,1564,1056,1045,657,456,742,1885,320,1028,693,2247
SLC25A16_6_4613,2755,1888,3777,2481,2311,695,2046,4614,1647,1753,3850,
2476
SLC25A16_6_4614,7899,7998,9508,8313,6695,7745,10510,13327,6080,10837,1
1941,7872
SLC25A16_6_4615,2384,2279,2086,2992,1628,3031,1420,2816,1027,2033,3334
,3781
SLC25A16_6_4616,430,368,615,668,588,1749,63,332,1108,32,615,615
SLC25A16_6_4617,2576,3327,2474,2781,1042,3823,3534,3793,1807,3194,2498
,3563
SLC25A16_6_4618,258,597,435,445,119,291,297,753,205,292,454,634
SLC25A21_6_4619,14594,12437,15361,15713,11187,13798,12903,15227,10401,
16957,18266,15049
SLC25A21_6_4620,3587,3362,4248,3989,4445,2597,5247,2219,4580,8238,5087
,4285
SLC25A21_6_4621,2590,3173,3503,3813,2529,2707,3178,4119,1838,5675,3900
,2649
SLC25A21_6_4622,5505,4697,6798,6693,3337,6511,6971,6989,4279,4041,9032
,7740
SLC25A21_6_4623,1314,753,1053,1107,1665,379,1069,563,1224,1035,1240,15
51
SLC25A21_6_4624,1951,2610,2625,3447,1809,1727,3667,1687,2216,3145,3439
,985
SLC25A21_6_4625,1349,772,1471,1501,1158,610,880,1651,494,1243,1786,215
2
SLC25A21_6_4626,975,945,1225,1085,904,2469,766,937,1202,849,2663,2456
SLC25A21_6_4627,2259,1992,2960,3121,4648,882,2581,1934,828,2248,1762,1
496
SLC25A21_6_4628,24645,20152,25472,21805,25256,30924,26660,26039,21380,
28067,25915,23425
SLC34A1_6_4629,741,1003,836,1033,1545,1669,1487,603,441,512,637,619
SLC34A1_6_4630,629,907,681,651,490,1502,435,459,1582,601,1016,1589
SLC34A1_6_4631,1185,1302,1387,1410,548,1719,743,2549,1289,2784,1924,22
50
SLC34A1_6_4632,499,395,645,686,1425,379,911,774,461,1262,531,1511
SLC34A1_6_4633,2674,2428,2893,3209,2931,4123,2026,4546,1782,1638,2684,
2071
SLC34A1_6_4634,3502,3570,4148,4245,5828,7631,4252,3897,4834,1441,6836,
2161
SLC34A1_6_4635,3863,4801,5330,5778,3753,6169,5023,5265,4809,6439,5008,
6350
SLC34A1_6_4636,2226,1724,2585,1781,2063,4637,2326,1504,2073,3058,2694,
850
SLC34A1_6_4637,366,253,449,562,80,266,7,761,276,224,873,1093
SLC34A1_6_4638,3956,4887,5205,5900,3698,5768,5205,5259,4816,6885,4990,
5926
SLC38A1_6_4639,10613,9645,10983,10252,11379,9873,8828,13842,7899,10900
,12213,9116
SLC38A1_6_4640,2556,2730,2296,3021,1618,3281,2287,1735,1002,1387,2739,

4453
SLC38A1_6_4641,1126,976,1037,1597,516,1961,1673,429,302,370,1849,1813
SLC38A1_6_4642,2923,2883,3633,3278,1366,1103,3525,4239,1852,1902,5155,
3804
SLC38A1_6_4643,2067,1738,3074,1656,1258,911,942,1597,1052,3615,1885,86
3
SLC38A1_6_4644,850,886,1256,1203,611,697,1180,51,3042,1667,2451,185
SLC38A1_6_4645,14997,14318,18019,16715,17803,11996,20964,17251,15311,1
6294,21596,16844
SLC38A1_6_4646,2561,1320,2782,3015,1954,2214,2273,1444,3254,3202,2793,
2588
SLC38A1_6_4647,201,136,194,62,2,1,62,2,55,1,162,3
SLC38A1_6_4648,1285,1695,1315,1650,1598,2197,1192,2421,1585,1569,2972,
1366
SLC38A1_6_4649,1675,1479,1854,1773,1498,2975,1212,2289,1758,934,2612,8
00
SLC38A1_6_4650,3602,3769,3749,3683,4171,4743,3211,3223,4862,6877,3466,
4106
SLC38A1_6_4651,413,1024,646,933,36,166,850,1077,98,1168,974,379
SLC38A1_6_4652,2349,1335,2119,2235,1824,1678,2836,2411,1426,1557,2719,
2830
SLC38A1_6_4653,5073,5904,6469,5546,4676,7884,5984,5560,4918,6290,9692,
7086
SLC38A1_6_4654,691,486,427,649,196,101,709,114,239,1030,971,1511
SLC38A1_6_4655,1099,948,1025,1620,2947,894,928,991,1637,1371,2074,1500
SLC38A1_6_4656,664,285,857,748,624,1144,779,2817,556,410,1300,401
SLC38A1_6_4657,1144,1567,1659,1769,1019,1545,1233,602,2362,1857,2229,5
27
SLC38A1_6_4658,1259,1595,1646,1445,2287,1082,1452,1340,1968,1809,1008,
2438
SLC38A2_6_4659,1302,1613,1636,1789,762,1460,2263,1186,1599,784,2739,12
18
SLC38A2_6_4660,1573,1252,2319,1978,1283,614,983,2009,792,1800,1016,306
8
SLC38A2_6_4661,1476,1611,2109,2185,2489,1462,2395,2071,1794,930,2573,2
569
SLC38A2_6_4662,3440,3025,3743,3669,2277,3428,3890,3189,1770,3576,3637,
3443
SLC38A2_6_4663,182,88,413,376,1,365,307,687,172,301,586,0
SLC38A2_6_4664,1246,1342,1459,1566,622,1197,1712,2058,1880,489,1530,18
77
SLC38A2_6_4665,2073,2148,2668,2315,2543,3960,2101,2664,2142,3144,2593,
3994
SLC38A2_6_4666,3818,3597,4792,4387,1783,2595,6739,4874,2612,3514,5856,
5428
SLC38A2_6_4667,1141,1041,1580,1708,685,952,683,1975,2352,724,2204,1889
SLC38A2_6_4668,1288,1024,2044,1472,523,546,981,1029,1334,2742,2447,101
1
