# Supplementary material for: Cross-species genetic screens identify transglutaminase 5 as a regulator of polyglutamine-expanded ataxin-1
Source: J Clin Invest. 2022 May 2;132(9):e156616. doi: 10.1172/JCI156616 (PMC9057624; doi:10.1172/JCI156616)
Supplement: Supplemental data set 8 [file jci-132-156616-s053.pdf]

```
sRNA,B1,B2,B3,B4,H1,H2,H3,H4,L1,L2,L3,L4
ABTB2_7_1,247,956,103,182,689,317,3,162,568,59,1987,387
AMBRA1_7_2,1285,1289,124,458,460,2752,1504,138,677,657,1124,1444
AMFR_7_3,171,1010,1025,727,1244,683,350,1377,891,933,2135,395
ANAPC10_7_4,281,1194,151,41,205,14,451,1120,0,8,1461,147
ANAPC1_7_5,374,513,244,411,94,1195,166,1467,1070,454,0,444
ANAPC2_7_6,0,0,1,93,0,26,104,11,0,2,455,0
ANAPC4_7_7,124,73,2186,18,197,715,488,1078,306,139,1738,236
ANKIB1_7_8,140,95,536,43,89,393,62,413,0,411,858,380
ANKRD13A_7_9,14,482,0,39,0,50,1,0,179,199,0,24
ANKRD13D_7_10,0,0,0,0,0,0,0,0,27,97,1,39
ARIH1_7_11,425,0,13,262,122,91,158,19,21,2,0,129
ARIH2_7_12,242,326,2,672,108,95,1164,32,752,503,298,311
ASB12_7_13,988,526,230,372,30,467,2054,70,1075,2335,298,769
ASB13_7_14,6,31,12,88,17,91,124,79,15,74,1035,2
ASB15_7_15,552,1,32,15,0,44,9,0,20,32,0,13
ASB16_7_16,0,0,1,0,0,1,0,0,0,0,0,0
ASB17_7_17,1417,1262,119,359,3429,1868,1140,1352,1332,1635,1119,1699
ASB5_7_18,1272,310,148,118,452,817,231,297,287,395,247,1645
ASB8_7_19,21,527,14,15,3,455,1512,19,128,4,1282,464
ASH1L_7_20,63,235,9,242,0,233,359,141,221,58,6,25
ASPSCR1_7_21,1725,2088,1975,1337,2651,4170,876,2545,1715,819,2315,1030
ATG12_7_22,4564,3073,2812,1416,1798,3348,5565,6237,2708,6093,4404,5635
ATG3_7_23,186,493,642,151,742,105,211,53,117,458,5,135
ATG5_7_24,382,1637,1267,314,108,223,2509,751,430,1276,357,784
ATXN1L_7_25,55,735,1239,138,524,873,17,1928,871,12,616,128
ATXN3L_7_26,222,21,224,1126,232,253,362,332,51,350,86,42
BARD1_7_27,0,0,0,0,0,0,0,0,0,0,0,0
BAZ1B_7_28,5245,3629,1549,3362,5595,4215,1735,3329,3756,3235,5671,4634
BAZ2A_7_29,355,708,148,564,464,126,2256,1120,476,895,0,896
BAZ2B_7_30,1033,1154,1102,867,691,418,1394,777,1073,2174,1399,250
BECN1_7_31,183,99,305,3,1,901,765,1,240,87,214,127
BIRC2_7_32,676,1033,334,244,272,1052,188,2837,1196,536,1404,429
BIRC6_7_33,0,1285,795,158,590,862,0,685,435,1220,478,482
BIRC8_7_34,520,922,285,1547,2564,614,1083,1498,2198,2166,1929,1534
BMI1_7_35,10,5,353,798,700,420,263,979,336,349,563,691
BRAP_7_36,1111,76,0,149,2022,15,47,283,130,1537,1,342
BRD1_7_37,1,441,620,0,188,198,0,9,795,631,671,975
BRPF3_7_38,38,367,133,4,214,0,56,2,0,0,25,331
BTBD2_7_39,62,10,361,573,162,0,170,94,208,150,10,726
BTBD6_7_40,1,34,2,0,170,146,18,0,637,36,2563,559
CAND1_7_41,1065,916,2064,1478,1254,1928,1944,3647,2249,2711,1444,3776
CBLB_7_42,58,0,0,32,0,0,0,12,0,0,54,1027
CBL_7_43,936,560,1038,890,865,664,676,620,671,733,39,367
CBLL1_7_44,91,62,979,230,152,4,253,56,12,686,55,459
CCIN_7_45,0,16,106,0,89,100,274,0,1,471,31,108
CCNF_7_46,0,0,227,11,0,32,0,3,277,4,956,37
CDC20_7_47,96,60,143,0,0,504,0,0,0,256,537,1
CDC23_7_48,0,0,0,0,0,0,0,0,0,0,0,0
CDC26_7_49,58,10,40,73,138,289,28,756,35,70,68,58
```

CDC34_7_50,19,780,613,373,627,500,0,663,74,453,1088,35
CGRRF1_7_51,1802,599,1554,1384,1999,854,811,1733,2174,1325,2735,1814
CHD4_7_52,722,46,0,73,0,3,0,586,0,23,0,353
CIAO1_7_53,295,838,28,24,4,1303,1611,1416,1131,238,610,311
COPS4_7_54,1035,514,650,959,784,276,2280,286,267,365,81,1221
COPS5_7_55,890,439,332,393,380,324,964,534,401,378,0,759
COPS6_7_56,206,271,831,35,241,240,383,839,399,1270,1376,1491
COPS7B_7_57,6,425,83,231,473,1016,74,574,606,182,176,67
CORO6_7_58,1159,19,314,0,208,2,1,0,255,6,7,31
CUEDC1_7_59,33,25,0,76,67,176,6,2,171,7,0,159
CUL1_7_60,1072,1585,675,277,1904,2838,1022,1814,2026,2798,1108,1756
CUL3_7_61,557,1,47,97,1988,112,407,1062,298,386,3,515
CUL5_7_62,2787,1307,1306,1594,3737,2056,2820,2441,1748,899,3276,2177
CUL9_7_63,2393,695,886,1095,1659,952,297,1063,1109,1466,997,665
DCAF10_7_64,92,943,43,14,226,752,8,246,340,568,569,1176
DCAF12_7_65,582,175,45,1974,1928,2828,90,225,390,1616,615,2450
DCAF13_7_66,359,871,367,103,234,1473,455,961,256,549,971,691
DCAF16_7_67,0,6,0,1,135,31,324,340,2,1002,0,3
DCAF5_7_68,67,96,200,0,306,246,7,265,293,858,109,348
DCAF7_7_69,1256,1165,2533,341,1037,22,224,279,694,1035,843,1205
DCAF8_7_70,368,0,181,0,0,1,213,432,28,0,125,1
DDA1_7_71,236,144,34,407,131,194,156,666,76,147,752,241
DDB1_7_72,0,0,0,19,352,0,378,185,148,63,0,19
DDB2_7_73,44,56,0,0,0,0,6,79,31,152,0,3
DPF2_7_74,398,493,2,858,596,235,1497,356,996,42,726,120
DTX1_7_75,0,0,170,3,0,5,0,96,0,0,6,3
DTX3L_7_76,2,1131,364,732,1,101,904,156,62,8,377,169
DTX4_7_77,48,0,1152,0,1,9,1,0,517,8,924,836
DZIP3_7_78,1090,412,5,47,53,116,947,90,1072,1061,1583,1313
EIF3D_7_79,341,1,56,1,261,740,0,20,34,392,1851,230
EIF3E_7_80,206,249,341,136,685,249,490,386,561,561,242,1118
EIF3F_7_81,0,398,0,408,0,565,46,779,67,365,0,1020
EIF3G_7_82,0,4,619,94,4,204,116,122,12,22,28,416
EIF3H_7_83,382,1418,814,1238,857,428,988,1067,1408,1346,1093,396
EIF3I_7_84,193,71,387,0,57,601,151,0,56,3,255,229
EIF3J_7_85,142,562,19,237,9,339,0,96,92,516,42,60
EIF3K_7_86,131,1,450,76,459,19,231,120,51,43,33,222
ENC1_7_87,2156,2652,2000,1548,1462,1393,3292,2029,2894,1934,2681,4759
EPN3_7_88,892,185,455,720,0,385,1061,182,39,92,315,146
EPOR_7_89,932,397,418,13,5,38,1,810,350,407,10,430
ERCC8_7_90,0,43,29,611,133,287,0,70,45,4,223,4
FAF1_7_91,1684,5086,2878,2018,4532,1602,1048,1977,3031,2017,4367,3383
FBXL12_7_92,446,0,0,0,0,0,0,0,0,0,0,0
FBXL14_7_93,596,610,352,803,583,222,0,351,631,1021,78,497
FBXL15_7_94,9,0,931,0,113,19,0,2,37,0,590,19
FBXL16_7_95,34,2,19,27,140,6,2,142,38,5,115,1855
FBXL17_7_96,323,201,5,224,51,291,1000,821,774,791,117,350
FBXL18_7_97,0,102,586,1,0,0,67,4,0,0,5,0
FBXL19_7_98,0,6,0,46,0,299,0,106,756,258,753,297
FBXL21_7_99,1912,1221,692,1453,2699,1763,264,630,1696,1205,607,2397

FBXL3_7_100,2113,1564,3512,1523,1324,1895,1058,4156,1277,2905,3365,283
6
FBXL4_7_101,1367,1810,3114,1603,1482,3573,1811,1832,814,2431,5271,187
FBXL7_7_102,11,411,858,0,387,0,0,5,0,0,910,0
FBXO10_7_103,403,15,153,153,236,93,1461,389,276,13,1611,308
FBXO16_7_104,645,1497,299,475,820,950,1,697,114,994,1142,16
FBXO27_7_105,0,307,1,584,0,0,0,1321,0,0,0,0
FBXO2_7_106,85,84,0,0,40,46,852,14,47,14,109,0
FBXO30_7_107,0,0,55,7,1,622,8,0,446,433,263,524
FBXO33_7_108,1,174,181,0,0,275,258,93,198,28,96,1275
FBXO36_7_109,22,1,342,29,527,216,73,361,23,15,0,4
FBXO39_7_110,58,0,0,0,0,186,337,26,677,13,0,78
FBXO40_7_111,174,554,979,351,136,653,195,786,1130,762,877,151
FBXO41_7_112,23,1,0,10,27,1,86,231,112,17,970,28
FBXO42_7_113,468,1395,957,1185,462,1094,1480,1233,252,441,992,622
FBXO43_7_114,617,2309,902,319,41,1056,6,2934,1271,924,1278,1687
FBXO46_7_115,14,330,52,189,126,21,64,795,173,21,192,1114
FBXO6_7_116,168,624,0,44,0,0,0,46,129,20,459,335
FBXO8_7_117,307,122,903,183,864,619,20,2495,1086,1848,1122,298
FBXW10_7_118,643,67,750,829,678,356,109,42,255,25,17,194
FBXW2_7_119,2742,1097,2105,2240,2293,1954,1166,1920,1100,1222,2995,140
6
FBXW5_7_120,216,0,0,594,0,16,0,0,66,154,0,27
FBXW9_7_121,0,0,0,0,0,0,0,2,0,1,0,2
G2E3_7_122,6,1533,138,399,674,217,1307,742,464,783,561,1382
GAN_7_123,1429,754,8,117,4,1044,143,1619,708,2231,376,118
GMCL1_7_124,0,0,0,9,58,0,2,0,0,9,0,0
GNB2_7_125,349,38,167,59,9,2,100,457,35,32,51,326
GRWD1_7_126,0,0,0,0,0,0,0,0,0,0,7,0
GZF1_7_127,1,91,0,90,2,16,0,1,23,0,199,2
HACE1_7_128,14,120,254,74,0,539,332,0,44,6,491,10
HDAC6_7_129,2718,660,3077,1670,659,994,2541,1075,961,1244,2024,1541
HECTD1_7_130,2,244,0,222,71,179,0,312,519,20,8,120
HECTD3_7_131,61,43,761,0,61,8,1,0,9,94,1298,839
HECW1_7_132,12,1,534,0,995,0,965,281,0,1,0,511
HECW2_7_133,87,0,1482,65,0,0,103,0,67,2,0,204
HERC1_7_134,176,294,1185,1220,1164,1697,2425,90,604,494,539,897
HERC2_7_135,0,0,2,61,557,20,0,0,218,7,0,24
HERC3_7_136,326,0,0,0,0,31,0,3,0,440,452,0
HERC5_7_137,1548,3789,5334,589,2261,2913,2064,2075,1338,1112,4009,1864
HGS_7_138,1,5,25,9,0,31,7,64,11,243,466,28
HIC2_7_139,1118,564,57,16,0,0,1,0,0,0,0,523
HUWE1_7_140,0,0,0,0,0,0,0,96,0,462,1034,0
IBTK_7_141,1342,39,542,1202,140,222,784,2029,1185,1280,1419,2981
IL10RA_7_142,419,107,1,493,100,521,410,491,800,552,62,961
IL6_7_143,614,1739,742,49,741,514,1927,1035,1456,551,2184,756
IRF9_7_144,0,0,0,1,1,8,0,837,1,0,50,286
ITCH_7_145,20,905,3,96,912,32,0,563,19,606,242,600
IVNS1ABP_7_146,129,0,0,0,19,0,0,1,0,0,872,0
JHDM1D_7_147,13,488,2,17,6,406,604,41,272,428,4,21

JOSD1_7_148,742,543,62,87,0,94,7,72,595,8,528,882
JOSD2_7_149,0,0,1,1,0,1,0,1422,11,86,385,9
KAT6B_7_150,459,766,1060,493,21,1242,1183,2078,352,358,1112,732
KATNB1_7_151,0,0,855,0,0,0,1,0,75,83,0,10
KBTBD10_7_152,1016,55,695,200,104,499,913,218,703,675,422,232
KBTBD11_7_153,0,0,0,443,0,401,0,329,0,1,977,0
KBTBD2_7_154,529,322,960,348,179,716,143,211,193,179,162,1629
KBTBD5_7_155,652,123,0,27,8,339,160,63,78,231,332,213
KBTBD7_7_156,1641,23,33,0,166,273,511,147,16,622,329,640
KBTBD8_7_157,440,876,1481,1220,795,1301,236,966,489,1107,313,932
KCTD10_7_158,2388,1250,3211,2323,2942,2071,1689,2392,3661,1382,3287,40
96
KCTD11_7_159,607,0,793,0,302,2,5,44,1,394,2,142
KCTD12_7_160,5,0,0,8,0,26,0,0,2,57,0,0
KCTD13_7_161,615,0,173,1,15,682,0,0,2,507,1167,2
KCTD16_7_162,19,75,0,37,111,168,75,0,81,46,39,3
KCTD17_7_163,490,0,42,0,0,24,36,31,478,242,470,90
KCTD18_7_164,66,0,14,49,209,710,146,24,8,12,184,211
KCTD3_7_165,286,731,350,851,1175,1603,1476,4147,2150,686,504,310
KCTD5_7_166,157,0,7,0,16,23,36,340,475,170,472,856
KCTD9_7_167,117,983,200,346,924,332,339,76,461,365,781,641
KDM2A_7_168,3,0,0,44,55,3,0,778,0,7,0,0
KDM4B_7_169,0,0,0,5,1159,240,29,216,295,6,0,30
KDM5B_7_170,805,294,1324,756,1279,178,96,250,1234,976,1579,445
KLHDC5_7_171,505,40,14,4,0,163,46,2,180,86,35,111
KLHL10_7_172,5,0,0,6,0,58,0,863,0,8,521,0
KLHL11_7_173,585,3017,1627,1456,394,677,880,810,1052,560,3196,1355
KLHL12_7_174,0,441,27,508,813,1011,547,258,914,558,6,439
KLHL14_7_175,124,262,2,0,0,413,0,557,97,82,735,17
KLHL15_7_176,349,81,0,104,244,8,987,52,82,25,0,7
KLHL17_7_177,24,878,21,8,19,3,0,478,12,70,1,54
KLHL18_7_178,628,226,0,191,1801,71,2008,61,22,45,976,612
KLHL1_7_179,2184,2223,1742,3481,72,4528,2980,1119,3355,3407,3448,4042
KLHL20_7_180,188,6,5,107,123,51,155,173,174,5,501,174
KLHL21_7_181,0,439,0,3,3,0,345,56,36,290,217,9
KLHL22_7_182,858,979,351,67,10,93,84,737,58,477,1382,308
KLHL23_7_183,527,208,1,1635,714,153,676,183,0,273,301,321
KLHL24_7_184,4,0,50,160,0,303,0,7,0,559,87,452
KLHL25_7_185,0,1,1,0,244,0,589,0,20,94,1,2
KLHL26_7_186,0,3,0,15,0,2,0,0,131,1,0,14
KLHL28_7_187,96,450,709,1,493,35,831,289,869,472,46,426
KLHL29_7_188,1,13,821,629,0,892,35,462,113,206,0,781
KLHL31_7_189,66,2,0,42,0,170,0,0,68,4,0,15
KLHL32_7_190,928,300,96,883,768,1516,589,1489,1151,1142,1307,1141
KLHL33_7_191,1069,683,2607,73,887,562,96,1432,430,10,8,477
KLHL34_7_192,1,0,1,45,45,0,0,623,509,94,1,70
KLHL36_7_193,19,0,110,0,0,513,4,2,588,518,11,463
KLHL3_7_194,63,129,106,268,0,198,0,166,204,160,0,27
KLHL8_7_195,342,296,1041,342,127,273,940,99,50,359,1682,462
LATS1_7_196,616,464,426,1517,698,619,281,824,169,332,146,646

LATS2_7_197,177,2,0,238,34,209,405,119,92,17,0,835
LIF_7_198,229,82,19,172,108,49,21,12,0,605,312,109
LNX2_7_199,699,80,943,452,440,24,415,105,580,123,413,1680
LOC283116_7_200,1080,347,492,1157,651,277,10,2207,471,311,1047,1828
LONRF1_7_201,1043,511,663,254,870,891,2,846,200,444,182,383
LTN1_7_202,21,675,1,435,82,5,512,560,555,956,123,230
LZTR1_7_203,212,403,15,180,0,725,778,298,4,647,259,2051
MAP1LC3B_7_204,0,1,0,331,55,0,146,5,716,9,12,90
MAP3K1_7_205,4,2,107,32,19,41,8,0,168,35,0,142
MARK1_7_206,8200,10479,6809,8507,6051,11362,7780,8044,9872,8878,7675,1
1621
MDM2_7_207,66,67,214,886,1794,620,1582,485,553,90,871,603
MED20_7_208,753,599,1190,607,213,218,48,1103,108,619,404,276
MEX3B_7_209,2,9,20,44,8,29,4,39,151,2,10,109
MIB1_7_210,1146,294,1564,421,366,466,1716,1419,1234,2137,564,1404
MKRN2_7_211,93,1391,769,17,645,484,717,1912,415,432,949,69
MKRN3_7_212,113,262,407,168,517,0,16,169,60,39,287,14
MLL2_7_213,977,1,0,607,0,974,827,126,12,1,905,169
MLLT6_7_214,0,1484,1036,199,961,1010,863,575,949,1408,2064,1852
MOCS3_7_215,92,237,440,543,2,433,1035,689,221,571,1059,92
MRPL49_7_216,256,16,567,51,0,316,0,147,178,783,72,26
MUL1_7_217,913,1061,843,104,152,72,998,0,430,64,773,46
MYCBP2_7_218,1273,2474,3759,3586,2347,2391,1918,1353,1005,2853,1500,28
45
MYLIP_7_219,2,270,47,791,905,706,12,149,433,179,884,1030
MYSM1_7_220,242,338,374,93,246,792,1098,537,387,183,1413,1117
NACC1_7_221,1340,8,1,304,253,785,0,0,16,1,352,488
NACC2_7_222,0,282,0,1,1,304,122,0,490,11,887,80
NEURL1B_7_223,0,0,0,0,0,0,0,0,0,0,0,0
NEURL_7_224,1701,1757,1004,207,1139,1311,261,1579,1057,776,34,208
NHLRC1_7_225,2127,1293,497,437,1158,2088,3640,2051,1569,1700,1737,1280
NUP43_7_226,573,179,1270,800,573,527,0,10,614,216,75,79
OTUB1_7_227,0,0,0,0,0,0,0,0,0,0,0,0
OTUB2_7_228,1867,1057,315,1958,678,1165,252,628,1351,563,667,836
OTUD1_7_229,1,0,401,0,0,6,0,0,0,0,0,0
OTUD6A_7_230,0,0,8,12,0,97,0,0,94,8,0,18
OTUD6B_7_231,3237,739,312,234,2525,410,2843,2026,1195,2656,733,1669
OTUD7A_7_232,16,237,116,51,0,240,0,85,3,134,8,315
OTUD7B_7_233,79,753,1,489,1462,16,294,456,41,636,26,36
PAFAH1B1_7_234,902,289,516,745,502,129,1678,1493,776,856,1249,109
PARP10_7_235,0,730,21,29,265,19,395,515,542,222,381,89
PARP11_7_236,374,310,550,515,0,26,7,8,457,100,76,45
PARP14_7_237,832,439,142,185,667,115,341,189,1034,151,1157,1017
PCGF1_7_238,324,42,56,266,271,0,10,261,47,233,625,22
PCGF2_7_239,0,0,0,0,0,0,0,456,0,0,0,0
PCGF3_7_240,52,345,1,703,995,32,0,725,835,44,654,148
PDZRN3_7_241,86,2,0,557,356,0,268,624,245,90,31,27
PEBP4_7_242,500,160,67,950,13,27,6,202,781,284,99,492
PEX12_7_243,223,66,10,56,719,178,0,65,375,611,441,964
PHF14_7_244,1181,266,1356,833,73,528,584,932,1462,1066,678,185

PHF15_7_245,1012,1388,1034,82,15,855,1606,557,87,35,412,120
PHF20_7_246,1540,458,896,435,1619,814,902,187,196,617,908,977
PHF2_7_247,28,0,13,0,267,17,309,560,78,32,0,237
PHF3_7_248,894,279,427,577,1323,978,295,453,523,705,1051,671
PHIP_7_249,965,1469,1638,349,3266,2486,944,1592,1776,4512,2080,2795
PHRF1_7_250,504,800,287,37,412,909,564,518,318,404,1008,674
PJA2_7_251,437,1414,2572,449,1332,318,578,1378,958,1556,1167,1524
PRPF19_7_252,14,58,43,329,0,6,0,0,0,0,0,0
PRPF8_7_253,25,0,0,13,25,0,121,161,31,4,18,5
PSMD14_7_254,260,341,0,0,336,5,27,883,238,138,805,110
PSMD2_7_255,55,174,302,4,861,1311,1264,596,13,858,1,169
PSMD4_7_256,0,100,2,164,3,711,0,603,0,242,20,119
PSMD7_7_257,0,0,0,220,0,406,148,0,2,30,13,253
PWP1_7_258,1196,699,559,1309,2341,508,704,379,1247,810,1649,1219
RAB40A_7_259,1559,1200,2199,1884,3442,4180,3293,1892,3357,1400,5244,32
36
RAB40AL_7_260,1559,1200,2199,1884,3442,4180,3293,1892,3357,1400,5244,3
236
RAB40B_7_261,2056,1626,1759,1161,594,675,848,1485,2445,1964,1483,1994
RAD18_7_262,492,447,335,813,1173,1763,782,2411,1473,1025,2643,3240
RAD23A_7_263,0,286,92,1152,116,0,0,0,0,981,0,4
RAD23B_7_264,1154,744,599,751,1971,98,491,509,1388,418,804,719
RAG1_7_265,555,501,465,63,108,717,1118,1131,1330,592,1543,154
RAI1_7_266,84,52,915,0,0,184,229,0,49,14,147,166
RBX1_7_267,863,1990,1431,790,50,1098,419,446,1331,645,2524,668
RCBTB1_7_268,44,40,1,44,280,617,573,70,5,292,1615,32
RCBTB2_7_269,213,875,789,590,573,646,603,458,2986,1020,1483,1347
RFFL_7_270,1358,159,2,162,85,691,0,0,259,1053,77,41
RFPL1_7_271,1367,1396,1041,241,122,637,87,1920,2283,1812,1703,845
RFWD3_7_272,46,158,22,69,0,0,49,20,339,6,489,94
RHOBTB3_7_273,578,17,16,225,225,32,10,538,78,253,122,4
RING1_7_274,513,1595,98,44,0,27,809,337,234,271,0,79
RNF10_7_275,0,0,0,0,84,0,1,0,71,77,0,358
RNF111_7_276,1,565,698,24,0,437,0,957,171,38,475,49
RNF112_7_277,136,584,143,172,1026,104,307,148,463,1870,1897,911
RNF113A_7_278,13,63,234,2,112,139,660,1,22,43,127,704
RNF113B_7_279,1,0,0,0,815,0,0,788,8,7,59,0
RNF114_7_280,8,49,0,64,457,857,0,0,1,118,1077,0
RNF115_7_281,1072,698,1288,1005,119,398,2158,1390,1015,888,812,1033
RNF11_7_282,338,833,0,657,1180,110,1,456,766,1197,745,677
RNF121_7_283,115,852,185,284,233,900,0,504,0,42,12,129
RNF122_7_284,800,2348,1574,1150,3751,2119,2748,2013,2259,2204,1484,209
6
RNF123_7_285,12,220,0,579,3,4,219,0,766,11,23,116
RNF125_7_286,8,10,44,127,0,0,0,37,41,86,0,89
RNF126_7_287,609,498,765,490,1225,909,942,746,1618,612,1303,1627
RNF130_7_288,218,253,1338,1,799,1630,113,634,1664,3314,446,1538
RNF133_7_289,375,217,124,517,0,1,689,23,82,1,117,18
RNF139_7_290,192,68,523,230,283,2,0,66,67,297,0,6
RNF141_7_291,0,70,155,0,0,0,142,117,33,6,356,232

RNF144A_7_292,0,8,70,3,0,25,0,1,0,149,0,0
RNF144B_7_293,38,84,467,31,22,7,267,0,0,60,566,336
RNF149_7_294,5,296,11,31,23,256,0,36,74,437,507,57
RNF150_7_295,661,167,926,500,279,537,2525,683,795,2126,2099,1918
RNF152_7_296,297,4,1390,3,0,147,0,852,1,102,0,0
RNF157_7_297,108,75,1173,480,519,531,101,30,1,1459,75,1075
RNF167_7_298,384,100,1047,706,2,341,40,0,880,28,324,2351
RNF168_7_299,9,93,24,178,181,354,791,1080,187,405,1470,34
RNF169_7_300,1063,930,876,696,90,796,1030,1066,453,1373,116,241
RNF181_7_301,0,0,0,0,0,0,0,0,152,3,370,12
RNF183_7_302,427,0,962,30,0,0,6,0,0,77,12,1173
RNF186_7_303,8,12,0,710,6,0,28,9,0,157,0,1
RNF187_7_304,2,26,0,0,333,5,0,134,318,307,19,50
RNF20_7_305,813,636,1642,1481,1115,628,1168,73,871,322,161,1050
RNF217_7_306,4,212,490,791,1974,99,439,1303,141,27,155,117
RNF220_7_307,57,12,142,243,277,129,27,564,81,293,7,718
RNF25_7_308,1023,391,152,100,2183,3,192,5,359,356,865,40
RNF26_7_309,23,10,105,33,696,59,0,0,0,0,0,0
RNF2_7_310,395,1281,675,187,1375,525,236,2471,590,717,1163,815
RNF31_7_311,1,485,0,432,944,183,764,1407,257,857,274,1062
RNF43_7_312,12,252,0,320,254,11,9,348,2,133,165,525
RNF44_7_313,163,192,129,293,28,651,125,542,103,319,993,774
RNF5_7_314,1626,1994,935,168,584,979,1507,1465,1008,2065,1151,1567
RSC1A1_7_315,2213,642,95,172,793,1019,121,164,420,376,1263,402
RSF1_7_316,1808,916,3596,1208,0,2797,693,320,533,1355,682,2147
RSPRY1_7_317,8,297,145,5,390,155,0,0,30,41,455,875
SCLY_7_318,0,38,1,0,0,326,0,0,0,0,32,2
SENP1_7_319,2797,1924,5564,4032,2579,1508,3276,3719,3456,3566,3857,254
8
SENP2_7_320,46,2091,935,199,118,1974,433,878,392,1608,242,888
SENP3_7_321,140,51,63,0,46,142,0,12,0,0,0,0
SENP5_7_322,456,1025,40,200,442,532,1353,1386,711,278,75,1157
SH3RF1_7_323,595,1169,1731,422,153,18,0,12,1704,1100,247,487
SH3RF2_7_324,0,128,356,113,0,145,0,4,125,64,230,140
SH3RF3_7_325,1898,2142,1342,1203,452,426,322,1017,1644,561,980,509
SHKBP1_7_326,0,29,0,5,32,4,14,169,51,25,2,7
SIAH2_7_327,531,422,657,310,321,2557,133,774,1104,1803,1134,722
SIK1_7_328,1987,63,0,27,0,78,8,34,9,328,0,4
SLX4_7_329,3015,159,5,578,237,526,767,761,917,14,1108,194
SMU1_7_330,1231,763,854,1039,20,801,4192,555,1052,1293,1109,1371
SMURF2_7_331,0,0,281,66,107,422,775,41,190,3,656,23
SNRNP40_7_332,2939,978,103,360,2389,1209,1164,0,726,909,398,482
SOCS1_7_333,986,0,977,7,0,0,511,0,0,0,1,484
SOCS2_7_334,3141,2183,2257,2063,1968,862,877,1223,1030,1980,2230,489
SOCS3_7_335,0,0,0,0,0,0,0,233,0,126,0,0
SOCS6_7_336,0,254,0,20,69,4,0,53,3,525,44,0
SPOPL_7_337,9,9,3,25,1,0,143,11,30,149,50,450
SPSB1_7_338,84,3,258,0,119,1,224,36,2,41,3,0
SPSB3_7_339,160,163,1474,542,214,1194,3,0,200,173,5,253
SPSB4_7_340,0,0,0,0,388,0,0,0,0,0,0,0

STAM2_7_341,61,162,44,138,2114,170,2,150,39,0,827,88
STAMBPL1_7_342,30,147,11,168,1550,572,736,30,734,555,162,110
STAM_7_343,2666,1795,6135,3275,1706,2491,146,2682,1914,1888,2247,2482
STUB1_7_344,0,0,0,707,0,82,0,469,0,0,0,499
SUMO3_7_345,33,1,0,23,282,0,0,0,222,13,10,26
SYNGAP1_7_346,0,0,61,0,0,19,0,1,247,10,72,47
TAB2_7_347,67,14,9,114,7,92,58,111,0,3,1155,144
TAB3_7_348,262,15,144,17,462,269,43,319,41,348,2,279
TAF1D_7_349,1064,1739,1843,463,328,1105,2587,570,806,1318,605,1048
TBC1D1_7_350,932,1055,373,1804,562,800,79,497,730,1133,2680,249
TLE1_7_351,67,0,38,101,0,41,100,0,402,541,0,992
TNFAIP3_7_352,20,830,1,182,621,13,680,33,383,119,121,84
TOLLIP_7_353,80,57,6,1841,0,0,0,0,381,663,430,52
TRAF7_7_354,0,0,8,9,2,0,0,0,241,114,2,35
TRAIP_7_355,1969,1494,1897,612,1553,296,495,2786,983,1121,711,1881
TRIM11_7_356,804,304,900,524,0,566,75,0,73,1051,0,551
TRIM15_7_357,0,0,0,0,223,0,182,0,29,22,392,1
TRIM25_7_358,0,0,6,0,0,0,0,0,5,0,0,0
TRIM27_7_359,16,0,4,0,49,0,0,21,84,205,279,13
TRIM28_7_360,65,0,31,1,0,28,0,0,705,355,84,84
TRIM31_7_361,11,2,13,658,215,110,0,12,9,0,96,0
TRIM35_7_362,116,3,16,1,2,156,37,1,13,7,0,0
TRIM42_7_363,419,302,539,183,2427,1105,25,93,714,374,534,658
TRIM46_7_364,132,580,33,48,5,0,1653,5,491,13,318,69
TRIM47_7_365,56,106,0,53,50,171,102,138,145,363,0,21
TRIM48_7_366,96,758,0,66,0,445,3,603,44,294,1016,2
TRIM52_7_367,61,1133,544,648,391,633,107,41,482,202,788,1288
TRIM56_7_368,328,5,0,221,1095,204,642,1,18,78,10,87
TRIM62_7_369,20,37,0,195,4,0,33,1,8,772,26,332
TRIM63_7_370,592,1,0,0,155,247,60,0,4,0,195,709
TRIM65_7_371,0,624,0,258,3,33,3,31,874,121,656,110
TRIM67_7_372,44,42,80,143,68,39,253,320,82,73,547,52
TRIM68_7_373,0,0,857,11,1,0,0,30,6,1,0,0
TRIM8_7_374,681,3,169,46,1,505,254,468,1256,761,752,395
TRIP12_7_375,684,945,1437,1308,1296,988,1254,180,935,508,744,398
UBA2_7_376,390,642,438,73,809,381,730,665,9,159,27,267
UBA6_7_377,26,726,582,832,75,1,445,916,806,114,1252,92
UBA7_7_378,7,1,63,548,2,721,1,684,0,0,168,802
UBAC1_7_379,52,2,725,5,4,250,6,834,311,9,6,122
UBAP2_7_380,276,1669,122,568,13,427,17,455,160,306,42,937
UBASH3B_7_381,0,0,250,0,0,221,827,0,0,0,0,1
UBC_7_382,10,0,0,0,10,28,0,0,0,0,323,0
UBE2B_7_383,471,1614,1565,890,471,855,137,36,334,447,384,985
UBE2D4_7_384,183,26,727,98,421,424,39,526,584,137,38,352
UBE2E2_7_385,2172,2278,38,162,24,1068,1004,1096,242,987,0,375
UBE2F_7_386,1274,226,1533,17,2180,320,350,721,280,257,449,1121
UBE2G1_7_387,2724,1673,1307,2536,2066,1903,1377,2173,1944,4413,2803,21
13
UBE2J1_7_388,3374,796,1035,1920,4452,3362,2608,2707,3972,5755,3318,253
7

UBE2L3_7_389,564,1561,189,1159,1058,314,590,2533,297,1234,1118,1174
UBE2M_7_390,2376,556,620,844,1224,922,692,872,533,1318,5,406
UBE2N_7_391,83,335,143,48,34,58,209,222,146,126,1074,816
UBE2NL_7_392,83,335,143,48,34,58,209,222,146,126,1074,816
UBE2O_7_393,0,0,2,0,43,0,0,0,0,0,736,0
UBE2Q1_7_394,333,63,396,402,267,198,53,74,423,66,0,32
UBE2QL1_7_395,3,0,0,0,745,77,1546,0,372,10,2,34
UBE2R2_7_396,94,241,160,9,916,392,53,20,0,75,351,717
UBE2S_7_397,228,0,1,289,197,5,519,1,67,37,0,26
UBE2T_7_398,2508,1085,1470,866,456,804,1173,1710,702,1027,1947,1982
UBE2U_7_399,1617,2297,1630,485,724,639,849,667,1229,499,858,3665
UBE2V2_7_400,565,401,3615,57,78,1336,1807,1149,978,322,1451,142
UBE2Z_7_401,446,222,895,239,1348,1260,652,538,393,518,469,1913
UBE3C_7_402,111,458,1474,311,1454,814,749,348,294,415,185,153
UBQLN2_7_403,1078,610,325,172,340,487,234,28,29,275,67,230
UBQLN3_7_404,292,34,0,0,1,0,0,7,613,11,661,67
UBQLN4_7_405,341,447,77,31,1197,256,121,266,457,320,37,456
UBR1_7_406,5,127,8,59,111,240,1,439,119,219,777,1657
UBR3_7_407,1592,1929,3224,1111,1708,1591,1070,1600,1057,2220,1319,1772
UBR4_7_408,463,1221,354,719,848,888,547,1320,797,1915,2124,1519
UBR5_7_409,632,0,25,9,0,0,127,623,172,1015,293,149
UBR7_7_410,1901,339,705,539,1448,842,585,272,1074,69,705,386
UBXN10_7_411,289,95,224,1,0,69,4,65,259,66,108,136
UBXN1_7_412,1649,1112,1192,605,1190,194,407,153,537,688,1311,402
UBXN2A_7_413,339,600,2716,597,316,778,1369,301,184,241,547,1973
UBXN4_7_414,750,139,425,33,595,397,11,349,102,9,114,26
UBXN7_7_415,0,1,0,1,1,7,469,3,0,175,385,278
UBXN8_7_416,400,1298,3933,870,369,1778,2270,1703,66,44,525,993
UCHL1_7_417,1837,1445,697,1581,1115,1075,215,829,1291,1010,803,1596
UCHL3_7_418,6,0,1672,0,17,250,2114,0,152,1148,346,24
UFC1_7_419,1,826,152,1039,78,48,1,220,134,1056,720,891
UHRF2_7_420,1618,172,1458,776,1765,1292,581,1466,1985,1472,3440,1769
UNK_7_421,129,804,591,189,3,654,261,0,533,178,790,816
USP11_7_422,0,11,0,49,0,95,0,430,69,117,0,7
USP13_7_423,4,21,31,0,0,381,0,1204,26,336,604,4
USP15_7_424,972,897,841,1007,779,181,527,1841,336,1176,454,1531
USP17_7_425,255,4,269,173,470,154,14,209,451,18,1282,73
USP17L2_7_426,255,4,269,173,470,154,14,209,451,18,1282,73
USP17L5_7_427,255,4,269,173,470,154,14,209,451,18,1282,73
USP18_7_428,568,0,67,16,18,154,32,586,0,143,0,23
USP22_7_429,1621,1374,420,1719,987,776,367,1255,1412,1050,1168,1537
USP24_7_430,87,24,3,19,12,58,904,182,387,500,173,131
USP25_7_431,1048,243,0,602,211,0,556,56,89,8,0,7
USP26_7_432,95,560,749,1001,240,404,352,1323,3,252,1180,157
USP27X_7_433,1117,1024,124,512,682,647,597,536,909,68,648,2293
USP28_7_434,699,1798,691,865,281,2632,4051,2497,1561,1272,2429,778
USP29_7_435,1754,716,1658,1934,357,231,962,419,1752,733,294,798
USP30_7_436,276,331,18,2,29,582,23,708,0,111,315,268
USP32_7_437,777,1250,499,1107,1343,1857,206,115,1249,1523,1211,1106
USP34_7_438,844,775,34,1315,0,1402,166,17,980,36,283,313

USP35_7_439,484,498,73,605,129,76,14,0,64,0,198,90
USP36_7_440,552,324,419,68,155,28,0,100,0,1,279,0
USP37_7_441,32,337,10,195,559,0,17,405,238,400,86,261
USP38_7_442,1150,2904,1244,3367,2460,2624,2405,2428,2409,2720,3400,155
2
USP39_7_443,174,19,161,36,0,177,0,0,1,8,0,0
USP3_7_444,1261,2151,2603,4814,4942,3066,1803,3071,2078,960,2628,1081
USP40_7_445,61,494,6,972,257,2131,1110,403,412,787,532,179
USP42_7_446,0,0,0,0,2,0,0,0,0,321,714,1
USP43_7_447,55,0,434,72,842,0,911,1,362,1167,2,1435
USP45_7_448,13,1895,370,42,5,117,19,424,250,194,18,277
USP47_7_449,1797,1520,2589,2615,386,841,2150,1291,1543,1961,2079,905
USP49_7_450,859,59,334,1,0,259,77,309,886,93,493,334
USP50_7_451,325,8,3,94,174,0,40,934,103,80,0,441
USP51_7_452,304,0,165,121,400,42,4,0,168,907,37,55
USP53_7_453,1272,232,327,7,214,982,873,268,11,114,414,717
USP54_7_454,798,687,1044,1761,2267,2114,736,1603,1705,1597,2061,1764
USP6_7_455,593,193,28,446,646,41,0,4,190,19,94,368
USP7_7_456,1594,854,605,1075,1230,1152,1199,1664,2203,1581,2314,1062
USP9Y_7_457,1882,1708,1890,1921,1912,1774,1935,2524,3398,3183,3419,448
9
USPL1_7_458,101,11,256,92,1032,636,3,243,611,115,439,574
VCPIP1_7_459,1692,712,178,313,466,1168,814,1045,352,814,696,310
VPS11_7_460,168,114,1586,59,536,316,38,654,1458,465,545,878
WDR12_7_461,573,881,106,2039,800,726,1344,3069,1109,1053,1488,2366
WDR53_7_462,531,16,751,1218,484,352,1101,1214,285,90,683,544
WDR59_7_463,16,639,535,99,2198,3,1,894,65,455,1055,1324
WDR5B_7_464,0,24,0,70,422,55,157,287,0,258,67,1
WDR61_7_465,190,350,135,110,0,831,307,171,403,343,0,268
WDTC1_7_466,0,76,0,0,0,0,0,12,0,0,1,0
WSB2_7_467,2093,301,194,231,1505,1974,52,506,1206,131,865,164
WWP1_7_468,3111,2347,5411,2651,2065,1442,1413,1408,3343,2936,2014,1919
YOD1_7_469,21,0,10,0,24,219,14,56,452,108,9,62
ZBTB11_7_470,1661,1241,1155,1649,1461,838,787,868,995,1245,987,265
ZBTB25_7_471,320,62,0,8,2,9,458,141,124,71,225,170
ZBTB2_7_472,25,600,155,740,1018,767,2,503,447,377,747,238
ZBTB32_7_473,0,13,0,0,0,0,0,59,16,0,0,0
ZBTB34_7_474,0,18,32,0,109,1,2,0,56,192,0,58
ZBTB39_7_475,312,549,772,336,784,5,409,317,1327,960,9,173
ZBTB3_7_476,0,69,0,5,1,49,0,0,20,0,497,2
ZBTB41_7_477,696,819,1278,627,1990,1276,1927,308,925,3531,929,1209
ZBTB44_7_478,195,822,1078,713,183,144,207,670,674,1172,705,1470
ZBTB45_7_479,515,555,1,0,0,187,2468,226,82,212,2,9
ZBTB46_7_480,12,868,54,1,236,260,1,44,67,237,497,307
ZBTB47_7_481,0,222,68,0,2,179,0,4,135,630,125,1256
ZBTB48_7_482,5,8,8,63,60,30,562,1,0,0,15,4
ZBTB49_7_483,2275,885,1046,948,435,1876,392,2563,2298,981,1346,1604
ZBTB5_7_484,13,392,15,207,0,17,25,46,514,87,162,76
ZBTB7A_7_485,802,738,0,226,255,0,69,4,197,639,145,609
ZBTB7B_7_486,0,0,12,2,440,153,9,41,573,443,887,72

ZBTB7C_7_487,246,0,28,553,27,137,79,91,106,396,21,136
ZBTB8A_7_488,187,3,33,690,164,1311,708,340,1149,653,651,262
ZFAND2B_7_489,74,38,197,99,12,241,45,1,78,133,101,37
ZFPL1_7_490,0,0,113,14,41,21,0,187,0,39,33,21
ZMYND10_7_491,32,0,962,42,48,0,88,0,496,731,0,915
ZNF131_7_492,769,2572,786,1427,2,679,304,1080,1043,277,801,873
ZNF598_7_493,117,117,7,640,24,292,18,1032,61,568,207,75
ZNF645_7_494,389,159,287,48,127,242,1090,1263,2,156,212,155
ZNRF1_7_495,6,25,1236,409,47,78,53,292,316,644,972,592
ZNRF2_7_496,9,38,36,0,322,174,237,603,189,652,485,207
ZNRF4_7_497,3,14,56,291,95,14,42,12,17,16,0,0
ZRANB1_7_498,721,38,0,190,11,276,396,1634,544,86,20,959
ZSWIM2_7_499,1471,2235,2601,3821,183,538,1250,3128,526,1238,1374,1429
AIRE_7_500,1,2,317,597,509,0,2,157,0,90,714,270
ANAPC5_7_501,83,384,866,61,0,30,6,12,965,41,712,141
ANAPC7_7_502,161,571,4,526,1721,688,7,1335,833,849,146,254
ANKFY1_7_503,61,385,35,165,346,20,147,399,371,88,190,281
ASB10_7_504,457,1142,1305,141,734,1129,1323,1,842,42,23,465
ASB11_7_505,363,65,56,0,523,73,258,64,76,274,25,15
ASB14_7_506,445,15,13,0,329,78,40,2,20,88,134,9
ASB2_7_507,0,0,0,0,657,185,0,85,0,0,1,0
ASB3_7_508,353,437,895,702,1149,510,1659,13,1258,277,1848,167
ASB4_7_509,0,230,1,0,1,27,59,583,0,0,3,0
ASB6_7_510,0,4,11,0,0,0,0,0,10,0,3,0
ASB7_7_511,76,325,0,558,651,300,869,13,334,118,4,41
ASB9_7_512,49,0,0,0,0,20,688,320,573,108,0,78
ASCC2_7_513,1533,11,0,262,512,344,132,81,13,74,884,246
ATG10_7_514,2236,0,27,327,0,1137,2,1139,1602,812,1967,339
ATG16L1_7_515,436,0,0,0,183,0,0,1105,98,491,1230,799
ATG7_7_516,883,363,1,213,0,11,759,7,800,193,2,188
ATRX_7_517,1266,832,1501,1620,1537,2167,326,638,1186,2051,2198,2680
BACH2_7_518,518,7,688,258,89,665,491,0,616,215,323,118
BAG6_7_519,683,23,488,250,679,195,26,35,360,537,232,492
BAZ1A_7_520,3179,1871,1031,3635,1073,796,1527,970,2900,1197,1324,1865
BCL6_7_521,894,1419,121,508,3,1475,320,190,261,46,9,482
BIRC3_7_522,10,91,1373,409,379,975,1210,1162,797,1071,1268,149
BIRC7_7_523,219,659,109,53,143,76,736,185,596,13,290,351
BPTF_7_524,1041,948,1084,107,228,819,55,490,757,488,570,840
BRCA1_7_525,335,5,0,279,7,238,5,103,249,297,576,35
BRPF1_7_526,2955,2188,2875,1037,2572,2377,929,3457,2039,3909,1916,1100
BRWD1_7_527,112,57,803,1168,1843,484,1578,373,2,65,155,80
BTBD11_7_528,1062,97,793,1174,2,953,687,196,255,349,594,327
BTBD1_7_529,34,846,2,0,278,794,1038,63,395,94,768,218
BTBD3_7_530,12,419,11,244,680,82,47,5,407,5,815,1364
BTBD7_7_531,0,0,0,239,0,0,0,65,213,9,0,329
BTBD9_7_532,1616,110,175,466,1925,832,1367,471,514,1287,617,276
BTRC_7_533,162,455,4,3,472,645,0,3,1022,297,1642,205
C3orf26_7_534,739,1,1528,874,1015,175,327,131,476,732,112,984
CBLC_7_535,1315,618,0,16,0,157,352,39,9,84,0,17
CCNB1IP1_7_536,23,0,2,15,0,92,8,11,61,1,9,42

CDC16_7_537,830,245,1783,531,3564,558,37,500,709,467,1484,163
CDC27_7_538,0,242,304,213,66,125,0,42,143,466,0,172
CHFR_7_539,398,456,36,15,20,246,55,5,455,9,67,527
CISH_7_540,177,198,0,340,252,73,965,678,441,624,39,58
CNOT4_7_541,773,1094,2,55,275,8,142,452,672,733,2,722
COPS2_7_542,5403,6178,3223,1870,2173,4851,3244,4377,5628,4931,6474,282
8
COPS3_7_543,1917,1851,781,1831,2280,1710,3802,1910,952,1475,2534,1326
COPS7A_7_544,162,67,69,268,1,74,173,11,369,6,370,793
COPS8_7_545,421,1101,24,240,164,381,73,0,29,24,2,76
CRBN_7_546,98,1371,7,0,3718,360,842,186,926,433,62,379
CUL2_7_547,288,1059,812,627,226,150,133,1341,550,1428,586,1405
CUL4A_7_548,847,868,471,1259,1358,346,790,194,284,374,686,695
CUL4B_7_549,1023,417,3211,2006,2495,663,112,1791,2006,2372,938,1425
CUL7_7_550,1854,1372,470,1379,1452,1042,1123,1385,300,134,1146,930
CXXC1_7_551,4,16,0,6,39,51,1,4,29,2,1,6
CYLD_7_552,517,1976,237,610,2303,760,32,972,1442,1152,599,1377
DCAF11_7_553,401,607,106,1074,4,950,149,8,525,136,388,1935
DCAF17_7_554,346,768,167,338,422,171,1203,853,1451,724,273,297
DCAF4_7_555,0,0,0,115,0,24,0,0,20,0,0,4
DCAF6_7_556,619,177,603,312,128,1916,0,18,926,522,228,526
DCST1_7_557,463,940,22,406,262,67,1041,1056,743,1021,840,655
DEPDC1B_7_558,1641,895,725,199,1256,749,578,701,288,874,58,1210
DET1_7_559,26,85,38,130,231,0,858,1406,1,4,116,0
DIDO1_7_560,102,158,17,0,93,25,0,0,0,1,0,0
DNAJB2_7_561,161,25,98,271,0,3,79,87,2,0,3,0
DTX2_7_562,35,110,0,600,3,9,0,0,0,0,0,0
EED_7_563,16,3,0,4,0,32,0,0,495,531,6,811
EIF3B_7_564,220,0,14,0,0,1102,612,3,197,63,0,38
EIF3C_7_565,69,15,1057,2,0,224,229,272,1,832,84,169
EIF6_7_566,291,0,0,10,0,76,0,31,0,31,0,106
EPN1_7_567,0,0,0,0,204,0,0,23,0,0,179,20
EPN2_7_568,391,34,16,62,26,392,331,387,15,691,10,24
EPS15_7_569,1366,0,269,0,869,0,0,0,107,1,272,660
FAM70A_7_570,603,140,66,354,27,259,570,653,22,1,737,193
FANCL_7_571,1300,1537,1783,1170,1914,213,617,2002,1582,1361,424,1993
FBXL13_7_572,1382,2159,1638,303,245,436,2983,1746,1187,215,408,1031
FBXL20_7_573,387,334,1077,37,185,844,885,203,299,375,33,445
FBXL2_7_574,69,1219,400,118,138,388,1217,1976,739,832,833,481
FBXL5_7_575,49,64,592,448,20,94,101,367,13,12,777,227
FBXL6_7_576,0,61,8,0,0,0,46,0,488,43,223,95
FBXO11_7_577,3833,1919,2652,4110,2095,2656,4410,2998,3649,2719,4534,25
77
FBXO15_7_578,1772,108,390,948,164,926,613,173,1252,1380,647,614
FBXO17_7_579,0,0,0,0,0,473,0,0,7,0,1,3
FBXO18_7_580,924,1276,1869,771,423,2667,2377,1832,803,1196,2522,227
FBXO21_7_581,793,24,167,753,144,613,64,206,1712,197,771,1727
FBXO22_7_582,282,976,680,653,800,1109,363,246,1427,238,1946,1393
FBXO24_7_583,12,1151,337,297,1669,109,183,531,418,502,2,998
FBXO25_7_584,347,154,489,12,0,284,114,701,534,280,421,340

FBXO28_7_585,258,204,331,12,1110,334,0,32,192,86,415,28
FBXO32_7_586,604,672,318,296,785,719,33,796,428,43,398,536
FBXO34_7_587,339,579,328,120,190,147,7,170,59,669,47,141
FBXO38_7_588,280,533,0,165,408,429,1508,140,897,689,173,1229
FBXO3_7_589,2956,695,3727,1615,1021,1260,214,1340,799,256,3012,1117
FBXO44_7_590,693,720,14,0,257,344,14,1318,185,343,573,700
FBXO4_7_591,385,713,47,112,3156,0,0,21,88,237,561,599
FBXO5_7_592,963,23,313,537,0,17,485,231,522,35,112,54
FBXO7_7_593,38,86,47,0,861,10,75,106,158,740,303,132
FBXO9_7_594,19,298,60,433,57,20,400,283,207,527,24,67
FBXW11_7_595,625,16,133,517,0,168,29,107,0,251,774,1
FBXW7_7_596,463,27,76,1,0,292,143,324,61,284,290,6
FBXW8_7_597,179,136,231,218,1786,416,332,129,306,231,555,618
GPS1_7_598,742,60,283,520,709,1,168,19,211,402,0,66
HECTD2_7_599,1693,849,611,669,406,1261,106,2499,735,1003,1614,723
HERC4_7_600,54,0,46,106,36,397,0,4,526,257,115,1082
HERC6_7_601,1043,1795,1685,578,1223,586,664,104,759,306,1303,1811
HIC1_7_602,136,118,0,0,0,1,0,192,0,0,0,645
HLTF_7_603,931,1270,2731,1239,1532,1820,1456,3331,1058,927,2892,1423
HSF4_7_604,0,128,272,0,1,10,739,248,582,8,278,207
IPP_7_605,674,360,616,545,296,446,550,24,2039,614,830,2449
KAT6A_7_606,416,688,303,1439,898,40,89,456,97,778,9,178
KBTBD3_7_607,2171,1423,517,1702,547,3186,3398,3910,1061,2465,1298,1386
KCTD6_7_608,670,0,4,6,312,30,620,20,201,8,8,76
KCTD7_7_609,18,6,23,41,233,565,198,23,75,296,132,7
KDM2B_7_610,400,155,246,119,1249,507,998,680,158,1731,34,1235
KDM4C_7_611,32,1151,2,0,767,501,363,0,110,1030,0,12
KDM5C_7_612,414,822,2329,489,586,1562,814,3013,586,431,1038,1032
KEAP1_7_613,588,0,0,12,350,37,77,12,120,6,86,582
KIAA1841_7_614,156,215,3,49,91,113,877,344,78,97,141,117
KLHL13_7_615,384,356,313,161,788,24,0,433,903,51,166,793
KLHL2_7_616,185,0,83,176,913,78,452,126,189,133,183,397
KLHL4_7_617,1718,490,244,1,0,1112,333,1924,547,1347,1353,388
KLHL5_7_618,1174,504,958,1331,1108,890,913,749,374,809,1462,267
KLHL7_7_619,869,464,357,910,1531,870,2362,1584,300,2228,3429,1212
LNX1_7_620,648,1869,15,180,827,289,40,909,957,438,1,567
LONRF3_7_621,498,1830,287,406,250,482,708,269,544,1641,1265,540
LRRC29_7_622,138,30,354,0,0,52,235,31,670,59,0,82
LRSAM1_7_623,783,0,0,1,0,311,479,0,23,846,1,9
MARK2_7_624,0,0,0,562,0,1,28,608,135,42,1,18
MARK3_7_625,722,798,596,229,148,215,429,903,297,233,1272,46
MARK4_7_626,1024,381,6,98,79,204,119,30,512,90,842,70
MDM4_7_627,632,197,795,339,567,310,218,202,483,1629,1356,2742
MGRN1_7_628,358,438,101,20,148,10,80,451,8,122,14,3
MIB2_7_629,13,18,0,0,0,0,1,0,2,0,1,0
MID1_7_630,3,12,1174,660,26,112,49,0,57,9,84,52
MID2_7_631,420,509,1463,1817,1036,691,587,69,250,1086,419,233
MKRN1_7_632,59,76,102,19,1082,151,52,278,462,494,275,531
MLL5_7_633,905,550,708,463,239,1016,515,692,1122,552,772,1060
MLL_7_634,698,382,519,672,1447,685,852,113,874,469,893,809

MNAT1_7_635,0,0,0,0,0,0,0,0,0,0,0,0
MPND_7_636,531,48,210,3,0,12,11,15,442,22,228,44
MTF2_7_637,446,381,1404,315,196,761,762,352,361,584,1251,1086
MYNN_7_638,798,714,5,488,50,670,2006,618,1,539,60,1063
NAE1_7_639,23,349,130,167,0,253,578,30,26,167,187,23
NDUFC2_7_640,308,338,290,759,930,1372,1845,579,742,1181,650,382
NEDD4_7_641,1,1374,36,14,678,992,0,715,29,35,48,21
NEDD4L_7_642,213,1129,304,51,7,891,65,2,645,1104,167,374
NFX1_7_643,1104,3,0,170,0,6,381,28,942,22,1625,592
NLE1_7_644,809,19,32,7,3,850,171,1572,24,398,120,95
NSD1_7_645,167,133,592,292,25,93,1215,567,477,414,0,1335
NSFL1C_7_646,13,96,18,0,8,171,45,66,135,27,349,299
NUB1_7_647,77,28,0,438,194,130,11,299,308,1,357,281
ODF2_7_648,897,25,0,0,184,140,652,0,119,186,522,123
OTUD5_7_649,399,652,0,178,306,291,0,198,619,82,58,437
PARK2_7_650,84,1098,1,106,210,36,889,245,123,21,41,274
PARP9_7_651,821,739,1771,1434,796,2454,1435,1110,1181,1668,920,614
PATZ1_7_652,237,512,872,2,309,143,7,3,70,928,82,556
PCGF6_7_653,475,65,19,0,11,0,648,11,151,171,266,21
PEX10_7_654,1227,32,376,124,5,3,0,259,185,4,0,146
PEX2_7_655,192,139,624,1,1257,2548,196,162,480,434,183,68
PHF12_7_656,0,583,686,19,356,130,0,1045,224,1027,587,43
PHF16_7_657,529,1287,42,176,1069,172,3,745,360,363,181,62
PHF17_7_658,58,1,17,23,47,20,24,0,13,40,86,61
PHF1_7_659,3069,259,4620,878,1598,103,486,794,965,721,2554,1347
PHF21A_7_660,0,104,4,5,0,0,358,20,0,240,202,20
PHF7_7_661,2,61,22,34,0,5,277,6,340,17,1,193
PHF8_7_662,13,13,302,142,1556,730,259,154,282,4,344,831
PJA1_7_663,723,178,5,588,24,94,0,4,0,207,220,1118
PML_7_664,32,0,0,0,0,160,0,0,0,0,0,12
POC1B_7_665,693,458,217,196,802,72,253,0,472,195,35,90
PSMD1_7_666,1510,443,900,814,0,898,0,687,440,2060,435,343
RAPSN_7_667,16,3,1084,3,410,267,76,526,115,37,233,394
RBBP4_7_668,179,373,443,75,0,337,312,2,170,632,0,11
RBBP5_7_669,808,2200,1154,660,729,1090,574,1052,1047,481,861,2158
RBBP6_7_670,0,0,0,0,0,0,0,0,0,0,0,0
RBBP7_7_671,276,6,0,0,13,0,466,1,264,186,14,34
RBCK1_7_672,32,550,326,129,105,66,98,539,316,45,107,51
RC3H2_7_673,303,422,0,0,0,30,0,4,353,161,0,72
RCHY1_7_674,611,27,39,548,9,231,2227,1153,40,225,48,642
RFPL2_7_675,1788,327,957,0,190,2,841,4,532,1211,89,427
RFPL3_7_676,1788,327,957,0,190,2,841,4,532,1211,89,427
RFWD2_7_677,170,411,1584,1823,1,1039,1054,1401,969,1061,1114,237
RHOBTB1_7_678,1406,1012,441,617,1143,305,50,836,728,1478,1328,365
RHOBTB2_7_679,60,36,94,497,0,21,181,240,579,1603,57,1048
RLIM_7_680,0,444,74,7,348,341,1473,1423,657,10,0,546
RNF103_7_681,4513,2207,214,2782,978,4114,3775,3852,1727,1845,1395,2302
RNF128_7_682,714,1173,433,392,2003,1865,63,812,1513,2065,773,1465
RNF135_7_683,0,1,3,0,59,0,0,0,0,10,0,0
RNF138_7_684,1274,345,194,86,109,65,1866,12,505,434,22,644

RNF13_7_685,39,819,266,84,477,26,1,176,815,1244,433,1379
RNF145_7_686,434,556,765,377,5,21,299,540,97,249,33,11
RNF146_7_687,556,566,3340,17,277,216,390,970,848,1342,2286,1289
RNF14_7_688,975,0,961,11,23,466,3,353,26,402,0,1751
RNF166_7_689,0,0,846,0,0,0,0,0,0,3,0,0
RNF170_7_690,0,1400,0,303,1396,75,254,1295,66,96,2502,630
RNF17_7_691,1155,13,2532,705,1768,898,1189,1021,745,855,800,708
RNF180_7_692,961,2239,1054,427,1271,769,0,2401,830,917,808,414
RNF182_7_693,55,350,0,360,1328,1114,543,18,592,759,984,1213
RNF185_7_694,25,570,0,598,27,377,516,288,454,342,968,500
RNF19A_7_695,4,898,1013,4,1478,171,101,413,51,311,132,14
RNF19B_7_696,928,1195,530,385,805,154,1079,2,530,517,662,821
RNF213_7_697,1308,918,536,173,1704,578,1000,252,636,724,1590,273
RNF214_7_698,0,5,1,111,821,0,694,0,847,18,129,113
RNF216_7_699,1190,491,378,304,1300,1941,166,1764,1312,577,151,1124
RNF24_7_700,2936,1317,2002,1810,2923,2331,1715,3956,2957,1925,3866,217
6
RNF32_7_701,267,303,0,197,551,199,165,224,418,431,0,43
RNF34_7_702,1823,499,1668,132,169,899,815,137,769,51,671,142
RNF38_7_703,1742,1668,164,2015,641,1522,3687,1658,1684,439,2518,222
RNF40_7_704,25,0,10,0,35,4,35,13,42,168,0,8
RNF41_7_705,0,14,0,18,0,0,4,6,35,423,2077,4
RNF6_7_706,416,113,0,84,45,1595,948,172,328,627,1373,546
RNF7_7_707,0,1,0,0,0,0,0,0,0,42,41,0
RNF8_7_708,674,0,0,589,0,0,682,3,146,64,54,31
SAE1_7_709,489,211,0,485,1038,741,0,652,334,41,433,1352
SENP6_7_710,113,550,79,28,499,3,9,89,216,15,5,100
SENP7_7_711,583,197,0,106,554,1566,1159,979,586,209,796,172
SENP8_7_712,69,200,377,572,308,81,1948,4,141,1554,0,27
SF3A1_7_713,573,3,0,0,25,0,263,0,434,113,0,106
SHPRH_7_714,911,376,760,1480,3478,1027,1497,497,1328,962,1319,1869
SIAH1_7_715,679,2,1088,165,309,0,0,79,339,90,1,54
SKP2_7_716,6,272,405,1127,213,664,37,327,642,207,679,1128
SMURF1_7_717,219,631,682,385,934,5,0,224,11,30,1,624
SOCS5_7_718,165,273,48,2,0,344,338,669,21,110,152,237
SP100_7_719,48,441,3,170,390,1017,548,180,376,667,455,79
SP110_7_720,8,18,1,84,0,631,0,11,960,296,185,661
SPOP_7_721,28,8,172,0,718,6,262,0,521,21,157,66
SPSB2_7_722,0,0,0,64,0,0,0,8,0,86,0,234
SQSTM1_7_723,0,0,2,0,0,2,0,0,4,95,107,1079
STAMBP_7_724,87,777,574,1022,4,550,424,1171,381,182,177,1493
SUMO1_7_725,0,0,0,0,0,0,7,0,114,2,8,246
SUMO2_7_726,51,925,312,464,2854,594,1986,421,1347,1610,1358,473
SYTL4_7_727,1439,317,1683,1273,533,975,10,156,862,75,1357,512
SYVN1_7_728,442,540,32,4,70,701,386,109,840,83,401,183
TCF20_7_729,860,1605,1383,197,1644,210,1525,1867,1167,1458,1153,799
TDRD3_7_730,2,169,0,76,0,12,91,17,613,17,730,194
TIPARP_7_731,1125,14,300,24,267,104,1,674,637,514,1202,217
TLE2_7_732,20,26,0,0,467,0,6,89,0,112,1,4
TLE3_7_733,0,86,72,95,0,209,187,276,310,6,935,339

TNK2_7_734,1237,184,9,402,459,160,993,274,326,49,525,125
TNRC6C_7_735,786,344,638,0,52,351,0,128,161,721,495,76
TOPORS_7_736,206,80,2131,605,411,880,758,230,8,567,27,35
TOR1AIP2_7_737,204,837,24,0,1,147,265,0,0,0,9,0
TRAF3_7_738,7,3,0,176,67,2,505,0,227,24,8,30
TRAF5_7_739,799,279,36,578,12,403,1259,231,1197,545,1196,1098
TRAF6_7_740,272,101,707,349,16,513,0,1734,737,138,161,2754
TRIM10_7_741,22,529,0,113,411,225,736,598,518,25,153,140
TRIM13_7_742,1314,100,510,196,58,1081,599,3022,1023,712,645,282
TRIM17_7_743,5,1526,39,810,88,67,1982,751,1295,315,39,208
TRIM22_7_744,120,1182,2369,127,108,297,1701,1288,16,321,189,1510
TRIM23_7_745,734,502,1111,841,1541,520,525,415,1148,456,2034,276
TRIM24_7_746,805,712,0,2001,470,402,2192,532,226,295,195,57
TRIM26_7_747,0,0,0,267,3,0,2,0,0,168,0,0
TRIM2_7_748,853,1612,1609,673,2000,1241,476,650,504,1606,2108,1095
TRIM32_7_749,2402,271,303,279,969,594,1234,561,417,453,786,783
TRIM33_7_750,706,723,536,28,590,1482,21,1121,165,384,1,35
TRIM34_7_751,706,988,414,1181,42,348,652,22,415,54,809,1294
TRIM37_7_752,159,287,709,708,1,37,42,1203,192,78,355,451
TRIM39_7_753,241,79,41,431,686,774,48,30,840,897,41,305
TRIM3_7_754,205,200,19,181,0,58,762,191,81,228,167,48
TRIM41_7_755,22,30,32,347,15,475,0,9,315,32,166,1170
TRIM45_7_756,3266,1,0,1,1838,459,1126,443,0,1468,13,0
TRIM4_7_757,105,110,1066,5,25,410,323,34,319,507,12,1030
TRIM54_7_758,0,0,0,0,168,0,926,0,251,1,478,24
TRIM55_7_759,0,0,0,0,0,0,0,0,0,0,0,0
TRIM5_7_760,128,0,0,210,0,19,0,1,0,0,0,2
TRIM7_7_761,0,0,813,37,44,372,0,0,0,50,5,1
TRIM9_7_762,0,0,0,31,21,535,940,0,0,4,451,4
TRPC4AP_7_763,6,364,140,0,383,10,440,643,354,170,368,303
TSPAN17_7_764,1,135,7,27,225,383,103,7,17,204,312,444
TTC3_7_765,149,316,355,608,263,438,75,209,7,272,995,353
TULP4_7_766,195,233,1463,294,640,333,77,183,569,16,312,918
UBA1_7_767,202,23,257,163,540,24,713,62,516,249,23,60
UBA3_7_768,2140,1044,1990,1866,1082,3722,1727,1987,1861,2489,1620,3886
UBA5_7_769,869,1479,1073,2296,2114,485,489,760,1042,1124,1760,4048
UBAC2_7_770,0,0,15,101,4,17,33,1,21,43,4,4
UBAP2L_7_771,82,143,27,19,82,441,211,170,47,501,215,403
UBASH3A_7_772,862,0,0,4,123,722,0,1301,28,641,0,0
UBE2A_7_773,1135,181,1637,402,772,485,73,168,228,509,558,343
UBE2D1_7_774,1352,130,0,621,0,8,295,885,307,263,0,926
UBE2D2_7_775,484,844,741,876,1459,1200,861,3362,494,1715,1150,2750
UBE2D3_7_776,0,761,54,233,1898,936,819,79,0,67,723,0
UBE2E1_7_777,0,801,42,4,41,44,60,1,8,0,1063,742
UBE2E3_7_778,1,187,842,0,74,6,377,0,49,0,689,7
UBE2G2_7_779,0,134,292,0,0,8,0,0,4,227,0,0
UBE2H_7_780,2061,1769,2928,4248,2642,4188,4529,3025,3181,3397,4972,462
4
UBE2I_7_781,11,0,161,235,0,33,36,548,8,0,24,320
UBE2J2_7_782,309,96,145,788,53,72,43,347,603,112,1308,452

UBE2K_7_783,40,9,0,190,13,1436,795,1083,34,871,479,43
UBE2Q2_7_784,2649,2200,651,725,1266,2388,971,2262,2370,1677,1720,1309
UBE2V1_7_785,260,2777,680,1167,2102,1605,273,2282,867,1571,2224,1179
UBE2W_7_786,1064,903,1130,1109,474,641,413,366,1402,1235,501,1133
UBE3A_7_787,2774,340,506,1005,1306,1063,359,637,1044,475,1991,1058
UBE3B_7_788,0,50,139,45,762,392,700,259,809,763,293,964
UBE4A_7_789,451,1299,35,540,784,454,2324,1767,649,2019,413,578
UBE4B_7_790,776,436,230,422,78,1177,977,859,198,726,889,663
UBL7_7_791,8,41,524,51,0,249,0,195,75,157,0,10
UBOX5_7_792,0,34,49,3,47,776,150,1,62,861,783,424
UBQLN1_7_793,686,441,1575,842,590,2887,2274,1339,152,183,2000,70
UBR2_7_794,731,0,407,390,670,1149,727,200,426,243,439,1613
UBXN11_7_795,9,209,0,137,0,0,2,0,0,393,37,0
UBXN6_7_796,10,575,0,7,0,0,137,36,140,313,338,40
UCHL5_7_797,549,854,904,454,743,217,2760,916,517,228,931,559
UHRF1_7_798,17,605,17,22,311,0,418,237,150,60,2,66
UIMC1_7_799,57,381,0,341,573,0,1,2320,89,28,0,7
UNKL_7_800,774,93,0,0,264,0,1,2,0,39,0,0
USP14_7_801,128,88,1469,1125,288,684,438,120,51,1191,1258,1207
USP19_7_802,1553,172,269,381,203,0,117,257,13,104,9,875
USP1_7_803,1071,1067,353,556,498,201,1697,1655,585,397,1520,1096
USP20_7_804,0,128,351,74,9,77,115,0,256,190,398,407
USP2_7_805,0,3,1,12,0,0,0,207,0,1,11,0
USP33_7_806,1605,1233,1525,413,506,1817,2142,886,1360,2419,1089,1637
USP44_7_807,1,2,190,129,536,516,5,50,56,0,60,250
USP46_7_808,397,813,17,19,656,125,14,8,81,89,35,318
USP48_7_809,2,263,0,143,473,317,0,1076,0,11,8,462
USP4_7_810,9,406,1,0,3,0,326,0,462,12,0,488
USP5_7_811,1,491,21,0,9,669,32,26,14,1,31,652
USP6NL_7_812,1161,845,813,866,1444,1774,2974,913,2739,1295,823,990
USP8_7_813,465,60,285,47,377,404,713,1211,182,376,803,424
USP9X_7_814,1882,1708,1890,1921,1912,1774,1935,2524,3398,3183,3419,448
9
VHL_7_815,65,885,231,1025,321,101,98,45,665,323,268,437
VPRBP_7_816,158,0,0,171,183,147,508,5,1,525,340,1
VPS13D_7_817,365,552,2727,1374,587,117,159,134,838,698,9,149
VPS41_7_818,168,1438,1224,1182,350,1773,18,184,438,411,292,431
VPS8_7_819,997,530,221,1913,69,1123,2587,539,675,462,3,225
WDR26_7_820,407,853,1478,971,591,650,1205,1569,525,1020,635,1334
WDR5_7_821,0,1,0,0,0,0,0,0,0,0,0,0
WDR76_7_822,98,8,16,44,0,1108,93,1152,44,918,14,503
WHSC1_7_823,0,218,1,710,316,47,481,60,446,159,815,108
WHSC1L1_7_824,26,13,11,0,0,481,816,6,8,147,0,3
WSB1_7_825,203,41,74,27,0,1133,810,537,937,215,278,500
WWP2_7_826,12,1563,311,429,1008,1206,1430,1689,978,849,309,365
XIAP_7_827,1,8,2283,192,257,98,275,934,181,101,1691,327
ZBTB10_7_828,458,883,204,551,1460,1481,1599,609,1871,517,1321,927
ZBTB16_7_829,1401,129,100,1079,857,23,35,561,838,216,736,1473
ZBTB17_7_830,124,162,4,711,1446,332,501,127,131,203,513,471
ZBTB1_7_831,40,810,1295,123,39,965,205,442,49,422,440,970

ZBTB20_7_832,0,0,0,2,0,11,0,861,7,72,14,7
ZBTB22_7_833,0,242,0,4,0,24,0,0,67,36,200,339
ZBTB24_7_834,1248,176,371,1365,209,246,105,1136,225,837,196,519
ZBTB33_7_835,1333,33,1096,271,465,1330,737,1189,425,1086,1367,108
ZBTB37_7_836,1,369,278,59,0,0,782,90,93,221,78,560
ZBTB40_7_837,122,108,0,39,614,688,0,633,0,148,2,295
ZBTB4_7_838,2,68,426,0,1408,0,27,0,0,450,0,0
ZFP161_7_839,0,0,0,0,42,0,0,0,4,271,294,0
ZMYND11_7_840,523,110,0,101,647,0,41,2,316,193,11,62
ZMYND8_7_841,302,758,691,0,997,85,63,187,810,629,755,489
ZNF238_7_842,752,733,468,67,0,246,122,624,19,2,65,499
ZNF295_7_843,1094,1598,2724,396,41,1608,387,0,355,159,0,1784
ZNRF3_7_844,1,107,27,218,50,113,0,328,55,23,950,332
ANAPC11_7_845,84,492,315,1,40,30,61,387,34,2,0,253
ATXN3_7_846,241,278,29,20,1,76,401,35,783,806,421,545
MLLT10_7_847,906,2441,1673,1143,228,828,1243,1033,477,363,1753,683
OTUD4_7_848,1826,3163,1488,835,1930,1626,3460,888,2147,1315,953,848
PHF19_7_849,477,2047,1774,607,84,788,1144,1372,759,549,1979,1118
SP140_7_850,1343,2305,135,349,1914,558,18,7,478,388,804,663
TRIM36_7_851,454,1370,393,671,1517,734,1132,65,530,1152,208,562
UBE2C_7_852,553,1652,0,1048,18,1099,877,0,547,1208,252,800
UBE2L6_7_853,4,4,159,20,25,3,2,504,361,170,0,56
ABTB2_7_854,215,1029,420,179,1438,404,3,5,1383,833,1989,498
AMBRA1_7_855,153,17,485,215,862,427,493,122,496,1055,429,670
AMFR_7_856,872,220,2729,508,756,483,1404,112,249,626,695,58
ANAPC10_7_857,591,90,8,645,167,471,372,225,72,506,227,16
ANAPC1_7_858,0,376,0,0,718,138,0,0,0,585,25,0
ANAPC2_7_859,150,0,0,0,0,0,0,0,37,1193,0,4
ANAPC4_7_860,1163,752,2,481,0,482,1827,0,136,192,51,1212
ANKIB1_7_861,266,169,124,0,3,429,0,1,318,394,577,661
ANKRD13A_7_862,1039,277,1642,399,2026,225,653,1327,912,411,283,136
ANKRD13D_7_863,2620,30,1,26,299,431,674,37,59,924,236,11
ARIH1_7_864,139,0,774,89,683,523,269,1263,45,358,640,12
ARIH2_7_865,670,1584,62,14,589,1089,1203,2698,828,632,9,671
ASB12_7_866,542,722,944,662,124,1003,1344,748,635,69,634,578
ASB13_7_867,5,16,262,35,0,46,134,0,0,10,115,201
ASB15_7_868,12,942,936,56,616,1106,122,0,728,433,345,440
ASB16_7_869,0,0,5,52,11,489,0,0,0,16,0,0
ASB17_7_870,364,1280,3550,821,851,932,1939,824,827,644,58,462
ASB5_7_871,1,37,0,24,181,0,0,0,50,22,360,10
ASB8_7_872,8,504,0,89,0,0,1704,155,83,252,35,9
ASH1L_7_873,94,0,36,20,60,88,0,306,86,1,21,16
ASPSCR1_7_874,0,0,0,0,0,0,0,0,0,0,0,0
ATG12_7_875,186,161,487,27,9,27,31,134,193,559,252,129
ATG3_7_876,1680,0,0,555,1675,8,70,1603,632,879,1121,327
ATG5_7_877,296,950,2,233,334,51,0,519,161,186,212,454
ATXN1L_7_878,0,86,0,369,1,69,7,1327,246,335,104,30
ATXN3L_7_879,1036,7,0,611,1733,514,0,95,631,361,279,829
BARD1_7_880,166,271,32,642,979,758,328,30,0,285,331,9
BAZ1B_7_881,115,0,7,0,736,6,0,244,873,18,1,109

BAZ2A_7_882,418,560,3,62,33,80,1051,81,345,1222,1,981
BAZ2B_7_883,74,25,518,721,1,176,135,277,40,64,89,11
BECN1_7_884,413,56,284,248,0,403,271,29,561,876,1208,548
BIRC2_7_885,28,989,3,83,0,1084,1237,733,558,366,687,1092
BIRC6_7_886,1025,160,1005,881,273,29,336,106,984,869,783,530
BIRC8_7_887,623,314,189,432,47,1198,101,606,1308,75,1213,699
BMI1_7_888,1258,560,175,124,49,916,136,645,61,790,1459,518
BRAP_7_889,35,0,692,41,0,0,0,482,55,6,39,197
BRD1_7_890,93,737,1523,222,46,99,83,258,5,368,56,0
BRPF3_7_891,14,4,29,599,0,0,125,43,0,0,0,0
BTBD2_7_892,1,78,148,87,6,72,0,0,13,0,0,1
BTBD6_7_893,0,0,9,333,7,1,0,627,5,0,0,0
CAND1_7_894,45,1181,69,493,502,2030,151,1242,1293,404,1145,1073
CBLB_7_895,485,11,142,0,0,396,383,0,90,123,61,10
CBL_7_896,0,30,105,540,0,0,22,6,1126,280,0,119
CBLL1_7_897,2103,597,526,834,0,376,208,331,296,834,11,519
CCIN_7_898,38,366,334,11,51,95,7,23,143,21,0,388
CCNF_7_899,946,250,29,144,11,349,28,219,258,97,908,415
CDC20_7_900,42,0,244,17,0,65,0,0,155,542,1,131
CDC23_7_901,442,217,469,238,1331,275,42,1548,1208,558,141,160
CDC26_7_902,300,488,777,770,22,71,470,13,170,129,463,28
CDC34_7_903,23,0,0,1,151,789,0,0,427,9,86,50
CGRRF1_7_904,1046,445,1,0,468,401,11,0,599,344,18,358
CHD4_7_905,17,0,0,91,0,2,0,298,755,101,0,100
CIAO1_7_906,59,1,558,0,2,0,1,0,65,11,311,701
COPS4_7_907,0,0,0,0,371,105,0,0,4,256,17,3
COPS5_7_908,144,52,143,965,111,48,9,0,5,213,18,2
COPS6_7_909,0,0,0,36,1,9,0,0,0,0,0,12
COPS7B_7_910,0,0,0,0,0,9,0,0,1,71,0,0
CORO6_7_911,1,0,0,24,0,39,0,282,0,0,9,46
CUEDC1_7_912,1,83,48,17,2,36,831,0,0,52,919,795
CUL1_7_913,30,235,220,1237,711,550,75,95,1091,1219,9,494
CUL3_7_914,633,484,799,527,585,387,570,916,836,26,3098,479
CUL5_7_915,926,86,1878,49,1947,871,616,175,398,1215,1100,528
CUL9_7_916,5,158,11,18,6,5,4,535,49,13,461,1491
DCAF10_7_917,616,352,27,413,442,525,109,199,148,335,267,108
DCAF12_7_918,294,27,683,65,72,283,39,707,230,276,645,96
DCAF13_7_919,864,1235,247,22,1141,1128,165,548,85,660,303,40
DCAF16_7_920,387,11,0,0,18,70,520,0,0,0,691,55
DCAF5_7_921,0,303,0,0,0,378,0,1,0,0,0,267
DCAF7_7_922,16,762,1018,412,871,505,303,453,408,378,617,105
DCAF8_7_923,39,0,40,111,2,0,0,2,156,3,26,27
DDA1_7_924,318,1059,29,39,5,13,40,27,148,856,275,293
DDB1_7_925,918,343,398,742,932,239,13,1630,1053,526,65,477
DDB2_7_926,696,291,0,9,50,60,4,142,740,90,462,71
DPF2_7_927,18,158,0,5,750,141,40,669,187,155,5,123
DTX1_7_928,76,0,16,0,0,3,0,774,192,36,1089,25
DTX3L_7_929,18,0,351,481,442,637,0,415,200,396,319,36
DTX4_7_930,1624,567,1077,549,1421,3425,607,1216,1278,1512,831,1446
DZIP3_7_931,253,437,125,143,43,5,234,690,422,1273,120,67

EIF3D_7_932,238,247,270,5,126,1435,294,571,287,168,296,252
EIF3E_7_933,546,1356,2,762,63,1531,360,1161,423,549,666,511
EIF3F_7_934,0,0,63,0,73,1,0,394,250,4,0,32
EIF3G_7_935,376,11,784,46,983,197,594,197,34,3,20,837
EIF3H_7_936,1479,1714,2295,1432,858,2286,2513,1251,1250,342,1869,3781
EIF3I_7_937,27,56,477,28,12,594,249,264,67,816,270,177
EIF3J_7_938,504,15,132,56,286,42,151,530,62,510,951,268
EIF3K_7_939,213,210,181,408,3366,161,1315,2124,1458,261,1751,778
ENC1_7_940,923,1,0,0,0,16,0,108,0,152,0,0
EPN3_7_941,1,0,711,0,0,0,45,0,0,0,7,0
EPOR_7_942,242,15,621,89,88,1,0,432,119,12,442,46
ERCC8_7_943,207,773,328,45,0,485,1,1554,197,833,1463,1686
FAF1_7_944,534,517,558,1451,278,514,27,435,631,1508,1293,910
FBXL12_7_945,86,1,0,232,0,102,212,38,0,40,1,165
FBXL14_7_946,149,562,307,0,395,432,150,113,0,65,1,0
FBXL15_7_947,0,0,0,0,0,0,0,0,0,0,0,0
FBXL16_7_948,0,0,0,0,0,0,10,0,0,0,0,0
FBXL17_7_949,1667,467,595,6,1429,17,0,0,0,240,598,820
FBXL18_7_950,18,0,0,0,111,0,0,0,12,0,8,62
FBXL19_7_951,0,0,7,0,0,27,0,0,32,1103,15,497
FBXL21_7_952,512,137,5,0,7,762,136,223,1,14,297,1153
FBXL3_7_953,247,428,384,217,361,1050,800,137,244,358,256,1803
FBXL4_7_954,108,0,0,12,44,265,55,233,3,51,1,35
FBXL7_7_955,1,0,0,71,0,2,92,11,1,146,789,1
FBXO10_7_956,52,391,0,875,607,444,9,230,49,378,1535,639
FBXO16_7_957,424,452,343,18,145,30,64,29,778,217,312,83
FBXO27_7_958,809,588,385,192,519,656,299,1490,525,1339,502,1923
FBXO2_7_959,500,7,48,0,235,0,246,1,5,0,0,18
FBXO30_7_960,426,130,1790,609,7,1020,2237,1070,2056,398,845,986
FBXO33_7_961,34,1122,44,366,614,875,605,1171,675,585,744,380
FBXO36_7_962,224,358,16,0,552,213,642,312,34,159,230,76
FBXO39_7_963,0,0,0,0,0,0,0,0,0,0,0,0
FBXO40_7_964,783,62,3059,405,486,1098,1521,411,2510,92,81,780
FBXO41_7_965,0,349,44,110,0,536,233,1153,205,4,0,20
FBXO42_7_966,0,0,0,0,0,63,0,580,416,11,175,54
FBXO43_7_967,996,24,1463,792,1829,238,274,382,520,51,304,1508
FBXO46_7_968,473,5,0,541,0,192,1485,581,23,5,14,5
FBXO6_7_969,77,73,2,249,1,1088,0,42,0,57,0,79
FBXO8_7_970,1323,962,548,842,231,1183,442,523,2293,1340,2443,3169
FBXW10_7_971,548,1987,2020,72,288,949,724,30,631,2201,962,684
FBXW2_7_972,16,658,10,148,2,1047,508,784,801,1059,22,177
FBXW5_7_973,27,0,0,635,523,72,72,239,40,10,11,256
FBXW9_7_974,8,299,404,1002,92,468,343,541,696,119,1018,684
G2E3_7_975,1008,490,882,407,969,414,1071,929,440,838,308,140
GAN_7_976,47,281,21,133,109,38,1124,0,26,145,0,2
GMCL1_7_977,24,189,0,469,0,701,19,0,135,3,545,14
GNB2_7_978,15,0,32,0,63,6,98,54,95,5,62,5
GRWD1_7_979,0,86,901,853,0,1022,279,0,0,46,181,16
GZF1_7_980,159,0,21,8,0,4,398,0,1,121,2,308
HACE1_7_981,1,6,0,73,0,253,84,105,669,154,6,88

HDAC6_7_982,1,0,4,0,17,64,1108,0,60,43,8,7
HECTD1_7_983,29,1,0,0,1,78,0,0,10,562,2,7
HECTD3_7_984,1,19,66,24,128,158,40,23,4,6,195,161
HECW1_7_985,0,0,0,0,0,28,0,9,429,43,0,62
HECW2_7_986,30,23,267,0,532,0,0,803,0,0,23,0
HERC1_7_987,46,208,1589,360,436,442,718,1460,176,602,74,501
HERC2_7_988,41,1,729,80,756,828,11,0,495,696,674,60
HERC3_7_989,2316,1397,1256,2317,1621,1784,3178,1883,2036,2992,1772,111
0
HERC5_7_990,310,12,25,51,553,2098,264,226,132,225,584,1951
HGS_7_991,825,294,947,472,13,2,319,84,198,65,140,854
HIC2_7_992,214,61,25,122,722,72,2,152,15,113,511,103
HUWE1_7_993,963,1132,1492,680,4,855,170,252,1848,1047,503,293
IBTK_7_994,53,144,110,123,1288,95,1118,503,844,770,1192,1289
IL10RA_7_995,316,1010,3,0,535,712,150,137,85,297,267,14
IL6_7_996,407,15,43,180,395,8,3,291,331,344,104,212
IRF9_7_997,165,411,85,0,0,43,1112,0,21,0,1,28
ITCH_7_998,412,167,0,14,225,382,51,358,342,469,417,126
IVNS1ABP_7_999,147,445,221,251,220,610,185,678,750,364,72,680
JHDM1D_7_1000,264,186,207,0,0,287,782,1,923,47,11,1055
JOSD1_7_1001,202,401,0,250,0,0,993,796,519,20,176,62
JOSD2_7_1002,0,515,0,0,559,0,0,0,0,382,721,0
KAT6B_7_1003,430,167,1341,386,124,534,331,901,70,701,1367,508
KATNB1_7_1004,57,85,1086,158,48,226,297,13,165,3,639,20
KBTBD10_7_1005,26,0,1,102,1812,460,1166,42,653,932,827,216
KBTBD11_7_1006,1082,66,0,2299,76,289,450,0,464,1424,75,325
KBTBD2_7_1007,501,416,1331,577,113,44,9,590,671,984,470,820
KBTBD5_7_1008,383,14,0,0,0,555,10,0,296,8,0,57
KBTBD7_7_1009,194,82,659,773,1006,876,653,81,510,208,724,103
KBTBD8_7_1010,1287,1178,33,1796,956,1212,98,1421,243,1321,75,946
KCTD10_7_1011,16,133,15,0,54,90,17,31,0,0,15,12
KCTD11_7_1012,135,144,110,0,1446,27,7,574,8,45,0,18
KCTD12_7_1013,79,2,3,0,20,30,13,66,1,79,238,250
KCTD13_7_1014,0,0,0,0,0,0,0,0,13,0,0,2
KCTD16_7_1015,0,0,0,0,3,342,0,402,0,0,0,1
KCTD17_7_1016,0,0,7,0,0,0,0,0,48,0,0,0,0
KCTD18_7_1017,295,1352,1578,648,1415,380,272,83,353,347,2105,429
KCTD3_7_1018,802,432,11,8,1420,962,452,1,754,809,707,103
KCTD5_7_1019,0,0,34,6,0,130,10,0,0,0,0,0
KCTD9_7_1020,1132,529,725,69,25,529,971,2438,1438,1539,1318,597
KDM2A_7_1021,0,0,0,28,0,1009,0,0,0,0,0,0
KDM4B_7_1022,0,0,0,21,0,0,0,0,1,158,0,410
KDM5B_7_1023,359,335,284,29,53,18,1,178,146,125,4,61
KLHDC5_7_1024,56,0,2,755,96,9,0,66,2,4,0,0
KLHL10_7_1025,617,207,10,548,0,1,0,8,156,1,155,20
KLHL11_7_1026,563,0,10,12,0,0,46,42,12,335,330,28
KLHL12_7_1027,880,201,1,53,346,1126,295,122,19,600,0,788
KLHL14_7_1028,1147,273,1074,653,137,534,823,1011,1130,1199,2293,1751
KLHL15_7_1029,703,502,0,9,1778,355,70,305,806,563,958,640
KLHL17_7_1030,0,0,0,0,0,0,0,0,0,0,0,0

KLHL18_7_1031,100,162,0,14,193,43,196,679,404,72,173,246
KLHL1_7_1032,78,78,69,0,0,27,98,131,132,703,166,60
KLHL20_7_1033,1,373,377,442,190,1058,1025,403,135,438,448,107
KLHL21_7_1034,26,0,0,0,0,0,0,0,9,19,0,2
KLHL22_7_1035,30,7,0,0,0,1,0,8,20,1,0,179
KLHL23_7_1036,1389,1348,938,1398,2030,725,82,222,1630,1740,1087,333
KLHL24_7_1037,700,872,526,856,819,1322,1414,3000,148,2143,1049,1555
KLHL25_7_1038,95,0,0,10,39,0,61,72,0,182,1,148
KLHL26_7_1039,1,3,0,2,1,26,3,3,58,0,60,45
KLHL28_7_1040,8,19,137,277,756,654,1581,2149,182,2438,425,1213
KLHL29_7_1041,472,521,8,1108,795,990,194,1458,2618,810,1343,811
KLHL31_7_1042,3,17,10,728,0,0,0,4,8,0,0,1
KLHL32_7_1043,79,96,245,7,47,30,451,1,233,53,0,218
KLHL33_7_1044,508,313,40,483,0,3,1,115,149,382,276,25
KLHL34_7_1045,0,93,31,4,0,0,0,0,1,271,408,98
KLHL36_7_1046,1,0,0,27,0,0,622,749,333,34,100,45
KLHL3_7_1047,1,266,0,1050,1,1,451,375,794,12,278,98
KLHL8_7_1048,17,32,0,566,94,126,736,12,126,233,55,235
LATS1_7_1049,2,15,4,0,65,0,0,85,79,1131,6,12
LATS2_7_1050,23,0,14,609,20,0,1,70,0,1,8,12
LIF_7_1051,502,94,0,554,2,0,240,0,0,1,0,127
LNX2_7_1052,239,0,0,0,0,145,873,1,3,12,4,1
LOC283116_7_1053,1196,1,1381,617,127,412,160,62,700,455,1425,558
LONRF1_7_1054,1810,1441,1312,1911,1846,565,112,1372,1327,2734,1253,368
5
LTN1_7_1055,9825,9361,10360,7142,2868,7742,8789,8065,9225,10515,6006,5
567
LZTR1_7_1056,495,299,217,52,315,134,0,1202,257,72,538,64
MAP1LC3B_7_1057,0,0,33,2,9,0,6,62,44,130,0,6
MAP3K1_7_1058,4049,1250,2395,1675,3120,1484,3426,2043,1843,1433,1885,2
272
MARK1_7_1059,394,1333,1300,65,403,429,196,1386,335,1304,59,97
MDM2_7_1060,106,942,67,13,118,742,730,522,23,257,1284,156
MED20_7_1061,125,0,297,0,0,151,0,0,46,239,0,3
MEX3B_7_1062,59,132,59,246,40,694,0,0,1,214,42,20
MIB1_7_1063,61,64,659,383,63,159,185,17,486,129,51,381
MKRN2_7_1064,723,1331,164,178,2210,1173,1834,0,630,1020,817,1011
MKRN3_7_1065,473,20,13,0,1,0,55,0,1,12,5,0
MLL2_7_1066,0,0,0,24,0,22,20,0,3,0,0,0
MLLT6_7_1067,0,70,0,3,0,183,0,0,24,93,45,68
MOCS3_7_1068,0,0,664,2,0,3,71,0,0,0,0,0
MRPL49_7_1069,2,10,0,298,314,0,0,21,63,4,3,2
MUL1_7_1070,72,501,146,3,28,62,115,6,5,1,0,0
MYCBP2_7_1071,0,14,52,30,13,0,0,0,64,200,199,118
MYLIP_7_1072,1229,1729,3976,3510,1600,1630,1784,1716,1725,221,951,2802
MYSM1_7_1073,1657,1362,5618,2337,1336,1653,2069,3659,2148,5113,1670,39
76
NACC1_7_1074,200,29,0,0,2,0,1555,17,150,3,0,24
NACC2_7_1075,5,358,52,710,739,0,0,47,341,88,58,457
NEURL1B_7_1076,0,0,0,0,0,0,0,0,0,0,0,0

NEURL_7_1077,2,1,0,80,1,0,6,209,10,0,0,75
NHLRC1_7_1078,587,839,0,646,5,827,1190,0,488,5,22,225
NUP43_7_1079,1,217,0,28,0,2,932,263,187,29,0,41
OTUB1_7_1080,99,339,4,138,2,0,217,88,57,3,302,81
OTUB2_7_1081,0,0,0,0,0,323,0,0,0,25,0,0
OTUD1_7_1082,49,48,0,3,108,467,0,0,47,1,3,48
OTUD6A_7_1083,110,0,130,71,0,0,0,61,28,13,0,22
OTUD6B_7_1084,1737,976,1451,351,147,278,1537,152,388,950,2153,943
OTUD7A_7_1085,0,0,0,1071,0,2,986,17,0,0,0,1536
OTUD7B_7_1086,708,1098,0,610,507,465,1115,1195,472,782,1229,126
PAFAH1B1_7_1087,427,261,600,466,121,415,14,465,746,478,1050,470
PARP10_7_1088,24,205,2,5,3,0,0,0,17,381,42,50
PARP11_7_1089,1256,2785,451,1019,2832,1551,1195,1092,1170,953,617,2537
PARP14_7_1090,223,9,0,8,0,0,905,644,0,455,0,0
PCGF1_7_1091,244,49,44,552,109,218,5,716,19,1,166,1
PCGF2_7_1092,0,73,0,0,0,667,26,154,133,15,1,608
PCGF3_7_1093,170,0,0,271,28,0,0,0,0,1,1,200
PDZRN3_7_1094,769,393,1203,38,1123,189,149,345,626,599,667,676
PEBP4_7_1095,0,1018,0,211,82,33,39,0,0,494,2,0
PEX12_7_1096,63,0,151,82,0,727,274,0,53,2,53,801
PHF14_7_1097,340,494,400,237,0,0,565,29,269,255,27,218
PHF15_7_1098,0,523,0,86,0,21,52,11,30,2,0,3
PHF20_7_1099,7,8,1,5,6,394,64,4,3,223,0,7
PHF2_7_1100,0,0,0,0,0,0,0,31,0,0,0,0
PHF3_7_1101,136,345,0,0,471,622,7,92,547,11,1,71
PHIP_7_1102,12,465,8,116,1683,501,4,701,25,589,7,65
PHRF1_7_1103,209,874,0,87,0,145,660,445,520,317,233,763
PJA2_7_1104,1022,54,593,634,4,312,27,50,778,133,1505,212
PRPF19_7_1105,0,43,136,202,289,49,2,28,5,367,0,1
PRPF8_7_1106,0,27,0,784,849,1647,0,242,575,84,0,878
PSMD14_7_1107,4,0,29,0,726,832,283,1,77,20,148,42
PSMD2_7_1108,61,11,2,83,1,0,102,35,50,98,457,33
PSMD4_7_1109,75,338,2,1,0,394,867,330,531,1383,915,623
PSMD7_7_1110,0,0,536,41,960,601,213,290,197,312,633,211
PWP1_7_1111,382,71,0,6,660,0,0,5,0,178,1,252
RAB40A_7_1112,1921,1445,1048,1566,967,194,599,417,476,279,575,652
RAB40AL_7_1113,1921,1445,1048,1566,967,194,599,417,476,279,575,652
RAB40B_7_1114,27,377,6,38,1521,110,255,0,313,83,52,50
RAD18_7_1115,289,738,373,279,185,390,0,64,0,1056,195,701
RAD23A_7_1116,0,13,0,0,0,0,0,760,0,1,0,0
RAD23B_7_1117,348,745,756,0,822,541,0,563,608,374,20,248
RAG1_7_1118,1696,16,1577,0,74,325,0,654,0,79,909,247
RAI1_7_1119,854,506,551,166,1036,1014,1935,3312,197,1743,674,476
RBX1_7_1120,8,912,689,250,69,758,22,232,377,2,565,50
RCBTB1_7_1121,1,0,0,0,0,0,781,0,25,464,6,6
RCBTB2_7_1122,376,81,120,173,13,169,2,316,224,934,34,344
RFFL_7_1123,87,0,15,83,144,12,251,15,317,29,4,162
RFPL1_7_1124,0,0,0,0,11,0,1,0,462,86,0,45
RFWD3_7_1125,515,324,1057,162,1661,1562,967,335,676,1271,35,118
RHOBTB3_7_1126,272,341,3,180,510,1002,572,176,558,314,474,347

RING1_7_1127,132,274,157,35,34,653,115,148,90,543,124,150
RNF10_7_1128,12,63,151,185,0,6,0,33,9,75,241,800
RNF111_7_1129,601,30,213,472,884,102,166,393,281,1335,1450,1345
RNF112_7_1130,81,9,252,3,387,257,410,10,432,12,301,156
RNF113A_7_1131,0,0,84,0,0,0,0,2,4,0,0,0
RNF113B_7_1132,390,16,509,336,13,43,0,121,41,397,115,587
RNF114_7_1133,68,111,785,16,97,376,218,0,405,70,659,159
RNF115_7_1134,993,367,2,287,10,180,33,2015,587,588,1028,1122
RNF11_7_1135,0,337,328,396,60,185,310,364,119,16,604,317
RNF121_7_1136,361,316,68,651,577,797,1,48,0,44,0,124
RNF122_7_1137,22,597,3,1563,347,620,40,161,145,79,79,53
RNF123_7_1138,6,541,6,0,0,0,0,0,0,176,0,0
RNF125_7_1139,656,75,647,271,16,97,686,0,850,101,1400,112
RNF126_7_1140,420,5,61,18,0,10,2,90,3,431,4,16
RNF130_7_1141,858,921,16,1174,1861,414,439,1224,902,216,7,1670
RNF133_7_1142,3,61,14,9,147,315,120,0,879,1002,30,811
RNF139_7_1143,735,13,12,97,0,7,35,8,9,41,306,414
RNF141_7_1144,3477,4136,2559,2419,2251,1682,2558,5370,2852,757,2239,22
12
RNF144A_7_1145,735,1269,80,264,7,248,99,195,509,840,65,313
RNF144B_7_1146,158,4,0,8,1728,4,62,1,522,58,49,80
RNF149_7_1147,650,589,453,6,14,402,3,376,16,874,933,59
RNF150_7_1148,4,69,73,101,0,241,263,53,511,482,723,725
RNF152_7_1149,0,17,109,173,26,113,422,1431,47,216,21,93
RNF157_7_1150,0,11,0,0,175,0,9,106,13,0,6,4
RNF167_7_1151,0,150,0,85,55,277,328,2,1138,473,489,589
RNF168_7_1152,1040,1825,1865,1172,1600,1418,1423,1237,2357,1119,1914,1
617
RNF169_7_1153,1447,610,496,106,95,1042,455,541,619,1130,1526,204
RNF181_7_1154,93,0,0,11,24,3,0,6,1,3,40,673
RNF183_7_1155,15,0,211,0,1,45,0,10,111,583,0,524
RNF186_7_1156,3,69,209,1,0,370,93,31,329,170,8,657
RNF187_7_1157,418,620,1462,117,828,1714,1172,957,253,2035,2,62
RNF20_7_1158,0,482,0,0,5,332,0,1,7,117,0,18
RNF217_7_1159,2,35,179,0,106,325,111,126,59,66,722,291
RNF220_7_1160,381,1303,2291,125,250,531,115,12,802,238,211,880
RNF25_7_1161,1405,468,1367,1314,156,537,530,709,22,498,764,680
RNF26_7_1162,101,2681,1177,1482,2258,1503,953,394,1800,755,697,669
RNF2_7_1163,1830,281,1137,2,746,392,1,1366,931,310,596,1126
RNF31_7_1164,1,0,0,32,642,174,663,0,51,615,24,305
RNF43_7_1165,33,1,435,0,428,3,308,0,516,169,1,55
RNF44_7_1166,0,533,0,0,163,0,0,5,527,5,1077,104
RNF5_7_1167,68,71,0,2,7,115,0,14,56,24,439,9
RSC1A1_7_1168,249,0,22,106,146,0,0,44,360,198,0,353
RSF1_7_1169,895,1044,1801,1162,215,2549,318,580,1719,1160,1061,708
RSPRY1_7_1170,1081,249,1,31,1347,22,189,134,20,16,35,36
SCLY_7_1171,482,7,305,52,0,4,31,159,257,434,0,28
SENP1_7_1172,2,44,827,92,2257,412,6,451,52,345,294,22
SENP2_7_1173,440,1772,382,1202,1610,340,742,2960,1251,555,111,151
SENP3_7_1174,0,53,1,16,251,146,0,0,104,1,428,13

SENP5_7_1175,0,0,0,0,0,0,0,0,0,0,0,0
SH3RF1_7_1176,198,19,779,392,945,214,214,0,7,442,1,136
SH3RF2_7_1177,0,0,1715,415,0,974,376,237,151,1033,139,570
SH3RF3_7_1178,126,3,21,654,0,21,0,11,226,7,0,28
SHKBP1_7_1179,318,5,19,0,104,461,350,480,20,264,749,404
SIAH2_7_1180,2,0,39,0,1,78,315,0,0,1,0,0
SIK1_7_1181,415,20,11,69,259,174,56,903,1091,773,8,197
SLX4_7_1182,650,1040,1644,357,337,174,934,264,485,745,677,300
SMU1_7_1183,482,533,735,267,4,0,913,44,916,17,55,1146
SMURF2_7_1184,3400,1812,1056,1662,3249,2701,1699,891,2633,2347,2318,11
44
SNRNP40_7_1185,497,444,319,792,81,669,1096,163,228,554,1709,182
SOCS1_7_1186,0,0,0,0,0,0,0,0,0,0,0,0
SOCS2_7_1187,864,1807,1234,817,526,1478,1805,1842,1041,1911,2083,909
SOCS3_7_1188,381,155,64,15,6,0,231,926,56,133,102,1138
SOCS6_7_1189,20,169,340,523,43,501,424,527,880,566,113,1623
SPOPL_7_1190,13,0,0,13,1,406,43,695,73,1,0,20
SPSB1_7_1191,0,1,0,570,334,0,0,1,50,143,94,9
SPSB3_7_1192,49,0,48,3,0,0,6,78,162,4,0,141
SPSB4_7_1193,0,0,4,0,54,190,0,0,65,197,0,6
STAM2_7_1194,832,2493,1019,268,72,2060,2210,1792,1771,819,861,2822
STAMBPL1_7_1195,569,549,191,780,1124,638,0,911,1258,55,499,291
STAM_7_1196,982,629,1112,777,2337,597,260,0,1211,550,1090,196
STUB1_7_1197,0,14,1,0,0,90,0,350,8,1,0,10
SUMO3_7_1198,12,26,0,18,2,810,885,0,79,95,65,5
SYNGAP1_7_1199,105,222,0,10,69,688,8,98,122,748,8,1168
TAB2_7_1200,25,2,415,296,692,668,514,520,427,988,1,269
TAB3_7_1201,100,121,3,170,181,395,343,917,589,19,638,434
TAF1D_7_1202,528,2,11,26,196,13,69,47,7,3,0,460
TBC1D1_7_1203,571,1982,3563,1992,746,1663,1603,1064,2991,1784,4104,351
2
TLE1_7_1204,0,1,80,182,0,43,17,801,104,359,0,11
TNFAIP3_7_1205,851,633,240,337,160,815,27,673,46,49,215,50
TOLLIP_7_1206,0,33,0,14,0,160,0,274,0,2,0,22
TRAF7_7_1207,629,72,4,367,2,0,1215,0,0,1,0,71
TRAIP_7_1208,0,215,0,25,258,212,0,0,120,4,7,42
TRIM11_7_1209,0,101,14,18,106,2,391,355,143,48,6,16
TRIM15_7_1210,5,607,4,756,208,355,1898,459,995,775,1458,252
TRIM25_7_1211,80,1123,32,46,670,690,53,791,197,832,138,22
TRIM27_7_1212,306,354,1772,317,932,603,699,382,495,184,695,578
TRIM28_7_1213,46,181,3,770,840,264,94,0,170,470,447,275
TRIM31_7_1214,0,85,26,1,0,12,0,75,150,21,1068,187
TRIM35_7_1215,352,257,2533,270,971,200,676,640,152,233,104,394
TRIM42_7_1216,80,566,4,7,240,0,41,813,409,173,31,112
TRIM46_7_1217,0,237,137,0,319,10,0,401,36,40,942,450
TRIM47_7_1218,0,0,0,0,0,0,0,0,0,0,0,0
TRIM48_7_1219,468,769,332,1540,1114,1150,595,653,247,385,487,1868
TRIM52_7_1220,108,450,1122,654,353,1766,1381,1622,795,798,54,366
TRIM56_7_1221,0,0,0,0,0,0,0,0,0,0,2,1
TRIM62_7_1222,0,5,0,46,383,26,0,469,0,39,12,438

TRIM63_7_1223,409,0,0,248,81,0,85,38,155,2,0,56
TRIM65_7_1224,0,773,0,0,0,0,0,0,423,7,0,58
TRIM67_7_1225,56,32,7,1,29,19,11,96,79,0,1,14
TRIM68_7_1226,71,529,0,0,0,51,8,614,0,225,0,19
TRIM8_7_1227,0,27,77,8,12,0,0,188,49,6,60,866
TRIP12_7_1228,171,342,0,70,23,19,0,84,17,765,10,496
UBA2_7_1229,41,232,132,107,74,21,202,113,0,14,150,0
UBA6_7_1230,1343,1626,954,119,1833,1619,949,789,1375,655,1183,1257
UBA7_7_1231,184,220,46,0,0,252,378,242,1,56,0,163
UBAC1_7_1232,45,3,12,540,206,617,26,448,10,402,267,89
UBAP2_7_1233,0,41,0,999,0,130,314,96,640,853,59,456
UBASH3B_7_1234,223,0,945,80,0,27,206,2,61,5,1,425
UBC_7_1235,19,172,186,0,715,3,0,0,0,5,0,0
UBE2B_7_1236,1129,95,4,66,877,2,566,16,169,34,115,37
UBE2D4_7_1237,449,336,275,76,108,100,56,389,683,577,112,140
UBE2E2_7_1238,1,146,53,19,2,15,6,36,382,149,1210,105
UBE2F_7_1239,48,46,8,1,737,177,68,92,262,365,7,45
UBE2G1_7_1240,1624,435,1285,3644,872,1978,832,1170,2203,853,2029,1188
UBE2J1_7_1241,76,74,21,387,19,116,5,299,156,431,437,56
UBE2L3_7_1242,303,311,41,658,177,112,5,914,406,44,5,335
UBE2M_7_1243,447,546,201,1448,856,3,42,16,742,51,243,649
UBE2N_7_1244,750,291,390,1042,1452,388,491,197,446,407,942,185
UBE2NL_7_1245,336,418,48,74,132,840,25,175,1004,430,659,557
UBE2O_7_1246,8,7,0,0,13,4,0,216,0,1,0,694
UBE2Q1_7_1247,0,0,0,0,0,0,0,0,0,0,0,0
UBE2QL1_7_1248,150,25,1059,47,8,172,35,1235,270,581,15,1157
UBE2R2_7_1249,1,0,14,51,0,3,132,11,628,15,635,92
UBE2S_7_1250,0,531,610,338,0,989,0,406,350,511,0,48
UBE2T_7_1251,119,306,33,0,4,0,2152,2,34,502,880,6
UBE2U_7_1252,971,254,1570,913,698,1426,1802,184,396,979,1472,481
UBE2V2_7_1253,1248,13,40,234,25,982,0,373,3,36,211,995
UBE2Z_7_1254,1472,104,39,125,1,533,1308,1102,585,285,880,447
UBE3C_7_1255,0,0,0,0,0,0,0,0,0,0,0,0
UBQLN2_7_1256,266,406,2,11,69,548,0,169,12,406,112,573
UBQLN3_7_1257,932,624,503,823,70,670,535,149,880,1056,928,975
UBQLN4_7_1258,0,0,1,6,0,4,0,431,13,158,128,114
UBR1_7_1259,21,115,8,1,5,18,577,553,47,57,32,69
UBR3_7_1260,1206,2343,752,1818,1988,1120,714,870,903,1618,807,1521
UBR4_7_1261,698,29,0,912,52,583,369,103,80,367,65,161
UBR5_7_1262,42,0,59,305,99,0,0,101,1,309,1,35
UBR7_7_1263,5,286,49,0,0,5,0,0,0,0,800,0
UBXN10_7_1264,41,5,0,248,7,2,291,0,571,481,949,183
UBXN1_7_1265,550,72,1756,472,18,990,291,0,538,703,12,1817
UBXN2A_7_1266,1598,1056,146,1118,677,1702,651,1596,1774,917,258,1744
UBXN4_7_1267,654,274,907,1613,889,302,2405,487,483,809,345,1413
UBXN7_7_1268,114,5,1303,1,85,564,759,628,229,274,428,503
UBXN8_7_1269,110,0,696,146,541,66,719,299,678,355,63,84
UCHL1_7_1270,1823,901,697,1575,800,636,214,583,1047,827,801,1549
UCHL3_7_1271,1203,17,753,675,4,67,0,788,55,373,0,859
UFC1_7_1272,243,44,0,247,80,13,159,302,145,94,307,864

UHRF2_7_1273,265,44,0,1024,297,5,99,878,383,365,1,53
UNK_7_1274,0,19,8,277,765,18,619,278,414,281,155,88
USP11_7_1275,1,4,1,14,44,0,522,88,0,194,601,0
USP13_7_1276,99,1459,718,215,28,257,363,1473,1389,162,388,201
USP15_7_1277,670,1372,3060,2773,1191,1272,3484,951,1443,2062,1697,1431
USP17_7_1278,136,392,7,713,231,451,13,761,84,375,462,13
USP17L2_7_1279,136,392,7,713,231,451,13,761,84,375,462,13
USP17L5_7_1280,196,413,19,58,12,274,5,855,255,256,0,1201
USP18_7_1281,382,0,997,661,45,874,245,40,112,1156,29,480
USP22_7_1282,28,551,532,17,193,324,1625,513,592,856,1073,505
USP24_7_1283,817,991,626,53,198,82,469,1758,2357,156,272,409
USP25_7_1284,617,0,0,558,568,351,74,483,212,73,3,46
USP26_7_1285,5,404,1537,434,698,483,562,53,235,404,12,81
USP27X_7_1286,2,0,1,0,0,1,2,0,7,2,163,16
USP28_7_1287,197,117,127,422,2277,1577,360,356,632,25,463,326
USP29_7_1288,789,293,980,120,508,1582,424,1086,1536,70,616,1259
USP30_7_1289,134,300,240,252,1,18,1017,361,633,42,1400,90
USP32_7_1290,1656,9,140,256,1199,537,1511,81,894,284,36,489
USP34_7_1291,45,444,606,25,58,17,0,626,355,31,0,770
USP35_7_1292,236,112,2,259,0,4,1025,270,108,286,115,530
USP36_7_1293,1021,0,0,13,0,2,0,0,980,24,0,115
USP37_7_1294,230,257,79,14,1738,317,401,9,346,73,29,54
USP38_7_1295,13,315,5,154,20,11,0,0,102,120,0,11
USP39_7_1296,12,51,161,2,0,4,0,130,0,8,17,58
USP3_7_1297,389,1278,1540,758,771,1843,946,1071,834,1208,1045,2518
USP40_7_1298,246,1566,1659,2566,2746,2067,4026,1480,1539,2498,2366,321
1
USP42_7_1299,515,364,2180,730,1417,84,2755,1080,350,1983,4925,48
USP43_7_1300,22,1153,140,71,1259,110,114,797,694,1216,540,128
USP45_7_1301,88,148,563,7,0,5,1239,1127,21,144,1155,37
USP47_7_1302,239,2012,55,898,125,269,239,727,522,1055,757,1853
USP49_7_1303,359,513,851,286,53,331,699,1542,768,304,369,138
USP50_7_1304,638,90,170,92,33,28,320,618,557,626,9,466
USP51_7_1305,1268,650,463,85,679,1033,378,288,1039,1192,845,1052
USP53_7_1306,442,1300,1462,878,2331,904,983,736,1700,777,1272,1798
USP54_7_1307,0,31,255,1318,32,18,226,252,775,209,298,204
USP6_7_1308,606,41,127,295,451,202,570,1517,279,1076,470,164
USP7_7_1309,0,0,0,0,0,0,0,0,0,0,0,0
USP9Y_7_1310,25,0,650,1,3,0,6,0,79,2,900,18
USPL1_7_1311,1361,0,2,0,0,29,0,0,14,250,0,4
VCPIP1_7_1312,0,0,0,9,298,143,0,0,0,192,2,68
VPS11_7_1313,27,0,394,80,15,697,572,29,117,1,0,15
WDR12_7_1314,0,46,481,36,189,22,45,37,46,402,5,796
WDR53_7_1315,631,4,112,3,268,129,91,45,3,17,6,1
WDR59_7_1316,0,1,61,0,0,0,190,363,12,91,64,12
WDR5B_7_1317,434,653,294,24,112,1225,653,770,537,626,507,991
WDR61_7_1318,424,80,2,390,0,253,0,107,34,234,152,16
WDTC1_7_1319,0,0,0,0,0,0,0,0,0,0,0,0
WSB2_7_1320,8,159,2919,1249,34,421,385,244,389,315,60,643
WWP1_7_1321,818,1356,1562,311,23,892,893,656,464,1198,1231,732

YOD1_7_1322,971,718,828,472,974,526,1343,99,310,204,59,228
ZBTB11_7_1323,56,457,709,280,1224,214,1020,77,26,1590,1352,676
ZBTB25_7_1324,444,0,251,404,0,0,0,0,409,9,344,111
ZBTB2_7_1325,4,0,2,0,19,4,2,29,108,91,56,98
ZBTB32_7_1326,158,560,344,561,39,1,29,44,12,182,153,209
ZBTB34_7_1327,244,7,156,8,0,985,798,215,4,2,631,5
ZBTB39_7_1328,0,2,0,0,6,28,499,0,127,81,0,82
ZBTB3_7_1329,514,327,30,12,836,609,43,0,394,29,28,285
ZBTB41_7_1330,686,572,108,1062,0,1997,2767,2038,674,1420,719,1343
ZBTB44_7_1331,58,193,721,131,578,890,409,703,603,130,501,952
ZBTB45_7_1332,2,596,0,77,0,201,875,0,0,58,353,0
ZBTB46_7_1333,47,891,0,4,0,26,0,0,49,0,18,131
ZBTB47_7_1334,274,82,558,34,128,636,6,116,378,428,371,96
ZBTB48_7_1335,52,20,1037,720,481,529,679,1013,319,345,439,125
ZBTB49_7_1336,2452,937,2273,1470,1120,1434,767,922,721,2153,1231,1428
ZBTB5_7_1337,99,4,13,173,34,17,17,583,305,111,3,72
ZBTB7A_7_1338,0,6,0,335,78,597,695,36,937,1187,0,209
ZBTB7B_7_1339,0,10,0,1,42,25,0,0,5,1,42,0
ZBTB7C_7_1340,0,614,0,0,1597,0,0,0,307,10,2,25
ZBTB8A_7_1341,250,0,10,265,82,16,180,537,390,4,0,1136
ZFAND2B_7_1342,482,57,713,266,0,183,460,997,519,1004,361,667
ZFPL1_7_1343,1038,195,428,59,1196,50,1108,602,593,1439,2583,83
ZMYND10_7_1344,547,0,700,52,253,150,0,0,125,228,792,21
ZNF131_7_1345,1402,4,1552,400,540,1030,11,1050,350,727,519,1358
ZNF598_7_1346,618,603,0,752,993,88,50,506,0,317,121,403
ZNF645_7_1347,0,176,3,0,0,162,0,0,510,5,0,59
ZNRF1_7_1348,78,26,558,181,231,34,543,692,61,100,551,4
ZNRF2_7_1349,58,459,22,0,0,464,11,1010,1049,495,1169,137
ZNRF4_7_1350,1,48,0,0,42,68,120,6,27,3,293,19
ZRANB1_7_1351,61,0,5,15,87,57,5,315,1,856,0,0
ZSWIM2_7_1352,432,4,1978,69,221,996,3507,2195,822,1492,278,712
AIRE_7_1353,23,33,0,39,0,22,0,0,25,0,0,2
ANAPC5_7_1354,0,5,6,627,34,80,0,189,61,42,476,12
ANAPC7_7_1355,80,514,0,106,193,197,108,9,11,16,0,5
ANKFY1_7_1356,45,755,151,55,55,2152,0,182,144,93,83,51
ASB10_7_1357,1197,695,1359,182,1203,242,0,195,812,769,50,699
ASB11_7_1358,0,0,0,12,3,98,0,0,0,0,8,64
ASB14_7_1359,0,1,0,0,0,0,0,0,352,14,0,36
ASB2_7_1360,103,0,1,0,0,39,0,0,22,0,18,7
ASB3_7_1361,152,148,1063,279,627,126,28,395,468,570,548,275
ASB4_7_1362,41,499,146,350,25,268,49,117,262,30,643,554
ASB6_7_1363,0,66,0,0,0,0,0,1,0,0,0,0
ASB7_7_1364,31,107,352,32,246,0,0,303,325,6,42,286
ASB9_7_1365,48,16,82,1236,1,134,7,255,90,232,369,19
ASCC2_7_1366,217,99,837,119,0,109,788,164,1060,66,5,137
ATG10_7_1367,0,0,0,0,0,0,0,39,0,0,0,0
ATG16L1_7_1368,141,128,282,100,4,75,0,57,118,496,128,15
ATG7_7_1369,139,74,0,158,378,993,0,394,599,54,42,786
ATRX_7_1370,562,1192,405,0,90,251,220,86,776,30,30,106
BACH2_7_1371,0,0,0,0,0,0,0,0,0,101,236,0

BAG6_7_1372,132,1053,450,85,1046,581,190,84,1588,711,434,802
BAZ1A_7_1373,2057,1250,943,253,1064,2054,285,884,831,996,642,887
BCL6_7_1374,24,176,0,0,10,3,82,6,0,30,19,974
BIRC3_7_1375,14,61,9,574,165,211,835,151,647,35,871,176
BIRC7_7_1376,537,710,968,451,104,1171,87,1551,236,324,1424,1821
BPTF_7_1377,46,198,0,133,0,0,0,0,214,712,107,73
BRCA1_7_1378,447,70,728,66,0,420,198,763,374,10,413,629
BRPF1_7_1379,353,137,5,8,23,399,394,1,2,90,188,114
BRWD1_7_1380,5,0,0,11,0,302,1792,18,51,73,53,42
BTBD11_7_1381,76,121,12,0,2,41,486,0,27,57,0,2
BTBD1_7_1382,23,21,1015,392,7,58,146,437,115,170,29,241
BTBD3_7_1383,512,260,154,1,100,763,0,417,566,399,284,310
BTBD7_7_1384,0,9,31,0,1471,615,2,0,0,0,1,16
BTBD9_7_1385,242,170,5,187,0,320,170,135,329,302,346,203
BTRC_7_1386,1048,1602,19,372,929,259,2547,2429,814,2884,2768,337
C3orf26_7_1387,304,63,52,263,227,3,890,116,533,454,17,728
CBLC_7_1388,0,0,0,16,0,0,0,0,19,0,0,116
CCNB1IP1_7_1389,730,4547,2091,882,2632,862,1182,2147,1578,3506,2290,19
56
CDC16_7_1390,1298,883,1950,1132,3382,2186,1233,761,830,1050,3184,758
CDC27_7_1391,382,1224,505,910,2431,73,426,3525,778,1559,1132,1326
CHFR_7_1392,156,556,11,12,0,92,0,682,51,425,473,654
CISH_7_1393,0,4,0,0,0,601,0,9,157,0,47,23
CNOT4_7_1394,14,10,17,89,139,617,0,30,101,34,313,20
COPS2_7_1395,30,101,218,318,102,266,1420,0,173,565,612,848
COPS3_7_1396,761,741,903,99,270,395,933,928,237,333,300,291
COPS7A_7_1397,1,10,25,0,4,55,157,551,454,33,11,84
COPS8_7_1398,553,145,217,265,3067,16,485,326,403,6,1,1396
CRBN_7_1399,114,440,7,303,1089,1123,825,1017,322,941,874,979
CUL2_7_1400,0,277,0,37,0,0,0,17,5,173,1203,114
CUL4A_7_1401,534,27,689,149,0,215,0,1341,418,19,122,751
CUL4B_7_1402,30,231,228,0,0,0,75,267,174,252,4,209
CUL7_7_1403,167,0,0,0,52,5,0,0,211,4,4,16
CXXC1_7_1404,25,27,0,0,27,2,57,3,34,550,925,19
CYLD_7_1405,1071,309,0,27,403,0,2,1,248,28,78,331
DCAF11_7_1406,1666,570,0,426,0,282,152,287,1247,19,352,454
DCAF17_7_1407,706,476,1304,0,0,46,774,0,920,745,0,436
DCAF4_7_1408,1895,685,1901,876,531,2158,1772,1465,1130,1972,1534,661
DCAF6_7_1409,11,15,641,155,100,193,263,967,28,39,0,417
DCST1_7_1410,1243,6,109,5,0,254,235,23,257,822,744,33
DEPDC1B_7_1411,59,52,75,0,36,1164,0,0,23,5,804,3
DET1_7_1412,117,418,1232,188,162,3,0,761,825,654,17,534
DIDO1_7_1413,500,117,248,40,203,1420,421,213,1079,192,194,1093
DNAJB2_7_1414,49,356,0,580,190,296,0,667,1414,607,1,164
DTX2_7_1415,475,44,222,654,4,35,308,0,874,998,334,96
EED_7_1416,246,171,1113,1318,329,986,1667,60,40,492,1294,893
EIF3B_7_1417,3,0,901,590,20,524,7,0,3,350,394,158
EIF3C_7_1418,47,110,0,503,378,98,463,0,295,6,1510,2059
EIF6_7_1419,0,683,466,0,238,699,1660,177,19,1389,113,813
EPN1_7_1420,98,254,0,20,0,0,3,0,269,5,0,40

EPN2_7_1421,22,0,3,0,41,0,0,3,4,6,365,0
EPS15_7_1422,0,1090,34,51,7,449,125,1105,1283,46,100,910
FAM70A_7_1423,131,194,0,0,524,247,0,15,0,81,277,727
FANCL_7_1424,1819,2290,788,1412,512,1831,886,1241,1527,1972,399,1137
FBXL13_7_1425,1063,1092,903,603,438,1984,2215,1098,1022,1979,1998,122
FBXL20_7_1426,2,218,12,28,1710,189,0,631,135,232,656,786
FBXL2_7_1427,0,6,0,0,0,545,0,0,4,2,0,56
FBXL5_7_1428,0,5,2,43,141,4,8,89,10,808,182,99
FBXL6_7_1429,9,1508,590,1595,1757,731,1942,759,532,18,579,642
FBXO11_7_1430,715,1450,304,77,389,1161,207,0,31,344,23,10
FBXO15_7_1431,48,36,1768,4,613,133,330,236,137,32,159,178
FBXO17_7_1432,4,181,1044,0,293,0,13,3,38,2,303,226
FBXO18_7_1433,130,683,3,187,436,564,149,0,148,75,514,701
FBXO21_7_1434,571,541,32,231,7,4,0,11,72,105,1,8
FBXO22_7_1435,1,38,690,13,74,148,73,134,116,54,77,581
FBXO24_7_1436,175,84,323,259,377,55,988,1062,393,10,956,192
FBXO25_7_1437,1369,117,537,853,614,386,3,464,766,476,491,254
FBXO28_7_1438,433,10,822,38,7,970,221,3,548,570,1011,949
FBXO32_7_1439,562,0,858,2530,298,558,0,0,227,52,0,773
FBXO34_7_1440,545,1154,489,1743,28,616,832,403,983,959,1037,1546
FBXO38_7_1441,402,492,136,245,365,60,89,289,102,1,195,779
FBXO3_7_1442,0,0,0,0,0,0,0,0,0,184,0,0
FBXO44_7_1443,0,0,0,0,0,0,0,53,0,0,0,0
FBXO4_7_1444,151,122,187,12,626,972,180,580,670,125,399,1404
FBXO5_7_1445,283,10,133,71,487,224,58,157,36,258,60,9
FBXO7_7_1446,2,403,93,113,1456,100,223,0,0,95,0,873
FBXO9_7_1447,492,1842,1223,415,1425,811,27,588,109,1503,2202,1907
FBXW11_7_1448,0,22,767,0,212,22,851,134,705,345,123,84
FBXW7_7_1449,771,659,59,186,168,1295,709,189,108,393,430,664
FBXW8_7_1450,55,940,258,36,1469,1098,1219,125,11,1216,1,245
GPS1_7_1451,0,1,7,1,22,0,0,0,21,34,0,2
HECTD2_7_1452,564,1858,217,40,15,212,280,66,5,469,944,472
HERC4_7_1453,184,6,32,7,260,330,63,138,273,16,41,249
HERC6_7_1454,1,842,518,1,237,10,15,661,143,417,43,806
HIC1_7_1455,0,0,691,0,3,0,611,0,319,4,0,41
HLTF_7_1456,19,46,340,961,113,54,0,1084,426,457,148,630
HSF4_7_1457,250,0,0,50,0,362,634,76,0,43,0,1
IPP_7_1458,0,1,35,0,382,45,256,0,222,101,818,328
KAT6A_7_1459,272,248,229,49,73,423,355,1428,385,308,1439,431
KBTBD3_7_1460,655,1299,639,2606,2546,557,345,1850,5037,3679,2204,2706
KCTD6_7_1461,1723,729,457,1430,172,1069,855,2075,886,615,2376,1557
KCTD7_7_1462,1737,907,741,1144,726,830,2341,167,469,892,2938,567
KDM2B_7_1463,256,124,8,28,1152,283,8,27,118,516,220,1810
KDM4C_7_1464,7537,11368,9556,4248,7002,8327,10873,6974,10614,7168,5955
,4451
KDM5C_7_1465,0,1,65,0,0,0,0,0,0,0,0,47
KEAP1_7_1466,0,1,0,0,86,344,0,0,1,0,0,58
KIAA1841_7_1467,390,208,421,6,28,112,77,753,398,1766,714,749
KLHL13_7_1468,1056,1080,729,2,494,480,492,18,1690,404,197,1194
KLHL2_7_1469,1508,357,1861,950,644,91,120,94,227,371,822,48

KLHL4_7_1470,1659,737,177,0,2,614,715,105,31,53,51,731
KLHL5_7_1471,122,243,135,587,56,329,584,82,44,68,32,24
KLHL7_7_1472,101,892,1091,53,6,142,152,5,12,60,83,586
LNX1_7_1473,0,244,0,0,0,142,0,188,135,14,0,304
LONRF3_7_1474,407,148,0,27,1,19,59,0,141,1151,913,1820
LRRC29_7_1475,0,0,42,0,0,1,0,0,0,288,0,0
LRSAM1_7_1476,143,6,0,50,290,0,131,21,205,144,76,21
MARK2_7_1477,0,117,0,1,0,1,0,1,182,3,0,30
MARK3_7_1478,1102,831,516,490,1624,834,284,523,390,719,1131,669
MARK4_7_1479,1240,63,545,119,1127,67,309,184,334,233,1763,107
MDM4_7_1480,794,2013,3600,1894,26,2049,1621,1574,1548,818,2899,454
MGRN1_7_1481,0,256,317,156,964,1004,954,217,707,31,466,503
MIB2_7_1482,443,0,1754,0,673,320,580,9,1,3,507,44
MID1_7_1483,289,72,600,37,527,240,48,712,272,124,712,113
MID2_7_1484,717,1102,693,150,100,469,16,1401,817,416,414,371
MKRN1_7_1485,99,26,1083,1269,150,767,1347,391,152,265,18,121
MLL5_7_1486,1,4,2,3,4,0,0,0,44,196,14,184
MLL_7_1487,894,2529,29,5,2323,357,594,862,578,1704,388,938
MNAT1_7_1488,203,96,10,31,0,0,43,333,16,268,2,337
MPND_7_1489,44,0,0,0,0,0,0,0,0,0,0,2
MTF2_7_1490,508,521,1838,306,200,1565,761,1446,930,1310,2238,804
MYNN_7_1491,281,198,185,106,8,262,0,259,60,0,364,331
NAE1_7_1492,673,2,0,1734,0,0,0,2,0,27,0,852
NDUFC2_7_1493,646,95,608,348,1061,458,222,453,767,1064,564,386
NEDD4_7_1494,0,378,0,107,0,153,7,0,228,147,0,30
NEDD4L_7_1495,33,0,30,254,21,435,1,71,25,270,148,9
NFX1_7_1496,1542,1455,1321,404,2554,1768,3563,956,3578,4216,3173,2956
NLE1_7_1497,339,167,4,11,5,732,622,188,73,333,615,24
NSD1_7_1498,140,563,0,3,13,228,0,171,424,12,2,43
NSFL1C_7_1499,235,655,673,792,781,160,640,605,1120,878,669,921
NUB1_7_1500,42,94,811,549,1,116,0,583,140,79,596,204
ODF2_7_1501,0,1,0,250,217,1,0,69,983,21,9,199
OTUD5_7_1502,210,226,47,94,283,4,52,143,294,365,200,1346
PARK2_7_1503,22,521,124,599,551,563,485,31,515,342,788,1118
PARP9_7_1504,170,8,1012,44,424,250,61,6,12,77,142,393
PATZ1_7_1505,1,290,1503,214,437,208,165,586,103,122,676,458
PCGF6_7_1506,298,247,60,893,2317,1678,69,97,1024,136,974,894
PEX10_7_1507,2,1,981,0,0,0,937,0,0,0,3,0
PEX2_7_1508,1771,1676,339,1233,1966,967,2238,2226,1279,2289,1504,706
PHF12_7_1509,5,306,52,73,11,610,13,653,135,514,0,36
PHF16_7_1510,206,109,30,248,1,0,0,75,361,20,5,54
PHF17_7_1511,236,1,345,1005,1181,324,658,524,503,364,1347,471
PHF1_7_1512,0,18,0,0,2,4,3,0,7,391,0,0
PHF21A_7_1513,817,2040,188,1109,389,938,3007,1402,1235,237,34,270
PHF7_7_1514,20,50,1,216,10,4,336,0,219,41,256,85
PHF8_7_1515,6,78,0,0,21,0,61,178,18,69,34,29
PJA1_7_1516,27,0,7,155,91,136,268,4,5,0,700,589
PML_7_1517,0,10,790,32,0,8,0,178,0,86,374,40
POC1B_7_1518,1448,2014,666,770,1456,857,489,2103,352,1582,296,332
PSMD1_7_1519,0,1,111,0,0,173,17,399,24,594,0,965

RAPSN_7_1520,188,1,838,7,189,110,1209,647,387,169,39,459
RBBP4_7_1521,1924,2553,352,148,753,1831,1860,4773,2633,931,4001,2327
RBBP5_7_1522,63,431,59,136,430,48,29,1,151,4,0,33
RBBP6_7_1523,2414,164,419,577,2152,1001,1170,753,710,1141,2849,864
RBBP7_7_1524,402,359,55,186,589,31,487,362,244,366,16,1168
RBCK1_7_1525,0,369,0,0,0,0,0,0,1,86,0,0
RC3H2_7_1526,414,374,1722,349,92,428,4,413,120,443,246,20
RCHY1_7_1527,1,932,3173,201,471,1978,75,55,2035,744,460,526
RFPL2_7_1528,1755,889,1848,12,765,370,1374,219,1040,2056,1328,907
RFPL3_7_1529,1773,1992,40,780,31,1553,885,387,1238,507,2737,2213
RFWD2_7_1530,499,208,0,0,2,0,330,307,745,23,3,352
RHOBTB1_7_1531,0,0,0,0,0,0,0,0,0,0,0,0
RHOBTB2_7_1532,0,273,381,234,0,562,220,480,707,404,1144,238
RLIM_7_1533,855,16,593,574,239,173,881,1136,1071,136,876,1189
RNF103_7_1534,0,515,2146,0,14,28,189,7,355,8,495,42
RNF128_7_1535,212,47,0,3,77,142,132,393,1,18,784,41
RNF135_7_1536,101,6,1,0,893,11,577,230,50,22,746,4
RNF138_7_1537,2841,1037,3068,312,796,503,3331,1843,892,836,874,1422
RNF13_7_1538,473,572,651,46,1501,523,570,909,177,882,714,19
RNF145_7_1539,1129,146,158,250,708,555,0,63,169,1088,194,341
RNF146_7_1540,93,244,0,0,3,11,639,79,46,361,41,18
RNF14_7_1541,10,0,0,0,0,0,0,11,670,25,0,201
RNF166_7_1542,144,0,1209,0,781,879,0,1,3,0,16,593
RNF170_7_1543,408,649,1102,87,1056,329,737,979,1435,1599,1790,2467
RNF17_7_1544,77,206,161,307,21,13,285,279,1806,46,672,469
RNF180_7_1545,1378,850,1911,998,1580,298,769,1322,2756,895,105,1589
RNF182_7_1546,957,14,1238,1666,929,331,911,1021,1292,1163,2686,357
RNF185_7_1547,170,356,0,21,0,59,0,104,335,35,11,76
RNF19A_7_1548,29,0,0,0,0,0,1029,0,225,6,0,18
RNF19B_7_1549,1575,755,141,1325,1412,2242,1233,22,1718,1208,581,1471
RNF213_7_1550,583,426,111,448,829,1023,1020,119,376,12,308,1072
RNF214_7_1551,22,246,0,92,4,32,370,11,104,2,40,16
RNF216_7_1552,596,24,944,33,3,419,1774,733,64,283,0,37
RNF24_7_1553,2364,3761,2193,934,1182,1289,1062,1603,3427,2210,1433,141
0
RNF32_7_1554,1360,452,15,877,215,220,86,950,478,1010,796,599
RNF34_7_1555,315,162,8,138,10,3,198,9,989,22,13,288
RNF38_7_1556,625,1,512,291,267,77,54,442,843,15,204,238
RNF40_7_1557,6,551,348,0,200,588,352,408,320,166,3,223
RNF41_7_1558,36,533,0,196,0,54,0,0,170,48,0,17
RNF6_7_1559,1900,2186,1713,2164,1547,1441,1668,2187,3252,1744,8302,359
1
RNF7_7_1560,554,674,0,0,0,0,0,49,8,21,78,14
RNF8_7_1561,0,371,1030,68,0,544,170,561,924,347,352,159
SAE1_7_1562,1467,695,432,727,811,494,1096,278,260,541,709,59
SENP6_7_1563,634,760,33,211,852,1,530,1,1,267,613,377
SENP7_7_1564,1119,274,390,128,1848,217,739,430,554,1380,675,1031
SENP8_7_1565,14,448,27,0,681,0,52,769,464,1060,0,301
SF3A1_7_1566,750,70,0,547,3,0,732,589,55,2411,0,407
SHPRH_7_1567,201,0,26,489,67,175,2440,734,296,2125,574,826

SIAH1_7_1568,21,0,0,56,1148,44,266,0,14,44,599,18
SKP2_7_1569,864,825,149,1327,903,1081,690,745,599,653,27,647
SMURF1_7_1570,2,6,0,0,0,9,94,2,897,22,714,171
SOCS5_7_1571,248,0,1,62,32,0,1666,9,39,156,539,9
SP100_7_1572,3477,1819,1045,3754,2210,3460,4394,2192,4420,1942,1339,37
10
SP110_7_1573,54,4,201,16,289,5,1047,179,710,364,202,854
SPOP_7_1574,1225,423,211,1065,714,1101,430,3139,1082,457,589,1200
SPSB2_7_1575,971,1160,808,440,740,736,119,2940,1237,928,975,222
SQSTM1_7_1576,15,416,464,383,181,3,114,3,190,10,619,33
STAMBP_7_1577,227,0,108,329,769,745,45,588,0,527,503,471
SUMO1_7_1578,79,21,261,67,166,70,12,413,319,607,457,64
SUMO2_7_1579,3431,2098,2235,1875,1770,5513,821,1540,2163,1047,3744,276
9
SYTL4_7_1580,30,456,58,6,151,183,524,358,1154,279,51,1132
SYVN1_7_1581,280,0,0,3,2,0,30,1,7,88,0,9
TCF20_7_1582,0,0,0,0,371,0,0,52,1,0,0,1
TDRD3_7_1583,0,453,0,2,0,0,0,0,0,37,147,0
TIPARP_7_1584,1307,963,1486,1193,59,4796,775,2073,4334,1882,3370,2112
TLE2_7_1585,439,888,160,1703,131,849,604,1417,199,297,1044,349
TLE3_7_1586,182,121,2,24,108,124,51,82,344,33,482,125
TNK2_7_1587,810,4,9,142,51,0,512,609,0,5,39,17
TNRC6C_7_1588,30,18,32,71,0,62,594,1014,660,469,2,250
TOPORS_7_1589,1761,2938,2926,2615,3208,1827,3756,1932,3290,3566,2247,3
953
TOR1AIP2_7_1590,2091,673,828,1051,129,2392,23,794,1382,1480,490,244
TRAF3_7_1591,1989,2993,3207,1961,1036,2397,2888,3316,2532,5106,2981,31
61
TRAF5_7_1592,553,40,643,850,265,588,75,163,1064,334,1351,664
TRAF6_7_1593,2841,736,3154,382,4400,1484,632,1279,2588,877,1985,823
TRIM10_7_1594,79,135,0,12,0,6,9,150,147,229,1,42
TRIM13_7_1595,674,487,403,519,145,128,1188,859,97,888,1,81
TRIM17_7_1596,110,19,577,260,628,173,177,221,211,39,37,261
TRIM22_7_1597,611,54,116,268,147,14,182,1413,805,171,7,1470
TRIM23_7_1598,964,980,59,811,1238,716,99,578,601,549,46,1373
TRIM24_7_1599,0,0,0,0,0,0,0,0,0,0,0,0
TRIM26_7_1600,0,113,0,0,193,0,413,2,0,0,1,0
TRIM2_7_1601,477,131,1,0,0,36,0,15,47,41,0,5
TRIM32_7_1602,9,0,0,1,62,108,0,0,14,23,0,0
TRIM33_7_1603,0,188,839,4,4,1015,172,157,119,244,344,235
TRIM34_7_1604,1687,894,1150,1248,549,1293,856,753,1196,1300,1708,1441
TRIM37_7_1605,509,104,773,25,0,0,255,23,462,149,226,229
TRIM39_7_1606,0,134,6,600,610,207,285,20,2,785,0,1
TRIM3_7_1607,0,2,0,2,0,0,171,18,0,2,1010,0
TRIM41_7_1608,0,0,0,0,0,0,1,0,0,0,0,0
TRIM45_7_1609,0,0,0,0,0,0,0,0,0,0,0,237
TRIM4_7_1610,130,49,525,331,6,221,1626,105,0,220,158,19
TRIM54_7_1611,0,0,114,0,0,0,49,9,530,15,0,61
TRIM55_7_1612,0,157,33,459,1086,1,177,591,17,26,51,2
TRIM5_7_1613,0,0,0,0,0,83,0,1,0,283,3,122

TRIM7_7_1614,56,0,3,136,497,654,488,568,4,391,828,273
TRIM9_7_1615,49,35,13,874,6,6,7,117,1,249,39,128
TRPC4AP_7_1616,111,134,25,3,569,243,9,0,65,0,85,5
TSPAN17_7_1617,648,330,936,15,1559,488,25,110,986,486,916,325
TTC3_7_1618,0,0,0,0,307,640,0,0,521,9,0,77
TULP4_7_1619,137,328,2,21,40,35,132,187,80,241,18,154
UBA1_7_1620,26,362,249,38,1092,457,262,2153,542,82,156,69
UBA3_7_1621,22,235,48,281,989,538,586,162,482,338,589,680
UBA5_7_1622,661,480,11,303,893,0,0,253,832,21,233,93
UBAC2_7_1623,23,332,7,45,28,0,210,455,148,516,532,25
UBAP2L_7_1624,1,0,43,30,0,116,0,8,0,0,0,0
UBASH3A_7_1625,0,1,0,340,0,122,534,143,136,539,196,44
UBE2A_7_1626,6,27,2,174,0,20,0,277,64,14,0,36
UBE2D1_7_1627,484,527,176,98,306,569,184,4,1466,548,768,1413
UBE2D2_7_1628,10,4,0,355,1,0,0,212,86,7,0,422
UBE2D3_7_1629,11,0,0,86,0,12,11,13,72,0,987,9
UBE2E1_7_1630,4814,3697,5479,2566,3993,3006,4483,4751,2868,2257,4161,4
855
UBE2E3_7_1631,228,443,0,240,113,206,998,145,241,376,327,430
UBE2G2_7_1632,7,0,20,0,66,0,10,0,6,5,7,1
UBE2H_7_1633,730,577,2072,654,79,27,314,426,568,86,90,1253
UBE2I_7_1634,2031,1837,1836,1813,1721,3099,4281,2253,1614,1432,1118,19
77
UBE2J2_7_1635,306,84,141,388,52,21,26,345,594,95,373,452
UBE2K_7_1636,781,1492,1934,1808,2841,744,956,868,1369,1759,53,3395
UBE2Q2_7_1637,4,575,36,9,1215,757,0,0,0,9,60,32
UBE2V1_7_1638,0,431,675,14,17,420,72,644,230,37,398,93
UBE2W_7_1639,568,1243,409,654,1273,1250,364,853,1570,1789,2476,1144
UBE3A_7_1640,3387,1866,46,300,1518,27,48,0,1097,480,762,1509
UBE3B_7_1641,120,94,152,978,0,45,21,42,45,86,231,562
UBE4A_7_1642,5207,591,1546,1027,1274,2462,788,2228,2494,2055,3612,3712
UBE4B_7_1643,0,86,11,1,118,0,2,63,25,35,100,3
UBL7_7_1644,0,167,902,31,0,34,682,224,37,177,231,325
UBOX5_7_1645,215,0,532,436,518,321,4,5,408,380,698,184
UBQLN1_7_1646,118,58,1221,331,1,439,0,1146,583,13,709,103
UBR2_7_1647,116,5,81,266,1021,330,5,254,118,474,522,754
UBXN11_7_1648,615,719,160,1220,808,383,1160,2137,965,247,689,2067
UBXN6_7_1649,0,0,0,21,3,0,14,686,5,292,0,0
UCHL5_7_1650,0,129,61,40,99,114,1,10,7,78,23,34
UHRF1_7_1651,69,0,0,0,0,0,0,144,0,0,1,0
UIMC1_7_1652,646,1841,2448,2186,171,2250,1005,260,455,926,1279,724
UNKL_7_1653,862,2,6,2,497,75,0,332,0,1,1,11
USP14_7_1654,0,2,165,212,108,1,2,0,4,6,762,0
USP19_7_1655,18,233,27,0,155,2,120,0,156,75,0,22
USP1_7_1656,1216,407,679,503,3239,263,994,512,1208,1222,1536,807
USP20_7_1657,92,139,0,91,0,153,0,0,1,239,0,811
USP2_7_1658,21,162,815,165,0,0,0,20,1,12,0,4
USP33_7_1659,376,50,87,385,2075,490,598,1746,650,587,286,942
USP44_7_1660,92,1468,296,380,80,163,396,247,261,377,3,399
USP46_7_1661,1864,131,116,1232,986,2287,94,640,769,407,34,1730

USP48_7_1662,19,28,14,0,506,0,33,18,126,30,297,46
USP4_7_1663,532,490,0,1,3,0,263,1,456,256,0,110
USP5_7_1664,259,136,48,777,0,4,3,9,3,8,39,37
USP6NL_7_1665,7,1,20,82,0,2,6,30,63,252,0,14
USP8_7_1666,2945,1771,1602,662,4676,1446,40,2812,1220,1802,901,336
USP9X_7_1667,1808,745,1379,53,643,1609,56,651,848,847,228,776
VHL_7_1668,0,150,0,13,0,0,0,0,12,2,0,1
VPRBP_7_1669,110,479,839,100,2238,50,174,737,361,947,1344,192
VPS13D_7_1670,81,0,58,273,0,869,0,250,2,209,57,0
VPS41_7_1671,5,140,0,0,0,4,0,0,0,375,0,0
VPS8_7_1672,0,4,246,46,451,14,113,137,164,137,358,846
WDR26_7_1673,479,80,207,190,0,353,464,234,95,57,33,18
WDR5_7_1674,8,37,0,8,223,19,0,1183,0,208,351,0
WDR76_7_1675,633,358,24,170,626,612,0,0,0,432,84,487
WHSC1_7_1676,9,1372,125,767,400,1987,619,1880,960,1700,1619,1591
WHSC1L1_7_1677,625,697,42,484,807,716,15,1395,42,2813,368,258
WSB1_7_1678,1964,131,127,706,28,1259,3080,2886,1331,2033,3123,935
WWP2_7_1679,0,0,494,0,0,8,117,2,10,88,543,577
XIAP_7_1680,352,682,19,181,1,2036,1473,1031,1172,195,517,1919
ZBTB10_7_1681,11,10,10,76,4,273,0,0,164,9,160,26
ZBTB16_7_1682,1,13,401,221,16,673,0,113,312,588,5,2896
ZBTB17_7_1683,85,1,585,0,0,431,140,0,26,2,0,6
ZBTB1_7_1684,1883,1,885,0,725,43,920,888,1170,2285,180,1468
ZBTB20_7_1685,609,0,0,0,0,15,0,0,0,0,0,0
ZBTB22_7_1686,1,75,0,3,14,160,0,291,35,4,0,6
ZBTB24_7_1687,2348,1057,2036,669,5660,1981,3735,1242,1647,1898,4851,40
70
ZBTB33_7_1688,69,26,8,853,181,220,1000,71,276,219,224,530
ZBTB37_7_1689,956,234,102,656,945,1070,1785,1548,437,1305,1120,899
ZBTB40_7_1690,131,18,0,216,1,238,0,46,150,2,459,20
ZBTB4_7_1691,8,0,1913,45,881,256,1,93,385,25,1,158
ZFP161_7_1692,23,2280,4,10,84,1367,1244,0,0,531,65,21
ZMYND11_7_1693,109,75,193,147,12,7,0,0,135,33,83,136
ZMYND8_7_1694,540,352,0,432,492,548,37,0,4,479,569,153
ZNF238_7_1695,58,16,420,1,67,241,1396,2,188,138,627,149
ZNF295_7_1696,0,0,0,130,0,296,0,37,500,46,0,61
ZNRF3_7_1697,311,0,686,584,176,92,385,482,663,310,326,157
ANAPC11_7_1698,4,404,72,24,184,353,25,1377,15,404,61,362
ATXN3_7_1699,667,252,71,67,68,348,1057,0,242,1793,157,1505
MLLT10_7_1700,1847,796,2853,3185,994,1805,60,2781,2491,3536,2705,1156
OTUD4_7_1701,5,7,0,0,0,24,0,0,31,0,0,3
PHF19_7_1702,2453,447,1232,565,1858,773,1596,2452,2138,1423,1929,757
SP140_7_1703,58,0,0,0,0,7,130,33,558,241,319,231
TRIM36_7_1704,2298,3167,2304,2394,3061,1890,1467,4081,3196,2053,2517,2
849
UBE2C_7_1705,1240,841,1585,1458,1735,1666,216,1243,171,756,330,1145
UBE2L6_7_1706,26,1,1,0,0,41,333,0,2,4,0,182
ABTB2_7_1707,439,555,1167,979,128,986,1286,440,1072,341,513,1850
AMBRA1_7_1708,0,73,469,2,45,0,0,151,389,2,0,144
AMFR_7_1709,170,855,1397,726,256,68,0,94,36,582,289,281

ANAPC10_7_1710,1059,753,78,543,1228,3697,1135,1069,2717,1703,1489,1675
ANAPC1_7_1711,132,66,1813,66,219,0,676,35,367,28,1093,58
ANAPC2_7_1712,0,0,0,0,2,74,0,3,250,6,0,37
ANAPC4_7_1713,8,523,693,455,0,432,37,0,6,5,1029,0
ANKIB1_7_1714,191,629,1520,0,0,522,0,765,500,273,24,1092
ANKRD13A_7_1715,293,629,276,604,1324,2057,1386,15,404,1182,1578,1160
ANKRD13D_7_1716,13,562,0,2,0,0,771,0,32,3,0,265
ARIH1_7_1717,1323,976,1790,2261,1314,995,1062,2141,1014,1605,2490,978
ARIH2_7_1718,677,232,24,77,537,463,164,655,539,783,772,321
ASB12_7_1719,1547,1939,1506,237,1857,915,1549,734,408,1883,1412,2032
ASB13_7_1720,0,23,0,11,0,0,0,18,570,14,55,79
ASB15_7_1721,253,160,217,84,13,39,5,0,132,2,861,156
ASB16_7_1722,14,3,0,586,0,0,527,0,0,0,0,349
ASB17_7_1723,595,59,18,161,75,824,43,344,236,583,37,83
ASB5_7_1724,6,105,623,0,166,358,1423,0,72,110,736,158
ASB8_7_1725,556,1513,564,562,22,1508,2489,1349,1291,741,2074,1133
ASH1L_7_1726,75,13,3,17,0,141,0,820,26,291,9,654
ASPSCR1_7_1727,119,1,507,0,0,0,0,14,0,632,37,0
ATG12_7_1728,885,114,17,521,273,180,71,356,543,32,198,861
ATG3_7_1729,2377,4153,2131,1845,2269,2288,1290,2758,1667,2009,1977,139
6
ATG5_7_1730,172,418,935,765,1315,42,534,1753,1109,1759,558,1752
ATXN1L_7_1731,163,217,169,98,469,1040,0,78,9,457,93,551
ATXN3L_7_1732,3,0,29,0,0,0,0,44,63,22,298,18
BARD1_7_1733,916,553,625,260,30,748,895,535,228,281,108,353
BAZ1B_7_1734,995,1942,135,535,142,843,1064,1580,1280,1521,947,1020
BAZ2A_7_1735,467,205,615,913,1031,172,121,1012,735,1390,984,149
BAZ2B_7_1736,1145,1213,2275,1531,1383,1612,1273,1289,1803,1044,1065,12
53
BECN1_7_1737,292,79,0,98,165,5,36,0,61,1,305,5
BIRC2_7_1738,701,2841,1773,42,180,588,2148,1329,1572,1375,842,750
BIRC6_7_1739,459,177,40,1173,151,362,2247,291,147,423,1207,1701
BIRC8_7_1740,188,811,450,447,334,881,39,408,197,563,380,1006
BMI1_7_1741,40,2,458,882,12,12,46,505,2,130,214,101
BRAP_7_1742,1609,857,649,948,1133,754,1287,599,550,187,270,541
BRD1_7_1743,0,0,193,4,360,19,32,1059,872,18,402,110
BRPF3_7_1744,1370,854,91,514,128,572,49,11,1,566,55,108
BTBD2_7_1745,457,0,301,0,0,0,965,0,0,15,3,1
BTBD6_7_1746,49,660,15,240,0,0,0,22,4,30,0,2
CAND1_7_1747,47,920,39,747,251,897,0,549,661,51,2,360
CBLB_7_1748,341,556,51,1,23,582,16,279,22,760,384,5
CBL_7_1749,1140,564,1077,848,194,392,392,346,1167,770,426,2379
CBLL1_7_1750,81,270,0,258,77,338,35,660,29,356,1122,2
CCIN_7_1751,0,0,0,0,20,0,138,135,0,0,44,64
CCNF_7_1752,0,76,1,54,0,0,30,0,165,212,0,22
CDC20_7_1753,764,1346,1246,1187,2258,510,730,250,508,830,1948,2464
CDC23_7_1754,0,47,6,0,9,0,10,0,22,0,5,5
CDC26_7_1755,1,0,0,6,0,9,0,0,0,0,0,0
CDC34_7_1756,5,0,321,459,0,86,522,146,8,13,0,25
CGRRF1_7_1757,3852,4140,3241,1826,1655,1399,1008,3191,1298,3070,4714,2

492
CHD4_7_1758,83,120,470,414,0,1,629,475,103,259,80,581
CIAO1_7_1759,1,403,1097,112,1294,240,268,224,1707,783,1636,687
COPS4_7_1760,0,0,0,29,0,0,0,0,0,0,223,0
COPS5_7_1761,111,851,633,902,413,295,824,1,450,115,756,1830
COPS6_7_1762,0,34,35,0,71,0,85,0,6,306,229,183
COPS7B_7_1763,18,17,1,82,3,0,9,47,44,156,41,59
CORO6_7_1764,13,547,207,486,0,171,0,100,763,11,0,445
CUEDC1_7_1765,798,17,2,44,2,68,14,44,917,206,137,114
CUL1_7_1766,1016,796,0,1517,63,468,22,627,374,368,863,819
CUL3_7_1767,381,1325,0,183,2029,752,69,259,873,764,540,1000
CUL5_7_1768,2926,4364,2942,1571,2282,1320,4185,1974,2010,3111,3046,245
0
CUL9_7_1769,5,100,626,68,130,3,26,364,28,18,875,300
DCAF10_7_1770,1326,2886,522,2874,1878,4387,4502,2957,2722,2845,6091,28
92
DCAF12_7_1771,194,47,282,159,353,111,46,96,97,341,0,13
DCAF13_7_1772,3980,746,50,987,865,65,245,186,672,449,43,673
DCAF16_7_1773,1966,2558,2040,1067,4481,828,1223,2503,2380,3000,1630,25
45
DCAF5_7_1774,102,648,1,859,1034,1774,334,504,573,716,693,999
DCAF7_7_1775,0,211,0,0,0,0,0,91,0,231,0,231
DCAF8_7_1776,0,1,193,0,0,62,336,13,4,0,0,13
DDA1_7_1777,0,0,0,15,0,0,3,0,0,4,0,0
DDB1_7_1778,524,317,1571,910,1466,1365,1093,679,869,680,1283,547
DDB2_7_1779,0,175,0,9,0,3,0,0,0,0,22,0
DPF2_7_1780,184,0,2,0,19,2,29,0,304,811,23,808
DTX1_7_1781,0,4,0,22,3,2,0,140,742,142,822,211
DTX3L_7_1782,55,254,103,1015,123,304,544,286,729,1090,92,242
DTX4_7_1783,1640,543,280,361,1466,772,1021,222,73,450,1154,56
DZIP3_7_1784,2418,2773,2363,1964,1792,2680,1421,1235,1448,1828,2601,15
68
EIF3D_7_1785,479,248,278,5,148,1415,297,1196,639,193,300,287
EIF3E_7_1786,611,0,191,169,105,0,31,184,133,96,704,130
EIF3F_7_1787,213,598,427,661,158,313,338,543,724,1102,809,1190
EIF3G_7_1788,555,343,26,939,5,6,162,20,31,1128,2,710
EIF3H_7_1789,3557,2074,3519,2144,1742,3614,3847,4214,1033,2521,2564,24
60
EIF3I_7_1790,1,780,0,0,0,0,715,0,106,2,0,7
EIF3J_7_1791,564,1319,1251,1910,855,738,1113,1382,366,847,559,1193
EIF3K_7_1792,81,0,153,0,115,77,19,58,1,25,852,86
ENC1_7_1793,561,412,1229,166,318,646,56,181,552,288,703,66
EPN3_7_1794,144,61,112,0,3,0,0,0,505,508,0,758
EPOR_7_1795,494,314,8,841,0,18,1,24,1046,322,50,520
ERCC8_7_1796,737,2825,3088,849,1143,609,1570,421,2353,1832,2063,487
FAF1_7_1797,137,0,0,0,82,786,0,128,9,67,261,3
FBXL12_7_1798,91,543,1,148,9,0,0,0,252,79,1007,53
FBXL14_7_1799,202,438,41,162,545,327,9,43,305,67,31,33
FBXL15_7_1800,619,1,1,593,1259,47,0,58,1005,168,0,138
FBXL16_7_1801,249,123,245,65,645,774,1850,5,369,385,134,480

FBXL17_7_1802,724,122,31,934,3819,1349,1579,194,640,403,257,1010
FBXL18_7_1803,842,1298,2266,2258,2877,1705,1098,672,1463,710,1115,2471
FBXL19_7_1804,60,0,0,2,8,39,125,0,39,0,0,4
FBXL21_7_1805,0,0,0,0,0,0,0,0,0,0,0,0
FBXL3_7_1806,651,164,453,55,860,35,10,275,482,1122,378,64
FBXL4_7_1807,1340,1778,735,1249,1752,1607,2047,3267,740,2787,351,3219
FBXL7_7_1808,0,0,0,123,0,0,0,0,0,0,0,0
FBXO10_7_1809,0,103,0,79,0,35,0,0,0,50,57,265
FBXO16_7_1810,206,557,0,37,5,54,8,164,146,1,827,103
FBXO27_7_1811,6,0,0,127,34,2,844,3,30,1,30,627
FBXO2_7_1812,4,2,0,32,3,4,15,0,0,6,0,0
FBXO30_7_1813,343,537,1979,1016,869,1520,1093,640,1660,1199,181,325
FBXO33_7_1814,371,651,1385,636,464,232,1,47,563,1043,387,1428
FBXO36_7_1815,1422,2119,1423,412,2324,344,1994,594,1214,645,1600,1439
FBXO39_7_1816,195,1005,0,9,45,4,675,114,445,175,0,229
FBXO40_7_1817,0,65,407,9,7,0,0,0,485,22,0,55
FBXO41_7_1818,5,0,0,2,0,103,0,1,1,0,615,0
FBXO42_7_1819,472,0,12,180,285,336,240,123,26,24,1753,447
FBXO43_7_1820,697,233,45,453,103,1126,453,2321,626,556,547,436
FBXO46_7_1821,28,1796,1747,455,20,980,2551,1675,632,828,815,375
FBXO6_7_1822,544,612,14,0,293,320,0,537,361,530,509,845
FBXO8_7_1823,932,404,2,266,0,4,1310,687,225,1207,7,768
FBXW10_7_1824,605,888,89,98,770,1691,46,129,210,962,478,1519
FBXW2_7_1825,1099,1102,1296,1207,1004,863,1862,806,1896,839,335,1905
FBXW5_7_1826,112,1,1379,145,0,650,584,398,160,142,608,700
FBXW9_7_1827,5,0,0,64,0,0,0,15,0,171,310,14
G2E3_7_1828,656,1308,0,654,9,513,29,173,0,621,680,489
GAN_7_1829,0,0,0,0,166,0,279,0,2,0,12,0
GMCL1_7_1830,140,83,1971,662,29,162,1163,420,516,876,586,407
GNB2_7_1831,0,0,0,1,3,3,0,0,43,0,23,222
GRWD1_7_1832,520,328,143,1406,286,914,147,196,808,531,578,786
GZF1_7_1833,554,367,92,1414,328,1123,401,2635,1062,812,596,499
HACE1_7_1834,1395,452,1073,1574,588,581,739,2606,1103,1054,1431,2084
HDAC6_7_1835,1836,433,2982,1332,518,323,1070,435,1012,1456,2050,1056
HECTD1_7_1836,0,577,0,592,0,521,2,1465,5,661,0,788
HECTD3_7_1837,916,267,545,847,1217,904,350,1410,141,36,378,65
HECW1_7_1838,0,1,0,0,0,0,0,486,99,1,0,22
HECW2_7_1839,15,1,292,418,108,560,285,672,332,175,897,143
HERC1_7_1840,983,4,229,590,708,970,238,435,905,473,352,1740
HERC2_7_1841,1255,686,1178,1607,294,1331,1296,541,845,1252,1569,327
HERC3_7_1842,734,846,649,203,775,784,264,437,1068,980,395,1910
HERC5_7_1843,690,1238,273,962,301,602,1172,703,709,935,201,1564
HGS_7_1844,0,24,0,589,1,0,0,148,7,86,16,5
HIC2_7_1845,22,10,0,4,17,249,100,0,623,20,1,84
HUWE1_7_1846,137,92,0,620,206,373,0,13,80,100,546,57
IBTK_7_1847,711,231,34,883,182,123,1206,459,424,314,977,1128
IL10RA_7_1848,65,0,0,168,247,77,0,0,0,201,2,15
IL6_7_1849,430,628,740,316,218,230,721,1498,683,360,39,1063
IRF9_7_1850,1445,690,2582,417,481,708,705,56,281,31,980,33
ITCH_7_1851,997,81,3704,591,219,119,2753,222,957,126,1390,206

IVNS1ABP_7_1852,0,0,0,0,0,0,0,0,0,0,0,0
JHDM1D_7_1853,162,1837,779,1157,541,1727,926,988,1685,1188,924,638
JOSD1_7_1854,222,2,453,99,667,256,1164,37,744,438,1599,339
JOSD2_7_1855,114,62,18,398,18,15,131,185,232,6,365,44
KAT6B_7_1856,748,293,84,821,487,924,1837,1028,333,957,473,399
KATNB1_7_1857,30,485,26,116,44,507,155,792,115,673,62,417
KBTBD10_7_1858,208,601,2,191,355,8,6,454,431,783,2,56
KBTBD11_7_1859,30,4,5,64,415,1,65,265,657,45,0,748
KBTBD2_7_1860,58,1,9,204,2,104,509,149,0,5,9,735
KBTBD5_7_1861,0,0,460,0,62,14,0,134,0,0,0,10
KBTBD7_7_1862,282,126,59,56,324,91,750,126,52,70,533,30
KBTBD8_7_1863,628,576,435,511,1,153,253,983,417,164,1975,505
KCTD10_7_1864,916,447,1117,150,2274,1215,1440,715,871,1828,813,495
KCTD11_7_1865,0,26,0,2,4,69,0,22,330,4,0,52
KCTD12_7_1866,0,0,1,0,2,0,0,62,5,0,157,1
KCTD13_7_1867,493,518,0,36,17,1072,561,2,3,403,0,1
KCTD16_7_1868,129,20,13,0,0,178,37,0,589,178,815,63
KCTD17_7_1869,0,92,75,0,0,16,26,0,0,0,976,26
KCTD18_7_1870,548,4,14,315,395,340,2,1636,27,23,164,441
KCTD3_7_1871,1802,4046,2989,1161,1544,3459,2230,1481,2990,4529,2308,25
73
KCTD5_7_1872,340,67,0,147,18,0,539,42,0,37,465,35
KCTD9_7_1873,113,55,584,454,44,1383,3557,270,1610,711,1054,878
KDM2A_7_1874,16,467,45,168,1,436,9,44,0,0,33,65
KDM4B_7_1875,1,35,7,3,39,36,1143,0,15,4,78,6
KDM5B_7_1876,935,159,58,321,9,543,241,562,227,106,377,64
KLHDC5_7_1877,2184,1990,2862,1300,1673,1010,1346,1510,1722,2752,2053,2
110
KLHL10_7_1878,24,8,0,65,0,310,0,0,8,0,106,211
KLHL11_7_1879,5,146,267,0,89,42,619,256,105,205,1303,522
KLHL12_7_1880,409,10,0,866,306,497,9,1,553,586,932,481
KLHL14_7_1881,74,93,1,8,13,0,1169,0,0,89,63,0
KLHL15_7_1882,858,1,429,0,14,222,131,10,171,58,0,47
KLHL17_7_1883,0,970,0,40,0,4,245,0,31,2,840,2
KLHL18_7_1884,0,0,6,61,169,5,0,266,392,11,111,63
KLHL1_7_1885,88,2,2974,945,623,315,319,220,27,751,984,1448
KLHL20_7_1886,88,357,0,0,192,43,366,0,411,92,1182,185
KLHL21_7_1887,92,310,0,83,21,421,0,0,0,8,55,48
KLHL22_7_1888,173,38,85,123,112,193,36,141,222,51,152,24
KLHL23_7_1889,108,54,0,334,230,301,263,192,30,6,1,47
KLHL24_7_1890,111,82,53,240,423,0,34,470,105,8,1,8
KLHL25_7_1891,1,0,0,10,0,3,1021,655,11,0,0,210
KLHL26_7_1892,1008,0,0,9,1,0,0,0,12,5,722,2
KLHL28_7_1893,297,242,67,564,0,397,1716,247,797,1253,359,902
KLHL29_7_1894,201,13,383,35,500,12,770,0,431,104,3,315
KLHL31_7_1895,32,130,0,139,0,701,0,616,197,11,34,53
KLHL32_7_1896,0,73,307,2,44,0,350,10,76,1,238,262
KLHL33_7_1897,5,0,3,9,522,0,2,0,125,12,7,20
KLHL34_7_1898,113,0,61,0,0,225,0,95,0,12,279,0
KLHL36_7_1899,12,544,0,1109,168,18,0,754,16,93,9,486

KLHL3_7_1900,279,49,183,430,671,490,1389,11,313,30,3,1020
KLHL8_7_1901,2115,1689,542,1350,4363,1103,2505,2032,1405,1453,2442,176
6
LATS1_7_1902,117,7,720,942,892,1218,5,358,1552,150,867,267
LATS2_7_1903,341,614,8,193,1622,13,866,2,210,269,15,85
LIF_7_1904,533,43,969,124,154,1,109,109,101,164,203,594
LNX2_7_1905,576,437,934,738,3,0,1062,1264,308,253,562,923
LOC283116_7_1906,1,42,493,466,0,298,0,2,766,237,23,271
LONRF1_7_1907,3520,3952,1894,2591,730,2099,2634,2258,1325,2675,1809,27
75
LTN1_7_1908,1891,539,387,629,413,698,226,1154,556,1010,915,778
LZTR1_7_1909,470,247,1095,145,841,1346,360,614,577,429,159,752
MAP1LC3B_7_1910,567,512,118,762,730,0,21,13,20,575,526,124
MAP3K1_7_1911,0,0,43,0,61,260,65,836,158,205,37,38
MARK1_7_1912,7,443,293,199,880,413,961,50,80,296,157,23
MDM2_7_1913,27,53,118,73,461,1637,88,141,398,445,1298,489
MED20_7_1914,631,98,0,155,346,490,0,669,3,309,604,0
MEX3B_7_1915,0,0,1,9,0,0,1,0,0,0,0,27
MIB1_7_1916,622,55,301,445,17,21,2,35,95,110,17,194
MKRN2_7_1917,135,10,821,9,608,649,418,2,204,908,327,558
MKRN3_7_1918,625,594,688,562,71,265,22,805,93,1238,1493,912
MLL2_7_1919,155,268,985,151,0,29,0,0,270,105,7,50
MLLT6_7_1920,0,395,423,27,350,176,47,36,1484,141,93,1133
MOCS3_7_1921,13,19,224,927,791,1002,29,2,345,5,976,477
MRPL49_7_1922,66,67,1,75,0,848,0,332,335,465,209,177
MUL1_7_1923,210,15,54,194,128,437,0,70,253,49,221,54
MYCBP2_7_1924,183,410,483,715,771,1633,432,474,398,1393,1070,84
MYLIP_7_1925,3,0,0,21,147,28,0,28,0,7,0,19
MYSM1_7_1926,149,677,1268,1002,78,1678,1782,586,171,876,57,392
NACC1_7_1927,0,0,0,0,43,10,162,0,2,92,12,239
NACC2_7_1928,347,286,457,51,667,0,681,664,212,51,337,21
NEURL1B_7_1929,604,485,2,0,0,1,0,0,847,11,0,96
NEURL_7_1930,1276,767,633,655,539,372,988,615,293,708,248,839
NHLRC1_7_1931,290,329,833,7,138,197,572,44,319,157,447,512
NUP43_7_1932,0,357,24,87,8,102,0,806,82,561,0,20
OTUB1_7_1933,7,24,147,85,24,0,1,7,4,207,0,0
OTUB2_7_1934,509,12,25,0,1756,287,0,7,336,366,253,56
OTUD1_7_1935,0,1,963,595,0,0,0,856,0,1,0,0
OTUD6A_7_1936,442,13,0,252,0,136,11,28,0,0,1065,1
OTUD6B_7_1937,0,6,0,0,0,38,0,10,0,0,0,0
OTUD7A_7_1938,17,8,439,0,354,2,0,0,0,0,0,0
OTUD7B_7_1939,45,54,113,3,26,171,68,747,90,362,0,11
PAFAH1B1_7_1940,0,1,0,0,0,10,0,770,0,0,550,0
PARP10_7_1941,24,200,26,0,3,0,0,20,15,378,43,49
PARP11_7_1942,907,16,899,462,180,764,129,122,1241,1294,1183,1036
PARP14_7_1943,423,168,1,104,775,214,21,553,12,880,766,315
PCGF1_7_1944,0,42,0,0,1,0,0,0,0,0,0,12
PCGF2_7_1945,54,373,1573,935,1553,473,89,1278,844,1134,321,300
PCGF3_7_1946,9,0,3,0,2,1,0,0,37,0,0,32
PDZRN3_7_1947,4,42,7,16,119,0,1,33,176,580,1,291

PEBP4_7_1948,314,733,2,160,21,30,0,928,1041,570,139,244
PEX12_7_1949,117,0,841,0,331,0,24,0,0,5,0,138
PHF14_7_1950,1783,273,76,894,1209,389,656,214,1181,519,623,3665
PHF15_7_1951,1233,130,855,107,6,322,5,0,268,618,212,718
PHF20_7_1952,211,143,368,687,642,598,297,990,309,251,0,664
PHF2_7_1953,0,0,3,10,75,43,31,23,8,23,122,98
PHF3_7_1954,142,734,1589,238,344,159,450,1684,923,513,117,397
PHIP_7_1955,927,1847,2095,1878,786,2161,2391,365,594,685,551,1986
PHRF1_7_1956,0,158,879,296,50,0,332,147,0,738,0,0
PJA2_7_1957,1710,299,1263,92,1009,939,258,0,346,170,753,717
PRPF19_7_1958,105,149,200,1,64,515,299,482,177,726,159,774
PRPF8_7_1959,914,352,482,761,111,1478,282,55,625,294,575,295
PSMD14_7_1960,1997,3645,2468,2506,4752,2554,4273,1578,4435,1576,4820,3
699
PSMD2_7_1961,511,48,237,0,148,17,258,7,103,55,42,15
PSMD4_7_1962,670,143,159,712,281,1082,812,555,1112,1042,121,757
PSMD7_7_1963,680,1,504,847,906,143,1255,430,975,442,139,583
PWP1_7_1964,2366,1248,2587,319,2046,1414,3136,1444,1805,1311,1219,1455
RAB40A_7_1965,1893,1632,1388,463,766,1668,2278,1491,2697,2451,1489,115
2
RAB40AL_7_1966,1893,1632,1388,463,766,1668,2278,1491,2697,2451,1489,11
52
RAB40B_7_1967,593,0,74,493,712,745,58,880,838,1077,114,179
RAD18_7_1968,56,46,270,57,62,19,62,24,67,30,6,748
RAD23A_7_1969,69,0,0,55,0,49,0,0,0,0,0,0
RAD23B_7_1970,463,2719,3867,766,829,1147,2028,177,1060,1624,636,1359
RAG1_7_1971,727,1057,299,400,1081,4,39,233,1054,336,1771,201
RAI1_7_1972,618,275,1168,274,368,6,227,0,379,12,623,205
RBX1_7_1973,0,854,0,0,0,0,0,0,0,0,0,0
RCBTB1_7_1974,1289,341,453,1795,1,1133,712,1295,761,832,931,3259
RCBTB2_7_1975,48,233,735,206,64,300,9,4,52,28,890,9
RFFL_7_1976,0,0,404,0,0,5,0,0,0,0,0,0
RFPL1_7_1977,264,375,0,2,27,157,30,17,725,27,1355,98
RFWD3_7_1978,190,0,0,81,31,165,25,741,132,2,0,15
RHOBTB3_7_1979,123,225,0,141,606,0,28,558,15,46,3,115
RING1_7_1980,16,464,637,532,132,145,120,32,278,762,380,593
RNF10_7_1981,401,328,452,220,525,337,35,10,35,582,427,845
RNF111_7_1982,1072,415,79,5,846,969,10,9,106,60,307,26
RNF112_7_1983,0,0,0,522,0,589,0,33,33,0,0,4
RNF113A_7_1984,205,606,711,662,962,566,34,208,2167,1104,136,1334
RNF113B_7_1985,0,35,0,2,0,0,0,72,350,11,0,526
RNF114_7_1986,0,37,0,3,766,19,0,0,11,2,5,5
RNF115_7_1987,2407,668,1456,418,1518,1217,230,1635,957,1437,2091,188
RNF11_7_1988,30,1168,26,198,0,17,417,206,17,30,388,126
RNF121_7_1989,3,0,0,0,0,0,0,0,0,0,0,0
RNF122_7_1990,0,58,546,0,541,243,1,640,511,6,203,48
RNF123_7_1991,217,66,1326,5,298,823,213,545,233,118,65,592
RNF125_7_1992,462,2254,795,256,1477,472,250,1692,869,832,568,1642
RNF126_7_1993,2,0,457,0,0,81,0,0,0,0,20,0
RNF130_7_1994,733,812,1374,698,701,175,1234,854,1858,791,574,1013

RNF133_7_1995,236,809,99,785,341,958,764,1055,84,599,354,42
RNF139_7_1996,701,53,114,637,1,106,1,37,10,301,162,4
RNF141_7_1997,0,3,564,1132,0,163,47,0,1,0,9,417
RNF144A_7_1998,353,41,663,426,0,276,192,0,687,972,417,95
RNF144B_7_1999,86,198,1641,1128,0,0,640,80,942,619,1855,677
RNF149_7_2000,25,155,238,15,0,249,2,53,0,3,1,51
RNF150_7_2001,16,143,1,5,31,17,0,0,21,4,22,15
RNF152_7_2002,481,242,862,1139,299,67,78,420,282,314,997,1050
RNF157_7_2003,840,89,35,52,69,203,154,510,515,305,559,134
RNF167_7_2004,0,0,0,0,0,459,0,0,0,0,186,0
RNF168_7_2005,0,879,0,0,1,0,4,0,7,0,0,2
RNF169_7_2006,27,624,28,891,0,265,219,112,225,25,496,25
RNF181_7_2007,596,3,73,162,0,117,411,953,20,1264,0,327
RNF183_7_2008,45,0,192,4,71,3,755,7,32,24,111,7
RNF186_7_2009,264,41,265,45,0,25,29,0,80,87,2,4
RNF187_7_2010,72,0,0,38,277,442,661,346,41,0,0,942
RNF20_7_2011,44,2,68,0,7,0,0,0,0,4,0,0
RNF217_7_2012,0,2,86,0,0,3,59,29,1010,40,51,112
RNF220_7_2013,105,1001,20,605,23,30,28,183,663,381,46,686
RNF25_7_2014,88,843,30,614,768,100,1512,1123,422,179,54,428
RNF26_7_2015,160,6,21,190,0,469,0,170,795,417,827,113
RNF2_7_2016,0,205,1013,0,21,176,0,383,21,1,0,5
RNF31_7_2017,484,482,629,706,435,1996,210,331,1923,484,1716,854
RNF43_7_2018,130,12,259,1,310,103,79,0,103,420,233,32
RNF44_7_2019,271,2,0,0,3,0,125,151,180,201,0,23
RNF5_7_2020,0,11,1,273,0,0,1771,0,142,6,9,21
RSC1A1_7_2021,1342,293,1058,1649,902,2005,1456,3687,969,2782,507,1305
RSF1_7_2022,355,525,567,81,1025,1087,486,2352,911,1519,834,1352
RSPRY1_7_2023,13,2,454,0,7,153,0,790,1,71,20,952
SCLY_7_2024,160,36,0,49,0,148,190,9,128,27,44,14
SENP1_7_2025,1882,754,178,44,9,492,0,0,448,32,379,63
SENP2_7_2026,106,0,3,0,394,886,281,228,339,7,521,46
SENP3_7_2027,1094,495,500,228,2589,814,157,144,694,1031,1039,200
SENP5_7_2028,103,604,317,340,238,938,309,922,867,833,146,1716
SH3RF1_7_2029,33,0,1,0,0,0,48,88,1,62,1,0
SH3RF2_7_2030,55,19,558,213,2,338,177,851,748,300,489,95
SH3RF3_7_2031,37,16,10,0,7,152,0,22,31,222,0,3
SHKBP1_7_2032,0,0,0,0,1,0,0,0,60,73,0,3
SIAH2_7_2033,34,29,756,506,157,326,75,2086,69,417,652,618
SIK1_7_2034,2,0,32,579,7,0,0,0,13,0,0,0
SLX4_7_2035,606,81,0,1,0,22,89,178,14,0,0,342
SMU1_7_2036,231,540,0,894,2527,2,635,61,42,171,521,425
SMURF2_7_2037,369,569,0,41,19,81,0,0,0,35,0,0
SNRNP40_7_2038,28,13,0,38,13,489,0,16,12,12,134,2
SOCS1_7_2039,32,0,0,28,0,0,5,0,401,9,0,88
SOCS2_7_2040,65,0,0,308,78,156,319,109,173,149,96,21
SOCS3_7_2041,298,479,10,182,58,278,10,18,483,1110,836,575
SOCS6_7_2042,1,9,78,0,413,0,1913,5,62,501,6,10
SPOPL_7_2043,40,1,181,410,255,533,548,586,538,12,1043,126
SPSB1_7_2044,3,0,0,0,1683,146,1530,0,87,0,0,16

SPSB3_7_2045,16,438,13,59,0,17,0,78,4,0,0,4
SPSB4_7_2046,0,7,30,0,0,22,113,3,127,3,5,20
STAM2_7_2047,219,287,10,335,391,426,15,453,100,141,889,78
STAMBPL1_7_2048,654,348,1,580,652,251,901,19,318,139,138,338
STAM_7_2049,2096,875,1241,1668,2885,183,1055,1322,1821,3003,1638,2460
STUB1_7_2050,1,5,269,172,21,331,0,295,218,174,2,23
SUMO3_7_2051,70,917,32,423,103,159,1071,81,528,542,376,640
SYNGAP1_7_2052,850,61,546,707,868,564,3,274,854,753,420,3190
TAB2_7_2053,1151,570,257,394,274,541,524,1225,487,697,889,745
TAB3_7_2054,265,34,13,89,61,26,247,2,308,1510,63,29
TAF1D_7_2055,2,168,72,520,23,3,65,491,258,59,1018,1137
TBC1D1_7_2056,26,57,595,658,27,96,0,173,12,581,2551,96
TLE1_7_2057,8,1,0,10,0,0,0,0,0,6,0,1
TNFAIP3_7_2058,512,313,218,629,429,93,429,1611,650,964,715,793
TOLLIP_7_2059,26,215,164,49,0,8,159,633,1763,211,1607,403
TRAF7_7_2060,856,505,495,91,472,33,312,49,269,151,198,904
TRAIP_7_2061,33,270,3,27,2,0,4,0,112,11,202,21
TRIM11_7_2062,452,21,0,673,570,676,1131,1018,1399,1085,1557,358
TRIM15_7_2063,1,0,0,0,0,0,0,0,0,19,0,282
TRIM25_7_2064,0,23,0,0,0,0,3,10,16,307,0,5
TRIM27_7_2065,180,146,327,954,33,171,29,1530,85,20,207,673
TRIM28_7_2066,297,0,1,9,2,529,12,337,47,15,56,8
TRIM31_7_2067,1326,1762,1656,348,2908,2046,1098,2653,1299,1859,1011,21
33
TRIM35_7_2068,0,1,0,0,0,0,0,597,11,405,119,2
TRIM42_7_2069,251,3,764,12,265,102,39,19,65,123,3,854
TRIM46_7_2070,0,55,0,0,0,0,4,6,4,9,0,1
TRIM47_7_2071,0,71,91,0,755,2,0,0,72,10,605,58
TRIM48_7_2072,38,174,0,1,324,0,940,1,0,0,758,1
TRIM52_7_2073,905,408,470,892,977,999,345,1515,1065,449,1088,2351
TRIM56_7_2074,336,370,976,938,202,221,1016,1619,830,818,655,836
TRIM62_7_2075,0,3,0,27,0,0,0,0,12,0,0,0
TRIM63_7_2076,84,1209,413,939,3254,1144,1854,743,1428,2615,1847,313
TRIM65_7_2077,24,0,0,0,0,0,0,0,0,0,35,0
TRIM67_7_2078,186,294,472,645,0,85,564,0,134,324,664,285
TRIM68_7_2079,286,298,1000,1423,120,380,185,180,471,754,51,834
TRIM8_7_2080,23,370,423,1235,59,30,616,13,53,9,0,833
TRIP12_7_2081,6,468,69,930,7,414,175,37,9,62,140,1053
UBA2_7_2082,908,991,1995,1921,679,1068,596,502,2086,1891,278,1238
UBA6_7_2083,1752,1825,649,822,1769,2606,2763,2608,2747,2442,1823,559
UBA7_7_2084,0,0,0,337,0,0,0,0,0,4,0,3
UBAC1_7_2085,0,1,96,8,0,21,38,700,12,26,257,3
UBAP2_7_2086,18,124,2,29,1991,787,0,104,1135,265,410,192
UBASH3B_7_2087,1164,1035,250,85,0,688,866,752,264,416,119,869
UBE2B_7_2088,1339,643,3089,1409,1133,686,1137,84,351,801,486,60
UBE2D4_7_2089,1,17,0,24,8,0,0,408,2,0,2,1
UBE2E2_7_2090,33,269,0,217,0,69,737,49,1,89,22,5
UBE2F_7_2091,148,801,95,324,499,58,482,34,388,237,1417,890
UBE2G1_7_2092,239,466,625,888,1143,231,545,463,755,1204,272,78
UBE2J1_7_2093,0,62,0,0,412,49,0,105,36,38,245,5

UBE2L3_7_2094,574,35,0,0,46,97,68,458,23,96,518,305
UBE2M_7_2095,0,71,2,627,60,29,0,152,6,91,2,166
UBE2N_7_2096,51,72,125,694,76,97,29,20,37,178,12,10
UBE2NL_7_2097,141,1,249,13,255,654,291,1582,844,423,34,1169
UBE2O_7_2098,73,3,0,6,0,21,0,300,161,3,1,34
UBE2Q1_7_2099,764,635,2087,914,107,924,775,418,554,1067,561,251
UBE2QL1_7_2100,0,26,0,1,0,0,1,0,0,36,0,287
UBE2R2_7_2101,1439,182,28,34,0,754,1477,155,362,312,2179,1187
UBE2S_7_2102,7,55,4,0,0,0,1,0,368,4,21,37
UBE2T_7_2103,6,9,143,846,394,775,72,494,2,284,230,383
UBE2U_7_2104,84,518,39,718,9,754,472,378,1545,855,23,207
UBE2V2_7_2105,157,539,748,11,5,11,16,7,1150,29,16,529
UBE2Z_7_2106,167,315,286,684,0,11,110,0,243,641,1,448
UBE3C_7_2107,22,392,11,376,0,31,0,1,76,32,0,303
UBQLN2_7_2108,1006,245,222,40,162,39,199,1413,73,728,502,83
UBQLN3_7_2109,0,0,1,0,0,0,1,0,874,12,15,115
UBQLN4_7_2110,1,346,1,0,346,164,23,0,443,41,506,444
UBR1_7_2111,1722,1109,277,671,817,818,1549,1045,2259,2146,551,1775
UBR3_7_2112,3,545,6,2,1,2,4,7,669,21,3,69
UBR4_7_2113,2040,45,512,520,978,597,1088,853,1209,382,337,550
UBR5_7_2114,2513,3279,7627,1717,1373,668,1218,1861,1227,1192,2079,1949
UBR7_7_2115,1141,472,56,1516,1375,240,855,213,175,494,488,455
UBXN10_7_2116,0,9,4,69,0,42,0,41,0,169,15,0
UBXN1_7_2117,0,358,0,2,2,803,49,324,538,241,435,635
UBXN2A_7_2118,273,814,12,107,706,1003,255,328,286,169,254,31
UBXN4_7_2119,679,0,29,103,176,33,0,143,823,79,5,97
UBXN7_7_2120,238,34,164,132,3,44,4,10,432,573,88,625
UBXN8_7_2121,2197,2211,585,658,616,1996,2644,1557,2026,1905,2655,1139
UCHL1_7_2122,1872,2422,1658,774,1294,945,347,1002,2144,877,1366,759
UCHL3_7_2123,2014,619,2896,1606,1053,2092,1957,890,3056,1850,648,3979
UFC1_7_2124,47,18,15,5,110,7,1,489,8,76,0,89
UHRF2_7_2125,762,106,733,573,311,77,654,0,870,622,134,93
UNK_7_2126,9,2,467,33,0,1,0,17,315,5,2,36
USP11_7_2127,531,274,0,414,0,0,0,273,11,431,0,0
USP13_7_2128,5,0,878,5,92,370,842,277,363,599,836,135
USP15_7_2129,1416,297,871,1028,3020,744,1669,1135,2994,1510,1709,2898
USP17_7_2130,1399,301,267,323,310,215,185,376,1826,67,114,1342
USP17L2_7_2131,704,3088,1430,529,1291,1348,1882,803,1097,1281,1033,787
USP17L5_7_2132,1399,301,267,323,310,215,185,376,1826,67,114,1342
USP18_7_2133,1141,500,1345,798,694,1242,2107,1276,1359,1445,1117,617
USP22_7_2134,2,189,569,10,5,2,260,21,140,28,14,568
USP24_7_2135,734,220,625,425,704,829,480,901,1600,614,1633,219
USP25_7_2136,357,230,390,260,116,162,189,956,1093,183,1192,1097
USP26_7_2137,578,404,238,1770,1185,237,1026,2139,2131,971,838,1636
USP27X_7_2138,10,12,133,524,56,50,3,155,33,412,7,132
USP28_7_2139,212,439,6,1040,1479,61,406,575,921,718,2906,215
USP29_7_2140,234,42,1796,777,1091,407,1223,1198,1186,765,1052,423
USP30_7_2141,17,3,77,389,19,360,370,156,212,26,534,55
USP32_7_2142,323,332,273,206,172,167,996,746,1804,562,1227,1493
USP34_7_2143,25,38,203,276,1521,758,0,330,95,105,47,16

USP35_7_2144,1512,1299,1466,2240,3434,962,1469,1134,1125,2241,1144,198
9
USP36_7_2145,0,0,0,1,0,7,0,0,477,571,0,55
USP37_7_2146,159,125,409,42,73,231,17,25,987,488,0,486
USP38_7_2147,587,829,35,592,22,134,446,286,660,652,2075,817
USP39_7_2148,33,622,31,89,0,0,14,5,3,0,28,8
USP3_7_2149,188,1653,1452,2239,1759,1243,1550,686,1063,1077,3355,714
USP40_7_2150,878,1796,1142,518,828,910,123,565,1042,1755,86,1315
USP42_7_2151,94,722,569,311,6,1096,533,1610,268,629,692,333
USP43_7_2152,1095,0,0,221,92,63,4,6,39,0,135,3
USP45_7_2153,848,176,156,1336,654,119,1352,142,749,835,486,774
USP47_7_2154,638,326,1150,2039,598,528,1294,91,403,1080,414,373
USP49_7_2155,701,62,332,1,0,260,373,324,394,201,492,274
USP50_7_2156,0,0,0,0,0,0,0,0,0,0,0,0
USP51_7_2157,0,578,2,116,0,0,0,0,6,0,97,0
USP53_7_2158,1480,2065,633,2672,1153,1226,1940,1078,2077,1981,1269,235
1
USP54_7_2159,1,33,403,0,0,126,176,172,318,351,0,241
USP6_7_2160,302,690,54,147,688,374,0,206,275,750,162,130
USP7_7_2161,1271,2018,2889,1023,885,733,916,1411,240,1635,2024,1596
USP9Y_7_2162,0,97,700,414,0,530,0,0,96,2,0,36
USPL1_7_2163,770,875,2080,319,54,162,46,1967,1024,395,164,330
VCPIP1_7_2164,2269,2793,1635,743,1134,891,702,5327,1436,1647,2407,2416
VPS11_7_2165,0,104,213,1,2,0,182,0,29,2,251,4
WDR12_7_2166,581,66,350,268,11,326,969,202,1103,1795,50,235
WDR53_7_2167,634,592,16,74,331,874,0,1289,178,1026,56,247
WDR59_7_2168,0,9,0,0,1,12,147,0,4,0,3,1
WDR5B_7_2169,1852,660,1146,988,578,1853,684,932,1213,1305,3178,465
WDR61_7_2170,36,406,160,7,307,634,169,363,44,1406,116,214
WDTC1_7_2171,0,42,72,20,0,973,95,192,26,93,0,839
WSB2_7_2172,73,0,0,0,0,0,0,0,0,7,29,2
WWP1_7_2173,0,404,0,0,0,7,1227,620,71,579,100,244
YOD1_7_2174,91,280,1059,689,182,0,785,0,1049,151,0,143
ZBTB11_7_2175,750,350,33,570,0,2774,983,81,209,409,1113,26
ZBTB25_7_2176,320,1028,1114,175,1162,1730,1982,744,1660,112,1287,841
ZBTB2_7_2177,95,1,0,0,0,471,0,0,0,0,0,19
ZBTB32_7_2178,11,0,0,0,0,0,0,0,0,0,0,1
ZBTB34_7_2179,173,824,300,494,0,0,325,561,867,599,149,148
ZBTB39_7_2180,18,187,282,52,0,71,0,0,167,152,27,21
ZBTB3_7_2181,12,192,16,6,11,18,0,12,345,228,3,174
ZBTB41_7_2182,200,1993,2734,644,2811,495,1687,700,1636,2359,2723,1015
ZBTB44_7_2183,2272,17,0,47,703,196,7,9,82,680,27,28
ZBTB45_7_2184,213,384,493,24,964,347,125,232,300,106,329,1506
ZBTB46_7_2185,128,56,0,163,6,139,35,28,158,42,3,20
ZBTB47_7_2186,0,101,38,365,18,734,72,648,406,158,0,544
ZBTB48_7_2187,0,3,45,32,10,75,44,27,137,329,0,181
ZBTB49_7_2188,0,239,0,17,0,20,0,2,1,429,0,26
ZBTB5_7_2189,878,1,0,259,0,727,453,1424,94,1496,5,309
ZBTB7A_7_2190,16,0,0,0,1,30,11,0,0,524,658,3
ZBTB7B_7_2191,682,91,23,12,430,764,0,876,1034,646,155,303

ZBTB7C_7_2192,1,0,33,6,0,5,0,0,0,2,252,131
ZBTB8A_7_2193,143,122,120,604,11,577,232,452,58,461,0,299
ZFAND2B_7_2194,279,1135,429,143,656,224,416,193,518,1644,19,164
ZFPL1_7_2195,2,73,7,5,55,0,1072,531,1,294,442,0
ZMYND10_7_2196,468,199,1043,88,170,498,917,1181,272,678,1155,40
ZNF131_7_2197,43,0,0,0,0,0,0,0,1021,62,124,113
ZNF598_7_2198,1415,1014,832,69,2066,1648,392,856,1558,1317,1635,1988
ZNF645_7_2199,625,0,0,0,1106,0,0,18,300,6,74,463
ZNRF1_7_2200,52,0,0,0,40,2,156,0,29,0,0,6
ZNRF2_7_2201,27,114,0,3,0,49,0,131,5,124,1421,0
ZNRF4_7_2202,0,0,0,0,0,0,0,0,0,453,25,0
ZRANB1_7_2203,0,0,5,0,12,409,0,0,0,13,158,3
ZSWIM2_7_2204,561,1028,1018,152,831,678,2324,1237,1718,539,872,798
AIRE_7_2205,0,0,0,9,0,0,0,0,0,4,150,0
ANAPC5_7_2206,61,591,536,450,22,172,418,205,708,959,326,177
ANAPC7_7_2207,101,263,226,21,91,281,711,49,1578,919,3,193
ANKFY1_7_2208,188,0,1030,901,383,1341,3,32,180,97,377,1318
ASB10_7_2209,455,1145,978,111,515,637,1413,1,626,161,67,944
ASB11_7_2210,1052,777,57,545,982,936,882,1407,478,633,288,545
ASB14_7_2211,0,57,503,2,418,43,161,110,374,8,0,60
ASB2_7_2212,0,70,0,195,0,11,0,0,80,60,153,276
ASB3_7_2213,447,389,743,217,37,4,0,16,416,108,0,72
ASB4_7_2214,0,326,222,0,0,6,0,0,0,16,0,0
ASB6_7_2215,870,2491,2083,471,858,481,974,941,1655,2766,1306,643
ASB7_7_2216,0,0,407,101,353,0,0,0,469,12,0,73
ASB9_7_2217,91,1310,25,2,0,351,449,167,7,38,0,162
ASCC2_7_2218,1185,627,1546,144,830,995,456,483,800,424,155,79
ATG10_7_2219,912,532,129,579,59,411,888,1,658,1100,180,483
ATG16L1_7_2220,1138,468,4,53,323,8,0,100,368,42,769,592
ATG7_7_2221,1,336,0,0,0,7,117,127,119,22,9,28
ATRX_7_2222,2256,2138,700,1504,3476,758,398,850,1681,1471,2076,1050
BACH2_7_2223,639,124,0,94,0,766,314,228,140,188,439,13
BAG6_7_2224,697,158,123,684,51,0,17,0,86,91,2,173
BAZ1A_7_2225,1463,210,968,268,697,112,629,815,1473,589,1061,322
BCL6_7_2226,0,49,0,0,0,0,0,317,188,4,2,25
BIRC3_7_2227,823,1821,79,485,825,541,1045,759,468,1796,193,486
BIRC7_7_2228,319,325,111,122,209,172,708,541,576,72,262,215
BPTF_7_2229,33,929,746,524,3723,510,78,779,813,838,934,2123
BRCA1_7_2230,601,437,840,190,350,853,0,8,23,80,390,45
BRPF1_7_2231,0,1,68,3,11,5,0,2,101,20,3,18
BRWD1_7_2232,683,465,711,181,0,870,158,33,189,3,468,26
BTBD11_7_2233,52,0,36,696,712,39,537,1179,327,153,8,737
BTBD1_7_2234,58,1493,1851,56,936,618,29,0,13,126,430,58
BTBD3_7_2235,115,148,787,39,12,218,862,1676,52,133,1644,260
BTBD7_7_2236,1410,196,1980,877,837,480,1071,998,688,633,487,1206
BTBD9_7_2237,97,709,367,533,1326,500,1446,536,226,622,40,496
BTRC_7_2238,1131,508,686,449,2717,1261,1657,652,19,1108,575,5
C3orf26_7_2239,253,437,2,389,277,324,6,458,28,243,1268,133
CBLC_7_2240,0,0,0,0,1,0,0,2,108,3,0,15
CCNB1IP1_7_2241,264,315,194,26,699,82,0,78,259,507,903,940

CDC16_7_2242,505,364,294,1768,1131,56,483,0,1,195,1357,844
CDC27_7_2243,1942,823,140,1677,474,531,412,1811,563,136,1453,1266
CHFR_7_2244,305,0,0,610,35,0,474,0,419,63,217,49
CISH_7_2245,0,1,0,0,0,0,0,501,0,0,29,0
CNOT4_7_2246,643,165,323,122,573,1401,22,1277,88,793,1199,982
COPS2_7_2247,2,0,0,0,0,495,636,0,954,10,2,105
COPS3_7_2248,2531,269,521,1505,470,888,1497,798,1082,533,1107,133
COPS7A_7_2249,26,4,53,1,38,28,147,57,0,217,46,75
COPS8_7_2250,477,476,80,1091,769,258,1,11,46,81,409,1133
CRBN_7_2251,0,1,910,623,918,411,72,367,101,0,9,17
CUL2_7_2252,293,262,3,572,279,879,235,901,751,913,1691,1296
CUL4A_7_2253,151,667,0,662,478,469,264,1616,222,191,276,20
CUL4B_7_2254,579,321,0,2,0,0,3,15,10,19,38,542
CUL7_7_2255,0,27,27,13,0,441,8,7,0,0,587,0
CXXC1_7_2256,58,33,75,66,199,138,17,0,214,489,0,1649
CYLD_7_2257,33,525,0,8,376,89,548,2,460,614,2,235
DCAF11_7_2258,192,708,231,0,62,793,5,2,174,217,355,102
DCAF17_7_2259,17,259,199,537,40,43,0,0,1,167,43,0
DCAF4_7_2260,356,0,17,0,0,0,701,496,287,192,0,29
DCAF6_7_2261,653,2194,11,916,656,772,615,2,775,1867,1057,796
DCST1_7_2262,33,151,160,0,2,4,31,137,50,94,12,8
DEPDC1B_7_2263,15,142,166,1,0,2,0,4,30,90,180,244
DET1_7_2264,172,67,1,454,131,729,76,94,98,137,78,801
DIDO1_7_2265,0,0,417,165,80,1,0,15,189,3,0,27
DNAJB2_7_2266,0,45,0,0,0,1,0,1,0,64,1,0
DTX2_7_2267,1552,2182,157,246,300,155,320,349,1147,119,772,244
EED_7_2268,163,6,0,23,0,1,299,459,105,64,82,248
EIF3B_7_2269,615,144,1469,509,540,358,200,11,487,223,234,292
EIF3C_7_2270,9,190,39,1412,629,15,588,3,351,1234,664,45
EIF6_7_2271,33,0,0,0,0,31,12,2,19,6,1,200
EPN1_7_2272,3,18,32,677,758,11,308,0,1195,22,2,238
EPN2_7_2273,42,28,1,33,387,0,933,0,209,41,1,35
EPS15_7_2274,1399,965,4525,1888,1369,1462,2055,512,1236,938,989,554
FAM70A_7_2275,2,34,977,0,0,137,0,3,4,48,1250,2
FANCL_7_2276,1039,1307,1666,565,2408,250,1454,599,816,661,3779,3406
FBXL13_7_2277,298,48,0,374,49,3,462,1268,911,843,34,113
FBXL20_7_2278,0,0,0,0,56,0,0,0,1,0,0,0
FBXL2_7_2279,1531,163,884,133,26,944,401,370,620,521,1081,259
FBXL5_7_2280,648,2101,967,27,650,1840,79,457,839,1141,148,473
FBXL6_7_2281,90,363,383,3,476,2,113,5,4,96,0,94
FBXO11_7_2282,1965,1348,1639,1346,0,865,897,656,2084,997,1380,1566
FBXO15_7_2283,68,270,838,54,208,18,317,1258,681,304,2,80
FBXO17_7_2284,2627,829,15,140,100,69,1505,535,303,1176,1716,459
FBXO18_7_2285,0,5,35,0,0,0,913,780,380,56,0,118
FBXO21_7_2286,8,1022,794,294,152,1853,68,798,1020,773,1050,1384
FBXO22_7_2287,155,1,11,27,114,43,136,13,144,595,0,105
FBXO24_7_2288,0,7,0,11,120,275,27,0,511,29,319,218
FBXO25_7_2289,219,501,504,418,739,327,322,87,937,186,26,422
FBXO28_7_2290,857,73,68,303,884,811,55,444,392,1089,404,818
FBXO32_7_2291,141,241,0,13,201,54,0,976,114,372,21,15

FBXO34_7_2292,59,8,87,10,0,8,1,19,22,160,0,56
FBXO38_7_2293,814,17,273,1,160,1,135,12,2,480,33,980
FBXO3_7_2294,436,1275,262,572,504,113,1536,226,615,836,1256,1555
FBXO44_7_2295,0,0,118,141,11,0,257,83,514,12,173,567
FBXO4_7_2296,0,364,75,0,0,0,0,1,880,15,757,97
FBXO5_7_2297,484,397,1559,2764,669,423,330,1331,2256,1520,2112,1464
FBXO7_7_2298,44,2,1,0,127,0,0,0,42,1029,613,126
FBXO9_7_2299,513,631,990,489,1824,1706,987,620,587,684,822,1039
FBXW11_7_2300,1143,2,537,2,128,19,628,23,196,25,0,20
FBXW7_7_2301,1066,134,20,567,523,619,1,388,441,832,1038,52
FBXW8_7_2302,27,167,0,101,746,16,132,22,58,515,880,4
GPS1_7_2303,50,167,11,387,364,224,4,53,344,27,0,38
HECTD2_7_2304,114,462,502,41,102,46,486,360,637,175,256,1278
HERC4_7_2305,79,88,530,481,295,1679,492,163,564,173,921,1302
HERC6_7_2306,107,33,577,97,905,179,230,55,39,647,16,759
HIC1_7_2307,22,10,0,4,17,249,100,0,623,20,1,84
HLTF_7_2308,287,170,948,39,23,578,821,18,0,500,6,833
HSF4_7_2309,412,22,5,15,185,0,21,348,1166,18,664,155
IPP_7_2310,416,0,356,118,0,0,202,0,0,353,0,474
KAT6A_7_2311,1217,552,48,952,105,1040,649,309,130,258,19,1218
KBTBD3_7_2312,4535,4921,5356,4181,2215,7909,4855,4993,4050,2640,2525,2
303
KCTD6_7_2313,607,715,487,160,39,1622,1163,433,1296,1981,1368,432
KCTD7_7_2314,157,397,204,869,267,191,67,452,1249,687,945,3606
KDM2B_7_2315,134,137,1503,19,912,704,613,182,126,1556,1304,589
KDM4C_7_2316,654,213,4,42,767,161,12,796,575,448,198,271
KDM5C_7_2317,436,363,838,288,91,155,0,891,900,248,35,341
KEAP1_7_2318,36,313,147,412,55,187,518,667,665,435,247,479
KIAA1841_7_2319,100,16,648,414,1,533,6,412,313,435,288,213
KLHL13_7_2320,17,208,1003,46,2864,380,1833,429,44,314,922,325
KLHL2_7_2321,1097,172,30,16,192,714,2,50,92,66,19,277
KLHL4_7_2322,1547,327,676,572,2212,396,1680,103,1705,972,516,1095
KLHL5_7_2323,327,517,265,0,151,202,561,596,499,25,881,66
KLHL7_7_2324,249,647,262,590,110,192,70,21,124,792,510,1149
LNX1_7_2325,0,0,32,79,146,0,99,182,273,7,47,61
LONRF3_7_2326,0,0,0,0,0,12,0,109,5,87,0,513
LRRC29_7_2327,1304,1115,771,1151,257,1022,3480,1755,481,1000,2008,968
LRSAM1_7_2328,83,599,158,6,185,9,0,1,459,100,7,59
MARK2_7_2329,95,25,47,153,189,394,290,777,209,80,714,724
MARK3_7_2330,151,323,6,312,25,231,2426,972,1401,667,1081,1820
MARK4_7_2331,0,5,0,2,174,93,1,365,4,45,20,9
MDM4_7_2332,290,335,912,1376,0,327,101,885,366,318,2107,68
MGRN1_7_2333,4,1,194,66,1690,119,0,0,420,198,51,114
MIB2_7_2334,4,53,23,352,0,127,257,2,499,14,134,242
MID1_7_2335,1043,962,0,28,1176,669,366,749,206,936,1840,165
MID2_7_2336,286,37,0,1398,0,509,0,937,0,988,896,0
MKRN1_7_2337,222,16,35,286,2,193,20,1173,628,392,5,276
MLL5_7_2338,133,562,3,105,0,26,1247,705,473,22,1,210
MLL_7_2339,718,1379,162,375,528,485,109,0,376,443,709,117
MNAT1_7_2340,638,0,1,238,167,1,132,23,488,8,723,70

MPND_7_2341,0,196,2,0,1,27,336,350,1560,250,20,187
MTF2_7_2342,2369,1611,4902,2306,3760,2684,2753,2575,2354,3442,1238,180
1
MYNN_7_2343,1599,598,344,1243,1541,966,1122,2005,1011,1371,467,1225
NAE1_7_2344,37,4,12,790,531,59,487,0,481,93,274,239
NDUFC2_7_2345,145,33,967,520,872,209,134,10,199,700,292,347
NEDD4_7_2346,435,143,914,182,1578,476,982,825,445,8,35,46
NEDD4L_7_2347,2263,479,1748,1302,604,1225,1177,988,1843,455,2786,2957
NFX1_7_2348,1454,582,173,229,327,33,177,267,384,53,3,1372
NLE1_7_2349,0,410,0,122,19,470,0,0,18,0,0,96
NSD1_7_2350,1544,2113,3798,1892,3836,1206,2127,1107,2933,3148,1186,194
1
NSFL1C_7_2351,198,0,2,141,0,503,0,0,17,116,3,5
NUB1_7_2352,1,301,73,836,856,764,10,45,579,861,1508,237
ODF2_7_2353,1196,9,454,0,1136,10,0,19,50,515,134,11
OTUD5_7_2354,0,504,711,0,207,15,9,57,2,42,1,7
PARK2_7_2355,97,155,99,201,182,292,156,147,298,100,36,74
PARP9_7_2356,0,84,77,715,541,24,15,901,152,467,611,829
PATZ1_7_2357,90,540,235,254,187,202,52,353,85,822,653,1184
PCGF6_7_2358,0,455,4,271,644,530,246,113,1507,352,425,675
PEX10_7_2359,98,8,21,0,88,2,129,275,1,282,31,2
PEX2_7_2360,50,18,14,8,0,431,10,0,0,0,18,0
PHF12_7_2361,0,4,19,0,26,438,0,244,0,15,0,29
PHF16_7_2362,2674,1344,1848,2085,876,2362,1796,5161,967,3479,2100,607
PHF17_7_2363,2277,1538,88,1386,2294,773,267,2309,1573,1453,2635,750
PHF1_7_2364,1,0,131,188,44,90,27,4,274,794,413,33
PHF21A_7_2365,2865,131,352,5,336,31,1509,3,139,968,523,435
PHF7_7_2366,32,81,3,93,1,196,0,779,378,1654,1021,327
PHF8_7_2367,1656,1523,147,103,185,638,617,280,770,861,583,652
PJA1_7_2368,655,42,3,0,357,373,149,0,390,47,0,181
PML_7_2369,15,275,0,382,0,0,102,0,0,0,44,22
POC1B_7_2370,482,547,50,1248,3297,1832,576,800,717,764,831,3041
PSMD1_7_2371,2,93,0,241,470,100,39,199,0,63,0,72
RAPSN_7_2372,4,2,1465,277,527,460,2230,1178,1523,664,99,312
RBBP4_7_2373,343,415,1685,566,3789,2539,728,1885,731,760,1376,1073
RBBP5_7_2374,367,93,69,48,3,908,239,90,18,179,75,211
RBBP6_7_2375,183,257,638,1572,1140,528,0,247,229,220,552,502
RBBP7_7_2376,53,109,1392,44,209,1062,550,761,181,749,139,807
RBCK1_7_2377,21,0,0,209,0,1200,0,32,321,100,79,354
RC3H2_7_2378,572,34,0,433,13,16,892,865,4,430,258,1
RCHY1_7_2379,0,4,510,542,0,0,0,1565,710,295,408,498
RFPL2_7_2380,0,6,250,0,3,232,0,267,409,13,144,465
RFPL3_7_2381,468,198,2250,833,869,15,155,382,1581,1231,1788,572
RFWD2_7_2382,1,24,0,90,778,0,0,0,35,3,52,3
RHOBTB1_7_2383,42,104,8,395,1027,93,2,804,246,226,253,33
RHOBTB2_7_2384,2,0,722,13,0,0,0,0,0,0,0,18
RLIM_7_2385,1886,2247,93,790,1196,2292,2147,1226,22,763,3250,1898
RNF103_7_2386,0,522,123,1,105,258,0,57,842,244,373,708
RNF128_7_2387,279,1180,0,48,304,3,921,352,684,378,172,446
RNF135_7_2388,158,10,1,64,0,163,141,63,24,341,15,196

RNF138_7_2389,321,474,2012,354,1744,708,441,805,962,1190,1114,2152
RNF13_7_2390,75,61,301,74,0,7,27,0,751,18,1,76
RNF145_7_2391,329,67,375,276,1683,446,0,21,951,106,1,472
RNF146_7_2392,0,3,0,246,0,16,661,147,23,66,705,11
RNF14_7_2393,0,6,0,43,0,235,132,0,0,15,244,0
RNF166_7_2394,308,1414,66,0,18,10,203,0,70,47,133,574
RNF170_7_2395,727,174,0,314,59,691,452,299,84,211,0,52
RNF17_7_2396,100,2246,3081,3019,185,3678,945,784,1860,2044,2209,1069
RNF180_7_2397,28,505,2450,551,1574,554,559,369,530,279,57,1214
RNF182_7_2398,98,68,0,749,0,235,114,0,246,7,21,36
RNF185_7_2399,87,163,51,60,678,72,178,42,187,93,7,25
RNF19A_7_2400,3,132,0,1,0,45,0,0,2,14,182,2
RNF19B_7_2401,12,36,339,272,268,71,437,499,102,864,4,525
RNF213_7_2402,288,21,124,110,231,40,325,78,503,101,404,482
RNF214_7_2403,1593,1164,1269,935,668,1679,4151,1420,1130,729,1680,2661
RNF216_7_2404,100,180,47,172,108,25,3,173,1,7,509,22
RNF24_7_2405,0,0,0,0,0,0,0,92,33,159,1798,2
RNF32_7_2406,810,58,496,2016,770,263,1341,3654,1163,979,1067,1890
RNF34_7_2407,519,59,214,20,67,50,71,146,86,173,503,38
RNF38_7_2408,2,56,426,31,313,1092,815,1126,869,1324,581,295
RNF40_7_2409,0,2,0,0,0,4,302,0,0,0,14,62
RNF41_7_2410,11,609,299,75,121,14,84,10,91,117,208,819
RNF6_7_2411,19,25,50,14,5,3,36,257,58,47,144,157
RNF7_7_2412,57,76,512,404,484,423,0,65,1032,238,0,140
RNF8_7_2413,38,616,67,55,1198,753,74,975,7,761,17,9
SAE1_7_2414,40,0,464,0,334,18,0,0,0,0,0,0
SENP6_7_2415,676,855,299,347,791,520,1431,861,247,1079,1016,580
SENP7_7_2416,2724,3533,6644,2231,3253,2840,3766,2793,3694,3567,2309,23
88
SENP8_7_2417,177,34,8,0,0,1106,0,0,5,726,855,52
SF3A1_7_2418,4,4,0,0,93,3,0,0,5,2,3,0
SHPRH_7_2419,51,1579,1501,2065,437,396,518,574,47,1055,654,574
SIAH1_7_2420,2,8,33,262,3,6,2,80,283,47,88,43
SKP2_7_2421,65,334,1153,10,0,59,34,783,252,15,822,272
SMURF1_7_2422,57,0,45,75,114,6,213,0,40,327,1,13
SOCS5_7_2423,1781,1161,689,843,686,1151,1399,338,1078,571,761,561
SP100_7_2424,210,28,0,122,292,1231,722,2,102,232,152,426
SP110_7_2425,43,103,0,0,0,19,0,0,598,10,2,359
SPOP_7_2426,0,5,0,4,363,0,217,1,395,651,0,26
SPSB2_7_2427,203,46,973,2,414,9,1,16,1577,130,6,216
SQSTM1_7_2428,0,881,0,302,21,35,0,437,954,10,2123,1003
STAMBP_7_2429,0,508,0,0,1,0,0,583,0,609,0,0
SUMO1_7_2430,16,543,12,0,0,233,262,65,811,117,127,719
SUMO2_7_2431,1400,382,3,0,0,27,507,32,7,1,1453,257
SYTL4_7_2432,0,4,0,74,0,1,78,211,754,304,11,834
SYVN1_7_2433,60,12,333,0,1,0,0,0,0,0,0,0
TCF20_7_2434,677,861,335,1226,12,582,37,969,205,1216,1099,3016
TDRD3_7_2435,2410,648,284,190,762,1212,2370,11,1741,341,675,457
TIPARP_7_2436,234,1,75,308,81,625,150,106,967,112,79,301
TLE2_7_2437,0,69,854,0,0,0,0,18,0,369,18,0

TLE3_7_2438,414,4,86,116,0,63,45,15,0,0,1286,47
TNK2_7_2439,991,325,851,315,7,62,16,1369,802,335,27,945
TNRC6C_7_2440,37,11,12,25,508,1,0,458,35,56,121,252
TOPORS_7_2441,406,771,849,985,1088,391,330,258,111,420,419,668
TOR1AIP2_7_2442,47,1,43,2,0,10,293,0,2,0,117,0
TRAF3_7_2443,0,48,1,84,0,2,1165,1,0,136,596,0
TRAF5_7_2444,170,32,23,112,0,54,49,24,19,0,241,182
TRAF6_7_2445,517,597,8,466,460,533,243,1595,640,339,282,155
TRIM10_7_2446,942,1330,1089,1935,1101,686,866,2342,934,301,2242,1705
TRIM13_7_2447,392,736,633,14,47,560,6,1017,524,66,119,251
TRIM17_7_2448,1,639,1151,39,466,977,963,702,276,613,67,828
TRIM22_7_2449,785,1426,33,733,364,1208,162,999,567,1405,691,679
TRIM23_7_2450,272,0,344,154,712,99,19,45,957,465,1133,621
TRIM24_7_2451,2999,694,177,1114,110,1758,445,1398,725,1191,1204,2568
TRIM26_7_2452,92,301,0,240,2,215,30,205,194,196,820,121
TRIM2_7_2453,556,8,88,247,289,165,136,42,1105,901,105,145
TRIM32_7_2454,52,0,0,5,169,31,137,12,408,341,2,396
TRIM33_7_2455,6,372,220,66,0,182,724,714,53,532,150,411
TRIM34_7_2456,319,854,0,570,137,319,10,278,1,69,4,580
TRIM37_7_2457,4181,1684,1654,1710,2932,1356,1162,2649,3607,3082,1972,4
096
TRIM39_7_2458,491,201,37,69,1188,44,27,234,0,54,1,347
TRIM3_7_2459,257,105,112,99,114,6,0,267,393,35,219,38
TRIM41_7_2460,0,30,186,420,30,8,272,0,30,1,5,279
TRIM45_7_2461,399,73,0,24,4,2,0,0,71,113,33,514
TRIM4_7_2462,305,769,712,434,236,238,124,164,29,807,929,705
TRIM54_7_2463,2,90,150,1,110,111,0,59,0,370,169,109
TRIM55_7_2464,92,400,150,0,0,81,0,7,56,836,29,1247
TRIM5_7_2465,1,29,2,9,29,0,0,123,59,99,0,8
TRIM7_7_2466,353,0,0,39,0,0,0,30,0,1091,34,199
TRIM9_7_2467,20,234,1083,19,49,503,0,415,102,2,29,280
TRPC4AP_7_2468,1885,1803,4010,519,1676,1132,1648,1028,724,2413,1597,12
26
TSPAN17_7_2469,3,552,193,16,0,0,0,10,160,7,0,86
TTC3_7_2470,1311,2043,61,1253,2024,866,380,1154,2155,1935,3446,2092
TULP4_7_2471,191,515,184,44,0,547,335,0,236,796,2,649
UBA1_7_2472,536,3,0,283,43,11,51,1559,546,89,450,1439
UBA3_7_2473,2510,2737,3158,1696,3654,2512,4271,2120,2037,2221,5872,368
0
UBA5_7_2474,32,153,81,144,272,29,14,509,54,3,0,16
UBAC2_7_2475,282,666,795,863,974,1112,15,474,1084,1711,391,137
UBAP2L_7_2476,257,30,0,40,90,117,2,34,0,18,16,20
UBASH3A_7_2477,100,22,245,111,0,245,20,599,2,10,0,4
UBE2A_7_2478,1144,2400,1509,267,487,679,575,1794,668,2051,599,1413
UBE2D1_7_2479,5435,4325,1262,4556,2397,5229,4995,4447,1828,3458,5836,5
933
UBE2D2_7_2480,3130,1429,2995,467,2036,2546,57,1271,1356,3121,3847,2496
UBE2D3_7_2481,3308,1539,924,470,2568,2373,58,1621,1745,3545,3800,2552
UBE2E1_7_2482,152,0,0,0,0,2,0,12,0,415,2,0
UBE2E3_7_2483,66,887,271,128,262,0,439,289,2,0,361,781

UBE2G2_7_2484,760,800,151,746,1518,735,1098,283,132,883,1407,1595
UBE2H_7_2485,203,109,394,204,156,523,297,191,35,257,451,8
UBE2I_7_2486,480,1594,1363,79,739,508,216,975,1224,2066,445,1185
UBE2J2_7_2487,10,18,568,143,0,465,21,550,264,481,333,82
UBE2K_7_2488,1378,383,852,448,928,6,1161,2013,1545,491,725,1180
UBE2Q2_7_2489,50,180,374,50,1102,644,109,9,198,574,160,53
UBE2V1_7_2490,410,299,322,6,67,119,692,94,325,299,410,52
UBE2W_7_2491,1997,818,370,339,318,2583,1125,1241,485,233,2215,138
UBE3A_7_2492,1423,317,3028,190,26,1567,2344,4530,562,756,835,1119
UBE3B_7_2493,0,87,6,456,436,0,0,0,62,64,18,43
UBE4A_7_2494,0,95,688,206,179,646,285,788,131,970,102,929
UBE4B_7_2495,558,0,433,13,1507,159,119,52,1627,27,1271,271
UBL7_7_2496,707,831,35,717,692,1462,635,441,138,699,365,877
UBOX5_7_2497,686,0,0,91,0,16,298,84,357,6,0,584
UBQLN1_7_2498,206,180,7,556,718,0,59,325,154,140,617,30
UBR2_7_2499,51,460,552,714,305,547,5,2619,1160,39,464,1727
UBXN11_7_2500,591,362,112,721,498,220,3,5,252,5,220,566
UBXN6_7_2501,0,559,232,355,0,246,0,321,51,5,305,7
UCHL5_7_2502,190,0,0,54,0,88,0,0,0,86,0,142
UHRF1_7_2503,0,997,0,8,19,0,1,41,24,0,10,4
UIMC1_7_2504,765,1931,3473,2935,1426,2253,1194,419,862,1026,1282,759
UNKL_7_2505,169,22,16,245,14,1,23,160,5,100,8,83
USP14_7_2506,1350,56,901,1215,1320,1457,9,477,608,178,611,1828
USP19_7_2507,161,14,38,14,16,71,0,17,65,291,0,237
USP1_7_2508,0,0,0,0,0,0,0,0,0,0,0,0
USP20_7_2509,326,82,968,0,702,21,0,0,19,0,0,1
USP2_7_2510,0,0,0,0,0,5,0,0,0,0,0,0
USP33_7_2511,447,48,90,316,47,118,73,160,135,602,126,94
USP44_7_2512,15,10,941,211,58,162,0,0,21,15,107,289
USP46_7_2513,509,3,87,189,380,77,245,657,369,167,1167,439
USP48_7_2514,591,631,1514,855,1360,122,19,1320,158,483,2203,285
USP4_7_2515,310,277,0,543,0,52,332,62,22,1,265,44
USP5_7_2516,0,136,2,8,0,556,0,0,0,105,0,0
USP6NL_7_2517,53,300,8,10,266,0,13,321,17,102,224,93
USP8_7_2518,747,858,311,134,423,659,250,380,857,661,275,96
USP9X_7_2519,0,0,0,456,0,0,0,0,0,1,0,0
VHL_7_2520,1,8,0,0,202,1,0,0,0,0,136,0
VPRBP_7_2521,1423,44,0,536,363,768,524,878,817,258,605,1035
VPS13D_7_2522,362,153,565,207,584,27,112,446,39,1,456,904
VPS41_7_2523,231,369,601,184,125,538,0,187,6,395,646,363
VPS8_7_2524,464,10,5,477,11,317,61,833,180,357,183,22
WDR26_7_2525,973,44,339,869,1633,1152,1869,1714,1634,2131,2662,1841
WDR5_7_2526,1852,660,1146,988,578,1853,684,932,1213,1305,3178,465
WDR76_7_2527,130,67,0,232,173,338,208,1,221,833,585,34
WHSC1_7_2528,0,1,15,0,0,0,14,2,25,7,0,242
WHSC1L1_7_2529,226,138,0,278,810,494,865,87,1578,425,1,1165
WSB1_7_2530,230,449,2016,444,141,664,49,916,1133,1550,570,374
WWP2_7_2531,0,0,0,494,3,267,874,993,208,531,283,178
XIAP_7_2532,211,100,1456,278,59,214,443,939,174,475,86,779
ZBTB10_7_2533,0,391,0,0,60,269,112,0,233,323,1,29

ZBTB16_7_2534,200,0,1,403,21,1,12,0,0,2,0,0
ZBTB17_7_2535,513,8,691,3,3,288,0,9,70,11,0,5
ZBTB1_7_2536,136,208,104,531,569,768,1152,80,925,951,68,467
ZBTB20_7_2537,38,136,1090,184,2,0,575,264,844,255,200,593
ZBTB22_7_2538,20,0,0,4,0,155,7,0,0,2,5,13
ZBTB24_7_2539,889,1389,146,915,537,1137,469,644,1789,2654,1468,1620
ZBTB33_7_2540,354,1711,2586,3254,1545,2028,1097,2025,2849,2127,1140,17
17
ZBTB37_7_2541,38,3,0,19,2,0,0,0,186,578,187,460
ZBTB40_7_2542,0,0,0,0,0,0,4,0,0,2,0,0
ZBTB4_7_2543,530,11,780,272,316,642,819,333,32,433,274,437
ZFP161_7_2544,1882,90,329,454,113,164,2,969,56,238,1022,876
ZMYND11_7_2545,51,1057,156,123,1505,33,292,289,0,359,242,0
ZMYND8_7_2546,118,0,0,2,258,481,18,637,233,26,607,18
ZNF238_7_2547,953,200,703,228,1906,941,922,333,361,968,1153,185
ZNF295_7_2548,284,1,0,0,719,0,0,0,121,296,0,1064
ZNRF3_7_2549,507,1465,322,1316,708,1147,300,1668,621,978,1788,959
ANAPC11_7_2550,724,1213,636,474,186,379,688,28,1150,598,641,1259
ATXN3_7_2551,1,936,64,599,0,4,14,26,216,81,24,30
MLLT10_7_2552,202,625,5,749,0,245,66,0,108,578,40,21
OTUD4_7_2553,103,440,103,233,530,6,636,397,1121,398,873,146
PHF19_7_2554,1949,481,1129,649,1631,1521,1369,1589,1150,4414,174,1651
SP140_7_2555,0,443,0,0,581,0,0,1185,360,3,1,822
TRIM36_7_2556,693,0,1093,1027,0,414,1,74,807,612,1,175
UBE2C_7_2557,174,0,4,0,82,16,2,0,36,37,654,146
UBE2L6_7_2558,223,225,39,1,16,436,1,56,32,403,24,4
ABTB2_7_2559,275,0,0,729,0,0,0,0,8,1,0,3
AMBRA1_7_2560,45,35,24,6,0,5,0,128,4,3,60,11
AMFR_7_2561,551,1399,0,0,0,0,0,1,752,238,1,63
ANAPC10_7_2562,118,596,3,245,138,614,588,327,589,49,867,78
ANAPC1_7_2563,704,76,1209,634,0,45,4,1,758,1644,38,608
ANAPC2_7_2564,0,492,197,0,0,0,1,3,0,0,1,0
ANAPC4_7_2565,311,453,2844,1925,354,3021,1076,425,1974,1397,1583,796
ANKIB1_7_2566,184,907,1992,188,196,0,594,139,811,22,950,95
ANKRD13A_7_2567,496,907,2332,785,679,181,931,161,746,418,75,495
ANKRD13D_7_2568,7,26,38,1,3,33,0,33,394,450,45,74
ARIH1_7_2569,127,533,1,45,0,29,662,312,190,353,28,315
ARIH2_7_2570,314,28,141,0,1019,22,266,792,1195,1126,1130,165
ASB12_7_2571,906,1084,835,182,977,983,1559,338,650,1115,1155,1779
ASB13_7_2572,8,1029,0,3,683,549,0,318,122,636,43,22
ASB15_7_2573,1270,533,12,287,477,259,30,134,8,179,295,194
ASB16_7_2574,0,0,0,0,0,0,0,0,0,0,2,0
ASB17_7_2575,1639,1955,2019,2277,811,834,31,3001,1753,1619,2270,397
ASB5_7_2576,8,0,0,0,0,0,385,47,0,202,0,0
ASB8_7_2577,0,8,2,1,6,0,0,205,0,3,39,1020
ASH1L_7_2578,99,890,103,532,8,19,336,200,227,344,1,606
ASPSCR1_7_2579,378,11,1,36,86,24,33,39,1220,776,38,285
ATG12_7_2580,61,64,0,93,0,115,1635,775,225,6,0,755
ATG3_7_2581,5000,8307,11974,4418,3756,5195,9255,5957,4692,9841,7035,58
90

ATG5_7_2582,588,309,420,575,46,1596,572,229,108,957,1022,900
ATXN1L_7_2583,34,169,935,33,133,385,15,310,113,372,1279,18
ATXN3L_7_2584,1223,231,564,45,222,223,955,1023,560,209,816,1509
BARD1_7_2585,6,2,0,0,0,1029,0,0,112,4,119,9
BAZ1B_7_2586,2566,2128,3327,941,4340,2729,1592,2125,3135,2225,1515,261
8
BAZ2A_7_2587,701,1953,695,600,504,21,535,177,752,1509,290,888
BAZ2B_7_2588,502,1316,2,1034,47,894,272,1062,1090,385,2055,1052
BECN1_7_2589,829,1670,813,677,1050,906,228,2978,649,2861,1090,1888
BIRC2_7_2590,1649,2490,1652,1590,380,2335,1945,3104,1692,689,3072,1092
BIRC6_7_2591,1610,563,118,63,502,368,1208,525,1228,1847,120,893
BIRC8_7_2592,1,68,125,4,56,1030,998,16,806,569,1049,128
BMI1_7_2593,607,622,469,854,437,25,49,3556,1053,704,196,346
BRAP_7_2594,438,214,967,1791,1214,511,2395,591,432,470,2229,895
BRD1_7_2595,184,21,397,8,36,76,25,246,88,1905,0,12
BRPF3_7_2596,1,119,0,68,336,506,161,190,45,28,448,15
BTBD2_7_2597,423,1274,438,510,201,287,1155,39,261,429,478,384
BTBD6_7_2598,284,1,0,226,214,0,0,60,205,390,463,210
CAND1_7_2599,925,1007,1377,1367,1466,3328,1117,2865,1322,2235,1134,245
5
CBLB_7_2600,613,1878,71,728,827,998,1287,898,744,1154,1260,1100
CBL_7_2601,184,4,854,243,668,751,907,0,990,849,205,1023
CBLL1_7_2602,0,0,34,18,1,25,0,25,959,33,953,112
CCIN_7_2603,57,6,781,0,0,1006,30,226,848,530,429,114
CCNF_7_2604,1,399,0,423,32,72,0,226,5,0,1,12
CDC20_7_2605,20,12,939,0,33,0,13,0,0,0,284,37
CDC23_7_2606,24,197,1,103,404,14,0,14,217,195,329,23
CDC26_7_2607,330,22,3,4,5,21,146,173,169,108,139,43
CDC34_7_2608,0,0,0,48,0,0,0,0,0,0,309,22
CGRRF1_7_2609,1642,1459,2053,1834,1685,1031,1732,712,1893,1098,747,243
CHD4_7_2610,92,170,0,80,1023,438,0,598,583,877,176,712
CIAO1_7_2611,525,620,226,2,292,828,351,258,451,126,60,286
COPS4_7_2612,2,5,0,382,151,319,322,0,50,5,0,9
COPS5_7_2613,9,0,0,436,475,87,84,946,5,62,385,651
COPS6_7_2614,103,189,200,84,76,11,1266,498,310,261,678,452
COPS7B_7_2615,247,17,42,71,569,609,716,1030,505,259,282,1228
CORO6_7_2616,43,96,4,3,155,16,127,35,0,96,394,223
CUEDC1_7_2617,598,48,71,360,599,2221,829,48,360,2958,1532,32
CUL1_7_2618,1241,91,362,12,100,0,3,734,55,62,69,21
CUL3_7_2619,0,747,0,117,384,30,1936,855,398,421,1,130
CUL5_7_2620,27,47,5,22,48,37,297,5,46,6,34,101
CUL9_7_2621,1683,13,1324,2333,241,2,236,207,351,1281,331,57
DCAF10_7_2622,3006,2884,2860,474,2834,2274,390,403,552,686,1070,1103
DCAF12_7_2623,342,1099,545,139,436,0,530,558,369,661,1145,465
DCAF13_7_2624,901,995,800,577,1229,587,49,818,1349,734,819,718
DCAF16_7_2625,1569,416,679,1269,142,592,289,491,710,1546,1601,1441
DCAF5_7_2626,1662,1667,3057,1217,2217,2494,2308,782,3662,4048,2885,310
8
DCAF7_7_2627,75,106,63,82,0,96,1,36,95,690,90,657
DCAF8_7_2628,3545,1487,1658,618,1715,220,1348,449,37,861,1202,1116

DDA1_7_2629,23,145,739,740,22,116,115,351,36,6,62,561
DDB1_7_2630,50,5,608,17,0,50,0,51,250,39,0,35
DDB2_7_2631,494,991,1258,622,1055,1743,2075,400,3129,2620,3285,1547
DPF2_7_2632,112,229,422,154,1569,217,650,240,1263,423,736,728
DTX1_7_2633,1,777,277,60,1033,211,388,147,46,14,17,408
DTX3L_7_2634,294,708,674,778,1359,282,356,526,1742,1380,1412,1035
DTX4_7_2635,969,0,0,0,0,0,0,0,0,0,0,0
DZIP3_7_2636,519,520,1052,500,1521,666,1974,911,857,987,2529,1059
EIF3D_7_2637,0,1,7,35,175,1,52,0,47,25,2,8
EIF3E_7_2638,3474,2052,1618,2082,1570,1578,1553,1766,1906,1614,880,331
5
EIF3F_7_2639,118,60,325,3,71,188,247,647,9,121,0,239
EIF3G_7_2640,828,553,395,305,1387,122,369,628,683,1034,96,169
EIF3H_7_2641,697,58,425,1399,391,1534,519,704,772,1071,1068,758
EIF3I_7_2642,0,0,0,0,0,0,0,0,23,0,134,2
EIF3J_7_2643,112,77,182,517,375,311,223,22,544,939,14,80
EIF3K_7_2644,242,1,50,1802,832,24,3092,26,28,585,10,0
ENC1_7_2645,433,8,60,20,452,1,1034,1096,831,100,25,134
EPN3_7_2646,0,0,0,552,1,0,726,37,0,4,614,0
EPOR_7_2647,393,0,0,187,0,0,0,121,277,10,1167,39
ERCC8_7_2648,2671,296,2016,2432,4574,1576,989,1377,1874,1130,2264,371
FAF1_7_2649,1019,476,623,486,879,981,511,541,112,185,515,13
FBXL12_7_2650,568,0,0,0,0,0,0,0,0,0,0,0
FBXL14_7_2651,17,516,0,0,388,496,0,1,1039,437,21,118
FBXL15_7_2652,2,5,0,1,690,1,184,498,0,8,857,1401
FBXL16_7_2653,1,174,204,0,0,66,760,363,90,126,64,654
FBXL17_7_2654,529,269,64,277,143,7,107,898,55,48,24,219
FBXL18_7_2655,0,345,89,73,0,0,0,59,164,8,9,8
FBXL19_7_2656,234,0,0,817,0,1155,128,808,111,5,1271,765
FBXL21_7_2657,1,709,1,242,860,786,0,544,93,283,5,1541
FBXL3_7_2658,270,345,316,611,83,21,147,937,160,552,181,1321
FBXL4_7_2659,585,12,104,92,159,63,22,21,60,1603,33,50
FBXL7_7_2660,0,0,0,0,0,0,0,3,0,0,0,5
FBXO10_7_2661,141,552,140,30,241,131,36,206,33,17,35,7
FBXO16_7_2662,70,112,211,8,1546,594,47,465,413,433,958,894
FBXO27_7_2663,0,0,0,91,1,0,7,6,607,13,9,98
FBXO2_7_2664,146,267,514,99,0,364,1,12,2,93,1274,54
FBXO30_7_2665,0,0,855,537,0,11,2,4,0,0,0,774
FBXO33_7_2666,625,431,26,848,1160,66,945,251,226,485,251,706
FBXO36_7_2667,0,2,78,0,0,0,1,1169,0,13,0,0
FBXO39_7_2668,412,12,1023,0,9,688,229,11,462,21,39,48
FBXO40_7_2669,1460,424,1506,431,922,363,579,925,362,929,77,581
FBXO41_7_2670,0,0,72,0,66,228,0,111,30,1,10,4
FBXO42_7_2671,1469,1733,3767,2717,932,594,2208,2085,1853,1994,3440,116
0
FBXO43_7_2672,355,291,82,0,710,17,0,67,1,0,0,1
FBXO46_7_2673,1056,1715,253,193,799,1585,5,112,542,75,610,825
FBXO6_7_2674,43,0,24,7,18,1054,660,2,2,451,285,1
FBXO8_7_2675,1604,2835,2518,834,810,1065,1046,2707,1666,951,1242,363
FBXW10_7_2676,0,71,12,304,0,0,7,73,310,164,1,666

FBXW2_7_2677,2139,638,1523,796,2154,995,2412,1303,2004,2609,922,802
FBXW5_7_2678,7,410,151,603,916,143,596,326,64,52,290,91
FBXW9_7_2679,72,0,0,0,592,0,405,0,3,2,0,9
G2E3_7_2680,862,1538,83,1142,105,595,1182,19,1133,1199,1652,1363
GAN_7_2681,43,924,1,91,743,744,1086,64,1149,855,942,167
GMCL1_7_2682,936,8,6,626,14,0,643,355,330,373,173,193
GNB2_7_2683,74,6,0,636,188,3,794,556,835,664,0,122
GRWD1_7_2684,39,0,61,0,677,1,86,2,6,656,94,151
GZF1_7_2685,548,364,1319,555,0,555,348,152,204,259,72,354
HACE1_7_2686,0,276,19,340,246,2,98,237,159,8,0,2011
HDAC6_7_2687,0,0,0,0,0,0,0,0,0,0,0,0
HECTD1_7_2688,727,710,363,1100,461,968,2384,2082,350,1935,1535,1449
HECTD3_7_2689,84,27,0,0,0,45,0,0,33,436,54,3
HECW1_7_2690,2,76,14,170,204,80,104,402,303,12,34,84
HECW2_7_2691,1912,134,272,1142,0,17,757,7,671,169,40,1437
HERC1_7_2692,520,1424,2867,462,307,265,810,590,880,561,4,252
HERC2_7_2693,14,0,390,2,241,155,895,428,333,98,928,403
HERC3_7_2694,1,50,29,0,94,0,0,102,139,330,1023,10
HERC5_7_2695,1118,578,46,496,97,301,1046,0,36,99,172,871
HGS_7_2696,41,2,161,5,1,0,0,37,19,270,2,379
HIC2_7_2697,1410,1752,1212,372,771,482,122,241,909,1040,76,552
HUWE1_7_2698,5,981,0,797,166,437,587,1956,620,577,574,81
IBTK_7_2699,190,1078,764,547,492,1360,1158,167,685,160,1653,863
IL10RA_7_2700,88,2,3,118,0,0,0,240,242,6,33,584
IL6_7_2701,76,22,846,590,532,434,182,147,428,256,31,267
IRF9_7_2702,14,41,436,166,699,496,54,261,901,40,1213,779
ITCH_7_2703,1576,1389,22,167,339,765,1315,2687,1147,1109,91,190
IVNS1ABP_7_2704,1000,856,193,15,194,119,276,339,903,140,1013,717
JHDM1D_7_2705,477,24,1,57,504,838,565,242,844,740,87,120
JOSD1_7_2706,0,7,41,115,0,451,0,0,9,280,225,225
JOSD2_7_2707,0,0,0,0,0,20,0,103,1,18,999,0
KAT6B_7_2708,412,687,301,1138,902,39,119,315,57,732,9,166
KATNB1_7_2709,8,9,1,26,112,57,547,1273,2,0,287,67
KBTBD10_7_2710,425,521,35,32,0,3,1124,1,241,1021,1102,58
KBTBD11_7_2711,0,9,99,187,441,32,112,61,22,8,339,5
KBTBD2_7_2712,731,1519,836,591,39,1219,577,705,1261,869,732,1329
KBTBD5_7_2713,868,55,83,31,1,720,439,790,200,28,244,120
KBTBD7_7_2714,685,83,1107,871,576,221,67,934,15,900,542,371
KBTBD8_7_2715,194,0,0,0,112,0,0,0,0,38,633,0
KCTD10_7_2716,98,356,0,0,0,1137,284,1003,4,511,325,0
KCTD11_7_2717,3,32,0,31,510,233,141,230,379,7,168,248
KCTD12_7_2718,1107,24,0,0,0,49,1100,1132,2,350,116,51
KCTD13_7_2719,165,1,0,509,183,825,80,533,303,215,256,33
KCTD16_7_2720,1,201,918,117,575,0,0,751,2,0,0,80
KCTD17_7_2721,0,12,5,51,1,0,19,0,90,12,4,26
KCTD18_7_2722,331,441,283,384,150,498,11,483,1269,1172,23,1462
KCTD3_7_2723,45,1,240,7,1,543,88,523,82,1038,2,17
KCTD5_7_2724,5,0,0,1,0,0,1,0,0,0,150,0
KCTD9_7_2725,1640,1639,1542,1324,1206,887,1790,4062,1749,1452,1133,322
8

KDM2A_7_2726,0,7,43,0,72,401,0,344,512,25,23,74
KDM4B_7_2727,0,0,0,0,0,0,0,0,0,0,0,0
KDM5B_7_2728,159,163,888,838,110,531,8,1192,0,11,4,717
KLHDC5_7_2729,40,65,0,1,0,26,187,0,213,42,0,26
KLHL10_7_2730,136,131,402,69,31,117,1307,118,295,98,0,30
KLHL11_7_2731,124,63,129,434,5,22,0,423,4,106,640,0
KLHL12_7_2732,48,382,517,8,458,1238,375,3,167,255,8,185
KLHL14_7_2733,128,14,2,1,417,5,5,132,13,0,5,860
KLHL15_7_2734,7,307,70,105,220,922,126,560,435,2029,4,454
KLHL17_7_2735,3,0,0,0,39,0,0,1,468,2,0,51
KLHL18_7_2736,333,49,670,0,1772,241,0,6,659,8,7,77
KLHL1_7_2737,346,74,145,184,1073,299,154,213,226,662,340,707
KLHL20_7_2738,361,288,9,284,153,692,513,312,38,733,410,208
KLHL21_7_2739,240,3,0,286,13,0,1573,0,0,377,0,764
KLHL22_7_2740,40,552,121,97,611,16,907,48,261,386,5,215
KLHL23_7_2741,471,105,578,3,0,0,80,240,207,686,899,749
KLHL24_7_2742,799,473,12,867,17,43,346,20,605,149,462,388
KLHL25_7_2743,37,137,80,1,545,19,0,13,130,2,0,35
KLHL26_7_2744,22,0,20,0,178,22,8,0,11,2,351,867
KLHL28_7_2745,4262,3973,2803,2158,966,2075,1745,4607,3368,1746,3323,26
34
KLHL29_7_2746,0,125,12,109,0,326,2,0,194,0,0,216
KLHL31_7_2747,375,15,1383,771,166,963,2299,1759,336,1767,2973,649
KLHL32_7_2748,502,155,0,32,166,93,4,99,249,158,6,43
KLHL33_7_2749,835,95,9,0,110,66,132,547,228,57,18,188
KLHL34_7_2750,78,1,0,427,1090,313,4,223,240,6,2,387
KLHL36_7_2751,542,293,31,263,237,318,1291,762,231,983,460,38
KLHL3_7_2752,240,0,880,71,536,94,0,237,3,360,274,18
KLHL8_7_2753,77,282,0,413,0,115,5,1,296,35,4,47
LATS1_7_2754,511,739,2438,2403,877,975,1457,1194,1894,760,1273,1166
LATS2_7_2755,3,5,0,12,5,12,171,0,0,0,0,10
LIF_7_2756,169,0,2,0,401,1,46,479,306,21,17,37
LNX2_7_2757,91,188,87,0,471,201,134,0,126,37,905,50
LOC283116_7_2758,0,15,30,46,325,0,31,574,19,0,20,0
LONRF1_7_2759,918,1555,1443,1414,936,513,2291,453,565,1074,1242,922
LTN1_7_2760,758,143,1412,1084,185,791,1202,2100,2192,991,391,2341
LZTR1_7_2761,278,18,1081,598,5,4,1568,944,45,0,8,86
MAP1LC3B_7_2762,1,7,6,77,27,0,4,1,0,2,112,0
MAP3K1_7_2763,914,21,271,2217,404,592,1672,346,560,323,454,175
MARK1_7_2764,139,188,1,265,224,5,12,1,0,131,583,0
MDM2_7_2765,1080,931,1072,1024,223,1459,2174,701,1023,570,1499,2389
MED20_7_2766,0,0,150,411,0,0,0,277,171,205,20,22
MEX3B_7_2767,0,1,0,1,0,48,0,0,0,0,10,241
MIB1_7_2768,148,1775,19,576,818,57,27,453,1069,358,111,1273
MKRN2_7_2769,22,31,1253,274,366,566,2,916,578,954,1532,718
MKRN3_7_2770,208,686,702,496,186,232,62,416,647,36,40,356
MLL2_7_2771,3,583,49,0,5,7,0,194,3,6,0,0
MLLT6_7_2772,83,0,6,0,352,86,889,1,3,1,0,2
MOCS3_7_2773,382,375,0,135,62,786,129,48,75,197,228,95
MRPL49_7_2774,0,62,0,537,0,9,0,0,31,0,33,3

MUL1_7_2775,422,46,0,44,35,3,208,0,286,168,15,35
MYCBP2_7_2776,545,254,19,1204,51,738,36,1088,71,997,1282,341
MYLIP_7_2777,7,698,0,363,124,18,0,225,64,138,25,8
MYSM1_7_2778,677,1602,1523,238,1114,1281,1028,2347,1006,687,667,1585
NACC1_7_2779,372,271,684,581,7,362,149,22,188,118,10,25
NACC2_7_2780,16,136,7,131,0,50,897,694,170,6,1,27
NEURL1B_7_2781,565,247,827,1014,3505,1387,532,2072,1037,1062,981,782
NEURL_7_2782,91,4,33,0,61,2,0,0,5,1,1,1
NHLRC1_7_2783,0,492,88,11,27,26,343,61,83,17,22,277
NUP43_7_2784,313,0,178,605,1542,560,262,528,1250,1794,1009,490
OTUB1_7_2785,235,863,0,0,2257,1040,1227,1537,451,131,3,578
OTUB2_7_2786,9,293,1,1077,1184,0,179,1263,3,22,1,30
OTUD1_7_2787,98,25,409,249,589,473,48,162,598,187,251,79
OTUD6A_7_2788,106,15,4,7,517,183,1771,1014,171,38,11,418
OTUD6B_7_2789,0,4,15,29,30,0,56,4,0,13,167,6
OTUD7A_7_2790,5,1232,3,0,66,0,75,688,20,0,13,4
OTUD7B_7_2791,50,43,33,345,178,35,8,750,559,29,121,495
PAFAH1B1_7_2792,203,279,714,233,704,1400,0,10,327,1286,2,50
PARP10_7_2793,0,0,0,317,1,403,0,38,0,0,1694,0
PARP11_7_2794,1245,2860,451,1025,2835,1553,1195,1102,1163,1036,1386,25
34
PARP14_7_2795,524,159,33,428,191,114,1345,127,37,176,767,29
PCGF1_7_2796,3248,2038,1623,769,1297,959,660,821,1175,1260,1368,523
PCGF2_7_2797,7,293,1453,418,2113,630,0,76,982,163,3,369
PCGF3_7_2798,339,0,0,0,0,382,1,625,2,6,34,1
PDZRN3_7_2799,0,55,59,0,221,2,62,450,31,90,60,4
PEBP4_7_2800,0,1062,852,0,0,402,0,2,390,4,4,44
PEX12_7_2801,12,368,0,28,0,25,30,1,460,378,51,47
PHF14_7_2802,137,579,1157,418,85,1339,672,6,180,7,468,839
PHF15_7_2803,319,269,1,213,0,639,6,49,568,25,408,92
PHF20_7_2804,1544,923,1316,1103,650,2436,25,837,309,1277,2829,449
PHF2_7_2805,268,312,1,73,904,166,641,4,439,24,1142,1006
PHF3_7_2806,1426,633,1267,1022,867,982,90,928,768,1206,1392,1003
PHIP_7_2807,213,1013,472,900,162,1934,16,675,1141,460,3657,2591
PHRF1_7_2808,0,0,0,0,0,52,0,11,254,171,0,35
PJA2_7_2809,39,1,162,546,0,299,848,32,173,786,1218,236
PRPF19_7_2810,3224,1372,581,1397,1771,888,1503,2348,1963,3391,1704,103
0
PRPF8_7_2811,0,5,844,0,33,0,34,719,87,7,48,5
PSMD14_7_2812,267,55,197,8,769,0,11,546,76,621,638,670
PSMD2_7_2813,971,29,2,0,222,507,396,657,641,869,176,766
PSMD4_7_2814,0,147,0,620,126,212,44,0,107,302,201,346
PSMD7_7_2815,179,221,16,569,302,731,168,58,105,136,179,180
PWP1_7_2816,22,1205,1379,1354,2066,1009,178,1153,1130,1149,1729,773
RAB40A_7_2817,8,74,278,141,100,16,9,13,9,0,693,56
RAB40AL_7_2818,8,74,278,141,100,16,9,13,9,0,693,56
RAB40B_7_2819,0,2,0,0,9,0,0,387,331,7,0,44
RAD18_7_2820,16,140,0,247,332,705,795,576,835,489,59,97
RAD23A_7_2821,345,226,0,219,597,211,351,743,97,506,57,284
RAD23B_7_2822,0,0,0,1,0,0,0,12,0,0,0,0

RAG1_7_2823,139,92,51,537,543,1,715,0,30,0,0,55
RAI1_7_2824,2,238,1148,303,461,3,281,361,91,199,108,34
RBX1_7_2825,137,93,742,857,8,713,140,1297,6,21,3,50
RCBTB1_7_2826,636,656,770,1038,12,1024,800,555,789,1338,563,1809
RCBTB2_7_2827,2194,165,1353,2735,7,940,821,0,487,31,613,70
RFFL_7_2828,776,455,230,270,1020,887,1136,156,631,715,38,879
RFPL1_7_2829,0,91,2,420,0,2,21,206,321,288,250,546
RFWD3_7_2830,73,20,8,434,1081,15,273,0,11,67,0,261
RHOBTB3_7_2831,1901,2937,2447,702,1753,1152,3680,2088,2826,1456,3525,2
744
RING1_7_2832,242,715,1,104,21,426,644,0,38,408,17,262
RNF10_7_2833,601,459,7,611,252,1159,674,635,123,369,11,824
RNF111_7_2834,79,17,0,38,813,304,1286,193,732,105,96,229
RNF112_7_2835,308,876,397,269,17,809,29,14,178,2090,886,625
RNF113A_7_2836,0,1,0,7,0,151,0,0,0,0,0,0
RNF113B_7_2837,1392,582,1023,186,395,1013,1491,357,576,575,269,126
RNF114_7_2838,16,1,8,337,1247,11,852,723,237,3,38,947
RNF115_7_2839,1255,165,1322,987,203,609,654,780,234,946,504,177
RNF11_7_2840,644,1605,963,133,44,702,784,1099,621,331,1531,786
RNF121_7_2841,0,824,235,1188,60,374,751,761,324,843,1007,247
RNF122_7_2842,9,51,19,0,183,233,10,103,252,9,209,45
RNF123_7_2843,71,43,41,33,635,22,1258,8,231,103,1419,27
RNF125_7_2844,26,39,153,385,860,375,22,173,17,1,657,28
RNF126_7_2845,305,15,93,136,441,373,625,400,590,564,266,87
RNF130_7_2846,719,676,8,803,96,166,538,388,928,490,738,706
RNF133_7_2847,2061,1607,3488,643,686,1603,3509,2216,900,1881,1899,2742
RNF139_7_2848,45,783,1432,705,614,196,394,1243,162,330,33,148
RNF141_7_2849,969,3016,1630,1085,156,735,180,988,1282,1089,1944,311
RNF144A_7_2850,336,0,13,0,0,51,0,124,0,0,0,151
RNF144B_7_2851,49,406,0,633,8,0,37,0,31,310,1,72
RNF149_7_2852,1,513,683,0,0,3,0,0,1,17,0,0
RNF150_7_2853,20,1049,1306,1407,866,898,1829,929,1199,330,2113,1494
RNF152_7_2854,980,0,198,492,19,8,0,55,340,66,454,625
RNF157_7_2855,128,0,367,0,1,0,0,0,137,26,296,15
RNF167_7_2856,257,461,79,469,626,920,4,1239,143,1005,574,794
RNF168_7_2857,608,1057,504,1471,1385,1424,639,124,1801,355,380,1444
RNF169_7_2858,1095,568,61,622,1012,291,306,159,1213,450,582,842
RNF181_7_2859,0,412,0,1,473,246,0,8,10,27,1,388
RNF183_7_2860,137,1,445,0,4,61,4,448,50,17,11,5
RNF186_7_2861,2575,2948,511,617,1370,881,701,1128,848,1641,505,509
RNF187_7_2862,1885,171,630,366,1916,328,2001,2617,1841,957,737,1053
RNF20_7_2863,66,835,0,239,107,185,287,128,41,407,1877,255
RNF217_7_2864,235,5,0,830,28,0,669,2,419,608,13,274
RNF220_7_2865,236,0,0,1,5,217,0,0,0,20,0,0
RNF25_7_2866,256,0,0,0,0,1,1258,0,7,299,0,0
RNF26_7_2867,0,0,14,15,289,357,0,0,35,67,0,71
RNF2_7_2868,260,20,0,38,118,236,0,32,102,1079,0,82
RNF31_7_2869,237,200,1069,3,366,535,430,108,357,980,1,185
RNF43_7_2870,308,896,196,293,1035,1208,667,1174,269,207,0,671
RNF44_7_2871,253,268,33,91,24,58,5,25,127,3,151,19

RNF5_7_2872,0,87,0,88,43,0,0,0,69,2,0,13
RSC1A1_7_2873,1233,424,3348,1234,1599,1603,1001,2664,3930,2706,3104,19
86
RSF1_7_2874,61,1,12,0,0,0,0,1075,75,165,346,6
RSPRY1_7_2875,1165,142,56,7,394,1094,0,624,161,43,32,148
SCLY_7_2876,299,1561,235,2312,1022,1308,1613,3459,2758,2056,1007,3602
SENP1_7_2877,430,161,804,295,709,75,321,77,1426,212,0,494
SENP2_7_2878,1418,1067,1769,191,107,229,1780,1952,113,572,1003,69
SENP3_7_2879,45,0,0,9,0,17,0,56,0,0,0,0
SENP5_7_2880,234,344,350,212,250,369,1712,711,1618,644,216,1098
SH3RF1_7_2881,0,180,0,0,0,0,0,0,0,0,0,0
SH3RF2_7_2882,0,0,0,0,742,0,1,0,0,441,0,0
SH3RF3_7_2883,958,622,103,1193,73,1506,1468,637,1501,1142,606,1305
SHKBP1_7_2884,2,2,0,0,11,306,115,0,0,30,99,57
SIAH2_7_2885,0,4,5,0,0,1,0,690,410,226,0,52
SIK1_7_2886,1,0,116,333,25,22,4,367,513,219,0,112
SLX4_7_2887,1035,496,89,874,342,885,2799,1620,819,1832,447,1868
SMU1_7_2888,16,89,17,29,0,1,22,0,177,59,745,722
SMURF2_7_2889,628,1,7,202,0,361,775,264,81,1182,3,44
SNRNP40_7_2890,203,305,45,15,369,337,18,1420,552,34,40,57
SOCS1_7_2891,7,0,0,61,16,0,0,0,8,0,0,2
SOCS2_7_2892,7,74,136,424,1,25,55,399,1003,13,582,288
SOCS3_7_2893,1,0,438,0,0,0,0,0,0,0,0,0
SOCS6_7_2894,498,560,215,952,14,2053,1,1348,1853,1063,625,1010
SPOPL_7_2895,1156,1194,33,519,2067,1253,1117,415,2374,1496,1648,2519
SPSB1_7_2896,543,34,145,8,845,634,425,1,228,122,84,24
SPSB3_7_2897,0,0,0,0,0,0,0,0,0,0,0,0
SPSB4_7_2898,37,272,39,485,345,11,353,23,131,95,983,104
STAM2_7_2899,1387,243,639,1795,533,973,61,500,1215,865,291,536
STAMBPL1_7_2900,1898,698,0,725,1641,926,618,1220,181,211,169,246
STAM_7_2901,519,177,351,0,0,0,0,1,0,0,0,0
STUB1_7_2902,95,3,23,73,0,145,669,683,490,140,585,311
SUMO3_7_2903,0,0,14,0,0,1,0,0,195,30,0,21
SYNGAP1_7_2904,563,2971,219,355,555,1879,1239,1148,208,1432,24,780
TAB2_7_2905,396,1297,157,375,2759,836,998,484,1289,204,1788,1127
TAB3_7_2906,92,30,30,264,65,35,7,679,118,875,25,1318
TAF1D_7_2907,16,564,6,275,846,1066,1214,201,191,617,657,43
TBC1D1_7_2908,7,90,603,893,727,850,983,101,369,224,2758,1045
TLE1_7_2909,33,92,1339,408,1542,647,908,50,772,655,331,306
TNFAIP3_7_2910,125,50,1296,91,151,480,727,0,280,655,906,26
TOLLIP_7_2911,0,0,0,0,0,0,0,0,0,0,0,0
TRAF7_7_2912,486,124,1166,155,146,683,872,526,36,1735,2075,279
TRAIP_7_2913,63,201,39,2,20,63,63,137,26,2,10,24
TRIM11_7_2914,0,0,0,0,0,0,0,0,0,0,0,0
TRIM15_7_2915,627,14,25,157,427,412,45,1469,439,278,449,415
TRIM25_7_2916,73,1884,1111,227,541,1170,397,745,988,927,76,1166
TRIM27_7_2917,278,4,8,3,0,167,743,772,319,8,49,52
TRIM28_7_2918,42,4,0,15,144,33,567,0,0,246,254,51
TRIM31_7_2919,271,1,59,0,129,45,34,98,0,4,43,0
TRIM35_7_2920,29,275,348,467,0,168,368,17,154,143,0,54

TRIM42_7_2921,36,328,152,59,13,1758,97,0,74,3,6,12
TRIM46_7_2922,264,394,131,117,279,1,128,365,0,467,1068,286
TRIM47_7_2923,0,0,1038,0,25,0,24,9,0,0,0,0
TRIM48_7_2924,1531,2360,1025,379,156,583,370,3139,1269,2329,547,2430
TRIM52_7_2925,775,1126,71,195,58,576,665,56,159,756,15,624
TRIM56_7_2926,1239,541,373,1040,0,67,633,854,305,607,786,827
TRIM62_7_2927,0,2,0,49,0,14,0,0,0,33,980,6
TRIM63_7_2928,21,561,406,1,0,288,0,0,1,849,34,0
TRIM65_7_2929,1,786,0,0,0,0,3,0,429,7,0,59
TRIM67_7_2930,23,8,457,0,979,403,1053,0,2,0,183,23
TRIM68_7_2931,2146,300,423,34,2,493,211,197,793,227,1464,100
TRIM8_7_2932,0,0,0,0,0,0,2,212,47,490,33,6
TRIP12_7_2933,674,61,718,0,543,63,89,279,340,789,599,41
UBA2_7_2934,386,10,10,143,18,862,0,269,58,88,0,10
UBA6_7_2935,474,279,0,605,0,282,459,93,795,668,29,394
UBA7_7_2936,0,0,0,2,36,0,0,0,8,0,1,0
UBAC1_7_2937,213,5,104,207,114,777,569,568,394,133,522,440
UBAP2_7_2938,396,190,688,165,122,0,177,270,306,199,80,929
UBASH3B_7_2939,0,15,0,0,10,31,139,199,13,124,6,5
UBC_7_2940,53,1059,278,356,817,738,837,805,7,263,618,194
UBE2B_7_2941,2219,637,2074,1358,926,686,1004,35,744,1064,494,231
UBE2D4_7_2942,465,1244,0,114,1032,182,257,104,333,146,729,41
UBE2E2_7_2943,646,1510,107,487,390,1434,5009,637,1279,2652,1112,792
UBE2F_7_2944,2,0,0,18,0,79,0,0,29,1,0,4
UBE2G1_7_2945,0,0,720,0,1,19,11,155,4,161,0,1
UBE2J1_7_2946,1084,621,197,419,284,772,0,35,225,359,311,43
UBE2L3_7_2947,75,667,1043,972,2404,193,267,493,536,395,235,1432
UBE2M_7_2948,2211,401,524,838,564,921,270,964,546,1323,723,406
UBE2N_7_2949,5,28,0,643,226,337,81,11,28,248,40,86
UBE2NL_7_2950,1060,18,0,7,3,77,205,55,140,5,96,29
UBE2O_7_2951,352,586,211,35,1109,218,459,192,413,1068,935,724
UBE2Q1_7_2952,0,3,0,0,0,0,19,15,0,0,0,0
UBE2QL1_7_2953,18,432,174,30,12,757,612,10,111,325,100,61
UBE2R2_7_2954,192,1799,191,353,128,8,1724,1952,1235,131,602,537
UBE2S_7_2955,0,524,3,71,0,35,515,109,302,3,0,22
UBE2T_7_2956,321,408,396,313,218,119,368,0,216,764,604,68
UBE2U_7_2957,791,517,127,935,635,1162,1019,842,3002,2286,1767,691
UBE2V2_7_2958,110,4,116,0,0,60,0,0,222,10,0,464
UBE2Z_7_2959,332,13,247,41,0,211,162,0,66,0,0,394
UBE3C_7_2960,3054,2465,1484,1681,3649,3419,2911,2822,4505,4294,4272,57
54
UBQLN2_7_2961,186,486,4,701,518,183,126,223,470,107,28,1441
UBQLN3_7_2962,1250,130,0,534,1046,10,64,134,376,394,323,721
UBQLN4_7_2963,0,70,3,0,34,180,0,27,70,5,33,6
UBR1_7_2964,2008,1457,1022,451,1668,1146,1073,868,1785,2540,1522,2183
UBR3_7_2965,4787,2179,4515,5419,3040,2692,6849,3454,5728,3808,1585,390
7
UBR4_7_2966,0,0,0,3,407,264,28,15,27,0,3,16
UBR5_7_2967,61,182,0,24,1873,0,85,0,895,12,561,555
UBR7_7_2968,62,267,124,48,247,9,362,89,113,172,271,16

UBXN10_7_2969,0,1135,0,109,0,0,1,3,9,10,0,740
UBXN1_7_2970,642,1264,2,7,502,278,1880,1583,32,1,172,1457
UBXN2A_7_2971,12,13,0,6,0,199,0,889,42,247,148,29
UBXN4_7_2972,305,1644,602,271,0,168,2039,59,1386,1426,2338,174
UBXN7_7_2973,154,15,81,80,13,86,247,353,543,493,774,568
UBXN8_7_2974,303,61,0,116,0,0,8,0,0,10,0,651
UCHL1_7_2975,0,10,458,4,0,0,0,246,0,82,0,409
UCHL3_7_2976,195,503,2,24,44,851,284,3,1120,608,626,612
UFC1_7_2977,1,2230,826,964,2247,362,717,465,58,191,234,45
UHRF2_7_2978,2818,898,3357,1299,1736,786,2986,2407,960,1263,466,2571
UNK_7_2979,0,0,1,0,222,20,0,1,3,99,0,1620
USP11_7_2980,0,2,158,1,2,657,0,8,0,169,257,52
USP13_7_2981,337,2083,435,27,13,1960,2,833,403,516,191,459
USP15_7_2982,3718,4822,3246,2102,3691,3568,3287,3927,5117,4538,4314,46
26
USP17_7_2983,108,411,16,1,12,77,4,522,197,124,0,409
USP17L2_7_2984,1111,1733,487,1334,2043,1777,1557,1925,2329,720,2129,12
75
USP17L5_7_2985,704,3088,1430,529,1291,1348,1882,803,1097,1281,1033,787
USP18_7_2986,1388,308,779,621,970,738,14,177,1282,1715,971,2262
USP22_7_2987,1411,1065,792,1478,991,1170,407,1876,1328,1475,1555,1520
USP24_7_2988,300,158,165,143,1534,748,1028,1184,1374,1184,1066,2813
USP25_7_2989,603,814,1106,135,0,241,47,1125,521,324,3,100
USP26_7_2990,635,999,899,345,1431,896,1909,1,1121,58,909,834
USP27X_7_2991,9,331,0,144,0,387,0,126,2,608,13,4
USP28_7_2992,744,2637,1830,1099,3380,1294,1366,1060,1797,1496,582,2885
USP29_7_2993,2166,169,18,869,354,92,1077,143,69,1161,672,979
USP30_7_2994,283,11,3,3,0,1,12,30,22,14,5,2
USP32_7_2995,0,87,3,33,529,25,1337,178,84,3,410,399
USP34_7_2996,166,164,779,1314,783,733,1182,3057,136,1177,690,297
USP35_7_2997,1,0,0,0,0,0,0,0,290,1,0,36
USP36_7_2998,0,1,0,1,0,0,0,0,0,0,0,0
USP37_7_2999,188,534,104,856,1635,224,0,140,18,654,461,803
USP38_7_3000,13,553,172,50,0,0,0,2,0,2,0,86
USP39_7_3001,533,192,306,148,973,299,499,286,18,53,616,443
USP3_7_3002,1077,98,8,109,91,349,348,554,351,475,321,47
USP40_7_3003,212,1,0,108,114,4,277,6,4,209,395,4
USP42_7_3004,12,45,22,71,313,159,38,0,0,119,222,18
USP43_7_3005,835,337,301,475,1110,41,1269,127,153,922,98,741
USP45_7_3006,0,1,0,17,0,0,0,0,0,661,15,1
USP47_7_3007,12,0,0,11,1,32,1016,220,170,37,3,119
USP49_7_3008,1097,1347,488,262,1734,412,677,450,689,242,618,2124
USP50_7_3009,101,987,173,195,654,1156,504,62,2232,436,1,270
USP51_7_3010,1369,99,10,1958,947,116,293,1,685,1278,18,80
USP53_7_3011,858,270,357,23,1313,238,427,690,1028,115,41,1066
USP54_7_3012,376,7,982,1,1005,31,108,1091,24,48,310,187
USP6_7_3013,2275,3082,1295,2959,2490,1991,1103,1990,2808,3966,250,4385
USP7_7_3014,1754,1436,1023,1007,2827,674,1530,2904,1043,1232,2445,2227
USP9Y_7_3015,333,799,8,65,471,1360,106,1,204,266,1,352
USPL1_7_3016,381,1,1,173,0,752,0,0,1,90,305,1

VCPIP1_7_3017,757,520,400,238,1005,79,250,45,285,595,203,854
VPS11_7_3018,288,102,169,82,18,247,1011,15,41,15,81,843
WDR12_7_3019,18,449,13,282,102,120,588,66,81,233,3,12
WDR53_7_3020,542,655,823,108,2570,1279,1625,1074,1484,491,81,369
WDR59_7_3021,0,0,0,171,0,1,0,37,148,219,351,223
WDR5B_7_3022,1258,604,669,514,516,151,559,1938,274,947,1362,1125
WDR61_7_3023,1454,1678,325,530,894,375,538,185,1226,937,1117,829
WDTC1_7_3024,0,21,0,105,0,0,17,0,45,35,341,38
WSB2_7_3025,232,258,87,748,375,485,805,27,596,968,154,895
WWP1_7_3026,684,632,832,452,1966,1201,79,413,956,731,46,1833
YOD1_7_3027,877,1299,249,24,47,1084,99,351,50,2135,278,274
ZBTB11_7_3028,0,206,85,0,0,245,3,0,0,0,0,0
ZBTB25_7_3029,21,218,631,332,568,449,0,658,243,233,21,166
ZBTB2_7_3030,87,186,243,68,11,65,43,115,275,7,663,471
ZBTB32_7_3031,54,0,1671,0,268,0,115,0,297,463,56,248
ZBTB34_7_3032,90,281,22,0,597,1,270,193,25,255,1149,650
ZBTB39_7_3033,514,281,167,9,0,738,1002,761,143,710,12,52
ZBTB3_7_3034,705,202,1162,1186,681,222,1377,1620,2336,936,287,1567
ZBTB41_7_3035,50,288,1050,646,1082,844,295,612,471,1131,162,1214
ZBTB44_7_3036,53,49,47,68,8,0,18,4,519,65,480,256
ZBTB45_7_3037,60,203,888,167,0,9,6,424,294,543,1,66
ZBTB46_7_3038,277,59,294,732,458,98,505,740,1129,36,319,304
ZBTB47_7_3039,64,159,2,2,2,3,0,2,165,4,1,14
ZBTB48_7_3040,120,6,0,0,57,0,129,1569,17,1,0,12
ZBTB49_7_3041,701,1052,0,223,480,354,1999,403,739,725,69,1447
ZBTB5_7_3042,1,6,51,246,789,544,1323,208,833,776,225,1058
ZBTB7A_7_3043,2,402,82,71,0,11,27,0,0,10,0,349
ZBTB7B_7_3044,1709,90,0,3,45,14,1,212,32,3,0,5
ZBTB7C_7_3045,108,382,28,5,500,301,17,27,420,20,13,55
ZBTB8A_7_3046,814,756,23,345,292,364,756,594,1651,410,494,698
ZFAND2B_7_3047,94,239,0,0,147,156,0,21,114,153,29,22
ZFPL1_7_3048,480,58,799,103,467,18,316,210,76,52,466,196
ZMYND10_7_3049,864,848,600,302,552,1682,2243,1893,1679,1497,868,993
ZNF131_7_3050,312,14,196,2,851,2,0,495,4,13,1,46
ZNF598_7_3051,4,486,153,1136,1158,22,529,10,237,903,1830,774
ZNF645_7_3052,838,252,116,269,3199,468,1136,499,966,862,1068,1649
ZNRF1_7_3053,888,1802,1095,1137,438,1148,745,1942,302,2512,612,533
ZNRF2_7_3054,518,63,500,863,17,446,6,271,153,93,309,1429
ZNRF4_7_3055,653,36,9,47,118,392,783,348,2067,182,36,456
ZRANB1_7_3056,29,0,488,15,0,132,0,263,1048,47,306,121
ZSWIM2_7_3057,697,315,650,42,874,385,2241,1507,712,65,768,638
AIRE_7_3058,430,0,0,0,0,0,0,0,0,0,0,0
ANAPC5_7_3059,1,145,350,98,1018,488,734,1849,824,1464,287,294
ANAPC7_7_3060,941,837,1207,1711,1384,1562,2319,908,1409,2336,469,2399
ANKFY1_7_3061,186,102,164,43,461,1297,131,279,390,126,93,871
ASB10_7_3062,802,44,159,0,206,279,38,805,176,419,90,1222
ASB11_7_3063,9,14,0,78,0,3,24,0,6,227,20,2
ASB14_7_3064,953,350,515,503,354,366,520,608,149,2041,508,1079
ASB2_7_3065,113,556,57,466,0,599,327,475,418,1181,373,769
ASB3_7_3066,51,128,0,18,16,10,95,408,0,167,98,453

ASB4_7_3067,2277,1183,350,710,0,89,84,0,57,358,844,10
ASB6_7_3068,1262,146,71,5,460,340,323,12,232,164,0,28
ASB7_7_3069,56,12,37,52,1311,36,24,50,15,12,1,203
ASB9_7_3070,0,88,0,214,0,21,0,531,217,1,0,22
ASCC2_7_3071,13,310,173,148,55,397,290,0,56,587,231,10
ATG10_7_3072,0,194,0,0,0,190,0,65,55,231,22,7
ATG16L1_7_3073,1,118,0,114,14,5,0,0,378,257,19,220
ATG7_7_3074,1999,1681,262,1166,1605,2593,4697,3237,2961,535,1458,2569
ATRX_7_3075,0,249,0,0,0,556,0,0,0,0,304,0
BACH2_7_3076,1102,52,922,702,31,150,1183,660,524,206,382,54
BAG6_7_3077,374,389,646,2410,296,551,1644,694,888,1473,1285,339
BAZ1A_7_3078,2834,4082,4495,2374,1360,4432,3534,5460,5146,4422,2385,57
40
BCL6_7_3079,322,598,196,122,1152,381,783,666,123,711,1126,105
BIRC3_7_3080,297,35,1103,66,741,834,32,282,906,370,455,131
BIRC7_7_3081,79,0,1,515,20,627,0,0,39,0,109,3
BPTF_7_3082,186,544,138,991,1044,587,378,906,739,671,1644,389
BRCA1_7_3083,38,1499,40,240,26,193,616,1016,370,34,50,345
BRPF1_7_3084,95,9,142,34,521,56,863,22,289,27,110,709
BRWD1_7_3085,9,125,0,182,1206,433,43,440,352,299,117,95
BTBD11_7_3086,23,0,102,9,0,1565,1005,0,232,585,0,23
BTBD1_7_3087,262,616,278,2,482,598,674,800,403,918,403,360
BTBD3_7_3088,51,362,321,2,7,122,260,270,104,456,132,5
BTBD7_7_3089,83,450,11,801,902,1410,1228,159,1154,726,195,593
BTBD9_7_3090,3,1,0,1,142,1,78,1251,46,9,2,4
BTRC_7_3091,1676,0,0,83,447,419,0,0,79,15,1,13
C3orf26_7_3092,2442,413,375,26,576,458,1155,59,578,502,374,506
CBLC_7_3093,141,0,151,0,105,605,0,406,30,839,1,518
CCNB1IP1_7_3094,1053,1,1,241,0,59,10,0,17,6,1,1038
CDC16_7_3095,1653,1487,1154,483,1741,689,536,3236,616,2205,1901,1648
CDC27_7_3096,1426,1032,1982,783,4256,1347,1364,614,903,1815,2715,781
CHFR_7_3097,266,556,313,15,0,448,0,184,77,8,2176,34
CISH_7_3098,0,502,0,548,59,13,0,0,676,5,0,105
CNOT4_7_3099,1,0,4,0,34,29,0,0,45,8,9,91
COPS2_7_3100,370,177,556,726,834,1324,223,1166,1289,772,893,1177
COPS3_7_3101,1297,1189,783,1503,2050,1827,2481,480,257,2367,668,315
COPS7A_7_3102,350,146,0,16,0,95,1,3,4,176,0,0
COPS8_7_3103,3,162,510,99,248,279,322,4,21,425,2,149
CRBN_7_3104,221,261,1089,1024,0,163,626,971,538,2877,5,302
CUL2_7_3105,63,7,2,76,327,22,85,246,0,166,9,1074
CUL4A_7_3106,1443,419,3103,1352,4004,494,354,1765,2022,2567,1398,1422
CUL4B_7_3107,1663,2576,2479,1941,2002,3596,2005,4461,3124,3975,7796,34
10
CUL7_7_3108,0,50,4,0,0,59,0,0,7,0,54,0
CXXC1_7_3109,0,450,0,2,1,60,0,54,24,711,20,0
CYLD_7_3110,0,0,0,0,0,0,0,0,0,0,0,0
DCAF11_7_3111,0,0,0,0,0,0,0,0,0,0,0,0
DCAF17_7_3112,1386,330,983,370,38,0,0,102,245,335,10,709
DCAF4_7_3113,969,44,33,1086,478,809,472,182,710,84,53,164
DCAF6_7_3114,61,1,1118,475,270,495,29,6,165,75,35,44

DCST1_7_3115,0,611,0,0,13,0,897,14,0,0,0,0
DEPDC1B_7_3116,1012,1626,3998,592,631,2783,945,819,800,1815,1196,770
DET1_7_3117,0,22,27,5,60,0,6,55,0,170,9,0
DIDO1_7_3118,87,35,0,0,1623,0,0,0,0,158,1,0
DNAJB2_7_3119,19,634,1213,139,26,131,27,79,15,280,97,321
DTX2_7_3120,14,148,16,148,0,222,249,8,128,434,202,344
EED_7_3121,608,953,0,1592,693,5,1684,608,622,1421,350,1727
EIF3B_7_3122,0,5,0,581,381,6,0,447,927,1902,44,296
EIF3C_7_3123,2,38,133,5,100,25,2,362,140,4,161,145
EIF6_7_3124,0,32,2,4,6,19,251,190,826,756,186,818
EPN1_7_3125,7,121,0,13,267,34,2,127,0,57,2,27
EPN2_7_3126,659,175,342,70,471,656,488,575,342,1011,1404,224
EPS15_7_3127,359,2199,835,2214,89,599,1170,583,1195,2072,3433,1105
FAM70A_7_3128,281,42,54,10,10,78,4,226,336,327,166,33
FANCL_7_3129,741,1552,700,99,6,315,351,813,1968,683,948,940
FBXL13_7_3130,7,2,1101,679,0,0,13,362,600,10,5,576
FBXL20_7_3131,10,0,31,12,0,67,0,0,119,2,766,21
FBXL2_7_3132,0,27,1889,0,0,1,748,0,55,0,2,135
FBXL5_7_3133,279,11,17,159,11,747,871,158,42,744,9,348
FBXL6_7_3134,0,16,0,0,0,0,0,0,0,0,0,3
FBXO11_7_3135,2560,2661,5760,1373,1437,1678,2619,2760,2532,2684,1644,1
487
FBXO15_7_3136,285,275,356,383,568,144,720,287,239,51,160,47
FBXO17_7_3137,5,323,7,28,263,386,387,53,40,446,273,36
FBXO18_7_3138,1051,575,771,1300,83,176,287,10,374,506,665,60
FBXO21_7_3139,676,181,957,787,348,822,16,143,405,1473,935,142
FBXO22_7_3140,0,1589,973,0,416,46,0,622,437,104,771,170
FBXO24_7_3141,519,5,0,340,739,1269,239,805,844,235,248,256
FBXO25_7_3142,51,1471,134,232,127,276,38,409,98,267,5,36
FBXO28_7_3143,522,464,231,407,751,982,712,779,1715,1623,143,837
FBXO32_7_3144,264,473,1715,123,32,417,319,18,373,1061,2030,1007
FBXO34_7_3145,577,1729,542,822,111,101,53,514,765,927,1299,1487
FBXO38_7_3146,161,229,698,1468,299,379,344,231,2060,932,1488,1604
FBXO3_7_3147,620,1508,1045,1729,2517,1243,1011,2208,2352,1031,2371,186
9
FBXO44_7_3148,224,1088,1921,771,2809,1707,249,1346,932,810,1579,3001
FBXO4_7_3149,329,915,194,1381,216,368,321,213,213,753,1279,28
FBXO5_7_3150,183,627,1065,59,199,166,0,0,0,4,680,8
FBXO7_7_3151,989,735,2,88,95,591,486,2,942,259,82,359
FBXO9_7_3152,160,140,26,687,266,24,715,327,278,979,16,355
FBXW11_7_3153,546,0,0,1,52,0,3,27,275,59,0,763
FBXW7_7_3154,126,483,0,0,20,815,302,521,568,820,54,724
FBXW8_7_3155,0,0,0,0,0,93,511,43,310,4,0,46
GPS1_7_3156,0,127,0,0,0,8,0,0,0,514,0,2
HECTD2_7_3157,1,3,1,0,339,275,412,617,241,227,0,707
HERC4_7_3158,1168,993,1690,2517,1050,744,1249,251,1889,1149,1441,1842
HERC6_7_3159,1381,1100,2109,111,91,980,670,192,870,896,20,1507
HIC1_7_3160,0,4,12,348,0,0,0,7,0,0,0,0
HLTF_7_3161,1343,423,563,760,706,1398,4,23,460,479,713,692
HSF4_7_3162,4,1747,929,719,521,522,442,44,1291,394,818,193

IPP_7_3163,1014,1161,357,474,784,364,818,591,738,2503,2662,1121
KAT6A_7_3164,550,492,564,47,1,1112,134,208,116,957,1267,1185
KBTBD3_7_3165,1163,581,4802,1324,2312,1347,781,2083,1104,1286,662,1412
KCTD6_7_3166,61,63,1,1004,0,84,94,345,1,84,228,0
KCTD7_7_3167,0,56,436,0,43,2,0,653,0,0,100,4
KDM2B_7_3168,0,0,0,0,0,0,630,0,2,0,0,0
KDM4C_7_3169,120,1,544,0,1172,1177,284,679,409,208,282,92
KDM5C_7_3170,479,5,89,668,35,340,683,55,500,1283,1449,1142
KEAP1_7_3171,0,2,0,147,0,244,0,389,9,4,79,0
KIAA1841_7_3172,0,0,0,0,0,0,0,0,0,0,0,0
KLHL13_7_3173,392,578,1513,1184,459,152,1198,1199,1354,2232,1398,2119
KLHL2_7_3174,5,241,1,10,1,15,0,1123,42,8,2,2127
KLHL4_7_3175,746,83,662,295,273,631,0,482,32,51,897,783
KLHL5_7_3176,1437,667,1042,1563,3961,530,205,760,2140,1677,1280,2105
KLHL7_7_3177,1133,423,105,195,406,43,1310,474,1127,569,162,286
LNX1_7_3178,2,69,225,705,90,156,0,20,5,148,29,0
LONRF3_7_3179,1406,1531,1543,84,1888,1416,264,905,545,198,1439,77
LRRC29_7_3180,0,6,146,5,0,0,0,0,10,35,1,0
LRSAM1_7_3181,4,0,7,127,1542,84,1948,27,590,15,534,73
MARK2_7_3182,1859,589,962,352,1157,117,857,1023,1493,798,90,1047
MARK3_7_3183,456,161,82,391,318,126,0,0,27,719,440,5
MARK4_7_3184,34,49,423,192,0,429,309,0,2,0,12,36
MDM4_7_3185,306,36,0,0,147,13,0,833,1296,66,1209,530
MGRN1_7_3186,971,34,353,225,728,477,206,1100,196,4,83,383
MIB2_7_3187,0,0,0,0,10,0,0,0,0,0,1,0
MID1_7_3188,0,0,767,703,993,0,0,183,439,309,1,33
MID2_7_3189,2,301,59,186,22,110,40,32,620,1427,476,137
MKRN1_7_3190,1307,781,960,349,618,1044,1602,261,1257,1564,345,2386
MLL5_7_3191,1163,206,997,107,2737,611,633,758,1072,716,384,1269
MLL_7_3192,125,1,54,166,362,462,1699,733,694,715,633,89
MNAT1_7_3193,107,396,683,96,549,102,124,20,131,36,25,301
MPND_7_3194,73,0,0,0,0,0,0,3,8,1,0,0
MTF2_7_3195,96,1360,1194,282,146,279,16,725,349,172,1325,421
MYNN_7_3196,4,103,0,13,0,48,667,0,0,419,0,0
NAE1_7_3197,499,1933,21,23,178,217,785,184,293,165,929,659
NDUFC2_7_3198,316,500,604,791,538,408,632,421,551,973,481,696
NEDD4_7_3199,19,234,1658,136,1127,256,1190,152,214,2309,251,234
NEDD4L_7_3200,293,637,1,12,9,218,27,17,206,210,0,64
NFX1_7_3201,238,0,245,2,150,40,1,222,3,93,66,445
NLE1_7_3202,19,3,1829,83,0,0,17,765,0,37,19,0
NSD1_7_3203,382,168,852,0,0,6,1,1,74,3,6,299
NSFL1C_7_3204,8,335,813,173,1,448,57,502,291,981,0,131
NUB1_7_3205,0,0,378,0,0,0,0,0,0,0,0,0
ODF2_7_3206,4,7,1,2,0,161,212,1,142,4,8,27
OTUD5_7_3207,18,0,0,44,0,538,250,0,373,23,261,41
PARK2_7_3208,127,670,12,18,4,304,90,9,832,277,401,447
PARP9_7_3209,303,227,273,0,262,228,17,0,439,165,15,342
PATZ1_7_3210,1337,2,147,95,106,1,991,1,297,4,0,38
PCGF6_7_3211,27,0,9,0,0,0,0,0,11,297,69,0
PEX10_7_3212,0,12,6,0,0,77,241,154,0,0,0,0

PEX2_7_3213,2776,3725,3290,3323,4476,4341,5645,3442,3811,3636,4501,352
4
PHF12_7_3214,430,177,996,176,311,1369,107,20,199,601,496,65
PHF16_7_3215,1013,46,482,720,113,9,382,0,350,773,229,1391
PHF17_7_3216,0,37,0,0,0,0,1,551,0,32,257,0
PHF1_7_3217,20,381,1039,0,0,19,1,0,29,650,54,6
PHF21A_7_3218,15,71,984,103,1,4,162,3,0,207,979,4
PHF7_7_3219,352,0,0,5,1,0,0,0,10,279,140,24
PHF8_7_3220,0,21,0,4,88,0,0,0,0,1,0,0
PJA1_7_3221,308,0,298,129,23,264,0,586,35,620,109,2
PML_7_3222,80,10,7,9,0,0,89,0,2,69,6,0
POC1B_7_3223,1178,702,165,673,1625,1453,147,1399,1978,1057,3444,2124
PSMD1_7_3224,253,240,1353,503,1430,510,1778,230,746,320,1742,765
RAPSN_7_3225,86,146,32,4,2,20,0,0,18,618,0,4
RBBP4_7_3226,0,252,834,495,500,258,16,29,43,176,205,24
RBBP5_7_3227,707,549,4,440,0,60,447,54,538,170,451,433
RBBP6_7_3228,749,686,1157,315,850,361,466,919,903,491,777,578
RBBP7_7_3229,780,98,451,581,464,127,217,993,1027,1094,1099,910
RBCK1_7_3230,1078,1506,1603,1752,246,551,155,1,739,41,61,887
RC3H2_7_3231,1454,914,521,369,1645,323,1191,567,149,355,374,1469
RCHY1_7_3232,1414,493,993,1209,136,677,687,610,1557,131,431,606
RFPL2_7_3233,468,198,2250,833,869,15,155,382,1581,1231,1788,572
RFPL3_7_3234,359,793,546,100,556,0,625,64,721,342,1,474
RFWD2_7_3235,314,1027,63,617,925,116,0,111,1095,1345,1520,284
RHOBTB1_7_3236,1,26,0,0,129,3,0,348,14,0,802,4
RHOBTB2_7_3237,159,0,412,21,0,76,24,35,61,554,385,394
RLIM_7_3238,1468,112,529,1072,2156,964,723,662,815,581,210,188
RNF103_7_3239,369,336,629,1,181,2,0,0,320,41,288,32
RNF128_7_3240,0,80,0,137,0,26,152,230,0,0,0,618
RNF135_7_3241,59,14,0,610,653,144,68,5,0,8,1,78
RNF138_7_3242,1266,1615,783,1885,0,1076,3197,46,652,1118,4948,1956
RNF13_7_3243,952,61,259,1256,875,1375,2371,1725,689,955,2903,523
RNF145_7_3244,255,3,8,45,541,329,218,4,66,591,656,30
RNF146_7_3245,550,676,443,686,973,152,1774,1427,1935,382,1701,914
RNF14_7_3246,1,250,1,78,193,230,729,6,325,470,1,256
RNF166_7_3247,228,1437,0,25,19,10,115,1,71,46,34,150
RNF170_7_3248,260,132,0,71,361,0,109,664,2,115,222,71
RNF17_7_3249,0,0,0,326,304,79,0,0,236,5,80,34
RNF180_7_3250,185,257,44,204,0,134,246,13,23,116,382,488
RNF182_7_3251,0,0,0,0,0,48,0,0,404,2,0,292
RNF185_7_3252,0,0,0,0,545,78,0,0,72,10,1,3
RNF19A_7_3253,1000,34,3620,1055,641,969,43,255,2753,330,271,2896
RNF19B_7_3254,106,214,132,22,0,59,228,117,125,880,522,308
RNF213_7_3255,1006,464,299,779,763,1119,2285,1278,1781,257,1082,792
RNF214_7_3256,589,0,16,6,0,0,0,34,0,0,4,37
RNF216_7_3257,1096,405,1143,1045,7,777,450,940,550,43,664,1518
RNF24_7_3258,0,0,0,4,0,0,0,0,69,205,0,11
RNF32_7_3259,155,0,0,4,95,0,0,103,0,18,6,7
RNF34_7_3260,120,14,339,2,2,37,26,2,325,321,63,77
RNF38_7_3261,253,17,16,763,92,0,911,1432,103,61,299,10

RNF40_7_3262,206,245,2032,706,401,439,19,0,265,194,1103,36
RNF41_7_3263,331,6,0,3,2,10,153,364,422,56,33,277
RNF6_7_3264,355,11,0,739,0,0,653,31,208,6,5,36
RNF7_7_3265,569,3,0,159,42,96,0,157,0,41,0,305
RNF8_7_3266,1806,271,952,809,434,967,97,922,431,249,694,352
SAE1_7_3267,359,832,0,203,436,27,908,169,209,229,414,1174
SENP6_7_3268,1447,868,84,24,200,72,508,353,862,122,216,341
SENP7_7_3269,1,134,0,0,0,0,0,0,0,102,0,4
SENP8_7_3270,707,199,302,225,1879,1520,382,463,178,399,1007,1475
SF3A1_7_3271,0,0,0,0,0,132,1,0,79,551,10,308
SHPRH_7_3272,0,0,57,4,2,11,0,20,291,65,0,37
SIAH1_7_3273,114,23,295,103,402,222,69,112,1,3,32,15
SKP2_7_3274,65,14,2,5,0,1,22,11,423,30,3,66
SMURF1_7_3275,4,18,235,70,137,6,0,768,17,441,13,20
SOCS5_7_3276,2119,109,0,131,287,54,1,217,45,551,4,186
SP100_7_3277,872,731,4248,2372,2414,2036,1399,3815,2725,3333,1484,2139
SP110_7_3278,0,0,0,1,6,71,283,0,7,148,130,7
SPOP_7_3279,1,244,57,77,270,287,16,4,79,447,11,237
SPSB2_7_3280,19,124,80,33,413,156,556,11,28,19,60,35
SQSTM1_7_3281,476,2,795,640,19,1749,125,0,220,552,37,51
STAMBP_7_3282,228,298,61,0,617,492,262,537,619,1555,732,772
SUMO1_7_3283,0,0,0,0,0,0,0,0,0,0,0,0
SUMO2_7_3284,913,858,226,1055,1428,165,884,970,1119,1001,1115,1227
SYTL4_7_3285,1778,1980,1217,922,818,726,2220,1823,918,1559,2089,1775
SYVN1_7_3286,183,0,281,0,0,21,0,10,440,7,140,746
TCF20_7_3287,0,430,0,0,222,160,21,819,42,5,0,29
TDRD3_7_3288,23,44,63,159,50,59,20,240,62,647,863,88
TIPARP_7_3289,604,172,0,1198,681,10,507,924,720,426,691,1301
TLE2_7_3290,417,379,273,301,1829,1005,153,168,81,76,1681,1057
TLE3_7_3291,59,0,0,32,175,135,357,478,104,70,371,108
TNK2_7_3292,126,622,2155,122,1241,777,410,18,219,102,50,214
TNRC6C_7_3293,0,60,17,2,0,305,4,0,11,240,1174,2075
TOPORS_7_3294,0,68,0,0,0,168,1813,0,579,600,1,644
TOR1AIP2_7_3295,303,277,10,106,61,148,2,0,147,2,0,1708
TRAF3_7_3296,0,353,15,16,31,92,1,399,485,1000,0,94
TRAF5_7_3297,0,3,1319,200,1,44,4,0,59,119,70,23
TRAF6_7_3298,1209,0,8,0,0,0,0,0,43,1,0,6
TRIM10_7_3299,662,381,28,686,696,571,48,1457,446,283,735,769
TRIM13_7_3300,0,196,239,10,8,261,0,0,4,2,194,0
TRIM17_7_3301,0,4,0,52,0,90,0,1,15,163,16,1026
TRIM22_7_3302,76,53,217,329,1272,684,933,1267,1369,1325,2032,432
TRIM23_7_3303,554,346,520,802,751,897,235,141,6,2010,972,1305
TRIM24_7_3304,2161,267,1429,2407,1471,109,3078,397,1516,1344,1060,773
TRIM26_7_3305,643,0,0,46,0,0,0,200,20,0,0,14
TRIM2_7_3306,102,22,0,75,29,16,0,30,179,3,0,37
TRIM32_7_3307,4,661,0,555,680,714,2469,1352,16,826,911,152
TRIM33_7_3308,88,466,1384,1308,1460,982,112,2381,1070,353,1567,948
TRIM34_7_3309,0,0,400,0,0,303,0,0,0,43,0,0
TRIM37_7_3310,322,339,441,293,538,646,371,408,352,781,180,335
TRIM39_7_3311,0,0,0,79,0,334,0,0,0,111,0,0

TRIM3_7_3312,912,1993,1634,23,1968,1661,3233,502,582,1619,921,1189
TRIM41_7_3313,32,103,0,393,0,1146,203,763,0,200,761,13
TRIM45_7_3314,34,52,240,170,1,548,122,821,175,18,1,15
TRIM4_7_3315,285,305,293,576,25,24,5,572,886,25,706,1925
TRIM54_7_3316,751,552,1757,740,1162,1094,196,1674,848,1346,991,2220
TRIM55_7_3317,53,126,39,555,78,0,136,192,614,785,677,473
TRIM5_7_3318,1,12,22,9,29,0,0,66,56,100,0,42
TRIM7_7_3319,831,807,361,703,658,1129,97,591,619,893,586,1000
TRIM9_7_3320,623,0,652,418,730,69,975,717,53,46,1064,18
TRPC4AP_7_3321,147,423,77,687,0,890,287,851,392,478,904,300
TSPAN17_7_3322,681,306,914,572,2371,810,20,332,945,783,1823,394
TTC3_7_3323,13,1021,497,0,30,752,924,1420,325,6,638,289
TULP4_7_3324,164,408,1290,1404,67,1125,2627,566,713,911,61,934
UBA1_7_3325,726,629,692,350,837,894,983,3,399,946,1059,625
UBA3_7_3326,714,753,1074,623,1231,1130,524,804,834,651,3152,134
UBA5_7_3327,3171,2315,1267,2782,1670,1996,2095,3081,4445,1849,1678,247
8
UBAC2_7_3328,269,0,8,648,57,22,641,36,41,462,959,6
UBAP2L_7_3329,0,0,0,0,0,0,0,0,0,26,0,0
UBASH3A_7_3330,1030,264,373,120,822,491,817,1533,438,1171,1120,1932
UBE2A_7_3331,3042,2730,3232,200,1626,1337,1284,2682,2880,1231,3487,161
8
UBE2D1_7_3332,218,48,0,1,781,447,77,4,120,348,733,63
UBE2D2_7_3333,96,435,1512,220,2284,242,73,76,1193,896,2,357
UBE2D3_7_3334,0,160,35,117,0,0,6,0,149,645,0,506
UBE2E1_7_3335,0,1079,0,366,0,1,0,1,170,347,0,24
UBE2E3_7_3336,444,0,0,456,0,0,0,0,0,0,666,501
UBE2G2_7_3337,322,308,0,431,10,0,124,813,411,9,103,120
UBE2H_7_3338,5,175,12,752,7,319,756,81,303,8,28,784
UBE2I_7_3339,186,5,948,634,1620,26,64,1248,297,426,499,770
UBE2J2_7_3340,42,81,109,421,1092,458,636,929,1036,777,324,735
UBE2K_7_3341,488,440,20,5,1227,596,193,126,2,488,34,3
UBE2Q2_7_3342,1357,116,711,514,371,984,502,485,304,953,56,1066
UBE2V1_7_3343,1390,170,878,140,848,280,11,605,657,440,1420,91
UBE2W_7_3344,1500,1948,4459,3196,6134,2781,2816,3008,1546,3438,5183,11
87
UBE3A_7_3345,2098,1515,228,85,270,2974,625,1356,1632,385,1242,1888
UBE3B_7_3346,361,0,63,0,188,316,48,243,375,14,60,164
UBE4A_7_3347,1036,1073,503,2556,1520,2032,1324,2853,1394,2535,2920,176
2
UBE4B_7_3348,2,855,827,1326,236,1448,406,898,690,459,891,800
UBL7_7_3349,13,2,66,202,0,9,175,50,0,142,268,57
UBOX5_7_3350,1063,1136,185,485,130,769,149,1450,1666,461,464,2076
UBQLN1_7_3351,835,2403,1087,855,931,246,726,892,1052,697,330,1103
UBR2_7_3352,371,0,34,1,1479,1035,14,0,0,268,2,1
UBXN11_7_3353,7,53,410,81,0,16,65,516,78,40,3,47
UBXN6_7_3354,17,0,0,90,0,5,703,0,31,14,0,0
UCHL5_7_3355,3463,1858,2579,1631,2674,990,2152,2120,1909,2585,1435,240
5
UHRF1_7_3356,41,0,0,347,0,0,0,0,121,435,35,157

UIMC1_7_3357,178,484,0,48,41,426,49,40,107,1045,314,348
UNKL_7_3358,3,579,27,0,108,670,0,147,868,538,1,191
USP14_7_3359,139,216,16,145,430,667,1115,289,560,541,582,250
USP19_7_3360,487,228,0,0,0,0,0,0,0,0,0,0
USP1_7_3361,4422,2240,1105,991,810,1132,3047,1886,2097,3102,3084,940
USP20_7_3362,313,11,0,50,0,13,437,27,0,0,1923,319
USP2_7_3363,8,3,233,65,0,17,50,0,0,1115,289,28
USP33_7_3364,328,539,769,1061,206,382,216,30,1342,1941,1878,641
USP44_7_3365,83,119,0,618,174,0,24,813,111,7,15,8
USP46_7_3366,1413,155,372,352,368,348,455,28,39,462,0,1142
USP48_7_3367,7,601,68,63,131,12,452,977,979,507,608,900
USP4_7_3368,132,444,54,44,559,139,23,988,392,913,48,138
USP5_7_3369,1,8,421,1286,120,583,80,144,16,3,552,240
USP6NL_7_3370,207,512,40,0,1365,102,160,81,338,117,10,40
USP8_7_3371,207,160,583,97,219,100,86,357,3,150,738,1
USP9X_7_3372,3,198,159,418,1631,701,137,385,103,454,78,398
VHL_7_3373,50,8,9,11,5,0,11,182,1,0,105,0
VPRBP_7_3374,282,0,0,0,0,0,0,31,0,215,0,43
VPS13D_7_3375,447,446,5,616,22,238,562,15,19,0,1054,9
VPS41_7_3376,1068,432,2379,216,516,1244,31,405,683,1408,303,809
VPS8_7_3377,6,319,1,403,5,240,910,171,766,113,376,141
WDR26_7_3378,512,3,784,29,0,0,3,205,9,668,517,2
WDR5_7_3379,637,719,31,566,584,1449,539,258,11,43,492,245
WDR76_7_3380,1091,348,2322,970,1800,1285,2448,3727,1620,1402,436,2539
WHSC1_7_3381,102,28,633,316,503,1463,291,17,265,982,150,967
WHSC1L1_7_3382,4,0,0,4,0,2,273,1,214,5,277,177
WSB1_7_3383,0,2,0,6,3,0,469,12,0,0,800,0
WWP2_7_3384,12,64,0,166,0,299,0,5,407,288,100,521
XIAP_7_3385,637,167,0,0,1,543,43,0,572,71,1117,1063
ZBTB10_7_3386,2108,1083,58,518,474,1609,1907,239,1425,2465,2240,734
ZBTB16_7_3387,0,0,229,0,122,55,22,2,2,1,0,63
ZBTB17_7_3388,0,0,0,454,4,1,689,10,1,10,15,4
ZBTB1_7_3389,537,400,711,239,43,1242,309,18,476,194,373,149
ZBTB20_7_3390,1583,358,0,284,2,617,0,484,579,675,0,78
ZBTB22_7_3391,0,349,0,39,310,259,0,548,299,11,0,166
ZBTB24_7_3392,547,0,36,4,470,0,2,342,9,3,63,40
ZBTB33_7_3393,1629,189,1708,1507,321,341,253,195,819,599,61,290
ZBTB37_7_3394,304,53,703,216,0,0,0,0,1121,117,15,1059
ZBTB40_7_3395,1808,2089,1352,213,1373,1113,987,803,1538,22,835,1331
ZBTB4_7_3396,1,0,18,395,446,1414,915,55,0,123,3,0
ZFP161_7_3397,1413,2262,1843,208,1091,1480,128,1499,1523,441,1417,1404
ZMYND11_7_3398,623,277,190,346,1013,467,115,1,157,360,152,130
ZMYND8_7_3399,604,93,702,99,6,758,853,166,798,327,18,369
ZNF238_7_3400,314,224,0,0,0,163,0,10,0,0,13,22
ZNF295_7_3401,22,1603,364,405,533,181,278,262,0,117,198,657
ZNRF3_7_3402,1467,160,1076,480,1397,764,1637,800,961,789,554,609
ANAPC11_7_3403,10,0,399,2,122,12,22,0,399,635,0,36
ATXN3_7_3404,165,344,2,363,16,162,1,879,142,388,797,25
MLLT10_7_3405,82,15,124,23,0,0,7,138,153,81,578,34
OTUD4_7_3406,664,1115,1181,1042,2303,1904,2976,3090,4246,2481,4126,172

5
PHF19_7_3407,0,0,0,0,0,0,0,0,0,146,0,0
SP140_7_3408,1914,2611,4596,2463,1422,2866,989,1848,2272,4124,2097,252
4
TRIM36_7_3409,200,514,1,42,26,0,1328,0,456,87,963,535
UBE2C_7_3410,598,1,561,100,1,4,239,1070,12,1125,668,306
UBE2L6_7_3411,32,17,0,13,0,143,8,19,18,1,0,52
ABTB2_7_3412,0,0,0,0,129,393,157,0,1,15,1113,556
AMBRA1_7_3413,2,51,0,0,0,325,0,153,0,0,2,0
AMFR_7_3414,2,0,0,12,0,44,32,0,0,0,0,6
ANAPC10_7_3415,32,43,594,673,887,14,1471,51,501,393,726,67
ANAPC1_7_3416,879,1137,384,1012,1270,1969,570,921,127,736,1120,712
ANAPC2_7_3417,880,246,0,583,0,1571,958,766,72,690,1023,1076
ANAPC4_7_3418,2198,598,904,902,518,941,1222,673,811,370,908,1264
ANKIB1_7_3419,555,333,539,1849,641,327,1577,474,1536,1208,1009,688
ANKRD13A_7_3420,1,0,39,305,199,2,2,737,842,565,394,143
ANKRD13D_7_3421,158,2,430,154,119,343,1,81,76,34,92,6
ARIH1_7_3422,418,0,547,811,8,321,6,126,10,7,373,579
ARIH2_7_3423,392,1766,116,933,1053,1836,522,1604,683,1030,701,924
ASB12_7_3424,10,583,0,2,94,0,0,0,27,62,282,439
ASB13_7_3425,587,65,256,152,1116,38,278,990,35,108,42,93
ASB15_7_3426,1925,2211,4443,2307,457,2176,750,2229,3174,863,1307,1130
ASB16_7_3427,0,0,1,0,6,5,1001,20,487,34,94,58
ASB17_7_3428,79,551,304,845,548,362,444,1572,525,676,639,122
ASB5_7_3429,880,1097,516,360,1197,741,969,667,428,1150,3132,1205
ASB8_7_3430,49,108,9,138,7,0,37,19,159,337,104,81
ASH1L_7_3431,0,14,49,7,243,27,788,0,650,179,0,127
ASPSCR1_7_3432,0,29,0,6,0,7,0,0,0,0,0,0
ATG12_7_3433,156,224,0,0,0,0,0,0,13,16,15,435
ATG3_7_3434,1113,441,16,1499,80,295,80,790,664,878,1037,1589
ATG5_7_3435,3,446,0,27,77,23,0,250,26,631,136,64
ATXN1L_7_3436,0,0,0,0,0,0,82,0,0,0,2,625
ATXN3L_7_3437,35,59,6,330,72,401,633,524,50,115,445,539
BARD1_7_3438,4,7,28,4,0,228,9,0,170,126,0,24
BAZ1B_7_3439,370,31,427,564,398,363,540,289,419,1416,345,1353
BAZ2A_7_3440,314,903,776,1034,856,2279,867,1419,2363,1768,1409,2146
BAZ2B_7_3441,1,886,501,1081,52,553,1393,1478,459,758,378,169
BECN1_7_3442,93,512,6,327,309,122,314,807,821,943,848,1122
BIRC2_7_3443,3773,2852,4472,7581,8314,6303,3475,6881,5538,3707,6448,82
83
BIRC6_7_3444,4414,769,48,408,2096,484,2609,1557,1563,923,1324,1357
BIRC8_7_3445,1839,2942,1751,119,1350,1108,407,905,1249,1527,121,1728
BMI1_7_3446,456,523,518,668,3,279,20,45,50,276,279,390
BRAP_7_3447,13,380,29,504,1088,35,210,434,184,282,4,23
BRD1_7_3448,2842,1517,1098,1494,844,1939,1229,1929,1001,1678,226,4236
BRPF3_7_3449,36,10,11,10,0,206,0,4,33,9,76,10
BTBD2_7_3450,377,301,169,0,66,0,0,0,335,104,0,41
BTBD6_7_3451,0,2,4,2,0,12,1,0,0,0,7,0
CAND1_7_3452,580,93,1598,1120,2034,696,165,1073,323,428,175,362
CBLB_7_3453,25,21,0,0,454,187,0,792,464,266,0,51

CBL_7_3454,0,287,873,453,0,588,1,21,0,9,0,4
CBLL1_7_3455,441,2,511,145,0,1180,4,1184,121,319,2,565
CCIN_7_3456,1121,65,3,68,903,9,8,18,26,333,0,6
CCNF_7_3457,947,250,29,144,11,359,29,72,244,104,917,379
CDC20_7_3458,0,73,715,25,0,25,4,85,239,526,509,32
CDC23_7_3459,32,330,893,58,533,33,134,151,17,555,227,423
CDC26_7_3460,169,699,432,328,60,19,1347,472,566,1308,408,2338
CDC34_7_3461,0,2,0,323,0,0,0,376,804,19,0,100
CGRRF1_7_3462,1159,281,230,25,774,431,81,355,74,251,297,417
CHD4_7_3463,2,202,1,396,129,391,6,0,19,0,46,2
CIAO1_7_3464,0,0,3,1301,793,488,1097,0,0,0,0,0
COPS4_7_3465,204,105,812,18,152,1150,2,486,1152,435,475,168
COPS5_7_3466,498,207,212,205,120,735,602,114,938,649,288,450
COPS6_7_3467,100,204,109,298,2,84,45,43,237,150,2,226
COPS7B_7_3468,200,23,329,194,2588,279,32,422,241,314,1725,122
CORO6_7_3469,235,1,27,213,1,8,18,0,9,82,794,506
CUEDC1_7_3470,590,106,61,339,1608,494,5,173,167,847,463,610
CUL1_7_3471,2,31,0,0,2,311,0,0,242,17,350,36
CUL3_7_3472,0,41,0,0,0,1,9,0,0,1,0,0
CUL5_7_3473,138,334,0,1032,164,512,327,72,733,170,118,122
CUL9_7_3474,420,546,1401,10,4,27,17,887,41,43,46,1460
DCAF10_7_3475,9,205,0,146,16,634,0,22,64,100,0,139
DCAF12_7_3476,14,180,0,0,0,497,92,5,0,31,2115,0
DCAF13_7_3477,350,4,199,308,45,759,327,1967,518,321,1404,116
DCAF16_7_3478,0,65,350,0,0,6,6,0,1,0,860,177
DCAF5_7_3479,284,223,285,28,949,1077,102,2,503,434,959,71
DCAF7_7_3480,0,0,0,0,0,30,0,0,0,0,478,0
DCAF8_7_3481,87,876,434,4,21,23,218,25,31,559,1123,10
DDA1_7_3482,491,722,276,48,36,218,466,1139,273,1012,53,1035
DDB1_7_3483,931,58,20,904,1594,307,77,18,152,210,763,234
DDB2_7_3484,4,123,0,21,0,44,0,12,16,23,117,33
DPF2_7_3485,1706,0,53,556,24,35,33,67,855,129,59,205
DTX1_7_3486,1,0,1,0,17,395,23,1,144,17,1012,14
DTX3L_7_3487,347,178,1522,763,702,455,675,47,2,177,905,492
DTX4_7_3488,1316,925,2371,1597,2308,233,946,1350,682,947,945,1445
DZIP3_7_3489,226,881,72,756,264,596,142,798,771,947,1089,513
EIF3D_7_3490,2,98,0,0,30,0,0,127,3,18,7,0
EIF3E_7_3491,20,15,0,0,0,82,18,442,245,3,0,34
EIF3F_7_3492,47,67,0,488,246,0,138,154,389,89,818,139
EIF3G_7_3493,616,929,4,0,294,894,1979,139,58,551,1,48
EIF3H_7_3494,45,0,473,136,50,0,5,0,161,6,402,469
EIF3I_7_3495,6,342,0,40,1054,8,1507,29,47,228,1252,849
EIF3J_7_3496,361,158,41,223,1226,219,675,23,108,278,587,945
EIF3K_7_3497,8,464,2,25,27,0,5,337,38,298,2,7
ENC1_7_3498,63,526,976,94,1959,596,463,711,991,443,21,314
EPN3_7_3499,1,263,10,57,113,3,627,0,5,0,26,165
EPOR_7_3500,91,70,843,624,0,33,0,3,164,154,640,84
ERCC8_7_3501,189,139,103,766,26,376,361,498,613,405,142,166
FAF1_7_3502,529,1,34,574,0,59,9,208,207,7,273,131
FBXL12_7_3503,16,276,376,9,1723,312,452,27,165,619,479,321

FBXL14_7_3504,83,26,687,169,858,223,1290,354,70,590,700,320
FBXL15_7_3505,7,0,0,1,738,0,833,0,0,0,1,0
FBXL16_7_3506,555,261,2,187,119,325,589,404,892,1034,77,497
FBXL17_7_3507,4,0,89,96,230,339,177,12,109,371,947,39
FBXL18_7_3508,5,28,0,0,0,16,0,0,178,6,0,16
FBXL19_7_3509,0,0,0,0,0,0,11,0,0,0,0,0
FBXL21_7_3510,0,0,0,0,0,0,0,0,0,0,0,0
FBXL3_7_3511,114,1,361,32,5,355,1,64,188,39,1143,12
FBXL4_7_3512,91,1981,1704,6,343,49,1323,1249,233,793,1514,1031
FBXL7_7_3513,179,0,6,22,0,0,2,38,86,100,1,9
FBXO10_7_3514,122,56,20,1071,251,709,79,119,0,20,259,6
FBXO16_7_3515,1466,2475,1773,3139,2212,2271,2048,652,2291,4628,332,298
9
FBXO27_7_3516,31,283,0,149,140,47,0,746,329,1805,184,92
FBXO2_7_3517,496,187,14,6,737,26,364,791,228,579,214,223
FBXO30_7_3518,39,2,0,1,0,0,16,421,0,4,0,0
FBXO33_7_3519,577,209,372,320,1638,1520,0,124,845,1131,50,212
FBXO36_7_3520,63,7,10,688,83,20,54,98,161,2180,759,23
FBXO39_7_3521,0,315,0,1171,122,165,63,38,7,371,0,393
FBXO40_7_3522,245,493,866,1302,100,58,3,912,622,129,1080,221
FBXO41_7_3523,1,523,1752,0,149,1102,421,0,49,372,9,132
FBXO42_7_3524,213,0,0,3,792,15,0,154,0,860,104,948
FBXO43_7_3525,211,25,60,3,797,609,841,77,623,141,1144,1938
FBXO46_7_3526,166,74,28,6,11,569,112,17,927,231,625,100
FBXO6_7_3527,193,2146,1194,509,1842,1298,247,1299,783,973,4864,2075
FBXO8_7_3528,1555,1313,1065,2245,1012,280,1853,2338,940,1790,2152,836
FBXW10_7_3529,605,687,3709,1520,1008,245,1428,629,1751,1429,285,766
FBXW2_7_3530,615,225,176,5,1304,736,14,21,1296,1500,913,670
FBXW5_7_3531,798,570,1,56,398,54,705,0,39,23,1566,506
FBXW9_7_3532,0,43,0,0,495,0,0,33,0,12,1399,0
G2E3_7_3533,0,0,60,16,1,169,257,0,1,79,17,6
GAN_7_3534,1039,111,722,192,139,221,280,1506,1065,619,1008,1419
GMCL1_7_3535,859,2316,1041,56,610,985,278,1259,1655,775,862,1789
GNB2_7_3536,270,20,0,90,40,255,367,803,3,289,294,236
GRWD1_7_3537,0,0,479,0,356,0,0,0,5,0,1,2
GZF1_7_3538,2,453,143,549,1800,175,268,437,224,277,1,618
HACE1_7_3539,424,624,874,537,456,509,477,870,105,222,775,79
HDAC6_7_3540,373,39,266,16,1443,94,8,324,477,85,19,53
HECTD1_7_3541,144,4,0,0,44,9,0,0,6,0,12,11
HECTD3_7_3542,1,78,0,55,0,76,356,39,317,520,1,47
HECW1_7_3543,4,154,57,61,1357,547,2,0,0,630,0,27
HECW2_7_3544,247,1,697,18,417,358,680,1069,488,9,2,680
HERC1_7_3545,0,0,0,0,0,0,0,0,0,0,91,0
HERC2_7_3546,0,0,0,0,0,0,0,0,0,0,0,0
HERC3_7_3547,0,0,0,0,0,536,0,0,0,12,0,0
HERC5_7_3548,2171,1310,910,1670,2068,3998,723,1507,1312,1762,1878,1029
HGS_7_3549,69,276,939,467,0,95,187,75,189,16,128,842
HIC2_7_3550,0,0,0,0,745,0,0,0,283,4,0,1431
HUWE1_7_3551,40,0,161,0,0,0,44,381,16,15,56,1
IBTK_7_3552,203,229,79,0,842,71,1,1201,221,11,359,33

IL10RA_7_3553,34,270,16,13,543,7,308,271,323,652,0,714
IL6_7_3554,70,309,0,0,23,123,5,2,272,685,384,742
IRF9_7_3555,55,8,0,5,20,28,1,0,333,201,164,48
ITCH_7_3556,1853,843,64,421,657,1278,3118,1886,1209,1861,478,1597
IVNS1ABP_7_3557,30,47,4,74,1621,453,25,325,130,65,1,157
JHDM1D_7_3558,0,4,0,0,69,241,0,0,585,75,1,76
JOSD1_7_3559,111,0,1,2,400,1,395,0,0,245,204,4
JOSD2_7_3560,54,0,42,0,78,3,632,0,635,41,0,78
KAT6B_7_3561,26,10,130,55,0,672,0,456,5,1,606,15
KATNB1_7_3562,267,3,1,1,167,180,56,2,65,1,389,10
KBTBD10_7_3563,369,147,474,156,0,683,4,542,11,81,39,506
KBTBD11_7_3564,0,0,122,0,0,0,0,0,0,0,0,0
KBTBD2_7_3565,1,23,19,149,249,11,828,58,1,31,0,0
KBTBD5_7_3566,37,242,242,454,215,448,587,360,865,451,453,142
KBTBD7_7_3567,0,1,0,8,0,97,23,0,18,0,489,35
KBTBD8_7_3568,223,10,1052,30,77,447,913,0,35,9,27,116
KCTD10_7_3569,1424,2444,2710,1268,1859,2824,1588,2249,2061,1746,2433,2
263
KCTD11_7_3570,0,1,98,0,0,342,2,0,218,6,0,507
KCTD12_7_3571,48,8,500,215,243,61,0,555,3,42,95,99
KCTD13_7_3572,15,188,77,279,0,3,3,0,117,15,0,128
KCTD16_7_3573,1767,2210,1267,1485,1832,2543,1454,699,1453,963,2153,391
5
KCTD17_7_3574,102,43,152,0,0,144,0,41,0,58,14,41
KCTD18_7_3575,1171,688,838,1184,695,1178,247,1377,2156,1535,1938,2251
KCTD3_7_3576,635,479,9,1,43,101,61,79,118,808,598,183
KCTD5_7_3577,568,7,30,15,343,414,398,233,96,33,192,278
KCTD9_7_3578,812,2221,1054,421,1116,113,384,24,607,350,365,805
KDM2A_7_3579,1,57,13,238,147,614,995,77,9,341,137,2
KDM4B_7_3580,211,1093,471,1140,104,692,495,936,49,617,76,62
KDM5B_7_3581,798,802,8,568,1998,699,281,463,815,833,380,678
KLHDC5_7_3582,80,0,0,0,0,23,4,415,141,29,0,21
KLHL10_7_3583,0,295,48,164,0,0,22,810,1,0,342,0
KLHL11_7_3584,521,227,0,0,0,57,0,0,5,16,0,0
KLHL12_7_3585,384,163,1178,112,234,201,51,10,344,191,80,121
KLHL14_7_3586,121,0,0,46,0,0,0,32,33,6,0,5
KLHL15_7_3587,532,4,0,46,1158,4,1006,414,380,45,9,48
KLHL17_7_3588,1,80,0,0,0,0,0,0,0,0,1062,0
KLHL18_7_3589,514,0,0,54,0,0,927,531,56,1171,1,909
KLHL1_7_3590,1222,0,3632,867,0,1266,585,100,762,996,721,314
KLHL20_7_3591,2,269,0,0,0,11,1,0,3,5,0,0
KLHL21_7_3592,0,10,4,0,220,343,3,710,91,40,95,1055
KLHL22_7_3593,32,498,219,176,250,3,0,0,434,63,0,111
KLHL23_7_3594,731,1279,3675,1653,799,1331,2136,2307,3780,2080,698,1958
KLHL24_7_3595,54,492,2,40,1,141,0,1,9,351,861,133
KLHL25_7_3596,0,1,0,0,0,0,2093,0,0,166,573,10
KLHL26_7_3597,526,699,186,10,179,774,619,0,515,199,0,60
KLHL28_7_3598,1140,816,196,0,305,47,1003,67,821,204,387,324
KLHL29_7_3599,15,106,1,0,21,72,2,0,104,106,314,257
KLHL31_7_3600,1285,1400,2478,210,1293,535,1501,2606,1106,825,1708,1831

KLHL32_7_3601,26,73,127,153,52,149,482,28,388,345,0,316
KLHL33_7_3602,28,582,43,1020,126,257,277,217,476,182,970,75
KLHL34_7_3603,2,4,0,0,0,0,426,13,3,20,45,2
KLHL36_7_3604,65,3,0,4,9,0,1046,13,1,280,14,0
KLHL3_7_3605,0,173,0,686,0,0,63,884,94,55,265,8
KLHL8_7_3606,923,675,947,1612,1119,1211,309,802,523,1140,1348,287
LATS1_7_3607,35,85,0,17,558,24,402,6,0,0,0,539
LATS2_7_3608,7,2,24,395,34,210,960,183,89,180,114,1357
LIF_7_3609,78,260,65,0,7,129,733,0,1,8,0,4
LNX2_7_3610,807,854,896,831,1189,1555,171,1625,284,66,572,833
LOC283116_7_3611,923,1980,2227,996,525,804,1130,1613,2496,1147,110,168
0
LONRF1_7_3612,235,383,126,526,307,397,1058,469,652,324,145,71
LTN1_7_3613,1,20,0,3,28,351,0,233,8,5,902,135
LZTR1_7_3614,263,256,10,10,369,220,60,0,57,11,1,3
MAP1LC3B_7_3615,122,1144,240,158,1482,0,533,73,321,337,0,82
MAP3K1_7_3616,99,65,0,0,0,17,0,0,97,7,0,10
MARK1_7_3617,257,35,631,260,0,404,28,36,57,113,0,175
MDM2_7_3618,1783,348,2111,701,96,1067,1168,448,394,603,778,1255
MED20_7_3619,34,481,102,33,24,735,82,33,52,2,51,592
MEX3B_7_3620,802,413,104,4,479,657,704,916,528,365,25,856
MIB1_7_3621,13,7,1,8,5,259,156,1,112,12,4,178
MKRN2_7_3622,517,268,225,16,120,71,17,0,7,0,64,3
MKRN3_7_3623,974,481,1416,1270,991,386,744,1475,931,1263,1260,2784
MLL2_7_3624,14,10,0,374,0,17,0,0,92,22,71,115
MLLT6_7_3625,0,0,50,31,74,2227,900,108,5,95,443,197
MOCS3_7_3626,0,0,0,0,0,0,1,0,1,0,0,0
MRPL49_7_3627,0,0,0,0,0,19,5,0,461,182,0,49
MUL1_7_3628,988,116,157,721,3,327,0,20,542,49,1763,438
MYCBP2_7_3629,115,1068,0,0,5,186,62,1,48,58,32,13
MYLIP_7_3630,430,648,107,515,426,950,14,465,270,826,115,270
MYSM1_7_3631,0,179,71,41,1,34,89,249,232,214,172,1390
NACC1_7_3632,0,12,913,69,1,170,4,7,221,4,131,57
NACC2_7_3633,354,500,1,594,0,527,0,688,470,500,1099,451
NEURL1B_7_3634,0,0,0,0,250,42,0,282,18,6,62,4
NEURL_7_3635,164,49,0,0,21,103,95,51,198,56,142,235
NHLRC1_7_3636,22,1384,628,66,334,367,19,0,377,441,230,435
NUP43_7_3637,1554,1428,815,1342,2340,1479,1640,3454,1041,1723,523,289
OTUB1_7_3638,1289,5,0,41,0,0,0,0,1012,15,51,132
OTUB2_7_3639,44,32,2,70,244,700,2,0,36,370,657,145
OTUD1_7_3640,337,1172,1076,612,972,960,2259,1420,2754,991,1668,1201
OTUD6A_7_3641,106,126,144,7,527,185,1758,1005,308,35,95,435
OTUD6B_7_3642,107,190,142,0,63,1,64,107,1060,78,535,1081
OTUD7A_7_3643,0,22,677,0,11,0,0,121,90,90,0,14
OTUD7B_7_3644,640,161,0,69,534,1007,279,7,240,11,1,39
PAFAH1B1_7_3645,444,212,753,513,776,78,21,221,149,119,227,166
PARP10_7_3646,0,14,60,0,5,79,0,40,708,12,0,64
PARP11_7_3647,53,180,817,16,956,253,419,616,439,468,687,420
PARP14_7_3648,1108,474,573,1084,901,867,8,46,1150,713,210,492
PCGF1_7_3649,202,196,135,301,728,39,48,37,4,86,6,44

PCGF2_7_3650,25,9,12,63,22,3,3,5,64,0,0,10
PCGF3_7_3651,0,550,574,578,1778,138,83,0,120,1,1086,761
PDZRN3_7_3652,0,69,0,11,0,0,0,0,491,4,1,53
PEBP4_7_3653,606,866,6,755,80,309,203,367,327,233,1191,40
PEX12_7_3654,48,284,995,838,579,649,80,432,484,1051,372,1135
PHF14_7_3655,227,287,1128,684,762,34,238,170,2182,1051,499,497
PHF15_7_3656,18,277,239,215,546,13,375,161,1145,66,1324,146
PHF20_7_3657,102,0,0,0,0,0,0,0,0,230,0,0
PHF2_7_3658,269,528,0,0,0,3,86,0,5,326,0,1
PHF3_7_3659,35,5,0,53,687,428,0,3,230,157,254,501
PHIP_7_3660,16,0,73,22,0,0,0,0,0,1,42,2
PHRF1_7_3661,86,0,0,160,0,0,0,61,386,306,2,284
PJA2_7_3662,1621,963,1213,1776,2522,1488,1568,995,1437,1160,2796,4319
PRPF19_7_3663,6,1,199,9,0,128,769,61,255,845,178,74
PRPF8_7_3664,1599,1364,239,899,1146,293,2039,3780,715,2582,382,1905
PSMD14_7_3665,385,0,35,451,2,111,154,17,986,336,10,1152
PSMD2_7_3666,73,154,24,0,1,0,139,18,131,169,318,52
PSMD4_7_3667,6,1031,71,0,98,186,0,22,6,10,3,101
PSMD7_7_3668,0,199,314,7,0,120,1048,2,0,416,302,161
PWP1_7_3669,11,13,167,130,30,199,488,2,1271,65,363,188
RAB40A_7_3670,593,0,74,493,712,745,58,880,838,1077,114,179
RAB40AL_7_3671,593,0,74,493,712,745,58,880,838,1077,114,179
RAB40B_7_3672,0,77,0,0,8,0,0,19,252,5,0,32
RAD18_7_3673,2304,388,555,2315,1562,535,667,73,1504,1411,2122,191
RAD23A_7_3674,0,43,10,216,338,4,22,760,315,29,5,48
RAD23B_7_3675,41,13,19,568,107,51,274,7,39,18,21,21
RAG1_7_3676,6,271,198,260,28,0,248,242,20,404,25,7
RAI1_7_3677,2606,2042,2783,1167,2919,537,1912,1621,835,2028,2672,1311
RBX1_7_3678,807,995,576,562,1313,89,641,1154,1026,796,724,554
RCBTB1_7_3679,100,539,282,0,96,3,180,0,165,16,0,75
RCBTB2_7_3680,130,146,36,0,33,495,26,108,205,557,0,34
RFFL_7_3681,0,103,0,21,25,0,0,8,5,463,7,96
RFPL1_7_3682,1788,327,957,0,190,2,841,4,532,1211,89,427
RFWD3_7_3683,10,64,0,121,2,0,11,30,69,621,774,882
RHOBTB3_7_3684,278,109,186,813,322,10,169,2,42,490,122,10
RING1_7_3685,6,314,1,1,495,186,486,9,330,190,1017,208
RNF10_7_3686,28,130,611,150,402,1174,325,0,384,100,971,108
RNF111_7_3687,462,779,52,719,660,1383,851,1153,2034,675,503,1164
RNF112_7_3688,0,31,0,94,0,23,6,0,169,21,136,35
RNF113A_7_3689,105,114,555,658,1442,8,9,789,252,220,753,457
RNF113B_7_3690,65,72,97,352,729,87,501,686,274,356,12,187
RNF114_7_3691,2,1034,2,594,710,986,126,169,97,1413,982,48
RNF115_7_3692,133,678,196,191,774,554,1973,406,538,739,458,605
RNF11_7_3693,192,1071,1256,122,732,220,1056,1020,1624,989,455,670
RNF121_7_3694,11,52,0,114,0,0,0,34,4,0,307,951
RNF122_7_3695,264,797,283,413,1186,426,170,712,106,304,1890,177
RNF123_7_3696,247,1,0,28,74,22,117,26,13,2,0,929
RNF125_7_3697,2208,925,1133,3302,3384,3615,2597,2534,1102,3606,3766,23
91
RNF126_7_3698,19,16,140,0,0,0,0,171,36,4,0,125

RNF130_7_3699,459,1364,39,392,688,421,617,17,1381,301,1665,626
RNF133_7_3700,140,0,43,39,0,108,373,1,100,356,4,60
RNF139_7_3701,1224,1352,3284,727,1153,1678,1666,1046,1943,678,1059,600
RNF141_7_3702,266,250,0,368,389,55,1,0,863,126,638,112
RNF144A_7_3703,323,3,368,157,48,608,527,1281,58,833,131,263
RNF144B_7_3704,176,92,2,70,0,113,411,158,567,14,139,88
RNF149_7_3705,1390,287,605,1381,3156,307,436,174,224,1036,158,412
RNF150_7_3706,25,29,1,32,345,543,241,4,445,325,42,77
RNF152_7_3707,546,360,769,1129,296,481,53,412,152,220,818,1093
RNF157_7_3708,322,434,661,4,209,25,8,149,409,71,229,377
RNF167_7_3709,0,0,0,0,0,0,0,0,0,0,0,0
RNF168_7_3710,709,474,534,72,896,864,489,1625,88,1019,794,563
RNF169_7_3711,577,1552,15,480,1010,693,155,193,310,567,839,557
RNF181_7_3712,1264,1,0,17,3,0,96,826,5,0,0,8
RNF183_7_3713,44,0,0,0,0,0,0,0,0,0,105,0
RNF186_7_3714,2,668,0,226,33,237,74,323,15,52,38,176
RNF187_7_3715,36,71,42,7,0,87,392,0,304,14,0,68
RNF20_7_3716,120,40,6,511,0,97,0,80,81,69,98,378
RNF217_7_3717,677,94,1322,30,1,127,1143,187,457,304,303,621
RNF220_7_3718,561,146,194,592,0,240,2388,511,134,35,346,221
RNF25_7_3719,130,0,248,0,8,24,340,96,132,127,53,317
RNF26_7_3720,251,570,14,36,621,120,0,435,0,263,1057,0
RNF2_7_3721,505,721,1186,1128,203,401,969,886,602,1353,725,2477
RNF31_7_3722,2035,1052,1419,879,1331,1817,515,269,1431,525,2197,537
RNF43_7_3723,369,240,806,413,76,52,24,542,47,223,44,43
RNF44_7_3724,283,507,133,135,13,88,2423,780,13,421,1029,618
RNF5_7_3725,1673,2151,1165,622,994,764,901,1460,319,1200,1151,878
RSC1A1_7_3726,0,1259,151,1,0,187,10,71,0,15,1,0
RSF1_7_3727,1585,1013,1695,76,953,674,516,2937,1190,1746,2657,2959
RSPRY1_7_3728,4,4,619,21,13,211,67,7,674,177,35,397
SCLY_7_3729,152,144,10,40,70,10,17,5,14,9,256,69
SENP1_7_3730,1419,2719,702,768,1936,2156,2078,1618,2624,1376,1413,2625
SENP2_7_3731,85,30,2,104,751,845,229,458,1458,283,226,163
SENP3_7_3732,210,47,84,5,11,143,0,501,550,661,372,297
SENP5_7_3733,153,6,77,0,0,20,28,737,281,498,3,27
SH3RF1_7_3734,372,274,657,110,416,17,918,311,264,824,953,188
SH3RF2_7_3735,2122,2301,721,1650,602,2053,1175,1036,2343,1973,2016,123
5
SH3RF3_7_3736,17,121,9,62,413,192,450,13,518,255,245,83
SHKBP1_7_3737,123,31,20,3,807,6,0,0,58,20,25,7
SIAH2_7_3738,70,142,122,358,519,313,6,300,236,220,217,147
SIK1_7_3739,8,413,111,39,4,566,503,227,504,1485,879,897
SLX4_7_3740,151,29,33,17,35,426,72,339,916,138,6,122
SMU1_7_3741,2,0,2,353,2843,14,4,369,320,288,238,48
SMURF2_7_3742,135,0,0,0,266,1,278,0,18,0,0,0
SNRNP40_7_3743,89,1589,1531,993,639,824,341,742,553,996,1573,698
SOCS1_7_3744,9,0,0,91,0,1,0,0,0,410,0,19
SOCS2_7_3745,579,246,2,903,222,4,0,614,350,494,7,1140
SOCS3_7_3746,132,0,124,0,0,0,0,267,3,399,0,135
SOCS6_7_3747,0,0,0,5,8,0,0,61,246,7,259,790

SPOPL_7_3748,957,462,118,197,3489,1727,5,729,83,1163,1315,243
SPSB1_7_3749,21,10,25,305,327,48,571,233,179,27,50,202
SPSB3_7_3750,5,0,0,0,0,0,0,466,3,0,0,0
SPSB4_7_3751,2,210,0,85,0,604,180,13,5,119,127,489
STAM2_7_3752,1172,160,688,1942,140,992,438,610,852,158,105,513
STAMBPL1_7_3753,6,179,7,295,626,1,176,54,103,5,2,14
STAM_7_3754,0,187,0,813,870,372,359,222,869,20,0,129
STUB1_7_3755,44,70,0,298,57,0,1320,52,0,2,329,30
SUMO3_7_3756,391,53,8,633,562,168,409,177,453,883,141,72
SYNGAP1_7_3757,0,0,0,0,0,0,0,0,0,0,0,0
TAB2_7_3758,224,205,463,241,283,13,1303,0,616,69,192,71
TAB3_7_3759,614,3,0,0,95,0,13,5,1080,25,55,249
TAF1D_7_3760,73,330,997,241,798,10,1005,604,533,424,0,456
TBC1D1_7_3761,0,341,1245,11,0,14,271,0,490,1005,184,948
TLE1_7_3762,1520,756,259,924,2662,1421,194,356,249,1863,666,1407
TNFAIP3_7_3763,0,1,0,610,48,141,0,15,0,1,0,48
TOLLIP_7_3764,0,11,0,276,389,21,249,723,396,51,38,44
TRAF7_7_3765,17,10,42,0,0,31,43,4,13,157,138,94
TRAIP_7_3766,1202,684,157,289,1840,760,899,98,757,571,746,823
TRIM11_7_3767,30,516,291,169,326,145,14,90,371,642,38,72
TRIM15_7_3768,0,1,0,1,0,0,0,2,0,475,1,280
TRIM25_7_3769,0,0,0,0,0,101,0,0,0,370,0,0
TRIM27_7_3770,0,0,0,930,2049,0,0,0,1211,15,4,148
TRIM28_7_3771,117,0,1038,653,12,255,1056,0,1105,23,877,149
TRIM31_7_3772,458,172,15,0,88,0,158,5,0,151,1,393
TRIM35_7_3773,2,393,0,54,1912,2,954,790,119,485,0,21
TRIM42_7_3774,1968,1777,380,408,1098,163,477,713,365,410,1418,1811
TRIM46_7_3775,1035,1532,227,50,207,1404,816,169,199,473,277,1692
TRIM47_7_3776,89,1,0,0,86,0,0,0,30,3,6,3
TRIM48_7_3777,2,14,0,5,781,8,0,56,82,3,151,78
TRIM52_7_3778,852,195,1,329,1,716,0,294,85,65,161,9
TRIM56_7_3779,6,11,9,1,0,5,241,0,1,169,1,12
TRIM62_7_3780,148,6,744,0,91,1,0,611,231,66,22,29
TRIM63_7_3781,199,842,675,12,2,5,0,100,306,12,640,143
TRIM65_7_3782,20,461,214,0,392,0,98,4,120,70,402,477
TRIM67_7_3783,168,36,257,517,127,5,54,437,368,2,1,49
TRIM68_7_3784,117,572,0,43,231,26,67,1,226,74,127,601
TRIM8_7_3785,0,0,6,0,0,4,0,0,9,7,0,0
TRIP12_7_3786,894,338,44,475,624,6,2265,749,1035,222,4,164
UBA2_7_3787,0,0,0,22,0,68,11,59,1,26,0,0
UBA6_7_3788,1,435,578,309,325,145,77,1355,211,45,1092,36
UBA7_7_3789,0,112,77,0,462,0,156,0,8,14,581,22
UBAC1_7_3790,3,717,69,0,805,686,14,69,412,105,1005,43
UBAP2_7_3791,982,54,787,32,491,642,217,1131,1520,717,2001,627
UBASH3B_7_3792,821,1166,57,777,512,18,380,308,164,91,529,697
UBC_7_3793,66,848,25,57,851,700,819,781,7,104,60,145
UBE2B_7_3794,54,558,555,177,400,654,73,12,1264,903,165,661
UBE2D4_7_3795,1389,59,0,668,1252,685,0,2432,590,1173,1455,77
UBE2E2_7_3796,50,505,1275,179,6,186,142,582,124,395,287,774
UBE2F_7_3797,1,510,137,189,0,28,0,6,126,16,33,18

UBE2G1_7_3798,0,123,0,565,252,224,452,217,372,7,80,671
UBE2J1_7_3799,0,0,0,0,0,0,0,0,0,0,0,0
UBE2L3_7_3800,4,26,398,2,365,129,41,106,581,213,3,492
UBE2M_7_3801,2,78,41,0,735,83,0,5,0,18,1,196
UBE2N_7_3802,162,1,250,252,257,615,290,1574,845,1020,35,387
UBE2NL_7_3803,14,201,0,1112,653,430,5,0,489,6,39,36
UBE2O_7_3804,8,13,2,63,19,81,6,138,87,269,0,18
UBE2Q1_7_3805,81,5,0,0,0,984,3,0,122,1019,526,13
UBE2QL1_7_3806,44,3,0,3,30,259,64,10,16,0,84,7
UBE2R2_7_3807,523,126,0,1361,2,450,2587,192,284,268,299,120
UBE2S_7_3808,22,26,0,0,103,0,14,51,2,23,0,0
UBE2T_7_3809,427,14,641,244,565,674,85,0,122,396,614,130
UBE2U_7_3810,1929,3703,1901,1544,210,705,697,1471,1131,3868,1946,289
UBE2V2_7_3811,2225,89,1114,494,1623,238,3060,320,1901,678,472,573
UBE2Z_7_3812,0,0,0,1204,0,0,1151,1,0,0,764,44
UBE3C_7_3813,1594,989,1604,777,758,373,975,1112,1094,330,982,1107
UBQLN2_7_3814,204,29,6,621,441,22,190,91,69,278,164,545
UBQLN3_7_3815,258,0,0,4,720,42,60,102,0,7,810,633
UBQLN4_7_3816,2,18,0,0,49,283,0,16,55,1,0,201
UBR1_7_3817,189,0,1,155,23,25,277,0,277,42,1,556
UBR3_7_3818,324,507,2292,549,303,879,138,295,635,1003,143,217
UBR4_7_3819,499,637,43,380,1139,5,97,268,538,599,95,562
UBR5_7_3820,107,183,301,498,430,29,818,79,1140,668,978,654
UBR7_7_3821,0,709,0,574,441,1350,0,378,581,249,46,468
UBXN10_7_3822,496,167,849,1615,876,1571,359,1775,631,263,1,193
UBXN1_7_3823,0,0,1,158,120,498,2,1,0,442,19,41
UBXN2A_7_3824,1467,1019,624,612,6,256,209,296,210,672,996,1960
UBXN4_7_3825,0,0,0,0,0,0,0,0,0,0,0,0
UBXN7_7_3826,3,586,146,0,261,350,1030,319,1113,416,203,184
UBXN8_7_3827,160,425,554,746,1057,217,383,1134,2024,526,3,483
UCHL1_7_3828,480,398,1622,221,187,1,891,4,212,314,691,1196
UCHL3_7_3829,539,817,1845,229,706,1082,1,648,89,666,767,335
UFC1_7_3830,603,561,0,202,30,88,579,15,36,27,0,6
UHRF2_7_3831,963,1115,1092,498,2939,1329,369,2027,860,1634,2328,1995
UNK_7_3832,480,18,478,3,1304,581,4,58,424,384,551,445
USP11_7_3833,1,580,0,89,91,474,0,0,0,0,0,1
USP13_7_3834,1690,741,85,643,2413,2509,115,372,1978,1062,570,887
USP15_7_3835,1327,139,1068,2175,1006,2294,1,780,1493,129,1572,2391
USP17_7_3836,704,3088,1430,529,1291,1348,1882,803,1097,1281,1033,787
USP17L2_7_3837,2647,1014,2976,1658,2862,2962,2172,1778,1527,2029,1391,
1595
USP17L5_7_3838,1111,1733,487,1334,2043,1777,1557,1925,2329,720,2129,12
75
USP18_7_3839,534,0,600,294,1545,184,534,1337,804,659,455,338
USP22_7_3840,192,332,1423,91,22,52,821,867,305,627,158,418
USP24_7_3841,42,512,276,890,236,1415,1128,991,305,442,373,73
USP25_7_3842,0,0,0,0,0,0,0,0,0,0,0,0
USP26_7_3843,514,1702,10,1324,1452,1109,593,889,784,728,1476,1078
USP27X_7_3844,1118,473,1183,501,687,803,1314,1065,445,248,772,1521
USP28_7_3845,412,541,15,415,245,2574,524,554,153,340,231,52

USP29_7_3846,0,37,161,14,275,2,0,0,0,345,0,0
USP30_7_3847,258,253,1162,706,143,64,311,1247,385,51,423,570
USP32_7_3848,3873,2265,513,1187,1129,933,2728,1291,1533,1548,1793,1430
USP34_7_3849,138,0,276,22,0,0,228,57,355,270,614,491
USP35_7_3850,23,0,0,568,0,0,877,0,47,167,50,13
USP36_7_3851,338,127,1126,182,186,1041,998,288,541,577,350,62
USP37_7_3852,0,0,121,0,0,1089,1,133,278,424,109,719
USP38_7_3853,1051,670,447,999,410,1509,505,2007,1020,896,637,645
USP39_7_3854,731,87,389,747,718,329,237,446,478,522,664,509
USP3_7_3855,575,782,376,2168,19,385,7,2391,129,379,66,15
USP40_7_3856,6246,7498,5881,2808,6741,5396,5440,3744,6096,6013,7439,55
00
USP42_7_3857,85,0,1289,580,578,1006,68,251,1775,645,845,395
USP43_7_3858,39,188,300,341,44,0,321,39,200,381,172,603
USP45_7_3859,3,27,0,0,54,690,309,0,127,429,366,14
USP47_7_3860,96,471,787,172,1105,718,769,835,1483,1369,1810,1256
USP49_7_3861,78,0,0,7,0,3,0,13,0,0,0,0
USP50_7_3862,37,215,247,51,223,152,499,0,558,153,398,115
USP51_7_3863,474,1328,1893,415,2426,1139,1760,346,447,658,96,518
USP53_7_3864,275,820,170,517,411,199,1374,103,565,963,70,392
USP54_7_3865,27,1,0,54,0,0,216,1558,529,1093,633,827
USP6_7_3866,3873,2265,513,1187,1129,933,2728,1291,1533,1548,1793,1430
USP7_7_3867,483,1,0,658,232,51,275,0,0,1,9,0
USP9Y_7_3868,352,340,1340,1360,365,125,647,939,520,342,921,900
USPL1_7_3869,902,943,3693,742,733,2367,955,3921,1948,524,4191,837
VCPIP1_7_3870,323,38,0,374,487,131,36,77,153,256,0,462
VPS11_7_3871,12,0,0,0,1,730,32,1073,1,53,0,2
WDR12_7_3872,999,485,1015,374,339,1312,953,0,124,25,223,39
WDR53_7_3873,63,6,1,408,762,422,213,703,91,7,33,377
WDR59_7_3874,0,0,0,0,0,0,0,0,0,0,0,0
WDR5B_7_3875,931,1619,1160,1084,1171,3737,2887,1511,2199,1524,492,1212
WDR61_7_3876,560,159,338,323,70,1080,194,810,505,1282,24,800
WDTC1_7_3877,11,34,68,0,1467,9,0,2,0,314,10,5
WSB2_7_3878,5,0,3,2,0,530,0,4,33,1,12,11
WWP1_7_3879,1627,36,1443,270,1781,1662,691,466,707,947,2126,141
YOD1_7_3880,1296,1100,57,222,1111,503,3,144,455,800,287,737
ZBTB11_7_3881,423,597,1010,273,1274,948,663,555,621,59,175,881
ZBTB25_7_3882,745,1296,1455,1195,2457,1224,1026,332,514,553,1410,544
ZBTB2_7_3883,487,0,253,41,0,4,227,502,41,314,284,0
ZBTB32_7_3884,570,144,547,141,167,0,0,161,57,32,23,7
ZBTB34_7_3885,133,360,280,273,41,168,27,693,10,915,261,608
ZBTB39_7_3886,395,60,775,159,15,200,123,170,87,14,193,61
ZBTB3_7_3887,0,2675,0,224,0,52,22,801,259,623,0,144
ZBTB41_7_3888,1250,35,2405,610,1534,30,897,318,1195,398,85,239
ZBTB44_7_3889,1922,467,806,461,1486,2364,87,2601,1405,1718,1147,3207
ZBTB45_7_3890,956,2,5,528,7,379,5,87,1417,76,309,558
ZBTB46_7_3891,585,261,153,80,647,167,70,2060,504,685,1026,1085
ZBTB47_7_3892,37,586,786,675,2,97,603,1416,319,162,3,808
ZBTB48_7_3893,226,138,950,0,84,525,14,228,414,139,444,955
ZBTB49_7_3894,0,0,0,0,784,0,0,0,9,0,1,676

ZBTB5_7_3895,33,2521,359,358,503,735,797,393,437,390,287,189
ZBTB7A_7_3896,249,444,165,349,0,92,190,1,1409,687,1103,321
ZBTB7B_7_3897,0,2,513,43,1,0,0,88,3,399,0,0
ZBTB7C_7_3898,13,454,663,318,213,14,139,2,108,338,88,16
ZBTB8A_7_3899,0,59,868,1112,63,676,9,207,253,254,1099,538
ZFAND2B_7_3900,0,45,116,313,311,539,0,251,72,463,274,250
ZFPL1_7_3901,598,198,668,115,711,51,1070,604,700,1429,1280,201
ZMYND10_7_3902,179,498,592,102,701,70,301,147,281,293,132,734
ZNF131_7_3903,141,105,0,543,1,163,221,130,169,4,535,293
ZNF598_7_3904,0,313,0,567,40,0,9,0,6,131,8,306
ZNF645_7_3905,117,49,963,144,217,110,0,918,2,56,0,0
ZNRF1_7_3906,4,27,1546,414,90,104,53,294,322,659,1035,607
ZNRF2_7_3907,1541,740,1692,1070,1541,2090,1368,254,446,1610,2267,785
ZNRF4_7_3908,0,63,55,0,91,223,0,12,247,574,0,161
ZRANB1_7_3909,187,65,469,594,6,0,218,0,306,10,9,124
ZSWIM2_7_3910,1512,926,776,1868,734,221,916,1193,908,1441,385,394
AIRE_7_3911,140,1401,832,121,2535,295,1782,754,568,1296,1361,474
ANAPC5_7_3912,7,110,152,212,330,22,0,0,39,45,133,38
ANAPC7_7_3913,1113,524,884,2159,1456,1218,403,2490,1350,967,3183,1546
ANKFY1_7_3914,40,19,114,137,230,74,904,413,120,445,437,92
ASB10_7_3915,203,190,1711,348,31,37,233,873,115,175,319,151
ASB11_7_3916,592,1146,1973,1662,1170,1157,1578,1258,1045,626,1994,340
ASB14_7_3917,289,13,908,1463,2112,598,60,527,492,1166,785,2729
ASB2_7_3918,0,0,0,125,0,0,395,0,0,6,0,0
ASB3_7_3919,137,542,1009,484,1848,929,317,733,238,333,4,753
ASB4_7_3920,9,6,5,187,31,513,1,4,2,256,581,737
ASB6_7_3921,252,375,0,46,94,602,27,1,0,387,90,26
ASB7_7_3922,1,107,2,866,0,40,767,48,2,28,147,91
ASB9_7_3923,0,0,333,0,20,28,0,0,0,0,2,18
ASCC2_7_3924,171,23,8,0,59,36,32,240,776,362,313,149
ATG10_7_3925,551,669,491,1555,346,547,749,603,793,823,875,107
ATG16L1_7_3926,1092,7,159,5,14,958,4,602,137,1044,864,520
ATG7_7_3927,11,623,0,555,1383,867,86,457,0,675,748,1099
ATRX_7_3928,171,453,481,11,236,1138,95,493,824,945,616,1080
BACH2_7_3929,0,62,40,16,39,100,108,4,6,340,18,67
BAG6_7_3930,83,0,0,0,0,26,174,219,522,3,1,103
BAZ1A_7_3931,386,487,96,33,754,332,962,952,1290,397,441,1271
BCL6_7_3932,502,0,0,264,49,0,0,0,0,10,0,655
BIRC3_7_3933,299,0,1752,30,0,0,802,209,376,108,155,817
BIRC7_7_3934,0,0,1052,136,0,108,0,0,0,0,0,11
BPTF_7_3935,1980,2551,2149,828,1921,2312,1513,1248,1952,1999,2061,2233
BRCA1_7_3936,159,0,31,0,104,108,0,133,0,5,3,38
BRPF1_7_3937,8,318,1655,991,337,524,146,432,414,105,1362,72
BRWD1_7_3938,1602,454,417,487,762,1173,3,974,1004,878,362,1550
BTBD11_7_3939,283,214,48,976,637,1516,1,126,1664,117,1488,1020
BTBD1_7_3940,43,170,256,5,312,311,1893,0,89,870,52,18
BTBD3_7_3941,0,212,0,582,2395,80,1,144,353,8,222,936
BTBD7_7_3942,3265,1744,1142,3050,1000,2743,549,1523,4390,2808,5165,478
4
BTBD9_7_3943,32,609,2471,1013,2609,1100,251,490,222,606,1113,956

BTRC_7_3944,38,359,0,1,0,7,0,13,330,236,0,35
C3orf26_7_3945,326,2,237,46,36,551,0,1083,499,13,787,63
CBLC_7_3946,151,0,223,150,0,41,578,82,105,4,1294,93
CCNB1IP1_7_3947,404,10,161,759,82,290,219,0,236,1281,795,208
CDC16_7_3948,133,222,449,12,513,9,1049,6,338,49,503,228
CDC27_7_3949,1269,586,0,0,0,0,0,0,353,6,198,513
CHFR_7_3950,44,142,0,9,0,7,118,0,67,11,41,88
CISH_7_3951,593,253,915,191,296,465,365,1783,354,411,846,514
CNOT4_7_3952,0,61,0,0,15,48,0,0,0,2,0,0
COPS2_7_3953,380,554,233,335,592,757,67,154,721,384,307,328
COPS3_7_3954,190,12,0,402,0,97,6,1005,368,271,0,42
COPS7A_7_3955,179,137,169,76,656,522,716,435,329,27,441,404
COPS8_7_3956,2,5,1627,67,156,81,0,96,721,118,557,93
CRBN_7_3957,1564,518,624,896,1601,361,1376,185,530,669,379,1235
CUL2_7_3958,3,123,502,120,695,160,156,183,479,598,891,331
CUL4A_7_3959,0,573,248,0,0,19,877,253,44,170,1037,949
CUL4B_7_3960,794,979,277,272,440,362,876,494,310,513,514,494
CUL7_7_3961,109,69,197,371,35,0,412,20,501,251,2,157
CXXC1_7_3962,1772,494,1025,1448,1894,2537,1817,715,44,1440,391,131
CYLD_7_3963,3,1,597,4,0,0,0,123,2,0,0,21
DCAF11_7_3964,0,0,0,0,0,0,0,0,0,0,0,0
DCAF17_7_3965,288,1327,2,636,310,781,84,20,667,1014,1423,192
DCAF4_7_3966,0,1,470,514,102,46,0,148,108,94,113,452
DCAF6_7_3967,24,693,1416,868,289,940,637,2239,292,448,2125,3108
DCST1_7_3968,312,179,3,561,59,63,0,0,853,440,1568,622
DEPDC1B_7_3969,0,0,0,9,43,0,0,9,245,487,133,229
DET1_7_3970,0,0,554,39,0,0,0,440,137,40,106,19
DIDO1_7_3971,1219,3190,1700,541,1000,455,2678,2649,1554,1016,1769,2927
DNAJB2_7_3972,1012,1278,214,297,8,568,1017,834,930,1632,1141,1126
DTX2_7_3973,162,1318,56,0,621,0,690,1166,366,1046,0,542
EED_7_3974,106,9,711,3,0,1,1506,435,97,230,0,17
EIF3B_7_3975,1597,323,1743,972,0,715,830,2466,1266,1276,1935,812
EIF3C_7_3976,25,434,139,411,324,23,380,836,65,287,651,856
EIF6_7_3977,11,131,2444,365,819,399,62,0,162,46,0,382
EPN1_7_3978,234,60,0,532,0,632,300,66,477,397,29,119
EPN2_7_3979,17,101,501,1,30,45,142,834,0,934,0,0
EPS15_7_3980,218,596,148,27,156,551,250,3,263,850,312,29
FAM70A_7_3981,1220,136,1,302,560,838,34,83,732,81,276,176
FANCL_7_3982,139,138,420,104,88,1,0,283,8,55,800,512
FBXL13_7_3983,1082,626,116,921,1314,18,235,485,226,627,0,612
FBXL20_7_3984,256,496,675,126,160,80,25,1085,15,800,1030,73
FBXL2_7_3985,0,0,0,0,0,0,0,0,17,0,0,3
FBXL5_7_3986,6,643,61,381,2,514,340,375,121,180,97,35
FBXL6_7_3987,0,0,42,101,11,0,0,0,1,0,0,18
FBXO11_7_3988,1,1496,240,145,347,200,33,0,115,240,556,315
FBXO15_7_3989,0,724,206,849,0,0,0,1686,1178,24,720,137
FBXO17_7_3990,5,255,7,28,163,466,70,276,5,419,242,474
FBXO18_7_3991,75,0,0,71,132,27,4,0,0,0,28,46
FBXO21_7_3992,1930,17,1260,460,690,175,12,527,535,10,240,189
FBXO22_7_3993,358,565,124,752,1043,242,905,181,1075,250,359,538

FBXO24_7_3994,82,2,60,112,5,238,0,17,5,64,289,3
FBXO25_7_3995,387,214,814,48,25,116,193,42,68,22,871,13
FBXO28_7_3996,375,1585,142,88,1192,83,471,365,176,121,1265,1109
FBXO32_7_3997,0,0,876,346,0,0,8,0,373,106,0,33
FBXO34_7_3998,449,10,70,584,1,66,372,24,308,179,881,1585
FBXO38_7_3999,408,484,0,3,1006,0,564,796,469,307,146,76
FBXO3_7_4000,54,251,98,187,149,12,362,117,16,1,238,544
FBXO44_7_4001,431,255,305,301,123,41,0,5,560,19,423,1717
FBXO4_7_4002,458,2,412,696,475,26,659,16,677,80,836,338
FBXO5_7_4003,475,203,747,24,133,1177,386,1566,76,729,0,24
FBXO7_7_4004,777,972,0,3,234,694,0,0,534,324,1,98
FBXO9_7_4005,0,59,493,25,264,26,403,282,191,607,532,129
FBXW11_7_4006,488,0,0,0,9,0,960,354,6,0,0,0
FBXW7_7_4007,14,18,0,212,2,0,0,0,0,1,0,0
FBXW8_7_4008,0,27,16,107,0,416,0,24,6,0,27,91
GPS1_7_4009,20,404,173,10,19,293,59,26,463,105,356,88
HECTD2_7_4010,867,1,990,824,1720,737,1806,1229,320,1638,277,327
HERC4_7_4011,200,81,0,5,22,17,44,0,31,490,419,38
HERC6_7_4012,171,25,59,0,199,377,1040,0,440,569,402,61
HIC1_7_4013,0,0,32,0,0,0,0,0,0,0,0,0
HLTF_7_4014,1781,480,1829,1310,1344,1745,903,1106,2236,1446,2390,363
HSF4_7_4015,204,33,110,372,1,1705,40,91,358,971,174,171
IPP_7_4016,1279,69,976,19,679,551,979,1521,611,573,714,121
KAT6A_7_4017,1676,877,254,78,964,395,5,951,308,266,1703,218
KBTBD3_7_4018,656,801,889,306,384,519,830,1436,201,81,981,372
KCTD6_7_4019,828,1879,88,573,770,1354,594,1178,686,1106,2559,508
KCTD7_7_4020,1,40,0,0,0,121,0,0,19,1,0,77
KDM2B_7_4021,1,0,0,37,17,10,301,241,1,425,769,62
KDM4C_7_4022,0,0,0,0,0,0,0,0,0,0,0,0
KDM5C_7_4023,347,1422,551,571,1237,693,135,454,1481,631,1,184
KEAP1_7_4024,57,0,0,45,0,0,0,123,0,0,503,11
KIAA1841_7_4025,762,681,446,215,1249,2277,170,1088,1974,630,882,925
KLHL13_7_4026,2541,1478,4,709,1163,700,414,756,1054,1554,2025,2348
KLHL2_7_4027,188,582,314,307,574,326,439,2645,711,349,983,1747
KLHL4_7_4028,160,160,1,486,0,44,1,18,0,0,777,27
KLHL5_7_4029,557,19,591,545,0,1137,1874,52,3,408,166,498
KLHL7_7_4030,171,2,600,84,2,513,0,1182,3,112,1167,0
LNX1_7_4031,1318,994,1969,1313,3115,3591,2044,2347,772,930,1511,1458
LONRF3_7_4032,1120,860,332,262,30,210,776,913,2176,905,1620,1488
LRRC29_7_4033,0,0,1725,0,0,0,0,0,0,1,420,0
LRSAM1_7_4034,1833,222,417,644,1335,773,1098,1349,0,536,470,499
MARK2_7_4035,400,15,517,11,1265,8,2986,682,332,1314,5,306
MARK3_7_4036,0,160,2,0,5,0,0,352,492,14,2,138
MARK4_7_4037,34,120,49,143,0,379,835,822,0,0,14,1
MDM4_7_4038,151,619,122,584,140,130,3,838,1296,92,146,1064
MGRN1_7_4039,86,336,45,83,1518,1058,0,117,135,521,16,426
MIB2_7_4040,2,47,0,9,0,16,0,411,0,0,1115,218
MID1_7_4041,485,249,406,127,44,786,531,222,456,63,722,750
MID2_7_4042,0,0,0,0,14,0,65,0,0,0,190,81
MKRN1_7_4043,1584,341,1001,613,1451,483,43,551,863,273,9,138

MLL5_7_4044,40,286,762,523,904,0,58,276,155,445,846,197
MLL_7_4045,0,7,12,6,178,88,34,19,31,3,0,12
MNAT1_7_4046,277,85,527,65,773,381,63,417,393,192,2062,274
MPND_7_4047,343,0,0,463,0,0,1525,154,0,0,0,0
MTF2_7_4048,1,0,0,0,0,0,154,0,207,6,0,37
MYNN_7_4049,193,149,0,61,521,433,0,363,271,293,121,24
NAE1_7_4050,2175,1691,3003,1624,1539,1705,411,2427,3739,2635,3808,4595
NDUFC2_7_4051,4,0,52,14,33,377,200,560,404,772,820,1001
NEDD4_7_4052,22,1394,0,328,217,647,1240,33,497,662,39,72
NEDD4L_7_4053,1,0,57,501,0,92,51,911,127,33,69,367
NFX1_7_4054,1,0,0,133,0,0,18,121,64,1,17,442
NLE1_7_4055,2,292,384,282,306,280,35,132,273,207,56,510
NSD1_7_4056,79,3,38,119,93,47,38,50,386,16,0,76
NSFL1C_7_4057,956,2370,755,1834,1623,2527,2512,4879,3587,4442,4322,343
4
NUB1_7_4058,920,1733,990,455,609,432,746,1471,826,1929,993,608
ODF2_7_4059,300,28,334,118,13,4,13,943,71,106,398,34
OTUD5_7_4060,99,5,309,14,218,22,112,0,124,35,223,91
PARK2_7_4061,5,73,2150,153,2568,19,137,848,32,120,252,119
PARP9_7_4062,467,727,1162,1412,783,1675,674,685,1164,932,913,607
PATZ1_7_4063,454,308,1498,201,332,393,30,663,213,1262,302,72
PCGF6_7_4064,1,0,3,360,43,154,0,3,0,605,0,28
PEX10_7_4065,850,248,136,284,211,731,57,2,746,611,698,120
PEX2_7_4066,3192,2107,593,2546,1359,2165,1584,1289,3716,2264,1641,3360
PHF12_7_4067,936,634,76,109,142,1,444,169,0,0,0,79
PHF16_7_4068,272,272,576,816,503,511,25,863,1151,450,385,368
PHF17_7_4069,18,277,239,215,546,13,375,161,1145,66,1324,146
PHF1_7_4070,447,19,0,73,411,0,422,8,420,17,6,259
PHF21A_7_4071,70,27,268,3,0,59,0,35,74,17,37,10
PHF7_7_4072,610,680,116,163,871,967,443,23,561,8,521,370
PHF8_7_4073,57,40,12,98,71,203,772,318,121,661,300,109
PJA1_7_4074,2410,2863,26,22,209,2726,711,1654,796,708,1710,603
PML_7_4075,89,49,48,83,10,10,133,27,0,0,0,9
POC1B_7_4076,32,3,12,0,96,259,513,2,87,651,19,172
PSMD1_7_4077,101,161,0,654,0,128,694,429,84,44,0,1064
RAPSN_7_4078,3,0,0,8,1475,4,0,180,397,7,1,39
RBBP4_7_4079,99,123,161,236,9,454,1,139,1,783,39,32
RBBP5_7_4080,0,14,1,99,0,15,382,0,515,41,0,61
RBBP6_7_4081,1573,828,914,1142,1608,75,364,708,2144,795,2033,659
RBBP7_7_4082,46,141,44,416,116,12,91,9,1,111,0,344
RBCK1_7_4083,9,0,1,0,21,0,0,0,47,0,0,3
RC3H2_7_4084,1693,12,82,5,249,269,221,937,585,300,973,801
RCHY1_7_4085,1641,644,336,1046,2513,73,48,401,140,838,363,728
RFPL2_7_4086,359,793,546,100,556,0,625,64,721,342,1,474
RFPL3_7_4087,957,1207,567,1345,333,1126,935,893,979,876,1206,523
RFWD2_7_4088,878,496,131,16,5,12,12,55,584,79,1319,489
RHOBTB1_7_4089,1015,755,1642,224,662,1097,776,525,577,759,1430,2358
RHOBTB2_7_4090,244,6,0,460,79,720,18,2,61,610,765,106
RLIM_7_4091,186,1,0,0,0,525,1,2250,252,6,0,36
RNF103_7_4092,973,2301,487,475,1066,2390,94,490,459,3179,1386,1168

RNF128_7_4093,882,60,2136,155,0,705,152,74,193,250,103,264
RNF135_7_4094,12,0,0,0,0,0,0,0,0,47,40,0
RNF138_7_4095,1915,1123,2176,1762,2304,527,1521,1752,1715,2469,2711,41
25
RNF13_7_4096,441,277,82,1474,499,1219,466,167,612,441,927,1024
RNF145_7_4097,5,110,0,30,1663,1,623,528,1,0,923,0
RNF146_7_4098,519,657,3,257,1,43,50,24,1639,22,780,2704
RNF14_7_4099,13,1,35,450,236,597,0,601,40,339,7,22
RNF166_7_4100,1081,341,131,15,0,89,27,611,9,206,1249,145
RNF170_7_4101,730,862,448,59,29,754,382,2,376,326,334,1161
RNF17_7_4102,370,0,251,214,282,211,15,0,285,130,12,341
RNF180_7_4103,1447,18,661,328,720,702,275,360,42,20,0,3
RNF182_7_4104,19,119,818,100,1921,105,69,209,188,216,127,215
RNF185_7_4105,23,0,0,84,849,0,0,473,454,7,129,49
RNF19A_7_4106,717,576,1145,491,1727,2158,1752,535,1688,1233,2971,1641
RNF19B_7_4107,0,0,0,0,0,0,5,0,15,180,8,3
RNF213_7_4108,94,302,2497,669,1879,785,895,17,1083,592,455,151
RNF214_7_4109,618,125,746,698,88,179,879,753,767,21,1227,767
RNF216_7_4110,15,256,0,675,18,1658,154,133,46,52,0,447
RNF24_7_4111,3279,2863,2300,386,1725,806,890,1631,3696,2344,1381,2362
RNF32_7_4112,16,0,963,38,4,36,380,1,1,1168,265,340
RNF34_7_4113,21,13,67,108,1,3,247,516,329,61,14,60
RNF38_7_4114,318,68,622,23,20,142,86,13,233,134,0,392
RNF40_7_4115,1,0,0,121,346,0,1395,0,109,2,6,1518
RNF41_7_4116,333,6,0,77,56,10,160,366,422,70,37,277
RNF6_7_4117,978,2621,49,829,945,1877,1440,1392,1148,1331,850,1283
RNF7_7_4118,0,18,0,0,0,0,8,0,185,7,0,23
RNF8_7_4119,0,353,0,0,466,87,714,160,144,315,555,185
SAE1_7_4120,184,1,38,518,88,701,0,114,59,3,392,6
SENP6_7_4121,418,752,1703,832,811,548,318,986,1018,1500,195,1298
SENP7_7_4122,298,1058,57,613,62,252,62,322,89,417,0,796
SENP8_7_4123,44,0,1,0,0,511,1,212,43,33,7,6
SF3A1_7_4124,330,102,0,57,0,0,9,401,0,19,0,48
SHPRH_7_4125,585,255,115,359,1340,742,1785,880,301,104,444,74
SIAH1_7_4126,220,57,103,46,62,17,208,39,647,23,95,363
SKP2_7_4127,670,262,165,1038,829,762,2233,1951,1461,603,245,244
SMURF1_7_4128,211,49,0,468,162,6,3,422,1,100,0,79
SOCS5_7_4129,160,287,1128,673,189,379,800,1492,484,628,646,176
SP100_7_4130,21,12,0,705,610,394,0,50,73,0,0,12
SP110_7_4131,10,0,6,0,14,208,8,63,8,522,1518,50
SPOP_7_4132,506,534,510,0,0,1409,1329,588,432,176,2410,570
SPSB2_7_4133,336,288,895,41,0,34,1,0,18,409,187,353
SQSTM1_7_4134,0,0,28,2,121,208,0,0,54,1,590,295
STAMBP_7_4135,786,1145,1367,889,1,1604,819,604,1129,2415,1848,1371
SUMO1_7_4136,664,0,15,0,221,21,0,44,3,16,0,167
SUMO2_7_4137,1,449,0,268,347,2,178,3,461,82,110,148
SYTL4_7_4138,495,61,761,595,810,456,452,56,461,876,247,606
SYVN1_7_4139,343,251,547,762,35,242,417,340,53,465,625,337
TCF20_7_4140,357,1657,907,1557,81,656,590,794,833,647,214,902
TDRD3_7_4141,175,2924,2919,1147,1119,1768,1475,4151,3831,2746,2455,376

8
TIPARP_7_4142,1286,513,2092,911,32,1857,1916,686,1464,2213,1295,520
TLE2_7_4143,9,493,813,119,221,652,0,65,428,352,26,70
TLE3_7_4144,485,0,39,3,0,0,0,4,9,0,25,159
TNK2_7_4145,0,0,0,79,26,0,0,122,1,36,0,0
TNRC6C_7_4146,216,232,32,128,298,428,109,256,39,295,463,1043
TOPORS_7_4147,24,3,0,255,216,124,19,0,57,639,492,64
TOR1AIP2_7_4148,9,358,11,192,832,7,2,1531,410,232,17,902
TRAF3_7_4149,29,16,31,0,0,4,1028,5,142,85,103,22
TRAF5_7_4150,86,561,1016,797,828,30,60,516,385,1241,476,136
TRAF6_7_4151,764,933,105,1388,973,4,1138,1976,390,548,621,76
TRIM10_7_4152,715,1289,2,55,35,54,541,699,204,223,1,43
TRIM13_7_4153,1,586,303,166,15,70,0,121,113,99,281,626
TRIM17_7_4154,0,0,0,0,0,1,0,0,119,1,0,40
TRIM22_7_4155,9,526,1,195,93,0,85,100,911,720,54,659
TRIM23_7_4156,0,597,57,337,2,1,0,0,350,149,0,28
TRIM24_7_4157,3326,722,44,1457,1685,2183,1874,819,678,1688,4221,1400
TRIM26_7_4158,710,1134,198,1134,635,512,238,534,1242,195,575,783
TRIM2_7_4159,200,14,12,0,337,5,532,281,101,51,61,136
TRIM32_7_4160,1529,310,288,173,191,171,0,1210,581,580,126,957
TRIM33_7_4161,4,311,426,428,456,175,232,650,98,99,59,52
TRIM34_7_4162,63,247,0,411,0,153,558,31,30,226,314,15
TRIM37_7_4163,257,23,1249,101,7,568,83,160,561,47,264,636
TRIM39_7_4164,0,126,0,34,0,2,25,20,295,9,4,555
TRIM3_7_4165,349,1140,75,529,49,70,6,0,84,513,6,905
TRIM41_7_4166,548,0,882,0,51,577,48,563,1,0,436,835
TRIM45_7_4167,0,0,3,57,0,0,618,0,0,0,1,0
TRIM4_7_4168,1484,787,775,1200,1681,201,1379,2286,830,697,817,540
TRIM54_7_4169,350,139,1247,78,1125,1592,139,335,826,1031,784,177
TRIM55_7_4170,671,172,653,321,1489,753,860,541,978,191,94,1113
TRIM5_7_4171,0,1007,452,0,0,0,0,687,2,0,7,0
TRIM7_7_4172,136,148,353,240,246,725,64,530,95,673,540,964
TRIM9_7_4173,47,12,79,875,1,0,415,0,455,13,4,300
TRPC4AP_7_4174,120,84,38,93,0,44,57,904,2,69,20,187
TSPAN17_7_4175,0,0,0,0,0,172,0,7,0,0,0,983
TTC3_7_4176,0,982,0,58,0,1,0,54,451,7,0,59
TULP4_7_4177,306,856,473,977,728,321,1280,107,1565,1538,1938,1363
UBA1_7_4178,0,0,0,4,4,0,0,0,0,73,4,0
UBA3_7_4179,852,1241,1947,1364,1225,1856,530,780,2451,2150,710,548
UBA5_7_4180,2041,1597,2512,484,2037,89,1255,464,1874,1539,2555,1574
UBAC2_7_4181,759,0,86,1146,662,1652,479,16,307,985,393,74
UBAP2L_7_4182,333,0,49,8,170,386,0,0,323,64,227,46
UBASH3A_7_4183,15,225,0,589,136,39,31,2,39,1,1,55
UBE2A_7_4184,1058,272,778,360,401,212,649,758,857,1037,1929,271
UBE2D1_7_4185,1437,1307,1305,2136,1852,1374,3533,1916,2232,1365,1403,2
777
UBE2D2_7_4186,197,326,24,656,183,6,170,0,220,502,188,461
UBE2D3_7_4187,0,0,0,0,1,724,0,0,0,63,2475,2
UBE2E1_7_4188,153,0,0,0,28,281,2,663,66,53,0,19
UBE2E3_7_4189,2,439,547,12,268,0,680,843,0,2,200,0

UBE2G2_7_4190,0,112,1,0,8,391,0,0,2,1035,947,6
UBE2H_7_4191,150,1164,462,749,0,54,804,12,844,103,0,2010
UBE2I_7_4192,11,461,0,1,0,0,1509,158,0,166,20,152
UBE2J2_7_4193,64,16,386,126,142,5,99,51,530,35,67,804
UBE2K_7_4194,566,1375,1238,705,218,416,690,317,790,186,264,534
UBE2Q2_7_4195,297,502,1313,92,30,69,534,791,25,981,1092,28
UBE2V1_7_4196,391,271,4,0,0,12,577,18,209,36,20,33
UBE2W_7_4197,0,2,64,584,445,74,600,1219,387,1360,320,365
UBE3A_7_4198,2056,1264,554,3798,658,1426,3806,1106,3595,2459,505,1744
UBE3B_7_4199,123,0,0,0,0,58,7,60,2,435,21,91
UBE4A_7_4200,217,0,1928,1253,33,106,465,340,719,875,672,1843
UBE4B_7_4201,924,277,586,1153,744,5,0,549,446,210,1289,2726
UBL7_7_4202,1435,337,139,4,269,188,468,43,388,252,1529,49
UBOX5_7_4203,4,0,0,41,0,0,4,0,0,0,0,0
UBQLN1_7_4204,233,5,1,0,60,2,89,5,91,194,1,116
UBR2_7_4205,77,160,34,59,9,267,16,198,220,4,217,43
UBXN11_7_4206,1226,1173,872,276,43,291,671,386,529,96,96,2017
UBXN6_7_4207,53,2,6,0,0,0,0,0,0,0,0,0
UCHL5_7_4208,1080,911,1125,2479,1107,399,1144,2958,1388,935,4506,893
UHRF1_7_4209,0,0,0,0,0,0,0,0,0,0,133,8
UIMC1_7_4210,0,251,0,245,0,534,0,35,0,126,0,282
UNKL_7_4211,0,180,0,0,0,0,0,0,0,220,0,0
USP14_7_4212,364,65,481,57,170,249,428,118,207,229,66,31
USP19_7_4213,12,0,0,294,250,1,339,6,8,116,301,1
USP1_7_4214,2025,523,441,996,338,883,2082,1859,203,803,981,1722
USP20_7_4215,0,1,2,2,0,0,5,2,6,0,0,3
USP2_7_4216,719,55,768,2,93,16,637,0,0,2,21,1492
USP33_7_4217,1399,471,1116,649,1283,700,2347,567,1839,1143,953,792
USP44_7_4218,286,70,35,1,648,84,1188,74,1119,369,926,137
USP46_7_4219,631,19,22,476,1274,410,1322,776,427,1856,132,839
USP48_7_4220,614,1045,113,768,250,1158,497,679,230,1281,2044,115
USP4_7_4221,21,824,0,23,245,190,0,443,1,69,1496,76
USP5_7_4222,648,0,0,0,0,0,1037,0,0,6,0,1
USP6NL_7_4223,475,39,73,151,1584,107,265,486,331,29,1288,818
USP8_7_4224,331,612,481,1447,188,475,996,1364,958,997,1003,956
USP9X_7_4225,1109,544,979,343,41,1182,106,1396,1155,847,51,184
VHL_7_4226,363,630,347,172,911,213,488,604,239,64,935,320
VPRBP_7_4227,0,1,297,1,119,0,0,55,27,6,667,818
VPS13D_7_4228,0,191,0,0,42,311,89,59,213,992,0,27
VPS41_7_4229,997,0,4,572,741,357,2,656,1308,870,12,1461
VPS8_7_4230,2210,1195,644,175,2313,265,450,131,2433,974,307,2077
WDR26_7_4231,3354,2597,3090,1562,1079,2821,3581,1780,1751,2295,992,202
0
WDR5_7_4232,0,116,167,0,0,576,0,0,161,2,0,14
WDR76_7_4233,0,1036,23,183,0,175,290,0,0,0,0,0
WHSC1_7_4234,0,120,153,39,0,173,134,0,19,702,9,86
WHSC1L1_7_4235,531,229,0,134,3,434,310,7,25,102,47,4
WSB1_7_4236,1839,1116,987,891,1180,270,1286,391,760,287,1532,1817
WWP2_7_4237,1,4,1,0,0,0,5,199,0,0,0,0
XIAP_7_4238,203,113,1482,215,118,337,341,960,179,528,60,701

ZBTB10_7_4239,429,1491,1183,381,1766,1331,1400,1485,1040,1420,1995,119
0
ZBTB16_7_4240,2,210,1047,80,0,0,0,0,891,38,257,144
ZBTB17_7_4241,0,0,2060,94,12,333,0,59,1,470,0,0
ZBTB1_7_4242,1,1,1017,671,147,635,463,299,0,0,12,0
ZBTB20_7_4243,233,562,445,1278,1427,3,2446,756,30,145,515,108
ZBTB22_7_4244,22,0,0,78,62,46,29,6,3,0,0,48
ZBTB24_7_4245,791,575,1448,813,1351,870,2049,1020,540,695,39,744
ZBTB33_7_4246,594,1261,69,412,1084,272,108,602,416,327,351,804
ZBTB37_7_4247,868,0,0,835,1317,1,1,777,37,470,711,847
ZBTB40_7_4248,352,734,20,370,221,302,20,542,85,518,290,532
ZBTB4_7_4249,2005,1164,534,434,291,1102,861,494,802,2006,1262,461
ZFP161_7_4250,8,426,4,11,100,0,0,0,13,17,0,1
ZMYND11_7_4251,769,1716,382,1521,1977,234,852,2547,846,849,2628,1466
ZMYND8_7_4252,3,26,288,238,1367,209,425,0,5,3,7,0
ZNF238_7_4253,17,367,4,6,0,7,0,4,383,132,0,440
ZNF295_7_4254,2224,802,599,1400,1222,1311,1746,1098,2086,165,1506,1664
ZNRF3_7_4255,21,0,424,1,38,47,5,13,193,75,18,22
ANAPC11_7_4256,600,843,509,1255,6,759,97,1058,625,221,2018,797
ATXN3_7_4257,20,20,818,48,45,220,1790,292,180,219,56,247
MLLT10_7_4258,591,20,0,46,0,80,5,18,180,79,227,66
OTUD4_7_4259,0,1,2,6,281,4,87,61,88,36,53,195
PHF19_7_4260,0,0,0,0,105,0,24,1,456,40,0,49
SP140_7_4261,431,0,1,48,6,0,843,0,127,226,1276,12
TRIM36_7_4262,313,0,0,0,900,1,0,1,402,701,387,52
UBE2C_7_4263,0,29,46,0,0,131,149,260,50,73,336,41
UBE2L6_7_4264,78,590,420,38,744,54,1014,22,952,80,210,355
ABTB2_7_4265,0,28,0,0,33,0,54,59,229,78,737,29
AMBRA1_7_4266,0,0,8,4,0,4,21,19,0,371,0,16
AMFR_7_4267,639,589,0,649,244,42,0,506,371,8,0,46
ANAPC10_7_4268,554,375,0,0,0,0,572,0,554,11,0,90
ANAPC1_7_4269,93,3,1257,553,15,691,0,747,360,1081,93,1935
ANAPC2_7_4270,53,2,37,11,8,0,197,80,176,7,2,250
ANAPC4_7_4271,209,1250,1569,911,283,167,387,910,764,482,1566,1222
ANKIB1_7_4272,61,380,1864,1013,2194,551,527,187,221,1314,1415,1396
ANKRD13A_7_4273,25,7,1,534,0,0,100,199,43,2,1,6
ANKRD13D_7_4274,17,49,0,997,757,16,1,0,1273,64,0,213
ARIH1_7_4275,1079,232,2510,1416,1219,922,3143,2208,2978,1235,2018,2603
ARIH2_7_4276,575,1017,391,1508,2503,1457,515,1750,687,656,293,649
ASB12_7_4277,218,561,1819,1506,246,1108,267,144,422,1016,672,77
ASB13_7_4278,205,0,575,0,1117,100,0,0,120,1,77,12
ASB15_7_4279,1494,2007,3630,1469,1598,1404,292,1941,1184,2195,1832,202
0
ASB16_7_4280,1,0,0,7,18,0,0,0,2,0,0,1
ASB17_7_4281,347,2381,355,716,0,415,1240,232,425,516,592,657
ASB5_7_4282,1260,257,150,455,797,328,24,1042,640,809,739,206
ASB8_7_4283,352,890,1610,2744,1224,187,358,12,315,1756,1326,2189
ASH1L_7_4284,248,182,0,0,586,288,326,54,845,109,1,141
ASPSCR1_7_4285,1297,1947,2204,949,1923,2997,541,1825,788,334,2231,1044
ATG12_7_4286,0,0,0,0,0,0,0,0,42,9,794,5

ATG3_7_4287,777,164,12,290,753,635,734,582,26,215,1491,149
ATG5_7_4288,892,241,255,551,304,700,1085,207,1051,698,126,1737
ATXN1L_7_4289,104,265,20,111,921,293,186,70,129,473,1146,349
ATXN3L_7_4290,141,164,1291,460,691,844,227,63,278,9,242,1252
BARD1_7_4291,101,0,0,99,0,522,251,55,13,3,0,105
BAZ1B_7_4292,685,98,2156,328,328,765,1288,1562,935,1157,889,488
BAZ2A_7_4293,691,1183,442,283,1808,431,1222,238,237,817,862,476
BAZ2B_7_4294,594,462,19,534,936,498,1194,1210,976,204,2088,1314
BECN1_7_4295,271,1,9,25,334,474,33,218,228,276,59,507
BIRC2_7_4296,646,496,143,736,754,98,688,3001,423,440,2207,3093
BIRC6_7_4297,1588,701,475,278,504,797,1214,529,1540,1874,611,956
BIRC8_7_4298,202,186,670,379,192,256,182,1265,1433,329,972,1027
BMI1_7_4299,661,1,0,110,6,0,0,209,38,111,29,2
BRAP_7_4300,252,69,7,44,19,47,305,423,391,834,313,277
BRD1_7_4301,0,431,0,0,56,0,0,6,460,518,0,41
BRPF3_7_4302,0,300,40,0,0,0,0,16,0,0,0,55
BTBD2_7_4303,0,10,507,0,26,569,266,198,7,0,6,16
BTBD6_7_4304,1,133,0,252,0,10,1152,15,32,127,0,575
CAND1_7_4305,1313,1955,975,1020,2342,787,956,1740,881,594,1509,117
CBLB_7_4306,713,711,442,132,728,1020,332,253,836,1616,1146,1100
CBL_7_4307,61,422,911,312,704,1455,327,482,207,528,516,1086
CBLL1_7_4308,1054,142,977,685,152,4,250,59,153,1326,84,243
CCIN_7_4309,281,353,180,385,0,257,197,167,267,157,428,91
CCNF_7_4310,1069,788,2252,1237,754,2593,1414,681,593,1194,1621,613
CDC20_7_4311,328,0,0,0,7,0,0,64,159,72,112,87
CDC23_7_4312,629,131,1,14,984,54,1,81,5,27,5,55
CDC26_7_4313,447,318,958,716,1129,15,688,23,199,459,2674,326
CDC34_7_4314,163,2,368,1,13,0,148,3,0,0,25,329
CGRRF1_7_4315,0,202,2,84,0,0,51,26,12,3,10,138
CHD4_7_4316,4,47,237,6,10,6,117,26,184,309,11,15
CIAO1_7_4317,1593,43,26,1208,745,220,505,177,36,470,249,327
COPS4_7_4318,520,0,17,0,0,2,1,580,0,0,92,14
COPS5_7_4319,996,7,3976,217,844,1021,3455,626,831,2174,950,1379
COPS6_7_4320,164,137,185,8,0,0,344,0,551,236,211,291
COPS7B_7_4321,0,4,0,0,0,118,242,0,22,214,503,306
CORO6_7_4322,224,72,20,2,0,900,0,0,0,44,16,127
CUEDC1_7_4323,1470,10,743,1003,26,354,1461,319,131,951,14,11
CUL1_7_4324,650,355,128,551,253,0,559,7,4,616,0,5
CUL3_7_4325,95,273,0,110,0,36,1046,471,174,8,1212,22
CUL5_7_4326,1664,1603,1084,2456,2256,849,1227,230,530,827,1,2354
CUL9_7_4327,0,8,0,0,6,13,156,481,136,192,0,8
DCAF10_7_4328,1061,317,85,1163,2649,1214,667,1028,1851,1497,93,410
DCAF12_7_4329,616,31,0,31,147,180,1155,233,347,111,21,46
DCAF13_7_4330,616,1,17,277,137,1279,72,80,613,66,1002,343
DCAF16_7_4331,1632,1204,480,287,1687,1037,1115,1045,626,406,1241,1251
DCAF5_7_4332,58,24,128,0,5,21,30,0,40,215,42,653
DCAF7_7_4333,0,0,908,0,0,0,0,0,428,9,0,41
DCAF8_7_4334,92,398,38,721,32,163,209,899,881,481,715,354
DDA1_7_4335,277,645,295,220,1836,341,74,1200,285,562,24,1242
DDB1_7_4336,89,551,364,0,0,30,75,0,45,27,1364,62

DDB2_7_4337,9,2,7,8,0,22,26,0,19,123,0,27
DPF2_7_4338,20,160,14,155,138,661,1137,273,59,74,325,215
DTX1_7_4339,8,9,127,134,265,27,90,163,562,84,57,135
DTX3L_7_4340,797,132,288,369,45,36,1084,1570,367,291,1609,309
DTX4_7_4341,0,0,0,10,0,2,0,11,0,5,0,0
DZIP3_7_4342,518,183,52,2,0,181,659,0,162,136,5,14
EIF3D_7_4343,1760,67,586,1484,1838,337,917,642,1008,1691,174,1260
EIF3E_7_4344,96,20,60,51,173,559,134,540,311,107,28,108
EIF3F_7_4345,766,458,455,126,237,527,713,559,228,253,133,110
EIF3G_7_4346,1073,347,772,356,376,268,325,508,781,857,446,902
EIF3H_7_4347,0,510,108,7,10,550,537,15,26,0,131,343
EIF3I_7_4348,0,259,0,521,126,106,1,1,435,31,0,749
EIF3J_7_4349,985,833,0,1550,2422,2269,2175,2231,2202,792,595,5145
EIF3K_7_4350,551,12,162,346,939,11,237,53,10,103,198,238
ENC1_7_4351,1653,44,21,12,591,1143,3,0,1388,46,915,167
EPN3_7_4352,273,47,109,186,338,289,0,818,188,79,258,44
EPOR_7_4353,99,2,59,0,9,881,172,118,492,138,522,49
ERCC8_7_4354,0,72,167,0,0,0,0,5,165,99,600,26
FAF1_7_4355,902,3606,2193,1174,3862,1815,1043,1965,2708,1527,3119,1073
FBXL12_7_4356,27,0,560,54,0,0,1040,0,1,0,0,908
FBXL14_7_4357,7,620,117,6,429,254,732,1582,466,693,814,1988
FBXL15_7_4358,0,5,0,124,0,0,0,1,0,0,0,0
FBXL16_7_4359,263,802,0,128,122,500,64,754,118,58,531,127
FBXL17_7_4360,196,463,0,277,12,83,0,0,0,655,0,8
FBXL18_7_4361,186,385,1542,549,1284,1096,1879,478,346,333,362,1794
FBXL19_7_4362,18,0,7,588,80,1,0,108,418,308,1,288
FBXL21_7_4363,85,34,0,5,2,2,9,0,332,364,170,37
FBXL3_7_4364,2069,1346,1358,1951,576,838,461,2665,368,1398,1592,1092
FBXL4_7_4365,1,14,0,10,0,686,3,0,43,1,275,231
FBXL7_7_4366,0,0,51,10,3,134,5,103,4,249,82,302
FBXO10_7_4367,152,77,4,493,0,36,11,357,86,70,196,14
FBXO16_7_4368,3379,2580,5971,6091,3826,1615,2283,2291,2475,3240,2160,3
401
FBXO27_7_4369,194,293,0,489,111,95,722,0,16,661,822,161
FBXO2_7_4370,0,0,117,0,156,0,0,0,143,304,25,23
FBXO30_7_4371,111,157,124,375,450,500,0,626,455,438,103,74
FBXO33_7_4372,4,3,21,297,58,674,1,0,31,0,0,4
FBXO36_7_4373,80,477,13,33,529,676,953,437,312,17,0,29
FBXO39_7_4374,24,689,307,323,649,311,72,15,1,125,5,681
FBXO40_7_4375,82,171,1,82,23,11,54,17,79,76,71,200
FBXO41_7_4376,1,0,0,3,0,87,1,0,0,0,0,0
FBXO42_7_4377,2,0,1,212,0,55,89,14,663,134,388,491
FBXO43_7_4378,698,127,629,0,235,605,0,0,456,8,128,1146
FBXO46_7_4379,0,0,0,0,0,0,0,190,374,7,0,42
FBXO6_7_4380,93,24,0,220,0,147,0,40,17,46,0,1
FBXO8_7_4381,1,162,65,181,145,926,0,0,97,57,417,75
FBXW10_7_4382,200,720,123,20,154,40,0,12,464,229,0,458
FBXW2_7_4383,449,610,265,1316,0,1398,39,930,258,249,1712,45
FBXW5_7_4384,176,54,0,4,244,0,48,2,139,7,125,837
FBXW9_7_4385,0,5,0,0,0,0,4,0,1,0,42,11

G2E3_7_4386,649,1391,0,345,0,512,36,52,0,0,182,227
GAN_7_4387,427,451,310,417,92,1100,446,1505,1079,1275,1831,1407
GMCL1_7_4388,53,11,10,0,0,41,0,246,43,84,0,3
GNB2_7_4389,162,22,659,121,16,142,577,369,135,316,1201,794
GRWD1_7_4390,92,0,202,9,1,42,5,1,17,11,0,15
GZF1_7_4391,833,839,867,1862,669,205,917,694,751,544,1246,1468
HACE1_7_4392,19,0,0,0,0,0,0,0,9,45,0,2
HDAC6_7_4393,0,0,0,0,0,0,8,0,0,0,0,0
HECTD1_7_4394,7,622,515,14,1120,91,4,243,0,326,972,1405
HECTD3_7_4395,259,1570,1143,1917,1161,1650,962,381,741,2050,1706,2759
HECW1_7_4396,1,320,1404,92,256,288,311,1014,63,334,882,30
HECW2_7_4397,324,182,166,161,29,603,616,0,632,155,439,623
HERC1_7_4398,114,1,231,424,28,9,225,797,41,279,0,11
HERC2_7_4399,1,3,8,101,9,469,0,120,27,142,198,27
HERC3_7_4400,0,0,0,318,12,0,14,0,81,1,22,4
HERC5_7_4401,510,696,640,379,1507,39,31,524,727,203,266,760
HGS_7_4402,13,1257,89,139,0,885,124,1299,61,252,7,1008
HIC2_7_4403,28,1,42,74,0,15,381,149,109,169,78,265
HUWE1_7_4404,2905,1764,904,1517,3076,1256,2269,2049,2806,1942,3599,155
2
IBTK_7_4405,727,106,1028,124,7,1365,2916,493,577,807,481,1781
IL10RA_7_4406,141,542,276,351,3,735,1532,0,727,698,1454,91
IL6_7_4407,66,67,194,213,835,154,262,630,237,95,183,59
IRF9_7_4408,698,94,260,714,1120,406,66,37,890,477,44,1916
ITCH_7_4409,993,143,770,244,1308,759,74,1514,35,691,1254,1357
IVNS1ABP_7_4410,0,224,916,170,6,47,156,0,365,7,182,49
JHDM1D_7_4411,489,273,1073,916,379,655,2034,777,454,783,1049,845
JOSD1_7_4412,24,12,21,8,36,411,27,9,723,25,1101,1026
JOSD2_7_4413,0,81,5,0,6,5,123,902,156,385,35,17
KAT6B_7_4414,7,49,0,96,96,1568,2,0,24,12,20,255
KATNB1_7_4415,44,568,5,129,0,783,2,293,7,7,0,2
KBTBD10_7_4416,2598,806,2037,217,894,287,2671,1184,658,887,263,197
KBTBD11_7_4417,0,0,63,78,0,0,0,0,0,226,0,0
KBTBD2_7_4418,99,0,1,19,336,94,5,148,27,0,36,5
KBTBD5_7_4419,72,185,21,103,0,0,236,91,64,539,37,210
KBTBD7_7_4420,484,0,264,0,0,0,0,0,0,0,53,0
KBTBD8_7_4421,583,695,428,514,1,155,456,495,712,553,1943,713
KCTD10_7_4422,59,223,14,107,91,25,0,138,40,236,0,73
KCTD11_7_4423,646,1,806,0,302,12,63,321,1,539,2,144
KCTD12_7_4424,0,308,5,0,368,1,43,58,80,89,1,9
KCTD13_7_4425,286,328,1267,1045,1279,287,462,1794,423,615,1982,1561
KCTD16_7_4426,88,200,0,157,8,1,39,0,485,19,54,56
KCTD17_7_4427,1,317,242,0,1,0,557,0,349,224,7,277
KCTD18_7_4428,288,217,440,630,579,181,1555,1492,1172,1143,2178,3223
KCTD3_7_4429,0,420,0,168,18,59,141,129,0,164,470,0
KCTD5_7_4430,571,7,30,15,340,324,366,231,96,33,192,273
KCTD9_7_4431,674,6,150,608,478,384,17,14,136,54,435,15
KDM2A_7_4432,0,283,0,0,1,0,201,293,283,8,0,31
KDM4B_7_4433,1019,0,212,0,0,42,5,112,508,850,0,104
KDM5B_7_4434,1,0,0,1,436,0,301,18,114,512,18,461

KLHDC5_7_4435,1,0,0,577,364,0,0,3,184,38,582,15
KLHL10_7_4436,842,638,635,170,146,737,21,604,548,88,199,441
KLHL11_7_4437,1453,526,1184,157,1914,78,1221,1488,1909,933,1384,472
KLHL12_7_4438,46,0,0,0,0,0,0,0,0,5,34,0
KLHL14_7_4439,0,0,7,468,0,62,19,2,146,3,0,34
KLHL15_7_4440,338,125,4,108,564,1132,1407,98,109,114,222,1228
KLHL17_7_4441,1,16,0,0,0,154,1455,1,0,45,0,0
KLHL18_7_4442,94,462,0,0,11,449,600,378,68,155,9,363
KLHL1_7_4443,42,58,2,39,994,48,9,703,125,440,285,37
KLHL20_7_4444,0,254,8,10,2799,119,214,939,701,412,2,695
KLHL21_7_4445,0,365,0,1116,0,20,0,0,46,0,0,13
KLHL22_7_4446,229,350,4,6,219,1042,125,215,297,1288,38,90
KLHL23_7_4447,520,149,58,0,1175,315,8,533,832,212,654,109
KLHL24_7_4448,3,71,0,377,170,4,285,0,149,88,12,136
KLHL25_7_4449,736,29,365,83,198,41,33,186,260,114,613,212
KLHL26_7_4450,3,0,0,0,0,0,0,0,0,2,41,0
KLHL28_7_4451,751,116,3,599,1117,30,1300,211,131,515,581,404
KLHL29_7_4452,1,0,0,81,2,32,582,145,609,402,879,59
KLHL31_7_4453,674,62,0,12,0,2,5,3,309,420,0,43
KLHL32_7_4454,0,0,3,0,0,0,1,92,247,638,0,70
KLHL33_7_4455,641,199,0,0,0,0,246,0,922,54,1110,711
KLHL34_7_4456,110,5,189,74,125,252,415,132,0,186,1,472
KLHL36_7_4457,240,7,760,0,175,0,30,1,15,16,135,11
KLHL3_7_4458,89,1590,1626,209,17,335,994,905,515,603,1199,397
KLHL8_7_4459,27,4,68,136,322,1,456,61,43,110,67,502
LATS1_7_4460,1290,640,571,173,1152,285,585,1249,1497,1801,288,223
LATS2_7_4461,284,16,141,338,1224,336,81,0,222,778,586,1335
LIF_7_4462,15,434,0,5,0,622,0,0,126,4,825,19
LNX2_7_4463,874,176,78,547,1,344,205,590,285,422,145,77
LOC283116_7_4464,66,306,174,0,435,70,282,0,8,0,178,0
LONRF1_7_4465,313,523,38,228,0,55,30,82,747,652,663,681
LTN1_7_4466,126,88,378,146,2056,998,1448,106,422,666,581,63
LZTR1_7_4467,545,16,100,933,92,763,1186,268,946,326,772,622
MAP1LC3B_7_4468,1208,344,26,281,1077,41,892,0,695,47,0,635
MAP3K1_7_4469,334,242,341,501,320,284,884,314,226,78,36,608
MARK1_7_4470,21,0,2,0,113,299,132,0,559,49,484,57
MDM2_7_4471,745,2048,1969,1305,1455,989,830,1057,1253,1140,1854,2490
MED20_7_4472,1179,955,656,933,227,1016,1012,523,1362,2152,1749,898
MEX3B_7_4473,0,536,0,1,0,1,0,0,0,1,0,2
MIB1_7_4474,197,409,1091,1222,353,128,66,232,1708,526,1258,2031
MKRN2_7_4475,642,21,2157,23,554,156,24,977,187,2096,890,981
MKRN3_7_4476,715,475,2043,176,322,450,269,652,344,912,569,1517
MLL2_7_4477,0,113,99,0,0,10,610,281,0,1,15,240
MLLT6_7_4478,679,38,1017,227,0,96,126,90,302,90,90,1348
MOCS3_7_4479,0,0,0,0,37,2,3,13,2,0,0,0
MRPL49_7_4480,0,170,0,329,334,118,0,90,15,271,0,92
MUL1_7_4481,538,2,0,15,3,33,0,13,42,119,122,20
MYCBP2_7_4482,142,270,430,0,0,1051,0,0,417,142,1290,458
MYLIP_7_4483,1271,948,510,626,1302,1100,244,1091,1093,1227,614,1457
MYSM1_7_4484,799,2256,1504,986,3651,758,1141,607,577,1360,2850,113

NACC1_7_4485,292,5,47,0,383,64,0,17,4,501,132,67
NACC2_7_4486,0,0,0,0,0,53,0,0,0,0,0,68
NEURL1B_7_4487,176,29,3,7,0,22,59,0,6,2,13,3
NEURL_7_4488,10,11,1,0,0,0,2,0,318,6,0,35
NHLRC1_7_4489,637,1024,148,1373,929,1714,145,122,497,1853,1047,1841
NUP43_7_4490,0,43,8,303,2,23,1,28,0,5,0,34
OTUB1_7_4491,84,316,254,6,0,316,0,7,166,503,16,269
OTUB2_7_4492,0,4,0,8,11,112,0,29,39,0,1,33
OTUD1_7_4493,169,227,134,15,29,156,0,493,1489,592,59,168
OTUD6A_7_4494,0,63,112,580,104,8,53,104,32,141,158,405
OTUD6B_7_4495,333,62,497,118,7,124,0,148,1277,880,85,228
OTUD7A_7_4496,0,0,0,2,0,0,146,0,14,1,0,286
OTUD7B_7_4497,3865,5020,3309,3705,3365,5073,2724,3749,2292,4004,2923,6
780
PAFAH1B1_7_4498,367,881,1592,505,218,294,158,92,94,613,393,12
PARP10_7_4499,0,0,6,4,0,0,0,66,20,167,3,2
PARP11_7_4500,11,34,330,77,7,978,1817,0,388,36,1016,44
PARP14_7_4501,580,82,11,220,41,346,40,320,100,42,216,473
PCGF1_7_4502,247,22,512,0,240,1094,57,0,291,1103,19,269
PCGF2_7_4503,213,6,16,293,328,24,83,121,1,1,202,221
PCGF3_7_4504,0,18,17,0,1256,0,519,35,286,6,1,147
PDZRN3_7_4505,386,207,10,26,426,0,58,191,196,110,1,40
PEBP4_7_4506,1289,231,36,3,1646,2638,238,297,527,318,406,1289
PEX12_7_4507,1535,3697,1039,493,220,607,416,734,1597,1978,582,888
PHF14_7_4508,127,5,171,82,0,0,0,17,87,19,0,13
PHF15_7_4509,1864,1556,431,378,575,865,595,1108,877,410,1378,502
PHF20_7_4510,0,0,1,128,228,0,0,235,12,236,589,1548
PHF2_7_4511,62,310,1023,73,74,188,145,871,776,659,56,517
PHF3_7_4512,267,292,24,1511,278,0,9,1,2,1,0,298
PHIP_7_4513,847,902,1642,1040,261,527,1089,831,1358,700,3934,1051
PHRF1_7_4514,0,0,0,0,0,0,0,0,0,0,0,0
PJA2_7_4515,511,272,349,1537,878,1730,4529,1878,1651,899,766,688
PRPF19_7_4516,2,176,8,18,379,254,13,165,190,469,18,546
PRPF8_7_4517,375,15,2,0,12,4,0,2864,9,27,141,0
PSMD14_7_4518,5,0,172,135,122,55,194,0,245,443,1,249
PSMD2_7_4519,2031,1260,2057,366,2137,1568,442,1152,713,998,928,98
PSMD4_7_4520,354,1584,442,316,844,389,22,545,795,186,29,109
PSMD7_7_4521,68,581,412,165,81,1077,977,158,159,325,6,129
PWP1_7_4522,0,4,4,202,397,361,52,15,345,50,147,596
RAB40A_7_4523,0,0,0,0,0,0,0,14,0,0,0,0
RAB40AL_7_4524,0,0,0,0,0,0,0,14,0,0,0,0
RAB40B_7_4525,4,0,0,0,0,0,0,692,2,0,631,0
RAD18_7_4526,135,516,256,0,53,545,672,5,727,151,23,143
RAD23A_7_4527,176,0,13,35,1,68,0,402,92,3,0,125
RAD23B_7_4528,60,38,70,130,63,714,38,0,196,36,6,40
RAG1_7_4529,473,860,2858,899,382,1210,1365,233,1498,503,1668,561
RAI1_7_4530,108,389,0,0,0,8,51,1,39,226,1,4
RBX1_7_4531,533,10,1,267,0,0,992,0,0,123,871,1900
RCBTB1_7_4532,641,197,15,205,948,634,81,48,87,252,156,706
RCBTB2_7_4533,261,714,469,348,185,3,430,0,35,1325,527,401

RFFL_7_4534,9,37,10,58,414,77,390,206,223,277,3,96
RFPL1_7_4535,184,136,117,668,16,38,2,355,751,307,257,725
RFWD3_7_4536,420,126,0,436,9,733,799,0,178,29,593,21
RHOBTB3_7_4537,242,294,99,365,29,3,460,217,143,277,625,426
RING1_7_4538,0,5,0,0,0,478,0,0,1,8,0,461
RNF10_7_4539,931,1203,1064,797,0,971,1719,141,1148,87,2658,261
RNF111_7_4540,38,593,144,415,195,1522,996,1106,1610,217,77,832
RNF112_7_4541,0,0,3,3,0,1,0,6,0,10,0,0
RNF113A_7_4542,1370,306,1011,354,62,1029,1360,481,968,590,240,137
RNF113B_7_4543,181,715,235,12,1476,142,373,8,22,561,130,734
RNF114_7_4544,271,621,3,136,4,257,1239,903,514,124,22,260
RNF115_7_4545,13,4,1331,0,735,402,131,591,1131,655,128,278
RNF11_7_4546,77,28,196,6,61,517,208,0,0,396,0,395
RNF121_7_4547,152,72,0,441,27,31,607,133,475,1217,317,452
RNF122_7_4548,347,133,1111,238,316,83,55,275,114,592,44,22
RNF123_7_4549,1,3,36,80,0,136,439,91,0,234,258,60
RNF125_7_4550,2,0,0,1101,57,0,78,0,92,63,61,670
RNF126_7_4551,0,4,0,2,0,0,0,0,0,0,1,0
RNF130_7_4552,3,109,0,1,25,64,0,4,95,2,66,126
RNF133_7_4553,48,238,243,296,4,117,51,882,131,155,360,15
RNF139_7_4554,221,498,227,1629,445,1293,73,2564,2771,338,564,2562
RNF141_7_4555,548,635,873,101,502,486,939,882,1954,1685,373,2636
RNF144A_7_4556,393,219,1077,1695,341,644,132,1393,618,911,1458,532
RNF144B_7_4557,0,0,3,12,12,5,44,629,11,191,548,104
RNF149_7_4558,703,11,1786,465,268,63,42,78,403,157,3,117
RNF150_7_4559,421,126,166,12,279,290,899,108,104,253,14,344
RNF152_7_4560,1,0,966,50,691,0,1369,0,215,4,3,35
RNF157_7_4561,0,0,1,547,227,487,0,57,105,129,5,269
RNF167_7_4562,620,265,1331,851,163,1088,58,2277,1610,1677,1503,2733
RNF168_7_4563,217,658,0,124,69,21,65,0,1023,1144,343,216
RNF169_7_4564,0,1124,12,0,436,5,0,3,25,228,148,13
RNF181_7_4565,1,3,122,67,4,73,154,1143,260,8,277,302
RNF183_7_4566,1,52,32,0,46,62,0,0,0,0,0,0
RNF186_7_4567,64,108,0,106,37,197,606,0,90,401,26,954
RNF187_7_4568,20,63,148,7,115,139,281,11,33,147,10,296
RNF20_7_4569,2059,1099,1356,198,2472,1224,130,554,620,201,408,850
RNF217_7_4570,22,1445,268,1392,37,849,1078,327,1427,818,140,1664
RNF220_7_4571,2,86,0,0,0,465,5,0,2,0,104,0
RNF25_7_4572,190,1236,739,728,875,162,172,1,1208,480,15,200
RNF26_7_4573,21,1,1,0,0,21,2,78,0,126,28,0
RNF2_7_4574,566,250,1333,19,1818,597,2,708,175,635,141,2380
RNF31_7_4575,2,43,112,25,35,17,45,51,79,432,0,74
RNF43_7_4576,189,613,6,93,3,8,324,8,35,645,500,3
RNF44_7_4577,8,0,1,0,0,661,0,1,247,1,1150,163
RNF5_7_4578,178,1387,1058,156,825,845,2996,1467,1144,364,8,547
RSC1A1_7_4579,82,269,0,470,0,68,5,147,252,6,72,332
RSF1_7_4580,2,2507,1606,679,1682,500,1256,65,243,1339,711,309
RSPRY1_7_4581,10,782,215,30,1447,746,1674,682,147,1463,538,785
SCLY_7_4582,1044,746,520,1826,37,2883,1855,2145,1071,574,751,1514
SENP1_7_4583,17,34,130,0,0,50,152,114,16,41,23,550

SENP2_7_4584,0,48,26,320,95,589,570,607,314,255,0,132
SENP3_7_4585,0,2,0,500,0,1,0,0,537,644,488,63
SENP5_7_4586,2,82,0,20,0,0,18,0,0,752,0,3
SH3RF1_7_4587,88,4,6,6,17,0,840,0,408,19,5,364
SH3RF2_7_4588,9,76,1019,24,87,275,417,59,402,72,250,375
SH3RF3_7_4589,1562,960,426,939,3904,1662,701,437,2142,1963,2513,2305
SHKBP1_7_4590,38,499,4,170,0,32,3,550,0,46,36,467
SIAH2_7_4591,2,8,32,80,1,6,0,80,163,35,85,32
SIK1_7_4592,20,16,389,1,60,0,0,0,0,412,1,0
SLX4_7_4593,93,83,0,359,18,137,674,1,44,17,43,13
SMU1_7_4594,5,640,1045,827,1,869,0,111,14,436,700,751
SMURF2_7_4595,129,359,41,0,152,697,104,68,592,580,142,94
SNRNP40_7_4596,0,3,0,192,1480,1049,227,2331,1087,48,18,239
SOCS1_7_4597,0,0,0,0,0,0,0,531,0,0,0,0
SOCS2_7_4598,31,5,2,4,32,108,4,880,89,390,0,12
SOCS3_7_4599,8,652,0,147,10,311,0,5,55,523,618,72
SOCS6_7_4600,779,459,87,2366,0,830,1011,225,983,1104,135,1031
SPOPL_7_4601,21,8,983,459,0,652,0,0,400,595,45,382
SPSB1_7_4602,2577,352,109,65,456,122,1019,331,174,272,331,242
SPSB3_7_4603,0,3,85,0,0,23,0,0,1,0,3,1
SPSB4_7_4604,3,803,8,619,0,814,209,1132,27,4,129,490
STAM2_7_4605,1417,110,412,675,412,1910,1210,60,980,624,471,650
STAMBPL1_7_4606,4,41,194,119,53,240,32,11,611,6,0,133
STAM_7_4607,2163,501,345,648,312,690,666,1229,77,232,477,589
STUB1_7_4608,0,0,19,0,193,306,0,0,0,6,51,0
SUMO3_7_4609,3,538,0,0,1548,2,518,0,0,1,64,0
SYNGAP1_7_4610,42,4,69,112,0,0,0,18,299,499,0,67
TAB2_7_4611,67,105,190,25,216,256,248,0,225,158,36,415
TAB3_7_4612,0,488,65,303,878,618,6,179,422,319,1225,2209
TAF1D_7_4613,1322,2034,1830,461,331,1177,2393,572,481,740,613,1007
TBC1D1_7_4614,10,0,0,1,197,981,907,0,391,143,992,202
TLE1_7_4615,410,0,38,52,0,619,233,0,49,16,264,4
TNFAIP3_7_4616,98,87,41,145,3,291,6,64,3,333,20,13
TOLLIP_7_4617,11,0,0,494,0,85,0,1,0,6,758,0
TRAF7_7_4618,155,457,78,224,215,102,236,834,726,1081,61,623
TRAIP_7_4619,44,0,0,173,4,223,0,59,623,20,43,63
TRIM11_7_4620,0,0,33,0,0,14,0,13,0,0,0,0
TRIM15_7_4621,1330,460,1655,962,1468,1939,1516,1854,990,868,1994,1902
TRIM25_7_4622,368,696,1365,1588,757,531,867,446,1076,1595,170,493
TRIM27_7_4623,24,147,198,31,28,1175,16,512,42,9,0,645
TRIM28_7_4624,443,1094,104,359,1227,323,177,1,340,69,3,100
TRIM31_7_4625,40,0,10,53,127,3,0,119,11,613,3,516
TRIM35_7_4626,0,0,0,6,0,0,101,0,6,0,1,0
TRIM42_7_4627,0,0,0,0,0,0,0,0,0,358,0,0
TRIM46_7_4628,0,146,678,248,101,17,114,77,283,17,0,378
TRIM47_7_4629,5,0,11,265,1,75,143,147,71,8,10,122
TRIM48_7_4630,2,4,0,411,0,528,31,203,13,193,17,358
TRIM52_7_4631,480,466,0,397,866,569,83,556,1297,765,14,1779
TRIM56_7_4632,12,14,107,10,98,2,53,1,514,691,0,1056
TRIM62_7_4633,173,959,47,115,0,178,384,0,367,12,112,976

TRIM63_7_4634,0,0,0,0,0,0,0,0,0,0,0,0
TRIM65_7_4635,0,1,0,0,0,0,0,157,119,4,0,32
TRIM67_7_4636,81,461,10,78,333,121,0,0,277,147,913,82
TRIM68_7_4637,0,1,451,2,219,1,2,0,0,0,54,245
TRIM8_7_4638,79,1211,24,0,2,0,16,0,1091,35,184,256
TRIP12_7_4639,925,205,20,198,179,348,942,446,404,101,436,764
UBA2_7_4640,35,101,42,0,3,0,134,150,208,62,0,236
UBA6_7_4641,778,1088,841,141,492,618,37,153,1158,1050,279,781
UBA7_7_4642,56,322,475,517,0,7,0,16,454,83,137,615
UBAC1_7_4643,974,19,560,482,593,696,36,111,72,3,607,913
UBAP2_7_4644,3318,87,2071,82,0,164,149,131,18,725,1016,294
UBASH3B_7_4645,98,336,306,259,0,0,944,616,5,6,405,0
UBC_7_4646,47,851,18,87,50,849,818,782,7,105,31,145
UBE2B_7_4647,6,0,438,30,0,9,0,0,0,1,0,261
UBE2D4_7_4648,414,1183,2116,1028,857,1403,95,353,323,686,2315,1039
UBE2E2_7_4649,179,1341,1060,199,278,273,430,332,426,1064,605,412
UBE2F_7_4650,1746,673,1515,279,1444,1248,1142,161,1496,881,674,347
UBE2G1_7_4651,138,39,471,0,102,12,0,1542,470,8,0,52
UBE2J1_7_4652,2798,233,2665,440,1276,1662,1063,1123,2155,730,2742,3376
UBE2L3_7_4653,678,1168,783,336,40,367,125,8,240,570,536,1091
UBE2M_7_4654,43,219,394,46,3,111,148,473,119,260,37,125
UBE2N_7_4655,0,0,0,196,0,303,0,416,0,13,0,134
UBE2NL_7_4656,5,28,0,643,226,337,81,11,28,248,40,86
UBE2O_7_4657,376,519,0,68,0,819,1300,1287,106,90,659,36
UBE2Q1_7_4658,232,279,501,0,144,397,860,502,53,9,4,62
UBE2QL1_7_4659,0,0,0,0,6,0,47,32,438,14,68,43
UBE2R2_7_4660,2081,762,3319,2077,7,704,1213,474,228,2386,1391,367
UBE2S_7_4661,274,249,146,301,1182,2,526,217,434,295,14,51
UBE2T_7_4662,323,752,374,14,66,874,1445,887,613,822,1468,1185
UBE2U_7_4663,1,0,0,248,0,0,0,0,0,0,0,0
UBE2V2_7_4664,311,289,81,156,10,653,902,862,573,159,1,155
UBE2Z_7_4665,0,114,123,0,388,184,0,0,47,70,21,0
UBE3C_7_4666,714,311,50,475,0,171,1113,579,156,608,1932,275
UBQLN2_7_4667,17,234,94,55,107,62,342,5,170,205,197,32
UBQLN3_7_4668,166,1,1307,122,1413,233,1,803,551,70,1085,59
UBQLN4_7_4669,0,0,0,29,0,36,1,770,68,2,78,6
UBR1_7_4670,1196,147,414,1247,568,1151,49,1972,835,303,1287,1202
UBR3_7_4671,1300,1395,1933,3083,801,1072,995,2202,2701,2127,2967,2589
UBR4_7_4672,890,1596,3809,1115,823,778,955,998,3316,3572,2873,1611
UBR5_7_4673,176,260,616,541,475,1,69,3225,2012,1610,1063,1668
UBR7_7_4674,92,22,0,251,1695,8,34,105,184,66,6,546
UBXN10_7_4675,151,341,1,676,1961,1552,1535,1241,390,669,107,82
UBXN1_7_4676,1291,474,586,839,516,870,1662,1121,1253,637,778,1169
UBXN2A_7_4677,851,41,1295,1205,740,195,187,907,451,1942,1409,782
UBXN4_7_4678,4,585,92,0,275,3,62,13,521,67,0,78
UBXN7_7_4679,281,267,428,1149,332,724,598,114,379,483,339,365
UBXN8_7_4680,175,7,394,956,1153,83,139,0,159,495,760,638
UCHL1_7_4681,407,0,481,340,0,673,1077,302,118,502,0,820
UCHL3_7_4682,465,28,1117,140,90,15,1049,1,226,13,117,38
UFC1_7_4683,109,206,287,94,1836,184,1188,2,129,368,324,42

UHRF2_7_4684,614,482,1023,351,1000,1393,332,1468,718,1262,463,257
UNK_7_4685,76,16,13,36,12,124,59,214,14,0,52,3
USP11_7_4686,66,235,585,0,118,227,1083,674,200,744,21,530
USP13_7_4687,347,594,407,54,1263,767,814,2054,149,107,46,695
USP15_7_4688,39,95,9,0,120,79,28,96,394,171,199,334
USP17_7_4689,1111,1733,487,1334,2043,1777,1557,1925,2329,720,2129,1275
USP17L2_7_4690,486,452,1654,492,1136,1025,550,389,404,473,306,1373
USP17L5_7_4691,2647,1014,2976,1658,2862,2962,2172,1778,1527,2029,1391,
1595
USP18_7_4692,6,26,371,0,0,180,0,117,525,435,2,62
USP22_7_4693,0,96,0,465,0,0,302,807,0,616,209,1
USP24_7_4694,1885,32,503,224,1605,1231,0,1,819,780,622,2458
USP25_7_4695,723,515,21,65,472,645,164,486,499,929,375,397
USP26_7_4696,345,435,0,5,875,720,0,98,0,64,480,0
USP27X_7_4697,39,19,40,0,11,7,787,0,0,0,0,0
USP28_7_4698,1445,663,1746,244,481,552,964,1760,90,702,72,1285
USP29_7_4699,1108,181,9,1146,0,597,252,654,599,224,63,1027
USP30_7_4700,14,0,90,0,0,0,105,0,87,7,1,29
USP32_7_4701,32,0,142,1000,218,1030,499,57,396,219,9,1282
USP34_7_4702,374,996,23,209,742,933,89,1614,1616,654,1571,346
USP35_7_4703,2,0,4,2,0,16,0,1129,6,0,27,2
USP36_7_4704,0,0,2,0,18,0,0,0,65,0,0,7
USP37_7_4705,790,1609,1825,1109,2122,1226,2313,615,1178,818,2296,2525
USP38_7_4706,164,0,142,118,0,142,1128,40,370,612,97,563
USP39_7_4707,52,603,2110,647,8,549,574,1636,184,703,748,452
USP3_7_4708,180,50,26,253,0,0,1839,1092,604,596,1381,78
USP40_7_4709,0,7,0,0,11,84,32,7,0,552,0,0
USP42_7_4710,336,1521,4001,42,1179,2070,825,1849,2857,1469,5399,1667
USP43_7_4711,5,0,63,5,0,0,0,4,0,0,1,0
USP45_7_4712,439,307,161,510,598,1467,263,1486,240,657,661,1087
USP47_7_4713,0,508,4,0,746,38,20,726,3,1,368,0
USP49_7_4714,366,457,1981,235,1477,451,1273,16,10,30,25,120
USP50_7_4715,566,97,1336,496,280,1552,515,38,626,913,854,350
USP51_7_4716,754,111,0,483,642,1328,1317,214,1007,105,1155,135
USP53_7_4717,806,867,849,1117,351,1292,3939,2579,838,1436,1764,1577
USP54_7_4718,1103,1098,258,368,1558,19,568,12,316,456,1077,845
USP6_7_4719,172,589,1168,76,1616,7,1587,1380,361,776,1372,616
USP7_7_4720,1527,759,132,316,324,130,273,1297,820,1176,341,1657
USP9Y_7_4721,905,2214,278,1395,2055,2826,1336,786,473,858,1213,229
USPL1_7_4722,1505,2675,1481,2226,495,2130,2070,552,509,1038,1359,1116
VCPIP1_7_4723,240,57,6,2,211,27,1092,658,16,0,0,30
VPS11_7_4724,363,271,0,395,46,14,545,0,128,1424,783,330
WDR12_7_4725,78,0,1,0,1064,0,0,0,0,44,0,5
WDR53_7_4726,175,21,174,18,240,77,68,50,396,144,48,67
WDR59_7_4727,3,4,100,0,79,0,112,362,64,221,0,46
WDR5B_7_4728,352,1310,74,320,194,53,249,128,497,118,338,73
WDR61_7_4729,1132,395,1149,678,139,1554,24,1384,640,535,1104,1959
WDTC1_7_4730,36,0,525,1827,297,36,1,21,155,414,0,165
WSB2_7_4731,40,0,281,7,66,189,432,0,14,0,32,118
WWP1_7_4732,405,310,68,133,258,604,804,371,1,583,448,608

YOD1_7_4733,0,0,0,49,94,0,160,0,0,0,0,0
ZBTB11_7_4734,0,2,9,45,208,304,0,1419,192,24,46,844
ZBTB25_7_4735,13,361,168,12,1462,0,114,127,0,10,1223,14
ZBTB2_7_4736,811,632,1760,0,894,57,987,759,329,801,24,355
ZBTB32_7_4737,66,225,271,7,342,46,126,57,391,53,1,88
ZBTB34_7_4738,103,5,0,78,175,9,457,160,0,803,338,609
ZBTB39_7_4739,275,424,0,143,318,21,664,696,117,541,1139,15
ZBTB3_7_4740,304,826,299,217,173,736,1,277,0,10,492,635
ZBTB41_7_4741,265,157,0,324,243,150,460,166,138,519,1450,781
ZBTB44_7_4742,537,151,1308,958,102,120,117,963,1475,825,975,1059
ZBTB45_7_4743,46,83,270,32,0,28,2,56,233,82,1023,27
ZBTB46_7_4744,183,0,40,0,0,0,0,2,0,194,46,0
ZBTB47_7_4745,247,161,842,69,33,483,37,468,419,104,548,48
ZBTB48_7_4746,159,602,26,142,396,1341,540,707,410,390,912,628
ZBTB49_7_4747,54,36,0,147,0,13,58,324,178,185,435,45
ZBTB5_7_4748,218,502,21,292,433,0,0,407,83,467,1358,1010
ZBTB7A_7_4749,158,12,9,19,204,637,1751,13,51,754,47,615
ZBTB7B_7_4750,53,0,0,0,19,0,508,8,496,15,3,68
ZBTB7C_7_4751,243,483,1128,358,1,3,193,1,542,93,581,73
ZBTB8A_7_4752,4657,3668,3751,2308,1157,3553,6057,2018,2113,5642,2834,2
842
ZFAND2B_7_4753,3,0,2,73,1,0,0,649,7,7,18,106
ZFPL1_7_4754,0,85,7,5,36,0,61,73,277,293,426,26
ZMYND10_7_4755,45,419,1071,16,384,248,104,28,551,162,131,472
ZNF131_7_4756,1362,713,0,6,648,255,65,547,275,791,304,270
ZNF598_7_4757,29,278,844,116,0,0,159,3,192,6,5,278
ZNF645_7_4758,20,107,52,219,1387,292,59,1169,1323,666,171,423
ZNRF1_7_4759,410,190,110,373,48,376,105,3,491,18,0,65
ZNRF2_7_4760,1512,916,995,463,497,1291,1199,254,451,1385,1970,505
ZNRF4_7_4761,36,0,33,0,0,5,610,390,382,880,140,1117
ZRANB1_7_4762,48,118,85,0,0,461,1,0,6,705,125,0
ZSWIM2_7_4763,0,139,0,68,1,1451,69,605,515,271,30,60
AIRE_7_4764,1,0,0,0,193,0,0,0,0,1,0,3
ANAPC5_7_4765,699,741,0,0,245,235,553,437,999,472,1440,125
ANAPC7_7_4766,136,536,454,3,58,456,256,427,673,949,0,1397
ANKFY1_7_4767,344,26,622,225,9,266,0,913,30,112,491,1481
ASB10_7_4768,538,433,120,31,17,213,169,418,51,923,886,269
ASB11_7_4769,792,896,120,349,174,952,330,1787,602,456,293,265
ASB14_7_4770,648,964,2060,497,275,1268,1374,860,1212,940,1731,751
ASB2_7_4771,11,3,0,4,268,14,0,3,31,787,3,6
ASB3_7_4772,213,135,93,321,138,576,2260,1191,244,981,3002,111
ASB4_7_4773,0,42,1521,65,0,990,445,0,18,1224,856,1198
ASB6_7_4774,198,123,3,407,0,133,0,0,123,320,727,637
ASB7_7_4775,89,0,0,0,0,19,0,0,129,8,653,15
ASB9_7_4776,2,251,28,482,14,10,0,2,626,40,0,77
ASCC2_7_4777,1,1,0,73,3,429,31,40,18,8,573,26
ATG10_7_4778,906,739,830,939,802,1063,678,199,568,2194,129,717
ATG16L1_7_4779,13,281,96,493,528,232,302,31,111,50,955,240
ATG7_7_4780,232,21,449,1724,1743,40,730,200,126,272,0,379
ATRX_7_4781,51,5,769,225,457,0,1045,608,56,11,1,283

BACH2_7_4782,1143,501,1337,1714,1063,1751,1326,384,1118,1080,1290,1717
BAG6_7_4783,47,158,4,67,0,283,3,2,42,179,28,12
BAZ1A_7_4784,18,365,51,1230,29,106,6,75,553,1255,566,217
BCL6_7_4785,0,2,0,0,0,66,0,2,66,10,0,615
BIRC3_7_4786,0,136,23,0,301,1,0,5,378,111,238,123
BIRC7_7_4787,91,0,0,10,0,0,0,0,19,169,0,3
BPTF_7_4788,104,4,0,442,787,633,77,396,15,615,371,964
BRCA1_7_4789,518,1,0,197,276,1384,6,987,446,159,373,304
BRPF1_7_4790,235,1,555,43,259,737,0,65,112,226,539,43
BRWD1_7_4791,18,251,247,26,146,33,776,113,924,1595,1389,1039
BTBD11_7_4792,1786,3659,2963,1607,4722,953,881,1939,1061,1879,2731,832
BTBD1_7_4793,2,549,1263,710,2708,500,230,1943,1585,589,199,1474
BTBD3_7_4794,637,830,86,338,272,1380,1784,52,1076,662,738,568
BTBD7_7_4795,2,532,0,0,0,1,1172,565,79,4,4,667
BTBD9_7_4796,257,40,462,20,498,151,860,108,44,21,61,169
BTRC_7_4797,1539,398,1253,42,211,13,1046,40,162,310,213,109
C3orf26_7_4798,37,20,616,244,489,46,1155,182,22,528,720,196
CBLC_7_4799,70,0,105,72,0,697,423,10,380,150,230,40
CCNB1IP1_7_4800,1282,718,774,942,1633,187,1738,475,61,822,124,140
CDC16_7_4801,2,48,1,103,256,93,30,416,491,287,151,162
CDC27_7_4802,951,0,8,367,10,18,470,2,3,1,626,81
CHFR_7_4803,5,375,458,559,185,64,429,427,338,1362,898,574
CISH_7_4804,0,11,0,1,18,0,0,0,152,24,0,22
CNOT4_7_4805,2192,217,1,69,12,72,707,0,112,905,44,15
COPS2_7_4806,11,68,619,760,55,1062,1013,76,0,391,0,416
COPS3_7_4807,1,1,0,3,0,246,25,721,0,212,1686,4
COPS7A_7_4808,356,376,269,608,749,35,566,32,33,349,865,9
COPS8_7_4809,419,1149,18,250,164,380,10,56,36,164,2,230
CRBN_7_4810,10,417,3,1,526,0,0,1,3,27,331,0
CUL2_7_4811,113,109,202,324,780,18,1304,468,408,466,129,870
CUL4A_7_4812,0,0,0,0,15,26,355,2,299,24,26,20
CUL4B_7_4813,0,1117,423,441,501,212,12,510,155,22,2,20
CUL7_7_4814,7,11,1,73,19,108,1,349,0,0,2,55
CXXC1_7_4815,1644,10,66,0,17,4,864,5,518,386,159,65
CYLD_7_4816,250,53,832,1008,580,0,336,463,264,2,177,145
DCAF11_7_4817,47,1168,133,0,242,1807,1064,149,279,378,748,717
DCAF17_7_4818,1,148,5,201,0,188,73,367,1114,609,0,182
DCAF4_7_4819,0,13,490,512,0,571,0,167,6,547,0,0
DCAF6_7_4820,103,199,480,527,479,1491,227,225,483,14,291,2603
DCST1_7_4821,1459,35,594,0,0,447,241,144,521,895,766,71
DEPDC1B_7_4822,12,378,0,4,0,447,63,23,55,49,1,556
DET1_7_4823,1024,78,1296,1130,302,1013,1486,0,387,3339,2873,794
DIDO1_7_4824,121,426,395,135,42,22,646,2,572,60,1460,724
DNAJB2_7_4825,370,43,0,78,36,43,0,0,42,20,0,90
DTX2_7_4826,9,35,77,9,185,119,570,32,5,155,0,196
EED_7_4827,24,620,2,93,917,371,324,183,2,92,881,989
EIF3B_7_4828,760,16,74,36,245,653,285,0,401,15,229,54
EIF3C_7_4829,308,88,6,1398,716,84,314,8,691,562,732,511
EIF6_7_4830,223,1276,784,46,285,692,713,807,898,779,2330,102
EPN1_7_4831,0,24,0,0,242,0,2,0,0,0,288,229

EPN2_7_4832,0,28,0,44,48,0,1,0,0,5,0,0
EPS15_7_4833,0,0,580,38,0,235,811,257,254,8,1,36
FAM70A_7_4834,1490,1047,122,74,594,990,762,119,1543,73,1459,450
FANCL_7_4835,2520,1912,2490,2966,3104,1525,3666,3783,3755,2369,3135,29
51
FBXL13_7_4836,230,395,551,137,3,26,425,221,417,702,187,107
FBXL20_7_4837,2,0,0,492,171,297,856,843,552,19,2,166
FBXL2_7_4838,769,84,26,28,0,482,0,154,1756,34,0,226
FBXL5_7_4839,1203,19,171,597,1277,138,1403,1545,643,170,924,163
FBXL6_7_4840,316,816,358,405,0,116,770,474,112,62,0,9
FBXO11_7_4841,265,271,3,134,1235,369,1477,1119,28,424,17,141
FBXO15_7_4842,684,2955,1766,1849,771,2630,3482,3046,1999,2467,1432,294
4
FBXO17_7_4843,0,0,0,0,0,0,439,114,3,0,0,0
FBXO18_7_4844,237,397,393,56,1,291,328,381,337,308,674,798
FBXO21_7_4845,3001,3099,3598,3260,1560,1781,2980,2263,3815,3927,1756,2
568
FBXO22_7_4846,20,608,39,708,1504,28,441,1,16,699,192,3
FBXO24_7_4847,175,950,1177,690,2244,1613,1309,4246,2916,72,1,1085
FBXO25_7_4848,27,41,136,571,233,155,25,9,6,1,0,91
FBXO28_7_4849,766,803,401,92,1201,141,477,368,178,138,2274,1116
FBXO32_7_4850,1150,497,1871,303,366,1461,1104,3050,1461,620,1287,525
FBXO34_7_4851,2298,998,2797,899,723,538,450,1026,557,3222,1359,1997
FBXO38_7_4852,94,837,1347,707,235,240,635,39,76,627,443,671
FBXO3_7_4853,591,1759,364,1016,1532,1629,1014,1601,1154,750,1962,243
FBXO44_7_4854,23,0,541,94,992,0,0,62,655,22,46,74
FBXO4_7_4855,2219,572,1217,780,55,2084,2861,1381,1498,2954,1195,1256
FBXO5_7_4856,1393,164,1816,835,552,505,58,1131,297,813,165,882
FBXO7_7_4857,1219,86,610,72,2493,280,159,755,77,662,53,520
FBXO9_7_4858,42,43,307,401,372,65,793,122,222,561,250,749
FBXW11_7_4859,84,28,457,11,90,404,259,160,277,24,262,480
FBXW7_7_4860,77,93,187,45,16,264,541,95,81,388,94,16
FBXW8_7_4861,502,1352,1491,15,259,1029,21,1402,827,614,500,1579
GPS1_7_4862,0,9,62,0,26,55,0,0,201,2,16,23
HECTD2_7_4863,183,9,1,0,192,1159,0,0,0,6,958,798
HERC4_7_4864,10,6,0,32,49,14,0,0,0,0,678,0
HERC6_7_4865,1789,673,1368,1338,757,350,846,3922,2590,707,1526,540
HIC1_7_4866,0,0,0,0,0,0,0,0,234,1,0,29
HLTF_7_4867,0,175,6,3,1,49,65,0,547,49,0,216
HSF4_7_4868,0,561,0,2,43,12,0,7,600,8,1043,64
IPP_7_4869,224,234,612,81,73,378,363,18,569,1428,33,2009
KAT6A_7_4870,2,276,724,156,57,192,0,0,114,801,80,328
KBTBD3_7_4871,1840,1928,1842,1199,3106,2856,2415,1586,3183,1266,2666,1
437
KCTD6_7_4872,708,1737,2322,0,0,339,0,765,474,801,1332,585
KCTD7_7_4873,0,0,0,0,0,0,0,0,0,0,0,0
KDM2B_7_4874,105,3,465,6,37,4,16,1000,1,1,534,22
KDM4C_7_4875,630,53,29,50,741,52,8,0,0,112,0,1
KDM5C_7_4876,0,1,0,0,472,4,883,0,0,0,4,13
KEAP1_7_4877,0,0,0,0,0,0,15,0,0,0,0,73

KIAA1841_7_4878,41,186,759,91,183,0,34,931,822,588,24,161
KLHL13_7_4879,209,546,1937,110,529,88,713,183,0,0,1453,188
KLHL2_7_4880,0,255,15,1032,612,163,0,160,279,399,1002,686
KLHL4_7_4881,898,366,944,958,178,300,210,904,356,230,513,390
KLHL5_7_4882,1,101,31,1314,264,110,47,143,42,462,362,321
KLHL7_7_4883,0,579,0,25,0,6,0,13,29,51,29,1882
LNX1_7_4884,374,0,89,772,25,0,148,918,2,830,6,2
LONRF3_7_4885,4,20,983,34,0,0,6,94,658,271,21,98
LRRC29_7_4886,0,4,0,0,92,465,0,769,0,0,0,0
LRSAM1_7_4887,0,39,593,11,13,72,67,319,10,0,65,184
MARK2_7_4888,223,7,0,86,526,0,117,124,54,623,272,399
MARK3_7_4889,268,374,134,684,78,637,440,0,702,61,154,622
MARK4_7_4890,1,0,41,7,21,72,147,76,19,78,14,53
MDM4_7_4891,0,0,0,0,439,0,29,0,4,0,0,2
MGRN1_7_4892,365,25,101,21,99,0,80,438,2,122,2,4
MIB2_7_4893,125,3,0,0,0,119,0,0,28,2,4,2
MID1_7_4894,0,255,193,0,14,5,0,2,0,1,214,202
MID2_7_4895,981,535,1408,352,1229,0,109,1676,558,659,2143,272
MKRN1_7_4896,15,589,945,72,119,216,802,1226,341,288,311,975
MLL5_7_4897,327,0,625,399,0,518,0,0,0,588,0,598
MLL_7_4898,92,414,72,56,0,1,60,2,207,8,65,980
MNAT1_7_4899,126,460,337,521,831,852,511,796,57,301,212,744
MPND_7_4900,1,0,0,1,0,0,503,0,0,0,108,0
MTF2_7_4901,2196,2776,288,1220,335,338,519,1276,2248,2447,1203,477
MYNN_7_4902,68,39,182,0,6,877,127,74,0,242,437,1
NAE1_7_4903,222,414,133,31,501,1324,814,2357,395,315,759,80
NDUFC2_7_4904,924,414,317,705,91,526,737,129,261,948,398,116
NEDD4_7_4905,674,51,92,169,1350,656,1161,251,369,228,157,556
NEDD4L_7_4906,274,469,423,76,60,759,78,23,830,1326,578,173
NFX1_7_4907,17,394,103,158,231,529,153,0,270,28,53,80
NLE1_7_4908,22,0,497,82,0,0,0,0,0,0,0,0
NSD1_7_4909,2045,281,1346,1102,602,1187,407,579,1301,154,630,1689
NSFL1C_7_4910,2,1,1,9,553,13,0,0,0,1,971,1
NUB1_7_4911,799,550,9,314,2865,1695,0,987,56,467,1501,796
ODF2_7_4912,0,0,0,31,2,87,20,532,16,1,0,222
OTUD5_7_4913,178,121,0,0,0,0,0,0,0,0,0,0
PARK2_7_4914,83,0,1,311,0,9,8,0,51,20,1,16
PARP9_7_4915,1888,192,1387,2519,131,4197,120,1279,895,4206,3578,1047
PATZ1_7_4916,0,0,1,1,0,115,0,0,0,53,0,0
PCGF6_7_4917,0,109,994,196,34,715,1354,196,1057,614,1072,123
PEX10_7_4918,0,34,18,0,65,0,0,0,0,0,0,40
PEX2_7_4919,179,1656,434,251,43,1292,760,470,714,1251,119,179
PHF12_7_4920,123,311,0,32,0,617,2443,727,0,562,478,1
PHF16_7_4921,58,59,16,408,79,253,325,121,1,602,64,0
PHF17_7_4922,83,38,92,109,43,91,235,159,51,976,52,142
PHF1_7_4923,0,0,0,734,131,0,0,9,152,12,2,25
PHF21A_7_4924,143,173,843,27,0,11,798,1149,224,513,0,264
PHF7_7_4925,4,465,436,304,204,228,1134,453,560,702,760,483
PHF8_7_4926,96,94,28,30,4,23,3,250,436,145,0,229
PJA1_7_4927,64,136,43,110,174,17,50,385,19,44,48,243

PML_7_4928,514,0,291,0,295,0,0,95,249,3,0,110
POC1B_7_4929,21,1243,7,599,116,46,32,50,276,10,410,223
PSMD1_7_4930,65,0,0,0,362,0,0,10,11,28,102,1
RAPSN_7_4931,3,3,1480,250,565,464,2131,1178,1514,673,98,310
RBBP4_7_4932,155,465,0,824,928,917,197,10,77,1065,73,333
RBBP5_7_4933,0,98,4,270,211,155,65,1,3,243,1,6
RBBP6_7_4934,785,166,177,732,108,212,0,866,1276,211,213,177
RBBP7_7_4935,1142,999,1483,2226,1922,2419,4425,2704,1810,1708,3410,564
3
RBCK1_7_4936,279,236,138,566,76,169,1010,979,89,103,4,790
RC3H2_7_4937,1063,280,2409,309,1034,479,437,733,364,431,669,141
RCHY1_7_4938,604,61,44,545,9,267,3212,1131,42,256,47,636
RFPL2_7_4939,957,1207,567,1345,333,1126,935,893,979,876,1206,523
RFPL3_7_4940,0,0,0,0,0,0,0,0,0,0,0,0
RFWD2_7_4941,935,1291,746,599,2026,1142,158,450,492,778,958,154
RHOBTB1_7_4942,5,0,0,0,26,0,0,2,7,2,0,0
RHOBTB2_7_4943,373,79,360,99,11,12,340,364,617,398,680,244
RLIM_7_4944,0,452,268,54,344,168,309,1,105,350,0,35
RNF103_7_4945,303,539,113,0,956,494,78,131,422,382,6,83
RNF128_7_4946,359,187,180,498,27,276,431,1574,528,448,1493,878
RNF135_7_4947,0,21,30,0,0,425,462,0,75,6,0,518
RNF138_7_4948,403,1212,304,196,656,342,2177,12,986,199,694,717
RNF13_7_4949,747,830,2497,284,553,490,635,625,371,272,1410,735
RNF145_7_4950,147,58,168,41,290,25,4,3,358,7,0,654
RNF146_7_4951,0,0,0,0,0,4,22,53,0,0,0,61
RNF14_7_4952,1340,0,435,377,0,66,9,3,538,278,0,69
RNF166_7_4953,393,215,98,1201,80,637,635,644,430,293,1135,204
RNF170_7_4954,12,54,0,426,7,0,74,2,61,1,0,10
RNF17_7_4955,143,35,549,0,378,34,239,5,258,492,360,499
RNF180_7_4956,174,0,1,418,715,6,7,16,301,5,0,661
RNF182_7_4957,153,0,652,561,232,349,0,178,131,715,906,9
RNF185_7_4958,0,0,795,0,87,84,0,4,0,440,0,0
RNF19A_7_4959,3,94,0,0,0,19,1046,33,2,0,0,186
RNF19B_7_4960,2315,537,747,1570,2825,2651,211,569,2343,1202,1993,1005
RNF213_7_4961,410,691,352,517,402,302,1,793,531,537,764,642
RNF214_7_4962,13,184,0,0,0,74,5,777,0,4,0,0
RNF216_7_4963,59,667,53,426,4,389,18,52,1530,220,284,191
RNF24_7_4964,269,848,1340,744,652,2592,797,802,1823,2094,1942,1277
RNF32_7_4965,1696,941,1843,179,476,867,1048,1014,2946,496,1904,2976
RNF34_7_4966,14,50,0,57,300,46,6,0,43,82,36,40
RNF38_7_4967,11,0,0,0,216,11,6,209,203,529,9,395
RNF40_7_4968,4,344,0,0,0,68,576,27,671,382,0,77
RNF41_7_4969,24,324,0,2,1,1,0,0,4,38,0,60
RNF6_7_4970,75,871,615,377,0,623,66,28,53,567,74,11
RNF7_7_4971,0,0,0,123,0,0,0,32,0,0,0,0
RNF8_7_4972,27,185,1111,174,136,366,218,622,1075,156,0,174
SAE1_7_4973,171,139,0,11,739,28,58,400,5,0,199,4
SENP6_7_4974,682,408,35,45,797,815,1398,1397,810,839,2018,798
SENP7_7_4975,3,1,60,44,178,0,7,172,983,14,0,115
SENP8_7_4976,307,481,1001,433,482,355,72,523,639,1995,533,200

SF3A1_7_4977,3093,1193,1847,1819,829,1170,466,217,1214,842,26,1083
SHPRH_7_4978,245,70,0,6,240,456,119,47,78,405,583,13
SIAH1_7_4979,454,0,56,16,286,2,1,83,55,231,94,7
SKP2_7_4980,669,1744,81,950,0,1313,287,663,605,189,329,329
SMURF1_7_4981,1606,321,1376,16,1,76,6,0,801,241,182,91
SOCS5_7_4982,724,0,374,0,753,593,0,888,676,22,369,938
SP100_7_4983,125,27,7,38,67,108,48,0,6,7,31,1
SP110_7_4984,139,10,125,179,14,28,1141,4,90,533,3,28
SPOP_7_4985,322,56,2,7,0,0,553,36,420,83,141,73
SPSB2_7_4986,0,0,0,0,0,0,0,0,0,20,0,2
SQSTM1_7_4987,130,787,709,384,61,618,260,453,244,669,222,32
STAMBP_7_4988,906,554,614,149,2582,0,226,24,677,650,1620,879
SUMO1_7_4989,480,989,223,312,265,983,1019,1528,816,605,199,542
SUMO2_7_4990,2168,1561,2271,683,2392,1816,818,1508,789,2341,1533,769
SYTL4_7_4991,142,1,306,10,312,251,765,953,1,508,241,6
SYVN1_7_4992,81,832,607,314,526,0,1014,308,307,212,442,814
TCF20_7_4993,7,1087,904,190,209,651,216,290,70,332,269,964
TDRD3_7_4994,2540,2903,188,63,627,621,210,1608,705,289,1057,709
TIPARP_7_4995,52,10,41,57,590,54,81,230,97,79,2,20
TLE2_7_4996,0,12,0,149,0,0,613,0,0,2,0,0
TLE3_7_4997,56,11,28,4,5,398,0,0,238,200,998,723
TNK2_7_4998,25,4,0,335,0,578,0,279,9,209,342,1
TNRC6C_7_4999,4,14,61,17,0,235,0,4,2,201,446,523
TOPORS_7_5000,477,521,215,0,441,0,0,283,0,0,329,0
TOR1AIP2_7_5001,162,32,1,163,39,635,2612,88,343,1818,84,88
TRAF3_7_5002,5,12,0,43,101,388,510,0,231,5,9,32
TRAF5_7_5003,294,322,320,150,1447,322,875,116,408,530,1909,78
TRAF6_7_5004,201,171,7,0,253,679,513,169,280,890,41,282
TRIM10_7_5005,248,225,206,395,79,252,863,305,350,508,1603,1461
TRIM13_7_5006,1066,21,39,556,222,293,769,763,724,312,2928,1561
TRIM17_7_5007,157,0,388,38,2,1,72,0,285,58,15,40
TRIM22_7_5008,3,376,592,74,1548,69,416,500,56,138,333,13
TRIM23_7_5009,275,896,802,1246,926,668,2968,2398,2108,759,1761,331
TRIM24_7_5010,513,99,1248,376,0,79,469,546,17,481,40,320
TRIM26_7_5011,324,67,235,241,14,0,39,0,313,4,223,51
TRIM2_7_5012,0,0,0,0,19,0,0,0,0,0,0,0
TRIM32_7_5013,1111,321,426,78,38,2,28,197,63,203,241,152
TRIM33_7_5014,2179,1918,1306,1546,1219,1197,744,760,2801,2882,2362,213
6
TRIM34_7_5015,247,775,622,330,337,162,1866,334,269,149,1862,64
TRIM37_7_5016,3766,1638,1714,154,1396,2158,2672,1799,1111,1885,2596,24
65
TRIM39_7_5017,3,10,0,343,5,1,0,907,0,40,4,54
TRIM3_7_5018,0,69,710,184,0,10,12,0,4,1,6,496
TRIM41_7_5019,5,0,593,0,23,4,0,65,66,44,410,4
TRIM45_7_5020,1156,612,50,575,0,13,35,222,548,789,145,72
TRIM4_7_5021,1059,965,1283,2,3,61,1186,64,980,226,18,455
TRIM54_7_5022,9,165,101,427,0,0,71,622,17,429,0,2
TRIM55_7_5023,1629,288,1193,908,2380,2799,2148,1649,1064,1305,699,486
TRIM5_7_5024,0,0,35,15,0,0,40,2,4,603,0,256

TRIM7_7_5025,34,302,359,345,193,680,0,421,73,128,9,140
TRIM9_7_5026,1114,0,0,0,0,0,0,0,0,0,0,1
TRPC4AP_7_5027,334,1364,261,1126,120,160,1149,371,1008,491,917,453
TSPAN17_7_5028,979,115,554,200,472,171,172,65,149,429,403,614
TTC3_7_5029,82,307,12,439,116,21,351,15,896,471,5,121
TULP4_7_5030,180,55,8,19,110,88,140,75,289,232,501,78
UBA1_7_5031,249,365,0,55,614,213,494,484,18,2,4,1
UBA3_7_5032,1732,1680,1712,1502,1624,2716,909,3142,1941,2893,2990,1263
UBA5_7_5033,1423,671,2711,486,2539,294,2382,1238,1955,1965,1086,1582
UBAC2_7_5034,51,1,3,0,90,1144,362,0,693,11,759,609
UBAP2L_7_5035,99,633,26,520,621,861,181,80,463,311,170,204
UBASH3A_7_5036,149,329,34,55,1893,190,1019,874,553,1311,1009,75
UBE2A_7_5037,27,256,96,1398,400,621,1,68,1130,23,147,157
UBE2D1_7_5038,337,378,177,288,350,571,589,12,1944,690,601,1055
UBE2D2_7_5039,239,35,32,90,0,149,0,35,261,170,231,823
UBE2D3_7_5040,28,0,0,0,22,8,0,4,0,0,0,0
UBE2E1_7_5041,142,26,73,22,313,28,403,432,39,605,297,173
UBE2E3_7_5042,1397,2267,1079,373,308,644,558,922,106,1024,708,937
UBE2G2_7_5043,14,6,17,18,350,187,45,509,278,285,103,44
UBE2H_7_5044,24,1,629,130,28,343,2,252,687,23,1,210
UBE2I_7_5045,2,29,0,1,5,0,0,0,66,15,0,21
UBE2J2_7_5046,0,0,0,0,0,0,0,0,0,286,50,0
UBE2K_7_5047,0,0,0,0,0,12,0,0,0,0,0,801
UBE2Q2_7_5048,144,1419,1805,529,761,376,534,250,980,719,726,572
UBE2V1_7_5049,1081,384,28,215,577,333,0,4,725,312,859,650
UBE2W_7_5050,71,792,3281,848,203,477,146,447,1184,1201,497,513
UBE3A_7_5051,1258,809,2729,2029,2889,2211,1485,1659,2237,2661,937,1644
UBE3B_7_5052,193,68,5,0,9,889,0,3,93,6,801,45
UBE4A_7_5053,3,96,458,655,0,827,906,700,607,132,40,90
UBE4B_7_5054,0,0,0,0,0,0,0,0,0,16,44,0
UBL7_7_5055,226,907,100,729,1622,51,5,1,459,339,766,66
UBOX5_7_5056,518,1202,839,194,3001,997,1796,1335,718,2111,1317,1782
UBQLN1_7_5057,945,975,2512,2336,595,2120,1419,958,668,1568,580,1181
UBR2_7_5058,7,322,0,506,120,4,23,0,310,145,2,245
UBXN11_7_5059,124,0,0,0,1,0,452,14,0,0,837,1
UBXN6_7_5060,0,0,3,0,9,0,498,0,0,471,0,5
UCHL5_7_5061,91,25,194,218,222,97,453,540,73,82,356,559
UHRF1_7_5062,62,494,204,421,0,8,0,1,435,72,388,50
UIMC1_7_5063,480,349,524,224,342,1699,185,1012,320,859,202,596
UNKL_7_5064,0,0,1,0,5,0,5,0,0,132,1,0
USP14_7_5065,471,206,5,1021,344,219,236,749,479,203,395,780
USP19_7_5066,7,8,0,50,0,1520,953,826,299,86,959,300
USP1_7_5067,254,608,292,614,604,541,749,391,368,277,1073,730
USP20_7_5068,424,3,44,1,0,111,299,0,37,1,0,11
USP2_7_5069,319,6,0,335,1252,469,1392,39,446,740,1,54
USP33_7_5070,1395,2455,804,1830,2656,3123,1965,1166,756,3666,2401,4205
USP44_7_5071,868,63,1349,437,0,407,15,0,123,46,0,407
USP46_7_5072,1965,137,77,78,360,219,3,221,699,490,407,1462
USP48_7_5073,2,2,153,0,165,55,0,500,0,41,0,16
USP4_7_5074,14,106,511,388,308,244,4,83,90,206,458,77

USP5_7_5075,495,823,430,302,1601,537,1987,201,96,701,1203,886
USP6NL_7_5076,200,911,706,698,263,1132,428,545,1380,258,858,901
USP8_7_5077,721,1975,397,465,788,287,1321,322,827,656,769,449
USP9X_7_5078,27,12,2896,490,787,1010,946,641,821,521,74,702
VHL_7_5079,1036,982,0,605,156,11,792,197,14,29,50,0
VPRBP_7_5080,717,366,708,3,264,169,462,0,359,194,48,125
VPS13D_7_5081,2428,3086,3766,3462,2104,3065,5219,1296,2513,3368,4429,3
004
VPS41_7_5082,1640,1381,1265,1321,144,845,1793,3691,2024,1311,1979,1192
VPS8_7_5083,450,53,191,25,536,612,1238,206,617,44,153,202
WDR26_7_5084,160,543,2,1,72,661,138,194,83,1066,95,536
WDR5_7_5085,0,0,1,0,0,3,0,0,0,0,2227,0
WDR76_7_5086,1632,1556,1449,1338,3005,1005,586,1527,1105,392,168,2227
WHSC1_7_5087,1,1269,0,409,0,0,663,398,539,354,131,82
WHSC1L1_7_5088,4934,1065,2084,2787,2088,2872,1463,2911,1795,2098,2096,
1785
WSB1_7_5089,0,106,13,1,0,19,82,106,54,73,706,119
WWP2_7_5090,0,889,2,58,331,517,0,683,2,811,76,35
XIAP_7_5091,1817,1049,964,351,1627,1607,2860,1614,2350,2304,1968,1974
ZBTB10_7_5092,1812,366,1966,1197,2512,3000,2720,1437,2696,5549,4442,42
91
ZBTB16_7_5093,409,366,17,0,282,14,146,286,11,294,1528,181
ZBTB17_7_5094,0,0,0,0,0,0,0,0,266,44,0,25
ZBTB1_7_5095,1891,333,208,1837,1213,1026,1557,1310,979,974,1081,208
ZBTB20_7_5096,0,288,30,163,608,373,108,1153,91,252,1,162
ZBTB22_7_5097,93,1365,39,293,0,144,151,160,295,1005,589,387
ZBTB24_7_5098,340,3,477,0,0,0,0,54,213,3,0,31
ZBTB33_7_5099,1467,863,1021,1873,750,679,1681,3575,2508,2425,2109,930
ZBTB37_7_5100,669,724,1586,1577,1258,992,1683,1022,1345,1240,528,808
ZBTB40_7_5101,467,893,1459,7,3,679,6,4,13,1227,2,896
ZBTB4_7_5102,18,572,16,164,65,1,0,3,8,968,360,0
ZFP161_7_5103,0,0,115,0,0,0,348,100,0,0,0,991
ZMYND11_7_5104,887,257,855,89,17,692,48,674,85,35,111,706
ZMYND8_7_5105,0,151,823,26,0,126,0,12,0,0,76,21
ZNF238_7_5106,288,80,137,84,0,381,3,0,34,346,36,14
ZNF295_7_5107,747,229,486,515,214,753,4,725,514,8,0,739
ZNRF3_7_5108,454,87,2,56,678,183,115,481,263,10,1040,395
ANAPC11_7_5109,864,462,762,732,1068,470,441,74,91,641,993,241
ATXN3_7_5110,583,8,135,0,0,4,384,490,313,1129,31,457
MLLT10_7_5111,77,1,0,7,0,275,1008,1176,0,121,186,0
OTUD4_7_5112,103,33,114,0,15,138,178,464,11,17,31,414
PHF19_7_5113,0,49,276,28,74,141,0,26,0,35,7,3
SP140_7_5114,884,114,522,830,236,282,115,642,537,791,5,1669
TRIM36_7_5115,1378,473,1556,1238,0,652,108,975,1038,501,300,624
UBE2C_7_5116,1626,2,89,1229,91,770,298,42,980,1087,1024,130
UBE2L6_7_5117,0,0,152,565,1,0,20,0,591,15,0,73
ABTB2_7_5118,0,0,0,8,0,0,1,9,0,2,0,0
AMBRA1_7_5119,254,382,46,0,606,921,94,28,0,176,269,1
AMFR_7_5120,726,1148,70,61,1100,713,2,780,217,553,714,845
ANAPC10_7_5121,741,51,144,351,0,3,4,520,533,127,562,937

ANAPC1_7_5122,454,140,84,154,0,552,1144,0,15,500,0,34
ANAPC2_7_5123,1,8,0,0,0,731,496,603,81,4,0,9
ANAPC4_7_5124,213,203,407,113,672,24,398,689,1050,601,633,173
ANKIB1_7_5125,201,312,1644,706,840,543,283,589,1134,674,3124,1213
ANKRD13A_7_5126,796,791,562,592,439,1206,138,1010,1031,2191,135,923
ANKRD13D_7_5127,139,13,0,434,468,489,1858,131,0,216,379,266
ARIH1_7_5128,1706,633,1090,378,1144,1036,1238,856,2480,1142,2772,1497
ARIH2_7_5129,411,447,30,7,0,599,22,106,30,190,0,16
ASB12_7_5130,546,504,25,1103,296,48,3408,1529,332,782,2552,123
ASB13_7_5131,0,59,0,0,0,0,0,396,0,0,0,2
ASB15_7_5132,928,1017,2589,845,2093,773,461,1264,936,1648,1044,1994
ASB16_7_5133,0,147,1,0,0,3,0,0,0,50,0,0
ASB17_7_5134,1545,500,7,0,403,1,56,1452,321,1068,458,37
ASB5_7_5135,419,1090,662,943,62,2,296,562,260,877,1521,1372
ASB8_7_5136,1323,30,301,46,2032,42,110,835,475,125,485,60
ASH1L_7_5137,216,1258,348,436,1208,679,0,723,825,570,122,1783
ASPSCR1_7_5138,0,0,13,0,0,0,0,0,0,0,8,6
ATG12_7_5139,17,7,9,10,645,279,93,1,832,50,1,766
ATG3_7_5140,0,0,0,40,64,0,0,8,26,2,0,126
ATG5_7_5141,170,454,847,100,430,136,14,26,152,98,662,23
ATXN1L_7_5142,0,0,0,1,0,0,15,0,0,0,189,561
ATXN3L_7_5143,1313,222,1059,404,102,888,1436,473,1156,776,1530,1792
BARD1_7_5144,67,152,4,19,0,427,1,8,15,107,899,26
BAZ1B_7_5145,277,865,657,1731,514,18,3299,22,40,168,472,400
BAZ2A_7_5146,28,21,8,0,143,178,0,229,96,16,123,20
BAZ2B_7_5147,0,0,320,564,0,0,1001,0,342,4,0,41
BECN1_7_5148,1053,2020,2801,971,2732,1875,2002,2028,785,1680,817,504
BIRC2_7_5149,323,941,715,226,2416,1449,829,636,2592,1653,2859,2727
BIRC6_7_5150,2080,1116,2240,1079,2697,1469,1290,2512,1919,1425,9,1629
BIRC8_7_5151,153,268,98,198,799,986,680,189,416,404,196,111
BMI1_7_5152,2559,744,517,1309,2138,2352,1508,1170,1351,2199,1729,1822
BRAP_7_5153,4,870,2296,327,619,14,694,14,384,87,97,35
BRD1_7_5154,0,155,144,0,0,0,0,5,0,56,0,0
BRPF3_7_5155,192,206,419,116,0,561,139,28,202,296,981,2368
BTBD2_7_5156,2,143,7,12,1,50,3,5,215,4,22,29
BTBD6_7_5157,616,1145,213,13,990,83,215,1360,305,353,752,1637
CAND1_7_5158,219,352,804,978,791,90,155,517,284,111,1113,366
CBLB_7_5159,272,1501,1220,835,3732,126,1082,2054,1968,985,309,733
CBL_7_5160,0,54,0,3,7,0,26,18,25,52,0,3
CBLL1_7_5161,321,44,1844,522,953,597,860,1688,200,668,171,604
CCIN_7_5162,1,494,295,49,17,84,53,45,463,132,648,255
CCNF_7_5163,520,207,474,683,49,662,80,435,722,52,170,190
CDC20_7_5164,771,1190,2469,310,763,645,1916,646,1168,642,1381,237
CDC23_7_5165,357,218,703,665,81,68,0,556,124,1052,6,450
CDC26_7_5166,742,473,799,411,19,922,1113,59,784,739,457,368
CDC34_7_5167,0,0,0,15,93,0,1,3,20,1,144,3
CGRRF1_7_5168,541,796,83,251,1590,1291,1503,483,678,950,1032,1997
CHD4_7_5169,42,206,240,84,77,47,843,31,1015,46,19,93
CIAO1_7_5170,222,1,44,157,70,172,1737,551,585,46,1121,423
COPS4_7_5171,2,586,631,31,74,665,239,821,618,8,1,318

COPS5_7_5172,2,0,758,0,1,72,4,3,0,1,107,257
COPS6_7_5173,0,1,0,199,55,23,0,7,421,12,134,64
COPS7B_7_5174,67,471,709,586,59,0,69,0,4,0,0,0
CORO6_7_5175,218,5,923,10,0,464,331,764,785,566,154,100
CUEDC1_7_5176,369,110,90,95,1462,0,277,96,5,1026,16,0
CUL1_7_5177,454,0,20,0,1,549,1616,4,396,13,98,393
CUL3_7_5178,8,142,1443,584,29,724,1271,1058,656,350,62,103
CUL5_7_5179,0,0,1,0,0,45,9,26,0,154,0,14
CUL9_7_5180,205,49,445,225,663,421,383,170,148,145,90,667
DCAF10_7_5181,0,418,0,15,10,0,0,6,6,39,58,0
DCAF12_7_5182,482,287,12,914,45,2001,63,546,1118,442,733,1017
DCAF13_7_5183,1,382,10,145,84,81,273,1,87,288,478,144
DCAF16_7_5184,1446,413,267,1180,44,1254,259,1014,203,78,948,644
DCAF5_7_5185,13,301,758,65,9,154,482,7,3,341,0,1
DCAF7_7_5186,87,0,0,1,0,0,13,171,25,550,0,21
DCAF8_7_5187,0,326,1077,83,19,3,0,3,0,73,1036,0
DDA1_7_5188,1,7,0,9,2,29,107,9,0,0,127,0
DDB1_7_5189,1228,173,0,185,894,0,4,489,1616,416,2,212
DDB2_7_5190,8,0,0,0,10,23,311,0,4,48,0,0
DPF2_7_5191,0,298,0,0,18,0,95,0,0,1,0,0
DTX1_7_5192,0,0,0,0,0,613,0,3,0,0,0,0
DTX3L_7_5193,571,6,511,17,1228,428,5,75,8,50,2,90
DTX4_7_5194,336,852,1310,159,12,1453,1458,351,1410,676,333,582
DZIP3_7_5195,0,0,0,36,725,0,76,10,0,0,0,0
EIF3D_7_5196,75,1241,354,98,405,811,1252,750,303,228,313,37
EIF3E_7_5197,48,288,0,6,83,0,53,651,136,19,12,34
EIF3F_7_5198,213,485,436,658,156,315,338,186,754,946,807,1187
EIF3G_7_5199,703,88,18,502,1336,123,380,648,643,1023,103,171
EIF3H_7_5200,191,82,0,158,103,50,315,423,87,0,316,453
EIF3I_7_5201,19,0,545,0,547,361,0,171,139,348,35,120
EIF3J_7_5202,1,38,1,380,388,287,75,0,5,140,35,0
EIF3K_7_5203,0,0,110,21,0,5,1,0,73,2,0,5
ENC1_7_5204,0,47,288,142,371,837,112,334,20,10,24,518
EPN3_7_5205,154,0,0,79,853,742,456,22,443,11,0,73
EPOR_7_5206,7,0,422,1306,0,156,0,0,605,8,0,77
ERCC8_7_5207,2270,801,1236,201,452,545,333,1959,2250,550,740,632
FAF1_7_5208,276,10,133,116,0,6,0,120,456,896,1,94
FBXL12_7_5209,25,21,257,858,0,0,0,60,32,149,10,3
FBXL14_7_5210,37,1,411,0,4,0,0,2,0,19,0,0
FBXL15_7_5211,524,74,0,0,0,8,0,0,30,7,4,2
FBXL16_7_5212,211,9,143,86,0,8,1348,38,0,773,7,8
FBXL17_7_5213,160,21,67,278,2077,248,93,52,325,878,685,615
FBXL18_7_5214,752,980,2260,983,2068,1408,899,448,1461,710,1054,2339
FBXL19_7_5215,1,0,83,54,683,0,811,118,226,18,1,41
FBXL21_7_5216,222,0,0,0,0,21,1,13,0,0,0,0
FBXL3_7_5217,445,131,39,394,674,77,363,489,295,1095,458,66
FBXL4_7_5218,1,942,14,0,1055,3,794,1005,443,6,5,48
FBXL7_7_5219,951,8,1026,0,0,134,663,99,317,200,1,106
FBXO10_7_5220,218,58,14,3,21,27,300,11,193,24,342,1011
FBXO16_7_5221,483,38,0,196,193,407,1495,814,15,455,19,7

FBXO27_7_5222,745,468,670,338,762,573,267,756,247,280,240,816
FBXO2_7_5223,156,462,503,167,6,416,4,131,2,308,1318,1048
FBXO30_7_5224,449,181,251,165,1132,10,346,105,583,32,1552,481
FBXO33_7_5225,2050,2353,2741,1792,93,1333,2102,1716,2783,1530,3795,308
2
FBXO36_7_5226,949,302,716,769,296,2711,0,916,1497,1132,151,1274
FBXO39_7_5227,191,248,242,0,23,177,16,120,110,46,386,1215
FBXO40_7_5228,2337,306,528,621,650,1198,1136,1698,1806,976,1875,750
FBXO41_7_5229,707,1151,2813,33,1,1352,2699,1204,148,871,688,513
FBXO42_7_5230,0,84,0,0,1258,6,0,0,4,0,1006,54
FBXO43_7_5231,443,231,944,860,340,263,542,1371,1475,1444,1907,913
FBXO46_7_5232,9,5,1,0,0,149,58,9,428,173,282,40
FBXO6_7_5233,0,0,118,141,11,0,257,83,514,12,173,567
FBXO8_7_5234,917,40,619,331,0,838,444,670,2,383,499,11
FBXW10_7_5235,215,68,0,6,0,118,0,1,39,360,13,62
FBXW2_7_5236,23,92,506,331,108,108,36,246,271,153,10,154
FBXW5_7_5237,0,1,0,69,1,489,1511,0,4,159,0,1
FBXW9_7_5238,235,1183,0,0,39,62,1,0,65,23,4,12
G2E3_7_5239,2,0,0,10,0,0,8,0,9,0,10,738
GAN_7_5240,996,391,361,13,14,1394,387,79,798,463,149,317
GMCL1_7_5241,40,887,889,740,201,331,791,544,684,1554,1692,1346
GNB2_7_5242,3,41,0,0,2,9,0,190,346,159,391,273
GRWD1_7_5243,0,2,0,0,1,0,0,0,437,409,0,52
GZF1_7_5244,9,0,78,73,1,531,1305,340,422,832,3,73
HACE1_7_5245,1,1218,17,0,0,88,0,86,12,2,89,41
HDAC6_7_5246,169,8,42,200,3,66,5,129,11,95,437,2
HECTD1_7_5247,229,1,0,0,24,78,14,11,10,571,2,7
HECTD3_7_5248,75,410,0,9,388,20,75,742,329,74,130,54
HECW1_7_5249,4,46,11,33,3,22,17,32,17,31,61,6
HECW2_7_5250,996,312,1096,470,1306,1076,448,396,1349,1579,1017,3115
HERC1_7_5251,507,2868,1044,394,1,365,1948,896,1820,948,1356,798
HERC2_7_5252,50,473,1151,197,594,363,1054,26,31,489,139,599
HERC3_7_5253,0,132,218,7,217,22,314,47,476,533,1247,933
HERC5_7_5254,1162,1200,755,1455,1469,1616,835,3211,1123,335,619,1601
HGS_7_5255,2120,1413,1959,297,695,186,713,1536,589,700,2978,3222
HIC2_7_5256,557,61,32,692,730,72,2,271,15,115,1445,102
HUWE1_7_5257,437,106,454,19,192,52,840,3,238,270,97,584
IBTK_7_5258,194,0,435,175,0,0,1453,11,99,2,847,10
IL10RA_7_5259,207,198,0,0,256,0,0,1192,16,1083,332,3
IL6_7_5260,197,403,255,196,2,317,0,244,117,861,1266,16
IRF9_7_5261,0,290,0,407,0,0,105,0,630,13,0,59
ITCH_7_5262,433,148,1017,49,274,300,878,1059,94,1274,228,107
IVNS1ABP_7_5263,60,27,2,0,704,41,28,42,11,31,154,2
JHDM1D_7_5264,4,265,32,352,235,87,610,0,568,1466,6,263
JOSD1_7_5265,1862,280,826,1104,1558,854,1850,1160,1899,644,1059,518
JOSD2_7_5266,8,0,3,0,0,0,0,0,0,0,0,0
KAT6B_7_5267,253,204,42,1242,134,1178,721,297,544,2957,1482,993
KATNB1_7_5268,739,167,240,540,306,169,568,110,1444,637,350,615
KBTBD10_7_5269,263,1407,90,172,738,571,766,687,444,313,946,85
KBTBD11_7_5270,0,0,0,0,1016,0,0,559,0,0,1,0

KBTBD2_7_5271,36,55,392,526,451,223,1114,962,589,163,1432,159
KBTBD5_7_5272,0,0,0,537,124,27,354,808,268,223,0,453
KBTBD7_7_5273,2503,0,0,809,573,324,1124,688,239,160,2370,479
KBTBD8_7_5274,1303,642,1963,818,1918,1449,438,650,516,1114,1028,1020
KCTD10_7_5275,2014,1232,2487,2357,2913,1516,1667,2386,2837,1354,2670,3
050
KCTD11_7_5276,3,3,104,18,88,0,0,1,30,503,0,1291
KCTD12_7_5277,45,0,5,0,0,0,0,0,0,725,996,0
KCTD13_7_5278,460,592,899,90,197,3571,350,283,194,474,712,170
KCTD16_7_5279,0,0,0,0,0,0,0,0,0,0,0,0
KCTD17_7_5280,16,550,144,113,676,510,36,754,1238,471,1196,1082
KCTD18_7_5281,122,33,912,81,722,48,5,42,89,167,69,311
KCTD3_7_5282,500,488,2,23,416,102,15,70,157,1018,589,189
KCTD5_7_5283,88,79,0,884,0,0,0,0,3,0,0,0
KCTD9_7_5284,809,385,972,647,3462,1503,438,2503,1548,1089,1349,1209
KDM2A_7_5285,612,0,362,0,39,0,0,0,0,14,6,0
KDM4B_7_5286,0,253,0,14,3,0,214,6,206,8,25,19
KDM5B_7_5287,2013,160,1023,1531,4553,1337,240,1552,970,1298,110,2205
KLHDC5_7_5288,76,626,112,0,0,29,0,77,15,0,2,3
KLHL10_7_5289,29,579,1,106,38,1147,103,18,13,722,181,932
KLHL11_7_5290,86,104,770,0,202,213,107,53,181,54,13,197
KLHL12_7_5291,247,481,0,0,380,262,0,218,797,624,2,346
KLHL14_7_5292,0,89,1,495,0,574,18,290,8,764,26,89
KLHL15_7_5293,653,1762,956,1653,3091,718,682,208,1382,610,776,1142
KLHL17_7_5294,111,0,136,0,0,0,0,754,0,5,0,0
KLHL18_7_5295,179,1,86,142,166,202,0,0,9,93,59,262
KLHL1_7_5296,75,4,28,124,0,277,0,0,562,5,6,425
KLHL20_7_5297,0,30,0,0,0,2,281,0,6,194,153,504
KLHL21_7_5298,641,1509,97,32,0,110,408,1305,244,98,48,1642
KLHL22_7_5299,93,109,333,583,0,25,762,1422,771,272,69,105
KLHL23_7_5300,311,938,894,723,1233,431,915,1236,409,875,954,680
KLHL24_7_5301,1724,1563,3765,1629,2276,1667,2747,2543,2795,2910,2571,4
724
KLHL25_7_5302,611,16,8,27,0,117,136,2117,16,0,12,340
KLHL26_7_5303,1300,33,247,1145,1790,538,138,731,1009,205,254,1287
KLHL28_7_5304,58,386,951,1188,129,400,610,277,233,529,114,1129
KLHL29_7_5305,2418,54,124,247,532,31,257,897,448,6,98,180
KLHL31_7_5306,560,5,10,25,522,1258,469,452,1005,234,24,450
KLHL32_7_5307,2,0,0,205,0,0,0,43,0,12,862,421
KLHL33_7_5308,146,185,158,496,12,774,338,229,144,173,707,53
KLHL34_7_5309,8,0,0,8,0,0,0,0,0,0,1,0
KLHL36_7_5310,0,58,13,0,0,0,0,0,1,43,34,255
KLHL3_7_5311,2,134,16,1,1757,53,0,233,0,953,1,0
KLHL8_7_5312,0,0,0,0,697,46,0,0,0,0,0,0
LATS1_7_5313,605,928,341,54,25,95,523,607,68,474,556,417
LATS2_7_5314,1,596,0,0,0,524,0,0,1057,153,0,508
LIF_7_5315,0,85,0,0,2,30,0,511,199,24,0,79
LNX2_7_5316,32,221,2431,119,1922,519,441,344,496,1101,112,60
LOC283116_7_5317,60,4,0,420,0,527,32,205,12,197,17,364
LONRF1_7_5318,209,282,1666,79,1004,157,82,148,781,1189,745,1089

LTN1_7_5319,1602,948,31,2291,1620,1479,231,905,482,316,817,1908
LZTR1_7_5320,17,0,2,505,8,0,0,0,0,1,2,0
MAP1LC3B_7_5321,617,299,526,45,250,64,654,469,330,339,275,159
MAP3K1_7_5322,135,676,14,387,40,582,1036,782,698,232,1608,754
MARK1_7_5323,16,50,21,35,0,0,3,0,32,68,0,12
MDM2_7_5324,1857,375,1021,931,507,1648,1513,668,2298,320,563,2371
MED20_7_5325,33,170,4163,764,793,404,894,410,1659,787,815,198
MEX3B_7_5326,19,498,24,0,595,426,232,3,495,23,143,310
MIB1_7_5327,2935,1322,1619,220,2010,896,1222,1379,841,999,1029,996
MKRN2_7_5328,602,153,303,589,170,112,545,547,838,85,171,448
MKRN3_7_5329,3487,3425,3596,1859,2260,2186,2106,2802,1898,2677,3366,48
36
MLL2_7_5330,317,1340,277,226,314,593,10,379,381,262,459,254
MLLT6_7_5331,77,234,0,67,294,733,59,0,313,5,0,623
MOCS3_7_5332,133,132,0,2,448,452,10,177,101,537,0,51
MRPL49_7_5333,27,491,519,174,627,538,0,1,396,505,0,64
MUL1_7_5334,4,204,167,0,18,337,105,1,0,1,0,212
MYCBP2_7_5335,83,539,1790,724,1261,10,1209,898,350,696,1977,455
MYLIP_7_5336,585,1457,1807,61,55,2,76,916,434,263,385,118
MYSM1_7_5337,476,597,144,24,173,370,476,26,294,241,44,33
NACC1_7_5338,1005,792,1797,42,213,1807,873,650,1908,681,678,751
NACC2_7_5339,0,0,13,0,0,0,0,0,1,621,9,0
NEURL1B_7_5340,281,237,154,42,58,13,243,587,2,566,75,259
NEURL_7_5341,21,435,64,89,718,563,1,0,24,695,1289,2
NHLRC1_7_5342,2,0,807,700,0,16,94,0,2,20,837,0
NUP43_7_5343,1763,1963,2299,585,88,678,167,390,1284,105,259,1800
OTUB1_7_5344,38,27,19,154,107,202,112,177,680,64,11,102
OTUB2_7_5345,674,901,638,439,35,55,2002,42,327,111,25,110
OTUD1_7_5346,2,858,0,5,121,0,0,0,21,0,1,10
OTUD6A_7_5347,0,0,0,0,23,399,0,0,0,0,0,0
OTUD6B_7_5348,536,57,11,1181,39,33,9,701,430,190,145,106
OTUD7A_7_5349,2,44,157,0,0,236,182,337,23,75,194,1
OTUD7B_7_5350,267,250,75,628,29,124,469,423,378,895,668,76
PAFAH1B1_7_5351,13,441,550,702,0,1,409,408,359,231,591,526
PARP10_7_5352,693,1,107,164,0,0,512,0,0,367,1824,865
PARP11_7_5353,374,146,0,0,0,281,286,514,0,0,19,2
PARP14_7_5354,4,0,234,8,516,19,968,3,0,346,147,38
PCGF1_7_5355,59,426,0,322,15,102,793,2,577,8,0,559
PCGF2_7_5356,1801,440,713,0,85,977,45,186,255,1142,285,315
PCGF3_7_5357,0,19,20,14,310,911,122,328,756,308,446,92
PDZRN3_7_5358,15,472,769,0,0,0,16,371,0,0,1,0
PEBP4_7_5359,0,0,0,464,4,0,0,0,0,4,760,0
PEX12_7_5360,0,24,0,8,0,1,0,0,1,14,0,0
PHF14_7_5361,103,569,1173,830,567,1899,182,754,978,1993,789,905
PHF15_7_5362,554,123,1,328,199,0,56,0,11,35,0,3
PHF20_7_5363,109,215,344,2,185,469,0,328,782,497,0,360
PHF2_7_5364,2,3,939,0,3,304,0,11,416,12,1110,42
PHF3_7_5365,315,131,295,0,367,440,895,632,824,60,515,895
PHIP_7_5366,22,0,713,11,2,270,3,0,1,31,17,48
PHRF1_7_5367,75,0,1,12,43,0,56,1,0,85,0,0

PJA2_7_5368,88,2,37,26,772,5,116,321,37,110,485,14
PRPF19_7_5369,157,507,2,277,0,467,6,72,52,10,498,5
PRPF8_7_5370,210,0,224,760,0,0,180,18,0,217,303,21
PSMD14_7_5371,1129,144,136,559,732,881,803,195,2170,405,1240,929
PSMD2_7_5372,78,60,708,292,696,297,8,859,532,72,1284,61
PSMD4_7_5373,4,66,174,389,273,485,245,0,546,68,168,153
PSMD7_7_5374,406,466,724,3,0,313,9,1020,326,978,455,33
PWP1_7_5375,317,640,928,850,0,930,1116,641,171,311,3389,1170
RAB40A_7_5376,1741,1857,1754,1840,1430,1701,4827,2030,2600,3399,5225,3
166
RAB40AL_7_5377,1741,1857,1754,1840,1430,1701,4827,2030,2600,3399,5225,
3166
RAB40B_7_5378,493,526,29,588,2184,459,473,659,100,1444,110,562
RAD18_7_5379,0,0,579,201,4,238,0,0,79,525,876,7
RAD23A_7_5380,295,280,611,141,285,938,385,39,8,1386,262,27
RAD23B_7_5381,69,531,0,706,3117,72,22,231,1361,527,6,819
RAG1_7_5382,1169,1988,1403,615,411,714,878,1754,503,408,828,1059
RAI1_7_5383,5,274,37,23,0,40,0,15,0,1,37,93
RBX1_7_5384,282,1657,739,79,42,190,52,757,29,470,129,378
RCBTB1_7_5385,145,1001,1413,0,0,233,571,0,278,150,952,349
RCBTB2_7_5386,1,0,0,1,25,5,0,0,6,0,0,29
RFFL_7_5387,50,0,0,282,18,186,0,752,0,28,29,0
RFPL1_7_5388,468,198,2250,833,869,15,155,382,1581,1231,1788,572
RFWD3_7_5389,3,37,529,718,0,40,230,293,5,4,164,64
RHOBTB3_7_5390,643,527,1850,279,1234,2463,2125,359,1336,2081,1550,329
RING1_7_5391,42,92,425,127,1,1386,939,11,5,163,65,23
RNF10_7_5392,0,0,1,12,232,406,1,462,917,172,57,103
RNF111_7_5393,3133,3990,1566,5682,5264,3085,4660,4070,5475,5063,7920,3
972
RNF112_7_5394,21,84,1752,439,0,71,23,208,112,203,12,29
RNF113A_7_5395,0,1,0,0,1,0,0,0,0,0,0,580
RNF113B_7_5396,0,0,0,0,13,0,0,0,27,1,73,4
RNF114_7_5397,0,0,0,0,0,0,0,126,1157,34,0,102
RNF115_7_5398,44,165,1,182,219,292,148,31,15,210,8,139
RNF11_7_5399,6,301,0,2061,710,18,66,211,166,1291,60,1222
RNF121_7_5400,77,587,0,135,0,335,86,228,549,195,1,259
RNF122_7_5401,1968,482,183,726,53,1803,838,1727,495,388,382,1360
RNF123_7_5402,24,0,0,0,0,0,0,28,0,6,0,0
RNF125_7_5403,25,9,33,476,2,237,1999,424,0,0,869,54
RNF126_7_5404,1679,1443,403,2401,807,847,1712,543,3112,2484,1867,1684
RNF130_7_5405,75,70,3,470,805,706,5,447,21,398,5,419
RNF133_7_5406,111,757,25,752,7,674,1374,1270,259,651,612,619
RNF139_7_5407,1808,2278,1722,917,784,238,1187,2267,2039,1016,2173,2715
RNF141_7_5408,0,9,167,0,8,148,164,68,1,0,265,1
RNF144A_7_5409,103,5,99,92,0,72,2,209,77,67,111,91
RNF144B_7_5410,345,223,424,1074,587,424,2088,1413,854,1014,391,1483
RNF149_7_5411,2,5,62,110,0,1,1283,105,35,0,58,473
RNF150_7_5412,497,188,1462,325,1892,545,16,27,591,1010,1372,572
RNF152_7_5413,340,175,110,356,596,574,1829,386,230,235,571,1699
RNF157_7_5414,73,0,238,24,145,584,387,304,732,100,302,92

RNF167_7_5415,101,0,0,364,0,1527,361,338,247,1054,0,30
RNF168_7_5416,151,22,320,39,337,699,731,1270,130,312,114,342
RNF169_7_5417,16,0,399,290,86,178,104,756,104,348,248,16
RNF181_7_5418,17,7,0,3,48,0,34,0,41,3,2,2
RNF183_7_5419,0,6,45,81,185,73,12,20,82,321,524,145
RNF186_7_5420,77,152,66,231,38,59,248,12,112,135,762,26
RNF187_7_5421,0,9,0,355,502,27,0,0,0,58,152,173
RNF20_7_5422,551,306,0,0,556,8,47,138,799,109,2144,1016
RNF217_7_5423,22,1167,269,1405,35,1567,1027,325,1369,1365,50,1587
RNF220_7_5424,1186,0,51,220,14,53,25,0,21,951,241,0
RNF25_7_5425,6,0,0,0,109,52,0,0,2,1,0,1
RNF26_7_5426,0,0,0,0,0,0,464,0,0,0,0,0
RNF2_7_5427,1442,3082,4331,615,1845,3057,166,1146,1116,85,1903,1872
RNF31_7_5428,1178,842,211,1,35,768,1323,612,421,289,400,569
RNF43_7_5429,1006,121,13,17,6,0,542,197,3,333,8,126
RNF44_7_5430,17,361,79,11,9,372,0,722,496,114,1388,130
RNF5_7_5431,1,0,0,0,0,0,0,0,0,158,0,0
RSC1A1_7_5432,620,200,235,337,869,387,866,6,246,545,662,446
RSF1_7_5433,1,453,70,0,800,1,885,0,128,886,16,65
RSPRY1_7_5434,28,307,32,4,0,0,0,18,227,415,11,284
SCLY_7_5435,230,145,964,16,3,356,31,107,492,26,0,69
SENP1_7_5436,1519,24,1,539,125,587,157,398,149,197,5,1005
SENP2_7_5437,529,20,37,575,67,224,1557,27,400,12,31,58
SENP3_7_5438,4,0,0,0,0,0,0,0,1,0,0,0
SENP5_7_5439,221,8,11,851,444,0,265,482,175,5,45,27
SH3RF1_7_5440,0,45,5,9,0,158,0,259,2,239,461,0
SH3RF2_7_5441,0,0,911,4,0,0,0,544,0,0,386,0
SH3RF3_7_5442,0,3,3,0,0,0,17,0,408,673,507,87
SHKBP1_7_5443,400,409,1,719,538,194,0,0,600,338,834,313
SIAH2_7_5444,290,3,104,32,6,14,209,39,512,83,17,352
SIK1_7_5445,17,119,165,10,2,1,0,8,643,15,39,71
SLX4_7_5446,1086,236,1207,448,1235,578,30,1,412,498,1822,1381
SMU1_7_5447,729,1916,59,569,1540,766,955,39,1447,498,569,1334
SMURF2_7_5448,379,498,266,137,706,397,1352,364,877,851,539,1907
SNRNP40_7_5449,0,243,0,264,696,0,6,234,0,0,0,0
SOCS1_7_5450,24,1,50,0,733,13,3,412,25,1,2,5
SOCS2_7_5451,1,3,157,0,0,18,0,86,0,0,0,0
SOCS3_7_5452,3,0,0,0,0,0,0,0,2,0,0,1
SOCS6_7_5453,164,90,0,411,10,229,1014,37,125,200,121,471
SPOPL_7_5454,107,0,544,51,122,79,105,35,110,60,557,94
SPSB1_7_5455,542,41,275,213,2154,664,425,2,230,152,84,24
SPSB3_7_5456,27,560,412,581,203,176,865,444,1155,1244,877,394
SPSB4_7_5457,1,787,8,410,0,808,211,1139,4,3,127,485
STAM2_7_5458,213,3,25,56,843,39,38,222,486,16,5,212
STAMBPL1_7_5459,2457,1724,1114,1059,919,2424,1715,1795,2727,2168,2298,
2457
STAM_7_5460,31,192,89,275,327,115,332,228,897,66,422,460
STUB1_7_5461,0,417,0,3,6,16,0,0,44,0,0,6
SUMO3_7_5462,1,78,0,560,0,138,0,0,51,2,30,16
SYNGAP1_7_5463,172,2,36,0,196,375,496,1277,0,1,33,19

TAB2_7_5464,971,643,389,124,726,2500,3208,1832,1737,2074,1669,698
TAB3_7_5465,4454,3698,5027,5042,3116,2801,5839,4182,4188,6565,3941,147
6
TAF1D_7_5466,102,0,0,594,0,66,39,0,316,19,563,464
TBC1D1_7_5467,628,83,803,214,733,363,842,88,825,731,845,242
TLE1_7_5468,1078,629,1171,166,1180,7,871,136,734,870,329,623
TNFAIP3_7_5469,94,579,306,0,33,26,152,116,129,43,856,28
TOLLIP_7_5470,0,0,909,0,0,11,10,19,2,15,0,0
TRAF7_7_5471,75,10,670,192,408,6,0,0,592,43,133,63
TRAIP_7_5472,530,49,319,367,101,0,1033,295,179,89,257,30
TRIM11_7_5473,419,132,20,8,81,1,27,10,109,1,0,13
TRIM15_7_5474,16,1134,312,9,182,4,848,9,532,767,1,637
TRIM25_7_5475,2304,1171,113,730,596,1332,333,1853,235,184,1039,734
TRIM27_7_5476,17,41,64,0,0,204,6,28,0,6,1217,457
TRIM28_7_5477,19,21,23,501,0,79,21,2,3,1,20,672
TRIM31_7_5478,512,492,1812,603,94,0,458,92,375,748,1170,553
TRIM35_7_5479,555,5,0,453,0,76,90,347,792,22,0,101
TRIM42_7_5480,653,136,86,761,469,40,0,300,0,1439,161,6
TRIM46_7_5481,43,60,397,0,723,275,0,11,0,1,2,464
TRIM47_7_5482,134,0,1,84,0,99,0,16,3,1,397,0
TRIM48_7_5483,50,858,340,1447,316,520,485,425,606,2520,716,414
TRIM52_7_5484,0,367,201,162,772,289,135,135,52,353,35,8
TRIM56_7_5485,1,0,776,0,13,173,0,0,2,10,393,0
TRIM62_7_5486,37,0,0,0,0,0,3,2,0,209,0,0
TRIM63_7_5487,688,429,678,45,2562,29,769,219,972,475,806,231
TRIM65_7_5488,0,1,0,0,0,0,0,121,129,10,0,13
TRIM67_7_5489,0,0,0,0,2,0,8,0,4,590,0,0
TRIM68_7_5490,6,45,0,68,0,0,1534,0,13,1,0,3
TRIM8_7_5491,16,146,141,440,447,0,203,49,593,58,14,163
TRIP12_7_5492,668,138,7,1731,0,180,0,1344,1138,1319,1299,203
UBA2_7_5493,3641,3523,5463,3874,5523,4002,5660,3281,3847,2019,5740,349
4
UBA6_7_5494,125,22,0,0,27,216,1985,0,71,40,228,7
UBA7_7_5495,0,0,35,0,24,0,2,86,15,0,4,386
UBAC1_7_5496,163,119,198,2,89,401,1404,60,37,165,147,55
UBAP2_7_5497,755,0,0,167,0,12,0,0,0,275,0,0
UBASH3B_7_5498,11,147,0,32,0,0,0,239,494,106,0,1365
UBE2B_7_5499,349,770,12,1110,14,891,2247,629,348,1288,2326,825
UBE2D4_7_5500,366,1631,537,661,619,15,1694,260,303,370,485,2523
UBE2E2_7_5501,16,412,0,105,0,0,92,80,218,267,75,20
UBE2F_7_5502,414,39,918,12,133,444,1175,3,242,251,54,291
UBE2G1_7_5503,237,448,376,289,864,233,544,332,338,563,105,34
UBE2J1_7_5504,320,397,152,444,804,41,23,833,332,111,0,180
UBE2L3_7_5505,843,1247,205,1369,989,1803,312,444,513,1964,260,712
UBE2M_7_5506,8,621,1,13,0,505,0,316,246,33,0,215
UBE2N_7_5507,1,83,221,333,1,0,616,1254,341,683,202,164
UBE2NL_7_5508,51,25,121,711,79,94,26,85,36,173,14,10
UBE2O_7_5509,20,425,23,1,22,218,90,190,22,271,7,471
UBE2Q1_7_5510,3250,2286,3385,579,1231,1605,3960,2372,4462,3180,3082,50
86

UBE2QL1_7_5511,0,0,0,0,0,200,223,0,0,8,0,9
UBE2R2_7_5512,571,32,37,330,545,255,55,775,440,5,1,333
UBE2S_7_5513,181,255,148,19,241,0,1731,44,398,305,153,41
UBE2T_7_5514,3214,1040,3041,1068,1368,987,1964,2042,2482,1982,3526,302
7
UBE2U_7_5515,319,761,3,913,425,575,709,765,677,1582,977,620
UBE2V2_7_5516,138,444,2,1366,684,335,2354,935,317,711,1804,450
UBE2Z_7_5517,178,259,102,196,0,782,45,306,0,576,283,156
UBE3C_7_5518,462,405,143,332,1393,188,370,142,795,281,398,89
UBQLN2_7_5519,0,205,20,317,0,32,6,0,7,1,7,27
UBQLN3_7_5520,0,0,75,0,315,0,16,34,0,1,320,0
UBQLN4_7_5521,324,19,40,112,605,312,2,0,889,1228,644,122
UBR1_7_5522,982,486,1204,324,1,1413,2,316,88,507,1306,827
UBR3_7_5523,14,64,225,2,3,57,119,14,303,89,83,54
UBR4_7_5524,0,1,0,401,5,9,120,32,917,29,0,111
UBR5_7_5525,92,295,0,452,9,902,28,399,802,71,338,947
UBR7_7_5526,925,807,0,13,8,7,0,759,804,749,221,226
UBXN10_7_5527,734,137,298,610,130,72,83,513,34,401,936,113
UBXN1_7_5528,0,0,0,0,0,0,0,0,0,112,0,0
UBXN2A_7_5529,1032,849,665,364,907,986,308,224,370,188,794,296
UBXN4_7_5530,16,5,0,0,0,0,321,0,12,246,41,150
UBXN7_7_5531,7,1,0,69,0,520,0,769,190,138,82,22
UBXN8_7_5532,198,629,199,558,0,356,0,0,376,8,0,36
UCHL1_7_5533,139,37,1259,332,169,226,1434,1568,969,51,558,618
UCHL3_7_5534,4463,2341,4553,3402,2407,1999,4446,5121,4456,4722,2046,33
24
UFC1_7_5535,1293,1060,736,327,698,1346,1593,820,959,470,245,1447
UHRF2_7_5536,619,508,550,454,1811,2603,847,1079,788,342,1107,843
UNK_7_5537,0,0,0,0,135,0,0,44,24,0,0,1
USP11_7_5538,47,0,813,23,0,0,213,18,0,40,521,0
USP13_7_5539,95,72,367,84,0,133,712,368,733,668,845,422
USP15_7_5540,486,374,50,922,3275,987,393,335,289,1631,247,2330
USP17_7_5541,2647,1014,2976,1658,2862,2962,2172,1778,1527,2029,1391,15
95
USP17L2_7_5542,3,13,7,8,1165,494,101,414,76,27,316,20
USP17L5_7_5543,486,452,1654,492,1136,1025,550,389,404,473,306,1373
USP18_7_5544,3,0,0,579,0,0,0,0,355,469,897,39
USP22_7_5545,33,210,8,3,0,345,1,6,6,14,13,3
USP24_7_5546,102,526,3,19,12,56,915,212,447,109,285,183
USP25_7_5547,1343,820,2147,574,795,1099,786,37,670,1215,393,525
USP26_7_5548,211,26,463,68,953,1199,320,14,0,1203,912,447
USP27X_7_5549,297,3,0,0,0,3,0,0,17,2,0,5
USP28_7_5550,47,3,179,12,229,108,3,295,71,62,111,674
USP29_7_5551,366,531,2,529,658,1078,310,655,145,61,391,242
USP30_7_5552,3,2,0,218,300,92,2,0,1,104,1,10
USP32_7_5553,91,567,238,87,63,157,965,3,61,821,1370,969
USP34_7_5554,162,1362,427,1004,274,40,711,322,404,6,887,580
USP35_7_5555,25,1156,0,15,0,4,24,67,705,13,192,165
USP36_7_5556,13,0,0,124,0,0,0,0,0,0,0,0
USP37_7_5557,0,0,3,6,0,0,0,5,13,187,13,848

USP38_7_5558,1636,962,335,2774,1394,1173,2792,1410,1437,3561,4216,3007
USP39_7_5559,174,601,675,411,8,448,876,1665,581,705,751,500
USP3_7_5560,1912,0,31,671,367,1001,430,50,62,981,61,52
USP40_7_5561,1035,0,6,9,0,1,373,0,128,374,3,103
USP42_7_5562,0,56,22,0,0,31,154,4,110,7,387,11
USP43_7_5563,7,451,486,58,737,513,13,51,348,226,1834,175
USP45_7_5564,1522,583,66,117,458,235,646,293,118,690,250,102
USP47_7_5565,473,17,444,331,2832,137,455,243,929,1067,1112,1210
USP49_7_5566,3739,689,1585,474,1479,740,1308,3140,1283,1846,3409,1896
USP50_7_5567,2352,323,805,663,199,774,2113,1032,1622,947,1757,1727
USP51_7_5568,3,862,159,1128,209,973,6,807,1025,1382,319,118
USP53_7_5569,231,158,204,278,984,465,195,462,363,40,1023,197
USP54_7_5570,228,199,654,522,116,95,444,1139,743,707,1,666
USP6_7_5571,91,567,238,87,63,157,965,3,61,821,1370,969
USP7_7_5572,136,0,1351,526,56,25,1470,362,223,33,45,935
USP9Y_7_5573,906,341,643,395,0,654,141,1,1544,331,467,646
USPL1_7_5574,607,127,467,149,143,251,3,518,540,233,1,127
VCPIP1_7_5575,317,41,0,306,499,3,36,43,101,447,0,478
VPS11_7_5576,622,623,990,319,189,1271,268,1509,1022,411,1190,1090
WDR12_7_5577,22,119,2,60,738,0,0,6,0,0,77,148
WDR53_7_5578,0,33,0,0,483,279,0,8,0,5,0,0
WDR59_7_5579,43,331,2,0,0,0,30,0,125,3,857,90
WDR5B_7_5580,1322,81,0,722,73,6,377,567,658,1483,8,792
WDR61_7_5581,24,244,78,746,97,193,1464,1795,1047,707,164,472
WDTC1_7_5582,259,83,47,7,21,3,618,4,27,741,138,4
WSB2_7_5583,879,65,0,292,643,102,18,455,299,434,882,181
WWP1_7_5584,66,2,0,308,1105,28,0,1453,94,18,134,902
YOD1_7_5585,1001,580,1058,1825,2854,890,142,940,253,722,336,2567
ZBTB11_7_5586,1180,22,372,678,1215,659,661,1038,432,1635,1013,151
ZBTB25_7_5587,107,7,0,2,739,387,29,685,25,748,836,1203
ZBTB2_7_5588,113,331,832,1,706,746,0,324,141,137,135,891
ZBTB32_7_5589,0,14,0,335,0,0,0,778,501,9,37,75
ZBTB34_7_5590,0,275,63,78,0,64,357,601,286,211,33,29
ZBTB39_7_5591,0,0,0,0,0,0,0,0,0,0,0,0
ZBTB3_7_5592,82,1,8,90,0,760,10,717,90,5,0,86
ZBTB41_7_5593,229,7,3,529,217,540,45,121,511,47,27,110
ZBTB44_7_5594,373,341,476,705,648,1170,46,112,151,284,145,44
ZBTB45_7_5595,23,5,633,0,0,41,35,0,169,3,0,58
ZBTB46_7_5596,0,42,117,62,135,8,0,193,129,514,1,11
ZBTB47_7_5597,28,0,0,4,433,383,0,151,1,0,0,0
ZBTB48_7_5598,221,43,954,0,6,481,14,227,327,134,438,951
ZBTB49_7_5599,6,0,0,0,0,508,0,548,2,0,512,39
ZBTB5_7_5600,23,234,7,1,19,337,5,117,31,110,303,252
ZBTB7A_7_5601,627,466,325,1351,681,63,1234,413,554,255,1781,89
ZBTB7B_7_5602,48,79,1,25,0,4,129,25,0,1,0,1
ZBTB7C_7_5603,0,4,0,0,0,0,0,394,36,1,90,3
ZBTB8A_7_5604,1128,1279,1260,313,1685,241,1541,83,1642,1726,994,1068
ZFAND2B_7_5605,0,97,33,1,0,38,4,94,0,35,5,5
ZFPL1_7_5606,10,0,0,643,0,16,484,0,36,45,566,12
ZMYND10_7_5607,0,3,0,0,0,0,0,617,0,7,0,0

ZNF131_7_5608,0,778,631,0,573,0,30,0,121,1225,784,17
ZNF598_7_5609,825,170,1160,5,660,637,0,72,917,214,173,521
ZNF645_7_5610,73,0,0,256,0,0,823,0,106,86,326,126
ZNRF1_7_5611,0,2,143,1233,1610,77,30,402,4,0,120,0
ZNRF2_7_5612,15,230,49,28,250,336,171,1004,153,400,163,1571
ZNRF4_7_5613,0,0,488,0,0,839,0,0,474,14,853,62
ZRANB1_7_5614,38,1308,27,1040,73,1734,860,1004,791,528,2720,300
ZSWIM2_7_5615,792,431,391,1,293,294,958,430,5,301,1,160
AIRE_7_5616,802,348,1135,108,2492,114,1743,762,429,1378,1900,1186
ANAPC5_7_5617,174,0,956,82,2,110,0,14,246,74,184,33
ANAPC7_7_5618,602,1333,259,684,2685,582,509,296,1224,756,1266,1030
ANKFY1_7_5619,457,0,7,102,0,29,43,0,167,3,61,20
ASB10_7_5620,1,0,0,0,0,0,0,0,1,0,31,0
ASB11_7_5621,308,12,5,15,173,350,69,7,531,800,1,157
ASB14_7_5622,146,17,157,8,24,8,74,367,548,83,284,630
ASB2_7_5623,12,0,0,159,0,1,0,0,0,0,26,0
ASB3_7_5624,52,0,0,0,0,0,41,0,51,44,41,31
ASB4_7_5625,90,0,1,0,0,26,6,0,0,9,0,4
ASB6_7_5626,869,2484,2079,469,852,481,900,939,1638,2737,1303,663
ASB7_7_5627,388,236,1584,179,84,26,1183,5,466,35,364,63
ASB9_7_5628,18,963,38,0,0,143,279,185,35,237,186,23
ASCC2_7_5629,29,279,980,1,79,0,0,0,0,524,1106,116
ATG10_7_5630,155,465,0,0,0,23,1053,20,23,0,0,3
ATG16L1_7_5631,2677,1510,1273,377,959,1419,825,1180,1151,1190,1389,152
7
ATG7_7_5632,851,855,295,150,2719,550,1239,268,1801,782,1880,861
ATRX_7_5633,799,327,940,900,1271,705,115,774,558,246,2376,715
BACH2_7_5634,0,0,1,0,34,42,0,0,6,892,0,679
BAG6_7_5635,0,0,0,501,0,0,17,663,10,0,0,0
BAZ1A_7_5636,24,383,728,72,245,1397,23,1151,375,302,44,161
BCL6_7_5637,0,103,15,1,8,33,30,52,0,281,406,387
BIRC3_7_5638,4,507,211,94,2174,739,1738,1005,708,227,119,479
BIRC7_7_5639,15,0,0,0,0,29,61,0,0,0,0,809
BPTF_7_5640,96,0,42,27,345,0,247,81,15,72,36,1
BRCA1_7_5641,27,628,317,176,5,116,176,23,134,66,0,611
BRPF1_7_5642,4426,2721,5928,2858,4240,2585,1871,2201,2542,4353,3996,16
36
BRWD1_7_5643,87,475,99,18,730,3,277,194,622,288,5,176
BTBD11_7_5644,1059,97,1706,1283,2,1176,1383,1286,880,1011,2794,1082
BTBD1_7_5645,554,1,0,394,0,0,0,667,35,316,0,220
BTBD3_7_5646,574,457,541,770,1121,430,99,1889,458,574,2120,2262
BTBD7_7_5647,888,1426,1017,512,140,127,783,224,1527,580,386,801
BTBD9_7_5648,207,17,0,406,48,337,22,251,207,309,643,387
BTRC_7_5649,569,0,1,93,0,81,70,7,697,230,109,80
C3orf26_7_5650,1035,3972,2075,2498,3332,1282,3267,3340,1278,2587,1953,
2480
CBLC_7_5651,34,40,0,569,0,0,13,17,2,1,0,0
CCNB1IP1_7_5652,87,199,114,110,1093,167,244,803,484,70,0,337
CDC16_7_5653,64,705,59,236,913,426,62,561,874,909,477,953
CDC27_7_5654,349,246,592,490,716,411,33,17,245,745,156,163

CHFR_7_5655,0,68,32,0,130,0,0,158,754,213,0,91
CISH_7_5656,131,398,102,67,1792,203,1179,14,240,1246,1672,462
CNOT4_7_5657,107,1,0,60,0,0,3,0,332,31,0,372
COPS2_7_5658,4,3,1057,91,31,174,959,4,153,987,82,692
COPS3_7_5659,574,302,727,480,2614,13,0,52,510,47,74,107
COPS7A_7_5660,59,5,0,3,0,1,0,0,0,0,0,343
COPS8_7_5661,21,35,144,0,355,0,0,190,0,24,1,3
CRBN_7_5662,132,0,41,559,1262,306,1253,777,1191,800,430,624
CUL2_7_5663,0,80,824,76,1,51,0,27,275,454,0,153
CUL4A_7_5664,0,238,5,63,854,58,2,11,104,36,24,16
CUL4B_7_5665,339,0,1121,21,614,495,683,592,337,754,436,252
CUL7_7_5666,41,10,477,7,90,22,183,909,160,4,8,276
CXXC1_7_5667,0,526,35,554,125,24,4,1,0,0,7,0
CYLD_7_5668,3188,2855,1174,2257,3676,2849,1889,4236,2648,3183,674,5477
DCAF11_7_5669,22,3,73,0,1155,349,0,0,0,11,315,124
DCAF17_7_5670,0,0,0,0,0,0,0,0,0,3,0,2
DCAF4_7_5671,603,0,0,0,0,17,0,13,0,16,2,3
DCAF6_7_5672,494,742,481,42,2,781,938,1754,77,129,1595,252
DCST1_7_5673,501,18,815,236,110,10,115,469,19,152,20,9
DEPDC1B_7_5674,527,430,196,501,977,107,387,407,8,104,1051,368
DET1_7_5675,0,562,0,9,0,705,39,745,245,1201,280,202
DIDO1_7_5676,0,212,1,0,22,2,0,25,0,18,156,0
DNAJB2_7_5677,0,0,927,0,0,0,0,0,0,126,0,0
DTX2_7_5678,211,14,160,0,1296,0,117,68,303,145,1255,474
EED_7_5679,2601,1982,1688,1445,2500,2311,434,796,1230,713,2542,396
EIF3B_7_5680,488,0,0,95,578,885,154,0,0,28,19,11
EIF3C_7_5681,1,1,196,5,1,138,2,236,144,302,593,409
EIF6_7_5682,374,0,0,1530,904,2,1,509,0,466,0,3
EPN1_7_5683,583,481,619,138,423,503,197,45,64,152,844,314
EPN2_7_5684,706,1538,695,285,342,538,737,142,953,1515,1043,2905
EPS15_7_5685,190,1675,1083,659,191,406,170,1188,1088,713,684,651
FAM70A_7_5686,1,0,0,101,0,0,1,13,176,13,0,366
FANCL_7_5687,3,37,878,147,0,197,51,184,431,40,1175,84
FBXL13_7_5688,133,968,37,0,8,285,1025,595,70,255,2055,115
FBXL20_7_5689,29,126,27,57,0,131,0,1,215,276,223,129
FBXL2_7_5690,15,0,0,23,0,9,343,0,0,111,41,0
FBXL5_7_5691,261,569,1646,352,690,346,1238,847,1298,814,113,949
FBXL6_7_5692,275,553,0,962,2972,420,287,390,253,274,98,164
FBXO11_7_5693,1375,1295,1428,1325,314,668,1915,1206,1456,1868,2135,754
FBXO15_7_5694,2,185,121,29,0,242,2,30,12,180,0,350
FBXO17_7_5695,207,834,0,0,0,0,0,1860,471,589,7,623
FBXO18_7_5696,0,0,0,0,0,0,0,0,0,0,0,0
FBXO21_7_5697,142,182,733,88,50,844,5,49,22,456,4,47
FBXO22_7_5698,667,2,1151,4,461,538,1184,57,271,44,438,91
FBXO24_7_5699,22,49,27,15,640,5,162,0,14,188,0,12
FBXO25_7_5700,2674,3394,2036,2244,768,3090,979,1941,1673,1522,1358,191
1
FBXO28_7_5701,185,0,1,692,16,506,0,0,6,445,5,1621
FBXO32_7_5702,0,28,36,15,15,87,7,107,5,150,23,44
FBXO34_7_5703,585,18,0,0,0,359,199,658,474,551,931,394

FBXO38_7_5704,24,279,42,7,328,328,2,132,653,155,1407,529
FBXO3_7_5705,332,116,204,11,122,53,81,0,194,1,180,18
FBXO44_7_5706,168,3,12,413,73,38,733,18,20,103,25,1740
FBXO4_7_5707,382,568,654,28,16,17,1240,170,7,344,605,984
FBXO5_7_5708,1388,165,1828,839,552,764,58,1145,653,1232,166,1426
FBXO7_7_5709,1833,2352,961,1155,2195,1672,2036,1928,1539,2097,1117,271
8
FBXO9_7_5710,554,93,40,27,216,847,957,53,778,203,497,229
FBXW11_7_5711,910,292,540,341,1517,176,1590,134,245,812,2322,1061
FBXW7_7_5712,989,2232,805,1144,1665,969,1177,1480,1201,509,1722,183
FBXW8_7_5713,27,413,0,569,1,237,396,430,50,5,52,153
GPS1_7_5714,102,33,5,132,399,131,108,16,77,559,37,14
HECTD2_7_5715,264,5,1,33,85,0,0,55,13,6,180,3
HERC4_7_5716,402,536,1450,1709,1545,292,1727,789,1058,1594,909,725
HERC6_7_5717,358,213,133,310,213,117,0,668,449,652,1551,166
HIC1_7_5718,0,12,10,17,0,1,5,92,5,250,0,249
HLTF_7_5719,968,593,1886,572,1740,111,779,38,1349,821,2368,1324
HSF4_7_5720,13,1042,391,0,0,0,1376,0,389,13,1,45
IPP_7_5721,12,0,0,172,269,214,0,0,6,82,132,1
KAT6A_7_5722,30,574,20,178,1343,457,441,984,26,1005,1171,179
KBTBD3_7_5723,67,1169,209,871,2061,184,654,1071,1243,392,649,1145
KCTD6_7_5724,2527,1201,3058,767,2153,775,2248,2633,1792,1126,4355,722
KCTD7_7_5725,103,0,25,468,0,0,104,111,96,239,0,636
KDM2B_7_5726,0,0,0,3,0,179,20,11,1,244,55,62
KDM4C_7_5727,203,296,494,60,351,189,1291,369,659,655,744,697
KDM5C_7_5728,16,1,3,0,916,0,0,0,8,89,21,2
KEAP1_7_5729,38,548,0,92,151,162,11,0,58,167,1,98
KIAA1841_7_5730,100,0,67,1,0,1,306,217,267,55,151,15
KLHL13_7_5731,87,356,901,1,11,705,337,1283,605,661,279,248
KLHL2_7_5732,0,3,1,0,117,0,216,0,631,320,195,588
KLHL4_7_5733,331,469,662,0,951,262,161,27,560,248,1231,761
KLHL5_7_5734,932,103,1001,1436,2009,1227,294,648,2924,1431,679,2357
KLHL7_7_5735,583,17,58,31,146,138,472,26,26,2,1,983
LNX1_7_5736,684,1165,1237,1233,3170,2224,759,1365,1169,1138,643,806
LONRF3_7_5737,2156,1861,2090,2061,1010,1940,2780,3681,591,2056,1682,26
80
LRRC29_7_5738,48,56,110,2,181,194,9,1546,981,105,26,461
LRSAM1_7_5739,7,0,0,12,0,0,2,0,5,0,0,1
MARK2_7_5740,5,0,0,6,180,0,19,3,4,8,13,22
MARK3_7_5741,2,0,779,548,1279,837,1505,220,459,139,211,587
MARK4_7_5742,1,381,106,377,54,50,0,673,173,645,325,799
MDM4_7_5743,0,0,0,0,441,0,30,0,5,0,0,2
MGRN1_7_5744,0,0,1,25,203,3,0,0,591,8,0,86
MIB2_7_5745,0,14,643,12,275,41,169,77,2,1,116,172
MID1_7_5746,4,1171,0,48,2,129,393,2,456,178,5,651
MID2_7_5747,334,190,0,151,957,556,500,191,284,1542,411,838
MKRN1_7_5748,64,128,842,47,322,228,122,476,1197,323,476,407
MLL5_7_5749,943,1247,1465,855,865,1506,738,140,1109,2599,650,914
MLL_7_5750,59,319,15,387,1,45,756,92,1319,20,104,453
MNAT1_7_5751,504,1107,1230,355,1206,554,1209,395,523,692,328,1709

MPND_7_5752,0,136,8,429,1397,216,159,1060,680,451,15,207
MTF2_7_5753,0,0,218,51,13,968,57,1240,0,2,0,1
MYNN_7_5754,120,368,645,1,181,893,623,208,133,1061,442,183
NAE1_7_5755,439,271,380,173,570,782,539,595,605,481,861,340
NDUFC2_7_5756,1130,9,26,95,17,9,24,5,22,31,16,170
NEDD4_7_5757,18,330,454,114,406,280,630,26,64,390,55,367
NEDD4L_7_5758,627,233,1310,806,743,754,1354,127,313,623,297,1842
NFX1_7_5759,136,322,349,227,103,397,790,236,820,807,160,372
NLE1_7_5760,230,85,4,544,4,22,6,0,281,326,10,110
NSD1_7_5761,0,0,0,0,0,0,0,0,0,0,0,0
NSFL1C_7_5762,615,3,225,592,0,129,139,2,251,268,11,26
NUB1_7_5763,4,10,1,70,0,2,0,0,5,487,11,1
ODF2_7_5764,0,0,0,0,0,0,0,0,0,0,0,0
OTUD5_7_5765,723,164,727,53,435,512,0,130,209,134,128,465
PARK2_7_5766,131,252,749,660,295,468,113,306,440,522,0,66
PARP9_7_5767,76,220,100,333,622,60,103,540,394,185,256,402
PATZ1_7_5768,655,0,0,0,0,0,4,0,10,0,148,0
PCGF6_7_5769,9,25,3,16,332,14,3,16,128,9,9,21
PEX10_7_5770,0,104,1083,270,212,58,28,872,27,116,24,268
PEX2_7_5771,651,57,258,2,634,1640,887,994,2108,1315,105,730
PHF12_7_5772,89,51,1476,41,281,36,1,516,204,954,321,246
PHF16_7_5773,1617,1251,986,81,288,381,37,175,392,319,755,735
PHF17_7_5774,29,115,535,14,0,164,694,210,231,263,0,368
PHF1_7_5775,5,272,0,985,0,0,0,0,63,500,0,617
PHF21A_7_5776,191,18,165,92,569,0,203,19,0,508,420,39
PHF7_7_5777,0,0,3,58,0,0,0,0,76,561,14,5
PHF8_7_5778,300,829,156,328,930,491,1255,42,472,293,414,602
PJA1_7_5779,1952,1451,222,998,437,1415,958,2034,1238,732,34,654
PML_7_5780,0,0,0,0,0,0,0,0,0,47,0,7
POC1B_7_5781,260,533,550,11,253,768,878,829,1123,554,11,815
PSMD1_7_5782,8,3,40,269,0,1102,883,10,746,23,370,127
RAPSN_7_5783,122,57,273,1,67,2,301,458,1,45,22,0
RBBP4_7_5784,146,598,358,189,1026,1876,246,338,459,1281,213,2786
RBBP5_7_5785,1166,3,1250,635,1450,432,297,3237,5,797,27,8
RBBP6_7_5786,3270,700,500,1544,89,384,1074,225,931,1686,1717,2296
RBBP7_7_5787,2679,657,1293,1087,2247,1737,952,916,1429,1006,561,1175
RBCK1_7_5788,689,0,277,12,790,24,0,244,65,0,543,121
RC3H2_7_5789,201,295,130,140,0,64,455,36,0,585,431,583
RCHY1_7_5790,414,2815,543,376,2158,618,494,23,1253,1662,257,336
RFPL2_7_5791,807,26,143,789,1496,0,47,138,85,84,1,331
RFPL3_7_5792,361,1276,1351,1585,1035,1568,1280,2163,634,1039,895,991
RFWD2_7_5793,473,102,0,544,617,1264,326,2336,891,441,2430,1371
RHOBTB1_7_5794,756,161,15,507,29,160,272,604,9,714,269,730
RHOBTB2_7_5795,867,149,576,1906,436,32,1041,43,339,74,40,637
RLIM_7_5796,3290,1003,1202,1101,766,455,916,817,1950,1257,1890,465
RNF103_7_5797,9,14,127,572,107,52,287,870,107,406,639,562
RNF128_7_5798,1195,302,1883,808,570,180,158,245,849,900,1423,198
RNF135_7_5799,399,43,589,132,24,280,437,179,45,75,397,6
RNF138_7_5800,257,560,1227,1110,346,1855,1153,663,1023,1617,771,1319
RNF13_7_5801,19,1324,143,327,0,1,697,682,41,73,1016,182

RNF145_7_5802,8,30,2,5,47,1103,22,694,111,693,95,95
RNF146_7_5803,1258,1148,404,1753,279,1458,582,969,531,937,1293,2330
RNF14_7_5804,1281,525,1383,111,162,1246,776,728,853,1213,1009,719
RNF166_7_5805,4,0,86,59,426,15,1,0,0,0,89,13
RNF170_7_5806,129,0,0,0,410,54,875,0,127,1052,665,14
RNF17_7_5807,6,32,0,168,0,226,0,0,376,89,349,236
RNF180_7_5808,10,345,1049,995,0,1284,41,6,133,369,0,30
RNF182_7_5809,0,0,0,0,217,0,0,0,0,0,0,0
RNF185_7_5810,0,0,255,182,833,252,7,53,52,72,1,16
RNF19A_7_5811,2347,1910,1027,1494,1749,182,209,2191,1825,2341,2474,131
1
RNF19B_7_5812,121,101,463,0,2,0,69,17,0,647,158,141
RNF213_7_5813,357,104,0,248,46,192,543,843,117,84,0,10
RNF214_7_5814,283,249,349,176,336,906,914,1670,859,942,992,197
RNF216_7_5815,641,842,409,1029,1479,872,1551,673,985,1804,781,1694
RNF24_7_5816,0,0,0,537,0,50,274,0,498,85,0,47
RNF32_7_5817,1090,594,1107,658,290,1168,514,739,1182,247,273,1126
RNF34_7_5818,0,4,0,30,622,0,0,848,0,0,8,104
RNF38_7_5819,25,107,909,1,208,30,1,3,247,198,155,44
RNF40_7_5820,3,138,565,697,311,1039,260,56,450,1222,424,819
RNF41_7_5821,3,0,0,0,0,0,0,0,0,0,0,0
RNF6_7_5822,2513,586,526,871,1890,644,1227,2261,2033,390,1831,2145
RNF7_7_5823,1679,789,1625,791,1198,170,190,24,964,464,924,234
RNF8_7_5824,543,6,183,0,16,161,0,34,110,138,0,12
SAE1_7_5825,583,147,681,1,221,32,1488,195,951,190,157,792
SENP6_7_5826,89,507,122,0,0,0,0,96,0,530,0,0
SENP7_7_5827,21,357,76,650,345,5,449,754,541,307,0,1148
SENP8_7_5828,986,1712,175,2658,1439,1329,1565,1171,135,1154,611,632
SF3A1_7_5829,275,1062,77,372,1595,69,994,1330,29,417,458,9
SHPRH_7_5830,2354,2819,1739,2373,823,3048,3238,2594,2503,1498,5372,247
4
SIAH1_7_5831,553,5,552,417,723,135,0,129,3,1229,1,422
SKP2_7_5832,544,145,1034,8,866,697,58,0,482,486,617,830
SMURF1_7_5833,1,45,387,132,0,940,1333,0,392,34,0,508
SOCS5_7_5834,570,952,133,24,319,0,2257,704,489,184,495,78
SP100_7_5835,989,1704,47,881,1794,684,1105,631,1845,551,630,774
SP110_7_5836,153,0,342,0,140,136,182,102,100,194,53,220
SPOP_7_5837,1811,537,343,524,2611,1374,530,1495,1408,410,1117,619
SPSB2_7_5838,576,46,1198,0,155,1,0,265,1576,411,6,195
SQSTM1_7_5839,28,61,26,712,58,0,27,0,32,7,808,10
STAMBP_7_5840,2,0,51,0,1067,338,0,301,0,1047,23,1
SUMO1_7_5841,36,392,420,414,34,335,447,830,349,554,1,1172
SUMO2_7_5842,6,243,62,711,1037,324,75,127,132,523,454,29
SYTL4_7_5843,1,311,61,140,80,421,13,6,100,1005,112,829
SYVN1_7_5844,1,109,644,386,1,470,149,782,323,9,204,852
TCF20_7_5845,11,8,2,157,242,109,0,245,441,13,48,64
TDRD3_7_5846,424,1005,1558,76,1078,1330,679,1053,365,759,1115,51
TIPARP_7_5847,612,530,0,388,204,468,224,0,517,41,1,82
TLE2_7_5848,1091,0,0,1060,56,99,177,6,278,696,826,787
TLE3_7_5849,312,58,8,85,406,424,128,70,646,25,520,298

TNK2_7_5850,598,65,33,44,265,515,0,0,254,189,815,460
TNRC6C_7_5851,44,134,9,0,539,11,65,7,217,4,134,46
TOPORS_7_5852,283,0,0,364,0,5,0,101,72,633,904,4
TOR1AIP2_7_5853,1863,959,3387,701,1408,285,858,1285,1295,988,2182,1257
TRAF3_7_5854,0,6,0,637,28,0,0,2,0,0,0,0
TRAF5_7_5855,861,380,293,922,12,1569,159,222,1136,821,299,968
TRAF6_7_5856,192,808,913,58,0,222,348,49,120,598,658,10
TRIM10_7_5857,50,9,672,217,84,0,0,0,496,77,32,766
TRIM13_7_5858,651,274,422,0,1907,0,29,114,4,310,650,59
TRIM17_7_5859,0,0,0,0,229,0,6,0,0,299,1,0
TRIM22_7_5860,1,175,76,541,158,196,788,43,68,209,1,47
TRIM23_7_5861,178,471,194,176,882,318,897,323,354,170,80,115
TRIM24_7_5862,579,13,59,3,66,135,40,248,279,515,0,43
TRIM26_7_5863,72,127,125,42,5,0,227,9,69,332,0,441
TRIM2_7_5864,5,0,0,0,0,1,0,0,0,0,0,0
TRIM32_7_5865,1810,1035,1919,190,297,1205,670,894,559,2099,508,900
TRIM33_7_5866,339,621,839,587,1395,1199,79,874,887,876,1577,881
TRIM34_7_5867,280,0,0,8,981,167,5,115,53,628,160,561
TRIM37_7_5868,0,16,1418,0,1,903,188,120,24,10,241,371
TRIM39_7_5869,1261,2114,382,2025,208,1313,2262,616,902,2389,2066,1663
TRIM3_7_5870,0,0,0,0,55,0,0,0,0,0,0,0
TRIM41_7_5871,0,3,0,0,0,0,79,304,151,15,13,68
TRIM45_7_5872,1263,0,0,155,433,586,0,0,7,12,0,0
TRIM4_7_5873,15,460,19,807,56,237,0,425,81,412,663,206
TRIM54_7_5874,191,932,5,230,0,528,18,0,527,579,193,45
TRIM55_7_5875,50,575,4,459,6,1,116,10,63,0,0,13
TRIM5_7_5876,555,19,560,468,554,81,439,660,612,153,541,226
TRIM7_7_5877,0,3,2,33,0,19,0,34,313,48,4,26
TRIM9_7_5878,33,305,0,5,19,24,0,7,0,6,0,267
TRPC4AP_7_5879,1849,2748,5099,1048,1158,1526,1661,1956,1526,2514,1483,
1530
TSPAN17_7_5880,608,725,938,320,0,157,0,1342,861,349,979,114
TTC3_7_5881,951,2147,1511,745,389,1708,106,739,181,658,788,732
TULP4_7_5882,241,732,94,756,648,751,515,670,481,702,1456,1100
UBA1_7_5883,179,18,63,4,225,14,633,920,80,473,1697,16
UBA3_7_5884,1,0,0,113,6,0,462,29,1,0,7,0
UBA5_7_5885,1944,129,233,33,471,100,91,144,600,225,148,586
UBAC2_7_5886,173,1119,3,167,264,822,442,655,66,717,505,566
UBAP2L_7_5887,324,134,718,6,148,503,69,95,639,6,220,102
UBASH3A_7_5888,0,0,0,0,0,0,0,0,0,0,0,0
UBE2A_7_5889,1355,1955,2649,432,1131,1926,1843,2075,2251,1717,1747,114
9
UBE2D1_7_5890,2908,1718,1544,1893,1408,3041,2869,2667,1324,1890,4134,1
157
UBE2D2_7_5891,3536,2924,1417,3232,3140,1530,4281,2198,2877,1652,1833,2
177
UBE2D3_7_5892,1148,486,61,677,77,582,587,557,218,791,662,678
UBE2E1_7_5893,444,293,983,360,987,847,215,547,164,318,7,49
UBE2E3_7_5894,1327,508,1117,1016,1238,562,969,797,874,890,625,335
UBE2G2_7_5895,2,414,469,16,0,2,104,64,556,224,106,94

UBE2H_7_5896,729,872,31,1168,1163,250,68,1304,689,1196,1025,619
UBE2I_7_5897,61,121,3,104,9,2,34,1384,934,529,6,797
UBE2J2_7_5898,0,15,0,0,38,1201,2,524,37,50,2014,177
UBE2K_7_5899,307,164,397,72,305,267,138,201,473,574,285,129
UBE2Q2_7_5900,3692,2602,4455,602,1849,1609,2558,3919,3179,3489,2518,60
32
UBE2V1_7_5901,1507,1736,2881,996,2600,2008,2044,3346,1901,839,2636,250
9
UBE2W_7_5902,1,6,0,2,579,0,354,0,879,102,15,742
UBE3A_7_5903,1486,602,1034,107,652,583,926,1272,1148,107,240,218
UBE3B_7_5904,541,48,0,46,321,172,832,0,611,20,135,478
UBE4A_7_5905,108,531,294,337,210,262,79,716,91,278,861,15
UBE4B_7_5906,212,482,10,377,12,234,587,13,28,183,88,310
UBL7_7_5907,911,460,423,129,1394,934,16,197,318,177,50,451
UBOX5_7_5908,1823,192,57,10,64,639,1703,0,116,41,237,672
UBQLN1_7_5909,402,291,443,598,877,301,798,396,1025,1387,1431,1119
UBR2_7_5910,244,0,14,3,624,698,40,68,672,568,17,79
UBXN11_7_5911,102,251,0,18,0,0,0,2,71,6,1,7
UBXN6_7_5912,0,0,0,0,0,30,0,122,5,4,0,31
UCHL5_7_5913,415,491,157,58,341,709,99,1,234,265,588,857
UHRF1_7_5914,17,605,706,22,422,0,426,235,149,59,56,33
UIMC1_7_5915,203,89,1229,384,0,1613,3,1132,755,45,359,618
UNKL_7_5916,56,3,0,19,9,18,0,366,75,368,648,18
USP14_7_5917,183,111,0,121,0,26,246,142,246,281,23,387
USP19_7_5918,0,6,6,8,0,0,8,268,1,0,159,0
USP1_7_5919,461,1256,2356,943,1759,1892,3247,1620,1664,1147,1278,1523
USP20_7_5920,220,428,782,148,0,21,0,15,286,121,67,640
USP2_7_5921,695,3079,974,402,668,688,309,2243,933,102,2257,235
USP33_7_5922,256,406,228,501,1050,456,423,88,105,1614,20,91
USP44_7_5923,1725,1282,1154,929,1927,877,301,2055,1111,1268,1205,3061
USP46_7_5924,160,0,1,397,260,220,0,675,2,224,492,183
USP48_7_5925,657,675,470,242,538,507,161,1133,1427,485,1660,915
USP4_7_5926,1021,1770,0,382,1291,493,782,88,1083,422,1243,1023
USP5_7_5927,998,783,424,359,1621,574,1985,443,96,595,1245,798
USP6NL_7_5928,335,0,0,383,0,0,1639,536,0,0,0,744
USP8_7_5929,202,19,1065,7,0,121,0,73,152,8,1057,24
USP9X_7_5930,1807,1381,1597,903,2172,1247,2643,1928,1570,1372,2686,513
VHL_7_5931,381,930,45,16,1017,109,0,344,45,482,136,8
VPRBP_7_5932,66,0,0,163,569,46,33,390,696,116,0,964
VPS13D_7_5933,281,1018,637,288,291,152,374,342,261,414,1662,1260
VPS41_7_5934,948,5985,2918,963,298,2341,712,4249,2080,1328,1290,1725
VPS8_7_5935,76,33,10,67,38,1,150,42,5,1,0,173
WDR26_7_5936,1880,550,262,880,276,827,546,383,152,473,2014,837
WDR5_7_5937,3723,2510,2421,4088,3969,3649,5355,4366,2497,4219,7055,462
7
WDR76_7_5938,1248,2391,2575,908,48,765,1231,17,1388,1217,759,1257
WHSC1_7_5939,0,0,0,305,1120,0,0,0,0,17,0,0
WHSC1L1_7_5940,0,507,501,1359,999,23,0,0,87,345,1033,388
WSB1_7_5941,507,1547,0,419,1049,820,654,992,1188,1766,721,144
WWP2_7_5942,1,0,59,0,0,0,0,0,6,221,0,0

XIAP_7_5943,286,2099,28,897,1134,1139,1433,1964,694,773,1754,398
ZBTB10_7_5944,3,493,861,23,24,525,146,81,26,9,856,928
ZBTB16_7_5945,24,263,2,527,251,0,0,303,210,31,65,21
ZBTB17_7_5946,0,0,0,0,0,0,0,0,0,0,57,0
ZBTB1_7_5947,2372,1860,1793,469,773,580,723,1781,1861,1409,844,2172
ZBTB20_7_5948,109,3,163,34,192,112,571,7,397,169,339,297
ZBTB22_7_5949,39,53,45,0,0,1,270,0,0,100,1074,2
ZBTB24_7_5950,13,243,1,516,450,180,1271,34,1126,733,1093,774
ZBTB33_7_5951,30,38,1488,1190,13,1115,105,1457,1080,381,484,1264
ZBTB37_7_5952,1973,1034,1226,76,1223,1277,4138,1766,1517,898,1166,771
ZBTB40_7_5953,7,27,0,0,3,1,0,270,1,8,850,55
ZBTB4_7_5954,22,194,1,648,2,24,3,0,476,72,0,62
ZFP161_7_5955,0,14,96,161,5,2,39,0,7,398,1189,562
ZMYND11_7_5956,283,0,82,0,68,297,68,224,644,227,107,233
ZMYND8_7_5957,0,169,157,8,0,3,1,735,24,309,14,80
ZNF238_7_5958,252,1118,803,2335,440,1335,750,374,2284,2444,1006,351
ZNF295_7_5959,181,663,796,190,0,906,759,262,61,108,0,24
ZNRF3_7_5960,101,269,282,66,858,205,336,0,1210,1119,959,146
ANAPC11_7_5961,233,205,53,200,0,85,0,134,75,29,435,294
ATXN3_7_5962,0,0,446,0,228,306,62,14,525,16,1,112
MLLT10_7_5963,0,0,0,0,33,4,0,2,483,5,0,264
OTUD4_7_5964,524,510,8,85,1320,570,1202,771,972,142,1458,320
PHF19_7_5965,1010,416,197,461,177,1084,714,1434,178,1155,2157,937
SP140_7_5966,33,19,25,3,969,519,244,93,752,798,14,135
TRIM36_7_5967,0,0,0,51,0,0,0,0,3,18,0,0
UBE2C_7_5968,219,115,1479,20,722,813,868,1235,379,106,176,195
UBE2L6_7_5969,0,8,2,58,11,0,0,2,168,4,0,15
ABTB2_7_5970,259,657,1195,1063,1179,396,992,1921,1430,852,1746,502
AMBRA1_7_5971,63,70,1000,75,53,67,290,1,127,16,21,10
AMFR_7_5972,82,196,0,811,53,97,45,22,121,432,303,182
ANAPC10_7_5973,555,95,441,616,660,18,0,220,2,249,1,152
ANAPC1_7_5974,51,1041,556,985,779,1335,657,115,663,214,1671,608
ANAPC2_7_5975,0,0,0,0,0,291,1,0,0,0,225,0
ANAPC4_7_5976,2445,979,248,833,650,325,49,1227,1243,2532,1013,294
ANKIB1_7_5977,754,17,799,108,613,174,23,0,3,258,10,293
ANKRD13A_7_5978,376,1273,77,310,976,943,21,1808,595,1081,104,865
ANKRD13D_7_5979,4,543,0,0,70,5,29,1,1,142,0,0
ARIH1_7_5980,10,808,0,93,197,24,0,0,561,53,141,44
ARIH2_7_5981,166,46,1587,498,1922,83,40,551,155,537,77,230
ASB12_7_5982,473,0,0,261,0,367,0,544,72,2,120,812
ASB13_7_5983,105,5,0,12,36,4,148,1,52,23,99,6
ASB15_7_5984,480,97,5,932,0,350,0,625,908,1076,117,810
ASB16_7_5985,36,133,1,0,4,589,0,149,74,4,110,75
ASB17_7_5986,466,72,156,523,367,96,0,0,395,572,1190,1738
ASB5_7_5987,913,9,2678,439,914,1406,16,746,1401,804,704,1044
ASB8_7_5988,431,321,339,916,251,266,721,727,525,1041,976,734
ASH1L_7_5989,1236,371,1183,633,1387,789,2020,885,1113,1176,1666,1155
ASPSCR1_7_5990,490,447,6,31,0,24,0,0,31,169,21,54
ATG12_7_5991,92,5,31,0,2,0,23,792,436,11,0,64
ATG3_7_5992,377,263,318,368,83,411,760,1390,411,620,349,115

ATG5_7_5993,110,19,1441,0,4,130,0,24,1225,120,104,515
ATXN1L_7_5994,94,0,1041,0,0,0,0,0,6,0,1385,1
ATXN3L_7_5995,1626,821,153,93,392,727,28,0,1199,420,2479,744
BARD1_7_5996,2066,316,62,1529,1211,594,1471,378,492,621,193,1020
BAZ1B_7_5997,907,809,18,631,1448,1166,1717,181,566,681,975,918
BAZ2A_7_5998,53,0,1,246,1,55,3,1,33,299,27,86
BAZ2B_7_5999,120,434,24,445,302,528,8,523,76,983,129,609
BECN1_7_6000,275,316,90,387,53,462,1759,148,62,84,375,605
BIRC2_7_6001,4,282,349,193,2132,752,2327,1020,26,229,89,261
BIRC6_7_6002,344,1430,131,383,809,97,289,376,646,1386,1973,942
BIRC8_7_6003,320,1369,159,922,1157,1149,1417,2026,675,799,1874,569
BMI1_7_6004,1506,1255,1887,479,1040,0,3,353,1231,955,3037,1270
BRAP_7_6005,41,1,0,43,42,24,160,857,271,13,14,63
BRD1_7_6006,0,503,17,99,105,162,0,150,468,2,0,122
BRPF3_7_6007,336,625,728,863,399,334,809,514,644,739,183,429
BTBD2_7_6008,0,0,0,0,0,0,0,0,0,49,825,10
BTBD6_7_6009,97,254,6,10,201,283,618,72,544,53,0,608
CAND1_7_6010,822,913,24,136,350,493,2614,515,638,854,1230,700
CBLB_7_6011,1613,1850,2400,1468,843,2434,2077,1684,1770,2534,2053,2428
CBL_7_6012,0,0,0,3,237,1,105,0,98,1,1,15
CBLL1_7_6013,309,66,2014,119,26,442,280,0,0,486,1638,1118
CCIN_7_6014,293,407,29,471,170,275,941,84,167,94,452,1038
CCNF_7_6015,1361,132,10,216,777,39,1193,1034,970,150,1532,580
CDC20_7_6016,934,1846,1431,2158,9,295,892,1152,1249,203,1606,3182
CDC23_7_6017,0,0,0,0,0,0,0,0,0,0,0,0
CDC26_7_6018,1022,329,1083,477,622,142,203,181,597,161,1406,507
CDC34_7_6019,1,43,0,5,800,0,0,0,89,80,226,15
CGRRF1_7_6020,1428,346,62,1807,2331,919,64,1506,1032,1918,864,3238
CHD4_7_6021,97,398,10,3,171,511,0,126,175,7,15,773
CIAO1_7_6022,539,0,40,0,0,82,3,2,16,0,34,627
COPS4_7_6023,500,172,0,29,312,113,403,983,1082,1215,844,1149
COPS5_7_6024,0,78,0,594,515,182,138,436,57,345,575,12
COPS6_7_6025,0,443,726,473,1,313,687,1,1,0,310,0
COPS7B_7_6026,16,42,38,32,253,9,457,102,212,6,15,22
CORO6_7_6027,256,4,216,65,0,57,0,0,558,102,535,980
CUEDC1_7_6028,0,647,923,2,68,24,2,83,2,602,168,0
CUL1_7_6029,61,0,2,165,0,904,24,310,34,507,288,11
CUL3_7_6030,107,23,428,99,149,228,620,91,421,316,788,1291
CUL5_7_6031,0,680,33,174,438,163,13,292,157,310,227,743
CUL9_7_6032,141,0,0,6,0,111,0,0,27,82,85,46
DCAF10_7_6033,0,52,72,0,25,3,480,39,0,671,14,143
DCAF12_7_6034,0,412,967,128,202,3,383,4,142,344,73,20
DCAF13_7_6035,790,10,214,942,1248,33,97,1368,527,813,572,1122
DCAF16_7_6036,393,1165,327,515,106,62,395,144,40,138,339,45
DCAF5_7_6037,0,1,0,0,1,1,0,0,0,0,0,0
DCAF7_7_6038,941,348,74,39,415,75,358,455,267,1765,881,35
DCAF8_7_6039,438,351,2296,1829,855,1,315,7,1182,1412,370,1783
DDA1_7_6040,254,0,264,374,200,90,485,0,0,429,1849,0
DDB1_7_6041,0,112,0,0,11,179,2,4,465,106,56,246
DDB2_7_6042,268,0,0,0,404,1,542,0,0,61,0,0

DPF2_7_6043,835,280,735,1593,1494,908,1,546,343,644,1757,695
DTX1_7_6044,1,16,0,28,0,176,20,11,476,601,0,51
DTX3L_7_6045,92,316,572,56,3564,602,933,479,1519,1242,1731,966
DTX4_7_6046,2,3,0,53,0,0,22,0,1,42,0,1
DZIP3_7_6047,699,0,1,181,0,135,0,1000,0,21,0,7
EIF3D_7_6048,0,53,2,65,92,372,468,0,104,587,28,31
EIF3E_7_6049,258,63,0,0,488,268,1311,71,64,357,20,37
EIF3F_7_6050,274,68,0,25,689,1,2,50,1,658,38,0
EIF3G_7_6051,868,569,1576,774,250,3735,1032,512,2609,539,2894,2333
EIF3H_7_6052,208,43,0,154,0,11,0,584,77,111,557,11
EIF3I_7_6053,128,224,1477,1,790,21,6,0,165,485,23,573
EIF3J_7_6054,440,184,665,399,1008,351,923,822,220,1162,1034,291
EIF3K_7_6055,215,384,181,409,3368,161,1480,2133,1466,287,1799,787
ENC1_7_6056,234,45,17,122,10,124,73,91,193,138,193,55
EPN3_7_6057,42,965,1,839,0,107,161,90,179,174,139,276
EPOR_7_6058,1,845,296,641,133,21,767,72,207,12,31,29
ERCC8_7_6059,72,53,26,28,48,29,120,0,16,440,626,145
FAF1_7_6060,301,320,20,29,346,23,1471,161,11,529,808,316
FBXL12_7_6061,665,493,832,25,0,139,294,133,133,269,672,579
FBXL14_7_6062,0,251,913,93,9,11,24,173,88,180,58,10
FBXL15_7_6063,0,10,865,14,26,86,41,39,6,329,2142,34
FBXL16_7_6064,0,0,0,0,0,0,0,0,0,0,0,0
FBXL17_7_6065,0,2,0,0,0,276,0,0,0,0,0,0
FBXL18_7_6066,0,0,0,0,0,0,0,0,0,0,0,0
FBXL19_7_6067,44,66,832,221,14,549,0,98,68,38,104,609
FBXL21_7_6068,3,1092,292,130,43,785,92,765,1051,824,546,159
FBXL3_7_6069,884,495,27,72,1299,1,17,55,35,13,31,87
FBXL4_7_6070,0,72,5,8,139,2,209,434,167,43,55,28
FBXL7_7_6071,427,531,0,1,20,90,0,220,213,260,654,45
FBXO10_7_6072,0,0,0,0,77,0,95,2,0,154,0,0
FBXO16_7_6073,2708,1684,464,822,414,1954,120,83,1005,835,712,681
FBXO27_7_6074,0,12,716,160,657,212,22,4,15,1,335,728
FBXO2_7_6075,41,58,630,119,4,496,20,32,140,5,48,412
FBXO30_7_6076,1289,155,884,224,275,1272,748,8,208,1512,577,1080
FBXO33_7_6077,83,149,919,41,9,241,442,13,2,224,977,273
FBXO36_7_6078,13,8,611,228,693,829,806,803,437,1639,900,756
FBXO39_7_6079,21,7,9,0,0,248,35,0,0,0,0,202
FBXO40_7_6080,1640,1089,3630,1342,953,1294,2551,2743,1128,1262,1990,15
05
FBXO41_7_6081,7,0,13,9,1014,341,0,687,271,109,55,153
FBXO42_7_6082,663,184,758,228,1103,338,1,6,440,102,937,394
FBXO43_7_6083,127,743,535,1189,1052,197,685,997,913,1164,1060,243
FBXO46_7_6084,191,1,439,499,1043,202,2,8,511,712,12,879
FBXO6_7_6085,390,316,1011,800,23,28,234,10,606,266,77,783
FBXO8_7_6086,71,603,672,114,6,173,969,0,210,580,876,220
FBXW10_7_6087,325,323,3,659,914,1160,1284,570,433,1289,802,1209
FBXW2_7_6088,4020,1347,1663,1404,3998,1203,4057,3313,3421,3371,1055,15
16
FBXW5_7_6089,0,0,0,0,251,0,0,597,0,47,49,0
FBXW9_7_6090,0,0,0,0,18,0,793,0,0,0,66,0

G2E3_7_6091,18,119,22,44,635,99,520,0,0,694,1064,58
GAN_7_6092,1307,781,1056,1504,380,1894,2169,1803,423,215,1076,114
GMCL1_7_6093,129,11,0,66,203,177,2,559,475,86,68,222
GNB2_7_6094,33,0,0,122,0,3,61,0,195,73,2,32
GRWD1_7_6095,30,56,106,9,61,0,1,72,0,42,0,8
GZF1_7_6096,1761,365,406,760,311,550,134,59,19,167,26,4
HACE1_7_6097,1507,2,159,419,305,260,896,319,309,18,1060,81
HDAC6_7_6098,181,302,965,0,1,485,83,249,450,371,329,50
HECTD1_7_6099,141,32,136,26,29,0,0,100,435,103,361,164
HECTD3_7_6100,1,2,0,0,0,105,59,527,411,7,0,53
HECW1_7_6101,0,0,0,0,0,0,1071,18,0,0,25,1
HECW2_7_6102,10,10,0,215,0,39,0,0,0,0,0,0
HERC1_7_6103,144,483,870,18,693,409,110,331,228,56,416,2095
HERC2_7_6104,1057,1249,1649,1408,4200,905,925,217,821,165,428,141
HERC3_7_6105,0,208,165,0,16,106,759,39,653,1110,324,405
HERC5_7_6106,0,1455,0,0,0,0,0,1,318,1126,0,448
HGS_7_6107,810,101,1803,748,685,55,1512,0,1424,313,573,200
HIC2_7_6108,0,0,0,0,4,0,2,24,0,1,0,0
HUWE1_7_6109,3,9,0,0,89,0,0,34,62,1,0,6
IBTK_7_6110,237,2,927,29,291,95,864,816,118,1,383,10
IL10RA_7_6111,924,790,1087,655,263,1175,388,160,921,299,191,2153
IL6_7_6112,9,557,249,108,1080,137,75,779,172,68,24,866
IRF9_7_6113,0,69,2,452,369,11,63,86,10,0,0,24
ITCH_7_6114,180,449,1,292,582,1709,13,40,56,165,786,667
IVNS1ABP_7_6115,180,135,805,0,98,1430,0,0,4,214,1776,1355
JHDM1D_7_6116,445,568,202,133,53,60,185,48,492,117,13,131
JOSD1_7_6117,621,5,10,487,0,0,103,194,100,157,732,11
JOSD2_7_6118,369,448,730,1466,634,359,297,581,921,736,925,304
KAT6B_7_6119,31,13,57,0,4,468,0,712,36,8,0,6
KATNB1_7_6120,23,262,899,33,148,80,0,495,88,519,197,28
KBTBD10_7_6121,173,327,1168,215,702,326,118,934,808,313,451,1266
KBTBD11_7_6122,0,120,174,0,0,47,0,456,0,31,0,3
KBTBD2_7_6123,1,220,120,18,371,8,4,1189,1154,421,133,147
KBTBD5_7_6124,432,2,0,0,0,666,0,1882,15,47,0,1
KBTBD7_7_6125,3,0,1054,803,2,196,926,0,110,737,237,9
KBTBD8_7_6126,1220,1480,0,57,63,679,130,174,548,38,0,534
KCTD10_7_6127,41,0,0,496,144,7,98,789,585,16,779,737
KCTD11_7_6128,61,234,0,0,2,0,1,4,305,64,318,42
KCTD12_7_6129,1,1,119,331,310,42,957,37,261,462,178,555
KCTD13_7_6130,544,49,249,738,618,298,0,868,177,5,556,226
KCTD16_7_6131,1379,57,315,55,881,205,929,240,406,336,797,1770
KCTD17_7_6132,305,10,0,78,0,4,11,0,156,9,0,30
KCTD18_7_6133,0,0,895,0,13,226,12,0,211,1043,0,45
KCTD3_7_6134,287,0,478,29,948,28,54,70,29,1705,144,292
KCTD5_7_6135,454,148,0,78,432,6,0,106,92,479,41,6
KCTD9_7_6136,95,1496,318,437,2019,500,690,510,292,1580,1120,1124
KDM2A_7_6137,144,30,624,577,262,443,420,278,661,9,0,108
KDM4B_7_6138,284,0,0,5,0,0,0,0,0,0,0,0
KDM5B_7_6139,325,114,166,1,131,200,317,0,782,11,53,98
KLHDC5_7_6140,0,0,0,0,0,0,0,0,0,0,0,0

KLHL10_7_6141,547,1148,1255,393,628,999,190,334,1020,89,2012,1183
KLHL11_7_6142,85,0,61,185,813,297,22,9,202,226,23,278
KLHL12_7_6143,3,0,74,0,0,3,464,0,141,457,0,27
KLHL14_7_6144,922,684,837,519,458,30,691,170,640,241,456,659
KLHL15_7_6145,53,335,2,1763,935,1726,465,107,769,821,514,414
KLHL17_7_6146,1943,521,1417,466,483,0,292,592,1277,804,38,215
KLHL18_7_6147,206,590,142,45,542,34,36,465,118,3,45,330
KLHL1_7_6148,905,2816,480,94,2128,2143,362,2162,896,1210,1966,1749
KLHL20_7_6149,25,56,0,6,545,23,163,581,0,689,1,388
KLHL21_7_6150,371,121,190,17,44,5,3,408,16,489,27,5
KLHL22_7_6151,0,563,39,12,347,175,0,3,13,23,24,122
KLHL23_7_6152,4,633,799,730,350,111,0,41,1219,255,1,787
KLHL24_7_6153,332,93,0,13,1454,0,220,497,76,3,920,345
KLHL25_7_6154,3,0,22,0,0,13,8,2,3,10,96,0
KLHL26_7_6155,17,0,0,4,27,0,0,2,16,0,75,0
KLHL28_7_6156,18,12,859,0,0,0,274,0,3,0,50,0
KLHL29_7_6157,1022,41,944,25,55,1,6,145,0,202,0,37
KLHL31_7_6158,1473,1248,538,300,1052,733,560,1151,1575,1709,383,729
KLHL32_7_6159,1505,196,362,188,4787,172,1,1692,2050,656,506,636
KLHL33_7_6160,0,0,0,0,0,0,0,0,0,0,0,23
KLHL34_7_6161,0,556,0,0,0,8,0,0,0,0,0,0
KLHL36_7_6162,69,67,638,281,542,0,265,4,226,157,36,34
KLHL3_7_6163,0,130,0,37,780,257,0,6,1007,546,1,107
KLHL8_7_6164,177,176,458,856,141,1657,774,1558,1397,2508,3069,1316
LATS1_7_6165,289,756,1283,501,709,532,1661,169,1124,449,215,340
LATS2_7_6166,0,0,0,3,4,10,136,12,0,0,0,0
LIF_7_6167,11,0,10,489,0,144,1106,18,273,19,844,42
LNX2_7_6168,0,4,484,16,785,84,43,546,25,90,1073,1
LOC283116_7_6169,5,140,502,1,28,0,6,203,541,10,1265,77
LONRF1_7_6170,453,34,118,419,35,525,46,326,238,308,352,114
LTN1_7_6171,0,0,0,0,58,0,0,17,0,31,1,0
LZTR1_7_6172,5,252,52,85,308,930,0,828,235,617,0,584
MAP1LC3B_7_6173,211,1439,88,0,13,411,919,18,306,279,22,454
MAP3K1_7_6174,1,10,10,72,9,69,65,2,885,141,1436,133
MARK1_7_6175,295,627,582,257,615,106,512,33,505,125,576,2644
MDM2_7_6176,147,982,0,264,1526,936,1078,0,717,59,950,80
MED20_7_6177,0,6,0,31,0,2,0,38,29,0,52,72
MEX3B_7_6178,32,170,0,0,0,7,0,855,11,280,1045,121
MIB1_7_6179,0,0,0,0,764,0,426,0,0,253,0,0
MKRN2_7_6180,2,30,17,354,25,0,395,71,0,57,217,33
MKRN3_7_6181,63,27,50,75,27,2,834,59,155,8,5,110
MLL2_7_6182,0,0,0,0,0,1,0,0,0,1,0,163
MLLT6_7_6183,75,107,25,369,1251,230,372,626,211,51,38,286
MOCS3_7_6184,292,423,0,29,5,0,41,0,33,0,208,555
MRPL49_7_6185,2,21,3,0,0,0,0,467,44,3,7,6
MUL1_7_6186,30,23,259,100,128,597,523,31,24,42,0,152
MYCBP2_7_6187,453,1105,99,65,508,963,1170,774,1389,713,192,191
MYLIP_7_6188,441,792,107,666,608,954,263,624,453,1218,116,308
MYSM1_7_6189,393,178,1,221,334,309,48,236,478,489,627,824
NACC1_7_6190,12,0,0,0,7,0,1,0,0,2,0,1

NACC2_7_6191,432,419,0,22,0,254,605,161,3,44,19,54
NEURL1B_7_6192,0,0,287,0,0,0,0,0,0,0,0,0
NEURL_7_6193,0,0,0,0,0,0,0,0,0,0,0,0
NHLRC1_7_6194,81,0,656,115,0,343,77,1,5,39,261,48
NUP43_7_6195,323,66,163,136,734,211,48,685,115,312,128,286
OTUB1_7_6196,88,1,362,40,35,205,0,80,560,227,169,540
OTUB2_7_6197,5,567,0,0,390,23,30,23,171,248,220,91
OTUD1_7_6198,114,481,0,137,1703,209,1,1059,10,125,1904,4
OTUD6A_7_6199,2,0,11,0,111,36,48,73,18,87,0,95
OTUD6B_7_6200,509,817,1695,841,1343,653,1101,478,258,1071,1011,1246
OTUD7A_7_6201,350,452,16,298,1667,565,231,769,1231,667,1029,1657
OTUD7B_7_6202,328,0,206,655,166,19,122,1,131,262,59,35
PAFAH1B1_7_6203,803,1497,981,2436,1126,865,4240,1370,1640,2017,4042,11
07
PARP10_7_6204,234,38,0,0,0,2,0,0,1,0,0,0
PARP11_7_6205,303,968,1275,51,297,483,591,285,381,265,293,485
PARP14_7_6206,159,164,133,106,444,228,296,0,140,92,328,447
PCGF1_7_6207,1122,285,1408,42,187,1068,10,548,627,543,362,366
PCGF2_7_6208,348,164,2,131,22,0,1148,286,208,6,23,30
PCGF3_7_6209,173,0,0,267,29,0,0,0,134,4,3,211
PDZRN3_7_6210,345,364,0,2,0,4,0,40,0,355,0,0
PEBP4_7_6211,26,313,123,56,93,0,756,3,0,614,1181,8
PEX12_7_6212,38,311,52,2,282,1981,867,424,435,1477,2033,429
PHF14_7_6213,0,0,205,3,151,664,641,10,119,386,1,74
PHF15_7_6214,3,30,0,0,294,3,49,0,871,108,231,982
PHF20_7_6215,562,459,0,754,250,1068,0,33,0,259,301,99
PHF2_7_6216,0,536,0,0,0,496,0,1,0,2,2383,0
PHF3_7_6217,777,88,0,1305,634,831,231,16,848,376,3916,604
PHIP_7_6218,534,1,0,32,0,8,0,0,21,0,0,4
PHRF1_7_6219,0,17,15,0,0,67,0,769,2,94,475,8
PJA2_7_6220,751,1135,3041,1740,996,964,1577,1089,480,552,634,1718
PRPF19_7_6221,140,345,0,64,0,1,25,46,0,3,57,17
PRPF8_7_6222,100,181,0,2,6,344,0,0,243,55,563,841
PSMD14_7_6223,206,335,262,1354,1102,810,1433,310,838,501,1686,1088
PSMD2_7_6224,1300,16,264,278,288,35,0,191,59,108,40,24
PSMD4_7_6225,453,131,0,1,0,29,0,0,427,71,24,278
PSMD7_7_6226,5,7,2,0,0,4,88,194,552,3,678,1428
PWP1_7_6227,1092,254,356,235,1769,776,1593,1661,995,181,691,1298
RAB40A_7_6228,24,5,0,0,0,503,0,0,290,408,0,37
RAB40AL_7_6229,24,5,0,0,0,503,0,0,290,408,0,37
RAB40B_7_6230,1921,1445,1048,1566,967,194,599,417,476,279,575,652
RAD18_7_6231,50,128,972,54,0,485,254,266,178,867,1208,109
RAD23A_7_6232,1055,2389,116,1214,1094,383,526,2711,2867,1242,158,2867
RAD23B_7_6233,72,0,1,0,0,50,78,210,565,43,178,72
RAG1_7_6234,702,33,317,521,69,59,231,1390,1126,753,966,254
RAI1_7_6235,27,0,0,0,0,355,0,0,628,30,15,73
RBX1_7_6236,178,836,259,648,88,428,2887,183,1429,88,196,1135
RCBTB1_7_6237,46,0,17,4,459,488,540,795,444,12,23,47
RCBTB2_7_6238,591,2,36,0,213,448,588,0,15,0,1,9
RFFL_7_6239,93,0,0,10,0,0,163,0,2,10,5,255

RFPL1_7_6240,957,1207,567,1345,333,1126,935,893,979,876,1206,523
RFWD3_7_6241,172,400,209,41,1916,512,713,261,48,292,2211,3
RHOBTB3_7_6242,26,711,0,1003,14,62,0,0,46,0,8,534
RING1_7_6243,2770,4016,127,2179,2454,1225,3664,3108,1477,3540,3078,275
3
RNF10_7_6244,338,201,558,219,870,520,351,1031,279,550,1290,693
RNF111_7_6245,570,14,11,42,69,344,95,677,107,118,65,32
RNF112_7_6246,134,141,134,0,853,153,981,246,124,11,1,31
RNF113A_7_6247,0,30,0,610,0,0,0,777,180,4,0,12
RNF113B_7_6248,57,1,0,0,3,236,232,5,21,29,3,747
RNF114_7_6249,366,567,823,211,376,203,282,64,142,1043,465,137
RNF115_7_6250,124,1732,222,509,220,636,735,369,1042,470,127,264
RNF11_7_6251,0,172,0,0,143,5,24,2,1,0,193,0
RNF121_7_6252,528,202,336,26,168,341,21,0,74,665,137,31
RNF122_7_6253,6,6,28,0,2,314,0,232,2,14,43,60
RNF123_7_6254,331,508,253,513,350,181,15,0,6,335,0,295
RNF125_7_6255,22,123,156,390,981,373,22,175,17,302,666,27
RNF126_7_6256,0,1,0,0,0,0,0,821,0,0,0,2
RNF130_7_6257,0,979,0,906,224,71,715,446,752,24,285,83
RNF133_7_6258,1367,1256,2222,1586,905,1299,3084,1965,981,780,1587,693
RNF139_7_6259,67,112,1582,526,162,13,0,1066,770,836,7,806
RNF141_7_6260,278,475,46,166,109,193,760,813,491,165,302,90
RNF144A_7_6261,2234,1958,2660,2333,1418,3530,1516,1599,1754,4770,1621,
3158
RNF144B_7_6262,0,16,0,0,44,542,28,6,41,603,0,6
RNF149_7_6263,545,300,2040,304,62,0,1269,426,656,209,0,932
RNF150_7_6264,297,131,1,0,3,724,209,1,79,316,577,7
RNF152_7_6265,26,990,559,493,469,656,1130,1095,633,1024,3588,333
RNF157_7_6266,946,2276,1717,781,3375,2683,3257,1768,1999,1732,1254,196
4
RNF167_7_6267,0,0,5,0,0,0,0,0,0,0,0,0
RNF168_7_6268,0,0,0,0,0,0,0,0,0,0,0,0
RNF169_7_6269,1309,320,1513,800,1,1795,257,319,387,372,0,122
RNF181_7_6270,9,308,8,0,52,251,0,341,0,6,0,1
RNF183_7_6271,1388,940,1254,986,463,1449,1564,2558,1743,1104,2040,1843
RNF186_7_6272,42,54,142,111,133,0,1,143,58,147,0,255
RNF187_7_6273,25,0,0,27,5,982,0,4,7,3,559,0
RNF20_7_6274,244,610,564,1583,1207,234,673,119,519,964,68,747
RNF217_7_6275,216,351,570,4,299,0,846,18,1075,392,531,361
RNF220_7_6276,11,8,21,0,9,26,0,0,0,0,10,148
RNF25_7_6277,519,15,1474,169,20,558,1124,1690,640,270,792,944
RNF26_7_6278,307,6,220,36,467,2,213,35,177,170,337,531
RNF2_7_6279,272,6,553,159,92,1005,177,14,168,93,0,19
RNF31_7_6280,35,222,126,25,0,1,482,0,271,226,342,32
RNF43_7_6281,0,332,0,4,593,3,12,382,51,14,3,574
RNF44_7_6282,6,536,0,223,24,4,0,0,333,25,0,38
RNF5_7_6283,1153,13,204,693,527,148,300,41,123,567,6,16
RSC1A1_7_6284,1044,1018,532,520,338,721,0,866,293,786,1018,1162
RSF1_7_6285,1614,985,509,187,453,340,1577,2828,1016,1198,730,654
RSPRY1_7_6286,0,0,0,0,0,249,0,9,0,0,0,0

SCLY_7_6287,95,158,43,778,830,438,42,84,68,160,74,75
SENP1_7_6288,467,137,107,471,686,167,114,363,659,185,766,612
SENP2_7_6289,877,926,2587,2497,1120,298,287,2926,825,689,2062,882
SENP3_7_6290,47,158,38,551,742,18,0,69,80,19,652,34
SENP5_7_6291,0,48,403,29,3,35,64,0,82,672,0,153
SH3RF1_7_6292,365,347,163,234,45,348,481,59,70,662,1439,930
SH3RF2_7_6293,61,153,4,26,631,108,335,12,43,22,1,219
SH3RF3_7_6294,0,0,0,0,757,0,0,0,0,0,1,0
SHKBP1_7_6295,136,362,898,306,129,699,2193,1393,72,89,301,265
SIAH2_7_6296,268,0,0,80,23,0,0,50,0,0,0,0
SIK1_7_6297,0,0,0,0,106,0,14,9,0,0,0,1
SLX4_7_6298,1,0,34,6,101,0,22,73,199,10,0,18
SMU1_7_6299,2545,2785,3941,4403,1957,1824,644,2006,2649,3253,3801,2564
SMURF2_7_6300,28,0,0,520,64,195,283,7,183,100,1,23
SNRNP40_7_6301,0,0,52,1,0,1,14,51,65,51,1,1164
SOCS1_7_6302,0,0,0,0,4,0,964,3,436,9,0,52
SOCS2_7_6303,0,0,0,0,0,0,0,0,0,0,0,0
SOCS3_7_6304,537,3,64,32,6,0,5,937,56,112,101,1126
SOCS6_7_6305,211,828,553,1006,1054,845,30,859,96,155,1179,825
SPOPL_7_6306,46,235,3,40,481,168,774,124,107,78,34,36
SPSB1_7_6307,0,0,0,1,46,3,0,50,1,362,17,0
SPSB3_7_6308,49,322,276,0,317,203,176,19,387,373,210,105
SPSB4_7_6309,1,0,16,30,5,0,67,0,22,26,971,307
STAM2_7_6310,70,245,68,140,2275,172,34,386,153,2,833,105
STAMBPL1_7_6311,1143,1677,1065,317,296,784,1603,452,1793,297,3216,418
STAM_7_6312,303,1169,144,276,565,185,1042,168,333,546,0,722
STUB1_7_6313,373,386,560,736,620,198,542,1480,615,208,737,2072
SUMO3_7_6314,42,97,6,183,28,139,531,0,1006,30,12,286
SYNGAP1_7_6315,1241,10,1082,404,706,135,411,0,50,916,653,7
TAB2_7_6316,36,60,108,229,1067,197,566,62,364,119,102,335
TAB3_7_6317,663,1719,882,2025,1799,648,1619,2441,437,463,49,1212
TAF1D_7_6318,820,740,532,762,922,1012,1293,2829,1495,46,2281,630
TBC1D1_7_6319,104,78,0,57,0,118,0,46,7,649,0,1
TLE1_7_6320,26,6,7,0,0,0,407,7,25,320,0,3
TNFAIP3_7_6321,0,0,365,199,0,0,0,1,603,8,0,91
TOLLIP_7_6322,0,0,0,0,0,0,11,0,0,0,0,0
TRAF7_7_6323,7,53,4,7,11,25,0,8,127,18,229,915
TRAIP_7_6324,1,8,0,677,25,11,0,0,0,0,0,257
TRIM11_7_6325,332,395,1817,271,336,723,43,47,706,606,574,752
TRIM15_7_6326,34,0,223,184,0,19,0,7,0,2,77,1
TRIM25_7_6327,549,2579,785,916,966,1939,1410,851,1166,821,346,1020
TRIM27_7_6328,542,246,382,301,1004,64,546,742,1410,201,76,263
TRIM28_7_6329,0,0,0,13,1,139,0,0,0,0,0,310
TRIM31_7_6330,299,14,7,3,72,44,5,70,109,385,425,72
TRIM35_7_6331,111,0,0,0,0,642,0,2,0,0,0,1
TRIM42_7_6332,28,21,794,0,3,132,0,0,22,0,276,5
TRIM46_7_6333,0,0,45,2,0,0,0,0,307,11,161,34
TRIM47_7_6334,0,110,0,0,48,139,0,0,0,0,0,178
TRIM48_7_6335,34,411,0,1142,0,464,448,53,43,2,7,72
TRIM52_7_6336,0,0,357,479,367,0,552,212,582,484,261,61

TRIM56_7_6337,0,199,4,3,205,35,1,444,12,0,4,397
TRIM62_7_6338,327,1246,124,0,62,681,0,28,668,641,1045,70
TRIM63_7_6339,3,16,35,452,84,185,709,48,470,5,13,504
TRIM65_7_6340,0,0,0,0,7,160,46,0,160,5,0,14
TRIM67_7_6341,1416,39,0,135,20,638,0,392,750,104,170,145
TRIM68_7_6342,14,234,8,132,0,1,0,0,38,459,0,364
TRIM8_7_6343,0,528,0,0,7,920,0,0,0,554,130,0
TRIP12_7_6344,0,0,0,0,0,0,0,0,0,0,0,0
UBA2_7_6345,1278,133,383,622,320,520,487,964,886,245,574,1453
UBA6_7_6346,5036,5117,6675,3017,1900,6684,3059,7319,3921,5303,7949,327
9
UBA7_7_6347,82,0,125,8,1165,244,36,0,540,321,217,127
UBAC1_7_6348,659,0,0,31,524,0,427,0,393,5,13,55
UBAP2_7_6349,531,2140,1128,92,1034,345,1064,74,1518,1163,2320,1019
UBASH3B_7_6350,1372,2199,1228,498,1628,1445,2027,892,1011,1165,712,142
7
UBE2B_7_6351,2,715,396,482,11,218,1,70,1086,815,636,152
UBE2D4_7_6352,412,9,0,0,854,90,0,0,281,156,1,953
UBE2E2_7_6353,3095,1355,203,1812,2709,1820,532,1631,1613,2291,1162,129
2
UBE2F_7_6354,1202,1554,2272,1630,261,1038,1810,1320,254,746,3151,445
UBE2G1_7_6355,68,253,251,24,377,8,141,331,269,76,0,353
UBE2J1_7_6356,581,743,1660,614,0,424,243,410,90,646,247,660
UBE2L3_7_6357,3839,1454,1917,1533,2362,1240,2548,1788,1594,2204,2897,1
703
UBE2M_7_6358,0,0,15,0,1,0,0,42,0,122,0,0
UBE2N_7_6359,401,1012,840,170,145,96,729,425,780,372,13,944
UBE2NL_7_6360,401,1012,840,170,145,96,729,425,780,372,13,944
UBE2O_7_6361,0,598,0,162,0,0,1041,0,1,350,550,16
UBE2Q1_7_6362,363,468,0,0,2,0,0,56,506,5,37,540
UBE2QL1_7_6363,0,0,0,0,3,224,0,0,0,0,0,1
UBE2R2_7_6364,212,526,383,719,1174,33,1172,821,113,984,356,1094
UBE2S_7_6365,0,0,0,0,0,0,0,0,0,0,0,1
UBE2T_7_6366,8,77,313,226,9,627,72,832,220,88,759,301
UBE2U_7_6367,782,513,127,844,635,1158,943,718,2638,2117,1690,663
UBE2V2_7_6368,119,24,606,75,14,837,2019,437,57,606,1264,238
UBE2Z_7_6369,744,0,219,1863,1681,564,288,491,472,66,36,340
UBE3C_7_6370,864,746,1152,708,179,484,308,540,67,473,1293,1993
UBQLN2_7_6371,128,286,572,559,126,547,774,100,124,3,51,27
UBQLN3_7_6372,647,25,271,0,241,14,1,0,44,164,0,226
UBQLN4_7_6373,0,0,0,0,57,45,3,0,15,1,15,2
UBR1_7_6374,812,665,58,724,6,125,295,265,622,605,179,544
UBR3_7_6375,269,495,25,25,183,146,403,285,76,98,140,341
UBR4_7_6376,146,4781,8,679,460,1605,53,1583,401,1191,789,938
UBR5_7_6377,973,457,5,231,435,907,643,363,387,703,587,1018
UBR7_7_6378,1725,1513,1434,2122,1430,2189,227,1281,577,1097,1135,299
UBXN10_7_6379,3,0,1476,0,0,0,8,15,120,83,338,493
UBXN1_7_6380,181,398,288,348,50,556,661,364,642,21,681,88
UBXN2A_7_6381,432,3315,1847,1481,1358,1012,970,1705,401,1395,1961,1212
UBXN4_7_6382,652,220,1923,1637,890,335,2440,484,651,857,1520,923

UBXN7_7_6383,2669,4558,2337,6293,4977,5621,5017,4827,4342,5169,9634,39
64
UBXN8_7_6384,242,0,381,29,26,546,0,10,588,76,50,95
UCHL1_7_6385,678,1,349,748,1309,408,794,408,39,556,1006,444
UCHL3_7_6386,599,1140,1342,341,168,135,789,39,7,1288,386,1077
UFC1_7_6387,2,113,8,363,4,522,11,413,169,186,461,27
UHRF2_7_6388,419,196,911,1007,493,103,958,682,182,552,363,28
UNK_7_6389,156,0,1,142,0,5,1,3,214,5,17,37
USP11_7_6390,439,119,555,310,14,103,466,979,347,173,88,184
USP13_7_6391,91,1,0,0,303,54,21,1021,0,379,477,399
USP15_7_6392,567,0,0,5,0,0,209,0,0,0,0,137
USP17_7_6393,486,452,1654,492,1136,1025,550,389,404,473,306,1373
USP17L2_7_6394,610,370,1,1245,788,706,1,1035,3,350,1071,826
USP17L5_7_6395,3,13,7,8,1165,494,101,414,76,27,316,20
USP18_7_6396,0,10,50,1,0,0,26,266,436,4,388,475
USP22_7_6397,2,188,569,10,5,2,12,19,127,44,14,544
USP24_7_6398,0,0,0,0,21,0,0,0,309,10,2,475
USP25_7_6399,921,338,3078,1323,3330,1596,1526,3834,1588,1369,686,2868
USP26_7_6400,285,435,720,920,110,962,7,1,186,858,1003,959
USP27X_7_6401,1,6,0,49,0,30,0,0,26,246,273,9
USP28_7_6402,114,99,152,225,111,176,49,82,9,286,344,2
USP29_7_6403,1170,1123,1745,379,763,1604,1025,3094,1449,214,425,640
USP30_7_6404,1033,245,247,6,213,155,922,255,538,112,215,94
USP32_7_6405,390,40,402,0,0,0,0,0,0,0,0,0
USP34_7_6406,141,360,792,232,44,1556,60,687,1226,61,980,161
USP35_7_6407,255,282,551,834,0,3,741,930,518,377,942,905
USP36_7_6408,19,508,1233,128,626,53,56,224,54,75,274,795
USP37_7_6409,233,590,146,315,836,412,95,1643,38,837,633,937
USP38_7_6410,386,0,1,858,0,293,1275,189,32,2,0,41
USP39_7_6411,535,238,308,151,983,335,689,282,24,53,622,474
USP3_7_6412,0,0,0,0,0,0,0,0,0,0,0,0
USP40_7_6413,341,793,41,18,640,104,0,492,31,725,1,175
USP42_7_6414,0,0,0,0,0,0,0,0,0,0,0,0
USP43_7_6415,711,0,159,1,112,18,207,9,1,107,299,219
USP45_7_6416,509,213,195,1,401,61,118,1,495,405,413,137
USP47_7_6417,2737,1666,1230,423,1404,434,1390,1860,658,1348,1545,169
USP49_7_6418,0,2,1,16,0,0,310,0,0,34,0,0
USP50_7_6419,417,0,0,109,23,17,182,0,378,58,0,169
USP51_7_6420,1,758,1098,0,245,509,128,20,28,106,2,125
USP53_7_6421,1429,4,942,600,989,489,1702,874,11,882,163,3
USP54_7_6422,2,1133,11,534,1385,19,392,481,845,1059,903,304
USP6_7_6423,390,40,402,0,0,0,0,0,0,0,0,0
USP7_7_6424,169,926,47,372,2326,485,31,2945,759,1737,1324,970
USP9Y_7_6425,0,0,0,0,0,0,0,0,0,0,0,0
USPL1_7_6426,301,99,4,264,23,57,1,969,564,492,610,1275
VCPIP1_7_6427,1977,576,1308,233,23,549,1076,2480,91,130,65,242
VPS11_7_6428,0,48,0,0,0,0,0,22,0,0,30,31
WDR12_7_6429,201,185,883,5,257,20,5,753,155,14,449,16
WDR53_7_6430,26,656,603,689,11,522,326,1457,315,568,591,422
WDR59_7_6431,5016,2335,3009,17,902,579,167,2435,976,3353,2101,1095

WDR5B_7_6432,0,4,29,9,123,417,2,7,344,6,1145,43
WDR61_7_6433,83,350,0,0,90,0,0,0,136,80,664,70
WDTC1_7_6434,619,11,12,1108,3143,247,682,52,347,171,412,164
WSB2_7_6435,0,153,0,76,0,3,317,400,5,32,203,425
WWP1_7_6436,1075,959,2521,785,3239,2221,503,1251,2187,3368,2948,2827
YOD1_7_6437,112,797,318,1160,738,270,1009,469,729,279,283,428
ZBTB11_7_6438,115,532,259,93,601,462,0,175,428,112,264,206
ZBTB25_7_6439,732,48,360,39,8,427,41,64,1017,1859,1277,956
ZBTB2_7_6440,290,31,317,537,829,244,689,6,175,67,790,816
ZBTB32_7_6441,6,0,7,42,130,37,857,5,0,0,44,2
ZBTB34_7_6442,0,0,2,0,13,6,0,0,0,0,0,0
ZBTB39_7_6443,0,483,0,0,0,0,0,91,0,567,0,1
ZBTB3_7_6444,448,268,610,605,1658,929,274,738,457,839,1027,1467
ZBTB41_7_6445,1735,193,968,1488,137,524,2829,1769,786,651,2890,2420
ZBTB44_7_6446,298,60,0,386,0,494,9,28,525,10,0,826
ZBTB45_7_6447,61,7,2,368,414,173,734,178,76,137,106,562
ZBTB46_7_6448,107,12,1737,106,3494,1408,1258,1315,497,395,1098,580
ZBTB47_7_6449,56,0,446,29,392,51,4,1,110,87,161,174
ZBTB48_7_6450,0,5,342,0,90,258,63,365,54,130,77,11
ZBTB49_7_6451,0,546,69,3,0,0,0,14,0,0,0,0
ZBTB5_7_6452,2,0,5,7,0,20,0,0,7,0,0,136
ZBTB7A_7_6453,0,0,0,0,1,1,0,0,0,0,1753,546
ZBTB7B_7_6454,2,0,0,110,0,3,0,9,1,78,2,0
ZBTB7C_7_6455,123,69,999,234,19,550,38,0,767,1079,1083,300
ZBTB8A_7_6456,767,357,189,155,170,660,1309,977,377,63,0,552
ZFAND2B_7_6457,278,39,138,3,27,13,303,106,72,136,6,14
ZFPL1_7_6458,0,318,21,33,0,51,76,0,1,96,0,47
ZMYND10_7_6459,126,0,666,13,0,0,688,363,0,0,0,36
ZNF131_7_6460,6,604,152,276,218,58,219,1025,99,263,1300,300
ZNF598_7_6461,128,32,7,646,3,271,16,1026,61,235,67,76
ZNF645_7_6462,0,80,0,524,114,11,88,373,440,387,20,63
ZNRF1_7_6463,0,0,255,564,251,420,156,1,0,10,893,57
ZNRF2_7_6464,0,0,0,0,0,0,6,0,0,0,0,0
ZNRF4_7_6465,3,627,4,664,19,171,35,368,285,418,171,26
ZRANB1_7_6466,670,909,358,755,230,253,1324,49,153,445,494,23
ZSWIM2_7_6467,13,1,44,76,115,197,784,331,757,660,43,943
AIRE_7_6468,804,348,1134,136,2679,111,1742,749,511,1401,1922,1217
ANAPC5_7_6469,218,761,379,571,0,2474,7,256,7,350,1869,241
ANAPC7_7_6470,1136,1032,423,291,63,1074,779,327,705,702,1005,96
ANKFY1_7_6471,1292,305,5,474,2,306,108,1294,213,88,6,713
ASB10_7_6472,0,0,0,0,0,0,0,0,0,0,68,0
ASB11_7_6473,180,334,1,289,179,15,9,448,141,239,12,400
ASB14_7_6474,240,13,908,1387,2112,588,60,362,494,1124,788,1806
ASB2_7_6475,0,0,47,89,11,87,0,0,94,4,7,107
ASB3_7_6476,852,245,332,269,412,97,0,695,56,59,229,457
ASB4_7_6477,365,753,246,132,460,1194,1199,3629,1033,82,1756,387
ASB6_7_6478,3071,498,144,467,2420,881,951,284,1319,937,175,1109
ASB7_7_6479,776,671,1402,2211,1146,2080,1088,2277,671,1121,2629,213
ASB9_7_6480,46,457,3,37,378,14,744,23,226,39,437,706
ASCC2_7_6481,11,0,0,0,453,399,1,0,26,505,233,6

ATG10_7_6482,0,0,0,0,0,0,0,0,0,0,0,0
ATG16L1_7_6483,368,60,436,178,156,276,1,213,11,1,1279,178
ATG7_7_6484,426,924,108,642,1840,1891,1573,625,1140,661,15,179
ATRX_7_6485,16,15,201,63,142,1118,353,1167,926,148,1187,1077
BACH2_7_6486,837,12,128,12,885,685,1168,1,532,173,2,484
BAG6_7_6487,466,39,2,368,86,3,528,568,1153,13,102,459
BAZ1A_7_6488,3117,3182,2508,3025,1627,2426,2188,1446,2149,2439,3707,26
43
BCL6_7_6489,143,150,0,0,591,942,947,4,265,628,25,228
BIRC3_7_6490,0,0,111,0,245,0,385,5,9,335,1,0
BIRC7_7_6491,0,32,0,0,0,0,0,0,0,14,0,0
BPTF_7_6492,663,782,6,281,276,263,992,5,170,1220,108,555
BRCA1_7_6493,93,1058,16,186,334,115,19,709,225,130,83,181
BRPF1_7_6494,72,32,850,214,189,270,0,387,510,669,230,77
BRWD1_7_6495,235,738,51,653,30,1208,0,1107,785,266,327,308
BTBD11_7_6496,3456,1778,494,2003,667,352,607,1144,1690,1310,3172,876
BTBD1_7_6497,5,220,110,53,367,24,3,11,277,86,722,81
BTBD3_7_6498,39,37,0,251,0,65,140,19,0,2,59,0
BTBD7_7_6499,3587,1049,944,423,1625,574,836,61,1587,1953,888,507
BTBD9_7_6500,1015,644,235,220,572,682,251,1333,234,1113,664,1061
BTRC_7_6501,141,443,347,137,1085,390,183,68,274,962,28,56
C3orf26_7_6502,22,0,31,0,0,17,2,0,0,0,0,0
CBLC_7_6503,48,16,0,9,4,85,0,0,18,343,0,2
CCNB1IP1_7_6504,665,439,234,897,1580,339,434,1329,615,460,315,939
CDC16_7_6505,0,0,0,0,168,13,66,9,441,274,424,865
CDC27_7_6506,1671,892,3875,2108,3440,1475,5632,1085,966,2633,3226,2477
CHFR_7_6507,33,57,36,40,83,0,46,481,61,161,172,313
CISH_7_6508,177,198,0,342,245,74,944,661,450,624,39,58
CNOT4_7_6509,78,206,925,0,32,1,0,27,44,566,3,763
COPS2_7_6510,204,201,1293,25,905,0,147,1114,1426,549,1789,498
COPS3_7_6511,24,158,197,17,203,7,87,613,996,188,12,813
COPS7A_7_6512,0,0,0,0,0,0,0,145,0,0,0,0
COPS8_7_6513,978,75,0,268,53,5,74,54,148,496,1095,868
CRBN_7_6514,980,140,612,52,50,54,0,808,714,258,230,1388
CUL2_7_6515,287,83,192,80,865,6,245,1185,619,554,689,113
CUL4A_7_6516,1181,1760,355,687,268,204,297,499,524,442,1,1676
CUL4B_7_6517,2036,1524,288,1548,2059,1190,3515,2078,952,1617,3056,1654
CUL7_7_6518,1,0,0,0,8,163,808,0,188,75,7,20
CXXC1_7_6519,386,122,148,156,575,446,871,314,70,475,769,13
CYLD_7_6520,5065,4716,4375,3045,6524,5948,4721,2860,4612,5587,2330,398
5
DCAF11_7_6521,0,0,0,0,161,240,0,0,0,11,1,0
DCAF17_7_6522,127,0,11,158,51,0,0,0,0,6,101,0
DCAF4_7_6523,20,547,118,115,682,70,80,42,590,862,47,999
DCAF6_7_6524,150,81,1,0,0,91,106,31,0,311,127,1
DCST1_7_6525,3,0,2,1,0,92,0,214,39,235,1,439
DEPDC1B_7_6526,961,915,362,2132,963,448,1735,3062,1812,948,951,1126
DET1_7_6527,1959,448,1875,24,373,344,1238,7,276,796,1133,58
DIDO1_7_6528,0,0,155,0,11,0,6,0,165,149,0,19
DNAJB2_7_6529,217,293,166,720,838,245,307,363,178,507,57,123

DTX2_7_6530,1440,68,802,796,35,485,2274,1314,243,48,442,454
EED_7_6531,991,435,2648,973,1399,413,2237,967,1289,1709,612,763
EIF3B_7_6532,0,0,0,0,6,0,0,0,0,0,0,1
EIF3C_7_6533,1638,2086,1390,1871,1717,1420,600,1168,3302,1728,2181,124
7
EIF6_7_6534,531,0,0,75,1,76,1032,656,317,4,749,612
EPN1_7_6535,519,644,399,205,420,697,198,136,218,164,1252,352
EPN2_7_6536,26,80,453,156,49,233,371,203,695,28,37,143
EPS15_7_6537,1241,428,321,191,434,543,764,172,734,1078,160,1160
FAM70A_7_6538,90,0,4,0,0,0,0,0,0,0,339,1
FANCL_7_6539,2643,1228,108,38,1100,395,2502,492,461,787,423,1728
FBXL13_7_6540,253,650,682,701,0,955,916,1190,770,246,149,86
FBXL20_7_6541,15,0,12,3,0,4,0,14,0,1,0,0
FBXL2_7_6542,2799,2010,1848,1828,2402,2644,1070,2703,1517,2709,2669,24
17
FBXL5_7_6543,512,48,1156,1137,1,207,564,12,1127,1150,398,584
FBXL6_7_6544,0,1,0,39,0,0,0,0,60,1,8,24
FBXO11_7_6545,0,0,0,0,0,0,0,0,0,0,0,0
FBXO15_7_6546,655,1196,139,429,172,84,627,939,1439,1024,345,787
FBXO17_7_6547,427,0,140,0,17,0,207,925,2,491,306,0
FBXO18_7_6548,17,36,0,0,792,4,8,0,0,0,316,1
FBXO21_7_6549,321,1020,1787,821,1097,2207,1473,2264,1980,1254,1658,134
0
FBXO22_7_6550,392,189,120,898,895,119,897,263,1724,1264,193,630
FBXO24_7_6551,1631,902,3170,196,351,81,757,865,101,439,820,716
FBXO25_7_6552,1,48,83,99,64,284,632,41,1093,612,522,608
FBXO28_7_6553,85,117,9,71,2,554,15,133,75,358,183,9
FBXO32_7_6554,55,0,0,44,50,141,1,0,648,197,1503,88
FBXO34_7_6555,608,296,312,36,1206,84,932,275,522,732,2540,1169
FBXO38_7_6556,1,113,225,267,128,449,241,484,296,322,7,668
FBXO3_7_6557,380,1869,145,745,421,557,1635,111,484,669,77,791
FBXO44_7_6558,0,0,0,3,241,112,0,0,0,6,2,26
FBXO4_7_6559,586,110,1400,218,114,20,878,1,7,66,530,708
FBXO5_7_6560,4,167,291,472,0,376,0,892,207,145,1,21
FBXO7_7_6561,1767,32,884,1014,766,1145,834,96,1332,1933,1155,2349
FBXO9_7_6562,61,236,375,76,479,71,574,792,498,413,32,474
FBXW11_7_6563,183,1192,313,332,561,250,681,256,801,1719,23,243
FBXW7_7_6564,136,550,79,202,1808,1085,437,53,720,1566,546,153
FBXW8_7_6565,764,261,518,761,133,160,872,205,508,363,1133,790
GPS1_7_6566,486,84,0,8,0,75,0,610,593,8,459,90
HECTD2_7_6567,70,175,39,360,364,448,0,261,7,23,132,87
HERC4_7_6568,694,718,1181,1042,1475,2305,843,1425,950,822,1713,947
HERC6_7_6569,838,1344,1267,594,649,1415,208,1663,824,2835,1082,1572
HIC1_7_6570,273,0,0,0,5,0,50,0,10,0,292,3
HLTF_7_6571,90,86,380,16,94,22,347,9,58,237,79,366
HSF4_7_6572,12,4,1,379,0,0,105,0,265,2,308,431
IPP_7_6573,0,57,0,0,0,0,73,0,0,362,0,7
KAT6A_7_6574,656,537,1437,626,1044,1026,578,194,814,1222,703,1831
KBTBD3_7_6575,305,141,208,1,447,239,191,723,211,1175,534,2126
KCTD6_7_6576,624,1349,1184,1070,2108,811,1041,577,671,695,959,87

KCTD7_7_6577,7,574,198,701,2114,233,135,309,1147,151,656,1613
KDM2B_7_6578,2820,1053,1327,1127,2457,1466,1641,2694,2586,1898,1004,48
72
KDM4C_7_6579,7,34,669,36,0,0,415,0,380,54,0,50
KDM5C_7_6580,1883,165,59,1300,2003,850,583,973,837,749,1092,885
KEAP1_7_6581,130,187,17,137,97,146,1,0,530,50,1008,246
KIAA1841_7_6582,78,171,0,335,55,754,67,553,772,249,1,133
KLHL13_7_6583,896,350,891,340,1284,7,1584,46,38,47,469,373
KLHL2_7_6584,651,177,30,22,195,187,2,431,94,18,28,264
KLHL4_7_6585,0,282,0,292,6,459,177,0,11,41,1,1
KLHL5_7_6586,255,202,0,81,45,742,87,0,296,36,202,1387
KLHL7_7_6587,95,1,588,418,23,60,0,1072,158,1202,19,1970
LNX1_7_6588,944,1751,108,2038,2811,452,1401,1561,2238,1139,1501,1154
LONRF3_7_6589,139,593,1,132,204,33,0,10,2,0,0,123
LRRC29_7_6590,1,0,0,2,195,10,0,0,322,5,0,398
LRSAM1_7_6591,0,1,2,2,0,5,70,11,826,21,38,95
MARK2_7_6592,90,172,0,65,0,2,0,69,25,7,63,4
MARK3_7_6593,72,335,0,1,0,7,361,480,159,584,0,226
MARK4_7_6594,100,188,7,156,702,35,242,311,309,192,23,696
MDM4_7_6595,4376,1687,2719,763,1615,1035,291,2883,1204,1235,1492,2520
MGRN1_7_6596,433,0,0,0,0,331,0,0,379,240,0,36
MIB2_7_6597,16,7,20,1022,359,614,59,306,360,1200,148,878
MID1_7_6598,42,0,0,6,304,24,954,67,326,1,584,659
MID2_7_6599,98,85,0,60,429,374,52,72,96,12,58,39
MKRN1_7_6600,869,5,464,28,0,329,67,0,225,4,73,177
MLL5_7_6601,229,327,60,848,798,56,1361,35,645,829,60,797
MLL_7_6602,119,13,0,0,400,4,81,1298,0,0,0,0
MNAT1_7_6603,393,327,1129,7,16,49,3,1,265,112,1614,312
MPND_7_6604,3,323,364,90,4,439,400,4,366,460,604,39
MTF2_7_6605,27,304,75,352,18,11,148,322,449,1067,956,604
MYNN_7_6606,283,864,681,502,151,770,0,403,4,1552,585,1102
NAE1_7_6607,765,1041,1848,1105,545,3590,612,444,336,1582,1969,728
NDUFC2_7_6608,0,0,0,0,688,0,0,0,0,0,0,685
NEDD4_7_6609,401,386,162,232,620,354,144,48,228,181,748,30
NEDD4L_7_6610,174,485,672,76,837,1311,790,9,425,112,1267,75
NFX1_7_6611,172,279,1445,558,0,3,1,664,323,618,666,440
NLE1_7_6612,0,45,46,0,109,555,4,0,618,13,106,73
NSD1_7_6613,101,39,16,271,8,227,138,411,858,142,697,109
NSFL1C_7_6614,102,897,813,176,23,585,58,504,479,1020,0,323
NUB1_7_6615,11,287,351,672,165,188,610,622,58,128,111,76
ODF2_7_6616,94,4,1143,581,127,237,8,10,128,610,15,50
OTUD5_7_6617,3,3,1,0,3,11,0,0,138,209,82,21
PARK2_7_6618,0,0,15,0,0,0,1,11,0,26,40,0
PARP9_7_6619,84,161,5,163,563,452,183,579,544,103,428,132
PATZ1_7_6620,537,337,28,331,38,373,101,896,101,334,642,953
PCGF6_7_6621,0,0,0,0,106,0,0,0,1,6,10,0
PEX10_7_6622,18,956,0,0,65,6,59,21,2,0,0,1
PEX2_7_6623,154,135,588,862,818,1309,1770,167,1747,161,1619,1093
PHF12_7_6624,0,54,0,0,41,0,5,0,1,0,1,23
PHF16_7_6625,56,276,1563,42,7,4,0,0,250,726,616,26

PHF17_7_6626,29,746,6,202,1176,53,226,1,397,364,743,76
PHF1_7_6627,53,144,949,353,0,0,937,0,0,344,938,703
PHF21A_7_6628,0,6,0,6,13,6,1,0,76,52,325,11
PHF7_7_6629,320,116,0,1,1462,324,1326,0,585,13,1,696
PHF8_7_6630,3364,2651,618,1594,1014,3383,1196,746,599,1393,1429,1423
PJA1_7_6631,157,17,148,255,680,354,60,147,192,264,29,591
PML_7_6632,14,20,2,490,0,0,14,0,21,0,138,30
POC1B_7_6633,2311,832,2001,675,3477,2485,207,3437,1181,2219,852,1163
PSMD1_7_6634,387,497,338,1110,56,508,167,218,926,529,43,247
RAPSN_7_6635,89,326,0,0,1296,0,0,128,0,0,1,0
RBBP4_7_6636,694,1705,104,975,149,644,1480,1188,671,539,1113,139
RBBP5_7_6637,147,80,1030,287,822,639,0,0,608,827,179,851
RBBP6_7_6638,0,99,0,326,0,15,0,737,6,69,0,117
RBBP7_7_6639,194,21,68,42,31,233,362,331,27,205,518,692
RBCK1_7_6640,896,80,1091,427,1,3,862,654,504,45,10,1322
RC3H2_7_6641,0,305,1,3,2,18,75,72,21,15,0,76
RCHY1_7_6642,248,140,472,85,133,8,179,50,269,355,297,1518
RFPL2_7_6643,17,18,24,7,0,158,48,253,287,60,23,43
RFPL3_7_6644,1755,889,1848,12,765,370,1374,219,1040,2056,1328,907
RFWD2_7_6645,626,146,1052,263,103,1227,332,165,315,160,1361,521
RHOBTB1_7_6646,0,0,0,0,0,0,0,0,0,0,0,0
RHOBTB2_7_6647,13,0,960,0,812,0,11,596,0,0,4,0
RLIM_7_6648,499,1154,176,6,623,592,30,105,459,1139,59,108
RNF103_7_6649,570,1313,656,1233,734,502,856,175,825,723,1496,1701
RNF128_7_6650,1154,301,1790,326,568,180,158,179,793,617,1422,195
RNF135_7_6651,518,0,1600,242,331,230,0,54,184,367,805,1075
RNF138_7_6652,9,97,309,526,415,746,530,44,14,27,439,80
RNF13_7_6653,1,0,13,6,23,1,0,6,30,254,0,6
RNF145_7_6654,0,21,676,0,190,0,0,13,0,0,2,0
RNF146_7_6655,1124,121,1539,862,1470,1378,1283,1169,810,1490,423,105
RNF14_7_6656,73,49,17,1,0,1,435,6,0,13,122,0
RNF166_7_6657,142,144,92,167,1230,0,271,204,411,265,209,194
RNF170_7_6658,193,2106,1810,423,2880,1337,3625,343,1188,2461,892,161
RNF17_7_6659,2988,1353,2818,1363,943,2432,1015,2225,4732,3658,1010,119
6
RNF180_7_6660,93,9,181,207,9,112,256,2168,383,10,307,119
RNF182_7_6661,24,20,0,189,75,70,0,10,461,30,21,523
RNF185_7_6662,245,84,521,251,0,27,20,1130,302,6,132,110
RNF19A_7_6663,80,217,216,24,101,291,194,2580,348,139,81,692
RNF19B_7_6664,1458,359,1017,364,2531,1551,211,980,1264,739,760,731
RNF213_7_6665,621,1114,605,793,820,1167,854,1865,1786,1137,2182,468
RNF214_7_6666,8,5,3,335,95,0,53,583,105,2,819,81
RNF216_7_6667,483,3,416,244,570,299,432,77,322,72,449,398
RNF24_7_6668,326,236,1,10,852,211,718,494,238,24,82,658
RNF32_7_6669,923,54,1865,33,732,97,367,1488,188,465,445,1031
RNF34_7_6670,19,53,260,58,379,46,466,17,45,130,35,287
RNF38_7_6671,612,5,0,240,174,23,61,489,39,5,42,6
RNF40_7_6672,0,0,4,0,129,4,2,0,0,0,0,0
RNF41_7_6673,24,0,0,17,1,0,0,5,2,50,0,0
RNF6_7_6674,713,1754,307,414,903,1653,291,2148,1280,683,1003,678

RNF7_7_6675,1750,264,2469,901,768,595,43,107,1018,492,943,1018
RNF8_7_6676,846,1343,13,2778,702,1081,1306,11,167,687,1252,2005
SAE1_7_6677,0,7,0,0,0,0,0,0,0,0,35,0
SENP6_7_6678,115,74,850,68,0,62,33,646,22,1,0,6
SENP7_7_6679,454,1991,326,268,1895,805,1659,844,501,1282,325,1803
SENP8_7_6680,73,0,0,24,258,0,229,20,13,501,2,234
SF3A1_7_6681,2,0,142,32,0,0,23,1,0,3,0,0
SHPRH_7_6682,36,38,11,151,256,392,0,294,426,316,187,567
SIAH1_7_6683,34,0,0,16,510,0,214,0,34,0,0,2
SKP2_7_6684,0,2,0,326,319,132,0,17,123,154,375,74
SMURF1_7_6685,328,172,6,1462,1193,52,7,365,1057,162,986,131
SOCS5_7_6686,730,157,179,27,2,63,87,234,216,24,315,32
SP100_7_6687,536,252,504,51,1,138,152,516,223,378,1,124
SP110_7_6688,395,876,455,230,233,613,1123,888,147,1709,132,23
SPOP_7_6689,81,148,210,564,0,6,15,3,77,18,3,162
SPSB2_7_6690,161,1,5,6,6,38,0,57,0,17,11,103
SQSTM1_7_6691,196,414,1851,16,675,15,3477,212,48,273,2589,941
STAMBP_7_6692,0,645,0,0,21,0,0,0,0,4,127,0
SUMO1_7_6693,1692,1800,1763,950,1617,2278,425,1549,1351,906,225,1609
SUMO2_7_6694,2513,119,1738,832,1325,590,2090,1321,1440,1601,483,1120
SYTL4_7_6695,530,827,1097,1276,1325,290,2507,1882,1360,1801,1073,1675
SYVN1_7_6696,1533,2198,616,320,1416,887,778,619,1003,423,879,118
TCF20_7_6697,348,420,1074,248,1675,350,114,774,483,1602,777,235
TDRD3_7_6698,32,8,303,53,189,146,0,222,11,57,441,4
TIPARP_7_6699,29,168,1230,0,47,88,0,0,16,158,1185,58
TLE2_7_6700,0,0,0,0,0,0,0,0,0,0,0,0
TLE3_7_6701,69,602,16,6,445,125,5,0,2,275,2,9
TNK2_7_6702,2,794,126,1,476,170,150,0,10,172,4,80
TNRC6C_7_6703,0,0,0,9,0,222,0,0,177,1,0,448
TOPORS_7_6704,111,307,582,654,172,604,597,1440,1342,485,1750,806
TOR1AIP2_7_6705,272,0,0,572,153,1225,490,234,39,359,91,36
TRAF3_7_6706,389,19,2,100,513,963,246,977,1155,43,1498,169
TRAF5_7_6707,1,78,892,44,8,116,10,154,0,0,0,2
TRAF6_7_6708,1208,74,234,4,416,1132,0,210,430,339,40,286
TRIM10_7_6709,24,0,11,3,3,37,0,238,280,164,0,39
TRIM13_7_6710,834,1307,1014,774,1926,2033,4631,687,1439,838,2506,1598
TRIM17_7_6711,151,569,0,0,125,32,58,14,24,0,0,0
TRIM22_7_6712,977,62,341,1197,517,487,173,75,213,433,32,649
TRIM23_7_6713,1235,1683,112,110,230,871,203,17,557,70,67,1048
TRIM24_7_6714,1300,1965,133,733,1122,1250,495,1682,2013,1178,930,1254
TRIM26_7_6715,611,16,1085,59,1827,1631,42,397,608,1474,465,103
TRIM2_7_6716,1312,526,916,1406,0,1288,962,1020,1025,714,1187,957
TRIM32_7_6717,1548,1022,1543,1141,1703,277,688,1430,1534,560,1342,1526
TRIM33_7_6718,1318,1006,0,669,30,950,426,182,672,983,8,538
TRIM34_7_6719,187,5,157,33,561,41,31,36,726,215,542,809
TRIM37_7_6720,52,39,0,272,45,18,1,738,14,717,310,14
TRIM39_7_6721,65,0,0,76,42,395,355,350,437,807,3,287
TRIM3_7_6722,153,353,643,346,2,128,46,254,46,1601,1574,2164
TRIM41_7_6723,212,0,68,168,14,474,0,0,20,266,1631,44
TRIM45_7_6724,0,0,1,0,0,2,0,1,0,86,2,820

TRIM4_7_6725,85,108,0,3,18,526,0,34,990,13,0,103
TRIM54_7_6726,836,2305,4274,655,2418,1315,4236,2947,2548,2441,1845,140
4
TRIM55_7_6727,312,530,0,587,18,386,4,1269,4,429,683,337
TRIM5_7_6728,92,148,67,17,346,717,144,1287,0,0,153,539
TRIM7_7_6729,0,0,0,0,0,242,11,26,0,12,0,0
TRIM9_7_6730,1534,234,500,142,29,149,10,1575,585,798,179,333
TRPC4AP_7_6731,81,407,2739,939,981,617,508,336,250,686,2173,1083
TSPAN17_7_6732,375,156,9,594,1,0,13,6,120,2,488,259
TTC3_7_6733,1572,926,122,335,288,301,58,373,502,42,858,1235
TULP4_7_6734,897,25,0,1,0,65,69,0,157,118,0,13
UBA1_7_6735,440,1209,569,152,373,628,1292,597,967,585,1382,491
UBA3_7_6736,0,0,2,101,6,182,0,0,0,701,594,1279
UBA5_7_6737,1,2,655,1,12,131,0,746,4,2,393,1
UBAC2_7_6738,1738,2361,3986,2949,1072,1484,3430,1991,6385,1900,2307,38
81
UBAP2L_7_6739,142,813,690,1690,1155,41,343,8,1129,120,625,282
UBASH3A_7_6740,3,15,16,0,29,24,0,334,4,0,269,6
UBE2A_7_6741,387,79,659,23,2244,0,34,50,505,39,1240,64
UBE2D1_7_6742,697,295,1065,263,1283,464,10,1038,1167,823,1080,774
UBE2D2_7_6743,469,1206,573,624,622,15,1346,260,306,307,144,1675
UBE2D3_7_6744,1277,476,364,97,1589,489,605,5,498,126,1243,450
UBE2E1_7_6745,460,625,0,373,558,153,0,435,160,89,1853,913
UBE2E3_7_6746,566,1313,514,64,271,574,549,571,1002,289,346,210
UBE2G2_7_6747,224,0,0,173,0,0,0,554,0,631,0,0
UBE2H_7_6748,985,520,1316,392,529,437,418,1092,354,1063,126,60
UBE2I_7_6749,37,0,0,552,652,479,14,0,56,786,853,851
UBE2J2_7_6750,0,705,1,42,32,86,0,3,13,4,515,2
UBE2K_7_6751,349,810,3,245,1238,608,167,5,152,634,441,232
UBE2Q2_7_6752,557,49,1300,10,17,1674,1116,246,1310,460,480,813
UBE2V1_7_6753,300,165,175,0,138,0,16,0,0,0,284,0
UBE2W_7_6754,928,1482,390,728,1803,1392,633,720,1842,1583,2236,1188
UBE3A_7_6755,71,0,13,322,0,0,0,0,603,42,0,128
UBE3B_7_6756,0,7,0,0,99,0,0,16,14,0,0,2
UBE4A_7_6757,303,17,772,317,17,704,3,703,103,558,217,856
UBE4B_7_6758,11,13,0,77,626,0,928,866,961,214,803,124
UBL7_7_6759,1176,509,1256,498,288,745,725,527,1507,626,748,1804
UBOX5_7_6760,646,127,145,997,1505,255,481,9,120,414,15,328
UBQLN1_7_6761,3729,1599,2617,1036,545,2458,1410,1366,1346,2237,5560,12
52
UBR2_7_6762,326,259,248,27,29,722,62,1102,459,850,32,1468
UBXN11_7_6763,672,57,0,0,34,0,1002,29,202,2,33,30
UBXN6_7_6764,217,0,0,0,117,67,0,57,94,455,58,275
UCHL5_7_6765,859,484,15,90,433,1111,11,508,759,277,335,1230
UHRF1_7_6766,0,1085,877,0,1468,161,817,34,778,869,24,96
UIMC1_7_6767,375,0,0,161,205,2,82,355,639,36,2,983
UNKL_7_6768,3,0,0,50,0,9,0,0,33,1,0,4
USP14_7_6769,496,351,31,1551,561,965,1874,1359,75,1261,833,181
USP19_7_6770,0,0,0,0,0,0,0,0,0,0,0,0
USP1_7_6771,65,131,760,139,1005,145,0,199,972,209,1,157

USP20_7_6772,491,361,547,287,591,1389,177,1973,268,31,2398,359
USP2_7_6773,50,18,0,61,841,233,1,17,162,11,166,36
USP33_7_6774,0,129,0,1,26,0,0,0,16,8,32,4
USP44_7_6775,1757,1146,25,625,1435,0,2805,677,1757,82,1,1008
USP46_7_6776,47,167,37,26,160,713,584,370,70,597,626,8
USP48_7_6777,187,57,368,223,233,15,176,541,827,371,92,125
USP4_7_6778,93,98,0,942,80,331,47,678,6,709,0,1
USP5_7_6779,0,266,56,666,64,38,604,243,673,168,0,666
USP6NL_7_6780,0,0,10,1,167,0,0,576,32,13,17,3
USP8_7_6781,78,2,0,0,588,54,7,0,28,142,10,233
USP9X_7_6782,1885,1134,1494,688,1173,1879,1383,1894,1294,469,713,455
VHL_7_6783,27,5,0,55,707,354,22,2255,0,30,5,742
VPRBP_7_6784,16,402,58,0,5,233,0,538,38,114,132,174
VPS13D_7_6785,550,166,679,1606,876,4,175,123,507,757,3,916
VPS41_7_6786,258,298,113,99,634,89,351,2008,643,5,297,1002
VPS8_7_6787,2675,3168,7565,1736,3287,3322,4507,4449,4899,2936,3713,340
4
WDR26_7_6788,5,387,101,540,1718,518,9,222,712,99,186,106
WDR5_7_6789,708,207,61,897,580,1621,1809,330,12,29,503,83
WDR76_7_6790,1161,1435,987,40,4131,232,160,1131,119,221,2035,1452
WHSC1_7_6791,11,1,1096,191,0,22,641,993,222,482,168,114
WHSC1L1_7_6792,1581,1211,1012,677,1095,408,1456,159,285,900,646,425
WSB1_7_6793,88,379,89,1007,183,277,1502,366,1324,768,675,334
WWP2_7_6794,4428,3223,6516,4478,5113,5126,3291,4696,4034,6184,4457,104
15
XIAP_7_6795,1,351,0,80,137,547,208,0,0,0,38,1
ZBTB10_7_6796,61,244,181,595,51,586,655,82,145,948,198,196
ZBTB16_7_6797,4,111,266,4,0,0,457,0,2,273,0,1
ZBTB17_7_6798,0,278,0,359,0,151,24,0,388,4,1,594
ZBTB1_7_6799,1405,528,16,1155,951,56,383,1289,667,872,922,1984
ZBTB20_7_6800,0,0,0,10,0,1010,0,196,524,7,1,52
ZBTB22_7_6801,316,303,213,6,245,97,195,263,307,11,61,192
ZBTB24_7_6802,585,168,2338,251,523,41,265,9,1095,1052,335,357
ZBTB33_7_6803,1678,802,1834,1253,900,1534,3344,312,2510,1742,1064,2681
ZBTB37_7_6804,453,215,153,1211,66,0,0,84,3,135,424,1554
ZBTB40_7_6805,312,129,11,7,537,0,118,15,15,175,147,2
ZBTB4_7_6806,65,525,9,613,6,36,18,14,521,28,1,64
ZFP161_7_6807,2,114,0,0,112,0,58,42,17,26,10,8
ZMYND11_7_6808,267,516,17,120,70,175,422,786,689,292,2,142
ZMYND8_7_6809,49,626,52,0,0,85,636,174,0,617,153,139
ZNF238_7_6810,5,2,0,0,8,7,16,531,506,67,0,65
ZNF295_7_6811,1313,37,7,1,147,936,696,0,40,352,1937,130
ZNRF3_7_6812,982,0,415,21,469,45,4,13,533,70,71,168
ANAPC11_7_6813,30,1,431,760,0,340,734,163,392,100,36,1361
ATXN3_7_6814,997,1472,2074,1953,719,1658,2515,2230,1895,4925,1197,2698
MLLT10_7_6815,468,893,1136,602,477,509,1437,924,1018,923,1310,1515
OTUD4_7_6816,45,88,27,272,33,151,400,44,3,106,186,152
PHF19_7_6817,10,160,20,44,919,478,269,898,405,313,430,778
SP140_7_6818,2179,1039,1276,1462,1009,1280,1856,708,1975,2819,1530,273
1

TRIM36_7_6819,153,162,26,69,27,84,246,205,638,511,378,652
UBE2C_7_6820,0,152,0,1,0,129,0,0,2,21,0,2
UBE2L6_7_6821,43,24,0,79,371,0,134,0,2,0,24,592
ABTB2_7_6822,0,1,9,0,0,0,0,9,0,39,3,27
AMBRA1_7_6823,15,13,760,11,573,0,776,491,0,0,0,0
AMFR_7_6824,2960,3570,1247,2167,2752,2409,2786,2834,3745,1849,2230,352
8
ANAPC10_7_6825,80,256,403,1037,54,434,516,1014,965,188,1133,138
ANAPC1_7_6826,773,1120,381,558,1073,1160,564,910,125,624,1046,120
ANAPC2_7_6827,18,34,0,0,417,715,964,0,384,405,780,51
ANAPC4_7_6828,1,9,710,32,0,0,667,767,0,3,862,16
ANKIB1_7_6829,1125,1810,159,1150,602,405,400,552,752,356,2121,2322
ANKRD13A_7_6830,0,95,0,1,189,0,0,46,438,212,220,53
ANKRD13D_7_6831,0,162,0,3,0,1,249,0,0,0,91,36
ARIH1_7_6832,0,106,644,162,1493,722,996,1076,1174,257,65,556
ARIH2_7_6833,913,67,137,846,1091,911,318,525,400,152,980,742
ASB12_7_6834,188,65,311,411,0,83,975,31,238,821,971,151
ASB13_7_6835,0,0,0,0,710,0,0,0,540,89,0,62
ASB15_7_6836,1422,151,715,491,728,744,625,1219,555,871,72,928
ASB16_7_6837,1,131,15,0,101,6,463,1,0,49,130,54
ASB17_7_6838,1132,22,199,84,970,1024,292,524,671,226,373,64
ASB5_7_6839,2069,1755,1758,2092,4067,2661,1116,2048,1494,2504,1701,909
ASB8_7_6840,64,497,288,510,300,0,882,34,680,778,97,99
ASH1L_7_6841,42,0,1,0,29,0,0,62,0,0,0,37
ASPSCR1_7_6842,0,0,0,0,0,0,0,0,0,0,0,0
ATG12_7_6843,154,11,8,5,90,29,505,0,0,1,42,0
ATG3_7_6844,0,0,0,566,0,0,0,333,501,8,0,56
ATG5_7_6845,151,1052,1070,1212,802,1070,2677,2319,1847,979,2361,324
ATXN1L_7_6846,64,1783,758,51,51,36,127,812,681,575,193,115
ATXN3L_7_6847,702,66,86,296,0,0,0,17,278,467,1671,29
BARD1_7_6848,501,303,175,54,59,318,490,23,33,226,0,832
BAZ1B_7_6849,17,0,0,536,467,91,0,200,666,10,40,439
BAZ2A_7_6850,181,209,158,29,474,75,44,13,450,738,701,144
BAZ2B_7_6851,0,8,0,0,8,18,124,263,6,8,1,2
BECN1_7_6852,1039,939,1256,23,360,1463,1157,669,1323,1531,2158,1606
BIRC2_7_6853,53,438,0,99,661,712,1083,552,0,237,285,25
BIRC6_7_6854,1928,96,443,322,2788,85,2654,453,1801,1084,2626,1877
BIRC8_7_6855,31,374,162,412,0,192,11,0,278,191,699,449
BMI1_7_6856,1147,1543,742,1237,1222,1291,660,737,1860,1256,27,1417
BRAP_7_6857,1299,728,2142,1789,310,889,2555,265,402,540,1154,1506
BRD1_7_6858,3,159,0,0,6,34,0,676,1,0,109,20
BRPF3_7_6859,41,201,0,0,0,0,1082,438,9,0,105,172
BTBD2_7_6860,456,0,15,0,0,0,2,0,271,2,0,32
BTBD6_7_6861,207,336,0,0,0,0,333,0,1833,32,0,269
CAND1_7_6862,1614,3267,1283,2374,1965,798,2026,2987,1888,3284,3412,171
2
CBLB_7_6863,290,312,577,198,413,528,337,133,380,355,31,1416
CBL_7_6864,292,369,462,515,71,1318,576,19,236,715,259,137
CBLL1_7_6865,42,6,8,61,0,0,8,2,58,341,13,8
CCIN_7_6866,935,0,0,0,43,12,63,203,247,192,0,51

CCNF_7_6867,11,230,0,3,802,1,0,0,0,0,0,0
CDC20_7_6868,215,59,411,68,0,472,793,896,1053,1055,621,451
CDC23_7_6869,69,4,0,4,0,154,0,0,8,265,1,893
CDC26_7_6870,1139,341,1838,889,860,365,796,2110,1014,967,1333,1689
CDC34_7_6871,385,0,0,0,17,159,0,0,0,9,0,0
CGRRF1_7_6872,393,60,57,0,1,0,39,67,199,29,5,32
CHD4_7_6873,0,0,119,0,0,0,0,0,0,0,0,0
CIAO1_7_6874,389,8,0,212,0,106,1,0,79,0,10,77
COPS4_7_6875,240,3,12,0,1069,1,171,0,270,24,2,26
COPS5_7_6876,627,745,508,169,625,846,0,324,1136,75,1110,666
COPS6_7_6877,601,81,18,43,808,9,3,0,68,8,262,3
COPS7B_7_6878,618,235,75,1141,114,288,37,461,158,5,391,131
CORO6_7_6879,4,7,195,10,1446,0,20,11,4,0,35,18
CUEDC1_7_6880,891,291,27,73,506,3472,39,466,205,1100,324,261
CUL1_7_6881,701,147,107,325,488,100,412,690,6,794,588,153
CUL3_7_6882,6,87,165,0,1047,441,277,590,655,79,2,353
CUL5_7_6883,611,1794,2241,1736,620,684,866,1463,2429,1412,2319,1749
CUL9_7_6884,0,169,276,0,901,2,78,14,14,29,1139,360
DCAF10_7_6885,391,213,551,552,526,1303,227,313,2038,1441,583,1230
DCAF12_7_6886,342,100,0,459,331,308,110,269,430,36,535,535
DCAF13_7_6887,2484,495,1240,1376,1706,1450,286,240,1459,1867,394,715
DCAF16_7_6888,277,1054,0,395,735,4,51,579,1162,16,644,669
DCAF5_7_6889,1,0,291,0,0,0,39,56,3,20,8,0
DCAF7_7_6890,0,1130,0,0,0,160,308,0,262,103,0,32
DCAF8_7_6891,219,4,0,121,0,0,460,332,0,300,0,166
DDA1_7_6892,362,796,291,191,36,217,315,1441,273,710,871,1021
DDB1_7_6893,724,580,797,263,2519,374,569,979,83,483,436,369
DDB2_7_6894,10,221,143,0,26,11,37,2,153,112,413,7
DPF2_7_6895,23,65,44,12,32,0,278,548,398,177,0,989
DTX1_7_6896,6,41,74,67,388,2,19,196,1,1,32,0
DTX3L_7_6897,15,79,209,0,175,1290,0,432,194,71,908,416
DTX4_7_6898,78,109,113,67,949,352,741,73,750,153,479,429
DZIP3_7_6899,516,163,22,593,0,588,0,141,841,326,38,1024
EIF3D_7_6900,710,642,823,1416,1250,619,2005,1056,2125,1781,1333,2479
EIF3E_7_6901,13,91,0,10,77,2,239,0,58,51,260,84
EIF3F_7_6902,0,151,0,3,36,97,5,107,223,416,0,571
EIF3G_7_6903,0,0,0,8,0,0,335,74,0,170,869,56
EIF3H_7_6904,234,23,108,5,817,69,2360,0,55,5,389,1045
EIF3I_7_6905,62,0,0,0,818,0,9,1,41,110,223,9
EIF3J_7_6906,297,299,773,0,982,35,224,726,9,65,29,804
EIF3K_7_6907,1,64,0,0,0,51,0,227,808,48,24,116
ENC1_7_6908,0,269,11,4,1094,141,822,0,705,153,956,1548
EPN3_7_6909,0,0,0,0,49,650,0,634,100,3,0,17
EPOR_7_6910,0,4,0,0,0,0,28,4,0,1,0,2
ERCC8_7_6911,1355,80,1268,271,246,947,176,1242,902,1207,51,1333
FAF1_7_6912,50,0,1,252,592,114,835,741,1066,241,37,564
FBXL12_7_6913,0,249,1,0,0,0,340,530,0,1,0,0
FBXL14_7_6914,139,229,0,0,0,0,0,32,14,2,102,1
FBXL15_7_6915,19,0,10,0,0,334,0,185,0,0,0,229
FBXL16_7_6916,0,0,0,0,0,0,196,0,0,0,0,0

FBXL17_7_6917,545,389,345,538,2014,1545,267,32,1607,589,407,543
FBXL18_7_6918,39,156,0,1,96,60,24,106,6,662,213,78
FBXL19_7_6919,195,187,0,126,0,0,26,1,24,100,0,8
FBXL21_7_6920,1810,9,14,3,4,337,0,365,300,84,292,104
FBXL3_7_6921,1234,1286,1895,1436,6203,1649,2307,2208,1476,1329,3221,16
74
FBXL4_7_6922,140,19,0,136,47,14,15,0,62,56,268,977
FBXL7_7_6923,194,1,23,197,53,31,19,503,5,89,950,160
FBXO10_7_6924,6,492,930,143,280,179,604,3,358,111,1601,944
FBXO16_7_6925,729,939,1425,895,993,1354,856,765,1193,482,1588,477
FBXO27_7_6926,65,0,165,1014,152,40,1,0,55,171,331,18
FBXO2_7_6927,505,202,182,29,687,340,363,783,278,584,280,192
FBXO30_7_6928,433,0,0,864,47,520,0,655,565,361,569,82
FBXO33_7_6929,642,0,107,50,1080,344,6,0,276,85,2,83
FBXO36_7_6930,165,24,68,120,393,147,144,347,485,252,427,891
FBXO39_7_6931,22,228,884,37,1111,156,132,696,27,3,46,72
FBXO40_7_6932,1005,442,556,756,380,291,594,740,199,833,1269,768
FBXO41_7_6933,167,607,0,0,11,73,751,43,61,1,0,3
FBXO42_7_6934,2065,932,191,44,10,172,298,154,326,562,426,800
FBXO43_7_6935,37,348,574,195,789,559,17,16,92,21,11,13
FBXO46_7_6936,418,1,13,0,0,392,0,1,27,244,0,5
FBXO6_7_6937,0,69,53,40,0,528,32,110,480,24,687,635
FBXO8_7_6938,431,1357,128,977,834,1509,278,2198,579,1358,2098,558
FBXW10_7_6939,897,287,41,152,470,257,69,268,398,153,1,55
FBXW2_7_6940,330,6,0,0,0,30,409,745,1,0,0,595
FBXW5_7_6941,0,610,0,487,0,0,609,0,0,29,0,0
FBXW9_7_6942,0,257,32,6,745,318,331,228,218,107,16,45
G2E3_7_6943,792,1750,880,341,1772,628,271,641,1545,1430,246,1476
GAN_7_6944,363,178,24,1,562,764,10,1828,1306,51,971,535
GMCL1_7_6945,134,143,312,23,1,444,817,412,692,366,6,585
GNB2_7_6946,0,0,3,0,733,0,0,159,1,0,0,0
GRWD1_7_6947,0,21,288,198,107,160,6,0,64,64,56,62
GZF1_7_6948,206,396,583,44,1,53,15,0,0,125,0,205
HACE1_7_6949,2375,865,2793,1801,76,1229,2332,2527,987,462,1008,2057
HDAC6_7_6950,2,630,0,9,3,80,0,700,0,14,210,0
HECTD1_7_6951,1974,221,156,739,848,798,461,42,808,1058,1620,379
HECTD3_7_6952,0,0,10,46,36,1118,32,3,304,94,143,39
HECW1_7_6953,145,22,47,0,56,726,1643,849,51,79,4,22
HECW2_7_6954,318,64,123,557,288,16,20,0,803,364,11,122
HERC1_7_6955,570,243,95,533,363,679,2398,973,754,358,1304,701
HERC2_7_6956,688,219,410,31,104,276,0,205,759,404,161,97
HERC3_7_6957,640,3,11,0,27,99,113,104,0,97,14,935
HERC5_7_6958,434,74,29,22,201,146,23,308,102,1,277,137
HGS_7_6959,23,363,243,162,0,67,1323,1410,289,589,1054,185
HIC2_7_6960,1894,170,1275,480,52,1112,720,221,427,1394,1761,2042
HUWE1_7_6961,25,9,278,75,1386,328,1182,169,150,19,357,521
IBTK_7_6962,474,48,388,0,156,10,551,395,9,6,44,15
IL10RA_7_6963,32,16,18,4,4,54,966,2,286,25,1,43
IL6_7_6964,1605,147,720,93,498,1213,1,8,662,494,957,975
IRF9_7_6965,0,461,0,150,0,0,37,0,420,8,597,666

ITCH_7_6966,71,183,120,1385,1684,19,700,1151,1247,634,904,728
IVNS1ABP_7_6967,247,486,965,366,938,860,241,1035,0,78,168,704
JHDM1D_7_6968,1,42,0,326,861,0,267,772,122,427,878,348
JOSD1_7_6969,13,329,257,342,41,554,0,7,208,82,658,350
JOSD2_7_6970,5,1171,267,24,0,2,0,3,106,2,4,44
KAT6B_7_6971,347,14,0,0,132,44,26,689,0,55,1,0
KATNB1_7_6972,2,310,173,19,236,1,0,0,8,360,34,2
KBTBD10_7_6973,9,0,0,0,5,89,16,0,0,2,0,12
KBTBD11_7_6974,14,193,686,318,0,5,0,1,1,94,10,1
KBTBD2_7_6975,111,641,106,401,489,214,205,1151,166,836,271,803
KBTBD5_7_6976,373,475,584,949,465,180,43,7,135,1167,67,803
KBTBD7_7_6977,0,159,0,239,0,0,0,0,0,4,0,7
KBTBD8_7_6978,569,45,1854,6,0,295,0,233,232,750,430,46
KCTD10_7_6979,230,110,57,22,23,88,71,214,103,18,489,6
KCTD11_7_6980,265,106,118,68,0,16,539,343,122,60,13,370
KCTD12_7_6981,120,79,62,0,19,30,28,58,10,15,218,5
KCTD13_7_6982,0,0,0,14,0,2,0,0,0,39,0,17
KCTD16_7_6983,74,10,535,42,185,531,0,1219,440,163,535,76
KCTD17_7_6984,0,0,0,32,0,0,0,0,0,21,0,0
KCTD18_7_6985,26,135,0,235,1075,61,14,176,164,551,3,148
KCTD3_7_6986,1149,1076,916,492,2567,414,986,952,786,567,932,125
KCTD5_7_6987,0,0,502,0,0,0,246,0,0,0,0,0
KCTD9_7_6988,761,1249,0,87,1255,1049,0,13,666,596,298,584
KDM2A_7_6989,1194,2723,2737,801,860,497,2299,2660,821,602,2340,1606
KDM4B_7_6990,206,158,986,3,10,533,842,816,268,344,56,156
KDM5B_7_6991,96,5,23,4,138,126,5,38,652,292,98,91
KLHDC5_7_6992,1529,511,221,381,1816,579,9,1706,1263,347,862,524
KLHL10_7_6993,324,5,1431,488,735,6,18,798,33,213,74,7
KLHL11_7_6994,265,892,1938,1248,629,1168,1327,1789,995,241,118,783
KLHL12_7_6995,651,0,1,260,577,44,141,203,160,226,284,635
KLHL14_7_6996,146,104,0,50,426,568,721,290,194,880,730,729
KLHL15_7_6997,512,30,0,19,22,582,220,269,4,29,72,1081
KLHL17_7_6998,2,0,0,11,207,0,0,0,150,184,27,623
KLHL18_7_6999,6,0,3,0,0,658,0,0,0,0,192,1
KLHL1_7_7000,0,328,0,7,0,0,16,0,15,970,1,5
KLHL20_7_7001,894,83,934,0,1,40,0,428,403,5,1169,37
KLHL21_7_7002,8,0,36,0,2,0,0,129,13,1,0,4
KLHL22_7_7003,0,1,0,2,0,0,23,139,91,3,13,14
KLHL23_7_7004,1413,2650,2272,610,777,1504,2951,2663,2613,2998,781,1565
KLHL24_7_7005,1228,934,672,708,965,596,1155,602,1123,738,2222,2117
KLHL25_7_7006,163,325,543,153,245,267,430,258,479,843,641,1703
KLHL26_7_7007,1192,562,72,379,886,729,2606,658,764,623,2075,978
KLHL28_7_7008,450,206,317,80,655,1,61,0,81,418,0,251
KLHL29_7_7009,31,501,1045,563,0,190,0,1403,138,29,2,9
KLHL31_7_7010,1800,1238,3035,1274,1090,2472,2217,3034,1847,719,612,295
8
KLHL32_7_7011,21,0,0,0,0,0,0,8,40,0,0,5
KLHL33_7_7012,615,435,1183,976,139,999,540,440,0,169,2135,872
KLHL34_7_7013,0,94,0,0,867,1,0,0,32,257,48,6
KLHL36_7_7014,601,752,1741,54,1,528,2401,2237,1150,869,19,642

KLHL3_7_7015,108,9,0,1066,0,88,4,0,146,51,124,816
KLHL8_7_7016,774,1187,857,656,769,1606,2675,219,1505,2100,1454,932
LATS1_7_7017,172,317,1566,223,119,672,0,665,1381,1112,474,960
LATS2_7_7018,176,41,14,1,10,60,20,1,4,7,116,22
LIF_7_7019,172,0,0,35,434,38,3,0,190,13,3,147
LNX2_7_7020,0,0,0,0,2,5,0,0,7,0,0,0
LOC283116_7_7021,1531,2360,1025,379,156,583,370,3139,1269,2329,547,243
0
LONRF1_7_7022,2285,2204,2295,1575,1489,1871,1611,3076,2761,3315,3299,1
619
LTN1_7_7023,18,0,177,185,327,144,772,23,180,32,0,27
LZTR1_7_7024,0,6,104,96,212,9,18,55,38,1,20,10
MAP1LC3B_7_7025,291,642,403,564,85,785,32,121,336,159,6,1118
MAP3K1_7_7026,375,1650,0,2479,228,628,537,2,55,173,52,7
MARK1_7_7027,828,427,114,99,1318,468,1089,929,949,1031,575,102
MDM2_7_7028,166,300,103,174,46,1104,1164,705,636,397,466,597
MED20_7_7029,0,0,11,0,17,3,0,7,0,172,141,839
MEX3B_7_7030,0,0,0,0,0,31,0,0,0,0,6,0
MIB1_7_7031,0,0,70,0,6,0,0,0,12,0,2,1
MKRN2_7_7032,48,127,0,835,0,0,0,297,400,369,0,50
MKRN3_7_7033,730,54,74,645,108,1,792,815,228,8,0,1102
MLL2_7_7034,13,46,307,63,23,705,1001,1629,42,389,153,8
MLLT6_7_7035,23,0,0,0,0,5,235,50,33,0,294,4
MOCS3_7_7036,377,68,373,479,54,994,1372,658,793,895,321,318
MRPL49_7_7037,0,0,0,20,8,0,0,1,0,0,0,0
MUL1_7_7038,2,0,0,0,0,0,1002,210,0,0,0,0
MYCBP2_7_7039,999,659,101,783,578,823,215,702,434,1375,665,513
MYLIP_7_7040,979,220,498,529,44,10,64,2365,201,82,234,82
MYSM1_7_7041,42,13,28,20,662,430,0,26,147,13,67,40
NACC1_7_7042,39,0,0,7,0,0,435,0,416,102,0,55
NACC2_7_7043,288,1,0,11,44,21,0,282,8,4,0,15
NEURL1B_7_7044,909,723,609,638,259,199,1294,432,279,182,249,918
NEURL_7_7045,1183,1137,1667,1017,1620,722,188,122,1024,793,73,209
NHLRC1_7_7046,0,960,0,232,0,875,12,0,492,578,1,319
NUP43_7_7047,74,1692,544,92,823,920,56,802,3,156,274,31
OTUB1_7_7048,345,1000,807,656,380,273,4,433,109,61,93,16
OTUB2_7_7049,2,273,84,31,108,180,886,1074,26,95,59,189
OTUD1_7_7050,0,0,0,606,2,0,0,0,45,2,8,2
OTUD6A_7_7051,1,1,0,353,0,478,1,54,0,5,50,0
OTUD6B_7_7052,0,23,3,37,0,30,0,97,0,14,0,16
OTUD7A_7_7053,0,16,0,0,313,0,0,0,0,129,0,80
OTUD7B_7_7054,659,1794,1754,18,431,412,97,0,0,150,66,412
PAFAH1B1_7_7055,684,697,0,447,578,1,871,839,256,744,0,119
PARP10_7_7056,454,0,4,555,193,372,74,6,15,64,533,254
PARP11_7_7057,0,19,555,72,0,0,245,1,489,105,18,44
PARP14_7_7058,0,55,0,4,0,1,0,0,58,187,4,3
PCGF1_7_7059,321,0,0,0,13,3,31,0,1390,23,0,469
PCGF2_7_7060,407,280,1313,368,512,1620,1725,410,622,930,2843,94
PCGF3_7_7061,36,351,142,7,118,369,1,130,15,40,49,899
PDZRN3_7_7062,0,83,17,13,0,0,2,90,562,26,13,55

PEBP4_7_7063,0,0,0,0,0,0,1,0,0,49,0,0
PEX12_7_7064,1,26,227,24,399,0,0,0,1,79,1,0
PHF14_7_7065,50,1782,972,757,1879,513,754,177,749,613,1871,1233
PHF15_7_7066,1,10,30,0,39,516,3,6,16,89,253,1054
PHF20_7_7067,745,505,169,189,682,511,598,918,1306,1249,1543,270
PHF2_7_7068,132,0,387,0,0,0,0,28,0,1,0,0
PHF3_7_7069,657,1153,527,1438,488,1524,9,351,1669,1498,1074,807
PHIP_7_7070,704,33,126,1187,1102,1791,834,377,33,606,703,32
PHRF1_7_7071,1233,631,12,0,24,655,2037,1390,939,896,14,390
PJA2_7_7072,274,410,48,209,1,141,683,333,747,321,579,520
PRPF19_7_7073,0,0,0,838,1,1,0,0,0,1902,0,0
PRPF8_7_7074,585,182,154,493,0,169,75,80,28,349,470,3
PSMD14_7_7075,17,18,0,6,16,5,0,17,738,11,146,107
PSMD2_7_7076,0,0,24,1,81,0,1,33,157,276,5,20
PSMD4_7_7077,305,36,4,40,0,528,445,60,21,2,0,3
PSMD7_7_7078,367,1655,1055,134,44,2442,313,3313,412,2748,1128,1154
PWP1_7_7079,0,2,436,0,0,0,0,0,0,0,0,0
RAB40A_7_7080,163,2,1078,5,973,455,636,668,38,4,294,105
RAB40AL_7_7081,163,2,1078,5,973,455,636,668,38,4,294,105
RAB40B_7_7082,0,0,0,7,1074,14,0,5,6,640,0,258
RAD18_7_7083,503,689,2252,141,813,644,728,326,752,520,842,1018
RAD23A_7_7084,0,0,0,546,0,0,0,0,4,0,689,0
RAD23B_7_7085,67,0,0,14,0,249,24,17,6,36,270,96
RAG1_7_7086,0,3,1210,1,0,0,338,30,60,7,1,1012
RAI1_7_7087,118,256,13,161,111,143,197,3,273,5,34,585
RBX1_7_7088,0,39,0,0,0,0,0,0,0,0,0,0
RCBTB1_7_7089,363,1183,189,1033,153,640,155,38,0,89,405,757
RCBTB2_7_7090,303,7,405,19,336,75,784,22,65,204,432,1331
RFFL_7_7091,287,7,70,51,33,715,285,113,572,643,559,80
RFPL1_7_7092,168,2,44,132,41,601,375,193,295,252,772,635
RFWD3_7_7093,0,0,0,4,0,0,0,0,502,13,340,45
RHOBTB3_7_7094,11,132,0,13,445,513,1802,0,42,46,74,9
RING1_7_7095,198,1239,522,192,2854,864,318,562,867,22,483,526
RNF10_7_7096,0,0,834,0,58,276,0,0,0,0,0,13
RNF111_7_7097,201,528,550,544,1539,1143,2203,1697,417,942,1561,1487
RNF112_7_7098,6,82,1348,56,159,117,85,516,0,3,265,18
RNF113A_7_7099,0,84,0,0,0,1,0,39,0,0,0,709
RNF113B_7_7100,2,313,0,79,418,34,6,9,216,7,49,226
RNF114_7_7101,499,5,34,40,80,446,0,5,167,45,86,20
RNF115_7_7102,1095,81,141,235,827,130,1105,1325,532,204,685,1043
RNF11_7_7103,1305,1728,962,283,49,725,779,1067,722,624,830,1476
RNF121_7_7104,64,22,327,102,230,222,199,460,9,115,77,2
RNF122_7_7105,7,915,1034,1030,50,840,8,1034,1432,1006,1230,1295
RNF123_7_7106,0,0,0,0,0,32,2,0,0,0,26,0
RNF125_7_7107,4,477,271,0,0,0,3,75,5,20,77,3
RNF126_7_7108,0,305,557,195,76,0,24,27,2,20,4,14
RNF130_7_7109,157,791,34,265,837,29,1,157,958,22,810,752
RNF133_7_7110,704,357,1013,444,295,216,149,712,167,1490,791,774
RNF139_7_7111,3195,2116,1553,2833,7742,2483,1934,2230,3796,3926,663,22
48

RNF141_7_7112,233,28,296,23,1415,924,535,396,4,594,1055,97
RNF144A_7_7113,990,60,115,106,198,0,168,533,50,243,161,109
RNF144B_7_7114,10,80,0,2,30,0,0,98,0,96,0,0
RNF149_7_7115,995,0,0,103,3,45,310,0,846,553,0,98
RNF150_7_7116,58,58,1871,394,165,590,55,298,16,48,658,95
RNF152_7_7117,129,7,0,223,0,0,32,1,1,39,0,426
RNF157_7_7118,3,239,3448,1163,351,6,767,118,112,488,427,1207
RNF167_7_7119,1092,920,1940,443,3255,639,1174,1141,1808,1077,1540,1758
RNF168_7_7120,319,1584,50,1401,786,1132,1874,113,407,1174,1299,801
RNF169_7_7121,218,0,564,0,7,0,619,4,13,390,1304,24
RNF181_7_7122,475,3,24,523,80,56,7,29,12,1,116,572
RNF183_7_7123,129,654,0,11,8,43,7,60,102,47,29,462
RNF186_7_7124,30,0,0,0,7,15,0,0,0,0,30,11
RNF187_7_7125,1535,165,1206,365,1612,150,2380,1846,2277,1384,1471,1104
RNF20_7_7126,0,19,1,197,0,0,0,602,0,0,0,0
RNF217_7_7127,163,378,0,552,0,1,1,832,849,228,0,1772
RNF220_7_7128,38,490,1524,535,63,1269,4,1269,159,545,9,15
RNF25_7_7129,8,432,0,0,0,0,70,302,337,27,40,52
RNF26_7_7130,13,112,692,580,0,775,211,67,387,37,1,184
RNF2_7_7131,91,245,281,2,172,81,0,339,440,195,292,446
RNF31_7_7132,78,23,67,0,1650,96,976,0,166,342,343,426
RNF43_7_7133,218,197,0,457,19,44,10,835,197,74,366,361
RNF44_7_7134,73,774,179,0,0,548,99,156,3,0,737,2
RNF5_7_7135,506,264,1188,396,1948,42,32,208,111,834,505,499
RSC1A1_7_7136,180,398,761,106,88,84,571,247,33,27,601,217
RSF1_7_7137,42,0,1,14,739,723,0,15,308,266,1,48
RSPRY1_7_7138,1,0,26,0,48,707,891,1,213,3,0,24
SCLY_7_7139,0,0,0,361,0,0,0,1934,782,14,14,568
SENP1_7_7140,461,28,0,381,32,1412,1081,116,515,997,303,541
SENP2_7_7141,165,117,0,116,1549,21,9,0,98,23,1,39
SENP3_7_7142,325,11,0,474,0,399,16,1,24,88,0,433
SENP5_7_7143,1272,0,0,505,1597,536,0,192,87,3,1183,749
SH3RF1_7_7144,2389,4738,4236,3810,2871,1909,3936,3111,2961,2274,5471,6
755
SH3RF2_7_7145,165,3,0,273,127,14,1131,0,124,186,288,10
SH3RF3_7_7146,0,366,56,0,0,0,0,1000,4,44,694,4
SHKBP1_7_7147,0,0,0,0,0,501,0,0,0,0,0,0
SIAH2_7_7148,753,101,161,2,243,176,34,27,88,92,440,71
SIK1_7_7149,339,7,120,520,169,4,0,12,186,55,1018,146
SLX4_7_7150,14,26,0,20,0,0,19,0,0,84,0,3
SMU1_7_7151,668,74,441,328,74,884,798,1441,135,1048,879,612
SMURF2_7_7152,102,290,0,593,1368,176,226,836,270,212,242,30
SNRNP40_7_7153,231,0,1634,359,1,0,0,0,517,94,453,99
SOCS1_7_7154,1,0,50,0,729,0,0,424,5,41,2,2
SOCS2_7_7155,433,189,735,291,31,129,2,148,6,77,65,20
SOCS3_7_7156,0,0,0,0,0,0,0,0,0,0,0,0
SOCS6_7_7157,219,197,0,0,41,34,0,23,370,41,37,41
SPOPL_7_7158,909,67,1300,239,124,567,43,817,395,1600,384,593
SPSB1_7_7159,0,225,0,1,0,8,0,533,93,1,0,5
SPSB3_7_7160,0,12,409,0,0,403,0,2,7,79,0,6

SPSB4_7_7161,1,21,287,8,0,0,0,14,7,58,16,229
STAM2_7_7162,189,331,496,587,298,156,424,105,40,900,495,6
STAMBPL1_7_7163,0,0,0,0,0,0,0,0,0,0,0,0
STAM_7_7164,327,0,0,0,0,1,52,784,1,3,4,2608
STUB1_7_7165,312,203,0,114,602,107,789,632,376,327,151,993
SUMO3_7_7166,32,12,97,108,6,604,76,14,58,16,0,35
SYNGAP1_7_7167,132,786,51,160,1396,461,4,1009,777,508,475,1167
TAB2_7_7168,340,788,710,286,10,330,295,340,582,197,77,475
TAB3_7_7169,102,370,23,0,677,403,0,0,0,0,1,8
TAF1D_7_7170,14,0,5,228,0,0,0,0,16,0,32,2
TBC1D1_7_7171,779,84,121,2,614,298,166,427,1606,1119,17,190
TLE1_7_7172,501,61,656,1383,132,24,442,7,875,867,0,816
TNFAIP3_7_7173,0,51,23,230,401,0,149,1058,319,31,561,99
TOLLIP_7_7174,0,84,0,0,0,0,0,0,0,0,0,0
TRAF7_7_7175,0,0,3,0,0,41,0,0,0,0,0,0
TRAIP_7_7176,51,211,863,465,194,118,1335,292,798,321,23,271
TRIM11_7_7177,24,4,0,16,225,0,59,2,140,950,10,102
TRIM15_7_7178,680,111,209,109,75,827,894,1306,687,1041,1294,1518
TRIM25_7_7179,38,109,0,1,652,31,96,331,247,3,240,33
TRIM27_7_7180,234,1538,273,305,681,781,577,650,736,578,1173,593
TRIM28_7_7181,513,16,1009,0,114,246,109,222,439,887,1896,1014
TRIM31_7_7182,115,3,0,0,621,15,0,1077,0,0,1,839
TRIM35_7_7183,337,247,879,229,489,52,682,701,164,289,103,113
TRIM42_7_7184,0,0,0,0,41,14,89,0,0,4,3,0
TRIM46_7_7185,239,23,24,10,4,631,30,2,31,103,1532,16
TRIM47_7_7186,656,0,298,338,7,94,0,165,216,167,329,31
TRIM48_7_7187,0,37,0,615,0,186,0,1008,0,4,42,2
TRIM52_7_7188,1,297,163,542,1,33,155,8,0,3,142,16
TRIM56_7_7189,34,0,0,0,0,0,0,0,0,0,0,0
TRIM62_7_7190,19,186,308,0,0,0,0,0,617,578,0,76
TRIM63_7_7191,10,1,580,60,360,20,0,636,1,31,158,170
TRIM65_7_7192,0,279,0,0,162,0,0,149,738,10,2,82
TRIM67_7_7193,594,19,131,17,277,146,64,41,17,546,776,2
TRIM68_7_7194,609,128,256,36,3,99,1263,107,352,671,1,270
TRIM8_7_7195,1253,592,698,680,99,588,995,911,924,2027,636,206
TRIP12_7_7196,656,940,686,1545,1748,1059,405,2064,1975,1469,117,801
UBA2_7_7197,405,253,64,19,454,264,1003,61,546,232,717,78
UBA6_7_7198,451,1055,1827,830,2386,354,2055,1567,528,1041,257,1056
UBA7_7_7199,301,634,157,753,441,616,716,282,642,219,4,87
UBAC1_7_7200,645,404,0,0,343,368,754,60,0,31,0,0
UBAP2_7_7201,71,0,427,861,78,618,684,156,30,1,60,17
UBASH3B_7_7202,5415,2622,1898,1901,4125,2696,6518,2734,5990,6107,7292,
3729
UBE2B_7_7203,105,214,52,551,378,257,788,1263,135,140,574,103
UBE2D4_7_7204,267,1309,526,857,2320,311,266,4,2110,1948,931,1402
UBE2E2_7_7205,308,13,35,289,145,0,776,5,211,327,139,368
UBE2F_7_7206,129,204,184,610,8,724,177,1742,3,1268,117,247
UBE2G1_7_7207,1961,243,389,886,606,1888,274,1663,443,732,1103,384
UBE2J1_7_7208,625,1221,2675,532,2658,213,1324,260,1503,1200,1075,339
UBE2L3_7_7209,3754,1849,2292,1537,4115,2695,873,3194,3844,2229,4653,40

39
UBE2M_7_7210,225,137,0,32,178,6,15,1,176,56,0,66
UBE2N_7_7211,336,418,48,74,132,840,25,175,1004,430,659,557
UBE2NL_7_7212,483,11,0,0,3,72,0,50,7,29,95,4
UBE2O_7_7213,634,1,12,17,60,1,759,0,300,134,865,1180
UBE2Q1_7_7214,3628,1922,3651,2270,2604,1831,4319,1722,4562,2326,4613,3
171
UBE2QL1_7_7215,0,0,0,0,0,32,0,0,7,412,0,1
UBE2R2_7_7216,454,32,178,395,178,97,45,377,200,458,118,236
UBE2S_7_7217,0,18,22,10,677,273,0,16,21,15,848,3
UBE2T_7_7218,1934,620,585,4682,2206,3169,766,733,1157,2521,1603,2727
UBE2U_7_7219,12,2,8,0,9,591,1,0,13,4,500,1
UBE2V2_7_7220,340,2700,217,653,689,1379,173,3263,486,2092,892,629
UBE2Z_7_7221,0,1583,33,0,3,50,411,9,78,0,0,6
UBE3C_7_7222,842,181,81,88,874,1342,0,1369,676,210,17,122
UBQLN2_7_7223,13,239,24,0,4,0,0,0,0,33,6,0
UBQLN3_7_7224,797,26,692,505,210,426,481,683,419,794,652,277
UBQLN4_7_7225,129,0,0,0,0,1013,1,349,0,0,158,1
UBR1_7_7226,2863,1983,2822,930,1,1365,5,807,204,1902,1859,1993
UBR3_7_7227,493,177,1551,498,1306,1908,2529,2230,1876,485,1174,541
UBR4_7_7228,477,305,251,309,719,872,637,1257,317,590,712,863
UBR5_7_7229,480,753,2,43,795,463,0,0,243,7,2,29
UBR7_7_7230,653,851,12,563,743,469,72,934,330,347,525,375
UBXN10_7_7231,22,0,0,2,0,110,0,1,0,1,1,0
UBXN1_7_7232,325,134,0,0,2,0,0,9,61,12,0,16
UBXN2A_7_7233,1080,615,1224,1109,2702,1142,342,2441,653,962,1259,657
UBXN4_7_7234,305,701,580,28,748,88,121,54,101,98,14,158
UBXN7_7_7235,228,249,653,2546,1371,662,1088,295,359,441,3224,847
UBXN8_7_7236,129,460,0,215,172,912,414,548,153,184,112,166
UCHL1_7_7237,0,158,4,4,0,8,542,408,446,5,0,145
UCHL3_7_7238,566,1708,3052,2061,944,662,3783,957,2218,1717,5110,2330
UFC1_7_7239,538,240,33,244,113,53,630,513,832,551,190,358
UHRF2_7_7240,35,1778,1086,886,1332,2654,1033,1365,1356,475,303,828
UNK_7_7241,2,294,0,0,30,510,11,19,0,69,259,0
USP11_7_7242,384,121,65,154,410,27,569,429,397,47,297,449
USP13_7_7243,285,221,124,317,1445,116,208,533,263,885,11,97
USP15_7_7244,2004,937,940,1287,3406,1272,5014,2761,1245,2571,923,733
USP17_7_7245,3,13,7,8,1165,494,101,414,76,27,316,20
USP17L2_7_7246,45,408,409,4,175,193,829,176,41,23,36,713
USP17L5_7_7247,610,370,1,1245,788,706,1,1035,3,350,1071,826
USP18_7_7248,1,231,0,1,71,849,6,0,34,6,0,5
USP22_7_7249,515,1568,83,690,718,195,146,144,734,1129,775,364
USP24_7_7250,0,0,0,0,0,0,0,0,0,0,0,0
USP25_7_7251,2,774,520,31,1,310,246,186,612,9,193,82
USP26_7_7252,0,166,727,451,55,125,9,0,2,671,118,0
USP27X_7_7253,299,88,1564,229,213,977,562,796,689,390,298,177
USP28_7_7254,118,3,0,0,70,3,113,0,72,0,10,140
USP29_7_7255,55,30,957,10,1402,357,731,0,323,481,0,143
USP30_7_7256,83,10,3,1,235,27,23,142,156,702,51,466
USP32_7_7257,1586,0,0,0,2,217,625,0,1,253,0,1

USP34_7_7258,264,59,382,245,274,239,59,148,584,100,1403,1185
USP35_7_7259,1539,596,1,0,61,1650,646,539,196,2684,734,1448
USP36_7_7260,482,761,2,4,0,0,3,0,0,500,0,0
USP37_7_7261,775,133,394,0,1,66,33,250,630,349,371,254
USP38_7_7262,222,216,14,1,268,0,30,0,32,305,319,404
USP39_7_7263,0,0,118,1260,0,0,0,0,0,489,8,593
USP3_7_7264,665,212,0,76,49,400,3,1417,472,401,1541,61
USP40_7_7265,1194,381,0,236,245,50,839,1345,300,8,99,27
USP42_7_7266,28,38,337,38,0,250,6,199,218,11,0,103
USP43_7_7267,1567,675,823,282,1645,548,535,153,1161,692,1717,1753
USP45_7_7268,5,0,0,168,0,8,47,0,475,45,0,163
USP47_7_7269,67,222,436,636,11,1707,0,555,45,380,42,108
USP49_7_7270,34,234,281,262,464,541,130,593,4,609,48,84
USP50_7_7271,373,31,293,15,379,803,89,0,10,1,117,1912
USP51_7_7272,2204,448,1863,1285,34,1266,1116,2629,1269,647,1058,1089
USP53_7_7273,101,1,1465,274,7,0,0,516,110,295,92,960
USP54_7_7274,370,171,480,433,370,21,760,0,2,302,774,1
USP6_7_7275,45,1014,371,0,0,226,992,44,255,16,7,31
USP7_7_7276,0,15,0,22,0,1165,50,69,185,219,71,176
USP9Y_7_7277,1402,2509,363,965,161,1699,1399,2378,680,530,1149,295
USPL1_7_7278,3,474,127,11,390,384,23,11,282,414,0,40
VCPIP1_7_7279,423,468,24,296,284,43,438,352,346,752,1503,151
VPS11_7_7280,579,232,199,213,107,96,129,0,63,586,0,561
WDR12_7_7281,225,348,352,393,583,698,61,262,352,424,1785,622
WDR53_7_7282,1,287,1,3,451,0,950,0,1595,40,29,253
WDR59_7_7283,130,698,114,954,1547,1132,1142,0,463,1356,967,159
WDR5B_7_7284,2343,4428,2194,4056,3697,3556,3347,3111,2382,4533,7022,49
26
WDR61_7_7285,37,404,31,10,0,181,1,39,138,222,7,53
WDTC1_7_7286,5,58,0,32,346,594,0,187,0,243,345,123
WSB2_7_7287,398,1185,337,188,439,244,13,708,362,293,1100,38
WWP1_7_7288,131,419,1556,534,1,251,172,123,813,53,844,349
YOD1_7_7289,1,0,0,0,0,14,0,28,0,0,131,0
ZBTB11_7_7290,53,409,191,42,134,791,1,818,8,613,980,5
ZBTB25_7_7291,9,24,921,10,1,170,0,0,9,178,1287,64
ZBTB2_7_7292,1,16,1007,1,0,0,0,8,566,28,928,55
ZBTB32_7_7293,250,210,713,621,94,464,298,836,168,458,351,80
ZBTB34_7_7294,18,346,0,5,10,256,17,16,0,457,35,7
ZBTB39_7_7295,304,17,470,5,1579,368,175,436,15,1328,0,184
ZBTB3_7_7296,199,21,1142,5,0,26,1,517,86,61,0,43
ZBTB41_7_7297,286,0,1233,0,0,0,1,540,0,0,143,455
ZBTB44_7_7298,776,164,794,203,1440,286,153,876,282,59,657,943
ZBTB45_7_7299,3,0,0,0,0,0,0,1,1,193,0,51
ZBTB46_7_7300,0,357,34,193,52,85,19,67,84,54,161,15
ZBTB47_7_7301,1050,889,1764,1356,189,1424,1477,1081,1828,1971,2507,502
ZBTB48_7_7302,699,258,16,134,900,236,899,694,283,1511,632,426
ZBTB49_7_7303,243,0,73,527,26,130,16,0,0,0,0,113
ZBTB5_7_7304,31,0,0,0,658,54,1158,0,641,1028,953,234
ZBTB7A_7_7305,2,20,104,554,34,157,0,1,37,4,0,1659
ZBTB7B_7_7306,10,0,0,3,35,4,96,0,96,152,0,9

ZBTB7C_7_7307,114,38,556,49,532,526,292,231,89,6,36,439
ZBTB8A_7_7308,0,4,88,52,0,5,0,6,77,0,178,45
ZFAND2B_7_7309,665,36,0,29,199,2,74,0,65,299,0,70
ZFPL1_7_7310,66,49,856,4,133,62,2520,369,371,1077,2,74
ZMYND10_7_7311,0,0,0,489,777,0,0,0,1,0,0,766
ZNF131_7_7312,27,174,150,409,29,741,658,463,967,250,1240,833
ZNF598_7_7313,909,255,814,114,517,430,433,702,728,270,184,897
ZNF645_7_7314,809,361,230,46,484,316,252,33,494,1272,112,160
ZNRF1_7_7315,1270,3,2,115,586,25,473,1481,77,87,62,118
ZNRF2_7_7316,238,814,209,903,1285,156,25,1216,749,997,572,303
ZNRF4_7_7317,484,908,56,15,0,5,79,1191,558,625,1122,364
ZRANB1_7_7318,4,186,58,156,2047,32,921,70,703,240,258,993
ZSWIM2_7_7319,115,2466,1265,505,688,440,2,618,363,379,657,1342
AIRE_7_7320,0,634,132,31,6,0,12,49,0,1,0,0
ANAPC5_7_7321,570,3,17,93,47,19,53,36,377,58,11,373
ANAPC7_7_7322,83,127,65,903,1,377,4,23,399,98,339,209
ANKFY1_7_7323,29,203,702,174,587,40,39,22,335,794,543,41
ASB10_7_7324,789,0,0,0,0,1,0,352,0,0,0,640
ASB11_7_7325,0,0,0,0,0,0,0,0,0,71,0,0
ASB14_7_7326,283,517,423,150,1137,1091,902,88,427,687,274,1065
ASB2_7_7327,23,33,16,6,1,1,0,0,10,23,29,665
ASB3_7_7328,171,0,32,239,650,0,14,0,5,90,15,337
ASB4_7_7329,2102,221,1651,766,545,1070,27,289,1036,626,970,3251
ASB6_7_7330,0,4,338,801,940,0,337,378,462,5,0,975
ASB7_7_7331,93,12,956,52,1293,38,68,32,15,155,20,390
ASB9_7_7332,0,5,0,12,0,2,137,2,168,5,240,255
ASCC2_7_7333,0,3,0,0,8,5,0,0,0,0,0,0
ATG10_7_7334,0,0,0,0,0,0,0,0,0,0,0,0
ATG16L1_7_7335,31,1094,679,37,184,458,582,545,16,159,0,195
ATG7_7_7336,671,710,0,26,0,284,0,144,487,517,276,83
ATRX_7_7337,2314,1374,2118,1038,896,1500,975,1196,1260,1157,1008,4737
BACH2_7_7338,0,152,189,37,0,128,135,18,676,948,1033,179
BAG6_7_7339,1796,47,0,0,154,298,762,49,775,323,633,88
BAZ1A_7_7340,1184,2038,1523,1398,2434,1573,3757,2953,3490,3671,4185,38
79
BCL6_7_7341,80,0,38,212,484,568,1057,294,521,327,9,760
BIRC3_7_7342,308,884,831,122,1795,1111,1052,684,1119,1006,330,1261
BIRC7_7_7343,0,0,0,105,0,0,0,0,0,0,0,0
BPTF_7_7344,10,34,58,43,0,1,133,93,115,94,0,27
BRCA1_7_7345,1162,923,2068,859,431,1999,1619,3883,2437,1853,2565,1674
BRPF1_7_7346,8,40,27,110,224,23,56,728,23,3,12,2
BRWD1_7_7347,200,13,0,521,1469,0,713,26,285,9,384,34
BTBD11_7_7348,0,295,0,0,0,0,0,1,5,79,806,0
BTBD1_7_7349,0,26,0,31,70,70,0,49,0,529,52,0
BTBD3_7_7350,114,147,782,40,12,90,867,1171,51,89,1199,341
BTBD7_7_7351,0,1,0,0,0,4,72,388,0,0,201,0
BTBD9_7_7352,0,0,0,0,0,0,590,0,276,8,669,605
BTRC_7_7353,5850,4951,5234,2393,3856,2964,4158,4765,4967,6661,4358,750
8
C3orf26_7_7354,12,375,121,238,723,429,531,99,195,1045,812,46

CBLC_7_7355,0,25,0,0,2,0,0,2,1,0,0,2
CCNB1IP1_7_7356,686,1753,1129,1161,32,978,2089,805,0,3,0,158
CDC16_7_7357,14,0,155,719,256,170,24,0,75,2,0,496
CDC27_7_7358,670,678,313,583,1287,638,1018,1933,648,1021,2300,582
CHFR_7_7359,48,112,303,12,0,6,245,3,168,13,78,555
CISH_7_7360,9,277,2065,64,3,94,0,26,196,6,947,13
CNOT4_7_7361,2933,1248,3,2511,297,1661,758,1484,1320,1092,1846,957
COPS2_7_7362,1171,2583,3005,1591,1816,3297,3521,4234,2839,6269,3913,12
54
COPS3_7_7363,0,1,25,238,17,378,36,5,143,117,1,210
COPS7A_7_7364,0,59,0,0,23,0,18,1,0,169,0,12
COPS8_7_7365,41,22,0,14,238,364,0,0,0,38,0,55
CRBN_7_7366,339,191,1212,84,109,462,401,1272,263,526,231,762
CUL2_7_7367,348,35,0,320,737,61,411,581,123,1440,445,683
CUL4A_7_7368,1396,2392,3345,1576,3432,1959,1314,2688,3429,3199,3920,29
74
CUL4B_7_7369,431,284,404,4,1409,8,184,553,265,16,467,50
CUL7_7_7370,347,10,100,563,635,141,34,342,174,63,1679,26
CXXC1_7_7371,23,2,224,111,49,0,1,15,625,11,3,71
CYLD_7_7372,490,695,368,374,193,268,1194,818,350,551,1323,887
DCAF11_7_7373,0,0,0,0,0,0,0,0,0,0,0,0
DCAF17_7_7374,527,845,137,280,2,454,440,297,328,282,4,117
DCAF4_7_7375,0,0,0,0,0,0,0,0,0,93,0,0
DCAF6_7_7376,9,32,252,89,35,10,9,0,522,596,0,91
DCST1_7_7377,0,0,1,0,0,27,5,39,557,12,37,88
DEPDC1B_7_7378,5,60,13,14,1096,192,644,211,408,103,287,111
DET1_7_7379,73,575,100,0,361,164,16,1139,1356,35,934,154
DIDO1_7_7380,0,13,4,0,10,61,0,0,0,0,0,0
DNAJB2_7_7381,0,195,0,150,0,597,449,1,221,139,0,34
DTX2_7_7382,0,0,0,0,0,145,0,12,35,2,17,3
EED_7_7383,211,60,42,0,0,0,162,263,488,75,129,142
EIF3B_7_7384,563,51,2,425,621,887,351,7,1468,228,0,302
EIF3C_7_7385,108,32,240,0,208,471,482,102,153,209,20,134
EIF6_7_7386,612,22,1785,47,499,131,57,1212,147,185,831,542
EPN1_7_7387,106,0,17,0,1,10,0,0,0,0,0,0
EPN2_7_7388,290,148,0,280,152,272,1,1351,21,299,341,122
EPS15_7_7389,0,0,6,37,0,0,243,0,20,0,0,5
FAM70A_7_7390,350,109,1083,786,560,136,1027,1,597,16,557,725
FANCL_7_7391,411,314,320,303,345,474,79,299,424,725,168,56
FBXL13_7_7392,31,295,0,0,156,1147,66,269,429,351,28,62
FBXL20_7_7393,14,384,114,772,35,74,0,16,719,17,43,110
FBXL2_7_7394,1176,168,2214,320,467,559,741,2070,646,1675,875,1726
FBXL5_7_7395,301,0,1446,317,93,107,910,0,866,1108,886,788
FBXL6_7_7396,1,0,14,2,0,3,5,1,477,45,0,37
FBXO11_7_7397,300,27,253,99,217,32,42,271,898,422,7,177
FBXO15_7_7398,1469,754,223,1108,53,1254,30,257,1319,51,109,561
FBXO17_7_7399,15,1,185,401,0,1,2,164,2,327,997,60
FBXO18_7_7400,960,732,861,25,415,148,1156,431,1446,369,695,1829
FBXO21_7_7401,113,42,764,658,0,577,0,0,1,172,0,0
FBXO22_7_7402,689,1094,98,743,9,777,128,395,290,374,295,390

FBXO24_7_7403,0,0,38,0,0,1,0,0,19,0,0,3
FBXO25_7_7404,724,226,658,739,99,328,0,427,738,52,661,1341
FBXO28_7_7405,811,608,367,580,135,222,521,1186,765,409,218,141
FBXO32_7_7406,244,189,265,210,182,196,59,492,8,15,25,217
FBXO34_7_7407,139,1434,169,391,1196,547,2041,848,665,709,1142,1389
FBXO38_7_7408,63,558,1,229,3,327,1143,8,628,69,194,83
FBXO3_7_7409,93,457,143,574,450,82,1554,454,573,1297,1212,2023
FBXO44_7_7410,22,20,28,0,4,197,0,0,211,2,0,39
FBXO4_7_7411,12,2,179,33,295,98,285,114,16,99,0,4
FBXO5_7_7412,99,78,4,323,0,254,145,55,0,249,100,794
FBXO7_7_7413,31,53,1097,0,0,168,2827,1061,423,656,26,1319
FBXO9_7_7414,0,0,0,0,0,0,0,0,0,0,0,0
FBXW11_7_7415,919,581,545,349,1179,117,1615,256,573,255,1901,1114
FBXW7_7_7416,33,779,0,362,0,536,0,14,511,660,778,323
FBXW8_7_7417,184,1097,118,234,267,148,665,325,56,774,505,43
GPS1_7_7418,1,474,262,590,2,48,4,5,481,19,21,66
HECTD2_7_7419,1682,2820,1090,396,797,2228,0,2114,1432,1696,266,1574
HERC4_7_7420,3,591,154,218,108,0,18,2,12,91,354,125
HERC6_7_7421,1080,735,175,1,11,5,1,25,429,813,0,89
HIC1_7_7422,0,0,0,55,0,466,17,0,1261,363,62,129
HLTF_7_7423,163,334,595,926,1701,737,55,844,977,515,97,1019
HSF4_7_7424,729,3,10,27,15,376,171,202,284,400,26,57
IPP_7_7425,317,462,1043,454,147,605,1323,646,1821,338,251,780
KAT6A_7_7426,579,1455,1602,539,1,678,1137,726,377,2500,2602,2385
KBTBD3_7_7427,491,1376,472,744,697,1350,592,1857,1226,1870,1280,1056
KCTD6_7_7428,73,319,20,19,7,17,125,25,75,179,352,55
KCTD7_7_7429,46,0,0,0,0,1,0,0,0,94,10,10
KDM2B_7_7430,0,0,0,0,0,458,401,778,434,6,0,32
KDM4C_7_7431,527,1161,1144,347,172,903,4,359,383,29,1359,442
KDM5C_7_7432,131,47,2,104,7,8,225,50,1398,26,987,471
KEAP1_7_7433,284,0,138,1038,1086,183,476,103,0,0,375,425
KIAA1841_7_7434,893,1487,0,579,0,1417,1659,387,1510,1267,137,698
KLHL13_7_7435,210,6,0,4,3,0,0,328,723,584,13,101
KLHL2_7_7436,157,1823,1416,703,133,601,262,0,152,226,1663,192
KLHL4_7_7437,1658,124,1,248,351,292,28,186,4,127,104,668
KLHL5_7_7438,542,87,313,17,266,765,817,152,73,991,428,984
KLHL7_7_7439,59,0,23,44,0,3,0,0,94,153,44,31
LNX1_7_7440,402,733,1395,1285,3420,1592,769,1523,1306,1138,355,303
LONRF3_7_7441,0,11,1,13,0,0,65,1,290,10,1,34
LRRC29_7_7442,0,0,0,0,0,0,0,0,8,0,14,0
LRSAM1_7_7443,32,518,304,441,959,57,602,1214,31,397,2,108
MARK2_7_7444,95,53,23,457,296,809,264,750,1410,585,367,722
MARK3_7_7445,193,1,18,32,0,44,0,63,80,161,101,115
MARK4_7_7446,1479,458,1193,168,33,12,0,642,1012,663,16,132
MDM4_7_7447,151,679,22,4,262,237,681,177,95,913,1017,328
MGRN1_7_7448,433,0,34,79,0,0,0,0,227,12,1,30
MIB2_7_7449,41,1,0,59,0,129,0,1,602,13,811,672
MID1_7_7450,103,75,10,0,1,197,0,24,53,9,0,9
MID2_7_7451,147,70,0,0,0,58,2,492,20,1,1013,40
MKRN1_7_7452,407,54,0,172,1277,16,1,108,92,6,4,9

MLL5_7_7453,213,1,930,322,384,369,265,186,501,13,213,108
MLL_7_7454,502,335,317,336,0,0,29,98,57,315,18,36
MNAT1_7_7455,953,717,1016,400,341,283,1188,883,802,211,4,495
MPND_7_7456,511,163,243,3,0,613,0,0,0,2,0,0
MTF2_7_7457,0,0,0,0,8,3,0,2,74,293,0,94
MYNN_7_7458,777,291,2,588,114,719,18,2069,882,1371,735,114
NAE1_7_7459,586,134,917,573,1,6,1976,17,1494,609,767,241
NDUFC2_7_7460,933,381,386,1140,210,490,926,281,456,990,307,339
NEDD4_7_7461,712,1135,0,840,521,1382,2137,751,2,1429,1221,698
NEDD4L_7_7462,10,349,0,126,12,114,169,30,78,115,191,89
NFX1_7_7463,114,21,746,1,0,601,0,115,885,373,25,426
NLE1_7_7464,1302,727,1658,283,501,148,151,189,785,127,1830,218
NSD1_7_7465,212,959,2,257,52,2222,156,297,476,837,2,827
NSFL1C_7_7466,565,330,73,15,267,45,197,74,0,317,0,1
NUB1_7_7467,0,0,0,654,0,0,0,0,0,0,0,0
ODF2_7_7468,25,411,7,4,0,144,0,143,412,5,0,48
OTUD5_7_7469,161,1889,1493,802,64,1151,705,501,306,58,410,35
PARK2_7_7470,681,390,401,465,15,813,115,32,243,804,18,706
PARP9_7_7471,744,911,386,847,1239,1323,886,259,284,618,1058,40
PATZ1_7_7472,0,315,0,0,0,0,39,0,151,2,599,28
PCGF6_7_7473,0,443,31,1550,5,726,1173,429,13,1402,47,1078
PEX10_7_7474,0,0,1,4,8,4,57,90,0,6,92,1
PEX2_7_7475,786,835,7,45,252,5,37,669,351,16,42,45
PHF12_7_7476,1,0,740,0,24,44,652,57,249,38,582,178
PHF16_7_7477,2,375,0,284,535,43,0,0,0,4,0,12
PHF17_7_7478,48,22,419,50,0,169,436,20,342,6,2,230
PHF1_7_7479,33,9,7,0,0,0,32,2,535,3,1,69
PHF21A_7_7480,16,836,51,0,0,216,12,0,17,136,0,32
PHF7_7_7481,1531,878,33,572,1,438,1836,1650,975,1937,832,994
PHF8_7_7482,725,23,868,356,1565,1005,255,528,413,677,394,682
PJA1_7_7483,476,259,786,320,591,1284,299,118,49,488,226,209
PML_7_7484,95,374,0,12,254,161,0,689,757,505,0,101
POC1B_7_7485,490,1080,501,0,15,86,78,1332,142,3,4,58
PSMD1_7_7486,606,11,15,25,437,299,579,10,1,442,1,25
RAPSN_7_7487,0,20,3047,0,104,46,162,7,71,262,1467,142
RBBP4_7_7488,863,645,512,118,81,240,1304,769,235,269,6,715
RBBP5_7_7489,75,88,920,1059,1056,20,169,1719,634,99,144,889
RBBP6_7_7490,583,217,1812,446,699,767,430,742,743,1279,2437,773
RBBP7_7_7491,694,1705,104,975,149,644,1480,1188,671,539,1113,139
RBCK1_7_7492,23,1,1205,0,0,0,0,110,265,4,2,35
RC3H2_7_7493,396,675,660,656,476,526,1688,1292,380,415,2161,894
RCHY1_7_7494,185,115,15,181,480,239,275,11,493,299,899,831
RFPL2_7_7495,6,261,0,236,0,42,5,3,2,0,11,0
RFPL3_7_7496,288,962,1560,1124,444,629,879,619,1124,955,1584,592
RFWD2_7_7497,1586,1294,1399,1954,870,414,1675,1241,1835,3016,1566,1371
RHOBTB1_7_7498,0,0,0,89,0,10,0,0,0,5,44,0
RHOBTB2_7_7499,214,261,57,226,523,20,403,454,50,14,15,601
RLIM_7_7500,15,430,1775,111,2,204,220,0,0,1,0,142
RNF103_7_7501,1809,1009,1140,1288,831,2794,3018,189,4485,4237,4114,406
6

RNF128_7_7502,409,535,1019,32,2064,197,67,346,349,209,993,956
RNF135_7_7503,720,151,1,22,1,265,8,167,35,109,753,8
RNF138_7_7504,3,0,15,0,0,2,0,4,384,8,1380,116
RNF13_7_7505,52,459,840,301,0,244,504,952,480,530,1341,369
RNF145_7_7506,777,331,7,139,0,1224,214,0,332,708,95,105
RNF146_7_7507,99,124,743,292,693,381,74,171,324,452,1270,723
RNF14_7_7508,414,446,1684,2557,115,350,705,0,673,807,390,121
RNF166_7_7509,0,0,0,6,0,5,0,0,0,1,0,35
RNF170_7_7510,67,44,0,21,26,0,20,0,4,2,57,110
RNF17_7_7511,3,20,399,740,7,0,0,5,25,287,714,647
RNF180_7_7512,184,30,0,105,114,0,0,0,395,44,64,46
RNF182_7_7513,381,171,598,974,184,269,119,243,380,1506,3009,469
RNF185_7_7514,343,114,893,213,108,761,280,115,38,51,9,190
RNF19A_7_7515,836,29,46,1008,150,1224,192,2092,225,509,1656,320
RNF19B_7_7516,1148,97,440,14,1123,444,195,340,188,328,15,88
RNF213_7_7517,9,1,0,0,0,0,0,0,20,0,13,2
RNF214_7_7518,0,584,437,1040,3187,629,771,157,1256,523,25,2191
RNF216_7_7519,2101,647,2005,1002,867,698,1433,528,3142,401,1166,1926
RNF24_7_7520,2436,1937,965,774,1484,1390,1526,4889,1936,2756,1463,720
RNF32_7_7521,43,0,158,77,0,1,0,10,133,48,0,28
RNF34_7_7522,3,233,0,437,0,13,56,0,577,94,128,1160
RNF38_7_7523,22,499,1473,253,560,513,1044,1075,263,292,1506,617
RNF40_7_7524,10,459,922,38,18,0,211,52,10,16,306,0
RNF41_7_7525,660,1100,266,0,354,508,52,3,66,81,6,12
RNF6_7_7526,117,187,675,1,736,7,0,0,885,334,112,670
RNF7_7_7527,3,6,503,24,0,143,18,545,34,15,256,120
RNF8_7_7528,716,370,107,10,639,6,136,121,251,475,274,151
SAE1_7_7529,3,9,0,1115,7,969,0,0,0,113,582,31
SENP6_7_7530,216,131,678,482,354,1340,605,858,141,112,249,49
SENP7_7_7531,757,1650,1081,8,1925,1511,131,458,2759,3342,106,963
SENP8_7_7532,556,172,843,234,904,150,64,943,645,926,203,94
SF3A1_7_7533,626,584,3162,732,1143,285,831,20,325,427,3,377
SHPRH_7_7534,1031,1341,401,671,78,944,2418,1237,1231,1621,1288,466
SIAH1_7_7535,242,246,30,327,401,128,24,600,467,256,121,280
SKP2_7_7536,94,24,42,1,0,124,10,29,654,30,318,63
SMURF1_7_7537,523,356,383,18,394,337,1,0,249,490,236,263
SOCS5_7_7538,612,0,0,0,75,12,0,812,162,972,744,32
SP100_7_7539,423,0,274,1136,1883,194,327,49,1531,613,488,526
SP110_7_7540,70,1,2,302,22,33,8,185,5,63,2,46
SPOP_7_7541,1535,1463,1516,839,1258,294,1804,431,776,293,1953,1008
SPSB2_7_7542,1,30,0,214,13,0,1,622,462,618,0,398
SQSTM1_7_7543,0,24,0,0,0,494,0,0,0,21,127,1
STAMBP_7_7544,1684,607,138,115,2300,1698,77,456,1567,45,511,847
SUMO1_7_7545,19,106,0,176,0,0,242,398,76,1,674,7
SUMO2_7_7546,1033,125,1,289,1004,897,525,509,470,370,1076,57
SYTL4_7_7547,585,1443,922,1617,1725,673,1395,226,169,775,279,365
SYVN1_7_7548,0,469,103,322,6,367,0,246,315,631,14,179
TCF20_7_7549,242,15,2,278,6,882,11,0,611,197,278,77
TDRD3_7_7550,688,15,68,343,722,1,563,109,455,111,4,231
TIPARP_7_7551,618,131,0,20,200,0,845,39,122,22,1199,113

TLE2_7_7552,387,115,0,0,757,167,26,226,1034,623,1022,127
TLE3_7_7553,474,0,0,3,306,0,31,0,0,0,998,0
TNK2_7_7554,0,5,765,1,0,0,2,604,4,0,0,495
TNRC6C_7_7555,182,0,0,567,967,54,0,2,0,0,130,96
TOPORS_7_7556,364,201,25,270,222,183,110,126,244,1850,584,888
TOR1AIP2_7_7557,0,105,45,9,28,3,110,356,0,463,217,30
TRAF3_7_7558,615,700,3637,334,58,1309,5092,815,1813,1325,2203,1439
TRAF5_7_7559,510,1499,669,582,16,1,422,53,747,978,3,862
TRAF6_7_7560,310,38,445,98,1067,299,3,786,607,591,591,967
TRIM10_7_7561,592,317,360,770,230,171,624,145,250,584,0,106
TRIM13_7_7562,834,1308,1014,774,1922,1419,4618,687,1207,832,2506,1563
TRIM17_7_7563,1,168,38,0,11,114,149,234,56,773,949,10
TRIM22_7_7564,0,46,2,0,0,117,0,0,0,0,0,0
TRIM23_7_7565,792,311,16,1441,710,9,1,682,1348,578,26,712
TRIM24_7_7566,211,181,2445,260,815,759,449,814,538,22,29,731
TRIM26_7_7567,25,175,791,100,20,78,123,1224,212,274,5,25
TRIM2_7_7568,43,13,3,0,0,0,0,0,515,9,0,84
TRIM32_7_7569,1539,1027,1540,1140,1694,277,692,1436,1399,560,1302,1496
TRIM33_7_7570,828,1046,370,0,5,451,636,409,3,446,720,1
TRIM34_7_7571,0,0,0,0,0,0,0,148,7,0,0,1
TRIM37_7_7572,2373,1578,872,593,729,190,482,429,926,2562,3405,1478
TRIM39_7_7573,7,155,321,180,1140,10,0,131,732,163,0,527
TRIM3_7_7574,55,321,303,101,1262,527,107,14,183,31,76,306
TRIM41_7_7575,262,469,1498,775,119,664,700,707,1359,189,749,1074
TRIM45_7_7576,1,9,0,0,1291,0,20,0,0,50,0,0
TRIM4_7_7577,196,1432,724,752,22,708,0,555,928,2285,1152,1093
TRIM54_7_7578,6,88,72,98,37,0,2,839,102,537,17,396
TRIM55_7_7579,246,40,0,813,2,573,129,0,752,577,193,846
TRIM5_7_7580,264,636,530,76,39,142,266,542,315,806,340,133
TRIM7_7_7581,29,4,7,271,0,61,0,60,341,26,642,45
TRIM9_7_7582,139,0,0,0,0,0,151,15,254,299,0,179
TRPC4AP_7_7583,22,97,2,0,205,0,7,144,429,605,17,62
TSPAN17_7_7584,507,55,0,100,150,420,266,911,547,454,326,239
TTC3_7_7585,1694,534,2711,858,2121,1734,2716,578,2468,782,1949,1057
TULP4_7_7586,12,0,4,0,43,167,0,0,0,1,596,0
UBA1_7_7587,1412,525,1277,2398,2217,39,140,1944,820,838,7,429
UBA3_7_7588,1,527,179,172,27,432,1090,24,331,26,160,40
UBA5_7_7589,202,1044,1316,537,0,7,791,369,353,722,763,825
UBAC2_7_7590,474,712,234,395,850,730,581,649,671,29,1036,842
UBAP2L_7_7591,17,837,555,551,533,1324,362,141,657,2321,517,86
UBASH3A_7_7592,119,93,0,306,167,430,32,750,652,268,734,105
UBE2A_7_7593,74,177,0,89,426,92,44,2,28,608,219,250
UBE2D1_7_7594,60,83,24,165,55,661,71,182,170,27,1,893
UBE2D2_7_7595,593,498,839,919,346,350,1239,0,398,338,10,852
UBE2D3_7_7596,243,707,419,364,285,32,170,24,309,254,287,489
UBE2E1_7_7597,1,45,146,15,593,262,309,257,501,26,146,192
UBE2E3_7_7598,114,515,1131,136,516,1077,275,67,386,446,367,767
UBE2G2_7_7599,254,351,0,367,2,0,121,442,284,20,17,101
UBE2H_7_7600,26,8,0,55,1,654,41,1,142,16,4,113
UBE2I_7_7601,1654,103,100,300,831,212,88,4,251,38,1207,293

UBE2J2_7_7602,50,523,495,9,146,444,1772,3,8,237,2,25
UBE2K_7_7603,2194,645,940,1023,1826,261,1418,740,1097,1344,721,2167
UBE2Q2_7_7604,0,44,0,202,0,0,0,0,96,219,183,409
UBE2V1_7_7605,13,104,0,5,0,6,18,0,88,54,720,17
UBE2W_7_7606,327,298,579,159,5,645,377,371,108,73,642,11
UBE3A_7_7607,167,120,25,126,32,241,411,269,79,586,192,830
UBE3B_7_7608,54,3,0,574,24,0,58,671,2,26,113,331
UBE4A_7_7609,399,124,213,1138,1459,1200,1533,656,663,784,2,1354
UBE4B_7_7610,0,269,545,58,0,2,35,65,95,3,2,35
UBL7_7_7611,29,704,147,9,1398,1247,0,391,442,83,347,55
UBOX5_7_7612,4,2,585,521,1779,363,967,64,532,318,892,219
UBQLN1_7_7613,1298,1080,1316,1089,1367,2038,605,1275,1266,455,660,1968
UBR2_7_7614,2535,635,1546,468,1364,222,1064,2990,863,456,228,2122
UBXN11_7_7615,9,0,0,74,0,471,0,0,418,7,0,68
UBXN6_7_7616,91,7,63,152,0,307,30,9,73,420,235,16
UCHL5_7_7617,0,50,1280,357,3,41,1221,349,50,11,0,1098
UHRF1_7_7618,3,1349,0,965,752,110,675,998,424,2740,1062,573
UIMC1_7_7619,23,511,738,9,0,0,37,452,373,32,137,74
UNKL_7_7620,0,1,0,0,0,9,0,0,63,1,0,10
USP14_7_7621,210,1114,10,528,585,657,483,752,751,618,144,1394
USP19_7_7622,493,553,1925,1230,193,1829,372,2138,123,1095,1,427
USP1_7_7623,757,1067,1092,481,61,298,170,1441,1122,453,787,248
USP20_7_7624,383,0,0,1,1020,3,323,80,1,0,2,35
USP2_7_7625,34,319,8,704,1347,5,373,228,1366,1427,207,1070
USP33_7_7626,399,374,0,161,1976,360,20,0,616,622,272,318
USP44_7_7627,2,14,30,1,763,117,610,0,15,1,14,0
USP46_7_7628,1489,2546,776,747,1547,2352,1037,606,942,225,919,724
USP48_7_7629,417,92,17,21,92,19,100,386,320,1302,18,43
USP4_7_7630,0,13,870,45,0,9,929,70,391,22,0,47
USP5_7_7631,10,0,0,136,70,9,3,13,0,0,22,0
USP6NL_7_7632,0,2,0,0,1129,0,0,422,0,69,2,0
USP8_7_7633,4,127,0,12,0,0,0,1,993,41,380,121
USP9X_7_7634,244,40,469,54,1,36,833,76,216,74,248,44
VHL_7_7635,297,391,351,107,1346,248,471,582,190,102,929,273
VPRBP_7_7636,194,1772,1539,158,1003,994,2095,632,712,787,950,654
VPS13D_7_7637,1165,50,1439,144,1,196,1035,497,27,69,3,714
VPS41_7_7638,577,261,572,275,232,211,19,477,188,283,2061,748
VPS8_7_7639,71,2137,2562,1596,2197,1060,1575,356,1302,3046,1811,716
WDR26_7_7640,5,1005,100,937,703,432,9,293,460,97,185,295
WDR5_7_7641,6,42,0,75,9,0,477,2,92,0,42,80
WDR76_7_7642,14,0,0,0,275,0,0,0,0,575,16,7
WHSC1_7_7643,1504,14,4,456,347,156,0,499,163,829,58,47
WHSC1L1_7_7644,0,14,960,10,2,0,20,0,0,0,0,18
WSB1_7_7645,60,85,413,55,465,479,162,41,132,442,35,513
WWP2_7_7646,0,0,0,8,0,1145,26,18,128,454,0,15
XIAP_7_7647,1983,1039,1113,2300,2145,1249,2908,2231,2481,2183,2893,395
8
ZBTB10_7_7648,245,330,116,844,1504,405,1324,4,1297,2206,484,177
ZBTB16_7_7649,0,0,0,155,0,0,804,61,0,101,0,63
ZBTB17_7_7650,39,508,34,281,329,257,0,2,0,142,556,1

ZBTB1_7_7651,473,0,0,0,0,0,0,0,317,7,0,27
ZBTB20_7_7652,85,81,351,443,408,689,96,1788,1,502,84,125
ZBTB22_7_7653,211,169,0,416,125,0,93,365,368,14,30,124
ZBTB24_7_7654,1900,469,2419,1172,920,923,2127,559,2605,1461,1989,3598
ZBTB33_7_7655,96,81,8,636,1,0,30,0,6,342,468,0
ZBTB37_7_7656,0,237,39,769,15,169,1050,113,334,327,30,214
ZBTB40_7_7657,1002,825,419,14,54,660,3,454,885,377,4,891
ZBTB4_7_7658,657,1100,1130,1290,4191,3616,3209,549,1612,864,1052,2699
ZFP161_7_7659,0,0,0,0,0,0,0,0,276,5,0,27
ZMYND11_7_7660,4733,564,2128,2449,971,1058,2165,745,945,1727,1222,1333
ZMYND8_7_7661,230,166,122,1036,387,166,1138,240,1056,2116,2033,861
ZNF238_7_7662,0,0,0,0,0,0,0,0,0,0,0,0
ZNF295_7_7663,0,24,0,305,457,665,16,82,2,378,0,183
ZNRF3_7_7664,1684,389,211,760,1057,20,971,1138,1173,888,1735,220
ANAPC11_7_7665,489,653,9,32,0,343,1359,822,734,77,0,86
ATXN3_7_7666,810,2163,2712,1411,4153,971,1694,827,1015,873,1219,1024
MLLT10_7_7667,991,248,2,634,0,365,1305,870,529,1440,1124,749
OTUD4_7_7668,184,1521,8,28,447,985,99,123,2,263,112,1137
PHF19_7_7669,555,310,21,109,514,483,284,21,722,528,651,401
SP140_7_7670,17,545,111,64,20,83,11,0,12,9,307,67
TRIM36_7_7671,20,67,5,150,224,20,0,0,2,67,99,10
UBE2C_7_7672,1,59,0,761,1374,550,0,0,37,625,0,43
UBE2L6_7_7673,21,249,820,258,212,468,120,345,552,227,77,600
ABTB2_7_7674,0,0,0,0,0,0,5,0,0,0,6,0
AMBRA1_7_7675,329,2,0,0,0,0,0,0,3,2,0,698
AMFR_7_7676,203,1010,1670,918,1279,695,1054,2023,1017,1769,2605,741
ANAPC10_7_7677,110,19,1,47,67,331,809,57,90,11,135,74
ANAPC1_7_7678,0,0,397,0,0,0,0,0,0,21,0,0
ANAPC2_7_7679,27,231,0,21,676,137,1321,525,200,17,484,36
ANAPC4_7_7680,475,3,56,5,2,14,1546,1134,242,12,5,903
ANKIB1_7_7681,477,12,68,2,1160,35,0,220,393,15,195,199
ANKRD13A_7_7682,1012,0,0,0,701,28,3,698,124,39,4,428
ANKRD13D_7_7683,184,647,461,188,103,841,314,59,66,5,732,161
ARIH1_7_7684,198,57,162,428,213,1600,1042,1023,210,375,226,248
ARIH2_7_7685,1145,61,317,1176,899,417,1774,379,316,877,1448,797
ASB12_7_7686,1111,607,894,354,927,362,35,420,393,1148,346,1001
ASB13_7_7687,0,0,0,3,0,3,49,0,196,37,0,16
ASB15_7_7688,788,593,26,494,1044,331,912,1,436,72,1965,886
ASB16_7_7689,10,41,41,0,43,0,0,0,61,12,832,8
ASB17_7_7690,2875,2971,2456,1949,3765,3959,3127,2658,4219,3387,3747,42
50
ASB5_7_7691,271,397,0,633,524,483,256,40,536,146,148,1574
ASB8_7_7692,602,372,165,318,4,300,342,739,944,418,375,1261
ASH1L_7_7693,290,249,19,524,470,1146,1053,24,370,1045,641,61
ASPSCR1_7_7694,0,0,0,0,0,0,0,0,0,0,0,0
ATG12_7_7695,125,329,66,770,914,158,151,593,324,239,150,168
ATG3_7_7696,2088,821,637,1327,2212,988,28,1468,978,1104,1152,1622
ATG5_7_7697,72,44,0,53,173,3,5,3,3,190,838,453
ATXN1L_7_7698,30,289,373,169,1,318,485,327,182,635,244,81
ATXN3L_7_7699,402,0,94,0,0,0,57,0,3,0,0,0

BARD1_7_7700,71,123,61,52,255,456,25,3,352,63,180,938
BAZ1B_7_7701,682,128,110,2062,468,754,1141,713,1399,761,1194,557
BAZ2A_7_7702,2130,1622,465,496,269,850,3673,1007,973,190,2063,449
BAZ2B_7_7703,1966,1465,1561,570,1720,1870,363,630,1751,411,1068,237
BECN1_7_7704,12,11,0,76,418,11,422,488,307,106,0,130
BIRC2_7_7705,0,1204,59,176,232,26,0,661,62,641,18,15
BIRC6_7_7706,142,307,58,805,1638,344,216,255,627,256,34,126
BIRC8_7_7707,1231,15,61,13,209,126,288,230,52,21,28,53
BMI1_7_7708,278,1232,83,663,71,747,183,240,9,488,471,55
BRAP_7_7709,369,279,1013,968,384,1170,550,1897,577,294,334,799
BRD1_7_7710,603,811,106,370,1,451,652,915,704,1073,564,831
BRPF3_7_7711,63,59,0,377,0,712,87,0,170,0,0,19
BTBD2_7_7712,0,7,372,5,216,0,0,4,5,492,447,0
BTBD6_7_7713,4,4,320,0,43,661,10,0,0,0,4,0
CAND1_7_7714,42,514,1674,0,0,358,207,145,258,311,1100,1118
CBLB_7_7715,0,124,0,381,1328,120,811,156,5,180,32,241
CBL_7_7716,770,712,18,1236,1460,344,258,4,300,92,2011,455
CBLL1_7_7717,1097,313,269,541,433,349,354,194,225,576,45,367
CCIN_7_7718,0,19,0,496,0,0,0,23,0,0,0,0
CCNF_7_7719,1363,415,14,34,2,319,138,409,519,505,644,154
CDC20_7_7720,298,87,412,515,187,35,96,1112,97,20,35,98
CDC23_7_7721,968,120,2,496,1933,400,299,398,955,887,81,1181
CDC26_7_7722,538,5,0,9,0,413,11,41,138,141,7,491
CDC34_7_7723,0,4,73,39,1,77,166,127,38,14,546,68
CGRRF1_7_7724,296,7,231,488,138,71,502,763,446,20,57,61
CHD4_7_7725,0,0,0,0,0,0,0,0,0,0,0,0
CIAO1_7_7726,740,2661,1557,551,707,1511,1137,678,787,591,2174,778
COPS4_7_7727,281,204,101,458,78,170,475,402,1177,673,833,747
COPS5_7_7728,0,1085,493,4,538,880,7,2,30,33,723,976
COPS6_7_7729,0,0,0,0,0,1,0,0,1,0,277,0
COPS7B_7_7730,1095,361,452,310,1981,1575,899,198,599,1597,3379,1494
CORO6_7_7731,22,5,1429,63,209,468,325,777,790,548,49,100
CUEDC1_7_7732,367,5,131,15,0,154,253,0,175,725,62,110
CUL1_7_7733,41,62,396,593,604,798,158,1166,25,107,540,1156
CUL3_7_7734,1369,438,4,128,1399,2204,374,2919,647,1290,1645,4134
CUL5_7_7735,671,700,440,10,729,230,178,2222,162,461,1289,226
CUL9_7_7736,75,0,0,0,246,0,0,512,0,0,1,368
DCAF10_7_7737,79,72,139,542,176,54,146,6,83,41,73,10
DCAF12_7_7738,195,182,44,15,10,120,7,11,374,281,42,931
DCAF13_7_7739,906,2095,1213,884,858,745,816,1118,1069,589,1388,886
DCAF16_7_7740,14,22,6,0,44,0,94,6,2,296,0,0
DCAF5_7_7741,79,0,0,0,0,0,213,0,81,4,0,37
DCAF7_7_7742,486,816,50,6,1039,950,1325,509,524,107,476,327
DCAF8_7_7743,263,4,6,2309,855,320,0,556,1051,647,617,445
DDA1_7_7744,0,1,0,1,3,0,0,350,500,8,0,56
DDB1_7_7745,98,0,13,13,142,11,0,0,35,0,11,129
DDB2_7_7746,1021,577,42,0,1264,310,215,86,853,739,31,512
DPF2_7_7747,1672,378,32,356,107,588,229,374,13,733,1590,178
DTX1_7_7748,29,0,0,58,80,801,150,0,161,571,18,77
DTX3L_7_7749,662,677,948,2,3,401,732,609,440,628,323,998

DTX4_7_7750,160,93,550,0,14,694,439,21,37,80,5,403
DZIP3_7_7751,179,722,0,0,47,0,326,46,273,1122,640,26
EIF3D_7_7752,75,349,450,491,7,165,513,95,376,322,0,741
EIF3E_7_7753,161,353,0,107,643,1322,0,236,0,771,0,716
EIF3F_7_7754,47,14,386,211,0,718,114,85,19,555,82,0
EIF3G_7_7755,145,95,0,0,125,172,28,217,10,6,2067,2
EIF3H_7_7756,852,202,47,535,285,439,0,727,326,4,371,285
EIF3I_7_7757,0,0,0,0,0,216,36,359,0,0,38,0
EIF3J_7_7758,0,1,0,1,159,7,19,113,11,2,0,15
EIF3K_7_7759,537,158,373,320,429,256,129,225,107,204,358,18
ENC1_7_7760,0,0,1,2,0,0,0,0,0,0,0,0
EPN3_7_7761,1,466,18,0,0,2,0,6,0,9,1,0
EPOR_7_7762,97,415,374,9,8,699,240,13,1355,630,0,534
ERCC8_7_7763,2198,2238,1654,3940,2046,1512,4032,1377,3346,2348,1633,26
93
FAF1_7_7764,0,13,0,1,2,11,75,60,655,426,349,436
FBXL12_7_7765,0,0,0,0,0,0,9,191,10,0,0,2
FBXL14_7_7766,549,181,4,154,0,166,125,344,507,597,4,121
FBXL15_7_7767,95,0,0,8,7,0,0,0,0,0,0,36
FBXL16_7_7768,0,0,0,63,0,139,0,0,0,0,0,11
FBXL17_7_7769,332,1,249,5,1,32,29,0,525,640,488,110
FBXL18_7_7770,130,105,198,304,394,42,908,525,755,104,344,364
FBXL19_7_7771,206,226,260,226,0,11,114,0,490,51,20,240
FBXL21_7_7772,0,0,0,0,0,37,0,0,0,0,87,19
FBXL3_7_7773,1755,1335,440,1898,1173,683,164,3048,359,1618,611,863
FBXL4_7_7774,35,1076,0,0,74,5,0,0,0,0,0,822
FBXL7_7_7775,0,157,44,0,719,87,2017,274,0,54,295,508
FBXO10_7_7776,423,1631,919,896,188,546,1123,228,286,888,1352,136
FBXO16_7_7777,4,435,0,317,12,116,452,453,694,350,530,770
FBXO27_7_7778,362,302,0,641,111,100,714,5,22,707,823,381
FBXO2_7_7779,0,0,0,0,24,0,0,0,43,155,79,8
FBXO30_7_7780,1143,29,1306,20,1750,35,503,50,618,43,755,241
FBXO33_7_7781,943,246,2746,130,1472,85,615,14,151,312,469,57
FBXO36_7_7782,151,86,2,156,39,9,770,157,3,13,67,121
FBXO39_7_7783,1,0,0,0,768,0,294,0,0,1,0,0
FBXO40_7_7784,74,141,484,1006,0,481,49,879,222,109,0,39
FBXO41_7_7785,596,23,0,8,1,737,97,0,73,1,278,820
FBXO42_7_7786,2792,1969,1548,747,1906,1785,2679,1753,1379,2526,915,115
2
FBXO43_7_7787,1721,246,2,352,591,59,1437,0,648,709,1730,1233
FBXO46_7_7788,0,14,0,0,0,0,0,0,11,0,0,75
FBXO6_7_7789,168,3,12,413,73,38,733,18,20,103,25,1740
FBXO8_7_7790,2614,1412,994,1871,1014,502,1884,1610,1755,1710,1797,1562
FBXW10_7_7791,232,153,39,33,97,1,739,14,39,25,0,602
FBXW2_7_7792,412,306,54,239,75,878,440,212,340,66,0,264
FBXW5_7_7793,327,86,1,0,111,259,96,22,372,114,98,87
FBXW9_7_7794,44,886,276,0,487,15,879,1,128,3,676,22
G2E3_7_7795,0,791,1393,668,314,211,25,1383,460,1137,11,1450
GAN_7_7796,1377,927,140,319,1202,1253,460,902,1150,846,178,297
GMCL1_7_7797,250,43,1948,169,33,0,1089,1607,0,6,256,630

GNB2_7_7798,2,2,86,27,9,1,130,2,80,3,3,7
GRWD1_7_7799,0,22,0,0,188,0,10,104,5,1,1,2
GZF1_7_7800,5,3,71,2,146,179,3384,0,0,894,2,0
HACE1_7_7801,3959,3706,5518,2833,3344,1141,5071,4459,2888,3150,3902,39
39
HDAC6_7_7802,357,38,22,210,1528,241,84,125,132,1142,285,54
HECTD1_7_7803,9,483,0,413,0,0,70,852,16,349,59,0
HECTD3_7_7804,17,51,6,5,0,6,17,292,380,49,479,794
HECW1_7_7805,610,3,85,0,401,4,26,105,574,18,63,75
HECW2_7_7806,201,1195,42,565,1,1327,30,205,99,10,7,262
HERC1_7_7807,32,580,0,0,725,49,122,527,506,550,18,333
HERC2_7_7808,263,905,520,18,5,28,67,1135,913,170,388,242
HERC3_7_7809,1021,19,0,693,1,0,0,0,1,0,29,0
HERC5_7_7810,25,182,640,408,491,558,377,235,177,462,1983,804
HGS_7_7811,21,438,4,132,20,186,0,0,15,4,732,1
HIC2_7_7812,241,34,207,48,124,4,0,421,415,253,93,390
HUWE1_7_7813,11,3,0,347,0,35,123,199,42,11,951,8
IBTK_7_7814,400,18,0,2,0,0,0,17,0,3,0,46
IL10RA_7_7815,1512,0,0,0,15,0,500,2,245,742,18,43
IL6_7_7816,1120,63,420,782,0,81,344,760,19,263,1,1062
IRF9_7_7817,1,24,240,6,162,201,74,115,6,263,96,2
ITCH_7_7818,0,0,606,383,1336,0,0,0,2,376,17,0
IVNS1ABP_7_7819,50,6,4,0,0,674,467,0,57,89,360,1106
JHDM1D_7_7820,229,931,507,905,406,1259,854,1746,1090,734,42,2058
JOSD1_7_7821,1,45,0,0,206,0,0,1089,1,77,0,105
JOSD2_7_7822,124,317,0,0,0,0,0,145,0,0,0,0
KAT6B_7_7823,654,107,71,408,558,74,30,124,279,306,591,143
KATNB1_7_7824,0,0,0,0,0,0,0,0,0,0,0,0
KBTBD10_7_7825,257,156,303,420,364,301,764,120,24,785,287,418
KBTBD11_7_7826,0,0,0,0,0,66,0,0,13,5,0,121
KBTBD2_7_7827,103,0,0,83,0,0,0,0,0,0,0,0
KBTBD5_7_7828,47,0,0,214,0,100,0,168,11,0,76,0
KBTBD7_7_7829,486,114,121,342,475,316,759,473,541,761,37,1070
KBTBD8_7_7830,262,26,3,85,0,109,0,0,53,837,0,55
KCTD10_7_7831,220,386,919,286,29,293,0,11,209,195,14,315
KCTD11_7_7832,623,149,321,473,702,115,138,23,152,776,159,547
KCTD12_7_7833,28,26,0,0,119,292,10,3,0,242,579,1
KCTD13_7_7834,0,1,1,1,299,0,1,162,30,7,2,3
KCTD16_7_7835,399,299,1430,91,0,767,3,277,19,703,1,189
KCTD17_7_7836,0,197,0,0,0,0,0,0,1,86,46,36
KCTD18_7_7837,93,898,1465,1068,29,383,49,370,148,1412,158,1448
KCTD3_7_7838,540,12,442,235,74,548,1674,236,36,986,104,855
KCTD5_7_7839,177,0,0,333,1,279,17,4,382,170,375,38
KCTD9_7_7840,0,3,83,0,2,611,2,0,109,1,101,42
KDM2A_7_7841,148,4,0,0,5,28,0,363,19,213,132,653
KDM4B_7_7842,9,592,505,41,96,594,2,361,1,238,100,872
KDM5B_7_7843,43,469,0,1,911,87,89,0,57,10,19,7
KLHDC5_7_7844,2124,452,1407,117,502,433,851,223,1033,835,987,275
KLHL10_7_7845,1228,1132,1581,962,1180,79,1686,693,1168,1076,1279,1175
KLHL11_7_7846,11,7,182,65,408,289,446,126,0,389,1205,172

KLHL12_7_7847,13,442,550,3,1,3,0,1,3,6,0,1
KLHL14_7_7848,146,530,457,314,448,1470,388,743,953,536,2292,229
KLHL15_7_7849,20,168,1831,675,180,213,0,0,502,209,0,61
KLHL17_7_7850,27,5,0,0,0,1,0,23,494,7,0,61
KLHL18_7_7851,297,254,587,9,0,146,0,0,11,19,586,223
KLHL1_7_7852,463,418,0,24,965,219,779,19,307,210,687,982
KLHL20_7_7853,5,201,40,16,3,101,923,5,22,154,146,2
KLHL21_7_7854,847,43,2219,2,0,308,1307,587,364,166,218,59
KLHL22_7_7855,385,638,3134,210,510,3141,3619,1553,2065,1010,248,451
KLHL23_7_7856,383,228,828,35,870,340,44,406,71,202,158,212
KLHL24_7_7857,1166,688,350,322,45,599,527,1001,932,663,1851,760
KLHL25_7_7858,753,15,8,221,0,121,0,2022,8,4,22,18
KLHL26_7_7859,197,60,0,83,0,1,1,70,1,129,0,8
KLHL28_7_7860,3124,411,1722,813,1137,1824,41,435,1473,1022,656,1376
KLHL29_7_7861,0,211,36,13,295,347,14,35,4,48,5,115
KLHL31_7_7862,1446,979,1538,1581,3810,1714,463,1283,816,1981,694,671
KLHL32_7_7863,131,2,99,19,790,50,990,76,15,6,1230,1736
KLHL33_7_7864,2,36,0,0,2,72,0,111,0,21,0,0
KLHL34_7_7865,0,11,591,8,0,12,0,1241,2,160,0,5
KLHL36_7_7866,3,22,245,25,163,1,0,7,294,285,420,43
KLHL3_7_7867,128,31,3,331,92,36,2,6,13,333,0,5
KLHL8_7_7868,1153,1159,1824,218,1498,629,1056,1085,1118,1937,1823,129
LATS1_7_7869,0,0,0,0,0,0,0,0,0,0,0,0
LATS2_7_7870,512,11,112,1151,762,432,178,506,691,36,378,424
LIF_7_7871,0,12,0,0,0,0,0,119,177,416,0,17
LNX2_7_7872,863,325,495,886,1344,319,1263,941,1015,602,114,251
LOC283116_7_7873,1148,265,267,0,191,3,74,200,65,111,79,9
LONRF1_7_7874,855,389,1175,374,527,355,271,449,245,773,357,147
LTN1_7_7875,189,434,729,55,1008,271,975,1298,418,507,669,124
LZTR1_7_7876,81,11,222,782,578,116,392,471,10,6,2,315
MAP1LC3B_7_7877,467,119,524,0,1776,862,843,538,281,466,1599,1508
MAP3K1_7_7878,4,4,472,1,0,514,0,87,98,363,90,242
MARK1_7_7879,540,87,1467,5,130,475,3267,334,643,1043,748,439
MDM2_7_7880,14,293,97,174,46,987,954,89,507,84,132,312
MED20_7_7881,18,26,78,68,352,45,11,57,187,81,199,1042
MEX3B_7_7882,0,0,0,1,0,0,0,1,20,0,0,0
MIB1_7_7883,902,1591,900,463,89,109,683,446,618,816,2400,2185
MKRN2_7_7884,3,0,1,0,0,0,1,0,85,1,0,13
MKRN3_7_7885,1439,1275,651,271,1351,2,912,1203,989,977,473,182
MLL2_7_7886,0,90,1013,291,0,492,129,262,15,298,480,36
MLLT6_7_7887,109,61,789,0,5,6,233,1,140,0,463,340
MOCS3_7_7888,213,65,0,9,8,296,336,764,227,475,38,27
MRPL49_7_7889,895,2751,12,17,333,514,0,53,128,460,31,62
MUL1_7_7890,5,0,98,0,106,5,39,0,16,1,123,14
MYCBP2_7_7891,9,195,933,12,0,178,45,338,183,6,72,687
MYLIP_7_7892,0,0,0,0,0,0,0,52,0,0,0,0
MYSM1_7_7893,117,481,20,357,40,664,307,197,14,258,104,2
NACC1_7_7894,0,2,0,0,0,0,0,0,0,0,0,4
NACC2_7_7895,16,0,0,0,4,3,0,0,0,0,0,56
NEURL1B_7_7896,0,0,0,9,673,79,0,0,4,666,1,1

NEURL_7_7897,2,238,0,43,0,569,0,3,0,0,263,115
NHLRC1_7_7898,0,8,0,0,0,1,0,0,0,0,0,0
NUP43_7_7899,559,369,821,584,1714,204,591,979,929,329,976,421
OTUB1_7_7900,0,356,0,0,0,1191,0,0,0,207,956,1131
OTUB2_7_7901,249,44,927,11,48,3,1,524,73,513,419,628
OTUD1_7_7902,768,1178,1600,613,990,978,2964,1836,2978,1004,1988,1242
OTUD6A_7_7903,44,0,0,0,0,239,0,0,1,0,1,8
OTUD6B_7_7904,211,287,171,572,1519,664,183,607,828,776,191,928
OTUD7A_7_7905,0,74,0,260,0,0,0,5,0,0,0,0
OTUD7B_7_7906,756,396,1878,767,1066,978,290,1323,333,1147,3990,722
PAFAH1B1_7_7907,0,292,0,0,503,164,100,0,1,34,2,0
PARP10_7_7908,152,492,365,3,877,99,477,577,376,494,55,60
PARP11_7_7909,648,1283,954,1618,101,735,402,491,855,736,1992,1100
PARP14_7_7910,359,1,270,447,0,832,1204,0,482,540,455,338
PCGF1_7_7911,800,752,941,852,197,1105,1983,3410,2179,1051,1452,1046
PCGF2_7_7912,0,0,0,0,0,0,0,0,0,509,0,4
PCGF3_7_7913,346,97,277,186,768,42,36,0,0,193,13,0
PDZRN3_7_7914,160,1,405,107,1,71,364,8,121,580,154,507
PEBP4_7_7915,389,0,0,0,2,0,96,30,50,1,0,164
PEX12_7_7916,235,885,1142,96,2047,1880,34,570,824,1460,2026,980
PHF14_7_7917,1431,2828,613,185,898,758,260,566,877,91,36,121
PHF15_7_7918,344,43,0,0,0,1,263,22,440,35,1668,237
PHF20_7_7919,586,58,46,207,1178,1077,653,497,2,354,41,500
PHF2_7_7920,60,29,110,1002,64,339,0,387,61,401,561,146
PHF3_7_7921,69,739,288,516,846,2021,1623,253,1766,1916,828,940
PHIP_7_7922,3,0,0,0,0,216,0,1,165,195,28,159
PHRF1_7_7923,394,21,78,1199,2,182,268,0,567,211,399,85
PJA2_7_7924,526,200,1090,874,1752,1432,65,151,999,606,1333,1109
PRPF19_7_7925,152,0,484,240,2,302,342,47,38,588,16,280
PRPF8_7_7926,1,22,0,0,0,6,13,23,0,202,11,36
PSMD14_7_7927,203,338,2275,1330,46,1921,1609,1852,1498,2632,1614,1194
PSMD2_7_7928,623,0,313,96,20,190,416,0,637,390,190,82
PSMD4_7_7929,1,1,0,0,0,0,0,0,2,0,2,0
PSMD7_7_7930,6,608,285,178,229,291,16,29,3,177,8,399
PWP1_7_7931,1286,420,1953,4,310,689,1,526,191,61,154,510
RAB40A_7_7932,493,526,29,588,2184,459,473,659,100,1444,110,562
RAB40AL_7_7933,493,526,29,588,2184,459,473,659,100,1444,110,562
RAB40B_7_7934,0,0,6,0,276,0,0,0,3,0,17,0
RAD18_7_7935,3589,127,1210,156,1149,557,241,1322,1531,620,111,1584
RAD23A_7_7936,2,5,942,54,179,0,835,51,31,121,155,4
RAD23B_7_7937,89,0,0,177,0,550,0,3,209,52,389,20
RAG1_7_7938,1,3,54,21,1479,26,17,26,0,655,2,192
RAI1_7_7939,354,559,0,528,2716,0,0,0,0,808,483,1
RBX1_7_7940,1379,868,345,1053,0,842,83,16,248,514,122,394
RCBTB1_7_7941,748,1388,1353,651,1172,2167,1723,2440,534,1020,2412,2260
RCBTB2_7_7942,627,2615,1387,813,2093,706,3191,693,1546,2588,1474,553
RFFL_7_7943,601,157,0,0,138,0,0,4,186,10,35,15
RFPL1_7_7944,807,26,143,789,1496,0,47,138,85,84,1,331
RFWD3_7_7945,48,44,34,202,528,22,992,0,31,41,0,496
RHOBTB3_7_7946,8,14,613,32,141,204,220,474,5,40,38,276

RING1_7_7947,55,13,1289,123,1,1370,151,0,5,491,706,23
RNF10_7_7948,0,13,2,0,2,352,273,1114,235,280,0,562
RNF111_7_7949,151,276,812,768,164,435,1,234,350,372,2015,147
RNF112_7_7950,0,0,0,117,0,0,0,0,0,0,0,0
RNF113A_7_7951,1,4,0,1,0,0,0,364,13,1,403,1
RNF113B_7_7952,5,0,0,0,202,0,0,12,1,4,294,0
RNF114_7_7953,334,96,1246,890,0,379,0,254,95,1309,529,1145
RNF115_7_7954,176,108,42,117,0,1,653,2,292,445,279,94
RNF11_7_7955,223,586,1095,0,19,1,4,336,170,106,462,1629
RNF121_7_7956,0,1,0,0,0,0,53,0,0,0,0,0
RNF122_7_7957,30,0,0,0,498,0,337,7,3,0,0,21
RNF123_7_7958,1612,0,0,0,1502,504,387,14,415,681,1,63
RNF125_7_7959,115,327,135,26,228,180,702,294,542,72,342,134
RNF126_7_7960,0,0,23,0,0,0,0,0,0,0,0,0
RNF130_7_7961,231,105,51,326,388,411,98,99,54,145,225,195
RNF133_7_7962,468,1001,6,393,633,602,1243,977,365,1050,2224,899
RNF139_7_7963,138,1829,79,1022,11,333,4,287,97,1500,464,51
RNF141_7_7964,329,256,0,203,695,323,60,548,196,69,2,397
RNF144A_7_7965,0,0,0,0,0,0,0,0,0,3,0,0
RNF144B_7_7966,93,0,60,50,0,0,4,5,0,0,1,157
RNF149_7_7967,1501,1467,937,2710,1331,732,1821,972,2032,901,1388,2827
RNF150_7_7968,1166,1157,333,159,561,82,535,970,235,648,1203,248
RNF152_7_7969,59,380,715,164,97,76,154,554,770,225,28,284
RNF157_7_7970,3,69,909,133,350,6,760,114,83,403,275,1049
RNF167_7_7971,910,775,1305,440,3962,756,448,949,1507,1112,1526,1207
RNF168_7_7972,16,206,534,410,0,175,13,0,0,26,78,156
RNF169_7_7973,1630,412,2081,0,233,812,62,698,359,152,1035,1627
RNF181_7_7974,464,2,0,52,7,327,115,0,624,928,0,591
RNF183_7_7975,813,777,769,1899,753,831,1185,1633,557,382,429,544
RNF186_7_7976,0,0,0,540,0,7,0,0,556,29,0,75
RNF187_7_7977,0,24,0,114,0,31,13,0,481,310,30,54
RNF20_7_7978,1331,2,160,280,0,0,0,851,592,295,2,288
RNF217_7_7979,54,6,41,26,8,50,0,0,149,0,1,113
RNF220_7_7980,1669,271,109,270,318,39,1253,60,555,503,476,60
RNF25_7_7981,132,0,79,9,0,6,1027,4,14,0,26,2
RNF26_7_7982,274,6,0,64,9,167,2,19,82,148,131,16
RNF2_7_7983,528,557,427,8,0,636,16,114,172,1041,35,662
RNF31_7_7984,14,11,989,28,0,47,36,2,0,0,151,9
RNF43_7_7985,0,0,0,0,32,0,14,463,86,97,17,9
RNF44_7_7986,0,281,351,3,192,192,665,553,67,261,1201,876
RNF5_7_7987,4,44,11,177,137,150,135,28,316,8,0,131
RSC1A1_7_7988,45,16,0,4,0,192,0,0,0,149,6,0
RSF1_7_7989,694,351,1407,752,1203,1189,862,609,1193,234,611,1268
RSPRY1_7_7990,810,1594,1971,27,954,387,46,81,831,1091,1382,212
SCLY_7_7991,0,16,2,0,0,0,252,0,0,31,681,0
SENP1_7_7992,22,549,0,208,38,559,825,80,343,338,63,54
SENP2_7_7993,407,522,717,345,13,9,9,22,470,150,778,240
SENP3_7_7994,1,0,72,77,3,182,460,0,220,17,6,151
SENP5_7_7995,4,0,253,369,100,5,1806,266,83,319,731,1041
SH3RF1_7_7996,206,308,0,51,702,110,366,285,237,605,194,50

SH3RF2_7_7997,1540,300,435,1217,905,1820,1362,1654,3133,1604,830,3336
SH3RF3_7_7998,72,0,0,0,0,0,826,582,0,0,80,3
SHKBP1_7_7999,15,659,5,6,122,105,0,0,37,16,890,5
SIAH2_7_8000,2,0,0,14,81,4,158,187,2,413,107,87
SIK1_7_8001,798,530,539,198,1123,1433,308,353,767,344,1602,964
SLX4_7_8002,0,0,0,0,0,0,0,0,0,0,30,0
SMU1_7_8003,572,104,19,5,445,173,10,47,287,35,1,34
SMURF2_7_8004,22,66,0,7,648,728,72,551,0,313,1430,476
SNRNP40_7_8005,397,559,2,634,425,74,930,97,114,359,789,114
SOCS1_7_8006,1,187,887,442,8,62,7,18,85,511,879,23
SOCS2_7_8007,1389,1105,1302,41,52,1040,894,752,1269,1769,1593,2564
SOCS3_7_8008,31,19,0,0,0,0,17,9,134,6,4,30
SOCS6_7_8009,217,210,0,574,294,532,7,704,738,779,29,475
SPOPL_7_8010,550,637,0,516,658,0,1273,105,201,158,758,190
SPSB1_7_8011,0,0,0,2,0,0,0,0,0,0,4,53
SPSB3_7_8012,174,0,578,1,0,3,92,475,0,509,30,789
SPSB4_7_8013,5,226,146,235,1,326,0,13,0,46,0,0
STAM2_7_8014,53,0,0,19,0,5,106,1,70,2,0,11
STAMBPL1_7_8015,8,0,0,0,719,9,19,5,75,2,2,91
STAM_7_8016,1348,2889,472,506,5187,2556,4969,2385,1625,2310,2821,2953
STUB1_7_8017,0,0,497,59,1,2,0,1,443,10,7,58
SUMO3_7_8018,393,110,8,456,600,7,402,173,613,847,64,93
SYNGAP1_7_8019,276,32,296,202,510,635,71,0,312,49,1,850
TAB2_7_8020,1,0,1,192,23,1,0,52,85,483,689,182
TAB3_7_8021,505,5,268,1224,12,484,285,4,0,230,58,604
TAF1D_7_8022,7,80,417,3,0,0,580,0,50,0,0,3
TBC1D1_7_8023,218,7,1115,144,30,8,546,192,480,382,0,191
TLE1_7_8024,0,1,0,0,0,0,12,177,1,0,33,6
TNFAIP3_7_8025,1,284,328,170,852,287,443,551,382,734,420,1078
TOLLIP_7_8026,0,1,6,0,0,0,252,0,0,93,951,0
TRAF7_7_8027,266,29,128,72,325,88,146,306,28,293,5,52
TRAIP_7_8028,2,774,0,5,9,113,1558,40,713,423,1772,134
TRIM11_7_8029,0,0,386,0,0,0,0,0,0,0,0,0
TRIM15_7_8030,75,0,65,0,80,273,42,259,179,10,23,316
TRIM25_7_8031,1,1,372,624,7,258,767,2,1,98,0,100
TRIM27_7_8032,216,953,157,1018,623,1880,323,690,3310,1691,1641,1226
TRIM28_7_8033,0,1,246,3,0,0,9,791,0,6,917,0
TRIM31_7_8034,227,2,263,531,979,425,58,1645,382,22,0,633
TRIM35_7_8035,215,0,0,3,0,0,76,231,250,119,0,38
TRIM42_7_8036,241,48,30,276,420,0,0,0,0,9,134,23
TRIM46_7_8037,20,0,156,110,798,107,335,834,147,677,48,18
TRIM47_7_8038,0,0,0,0,0,0,0,0,0,0,0,0
TRIM48_7_8039,329,0,0,0,10,0,0,0,38,0,0,3
TRIM52_7_8040,176,7,0,122,1192,228,1254,243,25,95,0,79
TRIM56_7_8041,1,24,242,55,0,415,433,328,390,5,203,414
TRIM62_7_8042,0,0,0,0,147,0,0,2,0,9,7,0
TRIM63_7_8043,100,34,435,63,146,0,0,337,541,133,854,48
TRIM65_7_8044,4,0,3,0,53,0,0,9,0,0,16,0
TRIM67_7_8045,588,91,66,49,21,539,162,1091,134,144,113,629
TRIM68_7_8046,0,0,0,76,0,0,0,1,0,9,0,0

TRIM8_7_8047,0,313,0,3,0,24,0,0,0,205,393,0
TRIP12_7_8048,696,2668,1047,146,292,1126,1439,751,2140,807,462,1145
UBA2_7_8049,9,40,13,0,1565,74,2626,21,80,0,12,6
UBA6_7_8050,68,823,483,123,1560,529,811,214,169,230,29,109
UBA7_7_8051,0,0,1,0,0,0,2090,0,321,8,0,45
UBAC1_7_8052,71,2,143,17,46,134,1,220,4,36,143,323
UBAP2_7_8053,4,5,0,0,3,0,0,127,2,26,0,22
UBASH3B_7_8054,46,360,161,0,662,2113,652,0,534,397,101,833
UBC_7_8055,72,33,0,2,2,543,16,299,0,36,30,264
UBE2B_7_8056,962,1835,2368,1620,547,1093,1104,543,644,1549,801,2249
UBE2D4_7_8057,364,150,1309,33,0,507,9,173,107,3,34,229
UBE2E2_7_8058,0,1,0,1,0,11,18,5,15,174,0,0
UBE2F_7_8059,601,893,953,331,577,2144,1785,379,1614,733,1569,2146
UBE2G1_7_8060,1087,480,954,874,419,207,1087,474,1222,1189,723,1062
UBE2J1_7_8061,125,417,764,526,777,1703,377,898,810,1075,1074,2769
UBE2L3_7_8062,1532,262,547,1080,889,190,2129,469,948,1433,536,755
UBE2M_7_8063,0,10,0,0,1,66,0,0,0,0,575,0
UBE2N_7_8064,1173,519,190,479,121,132,1461,8,2062,633,1862,483
UBE2NL_7_8065,554,3,948,540,59,0,0,0,28,83,0,2
UBE2O_7_8066,205,0,0,0,29,0,0,0,0,0,29,0
UBE2Q1_7_8067,302,128,6,135,307,17,267,6,429,1393,0,185
UBE2QL1_7_8068,292,20,11,23,3,0,1830,2,160,83,39,27
UBE2R2_7_8069,369,684,161,614,124,639,291,56,40,66,964,458
UBE2S_7_8070,0,0,0,0,0,421,1,0,65,294,0,9
UBE2T_7_8071,47,97,1,655,101,28,118,1126,277,920,803,326
UBE2U_7_8072,2086,2418,4934,3295,3437,2409,3574,1956,3469,4354,2655,26
26
UBE2V2_7_8073,156,3,1072,34,11,96,1148,30,4,739,401,3
UBE2Z_7_8074,632,1228,1546,247,260,265,800,119,868,1292,2684,573
UBE3C_7_8075,352,88,49,64,69,8,4,238,561,195,170,151
UBQLN2_7_8076,776,1420,983,438,715,654,392,471,795,442,1658,379
UBQLN3_7_8077,568,1046,1244,70,2360,0,5,931,536,215,170,79
UBQLN4_7_8078,17,45,476,262,14,7,737,10,166,36,1,233
UBR1_7_8079,341,242,1,79,266,183,11,86,610,801,305,596
UBR3_7_8080,793,407,934,1039,1484,1114,1112,527,1537,464,1288,279
UBR4_7_8081,444,0,0,0,0,406,0,7,0,312,27,150
UBR5_7_8082,7,346,315,297,0,493,0,492,515,506,435,52
UBR7_7_8083,123,0,861,18,58,150,639,456,641,57,143,574
UBXN10_7_8084,0,0,0,0,34,20,12,4,0,49,4,0
UBXN1_7_8085,0,4,116,141,116,119,26,119,179,125,12,68
UBXN2A_7_8086,1140,1241,2910,589,1207,549,1461,503,763,351,1098,2263
UBXN4_7_8087,1548,99,1644,409,1309,960,58,1543,248,109,434,275
UBXN7_7_8088,647,758,1097,946,145,556,908,709,540,551,1476,129
UBXN8_7_8089,99,381,1,334,0,457,0,19,2,190,85,132
UCHL1_7_8090,21,0,138,442,716,1,0,305,671,387,6,74
UCHL3_7_8091,714,11,1000,694,7,292,960,712,1026,738,21,1628
UFC1_7_8092,633,234,157,535,0,565,266,178,31,498,0,1
UHRF2_7_8093,4,26,605,2,144,874,1579,318,271,505,777,959
UNK_7_8094,8,13,0,5,46,2,47,8,547,20,271,57
USP11_7_8095,74,27,0,24,431,2,0,1,0,9,116,3

USP13_7_8096,222,7,7,23,65,14,11,426,5,12,0,283
USP15_7_8097,8,15,0,86,634,20,360,209,6,0,37,646
USP17_7_8098,45,408,409,4,175,193,829,176,41,23,36,713
USP17L2_7_8099,196,413,19,58,12,274,5,855,255,256,0,1201
USP17L5_7_8100,45,408,409,4,175,193,829,176,41,23,36,713
USP18_7_8101,4,48,434,6,124,245,0,88,206,338,734,114
USP22_7_8102,0,9,0,0,18,0,0,0,2,0,0,1
USP24_7_8103,547,1142,2677,898,2422,1620,754,4074,1356,997,627,2813
USP25_7_8104,114,99,152,225,111,176,49,82,9,286,344,2
USP26_7_8105,733,2258,478,445,248,1573,1813,63,1328,1328,1190,1539
USP27X_7_8106,78,2,53,432,896,51,0,161,68,13,0,810
USP28_7_8107,487,617,196,1,1355,481,80,1326,445,595,694,342
USP29_7_8108,0,0,1240,11,0,0,0,0,873,12,1,1185
USP30_7_8109,199,286,114,246,1519,38,0,209,516,6,840,94
USP32_7_8110,62,96,52,7,1473,122,1,95,600,1850,790,73
USP34_7_8111,723,646,689,1803,205,381,801,418,1766,2050,3782,2212
USP35_7_8112,422,111,574,1616,0,15,209,1056,676,823,973,1572
USP36_7_8113,640,286,1,0,1145,568,36,243,191,8,59,746
USP37_7_8114,611,857,385,586,1838,627,4,220,219,550,856,39
USP38_7_8115,357,75,163,1164,224,856,16,1306,69,508,555,11
USP39_7_8116,301,617,253,42,1313,1362,1638,794,797,565,497,1531
USP3_7_8117,511,241,14,522,172,457,359,260,231,365,43,111
USP40_7_8118,299,510,4,221,1,370,1123,458,739,401,0,415
USP42_7_8119,2590,825,1629,2150,1707,2156,883,2307,1252,1657,901,3806
USP43_7_8120,0,0,0,17,0,3,0,110,0,0,0,1
USP45_7_8121,1809,166,772,1285,992,181,1834,1474,2076,1413,172,1048
USP47_7_8122,20,79,1,19,1391,240,0,0,107,65,239,86
USP49_7_8123,1,0,305,220,0,8,0,1173,241,260,0,34
USP50_7_8124,495,540,126,339,39,49,3,112,0,339,77,20
USP51_7_8125,3064,2000,216,908,833,0,218,415,866,382,454,1085
USP53_7_8126,57,0,8,2,0,102,159,0,47,159,0,6
USP54_7_8127,1183,1314,1323,956,3308,904,892,291,604,1778,2719,63
USP6_7_8128,0,1,1,98,0,510,63,1,84,626,0,26
USP7_7_8129,803,19,279,1094,1093,166,2,594,388,23,461,101
USP9Y_7_8130,498,730,378,1842,1827,769,1779,362,648,249,725,636
USPL1_7_8131,557,273,208,21,0,181,440,949,41,163,275,6
VCPIP1_7_8132,274,138,552,1,577,18,63,279,271,474,1418,81
VPS11_7_8133,84,0,0,0,0,89,0,0,16,0,0,10
WDR12_7_8134,211,331,327,18,4,123,336,1176,24,0,602,775
WDR53_7_8135,224,34,355,206,665,176,2,0,269,195,274,993
WDR59_7_8136,688,370,1,364,4,650,254,0,242,147,14,298
WDR5B_7_8137,0,30,2,570,225,78,33,100,37,43,197,23
WDR61_7_8138,10,8,6,53,5,2,453,3,59,57,724,12
WDTC1_7_8139,0,0,0,0,0,0,0,746,0,139,0,0
WSB2_7_8140,0,1,0,0,0,0,0,2,184,338,19,15
WWP1_7_8141,2131,747,1860,764,1208,1192,2936,1421,1359,4454,2868,473
YOD1_7_8142,3,0,0,16,0,0,28,204,0,0,0,444
ZBTB11_7_8143,512,348,955,570,0,2133,1423,280,239,576,876,35
ZBTB25_7_8144,0,85,205,136,766,138,59,86,2,0,8,614
ZBTB2_7_8145,0,0,5,506,0,35,0,0,16,1231,215,690

ZBTB32_7_8146,6,14,18,508,0,0,0,1408,551,176,0,56
ZBTB34_7_8147,0,462,0,78,0,172,0,0,1015,82,0,115
ZBTB39_7_8148,181,116,92,138,70,104,0,111,178,75,538,1536
ZBTB3_7_8149,0,358,403,64,45,4,31,1,306,37,163,42
ZBTB41_7_8150,1093,574,643,307,591,220,1361,1747,569,1038,1612,2086
ZBTB44_7_8151,286,193,723,22,0,48,47,105,221,713,6,64
ZBTB45_7_8152,0,0,388,0,255,0,31,22,2,1,2,1
ZBTB46_7_8153,1,0,39,493,10,51,843,716,0,154,6,0
ZBTB47_7_8154,40,149,4,208,1410,10,0,169,44,0,117,26
ZBTB48_7_8155,0,0,0,0,0,0,0,0,0,0,0,0
ZBTB49_7_8156,50,11,51,0,0,133,689,7,254,146,2,34
ZBTB5_7_8157,303,23,109,91,35,3,40,90,23,4,0,100
ZBTB7A_7_8158,1,0,3,189,0,309,0,141,0,1,0,2
ZBTB7B_7_8159,135,1,52,3,268,4,0,0,3,34,173,4
ZBTB7C_7_8160,148,720,283,5,125,684,0,9,13,537,13,18
ZBTB8A_7_8161,805,338,1066,31,460,839,1163,1352,1044,464,346,850
ZFAND2B_7_8162,314,9,4,17,0,366,1,1,51,58,9,188
ZFPL1_7_8163,0,1,0,92,99,4,0,0,718,20,0,91
ZMYND10_7_8164,42,0,15,96,0,601,0,23,28,5,54,367
ZNF131_7_8165,59,582,39,49,656,49,0,11,192,20,233,555
ZNF598_7_8166,0,40,0,413,825,11,0,199,546,61,35,98
ZNF645_7_8167,257,395,252,861,865,0,4,141,906,775,14,1931
ZNRF1_7_8168,421,190,110,403,53,376,105,3,551,18,0,70
ZNRF2_7_8169,55,1,1,98,0,8,0,74,158,2,167,119
ZNRF4_7_8170,0,0,0,0,0,249,0,0,296,3,87,37
ZRANB1_7_8171,5,206,402,105,64,948,1242,331,112,43,159,813
ZSWIM2_7_8172,674,275,18,397,210,285,895,290,158,369,622,638
AIRE_7_8173,138,72,35,146,0,75,5,354,174,16,74,24
ANAPC5_7_8174,120,146,1112,624,58,999,1962,1537,20,162,1,295
ANAPC7_7_8175,924,792,1219,1694,612,1553,2258,906,1370,1925,461,2399
ANKFY1_7_8176,0,0,0,0,222,0,0,34,239,95,423,30
ASB10_7_8177,792,642,1212,162,277,225,0,183,542,703,51,622
ASB11_7_8178,605,5,156,61,11,7,992,103,269,10,304,110
ASB14_7_8179,952,1481,987,384,4055,1558,2979,2556,751,3241,1259,1322
ASB2_7_8180,0,321,841,0,314,114,4,162,518,10,0,57
ASB3_7_8181,247,318,2917,104,269,181,4,1166,671,867,2581,780
ASB4_7_8182,0,175,0,0,1083,146,2,87,0,0,363,50
ASB6_7_8183,326,190,295,640,20,120,14,0,704,567,641,155
ASB7_7_8184,0,0,34,0,0,0,0,0,4,59,21,0
ASB9_7_8185,0,0,57,660,8,665,0,0,45,39,0,279
ASCC2_7_8186,1046,84,101,50,251,64,0,560,629,202,0,1185
ATG10_7_8187,259,114,324,76,487,360,364,86,688,281,360,252
ATG16L1_7_8188,254,299,1,20,642,207,261,1429,285,1234,1329,1362
ATG7_7_8189,26,38,26,227,1465,1796,634,585,272,27,329,36
ATRX_7_8190,14,87,6,288,0,46,73,101,526,223,65,295
BACH2_7_8191,0,1039,1100,33,0,210,339,826,659,10,63,85
BAG6_7_8192,185,326,685,8,277,44,973,1028,826,415,658,387
BAZ1A_7_8193,0,0,0,4,250,9,0,0,0,0,0,0
BCL6_7_8194,810,321,775,2181,838,862,3290,551,1299,1534,1024,1010
BIRC3_7_8195,165,259,77,166,586,351,1039,1208,484,683,193,468

BIRC7_7_8196,19,0,0,0,15,0,0,0,0,0,0,26
BPTF_7_8197,304,156,1175,31,48,0,0,102,1,10,2,502
BRCA1_7_8198,1222,86,482,0,1087,270,1512,392,576,176,901,811
BRPF1_7_8199,62,0,270,284,4,96,38,0,63,504,0,22
BRWD1_7_8200,339,179,1814,20,10,269,2287,0,466,1422,0,597
BTBD11_7_8201,13,32,8,12,575,318,42,36,90,239,1,12
BTBD1_7_8202,1093,491,33,68,320,132,13,421,1024,1238,5,710
BTBD3_7_8203,40,819,70,0,120,101,715,351,1007,51,1611,135
BTBD7_7_8204,249,83,73,142,39,82,31,64,753,147,219,558
BTBD9_7_8205,1950,222,955,560,942,1080,1306,269,2119,1291,2571,2508
BTRC_7_8206,848,64,661,87,0,378,1731,35,278,79,29,53
C3orf26_7_8207,662,15,1065,2,45,332,361,910,0,387,1744,149
CBLC_7_8208,0,1,0,0,0,0,0,0,0,60,0,0
CCNB1IP1_7_8209,730,1576,793,282,1773,1220,106,62,667,407,1585,334
CDC16_7_8210,849,978,911,720,526,412,804,13,335,217,355,604
CDC27_7_8211,1080,308,505,170,35,285,1398,464,569,1057,656,653
CHFR_7_8212,183,64,408,1010,772,151,509,306,909,2133,1159,1085
CISH_7_8213,718,0,0,7,0,679,0,0,1,1,477,1091
CNOT4_7_8214,2410,1154,2016,1494,3133,2672,5081,3168,3295,508,4671,247
5
COPS2_7_8215,1295,919,824,711,1028,279,573,805,997,468,1229,765
COPS3_7_8216,0,1260,618,120,531,314,701,443,366,139,105,185
COPS7A_7_8217,253,404,1186,100,5,99,1,47,172,4,844,34
COPS8_7_8218,1,919,20,0,1030,6,0,0,102,118,5,12
CRBN_7_8219,121,1252,514,404,2311,911,614,802,149,602,350,1326
CUL2_7_8220,232,0,0,0,0,104,14,35,287,5,337,26
CUL4A_7_8221,23,186,41,0,165,79,0,0,0,140,82,0
CUL4B_7_8222,322,1328,495,448,270,487,87,732,804,2271,2431,1073
CUL7_7_8223,0,124,0,10,31,0,73,58,0,107,4,0
CXXC1_7_8224,878,142,947,116,335,640,1194,17,790,719,1037,373
CYLD_7_8225,0,0,0,0,453,0,0,0,0,0,37,0
DCAF11_7_8226,531,48,0,41,1074,650,0,193,64,1,13,338
DCAF17_7_8227,111,0,6,180,295,479,0,366,0,55,0,402
DCAF4_7_8228,136,385,18,458,323,2,197,689,303,3,91,40
DCAF6_7_8229,113,202,2522,498,0,1497,92,0,1053,721,702,1003
DCST1_7_8230,1,761,25,65,206,156,10,251,137,1825,340,125
DEPDC1B_7_8231,866,20,1262,1489,587,858,3361,1519,991,1080,1533,1736
DET1_7_8232,22,124,53,141,87,657,179,39,629,412,835,818
DIDO1_7_8233,557,14,0,12,31,2,23,0,16,405,6,5
DNAJB2_7_8234,578,143,3,4,215,156,988,1,0,600,2,701
DTX2_7_8235,400,0,1017,0,0,0,0,0,92,2,0,8
EED_7_8236,1775,2442,1175,672,1180,1716,1652,3931,2836,1080,740,2118
EIF3B_7_8237,1641,13,531,924,691,264,535,0,35,247,259,697
EIF3C_7_8238,206,10,0,20,71,40,1,1,29,14,7,2
EIF6_7_8239,981,90,0,200,360,241,453,127,1153,724,684,159
EPN1_7_8240,108,157,1937,825,725,467,23,400,154,28,607,406
EPN2_7_8241,244,31,93,3,261,258,107,175,134,84,179,19
EPS15_7_8242,15,198,4,238,362,46,81,55,139,43,105,115
FAM70A_7_8243,16,0,11,290,0,2,0,613,67,3,617,12
FANCL_7_8244,43,429,224,82,589,24,43,3,161,318,547,28

FBXL13_7_8245,994,557,682,1011,1040,1265,185,871,914,476,1066,1375
FBXL20_7_8246,894,920,193,491,139,514,149,9,492,186,67,123
FBXL2_7_8247,1451,586,953,2655,232,707,790,2156,2257,1707,3495,1664
FBXL5_7_8248,802,449,2041,322,1168,70,396,1534,798,1499,4,178
FBXL6_7_8249,31,66,0,0,0,20,0,92,0,1025,0,8
FBXO11_7_8250,37,0,0,6,1493,0,120,1,0,0,0,68
FBXO15_7_8251,145,507,15,391,1179,645,0,0,330,583,98,72
FBXO17_7_8252,14,0,33,382,343,96,304,58,561,62,317,266
FBXO18_7_8253,4,0,0,526,1718,604,4,0,2,1,5,1
FBXO21_7_8254,27,256,0,20,0,0,0,0,21,2,0,0
FBXO22_7_8255,376,366,125,596,1354,0,57,353,0,1059,800,563
FBXO24_7_8256,896,566,2821,353,0,1330,1245,63,627,782,281,2625
FBXO25_7_8257,100,381,134,635,336,640,5,642,663,573,304,81
FBXO28_7_8258,1,0,0,0,162,0,278,773,13,0,347,313
FBXO32_7_8259,329,1096,865,87,2068,1244,43,485,179,773,819,22
FBXO34_7_8260,1042,0,1506,575,1,26,372,156,12,201,814,22
FBXO38_7_8261,725,53,743,75,1032,700,0,476,1149,597,1077,1712
FBXO3_7_8262,800,701,977,515,2481,36,307,1959,1638,1150,600,1900
FBXO44_7_8263,175,198,519,20,104,767,970,275,443,217,1474,1017
FBXO4_7_8264,135,115,388,90,26,266,16,941,7,633,779,4
FBXO5_7_8265,0,0,87,66,6,0,1,38,68,1,0,106
FBXO7_7_8266,262,1681,0,0,0,0,0,35,21,32,126,6
FBXO9_7_8267,349,22,7,40,0,1,1,813,80,1182,0,132
FBXW11_7_8268,1372,2374,956,167,294,1662,1535,513,1298,1051,2289,1138
FBXW7_7_8269,54,3,1001,9,36,4,0,0,49,94,612,6
FBXW8_7_8270,69,118,293,233,106,250,3,29,263,90,95,517
GPS1_7_8271,0,0,0,175,0,12,9,60,0,665,350,145
HECTD2_7_8272,1252,764,281,5,0,1435,1564,173,691,1961,844,127
HERC4_7_8273,514,586,110,173,1821,1396,699,631,541,1828,1301,869
HERC6_7_8274,379,0,0,0,52,0,0,2,0,0,21,0
HIC1_7_8275,0,0,0,9,0,283,0,0,0,258,0,11
HLTF_7_8276,349,307,962,2417,2554,1644,2728,2165,1337,114,984,1805
HSF4_7_8277,238,431,42,196,23,741,10,123,876,180,29,115
IPP_7_8278,1386,71,19,614,161,94,729,388,407,69,681,640
KAT6A_7_8279,0,0,0,0,0,5,1,0,1,45,7,0
KBTBD3_7_8280,118,8,259,663,549,526,26,61,253,74,462,53
KCTD6_7_8281,1711,427,214,794,171,819,1065,1867,180,580,1870,1320
KCTD7_7_8282,568,515,903,0,145,513,0,2,0,1,0,0
KDM2B_7_8283,23,1,0,130,0,1,1,208,1,50,261,13
KDM4C_7_8284,594,25,39,75,0,176,0,0,645,288,821,1020
KDM5C_7_8285,0,461,1,571,3,291,0,1118,201,4,0,321
KEAP1_7_8286,0,0,0,10,4,0,285,67,0,0,129,934
KIAA1841_7_8287,824,1303,788,1231,1365,497,681,1215,1248,1338,1140,636
KLHL13_7_8288,0,14,0,282,0,0,196,0,0,0,0,0
KLHL2_7_8289,155,1,114,0,639,3,31,0,56,79,7,883
KLHL4_7_8290,24,317,0,131,477,2,348,304,20,104,556,509
KLHL5_7_8291,191,948,15,418,296,298,191,73,5,694,834,5
KLHL7_7_8292,51,106,1,0,165,228,4,288,83,16,1306,838
LNX1_7_8293,103,88,1653,175,0,348,38,4,204,35,9,946
LONRF3_7_8294,0,6,11,206,0,536,4,618,0,0,591,0

LRRC29_7_8295,0,177,0,184,469,691,4,116,0,0,1,89
LRSAM1_7_8296,747,197,115,18,220,17,161,124,589,269,879,164
MARK2_7_8297,704,854,290,417,433,424,15,50,48,122,18,13
MARK3_7_8298,60,0,71,1,152,4,0,81,122,22,1,111
MARK4_7_8299,294,1,62,137,76,1,27,704,730,327,51,100
MDM4_7_8300,292,309,647,92,0,222,1278,266,21,73,384,303
MGRN1_7_8301,161,6,457,911,1,0,49,466,158,128,34,473
MIB2_7_8302,151,0,82,0,307,38,797,0,143,0,0,16
MID1_7_8303,0,636,193,0,0,16,467,0,229,22,114,336
MID2_7_8304,3,351,158,229,30,87,808,21,678,19,0,95
MKRN1_7_8305,252,1207,0,5,0,0,0,683,0,0,1225,47
MLL5_7_8306,7,398,203,654,158,98,762,0,1720,339,1,298
MLL_7_8307,662,1176,271,426,194,341,219,5,88,705,182,704
MNAT1_7_8308,610,79,395,613,83,804,304,10,1261,32,1070,282
MPND_7_8309,548,1,2,0,0,0,0,0,0,253,0,336
MTF2_7_8310,29,139,421,68,920,748,19,102,36,820,572,112
MYNN_7_8311,49,3,0,6,152,17,2,423,273,42,6,106
NAE1_7_8312,542,1037,245,1530,632,243,736,1243,1871,781,1060,438
NDUFC2_7_8313,291,639,1563,736,557,640,632,422,881,1174,488,590
NEDD4_7_8314,160,10,264,257,0,1003,1040,376,199,41,50,704
NEDD4L_7_8315,674,926,1207,894,2157,450,38,2027,933,699,378,944
NFX1_7_8316,0,220,82,578,0,0,285,681,99,3,0,15
NLE1_7_8317,1294,718,2281,276,1125,1391,154,355,1012,148,2244,2596
NSD1_7_8318,157,0,0,1,0,0,0,0,2,0,1,19
NSFL1C_7_8319,68,71,39,123,20,628,84,366,16,158,13,114
NUB1_7_8320,435,1393,1,27,254,644,751,928,998,1568,1073,631
ODF2_7_8321,6,10,0,1,0,249,156,834,68,257,3,6
OTUD5_7_8322,0,0,0,135,5,13,0,0,27,159,0,465
PARK2_7_8323,1608,2563,2851,1209,888,1441,2301,3513,1316,2153,3818,163
0
PARP9_7_8324,670,1,1812,237,642,1463,4391,188,880,1453,133,715
PATZ1_7_8325,0,0,0,0,2,0,0,25,0,0,0,0
PCGF6_7_8326,44,13,437,255,397,729,135,421,0,1,222,293
PEX10_7_8327,0,0,328,48,61,196,343,180,115,167,22,147
PEX2_7_8328,1,0,46,32,111,441,8,434,8,0,0,17
PHF12_7_8329,375,1017,422,671,488,486,820,562,1039,1334,811,972
PHF16_7_8330,0,0,0,0,0,0,19,0,0,58,660,531
PHF17_7_8331,830,746,972,1279,493,1002,628,2173,593,575,950,3053
PHF1_7_8332,39,589,332,485,235,119,21,3,158,463,771,969
PHF21A_7_8333,579,828,766,1671,465,131,2299,316,769,510,445,870
PHF7_7_8334,507,406,1315,1336,1170,992,279,408,595,989,245,867
PHF8_7_8335,1091,1604,1258,491,301,240,1737,936,1632,1601,2476,2238
PJA1_7_8336,0,0,0,0,0,0,0,158,0,0,0,0
PML_7_8337,93,346,38,12,254,9,0,674,743,496,10,100
POC1B_7_8338,268,20,1,166,179,0,376,0,12,114,1,306
PSMD1_7_8339,62,64,272,0,0,9,0,0,89,4,1,7
RAPSN_7_8340,0,1,1,0,0,0,0,0,3,1,0,1
RBBP4_7_8341,247,1578,1214,1429,1354,813,1288,1145,1575,1375,1060,2342
RBBP5_7_8342,33,114,908,0,407,250,0,175,46,375,275,76
RBBP6_7_8343,0,60,0,603,0,20,0,552,658,392,0,93

RBBP7_7_8344,2378,2639,1249,1784,4411,2138,4129,1703,994,1008,2986,262
2
RBCK1_7_8345,43,66,0,8,0,6,29,3,234,5,4,467
RC3H2_7_8346,41,1212,7,487,768,232,68,114,23,105,506,787
RCHY1_7_8347,0,33,105,0,401,237,0,8,32,43,110,20
RFPL2_7_8348,361,1276,1351,1585,1035,1568,1280,2163,634,1039,895,991
RFPL3_7_8349,283,1189,2238,580,852,520,1417,816,329,641,95,295
RFWD2_7_8350,941,1543,765,519,2031,1150,136,181,253,670,1364,370
RHOBTB1_7_8351,1267,378,390,1521,537,718,1303,1229,1601,2917,1085,1968
RHOBTB2_7_8352,1,0,738,8,7,2,0,1,0,0,0,274
RLIM_7_8353,368,1391,745,594,461,704,1249,0,111,2179,735,929
RNF103_7_8354,6,165,0,172,38,35,39,0,83,25,2100,8
RNF128_7_8355,1881,2112,494,1975,2180,1453,1279,1349,2890,1216,2642,19
26
RNF135_7_8356,0,0,559,0,0,0,0,0,0,1,0,0
RNF138_7_8357,3496,3292,957,2399,444,909,1626,1081,2934,2570,2355,1839
RNF13_7_8358,692,399,159,40,98,363,261,453,526,236,56,919
RNF145_7_8359,356,62,890,150,1,11,737,0,1431,346,18,947
RNF146_7_8360,41,62,1347,191,0,138,192,213,584,12,342,328
RNF14_7_8361,1336,272,663,555,835,172,0,385,1274,2134,615,380
RNF166_7_8362,588,256,110,1251,80,777,701,1281,437,286,1953,287
RNF170_7_8363,191,17,0,31,317,27,2,9,32,2,7,13
RNF17_7_8364,562,568,270,760,175,969,172,2090,598,1615,5,507
RNF180_7_8365,53,214,73,0,179,1976,1,828,495,50,2,66
RNF182_7_8366,20,74,45,266,29,734,1123,336,8,507,559,27
RNF185_7_8367,1,0,6,0,1045,36,252,0,257,212,1,321
RNF19A_7_8368,987,983,1938,1660,1328,1862,2234,2442,2064,713,2607,991
RNF19B_7_8369,15,0,0,221,0,52,458,0,281,653,0,33
RNF213_7_8370,29,9,303,456,780,0,512,27,9,2,153,977
RNF214_7_8371,449,446,1955,753,566,304,375,380,61,116,1239,1704
RNF216_7_8372,284,458,671,7,164,166,507,58,210,329,444,394
RNF24_7_8373,0,45,23,170,2119,112,6,284,20,107,103,1
RNF32_7_8374,364,772,252,152,1075,35,874,137,543,33,319,220
RNF34_7_8375,0,0,0,0,0,0,0,0,0,0,0,0
RNF38_7_8376,0,4,663,2,2,0,7,1383,176,470,150,63
RNF40_7_8377,1270,106,1279,188,179,726,1830,690,683,236,598,968
RNF41_7_8378,2008,1120,180,221,922,526,149,862,2209,1708,1189,1225
RNF6_7_8379,91,84,20,3,232,621,182,6,810,274,237,87
RNF7_7_8380,535,340,1193,386,199,26,1215,1011,89,547,443,757
RNF8_7_8381,6,220,625,22,155,0,956,459,314,29,1282,68
SAE1_7_8382,6,151,0,0,7,0,0,0,476,12,770,52
SENP6_7_8383,1395,1814,1233,1801,8608,3426,5427,1317,2098,2000,1987,16
17
SENP7_7_8384,100,706,2,112,391,303,112,148,542,296,603,328
SENP8_7_8385,196,415,10,10,257,5,0,214,10,9,13,2
SF3A1_7_8386,107,106,316,355,3,1,518,3,366,13,0,798
SHPRH_7_8387,29,6,0,55,0,2,940,22,0,5,722,89
SIAH1_7_8388,61,416,0,120,214,0,332,0,128,584,0,11
SKP2_7_8389,0,76,1,7,0,0,0,83,13,120,62,5
SMURF1_7_8390,34,1,0,328,0,0,0,62,0,0,0,0

SOCS5_7_8391,3,305,0,15,1,316,101,237,82,216,129,6
SP100_7_8392,1165,3469,1338,695,312,420,615,1351,339,1395,1609,2719
SP110_7_8393,0,6,0,40,218,422,1,568,166,1,1257,16
SPOP_7_8394,143,84,553,463,768,254,357,637,156,100,562,647
SPSB2_7_8395,126,21,120,950,137,971,1145,45,31,42,0,6
SQSTM1_7_8396,25,540,161,4,161,62,375,572,349,122,17,51
STAMBP_7_8397,7,229,525,0,311,355,40,373,617,1471,753,769
SUMO1_7_8398,1383,98,20,6,22,339,57,150,439,595,22,1345
SUMO2_7_8399,565,56,0,0,0,23,0,89,142,9,0,1004
SYTL4_7_8400,2021,381,1842,980,512,853,10,153,827,151,878,240
SYVN1_7_8401,3001,4307,750,5435,2824,1935,2975,3595,3710,2367,2260,119
9
TCF20_7_8402,0,430,0,0,222,160,21,791,42,5,0,29
TDRD3_7_8403,1004,1136,323,1727,2726,1180,1220,2570,1879,1175,828,1413
TIPARP_7_8404,1358,256,951,21,1066,238,390,721,1684,249,3056,1017
TLE2_7_8405,0,0,0,0,0,0,0,0,0,0,0,0
TLE3_7_8406,3,0,317,2,28,8,11,0,80,4,145,7
TNK2_7_8407,0,0,0,0,0,24,0,25,0,538,269,11
TNRC6C_7_8408,63,0,183,563,216,576,32,301,2,157,50,3
TOPORS_7_8409,282,94,35,1547,264,239,254,845,627,308,909,153
TOR1AIP2_7_8410,0,1,0,0,18,605,0,0,500,52,20,59
TRAF3_7_8411,22,195,48,106,0,0,280,0,2,0,138,4
TRAF5_7_8412,226,24,0,0,0,0,0,7,14,0,0,4
TRAF6_7_8413,1598,1253,2115,2555,2320,2026,1722,3199,3188,2514,922,132
9
TRIM10_7_8414,620,5,0,2,503,4,11,264,204,4,6,173
TRIM13_7_8415,0,1162,24,356,1127,152,999,97,2,63,1063,36
TRIM17_7_8416,121,194,0,259,1017,15,1,339,2,69,69,73
TRIM22_7_8417,218,0,306,567,34,723,338,55,292,475,0,424
TRIM23_7_8418,34,786,0,476,376,13,588,2,313,679,513,168
TRIM24_7_8419,203,50,0,162,164,680,1702,3,88,330,12,145
TRIM26_7_8420,1107,1456,1032,1258,1017,886,1342,1543,1348,1529,839,371
TRIM2_7_8421,595,0,67,3,0,476,342,197,12,0,305,422
TRIM32_7_8422,8,139,306,3,496,5,10,5,685,51,1,109
TRIM33_7_8423,126,1,141,28,0,1,309,314,128,4,24,21
TRIM34_7_8424,200,75,103,990,60,109,1118,406,372,53,1177,368
TRIM37_7_8425,284,263,840,36,779,596,333,1352,594,135,695,828
TRIM39_7_8426,678,1083,600,0,505,1047,83,3,69,976,1655,25
TRIM3_7_8427,0,0,0,0,0,0,0,0,0,0,0,0
TRIM41_7_8428,158,0,0,2,7,0,579,0,2,13,0,1
TRIM45_7_8429,303,2235,108,266,934,772,1088,1663,941,957,109,1225
TRIM4_7_8430,379,897,1176,1723,1397,1116,1419,1251,1318,1009,816,1691
TRIM54_7_8431,182,13,1001,280,807,521,928,370,1021,707,31,151
TRIM55_7_8432,507,170,319,180,7,17,38,384,110,101,0,33
TRIM5_7_8433,78,1005,1,0,0,492,0,90,68,734,606,6
TRIM7_7_8434,146,53,30,175,7,198,317,31,670,713,0,77
TRIM9_7_8435,0,6,0,0,18,514,0,10,61,557,6,7
TRPC4AP_7_8436,114,1201,1258,491,923,58,100,173,1140,1655,683,650
TSPAN17_7_8437,1,0,0,1,78,63,0,0,2,329,82,0
TTC3_7_8438,0,0,0,0,0,0,0,0,0,0,0,0

TULP4_7_8439,352,0,18,469,29,336,0,1583,1,225,154,211
UBA1_7_8440,40,0,0,98,0,9,105,17,71,4,0,12
UBA3_7_8441,3376,1991,300,2320,706,975,1052,1763,758,1186,4012,1201
UBA5_7_8442,404,703,31,501,179,525,14,0,79,149,639,18
UBAC2_7_8443,600,3541,1989,1617,1004,1648,1631,654,2099,1149,2138,5000
UBAP2L_7_8444,180,0,0,20,0,7,0,26,80,2,67,6
UBASH3A_7_8445,22,242,2,14,20,438,3,223,227,234,16,67
UBE2A_7_8446,76,1188,1051,685,304,804,543,590,903,1183,789,162
UBE2D1_7_8447,503,22,633,76,640,171,27,738,226,632,1366,506
UBE2D2_7_8448,424,106,83,22,0,308,0,0,0,0,296,0
UBE2D3_7_8449,3536,2924,1417,3232,3140,1530,4281,2198,2877,1652,1833,2
177
UBE2E1_7_8450,143,0,326,22,312,30,404,468,39,612,309,160
UBE2E3_7_8451,363,263,660,369,159,569,432,148,785,284,330,570
UBE2G2_7_8452,24,0,0,0,130,11,253,645,180,31,0,33
UBE2H_7_8453,852,79,45,12,1274,646,231,784,634,23,454,1095
UBE2I_7_8454,116,0,13,242,1,68,0,96,238,14,0,151
UBE2J2_7_8455,19,36,103,2,5,0,0,254,484,88,57,53
UBE2K_7_8456,1214,627,330,776,1620,880,2269,1439,564,447,785,962
UBE2Q2_7_8457,0,0,13,180,0,26,4,0,913,18,0,122
UBE2V1_7_8458,1890,429,1548,1951,465,400,1667,1592,2228,1437,611,395
UBE2W_7_8459,104,796,1541,232,132,1426,1046,1017,2377,655,2458,2023
UBE3A_7_8460,1601,214,721,1704,1435,767,1228,1053,267,595,1523,1686
UBE3B_7_8461,1,126,0,288,74,32,712,0,64,661,513,268
UBE4A_7_8462,34,505,613,231,9,995,365,175,50,350,0,6
UBE4B_7_8463,148,0,174,0,741,158,83,186,785,10,418,375
UBL7_7_8464,0,105,0,0,0,0,937,0,375,149,203,41
UBOX5_7_8465,575,36,141,0,41,25,0,102,114,91,212,286
UBQLN1_7_8466,613,808,973,194,329,296,1698,1082,552,2349,569,545
UBR2_7_8467,0,4,0,145,0,678,0,72,17,492,0,428
UBXN11_7_8468,76,70,809,0,0,5,17,0,10,0,6,517
UBXN6_7_8469,217,33,134,18,0,24,0,294,302,33,362,539
UCHL5_7_8470,1361,134,249,520,1025,717,75,354,1531,605,1388,219
UHRF1_7_8471,358,392,1145,4,1050,270,2,1383,554,308,268,893
UIMC1_7_8472,0,1,404,19,0,71,7,232,69,3,0,6
UNKL_7_8473,0,5,7,96,14,20,0,0,29,26,1076,2
USP14_7_8474,475,190,114,36,1827,780,75,592,292,100,596,31
USP19_7_8475,387,0,210,643,1525,620,0,20,399,648,971,1072
USP1_7_8476,10051,4020,3511,2773,1094,2757,3236,7546,4186,4151,5016,21
48
USP20_7_8477,277,63,8,281,106,281,227,454,129,1054,308,715
USP2_7_8478,12,26,31,63,201,281,557,189,251,512,554,74
USP33_7_8479,994,42,89,155,162,197,534,157,34,44,29,6
USP44_7_8480,42,16,0,151,0,53,0,56,5,1,0,0
USP46_7_8481,99,22,693,0,0,0,0,444,174,26,0,40
USP48_7_8482,7,132,24,0,113,49,605,79,259,38,302,41
USP4_7_8483,7,11,391,0,67,40,0,0,14,9,174,4
USP5_7_8484,259,136,48,845,0,4,3,9,3,8,39,37
USP6NL_7_8485,1555,2188,5133,1456,1483,950,1781,2262,1117,855,1498,903
USP8_7_8486,2164,1005,4760,926,2605,1873,2368,3591,2111,3449,4026,1875

USP9X_7_8487,11,1567,1055,0,237,26,0,264,2,27,5,0
VHL_7_8488,646,968,0,602,177,2,794,60,0,22,1,451
VPRBP_7_8489,190,872,193,1085,284,2104,585,499,466,1049,1160,857
VPS13D_7_8490,48,1,0,595,12,71,0,240,83,79,25,297
VPS41_7_8491,523,204,883,70,343,754,358,7,9,402,77,269
VPS8_7_8492,19,186,203,564,374,479,37,802,652,351,689,246
WDR26_7_8493,504,202,2639,1180,3216,3151,2015,1701,3153,1461,1620,2735
WDR5_7_8494,67,103,1000,381,0,440,480,44,44,142,0,563
WDR76_7_8495,280,17,1135,305,11,594,916,622,753,75,423,1260
WHSC1_7_8496,529,502,1044,1570,1174,2629,3310,2714,1387,1937,642,2558
WHSC1L1_7_8497,2927,1036,1343,791,1384,250,326,300,1498,1130,3669,203
WSB1_7_8498,1,972,929,1239,250,1089,341,637,245,343,5,102
WWP2_7_8499,539,241,362,0,0,23,73,0,157,12,50,750
XIAP_7_8500,31,534,5,410,0,94,258,0,273,546,691,473
ZBTB10_7_8501,36,3,353,354,43,0,56,0,150,177,15,82
ZBTB16_7_8502,1,119,0,80,0,21,9,0,0,19,0,21
ZBTB17_7_8503,194,347,280,10,44,20,0,1,174,1,893,44
ZBTB1_7_8504,1225,681,440,527,816,434,274,1939,1887,148,4320,1232
ZBTB20_7_8505,0,0,0,56,0,0,70,0,18,56,58,0
ZBTB22_7_8506,0,548,0,1124,0,93,31,11,1,313,0,3
ZBTB24_7_8507,639,416,890,13,455,115,1413,390,1324,1857,199,1149
ZBTB33_7_8508,0,0,3,0,185,2,20,835,142,4,118,159
ZBTB37_7_8509,45,1042,3,25,0,643,131,57,244,100,347,155
ZBTB40_7_8510,355,11,0,325,422,0,30,0,363,25,173,1827
ZBTB4_7_8511,369,511,15,1,1179,975,0,1221,1440,367,2502,1421
ZFP161_7_8512,999,135,17,38,23,697,1793,803,1184,653,31,1142
ZMYND11_7_8513,0,4,13,6,458,17,8,293,0,80,977,46
ZMYND8_7_8514,168,26,0,75,0,0,0,7,1,0,0,0
ZNF238_7_8515,23,2,0,0,0,529,0,43,3,0,102,60
ZNF295_7_8516,1164,71,0,52,29,11,463,0,36,2,0,4
ZNRF3_7_8517,559,895,1716,283,189,813,1296,534,737,1445,981,1077
ATXN3_7_8518,1,1981,0,8,0,0,286,278,286,322,40,736
MLLT10_7_8519,7,265,422,304,39,651,162,307,450,744,206,628
OTUD4_7_8520,286,490,0,283,0,138,137,372,257,503,416,1248
PHF19_7_8521,2205,446,269,583,1106,709,406,2484,2214,1086,959,838
SP140_7_8522,1881,161,19,274,1390,3791,187,953,726,980,759,1426
TRIM36_7_8523,15,0,345,613,13,122,148,18,36,0,0,103
UBE2C_7_8524,0,443,4,0,118,63,27,0,4,0,22,0
UBE2L6_7_8525,319,257,1209,276,212,548,119,336,1002,815,416,1237
ANAPC2_7_8526,644,0,0,23,49,0,0,0,1,0,0,0
ANAPC2_7_8527,1,51,21,6,0,110,0,0,99,8,0,24
ANAPC2_7_8528,92,2,0,36,516,533,1,2,143,2,29,22
ANAPC2_7_8529,0,0,0,0,0,0,0,0,0,0,75,137
ANAPC2_7_8530,0,422,0,0,0,0,0,0,0,1,0,0
ANAPC2_7_8531,80,0,10,1236,0,672,88,475,206,25,258,395
ANAPC2_7_8532,0,1,23,75,0,3,128,0,426,162,215,541
ANAPC2_7_8533,32,86,62,0,98,0,0,0,0,0,57,653
ANAPC2_7_8534,0,0,0,0,0,0,8,0,0,318,0,0
ANAPC2_7_8535,0,6,181,23,3,21,31,726,206,2,0,312
BRIP1_7_8536,257,0,313,0,582,0,81,0,220,164,1221,18

BRIP1_7_8537,1959,3956,2575,1368,3141,3282,4367,5064,2503,3904,4654,24
43
BRIP1_7_8538,308,327,194,250,151,4,5,669,790,28,60,164
BRIP1_7_8539,767,486,1522,399,632,611,777,75,452,877,83,705
BRIP1_7_8540,210,185,479,528,854,58,815,5,17,256,270,469
BRIP1_7_8541,1822,1295,2617,1657,1541,1118,2085,4118,1400,894,2977,152
7
BRIP1_7_8542,529,726,286,1860,174,872,1790,636,1481,1787,1609,503
BRIP1_7_8543,0,61,523,10,8,1130,135,4,0,6,323,46
BRIP1_7_8544,76,614,1369,3,15,48,1826,617,1676,106,695,1368
BRIP1_7_8545,249,847,1377,2395,930,1421,319,330,2105,402,2008,1432
COPS2_7_8546,463,311,150,577,355,152,485,700,411,853,355,224
COPS2_7_8547,12,44,265,105,26,78,33,4,19,47,0,30
COPS2_7_8548,626,500,67,75,417,1024,1082,29,215,230,315,338
COPS2_7_8549,307,1023,1294,586,1874,1397,11,5,226,4,1164,376
COPS2_7_8550,177,231,663,0,0,430,0,0,267,686,467,174
COPS2_7_8551,378,776,155,146,461,99,806,49,263,298,152,278
COPS2_7_8552,1356,1491,3772,1051,2091,739,7,1418,850,1466,1498,1509
COPS2_7_8553,12,293,1134,0,1719,756,24,1356,858,283,308,197
COPS2_7_8554,1119,1683,421,1012,750,1458,1268,2401,1113,2518,2228,460
COPS2_7_8555,0,8,0,9,21,152,0,0,3,195,302,1267
COPS3_7_8556,626,246,14,8,2123,3,331,112,164,451,246,787
COPS3_7_8557,0,0,35,47,27,25,60,51,2,292,573,1
COPS3_7_8558,3,0,0,0,87,6,271,116,0,12,37,1
COPS3_7_8559,234,87,487,209,736,315,0,246,477,137,854,1304
COPS3_7_8560,12,75,484,200,0,444,0,202,482,72,844,1096
COPS3_7_8561,85,44,0,0,0,11,1150,639,539,326,0,768
COPS3_7_8562,545,55,1,348,0,257,1407,30,163,33,563,509
COPS3_7_8563,569,602,0,248,42,19,64,219,625,1155,243,158
COPS3_7_8564,384,592,322,589,303,1626,31,2124,645,149,590,89
COPS3_7_8565,0,228,61,0,32,111,3,1,44,0,1,1
CXXC1_7_8566,452,274,37,0,465,11,0,0,138,3,0,11
CXXC1_7_8567,1308,319,5,693,12,258,1167,245,4,46,1403,566
CXXC1_7_8568,47,850,12,473,130,25,247,103,1186,803,358,598
CXXC1_7_8569,170,3,525,5,4,39,61,0,5,6,54,240
CXXC1_7_8570,7,151,0,0,195,56,799,0,502,52,206,702
CXXC1_7_8571,774,989,6,319,103,3,602,40,38,1013,416,667
CXXC1_7_8572,40,263,172,653,6,298,814,72,141,39,370,108
CXXC1_7_8573,990,1650,53,85,522,215,1568,776,756,875,932,2068
CXXC1_7_8574,0,741,706,257,0,413,0,281,433,270,383,135
CXXC1_7_8575,0,305,0,4,9,0,55,0,266,129,0,26
DEPDC1B_7_8576,0,0,775,1,0,654,7,23,5,6,245,0
DEPDC1B_7_8577,908,1050,199,1561,274,467,2989,1222,800,573,1195,399
DEPDC1B_7_8578,657,657,47,509,972,25,541,1368,140,881,715,456
DEPDC1B_7_8579,1173,1155,3018,2172,749,1314,2192,1335,1829,2388,1781,1
607
DEPDC1B_7_8580,115,0,1,0,0,0,18,485,0,1,68,822
DEPDC1B_7_8581,579,627,1066,645,58,111,0,967,488,786,678,299
DEPDC1B_7_8582,1789,503,237,879,2677,2568,2954,1996,1524,234,2305,2522
DEPDC1B_7_8583,799,167,357,0,0,364,8,84,141,128,16,13

DEPDC1B_7_8584,1951,2808,2204,1904,1045,2146,2823,1224,697,837,2097,2104
DEPDC1B_7_8585,0,1214,497,564,1,265,17,0,0,232,9,0
DIDO1_7_8586,928,45,9,700,34,124,134,453,490,1198,1258,414
DIDO1_7_8587,535,76,78,865,365,39,38,806,301,841,33,75
DIDO1_7_8588,1436,2424,810,967,2059,3545,540,68,1000,460,1765,2669
DIDO1_7_8589,741,231,722,723,267,1582,175,869,1135,1102,1256,1354
DIDO1_7_8590,458,0,0,0,0,0,1444,435,79,4,0,14
DIDO1_7_8591,1413,375,1770,1520,270,2171,578,1078,1501,1105,1264,1406
DIDO1_7_8592,0,0,0,0,0,0,476,3,0,0,2,1
DIDO1_7_8593,436,79,440,293,1159,234,101,8,69,125,48,2737
DIDO1_7_8594,38,117,20,20,13,264,203,106,49,667,0,534
DIDO1_7_8595,469,128,20,21,13,627,203,16,119,674,0,543
EIF6_7_8596,1156,1225,550,426,1556,2042,1114,1915,1595,1105,1307,1633
EIF6_7_8597,0,5,0,6,74,0,0,6,6,0,0,0
EIF6_7_8598,592,2805,1337,1201,804,576,285,195,658,406,0,333
EIF6_7_8599,25,179,0,1,66,1,0,0,56,134,31,10
EIF6_7_8600,661,2452,449,370,306,1256,512,164,888,582,35,906
EIF6_7_8601,413,918,779,616,353,681,877,1304,608,818,580,511
EIF6_7_8602,0,45,0,0,0,0,0,0,0,1,4,0
EIF6_7_8603,406,896,1469,42,668,1675,9,682,272,344,565,163
EIF6_7_8604,315,82,23,0,0,642,58,8,35,10,0,4
EIF6_7_8605,243,13,710,474,300,264,1701,127,761,459,0,818
IL6_7_8606,1838,1426,3278,1064,3456,1475,3172,1541,4859,3788,4039,2987
IL6_7_8607,1389,1892,2932,2314,1792,980,749,1278,619,3033,1402,1126
IL6_7_8608,237,0,24,19,0,5,0,0,2,18,0,0
IL6_7_8609,963,0,205,0,836,116,659,13,115,412,288,49
IL6_7_8610,75,643,783,255,3,617,139,0,183,37,1296,646
IL6_7_8611,524,147,40,0,33,380,13,46,194,5,288,19
IL6_7_8612,6,1269,130,9,13,1340,8,380,365,734,769,67
IL6_7_8613,41,0,836,413,29,16,3,0,58,2,0,18
IL6_7_8614,249,465,18,49,356,930,681,234,258,223,316,92
IL6_7_8615,146,116,151,448,62,2,3,111,5,560,162,4
KCTD11_7_8616,0,1492,30,0,0,6,0,0,63,410,372,30
KCTD11_7_8617,507,196,452,230,36,511,41,1053,757,212,365,944
KCTD11_7_8618,141,0,231,179,1193,472,1934,136,611,337,455,259
KCTD11_7_8619,150,149,277,7,44,415,639,63,99,1235,549,786
KCTD11_7_8620,136,514,1331,307,355,70,493,386,0,7,94,1134
KCTD11_7_8621,1164,1379,1948,944,1047,556,2209,2277,1212,1935,692,1672
KCTD11_7_8622,281,3,264,76,0,90,0,389,16,5,1081,0
KCTD11_7_8623,0,0,0,0,0,100,0,0,203,23,415,617
KCTD11_7_8624,893,1045,55,182,0,206,1615,76,27,88,48,32
KCTD11_7_8625,117,194,242,0,265,392,0,8,178,6,1043,709
KLHL7_7_8626,54,27,63,179,951,66,837,905,544,620,1632,400
KLHL7_7_8627,169,235,64,524,954,393,830,244,318,447,61,181
KLHL7_7_8628,467,155,142,533,1178,615,485,581,626,675,493,284
KLHL7_7_8629,0,0,0,0,0,0,0,0,0,0,0,0
KLHL7_7_8630,6,0,9,1,0,177,117,0,252,210,1122,51
KLHL7_7_8631,47,27,63,183,959,5,821,902,531,459,1596,390
KLHL7_7_8632,299,155,233,138,1227,284,481,725,627,706,189,284

KLHL7_7_8633,246,13,0,511,314,113,6,0,212,363,15,40
KLHL7_7_8634,135,42,65,502,961,183,785,219,212,589,287,872
KLHL7_7_8635,1036,121,1,1123,198,758,30,140,479,862,1577,300
LNX1_7_8636,1445,305,103,797,454,446,50,389,53,27,1713,1043
LNX1_7_8637,99,487,1544,231,1762,375,448,8,561,830,579,1014
LNX1_7_8638,0,269,183,583,277,123,0,1,2,500,0,136
LNX1_7_8639,1446,303,83,798,454,450,49,393,53,27,1235,1043
LNX1_7_8640,4,85,81,180,0,0,420,389,518,45,23,45
LNX1_7_8641,9,103,312,423,1025,406,157,201,263,211,741,88
LNX1_7_8642,147,719,844,355,166,10,674,734,27,341,154,28
LNX1_7_8643,1872,563,398,554,479,815,493,1190,319,1033,1727,747
LNX1_7_8644,350,430,1912,102,27,915,221,682,737,649,168,879
LNX1_7_8645,0,38,139,13,0,216,1446,124,138,94,78,47
RNF2_7_8646,303,32,0,942,181,78,0,192,552,31,7,57
RNF2_7_8647,154,28,0,788,0,7,0,36,40,52,14,11
RNF2_7_8648,1130,146,201,401,1101,858,734,657,1182,96,914,2076
RNF2_7_8649,96,141,1,14,69,20,0,38,15,75,118,3
RNF2_7_8650,82,42,122,2,0,0,0,425,17,2,0,96
RNF2_7_8651,48,2,0,297,0,8,0,0,613,12,791,202
RNF2_7_8652,681,826,317,15,165,206,25,302,181,548,883,1245
RNF2_7_8653,273,39,966,1,11,212,48,1009,199,631,0,109
RNF2_7_8654,39,511,87,983,0,0,91,3,70,434,626,16
RNF2_7_8655,579,770,116,209,552,362,1672,1821,648,187,543,400
MLL2_7_8656,0,0,12,0,0,14,0,432,161,2,9,27
MLL2_7_8657,0,155,0,0,0,0,1,79,14,91,182,0
MLL2_7_8658,35,666,18,245,71,515,0,798,570,485,338,72
MLL2_7_8659,0,913,42,102,115,164,0,392,254,0,125,2932
MLL2_7_8660,0,0,0,0,0,94,0,0,0,0,0,15
MLL2_7_8661,655,506,270,75,562,937,1000,469,178,470,689,191
MLL2_7_8662,0,14,2,0,15,0,0,0,0,76,0,0
MLL2_7_8663,15,0,1950,5,24,1,0,300,30,2,1243,645
MLL2_7_8664,34,10,0,1,0,0,0,38,0,606,61,0
MLL2_7_8665,688,486,71,768,22,29,1090,707,1026,210,974,678
MYCBP2_7_8666,932,666,2908,1108,3,1,188,1153,1241,1637,1701,2698
MYCBP2_7_8667,1891,133,600,886,1751,495,890,657,386,408,772,46
MYCBP2_7_8668,574,1215,3093,803,545,1977,1242,3321,1870,1088,468,320
MYCBP2_7_8669,16,2410,1471,345,1475,2276,983,2010,1614,1295,2359,938
MYCBP2_7_8670,2744,2473,6191,3186,3059,4103,1651,2536,4256,2966,3847,1
671
MYCBP2_7_8671,35,16,0,0,0,33,3,1293,41,0,41,84
MYCBP2_7_8672,1163,395,40,829,698,548,680,1430,137,1342,609,2281
MYCBP2_7_8673,1465,1172,780,725,2853,3307,1074,1201,1472,2879,1832,104
8
MYCBP2_7_8674,50,0,0,0,63,15,401,143,548,15,0,772
MYCBP2_7_8675,60,231,300,4,491,57,0,0,0,23,1,411
NEDD4_7_8676,32,0,0,23,64,153,7,67,121,633,0,476
NEDD4_7_8677,15,0,442,300,286,95,634,821,25,210,786,1397
NEDD4_7_8678,898,131,103,2451,1166,214,1019,285,193,158,1209,29
NEDD4_7_8679,20,0,0,0,0,0,0,0,6,1,1,13
NEDD4_7_8680,286,0,2,54,0,334,253,1142,0,301,475,8

NEDD4_7_8681,677,173,915,832,177,381,727,906,21,646,1691,238
NEDD4_7_8682,179,98,20,1,0,213,185,23,0,12,2,0
NEDD4_7_8683,1460,1157,2361,1348,2176,757,1643,732,2082,2294,3173,1692
NEDD4_7_8684,550,339,84,1453,318,83,5,1307,568,153,322,1140
NEDD4_7_8685,146,745,1423,2279,543,651,312,360,398,1831,1711,948
PHIP_7_8686,4406,4245,2675,6221,3249,3763,3581,3961,5465,7229,6293,598
5
PHIP_7_8687,582,585,353,544,932,675,227,1070,124,862,1023,544
PHIP_7_8688,270,166,0,771,58,1109,0,172,41,573,452,102
PHIP_7_8689,3,7,34,22,0,0,102,9,536,9,0,55
PHIP_7_8690,4366,5221,2566,4222,2503,2635,3573,2949,5401,5420,3356,600
6
PHIP_7_8691,35,462,991,1208,1094,352,779,2320,703,304,5,477
PHIP_7_8692,37,1,0,0,155,1,0,0,386,25,16,43
PHIP_7_8693,1,340,0,0,0,0,0,764,839,21,16,105
PHIP_7_8694,969,1570,300,1380,1136,864,163,1554,755,629,71,348
PHIP_7_8695,5695,4376,2575,4117,5902,5744,1964,5039,5553,4726,3196,727
1
RAI1_7_8696,509,917,322,1036,145,1498,803,668,383,896,516,1002
RAI1_7_8697,706,42,48,114,352,41,961,81,89,0,417,24
RAI1_7_8698,913,1045,3419,1275,777,397,559,631,1003,793,89,717
RAI1_7_8699,0,0,0,0,0,69,0,0,0,6,0,0
RAI1_7_8700,2,691,4,100,794,177,36,477,15,1,147,2
RAI1_7_8701,915,1024,985,1292,2458,871,215,3010,1193,1876,1392,658
RAI1_7_8702,802,1259,1204,120,1809,1013,1638,1212,359,602,3448,1647
RAI1_7_8703,882,259,937,161,316,686,470,722,495,329,458,663
RAI1_7_8704,19,165,26,174,9,638,55,491,937,1721,640,115
RAI1_7_8705,801,163,912,613,348,227,1034,99,263,738,1293,1102
RNF7_7_8706,2,77,0,4,46,8,1,0,151,5,0,25
RNF7_7_8707,619,132,3026,987,936,1004,660,994,941,874,596,1551
RNF7_7_8708,0,53,0,0,0,0,0,27,0,526,0,93
RNF7_7_8709,6,533,72,23,0,0,126,268,201,7,0,20
RNF7_7_8710,1452,22,861,2,0,2,692,0,158,141,142,559
RNF7_7_8711,18,0,0,0,0,0,0,0,0,0,0,98
RNF7_7_8712,1270,114,1,900,27,483,18,349,363,637,205,376
RNF7_7_8713,1418,794,527,474,2430,2256,253,1862,1330,938,2217,1585
RNF7_7_8714,43,409,0,63,0,839,5,0,88,3,0,11
RNF7_7_8715,0,0,0,0,0,0,0,0,0,10,0,0
RNF141_7_8716,19,36,807,0,0,94,0,5,21,211,0,455
RNF141_7_8717,0,260,0,342,925,42,0,0,0,93,240,212
RNF141_7_8718,0,236,105,0,18,153,70,352,333,743,0,468
RNF141_7_8719,25,375,120,253,13,32,163,468,146,90,0,908
RNF141_7_8720,2,428,0,79,477,0,154,0,4,295,572,0
RNF141_7_8721,39,2830,23,182,26,77,85,268,1010,517,0,538
RNF141_7_8722,1050,177,36,36,414,136,23,158,12,175,33,1
RNF141_7_8723,341,387,854,490,30,149,195,356,395,81,134,222
RNF141_7_8724,10,0,0,0,553,745,11,583,159,483,94,45
RNF141_7_8725,0,402,0,106,0,32,172,482,559,92,0,1240
RSC1A1_7_8726,4,45,17,46,560,34,0,29,95,20,20,16
RSC1A1_7_8727,1,944,161,0,91,0,256,0,89,7,0,9

RSC1A1_7_8728,0,169,246,84,44,0,198,60,1,107,0,317
RSC1A1_7_8729,83,30,0,37,13,9,4,26,416,236,34,47
RSC1A1_7_8730,472,422,420,274,401,295,6,807,259,577,523,213
RSC1A1_7_8731,848,97,710,1,467,0,794,454,710,453,1,616
RSC1A1_7_8732,900,139,652,466,1223,399,152,1892,152,1712,1827,2165
RSC1A1_7_8733,96,136,0,0,0,0,9,62,71,0,7,42
RSC1A1_7_8734,0,222,0,0,0,147,0,0,0,6,0,1
RSC1A1_7_8735,1956,2148,1483,470,2163,888,1939,279,1190,997,2163,1943
SOCS4_7_8736,0,13,0,89,66,460,25,0,152,880,61,1279
SOCS4_7_8737,285,1054,46,451,105,1237,188,240,8,4,76,294
SOCS4_7_8738,279,0,0,0,20,0,0,11,0,1,1,0
SOCS4_7_8739,241,166,0,35,584,1,11,406,26,103,708,6
SOCS4_7_8740,1,16,2676,470,930,8,728,11,438,93,5,1131
SOCS4_7_8741,434,15,0,741,52,136,808,810,1120,559,95,274
SOCS4_7_8742,197,4,28,291,0,533,203,8,67,772,9,648
SOCS4_7_8743,0,0,0,615,552,28,2,0,367,29,2,85
SOCS4_7_8744,316,1,5,149,0,150,0,26,190,132,6,1344
SOCS4_7_8745,0,0,0,84,449,96,642,212,150,27,25,19
SOCS6_7_8746,0,27,10,45,33,31,0,0,537,21,182,89
SOCS6_7_8747,1,0,925,0,0,204,0,394,0,442,0,586
SOCS6_7_8748,310,19,47,56,15,0,0,0,0,378,0,0
SOCS6_7_8749,2410,1080,1132,1736,3040,2535,3038,2650,2468,2051,3189,39
60
SOCS6_7_8750,1054,2644,1239,2735,2477,1545,2683,2564,2393,1008,5760,21
38
SOCS6_7_8751,999,2332,1325,1637,1949,1413,253,1554,1663,1699,1506,775
SOCS6_7_8752,235,262,281,142,1390,3,42,8,4,153,4,35
SOCS6_7_8753,1565,477,1553,113,45,391,1402,476,1320,415,4476,2929
SOCS6_7_8754,490,213,12,846,0,66,3,650,553,218,144,86
SOCS6_7_8755,0,332,0,0,562,710,483,941,1264,517,890,926
UBA2_7_8756,526,0,76,1,3,8,125,54,71,17,71,8
UBA2_7_8757,48,1396,1113,359,86,455,586,673,1066,451,737,128
UBA2_7_8758,1716,559,35,1587,1101,1583,138,1660,828,2509,584,1106
UBA2_7_8759,0,0,0,0,0,0,0,0,0,8,0,0
UBA2_7_8760,0,325,0,0,0,102,0,0,0,0,0,1
UBA2_7_8761,6,0,85,0,12,7,0,121,512,16,873,76
UBA2_7_8762,0,24,1,36,17,0,26,585,0,39,67,34
UBA2_7_8763,6,0,86,561,316,7,427,183,555,175,994,84
UBA2_7_8764,91,0,0,0,0,219,1,0,9,58,8,73
UBA2_7_8765,32,0,0,418,0,0,6,0,0,58,23,0
UBASH3A_7_8766,119,245,0,1,62,127,0,0,81,37,28,8
UBASH3A_7_8767,441,16,461,148,148,0,3,51,1697,931,1799,396
UBASH3A_7_8768,194,216,19,345,209,256,388,65,45,212,270,72
UBASH3A_7_8769,249,255,89,736,3394,1233,58,1174,1403,1758,1202,1459
UBASH3A_7_8770,967,1841,2313,4054,2259,1962,1793,1360,1790,1819,1414,2
865
UBASH3A_7_8771,127,309,18,89,1,360,43,756,495,29,261,278
UBASH3A_7_8772,966,1664,1548,3373,2860,2708,1199,927,1881,2301,3182,26
03
UBASH3A_7_8773,1725,963,87,44,121,24,765,506,138,409,880,192

UBASH3A_7_8774,138,44,114,160,53,309,17,4,196,94,271,54
UBASH3A_7_8775,998,205,3,1059,784,522,4,4,63,590,790,241
UBE2Z_7_8776,99,0,0,102,542,0,1,0,568,62,1,76
UBE2Z_7_8777,343,449,796,161,412,1,277,50,137,157,71,23
UBE2Z_7_8778,58,0,0,0,0,36,3,370,167,291,1,75
UBE2Z_7_8779,0,2,144,0,0,1,125,396,0,1,28,0
UBE2Z_7_8780,565,1262,0,75,496,192,265,840,436,392,2,310
UBE2Z_7_8781,474,42,0,11,81,249,4,204,1131,248,399,1073
UBE2Z_7_8782,43,410,0,0,0,0,0,0,0,179,206,0
UBE2Z_7_8783,19,0,118,0,390,30,184,17,466,7,34,73
UBE2Z_7_8784,263,350,24,24,713,213,486,381,382,82,1,255
UBE2Z_7_8785,879,883,406,1651,628,1199,649,836,836,952,879,472
USP12P1_7_8786,99,67,445,581,334,304,529,169,444,546,21,64
USP12P1_7_8787,995,1173,2267,638,1753,3092,2821,828,2392,2701,4393,152
3
USP12P1_7_8788,12,52,97,21,1,0,99,7,1,474,0,0
USP12P1_7_8789,504,10,564,755,26,4,0,3,37,526,118,748
USP12P1_7_8790,80,0,0,0,2,843,0,9,31,431,67,6
USP12P1_7_8791,338,126,216,0,698,11,1603,206,542,1002,305,97
USP12P1_7_8792,851,2322,1469,2472,3109,1584,1555,1487,2507,1413,1980,7
47
USP12P1_7_8793,56,912,0,190,1209,156,693,240,351,132,135,1272
USP12P1_7_8794,0,144,283,3,14,1,322,443,1,25,478,822
USP12P1_7_8795,427,103,27,232,308,296,21,269,608,292,38,582
USP21_7_8796,980,346,191,383,70,299,597,863,405,176,219,1029
USP21_7_8797,224,2,1,81,32,21,77,224,197,316,0,229
USP21_7_8798,145,433,2,416,427,630,1579,930,360,1836,679,370
USP21_7_8799,123,127,707,5,108,111,237,14,426,269,0,131
USP21_7_8800,2438,110,1092,267,270,186,381,106,373,269,1,276
USP21_7_8801,2572,959,2811,2237,4556,2598,2594,960,2043,1730,3029,1691
USP21_7_8802,1431,12,1843,2,52,10,141,231,52,1,57,794
USP21_7_8803,627,1245,808,1532,2056,476,1763,1059,435,754,2415,799
USP21_7_8804,763,489,688,48,0,294,2271,430,597,30,308,83
USP21_7_8805,101,2145,27,653,274,615,55,133,1329,230,690,520
USP25_7_8806,253,26,211,0,0,0,0,177,48,178,9,4
USP25_7_8807,2,10,64,0,24,124,281,29,20,0,31,209
USP25_7_8808,557,1263,856,331,2197,649,870,1502,471,969,1186,711
USP25_7_8809,2,5,84,0,0,3,73,0,11,4,390,2
USP25_7_8810,0,1,0,409,375,0,363,0,0,0,0,0
USP25_7_8811,10,3,1,468,6,323,57,256,148,122,252,181
USP25_7_8812,23,0,1,0,0,4,0,1,9,0,0,1
USP25_7_8813,0,0,0,0,1,11,0,0,1,12,0,0
USP25_7_8814,148,1,139,693,149,95,75,2,0,0,52,158
USP25_7_8815,382,208,20,493,386,1394,0,1,0,9,26,81
USP32_7_8816,720,795,1664,894,1008,2336,949,3940,735,818,1765,181
USP32_7_8817,206,4,21,2,142,704,568,76,514,893,160,481
USP32_7_8818,0,144,0,865,655,24,4,844,139,1,0,750
USP32_7_8819,23,227,293,628,96,258,1846,661,537,238,89,469
USP32_7_8820,61,3,1196,0,413,153,634,584,175,392,1,41
USP32_7_8821,235,2,32,1,0,309,22,0,12,70,1097,889

USP32_7_8822,231,485,258,42,0,2,308,121,120,618,0,85
USP32_7_8823,155,242,1,0,335,295,46,443,749,130,0,93
USP32_7_8824,0,0,0,0,0,2,0,0,0,73,0,0
USP32_7_8825,854,283,1715,299,59,133,598,778,46,638,312,1274
USP48_7_8826,275,54,59,16,21,0,62,37,2,77,166,0
USP48_7_8827,13,478,588,465,11,154,0,98,617,16,713,105
USP48_7_8828,228,350,507,403,22,1135,797,1,108,26,560,529
USP48_7_8829,4176,4514,3811,1768,5824,4274,4955,3980,6363,6559,5584,61
84
USP48_7_8830,685,65,299,129,13,82,2210,736,1220,1230,1362,608
USP48_7_8831,344,422,371,933,224,345,345,231,126,189,144,653
USP48_7_8832,2,586,313,558,2347,625,614,484,64,118,241,14
USP48_7_8833,491,227,700,256,0,125,0,4,39,1,229,8
USP48_7_8834,654,60,0,33,59,117,0,0,96,40,276,15
USP48_7_8835,754,6,0,442,0,151,342,0,21,471,812,35
ZFAND4_7_8836,669,1292,1706,2204,412,1707,1729,827,929,654,2329,1406
ZFAND4_7_8837,208,951,189,775,499,202,541,451,510,223,3,924
ZFAND4_7_8838,6,598,14,0,0,0,2,23,36,171,44,114
ZFAND4_7_8839,39,486,481,70,132,1079,7,451,213,415,760,613
ZFAND4_7_8840,239,286,147,26,2,472,7,493,245,139,0,805
ZFAND4_7_8841,971,28,1431,946,1344,367,1324,1098,414,1337,77,398
ZFAND4_7_8842,96,166,243,987,899,712,182,1236,474,1044,677,1165
ZFAND4_7_8843,43,96,680,127,64,663,157,471,0,130,239,56
ZFAND4_7_8844,143,844,187,578,508,59,9,306,517,44,3,821
ZFAND4_7_8845,60,193,628,527,61,176,72,318,13,608,554,43
